# Supplementary material for: Linking gene expression to clinical outcomes in pediatric Crohn’s disease using machine learning
Source: Sci Rep. 2024 Feb 1;14:2667. doi: 10.1038/s41598-024-52678-0 (PMC10834600; doi:10.1038/s41598-024-52678-0)
Supplement: Supplementary file 4 — Supplementary Table 4. [file 41598_2024_52678_MOESM4_ESM.pdf]

		model	AUROC	AUROC 95% CI	AUPRC	AUPRC 95%
Clinical variables<br>No RSI	B2	LASSO	0.5	0.434 - 0.566	0.288	0.191 - 0.3[illegible]
		RF	0.482	0.416 - 0.549	0.37	0.292 - 0.4[illegible]
		XGB	0.507	0.441 - 0.574	0.33	0.228 - 0.3[illegible]
		NN	0.583	0.518 - 0.649	0.284	0.188 - 0.3[illegible]
	Remission	LASSO	0.427	0.361 - 0.492	0.767	0.705 - 0.8[illegible]
		RF	0.531	0.465 - 0.598	0.811	0.754 - 0.8[illegible]
		XGB	0.561	0.495 - 0.627	0.84	0.786 - 0.8[illegible]
		NN	0.395	0.33 - 0.46	0.754	0.683 - 0.7[illegible]
	Surgery	LASSO	0.519	0.453 - 0.586	0.342	0.26 - 0.38[illegible]
		RF	0.435	0.369 - 0.501	0.339	0.249 - 0.3[illegible]
		XGB	0.564	0.498 - 0.629	0.357	0.275 - 0.4
		NN	0.573	0.508 - 0.639	0.409	0.313 - 0.4[illegible]
Clinical variables<br>With RSI	B2	LASSO	0.78	0.725 - 0.835	0.468	0.364 - 0.4[illegible]
		RF	0.74	0.682 - 0.798	0.509	0.429 - 0.5[illegible]
		XGB	0.817	0.765 - 0.868	0.567	0.429 - 0.5[illegible]
		NN	0.754	0.697 - 0.811	0.447	0.337 - 0.4[illegible]
	Remission	LASSO	0.703	0.642 - 0.763	0.889	0.844 - 0.9[illegible]
		RF	0.755	0.698 - 0.812	0.911	0.873 - 0.9[illegible]
		XGB	0.811	0.759 - 0.863	0.926	0.888 - 0.9[illegible]
		NN	0.673	0.611 - 0.735	0.847	0.785 - 0.8[illegible]
	Surgery	LASSO	0.633	0.569 - 0.697	0.488	0.39 - 0.52[illegible]
		RF	0.602	0.538 - 0.667	0.51	0.427 - 0.5[illegible]
		XGB	0.751	0.693 - 0.808	0.635	0.556 - 0.6[illegible]
		NN	0.713	0.653 - 0.773	0.524	0.426 - 0.5[illegible]
Clinical and gene expression<br>No RSI	B2	LASSO	0.79	0.735 - 0.844	0.468	0.354 - 0.4[illegible]
		RF	0.625	0.561 - 0.69	0.369	0.284 - 0.4[illegible]
		XGB	0.659	0.595 - 0.722	0.373	0.286 - 0.4[illegible]
		NN	0.806	0.753 - 0.859	0.589	0.509 - 0.6[illegible]
	Remission	LASSO	0.742	0.684 - 0.801	0.907	0.867 - 0.9[illegible]
		RF	0.716	0.656 - 0.777	0.902	0.862 - 0.9[illegible]
		XGB	0.68	0.617 - 0.742	0.867	0.817 - 0.9[illegible]
		NN	0.834	0.784 - 0.883	0.931	0.895 - 0.9[illegible]
	Surgery	LASSO	0.523	0.457 - 0.59	0.363	0.274 - 0.4[illegible]
		RF	0.656	0.592 - 0.719	0.559	0.482 - 0.6[illegible]
		XGB	0.577	0.511 - 0.643	0.356	0.269 - 0.3[illegible]
		NN	0.732	0.673 - 0.792	0.654	0.58 - 0.70[illegible]
Clinical and gene expression<br>With RSI	B2	LASSO	0.782	0.727 - 0.837	0.61	0.532 - 0.6[illegible]
		RF	0.647	0.584 - 0.711	0.329	0.222 - 0.3[illegible]
		XGB	0.814	0.762 - 0.866	0.517	0.424 - 0.5[illegible]
		NN	0.836	0.787 - 0.886	0.609	0.529 - 0.6[illegible]
	Remission	LASSO	0.828	0.778 - 0.879	0.94	0.907 - 0.9[illegible]
		RF	0.766	0.709 - 0.822	0.916	0.878 - 0.9[illegible]
		XGB	0.834	0.784 - 0.883	0.945	0.913 - 0.9[illegible]
		NN	0.755	0.697 - 0.812	0.899	0.87 - 0.94[illegible]
	Surgery	LASSO	0.599	0.534 - 0.665	0.517	0.437 - 0.5[illegible]
		RF	0.658	0.594 - 0.721	0.505	0.423 - 0.5[illegible]

	XGB	0.64	0.576 - 0.704		0.466	0.367 - 0.4[illegible]
	NN	0.731	0.672 - 0.79		0.603	0.525 - 0.6[illegible]

Supplementary Table 4. Full Results for AUROC and AUPRC for All Models and Outcomes

AUROC - area under the receiver operating characteristic curve,
AUPRC - area under the precision-recall curve

6 CI
05
18
48
02
18
59
34
99
3
72

42
95
52
51
57
28
49
59
34
2
6
34
58
36
11
14
41
45
41
09
54
01
15
95
3
53
42
58
6
71
52
75
7
7
57

99
57
