# Supplementary material for: Linking gene expression to clinical outcomes in pediatric Crohn’s disease using machine learning
Source: Sci Rep. 2024 Feb 1;14:2667. doi: 10.1038/s41598-024-52678-0 (PMC10834600; doi:10.1038/s41598-024-52678-0)
Supplement: Supplementary file 1 — Supplementary Table 1. [file 41598_2024_52678_MOESM1_ESM.pdf]

ensembl	gene	baseMean	log2FoldCh	pvalue	padj	biotype
ENSG0000(	AQP9	212.3146	6.479299	2.10E-28	6.57E-24	protein_coding
ENSG0000(	CXCL5	602.2539	5.797342	5.55E-28	8.67E-24	protein_coding
ENSG0000(	CXCL8	352.5058	4.940754	1.06E-26	1.10E-22	protein_coding
ENSG0000(	S100A9	124.6617	4.867321	1.16E-23	9.04E-20	protein_coding
ENSG0000(	S100A8	105.7021	5.831763	3.96E-23	2.48E-19	protein_coding
ENSG0000(	CHI3L1	835.5755	3.855663	5.29E-23	2.50E-19	protein_coding
ENSG0000(	OSM	97.61954	4.307992	5.60E-23	2.50E-19	protein_coding
ENSG0000(	REG1B	268.6374	6.102726	9.55E-23	3.73E-19	protein_coding
ENSG0000(	DUOX2	6412.8	4.180778	1.29E-21	4.05E-18	protein_coding
ENSG0000(	FCGR1B	179.971	3.137374	1.21E-21	4.05E-18	protein_coding
ENSG0000(	IL1B	771.6228	3.868796	1.57E-21	4.47E-18	protein_coding
ENSG0000(	TREM1	153.0155	4.429532	1.23E-20	3.21E-17	protein_coding
ENSG0000(	UCN2	91.62877	5.092998	3.88E-20	9.33E-17	protein_coding
ENSG0000(	HCAR2	169.8925	4.776189	4.84E-20	1.08E-16	protein_coding
ENSG0000(	CXCL1	482.1647	2.585821	1.46E-19	3.04E-16	protein_coding
ENSG0000(	ATXN1	4441.133	0.495897	1.75E-19	3.22E-16	protein_coding
ENSG0000(	TP53INP1	3404.961	0.947784	1.75E-19	3.22E-16	protein_coding
ENSG0000(	ENSG0000(	311.9418	2.561845	1.87E-19	3.24E-16	lncRNA
ENSG0000(	DUOXA2	281.8285	4.568564	4.63E-19	7.63E-16	protein_coding
ENSG0000(	INHBA	626.8933	3.583748	6.65E-19	1.04E-15	protein_coding
ENSG0000(	PRDM1	2311.652	1.229644	7.10E-19	1.06E-15	protein_coding
ENSG0000(	FCGR1A	95.87634	2.897471	1.02E-18	1.44E-15	protein_coding
ENSG0000(	APEH	887.5975	-0.6737	3.61E-18	4.91E-15	protein_coding
ENSG0000(	ENSG0000(	228.4436	0.704885	4.32E-18	5.63E-15	TEC
ENSG0000(	FCGR3A	390.8108	2.445528	7.08E-18	8.85E-15	protein_coding
ENSG0000(	GRSF1	2417.012	-0.42095	8.67E-18	1.04E-14	protein_coding
ENSG0000(	CCN4	459.14	2.611052	2.26E-17	2.61E-14	protein_coding
ENSG0000(	ETV6	3091.616	0.433217	2.43E-17	2.71E-14	protein_coding
ENSG0000(	CXCL6	93.72116	3.832095	2.58E-17	2.75E-14	protein_coding
ENSG0000(	MAZ	1794.613	-0.53245	2.64E-17	2.75E-14	protein_coding
ENSG0000(	SLED1	64.6498	2.709448	3.98E-17	4.01E-14	transcribed_processed_pseudoge
ENSG0000(	SAA1	39.85757	3.980185	4.87E-17	4.76E-14	protein_coding
ENSG0000(	ENSG0000(	331.2351	1.188265	6.05E-17	5.73E-14	lncRNA
ENSG0000(	GBP1	2137.331	1.920685	8.44E-17	7.77E-14	protein_coding
ENSG0000(	NOS2	1866.585	2.76503	1.04E-16	9.29E-14	protein_coding
ENSG0000(	CXCL3	367.1203	2.09073	1.36E-16	1.18E-13	protein_coding
ENSG0000(	ENSG0000(	1050.349	0.719255	1.46E-16	1.23E-13	lncRNA
ENSG0000(	CXCL2	155.5218	2.085072	1.74E-16	1.40E-13	protein_coding
ENSG0000(	RAB1AP1	259.6699	1.740719	1.74E-16	1.40E-13	processed_pseudogene
ENSG0000(	ENSG0000(	205.7053	0.779586	2.50E-16	1.96E-13	lncRNA
ENSG0000(	CXCL10	205.2695	3.038737	2.82E-16	2.10E-13	protein_coding
ENSG0000(	ENSG0000(	35.5304	3.143926	2.78E-16	2.10E-13	lncRNA
ENSG0000(	ENSG0000(	201.116	0.96634	3.08E-16	2.24E-13	TEC
ENSG0000(	CYTOR	1848.989	1.391568	3.34E-16	2.38E-13	lncRNA
ENSG0000(	ENSG0000(	117.0442	1.174626	3.48E-16	2.42E-13	lncRNA
ENSG0000(	DUSP4	291.3301	1.627276	4.13E-16	2.81E-13	protein_coding

ENSG0000(	IDH3B	624.1663	-0.60318	5.20E-16	3.46E-13	protein_coding
ENSG0000(	CCN1	536.5416	2.458004	6.21E-16	4.05E-13	protein_coding
ENSG0000(	CLEC4D	32.22037	3.859848	7.76E-16	4.95E-13	protein_coding
ENSG0000(	PIM2	2193.28	1.270576	1.02E-15	6.39E-13	protein_coding
ENSG0000(	KCND3	693.1978	1.054261	1.15E-15	7.03E-13	protein_coding
ENSG0000(	APOL4	593.8827	0.860882	1.29E-15	7.79E-13	protein_coding
ENSG0000(	ENSG0000(	74.9977	1.16867	1.38E-15	8.06E-13	lncRNA
ENSG0000(	PAQR9	409.7917	1.952728	1.39E-15	8.06E-13	protein_coding
ENSG0000(	ENSG0000(	864.9395	0.305195	1.68E-15	9.36E-13	lncRNA
ENSG0000(	THEMIS2	1065.41	1.141262	1.65E-15	9.36E-13	protein_coding
ENSG0000(	MED13L	8917.219	0.292133	1.93E-15	1.06E-12	protein_coding
ENSG0000(	TLR2	915.286	1.256925	2.05E-15	1.11E-12	protein_coding
ENSG0000(	TIMM21	612.7558	-0.44542	2.12E-15	1.12E-12	protein_coding
ENSG0000(	DSE	1461.372	0.698141	2.45E-15	1.27E-12	protein_coding
ENSG0000(	CSF3R	1330.277	2.210606	3.48E-15	1.78E-12	protein_coding
ENSG0000(	ENSG0000(	153.7514	0.784383	3.70E-15	1.87E-12	lncRNA
ENSG0000(	ENSG0000(	218.1485	0.808028	4.69E-15	2.29E-12	lncRNA
ENSG0000(	ERN1	4359.084	0.594207	4.66E-15	2.29E-12	protein_coding
ENSG0000(	GBP5	2589.825	1.973201	5.59E-15	2.69E-12	protein_coding
ENSG0000(	ENSG0000(	973.8588	1.076411	6.54E-15	3.10E-12	lncRNA
ENSG0000(	FKBP4	1000.57	-0.63819	7.50E-15	3.49E-12	protein_coding
ENSG0000(	SLC1A3	852.9051	1.792719	7.59E-15	3.49E-12	protein_coding
ENSG0000(	CHST15	1610.545	1.05247	8.93E-15	4.05E-12	protein_coding
ENSG0000(	ENSG0000(	488.7458	0.396199	9.30E-15	4.12E-12	lncRNA
ENSG0000(	SLC6A14	378.8244	2.480896	9.36E-15	4.12E-12	protein_coding
ENSG0000(	TNPO3	1651.13	-0.30904	1.09E-14	4.73E-12	protein_coding
ENSG0000(	IGHG3	2370.43	2.965262	1.14E-14	4.89E-12	IG_C_gene
ENSG0000(	LILRA1	56.32177	1.794148	1.20E-14	5.05E-12	protein_coding
ENSG0000(	APOL2	1718.457	0.933803	1.37E-14	5.73E-12	protein_coding
ENSG0000(	NDUFA13	557.7299	-0.57628	1.43E-14	5.88E-12	protein_coding
ENSG0000(	IRF1-AS1	4216.505	0.609689	1.56E-14	6.25E-12	lncRNA
ENSG0000(	SAA2	300.1715	1.790665	1.54E-14	6.25E-12	protein_coding
ENSG0000(	ENSG0000(	339.5816	0.521771	1.74E-14	6.71E-12	lncRNA
ENSG0000(	LAMTOR4	534.3547	-0.43374	1.70E-14	6.71E-12	protein_coding
ENSG0000(	SOCS1	136.6132	1.521641	1.74E-14	6.71E-12	protein_coding
ENSG0000(	IL1RN	265.1698	3.341222	1.99E-14	7.51E-12	protein_coding
ENSG0000(	QARS1	2005.333	-0.45999	1.97E-14	7.51E-12	protein_coding
ENSG0000(	ADAMTSL4	647.9727	1.064355	2.09E-14	7.67E-12	lncRNA
ENSG0000(	RAB31	977.0462	1.356758	2.08E-14	7.67E-12	protein_coding
ENSG0000(	H2BC9	196.0378	-0.89652	2.21E-14	8.04E-12	protein_coding
ENSG0000(	PLEKHS1	556.6088	1.476969	2.28E-14	8.18E-12	protein_coding
ENSG0000(	NEURL4	945.4444	-0.54081	2.57E-14	9.15E-12	protein_coding
ENSG0000(	SUPT20HL2	2144.224	1.608234	2.84E-14	9.98E-12	protein_coding
ENSG0000(	ZNF449	357.4799	0.501689	2.90E-14	1.01E-11	protein_coding
ENSG0000(	IRAK3	1742.995	1.141289	3.15E-14	1.08E-11	protein_coding
ENSG0000(	GBP4	2445.502	1.044437	3.19E-14	1.09E-11	protein_coding
ENSG0000(	MIR34AHG	570.6522	1.172928	3.39E-14	1.14E-11	lncRNA

ENSG0000(	GLT1D1	47.47456	3.672668	3.71E-14	1.23E-11	protein_coding
ENSG0000(	CELF5	3109.582	1.323591	3.93E-14	1.28E-11	protein_coding
ENSG0000(	LCN2	882.3357	2.443968	3.92E-14	1.28E-11	protein_coding
ENSG0000(	NUP88	1495.165	-0.37892	3.98E-14	1.28E-11	protein_coding
ENSG0000(	ENSG0000(	252.5534	0.718451	4.14E-14	1.32E-11	lncRNA
ENSG0000(	U2AF2	1713.903	-0.33366	4.22E-14	1.33E-11	protein_coding
ENSG0000(	INTS11	1868.921	-0.31257	4.29E-14	1.34E-11	protein_coding
ENSG0000(	STAT1	7069.108	1.305472	4.32E-14	1.34E-11	protein_coding
ENSG0000(	HNRNPUL2	2935.327	-0.32434	4.53E-14	1.39E-11	protein_coding
ENSG0000(	CFB	734.0856	1.451258	6.64E-14	2.02E-11	protein_coding
ENSG0000(	TAGAP-AS1	1527.64	0.257267	7.30E-14	2.19E-11	lncRNA
ENSG0000(	ALPL	136.4578	2.376205	7.44E-14	2.22E-11	protein_coding
ENSG0000(	LINC02384	551.2044	0.827229	8.76E-14	2.58E-11	lncRNA
ENSG0000(	IGHG2	3421.661	2.707075	8.96E-14	2.61E-11	IG_C_gene
ENSG0000(	SLC31A2	264.942	0.96378	9.03E-14	2.61E-11	protein_coding
ENSG0000(	STIP1	824.1867	-0.37201	9.14E-14	2.62E-11	protein_coding
ENSG0000(	ENSG0000(	36.41306	1.717567	1.02E-13	2.89E-11	lncRNA
ENSG0000(	CEACAM4	34.23367	2.005497	1.10E-13	3.07E-11	protein_coding
ENSG0000(	ENSG0000(	383.281	0.422903	1.10E-13	3.07E-11	lncRNA
ENSG0000(	TOMM40	912.6478	-0.62186	1.11E-13	3.07E-11	protein_coding
ENSG0000(	PPP5C	1017.32	-0.3576	1.15E-13	3.15E-11	protein_coding
ENSG0000(	MTCO1P11	301.3873	0.882694	1.23E-13	3.33E-11	unprocessed_pseudogene
ENSG0000(	ATP5F1A	6902.756	-0.58167	1.28E-13	3.44E-11	protein_coding
ENSG0000(	CADM1	436.216	0.942935	1.29E-13	3.44E-11	protein_coding
ENSG0000(	IRF1	6172.502	0.880276	1.33E-13	3.54E-11	protein_coding
ENSG0000(	ATP5MC1	840.9797	-0.91054	1.42E-13	3.72E-11	protein_coding
ENSG0000(	LRRK2	1524.442	1.269438	1.43E-13	3.72E-11	protein_coding
ENSG0000(	REG1A	1460.915	3.724275	1.45E-13	3.74E-11	protein_coding
ENSG0000(	ELL2	1599.279	0.952956	1.51E-13	3.85E-11	protein_coding
ENSG0000(	GUSB	1094.149	-0.29451	1.50E-13	3.85E-11	protein_coding
ENSG0000(	USP7	4035.005	-0.32199	1.58E-13	3.98E-11	protein_coding
ENSG0000(	TECR	1249.476	-0.32449	1.62E-13	4.04E-11	protein_coding
ENSG0000(	CPZ	40.91066	2.37028	1.67E-13	4.14E-11	protein_coding
ENSG0000(	ENSG0000(	15.76126	1.623614	1.71E-13	4.18E-11	lncRNA
ENSG0000(	IL6	61.73142	3.41969	1.71E-13	4.18E-11	protein_coding
ENSG0000(	COL7A1	2658.403	2.348303	1.80E-13	4.37E-11	protein_coding
ENSG0000(	UBE2L6	613.6959	1.125379	1.87E-13	4.50E-11	protein_coding
ENSG0000(	COL6A3	8909.308	1.266843	1.93E-13	4.62E-11	protein_coding
ENSG0000(	GAS1	99.55759	3.168092	2.02E-13	4.78E-11	protein_coding
ENSG0000(	GBP1P1	129.4709	1.715477	2.20E-13	5.18E-11	transcribed_unprocessed_pseudo
ENSG0000(	WARS1	3173.051	1.263519	2.22E-13	5.19E-11	protein_coding
ENSG0000(	PA2G4	971.8458	-0.50664	2.32E-13	5.37E-11	protein_coding
ENSG0000(	TRIM38	3999.158	0.360844	2.45E-13	5.63E-11	protein_coding
ENSG0000(	CXCL11	58.33914	3.117564	2.71E-13	6.18E-11	protein_coding
ENSG0000(	TNIP3	477.2816	2.118649	3.49E-13	7.92E-11	protein_coding
ENSG0000(	VNN1	207.4842	2.208656	3.62E-13	8.15E-11	protein_coding
ENSG0000(	ATP5ME	356.0908	-0.93788	3.74E-13	8.36E-11	protein_coding

ENSG0000(	CISD3	686.4598	-0.69285	4.22E-13	9.30E-11	protein_coding
ENSG0000(	EIF3K	1296.839	-0.42059	4.20E-13	9.30E-11	protein_coding
ENSG0000(	LILRA6	139.5845	1.648486	4.48E-13	9.79E-11	protein_coding
ENSG0000(	ADM	394.9876	1.521263	4.72E-13	1.02E-10	protein_coding
ENSG0000(	P2RY13	250.7954	1.381812	4.86E-13	1.05E-10	protein_coding
ENSG0000(	DOCK4	1381.98	0.841273	5.06E-13	1.08E-10	protein_coding
ENSG0000(	FAP	139.6939	2.553874	5.34E-13	1.14E-10	protein_coding
ENSG0000(	CXCR2P1	234.1015	1.55362	5.42E-13	1.15E-10	transcribed_unprocessed_pseudo
ENSG0000(	N4BP2L2	14882.09	0.319896	5.54E-13	1.16E-10	protein_coding
ENSG0000(	APOL1	1397.789	1.062739	6.34E-13	1.31E-10	protein_coding
ENSG0000(	TDO2	213.5707	1.825952	6.33E-13	1.31E-10	protein_coding
ENSG0000(	ADGRG3	121.4504	2.387294	6.59E-13	1.36E-10	protein_coding
ENSG0000(	SAMD9L	3931.333	1.212978	6.75E-13	1.37E-10	protein_coding
ENSG0000(	WSB1	7917.935	0.395601	6.76E-13	1.37E-10	protein_coding
ENSG0000(	MTND5P14	111.9638	1.032271	7.53E-13	1.52E-10	unprocessed_pseudogene
ENSG0000(	AUP1	1524.115	-0.38077	8.02E-13	1.61E-10	protein_coding
ENSG0000(	MIR4435-2	4014.775	1.185694	8.16E-13	1.62E-10	lncRNA
ENSG0000(	ENSG0000(	1280.05	0.827061	8.26E-13	1.63E-10	TEC
ENSG0000(	ENSG0000(	5856.216	2.322711	8.39E-13	1.65E-10	processed_pseudogene
ENSG0000(	ENSG0000(	5243.771	1.88072	8.57E-13	1.68E-10	lncRNA
ENSG0000(	ENSG0000(	6351.621	1.228248	9.40E-13	1.82E-10	lncRNA
ENSG0000(	PHB2	1390.644	-0.49949	9.35E-13	1.82E-10	protein_coding
ENSG0000(	PCMTD1	2816.248	0.411639	1.04E-12	1.99E-10	protein_coding
ENSG0000(	THBS2	330.7482	3.172335	1.06E-12	2.02E-10	protein_coding
ENSG0000(	TGM2	1860.593	1.174112	1.15E-12	2.17E-10	protein_coding
ENSG0000(	IL18R1	475.1982	0.847485	1.16E-12	2.19E-10	protein_coding
ENSG0000(	DDX17	41253.8	0.206515	1.22E-12	2.26E-10	protein_coding
ENSG0000(	FAM214A	3623.37	0.372049	1.21E-12	2.26E-10	protein_coding
ENSG0000(	ENSG0000(	493.0702	0.617421	1.23E-12	2.27E-10	lncRNA
ENSG0000(	PDPN	316.3726	1.38629	1.34E-12	2.47E-10	protein_coding
ENSG0000(	FPR1	141.4951	2.093356	1.36E-12	2.48E-10	protein_coding
ENSG0000(	TXN	543.0516	-0.81318	1.36E-12	2.48E-10	protein_coding
ENSG0000(	SP100	9642.275	0.672376	1.51E-12	2.72E-10	protein_coding
ENSG0000(	CD274	382.3523	1.740268	1.68E-12	3.02E-10	protein_coding
ENSG0000(	BTN3A1	3341.886	0.665461	1.96E-12	3.49E-10	protein_coding
ENSG0000(	ENSG0000(	417.0744	0.51367	1.98E-12	3.49E-10	lncRNA
ENSG0000(	ENSG0000(	43.31613	1.193968	1.97E-12	3.49E-10	lncRNA
ENSG0000(	MRPL11	203.3182	-0.76372	2.02E-12	3.51E-10	protein_coding
ENSG0000(	OCIAD1	1625.506	-0.24492	2.01E-12	3.51E-10	protein_coding
ENSG0000(	SOCS3	940.3458	1.973968	2.02E-12	3.51E-10	protein_coding
ENSG0000(	HSPE1	472.4327	-0.71448	2.05E-12	3.54E-10	protein_coding
ENSG0000(	PDE4B	918.9307	1.582198	2.08E-12	3.57E-10	protein_coding
ENSG0000(	ENSG0000(	49.92261	1.639386	2.10E-12	3.59E-10	TEC
ENSG0000(	CEMIP	694.0137	2.457757	2.15E-12	3.66E-10	protein_coding
ENSG0000(	HSPD1	3208.507	-0.66197	2.36E-12	3.98E-10	protein_coding
ENSG0000(	BTG2	2576.617	0.879292	2.44E-12	4.10E-10	protein_coding
ENSG0000(	KLF7-IT1	102.8957	1.237704	2.52E-12	4.21E-10	lncRNA

ENSG0000(	GBP2	1894.153	1.131253	2.79E-12	4.63E-10	protein_coding
ENSG0000(	SDC3	1508.48	0.678058	2.81E-12	4.65E-10	protein_coding
ENSG0000(	FER1L4	1310.654	0.713074	2.86E-12	4.69E-10	transcribed_unitary_pseudogene
ENSG0000(	TFAP2C	39.17069	2.180272	2.86E-12	4.69E-10	protein_coding
ENSG0000(	MINK1	4096.358	-0.51223	3.16E-12	5.06E-10	protein_coding
ENSG0000(	NOD2	333.904	1.158891	3.15E-12	5.06E-10	protein_coding
ENSG0000(	PRRG3	8573.681	1.680933	3.16E-12	5.06E-10	protein_coding
ENSG0000(	RIPOR3	752.481	1.008002	3.15E-12	5.06E-10	protein_coding
ENSG0000(	FH	1125.738	-0.49018	3.26E-12	5.21E-10	protein_coding
ENSG0000(	UGDH	3343.501	-1.0287	3.28E-12	5.21E-10	protein_coding
ENSG0000(	SF3A2	602.5729	-0.31412	3.44E-12	5.43E-10	protein_coding
ENSG0000(	ETV7	151.4208	1.054861	3.47E-12	5.46E-10	protein_coding
ENSG0000(	FCRL5	4156.152	1.009548	3.50E-12	5.48E-10	protein_coding
ENSG0000(	MMP25	375.4299	1.712429	3.73E-12	5.77E-10	protein_coding
ENSG0000(	NAMPT	12509.3	1.596047	3.74E-12	5.77E-10	protein_coding
ENSG0000(	STAG3L3	2348.613	-0.30543	3.74E-12	5.77E-10	transcribed_unprocessed_pseudo
ENSG0000(	CTSK	1651.49	0.56571	3.88E-12	5.95E-10	protein_coding
ENSG0000(	CNOT1	8392.465	-0.25519	3.93E-12	6.00E-10	protein_coding
ENSG0000(	FCGR2A	976.1871	1.64444	4.14E-12	6.28E-10	protein_coding
ENSG0000(	CEP128	884.4455	0.931089	4.47E-12	6.75E-10	protein_coding
ENSG0000(	MTND4P14	148.2135	0.868537	4.57E-12	6.87E-10	unprocessed_pseudogene
ENSG0000(	PPID	430.8991	-0.38904	4.87E-12	7.29E-10	protein_coding
ENSG0000(	TMEM140	508.674	0.769771	5.01E-12	7.45E-10	protein_coding
ENSG0000(	PARP14	10442.06	0.942314	5.08E-12	7.50E-10	protein_coding
ENSG0000(	SLAMF8	421.6852	1.127871	5.07E-12	7.50E-10	protein_coding
ENSG0000(	ADAMTSL4	118.0916	0.901325	5.17E-12	7.60E-10	lncRNA
ENSG0000(	COX7C	1309.299	-0.7972	5.24E-12	7.64E-10	protein_coding
ENSG0000(	SFMBT2	1812.669	0.602578	5.25E-12	7.64E-10	protein_coding
ENSG0000(	PLEK	1297.128	1.990357	5.33E-12	7.71E-10	protein_coding
ENSG0000(	WDR12	1588.67	-0.4627	5.50E-12	7.93E-10	protein_coding
ENSG0000(	DPPA4	2598.037	1.173907	5.93E-12	8.50E-10	protein_coding
ENSG0000(	TRAFD1	864.5626	0.601461	6.69E-12	9.55E-10	protein_coding
ENSG0000(	TUFM	1077.964	-0.60947	7.15E-12	1.02E-09	protein_coding
ENSG0000(	ENSG0000(	26.76636	1.450322	7.50E-12	1.06E-09	lncRNA
ENSG0000(	CFLAR	22168.94	0.394542	7.64E-12	1.08E-09	protein_coding
ENSG0000(	ATP5MG	2086.181	-0.52282	8.03E-12	1.13E-09	protein_coding
ENSG0000(	LINC02605	1958.637	0.748415	8.30E-12	1.16E-09	lncRNA
ENSG0000(	DDX1	2022.124	-0.22173	8.51E-12	1.18E-09	protein_coding
ENSG0000(	RN7SL473F	163.4774	0.955316	8.64E-12	1.20E-09	misc_RNA
ENSG0000(	SNRPA	561.1952	-0.44269	8.88E-12	1.22E-09	protein_coding
ENSG0000(	ENSG0000(	4454.716	0.743123	9.00E-12	1.23E-09	lncRNA
ENSG0000(	IGHG1	6934.442	2.527508	9.35E-12	1.28E-09	IG_C_gene
ENSG0000(	FAM13A-A!	1948.045	0.397812	9.45E-12	1.29E-09	lncRNA
ENSG0000(	LINC01278	1776.401	0.280149	9.64E-12	1.31E-09	lncRNA
ENSG0000(	SLC7A5	424.4424	1.208509	1.00E-11	1.35E-09	protein_coding
ENSG0000(	ENSG0000(	367.2771	0.621221	1.01E-11	1.35E-09	lncRNA
ENSG0000(	G0S2	130.448	2.71453	1.02E-11	1.36E-09	protein_coding

ENSG0000(	ATP5MC3	1893.553	-0.77661	1.03E-11	1.37E-09	protein_coding
ENSG0000(	CACNA1E	135.7905	2.057986	1.07E-11	1.42E-09	protein_coding
ENSG0000(	ENSG0000(	43.22184	1.673535	1.09E-11	1.44E-09	lncRNA
ENSG0000(	CLUH	2558.521	-0.73015	1.14E-11	1.49E-09	protein_coding
ENSG0000(	LINC02035	519.8011	0.451804	1.14E-11	1.49E-09	lncRNA
ENSG0000(	ENSG0000(	681.0067	0.633544	1.19E-11	1.55E-09	processed_pseudogene
ENSG0000(	MIRLET7IH	991.9452	0.704632	1.19E-11	1.55E-09	lncRNA
ENSG0000(	SLC5A6	601.3437	-0.48007	1.20E-11	1.56E-09	protein_coding
ENSG0000(	ENSG0000(	353.3211	0.627737	1.21E-11	1.56E-09	lncRNA
ENSG0000(	TMEM132/	422.8358	1.436152	1.25E-11	1.60E-09	protein_coding
ENSG0000(	ZEB2	7137.078	0.681	1.27E-11	1.62E-09	protein_coding
ENSG0000(	HRH2	187.062	1.534493	1.28E-11	1.63E-09	protein_coding
ENSG0000(	APOL3	1290.055	0.887453	1.30E-11	1.64E-09	protein_coding
ENSG0000(	THOP1	662.3889	-0.47947	1.30E-11	1.64E-09	protein_coding
ENSG0000(	SLC25A39	1276.847	-0.44089	1.32E-11	1.65E-09	protein_coding
ENSG0000(	TMC3-AS1	1211.194	0.72874	1.33E-11	1.67E-09	lncRNA
ENSG0000(	ENSG0000(	104.588	2.470697	1.36E-11	1.69E-09	lncRNA
ENSG0000(	TENT4B	766.4129	-0.36577	1.39E-11	1.72E-09	protein_coding
ENSG0000(	CPEB4	2599.853	0.743788	1.41E-11	1.74E-09	protein_coding
ENSG0000(	MAP3K8	540.2608	0.829675	1.42E-11	1.75E-09	protein_coding
ENSG0000(	CCN2	669.3477	1.355003	1.44E-11	1.77E-09	protein_coding
ENSG0000(	ARSA	6220.349	0.974806	1.45E-11	1.77E-09	protein_coding
ENSG0000(	ENSG0000(	903.4999	0.302864	1.49E-11	1.82E-09	lncRNA
ENSG0000(	IL1RAP	1107.025	0.463791	1.60E-11	1.93E-09	protein_coding
ENSG0000(	LRRC69	2224.752	0.888269	1.60E-11	1.93E-09	protein_coding
ENSG0000(	FCGR1CP	14.04077	3.513175	1.68E-11	2.02E-09	unprocessed_pseudogene
ENSG0000(	IMMT	1374.716	-0.29717	1.74E-11	2.09E-09	protein_coding
ENSG0000(	MTND1P11	119.915	0.970885	1.90E-11	2.27E-09	unprocessed_pseudogene
ENSG0000(	VOPP1	2612.892	0.500192	1.92E-11	2.28E-09	protein_coding
ENSG0000(	ENSG0000(	43.1593	1.37862	1.96E-11	2.33E-09	lncRNA
ENSG0000(	ENSG0000(	30.73633	1.152308	2.16E-11	2.54E-09	lncRNA
ENSG0000(	ENSG0000(	799.884	0.499101	2.23E-11	2.62E-09	transcribed_unprocessed_pseudo
ENSG0000(	RN7SL834P	177.6739	0.85364	2.23E-11	2.62E-09	misc_RNA
ENSG0000(	H2AC6	784.6285	-0.62744	2.24E-11	2.62E-09	protein_coding
ENSG0000(	BEST3	415.1962	0.447206	2.27E-11	2.63E-09	protein_coding
ENSG0000(	NFE2	28.55756	2.917766	2.27E-11	2.63E-09	protein_coding
ENSG0000(	TNC	1915.954	2.263785	2.33E-11	2.69E-09	protein_coding
ENSG0000(	RHOQP1	54.55523	-0.80284	2.38E-11	2.74E-09	processed_pseudogene
ENSG0000(	PRRX1	443.9821	2.359655	2.41E-11	2.76E-09	protein_coding
ENSG0000(	CENPV	725.316	-0.52604	2.42E-11	2.76E-09	protein_coding
ENSG0000(	RBPJ	2990.203	0.324705	2.47E-11	2.81E-09	protein_coding
ENSG0000(	ATP5F1D	669.7452	-0.5969	2.58E-11	2.93E-09	protein_coding
ENSG0000(	LINC01146	228.1695	0.640916	2.68E-11	3.02E-09	lncRNA
ENSG0000(	IGDCC4	488.8125	1.050574	2.71E-11	3.05E-09	protein_coding
ENSG0000(	ENSG0000(	123.7814	0.627604	2.75E-11	3.09E-09	TEC
ENSG0000(	AKR1B10	1209.165	-1.29471	2.80E-11	3.13E-09	protein_coding
ENSG0000(	PML	2693.163	0.641713	2.82E-11	3.14E-09	protein_coding

ENSG0000(	CXCL9	852.801	2.790363	2.87E-11	3.18E-09	protein_coding
ENSG0000(	ENSG0000(	306.7723	0.387026	2.90E-11	3.18E-09	lncRNA
ENSG0000(	HSF1	1012.742	-0.30776	2.90E-11	3.18E-09	protein_coding
ENSG0000(	HSP90AA1	5413.906	-0.59903	2.90E-11	3.18E-09	protein_coding
ENSG0000(	ADAM12	306.5867	1.882937	3.00E-11	3.28E-09	protein_coding
ENSG0000(	CCL3	88.46617	1.840057	3.02E-11	3.29E-09	protein_coding
ENSG0000(	MRPL12	280.12	-0.75435	3.03E-11	3.29E-09	protein_coding
ENSG0000(	NAA50	2697.884	-0.41094	3.13E-11	3.39E-09	protein_coding
ENSG0000(	ATXN7	5171.764	0.242463	3.23E-11	3.48E-09	protein_coding
ENSG0000(	PEX11A	742.9444	-0.51418	3.25E-11	3.49E-09	protein_coding
ENSG0000(	ENSG0000(	53.67756	-0.72917	3.32E-11	3.56E-09	processed_pseudogene
ENSG0000(	NACA	3884.967	-0.49793	3.34E-11	3.56E-09	protein_coding
ENSG0000(	DAPP1	816.8018	1.151717	3.36E-11	3.57E-09	protein_coding
ENSG0000(	LINC01537	851.5926	0.328938	3.43E-11	3.64E-09	lncRNA
ENSG0000(	TET2	6136.603	0.286216	3.64E-11	3.84E-09	protein_coding
ENSG0000(	ENSG0000(	819.1463	0.256279	3.77E-11	3.97E-09	processed_pseudogene
ENSG0000(	ENSG0000(	235.8506	0.577704	3.81E-11	3.99E-09	lncRNA
ENSG0000(	NEUROD2	4423.197	1.442619	3.81E-11	3.99E-09	protein_coding
ENSG0000(	FIBIN	53.61161	1.663044	3.84E-11	4.00E-09	protein_coding
ENSG0000(	ODF3B	259.0151	0.957697	3.87E-11	4.02E-09	protein_coding
ENSG0000(	FOSB	880.8865	2.614665	3.93E-11	4.07E-09	protein_coding
ENSG0000(	ATP5IF1	1189.3	-0.58063	4.04E-11	4.16E-09	protein_coding
ENSG0000(	TSEN15	1657.539	0.29696	4.05E-11	4.16E-09	protein_coding
ENSG0000(	CREB3L2	4766.743	0.648737	4.08E-11	4.17E-09	protein_coding
ENSG0000(	ENSG0000(	2526.285	0.443506	4.08E-11	4.17E-09	lncRNA
ENSG0000(	CSNK2B	1073.054	-0.40232	4.13E-11	4.19E-09	protein_coding
ENSG0000(	DIPK2B	575.678	0.778575	4.12E-11	4.19E-09	protein_coding
ENSG0000(	ENSG0000(	55.91683	0.834214	4.14E-11	4.19E-09	lncRNA
ENSG0000(	DCC	136.4231	1.29944	4.17E-11	4.20E-09	protein_coding
ENSG0000(	VMP1	16213.19	0.55754	4.16E-11	4.20E-09	protein_coding
ENSG0000(	ENSG0000(	202.6524	1.576501	4.22E-11	4.22E-09	lncRNA
ENSG0000(	IL1A	50.80293	3.068651	4.32E-11	4.31E-09	protein_coding
ENSG0000(	CLDN2	73.06539	2.806072	4.55E-11	4.53E-09	protein_coding
ENSG0000(	AGMAT	1034.754	-0.3548	4.57E-11	4.54E-09	protein_coding
ENSG0000(	HNRNPF	2335.915	-0.3937	4.66E-11	4.61E-09	protein_coding
ENSG0000(	IGHGP	303.2272	2.669445	4.75E-11	4.68E-09	IG_C_pseudogene
ENSG0000(	ENSG0000(	925.4564	0.702693	4.78E-11	4.71E-09	lncRNA
ENSG0000(	VPS72	230.7584	-0.34933	4.81E-11	4.71E-09	protein_coding
ENSG0000(	CCDC88B	2575.63	0.612206	5.00E-11	4.87E-09	protein_coding
ENSG0000(	CKS2	91.4502	-0.83959	5.00E-11	4.87E-09	protein_coding
ENSG0000(	NDUFS5	327.8558	-0.70937	5.01E-11	4.87E-09	protein_coding
ENSG0000(	IDO1	560.2293	1.607956	5.06E-11	4.90E-09	protein_coding
ENSG0000(	DISC1	1097.097	0.457218	5.24E-11	5.06E-09	protein_coding
ENSG0000(	H2BC8	210.2657	-0.79429	5.37E-11	5.17E-09	protein_coding
ENSG0000(	MRPS35	457.8505	-0.66177	5.74E-11	5.51E-09	protein_coding
ENSG0000(	ANKRD36B	4627.767	0.737289	6.21E-11	5.94E-09	transcribed_unprocessed_pseudo
ENSG0000(	MPC2	920.0582	-0.54538	6.43E-11	6.13E-09	protein_coding

ENSG0000(	ENSG0000(	78.32992	0.759103	6.46E-11	6.14E-09	lncRNA
ENSG0000(	ACSL1	1241.64	1.689719	6.56E-11	6.20E-09	protein_coding
ENSG0000(	RGS1	738.3714	1.742568	6.54E-11	6.20E-09	protein_coding
ENSG0000(	FGR	306.059	1.42061	6.61E-11	6.23E-09	protein_coding
ENSG0000(	SLC25A3	5152.871	-0.51486	6.80E-11	6.38E-09	protein_coding
ENSG0000(	ENSG0000(	121.3555	0.504313	6.82E-11	6.39E-09	lncRNA
ENSG0000(	COX5B	841.0199	-0.63085	7.00E-11	6.48E-09	protein_coding
ENSG0000(	ERAL1	302.4641	-0.50929	7.01E-11	6.48E-09	protein_coding
ENSG0000(	FAM210A	738.9714	-0.4007	6.99E-11	6.48E-09	protein_coding
ENSG0000(	GLCCI1	1406.384	0.657525	6.97E-11	6.48E-09	protein_coding
ENSG0000(	SEC22B	3350.793	0.296892	7.15E-11	6.60E-09	protein_coding
ENSG0000(	RTEL1P1	236.7869	0.549837	7.18E-11	6.60E-09	transcribed_processed_pseudoge
ENSG0000(	ENSG0000(	2889.374	0.203482	7.46E-11	6.77E-09	lncRNA
ENSG0000(	ENSG0000(	128.168	0.678318	7.47E-11	6.77E-09	TEC
ENSG0000(	OR1I1	3017.958	0.950677	7.41E-11	6.77E-09	protein_coding
ENSG0000(	RPSAP48	197.0869	1.116634	7.40E-11	6.77E-09	processed_pseudogene
ENSG0000(	TUBB4B	1793.431	-0.77233	7.44E-11	6.77E-09	protein_coding
ENSG0000(	GTPBP6	470.2706	-0.44119	7.66E-11	6.92E-09	protein_coding
ENSG0000(	NDUFB7	214.4841	-0.82638	8.03E-11	7.24E-09	protein_coding
ENSG0000(	GOSR2	3546.633	0.207191	8.15E-11	7.33E-09	protein_coding
ENSG0000(	H4C2	196.5889	-0.89359	8.30E-11	7.43E-09	protein_coding
ENSG0000(	BIRC3	6192.525	0.871999	8.65E-11	7.73E-09	protein_coding
ENSG0000(	ENSG0000(	209.6115	0.69163	8.83E-11	7.86E-09	TEC
ENSG0000(	PRKCSH	1583.776	-0.3155	9.08E-11	8.07E-09	protein_coding
ENSG0000(	CD9	2830.85	-0.83278	9.36E-11	8.29E-09	protein_coding
ENSG0000(	SUSD6	3210.596	0.579034	9.38E-11	8.29E-09	protein_coding
ENSG0000(	HCG27	551.4436	0.583944	9.74E-11	8.55E-09	lncRNA
ENSG0000(	POU2AF1	2548.656	0.929473	9.73E-11	8.55E-09	protein_coding
ENSG0000(	ITGAX	2499.985	1.042095	1.00E-10	8.76E-09	protein_coding
ENSG0000(	MTIF2	890.4801	-0.39638	1.01E-10	8.79E-09	protein_coding
ENSG0000(	TNFRSF6B	84.34609	7.484175	1.01E-10	8.79E-09	protein_coding
ENSG0000(	LILRA5	215.7367	0.906794	1.02E-10	8.82E-09	protein_coding
ENSG0000(	ZNF283	1497.415	-0.28564	1.02E-10	8.82E-09	protein_coding
ENSG0000(	RPS20P20	16.04635	1.637557	1.03E-10	8.88E-09	processed_pseudogene
ENSG0000(	AHSA1	727.7605	-0.42829	1.04E-10	8.94E-09	protein_coding
ENSG0000(	ENSG0000(	767.9294	-0.43976	1.06E-10	9.12E-09	lncRNA
ENSG0000(	ARHGAP29	1538.297	0.369477	1.08E-10	9.21E-09	protein_coding
ENSG0000(	YPEL2	2251.051	0.60563	1.09E-10	9.27E-09	protein_coding
ENSG0000(	NABP1	6999.73	0.795619	1.09E-10	9.33E-09	protein_coding
ENSG0000(	PTPN13	709.7429	0.756747	1.10E-10	9.36E-09	protein_coding
ENSG0000(	C9orf72	1006.57	0.577275	1.12E-10	9.47E-09	protein_coding
ENSG0000(	GJB1	926.7109	-0.67715	1.12E-10	9.47E-09	protein_coding
ENSG0000(	MIR223HG	903.0441	1.353015	1.12E-10	9.47E-09	lncRNA
ENSG0000(	SLC22A25	2801.451	0.218756	1.21E-10	1.01E-08	protein_coding
ENSG0000(	NOS2P1	245.1454	1.171349	1.21E-10	1.02E-08	transcribed_unprocessed_pseudo
ENSG0000(	ENSG0000(	393.3938	0.666918	1.22E-10	1.02E-08	TEC
ENSG0000(	LINC01232	2628.276	0.270957	1.22E-10	1.02E-08	lncRNA

ENSG0000(	FASTK	1138.477	-0.39328	1.24E-10	1.03E-08	protein_coding
ENSG0000(	NLRP14	70.20232	1.034855	1.27E-10	1.05E-08	protein_coding
ENSG0000(	PRSS22	71.84578	2.124956	1.27E-10	1.05E-08	protein_coding
ENSG0000(	CDC42EP3	1569.836	0.365555	1.28E-10	1.06E-08	protein_coding
ENSG0000(	C2CD4B	33.1246	1.610356	1.29E-10	1.06E-08	protein_coding
ENSG0000(	SRSF3	5359.299	-0.18909	1.34E-10	1.10E-08	protein_coding
ENSG0000(	TEX35	262.589	0.484339	1.35E-10	1.10E-08	protein_coding
ENSG0000(	PDZK1IP1	236.2092	1.719169	1.36E-10	1.11E-08	protein_coding
ENSG0000(	KIF26B	202.5225	1.81862	1.38E-10	1.12E-08	protein_coding
ENSG0000(	SLC35F2	689.356	-0.51186	1.38E-10	1.12E-08	protein_coding
ENSG0000(	ENSG0000(	199.625	0.953957	1.39E-10	1.12E-08	lncRNA
ENSG0000(	NDUFS4	277.5983	-0.53082	1.41E-10	1.13E-08	protein_coding
ENSG0000(	STRADA	1735.62	0.316394	1.40E-10	1.13E-08	protein_coding
ENSG0000(	HLA-L	1316.435	0.329658	1.42E-10	1.14E-08	transcribed_unprocessed_pseudo
ENSG0000(	NDUFB1	708.5712	-0.51751	1.43E-10	1.15E-08	protein_coding
ENSG0000(	CCT3	1195.256	-0.34618	1.45E-10	1.16E-08	protein_coding
ENSG0000(	ICAM1	1067.629	1.230546	1.46E-10	1.17E-08	protein_coding
ENSG0000(	QTRT1	890.5366	-0.41529	1.47E-10	1.17E-08	protein_coding
ENSG0000(	MT-CO3	56152.33	-1.06492	1.52E-10	1.21E-08	protein_coding
ENSG0000(	H4C8	517.2751	-0.84068	1.54E-10	1.22E-08	protein_coding
ENSG0000(	CCR1	197.5719	1.185665	1.57E-10	1.24E-08	protein_coding
ENSG0000(	DYSF	721.1736	1.509698	1.65E-10	1.30E-08	protein_coding
ENSG0000(	GLOD4	1071.163	-0.31733	1.68E-10	1.32E-08	protein_coding
ENSG0000(	LILRB1	996.3874	0.834442	1.69E-10	1.32E-08	protein_coding
ENSG0000(	SNX10	211.4992	1.337134	1.70E-10	1.33E-08	protein_coding
ENSG0000(	ENSG0000(	212.879	0.453816	1.71E-10	1.33E-08	TEC
ENSG0000(	SLC27A4	772.1047	-0.96589	1.72E-10	1.33E-08	protein_coding
ENSG0000(	TENM4	345.8391	1.743715	1.74E-10	1.35E-08	protein_coding
ENSG0000(	ENSG0000(	2393.633	0.45082	1.78E-10	1.38E-08	lncRNA
ENSG0000(	RPL17P10	33.25721	1.121031	1.79E-10	1.38E-08	processed_pseudogene
ENSG0000(	RRP9	111.6263	-0.62895	1.80E-10	1.39E-08	protein_coding
ENSG0000(	CASP5	463.6296	1.408276	1.82E-10	1.40E-08	protein_coding
ENSG0000(	PFDN6	128.5974	-0.62157	1.82E-10	1.40E-08	protein_coding
ENSG0000(	CEBPB	341.7278	1.002094	1.94E-10	1.48E-08	protein_coding
ENSG0000(	H2BC5	378.6022	-0.71292	2.02E-10	1.54E-08	protein_coding
ENSG0000(	ENSG0000(	20.48661	2.255086	2.06E-10	1.57E-08	lncRNA
ENSG0000(	MYOSLID	167.4006	0.550338	2.17E-10	1.64E-08	lncRNA
ENSG0000(	NCF2	283.467	1.366228	2.18E-10	1.65E-08	protein_coding
ENSG0000(	SRSF1	6151.243	-0.26279	2.25E-10	1.70E-08	protein_coding
ENSG0000(	ENSG0000(	271.3391	-0.4241	2.33E-10	1.75E-08	lncRNA
ENSG0000(	NDUFS3	936.5512	-0.6092	2.35E-10	1.77E-08	protein_coding
ENSG0000(	LINC02362	1959.579	0.726146	2.39E-10	1.79E-08	lncRNA
ENSG0000(	C2	910.6064	0.543715	2.44E-10	1.82E-08	protein_coding
ENSG0000(	MMP19	564.0053	1.412029	2.44E-10	1.82E-08	protein_coding
ENSG0000(	MRPL45P2	811.8423	-0.29837	2.50E-10	1.86E-08	transcribed_unprocessed_pseudo
ENSG0000(	RPL21P123	208.5457	0.689853	2.53E-10	1.88E-08	transcribed_processed_pseudoge
ENSG0000(	BICD1	840.6908	0.83397	2.53E-10	1.88E-08	protein_coding

ENSG0000(	GALNT2	1293.368	0.450152	2.57E-10	1.90E-08	protein_coding
ENSG0000(	CACYBP	555.6368	-0.55627	2.68E-10	1.97E-08	protein_coding
ENSG0000(	ENSG0000(	90.58023	0.61443	2.68E-10	1.97E-08	lncRNA
ENSG0000(	CHID1	629.5296	-0.39506	2.71E-10	1.99E-08	protein_coding
ENSG0000(	PHB	951.3086	-0.59397	2.71E-10	1.99E-08	protein_coding
ENSG0000(	ENSG0000(	2048.179	0.635486	2.86E-10	2.09E-08	lncRNA
ENSG0000(	RASGEF1B	1806.015	0.537947	2.86E-10	2.09E-08	protein_coding
ENSG0000(	LDLRAD2	82.28801	1.130831	2.92E-10	2.12E-08	protein_coding
ENSG0000(	LYN	1591.727	0.775992	2.98E-10	2.16E-08	protein_coding
ENSG0000(	SPACA6P-A	366.9865	0.319792	3.01E-10	2.18E-08	lncRNA
ENSG0000(	ENSG0000(	9.440062	1.709016	3.10E-10	2.24E-08	lncRNA
ENSG0000(	LINC01623	267.1291	0.469456	3.10E-10	2.24E-08	lncRNA
ENSG0000(	NFKBIZ	3276.575	0.710926	3.12E-10	2.24E-08	protein_coding
ENSG0000(	KIAA0100	5381.173	-0.22172	3.14E-10	2.25E-08	protein_coding
ENSG0000(	LINC01709	35.20029	2.37545	3.23E-10	2.31E-08	lncRNA
ENSG0000(	ALPK1	2897.236	0.353964	3.24E-10	2.31E-08	protein_coding
ENSG0000(	UQCRC1	1240.713	-0.68884	3.30E-10	2.35E-08	protein_coding
ENSG0000(	LAX1	709.151	0.873767	3.38E-10	2.40E-08	protein_coding
ENSG0000(	RNU7-45P	73.81849	0.829638	3.40E-10	2.41E-08	snRNA
ENSG0000(	VPS50	1046.324	-0.30996	3.41E-10	2.41E-08	protein_coding
ENSG0000(	ENSG0000(	39.63083	1.396682	3.43E-10	2.42E-08	lncRNA
ENSG0000(	ENSG0000(	839.1668	0.40229	3.44E-10	2.42E-08	lncRNA
ENSG0000(	FAM225A	28.40349	2.917586	3.49E-10	2.45E-08	lncRNA
ENSG0000(	MTCO2P11	114.9098	0.830357	3.51E-10	2.46E-08	unprocessed_pseudogene
ENSG0000(	SLC25A37	5481.393	0.590007	3.52E-10	2.46E-08	protein_coding
ENSG0000(	LILRB3	472.7661	1.407411	3.55E-10	2.48E-08	protein_coding
ENSG0000(	MAPKAPK5	661.5823	-0.31771	3.63E-10	2.52E-08	lncRNA
ENSG0000(	SRGAP2	2313.971	0.472599	3.63E-10	2.52E-08	protein_coding
ENSG0000(	VENTXP1	444.2607	0.320108	3.66E-10	2.54E-08	transcribed_processed_pseudoge
ENSG0000(	VPS28	976.5335	-0.39101	3.67E-10	2.54E-08	protein_coding
ENSG0000(	DAZAP1	3926.2	-0.23034	3.69E-10	2.55E-08	protein_coding
ENSG0000(	PLAAT4	282.1034	1.045315	3.83E-10	2.64E-08	protein_coding
ENSG0000(	CD38	1553.353	0.852676	3.88E-10	2.66E-08	protein_coding
ENSG0000(	ZDHHC6	2141.588	-0.17351	3.91E-10	2.68E-08	protein_coding
ENSG0000(	GOLGA4-A!	483.3191	0.313425	3.94E-10	2.70E-08	lncRNA
ENSG0000(	RPS3AP32	16.59455	1.581557	3.97E-10	2.71E-08	processed_pseudogene
ENSG0000(	CRTC3	1758.702	0.423503	4.00E-10	2.72E-08	protein_coding
ENSG0000(	LILRB4	361.4824	1.235674	4.01E-10	2.72E-08	protein_coding
ENSG0000(	PRDX5	850.6017	-0.75959	4.01E-10	2.72E-08	protein_coding
ENSG0000(	SLA	1460.967	1.202828	4.01E-10	2.72E-08	protein_coding
ENSG0000(	N4BP2L2-IT	1539.772	0.353596	4.02E-10	2.72E-08	lncRNA
ENSG0000(	HK3	249.175	1.447037	4.20E-10	2.83E-08	protein_coding
ENSG0000(	BTN3A3	2277.425	0.656822	4.26E-10	2.86E-08	protein_coding
ENSG0000(	CCL4	92.24931	1.522431	4.31E-10	2.89E-08	protein_coding
ENSG0000(	CPXM1	78.532	1.678396	4.32E-10	2.89E-08	protein_coding
ENSG0000(	RN7SL600F	242.646	0.908881	4.33E-10	2.89E-08	misc_RNA
ENSG0000(	TPK1	857.5187	0.683232	4.35E-10	2.90E-08	protein_coding

ENSG0000(	TMEM165	2167.834	0.495305	4.42E-10	2.94E-08	protein_coding
ENSG0000(	FAM200B	666.5368	-0.29807	4.46E-10	2.96E-08	protein_coding
ENSG0000(	LYPLA2	561.3359	-0.51048	4.47E-10	2.96E-08	protein_coding
ENSG0000(	PTAFR	667.8391	0.700185	4.47E-10	2.96E-08	protein_coding
ENSG0000(	ENSG0000(	242.6789	0.556633	4.51E-10	2.97E-08	lncRNA
ENSG0000(	ENSG0000(	2017.203	0.36781	4.56E-10	3.01E-08	lncRNA
ENSG0000(	SCFD1	5696.519	0.225462	4.58E-10	3.01E-08	protein_coding
ENSG0000(	MTFMT	922.9122	-0.29474	4.67E-10	3.06E-08	protein_coding
ENSG0000(	WWC3	1206.237	0.548063	4.69E-10	3.07E-08	protein_coding
ENSG0000(	ZC3H14	3760.189	-0.29103	4.73E-10	3.09E-08	protein_coding
ENSG0000(	MTATP6P1	99.81207	0.893785	4.75E-10	3.09E-08	unprocessed_pseudogene
ENSG0000(	SAMSN1	402.103	1.199636	4.78E-10	3.11E-08	protein_coding
ENSG0000(	SEC14L1	2279.373	0.648054	4.80E-10	3.11E-08	protein_coding
ENSG0000(	CXCR2	208.0442	1.472374	4.87E-10	3.16E-08	protein_coding
ENSG0000(	STK11	1594.286	-0.33613	5.05E-10	3.27E-08	protein_coding
ENSG0000(	CYSLTR2	285.3311	0.668802	5.15E-10	3.32E-08	protein_coding
ENSG0000(	HCK	466.1182	0.865207	5.15E-10	3.32E-08	protein_coding
ENSG0000(	ENSG0000(	365.7298	1.509687	5.28E-10	3.39E-08	lncRNA
ENSG0000(	SLC8A1	1347.108	0.790169	5.27E-10	3.39E-08	protein_coding
ENSG0000(	RNF213	29037.3	0.671491	5.36E-10	3.43E-08	protein_coding
ENSG0000(	SMARCD2	943.9932	-0.29623	5.38E-10	3.44E-08	protein_coding
ENSG0000(	CDH11	1338.845	0.757803	5.44E-10	3.46E-08	protein_coding
ENSG0000(	DPH2	358.0671	-0.52991	5.43E-10	3.46E-08	protein_coding
ENSG0000(	TBCEL	983.4214	0.391496	5.50E-10	3.48E-08	protein_coding
ENSG0000(	TYMP	1403.79	0.939231	5.49E-10	3.48E-08	protein_coding
ENSG0000(	SLC25A33	661.2589	-0.47647	5.52E-10	3.49E-08	protein_coding
ENSG0000(	KHSRP	1904.701	-0.43472	5.58E-10	3.52E-08	protein_coding
ENSG0000(	NNMT	428.0232	1.25224	5.78E-10	3.64E-08	protein_coding
ENSG0000(	KLF7	1138.847	0.818588	5.79E-10	3.64E-08	protein_coding
ENSG0000(	CDK16	1239.5	-0.35397	5.84E-10	3.66E-08	protein_coding
ENSG0000(	EIF6	672.8826	-0.61889	5.85E-10	3.66E-08	protein_coding
ENSG0000(	CYC1	918.8804	-0.66494	6.05E-10	3.78E-08	protein_coding
ENSG0000(	ENTPD1	5494.328	0.572246	6.09E-10	3.78E-08	protein_coding
ENSG0000(	FAM20A	408.0965	1.278765	6.09E-10	3.78E-08	protein_coding
ENSG0000(	BTN2A2	1097.923	0.658246	6.11E-10	3.79E-08	protein_coding
ENSG0000(	EPSTI1	1120.29	1.001926	6.23E-10	3.85E-08	protein_coding
ENSG0000(	VPS51	1252.211	-0.35385	6.23E-10	3.85E-08	protein_coding
ENSG0000(	BACE2-IT1	59.44352	0.827839	6.39E-10	3.94E-08	lncRNA
ENSG0000(	UQCC3	275.9184	-0.31036	6.42E-10	3.95E-08	protein_coding
ENSG0000(	OR4K17	1109.956	0.356715	6.57E-10	4.04E-08	protein_coding
ENSG0000(	GPS2	472.3745	-0.45983	6.61E-10	4.06E-08	protein_coding
ENSG0000(	PFKFB3	1573.8	0.966298	6.66E-10	4.07E-08	protein_coding
ENSG0000(	EIF3D	1301.092	-0.31568	7.10E-10	4.34E-08	protein_coding
ENSG0000(	POU5F2	1611.761	0.217548	7.15E-10	4.36E-08	protein_coding
ENSG0000(	ENSG0000(	111.1958	1.074342	7.31E-10	4.45E-08	processed_pseudogene
ENSG0000(	RIC1	2511.127	0.263914	7.34E-10	4.45E-08	protein_coding
ENSG0000(	XBP1	4928.638	0.819612	7.45E-10	4.51E-08	protein_coding

ENSG0000(	E2F4	1113.613	-0.32722	7.53E-10	4.55E-08	protein_coding
ENSG0000(	MTREX	1824.959	-0.31751	7.57E-10	4.57E-08	protein_coding
ENSG0000(	ENSG0000(	635.0865	0.515855	7.69E-10	4.64E-08	lncRNA
ENSG0000(	JPT2	1215.849	-0.4059	7.72E-10	4.64E-08	protein_coding
ENSG0000(	RBM10	938.9133	-0.37113	7.90E-10	4.74E-08	protein_coding
ENSG0000(	ACOXL	255.4248	0.557514	8.23E-10	4.93E-08	protein_coding
ENSG0000(	ENSG0000(	40.00986	1.878657	8.43E-10	5.04E-08	lncRNA
ENSG0000(	KLF7P1	16.48589	1.397468	8.56E-10	5.11E-08	processed_pseudogene
ENSG0000(	KIF23	546.0387	-0.41226	8.66E-10	5.16E-08	protein_coding
ENSG0000(	ENSG0000(	72.89739	1.587567	8.92E-10	5.30E-08	lncRNA
ENSG0000(	GLB1L3	726.5059	0.353936	9.14E-10	5.43E-08	protein_coding
ENSG0000(	TRABD2A	4919.001	-0.76452	9.23E-10	5.47E-08	protein_coding
ENSG0000(	HSPA1B	1801.676	-0.88507	9.40E-10	5.55E-08	protein_coding
ENSG0000(	LINC01484	665.1287	0.643699	9.39E-10	5.55E-08	lncRNA
ENSG0000(	ENSG0000(	290.1629	0.339659	9.43E-10	5.56E-08	lncRNA
ENSG0000(	H2AC11	199.3939	-0.86413	9.63E-10	5.66E-08	protein_coding
ENSG0000(	SYNE3	2364.057	0.441234	9.80E-10	5.75E-08	protein_coding
ENSG0000(	PGAM5	1162.441	-0.29485	9.85E-10	5.77E-08	protein_coding
ENSG0000(	TCAF2	1270.981	0.377128	9.97E-10	5.83E-08	protein_coding
ENSG0000(	MRPL13	287.306	-0.6118	1.00E-09	5.86E-08	protein_coding
ENSG0000(	ATP5PD	1095.968	-0.55694	1.02E-09	5.93E-08	protein_coding
ENSG0000(	THEM6	247.5567	-0.70891	1.04E-09	6.06E-08	protein_coding
ENSG0000(	ENSG0000(	23.1564	1.303163	1.06E-09	6.13E-08	processed_pseudogene
ENSG0000(	SSB	595.2608	-0.38319	1.07E-09	6.19E-08	protein_coding
ENSG0000(	EEF1B2	1757.526	-0.57735	1.08E-09	6.22E-08	protein_coding
ENSG0000(	BIN1	944.6981	-0.34663	1.09E-09	6.29E-08	protein_coding
ENSG0000(	H2BC7	140.6029	-0.84953	1.13E-09	6.48E-08	protein_coding
ENSG0000(	LINC00271	1080.163	0.301281	1.15E-09	6.61E-08	lncRNA
ENSG0000(	ZNF469	272.4701	1.361315	1.17E-09	6.69E-08	protein_coding
ENSG0000(	AIFM1	2260.668	-0.22809	1.19E-09	6.80E-08	protein_coding
ENSG0000(	STX11	371.7263	0.651439	1.19E-09	6.80E-08	protein_coding
ENSG0000(	GSEC	241.3745	0.522417	1.20E-09	6.88E-08	lncRNA
ENSG0000(	TCP1	1735.987	-0.3989	1.22E-09	6.93E-08	protein_coding
ENSG0000(	INTS1	2373.489	-0.34999	1.26E-09	7.14E-08	protein_coding
ENSG0000(	ADAMTS4	1315.107	1.041318	1.28E-09	7.24E-08	protein_coding
ENSG0000(	TRIP10	1211.904	-0.4885	1.28E-09	7.25E-08	protein_coding
ENSG0000(	LINC01291	51.41782	2.047648	1.29E-09	7.29E-08	lncRNA
ENSG0000(	SLC6A6	1445.577	0.834123	1.31E-09	7.37E-08	protein_coding
ENSG0000(	RPL5	3724.731	-0.3837	1.32E-09	7.44E-08	protein_coding
ENSG0000(	ENSG0000(	3162.248	0.337422	1.32E-09	7.45E-08	lncRNA
ENSG0000(	DGAT1	1278.137	-0.6057	1.34E-09	7.54E-08	protein_coding
ENSG0000(	HSPBP1	163.9062	-0.65788	1.35E-09	7.54E-08	protein_coding
ENSG0000(	UQCRB	1525.944	-0.50467	1.36E-09	7.63E-08	protein_coding
ENSG0000(	COX6C	926.3679	-0.56168	1.40E-09	7.79E-08	protein_coding
ENSG0000(	LINC01409	1423.796	0.341664	1.41E-09	7.87E-08	lncRNA
ENSG0000(	ALYREF	237.3231	-0.58503	1.46E-09	8.10E-08	protein_coding
ENSG0000(	LILRB2	413.3727	1.212583	1.48E-09	8.23E-08	protein_coding

ENSG0000(	ENSG0000(	236.7018	0.44284	1.53E-09	8.50E-08	lncRNA
ENSG0000(	KCNJ10	205.2759	1.115165	1.55E-09	8.56E-08	protein_coding
ENSG0000(	ZBP1	874.4765	0.82651	1.56E-09	8.62E-08	protein_coding
ENSG0000(	LSAMP	570.8056	0.785058	1.56E-09	8.63E-08	protein_coding
ENSG0000(	PLEKHJ1	1424.506	-0.52227	1.57E-09	8.66E-08	protein_coding
ENSG0000(	FTX	21019.68	0.157763	1.61E-09	8.85E-08	lncRNA
ENSG0000(	MRPL39	150.1733	-0.54041	1.64E-09	8.97E-08	protein_coding
ENSG0000(	ENSG0000(	226.219	0.439771	1.74E-09	9.53E-08	lncRNA
ENSG0000(	KRTCAP3	218.4156	-0.69383	1.74E-09	9.53E-08	protein_coding
ENSG0000(	TCEAL9	223.8116	-0.65001	1.76E-09	9.62E-08	protein_coding
ENSG0000(	BACH1-IT2	192.8058	0.779052	1.77E-09	9.64E-08	lncRNA
ENSG0000(	ANXA1	600.7201	1.160599	1.78E-09	9.68E-08	protein_coding
ENSG0000(	MAFF	1198.109	0.591222	1.78E-09	9.68E-08	protein_coding
ENSG0000(	C6orf141	637.0987	-0.68273	1.81E-09	9.82E-08	protein_coding
ENSG0000(	TPST2	952.1612	0.458789	1.82E-09	9.84E-08	protein_coding
ENSG0000(	CIAO2B	222.1515	-0.52121	1.83E-09	9.88E-08	protein_coding
ENSG0000(	TSTD1	346.5437	-0.68107	1.84E-09	9.90E-08	protein_coding
ENSG0000(	SERPINE1	332.8936	1.263253	1.85E-09	9.94E-08	protein_coding
ENSG0000(	PANK4	489.8042	-0.33765	1.85E-09	9.94E-08	protein_coding
ENSG0000(	ADAMTS2	645.2957	1.06437	1.87E-09	1.00E-07	protein_coding
ENSG0000(	CTHRC1	34.63093	1.790285	1.91E-09	1.02E-07	protein_coding
ENSG0000(	H4C5	492.5551	-0.71115	1.91E-09	1.02E-07	protein_coding
ENSG0000(	NDUFA3	578.3598	-0.36642	1.92E-09	1.02E-07	protein_coding
ENSG0000(	GDPD3	404.4443	-0.90999	1.93E-09	1.03E-07	protein_coding
ENSG0000(	NDUFB9	3192.131	-0.47574	1.93E-09	1.03E-07	protein_coding
ENSG0000(	FABP1	11650.32	-1.36216	1.97E-09	1.04E-07	protein_coding
ENSG0000(	FIS1	464.4186	-0.30957	1.97E-09	1.04E-07	protein_coding
ENSG0000(	YWHAE	2572.158	-0.34572	1.97E-09	1.04E-07	protein_coding
ENSG0000(	EIF3A	4820.56	-0.29023	2.01E-09	1.06E-07	protein_coding
ENSG0000(	ST6GAL1	3762.992	0.794373	2.02E-09	1.07E-07	protein_coding
ENSG0000(	RAC3	40.7826	-0.96857	2.03E-09	1.07E-07	protein_coding
ENSG0000(	H3C2	224.2972	-1.04872	2.05E-09	1.08E-07	protein_coding
ENSG0000(	ENSG0000(	113.6102	0.721791	2.05E-09	1.08E-07	lncRNA
ENSG0000(	ALDH2	5943.021	-0.52456	2.18E-09	1.14E-07	protein_coding
ENSG0000(	MIAT	3455.028	0.63074	2.19E-09	1.14E-07	lncRNA
ENSG0000(	ENSG0000(	74.35193	0.811276	2.20E-09	1.15E-07	lncRNA
ENSG0000(	ENSG0000(	279.6882	0.395932	2.24E-09	1.17E-07	transcribed_processed_pseudoge
ENSG0000(	ENSG0000(	146.6641	0.446424	2.27E-09	1.18E-07	lncRNA
ENSG0000(	HAPLN3	364.4575	1.427243	2.32E-09	1.21E-07	protein_coding
ENSG0000(	ENSG0000(	797.3544	0.270937	2.37E-09	1.23E-07	lncRNA
ENSG0000(	TNFRSF10C	114.3645	1.607329	2.44E-09	1.26E-07	protein_coding
ENSG0000(	MIR503HG	73.4939	1.203109	2.46E-09	1.27E-07	lncRNA
ENSG0000(	TRIM28	1514.035	-0.40694	2.54E-09	1.31E-07	protein_coding
ENSG0000(	SIGLEC9	110.3011	0.961976	2.56E-09	1.32E-07	protein_coding
ENSG0000(	C7orf31	380.5902	-0.70589	2.57E-09	1.32E-07	protein_coding
ENSG0000(	ETHE1	1957.895	-0.81988	2.62E-09	1.34E-07	protein_coding
ENSG0000(	ENSG0000(	340.144	0.678369	2.63E-09	1.35E-07	TEC

ENSG0000(	TIMM13	471.9018	-0.56701	2.63E-09	1.35E-07	protein_coding
ENSG0000(	CASP4	1521.193	0.702991	2.64E-09	1.35E-07	protein_coding
ENSG0000(	KIFC3	834.2734	0.625724	2.66E-09	1.36E-07	protein_coding
ENSG0000(	PFN1	2954.321	-0.55731	2.71E-09	1.38E-07	protein_coding
ENSG0000(	MRPL18	291.9187	-0.53955	2.73E-09	1.39E-07	protein_coding
ENSG0000(	MCM7	1204.129	-0.33644	2.75E-09	1.39E-07	protein_coding
ENSG0000(	NDUFA11	904.2462	-0.35828	2.75E-09	1.39E-07	protein_coding
ENSG0000(	ALG12	762.169	-0.46473	2.76E-09	1.39E-07	protein_coding
ENSG0000(	CDKN2A	57.24377	0.812431	2.78E-09	1.40E-07	protein_coding
ENSG0000(	GPR84	38.22553	2.421566	2.78E-09	1.40E-07	protein_coding
ENSG0000(	ENSG0000(	146.8433	1.046531	2.80E-09	1.41E-07	lncRNA
ENSG0000(	SH3BP5-AS	1837.485	0.310709	2.80E-09	1.41E-07	lncRNA
ENSG0000(	CDK2AP2	480.6578	-0.52121	2.81E-09	1.41E-07	protein_coding
ENSG0000(	PDCD5	428.0501	-0.45943	2.80E-09	1.41E-07	protein_coding
ENSG0000(	ENSG0000(	343.475	0.3141	2.83E-09	1.41E-07	lncRNA
ENSG0000(	ENSG0000(	561.0041	0.304685	2.83E-09	1.42E-07	lncRNA
ENSG0000(	TRIB2	1039.678	1.064119	2.94E-09	1.47E-07	protein_coding
ENSG0000(	CD2BP2	874.0374	-0.29045	3.04E-09	1.51E-07	protein_coding
ENSG0000(	HPDL	50.70877	-1.14447	3.04E-09	1.51E-07	protein_coding
ENSG0000(	DENND3	4041.82	0.606919	3.09E-09	1.53E-07	protein_coding
ENSG0000(	SNRPD3	1500.716	-0.27346	3.10E-09	1.54E-07	protein_coding
ENSG0000(	CFI	567.4789	0.6222	3.16E-09	1.56E-07	protein_coding
ENSG0000(	ENSG0000(	393.5475	0.37566	3.16E-09	1.56E-07	lncRNA
ENSG0000(	SGMS1-AS1	817.1964	0.364204	3.16E-09	1.56E-07	lncRNA
ENSG0000(	MTCH2	1242.308	-0.54532	3.18E-09	1.57E-07	protein_coding
ENSG0000(	GRIN3A	147.2593	1.320367	3.19E-09	1.57E-07	protein_coding
ENSG0000(	DPP10-AS1	72.37458	-1.01508	3.28E-09	1.61E-07	lncRNA
ENSG0000(	EDF1	1035.187	-0.47142	3.29E-09	1.61E-07	protein_coding
ENSG0000(	ENSG0000(	1140.001	0.243316	3.31E-09	1.62E-07	lncRNA
ENSG0000(	ENSG0000(	639.141	-0.3757	3.35E-09	1.63E-07	lncRNA
ENSG0000(	ENSG0000(	2813.633	0.237256	3.35E-09	1.63E-07	lncRNA
ENSG0000(	SUGT1	3699.569	-0.22195	3.37E-09	1.64E-07	protein_coding
ENSG0000(	TMEM158	96.69292	1.6755	3.38E-09	1.64E-07	protein_coding
ENSG0000(	CLDN1	101.266	1.789882	3.48E-09	1.69E-07	protein_coding
ENSG0000(	RAB12	824.3103	0.629558	3.50E-09	1.70E-07	protein_coding
ENSG0000(	SETD3	1117.626	-0.34315	3.50E-09	1.70E-07	protein_coding
ENSG0000(	BRINP2	19.84891	-2.67235	3.53E-09	1.71E-07	protein_coding
ENSG0000(	CCT5	1161.11	-0.36018	3.56E-09	1.71E-07	protein_coding
ENSG0000(	HADHA	3027.936	-0.4449	3.55E-09	1.71E-07	protein_coding
ENSG0000(	H2AC17	137.3913	-0.89321	3.59E-09	1.73E-07	protein_coding
ENSG0000(	GLB1L2	1039.036	-0.88399	3.64E-09	1.75E-07	protein_coding
ENSG0000(	RGS17	419.9241	0.276186	3.67E-09	1.76E-07	protein_coding
ENSG0000(	CBLB	3020.788	0.2864	3.68E-09	1.76E-07	protein_coding
ENSG0000(	MGST1	2048.384	-0.67049	3.74E-09	1.79E-07	protein_coding
ENSG0000(	ENSG0000(	584.5764	0.346006	3.81E-09	1.82E-07	TEC
ENSG0000(	SLC52A2	381.5767	-0.59026	3.83E-09	1.82E-07	protein_coding
ENSG0000(	TANK	1391.592	0.376997	3.83E-09	1.82E-07	protein_coding

ENSG0000(	H2AC8	168.1239	-0.80272	3.86E-09	1.84E-07	protein_coding
ENSG0000(	ATG7	1734.936	0.281047	3.87E-09	1.84E-07	protein_coding
ENSG0000(	SPAG16-DT	1041.817	0.255329	3.90E-09	1.85E-07	lncRNA
ENSG0000(	TNFAIP2	3206.547	0.858558	3.91E-09	1.85E-07	protein_coding
ENSG0000(	LINC01016	975.028	0.38171	3.95E-09	1.86E-07	lncRNA
ENSG0000(	FAM111A	3837.161	0.286092	3.99E-09	1.88E-07	protein_coding
ENSG0000(	ARID1B	10667.9	0.244865	4.13E-09	1.95E-07	protein_coding
ENSG0000(	FBL	594.9564	-0.50823	4.16E-09	1.96E-07	protein_coding
ENSG0000(	ATXN1-AS1	95.89978	0.574617	4.17E-09	1.96E-07	lncRNA
ENSG0000(	SMIM15	980.1239	-0.47836	4.25E-09	1.99E-07	protein_coding
ENSG0000(	IGHJ3P	428.0564	0.877053	4.32E-09	2.02E-07	IG_J_pseudogene
ENSG0000(	NDUFAB1	361.7269	-0.5449	4.36E-09	2.04E-07	protein_coding
ENSG0000(	ACTR1A	1401.683	-0.31022	4.38E-09	2.05E-07	protein_coding
ENSG0000(	ENSG0000(	37.21515	1.036939	4.50E-09	2.10E-07	TEC
ENSG0000(	EIF2AK3	4332.66	0.21312	4.52E-09	2.10E-07	protein_coding
ENSG0000(	LINC02013	682.9194	0.386139	4.62E-09	2.15E-07	lncRNA
ENSG0000(	TNFSF15	735.9889	0.519431	4.65E-09	2.16E-07	protein_coding
ENSG0000(	LINC01356	820.2067	0.339191	4.68E-09	2.17E-07	lncRNA
ENSG0000(	ENSG0000(	134.1482	0.719517	4.74E-09	2.19E-07	TEC
ENSG0000(	UBE2K	2340.46	-0.35982	4.77E-09	2.20E-07	protein_coding
ENSG0000(	BCL6	1193.306	1.376309	4.80E-09	2.21E-07	protein_coding
ENSG0000(	FSTL1	2194.562	0.968767	4.82E-09	2.22E-07	protein_coding
ENSG0000(	PHLDA1	1223.963	0.958008	4.85E-09	2.23E-07	protein_coding
ENSG0000(	PATL2	452.337	0.666106	4.88E-09	2.24E-07	protein_coding
ENSG0000(	S100A14	1174.458	-0.97271	4.98E-09	2.28E-07	protein_coding
ENSG0000(	COX7B	1287.545	-0.67821	5.13E-09	2.35E-07	protein_coding
ENSG0000(	ENSG0000(	202.2247	0.726027	5.18E-09	2.36E-07	lncRNA
ENSG0000(	POM121L7	139.6887	0.756273	5.19E-09	2.36E-07	processed_pseudogene
ENSG0000(	RPL14	3464.553	-0.54368	5.18E-09	2.36E-07	protein_coding
ENSG0000(	MT-CYB	32929.2	-0.88409	5.21E-09	2.37E-07	protein_coding
ENSG0000(	ENSG0000(	268.3696	0.782974	5.30E-09	2.41E-07	lncRNA
ENSG0000(	H3C3	143.4972	-0.91496	5.37E-09	2.44E-07	protein_coding
ENSG0000(	ATP5PO	1024.51	-0.65712	5.39E-09	2.45E-07	protein_coding
ENSG0000(	CCDC170	231.8884	0.44604	5.45E-09	2.47E-07	protein_coding
ENSG0000(	GARS1-DT	11032.51	0.232362	5.66E-09	2.56E-07	lncRNA
ENSG0000(	SUPV3L1	740.4805	-0.30343	5.75E-09	2.59E-07	protein_coding
ENSG0000(	ENSG0000(	103.8263	-0.41876	5.81E-09	2.62E-07	lncRNA
ENSG0000(	RNF149	2229.169	0.546334	5.89E-09	2.65E-07	protein_coding
ENSG0000(	WDR82	2877.01	-0.21102	5.92E-09	2.66E-07	protein_coding
ENSG0000(	ASPHD2	157.1429	0.753444	5.94E-09	2.66E-07	protein_coding
ENSG0000(	CYBB	2107.804	0.872085	5.95E-09	2.66E-07	protein_coding
ENSG0000(	DMBT1	8571.492	2.197884	6.00E-09	2.69E-07	protein_coding
ENSG0000(	ENSG0000(	1159.394	0.234395	6.04E-09	2.70E-07	lncRNA
ENSG0000(	MIR4300H(	168.7938	-0.35872	6.11E-09	2.73E-07	lncRNA
ENSG0000(	ENSG0000(	32.85592	1.228113	6.20E-09	2.76E-07	lncRNA
ENSG0000(	H2AC4	52.31992	-1.06585	6.23E-09	2.77E-07	protein_coding
ENSG0000(	SLC25A10	397.0571	-0.75152	6.35E-09	2.82E-07	protein_coding

ENSG0000(	ENSG0000(	214.2249	0.435099	6.39E-09	2.83E-07	TEC
ENSG0000(	DPYD	1494.505	0.531371	6.46E-09	2.86E-07	protein_coding
ENSG0000(	H3C7	138.5423	-1.04852	6.48E-09	2.87E-07	protein_coding
ENSG0000(	GPR176	137.6433	1.309047	6.54E-09	2.89E-07	protein_coding
ENSG0000(	MDK	304.5851	1.116371	6.57E-09	2.89E-07	protein_coding
ENSG0000(	PDSS1	329.808	-0.55273	6.57E-09	2.89E-07	protein_coding
ENSG0000(	MRPS12	211.1708	-0.41496	6.61E-09	2.91E-07	protein_coding
ENSG0000(	NUDT15	560.4497	-0.45676	6.65E-09	2.92E-07	protein_coding
ENSG0000(	GRINA	757.4954	0.646775	6.67E-09	2.93E-07	protein_coding
ENSG0000(	TSC22D3	1249.105	1.436176	6.73E-09	2.95E-07	protein_coding
ENSG0000(	COPS5	2205.556	-0.21918	6.75E-09	2.95E-07	protein_coding
ENSG0000(	PSMB9	1034.092	0.61449	6.77E-09	2.96E-07	protein_coding
ENSG0000(	ADAMTS6	212.0021	0.960234	6.84E-09	2.98E-07	protein_coding
ENSG0000(	TCF4	5505.608	0.523625	6.84E-09	2.98E-07	protein_coding
ENSG0000(	MDH2	994.924	-0.46991	6.89E-09	3.00E-07	protein_coding
ENSG0000(	H2BC3	59.13519	-1.10174	6.93E-09	3.01E-07	protein_coding
ENSG0000(	STX16	4783.701	0.194769	7.08E-09	3.07E-07	protein_coding
ENSG0000(	PHF21A	4547.964	0.42904	7.10E-09	3.08E-07	protein_coding
ENSG0000(	HSP90AB1	3525.495	-0.5889	7.17E-09	3.10E-07	protein_coding
ENSG0000(	SLC39A4	525.7669	-0.47891	7.16E-09	3.10E-07	protein_coding
ENSG0000(	RCC1L	478.9119	-0.3337	7.32E-09	3.16E-07	protein_coding
ENSG0000(	SUCNR1	45.53647	1.300901	7.38E-09	3.18E-07	protein_coding
ENSG0000(	APOL6	6268.634	0.486556	7.53E-09	3.24E-07	protein_coding
ENSG0000(	FAU	800.1334	-0.55928	7.59E-09	3.26E-07	protein_coding
ENSG0000(	ENSG0000(	434.1722	0.404518	7.79E-09	3.34E-07	lncRNA
ENSG0000(	PLXNC1	1319.095	0.873477	7.91E-09	3.39E-07	protein_coding
ENSG0000(	ENSG0000(	2256.787	0.437181	7.94E-09	3.39E-07	transcribed_unprocessed_pseudo
ENSG0000(	SLAMF7	1737.697	0.770929	7.93E-09	3.39E-07	protein_coding
ENSG0000(	H4C4	206.0452	-0.8367	8.10E-09	3.46E-07	protein_coding
ENSG0000(	STAT3	6723.172	0.406054	8.16E-09	3.48E-07	protein_coding
ENSG0000(	MNDA	101.537	1.681437	8.19E-09	3.48E-07	protein_coding
ENSG0000(	C4orf50	92.35741	0.737687	8.34E-09	3.54E-07	protein_coding
ENSG0000(	DEPDC1P2	83.21389	-0.66039	8.44E-09	3.58E-07	processed_pseudogene
ENSG0000(	ZNF275	1049.929	0.415101	8.67E-09	3.67E-07	protein_coding
ENSG0000(	ATF3	659.2952	1.609969	8.70E-09	3.68E-07	protein_coding
ENSG0000(	AAMP	751.6508	-0.49708	8.77E-09	3.71E-07	protein_coding
ENSG0000(	RNF24	1067.577	0.767464	8.89E-09	3.75E-07	protein_coding
ENSG0000(	ORMDL1	1272.636	0.289372	8.93E-09	3.76E-07	protein_coding
ENSG0000(	PRELID3B	1461.919	-0.65135	9.01E-09	3.79E-07	protein_coding
ENSG0000(	CIITA	9294.734	0.854778	9.28E-09	3.90E-07	protein_coding
ENSG0000(	SLC16A5	787.3464	-0.7148	9.27E-09	3.90E-07	protein_coding
ENSG0000(	PABPN1	2863.366	-0.20384	9.31E-09	3.90E-07	protein_coding
ENSG0000(	RNF168	1545.594	-0.21436	9.32E-09	3.90E-07	protein_coding
ENSG0000(	MANEA	738.3721	0.517135	9.34E-09	3.91E-07	protein_coding
ENSG0000(	SAE1	777.1937	-0.31884	9.46E-09	3.95E-07	protein_coding
ENSG0000(	KRCC1	728.1888	-0.51148	9.49E-09	3.95E-07	protein_coding
ENSG0000(	SLCO1B3	19.97196	2.126644	9.49E-09	3.95E-07	protein_coding

ENSG0000(	NDRG2	1934.401	-0.43475	9.57E-09	3.98E-07	protein_coding
ENSG0000(	PRECSIT	877.5503	0.292668	9.87E-09	4.10E-07	lncRNA
ENSG0000(	FARSA	340.0955	-0.50541	9.90E-09	4.11E-07	protein_coding
ENSG0000(	ENSG0000(	521.355	0.304044	9.95E-09	4.11E-07	lncRNA
ENSG0000(	PLEKHO1	1321	0.724789	9.94E-09	4.11E-07	protein_coding
ENSG0000(	ENSG0000(	236.3894	0.621695	1.01E-08	4.17E-07	lncRNA
ENSG0000(	ENSG0000(	236.1553	-0.4596	1.01E-08	4.17E-07	lncRNA
ENSG0000(	CASC15	2718.022	0.263307	1.01E-08	4.17E-07	lncRNA
ENSG0000(	H2AC12	126.9758	-1.11233	1.02E-08	4.21E-07	protein_coding
ENSG0000(	TNS3	5203.877	0.353003	1.03E-08	4.22E-07	protein_coding
ENSG0000(	NDUFA6	429.7241	-0.55875	1.04E-08	4.25E-07	protein_coding
ENSG0000(	HDAC9	990.6899	0.613471	1.05E-08	4.31E-07	protein_coding
ENSG0000(	STARD10	1085.854	-0.62948	1.06E-08	4.36E-07	protein_coding
ENSG0000(	HSPH1	1599.616	-0.55382	1.07E-08	4.36E-07	protein_coding
ENSG0000(	CDK14	545.7666	0.826897	1.08E-08	4.40E-07	protein_coding
ENSG0000(	ENSG0000(	24.57191	0.821945	1.10E-08	4.48E-07	lncRNA
ENSG0000(	ADAP1	1612.274	-0.68722	1.10E-08	4.49E-07	protein_coding
ENSG0000(	FAM114A2	512.6809	0.386249	1.11E-08	4.51E-07	protein_coding
ENSG0000(	ARNTL2	762.764	0.794088	1.11E-08	4.52E-07	protein_coding
ENSG0000(	RPIA	330.7834	-0.36437	1.11E-08	4.52E-07	protein_coding
ENSG0000(	ARIH2	3065.545	-0.18389	1.12E-08	4.52E-07	protein_coding
ENSG0000(	SLC25A20	487.8842	-0.74677	1.12E-08	4.54E-07	protein_coding
ENSG0000(	AIFM2	701.6978	-0.54175	1.16E-08	4.69E-07	protein_coding
ENSG0000(	PTGES	292.0314	0.823176	1.16E-08	4.69E-07	protein_coding
ENSG0000(	CDC20	182.0415	-0.88364	1.16E-08	4.69E-07	protein_coding
ENSG0000(	AHCY	757.5788	-0.44337	1.17E-08	4.70E-07	protein_coding
ENSG0000(	CCL18	137.5309	2.577799	1.17E-08	4.71E-07	protein_coding
ENSG0000(	ENSG0000(	1081.869	0.253576	1.17E-08	4.71E-07	lncRNA
ENSG0000(	ENSG0000(	1211.072	0.293262	1.18E-08	4.75E-07	TEC
ENSG0000(	POLRMT	660.2348	-0.43432	1.20E-08	4.79E-07	protein_coding
ENSG0000(	HSH2D	1397.11	0.394763	1.20E-08	4.79E-07	protein_coding
ENSG0000(	MRPL37	432.3606	-0.43635	1.24E-08	4.95E-07	protein_coding
ENSG0000(	AGPAT2	1337.656	-1.02332	1.26E-08	5.03E-07	protein_coding
ENSG0000(	MTA2	1182.545	-0.33258	1.27E-08	5.05E-07	protein_coding
ENSG0000(	ACTR1B	863.2007	-0.26385	1.27E-08	5.07E-07	protein_coding
ENSG0000(	ENSG0000(	187.2089	0.422095	1.29E-08	5.13E-07	lncRNA
ENSG0000(	MUC1	2772.296	0.917041	1.31E-08	5.18E-07	protein_coding
ENSG0000(	NDUFS1	5490.919	-0.37462	1.31E-08	5.20E-07	protein_coding
ENSG0000(	ENSG0000(	883.5457	0.248946	1.34E-08	5.29E-07	lncRNA
ENSG0000(	SLFN11	1233.505	0.700722	1.34E-08	5.29E-07	protein_coding
ENSG0000(	ZRANB2	3369.739	-0.31937	1.35E-08	5.35E-07	protein_coding
ENSG0000(	SLC27A1	1765.314	0.26889	1.36E-08	5.38E-07	protein_coding
ENSG0000(	SPON2	785.9741	0.567357	1.37E-08	5.40E-07	protein_coding
ENSG0000(	RPS24	6474.059	-0.49582	1.38E-08	5.42E-07	protein_coding
ENSG0000(	ENSG0000(	19.82246	0.942313	1.39E-08	5.46E-07	lncRNA
ENSG0000(	NR2F1-AS1	2965.523	0.225642	1.42E-08	5.59E-07	lncRNA
ENSG0000(	OR52K3P	149.3872	0.594958	1.46E-08	5.73E-07	transcribed_unprocessed_pseudo

ENSG0000(	ENSG0000(	740.8155	0.266461	1.47E-08	5.73E-07	lncRNA
ENSG0000(	OSBPL7	1699.2	-0.54725	1.47E-08	5.73E-07	protein_coding
ENSG0000(	CHCHD4	157.5095	-0.54843	1.47E-08	5.74E-07	protein_coding
ENSG0000(	ENSG0000(	644.6766	0.452805	1.50E-08	5.87E-07	TEC
ENSG0000(	VNN3	420.4707	0.569695	1.52E-08	5.92E-07	protein_coding
ENSG0000(	ENSG0000(	101.0676	0.601836	1.53E-08	5.95E-07	lncRNA
ENSG0000(	SLC25A6	2664.193	-0.40916	1.54E-08	5.97E-07	protein_coding
ENSG0000(	MCL1	12411.72	0.5224	1.54E-08	5.97E-07	protein_coding
ENSG0000(	MRPL14	201.6245	-0.49571	1.55E-08	6.01E-07	protein_coding
ENSG0000(	PCDH17	278.5252	1.178847	1.56E-08	6.02E-07	protein_coding
ENSG0000(	NDUFV1	1769.027	-0.37431	1.56E-08	6.03E-07	protein_coding
ENSG0000(	MITF	439.0714	0.444172	1.57E-08	6.04E-07	protein_coding
ENSG0000(	PCNA	281.3379	-0.69453	1.57E-08	6.04E-07	protein_coding
ENSG0000(	H2BC13	82.06523	-1.05411	1.58E-08	6.07E-07	protein_coding
ENSG0000(	NAV2-AS5	29.38054	0.913414	1.58E-08	6.08E-07	lncRNA
ENSG0000(	MACF1	29466.57	0.29998	1.59E-08	6.11E-07	protein_coding
ENSG0000(	WTIP	3071.312	0.312011	1.60E-08	6.12E-07	protein_coding
ENSG0000(	HMGA1	1221.515	-0.52284	1.60E-08	6.13E-07	protein_coding
ENSG0000(	ENSG0000(	376.7257	0.450974	1.63E-08	6.24E-07	lncRNA
ENSG0000(	CASP1	1265.738	0.632512	1.65E-08	6.30E-07	protein_coding
ENSG0000(	GRPEL1	369.9663	-0.38785	1.65E-08	6.30E-07	protein_coding
ENSG0000(	AMN1	897.7446	0.223293	1.65E-08	6.31E-07	protein_coding
ENSG0000(	H2BC4	351.8874	-0.64969	1.66E-08	6.31E-07	protein_coding
ENSG0000(	MRPL15	345.2351	-0.55531	1.69E-08	6.41E-07	protein_coding
ENSG0000(	ENSG0000(	107.0614	0.868907	1.69E-08	6.43E-07	lncRNA
ENSG0000(	COL4A1	3052.203	1.072411	1.72E-08	6.52E-07	protein_coding
ENSG0000(	IGSF8	381.1574	-0.4853	1.73E-08	6.54E-07	protein_coding
ENSG0000(	TMEM18-D	387.1275	0.359939	1.73E-08	6.57E-07	lncRNA
ENSG0000(	RGS16	107.383	1.299527	1.74E-08	6.59E-07	protein_coding
ENSG0000(	TRAF3IP2	2760.023	-0.47618	1.77E-08	6.70E-07	protein_coding
ENSG0000(	ENSG0000(	644.1653	-0.3946	1.78E-08	6.70E-07	lncRNA
ENSG0000(	NDUFA8	160.4859	-0.67541	1.79E-08	6.75E-07	protein_coding
ENSG0000(	ROCK2	4648.996	-0.41452	1.80E-08	6.78E-07	protein_coding
ENSG0000(	ENSG0000(	472.0396	0.346178	1.82E-08	6.85E-07	lncRNA
ENSG0000(	ENSG0000(	277.5645	-0.36322	1.83E-08	6.85E-07	lncRNA
ENSG0000(	ENSG0000(	1862.423	0.186606	1.84E-08	6.89E-07	lncRNA
ENSG0000(	CCR8	10.78248	1.776355	1.84E-08	6.90E-07	protein_coding
ENSG0000(	SLC39A12-/	335.1826	0.336575	1.84E-08	6.90E-07	lncRNA
ENSG0000(	RNF115	3473.228	0.234943	1.87E-08	6.98E-07	protein_coding
ENSG0000(	TRAPPC3L	498.8073	0.266655	1.88E-08	7.01E-07	protein_coding
ENSG0000(	MAT2A	8978.929	-0.43169	1.89E-08	7.05E-07	protein_coding
ENSG0000(	FAM157A	628.7963	1.53954	1.90E-08	7.07E-07	transcribed_unprocessed_pseudo
ENSG0000(	ENSG0000(	541.2769	0.414179	1.90E-08	7.08E-07	lncRNA
ENSG0000(	NMT2	1590.107	-0.35978	1.92E-08	7.13E-07	protein_coding
ENSG0000(	FNDC3B	6264.392	0.477503	1.93E-08	7.16E-07	protein_coding
ENSG0000(	DUSP1	1170.809	1.063244	1.94E-08	7.18E-07	protein_coding
ENSG0000(	SEC24B	1665.65	-0.22171	1.96E-08	7.26E-07	protein_coding

ENSG0000(	UQCRH	1095.564	-0.51312	1.98E-08	7.33E-07	protein_coding
ENSG0000(	UNG	396.1008	-0.50109	1.99E-08	7.35E-07	protein_coding
ENSG0000(	ENSG0000(	188.6968	0.90763	2.01E-08	7.41E-07	lncRNA
ENSG0000(	H3C10	212.9789	-0.8489	2.01E-08	7.41E-07	protein_coding
ENSG0000(	RNF175	21.73283	1.447393	2.02E-08	7.43E-07	protein_coding
ENSG0000(	CSF2RB	1379.832	0.972555	2.03E-08	7.46E-07	protein_coding
ENSG0000(	ENSG0000(	46.64473	1.165196	2.05E-08	7.52E-07	processed_pseudogene
ENSG0000(	KIAA0825	1416.779	0.280315	2.05E-08	7.52E-07	protein_coding
ENSG0000(	SEPTIN7P1:	1023.143	0.322251	2.05E-08	7.52E-07	transcribed_unprocessed_pseudo
ENSG0000(	BOC	523.4356	0.711566	2.09E-08	7.64E-07	protein_coding
ENSG0000(	DNM3OS	319.4572	0.751749	2.09E-08	7.64E-07	lncRNA
ENSG0000(	NOTCH4	1157.678	0.377682	2.10E-08	7.65E-07	protein_coding
ENSG0000(	LAD1	1760.864	-0.82531	2.11E-08	7.68E-07	protein_coding
ENSG0000(	PTGES3P1	71.47641	1.041664	2.12E-08	7.71E-07	processed_pseudogene
ENSG0000(	COX5A	1187.272	-0.70765	2.13E-08	7.75E-07	protein_coding
ENSG0000(	SNHG29	3018.496	-0.48934	2.18E-08	7.91E-07	lncRNA
ENSG0000(	CNIH3-AS2	1075.076	0.325654	2.20E-08	7.98E-07	lncRNA
ENSG0000(	IFT74	804.18	-0.36507	2.21E-08	8.01E-07	protein_coding
ENSG0000(	SYNJ2	2655.443	-0.20512	2.21E-08	8.01E-07	protein_coding
ENSG0000(	ADAR	9175.389	0.319716	2.22E-08	8.03E-07	protein_coding
ENSG0000(	GPAA1	578.1567	-0.38504	2.24E-08	8.09E-07	protein_coding
ENSG0000(	DGKD	3764.755	-0.40145	2.24E-08	8.10E-07	protein_coding
ENSG0000(	STC2	68.88893	2.057975	2.27E-08	8.17E-07	protein_coding
ENSG0000(	CFP	408.0471	0.534773	2.27E-08	8.17E-07	protein_coding
ENSG0000(	SUCLG1	1313.456	-0.44809	2.29E-08	8.22E-07	protein_coding
ENSG0000(	CLK2	1351.189	-0.23219	2.33E-08	8.37E-07	protein_coding
ENSG0000(	PLAA	742.3586	-0.39022	2.34E-08	8.40E-07	protein_coding
ENSG0000(	LINC00691	269.5367	0.34358	2.35E-08	8.44E-07	lncRNA
ENSG0000(	BAHD1	975.6562	-0.42324	2.40E-08	8.58E-07	protein_coding
ENSG0000(	ENSG0000(	22.83336	1.087964	2.43E-08	8.67E-07	misc_RNA
ENSG0000(	ASAP1-IT2	350.0363	0.393488	2.43E-08	8.69E-07	lncRNA
ENSG0000(	DENND5A	2693.978	0.554884	2.47E-08	8.78E-07	protein_coding
ENSG0000(	MIR5690	40.57572	1.732145	2.46E-08	8.78E-07	miRNA
ENSG0000(	GJD3	105.0013	0.847705	2.49E-08	8.86E-07	protein_coding
ENSG0000(	FOXP3	67.40276	1.221356	2.50E-08	8.88E-07	protein_coding
ENSG0000(	NME2	949.1981	-0.49549	2.50E-08	8.88E-07	protein_coding
ENSG0000(	ITGA5	1152.276	1.062461	2.51E-08	8.90E-07	protein_coding
ENSG0000(	STXBP1	727.065	0.441211	2.53E-08	8.95E-07	protein_coding
ENSG0000(	ECH1	829.1347	-0.47643	2.54E-08	8.98E-07	protein_coding
ENSG0000(	PEBP1	1806.152	-0.52544	2.55E-08	8.99E-07	protein_coding
ENSG0000(	EGFLAM	150.5162	0.585413	2.56E-08	9.00E-07	protein_coding
ENSG0000(	ENSG0000(	227.5771	0.763325	2.56E-08	9.00E-07	lncRNA
ENSG0000(	FAAH	743.996	-0.72069	2.56E-08	9.00E-07	protein_coding
ENSG0000(	RPS26P21	59.52098	0.629481	2.55E-08	9.00E-07	processed_pseudogene
ENSG0000(	CPNE5	553.4757	0.770024	2.58E-08	9.06E-07	protein_coding
ENSG0000(	ALDH1B1	955.3316	-0.52525	2.60E-08	9.13E-07	protein_coding
ENSG0000(	TNPO2	1728.261	-0.3576	2.65E-08	9.29E-07	protein_coding

ENSG0000(	HINT1	3387.226	-0.4988	2.70E-08	9.46E-07	protein_coding
ENSG0000(	GAS5	2075.406	-0.3403	2.75E-08	9.61E-07	lncRNA
ENSG0000(	LINC01001	1335.314	0.669379	2.76E-08	9.66E-07	lncRNA
ENSG0000(	SLC25A48	216.1789	0.76438	2.81E-08	9.82E-07	protein_coding
ENSG0000(	ENSG0000(	79.20325	0.997841	2.83E-08	9.85E-07	lncRNA
ENSG0000(	ENTPD1-AS	3320.548	0.144639	2.83E-08	9.85E-07	lncRNA
ENSG0000(	H2AC13	162.8394	-0.86127	2.83E-08	9.85E-07	protein_coding
ENSG0000(	GCNT1P3	12.91072	1.763884	2.85E-08	9.90E-07	processed_pseudogene
ENSG0000(	TAP1	2274.192	0.698177	2.86E-08	9.91E-07	protein_coding
ENSG0000(	ENSG0000(	37.50298	1.480025	2.88E-08	9.99E-07	lncRNA
ENSG0000(	NR4A3	175.4554	1.586488	2.89E-08	1.00E-06	protein_coding
ENSG0000(	BHLHB9P1	28.56575	1.221572	2.91E-08	1.01E-06	processed_pseudogene
ENSG0000(	ATP5F1C	1510.097	-0.42645	2.93E-08	1.01E-06	protein_coding
ENSG0000(	TLR8	149.38	1.455039	2.93E-08	1.01E-06	protein_coding
ENSG0000(	ENSG0000(	415.8273	0.400692	2.94E-08	1.01E-06	TEC
ENSG0000(	RBPMS	2205.422	0.720505	3.02E-08	1.04E-06	protein_coding
ENSG0000(	CYLD-AS1	180.1023	0.627839	3.03E-08	1.04E-06	lncRNA
ENSG0000(	FABP5	741.3588	-0.8644	3.03E-08	1.04E-06	protein_coding
ENSG0000(	ENSG0000(	18.49619	1.734495	3.05E-08	1.05E-06	lncRNA
ENSG0000(	ZNF415P1	38.17017	1.258412	3.15E-08	1.08E-06	processed_pseudogene
ENSG0000(	FUT8-AS1	51.39524	0.790376	3.16E-08	1.08E-06	lncRNA
ENSG0000(	TPRKB	269.4427	-0.416	3.16E-08	1.08E-06	protein_coding
ENSG0000(	ZNF675	1358.72	-0.22035	3.17E-08	1.08E-06	protein_coding
ENSG0000(	ENSG0000(	170.8211	0.429184	3.19E-08	1.09E-06	TEC
ENSG0000(	IMP4	695.0608	-0.33406	3.20E-08	1.09E-06	protein_coding
ENSG0000(	MIR3142H(	409.878	0.844034	3.20E-08	1.09E-06	lncRNA
ENSG0000(	BICC1	491.8519	0.867899	3.24E-08	1.10E-06	protein_coding
ENSG0000(	SSU72	2728.475	-0.21369	3.28E-08	1.11E-06	protein_coding
ENSG0000(	ENSG0000(	15.13216	2.3729	3.29E-08	1.12E-06	lncRNA
ENSG0000(	TRPS1	1272.944	0.760788	3.31E-08	1.12E-06	protein_coding
ENSG0000(	IFIT2	430.9225	1.519362	3.34E-08	1.13E-06	protein_coding
ENSG0000(	TRIM69	1627.17	0.454616	3.39E-08	1.15E-06	protein_coding
ENSG0000(	KCND3-IT1	20.26353	1.17613	3.40E-08	1.15E-06	lncRNA
ENSG0000(	ENSG0000(	1161.037	-0.17004	3.45E-08	1.17E-06	lncRNA
ENSG0000(	C11orf86	193.9014	-1.52177	3.47E-08	1.17E-06	protein_coding
ENSG0000(	DNAJA3	1681.438	-0.27938	3.47E-08	1.17E-06	protein_coding
ENSG0000(	SERPINA1	1282.89	1.031801	3.51E-08	1.18E-06	protein_coding
ENSG0000(	TATDN1	616.36	-0.25535	3.56E-08	1.20E-06	protein_coding
ENSG0000(	NUDT19	699.8015	-0.2828	3.63E-08	1.22E-06	protein_coding
ENSG0000(	ZFYVE21	1388.146	-0.29363	3.63E-08	1.22E-06	protein_coding
ENSG0000(	ENSG0000(	120.8561	0.975956	3.64E-08	1.22E-06	TEC
ENSG0000(	GCN1	3209.777	-0.39701	3.64E-08	1.22E-06	protein_coding
ENSG0000(	ACADS	1033.433	-0.78673	3.64E-08	1.22E-06	protein_coding
ENSG0000(	MIR181A1I	450.8854	0.665771	3.65E-08	1.22E-06	lncRNA
ENSG0000(	CALHM6	52.62957	1.390727	3.67E-08	1.23E-06	protein_coding
ENSG0000(	ENSG0000(	122.114	-0.42355	3.71E-08	1.24E-06	unprocessed_pseudogene
ENSG0000(	ZNF292	7249.673	0.183409	3.72E-08	1.24E-06	protein_coding

ENSG0000(	KAT14	522.0174	-0.4257	3.78E-08	1.26E-06	protein_coding
ENSG0000(	SPAG4	87.05064	0.865733	3.79E-08	1.26E-06	protein_coding
ENSG0000(	CHRNE	29.74077	0.902897	3.82E-08	1.27E-06	protein_coding
ENSG0000(	CLEC4E	338.8607	0.670421	3.83E-08	1.27E-06	protein_coding
ENSG0000(	ENSG0000(	58.34001	1.425058	3.83E-08	1.27E-06	lncRNA
ENSG0000(	ODC1-DT	652.0637	0.324548	3.84E-08	1.27E-06	lncRNA
ENSG0000(	PSMC5	861.9736	-0.39043	3.85E-08	1.27E-06	protein_coding
ENSG0000(	H2BC6	234.3803	-0.95282	3.89E-08	1.28E-06	protein_coding
ENSG0000(	LINC02649	432.1798	0.477439	3.89E-08	1.28E-06	lncRNA
ENSG0000(	DLEU2	3066.495	0.272503	3.92E-08	1.29E-06	lncRNA
ENSG0000(	NOX4	308.1912	0.488518	3.93E-08	1.29E-06	protein_coding
ENSG0000(	ENSG0000(	23.27877	-0.92941	3.96E-08	1.30E-06	lncRNA
ENSG0000(	ENSG0000(	951.2361	-0.27913	4.01E-08	1.32E-06	TEC
ENSG0000(	PSTPIP2	578.8624	0.860811	4.02E-08	1.32E-06	protein_coding
ENSG0000(	RBMS1	1871.08	0.586787	4.04E-08	1.32E-06	protein_coding
ENSG0000(	SLC25A15	750.4296	-0.26797	4.04E-08	1.32E-06	protein_coding
ENSG0000(	ENSG0000(	593.374	0.546144	4.07E-08	1.33E-06	TEC
ENSG0000(	ENSG0000(	609.4047	0.268272	4.16E-08	1.36E-06	lncRNA
ENSG0000(	HCLS1	1694.197	0.700729	4.18E-08	1.36E-06	protein_coding
ENSG0000(	PSMC1	1018.441	-0.46031	4.21E-08	1.37E-06	protein_coding
ENSG0000(	CLEC12A	121.7727	0.961871	4.23E-08	1.38E-06	protein_coding
ENSG0000(	ALDH3A2	4372.032	-0.39067	4.25E-08	1.38E-06	protein_coding
ENSG0000(	IRF2-DT	660.5533	0.26758	4.25E-08	1.38E-06	lncRNA
ENSG0000(	PLCD3	3950.84	-1.05791	4.24E-08	1.38E-06	protein_coding
ENSG0000(	TMX2	1079.984	-0.32507	4.25E-08	1.38E-06	protein_coding
ENSG0000(	DMAC1	502.8769	-0.56539	4.26E-08	1.38E-06	protein_coding
ENSG0000(	ENSG0000(	115.3244	0.359352	4.31E-08	1.39E-06	lncRNA
ENSG0000(	ENSG0000(	394.3466	0.484864	4.35E-08	1.41E-06	TEC
ENSG0000(	TIGD1	5568.674	0.206628	4.40E-08	1.42E-06	protein_coding
ENSG0000(	TRAPPC2	1514.381	-0.22	4.41E-08	1.42E-06	protein_coding
ENSG0000(	DAPK1-IT1	118.2153	0.508131	4.42E-08	1.43E-06	lncRNA
ENSG0000(	POLDIP2	730.4837	-0.45897	4.43E-08	1.43E-06	protein_coding
ENSG0000(	NFS1	836.117	-0.26822	4.46E-08	1.44E-06	protein_coding
ENSG0000(	ENSG0000(	45.87271	1.021898	4.50E-08	1.45E-06	lncRNA
ENSG0000(	LINC00635	300.3614	0.328144	4.50E-08	1.45E-06	lncRNA
ENSG0000(	H4C14	460.0439	-0.97103	4.51E-08	1.45E-06	protein_coding
ENSG0000(	LIMS1	2014.522	0.346995	4.52E-08	1.45E-06	protein_coding
ENSG0000(	CAPS2	633.1589	0.299114	4.55E-08	1.46E-06	protein_coding
ENSG0000(	PSMD3	1283.497	-0.38016	4.62E-08	1.48E-06	protein_coding
ENSG0000(	ENSG0000(	310.5323	0.366092	4.70E-08	1.50E-06	lncRNA
ENSG0000(	RPL36AL	650.8346	-0.49115	4.73E-08	1.51E-06	protein_coding
ENSG0000(	RBBP5	1131.273	-0.23381	4.74E-08	1.51E-06	protein_coding
ENSG0000(	PARP9	2303.934	0.789826	4.79E-08	1.53E-06	protein_coding
ENSG0000(	SNORD68	42.83017	1.579187	4.83E-08	1.54E-06	snoRNA
ENSG0000(	ENSG0000(	212.0807	0.305567	4.88E-08	1.55E-06	lncRNA
ENSG0000(	RAVER1	872.5263	-0.4789	4.92E-08	1.56E-06	protein_coding
ENSG0000(	ENSG0000(	109.7463	0.748948	4.93E-08	1.56E-06	lncRNA

ENSG0000(	OSCAR	60.38853	0.94068	5.05E-08	1.60E-06	protein_coding
ENSG0000(	PRDX2	555.7504	-0.5266	5.05E-08	1.60E-06	protein_coding
ENSG0000(	ENSG0000(	1686.824	0.212512	5.08E-08	1.60E-06	lncRNA
ENSG0000(	ENSG0000(	86.34775	0.622546	5.12E-08	1.62E-06	processed_pseudogene
ENSG0000(	FCGR2B	1141.683	0.942867	5.12E-08	1.62E-06	protein_coding
ENSG0000(	ATF6B	1871.175	-0.17218	5.13E-08	1.62E-06	protein_coding
ENSG0000(	GATD1	2969.77	-0.22042	5.21E-08	1.64E-06	protein_coding
ENSG0000(	IMPA2	1044.199	-0.4563	5.22E-08	1.64E-06	protein_coding
ENSG0000(	ZNF90	728.6191	-0.33155	5.29E-08	1.66E-06	protein_coding
ENSG0000(	SAT1	6819.795	0.618281	5.33E-08	1.67E-06	protein_coding
ENSG0000(	UCKL1	766.3972	-0.40854	5.34E-08	1.68E-06	protein_coding
ENSG0000(	CDRT4	805.3866	0.508325	5.43E-08	1.70E-06	protein_coding
ENSG0000(	SGIP1	928.0419	1.221254	5.43E-08	1.70E-06	protein_coding
ENSG0000(	PPM1G	906.7505	-0.36932	5.44E-08	1.70E-06	protein_coding
ENSG0000(	STAP2	1317.205	-0.54564	5.44E-08	1.70E-06	protein_coding
ENSG0000(	MMP13	34.17842	2.396857	5.46E-08	1.70E-06	protein_coding
ENSG0000(	SIRPA	707.552	0.88125	5.46E-08	1.70E-06	protein_coding
ENSG0000(	GAR1	163.4227	-0.43233	5.47E-08	1.70E-06	protein_coding
ENSG0000(	FBXW5	1388.796	-0.4254	5.51E-08	1.72E-06	protein_coding
ENSG0000(	ATP5MK	474.8467	-0.70338	5.52E-08	1.72E-06	protein_coding
ENSG0000(	PAICS	1745.962	-0.32835	5.59E-08	1.74E-06	protein_coding
ENSG0000(	NARS1	1831.318	-0.37906	5.61E-08	1.74E-06	protein_coding
ENSG0000(	YAF2	1750.847	0.220445	5.66E-08	1.75E-06	protein_coding
ENSG0000(	ENSG0000(	58.9168	0.654709	5.69E-08	1.76E-06	lncRNA
ENSG0000(	ENSG0000(	148.9124	0.57148	5.69E-08	1.76E-06	TEC
ENSG0000(	DEPP1	346.3533	0.889507	5.70E-08	1.76E-06	protein_coding
ENSG0000(	RN7SL141F	112.643	0.611721	5.74E-08	1.77E-06	misc_RNA
ENSG0000(	CREB5	786.0031	1.959351	5.87E-08	1.81E-06	protein_coding
ENSG0000(	RPL11P3	330.5916	0.395451	5.95E-08	1.83E-06	processed_pseudogene
ENSG0000(	SNORD33	240.6677	0.699349	5.94E-08	1.83E-06	snoRNA
ENSG0000(	ENSG0000(	405.4345	0.39699	5.98E-08	1.84E-06	lncRNA
ENSG0000(	WFDC12	34.79382	0.661631	6.00E-08	1.84E-06	protein_coding
ENSG0000(	CES2	4255.874	-0.642	6.01E-08	1.84E-06	protein_coding
ENSG0000(	PDHA1	1931.244	-0.4352	6.06E-08	1.86E-06	protein_coding
ENSG0000(	USP22	3541.058	-0.22647	6.11E-08	1.87E-06	protein_coding
ENSG0000(	RPL13A	6580.445	-0.51899	6.14E-08	1.88E-06	protein_coding
ENSG0000(	ENSG0000(	18.87437	1.408516	6.15E-08	1.88E-06	lncRNA
ENSG0000(	ENSG0000(	150.6994	0.421673	6.16E-08	1.88E-06	lncRNA
ENSG0000(	CHST11	788.9456	0.765448	6.18E-08	1.89E-06	protein_coding
ENSG0000(	CD300E	566.11	1.24457	6.20E-08	1.89E-06	protein_coding
ENSG0000(	YTHDF1	878.6823	-0.23504	6.20E-08	1.89E-06	protein_coding
ENSG0000(	ENSG0000(	67.74384	1.146325	6.21E-08	1.89E-06	lncRNA
ENSG0000(	ENSG0000(	155.7982	0.542483	6.27E-08	1.90E-06	lncRNA
ENSG0000(	ENSG0000(	299.1581	0.303591	6.28E-08	1.91E-06	lncRNA
ENSG0000(	LRR1	309.009	-0.52588	6.30E-08	1.91E-06	protein_coding
ENSG0000(	SLC6A20	649.9884	0.703427	6.31E-08	1.91E-06	protein_coding
ENSG0000(	COL27A1	1951.728	0.611536	6.36E-08	1.93E-06	protein_coding

ENSG0000(	DEAF1	905.7948	-0.26567	6.38E-08	1.93E-06	protein_coding
ENSG0000(	NEDD9	3171.139	0.484457	6.42E-08	1.94E-06	protein_coding
ENSG0000(	ENSG0000(	112.2101	1.209836	6.50E-08	1.96E-06	lncRNA
ENSG0000(	BAX	650.7617	-0.34409	6.53E-08	1.97E-06	protein_coding
ENSG0000(	OSBPL3	1538.062	0.42086	6.56E-08	1.98E-06	protein_coding
ENSG0000(	KANSL1L	1079.906	0.264803	6.57E-08	1.98E-06	protein_coding
ENSG0000(	ENSG0000(	309.1221	-0.33963	6.63E-08	1.99E-06	TEC
ENSG0000(	ENSG0000(	1038.056	0.2866	6.77E-08	2.03E-06	lncRNA
ENSG0000(	ENSG0000(	62.957	0.519313	6.83E-08	2.05E-06	lncRNA
ENSG0000(	ATP5PB	1668.089	-0.6669	6.87E-08	2.06E-06	protein_coding
ENSG0000(	MTCO3P11	143.3542	0.64687	6.87E-08	2.06E-06	unprocessed_pseudogene
ENSG0000(	ENSG0000(	41.34126	1.505093	6.99E-08	2.09E-06	TEC
ENSG0000(	MRPS18B	403.3315	-0.36596	6.99E-08	2.09E-06	protein_coding
ENSG0000(	ENSG0000(	1541.69	0.248959	7.10E-08	2.12E-06	lncRNA
ENSG0000(	BACH1	2789.943	0.445315	7.14E-08	2.13E-06	protein_coding
ENSG0000(	ENSG0000(	141.0371	-0.44217	7.20E-08	2.14E-06	lncRNA
ENSG0000(	NDUFB6	173.6662	-0.6309	7.20E-08	2.14E-06	protein_coding
ENSG0000(	IQSEC1	2005.368	0.643367	7.23E-08	2.15E-06	protein_coding
ENSG0000(	ENSG0000(	239.8565	0.549957	7.32E-08	2.17E-06	lncRNA
ENSG0000(	ATP5MC2	1480.439	-0.48025	7.36E-08	2.19E-06	protein_coding
ENSG0000(	ENSG0000(	3091.159	0.427888	7.39E-08	2.19E-06	transcribed_unprocessed_pseudo
ENSG0000(	TUBA1C	1412.682	-0.68096	7.47E-08	2.21E-06	protein_coding
ENSG0000(	SNHG25	62.61143	-0.98542	7.48E-08	2.22E-06	lncRNA
ENSG0000(	APOOL	1055.099	-0.22555	7.62E-08	2.25E-06	protein_coding
ENSG0000(	PDGFRB	1735.706	0.946194	7.64E-08	2.26E-06	protein_coding
ENSG0000(	MRPS9	251.4934	-0.44065	7.73E-08	2.28E-06	protein_coding
ENSG0000(	STOML2	371.23	-0.48012	7.75E-08	2.29E-06	protein_coding
ENSG0000(	ADGRE2	1433.253	0.688627	7.78E-08	2.29E-06	protein_coding
ENSG0000(	LGALS3	6611.788	-0.81372	7.82E-08	2.30E-06	protein_coding
ENSG0000(	ALDH6A1	1970.792	-0.26802	7.89E-08	2.32E-06	protein_coding
ENSG0000(	PI3	123.7462	2.629183	7.91E-08	2.32E-06	protein_coding
ENSG0000(	DNAJC4	462.317	-0.36121	8.02E-08	2.36E-06	protein_coding
ENSG0000(	ENSG0000(	95.6286	0.796677	8.07E-08	2.37E-06	lncRNA
ENSG0000(	SIRPB2	429.2367	0.814151	8.10E-08	2.37E-06	protein_coding
ENSG0000(	POLR2L	200.7639	-0.5371	8.12E-08	2.38E-06	protein_coding
ENSG0000(	ENSG0000(	475.298	0.503939	8.16E-08	2.39E-06	lncRNA
ENSG0000(	MS4A6E	36.13202	1.007162	8.17E-08	2.39E-06	protein_coding
ENSG0000(	PWWP3A	2120.133	-0.2474	8.29E-08	2.42E-06	protein_coding
ENSG0000(	RPS12	2368.052	-0.56471	8.36E-08	2.44E-06	protein_coding
ENSG0000(	ECHS1	761.301	-0.70455	8.51E-08	2.48E-06	protein_coding
ENSG0000(	ENSG0000(	32.22932	1.810961	8.55E-08	2.49E-06	lncRNA
ENSG0000(	STARD7	2833.268	-0.30081	8.60E-08	2.50E-06	protein_coding
ENSG0000(	TONSL	977.0951	-0.27106	8.60E-08	2.50E-06	protein_coding
ENSG0000(	TOB2	1379.286	-0.39079	8.64E-08	2.51E-06	protein_coding
ENSG0000(	RHOQ	1233.496	0.726216	8.66E-08	2.51E-06	protein_coding
ENSG0000(	KMT5AP3	69.02008	-1.05672	8.71E-08	2.52E-06	processed_pseudogene
ENSG0000(	IL2RA	445.4453	1.016639	8.78E-08	2.54E-06	protein_coding

ENSG0000(	ENSG0000(	37.35128	1.570054	8.81E-08	2.55E-06	lncRNA
ENSG0000(	ENSG0000(	268.6701	0.204051	8.95E-08	2.59E-06	lncRNA
ENSG0000(	MARVELD2	1693.972	-0.44629	8.99E-08	2.60E-06	protein_coding
ENSG0000(	ZFP30	841.4115	-0.30517	9.03E-08	2.61E-06	protein_coding
ENSG0000(	STYXL1	346.0001	-0.44481	9.07E-08	2.61E-06	protein_coding
ENSG0000(	LINC01721	394.0487	0.31094	9.11E-08	2.62E-06	lncRNA
ENSG0000(	BCCIP	511.6071	-0.31706	9.14E-08	2.62E-06	protein_coding
ENSG0000(	ENSG0000(	121.9663	0.479479	9.13E-08	2.62E-06	transcribed_unprocessed_pseudo
ENSG0000(	LINC02207	424.8819	0.311271	9.13E-08	2.62E-06	lncRNA
ENSG0000(	ERI3	428.6332	-0.33706	9.21E-08	2.64E-06	protein_coding
ENSG0000(	LINC00456	237.1612	0.38274	9.29E-08	2.66E-06	lncRNA
ENSG0000(	FADS1	737.1547	0.583806	9.30E-08	2.66E-06	protein_coding
ENSG0000(	ENSG0000(	715.7385	0.292287	9.36E-08	2.67E-06	lncRNA
ENSG0000(	H2BC15	567.7536	-0.67716	9.36E-08	2.67E-06	protein_coding
ENSG0000(	NDUFA1	361.7303	-0.7535	9.36E-08	2.67E-06	protein_coding
ENSG0000(	RPL35	1293.14	-0.48282	9.43E-08	2.69E-06	protein_coding
ENSG0000(	ENSG0000(	56.7295	1.320487	9.44E-08	2.69E-06	lncRNA
ENSG0000(	KIRREL1	942.8004	0.679042	9.51E-08	2.71E-06	protein_coding
ENSG0000(	SUCLG2	1964.739	-0.54981	9.51E-08	2.71E-06	protein_coding
ENSG0000(	NDUFS2	2161.285	-0.33058	9.54E-08	2.71E-06	protein_coding
ENSG0000(	PHPT1	613.7341	-0.51134	9.57E-08	2.72E-06	protein_coding
ENSG0000(	TOR3A	916.6871	0.243378	9.59E-08	2.72E-06	protein_coding
ENSG0000(	NPL	530.9681	0.647957	9.67E-08	2.74E-06	protein_coding
ENSG0000(	DDX23	1456.605	-0.19627	9.68E-08	2.74E-06	protein_coding
ENSG0000(	SCART1	873.0685	0.374199	9.93E-08	2.81E-06	protein_coding
ENSG0000(	ENSG0000(	298.8958	0.289192	1.00E-07	2.83E-06	lncRNA
ENSG0000(	NPTX2	46.94744	1.76133	1.03E-07	2.90E-06	protein_coding
ENSG0000(	SLC2A3	1139.415	0.974216	1.03E-07	2.90E-06	protein_coding
ENSG0000(	SUPT4H1	400.2284	-0.34048	1.03E-07	2.90E-06	protein_coding
ENSG0000(	ZNF681	615.9996	-0.35471	1.03E-07	2.90E-06	protein_coding
ENSG0000(	FDPSP3	93.54304	0.736976	1.04E-07	2.93E-06	transcribed_processed_pseudoge
ENSG0000(	LINC02346	350.8041	0.396825	1.04E-07	2.93E-06	lncRNA
ENSG0000(	ELOVL1	858.6998	-0.52224	1.05E-07	2.94E-06	protein_coding
ENSG0000(	LINC02656	230.2311	0.749144	1.05E-07	2.94E-06	lncRNA
ENSG0000(	SPATS2	2308.307	0.230519	1.08E-07	3.02E-06	protein_coding
ENSG0000(	NXPE3	1627.755	0.376496	1.08E-07	3.02E-06	protein_coding
ENSG0000(	DUSP10	188.033	0.67933	1.08E-07	3.02E-06	protein_coding
ENSG0000(	FAM234A	1408.728	-0.42133	1.09E-07	3.04E-06	protein_coding
ENSG0000(	ENSG0000(	33.67148	0.984522	1.11E-07	3.09E-06	TEC
ENSG0000(	FAM110B	115.1705	0.827863	1.11E-07	3.09E-06	protein_coding
ENSG0000(	KMT5B	2577.519	-0.24865	1.11E-07	3.11E-06	protein_coding
ENSG0000(	IL18RAP	167.6072	1.085829	1.13E-07	3.15E-06	protein_coding
ENSG0000(	MSL3	1434.742	0.407786	1.13E-07	3.15E-06	protein_coding
ENSG0000(	MRPS17	710.6733	-0.20774	1.14E-07	3.16E-06	protein_coding
ENSG0000(	IRF9	1967.517	0.470724	1.14E-07	3.17E-06	protein_coding
ENSG0000(	ENSG0000(	27.69589	0.746023	1.16E-07	3.23E-06	lncRNA
ENSG0000(	HOOK3	4288.818	0.208717	1.16E-07	3.23E-06	protein_coding

ENSG0000(	RXFP4	23.06711	-1.01935	1.17E-07	3.24E-06	protein_coding
ENSG0000(	MIR29B2CI	7756.633	0.34005	1.17E-07	3.24E-06	lncRNA
ENSG0000(	PHGDH	391.5242	0.34554	1.17E-07	3.24E-06	protein_coding
ENSG0000(	NXN	579.5135	0.570206	1.18E-07	3.27E-06	protein_coding
ENSG0000(	IL15RA	412.8441	0.510954	1.21E-07	3.33E-06	protein_coding
ENSG0000(	SPHK1	139.7419	1.131748	1.21E-07	3.33E-06	protein_coding
ENSG0000(	ENSG0000(	25.16634	1.139908	1.21E-07	3.34E-06	lncRNA
ENSG0000(	FAM83A	83.60319	1.180461	1.21E-07	3.34E-06	protein_coding
ENSG0000(	PCCA-DT	419.7411	-0.30019	1.21E-07	3.34E-06	lncRNA
ENSG0000(	PCSK1	88.9326	1.367139	1.21E-07	3.34E-06	protein_coding
ENSG0000(	NME8	42.05601	1.292187	1.22E-07	3.34E-06	protein_coding
ENSG0000(	ARID3A	1189.9	0.409982	1.22E-07	3.35E-06	protein_coding
ENSG0000(	LINC00838	73.61819	0.528662	1.22E-07	3.35E-06	lncRNA
ENSG0000(	LYPD1	36.65023	1.326807	1.22E-07	3.35E-06	protein_coding
ENSG0000(	RPSA	3705.211	-0.46251	1.22E-07	3.35E-06	protein_coding
ENSG0000(	ARF5	537.1307	-0.43929	1.23E-07	3.35E-06	protein_coding
ENSG0000(	CDK12	4574.975	0.179093	1.23E-07	3.36E-06	protein_coding
ENSG0000(	GOT2	1234.59	-0.3153	1.23E-07	3.36E-06	protein_coding
ENSG0000(	PLAU	633.9209	1.459966	1.24E-07	3.39E-06	protein_coding
ENSG0000(	WDHD1	300.2869	-0.61267	1.25E-07	3.40E-06	protein_coding
ENSG0000(	ACSL4	1633.285	1.033967	1.26E-07	3.43E-06	protein_coding
ENSG0000(	ENSG0000(	322.1272	0.947281	1.26E-07	3.43E-06	TEC
ENSG0000(	CBLN3	174.9543	0.914913	1.26E-07	3.44E-06	protein_coding
ENSG0000(	LINC01270	111.4122	0.825877	1.26E-07	3.44E-06	lncRNA
ENSG0000(	SIK1B	514.126	1.350446	1.27E-07	3.44E-06	protein_coding
ENSG0000(	TLR1	609.3845	0.874336	1.27E-07	3.45E-06	protein_coding
ENSG0000(	STON1	528.0872	0.596423	1.28E-07	3.47E-06	protein_coding
ENSG0000(	THBS1-IT1	479.7832	1.109966	1.28E-07	3.47E-06	lncRNA
ENSG0000(	TMC3	112.7089	0.360879	1.29E-07	3.50E-06	protein_coding
ENSG0000(	ENSG0000(	221.8975	-0.34314	1.29E-07	3.50E-06	lncRNA
ENSG0000(	ENSG0000(	910.9739	-0.22448	1.31E-07	3.54E-06	lncRNA
ENSG0000(	RPS14	3932.666	-0.57291	1.31E-07	3.54E-06	protein_coding
ENSG0000(	RN7SKP56	21.66188	1.03438	1.32E-07	3.56E-06	misc_RNA
ENSG0000(	MAGOH	185.5109	-0.33421	1.32E-07	3.56E-06	protein_coding
ENSG0000(	DLAT	1185.707	-0.34752	1.33E-07	3.59E-06	protein_coding
ENSG0000(	SNRPB	1039.095	-0.3903	1.33E-07	3.59E-06	protein_coding
ENSG0000(	DENND5B	1816.296	0.410943	1.34E-07	3.60E-06	protein_coding
ENSG0000(	MPST	3776.46	-0.55309	1.35E-07	3.63E-06	protein_coding
ENSG0000(	PIH1D1	579.9172	-0.25014	1.36E-07	3.65E-06	protein_coding
ENSG0000(	MRPL47	180.5094	-0.45934	1.36E-07	3.66E-06	protein_coding
ENSG0000(	ARL6IP1	2282.462	-0.37378	1.37E-07	3.66E-06	protein_coding
ENSG0000(	CSTF2	255.3501	-0.44198	1.39E-07	3.71E-06	protein_coding
ENSG0000(	NEBL	766.3906	0.606098	1.39E-07	3.71E-06	protein_coding
ENSG0000(	LDLRAD4	7801.045	0.295321	1.40E-07	3.75E-06	protein_coding
ENSG0000(	KIF5B	4078.234	-0.3192	1.40E-07	3.75E-06	protein_coding
ENSG0000(	ENSG0000(	46.70122	0.966008	1.41E-07	3.75E-06	lncRNA
ENSG0000(	HDAC1	2879.756	-0.38857	1.41E-07	3.75E-06	protein_coding

ENSG0000(	LRRC47	722.8895	-0.32223	1.41E-07	3.75E-06	protein_coding
ENSG0000(	RACK1	5087.304	-0.36922	1.41E-07	3.75E-06	protein_coding
ENSG0000(	ENSG0000(	575.9054	0.332005	1.42E-07	3.76E-06	protein_coding
ENSG0000(	SAMHD1	3661.992	0.393509	1.42E-07	3.76E-06	protein_coding
ENSG0000(	ENSG0000(	282.3669	0.293658	1.43E-07	3.80E-06	lncRNA
ENSG0000(	GFPT1	6017.998	-0.43266	1.44E-07	3.81E-06	protein_coding
ENSG0000(	ZNF721	2001.426	-0.28571	1.44E-07	3.81E-06	protein_coding
ENSG0000(	PTGS2	310.6464	1.727628	1.45E-07	3.85E-06	protein_coding
ENSG0000(	GOLT1A	84.8722	-1.1298	1.46E-07	3.87E-06	protein_coding
ENSG0000(	AGPS	2610.461	-0.29534	1.48E-07	3.90E-06	protein_coding
ENSG0000(	RNF144B	483.9751	0.956786	1.49E-07	3.93E-06	protein_coding
ENSG0000(	MTA1	1361.794	-0.2654	1.49E-07	3.93E-06	protein_coding
ENSG0000(	CCDC142	1685.886	-0.22518	1.49E-07	3.94E-06	protein_coding
ENSG0000(	BCAP31	649.0415	-0.42285	1.50E-07	3.95E-06	protein_coding
ENSG0000(	EEF1D	3207.281	-0.23836	1.51E-07	3.96E-06	protein_coding
ENSG0000(	IGHG4	862.2066	2.095833	1.51E-07	3.96E-06	IG_C_gene
ENSG0000(	UQCR10	477.6608	-0.557	1.51E-07	3.96E-06	protein_coding
ENSG0000(	MAFB	411.3337	0.715776	1.51E-07	3.97E-06	protein_coding
ENSG0000(	TRIM56	7387.106	0.256601	1.52E-07	3.98E-06	protein_coding
ENSG0000(	ENSG0000(	59.58967	0.77579	1.53E-07	4.00E-06	lncRNA
ENSG0000(	HSPE1P18	88.51954	0.737811	1.53E-07	4.00E-06	processed_pseudogene
ENSG0000(	LGALS4	11756.83	-0.89894	1.53E-07	4.00E-06	protein_coding
ENSG0000(	ZNF787	540.3481	-0.28564	1.54E-07	4.01E-06	protein_coding
ENSG0000(	DBI	973.166	-0.54588	1.55E-07	4.05E-06	protein_coding
ENSG0000(	ENSG0000(	155.8425	0.365143	1.55E-07	4.05E-06	lncRNA
ENSG0000(	MT-ND3	3686.922	-0.7684	1.55E-07	4.05E-06	protein_coding
ENSG0000(	OLFML2B	103.1026	1.314146	1.56E-07	4.07E-06	protein_coding
ENSG0000(	PRAM1	276.8824	0.384871	1.57E-07	4.08E-06	protein_coding
ENSG0000(	PDCD6-DT	26.84686	0.832221	1.57E-07	4.09E-06	lncRNA
ENSG0000(	NELFE	340.9856	-0.3994	1.58E-07	4.10E-06	protein_coding
ENSG0000(	POP5	424.6512	-0.30105	1.58E-07	4.11E-06	protein_coding
ENSG0000(	H2BC17	78.65208	-0.97771	1.58E-07	4.11E-06	protein_coding
ENSG0000(	LCP2	1885.351	0.825321	1.59E-07	4.12E-06	protein_coding
ENSG0000(	ENSG0000(	237.9885	-0.40581	1.60E-07	4.13E-06	lncRNA
ENSG0000(	SPRED3	223.1286	0.640337	1.61E-07	4.16E-06	protein_coding
ENSG0000(	LINC02908	358.6086	0.51328	1.61E-07	4.17E-06	lncRNA
ENSG0000(	MRPS7	569.9995	-0.31456	1.62E-07	4.19E-06	protein_coding
ENSG0000(	PCGF1	181.0533	-0.4744	1.63E-07	4.22E-06	protein_coding
ENSG0000(	PNOC	64.18315	0.944911	1.64E-07	4.23E-06	protein_coding
ENSG0000(	SEC24D	1839.489	0.531655	1.65E-07	4.25E-06	protein_coding
ENSG0000(	WARS2-IT1	1470.994	0.228599	1.66E-07	4.28E-06	lncRNA
ENSG0000(	H4C13	98.78146	-0.90911	1.68E-07	4.31E-06	protein_coding
ENSG0000(	DVL1	814.9817	-0.45282	1.68E-07	4.33E-06	protein_coding
ENSG0000(	ENSG0000(	506.1872	0.230003	1.69E-07	4.34E-06	lncRNA
ENSG0000(	LINC00299	971.9014	0.369407	1.69E-07	4.34E-06	lncRNA
ENSG0000(	CACNA1C	1200.302	0.725066	1.70E-07	4.34E-06	protein_coding
ENSG0000(	RPL23	3668.892	-0.48203	1.70E-07	4.34E-06	protein_coding

ENSG0000(	ZNF738	1187.103	-0.22914	1.70E-07	4.34E-06	protein_coding
ENSG0000(	MCRIP2	1543.072	-0.60271	1.70E-07	4.36E-06	protein_coding
ENSG0000(	EHD3	225.3883	0.700329	1.71E-07	4.36E-06	protein_coding
ENSG0000(	KIF11	599.6522	-0.48688	1.71E-07	4.37E-06	protein_coding
ENSG0000(	ERVK3-1	2218.854	0.214752	1.72E-07	4.39E-06	protein_coding
ENSG0000(	ENSG0000(	84.192	0.545588	1.73E-07	4.40E-06	lncRNA
ENSG0000(	N4BP2L1	1067.797	0.416229	1.73E-07	4.41E-06	protein_coding
ENSG0000(	ENSG0000(	70.64573	0.540805	1.73E-07	4.41E-06	lncRNA
ENSG0000(	GPR141	988.4109	0.331144	1.74E-07	4.42E-06	protein_coding
ENSG0000(	RNU6-1003	19.00482	1.414384	1.74E-07	4.42E-06	snRNA
ENSG0000(	TMEM259	2602.648	-0.2982	1.75E-07	4.44E-06	protein_coding
ENSG0000(	ENSG0000(	674.1688	-0.28836	1.76E-07	4.45E-06	lncRNA
ENSG0000(	MT-RNR2	70764.18	-0.81832	1.76E-07	4.45E-06	Mt_rRNA
ENSG0000(	VDAC1	2593.368	-0.48496	1.77E-07	4.49E-06	protein_coding
ENSG0000(	DUSP12	177.9104	-0.42331	1.80E-07	4.54E-06	protein_coding
ENSG0000(	TAGAP	530.0035	1.292017	1.80E-07	4.56E-06	protein_coding
ENSG0000(	BLVRB	275.4773	-0.50471	1.82E-07	4.60E-06	protein_coding
ENSG0000(	ENSG0000(	167.4467	0.645107	1.83E-07	4.61E-06	lncRNA
ENSG0000(	ENSG0000(	169.0687	-0.44609	1.83E-07	4.61E-06	lncRNA
ENSG0000(	PTCSC1	37.57652	0.860176	1.83E-07	4.62E-06	lncRNA
ENSG0000(	TBC1D8	1307.674	0.262888	1.84E-07	4.64E-06	protein_coding
ENSG0000(	UQCRC2	2837.446	-0.29119	1.85E-07	4.65E-06	protein_coding
ENSG0000(	HECTD1	8885.799	-0.15592	1.86E-07	4.68E-06	protein_coding
ENSG0000(	CEBPA	883.9533	-0.66169	1.87E-07	4.69E-06	protein_coding
ENSG0000(	BRMS1	288.6507	-0.37944	1.88E-07	4.71E-06	protein_coding
ENSG0000(	RPL10A	2316.906	-0.51494	1.88E-07	4.71E-06	protein_coding
ENSG0000(	TTYH3	1661.403	0.471702	1.89E-07	4.73E-06	protein_coding
ENSG0000(	WHAMM	1243.67	-0.20675	1.89E-07	4.73E-06	protein_coding
ENSG0000(	PKD1P4	1034.468	-0.55542	1.90E-07	4.75E-06	transcribed_unprocessed_pseudo
ENSG0000(	PLSCR2	180.0968	0.669622	1.90E-07	4.76E-06	protein_coding
ENSG0000(	USP19	1344.046	-0.29279	1.91E-07	4.76E-06	protein_coding
ENSG0000(	ETV3	1256.658	0.318729	1.91E-07	4.77E-06	protein_coding
ENSG0000(	ANKRD49P	238.4099	0.344772	1.92E-07	4.79E-06	processed_pseudogene
ENSG0000(	GTPBP4	1055.548	-0.34747	1.93E-07	4.81E-06	protein_coding
ENSG0000(	MTND6P5	53.51981	0.920075	1.95E-07	4.86E-06	unprocessed_pseudogene
ENSG0000(	PTPRVP	151.7876	0.497321	1.96E-07	4.89E-06	unitary_pseudogene
ENSG0000(	EGR2	124.0305	1.185191	2.01E-07	4.99E-06	protein_coding
ENSG0000(	UBD	305.5697	1.696528	2.01E-07	4.99E-06	protein_coding
ENSG0000(	PDAP1	829.9863	-0.37212	2.02E-07	5.00E-06	protein_coding
ENSG0000(	DENND2B	2637.688	0.410874	2.03E-07	5.03E-06	protein_coding
ENSG0000(	DERL3	1235.275	0.768326	2.03E-07	5.03E-06	protein_coding
ENSG0000(	DZIP1L	250.9143	0.595871	2.04E-07	5.06E-06	protein_coding
ENSG0000(	ESR2	1090.363	0.404127	2.05E-07	5.08E-06	protein_coding
ENSG0000(	ENSG0000(	370.4019	0.700503	2.06E-07	5.09E-06	lncRNA
ENSG0000(	ENSG0000(	171.9351	0.603047	2.08E-07	5.14E-06	lncRNA
ENSG0000(	RPS14P4	36.16542	1.250304	2.08E-07	5.14E-06	processed_pseudogene
ENSG0000(	CASP4LP	540.3855	0.688265	2.09E-07	5.14E-06	transcribed_unprocessed_pseudo

ENSG0000(	H3C4	161.0181	-0.79923	2.09E-07	5.15E-06	protein_coding
ENSG0000(	ENSG0000(	10.51088	1.514172	2.09E-07	5.15E-06	lncRNA
ENSG0000(	PGGT1B	2299.099	-0.26774	2.09E-07	5.15E-06	protein_coding
ENSG0000(	TXN2	502.6852	-0.35842	2.10E-07	5.16E-06	protein_coding
ENSG0000(	ENSG0000(	741.6132	0.33856	2.11E-07	5.18E-06	lncRNA
ENSG0000(	COX7A2	751.8212	-0.49927	2.13E-07	5.23E-06	protein_coding
ENSG0000(	ACBD5	2326.165	-0.18066	2.14E-07	5.24E-06	protein_coding
ENSG0000(	ENSG0000(	60.72531	0.462609	2.15E-07	5.27E-06	processed_pseudogene
ENSG0000(	EBF3	49.62224	1.459513	2.18E-07	5.35E-06	protein_coding
ENSG0000(	LMO4	992.4911	0.407553	2.24E-07	5.48E-06	protein_coding
ENSG0000(	MFSD9	973.9174	-0.42735	2.24E-07	5.48E-06	protein_coding
ENSG0000(	SBDS	704.8503	-0.5284	2.24E-07	5.48E-06	protein_coding
ENSG0000(	ENSG0000(	84.81275	0.58969	2.26E-07	5.51E-06	lncRNA
ENSG0000(	RPS3	4252.783	-0.47637	2.26E-07	5.51E-06	protein_coding
ENSG0000(	PDHB	1314.674	-0.31569	2.27E-07	5.53E-06	protein_coding
ENSG0000(	KRT80	42.83853	0.808016	2.28E-07	5.55E-06	protein_coding
ENSG0000(	PCBP3	279.8858	0.717906	2.28E-07	5.55E-06	protein_coding
ENSG0000(	ENSG0000(	38.22284	-0.68634	2.30E-07	5.60E-06	lncRNA
ENSG0000(	SLC16A1	3183.724	-0.86539	2.30E-07	5.60E-06	protein_coding
ENSG0000(	ENSG0000(	145.3693	0.430131	2.33E-07	5.65E-06	TEC
ENSG0000(	MTCL1	431.4226	0.716758	2.35E-07	5.69E-06	protein_coding
ENSG0000(	LRPPRC	7842.651	-0.25572	2.35E-07	5.70E-06	protein_coding
ENSG0000(	NLRC5	7258.108	0.561765	2.38E-07	5.78E-06	protein_coding
ENSG0000(	COL12A1	2351.493	1.35842	2.39E-07	5.79E-06	protein_coding
ENSG0000(	MOB3C	1126.193	0.302005	2.40E-07	5.82E-06	protein_coding
ENSG0000(	ENSG0000(	126.2997	0.548916	2.41E-07	5.82E-06	lncRNA
ENSG0000(	HGH1	245.0252	-0.45744	2.41E-07	5.82E-06	protein_coding
ENSG0000(	ATP5PF	404.6237	-0.61411	2.42E-07	5.83E-06	protein_coding
ENSG0000(	ILVBL	770.8726	-0.43475	2.42E-07	5.84E-06	protein_coding
ENSG0000(	PNRC2	2882.948	-0.3169	2.42E-07	5.84E-06	protein_coding
ENSG0000(	MIR3945H(	138.1724	0.834462	2.43E-07	5.86E-06	lncRNA
ENSG0000(	DDX47	662.7441	-0.235	2.44E-07	5.87E-06	protein_coding
ENSG0000(	H3C12	80.16227	-0.9231	2.44E-07	5.87E-06	protein_coding
ENSG0000(	POLR2E	844.6231	-0.325	2.44E-07	5.87E-06	protein_coding
ENSG0000(	MT-ND5	30494.07	-0.72407	2.45E-07	5.87E-06	protein_coding
ENSG0000(	TMTC1	904.633	0.488045	2.49E-07	5.97E-06	protein_coding
ENSG0000(	DARS2	876.8964	-0.22246	2.50E-07	5.98E-06	protein_coding
ENSG0000(	EIF4G2	13424.11	-0.26794	2.50E-07	5.98E-06	protein_coding
ENSG0000(	CS	3994.886	-0.28117	2.50E-07	5.98E-06	protein_coding
ENSG0000(	FFAR2	367.4303	0.806204	2.50E-07	5.98E-06	protein_coding
ENSG0000(	DNAH11	319.8434	0.330305	2.51E-07	5.99E-06	protein_coding
ENSG0000(	SALRNA3	191.3968	0.357821	2.53E-07	6.03E-06	lncRNA
ENSG0000(	SLC27A2	409.8452	-0.78674	2.55E-07	6.08E-06	protein_coding
ENSG0000(	CDCA7	895.0226	-0.67133	2.55E-07	6.08E-06	protein_coding
ENSG0000(	NDUFS6	201.4507	-0.575	2.56E-07	6.09E-06	protein_coding
ENSG0000(	ENSG0000(	216.8678	0.505339	2.58E-07	6.12E-06	TEC
ENSG0000(	ENSG0000(	171.3931	0.338549	2.58E-07	6.12E-06	lncRNA

ENSG0000(	SLC40A1	3440.202	0.737579	2.58E-07	6.12E-06	protein_coding
ENSG0000(	THY1	527.8747	0.978907	2.57E-07	6.12E-06	protein_coding
ENSG0000(	ATXN8OS	330.2504	0.370087	2.59E-07	6.15E-06	lncRNA
ENSG0000(	LINC-PINT	3907.099	0.439873	2.60E-07	6.16E-06	lncRNA
ENSG0000(	SRPX2	704.8379	0.986764	2.61E-07	6.18E-06	protein_coding
ENSG0000(	BISPR	539.7615	0.468116	2.62E-07	6.21E-06	lncRNA
ENSG0000(	ZNF816	413.0075	-0.35831	2.64E-07	6.25E-06	protein_coding
ENSG0000(	HLA-E	8955.262	0.457811	2.66E-07	6.28E-06	protein_coding
ENSG0000(	LMCD1	658.409	0.947659	2.66E-07	6.29E-06	protein_coding
ENSG0000(	RAB2B	1087.01	0.225217	2.67E-07	6.30E-06	protein_coding
ENSG0000(	PUM3	775.7667	-0.39961	2.68E-07	6.32E-06	protein_coding
ENSG0000(	AACSP1	83.50252	0.530028	2.69E-07	6.33E-06	transcribed_unprocessed_pseudo
ENSG0000(	ENSG0000(	25.59033	1.051528	2.69E-07	6.34E-06	lncRNA
ENSG0000(	PMPCB	1253.726	-0.23621	2.69E-07	6.34E-06	protein_coding
ENSG0000(	BNC2	1046.508	0.689999	2.71E-07	6.37E-06	protein_coding
ENSG0000(	ENSG0000(	102.3573	0.424843	2.71E-07	6.37E-06	lncRNA
ENSG0000(	PRXL2B	691.0774	-0.45407	2.72E-07	6.38E-06	protein_coding
ENSG0000(	SMPD2	286.813	-0.3745	2.72E-07	6.38E-06	protein_coding
ENSG0000(	HACD2	2042.678	-0.30514	2.73E-07	6.40E-06	protein_coding
ENSG0000(	STAG3L5P-	726.3454	-0.77971	2.74E-07	6.42E-06	lncRNA
ENSG0000(	PLEKHO2	629.3398	0.641924	2.76E-07	6.45E-06	protein_coding
ENSG0000(	TTLL12	934.0357	-0.50477	2.76E-07	6.46E-06	protein_coding
ENSG0000(	ENSG0000(	115.2413	1.166523	2.77E-07	6.48E-06	lncRNA
ENSG0000(	HHIPL1	308.1983	0.469674	2.78E-07	6.49E-06	protein_coding
ENSG0000(	ACADVL	6926.054	-0.39497	2.79E-07	6.50E-06	protein_coding
ENSG0000(	ENSG0000(	130.6293	0.666296	2.79E-07	6.50E-06	lncRNA
ENSG0000(	H3C8	140.9977	-0.86263	2.79E-07	6.50E-06	protein_coding
ENSG0000(	ENSG0000(	470.1248	0.285818	2.81E-07	6.54E-06	lncRNA
ENSG0000(	KRT18	2960.349	-0.70617	2.81E-07	6.54E-06	protein_coding
ENSG0000(	UXT	219.152	-0.43562	2.81E-07	6.54E-06	protein_coding
ENSG0000(	PHLPP1	1100.443	-0.30823	2.83E-07	6.57E-06	protein_coding
ENSG0000(	PAQR5	2224.435	-0.76474	2.84E-07	6.59E-06	protein_coding
ENSG0000(	BHLHE22	34.40569	1.306973	2.85E-07	6.62E-06	protein_coding
ENSG0000(	MIXL1	113.3159	0.418555	2.88E-07	6.67E-06	protein_coding
ENSG0000(	MRPL48	736.7882	-0.25918	2.89E-07	6.69E-06	protein_coding
ENSG0000(	ENSG0000(	558.5779	0.269665	2.89E-07	6.70E-06	lncRNA
ENSG0000(	STEAP4	1250.861	0.985565	2.90E-07	6.71E-06	protein_coding
ENSG0000(	NUDC	604.9783	-0.2791	2.93E-07	6.78E-06	protein_coding
ENSG0000(	ENSG0000(	22.14798	0.688144	2.95E-07	6.82E-06	lncRNA
ENSG0000(	FAM184B	517.8175	0.559886	2.96E-07	6.83E-06	protein_coding
ENSG0000(	CENPX	309.5596	-0.54197	2.97E-07	6.83E-06	protein_coding
ENSG0000(	ENSG0000(	75.27628	0.60231	2.97E-07	6.83E-06	lncRNA
ENSG0000(	RAB3GAP2	4015.705	0.126455	2.96E-07	6.83E-06	protein_coding
ENSG0000(	LINC02289	61.33442	0.947764	2.97E-07	6.84E-06	lncRNA
ENSG0000(	ATP5MF	570.209	-0.52592	2.99E-07	6.87E-06	protein_coding
ENSG0000(	ARNILA	271.6731	0.446827	3.00E-07	6.88E-06	lncRNA
ENSG0000(	CCDC71L	716.6827	0.721716	3.00E-07	6.88E-06	protein_coding

ENSG0000(	EGR3	296.1858	0.992257	3.00E-07	6.88E-06	protein_coding
ENSG0000(	THRAP3	2541.491	-0.25241	3.00E-07	6.88E-06	protein_coding
ENSG0000(	SH3BP1	1225.515	-0.5235	3.01E-07	6.90E-06	protein_coding
ENSG0000(	SLFN13	1405.52	0.573601	3.04E-07	6.96E-06	protein_coding
ENSG0000(	BACH1-IT1	183.0827	0.606566	3.05E-07	6.98E-06	lncRNA
ENSG0000(	ENSG0000(	89.65909	0.573045	3.06E-07	6.99E-06	lncRNA
ENSG0000(	CALD1	2877.027	0.721558	3.06E-07	7.00E-06	protein_coding
ENSG0000(	ENSG0000(	552.0374	0.416147	3.07E-07	7.01E-06	lncRNA
ENSG0000(	ENSG0000(	494.3402	-0.28884	3.09E-07	7.04E-06	lncRNA
ENSG0000(	ENSG0000(	560.4169	-0.2887	3.10E-07	7.06E-06	lncRNA
ENSG0000(	HSPA9	3269.38	-0.39464	3.10E-07	7.06E-06	protein_coding
ENSG0000(	COX14	244.6405	-0.36448	3.10E-07	7.07E-06	protein_coding
ENSG0000(	CSE1L	767.6472	-0.39662	3.11E-07	7.07E-06	protein_coding
ENSG0000(	GRAMD1B	617.7425	0.651182	3.12E-07	7.09E-06	protein_coding
ENSG0000(	INSYN2B	17.55209	1.4549	3.13E-07	7.10E-06	protein_coding
ENSG0000(	MRPL46	492.0619	-0.30024	3.13E-07	7.10E-06	protein_coding
ENSG0000(	ATAD3A	308.9524	-0.64154	3.14E-07	7.11E-06	protein_coding
ENSG0000(	LRRK2-DT	46.98846	0.832308	3.15E-07	7.13E-06	lncRNA
ENSG0000(	ENC1	3203.679	0.443575	3.17E-07	7.17E-06	protein_coding
ENSG0000(	PCNX1	4703.29	0.433988	3.17E-07	7.17E-06	protein_coding
ENSG0000(	MPND	279.6691	-0.59936	3.19E-07	7.20E-06	protein_coding
ENSG0000(	ENSG0000(	66.18927	0.657206	3.23E-07	7.30E-06	lncRNA
ENSG0000(	RPS21	935.6568	-0.60657	3.24E-07	7.31E-06	protein_coding
ENSG0000(	MAIP1	128.8886	-0.52371	3.24E-07	7.31E-06	protein_coding
ENSG0000(	ENSG0000(	390.392	0.27411	3.26E-07	7.35E-06	lncRNA
ENSG0000(	PVR	1583.402	-0.45275	3.26E-07	7.35E-06	protein_coding
ENSG0000(	GOLGA8A	6265.841	0.507402	3.28E-07	7.39E-06	protein_coding
ENSG0000(	CCNB1	252.3899	-0.80832	3.29E-07	7.39E-06	protein_coding
ENSG0000(	SMAGP	1224.162	-0.40917	3.30E-07	7.41E-06	protein_coding
ENSG0000(	ENSG0000(	69.78969	0.603983	3.32E-07	7.47E-06	lncRNA
ENSG0000(	CLIC1	1788.098	-0.5841	3.33E-07	7.48E-06	protein_coding
ENSG0000(	FAM225B	127.2316	0.569667	3.34E-07	7.48E-06	lncRNA
ENSG0000(	SULF1	1104.934	0.759315	3.34E-07	7.48E-06	protein_coding
ENSG0000(	APRT	297.9739	-0.49699	3.34E-07	7.48E-06	protein_coding
ENSG0000(	TRIM22	3140.941	0.581235	3.36E-07	7.52E-06	protein_coding
ENSG0000(	CLIP4	1927.615	0.458632	3.37E-07	7.53E-06	protein_coding
ENSG0000(	CSGALNAC	342.742	0.826205	3.37E-07	7.53E-06	protein_coding
ENSG0000(	TINAGL1	2045.258	-0.50623	3.37E-07	7.53E-06	protein_coding
ENSG0000(	NOB1	276.5953	-0.46294	3.38E-07	7.55E-06	protein_coding
ENSG0000(	ENSG0000(	317.1619	0.277333	3.39E-07	7.56E-06	lncRNA
ENSG0000(	ENSG0000(	66.53497	0.943785	3.40E-07	7.57E-06	TEC
ENSG0000(	TMEM263	859.0695	0.39326	3.40E-07	7.57E-06	protein_coding
ENSG0000(	RNU6-101(	16.58483	1.081067	3.41E-07	7.58E-06	snRNA
ENSG0000(	MR1	1413.203	0.30419	3.43E-07	7.63E-06	protein_coding
ENSG0000(	ARFRP1	2102.777	-0.21937	3.44E-07	7.64E-06	protein_coding
ENSG0000(	ENSG0000(	475.5009	0.57921	3.44E-07	7.64E-06	TEC
ENSG0000(	FTSJ3	559.001	-0.30755	3.45E-07	7.65E-06	protein_coding

ENSG0000(	KCNJ15	2021.811	0.842825	3.48E-07	7.71E-06	protein_coding
ENSG0000(	OR13K1P	55.24595	0.578402	3.48E-07	7.71E-06	unprocessed_pseudogene
ENSG0000(	METTL7B	295.9644	-1.03161	3.49E-07	7.72E-06	protein_coding
ENSG0000(	GMPS	1941.06	-0.35664	3.49E-07	7.73E-06	protein_coding
ENSG0000(	CLEC7A	869.562	0.537871	3.50E-07	7.74E-06	protein_coding
ENSG0000(	TMEM223	158.1053	-0.39192	3.51E-07	7.76E-06	protein_coding
ENSG0000(	ITPRIP	644.0294	0.859177	3.53E-07	7.78E-06	protein_coding
ENSG0000(	TPX2	548.3213	-0.57557	3.53E-07	7.78E-06	protein_coding
ENSG0000(	SLC25A38	673.1564	-0.35036	3.54E-07	7.82E-06	protein_coding
ENSG0000(	ARSL	455.0769	-0.44634	3.60E-07	7.92E-06	protein_coding
ENSG0000(	DUSP23	112.2291	-0.57597	3.60E-07	7.92E-06	protein_coding
ENSG0000(	HLA-B	15510.16	0.574497	3.59E-07	7.92E-06	protein_coding
ENSG0000(	ENSG0000(	140.2133	0.394709	3.63E-07	7.97E-06	lncRNA
ENSG0000(	ENSG0000(	172.9917	0.31121	3.63E-07	7.97E-06	lncRNA
ENSG0000(	SAFB2	2433.928	-0.15656	3.64E-07	7.99E-06	protein_coding
ENSG0000(	RTN1	96.587	0.735456	3.69E-07	8.11E-06	protein_coding
ENSG0000(	ENSG0000(	56.40736	0.526654	3.70E-07	8.11E-06	lncRNA
ENSG0000(	H2BC18	263.9082	-0.71324	3.70E-07	8.11E-06	protein_coding
ENSG0000(	FCER1G	275.3976	0.717235	3.71E-07	8.12E-06	protein_coding
ENSG0000(	RPL41	5904.113	-0.49634	3.75E-07	8.20E-06	protein_coding
ENSG0000(	GGH	455.2503	-0.59126	3.75E-07	8.21E-06	protein_coding
ENSG0000(	PLXNB1	3203.589	-0.44049	3.80E-07	8.31E-06	protein_coding
ENSG0000(	RPL27A	4259.808	-0.46608	3.82E-07	8.34E-06	protein_coding
ENSG0000(	SLC2A6	250.7236	0.745005	3.82E-07	8.34E-06	protein_coding
ENSG0000(	NOP14	780.5432	-0.31311	3.83E-07	8.36E-06	protein_coding
ENSG0000(	ADIRF	244.509	-1.03039	3.85E-07	8.38E-06	protein_coding
ENSG0000(	EPOP	47.80538	0.911938	3.85E-07	8.38E-06	protein_coding
ENSG0000(	RPP21	182.0058	-0.47491	3.86E-07	8.41E-06	protein_coding
ENSG0000(	KIF20A	143.3882	-0.75347	3.87E-07	8.41E-06	protein_coding
ENSG0000(	LINC01750	45.60293	1.140628	3.87E-07	8.41E-06	lncRNA
ENSG0000(	PET100	345.7851	-0.49555	3.87E-07	8.41E-06	protein_coding
ENSG0000(	ENSG0000(	59.61792	0.832119	3.92E-07	8.51E-06	TEC
ENSG0000(	BATF3	95.55156	1.004326	3.95E-07	8.58E-06	protein_coding
ENSG0000(	DDA1	793.6599	-0.41795	3.96E-07	8.58E-06	protein_coding
ENSG0000(	RPL36	1445.24	-0.456	3.98E-07	8.63E-06	protein_coding
ENSG0000(	RAD52	936.8999	-0.46876	3.99E-07	8.64E-06	protein_coding
ENSG0000(	AURKAIP1	286.6617	-0.47791	4.02E-07	8.69E-06	protein_coding
ENSG0000(	BATF2	454.3363	0.867184	4.02E-07	8.69E-06	protein_coding
ENSG0000(	ENSG0000(	40.5463	0.761176	4.02E-07	8.69E-06	snoRNA
ENSG0000(	RYR1	234.6959	-0.58846	4.03E-07	8.70E-06	protein_coding
ENSG0000(	COX4I1	2648.927	-0.60897	4.06E-07	8.76E-06	protein_coding
ENSG0000(	HCP5	5691.165	0.502345	4.07E-07	8.76E-06	lncRNA
ENSG0000(	RPL7	4101.084	-0.49047	4.10E-07	8.82E-06	protein_coding
ENSG0000(	ENSG0000(	445.0077	-0.52837	4.15E-07	8.93E-06	protein_coding
ENSG0000(	ERVW-1	1722.513	0.306942	4.15E-07	8.93E-06	protein_coding
ENSG0000(	CDH1	7668.724	-0.61057	4.17E-07	8.95E-06	protein_coding
ENSG0000(	ENSG0000(	84.20918	0.451785	4.19E-07	8.99E-06	lncRNA

ENSG0000(	IL12RB2	347.6622	0.499235	4.19E-07	8.99E-06	protein_coding
ENSG0000(	TNFAIP3	2506.877	0.705858	4.19E-07	8.99E-06	protein_coding
ENSG0000(	GSDME	125.4824	0.961212	4.23E-07	9.07E-06	protein_coding
ENSG0000(	CTDSPL2	1860.154	-0.20295	4.24E-07	9.09E-06	protein_coding
ENSG0000(	HNRNPUL1	3155.091	-0.1693	4.26E-07	9.13E-06	protein_coding
ENSG0000(	ADAMTS12	240.0827	1.013592	4.28E-07	9.15E-06	protein_coding
ENSG0000(	H4C1	74.58992	-0.99024	4.28E-07	9.15E-06	protein_coding
ENSG0000(	ENSG0000(	77.7286	0.457938	4.30E-07	9.18E-06	lncRNA
ENSG0000(	TST	1469.398	-0.88334	4.30E-07	9.18E-06	protein_coding
ENSG0000(	PARP8	1738.025	0.624416	4.32E-07	9.22E-06	protein_coding
ENSG0000(	CCDC47	1532.395	-0.32598	4.33E-07	9.23E-06	protein_coding
ENSG0000(	XKR6	466.0002	0.410196	4.35E-07	9.26E-06	protein_coding
ENSG0000(	ENSG0000(	16.5275	1.946575	4.39E-07	9.35E-06	processed_pseudogene
ENSG0000(	VPS52	1223.088	-0.20537	4.41E-07	9.39E-06	protein_coding
ENSG0000(	MAILR	681.3893	0.434873	4.42E-07	9.39E-06	lncRNA
ENSG0000(	RPL37A	3809.465	-0.57818	4.42E-07	9.39E-06	protein_coding
ENSG0000(	STRN	3267.365	-0.29384	4.42E-07	9.39E-06	protein_coding
ENSG0000(	LINC00621	2564.648	0.19471	4.43E-07	9.39E-06	lncRNA
ENSG0000(	TTC34	601.2193	0.346169	4.43E-07	9.39E-06	protein_coding
ENSG0000(	EIF2AK4	1627.082	0.18743	4.44E-07	9.40E-06	protein_coding
ENSG0000(	ENSG0000(	290.9396	0.553517	4.45E-07	9.42E-06	protein_coding
ENSG0000(	MRPS34	249.1394	-0.43238	4.46E-07	9.42E-06	protein_coding
ENSG0000(	RNU6-60P	35.55501	0.794642	4.46E-07	9.42E-06	snRNA
ENSG0000(	YBX1	3653.152	-0.46995	4.46E-07	9.42E-06	protein_coding
ENSG0000(	ENSG0000(	1896.428	0.286208	4.47E-07	9.44E-06	lncRNA
ENSG0000(	WDR3	1162.899	-0.3064	4.49E-07	9.46E-06	protein_coding
ENSG0000(	RPS29	3658.161	-0.55191	4.49E-07	9.47E-06	protein_coding
ENSG0000(	DPP10	302.344	-0.67613	4.51E-07	9.50E-06	protein_coding
ENSG0000(	MTCO1P2	24.73694	-1.59518	4.53E-07	9.55E-06	unprocessed_pseudogene
ENSG0000(	RBM8A	1540.536	-0.32869	4.63E-07	9.73E-06	protein_coding
ENSG0000(	RPS6	6670.669	-0.4925	4.63E-07	9.73E-06	protein_coding
ENSG0000(	ENSG0000(	63.91103	-0.65827	4.66E-07	9.80E-06	lncRNA
ENSG0000(	TBL3	481.9514	-0.41373	4.67E-07	9.80E-06	protein_coding
ENSG0000(	LATS2	463.0103	0.558357	4.70E-07	9.87E-06	protein_coding
ENSG0000(	SLC2A11	1168.114	0.255432	4.71E-07	9.87E-06	protein_coding
ENSG0000(	UGT1A8	379.0997	-1.15515	4.72E-07	9.89E-06	protein_coding
ENSG0000(	IMPDH2	812.5587	-0.55064	4.75E-07	9.94E-06	protein_coding
ENSG0000(	ATP2B4	2676.94	0.507379	4.75E-07	9.95E-06	protein_coding
ENSG0000(	DDR1-DT	1706.174	0.219657	4.76E-07	9.95E-06	lncRNA
ENSG0000(	ENSG0000(	175.1723	0.46804	4.77E-07	9.97E-06	lncRNA
ENSG0000(	MSC-AS1	715.6703	0.516812	4.79E-07	1.00E-05	lncRNA
ENSG0000(	ERV3-1	1414.951	0.291416	4.80E-07	1.00E-05	protein_coding
ENSG0000(	GABPB1-IT:	1380.193	0.312086	4.82E-07	1.01E-05	lncRNA
ENSG0000(	MIR4453H(	498.2101	0.311413	4.84E-07	1.01E-05	lncRNA
ENSG0000(	ENSG0000(	295.8177	0.440289	4.86E-07	1.01E-05	lncRNA
ENSG0000(	ENSG0000(	95.35829	-0.45561	4.86E-07	1.01E-05	lncRNA
ENSG0000(	SLC25A11	561.0895	-0.34156	4.86E-07	1.01E-05	protein_coding

ENSG0000(	SNRPF	209.2873	-0.40954	4.87E-07	1.01E-05	protein_coding
ENSG0000(	PPP1CA	1176.663	-0.41714	4.92E-07	1.02E-05	protein_coding
ENSG0000(	AGTRAP	190.7933	0.525899	4.99E-07	1.03E-05	protein_coding
ENSG0000(	ENSG0000(	55.58436	0.81114	4.99E-07	1.03E-05	lncRNA
ENSG0000(	IL6-AS1	15.09724	1.818218	4.99E-07	1.03E-05	lncRNA
ENSG0000(	PKDCC	1068.574	-0.6562	4.99E-07	1.03E-05	protein_coding
ENSG0000(	BACE1	720.2425	0.277979	5.04E-07	1.04E-05	protein_coding
ENSG0000(	ENSG0000(	49.71131	1.562012	5.03E-07	1.04E-05	processed_pseudogene
ENSG0000(	SFN	1145.06	-0.83637	5.04E-07	1.04E-05	protein_coding
ENSG0000(	POLD2	381.0453	-0.44807	5.10E-07	1.05E-05	protein_coding
ENSG0000(	RPLP2	2155.798	-0.53461	5.10E-07	1.05E-05	protein_coding
ENSG0000(	NOL12	759.8092	-0.33013	5.11E-07	1.05E-05	protein_coding
ENSG0000(	POM121L1	9.553843	1.514762	5.16E-07	1.06E-05	transcribed_unprocessed_pseudo
ENSG0000(	HPRT1	194.4011	-0.54956	5.21E-07	1.07E-05	protein_coding
ENSG0000(	ENSG0000(	52.27473	0.588077	5.23E-07	1.08E-05	lncRNA
ENSG0000(	H2AC21	74.46939	-0.85806	5.24E-07	1.08E-05	protein_coding
ENSG0000(	PSME3	1266.994	-0.31973	5.24E-07	1.08E-05	protein_coding
ENSG0000(	TMC5	3449.035	0.777939	5.24E-07	1.08E-05	protein_coding
ENSG0000(	FLII	1944.894	-0.28815	5.26E-07	1.08E-05	protein_coding
ENSG0000(	TRAM2	2593.867	0.412893	5.27E-07	1.08E-05	protein_coding
ENSG0000(	ENSG0000(	424.0429	0.404459	5.29E-07	1.09E-05	transcribed_unprocessed_pseudo
ENSG0000(	GPI	5517.718	-0.22256	5.29E-07	1.09E-05	protein_coding
ENSG0000(	ZNF697	383.7416	0.523313	5.32E-07	1.09E-05	protein_coding
ENSG0000(	ENSG0000(	91.91304	0.772391	5.33E-07	1.09E-05	lncRNA
ENSG0000(	ENSG0000(	41.52196	0.850115	5.35E-07	1.09E-05	lncRNA
ENSG0000(	SOD2	43460.46	0.636293	5.36E-07	1.10E-05	protein_coding
ENSG0000(	PLSCR1	2173.95	0.477545	5.40E-07	1.10E-05	protein_coding
ENSG0000(	POLR1B	1458.246	-0.34449	5.40E-07	1.10E-05	protein_coding
ENSG0000(	ENSG0000(	248.9197	-0.31283	5.44E-07	1.11E-05	TEC
ENSG0000(	ENSG0000(	2373.61	0.36568	5.46E-07	1.11E-05	lncRNA
ENSG0000(	GSTA1	26.46232	-1.78016	5.50E-07	1.12E-05	protein_coding
ENSG0000(	ENSG0000(	122.1786	0.518409	5.51E-07	1.12E-05	lncRNA
ENSG0000(	ELMO2	778.819	0.421475	5.53E-07	1.13E-05	protein_coding
ENSG0000(	ORC4	10650.4	-0.17254	5.53E-07	1.13E-05	protein_coding
ENSG0000(	NUDT22	1120.499	-0.32117	5.54E-07	1.13E-05	protein_coding
ENSG0000(	TTC19	2440.424	-0.32234	5.55E-07	1.13E-05	protein_coding
ENSG0000(	PSMD2	1153.295	-0.40226	5.55E-07	1.13E-05	protein_coding
ENSG0000(	HIRIP3	124.7277	-0.49263	5.63E-07	1.14E-05	protein_coding
ENSG0000(	SNHG12	1004.459	-0.2447	5.64E-07	1.14E-05	lncRNA
ENSG0000(	GREM1	4746.682	1.214738	5.66E-07	1.15E-05	protein_coding
ENSG0000(	RPL34	2114.46	-0.48297	5.70E-07	1.15E-05	protein_coding
ENSG0000(	PRPF6	850.2026	-0.30176	5.72E-07	1.16E-05	protein_coding
ENSG0000(	DLEU2L	157.5995	0.423922	5.74E-07	1.16E-05	lncRNA
ENSG0000(	MT-CO2	32842.33	-0.79649	5.75E-07	1.16E-05	protein_coding
ENSG0000(	KCNJ8	31.13626	1.372593	5.76E-07	1.16E-05	protein_coding
ENSG0000(	MIR122HG	461.5077	0.217493	5.76E-07	1.16E-05	lncRNA
ENSG0000(	ENSG0000(	39.80658	0.705649	5.82E-07	1.17E-05	lncRNA

ENSG0000(	TRIM16L	501.1761	-0.37719	5.86E-07	1.18E-05	transcribed_unprocessed_pseudo
ENSG0000(	ENKUR	66.73074	1.489199	5.88E-07	1.18E-05	protein_coding
ENSG0000(	HMCES	451.546	-0.40373	5.88E-07	1.18E-05	protein_coding
ENSG0000(	DDAH2	537.7298	-0.6478	5.90E-07	1.19E-05	protein_coding
ENSG0000(	RASSF8	520.4651	0.69869	5.90E-07	1.19E-05	protein_coding
ENSG0000(	SMC3	2366.088	-0.19838	5.95E-07	1.20E-05	protein_coding
ENSG0000(	MSI2	4800.692	0.274411	5.96E-07	1.20E-05	protein_coding
ENSG0000(	IARS2	1161.776	-0.41741	5.97E-07	1.20E-05	protein_coding
ENSG0000(	TIMP1	310.3325	1.346214	5.97E-07	1.20E-05	protein_coding
ENSG0000(	MLEC	9366.538	-0.45575	5.99E-07	1.20E-05	protein_coding
ENSG0000(	NBPF3	1312.746	0.322452	6.00E-07	1.20E-05	protein_coding
ENSG0000(	FBXL6	456.5852	-0.46893	6.02E-07	1.20E-05	protein_coding
ENSG0000(	MKKS	939.902	-0.48548	6.04E-07	1.21E-05	protein_coding
ENSG0000(	SNORD14C	75.54518	0.785142	6.06E-07	1.21E-05	snoRNA
ENSG0000(	BHLHA15	220.2626	0.843147	6.11E-07	1.22E-05	protein_coding
ENSG0000(	RPS28P7	233.9025	-0.71137	6.12E-07	1.22E-05	processed_pseudogene
ENSG0000(	MT-ND4	51489.61	-0.77442	6.14E-07	1.22E-05	protein_coding
ENSG0000(	HOXB7	219.528	-0.43802	6.16E-07	1.23E-05	protein_coding
ENSG0000(	MIR23AHG	5831.762	0.591869	6.17E-07	1.23E-05	lncRNA
ENSG0000(	WNT5A	1089.299	1.098553	6.23E-07	1.24E-05	protein_coding
ENSG0000(	FAM171A1	1070.606	-0.42395	6.24E-07	1.24E-05	protein_coding
ENSG0000(	CD80	293.0079	1.105865	6.27E-07	1.25E-05	protein_coding
ENSG0000(	LENG1	96.38392	-0.45044	6.27E-07	1.25E-05	protein_coding
ENSG0000(	ENSG0000(	49.94854	0.853134	6.28E-07	1.25E-05	unprocessed_pseudogene
ENSG0000(	NRCAM	161.2451	1.561614	6.32E-07	1.25E-05	protein_coding
ENSG0000(	POLD1	379.6745	-0.44056	6.35E-07	1.26E-05	protein_coding
ENSG0000(	KYNU	5717.222	0.469783	6.36E-07	1.26E-05	protein_coding
ENSG0000(	RUNX1	3664.28	0.434138	6.36E-07	1.26E-05	protein_coding
ENSG0000(	LDB2	452.6021	0.556345	6.38E-07	1.26E-05	protein_coding
ENSG0000(	APLP2	4669.472	-0.29453	6.41E-07	1.27E-05	protein_coding
ENSG0000(	ENSG0000(	303.5186	0.690021	6.41E-07	1.27E-05	processed_pseudogene
ENSG0000(	EIF5	4627.759	-0.18553	6.48E-07	1.28E-05	protein_coding
ENSG0000(	CHMP6	384.0789	-0.38276	6.49E-07	1.28E-05	protein_coding
ENSG0000(	BACH1-IT3	54.04944	0.862031	6.51E-07	1.28E-05	lncRNA
ENSG0000(	DGKQ	1331.078	-0.48794	6.52E-07	1.28E-05	protein_coding
ENSG0000(	ENSG0000(	95.78076	0.874177	6.51E-07	1.28E-05	lncRNA
ENSG0000(	ENSG0000(	1673.753	0.273069	6.51E-07	1.28E-05	TEC
ENSG0000(	MYL3	222.3611	0.265455	6.51E-07	1.28E-05	protein_coding
ENSG0000(	RRN3	862.3808	-0.33457	6.51E-07	1.28E-05	protein_coding
ENSG0000(	ENSG0000(	899.1135	0.194172	6.54E-07	1.29E-05	lncRNA
ENSG0000(	ENSG0000(	258.2943	0.43966	6.57E-07	1.29E-05	lncRNA
ENSG0000(	TMEM198	54.28945	0.650396	6.59E-07	1.29E-05	protein_coding
ENSG0000(	MCMDC2	148.6535	-0.44764	6.63E-07	1.30E-05	protein_coding
ENSG0000(	MSC	72.24408	1.018896	6.66E-07	1.31E-05	protein_coding
ENSG0000(	MT-ATP6	20041.19	-0.72719	6.66E-07	1.31E-05	protein_coding
ENSG0000(	CLDN15	601.2099	-0.34108	6.69E-07	1.31E-05	protein_coding
ENSG0000(	HDGF	2248.284	-0.38193	6.70E-07	1.31E-05	protein_coding

ENSG0000(	AK4	843.8484	-0.5085	6.71E-07	1.31E-05	protein_coding
ENSG0000(	CD55	2128.273	1.057281	6.72E-07	1.31E-05	protein_coding
ENSG0000(	TIMM23	396.6911	-0.36263	6.72E-07	1.31E-05	protein_coding
ENSG0000(	GOLIM4	2434.282	-0.46466	6.72E-07	1.31E-05	protein_coding
ENSG0000(	ENSG0000(	39.56595	0.852406	6.74E-07	1.32E-05	lncRNA
ENSG0000(	CNPPD1	1080.717	-0.47168	6.76E-07	1.32E-05	protein_coding
ENSG0000(	ENSG0000(	167.0295	0.415962	6.76E-07	1.32E-05	lncRNA
ENSG0000(	DNM1P34	42.82441	0.877062	6.77E-07	1.32E-05	transcribed_unprocessed_pseudo
ENSG0000(	RAD51-AS1	1616.459	0.218142	6.81E-07	1.33E-05	lncRNA
ENSG0000(	IL18	1373.716	0.508501	6.82E-07	1.33E-05	protein_coding
ENSG0000(	NLRC4	100.2876	0.959497	6.83E-07	1.33E-05	protein_coding
ENSG0000(	ANP32B	758.4243	-0.32863	6.88E-07	1.34E-05	protein_coding
ENSG0000(	C6orf223	99.86389	1.113943	6.90E-07	1.34E-05	lncRNA
ENSG0000(	CEBPD	381.9141	0.61734	6.91E-07	1.34E-05	protein_coding
ENSG0000(	ENSG0000(	203.8038	0.330029	6.91E-07	1.34E-05	lncRNA
ENSG0000(	RETREG2	1697.967	-0.37608	6.94E-07	1.34E-05	protein_coding
ENSG0000(	ZNF732	32.93123	-1.37382	6.95E-07	1.35E-05	protein_coding
ENSG0000(	RBM42	287.4326	-0.34892	6.99E-07	1.35E-05	protein_coding
ENSG0000(	GMDS-DT	5354.901	0.280522	7.00E-07	1.35E-05	lncRNA
ENSG0000(	ENSG0000(	13.6843	1.037026	7.06E-07	1.37E-05	lncRNA
ENSG0000(	ENSG0000(	252.1634	0.271352	7.08E-07	1.37E-05	TEC
ENSG0000(	AGPAT5	872.9952	-0.40189	7.09E-07	1.37E-05	protein_coding
ENSG0000(	ENSG0000(	24.55014	1.76644	7.11E-07	1.37E-05	lncRNA
ENSG0000(	RBBP4P1	172.2152	-0.65972	7.11E-07	1.37E-05	processed_pseudogene
ENSG0000(	LMO3	153.1369	0.961687	7.13E-07	1.37E-05	protein_coding
ENSG0000(	TRAF7	1093.072	-0.36208	7.14E-07	1.38E-05	protein_coding
ENSG0000(	C1S	2484.095	0.536407	7.15E-07	1.38E-05	protein_coding
ENSG0000(	ENSG0000(	90.22706	1.321087	7.15E-07	1.38E-05	lncRNA
ENSG0000(	ENSG0000(	136.1691	0.460112	7.18E-07	1.38E-05	processed_pseudogene
ENSG0000(	PLCB1	545.6117	0.707152	7.25E-07	1.39E-05	protein_coding
ENSG0000(	RAMACL	157.5006	0.668717	7.25E-07	1.39E-05	protein_coding
ENSG0000(	SGF29	202.9547	-0.36018	7.29E-07	1.40E-05	protein_coding
ENSG0000(	SF3B6	254.9863	-0.41521	7.33E-07	1.41E-05	protein_coding
ENSG0000(	ENSG0000(	2199.929	0.380766	7.34E-07	1.41E-05	transcribed_unprocessed_pseudo
ENSG0000(	PFKL	4509.454	-0.48057	7.40E-07	1.42E-05	protein_coding
ENSG0000(	ENSG0000(	14.98026	1.384965	7.42E-07	1.42E-05	processed_pseudogene
ENSG0000(	LINC01204	249.4713	-0.41326	7.45E-07	1.43E-05	lncRNA
ENSG0000(	S100A16	902.9345	-0.85517	7.47E-07	1.43E-05	protein_coding
ENSG0000(	LINC01094	461.6054	0.236607	7.47E-07	1.43E-05	lncRNA
ENSG0000(	SNRPD2	324.6864	-0.42312	7.49E-07	1.43E-05	protein_coding
ENSG0000(	ENSG0000(	21.09948	1.284523	7.51E-07	1.43E-05	lncRNA
ENSG0000(	ATG4B	1610.367	-0.2703	7.54E-07	1.44E-05	protein_coding
ENSG0000(	ENSG0000(	261.2674	0.344151	7.59E-07	1.45E-05	TEC
ENSG0000(	LINC00641	1895.144	0.324949	7.60E-07	1.45E-05	lncRNA
ENSG0000(	SSRP1	1109.12	-0.39111	7.61E-07	1.45E-05	protein_coding
ENSG0000(	CD84	2121.518	0.659818	7.63E-07	1.45E-05	protein_coding
ENSG0000(	C16orf91	70.20068	-0.56627	7.65E-07	1.45E-05	protein_coding

ENSG0000(	H2BC20P	1279.531	0.291945	7.67E-07	1.46E-05	transcribed_unprocessed_pseudo
ENSG0000(	BLACAT1	60.74138	1.549306	7.69E-07	1.46E-05	protein_coding
ENSG0000(	NIPAL4-DT	12.29019	1.361165	7.68E-07	1.46E-05	lncRNA
ENSG0000(	SLC22A18	1507.959	-0.70992	7.69E-07	1.46E-05	protein_coding
ENSG0000(	ENSG0000(	259.2492	0.370268	7.70E-07	1.46E-05	lncRNA
ENSG0000(	CHCHD3	999.9758	-0.24912	7.75E-07	1.47E-05	protein_coding
ENSG0000(	KLHL6	3139.647	0.52569	7.74E-07	1.47E-05	protein_coding
ENSG0000(	LINC01679	24.63241	1.182606	7.74E-07	1.47E-05	lncRNA
ENSG0000(	MTRNR2L7	29.82659	0.791561	7.74E-07	1.47E-05	protein_coding
ENSG0000(	ENSG0000(	763.2984	-0.28841	7.78E-07	1.47E-05	lncRNA
ENSG0000(	BGN	487.0282	1.026403	7.81E-07	1.48E-05	protein_coding
ENSG0000(	NCOA7	1557.3	0.440918	7.83E-07	1.48E-05	protein_coding
ENSG0000(	ENSG0000(	50.27297	0.858782	7.84E-07	1.48E-05	lncRNA
ENSG0000(	TFEC	884.6264	1.089557	7.85E-07	1.48E-05	protein_coding
ENSG0000(	NEXMIF	136.717	-0.56426	7.88E-07	1.49E-05	protein_coding
ENSG0000(	PAIP1P1	151.5415	0.332049	7.91E-07	1.49E-05	processed_pseudogene
ENSG0000(	CAAP1	551.5561	-0.37568	7.93E-07	1.49E-05	protein_coding
ENSG0000(	ENSG0000(	15.0977	1.87054	7.93E-07	1.49E-05	lncRNA
ENSG0000(	FGD2	2184.607	0.565659	7.96E-07	1.49E-05	protein_coding
ENSG0000(	LINC02306	252.668	0.442592	7.96E-07	1.49E-05	lncRNA
ENSG0000(	NDC1	701.0692	-0.49301	7.95E-07	1.49E-05	protein_coding
ENSG0000(	SELP	111.8549	1.322347	7.96E-07	1.49E-05	protein_coding
ENSG0000(	KDM3AP1	17.80995	1.080131	8.07E-07	1.51E-05	processed_pseudogene
ENSG0000(	MIR325HG	250.2802	0.344832	8.08E-07	1.51E-05	lncRNA
ENSG0000(	TMEM141	720.8783	-0.61163	8.08E-07	1.51E-05	protein_coding
ENSG0000(	PSMD7-DT	603.593	0.229604	8.09E-07	1.52E-05	lncRNA
ENSG0000(	DTYMK	331.4269	-0.34129	8.12E-07	1.52E-05	protein_coding
ENSG0000(	CHCHD10	835.9301	-0.67673	8.19E-07	1.53E-05	protein_coding
ENSG0000(	LPCAT1	807.289	0.658354	8.20E-07	1.53E-05	protein_coding
ENSG0000(	NDC80	403.3187	-0.29501	8.20E-07	1.53E-05	protein_coding
ENSG0000(	NELFA	743.5675	-0.22459	8.23E-07	1.54E-05	protein_coding
ENSG0000(	PTGES2	1410.232	-0.24609	8.34E-07	1.56E-05	protein_coding
ENSG0000(	RND1	471.8303	0.630224	8.35E-07	1.56E-05	protein_coding
ENSG0000(	PTPRM	1629.219	0.389961	8.38E-07	1.56E-05	protein_coding
ENSG0000(	PRPF38A	1847.418	-0.22563	8.41E-07	1.57E-05	protein_coding
ENSG0000(	DHX36	2509.416	0.213969	8.44E-07	1.57E-05	protein_coding
ENSG0000(	ENSG0000(	485.4788	0.234917	8.46E-07	1.57E-05	lncRNA
ENSG0000(	GLUL	6772.378	0.526511	8.45E-07	1.57E-05	protein_coding
ENSG0000(	PELATON	247.8924	0.789402	8.49E-07	1.58E-05	lncRNA
ENSG0000(	EIF3L	2141.595	-0.48656	8.55E-07	1.59E-05	protein_coding
ENSG0000(	H2BC12	325.7563	-0.60634	8.56E-07	1.59E-05	protein_coding
ENSG0000(	MSX1	45.472	0.749983	8.59E-07	1.59E-05	protein_coding
ENSG0000(	PDK1	5615.817	0.539938	8.60E-07	1.59E-05	protein_coding
ENSG0000(	ATIC	603.9392	-0.40015	8.64E-07	1.60E-05	protein_coding
ENSG0000(	DVL2	565.9212	-0.2788	8.64E-07	1.60E-05	protein_coding
ENSG0000(	RPL26	2767.579	-0.45438	8.67E-07	1.60E-05	protein_coding
ENSG0000(	COLQ	1081.476	0.379659	8.75E-07	1.62E-05	protein_coding

ENSG0000(	CBLC	740.6739	-0.74623	8.76E-07	1.62E-05	protein_coding
ENSG0000(	RAB10	2322.169	-0.42372	8.79E-07	1.62E-05	protein_coding
ENSG0000(	DDR2	1761.747	0.600005	8.81E-07	1.62E-05	protein_coding
ENSG0000(	FBXO45	724.8801	-0.28127	8.80E-07	1.62E-05	protein_coding
ENSG0000(	TENT5C	3747.251	0.676236	8.83E-07	1.63E-05	protein_coding
ENSG0000(	H4C3	251.8157	-0.75644	8.87E-07	1.63E-05	protein_coding
ENSG0000(	CCL16	313.7996	-0.25776	8.89E-07	1.64E-05	protein_coding
ENSG0000(	CLN5	4540.227	0.163186	8.92E-07	1.64E-05	protein_coding
ENSG0000(	ENSG0000(	491.1723	0.221102	8.90E-07	1.64E-05	lncRNA
ENSG0000(	ENSG0000(	55.2101	0.621919	8.90E-07	1.64E-05	TEC
ENSG0000(	MRPS16	1424.362	-0.27405	8.89E-07	1.64E-05	protein_coding
ENSG0000(	NLN	1180.426	-0.28541	8.91E-07	1.64E-05	protein_coding
ENSG0000(	SRGN	509.5735	1.116478	8.92E-07	1.64E-05	protein_coding
ENSG0000(	ENSG0000(	63.04921	0.694431	8.94E-07	1.64E-05	lncRNA
ENSG0000(	ADORA2A	103.6497	0.570711	8.96E-07	1.64E-05	protein_coding
ENSG0000(	MRPS18A	294.4886	-0.38773	8.95E-07	1.64E-05	protein_coding
ENSG0000(	ANTKMT	96.25256	-0.54608	8.97E-07	1.64E-05	protein_coding
ENSG0000(	SAMD4A	1424.096	0.574082	8.97E-07	1.64E-05	protein_coding
ENSG0000(	PXDC1	166.837	0.604983	8.97E-07	1.64E-05	protein_coding
ENSG0000(	LINC02694	787.2547	0.31064	8.98E-07	1.64E-05	lncRNA
ENSG0000(	COP1	1636.46	-0.27999	9.01E-07	1.65E-05	protein_coding
ENSG0000(	ZCCHC14	2026.401	-0.3375	9.06E-07	1.65E-05	protein_coding
ENSG0000(	ENSG0000(	571.676	0.209678	9.09E-07	1.66E-05	lncRNA
ENSG0000(	HNRNPH3	2288.766	-0.16951	9.12E-07	1.66E-05	protein_coding
ENSG0000(	CMBL	2001.325	-0.67801	9.14E-07	1.66E-05	protein_coding
ENSG0000(	ENSG0000(	37.75086	0.994759	9.14E-07	1.67E-05	lncRNA
ENSG0000(	ENTPD6	2919.296	-0.39083	9.15E-07	1.67E-05	protein_coding
ENSG0000(	EPCAM	5552.018	-0.66908	9.16E-07	1.67E-05	protein_coding
ENSG0000(	NIFK	399.159	-0.3654	9.17E-07	1.67E-05	protein_coding
ENSG0000(	SERPING1	1555.124	0.516889	9.18E-07	1.67E-05	protein_coding
ENSG0000(	KLHDC3	490.4664	-0.34674	9.19E-07	1.67E-05	protein_coding
ENSG0000(	AMOTL2	401.6116	0.592886	9.31E-07	1.69E-05	protein_coding
ENSG0000(	LINC00528	102.8981	0.634648	9.34E-07	1.69E-05	lncRNA
ENSG0000(	EGR1	1557.884	1.314883	9.36E-07	1.70E-05	protein_coding
ENSG0000(	FIP1L1	1000.445	-0.2118	9.36E-07	1.70E-05	protein_coding
ENSG0000(	ENSG0000(	158.2671	-0.46112	9.39E-07	1.70E-05	unprocessed_pseudogene
ENSG0000(	DNAAF2	182.9461	-0.32191	9.41E-07	1.70E-05	protein_coding
ENSG0000(	ADGRV1	1633.857	-0.3204	9.50E-07	1.72E-05	protein_coding
ENSG0000(	DDX55	1288.162	-0.22722	9.51E-07	1.72E-05	protein_coding
ENSG0000(	ENSG0000(	77.57953	0.913758	9.49E-07	1.72E-05	lncRNA
ENSG0000(	SMCR5	111.9816	0.432453	9.51E-07	1.72E-05	lncRNA
ENSG0000(	ZNF124	1682.091	-0.26419	9.50E-07	1.72E-05	protein_coding
ENSG0000(	PSMA7	572.7688	-0.37402	9.53E-07	1.72E-05	protein_coding
ENSG0000(	ENSG0000(	57.07032	0.500383	9.58E-07	1.73E-05	lncRNA
ENSG0000(	PUF60	734.9896	-0.29261	9.62E-07	1.73E-05	protein_coding
ENSG0000(	ENSG0000(	39.84669	0.699308	9.68E-07	1.74E-05	lncRNA
ENSG0000(	ADAM19	2369.672	0.681223	9.70E-07	1.74E-05	protein_coding

ENSG0000(	PDP1	627.2092	0.526667	9.71E-07	1.75E-05	protein_coding
ENSG0000(	ENSG0000(	20.84407	1.074777	9.74E-07	1.75E-05	lncRNA
ENSG0000(	CCT8	924.2484	-0.30472	9.76E-07	1.75E-05	protein_coding
ENSG0000(	FCGR2C	561.4656	0.977182	9.78E-07	1.75E-05	polymorphic_pseudogene
ENSG0000(	GSAP	1351.432	0.5105	9.78E-07	1.75E-05	protein_coding
ENSG0000(	NOC4L	130.7741	-0.43825	9.81E-07	1.76E-05	protein_coding
ENSG0000(	ENSG0000(	462.0994	0.686086	9.84E-07	1.76E-05	lncRNA
ENSG0000(	MICOS13	402.3386	-0.26793	9.90E-07	1.77E-05	protein_coding
ENSG0000(	TSR2	578.307	-0.21901	9.91E-07	1.77E-05	protein_coding
ENSG0000(	MGST2	534.4921	-0.45974	9.93E-07	1.78E-05	protein_coding
ENSG0000(	PLEKHA1	2921.414	-0.22396	9.96E-07	1.78E-05	protein_coding
ENSG0000(	ANGPTL2	344.7308	1.182146	9.99E-07	1.78E-05	protein_coding
ENSG0000(	TIMM8B	214.3604	-0.4175	9.99E-07	1.78E-05	protein_coding
ENSG0000(	MTATP6P1	2642.781	-0.76577	1.00E-06	1.79E-05	unprocessed_pseudogene
ENSG0000(	NAPRT	737.7824	-0.49429	1.00E-06	1.79E-05	protein_coding
ENSG0000(	PECAM1	2304.55	0.655703	1.00E-06	1.79E-05	protein_coding
ENSG0000(	SMC2-DT	726.7409	0.249306	1.00E-06	1.79E-05	lncRNA
ENSG0000(	CAPN12	221.7247	0.614664	1.01E-06	1.79E-05	protein_coding
ENSG0000(	POLR1H	156.2123	-0.49609	1.01E-06	1.79E-05	protein_coding
ENSG0000(	ENSG0000(	118.6814	0.482595	1.01E-06	1.80E-05	lncRNA
ENSG0000(	SCARNA8	37.01155	0.614941	1.01E-06	1.80E-05	scaRNA
ENSG0000(	VIL1	3924.129	-0.76985	1.01E-06	1.80E-05	protein_coding
ENSG0000(	DNPEP	1721.719	-0.30369	1.02E-06	1.81E-05	protein_coding
ENSG0000(	PLA1A	32.3798	1.483749	1.03E-06	1.82E-05	protein_coding
ENSG0000(	TMSB4XP4	40.44463	-0.68023	1.03E-06	1.83E-05	processed_pseudogene
ENSG0000(	TTLL3	4118.569	0.207015	1.03E-06	1.83E-05	protein_coding
ENSG0000(	H3-2	504.8101	0.438753	1.03E-06	1.83E-05	protein_coding
ENSG0000(	QKI	2227.28	0.428113	1.04E-06	1.84E-05	protein_coding
ENSG0000(	ST3GAL5	2172.087	0.51108	1.04E-06	1.85E-05	protein_coding
ENSG0000(	HIGD1A	4134.654	-0.79252	1.06E-06	1.87E-05	protein_coding
ENSG0000(	NOL7	316.6018	-0.37587	1.06E-06	1.88E-05	protein_coding
ENSG0000(	ENSG0000(	475.6356	0.353797	1.07E-06	1.89E-05	lncRNA
ENSG0000(	THOC6	152.8369	-0.42793	1.07E-06	1.89E-05	protein_coding
ENSG0000(	CHIT1	132.4005	0.972846	1.08E-06	1.91E-05	protein_coding
ENSG0000(	LHFPL3-AS:	947.7923	-0.86	1.08E-06	1.91E-05	lncRNA
ENSG0000(	ARID5A	519.4409	0.722469	1.08E-06	1.91E-05	protein_coding
ENSG0000(	RNF113B	30.68661	0.886192	1.09E-06	1.93E-05	protein_coding
ENSG0000(	ENSG0000(	2535.101	0.287184	1.10E-06	1.94E-05	lncRNA
ENSG0000(	ENSG0000(	193.2252	0.586847	1.10E-06	1.94E-05	lncRNA
ENSG0000(	LINC02422	164.4763	1.220172	1.10E-06	1.94E-05	lncRNA
ENSG0000(	UQCRQ	1104.531	-0.63711	1.11E-06	1.95E-05	protein_coding
ENSG0000(	MEF2C	1891.095	0.611069	1.11E-06	1.96E-05	protein_coding
ENSG0000(	GOLGA6L5I	272.7505	0.451945	1.12E-06	1.96E-05	transcribed_unprocessed_pseudo
ENSG0000(	C1orf35	190.9064	-0.41426	1.12E-06	1.96E-05	protein_coding
ENSG0000(	PPP4C	609.5478	-0.33765	1.12E-06	1.97E-05	protein_coding
ENSG0000(	MYO1C	4008.746	-0.3282	1.13E-06	1.97E-05	protein_coding
ENSG0000(	RPS6KA4	516.8562	-0.37585	1.13E-06	1.98E-05	protein_coding

ENSG0000(	FOSL2	6221.571	0.511232	1.13E-06	1.98E-05	protein_coding
ENSG0000(	RPL8	3344.284	-0.48179	1.14E-06	1.99E-05	protein_coding
ENSG0000(	ENSG0000(	391.4376	0.303761	1.14E-06	2.00E-05	TEC
ENSG0000(	FKBP10	674.0258	0.410574	1.15E-06	2.00E-05	protein_coding
ENSG0000(	ENSG0000(	39.91244	0.671578	1.15E-06	2.00E-05	snoRNA
ENSG0000(	H2AC14	120.0289	-0.88216	1.15E-06	2.00E-05	protein_coding
ENSG0000(	TAF4	1040.372	-0.36146	1.15E-06	2.01E-05	protein_coding
ENSG0000(	RPL18	2296.663	-0.49263	1.16E-06	2.03E-05	protein_coding
ENSG0000(	RMRP	29152.61	-0.61756	1.17E-06	2.04E-05	lncRNA
ENSG0000(	DUXAP9	318.3077	0.525353	1.17E-06	2.04E-05	transcribed_processed_pseudoge
ENSG0000(	SVEP1	670.4503	0.645052	1.17E-06	2.04E-05	protein_coding
ENSG0000(	ZDBF2	747.228	0.402317	1.18E-06	2.05E-05	protein_coding
ENSG0000(	LINC01002	1537.744	0.680642	1.18E-06	2.05E-05	lncRNA
ENSG0000(	BCAT2	419.9472	-0.40778	1.19E-06	2.06E-05	protein_coding
ENSG0000(	CPN2	112.8638	0.533225	1.19E-06	2.07E-05	protein_coding
ENSG0000(	NDUFB2	1194.311	-0.26617	1.19E-06	2.07E-05	protein_coding
ENSG0000(	RPL17	2924.733	-0.42707	1.19E-06	2.07E-05	protein_coding
ENSG0000(	LRRC45	299.977	-0.46374	1.19E-06	2.07E-05	protein_coding
ENSG0000(	PAXX	221.7862	-0.43461	1.21E-06	2.09E-05	protein_coding
ENSG0000(	RGS11	378.447	-0.47778	1.21E-06	2.09E-05	protein_coding
ENSG0000(	HSDL2	828.4531	-0.46937	1.21E-06	2.09E-05	protein_coding
ENSG0000(	NDUFS7	1423.795	-0.26201	1.21E-06	2.10E-05	protein_coding
ENSG0000(	ENSG0000(	137.633	0.483598	1.22E-06	2.11E-05	lncRNA
ENSG0000(	ENSG0000(	47.68094	0.551875	1.22E-06	2.11E-05	TEC
ENSG0000(	H1-2	587.2949	-0.59031	1.22E-06	2.11E-05	protein_coding
ENSG0000(	LST1	513.4306	0.449825	1.22E-06	2.11E-05	protein_coding
ENSG0000(	ZNHIT1	1631.184	-0.27961	1.22E-06	2.11E-05	protein_coding
ENSG0000(	ENSG0000(	693.9669	0.230733	1.23E-06	2.11E-05	lncRNA
ENSG0000(	LIPT1	315.8905	-0.25514	1.23E-06	2.11E-05	protein_coding
ENSG0000(	TRPM5	120.8044	-0.98739	1.23E-06	2.12E-05	protein_coding
ENSG0000(	CXCR6	163.3063	0.739562	1.23E-06	2.13E-05	protein_coding
ENSG0000(	SEH1L	1842.753	-0.21435	1.24E-06	2.13E-05	protein_coding
ENSG0000(	ENSG0000(	102.4512	0.579885	1.24E-06	2.13E-05	processed_pseudogene
ENSG0000(	LTV1	250.2072	-0.4023	1.24E-06	2.13E-05	protein_coding
ENSG0000(	UBE2M	254.1893	-0.43015	1.24E-06	2.14E-05	protein_coding
ENSG0000(	BCL2A1	85.79696	1.47366	1.25E-06	2.14E-05	protein_coding
ENSG0000(	SNORA70E	14.17515	1.021796	1.25E-06	2.15E-05	snoRNA
ENSG0000(	TLR4	2109.641	0.714102	1.26E-06	2.15E-05	protein_coding
ENSG0000(	PTPN14	2272.469	0.254827	1.26E-06	2.16E-05	protein_coding
ENSG0000(	RBBP4	5018.948	-0.21783	1.26E-06	2.16E-05	protein_coding
ENSG0000(	ENSG0000(	285.9233	0.332811	1.27E-06	2.17E-05	lncRNA
ENSG0000(	GSTP1	824.2408	-0.52932	1.27E-06	2.17E-05	protein_coding
ENSG0000(	DDHD1	4174.604	0.258341	1.28E-06	2.19E-05	protein_coding
ENSG0000(	H1-5	330.6323	-0.86041	1.28E-06	2.19E-05	protein_coding
ENSG0000(	KAT2B	1225.849	0.358278	1.28E-06	2.19E-05	protein_coding
ENSG0000(	ENSG0000(	300.086	-0.34167	1.28E-06	2.19E-05	lncRNA
ENSG0000(	LAP3	911.949	0.661159	1.28E-06	2.19E-05	protein_coding

ENSG0000(	SLC45A3	554.8561	-0.48425	1.29E-06	2.21E-05	protein_coding
ENSG0000(	ENSG0000(	1404.935	0.343792	1.30E-06	2.21E-05	lncRNA
ENSG0000(	GPKOW	190.1522	-0.35684	1.30E-06	2.21E-05	protein_coding
ENSG0000(	CCP110	1343.915	0.318438	1.30E-06	2.22E-05	protein_coding
ENSG0000(	TSR3	161.2691	-0.42828	1.30E-06	2.22E-05	protein_coding
ENSG0000(	ENSG0000(	46.29706	0.775837	1.31E-06	2.22E-05	protein_coding
ENSG0000(	SIRPB1	423.4379	0.773638	1.31E-06	2.23E-05	protein_coding
ENSG0000(	PLCB3	1481.918	-0.49067	1.32E-06	2.24E-05	protein_coding
ENSG0000(	ABHD14B	580.7686	-0.39774	1.32E-06	2.25E-05	protein_coding
ENSG0000(	ENSG0000(	98.67543	0.411556	1.33E-06	2.25E-05	lncRNA
ENSG0000(	ZWINT	271.5836	-0.5352	1.34E-06	2.27E-05	protein_coding
ENSG0000(	LMAN2L	324.7669	-0.36224	1.34E-06	2.28E-05	protein_coding
ENSG0000(	TIFAB	303.5858	0.824829	1.35E-06	2.29E-05	protein_coding
ENSG0000(	VILL	2184.573	-0.5806	1.36E-06	2.30E-05	protein_coding
ENSG0000(	PSAT1	89.85791	1.100019	1.36E-06	2.30E-05	protein_coding
ENSG0000(	SH3RF3	411.4186	0.546152	1.37E-06	2.31E-05	protein_coding
ENSG0000(	ABCB1	992.2133	-0.97947	1.37E-06	2.31E-05	protein_coding
ENSG0000(	URM1	600.3785	-0.34639	1.37E-06	2.31E-05	protein_coding
ENSG0000(	WRNIP1	563.3344	-0.27601	1.38E-06	2.32E-05	protein_coding
ENSG0000(	TERB1	208.9648	-0.50639	1.38E-06	2.33E-05	protein_coding
ENSG0000(	COL4A2	3825.474	0.74695	1.38E-06	2.33E-05	protein_coding
ENSG0000(	SIGMAR1	775.1912	-0.32615	1.38E-06	2.33E-05	protein_coding
ENSG0000(	RUNX2	946.515	0.542891	1.39E-06	2.35E-05	protein_coding
ENSG0000(	LINC00939	1621.349	0.177025	1.40E-06	2.35E-05	lncRNA
ENSG0000(	ATRN	1238.945	-0.2472	1.40E-06	2.35E-05	protein_coding
ENSG0000(	ENSG0000(	366.9484	0.335445	1.40E-06	2.36E-05	lncRNA
ENSG0000(	COL1A1	6011.087	1.10917	1.41E-06	2.37E-05	protein_coding
ENSG0000(	IGKJ3	64.95987	1.115739	1.41E-06	2.37E-05	IG_J_gene
ENSG0000(	CRISPLD2	1794.917	0.590894	1.41E-06	2.37E-05	protein_coding
ENSG0000(	ENSG0000(	41.94692	1.062371	1.41E-06	2.37E-05	lncRNA
ENSG0000(	NCKIPSD	500.926	-0.47291	1.42E-06	2.38E-05	protein_coding
ENSG0000(	SLAMF1	388.2405	0.80988	1.42E-06	2.38E-05	protein_coding
ENSG0000(	ETFA	3540.53	-0.2706	1.42E-06	2.38E-05	protein_coding
ENSG0000(	PLEKHA6	4836.996	-0.60275	1.43E-06	2.39E-05	protein_coding
ENSG0000(	NDUFB10	309.1939	-0.59274	1.43E-06	2.39E-05	protein_coding
ENSG0000(	COL22A1	89.76529	1.301322	1.43E-06	2.40E-05	protein_coding
ENSG0000(	MAPRE3	510.9062	-0.66336	1.43E-06	2.40E-05	protein_coding
ENSG0000(	NOP56	1176.444	-0.40089	1.44E-06	2.40E-05	protein_coding
ENSG0000(	PLXND1	1974.525	0.573949	1.44E-06	2.41E-05	protein_coding
ENSG0000(	ELOB	1117.918	-0.55385	1.45E-06	2.43E-05	protein_coding
ENSG0000(	RPL11	3568.25	-0.42634	1.45E-06	2.43E-05	protein_coding
ENSG0000(	TCEANC2	2003.855	-0.20486	1.46E-06	2.43E-05	protein_coding
ENSG0000(	KLRK1-AS1	622.0799	0.293916	1.46E-06	2.43E-05	lncRNA
ENSG0000(	DYRK3	485.4401	0.572194	1.46E-06	2.44E-05	protein_coding
ENSG0000(	ENSG0000(	358.5521	0.2755	1.47E-06	2.45E-05	lncRNA
ENSG0000(	PFN1P2	101.1187	0.602106	1.47E-06	2.46E-05	transcribed_processed_pseudoge
ENSG0000(	B3GNT9	152.5766	0.485492	1.48E-06	2.46E-05	protein_coding

ENSG0000(	E4F1	770.5938	-0.34485	1.48E-06	2.46E-05	protein_coding
ENSG0000(	SHC3	136.241	1.802803	1.48E-06	2.46E-05	protein_coding
ENSG0000(	ZC3H12A	888.486	0.636324	1.48E-06	2.46E-05	protein_coding
ENSG0000(	C3orf33	224.4742	-0.43897	1.49E-06	2.47E-05	protein_coding
ENSG0000(	CCL20	192.7839	0.827041	1.50E-06	2.49E-05	protein_coding
ENSG0000(	SBNO2	2185.91	0.432389	1.50E-06	2.49E-05	protein_coding
ENSG0000(	HMGN2	3707.267	-0.31889	1.51E-06	2.50E-05	protein_coding
ENSG0000(	RAB27A	1571.542	0.351098	1.51E-06	2.51E-05	protein_coding
ENSG0000(	ENSG0000(	113.0106	0.84493	1.52E-06	2.51E-05	lncRNA
ENSG0000(	ARHGAP31	1584.264	0.437129	1.52E-06	2.52E-05	protein_coding
ENSG0000(	RTKN	689.4801	-0.43464	1.52E-06	2.52E-05	protein_coding
ENSG0000(	KLHL5	1970.341	0.53463	1.53E-06	2.52E-05	protein_coding
ENSG0000(	GOLGA8K	121.2128	0.54299	1.53E-06	2.52E-05	protein_coding
ENSG0000(	TLE5	2758.722	-0.2847	1.53E-06	2.53E-05	protein_coding
ENSG0000(	MRPS21	605.2447	-0.17935	1.53E-06	2.53E-05	protein_coding
ENSG0000(	RPS8	4194.605	-0.49448	1.53E-06	2.53E-05	protein_coding
ENSG0000(	HMX3	144.1326	-0.37261	1.53E-06	2.53E-05	protein_coding
ENSG0000(	HLA-DPB2	104.0452	0.48629	1.54E-06	2.54E-05	transcribed_unprocessed_pseudo
ENSG0000(	ENSG0000(	693.9252	0.410032	1.55E-06	2.55E-05	transcribed_unprocessed_pseudo
ENSG0000(	ATP5F1B	5137.522	-0.52524	1.55E-06	2.55E-05	protein_coding
ENSG0000(	MINAR1	93.97025	0.645995	1.55E-06	2.55E-05	protein_coding
ENSG0000(	SPI1	330.9346	0.839816	1.55E-06	2.55E-05	protein_coding
ENSG0000(	ENSG0000(	105.0588	-0.51224	1.56E-06	2.56E-05	lncRNA
ENSG0000(	ARSB	795.8928	0.323565	1.56E-06	2.57E-05	protein_coding
ENSG0000(	ENSG0000(	139.7015	-0.40568	1.56E-06	2.57E-05	lncRNA
ENSG0000(	NDUFC2	673.5612	-0.64641	1.57E-06	2.57E-05	protein_coding
ENSG0000(	HSPA8	6637.651	-0.4881	1.57E-06	2.58E-05	protein_coding
ENSG0000(	SUSD1	687.6856	-0.38986	1.57E-06	2.58E-05	protein_coding
ENSG0000(	CSGALNAC	901.5315	0.55835	1.58E-06	2.59E-05	protein_coding
ENSG0000(	ENSG0000(	465.3525	0.283648	1.58E-06	2.59E-05	TEC
ENSG0000(	ELAC2	1002.223	-0.27842	1.58E-06	2.59E-05	protein_coding
ENSG0000(	RBM3	3131.779	-0.27502	1.59E-06	2.60E-05	protein_coding
ENSG0000(	HSPB1	782.738	-0.65534	1.60E-06	2.61E-05	protein_coding
ENSG0000(	EPN3	357.8212	-0.80261	1.61E-06	2.63E-05	protein_coding
ENSG0000(	TRIR	728.6193	-0.24661	1.61E-06	2.63E-05	protein_coding
ENSG0000(	ENSG0000(	106.0379	0.526572	1.62E-06	2.64E-05	lncRNA
ENSG0000(	SLC25A4	335.7278	-0.45317	1.64E-06	2.67E-05	protein_coding
ENSG0000(	MAPK13	3158.546	-0.33548	1.64E-06	2.68E-05	protein_coding
ENSG0000(	STT3B	3353.188	-0.22197	1.64E-06	2.68E-05	protein_coding
ENSG0000(	KLHL29	547.7064	0.54339	1.65E-06	2.68E-05	protein_coding
ENSG0000(	ENSG0000(	33.38746	0.806322	1.66E-06	2.70E-05	lncRNA
ENSG0000(	SNORD66	16.40749	0.962025	1.66E-06	2.70E-05	snoRNA
ENSG0000(	NEK2	357.8969	-0.29743	1.66E-06	2.70E-05	protein_coding
ENSG0000(	H2AC16	84.68189	-0.93513	1.67E-06	2.71E-05	protein_coding
ENSG0000(	SND1-IT1	729.3868	0.379235	1.67E-06	2.71E-05	lncRNA
ENSG0000(	RPS23	2911.158	-0.59185	1.68E-06	2.74E-05	protein_coding
ENSG0000(	LINC00174	1926.067	0.22351	1.69E-06	2.75E-05	lncRNA

ENSG0000(	VAMP4	622.8196	0.240555	1.69E-06	2.75E-05	protein_coding
ENSG0000(	ENSG0000(	375.4084	0.377936	1.69E-06	2.75E-05	TEC
ENSG0000(	PIM3	1124.835	0.438178	1.69E-06	2.75E-05	protein_coding
ENSG0000(	WDFY3	4161.011	0.323057	1.70E-06	2.75E-05	protein_coding
ENSG0000(	HSPA6	230.7653	1.279252	1.70E-06	2.76E-05	protein_coding
ENSG0000(	ENSG0000(	319.8632	-0.29064	1.71E-06	2.76E-05	lncRNA
ENSG0000(	TERT	288.6458	-0.63883	1.71E-06	2.76E-05	protein_coding
ENSG0000(	SERPINA3	73.46503	1.749906	1.72E-06	2.78E-05	protein_coding
ENSG0000(	NOSIP	630.9347	-0.28865	1.72E-06	2.79E-05	protein_coding
ENSG0000(	ENSG0000(	105.2026	-0.50482	1.73E-06	2.79E-05	lncRNA
ENSG0000(	ENSG0000(	359.0768	0.255677	1.73E-06	2.80E-05	lncRNA
ENSG0000(	CCL2	288.638	0.87521	1.73E-06	2.80E-05	protein_coding
ENSG0000(	GPAT3	572.9028	-0.79302	1.73E-06	2.80E-05	protein_coding
ENSG0000(	OPA1	2597.01	-0.25033	1.74E-06	2.81E-05	protein_coding
ENSG0000(	ENSG0000(	358.0909	-0.39738	1.75E-06	2.83E-05	lncRNA
ENSG0000(	ME2	4107.991	-0.2732	1.76E-06	2.83E-05	protein_coding
ENSG0000(	ALDH1L2	808.7814	0.652957	1.77E-06	2.84E-05	protein_coding
ENSG0000(	DUXAP10	575.6589	0.689586	1.77E-06	2.84E-05	transcribed_processed_pseudoge
ENSG0000(	ENSG0000(	156.854	0.4194	1.78E-06	2.86E-05	lncRNA
ENSG0000(	TAF6	1021.062	-0.27408	1.78E-06	2.86E-05	protein_coding
ENSG0000(	C1orf131	712.6632	-0.22664	1.79E-06	2.87E-05	protein_coding
ENSG0000(	TFAM	819.2311	-0.33152	1.79E-06	2.87E-05	protein_coding
ENSG0000(	ENSG0000(	80.50606	0.548593	1.79E-06	2.87E-05	lncRNA
ENSG0000(	APH1A	1403.77	-0.36507	1.80E-06	2.88E-05	protein_coding
ENSG0000(	CDX1	1517.397	-0.70085	1.80E-06	2.89E-05	protein_coding
ENSG0000(	DCUN1D3	1016.003	0.575274	1.80E-06	2.89E-05	protein_coding
ENSG0000(	CLMP	996.6741	0.457249	1.81E-06	2.89E-05	protein_coding
ENSG0000(	RAPGEF4	193.4367	0.617803	1.81E-06	2.90E-05	protein_coding
ENSG0000(	USP1	808.2225	-0.29168	1.81E-06	2.90E-05	protein_coding
ENSG0000(	HLA-DMA	910.0357	0.630099	1.82E-06	2.90E-05	protein_coding
ENSG0000(	POM121L9	130.3048	0.921997	1.82E-06	2.90E-05	transcribed_unprocessed_pseudo
ENSG0000(	ENSG0000(	39.51078	0.714779	1.82E-06	2.90E-05	lncRNA
ENSG0000(	SNORD89	306.1616	0.469984	1.83E-06	2.91E-05	snoRNA
ENSG0000(	ENSG0000(	108.2773	0.451037	1.83E-06	2.91E-05	lncRNA
ENSG0000(	H2BC19P	694.0903	0.340583	1.83E-06	2.92E-05	transcribed_unprocessed_pseudo
ENSG0000(	ENSG0000(	13.6417	1.377041	1.84E-06	2.93E-05	lncRNA
ENSG0000(	FBXW7	2056.572	0.321815	1.84E-06	2.93E-05	protein_coding
ENSG0000(	LETM1	3803.919	-0.52196	1.84E-06	2.93E-05	protein_coding
ENSG0000(	RPS19BP1	642.9092	-0.25611	1.84E-06	2.93E-05	protein_coding
ENSG0000(	LINC02484	64.10536	-0.81827	1.85E-06	2.94E-05	lncRNA
ENSG0000(	CCNL1	5996.177	0.223174	1.85E-06	2.94E-05	protein_coding
ENSG0000(	NUSAP1	498.8357	-0.47962	1.85E-06	2.94E-05	protein_coding
ENSG0000(	ENSG0000(	63.74993	0.493708	1.87E-06	2.96E-05	lncRNA
ENSG0000(	RANBP1	511.1307	-0.44367	1.87E-06	2.97E-05	protein_coding
ENSG0000(	C4A-AS1	31.33688	0.663353	1.90E-06	3.01E-05	lncRNA
ENSG0000(	GOT1	728.2137	-0.53571	1.91E-06	3.03E-05	protein_coding
ENSG0000(	TMEM74B	134.2318	0.542908	1.91E-06	3.03E-05	protein_coding

ENSG0000(	CALU	2321.647	0.407024	1.91E-06	3.03E-05	protein_coding
ENSG0000(	NOTCH2NL	9342.031	0.345357	1.92E-06	3.04E-05	protein_coding
ENSG0000(	ST3GAL6	393.7164	0.46951	1.93E-06	3.06E-05	protein_coding
ENSG0000(	THAP4	549.3269	-0.3596	1.93E-06	3.06E-05	protein_coding
ENSG0000(	ENSG0000(	10.36976	1.293221	1.94E-06	3.07E-05	lncRNA
ENSG0000(	MRPL1	182.2083	-0.45523	1.96E-06	3.09E-05	protein_coding
ENSG0000(	RPS10	1770.048	-0.49886	1.96E-06	3.10E-05	protein_coding
ENSG0000(	ATP10D	1133.4	0.31499	1.97E-06	3.11E-05	protein_coding
ENSG0000(	CTLA4	190.8904	1.12847	1.98E-06	3.12E-05	protein_coding
ENSG0000(	KCNA2	371.475	0.609107	1.99E-06	3.13E-05	protein_coding
ENSG0000(	SLC28A3	136.2552	1.381859	1.99E-06	3.14E-05	protein_coding
ENSG0000(	ENSG0000(	178.5633	0.383413	2.00E-06	3.14E-05	TEC
ENSG0000(	TICAM2	186.3651	0.882656	2.00E-06	3.15E-05	protein_coding
ENSG0000(	YJEFN3	382.7827	-0.47034	2.01E-06	3.16E-05	protein_coding
ENSG0000(	TNFRSF1B	1228.38	0.531746	2.02E-06	3.18E-05	protein_coding
ENSG0000(	RASSF4	2409.264	0.505638	2.02E-06	3.18E-05	protein_coding
ENSG0000(	IFNG-AS1	454.2627	0.738499	2.03E-06	3.18E-05	lncRNA
ENSG0000(	LTBP4	2699.125	-0.33274	2.03E-06	3.18E-05	protein_coding
ENSG0000(	SLC25A51	689.63	-0.19175	2.03E-06	3.18E-05	protein_coding
ENSG0000(	CHST2	293.2501	0.772055	2.03E-06	3.19E-05	protein_coding
ENSG0000(	ANKRD44-I	534.0996	0.595299	2.06E-06	3.24E-05	lncRNA
ENSG0000(	CCDC91	1008.924	0.243787	2.06E-06	3.24E-05	protein_coding
ENSG0000(	H2BC14	95.55241	-0.82021	2.07E-06	3.24E-05	protein_coding
ENSG0000(	SELENOW	3246.232	-0.35965	2.07E-06	3.25E-05	protein_coding
ENSG0000(	DOCK8	4712.528	0.501317	2.10E-06	3.28E-05	protein_coding
ENSG0000(	NEU3	1285.893	0.321557	2.10E-06	3.28E-05	protein_coding
ENSG0000(	MIR31HG	20.49576	1.763548	2.13E-06	3.33E-05	lncRNA
ENSG0000(	PSG2	144.6703	0.693292	2.13E-06	3.33E-05	protein_coding
ENSG0000(	FCN1	723.3812	0.432605	2.14E-06	3.35E-05	protein_coding
ENSG0000(	VPS13C	12096.52	0.234707	2.15E-06	3.36E-05	protein_coding
ENSG0000(	RPL21	3223.343	-0.43408	2.16E-06	3.37E-05	protein_coding
ENSG0000(	ENSG0000(	207.4369	-0.31062	2.17E-06	3.38E-05	lncRNA
ENSG0000(	H2AC7	126.1814	-0.64871	2.18E-06	3.39E-05	protein_coding
ENSG0000(	ANGEL1	1211.094	0.277686	2.18E-06	3.39E-05	protein_coding
ENSG0000(	TREML2	238.3216	0.6343	2.20E-06	3.42E-05	protein_coding
ENSG0000(	ENSG0000(	25.23039	1.454369	2.20E-06	3.43E-05	lncRNA
ENSG0000(	HINT2	201.944	-0.55983	2.21E-06	3.45E-05	protein_coding
ENSG0000(	ATAT1	517.8344	-0.30127	2.22E-06	3.45E-05	protein_coding
ENSG0000(	BCS1L	555.1745	-0.29722	2.23E-06	3.46E-05	protein_coding
ENSG0000(	CUL2	2251.454	-0.21298	2.23E-06	3.46E-05	protein_coding
ENSG0000(	CFHR2	214.4103	-0.42879	2.24E-06	3.47E-05	protein_coding
ENSG0000(	EEF1G	7616.397	-0.46511	2.24E-06	3.48E-05	protein_coding
ENSG0000(	C4BPB	902.0358	0.596023	2.25E-06	3.48E-05	protein_coding
ENSG0000(	CCNB2	236.1645	-0.54888	2.25E-06	3.48E-05	protein_coding
ENSG0000(	DCAF8	4203.737	0.187742	2.25E-06	3.48E-05	protein_coding
ENSG0000(	MEP1B	233.9946	-1.28211	2.26E-06	3.50E-05	protein_coding
ENSG0000(	RPLP1	3163.721	-0.47499	2.26E-06	3.50E-05	protein_coding

ENSG0000(	CDKN2AIP	499.7583	-0.32237	2.27E-06	3.52E-05	protein_coding
ENSG0000(	PSMB1	982.7638	-0.34233	2.27E-06	3.52E-05	protein_coding
ENSG0000(	LINC00106	248.5434	-0.36291	2.29E-06	3.54E-05	lncRNA
ENSG0000(	ENSG0000(	153.5665	0.452205	2.30E-06	3.56E-05	lncRNA
ENSG0000(	ATP2B1	3685.622	-0.45215	2.30E-06	3.56E-05	protein_coding
ENSG0000(	LINC01303	64.53691	1.13207	2.31E-06	3.56E-05	lncRNA
ENSG0000(	MMP7	28.00743	1.59137	2.30E-06	3.56E-05	protein_coding
ENSG0000(	NR2C2AP	113.6265	-0.58097	2.31E-06	3.56E-05	protein_coding
ENSG0000(	PMPCA	1030.337	-0.20688	2.31E-06	3.56E-05	protein_coding
ENSG0000(	UBE2D2	1538.006	-0.23258	2.31E-06	3.56E-05	protein_coding
ENSG0000(	EAPP	342.6975	-0.35458	2.31E-06	3.56E-05	protein_coding
ENSG0000(	LINC02889	628.4772	0.292845	2.31E-06	3.56E-05	lncRNA
ENSG0000(	CFAP20	147.0749	-0.45022	2.32E-06	3.57E-05	protein_coding
ENSG0000(	NOP53	1760.79	-0.26632	2.33E-06	3.58E-05	protein_coding
ENSG0000(	SHFL	1105.569	0.277332	2.34E-06	3.59E-05	protein_coding
ENSG0000(	TPP1	2368.982	0.372643	2.35E-06	3.61E-05	protein_coding
ENSG0000(	MRPS33	1184.183	-0.26789	2.36E-06	3.63E-05	protein_coding
ENSG0000(	VASH1	983.0207	0.495664	2.37E-06	3.64E-05	protein_coding
ENSG0000(	ENSG0000(	909.2572	0.253253	2.37E-06	3.64E-05	lncRNA
ENSG0000(	ENSG0000(	93.1796	0.421821	2.38E-06	3.64E-05	lncRNA
ENSG0000(	HMGB3	549.419	-0.515	2.38E-06	3.64E-05	protein_coding
ENSG0000(	CALCOCO2	3923.874	-0.16642	2.38E-06	3.64E-05	protein_coding
ENSG0000(	EML2	1128.021	-0.22416	2.38E-06	3.64E-05	protein_coding
ENSG0000(	ENSG0000(	546.5669	0.268053	2.38E-06	3.64E-05	transcribed_processed_pseudoge
ENSG0000(	GK	1260.123	0.977965	2.38E-06	3.64E-05	protein_coding
ENSG0000(	NDUFAF4	369.7997	-0.3933	2.38E-06	3.64E-05	protein_coding
ENSG0000(	TBC1D9	1230.226	0.442174	2.38E-06	3.64E-05	protein_coding
ENSG0000(	POLR2B	1770.98	-0.2462	2.41E-06	3.68E-05	protein_coding
ENSG0000(	UQCC2	545.7321	-0.53254	2.41E-06	3.68E-05	protein_coding
ENSG0000(	ENSG0000(	15.04028	1.271806	2.42E-06	3.69E-05	lncRNA
ENSG0000(	ZNF829	1231.823	-0.25828	2.43E-06	3.70E-05	protein_coding
ENSG0000(	ENSG0000(	1681.061	0.229488	2.43E-06	3.71E-05	lncRNA
ENSG0000(	NT5C	374.8738	-0.40634	2.44E-06	3.72E-05	protein_coding
ENSG0000(	DEPDC5	3369.561	-0.17854	2.45E-06	3.73E-05	protein_coding
ENSG0000(	ENSG0000(	210.4055	0.400298	2.45E-06	3.73E-05	lncRNA
ENSG0000(	HDAC8	3913.559	0.245096	2.45E-06	3.73E-05	protein_coding
ENSG0000(	TWNK	225.2199	-0.52399	2.45E-06	3.73E-05	protein_coding
ENSG0000(	DOHH	94.90742	-0.48797	2.46E-06	3.73E-05	protein_coding
ENSG0000(	PM20D2	890.6862	-0.31916	2.46E-06	3.74E-05	protein_coding
ENSG0000(	CARMN	13150.53	0.599569	2.48E-06	3.76E-05	lncRNA
ENSG0000(	CHI3L2	138.5642	0.986541	2.48E-06	3.76E-05	protein_coding
ENSG0000(	TRPM7	5455.691	-0.17889	2.48E-06	3.76E-05	protein_coding
ENSG0000(	TRUB2	1896.399	-0.1519	2.49E-06	3.77E-05	protein_coding
ENSG0000(	AKAP6	684.6619	-0.30683	2.50E-06	3.78E-05	protein_coding
ENSG0000(	DYNLL1	1227.251	-0.39225	2.50E-06	3.79E-05	protein_coding
ENSG0000(	RAB11B	769.8627	-0.25914	2.50E-06	3.79E-05	protein_coding
ENSG0000(	RPS27A	2351.3	-0.46961	2.50E-06	3.79E-05	protein_coding

ENSG0000(	CALM1	7607.04	-0.3553	2.51E-06	3.80E-05	protein_coding
ENSG0000(	ENSG0000(	22.395	0.919374	2.52E-06	3.80E-05	lncRNA
ENSG0000(	SNX18	745.4277	0.40938	2.52E-06	3.80E-05	protein_coding
ENSG0000(	ENSG0000(	37.69084	0.592079	2.53E-06	3.81E-05	processed_pseudogene
ENSG0000(	PHOSPHO1	16.77449	1.587294	2.53E-06	3.82E-05	protein_coding
ENSG0000(	IDH1	1554.825	-0.47543	2.54E-06	3.83E-05	protein_coding
ENSG0000(	ACAA1	1989.559	-0.45028	2.54E-06	3.83E-05	protein_coding
ENSG0000(	SACM1L	1722.254	-0.14898	2.56E-06	3.85E-05	protein_coding
ENSG0000(	ENSG0000(	56.78318	0.75939	2.57E-06	3.86E-05	lncRNA
ENSG0000(	MRPS15	573.5362	-0.29333	2.57E-06	3.88E-05	protein_coding
ENSG0000(	CLCC1	5374.592	0.129534	2.59E-06	3.89E-05	protein_coding
ENSG0000(	IL4I1	153.5325	0.979827	2.59E-06	3.89E-05	protein_coding
ENSG0000(	ZNF20	382.8794	-0.25942	2.59E-06	3.89E-05	protein_coding
ENSG0000(	MEIS1	1032.073	0.651824	2.59E-06	3.90E-05	protein_coding
ENSG0000(	ACP3	241.5592	0.651667	2.60E-06	3.90E-05	protein_coding
ENSG0000(	LINC02869	73.95458	-0.61297	2.60E-06	3.91E-05	lncRNA
ENSG0000(	CASP1P2	22.97263	1.290829	2.61E-06	3.91E-05	unprocessed_pseudogene
ENSG0000(	FBXL16	41.15667	0.951884	2.61E-06	3.92E-05	protein_coding
ENSG0000(	TMEM69	300.1438	-0.31073	2.63E-06	3.95E-05	protein_coding
ENSG0000(	MT-ND2	18313.46	-0.67271	2.64E-06	3.96E-05	protein_coding
ENSG0000(	TWIST1	20.40904	1.595203	2.64E-06	3.96E-05	protein_coding
ENSG0000(	MIR155HG	118.3785	1.048651	2.66E-06	3.99E-05	lncRNA
ENSG0000(	SORCS2	113.94	1.016443	2.67E-06	3.99E-05	protein_coding
ENSG0000(	LINC02453	145.4403	-0.43721	2.69E-06	4.03E-05	lncRNA
ENSG0000(	ANTXR1	2249.389	0.658831	2.70E-06	4.03E-05	protein_coding
ENSG0000(	ENSG0000(	24.54111	0.649558	2.71E-06	4.04E-05	lncRNA
ENSG0000(	EEPD1	455.1205	-0.47625	2.74E-06	4.09E-05	protein_coding
ENSG0000(	AP1S2	399.8369	0.439502	2.75E-06	4.10E-05	protein_coding
ENSG0000(	CWC22	501.8862	-0.30907	2.75E-06	4.11E-05	protein_coding
ENSG0000(	HSPA5	3562.196	-0.53942	2.78E-06	4.14E-05	protein_coding
ENSG0000(	TOE1	248.7136	-0.3135	2.78E-06	4.14E-05	protein_coding
ENSG0000(	JAGN1	340.4819	-0.31086	2.78E-06	4.15E-05	protein_coding
ENSG0000(	INF2	5219.082	-0.41451	2.78E-06	4.15E-05	protein_coding
ENSG0000(	FBN1	1796.023	0.66392	2.80E-06	4.17E-05	protein_coding
ENSG0000(	DMAC2	513.7335	-0.27262	2.81E-06	4.18E-05	protein_coding
ENSG0000(	PTPN23-DT	175.7552	0.357716	2.81E-06	4.19E-05	lncRNA
ENSG0000(	UNC80	1460.26	0.201279	2.83E-06	4.21E-05	protein_coding
ENSG0000(	ENSG0000(	136.3797	0.420288	2.85E-06	4.24E-05	processed_pseudogene
ENSG0000(	BPNT1	1021.582	-0.38442	2.86E-06	4.25E-05	protein_coding
ENSG0000(	CD40	517.0796	0.693415	2.87E-06	4.26E-05	protein_coding
ENSG0000(	ENSG0000(	41.74659	0.791843	2.87E-06	4.26E-05	TEC
ENSG0000(	PLIN1	115.6703	-0.74644	2.87E-06	4.26E-05	protein_coding
ENSG0000(	GFER	318.8931	-0.49098	2.87E-06	4.26E-05	protein_coding
ENSG0000(	DDC	440.9764	-0.7923	2.88E-06	4.27E-05	protein_coding
ENSG0000(	RPL3	7299.384	-0.38793	2.88E-06	4.27E-05	protein_coding
ENSG0000(	DOC2B	469.1077	0.853041	2.90E-06	4.30E-05	protein_coding
ENSG0000(	ENSG0000(	175.806	-0.35222	2.90E-06	4.30E-05	lncRNA

ENSG0000(	SHISAL1	286.8487	0.636177	2.91E-06	4.30E-05	protein_coding
ENSG0000(	ID3	813.8947	-0.54933	2.91E-06	4.30E-05	protein_coding
ENSG0000(	LARP6	210.6098	0.54683	2.92E-06	4.30E-05	protein_coding
ENSG0000(	TSHZ2	831.0697	0.561255	2.91E-06	4.30E-05	protein_coding
ENSG0000(	GPSM2	2728.131	-0.21604	2.92E-06	4.31E-05	protein_coding
ENSG0000(	LRATD2	1055.57	-0.40175	2.92E-06	4.31E-05	protein_coding
ENSG0000(	PLA2G2D	417.0319	0.731566	2.93E-06	4.32E-05	protein_coding
ENSG0000(	ASL	2620.983	-0.33108	2.94E-06	4.34E-05	protein_coding
ENSG0000(	OVCH2	29.28927	0.741347	2.96E-06	4.36E-05	protein_coding
ENSG0000(	ELP5	269.9079	-0.40647	2.97E-06	4.36E-05	protein_coding
ENSG0000(	ENSG0000(	535.0757	0.268079	2.97E-06	4.36E-05	lncRNA
ENSG0000(	ESD	604.6999	-0.42492	2.96E-06	4.36E-05	protein_coding
ENSG0000(	SLC5A8	59.82675	0.939037	2.97E-06	4.36E-05	protein_coding
ENSG0000(	EIF3F	3412.658	-0.37691	2.97E-06	4.37E-05	protein_coding
ENSG0000(	CHST12	3731.802	0.193278	2.99E-06	4.39E-05	protein_coding
ENSG0000(	ARFGAP2	1605.319	-0.15336	3.00E-06	4.40E-05	protein_coding
ENSG0000(	UQCR11	563.8965	-0.50815	3.01E-06	4.42E-05	protein_coding
ENSG0000(	MCAT	140.9848	-0.54648	3.01E-06	4.42E-05	protein_coding
ENSG0000(	CRKL	1988.633	-0.20513	3.01E-06	4.42E-05	protein_coding
ENSG0000(	ENSG0000(	52.28265	0.552132	3.03E-06	4.44E-05	lncRNA
ENSG0000(	PIK3R3	1249.205	0.282006	3.04E-06	4.45E-05	protein_coding
ENSG0000(	HLA-DRB1	2133.188	0.708135	3.05E-06	4.46E-05	protein_coding
ENSG0000(	RPS28	1316.185	-0.52861	3.05E-06	4.46E-05	protein_coding
ENSG0000(	TRMT10C	214.9069	-0.36981	3.05E-06	4.46E-05	protein_coding
ENSG0000(	PRUNE1	424.8844	-0.33538	3.05E-06	4.46E-05	protein_coding
ENSG0000(	VWF	2113.099	0.786396	3.05E-06	4.46E-05	protein_coding
ENSG0000(	ENSG0000(	475.5934	0.278013	3.06E-06	4.47E-05	lncRNA
ENSG0000(	H4C12	249.5738	-0.90214	3.06E-06	4.47E-05	protein_coding
ENSG0000(	ENSG0000(	621.2943	0.300493	3.08E-06	4.50E-05	lncRNA
ENSG0000(	CLK3	3434.235	-0.21525	3.09E-06	4.51E-05	protein_coding
ENSG0000(	ENSG0000(	55.05661	0.850123	3.12E-06	4.54E-05	lncRNA
ENSG0000(	ENSG0000(	56.50622	0.530502	3.14E-06	4.57E-05	lncRNA
ENSG0000(	WWOX	1608.472	0.202173	3.16E-06	4.60E-05	protein_coding
ENSG0000(	PROB1	66.2733	0.453885	3.18E-06	4.62E-05	protein_coding
ENSG0000(	ENSG0000(	59.0876	0.774598	3.18E-06	4.63E-05	lncRNA
ENSG0000(	LDLRAD3	251.7265	0.644636	3.20E-06	4.65E-05	protein_coding
ENSG0000(	PRXL2A	1907.17	-0.5072	3.21E-06	4.66E-05	protein_coding
ENSG0000(	ENSG0000(	202.757	0.430627	3.21E-06	4.66E-05	lncRNA
ENSG0000(	CYP7B1	626.3425	0.854301	3.22E-06	4.68E-05	protein_coding
ENSG0000(	ENSG0000(	392.9873	0.299135	3.22E-06	4.68E-05	lncRNA
ENSG0000(	LINC02861	390.5379	-0.23764	3.23E-06	4.68E-05	lncRNA
ENSG0000(	PSMB8-AS1	534.6202	0.393029	3.26E-06	4.72E-05	lncRNA
ENSG0000(	SH3TC1	801.2282	0.670343	3.26E-06	4.72E-05	protein_coding
ENSG0000(	MFAP2	59.38426	1.120014	3.28E-06	4.75E-05	protein_coding
ENSG0000(	GANAB	2955.265	-0.22353	3.31E-06	4.80E-05	protein_coding
ENSG0000(	ENSG0000(	33.96716	1.206477	3.33E-06	4.82E-05	lncRNA
ENSG0000(	ENSG0000(	186.7923	-0.28861	3.33E-06	4.82E-05	lncRNA

ENSG0000(	CTSH	2507.514	0.600643	3.34E-06	4.83E-05	protein_coding
ENSG0000(	LRRC8C	897.3842	0.558563	3.36E-06	4.85E-05	protein_coding
ENSG0000(	GIMAP8	755.6088	0.501718	3.36E-06	4.85E-05	protein_coding
ENSG0000(	KLK11	189.546	0.36274	3.37E-06	4.86E-05	protein_coding
ENSG0000(	LINC00920	30.42055	0.741497	3.38E-06	4.88E-05	lncRNA
ENSG0000(	IGFBP5	4021.828	1.133356	3.39E-06	4.89E-05	protein_coding
ENSG0000(	LRP6	2883.903	-0.41761	3.40E-06	4.90E-05	protein_coding
ENSG0000(	RN7SL172F	50.64534	0.590466	3.40E-06	4.90E-05	misc_RNA
ENSG0000(	GRB10	1061.518	0.412937	3.41E-06	4.92E-05	protein_coding
ENSG0000(	MRPS24	305.4616	-0.42007	3.42E-06	4.92E-05	protein_coding
ENSG0000(	CYP2S1	428.0295	-0.68246	3.43E-06	4.93E-05	protein_coding
ENSG0000(	GPLD1	593.2257	0.217475	3.43E-06	4.94E-05	protein_coding
ENSG0000(	MRPL38	357.653	-0.26572	3.43E-06	4.94E-05	protein_coding
ENSG0000(	LINC02005	28.49796	0.723202	3.45E-06	4.95E-05	lncRNA
ENSG0000(	NOC2L	1265.481	-0.23802	3.44E-06	4.95E-05	protein_coding
ENSG0000(	ZNF683	42.80146	-0.80975	3.46E-06	4.98E-05	protein_coding
ENSG0000(	PFDN2	88.32092	-0.54139	3.47E-06	4.98E-05	protein_coding
ENSG0000(	PMEPA1	937.6344	0.587337	3.47E-06	4.98E-05	protein_coding
ENSG0000(	ENSG0000(	21.05187	0.674419	3.47E-06	4.98E-05	lncRNA
ENSG0000(	ENSG0000(	500.4613	0.294434	3.47E-06	4.98E-05	lncRNA
ENSG0000(	ENSG0000(	19.61913	1.347286	3.48E-06	4.99E-05	lncRNA
ENSG0000(	RN7SKP16	76.88101	0.606415	3.48E-06	4.99E-05	misc_RNA
ENSG0000(	RPL37	5930.109	-0.45445	3.49E-06	5.00E-05	protein_coding
ENSG0000(	ENSG0000(	60.17415	-0.40642	3.49E-06	5.00E-05	lncRNA
ENSG0000(	UBAP2	2020.949	-0.18327	3.49E-06	5.00E-05	protein_coding
ENSG0000(	LINC01344	215.9023	0.57785	3.51E-06	5.02E-05	lncRNA
ENSG0000(	ENSG0000(	181.3457	0.911024	3.52E-06	5.02E-05	lncRNA
ENSG0000(	PGF	518.7143	0.425491	3.52E-06	5.02E-05	protein_coding
ENSG0000(	CEBPB-AS1	427.7421	0.289747	3.52E-06	5.02E-05	lncRNA
ENSG0000(	ENSG0000(	30.85309	-0.80167	3.52E-06	5.03E-05	processed_pseudogene
ENSG0000(	ENSG0000(	1301.164	0.240354	3.54E-06	5.05E-05	transcribed_processed_pseudoge
ENSG0000(	TMEM253	119.8328	-0.74959	3.55E-06	5.06E-05	protein_coding
ENSG0000(	GRAMD4	2228.979	-0.50438	3.56E-06	5.08E-05	protein_coding
ENSG0000(	PSMB7	384.6506	-0.33983	3.56E-06	5.08E-05	protein_coding
ENSG0000(	NARF-IT1	104.9978	0.411738	3.56E-06	5.08E-05	lncRNA
ENSG0000(	ZNF618	727.4475	0.446695	3.57E-06	5.08E-05	protein_coding
ENSG0000(	ENSG0000(	40.55359	0.769895	3.57E-06	5.08E-05	lncRNA
ENSG0000(	SP110	2084.76	0.700722	3.57E-06	5.08E-05	protein_coding
ENSG0000(	PXMP2	310.2656	-0.61409	3.58E-06	5.09E-05	protein_coding
ENSG0000(	ENSG0000(	264.7251	0.315139	3.59E-06	5.10E-05	lncRNA
ENSG0000(	ENSG0000(	146.88	0.347685	3.59E-06	5.10E-05	lncRNA
ENSG0000(	PEX6	487.2497	-0.43698	3.60E-06	5.11E-05	protein_coding
ENSG0000(	ENSG0000(	22.64114	-1.16074	3.61E-06	5.12E-05	processed_pseudogene
ENSG0000(	DDX54	578.9338	-0.3585	3.62E-06	5.13E-05	protein_coding
ENSG0000(	ENSG0000(	817.3237	0.344898	3.62E-06	5.13E-05	TEC
ENSG0000(	ENSG0000(	164.935	0.32128	3.62E-06	5.13E-05	lncRNA
ENSG0000(	WNT5B	283.2347	0.409735	3.62E-06	5.13E-05	protein_coding

ENSG0000(	ATG16L1	1145.847	-0.25213	3.63E-06	5.14E-05	protein_coding
ENSG0000(	FOS	2715.888	1.307661	3.65E-06	5.16E-05	protein_coding
ENSG0000(	ST3GAL1	1850.727	0.598623	3.65E-06	5.16E-05	protein_coding
ENSG0000(	DHPS	550.833	-0.25271	3.65E-06	5.16E-05	protein_coding
ENSG0000(	ENSG0000(	198.3134	0.435992	3.66E-06	5.17E-05	lncRNA
ENSG0000(	HNRNPK	5039.115	-0.18481	3.67E-06	5.19E-05	protein_coding
ENSG0000(	PPME1	885.7441	-0.28147	3.67E-06	5.19E-05	protein_coding
ENSG0000(	ENSG0000(	136.273	0.448644	3.68E-06	5.20E-05	lncRNA
ENSG0000(	NOL11	705.033	-0.37799	3.68E-06	5.20E-05	protein_coding
ENSG0000(	COPS6	548.5583	-0.23385	3.69E-06	5.20E-05	protein_coding
ENSG0000(	NAA10	447.2868	-0.27679	3.69E-06	5.20E-05	protein_coding
ENSG0000(	ENSG0000(	170.3598	0.336506	3.69E-06	5.20E-05	lncRNA
ENSG0000(	DPYSL3	1089.911	0.714991	3.72E-06	5.23E-05	protein_coding
ENSG0000(	ENSG0000(	54.15699	0.759731	3.71E-06	5.23E-05	lncRNA
ENSG0000(	RNU7-40P	21.7916	0.940947	3.75E-06	5.28E-05	snRNA
ENSG0000(	TAF5	540.9332	-0.20191	3.77E-06	5.30E-05	protein_coding
ENSG0000(	PTBP3	5762.761	-0.28309	3.78E-06	5.31E-05	protein_coding
ENSG0000(	SOCS5	1084.309	0.155277	3.78E-06	5.31E-05	protein_coding
ENSG0000(	SERBP1	3656.929	-0.27466	3.79E-06	5.33E-05	protein_coding
ENSG0000(	COL24A1	187.1963	0.96559	3.82E-06	5.35E-05	protein_coding
ENSG0000(	ENSG0000(	17.33686	0.926847	3.82E-06	5.35E-05	lncRNA
ENSG0000(	MRPL43	404.6901	-0.2668	3.82E-06	5.35E-05	protein_coding
ENSG0000(	ENSG0000(	135.8222	0.357382	3.83E-06	5.36E-05	lncRNA
ENSG0000(	MAD2L1BP	328.1796	-0.3807	3.83E-06	5.37E-05	protein_coding
ENSG0000(	EFNB1	1108.124	-0.53176	3.84E-06	5.38E-05	protein_coding
ENSG0000(	TSPAN11	683.5204	0.672967	3.88E-06	5.43E-05	protein_coding
ENSG0000(	CMAS	678.2557	-0.4829	3.89E-06	5.44E-05	protein_coding
ENSG0000(	TRIM35	686.3378	-0.30647	3.90E-06	5.45E-05	protein_coding
ENSG0000(	OTC	35.04099	-1.13698	3.91E-06	5.46E-05	protein_coding
ENSG0000(	PTRHD1	137.2193	-0.49593	3.91E-06	5.46E-05	protein_coding
ENSG0000(	RPL36A	2183.017	-0.50622	3.91E-06	5.46E-05	protein_coding
ENSG0000(	NTHL1	116.982	-0.39563	3.92E-06	5.48E-05	protein_coding
ENSG0000(	ENSG0000(	41.37016	0.787988	3.93E-06	5.48E-05	lncRNA
ENSG0000(	ENSG0000(	1786.614	0.223576	3.93E-06	5.48E-05	lncRNA
ENSG0000(	CYCS	5013.116	-0.69866	3.95E-06	5.51E-05	protein_coding
ENSG0000(	BZW2	773.5135	-0.35872	3.95E-06	5.51E-05	protein_coding
ENSG0000(	ENSG0000(	50.41279	-0.60128	3.96E-06	5.51E-05	lncRNA
ENSG0000(	ENSG0000(	743.6017	0.240312	3.97E-06	5.53E-05	lncRNA
ENSG0000(	LIFR-AS1	742.082	0.256421	3.97E-06	5.53E-05	lncRNA
ENSG0000(	PTCD3	3586.739	-0.18228	3.98E-06	5.54E-05	protein_coding
ENSG0000(	TMA7	616.3142	-0.39031	3.98E-06	5.54E-05	protein_coding
ENSG0000(	ENSG0000(	32.98174	-0.68824	3.99E-06	5.55E-05	lncRNA
ENSG0000(	ENSG0000(	60.56759	0.565001	3.99E-06	5.55E-05	lncRNA
ENSG0000(	TMUB1	311.8031	-0.32711	4.00E-06	5.55E-05	protein_coding
ENSG0000(	TFCP2	1168.758	-0.19691	4.01E-06	5.57E-05	protein_coding
ENSG0000(	ATP6V0D1-	906.351	0.242332	4.02E-06	5.57E-05	lncRNA
ENSG0000(	TBX19	207.1376	0.406976	4.02E-06	5.57E-05	protein_coding

ENSG0000(	FJX1	19.22463	1.361197	4.03E-06	5.58E-05	protein_coding
ENSG0000(	ENSG0000(	656.3427	0.237176	4.03E-06	5.59E-05	processed_pseudogene
ENSG0000(	MOV10	2159.276	0.193335	4.05E-06	5.61E-05	protein_coding
ENSG0000(	ENSG0000(	209.847	0.543844	4.05E-06	5.61E-05	lncRNA
ENSG0000(	C1QBP	447.8216	-0.47456	4.06E-06	5.62E-05	protein_coding
ENSG0000(	ORAI2	3711.926	0.231	4.06E-06	5.62E-05	protein_coding
ENSG0000(	OSMR-DT	222.7356	0.340918	4.06E-06	5.62E-05	lncRNA
ENSG0000(	ENSG0000(	58.95271	0.726959	4.08E-06	5.64E-05	lncRNA
ENSG0000(	GCNT2	1391.267	-0.42151	4.08E-06	5.64E-05	protein_coding
ENSG0000(	TIPARP	1079.924	0.38269	4.09E-06	5.65E-05	protein_coding
ENSG0000(	INPP4A	3207.581	0.243276	4.10E-06	5.66E-05	protein_coding
ENSG0000(	ENSG0000(	34.77512	0.686787	4.10E-06	5.66E-05	lncRNA
ENSG0000(	FADS2	559.1475	0.550279	4.11E-06	5.66E-05	protein_coding
ENSG0000(	MMP28	339.9801	-0.84031	4.12E-06	5.67E-05	protein_coding
ENSG0000(	RNU6-16P	89.56794	0.610123	4.13E-06	5.69E-05	snRNA
ENSG0000(	LL22NC03-(	573.9181	0.197211	4.14E-06	5.70E-05	lncRNA
ENSG0000(	GBA2	3612.827	-0.37938	4.14E-06	5.70E-05	protein_coding
ENSG0000(	RETSAT	2912.024	-0.64151	4.19E-06	5.76E-05	protein_coding
ENSG0000(	BTN2A3P	601.0641	0.256226	4.20E-06	5.78E-05	transcribed_unprocessed_pseudo
ENSG0000(	PTGFR	147.1207	1.096778	4.25E-06	5.84E-05	protein_coding
ENSG0000(	ENSG0000(	156.8566	0.321295	4.25E-06	5.84E-05	lncRNA
ENSG0000(	RPS20P33	36.95086	-0.66668	4.26E-06	5.85E-05	processed_pseudogene
ENSG0000(	SYN3	1314.218	0.334633	4.26E-06	5.85E-05	protein_coding
ENSG0000(	PDCD1LG2	89.96329	0.996314	4.27E-06	5.86E-05	protein_coding
ENSG0000(	PTP4A1P3	15.11989	1.176025	4.27E-06	5.86E-05	processed_pseudogene
ENSG0000(	SLC25A36	2606.466	0.278186	4.30E-06	5.90E-05	protein_coding
ENSG0000(	ENSG0000(	71.59674	0.451293	4.30E-06	5.90E-05	TEC
ENSG0000(	ATL2	1534.787	-0.22093	4.31E-06	5.90E-05	protein_coding
ENSG0000(	CYBC1	3369.455	0.372132	4.32E-06	5.91E-05	protein_coding
ENSG0000(	ZNF468	760.8802	-0.28071	4.32E-06	5.91E-05	protein_coding
ENSG0000(	LINC02631	59.85342	0.473465	4.34E-06	5.94E-05	lncRNA
ENSG0000(	TMEM11	266.997	-0.309	4.34E-06	5.94E-05	protein_coding
ENSG0000(	ARMH3	1403.466	-0.25348	4.35E-06	5.95E-05	protein_coding
ENSG0000(	ENSG0000(	228.1845	0.316837	4.37E-06	5.96E-05	lncRNA
ENSG0000(	HNRNPA1P	9.223449	1.491194	4.37E-06	5.97E-05	processed_pseudogene
ENSG0000(	SUPT7L	2057.012	0.132321	4.40E-06	6.00E-05	protein_coding
ENSG0000(	CBX1	468.7594	-0.2839	4.40E-06	6.01E-05	protein_coding
ENSG0000(	PITPNM3	1303.599	-0.52254	4.41E-06	6.01E-05	protein_coding
ENSG0000(	HCAR1	590.5424	-0.19703	4.49E-06	6.12E-05	protein_coding
ENSG0000(	SH2D7	73.22396	-1.09221	4.50E-06	6.14E-05	protein_coding
ENSG0000(	AKAP7	434.3898	-0.30175	4.51E-06	6.15E-05	protein_coding
ENSG0000(	EMSLR	1129.723	-0.18808	4.52E-06	6.15E-05	lncRNA
ENSG0000(	ENSG0000(	197.1768	0.314297	4.52E-06	6.15E-05	lncRNA
ENSG0000(	ENSG0000(	144.508	0.291557	4.54E-06	6.18E-05	lncRNA
ENSG0000(	ANKRD54	691.3915	-0.17771	4.56E-06	6.20E-05	protein_coding
ENSG0000(	ENSG0000(	12.7836	1.099466	4.56E-06	6.20E-05	lncRNA
ENSG0000(	ENSG0000(	224.0287	0.331676	4.56E-06	6.20E-05	lncRNA

ENSG0000(	ENSG0000(	26.86121	0.629134	4.57E-06	6.20E-05	lncRNA
ENSG0000(	ENSG0000(	36.10021	0.728533	4.57E-06	6.21E-05	transcribed_processed_pseudoge
ENSG0000(	GABRP	863.6406	0.316936	4.59E-06	6.23E-05	protein_coding
ENSG0000(	ENSG0000(	32.10052	0.559261	4.63E-06	6.27E-05	lncRNA
ENSG0000(	ADCYAP1	26.48873	1.633038	4.63E-06	6.27E-05	protein_coding
ENSG0000(	HNRNPA1	10089.98	-0.25562	4.63E-06	6.28E-05	protein_coding
ENSG0000(	ENSG0000(	2200.228	0.293658	4.65E-06	6.29E-05	lncRNA
ENSG0000(	ENSG0000(	111.6056	0.397372	4.66E-06	6.31E-05	lncRNA
ENSG0000(	TMCO6	439.3344	-0.31774	4.67E-06	6.32E-05	protein_coding
ENSG0000(	RNU5F-1	10.47467	1.670001	4.68E-06	6.33E-05	snRNA
ENSG0000(	MRPL21	301.5043	-0.41103	4.71E-06	6.37E-05	protein_coding
ENSG0000(	THUMPD3-	3844.272	0.181096	4.72E-06	6.37E-05	lncRNA
ENSG0000(	IGHM	7279.831	1.061372	4.73E-06	6.39E-05	IG_C_gene
ENSG0000(	RPL30	2845.545	-0.41	4.73E-06	6.39E-05	protein_coding
ENSG0000(	PAK1IP1	130.2257	-0.50395	4.75E-06	6.41E-05	protein_coding
ENSG0000(	PRIM1	321.425	-0.32715	4.78E-06	6.45E-05	protein_coding
ENSG0000(	RPS15	1210.307	-0.46848	4.78E-06	6.45E-05	protein_coding
ENSG0000(	ELOVL6	1795.338	-0.64992	4.81E-06	6.48E-05	protein_coding
ENSG0000(	ENSG0000(	1719.595	0.414974	4.81E-06	6.48E-05	lncRNA
ENSG0000(	WIPF1	2929.614	0.538709	4.83E-06	6.50E-05	protein_coding
ENSG0000(	CTSO	1235.803	0.195917	4.84E-06	6.51E-05	protein_coding
ENSG0000(	MT-RNR1	15095.11	-0.78154	4.84E-06	6.51E-05	Mt_rRNA
ENSG0000(	ROMO1	123.6863	-0.64396	4.84E-06	6.52E-05	protein_coding
ENSG0000(	LINC02038	190.524	-0.82983	4.86E-06	6.53E-05	lncRNA
ENSG0000(	PALM2AKA	2406.08	0.500373	4.86E-06	6.53E-05	protein_coding
ENSG0000(	DDX49	255.3907	-0.42782	4.86E-06	6.53E-05	protein_coding
ENSG0000(	DNAJC11	3431.982	-0.24649	4.87E-06	6.53E-05	protein_coding
ENSG0000(	ENSG0000(	11.04484	1.317223	4.88E-06	6.56E-05	lncRNA
ENSG0000(	SLC16A7	1243.272	0.39109	4.90E-06	6.57E-05	protein_coding
ENSG0000(	PKIB	1112.884	-0.63414	4.90E-06	6.57E-05	protein_coding
ENSG0000(	THYN1	217.3186	-0.3416	4.90E-06	6.57E-05	protein_coding
ENSG0000(	ENSG0000(	40.30525	0.64569	4.92E-06	6.59E-05	TEC
ENSG0000(	SLC51A	927.995	-1.06849	4.93E-06	6.60E-05	protein_coding
ENSG0000(	ELL3	353.2171	-0.51732	4.97E-06	6.65E-05	protein_coding
ENSG0000(	ENSG0000(	87.42565	0.452877	4.99E-06	6.67E-05	lncRNA
ENSG0000(	ZNF480	1263.006	-0.24435	4.99E-06	6.67E-05	protein_coding
ENSG0000(	SNHG8	946.6208	-0.48502	5.00E-06	6.69E-05	lncRNA
ENSG0000(	EDEM1	3304.521	0.375583	5.02E-06	6.71E-05	protein_coding
ENSG0000(	CEP20	589.2437	-0.29165	5.04E-06	6.73E-05	protein_coding
ENSG0000(	FBXO39	17.51572	1.127857	5.04E-06	6.73E-05	protein_coding
ENSG0000(	ENSG0000(	385.9387	0.34718	5.04E-06	6.73E-05	lncRNA
ENSG0000(	SMCO2	12.72801	1.265717	5.05E-06	6.74E-05	protein_coding
ENSG0000(	ENSG0000(	225.622	0.2777	5.08E-06	6.77E-05	lncRNA
ENSG0000(	TBCEL-TEC	33.76032	0.688656	5.08E-06	6.78E-05	protein_coding
ENSG0000(	CANT1	3352.352	-0.43259	5.10E-06	6.79E-05	protein_coding
ENSG0000(	ZNFX1	3328.018	0.327333	5.10E-06	6.79E-05	protein_coding
ENSG0000(	MIR8061	14.7907	1.409468	5.12E-06	6.82E-05	miRNA

ENSG0000(	BMS1	1325.246	-0.26023	5.15E-06	6.85E-05	protein_coding
ENSG0000(	MT-ND4L	8091.302	-0.68257	5.17E-06	6.87E-05	protein_coding
ENSG0000(	ENSG0000(	279.5556	0.443993	5.19E-06	6.90E-05	lncRNA
ENSG0000(	TMEM98	1273.256	-0.33205	5.20E-06	6.91E-05	protein_coding
ENSG0000(	OSMR	1080.786	0.623133	5.21E-06	6.92E-05	protein_coding
ENSG0000(	ENSG0000(	206.1648	-0.2875	5.22E-06	6.92E-05	lncRNA
ENSG0000(	RAB39B	83.51092	0.699087	5.21E-06	6.92E-05	protein_coding
ENSG0000(	SF3B5	229.4422	-0.55567	5.22E-06	6.93E-05	protein_coding
ENSG0000(	AADACL3	25.63779	0.729589	5.25E-06	6.96E-05	protein_coding
ENSG0000(	ENSG0000(	91.66595	0.630271	5.25E-06	6.96E-05	lncRNA
ENSG0000(	HAS3	948.3964	-1.17268	5.25E-06	6.96E-05	protein_coding
ENSG0000(	ST6GALNA(	78.83019	1.231717	5.26E-06	6.97E-05	protein_coding
ENSG0000(	ENSG0000(	44.03866	0.859931	5.27E-06	6.98E-05	lncRNA
ENSG0000(	RPF2	402.5016	-0.34914	5.28E-06	6.98E-05	protein_coding
ENSG0000(	MTFP1	114.0147	-0.51096	5.28E-06	6.99E-05	protein_coding
ENSG0000(	SNX15	839.6672	-0.28611	5.31E-06	7.02E-05	protein_coding
ENSG0000(	WNK4	1044.661	-0.45527	5.31E-06	7.02E-05	protein_coding
ENSG0000(	MDGA1	344.0028	0.8161	5.32E-06	7.03E-05	protein_coding
ENSG0000(	SMIM22	383.3449	-0.67254	5.32E-06	7.03E-05	protein_coding
ENSG0000(	FBXO5	330.71	-0.31899	5.33E-06	7.03E-05	protein_coding
ENSG0000(	P2RX7	785.7478	0.371613	5.33E-06	7.03E-05	protein_coding
ENSG0000(	PLEK2	330.958	-0.67752	5.33E-06	7.03E-05	protein_coding
ENSG0000(	ENSG0000(	94.54066	-0.41758	5.36E-06	7.06E-05	lncRNA
ENSG0000(	FARSB	1477.277	-0.29461	5.36E-06	7.06E-05	protein_coding
ENSG0000(	UBE2T	69.02721	-0.7781	5.37E-06	7.07E-05	protein_coding
ENSG0000(	LINC02324	1098.85	0.178577	5.38E-06	7.09E-05	lncRNA
ENSG0000(	FANCE	175.5538	-0.37204	5.41E-06	7.11E-05	protein_coding
ENSG0000(	MICE	90.22411	0.607229	5.42E-06	7.12E-05	transcribed_unprocessed_pseudo
ENSG0000(	TMEM97	333.1884	-0.47778	5.42E-06	7.12E-05	protein_coding
ENSG0000(	ZBTB8A	1936.526	-0.19203	5.42E-06	7.13E-05	protein_coding
ENSG0000(	COL8A1	948.1564	0.457158	5.43E-06	7.13E-05	protein_coding
ENSG0000(	HSP90B2P	101.1113	-0.59281	5.43E-06	7.13E-05	processed_pseudogene
ENSG0000(	EBP	298.583	-0.47571	5.44E-06	7.14E-05	protein_coding
ENSG0000(	ENSG0000(	140.9474	0.325115	5.48E-06	7.19E-05	lncRNA
ENSG0000(	TXLNB	99.86629	0.861005	5.50E-06	7.22E-05	protein_coding
ENSG0000(	DDX27	1232.447	-0.26102	5.51E-06	7.22E-05	protein_coding
ENSG0000(	NCBP2AS2	155.4615	-0.35284	5.52E-06	7.24E-05	protein_coding
ENSG0000(	NEK4	1300.76	-0.26854	5.54E-06	7.25E-05	protein_coding
ENSG0000(	NDUFB3	335.1352	-0.58468	5.54E-06	7.26E-05	protein_coding
ENSG0000(	SELENOM	416.8852	0.494824	5.55E-06	7.26E-05	protein_coding
ENSG0000(	RHBDF2	1448.363	0.406414	5.57E-06	7.29E-05	protein_coding
ENSG0000(	PCBD1	287.2394	-0.44615	5.57E-06	7.29E-05	protein_coding
ENSG0000(	JADE1	1303.554	0.363076	5.58E-06	7.30E-05	protein_coding
ENSG0000(	AK3	2868.89	-0.39002	5.58E-06	7.30E-05	protein_coding
ENSG0000(	COX6B1	803.7235	-0.55276	5.61E-06	7.32E-05	protein_coding
ENSG0000(	MELK	280.3244	-0.41142	5.62E-06	7.34E-05	protein_coding
ENSG0000(	PTPN4	1778.222	-0.2911	5.63E-06	7.34E-05	protein_coding

ENSG0000(	SH3PXD2B	1035.483	0.771755	5.63E-06	7.35E-05	protein_coding
ENSG0000(	HIRA	907.7397	-0.28966	5.64E-06	7.35E-05	protein_coding
ENSG0000(	ENSG0000(	320.08	-0.25571	5.69E-06	7.42E-05	lncRNA
ENSG0000(	HSPA4	1742.311	-0.28412	5.70E-06	7.43E-05	protein_coding
ENSG0000(	ENSG0000(	354.2754	-0.37984	5.70E-06	7.43E-05	protein_coding
ENSG0000(	UBL7-DT	3028.265	0.225482	5.78E-06	7.52E-05	lncRNA
ENSG0000(	SLC6A13	16.5052	1.247908	5.78E-06	7.53E-05	protein_coding
ENSG0000(	ENSG0000(	232.4783	0.513441	5.80E-06	7.54E-05	lncRNA
ENSG0000(	SH2B1	3040.584	-0.24736	5.80E-06	7.54E-05	protein_coding
ENSG0000(	H2AX	256.8317	-0.44863	5.81E-06	7.55E-05	protein_coding
ENSG0000(	ZNF527	1084.597	-0.2115	5.81E-06	7.55E-05	protein_coding
ENSG0000(	CHPT1	2032.333	-0.4359	5.83E-06	7.58E-05	protein_coding
ENSG0000(	ENSG0000(	452.6101	0.309064	5.84E-06	7.58E-05	lncRNA
ENSG0000(	GPR78	127.7283	0.480586	5.84E-06	7.58E-05	protein_coding
ENSG0000(	ABCG2	1112.736	-1.38231	5.85E-06	7.59E-05	protein_coding
ENSG0000(	NDUFB8	711.339	-0.29596	5.86E-06	7.59E-05	protein_coding
ENSG0000(	COMTD1	165.9407	-0.50648	5.88E-06	7.62E-05	protein_coding
ENSG0000(	CHPF	342.8731	0.526395	5.91E-06	7.66E-05	protein_coding
ENSG0000(	KLHL22	671.3599	0.29107	5.91E-06	7.66E-05	protein_coding
ENSG0000(	TSIX	9168.822	0.255047	5.92E-06	7.66E-05	lncRNA
ENSG0000(	ENSG0000(	17.35463	1.181573	5.93E-06	7.68E-05	lncRNA
ENSG0000(	SMG5	2310.747	-0.24249	5.99E-06	7.74E-05	protein_coding
ENSG0000(	VWA1	341.6411	0.540334	5.99E-06	7.74E-05	protein_coding
ENSG0000(	ENSG0000(	278.5208	0.315031	5.99E-06	7.74E-05	TEC
ENSG0000(	FOXD2-AS1	470.0884	-0.64765	6.02E-06	7.78E-05	lncRNA
ENSG0000(	CCDC112	351.0925	-0.43794	6.02E-06	7.78E-05	protein_coding
ENSG0000(	ARHGDIA	2233.91	-0.42868	6.03E-06	7.78E-05	protein_coding
ENSG0000(	LINC01138	596.6731	0.398351	6.05E-06	7.80E-05	lncRNA
ENSG0000(	FGF7	545.7016	1.104194	6.06E-06	7.82E-05	protein_coding
ENSG0000(	IDH3G	503.5133	-0.25314	6.08E-06	7.84E-05	protein_coding
ENSG0000(	ENSG0000(	34.81914	0.571259	6.10E-06	7.86E-05	lncRNA
ENSG0000(	ZCCHC17	257.7468	-0.25367	6.10E-06	7.86E-05	protein_coding
ENSG0000(	DDOST	1136.257	-0.32674	6.11E-06	7.87E-05	protein_coding
ENSG0000(	SLC39A2	20.40509	-1.20902	6.13E-06	7.88E-05	protein_coding
ENSG0000(	ENSG0000(	184.9413	-0.34758	6.15E-06	7.90E-05	lncRNA
ENSG0000(	ZDHHC3	4760.292	-0.37674	6.14E-06	7.90E-05	protein_coding
ENSG0000(	RAB21	3483.114	-0.30444	6.15E-06	7.90E-05	protein_coding
ENSG0000(	ENSG0000(	284.2521	0.256753	6.17E-06	7.92E-05	TEC
ENSG0000(	SMARCC1	2397.177	-0.26035	6.16E-06	7.92E-05	protein_coding
ENSG0000(	ACSL5	4298.385	-0.46445	6.18E-06	7.93E-05	protein_coding
ENSG0000(	ENSG0000(	65.33418	-0.46801	6.18E-06	7.93E-05	lncRNA
ENSG0000(	ENSG0000(	218.3483	0.387908	6.19E-06	7.93E-05	lncRNA
ENSG0000(	ENSG0000(	153.3203	0.291643	6.19E-06	7.94E-05	TEC
ENSG0000(	JSRP1	15.14679	1.098208	6.20E-06	7.95E-05	protein_coding
ENSG0000(	PEX19	859.9781	-0.31258	6.20E-06	7.95E-05	protein_coding
ENSG0000(	RPPH1	33390.53	-0.58532	6.24E-06	7.98E-05	ribozyme
ENSG0000(	TSEN54	731.4987	-0.21748	6.24E-06	7.98E-05	protein_coding

ENSG0000(	RGS2	383.9746	0.826608	6.26E-06	8.01E-05	protein_coding
ENSG0000(	SERPINB9P	948.6072	0.293005	6.28E-06	8.03E-05	lncRNA
ENSG0000(	KIF19	244.0748	0.40709	6.29E-06	8.04E-05	protein_coding
ENSG0000(	H2AC20	195.574	-0.56776	6.29E-06	8.04E-05	protein_coding
ENSG0000(	APOBEC3A	304.7789	1.014012	6.31E-06	8.06E-05	protein_coding
ENSG0000(	TRAPPC1	240.857	-0.35462	6.34E-06	8.09E-05	protein_coding
ENSG0000(	DENND4C	4181.667	-0.30777	6.36E-06	8.11E-05	protein_coding
ENSG0000(	CEP170	1620.167	0.337811	6.36E-06	8.11E-05	protein_coding
ENSG0000(	CFAP298	571.0422	-0.22816	6.38E-06	8.13E-05	protein_coding
ENSG0000(	EMC1	3351.54	-0.10335	6.43E-06	8.20E-05	protein_coding
ENSG0000(	ENSG0000(	79.72003	0.813532	6.44E-06	8.20E-05	lncRNA
ENSG0000(	PARVB	1247.719	0.330994	6.43E-06	8.20E-05	protein_coding
ENSG0000(	RFX3	1126.412	0.333207	6.43E-06	8.20E-05	protein_coding
ENSG0000(	DPF1	183.5381	0.314973	6.45E-06	8.21E-05	protein_coding
ENSG0000(	ZNF302	2529.949	0.506244	6.46E-06	8.22E-05	protein_coding
ENSG0000(	ENSG0000(	156.7355	0.412749	6.48E-06	8.24E-05	lncRNA
ENSG0000(	HSD17B10	472.4428	-0.38993	6.49E-06	8.25E-05	protein_coding
ENSG0000(	CNNM3	1288.724	-0.26666	6.50E-06	8.26E-05	protein_coding
ENSG0000(	FOXD2	381.8363	-0.66163	6.51E-06	8.27E-05	protein_coding
ENSG0000(	MARS2	323.3559	-0.35362	6.52E-06	8.27E-05	protein_coding
ENSG0000(	BCL6B	155.4078	0.750614	6.53E-06	8.29E-05	protein_coding
ENSG0000(	ENSG0000(	638.9093	0.311406	6.54E-06	8.30E-05	lncRNA
ENSG0000(	NTNG2	123.7036	0.706426	6.55E-06	8.31E-05	protein_coding
ENSG0000(	ZNRF3	538.3386	-0.47561	6.55E-06	8.31E-05	protein_coding
ENSG0000(	XRCC6	1361.617	-0.26972	6.56E-06	8.31E-05	protein_coding
ENSG0000(	ENSG0000(	289.4065	0.237691	6.60E-06	8.35E-05	TEC
ENSG0000(	MSX2	19.56537	1.456616	6.61E-06	8.36E-05	protein_coding
ENSG0000(	RELT	727.2569	0.721221	6.62E-06	8.38E-05	protein_coding
ENSG0000(	KCNMB3	472.8744	0.278071	6.65E-06	8.41E-05	protein_coding
ENSG0000(	PLIN2	857.9727	-0.54734	6.65E-06	8.41E-05	protein_coding
ENSG0000(	TXNDC15	1752.391	0.317254	6.66E-06	8.41E-05	protein_coding
ENSG0000(	UBE2I	1914.893	-0.22416	6.65E-06	8.41E-05	protein_coding
ENSG0000(	APMAP	882.1329	-0.31053	6.67E-06	8.41E-05	protein_coding
ENSG0000(	ENSG0000(	177.9878	0.44481	6.66E-06	8.41E-05	lncRNA
ENSG0000(	MAF1	493.333	-0.30726	6.66E-06	8.41E-05	protein_coding
ENSG0000(	MIR8075	109.5989	-0.42328	6.67E-06	8.42E-05	miRNA
ENSG0000(	RPS6KB2	1006.377	-0.27341	6.69E-06	8.43E-05	protein_coding
ENSG0000(	ANKRD6	474.8123	0.291915	6.72E-06	8.47E-05	protein_coding
ENSG0000(	FASTKD1	1846.588	-0.35607	6.73E-06	8.47E-05	protein_coding
ENSG0000(	ENSG0000(	453.1856	0.275099	6.73E-06	8.47E-05	lncRNA
ENSG0000(	NOA1	373.3699	-0.36709	6.75E-06	8.49E-05	protein_coding
ENSG0000(	COL1A2	5737.871	0.973225	6.75E-06	8.50E-05	protein_coding
ENSG0000(	BATF	54.80487	1.041248	6.82E-06	8.58E-05	protein_coding
ENSG0000(	ENSG0000(	64.07823	0.965102	6.83E-06	8.58E-05	processed_pseudogene
ENSG0000(	FAM238C	223.4292	0.377042	6.83E-06	8.58E-05	transcribed_unprocessed_pseudo
ENSG0000(	MARK3	8389.188	0.131347	6.83E-06	8.58E-05	protein_coding
ENSG0000(	ZFYVE19	546.0133	-0.36493	6.83E-06	8.58E-05	protein_coding

ENSG0000(	ENSG0000(	156.8824	0.372505	6.84E-06	8.59E-05	lncRNA
ENSG0000(	ENSG0000(	2220.081	0.405401	6.87E-06	8.62E-05	lncRNA
ENSG0000(	ARMCX3	927.7627	0.330128	6.88E-06	8.62E-05	protein_coding
ENSG0000(	SIK1	251.713	1.308192	6.88E-06	8.62E-05	protein_coding
ENSG0000(	NPR2	685.4533	-0.31483	6.88E-06	8.63E-05	protein_coding
ENSG0000(	HSD17B2	3239.734	-0.86987	6.94E-06	8.69E-05	protein_coding
ENSG0000(	ANO4	23.5276	-1.37798	6.94E-06	8.69E-05	protein_coding
ENSG0000(	ITGA6-AS1	38.11718	0.747312	6.94E-06	8.69E-05	lncRNA
ENSG0000(	ENSG0000(	810.9654	0.344252	6.97E-06	8.73E-05	TEC
ENSG0000(	PPP1R14B	460.2434	-0.37861	7.03E-06	8.80E-05	protein_coding
ENSG0000(	TXNDC17	453.3136	-0.66236	7.04E-06	8.80E-05	protein_coding
ENSG0000(	LINC00565	423.4569	0.229044	7.04E-06	8.80E-05	lncRNA
ENSG0000(	CHORDC1P	233.6273	0.514876	7.05E-06	8.80E-05	transcribed_processed_pseudoge
ENSG0000(	ERGIC3	2053.119	-0.22849	7.05E-06	8.81E-05	protein_coding
ENSG0000(	CALHM5	152.8911	0.491589	7.08E-06	8.84E-05	protein_coding
ENSG0000(	LINC02150	51.58605	0.81986	7.09E-06	8.85E-05	lncRNA
ENSG0000(	KCNA3	436.3222	0.595475	7.10E-06	8.85E-05	protein_coding
ENSG0000(	RAB11A	2823.641	-0.40564	7.12E-06	8.88E-05	protein_coding
ENSG0000(	MIR5194	36.90222	0.699919	7.16E-06	8.92E-05	miRNA
ENSG0000(	SLC16A14	191.8687	0.534158	7.16E-06	8.93E-05	protein_coding
ENSG0000(	FRMD6-AS:	289.4074	0.241397	7.19E-06	8.95E-05	lncRNA
ENSG0000(	LINC00311	524.3488	0.247379	7.24E-06	9.02E-05	lncRNA
ENSG0000(	ATF1	317.0429	-0.30152	7.25E-06	9.02E-05	protein_coding
ENSG0000(	APOBEC3B	132.0716	-1.07468	7.26E-06	9.03E-05	protein_coding
ENSG0000(	CARD16	290.6182	0.577423	7.27E-06	9.04E-05	protein_coding
ENSG0000(	CYRIA	539.5281	0.63609	7.27E-06	9.04E-05	protein_coding
ENSG0000(	C11orf96	176.927	0.778645	7.30E-06	9.07E-05	protein_coding
ENSG0000(	NAA38	172.9985	-0.4135	7.31E-06	9.08E-05	protein_coding
ENSG0000(	NMNAT1	1632.266	-0.1898	7.32E-06	9.09E-05	protein_coding
ENSG0000(	DSC2	6846.082	-0.66157	7.36E-06	9.13E-05	protein_coding
ENSG0000(	ENGASE	3208.051	-0.32778	7.38E-06	9.16E-05	protein_coding
ENSG0000(	ENSG0000(	134.9173	0.494698	7.39E-06	9.16E-05	lncRNA
ENSG0000(	ECI1	423.4857	-0.36018	7.39E-06	9.16E-05	protein_coding
ENSG0000(	SLC7A14-A:	611.9484	0.200561	7.44E-06	9.21E-05	lncRNA
ENSG0000(	PKD1L3	51.29203	0.851915	7.44E-06	9.22E-05	protein_coding
ENSG0000(	GFPT2	118.8203	1.410864	7.51E-06	9.30E-05	protein_coding
ENSG0000(	CNTN4	578.6769	-0.68455	7.52E-06	9.31E-05	protein_coding
ENSG0000(	RPS7	3104.261	-0.45119	7.52E-06	9.31E-05	protein_coding
ENSG0000(	ENSG0000(	1160.475	0.19102	7.57E-06	9.36E-05	lncRNA
ENSG0000(	ENSG0000(	177.3839	0.331576	7.62E-06	9.42E-05	lncRNA
ENSG0000(	HMGB1P21	66.09923	-0.59227	7.62E-06	9.42E-05	processed_pseudogene
ENSG0000(	PCCB	791.9532	-0.29706	7.63E-06	9.42E-05	protein_coding
ENSG0000(	DPP4-DT	132.664	0.490537	7.63E-06	9.43E-05	lncRNA
ENSG0000(	RPL38	1918.661	-0.43495	7.64E-06	9.43E-05	protein_coding
ENSG0000(	HOOK1	1810.096	-0.48887	7.65E-06	9.43E-05	protein_coding
ENSG0000(	DHX35	798.2751	-0.24215	7.67E-06	9.46E-05	protein_coding
ENSG0000(	SSNA1	162.1689	-0.4324	7.67E-06	9.46E-05	protein_coding

ENSG0000(	ENSG0000(	180.5974	0.461285	7.71E-06	9.50E-05	TEC
ENSG0000(	ENSG0000(	70.54739	0.600969	7.71E-06	9.50E-05	processed_pseudogene
ENSG0000(	ENSG0000(	497.2184	0.296947	7.74E-06	9.53E-05	TEC
ENSG0000(	TTC3P1	184.5516	-0.43177	7.80E-06	9.60E-05	processed_pseudogene
ENSG0000(	RPL35P3	36.63501	-0.73725	7.80E-06	9.60E-05	processed_pseudogene
ENSG0000(	ALOX15B	60.02064	1.021756	7.82E-06	9.61E-05	protein_coding
ENSG0000(	ENSG0000(	79.45677	0.509056	7.83E-06	9.62E-05	lncRNA
ENSG0000(	CLEC4A	71.27855	0.785124	7.84E-06	9.64E-05	protein_coding
ENSG0000(	SHCBP1L	50.91544	-0.47433	7.85E-06	9.64E-05	protein_coding
ENSG0000(	TSHR	821.1386	0.268541	7.85E-06	9.64E-05	protein_coding
ENSG0000(	RPL12	3412.881	-0.45109	7.88E-06	9.67E-05	protein_coding
ENSG0000(	TRAP1	863.619	-0.22689	7.89E-06	9.68E-05	protein_coding
ENSG0000(	ADORA2B	763.3279	-0.64875	7.90E-06	9.69E-05	protein_coding
ENSG0000(	ENSG0000(	231.6357	0.244902	7.93E-06	9.73E-05	lncRNA
ENSG0000(	SETBP1	2102.344	0.341119	7.94E-06	9.73E-05	protein_coding
ENSG0000(	CD93	1374.454	0.661158	7.95E-06	9.74E-05	protein_coding
ENSG0000(	KRT19	2854.346	-0.84911	7.96E-06	9.75E-05	protein_coding
ENSG0000(	LINC01943	49.98396	1.330086	7.97E-06	9.76E-05	lncRNA
ENSG0000(	THUMPD3	778.7332	-0.31872	7.97E-06	9.76E-05	protein_coding
ENSG0000(	LSM4	525.065	-0.37203	7.98E-06	9.76E-05	protein_coding
ENSG0000(	CMIP	3698.186	0.198637	8.00E-06	9.78E-05	protein_coding
ENSG0000(	HMGN1P8	182.4823	0.354565	8.01E-06	9.79E-05	processed_pseudogene
ENSG0000(	PPP6R3	4080.505	-0.13965	8.03E-06	9.81E-05	protein_coding
ENSG0000(	ENSG0000(	13.79065	1.174947	8.04E-06	9.81E-05	lncRNA
ENSG0000(	ENSG0000(	23.5468	1.373544	8.05E-06	9.81E-05	lncRNA
ENSG0000(	NACC1	892.069	-0.34037	8.04E-06	9.81E-05	protein_coding
ENSG0000(	SPINT2	2823.653	-0.58269	8.05E-06	9.81E-05	protein_coding
ENSG0000(	XAB2	509.926	-0.25774	8.06E-06	9.82E-05	protein_coding
ENSG0000(	BHLHE40	1185.747	0.547633	8.06E-06	9.82E-05	protein_coding
ENSG0000(	DHX16	1088.826	-0.25285	8.07E-06	9.83E-05	protein_coding
ENSG0000(	ENSG0000(	332.5342	0.30358	8.08E-06	9.84E-05	lncRNA
ENSG0000(	PPP4R1	1444.481	0.305092	8.10E-06	9.86E-05	protein_coding
ENSG0000(	DHRS1	706.9813	-0.29225	8.12E-06	9.88E-05	protein_coding
ENSG0000(	CPT2	1439.059	-0.29348	8.20E-06	9.97E-05	protein_coding
ENSG0000(	RANBP3	1432.481	-0.1478	8.20E-06	9.97E-05	protein_coding
ENSG0000(	ABCC3	11031.16	-0.52634	8.24E-06	0.0001	protein_coding
ENSG0000(	UBE2Q1	809.7926	-0.40773	8.26E-06	0.0001	protein_coding
ENSG0000(	H2AJ	603.0593	-0.44607	8.30E-06	0.000101	protein_coding
ENSG0000(	KRIT1	5390.753	0.128926	8.30E-06	0.000101	protein_coding
ENSG0000(	TGIF2	608.884	0.449907	8.30E-06	0.000101	protein_coding
ENSG0000(	BORA	315.812	-0.37585	8.34E-06	0.000101	protein_coding
ENSG0000(	MAP2K2	2329.17	-0.37579	8.34E-06	0.000101	protein_coding
ENSG0000(	AK7	310.3372	-0.28966	8.35E-06	0.000101	protein_coding
ENSG0000(	NFYA	1669.078	-0.2128	8.34E-06	0.000101	protein_coding
ENSG0000(	ENSG0000(	358.0232	0.306841	8.36E-06	0.000101	lncRNA
ENSG0000(	LINC02887	472.5812	0.654309	8.38E-06	0.000101	lncRNA
ENSG0000(	LINC01182	688.6078	0.226465	8.41E-06	0.000102	lncRNA

ENSG0000(	PDCD11	1270.893	-0.26461	8.42E-06	0.000102	protein_coding
ENSG0000(	NCALD	969.0674	0.31921	8.46E-06	0.000102	protein_coding
ENSG0000(	ENSG0000(	37.32804	0.567994	8.50E-06	0.000103	lncRNA
ENSG0000(	ENSG0000(	941.2516	-0.4731	8.50E-06	0.000103	lncRNA
ENSG0000(	ESF1	493.1797	-0.30429	8.51E-06	0.000103	protein_coding
ENSG0000(	FNIP1	1968.965	0.181806	8.52E-06	0.000103	protein_coding
ENSG0000(	RPS3AP19	167.4285	-0.684	8.53E-06	0.000103	processed_pseudogene
ENSG0000(	ZNF182	457.2428	0.334858	8.53E-06	0.000103	protein_coding
ENSG0000(	TOMM22	660.9752	-0.47219	8.54E-06	0.000103	protein_coding
ENSG0000(	ENSG0000(	523.4989	0.323988	8.55E-06	0.000103	TEC
ENSG0000(	SBK1	615.2081	0.719824	8.55E-06	0.000103	protein_coding
ENSG0000(	VPS16	611.8208	-0.26172	8.55E-06	0.000103	protein_coding
ENSG0000(	BAG6	2484.68	-0.2295	8.56E-06	0.000103	protein_coding
ENSG0000(	NOXA1	339.4956	-0.58077	8.58E-06	0.000103	protein_coding
ENSG0000(	VPS33B-DT	394.6547	-0.26907	8.59E-06	0.000103	lncRNA
ENSG0000(	CPT1A	6035.916	-0.52485	8.61E-06	0.000104	protein_coding
ENSG0000(	SUFU	517.483	0.274162	8.65E-06	0.000104	protein_coding
ENSG0000(	SF3A1	1551.748	-0.2594	8.65E-06	0.000104	protein_coding
ENSG0000(	CA2	8155.796	-0.97872	8.66E-06	0.000104	protein_coding
ENSG0000(	ECSIT	1122.038	-0.22525	8.67E-06	0.000104	protein_coding
ENSG0000(	KLHL7-DT	688.8196	0.256804	8.68E-06	0.000104	lncRNA
ENSG0000(	MCTP1	669.1656	0.641232	8.72E-06	0.000105	protein_coding
ENSG0000(	ENSG0000(	171.9958	0.58317	8.73E-06	0.000105	lncRNA
ENSG0000(	ENSG0000(	391.476	0.227245	8.75E-06	0.000105	lncRNA
ENSG0000(	GABRR2	476.7429	0.38074	8.79E-06	0.000105	protein_coding
ENSG0000(	C2CD4A	209.8868	0.445457	8.85E-06	0.000106	protein_coding
ENSG0000(	FGFR4	742.2736	-0.5642	8.85E-06	0.000106	protein_coding
ENSG0000(	ENSG0000(	123.8726	0.555283	8.88E-06	0.000106	lncRNA
ENSG0000(	ACY1	735.0582	-0.32885	8.89E-06	0.000106	protein_coding
ENSG0000(	FGFR1OP2	1418.747	0.229082	8.91E-06	0.000107	protein_coding
ENSG0000(	SOX11	96.46152	0.566232	8.94E-06	0.000107	protein_coding
ENSG0000(	FAM124A	283.7536	0.462846	8.96E-06	0.000107	protein_coding
ENSG0000(	INTS8	1924.33	-0.21664	8.96E-06	0.000107	protein_coding
ENSG0000(	POLR2H	1261.286	-0.28339	8.97E-06	0.000107	protein_coding
ENSG0000(	SLC22A3	262.7449	-0.61793	8.98E-06	0.000107	protein_coding
ENSG0000(	FBXW9	70.14721	-0.44603	9.00E-06	0.000107	protein_coding
ENSG0000(	ATP5PBP1	37.8241	0.60948	9.02E-06	0.000108	processed_pseudogene
ENSG0000(	ZFP36	3220.72	0.572812	9.03E-06	0.000108	protein_coding
ENSG0000(	STK25	1858.342	-0.19002	9.04E-06	0.000108	protein_coding
ENSG0000(	ALDH7A1	2709.623	-0.16471	9.07E-06	0.000108	protein_coding
ENSG0000(	ELAVL3	84.38071	0.719092	9.07E-06	0.000108	protein_coding
ENSG0000(	RGS5	2214.654	0.391938	9.11E-06	0.000108	protein_coding
ENSG0000(	DHRS4	289.0286	-0.44989	9.13E-06	0.000109	protein_coding
ENSG0000(	TVP23CP1	31.94694	0.97742	9.17E-06	0.000109	processed_pseudogene
ENSG0000(	BOLA3	572.2956	-0.40478	9.21E-06	0.00011	protein_coding
ENSG0000(	ENSG0000(	1806.035	0.856268	9.21E-06	0.00011	lncRNA
ENSG0000(	LRFN4	307.5299	-0.477	9.22E-06	0.00011	protein_coding

ENSG0000(	ENSG0000(	343.4916	0.426802	9.25E-06	0.00011	lncRNA
ENSG0000(	FMO1	33.42568	1.465282	9.25E-06	0.00011	protein_coding
ENSG0000(	PGLS	485.5401	-0.26527	9.25E-06	0.00011	protein_coding
ENSG0000(	RPF1	282.2463	-0.3252	9.25E-06	0.00011	protein_coding
ENSG0000(	ENSG0000(	122.9926	0.347509	9.26E-06	0.00011	TEC
ENSG0000(	SARS2	252.5628	-0.40574	9.28E-06	0.00011	protein_coding
ENSG0000(	NCL	3643.106	-0.26656	9.29E-06	0.00011	protein_coding
ENSG0000(	SSBP1	1171.71	-0.19895	9.32E-06	0.000111	protein_coding
ENSG0000(	LINC01671	143.48	0.419732	9.35E-06	0.000111	lncRNA
ENSG0000(	ZEB1	1524.844	0.501833	9.38E-06	0.000111	protein_coding
ENSG0000(	ENSG0000(	1254.008	0.269138	9.38E-06	0.000111	lncRNA
ENSG0000(	PDK2	661.9731	-0.47455	9.42E-06	0.000111	protein_coding
ENSG0000(	WAPL	2532.832	-0.15956	9.47E-06	0.000112	protein_coding
ENSG0000(	RUNX1T1	845.6892	0.413982	9.50E-06	0.000112	protein_coding
ENSG0000(	TDGF1P3	75.98531	-0.44971	9.54E-06	0.000113	protein_coding
ENSG0000(	ENSG0000(	354.9102	0.220602	9.54E-06	0.000113	lncRNA
ENSG0000(	EXOG	909.3928	0.269082	9.58E-06	0.000113	protein_coding
ENSG0000(	ABCD1	307.4504	0.322907	9.60E-06	0.000113	protein_coding
ENSG0000(	H3C1	56.68638	-0.77605	9.60E-06	0.000113	protein_coding
ENSG0000(	ENSG0000(	35.25363	-0.68448	9.61E-06	0.000113	transcribed_processed_pseudoge
ENSG0000(	ENSG0000(	87.9818	0.552474	9.70E-06	0.000114	lncRNA
ENSG0000(	PHLDB3	696.1231	-0.49314	9.76E-06	0.000115	protein_coding
ENSG0000(	NELFCD	1140.7	-0.18696	9.79E-06	0.000115	protein_coding
ENSG0000(	COX6A1	1173.365	-0.6211	9.84E-06	0.000116	protein_coding
ENSG0000(	SLIRP	476.506	-0.3393	9.84E-06	0.000116	protein_coding
ENSG0000(	ENSG0000(	22.40721	1.122427	9.87E-06	0.000116	lncRNA
ENSG0000(	EBAG9	337.8582	-0.28666	9.90E-06	0.000116	protein_coding
ENSG0000(	AGPAT4	1050.729	0.694458	9.92E-06	0.000117	protein_coding
ENSG0000(	SLC30A7	2278.011	0.240115	1.00E-05	0.000118	protein_coding
ENSG0000(	ENSG0000(	140.8298	-0.34306	1.01E-05	0.000119	lncRNA
ENSG0000(	ADCY6	5330.435	-0.46605	1.01E-05	0.000119	protein_coding
ENSG0000(	WDR77	648.2639	-0.34207	1.02E-05	0.00012	protein_coding
ENSG0000(	TLR6	956.5338	0.641173	1.02E-05	0.00012	protein_coding
ENSG0000(	ENSG0000(	80.8825	0.34236	1.02E-05	0.00012	lncRNA
ENSG0000(	PWRN4	87.65167	0.958262	1.02E-05	0.00012	lncRNA
ENSG0000(	RORA	2154.252	0.353634	1.02E-05	0.00012	protein_coding
ENSG0000(	CHROMR	1312.889	0.239384	1.03E-05	0.00012	lncRNA
ENSG0000(	ZC3H12B	485.3758	-0.28747	1.03E-05	0.00012	protein_coding
ENSG0000(	CSRNP1	501.9805	0.589311	1.03E-05	0.00012	protein_coding
ENSG0000(	ENSG0000(	217.1284	0.281476	1.03E-05	0.00012	lncRNA
ENSG0000(	ENSG0000(	106.5759	0.373194	1.03E-05	0.000121	lncRNA
ENSG0000(	RUFY4	71.24857	1.015129	1.04E-05	0.000122	protein_coding
ENSG0000(	ARV1	309.7732	-0.33838	1.05E-05	0.000123	protein_coding
ENSG0000(	MIGA2	1642.833	-0.42425	1.05E-05	0.000123	protein_coding
ENSG0000(	TYW1	720.5728	-0.35282	1.05E-05	0.000123	protein_coding
ENSG0000(	SLBP	324.2333	-0.35084	1.06E-05	0.000123	protein_coding
ENSG0000(	ABCD3	2432.457	-0.38889	1.06E-05	0.000124	protein_coding

ENSG0000(	PIK3R5	718.7639	0.609468	1.06E-05	0.000124	protein_coding
ENSG0000(	ENSG0000(	46.50301	0.651481	1.06E-05	0.000124	lncRNA
ENSG0000(	IHH	797.0454	-0.77856	1.06E-05	0.000124	protein_coding
ENSG0000(	TRAF3	2289.468	0.406282	1.06E-05	0.000124	protein_coding
ENSG0000(	PC	541.6602	-0.64843	1.07E-05	0.000124	protein_coding
ENSG0000(	SEM1	898.4994	-0.19604	1.07E-05	0.000125	protein_coding
ENSG0000(	UBL4A	324.145	-0.39653	1.07E-05	0.000125	protein_coding
ENSG0000(	PAF1	312.6549	-0.31249	1.07E-05	0.000125	protein_coding
ENSG0000(	NINJ2-AS1	300.8567	0.371176	1.07E-05	0.000125	lncRNA
ENSG0000(	COBLL1	2095.046	0.271066	1.08E-05	0.000126	protein_coding
ENSG0000(	RNU6-638F	70.88806	0.682264	1.08E-05	0.000126	snRNA
ENSG0000(	ENSG0000(	45.47294	0.580449	1.08E-05	0.000126	processed_pseudogene
ENSG0000(	LRRCC1	523.0628	-0.42737	1.08E-05	0.000126	protein_coding
ENSG0000(	NXNL2	435.12	0.34794	1.08E-05	0.000126	protein_coding
ENSG0000(	ENSG0000(	10.31862	1.317094	1.09E-05	0.000126	lncRNA
ENSG0000(	DAGLB	771.1382	-0.22879	1.09E-05	0.000126	protein_coding
ENSG0000(	IDS	2608.16	0.264569	1.09E-05	0.000126	protein_coding
ENSG0000(	RBM44	120.18	0.641126	1.09E-05	0.000127	protein_coding
ENSG0000(	ELK3	786.2624	0.492039	1.09E-05	0.000127	protein_coding
ENSG0000(	ATG16L2	2721.911	0.279077	1.10E-05	0.000127	protein_coding
ENSG0000(	MCM4	1016.49	-0.38606	1.10E-05	0.000127	protein_coding
ENSG0000(	HS3ST3B1	539.6168	0.550429	1.10E-05	0.000127	protein_coding
ENSG0000(	PRNCR1	1253.071	0.200828	1.10E-05	0.000127	lncRNA
ENSG0000(	LINC01761	1149.57	0.192292	1.10E-05	0.000128	lncRNA
ENSG0000(	SNAPC5	351.0608	-0.23631	1.10E-05	0.000128	protein_coding
ENSG0000(	MRPS36	181.7658	-0.61969	1.10E-05	0.000128	protein_coding
ENSG0000(	LTBR	2646.038	-0.20423	1.11E-05	0.000128	protein_coding
ENSG0000(	ENSG0000(	1129.517	0.176909	1.11E-05	0.000128	lncRNA
ENSG0000(	PSG11	812.5771	0.692897	1.11E-05	0.000129	protein_coding
ENSG0000(	ENSG0000(	556.415	0.210228	1.11E-05	0.000129	lncRNA
ENSG0000(	ENSG0000(	135.9	0.540982	1.12E-05	0.000129	processed_pseudogene
ENSG0000(	CCT2	956.8183	-0.26916	1.12E-05	0.000129	protein_coding
ENSG0000(	C1GALT1P1	58.86742	0.518825	1.13E-05	0.00013	processed_pseudogene
ENSG0000(	COASY	925.7183	-0.27912	1.13E-05	0.00013	protein_coding
ENSG0000(	MANSC1	1711.751	-0.20635	1.13E-05	0.00013	protein_coding
ENSG0000(	HECTD3	1383.27	-0.37914	1.13E-05	0.00013	protein_coding
ENSG0000(	MTHFD2L	692.6308	0.268703	1.13E-05	0.00013	protein_coding
ENSG0000(	DCAF4L2	14.11698	0.773294	1.13E-05	0.00013	protein_coding
ENSG0000(	NLRP3	292.9924	0.693689	1.13E-05	0.00013	protein_coding
ENSG0000(	ENSG0000(	74.54451	-0.45246	1.13E-05	0.00013	transcribed_processed_pseudoge
ENSG0000(	ENSG0000(	68.04024	0.40022	1.13E-05	0.00013	lncRNA
ENSG0000(	HSPBAP1	764.6497	0.252927	1.14E-05	0.000131	protein_coding
ENSG0000(	MAPK3	1603.884	-0.34618	1.14E-05	0.000131	protein_coding
ENSG0000(	IGHV1-24	68.30708	1.733185	1.14E-05	0.000131	IG_V_gene
ENSG0000(	ITPR1	4140.992	0.515378	1.14E-05	0.000131	protein_coding
ENSG0000(	ENSG0000(	470.8097	0.317174	1.14E-05	0.000131	lncRNA
ENSG0000(	FAM126A	2208.566	0.414225	1.15E-05	0.000131	protein_coding

ENSG0000(	FOXQ1	88.84944	0.909496	1.14E-05	0.000131	protein_coding
ENSG0000(	TEX9	1376.066	0.243327	1.15E-05	0.000131	protein_coding
ENSG0000(	ENSG0000(	869.2411	0.239663	1.16E-05	0.000133	TEC
ENSG0000(	SEL1L3	4646.873	0.283813	1.16E-05	0.000133	protein_coding
ENSG0000(	ENSG0000(	72.58797	0.961696	1.16E-05	0.000133	TEC
ENSG0000(	HLA-DMB	1992.259	0.639138	1.17E-05	0.000133	protein_coding
ENSG0000(	UBE2L3	921.2862	-0.32863	1.17E-05	0.000133	protein_coding
ENSG0000(	CNBP	3413.907	-0.26373	1.17E-05	0.000134	protein_coding
ENSG0000(	FAM221A	1987.579	-0.18028	1.17E-05	0.000134	protein_coding
ENSG0000(	ENSG0000(	49.85237	0.548559	1.17E-05	0.000134	lncRNA
ENSG0000(	SLC7A11	910.6483	0.573218	1.17E-05	0.000134	protein_coding
ENSG0000(	ENSG0000(	228.2834	0.419102	1.17E-05	0.000134	lncRNA
ENSG0000(	ATP5MJ	1287.677	-0.33429	1.18E-05	0.000135	protein_coding
ENSG0000(	ENSG0000(	32.97444	-0.68362	1.18E-05	0.000135	lncRNA
ENSG0000(	PUS7	462.9407	-0.44345	1.18E-05	0.000135	protein_coding
ENSG0000(	MZB1	823.4048	0.641337	1.18E-05	0.000135	protein_coding
ENSG0000(	LINC01145	850.134	0.562562	1.18E-05	0.000135	transcribed_unprocessed_pseudo
ENSG0000(	STEAP1B	93.66943	1.136989	1.18E-05	0.000135	protein_coding
ENSG0000(	MTMR8	338.0481	-0.4784	1.19E-05	0.000135	protein_coding
ENSG0000(	TNFRSF10A	546.0828	-0.36341	1.19E-05	0.000135	protein_coding
ENSG0000(	ENSG0000(	1072.176	0.171289	1.19E-05	0.000135	TEC
ENSG0000(	DIABLO	1088.511	-0.14679	1.19E-05	0.000136	protein_coding
ENSG0000(	ROBO1	1365.467	0.526303	1.19E-05	0.000136	protein_coding
ENSG0000(	TAP2	2218.307	0.549634	1.20E-05	0.000137	protein_coding
ENSG0000(	BMS1P14	131.7699	0.727508	1.20E-05	0.000137	lncRNA
ENSG0000(	KRT18P31	227.0161	-0.34194	1.20E-05	0.000137	processed_pseudogene
ENSG0000(	PDRG1	127.9879	-0.34422	1.21E-05	0.000137	protein_coding
ENSG0000(	FGFR1	1872.225	0.323348	1.21E-05	0.000137	protein_coding
ENSG0000(	ENDOG	56.52577	-0.67797	1.22E-05	0.000138	protein_coding
ENSG0000(	ENSG0000(	39.62648	0.632315	1.23E-05	0.000139	lncRNA
ENSG0000(	MAPK1	3041.131	-0.24761	1.23E-05	0.000139	protein_coding
ENSG0000(	ADAMTS1	901.8071	0.701941	1.23E-05	0.000139	protein_coding
ENSG0000(	NAGK	3143.088	0.237594	1.23E-05	0.000139	protein_coding
ENSG0000(	SDAD1-AS1	278.6127	-0.32758	1.24E-05	0.00014	lncRNA
ENSG0000(	ECI2	802.0018	-0.41146	1.24E-05	0.000141	protein_coding
ENSG0000(	EMX2	482.4349	0.654482	1.24E-05	0.000141	protein_coding
ENSG0000(	MRPL27	587.0649	-0.34137	1.25E-05	0.000141	protein_coding
ENSG0000(	PKP3	1003.57	-0.61386	1.25E-05	0.000141	protein_coding
ENSG0000(	NR4A1	1322.663	1.014121	1.25E-05	0.000142	protein_coding
ENSG0000(	SURF6	1045.139	-0.23902	1.25E-05	0.000142	protein_coding
ENSG0000(	ENSG0000(	132.2404	-0.37609	1.26E-05	0.000142	pseudogene
ENSG0000(	UGT1A10	1014.097	-0.95239	1.26E-05	0.000142	protein_coding
ENSG0000(	ENSG0000(	25.40484	-0.72063	1.26E-05	0.000143	lncRNA
ENSG0000(	TBX15	11.45299	1.037623	1.27E-05	0.000143	protein_coding
ENSG0000(	ESR1	161.3493	0.832368	1.27E-05	0.000143	protein_coding
ENSG0000(	ABCC8	596.7743	0.185125	1.27E-05	0.000143	protein_coding
ENSG0000(	EIF1AY	327.0359	-0.37368	1.27E-05	0.000143	protein_coding

ENSG0000(	PLCB1-IT1	34.62291	0.80574	1.28E-05	0.000144	lncRNA
ENSG0000(	EPHB4	764.5386	-0.34188	1.28E-05	0.000144	protein_coding
ENSG0000(	PROM2	1426.348	-0.46364	1.28E-05	0.000144	protein_coding
ENSG0000(	ENSG0000(	40.16803	0.545208	1.28E-05	0.000145	lncRNA
ENSG0000(	MCFD2	4133.896	0.142303	1.29E-05	0.000145	protein_coding
ENSG0000(	GMNN	349.0446	-0.51514	1.29E-05	0.000145	protein_coding
ENSG0000(	PRKCB	1678.945	0.718049	1.29E-05	0.000145	protein_coding
ENSG0000(	COX6CP17	31.8655	0.738573	1.29E-05	0.000145	processed_pseudogene
ENSG0000(	GK-IT1	131.4393	0.734077	1.30E-05	0.000146	lncRNA
ENSG0000(	MGAT1	2405.884	0.239058	1.30E-05	0.000146	protein_coding
ENSG0000(	MYL6	5092.97	-0.41132	1.30E-05	0.000146	protein_coding
ENSG0000(	ZNRD2	74.72016	-0.48293	1.31E-05	0.000147	protein_coding
ENSG0000(	ENSG0000(	275.7355	0.283372	1.31E-05	0.000147	TEC
ENSG0000(	MIR4537	149.3699	0.833986	1.31E-05	0.000147	miRNA
ENSG0000(	ENSG0000(	10.81588	1.070903	1.32E-05	0.000148	lncRNA
ENSG0000(	TRIM7-AS1	283.6462	-0.37513	1.32E-05	0.000148	lncRNA
ENSG0000(	TRIOBP	2531.913	-0.2578	1.32E-05	0.000148	protein_coding
ENSG0000(	RNF2	369.6307	-0.29929	1.32E-05	0.000148	protein_coding
ENSG0000(	E2F2	903.8885	-0.47818	1.32E-05	0.000148	protein_coding
ENSG0000(	MTND2P12	226.0884	-0.28239	1.32E-05	0.000148	unprocessed_pseudogene
ENSG0000(	H2BC11	145.8404	-0.7643	1.32E-05	0.000148	protein_coding
ENSG0000(	AMZ1	169.8184	0.471254	1.33E-05	0.000149	protein_coding
ENSG0000(	LINC01267	90.46153	0.541268	1.33E-05	0.000149	lncRNA
ENSG0000(	HSD17B11	1571.259	-0.47134	1.33E-05	0.000149	protein_coding
ENSG0000(	CXXC1	753.4999	-0.29653	1.34E-05	0.000149	protein_coding
ENSG0000(	ZNF799	880.9132	-0.26093	1.34E-05	0.000149	protein_coding
ENSG0000(	ENSG0000(	1581.661	0.139633	1.34E-05	0.000149	lncRNA
ENSG0000(	CETN3	219.9227	-0.39671	1.34E-05	0.00015	protein_coding
ENSG0000(	NTAN1	918.3817	-0.30222	1.34E-05	0.00015	protein_coding
ENSG0000(	SKA1	757.4508	-0.24885	1.34E-05	0.00015	protein_coding
ENSG0000(	PDHX	611.7347	-0.28987	1.35E-05	0.00015	protein_coding
ENSG0000(	TBRG4	1426.589	-0.28269	1.35E-05	0.00015	protein_coding
ENSG0000(	TACO1	340.2439	-0.36589	1.35E-05	0.000151	protein_coding
ENSG0000(	JAKMIP3	77.37837	0.605494	1.35E-05	0.000151	protein_coding
ENSG0000(	THOC2	3202.633	-0.2082	1.36E-05	0.000151	protein_coding
ENSG0000(	FAM117B	1532.081	-0.27879	1.36E-05	0.000151	protein_coding
ENSG0000(	CCDC88A	4867.28	0.280267	1.36E-05	0.000151	protein_coding
ENSG0000(	DCPS	512.269	0.32326	1.36E-05	0.000151	protein_coding
ENSG0000(	MRS2	1249.144	-0.19748	1.36E-05	0.000151	protein_coding
ENSG0000(	LNCTAM34	22.40547	1.083486	1.36E-05	0.000152	lncRNA
ENSG0000(	GJB2	606.4286	-0.56081	1.36E-05	0.000152	protein_coding
ENSG0000(	GRIA1	133.9846	0.385992	1.36E-05	0.000152	protein_coding
ENSG0000(	GALNT15	596.909	0.315471	1.37E-05	0.000152	protein_coding
ENSG0000(	RNF181	187.1197	-0.35682	1.38E-05	0.000153	protein_coding
ENSG0000(	ENSG0000(	228.6826	0.527805	1.39E-05	0.000154	TEC
ENSG0000(	LINC00543	44.80968	-0.7686	1.39E-05	0.000154	lncRNA
ENSG0000(	ENSG0000(	317.8524	0.301351	1.40E-05	0.000155	lncRNA

ENSG0000(	PLP2	597.2859	-0.53116	1.40E-05	0.000156	protein_coding
ENSG0000(	SSTR1	115.4738	-0.67082	1.41E-05	0.000156	protein_coding
ENSG0000(	ENSG0000(	663.864	-0.28224	1.41E-05	0.000157	lncRNA
ENSG0000(	ENSG0000(	54.99626	1.547113	1.42E-05	0.000157	lncRNA
ENSG0000(	ILF2	621.2526	-0.38204	1.42E-05	0.000157	protein_coding
ENSG0000(	CDC34	342.8381	-0.3879	1.42E-05	0.000157	protein_coding
ENSG0000(	ENSG0000(	1431.412	0.270729	1.43E-05	0.000158	lncRNA
ENSG0000(	ENSG0000(	198.2057	0.285379	1.43E-05	0.000158	lncRNA
ENSG0000(	ST7-OT4	291.3553	0.321326	1.43E-05	0.000158	lncRNA
ENSG0000(	OR6Y1	580.0264	0.220629	1.43E-05	0.000158	protein_coding
ENSG0000(	ENSG0000(	272.8192	-0.32171	1.43E-05	0.000158	lncRNA
ENSG0000(	SURF2	44.26423	-0.56905	1.43E-05	0.000158	protein_coding
ENSG0000(	RPS3A	4908.004	-0.46401	1.44E-05	0.000159	protein_coding
ENSG0000(	DLEU7	557.5431	0.241692	1.45E-05	0.00016	protein_coding
ENSG0000(	ENSG0000(	244.1519	0.306538	1.45E-05	0.00016	lncRNA
ENSG0000(	PCDH9	2560.116	0.663766	1.45E-05	0.00016	protein_coding
ENSG0000(	OVOL1	603.2516	-0.64108	1.45E-05	0.00016	protein_coding
ENSG0000(	CDH7	227.3377	-0.26054	1.45E-05	0.00016	protein_coding
ENSG0000(	ENSG0000(	201.9679	0.342188	1.46E-05	0.000161	lncRNA
ENSG0000(	AP1S1	308.5242	-0.5007	1.47E-05	0.000162	protein_coding
ENSG0000(	LMAN1	2076.315	0.398338	1.47E-05	0.000162	protein_coding
ENSG0000(	USP49	1057.745	0.192087	1.47E-05	0.000162	protein_coding
ENSG0000(	EIF3B	2039.983	-0.22126	1.47E-05	0.000162	protein_coding
ENSG0000(	DLST	2267.73	-0.15344	1.48E-05	0.000162	protein_coding
ENSG0000(	ZBED2	17.37626	1.696537	1.48E-05	0.000162	protein_coding
ENSG0000(	ENSG0000(	24.21518	0.949966	1.48E-05	0.000162	lncRNA
ENSG0000(	C4BPA	66.60663	1.033622	1.48E-05	0.000163	protein_coding
ENSG0000(	ENSG0000(	9.680687	0.970872	1.49E-05	0.000163	lncRNA
ENSG0000(	SERPINB9	1444.058	0.608861	1.49E-05	0.000163	protein_coding
ENSG0000(	ENSG0000(	588.0721	-0.20988	1.49E-05	0.000163	lncRNA
ENSG0000(	UBAC2	1338.99	-0.18204	1.49E-05	0.000163	protein_coding
ENSG0000(	CD320	386.2373	-0.34103	1.49E-05	0.000164	protein_coding
ENSG0000(	PLAC4	138.936	0.56397	1.50E-05	0.000164	lncRNA
ENSG0000(	KCNN3	609.029	0.556353	1.50E-05	0.000165	protein_coding
ENSG0000(	IGHV3-15	828.405	1.033811	1.50E-05	0.000165	IG_V_gene
ENSG0000(	PSMD6-AS:	699.4019	0.290849	1.51E-05	0.000165	lncRNA
ENSG0000(	BTN3A2	4172.935	0.37744	1.51E-05	0.000165	protein_coding
ENSG0000(	ENSG0000(	89.5417	0.545186	1.51E-05	0.000165	lncRNA
ENSG0000(	SDHC	742.9435	-0.33552	1.51E-05	0.000166	protein_coding
ENSG0000(	ENSG0000(	64.96097	0.587419	1.53E-05	0.000167	lncRNA
ENSG0000(	TRPM4	7318.07	-0.61918	1.53E-05	0.000167	protein_coding
ENSG0000(	ADIPOQ-AS	254.2718	0.217115	1.53E-05	0.000168	lncRNA
ENSG0000(	ENSG0000(	478.9657	-0.49089	1.53E-05	0.000168	protein_coding
ENSG0000(	TAPBP	6548.671	0.259877	1.54E-05	0.000168	protein_coding
ENSG0000(	PCBP2	8313.366	-0.12978	1.54E-05	0.000168	protein_coding
ENSG0000(	CARNMT1	672.6089	-0.28113	1.54E-05	0.000169	protein_coding
ENSG0000(	ZNF724	525.9806	-0.25739	1.54E-05	0.000169	protein_coding

ENSG0000(	ENSG0000(	139.0378	0.338355	1.55E-05	0.000169	lncRNA
ENSG0000(	CDYLP1	60.37948	0.469972	1.55E-05	0.000169	unprocessed_pseudogene
ENSG0000(	PEX11B	285.4229	-0.29861	1.55E-05	0.000169	protein_coding
ENSG0000(	ENSG0000(	337.3545	0.305175	1.56E-05	0.00017	transcribed_processed_pseudoge
ENSG0000(	GHITM	3111.389	-0.51951	1.56E-05	0.00017	protein_coding
ENSG0000(	REXO4	370.7996	-0.31652	1.56E-05	0.00017	protein_coding
ENSG0000(	RPL23A	2416.135	-0.33322	1.56E-05	0.00017	protein_coding
ENSG0000(	SRPK1	2924.6	-0.26138	1.56E-05	0.00017	protein_coding
ENSG0000(	TMTC3	711.5318	-0.27905	1.56E-05	0.00017	protein_coding
ENSG0000(	LINC00891	1772.424	0.244916	1.57E-05	0.00017	lncRNA
ENSG0000(	ANKRD40	1341.072	-0.18306	1.57E-05	0.000171	protein_coding
ENSG0000(	EVA1A	44.95321	1.159548	1.57E-05	0.000171	protein_coding
ENSG0000(	IL1R1	2245.208	0.562597	1.58E-05	0.000171	protein_coding
ENSG0000(	JUNB	1396.788	0.548501	1.58E-05	0.000171	protein_coding
ENSG0000(	CLASRP	910.5745	-0.19435	1.58E-05	0.000172	protein_coding
ENSG0000(	GALNTL6	33.51856	1.00641	1.58E-05	0.000172	protein_coding
ENSG0000(	MGAT4B	3866.553	-0.49726	1.59E-05	0.000172	protein_coding
ENSG0000(	TG	131.3095	0.86888	1.59E-05	0.000172	protein_coding
ENSG0000(	KANK4	263.474	-0.46134	1.59E-05	0.000172	protein_coding
ENSG0000(	KCNE1	404.8198	0.493637	1.59E-05	0.000173	protein_coding
ENSG0000(	ENSG0000(	300.4211	0.28641	1.60E-05	0.000173	lncRNA
ENSG0000(	H4C9	63.86678	-0.99395	1.60E-05	0.000173	protein_coding
ENSG0000(	ACOT7	339.8841	-0.44383	1.60E-05	0.000173	protein_coding
ENSG0000(	ENSG0000(	29.19532	0.964236	1.60E-05	0.000174	lncRNA
ENSG0000(	SLK	3142.221	-0.3067	1.60E-05	0.000174	protein_coding
ENSG0000(	DKC1	635.6738	-0.32893	1.61E-05	0.000174	protein_coding
ENSG0000(	MRPL20	727.7652	-0.24781	1.61E-05	0.000174	protein_coding
ENSG0000(	PALMD	218.1135	0.594685	1.61E-05	0.000174	protein_coding
ENSG0000(	ENSG0000(	38.44174	0.771337	1.61E-05	0.000174	lncRNA
ENSG0000(	ENSG0000(	56.89045	0.708062	1.62E-05	0.000175	lncRNA
ENSG0000(	IGHV3-7	1278.655	0.902872	1.62E-05	0.000175	IG_V_gene
ENSG0000(	C11orf53	19.58827	-1.04289	1.63E-05	0.000176	protein_coding
ENSG0000(	MKI67	4280.646	-0.49772	1.63E-05	0.000176	protein_coding
ENSG0000(	ABR	9524.758	-0.40734	1.64E-05	0.000176	protein_coding
ENSG0000(	LINC01445	2097.641	0.267997	1.64E-05	0.000176	lncRNA
ENSG0000(	LINC00970	405.8312	-0.22404	1.64E-05	0.000177	lncRNA
ENSG0000(	NCKAP5	435.3195	-0.56932	1.64E-05	0.000177	protein_coding
ENSG0000(	ENSG0000(	289.6284	-0.32147	1.65E-05	0.000177	lncRNA
ENSG0000(	KSR1	2023.265	0.387919	1.65E-05	0.000177	protein_coding
ENSG0000(	MLF2	728.3518	-0.23979	1.65E-05	0.000177	protein_coding
ENSG0000(	FTLP14	77.22947	-0.63713	1.65E-05	0.000178	processed_pseudogene
ENSG0000(	CMTM7	346.1853	0.593605	1.65E-05	0.000178	protein_coding
ENSG0000(	ENSG0000(	358.5015	0.209315	1.65E-05	0.000178	lncRNA
ENSG0000(	PRSS51	15.90649	1.101469	1.65E-05	0.000178	protein_coding
ENSG0000(	PBDC1	154.2033	-0.40314	1.66E-05	0.000178	protein_coding
ENSG0000(	LINC01050	335.2767	0.312845	1.66E-05	0.000178	lncRNA
ENSG0000(	LINC02717	326.1906	0.248439	1.66E-05	0.000178	lncRNA

ENSG0000(	SEPTIN7P14	1211.097	0.291921	1.66E-05	0.000178	transcribed_unprocessed_pseudo
ENSG0000(	SNHG18	446.8236	-0.53753	1.67E-05	0.000179	lncRNA
ENSG0000(	GEM	411.4054	0.82497	1.67E-05	0.000179	protein_coding
ENSG0000(	PRPSAP1	1687.136	-0.17916	1.67E-05	0.000179	protein_coding
ENSG0000(	HLA-DQB1	1713.148	0.885263	1.68E-05	0.00018	protein_coding
ENSG0000(	LECT2	169.7289	0.356398	1.68E-05	0.00018	protein_coding
ENSG0000(	HMGB2	488.998	-0.41006	1.69E-05	0.000181	protein_coding
ENSG0000(	AOX1	101.4844	1.416595	1.69E-05	0.000181	protein_coding
ENSG0000(	ENSG0000(	28.93598	0.743852	1.69E-05	0.000181	lncRNA
ENSG0000(	SYDE2	682.9852	-0.3128	1.70E-05	0.000182	protein_coding
ENSG0000(	PPP2R5D	692.9598	-0.32908	1.71E-05	0.000182	protein_coding
ENSG0000(	FMC1	236.6108	-0.26838	1.71E-05	0.000182	protein_coding
ENSG0000(	ENSG0000(	203.0863	0.378557	1.71E-05	0.000182	TEC
ENSG0000(	TSPO	478.2163	-0.45431	1.71E-05	0.000183	protein_coding
ENSG0000(	ENSG0000(	143.5985	0.574047	1.71E-05	0.000183	lncRNA
ENSG0000(	KEAP1	552.6892	-0.34435	1.72E-05	0.000184	protein_coding
ENSG0000(	EIF4G1	5009.185	-0.22465	1.73E-05	0.000184	protein_coding
ENSG0000(	ENSG0000(	199.971	0.278627	1.73E-05	0.000184	lncRNA
ENSG0000(	PWP2	175.6013	-0.85834	1.73E-05	0.000184	protein_coding
ENSG0000(	TP53I11	1541.025	-0.29032	1.74E-05	0.000185	protein_coding
ENSG0000(	JPX	7301.495	0.176364	1.74E-05	0.000186	lncRNA
ENSG0000(	PDE12	2231.776	-0.15202	1.74E-05	0.000186	protein_coding
ENSG0000(	C1RL-AS1	1876.304	0.329388	1.74E-05	0.000186	lncRNA
ENSG0000(	ENSG0000(	75.07734	0.383103	1.74E-05	0.000186	lncRNA
ENSG0000(	C20orf202	17.04882	0.889164	1.74E-05	0.000186	protein_coding
ENSG0000(	ENSG0000(	41.21465	0.997241	1.75E-05	0.000186	lncRNA
ENSG0000(	NSMF	808.7389	-0.38585	1.75E-05	0.000186	protein_coding
ENSG0000(	WDFY2	1808.166	0.333956	1.75E-05	0.000186	protein_coding
ENSG0000(	RPS13	1740.474	-0.3916	1.75E-05	0.000186	protein_coding
ENSG0000(	ADPGK	2852.294	0.219861	1.75E-05	0.000186	protein_coding
ENSG0000(	RPS5	1443.292	-0.43697	1.75E-05	0.000186	protein_coding
ENSG0000(	XAF1	6511.892	0.74081	1.75E-05	0.000186	protein_coding
ENSG0000(	ZNF703	1301.87	-0.54652	1.76E-05	0.000187	protein_coding
ENSG0000(	ENSG0000(	204.3453	-0.35103	1.76E-05	0.000187	lncRNA
ENSG0000(	ENSG0000(	189.647	-0.28296	1.77E-05	0.000187	lncRNA
ENSG0000(	PSG6	233.2097	0.625983	1.77E-05	0.000188	protein_coding
ENSG0000(	ENSG0000(	17.7621	0.93186	1.77E-05	0.000188	lncRNA
ENSG0000(	HSBP1	1074.857	-0.27772	1.77E-05	0.000188	protein_coding
ENSG0000(	SLC35A4	1039.668	-0.32235	1.78E-05	0.000188	protein_coding
ENSG0000(	PPP1R16B	1242.313	0.702947	1.78E-05	0.000189	protein_coding
ENSG0000(	PDCL3	86.94759	-0.44229	1.79E-05	0.000189	protein_coding
ENSG0000(	GNG4	362.9781	-0.35133	1.79E-05	0.00019	protein_coding
ENSG0000(	ZW10	356.6668	-0.26899	1.80E-05	0.00019	protein_coding
ENSG0000(	DGCR8	1892.388	-0.25789	1.80E-05	0.00019	protein_coding
ENSG0000(	ENSG0000(	100.9887	0.450695	1.81E-05	0.000191	processed_pseudogene
ENSG0000(	CCNA2	276.6297	-0.45607	1.81E-05	0.000191	protein_coding
ENSG0000(	COQ4	2412.063	-0.22171	1.81E-05	0.000192	protein_coding

ENSG0000(	ENSG0000(	77.41071	0.513874	1.81E-05	0.000192	lncRNA
ENSG0000(	PSMD8	693.4712	-0.25193	1.81E-05	0.000192	protein_coding
ENSG0000(	NBPF15	1888.092	0.349164	1.82E-05	0.000192	protein_coding
ENSG0000(	NSMCE4A	731.4997	-0.26615	1.82E-05	0.000192	protein_coding
ENSG0000(	ORC1	229.3642	-0.38198	1.82E-05	0.000192	protein_coding
ENSG0000(	TJP2	4337.934	-0.37207	1.82E-05	0.000192	protein_coding
ENSG0000(	C14orf119	671.6481	-0.31685	1.82E-05	0.000192	protein_coding
ENSG0000(	C18orf15	403.5543	0.269801	1.82E-05	0.000192	TEC
ENSG0000(	RUSC1	737.4051	-0.37927	1.83E-05	0.000192	protein_coding
ENSG0000(	ATP6V0D2	268.3916	-0.39953	1.83E-05	0.000192	protein_coding
ENSG0000(	IPO5	2049.767	-0.29404	1.83E-05	0.000193	protein_coding
ENSG0000(	HNRNPA1P	89.82904	0.389104	1.84E-05	0.000193	processed_pseudogene
ENSG0000(	GIT2	3357.416	0.215505	1.85E-05	0.000195	protein_coding
ENSG0000(	PGP	414.0125	-0.29389	1.85E-05	0.000195	protein_coding
ENSG0000(	IGSF6	763.7337	0.442718	1.86E-05	0.000196	protein_coding
ENSG0000(	ADCY2	988.3858	0.254413	1.87E-05	0.000196	protein_coding
ENSG0000(	TBK1	2777.067	0.179442	1.87E-05	0.000196	protein_coding
ENSG0000(	ENSG0000(	171.1218	0.319373	1.87E-05	0.000196	lncRNA
ENSG0000(	REEP4	348.1297	-0.35884	1.89E-05	0.000198	protein_coding
ENSG0000(	ATAD3B	1237.087	-0.3137	1.89E-05	0.000198	protein_coding
ENSG0000(	WDR43	850.2255	-0.30456	1.89E-05	0.000198	protein_coding
ENSG0000(	MAP4K5	1781.437	-0.2503	1.91E-05	0.0002	protein_coding
ENSG0000(	VPS4B	1720.665	-0.24957	1.91E-05	0.0002	protein_coding
ENSG0000(	SIRT6	623.3815	-0.56903	1.91E-05	0.0002	protein_coding
ENSG0000(	ENSG0000(	113.042	-0.38533	1.91E-05	0.0002	lncRNA
ENSG0000(	ENSG0000(	157.2542	-0.4382	1.91E-05	0.0002	lncRNA
ENSG0000(	DRG1	394.6956	-0.29559	1.91E-05	0.0002	protein_coding
ENSG0000(	IRF4	2296.935	0.679258	1.91E-05	0.0002	protein_coding
ENSG0000(	KHK	101.2989	-0.65698	1.91E-05	0.0002	protein_coding
ENSG0000(	ENSG0000(	412.9938	-0.18424	1.92E-05	0.000201	lncRNA
ENSG0000(	RNASE1	404.3162	-0.55341	1.93E-05	0.000201	protein_coding
ENSG0000(	CSRNP2	1193.327	0.241369	1.93E-05	0.000201	protein_coding
ENSG0000(	TSHZ3	385.0883	0.626337	1.93E-05	0.000202	protein_coding
ENSG0000(	SNW1	575.993	-0.30617	1.93E-05	0.000202	protein_coding
ENSG0000(	ZNF793	2165.855	-0.17347	1.94E-05	0.000202	protein_coding
ENSG0000(	PCOLCE	251.8612	0.702796	1.94E-05	0.000202	protein_coding
ENSG0000(	NLRP7	15.49654	1.177273	1.94E-05	0.000202	protein_coding
ENSG0000(	RPP38	286.394	-0.25663	1.94E-05	0.000202	protein_coding
ENSG0000(	LINC02804	63.76697	0.424025	1.95E-05	0.000203	lncRNA
ENSG0000(	ENSG0000(	120.9156	0.358744	1.96E-05	0.000204	lncRNA
ENSG0000(	SLX9	111.294	-0.34588	1.96E-05	0.000204	protein_coding
ENSG0000(	LINC01011	234.1148	0.329677	1.97E-05	0.000206	lncRNA
ENSG0000(	TMEM201	384.9058	-0.3362	1.97E-05	0.000206	protein_coding
ENSG0000(	CRIPT	2378.339	0.15954	1.98E-05	0.000206	protein_coding
ENSG0000(	EPB41	5592.189	-0.16423	1.99E-05	0.000207	protein_coding
ENSG0000(	RNF126	490.3834	-0.28342	1.99E-05	0.000207	protein_coding
ENSG0000(	RGS18	81.16527	0.784314	1.99E-05	0.000207	protein_coding

ENSG0000(	LRRC14	980.9989	-0.26829	1.99E-05	0.000207	protein_coding
ENSG0000(	RPS18	4513.42	-0.47851	2.00E-05	0.000207	protein_coding
ENSG0000(	BSG	4369.222	-0.68215	2.00E-05	0.000208	protein_coding
ENSG0000(	MANEA-DT	81.37472	0.506677	2.00E-05	0.000208	lncRNA
ENSG0000(	SLC23A3	665.7105	-0.62439	2.00E-05	0.000208	protein_coding
ENSG0000(	ZNF229	166.3839	-0.51507	2.00E-05	0.000208	protein_coding
ENSG0000(	HAGLR	892.8041	-0.52683	2.00E-05	0.000208	lncRNA
ENSG0000(	JMJD8	420.2164	-0.36816	2.01E-05	0.000208	protein_coding
ENSG0000(	CAPN8	4639.856	-0.91227	2.02E-05	0.00021	protein_coding
ENSG0000(	CFL1P1	719.8271	0.266592	2.03E-05	0.00021	transcribed_unprocessed_pseudo
ENSG0000(	KIF22	1852.578	-0.2324	2.03E-05	0.00021	protein_coding
ENSG0000(	MOCS3	509.8776	-0.22477	2.04E-05	0.000211	protein_coding
ENSG0000(	RPL22	2698.766	-0.35858	2.04E-05	0.000211	protein_coding
ENSG0000(	NFAM1	557.4955	0.509291	2.04E-05	0.000211	protein_coding
ENSG0000(	AURKA	295.0051	-0.35786	2.08E-05	0.000215	protein_coding
ENSG0000(	PTGES2-AS	639.9206	0.224861	2.08E-05	0.000215	lncRNA
ENSG0000(	LINC02842	19.25519	1.003363	2.08E-05	0.000216	lncRNA
ENSG0000(	AMD1	3622.185	-0.23349	2.10E-05	0.000217	protein_coding
ENSG0000(	LIMK1	1135.061	0.264554	2.11E-05	0.000218	protein_coding
ENSG0000(	ENSG0000(	138.2957	0.280978	2.11E-05	0.000218	TEC
ENSG0000(	ENSG0000(	124.7622	-0.40776	2.11E-05	0.000218	pseudogene
ENSG0000(	H3C11	126.5572	-0.60825	2.11E-05	0.000218	protein_coding
ENSG0000(	TBL1XR1	6572.644	0.290685	2.12E-05	0.000219	protein_coding
ENSG0000(	ENSG0000(	14.17037	1.085384	2.12E-05	0.000219	lncRNA
ENSG0000(	ENSG0000(	1154.558	0.208074	2.12E-05	0.000219	lncRNA
ENSG0000(	NALF1-IT1	26.26934	1.278835	2.13E-05	0.00022	lncRNA
ENSG0000(	ENSG0000(	41.27525	0.549467	2.13E-05	0.00022	lncRNA
ENSG0000(	PSMB3	291.6304	-0.43216	2.13E-05	0.00022	protein_coding
ENSG0000(	CEBPG	2010.659	-0.40858	2.14E-05	0.00022	protein_coding
ENSG0000(	ENSG0000(	82.33267	0.450857	2.14E-05	0.000221	lncRNA
ENSG0000(	ENSG0000(	212.4994	0.315753	2.14E-05	0.000221	lncRNA
ENSG0000(	TSPAN19	322.9331	-0.42784	2.15E-05	0.000221	protein_coding
ENSG0000(	ENSG0000(	1339.836	0.184887	2.15E-05	0.000222	lncRNA
ENSG0000(	ENSG0000(	412.553	0.460392	2.16E-05	0.000222	lncRNA
ENSG0000(	AP1B1	2021.394	-0.33029	2.16E-05	0.000222	protein_coding
ENSG0000(	ENSG0000(	483.3963	0.352885	2.16E-05	0.000222	lncRNA
ENSG0000(	PNKD	814.8803	-0.31482	2.16E-05	0.000222	protein_coding
ENSG0000(	WDR86	56.97303	0.849181	2.16E-05	0.000222	protein_coding
ENSG0000(	SIGIRR	890.4781	-0.26391	2.17E-05	0.000222	protein_coding
ENSG0000(	ZNF460-AS	300.9232	0.236708	2.17E-05	0.000223	lncRNA
ENSG0000(	ADAMTS14	777.2003	0.550862	2.17E-05	0.000223	protein_coding
ENSG0000(	ENSG0000(	115.3351	0.629136	2.18E-05	0.000223	TEC
ENSG0000(	ENSG0000(	56.44901	0.725314	2.18E-05	0.000223	lncRNA
ENSG0000(	CRB3	353.6913	-0.65206	2.18E-05	0.000224	protein_coding
ENSG0000(	IFIT3	937.8712	0.775302	2.18E-05	0.000224	protein_coding
ENSG0000(	BRI3BP	2676.895	-0.28015	2.19E-05	0.000224	protein_coding
ENSG0000(	MCM2	284.5591	-0.5769	2.20E-05	0.000225	protein_coding

ENSG0000(	EVI5	2071.839	-0.20891	2.20E-05	0.000225	protein_coding
ENSG0000(	GASK1B	828.6763	0.562605	2.20E-05	0.000225	protein_coding
ENSG0000(	CTNNA1P1	40.30459	-0.77237	2.21E-05	0.000226	processed_pseudogene
ENSG0000(	SEMA5A	4351.902	-0.59099	2.21E-05	0.000226	protein_coding
ENSG0000(	DNAJB1	1483.113	-0.38079	2.21E-05	0.000226	protein_coding
ENSG0000(	CALML4	1610.263	-0.55841	2.22E-05	0.000227	protein_coding
ENSG0000(	COQ3	105.1595	-0.43074	2.22E-05	0.000227	protein_coding
ENSG0000(	ST3GAL4	628.455	0.72905	2.23E-05	0.000227	protein_coding
ENSG0000(	CEBPZ	881.5073	-0.33709	2.23E-05	0.000228	protein_coding
ENSG0000(	PTPN23	1309.711	-0.27901	2.24E-05	0.000228	protein_coding
ENSG0000(	ENSG0000(	2386.847	-0.2245	2.24E-05	0.000228	lncRNA
ENSG0000(	CDT1	271.4897	-0.34562	2.24E-05	0.000229	protein_coding
ENSG0000(	SULT1C2P1	125.58	0.382914	2.26E-05	0.00023	transcribed_unprocessed_pseudo
ENSG0000(	POLE	3191.548	-0.194	2.27E-05	0.000231	protein_coding
ENSG0000(	ENSG0000(	226.5462	0.317681	2.27E-05	0.000231	TEC
ENSG0000(	DDT	787.0118	-0.33514	2.27E-05	0.000232	protein_coding
ENSG0000(	ENSG0000(	16.90095	0.987657	2.28E-05	0.000232	lncRNA
ENSG0000(	FCHO2	1098.175	-0.27795	2.28E-05	0.000232	protein_coding
ENSG0000(	GATAD2A	2287.737	-0.22049	2.28E-05	0.000232	protein_coding
ENSG0000(	SNCAIP	295.9065	0.510713	2.28E-05	0.000232	protein_coding
ENSG0000(	ENSG0000(	41.89347	0.579579	2.30E-05	0.000234	lncRNA
ENSG0000(	WNT3	123.864	0.777154	2.30E-05	0.000234	protein_coding
ENSG0000(	CCDC125	4517.21	0.105365	2.30E-05	0.000234	protein_coding
ENSG0000(	GJA6P	32.01659	0.535979	2.30E-05	0.000234	processed_pseudogene
ENSG0000(	ENSG0000(	486.9669	-0.75968	2.31E-05	0.000234	lncRNA
ENSG0000(	DUSP22	1407.37	0.332541	2.31E-05	0.000235	protein_coding
ENSG0000(	VPS33A	1224.614	-0.14828	2.32E-05	0.000235	protein_coding
ENSG0000(	DGCR5	693.6505	0.241893	2.32E-05	0.000236	lncRNA
ENSG0000(	SLC35D1	3729.859	-0.47008	2.33E-05	0.000236	protein_coding
ENSG0000(	CLIP1	4368.132	0.152758	2.33E-05	0.000236	protein_coding
ENSG0000(	RTRAF	1302.11	-0.34143	2.34E-05	0.000238	protein_coding
ENSG0000(	HTRA2	249.3612	-0.32412	2.35E-05	0.000239	protein_coding
ENSG0000(	CD151	1781.602	-0.29826	2.36E-05	0.000239	protein_coding
ENSG0000(	FAM20B	1558.319	-0.19541	2.37E-05	0.00024	protein_coding
ENSG0000(	ENSG0000(	321.5061	0.367808	2.38E-05	0.000241	lncRNA
ENSG0000(	YPEL5	1142.45	0.330983	2.38E-05	0.000241	protein_coding
ENSG0000(	ENSG0000(	25.86684	-0.80296	2.38E-05	0.000241	lncRNA
ENSG0000(	TPTEP2	814.0688	0.274815	2.39E-05	0.000242	transcribed_unprocessed_pseudo
ENSG0000(	FGFBP1	79.77152	-1.15948	2.39E-05	0.000242	protein_coding
ENSG0000(	ENSG0000(	297.9861	-0.49267	2.39E-05	0.000242	protein_coding
ENSG0000(	TCIRG1	2147.79	0.329963	2.40E-05	0.000242	protein_coding
ENSG0000(	CDCA8	230.7111	-0.46261	2.41E-05	0.000243	protein_coding
ENSG0000(	HTATSF1	476.6681	-0.39471	2.41E-05	0.000243	protein_coding
ENSG0000(	LUM	684.4435	1.053921	2.41E-05	0.000243	protein_coding
ENSG0000(	INPP5E	689.2214	-0.2714	2.41E-05	0.000244	protein_coding
ENSG0000(	ENSG0000(	26.46783	0.625652	2.42E-05	0.000244	lncRNA
ENSG0000(	OXSM	188.3605	-0.3994	2.42E-05	0.000244	protein_coding

ENSG0000(	PSMB4	592.7003	-0.3468	2.42E-05	0.000244	protein_coding
ENSG0000(	ENSG0000(	737.7819	0.254302	2.42E-05	0.000244	lncRNA
ENSG0000(	ARHGAP25	797.708	0.52725	2.42E-05	0.000244	protein_coding
ENSG0000(	ENSG0000(	582.4165	0.22923	2.43E-05	0.000244	lncRNA
ENSG0000(	BTBD2	895.5535	-0.28066	2.43E-05	0.000245	protein_coding
ENSG0000(	PLPP2	1793.385	-0.41074	2.43E-05	0.000245	protein_coding
ENSG0000(	ENSG0000(	76.19331	0.493411	2.44E-05	0.000245	lncRNA
ENSG0000(	KCNK3	434.6925	0.237114	2.45E-05	0.000246	protein_coding
ENSG0000(	CREBRF	2444.318	0.202637	2.45E-05	0.000246	protein_coding
ENSG0000(	TFB2M	145.7858	-0.40813	2.46E-05	0.000248	protein_coding
ENSG0000(	RPL32P1	49.77491	0.912895	2.47E-05	0.000248	processed_pseudogene
ENSG0000(	RNY1P9	22.17704	0.85369	2.47E-05	0.000248	misc_RNA
ENSG0000(	IGHJ4	773.5655	0.988365	2.48E-05	0.000249	IG_J_gene
ENSG0000(	ADPRH	148.8065	0.572382	2.48E-05	0.000249	protein_coding
ENSG0000(	IFIT5	498.0378	0.523682	2.48E-05	0.000249	protein_coding
ENSG0000(	SPART	562.3619	0.380239	2.49E-05	0.000249	protein_coding
ENSG0000(	ENSG0000(	238.5309	0.285142	2.49E-05	0.00025	lncRNA
ENSG0000(	SCPEP1	538.0705	0.295546	2.49E-05	0.00025	protein_coding
ENSG0000(	CCDC198	181.6803	-0.30921	2.50E-05	0.000251	protein_coding
ENSG0000(	MAP7	1308.626	-0.42779	2.51E-05	0.000251	protein_coding
ENSG0000(	ROR2	283.1452	0.741808	2.51E-05	0.000251	protein_coding
ENSG0000(	TMEM121E	18.53957	0.973403	2.51E-05	0.000251	protein_coding
ENSG0000(	IFRD2	397.2003	-0.41342	2.51E-05	0.000252	protein_coding
ENSG0000(	ERVK9-11	850.5339	0.210393	2.52E-05	0.000252	lncRNA
ENSG0000(	TMEM120/	394.7912	-0.55721	2.53E-05	0.000253	protein_coding
ENSG0000(	HNRNPA1P	3307.181	0.177986	2.53E-05	0.000253	processed_pseudogene
ENSG0000(	RPS19	2613.212	-0.42924	2.54E-05	0.000254	protein_coding
ENSG0000(	ENSG0000(	157.4228	0.300511	2.55E-05	0.000255	processed_pseudogene
ENSG0000(	FKBP14	1643.562	0.205033	2.55E-05	0.000255	protein_coding
ENSG0000(	LINC02572	61.4998	-0.42134	2.56E-05	0.000256	lncRNA
ENSG0000(	KLRC2	99.04714	-0.77299	2.56E-05	0.000256	protein_coding
ENSG0000(	ATG4D	628.5011	-0.47154	2.56E-05	0.000256	protein_coding
ENSG0000(	ENSG0000(	101.0339	0.350009	2.57E-05	0.000256	lncRNA
ENSG0000(	ATXN7L3B	2030.948	-0.19522	2.57E-05	0.000256	protein_coding
ENSG0000(	MRPL44	361.0513	-0.33992	2.57E-05	0.000256	protein_coding
ENSG0000(	PRRG2	135.3235	-0.58338	2.58E-05	0.000257	protein_coding
ENSG0000(	FASTKD5	1007.952	-0.15965	2.59E-05	0.000258	protein_coding
ENSG0000(	NDUFA7	328.8551	-0.26858	2.59E-05	0.000258	protein_coding
ENSG0000(	ZNF521	507.3623	0.672823	2.59E-05	0.000258	protein_coding
ENSG0000(	TOMM5	712.4163	-0.33257	2.59E-05	0.000258	protein_coding
ENSG0000(	NR4A2	309.2945	0.866127	2.60E-05	0.000259	protein_coding
ENSG0000(	DNAAF5	771.3766	-0.33659	2.60E-05	0.000259	protein_coding
ENSG0000(	DUXAP8	355.5231	0.616254	2.61E-05	0.000259	lncRNA
ENSG0000(	ENSG0000(	200.2528	-0.36786	2.61E-05	0.000259	lncRNA
ENSG0000(	ENSG0000(	205.3656	0.364417	2.62E-05	0.000261	lncRNA
ENSG0000(	VLDLR	818.1612	-0.56396	2.62E-05	0.000261	protein_coding
ENSG0000(	ENSG0000(	164.0815	-0.62318	2.63E-05	0.000261	lncRNA

ENSG0000(	ENSG0000(	941.5526	0.755088	2.63E-05	0.000261	unprocessed_pseudogene
ENSG0000(	MT-ND1	18011.2	-0.58256	2.64E-05	0.000263	protein_coding
ENSG0000(	MAX	1696.356	0.271763	2.65E-05	0.000263	protein_coding
ENSG0000(	ENSG0000(	67.9593	0.628267	2.66E-05	0.000264	processed_pseudogene
ENSG0000(	ARHGEF3	565.0508	0.51488	2.66E-05	0.000264	protein_coding
ENSG0000(	CHSY1	1035.269	0.531224	2.66E-05	0.000264	protein_coding
ENSG0000(	CLTC	8268.664	-0.24684	2.66E-05	0.000264	protein_coding
ENSG0000(	FAS	4472.469	0.272853	2.66E-05	0.000264	protein_coding
ENSG0000(	LNX2	1342.843	-0.37255	2.66E-05	0.000264	protein_coding
ENSG0000(	ENSG0000(	208.3961	-0.42447	2.67E-05	0.000264	protein_coding
ENSG0000(	EDNRA	197.9796	0.91028	2.67E-05	0.000264	protein_coding
ENSG0000(	TXNDC5	6475.959	0.599228	2.67E-05	0.000264	protein_coding
ENSG0000(	GALNT10	2442.327	0.25179	2.68E-05	0.000265	protein_coding
ENSG0000(	TCTN3	1870.654	-0.20916	2.68E-05	0.000265	protein_coding
ENSG0000(	ENSG0000(	99.20879	0.3054	2.68E-05	0.000265	lncRNA
ENSG0000(	HDGFL3	1346.211	0.340249	2.69E-05	0.000265	protein_coding
ENSG0000(	ALKBH7	148.0957	-0.61438	2.69E-05	0.000266	protein_coding
ENSG0000(	HTRA3	134.7853	0.879549	2.69E-05	0.000266	protein_coding
ENSG0000(	TBC1D10B	1542.459	-0.16491	2.69E-05	0.000266	protein_coding
ENSG0000(	HDAC1P1	216.2176	-0.79076	2.70E-05	0.000266	processed_pseudogene
ENSG0000(	LYRM7	2235.058	-0.17955	2.70E-05	0.000266	protein_coding
ENSG0000(	LSR	1033.727	-0.50732	2.70E-05	0.000266	protein_coding
ENSG0000(	NPAP1	556.4815	0.207425	2.70E-05	0.000267	protein_coding
ENSG0000(	STAT2	4605.002	0.374028	2.70E-05	0.000267	protein_coding
ENSG0000(	ZNF627	555.1875	0.200137	2.70E-05	0.000267	protein_coding
ENSG0000(	AKR7A2	402.259	-0.38027	2.71E-05	0.000267	protein_coding
ENSG0000(	HNRNPAB	1802.348	-0.35195	2.71E-05	0.000267	protein_coding
ENSG0000(	ENSG0000(	2570.946	0.22015	2.72E-05	0.000267	lncRNA
ENSG0000(	LMNB2	825.5604	-0.37712	2.73E-05	0.000269	protein_coding
ENSG0000(	RPL13	3965.626	-0.43926	2.74E-05	0.000269	protein_coding
ENSG0000(	TGFBI	1738.622	0.64293	2.74E-05	0.000269	protein_coding
ENSG0000(	CYP4A22-A	426.0495	0.209812	2.74E-05	0.00027	lncRNA
ENSG0000(	VPS25	440.3924	-0.29881	2.76E-05	0.000271	protein_coding
ENSG0000(	CHAC1	61.40808	1.016457	2.76E-05	0.000272	protein_coding
ENSG0000(	ENSG0000(	813.7742	0.297373	2.77E-05	0.000272	lncRNA
ENSG0000(	ENSG0000(	11.11905	1.060949	2.77E-05	0.000272	lncRNA
ENSG0000(	FAHD1	502.6298	-0.40519	2.77E-05	0.000272	protein_coding
ENSG0000(	HSPE1P13	211.6165	0.317949	2.78E-05	0.000273	processed_pseudogene
ENSG0000(	INSL3	35.99593	0.598103	2.78E-05	0.000273	protein_coding
ENSG0000(	GCA	410.3556	0.654078	2.79E-05	0.000273	protein_coding
ENSG0000(	TMEM59	2898.986	-0.44418	2.79E-05	0.000273	protein_coding
ENSG0000(	MIR3690	12.5923	1.115429	2.80E-05	0.000275	miRNA
ENSG0000(	ENSG0000(	269.4014	-0.19448	2.81E-05	0.000275	lncRNA
ENSG0000(	RN7SL275F	579.3116	0.224689	2.81E-05	0.000275	misc_RNA
ENSG0000(	BID	703.968	0.231235	2.82E-05	0.000276	protein_coding
ENSG0000(	FBXO41	497.9419	0.515903	2.83E-05	0.000277	protein_coding
ENSG0000(	MTMR9	1393.687	0.226345	2.82E-05	0.000277	protein_coding

ENSG0000(	UBB	1939.603	-0.49875	2.83E-05	0.000277	protein_coding
ENSG0000(	MED18	535.0555	-0.2221	2.83E-05	0.000277	protein_coding
ENSG0000(	AATBC	161.0144	-1.31506	2.83E-05	0.000277	lncRNA
ENSG0000(	CILK1	1076.022	-0.25325	2.83E-05	0.000277	protein_coding
ENSG0000(	COX10	1028.143	-0.30315	2.85E-05	0.000279	protein_coding
ENSG0000(	TTC23	715.1441	-0.27753	2.86E-05	0.000279	protein_coding
ENSG0000(	BAIAP3	217.6029	-0.60761	2.87E-05	0.00028	protein_coding
ENSG0000(	METTL26	171.4178	-0.38868	2.87E-05	0.00028	protein_coding
ENSG0000(	MIR548O2	16.668	0.766622	2.87E-05	0.00028	miRNA
ENSG0000(	NCF4	334.9356	0.51757	2.87E-05	0.00028	protein_coding
ENSG0000(	POM121C	2028.54	-0.17919	2.89E-05	0.000282	protein_coding
ENSG0000(	ADAMTSL4	383.8029	0.54532	2.90E-05	0.000282	protein_coding
ENSG0000(	ENSG0000(	537.1135	0.250095	2.90E-05	0.000283	lncRNA
ENSG0000(	IL15	1033.238	0.335506	2.90E-05	0.000283	protein_coding
ENSG0000(	STARD13-A	124.9276	0.391627	2.91E-05	0.000283	lncRNA
ENSG0000(	RPS27AP12	77.59293	-0.5739	2.91E-05	0.000283	processed_pseudogene
ENSG0000(	SNF8	1001.788	-0.16708	2.91E-05	0.000283	protein_coding
ENSG0000(	ENSG0000(	44.62099	0.664517	2.91E-05	0.000284	lncRNA
ENSG0000(	CCR5	256.0581	0.582422	2.91E-05	0.000284	protein_coding
ENSG0000(	ARHGAP33	397.9833	-0.48524	2.92E-05	0.000284	protein_coding
ENSG0000(	ENSG0000(	226.3046	0.331158	2.93E-05	0.000284	transcribed_unprocessed_pseudo
ENSG0000(	ENSG0000(	573.7022	-0.31699	2.93E-05	0.000285	lncRNA
ENSG0000(	KCNIP4	49.99564	1.019638	2.93E-05	0.000285	protein_coding
ENSG0000(	ENSG0000(	388.1502	0.313018	2.94E-05	0.000286	lncRNA
ENSG0000(	SDHB	505.7309	-0.421	2.94E-05	0.000286	protein_coding
ENSG0000(	EEF2	12059.27	-0.29668	2.95E-05	0.000286	protein_coding
ENSG0000(	RPP25	312.8777	-0.58065	2.96E-05	0.000287	protein_coding
ENSG0000(	ASCC1	914.6458	0.294997	2.97E-05	0.000288	protein_coding
ENSG0000(	APH1B	365.5072	0.454834	2.97E-05	0.000288	protein_coding
ENSG0000(	ENSG0000(	629.0156	0.202092	2.97E-05	0.000288	lncRNA
ENSG0000(	NEDD8	1162.628	-0.18639	2.99E-05	0.00029	protein_coding
ENSG0000(	ENSG0000(	36.65144	-0.56158	2.99E-05	0.00029	processed_pseudogene
ENSG0000(	TNIK	2229.317	-0.26983	2.99E-05	0.00029	protein_coding
ENSG0000(	ENSG0000(	395.6848	0.384339	3.00E-05	0.00029	lncRNA
ENSG0000(	SLC30A3	290.6076	0.285612	2.99E-05	0.00029	protein_coding
ENSG0000(	B3GAT1	152.9003	0.486419	3.00E-05	0.00029	protein_coding
ENSG0000(	OXSR1	1725.762	-0.25368	3.00E-05	0.00029	protein_coding
ENSG0000(	RGP1	2874.245	-0.25137	3.00E-05	0.00029	protein_coding
ENSG0000(	RAB1B	1081.892	-0.35111	3.01E-05	0.000292	protein_coding
ENSG0000(	ELP4	2069.96	0.160308	3.02E-05	0.000292	protein_coding
ENSG0000(	GSR	1644.263	-0.38511	3.02E-05	0.000292	protein_coding
ENSG0000(	MMP2	1585.035	1.014718	3.03E-05	0.000292	protein_coding
ENSG0000(	NDUFB11	289.9577	-0.50013	3.04E-05	0.000293	protein_coding
ENSG0000(	SOBP	224.257	0.493912	3.04E-05	0.000293	protein_coding
ENSG0000(	STAT5A	922.4288	0.352523	3.04E-05	0.000293	protein_coding
ENSG0000(	BUD31	483.122	-0.24174	3.05E-05	0.000294	protein_coding
ENSG0000(	ENSG0000(	777.8094	0.476872	3.05E-05	0.000294	lncRNA

ENSG0000(	HLA-F	2544.468	0.507602	3.05E-05	0.000294	protein_coding
ENSG0000(	SLC17A9	747.4144	0.646989	3.05E-05	0.000294	protein_coding
ENSG0000(	NGRN	889.7334	-0.29038	3.06E-05	0.000295	protein_coding
ENSG0000(	SLC9B2	251.2646	0.568688	3.07E-05	0.000295	protein_coding
ENSG0000(	ELOVL2	13.6208	1.570339	3.07E-05	0.000296	protein_coding
ENSG0000(	CEP164	1194.668	0.235779	3.07E-05	0.000296	protein_coding
ENSG0000(	MOCS2-DT	914.8332	0.302803	3.08E-05	0.000296	lncRNA
ENSG0000(	HSPA1A	1046.92	-0.69873	3.08E-05	0.000296	protein_coding
ENSG0000(	SHANK2-AS	594.036	0.165826	3.08E-05	0.000296	lncRNA
ENSG0000(	MPZL2	1040.97	-0.3793	3.09E-05	0.000297	protein_coding
ENSG0000(	CD63	1614.004	-0.46585	3.10E-05	0.000298	protein_coding
ENSG0000(	ENSG0000(	687.7653	0.171447	3.11E-05	0.000299	lncRNA
ENSG0000(	ENSG0000(	151.3226	0.291741	3.12E-05	0.000299	lncRNA
ENSG0000(	BUB1B	383.905	-0.54022	3.14E-05	0.000301	protein_coding
ENSG0000(	ENSG0000(	122.5524	-0.27728	3.14E-05	0.000302	lncRNA
ENSG0000(	C6orf136	440.57	-0.46025	3.15E-05	0.000302	protein_coding
ENSG0000(	MT1L	11.21609	1.274486	3.15E-05	0.000302	transcribed_unprocessed_pseudo
ENSG0000(	PPP1R1B	1873.729	-0.65106	3.15E-05	0.000302	protein_coding
ENSG0000(	CCDC12	573.8146	-0.18747	3.16E-05	0.000303	protein_coding
ENSG0000(	SOX5	421.405	0.553399	3.16E-05	0.000303	protein_coding
ENSG0000(	ENSG0000(	62.53	-0.57305	3.16E-05	0.000303	processed_pseudogene
ENSG0000(	CD74	10678.4	0.659342	3.17E-05	0.000303	protein_coding
ENSG0000(	MIR4729	13.96592	0.903251	3.17E-05	0.000303	miRNA
ENSG0000(	SLC16A9	863.2088	-1.07181	3.17E-05	0.000303	protein_coding
ENSG0000(	MAP1LC3A	242.4832	-0.41854	3.17E-05	0.000304	protein_coding
ENSG0000(	ENSG0000(	30.87644	0.557455	3.20E-05	0.000306	processed_pseudogene
ENSG0000(	TBC1D15	1490.418	0.20639	3.20E-05	0.000306	protein_coding
ENSG0000(	AKAP1	3781.522	-0.49499	3.20E-05	0.000306	protein_coding
ENSG0000(	RAI14	977.0769	0.606901	3.20E-05	0.000306	protein_coding
ENSG0000(	ENSG0000(	45.24077	0.626115	3.21E-05	0.000306	lncRNA
ENSG0000(	ENSG0000(	93.06075	0.38925	3.21E-05	0.000307	lncRNA
ENSG0000(	PPIA	6696.965	-0.35864	3.21E-05	0.000307	protein_coding
ENSG0000(	MXRA5	1836.79	0.693458	3.22E-05	0.000308	protein_coding
ENSG0000(	RAX2	167.7981	0.373665	3.22E-05	0.000308	protein_coding
ENSG0000(	RIPK2	200.5297	0.530779	3.24E-05	0.000309	protein_coding
ENSG0000(	PIGO	958.5154	-0.23707	3.24E-05	0.000309	protein_coding
ENSG0000(	NDEL1	2247.269	0.214606	3.24E-05	0.000309	protein_coding
ENSG0000(	LOXL3	187.9165	0.398011	3.24E-05	0.000309	protein_coding
ENSG0000(	ENSG0000(	340.4096	-0.28151	3.25E-05	0.00031	lncRNA
ENSG0000(	BOLA2-SM(	1305.993	-0.33072	3.25E-05	0.00031	protein_coding
ENSG0000(	SMIM20	132.5315	-0.3662	3.25E-05	0.00031	protein_coding
ENSG0000(	TSPAN5-DT	200.8277	0.322761	3.26E-05	0.00031	lncRNA
ENSG0000(	C1QTNF6	1064.785	0.259066	3.26E-05	0.00031	protein_coding
ENSG0000(	ELMO3	467.9828	-0.5203	3.26E-05	0.00031	protein_coding
ENSG0000(	FGFR3	1220.54	-0.66336	3.26E-05	0.00031	protein_coding
ENSG0000(	SNORD13E	18.60465	0.793241	3.26E-05	0.00031	snoRNA
ENSG0000(	ENSG0000(	358.8206	0.741657	3.27E-05	0.00031	processed_pseudogene

ENSG0000(	MS4A4A	691.7505	0.247429	3.28E-05	0.000312	protein_coding
ENSG0000(	LSM12	964.6016	-0.21547	3.29E-05	0.000313	protein_coding
ENSG0000(	PTMS	225.5016	-0.39957	3.30E-05	0.000313	protein_coding
ENSG0000(	HPS1	1703.381	-0.1964	3.30E-05	0.000313	protein_coding
ENSG0000(	RAB3IP	2381.23	-0.30838	3.30E-05	0.000314	protein_coding
ENSG0000(	SMU1	1593.315	-0.15842	3.32E-05	0.000315	protein_coding
ENSG0000(	TUBG2	193.1453	0.373246	3.32E-05	0.000315	protein_coding
ENSG0000(	ENSG0000(	61.79132	0.432024	3.32E-05	0.000315	lncRNA
ENSG0000(	ST18	234.7451	-0.33485	3.33E-05	0.000316	protein_coding
ENSG0000(	DNAAF9	697.0705	0.282819	3.33E-05	0.000316	protein_coding
ENSG0000(	ENSG0000(	139.0729	-0.34358	3.34E-05	0.000316	lncRNA
ENSG0000(	ENSG0000(	505.4597	0.186169	3.34E-05	0.000316	lncRNA
ENSG0000(	RAP2C-AS1	340.1181	0.208178	3.35E-05	0.000318	lncRNA
ENSG0000(	ENSG0000(	532.9539	-0.17031	3.36E-05	0.000319	lncRNA
ENSG0000(	H2BC10	108.2219	-0.6862	3.38E-05	0.00032	protein_coding
ENSG0000(	MLH3	2454.051	-0.22538	3.39E-05	0.000321	protein_coding
ENSG0000(	TELO2	598.3624	-0.35736	3.39E-05	0.000321	protein_coding
ENSG0000(	TIE1	446.7149	0.379471	3.39E-05	0.000321	protein_coding
ENSG0000(	RPS2	4533.678	-0.47001	3.40E-05	0.000321	protein_coding
ENSG0000(	ENSG0000(	15.21276	0.85421	3.41E-05	0.000322	lncRNA
ENSG0000(	LINC00513	460.8083	0.248242	3.41E-05	0.000322	lncRNA
ENSG0000(	LINC01139	289.8583	0.316161	3.41E-05	0.000322	lncRNA
ENSG0000(	IL17RB	439.5447	-0.66141	3.43E-05	0.000324	protein_coding
ENSG0000(	ENSG0000(	86.33657	0.428055	3.43E-05	0.000324	lncRNA
ENSG0000(	ENSG0000(	12.4586	1.206067	3.46E-05	0.000327	unprocessed_pseudogene
ENSG0000(	PIK3R4	970.5123	-0.34327	3.48E-05	0.000328	protein_coding
ENSG0000(	ZEB2-AS1	55.91702	1.389245	3.48E-05	0.000328	lncRNA
ENSG0000(	DAP3	1267.449	-0.16455	3.50E-05	0.00033	protein_coding
ENSG0000(	ANKRD17	6403.492	-0.12487	3.51E-05	0.000331	protein_coding
ENSG0000(	RPS6KA2	828.6246	0.376483	3.51E-05	0.000331	protein_coding
ENSG0000(	PI15	507.5751	1.980421	3.53E-05	0.000333	protein_coding
ENSG0000(	TPRKBP2	44.03164	0.506862	3.53E-05	0.000333	processed_pseudogene
ENSG0000(	MEG3	4803.993	0.561812	3.53E-05	0.000333	lncRNA
ENSG0000(	NCBP2	1269.852	-0.18549	3.54E-05	0.000333	protein_coding
ENSG0000(	SNX6	1302.085	-0.2326	3.54E-05	0.000333	protein_coding
ENSG0000(	ZFC3H1	4636.005	-0.20039	3.54E-05	0.000333	protein_coding
ENSG0000(	MPHOSPH8	2300.137	-0.20061	3.56E-05	0.000334	protein_coding
ENSG0000(	ZNF566	889.8958	-0.19532	3.56E-05	0.000334	protein_coding
ENSG0000(	EEF1AKMT4	37.28582	-0.55631	3.56E-05	0.000335	protein_coding
ENSG0000(	JAKMIP1	26.52746	0.919433	3.57E-05	0.000335	protein_coding
ENSG0000(	ENSG0000(	316.4762	0.218695	3.57E-05	0.000336	lncRNA
ENSG0000(	HPS6	265.394	-0.28373	3.58E-05	0.000336	protein_coding
ENSG0000(	ENSG0000(	59.04505	0.685148	3.58E-05	0.000336	TEC
ENSG0000(	GTF2F1	653.7273	-0.37208	3.58E-05	0.000336	protein_coding
ENSG0000(	GTF2F2	533.0067	-0.26364	3.58E-05	0.000336	protein_coding
ENSG0000(	ENSG0000(	144.6394	0.661222	3.59E-05	0.000336	lncRNA
ENSG0000(	RN7SL288P	59.79466	0.615587	3.59E-05	0.000336	misc_RNA

ENSG0000(	RPA2	231.2309	-0.36748	3.60E-05	0.000337	protein_coding
ENSG0000(	FAM3A	1009.981	-0.26369	3.60E-05	0.000337	protein_coding
ENSG0000(	MX2	2370.119	0.558918	3.61E-05	0.000338	protein_coding
ENSG0000(	PRICKLE3	311.7776	-0.29418	3.61E-05	0.000338	protein_coding
ENSG0000(	ENSG0000(	10.5037	1.148067	3.62E-05	0.000339	lncRNA
ENSG0000(	NSDHL	130.5927	-0.37951	3.62E-05	0.000339	protein_coding
ENSG0000(	TIFA	361.5415	0.622949	3.62E-05	0.000339	protein_coding
ENSG0000(	EIF4H	3987.452	-0.26823	3.62E-05	0.000339	protein_coding
ENSG0000(	ENSG0000(	440.3353	-0.23111	3.63E-05	0.000339	lncRNA
ENSG0000(	PPT2	492.5005	-0.37043	3.63E-05	0.000339	protein_coding
ENSG0000(	ITPRIPL1	248.9854	0.28656	3.63E-05	0.000339	protein_coding
ENSG0000(	ENSG0000(	186.1516	-0.39742	3.63E-05	0.000339	lncRNA
ENSG0000(	ENSG0000(	37.00416	0.772568	3.64E-05	0.00034	lncRNA
ENSG0000(	BLOC1S1	484.4506	-0.41634	3.64E-05	0.00034	protein_coding
ENSG0000(	POR	615.9127	-0.32736	3.64E-05	0.00034	protein_coding
ENSG0000(	CYP2B6	752.3089	-0.89693	3.65E-05	0.00034	protein_coding
ENSG0000(	KCNC3	246.4425	0.57682	3.66E-05	0.000341	protein_coding
ENSG0000(	MORC4	6460.601	-0.24375	3.66E-05	0.000341	protein_coding
ENSG0000(	NOM1	1246.337	-0.27476	3.67E-05	0.000342	protein_coding
ENSG0000(	UGT2A3	697.9853	-0.97842	3.67E-05	0.000342	protein_coding
ENSG0000(	EIF3M	1621.977	-0.30428	3.68E-05	0.000343	protein_coding
ENSG0000(	FAM78B-A!	101.8425	-0.30926	3.68E-05	0.000343	lncRNA
ENSG0000(	CHD1L	920.914	-0.28591	3.69E-05	0.000344	protein_coding
ENSG0000(	ZNF157	300.8873	-0.29312	3.71E-05	0.000345	protein_coding
ENSG0000(	ENSG0000(	117.2417	-0.4873	3.72E-05	0.000346	lncRNA
ENSG0000(	CDH17	8683.657	-0.59582	3.73E-05	0.000347	protein_coding
ENSG0000(	NDUFS8	1058.625	-0.27322	3.73E-05	0.000347	protein_coding
ENSG0000(	CCT6A	1156.794	-0.26844	3.73E-05	0.000347	protein_coding
ENSG0000(	CYP27B1	64.62841	0.923629	3.74E-05	0.000347	protein_coding
ENSG0000(	MAN1A2	2673.648	0.227254	3.75E-05	0.000348	protein_coding
ENSG0000(	RPL21P135	454.8993	0.223211	3.75E-05	0.000348	processed_pseudogene
ENSG0000(	LINC01120	14.17452	1.038972	3.77E-05	0.00035	lncRNA
ENSG0000(	ENSG0000(	171.6322	-0.29272	3.77E-05	0.00035	lncRNA
ENSG0000(	CFL1	5267.03	-0.41477	3.77E-05	0.00035	protein_coding
ENSG0000(	CCNJL	1358.707	-0.41546	3.78E-05	0.00035	protein_coding
ENSG0000(	PTK7	843.4184	0.368525	3.78E-05	0.00035	protein_coding
ENSG0000(	CAT	1014.969	-0.33566	3.79E-05	0.000351	protein_coding
ENSG0000(	RNF128	316.2924	-0.66093	3.79E-05	0.000352	protein_coding
ENSG0000(	C1GALT1	1659.599	0.267182	3.80E-05	0.000352	protein_coding
ENSG0000(	ENSG0000(	20.06822	0.920189	3.80E-05	0.000352	lncRNA
ENSG0000(	ENSG0000(	62.89406	0.522219	3.81E-05	0.000352	lncRNA
ENSG0000(	ENSG0000(	119.0849	0.360628	3.81E-05	0.000353	lncRNA
ENSG0000(	NPRL3	533.8627	-0.25539	3.82E-05	0.000353	protein_coding
ENSG0000(	SGSM3-AS:	106.198	0.367858	3.82E-05	0.000354	lncRNA
ENSG0000(	VLDLR-AS1	601.1988	-0.58023	3.83E-05	0.000354	lncRNA
ENSG0000(	MAK16	461.7041	-0.35079	3.84E-05	0.000355	protein_coding
ENSG0000(	ACOT2	142.9543	-0.44472	3.84E-05	0.000355	protein_coding

ENSG0000(	RPL28	2475.281	-0.41384	3.84E-05	0.000355	protein_coding
ENSG0000(	HERPUD1	4006.668	0.318587	3.85E-05	0.000356	protein_coding
ENSG0000(	PITPNM1	1644.874	-0.35319	3.85E-05	0.000356	protein_coding
ENSG0000(	TERF1	2582.047	0.143126	3.86E-05	0.000357	protein_coding
ENSG0000(	CLSTN3	1103.331	0.518599	3.87E-05	0.000357	protein_coding
ENSG0000(	RPL24	1638.194	-0.40073	3.87E-05	0.000357	protein_coding
ENSG0000(	ENSG0000(	174.7463	-0.41511	3.88E-05	0.000358	lncRNA
ENSG0000(	GSTK1	3354.505	-0.21721	3.88E-05	0.000358	protein_coding
ENSG0000(	ADRM1	361.6907	-0.39452	3.89E-05	0.000359	protein_coding
ENSG0000(	ENSG0000(	153.862	0.277602	3.89E-05	0.000359	lncRNA
ENSG0000(	B4GALT5	2437.851	-0.44475	3.90E-05	0.000359	protein_coding
ENSG0000(	FAM83B	2668.1	-0.48021	3.90E-05	0.00036	protein_coding
ENSG0000(	MOSPD1	138.613	-0.39663	3.91E-05	0.00036	protein_coding
ENSG0000(	ENSG0000(	19.85409	-1.27172	3.92E-05	0.000361	processed_pseudogene
ENSG0000(	MRPL35	1044.183	-0.36284	3.92E-05	0.000361	protein_coding
ENSG0000(	LINC01284	400.1588	0.200994	3.93E-05	0.000361	lncRNA
ENSG0000(	ENSG0000(	565.0783	0.213918	3.93E-05	0.000362	lncRNA
ENSG0000(	WDR46	317.9628	-0.36098	3.93E-05	0.000362	protein_coding
ENSG0000(	TRAPPC6A	75.83552	-0.45181	3.94E-05	0.000363	protein_coding
ENSG0000(	C3orf35	1850.387	0.121706	3.95E-05	0.000363	lncRNA
ENSG0000(	RNF122	169.2064	0.408478	3.96E-05	0.000364	protein_coding
ENSG0000(	ANPEP	13702.27	-1.1529	3.98E-05	0.000366	protein_coding
ENSG0000(	CD69	239.4646	0.923582	3.98E-05	0.000366	protein_coding
ENSG0000(	PGRMC2	1185.65	-0.26371	3.98E-05	0.000366	protein_coding
ENSG0000(	DOT1L	2561.813	-0.27313	3.98E-05	0.000366	protein_coding
ENSG0000(	HDAC2	1550.899	-0.24635	3.99E-05	0.000366	protein_coding
ENSG0000(	EBNA1BP2	289.8057	-0.29216	4.01E-05	0.000368	protein_coding
ENSG0000(	ENSG0000(	44.66281	0.551383	4.01E-05	0.000368	TEC
ENSG0000(	ENSG0000(	1486.41	0.181362	4.02E-05	0.000368	transcribed_unprocessed_pseudo
ENSG0000(	ENSG0000(	225.9627	0.414389	4.02E-05	0.000368	lncRNA
ENSG0000(	MAMDC4	876.8626	-0.48014	4.02E-05	0.000368	protein_coding
ENSG0000(	PCYOX1	1437.117	-0.29659	4.02E-05	0.000368	protein_coding
ENSG0000(	PRPF18	476.2984	-0.26141	4.02E-05	0.000368	protein_coding
ENSG0000(	ENSG0000(	54.6496	0.603369	4.03E-05	0.000368	lncRNA
ENSG0000(	PDZD7	283.004	0.30179	4.04E-05	0.000369	protein_coding
ENSG0000(	TUBA3FP	320.6309	0.231599	4.05E-05	0.00037	lncRNA
ENSG0000(	EPS15L1	1448.367	-0.14466	4.07E-05	0.000372	protein_coding
ENSG0000(	ENSG0000(	358.0983	0.234947	4.07E-05	0.000372	lncRNA
ENSG0000(	TK1	169.893	-0.54932	4.08E-05	0.000373	protein_coding
ENSG0000(	SEMA6A-A!	110.8947	0.430771	4.08E-05	0.000373	lncRNA
ENSG0000(	CRLS1	1078.981	-0.25205	4.09E-05	0.000373	protein_coding
ENSG0000(	ZWILCH	354.6455	-0.36915	4.09E-05	0.000373	protein_coding
ENSG0000(	KDELR1	1467.511	-0.23709	4.11E-05	0.000375	protein_coding
ENSG0000(	ANKS4B	1945.771	-0.39809	4.11E-05	0.000375	protein_coding
ENSG0000(	KLHL30-AS:	162.8018	0.373515	4.11E-05	0.000375	lncRNA
ENSG0000(	PMCHL1	98.56094	-0.47376	4.11E-05	0.000375	transcribed_unprocessed_pseudo
ENSG0000(	ENSG0000(	680.2639	-0.166	4.11E-05	0.000375	lncRNA

ENSG0000(	GGT5	983.6918	0.383467	4.12E-05	0.000375	protein_coding
ENSG0000(	SNHG4	1270.328	-0.28195	4.13E-05	0.000376	lncRNA
ENSG0000(	SLFN12	308.1282	0.515976	4.13E-05	0.000376	protein_coding
ENSG0000(	TNK1	348.0149	-0.46243	4.13E-05	0.000376	protein_coding
ENSG0000(	ACTG1P20	24.60338	-0.66731	4.15E-05	0.000378	processed_pseudogene
ENSG0000(	ENSG0000(	351.3873	-0.24326	4.16E-05	0.000378	lncRNA
ENSG0000(	SIVA1	737.5639	-0.30078	4.16E-05	0.000378	protein_coding
ENSG0000(	ZDHHC7	2291.641	-0.26424	4.16E-05	0.000378	protein_coding
ENSG0000(	GALT	727.5268	-0.27312	4.16E-05	0.000378	protein_coding
ENSG0000(	DRAM1	938.2849	0.363229	4.17E-05	0.000379	protein_coding
ENSG0000(	IL11	299.0096	1.199583	4.17E-05	0.000379	protein_coding
ENSG0000(	H3C13	54.096	-0.86598	4.18E-05	0.000379	protein_coding
ENSG0000(	PFAS	864.9853	-0.3179	4.19E-05	0.000381	protein_coding
ENSG0000(	LINC02065	33.67142	0.476495	4.20E-05	0.000382	lncRNA
ENSG0000(	TMA16	495.4311	-0.29071	4.21E-05	0.000382	protein_coding
ENSG0000(	TMEM179E	319.2761	-0.30241	4.21E-05	0.000382	protein_coding
ENSG0000(	PARK7	704.2787	-0.23786	4.23E-05	0.000384	protein_coding
ENSG0000(	UQCRFS1	1090.739	-0.41764	4.24E-05	0.000384	protein_coding
ENSG0000(	HMGB3P9	166.2589	0.305939	4.24E-05	0.000384	processed_pseudogene
ENSG0000(	GNA15	65.19576	0.823277	4.25E-05	0.000385	protein_coding
ENSG0000(	LINC01632	13.54369	1.232889	4.25E-05	0.000385	lncRNA
ENSG0000(	UBAC1	550.8949	-0.33298	4.26E-05	0.000386	protein_coding
ENSG0000(	LRRC8C-DT	459.8125	0.423815	4.27E-05	0.000386	lncRNA
ENSG0000(	BTK	454.0297	0.720824	4.27E-05	0.000387	protein_coding
ENSG0000(	AK2	4661.24	-0.22454	4.27E-05	0.000387	protein_coding
ENSG0000(	PHGR1	6060.73	-0.87424	4.27E-05	0.000387	protein_coding
ENSG0000(	PARD3	1554.816	-0.3304	4.28E-05	0.000387	protein_coding
ENSG0000(	CTCFL	626.7058	0.155537	4.28E-05	0.000387	protein_coding
ENSG0000(	POLR2J	133.7911	-0.40523	4.29E-05	0.000388	protein_coding
ENSG0000(	ACTG1P17	351.1417	0.247135	4.29E-05	0.000388	transcribed_processed_pseudoge
ENSG0000(	NELFB	575.4382	-0.3132	4.30E-05	0.000388	protein_coding
ENSG0000(	ENSG0000(	109.5661	0.325569	4.30E-05	0.000388	processed_pseudogene
ENSG0000(	TMEM14A	55.44239	-0.61157	4.30E-05	0.000388	protein_coding
ENSG0000(	CRK	2668.278	-0.20691	4.31E-05	0.000389	protein_coding
ENSG0000(	ENSG0000(	90.92036	0.365585	4.31E-05	0.000389	lncRNA
ENSG0000(	LINC02352	240.7171	0.262947	4.31E-05	0.000389	lncRNA
ENSG0000(	ADAMTS16	38.83709	0.729141	4.32E-05	0.000389	protein_coding
ENSG0000(	TIGD5	312.6876	-0.25035	4.32E-05	0.000389	protein_coding
ENSG0000(	EPB41L1	1582.767	-0.57704	4.33E-05	0.00039	protein_coding
ENSG0000(	SNORA53	672.9904	-0.60017	4.35E-05	0.000392	snoRNA
ENSG0000(	ENSG0000(	420.2471	0.299252	4.35E-05	0.000392	lncRNA
ENSG0000(	METAP1	1163.022	-0.24376	4.35E-05	0.000392	protein_coding
ENSG0000(	PAM	1049.326	0.370012	4.36E-05	0.000393	protein_coding
ENSG0000(	OTOAP1	51.42091	0.618609	4.39E-05	0.000395	transcribed_unprocessed_pseudo
ENSG0000(	UTS2R	818.5298	0.393801	4.40E-05	0.000396	protein_coding
ENSG0000(	GLYR1	2022.622	-0.11939	4.41E-05	0.000397	protein_coding
ENSG0000(	SUCLA2	781.6905	-0.25253	4.41E-05	0.000397	protein_coding

ENSG0000(	TACC2	4087.84	-0.33448	4.42E-05	0.000397	protein_coding
ENSG0000(	RN7SL440F	269.077	-0.30566	4.42E-05	0.000397	misc_RNA
ENSG0000(	GPX7	85.50986	0.698465	4.42E-05	0.000398	protein_coding
ENSG0000(	CASP8	1917.791	0.266954	4.44E-05	0.000398	protein_coding
ENSG0000(	ZNF516	1408.854	0.295791	4.44E-05	0.000398	protein_coding
ENSG0000(	ATP8B1	6795.523	-0.46022	4.46E-05	0.0004	protein_coding
ENSG0000(	ENSG0000(	553.2681	0.353478	4.46E-05	0.0004	protein_coding
ENSG0000(	HELZ2	2227.932	0.467422	4.46E-05	0.0004	protein_coding
ENSG0000(	MEF2C-AS1	285.5852	0.462722	4.46E-05	0.0004	lncRNA
ENSG0000(	ENSG0000(	264.8448	0.250503	4.48E-05	0.000401	lncRNA
ENSG0000(	DCXR	182.9985	-0.47446	4.51E-05	0.000404	protein_coding
ENSG0000(	RDH5	487.2192	-0.40027	4.51E-05	0.000404	protein_coding
ENSG0000(	GPD1L	2186.204	-0.49015	4.52E-05	0.000405	protein_coding
ENSG0000(	GABARAPL	836.6601	0.36889	4.52E-05	0.000405	protein_coding
ENSG0000(	NSUN2	1484.559	-0.23766	4.52E-05	0.000405	protein_coding
ENSG0000(	CALM2	7284.939	-0.27283	4.54E-05	0.000407	protein_coding
ENSG0000(	ENSG0000(	25.09479	0.899287	4.55E-05	0.000407	lncRNA
ENSG0000(	MTHFR	2857.611	0.211208	4.56E-05	0.000408	protein_coding
ENSG0000(	KPNA2	1156.794	-0.31016	4.56E-05	0.000408	protein_coding
ENSG0000(	NOVA1-DT	109.5338	-0.91939	4.58E-05	0.000409	lncRNA
ENSG0000(	ENSG0000(	345.4454	-0.24565	4.58E-05	0.000409	processed_pseudogene
ENSG0000(	ENSG0000(	223.7586	-0.34113	4.58E-05	0.00041	lncRNA
ENSG0000(	TOMM70	1283.8	-0.24197	4.59E-05	0.00041	protein_coding
ENSG0000(	NR3C1	1922.516	0.396095	4.61E-05	0.000412	protein_coding
ENSG0000(	FHAD1	303.3282	0.428758	4.61E-05	0.000412	protein_coding
ENSG0000(	DDX10P1	68.17978	0.499599	4.62E-05	0.000412	processed_pseudogene
ENSG0000(	NAA11	31.08909	0.686318	4.62E-05	0.000412	protein_coding
ENSG0000(	RPS17	2680.629	-0.41085	4.62E-05	0.000412	protein_coding
ENSG0000(	ADD2	373.9106	0.567797	4.62E-05	0.000412	protein_coding
ENSG0000(	ENSG0000(	21.96121	0.888165	4.63E-05	0.000413	TEC
ENSG0000(	RPL29	2207.62	-0.35886	4.65E-05	0.000414	protein_coding
ENSG0000(	TRIM21	397.9698	0.397148	4.65E-05	0.000414	protein_coding
ENSG0000(	DGAT2	254.7174	0.717638	4.66E-05	0.000415	protein_coding
ENSG0000(	DTNB	320.5878	0.349456	4.67E-05	0.000416	protein_coding
ENSG0000(	ENSG0000(	145.4313	-0.25074	4.69E-05	0.000418	lncRNA
ENSG0000(	DOCK2	2628.606	0.564777	4.70E-05	0.000418	protein_coding
ENSG0000(	JTB	731.977	-0.3146	4.72E-05	0.00042	protein_coding
ENSG0000(	OR52K1	406.8181	0.237253	4.73E-05	0.000421	protein_coding
ENSG0000(	ATAD3C	495.0412	0.412944	4.73E-05	0.000421	protein_coding
ENSG0000(	VDAC2	1602.869	-0.4449	4.73E-05	0.000421	protein_coding
ENSG0000(	EXO1	155.641	-0.47853	4.74E-05	0.000421	protein_coding
ENSG0000(	LPCAT3	1269.042	-0.36882	4.74E-05	0.000421	protein_coding
ENSG0000(	LSM11	703.4321	-0.19046	4.74E-05	0.000421	protein_coding
ENSG0000(	NIPSNAP2	1144.788	-0.22973	4.75E-05	0.000422	protein_coding
ENSG0000(	PLK1	451.7184	-0.31885	4.76E-05	0.000422	protein_coding
ENSG0000(	RBMXL1	891.9893	0.187911	4.77E-05	0.000424	protein_coding
ENSG0000(	TMEM268	772.9272	0.319722	4.77E-05	0.000424	protein_coding

ENSG0000(	TMEM60	126.7466	-0.37099	4.77E-05	0.000424	protein_coding
ENSG0000(	IGF2	689.4306	0.757113	4.78E-05	0.000424	protein_coding
ENSG0000(	VTA1	922.4029	-0.36052	4.78E-05	0.000424	protein_coding
ENSG0000(	ENSG0000(	121.0174	0.549112	4.79E-05	0.000424	lncRNA
ENSG0000(	ST3GAL2	2138.341	0.387922	4.80E-05	0.000425	protein_coding
ENSG0000(	RFX3-AS1	382.2979	0.219714	4.80E-05	0.000425	lncRNA
ENSG0000(	SNX20	485.4001	0.600304	4.80E-05	0.000425	protein_coding
ENSG0000(	ATF7IP	5441.323	0.297979	4.82E-05	0.000427	protein_coding
ENSG0000(	HOXA6	377.9479	-0.47271	4.82E-05	0.000427	protein_coding
ENSG0000(	NBPF2P	113.8059	-0.50098	4.83E-05	0.000427	unprocessed_pseudogene
ENSG0000(	LINC01160	267.1449	0.213411	4.83E-05	0.000427	lncRNA
ENSG0000(	KRT8	12485.21	-0.63468	4.86E-05	0.000429	protein_coding
ENSG0000(	PQBP1	448.2911	-0.25708	4.86E-05	0.00043	protein_coding
ENSG0000(	EPPIN	501.1452	0.22734	4.89E-05	0.000432	protein_coding
ENSG0000(	IRS1	906.9254	0.566564	4.89E-05	0.000432	protein_coding
ENSG0000(	MIR4458H(	204.3957	-0.5373	4.90E-05	0.000433	lncRNA
ENSG0000(	NDUFV3	2413.413	-0.15663	4.91E-05	0.000434	protein_coding
ENSG0000(	URGCP	2380.92	-0.29818	4.91E-05	0.000434	protein_coding
ENSG0000(	NCF1	552.1632	0.531094	4.92E-05	0.000435	protein_coding
ENSG0000(	STMN1	963.8068	-0.348	4.92E-05	0.000435	protein_coding
ENSG0000(	NAXE	674.7305	-0.31305	4.93E-05	0.000435	protein_coding
ENSG0000(	TNFSF8	261.9439	0.921041	4.95E-05	0.000437	protein_coding
ENSG0000(	LINC02632	268.5274	0.287199	4.97E-05	0.000438	lncRNA
ENSG0000(	LINC01499	102.2427	-0.32307	5.00E-05	0.000441	lncRNA
ENSG0000(	STK39	1554.709	-0.39417	5.01E-05	0.000441	protein_coding
ENSG0000(	DQX1	808.734	-0.80814	5.01E-05	0.000441	protein_coding
ENSG0000(	OR56B1	11.08837	1.348948	5.02E-05	0.000442	protein_coding
ENSG0000(	ENSG0000(	17.61212	1.222212	5.05E-05	0.000444	lncRNA
ENSG0000(	PLCD1	630.2322	-0.68815	5.05E-05	0.000444	protein_coding
ENSG0000(	ENSG0000(	14.29604	0.795166	5.05E-05	0.000444	unprocessed_pseudogene
ENSG0000(	DEPDC1	353.1112	-0.40741	5.06E-05	0.000445	protein_coding
ENSG0000(	SMAP2	1434.069	0.641779	5.06E-05	0.000445	protein_coding
ENSG0000(	RPS25	1957.564	-0.43178	5.06E-05	0.000445	protein_coding
ENSG0000(	ACYP1	381.1388	-0.26611	5.07E-05	0.000446	protein_coding
ENSG0000(	AASS	1614.428	0.283978	5.09E-05	0.000448	protein_coding
ENSG0000(	ENSG0000(	30.05574	0.720167	5.10E-05	0.000448	lncRNA
ENSG0000(	ZFHX4	163.7128	0.401618	5.11E-05	0.000448	protein_coding
ENSG0000(	FZD4	1098.08	0.307966	5.12E-05	0.000449	protein_coding
ENSG0000(	SPAG5	662.318	-0.37352	5.12E-05	0.00045	protein_coding
ENSG0000(	FAM167A	109.7021	0.936315	5.13E-05	0.00045	protein_coding
ENSG0000(	PTMA	6553.051	-0.28808	5.13E-05	0.00045	protein_coding
ENSG0000(	ENSG0000(	87.52414	0.351114	5.13E-05	0.00045	lncRNA
ENSG0000(	CYP4F2	113.4653	-1.02463	5.14E-05	0.000451	protein_coding
ENSG0000(	TRAPPC4	909.1649	-0.22525	5.14E-05	0.000451	protein_coding
ENSG0000(	ENSG0000(	141.2834	0.438258	5.15E-05	0.000452	lncRNA
ENSG0000(	RABGAP1L-	1216.944	-0.16316	5.16E-05	0.000452	lncRNA
ENSG0000(	RPL19	4392.735	-0.40713	5.17E-05	0.000452	protein_coding

ENSG0000(	PFKFB4	580.8224	1.198248	5.19E-05	0.000454	protein_coding
ENSG0000(	ARF6	3365.673	-0.3221	5.21E-05	0.000456	protein_coding
ENSG0000(	ENSG0000(	141.3469	-0.30928	5.23E-05	0.000457	lncRNA
ENSG0000(	JDP2	773.9158	0.346548	5.29E-05	0.000463	protein_coding
ENSG0000(	RNU7-41P	13.29187	0.826243	5.29E-05	0.000463	snRNA
ENSG0000(	RAB4A	616.3731	-0.36404	5.30E-05	0.000464	protein_coding
ENSG0000(	CNN3	1018.419	-0.28782	5.31E-05	0.000464	protein_coding
ENSG0000(	IGKJ2	22.61942	1.205884	5.32E-05	0.000465	IG_J_gene
ENSG0000(	PCF11	4956.609	0.170529	5.32E-05	0.000465	protein_coding
ENSG0000(	AGT	171.79	0.759164	5.33E-05	0.000465	protein_coding
ENSG0000(	FRAT2	407.5348	-0.29671	5.33E-05	0.000465	protein_coding
ENSG0000(	DHRSX-IT1	102.3342	0.360504	5.36E-05	0.000468	lncRNA
ENSG0000(	TMEM185I	1025.371	0.319713	5.38E-05	0.000469	protein_coding
ENSG0000(	ENSG0000(	495.4993	0.209159	5.38E-05	0.000469	protein_coding
ENSG0000(	MRAS	310.1349	0.602972	5.38E-05	0.00047	protein_coding
ENSG0000(	LINC02351	11.67938	1.471659	5.40E-05	0.000471	lncRNA
ENSG0000(	ENSG0000(	101.0096	-0.35395	5.41E-05	0.000472	lncRNA
ENSG0000(	ACTR8	643.6756	-0.28327	5.42E-05	0.000472	protein_coding
ENSG0000(	CYTH4	1396.998	0.523826	5.43E-05	0.000473	protein_coding
ENSG0000(	EFNA1	660.6519	-0.66127	5.44E-05	0.000474	protein_coding
ENSG0000(	LINC02214	80.78776	-0.34725	5.45E-05	0.000474	lncRNA
ENSG0000(	NAE1	868.0533	-0.14734	5.45E-05	0.000474	protein_coding
ENSG0000(	ENSG0000(	27.99113	0.649544	5.46E-05	0.000475	lncRNA
ENSG0000(	USP5	567.986	-0.34377	5.47E-05	0.000476	protein_coding
ENSG0000(	CNOT11	867.2995	-0.2745	5.48E-05	0.000476	protein_coding
ENSG0000(	NEAT1	388432	0.434223	5.48E-05	0.000476	lncRNA
ENSG0000(	ZNF511	202.0528	-0.41276	5.48E-05	0.000477	protein_coding
ENSG0000(	RPL32	3494.54	-0.38971	5.49E-05	0.000477	protein_coding
ENSG0000(	C8orf82	366.8439	-0.37521	5.49E-05	0.000477	protein_coding
ENSG0000(	RPS4X	4827.204	-0.38461	5.50E-05	0.000478	protein_coding
ENSG0000(	CASP10	3064.943	0.31104	5.50E-05	0.000478	protein_coding
ENSG0000(	ENSG0000(	46.52022	0.584306	5.50E-05	0.000478	unprocessed_pseudogene
ENSG0000(	MRPS22	1394.528	-0.20892	5.51E-05	0.000478	protein_coding
ENSG0000(	SLC41A1	455.0849	0.515403	5.51E-05	0.000478	protein_coding
ENSG0000(	MCM3	830.8173	-0.32374	5.52E-05	0.000478	protein_coding
ENSG0000(	UBE2V2	1047.451	-0.18417	5.52E-05	0.000478	protein_coding
ENSG0000(	IL33	290.301	0.528221	5.54E-05	0.00048	protein_coding
ENSG0000(	CCDC113	350.3411	-0.26034	5.55E-05	0.000481	protein_coding
ENSG0000(	CHRAC1	331.2308	-0.27641	5.57E-05	0.000482	protein_coding
ENSG0000(	PRDX6	1808.733	-0.58783	5.57E-05	0.000482	protein_coding
ENSG0000(	ZNF33BP1	106.5572	0.493868	5.57E-05	0.000482	processed_pseudogene
ENSG0000(	RPLP0	6009.179	-0.45351	5.58E-05	0.000483	protein_coding
ENSG0000(	BOP1	241.021	-0.39516	5.60E-05	0.000484	protein_coding
ENSG0000(	ACBD4	517.9311	-0.2716	5.60E-05	0.000485	protein_coding
ENSG0000(	ENSG0000(	46.08159	0.436316	5.61E-05	0.000485	lncRNA
ENSG0000(	CT75	135.8526	0.302503	5.62E-05	0.000486	transcribed_unprocessed_pseudo
ENSG0000(	TMEM102	136.5527	-0.4217	5.62E-05	0.000486	protein_coding

ENSG0000(	ATP11A	1805.805	0.309836	5.63E-05	0.000487	protein_coding
ENSG0000(	ENSG0000(	97.18095	0.286498	5.64E-05	0.000487	lncRNA
ENSG0000(	ENSG0000(	55.43524	-1.29272	5.65E-05	0.000488	TEC
ENSG0000(	IRF2BP1	205.1838	-0.22413	5.67E-05	0.00049	protein_coding
ENSG0000(	TRERF1	942.3571	0.330883	5.68E-05	0.00049	protein_coding
ENSG0000(	PRR5	979.9376	-0.17771	5.68E-05	0.00049	protein_coding
ENSG0000(	TMEM92	125.8857	0.659685	5.75E-05	0.000496	protein_coding
ENSG0000(	CYP4B1	70.54109	-0.41828	5.76E-05	0.000497	protein_coding
ENSG0000(	LINC02603	38.19477	0.716875	5.76E-05	0.000497	lncRNA
ENSG0000(	MRPL4	453.9045	-0.21108	5.78E-05	0.000498	protein_coding
ENSG0000(	KDM4A	2001.421	-0.29523	5.79E-05	0.000499	protein_coding
ENSG0000(	ENSG0000(	251.1787	0.341246	5.79E-05	0.000499	TEC
ENSG0000(	ACSM2B	371.2593	0.388928	5.80E-05	0.0005	protein_coding
ENSG0000(	SMC2	647.354	-0.43581	5.81E-05	0.0005	protein_coding
ENSG0000(	ENSG0000(	84.1366	0.527379	5.84E-05	0.000503	lncRNA
ENSG0000(	LAMP3	509.9674	0.72747	5.84E-05	0.000503	protein_coding
ENSG0000(	LINC02363	269.5286	0.259548	5.84E-05	0.000503	lncRNA
ENSG0000(	ARHGEF2	4281.624	-0.22839	5.85E-05	0.000503	protein_coding
ENSG0000(	ENSG0000(	62.26141	0.469414	5.86E-05	0.000504	lncRNA
ENSG0000(	MYO1F	1341.041	0.416925	5.87E-05	0.000504	protein_coding
ENSG0000(	MFAP1	242.1839	-0.25327	5.87E-05	0.000505	protein_coding
ENSG0000(	VDR	2889.099	-0.46767	5.87E-05	0.000505	protein_coding
ENSG0000(	ENSG0000(	394.3939	0.293186	5.88E-05	0.000505	lncRNA
ENSG0000(	CD44	6117.93	0.55226	5.90E-05	0.000506	protein_coding
ENSG0000(	PDE1B	675.7951	0.2407	5.92E-05	0.000508	protein_coding
ENSG0000(	ENSG0000(	325.8126	0.373232	5.93E-05	0.000509	lncRNA
ENSG0000(	DAGLA	593.0695	-0.50979	5.95E-05	0.00051	protein_coding
ENSG0000(	PRKG2	319.7537	-1.08057	5.95E-05	0.00051	protein_coding
ENSG0000(	NRP2	1095.113	0.459819	5.95E-05	0.000511	protein_coding
ENSG0000(	MROH7-TT	388.0832	-0.47821	5.97E-05	0.000511	protein_coding
ENSG0000(	ENSG0000(	374.4675	0.229827	5.98E-05	0.000512	TEC
ENSG0000(	AFG3L2	2677.173	-0.29832	5.99E-05	0.000513	protein_coding
ENSG0000(	ENSG0000(	309.0649	0.566035	6.00E-05	0.000514	lncRNA
ENSG0000(	AXDND1	339.8031	-0.75719	6.01E-05	0.000515	protein_coding
ENSG0000(	GTF2IP12	2998.075	0.247561	6.01E-05	0.000515	transcribed_unprocessed_pseudo
ENSG0000(	ENSG0000(	56.10905	0.456765	6.02E-05	0.000515	lncRNA
ENSG0000(	DYNAP	291.813	0.226191	6.02E-05	0.000515	protein_coding
ENSG0000(	UCHL3	115.3996	-0.40065	6.02E-05	0.000515	protein_coding
ENSG0000(	EIF2B1	778.6979	-0.15047	6.04E-05	0.000516	protein_coding
ENSG0000(	FILNC1	134.4913	-0.34157	6.04E-05	0.000516	lncRNA
ENSG0000(	PRDX3	1016.507	-0.3382	6.04E-05	0.000516	protein_coding
ENSG0000(	ENSG0000(	1039.541	-0.38067	6.05E-05	0.000517	lncRNA
ENSG0000(	COA3	572.3009	-0.41925	6.07E-05	0.000519	protein_coding
ENSG0000(	FILIP1L	943.6147	0.428181	6.07E-05	0.000519	protein_coding
ENSG0000(	7SK	1687.202	-1.41493	6.08E-05	0.000519	misc_RNA
ENSG0000(	RNF19A	2944.676	0.223319	6.08E-05	0.000519	protein_coding
ENSG0000(	ENSG0000(	279.9455	0.241905	6.09E-05	0.00052	lncRNA

ENSG0000(	GGCT	345.9813	-0.35932	6.10E-05	0.00052	protein_coding
ENSG0000(	LMOD3	439.9124	0.273911	6.10E-05	0.00052	protein_coding
ENSG0000(	ENSG0000(	750.0676	0.211926	6.11E-05	0.000521	lncRNA
ENSG0000(	NRDC	2851.147	0.148846	6.11E-05	0.000521	protein_coding
ENSG0000(	CSF2RA	1007.826	0.334518	6.13E-05	0.000522	protein_coding
ENSG0000(	PHETA1	392.5438	-0.37	6.13E-05	0.000522	protein_coding
ENSG0000(	ENSG0000(	16.04224	0.871029	6.15E-05	0.000524	lncRNA
ENSG0000(	OSBPL5	1015.671	-0.28636	6.15E-05	0.000524	protein_coding
ENSG0000(	LMNB1	856.6412	-0.43451	6.15E-05	0.000524	protein_coding
ENSG0000(	SCAF11	7359.367	0.139658	6.15E-05	0.000524	protein_coding
ENSG0000(	TNFSF14	593.5708	0.57087	6.16E-05	0.000524	protein_coding
ENSG0000(	CNOT3	1204.713	-0.2778	6.16E-05	0.000524	protein_coding
ENSG0000(	ENSG0000(	10.82116	1.204214	6.16E-05	0.000524	lncRNA
ENSG0000(	ENSG0000(	96.19507	0.338967	6.19E-05	0.000527	TEC
ENSG0000(	ENSG0000(	107.0161	0.312695	6.20E-05	0.000527	lncRNA
ENSG0000(	TRIM13	1778.02	-0.18343	6.21E-05	0.000527	protein_coding
ENSG0000(	PREX1	1430.442	0.5334	6.22E-05	0.000528	protein_coding
ENSG0000(	RALY-AS1	1311.751	0.130581	6.22E-05	0.000528	lncRNA
ENSG0000(	ENSG0000(	423.3695	0.179517	6.23E-05	0.000529	TEC
ENSG0000(	LINC01307	148.6249	0.265223	6.27E-05	0.000532	lncRNA
ENSG0000(	NRP1	1911.385	0.373944	6.27E-05	0.000532	protein_coding
ENSG0000(	PVT1	2733.829	0.411221	6.27E-05	0.000532	lncRNA
ENSG0000(	AK6	220.3299	-0.3395	6.28E-05	0.000533	protein_coding
ENSG0000(	HAT1	950.1936	-0.24579	6.29E-05	0.000533	protein_coding
ENSG0000(	MCCC1	761.9045	-0.31047	6.29E-05	0.000533	protein_coding
ENSG0000(	TRPV4	41.42291	0.711402	6.30E-05	0.000533	protein_coding
ENSG0000(	PPP2R3B	542.3485	-0.30404	6.31E-05	0.000534	protein_coding
ENSG0000(	SULT1A4	145.6464	-0.9698	6.31E-05	0.000534	protein_coding
ENSG0000(	THSD1	99.15236	0.420913	6.31E-05	0.000534	protein_coding
ENSG0000(	ENSG0000(	150.831	-0.28985	6.31E-05	0.000534	lncRNA
ENSG0000(	ALCAM	578.0959	0.397314	6.32E-05	0.000535	protein_coding
ENSG0000(	FTLP10	31.47003	-0.59862	6.32E-05	0.000535	transcribed_processed_pseudoge
ENSG0000(	GLTP	1700.611	-0.34386	6.32E-05	0.000535	protein_coding
ENSG0000(	NOTCH2NL	3760.855	0.266576	6.34E-05	0.000535	protein_coding
ENSG0000(	PGAP3	696.4554	-0.41759	6.33E-05	0.000535	protein_coding
ENSG0000(	SMC6	2633.081	0.179874	6.33E-05	0.000535	protein_coding
ENSG0000(	UBA52	1778.185	-0.38717	6.33E-05	0.000535	protein_coding
ENSG0000(	WDR5	595.6998	-0.25361	6.34E-05	0.000535	protein_coding
ENSG0000(	ENSG0000(	13.68939	0.923644	6.34E-05	0.000535	lncRNA
ENSG0000(	APLN	14.0193	1.598333	6.34E-05	0.000536	protein_coding
ENSG0000(	RNU4-24P	36.14078	0.571424	6.35E-05	0.000536	snRNA
ENSG0000(	DZIP1	635.1097	0.392062	6.37E-05	0.000537	protein_coding
ENSG0000(	LENG8-AS1	817.22	-0.17134	6.37E-05	0.000537	lncRNA
ENSG0000(	TNFRSF21	2406.4	-0.50113	6.38E-05	0.000538	protein_coding
ENSG0000(	CYTIP	939.2079	0.698915	6.39E-05	0.000539	protein_coding
ENSG0000(	LRRC38	52.30232	0.44147	6.40E-05	0.00054	protein_coding
ENSG0000(	TMEM115	358.1737	-0.32311	6.42E-05	0.000541	protein_coding

ENSG0000(	DENND3-A:	388.5127	0.299281	6.43E-05	0.000541	lncRNA
ENSG0000(	MRPS26	198.4137	-0.35911	6.44E-05	0.000542	protein_coding
ENSG0000(	OSER1-DT	1028.308	0.140069	6.44E-05	0.000542	lncRNA
ENSG0000(	WWC2	507.8165	0.438756	6.45E-05	0.000543	protein_coding
ENSG0000(	ANKRD36	6234.143	0.28248	6.45E-05	0.000543	protein_coding
ENSG0000(	DCUN1D1	1486.001	-0.23081	6.47E-05	0.000544	protein_coding
ENSG0000(	ENSG0000(	36.2905	0.670636	6.48E-05	0.000545	lncRNA
ENSG0000(	TP73	34.69121	0.871014	6.49E-05	0.000546	protein_coding
ENSG0000(	ERG	328.7009	0.47551	6.51E-05	0.000547	protein_coding
ENSG0000(	ENSG0000(	287.717	0.333023	6.51E-05	0.000547	lncRNA
ENSG0000(	SCAI	2032.242	-0.25247	6.51E-05	0.000547	protein_coding
ENSG0000(	IRF2BPL	526.8692	0.261069	6.53E-05	0.000548	protein_coding
ENSG0000(	ERMN	167.9303	0.549111	6.54E-05	0.000549	protein_coding
ENSG0000(	MMUT	559.3958	-0.33786	6.54E-05	0.000549	protein_coding
ENSG0000(	USP32	1616.104	0.254323	6.54E-05	0.000549	protein_coding
ENSG0000(	ZNF317	935.6307	-0.20695	6.54E-05	0.000549	protein_coding
ENSG0000(	ZKSCAN8	2031.469	0.176396	6.54E-05	0.000549	protein_coding
ENSG0000(	MEI1	717.8641	0.312303	6.57E-05	0.000551	protein_coding
ENSG0000(	SNORA12	272.3319	-0.3957	6.60E-05	0.000553	snoRNA
ENSG0000(	SEPTIN7P9	433.5335	0.203463	6.62E-05	0.000555	transcribed_unprocessed_pseudo
ENSG0000(	ENSG0000(	68.62477	0.690056	6.63E-05	0.000556	lncRNA
ENSG0000(	SMIM26	170.595	-0.36854	6.64E-05	0.000556	protein_coding
ENSG0000(	RPL39	2188.396	-0.46574	6.64E-05	0.000556	protein_coding
ENSG0000(	SLC35G1	804.1775	-0.55908	6.64E-05	0.000556	protein_coding
ENSG0000(	FAM162A	2322.023	-0.33567	6.67E-05	0.000558	protein_coding
ENSG0000(	ENSG0000(	157.0767	0.263039	6.68E-05	0.000559	lncRNA
ENSG0000(	ENSG0000(	51.11064	0.620728	6.70E-05	0.00056	lncRNA
ENSG0000(	JPT1	926.8691	-0.39939	6.70E-05	0.00056	protein_coding
ENSG0000(	ENSG0000(	107.7591	0.349348	6.70E-05	0.00056	TEC
ENSG0000(	LAMA4	3319.876	0.326295	6.71E-05	0.000561	protein_coding
ENSG0000(	TPM1-AS	235.5325	0.727641	6.71E-05	0.000561	lncRNA
ENSG0000(	RNU6-396F	10.95621	0.871528	6.72E-05	0.000561	snRNA
ENSG0000(	YIPF2	316.8086	-0.39274	6.76E-05	0.000564	protein_coding
ENSG0000(	INPP4B	1981.796	0.343628	6.76E-05	0.000564	protein_coding
ENSG0000(	ENSG0000(	131.7812	0.298285	6.78E-05	0.000565	lncRNA
ENSG0000(	MTMR2	1356.812	-0.31257	6.78E-05	0.000565	protein_coding
ENSG0000(	OR9M1P	56.05507	0.520504	6.79E-05	0.000567	transcribed_unprocessed_pseudo
ENSG0000(	PLA2G4C-A	34.47217	0.569529	6.80E-05	0.000567	lncRNA
ENSG0000(	LINC02886	203.1125	-0.43182	6.80E-05	0.000567	lncRNA
ENSG0000(	ENSG0000(	74.34724	0.414883	6.83E-05	0.000569	processed_pseudogene
ENSG0000(	NAV1	3421.452	0.18176	6.83E-05	0.000569	protein_coding
ENSG0000(	NUP42	860.0178	-0.19646	6.84E-05	0.00057	protein_coding
ENSG0000(	PCYT2	569.123	-0.43287	6.84E-05	0.00057	protein_coding
ENSG0000(	ENSG0000(	46.50065	0.520502	6.85E-05	0.00057	lncRNA
ENSG0000(	NUTF2	587.4415	-0.29104	6.86E-05	0.000571	protein_coding
ENSG0000(	OR6M2P	64.68501	0.341219	6.87E-05	0.000572	transcribed_unprocessed_pseudo
ENSG0000(	IL1RL1	532.1313	0.678143	6.89E-05	0.000573	protein_coding

ENSG0000(	ENSG0000(	180.4425	-0.37145	6.89E-05	0.000573	protein_coding
ENSG0000(	KIF15	287.6612	-0.52838	6.89E-05	0.000573	protein_coding
ENSG0000(	STRN4	1263.898	-0.17768	6.90E-05	0.000574	protein_coding
ENSG0000(	TOMM40L	331.7119	-0.33478	6.91E-05	0.000574	protein_coding
ENSG0000(	MRM1	287.3125	-0.37484	6.93E-05	0.000576	protein_coding
ENSG0000(	ENSG0000(	286.9175	0.230692	6.93E-05	0.000576	lncRNA
ENSG0000(	CYP46A1	1077.47	0.204075	6.94E-05	0.000576	protein_coding
ENSG0000(	RHOF	1548.291	-0.42691	6.95E-05	0.000577	protein_coding
ENSG0000(	ENSG0000(	53.49921	-0.62256	6.96E-05	0.000578	lncRNA
ENSG0000(	HLA-DRA	3247.25	0.700707	6.97E-05	0.000578	protein_coding
ENSG0000(	CLEC5A	54.51799	1.078876	6.97E-05	0.000579	protein_coding
ENSG0000(	GUCY1B1	170.3001	0.737948	6.98E-05	0.000579	protein_coding
ENSG0000(	DDX19A	1697.426	-0.13528	6.99E-05	0.00058	protein_coding
ENSG0000(	ENSG0000(	238.1573	0.284156	7.00E-05	0.00058	lncRNA
ENSG0000(	MSH2	1079.421	-0.33051	7.01E-05	0.000581	protein_coding
ENSG0000(	LPIN1	2940.595	0.298173	7.01E-05	0.000581	protein_coding
ENSG0000(	FBXL15	129.1464	-0.3877	7.02E-05	0.000582	protein_coding
ENSG0000(	ENSG0000(	100.8669	-0.4864	7.03E-05	0.000582	pseudogene
ENSG0000(	PFDN5	1305.374	-0.30175	7.03E-05	0.000582	protein_coding
ENSG0000(	PIK3AP1	989.9778	0.646154	7.04E-05	0.000582	protein_coding
ENSG0000(	MOGAT3	1251.033	-0.48772	7.04E-05	0.000582	protein_coding
ENSG0000(	AIMP2	275.8642	-0.31284	7.06E-05	0.000584	protein_coding
ENSG0000(	ZNF366	482.98	0.487129	7.06E-05	0.000584	protein_coding
ENSG0000(	N4BP1	2033.242	0.286993	7.06E-05	0.000584	protein_coding
ENSG0000(	KLHL35	338.034	0.301283	7.07E-05	0.000585	protein_coding
ENSG0000(	MPRIP	8656.937	-0.21329	7.09E-05	0.000585	protein_coding
ENSG0000(	SMG6	2602.458	0.148315	7.09E-05	0.000585	protein_coding
ENSG0000(	STRIP2	520.0825	0.536767	7.09E-05	0.000585	protein_coding
ENSG0000(	CHST3	327.8415	0.417884	7.15E-05	0.00059	protein_coding
ENSG0000(	NOTCH1	3769.301	-0.29428	7.20E-05	0.000594	protein_coding
ENSG0000(	ENSG0000(	370.5285	0.373201	7.22E-05	0.000596	lncRNA
ENSG0000(	DOLPP1	377.5827	-0.48427	7.23E-05	0.000596	protein_coding
ENSG0000(	RGR	310.4866	0.27439	7.25E-05	0.000598	protein_coding
ENSG0000(	DCAF11	1920.306	-0.18852	7.26E-05	0.000598	protein_coding
ENSG0000(	PSMG1	240.6682	-0.31795	7.26E-05	0.000598	protein_coding
ENSG0000(	CISD2	616.0295	-0.2381	7.30E-05	0.000602	protein_coding
ENSG0000(	TMC2	585.0016	0.273742	7.31E-05	0.000602	protein_coding
ENSG0000(	ENSG0000(	11.469	1.136353	7.32E-05	0.000603	unprocessed_pseudogene
ENSG0000(	MYBPC3	29.44458	0.832661	7.34E-05	0.000605	protein_coding
ENSG0000(	NUB1	1610.096	0.224513	7.35E-05	0.000605	protein_coding
ENSG0000(	RAB33B-AS	2446.918	0.15546	7.35E-05	0.000605	lncRNA
ENSG0000(	ATG2B	2809.103	0.215924	7.36E-05	0.000606	protein_coding
ENSG0000(	TPPP2	13.97176	-1.16694	7.36E-05	0.000606	protein_coding
ENSG0000(	SERPINA10	858.3899	0.366286	7.37E-05	0.000606	protein_coding
ENSG0000(	C1R	1378.596	0.551562	7.38E-05	0.000607	protein_coding
ENSG0000(	OR7M1P	19.01536	0.669272	7.40E-05	0.000608	unprocessed_pseudogene
ENSG0000(	DRAXIN	738.9655	0.152599	7.41E-05	0.000609	protein_coding

ENSG0000(	INSIG1-DT	19.68834	0.660191	7.41E-05	0.000609	lncRNA
ENSG0000(	NDOR1	654.6797	-0.27234	7.44E-05	0.000611	protein_coding
ENSG0000(	ADH6	250.3863	-0.69333	7.46E-05	0.000612	protein_coding
ENSG0000(	B3GNT8	329.0536	-0.73604	7.46E-05	0.000612	protein_coding
ENSG0000(	TIMM17B	414.8871	-0.17321	7.47E-05	0.000613	protein_coding
ENSG0000(	RPL4	6606.109	-0.34984	7.57E-05	0.000621	protein_coding
ENSG0000(	TNFSF13B	327.4177	0.465163	7.58E-05	0.000621	protein_coding
ENSG0000(	ENSG0000(	171.1009	-0.26946	7.58E-05	0.000622	lncRNA
ENSG0000(	ENSG0000(	25.01395	0.629305	7.60E-05	0.000623	lncRNA
ENSG0000(	WDR90	1596.951	-0.26322	7.61E-05	0.000624	protein_coding
ENSG0000(	DNASE1L2	31.75535	-0.53696	7.62E-05	0.000624	protein_coding
ENSG0000(	LINC00700	91.30051	-0.3723	7.62E-05	0.000624	lncRNA
ENSG0000(	PDLIM3	924.6078	0.530882	7.62E-05	0.000624	protein_coding
ENSG0000(	PTPRE	2140.19	0.336861	7.64E-05	0.000625	protein_coding
ENSG0000(	PHF11	1120.514	0.242076	7.64E-05	0.000625	protein_coding
ENSG0000(	SF3A3	804.8753	-0.27343	7.66E-05	0.000627	protein_coding
ENSG0000(	TIMM17A	1176.356	-0.22572	7.66E-05	0.000627	protein_coding
ENSG0000(	SLC4A11	40.19792	1.154443	7.70E-05	0.000629	protein_coding
ENSG0000(	ENSG0000(	278.3885	-0.29142	7.71E-05	0.000631	transcribed_unprocessed_pseudo
ENSG0000(	CSNK1D	4603.157	-0.2166	7.73E-05	0.000631	protein_coding
ENSG0000(	RNF227	785.1921	-0.18305	7.74E-05	0.000632	protein_coding
ENSG0000(	TMEM273	475.3555	0.386266	7.75E-05	0.000633	protein_coding
ENSG0000(	ENSG0000(	38.00201	0.565539	7.76E-05	0.000633	lncRNA
ENSG0000(	OGDH	2505.441	-0.33583	7.76E-05	0.000633	protein_coding
ENSG0000(	ENSG0000(	399.2853	0.751147	7.78E-05	0.000635	lncRNA
ENSG0000(	RTEL1-TNFI	825.5494	0.363783	7.79E-05	0.000636	protein_coding
ENSG0000(	ENSG0000(	282.8803	0.235326	7.80E-05	0.000637	TEC
ENSG0000(	ENSG0000(	35.62394	-0.67877	7.81E-05	0.000637	unprocessed_pseudogene
ENSG0000(	PPARD	1776.246	-0.42333	7.82E-05	0.000638	protein_coding
ENSG0000(	TMEM106/	1320.279	0.24589	7.83E-05	0.000638	protein_coding
ENSG0000(	EDIL3	1080.173	-0.5479	7.85E-05	0.00064	protein_coding
ENSG0000(	FNDC3A	3565.932	0.217446	7.86E-05	0.000641	protein_coding
ENSG0000(	MRPS5	2989.19	-0.13032	7.86E-05	0.000641	protein_coding
ENSG0000(	PYGO2	746.1801	-0.18647	7.87E-05	0.000641	protein_coding
ENSG0000(	TIMM44	577.4727	-0.26971	7.88E-05	0.000642	protein_coding
ENSG0000(	TMCO1	1475.532	-0.35	7.89E-05	0.000642	protein_coding
ENSG0000(	CD300C	29.38433	0.74417	7.91E-05	0.000644	protein_coding
ENSG0000(	ENSG0000(	251.1804	0.224656	7.94E-05	0.000646	lncRNA
ENSG0000(	ENSG0000(	276.1561	-0.26603	7.95E-05	0.000647	lncRNA
ENSG0000(	LINC02604	317.8863	0.366166	7.95E-05	0.000647	lncRNA
ENSG0000(	KCNB1	2206.719	0.20841	7.97E-05	0.000648	protein_coding
ENSG0000(	ENSG0000(	158.0442	0.264332	7.97E-05	0.000648	lncRNA
ENSG0000(	DCTPP1	258.4508	-0.48346	7.99E-05	0.000649	protein_coding
ENSG0000(	FAM30A	2761.868	0.578721	7.99E-05	0.000649	lncRNA
ENSG0000(	IGHV3-74	683.5331	0.872646	8.00E-05	0.00065	IG_V_gene
ENSG0000(	LINC02132	38.25221	0.501665	8.01E-05	0.00065	lncRNA
ENSG0000(	POC1A	106.2906	-0.5534	8.00E-05	0.00065	protein_coding

ENSG0000(	ENSG0000(	39.77557	0.47572	8.07E-05	0.000655	lncRNA
ENSG0000(	CCDC51	105.5008	-0.31761	8.09E-05	0.000656	protein_coding
ENSG0000(	DNM1	1242.47	0.676872	8.10E-05	0.000657	protein_coding
ENSG0000(	JCAD	381.935	0.573584	8.10E-05	0.000657	protein_coding
ENSG0000(	ENSG0000(	13.10017	-0.98318	8.17E-05	0.000663	lncRNA
ENSG0000(	DUT	411.4569	-0.28565	8.20E-05	0.000664	protein_coding
ENSG0000(	LINC02012	182.2351	-0.39843	8.20E-05	0.000664	lncRNA
ENSG0000(	RFLNA	360.485	-0.6116	8.20E-05	0.000664	protein_coding
ENSG0000(	ENSG0000(	1240.8	0.133288	8.21E-05	0.000665	lncRNA
ENSG0000(	IGHV3-49	244.8516	0.997086	8.21E-05	0.000665	IG_V_gene
ENSG0000(	CCSAP	553.3131	0.244482	8.23E-05	0.000666	protein_coding
ENSG0000(	GET3	178.1699	-0.38071	8.23E-05	0.000666	protein_coding
ENSG0000(	USP15	4438.92	0.254001	8.30E-05	0.000671	protein_coding
ENSG0000(	ENSG0000(	69.49004	0.345879	8.30E-05	0.000671	lncRNA
ENSG0000(	ENSG0000(	867.6844	0.191084	8.31E-05	0.000672	lncRNA
ENSG0000(	RPS15A	3264.045	-0.44718	8.34E-05	0.000674	protein_coding
ENSG0000(	SPATA41	95.07119	0.444702	8.33E-05	0.000674	lncRNA
ENSG0000(	RSPO1	57.17277	0.581097	8.34E-05	0.000674	protein_coding
ENSG0000(	ENSG0000(	38.69836	-0.96228	8.35E-05	0.000675	processed_pseudogene
ENSG0000(	TRAK2	2092.608	-0.26601	8.36E-05	0.000675	protein_coding
ENSG0000(	ANKH	4001.859	-0.4087	8.37E-05	0.000676	protein_coding
ENSG0000(	ACTG1P3	64.99569	-0.53204	8.37E-05	0.000676	processed_pseudogene
ENSG0000(	ENSG0000(	95.49595	0.292971	8.39E-05	0.000677	lncRNA
ENSG0000(	GTF2IP20	1090.291	0.22335	8.40E-05	0.000677	transcribed_unprocessed_pseudo
ENSG0000(	MYG1	61.45551	-0.62686	8.40E-05	0.000677	protein_coding
ENSG0000(	TMPRSS3	110.8711	0.545791	8.40E-05	0.000677	protein_coding
ENSG0000(	LINC01126	124.0678	0.359609	8.41E-05	0.000677	lncRNA
ENSG0000(	RN7SL751F	52.40508	0.68622	8.41E-05	0.000678	misc_RNA
ENSG0000(	ENTPD5	5954.547	-0.54581	8.41E-05	0.000678	protein_coding
ENSG0000(	ENSG0000(	119.253	0.331181	8.42E-05	0.000679	lncRNA
ENSG0000(	OR2I1P	104.194	0.401356	8.45E-05	0.00068	protein_coding
ENSG0000(	GZMB	22.08534	1.154848	8.45E-05	0.00068	protein_coding
ENSG0000(	LINC01948	318.9601	0.258098	8.47E-05	0.000682	lncRNA
ENSG0000(	NLE1	430.3575	-0.2981	8.49E-05	0.000683	protein_coding
ENSG0000(	CHN1	397.6942	0.406865	8.51E-05	0.000684	protein_coding
ENSG0000(	ENSG0000(	34.83646	0.666821	8.52E-05	0.000685	lncRNA
ENSG0000(	ENSG0000(	147.4503	-0.45674	8.54E-05	0.000687	lncRNA
ENSG0000(	LINC02193	787.8127	0.373446	8.56E-05	0.000688	lncRNA
ENSG0000(	LYAR	121.5228	-0.43904	8.57E-05	0.000688	protein_coding
ENSG0000(	CKAP5	1942.922	-0.28546	8.59E-05	0.00069	protein_coding
ENSG0000(	MAGI2-AS3	1266.475	0.317163	8.59E-05	0.00069	lncRNA
ENSG0000(	ENSG0000(	61.04722	-0.47083	8.60E-05	0.00069	lncRNA
ENSG0000(	ENSG0000(	138.1394	-0.30455	8.60E-05	0.00069	lncRNA
ENSG0000(	WDR4	508.5501	-0.36739	8.60E-05	0.00069	protein_coding
ENSG0000(	RPSAP58	2356.83	-0.49659	8.61E-05	0.000691	protein_coding
ENSG0000(	RPL6	4061.431	-0.36099	8.63E-05	0.000693	protein_coding
ENSG0000(	HLA-DOA	955.231	0.55094	8.64E-05	0.000693	protein_coding

ENSG0000(	ENSG0000(	207.7751	0.274807	8.65E-05	0.000694	lncRNA
ENSG0000(	ENSG0000(	140.7042	0.325645	8.67E-05	0.000695	processed_pseudogene
ENSG0000(	ENSG0000(	261.5398	0.308129	8.67E-05	0.000695	lncRNA
ENSG0000(	CDK20	368.3604	-0.56629	8.67E-05	0.000695	protein_coding
ENSG0000(	CHMP2A	919.9128	-0.37322	8.69E-05	0.000696	protein_coding
ENSG0000(	RPL7A	4285.6	-0.40342	8.69E-05	0.000696	protein_coding
ENSG0000(	SAPCD2	290.3678	-0.52287	8.75E-05	0.0007	protein_coding
ENSG0000(	TAF13	1133.771	-0.22939	8.75E-05	0.0007	protein_coding
ENSG0000(	RABGGTA	287.8106	-0.2901	8.76E-05	0.000701	protein_coding
ENSG0000(	SLC25A5	2613.246	-0.57674	8.76E-05	0.000701	protein_coding
ENSG0000(	C15orf48	1371.269	-0.57216	8.77E-05	0.000701	protein_coding
ENSG0000(	USP14	1207.68	-0.25354	8.78E-05	0.000702	protein_coding
ENSG0000(	ENSG0000(	213.6608	-0.2763	8.79E-05	0.000703	lncRNA
ENSG0000(	TMEM30B	1544.744	-0.41899	8.79E-05	0.000703	protein_coding
ENSG0000(	ENSG0000(	35.00256	0.697821	8.83E-05	0.000705	lncRNA
ENSG0000(	OR10J1	137.9792	-0.32781	8.83E-05	0.000705	protein_coding
ENSG0000(	ENSG0000(	116.9573	-0.56064	8.86E-05	0.000707	processed_pseudogene
ENSG0000(	ENSG0000(	26.63762	1.13218	8.86E-05	0.000707	lncRNA
ENSG0000(	ENSG0000(	92.75144	-0.36353	8.86E-05	0.000707	lncRNA
ENSG0000(	ENSG0000(	10.89482	1.114968	8.86E-05	0.000707	lncRNA
ENSG0000(	PLA2G2A	606.2586	1.415647	8.87E-05	0.000707	protein_coding
ENSG0000(	ENSG0000(	50.16651	0.731681	8.88E-05	0.000708	lncRNA
ENSG0000(	FGF2	275.2777	0.578301	8.88E-05	0.000708	protein_coding
ENSG0000(	CDK17	1302.898	0.286982	8.90E-05	0.000709	protein_coding
ENSG0000(	NAT8B	8.840127	-1.15792	8.89E-05	0.000709	polymorphic_pseudogene
ENSG0000(	TNFRSF8	32.86014	1.058725	8.90E-05	0.000709	protein_coding
ENSG0000(	ZNF224	2610.083	0.124971	8.90E-05	0.000709	protein_coding
ENSG0000(	LINC01786	39.27486	-0.51062	8.90E-05	0.000709	lncRNA
ENSG0000(	PDE4DIPP7	53.03229	0.58479	8.91E-05	0.000709	transcribed_unprocessed_pseudo
ENSG0000(	ELF1	1940.633	-0.21319	8.91E-05	0.000709	protein_coding
ENSG0000(	ENSG0000(	185.9561	0.235505	8.95E-05	0.000712	lncRNA
ENSG0000(	ENSG0000(	77.84043	0.636166	8.95E-05	0.000712	lncRNA
ENSG0000(	ENSG0000(	374.5741	0.281071	8.98E-05	0.000714	lncRNA
ENSG0000(	DIRAS2	168.528	-0.32633	9.00E-05	0.000715	protein_coding
ENSG0000(	VSTM2B-D	218.5983	0.334468	9.01E-05	0.000716	lncRNA
ENSG0000(	ENSG0000(	62.20335	0.535591	9.02E-05	0.000716	lncRNA
ENSG0000(	NECTIN3	1419.19	-0.3673	9.02E-05	0.000716	protein_coding
ENSG0000(	ENSG0000(	731.0566	0.549065	9.02E-05	0.000717	lncRNA
ENSG0000(	ENSG0000(	67.00487	0.55857	9.03E-05	0.000717	lncRNA
ENSG0000(	ENSG0000(	287.9947	-0.27288	9.05E-05	0.000718	TEC
ENSG0000(	MDN1	5516.088	-0.28403	9.06E-05	0.000718	protein_coding
ENSG0000(	MTBP	492.2386	-0.21651	9.05E-05	0.000718	protein_coding
ENSG0000(	SETD5	11887.28	0.134256	9.06E-05	0.000718	protein_coding
ENSG0000(	ENSG0000(	27.74246	0.593186	9.07E-05	0.000719	lncRNA
ENSG0000(	EGFR-AS1	105.163	0.512932	9.08E-05	0.000719	lncRNA
ENSG0000(	GRHL1	301.1133	0.27768	9.07E-05	0.000719	protein_coding
ENSG0000(	ENSG0000(	13.82511	0.905051	9.09E-05	0.00072	lncRNA

ENSG0000(	FOSL1P1	40.12175	-0.55834	9.09E-05	0.00072	processed_pseudogene
ENSG0000(	NAIP	4102.8	0.367019	9.09E-05	0.00072	protein_coding
ENSG0000(	SH2B3	1460.924	0.376511	9.11E-05	0.000722	protein_coding
ENSG0000(	SLF2	2867.181	0.164525	9.12E-05	0.000722	protein_coding
ENSG0000(	RIPOR3-AS:	14.25785	1.019145	9.13E-05	0.000722	lncRNA
ENSG0000(	ENSG0000(	53.66314	0.450705	9.13E-05	0.000722	lncRNA
ENSG0000(	ENSG0000(	50.69936	0.355981	9.18E-05	0.000726	lncRNA
ENSG0000(	ANKRD27	1098.748	-0.26492	9.19E-05	0.000727	protein_coding
ENSG0000(	MTX1	322.249	-0.30669	9.19E-05	0.000727	protein_coding
ENSG0000(	ENSG0000(	36.60523	0.519346	9.22E-05	0.000728	lncRNA
ENSG0000(	LZTS1	159.2265	0.495319	9.22E-05	0.000728	protein_coding
ENSG0000(	CENPW	37.07608	-0.70657	9.23E-05	0.000729	protein_coding
ENSG0000(	MASP1	378.9388	0.783361	9.24E-05	0.00073	protein_coding
ENSG0000(	CENPA	73.80662	-0.48733	9.28E-05	0.000733	protein_coding
ENSG0000(	TSNAX-DIS(	30.15899	0.735309	9.30E-05	0.000734	protein_coding
ENSG0000(	HNRNPM	1802.465	-0.15591	9.31E-05	0.000735	protein_coding
ENSG0000(	SPAG1	604.758	-0.4549	9.39E-05	0.00074	protein_coding
ENSG0000(	AXIN1	1220.479	-0.29003	9.40E-05	0.000741	protein_coding
ENSG0000(	THBS3-AS1	39.14165	0.664924	9.45E-05	0.000745	lncRNA
ENSG0000(	FAM83D	197.3871	-0.37038	9.47E-05	0.000747	protein_coding
ENSG0000(	TTC38	1366.882	-0.27418	9.48E-05	0.000747	protein_coding
ENSG0000(	ENSG0000(	36.00208	0.610321	9.49E-05	0.000748	lncRNA
ENSG0000(	ENSG0000(	977.7959	0.181324	9.49E-05	0.000748	lncRNA
ENSG0000(	GEMIN5	403.8456	-0.49071	9.52E-05	0.000749	protein_coding
ENSG0000(	PTPRG	2586.92	0.230189	9.58E-05	0.000754	protein_coding
ENSG0000(	RAPGEF3	766.1967	0.448693	9.61E-05	0.000756	protein_coding
ENSG0000(	IGKJ4	155.022	0.783161	9.63E-05	0.000758	IG_J_gene
ENSG0000(	ENSG0000(	395.4236	-0.27443	9.63E-05	0.000758	lncRNA
ENSG0000(	TGFB3	215.7209	0.907274	9.64E-05	0.000758	protein_coding
ENSG0000(	EMC8	404.2336	-0.24595	9.67E-05	0.00076	protein_coding
ENSG0000(	ENSG0000(	14.1363	0.684662	9.69E-05	0.000762	lncRNA
ENSG0000(	AHNAK	48661	-0.38835	9.70E-05	0.000763	protein_coding
ENSG0000(	ANO10	1403.467	-0.3299	9.72E-05	0.000764	protein_coding
ENSG0000(	ENSG0000(	119.265	0.323472	9.73E-05	0.000764	lncRNA
ENSG0000(	GTF2E2	497.7661	-0.26488	9.73E-05	0.000764	protein_coding
ENSG0000(	EGFL8	629.9011	-0.37272	9.74E-05	0.000765	protein_coding
ENSG0000(	PTP4A1	6122.644	-0.5526	9.76E-05	0.000766	protein_coding
ENSG0000(	NDUFB4	402.849	-0.37094	9.76E-05	0.000766	protein_coding
ENSG0000(	CR1	1490.939	0.986884	9.77E-05	0.000766	protein_coding
ENSG0000(	C1RL	1416.734	0.260262	9.79E-05	0.000768	protein_coding
ENSG0000(	TPST1	242.9864	0.584646	9.79E-05	0.000768	protein_coding
ENSG0000(	ING4	384.3	-0.26754	9.81E-05	0.000769	protein_coding
ENSG0000(	MFSD3	348.165	-0.53293	9.80E-05	0.000769	protein_coding
ENSG0000(	ABCA8	1669.051	-0.63594	9.82E-05	0.000769	protein_coding
ENSG0000(	F5	335.4175	0.601102	9.83E-05	0.00077	protein_coding
ENSG0000(	SDF2L1	137.085	-0.54087	9.84E-05	0.00077	protein_coding
ENSG0000(	CLPTM1	798.1191	-0.2342	9.86E-05	0.000772	protein_coding

ENSG0000(	EIF3H	1448.944	-0.27809	9.86E-05	0.000772	protein_coding
ENSG0000(	GFM1	2604.634	-0.16198	9.86E-05	0.000772	protein_coding
ENSG0000(	RPL18A	2682.502	-0.36634	9.87E-05	0.000772	protein_coding
ENSG0000(	ANKRD30B	20173.12	-0.6665	9.87E-05	0.000772	protein_coding
ENSG0000(	ENSG0000(	32.40338	0.622585	9.90E-05	0.000774	lncRNA
ENSG0000(	RFC4	265.1317	-0.33082	9.93E-05	0.000777	protein_coding
ENSG0000(	MDM1	360.6174	0.324374	9.97E-05	0.000779	protein_coding
ENSG0000(	ENSG0000(	72.28442	0.451793	1.00E-04	0.000781	lncRNA
ENSG0000(	TGFBRAP1	1095.399	-0.15759	0.0001	0.000782	protein_coding
ENSG0000(	PLK5	487.5969	0.228938	0.0001	0.000783	protein_coding
ENSG0000(	LINC00839	410.5045	0.240389	0.0001	0.000784	lncRNA
ENSG0000(	ENSG0000(	109.153	0.501771	0.000101	0.000786	lncRNA
ENSG0000(	LINC02780	76.96242	0.434797	0.000101	0.000786	lncRNA
ENSG0000(	ADGRG2	547.1896	-0.2965	0.000101	0.000787	protein_coding
ENSG0000(	ENSG0000(	309.9104	0.275913	0.000101	0.000789	lncRNA
ENSG0000(	PAPLN	1857.92	0.569594	0.000101	0.000789	protein_coding
ENSG0000(	NWD1	590.6585	0.316212	0.000101	0.00079	protein_coding
ENSG0000(	SIGLEC10	886.5584	0.249218	0.000101	0.00079	protein_coding
ENSG0000(	PTPN1	1640.902	0.315075	0.000102	0.000792	protein_coding
ENSG0000(	ABHD11	856.701	-0.36668	0.000102	0.000793	protein_coding
ENSG0000(	ACSM2A	412.7553	0.331893	0.000102	0.000793	protein_coding
ENSG0000(	ENSG0000(	14.17908	1.176195	0.000102	0.000793	lncRNA
ENSG0000(	PHF20L1	3243.234	0.176058	0.000102	0.000797	protein_coding
ENSG0000(	ENSG0000(	877.693	0.341707	0.000103	0.000798	lncRNA
ENSG0000(	ENSG0000(	415.4904	0.241043	0.000103	0.000798	lncRNA
ENSG0000(	FBH1	2554.177	0.170072	0.000103	0.000798	protein_coding
ENSG0000(	RBL1	960.0228	-0.32103	0.000103	0.0008	protein_coding
ENSG0000(	SLC35A1	661.182	-0.26038	0.000103	0.0008	protein_coding
ENSG0000(	ST13	1434.917	-0.30958	0.000103	0.000801	protein_coding
ENSG0000(	PPFIA3	315.0236	-0.53518	0.000103	0.000801	protein_coding
ENSG0000(	CD99	1495.88	-0.22649	0.000103	0.000802	protein_coding
ENSG0000(	SHD	73.45325	-0.88359	0.000103	0.000802	protein_coding
ENSG0000(	CD86	225.0268	0.649726	0.000103	0.000803	protein_coding
ENSG0000(	NBPF25P	998.7847	0.244334	0.000104	0.000803	transcribed_unprocessed_pseudo
ENSG0000(	OR52N5	30.31046	1.026162	0.000103	0.000803	protein_coding
ENSG0000(	MIR7848	11.42317	1.112385	0.000104	0.000805	miRNA
ENSG0000(	COPB2-DT	554.4158	0.276443	0.000104	0.000807	lncRNA
ENSG0000(	LINC01470	58.97104	-0.51716	0.000104	0.000807	lncRNA
ENSG0000(	FLYWCH2	130.777	-0.38406	0.000104	0.000808	protein_coding
ENSG0000(	MFN2	2918.192	-0.22076	0.000104	0.00081	protein_coding
ENSG0000(	MOCS1	790.5329	-0.58401	0.000104	0.00081	protein_coding
ENSG0000(	CHEK1	457.1549	-0.31539	0.000105	0.000811	protein_coding
ENSG0000(	BBX	1897.723	0.288513	0.000105	0.000811	protein_coding
ENSG0000(	DMC1	298.5256	-0.29194	0.000105	0.000811	protein_coding
ENSG0000(	LINC01414	166.6117	0.284991	0.000105	0.000813	lncRNA
ENSG0000(	ZNF267	1069.67	0.336486	0.000105	0.000813	protein_coding
ENSG0000(	ZNF28	934.1777	-0.27366	0.000105	0.000813	protein_coding

ENSG0000(	FKBP2	262.6379	-0.54381	0.000105	0.000814	protein_coding
ENSG0000(	SPINT1	2433.803	-0.56929	0.000105	0.000814	protein_coding
ENSG0000(	PTCSC3	566.8818	0.298494	0.000105	0.000815	lncRNA
ENSG0000(	CSMD2	308.1998	0.577893	0.000106	0.000816	protein_coding
ENSG0000(	FILIP1	308.8736	0.297326	0.000106	0.000816	protein_coding
ENSG0000(	HR	1464.044	-0.56863	0.000106	0.000816	protein_coding
ENSG0000(	IGSF5	241.0619	0.328055	0.000106	0.000817	protein_coding
ENSG0000(	LYZ	3660.062	0.78441	0.000106	0.00082	protein_coding
ENSG0000(	SLC51B	307.8306	-0.89992	0.000106	0.00082	protein_coding
ENSG0000(	ADGRG7	318.2989	0.93643	0.000107	0.000823	protein_coding
ENSG0000(	LRRC8D	857.4465	-0.25989	0.000107	0.000823	protein_coding
ENSG0000(	POMK	2958.666	-0.25026	0.000107	0.000824	protein_coding
ENSG0000(	PSMC3	359.5319	-0.35045	0.000107	0.000829	protein_coding
ENSG0000(	RPS3P2	121.8334	0.258361	0.000108	0.00083	processed_pseudogene
ENSG0000(	KCNK5	1321.34	-0.59681	0.000108	0.00083	protein_coding
ENSG0000(	NCOA5	755.878	-0.18986	0.000108	0.00083	protein_coding
ENSG0000(	ENSG0000(	798.4612	0.359304	0.000108	0.000831	lncRNA
ENSG0000(	SLIT3-AS2	554.8032	-0.34842	0.000108	0.000831	lncRNA
ENSG0000(	ENSG0000(	241.8996	0.298674	0.000108	0.000834	lncRNA
ENSG0000(	SEMA4A	517.6909	0.544325	0.000108	0.000834	protein_coding
ENSG0000(	ACOT11	5239.181	-0.3614	0.000108	0.000834	protein_coding
ENSG0000(	OSBP	1779.581	-0.19644	0.000109	0.000836	protein_coding
ENSG0000(	UBR7	543.4544	-0.31591	0.000109	0.000837	protein_coding
ENSG0000(	RPL26L1	69.64366	-0.50175	0.000109	0.000837	protein_coding
ENSG0000(	CLN3	3588.187	-0.1978	0.00011	0.000842	protein_coding
ENSG0000(	RTL6	726.0467	-0.20469	0.00011	0.000843	protein_coding
ENSG0000(	ELOVL5	1007.255	0.584454	0.00011	0.000844	protein_coding
ENSG0000(	IFNG	10.3759	1.495667	0.00011	0.000844	protein_coding
ENSG0000(	TPI1	2379.929	-0.32903	0.00011	0.000844	protein_coding
ENSG0000(	UBE3C	3465.209	-0.156	0.00011	0.000844	protein_coding
ENSG0000(	CBX4	324.4359	0.370487	0.00011	0.000845	protein_coding
ENSG0000(	ZNF33A	4171.08	0.178805	0.00011	0.000845	protein_coding
ENSG0000(	ENSG0000(	338.3328	-0.18318	0.00011	0.000847	lncRNA
ENSG0000(	LINC01905	92.37255	0.307479	0.00011	0.000848	lncRNA
ENSG0000(	DCAF15	424.0555	-0.25428	0.000111	0.000852	protein_coding
ENSG0000(	TSSK4	43.54162	0.482456	0.000111	0.000853	protein_coding
ENSG0000(	STX8	602.3762	-0.14993	0.000111	0.000853	protein_coding
ENSG0000(	CTNNA2-AS	173.0252	-0.385	0.000111	0.000855	lncRNA
ENSG0000(	TFDP1	1070.543	-0.2707	0.000112	0.000856	protein_coding
ENSG0000(	MIATNB	1595.826	0.256956	0.000112	0.000857	lncRNA
ENSG0000(	C1orf52	510.0283	-0.26151	0.000112	0.00086	protein_coding
ENSG0000(	LINC00519	267.8528	0.262914	0.000112	0.00086	lncRNA
ENSG0000(	NAP1L4P1	126.0673	-0.43848	0.000113	0.000862	processed_pseudogene
ENSG0000(	DDIT3	213.5225	-0.31465	0.000113	0.000862	protein_coding
ENSG0000(	PHF20	1525.793	0.188363	0.000113	0.000863	protein_coding
ENSG0000(	NORAD	8225.159	-0.22256	0.000113	0.000863	lncRNA
ENSG0000(	PRKAG2-AS	41.6854	-0.62045	0.000113	0.000863	lncRNA

ENSG0000(	SRPK2	1135.996	0.207726	0.000113	0.000863	protein_coding
ENSG0000(	PAK4	1764.235	-0.26621	0.000113	0.000866	protein_coding
ENSG0000(	ENSG0000(	25.22577	0.730726	0.000113	0.000866	lncRNA
ENSG0000(	ENSG0000(	10.61974	1.111224	0.000113	0.000866	lncRNA
ENSG0000(	ZNF257	332.5965	-0.35963	0.000113	0.000866	protein_coding
ENSG0000(	FAM83H	1305.9	-0.40774	0.000114	0.000868	protein_coding
ENSG0000(	CACNA1C-I	45.244	1.03496	0.000114	0.00087	lncRNA
ENSG0000(	LGR6	98.50751	-0.63498	0.000114	0.00087	protein_coding
ENSG0000(	RHOB	939.8818	0.49483	0.000114	0.00087	protein_coding
ENSG0000(	ZYX	1325.552	0.38792	0.000114	0.00087	protein_coding
ENSG0000(	NDUFA5	2122.226	-0.28456	0.000114	0.000871	protein_coding
ENSG0000(	EBLN3P	5225.839	0.23105	0.000114	0.000871	lncRNA
ENSG0000(	GYPA	166.156	-0.29765	0.000114	0.000872	protein_coding
ENSG0000(	B2M	21888.56	0.497524	0.000115	0.000875	protein_coding
ENSG0000(	ATP9B	7128.208	-0.21701	0.000115	0.000875	protein_coding
ENSG0000(	C2-AS1	41.47388	0.447604	0.000115	0.00088	lncRNA
ENSG0000(	ENSG0000(	66.63865	0.397048	0.000115	0.00088	lncRNA
ENSG0000(	CISD1	269.438	-0.36006	0.000115	0.00088	protein_coding
ENSG0000(	ENSG0000(	413.0323	0.241042	0.000116	0.000884	lncRNA
ENSG0000(	RPL35A	1558.407	-0.40648	0.000116	0.000884	protein_coding
ENSG0000(	FEZ1	572.4209	0.179104	0.000117	0.000887	protein_coding
ENSG0000(	FLJ31104	355.734	0.236813	0.000117	0.000887	lncRNA
ENSG0000(	ENSG0000(	267.9813	0.270811	0.000117	0.000888	lncRNA
ENSG0000(	GSPT1	2813.522	-0.24053	0.000117	0.000889	protein_coding
ENSG0000(	FUT7	16.17483	1.374816	0.000117	0.00089	protein_coding
ENSG0000(	GINS2	246.7765	-0.40526	0.000117	0.00089	protein_coding
ENSG0000(	HPGDS	133.2665	-0.69052	0.000117	0.00089	protein_coding
ENSG0000(	ENSG0000(	243.9122	-0.23577	0.000117	0.00089	lncRNA
ENSG0000(	LY86-AS1	900.8328	0.196241	0.000118	0.000893	lncRNA
ENSG0000(	VAMP1	840.34	0.438675	0.000118	0.000896	protein_coding
ENSG0000(	ENSG0000(	113.1785	0.396621	0.000118	0.000899	lncRNA
ENSG0000(	SRSF5	5345.435	-0.20202	0.000118	0.000899	protein_coding
ENSG0000(	HOOK2	1607.538	-0.30679	0.000119	0.000902	protein_coding
ENSG0000(	UTP6	1638.541	-0.17161	0.000119	0.000903	protein_coding
ENSG0000(	ENSG0000(	309.3332	-0.28794	0.000119	0.000904	lncRNA
ENSG0000(	ENSG0000(	77.95597	0.566934	0.000119	0.000904	lncRNA
ENSG0000(	ENSG0000(	14.86572	1.194575	0.000119	0.000904	TEC
ENSG0000(	PRKAR1A	3479.033	0.250088	0.000119	0.000904	protein_coding
ENSG0000(	MYH10	1890.533	0.227271	0.00012	0.000906	protein_coding
ENSG0000(	ZNF408	180.0871	-0.31519	0.00012	0.000906	protein_coding
ENSG0000(	RPS23P6	35.95771	0.532647	0.00012	0.000907	processed_pseudogene
ENSG0000(	DUS1L	1166.52	-0.29616	0.00012	0.000907	protein_coding
ENSG0000(	ENSG0000(	921.156	0.205855	0.00012	0.00091	lncRNA
ENSG0000(	SETD9	139.6346	-0.51546	0.00012	0.00091	protein_coding
ENSG0000(	TOPBP1	1574.379	-0.24087	0.00012	0.00091	protein_coding
ENSG0000(	CSTB	1166.013	-0.57808	0.00012	0.00091	protein_coding
ENSG0000(	NDUFAF3	204.5321	-0.34403	0.00012	0.00091	protein_coding

ENSG0000(	SMIM19	355.9781	-0.37613	0.00012	0.00091	protein_coding
ENSG0000(	CAMK1	295.7833	-0.25982	0.000121	0.000912	protein_coding
ENSG0000(	ENSG0000(	145.7	-0.28338	0.000121	0.000913	lncRNA
ENSG0000(	ERAP2	2474.287	0.395752	0.000121	0.000913	protein_coding
ENSG0000(	OPRL1	49.0369	0.592309	0.000121	0.000913	protein_coding
ENSG0000(	RPS3AP39	32.12806	-0.73161	0.000121	0.000913	processed_pseudogene
ENSG0000(	TMEM163	123.0818	1.134076	0.000121	0.000913	protein_coding
ENSG0000(	MEOX1	87.53316	0.899989	0.000121	0.000914	protein_coding
ENSG0000(	ENSG0000(	262.2845	0.37213	0.000121	0.000915	lncRNA
ENSG0000(	ZNF333	1717.475	0.139096	0.000121	0.000916	protein_coding
ENSG0000(	WDR81	1341.212	0.284743	0.000122	0.000919	protein_coding
ENSG0000(	ENSG0000(	22.18322	1.122138	0.000122	0.00092	unprocessed_pseudogene
ENSG0000(	DHRS11	3081.169	-0.60654	0.000123	0.000924	protein_coding
ENSG0000(	ENSG0000(	343.2136	0.327523	0.000123	0.000927	lncRNA
ENSG0000(	RDX	720.254	0.409654	0.000123	0.000927	protein_coding
ENSG0000(	ENSG0000(	85.47787	-0.41009	0.000124	0.000932	lncRNA
ENSG0000(	ENSG0000(	10.43946	0.835886	0.000124	0.000932	TEC
ENSG0000(	DEPDC1P1	73.66267	-0.67823	0.000124	0.000934	processed_pseudogene
ENSG0000(	ENSG0000(	1999.914	0.157847	0.000124	0.000934	lncRNA
ENSG0000(	ENSG0000(	542.9036	0.200422	0.000124	0.000935	lncRNA
ENSG0000(	FUNDC2P4	10.1427	1.146472	0.000124	0.000936	processed_pseudogene
ENSG0000(	ENSG0000(	174.1519	0.354275	0.000124	0.000936	lncRNA
ENSG0000(	DICER1-AS:	1088.938	0.181537	0.000124	0.000936	lncRNA
ENSG0000(	MBNL2	1959.997	0.196707	0.000125	0.000937	protein_coding
ENSG0000(	PCSK1N	43.86194	0.59106	0.000125	0.000942	protein_coding
ENSG0000(	PLIN5	375.8938	0.226072	0.000126	0.000944	protein_coding
ENSG0000(	COMMD4	1362.824	-0.1895	0.000126	0.000945	protein_coding
ENSG0000(	ENSG0000(	467.916	0.296114	0.000126	0.000945	lncRNA
ENSG0000(	TSC2	4086.284	-0.19108	0.000126	0.000945	protein_coding
ENSG0000(	FKBP4P1	58.72035	-0.79324	0.000126	0.000947	processed_pseudogene
ENSG0000(	PYURF	424.792	-0.34961	0.000126	0.000948	protein_coding
ENSG0000(	PTBP1	3699.332	-0.14447	0.000126	0.000949	protein_coding
ENSG0000(	SLCO3A1	988.5288	0.378578	0.000126	0.000949	protein_coding
ENSG0000(	METTL13	644.9978	-0.2533	0.000127	0.000952	protein_coding
ENSG0000(	LNCOC1	311.3369	0.242155	0.000127	0.000953	lncRNA
ENSG0000(	PLOD1	816.7807	0.186615	0.000127	0.000953	protein_coding
ENSG0000(	RPL7L1	3475.591	-0.11982	0.000127	0.000953	protein_coding
ENSG0000(	ENSG0000(	122.6353	0.35039	0.000127	0.000954	lncRNA
ENSG0000(	XBP1P1	69.09311	0.394525	0.000127	0.000954	processed_pseudogene
ENSG0000(	SMIM14	3708.553	-0.19091	0.000128	0.000958	protein_coding
ENSG0000(	PPT2-EGFL:	209.8988	-0.20961	0.000128	0.000958	protein_coding
ENSG0000(	FGL2	1579.816	0.482186	0.000128	0.000958	protein_coding
ENSG0000(	TDRKH-AS1	193.6226	-0.26542	0.000128	0.000959	lncRNA
ENSG0000(	ADGRL1	777.0569	-0.39575	0.000128	0.000959	protein_coding
ENSG0000(	ENSG0000(	106.7612	0.486932	0.000128	0.000959	TEC
ENSG0000(	LIN7C	1532.039	-0.2377	0.000129	0.000961	protein_coding
ENSG0000(	C6orf132	1589.913	-0.35464	0.000129	0.000962	protein_coding

ENSG0000(	SVIL-AS1	1420.066	0.15481	0.000129	0.000963	transcribed_unprocessed_pseudo
ENSG0000(	C9orf85	830.208	-0.14781	0.000129	0.000964	protein_coding
ENSG0000(	ENSG0000(	200.6783	0.349418	0.000129	0.000965	lncRNA
ENSG0000(	HORMAD1	71.92862	0.700205	0.000129	0.000966	protein_coding
ENSG0000(	ENSG0000(	67.05794	0.416197	0.00013	0.000969	protein_coding
ENSG0000(	IL3RA	114.863	0.68199	0.00013	0.000971	protein_coding
ENSG0000(	ENSG0000(	208.5003	0.289704	0.00013	0.000973	TEC
ENSG0000(	PTP4A3	268.1175	0.642333	0.000131	0.000975	protein_coding
ENSG0000(	JAK3	2221.13	0.717056	0.000131	0.000976	protein_coding
ENSG0000(	RNU6-674F	9.612196	0.899548	0.000131	0.000976	snRNA
ENSG0000(	TBC1D12	1249.344	-0.25455	0.000131	0.000976	protein_coding
ENSG0000(	AHCYL2	7581.445	-0.6266	0.000131	0.000978	protein_coding
ENSG0000(	PDF	345.1146	-0.33738	0.000131	0.000979	protein_coding
ENSG0000(	ENSG0000(	545.1569	0.238341	0.000132	0.00098	lncRNA
ENSG0000(	ZSWIM8-AS	39.63726	0.900508	0.000132	0.000983	lncRNA
ENSG0000(	GUCY1A1	714.4108	0.77249	0.000132	0.000984	protein_coding
ENSG0000(	ABCC1	2639.778	0.23811	0.000132	0.000985	protein_coding
ENSG0000(	SLC16A3	1721.172	0.445768	0.000132	0.000985	protein_coding
ENSG0000(	CTSB	7063.396	0.44378	0.000132	0.000985	protein_coding
ENSG0000(	GXYLT2	640.6088	-0.39435	0.000132	0.000985	protein_coding
ENSG0000(	ENSG0000(	69.65452	0.540025	0.000132	0.000985	lncRNA
ENSG0000(	PLBD1	481.0718	-0.34563	0.000133	0.000986	protein_coding
ENSG0000(	ENSG0000(	19.99188	0.952834	0.000133	0.000986	lncRNA
ENSG0000(	EIF4B	6502.356	-0.21913	0.000133	0.000987	protein_coding
ENSG0000(	CNBD2	224.4145	-0.26002	0.000133	0.00099	protein_coding
ENSG0000(	P2RY6	2093.526	0.263477	0.000133	0.000991	protein_coding
ENSG0000(	ENSG0000(	122.4926	0.243764	0.000134	0.000993	TEC
ENSG0000(	ENSG0000(	316.1982	0.184119	0.000134	0.000994	lncRNA
ENSG0000(	LINC00884	3458.78	0.122605	0.000134	0.000995	lncRNA
ENSG0000(	ENDOD1	1362.443	-0.4488	0.000135	0.000999	protein_coding
ENSG0000(	LINC01762	146.6538	-0.28645	0.000135	0.001	lncRNA
ENSG0000(	RN7SKP78	95.74518	0.494966	0.000135	0.001	misc_RNA
ENSG0000(	VPS26BP1	144.7352	0.344142	0.000135	0.001	processed_pseudogene
ENSG0000(	PRKAR2B	402.0762	0.485607	0.000135	0.001	protein_coding
ENSG0000(	PPM1K	2564.217	0.399335	0.000135	0.001001	protein_coding
ENSG0000(	ENSG0000(	280.6969	0.332266	0.000136	0.001007	lncRNA
ENSG0000(	ENSG0000(	83.73443	0.348426	0.000136	0.001007	lncRNA
ENSG0000(	GRK3	1267.629	0.455879	0.000136	0.001009	protein_coding
ENSG0000(	ENSG0000(	252.3158	0.321562	0.000137	0.001012	lncRNA
ENSG0000(	FCHSD2	1502.733	0.450881	0.000137	0.001013	protein_coding
ENSG0000(	PEX13	1590.689	-0.1756	0.000137	0.001013	protein_coding
ENSG0000(	SYPL1	846.2405	-0.29866	0.000137	0.001013	protein_coding
ENSG0000(	PSMD4	700.1517	-0.19504	0.000137	0.001015	protein_coding
ENSG0000(	ENSG0000(	326.5454	0.303435	0.000137	0.001015	lncRNA
ENSG0000(	CMTR1	1327.9	0.244847	0.000138	0.00102	protein_coding
ENSG0000(	LINC02389	110.5002	0.357999	0.000138	0.00102	lncRNA
ENSG0000(	STEEP1	840.457	-0.18952	0.000138	0.00102	protein_coding

ENSG0000(	ENSG0000(	98.62265	0.306873	0.000138	0.001021	processed_pseudogene
ENSG0000(	ENSG0000(	37.70396	0.592605	0.000138	0.001023	lncRNA
ENSG0000(	UGT3A1	56.10235	0.428417	0.000139	0.001025	protein_coding
ENSG0000(	CHTF18	406.343	-0.47287	0.000139	0.001025	protein_coding
ENSG0000(	S100A10	1919.037	-0.46429	0.000139	0.001025	protein_coding
ENSG0000(	ZNF75D	1270.198	0.144292	0.000139	0.001026	protein_coding
ENSG0000(	ENSG0000(	338.5962	0.656299	0.000139	0.001026	lncRNA
ENSG0000(	RAMP3	172.2316	0.664327	0.000139	0.001026	protein_coding
ENSG0000(	ENSG0000(	275.3426	-0.2307	0.000139	0.001026	lncRNA
ENSG0000(	ENSG0000(	90.20804	0.570983	0.000139	0.001026	lncRNA
ENSG0000(	MCRS1	518.1723	-0.28873	0.000139	0.001027	protein_coding
ENSG0000(	POLDIP3	1176.152	-0.21318	0.000139	0.001028	protein_coding
ENSG0000(	CREM	341.6	0.270883	0.00014	0.00103	protein_coding
ENSG0000(	JMJD6	830.2161	-0.26778	0.00014	0.00103	protein_coding
ENSG0000(	ENSG0000(	506.8934	0.217995	0.00014	0.001031	lncRNA
ENSG0000(	TARS3	715.7253	-0.2492	0.00014	0.001031	protein_coding
ENSG0000(	ENSG0000(	590.0173	0.21129	0.000141	0.001036	lncRNA
ENSG0000(	ENSG0000(	3186.683	0.286529	0.000141	0.001036	lncRNA
ENSG0000(	MTMR9LP	417.0291	0.365427	0.000141	0.001036	transcribed_unprocessed_pseudo
ENSG0000(	ENSG0000(	43.32147	0.537689	0.000141	0.001037	TEC
ENSG0000(	ALDH3A1	79.21922	-0.86715	0.000141	0.001038	protein_coding
ENSG0000(	THRA	1102.42	-0.31234	0.000141	0.001038	protein_coding
ENSG0000(	BNIP3L	942.9262	0.31064	0.000141	0.001038	protein_coding
ENSG0000(	DYNC2I2	330.332	-0.33653	0.000141	0.00104	protein_coding
ENSG0000(	ARHGAP24	597.4206	0.524214	0.000142	0.001041	protein_coding
ENSG0000(	C1orf210	474.0561	-0.46335	0.000142	0.001043	protein_coding
ENSG0000(	TIMM10	76.64488	-0.54526	0.000142	0.001047	protein_coding
ENSG0000(	LINC02073	492.8093	0.162153	0.000143	0.001052	lncRNA
ENSG0000(	ENSG0000(	365.5015	-0.24715	0.000144	0.001056	lncRNA
ENSG0000(	PDE6C	46.80439	0.450821	0.000144	0.001058	protein_coding
ENSG0000(	PSMB5	352.924	-0.3549	0.000144	0.001059	protein_coding
ENSG0000(	ENSG0000(	13.15794	0.88107	0.000145	0.001068	TEC
ENSG0000(	TDP2	2848.273	-0.50173	0.000146	0.001069	protein_coding
ENSG0000(	GP6-AS1	1281.788	0.233584	0.000146	0.00107	lncRNA
ENSG0000(	SNX12	673.047	-0.32209	0.000146	0.001072	protein_coding
ENSG0000(	LINC01137	159.914	0.385045	0.000147	0.001074	lncRNA
ENSG0000(	MRTFA-AS1	333.7619	-0.20457	0.000147	0.001074	lncRNA
ENSG0000(	LHFPL5	1360.541	0.213222	0.000147	0.001077	protein_coding
ENSG0000(	MBNL3	2048.26	-0.31975	0.000147	0.001077	protein_coding
ENSG0000(	PTTG1	113.8764	-0.53969	0.000147	0.001077	protein_coding
ENSG0000(	RUNX3-AS1	8.975648	1.077001	0.000147	0.001077	lncRNA
ENSG0000(	SP140L	1753.52	0.246294	0.000147	0.001077	protein_coding
ENSG0000(	STARD4-AS	129.1718	0.317032	0.000147	0.001077	lncRNA
ENSG0000(	ENSG0000(	483.6366	0.267853	0.000147	0.001077	lncRNA
ENSG0000(	LZIC	1433.2	-0.22546	0.000147	0.001077	protein_coding
ENSG0000(	PPP1R26	585.3419	-0.3689	0.000147	0.001077	protein_coding
ENSG0000(	SUDS3	2120.975	-0.15379	0.000148	0.001079	protein_coding

ENSG0000(	ENSG0000(	165.6501	0.255323	0.000148	0.00108	lncRNA
ENSG0000(	ZNF295-AS	229.4723	0.284902	0.000148	0.00108	lncRNA
ENSG0000(	ZNF524	174.3569	-0.3211	0.000148	0.00108	protein_coding
ENSG0000(	NOP16	273.8929	-0.34469	0.000148	0.00108	protein_coding
ENSG0000(	LINC02395	530.7652	-0.23725	0.000148	0.001082	lncRNA
ENSG0000(	POLR1A	3213.853	-0.20714	0.000148	0.001082	protein_coding
ENSG0000(	FLRT2	2416.584	0.65774	0.000148	0.001083	protein_coding
ENSG0000(	SURF1	264.6013	-0.26978	0.000149	0.001084	protein_coding
ENSG0000(	PABIR3	1441.919	-0.15129	0.000149	0.001085	protein_coding
ENSG0000(	GRIK2	841.6921	-0.19187	0.000149	0.001085	protein_coding
ENSG0000(	RNU4-62P	34.17318	0.716123	0.000149	0.001087	snRNA
ENSG0000(	UNC5CL	259.4996	0.598653	0.000149	0.001087	protein_coding
ENSG0000(	SGTA	1354.063	-0.16034	0.000149	0.001087	protein_coding
ENSG0000(	DNPH1	170.0534	-0.44836	0.000149	0.00109	protein_coding
ENSG0000(	ENSG0000(	59.75232	-0.54688	0.00015	0.00109	lncRNA
ENSG0000(	LTBP2	697.0349	0.73024	0.00015	0.00109	protein_coding
ENSG0000(	RNVU1-4	21.95335	0.774077	0.00015	0.00109	snRNA
ENSG0000(	RPS6KA6	1213.842	-0.3854	0.00015	0.00109	protein_coding
ENSG0000(	LINC00352	374.829	-0.22963	0.00015	0.001091	lncRNA
ENSG0000(	ENSG0000(	15.34887	0.657306	0.00015	0.001092	snoRNA
ENSG0000(	RN7SL105F	43.70858	0.614838	0.00015	0.001092	misc_RNA
ENSG0000(	ZNF584	467.128	-0.26279	0.00015	0.001092	protein_coding
ENSG0000(	ASMTL-AS1	492.5671	-0.28338	0.00015	0.001093	lncRNA
ENSG0000(	TUBA1B	2650.929	-0.43155	0.000151	0.001096	protein_coding
ENSG0000(	BEND6	213.3276	0.270878	0.000151	0.001096	protein_coding
ENSG0000(	GRK6P1	15.24049	-0.76783	0.000151	0.0011	processed_pseudogene
ENSG0000(	CKAP2	476.1761	-0.36538	0.000152	0.001102	protein_coding
ENSG0000(	OR2T29	9.705607	2.18695	0.000152	0.001102	protein_coding
ENSG0000(	OR7A19P	87.06393	0.456393	0.000152	0.001102	unprocessed_pseudogene
ENSG0000(	SEC61G	398.7908	-0.43458	0.000152	0.001102	protein_coding
ENSG0000(	TMOD4	117.771	-0.31893	0.000152	0.001104	protein_coding
ENSG0000(	ENSG0000(	16.82918	1.026598	0.000152	0.001105	lncRNA
ENSG0000(	GON7	156.3818	-0.32999	0.000152	0.001106	protein_coding
ENSG0000(	LSM14A	2155.908	-0.18126	0.000153	0.001108	protein_coding
ENSG0000(	FERMT1	2297.12	-0.45657	0.000153	0.001109	protein_coding
ENSG0000(	ENSG0000(	9.448943	0.829734	0.000153	0.001109	lncRNA
ENSG0000(	RACGAP1	451.7616	-0.38717	0.000153	0.001109	protein_coding
ENSG0000(	CENPI	755.7647	-0.33549	0.000153	0.001109	protein_coding
ENSG0000(	CNKSR2	1134.792	0.234924	0.000153	0.001109	protein_coding
ENSG0000(	ENSG0000(	65.50674	0.371057	0.000153	0.001109	lncRNA
ENSG0000(	VCAN	2748.201	0.719332	0.000153	0.00111	protein_coding
ENSG0000(	RN7SL154F	52.18784	-0.40877	0.000153	0.001112	misc_RNA
ENSG0000(	C6orf47	740.1663	-0.20317	0.000154	0.001116	protein_coding
ENSG0000(	CINP	785.4415	-0.20874	0.000154	0.001116	protein_coding
ENSG0000(	COA7	1331.011	-0.18518	0.000154	0.001116	protein_coding
ENSG0000(	ASAP1	1628.977	0.456234	0.000154	0.001117	protein_coding
ENSG0000(	SVIP	674.6598	-0.22938	0.000155	0.001123	protein_coding

ENSG0000(	TMCC1	1162.939	0.154306	0.000155	0.001123	protein_coding
ENSG0000(	ENSG0000(	32.61719	-0.56088	0.000156	0.001124	lncRNA
ENSG0000(	SCO2	257.6715	0.566308	0.000156	0.001125	protein_coding
ENSG0000(	ZNF616	569.2977	-0.27306	0.000156	0.001126	protein_coding
ENSG0000(	CKMT1A	1721.752	-0.51906	0.000156	0.001126	protein_coding
ENSG0000(	CBX6	1523.262	0.372693	0.000156	0.001128	protein_coding
ENSG0000(	ENSG0000(	80.49535	0.521656	0.000156	0.00113	lncRNA
ENSG0000(	MYCBP	362.9178	-0.43663	0.000157	0.00113	protein_coding
ENSG0000(	ENSG0000(	284.6485	-0.20722	0.000157	0.001133	lncRNA
ENSG0000(	SPHK2	1012.238	-0.31795	0.000157	0.001134	protein_coding
ENSG0000(	XIAPP2	285.8952	-0.28117	0.000158	0.001138	processed_pseudogene
ENSG0000(	BLVRA	98.8165	0.559378	0.000158	0.001139	protein_coding
ENSG0000(	CCL3L3	39.43658	1.276557	0.000158	0.001139	protein_coding
ENSG0000(	ENSG0000(	27.59779	0.573254	0.000158	0.001139	lncRNA
ENSG0000(	EVI2B	736.3068	0.534131	0.000158	0.00114	protein_coding
ENSG0000(	PPP1CC	4042.942	-0.23635	0.000158	0.00114	protein_coding
ENSG0000(	ITPK1	1642.264	-0.27566	0.000158	0.001142	protein_coding
ENSG0000(	NIBAN1	1077.07	0.330201	0.000159	0.001143	protein_coding
ENSG0000(	RN7SL81P	72.26424	0.450155	0.000159	0.001144	misc_RNA
ENSG0000(	SEPTIN4-AS	213.7012	0.295354	0.000159	0.001144	lncRNA
ENSG0000(	MRPL49	543.413	-0.2418	0.000159	0.001145	protein_coding
ENSG0000(	FLNB	34721.98	-0.48384	0.000159	0.001147	protein_coding
ENSG0000(	SLFN12L	5080.421	0.265309	0.000159	0.001147	protein_coding
ENSG0000(	ENSG0000(	81.88434	0.510514	0.00016	0.001149	TEC
ENSG0000(	MRPL16	299.2422	-0.28475	0.00016	0.001149	protein_coding
ENSG0000(	MCTP2	5237.867	0.265427	0.00016	0.001149	protein_coding
ENSG0000(	CMKLR1	701.1078	0.528574	0.00016	0.001153	protein_coding
ENSG0000(	HMGCS2	5168.153	-1.2247	0.00016	0.001153	protein_coding
ENSG0000(	ENSG0000(	43.90105	0.476144	0.000161	0.001155	lncRNA
ENSG0000(	ENSG0000(	86.10248	0.567243	0.000161	0.001155	lncRNA
ENSG0000(	UNC50	491.2844	-0.26901	0.000161	0.001158	protein_coding
ENSG0000(	ENSG0000(	529.9307	0.145223	0.000162	0.00116	TEC
ENSG0000(	GABRE	3072.614	0.328657	0.000162	0.00116	protein_coding
ENSG0000(	FADD	237.7091	-0.32463	0.000162	0.001161	protein_coding
ENSG0000(	GDF7	180.3538	0.741832	0.000162	0.001161	protein_coding
ENSG0000(	EBLN2	352.029	0.217166	0.000162	0.001165	protein_coding
ENSG0000(	SHARPIN	411.9192	-0.18597	0.000163	0.001166	protein_coding
ENSG0000(	STK3	1439.945	0.252528	0.000163	0.001167	protein_coding
ENSG0000(	ABHD6	439.1046	-0.23768	0.000163	0.001168	protein_coding
ENSG0000(	ENSG0000(	36.66456	0.900931	0.000163	0.00117	TEC
ENSG0000(	C2CD2	1228.321	0.305058	0.000163	0.001171	protein_coding
ENSG0000(	CPNE2	1127.676	-0.3403	0.000163	0.001171	protein_coding
ENSG0000(	RPL36AP33	37.68398	0.712289	0.000164	0.001173	processed_pseudogene
ENSG0000(	ENSG0000(	40.73028	0.571583	0.000164	0.001173	lncRNA
ENSG0000(	FLRT3	206.9296	0.92884	0.000164	0.001174	protein_coding
ENSG0000(	HMBS	323.7875	-0.34769	0.000164	0.001174	protein_coding
ENSG0000(	MIER3	6382.165	-0.80556	0.000164	0.001174	protein_coding

ENSG0000(	ENSG0000(	1021.15	-0.38193	0.000164	0.001176	lncRNA
ENSG0000(	OR52U1P	12.50903	1.052977	0.000165	0.001179	unprocessed_pseudogene
ENSG0000(	DHFR	1064.759	-0.24594	0.000165	0.00118	protein_coding
ENSG0000(	ENSG0000(	57.87739	-0.50855	0.000165	0.001181	unprocessed_pseudogene
ENSG0000(	SMARCD3	197.1177	0.422989	0.000165	0.001182	protein_coding
ENSG0000(	ENSG0000(	238.5185	0.307832	0.000166	0.001183	lncRNA
ENSG0000(	ENSG0000(	20.13108	0.71491	0.000166	0.001183	lncRNA
ENSG0000(	STING1	1245.698	0.273101	0.000166	0.001185	protein_coding
ENSG0000(	FAM50A	349.4008	-0.2734	0.000166	0.001187	protein_coding
ENSG0000(	DCN	1638.405	0.504293	0.000167	0.001189	protein_coding
ENSG0000(	RAD23A	865.8503	-0.18842	0.000167	0.001191	protein_coding
ENSG0000(	SNORA74A	502.2168	-0.56515	0.000167	0.001191	snoRNA
ENSG0000(	ENSG0000(	330.0999	0.258316	0.000167	0.001193	lncRNA
ENSG0000(	LINC01735	16.18594	1.112311	0.000167	0.001193	lncRNA
ENSG0000(	TMEM184/	2756.742	-0.50525	0.000168	0.001195	protein_coding
ENSG0000(	CCDC3	81.11142	1.056148	0.000168	0.001196	protein_coding
ENSG0000(	ENSG0000(	247488.8	0.947238	0.000168	0.001197	processed_pseudogene
ENSG0000(	ENSG0000(	23.77378	0.675322	0.000168	0.0012	lncRNA
ENSG0000(	GPATCH2L	5206.319	0.176824	0.000168	0.0012	protein_coding
ENSG0000(	ENSG0000(	295.0446	-0.22031	0.000169	0.001203	lncRNA
ENSG0000(	ENSG0000(	13.08377	0.717779	0.000169	0.001207	processed_pseudogene
ENSG0000(	NAA15	2041.873	-0.21739	0.00017	0.001207	protein_coding
ENSG0000(	ZNF385D	326.3626	0.752917	0.00017	0.001207	protein_coding
ENSG0000(	ENSG0000(	319.3009	0.231059	0.00017	0.001207	TEC
ENSG0000(	GEMIN6	1270.604	-0.14067	0.00017	0.001207	protein_coding
ENSG0000(	TMEM44-A	166.7254	0.301898	0.00017	0.001208	lncRNA
ENSG0000(	PPP1R14C	434.6523	-0.62276	0.00017	0.001208	protein_coding
ENSG0000(	ENSG0000(	3074.742	-0.27928	0.00017	0.001209	lncRNA
ENSG0000(	ENSG0000(	64.23653	0.364299	0.00017	0.00121	lncRNA
ENSG0000(	APELA	75.78116	-0.45069	0.00017	0.001211	protein_coding
ENSG0000(	STX1B	201.4334	0.466695	0.000171	0.001212	protein_coding
ENSG0000(	LINC02701	44.26595	0.649553	0.000171	0.001214	lncRNA
ENSG0000(	TMEM82	61.89664	-0.74146	0.000171	0.001215	protein_coding
ENSG0000(	ENSG0000(	29.19824	-0.91926	0.000172	0.001218	processed_pseudogene
ENSG0000(	CLYBL	373.2457	-0.50749	0.000172	0.001218	protein_coding
ENSG0000(	TAS1R3	66.36821	0.719875	0.000172	0.001221	protein_coding
ENSG0000(	ENSG0000(	24.34679	0.95316	0.000172	0.001222	lncRNA
ENSG0000(	PPIAL4G	25.279	0.851989	0.000172	0.001223	protein_coding
ENSG0000(	ENSG0000(	88.35789	-0.54466	0.000173	0.001224	lncRNA
ENSG0000(	EFL1P1	69.94469	0.494896	0.000173	0.001228	transcribed_unprocessed_pseudo
ENSG0000(	ENSG0000(	372.4659	0.25923	0.000174	0.001234	lncRNA
ENSG0000(	CTBP2	3245.424	-0.25283	0.000175	0.001238	protein_coding
ENSG0000(	C5orf64	1509.363	0.1515	0.000175	0.00124	lncRNA
ENSG0000(	RPUSD1	256.745	-0.23862	0.000175	0.00124	protein_coding
ENSG0000(	ENSG0000(	428.928	-0.20205	0.000175	0.001241	TEC
ENSG0000(	SMPDL3B	210.9632	-0.47299	0.000175	0.001241	protein_coding
ENSG0000(	AP1G2-AS1	631.2671	0.224312	0.000175	0.001242	lncRNA

ENSG0000(	FSTL3	178.2658	0.571667	0.000176	0.001243	protein_coding
ENSG0000(	ZNF3	1695.897	-0.16933	0.000176	0.001245	protein_coding
ENSG0000(	DTWD1	1746.942	0.155405	0.000176	0.001245	protein_coding
ENSG0000(	WASH5P	15668.92	0.246845	0.000177	0.001249	transcribed_processed_pseudoge
ENSG0000(	IGFL2	89.25027	0.318397	0.000177	0.001249	protein_coding
ENSG0000(	CSNK1G2-A	23.54966	0.670833	0.000177	0.00125	lncRNA
ENSG0000(	GPR155	1625.538	0.331863	0.000177	0.001252	protein_coding
ENSG0000(	ENSG0000(	11.90495	0.753817	0.000177	0.001253	processed_pseudogene
ENSG0000(	BTF3	1813.318	-0.33796	0.000178	0.001255	protein_coding
ENSG0000(	TMEM131	5310.231	-0.22585	0.000178	0.00126	protein_coding
ENSG0000(	ENSG0000(	1551.583	0.180545	0.000179	0.001261	lncRNA
ENSG0000(	H4C6	83.86783	-0.77363	0.000179	0.001263	protein_coding
ENSG0000(	PHF5A	307.9116	-0.27268	0.000179	0.001264	protein_coding
ENSG0000(	ISCA1	617.6216	-0.20726	0.000179	0.001265	protein_coding
ENSG0000(	LINC02284	130.5144	0.404771	0.00018	0.001267	lncRNA
ENSG0000(	LINC02517	206.3322	-0.3244	0.00018	0.001268	lncRNA
ENSG0000(	SRP9	1830.35	-0.19903	0.00018	0.00127	protein_coding
ENSG0000(	ENSG0000(	29.1508	0.526403	0.000181	0.001273	lncRNA
ENSG0000(	OR5BA1P	627.6504	-0.15236	0.000181	0.001273	transcribed_unprocessed_pseudo
ENSG0000(	PHACTR4	3068.129	-0.14084	0.000181	0.001273	protein_coding
ENSG0000(	LINC01229	47.79609	0.736343	0.000181	0.001274	lncRNA
ENSG0000(	RNF183	51.06655	1.041131	0.000181	0.001274	protein_coding
ENSG0000(	ENSG0000(	124.845	0.478254	0.000181	0.001275	lncRNA
ENSG0000(	RNU6-1005	26.0872	0.665824	0.000181	0.001277	snRNA
ENSG0000(	ENSG0000(	265.2194	0.286229	0.000181	0.001277	transcribed_processed_pseudoge
ENSG0000(	ZNF648	14.7145	1.039383	0.000182	0.001279	protein_coding
ENSG0000(	DNAJC25-G	144.5557	0.809869	0.000182	0.001281	protein_coding
ENSG0000(	ENSG0000(	214.6487	0.229119	0.000182	0.001282	lncRNA
ENSG0000(	RBBP7	1904.182	-0.16573	0.000182	0.001282	protein_coding
ENSG0000(	EMC4	1165.522	-0.14843	0.000183	0.001284	protein_coding
ENSG0000(	SF3B4	1032.274	-0.42444	0.000183	0.001284	protein_coding
ENSG0000(	DUSP5	871.9346	0.592315	0.000183	0.001284	protein_coding
ENSG0000(	ADAM20	163.8812	0.356027	0.000183	0.001285	protein_coding
ENSG0000(	ENSG0000(	141.1844	-0.30218	0.000183	0.001285	lncRNA
ENSG0000(	ENSG0000(	12.34739	1.520866	0.000183	0.001286	processed_pseudogene
ENSG0000(	GPR137B	375.7554	0.297098	0.000183	0.001287	protein_coding
ENSG0000(	GSS	1255.46	-0.18869	0.000183	0.001287	protein_coding
ENSG0000(	L1CAM	151.2953	-0.54269	0.000183	0.001287	protein_coding
ENSG0000(	NETO2	1340.684	-0.47373	0.000183	0.001287	protein_coding
ENSG0000(	PRRG4	1198.281	-0.21174	0.000183	0.001287	protein_coding
ENSG0000(	MST1R	2889.51	-0.47651	0.000184	0.001287	protein_coding
ENSG0000(	RN7SL368F	105.4561	0.891326	0.000184	0.001293	misc_RNA
ENSG0000(	SPTLC1	2213.611	-0.14667	0.000185	0.001294	protein_coding
ENSG0000(	ARIH1	5241.773	-0.18687	0.000185	0.001294	protein_coding
ENSG0000(	LINC02336	239.0479	0.238183	0.000185	0.001296	lncRNA
ENSG0000(	TMX4	911.5261	0.274947	0.000185	0.001296	protein_coding
ENSG0000(	USF1P1	47.17638	0.519712	0.000185	0.001296	processed_pseudogene

ENSG0000(	IL17RE	1127.439	-0.4161	0.000185	0.001299	protein_coding
ENSG0000(	PIK3CG	1022.788	0.46569	0.000186	0.001301	protein_coding
ENSG0000(	NRBF2P6	39.96753	-0.61196	0.000186	0.001302	processed_pseudogene
ENSG0000(	ENSG0000(	295.0749	0.235493	0.000186	0.001303	lncRNA
ENSG0000(	SYT13	659.1211	-0.54654	0.000186	0.001303	protein_coding
ENSG0000(	BUD13	313.2479	-0.23186	0.000187	0.001305	protein_coding
ENSG0000(	ENSG0000(	31.9202	0.75115	0.000187	0.001305	lncRNA
ENSG0000(	PSG3	82.72176	0.606713	0.000187	0.001306	protein_coding
ENSG0000(	EEF1A1P28	9.744585	-1.13787	0.000187	0.001309	processed_pseudogene
ENSG0000(	ENSG0000(	2312.917	0.170503	0.000187	0.001309	lncRNA
ENSG0000(	ZMAT2	372.9607	-0.27231	0.000188	0.001314	protein_coding
ENSG0000(	NALCN	445.5947	-0.18134	0.000189	0.001317	protein_coding
ENSG0000(	CTCF	2396.711	-0.11881	0.000189	0.00132	protein_coding
ENSG0000(	PCNX3	2026.734	-0.29961	0.000189	0.001321	protein_coding
ENSG0000(	SLC45A1	15.47627	0.761368	0.000189	0.001322	protein_coding
ENSG0000(	OR5B21	15.66594	0.897993	0.000189	0.001322	protein_coding
ENSG0000(	RNU4-9P	11.30058	0.865551	0.000189	0.001322	snRNA
ENSG0000(	SNHG6	335.89	-0.36274	0.000189	0.001322	lncRNA
ENSG0000(	C8orf34-AS	500.3026	0.327057	0.00019	0.001322	lncRNA
ENSG0000(	MIR624	23.85246	0.680213	0.00019	0.001323	miRNA
ENSG0000(	ENSG0000(	86.30084	0.346315	0.00019	0.001325	lncRNA
ENSG0000(	ENSG0000(	27.70875	0.587246	0.00019	0.001326	lncRNA
ENSG0000(	ENSG0000(	169.4873	0.387596	0.00019	0.001326	lncRNA
ENSG0000(	MAD2L1-D	230.2655	0.231676	0.00019	0.001326	lncRNA
ENSG0000(	SETBP1-DT	97.24874	0.384272	0.00019	0.001326	lncRNA
ENSG0000(	OR10A3	134.141	-0.39075	0.000191	0.001327	protein_coding
ENSG0000(	KHDC1	340.775	0.168118	0.000191	0.001328	protein_coding
ENSG0000(	KDM1B	1318.371	-0.25947	0.000191	0.001328	protein_coding
ENSG0000(	JAG1	3668.526	-0.32198	0.000191	0.001328	protein_coding
ENSG0000(	ENSG0000(	621.4628	0.196001	0.000191	0.00133	processed_pseudogene
ENSG0000(	MIR3667H(	755.9743	0.556089	0.000191	0.001331	lncRNA
ENSG0000(	TYK2	2705.369	0.13561	0.000192	0.001333	protein_coding
ENSG0000(	PDZD3	1300.166	-0.71912	0.000192	0.001336	protein_coding
ENSG0000(	GUCA2A	879.4671	-1.18106	0.000192	0.001337	protein_coding
ENSG0000(	ENSG0000(	1106.453	-0.38072	0.000193	0.00134	lncRNA
ENSG0000(	EIF4A1	3121.654	-0.25786	0.000193	0.001342	protein_coding
ENSG0000(	CDHR4	386.7095	-0.22654	0.000194	0.001348	protein_coding
ENSG0000(	C1orf105	87.871	0.295829	0.000195	0.001351	protein_coding
ENSG0000(	MAST4	2021.818	0.260778	0.000195	0.001353	protein_coding
ENSG0000(	CHMP4BP1	128.7839	-0.5498	0.000195	0.001353	processed_pseudogene
ENSG0000(	STOM	2414.48	0.599138	0.000195	0.001355	protein_coding
ENSG0000(	LRFN5	112.2996	1.107906	0.000195	0.001356	protein_coding
ENSG0000(	ENSG0000(	162.9988	0.397949	0.000195	0.001356	lncRNA
ENSG0000(	ENSG0000(	103.1086	0.32288	0.000195	0.001356	lncRNA
ENSG0000(	ENSG0000(	11.93245	0.874799	0.000196	0.001357	lncRNA
ENSG0000(	PRPF31	326.1003	-0.33297	0.000196	0.001358	protein_coding
ENSG0000(	UFM1	1727.344	0.221354	0.000196	0.001358	protein_coding

ENSG0000(	HOXA7	337.124	-0.50649	0.000196	0.001358	protein_coding
ENSG0000(	RTL8C	289.4298	0.34036	0.000196	0.001358	protein_coding
ENSG0000(	SLC6A7	663.6373	-0.59747	0.000196	0.00136	protein_coding
ENSG0000(	SPARC	2995.023	0.703991	0.000196	0.00136	protein_coding
ENSG0000(	ENSG0000(	1244.994	0.198878	0.000196	0.00136	TEC
ENSG0000(	XDH	2363.392	0.699057	0.000196	0.00136	protein_coding
ENSG0000(	TKT	1518.097	-0.29188	0.000197	0.001361	protein_coding
ENSG0000(	COMMD1	239.3313	-0.2936	0.000197	0.001361	protein_coding
ENSG0000(	PRPF19	554.1174	-0.27743	0.000198	0.001366	protein_coding
ENSG0000(	RPS15AP1(	155.5505	-0.31085	0.000198	0.001366	processed_pseudogene
ENSG0000(	COPS3	363.7987	-0.32947	0.000198	0.001368	protein_coding
ENSG0000(	MICOS10	1360.893	-0.24791	0.000198	0.001369	protein_coding
ENSG0000(	MPDU1	658.7328	-0.2091	0.000198	0.001369	protein_coding
ENSG0000(	ENSG0000(	647.3633	0.244662	0.000198	0.00137	TEC
ENSG0000(	NTPCR	2070.979	-0.29917	0.000198	0.00137	protein_coding
ENSG0000(	ENSG0000(	445.1937	-0.62813	0.000199	0.001372	processed_pseudogene
ENSG0000(	FAF1	1375.083	-0.26643	0.000199	0.001373	protein_coding
ENSG0000(	ENSG0000(	47.3439	0.617526	0.000199	0.001374	TEC
ENSG0000(	ENSG0000(	322818.8	1.065846	0.000199	0.001374	lncRNA
ENSG0000(	CLP1	105.5699	-0.40476	0.000199	0.001375	protein_coding
ENSG0000(	NAPB	991.5918	0.157455	0.000199	0.001375	protein_coding
ENSG0000(	CD300A	288.6818	0.474073	0.0002	0.001379	protein_coding
ENSG0000(	PERP	1144.259	-0.50139	0.0002	0.00138	protein_coding
ENSG0000(	SS18L2	283.6993	-0.30835	0.0002	0.001381	protein_coding
ENSG0000(	SLC4A1	320.4219	0.24277	0.0002	0.001382	protein_coding
ENSG0000(	OR7E94P	381.4539	0.303102	0.000201	0.001389	transcribed_unprocessed_pseudo
ENSG0000(	HPSE	618.7223	0.208553	0.000202	0.00139	protein_coding
ENSG0000(	LCN15	687.421	0.272433	0.000202	0.001391	protein_coding
ENSG0000(	TM9SF3	5685.36	-0.22649	0.000202	0.001391	protein_coding
ENSG0000(	IGKV2D-30	11.75955	1.949972	0.000202	0.001392	IG_V_gene
ENSG0000(	FAM227A	987.2289	0.153809	0.000202	0.001393	protein_coding
ENSG0000(	HERC2P5	1297.19	0.253129	0.000203	0.001396	transcribed_unprocessed_pseudo
ENSG0000(	CIBAR1	242.6806	0.375569	0.000203	0.001397	protein_coding
ENSG0000(	H2AZ2	3414.882	-0.20728	0.000203	0.001398	protein_coding
ENSG0000(	TAFA2	253.7451	0.375002	0.000204	0.0014	protein_coding
ENSG0000(	RNU7-181F	14.52456	0.805545	0.000204	0.001401	snRNA
ENSG0000(	TTC7A	1059.708	0.321995	0.000204	0.001402	protein_coding
ENSG0000(	POLR2I	73.86099	-0.45254	0.000204	0.001403	protein_coding
ENSG0000(	WBP11P2	382.4929	-0.19056	0.000204	0.001404	processed_pseudogene
ENSG0000(	ENSG0000(	46.15518	0.638463	0.000204	0.001404	lncRNA
ENSG0000(	ENSG0000(	125.3353	0.323982	0.000204	0.001405	lncRNA
ENSG0000(	ENSG0000(	120.137	0.288377	0.000204	0.001405	lncRNA
ENSG0000(	ENSG0000(	69.00588	0.54711	0.000205	0.001405	lncRNA
ENSG0000(	ENSG0000(	51.61928	0.744501	0.000205	0.001405	TEC
ENSG0000(	ENSG0000(	209.434	-0.28412	0.000205	0.001405	lncRNA
ENSG0000(	TMEM129	735.8833	-0.29176	0.000205	0.001406	protein_coding
ENSG0000(	ZNF91	8241.725	-0.30318	0.000205	0.001406	protein_coding

ENSG0000(	COPG2	359.718	-0.26432	0.000205	0.001407	protein_coding
ENSG0000(	CEP104	2232.79	-0.14711	0.000205	0.001408	protein_coding
ENSG0000(	CPTP	482.1036	-0.4413	0.000206	0.001411	protein_coding
ENSG0000(	MAPK9	1361.378	-0.15921	0.000206	0.001411	protein_coding
ENSG0000(	SMIM32	63.85634	-0.67601	0.000206	0.001411	protein_coding
ENSG0000(	FDX1	622.0094	-0.25992	0.000206	0.001413	protein_coding
ENSG0000(	ENSG0000(	51.49973	0.577742	0.000206	0.001413	lncRNA
ENSG0000(	RAB5C	1061.409	-0.25162	0.000207	0.001417	protein_coding
ENSG0000(	ENSG0000(	496.4165	-0.22166	0.000207	0.001418	lncRNA
ENSG0000(	ENSG0000(	236.3348	-0.29117	0.000207	0.001419	TEC
ENSG0000(	LINC01919	136.0662	0.272319	0.000208	0.001424	lncRNA
ENSG0000(	ENSG0000(	70.6103	0.434657	0.000208	0.001424	TEC
ENSG0000(	FRZB	187.7928	-0.55064	0.000208	0.001424	protein_coding
ENSG0000(	INPP5A	582.3719	-0.31448	0.000209	0.001429	protein_coding
ENSG0000(	NCR1	138.5878	0.245939	0.000209	0.001429	protein_coding
ENSG0000(	TRMT2A	618.3302	-0.25895	0.000209	0.001429	protein_coding
ENSG0000(	ENSG0000(	67.77525	0.377606	0.00021	0.001438	lncRNA
ENSG0000(	CDH5	424.6247	0.52469	0.000211	0.001439	protein_coding
ENSG0000(	ENSG0000(	63.02671	2.907901	0.000211	0.00144	lncRNA
ENSG0000(	GTPBP10	1240.974	-0.2682	0.000211	0.001441	protein_coding
ENSG0000(	COL5A2	1696.573	0.549601	0.000211	0.001442	protein_coding
ENSG0000(	GPATCH4	392.7288	-0.28926	0.000211	0.001442	protein_coding
ENSG0000(	RNF167	701.2903	-0.26187	0.000211	0.001443	protein_coding
ENSG0000(	CCL4L2	36.52226	1.130496	0.000212	0.001444	protein_coding
ENSG0000(	CARD17	17.76334	1.118339	0.000212	0.001447	transcribed_unprocessed_pseudo
ENSG0000(	ACAN	200.2295	0.273918	0.000212	0.001449	protein_coding
ENSG0000(	LINC02223	69.62207	0.565551	0.000213	0.001449	lncRNA
ENSG0000(	ENSG0000(	522.0236	-0.2926	0.000213	0.00145	transcribed_unprocessed_pseudo
ENSG0000(	PRKX	1973.564	0.228308	0.000213	0.001452	protein_coding
ENSG0000(	RNU6-433F	23.38226	0.640582	0.000213	0.001453	snRNA
ENSG0000(	NBPF8	994.2667	0.401815	0.000214	0.001456	transcribed_unprocessed_pseudo
ENSG0000(	LINC02747	245.7298	-0.73476	0.000214	0.001458	lncRNA
ENSG0000(	B4GALNT2	9256.034	-0.52427	0.000214	0.00146	protein_coding
ENSG0000(	BEND3P1	21.81215	0.631271	0.000215	0.001463	transcribed_processed_pseudoge
ENSG0000(	PRMT3	644.1457	-0.2241	0.000215	0.001464	protein_coding
ENSG0000(	MTERF1	1025.598	0.158947	0.000215	0.001464	protein_coding
ENSG0000(	ENSG0000(	40.37884	0.453466	0.000215	0.001465	lncRNA
ENSG0000(	ETFB	2528.53	-0.17635	0.000215	0.001466	protein_coding
ENSG0000(	ENSG0000(	16.0624	-0.74774	0.000216	0.001467	lncRNA
ENSG0000(	MAGED4B	104.7004	0.696265	0.000216	0.001467	protein_coding
ENSG0000(	ENSG0000(	97.13211	0.28902	0.000216	0.001467	lncRNA
ENSG0000(	SNX32	61.55856	-0.77165	0.000216	0.001468	protein_coding
ENSG0000(	ENSG0000(	291.9023	-0.20601	0.000216	0.001468	lncRNA
ENSG0000(	RBM15B	1414.59	-0.17068	0.000216	0.001468	protein_coding
ENSG0000(	ENSG0000(	165.2455	0.371471	0.000216	0.001469	lncRNA
ENSG0000(	TBCE	1850.372	-0.12792	0.000216	0.00147	protein_coding
ENSG0000(	CCZ1B	1005.366	-0.20032	0.000217	0.00147	protein_coding

ENSG0000(	ENSG0000(	28.5837	-1.08581	0.000217	0.00147	lncRNA
ENSG0000(	ENSG0000(	38.47163	-0.43185	0.000217	0.00147	transcribed_processed_pseudoge
ENSG0000(	ENSG0000(	362.4858	0.229633	0.000217	0.00147	lncRNA
ENSG0000(	ENSG0000(	192.8441	0.233819	0.000217	0.00147	TEC
ENSG0000(	FAM168B	2352.06	-0.19027	0.000217	0.00147	protein_coding
ENSG0000(	OPRM1	1484.834	0.163859	0.000217	0.00147	protein_coding
ENSG0000(	SPATA5L1	231.5336	-0.30131	0.000217	0.00147	protein_coding
ENSG0000(	ENSG0000(	130.3532	-0.33553	0.000217	0.00147	lncRNA
ENSG0000(	SAV1	600.516	0.297507	0.000217	0.001472	protein_coding
ENSG0000(	ENSG0000(	685.7153	0.273545	0.000217	0.001473	lncRNA
ENSG0000(	CHMP4C	390.4267	-0.2768	0.000218	0.001474	protein_coding
ENSG0000(	ENSG0000(	38.83484	0.558891	0.000218	0.001474	processed_pseudogene
ENSG0000(	TGS1	854.2411	-0.24007	0.000218	0.001474	protein_coding
ENSG0000(	ARHGAP5-/	40.9048	-0.52862	0.000218	0.001476	lncRNA
ENSG0000(	RHOJ	404.8405	0.420064	0.000218	0.001477	protein_coding
ENSG0000(	IGFBP2	1010.034	-0.54659	0.000219	0.001479	protein_coding
ENSG0000(	LINC00336	132.7953	0.384515	0.000219	0.001479	lncRNA
ENSG0000(	ENSG0000(	16.36631	1.003126	0.000219	0.001481	lncRNA
ENSG0000(	CC2D1A	2953.629	-0.22755	0.00022	0.001486	protein_coding
ENSG0000(	ENSG0000(	56.3159	0.549596	0.00022	0.001489	lncRNA
ENSG0000(	FANCA	1038.779	-0.40607	0.000221	0.001491	protein_coding
ENSG0000(	ENSG0000(	17.65586	0.894019	0.000221	0.001492	misc_RNA
ENSG0000(	ENSG0000(	759.8661	0.22063	0.000221	0.001492	lncRNA
ENSG0000(	CHRDL2	38.10796	1.550951	0.000221	0.001493	protein_coding
ENSG0000(	F2RL1	1774.839	-0.56616	0.000221	0.001493	protein_coding
ENSG0000(	TCP10L	935.1027	0.166119	0.000221	0.001493	protein_coding
ENSG0000(	PPP2R5E	1763.389	-0.13702	0.000221	0.001495	protein_coding
ENSG0000(	FAM167B	24.54257	0.616154	0.000222	0.001495	protein_coding
ENSG0000(	TRAF6	1717.431	-0.19363	0.000222	0.001499	protein_coding
ENSG0000(	FAM41C	81.77581	0.825142	0.000222	0.001499	lncRNA
ENSG0000(	GSTA4	272.9867	-0.3582	0.000222	0.001499	protein_coding
ENSG0000(	SCP2	2059.591	-0.45293	0.000222	0.001499	protein_coding
ENSG0000(	ENSG0000(	94.92038	-1.11069	0.000223	0.001501	transcribed_unitary_pseudogene
ENSG0000(	ENSG0000(	63.30301	0.34896	0.000223	0.001501	lncRNA
ENSG0000(	ENSG0000(	84.89245	0.428843	0.000223	0.001501	lncRNA
ENSG0000(	PDXP	322.8487	-0.50848	0.000223	0.001502	protein_coding
ENSG0000(	LONRF3	286.0413	-0.4165	0.000223	0.001502	protein_coding
ENSG0000(	ENSG0000(	9.931141	0.722725	0.000223	0.001505	lncRNA
ENSG0000(	ENSG0000(	438.9991	0.30001	0.000224	0.001505	lncRNA
ENSG0000(	ARRDC3	1820.507	0.302105	0.000224	0.001508	protein_coding
ENSG0000(	KIFAP3	831.045	-0.28081	0.000224	0.001508	protein_coding
ENSG0000(	SLX1B-SUL	1119.36	-0.22391	0.000225	0.001514	lncRNA
ENSG0000(	ENSG0000(	157.2919	-0.27222	0.000225	0.001515	lncRNA
ENSG0000(	SBNO1	3345.733	-0.13703	0.000225	0.001515	protein_coding
ENSG0000(	NUP85	1108.86	-0.24123	0.000226	0.001518	protein_coding
ENSG0000(	PITHD1	499.2853	-0.19746	0.000226	0.001521	protein_coding
ENSG0000(	SYTL3	187.9464	0.45486	0.000227	0.001524	protein_coding

ENSG0000(	ENSG0000(	46.2291	0.388714	0.000227	0.001524	processed_pseudogene
ENSG0000(	LINC00472	1328.565	0.296217	0.000227	0.001527	lncRNA
ENSG0000(	BYSL	124.2752	-0.37672	0.000228	0.001528	protein_coding
ENSG0000(	TEX261	958.9816	-0.2793	0.000228	0.001528	protein_coding
ENSG0000(	ARHGEF17	1345.831	0.363421	0.000228	0.001529	protein_coding
ENSG0000(	ECE1-AS1	31.72922	0.705632	0.000228	0.00153	lncRNA
ENSG0000(	ENSG0000(	253.8569	0.312337	0.000229	0.001534	lncRNA
ENSG0000(	MATN1	28.09924	0.712681	0.000229	0.001534	protein_coding
ENSG0000(	TNK2	2016.25	0.291096	0.000229	0.001535	protein_coding
ENSG0000(	MED4	682.3126	-0.17948	0.000229	0.001537	protein_coding
ENSG0000(	SLC25A1	604.3455	-0.3922	0.00023	0.001541	protein_coding
ENSG0000(	PTGIR	452.3618	0.338766	0.00023	0.001541	protein_coding
ENSG0000(	LARS1	6486.688	-0.14378	0.00023	0.001545	protein_coding
ENSG0000(	RASSF5	1089.719	0.579202	0.000231	0.001546	protein_coding
ENSG0000(	ENSG0000(	35.27588	-0.5601	0.000231	0.001547	lncRNA
ENSG0000(	SNX29	2105.001	0.243221	0.000232	0.001551	protein_coding
ENSG0000(	CYB5A	1644.689	-0.2414	0.000232	0.001556	protein_coding
ENSG0000(	RPS16P5	72.11223	0.649848	0.000232	0.001556	transcribed_processed_pseudoge
ENSG0000(	B4GALT6	189.3306	-0.38005	0.000232	0.001556	protein_coding
ENSG0000(	ENSG0000(	107.2736	0.322791	0.000233	0.001557	TEC
ENSG0000(	ENSG0000(	145.9784	-0.34015	0.000233	0.001562	lncRNA
ENSG0000(	SGO1	326.7344	-0.26976	0.000233	0.001562	protein_coding
ENSG0000(	UST	426.8338	0.333241	0.000234	0.001563	protein_coding
ENSG0000(	FBLN5	297.1168	0.399174	0.000234	0.001565	protein_coding
ENSG0000(	MRO	242.8323	-0.26634	0.000234	0.001565	protein_coding
ENSG0000(	SAMM50	932.8518	-0.22896	0.000234	0.001565	protein_coding
ENSG0000(	TMEM238	89.14621	-0.45956	0.000234	0.001566	protein_coding
ENSG0000(	ENSG0000(	79.78924	-0.59395	0.000235	0.001569	lncRNA
ENSG0000(	NAA60	2354.918	-0.16193	0.000235	0.001571	protein_coding
ENSG0000(	ABRACL	270.8877	-0.41691	0.000235	0.001572	protein_coding
ENSG0000(	ACVR1	970.6413	0.235009	0.000236	0.001572	protein_coding
ENSG0000(	PDZD11	223.0986	-0.32507	0.000236	0.001572	protein_coding
ENSG0000(	VSIG10	2656.681	-0.35716	0.000235	0.001572	protein_coding
ENSG0000(	FDX2	56.19637	-0.49688	0.000236	0.001577	protein_coding
ENSG0000(	GOLGA8H	65.78011	0.699314	0.000236	0.001577	protein_coding
ENSG0000(	DNAJA2	1127.643	-0.1457	0.000237	0.001579	protein_coding
ENSG0000(	ELMO1	1875.815	0.396605	0.000237	0.001579	protein_coding
ENSG0000(	DCAF1	1072.165	-0.1762	0.000237	0.001582	protein_coding
ENSG0000(	ENSG0000(	108.5886	0.457601	0.000237	0.001582	lncRNA
ENSG0000(	SHLD2	1218.272	-0.19637	0.000237	0.001582	protein_coding
ENSG0000(	NPM1P3	37.52574	-1.03267	0.000238	0.001586	processed_pseudogene
ENSG0000(	SUMO4	151.1159	0.269391	0.000238	0.001586	protein_coding
ENSG0000(	NID2	517.3635	0.498985	0.000239	0.00159	protein_coding
ENSG0000(	SPDYE10P	329.6101	0.270358	0.000239	0.00159	protein_coding
ENSG0000(	ENSG0000(	29.9811	0.559461	0.000239	0.001594	lncRNA
ENSG0000(	SCAP	2346.966	-0.27719	0.000239	0.001594	protein_coding
ENSG0000(	ACCS	928.6243	0.391961	0.000239	0.001594	protein_coding

ENSG0000(	RNU6-920F	29.9415	0.607609	0.00024	0.001595	snRNA
ENSG0000(	ENSG0000(	98.04276	0.287683	0.00024	0.001598	lncRNA
ENSG0000(	ADM2	262.0182	0.304748	0.000241	0.0016	protein_coding
ENSG0000(	C3orf80	22.29604	0.733282	0.000241	0.0016	protein_coding
ENSG0000(	MYO18A	5163.191	-0.2126	0.000241	0.0016	protein_coding
ENSG0000(	ENSG0000(	163.5848	0.237672	0.000241	0.001603	lncRNA
ENSG0000(	ENSG0000(	25.09976	0.681827	0.000242	0.001605	lncRNA
ENSG0000(	CASS4	234.5494	0.36853	0.000243	0.001614	protein_coding
ENSG0000(	ENSG0000(	19.57476	0.812289	0.000243	0.001614	processed_pseudogene
ENSG0000(	ENSG0000(	260.2217	0.445844	0.000243	0.001614	lncRNA
ENSG0000(	TLE4	1210.23	0.232613	0.000243	0.001616	protein_coding
ENSG0000(	ENSG0000(	122.1701	-0.39533	0.000244	0.00162	lncRNA
ENSG0000(	ENSG0000(	304.1491	-0.26019	0.000245	0.001626	lncRNA
ENSG0000(	ATP5MGL	206.9953	0.265792	0.000246	0.00163	protein_coding
ENSG0000(	ENSG0000(	218.5867	0.334988	0.000246	0.00163	lncRNA
ENSG0000(	ITPA	371.8919	-0.20971	0.000246	0.001631	protein_coding
ENSG0000(	TSR1	1106.905	-0.22902	0.000246	0.001631	protein_coding
ENSG0000(	ENSG0000(	33.21153	0.647073	0.000247	0.001638	lncRNA
ENSG0000(	ZNF720	1540.272	0.16289	0.000247	0.00164	protein_coding
ENSG0000(	PTEN	6182.513	0.221636	0.000247	0.00164	protein_coding
ENSG0000(	KIF1C	4344.256	-0.29832	0.000248	0.001641	protein_coding
ENSG0000(	ENSG0000(	205.6576	0.246692	0.000248	0.001641	lncRNA
ENSG0000(	INTS4P1	94.52049	0.369618	0.000248	0.001642	transcribed_unprocessed_pseudo
ENSG0000(	ENSG0000(	568.0132	-0.18728	0.000249	0.001646	TEC
ENSG0000(	PSG7	131.4826	0.667151	0.000249	0.001646	protein_coding
ENSG0000(	ENSG0000(	1015.878	-0.24504	0.000249	0.001649	lncRNA
ENSG0000(	SDR42E1	1755.002	-0.55438	0.000249	0.001651	protein_coding
ENSG0000(	EIF5A	1881.658	-0.33311	0.00025	0.001652	protein_coding
ENSG0000(	INTS6-AS1	841.9742	0.190515	0.00025	0.001654	lncRNA
ENSG0000(	TFE3	995.5702	0.250981	0.00025	0.001655	protein_coding
ENSG0000(	MYCBP2-AS	50.90067	0.412083	0.000251	0.001659	lncRNA
ENSG0000(	VTI1B	1737.146	-0.26938	0.000251	0.001662	protein_coding
ENSG0000(	CLDN3	1152.783	-0.60046	0.000253	0.001669	protein_coding
ENSG0000(	ENSG0000(	369.4578	0.334155	0.000253	0.001671	transcribed_unitary_pseudogene
ENSG0000(	ARHGEF35-	2966.385	0.238863	0.000253	0.001675	lncRNA
ENSG0000(	COX8A	791.3798	-0.50103	0.000254	0.001676	protein_coding
ENSG0000(	ESRP1	3685.316	-0.50147	0.000254	0.001676	protein_coding
ENSG0000(	METAP2	758.3862	-0.23023	0.000254	0.001676	protein_coding
ENSG0000(	ENSG0000(	48.77969	0.387633	0.000254	0.001677	lncRNA
ENSG0000(	ESCO2	576.3551	-0.269	0.000254	0.001678	protein_coding
ENSG0000(	FAM174C	177.2927	-0.31434	0.000254	0.001679	protein_coding
ENSG0000(	WDR86-AS	49.57941	0.648628	0.000255	0.001683	lncRNA
ENSG0000(	ZBED9	213.6996	-0.40099	0.000255	0.001683	protein_coding
ENSG0000(	ADAMTS9	971.0619	0.487345	0.000255	0.001683	protein_coding
ENSG0000(	LINC01643	18.72849	0.705859	0.000255	0.001684	lncRNA
ENSG0000(	GHR	540.2244	-0.21178	0.000256	0.001685	protein_coding
ENSG0000(	IGFL4	2982.839	-0.34563	0.000256	0.001685	protein_coding

ENSG0000(	FAM157B	127.0528	1.306779	0.000256	0.001686	transcribed_unprocessed_pseudo
ENSG0000(	GPR183	198.2916	0.722857	0.000256	0.001688	protein_coding
ENSG0000(	ENSG0000(	512.4549	0.372298	0.000257	0.001689	lncRNA
ENSG0000(	PEX26	9955.299	-0.39518	0.000257	0.00169	protein_coding
ENSG0000(	TTLL6	415.4898	-0.50183	0.000257	0.00169	protein_coding
ENSG0000(	SULF2	1568.878	0.301934	0.000258	0.001699	protein_coding
ENSG0000(	PROCA1	144.6844	0.441353	0.000259	0.001704	protein_coding
ENSG0000(	HYOU1	2215.114	-0.34944	0.00026	0.001707	protein_coding
ENSG0000(	GMFB	1615.267	-0.23172	0.00026	0.001709	protein_coding
ENSG0000(	LINC00604	40.20905	-0.45153	0.00026	0.001709	lncRNA
ENSG0000(	CCDC107	108.3116	-0.3701	0.00026	0.00171	protein_coding
ENSG0000(	NPM1P40	75.25338	0.454496	0.00026	0.001711	processed_pseudogene
ENSG0000(	ENSG0000(	82.32702	0.369774	0.000261	0.001714	lncRNA
ENSG0000(	APCDD1	438.3411	0.373955	0.000261	0.001716	protein_coding
ENSG0000(	STUB1	338.9109	-0.38312	0.000262	0.001718	protein_coding
ENSG0000(	ENSG0000(	37.43399	0.574028	0.000262	0.001719	lncRNA
ENSG0000(	ENSG0000(	32.70902	0.497043	0.000262	0.001719	lncRNA
ENSG0000(	MTRNR2L1	30.22233	1.19877	0.000262	0.001719	protein_coding
ENSG0000(	ZNF804A	37.85626	0.663375	0.000262	0.001719	protein_coding
ENSG0000(	ENSG0000(	26.2625	0.555557	0.000262	0.001719	processed_pseudogene
ENSG0000(	RN7SL517F	164.9154	0.331949	0.000262	0.001719	misc_RNA
ENSG0000(	MTRF1	482.9773	-0.30747	0.000262	0.001721	protein_coding
ENSG0000(	EPHB1	98.45837	0.693523	0.000263	0.001723	protein_coding
ENSG0000(	ENSG0000(	714.359	0.329915	0.000263	0.001725	lncRNA
ENSG0000(	DUSP26	28.10377	0.78224	0.000264	0.001729	protein_coding
ENSG0000(	C3AR1	177.9863	0.641494	0.000264	0.00173	protein_coding
ENSG0000(	BAZ1B	2562.817	-0.14937	0.000264	0.001731	protein_coding
ENSG0000(	BCL7C	387.0075	-0.22429	0.000264	0.001731	protein_coding
ENSG0000(	HAVCR1	196.4045	-0.64767	0.000265	0.001735	protein_coding
ENSG0000(	ENSG0000(	39.26351	0.497689	0.000265	0.001737	unprocessed_pseudogene
ENSG0000(	RNF146	857.3622	0.198318	0.000266	0.001738	protein_coding
ENSG0000(	ABALON	17.55934	0.891184	0.000266	0.001739	lncRNA
ENSG0000(	HDAC3	703.9442	-0.17296	0.000266	0.001739	protein_coding
ENSG0000(	ING5	2172.818	-0.22764	0.000266	0.001739	protein_coding
ENSG0000(	STK17B	3613.206	0.408094	0.000266	0.001739	protein_coding
ENSG0000(	TMEM125	306.2817	-0.54127	0.000266	0.00174	protein_coding
ENSG0000(	YAE1	345.4006	-0.28741	0.000267	0.001746	protein_coding
ENSG0000(	SORBS3	841.3425	-0.36104	0.000267	0.001748	protein_coding
ENSG0000(	RUVBL2	597.5811	-0.19188	0.000268	0.001749	protein_coding
ENSG0000(	KIFC1	389.8827	-0.40599	0.000268	0.00175	protein_coding
ENSG0000(	CDC45	126.0892	-0.64259	0.000268	0.001751	protein_coding
ENSG0000(	ENSG0000(	713.3412	0.220395	0.000268	0.001751	lncRNA
ENSG0000(	CYFIP1	2630.225	-0.28028	0.000268	0.001753	protein_coding
ENSG0000(	DAW1	142.1295	0.530218	0.000269	0.001753	protein_coding
ENSG0000(	ENSG0000(	82.73773	0.639006	0.000269	0.001753	lncRNA
ENSG0000(	IL17RA	2115.301	0.262559	0.000269	0.001754	protein_coding
ENSG0000(	MMP9	290.6231	1.118657	0.000269	0.001755	protein_coding

ENSG0000(	TOMM20	1821.193	-0.22019	0.000269	0.001757	protein_coding
ENSG0000(	MRPL32	438.2319	-0.25608	0.000269	0.001757	protein_coding
ENSG0000(	NLRP12	334.6674	0.237479	0.00027	0.001758	protein_coding
ENSG0000(	NR1H2	650.1559	-0.22544	0.00027	0.001758	protein_coding
ENSG0000(	ENSG0000(	29.2258	0.763641	0.00027	0.001761	lncRNA
ENSG0000(	NUP54	564.8236	-0.21956	0.00027	0.001762	protein_coding
ENSG0000(	ARL6IP4	363.3263	-0.37716	0.000271	0.001763	protein_coding
ENSG0000(	DIS3L2	1802.849	-0.12884	0.000271	0.001763	protein_coding
ENSG0000(	ENSG0000(	15.93474	1.029096	0.000271	0.001763	lncRNA
ENSG0000(	ENSG0000(	122.7798	0.324368	0.000271	0.001763	lncRNA
ENSG0000(	ENSG0000(	4713.662	0.340056	0.000271	0.001766	lncRNA
ENSG0000(	TXNDC11	1552.941	0.290559	0.000271	0.001766	protein_coding
ENSG0000(	AKR7A3	358.0264	-0.5408	0.000271	0.001766	protein_coding
ENSG0000(	ADGRE1	117.3807	0.647856	0.000272	0.001766	protein_coding
ENSG0000(	KCNQ3	106.517	0.443363	0.000272	0.001771	protein_coding
ENSG0000(	CEP120	1448.304	0.177736	0.000273	0.001772	protein_coding
ENSG0000(	ENSG0000(	398.4419	0.196953	0.000273	0.001776	lncRNA
ENSG0000(	GPR132	424.2452	0.574355	0.000274	0.001778	protein_coding
ENSG0000(	ENSG0000(	153.5771	-0.37417	0.000274	0.001783	lncRNA
ENSG0000(	ISOC2	441.4634	-0.28953	0.000275	0.001786	protein_coding
ENSG0000(	AOC1	1765.619	-0.51932	0.000276	0.001794	protein_coding
ENSG0000(	ENSG0000(	88.02912	0.490456	0.000276	0.001794	lncRNA
ENSG0000(	ENSG0000(	818.4504	0.162464	0.000277	0.001796	lncRNA
ENSG0000(	ENSG0000(	318.9114	-0.23994	0.000277	0.001797	lncRNA
ENSG0000(	RBMX	4543.287	-0.13546	0.000277	0.001798	protein_coding
ENSG0000(	GNAT1	172.8952	0.448925	0.000278	0.001801	protein_coding
ENSG0000(	LINC00862	118.681	0.393292	0.000278	0.001804	lncRNA
ENSG0000(	PLXDC1	1007.176	0.356557	0.000278	0.001805	protein_coding
ENSG0000(	RPS27L	2500.089	-0.31315	0.000278	0.001806	protein_coding
ENSG0000(	SLC25A2	50.2766	0.495019	0.000278	0.001806	protein_coding
ENSG0000(	SRCAP	5022.067	-0.14073	0.000279	0.00181	protein_coding
ENSG0000(	ENSG0000(	86.39743	0.334324	0.00028	0.001812	lncRNA
ENSG0000(	ENSG0000(	225.6385	0.251796	0.00028	0.001812	lncRNA
ENSG0000(	MT-CO1	177402.6	-0.96893	0.00028	0.001812	protein_coding
ENSG0000(	SLC19A3	1345.065	-0.3849	0.00028	0.001813	protein_coding
ENSG0000(	ENSG0000(	78.50858	-0.68034	0.00028	0.001814	processed_pseudogene
ENSG0000(	PGM2	432.5329	-0.26143	0.00028	0.001814	protein_coding
ENSG0000(	SMDT1	408.7478	-0.35593	0.00028	0.001815	protein_coding
ENSG0000(	AP2S1	262.6833	-0.36529	0.00028	0.001815	protein_coding
ENSG0000(	RPP14	2050.865	-0.20485	0.000281	0.001816	protein_coding
ENSG0000(	DCLK3	28.00007	1.063395	0.000282	0.001824	protein_coding
ENSG0000(	MICALL2	2859.417	-0.27582	0.000282	0.001825	protein_coding
ENSG0000(	IGLV3-27	26.0161	1.44089	0.000283	0.001827	IG_V_gene
ENSG0000(	MIR4441	123.1576	0.428726	0.000283	0.001827	miRNA
ENSG0000(	PPWD1	1244.453	0.167727	0.000283	0.001828	protein_coding
ENSG0000(	RAN	1530.041	-0.24982	0.000283	0.001828	protein_coding
ENSG0000(	ANKDD1A	364.3614	0.331756	0.000283	0.00183	protein_coding

ENSG0000(	TPRG1L	888.9885	-0.37447	0.000285	0.001838	protein_coding
ENSG0000(	WDFY4	2794.617	0.676424	0.000285	0.001838	protein_coding
ENSG0000(	RPL15	5778.83	-0.40918	0.000285	0.001838	protein_coding
ENSG0000(	UBQLN1	2423.441	-0.16637	0.000285	0.00184	protein_coding
ENSG0000(	LINC02199	267.3419	0.255991	0.000286	0.001844	lncRNA
ENSG0000(	SNHG19	107.3804	-0.43521	0.000286	0.001848	lncRNA
ENSG0000(	SPATA31A7	70.06836	-0.32766	0.000286	0.001848	protein_coding
ENSG0000(	ST20-AS1	841.8133	0.180245	0.000287	0.00185	lncRNA
ENSG0000(	ARF1P2	99.0702	-0.26482	0.000288	0.001856	processed_pseudogene
ENSG0000(	PUS1	1058.82	-0.18448	0.000288	0.00186	protein_coding
ENSG0000(	TOMM7	615.0958	-0.31594	0.000289	0.001862	protein_coding
ENSG0000(	TOP2A	1270.273	-0.45739	0.000289	0.001862	protein_coding
ENSG0000(	HS6ST1	790.5402	0.293717	0.000289	0.001863	protein_coding
ENSG0000(	KMT2E-AS1	143.2734	-0.27534	0.000289	0.001863	lncRNA
ENSG0000(	PRR15	1229.863	-0.67336	0.000289	0.001863	protein_coding
ENSG0000(	FLG-AS1	500.351	0.189745	0.00029	0.001868	lncRNA
ENSG0000(	MAL2	2956.352	-0.62317	0.00029	0.001868	protein_coding
ENSG0000(	ZNF137P	348.4093	-0.40034	0.000291	0.001871	transcribed_unprocessed_pseudo
ENSG0000(	LINC00205	782.0523	0.345035	0.000292	0.001876	lncRNA
ENSG0000(	ENSG0000(	21.2164	0.58912	0.000292	0.001877	processed_pseudogene
ENSG0000(	JAK2	2083.332	0.386819	0.000292	0.001878	protein_coding
ENSG0000(	LINC02836	17.54167	0.915669	0.000292	0.00188	lncRNA
ENSG0000(	CX3CL1	560.6997	0.256814	0.000292	0.00188	protein_coding
ENSG0000(	ENSG0000(	76.17501	0.276007	0.000292	0.00188	TEC
ENSG0000(	ENSG0000(	59.11903	-0.51009	0.000293	0.001881	lncRNA
ENSG0000(	ITPR2	4676.084	0.285708	0.000294	0.001887	protein_coding
ENSG0000(	TPMT	1178.242	-0.38806	0.000294	0.001887	protein_coding
ENSG0000(	ENSG0000(	255.4138	0.215048	0.000294	0.001888	lncRNA
ENSG0000(	RIMKLB	796.9904	0.336015	0.000294	0.001889	protein_coding
ENSG0000(	ZNF141	3913.66	-0.18121	0.000295	0.001892	protein_coding
ENSG0000(	UBA6-DT	1419.661	0.190981	0.000295	0.001894	lncRNA
ENSG0000(	ENSG0000(	28.86881	0.656619	0.000296	0.001898	lncRNA
ENSG0000(	FBXO6	214.9299	0.284477	0.000296	0.001901	protein_coding
ENSG0000(	RING1	463.2166	-0.16699	0.000296	0.001901	protein_coding
ENSG0000(	ALDH9A1	660.9453	-0.25723	0.000297	0.001906	protein_coding
ENSG0000(	AMH	43.56511	-0.54713	0.000297	0.001906	protein_coding
ENSG0000(	THOC7	259.6565	-0.33038	0.000297	0.001906	protein_coding
ENSG0000(	ZNF219	513.4865	-0.39268	0.000297	0.001906	protein_coding
ENSG0000(	DSTN	1958.175	-0.3697	0.000298	0.001909	protein_coding
ENSG0000(	LINC02260	198.8827	0.229058	0.000298	0.001909	lncRNA
ENSG0000(	UGT1A3	309.7654	-0.76832	0.000298	0.001909	protein_coding
ENSG0000(	DNAJC16	1487.034	-0.19093	0.000298	0.001911	protein_coding
ENSG0000(	ENSG0000(	36.64975	0.672409	0.000298	0.001911	lncRNA
ENSG0000(	RRM1	626.7058	-0.24286	0.000299	0.001912	protein_coding
ENSG0000(	FAM89A	79.98855	0.56741	0.000299	0.001913	protein_coding
ENSG0000(	RAB25	622.1087	-0.44623	0.000299	0.001913	protein_coding
ENSG0000(	TERF2	1206.376	-0.19448	0.000299	0.001913	protein_coding

ENSG0000(	ENSG0000(	85.01976	0.42895	0.000299	0.001915	lncRNA
ENSG0000(	ECM2	65.37481	0.819989	0.000299	0.001915	protein_coding
ENSG0000(	LINC02785	86.26511	0.557655	0.000299	0.001915	lncRNA
ENSG0000(	OLA1	906.7982	-0.25753	0.0003	0.001919	protein_coding
ENSG0000(	STARD9	3434.968	0.154706	0.0003	0.001919	protein_coding
ENSG0000(	CSNK1A1	3941.338	-0.15761	0.0003	0.00192	protein_coding
ENSG0000(	RIN1	345.7981	-0.34436	0.000301	0.001921	protein_coding
ENSG0000(	GPR160P1	109.6987	-0.31799	0.000301	0.001922	processed_pseudogene
ENSG0000(	ABCF3	873.9051	-0.21314	0.000301	0.001925	protein_coding
ENSG0000(	ALOX5	624.6089	0.580861	0.000302	0.001928	protein_coding
ENSG0000(	BTBD19	763.2506	0.419324	0.000302	0.001928	protein_coding
ENSG0000(	ZFP36L1	4039.464	0.304296	0.000302	0.00193	protein_coding
ENSG0000(	FLACC1	182.631	-0.29961	0.000302	0.00193	protein_coding
ENSG0000(	ARRDC5	194.4715	0.623482	0.000303	0.001932	protein_coding
ENSG0000(	LINC01675	44.05327	0.436153	0.000303	0.001932	lncRNA
ENSG0000(	RANP4	31.28416	0.563586	0.000303	0.001932	processed_pseudogene
ENSG0000(	S1PR3	733.4459	0.531648	0.000303	0.001932	protein_coding
ENSG0000(	RASGRP1	510.6326	0.644834	0.000303	0.001933	protein_coding
ENSG0000(	ENSG0000(	358.7949	0.320214	0.000303	0.001934	lncRNA
ENSG0000(	SERINC2	2899.426	-0.5356	0.000303	0.001934	protein_coding
ENSG0000(	ENSG0000(	128.4509	0.372598	0.000304	0.001935	lncRNA
ENSG0000(	FAM3D	1418.66	-0.5341	0.000304	0.001936	protein_coding
ENSG0000(	ILF3	6284.177	-0.18731	0.000304	0.001936	protein_coding
ENSG0000(	SLC39A5	1168.966	-0.59382	0.000304	0.001936	protein_coding
ENSG0000(	SUB1	2208.557	0.187069	0.000304	0.001936	protein_coding
ENSG0000(	ENSG0000(	642.7069	0.186314	0.000305	0.001941	lncRNA
ENSG0000(	AMZ2	653.1657	0.234519	0.000305	0.001941	protein_coding
ENSG0000(	PCNPP1	37.36919	-0.55376	0.000305	0.001941	processed_pseudogene
ENSG0000(	ZNF684	301.5676	-0.20402	0.000305	0.001941	protein_coding
ENSG0000(	BAALC	52.52448	0.660428	0.000306	0.001944	protein_coding
ENSG0000(	ENSG0000(	337.2602	-0.29136	0.000306	0.001947	lncRNA
ENSG0000(	NAT10	895.2679	-0.23486	0.000307	0.001949	protein_coding
ENSG0000(	ENSG0000(	66.81656	-0.54579	0.000307	0.001953	protein_coding
ENSG0000(	MON1A	249.3461	-0.30983	0.000308	0.001955	protein_coding
ENSG0000(	NANOGP4	98.87806	-0.5091	0.000308	0.001955	transcribed_processed_pseudoge
ENSG0000(	EZR	5242.499	-0.39194	0.000308	0.001956	protein_coding
ENSG0000(	ZNF674-AS	333.653	-0.31057	0.000308	0.001958	lncRNA
ENSG0000(	EIF4A2	7179.99	-0.1217	0.000309	0.001959	protein_coding
ENSG0000(	MIR548AA1	12.26135	0.884142	0.000309	0.001959	miRNA
ENSG0000(	VIM-AS1	112.9497	0.484387	0.000309	0.001959	lncRNA
ENSG0000(	SIGLEC12	48.35781	0.816725	0.000309	0.001961	protein_coding
ENSG0000(	FRA10AC1	1141.637	0.248023	0.00031	0.001968	protein_coding
ENSG0000(	NEU4	471.5416	-0.831	0.00031	0.001968	protein_coding
ENSG0000(	TMEM251	420.774	-0.33311	0.000311	0.001973	protein_coding
ENSG0000(	ENSG0000(	244.27	0.212262	0.000312	0.001977	lncRNA
ENSG0000(	PTGES3	2980.337	-0.23346	0.000312	0.001977	protein_coding
ENSG0000(	RPS9	2433.998	-0.30174	0.000313	0.001983	protein_coding

ENSG0000(	WTAP	2420.85	0.169256	0.000313	0.001985	protein_coding
ENSG0000(	ENSG0000(	1112.249	-0.18954	0.000314	0.001987	lncRNA
ENSG0000(	IFI30	1015.861	0.665452	0.000314	0.001987	protein_coding
ENSG0000(	ENSG0000(	113.2162	0.306497	0.000314	0.001988	lncRNA
ENSG0000(	ENSG0000(	27.20793	0.553417	0.000314	0.001989	lncRNA
ENSG0000(	TRMU	1192.513	-0.16406	0.000314	0.00199	protein_coding
ENSG0000(	PPP2R2D	1676.837	-0.15613	0.000315	0.001992	protein_coding
ENSG0000(	RNU1-100F	24.1348	0.660056	0.000315	0.001992	snRNA
ENSG0000(	PPAN	544.8367	-0.25637	0.000315	0.001995	protein_coding
ENSG0000(	ENSG0000(	26.71609	0.770209	0.000315	0.001995	lncRNA
ENSG0000(	SLC17A7	177.8934	-0.36552	0.000316	0.001995	protein_coding
ENSG0000(	RNU4-51P	21.33002	0.78248	0.000316	0.001997	snRNA
ENSG0000(	NAT2	88.07913	-0.61599	0.000316	0.001997	protein_coding
ENSG0000(	NOLC1	973.3606	-0.28298	0.000318	0.002008	protein_coding
ENSG0000(	PIGU	293.9342	-0.31509	0.000318	0.002008	protein_coding
ENSG0000(	ENSG0000(	136.8141	0.415069	0.000318	0.002009	lncRNA
ENSG0000(	CHCHD2	859.3541	-0.36473	0.000318	0.002011	protein_coding
ENSG0000(	ST14	5131.263	-0.48573	0.000318	0.002011	protein_coding
ENSG0000(	FMNL3	2834.29	0.490056	0.000319	0.002015	protein_coding
ENSG0000(	MLKL	1562.81	0.346856	0.00032	0.002019	protein_coding
ENSG0000(	ENSG0000(	63.52977	-0.51374	0.00032	0.002019	lncRNA
ENSG0000(	ENSG0000(	412.6969	-0.25143	0.00032	0.00202	protein_coding
ENSG0000(	BAG5	1345.425	-0.18613	0.00032	0.002022	protein_coding
ENSG0000(	CENPK	648.8463	-0.23867	0.000321	0.002022	protein_coding
ENSG0000(	MRPL58	101.6395	-0.33602	0.000321	0.002024	protein_coding
ENSG0000(	CRYL1	1096.116	-0.43358	0.000321	0.002026	protein_coding
ENSG0000(	ENSG0000(	75.5366	-0.38956	0.000321	0.002026	lncRNA
ENSG0000(	P3H1	1572.592	0.186223	0.000322	0.002026	protein_coding
ENSG0000(	PARL	458.7386	-0.15908	0.000321	0.002026	protein_coding
ENSG0000(	TAGLN2	1767.721	-0.36107	0.000322	0.002026	protein_coding
ENSG0000(	UTS2B	186.1145	0.288052	0.000322	0.002028	protein_coding
ENSG0000(	DHCR24	1881.021	-0.49282	0.000322	0.002029	protein_coding
ENSG0000(	PRKD1	259.2703	0.326955	0.000322	0.002029	protein_coding
ENSG0000(	ENTR1	543.3843	-0.29946	0.000323	0.002031	protein_coding
ENSG0000(	FBLIM1	4491.509	-0.41362	0.000323	0.002032	protein_coding
ENSG0000(	ENSG0000(	65.22118	0.38541	0.000323	0.002035	lncRNA
ENSG0000(	ENSG0000(	18.6544	0.678713	0.000324	0.002037	lncRNA
ENSG0000(	ARL4C	767.0887	0.516755	0.000324	0.002037	protein_coding
ENSG0000(	LINC01906	11.67879	0.972973	0.000324	0.002038	lncRNA
ENSG0000(	POLR1E	248.9479	-0.33771	0.000325	0.002041	protein_coding
ENSG0000(	RN7SL69P	12.24288	0.838874	0.000325	0.002042	misc_RNA
ENSG0000(	MTND2P13	9.596789	0.932006	0.000325	0.002043	unprocessed_pseudogene
ENSG0000(	SLC25A29	1352.02	0.319746	0.000325	0.002044	protein_coding
ENSG0000(	VAMP5	96.00112	0.428594	0.000326	0.002046	protein_coding
ENSG0000(	IGBP1	492.717	-0.26983	0.000326	0.002048	protein_coding
ENSG0000(	ENSG0000(	22.88869	0.603632	0.000326	0.002049	lncRNA
ENSG0000(	ENSG0000(	539.8565	0.202471	0.000327	0.002051	lncRNA

ENSG0000(	NSUN5P2	341.0545	-0.31041	0.000327	0.002052	transcribed_unprocessed_pseudo
ENSG0000(	ENSG0000(	26.62436	0.599565	0.000327	0.002052	lncRNA
ENSG0000(	MTCH1	1895.133	-0.1926	0.000329	0.002064	protein_coding
ENSG0000(	DDIT4	659.2214	0.63688	0.000329	0.002066	protein_coding
ENSG0000(	EFEMP2	325.8927	0.403183	0.00033	0.002068	protein_coding
ENSG0000(	ENSG0000(	1047.303	0.202104	0.00033	0.00207	TEC
ENSG0000(	FITM2	1188.729	-0.15196	0.00033	0.002071	protein_coding
ENSG0000(	ADH1C	2609.822	-0.7439	0.00033	0.002072	protein_coding
ENSG0000(	OR9K1P	102.6959	-0.40398	0.000331	0.002074	transcribed_unitary_pseudogene
ENSG0000(	PTPN7	846.2063	0.377322	0.000331	0.002074	protein_coding
ENSG0000(	ZDHHC12	297.5499	-0.42909	0.000331	0.002074	protein_coding
ENSG0000(	MRPS35-D1	152.8277	0.31203	0.000331	0.002076	lncRNA
ENSG0000(	LINC02863	17.37451	0.955295	0.000331	0.002076	lncRNA
ENSG0000(	SIGLEC5	44.54844	1.140816	0.000332	0.00208	protein_coding
ENSG0000(	MESP1	31.53437	-0.93392	0.000333	0.002082	protein_coding
ENSG0000(	NSMCE3	327.7246	-0.18	0.000334	0.002092	protein_coding
ENSG0000(	DDX39A	717.7286	-0.17477	0.000335	0.002093	protein_coding
ENSG0000(	MSN	2777.332	0.353179	0.000335	0.002093	protein_coding
ENSG0000(	DOK3	608.5284	0.525253	0.000335	0.002098	protein_coding
ENSG0000(	SPR	240.3933	-0.49144	0.000336	0.002099	protein_coding
ENSG0000(	NUDT16L1	264.9387	-0.27962	0.000337	0.002107	protein_coding
ENSG0000(	IGHV3-62	24.73445	0.883086	0.000338	0.002111	IG_V_pseudogene
ENSG0000(	UGT2B15	254.3138	-1.00177	0.000339	0.002118	protein_coding
ENSG0000(	C21orf91	643.8721	0.274576	0.000339	0.00212	protein_coding
ENSG0000(	MRPS10	775.9661	-0.20578	0.000339	0.00212	protein_coding
ENSG0000(	HADH	3045.749	-0.3779	0.000339	0.00212	protein_coding
ENSG0000(	ENSG0000(	536.41	0.211701	0.000341	0.002127	lncRNA
ENSG0000(	SYNE1	5559.268	0.31775	0.000341	0.002129	protein_coding
ENSG0000(	LINC02882	660.4304	0.190165	0.000341	0.002129	lncRNA
ENSG0000(	TMEM170A	2269.115	-0.2666	0.000342	0.002132	protein_coding
ENSG0000(	GPX4	490.4444	-0.34156	0.000342	0.002133	protein_coding
ENSG0000(	EVA1A-AS	296.7386	0.20907	0.000342	0.002136	lncRNA
ENSG0000(	GALNT6	762.7926	0.358312	0.000343	0.002136	protein_coding
ENSG0000(	PDGFB	228.0778	0.487804	0.000342	0.002136	protein_coding
ENSG0000(	ZBTB32	34.03818	0.972824	0.000343	0.002136	protein_coding
ENSG0000(	ENSG0000(	316.4904	-0.29549	0.000343	0.002138	lncRNA
ENSG0000(	PSPN	476.8918	-0.17829	0.000343	0.00214	protein_coding
ENSG0000(	IKZF1	2774.77	0.597014	0.000343	0.00214	protein_coding
ENSG0000(	ENSG0000(	49.00651	0.680216	0.000344	0.002143	lncRNA
ENSG0000(	PEX1	1324.624	-0.19491	0.000346	0.002153	protein_coding
ENSG0000(	LPIN2	2290.672	0.354363	0.000346	0.002156	protein_coding
ENSG0000(	ENSG0000(	49.12938	-0.66432	0.000347	0.002157	lncRNA
ENSG0000(	SLC22A4	73.06313	0.782119	0.000347	0.002159	protein_coding
ENSG0000(	FAM177A1	2822.041	-0.1837	0.000347	0.00216	protein_coding
ENSG0000(	OR52N4	12.52621	1.048711	0.000347	0.00216	protein_coding
ENSG0000(	ENSG0000(	83.90679	0.461724	0.000348	0.002163	lncRNA
ENSG0000(	DDX56	1233.4	-0.23779	0.000348	0.002166	protein_coding

ENSG0000(	BRAF	4610.191	0.117741	0.000348	0.002167	protein_coding
ENSG0000(	HS3ST3A1	92.80532	0.823281	0.000349	0.002169	protein_coding
ENSG0000(	ADCY9	1602.557	-0.29635	0.000349	0.00217	protein_coding
ENSG0000(	ENSG0000(	291.6145	0.165739	0.000349	0.00217	lncRNA
ENSG0000(	AKT1	2792.1	-0.2026	0.000349	0.002171	protein_coding
ENSG0000(	ALPK2	393.65	0.918017	0.000349	0.002171	protein_coding
ENSG0000(	LINC02894	242.1198	-0.31217	0.00035	0.002172	lncRNA
ENSG0000(	TRDC	47.60893	-0.80838	0.00035	0.002172	TR_C_gene
ENSG0000(	SIKE1	1477.626	-0.17665	0.00035	0.002176	protein_coding
ENSG0000(	VSIG10L2	30.84887	0.607673	0.000351	0.002179	protein_coding
ENSG0000(	POPDC3	40.12568	-1.09619	0.000351	0.002179	protein_coding
ENSG0000(	SLC30A10	4660.537	-1.0381	0.000351	0.002179	protein_coding
ENSG0000(	ENSG0000(	303.9453	-0.16125	0.000352	0.002184	lncRNA
ENSG0000(	OSBPL1A	1148.041	-0.20279	0.000352	0.002184	protein_coding
ENSG0000(	TMEM184E	1267.862	0.289751	0.000353	0.002189	protein_coding
ENSG0000(	MYZAP	325.6116	-0.71419	0.000353	0.002191	protein_coding
ENSG0000(	RWDD2B	283.5375	-0.26259	0.000354	0.002194	protein_coding
ENSG0000(	FUT6	2224.44	-0.44129	0.000354	0.002194	protein_coding
ENSG0000(	CASP6	524.246	-0.29499	0.000355	0.002196	protein_coding
ENSG0000(	ZNF273	1082.848	-0.16216	0.000354	0.002196	protein_coding
ENSG0000(	HRAS	317.6035	-0.50908	0.000355	0.002197	protein_coding
ENSG0000(	WDR91	1408.706	0.145459	0.000355	0.002197	protein_coding
ENSG0000(	ENSG0000(	51.90259	-0.86028	0.000355	0.002199	protein_coding
ENSG0000(	C5	252.7414	-0.33208	0.000355	0.0022	protein_coding
ENSG0000(	H1-10-AS1	290.3386	0.315895	0.000356	0.002201	lncRNA
ENSG0000(	ERMARD	750.259	0.191174	0.000356	0.002202	protein_coding
ENSG0000(	ENSG0000(	291.1306	0.294301	0.000356	0.002203	lncRNA
ENSG0000(	LBR	1903.5	-0.24719	0.000356	0.002203	protein_coding
ENSG0000(	MRPL54	81.673	-0.51386	0.000356	0.002204	protein_coding
ENSG0000(	TMEM200(	87.30695	0.782265	0.000357	0.002204	protein_coding
ENSG0000(	ENSG0000(	79.29033	0.387879	0.000358	0.00221	lncRNA
ENSG0000(	REX1BD	818.6923	-0.17901	0.000358	0.002212	protein_coding
ENSG0000(	JAML	1987.686	0.369016	0.000358	0.002212	protein_coding
ENSG0000(	ENSG0000(	306.3031	0.257251	0.000359	0.002217	lncRNA
ENSG0000(	FBXL2	489.3635	0.210578	0.000359	0.002219	protein_coding
ENSG0000(	RTF2	669.0903	-0.19196	0.00036	0.002222	protein_coding
ENSG0000(	ECEL1P2	14.99378	0.719213	0.00036	0.002222	transcribed_unprocessed_pseudo
ENSG0000(	NSD2	6615.866	-0.2043	0.000361	0.002226	protein_coding
ENSG0000(	FYB1	2477.818	0.555712	0.000361	0.002227	protein_coding
ENSG0000(	ENSG0000(	396.7999	0.1898	0.000361	0.002228	lncRNA
ENSG0000(	PLEKHN1	62.46679	0.771567	0.000361	0.002229	protein_coding
ENSG0000(	STRA6	264.3733	0.537705	0.000362	0.002233	protein_coding
ENSG0000(	OMG	15.58865	0.816848	0.000362	0.002234	protein_coding
ENSG0000(	PRPF40A	2605.903	-0.21304	0.000363	0.002235	protein_coding
ENSG0000(	CD1D	364.9195	0.351042	0.000363	0.002236	protein_coding
ENSG0000(	FAM135B	662.0417	-0.59113	0.000363	0.002236	protein_coding
ENSG0000(	SPATA2	587.3468	-0.28035	0.000363	0.002237	protein_coding

ENSG0000(	DHX58	327.832	0.489898	0.000363	0.002239	protein_coding
ENSG0000(	MLIP	126.6434	-0.49638	0.000364	0.002242	protein_coding
ENSG0000(	GYG1	1010.762	0.212463	0.000364	0.002242	protein_coding
ENSG0000(	CCDC34	130.6101	-0.3528	0.000364	0.002242	protein_coding
ENSG0000(	SPOCD1	229.9268	0.384995	0.000364	0.002244	protein_coding
ENSG0000(	ABCB7	1056.835	-0.18764	0.000365	0.002246	protein_coding
ENSG0000(	WDR59	1967.657	-0.15886	0.000365	0.002248	protein_coding
ENSG0000(	SDHCP1	15.25162	0.993115	0.000365	0.002248	processed_pseudogene
ENSG0000(	FAM98C	1852.295	-0.20117	0.000366	0.002249	protein_coding
ENSG0000(	ENSG0000(	312.7464	-0.22608	0.000366	0.00225	lncRNA
ENSG0000(	HEATR1	1819.557	-0.26095	0.000366	0.00225	protein_coding
ENSG0000(	ENSG0000(	39.36377	0.427469	0.000366	0.002252	processed_pseudogene
ENSG0000(	ENSG0000(	22.07298	0.614023	0.000366	0.002252	lncRNA
ENSG0000(	LINC02844	59.25972	-0.38702	0.000367	0.002255	lncRNA
ENSG0000(	COQ9	1113.574	-0.331	0.000367	0.002255	protein_coding
ENSG0000(	ENSG0000(	15.47508	0.768834	0.000367	0.002255	lncRNA
ENSG0000(	PBK	73.51574	-0.72058	0.000367	0.002255	protein_coding
ENSG0000(	PRDM8	112.2478	0.721814	0.000367	0.002255	protein_coding
ENSG0000(	ENSG0000(	67.10509	0.434217	0.000367	0.002256	lncRNA
ENSG0000(	ENOX1	249.4564	0.439286	0.000368	0.002256	protein_coding
ENSG0000(	ENSG0000(	639.0528	-0.20529	0.000368	0.002257	lncRNA
ENSG0000(	ENSG0000(	27.17866	0.616636	0.000368	0.002257	lncRNA
ENSG0000(	ENSG0000(	38.28414	0.560563	0.000368	0.002257	processed_pseudogene
ENSG0000(	ENSG0000(	84.1491	0.781256	0.000368	0.002258	lncRNA
ENSG0000(	ADRB2	155.0634	0.347319	0.000369	0.00226	protein_coding
ENSG0000(	CYP2C9	746.689	-0.48869	0.000369	0.002261	protein_coding
ENSG0000(	LRIG2	4197.119	-0.20038	0.00037	0.002265	protein_coding
ENSG0000(	DAPL1	20.32384	-0.69513	0.00037	0.002266	protein_coding
ENSG0000(	PRKAR2A	2355.685	-0.22328	0.00037	0.002267	protein_coding
ENSG0000(	GABBR1	1541.387	0.30062	0.00037	0.002268	protein_coding
ENSG0000(	SRGAP2B	820.5249	0.274924	0.000371	0.00227	protein_coding
ENSG0000(	ADCY4	870.7891	0.355086	0.000371	0.002271	protein_coding
ENSG0000(	CDK10	2696.092	-0.21042	0.000371	0.002271	protein_coding
ENSG0000(	ENSG0000(	56.17885	0.420655	0.000371	0.002271	TEC
ENSG0000(	FSCN1	282.388	0.561029	0.000371	0.002271	protein_coding
ENSG0000(	LINC01914	9.773356	0.925614	0.000371	0.002271	lncRNA
ENSG0000(	NQO1	973.0537	-0.55954	0.000371	0.002271	protein_coding
ENSG0000(	TIMM8A	423.2901	-0.23526	0.000373	0.00228	protein_coding
ENSG0000(	EHBP1L1	6293.867	0.283236	0.000373	0.002281	protein_coding
ENSG0000(	ENSG0000(	1326.132	0.175023	0.000373	0.002282	lncRNA
ENSG0000(	ENSG0000(	247.7782	0.296721	0.000374	0.002285	lncRNA
ENSG0000(	GRIN2A	507.6376	-0.88468	0.000374	0.002285	protein_coding
ENSG0000(	PRPF8	5658.071	-0.14784	0.000374	0.002285	protein_coding
ENSG0000(	PPFIA2	104.6616	0.946004	0.000374	0.002286	protein_coding
ENSG0000(	SLC35B1	761.0922	-0.22043	0.000375	0.002288	protein_coding
ENSG0000(	LINC01359	1059.329	0.210853	0.000375	0.002291	lncRNA
ENSG0000(	LINC01465	21.48578	0.704428	0.000376	0.002295	lncRNA

ENSG0000(	CDK11A	872.9836	-0.29639	0.000376	0.002295	protein_coding
ENSG0000(	DMRT2	69.37478	-0.85951	0.000377	0.002301	protein_coding
ENSG0000(	FKBP15	2751.685	0.144736	0.000377	0.002301	protein_coding
ENSG0000(	ENSG0000(	15.85931	-1.1678	0.000377	0.002301	TEC
ENSG0000(	LINC00189	50.68317	0.491422	0.000378	0.002303	lncRNA
ENSG0000(	ENSG0000(	24.21672	0.670881	0.000378	0.002307	lncRNA
ENSG0000(	ENSG0000(	129.0928	-0.24845	0.000378	0.002307	lncRNA
ENSG0000(	ENSG0000(	202.2838	-0.27199	0.000378	0.002307	lncRNA
ENSG0000(	ACOX1	6810.611	-0.56633	0.000379	0.002307	protein_coding
ENSG0000(	ODF2L	4765.296	0.157201	0.000379	0.002307	protein_coding
ENSG0000(	ZBED4	1421.628	-0.1477	0.000379	0.002308	protein_coding
ENSG0000(	SHC2	195.3117	0.401008	0.00038	0.002313	protein_coding
ENSG0000(	CWH43	328.6838	-0.83928	0.00038	0.002314	protein_coding
ENSG0000(	BEST1	963.4016	0.361326	0.00038	0.002315	protein_coding
ENSG0000(	SMPD1	651.5638	-0.52354	0.000381	0.00232	protein_coding
ENSG0000(	CMTM4	3716.267	-0.41943	0.000381	0.00232	protein_coding
ENSG0000(	COL23A1	135.011	0.649367	0.000382	0.002322	protein_coding
ENSG0000(	ENSG0000(	183.532	0.314297	0.000382	0.002323	lncRNA
ENSG0000(	PELP1	827.2065	-0.27071	0.000382	0.002327	protein_coding
ENSG0000(	FCF1P5	25.93058	0.671679	0.000383	0.002327	processed_pseudogene
ENSG0000(	FPR3	469.6662	0.454406	0.000383	0.002328	protein_coding
ENSG0000(	TRGC2	136.7723	-0.63875	0.000383	0.00233	TR_C_gene
ENSG0000(	MRTO4	373.7194	-0.27508	0.000383	0.002331	protein_coding
ENSG0000(	GFRAL	78.75626	-0.35883	0.000384	0.002334	protein_coding
ENSG0000(	MUL1	302.7626	-0.3203	0.000384	0.002334	protein_coding
ENSG0000(	MOCS2	409.2513	-0.29512	0.000386	0.002342	protein_coding
ENSG0000(	TMEM30A-	365.0679	-0.27275	0.000386	0.002342	lncRNA
ENSG0000(	PWRN1	1086.772	0.565439	0.000386	0.002346	lncRNA
ENSG0000(	LSM7	472.3467	-0.22378	0.000387	0.00235	protein_coding
ENSG0000(	NDRG3	644.9464	-0.20786	0.000387	0.002351	protein_coding
ENSG0000(	ANXA5	841.9624	0.434524	0.000387	0.002351	protein_coding
ENSG0000(	LIX1L-AS1	225.6577	-0.24572	0.000388	0.002351	lncRNA
ENSG0000(	CCDC18	825.447	-0.32262	0.000388	0.002352	protein_coding
ENSG0000(	FOXA1	713.4178	-0.46638	0.000388	0.002353	protein_coding
ENSG0000(	DDX41	568.3063	-0.30002	0.000388	0.002354	protein_coding
ENSG0000(	ELL	763.8328	0.285896	0.00039	0.00236	protein_coding
ENSG0000(	EPHA6	615.5542	-0.2457	0.00039	0.00236	protein_coding
ENSG0000(	TDGF1	196.9504	-0.24926	0.00039	0.00236	protein_coding
ENSG0000(	TMEM87B	2143.413	-0.21908	0.000389	0.00236	protein_coding
ENSG0000(	VBP1	249.7682	-0.37472	0.00039	0.002361	protein_coding
ENSG0000(	ENSG0000(	200.5051	1.117175	0.000391	0.002366	processed_pseudogene
ENSG0000(	ENSG0000(	9.2858	0.966749	0.000391	0.002367	lncRNA
ENSG0000(	ENSG0000(	19.51629	1.051453	0.000391	0.002367	unprocessed_pseudogene
ENSG0000(	INO80	1748.206	-0.2214	0.000391	0.002367	protein_coding
ENSG0000(	DIRC3-AS1	31.5941	-0.92042	0.000392	0.00237	lncRNA
ENSG0000(	LINC00670	1105.232	0.277235	0.000392	0.002374	lncRNA
ENSG0000(	PHLDA1-DT	23.61178	0.632744	0.000392	0.002375	lncRNA

ENSG0000(	SEPTIN4	614.3947	0.381505	0.000393	0.00238	protein_coding
ENSG0000(	ENSG0000(	238.7357	0.214435	0.000395	0.002388	lncRNA
ENSG0000(	RNU6-652F	54.43161	0.590002	0.000395	0.002388	snRNA
ENSG0000(	EMILIN2	674.1351	0.400615	0.000396	0.002391	protein_coding
ENSG0000(	UNC119	740.6594	0.227728	0.000396	0.002395	protein_coding
ENSG0000(	ENSG0000(	72.59993	0.311216	0.000397	0.002397	lncRNA
ENSG0000(	ENSG0000(	191.5942	-0.36028	0.000397	0.002401	lncRNA
ENSG0000(	PELI1	1756.662	0.227509	0.000398	0.002401	protein_coding
ENSG0000(	PGM1	961.1474	-0.4145	0.000397	0.002401	protein_coding
ENSG0000(	EBPL	543.5052	-0.24253	0.000398	0.002401	protein_coding
ENSG0000(	NIF3L1	285.8516	-0.21267	0.000398	0.002404	protein_coding
ENSG0000(	IPO7	2216.575	-0.21444	0.000399	0.002408	protein_coding
ENSG0000(	COMMD6	251.2138	-0.35828	0.0004	0.002411	protein_coding
ENSG0000(	ENSG0000(	31.16499	0.478835	0.0004	0.002415	lncRNA
ENSG0000(	TRAPPC5	1484.972	-0.26824	0.0004	0.002415	protein_coding
ENSG0000(	DMAP1	1536.513	-0.15107	0.000401	0.002417	protein_coding
ENSG0000(	FAM13C	284.6793	0.405126	0.000401	0.002417	protein_coding
ENSG0000(	HMMR	186.1894	-0.51843	0.000401	0.002417	protein_coding
ENSG0000(	MYO1A	3148.458	-0.53413	0.000401	0.00242	protein_coding
ENSG0000(	C9orf152	767.6139	-0.57664	0.000402	0.002422	protein_coding
ENSG0000(	ENSG0000(	12.67081	0.764371	0.000402	0.002423	TEC
ENSG0000(	ENSG0000(	51.24233	0.361415	0.000402	0.002425	processed_pseudogene
ENSG0000(	ZNF136	929.7527	0.167352	0.000403	0.002429	protein_coding
ENSG0000(	ENSG0000(	21.35876	0.691387	0.000405	0.00244	lncRNA
ENSG0000(	RPS11	3959.167	-0.34221	0.000405	0.002441	protein_coding
ENSG0000(	C11orf45	34.43038	0.447808	0.000406	0.002441	protein_coding
ENSG0000(	NCAPG	338.1914	-0.53314	0.000406	0.002441	protein_coding
ENSG0000(	ENSG0000(	660.0914	0.271549	0.000406	0.002441	lncRNA
ENSG0000(	IFFO1	584.329	0.280571	0.000406	0.002441	protein_coding
ENSG0000(	ENSG0000(	10.21956	0.929733	0.000407	0.002447	TEC
ENSG0000(	PADI2	6017.667	-0.77504	0.000407	0.002449	protein_coding
ENSG0000(	APC	2932.003	-0.17474	0.000407	0.002449	protein_coding
ENSG0000(	ENSG0000(	38.98605	0.597831	0.000408	0.002451	lncRNA
ENSG0000(	DDAH1	1958.577	-0.41618	0.000408	0.002452	protein_coding
ENSG0000(	ACKR4	41.24059	0.872344	0.000409	0.002456	protein_coding
ENSG0000(	ARRDC1	1205.83	-0.28016	0.000409	0.002456	protein_coding
ENSG0000(	DHODH	888.1201	-0.1739	0.00041	0.002465	protein_coding
ENSG0000(	TPTE2P6	86.58423	-0.34108	0.000411	0.002469	transcribed_unprocessed_pseudo
ENSG0000(	CCT4	710.4976	-0.3002	0.000412	0.002472	protein_coding
ENSG0000(	EVC	687.8698	0.373285	0.000412	0.002476	protein_coding
ENSG0000(	ENSG0000(	34.19618	0.705575	0.000413	0.002477	processed_pseudogene
ENSG0000(	RPS20	3585.198	-0.34997	0.000413	0.002477	protein_coding
ENSG0000(	WDR76	612.7208	-0.24592	0.000413	0.002479	protein_coding
ENSG0000(	ENSG0000(	77.09193	-0.34389	0.000414	0.002482	lncRNA
ENSG0000(	RNU6-769F	24.3494	0.654901	0.000414	0.002482	snRNA
ENSG0000(	CLDN10	155.2634	0.287855	0.000414	0.002485	protein_coding
ENSG0000(	LAMB3	1330.25	-0.55913	0.000415	0.002485	protein_coding

ENSG0000(	AGTPBP1	727.6884	0.28877	0.000416	0.002492	protein_coding
ENSG0000(	CCDC25	583.0545	-0.18022	0.000416	0.002492	protein_coding
ENSG0000(	ENSG0000(	335.2768	0.346134	0.000416	0.002492	transcribed_unprocessed_pseudo
ENSG0000(	SDR42E2	454.6934	0.238402	0.000418	0.002502	protein_coding
ENSG0000(	PAFAH2	1027.889	-0.20156	0.000418	0.002503	protein_coding
ENSG0000(	RAB6B	172.2413	-0.94682	0.000418	0.002503	protein_coding
ENSG0000(	PACERR	33.711	0.613248	0.000418	0.002503	lncRNA
ENSG0000(	ENSG0000(	136.3213	0.284026	0.000418	0.002505	lncRNA
ENSG0000(	PGBD2	753.7226	0.178902	0.000418	0.002505	protein_coding
ENSG0000(	RHPN2	1515.48	-0.50008	0.000419	0.002505	protein_coding
ENSG0000(	LINC01203	613.6083	0.137311	0.000419	0.002507	lncRNA
ENSG0000(	RASA2-IT1	111.7298	0.286598	0.000419	0.002507	lncRNA
ENSG0000(	COIL	422.3949	-0.22592	0.00042	0.002513	protein_coding
ENSG0000(	ZC2HC1A	249.5561	0.351182	0.000421	0.002518	protein_coding
ENSG0000(	GOLGA5P1	87.14109	0.669343	0.000422	0.002521	processed_pseudogene
ENSG0000(	SPEN	6218.424	-0.14808	0.000422	0.002521	protein_coding
ENSG0000(	ZFP91	2762.017	-0.20278	0.000422	0.002521	protein_coding
ENSG0000(	PSMB6	383.9062	-0.25112	0.000422	0.002521	protein_coding
ENSG0000(	ENSG0000(	38.52378	0.7295	0.000422	0.002523	processed_pseudogene
ENSG0000(	MCPH1-DT	548.5653	0.278151	0.000422	0.002523	lncRNA
ENSG0000(	ENSG0000(	120.6868	0.559591	0.000423	0.002525	lncRNA
ENSG0000(	RCC1	521.5676	-0.34805	0.000423	0.002525	protein_coding
ENSG0000(	IGHV3-73	179.0354	0.932741	0.000423	0.002526	IG_V_gene
ENSG0000(	METTL17	1117.777	-0.18212	0.000423	0.002527	protein_coding
ENSG0000(	FXYD1	28.534	-0.55845	0.000424	0.002531	protein_coding
ENSG0000(	CSRP2	560.4257	0.436405	0.000424	0.002531	protein_coding
ENSG0000(	UBE2D1	287.0846	0.319905	0.000424	0.002531	protein_coding
ENSG0000(	CSK	1330.816	-0.23382	0.000425	0.002532	protein_coding
ENSG0000(	ENSG0000(	12.79585	0.787244	0.000425	0.002532	lncRNA
ENSG0000(	IGHV3-69-1	77.84846	0.986001	0.000425	0.002532	IG_V_pseudogene
ENSG0000(	MSH5	1592.719	-0.14603	0.000425	0.002532	protein_coding
ENSG0000(	ORC5	360.1547	-0.24159	0.000425	0.002532	protein_coding
ENSG0000(	ENSG0000(	13.13541	1.03836	0.000425	0.002533	lncRNA
ENSG0000(	ADCK1	343.8118	-0.24909	0.000425	0.002533	protein_coding
ENSG0000(	MS4A14	192.3531	0.61643	0.000425	0.002533	protein_coding
ENSG0000(	RABL3	758.1518	-0.22117	0.000425	0.002534	protein_coding
ENSG0000(	NFKBIA	1716.387	0.368401	0.000426	0.002537	protein_coding
ENSG0000(	ENSG0000(	33.01633	0.536714	0.000428	0.002545	lncRNA
ENSG0000(	IFI44L	1687.773	1.124341	0.000428	0.002546	protein_coding
ENSG0000(	PHKG2	1169.075	-0.19177	0.000428	0.002549	protein_coding
ENSG0000(	POLR2G	233.4653	-0.33653	0.000429	0.002553	protein_coding
ENSG0000(	ALG13	2344.45	0.335726	0.00043	0.002557	protein_coding
ENSG0000(	EIF2S2P2	33.05701	-0.6551	0.00043	0.002557	processed_pseudogene
ENSG0000(	PMAIP1	78.31057	0.584724	0.00043	0.002559	protein_coding
ENSG0000(	MPP3	112.4175	0.528637	0.000431	0.00256	protein_coding
ENSG0000(	MTPAP	1519.201	-0.16111	0.000431	0.002564	protein_coding
ENSG0000(	ARHGAP15	1230.067	0.450902	0.000432	0.002569	protein_coding

ENSG0000(	ZNF716	412.0711	-0.22825	0.000432	0.002569	protein_coding
ENSG0000(	SLC22A18A	334.6121	-0.63433	0.000433	0.002571	protein_coding
ENSG0000(	PKD2L2-DT	3344.663	0.141026	0.000433	0.002574	lncRNA
ENSG0000(	RCE1	308.597	-0.33509	0.000434	0.002575	protein_coding
ENSG0000(	INPP5D	2035.946	0.483475	0.000434	0.002575	protein_coding
ENSG0000(	ENSG0000(	565.6188	0.233452	0.000434	0.002578	lncRNA
ENSG0000(	RXYLT1-AS1	105.1517	0.263113	0.000435	0.00258	lncRNA
ENSG0000(	LINC02227	20.23763	0.943687	0.000435	0.002582	lncRNA
ENSG0000(	UCA1	250.4374	0.314952	0.000437	0.002592	lncRNA
ENSG0000(	TESK1	549.2354	-0.22521	0.000437	0.002592	protein_coding
ENSG0000(	LINC00877	511.3046	0.237935	0.000437	0.002593	lncRNA
ENSG0000(	DEDD2	628.715	-0.30737	0.000438	0.002594	protein_coding
ENSG0000(	ZNF571-AS	381.1776	-0.19909	0.000438	0.002594	lncRNA
ENSG0000(	UBL7	175.0799	-0.44249	0.000438	0.002595	protein_coding
ENSG0000(	AIFM3	1421.942	-0.65961	0.000438	0.002596	protein_coding
ENSG0000(	ENSG0000(	112.8347	0.309331	0.000438	0.002596	lncRNA
ENSG0000(	LINC01215	434.9135	0.346972	0.000438	0.002596	lncRNA
ENSG0000(	ENSG0000(	53.55363	0.40249	0.000439	0.002601	lncRNA
ENSG0000(	ZNF282	635.3981	-0.3226	0.00044	0.002604	protein_coding
ENSG0000(	CLDN4	3616.369	-0.55776	0.000441	0.002608	protein_coding
ENSG0000(	TAF12	2516.843	0.153818	0.000441	0.00261	protein_coding
ENSG0000(	RPL7P7	92.14699	-0.36983	0.000441	0.002611	processed_pseudogene
ENSG0000(	ENSG0000(	128.9061	-0.33544	0.000441	0.002612	processed_pseudogene
ENSG0000(	SYT11	434.5847	0.300042	0.000442	0.002613	protein_coding
ENSG0000(	STN1	887.7302	-0.23981	0.000443	0.002617	protein_coding
ENSG0000(	CDKN2AIPN	296.2168	-0.18509	0.000443	0.002619	protein_coding
ENSG0000(	ENSG0000(	783.9188	0.224092	0.000443	0.002619	lncRNA
ENSG0000(	ENSG0000(	143.7439	0.27763	0.000443	0.002619	TEC
ENSG0000(	ENSG0000(	216.1515	0.275222	0.000443	0.002619	lncRNA
ENSG0000(	ENSG0000(	299.6515	0.251524	0.000444	0.002622	lncRNA
ENSG0000(	LINC02054	347.1758	-0.19554	0.000444	0.002622	lncRNA
ENSG0000(	TRIML2	489.8126	0.230623	0.000444	0.002622	protein_coding
ENSG0000(	CLRN3	549.5719	-0.49788	0.000444	0.002622	protein_coding
ENSG0000(	TMEM54	2000.022	-0.52924	0.000445	0.002626	protein_coding
ENSG0000(	TMEM108	154.1549	0.591639	0.000445	0.002626	protein_coding
ENSG0000(	APOBEC3G	1149.498	0.227412	0.000446	0.002629	protein_coding
ENSG0000(	DTD2	609.4324	-0.17349	0.000446	0.002631	protein_coding
ENSG0000(	ENSG0000(	120.7371	0.406012	0.000446	0.002631	lncRNA
ENSG0000(	HMGA1P4	120.4496	0.372326	0.000446	0.002631	lncRNA
ENSG0000(	ENSG0000(	11.71714	1.076683	0.000448	0.00264	unprocessed_pseudogene
ENSG0000(	TEDC2	40.6554	-0.74533	0.000448	0.002641	protein_coding
ENSG0000(	SLC12A6	2643.4	0.284257	0.000448	0.002642	protein_coding
ENSG0000(	ENSG0000(	146.7083	-0.30843	0.000449	0.002649	lncRNA
ENSG0000(	ENSG0000(	154.3095	0.223011	0.00045	0.002651	lncRNA
ENSG0000(	ENSG0000(	79.77288	0.434314	0.000451	0.002654	lncRNA
ENSG0000(	ENSG0000(	636.0776	-0.16067	0.000451	0.002655	lncRNA
ENSG0000(	MPI	1329.678	-0.24537	0.000451	0.002655	protein_coding

ENSG0000(	YJU2	162.467	-0.23869	0.000451	0.002657	protein_coding
ENSG0000(	HADHB	1730.544	-0.24908	0.000452	0.00266	protein_coding
ENSG0000(	NDUFAF8	192.3184	-0.28191	0.000454	0.002673	protein_coding
ENSG0000(	SCMH1	1181.341	-0.21844	0.000454	0.002674	protein_coding
ENSG0000(	HIVEP3	1100.698	0.277135	0.000456	0.002681	protein_coding
ENSG0000(	TOMM34	167.4987	-0.28189	0.000456	0.002683	protein_coding
ENSG0000(	UPF1	2322.583	-0.15249	0.000456	0.002684	protein_coding
ENSG0000(	DNAJC2	1031.574	-0.16295	0.000457	0.002688	protein_coding
ENSG0000(	CCT7	771.2953	-0.22356	0.000459	0.002696	protein_coding
ENSG0000(	VWA8	1701.272	-0.25537	0.000459	0.002696	protein_coding
ENSG0000(	MFAP3	1524.275	0.134995	0.000459	0.002699	protein_coding
ENSG0000(	PRAL	1835.787	0.289522	0.000459	0.0027	TEC
ENSG0000(	ENSG0000(	45.55758	-0.49258	0.00046	0.0027	lncRNA
ENSG0000(	CASK	3359.498	-0.12746	0.000461	0.002708	protein_coding
ENSG0000(	ENSG0000(	359.2075	0.262099	0.000461	0.002709	lncRNA
ENSG0000(	OR14L1P	30.79553	0.543486	0.000461	0.002709	protein_coding
ENSG0000(	PTPRA	1204.871	-0.22439	0.000461	0.002709	protein_coding
ENSG0000(	TROAP	354.8947	-0.33112	0.000462	0.002711	protein_coding
ENSG0000(	LINC02523	85.3308	-0.30243	0.000462	0.002713	lncRNA
ENSG0000(	GNPAT	704.0964	-0.18497	0.000462	0.002713	protein_coding
ENSG0000(	ENSG0000(	50.19505	-0.30761	0.000463	0.002717	lncRNA
ENSG0000(	RALBP1	1889.889	-0.16135	0.000463	0.002717	protein_coding
ENSG0000(	IGHV3-23	1912.391	0.767639	0.000465	0.002729	IG_V_gene
ENSG0000(	SH3GL1P1	353.8498	0.333086	0.000466	0.002734	transcribed_processed_pseudoge
ENSG0000(	ZNF281	822.8936	0.329542	0.000467	0.00274	protein_coding
ENSG0000(	ADAM8	398.5698	0.630268	0.000468	0.002744	protein_coding
ENSG0000(	TRAPPC2L	256.3234	-0.23222	0.000468	0.002744	protein_coding
ENSG0000(	SUPT6H	3316.917	0.130565	0.000469	0.002746	protein_coding
ENSG0000(	CYP27C1	425.6144	0.174084	0.000469	0.002747	protein_coding
ENSG0000(	CLVS1	151.5752	-0.27959	0.000469	0.002748	protein_coding
ENSG0000(	PSMA2	1215.51	-0.32095	0.000469	0.002748	protein_coding
ENSG0000(	SNHG21	31.27326	-0.56324	0.000469	0.002748	lncRNA
ENSG0000(	CYLD	3250.71	0.294755	0.00047	0.002749	protein_coding
ENSG0000(	EPN1	7179.662	-0.25374	0.00047	0.00275	protein_coding
ENSG0000(	FANCL	440.419	-0.23039	0.00047	0.00275	protein_coding
ENSG0000(	LINC02913	231.6411	0.1927	0.00047	0.00275	lncRNA
ENSG0000(	ANKDD1B	512.9226	-0.50631	0.00047	0.00275	protein_coding
ENSG0000(	ENSG0000(	31.52856	-0.79212	0.00047	0.002751	lncRNA
ENSG0000(	AOPEP	3211.839	0.194162	0.000471	0.002751	protein_coding
ENSG0000(	ENSG0000(	16.42669	0.63305	0.000471	0.002751	lncRNA
ENSG0000(	TMEM126/	69.69991	-0.51056	0.000471	0.002755	protein_coding
ENSG0000(	LINC01150	49.9266	0.351403	0.000472	0.002756	lncRNA
ENSG0000(	ENSG0000(	174.5645	-0.61993	0.000472	0.002756	lncRNA
ENSG0000(	LINC01238	695.2245	0.253479	0.000473	0.00276	lncRNA
ENSG0000(	MSH6	1356.034	-0.18006	0.000473	0.00276	protein_coding
ENSG0000(	DGKI	93.5898	0.859926	0.000474	0.002767	protein_coding
ENSG0000(	PAFAH1B2I	82.86722	-0.47018	0.000474	0.002767	transcribed_processed_pseudoge

ENSG0000(	SNCA	74.97566	0.466221	0.000474	0.002767	protein_coding
ENSG0000(	CASP8AP2	1728.789	-0.15865	0.000475	0.00277	protein_coding
ENSG0000(	LGALS3BP	2878.416	-0.30988	0.000475	0.00277	protein_coding
ENSG0000(	NOTCH3	1063.845	0.275137	0.000476	0.002775	protein_coding
ENSG0000(	ENSG0000(	348.9814	0.25156	0.000476	0.002776	lncRNA
ENSG0000(	ANO2	135.1607	0.66885	0.000477	0.00278	protein_coding
ENSG0000(	CIT	1915.549	-0.25004	0.000478	0.002786	protein_coding
ENSG0000(	EIF2S1	874.9078	-0.27314	0.000479	0.002793	protein_coding
ENSG0000(	ST8SIA4	1244.486	0.418785	0.000479	0.002793	protein_coding
ENSG0000(	ENSG0000(	98.54945	-0.24867	0.00048	0.002794	lncRNA
ENSG0000(	SLC43A2	1341.142	0.308087	0.00048	0.002794	protein_coding
ENSG0000(	ENSG0000(	308.648	0.3456	0.000482	0.00281	TEC
ENSG0000(	TMEM106(	1225.562	-0.30148	0.000483	0.002813	protein_coding
ENSG0000(	ENSG0000(	161.7777	0.299075	0.000483	0.002814	lncRNA
ENSG0000(	NT5DC1	737.9738	-0.28194	0.000485	0.002823	protein_coding
ENSG0000(	ENSG0000(	240.4571	0.214137	0.000485	0.002826	lncRNA
ENSG0000(	ENSG0000(	222.9504	-0.28173	0.000486	0.002827	lncRNA
ENSG0000(	ITGA6	7408.208	-0.28578	0.000486	0.002827	protein_coding
ENSG0000(	ZFR	1912.616	-0.16489	0.000487	0.002831	protein_coding
ENSG0000(	PGM3	1679.639	0.370288	0.000487	0.002834	protein_coding
ENSG0000(	TSG101	701.3169	-0.20699	0.000488	0.002839	protein_coding
ENSG0000(	RAB3C	111.7138	0.77659	0.000488	0.00284	protein_coding
ENSG0000(	ENSG0000(	317.5604	-0.23505	0.000492	0.002857	lncRNA
ENSG0000(	PDS5A	4200.116	-0.13192	0.000492	0.002858	protein_coding
ENSG0000(	ENSG0000(	11.61032	0.900403	0.000492	0.002859	lncRNA
ENSG0000(	ENSG0000(	10.48343	0.964012	0.000492	0.00286	lncRNA
ENSG0000(	ENSG0000(	1398.378	0.189369	0.000493	0.002861	transcribed_unprocessed_pseudo
ENSG0000(	ENSG0000(	37.16419	-0.6508	0.000493	0.002864	processed_pseudogene
ENSG0000(	PPP1R9A-A	157.2928	0.256087	0.000494	0.002869	lncRNA
ENSG0000(	ENSG0000(	20.44479	0.606101	0.000494	0.002869	lncRNA
ENSG0000(	PET117	172.4449	-0.37647	0.000495	0.002872	protein_coding
ENSG0000(	ENSG0000(	214.1258	-0.35525	0.000495	0.002873	processed_pseudogene
ENSG0000(	ENSG0000(	10.5133	1.020324	0.000495	0.002873	protein_coding
ENSG0000(	IL12A-AS1	352.834	0.189827	0.000495	0.002873	lncRNA
ENSG0000(	GTF2H3	827.51	-0.16219	0.000496	0.002879	protein_coding
ENSG0000(	LINC01327	29.93254	0.47397	0.000497	0.002881	lncRNA
ENSG0000(	ENSG0000(	557.994	0.311445	0.000497	0.002882	protein_coding
ENSG0000(	ENSG0000(	126.4215	0.287221	0.000497	0.002884	lncRNA
ENSG0000(	MAN1B1-D	693.2797	0.180481	0.000498	0.002884	lncRNA
ENSG0000(	DEF6	669.7207	0.440831	0.000498	0.002884	protein_coding
ENSG0000(	DNA2	475.4587	-0.28466	0.000498	0.002884	protein_coding
ENSG0000(	HSDL2-AS1	459.6989	0.187866	0.000498	0.002885	lncRNA
ENSG0000(	ENSG0000(	30.31852	0.609745	0.000499	0.002887	lncRNA
ENSG0000(	ENSG0000(	437.5573	0.190347	0.000499	0.002892	lncRNA
ENSG0000(	ENSG0000(	75.38259	0.365662	0.0005	0.002894	lncRNA
ENSG0000(	ENSG0000(	12.21801	0.892878	0.000501	0.002899	lncRNA
ENSG0000(	BIRC2	2713.327	0.198188	0.000501	0.002899	protein_coding

ENSG0000(	SLC26A6	1887.98	-0.33913	0.000502	0.002904	protein_coding
ENSG0000(	ENSG0000(	62.4583	0.450235	0.000502	0.002904	TEC
ENSG0000(	CDKL5	4291.306	0.149208	0.000502	0.002905	protein_coding
ENSG0000(	ZNF785	1665.698	-0.11516	0.000503	0.002908	protein_coding
ENSG0000(	MCM8	1214.222	-0.20736	0.000504	0.002913	protein_coding
ENSG0000(	OLFM1	118.6011	0.721286	0.000504	0.002913	protein_coding
ENSG0000(	SLC7A4	18.60141	-0.7594	0.000504	0.002914	protein_coding
ENSG0000(	RANGAP1	1245.408	-0.20034	0.000504	0.002915	protein_coding
ENSG0000(	TRPV1	1161.639	-0.29054	0.000505	0.002919	protein_coding
ENSG0000(	NPIPB2	255.4693	-0.3553	0.000506	0.002921	protein_coding
ENSG0000(	UGT1A9	587.6953	-0.68412	0.000506	0.002921	protein_coding
ENSG0000(	ENSG0000(	28.45118	0.591116	0.000507	0.002925	lncRNA
ENSG0000(	LINC01727	678.4929	0.168807	0.000507	0.002926	lncRNA
ENSG0000(	CEP170B	4036.976	-0.46589	0.000508	0.002931	protein_coding
ENSG0000(	ENSG0000(	61.14373	0.406042	0.000508	0.002933	lncRNA
ENSG0000(	L3MBTL4	608.9469	0.287349	0.000509	0.002933	protein_coding
ENSG0000(	MLST8	289.5791	-0.23175	0.000508	0.002933	protein_coding
ENSG0000(	ZFHX2-AS1	701.1089	0.227545	0.000508	0.002933	lncRNA
ENSG0000(	COL5A1	3249.35	0.469516	0.00051	0.002942	protein_coding
ENSG0000(	CCDC115	447.6629	-0.28272	0.000511	0.002944	protein_coding
ENSG0000(	BCL2L11	1844.357	0.291734	0.000511	0.002944	protein_coding
ENSG0000(	ENSG0000(	747.4117	0.242152	0.000511	0.002944	transcribed_unprocessed_pseudo
ENSG0000(	CDC42BPB	3708.269	-0.22409	0.000511	0.002946	protein_coding
ENSG0000(	C1QA	513.2988	0.59817	0.000512	0.002947	protein_coding
ENSG0000(	NT5E	685.4328	-0.57707	0.000512	0.002948	protein_coding
ENSG0000(	NLGN2	587.8141	0.457685	0.000512	0.00295	protein_coding
ENSG0000(	COX16	407.1233	-0.32172	0.000513	0.002954	protein_coding
ENSG0000(	ENSG0000(	79.96149	0.441024	0.000513	0.002955	lncRNA
ENSG0000(	ENSG0000(	65.64248	0.37578	0.000513	0.002955	lncRNA
ENSG0000(	ZNF639	1435.32	-0.18974	0.000514	0.002956	protein_coding
ENSG0000(	ENSG0000(	55.35991	0.34856	0.000514	0.002958	lncRNA
ENSG0000(	TBX18	63.59609	1.316432	0.000514	0.002958	protein_coding
ENSG0000(	CYP4F12	3016.885	-0.59293	0.000514	0.002958	protein_coding
ENSG0000(	SOWAHB	575.8592	-0.52615	0.000515	0.002961	protein_coding
ENSG0000(	PCED1B	269.881	0.645696	0.000515	0.002963	protein_coding
ENSG0000(	RNPS1	2603.995	-0.11051	0.000516	0.002965	protein_coding
ENSG0000(	ENSG0000(	119.3991	0.274919	0.000516	0.002966	lncRNA
ENSG0000(	ENSG0000(	46.35953	0.503905	0.000517	0.002968	lncRNA
ENSG0000(	ENSG0000(	20.54781	0.564799	0.000516	0.002968	lncRNA
ENSG0000(	POLE3	356.6807	-0.26298	0.000517	0.00297	protein_coding
ENSG0000(	SGSM3	1564.036	-0.24553	0.000517	0.002972	protein_coding
ENSG0000(	ENSG0000(	63.3782	0.532282	0.000518	0.002975	lncRNA
ENSG0000(	BMPR1B	42.13501	0.757756	0.00052	0.002983	protein_coding
ENSG0000(	ENSG0000(	1062.091	0.169247	0.000519	0.002983	lncRNA
ENSG0000(	MAPKAPK2	1552.736	0.374428	0.00052	0.002984	protein_coding
ENSG0000(	ENSG0000(	176.1594	-0.30221	0.00052	0.002984	lncRNA
ENSG0000(	NBN	1185.511	0.293382	0.00052	0.002984	protein_coding

ENSG0000(	GFM2	536.959	-0.19319	0.00052	0.002985	protein_coding
ENSG0000(	FES	303.8664	0.349228	0.00052	0.002985	protein_coding
ENSG0000(	ENSG0000(	33.58442	0.468709	0.000521	0.002988	lncRNA
ENSG0000(	RAC1	3681.576	-0.30215	0.000521	0.002988	protein_coding
ENSG0000(	EEF1A1	58383.74	-0.33463	0.000523	0.002995	protein_coding
ENSG0000(	PRPF38B	2688.294	0.155941	0.000523	0.002995	protein_coding
ENSG0000(	C2orf27A	256.5284	0.630801	0.000523	0.002995	lncRNA
ENSG0000(	AQP7P1	29.7529	1.001284	0.000523	0.002997	unprocessed_pseudogene
ENSG0000(	TMOD2	1133.214	0.257771	0.000523	0.002997	protein_coding
ENSG0000(	CHCHD1	219.7895	-0.2751	0.000524	0.003003	protein_coding
ENSG0000(	GTF3C5	725.7537	-0.15033	0.000525	0.003003	protein_coding
ENSG0000(	RBMXL2	12.62419	1.025902	0.000524	0.003003	protein_coding
ENSG0000(	IGKC	24394.49	0.759632	0.000525	0.003003	IG_C_gene
ENSG0000(	NEK7	1038.293	0.258302	0.000526	0.003007	protein_coding
ENSG0000(	ENSG0000(	134.9068	0.277535	0.000526	0.003009	lncRNA
ENSG0000(	ZBTB47	311.3513	0.344474	0.000526	0.003009	protein_coding
ENSG0000(	TRAPPC8	2198.332	-0.13015	0.000527	0.003014	protein_coding
ENSG0000(	CBX7	1555.306	0.23806	0.000527	0.003015	protein_coding
ENSG0000(	ENSG0000(	29.84289	-0.4483	0.000528	0.003016	lncRNA
ENSG0000(	S100PBP	2613.374	0.170613	0.000528	0.003017	protein_coding
ENSG0000(	PTPN2	2881.487	0.185398	0.00053	0.003031	protein_coding
ENSG0000(	SFXN4	357.0973	-0.44012	0.000533	0.003043	protein_coding
ENSG0000(	IK	1191.446	-0.15297	0.000533	0.003045	protein_coding
ENSG0000(	NRAV	790.6568	-0.10946	0.000533	0.003045	lncRNA
ENSG0000(	ENSG0000(	120.4084	0.229378	0.000534	0.003049	lncRNA
ENSG0000(	CDC25A	215.3048	-0.58957	0.000534	0.003049	protein_coding
ENSG0000(	HTATIP2	588.4894	-0.4231	0.000535	0.003052	protein_coding
ENSG0000(	COX5AP2	16.51616	0.778619	0.000536	0.003057	processed_pseudogene
ENSG0000(	MRPL24	233.3723	-0.26089	0.000536	0.00306	protein_coding
ENSG0000(	PPP1R8	467.7213	-0.20087	0.000537	0.003064	protein_coding
ENSG0000(	CAMTA1	614.2712	-0.23999	0.000537	0.003064	protein_coding
ENSG0000(	POMT2	1176.79	-0.17852	0.000537	0.003064	protein_coding
ENSG0000(	ZNF385C	325.3125	0.201041	0.000539	0.003072	protein_coding
ENSG0000(	CDC14C	273.5358	0.215333	0.000539	0.003072	protein_coding
ENSG0000(	KANSL1L-A	620.0236	0.154617	0.00054	0.003079	lncRNA
ENSG0000(	OSBPL9	2024.32	0.146411	0.00054	0.003079	protein_coding
ENSG0000(	KCTD18	1082.719	0.118789	0.00054	0.00308	protein_coding
ENSG0000(	KLHDC7B	56.27095	0.889326	0.000541	0.003083	protein_coding
ENSG0000(	ENSG0000(	33.26111	1.054675	0.000542	0.003086	lncRNA
ENSG0000(	LINC00347	11.87118	0.740747	0.000542	0.003086	lncRNA
ENSG0000(	MICALL1	1140.676	0.146792	0.000542	0.003086	protein_coding
ENSG0000(	SCCPDH	365.595	-0.27378	0.000542	0.003086	protein_coding
ENSG0000(	C15orf61	250.3421	-0.30411	0.000542	0.003086	protein_coding
ENSG0000(	HGF	257.514	0.640437	0.000542	0.003087	protein_coding
ENSG0000(	LDLRAP1	1786.904	-0.29909	0.000543	0.003087	protein_coding
ENSG0000(	HBP1	1652.838	0.172891	0.000544	0.003094	protein_coding
ENSG0000(	ENSG0000(	11.38731	0.786656	0.000546	0.003105	lncRNA

ENSG0000(	MRPL52	740.851	-0.19811	0.000547	0.00311	protein_coding
ENSG0000(	TMSB10	4278.355	-0.37032	0.000548	0.003117	protein_coding
ENSG0000(	ADCK2	261.9454	-0.32764	0.000549	0.003118	protein_coding
ENSG0000(	ENSG0000(	23.48369	0.591059	0.000549	0.003118	lncRNA
ENSG0000(	EVC2	192.6093	0.400907	0.000549	0.003122	protein_coding
ENSG0000(	ENSG0000(	40.77901	0.465983	0.00055	0.003122	lncRNA
ENSG0000(	ENSG0000(	310.5377	-0.25448	0.00055	0.003125	lncRNA
ENSG0000(	MTRES1	450.1683	-0.18666	0.00055	0.003126	protein_coding
ENSG0000(	TINAG	248.6726	-0.65563	0.000551	0.003127	protein_coding
ENSG0000(	XPNPEP2	138.812	-1.14784	0.000551	0.003127	protein_coding
ENSG0000(	TIPIN	331.5192	-0.23644	0.000552	0.003133	protein_coding
ENSG0000(	CHPF2	2068.276	0.154715	0.000553	0.003137	protein_coding
ENSG0000(	LPCAT2	709.8768	0.322537	0.000553	0.003137	protein_coding
ENSG0000(	LYPD6B	42.21521	0.83998	0.000554	0.003145	protein_coding
ENSG0000(	ENSG0000(	25.73334	0.534265	0.000555	0.003146	lncRNA
ENSG0000(	ITCH	4025.575	-0.21818	0.000555	0.00315	protein_coding
ENSG0000(	GNB2	1257.712	-0.29245	0.000556	0.003152	protein_coding
ENSG0000(	HOATZ	206.6034	-0.30282	0.000556	0.003152	protein_coding
ENSG0000(	CHMP4B	1313.539	-0.39914	0.000556	0.003153	protein_coding
ENSG0000(	RN7SL752F	213.0041	0.505234	0.000557	0.003155	misc_RNA
ENSG0000(	HCCS	277.0879	-0.38316	0.000557	0.003156	protein_coding
ENSG0000(	ING1	757.9873	-0.13562	0.000557	0.003157	protein_coding
ENSG0000(	PKD1L2	895.0482	0.137776	0.000557	0.003157	polymorphic_pseudogene
ENSG0000(	TOM1L2	2330.545	-0.25406	0.000557	0.003157	protein_coding
ENSG0000(	HDLBP	7596.193	0.176453	0.000558	0.003159	protein_coding
ENSG0000(	ZGRF1	911.1123	-0.25724	0.000559	0.003162	protein_coding
ENSG0000(	MRPL28	261.5618	-0.23077	0.000559	0.003163	protein_coding
ENSG0000(	ENSG0000(	579.7323	0.177428	0.000561	0.003178	transcribed_processed_pseudoge
ENSG0000(	ENSG0000(	142.4356	0.276242	0.000562	0.003181	TEC
ENSG0000(	IPO13	527.9885	-0.24293	0.000563	0.003185	protein_coding
ENSG0000(	ENSG0000(	103.2214	0.387531	0.000563	0.003186	lncRNA
ENSG0000(	RILPL2	133.3958	0.50515	0.000563	0.003186	protein_coding
ENSG0000(	TYRO3P	338.0434	-0.23652	0.000565	0.003197	processed_pseudogene
ENSG0000(	TASL	212.9331	0.324685	0.000566	0.003198	protein_coding
ENSG0000(	ZBTB7B	3276.039	-0.43353	0.000566	0.0032	protein_coding
ENSG0000(	CDKN3	77.91756	-0.5369	0.000567	0.003207	protein_coding
ENSG0000(	FAM102A	2930.634	-0.31205	0.000568	0.003207	protein_coding
ENSG0000(	CHMP1A	1678.595	-0.23754	0.000569	0.003212	protein_coding
ENSG0000(	CNTROB	782.4338	-0.19605	0.000569	0.003216	protein_coding
ENSG0000(	ENSG0000(	756.886	0.202543	0.000571	0.003223	lncRNA
ENSG0000(	ZFAT	696.9503	0.289137	0.000571	0.003225	protein_coding
ENSG0000(	MRPL37P1	112.8501	0.367928	0.000571	0.003225	processed_pseudogene
ENSG0000(	ECT2	745.0973	-0.21435	0.000572	0.003228	protein_coding
ENSG0000(	ENSG0000(	76.06728	0.267778	0.000572	0.003229	lncRNA
ENSG0000(	ZNHIT3	1084.461	-0.15953	0.000572	0.003229	protein_coding
ENSG0000(	ABCA7	1089.101	0.314499	0.000573	0.003231	protein_coding
ENSG0000(	CLPP	652.5994	-0.17276	0.000573	0.003231	protein_coding

ENSG0000(	ENSG0000(	71.69101	-0.3513	0.000573	0.003231	lncRNA
ENSG0000(	ENSG0000(	447.8193	0.129866	0.000573	0.003231	lncRNA
ENSG0000(	IGBP1-AS2	14.51782	-0.72403	0.000573	0.003231	lncRNA
ENSG0000(	MLX	1602.889	-0.24873	0.000573	0.003231	protein_coding
ENSG0000(	MSRA	402.3196	0.197834	0.000573	0.003231	protein_coding
ENSG0000(	MANBA	959.989	0.252455	0.000574	0.003233	protein_coding
ENSG0000(	ENSG0000(	96.35227	0.659597	0.000576	0.003243	lncRNA
ENSG0000(	CRYBG1	5855.163	-0.256	0.000576	0.003245	protein_coding
ENSG0000(	ANKRD20A	117.6623	0.340041	0.000577	0.003247	transcribed_unprocessed_pseudo
ENSG0000(	TP53	1352.066	-0.14608	0.000577	0.00325	protein_coding
ENSG0000(	HMOX2	610.2801	-0.21814	0.000577	0.00325	protein_coding
ENSG0000(	SMYD5	302.2429	-0.35397	0.000578	0.003251	protein_coding
ENSG0000(	MSRA-DT	150.0592	0.225604	0.000579	0.003255	lncRNA
ENSG0000(	OAS2	1702.677	0.381787	0.000579	0.003257	protein_coding
ENSG0000(	TM4SF5	28.19997	-0.92351	0.00058	0.00326	protein_coding
ENSG0000(	ENSG0000(	1938.869	0.201673	0.00058	0.003262	lncRNA
ENSG0000(	ENSG0000(	99.15916	0.31148	0.00058	0.003262	lncRNA
ENSG0000(	MT-ATP8	1806.425	-0.54316	0.00058	0.003262	protein_coding
ENSG0000(	NAA35	1553.026	-0.16074	0.000581	0.003268	protein_coding
ENSG0000(	KDM3A	2313.998	0.20623	0.000582	0.003268	protein_coding
ENSG0000(	RHOT1	949.6209	0.192321	0.000582	0.003268	protein_coding
ENSG0000(	ENSG0000(	249.7093	-0.26014	0.000582	0.003268	lncRNA
ENSG0000(	ZC3HAV1L	339.8877	-0.19331	0.000582	0.003269	protein_coding
ENSG0000(	ENSG0000(	49.496	0.341642	0.000582	0.003271	lncRNA
ENSG0000(	GPR52	362.7364	0.265456	0.000583	0.003274	protein_coding
ENSG0000(	ANKRD49P	90.37027	0.36928	0.000583	0.003274	processed_pseudogene
ENSG0000(	LHFPL6	387.6502	0.366665	0.000584	0.003275	protein_coding
ENSG0000(	ANO6	931.6861	0.332095	0.000586	0.003287	protein_coding
ENSG0000(	RSBN1	1157.257	-0.21034	0.000587	0.003295	protein_coding
ENSG0000(	JAZF1	306.5393	0.448911	0.000588	0.0033	protein_coding
ENSG0000(	ENSG0000(	15.76555	0.644161	0.000589	0.003301	lncRNA
ENSG0000(	FABP7P1	23.24038	0.649942	0.000589	0.003301	processed_pseudogene
ENSG0000(	RIOK1	303.5547	-0.34892	0.000589	0.003301	protein_coding
ENSG0000(	SEL1L	4109.698	0.267197	0.000589	0.003304	protein_coding
ENSG0000(	KIAA1522	4823.596	-0.43948	0.000591	0.00331	protein_coding
ENSG0000(	HOMER1	183.3349	0.421401	0.000591	0.003311	protein_coding
ENSG0000(	CPNE4	22.69523	1.397855	0.000593	0.003319	protein_coding
ENSG0000(	ESS2	256.5827	-0.29931	0.000593	0.003319	protein_coding
ENSG0000(	IFITM2	314.1067	0.608393	0.000593	0.003319	protein_coding
ENSG0000(	CRYBG2	604.3359	-0.74752	0.000593	0.00332	protein_coding
ENSG0000(	MS4A10	240.3529	-0.21343	0.000594	0.003324	protein_coding
ENSG0000(	SFXN5	1531.965	-0.17492	0.000594	0.003325	protein_coding
ENSG0000(	MEST	573.0513	-0.41048	0.000595	0.003327	protein_coding
ENSG0000(	ENSG0000(	154.112	0.378153	0.000595	0.003329	transcribed_unprocessed_pseudo
ENSG0000(	ENSG0000(	18.10264	0.555301	0.000595	0.003329	lncRNA
ENSG0000(	NUBP2	594.3449	-0.13969	0.000596	0.003334	protein_coding
ENSG0000(	RP2	468.9429	-0.26638	0.000596	0.003334	protein_coding

ENSG0000(	ENSG0000(	34.00247	0.528499	0.000596	0.003334	lncRNA
ENSG0000(	AP2B1	3617.17	-0.21567	0.000596	0.003335	protein_coding
ENSG0000(	TAOK1	6831.223	-0.15387	0.000597	0.003338	protein_coding
ENSG0000(	PPP6C	970.2583	-0.21016	0.000599	0.003345	protein_coding
ENSG0000(	ENSG0000(	85.55747	0.393814	0.000599	0.003346	lncRNA
ENSG0000(	PLD5P1	495.597	0.194192	0.0006	0.00335	protein_coding
ENSG0000(	ENSG0000(	97.98214	-0.43637	0.0006	0.003353	lncRNA
ENSG0000(	ENSG0000(	670.3881	0.187566	0.0006	0.003353	lncRNA
ENSG0000(	KRT26	47.50921	0.594625	0.000601	0.003356	protein_coding
ENSG0000(	AFAP1-AS1	85.7029	0.398208	0.000602	0.003358	lncRNA
ENSG0000(	ENSG0000(	21.87356	0.606833	0.000602	0.003361	lncRNA
ENSG0000(	RGL1	781.7509	0.324071	0.000602	0.003361	protein_coding
ENSG0000(	NUDT21	1769.117	-0.14681	0.000603	0.003366	protein_coding
ENSG0000(	MIA	58.16884	-0.52344	0.000603	0.003367	protein_coding
ENSG0000(	GYPE	72.92902	0.389209	0.000605	0.003372	protein_coding
ENSG0000(	LINC02241	529.0188	0.218427	0.000605	0.003374	lncRNA
ENSG0000(	NAPA	1211.524	-0.23463	0.000606	0.003378	protein_coding
ENSG0000(	HSF4	239.0157	-0.44644	0.000607	0.003382	protein_coding
ENSG0000(	KLF16	426.8946	-0.29759	0.000607	0.003384	protein_coding
ENSG0000(	ENSG0000(	825.2856	0.221309	0.000609	0.003393	processed_pseudogene
ENSG0000(	TADA3	950.8911	-0.17472	0.000609	0.003393	protein_coding
ENSG0000(	TP53I13	499.4478	-0.29372	0.000609	0.003393	protein_coding
ENSG0000(	AKT3-IT1	94.2831	0.310145	0.000609	0.003394	lncRNA
ENSG0000(	EIF4E	1277.793	-0.16332	0.000612	0.003409	protein_coding
ENSG0000(	GVINP1	2979.044	0.486244	0.000612	0.003409	transcribed_unprocessed_pseudo
ENSG0000(	ADGRE5	1765.289	0.246544	0.000613	0.003411	protein_coding
ENSG0000(	ATP5F1E	1208.923	-0.23962	0.000613	0.003411	protein_coding
ENSG0000(	TXN2P1	20.83003	0.662879	0.000613	0.003412	processed_pseudogene
ENSG0000(	FOXC1	25.94982	0.760731	0.000614	0.003416	protein_coding
ENSG0000(	CROT	672.0044	-0.36531	0.000615	0.003422	protein_coding
ENSG0000(	LINC01140	217.9787	0.306873	0.000615	0.003422	lncRNA
ENSG0000(	PEDS1	1774.48	-0.21845	0.000615	0.003422	protein_coding
ENSG0000(	ENSG0000(	28.43479	0.647677	0.000616	0.003422	processed_pseudogene
ENSG0000(	PRKCZ	1216.113	-0.26962	0.000616	0.003424	protein_coding
ENSG0000(	ENSG0000(	167.0088	0.325233	0.000616	0.003425	lncRNA
ENSG0000(	ENSG0000(	214.7871	-0.23699	0.000617	0.003426	lncRNA
ENSG0000(	ENSG0000(	23.81081	0.692446	0.000617	0.003427	lncRNA
ENSG0000(	ISY1	619.1201	-0.20734	0.000617	0.003427	protein_coding
ENSG0000(	MCM10	258.6827	-0.50034	0.000617	0.003428	protein_coding
ENSG0000(	ENSG0000(	333.2427	0.221975	0.000618	0.003432	lncRNA
ENSG0000(	TAS2R19	248.8415	0.278116	0.000618	0.003432	protein_coding
ENSG0000(	HLA-C	8804.701	0.408597	0.000619	0.003434	protein_coding
ENSG0000(	ENSG0000(	226.1234	0.294643	0.000619	0.003435	TEC
ENSG0000(	ANKRD9	2314.381	-0.43916	0.00062	0.003439	protein_coding
ENSG0000(	LINC02117	62.92807	-0.31023	0.000621	0.003444	lncRNA
ENSG0000(	CCNT1	1868.24	-0.17767	0.000621	0.003446	protein_coding
ENSG0000(	DAND5	325.3922	-0.188	0.000622	0.003448	protein_coding

ENSG0000(	POU2F1	2437.787	0.222423	0.000622	0.00345	protein_coding
ENSG0000(	ENSG0000(	49.1442	-0.52559	0.000622	0.00345	lncRNA
ENSG0000(	MOGAT2	1493.991	-0.55842	0.000623	0.003452	protein_coding
ENSG0000(	HSD17B8	119.9169	-0.35998	0.000623	0.003455	protein_coding
ENSG0000(	H2AZ1	603.9842	-0.40986	0.000625	0.003462	protein_coding
ENSG0000(	LINC01546	629.8162	0.188349	0.000626	0.003468	lncRNA
ENSG0000(	NPFFR1	1190.125	0.165851	0.000627	0.003472	protein_coding
ENSG0000(	RNU6-890F	29.96166	0.510133	0.000627	0.003475	snRNA
ENSG0000(	FAM245B	120.3897	0.267916	0.000629	0.00348	lncRNA
ENSG0000(	KIF1C-AS1	11.57632	0.779233	0.000629	0.00348	lncRNA
ENSG0000(	PACRG-AS1	189.448	-0.25743	0.000629	0.00348	lncRNA
ENSG0000(	EGFEM1P	122.9012	-0.45235	0.00063	0.003489	transcribed_unitary_pseudogene
ENSG0000(	AMIGO2	153.2859	0.515287	0.000631	0.003493	protein_coding
ENSG0000(	NAPG	1611.119	0.141043	0.000632	0.003494	protein_coding
ENSG0000(	OR2A1-AS1	359.162	-0.32612	0.000632	0.003494	lncRNA
ENSG0000(	SUV39H1	108.5882	-0.37357	0.000633	0.0035	protein_coding
ENSG0000(	PCDHB9	229.3835	-0.18983	0.000633	0.0035	protein_coding
ENSG0000(	LYPD4	13.57153	0.65265	0.000633	0.0035	protein_coding
ENSG0000(	TLN2	3773.491	-0.38139	0.000633	0.0035	protein_coding
ENSG0000(	TBCA	375.5987	-0.3045	0.000633	0.0035	protein_coding
ENSG0000(	ADA2	1709.957	0.221441	0.000634	0.003502	protein_coding
ENSG0000(	POLA1	1776.888	-0.1742	0.000634	0.003503	protein_coding
ENSG0000(	HINT3	768.3594	-0.18297	0.000634	0.003503	protein_coding
ENSG0000(	CERNA2	424.0423	0.311451	0.000635	0.003508	lncRNA
ENSG0000(	PEX5	495.9234	-0.24567	0.000636	0.003511	protein_coding
ENSG0000(	HDHD3	1928.587	-0.40011	0.000637	0.003519	protein_coding
ENSG0000(	DNAJC21	1451.471	-0.17436	0.000638	0.00352	protein_coding
ENSG0000(	PSMG3	376.5589	-0.18947	0.000638	0.00352	protein_coding
ENSG0000(	MYL5	588.7064	-0.3656	0.000639	0.003526	protein_coding
ENSG0000(	POU2F3	298.6632	-0.28479	0.000639	0.003526	protein_coding
ENSG0000(	ARHGEF10	842.3038	0.314234	0.000639	0.003527	protein_coding
ENSG0000(	UCK1	333.9072	-0.28232	0.000639	0.003527	protein_coding
ENSG0000(	ENSG0000(	36.67352	-0.59835	0.00064	0.003529	lncRNA
ENSG0000(	ENSG0000(	186.5819	0.220242	0.000641	0.00353	lncRNA
ENSG0000(	ENSG0000(	129.7573	0.277956	0.00064	0.00353	lncRNA
ENSG0000(	LARP1B	1667.88	-0.21557	0.00064	0.00353	protein_coding
ENSG0000(	PCCA	2511.837	-0.20497	0.000641	0.00353	protein_coding
ENSG0000(	SLC44A3	782.045	-0.47552	0.000641	0.003533	protein_coding
ENSG0000(	ENSG0000(	748.7949	0.220562	0.000642	0.003536	lncRNA
ENSG0000(	SMARCC2	3860.163	-0.10401	0.000642	0.003536	protein_coding
ENSG0000(	BAIAP2L2	1923.594	-0.41045	0.000642	0.003537	protein_coding
ENSG0000(	CD163L1	2075.144	-0.56997	0.000643	0.003538	protein_coding
ENSG0000(	YARS1	2061.735	0.162904	0.000643	0.003538	protein_coding
ENSG0000(	ADAMDEC1	3544.899	-0.52801	0.000643	0.003538	protein_coding
ENSG0000(	LINC00652	958.3851	0.163711	0.000643	0.003538	lncRNA
ENSG0000(	ENSG0000(	20.80657	0.613577	0.000645	0.003549	lncRNA
ENSG0000(	DNAH12	108.2039	0.349197	0.000645	0.003549	protein_coding

ENSG0000(	TRMT61A	314.6667	-0.27252	0.000646	0.00355	protein_coding
ENSG0000(	DAPK2	3793.915	-0.27139	0.000647	0.003557	protein_coding
ENSG0000(	FDPS	1349.899	-0.2325	0.000647	0.003558	protein_coding
ENSG0000(	ST3GAL1-D	29.0959	0.625522	0.000648	0.003559	lncRNA
ENSG0000(	GOLGA6L4	245.0574	0.375452	0.000648	0.003559	protein_coding
ENSG0000(	PLEC	18695.08	-0.38961	0.000648	0.003562	protein_coding
ENSG0000(	DFFB	816.4698	-0.14564	0.000649	0.003563	protein_coding
ENSG0000(	RABL6	2735.706	-0.24447	0.000649	0.003563	protein_coding
ENSG0000(	SNHG7	767.6377	-0.21801	0.00065	0.003568	lncRNA
ENSG0000(	IFIH1	658.9052	0.468125	0.00065	0.003571	protein_coding
ENSG0000(	ENTPD4	3952.516	0.180654	0.000651	0.003572	protein_coding
ENSG0000(	SIGLEC14	916.5143	0.298685	0.000651	0.003573	protein_coding
ENSG0000(	CNOT4	1511.677	0.136805	0.000652	0.003576	protein_coding
ENSG0000(	ENSG0000(	12.95405	0.684267	0.000652	0.003578	scaRNA
ENSG0000(	SLC9A3R2	484.431	-0.27055	0.000653	0.003583	protein_coding
ENSG0000(	ENSG0000(	79.21941	0.324167	0.000655	0.003594	lncRNA
ENSG0000(	ENSG0000(	121.9537	-0.27832	0.000656	0.003595	lncRNA
ENSG0000(	ENSG0000(	756.5595	-0.98441	0.000656	0.003599	TEC
ENSG0000(	FADS3	818.4514	0.323673	0.000657	0.003599	protein_coding
ENSG0000(	ENSG0000(	56.52066	0.515784	0.000657	0.003599	lncRNA
ENSG0000(	FIBP	413.1636	-0.21241	0.000657	0.003599	protein_coding
ENSG0000(	RBM34	700.391	-0.19046	0.000657	0.003599	protein_coding
ENSG0000(	ERICH6-AS:	924.7156	0.166112	0.000657	0.0036	lncRNA
ENSG0000(	DIRC3	278.2255	0.427588	0.000659	0.003607	lncRNA
ENSG0000(	ENSG0000(	115.4868	0.439296	0.000659	0.003608	lncRNA
ENSG0000(	ENSG0000(	476.5734	0.153471	0.00066	0.003611	TEC
ENSG0000(	ENSG0000(	32.55408	0.783168	0.00066	0.003614	lncRNA
ENSG0000(	ENSG0000(	141.8081	-0.34775	0.00066	0.003614	lncRNA
ENSG0000(	ASPN	131.9332	0.405821	0.000661	0.003615	protein_coding
ENSG0000(	ENSG0000(	9.803627	-0.92121	0.000662	0.003623	processed_pseudogene
ENSG0000(	TBX21	55.28778	0.662732	0.000664	0.003633	protein_coding
ENSG0000(	CYP51A1-A	99.78566	-0.26268	0.000664	0.003633	lncRNA
ENSG0000(	RN7SKP30	57.66131	0.35183	0.000664	0.003633	misc_RNA
ENSG0000(	NTRK1	146.696	0.30897	0.000665	0.003635	protein_coding
ENSG0000(	SPATA4	50.71161	0.375376	0.000665	0.003635	protein_coding
ENSG0000(	IFI16	1659.556	0.505581	0.000667	0.003642	protein_coding
ENSG0000(	SQOR	1574.845	-0.37022	0.000667	0.003642	protein_coding
ENSG0000(	TTC41P	95.97443	0.440092	0.000667	0.003642	transcribed_unitary_pseudogene
ENSG0000(	ITGA4	2715.243	0.446229	0.000667	0.003645	protein_coding
ENSG0000(	LINC01702	90.28882	-0.35593	0.000668	0.003647	lncRNA
ENSG0000(	ATG13	1516.645	0.133711	0.000669	0.003653	protein_coding
ENSG0000(	SNRPB2	260.1201	-0.34631	0.000669	0.003653	protein_coding
ENSG0000(	MPP1	408.8522	0.332599	0.00067	0.003657	protein_coding
ENSG0000(	ENSG0000(	263.841	0.342982	0.00067	0.003658	lncRNA
ENSG0000(	ENSG0000(	56.57863	-0.40488	0.00067	0.003658	lncRNA
ENSG0000(	EIF3G	786.8696	-0.17567	0.000671	0.003659	protein_coding
ENSG0000(	ENSG0000(	875.58	-0.22399	0.000671	0.003659	TEC

ENSG0000(	BRAP	698.233	-0.14393	0.000671	0.003661	protein_coding
ENSG0000(	DLX6-AS1	504.5742	-0.23748	0.000672	0.003662	lncRNA
ENSG0000(	AGR3	264.8122	-0.5349	0.000672	0.003665	protein_coding
ENSG0000(	ENSG0000(	130.7945	0.303738	0.000672	0.003665	lncRNA
ENSG0000(	HAGHL	131.3444	-0.58387	0.000673	0.003666	protein_coding
ENSG0000(	ABLIM3	362.5538	0.499761	0.000673	0.003666	protein_coding
ENSG0000(	TTC28	1757.379	0.273249	0.000674	0.003671	protein_coding
ENSG0000(	RPL3L	308.5844	0.266496	0.000674	0.003673	protein_coding
ENSG0000(	UBTF	1841.969	-0.15208	0.000674	0.003673	protein_coding
ENSG0000(	ENSG0000(	284.4726	0.271687	0.000675	0.003675	lncRNA
ENSG0000(	ENSG0000(	344.782	-0.53717	0.000676	0.003681	processed_pseudogene
ENSG0000(	ENSG0000(	181.3723	0.255046	0.000676	0.003682	lncRNA
ENSG0000(	RPRD2	2048.738	-0.13805	0.000677	0.003683	protein_coding
ENSG0000(	MIR941-1	68.32071	-0.6597	0.000678	0.003688	miRNA
ENSG0000(	EIF3C	3521.822	-0.24208	0.00068	0.0037	protein_coding
ENSG0000(	FAM157C	1260.873	0.520483	0.00068	0.0037	lncRNA
ENSG0000(	MPC1	637.0701	-0.37548	0.00068	0.0037	protein_coding
ENSG0000(	LMAN2	919.3672	-0.25151	0.000681	0.003704	protein_coding
ENSG0000(	SRSF8	994.5891	-0.27089	0.000682	0.003706	protein_coding
ENSG0000(	GAK	5698.109	-0.17368	0.000683	0.003712	protein_coding
ENSG0000(	RBM17P4	35.29705	-0.48747	0.000683	0.003712	processed_pseudogene
ENSG0000(	NPHS1	354.6486	0.290448	0.000683	0.003714	protein_coding
ENSG0000(	TDRD15	28.91656	0.756941	0.000685	0.003723	protein_coding
ENSG0000(	CZIB-DT	670.4367	0.158103	0.000685	0.003724	lncRNA
ENSG0000(	ENSG0000(	120.4656	0.247485	0.000687	0.003729	lncRNA
ENSG0000(	ZNF780A	2268.409	0.139294	0.000687	0.00373	protein_coding
ENSG0000(	ORMDL2	671.519	-0.23053	0.000687	0.003732	protein_coding
ENSG0000(	PSMA3-AS:	5214.978	0.130056	0.000688	0.003733	lncRNA
ENSG0000(	MED25	1219.515	-0.13889	0.000688	0.003733	protein_coding
ENSG0000(	GYS1	907.3975	-0.24636	0.000688	0.003734	protein_coding
ENSG0000(	GNL3	884.5229	-0.28073	0.000688	0.003735	protein_coding
ENSG0000(	IVNS1ABP	4981.802	0.307665	0.000689	0.00374	protein_coding
ENSG0000(	LINC02318	157.1998	0.276991	0.000691	0.003748	lncRNA
ENSG0000(	SULT1A2	511.569	-0.63129	0.000691	0.003748	protein_coding
ENSG0000(	SLC22A5	5876.158	-0.54752	0.000691	0.003749	protein_coding
ENSG0000(	SNX27	3960.83	0.214444	0.000692	0.003751	protein_coding
ENSG0000(	SUCO	1335.957	0.214517	0.000693	0.003755	protein_coding
ENSG0000(	ENSG0000(	19.72868	0.7195	0.000693	0.003755	lncRNA
ENSG0000(	UQCRFS1-[	169.1651	0.20003	0.000693	0.003756	lncRNA
ENSG0000(	RAPGEFL1	4414.26	-0.52673	0.000695	0.003765	protein_coding
ENSG0000(	MPDZ	708.7239	0.436002	0.000696	0.003771	protein_coding
ENSG0000(	ERG28	267.6665	-0.23789	0.000697	0.003774	protein_coding
ENSG0000(	RPS6KA1	2358.89	-0.27069	0.000697	0.003774	protein_coding
ENSG0000(	ACTL6A	404.6943	-0.28064	0.000697	0.003774	protein_coding
ENSG0000(	MCM6	519.4813	-0.36542	0.000697	0.003775	protein_coding
ENSG0000(	ENSG0000(	105.7847	0.309882	0.000698	0.003775	lncRNA
ENSG0000(	ENSG0000(	17.31191	0.698637	0.000698	0.003777	lncRNA

ENSG0000(	NCOA4	3397.532	-0.2977	0.000698	0.003777	protein_coding
ENSG0000(	SRSF7	2042.005	-0.15255	0.000698	0.003777	protein_coding
ENSG0000(	SLC29A2	496.6025	-0.41415	0.000701	0.003792	protein_coding
ENSG0000(	SPATS1	436.2423	0.275599	0.000701	0.003792	protein_coding
ENSG0000(	ENSG0000(	67.24018	-0.30262	0.000703	0.003802	lncRNA
ENSG0000(	ZNF780B	2754.556	-0.16271	0.000703	0.003802	protein_coding
ENSG0000(	CKMT1B	1757.688	-0.48676	0.000704	0.003805	protein_coding
ENSG0000(	NBL1	2848.337	-0.31552	0.000706	0.003812	protein_coding
ENSG0000(	PANK3	8599.357	-0.30967	0.000707	0.00382	protein_coding
ENSG0000(	CAPN3	1998.134	0.222244	0.000707	0.00382	protein_coding
ENSG0000(	GGCX	2536.859	0.117342	0.000708	0.003821	protein_coding
ENSG0000(	TTTY8B	12.12407	0.711835	0.000708	0.003824	lncRNA
ENSG0000(	AGFG1	3034.74	-0.15585	0.000709	0.003828	protein_coding
ENSG0000(	C11orf98	101.7015	-0.59854	0.000711	0.003838	protein_coding
ENSG0000(	RLIMP2	16.5405	0.916073	0.000711	0.003838	processed_pseudogene
ENSG0000(	CAMSAP3	929.7179	-0.47035	0.000711	0.003839	protein_coding
ENSG0000(	PTPRC	4934.051	0.602613	0.000712	0.003841	protein_coding
ENSG0000(	DPP3	547.3001	-0.33627	0.000712	0.003841	protein_coding
ENSG0000(	RN7SL801F	86.82986	0.317522	0.000713	0.003845	misc_RNA
ENSG0000(	LINC01517	248.2394	0.192441	0.000713	0.003846	lncRNA
ENSG0000(	FAM224A	74.35796	2.710325	0.000714	0.003849	lncRNA
ENSG0000(	ANKS1A	1489.167	-0.21606	0.000714	0.00385	protein_coding
ENSG0000(	ANAPC7	1414.494	-0.16646	0.000715	0.003851	protein_coding
ENSG0000(	HDDC2	511.3845	-0.21073	0.000715	0.003855	protein_coding
ENSG0000(	TTC27	483.9437	-0.3659	0.000716	0.003855	protein_coding
ENSG0000(	TFDP2	3100.637	-0.20763	0.000717	0.003861	protein_coding
ENSG0000(	GPATCH2	1117.013	0.17315	0.000719	0.003873	protein_coding
ENSG0000(	CD53	1300.046	0.644434	0.00072	0.003875	protein_coding
ENSG0000(	SEC31A	4426.812	0.162428	0.00072	0.003877	protein_coding
ENSG0000(	ENSG0000(	193.5434	-0.2268	0.000722	0.003885	protein_coding
ENSG0000(	TGFA	809.2588	-0.51366	0.000723	0.003889	protein_coding
ENSG0000(	SERPINB5	120.6954	0.873424	0.000724	0.003894	protein_coding
ENSG0000(	TMEM164	2258.093	-0.31847	0.000725	0.003898	protein_coding
ENSG0000(	CYP2E1	127.374	0.515201	0.000725	0.003901	protein_coding
ENSG0000(	KRT10	78.11887	-0.35035	0.000727	0.003907	protein_coding
ENSG0000(	ENSG0000(	16.04343	0.830812	0.000727	0.003912	lncRNA
ENSG0000(	LUZP1	3377.19	-0.22575	0.000728	0.003912	protein_coding
ENSG0000(	VDAC1P8	209.4154	0.193406	0.000728	0.003912	transcribed_processed_pseudoge
ENSG0000(	SMIM36	213.1306	0.232677	0.000728	0.003915	protein_coding
ENSG0000(	ZNF230-DT	48.30205	0.307559	0.000729	0.003919	lncRNA
ENSG0000(	LASP1	6253.69	-0.24141	0.00073	0.003922	protein_coding
ENSG0000(	ZNF875	2483.52	-0.12791	0.00073	0.003922	protein_coding
ENSG0000(	BRCC3	914.5583	-0.17235	0.000731	0.003927	protein_coding
ENSG0000(	SLC35F1	181.4897	-0.21245	0.000733	0.003937	protein_coding
ENSG0000(	LRP12	885.026	-0.24118	0.000734	0.00394	protein_coding
ENSG0000(	BRD2	5661.149	-0.12612	0.000736	0.00395	protein_coding
ENSG0000(	GAS6-AS1	366.5651	0.545422	0.000736	0.00395	lncRNA

ENSG0000(	RPL22L1	215.4994	-0.3526	0.000738	0.003963	protein_coding
ENSG0000(	CASC3	1859.066	-0.10024	0.000739	0.003964	protein_coding
ENSG0000(	LOXL2	629.8527	0.324519	0.000739	0.003967	protein_coding
ENSG0000(	BTNL9	607.548	0.24459	0.000741	0.003972	protein_coding
ENSG0000(	PPP1R12C	2137.158	-0.1846	0.000741	0.003972	protein_coding
ENSG0000(	TIPRL	742.6994	-0.18077	0.000741	0.003972	protein_coding
ENSG0000(	EPS8L2	3219.438	-0.4943	0.000741	0.003973	protein_coding
ENSG0000(	TRAF4	1399.72	-0.26528	0.000742	0.003978	protein_coding
ENSG0000(	ENSG0000(	88.49199	0.317862	0.000743	0.003979	lncRNA
ENSG0000(	BCKDHA	232.6058	-0.27923	0.000743	0.003982	protein_coding
ENSG0000(	LRPAP1	2387.348	-0.16701	0.000743	0.003982	protein_coding
ENSG0000(	RN7SKP118	116.1739	-0.7465	0.000743	0.003982	misc_RNA
ENSG0000(	ENSG0000(	18.21006	1.296654	0.000744	0.003982	lncRNA
ENSG0000(	ARMC7	361.0888	-0.33831	0.000745	0.003988	protein_coding
ENSG0000(	H2AC15	43.65961	-0.5016	0.000745	0.003988	protein_coding
ENSG0000(	GPA33	4778.911	-0.56067	0.000746	0.003993	protein_coding
ENSG0000(	SYDE1	193.3334	0.419918	0.000747	0.003998	protein_coding
ENSG0000(	TMEM154	1345.92	0.449768	0.000748	0.004	protein_coding
ENSG0000(	LINC02641	103.2388	0.391465	0.00075	0.004014	lncRNA
ENSG0000(	LINC01791	17.46586	0.661024	0.000751	0.004019	lncRNA
ENSG0000(	PDE4DIPP6	215.3927	0.243002	0.000754	0.004032	transcribed_unprocessed_pseudo
ENSG0000(	ENSG0000(	118.7162	0.313593	0.000754	0.004034	lncRNA
ENSG0000(	IGHV3-35	25.77548	0.897094	0.000755	0.004037	IG_V_gene
ENSG0000(	ASGR1	261.5391	0.284072	0.000756	0.004039	protein_coding
ENSG0000(	ZCCHC24	502.9928	0.383026	0.000757	0.004043	protein_coding
ENSG0000(	BCAS3	1771.026	0.158458	0.000757	0.004046	protein_coding
ENSG0000(	ENSG0000(	74.23114	0.383075	0.000757	0.004046	lncRNA
ENSG0000(	LINC02466	63.82597	0.328995	0.000758	0.004048	lncRNA
ENSG0000(	ZNF99	225.0593	-0.2831	0.000758	0.004048	protein_coding
ENSG0000(	PBX3	525.8622	0.35306	0.000759	0.004055	protein_coding
ENSG0000(	MTMR11	5039.442	-0.43994	0.00076	0.004057	protein_coding
ENSG0000(	ENSG0000(	468.8511	0.398924	0.00076	0.004058	protein_coding
ENSG0000(	SLTM	3213.299	-0.14216	0.000761	0.004061	protein_coding
ENSG0000(	ENSG0000(	20.87328	-0.92434	0.000761	0.004062	lncRNA
ENSG0000(	LY75	2198.54	0.251117	0.000762	0.004065	protein_coding
ENSG0000(	ENSG0000(	17.50743	1.426684	0.000763	0.004067	processed_pseudogene
ENSG0000(	RN7SL233F	40.65999	0.442953	0.000763	0.004067	misc_RNA
ENSG0000(	ZNF254	1407.525	-0.16608	0.000763	0.004069	protein_coding
ENSG0000(	POLR3K	554.0423	-0.1674	0.000765	0.004078	protein_coding
ENSG0000(	LINC00534	1086.972	0.119655	0.000765	0.00408	lncRNA
ENSG0000(	FOXL2NB	786.8524	0.193124	0.000766	0.004084	protein_coding
ENSG0000(	GPR68	109.4389	0.63303	0.000767	0.004085	protein_coding
ENSG0000(	ENSG0000(	64.25014	-0.67469	0.000768	0.004089	miRNA
ENSG0000(	DGCR2	1927.742	-0.19119	0.000768	0.004091	protein_coding
ENSG0000(	AGL	1228.59	-0.22629	0.000769	0.004094	protein_coding
ENSG0000(	SNHG28	161.4712	-0.42571	0.000772	0.004111	lncRNA
ENSG0000(	PPP1R3D	289.0598	0.211484	0.000773	0.004115	protein_coding

ENSG0000(	ENSG0000(	276.853	0.204029	0.000774	0.004118	lncRNA
ENSG0000(	FUNDC2	613.4185	-0.19053	0.000774	0.004119	protein_coding
ENSG0000(	MCF2L-AS1	20.82905	-0.70187	0.000777	0.004132	lncRNA
ENSG0000(	MIEF2	435.175	-0.21347	0.000777	0.004134	protein_coding
ENSG0000(	CACNA1A	3582.483	0.102759	0.000778	0.004139	protein_coding
ENSG0000(	HYI	616.8819	-0.28784	0.000778	0.004139	protein_coding
ENSG0000(	ENSG0000(	198.1677	-0.23619	0.000779	0.004141	lncRNA
ENSG0000(	VASH1-AS1	478.5718	0.163907	0.000779	0.004142	lncRNA
ENSG0000(	SLITRK6	612.2617	-0.63301	0.00078	0.004145	protein_coding
ENSG0000(	A2ML1	299.5803	0.164959	0.00078	0.004146	protein_coding
ENSG0000(	FBXL7	305.1788	0.318288	0.00078	0.004146	protein_coding
ENSG0000(	SPINT1-AS1	164.6781	-0.44435	0.00078	0.004146	lncRNA
ENSG0000(	ANK1	142.8301	0.582508	0.000781	0.004149	protein_coding
ENSG0000(	SH2D6	212.9626	-0.92664	0.000782	0.004152	protein_coding
ENSG0000(	CDC123	355.4876	-0.28579	0.000783	0.004157	protein_coding
ENSG0000(	ZNF343	612.4808	-0.144	0.000783	0.004158	protein_coding
ENSG0000(	DNAJC17	849.9062	-0.1788	0.000784	0.004164	protein_coding
ENSG0000(	ASB13	539.782	-0.37424	0.000785	0.004168	protein_coding
ENSG0000(	ENSG0000(	11.81391	0.72096	0.000786	0.004171	lncRNA
ENSG0000(	ADPRHL1	316.9831	-0.26607	0.000787	0.004177	protein_coding
ENSG0000(	USP40	1428.98	0.200362	0.000788	0.004181	protein_coding
ENSG0000(	ENSG0000(	186.7419	-0.23362	0.00079	0.004189	TEC
ENSG0000(	RAB5IF	667.9966	-0.38782	0.00079	0.004189	protein_coding
ENSG0000(	BRME1	167.9425	-0.51544	0.000791	0.004196	protein_coding
ENSG0000(	ENSG0000(	13994.8	-0.30169	0.000793	0.004203	lncRNA
ENSG0000(	CDCA2	238.9617	-0.46745	0.000793	0.004206	protein_coding
ENSG0000(	MMP15	1362.099	-0.48519	0.000794	0.004206	protein_coding
ENSG0000(	ENSG0000(	30.58438	-0.53597	0.000795	0.00421	lncRNA
ENSG0000(	ENSG0000(	60.10047	0.387164	0.000796	0.004219	lncRNA
ENSG0000(	CEP192	3715.099	-0.22792	0.000798	0.004227	protein_coding
ENSG0000(	PIAS1	2709.103	0.149561	0.000798	0.004227	protein_coding
ENSG0000(	TRPM2	600.5776	0.415789	0.000798	0.004228	protein_coding
ENSG0000(	LZTS3	165.3213	0.423248	0.000799	0.00423	protein_coding
ENSG0000(	FXN	1095.912	-0.15639	0.0008	0.004234	protein_coding
ENSG0000(	RLIM	2850.839	0.159679	0.0008	0.004234	protein_coding
ENSG0000(	CA6	274.2192	-0.18621	0.000802	0.004242	protein_coding
ENSG0000(	DDX10	2021.866	-0.18817	0.000802	0.004245	protein_coding
ENSG0000(	STARD13-IT	70.025	0.425543	0.000803	0.004247	lncRNA
ENSG0000(	PTENP1-AS	130.9042	0.265976	0.000804	0.004255	lncRNA
ENSG0000(	ENSG0000(	366.0572	-0.24853	0.000805	0.004257	lncRNA
ENSG0000(	PARP12	1998.076	0.30064	0.000806	0.004261	protein_coding
ENSG0000(	SCAF1	782.8998	-0.23136	0.000806	0.004263	protein_coding
ENSG0000(	LRP8	1207.135	0.340751	0.000807	0.004263	protein_coding
ENSG0000(	ENSG0000(	27.27359	0.508882	0.000807	0.004265	snoRNA
ENSG0000(	ACTR6	335.8218	-0.18937	0.000807	0.004266	protein_coding
ENSG0000(	VSIG4	140.4524	0.616734	0.000808	0.004266	protein_coding
ENSG0000(	PEMT	68.27531	-0.50406	0.000808	0.004269	protein_coding

ENSG0000(	TECRL	129.92	0.275172	0.000809	0.004271	protein_coding
ENSG0000(	LSM14B	685.9566	-0.15306	0.000809	0.004272	protein_coding
ENSG0000(	PPP1R16A	1619.632	-0.29163	0.00081	0.004275	protein_coding
ENSG0000(	PABPC1	11667.65	-0.23518	0.000812	0.004287	protein_coding
ENSG0000(	ZNF384	1286.88	-0.1225	0.000813	0.00429	protein_coding
ENSG0000(	SLFN5	3226.682	0.476589	0.000814	0.004297	protein_coding
ENSG0000(	AGBL5	354.2656	-0.20208	0.000815	0.004297	protein_coding
ENSG0000(	COA8	870.3543	-0.18301	0.000815	0.004298	protein_coding
ENSG0000(	ENSG0000(	286.669	0.216628	0.000815	0.0043	processed_pseudogene
ENSG0000(	POLQ	1036.303	-0.2185	0.000816	0.0043	protein_coding
ENSG0000(	ENSG0000(	29.95896	-0.70811	0.000819	0.004317	processed_pseudogene
ENSG0000(	RN7SL670F	417.5206	-0.1789	0.000819	0.004317	misc_RNA
ENSG0000(	RCAN1	1566.095	0.4706	0.000821	0.004328	protein_coding
ENSG0000(	PRADC1	128.9696	-0.42996	0.000823	0.004334	protein_coding
ENSG0000(	ENSG0000(	51.67559	-0.42355	0.000824	0.00434	lncRNA
ENSG0000(	PHACTR1	1313.058	0.254836	0.000824	0.004343	protein_coding
ENSG0000(	DDR1	3701.37	-0.36351	0.000827	0.004357	protein_coding
ENSG0000(	SOCS6	1683.065	-0.31258	0.000827	0.004357	protein_coding
ENSG0000(	RNF157	1255.999	-0.35618	0.000829	0.004364	protein_coding
ENSG0000(	ARHGEF39	779.3877	-0.21511	0.00083	0.004366	protein_coding
ENSG0000(	OR7D2	562.7335	-0.15025	0.000831	0.004372	protein_coding
ENSG0000(	SLC43A3	411.7067	0.398885	0.000833	0.004382	protein_coding
ENSG0000(	LITAF	3809.143	0.441349	0.000836	0.0044	protein_coding
ENSG0000(	RGPD5	6154.663	0.16685	0.000837	0.0044	protein_coding
ENSG0000(	AP1M2	476.1068	-0.44131	0.000838	0.004405	protein_coding
ENSG0000(	PRMT5	501.7955	-0.34908	0.000838	0.004406	protein_coding
ENSG0000(	ZC3H8	1177.919	-0.21347	0.000839	0.004413	protein_coding
ENSG0000(	OR2L2	684.6366	-0.24766	0.00084	0.004414	protein_coding
ENSG0000(	ENSG0000(	83.51488	0.396406	0.000841	0.004417	lncRNA
ENSG0000(	SNORA40B	20.3473	0.679799	0.00084	0.004417	snoRNA
ENSG0000(	KLHL11	819.1174	-0.14478	0.000841	0.004417	protein_coding
ENSG0000(	HLTF-AS1	38.03179	0.522021	0.000841	0.004417	lncRNA
ENSG0000(	ENSG0000(	14.62746	0.764366	0.000841	0.004418	lncRNA
ENSG0000(	CCDC80	1465.169	0.463702	0.000842	0.004421	protein_coding
ENSG0000(	ASNSP3	12.44842	-0.77199	0.000842	0.004422	processed_pseudogene
ENSG0000(	GTPBP1	3434.218	0.248506	0.000844	0.004433	protein_coding
ENSG0000(	TEX11	63.65771	-0.56103	0.000845	0.004434	protein_coding
ENSG0000(	EFL1	933.7593	-0.32307	0.000845	0.004435	protein_coding
ENSG0000(	LINC02225	263.0959	0.221321	0.000846	0.004439	lncRNA
ENSG0000(	PSMD6	1194.476	-0.14934	0.000847	0.004444	protein_coding
ENSG0000(	ANKRD26P	43.38089	0.46275	0.000847	0.004445	processed_pseudogene
ENSG0000(	CYYR1-AS1	701.346	0.139798	0.000848	0.004446	lncRNA
ENSG0000(	PEX16	354.2531	-0.24806	0.000848	0.004447	protein_coding
ENSG0000(	DRAM2	877.9291	0.261233	0.000851	0.00446	protein_coding
ENSG0000(	ENSG0000(	137.2427	-0.24234	0.000852	0.004465	lncRNA
ENSG0000(	MMADHCP	196.4265	0.592538	0.000852	0.004465	processed_pseudogene
ENSG0000(	LINC02043	496.1557	0.227068	0.000852	0.004465	lncRNA

ENSG0000(	PLA2G12A	1610.523	-0.26483	0.000852	0.004465	protein_coding
ENSG0000(	ENSG0000(	10.4665	-0.80973	0.000853	0.004466	lncRNA
ENSG0000(	ARRB2	1026.085	0.371974	0.000853	0.004469	protein_coding
ENSG0000(	RPL27	1347.605	-0.40775	0.000854	0.00447	protein_coding
ENSG0000(	SDHD	1775.294	-0.28599	0.000854	0.00447	protein_coding
ENSG0000(	TUBB6	313.1436	0.446368	0.000854	0.004471	protein_coding
ENSG0000(	PLK4	277.4693	-0.47472	0.000855	0.004476	protein_coding
ENSG0000(	NFIB	3424.213	0.181878	0.000858	0.004488	protein_coding
ENSG0000(	INSIG1	685.3444	0.473671	0.000858	0.004492	protein_coding
ENSG0000(	ENSG0000(	161.5347	0.209981	0.000859	0.004494	TEC
ENSG0000(	NSFL1C	1083.25	0.175491	0.000859	0.004495	protein_coding
ENSG0000(	ENSG0000(	150.733	0.25805	0.00086	0.004495	lncRNA
ENSG0000(	LRG1	570.0817	0.236318	0.00086	0.004495	protein_coding
ENSG0000(	GPR65	376.7228	0.444123	0.000862	0.004506	protein_coding
ENSG0000(	WASF2	4656.236	-0.15614	0.000862	0.004507	protein_coding
ENSG0000(	ELAVL2	84.4733	-0.36213	0.000864	0.004514	protein_coding
ENSG0000(	GLRX5	373.5279	-0.32712	0.000864	0.004514	protein_coding
ENSG0000(	OVOL2	330.4953	-0.52362	0.000865	0.004516	protein_coding
ENSG0000(	TSPYL1	1978.335	-0.16061	0.000865	0.004516	protein_coding
ENSG0000(	ZNF25	417.7488	0.234941	0.000865	0.004516	protein_coding
ENSG0000(	ARL14	774.0146	-0.66911	0.000866	0.004521	protein_coding
ENSG0000(	ENSG0000(	150.0576	0.258053	0.000866	0.004521	lncRNA
ENSG0000(	LINC00703	27.93038	0.433949	0.000868	0.004531	lncRNA
ENSG0000(	ASGR2	16.9221	0.83594	0.000869	0.004533	protein_coding
ENSG0000(	LINC00314	33.25963	0.670188	0.000869	0.004535	lncRNA
ENSG0000(	PPIC	466.2275	-0.33019	0.00087	0.004538	protein_coding
ENSG0000(	CLCN4	128.7642	0.608106	0.00087	0.004538	protein_coding
ENSG0000(	ENSG0000(	23.14119	-0.58141	0.00087	0.004538	transcribed_unprocessed_pseudo
ENSG0000(	ENSG0000(	66.74515	0.296686	0.000872	0.004549	lncRNA
ENSG0000(	CELA3B	74.8676	-0.62714	0.000873	0.00455	protein_coding
ENSG0000(	EBF2	73.34986	0.408429	0.000873	0.00455	protein_coding
ENSG0000(	XRN2	1044.687	-0.19815	0.000873	0.00455	protein_coding
ENSG0000(	APEX2	449.163	-0.21899	0.000874	0.004553	protein_coding
ENSG0000(	ENSG0000(	169.3771	0.234498	0.000874	0.004554	TEC
ENSG0000(	ENSG0000(	27.79682	0.666207	0.000875	0.004555	lncRNA
ENSG0000(	ENSG0000(	22.46881	0.53068	0.000875	0.004555	lncRNA
ENSG0000(	RPA1	712.4086	-0.20259	0.000875	0.004555	protein_coding
ENSG0000(	ENSG0000(	343.2824	0.313319	0.000876	0.004561	lncRNA
ENSG0000(	RN7SL457F	27.94587	-0.45082	0.000876	0.004562	misc_RNA
ENSG0000(	NMI	455.939	0.304529	0.000877	0.004567	protein_coding
ENSG0000(	ENSG0000(	70.21475	0.562154	0.000878	0.004569	processed_pseudogene
ENSG0000(	PHF12	3004.576	0.130187	0.000878	0.004569	protein_coding
ENSG0000(	UTP18	309.3611	-0.25754	0.000878	0.00457	protein_coding
ENSG0000(	BRK1	538.3728	-0.327	0.00088	0.004575	protein_coding
ENSG0000(	ENSG0000(	295.7988	0.184918	0.00088	0.004577	processed_pseudogene
ENSG0000(	PPP2R1A	2560.037	-0.24324	0.00088	0.004577	protein_coding
ENSG0000(	TACSTD2	26.69716	0.98107	0.000881	0.00458	protein_coding

ENSG0000(	VANGL1	972.9577	-0.4269	0.000881	0.004582	protein_coding
ENSG0000(	SLC15A3	800.5012	0.447954	0.000883	0.004588	protein_coding
ENSG0000(	LINC01093	138.2185	0.300571	0.000884	0.004592	lncRNA
ENSG0000(	LINC00159	197.4288	0.252449	0.000884	0.004594	lncRNA
ENSG0000(	RB1	1046.587	0.268482	0.000884	0.004595	protein_coding
ENSG0000(	ENSG0000(	9.109887	0.883687	0.000885	0.004596	lncRNA
ENSG0000(	MMP2-AS1	119.0792	0.74596	0.000886	0.004601	lncRNA
ENSG0000(	ATF5	351.8509	0.274995	0.000886	0.004602	protein_coding
ENSG0000(	MVB12B	1009.668	-0.29187	0.000886	0.004602	protein_coding
ENSG0000(	ENSG0000(	22.53496	0.849218	0.000887	0.004604	TEC
ENSG0000(	ENSG0000(	335.9182	0.184515	0.000888	0.004609	lncRNA
ENSG0000(	LRIG2-DT	659.5506	-0.17161	0.000888	0.004609	lncRNA
ENSG0000(	SLC28A2	1145.861	-0.35381	0.000888	0.004609	protein_coding
ENSG0000(	HS6ST2	20.56191	1.018145	0.000889	0.004613	protein_coding
ENSG0000(	SNORA63E	9.919042	0.786686	0.00089	0.004615	snoRNA
ENSG0000(	PHLDB2	1092.957	0.38288	0.000891	0.00462	protein_coding
ENSG0000(	LINC02558	905.0178	0.160304	0.000892	0.004622	lncRNA
ENSG0000(	ENSG0000(	473.4665	-0.15562	0.000892	0.004624	lncRNA
ENSG0000(	ZSCAN2	465.7905	-0.24967	0.000892	0.004625	protein_coding
ENSG0000(	ENSG0000(	18.10207	1.018649	0.000895	0.004637	lncRNA
ENSG0000(	RPL21P44	87.3063	0.381317	0.000896	0.004642	processed_pseudogene
ENSG0000(	LAMB1	7600.899	0.264895	0.000898	0.004653	protein_coding
ENSG0000(	SYNPO2L-A	177.8063	0.256131	0.000899	0.004658	lncRNA
ENSG0000(	NIBAN2	2351.599	-0.30016	0.0009	0.004658	protein_coding
ENSG0000(	STAT4	351.756	0.381823	0.0009	0.004661	protein_coding
ENSG0000(	ENSG0000(	523.2629	0.170963	0.000901	0.004664	lncRNA
ENSG0000(	RBM39	10731.01	0.11024	0.000901	0.004665	protein_coding
ENSG0000(	ENSG0000(	36.92507	-0.84542	0.000902	0.004665	processed_pseudogene
ENSG0000(	TYMS	450.1794	-0.34386	0.000902	0.004666	protein_coding
ENSG0000(	ARPC1A	863.9282	-0.27829	0.000903	0.004669	protein_coding
ENSG0000(	XRCC1	285.8815	-0.21311	0.000905	0.00468	protein_coding
ENSG0000(	HAVCR2	546.6731	0.263824	0.000906	0.004683	protein_coding
ENSG0000(	LINC00664	566.514	-0.154	0.000905	0.004683	lncRNA
ENSG0000(	SETD6	1250.009	-0.18258	0.000906	0.004683	protein_coding
ENSG0000(	ZMYND15	80.07339	0.574794	0.000906	0.004683	protein_coding
ENSG0000(	GUCY2C	1579.856	-0.45269	0.000906	0.004683	protein_coding
ENSG0000(	UMPS	1259.479	-0.22969	0.000906	0.004683	protein_coding
ENSG0000(	ENSG0000(	450.6432	0.156467	0.000908	0.004688	lncRNA
ENSG0000(	PRAP1	276.11	-0.83862	0.000907	0.004688	protein_coding
ENSG0000(	ADGRF5	620.1057	0.482688	0.000909	0.004696	protein_coding
ENSG0000(	ATP2A2	8161.243	-0.1738	0.000909	0.004697	protein_coding
ENSG0000(	GNA13	3285.586	-0.29394	0.00091	0.004697	protein_coding
ENSG0000(	CDK9	1016.318	-0.1916	0.000911	0.004702	protein_coding
ENSG0000(	ENSG0000(	20.12901	0.569614	0.000911	0.004702	TEC
ENSG0000(	GNAI2	1471.551	0.366112	0.000911	0.004702	protein_coding
ENSG0000(	PSRC1	99.01153	-0.41136	0.000911	0.004702	protein_coding
ENSG0000(	ENSG0000(	23.903	0.493905	0.000912	0.004702	lncRNA

ENSG0000(	ENSG0000(	252.2633	0.207859	0.000913	0.004708	protein_coding
ENSG0000(	C3orf36	86.4911	0.649589	0.000913	0.004708	lncRNA
ENSG0000(	YARS2	422.5962	-0.14976	0.000913	0.004709	protein_coding
ENSG0000(	SCRIB	1274.539	-0.26818	0.000914	0.00471	protein_coding
ENSG0000(	DCAF12	941.3942	-0.18165	0.000914	0.004711	protein_coding
ENSG0000(	RWDD2A	228.3195	0.287535	0.000914	0.004713	protein_coding
ENSG0000(	ENSG0000(	295.785	0.18904	0.000916	0.00472	lncRNA
ENSG0000(	VNN2	185.875	0.659647	0.000916	0.00472	protein_coding
ENSG0000(	TBC1D30	506.5848	-0.22595	0.000916	0.004721	protein_coding
ENSG0000(	RPS6KA2-IT	34.16974	0.458475	0.000917	0.004723	lncRNA
ENSG0000(	CBLL1	747.9732	-0.16437	0.000918	0.004724	protein_coding
ENSG0000(	CD47	10490.74	0.374317	0.000918	0.004724	protein_coding
ENSG0000(	RUNX3	785.345	0.439686	0.00092	0.004735	protein_coding
ENSG0000(	ENSG0000(	112.1115	0.261172	0.000921	0.004739	lncRNA
ENSG0000(	ARL4D	28.22132	-0.60321	0.000921	0.004741	protein_coding
ENSG0000(	HTATSF1P2	26.33674	0.734677	0.000921	0.004741	processed_pseudogene
ENSG0000(	BCL7A	1060.829	-0.28381	0.000922	0.004743	protein_coding
ENSG0000(	PRRT3-AS1	47.29557	-0.4394	0.000922	0.004743	lncRNA
ENSG0000(	SLC12A7	3078.243	-0.31433	0.000924	0.004752	protein_coding
ENSG0000(	SUPT16H	1655.572	-0.14076	0.000924	0.004753	protein_coding
ENSG0000(	ZNF165	204.9243	-0.43575	0.000926	0.004762	protein_coding
ENSG0000(	RAB24	430.3493	0.294268	0.000926	0.004762	protein_coding
ENSG0000(	CD82	777.1804	0.454468	0.000929	0.004773	protein_coding
ENSG0000(	GHRL	33.96537	0.662266	0.000929	0.004776	protein_coding
ENSG0000(	ZFPM2	459.3457	0.240994	0.000933	0.004794	protein_coding
ENSG0000(	EPM2A	1033.363	0.160434	0.000934	0.0048	protein_coding
ENSG0000(	CELA3A	22.81446	-1.25567	0.000937	0.004812	protein_coding
ENSG0000(	INSIG2	702.0959	0.241722	0.000937	0.004812	protein_coding
ENSG0000(	SLC6A4	292.0832	-0.27158	0.000937	0.004812	protein_coding
ENSG0000(	ZGLP1	58.96036	-0.59247	0.000938	0.004813	protein_coding
ENSG0000(	PRIMPOL	282.6447	-0.27637	0.000938	0.004815	protein_coding
ENSG0000(	IGKJ5	86.57147	0.668236	0.000939	0.004817	IG_J_gene
ENSG0000(	ENSG0000(	521.5521	0.147781	0.00094	0.004824	lncRNA
ENSG0000(	FCGRT	3340.838	-0.41876	0.000941	0.004827	protein_coding
ENSG0000(	ENSG0000(	18.62651	0.646491	0.000942	0.004831	lncRNA
ENSG0000(	CYP26C1	13.00902	0.835656	0.000943	0.004839	protein_coding
ENSG0000(	POLD4	1666.603	-0.29488	0.000944	0.004842	protein_coding
ENSG0000(	SNRNP25	129.731	-0.40245	0.000945	0.004844	protein_coding
ENSG0000(	NCAPD2	1145.967	-0.26066	0.000947	0.004853	protein_coding
ENSG0000(	DUS3L	349.8058	-0.29241	0.000947	0.004854	protein_coding
ENSG0000(	SETD7	1916.56	0.203494	0.000949	0.004863	protein_coding
ENSG0000(	SLC36A3	113.1077	-0.24677	0.000951	0.00487	protein_coding
ENSG0000(	ENSG0000(	523.5649	0.129234	0.000951	0.00487	lncRNA
ENSG0000(	ENSG0000(	352.9765	0.294314	0.000954	0.004884	lncRNA
ENSG0000(	CICP14	26.71287	1.311263	0.000955	0.004889	processed_pseudogene
ENSG0000(	NKAP	1591.695	-0.18106	0.000957	0.004898	protein_coding
ENSG0000(	ENSG0000(	1255.093	0.358429	0.000959	0.004907	lncRNA

ENSG0000(	ENSG0000(	220.8978	0.392318	0.000959	0.004907	lncRNA
ENSG0000(	UBE2N	1398.993	-0.21561	0.000959	0.004909	protein_coding
ENSG0000(	NEIL3	142.1252	-0.30437	0.00096	0.004909	protein_coding
ENSG0000(	UGT1A4	387.3922	-0.71797	0.000959	0.004909	protein_coding
ENSG0000(	ENSG0000(	20.5874	0.838465	0.00096	0.00491	lncRNA
ENSG0000(	RILP	260.6893	-0.41403	0.000961	0.004913	protein_coding
ENSG0000(	ENSG0000(	72.20338	-0.66326	0.000961	0.004915	lncRNA
ENSG0000(	LY96	31.93638	0.752606	0.000961	0.004916	protein_coding
ENSG0000(	CCR3	71.90552	0.467183	0.000962	0.004917	protein_coding
ENSG0000(	TPM3P7	37.49666	-0.50243	0.000962	0.004918	processed_pseudogene
ENSG0000(	NLRP1	2148.444	0.369939	0.000964	0.004925	protein_coding
ENSG0000(	INHBB	43.7703	0.658192	0.000964	0.004928	protein_coding
ENSG0000(	RDM1	100.4938	0.576499	0.000965	0.004931	protein_coding
ENSG0000(	LINC00868	210.9518	0.355895	0.000967	0.004938	lncRNA
ENSG0000(	ENSG0000(	34.62798	0.488105	0.000968	0.004942	lncRNA
ENSG0000(	ENSG0000(	232.906	0.201562	0.000969	0.004945	lncRNA
ENSG0000(	MIR5192	20.08581	0.596068	0.000969	0.004945	miRNA
ENSG0000(	NRBP2	1274.071	0.28019	0.000973	0.004966	protein_coding
ENSG0000(	ENSG0000(	111.7676	-0.2964	0.000975	0.004976	lncRNA
ENSG0000(	SDAD1	674.198	-0.28318	0.000975	0.004976	protein_coding
ENSG0000(	AQR	2509.73	-0.13625	0.000976	0.004981	protein_coding
ENSG0000(	HNRNPD	3479.595	-0.16274	0.000977	0.004985	protein_coding
ENSG0000(	CDC37	1431.576	-0.22302	0.000978	0.004986	protein_coding
ENSG0000(	FRY	1084.347	0.461074	0.000978	0.004986	protein_coding
ENSG0000(	CASP2	2144.143	-0.14068	0.00098	0.004996	protein_coding
ENSG0000(	DBF4	430.3324	-0.35608	0.000981	0.005003	protein_coding
ENSG0000(	CREB1	2348.17	0.194139	0.000984	0.005015	protein_coding
ENSG0000(	TNKS2-AS1	80.61218	-0.3313	0.000984	0.005016	lncRNA
ENSG0000(	WSB2	1686.097	-0.27398	0.000985	0.005018	protein_coding
ENSG0000(	ENSG0000(	33.39953	0.548255	0.000986	0.005021	lncRNA
ENSG0000(	GOLGA8R	692.5782	0.195093	0.000986	0.005023	protein_coding
ENSG0000(	NR2F2-AS1	587.0635	0.342676	0.000987	0.005026	lncRNA
ENSG0000(	RAB34	175.3811	0.398917	0.000988	0.005031	protein_coding
ENSG0000(	SP2-DT	824.7979	0.215043	0.000989	0.005034	lncRNA
ENSG0000(	ENSG0000(	44.81026	-0.39675	0.000989	0.005035	lncRNA
ENSG0000(	CA12	10138.12	-0.46812	0.00099	0.005037	protein_coding
ENSG0000(	TRAPPC11	1407.239	-0.19782	0.00099	0.005037	protein_coding
ENSG0000(	ENSG0000(	166.3157	0.262529	0.000993	0.005053	lncRNA
ENSG0000(	IREB2	3300.776	-0.12653	0.000995	0.005061	protein_coding
ENSG0000(	H2BP1	64.59241	0.712006	0.000996	0.005063	transcribed_unprocessed_pseudo
ENSG0000(	MARCHF6	6608.98	0.137615	0.000996	0.005064	protein_coding
ENSG0000(	DCAF4L1	108.8082	0.295598	0.000999	0.005078	protein_coding
ENSG0000(	LDAH	657.0904	-0.22633	0.001	0.005081	protein_coding
ENSG0000(	SLC39A8	1618.686	0.373692	0.001	0.005081	protein_coding
ENSG0000(	TOGARAM:	761.7824	0.155293	0.001	0.005083	protein_coding
ENSG0000(	LINC02263	259.1621	0.208692	0.001001	0.005087	lncRNA
ENSG0000(	RN7SL443F	16.25785	0.757088	0.001003	0.005093	misc_RNA

ENSG0000(	OR55B1P	330.2753	0.281698	0.001005	0.005105	transcribed_unitary_pseudogene
ENSG0000(	STXBP2	1293.446	-0.23568	0.001005	0.005106	protein_coding
ENSG0000(	MAPK11	116.9268	0.520886	0.001007	0.005112	protein_coding
ENSG0000(	LINC02071	218.8012	0.184142	0.00101	0.005128	lncRNA
ENSG0000(	ACTG1	17914.86	-0.33557	0.001011	0.005132	protein_coding
ENSG0000(	ENSG0000(	120.4693	0.247392	0.001017	0.005161	lncRNA
ENSG0000(	GOLGA2P1	294.8686	0.195657	0.001019	0.00517	transcribed_unprocessed_pseudo
ENSG0000(	SNORA72	130.0865	0.35999	0.001019	0.00517	snoRNA
ENSG0000(	ENSG0000(	15.64221	0.742688	0.00102	0.005175	lncRNA
ENSG0000(	IGHV3-33-2	9.331039	1.255201	0.001021	0.005179	IG_V_pseudogene
ENSG0000(	GLIPR1	752.4334	0.476183	0.001023	0.005186	protein_coding
ENSG0000(	NPIPB11	648.7616	0.366261	0.001023	0.005186	protein_coding
ENSG0000(	PMVK	132.5183	-0.34818	0.001024	0.005192	protein_coding
ENSG0000(	ANKRD13C	960.4207	-0.17159	0.001029	0.005218	protein_coding
ENSG0000(	BBC3	198.7636	0.397529	0.00103	0.005221	protein_coding
ENSG0000(	QRICH2	311.8127	-0.23373	0.001031	0.005226	protein_coding
ENSG0000(	ENSG0000(	173.804	-0.25289	0.001032	0.005228	lncRNA
ENSG0000(	ENSG0000(	117.4932	0.29401	0.001032	0.005228	TEC
ENSG0000(	SLC25A13	739.2721	-0.2896	0.001032	0.005228	protein_coding
ENSG0000(	ENSG0000(	281.8109	0.316669	0.001037	0.005249	lncRNA
ENSG0000(	UBXN1	352.9237	-0.26387	0.001037	0.005249	protein_coding
ENSG0000(	CHMP5	522.79	-0.28837	0.001038	0.005255	protein_coding
ENSG0000(	LPAR6	796.5351	0.309966	0.00104	0.005263	protein_coding
ENSG0000(	PDE4DIP	5390.534	0.230046	0.001041	0.005267	protein_coding
ENSG0000(	ACSS3	361.4282	0.276573	0.001042	0.005271	protein_coding
ENSG0000(	LINC01733	211.8056	-0.27928	0.001042	0.005271	lncRNA
ENSG0000(	YDJC	181.5353	-0.24146	0.001042	0.005272	protein_coding
ENSG0000(	NOTCH2NL	112.2987	0.804288	0.001043	0.005273	protein_coding
ENSG0000(	RN7SL116F	75.30561	0.505119	0.001043	0.005273	misc_RNA
ENSG0000(	LINC01127	83.57397	0.421667	0.001045	0.00528	lncRNA
ENSG0000(	TRMT1	499.5336	-0.32017	0.001045	0.00528	protein_coding
ENSG0000(	CENATAC	1764.588	0.143784	0.001045	0.005281	protein_coding
ENSG0000(	CTR9	765.4012	-0.24185	0.001046	0.005287	protein_coding
ENSG0000(	TARID	330.6258	-0.30106	0.001047	0.005288	lncRNA
ENSG0000(	TAMALIN	210.9741	0.369315	0.001047	0.00529	protein_coding
ENSG0000(	ASCC3	3059.434	0.18373	0.001049	0.005297	protein_coding
ENSG0000(	SLC9A4	311.8927	0.257064	0.001049	0.005297	protein_coding
ENSG0000(	PIP4K2C	716.9258	-0.29924	0.001052	0.005309	protein_coding
ENSG0000(	TBXAS1	543.6328	0.319183	0.001052	0.00531	protein_coding
ENSG0000(	ARMT1	257.1322	-0.27344	0.001052	0.00531	protein_coding
ENSG0000(	CBR1	390.4388	-0.45029	0.001054	0.005318	protein_coding
ENSG0000(	ENSG0000(	66.41851	-0.33991	0.001054	0.005319	lncRNA
ENSG0000(	PARS2	128.1795	-0.3075	0.001054	0.005319	protein_coding
ENSG0000(	ENSG0000(	97.62109	0.419727	0.001055	0.00532	lncRNA
ENSG0000(	ENSG0000(	23.8505	0.687084	0.001056	0.005323	TEC
ENSG0000(	APOLD1	535.8746	0.375943	0.001058	0.005333	protein_coding
ENSG0000(	ENSG0000(	93.1307	0.349267	0.001058	0.005333	lncRNA

ENSG0000(	SLC14A2	606.6639	-0.93386	0.001058	0.005333	protein_coding
ENSG0000(	FSIP2	12817.77	-0.20085	0.001059	0.005337	protein_coding
ENSG0000(	ENSG0000(	770.4827	-0.14763	0.001059	0.005337	lncRNA
ENSG0000(	GLDC	361.8379	0.270287	0.00106	0.00534	protein_coding
ENSG0000(	STK33	1492.824	0.145174	0.00106	0.00534	protein_coding
ENSG0000(	MRPL51	242.759	-0.40591	0.00106	0.005341	protein_coding
ENSG0000(	PHIP	4893.794	0.1202	0.001061	0.005342	protein_coding
ENSG0000(	RNLS	111.1072	-0.42665	0.001063	0.00535	protein_coding
ENSG0000(	ZNF529-AS	660.1049	0.174752	0.001064	0.005355	lncRNA
ENSG0000(	ZFP2	140.2638	-0.29756	0.001064	0.005356	protein_coding
ENSG0000(	H1-10	471.2915	0.264056	0.001065	0.005361	protein_coding
ENSG0000(	ENSG0000(	60.84169	0.299478	0.001068	0.005373	TEC
ENSG0000(	ENSG0000(	60.38778	0.323387	0.001068	0.005373	lncRNA
ENSG0000(	LINC02848	617.7051	0.148338	0.001068	0.005373	lncRNA
ENSG0000(	CTNNBIP1	361.0892	-0.25468	0.00107	0.005379	protein_coding
ENSG0000(	SLC4A8	1113.847	0.35722	0.00107	0.005382	protein_coding
ENSG0000(	CDX2	2931.561	-0.5182	0.001072	0.00539	protein_coding
ENSG0000(	ENSG0000(	10.04766	1.143251	0.001074	0.005397	lncRNA
ENSG0000(	APOBEC3H	16.76004	0.596611	0.001074	0.005397	protein_coding
ENSG0000(	ENSG0000(	2447.14	-0.28703	0.001074	0.005397	TEC
ENSG0000(	MCUR1P1	134.7156	0.22415	0.001074	0.005399	processed_pseudogene
ENSG0000(	NRXN2	210.1606	0.489105	0.001075	0.005399	protein_coding
ENSG0000(	ENSG0000(	382.825	0.229984	0.001078	0.005413	lncRNA
ENSG0000(	PRDM10	781.2766	-0.25033	0.001079	0.005418	protein_coding
ENSG0000(	ENSG0000(	537.6734	-0.13642	0.001081	0.00543	lncRNA
ENSG0000(	CEP95	1896.092	-0.1415	0.001082	0.005432	protein_coding
ENSG0000(	EPRS1	1746.054	-0.25171	0.001082	0.005433	protein_coding
ENSG0000(	GDF6	47.75939	0.72245	0.001085	0.005445	protein_coding
ENSG0000(	SCRN1	557.2973	0.380065	0.001085	0.005445	protein_coding
ENSG0000(	CUL4A	1966.051	-0.11493	0.001086	0.005449	protein_coding
ENSG0000(	DTX3L	3272.77	0.246839	0.001088	0.005456	protein_coding
ENSG0000(	DIAPH3	449.584	-0.24881	0.001088	0.005458	protein_coding
ENSG0000(	MIR3176	23.75263	-0.75113	0.001088	0.005458	miRNA
ENSG0000(	ANXA11	7764.026	-0.27575	0.001089	0.005461	protein_coding
ENSG0000(	CYP1B1	78.3937	0.9341	0.00109	0.005465	protein_coding
ENSG0000(	MYBBP1A	1150.116	-0.2271	0.001091	0.005468	protein_coding
ENSG0000(	TYMSOS	185.9047	-0.29122	0.001091	0.005468	lncRNA
ENSG0000(	CCDC148	114.1111	0.277805	0.001092	0.005469	protein_coding
ENSG0000(	LINC01685	20.98082	0.747488	0.001092	0.005469	lncRNA
ENSG0000(	ENSG0000(	52.41935	0.303893	0.001094	0.005478	lncRNA
ENSG0000(	ENSG0000(	35.09881	0.540787	0.001095	0.005486	processed_pseudogene
ENSG0000(	ENSG0000(	103.6617	0.297948	0.001096	0.005488	lncRNA
ENSG0000(	MIB2	1132.512	-0.27871	0.001097	0.005491	protein_coding
ENSG0000(	TOB1	1178.245	-0.36607	0.001097	0.005492	protein_coding
ENSG0000(	NIPSNAP1	567.643	-0.20375	0.001097	0.005493	protein_coding
ENSG0000(	KHDRBS1	1868.267	-0.16642	0.001098	0.005493	protein_coding
ENSG0000(	ADPRS	179.7563	-0.23222	0.0011	0.005504	protein_coding

ENSG0000(	ENSG0000(	124.1503	0.284527	0.001102	0.005515	lncRNA
ENSG0000(	LNCARSR	318.2356	-0.24653	0.001103	0.005516	lncRNA
ENSG0000(	SLC25A25-/	5380.079	-0.26756	0.001103	0.005516	lncRNA
ENSG0000(	TIMMDC1-	50.75115	0.284995	0.001104	0.005522	lncRNA
ENSG0000(	ENSG0000(	121.7207	0.243545	0.001106	0.005529	lncRNA
ENSG0000(	LINC01993	292.2122	0.235385	0.001107	0.005535	lncRNA
ENSG0000(	BIN2	719.6815	0.375921	0.001107	0.005535	protein_coding
ENSG0000(	CLEC1A	126.3005	0.312628	0.001108	0.005536	protein_coding
ENSG0000(	NIPA2	1345.457	-0.23915	0.001108	0.005536	protein_coding
ENSG0000(	ENSG0000(	87.55384	0.328746	0.001108	0.005536	transcribed_processed_pseudoge
ENSG0000(	HOXA10	400.9293	-0.34705	0.001109	0.005538	protein_coding
ENSG0000(	TIMM23B-/	773.5784	0.191099	0.001109	0.00554	protein_coding
ENSG0000(	NPIPB10P	65.90136	0.456828	0.00111	0.005544	unprocessed_pseudogene
ENSG0000(	NUP35	402.8381	-0.21979	0.001111	0.005545	protein_coding
ENSG0000(	PRKAR1B-A	20.74975	0.670927	0.001112	0.005552	lncRNA
ENSG0000(	ENSG0000(	28.68816	0.661188	0.001113	0.005553	lncRNA
ENSG0000(	ENSG0000(	19.51628	0.668481	0.001114	0.00556	TEC
ENSG0000(	CENPB	629.3514	-0.22818	0.001116	0.005569	protein_coding
ENSG0000(	ATP8B2	1463.696	0.273826	0.001117	0.005573	protein_coding
ENSG0000(	MYO19	3274.621	-0.2043	0.001118	0.005578	protein_coding
ENSG0000(	ENSG0000(	1130.671	0.292946	0.001119	0.005579	TEC
ENSG0000(	FAM120A	6992.181	-0.17155	0.00112	0.005585	protein_coding
ENSG0000(	UGT1A13P	341.1084	-0.69099	0.00112	0.005585	unprocessed_pseudogene
ENSG0000(	DPH1	1419.782	-0.14768	0.001121	0.005585	protein_coding
ENSG0000(	BPHL	734.8392	-0.18424	0.001123	0.005597	protein_coding
ENSG0000(	ENSG0000(	109.4872	0.267461	0.001124	0.0056	lncRNA
ENSG0000(	LINC01903	64.34259	-0.36433	0.001124	0.0056	lncRNA
ENSG0000(	SLC25A34	623.4351	-0.36822	0.001124	0.0056	protein_coding
ENSG0000(	RN7SL3	4696.978	0.597147	0.001127	0.005611	misc_RNA
ENSG0000(	EFHC2	44.17266	-0.5045	0.001127	0.005612	protein_coding
ENSG0000(	IGHV3-53	181.9035	0.821899	0.001129	0.00562	IG_V_gene
ENSG0000(	CPQ	572.5014	0.210195	0.001132	0.005634	protein_coding
ENSG0000(	GSG1	466.5229	0.18744	0.001132	0.005636	protein_coding
ENSG0000(	MRPS30	1314.805	-0.16877	0.001133	0.005639	protein_coding
ENSG0000(	BCAS2	194.927	-0.26279	0.001134	0.005641	protein_coding
ENSG0000(	RNF187	894.4827	-0.16679	0.001135	0.005646	protein_coding
ENSG0000(	U3	209.6791	0.275016	0.001135	0.005647	snoRNA
ENSG0000(	ENSG0000(	104.2863	-0.50373	0.001138	0.005658	transcribed_unprocessed_pseudo
ENSG0000(	ENSG0000(	1841.589	0.096873	0.001138	0.005658	lncRNA
ENSG0000(	C14orf132	745.687	0.24338	0.001138	0.005659	protein_coding
ENSG0000(	SGO2	626.4552	-0.19702	0.001139	0.005662	protein_coding
ENSG0000(	ENSG0000(	1675.853	0.156684	0.00114	0.005665	TEC
ENSG0000(	SRGAP3	1188.16	0.136319	0.00114	0.005666	protein_coding
ENSG0000(	LINC00706	150.6249	0.294326	0.001142	0.005672	lncRNA
ENSG0000(	SMC1A	2350.385	-0.24122	0.001143	0.005677	protein_coding
ENSG0000(	MMP14	1399.915	0.434272	0.001145	0.005686	protein_coding
ENSG0000(	LINC00458	138.9377	0.227385	0.001147	0.005696	lncRNA

ENSG0000(	ENSG0000(	54.92911	0.398075	0.001148	0.0057	lncRNA
ENSG0000(	SRSF2	3145.568	-0.19714	0.001148	0.0057	protein_coding
ENSG0000(	RAE1	577.8298	-0.18557	0.001149	0.005701	protein_coding
ENSG0000(	SNAI1	27.8382	0.823645	0.001149	0.005701	protein_coding
ENSG0000(	WNT2B	2043.504	0.320659	0.001149	0.005703	protein_coding
ENSG0000(	AIM2	161.68	0.557663	0.00115	0.005706	protein_coding
ENSG0000(	LINC01934	1776.168	0.330526	0.001151	0.005708	lncRNA
ENSG0000(	ENSG0000(	539.1094	0.416602	0.001155	0.005727	lncRNA
ENSG0000(	KCNMB1	725.6702	0.204273	0.001155	0.005727	protein_coding
ENSG0000(	PDCD2L	46.30547	-0.53165	0.001156	0.005733	protein_coding
ENSG0000(	LINC00645	36.89816	0.501418	0.001157	0.005736	lncRNA
ENSG0000(	RCOR1	1959.016	-0.21329	0.001157	0.005737	protein_coding
ENSG0000(	PLEKHG1	2689.35	0.213912	0.001159	0.005742	protein_coding
ENSG0000(	HNF4A	9713.756	-0.43559	0.00116	0.005748	protein_coding
ENSG0000(	DGKE	1275.499	0.153017	0.00116	0.005749	protein_coding
ENSG0000(	SNORA74D	111.3558	-0.36864	0.001162	0.005755	snoRNA
ENSG0000(	TMED4	2158.232	-0.1065	0.001162	0.005755	protein_coding
ENSG0000(	TPRN	2175.783	-0.54533	0.001162	0.005755	protein_coding
ENSG0000(	LINC00707	216.1905	0.200687	0.001162	0.005755	lncRNA
ENSG0000(	PMM1	629.193	-0.37554	0.001163	0.005756	protein_coding
ENSG0000(	CFAP299	117.3474	-0.28565	0.001164	0.00576	protein_coding
ENSG0000(	PODNL1	255.1102	0.187888	0.001164	0.00576	protein_coding
ENSG0000(	THNSL1	215.6443	-0.29526	0.001165	0.005767	protein_coding
ENSG0000(	ENSG0000(	33.97462	-0.45031	0.001167	0.005775	lncRNA
ENSG0000(	ZNF845	741.5727	-0.16295	0.00117	0.005786	protein_coding
ENSG0000(	BRSK1	141.4975	0.410115	0.001171	0.005787	protein_coding
ENSG0000(	ENSG0000(	248.8686	-0.25755	0.001171	0.005787	lncRNA
ENSG0000(	MBD3	1165.248	-0.15856	0.001171	0.005787	protein_coding
ENSG0000(	PDLIM7	568.0099	0.493168	0.001171	0.005787	protein_coding
ENSG0000(	PRPF3	2286.537	-0.10412	0.001171	0.005787	protein_coding
ENSG0000(	CHCHD5	322.3214	-0.22144	0.001172	0.005789	protein_coding
ENSG0000(	NECAB3	515.8571	-0.28806	0.001172	0.005789	protein_coding
ENSG0000(	ENSG0000(	25.97402	0.702649	0.001173	0.005793	lncRNA
ENSG0000(	ZNF195	3167.504	-0.15547	0.001173	0.005793	protein_coding
ENSG0000(	HSPA14	255.3528	-0.2242	0.001173	0.005793	protein_coding
ENSG0000(	ENSG0000(	72.47455	-0.30867	0.001173	0.005794	lncRNA
ENSG0000(	SLC9C2	15.52433	1.520327	0.001175	0.005803	protein_coding
ENSG0000(	LMCD1-AS1	814.7535	0.152344	0.001178	0.005815	lncRNA
ENSG0000(	CCR2	256.135	0.543394	0.001178	0.005817	protein_coding
ENSG0000(	CAMLG	1373.074	-0.15862	0.00118	0.005824	protein_coding
ENSG0000(	PRTFDC1	163.3764	-0.4043	0.00118	0.005824	protein_coding
ENSG0000(	ENSG0000(	220.7034	0.212692	0.001181	0.005825	lncRNA
ENSG0000(	ENSG0000(	656.386	-0.32199	0.001182	0.005829	lncRNA
ENSG0000(	MYO1E	4877.258	-0.33829	0.001184	0.00584	protein_coding
ENSG0000(	WWOX-AS1	49.28937	0.35885	0.001185	0.005842	lncRNA
ENSG0000(	ZBTB7C	969.7235	-0.47454	0.001185	0.005842	protein_coding
ENSG0000(	METAP1D	289.7241	-0.33645	0.001186	0.005846	protein_coding

ENSG0000(	ADCK5	293.2594	-0.35067	0.001186	0.005846	protein_coding
ENSG0000(	ENSG0000(	14.89296	1.025313	0.001186	0.005846	lncRNA
ENSG0000(	SLC38A9	1234.49	0.134896	0.001189	0.005861	protein_coding
ENSG0000(	IGHJ5	502.0692	0.588379	0.00119	0.005863	IG_J_gene
ENSG0000(	RNU6-312F	14.55186	0.702362	0.001191	0.005865	snRNA
ENSG0000(	OR5A1	911.3469	0.168317	0.001192	0.005872	protein_coding
ENSG0000(	RAB40C	897.9983	-0.372	0.001194	0.005881	protein_coding
ENSG0000(	AQP6	125.4575	-0.23072	0.001195	0.005883	protein_coding
ENSG0000(	ACY3	64.62144	0.52575	0.001196	0.005886	protein_coding
ENSG0000(	GATA6	828.5252	-0.39854	0.001196	0.005886	protein_coding
ENSG0000(	HTR6	13.24418	0.968471	0.001196	0.005886	protein_coding
ENSG0000(	SYT2	319.8449	0.387409	0.001196	0.005886	protein_coding
ENSG0000(	ENSG0000(	213.4518	0.206817	0.001198	0.005892	lncRNA
ENSG0000(	GLI3	492.893	0.564971	0.001198	0.005893	protein_coding
ENSG0000(	LNC-LBCS	103.2057	0.429004	0.001203	0.005917	lncRNA
ENSG0000(	SP140	678.9265	0.58353	0.001204	0.005918	protein_coding
ENSG0000(	UNC5B	1376.981	-0.40055	0.001204	0.005919	protein_coding
ENSG0000(	DIO2	133.4164	0.743283	0.001204	0.00592	protein_coding
ENSG0000(	LRRN1	67.54744	0.634881	0.001207	0.005932	protein_coding
ENSG0000(	SLC9A1	1880.26	-0.3685	0.001207	0.005933	protein_coding
ENSG0000(	TMEM161E	2002.517	-0.14734	0.001208	0.005933	protein_coding
ENSG0000(	ENSG0000(	49.3833	-0.84334	0.001208	0.005935	lncRNA
ENSG0000(	SLC44A1	6896.684	-0.30838	0.001211	0.005947	protein_coding
ENSG0000(	BTBD10P2	48.39177	0.436541	0.001213	0.005957	processed_pseudogene
ENSG0000(	ZBED9-AS1	28.735	0.616792	0.001213	0.005957	lncRNA
ENSG0000(	BAZ1A	2290.037	0.183767	0.001214	0.005958	protein_coding
ENSG0000(	CEP290	2802.489	-0.14906	0.001214	0.005958	protein_coding
ENSG0000(	CORO1B	1591.839	-0.33616	0.001214	0.005959	protein_coding
ENSG0000(	ENSG0000(	38.07525	0.468803	0.001216	0.005965	lncRNA
ENSG0000(	CYP2D8P	59.86537	-0.45171	0.001216	0.005967	unprocessed_pseudogene
ENSG0000(	MKNK2	3063.993	-0.30833	0.001216	0.005967	protein_coding
ENSG0000(	TIMELESS	681.2843	-0.30322	0.001217	0.005967	protein_coding
ENSG0000(	TCEA3	1777.338	-0.5106	0.001217	0.005968	protein_coding
ENSG0000(	IER5	599.3218	0.209296	0.001217	0.005969	protein_coding
ENSG0000(	ENSG0000(	482.9518	0.175331	0.001218	0.005972	lncRNA
ENSG0000(	ENSG0000(	27.27665	0.385225	0.001219	0.005974	lncRNA
ENSG0000(	HTR3E	11.43673	-0.99722	0.001221	0.005982	protein_coding
ENSG0000(	UIMC1	1189.482	-0.16445	0.001221	0.005982	protein_coding
ENSG0000(	SELPLG	304.0432	0.573129	0.001222	0.005988	protein_coding
ENSG0000(	STAG3L5P	1787.165	-0.17134	0.001224	0.005998	transcribed_unprocessed_pseudo
ENSG0000(	LINC01106	1126.208	-0.41038	0.001226	0.006006	lncRNA
ENSG0000(	ENSG0000(	338.1813	0.206281	0.001227	0.006007	lncRNA
ENSG0000(	PAQR4	225.0083	-0.37587	0.001227	0.006007	protein_coding
ENSG0000(	TSN	963.4694	-0.1758	0.001227	0.006007	protein_coding
ENSG0000(	UBE2J2	1014.772	-0.13433	0.001227	0.006008	protein_coding
ENSG0000(	NUCKS1	3695.365	-0.23809	0.001228	0.006008	protein_coding
ENSG0000(	ENSG0000(	227.0094	-0.27049	0.00123	0.006016	lncRNA

ENSG0000(	MIR663B	1887.79	-0.6189	0.00123	0.006016	miRNA
ENSG0000(	LDLRAD1	123.9835	0.225585	0.001232	0.006025	protein_coding
ENSG0000(	SBDSP1	294.9353	-0.22515	0.001233	0.00603	transcribed_unprocessed_pseudo
ENSG0000(	LINC00705	23.12148	-0.60637	0.001233	0.006031	lncRNA
ENSG0000(	AP2A2	2724.536	-0.15235	0.001234	0.006034	protein_coding
ENSG0000(	DNAJC3-DT	338.5935	0.204549	0.001235	0.006034	lncRNA
ENSG0000(	ENSG0000(	35.82173	0.339427	0.001234	0.006034	lncRNA
ENSG0000(	PSMA4	929.8337	-0.29795	0.001236	0.006042	protein_coding
ENSG0000(	MYO5A	4843.039	0.215629	0.001237	0.006047	protein_coding
ENSG0000(	RNU1-140F	18.38359	-0.8503	0.001238	0.006049	snRNA
ENSG0000(	C4A	879.0003	0.535473	0.001239	0.006053	protein_coding
ENSG0000(	KBTBD11	1858.386	-0.43142	0.001239	0.006053	protein_coding
ENSG0000(	ENSG0000(	263.46	-0.15977	0.001241	0.006058	processed_pseudogene
ENSG0000(	HSPE1P11	171.1064	0.314474	0.001241	0.006058	processed_pseudogene
ENSG0000(	SIRT5	2012.331	-0.14343	0.001241	0.006058	protein_coding
ENSG0000(	ENSG0000(	211.6695	0.196785	0.001242	0.006063	lncRNA
ENSG0000(	MRM3	172.4156	-0.24369	0.001242	0.006063	protein_coding
ENSG0000(	GIT1	691.4434	-0.19818	0.001244	0.006071	protein_coding
ENSG0000(	ENSG0000(	227.4156	0.247923	0.001246	0.006078	lncRNA
ENSG0000(	GJA5	51.41052	0.772746	0.001246	0.006079	protein_coding
ENSG0000(	LINC02311	443.5994	-0.28093	0.001246	0.006079	lncRNA
ENSG0000(	ARHGEF16	1405.694	-0.4275	0.001248	0.006085	protein_coding
ENSG0000(	F11R	4227.971	-0.29244	0.001249	0.006088	protein_coding
ENSG0000(	KLRC4-KLRI	52.45108	-0.77233	0.001249	0.006091	protein_coding
ENSG0000(	PRPF4B	4815.533	-0.11602	0.00125	0.006094	protein_coding
ENSG0000(	RCSD1	1960.05	0.344891	0.001251	0.006095	protein_coding
ENSG0000(	LINC02728	157.8014	0.276135	0.001254	0.006109	lncRNA
ENSG0000(	CCDC32	1812.163	0.195098	0.001254	0.006109	protein_coding
ENSG0000(	MMP12	237.7333	1.113502	0.001255	0.006112	protein_coding
ENSG0000(	EIF3E	6439.353	-0.1611	0.001256	0.006112	protein_coding
ENSG0000(	ENSG0000(	117.9927	0.222702	0.001255	0.006112	lncRNA
ENSG0000(	ENSG0000(	605.0216	-0.30973	0.001256	0.006112	lncRNA
ENSG0000(	ENSG0000(	85.03989	0.241068	0.001255	0.006112	lncRNA
ENSG0000(	TSPAN15	1076.432	-0.30499	0.001258	0.006125	protein_coding
ENSG0000(	ENSG0000(	57.47192	0.440556	0.00126	0.006129	lncRNA
ENSG0000(	GRHL2	1594.249	-0.29375	0.001259	0.006129	protein_coding
ENSG0000(	LPAR5	529.9481	-0.24082	0.001261	0.006135	protein_coding
ENSG0000(	ENSG0000(	20.63293	0.595189	0.001263	0.006142	lncRNA
ENSG0000(	SOX18	55.53603	0.592798	0.001263	0.006142	protein_coding
ENSG0000(	EVA1B	45.04306	0.471506	0.001263	0.006143	protein_coding
ENSG0000(	SLC9A3R1	1274.115	-0.43186	0.001263	0.006143	protein_coding
ENSG0000(	SPTLC2	3858.398	-0.12728	0.001264	0.006144	protein_coding
ENSG0000(	PLA2G7	694.3927	0.438655	0.001264	0.006145	protein_coding
ENSG0000(	EIF2AK1	1750.661	-0.21636	0.001265	0.006149	protein_coding
ENSG0000(	ENSG0000(	10.72886	0.899055	0.001266	0.006152	lncRNA
ENSG0000(	CEACAM6	5992.03	0.716948	0.001267	0.006157	protein_coding
ENSG0000(	ENSG0000(	270.189	0.236833	0.001268	0.006157	lncRNA

ENSG0000(	P3H2	384.8876	-0.62901	0.001268	0.006157	protein_coding
ENSG0000(	TMPRSS12	187.0457	-0.22694	0.001269	0.006162	protein_coding
ENSG0000(	ARRDC4	1978.436	-0.4146	0.001277	0.006199	protein_coding
ENSG0000(	RTP4	129.5919	0.430323	0.001277	0.006199	protein_coding
ENSG0000(	NSA2	615.5519	-0.34454	0.001278	0.006205	protein_coding
ENSG0000(	DHX33	778.9691	-0.16667	0.001279	0.006206	protein_coding
ENSG0000(	LINC00989	165.2122	0.290195	0.00128	0.006211	lncRNA
ENSG0000(	MIEN1	305.5072	-0.37394	0.00128	0.006212	protein_coding
ENSG0000(	SLC38A6	649.6245	0.22601	0.001282	0.006217	protein_coding
ENSG0000(	ENSG0000(	321.6787	0.23244	0.001282	0.006218	lncRNA
ENSG0000(	EIF2D	1139.83	-0.19098	0.001284	0.006223	protein_coding
ENSG0000(	PNISR	8707.424	0.148326	0.001283	0.006223	protein_coding
ENSG0000(	HK2	6489.674	0.473234	0.001285	0.006227	protein_coding
ENSG0000(	TPM4	9388.71	0.318987	0.001285	0.006227	protein_coding
ENSG0000(	SNAP47	737.8541	0.163792	0.001287	0.006238	protein_coding
ENSG0000(	ENSG0000(	33.62533	0.496764	0.001288	0.00624	lncRNA
ENSG0000(	ACTR3B	750.803	-0.19303	0.001289	0.006245	protein_coding
ENSG0000(	SLC16A2	178.4598	0.352178	0.001293	0.006263	protein_coding
ENSG0000(	FAM177B	168.0595	0.593781	0.001295	0.006272	protein_coding
ENSG0000(	PES1	361.9139	-0.19193	0.001296	0.006275	protein_coding
ENSG0000(	ENSG0000(	507.7886	0.18923	0.001297	0.006279	TEC
ENSG0000(	ENSG0000(	323.4562	0.178117	0.0013	0.006293	TEC
ENSG0000(	ENSG0000(	131.7957	0.249621	0.0013	0.006293	lncRNA
ENSG0000(	GDE1	1611.506	-0.32843	0.0013	0.006293	protein_coding
ENSG0000(	NMRK1	705.3581	0.241721	0.001302	0.006301	protein_coding
ENSG0000(	FLI1	1163.165	0.331917	0.001303	0.006304	protein_coding
ENSG0000(	EGFLAM-A!	24.27612	0.590084	0.001303	0.006305	lncRNA
ENSG0000(	ENSG0000(	132.026	0.466674	0.001305	0.006308	protein_coding
ENSG0000(	GPR137	359.1842	-0.19656	0.001304	0.006308	protein_coding
ENSG0000(	GREB1	705.1867	0.276307	0.001307	0.006322	protein_coding
ENSG0000(	ENSG0000(	179.9105	-0.25221	0.001308	0.006323	lncRNA
ENSG0000(	ANKRD36C	6827.198	0.233348	0.001309	0.006326	protein_coding
ENSG0000(	ALG10B	1351.377	0.13583	0.001309	0.006326	protein_coding
ENSG0000(	PHF6	3004.012	-0.12583	0.001309	0.006326	protein_coding
ENSG0000(	TNPO1P1	148.0264	0.285889	0.00131	0.006327	processed_pseudogene
ENSG0000(	ENSG0000(	72.80423	0.416749	0.001311	0.006331	lncRNA
ENSG0000(	RNU6-522F	44.90976	0.438299	0.001312	0.006337	snRNA
ENSG0000(	BAGE2	61.65701	0.465129	0.001313	0.00634	transcribed_unprocessed_pseudo
ENSG0000(	ENSG0000(	129.3257	-0.38969	0.001313	0.00634	lncRNA
ENSG0000(	TUSC2	275.241	-0.24823	0.001313	0.00634	protein_coding
ENSG0000(	ENSG0000(	34.45614	1.787575	0.001314	0.006342	transcribed_unprocessed_pseudo
ENSG0000(	RNU6-82P	50.07862	0.398157	0.001314	0.006342	snRNA
ENSG0000(	ATRIP	331.4802	-0.28849	0.001318	0.006358	protein_coding
ENSG0000(	DDX51	1065.599	-0.16143	0.001318	0.006361	protein_coding
ENSG0000(	ESRP2	2061.469	-0.45806	0.00132	0.006367	protein_coding
ENSG0000(	NOTCH2	4894.245	0.193608	0.00132	0.006368	protein_coding
ENSG0000(	TFB1M	460.8036	-0.18678	0.001322	0.006377	protein_coding

ENSG0000(	ENSG0000(	109.454	0.264527	0.001323	0.00638	lncRNA
ENSG0000(	LINC01890	12.10257	0.752936	0.001325	0.006388	lncRNA
ENSG0000(	FUT8	1877.849	0.20423	0.001327	0.006395	protein_coding
ENSG0000(	LINC00847	540.7198	0.190724	0.001327	0.006395	lncRNA
ENSG0000(	NPRL2	287.9177	-0.23914	0.001327	0.006395	protein_coding
ENSG0000(	ENSG0000(	234.1043	0.532678	0.001329	0.006403	lncRNA
ENSG0000(	SULT1A1	2816.299	-0.40728	0.00133	0.006408	protein_coding
ENSG0000(	CLDN7	4868.759	-0.4564	0.001331	0.006411	protein_coding
ENSG0000(	YY2	125.8936	0.236707	0.001332	0.006415	protein_coding
ENSG0000(	RS1	44.03186	0.531735	0.001333	0.006418	protein_coding
ENSG0000(	GJA4	37.31999	0.637956	0.001334	0.006424	protein_coding
ENSG0000(	ENSG0000(	11.28184	0.991719	0.001335	0.006425	lncRNA
ENSG0000(	LINC02896	89.83577	0.26814	0.001335	0.006426	lncRNA
ENSG0000(	ENSG0000(	81.96972	-0.26541	0.001336	0.006429	lncRNA
ENSG0000(	ENSG0000(	250.9617	0.199941	0.001337	0.006433	lncRNA
ENSG0000(	ICOSLG	1624.311	0.135213	0.001338	0.006436	protein_coding
ENSG0000(	HCG11	932.5028	0.139003	0.001339	0.006442	lncRNA
ENSG0000(	DEFB105A	293.5073	-0.20222	0.00134	0.006445	protein_coding
ENSG0000(	ENSG0000(	15.51938	0.971419	0.001341	0.006447	lncRNA
ENSG0000(	ENSG0000(	10.23245	0.794427	0.001341	0.006449	processed_pseudogene
ENSG0000(	RNF6	1070.375	-0.18418	0.001343	0.006454	protein_coding
ENSG0000(	SAC3D1	51.70586	-0.4453	0.001346	0.006467	protein_coding
ENSG0000(	SH3BGRL2	3896.005	-0.46066	0.001347	0.006475	protein_coding
ENSG0000(	UNC13B	4581.51	-0.35736	0.00135	0.006484	protein_coding
ENSG0000(	ENSG0000(	182.0784	-0.20124	0.00135	0.006485	lncRNA
ENSG0000(	PACS2	2148.566	-0.17452	0.00135	0.006485	protein_coding
ENSG0000(	MSRB2	270.7327	-0.27563	0.001351	0.006486	protein_coding
ENSG0000(	MYO5C	3644.637	-0.3803	0.001352	0.00649	protein_coding
ENSG0000(	ADAMTS19	25.69687	1.025436	0.001352	0.006492	lncRNA
ENSG0000(	AKAP5	1035.974	-0.21718	0.001353	0.006492	protein_coding
ENSG0000(	SNU13	609.2084	-0.18441	0.001353	0.006492	protein_coding
ENSG0000(	COPS9	159.6907	-0.38567	0.001355	0.006503	protein_coding
ENSG0000(	MYOM3	538.8295	0.377997	0.001356	0.006505	protein_coding
ENSG0000(	ENSG0000(	12.07716	1.052039	0.001356	0.006506	lncRNA
ENSG0000(	MIR5587	13.27986	-1.13081	0.001356	0.006506	miRNA
ENSG0000(	KLF6	8367.394	0.404483	0.001357	0.006506	protein_coding
ENSG0000(	SEMA3B	1196.45	-0.41662	0.001359	0.006518	protein_coding
ENSG0000(	RIPK2-DT	14704.64	0.15374	0.00136	0.00652	lncRNA
ENSG0000(	DVL3	1993.054	-0.14843	0.00136	0.006521	protein_coding
ENSG0000(	CHDH	1596.868	-0.4017	0.001361	0.006523	protein_coding
ENSG0000(	ENSG0000(	343.8889	0.230037	0.001362	0.006527	lncRNA
ENSG0000(	KCTD5P1	44.3406	-0.48908	0.001362	0.006527	processed_pseudogene
ENSG0000(	LINC00910	1989.242	0.146684	0.001362	0.006527	lncRNA
ENSG0000(	SNORD83B	26.57057	0.865153	0.001364	0.006532	snoRNA
ENSG0000(	PTDSS1	876.8502	-0.2607	0.001366	0.006541	protein_coding
ENSG0000(	SPOUT1	241.8371	-0.33089	0.001366	0.006541	protein_coding
ENSG0000(	FMO2	61.46589	0.977938	0.001367	0.006544	protein_coding

ENSG0000(	ENSG0000(	61.13416	0.353797	0.001368	0.006548	TEC
ENSG0000(	WDSUB1	221.6893	-0.26574	0.00137	0.006556	protein_coding
ENSG0000(	SPPL3	1885.259	-0.12043	0.001374	0.006574	protein_coding
ENSG0000(	THRIL	155.8508	-0.22841	0.001374	0.006575	lncRNA
ENSG0000(	ENSG0000(	771.3954	0.150838	0.001375	0.006578	TEC
ENSG0000(	MCC	964.6853	0.384649	0.001377	0.006586	protein_coding
ENSG0000(	ENSG0000(	131.8713	-0.31846	0.001377	0.006587	pseudogene
ENSG0000(	NCBP1	2097.032	-0.15816	0.001378	0.006589	protein_coding
ENSG0000(	ARID5B	2553.654	0.325887	0.001379	0.006592	protein_coding
ENSG0000(	HEXA	3443.815	0.129802	0.00138	0.006594	protein_coding
ENSG0000(	KRTAP3-1	22.56145	0.501968	0.00138	0.006594	protein_coding
ENSG0000(	LINC02609	442.3042	0.152396	0.00138	0.006595	lncRNA
ENSG0000(	CDS1	1004.137	-0.45647	0.001381	0.006597	protein_coding
ENSG0000(	PPIAP10	12.86212	0.834712	0.001383	0.006605	processed_pseudogene
ENSG0000(	ENSG0000(	169.9015	-0.40935	0.001383	0.006605	lncRNA
ENSG0000(	HELB	823.6086	0.373871	0.001383	0.006605	protein_coding
ENSG0000(	PHAX	1311.638	-0.11952	0.001383	0.006605	protein_coding
ENSG0000(	ERLIN1	1099.455	-0.24732	0.001384	0.006607	protein_coding
ENSG0000(	MDM4	10017.36	0.174791	0.001384	0.006608	protein_coding
ENSG0000(	KCCAT198	47.24779	0.407626	0.001385	0.006609	lncRNA
ENSG0000(	LINC01772	602.1944	0.154993	0.001386	0.006613	lncRNA
ENSG0000(	ENSG0000(	26.19102	0.961662	0.001388	0.006621	lncRNA
ENSG0000(	P2RY14	256.0302	0.432469	0.001388	0.006623	protein_coding
ENSG0000(	MIER1	2651.653	-0.19227	0.00139	0.006629	protein_coding
ENSG0000(	AP5M1	2970.895	-0.20809	0.00139	0.006631	protein_coding
ENSG0000(	IL21R	687.4018	0.607562	0.001394	0.006649	protein_coding
ENSG0000(	RPL30P13	41.39794	0.479527	0.001395	0.006652	processed_pseudogene
ENSG0000(	LINC01247	307.7381	-0.18407	0.001396	0.006657	lncRNA
ENSG0000(	ENSG0000(	209.4287	0.261333	0.001398	0.006662	lncRNA
ENSG0000(	ENSG0000(	21.3021	0.820059	0.001398	0.006663	lncRNA
ENSG0000(	ENSG0000(	30.14727	0.504917	0.001398	0.006663	lncRNA
ENSG0000(	RORB	118.7172	-0.28767	0.001399	0.006667	protein_coding
ENSG0000(	TMEM200/	278.835	0.662477	0.0014	0.006667	protein_coding
ENSG0000(	TRDMT1	1228.866	0.247555	0.0014	0.006669	protein_coding
ENSG0000(	ENSG0000(	23.2433	-0.52281	0.001402	0.006676	lncRNA
ENSG0000(	KCNN4	548.9725	-0.33233	0.001402	0.006677	protein_coding
ENSG0000(	PLEKHG6	1437.143	-0.58508	0.001406	0.006694	protein_coding
ENSG0000(	MIR1302-9	54.12001	0.764354	0.001407	0.006695	lncRNA
ENSG0000(	ENSG0000(	818.7875	-0.25923	0.001408	0.006702	protein_coding
ENSG0000(	LINC00547	893.1569	0.165713	0.00141	0.006709	lncRNA
ENSG0000(	PTRH2	833.7096	-0.17181	0.00141	0.006709	protein_coding
ENSG0000(	LINC01123	619.6203	-0.53921	0.001411	0.006709	lncRNA
ENSG0000(	LINC02267	145.5398	-0.34789	0.001411	0.006709	lncRNA
ENSG0000(	LINC02390	38.98812	0.396014	0.00141	0.006709	lncRNA
ENSG0000(	MRPL45	246.7219	-0.24829	0.001411	0.006709	protein_coding
ENSG0000(	RSRP1	7473.626	0.148545	0.001411	0.006709	protein_coding
ENSG0000(	ENSG0000(	83.35496	0.383855	0.001412	0.006714	lncRNA

ENSG0000(	GPAM	561.6884	-0.22386	0.001414	0.00672	protein_coding
ENSG0000(	EEF1AKMT:	1010.794	-0.18295	0.001415	0.006725	protein_coding
ENSG0000(	LCP1	2618.911	0.612423	0.001417	0.006731	protein_coding
ENSG0000(	SSH1	4029.61	0.197543	0.001417	0.006733	protein_coding
ENSG0000(	GCDH	323.5237	-0.2429	0.001418	0.006734	protein_coding
ENSG0000(	NDUFAF1	739.157	-0.13139	0.00142	0.006742	protein_coding
ENSG0000(	ENG	774.2034	0.44025	0.00142	0.006745	protein_coding
ENSG0000(	SAMD14	130.6707	0.568072	0.001422	0.006751	protein_coding
ENSG0000(	MIS18A	109.1833	-0.36855	0.001423	0.006753	protein_coding
ENSG0000(	TMEM203	284.7273	-0.21986	0.001423	0.006753	protein_coding
ENSG0000(	ENSG0000(	9.499582	0.856592	0.001423	0.006753	lncRNA
ENSG0000(	EIF4A2P2	62.16838	0.372874	0.001426	0.006765	processed_pseudogene
ENSG0000(	RNASEH2B	3949.243	0.210711	0.001428	0.006774	protein_coding
ENSG0000(	STK38	3486.087	-0.23995	0.00143	0.006783	protein_coding
ENSG0000(	MPZL3	877.3263	-0.22427	0.001431	0.006788	protein_coding
ENSG0000(	NEO1	3430.291	-0.37658	0.001432	0.00679	protein_coding
ENSG0000(	TRAPPC14	803.8027	-0.28569	0.001432	0.006791	protein_coding
ENSG0000(	ENSG0000(	9.192645	0.981676	0.001437	0.006813	lncRNA
ENSG0000(	ZC3H13	1969.565	-0.14705	0.001437	0.006813	protein_coding
ENSG0000(	ENSG0000(	110.0024	0.497551	0.001439	0.006818	lncRNA
ENSG0000(	SCAND1	117.4222	-0.30409	0.001441	0.006827	protein_coding
ENSG0000(	SLC15A2	1186.301	0.347154	0.001443	0.006838	protein_coding
ENSG0000(	ENSG0000(	24.30688	-0.52532	0.001446	0.00685	snoRNA
ENSG0000(	LRRC63	31.34484	0.509059	0.001447	0.00685	protein_coding
ENSG0000(	NDFIP2	1104.688	-0.37854	0.001447	0.00685	protein_coding
ENSG0000(	ENSG0000(	86.89491	0.28802	0.001448	0.006853	lncRNA
ENSG0000(	LINC00235	199.4512	-0.25132	0.001448	0.006853	lncRNA
ENSG0000(	RPS27	3011.378	-0.36909	0.00145	0.006863	protein_coding
ENSG0000(	SPRY3	610.0018	0.230671	0.00145	0.006863	protein_coding
ENSG0000(	FAM189A1	247.4827	-0.70148	0.001451	0.006866	protein_coding
ENSG0000(	ZFP92	83.28755	0.43602	0.001451	0.006867	protein_coding
ENSG0000(	NR6A1	919.5332	-0.36646	0.001453	0.006871	protein_coding
ENSG0000(	RCBTB1	513.6666	0.27191	0.001453	0.006871	protein_coding
ENSG0000(	CGAS	792.8621	0.199231	0.001454	0.006874	protein_coding
ENSG0000(	ENSG0000(	24.04787	0.485703	0.001454	0.006875	lncRNA
ENSG0000(	RNU2-71P	9.694755	-1.07914	0.001454	0.006875	snRNA
ENSG0000(	NEDD4	8079.709	-0.32213	0.001456	0.006884	protein_coding
ENSG0000(	KATNA1	212.9972	-0.21619	0.001461	0.006902	protein_coding
ENSG0000(	SLC4A2	886.8308	-0.2582	0.00146	0.006902	protein_coding
ENSG0000(	BLID	16.39619	0.912941	0.001462	0.006909	protein_coding
ENSG0000(	ENSG0000(	176.5761	0.181469	0.001463	0.006909	lncRNA
ENSG0000(	TMEM248	2327.742	-0.15837	0.001463	0.006909	protein_coding
ENSG0000(	API5	1562.353	-0.17156	0.001465	0.006917	protein_coding
ENSG0000(	H19	72.98385	1.06271	0.001466	0.006923	lncRNA
ENSG0000(	CASKIN2	546.7962	-0.3908	0.001469	0.006933	protein_coding
ENSG0000(	GIPC2	905.558	-0.47446	0.001474	0.006959	protein_coding
ENSG0000(	RN7SL683F	107.6329	0.275196	0.001475	0.006963	misc_RNA

ENSG0000(	C5orf15	503.6937	0.260028	0.001476	0.006964	protein_coding
ENSG0000(	CARNS1	96.8877	0.534761	0.001477	0.006968	protein_coding
ENSG0000(	ENSG0000(	482.0272	-0.13744	0.001478	0.006971	lncRNA
ENSG0000(	ENSG0000(	33.95845	0.524631	0.001478	0.006971	lncRNA
ENSG0000(	ENSG0000(	46.01757	0.32674	0.001479	0.006974	lncRNA
ENSG0000(	ZNF638	6500.846	0.122236	0.00148	0.00698	protein_coding
ENSG0000(	SLC25A28	681.4316	0.221445	0.001482	0.006985	protein_coding
ENSG0000(	CLTA	984.9458	-0.22685	0.001484	0.006997	protein_coding
ENSG0000(	WDR64	21.83758	0.899339	0.001485	0.007001	protein_coding
ENSG0000(	GAL3ST2	275.7161	-0.66957	0.001487	0.007006	protein_coding
ENSG0000(	ENSG0000(	83.08463	0.349056	0.001489	0.007015	lncRNA
ENSG0000(	LAMB4	138.1111	-0.23512	0.001491	0.007023	protein_coding
ENSG0000(	ENSG0000(	15.2205	0.642717	0.001491	0.007025	lncRNA
ENSG0000(	SLC22A15	243.5104	0.469849	0.001493	0.007033	protein_coding
ENSG0000(	CCNF	458.3407	-0.26605	0.001497	0.007052	protein_coding
ENSG0000(	G2E3	991.5442	-0.15287	0.0015	0.007064	protein_coding
ENSG0000(	ENSG0000(	65.2379	-0.27432	0.001501	0.007068	lncRNA
ENSG0000(	SUSD5	101.53	-0.24257	0.001502	0.007068	protein_coding
ENSG0000(	IMPACT	523.2087	-0.17679	0.001502	0.007071	protein_coding
ENSG0000(	ENSG0000(	14.13327	0.747148	0.001505	0.00708	lncRNA
ENSG0000(	IL12B	33.46692	0.414253	0.001506	0.007086	protein_coding
ENSG0000(	SBF2	5952.325	0.112002	0.001507	0.007087	protein_coding
ENSG0000(	ENSG0000(	37.56908	0.561632	0.001508	0.007091	lncRNA
ENSG0000(	ENSG0000(	456.1999	0.222014	0.001511	0.007104	lncRNA
ENSG0000(	HTRA1	183.7877	0.651809	0.001511	0.007104	protein_coding
ENSG0000(	ENSG0000(	326.5696	0.178255	0.001511	0.007105	TEC
ENSG0000(	CHRD	345.4966	0.268726	0.001512	0.007107	protein_coding
ENSG0000(	TBX1	83.25156	0.345093	0.001513	0.007112	protein_coding
ENSG0000(	ENSG0000(	379.7514	-0.17411	0.001514	0.007116	lncRNA
ENSG0000(	ENSG0000(	11.64396	0.686042	0.001515	0.007117	lncRNA
ENSG0000(	GPATCH1	645.7429	-0.20363	0.001515	0.007117	protein_coding
ENSG0000(	KMO	277.8601	0.568587	0.001515	0.007118	protein_coding
ENSG0000(	SDK1	743.7602	0.552237	0.001516	0.007121	protein_coding
ENSG0000(	PKP2	4663.284	-0.38771	0.001517	0.007123	protein_coding
ENSG0000(	ENSG0000(	156.8218	0.253086	0.001518	0.007127	TEC
ENSG0000(	ITPR3	7071.344	-0.23122	0.00152	0.007135	protein_coding
ENSG0000(	ENSG0000(	20.59872	-0.60341	0.001521	0.007137	lncRNA
ENSG0000(	KIRREL1-IT:	37.03461	0.634454	0.001521	0.007139	lncRNA
ENSG0000(	DLGAP4	1631.757	0.171972	0.001521	0.007139	protein_coding
ENSG0000(	MAPK14	3003.727	0.183107	0.001522	0.007141	protein_coding
ENSG0000(	PYCR2	927.6742	-0.16991	0.001524	0.007148	protein_coding
ENSG0000(	SPPL2B	3062.639	-0.1996	0.001524	0.007148	protein_coding
ENSG0000(	C11orf80	723.2665	0.220824	0.001524	0.007148	protein_coding
ENSG0000(	HSCB	376.6328	-0.17117	0.001526	0.007154	protein_coding
ENSG0000(	EOGT	645.4628	0.276288	0.001527	0.007159	protein_coding
ENSG0000(	ENSG0000(	12.78162	-0.82637	0.001529	0.007165	unprocessed_pseudogene
ENSG0000(	DDX3X	10687.62	0.129606	0.00153	0.007166	protein_coding

ENSG0000(	IMPDH1	403.7182	0.315255	0.001529	0.007166	protein_coding
ENSG0000(	LINC00653	80.49816	0.275107	0.00153	0.007166	lncRNA
ENSG0000(	MACROD1	167.8297	-0.43157	0.001529	0.007166	protein_coding
ENSG0000(	TMEM147	200.2123	-0.36374	0.00153	0.007166	protein_coding
ENSG0000(	ACADM	2543.891	-0.1592	0.001531	0.00717	protein_coding
ENSG0000(	RNU1-46P	31.47347	0.620599	0.001531	0.00717	snRNA
ENSG0000(	EXOSC4	89.7216	-0.29035	0.001533	0.007178	protein_coding
ENSG0000(	KRT17P8	20.37521	-0.86332	0.001535	0.007186	processed_pseudogene
ENSG0000(	ENSG0000(	255.9669	-0.18268	0.001536	0.007187	lncRNA
ENSG0000(	SNHG5	3540.132	-0.23908	0.001536	0.007189	lncRNA
ENSG0000(	DIPK1B	60.73052	0.54838	0.00154	0.007205	protein_coding
ENSG0000(	S100A6	2407.194	-0.51167	0.00154	0.007205	protein_coding
ENSG0000(	TWF1	1269.593	-0.25631	0.001541	0.007207	protein_coding
ENSG0000(	STAMBP	3741.039	0.100935	0.001543	0.007216	protein_coding
ENSG0000(	MMACHC	793.9178	-0.16974	0.001546	0.00723	protein_coding
ENSG0000(	LINC02160	61.31625	-0.33585	0.001547	0.007233	lncRNA
ENSG0000(	FYN	1329.373	0.486511	0.001547	0.007233	protein_coding
ENSG0000(	OCLN	3783.495	-0.23409	0.001548	0.007234	protein_coding
ENSG0000(	ENSG0000(	89.48102	-0.28496	0.001548	0.007236	lncRNA
ENSG0000(	PHKA2-AS1	17.05682	0.729236	0.001549	0.00724	lncRNA
ENSG0000(	PSMD6-AS:	185.2526	0.201224	0.001555	0.007263	lncRNA
ENSG0000(	THG1L	466.6421	-0.16444	0.001556	0.007266	protein_coding
ENSG0000(	CDC14B	1007.285	-0.23414	0.001556	0.007268	protein_coding
ENSG0000(	TWSG1	421.1578	0.25528	0.001556	0.007268	protein_coding
ENSG0000(	NRK	57.02446	0.742834	0.001557	0.007268	protein_coding
ENSG0000(	ENSG0000(	22.76235	0.555567	0.001558	0.007272	lncRNA
ENSG0000(	FMNL2	1563.231	0.227401	0.001558	0.007272	protein_coding
ENSG0000(	MAFG-DT	106.594	0.251612	0.00156	0.007281	lncRNA
ENSG0000(	CAPN10-DT	547.5158	0.164931	0.001561	0.007284	lncRNA
ENSG0000(	ENSG0000(	93.55632	-0.42029	0.001561	0.007284	lncRNA
ENSG0000(	NDST4	35.04957	-0.56403	0.001563	0.007292	protein_coding
ENSG0000(	KRI1	639.1816	-0.22877	0.001564	0.007295	protein_coding
ENSG0000(	TPM4P1	27.15289	0.665795	0.001565	0.007296	processed_pseudogene
ENSG0000(	P2RY11	276.2544	-0.30873	0.001567	0.007305	protein_coding
ENSG0000(	ZDHHC5	2533.222	-0.19605	0.001567	0.007305	protein_coding
ENSG0000(	BRPF3	2180.949	-0.28237	0.001567	0.007306	protein_coding
ENSG0000(	PRSS36	53.25727	-0.56085	0.001569	0.007313	protein_coding
ENSG0000(	SLC25A21	220.6557	-0.18943	0.001572	0.007324	protein_coding
ENSG0000(	CDK11B	744.1775	-0.25177	0.001573	0.00733	protein_coding
ENSG0000(	SF3B3	3521.575	-0.16534	0.001575	0.007336	protein_coding
ENSG0000(	ENSG0000(	68.64111	0.291853	0.001576	0.007339	lncRNA
ENSG0000(	BST2	262.5662	0.501695	0.001577	0.007344	protein_coding
ENSG0000(	MTHFD1	1991.553	-0.1718	0.00158	0.007355	protein_coding
ENSG0000(	SNHG16	2108.21	-0.19667	0.001582	0.007365	lncRNA
ENSG0000(	STOX1	96.12773	-0.54168	0.001582	0.007365	protein_coding
ENSG0000(	TAS2R30	796.5628	0.272387	0.001582	0.007365	protein_coding
ENSG0000(	LINC02878	253.5696	0.18184	0.001584	0.007374	lncRNA

ENSG0000(	BCO1	84.51813	0.662364	0.001589	0.007394	protein_coding
ENSG0000(	VASN	99.5178	0.559248	0.001591	0.007401	protein_coding
ENSG0000(	GAPDHP14	12.07958	0.994338	0.001594	0.007414	processed_pseudogene
ENSG0000(	MGC27382	385.002	0.175192	0.001595	0.007419	lncRNA
ENSG0000(	APOBEC3C	483.9109	0.332063	0.001596	0.007421	protein_coding
ENSG0000(	ENSG0000(	86.49856	0.318254	0.001596	0.007423	processed_pseudogene
ENSG0000(	TRMT5	424.8353	-0.27915	0.001598	0.007428	protein_coding
ENSG0000(	TAS2R20	302.4003	0.28298	0.001598	0.007429	protein_coding
ENSG0000(	ENSG0000(	217.5343	0.338809	0.001602	0.007445	lncRNA
ENSG0000(	RNU6-43P	21.38049	0.537462	0.001602	0.007445	snRNA
ENSG0000(	FEN1	214.9232	-0.39479	0.001609	0.007474	protein_coding
ENSG0000(	MEG9	56.30575	0.545155	0.001609	0.007474	lncRNA
ENSG0000(	LINC02733	80.4837	0.3197	0.00161	0.00748	lncRNA
ENSG0000(	ENSG0000(	37.72589	0.407343	0.001612	0.007487	lncRNA
ENSG0000(	ENSG0000(	287.885	-0.22322	0.001615	0.007498	lncRNA
ENSG0000(	NAGPA	575.4027	-0.17634	0.001616	0.007501	protein_coding
ENSG0000(	ENSG0000(	220.5245	-0.72402	0.001621	0.007524	lncRNA
ENSG0000(	SRD5A3	630.6031	0.354507	0.001621	0.007524	protein_coding
ENSG0000(	DNAJB11	1714.61	-0.29932	0.001622	0.007525	protein_coding
ENSG0000(	F2R	684.0541	0.377982	0.001627	0.007549	protein_coding
ENSG0000(	RFX7	1513.807	0.202101	0.001627	0.007549	protein_coding
ENSG0000(	IGLC3	3317.329	0.86174	0.001631	0.007563	IG_C_gene
ENSG0000(	MIR6739	10.01784	-0.68764	0.00163	0.007563	miRNA
ENSG0000(	C1QTNF1	257.3585	0.427852	0.001632	0.007569	protein_coding
ENSG0000(	IATPR	142.5587	0.246767	0.001633	0.007572	lncRNA
ENSG0000(	ORAI3	279.9546	0.280507	0.001634	0.007574	protein_coding
ENSG0000(	GOLGA8IP	68.56736	0.306174	0.001634	0.007574	transcribed_unprocessed_pseudo
ENSG0000(	CEBPA-DT	182.1095	-0.4168	0.001636	0.007582	lncRNA
ENSG0000(	ENSG0000(	182.1862	0.246568	0.001636	0.007582	lncRNA
ENSG0000(	ENSG0000(	13.42459	0.94519	0.001637	0.007584	lncRNA
ENSG0000(	ADGRL4	194.4779	0.420554	0.001638	0.007587	protein_coding
ENSG0000(	TEF	539.1114	-0.49695	0.001639	0.00759	protein_coding
ENSG0000(	LEP	41.88973	0.297156	0.001639	0.007592	protein_coding
ENSG0000(	ARMC9	2299.002	0.220455	0.00164	0.007594	protein_coding
ENSG0000(	MEX3C	1231.97	0.17458	0.001642	0.007601	protein_coding
ENSG0000(	ABT1	366.9883	-0.15518	0.001643	0.007603	protein_coding
ENSG0000(	ENSG0000(	44.96202	0.523765	0.001644	0.00761	lncRNA
ENSG0000(	YPEL3	851.4771	0.263366	0.001645	0.007613	protein_coding
ENSG0000(	TBC1D1	2041.902	0.154218	0.001648	0.007626	protein_coding
ENSG0000(	GUCD1	1804.203	-0.25676	0.001649	0.007629	protein_coding
ENSG0000(	HDHD5	606.468	-0.2717	0.001649	0.007629	protein_coding
ENSG0000(	ST3GAL5-A	25.57962	0.558143	0.001651	0.007634	lncRNA
ENSG0000(	RASA4B	1250.9	0.346026	0.001653	0.007642	protein_coding
ENSG0000(	ALDH18A1	2425.618	-0.44218	0.001653	0.007643	protein_coding
ENSG0000(	PLA2G4A	125.4165	-0.43661	0.001653	0.007643	protein_coding
ENSG0000(	JPH1	279.0087	-0.44286	0.001654	0.007646	protein_coding
ENSG0000(	BASP1	362.6247	0.514555	0.001656	0.007651	protein_coding

ENSG0000(	ENSG0000(	38.48135	0.332576	0.001656	0.007651	lncRNA
ENSG0000(	MTATP8P1	11.19972	0.761312	0.001656	0.007651	unprocessed_pseudogene
ENSG0000(	ENSG0000(	418.7858	0.162989	0.001658	0.007657	lncRNA
ENSG0000(	IGKV3-20	2241.425	0.744992	0.00166	0.007665	IG_V_gene
ENSG0000(	GPR180	580.8788	0.280211	0.001661	0.007668	protein_coding
ENSG0000(	CARD6	421.7836	0.226282	0.001664	0.00768	protein_coding
ENSG0000(	ENSG0000(	453.8835	0.218339	0.001663	0.00768	lncRNA
ENSG0000(	RNU1-138F	10.10076	-0.86901	0.001664	0.007682	snRNA
ENSG0000(	PTGES3L	477.1716	-0.20129	0.001665	0.007683	protein_coding
ENSG0000(	ENSG0000(	1087.57	0.128809	0.001666	0.007688	lncRNA
ENSG0000(	REST	2277.323	-0.12501	0.001668	0.007695	protein_coding
ENSG0000(	TET1	543.4259	0.235565	0.001668	0.007695	protein_coding
ENSG0000(	ZNF773	1130.27	0.158034	0.00167	0.007702	protein_coding
ENSG0000(	ENSG0000(	102.8894	0.642398	0.001671	0.007704	transcribed_processed_pseudoge
ENSG0000(	SPDEF	262.3337	-0.55973	0.001671	0.007704	protein_coding
ENSG0000(	FGF12	236.0527	-0.29232	0.001672	0.007709	protein_coding
ENSG0000(	ENSG0000(	94.79065	0.318898	0.001673	0.007714	lncRNA
ENSG0000(	P2RY2	1258.82	-0.34519	0.001675	0.00772	protein_coding
ENSG0000(	PHKA1P1	45.88424	-0.40176	0.001675	0.007721	processed_pseudogene
ENSG0000(	PLEKHG7	215.3509	0.412963	0.001677	0.007727	protein_coding
ENSG0000(	AKR1E2	124.1848	-0.4743	0.001677	0.007728	protein_coding
ENSG0000(	NEURL2	74.31559	-0.43296	0.00168	0.007738	protein_coding
ENSG0000(	TBC1D2B	3232.466	0.199117	0.00168	0.007738	protein_coding
ENSG0000(	GRAMD1A	1359.032	0.350645	0.001682	0.007744	protein_coding
ENSG0000(	ENSG0000(	43.0128	-0.42218	0.001683	0.007747	lncRNA
ENSG0000(	PNPT1	1788.058	-0.22	0.001684	0.007754	protein_coding
ENSG0000(	ENSG0000(	206.6832	-0.24457	0.001685	0.007756	lncRNA
ENSG0000(	ENSG0000(	59.64386	0.350394	0.00169	0.007777	lncRNA
ENSG0000(	NFKB1	1445.327	0.190564	0.001692	0.007787	protein_coding
ENSG0000(	PCAT14	852.0126	0.204681	0.001693	0.007787	lncRNA
ENSG0000(	ENSG0000(	161.7389	-0.50865	0.001693	0.007788	processed_pseudogene
ENSG0000(	TMEM43	1066.935	-0.17088	0.001693	0.007789	protein_coding
ENSG0000(	PPP1R18	790.9838	0.518689	0.001694	0.007792	protein_coding
ENSG0000(	SEC23A	2984.457	-0.17388	0.001696	0.007797	protein_coding
ENSG0000(	ZBTB26	604.9774	0.137072	0.0017	0.007814	protein_coding
ENSG0000(	TESMIN	313.4674	-0.29354	0.001701	0.007818	protein_coding
ENSG0000(	YWHAG	2412.715	-0.25495	0.001701	0.007818	protein_coding
ENSG0000(	OR7G2	255.0392	-0.16063	0.001702	0.007821	protein_coding
ENSG0000(	BTBD6	635.2283	-0.21651	0.001702	0.007822	protein_coding
ENSG0000(	TACR2	88.79447	0.349057	0.001703	0.007822	protein_coding
ENSG0000(	RNF222	232.432	0.182652	0.001704	0.007826	protein_coding
ENSG0000(	ENSG0000(	11.07474	1.123638	0.001704	0.007828	unitary_pseudogene
ENSG0000(	TMEM63B	977.5174	-0.28836	0.001706	0.007834	protein_coding
ENSG0000(	HIF1A	2826.6	0.354587	0.001707	0.007837	protein_coding
ENSG0000(	UBE2V1P1	35.70103	0.439957	0.001707	0.007837	processed_pseudogene
ENSG0000(	ENSG0000(	15.78166	-0.63308	0.00171	0.007847	lncRNA
ENSG0000(	RNF14	1370.86	-0.13883	0.001713	0.007859	protein_coding

ENSG0000(	SEPTIN14	493.8368	0.236173	0.001712	0.007859	protein_coding
ENSG0000(	C1orf162	292.616	0.432612	0.001713	0.00786	protein_coding
ENSG0000(	Y_RNA	3890.518	0.235322	0.001714	0.007863	misc_RNA
ENSG0000(	RPL31	3548.648	-0.30991	0.001715	0.007867	protein_coding
ENSG0000(	LSM1	199.7191	-0.26431	0.001716	0.007869	protein_coding
ENSG0000(	ENSG0000(	52.47188	-0.34498	0.001717	0.007871	lncRNA
ENSG0000(	C3orf62	1002.963	0.156194	0.001717	0.007872	protein_coding
ENSG0000(	LCAL1	76.92387	0.471226	0.001718	0.007877	lncRNA
ENSG0000(	UTP20	1123.598	-0.27645	0.001721	0.007886	protein_coding
ENSG0000(	ENSG0000(	12.33937	0.839654	0.001721	0.007887	lncRNA
ENSG0000(	ENSG0000(	514.5489	0.205098	0.001722	0.007888	lncRNA
ENSG0000(	EXOSC2	2651.726	-0.12185	0.001722	0.007888	protein_coding
ENSG0000(	RRS1	174.5477	-0.35637	0.001722	0.007888	protein_coding
ENSG0000(	ENSG0000(	1161.099	0.137847	0.001722	0.007889	lncRNA
ENSG0000(	DPY30	348.6928	-0.35	0.001724	0.007896	protein_coding
ENSG0000(	ENSG0000(	154.9099	-0.27366	0.001725	0.007896	lncRNA
ENSG0000(	ZSCAN16	167.3078	-0.24349	0.001727	0.007908	protein_coding
ENSG0000(	LINC02754	2301.917	0.293609	0.001729	0.007917	lncRNA
ENSG0000(	ENSG0000(	239.704	-0.25787	0.001731	0.00792	lncRNA
ENSG0000(	RPL37A-DT	209.9744	0.235781	0.001734	0.007933	lncRNA
ENSG0000(	VASP	2000.951	-0.25423	0.001734	0.007933	protein_coding
ENSG0000(	DAXX	509.457	-0.24096	0.001736	0.007942	protein_coding
ENSG0000(	HNF1A-AS1	3825.227	-0.41093	0.001736	0.007942	lncRNA
ENSG0000(	MPP4	146.9038	0.260164	0.001738	0.00795	protein_coding
ENSG0000(	CICP27	144.5465	0.438772	0.001739	0.007952	processed_pseudogene
ENSG0000(	OSBPL11	1100.969	-0.33155	0.001739	0.007952	protein_coding
ENSG0000(	SAG	1153.118	-0.09988	0.001739	0.007952	protein_coding
ENSG0000(	ENSG0000(	60.57973	-0.37151	0.00174	0.007954	pseudogene
ENSG0000(	PATL1-DT	293.2763	0.224142	0.001741	0.007957	lncRNA
ENSG0000(	EMC10	2734.363	-0.14864	0.001742	0.007958	protein_coding
ENSG0000(	MAPKAPK5	4938.614	-0.18409	0.001744	0.007966	protein_coding
ENSG0000(	ZNF329	862.7531	-0.15144	0.001746	0.007977	protein_coding
ENSG0000(	ENSG0000(	102.0106	0.290214	0.001749	0.007988	lncRNA
ENSG0000(	SERF2	3299.223	-0.23164	0.001749	0.007988	protein_coding
ENSG0000(	TAB3	2777.658	0.120054	0.001752	0.007999	protein_coding
ENSG0000(	MIR4539	58.11403	0.78182	0.001756	0.008015	miRNA
ENSG0000(	SH3GL1	821.9868	-0.16059	0.001757	0.008021	protein_coding
ENSG0000(	ACAA2	2547.849	-0.41609	0.001758	0.008024	protein_coding
ENSG0000(	SRP14	1024.567	-0.24162	0.00176	0.008034	protein_coding
ENSG0000(	POLR3E	1482.445	-0.17841	0.001761	0.008034	protein_coding
ENSG0000(	SLC1A7	70.47728	-0.96852	0.001762	0.008036	protein_coding
ENSG0000(	RESF1	6467.452	0.328658	0.001763	0.008044	protein_coding
ENSG0000(	ENSG0000(	36.90668	0.432527	0.001765	0.008048	lncRNA
ENSG0000(	ENSG0000(	103.862	0.266366	0.001765	0.008048	lncRNA
ENSG0000(	PTP4A2	4704.068	-0.19858	0.001768	0.008063	protein_coding
ENSG0000(	NHSL2	934.4704	0.411601	0.001769	0.008067	protein_coding
ENSG0000(	SLC44A4	4259.652	-0.38306	0.001772	0.008078	protein_coding

ENSG0000(	ENSG0000(	54.24828	0.412188	0.001774	0.008084	lncRNA
ENSG0000(	FBXL13	62.73895	0.592707	0.001774	0.008084	protein_coding
ENSG0000(	POLR3B	374.7271	-0.24651	0.001774	0.008084	protein_coding
ENSG0000(	DNAJA4	1494.918	-0.27609	0.001775	0.008084	protein_coding
ENSG0000(	RANGRF	101.4587	-0.31001	0.001775	0.008084	protein_coding
ENSG0000(	HOXA3	765.508	-0.33494	0.001775	0.008084	protein_coding
ENSG0000(	ENSG0000(	285.4348	0.363362	0.001777	0.008089	lncRNA
ENSG0000(	ENSG0000(	33.56587	0.45369	0.001777	0.00809	lncRNA
ENSG0000(	REC8	356.4816	0.3966	0.001777	0.00809	protein_coding
ENSG0000(	ZNF607	451.9874	-0.32299	0.001777	0.00809	protein_coding
ENSG0000(	CORO6	73.39977	0.526497	0.001779	0.008095	protein_coding
ENSG0000(	ENSG0000(	265.8543	0.160746	0.001779	0.008095	lncRNA
ENSG0000(	ENSG0000(	215.2208	-0.22598	0.00178	0.008097	lncRNA
ENSG0000(	ENSG0000(	46.36607	0.444043	0.001782	0.008108	lncRNA
ENSG0000(	TMCC2	61.06118	0.470639	0.001784	0.008114	protein_coding
ENSG0000(	MARCHF1	756.475	0.533041	0.001785	0.008119	protein_coding
ENSG0000(	ZNF85	581.0959	-0.14815	0.001787	0.008127	protein_coding
ENSG0000(	OBSL1	541.1743	0.3094	0.001788	0.008129	protein_coding
ENSG0000(	OR51L1	150.3463	0.230371	0.001789	0.00813	protein_coding
ENSG0000(	VIM	3459.19	0.417769	0.001791	0.008142	protein_coding
ENSG0000(	WDR93	193.5386	0.236026	0.001794	0.008151	protein_coding
ENSG0000(	ITGA3	2933.807	-0.25397	0.001796	0.008158	protein_coding
ENSG0000(	USP48	2610.131	0.174258	0.001797	0.008162	protein_coding
ENSG0000(	ENSG0000(	55.56274	-0.80802	0.001797	0.008163	processed_pseudogene
ENSG0000(	LINC02347	81.4404	0.305601	0.001798	0.008167	lncRNA
ENSG0000(	RAD51	149.3477	-0.45054	0.001799	0.008167	protein_coding
ENSG0000(	ENSG0000(	18.85096	0.526628	0.001801	0.008178	TEC
ENSG0000(	MYO7A	470.3485	0.282821	0.001802	0.008179	protein_coding
ENSG0000(	ENSG0000(	13.72954	-0.60437	0.001803	0.008182	lncRNA
ENSG0000(	ARX	36.35557	1.483496	0.001804	0.008187	protein_coding
ENSG0000(	RNF139	644.3919	-0.24257	0.001804	0.008187	protein_coding
ENSG0000(	ENSG0000(	198.4042	-0.64391	0.001806	0.008193	unprocessed_pseudogene
ENSG0000(	CDC42EP5	328.4206	-0.43585	0.001807	0.008196	protein_coding
ENSG0000(	ENSG0000(	54.56599	-0.31617	0.001807	0.008196	transcribed_unprocessed_pseudo
ENSG0000(	OLFM4	7665.713	0.925289	0.001808	0.0082	protein_coding
ENSG0000(	GPR4	141.2325	0.421271	0.001809	0.008203	protein_coding
ENSG0000(	CDC42BPG	1844.456	-0.41191	0.00181	0.008204	protein_coding
ENSG0000(	AHDC1	903.7209	0.200248	0.001812	0.008211	protein_coding
ENSG0000(	ENSG0000(	132.911	-0.27045	0.001812	0.008213	lncRNA
ENSG0000(	OR2A3P	128.3781	0.248962	0.001812	0.008213	transcribed_unprocessed_pseudo
ENSG0000(	TVP23C	2761.612	0.181279	0.001816	0.00823	protein_coding
ENSG0000(	HDAC11	286.9206	-0.31282	0.001819	0.008241	protein_coding
ENSG0000(	IGHV3-63	10.98038	0.869493	0.001821	0.008246	IG_V_pseudogene
ENSG0000(	OTUD7B	2428.984	-0.20675	0.001821	0.008246	protein_coding
ENSG0000(	DGCR6L	122.3334	-0.30899	0.001821	0.008248	protein_coding
ENSG0000(	RFC2	502.7333	-0.17561	0.001824	0.008257	protein_coding
ENSG0000(	IL10RA	2437.85	0.407083	0.001826	0.008266	protein_coding

ENSG0000(	TSPYL4	417.4148	0.208408	0.001831	0.008288	protein_coding
ENSG0000(	ENSG0000(	20.49663	0.636279	0.001832	0.00829	lncRNA
ENSG0000(	RNF5	369.0293	-0.36946	0.001832	0.00829	protein_coding
ENSG0000(	ENSG0000(	83.60645	0.321957	0.001834	0.008298	lncRNA
ENSG0000(	SCAT1	468.3672	0.163006	0.001835	0.0083	lncRNA
ENSG0000(	NADK2	609.1735	-0.25098	0.001839	0.008318	protein_coding
ENSG0000(	RPL10	5398.929	-0.32245	0.001841	0.008325	protein_coding
ENSG0000(	ENSG0000(	324.8114	-0.1855	0.001842	0.008329	lncRNA
ENSG0000(	RN7SL487F	25.47319	0.521908	0.001842	0.008329	misc_RNA
ENSG0000(	PSD2	13.13618	1.217344	0.001843	0.00833	protein_coding
ENSG0000(	PDIA6	1710.846	-0.30421	0.001843	0.008332	protein_coding
ENSG0000(	ENSG0000(	23.99234	-0.45948	0.001845	0.008336	lncRNA
ENSG0000(	ANKRD28	2374.357	0.221423	0.001845	0.008337	protein_coding
ENSG0000(	ZNF641	1384.699	0.223117	0.001845	0.008337	protein_coding
ENSG0000(	BLCAP	2895.651	-0.16149	0.001846	0.008339	protein_coding
ENSG0000(	ZNF556	1463.836	-0.13374	0.001846	0.008339	protein_coding
ENSG0000(	NDRG1	9368.707	-0.48638	0.001847	0.008343	protein_coding
ENSG0000(	ZNF205	133.5938	-0.30322	0.001849	0.00835	protein_coding
ENSG0000(	OR52H2P	13.42837	0.66606	0.00185	0.008351	unitary_pseudogene
ENSG0000(	CCDC18-AS	3274.222	0.148883	0.001851	0.008355	lncRNA
ENSG0000(	HTN3	332.0406	-0.21562	0.001851	0.008355	protein_coding
ENSG0000(	PGAP4	673.5997	-0.38169	0.001853	0.008364	protein_coding
ENSG0000(	CUTA	292.1161	-0.32285	0.001856	0.008376	protein_coding
ENSG0000(	RN7SL655F	71.24043	0.281821	0.001857	0.008376	misc_RNA
ENSG0000(	COG2	1644.283	-0.20794	0.001859	0.008385	protein_coding
ENSG0000(	LINC00494	216.2634	0.552542	0.001859	0.008386	lncRNA
ENSG0000(	ENSG0000(	260.9748	0.171822	0.001861	0.008394	lncRNA
ENSG0000(	TRIM24	1389.92	-0.21498	0.001862	0.008394	protein_coding
ENSG0000(	ENSG0000(	33.03978	0.507863	0.001862	0.008394	lncRNA
ENSG0000(	ENSG0000(	261.5579	0.160752	0.001862	0.008394	lncRNA
ENSG0000(	LINC02344	125.3134	0.377467	0.001865	0.008404	lncRNA
ENSG0000(	PLAG1	288.5093	0.365598	0.001865	0.008404	protein_coding
ENSG0000(	MTRFR	4182.827	0.10243	0.001866	0.008407	protein_coding
ENSG0000(	UGT8	2057.332	-0.29548	0.001866	0.008408	protein_coding
ENSG0000(	ENSG0000(	96.39929	0.263957	0.001868	0.008414	lncRNA
ENSG0000(	ENSG0000(	183.1299	0.232162	0.001869	0.008416	lncRNA
ENSG0000(	ENSG0000(	242.6997	-0.27148	0.001873	0.008432	lncRNA
ENSG0000(	GPR34	130.999	0.408847	0.001873	0.008432	protein_coding
ENSG0000(	REPIN1	2404.917	-0.19345	0.001873	0.008432	protein_coding
ENSG0000(	ENSG0000(	230.1783	0.28757	0.001874	0.008433	lncRNA
ENSG0000(	RASSF2	1409.593	0.525558	0.001874	0.008433	protein_coding
ENSG0000(	ENSG0000(	1965.188	0.192436	0.001876	0.008439	lncRNA
ENSG0000(	ENSG0000(	1391.718	0.173246	0.001881	0.00846	lncRNA
ENSG0000(	FOXD4L5	124.9364	0.312974	0.001881	0.00846	protein_coding
ENSG0000(	LINC02441	95.06294	-0.6946	0.001881	0.00846	lncRNA
ENSG0000(	EFNA2	1103.17	0.495555	0.001882	0.008465	protein_coding
ENSG0000(	ENOPH1	317.428	-0.25696	0.001885	0.008474	protein_coding

ENSG0000(	POMP	323.192	-0.34709	0.001886	0.008479	protein_coding
ENSG0000(	SLC35C1	943.721	-0.28855	0.001887	0.008483	protein_coding
ENSG0000(	AKT2	3893.358	-0.09489	0.001888	0.008488	protein_coding
ENSG0000(	LINC02483	20.73052	0.570688	0.00189	0.008492	lncRNA
ENSG0000(	TM4SF20	220.8594	0.835502	0.00189	0.008492	protein_coding
ENSG0000(	UBE2A	1215.284	-0.29638	0.00189	0.008492	protein_coding
ENSG0000(	ENSG0000(	93.76214	0.378121	0.001893	0.008504	lncRNA
ENSG0000(	SYPL1P2	41.93199	0.402562	0.001893	0.008504	processed_pseudogene
ENSG0000(	CPT1B	615.0772	-0.30872	0.001894	0.008507	protein_coding
ENSG0000(	PAXBP1-AS	1217.38	0.115948	0.001896	0.008512	lncRNA
ENSG0000(	TMEM213	601.9091	0.118434	0.001897	0.008513	protein_coding
ENSG0000(	TNFSF10	2316.398	0.351127	0.001896	0.008513	protein_coding
ENSG0000(	GALNT1	1426.266	-0.23375	0.001898	0.008515	protein_coding
ENSG0000(	KLHL34	61.12355	-0.46451	0.001897	0.008515	protein_coding
ENSG0000(	ENSG0000(	44.19491	0.523485	0.001902	0.008534	lncRNA
ENSG0000(	SET	2977.1	-0.19473	0.001903	0.008536	protein_coding
ENSG0000(	GMEB1	1470.426	-0.11082	0.001904	0.008542	protein_coding
ENSG0000(	LINC00310	313.2929	0.218335	0.001905	0.008542	lncRNA
ENSG0000(	RNU6-1016	198.5788	0.344074	0.001908	0.008556	snRNA
ENSG0000(	TMEM222	569.3495	-0.15843	0.001908	0.008557	protein_coding
ENSG0000(	SLC39A7	1233.115	-0.2335	0.001911	0.008568	protein_coding
ENSG0000(	ENSG0000(	43.71283	-0.53539	0.001912	0.008571	lncRNA
ENSG0000(	LINC01492	342.1145	-0.22102	0.001912	0.008571	lncRNA
ENSG0000(	ENSG0000(	167.2593	0.210556	0.001913	0.008573	lncRNA
ENSG0000(	LINC01191	89.39945	0.474696	0.001914	0.008576	lncRNA
ENSG0000(	C11orf54	1679.978	-0.19483	0.001915	0.00858	protein_coding
ENSG0000(	CDCA5	168.7516	-0.41325	0.001916	0.008581	protein_coding
ENSG0000(	MPP7	1098.345	-0.4249	0.001916	0.008581	protein_coding
ENSG0000(	OASL	1163.618	-0.4322	0.001922	0.008606	protein_coding
ENSG0000(	ENSG0000(	22.40536	0.527917	0.001922	0.008607	lncRNA
ENSG0000(	FMNL1	1179.557	0.349916	0.001923	0.008607	protein_coding
ENSG0000(	PVRIG	66.79004	0.367596	0.001924	0.008609	protein_coding
ENSG0000(	ASB11	179.8544	-0.18237	0.001928	0.008626	protein_coding
ENSG0000(	SNX4	486.7619	-0.21841	0.00193	0.008634	protein_coding
ENSG0000(	PLCE1	11916.18	-0.38841	0.001931	0.008641	protein_coding
ENSG0000(	SCARNA22	89.23852	-0.42713	0.001932	0.008645	scaRNA
ENSG0000(	ENSG0000(	20.02154	0.444776	0.001933	0.008645	lncRNA
ENSG0000(	SDHAF1	145.8621	-0.20985	0.001933	0.008645	protein_coding
ENSG0000(	MEFV	721.6098	0.314556	0.001935	0.008651	protein_coding
ENSG0000(	PPM1E	19.21833	0.828339	0.001935	0.008651	protein_coding
ENSG0000(	DHRS4L2	216.3265	-0.32628	0.001935	0.008651	protein_coding
ENSG0000(	LUC7L2	3135.557	0.182181	0.001936	0.008653	protein_coding
ENSG0000(	ADIPOQ	291.8309	-0.21638	0.001939	0.008665	protein_coding
ENSG0000(	BNIP3P37	94.72705	-0.29647	0.001939	0.008666	processed_pseudogene
ENSG0000(	ENSG0000(	655.6854	0.165545	0.001942	0.008676	lncRNA
ENSG0000(	TBRG1	2115.121	0.111441	0.001943	0.008681	protein_coding
ENSG0000(	ENSG0000(	4583.51	0.139003	0.001947	0.008696	lncRNA

ENSG0000(	MED13	7136.258	-0.21301	0.001948	0.008697	protein_coding
ENSG0000(	MED24	1345.249	-0.11978	0.00195	0.008707	protein_coding
ENSG0000(	ENSG0000(	380.4561	0.156557	0.00195	0.008707	lncRNA
ENSG0000(	GNA12	987.9668	0.231078	0.001952	0.008714	protein_coding
ENSG0000(	SBF1	3729.113	-0.17206	0.001958	0.008738	protein_coding
ENSG0000(	BHMT2	424.8794	0.273532	0.00196	0.008748	protein_coding
ENSG0000(	LINC02475	78.41706	0.260223	0.001961	0.008748	lncRNA
ENSG0000(	ENSG0000(	14.40063	0.779985	0.001963	0.008757	lncRNA
ENSG0000(	EBF1	683.1723	0.473212	0.001967	0.008775	protein_coding
ENSG0000(	ACVR2B-AS	239.9443	0.152508	0.001968	0.008776	lncRNA
ENSG0000(	ENSG0000(	16.37547	1.101835	0.001968	0.008777	lncRNA
ENSG0000(	ENSG0000(	186.9615	-0.20319	0.001969	0.008778	lncRNA
ENSG0000(	MAPK10	3280.016	0.148104	0.001969	0.008778	protein_coding
ENSG0000(	SASH1	1262.054	0.210436	0.001971	0.008787	protein_coding
ENSG0000(	ZNF37BP	3152.59	0.178338	0.001972	0.00879	transcribed_processed_pseudoge
ENSG0000(	PITPNM2	1449.247	-0.22003	0.001975	0.008802	protein_coding
ENSG0000(	LY6E	278.3073	0.535995	0.001979	0.008816	protein_coding
ENSG0000(	RN7SKP97	47.06588	0.400554	0.001979	0.008817	misc_RNA
ENSG0000(	MRPL19	995.0334	-0.22985	0.001981	0.008825	protein_coding
ENSG0000(	CCDC59	414.3657	-0.16882	0.001982	0.008827	protein_coding
ENSG0000(	ENSG0000(	16.40055	0.562862	0.001982	0.008827	lncRNA
ENSG0000(	MCF2L2	1346.683	0.150489	0.001982	0.008827	protein_coding
ENSG0000(	ENSG0000(	74.72387	0.391253	0.001988	0.008849	lncRNA
ENSG0000(	ENSG0000(	405.3407	0.148603	0.001988	0.00885	lncRNA
ENSG0000(	ZFAND1	686.7848	-0.21558	0.001989	0.00885	protein_coding
ENSG0000(	MDM2	3600.657	0.264341	0.001994	0.008874	protein_coding
ENSG0000(	PKD1P6	1171.365	-0.19363	0.001995	0.008874	transcribed_unprocessed_pseudo
ENSG0000(	PLEKHG2	2026.325	0.337628	0.001997	0.008882	protein_coding
ENSG0000(	ENSG0000(	95.2274	0.833228	0.002	0.008892	lncRNA
ENSG0000(	PSMC4	618.7693	-0.20545	0.001999	0.008892	protein_coding
ENSG0000(	UBASH3B	444.6746	0.343838	0.002	0.008892	protein_coding
ENSG0000(	ZNF426	2751.214	-0.11785	0.002	0.008892	protein_coding
ENSG0000(	TMPRSS13	94.6314	0.409125	0.002002	0.008898	protein_coding
ENSG0000(	RECQL	753.9333	0.204462	0.002002	0.008899	protein_coding
ENSG0000(	UGT1A5	333.0146	-0.68267	0.002005	0.00891	protein_coding
ENSG0000(	FNTB	293.528	-0.26902	0.002006	0.008912	protein_coding
ENSG0000(	GDPD2	157.2785	-0.53776	0.002006	0.008912	protein_coding
ENSG0000(	LINC00629	302.8871	0.248554	0.002008	0.008922	lncRNA
ENSG0000(	ENSG0000(	3397.975	-0.28734	0.002009	0.008922	lncRNA
ENSG0000(	ENSG0000(	165.1857	0.246283	0.00201	0.008925	lncRNA
ENSG0000(	ZNF580	1040.486	0.226736	0.002012	0.008933	protein_coding
ENSG0000(	TFPI	3867.033	0.167602	0.002016	0.008948	protein_coding
ENSG0000(	TRAPPC10	3041.491	-0.16228	0.002016	0.00895	protein_coding
ENSG0000(	FUT9	147.633	-0.32915	0.002018	0.008954	protein_coding
ENSG0000(	NPIPB15	7632.567	0.185931	0.002018	0.008954	protein_coding
ENSG0000(	RPSAP6	29.53742	-0.56637	0.002019	0.008959	processed_pseudogene
ENSG0000(	ENSG0000(	390.8577	0.24705	0.00202	0.00896	lncRNA

ENSG0000(	ENSG0000(	15.84589	0.530882	0.00202	0.00896	lncRNA
ENSG0000(	NOL10	663.9871	-0.2319	0.002023	0.008974	protein_coding
ENSG0000(	ENSG0000(	207.1938	0.2341	0.002024	0.008976	lncRNA
ENSG0000(	MAPT-AS1	394.8908	-0.17365	0.002024	0.008976	lncRNA
ENSG0000(	TNS1	3934.858	0.356488	0.002025	0.008979	protein_coding
ENSG0000(	ENSG0000(	238.9931	-0.2043	0.002026	0.008982	lncRNA
ENSG0000(	ENSG0000(	11.62613	0.862979	0.00203	0.008999	unitary_pseudogene
ENSG0000(	ENSG0000(	336.9855	0.206885	0.002033	0.009007	lncRNA
ENSG0000(	C15orf40	4205.993	-0.11611	0.002033	0.009007	protein_coding
ENSG0000(	PLK3	419.3129	0.284396	0.002038	0.009027	protein_coding
ENSG0000(	ACO2	3763.707	-0.26069	0.002039	0.00903	protein_coding
ENSG0000(	ATG5	930.661	-0.15541	0.002039	0.00903	protein_coding
ENSG0000(	NFE4	12.76651	0.769918	0.002039	0.00903	lncRNA
ENSG0000(	PRKACB	2532.38	-0.25675	0.002039	0.00903	protein_coding
ENSG0000(	PRR13	3001.354	-0.28151	0.002043	0.009046	protein_coding
ENSG0000(	RBMS3-AS2	147.8407	-0.25034	0.002045	0.009054	lncRNA
ENSG0000(	EZH1	2606.951	-0.10482	0.002047	0.009059	protein_coding
ENSG0000(	HYKK	1095.367	-0.10904	0.002047	0.009059	protein_coding
ENSG0000(	LARP7P1	9.193705	1.028876	0.002047	0.009059	processed_pseudogene
ENSG0000(	CCDC120	632.7096	-0.20052	0.00205	0.009069	protein_coding
ENSG0000(	CIR1	641.6485	0.12749	0.00205	0.00907	protein_coding
ENSG0000(	PAN2	1971.425	-0.17365	0.002051	0.009071	protein_coding
ENSG0000(	ENSG0000(	896.6608	-0.44763	0.00206	0.009112	lncRNA
ENSG0000(	MASP2	63.67981	0.359841	0.002062	0.009116	protein_coding
ENSG0000(	MDFI	41.91691	0.895709	0.002062	0.009116	protein_coding
ENSG0000(	ENSG0000(	484.4198	0.203638	0.002063	0.009119	lncRNA
ENSG0000(	AARSD1	716.2256	-0.17096	0.002067	0.009136	protein_coding
ENSG0000(	YBX1P8	18.29007	0.786981	0.002067	0.009136	processed_pseudogene
ENSG0000(	EDC4	1309.277	-0.27158	0.002071	0.009151	protein_coding
ENSG0000(	ENSG0000(	99.52063	-0.34722	0.002072	0.009154	lncRNA
ENSG0000(	ENSG0000(	30.06219	0.488601	0.002075	0.009165	lncRNA
ENSG0000(	ZNF554	1543.839	-0.12145	0.002079	0.009183	protein_coding
ENSG0000(	RNF180	218.8888	0.209412	0.002081	0.00919	protein_coding
ENSG0000(	USH1C	2281.363	-0.41415	0.002083	0.009195	protein_coding
ENSG0000(	ENSG0000(	17.51448	0.613378	0.002083	0.009195	lncRNA
ENSG0000(	SNRPE	325.1924	-0.34	0.002084	0.009197	protein_coding
ENSG0000(	ATP6V0B	577.2963	-0.26535	0.002085	0.009202	protein_coding
ENSG0000(	ENSG0000(	15.5882	0.587833	0.002085	0.009202	lncRNA
ENSG0000(	IGSF3	1115.342	-0.54516	0.002086	0.009202	protein_coding
ENSG0000(	ZNF593	45.03814	-0.41276	0.002086	0.009202	protein_coding
ENSG0000(	ENSG0000(	221.458	0.223433	0.002089	0.009214	lncRNA
ENSG0000(	PRKCH	1408.083	0.247907	0.002095	0.009238	protein_coding
ENSG0000(	CICP18	1061.292	0.202296	0.002096	0.009245	transcribed_processed_pseudoge
ENSG0000(	ENSG0000(	22.46259	-0.97665	0.002101	0.009263	unprocessed_pseudogene
ENSG0000(	SEMA3G	134.939	-0.52913	0.002101	0.009263	protein_coding
ENSG0000(	ENSG0000(	42.43683	0.529626	0.002103	0.009267	lncRNA
ENSG0000(	IGSF9	1652.763	-0.69151	0.002103	0.009267	protein_coding

ENSG0000(	NPY1R	250.7765	-0.7526	0.002103	0.009267	protein_coding
ENSG0000(	ENSG0000(	26.69174	-0.72391	0.002104	0.009272	protein_coding
ENSG0000(	ENSG0000(	1417.422	-0.31019	0.002105	0.009275	lncRNA
ENSG0000(	KHDRBS2	54.89566	-0.64933	0.002108	0.009287	protein_coding
ENSG0000(	ENSG0000(	182.7165	0.300392	0.002109	0.009287	lncRNA
ENSG0000(	UGT1A6	1270.921	-0.65583	0.00211	0.00929	protein_coding
ENSG0000(	ZNF574	280.4063	-0.23431	0.002112	0.009299	protein_coding
ENSG0000(	ENSG0000(	75.0984	0.342691	0.002113	0.009302	lncRNA
ENSG0000(	PDGFC	297.4503	0.368453	0.002114	0.009304	protein_coding
ENSG0000(	ENSG0000(	89.06151	0.302497	0.002114	0.009305	lncRNA
ENSG0000(	ENSG0000(	19.03232	0.593463	0.002115	0.009307	lncRNA
ENSG0000(	RN7SL351F	81.35728	0.324206	0.002116	0.009308	misc_RNA
ENSG0000(	ENSG0000(	327.8082	0.185804	0.002117	0.009312	lncRNA
ENSG0000(	IGHMBP2	1312.829	-0.20787	0.002118	0.009317	protein_coding
ENSG0000(	ANKRD10	5190.487	0.267452	0.002119	0.009318	protein_coding
ENSG0000(	RBP7	79.50315	-0.30112	0.002119	0.009318	protein_coding
ENSG0000(	ERRFI1	2491.155	0.564255	0.00212	0.00932	protein_coding
ENSG0000(	PLD6	176.1772	0.196562	0.002121	0.009326	protein_coding
ENSG0000(	ESRRAP2	120.5668	0.288921	0.002122	0.009328	transcribed_processed_pseudoge
ENSG0000(	IMP3	476.1285	-0.18808	0.002122	0.009328	protein_coding
ENSG0000(	ENSG0000(	651.835	0.173867	0.002125	0.009339	lncRNA
ENSG0000(	ENSG0000(	26.95371	0.557085	0.002126	0.009341	lncRNA
ENSG0000(	OGFRP1	356.3121	-0.1928	0.002126	0.009341	lncRNA
ENSG0000(	PNRC1	2264.781	0.269422	0.002131	0.009362	protein_coding
ENSG0000(	TMEM14EF	21.33033	1.001816	0.002137	0.009387	transcribed_processed_pseudoge
ENSG0000(	OGFRL1	1274.294	0.325312	0.002138	0.009389	protein_coding
ENSG0000(	ENSG0000(	55.86424	-0.58075	0.002139	0.009389	protein_coding
ENSG0000(	DOP1A	2727.087	-0.18799	0.00214	0.009392	protein_coding
ENSG0000(	PUM2	4971.117	-0.1168	0.00214	0.009395	protein_coding
ENSG0000(	ENSG0000(	234.907	-0.21149	0.002141	0.009397	lncRNA
ENSG0000(	ENSG0000(	27.17099	0.541129	0.002142	0.009397	lncRNA
ENSG0000(	RAB8B	849.9263	0.423763	0.002142	0.009398	protein_coding
ENSG0000(	OMP	68.23309	-0.6407	0.002143	0.009401	protein_coding
ENSG0000(	ENSG0000(	15.76403	0.795725	0.002145	0.009406	lncRNA
ENSG0000(	GDI2	2844.831	-0.20124	0.002145	0.009406	protein_coding
ENSG0000(	ENSG0000(	76.30634	0.30232	0.002146	0.00941	lncRNA
ENSG0000(	FBXO10	334.2881	0.349439	0.002146	0.00941	protein_coding
ENSG0000(	C19orf54	584.3765	-0.24026	0.00215	0.009423	protein_coding
ENSG0000(	SERPINF2	52.67302	0.579747	0.00215	0.009423	protein_coding
ENSG0000(	ENSG0000(	159.7669	0.25667	0.00215	0.009424	lncRNA
ENSG0000(	PATE4	304.7448	0.202489	0.002151	0.009426	protein_coding
ENSG0000(	ENSG0000(	161.5401	-0.53777	0.002153	0.009432	processed_pseudogene
ENSG0000(	SDE2	841.7147	-0.16247	0.002153	0.009432	protein_coding
ENSG0000(	LINC00971	102.8807	-0.3405	0.002155	0.009439	lncRNA
ENSG0000(	USP30-AS1	40.59085	0.540118	0.002156	0.00944	lncRNA
ENSG0000(	MAVS	13541.54	-0.22503	0.002157	0.009444	protein_coding
ENSG0000(	SEC11C	684.7333	0.256341	0.002157	0.009444	protein_coding

ENSG0000(	PSMG2	355.3632	-0.23462	0.002159	0.00945	protein_coding
ENSG0000(	FOLH1	128.2027	0.788982	0.002163	0.009467	protein_coding
ENSG0000(	ENSG0000(	637.0379	-0.14389	0.002165	0.009473	lncRNA
ENSG0000(	RNF112	168.1489	-0.46886	0.002165	0.009473	protein_coding
ENSG0000(	VN1R83P	74.58282	0.326407	0.002165	0.009473	unprocessed_pseudogene
ENSG0000(	ENSG0000(	105.3255	-0.3047	0.002166	0.009473	lncRNA
ENSG0000(	ARGLU1	6094.994	0.123836	0.002168	0.009483	protein_coding
ENSG0000(	SLC35B3	890.5425	-0.18172	0.002168	0.009483	protein_coding
ENSG0000(	NTM-AS1	91.0845	-0.23662	0.00217	0.009488	lncRNA
ENSG0000(	ENSG0000(	22.15508	0.475195	0.002173	0.009498	lncRNA
ENSG0000(	ABCB9	911.7393	0.135546	0.002174	0.009503	protein_coding
ENSG0000(	ENSG0000(	29.54395	0.461524	0.002174	0.009503	lncRNA
ENSG0000(	FAM228A	15.01855	0.834461	0.002175	0.009503	protein_coding
ENSG0000(	RFC5	320.9313	-0.25292	0.002176	0.009507	protein_coding
ENSG0000(	SPIN1	2352.691	-0.18004	0.002176	0.009507	protein_coding
ENSG0000(	KDM5D	1906.058	0.243706	0.002179	0.009517	protein_coding
ENSG0000(	SLC15A4	887.0603	0.187351	0.002184	0.009537	protein_coding
ENSG0000(	MGST3	2967.578	-0.24096	0.002184	0.009538	protein_coding
ENSG0000(	ZBTB46	411.4694	0.292441	0.002187	0.00955	protein_coding
ENSG0000(	KCTD5	961.0743	-0.29038	0.002188	0.009553	protein_coding
ENSG0000(	ENSG0000(	292.2024	0.251566	0.002189	0.009557	transcribed_processed_pseudoge
ENSG0000(	FHIP2A	811.1616	0.261213	0.00219	0.009557	protein_coding
ENSG0000(	ENSG0000(	1325.922	0.11478	0.002191	0.00956	TEC
ENSG0000(	ENSG0000(	84.51993	0.304309	0.002191	0.009562	lncRNA
ENSG0000(	KIF21A	1032.952	-0.26418	0.002193	0.009565	protein_coding
ENSG0000(	RNF7	839.1257	-0.25963	0.002193	0.009565	protein_coding
ENSG0000(	ENSG0000(	22.24356	-0.85564	0.002194	0.009568	lncRNA
ENSG0000(	LINC02481	86.10854	0.33278	0.002194	0.00957	lncRNA
ENSG0000(	LINC01962	331.6489	-0.16821	0.002197	0.00958	lncRNA
ENSG0000(	ENSG0000(	132.6007	0.269384	0.002199	0.009584	lncRNA
ENSG0000(	ENSG0000(	16.28571	0.625977	0.002198	0.009584	lncRNA
ENSG0000(	FBXO27	567.6611	0.199008	0.002199	0.009584	protein_coding
ENSG0000(	PWWP2B	578.8592	-0.38558	0.002199	0.009584	protein_coding
ENSG0000(	EED	822.8052	-0.1596	0.0022	0.009586	protein_coding
ENSG0000(	ERH	433.7719	-0.30869	0.002204	0.009601	protein_coding
ENSG0000(	ENSG0000(	83.7032	0.351899	0.00221	0.009627	TEC
ENSG0000(	TGFB1	585.3545	0.361705	0.002211	0.009628	protein_coding
ENSG0000(	CDK3	96.67501	-0.57843	0.002212	0.009631	protein_coding
ENSG0000(	NEMP2	552.2751	0.351253	0.002212	0.009633	protein_coding
ENSG0000(	MARCKSL1	51.09139	0.319218	0.002214	0.009639	processed_pseudogene
ENSG0000(	DLG3	1983.173	-0.32709	0.002217	0.009652	protein_coding
ENSG0000(	ENSG0000(	55.52773	0.359068	0.002218	0.009653	lncRNA
ENSG0000(	ENSG0000(	102.4276	0.308566	0.00222	0.00966	TEC
ENSG0000(	ANKRD10-I	542.9047	0.213445	0.002221	0.009661	lncRNA
ENSG0000(	CEP43	2381.538	-0.19772	0.00222	0.009661	protein_coding
ENSG0000(	ANAPC1	2204.979	-0.16846	0.002223	0.009671	protein_coding
ENSG0000(	ZSWIM4	230.4211	0.411821	0.002225	0.009678	protein_coding

ENSG0000(	ENSG0000(	10.95964	0.620334	0.002229	0.009692	lncRNA
ENSG0000(	CDH24	197.2815	-0.34319	0.002229	0.009693	protein_coding
ENSG0000(	C5orf22	572.7021	-0.1475	0.002231	0.0097	protein_coding
ENSG0000(	TDH-AS1	298.8366	0.362563	0.002232	0.009702	lncRNA
ENSG0000(	CENPH	169.1486	-0.26992	0.002232	0.009704	protein_coding
ENSG0000(	ENSG0000(	34.15384	0.510589	0.002234	0.009707	lncRNA
ENSG0000(	COQ5	531.1504	-0.13892	0.002234	0.009708	protein_coding
ENSG0000(	ENSG0000(	171.4684	-0.20818	0.002239	0.009728	TEC
ENSG0000(	LINC01012	683.535	-0.11159	0.00224	0.009729	lncRNA
ENSG0000(	DEK	2818.155	-0.14773	0.002242	0.009736	protein_coding
ENSG0000(	NPTN-IT1	676.6029	0.175965	0.002242	0.009736	lncRNA
ENSG0000(	TM9SF2	2377.902	-0.20899	0.002242	0.009736	protein_coding
ENSG0000(	DCDC2	105.4518	-0.44917	0.002243	0.009737	protein_coding
ENSG0000(	TXNL4B	395.9691	-0.19214	0.002243	0.009737	protein_coding
ENSG0000(	NRROS	140.5041	0.485887	0.002244	0.009742	protein_coding
ENSG0000(	NCF1B	175.0172	0.609382	0.002246	0.009745	transcribed_unprocessed_pseudo
ENSG0000(	KIF23-AS1	143.4685	0.339237	0.002247	0.009748	lncRNA
ENSG0000(	CYP4X1	28.98224	0.755423	0.00225	0.009761	protein_coding
ENSG0000(	TAPT1	999.3784	-0.14068	0.00225	0.009761	protein_coding
ENSG0000(	DALRD3	202.4478	-0.27989	0.002251	0.009764	protein_coding
ENSG0000(	FURIN	1385.595	0.284503	0.002251	0.009764	protein_coding
ENSG0000(	PGM5P3-A	144.927	-0.35912	0.002252	0.009764	lncRNA
ENSG0000(	OR8H2	21.99575	-0.55194	0.002253	0.00977	protein_coding
ENSG0000(	CEP41	845.4763	-0.12112	0.002256	0.009779	protein_coding
ENSG0000(	MN1	125.0975	0.577879	0.002259	0.00979	protein_coding
ENSG0000(	ENSG0000(	75.75935	0.315731	0.002261	0.009801	lncRNA
ENSG0000(	ENSG0000(	52.6721	0.593712	0.002265	0.009816	lncRNA
ENSG0000(	RN7SL43P	30.75321	0.411744	0.002267	0.009824	misc_RNA
ENSG0000(	ENSG0000(	94.05793	0.365522	0.002268	0.009824	lncRNA
ENSG0000(	PYGB	3959.837	-0.39769	0.002269	0.009829	protein_coding
ENSG0000(	YWHAZ	10830.1	-0.21327	0.00227	0.009831	protein_coding
ENSG0000(	BOLA2B	135.4336	-0.54365	0.002271	0.009836	protein_coding
ENSG0000(	GTF2H5	855.0321	-0.21826	0.002273	0.00984	protein_coding
ENSG0000(	MAP3K21	1105.579	-0.3739	0.002273	0.00984	protein_coding
ENSG0000(	TBC1D5	2325.023	0.15039	0.002273	0.00984	protein_coding
ENSG0000(	ENSG0000(	1881.297	0.146859	0.002275	0.009845	lncRNA
ENSG0000(	STEAP3	806.9897	-0.40817	0.002283	0.009879	protein_coding
ENSG0000(	ENSG0000(	764.2616	0.113499	0.002285	0.009884	TEC
ENSG0000(	ZNF395	1661.471	-0.23178	0.002285	0.009884	protein_coding
ENSG0000(	ENSG0000(	354.6848	0.361563	0.002286	0.009885	TEC
ENSG0000(	KDF1	194.3055	-0.43948	0.002286	0.009885	protein_coding
ENSG0000(	PREP	1976.43	-0.18807	0.002286	0.009886	protein_coding
ENSG0000(	SFR1	180.3974	-0.26759	0.002286	0.009886	protein_coding
ENSG0000(	CDK4	680.3544	-0.19664	0.002288	0.00989	protein_coding
ENSG0000(	KCNAB2	1656.677	0.349357	0.00229	0.009899	protein_coding
ENSG0000(	ESRRA	2023.833	-0.33064	0.002293	0.009908	protein_coding
ENSG0000(	MANF	598.2576	-0.33667	0.002295	0.009916	protein_coding

ENSG0000(	RHBDD1	1069.286	0.184691	0.002295	0.009916	protein_coding
ENSG0000(	TRIM65	882.4146	-0.13272	0.002295	0.009916	protein_coding
ENSG0000(	CTSG	25.10641	-0.78928	0.002296	0.009918	protein_coding
ENSG0000(	ENSG0000(	83.30592	-0.29271	0.0023	0.009933	lncRNA
ENSG0000(	LRP5	2401.242	-0.33284	0.0023	0.009933	protein_coding
ENSG0000(	HHIP-AS1	20.16397	-0.60732	0.002305	0.009952	lncRNA
ENSG0000(	CCNH	2065.5	0.119167	0.002307	0.009956	protein_coding
ENSG0000(	ENSG0000(	61.62956	0.359539	0.002307	0.009956	lncRNA
ENSG0000(	GLO1	537.1531	-0.31804	0.002307	0.009956	protein_coding
ENSG0000(	AADAT	148.0785	-0.31397	0.002309	0.009965	protein_coding
ENSG0000(	ENSG0000(	237.1079	-0.26997	0.00231	0.009965	lncRNA
ENSG0000(	ENSG0000(	16.52238	0.636522	0.00231	0.009965	lncRNA
ENSG0000(	ENSG0000(	163.9579	0.240943	0.002311	0.009969	lncRNA
ENSG0000(	TCF7L1-IT1	25.71057	0.431529	0.002311	0.009969	lncRNA
ENSG0000(	PLPBP	942.1196	-0.2196	0.002315	0.009982	protein_coding
ENSG0000(	TSPAN9-IT1	20.09021	0.731453	0.002315	0.009982	lncRNA
ENSG0000(	PANX1	316.5665	0.345416	0.002316	0.009985	protein_coding
ENSG0000(	ITGAM	315.8364	0.627508	0.002317	0.009989	protein_coding
ENSG0000(	ENSG0000(	257.0897	0.241219	0.002318	0.009992	transcribed_unprocessed_pseudo
ENSG0000(	ENSG0000(	140.4284	0.185471	0.002321	0.010001	lncRNA
ENSG0000(	RBFA	929.2519	-0.17457	0.002322	0.010004	protein_coding
ENSG0000(	WAKMAR2	271.2573	0.248942	0.002323	0.010006	lncRNA
ENSG0000(	ENSG0000(	176.527	0.201418	0.002323	0.010008	lncRNA
ENSG0000(	OSBPL10-A	68.6992	-0.39547	0.002328	0.010028	lncRNA
ENSG0000(	ENSG0000(	227.5443	0.253263	0.002329	0.010028	protein_coding
ENSG0000(	RPS3AP30	42.22247	-0.53785	0.00233	0.010031	processed_pseudogene
ENSG0000(	SVOPL	89.21795	-0.86154	0.00233	0.010031	protein_coding
ENSG0000(	RBM20	257.1511	0.373029	0.002334	0.010046	protein_coding
ENSG0000(	TMEM121	13.0008	0.675849	0.002338	0.010063	protein_coding
ENSG0000(	YKT6	1552.933	-0.19958	0.002341	0.010073	protein_coding
ENSG0000(	ENSG0000(	11.90869	0.747216	0.002341	0.010074	lncRNA
ENSG0000(	ENSG0000(	25.70797	0.653024	0.002343	0.01008	lncRNA
ENSG0000(	ACAD11	1098.711	0.22487	0.002345	0.010085	protein_coding
ENSG0000(	ENSG0000(	437.3035	0.258626	0.002345	0.010085	lncRNA
ENSG0000(	ENSG0000(	127.2449	-0.23515	0.002346	0.010087	lncRNA
ENSG0000(	ENSG0000(	38.72208	0.357987	0.002346	0.010087	lncRNA
ENSG0000(	ENSG0000(	42.6916	0.423689	0.002346	0.010087	TEC
ENSG0000(	DUSP19	370.5779	-0.20643	0.002349	0.010095	protein_coding
ENSG0000(	DYNC1I2	1430.24	-0.20475	0.002349	0.010095	protein_coding
ENSG0000(	ARHGAP9	900.4069	0.507496	0.002353	0.010111	protein_coding
ENSG0000(	AP2A1	1471.834	-0.1549	0.002354	0.010114	protein_coding
ENSG0000(	TMEM184(	1101.599	-0.16757	0.002354	0.010114	protein_coding
ENSG0000(	ALS2CL	2777.755	-0.29105	0.002355	0.010116	protein_coding
ENSG0000(	IGHE	17.05694	0.728951	0.002355	0.010116	IG_C_gene
ENSG0000(	LINC01370	329.7747	0.254046	0.002358	0.010125	lncRNA
ENSG0000(	SLC41A2	1972.07	-0.30278	0.002359	0.010131	protein_coding
ENSG0000(	COMMD7	329.9942	-0.23143	0.002361	0.010137	protein_coding

ENSG0000(	SELENOK	366.9608	0.284286	0.002362	0.010139	protein_coding
ENSG0000(	IGHV3-36	11.77344	0.789776	0.002362	0.010139	IG_V_pseudogene
ENSG0000(	RNY4	166.7677	0.404232	0.002365	0.010149	misc_RNA
ENSG0000(	KIF2C	192.0929	-0.44789	0.002366	0.010151	protein_coding
ENSG0000(	ACAD9	2894.469	-0.12791	0.002367	0.010153	protein_coding
ENSG0000(	LINC01970	492.5123	0.144694	0.002367	0.010153	lncRNA
ENSG0000(	ENSG0000(	314.1623	0.190232	0.00237	0.010166	lncRNA
ENSG0000(	ENSG0000(	50.91612	0.303649	0.002371	0.010168	lncRNA
ENSG0000(	IFT88	802.1209	-0.17446	0.002372	0.010171	protein_coding
ENSG0000(	EMC9	104.9223	-0.39308	0.002373	0.010175	protein_coding
ENSG0000(	CAPNS1	1861.418	-0.25523	0.002376	0.010187	protein_coding
ENSG0000(	KPNA4P1	118.5239	-0.37177	0.002378	0.010193	processed_pseudogene
ENSG0000(	IPO8P1	13.5738	-0.86658	0.002379	0.010197	processed_pseudogene
ENSG0000(	SNHG26	800.3927	0.315781	0.002383	0.010213	lncRNA
ENSG0000(	MGAT4A	5491.365	-0.23313	0.002385	0.010218	protein_coding
ENSG0000(	NRN1	60.98211	0.473229	0.002385	0.010218	protein_coding
ENSG0000(	CLCA3P	39.79282	-1.49441	0.002385	0.010218	transcribed_unprocessed_pseudo
ENSG0000(	TICAM2-AS	56.31934	0.626469	0.002386	0.010219	lncRNA
ENSG0000(	ENSG0000(	8.802541	0.797513	0.002389	0.010228	transcribed_unitary_pseudogene
ENSG0000(	MYOF	2522.573	0.347227	0.002389	0.010228	protein_coding
ENSG0000(	VPS37C	629.6226	0.136168	0.002389	0.010228	protein_coding
ENSG0000(	C12orf57	260.8352	-0.30117	0.00239	0.010229	protein_coding
ENSG0000(	CLVS2	32.81604	0.763471	0.002396	0.010256	protein_coding
ENSG0000(	SLC38A1	8743.46	-0.18754	0.0024	0.010269	protein_coding
ENSG0000(	ALDOA	4459.542	-0.37098	0.002402	0.010276	protein_coding
ENSG0000(	NPC1	2035.823	0.154654	0.002404	0.010284	protein_coding
ENSG0000(	OGFOD3	772.5455	-0.17928	0.002406	0.01029	protein_coding
ENSG0000(	ENSG0000(	12.4159	-0.79944	0.002407	0.010295	processed_pseudogene
ENSG0000(	PIAS4	375.7852	-0.2312	0.002408	0.010296	protein_coding
ENSG0000(	C17orf97	109.915	-0.67314	0.002409	0.010299	protein_coding
ENSG0000(	GRIN2D	36.25705	0.569972	0.002413	0.010317	protein_coding
ENSG0000(	VSIG2	798.7859	-0.54572	0.002414	0.01032	protein_coding
ENSG0000(	HDAC7	1928.306	0.328781	0.002415	0.01032	protein_coding
ENSG0000(	GALNT3	890.311	-0.30293	0.002418	0.010332	protein_coding
ENSG0000(	IGHEP2	75.22436	0.42656	0.002418	0.010334	IG_C_pseudogene
ENSG0000(	ENSG0000(	1331.956	0.150924	0.00242	0.010339	lncRNA
ENSG0000(	ENSG0000(	20.94259	0.502937	0.002422	0.010345	lncRNA
ENSG0000(	ENSG0000(	110.6224	0.315299	0.002422	0.010345	lncRNA
ENSG0000(	FGF13	2806.327	0.128845	0.002422	0.010345	protein_coding
ENSG0000(	SPRING1	2177.382	-0.29043	0.002424	0.01035	protein_coding
ENSG0000(	CLEC2B	376.7402	0.407293	0.002429	0.010369	protein_coding
ENSG0000(	TRGC1	140.4768	-0.47716	0.002429	0.010369	TR_C_gene
ENSG0000(	PRR14L	5818.172	-0.20322	0.00243	0.010372	protein_coding
ENSG0000(	TFCP2L1	5082.838	-0.46601	0.002431	0.010373	protein_coding
ENSG0000(	MGRN1	2628.976	-0.1043	0.002434	0.010387	protein_coding
ENSG0000(	FXR2	646.5332	-0.15209	0.002437	0.010399	protein_coding
ENSG0000(	CTSC	8549.694	0.264174	0.002439	0.010404	protein_coding

ENSG0000(	ENSG0000(	302.6668	-0.17299	0.002439	0.010404	lncRNA
ENSG0000(	SHC4	324.1738	0.21524	0.002442	0.010416	protein_coding
ENSG0000(	BABAM1	639.8116	-0.12456	0.002444	0.010418	protein_coding
ENSG0000(	ENSG0000(	172.7476	0.233656	0.002444	0.010418	protein_coding
ENSG0000(	ENSG0000(	106.3378	-0.21008	0.002444	0.010418	lncRNA
ENSG0000(	PCDHA9	272.1945	-0.18676	0.002443	0.010418	protein_coding
ENSG0000(	STEAP2	2264.568	0.315171	0.002448	0.010434	protein_coding
ENSG0000(	CHMP2B	1237.298	-0.24469	0.00245	0.010439	protein_coding
ENSG0000(	MYO18B-A	13.01889	0.632966	0.002451	0.010441	lncRNA
ENSG0000(	RBM33	5273.972	0.092955	0.002452	0.010446	protein_coding
ENSG0000(	BCL2L1	1121.762	0.226196	0.002457	0.010464	protein_coding
ENSG0000(	SNORD95	35.1042	0.694998	0.00246	0.010475	snoRNA
ENSG0000(	TAS2R15P	210.5802	0.296158	0.002462	0.010484	unprocessed_pseudogene
ENSG0000(	CARD8	3350.196	0.273915	0.002464	0.01049	protein_coding
ENSG0000(	MAP3K12	1101.577	0.212759	0.002467	0.010503	protein_coding
ENSG0000(	ENSG0000(	29.97546	-0.9172	0.002468	0.010505	protein_coding
ENSG0000(	RNPEP	1055.428	-0.25586	0.00247	0.010512	protein_coding
ENSG0000(	ENSG0000(	491.8671	-0.17046	0.002474	0.010529	lncRNA
ENSG0000(	ENSG0000(	23.08928	0.66941	0.002475	0.01053	lncRNA
ENSG0000(	PRPF19-DT	13.45182	0.609993	0.002475	0.010531	lncRNA
ENSG0000(	RNU6-212F	41.49945	0.629894	0.00248	0.01055	snRNA
ENSG0000(	MYL12B	2039.445	-0.4019	0.002483	0.010562	protein_coding
ENSG0000(	ENSG0000(	233.1114	-0.2506	0.002484	0.010563	lncRNA
ENSG0000(	PPFIA2-AS1	27.10015	0.449214	0.002489	0.010581	lncRNA
ENSG0000(	USP30	595.4379	-0.25637	0.002489	0.010581	protein_coding
ENSG0000(	LINC01441	202.6227	-0.24885	0.00249	0.010585	lncRNA
ENSG0000(	NAT1	378.8974	-0.33196	0.002493	0.010597	protein_coding
ENSG0000(	TMEM202-	157.0301	0.252345	0.002494	0.010598	lncRNA
ENSG0000(	TTLL10-AS1	73.36366	-0.30676	0.002496	0.010607	lncRNA
ENSG0000(	FBP1	220.8629	-0.39025	0.002499	0.010617	protein_coding
ENSG0000(	HUNK	845.1194	-0.2532	0.002504	0.010638	protein_coding
ENSG0000(	LINC00392	40.9022	-0.76962	0.002507	0.010649	lncRNA
ENSG0000(	NEMF	2780.309	-0.14769	0.002508	0.010651	protein_coding
ENSG0000(	LINC01694	1090.957	0.226367	0.002509	0.010652	lncRNA
ENSG0000(	LINC00881	72.16799	0.316579	0.002511	0.010663	lncRNA
ENSG0000(	ENSG0000(	74.33252	-0.52101	0.002514	0.010673	lncRNA
ENSG0000(	KIF16B	3051.787	-0.37669	0.002515	0.010673	protein_coding
ENSG0000(	ENSG0000(	24.25524	0.759325	0.002516	0.010677	processed_pseudogene
ENSG0000(	AGFG2	2059.857	-0.34939	0.002518	0.010681	protein_coding
ENSG0000(	ENSG0000(	282.0052	0.211504	0.002518	0.010681	lncRNA
ENSG0000(	LINC01647	127.166	0.269414	0.002517	0.010681	lncRNA
ENSG0000(	ENSG0000(	306.6766	-0.20186	0.00252	0.010688	lncRNA
ENSG0000(	ALOX5AP	93.74641	0.588825	0.002522	0.010698	protein_coding
ENSG0000(	OSBPL8	1439.858	0.338458	0.002523	0.010698	protein_coding
ENSG0000(	ENSG0000(	294.4443	0.198926	0.002523	0.010698	TEC
ENSG0000(	PLRG1	622.6275	-0.15276	0.002524	0.0107	protein_coding
ENSG0000(	LRCH1	1459.59	0.186796	0.002525	0.010703	protein_coding

ENSG0000(	DGUOK	334.9674	-0.19938	0.002526	0.010703	protein_coding
ENSG0000(	GPBP1L1	4704.062	-0.15977	0.002525	0.010703	protein_coding
ENSG0000(	SLC5A1	744.3132	0.497643	0.002526	0.010703	protein_coding
ENSG0000(	ANXA8	24.44377	0.64838	0.002527	0.010705	protein_coding
ENSG0000(	SNORD55	28.00492	0.845281	0.002532	0.010723	snoRNA
ENSG0000(	PIK3CD-AS:	75.16062	0.261127	0.002532	0.010724	lncRNA
ENSG0000(	CAPN1	2536.127	-0.22958	0.002536	0.010738	protein_coding
ENSG0000(	RSL1D1	1710.89	-0.22164	0.002544	0.010771	protein_coding
ENSG0000(	PSMF1	1890.284	-0.18945	0.002549	0.01079	protein_coding
ENSG0000(	ENSG0000(	48.6609	0.431505	0.002551	0.010796	lncRNA
ENSG0000(	LINC02837	211.4095	-0.22062	0.002551	0.010796	lncRNA
ENSG0000(	RFTN1	1137.655	0.415451	0.002551	0.010796	protein_coding
ENSG0000(	MTCYBP32	55.36215	0.395129	0.002557	0.010821	processed_pseudogene
ENSG0000(	TUBG1	524.5385	-0.22093	0.002559	0.010825	protein_coding
ENSG0000(	ENSG0000(	121.9311	0.271136	0.002562	0.010837	lncRNA
ENSG0000(	TMEM255E	292.9257	0.463196	0.002564	0.010843	protein_coding
ENSG0000(	ENSG0000(	136.7166	0.318534	0.002564	0.010844	lncRNA
ENSG0000(	LGI4	310.9045	-0.64181	0.002565	0.010846	protein_coding
ENSG0000(	COL6A1	3929.671	0.372188	0.002567	0.010851	protein_coding
ENSG0000(	GCH1	578.3234	0.344178	0.002572	0.010869	protein_coding
ENSG0000(	MME	373.35	0.694265	0.002572	0.010869	protein_coding
ENSG0000(	OR52N1	144.8913	0.51374	0.002572	0.010869	protein_coding
ENSG0000(	SBSPON	221.177	-0.17708	0.002572	0.010869	protein_coding
ENSG0000(	ENSG0000(	135.2917	0.259626	0.002575	0.010879	TEC
ENSG0000(	CREG2	329.1659	-0.18516	0.002576	0.010883	protein_coding
ENSG0000(	RCCD1	543.9937	-0.22207	0.00258	0.010896	protein_coding
ENSG0000(	ANKRD13A	2774.366	0.134911	0.002581	0.010901	protein_coding
ENSG0000(	HAUS1	258.0554	-0.24695	0.002584	0.01091	protein_coding
ENSG0000(	SPRYD4	2286.712	0.090266	0.002587	0.010921	protein_coding
ENSG0000(	GYPC	357.6385	0.412317	0.002589	0.010929	protein_coding
ENSG0000(	ABRAXAS2	407.2092	-0.17584	0.002592	0.010937	protein_coding
ENSG0000(	ENSG0000(	131.6635	0.207471	0.002592	0.010937	lncRNA
ENSG0000(	ENSG0000(	119.8611	0.364205	0.002596	0.010956	lncRNA
ENSG0000(	NFIX	1139.849	-0.3086	0.002597	0.010956	protein_coding
ENSG0000(	ENSG0000(	119.2868	0.262878	0.002599	0.010966	lncRNA
ENSG0000(	SAMD9	2601.498	0.434526	0.002601	0.010972	protein_coding
ENSG0000(	RASGRP4	266.3758	0.27405	0.002603	0.010977	protein_coding
ENSG0000(	ZNF768	622.1157	-0.32975	0.002605	0.010984	protein_coding
ENSG0000(	CUEDC2	110.0864	-0.30508	0.002606	0.01099	protein_coding
ENSG0000(	TTC21A	447.0799	0.23892	0.002608	0.010995	protein_coding
ENSG0000(	TTN-AS1	2342.961	0.167195	0.002609	0.010996	lncRNA
ENSG0000(	ENSG0000(	186.6516	-0.28416	0.002611	0.011007	lncRNA
ENSG0000(	ENSG0000(	54.83852	0.308825	0.002615	0.011021	lncRNA
ENSG0000(	IL19	303.3096	0.203889	0.002619	0.011033	protein_coding
ENSG0000(	LINC02782	39.94603	-0.43668	0.002619	0.011033	lncRNA
ENSG0000(	TRAF1	943.9805	0.545011	0.002619	0.011033	protein_coding
ENSG0000(	SCARNA12	1146.725	-0.22891	0.002621	0.011038	snoRNA

ENSG0000(	DACT2	48.84756	-0.67324	0.002622	0.011041	protein_coding
ENSG0000(	ENSG0000(	267.8604	-0.53888	0.002622	0.011041	protein_coding
ENSG0000(	ENSG0000(	108.7771	-0.20593	0.002623	0.011042	lncRNA
ENSG0000(	ENSG0000(	155.019	0.243024	0.002624	0.011046	lncRNA
ENSG0000(	PTGDR	953.04	-0.51405	0.002624	0.011046	protein_coding
ENSG0000(	ENSG0000(	666.4091	0.13295	0.002625	0.011048	lncRNA
ENSG0000(	ENSG0000(	58.20101	-0.3784	0.002628	0.011058	processed_pseudogene
ENSG0000(	AQP8	4821.642	-1.26659	0.002629	0.01106	protein_coding
ENSG0000(	LINC02833	63.09673	0.377446	0.002629	0.01106	lncRNA
ENSG0000(	CHP1	5613.724	-0.40047	0.002632	0.011069	protein_coding
ENSG0000(	C19orf71	63.35857	-0.3784	0.002638	0.011094	protein_coding
ENSG0000(	ENSG0000(	267.7716	0.232182	0.002645	0.011121	lncRNA
ENSG0000(	DDX59-AS1	171.9333	-0.2932	0.002647	0.011128	lncRNA
ENSG0000(	ENSG0000(	9.841798	0.711549	0.002648	0.011133	processed_pseudogene
ENSG0000(	NOP58	916.0038	-0.2503	0.002649	0.011134	protein_coding
ENSG0000(	ENSG0000(	406.1056	0.152267	0.002649	0.011135	lncRNA
ENSG0000(	RBMS2P1	16.27826	-0.80005	0.00265	0.011136	processed_pseudogene
ENSG0000(	ENSG0000(	12.56882	0.601097	0.002652	0.011143	lncRNA
ENSG0000(	TRMT6	211.3716	-0.22347	0.002652	0.011143	protein_coding
ENSG0000(	ENSG0000(	40.52571	0.378227	0.002658	0.011165	lncRNA
ENSG0000(	KIF18B	749.0668	-0.23794	0.00266	0.011173	protein_coding
ENSG0000(	GPR199P	109.5597	0.343854	0.002664	0.011187	transcribed_unitary_pseudogene
ENSG0000(	MTURN	737.14	0.238554	0.002666	0.011193	protein_coding
ENSG0000(	SEZ6	24.27655	0.804371	0.002666	0.011193	protein_coding
ENSG0000(	INCENP	503.132	-0.28477	0.002667	0.011195	protein_coding
ENSG0000(	RNY3P2	16.12418	0.590072	0.002667	0.011195	misc_RNA
ENSG0000(	GPN3	113.478	-0.25721	0.002669	0.011199	protein_coding
ENSG0000(	MIR647	28.65492	-0.45941	0.002669	0.011199	miRNA
ENSG0000(	LINC01449	108.9173	0.253773	0.002672	0.011212	lncRNA
ENSG0000(	MALT1	2585.047	0.208707	0.002673	0.011215	protein_coding
ENSG0000(	ENSG0000(	87.16234	0.311034	0.002674	0.011216	lncRNA
ENSG0000(	FNDC4	22.13792	0.725115	0.002675	0.011219	protein_coding
ENSG0000(	EIF4E2	3191.245	-0.14368	0.002677	0.011224	protein_coding
ENSG0000(	ENSG0000(	35.68322	-0.66027	0.002677	0.011224	lncRNA
ENSG0000(	E2F1	163.8383	-0.37966	0.002679	0.01123	protein_coding
ENSG0000(	HOXB6	1999.954	-0.41614	0.002681	0.011238	protein_coding
ENSG0000(	CHAF1B	1114.949	-0.10648	0.002684	0.011246	protein_coding
ENSG0000(	ENSG0000(	212.2115	0.224712	0.002684	0.011246	lncRNA
ENSG0000(	LINC02895	6647.719	-0.27637	0.002684	0.011246	lncRNA
ENSG0000(	ENSG0000(	49.51461	0.395005	0.002685	0.01125	lncRNA
ENSG0000(	SMS	541.8581	-0.26812	0.002689	0.011263	protein_coding
ENSG0000(	ENSG0000(	106.6929	-0.51797	0.00269	0.011267	TEC
ENSG0000(	EPHX1	273.4981	-0.34485	0.002697	0.011292	protein_coding
ENSG0000(	HCP5B	64.88338	0.464441	0.002699	0.011301	lncRNA
ENSG0000(	FAM32A	775.9271	-0.20568	0.002701	0.011305	protein_coding
ENSG0000(	NCOR1	11567.02	-0.12788	0.0027	0.011305	protein_coding
ENSG0000(	RANBP9	811.4632	-0.15363	0.002702	0.011309	protein_coding

ENSG0000(	SPON1	784.6386	-0.43521	0.002702	0.011309	protein_coding
ENSG0000(	ENSG0000(	52.82887	-0.31206	0.002704	0.011312	transcribed_processed_pseudoge
ENSG0000(	TNFRSF9	917.7538	0.219823	0.002705	0.011315	protein_coding
ENSG0000(	MTND1P1(	103.2135	-0.24789	0.00271	0.011337	processed_pseudogene
ENSG0000(	LINC01877	162.5315	0.228178	0.002711	0.011339	lncRNA
ENSG0000(	SENCR	9.946677	0.812887	0.002712	0.01134	lncRNA
ENSG0000(	DACT3-AS1	20.46285	0.610377	0.002713	0.011341	lncRNA
ENSG0000(	MTLN	140.2862	-0.48185	0.002713	0.011341	protein_coding
ENSG0000(	PIGW	353.6532	-0.20172	0.002713	0.011341	protein_coding
ENSG0000(	RNU6-548F	15.12115	0.694233	0.002714	0.011344	snRNA
ENSG0000(	RDM1P1	251.186	0.395209	0.002715	0.011348	transcribed_unprocessed_pseudo
ENSG0000(	ENSG0000(	24.62012	0.47158	0.002717	0.011355	lncRNA
ENSG0000(	OR8G1	100.9457	0.246302	0.002722	0.01137	protein_coding
ENSG0000(	ZNF660	612.536	0.219125	0.002722	0.01137	protein_coding
ENSG0000(	ENSG0000(	719.8836	0.184068	0.002723	0.011372	lncRNA
ENSG0000(	ENSG0000(	601.742	-0.12044	0.00273	0.011403	TEC
ENSG0000(	YLPM1	4627.245	0.153406	0.002734	0.011419	protein_coding
ENSG0000(	RFK	951.2607	-0.3754	0.002735	0.011419	protein_coding
ENSG0000(	SSBP2	2922.145	0.128833	0.002735	0.011419	protein_coding
ENSG0000(	CCDC15-DT	112.2927	-0.2534	0.002739	0.011433	lncRNA
ENSG0000(	TARDBP	3692.959	-0.11797	0.002739	0.011433	protein_coding
ENSG0000(	ZMIZ1-AS1	112.4895	0.337723	0.002741	0.011438	lncRNA
ENSG0000(	ZBTB20-AS	10.11153	0.617561	0.002745	0.011453	lncRNA
ENSG0000(	ENSG0000(	35.45542	0.461532	0.002749	0.01147	lncRNA
ENSG0000(	EHHADH	851.2212	-0.33922	0.002751	0.011477	protein_coding
ENSG0000(	EFR3B	395.9156	-0.15379	0.002753	0.011479	protein_coding
ENSG0000(	ENSG0000(	17.65529	0.623318	0.002753	0.011479	processed_pseudogene
ENSG0000(	RPAIN	2854.51	-0.10479	0.002753	0.011479	protein_coding
ENSG0000(	BBS10	267.8661	0.246015	0.002759	0.011501	protein_coding
ENSG0000(	CDC25C	78.6168	-0.43159	0.002759	0.011501	protein_coding
ENSG0000(	ZC3H18	1299.788	-0.14555	0.002764	0.01152	protein_coding
ENSG0000(	ENSG0000(	29.72971	-0.91368	0.002765	0.011521	lncRNA
ENSG0000(	TMPRSS7	49.34312	-0.33437	0.002765	0.011522	protein_coding
ENSG0000(	BACE2	1656.153	0.389388	0.002767	0.011524	protein_coding
ENSG0000(	ENSG0000(	298.2029	0.246807	0.002766	0.011524	lncRNA
ENSG0000(	RPL26P30	184.9396	0.247057	0.002766	0.011524	transcribed_processed_pseudoge
ENSG0000(	ENSG0000(	179.2809	0.186719	0.002769	0.011531	lncRNA
ENSG0000(	C19orf53	204.7366	-0.20144	0.00277	0.011536	protein_coding
ENSG0000(	ENSG0000(	1622.036	0.125791	0.002774	0.01155	TEC
ENSG0000(	LINC02074	78.32919	0.273127	0.002774	0.01155	lncRNA
ENSG0000(	TRO	171.6271	0.432687	0.002778	0.011563	protein_coding
ENSG0000(	ENSG0000(	589.6313	-0.3883	0.00278	0.011571	lncRNA
ENSG0000(	COL3A1	10586.04	0.577359	0.002781	0.011573	protein_coding
ENSG0000(	PPP2R3A	1530.997	-0.21847	0.002786	0.011592	protein_coding
ENSG0000(	SNX7	387.4066	-0.39777	0.002786	0.011592	protein_coding
ENSG0000(	MPC1-DT	405.2848	0.144632	0.002787	0.011593	lncRNA
ENSG0000(	RHNO1	398.4291	-0.16983	0.002788	0.011594	protein_coding

ENSG0000(	EXOC3L2	410.8412	0.210343	0.00279	0.011601	protein_coding
ENSG0000(	KPNA5	6743.189	-0.24275	0.002792	0.01161	protein_coding
ENSG0000(	DCTN6	363.8328	-0.16892	0.002793	0.011612	protein_coding
ENSG0000(	FMOD	384.6851	0.457414	0.002793	0.011612	protein_coding
ENSG0000(	STAG2	5557.021	0.238097	0.002797	0.011624	protein_coding
ENSG0000(	KDM5A	4696.256	0.098358	0.002799	0.01163	protein_coding
ENSG0000(	GUCA2B	165.6002	-1.05291	0.002799	0.011631	protein_coding
ENSG0000(	C19orf33	258.0624	-0.52267	0.0028	0.011634	protein_coding
ENSG0000(	GTF2IP13	602.2385	0.30298	0.002801	0.011637	transcribed_unprocessed_pseudo
ENSG0000(	SLC11A1	786.9508	0.422935	0.002805	0.011649	protein_coding
ENSG0000(	CLIC5	5065.816	0.462507	0.002805	0.01165	protein_coding
ENSG0000(	ENSG0000(	260.5459	0.228635	0.002812	0.011676	lncRNA
ENSG0000(	ENSG0000(	327.1034	0.302833	0.002813	0.01168	lncRNA
ENSG0000(	BMX	56.71015	-0.57503	0.002815	0.011687	protein_coding
ENSG0000(	ASF1B	157.5065	-0.40281	0.002816	0.01169	protein_coding
ENSG0000(	APOBEC3F	852.634	0.243653	0.002819	0.011699	protein_coding
ENSG0000(	KCNJ14	534.0431	0.15678	0.002819	0.011699	protein_coding
ENSG0000(	NR2F6	968.9043	-0.38093	0.002824	0.011717	protein_coding
ENSG0000(	ENSG0000(	265.5533	-0.22823	0.002828	0.011731	lncRNA
ENSG0000(	ACTN4	8651.083	-0.33185	0.002837	0.011768	protein_coding
ENSG0000(	MUC12-AS	58.03426	0.446449	0.002842	0.011787	lncRNA
ENSG0000(	GSK3A	1090.038	-0.13977	0.002843	0.011788	protein_coding
ENSG0000(	MPHOSPH1	560.3503	-0.17834	0.002843	0.011788	protein_coding
ENSG0000(	ZNF461	468.5995	-0.1702	0.002843	0.011789	protein_coding
ENSG0000(	ENSG0000(	98.08874	-0.23288	0.002848	0.011806	lncRNA
ENSG0000(	NCKAP5L	670.789	0.229343	0.002848	0.011807	protein_coding
ENSG0000(	ENSG0000(	63.84595	-0.28466	0.002859	0.011848	lncRNA
ENSG0000(	ENSG0000(	9.133256	0.795248	0.00286	0.011849	lncRNA
ENSG0000(	CYP2C18	901.1904	-0.6061	0.00286	0.011851	protein_coding
ENSG0000(	RN7SL760F	38.8061	0.363005	0.002867	0.011879	misc_RNA
ENSG0000(	ENO3	212.3791	-0.20642	0.002873	0.011898	protein_coding
ENSG0000(	VARS1	682.9038	-0.30925	0.002879	0.011923	protein_coding
ENSG0000(	BLOC1S4	136.664	-0.23189	0.002881	0.011932	protein_coding
ENSG0000(	C9orf131	128.7841	0.225981	0.002882	0.011935	protein_coding
ENSG0000(	RPL11P1	38.01711	0.406266	0.002883	0.011935	processed_pseudogene
ENSG0000(	ENSG0000(	12.53726	0.811678	0.002884	0.011937	lncRNA
ENSG0000(	ENSG0000(	38.49658	0.379824	0.002887	0.011947	lncRNA
ENSG0000(	BZW1	3129.105	-0.27536	0.002888	0.011953	protein_coding
ENSG0000(	ZNF346	1054.569	0.168227	0.00289	0.011957	protein_coding
ENSG0000(	ENSG0000(	78.76268	-0.31291	0.002892	0.011965	lncRNA
ENSG0000(	SAP130	825.6227	-0.1638	0.002897	0.011985	protein_coding
ENSG0000(	TNFRSF10E	1814.123	0.192005	0.002899	0.01199	protein_coding
ENSG0000(	ZNF575	304.644	-0.57536	0.0029	0.011994	protein_coding
ENSG0000(	L2HGDH	2715.21	-0.11798	0.002905	0.012012	protein_coding
ENSG0000(	TAGLN	1293.832	0.425798	0.002905	0.012012	protein_coding
ENSG0000(	CLBA1	796.3248	0.492694	0.002906	0.012014	protein_coding
ENSG0000(	SULT1B1	20430.63	-0.22587	0.002908	0.012021	protein_coding

ENSG0000(	STARD4	1360.327	0.304492	0.00291	0.012026	protein_coding
ENSG0000(	MYCBP2	7089.31	0.300167	0.002911	0.012028	protein_coding
ENSG0000(	NPFFR2	59.48903	-0.34192	0.002915	0.012043	protein_coding
ENSG0000(	ALDH1A1	742.2405	-0.42409	0.002916	0.012047	protein_coding
ENSG0000(	STAG3L1	534.5175	-0.19407	0.002919	0.012058	transcribed_unprocessed_pseudo
ENSG0000(	ZC3H15	875.8979	-0.17089	0.00292	0.012058	protein_coding
ENSG0000(	DCTN3	409.9562	-0.22909	0.002924	0.012076	protein_coding
ENSG0000(	NFYC-AS1	261.7424	0.22403	0.002926	0.012081	lncRNA
ENSG0000(	PPFIBP1	2071.799	0.177834	0.002927	0.012084	protein_coding
ENSG0000(	ENSG0000(	24.01163	0.87787	0.002928	0.012085	lncRNA
ENSG0000(	FHL3	129.3809	0.500239	0.002929	0.012087	protein_coding
ENSG0000(	RPL9	3026.197	-0.38895	0.002929	0.012087	protein_coding
ENSG0000(	ENSG0000(	597.0074	0.182279	0.002932	0.012096	lncRNA
ENSG0000(	ATP8A2P3	20.63011	0.506416	0.002936	0.012111	unprocessed_pseudogene
ENSG0000(	CEP112	875.8871	0.137543	0.002936	0.012111	protein_coding
ENSG0000(	LINC00216	148.0259	0.293671	0.002936	0.012111	lncRNA
ENSG0000(	ENSG0000(	17.87139	-0.57248	0.002941	0.012128	lncRNA
ENSG0000(	CD276	586.9416	0.341938	0.002943	0.012134	protein_coding
ENSG0000(	CYYR1	198.8065	0.415797	0.002944	0.012138	protein_coding
ENSG0000(	NRBP1	1424.407	-0.12804	0.002948	0.012153	protein_coding
ENSG0000(	ENSG0000(	492.001	0.188373	0.002949	0.012155	lncRNA
ENSG0000(	GDF9	32.44495	0.460624	0.00295	0.012158	protein_coding
ENSG0000(	ZNF492	244.9988	-0.23269	0.002952	0.012164	protein_coding
ENSG0000(	KRT8P39	55.06146	-0.39629	0.002956	0.012181	processed_pseudogene
ENSG0000(	ENSG0000(	224.9791	-0.19663	0.002958	0.012185	lncRNA
ENSG0000(	IGLV1-51	405.201	0.829661	0.002969	0.012231	IG_V_gene
ENSG0000(	ACTG1P1	20.18684	0.617512	0.002971	0.012238	processed_pseudogene
ENSG0000(	TIMM9	169.2912	-0.32354	0.002972	0.012238	protein_coding
ENSG0000(	HOXB4	430.9518	-0.29908	0.002977	0.012258	protein_coding
ENSG0000(	WRAP73	644.1759	-0.19907	0.002977	0.012258	protein_coding
ENSG0000(	ENSG0000(	42.4935	0.481806	0.002981	0.012271	transcribed_processed_pseudoge
ENSG0000(	ENSG0000(	16.12247	0.478683	0.002983	0.012276	lncRNA
ENSG0000(	ENSG0000(	107.4466	0.267698	0.002988	0.012298	lncRNA
ENSG0000(	AEBP1	1206.476	0.480811	0.00299	0.012303	protein_coding
ENSG0000(	MTX2	506.2105	-0.21856	0.002991	0.012306	protein_coding
ENSG0000(	RTN4RL1	20.74131	-0.71957	0.002998	0.012334	protein_coding
ENSG0000(	ZNF850	1310.518	-0.15225	0.002999	0.012334	protein_coding
ENSG0000(	OXR1	1741.182	-0.17821	0.002999	0.012335	protein_coding
ENSG0000(	DIDO1	4309.006	-0.08275	0.003001	0.01234	protein_coding
ENSG0000(	TOM1L1	859.4999	-0.26124	0.003001	0.01234	protein_coding
ENSG0000(	CAVIN4	82.21788	0.274469	0.003002	0.01234	protein_coding
ENSG0000(	ENSG0000(	217.8605	0.174075	0.003002	0.01234	lncRNA
ENSG0000(	RAP1GAP	754.6406	-0.43414	0.003003	0.012342	protein_coding
ENSG0000(	LINC02432	424.5285	0.156131	0.003008	0.012361	lncRNA
ENSG0000(	HMGB1P41	60.60473	-0.41246	0.00301	0.012367	processed_pseudogene
ENSG0000(	OAZ1	2544.168	-0.25751	0.003012	0.012375	protein_coding
ENSG0000(	SRGAP2C	692.4112	0.254764	0.003013	0.012379	protein_coding

ENSG0000(	ENSG0000(	290.113	-0.14141	0.003015	0.012382	lncRNA
ENSG0000(	TNF	107.902	0.590172	0.003016	0.012388	protein_coding
ENSG0000(	ENSG0000(	18.0074	-0.70175	0.003018	0.012392	unprocessed_pseudogene
ENSG0000(	CNTN3	917.9269	-0.59815	0.00302	0.012398	protein_coding
ENSG0000(	EDA	224.5108	-0.37766	0.00302	0.012399	protein_coding
ENSG0000(	ENSG0000(	311.624	0.193091	0.003022	0.012403	transcribed_processed_pseudoge
ENSG0000(	ENSG0000(	114.6817	0.258031	0.003024	0.012412	lncRNA
ENSG0000(	ARSJ	178.3674	0.357165	0.003033	0.012445	protein_coding
ENSG0000(	RUBCNL	3052.848	0.260929	0.003033	0.012445	protein_coding
ENSG0000(	THSD7A	217.0297	0.599641	0.003034	0.012447	protein_coding
ENSG0000(	CYP2J2	413.4212	-0.45588	0.003035	0.01245	protein_coding
ENSG0000(	SNORD35A	23.58809	0.849507	0.00304	0.012467	snoRNA
ENSG0000(	TMEM168	3021.285	-0.18832	0.003046	0.012489	protein_coding
ENSG0000(	ZNF100	1093.778	-0.17501	0.003047	0.012492	protein_coding
ENSG0000(	LINC01185	153.8213	0.227846	0.003054	0.012521	lncRNA
ENSG0000(	ENSG0000(	108.0012	0.312716	0.003055	0.012525	TEC
ENSG0000(	HARS2	965.0172	-0.1223	0.003057	0.012531	protein_coding
ENSG0000(	ZP3	253.2843	-0.2291	0.003059	0.012536	protein_coding
ENSG0000(	RN7SL734F	21.25254	0.46739	0.00306	0.012537	misc_RNA
ENSG0000(	RN7SL297F	31.92935	0.504429	0.003061	0.01254	misc_RNA
ENSG0000(	KCTD17	211.3278	-0.28884	0.003062	0.012545	protein_coding
ENSG0000(	CTTNBP2	319.1956	-0.38866	0.003064	0.012549	protein_coding
ENSG0000(	ENSG0000(	24.61803	0.629882	0.003065	0.012553	transcribed_unprocessed_pseudo
ENSG0000(	CHP2	2737.853	-0.52895	0.003071	0.01257	protein_coding
ENSG0000(	ENSG0000(	224.6998	0.185333	0.003071	0.01257	lncRNA
ENSG0000(	ICOS	148.0386	0.64854	0.00307	0.01257	protein_coding
ENSG0000(	SLC37A4	894.8008	-0.27602	0.00307	0.01257	protein_coding
ENSG0000(	BMP8A	448.0013	0.301659	0.003072	0.012575	protein_coding
ENSG0000(	PHYH	379.7578	-0.37158	0.003073	0.012576	protein_coding
ENSG0000(	FAM53C	1366.069	0.114811	0.003075	0.012582	protein_coding
ENSG0000(	ENSG0000(	170.5277	0.243227	0.003076	0.012586	lncRNA
ENSG0000(	RNU6-757F	12.95643	0.723556	0.003077	0.012589	snRNA
ENSG0000(	ENSG0000(	692.6488	0.157956	0.003079	0.012594	lncRNA
ENSG0000(	SAMD12	5432.638	-0.14801	0.003081	0.012601	protein_coding
ENSG0000(	PPFIA4	102.4473	0.573216	0.003082	0.012604	protein_coding
ENSG0000(	ENSG0000(	15.55331	0.567392	0.003084	0.01261	lncRNA
ENSG0000(	PIGN	5648.202	-0.11657	0.003091	0.012634	protein_coding
ENSG0000(	PSME4	3095.849	0.16306	0.003096	0.012655	protein_coding
ENSG0000(	PRR3	1044.003	0.143699	0.003099	0.012665	protein_coding
ENSG0000(	TUNAR	146.7044	0.214102	0.003099	0.012665	protein_coding
ENSG0000(	LINC00426	844.759	0.16326	0.003103	0.012679	lncRNA
ENSG0000(	LRRC37A3	1340.771	0.335779	0.003105	0.012683	protein_coding
ENSG0000(	NCLN	1229.364	-0.25933	0.003105	0.012683	protein_coding
ENSG0000(	WEE1	1408.089	-0.23391	0.003105	0.012683	protein_coding
ENSG0000(	LINC01288	38.2279	-0.38136	0.003106	0.012683	lncRNA
ENSG0000(	ENSG0000(	50.28582	0.612447	0.003111	0.012702	TEC
ENSG0000(	PPP1R37	989.9328	-0.14235	0.003111	0.012702	protein_coding

ENSG0000(	XYLB	941.2426	-0.20572	0.003112	0.012703	protein_coding
ENSG0000(	ALOX15P1	492.0823	0.164455	0.003119	0.012731	transcribed_unprocessed_pseudo
ENSG0000(	MTA3	1840.235	-0.13566	0.003121	0.012736	protein_coding
ENSG0000(	ZACN	24.34371	1.036955	0.003121	0.012736	protein_coding
ENSG0000(	DNMT3A	3064.717	0.164136	0.003127	0.012759	protein_coding
ENSG0000(	OR5A2	656.607	0.13312	0.003128	0.012759	protein_coding
ENSG0000(	RPS25P9	16.47213	0.622059	0.003128	0.012759	processed_pseudogene
ENSG0000(	HLTF	707.6105	-0.21016	0.003129	0.012761	protein_coding
ENSG0000(	CDC42BPA	6333.861	-0.25175	0.003132	0.012769	protein_coding
ENSG0000(	TRIM29	76.58067	0.550535	0.003131	0.012769	protein_coding
ENSG0000(	ZNF84-DT	136.5195	-0.27663	0.003132	0.012769	lncRNA
ENSG0000(	GAD1	15.42227	1.10257	0.003135	0.012776	protein_coding
ENSG0000(	GPR160	1547.533	-0.39679	0.003134	0.012776	protein_coding
ENSG0000(	ENSG0000(	155.6611	-0.44116	0.003137	0.012783	protein_coding
ENSG0000(	INIP	1278.925	-0.1665	0.003137	0.012783	protein_coding
ENSG0000(	GCSH	412.7387	-0.18509	0.003138	0.012784	protein_coding
ENSG0000(	FNDC1	140.2383	0.891814	0.003139	0.012787	protein_coding
ENSG0000(	KLK15	17.79531	-0.95763	0.00314	0.012788	protein_coding
ENSG0000(	ENSG0000(	1014.962	0.237734	0.003141	0.012793	processed_pseudogene
ENSG0000(	MTERF2	518.858	0.192924	0.003145	0.012804	protein_coding
ENSG0000(	TRPC7-AS1	70.5	-0.50426	0.003144	0.012804	lncRNA
ENSG0000(	SLC35E2A	564.0436	-0.21453	0.003148	0.012813	transcribed_unprocessed_pseudo
ENSG0000(	ENSG0000(	107.209	-0.24333	0.003151	0.012822	lncRNA
ENSG0000(	LHX6	65.73074	0.634127	0.00315	0.012822	protein_coding
ENSG0000(	SLC47A2	84.19468	-0.53709	0.003154	0.012834	protein_coding
ENSG0000(	LINC00887	242.5455	-0.25698	0.003156	0.012842	lncRNA
ENSG0000(	ADGRA2	781.4785	0.365227	0.003161	0.01286	protein_coding
ENSG0000(	EIF4A3	946.1916	-0.13025	0.003162	0.012864	protein_coding
ENSG0000(	NDUFV1-DT	238.5484	0.274659	0.003166	0.012875	lncRNA
ENSG0000(	SLC2A3P2	87.17622	0.356144	0.003166	0.012875	processed_pseudogene
ENSG0000(	SNORA73A	4787.314	-0.3828	0.003166	0.012875	snoRNA
ENSG0000(	ENSG0000(	25.56046	0.442464	0.003168	0.012878	lncRNA
ENSG0000(	COL28A1	640.3062	0.233328	0.003169	0.012881	protein_coding
ENSG0000(	NVL	1151.66	-0.15064	0.003174	0.012901	protein_coding
ENSG0000(	OBI1	322.5412	-0.19914	0.003174	0.012901	protein_coding
ENSG0000(	DUSP11	642.329	-0.14518	0.003176	0.012907	protein_coding
ENSG0000(	ENSG0000(	10.61539	0.972815	0.003181	0.012924	lncRNA
ENSG0000(	TIAF1	1593.807	0.165058	0.003183	0.01293	protein_coding
ENSG0000(	ENSG0000(	27.40244	-0.45941	0.003183	0.01293	lncRNA
ENSG0000(	HNRNPA0	1460.234	-0.18034	0.003187	0.012944	protein_coding
ENSG0000(	NFASC	2782.493	0.321385	0.003187	0.012944	protein_coding
ENSG0000(	CIBAR1P2	148.7925	-0.26115	0.00319	0.012951	transcribed_processed_pseudoge
ENSG0000(	GBGT1	497.0363	0.285404	0.003193	0.012963	protein_coding
ENSG0000(	TENT2	2212.831	0.131156	0.003193	0.012963	protein_coding
ENSG0000(	L3MBTL4-A	914.3997	0.211797	0.003199	0.012983	lncRNA
ENSG0000(	NT5DC4	56.14878	0.876471	0.003202	0.012995	protein_coding
ENSG0000(	SH3RF3-AS	29.49548	0.39407	0.003202	0.012995	lncRNA

ENSG0000(	ENSG0000(	38.62065	0.331349	0.003206	0.013009	unprocessed_pseudogene
ENSG0000(	STBD1	370.0763	-0.73866	0.003207	0.013009	protein_coding
ENSG0000(	GAS7	1407.902	0.298241	0.003208	0.013013	protein_coding
ENSG0000(	ENSG0000(	14.92229	0.562686	0.003211	0.013023	lncRNA
ENSG0000(	ZFP3	1045.333	-0.1944	0.003212	0.013026	protein_coding
ENSG0000(	CATSPERE	61.47861	0.481843	0.003214	0.013033	protein_coding
ENSG0000(	ENSG0000(	32.56359	0.400271	0.003215	0.013035	lncRNA
ENSG0000(	KLF3-AS1	1299.891	0.139247	0.003217	0.013041	lncRNA
ENSG0000(	SEMA4D	2889.558	0.301406	0.003218	0.013044	protein_coding
ENSG0000(	SLC5A4	18.53218	0.805021	0.003219	0.013044	protein_coding
ENSG0000(	STRN3	1302.524	-0.12365	0.003223	0.013061	protein_coding
ENSG0000(	ENSG0000(	15.57356	-0.59756	0.003226	0.013068	snoRNA
ENSG0000(	ENSG0000(	286.7651	-0.17114	0.003226	0.013068	lncRNA
ENSG0000(	LINC01205	819.059	-0.12674	0.003227	0.013072	lncRNA
ENSG0000(	INTS5	302.5535	-0.22329	0.003228	0.013073	protein_coding
ENSG0000(	APOBEC3B-	19.11885	0.626539	0.003231	0.013081	lncRNA
ENSG0000(	ZNF532	2473.739	0.145279	0.003231	0.013081	protein_coding
ENSG0000(	SLC8A2	17.88045	-1.04108	0.003232	0.013085	protein_coding
ENSG0000(	ENSG0000(	39.82213	0.337948	0.003235	0.013094	transcribed_processed_pseudoge
ENSG0000(	ENSG0000(	240.7584	0.246512	0.003238	0.013103	lncRNA
ENSG0000(	MRPL10	450.1789	-0.16563	0.003239	0.013108	protein_coding
ENSG0000(	SH2D3A	2138.844	-0.17212	0.003246	0.013134	protein_coding
ENSG0000(	ENSG0000(	11.95932	0.580011	0.003248	0.013135	lncRNA
ENSG0000(	OPA3	2171.593	0.099683	0.003247	0.013135	protein_coding
ENSG0000(	ZFAND2B	499.4149	-0.22422	0.003247	0.013135	protein_coding
ENSG0000(	ZNF280B	202.5735	0.262747	0.003248	0.013135	protein_coding
ENSG0000(	SCAMP2	2441.226	-0.24087	0.00325	0.013141	protein_coding
ENSG0000(	SEC14L2	128.077	0.610074	0.003253	0.013152	protein_coding
ENSG0000(	LRRC27	898.754	0.215507	0.003258	0.01317	protein_coding
ENSG0000(	AMMECR1I	1171.697	-0.13041	0.003262	0.013184	protein_coding
ENSG0000(	IL10	151.8505	0.270258	0.003263	0.013188	protein_coding
ENSG0000(	LINC01619	133.1088	0.42503	0.003264	0.013191	lncRNA
ENSG0000(	CDRT15P3	12.02948	0.863112	0.003269	0.013206	transcribed_unprocessed_pseudo
ENSG0000(	UBE2E2	174.4236	0.418129	0.003271	0.013216	protein_coding
ENSG0000(	ARHGAP10	663.0478	0.286771	0.003274	0.013221	protein_coding
ENSG0000(	ENSG0000(	62.90458	0.671177	0.003274	0.013221	TEC
ENSG0000(	TMCO3	1395.547	0.163267	0.003274	0.013221	protein_coding
ENSG0000(	HMGB3P4	180.0775	0.232323	0.003275	0.013224	processed_pseudogene
ENSG0000(	RNF144A	1204.969	0.252614	0.003275	0.013224	protein_coding
ENSG0000(	RAET1K	29.80088	0.384532	0.003279	0.013235	transcribed_unprocessed_pseudo
ENSG0000(	SEMA4G	2116.925	-0.42273	0.003281	0.013243	protein_coding
ENSG0000(	EIF4EBP1	97.13333	-0.33693	0.003284	0.013254	protein_coding
ENSG0000(	HNF1A	537.1773	-0.24485	0.003289	0.013272	protein_coding
ENSG0000(	MYO1G	776.1106	0.415093	0.00329	0.013274	protein_coding
ENSG0000(	NFE2L1	3939.046	-0.20199	0.003294	0.01329	protein_coding
ENSG0000(	SEC14L6	544.9048	0.134036	0.003296	0.013293	protein_coding
ENSG0000(	ENSG0000(	795.1898	-0.16631	0.003296	0.013294	lncRNA

ENSG0000(	CUL7	858.6233	0.176358	0.003298	0.0133	protein_coding
ENSG0000(	EDA2R	68.88872	0.484913	0.003298	0.0133	protein_coding
ENSG0000(	ZNF674	872.1658	-0.13622	0.0033	0.013306	protein_coding
ENSG0000(	NRARP	1617.392	-0.41421	0.003301	0.013308	protein_coding
ENSG0000(	ZMYND19	216.8862	-0.24466	0.003306	0.013325	protein_coding
ENSG0000(	CLCN2	1741.824	-0.41371	0.003307	0.013328	protein_coding
ENSG0000(	LINC00342	7682.124	0.208187	0.003308	0.013328	lncRNA
ENSG0000(	WSCD1	912.8184	-0.59453	0.003308	0.013328	protein_coding
ENSG0000(	ENSG0000(	1350.281	-0.16842	0.003315	0.013351	lncRNA
ENSG0000(	GRAPLDR	169.5419	0.791294	0.003315	0.013351	lncRNA
ENSG0000(	ENSG0000(	241.1147	-0.18489	0.003316	0.013356	lncRNA
ENSG0000(	ZNF232	192.9764	-0.33758	0.003317	0.013356	protein_coding
ENSG0000(	SNRPD1	1364.993	-0.12388	0.003318	0.013359	protein_coding
ENSG0000(	ENSG0000(	65.97815	0.665837	0.00332	0.013364	unprocessed_pseudogene
ENSG0000(	ENSG0000(	30.56345	-0.37447	0.003324	0.013378	lncRNA
ENSG0000(	PTGDR2	107.7032	-0.62729	0.003324	0.013378	protein_coding
ENSG0000(	LINC00964	411.5279	0.172361	0.003326	0.013385	lncRNA
ENSG0000(	NPM1P27	37.30395	-0.58933	0.003328	0.013393	processed_pseudogene
ENSG0000(	LINC02447	325.9476	-0.1477	0.003329	0.013393	lncRNA
ENSG0000(	MED21	583.0595	0.2798	0.003331	0.013401	protein_coding
ENSG0000(	ENSG0000(	24.63746	0.487494	0.003341	0.013437	lncRNA
ENSG0000(	CLRN1-AS1	111.9332	-0.25304	0.003342	0.01344	lncRNA
ENSG0000(	ENSG0000(	5096.696	-0.05658	0.003343	0.013442	lncRNA
ENSG0000(	IP6K3	32.11762	0.641261	0.003344	0.013445	protein_coding
ENSG0000(	E2F3-IT1	105.869	0.281511	0.003349	0.013465	lncRNA
ENSG0000(	TRAF3IP1	608.073	-0.1395	0.003352	0.013475	protein_coding
ENSG0000(	RSPO3	104.7961	0.892975	0.003353	0.013477	protein_coding
ENSG0000(	GCFC2	524.3671	-0.2157	0.00336	0.013501	protein_coding
ENSG0000(	GOLM1	4038.615	-0.31736	0.003361	0.013502	protein_coding
ENSG0000(	RND2	147.181	0.227833	0.00336	0.013502	protein_coding
ENSG0000(	ERVFRD-3	51.86547	0.375618	0.003361	0.013503	transcribed_processed_pseudoge
ENSG0000(	IL7R	3001.777	0.414494	0.003366	0.013521	protein_coding
ENSG0000(	ENSG0000(	44.80661	-0.34639	0.003367	0.013521	lncRNA
ENSG0000(	CA3-AS1	100.5845	-0.55288	0.003372	0.013539	lncRNA
ENSG0000(	PPIL3	706.3229	-0.10979	0.003374	0.013545	protein_coding
ENSG0000(	DDRGK1	334.8699	-0.17214	0.003374	0.013546	protein_coding
ENSG0000(	XK	1165.363	-0.47185	0.003377	0.013557	protein_coding
ENSG0000(	ENSG0000(	154.7505	-0.2244	0.003378	0.013558	lncRNA
ENSG0000(	PDIA5	642.3509	-0.27082	0.00338	0.013562	protein_coding
ENSG0000(	ENSG0000(	671.1298	0.186524	0.003382	0.01357	lncRNA
ENSG0000(	IGFL2-AS1	142.304	0.250483	0.003382	0.01357	lncRNA
ENSG0000(	ENSG0000(	44.2507	0.40713	0.003385	0.013578	lncRNA
ENSG0000(	ENSG0000(	34.04063	0.408169	0.003386	0.013578	lncRNA
ENSG0000(	POLI	1126.929	0.217196	0.003386	0.013578	protein_coding
ENSG0000(	APP	5088.877	-0.19499	0.003389	0.013586	protein_coding
ENSG0000(	CRX	527.3202	0.112066	0.003389	0.013586	protein_coding
ENSG0000(	LINC01184	3210.375	0.174371	0.003388	0.013586	lncRNA

ENSG0000(	ENSG0000(	122.644	0.21328	0.003391	0.013591	lncRNA
ENSG0000(	LINC02275	857.2781	0.14021	0.003397	0.013614	lncRNA
ENSG0000(	RNU2-30P	20.23674	-0.44003	0.003403	0.013637	snRNA
ENSG0000(	ESYT1	1201.491	-0.27136	0.003404	0.013641	protein_coding
ENSG0000(	ALDH1A3	295.6325	0.383121	0.003406	0.013645	protein_coding
ENSG0000(	GOLGA4	6958.154	-0.15628	0.003409	0.013656	protein_coding
ENSG0000(	CYB561D1	725.6967	-0.12591	0.003411	0.013661	protein_coding
ENSG0000(	OR2H2	153.277	0.27709	0.003413	0.013667	protein_coding
ENSG0000(	CAPN2	6360.749	-0.25572	0.003415	0.013674	protein_coding
ENSG0000(	BUB3	2053.231	-0.16022	0.003417	0.013681	protein_coding
ENSG0000(	SCN1B	615.5134	0.286377	0.003418	0.013684	protein_coding
ENSG0000(	AGO2	5177.215	-0.17007	0.003426	0.013712	protein_coding
ENSG0000(	THAP3	176.9043	-0.23845	0.003427	0.013716	protein_coding
ENSG0000(	ENSG0000(	123.3364	0.205799	0.00343	0.013727	lncRNA
ENSG0000(	AJM1	431.1416	-0.37133	0.003431	0.013727	protein_coding
ENSG0000(	ERFE	71.1306	0.5221	0.003431	0.013727	protein_coding
ENSG0000(	NF2	1524.033	-0.12125	0.003432	0.013727	protein_coding
ENSG0000(	ZNF587B	3213.062	0.206506	0.003432	0.013727	protein_coding
ENSG0000(	PKD1	4821.975	-0.14852	0.003437	0.013746	protein_coding
ENSG0000(	LINC02582	117.1825	-0.70227	0.003441	0.013761	lncRNA
ENSG0000(	ENSG0000(	165.3154	0.264307	0.003443	0.013765	lncRNA
ENSG0000(	NKTR	12726.37	0.106888	0.003445	0.013772	protein_coding
ENSG0000(	GABRD	480.5557	0.163072	0.003446	0.013773	protein_coding
ENSG0000(	CACFD1	798.9934	-0.33096	0.003448	0.013779	protein_coding
ENSG0000(	LNPK	1719.67	-0.23883	0.003448	0.013779	protein_coding
ENSG0000(	ENSG0000(	320.4431	0.236285	0.003451	0.013787	lncRNA
ENSG0000(	ENSG0000(	75.98308	-0.82899	0.003453	0.013797	processed_pseudogene
ENSG0000(	CTH	147.6408	-0.2879	0.003457	0.013809	protein_coding
ENSG0000(	MYO10	5886.26	-0.24683	0.003458	0.01381	protein_coding
ENSG0000(	ADGRE3	55.88399	0.729758	0.00346	0.013816	protein_coding
ENSG0000(	ENSG0000(	636.9456	-0.17239	0.00346	0.013816	lncRNA
ENSG0000(	ENSG0000(	112.822	-0.26176	0.003461	0.013816	lncRNA
ENSG0000(	ZMPSTE24	878.5473	-0.36829	0.003461	0.013816	protein_coding
ENSG0000(	C1orf174	806.7301	-0.15636	0.003464	0.013822	protein_coding
ENSG0000(	FUBP3	1362.844	-0.13896	0.003464	0.013822	protein_coding
ENSG0000(	POLR3DP1	27.88255	-0.52863	0.003464	0.013822	processed_pseudogene
ENSG0000(	ACLY	1500.606	-0.16651	0.003466	0.01383	protein_coding
ENSG0000(	SNRPA1	857.8673	-0.16319	0.003469	0.013839	protein_coding
ENSG0000(	COPZ2	61.68789	0.48329	0.003472	0.013848	protein_coding
ENSG0000(	ENSG0000(	16.97671	0.604824	0.003477	0.013866	lncRNA
ENSG0000(	CATSPERB	634.0998	0.38401	0.003479	0.013873	protein_coding
ENSG0000(	KIF25	99.49396	0.403136	0.003482	0.013884	protein_coding
ENSG0000(	ENSG0000(	730.6701	-0.1077	0.003485	0.013893	lncRNA
ENSG0000(	PAPPA	704.4848	0.505462	0.003488	0.013901	protein_coding
ENSG0000(	ZNF737	2251.185	-0.1842	0.003488	0.013901	protein_coding
ENSG0000(	NHSL1	4140.314	-0.38003	0.003491	0.013912	protein_coding
ENSG0000(	ENSG0000(	9.332426	0.863033	0.003496	0.013932	transcribed_unprocessed_pseudo

ENSG0000(	ENSG0000(	355.5981	0.187608	0.003497	0.013932	lncRNA
ENSG0000(	ENSG0000(	47.18517	-0.3751	0.003498	0.013937	lncRNA
ENSG0000(	ENSG0000(	598.9418	0.245411	0.0035	0.013941	lncRNA
ENSG0000(	ENSG0000(	160.3794	-0.22534	0.003506	0.013965	processed_pseudogene
ENSG0000(	GLRX2	616.5389	-0.21709	0.003508	0.013968	protein_coding
ENSG0000(	SPIRE2	3135.311	-0.29219	0.003507	0.013968	protein_coding
ENSG0000(	ATP5F1AP1	106.1675	0.303569	0.003517	0.014	processed_pseudogene
ENSG0000(	NDUFC1	383.8911	-0.33697	0.003516	0.014	protein_coding
ENSG0000(	NUGGC	783.8424	0.190946	0.003518	0.014001	protein_coding
ENSG0000(	SOCAR	14.80273	0.836964	0.003521	0.014012	lncRNA
ENSG0000(	SNHG30	377.5551	-0.15422	0.003522	0.014013	lncRNA
ENSG0000(	ENSG0000(	270.6679	0.195403	0.003525	0.014021	lncRNA
ENSG0000(	ENSG0000(	14.97844	0.546997	0.003525	0.014021	TEC
ENSG0000(	ENSG0000(	140.5578	-0.21247	0.003525	0.014021	lncRNA
ENSG0000(	ENSG0000(	70.64847	0.272307	0.003524	0.014021	lncRNA
ENSG0000(	TAS2R62P	11.41973	-0.78831	0.003529	0.014032	unitary_pseudogene
ENSG0000(	LINC01318	56.0142	0.36122	0.003533	0.014046	lncRNA
ENSG0000(	TLE2	451.3725	-0.28485	0.003533	0.014046	protein_coding
ENSG0000(	MTMR12	1135.136	-0.13647	0.003536	0.014057	protein_coding
ENSG0000(	ENSG0000(	488.0765	0.410788	0.003537	0.014059	TEC
ENSG0000(	ENSG0000(	12.60663	0.697851	0.00354	0.014069	lncRNA
ENSG0000(	CD33	297.6614	0.290881	0.003543	0.014079	protein_coding
ENSG0000(	ENSG0000(	242.739	0.324022	0.003547	0.014091	lncRNA
ENSG0000(	C4B	525.1201	0.678797	0.003551	0.014107	protein_coding
ENSG0000(	ENSG0000(	61.98712	0.333965	0.003553	0.014112	lncRNA
ENSG0000(	LAYN	100.4428	0.380325	0.003556	0.014122	protein_coding
ENSG0000(	ESYT2	2791.8	-0.12461	0.003557	0.014126	protein_coding
ENSG0000(	ENSG0000(	38.5353	-0.6285	0.003558	0.014129	unprocessed_pseudogene
ENSG0000(	COL4A4	745.4962	0.274667	0.003559	0.014129	protein_coding
ENSG0000(	MTFR2P1	44.98694	0.370898	0.003559	0.014129	processed_pseudogene
ENSG0000(	STX5-DT	57.29418	0.331939	0.003564	0.014147	lncRNA
ENSG0000(	ITGB3	846.4602	0.324468	0.003566	0.014153	protein_coding
ENSG0000(	ENSG0000(	300.324	0.186674	0.003571	0.014171	lncRNA
ENSG0000(	ENSG0000(	149.8806	0.236549	0.00358	0.014203	lncRNA
ENSG0000(	SNPH	148.9003	0.476425	0.003582	0.014209	protein_coding
ENSG0000(	ENSG0000(	107.3635	0.243251	0.003587	0.014226	lncRNA
ENSG0000(	NINJ1	367.1111	0.342708	0.003586	0.014226	protein_coding
ENSG0000(	ULK2	749.508	-0.25322	0.003587	0.014226	protein_coding
ENSG0000(	FREM2	202.2104	-0.41707	0.003592	0.014242	protein_coding
ENSG0000(	TRAIP	98.97853	-0.40704	0.003592	0.014243	protein_coding
ENSG0000(	SHISA5	1127.083	0.229076	0.003597	0.014257	protein_coding
ENSG0000(	SLC9A9	578.2508	0.362232	0.003601	0.014271	protein_coding
ENSG0000(	CFAP161	19.84153	0.528869	0.003602	0.014275	protein_coding
ENSG0000(	ENSG0000(	165.4085	-0.29981	0.003607	0.014293	processed_pseudogene
ENSG0000(	CTDSPL	1367.343	-0.27974	0.00361	0.014305	protein_coding
ENSG0000(	LINC00578	501.2833	0.223671	0.003612	0.014309	lncRNA
ENSG0000(	CCDC152	133.0929	0.414141	0.003614	0.014316	protein_coding

ENSG0000(	KIAA1755	742.0104	0.419473	0.003617	0.014326	protein_coding
ENSG0000(	WNT10B	65.92824	0.514679	0.00362	0.014336	protein_coding
ENSG0000(	PPP1R15A	883.203	0.321353	0.003624	0.014349	protein_coding
ENSG0000(	ADH5	1669.934	-0.21387	0.003625	0.014352	protein_coding
ENSG0000(	ENSG0000(	779.1299	-0.20061	0.003626	0.014352	lncRNA
ENSG0000(	SOS1	6091.491	0.111583	0.003626	0.014352	protein_coding
ENSG0000(	ENSG0000(	367.4802	0.166834	0.00363	0.014365	unprocessed_pseudogene
ENSG0000(	ENSG0000(	201.5056	0.212189	0.003634	0.01438	lncRNA
ENSG0000(	STK32B	42.89028	0.576406	0.003634	0.01438	protein_coding
ENSG0000(	MIR3136	11.68601	0.636272	0.003645	0.01442	miRNA
ENSG0000(	ENSG0000(	208.8861	0.185874	0.003646	0.014422	lncRNA
ENSG0000(	ENSG0000(	169.553	0.208452	0.003646	0.014422	lncRNA
ENSG0000(	ZNF296	54.03393	-0.33632	0.003647	0.014423	protein_coding
ENSG0000(	SNHG15	475.9093	-0.26981	0.003652	0.014441	lncRNA
ENSG0000(	G6PC3	711.6831	-0.13392	0.003655	0.01445	protein_coding
ENSG0000(	FUS	10826.78	0.178259	0.003662	0.014475	protein_coding
ENSG0000(	IGFBP7	731.4644	0.450547	0.003662	0.014475	protein_coding
ENSG0000(	ENSG0000(	131.3773	0.329159	0.003666	0.014487	lncRNA
ENSG0000(	B3GNT3	1692.518	-0.421	0.003666	0.014488	protein_coding
ENSG0000(	IGKJ1	115.5441	0.669486	0.00367	0.014499	IG_J_gene
ENSG0000(	KPNA3	835.3819	-0.17623	0.003671	0.014504	protein_coding
ENSG0000(	ENSG0000(	379.1646	-0.18623	0.003673	0.014506	lncRNA
ENSG0000(	LINC00482	263.3738	-0.40588	0.003673	0.014506	lncRNA
ENSG0000(	ENSG0000(	1541.473	-0.10542	0.003677	0.014519	lncRNA
ENSG0000(	FAH	491.1365	-0.17624	0.003678	0.014522	protein_coding
ENSG0000(	TRNP1	33.7941	0.762888	0.003678	0.014522	protein_coding
ENSG0000(	AKT3	2519.457	0.201046	0.003679	0.014523	protein_coding
ENSG0000(	CNNM4	3553.744	-0.45173	0.003679	0.014523	protein_coding
ENSG0000(	UBTFL11	64.54376	0.33207	0.003681	0.014527	processed_pseudogene
ENSG0000(	ANAPC11	257.3269	-0.21375	0.003685	0.014539	protein_coding
ENSG0000(	IQGAP2	3398.633	-0.21824	0.003685	0.014539	protein_coding
ENSG0000(	CD48	568.6569	0.510791	0.003688	0.014551	protein_coding
ENSG0000(	AKT1S1	865.4413	0.248336	0.003689	0.014551	protein_coding
ENSG0000(	TRIM23	395.4055	0.227951	0.003689	0.014551	protein_coding
ENSG0000(	IGHV3-64D	129.9749	1.223193	0.003691	0.014556	IG_V_gene
ENSG0000(	EIF2S2	758.1479	-0.23823	0.003694	0.014565	protein_coding
ENSG0000(	ENSG0000(	49.04579	0.377966	0.003698	0.014578	lncRNA
ENSG0000(	PRAG1	1005.535	-0.40151	0.003708	0.014618	protein_coding
ENSG0000(	DTWD2	991.3351	-0.12965	0.003711	0.014626	protein_coding
ENSG0000(	PPP4R3B	3084.034	-0.14455	0.003712	0.014629	protein_coding
ENSG0000(	DNM1P41	192.4122	0.247924	0.003715	0.01464	TEC
ENSG0000(	RSU1	982.1083	-0.22262	0.003716	0.01464	protein_coding
ENSG0000(	TCF20	4087.362	-0.13934	0.003716	0.014641	protein_coding
ENSG0000(	ENSG0000(	893.4816	0.139136	0.003719	0.014647	lncRNA
ENSG0000(	SLC22A23	4032.992	-0.34152	0.003718	0.014647	protein_coding
ENSG0000(	GAPDHP54	20.57374	-0.68286	0.003725	0.014668	processed_pseudogene
ENSG0000(	ENSG0000(	182.098	0.288384	0.003726	0.014672	lncRNA

ENSG0000(	ENSG0000(	28.11427	0.351582	0.00373	0.014685	lncRNA
ENSG0000(	KIF5C	2321.799	0.106087	0.003734	0.014698	protein_coding
ENSG0000(	SUN1	4094.927	-0.18875	0.003735	0.0147	protein_coding
ENSG0000(	DOK2	153.0335	0.343353	0.003736	0.014705	protein_coding
ENSG0000(	ENSG0000(	289.8015	0.168751	0.003737	0.014705	lncRNA
ENSG0000(	GALM	762.9139	-0.21454	0.00374	0.014715	protein_coding
ENSG0000(	ENSG0000(	18.43847	0.710846	0.003753	0.014763	lncRNA
ENSG0000(	ENSG0000(	10.86056	0.68011	0.003754	0.014763	lncRNA
ENSG0000(	ENSG0000(	94.7151	0.342717	0.003754	0.014763	TEC
ENSG0000(	EPM2AIP1	2448.831	0.144393	0.003754	0.014763	protein_coding
ENSG0000(	OXTR	132.258	0.295361	0.003754	0.014763	protein_coding
ENSG0000(	BROX	1750.221	0.206599	0.003756	0.014768	protein_coding
ENSG0000(	ASIC4	98.85418	0.238805	0.003764	0.014798	protein_coding
ENSG0000(	DNAJC30	369.6861	-0.14762	0.003765	0.0148	protein_coding
ENSG0000(	ENSG0000(	27.62637	0.403859	0.003768	0.014809	lncRNA
ENSG0000(	PCDH19	304.8645	-0.54301	0.003768	0.014809	protein_coding
ENSG0000(	ATP1A2	315.2209	-0.3172	0.00377	0.014814	protein_coding
ENSG0000(	ENSG0000(	112.1165	-0.22861	0.003774	0.014825	lncRNA
ENSG0000(	ATRAID	396.1648	-0.22574	0.003774	0.014827	protein_coding
ENSG0000(	ENSG0000(	127.5962	0.252269	0.003775	0.014828	lncRNA
ENSG0000(	ARHGEF6	1335.419	0.297826	0.003776	0.014828	protein_coding
ENSG0000(	CACNG8	819.4662	0.138781	0.003777	0.014828	protein_coding
ENSG0000(	ENSG0000(	104.3767	-0.23517	0.003776	0.014828	lncRNA
ENSG0000(	EPG5	2883.471	0.154858	0.003782	0.014845	protein_coding
ENSG0000(	KIAA2013	1023.651	-0.2244	0.003781	0.014845	protein_coding
ENSG0000(	HLA-DPA1	2562.194	0.458658	0.003783	0.014846	protein_coding
ENSG0000(	AURKAP1	75.15145	-0.57221	0.003789	0.014869	transcribed_processed_pseudoge
ENSG0000(	TGFBR1	1286.152	0.27691	0.003793	0.014883	protein_coding
ENSG0000(	ENSG0000(	30.22383	0.482736	0.003797	0.014896	lncRNA
ENSG0000(	RN7SL483F	37.3085	0.357303	0.003797	0.014896	misc_RNA
ENSG0000(	C3orf86	41.22414	0.616727	0.003803	0.014911	transcribed_unitary_pseudogene
ENSG0000(	DSG2	6542.442	-0.3395	0.003803	0.014911	protein_coding
ENSG0000(	IGHJ2P	15.60861	0.704315	0.003803	0.014911	IG_J_pseudogene
ENSG0000(	TIMP2	2182.809	0.420141	0.003802	0.014911	protein_coding
ENSG0000(	ZNF844	2071.347	-0.14022	0.003804	0.014911	protein_coding
ENSG0000(	ENSG0000(	31.43831	-0.41277	0.003806	0.014916	lncRNA
ENSG0000(	VN1R2	54.59767	0.331747	0.003806	0.014916	protein_coding
ENSG0000(	ENSG0000(	199.6709	-0.24329	0.003807	0.014918	lncRNA
ENSG0000(	FAM47E	1655.302	0.152197	0.00381	0.014928	protein_coding
ENSG0000(	ENSG0000(	64.86169	0.281255	0.003811	0.01493	lncRNA
ENSG0000(	GADD45GII	108.6594	-0.32272	0.003812	0.014935	protein_coding
ENSG0000(	LINC02577	180.4096	0.213575	0.003815	0.014943	lncRNA
ENSG0000(	ENSG0000(	50.27039	0.357878	0.003817	0.014948	TEC
ENSG0000(	PRELID1P4	15.16704	-0.65204	0.003819	0.014955	processed_pseudogene
ENSG0000(	RASSF8-AS1	747.5347	0.183998	0.003822	0.014964	lncRNA
ENSG0000(	RHOH	1424.461	0.470382	0.003825	0.014973	protein_coding
ENSG0000(	VSNL1	215.4109	-0.27227	0.003827	0.01498	protein_coding

ENSG0000(	ENSG0000(	175.3369	-0.19662	0.003829	0.014985	lncRNA
ENSG0000(	DNAJC19	826.0112	-0.17448	0.003831	0.014993	protein_coding
ENSG0000(	SCAF4	1344.312	-0.10867	0.003836	0.015009	protein_coding
ENSG0000(	COG4	1391.408	-0.14521	0.003839	0.015009	protein_coding
ENSG0000(	DTL	311.383	-0.41467	0.003836	0.015009	protein_coding
ENSG0000(	ENSG0000(	14.83466	0.795528	0.003838	0.015009	lncRNA
ENSG0000(	HEG1	1508.422	0.353669	0.003839	0.015009	protein_coding
ENSG0000(	SLC44A2	2012.773	-0.18221	0.003838	0.015009	protein_coding
ENSG0000(	TC2N	3181.425	0.247734	0.003838	0.015009	protein_coding
ENSG0000(	ENSG0000(	59.07271	-0.28962	0.003842	0.01502	lncRNA
ENSG0000(	AIF1	125.1034	0.473259	0.003843	0.015022	protein_coding
ENSG0000(	UFC1	606.4725	-0.17104	0.003843	0.015022	protein_coding
ENSG0000(	ENSG0000(	98.89613	-0.26989	0.003845	0.015025	lncRNA
ENSG0000(	ENSG0000(	27.8853	-0.58979	0.00386	0.015085	processed_pseudogene
ENSG0000(	ENSG0000(	131.1365	-0.43148	0.003865	0.015101	lncRNA
ENSG0000(	ATOX1	739.1582	0.230558	0.003872	0.015125	protein_coding
ENSG0000(	FBXO28	1151.235	-0.14399	0.003873	0.015129	protein_coding
ENSG0000(	SEC24A	1720.618	0.223967	0.003875	0.015134	protein_coding
ENSG0000(	LINC01506	168.623	-0.24547	0.003884	0.015167	lncRNA
ENSG0000(	ENSG0000(	51.23005	0.352839	0.003886	0.015174	lncRNA
ENSG0000(	MDFIC	928.5849	0.281363	0.003888	0.015178	protein_coding
ENSG0000(	YBX2	1642.327	-0.47121	0.00389	0.015186	protein_coding
ENSG0000(	ENSG0000(	37.30691	0.570339	0.003892	0.015192	lncRNA
ENSG0000(	PLP1	127.0495	-0.5869	0.003894	0.015197	protein_coding
ENSG0000(	STC1	137.8934	1.001872	0.003894	0.015197	protein_coding
ENSG0000(	ENSG0000(	27.58583	0.437194	0.003896	0.015201	TEC
ENSG0000(	PAUPAR	193.279	0.192855	0.003901	0.015219	lncRNA
ENSG0000(	ENSG0000(	46.33553	-0.37742	0.003903	0.015223	lncRNA
ENSG0000(	LLGL2	6118.691	-0.3703	0.003904	0.015228	protein_coding
ENSG0000(	RGPD4	44.25922	0.89187	0.003907	0.015237	protein_coding
ENSG0000(	FAM111A-I	259.0285	0.255003	0.003908	0.015239	lncRNA
ENSG0000(	CDK8	495.6296	-0.1674	0.00391	0.015243	protein_coding
ENSG0000(	UBFD1	1216.292	0.142772	0.003913	0.015254	protein_coding
ENSG0000(	DPF3	803.198	-0.34422	0.003914	0.015258	protein_coding
ENSG0000(	ENSG0000(	165.2782	0.692176	0.003917	0.015264	lncRNA
ENSG0000(	NFILZ	353.543	0.160972	0.003917	0.015264	protein_coding
ENSG0000(	LZTS2	979.6794	-0.19523	0.003919	0.015272	protein_coding
ENSG0000(	AKR1C2	261.5299	-0.4656	0.003923	0.015284	protein_coding
ENSG0000(	ESRRG	327.6589	-0.49176	0.003925	0.015291	protein_coding
ENSG0000(	HLA-DPB1	1941.769	0.374687	0.003927	0.015294	protein_coding
ENSG0000(	KDM2A	4982.887	0.134714	0.003929	0.015302	protein_coding
ENSG0000(	ENSG0000(	272.5724	0.17001	0.003931	0.015304	lncRNA
ENSG0000(	TWIST2	56.02234	0.706532	0.00393	0.015304	protein_coding
ENSG0000(	CCDC144N	311.7426	0.226434	0.003932	0.015307	transcribed_unprocessed_pseudo
ENSG0000(	ENSG0000(	16.73632	0.649206	0.003935	0.015315	lncRNA
ENSG0000(	ST7-AS1	24.31823	0.531912	0.003935	0.015315	lncRNA
ENSG0000(	ENSG0000(	13.12584	0.692214	0.003938	0.015324	TEC

ENSG0000(	ENSG0000(	40.3636	0.31721	0.003939	0.015327	lncRNA
ENSG0000(	LINC01600	13.18392	0.818405	0.00394	0.015328	lncRNA
ENSG0000(	THBS4	22.10941	0.637396	0.00395	0.015366	protein_coding
ENSG0000(	CST3	1365.086	-0.31396	0.003952	0.01537	protein_coding
ENSG0000(	TRPT1	370.21	-0.30136	0.003952	0.01537	protein_coding
ENSG0000(	S100P	96.21586	0.994877	0.003954	0.015376	protein_coding
ENSG0000(	LINC01151	665.1425	0.12796	0.003956	0.01538	lncRNA
ENSG0000(	SNORA47	114.0347	-0.43038	0.003961	0.015399	snoRNA
ENSG0000(	LINC02541	116.6041	0.367798	0.003963	0.015405	lncRNA
ENSG0000(	ENSG0000(	40.80704	0.358258	0.003972	0.015439	lncRNA
ENSG0000(	LINC01474	80.03583	0.313017	0.003978	0.015461	lncRNA
ENSG0000(	CECR7	425.8468	0.242126	0.00398	0.015464	transcribed_unprocessed_pseudo
ENSG0000(	NFATC2	1085.359	0.289051	0.003983	0.015473	protein_coding
ENSG0000(	TBC1D9B	3790.95	-0.1295	0.003983	0.015473	protein_coding
ENSG0000(	UBE2E3	625.4396	-0.19633	0.003983	0.015473	protein_coding
ENSG0000(	ENSG0000(	363.402	-0.50602	0.003987	0.015487	lncRNA
ENSG0000(	MBOAT1	2473.289	-0.32087	0.003994	0.015511	protein_coding
ENSG0000(	MBP	1833.15	0.118553	0.003996	0.015516	protein_coding
ENSG0000(	CEP76	617.1585	-0.13424	0.003999	0.015524	protein_coding
ENSG0000(	NT5C1A	486.8645	0.252454	0.003999	0.015525	protein_coding
ENSG0000(	RNU7-119F	18.33388	0.644303	0.004006	0.015548	snRNA
ENSG0000(	DCAF5	2325.673	0.100023	0.004009	0.015558	protein_coding
ENSG0000(	PHKG1	284.9968	0.198598	0.004011	0.015561	protein_coding
ENSG0000(	ZNF567	518.1836	-0.19444	0.00401	0.015561	protein_coding
ENSG0000(	CDC26	113.5284	-0.39952	0.004012	0.015563	protein_coding
ENSG0000(	ENSG0000(	42.37614	-0.66522	0.004012	0.015563	lncRNA
ENSG0000(	ZSWIM7	534.6581	-0.14494	0.004013	0.015563	protein_coding
ENSG0000(	OIP5	38.54193	-0.52956	0.004015	0.015569	protein_coding
ENSG0000(	CCDC71	257.5696	-0.20501	0.004016	0.015571	protein_coding
ENSG0000(	PDLIM4	83.38608	0.604628	0.004016	0.015571	protein_coding
ENSG0000(	HPN	168.7155	0.246233	0.004017	0.015572	protein_coding
ENSG0000(	RFC1	1237.37	-0.16302	0.004019	0.015577	protein_coding
ENSG0000(	LINC01961	54.50504	-0.35775	0.004021	0.015583	lncRNA
ENSG0000(	OR2A42	125.4944	0.255565	0.004022	0.015587	protein_coding
ENSG0000(	ENSG0000(	205.9193	0.197983	0.004024	0.01559	lncRNA
ENSG0000(	OR6C66P	150.1124	-0.29436	0.004026	0.0156	transcribed_unprocessed_pseudo
ENSG0000(	RN7SL622F	202.3358	-0.21133	0.004029	0.015608	misc_RNA
ENSG0000(	CAV1	510.8862	0.469004	0.00403	0.015609	protein_coding
ENSG0000(	ZBTB17	358.2837	-0.20183	0.004037	0.015633	protein_coding
ENSG0000(	KRT73-AS1	117.6821	-0.2669	0.004038	0.015636	lncRNA
ENSG0000(	GRID1	198.9608	0.266961	0.004045	0.01566	protein_coding
ENSG0000(	LINC02789	138.2475	0.198345	0.004045	0.01566	lncRNA
ENSG0000(	CMYA5	246.7056	0.393392	0.00405	0.015674	protein_coding
ENSG0000(	CRISPLD1	21.24026	1.013943	0.00405	0.015674	protein_coding
ENSG0000(	SKP1	3768.928	-0.15581	0.00405	0.015674	protein_coding
ENSG0000(	PLPP5	1552.741	0.190572	0.004055	0.015689	protein_coding
ENSG0000(	MIPEP	281.789	-0.25972	0.004058	0.015699	protein_coding

ENSG0000(	APBB1IP	503.6805	0.485575	0.00406	0.015706	protein_coding
ENSG0000(	ENSG0000(	848.9426	0.143335	0.004062	0.015712	TEC
ENSG0000(	EXOSC3	1180.672	-0.14045	0.00407	0.015739	protein_coding
ENSG0000(	EPB41L4B	2607.314	-0.52693	0.004072	0.015748	protein_coding
ENSG0000(	THOC3	399.7696	-0.27566	0.004075	0.015758	protein_coding
ENSG0000(	ETFDH	4463.213	-0.14404	0.004077	0.01576	protein_coding
ENSG0000(	ENSG0000(	160.715	-0.22906	0.004078	0.015765	lncRNA
ENSG0000(	ENSG0000(	232.0059	0.209453	0.004082	0.015775	TEC
ENSG0000(	LINC02542	82.41917	0.328735	0.004082	0.015775	lncRNA
ENSG0000(	ENSG0000(	64.81482	-0.42636	0.004084	0.01578	lncRNA
ENSG0000(	ENSG0000(	160.7549	0.193793	0.004084	0.01578	lncRNA
ENSG0000(	MIR583HG	15.12527	0.548124	0.004085	0.01578	lncRNA
ENSG0000(	ID2-AS1	368.5889	-0.76058	0.004086	0.015783	lncRNA
ENSG0000(	ECHDC1	1460.898	0.144388	0.004093	0.015809	protein_coding
ENSG0000(	TNFAIP8L2	78.27437	0.452748	0.004108	0.015866	protein_coding
ENSG0000(	ENSG0000(	16.46048	0.586022	0.004114	0.015885	lncRNA
ENSG0000(	ARRDC3-AS	924.5161	0.148747	0.004121	0.015906	lncRNA
ENSG0000(	POLR2F	517.8932	-0.14366	0.004121	0.015906	protein_coding
ENSG0000(	THAP7	239.5179	-0.21521	0.00412	0.015906	protein_coding
ENSG0000(	HSPA7	252.1335	0.558267	0.004124	0.015918	unprocessed_pseudogene
ENSG0000(	CDK18	988.8495	-0.2519	0.004126	0.01592	protein_coding
ENSG0000(	GPR1-AS	101.439	-0.32576	0.004128	0.015927	lncRNA
ENSG0000(	ENSG0000(	18.50394	0.475985	0.004128	0.015927	lncRNA
ENSG0000(	PDLIM1P4	37.96351	0.425899	0.004129	0.015929	processed_pseudogene
ENSG0000(	PYM1	435.559	-0.17577	0.004133	0.015941	protein_coding
ENSG0000(	PIGBOS1	234.5518	-0.19367	0.004134	0.015943	protein_coding
ENSG0000(	TOPORS	677.5802	-0.18765	0.00414	0.015964	protein_coding
ENSG0000(	ENSG0000(	22.74539	0.519107	0.004144	0.015978	lncRNA
ENSG0000(	ENSG0000(	34.03462	0.44716	0.004147	0.015987	processed_pseudogene
ENSG0000(	SLPI	54.7057	0.800188	0.00415	0.015996	protein_coding
ENSG0000(	SUV39H2	419.4714	-0.24364	0.00415	0.015996	protein_coding
ENSG0000(	KLK1	238.5634	-0.59406	0.004151	0.015997	protein_coding
ENSG0000(	ENSG0000(	44.59472	0.409154	0.004152	0.015997	unprocessed_pseudogene
ENSG0000(	ARHGAP5	5268.591	-0.18164	0.004155	0.016006	protein_coding
ENSG0000(	COL18A1	1722.174	0.411212	0.004155	0.016006	protein_coding
ENSG0000(	ENSG0000(	360.5131	0.13789	0.004161	0.016028	lncRNA
ENSG0000(	ENSG0000(	192.0829	-0.22597	0.004163	0.016032	lncRNA
ENSG0000(	FGD5P1	176.0552	0.198682	0.004168	0.016049	transcribed_unprocessed_pseudo
ENSG0000(	ENSG0000(	125.7706	0.300909	0.004169	0.016052	lncRNA
ENSG0000(	ENSG0000(	94.54812	0.278629	0.00417	0.016052	lncRNA
ENSG0000(	SPATA6L	1414.777	0.198715	0.00417	0.016052	protein_coding
ENSG0000(	UBR5-DT	287.7288	-0.26043	0.004169	0.016052	lncRNA
ENSG0000(	CLDN12	1238.877	0.179745	0.004179	0.016082	protein_coding
ENSG0000(	UBXN2A	1517.73	-0.1257	0.004179	0.016082	protein_coding
ENSG0000(	ENSG0000(	29.39152	0.665828	0.004181	0.016087	lncRNA
ENSG0000(	ENSG0000(	14.15726	0.704316	0.004189	0.016115	TEC
ENSG0000(	GEN1	3166.89	0.244842	0.004192	0.016126	protein_coding

ENSG0000(	IRAK2	322.4004	0.337954	0.004194	0.016129	protein_coding
ENSG0000(	MACO1	687.7604	-0.17041	0.004194	0.016129	protein_coding
ENSG0000(	ENSG0000(	137.9741	0.20231	0.004195	0.016132	lncRNA
ENSG0000(	SLC35G2	194.8656	0.250532	0.004196	0.016132	protein_coding
ENSG0000(	CDH12	332.3473	-0.21465	0.004198	0.016137	protein_coding
ENSG0000(	ENSG0000(	633.0094	-0.15848	0.0042	0.016143	lncRNA
ENSG0000(	ZBTB37	3585.402	0.14829	0.004203	0.016153	protein_coding
ENSG0000(	IL34	113.1103	0.361788	0.004204	0.016155	protein_coding
ENSG0000(	PMS2P3	321.2957	-0.30573	0.004207	0.016163	transcribed_unprocessed_pseudo
ENSG0000(	UEVLD	1460.548	-0.1198	0.004207	0.016163	protein_coding
ENSG0000(	MYO15A	412.8875	0.157449	0.00421	0.016171	protein_coding
ENSG0000(	LINC02904	332.3774	0.171586	0.004232	0.016255	lncRNA
ENSG0000(	SLCO5A1	517.9978	0.360691	0.004235	0.016263	protein_coding
ENSG0000(	SYF2	609.4862	-0.16073	0.004236	0.016266	protein_coding
ENSG0000(	ENSG0000(	23.13557	0.45376	0.004238	0.016271	lncRNA
ENSG0000(	IGKV1-5	1233.312	0.634904	0.004238	0.016271	IG_V_gene
ENSG0000(	INSL4	22.28416	0.497085	0.004243	0.016285	protein_coding
ENSG0000(	ENSG0000(	1049.223	0.287968	0.004244	0.016288	lncRNA
ENSG0000(	SIAE	1857.754	-0.27577	0.004244	0.016289	protein_coding
ENSG0000(	LINC02544	10.6026	0.60646	0.004246	0.016289	lncRNA
ENSG0000(	POLN	533.7293	0.220605	0.004245	0.016289	protein_coding
ENSG0000(	RBFOX2	2906.558	0.18402	0.004249	0.0163	protein_coding
ENSG0000(	ENSG0000(	15.28539	-0.68071	0.00425	0.016302	lncRNA
ENSG0000(	ENSG0000(	116.7398	-0.22618	0.004255	0.016321	TEC
ENSG0000(	ENSG0000(	45.03478	0.817836	0.004263	0.016349	unprocessed_pseudogene
ENSG0000(	ENSG0000(	36.00641	0.302418	0.004269	0.01637	processed_pseudogene
ENSG0000(	ATP7B	2168.811	-0.20668	0.004272	0.016377	protein_coding
ENSG0000(	ENSG0000(	106.6709	0.257977	0.004276	0.016391	lncRNA
ENSG0000(	TMEM14B	1022	-0.13784	0.004278	0.016397	protein_coding
ENSG0000(	REEP3	1185.076	-0.21375	0.004279	0.0164	protein_coding
ENSG0000(	ENSG0000(	21.44144	0.693766	0.004282	0.016409	transcribed_unprocessed_pseudo
ENSG0000(	PRDM4	1261.199	-0.17518	0.004283	0.016409	protein_coding
ENSG0000(	WDR61	698.8578	-0.12734	0.004284	0.016411	protein_coding
ENSG0000(	CENPS	76.79593	-0.32117	0.004285	0.016413	protein_coding
ENSG0000(	LAPTM4A	872.6802	-0.28692	0.004288	0.016421	protein_coding
ENSG0000(	TMEM134	932.0792	-0.12507	0.004288	0.016421	protein_coding
ENSG0000(	ADAM3B	27.20209	0.530383	0.004293	0.016437	processed_pseudogene
ENSG0000(	EIF3FP3	56.38928	-0.85147	0.004298	0.016454	processed_pseudogene
ENSG0000(	ENSG0000(	170.7323	-0.18355	0.004299	0.016456	lncRNA
ENSG0000(	IFI44	424.2363	0.686671	0.0043	0.01646	protein_coding
ENSG0000(	RAB11FIP4	2098.008	-0.18065	0.004304	0.016471	protein_coding
ENSG0000(	LSG1	1286.548	-0.19199	0.004307	0.016484	protein_coding
ENSG0000(	LINC01842	86.099	0.320477	0.004318	0.016523	lncRNA
ENSG0000(	PNP	1030.967	-0.26078	0.004321	0.01653	protein_coding
ENSG0000(	SMIM7	1818.622	-0.08236	0.004326	0.016549	protein_coding
ENSG0000(	HOXD1	84.80134	-0.5313	0.004341	0.016603	protein_coding
ENSG0000(	ZFP62	1612.091	-0.16579	0.004341	0.016603	protein_coding

ENSG0000(	LINC01800	24.54011	0.74309	0.004345	0.016616	lncRNA
ENSG0000(	ENSG0000(	55.78433	-0.30117	0.004347	0.016621	lncRNA
ENSG0000(	PCCA-AS1	19.09961	0.472976	0.004348	0.016621	lncRNA
ENSG0000(	ANGPT2	509.3482	0.176311	0.004351	0.016629	protein_coding
ENSG0000(	ENSG0000(	190.7002	0.167754	0.004351	0.016629	lncRNA
ENSG0000(	TACR1	46.79779	1.094975	0.004361	0.016668	protein_coding
ENSG0000(	DTHD1	147.6543	0.650131	0.004363	0.016672	protein_coding
ENSG0000(	ITGB2	1025.814	0.465764	0.004368	0.016687	protein_coding
ENSG0000(	C7orf25	212.4802	0.303831	0.004371	0.016697	protein_coding
ENSG0000(	MIEF1	1469.482	-0.15759	0.004376	0.016717	protein_coding
ENSG0000(	ARHGAP44	2540.569	-0.44677	0.004378	0.01672	protein_coding
ENSG0000(	FOXG1	14.01227	0.713245	0.004378	0.01672	protein_coding
ENSG0000(	E2F8	332.5337	-0.39388	0.004379	0.01672	protein_coding
ENSG0000(	ENSG0000(	49.46693	-0.30711	0.004379	0.01672	processed_pseudogene
ENSG0000(	ATG101	255.0794	-0.17695	0.00438	0.01672	protein_coding
ENSG0000(	MAS1	378.6846	0.191884	0.004386	0.016742	protein_coding
ENSG0000(	FRMD4B	1687.456	-0.12791	0.004387	0.016745	protein_coding
ENSG0000(	LINC02595	162.9864	0.432061	0.004393	0.016763	lncRNA
ENSG0000(	SOX17	28.40985	0.626435	0.004395	0.016771	protein_coding
ENSG0000(	ENSG0000(	10.72717	0.690814	0.004398	0.016777	lncRNA
ENSG0000(	LINC01737	16.70289	0.611552	0.004403	0.016796	lncRNA
ENSG0000(	MIR200CH(	116.1642	-0.43693	0.004405	0.016802	lncRNA
ENSG0000(	CCL15-CCL:	52.57345	-0.86096	0.00441	0.016819	protein_coding
ENSG0000(	ENSG0000(	423.9904	0.128087	0.004412	0.016825	lncRNA
ENSG0000(	ENSG0000(	467.0671	-0.1788	0.004414	0.016828	lncRNA
ENSG0000(	IQCK	415.7936	0.196758	0.004414	0.016828	protein_coding
ENSG0000(	SPATA25	65.75482	-0.442	0.004416	0.016832	protein_coding
ENSG0000(	ENSG0000(	267.9981	0.204429	0.004417	0.016836	lncRNA
ENSG0000(	LAIR1	1525.609	0.298759	0.004425	0.016862	protein_coding
ENSG0000(	SNORA80D	36.33967	-0.51932	0.004432	0.016889	snoRNA
ENSG0000(	ARL10	1753.001	0.200894	0.004434	0.016895	protein_coding
ENSG0000(	FAR2P4	128.057	0.360611	0.004437	0.016901	transcribed_unprocessed_pseudo
ENSG0000(	HERC5	251.8418	0.49007	0.004437	0.016902	protein_coding
ENSG0000(	GTF3C1	2005.7	-0.12948	0.004442	0.016916	protein_coding
ENSG0000(	ENSG0000(	41.54691	0.461135	0.004444	0.016922	processed_pseudogene
ENSG0000(	HAUS5	478.5348	-0.22832	0.004452	0.016951	protein_coding
ENSG0000(	SLC7A6OS	1026.141	0.141253	0.004456	0.016965	protein_coding
ENSG0000(	DYNC2LI1	741.2392	-0.17139	0.004465	0.016997	protein_coding
ENSG0000(	ZNF731P	2657.203	0.114168	0.004466	0.016997	transcribed_unprocessed_pseudo
ENSG0000(	LPIN3	2185.263	-0.33017	0.004466	0.016999	protein_coding
ENSG0000(	UNC119B	906.2847	-0.22029	0.00447	0.01701	protein_coding
ENSG0000(	RBM6	6416.779	0.118498	0.004471	0.017012	protein_coding
ENSG0000(	TEN1-CDK3	645.203	-0.19525	0.004472	0.017012	protein_coding
ENSG0000(	ENSG0000(	15.3164	0.678497	0.004473	0.017014	TEC
ENSG0000(	ARL11	520.0753	0.386688	0.004473	0.017014	protein_coding
ENSG0000(	TRHDE-AS1	145.4304	0.337578	0.004478	0.01703	lncRNA
ENSG0000(	ANXA6	1172.107	0.349288	0.004478	0.01703	protein_coding

ENSG0000(	ENSG0000(	175.4853	0.302057	0.00448	0.017033	lncRNA
ENSG0000(	ENSG0000(	164.149	0.24018	0.004482	0.017039	lncRNA
ENSG0000(	ENSG0000(	22.20285	-0.74313	0.004487	0.017056	processed_pseudogene
ENSG0000(	ENSG0000(	40.96572	0.350518	0.004488	0.017057	lncRNA
ENSG0000(	GRK5-IT1	177.4656	0.316812	0.004488	0.017057	lncRNA
ENSG0000(	RN7SL449F	23.71515	0.472509	0.004489	0.017057	misc_RNA
ENSG0000(	ENSG0000(	75.95667	-0.27711	0.00449	0.017058	lncRNA
ENSG0000(	JOSD2	90.00941	-0.30763	0.004491	0.017058	protein_coding
ENSG0000(	LINC00958	368.1622	-0.13615	0.004491	0.017058	lncRNA
ENSG0000(	ENSG0000(	132.7793	0.274675	0.004493	0.017063	lncRNA
ENSG0000(	UFL1-AS1	157.9887	0.237295	0.004502	0.017096	lncRNA
ENSG0000(	MIR548C	59.9338	0.369042	0.004506	0.017111	miRNA
ENSG0000(	RPL5P13	62.4104	0.337464	0.004507	0.017111	processed_pseudogene
ENSG0000(	ENSG0000(	125.9963	0.417266	0.004509	0.017115	lncRNA
ENSG0000(	ENSG0000(	149.1006	0.298742	0.00451	0.017119	lncRNA
ENSG0000(	PHKB	3749.949	0.091816	0.004511	0.017119	protein_coding
ENSG0000(	CAMSAP1	846.2365	-0.2389	0.004513	0.017127	protein_coding
ENSG0000(	ENSG0000(	50.79725	0.405691	0.004518	0.017143	lncRNA
ENSG0000(	LGMN	1151.104	-0.21792	0.004519	0.017146	protein_coding
ENSG0000(	EIF5A2P1	14.55147	-0.61663	0.004521	0.017149	processed_pseudogene
ENSG0000(	LINC01405	412.6827	0.170937	0.00453	0.017181	lncRNA
ENSG0000(	WDR55	1969.301	-0.12016	0.004534	0.017193	protein_coding
ENSG0000(	WHAMMP:	1122.901	0.143264	0.004534	0.017194	transcribed_unprocessed_pseudo
ENSG0000(	ENSG0000(	57.20943	0.326303	0.004537	0.017203	processed_pseudogene
ENSG0000(	RBM22	814.851	-0.11916	0.004538	0.017203	protein_coding
ENSG0000(	KIAA1191	1394.334	-0.15842	0.00454	0.017208	protein_coding
ENSG0000(	LINC00934	84.81149	-0.2337	0.004541	0.017211	lncRNA
ENSG0000(	RN7SL47P	47.08151	0.377827	0.004543	0.017217	misc_RNA
ENSG0000(	LINC00343	1958.072	-0.24092	0.004548	0.017234	lncRNA
ENSG0000(	ANXA7	825.3712	-0.19399	0.004549	0.017235	protein_coding
ENSG0000(	BOLL	11.87163	0.778462	0.00455	0.017235	protein_coding
ENSG0000(	MFSD13A	421.926	-0.19198	0.004551	0.017235	protein_coding
ENSG0000(	NDST1	2369.387	0.199171	0.004551	0.017235	protein_coding
ENSG0000(	TMCC1-DT	2022.595	0.149105	0.00455	0.017235	lncRNA
ENSG0000(	AGR2	2892.91	-0.58594	0.004566	0.017288	protein_coding
ENSG0000(	ENSG0000(	305.6529	0.13922	0.004568	0.01729	lncRNA
ENSG0000(	ENSG0000(	292.0501	0.186307	0.004567	0.01729	lncRNA
ENSG0000(	OCRL	1837.493	-0.22486	0.004567	0.01729	protein_coding
ENSG0000(	ENSG0000(	396.4628	0.152854	0.004573	0.017307	lncRNA
ENSG0000(	INVS	1339.251	0.119295	0.004575	0.01731	protein_coding
ENSG0000(	POLR1C	668.1324	-0.13374	0.004574	0.01731	protein_coding
ENSG0000(	ENSG0000(	25.75228	0.446513	0.004575	0.01731	transcribed_processed_pseudoge
ENSG0000(	ZMYM3	767.7343	0.18233	0.004577	0.017315	protein_coding
ENSG0000(	RPS6P20	174.5801	-0.30213	0.004579	0.017319	unprocessed_pseudogene
ENSG0000(	BRF1	1184.081	-0.11479	0.004583	0.017328	protein_coding
ENSG0000(	ENSG0000(	204.421	-0.20562	0.004583	0.017328	lncRNA
ENSG0000(	RDH13	2465.956	-0.24305	0.004583	0.017328	protein_coding

ENSG0000(	RPS12P3	35.1104	-0.43113	0.004583	0.017328	processed_pseudogene
ENSG0000(	ENSG0000(	46.51769	0.326283	0.004587	0.017341	unprocessed_pseudogene
ENSG0000(	MIR378D2I	13.60421	0.674938	0.004592	0.017356	lncRNA
ENSG0000(	RHBDL2	594.3733	-0.24268	0.004592	0.017356	protein_coding
ENSG0000(	CXCR4	842.9806	0.76053	0.004594	0.017358	protein_coding
ENSG0000(	ENSG0000(	30.80902	0.439583	0.004596	0.017364	lncRNA
ENSG0000(	RRP8	1374.13	-0.15051	0.0046	0.017376	protein_coding
ENSG0000(	ZSCAN16-A	586.3492	0.191431	0.004604	0.017391	lncRNA
ENSG0000(	LIN9	158.5313	-0.34559	0.004606	0.017396	protein_coding
ENSG0000(	ENSG0000(	49.55212	-0.37215	0.004616	0.017432	lncRNA
ENSG0000(	PPDPF	1199.922	-0.37706	0.004616	0.017432	protein_coding
ENSG0000(	TPRA1	464.7151	-0.15108	0.004619	0.017437	protein_coding
ENSG0000(	RCAN2	428.2544	-0.36625	0.004638	0.017508	protein_coding
ENSG0000(	ENSG0000(	61.44949	0.385076	0.004641	0.017517	lncRNA
ENSG0000(	DNAJB9	445.5635	0.309971	0.004642	0.017519	protein_coding
ENSG0000(	ENSG0000(	323.3964	-0.20483	0.004642	0.017519	lncRNA
ENSG0000(	MIR99AHG	2200.148	0.133755	0.004643	0.017519	lncRNA
ENSG0000(	DNAH5	266.7014	0.242684	0.004646	0.017523	protein_coding
ENSG0000(	GCC2	10869.03	0.188055	0.004645	0.017523	protein_coding
ENSG0000(	SS18	2671.971	0.144334	0.004646	0.017523	protein_coding
ENSG0000(	ZNF665	1202.714	-0.11839	0.004647	0.017528	protein_coding
ENSG0000(	ENSG0000(	15.52087	0.941355	0.004652	0.017543	lncRNA
ENSG0000(	RNU6-272F	23.82001	0.548801	0.004654	0.017547	snRNA
ENSG0000(	GLP2R	332.285	-0.56285	0.004657	0.017559	protein_coding
ENSG0000(	ENSG0000(	74.65051	-0.28999	0.004675	0.01762	lncRNA
ENSG0000(	ZPLD2P	304.543	0.19838	0.004674	0.01762	transcribed_unprocessed_pseudo
ENSG0000(	DUOX1	421.0099	0.210717	0.004676	0.017622	protein_coding
ENSG0000(	ENSG0000(	230.9414	-0.1858	0.004683	0.017646	lncRNA
ENSG0000(	ENSG0000(	114.4529	-0.24828	0.004685	0.017651	lncRNA
ENSG0000(	ENSG0000(	81.41621	0.285444	0.004686	0.017656	lncRNA
ENSG0000(	ENSG0000(	137.5621	0.201268	0.004694	0.017681	lncRNA
ENSG0000(	RBMS3	1501.161	0.231383	0.004695	0.017682	protein_coding
ENSG0000(	RN7SL237F	57.83735	0.320032	0.004695	0.017682	misc_RNA
ENSG0000(	ENSG0000(	41.48237	-0.30645	0.004696	0.017683	lncRNA
ENSG0000(	SDHAP1	1651.743	-0.29405	0.004697	0.017683	transcribed_unprocessed_pseudo
ENSG0000(	ANKRD1	11.48398	0.850213	0.004699	0.017692	protein_coding
ENSG0000(	ENSG0000(	191.7797	-0.18155	0.004704	0.017706	lncRNA
ENSG0000(	ENSG0000(	97.8724	0.531145	0.004704	0.017706	TEC
ENSG0000(	COL9A2	290.4724	0.398303	0.004706	0.017708	protein_coding
ENSG0000(	ENSG0000(	139.1784	0.312547	0.004706	0.017708	TEC
ENSG0000(	ENSG0000(	116.7249	0.558295	0.004714	0.017732	lncRNA
ENSG0000(	LINC01586	94.61707	0.373398	0.004714	0.017732	lncRNA
ENSG0000(	SERINC3	2586.4	-0.12766	0.004714	0.017732	protein_coding
ENSG0000(	GLCCI1-DT	10.1098	0.631179	0.004715	0.017735	lncRNA
ENSG0000(	CKB	1965.102	-0.85645	0.004717	0.017738	protein_coding
ENSG0000(	SLC19A1	471.8153	-0.24853	0.004722	0.017754	protein_coding
ENSG0000(	ENSG0000(	2462.825	-0.19127	0.004729	0.017776	transcribed_unprocessed_pseudo

ENSG0000(	ENSG0000(	16.52198	0.441358	0.004728	0.017776	lncRNA
ENSG0000(	ERCC6L	112.3153	-0.31672	0.004736	0.017802	protein_coding
ENSG0000(	COPDA1	20.02637	0.886086	0.004742	0.017824	lncRNA
ENSG0000(	LRRC57	2040.251	-0.11791	0.004744	0.017829	protein_coding
ENSG0000(	C1QB	503.4647	0.514838	0.004746	0.01783	protein_coding
ENSG0000(	RITA1	210.3562	-0.2161	0.004745	0.01783	protein_coding
ENSG0000(	ZBTB1	2002.513	0.166251	0.00475	0.017843	protein_coding
ENSG0000(	ENSG0000(	2869.649	-0.25744	0.004752	0.017847	lncRNA
ENSG0000(	ATL1	2268.48	0.103005	0.004754	0.017853	protein_coding
ENSG0000(	ENSG0000(	52.83161	0.296016	0.004754	0.017853	lncRNA
ENSG0000(	ENSG0000(	109.9685	0.207891	0.004755	0.017854	TEC
ENSG0000(	PAN3-AS1	131.4151	0.237374	0.004764	0.017883	lncRNA
ENSG0000(	SHROOM3-	23.11931	0.761332	0.004764	0.017883	lncRNA
ENSG0000(	RNF123	1009.936	-0.18333	0.004768	0.017895	protein_coding
ENSG0000(	RPL35AP2	90.20133	0.213086	0.004768	0.017895	processed_pseudogene
ENSG0000(	THBS1	7102.227	0.5028	0.004773	0.017909	protein_coding
ENSG0000(	TM7SF3	1489.258	-0.134	0.004779	0.017931	protein_coding
ENSG0000(	GPR161	427.6529	0.424216	0.00478	0.017934	protein_coding
ENSG0000(	HNRNPA2B	15628.59	0.101128	0.004784	0.017946	protein_coding
ENSG0000(	ENSG0000(	21.07914	0.567106	0.00479	0.017965	lncRNA
ENSG0000(	GAU1	128.258	-0.339	0.004794	0.017977	lncRNA
ENSG0000(	RHOBTB1	412.1396	0.235747	0.004794	0.017977	protein_coding
ENSG0000(	PRKACB-DT	15.96474	0.57177	0.004801	0.018	lncRNA
ENSG0000(	ENSG0000(	45.67122	0.326543	0.004804	0.018008	unprocessed_pseudogene
ENSG0000(	C9orf24	13.80099	-0.87003	0.004807	0.018014	protein_coding
ENSG0000(	GRWD1	573.0298	-0.17297	0.004807	0.018014	protein_coding
ENSG0000(	MACC1	1968.19	-0.28943	0.004807	0.018014	protein_coding
ENSG0000(	TMED8	1540.413	0.178624	0.004806	0.018014	protein_coding
ENSG0000(	ALKAL2	78.79578	-0.57885	0.004809	0.018017	protein_coding
ENSG0000(	ADD3	3354.537	-0.17441	0.004813	0.018031	protein_coding
ENSG0000(	PRSS27	518.3243	0.208994	0.004816	0.01804	protein_coding
ENSG0000(	LINC01936	310.5362	-0.15627	0.004819	0.018049	lncRNA
ENSG0000(	CPOX	401.5526	-0.19238	0.004821	0.018054	protein_coding
ENSG0000(	N6AMT1	729.8986	-0.19591	0.004823	0.018059	protein_coding
ENSG0000(	LINC02157	62.59143	-0.33481	0.004824	0.018059	lncRNA
ENSG0000(	MAP3K10	467.0413	0.186779	0.004827	0.018072	protein_coding
ENSG0000(	ENSG0000(	10.57075	0.629968	0.00484	0.018115	TEC
ENSG0000(	HMGN1P1!	19.73095	-0.5737	0.004841	0.018118	processed_pseudogene
ENSG0000(	RNU6-577F	23.70091	0.464992	0.004844	0.018127	snRNA
ENSG0000(	ENSG0000(	411.0418	-0.12663	0.004845	0.01813	processed_pseudogene
ENSG0000(	ENSG0000(	137.817	0.233215	0.004854	0.018157	lncRNA
ENSG0000(	UGT1A7	1630.19	-0.51857	0.004854	0.018157	protein_coding
ENSG0000(	FAM200A	149.3988	-0.24217	0.004857	0.018167	protein_coding
ENSG0000(	ENSG0000(	475.2767	0.258858	0.004862	0.018184	lncRNA
ENSG0000(	ENSG0000(	33.79231	0.477512	0.004865	0.018191	lncRNA
ENSG0000(	ZMYM5	805.9486	0.174002	0.004865	0.018191	protein_coding
ENSG0000(	ENSG0000(	77.92531	-0.27504	0.004875	0.018225	lncRNA

ENSG0000(	CD2AP	3756.45	-0.29532	0.004876	0.018228	protein_coding
ENSG0000(	RHOXF1-AS	179.911	0.211508	0.004877	0.018228	lncRNA
ENSG0000(	IGKV4-1	1096.251	0.625103	0.004879	0.018233	IG_V_gene
ENSG0000(	DDX52	1907.619	-0.08166	0.004881	0.018238	protein_coding
ENSG0000(	KIF9	127.2348	-0.35668	0.004882	0.018242	protein_coding
ENSG0000(	IGKV2-29	50.8521	1.57752	0.004885	0.018248	IG_V_pseudogene
ENSG0000(	ENSG0000(	279.9741	0.237988	0.004889	0.018262	unprocessed_pseudogene
ENSG0000(	APEX1	733.0382	-0.1924	0.004893	0.018274	protein_coding
ENSG0000(	ENSG0000(	24.45623	-0.8236	0.004896	0.018284	lncRNA
ENSG0000(	ENSG0000(	487.5521	0.150564	0.004907	0.01832	lncRNA
ENSG0000(	POLR1G	417.1638	-0.1361	0.004907	0.01832	protein_coding
ENSG0000(	TOMM6	331.3344	-0.21895	0.004906	0.01832	protein_coding
ENSG0000(	ENSG0000(	281.606	0.209035	0.004909	0.018323	lncRNA
ENSG0000(	ENSG0000(	84.30584	-0.95641	0.00491	0.018323	lncRNA
ENSG0000(	KIAA1549L	62.96032	-1.13865	0.004911	0.018323	protein_coding
ENSG0000(	RPL35AP9	31.37307	0.392197	0.004911	0.018323	processed_pseudogene
ENSG0000(	RN7SL444F	90.77225	0.24304	0.004914	0.018333	misc_RNA
ENSG0000(	ZNF676	372.1395	-0.11704	0.004914	0.018333	protein_coding
ENSG0000(	PREPL	1856.067	-0.13073	0.004919	0.018348	protein_coding
ENSG0000(	ENSG0000(	26.36115	0.518326	0.004921	0.018353	TEC
ENSG0000(	SLC35E2B	5844.275	0.119072	0.004928	0.018379	protein_coding
ENSG0000(	ACOT13	714.8897	-0.28867	0.004931	0.018385	protein_coding
ENSG0000(	ENSG0000(	12.0817	0.553662	0.004931	0.018385	lncRNA
ENSG0000(	SNORA77	19.09072	0.484069	0.004932	0.018386	snoRNA
ENSG0000(	LILRA2	451.8821	0.192507	0.004938	0.018407	protein_coding
ENSG0000(	ENSG0000(	143.4296	0.234966	0.00494	0.018409	lncRNA
ENSG0000(	LINS1	2091.659	0.225011	0.00494	0.018409	protein_coding
ENSG0000(	MYO5B	4424.678	-0.35527	0.004944	0.018421	protein_coding
ENSG0000(	ENSG0000(	64.83152	0.629185	0.004945	0.018423	lncRNA
ENSG0000(	PLA2G4D	205.3578	0.254678	0.004949	0.018434	protein_coding
ENSG0000(	SMIM15-AS	113.4951	0.254282	0.004955	0.018455	lncRNA
ENSG0000(	APBA2	500.9482	0.32351	0.004959	0.018462	protein_coding
ENSG0000(	CD34	904.1944	0.31127	0.004959	0.018462	protein_coding
ENSG0000(	DAD1	405.9891	-0.29722	0.004958	0.018462	protein_coding
ENSG0000(	NRSN2	101.7876	0.347807	0.004958	0.018462	protein_coding
ENSG0000(	RN7SL398F	80.36373	0.298639	0.00496	0.018463	misc_RNA
ENSG0000(	ENSG0000(	60.98649	-0.36865	0.004968	0.018491	lncRNA
ENSG0000(	LINC01837	53.86502	0.400572	0.004972	0.018503	lncRNA
ENSG0000(	COL15A1	905.8155	0.387309	0.004983	0.018541	protein_coding
ENSG0000(	ENSG0000(	708.6221	0.199323	0.004983	0.018541	lncRNA
ENSG0000(	ENSG0000(	915.0523	0.127731	0.004984	0.018541	lncRNA
ENSG0000(	CCDC22	202.4599	-0.24816	0.004986	0.018548	protein_coding
ENSG0000(	UTRN	11164.64	0.172265	0.004988	0.018553	protein_coding
ENSG0000(	ENSG0000(	19.27912	-0.51395	0.004989	0.018554	processed_pseudogene
ENSG0000(	RNF32-AS1	136.0199	0.34458	0.00499	0.018557	lncRNA
ENSG0000(	PGRMC1	1028.139	-0.32781	0.004996	0.018576	protein_coding
ENSG0000(	ZNF746	1646.647	0.101984	0.004997	0.018576	protein_coding

ENSG0000(	PANTR1	274.1902	-0.14482	0.005004	0.0186	lncRNA
ENSG0000(	ENSG0000(	997.4746	0.149371	0.005008	0.018613	lncRNA
ENSG0000(	RCN2	1949.933	-0.14417	0.005012	0.018626	protein_coding
ENSG0000(	ANLN	524.6885	-0.35427	0.005014	0.018633	protein_coding
ENSG0000(	OR51M1	291.7689	-0.20176	0.005015	0.018634	protein_coding
ENSG0000(	ENSG0000(	610.0141	0.125399	0.005017	0.018638	lncRNA
ENSG0000(	METTL16	1632.869	-0.11172	0.005018	0.018638	protein_coding
ENSG0000(	TRGV9	15.37197	-0.60435	0.005018	0.018638	TR_V_gene
ENSG0000(	CDC42EP4	1203.069	-0.18953	0.005023	0.018651	protein_coding
ENSG0000(	ENSG0000(	139.6046	-0.14677	0.005022	0.018651	lncRNA
ENSG0000(	GPT	1033.044	-0.50548	0.005024	0.018655	protein_coding
ENSG0000(	ENSG0000(	115.0062	0.220692	0.005028	0.018667	TEC
ENSG0000(	FBXO31	1499.044	-0.23452	0.00503	0.018672	protein_coding
ENSG0000(	CCDC141	900.7752	0.437853	0.005035	0.018686	protein_coding
ENSG0000(	LRRC4	50.7181	0.772554	0.005039	0.0187	protein_coding
ENSG0000(	NCAPH2	580.0025	-0.19805	0.005042	0.018708	protein_coding
ENSG0000(	ENSG0000(	13.68721	0.589676	0.005052	0.018744	lncRNA
ENSG0000(	ZFAND6	2011.579	0.137868	0.005056	0.018755	protein_coding
ENSG0000(	DUBR	386.6501	0.268915	0.005057	0.018758	lncRNA
ENSG0000(	IMPDH1P1(	10.30263	1.066213	0.00506	0.018766	processed_pseudogene
ENSG0000(	ENSG0000(	58.2518	-0.35323	0.005066	0.018787	pseudogene
ENSG0000(	CTNNA2	129.4022	0.266825	0.005072	0.018805	protein_coding
ENSG0000(	LINC01389	21.40948	-0.73139	0.005072	0.018805	lncRNA
ENSG0000(	CARD19	485.5625	-0.24163	0.00508	0.018833	protein_coding
ENSG0000(	RPS3P7	57.41016	0.322165	0.005081	0.018833	processed_pseudogene
ENSG0000(	ENSG0000(	32.50245	0.384078	0.005084	0.01884	lncRNA
ENSG0000(	KRT8P52	31.36193	-0.48654	0.005085	0.01884	processed_pseudogene
ENSG0000(	LMBR1	1722.545	-0.13844	0.005084	0.01884	protein_coding
ENSG0000(	TSPAN17	666.2303	-0.19066	0.00509	0.018859	protein_coding
ENSG0000(	CHRM4	21.63877	0.852872	0.005099	0.018881	protein_coding
ENSG0000(	DIAPH2	3012.22	-0.17206	0.005098	0.018881	protein_coding
ENSG0000(	OR2V1	49.37291	0.379427	0.005097	0.018881	protein_coding
ENSG0000(	VPS11	1040.351	-0.1176	0.005098	0.018881	protein_coding
ENSG0000(	ENSG0000(	938.6465	-0.33524	0.005103	0.018894	lncRNA
ENSG0000(	MAN2A1	3289.391	0.113595	0.005106	0.018903	protein_coding
ENSG0000(	ENSG0000(	19.38526	0.618393	0.005108	0.01891	lncRNA
ENSG0000(	PTN	40.47905	-0.56872	0.005116	0.018932	protein_coding
ENSG0000(	RMDN2-AS	50.19245	0.355265	0.005115	0.018932	lncRNA
ENSG0000(	LINC01705	38.70589	0.488038	0.005121	0.018947	lncRNA
ENSG0000(	SLC36A2	324.418	-0.1527	0.005121	0.018947	protein_coding
ENSG0000(	AIMP1	1929.707	-0.11292	0.005121	0.018947	protein_coding
ENSG0000(	PIDD1	596.5255	-0.15423	0.005126	0.018962	protein_coding
ENSG0000(	ENSG0000(	155.4463	0.203944	0.005131	0.01898	lncRNA
ENSG0000(	AMDHD2	668.0541	-0.16887	0.005133	0.018982	protein_coding
ENSG0000(	IQCH-AS1	1208.137	-0.17398	0.005135	0.018988	lncRNA
ENSG0000(	PAOX	100.8356	0.348713	0.005136	0.018991	protein_coding
ENSG0000(	SRFBP1	517.4652	-0.18649	0.005141	0.019007	protein_coding

ENSG0000(	HS1BP3-IT1	17.85616	0.545003	0.005144	0.019013	lncRNA
ENSG0000(	ST8SIA2	19.37319	-0.94648	0.005144	0.019013	protein_coding
ENSG0000(	EXOSC10	1450.177	-0.14594	0.005145	0.019015	protein_coding
ENSG0000(	HCG4B	34.47433	0.438974	0.005155	0.01905	unprocessed_pseudogene
ENSG0000(	LINC01886	145.4674	0.279667	0.005158	0.019057	lncRNA
ENSG0000(	SCARNA6	719.1329	-0.26771	0.005159	0.019061	scaRNA
ENSG0000(	CHD4	4576.744	-0.12545	0.005168	0.019089	protein_coding
ENSG0000(	XRCC5	2941.709	-0.12039	0.005176	0.019119	protein_coding
ENSG0000(	ENSG0000(	11.498	0.680925	0.005182	0.019137	lncRNA
ENSG0000(	MID1IP1	684.2674	-0.25942	0.005183	0.019137	protein_coding
ENSG0000(	RPA4	119.3118	-0.20904	0.005188	0.019154	protein_coding
ENSG0000(	ENSG0000(	242.6376	0.16179	0.005192	0.019167	lncRNA
ENSG0000(	FAM71F2	1537.554	0.16822	0.005193	0.019168	protein_coding
ENSG0000(	RNU6-181F	20.22228	0.635185	0.005194	0.019168	snRNA
ENSG0000(	TMEM171	540.1695	-0.59368	0.005193	0.019168	protein_coding
ENSG0000(	GASK1A	9902.912	-0.23434	0.005195	0.019168	protein_coding
ENSG0000(	ENSG0000(	10.97949	0.674019	0.005204	0.019201	lncRNA
ENSG0000(	TRIP13	224.5933	-0.23975	0.005207	0.019208	protein_coding
ENSG0000(	SLC18A2	149.5938	0.43923	0.005207	0.019208	protein_coding
ENSG0000(	VN1R5	13.87083	0.488498	0.005209	0.019211	polymorphic_pseudogene
ENSG0000(	YEATS4	112.3012	-0.27917	0.005209	0.019211	protein_coding
ENSG0000(	ENSG0000(	10.98045	-0.65828	0.005212	0.019219	lncRNA
ENSG0000(	ARGFXP1	25.81766	-0.39213	0.005214	0.019221	processed_pseudogene
ENSG0000(	KIAA1958	1402.284	-0.17446	0.005214	0.019221	protein_coding
ENSG0000(	RERGL	9.925352	-1.03509	0.005215	0.019221	protein_coding
ENSG0000(	ZNF621	3085.574	0.098495	0.005215	0.019221	protein_coding
ENSG0000(	EIF1AD	660.4035	-0.12991	0.005217	0.019225	protein_coding
ENSG0000(	LGALS17A	27.67098	0.645333	0.005218	0.019225	transcribed_unprocessed_pseudo
ENSG0000(	OR5T2	43.23287	0.278876	0.005218	0.019225	protein_coding
ENSG0000(	PWP1	442.8535	-0.1693	0.005221	0.019234	protein_coding
ENSG0000(	ENSG0000(	59.95707	-0.30505	0.005222	0.019234	lncRNA
ENSG0000(	RTL8B	46.07396	0.439203	0.005223	0.019235	protein_coding
ENSG0000(	EZH2	1476.693	-0.19588	0.005226	0.019242	protein_coding
ENSG0000(	HLA-DQA1	2267.881	0.426399	0.005226	0.019242	protein_coding
ENSG0000(	IPO4	318.8786	-0.32666	0.005226	0.019242	protein_coding
ENSG0000(	LRP3	155.7624	-0.3319	0.005227	0.019244	protein_coding
ENSG0000(	LINC00488	58.07933	-0.35233	0.005234	0.019266	lncRNA
ENSG0000(	PPCS	854.4064	-0.17117	0.005236	0.01927	protein_coding
ENSG0000(	LINC01828	169.656	0.209623	0.005236	0.019271	lncRNA
ENSG0000(	ENSG0000(	19.50949	0.452057	0.00524	0.019281	lncRNA
ENSG0000(	NANS	1845.692	-0.23884	0.00524	0.019281	protein_coding
ENSG0000(	CC2D2B	161.4528	0.483222	0.005251	0.019313	protein_coding
ENSG0000(	ME3	938.2163	-0.38248	0.005251	0.019313	protein_coding
ENSG0000(	SLC1A5	1248.563	-0.23656	0.00525	0.019313	protein_coding
ENSG0000(	PPM1F-AS1	1996.82	0.091121	0.005255	0.019326	lncRNA
ENSG0000(	ENSG0000(	116.8285	0.358713	0.00526	0.019337	lncRNA
ENSG0000(	PEA15	881.1718	0.340433	0.005259	0.019337	protein_coding

ENSG0000(	ENSG0000(	12.28448	0.893724	0.005264	0.01935	processed_pseudogene
ENSG0000(	NAMA	12.44483	0.832781	0.005264	0.01935	lncRNA
ENSG0000(	ARMC10	1213.588	-0.11378	0.005266	0.019353	protein_coding
ENSG0000(	DNAJB14	3140.534	0.123397	0.00527	0.019366	protein_coding
ENSG0000(	ENSG0000(	23.72038	0.441219	0.005276	0.019388	lncRNA
ENSG0000(	ALG1L2	308.346	0.30798	0.005278	0.019389	protein_coding
ENSG0000(	GRM1	72.93246	0.447048	0.005278	0.019389	protein_coding
ENSG0000(	SCO1	2827.919	-0.18665	0.005279	0.019389	protein_coding
ENSG0000(	TNFRSF1A	2192.097	-0.20318	0.005283	0.019404	protein_coding
ENSG0000(	VARS2	631.8293	-0.20946	0.005287	0.019414	protein_coding
ENSG0000(	PDE3A	2409.622	-0.39944	0.005288	0.019416	protein_coding
ENSG0000(	ENSG0000(	19.98355	0.707283	0.005289	0.019417	processed_pseudogene
ENSG0000(	TFPT	88.89411	-0.24289	0.005293	0.019431	protein_coding
ENSG0000(	RSKR	1923.835	-0.09811	0.005297	0.019442	protein_coding
ENSG0000(	ENSG0000(	498.3679	-0.12387	0.005298	0.019444	lncRNA
ENSG0000(	HERC2	5513.451	-0.10505	0.005301	0.019454	protein_coding
ENSG0000(	BAD	240.724	-0.27518	0.005302	0.019455	protein_coding
ENSG0000(	PRELID3A	105.3973	-0.24786	0.005317	0.019506	protein_coding
ENSG0000(	ENSG0000(	25.93596	0.4652	0.005318	0.019507	lncRNA
ENSG0000(	H1-4	627.7446	-0.33092	0.005319	0.01951	protein_coding
ENSG0000(	ENSG0000(	94.10806	-0.30285	0.005321	0.019515	lncRNA
ENSG0000(	MKRN2OS	386.3096	-0.13191	0.005323	0.019519	protein_coding
ENSG0000(	PER2	1626.279	-0.27473	0.005324	0.019521	protein_coding
ENSG0000(	AMPH	82.78247	0.600585	0.005328	0.019528	protein_coding
ENSG0000(	LCNL1	116.3875	0.711845	0.005328	0.019528	protein_coding
ENSG0000(	MTND2P28	1336.511	-0.60937	0.005328	0.019528	unprocessed_pseudogene
ENSG0000(	SNX11	631.0159	-0.14533	0.005327	0.019528	protein_coding
ENSG0000(	ENSG0000(	14.5501	-0.56753	0.005337	0.019556	lncRNA
ENSG0000(	FCRL3	833.8641	0.375777	0.005337	0.019556	protein_coding
ENSG0000(	RPS3AP38	353.3714	0.24568	0.005338	0.019556	processed_pseudogene
ENSG0000(	DBR1	395.5529	-0.18792	0.00534	0.019562	protein_coding
ENSG0000(	ENSG0000(	340.2161	0.294634	0.005341	0.019562	lncRNA
ENSG0000(	PLEKHA3P1	9.508877	0.684558	0.005343	0.019568	processed_pseudogene
ENSG0000(	EFCAB13	641.0006	0.248452	0.005344	0.01957	protein_coding
ENSG0000(	ENSG0000(	27.14488	0.387699	0.005345	0.019571	lncRNA
ENSG0000(	SNHG32	509.3594	-0.22592	0.005346	0.019572	lncRNA
ENSG0000(	ENSG0000(	124.0788	0.315382	0.005348	0.019577	lncRNA
ENSG0000(	ENSG0000(	684.9559	-0.13722	0.00535	0.019579	lncRNA
ENSG0000(	ENSG0000(	149.189	-0.40702	0.005351	0.019579	lncRNA
ENSG0000(	RPAP3-DT	274.1756	0.264609	0.00535	0.019579	lncRNA
ENSG0000(	RGS9	500.2757	-0.1722	0.005353	0.019585	protein_coding
ENSG0000(	DIP2C-AS1	564.158	-0.3819	0.005357	0.019593	lncRNA
ENSG0000(	ENSG0000(	30.99905	0.383781	0.005356	0.019593	lncRNA
ENSG0000(	ENSG0000(	28.85924	0.434891	0.005357	0.019593	lncRNA
ENSG0000(	CT55	10.89196	0.730744	0.005358	0.019594	protein_coding
ENSG0000(	TMEM167E	544.8517	-0.14932	0.005362	0.019609	lncRNA
ENSG0000(	MORC2-AS	137.121	0.297199	0.005365	0.019616	lncRNA

ENSG0000(	FAM106C	60.49506	0.845884	0.005368	0.019624	lncRNA
ENSG0000(	ENSG0000(	16.27152	0.474895	0.00537	0.019628	lncRNA
ENSG0000(	HNF4A-AS1	27.40861	0.414958	0.00537	0.019628	lncRNA
ENSG0000(	CYCSP23	15.20281	0.591736	0.005371	0.019628	processed_pseudogene
ENSG0000(	ENSG0000(	796.5452	-0.24727	0.005371	0.019629	lncRNA
ENSG0000(	LL22NC01-8	107.5701	-0.25721	0.005387	0.019685	lncRNA
ENSG0000(	PDILT	23.84481	-0.42267	0.005388	0.019686	protein_coding
ENSG0000(	ENSG0000(	116.8289	-0.18619	0.005391	0.019693	lncRNA
ENSG0000(	STK35	1209.564	-0.13505	0.005392	0.019693	protein_coding
ENSG0000(	ENSG0000(	89.26649	0.284096	0.005393	0.019695	lncRNA
ENSG0000(	RN7SL364F	55.63397	0.29085	0.005394	0.019697	misc_RNA
ENSG0000(	ACHE	328.6937	0.48278	0.005395	0.019697	protein_coding
ENSG0000(	ENSG0000(	796.1208	0.189794	0.005408	0.019745	lncRNA
ENSG0000(	RNVU1-1	114.2273	0.712408	0.00541	0.019749	snRNA
ENSG0000(	RAD23B	1931.684	-0.16111	0.005423	0.019793	protein_coding
ENSG0000(	SNX25	837.7467	0.164099	0.005425	0.0198	protein_coding
ENSG0000(	KIF21B	1395.183	0.232287	0.00543	0.019814	protein_coding
ENSG0000(	ZNF429	1610.697	-0.12493	0.00543	0.019814	protein_coding
ENSG0000(	ENSG0000(	66.63867	0.242605	0.005431	0.019815	lncRNA
ENSG0000(	ENSG0000(	165.8268	0.260123	0.005437	0.019835	lncRNA
ENSG0000(	P2RY10	1279.451	0.193434	0.005441	0.019847	protein_coding
ENSG0000(	KCNK10	463.7432	-0.39477	0.005443	0.01985	protein_coding
ENSG0000(	ENSG0000(	58.8369	-0.46042	0.00545	0.019871	lncRNA
ENSG0000(	MRC1	626.2902	0.377368	0.00545	0.019871	protein_coding
ENSG0000(	MTAPP2	10.87506	-0.97475	0.005452	0.019877	processed_pseudogene
ENSG0000(	LCLAT1	2481.495	-0.10955	0.005455	0.019884	protein_coding
ENSG0000(	ENSG0000(	581.5124	-0.13008	0.005463	0.019913	lncRNA
ENSG0000(	WEE2-AS1	204.1933	0.267597	0.005465	0.019915	lncRNA
ENSG0000(	ENSG0000(	24.96056	0.461351	0.005467	0.019923	lncRNA
ENSG0000(	RN7SL50P	68.60554	0.232385	0.005469	0.019926	misc_RNA
ENSG0000(	KHDC4	3408.174	-0.12301	0.00547	0.019927	protein_coding
ENSG0000(	KLHL2	410.2857	0.235277	0.00547	0.019927	protein_coding
ENSG0000(	EIF2S3	3485.743	-0.13127	0.005473	0.019935	protein_coding
ENSG0000(	RAD21	3686.304	-0.13933	0.00548	0.019957	protein_coding
ENSG0000(	ETFRF1	193.5632	0.223753	0.005481	0.019958	protein_coding
ENSG0000(	SATB2-AS1	1300.814	-0.48794	0.005483	0.019965	lncRNA
ENSG0000(	HGSNAT	1987.173	0.104918	0.005484	0.019966	protein_coding
ENSG0000(	ENSG0000(	58.05027	0.401772	0.005486	0.01997	lncRNA
ENSG0000(	ECSCR	43.67281	0.630213	0.005488	0.019975	protein_coding
ENSG0000(	CDCA4P4	54.39255	-0.36008	0.005494	0.019994	unprocessed_pseudogene
ENSG0000(	ENSG0000(	33.8298	0.48702	0.005495	0.019994	lncRNA
ENSG0000(	KIDINS220	6268.463	0.08651	0.005495	0.019994	protein_coding
ENSG0000(	SNHG1	1405.648	-0.21224	0.005502	0.020016	lncRNA
ENSG0000(	TSNAXIP1	26.22717	0.613055	0.005502	0.020016	protein_coding
ENSG0000(	ENSG0000(	144.947	-0.24598	0.005505	0.020024	lncRNA
ENSG0000(	CDNF	48.89712	0.367313	0.005516	0.020062	protein_coding
ENSG0000(	EFHD1	103.4946	-0.23686	0.005523	0.020085	protein_coding

ENSG0000(	ARHGAP27	1325.098	-0.22527	0.005525	0.020087	lncRNA
ENSG0000(	NHP2	238.2466	-0.21122	0.005526	0.020088	protein_coding
ENSG0000(	LINC01231	70.8136	0.216766	0.005532	0.020108	lncRNA
ENSG0000(	MLANA	329.4908	-0.21174	0.005532	0.020108	protein_coding
ENSG0000(	ACYP2	502.0983	0.137503	0.005537	0.020119	protein_coding
ENSG0000(	OGT	17296.84	0.154911	0.005536	0.020119	protein_coding
ENSG0000(	ENSG0000(	245.9985	-0.74866	0.005541	0.02013	lncRNA
ENSG0000(	ENSG0000(	35.70462	0.406023	0.005541	0.02013	lncRNA
ENSG0000(	STK32A	110.973	0.525467	0.005541	0.02013	protein_coding
ENSG0000(	ATF4P3	35.12905	-0.56552	0.005545	0.02014	processed_pseudogene
ENSG0000(	ZNF497	101.7583	-0.26757	0.005549	0.020151	protein_coding
ENSG0000(	ENSG0000(	65.09164	-0.28319	0.005551	0.020155	lncRNA
ENSG0000(	RNU6-1045	25.56895	0.540411	0.005553	0.020155	snRNA
ENSG0000(	SLC2A1-AS:	487.2482	0.130991	0.005553	0.020155	lncRNA
ENSG0000(	TMEM51	339.0076	-0.20377	0.005552	0.020155	protein_coding
ENSG0000(	TPO	180.0989	-0.49942	0.005551	0.020155	protein_coding
ENSG0000(	ENSG0000(	118.8569	0.294338	0.005557	0.020166	lncRNA
ENSG0000(	ENSG0000(	243.5139	0.333333	0.005561	0.020173	lncRNA
ENSG0000(	FBXW2	3880.372	0.068362	0.00556	0.020173	protein_coding
ENSG0000(	ZBTB20-AS	79.90833	0.494059	0.00556	0.020173	lncRNA
ENSG0000(	HOXA10-AS	76.54897	-0.27043	0.005564	0.020184	lncRNA
ENSG0000(	C1orf74	287.9211	0.435804	0.005568	0.020195	protein_coding
ENSG0000(	BTLA	421.5169	0.669868	0.00557	0.020198	protein_coding
ENSG0000(	ENSG0000(	83.46196	-0.59703	0.00557	0.020198	lncRNA
ENSG0000(	ZNF438	315.0876	0.222465	0.005571	0.020201	protein_coding
ENSG0000(	RPL32P3	1848.404	0.115729	0.005572	0.020202	transcribed_unprocessed_pseudo
ENSG0000(	FAM167A-/	176.1924	0.289645	0.005577	0.020217	lncRNA
ENSG0000(	HLA-DOB	223.0064	0.646688	0.005585	0.020245	protein_coding
ENSG0000(	ENSG0000(	190.7581	0.405927	0.005587	0.020247	lncRNA
ENSG0000(	RGN	137.9176	0.185542	0.00559	0.020255	protein_coding
ENSG0000(	MKLN1	5745.307	0.114209	0.005591	0.020258	protein_coding
ENSG0000(	CAGE1	93.47865	-0.25621	0.005593	0.020265	protein_coding
ENSG0000(	ENSG0000(	708.6971	-0.34028	0.005596	0.020271	protein_coding
ENSG0000(	ENSG0000(	719.7136	0.156928	0.005602	0.020292	lncRNA
ENSG0000(	ENSG0000(	290.6813	-0.32077	0.005605	0.020299	lncRNA
ENSG0000(	ENSG0000(	32.49168	0.50771	0.00561	0.020311	lncRNA
ENSG0000(	ENSG0000(	17.4814	0.565518	0.005609	0.020311	snoRNA
ENSG0000(	ENSG0000(	23.87459	0.687144	0.00561	0.020311	lncRNA
ENSG0000(	ENSG0000(	18.64752	0.631365	0.005613	0.020318	lncRNA
ENSG0000(	STX5	649.3041	0.117503	0.005615	0.020324	protein_coding
ENSG0000(	ENSG0000(	46.41026	0.296332	0.005617	0.02033	lncRNA
ENSG0000(	ENSG0000(	450.8318	-0.12406	0.005619	0.020333	lncRNA
ENSG0000(	LINC01224	1140.139	-0.21978	0.005619	0.020333	lncRNA
ENSG0000(	CDC42	8092.385	-0.17304	0.005626	0.020355	protein_coding
ENSG0000(	ENSG0000(	39.07385	0.358823	0.005631	0.020371	lncRNA
ENSG0000(	HEATR6-DT	246.9473	0.159461	0.005632	0.020371	lncRNA
ENSG0000(	CYP21A2	24.57874	0.600453	0.005634	0.020376	protein_coding

ENSG0000(	CABP1	276.6034	-0.37756	0.005638	0.020389	protein_coding
ENSG0000(	ENSG0000(	86.13672	0.499415	0.005641	0.020398	lncRNA
ENSG0000(	IFI6	148.4951	0.727448	0.005643	0.020399	protein_coding
ENSG0000(	LINC02325	168.9611	0.390075	0.005643	0.020399	lncRNA
ENSG0000(	GNB4	1381.553	0.204895	0.005644	0.020401	protein_coding
ENSG0000(	MED19	98.68644	-0.26242	0.005649	0.020417	protein_coding
ENSG0000(	POMGNT1	1109.943	-0.20528	0.005653	0.020427	protein_coding
ENSG0000(	ENSG0000(	199.1968	-0.23941	0.005659	0.020447	lncRNA
ENSG0000(	ENSG0000(	69.35251	0.305286	0.005663	0.020458	lncRNA
ENSG0000(	RNVU1-30	40.88793	0.34914	0.005667	0.020472	snRNA
ENSG0000(	THBD	203.7517	0.543869	0.005674	0.020496	protein_coding
ENSG0000(	ENSG0000(	29.88769	-0.41703	0.005676	0.020496	lncRNA
ENSG0000(	FNBP1	3482.962	0.215981	0.005676	0.020496	protein_coding
ENSG0000(	HMGN1	3658.067	-0.16922	0.005678	0.020503	protein_coding
ENSG0000(	ENSG0000(	293.1624	0.168817	0.005682	0.020511	transcribed_unprocessed_pseudo
ENSG0000(	FRG1BP	231.3049	-0.3167	0.005681	0.020511	unprocessed_pseudogene
ENSG0000(	ENSG0000(	31.785	0.432895	0.005689	0.020529	lncRNA
ENSG0000(	ENSG0000(	90.771	0.29558	0.005688	0.020529	lncRNA
ENSG0000(	RN7SL258P	61.13543	0.264848	0.005688	0.020529	misc_RNA
ENSG0000(	ENSG0000(	294.7126	-0.1756	0.005691	0.020534	TEC
ENSG0000(	CFTR	4999.794	-0.32772	0.005693	0.02054	protein_coding
ENSG0000(	ATP1B4	163.3469	-0.19654	0.005695	0.020545	protein_coding
ENSG0000(	BRD7	1553.352	-0.09558	0.005698	0.020553	protein_coding
ENSG0000(	ENSG0000(	58.72068	0.264294	0.005703	0.020569	lncRNA
ENSG0000(	IGKV2-30	379.7355	0.650146	0.005704	0.020569	IG_V_gene
ENSG0000(	TAT	186.1025	-0.36104	0.005704	0.020569	protein_coding
ENSG0000(	ENSG0000(	38.5712	0.50422	0.005706	0.020571	lncRNA
ENSG0000(	CD226	985.932	0.332368	0.005712	0.020591	protein_coding
ENSG0000(	OSTF1	462.0086	-0.24911	0.005713	0.020591	protein_coding
ENSG0000(	LINC01857	120.1483	0.29619	0.005725	0.020635	lncRNA
ENSG0000(	ENSG0000(	17.52587	0.580267	0.00573	0.020649	snoRNA
ENSG0000(	CTAGE3P	49.16325	-0.57317	0.005732	0.020654	processed_pseudogene
ENSG0000(	ENSG0000(	352.3173	0.177307	0.005739	0.020677	TEC
ENSG0000(	GGT2	26.61774	-0.8202	0.00574	0.020677	protein_coding
ENSG0000(	EIF1AX	1080.243	-0.1969	0.005742	0.020682	protein_coding
ENSG0000(	ENSG0000(	86.76272	0.315879	0.005742	0.020682	protein_coding
ENSG0000(	NPIPA8	72.33265	-0.68048	0.005744	0.020687	protein_coding
ENSG0000(	ZNF443	423.6134	-0.23511	0.005746	0.020688	protein_coding
ENSG0000(	PSMD10P2	132.088	-0.26611	0.005746	0.020689	transcribed_processed_pseudoge
ENSG0000(	CROCCP3	930.7816	0.141706	0.00575	0.020698	transcribed_unprocessed_pseudo
ENSG0000(	MFSD1	1119.162	0.155259	0.005755	0.020716	protein_coding
ENSG0000(	ENSG0000(	253.6025	-0.81235	0.005758	0.020723	lncRNA
ENSG0000(	LINC00211	76.21829	0.254256	0.005758	0.020723	lncRNA
ENSG0000(	NXF1	2601.566	0.158042	0.005772	0.020768	protein_coding
ENSG0000(	ENSG0000(	113.1959	-0.49065	0.005774	0.020773	protein_coding
ENSG0000(	BRIX1	433.2323	-0.18709	0.00579	0.020825	protein_coding
ENSG0000(	CHAT	78.10771	-0.53112	0.005789	0.020825	protein_coding

ENSG0000(	NRIP3	408.583	-0.17062	0.00579	0.020825	protein_coding
ENSG0000(	ARHGAP22	17.1249	0.656647	0.005793	0.020832	lncRNA
ENSG0000(	ENSG0000(	220.6049	0.200192	0.005793	0.020832	lncRNA
ENSG0000(	ENSG0000(	20.49996	-0.5727	0.005795	0.020834	unprocessed_pseudogene
ENSG0000(	TOR4A	874.4353	-0.24892	0.005796	0.020837	protein_coding
ENSG0000(	EXOC4	3295.013	0.107192	0.005801	0.020853	protein_coding
ENSG0000(	SIL1	499.1159	0.168391	0.005804	0.020861	protein_coding
ENSG0000(	GAS6-DT	270.1072	-0.17921	0.005805	0.020861	lncRNA
ENSG0000(	PAPSS1	370.022	0.28515	0.005809	0.020875	protein_coding
ENSG0000(	CKLF	214.0539	0.279023	0.005821	0.020912	protein_coding
ENSG0000(	RAD51C	855.9029	-0.13741	0.005821	0.020912	protein_coding
ENSG0000(	ENSG0000(	110.1183	-0.21162	0.005824	0.020919	lncRNA
ENSG0000(	POLR3GL	180.2159	-0.23714	0.005824	0.020919	protein_coding
ENSG0000(	INE1	557.9099	0.138407	0.005826	0.020923	lncRNA
ENSG0000(	ENSG0000(	18.76698	-0.56659	0.005831	0.020937	lncRNA
ENSG0000(	LINC01825	71.55864	0.263414	0.005833	0.020943	lncRNA
ENSG0000(	MIR548D2	74.94175	0.415799	0.005833	0.020943	miRNA
ENSG0000(	ENSG0000(	489.8461	0.147406	0.005836	0.02095	lncRNA
ENSG0000(	ERBB2	4756.968	-0.34214	0.005841	0.020966	protein_coding
ENSG0000(	ENSG0000(	38.51739	-0.46929	0.005843	0.020968	lncRNA
ENSG0000(	YIPF6	2083.883	-0.10776	0.005846	0.020979	protein_coding
ENSG0000(	ENSG0000(	33.38996	0.401606	0.005849	0.020988	transcribed_processed_pseudoge
ENSG0000(	RGPD3	39.90694	0.720271	0.005854	0.021001	protein_coding
ENSG0000(	ENSG0000(	45.94297	0.384815	0.005862	0.021028	lncRNA
ENSG0000(	ENSG0000(	174.3402	0.199237	0.005865	0.021035	lncRNA
ENSG0000(	RNU6-98P	23.03281	0.621025	0.005866	0.021036	snRNA
ENSG0000(	PANK1	382.4844	-0.4163	0.005867	0.021039	protein_coding
ENSG0000(	SHISA9	7265.044	0.180851	0.00587	0.021047	protein_coding
ENSG0000(	CXCL16	536.4456	0.279491	0.005873	0.021049	protein_coding
ENSG0000(	ERLIN2	1269.618	-0.13382	0.005873	0.021049	protein_coding
ENSG0000(	RHOA	4178.492	-0.24106	0.005872	0.021049	protein_coding
ENSG0000(	TMEM38B	298.0933	-0.24098	0.005873	0.021049	protein_coding
ENSG0000(	YPEL3-DT	22.54461	0.529611	0.005874	0.02105	lncRNA
ENSG0000(	AP4M1	460.5108	-0.16443	0.005875	0.021052	protein_coding
ENSG0000(	ENSG0000(	14.22715	0.665342	0.005878	0.021058	lncRNA
ENSG0000(	ENSG0000(	12.96657	0.643285	0.005879	0.021059	lncRNA
ENSG0000(	UBE2J1	2653.907	0.225351	0.005882	0.021069	protein_coding
ENSG0000(	LMO2	189.5307	0.39211	0.005884	0.021074	protein_coding
ENSG0000(	DNAH8	123.4085	0.864147	0.00589	0.021091	protein_coding
ENSG0000(	RNF26	315.3973	-0.2127	0.005896	0.021111	protein_coding
ENSG0000(	ENSG0000(	12.00378	0.661284	0.005902	0.02112	lncRNA
ENSG0000(	FTH1P3	31.33981	-0.45021	0.005901	0.02112	processed_pseudogene
ENSG0000(	PHF23	517.0959	-0.20623	0.005901	0.02112	protein_coding
ENSG0000(	RNU6-928F	8.98758	0.618968	0.005902	0.02112	snRNA
ENSG0000(	SPDYE2	16.23219	1.340337	0.005899	0.02112	protein_coding
ENSG0000(	UPF3AP3	36.40392	-0.40744	0.005906	0.021131	transcribed_processed_pseudoge
ENSG0000(	P2RY8	592.6848	0.505564	0.005908	0.021138	protein_coding

ENSG0000(	ENSG0000(	125.0125	-0.23503	0.005912	0.021148	lncRNA
ENSG0000(	RFX5	1409.232	0.225909	0.005913	0.021152	protein_coding
ENSG0000(	GABARAPL:	664.1758	-0.21637	0.00592	0.021173	protein_coding
ENSG0000(	ENSG0000(	131.582	-0.16639	0.005924	0.021185	TEC
ENSG0000(	YWHAH	1098.103	-0.22094	0.005925	0.021186	protein_coding
ENSG0000(	CPSF6	3648.932	-0.12767	0.005937	0.021227	protein_coding
ENSG0000(	ENSG0000(	101.1241	0.347686	0.00594	0.021236	lncRNA
ENSG0000(	DLGAP5	225.1497	-0.39013	0.005944	0.021247	protein_coding
ENSG0000(	P4HA3	112.7921	0.366392	0.005956	0.021286	protein_coding
ENSG0000(	ERI3-IT1	121.3875	-0.21538	0.005957	0.021287	lncRNA
ENSG0000(	RNU6-110(	9.862145	1.124374	0.005958	0.021289	snRNA
ENSG0000(	CNTNAP5	45.64506	0.566319	0.005961	0.021296	protein_coding
ENSG0000(	GNG7	453.3304	0.226813	0.005962	0.0213	protein_coding
ENSG0000(	LINC02182	18.47918	0.510219	0.005967	0.021314	lncRNA
ENSG0000(	IGKV3-15	794.9659	0.73027	0.005968	0.021317	IG_V_gene
ENSG0000(	ZDHHC15	452.5623	0.150205	0.005969	0.021317	protein_coding
ENSG0000(	CYB5D1	78.15652	-0.48558	0.005987	0.021374	protein_coding
ENSG0000(	PARP16	235.8835	-0.24622	0.005986	0.021374	protein_coding
ENSG0000(	BNIP3	239.6184	0.325259	0.005988	0.021376	protein_coding
ENSG0000(	GID8	924.893	-0.16936	0.005995	0.0214	protein_coding
ENSG0000(	PPP4R4	21.21678	0.78687	0.005996	0.0214	protein_coding
ENSG0000(	CRACR2B	777.4969	-0.35206	0.005999	0.021406	protein_coding
ENSG0000(	DAG1	1501.558	-0.1919	0.006	0.021406	protein_coding
ENSG0000(	ENSG0000(	278.958	0.15489	0.005999	0.021406	processed_pseudogene
ENSG0000(	ENSG0000(	49.98541	-0.30423	0.006011	0.021446	processed_pseudogene
ENSG0000(	KLF15	70.75724	0.495517	0.006012	0.021446	protein_coding
ENSG0000(	SLITRK1	8.637311	0.907301	0.006014	0.02145	protein_coding
ENSG0000(	RNF43	2751.732	-0.31958	0.006018	0.021463	protein_coding
ENSG0000(	ENSG0000(	237.6315	-0.1494	0.00602	0.021465	lncRNA
ENSG0000(	ENSG0000(	164.3548	0.190931	0.006022	0.021465	lncRNA
ENSG0000(	NUDT3	3465.044	0.124496	0.006022	0.021465	protein_coding
ENSG0000(	PPIAP30	29.48596	-0.4748	0.00602	0.021465	processed_pseudogene
ENSG0000(	CLEC3B	40.08772	-0.45891	0.006025	0.021475	protein_coding
ENSG0000(	TRIAP1	128.8127	-0.3037	0.006026	0.021475	protein_coding
ENSG0000(	CD300LF	145.2979	0.379523	0.006028	0.02148	protein_coding
ENSG0000(	MKRN9P	122.7229	-0.2283	0.006029	0.02148	transcribed_processed_pseudoge
ENSG0000(	DOCK8-AS1	210.2509	-0.17235	0.006036	0.021504	lncRNA
ENSG0000(	ENSG0000(	150.8472	-0.25587	0.006037	0.021505	lncRNA
ENSG0000(	ENSG0000(	26.07542	0.507793	0.006041	0.021516	lncRNA
ENSG0000(	TRIM36	952.8763	-0.4258	0.006041	0.021516	protein_coding
ENSG0000(	SLC2A5	677.8939	0.226998	0.006049	0.021542	protein_coding
ENSG0000(	ASAP2	2535.503	-0.25892	0.006052	0.021548	protein_coding
ENSG0000(	ENSG0000(	65.18855	0.295687	0.006056	0.021562	lncRNA
ENSG0000(	ENSG0000(	108.6992	0.289824	0.006064	0.021586	lncRNA
ENSG0000(	TMED2	2791.053	-0.21754	0.00607	0.021607	protein_coding
ENSG0000(	DNAH10OS	297.832	0.143655	0.006074	0.021619	lncRNA
ENSG0000(	RBPMS-AS:	82.60532	0.248957	0.006087	0.02166	lncRNA

ENSG0000(	NIP7	581.8728	-0.17051	0.006089	0.021664	protein_coding
ENSG0000(	EIF4EBP2	5496.107	-0.28758	0.006101	0.021703	protein_coding
ENSG0000(	ENSG0000(	9.86468	2.23905	0.006101	0.021703	lncRNA
ENSG0000(	ENSG0000(	8.684026	0.65485	0.0061	0.021703	lncRNA
ENSG0000(	RNA5SP39!	9.164788	1.005506	0.006106	0.021718	rRNA_pseudogene
ENSG0000(	ENSG0000(	72.24602	-0.31027	0.00611	0.021719	lncRNA
ENSG0000(	LAGE3	50.78643	-0.31988	0.00611	0.021719	protein_coding
ENSG0000(	NOD1	886.9371	0.219197	0.00611	0.021719	protein_coding
ENSG0000(	NOXRED1	147.1084	0.243505	0.006108	0.021719	protein_coding
ENSG0000(	RXRG	19.05261	-0.64074	0.006109	0.021719	protein_coding
ENSG0000(	ENSG0000(	9.550025	0.577429	0.006112	0.021722	lncRNA
ENSG0000(	ENSG0000(	58.63054	0.391174	0.006116	0.021736	lncRNA
ENSG0000(	ENSG0000(	1828.618	-0.29963	0.006142	0.021819	protein_coding
ENSG0000(	GEMIN7-A!	150.7715	0.198832	0.006142	0.021819	lncRNA
ENSG0000(	MARCKSL1	1092.12	-0.24668	0.006142	0.021819	protein_coding
ENSG0000(	GTF3C6	295.1772	-0.27405	0.006144	0.021823	protein_coding
ENSG0000(	PRC1	413.4905	-0.19019	0.006149	0.021841	protein_coding
ENSG0000(	DPY19L2P4	11.60859	0.79905	0.006154	0.021854	transcribed_unprocessed_pseudo
ENSG0000(	RPL29P25	59.08798	-0.26066	0.006154	0.021854	processed_pseudogene
ENSG0000(	PRR27	128.8989	-0.244	0.006155	0.021855	protein_coding
ENSG0000(	SCYL1	630.9217	-0.15524	0.006161	0.021873	protein_coding
ENSG0000(	SHOX2	13.60906	0.763688	0.006162	0.021875	protein_coding
ENSG0000(	DUXAP7	63.06984	0.216687	0.006166	0.021884	processed_pseudogene
ENSG0000(	DSP	8653.143	-0.32038	0.006166	0.021884	protein_coding
ENSG0000(	C16orf74	134.026	0.281939	0.00617	0.021895	protein_coding
ENSG0000(	TRIM17	29.17694	0.50478	0.006173	0.021901	protein_coding
ENSG0000(	ENSG0000(	1712.305	0.140408	0.006175	0.021904	protein_coding
ENSG0000(	TRIP6	375.5145	-0.24183	0.006175	0.021904	protein_coding
ENSG0000(	C5orf24	1345.891	-0.09483	0.006178	0.021911	protein_coding
ENSG0000(	ENSG0000(	12.78703	0.51434	0.006179	0.021912	lncRNA
ENSG0000(	RABGAP1L	5482.182	0.119006	0.006179	0.021912	protein_coding
ENSG0000(	Telomerase	299.0779	-0.32321	0.006181	0.021916	misc_RNA
ENSG0000(	CDC5L	1311.79	-0.1201	0.006185	0.021927	protein_coding
ENSG0000(	ENSG0000(	36.68127	0.366904	0.00619	0.021942	lncRNA
ENSG0000(	LINC02910	33.11366	0.486133	0.00619	0.021942	lncRNA
ENSG0000(	MRPL49P1	10.84355	0.651111	0.006191	0.021942	processed_pseudogene
ENSG0000(	B4GALT2	382.5782	-0.26411	0.006195	0.021952	protein_coding
ENSG0000(	PKP4-AS1	10.34609	0.872019	0.006195	0.021952	lncRNA
ENSG0000(	ARL6IP6	3899.828	0.160417	0.006197	0.021953	protein_coding
ENSG0000(	HYAL1	261.123	-0.27679	0.006197	0.021953	protein_coding
ENSG0000(	EPHA1	527.8714	-0.14339	0.006199	0.021956	protein_coding
ENSG0000(	ENSG0000(	10.26251	0.634181	0.0062	0.021958	lncRNA
ENSG0000(	HSPA2	520.1137	-0.24137	0.006206	0.021979	protein_coding
ENSG0000(	ENSG0000(	10.13991	0.694258	0.006208	0.021983	lncRNA
ENSG0000(	ENSG0000(	37.93937	0.401269	0.006215	0.022004	lncRNA
ENSG0000(	FAM86B1	165.8656	-0.35376	0.006222	0.022028	protein_coding
ENSG0000(	SGMS1	682.2247	0.222651	0.00623	0.022051	protein_coding

ENSG0000(	ENSG0000(	380.3184	0.172963	0.006231	0.022052	TEC
ENSG0000(	LRRC25	234.1506	0.241774	0.006236	0.022067	protein_coding
ENSG0000(	C19orf25	1000.988	-0.12325	0.006239	0.022076	protein_coding
ENSG0000(	WDR7	1887.284	0.151432	0.006247	0.022102	protein_coding
ENSG0000(	SELL	481.1383	0.924783	0.006252	0.022119	protein_coding
ENSG0000(	ENSG0000(	322.6113	-0.18369	0.006261	0.022147	TEC
ENSG0000(	ITPK1-AS1	59.07481	0.248929	0.006263	0.02215	lncRNA
ENSG0000(	PCDH1	2530.818	-0.31642	0.006263	0.02215	protein_coding
ENSG0000(	RWDD1	815.3041	-0.17078	0.006273	0.02218	protein_coding
ENSG0000(	ENSG0000(	9.250874	0.862273	0.006276	0.022191	lncRNA
ENSG0000(	ENSG0000(	134.3451	-0.2004	0.006277	0.022191	lncRNA
ENSG0000(	NPTN	1341.549	-0.15511	0.006278	0.022194	protein_coding
ENSG0000(	PCIF1	454.7741	-0.1463	0.006281	0.022199	protein_coding
ENSG0000(	YTHDF2	1363.024	-0.12629	0.006282	0.022201	protein_coding
ENSG0000(	LINC01519	66.52057	0.22545	0.006286	0.022211	lncRNA
ENSG0000(	SPINK8	39.32236	0.371582	0.006286	0.022211	protein_coding
ENSG0000(	RPS3AP48	42.41087	-0.67204	0.006297	0.022246	processed_pseudogene
ENSG0000(	KIF4CP	9.863874	0.848768	0.006299	0.022249	processed_pseudogene
ENSG0000(	SYNGAP1	976.5233	0.469372	0.006299	0.022249	protein_coding
ENSG0000(	SERPINF1	622.5613	0.23527	0.006301	0.022251	protein_coding
ENSG0000(	RN7SL386F	47.14928	-0.38119	0.006302	0.022256	misc_RNA
ENSG0000(	ENSG0000(	147.6122	0.237537	0.006307	0.022267	lncRNA
ENSG0000(	TAS2R46	109.6636	0.265231	0.006307	0.022267	protein_coding
ENSG0000(	MIR4296	58.41676	-0.36355	0.006308	0.022268	miRNA
ENSG0000(	DTNB-AS1	34.0132	0.610004	0.006325	0.022327	lncRNA
ENSG0000(	CNGB3	35.57987	0.604719	0.00633	0.022335	protein_coding
ENSG0000(	HEXA-AS1	116.3022	0.231507	0.00633	0.022335	lncRNA
ENSG0000(	KIF18A	236.8937	-0.27864	0.006329	0.022335	protein_coding
ENSG0000(	RN7SL653F	18.0503	0.499032	0.006331	0.022335	misc_RNA
ENSG0000(	GLDN	106.1988	0.629265	0.006335	0.022346	protein_coding
ENSG0000(	SCN1A	270.2699	-0.21696	0.006335	0.022346	protein_coding
ENSG0000(	ANO9	3933.372	-0.31277	0.006336	0.022348	protein_coding
ENSG0000(	CORO2B	64.46412	-0.392	0.006346	0.022378	protein_coding
ENSG0000(	ENSG0000(	345.6802	0.132302	0.006356	0.022411	lncRNA
ENSG0000(	STK16	358.3308	-0.19053	0.00636	0.022423	protein_coding
ENSG0000(	ZNF667	906.6651	0.138985	0.006362	0.022424	protein_coding
ENSG0000(	ZNF747	689.5142	-0.10212	0.006362	0.022424	protein_coding
ENSG0000(	GRK2	2476.064	-0.15111	0.00637	0.022449	protein_coding
ENSG0000(	ZNF132	251.1005	-0.26189	0.00637	0.022449	protein_coding
ENSG0000(	EIF5B	1523.839	-0.17292	0.00638	0.022481	protein_coding
ENSG0000(	RNF39	12.54341	0.945728	0.006381	0.022482	protein_coding
ENSG0000(	ACTR3	5539.687	-0.2072	0.006389	0.022501	protein_coding
ENSG0000(	ENSG0000(	42.17877	0.340598	0.006389	0.022501	lncRNA
ENSG0000(	SLC49A4	419.3438	0.237949	0.006389	0.022501	protein_coding
ENSG0000(	SOS1-IT1	45.27015	1.845126	0.006388	0.022501	lncRNA
ENSG0000(	ENSG0000(	14.24278	0.511581	0.006398	0.02253	processed_pseudogene
ENSG0000(	C2orf50	750.2491	-0.1161	0.006404	0.022538	protein_coding

ENSG0000(	ENSG0000(	9.694654	0.70369	0.006402	0.022538	snoRNA
ENSG0000(	ENSG0000(	144.972	0.196252	0.006403	0.022538	lncRNA
ENSG0000(	NUP43	1723.754	-0.12785	0.006401	0.022538	protein_coding
ENSG0000(	RARS1	1048.961	-0.19504	0.006403	0.022538	protein_coding
ENSG0000(	PIGB	829.6216	0.11282	0.006406	0.022541	protein_coding
ENSG0000(	INPP5J	619.7392	-0.59061	0.006411	0.022558	protein_coding
ENSG0000(	CYP4F35P	120.5053	0.425107	0.006416	0.022573	transcribed_unprocessed_pseudo
ENSG0000(	KCNAB3	288.656	-0.17399	0.006421	0.022584	protein_coding
ENSG0000(	LRRC36	289.3494	-0.17177	0.00642	0.022584	protein_coding
ENSG0000(	NSMCE1	303.7371	-0.20729	0.006425	0.022593	protein_coding
ENSG0000(	RHOQ-AS1	10.19029	0.595124	0.006425	0.022593	lncRNA
ENSG0000(	DNAH2	310.596	0.161034	0.006428	0.022601	protein_coding
ENSG0000(	ZNF221	324.8638	-0.16607	0.006434	0.022622	protein_coding
ENSG0000(	ENO1	3260.643	-0.21635	0.006436	0.022624	protein_coding
ENSG0000(	ENSG0000(	121.2358	0.219891	0.006438	0.02263	lncRNA
ENSG0000(	ATP6V1E2	97.31091	0.331056	0.006444	0.022648	protein_coding
ENSG0000(	RGS4	42.72697	0.615325	0.006451	0.022669	protein_coding
ENSG0000(	ENSG0000(	236.6325	0.324684	0.006457	0.022683	lncRNA
ENSG0000(	FBXO17	265.5965	0.215754	0.006457	0.022683	protein_coding
ENSG0000(	LINC00622	69.2662	0.340167	0.006457	0.022683	lncRNA
ENSG0000(	NXT1	67.41231	-0.38946	0.006455	0.022683	protein_coding
ENSG0000(	CFAP69	2287.281	0.131648	0.006463	0.022699	protein_coding
ENSG0000(	ENSG0000(	28.52759	0.414205	0.006466	0.022708	lncRNA
ENSG0000(	RN7SL581F	15.9628	0.476535	0.006473	0.022727	misc_RNA
ENSG0000(	ZNF488	24.03766	0.737288	0.006473	0.022727	protein_coding
ENSG0000(	MIR4307H(	168.6746	-0.33388	0.006476	0.022735	lncRNA
ENSG0000(	GGPS1	729.2149	-0.15215	0.006481	0.022749	protein_coding
ENSG0000(	TM6SF1	60.4982	0.469089	0.00649	0.022778	protein_coding
ENSG0000(	GCNT1	1831.747	-0.29259	0.006492	0.022783	protein_coding
ENSG0000(	CHASERR	3329.729	0.147283	0.006498	0.022803	lncRNA
ENSG0000(	PKN2	3157.502	-0.15893	0.006502	0.022815	protein_coding
ENSG0000(	NDUFA10	4388.602	-0.12884	0.006505	0.022822	protein_coding
ENSG0000(	CCNG2	1633.398	0.236618	0.006506	0.022824	protein_coding
ENSG0000(	LEMD3	1417.542	-0.15032	0.006509	0.022829	protein_coding
ENSG0000(	WDR13	1968.912	-0.17673	0.006514	0.022845	protein_coding
ENSG0000(	ENSG0000(	247.534	0.177714	0.006527	0.022887	lncRNA
ENSG0000(	DENND1A	1904.935	0.163437	0.006533	0.022908	protein_coding
ENSG0000(	CYSRT1	22.99705	0.467605	0.006537	0.022914	protein_coding
ENSG0000(	ENSG0000(	91.60852	-0.34384	0.006536	0.022914	lncRNA
ENSG0000(	ST7-AS2	10.05466	0.706635	0.006539	0.02292	lncRNA
ENSG0000(	CRYM-AS1	517.0772	-0.23527	0.00654	0.022921	transcribed_unitary_pseudogene
ENSG0000(	GPHN	753.0478	-0.21216	0.006542	0.022927	protein_coding
ENSG0000(	ENSG0000(	633.2471	0.167776	0.006554	0.022966	protein_coding
ENSG0000(	SLC30A9	2050.989	-0.18735	0.006558	0.022977	protein_coding
ENSG0000(	SRPK3	114.9916	-0.30579	0.006563	0.022993	protein_coding
ENSG0000(	MOSPD2	812.6704	0.30148	0.006565	0.022996	protein_coding
ENSG0000(	ENSG0000(	6791.463	-0.23588	0.006566	0.022996	lncRNA

ENSG0000(	ARSG	990.5101	0.118393	0.006569	0.023003	protein_coding
ENSG0000(	POLR3GP1	11.43586	0.901731	0.006569	0.023003	transcribed_processed_pseudoge
ENSG0000(	REXO5	166.3965	-0.23097	0.006573	0.023012	protein_coding
ENSG0000(	PIGS	1070.529	-0.13248	0.006574	0.023014	protein_coding
ENSG0000(	NCOA1	2383.72	0.149292	0.006576	0.02302	protein_coding
ENSG0000(	EHD4-AS1	69.3643	0.310078	0.006578	0.023024	lncRNA
ENSG0000(	PPIG	2443.323	-0.082	0.00658	0.023028	protein_coding
ENSG0000(	IL1RL2	57.13	-0.53284	0.006582	0.023032	protein_coding
ENSG0000(	RGS19	207.8134	0.390906	0.006589	0.023053	protein_coding
ENSG0000(	AAMDC	448.7184	-0.14453	0.00659	0.023054	protein_coding
ENSG0000(	RAMAC	164.2798	-0.3223	0.006608	0.023117	protein_coding
ENSG0000(	IGHV3-66	150.1605	0.688529	0.006611	0.023123	IG_V_gene
ENSG0000(	DUSP2	102.542	0.592077	0.006617	0.023143	protein_coding
ENSG0000(	ENSG0000(	15.82249	0.732874	0.006618	0.023143	processed_pseudogene
ENSG0000(	DHX38	1184.198	-0.11515	0.006626	0.023169	protein_coding
ENSG0000(	SH2D2A	129.2291	0.365506	0.00663	0.023179	protein_coding
ENSG0000(	HINT2P1	31.41733	-0.67938	0.006632	0.023182	processed_pseudogene
ENSG0000(	BLOC1S6	17898.83	-0.19137	0.006637	0.0232	protein_coding
ENSG0000(	LINC02186	17.43118	-0.47786	0.006639	0.023204	lncRNA
ENSG0000(	ERCC5	3187.363	-0.11326	0.006641	0.023206	protein_coding
ENSG0000(	HMGB1	4015.552	-0.15541	0.006641	0.023206	protein_coding
ENSG0000(	ENSG0000(	90.63712	0.259538	0.006646	0.02322	lncRNA
ENSG0000(	PHLDA2	82.35566	-0.66875	0.006648	0.023223	protein_coding
ENSG0000(	RAB30-DT	2786.045	0.118825	0.00665	0.023226	lncRNA
ENSG0000(	ZNF598	676.6647	-0.20974	0.006649	0.023226	protein_coding
ENSG0000(	ENSG0000(	523.044	0.144233	0.006654	0.023236	lncRNA
ENSG0000(	RNU6-1284	18.75601	0.522801	0.006668	0.023284	snRNA
ENSG0000(	PCDHGA6	648.8669	-0.11113	0.006676	0.023311	protein_coding
ENSG0000(	ENSG0000(	384.6561	-0.37037	0.006678	0.023314	lncRNA
ENSG0000(	NPY6R	317.2336	-0.4382	0.006681	0.02332	transcribed_unitary_pseudogene
ENSG0000(	ENSG0000(	70.44687	0.24141	0.006692	0.023356	lncRNA
ENSG0000(	SNX31	25.38625	-0.42227	0.006694	0.023359	protein_coding
ENSG0000(	ZNF765	1773.803	-0.12357	0.006694	0.023359	protein_coding
ENSG0000(	CILP	63.37498	0.47774	0.006695	0.023359	protein_coding
ENSG0000(	ENSG0000(	9.765691	0.730236	0.006696	0.023361	lncRNA
ENSG0000(	HOXA4	171.2751	-0.30613	0.006701	0.023376	protein_coding
ENSG0000(	POC1B-AS1	940.1285	0.258676	0.006705	0.023387	lncRNA
ENSG0000(	PDZRN3	639.68	0.267115	0.006709	0.023397	protein_coding
ENSG0000(	PICK1	540.7126	-0.14635	0.006709	0.023397	protein_coding
ENSG0000(	ENSG0000(	310.9655	0.14627	0.006718	0.023425	TEC
ENSG0000(	IGLC7	71.60259	1.018983	0.006722	0.023435	IG_C_gene
ENSG0000(	PLIN4	59.47364	0.799183	0.006722	0.023435	protein_coding
ENSG0000(	SYAP1	2763.324	-0.11254	0.006725	0.023442	protein_coding
ENSG0000(	ENSG0000(	12.12858	0.652596	0.006734	0.023469	lncRNA
ENSG0000(	ENSG0000(	3503.659	0.137117	0.006737	0.023476	lncRNA
ENSG0000(	VAC14	1466.448	0.183896	0.006737	0.023476	protein_coding
ENSG0000(	TATDN2P2	13.86223	0.68149	0.006739	0.023479	processed_pseudogene

ENSG0000(	ENSG0000(	81.42758	-0.90921	0.006741	0.023484	lncRNA
ENSG0000(	CCDC158	279.7516	-0.18729	0.006744	0.023493	protein_coding
ENSG0000(	EMC3	974.6431	-0.10522	0.006748	0.023502	protein_coding
ENSG0000(	ENSG0000(	437.6635	0.376082	0.00675	0.023506	lncRNA
ENSG0000(	TLDC2	694.9516	-0.44584	0.006752	0.023512	protein_coding
ENSG0000(	ENSG0000(	31.73732	0.344078	0.006753	0.023512	lncRNA
ENSG0000(	ENSG0000(	169.2889	-0.27897	0.006754	0.023512	lncRNA
ENSG0000(	ENSG0000(	33.80702	0.357487	0.006758	0.023524	lncRNA
ENSG0000(	PAFAH1B3	56.31705	-0.36212	0.006765	0.023546	protein_coding
ENSG0000(	ENSG0000(	270.7302	0.370959	0.006768	0.02355	lncRNA
ENSG0000(	ESRG	553.7101	-0.44326	0.006768	0.02355	lncRNA
ENSG0000(	HNRNPH1F	179.932	0.333829	0.00677	0.023557	processed_pseudogene
ENSG0000(	ENSG0000(	3053.212	-0.20768	0.006771	0.023558	lncRNA
ENSG0000(	ENSG0000(	13.03723	0.634654	0.006777	0.023575	lncRNA
ENSG0000(	ENSG0000(	17.8704	0.737128	0.006779	0.023576	lncRNA
ENSG0000(	UPF2	1796.501	-0.12182	0.006779	0.023576	protein_coding
ENSG0000(	ENSG0000(	28.91354	-0.50665	0.006781	0.023579	lncRNA
ENSG0000(	NOP9	1339.89	-0.14185	0.006789	0.023606	protein_coding
ENSG0000(	ENSG0000(	227.4715	0.413603	0.006799	0.023636	lncRNA
ENSG0000(	PNPLA5	58.01574	0.2521	0.006799	0.023636	protein_coding
ENSG0000(	ZNF467	102.4333	0.358267	0.006801	0.023638	protein_coding
ENSG0000(	VDAC3	1055.277	-0.16313	0.006802	0.023642	protein_coding
ENSG0000(	RCVRN	23.77992	0.520447	0.006814	0.023679	protein_coding
ENSG0000(	BIN3	884.3132	-0.15108	0.006815	0.023679	protein_coding
ENSG0000(	MFSD10	462.9143	-0.19039	0.006823	0.023704	protein_coding
ENSG0000(	PDPR2P	1492.88	-0.13196	0.006823	0.023704	transcribed_unprocessed_pseudo
ENSG0000(	CCPG1	2301.991	-0.1335	0.006825	0.023707	protein_coding
ENSG0000(	CLEC19A	336.363	0.148063	0.006832	0.023726	protein_coding
ENSG0000(	ENSG0000(	17.26928	1.05486	0.006833	0.023726	lncRNA
ENSG0000(	ENSG0000(	34.82204	0.541609	0.006833	0.023726	TEC
ENSG0000(	ZDHHC11	216.1612	-0.39892	0.006833	0.023726	protein_coding
ENSG0000(	ENSG0000(	57.89165	0.286441	0.006835	0.023729	TEC
ENSG0000(	ENSG0000(	26.95957	0.395962	0.006844	0.023753	lncRNA
ENSG0000(	LOXL1	102.8705	0.570064	0.006844	0.023753	protein_coding
ENSG0000(	ENSG0000(	30.794	-0.39306	0.006849	0.023767	processed_pseudogene
ENSG0000(	ENSG0000(	187.2467	0.148413	0.00685	0.023767	lncRNA
ENSG0000(	ENSG0000(	37.75535	0.420439	0.00685	0.023767	lncRNA
ENSG0000(	VSTM2A	224.2865	-0.49068	0.00685	0.023767	protein_coding
ENSG0000(	WASF3	124.0445	0.4158	0.006875	0.023849	protein_coding
ENSG0000(	ENSG0000(	353.7876	-0.14338	0.006882	0.023871	lncRNA
ENSG0000(	PLAUR	1198.252	0.453464	0.006887	0.023884	protein_coding
ENSG0000(	CRYBG3	4522.205	0.135608	0.006888	0.023886	protein_coding
ENSG0000(	ZNF658B	118.1886	0.367024	0.006892	0.023897	transcribed_unprocessed_pseudo
ENSG0000(	ENSG0000(	391.0545	-0.15962	0.006898	0.02391	lncRNA
ENSG0000(	KLHL3	720.2375	0.254032	0.006897	0.02391	protein_coding
ENSG0000(	STEAP3-AS	20.9184	0.449584	0.006898	0.02391	lncRNA
ENSG0000(	ENSG0000(	108.1806	0.191667	0.006903	0.023926	lncRNA

ENSG0000(	CDH3	408.9727	0.22226	0.006905	0.02393	protein_coding
ENSG0000(	TMEM41B	1525.115	-0.13213	0.006906	0.02393	protein_coding
ENSG0000(	LINC02890	74.91822	-0.29555	0.006914	0.023957	lncRNA
ENSG0000(	GDPD5	392.3883	0.327696	0.006916	0.02396	protein_coding
ENSG0000(	CDK7	220.9532	-0.22464	0.006918	0.023963	protein_coding
ENSG0000(	ZNF57	343.6671	-0.3566	0.00692	0.023967	protein_coding
ENSG0000(	ADAM9	2727.708	0.243166	0.006922	0.023973	protein_coding
ENSG0000(	ENSG0000(	52.28136	0.54836	0.00693	0.023998	lncRNA
ENSG0000(	AFTPH-DT	28.55042	0.488408	0.006936	0.024013	lncRNA
ENSG0000(	ENSG0000(	236.192	0.742441	0.006935	0.024013	TEC
ENSG0000(	C10orf55	20.58923	0.47379	0.006937	0.024015	lncRNA
ENSG0000(	HMGN1P1	84.23426	0.265794	0.006939	0.024016	processed_pseudogene
ENSG0000(	MCAM	864.5298	0.37241	0.006944	0.024033	protein_coding
ENSG0000(	ZBTB10	1560.832	-0.18916	0.006951	0.024053	protein_coding
ENSG0000(	FHIP1A	2488.656	-0.38289	0.006956	0.024068	protein_coding
ENSG0000(	ENSG0000(	346.113	-0.13255	0.006963	0.02409	processed_pseudogene
ENSG0000(	BDH1	4223.451	-0.26797	0.006964	0.024091	protein_coding
ENSG0000(	LAT2	976.4254	0.266869	0.006965	0.024091	protein_coding
ENSG0000(	ZNF687	1021.271	-0.11601	0.006966	0.024092	protein_coding
ENSG0000(	DCBLD1	1181.473	0.264442	0.006975	0.024122	protein_coding
ENSG0000(	ALAD	1241.381	-0.23856	0.006988	0.024163	protein_coding
ENSG0000(	BCL2L13	1377.36	-0.13736	0.006996	0.024186	protein_coding
ENSG0000(	LRRC19	1876.857	-0.43612	0.006996	0.024186	protein_coding
ENSG0000(	ASPG	320.7463	-0.83379	0.006997	0.024187	protein_coding
ENSG0000(	SOD3	116.7813	0.346754	0.007	0.024194	protein_coding
ENSG0000(	ENSG0000(	154.1448	0.176669	0.007003	0.024202	lncRNA
ENSG0000(	ALDOB	509.0078	0.89656	0.007005	0.024203	protein_coding
ENSG0000(	SARNP	641.3717	-0.143	0.007004	0.024203	protein_coding
ENSG0000(	SGK2	3368.37	-0.59728	0.007011	0.024222	protein_coding
ENSG0000(	ENSG0000(	99.6748	0.441857	0.007026	0.024272	protein_coding
ENSG0000(	STX6	1163.498	0.164917	0.007029	0.024279	protein_coding
ENSG0000(	ENSG0000(	613.1635	0.120731	0.007033	0.02429	lncRNA
ENSG0000(	ENSG0000(	18.07604	0.571888	0.007039	0.024308	lncRNA
ENSG0000(	GPR108	527.1951	-0.17966	0.007043	0.024313	protein_coding
ENSG0000(	NPIPB4	7258.801	0.148936	0.007043	0.024313	protein_coding
ENSG0000(	ZNF346-IT1	218.2827	0.150667	0.007042	0.024313	lncRNA
ENSG0000(	LINC02455	25.66472	-0.38929	0.007045	0.024317	lncRNA
ENSG0000(	ENSG0000(	123.2261	0.245142	0.007046	0.024317	lncRNA
ENSG0000(	RNU1-109F	23.43679	0.525933	0.007046	0.024317	snRNA
ENSG0000(	RPS6KA3	5077.294	-0.12663	0.007048	0.024319	protein_coding
ENSG0000(	GRIPAP1	1688.133	0.100939	0.00705	0.024323	protein_coding
ENSG0000(	PARP15	4221.127	0.27753	0.007051	0.024323	protein_coding
ENSG0000(	RNF170	1184.06	0.130045	0.007058	0.024346	protein_coding
ENSG0000(	PLXNA1	1436.82	0.272873	0.007062	0.024357	protein_coding
ENSG0000(	ALKBH3	233.8039	-0.24188	0.007064	0.024362	protein_coding
ENSG0000(	TNFRSF10A	445.8911	-0.12992	0.007086	0.024434	lncRNA
ENSG0000(	ASXL1	3371.772	0.153943	0.007106	0.024499	protein_coding

ENSG0000(	HIGD2A	573.9917	-0.27969	0.007106	0.024499	protein_coding
ENSG0000(	SMG7	2674.989	-0.14836	0.007109	0.024508	protein_coding
ENSG0000(	ZNF891	3013.058	-0.10441	0.007116	0.024526	protein_coding
ENSG0000(	ATXN1L	1971.087	0.142614	0.007119	0.024533	protein_coding
ENSG0000(	LRFN1	151.7471	0.349626	0.007119	0.024533	protein_coding
ENSG0000(	IL17RD	669.4687	-0.26756	0.007122	0.024541	protein_coding
ENSG0000(	POGLUT1	1092.281	0.191133	0.007124	0.024545	protein_coding
ENSG0000(	LINC00960	901.4376	0.212857	0.007125	0.024546	lncRNA
ENSG0000(	SCUBE2	385.7046	-0.28313	0.007126	0.024546	protein_coding
ENSG0000(	ENSG0000(	105.9863	-0.22049	0.007129	0.024554	lncRNA
ENSG0000(	ENSG0000(	39.666	0.411051	0.007136	0.024568	lncRNA
ENSG0000(	ENSG0000(	221.5537	-0.1956	0.007135	0.024568	lncRNA
ENSG0000(	RNU1-61P	9.930381	0.8526	0.007135	0.024568	snRNA
ENSG0000(	UBR5	5795.081	0.098625	0.007151	0.024619	protein_coding
ENSG0000(	SOCS5P4	99.27385	-0.26077	0.007155	0.024631	processed_pseudogene
ENSG0000(	NSRP1	1943.39	-0.11441	0.007159	0.024639	protein_coding
ENSG0000(	ENSG0000(	13960.66	-0.46143	0.00716	0.024642	lncRNA
ENSG0000(	COPS4	304.9955	-0.20243	0.007165	0.024655	protein_coding
ENSG0000(	ADGRF5P1	116.8288	-0.27527	0.007167	0.024659	transcribed_processed_pseudoge
ENSG0000(	AP1AR	905.7549	-0.25928	0.007177	0.024691	protein_coding
ENSG0000(	UBXN4	3694.944	0.108032	0.00718	0.0247	protein_coding
ENSG0000(	MRPS25	3116.188	-0.19234	0.007187	0.024721	protein_coding
ENSG0000(	ENSG0000(	185.5746	0.236577	0.007189	0.024726	TEC
ENSG0000(	LRRC37A11	84.33957	0.278425	0.007195	0.024741	transcribed_unprocessed_pseudo
ENSG0000(	ZNF512B	393.285	0.262629	0.007196	0.024743	protein_coding
ENSG0000(	PLPP7	151.6842	0.283164	0.007197	0.024744	protein_coding
ENSG0000(	ANKRD22	660.2836	0.420642	0.007207	0.024776	protein_coding
ENSG0000(	ADGRL1-AS	309.7298	0.238718	0.007212	0.024787	lncRNA
ENSG0000(	ENSG0000(	278.5997	0.187882	0.007211	0.024787	lncRNA
ENSG0000(	CALR	3337.039	-0.24483	0.007216	0.024797	protein_coding
ENSG0000(	ENSG0000(	176.6517	-0.43606	0.007226	0.024831	lncRNA
ENSG0000(	RN7SL681P	40.50991	-0.43251	0.007229	0.024837	misc_RNA
ENSG0000(	TRABD	792.2697	-0.20824	0.00723	0.024838	protein_coding
ENSG0000(	ENSG0000(	52.11585	-0.33492	0.007234	0.02485	lncRNA
ENSG0000(	POLE2	129.0402	-0.33679	0.007236	0.024855	protein_coding
ENSG0000(	FOXJ3	2411.556	-0.12607	0.007238	0.024856	protein_coding
ENSG0000(	PLA2G4C	855.862	-0.15823	0.007238	0.024856	protein_coding
ENSG0000(	GDPD1	956.7162	-0.13253	0.00724	0.02486	protein_coding
ENSG0000(	DHRS9	3351.16	-0.50042	0.007247	0.024882	protein_coding
ENSG0000(	PA2G4P4	121.8839	-0.30872	0.007249	0.024883	processed_pseudogene
ENSG0000(	ENSG0000(	107.9449	0.27216	0.007252	0.024891	lncRNA
ENSG0000(	ENSG0000(	177.825	0.180648	0.007252	0.024891	lncRNA
ENSG0000(	AGAP10P	34.15573	-0.79281	0.007253	0.024891	unprocessed_pseudogene
ENSG0000(	FANCD2OS	99.40202	-0.28787	0.00726	0.024906	protein_coding
ENSG0000(	GS1-124K5	137.5625	0.212395	0.00726	0.024906	lncRNA
ENSG0000(	HLA-DRB5	649.1825	0.643412	0.007258	0.024906	protein_coding
ENSG0000(	ANKRD40C	1341.178	-0.29675	0.007263	0.024915	protein_coding

ENSG0000(	ENSG0000(	28.01893	-0.48732	0.007267	0.02492	lncRNA
ENSG0000(	LINC01258	26.5445	0.456062	0.007266	0.02492	lncRNA
ENSG0000(	SETP17	9.869317	-0.63536	0.007274	0.024944	processed_pseudogene
ENSG0000(	FAAP24	717.7326	0.107421	0.007276	0.024947	protein_coding
ENSG0000(	ENSG0000(	31.43392	-0.39708	0.007283	0.024959	lncRNA
ENSG0000(	ENSG0000(	137.8538	0.243146	0.007281	0.024959	lncRNA
ENSG0000(	JAK1	4526.958	0.160388	0.007283	0.024959	protein_coding
ENSG0000(	OGFR	1156.482	0.18862	0.007282	0.024959	protein_coding
ENSG0000(	MTHFD2	2694.303	0.173824	0.00729	0.024981	protein_coding
ENSG0000(	CAPRIN1	4528.394	-0.09981	0.007293	0.024989	protein_coding
ENSG0000(	CCDC93	2769.094	-0.12967	0.007298	0.025005	protein_coding
ENSG0000(	KALRN	2633.974	-0.16553	0.007304	0.025019	protein_coding
ENSG0000(	CADM2-AS	91.66822	0.226955	0.007305	0.025021	lncRNA
ENSG0000(	KLRC4	72.22266	-0.58312	0.007308	0.025028	protein_coding
ENSG0000(	ENSG0000(	10.22875	0.685723	0.007311	0.025035	transcribed_unprocessed_pseudo
ENSG0000(	ENSG0000(	236.0712	0.188428	0.007311	0.025035	lncRNA
ENSG0000(	ENSG0000(	145.4425	-0.22936	0.007313	0.025037	lncRNA
ENSG0000(	ENSG0000(	137.1503	0.181556	0.007314	0.025038	lncRNA
ENSG0000(	ENSG0000(	17.97492	0.568174	0.007316	0.025043	processed_pseudogene
ENSG0000(	ZDHHC22	57.37012	0.406655	0.007317	0.025043	protein_coding
ENSG0000(	SPATA9	28.34918	0.415288	0.007318	0.025044	protein_coding
ENSG0000(	ASPH	4482.307	0.186883	0.007321	0.025051	protein_coding
ENSG0000(	TUBAL3	256.8413	-0.58232	0.007322	0.025053	protein_coding
ENSG0000(	MSANTD4	468.4992	0.182444	0.007324	0.025055	protein_coding
ENSG0000(	IRS3P	170.7811	0.287225	0.007325	0.025058	processed_pseudogene
ENSG0000(	PSMD14	636.9355	-0.23583	0.007329	0.025068	protein_coding
ENSG0000(	SRA1	516.6392	0.163207	0.007331	0.025071	protein_coding
ENSG0000(	NUP62CL	84.34916	-0.39788	0.007332	0.025075	protein_coding
ENSG0000(	IFFO2	446.7012	0.270663	0.007338	0.025088	protein_coding
ENSG0000(	LINC00491	292.043	0.215889	0.007338	0.025088	lncRNA
ENSG0000(	SPRYD7	487.6961	-0.21898	0.007339	0.02509	protein_coding
ENSG0000(	NCBP2L	16.19504	0.596833	0.007346	0.025109	protein_coding
ENSG0000(	REL	4701.155	0.168912	0.00735	0.025121	protein_coding
ENSG0000(	NDUFA4L2	44.71429	0.620195	0.007352	0.025124	protein_coding
ENSG0000(	TRMT10B	620.9456	-0.12078	0.007371	0.025186	protein_coding
ENSG0000(	FAM218A	45.33968	0.398254	0.007375	0.025198	lncRNA
ENSG0000(	RN7SL749F	100.3184	0.353461	0.007384	0.025227	misc_RNA
ENSG0000(	ENSG0000(	18.81293	0.821518	0.007388	0.025236	lncRNA
ENSG0000(	MIR186	19.28028	0.765935	0.007389	0.025239	miRNA
ENSG0000(	SFRP4	15.64463	1.049193	0.007391	0.02524	protein_coding
ENSG0000(	PLCG1	2654.512	-0.16191	0.007391	0.025241	protein_coding
ENSG0000(	NPHP1	789.6934	0.112633	0.007406	0.025288	protein_coding
ENSG0000(	ADAM5	37.2182	-0.36258	0.007412	0.025305	transcribed_unitary_pseudogene
ENSG0000(	PSMB2	1235.295	-0.20656	0.007413	0.025305	protein_coding
ENSG0000(	ENSG0000(	42.07055	0.3479	0.007415	0.025311	lncRNA
ENSG0000(	SPATC1	20.1127	0.679059	0.00742	0.025323	protein_coding
ENSG0000(	MRC2	756.7848	0.381339	0.007421	0.025326	protein_coding

ENSG0000(	ENSG0000(	81.76394	0.283965	0.007426	0.025341	lncRNA
ENSG0000(	LINC01346	29.9522	0.444841	0.007434	0.025363	lncRNA
ENSG0000(	GMEB2	443.3354	-0.13272	0.007437	0.02537	protein_coding
ENSG0000(	ENSG0000(	18.12464	0.432866	0.007438	0.025372	lncRNA
ENSG0000(	PRELID1	839.5081	-0.32559	0.00744	0.025374	protein_coding
ENSG0000(	FABP6-AS1	474.8561	0.255891	0.007444	0.025386	lncRNA
ENSG0000(	ENSG0000(	232.0081	-0.18781	0.00745	0.025406	lncRNA
ENSG0000(	USP9YP3	62.88087	-0.25218	0.007452	0.02541	unprocessed_pseudogene
ENSG0000(	IFT57	466.9566	-0.27509	0.007454	0.025411	protein_coding
ENSG0000(	HSD17B4	5756.69	-0.10395	0.007455	0.025415	protein_coding
ENSG0000(	ZNF823	509.6219	-0.28597	0.007465	0.025446	protein_coding
ENSG0000(	EPHA10	2276.656	-0.37435	0.007472	0.025464	protein_coding
ENSG0000(	LINGO1	24.1657	0.724545	0.007477	0.025479	protein_coding
ENSG0000(	LINC00968	11.37936	0.922006	0.007481	0.025492	lncRNA
ENSG0000(	ENSG0000(	211.9328	-0.19379	0.007483	0.025496	lncRNA
ENSG0000(	NETO1-DT	24.74816	0.81139	0.007489	0.025512	lncRNA
ENSG0000(	PKMYT1	175.9053	-0.37864	0.00749	0.025512	protein_coding
ENSG0000(	SUMO3	732.9417	-0.13954	0.0075	0.025543	protein_coding
ENSG0000(	ENSG0000(	19.68332	-0.50272	0.007506	0.02556	lncRNA
ENSG0000(	CLNK	390.0934	0.185651	0.007508	0.025564	protein_coding
ENSG0000(	SCGB3A2	431.3598	0.244583	0.007508	0.025564	protein_coding
ENSG0000(	ENSG0000(	13.5779	0.665947	0.007518	0.025588	lncRNA
ENSG0000(	ENSG0000(	72.50479	-0.32957	0.007517	0.025588	lncRNA
ENSG0000(	TNFRSF18	53.60419	0.41652	0.007517	0.025588	protein_coding
ENSG0000(	ENSG0000(	32.88289	0.344361	0.007522	0.025597	lncRNA
ENSG0000(	ENSG0000(	31.88433	0.467017	0.007524	0.025597	processed_pseudogene
ENSG0000(	ENSG0000(	10.89699	-0.78156	0.007525	0.025597	lncRNA
ENSG0000(	FOXP1-AS1	222.916	-0.15023	0.007523	0.025597	lncRNA
ENSG0000(	TBX4	55.13529	0.289736	0.007524	0.025597	protein_coding
ENSG0000(	ZNF106	4481.227	0.092898	0.007525	0.025597	protein_coding
ENSG0000(	PKIG	580.1003	0.209026	0.007528	0.025603	protein_coding
ENSG0000(	UNGP3	15.07408	-0.60146	0.007535	0.025623	processed_pseudogene
ENSG0000(	TCOF1	1414.01	-0.16678	0.00754	0.025637	protein_coding
ENSG0000(	ENSG0000(	25.42238	0.462379	0.007551	0.025671	TEC
ENSG0000(	ENSG0000(	277.6061	0.25495	0.007551	0.025671	lncRNA
ENSG0000(	CNIH4	1270.359	-0.23112	0.00756	0.025695	protein_coding
ENSG0000(	ENSG0000(	4220.056	0.231125	0.007559	0.025695	TEC
ENSG0000(	FTCDNL1	359.1071	0.26407	0.007561	0.025696	protein_coding
ENSG0000(	ENSG0000(	165.406	0.209531	0.007567	0.025715	lncRNA
ENSG0000(	ARHGAP39	320.6418	-0.32722	0.00757	0.025722	protein_coding
ENSG0000(	KDELR2	2819.072	-0.20314	0.007572	0.025725	protein_coding
ENSG0000(	ENSG0000(	25.96835	0.441826	0.007584	0.025762	lncRNA
ENSG0000(	TPRXL	199.1653	0.173052	0.007585	0.025762	transcribed_processed_pseudoge
ENSG0000(	ENSG0000(	47.22886	0.349594	0.007593	0.025787	lncRNA
ENSG0000(	DLD	1276.717	-0.17504	0.007596	0.025794	protein_coding
ENSG0000(	RNU6-130F	73.77396	0.340157	0.007596	0.025794	snRNA
ENSG0000(	SERPINA6	18.90333	-0.78488	0.007603	0.025814	protein_coding

ENSG0000(	PLCL1	297.5781	0.301843	0.007608	0.025827	protein_coding
ENSG0000(	ENSG0000(	3741.407	-0.11972	0.00761	0.025828	lncRNA
ENSG0000(	SCARNA16	430.4552	-0.26351	0.007609	0.025828	scaRNA
ENSG0000(	SLC66A3	228.0834	0.285191	0.007615	0.025842	protein_coding
ENSG0000(	METTL24	297.5187	0.174945	0.007624	0.025872	protein_coding
ENSG0000(	SLC38A2	4239.329	0.217262	0.007636	0.025909	protein_coding
ENSG0000(	FAM66C	452.8599	0.149214	0.007646	0.025933	lncRNA
ENSG0000(	NAA25	2166.633	-0.10931	0.007646	0.025933	protein_coding
ENSG0000(	OR4F3	22.41298	0.94226	0.007646	0.025933	protein_coding
ENSG0000(	ZFTA	431.0404	0.256334	0.007646	0.025933	protein_coding
ENSG0000(	ENSG0000(	124.3153	-0.27375	0.00765	0.025941	lncRNA
ENSG0000(	INPP5B	2490.155	-0.12309	0.007656	0.025959	protein_coding
ENSG0000(	ABCC12	59.17047	0.334891	0.007662	0.025978	protein_coding
ENSG0000(	NXNL1	18.70118	0.528291	0.007684	0.026048	protein_coding
ENSG0000(	ENSG0000(	191.2547	-0.22236	0.007685	0.026048	lncRNA
ENSG0000(	ENSG0000(	48.81638	-0.37413	0.007687	0.026055	lncRNA
ENSG0000(	ENSG0000(	1546.185	0.120208	0.007701	0.026092	lncRNA
ENSG0000(	F8	226.6561	0.230043	0.007701	0.026092	protein_coding
ENSG0000(	LINC02381	148.8951	0.218553	0.007701	0.026092	lncRNA
ENSG0000(	MRRF	2268.738	-0.15748	0.007702	0.026093	protein_coding
ENSG0000(	ACTN1	4042.334	0.23898	0.007708	0.026111	protein_coding
ENSG0000(	ENSG0000(	123.975	0.232294	0.007717	0.026138	lncRNA
ENSG0000(	SLC30A5	1228.118	-0.13653	0.00772	0.026145	protein_coding
ENSG0000(	CDKL1	2255.136	0.366001	0.007727	0.026165	protein_coding
ENSG0000(	ATE1-AS1	42.69002	-0.55624	0.007729	0.026172	transcribed_unitary_pseudogene
ENSG0000(	TAFA5	21.20101	0.7138	0.00773	0.026172	protein_coding
ENSG0000(	CSTA	21.52493	0.705063	0.007732	0.026173	protein_coding
ENSG0000(	ARID4B	2579.474	0.133221	0.007737	0.026186	protein_coding
ENSG0000(	ENSG0000(	18.44575	0.522915	0.007738	0.026186	snoRNA
ENSG0000(	ENSG0000(	29.64502	0.333716	0.007738	0.026186	lncRNA
ENSG0000(	TRIP4	593.2263	-0.18555	0.007745	0.026206	protein_coding
ENSG0000(	GNMT	15.18735	0.902059	0.007749	0.026219	protein_coding
ENSG0000(	ENSG0000(	44.03314	0.346432	0.007751	0.026222	lncRNA
ENSG0000(	S1PR2	794.6247	0.253392	0.007754	0.026229	protein_coding
ENSG0000(	EPB41L4A-/	575.5017	-0.2248	0.007758	0.026237	lncRNA
ENSG0000(	SNRPC	250.1796	-0.21448	0.007757	0.026237	protein_coding
ENSG0000(	COL9A1	346.505	-0.15581	0.00777	0.026276	protein_coding
ENSG0000(	FAR2P1	368.8493	-0.16473	0.007779	0.0263	transcribed_unprocessed_pseudo
ENSG0000(	TAL1	167.4921	0.387755	0.00778	0.0263	protein_coding
ENSG0000(	TRDN-AS1	1150.034	0.158748	0.00778	0.0263	lncRNA
ENSG0000(	HACD4	725.6797	0.200927	0.007785	0.026314	protein_coding
ENSG0000(	ENSG0000(	16.19161	0.490681	0.007788	0.026321	lncRNA
ENSG0000(	ENSG0000(	19.41635	0.537676	0.007792	0.026328	lncRNA
ENSG0000(	LINC02033	192.2924	0.406616	0.007791	0.026328	lncRNA
ENSG0000(	ENSG0000(	87.69159	0.346417	0.007797	0.026344	lncRNA
ENSG0000(	SCTR	76.40777	0.25695	0.0078	0.026352	protein_coding
ENSG0000(	NID1	1460.353	0.338465	0.007803	0.026358	protein_coding

ENSG0000(	CLCA1	9077.282	-0.60663	0.007813	0.026383	protein_coding
ENSG0000(	ENSG0000(	114.4134	0.235049	0.007813	0.026383	lncRNA
ENSG0000(	MS4A6A	1789.933	0.263855	0.007813	0.026383	protein_coding
ENSG0000(	ST8SIA5-DT	195.4838	-0.16809	0.007814	0.026384	lncRNA
ENSG0000(	DMTF1	5058.06	0.172636	0.007816	0.026388	protein_coding
ENSG0000(	ENSG0000(	377.3718	-0.1996	0.007821	0.026393	lncRNA
ENSG0000(	MYBL1	274.4233	-0.47754	0.007822	0.026393	protein_coding
ENSG0000(	PALLD	2483.941	0.198457	0.00782	0.026393	protein_coding
ENSG0000(	PAPLN-AS1	427.6362	0.13702	0.007821	0.026393	lncRNA
ENSG0000(	STPG2-AS1	577.3487	0.153852	0.00782	0.026393	lncRNA
ENSG0000(	PEAK1	8519.69	-0.1399	0.007825	0.026402	protein_coding
ENSG0000(	ENSG0000(	279.7275	0.211305	0.007827	0.026403	lncRNA
ENSG0000(	HMGB3P22	9.175098	-0.83428	0.007837	0.026435	transcribed_processed_pseudoge
ENSG0000(	TIGIT	277.0497	0.540894	0.007838	0.026435	protein_coding
ENSG0000(	CASP16P	199.1107	0.223292	0.007842	0.026447	transcribed_unitary_pseudogene
ENSG0000(	GRTP1	782.972	-0.28832	0.007843	0.026447	protein_coding
ENSG0000(	AREL1	1857.265	-0.14408	0.007848	0.026462	protein_coding
ENSG0000(	ENSG0000(	131.6663	0.400805	0.007851	0.026467	lncRNA
ENSG0000(	ENSG0000(	239.5581	-0.17958	0.007851	0.026467	lncRNA
ENSG0000(	MDH1	1102.955	-0.15438	0.007852	0.026467	protein_coding
ENSG0000(	LINC02159	21.27972	0.568736	0.007858	0.026483	lncRNA
ENSG0000(	ENSG0000(	86.79068	0.258511	0.007862	0.026494	lncRNA
ENSG0000(	KDSR	1266.799	0.155439	0.007865	0.026502	protein_coding
ENSG0000(	SPRN	10.05358	0.863335	0.007868	0.02651	protein_coding
ENSG0000(	ENSG0000(	189.6224	-0.21138	0.007873	0.026522	lncRNA
ENSG0000(	KLRB1	105.1745	-0.43374	0.007879	0.026541	protein_coding
ENSG0000(	BAZ2B	8031.144	0.113881	0.007886	0.026561	protein_coding
ENSG0000(	ITK	1683.167	0.318291	0.007887	0.026563	protein_coding
ENSG0000(	GPRC5B	280.6085	0.396467	0.007899	0.0266	protein_coding
ENSG0000(	GCOM1	655.3913	-0.48633	0.007913	0.026644	protein_coding
ENSG0000(	HTR2A	99.22164	0.347083	0.007915	0.026646	protein_coding
ENSG0000(	FAM20C	576.7574	0.462299	0.007922	0.026668	protein_coding
ENSG0000(	RASSF7	779.5045	-0.29647	0.007925	0.026674	protein_coding
ENSG0000(	ENSG0000(	44.51609	0.471399	0.00793	0.026686	lncRNA
ENSG0000(	RNU6-137F	15.68021	0.551646	0.007929	0.026686	snRNA
ENSG0000(	RIPOR1	735.0455	0.300812	0.007935	0.026699	protein_coding
ENSG0000(	ENSG0000(	769.6744	0.146956	0.007946	0.026731	lncRNA
ENSG0000(	RSPH3	1574.007	-0.14285	0.007946	0.026731	protein_coding
ENSG0000(	TPPP	141.243	-0.33844	0.007949	0.026737	protein_coding
ENSG0000(	ENSG0000(	43.6478	0.327203	0.007956	0.02676	lncRNA
ENSG0000(	NOMO1	1000.397	-0.24362	0.00797	0.026804	protein_coding
ENSG0000(	SNORA67	93.59481	-0.35749	0.007972	0.026809	snoRNA
ENSG0000(	ZBTB8OS	1125.857	-0.10288	0.007975	0.026816	protein_coding
ENSG0000(	ANKMY2	565.408	-0.22841	0.007988	0.026855	protein_coding
ENSG0000(	KCNE3	1384.373	0.368032	0.007995	0.026877	protein_coding
ENSG0000(	ENSG0000(	46.41093	-0.52888	0.008001	0.026895	lncRNA
ENSG0000(	IGKV2D-29	95.06535	0.716241	0.008005	0.026904	IG_V_gene

ENSG0000(	CDC42EP2	209.1723	0.30047	0.008007	0.026909	protein_coding
ENSG0000(	ENSG0000(	87.7906	-0.30641	0.008011	0.026917	lncRNA
ENSG0000(	NWD2	53.66494	-0.84031	0.008014	0.026924	protein_coding
ENSG0000(	CCNK	1637.494	-0.13093	0.008022	0.026951	protein_coding
ENSG0000(	GPC3	135.9842	-0.31418	0.008038	0.027	protein_coding
ENSG0000(	VPS33B	340.0803	-0.14188	0.008039	0.027	protein_coding
ENSG0000(	ATP2C2	2750.295	-0.45846	0.00804	0.027	protein_coding
ENSG0000(	C19orf12	814.362	0.172678	0.008042	0.027001	protein_coding
ENSG0000(	RPL36AP43	82.45972	-0.39081	0.008041	0.027001	processed_pseudogene
ENSG0000(	USP34-DT	358.3847	0.157087	0.008045	0.027008	lncRNA
ENSG0000(	ENSG0000(	26.29641	0.397884	0.008055	0.027037	lncRNA
ENSG0000(	RNU6-1283	30.46454	0.389248	0.008055	0.027037	snRNA
ENSG0000(	IGKV2-24	192.2962	0.744162	0.008061	0.027056	IG_V_gene
ENSG0000(	NANOG	800.9278	-0.13275	0.008063	0.027058	protein_coding
ENSG0000(	ATAD5	865.3925	-0.22969	0.008066	0.027064	protein_coding
ENSG0000(	LAMTOR5	691.2939	-0.23968	0.008066	0.027064	protein_coding
ENSG0000(	LINC02377	38.64929	-0.39612	0.008078	0.027099	lncRNA
ENSG0000(	RNGTT	825.3981	-0.13125	0.008079	0.027099	protein_coding
ENSG0000(	DTX4	3193.015	-0.28983	0.00808	0.027102	protein_coding
ENSG0000(	RN7SL657F	24.33703	1.493795	0.008086	0.027117	misc_RNA
ENSG0000(	DCLRE1B	159.7666	-0.30199	0.008087	0.027118	protein_coding
ENSG0000(	ATG10	626.3126	-0.16787	0.008089	0.027121	protein_coding
ENSG0000(	RETREG1-A	103.0343	0.385178	0.00809	0.027121	lncRNA
ENSG0000(	HECW2	858.1745	0.263788	0.008098	0.027145	protein_coding
ENSG0000(	TMEM249	19.1577	-0.5849	0.008098	0.027145	protein_coding
ENSG0000(	ENSG0000(	37.76166	0.413967	0.008114	0.027194	lncRNA
ENSG0000(	ENSG0000(	21.92284	-0.47146	0.008123	0.027221	lncRNA
ENSG0000(	KLRG1	473.8104	0.147135	0.008124	0.027221	protein_coding
ENSG0000(	RPLP0P2	460.0246	0.26606	0.008127	0.027228	transcribed_processed_pseudoge
ENSG0000(	ENSG0000(	30.93909	0.388281	0.008129	0.02723	snoRNA
ENSG0000(	KIAA0586	2262.599	-0.11166	0.008129	0.02723	protein_coding
ENSG0000(	MS4A7	1334.548	0.32235	0.00813	0.02723	protein_coding
ENSG0000(	CCDC194	63.17962	0.327258	0.008132	0.027233	protein_coding
ENSG0000(	LINC02392	84.65323	0.241945	0.00814	0.027258	lncRNA
ENSG0000(	MAP4K4	2837.864	0.277868	0.008148	0.027275	protein_coding
ENSG0000(	PRKAR1B	303.9988	-0.31194	0.008147	0.027275	protein_coding
ENSG0000(	STARD13	1326.468	0.173989	0.008147	0.027275	protein_coding
ENSG0000(	ENSG0000(	52.93549	0.353281	0.00815	0.02728	TEC
ENSG0000(	ENSG0000(	11.73489	0.682416	0.008178	0.027372	lncRNA
ENSG0000(	MTCO1P22	20.22065	-0.74168	0.008188	0.0274	processed_pseudogene
ENSG0000(	LINC02293	110.5718	-0.84692	0.008189	0.027401	lncRNA
ENSG0000(	CCR5AS	262.3848	0.169472	0.00819	0.027403	lncRNA
ENSG0000(	UBAP1	775.0211	-0.14386	0.008192	0.027406	protein_coding
ENSG0000(	ENSG0000(	230.9228	-0.17154	0.008201	0.027432	TEC
ENSG0000(	SF3B2	2215.081	-0.11753	0.008202	0.027432	protein_coding
ENSG0000(	CIAO3	1143.882	-0.10569	0.008208	0.02745	protein_coding
ENSG0000(	MYH14	8099.21	-0.3886	0.008209	0.027451	protein_coding

ENSG0000(	HCG20	178.6628	-0.16727	0.00821	0.027452	lncRNA
ENSG0000(	GNA15-DT	12.31645	0.747762	0.008213	0.027457	lncRNA
ENSG0000(	ABCC10	1893.231	-0.10249	0.008223	0.027483	protein_coding
ENSG0000(	FZD3	774.5163	-0.18982	0.008224	0.027483	protein_coding
ENSG0000(	RUSC2	414.3108	0.28348	0.008225	0.027483	protein_coding
ENSG0000(	TMPPE	629.5352	-0.12524	0.008225	0.027483	protein_coding
ENSG0000(	ZG16	3582.8	-0.6229	0.008225	0.027483	protein_coding
ENSG0000(	COA6	148.8379	-0.292	0.008231	0.027501	protein_coding
ENSG0000(	DYDC1	81.77583	-0.2876	0.008241	0.02753	protein_coding
ENSG0000(	ENSG0000(	145.466	0.237742	0.008246	0.027546	lncRNA
ENSG0000(	DPH5	747.3133	-0.15538	0.008252	0.027562	protein_coding
ENSG0000(	TXNL1	1043.739	-0.13933	0.008259	0.027581	protein_coding
ENSG0000(	ENSG0000(	35.77971	0.646531	0.008266	0.027604	transcribed_unprocessed_pseudo
ENSG0000(	ENSG0000(	217.2671	-0.18389	0.00827	0.027612	lncRNA
ENSG0000(	BTN2A1	1228.602	0.139545	0.008277	0.027631	protein_coding
ENSG0000(	KRT20	9335.354	-0.52703	0.008277	0.027631	protein_coding
ENSG0000(	SUGP2	5767.559	0.134481	0.008278	0.027632	protein_coding
ENSG0000(	RPS12P5	37.33547	0.454997	0.008286	0.027655	processed_pseudogene
ENSG0000(	ENSG0000(	63.31651	0.269697	0.008295	0.02768	lncRNA
ENSG0000(	UBE2C	137.9167	-0.34306	0.008295	0.02768	protein_coding
ENSG0000(	ARHGAP23	457.2528	0.399348	0.008301	0.027694	protein_coding
ENSG0000(	MIR605	14.42921	0.613177	0.008301	0.027694	miRNA
ENSG0000(	BMS1P17	101.0631	0.300696	0.008303	0.027696	transcribed_unprocessed_pseudo
ENSG0000(	PPARA	5790.255	-0.19679	0.008306	0.027705	protein_coding
ENSG0000(	ENSG0000(	71.45231	0.355601	0.00831	0.027714	lncRNA
ENSG0000(	RN7SKP124	21.43964	-0.49754	0.008321	0.027745	misc_RNA
ENSG0000(	ZKSCAN2-D	1152.122	0.138456	0.00832	0.027745	lncRNA
ENSG0000(	ENSG0000(	290.9778	0.179852	0.008332	0.027779	lncRNA
ENSG0000(	IGLV3-21	284.9108	0.831957	0.008339	0.027798	IG_V_gene
ENSG0000(	DENND11	3686.552	0.166341	0.008345	0.027818	protein_coding
ENSG0000(	CCL28	2257.299	0.283106	0.008347	0.02782	protein_coding
ENSG0000(	ENSG0000(	35.91874	0.468656	0.008351	0.027825	lncRNA
ENSG0000(	SCARNA4	174.2907	0.241222	0.00835	0.027825	scaRNA
ENSG0000(	STRAP	630.9839	-0.24089	0.008351	0.027825	protein_coding
ENSG0000(	PFDN1	783.9237	-0.26766	0.008354	0.027832	protein_coding
ENSG0000(	ENSG0000(	136.052	0.233415	0.008357	0.027837	processed_pseudogene
ENSG0000(	ANOS1	224.2462	0.434872	0.008367	0.02787	protein_coding
ENSG0000(	ABCE1	990.7848	-0.21087	0.008373	0.027886	protein_coding
ENSG0000(	ENSG0000(	35.76445	0.448169	0.008376	0.027893	lncRNA
ENSG0000(	ENSG0000(	21.69138	0.457181	0.008379	0.027901	lncRNA
ENSG0000(	LAPTM5	2011.04	0.452414	0.008382	0.027905	protein_coding
ENSG0000(	NET1	2866.991	-0.25847	0.008382	0.027905	protein_coding
ENSG0000(	LINC02766	137.6963	0.197108	0.008386	0.027915	lncRNA
ENSG0000(	KRTCAP2	894.318	-0.23487	0.008388	0.027919	protein_coding
ENSG0000(	N4BP2	1876.308	0.159758	0.008393	0.027932	protein_coding
ENSG0000(	PICART1	19.32895	0.504958	0.008407	0.027976	lncRNA
ENSG0000(	GNPTAB	2582.765	0.130623	0.008411	0.027987	protein_coding

ENSG0000(	ENSG0000(	56.07596	-0.27878	0.008416	0.027998	lncRNA
ENSG0000(	PPP4R3B-D	33.78254	-0.37611	0.008417	0.028	lncRNA
ENSG0000(	TRIM33	2527.728	0.089863	0.00842	0.028006	protein_coding
ENSG0000(	SPECC1	5019.447	0.181744	0.008421	0.028008	protein_coding
ENSG0000(	ENSG0000(	46.19562	-0.2657	0.008422	0.028008	lncRNA
ENSG0000(	HHEX	152.8288	0.450519	0.008423	0.028008	protein_coding
ENSG0000(	ZNF398	946.5346	-0.11851	0.008425	0.02801	protein_coding
ENSG0000(	GTF3C4	1086.219	-0.13367	0.008428	0.028018	protein_coding
ENSG0000(	SHANK3	2063.779	0.284798	0.008431	0.028026	protein_coding
ENSG0000(	DFFBP1	38.68461	-0.40007	0.008434	0.028033	processed_pseudogene
ENSG0000(	AKR1C3	601.171	-0.4533	0.008439	0.028045	protein_coding
ENSG0000(	FBXO44	584.3637	0.236823	0.00844	0.028047	protein_coding
ENSG0000(	SH3BP5	798.2726	0.208987	0.008464	0.02812	protein_coding
ENSG0000(	TSPAN10	870.3025	0.120636	0.008464	0.02812	protein_coding
ENSG0000(	ACKR2	661.8539	-0.34326	0.008472	0.028143	protein_coding
ENSG0000(	CLEC12B	25.05799	0.739269	0.00848	0.028163	protein_coding
ENSG0000(	ENSG0000(	15.91992	0.546382	0.008479	0.028163	TEC
ENSG0000(	PRXL2C	888.3925	-0.15623	0.008485	0.028176	protein_coding
ENSG0000(	SLCO1B7	43.94211	0.394172	0.008485	0.028176	transcribed_unprocessed_pseudo
ENSG0000(	EFCAB12	507.6882	0.122303	0.008488	0.028179	protein_coding
ENSG0000(	FOXO4	441.9928	-0.29554	0.008488	0.028179	protein_coding
ENSG0000(	KIZ	600.14	0.23178	0.008497	0.028205	protein_coding
ENSG0000(	ENSG0000(	170.3918	0.248197	0.008504	0.028225	lncRNA
ENSG0000(	ENSG0000(	92.20461	0.288904	0.008507	0.028233	lncRNA
ENSG0000(	ABCC5	4114.454	-0.28105	0.008509	0.028236	protein_coding
ENSG0000(	RPS26P18	16.56769	0.492565	0.008511	0.02824	processed_pseudogene
ENSG0000(	SPINDOC	154.5998	0.271837	0.008513	0.028245	protein_coding
ENSG0000(	ENSG0000(	10.21623	0.894212	0.008518	0.028256	lncRNA
ENSG0000(	TENT5A	1834.479	0.318574	0.008523	0.02827	protein_coding
ENSG0000(	COLEC12	1384.363	-0.1604	0.008527	0.028282	protein_coding
ENSG0000(	MISP	4378.168	-0.42631	0.008531	0.02829	protein_coding
ENSG0000(	ZNF775	89.97302	-0.39878	0.008533	0.028296	protein_coding
ENSG0000(	SLC36A4	544.2487	0.238945	0.008535	0.028299	protein_coding
ENSG0000(	RFXANK	227.6724	-0.21806	0.008536	0.0283	protein_coding
ENSG0000(	ENSG0000(	25.97978	-0.58355	0.008539	0.028307	TEC
ENSG0000(	KIZ-AS1	232.5973	0.185743	0.008543	0.028316	lncRNA
ENSG0000(	DYRK1B	250.022	-0.23713	0.008551	0.028341	protein_coding
ENSG0000(	GTF2H1	1228.007	0.125956	0.008561	0.028369	protein_coding
ENSG0000(	RNF125	1589.555	-0.145	0.008566	0.028383	protein_coding
ENSG0000(	ENSG0000(	173.1679	-0.2111	0.008568	0.028387	lncRNA
ENSG0000(	TMED2-DT	2734.427	-0.07254	0.008569	0.028387	lncRNA
ENSG0000(	ENSG0000(	40.33828	1.163418	0.008578	0.028414	lncRNA
ENSG0000(	FABP4	15.36445	-0.70552	0.008581	0.028421	protein_coding
ENSG0000(	HOXB5	942.806	-0.36054	0.008583	0.028423	protein_coding
ENSG0000(	ENSG0000(	144.392	0.176754	0.008588	0.028437	lncRNA
ENSG0000(	KAT2A	969.5719	-0.20662	0.008608	0.028501	protein_coding
ENSG0000(	ENSG0000(	172.0826	0.188453	0.008612	0.028512	lncRNA

ENSG0000(	ENSG0000(	280.4397	0.187208	0.008614	0.028515	TEC
ENSG0000(	LFNG	868.3092	-0.25061	0.00862	0.028531	protein_coding
ENSG0000(	KRT8P33	98.992	-0.30825	0.008625	0.028547	processed_pseudogene
ENSG0000(	CRPPA	540.987	-0.15761	0.008629	0.028555	protein_coding
ENSG0000(	CASP7	2190.124	-0.30128	0.008632	0.028556	protein_coding
ENSG0000(	ENSG0000(	47.93798	-0.49885	0.008631	0.028556	transcribed_unprocessed_pseudo
ENSG0000(	GGT1	429.7337	-0.38856	0.008631	0.028556	protein_coding
ENSG0000(	GRHPR	1241.145	-0.13881	0.008646	0.028601	protein_coding
ENSG0000(	KIF4A	253.5003	-0.39685	0.008649	0.028606	protein_coding
ENSG0000(	EMC6	74.59	-0.22371	0.008657	0.028626	protein_coding
ENSG0000(	GOLGA8J	111.4573	0.236907	0.008657	0.028626	protein_coding
ENSG0000(	RAB32	159.8018	-0.3035	0.008667	0.028656	protein_coding
ENSG0000(	RNF166	559.1454	0.312384	0.008668	0.028657	protein_coding
ENSG0000(	TRBV29OR9	55.69311	-0.46344	0.008682	0.028703	TR_V_pseudogene
ENSG0000(	BUD13P1	18.30936	-0.4931	0.00869	0.028719	processed_pseudogene
ENSG0000(	ENSG0000(	53.38096	0.285841	0.00869	0.028719	lncRNA
ENSG0000(	FALEC	38.197	-0.29954	0.00869	0.028719	lncRNA
ENSG0000(	COL19A1	537.935	0.289273	0.008695	0.028732	protein_coding
ENSG0000(	ENSG0000(	133.1225	-0.29153	0.008705	0.028753	lncRNA
ENSG0000(	ENSG0000(	15.52516	0.571726	0.008704	0.028753	lncRNA
ENSG0000(	HTRA4	351.5295	0.205178	0.008705	0.028753	protein_coding
ENSG0000(	ZNF154	1241.507	0.233382	0.008705	0.028753	protein_coding
ENSG0000(	FAM240B	60.26614	-0.26715	0.008718	0.028791	protein_coding
ENSG0000(	RGS13	215.2087	-0.5093	0.008718	0.028791	protein_coding
ENSG0000(	CYS1	60.97479	-0.45898	0.008721	0.028793	protein_coding
ENSG0000(	ENSG0000(	26.92227	0.380008	0.00872	0.028793	lncRNA
ENSG0000(	PDE6A	1270.322	0.130809	0.008731	0.028822	protein_coding
ENSG0000(	TULP1	29.26531	0.393901	0.008732	0.028822	protein_coding
ENSG0000(	LINC02371	367.4873	0.141454	0.008734	0.028823	lncRNA
ENSG0000(	OR2A5	640.5839	0.1921	0.008733	0.028823	protein_coding
ENSG0000(	ENSG0000(	193.3255	-0.16505	0.00875	0.028872	lncRNA
ENSG0000(	CRYZP1	70.57771	0.435597	0.008753	0.028875	processed_pseudogene
ENSG0000(	ZPR1	1641.376	0.273443	0.008752	0.028875	protein_coding
ENSG0000(	ZXDA	365.5961	0.17706	0.008753	0.028875	protein_coding
ENSG0000(	ENSG0000(	102.4282	0.250523	0.008755	0.028877	lncRNA
ENSG0000(	ENSG0000(	179.0661	-0.2114	0.008757	0.028883	lncRNA
ENSG0000(	GPR39	553.6443	-0.41952	0.008758	0.028883	protein_coding
ENSG0000(	ENSG0000(	40.03435	-0.33491	0.008767	0.02891	transcribed_unprocessed_pseudo
ENSG0000(	ENSG0000(	982.2831	0.139937	0.008782	0.028953	lncRNA
ENSG0000(	REEP2	165.6119	-0.16056	0.008782	0.028953	protein_coding
ENSG0000(	ENSG0000(	113.0708	-0.26933	0.008787	0.028965	lncRNA
ENSG0000(	LAMTOR3	798.857	-0.27043	0.008791	0.028974	protein_coding
ENSG0000(	PRICKLE2-A	64.3895	0.307283	0.008791	0.028974	lncRNA
ENSG0000(	ENSG0000(	22.42035	0.451901	0.008802	0.029004	lncRNA
ENSG0000(	ENSG0000(	169.3802	-0.22369	0.008804	0.02901	protein_coding
ENSG0000(	SPTSSB	18.60409	0.750679	0.008807	0.029015	protein_coding
ENSG0000(	PRKDC	6324.255	-0.20501	0.008808	0.029016	protein_coding

ENSG0000(	ENSG0000(	35.6732	0.398421	0.008812	0.029027	TEC
ENSG0000(	LINC01558	178.43	-0.19607	0.008819	0.029047	lncRNA
ENSG0000(	CHST10	64.73826	0.460878	0.008823	0.029058	protein_coding
ENSG0000(	ENSG0000(	257.154	0.342584	0.008828	0.029071	lncRNA
ENSG0000(	CARMAL	102.8067	-0.20639	0.008843	0.029117	lncRNA
ENSG0000(	ENSG0000(	646.9504	-0.16807	0.008849	0.029134	lncRNA
ENSG0000(	SETD1A	678.9832	-0.2167	0.008851	0.029137	protein_coding
ENSG0000(	HCAR3	226.6733	4.084951	0.008862	0.029169	protein_coding
ENSG0000(	COQ10A	319.162	0.134117	0.008878	0.029218	protein_coding
ENSG0000(	ENSG0000(	141.0125	-0.20936	0.008878	0.029218	lncRNA
ENSG0000(	ENSG0000(	9.953509	0.696413	0.008885	0.029237	processed_pseudogene
ENSG0000(	CYSTM1	945.8381	-0.34391	0.008888	0.029241	protein_coding
ENSG0000(	LSM2	160.9672	-0.23357	0.008888	0.029241	protein_coding
ENSG0000(	ZBTB7A	3481.143	-0.19831	0.008893	0.029253	protein_coding
ENSG0000(	ENSG0000(	97.40388	-0.2519	0.008897	0.029265	transcribed_processed_pseudoge
ENSG0000(	TMBIM6	8659.722	-0.20328	0.008901	0.029272	protein_coding
ENSG0000(	ZNF770	1226.795	-0.14302	0.008903	0.029276	protein_coding
ENSG0000(	ENSG0000(	534.5384	0.113347	0.008907	0.029286	lncRNA
ENSG0000(	CIPC	1722.594	-0.33033	0.008914	0.029307	protein_coding
ENSG0000(	ZNF682	414.6832	-0.25581	0.008917	0.029313	protein_coding
ENSG0000(	KCNMA1-A	310.6288	0.117512	0.008919	0.029316	lncRNA
ENSG0000(	STIMATE	639.0822	0.251638	0.008923	0.029326	protein_coding
ENSG0000(	LYRM2	1381.351	-0.09669	0.008926	0.029335	protein_coding
ENSG0000(	PDIK1L	528.3199	-0.23804	0.008937	0.029366	protein_coding
ENSG0000(	ENSG0000(	315.4986	-0.21974	0.008945	0.02939	lncRNA
ENSG0000(	ACTR10	679.1047	-0.13946	0.00895	0.029403	protein_coding
ENSG0000(	PTPRU	113.8276	0.56017	0.008951	0.029404	protein_coding
ENSG0000(	LINCR-0001	156.6154	0.1917	0.008955	0.029413	lncRNA
ENSG0000(	ENSG0000(	62.47224	0.333082	0.008957	0.029417	lncRNA
ENSG0000(	MED12L	143.5649	0.285327	0.00896	0.029424	protein_coding
ENSG0000(	GRIP2	146.6802	0.449941	0.008975	0.029469	protein_coding
ENSG0000(	LINC00366	112.8132	0.198614	0.008976	0.029469	lncRNA
ENSG0000(	PRUNE2	3916.57	0.328097	0.00898	0.029482	protein_coding
ENSG0000(	CFAP418-A	107.5713	0.265714	0.008991	0.029513	lncRNA
ENSG0000(	SLC9A2	2559.699	-0.48672	0.008992	0.029513	protein_coding
ENSG0000(	ENSG0000(	972.4415	-0.09616	0.008996	0.029522	protein_coding
ENSG0000(	FN3KRP	498.6762	-0.18176	0.009001	0.029537	protein_coding
ENSG0000(	ENSG0000(	197.4429	0.17398	0.009003	0.02954	lncRNA
ENSG0000(	MAMLD1	129.1411	0.361007	0.009005	0.029544	protein_coding
ENSG0000(	ENSG0000(	297.2737	-0.18991	0.009013	0.029568	lncRNA
ENSG0000(	ACSM6	51.74257	0.290592	0.009023	0.029597	protein_coding
ENSG0000(	FAR2	2291.657	-0.25786	0.009038	0.029644	protein_coding
ENSG0000(	LINC02009	57.93912	0.384767	0.009039	0.029644	lncRNA
ENSG0000(	RIPOR2	2360.328	0.574398	0.00904	0.029644	protein_coding
ENSG0000(	PTPRH	2883.313	-0.3974	0.009046	0.02966	protein_coding
ENSG0000(	ENSG0000(	49.05421	0.276822	0.009051	0.029668	lncRNA
ENSG0000(	ZNF595	781.3444	-0.17512	0.00905	0.029668	protein_coding

ENSG0000(	SYNRG	3972.437	0.12121	0.009054	0.029678	protein_coding
ENSG0000(	LINC02008	114.3717	0.205629	0.009061	0.029695	lncRNA
ENSG0000(	TMEM237	337.6518	0.225506	0.009063	0.029699	protein_coding
ENSG0000(	ENSG0000(	55.04734	0.310811	0.009072	0.029723	lncRNA
ENSG0000(	RALA	915.746	-0.18495	0.009072	0.029723	protein_coding
ENSG0000(	ENSG0000(	9.373382	0.590911	0.009104	0.029824	lncRNA
ENSG0000(	ENSG0000(	20.63265	0.554551	0.009107	0.029828	TEC
ENSG0000(	PALS2	383.4115	0.312498	0.009106	0.029828	protein_coding
ENSG0000(	ODAPH	253.5963	-0.25355	0.009115	0.029851	protein_coding
ENSG0000(	MIR3131	14.80638	-0.69083	0.009117	0.029857	miRNA
ENSG0000(	CHRNA10	109.9096	-0.22762	0.009126	0.029882	protein_coding
ENSG0000(	ENSG0000(	430.9427	0.14515	0.009129	0.029885	lncRNA
ENSG0000(	TMEM238L	598.3801	-0.43016	0.009129	0.029885	protein_coding
ENSG0000(	ENSG0000(	104.9565	-0.2108	0.009138	0.02991	lncRNA
ENSG0000(	ENSG0000(	26.68988	0.494628	0.009144	0.029929	lncRNA
ENSG0000(	N4BP3	138.1389	-0.37188	0.00915	0.029942	protein_coding
ENSG0000(	PARD6G-AS	129.7307	-0.30764	0.00915	0.029942	lncRNA
ENSG0000(	ENSG0000(	333.2045	0.159284	0.009156	0.029958	lncRNA
ENSG0000(	TMED7-TIC	94.7915	-0.36352	0.009161	0.029971	protein_coding
ENSG0000(	C9orf40	147.9129	-0.31619	0.009163	0.029976	protein_coding
ENSG0000(	CCN5	349.6583	0.244495	0.009166	0.02998	protein_coding
ENSG0000(	ENSG0000(	28.4718	0.39922	0.00917	0.029993	lncRNA
ENSG0000(	ACTN1-DT	15.83643	0.635954	0.009182	0.030028	lncRNA
ENSG0000(	GAPDH	7275.676	-0.27582	0.009191	0.030054	protein_coding
ENSG0000(	ADGRA3	3158.236	-0.25684	0.009196	0.030068	protein_coding
ENSG0000(	MAEL	84.93767	-0.28788	0.009197	0.030068	protein_coding
ENSG0000(	RAB30	1299.53	0.283244	0.009201	0.030075	protein_coding
ENSG0000(	ENSG0000(	105.0478	0.245846	0.009203	0.030079	lncRNA
ENSG0000(	LINC02843	178.9312	-0.22398	0.009215	0.030118	lncRNA
ENSG0000(	IFITM3	846.8832	0.275178	0.009235	0.030178	protein_coding
ENSG0000(	KCTD13-DT	315.0969	-0.13618	0.009237	0.030178	lncRNA
ENSG0000(	OR2T7	65.7531	0.286727	0.009237	0.030178	polymorphic_pseudogene
ENSG0000(	TEX264	733.05	-0.17206	0.009239	0.030182	protein_coding
ENSG0000(	ENSG0000(	135.0713	0.228277	0.00924	0.030183	lncRNA
ENSG0000(	ENSG0000(	24.71327	0.504117	0.009242	0.030186	lncRNA
ENSG0000(	ENSG0000(	58.75967	0.289893	0.009254	0.030221	lncRNA
ENSG0000(	TICAM1	454.8035	-0.33296	0.009263	0.030247	protein_coding
ENSG0000(	ENSG0000(	212.5741	0.171911	0.009265	0.030252	lncRNA
ENSG0000(	ENSG0000(	10.78507	0.757234	0.009267	0.030256	unprocessed_pseudogene
ENSG0000(	SERF1A	201.8258	-0.76046	0.009269	0.030256	protein_coding
ENSG0000(	LOXL4	70.01071	0.322482	0.009271	0.030261	protein_coding
ENSG0000(	GINM1	707.1966	-0.15552	0.009287	0.030307	protein_coding
ENSG0000(	ZPBP2	99.1321	0.268261	0.009287	0.030307	protein_coding
ENSG0000(	UBN2	4690.487	0.068788	0.00929	0.030314	protein_coding
ENSG0000(	IFITM3P3	8.715452	-0.6796	0.009291	0.030315	processed_pseudogene
ENSG0000(	ZBTB20	8637.662	0.160603	0.009292	0.030315	protein_coding
ENSG0000(	CCAR1	2627.173	-0.12729	0.009297	0.030321	protein_coding

ENSG0000(	FBXL17	889.8759	-0.19337	0.009296	0.030321	protein_coding
ENSG0000(	PODN	208.5504	0.318018	0.009296	0.030321	protein_coding
ENSG0000(	ENSG0000(	1171.342	0.098963	0.009303	0.030335	lncRNA
ENSG0000(	RPL22P1	234.4881	-0.62553	0.009303	0.030335	processed_pseudogene
ENSG0000(	TRIM62	266.7064	0.184562	0.009308	0.030349	protein_coding
ENSG0000(	PACS1	1801.976	0.213631	0.009311	0.030355	protein_coding
ENSG0000(	ENSG0000(	385.8224	0.135656	0.009313	0.030356	transcribed_unprocessed_pseudo
ENSG0000(	ENSG0000(	65.15517	-0.37384	0.009316	0.030364	TEC
ENSG0000(	KCNQ1	559.4844	-0.28145	0.009323	0.030383	protein_coding
ENSG0000(	GAL3ST4	205.183	-0.2774	0.009327	0.030395	protein_coding
ENSG0000(	ENSG0000(	154.7767	0.196417	0.009331	0.030404	lncRNA
ENSG0000(	SPANXA2-(	1413.737	0.092779	0.009335	0.030414	lncRNA
ENSG0000(	CENPE	806.8261	-0.31439	0.009338	0.030421	protein_coding
ENSG0000(	CFAP45	12.71959	0.658952	0.009343	0.030433	protein_coding
ENSG0000(	SERPINB12	187.7107	0.170328	0.009348	0.030446	protein_coding
ENSG0000(	NMNAT3	16128.92	-0.16484	0.009351	0.030453	protein_coding
ENSG0000(	KIAA1671	9240.778	-0.34319	0.009355	0.03046	protein_coding
ENSG0000(	SLC35A2	341.8898	-0.2253	0.009355	0.03046	protein_coding
ENSG0000(	CD24	25323.6	-0.38283	0.009365	0.030487	protein_coding
ENSG0000(	RNU1-91P	52.0523	0.37454	0.00937	0.0305	snRNA
ENSG0000(	ENSG0000(	142.3813	0.183	0.009391	0.030567	lncRNA
ENSG0000(	ADCY3	761.915	0.265517	0.009393	0.03057	protein_coding
ENSG0000(	AZGP1	80.37814	-0.38448	0.009407	0.030611	protein_coding
ENSG0000(	CHAMP1	785.9721	-0.18487	0.009412	0.030625	protein_coding
ENSG0000(	ACSF2	1317.502	-0.39121	0.009415	0.030633	protein_coding
ENSG0000(	RN7SKP151	39.30949	0.39281	0.009416	0.030633	misc_RNA
ENSG0000(	ENSG0000(	52.65337	-0.37085	0.009419	0.03064	lncRNA
ENSG0000(	HPAT5	106.8075	0.196716	0.009431	0.030675	lncRNA
ENSG0000(	LRRN3	90.51718	-0.58511	0.009434	0.030681	protein_coding
ENSG0000(	LINC00539	113.2178	0.294784	0.009446	0.030715	lncRNA
ENSG0000(	UBL5	777.6873	-0.25795	0.009446	0.030715	protein_coding
ENSG0000(	GALE	848.4629	-0.29798	0.00945	0.030723	protein_coding
ENSG0000(	ENSG0000(	19.56174	0.508871	0.009454	0.030732	lncRNA
ENSG0000(	SLC4A9	63.30171	-0.82862	0.009454	0.030732	protein_coding
ENSG0000(	RAB14	2077.699	-0.1719	0.009456	0.030732	protein_coding
ENSG0000(	CHSY3	48.34166	0.54781	0.009457	0.030732	protein_coding
ENSG0000(	TYRP1	13.97946	1.021721	0.009458	0.030732	protein_coding
ENSG0000(	GLB1	722.5074	0.175619	0.009469	0.030767	protein_coding
ENSG0000(	ENSG0000(	342.3269	0.215355	0.009473	0.030776	lncRNA
ENSG0000(	RAP2A	925.5217	0.178938	0.009474	0.030776	protein_coding
ENSG0000(	CYP24A1	50.8235	1.157135	0.009478	0.030787	protein_coding
ENSG0000(	LINC01588	1960.79	0.146397	0.009484	0.030801	lncRNA
ENSG0000(	PRKCH-AS1	503.173	0.16077	0.009487	0.030804	lncRNA
ENSG0000(	RPL9P16	121.8003	0.375567	0.009487	0.030804	processed_pseudogene
ENSG0000(	KDM5B	5518.824	-0.09376	0.009488	0.030805	protein_coding
ENSG0000(	COPS2	1082.366	-0.137	0.009492	0.030814	protein_coding
ENSG0000(	GAPVD1	3002.447	0.103663	0.009494	0.030819	protein_coding

ENSG0000(	ENSG0000(	322.0304	0.400796	0.009495	0.030819	lncRNA
ENSG0000(	EXD1	242.1633	-0.17124	0.009497	0.030823	protein_coding
ENSG0000(	ABI3	386.9485	0.265915	0.009506	0.030842	protein_coding
ENSG0000(	IL22RA2	24.77371	1.041606	0.009506	0.030842	protein_coding
ENSG0000(	TRIQK	670.5815	-0.16487	0.009506	0.030842	protein_coding
ENSG0000(	KIF17	45.12551	0.419806	0.009509	0.030847	protein_coding
ENSG0000(	HCFC1R1	99.97418	-0.24455	0.009514	0.03086	protein_coding
ENSG0000(	IP6K2	2826.096	-0.20304	0.009515	0.03086	protein_coding
ENSG0000(	PDCD10	604.264	-0.18536	0.009517	0.030865	protein_coding
ENSG0000(	CEACAM3	447.7007	0.38459	0.009525	0.030882	protein_coding
ENSG0000(	ENSG0000(	189.3238	-0.16985	0.009524	0.030882	lncRNA
ENSG0000(	FAM83E	4152.314	-0.32511	0.009525	0.030882	protein_coding
ENSG0000(	ENSG0000(	103.5701	0.319506	0.009529	0.030891	lncRNA
ENSG0000(	ADNP	3849.614	-0.10228	0.009533	0.030901	protein_coding
ENSG0000(	KLC4	1248.641	-0.17588	0.009536	0.030907	protein_coding
ENSG0000(	ENSG0000(	19.17247	0.476788	0.009546	0.030937	lncRNA
ENSG0000(	ICMT	1277.383	-0.20118	0.009547	0.030937	protein_coding
ENSG0000(	ENSG0000(	98.5329	0.209753	0.009557	0.03096	lncRNA
ENSG0000(	ENSG0000(	494.1998	-0.29336	0.009559	0.03096	lncRNA
ENSG0000(	FBXO16	73.44985	0.264775	0.009559	0.03096	protein_coding
ENSG0000(	GAB3	669.2183	0.226205	0.009557	0.03096	protein_coding
ENSG0000(	LINC00200	165.4744	0.220828	0.009556	0.03096	lncRNA
ENSG0000(	FKBPL	45.99765	-0.35189	0.009566	0.030979	protein_coding
ENSG0000(	FAXDC2	709.4625	0.161112	0.009569	0.030986	protein_coding
ENSG0000(	FLJ46284	203.1337	0.230072	0.009571	0.030987	lncRNA
ENSG0000(	IDE	2594.706	-0.12935	0.009585	0.03103	protein_coding
ENSG0000(	ALDH3B1	719.8554	0.221632	0.0096	0.031077	protein_coding
ENSG0000(	BLZF2P	90.05982	0.351945	0.009606	0.031093	unprocessed_pseudogene
ENSG0000(	HNRNPA1P	53.30294	0.302601	0.009609	0.031099	processed_pseudogene
ENSG0000(	ENSG0000(	25.64777	-0.48626	0.009622	0.031138	protein_coding
ENSG0000(	ENSG0000(	1313.335	0.135864	0.009624	0.031138	TEC
ENSG0000(	TMEM100	76.9408	-0.45088	0.009624	0.031138	protein_coding
ENSG0000(	SLC17A8	52.33953	-0.66929	0.009629	0.031149	protein_coding
ENSG0000(	SNORA84	67.64047	-0.33557	0.00964	0.031182	snoRNA
ENSG0000(	ENSG0000(	136.5692	-0.25579	0.009641	0.031183	lncRNA
ENSG0000(	NADK	2363.582	-0.13982	0.009647	0.031198	protein_coding
ENSG0000(	PPIAP11	9.694055	0.717373	0.009648	0.031198	processed_pseudogene
ENSG0000(	ENSG0000(	114.8536	0.19346	0.009651	0.031201	lncRNA
ENSG0000(	HSPA12A	2121.615	0.142214	0.009651	0.031201	protein_coding
ENSG0000(	MRPS28	437.8626	-0.1368	0.009652	0.031201	protein_coding
ENSG0000(	RN7SL543F	28.42128	0.34622	0.009661	0.031229	misc_RNA
ENSG0000(	CEP85L	1106.757	0.3401	0.009668	0.031248	protein_coding
ENSG0000(	ZDHHC17	1507.903	0.182162	0.009682	0.031289	protein_coding
ENSG0000(	SEC61G-DT	171.7384	-0.18296	0.009685	0.031295	lncRNA
ENSG0000(	USP45	925.3749	-0.15315	0.009692	0.031315	protein_coding
ENSG0000(	TBCCD1	728.3063	-0.17412	0.0097	0.031339	protein_coding
ENSG0000(	OPLAH	449.6619	-0.37112	0.009703	0.031344	protein_coding

ENSG0000(	FTH1P11	32.55426	0.508622	0.009707	0.031352	processed_pseudogene
ENSG0000(	SLC9A3	21106.88	-0.70106	0.009707	0.031352	protein_coding
ENSG0000(	NR1D1	177.158	-0.51144	0.00971	0.031356	protein_coding
ENSG0000(	LINC02397	760.4221	0.656027	0.00972	0.031385	lncRNA
ENSG0000(	PHC2	1123.185	0.239334	0.009727	0.031407	protein_coding
ENSG0000(	PTRH1	333.8157	0.164246	0.009734	0.031423	protein_coding
ENSG0000(	TTC14	3235.776	0.115463	0.009734	0.031423	protein_coding
ENSG0000(	ENSG0000(	51.7422	0.343279	0.009736	0.031426	lncRNA
ENSG0000(	SLC25A43	610.5429	-0.17861	0.009738	0.031426	protein_coding
ENSG0000(	VPS35	2348.249	-0.13252	0.009746	0.031451	protein_coding
ENSG0000(	ENSG0000(	287.8357	0.159265	0.009766	0.031511	lncRNA
ENSG0000(	MMP24	20.43371	0.623583	0.00977	0.031523	protein_coding
ENSG0000(	ENSG0000(	350.5847	-0.25835	0.009772	0.031525	lncRNA
ENSG0000(	DBNDD1	72.03459	0.436184	0.009776	0.031533	protein_coding
ENSG0000(	CYP2C19	1162.686	-0.37767	0.009777	0.031535	protein_coding
ENSG0000(	RNU5E-3P	10.34792	0.765925	0.009785	0.031555	snRNA
ENSG0000(	ENSG0000(	370.552	-0.24934	0.009788	0.03156	transcribed_unprocessed_pseudo
ENSG0000(	MIR3140	22.15022	0.497758	0.009788	0.03156	miRNA
ENSG0000(	WDR35	391.7778	0.287676	0.009791	0.031568	protein_coding
ENSG0000(	ENSG0000(	33.83033	0.332865	0.009795	0.031577	lncRNA
ENSG0000(	MRPS17P1	583.5015	-0.15685	0.009798	0.031584	processed_pseudogene
ENSG0000(	ENSG0000(	45.4776	0.379437	0.009801	0.03159	lncRNA
ENSG0000(	RALY	2820.856	-0.15784	0.009803	0.031593	protein_coding
ENSG0000(	SNRPG	292.4394	-0.18885	0.009806	0.031598	protein_coding
ENSG0000(	ENSG0000(	72.16112	-0.24593	0.009809	0.031603	lncRNA
ENSG0000(	KCND1	120.6339	0.304509	0.009809	0.031603	protein_coding
ENSG0000(	POF1B	2883.648	-0.35655	0.009814	0.031615	protein_coding
ENSG0000(	PKN2-AS1	339.4997	-0.1554	0.009818	0.031625	lncRNA
ENSG0000(	ENSG0000(	12.83723	0.45187	0.00983	0.03166	lncRNA
ENSG0000(	ENSG0000(	3828.428	-0.07501	0.009841	0.031686	transcribed_unprocessed_pseudo
ENSG0000(	ENSG0000(	43.12351	-0.58156	0.00984	0.031686	lncRNA
ENSG0000(	WASHC5-A	14.7829	0.528001	0.009841	0.031686	lncRNA
ENSG0000(	ARHGAP27	5696.559	-0.19483	0.009843	0.031689	protein_coding
ENSG0000(	FAM9C	417.7447	-0.16835	0.009845	0.031693	protein_coding
ENSG0000(	BRAT1	741.3854	-0.16763	0.009851	0.031706	protein_coding
ENSG0000(	MT1JP	34.07559	0.772399	0.009857	0.031722	transcribed_unprocessed_pseudo
ENSG0000(	ENSG0000(	11.48145	0.489434	0.009861	0.031734	snoRNA
ENSG0000(	SMIM17	255.9962	-0.19538	0.009868	0.031754	protein_coding
ENSG0000(	SLC35F4	42.68567	0.381712	0.009875	0.031771	protein_coding
ENSG0000(	ENSG0000(	176.729	-0.18502	0.009876	0.031773	lncRNA
ENSG0000(	SNORD3B-2	105.7125	0.552543	0.009879	0.031778	snoRNA
ENSG0000(	EAF2	284.3469	0.295958	0.009885	0.031792	protein_coding
ENSG0000(	UBE2S	199.3962	-0.28257	0.009895	0.031821	protein_coding
ENSG0000(	CD8B	148.8828	-0.54075	0.009902	0.031842	protein_coding
ENSG0000(	RN7SL842P	46.81418	0.313881	0.009905	0.031847	misc_RNA
ENSG0000(	LINC01634	102.1982	-0.25674	0.009906	0.03185	lncRNA
ENSG0000(	ENSG0000(	78.7089	0.37862	0.009921	0.031892	lncRNA

ENSG0000(	WFDC11	123.0354	0.224224	0.009926	0.031907	protein_coding
ENSG0000(	HMGB1P3	49.74162	-0.52238	0.009932	0.031923	processed_pseudogene
ENSG0000(	GRIA3	177.2266	-0.45829	0.009935	0.031927	protein_coding
ENSG0000(	DDX50	471.9959	-0.21531	0.009941	0.031944	protein_coding
ENSG0000(	IGHV3-11	356.4551	0.566977	0.009956	0.031989	IG_V_gene
ENSG0000(	SLC6A2	42.2263	0.360333	0.00996	0.031998	protein_coding
ENSG0000(	OPTN	1797.746	0.210273	0.009962	0.032002	protein_coding
ENSG0000(	LINC00368	53.46295	0.260578	0.009965	0.03201	lncRNA
ENSG0000(	ENSG0000(	24.09591	0.461213	0.009968	0.032015	lncRNA
ENSG0000(	ENSG0000(	161.7108	0.208419	0.009971	0.032021	lncRNA
ENSG0000(	AMD1P3	22.13411	0.462237	0.009977	0.032039	processed_pseudogene
ENSG0000(	ENSG0000(	44.10246	0.313388	0.00998	0.032042	lncRNA
ENSG0000(	ENSG0000(	19.81459	0.614017	0.009986	0.032057	lncRNA
ENSG0000(	FAM8A1	1250.566	0.203912	0.009985	0.032057	protein_coding
ENSG0000(	SHH	284.1794	0.394672	0.009988	0.032059	protein_coding
ENSG0000(	ENSG0000(	120.2256	-0.291	0.009989	0.032061	lncRNA
ENSG0000(	SNAP25-AS	725.3007	-0.16705	0.009991	0.032062	lncRNA
ENSG0000(	MZT2B	209.3534	-0.23244	0.010005	0.032105	protein_coding
ENSG0000(	RFX4	130.9877	0.290125	0.010016	0.032133	protein_coding
ENSG0000(	ZNF774	1512.151	-0.17674	0.010016	0.032133	protein_coding
ENSG0000(	ENSG0000(	4565.316	0.20175	0.010025	0.03216	lncRNA
ENSG0000(	SLC38A4	403.0186	-0.795	0.010032	0.032178	protein_coding
ENSG0000(	OR4D1	142.5819	-0.14611	0.010049	0.032229	protein_coding
ENSG0000(	CHRNA2	72.44469	0.356717	0.010059	0.032258	protein_coding
ENSG0000(	RNF44	1841.142	-0.15705	0.010066	0.032277	protein_coding
ENSG0000(	ENSG0000(	311.2514	0.154	0.010078	0.032312	lncRNA
ENSG0000(	MVP	5897.417	-0.19013	0.010085	0.032327	protein_coding
ENSG0000(	THTPA	403.9486	-0.16988	0.010085	0.032327	protein_coding
ENSG0000(	RPS3AP29	21.42303	-0.53939	0.010089	0.032337	processed_pseudogene
ENSG0000(	FRMD6	473.9141	0.319413	0.010094	0.032351	protein_coding
ENSG0000(	ENSG0000(	159.5437	0.210047	0.010113	0.032407	protein_coding
ENSG0000(	ZNF503-AS	24.81702	0.409441	0.010118	0.032419	lncRNA
ENSG0000(	AARS2	990.7714	-0.1572	0.010121	0.032426	protein_coding
ENSG0000(	ENSG0000(	108.0411	0.335433	0.010142	0.032488	lncRNA
ENSG0000(	ENSG0000(	273.0975	-0.14408	0.010143	0.032488	lncRNA
ENSG0000(	TNFSF11	69.42838	0.719723	0.010143	0.032488	protein_coding
ENSG0000(	ENSG0000(	40.81883	-0.3682	0.010146	0.032493	lncRNA
ENSG0000(	RAD18	708.1219	-0.20831	0.010148	0.032495	protein_coding
ENSG0000(	KCNN1	156.2624	0.200218	0.01015	0.032498	protein_coding
ENSG0000(	LINC01447	255.3805	0.143168	0.010157	0.032517	lncRNA
ENSG0000(	ENSG0000(	177.1658	0.194729	0.010164	0.032533	lncRNA
ENSG0000(	LINC00963	2544.89	0.12215	0.010164	0.032533	lncRNA
ENSG0000(	ENSG0000(	314.2699	0.148728	0.010169	0.032547	lncRNA
ENSG0000(	ENSG0000(	37.09067	0.30898	0.010171	0.03255	lncRNA
ENSG0000(	RRP7BP	1008.864	-0.1955	0.010181	0.032579	transcribed_unprocessed_pseudo
ENSG0000(	NPIPB13	1277.683	0.235142	0.010185	0.032587	protein_coding
ENSG0000(	PPM1H	357.6084	0.312139	0.010188	0.032595	protein_coding

ENSG0000(	HOXA5	640.3519	-0.32822	0.010192	0.032604	protein_coding
ENSG0000(	CYP2AB1P	26.29763	0.396206	0.010196	0.032612	transcribed_unitary_pseudogene
ENSG0000(	ENSG0000(	2046.392	0.290241	0.010211	0.032656	TEC
ENSG0000(	ENSG0000(	703.8649	-0.10598	0.010211	0.032656	lncRNA
ENSG0000(	TFAP2E	122.2757	0.301841	0.010213	0.032657	protein_coding
ENSG0000(	FCAMR	61.43097	-0.73197	0.010218	0.03267	protein_coding
ENSG0000(	ENSG0000(	112.4131	0.20437	0.010219	0.03267	processed_pseudogene
ENSG0000(	IGKV3D-20	84.96308	0.75582	0.010228	0.03269	IG_V_gene
ENSG0000(	RNU6-343F	16.35423	0.468736	0.010228	0.03269	snRNA
ENSG0000(	RPS6KB1	1641.612	-0.0995	0.010228	0.03269	protein_coding
ENSG0000(	CCDC15	208.9396	-0.21887	0.010231	0.032695	protein_coding
ENSG0000(	CAMK2D	2847.458	-0.16322	0.010235	0.032705	protein_coding
ENSG0000(	ENSG0000(	248.0156	-0.22753	0.010237	0.032707	lncRNA
ENSG0000(	SLC38A10	2637.943	-0.12266	0.010239	0.03271	protein_coding
ENSG0000(	ENSG0000(	64.32149	0.250695	0.010242	0.032717	processed_pseudogene
ENSG0000(	CGRRF1	200.3335	-0.21914	0.010253	0.03275	protein_coding
ENSG0000(	CSPG5	19.8379	0.621002	0.010264	0.032779	protein_coding
ENSG0000(	MORN2	38.75539	-0.3402	0.010266	0.032783	protein_coding
ENSG0000(	CYB5R4	3191.483	0.109754	0.01027	0.032788	protein_coding
ENSG0000(	PKNOX2	844.3427	0.366509	0.010269	0.032788	protein_coding
ENSG0000(	FOXP4	970.7218	-0.18941	0.010275	0.032803	protein_coding
ENSG0000(	ZNF320	2717.176	-0.14483	0.010287	0.032838	protein_coding
ENSG0000(	DNASE1	2134.619	-0.12548	0.010289	0.032841	protein_coding
ENSG0000(	GBP6	433.4686	-0.12087	0.010293	0.032851	protein_coding
ENSG0000(	KLRK1	516.733	-0.31715	0.010295	0.032854	protein_coding
ENSG0000(	FAM204BP	83.61053	-0.38316	0.010303	0.032874	processed_pseudogene
ENSG0000(	ZNF571	654.3158	0.167832	0.010311	0.032896	protein_coding
ENSG0000(	BSG-AS1	50.32147	-0.46761	0.010321	0.032925	lncRNA
ENSG0000(	ENSG0000(	35.58875	0.377843	0.010326	0.032939	TEC
ENSG0000(	TMEM229E	473.4664	0.286962	0.010335	0.032963	protein_coding
ENSG0000(	DNM1L	1934.97	-0.12484	0.010337	0.032964	protein_coding
ENSG0000(	FAHD2B	172.1012	0.223776	0.010337	0.032964	protein_coding
ENSG0000(	POLM	1101.589	0.113646	0.010352	0.033008	protein_coding
ENSG0000(	P3H4	728.4286	0.136267	0.01036	0.033031	protein_coding
ENSG0000(	ABHD15	681.7517	-0.13879	0.01037	0.033057	protein_coding
ENSG0000(	ISG20	2224.176	0.361525	0.010373	0.033064	protein_coding
ENSG0000(	OR4F17	2217.064	-0.22323	0.010375	0.033068	protein_coding
ENSG0000(	PTPRF	10410.82	-0.37206	0.010377	0.033069	protein_coding
ENSG0000(	IGKV3-11	1131.628	0.714569	0.010378	0.03307	IG_V_gene
ENSG0000(	RANBP10	1115.227	-0.09641	0.010398	0.03313	protein_coding
ENSG0000(	ENSG0000(	316.8303	0.162881	0.010404	0.033148	lncRNA
ENSG0000(	ENSG0000(	13.07222	0.827474	0.010413	0.033171	lncRNA
ENSG0000(	METTL2B	2802.738	-0.10427	0.010416	0.033177	protein_coding
ENSG0000(	ZMAT3	1428.282	0.184772	0.010422	0.033194	protein_coding
ENSG0000(	ENSG0000(	84.37171	0.250811	0.010424	0.033197	lncRNA
ENSG0000(	ENSG0000(	1854.996	0.101423	0.010425	0.033197	lncRNA
ENSG0000(	LPA	15.61604	-0.76292	0.01044	0.033242	protein_coding

ENSG0000(	CSF1R	983.2302	0.231568	0.010444	0.033247	protein_coding
ENSG0000(	SNORA2A	49.28124	0.271721	0.010444	0.033247	snoRNA
ENSG0000(	BCAR1	1919.917	-0.21655	0.010451	0.033265	protein_coding
ENSG0000(	ENSG0000(	777.0396	-0.18644	0.010453	0.03327	transcribed_unprocessed_pseudo
ENSG0000(	RPS16	2280.829	-0.26093	0.010458	0.033278	protein_coding
ENSG0000(	WAS	273.6055	0.334723	0.010457	0.033278	protein_coding
ENSG0000(	SLX1A-SUL1	488.8505	-0.48071	0.010464	0.033294	lncRNA
ENSG0000(	DHX57	961.0293	0.140716	0.010466	0.033296	protein_coding
ENSG0000(	ENSG0000(	10.51587	1.016011	0.010468	0.033299	TEC
ENSG0000(	RELB	519.6454	0.279497	0.010483	0.033344	protein_coding
ENSG0000(	RPSAP21	28.05481	-0.41988	0.010484	0.033344	processed_pseudogene
ENSG0000(	RNU6-268F	35.80649	0.459458	0.010486	0.033348	snRNA
ENSG0000(	ENSG0000(	65.33458	0.267228	0.010505	0.033405	lncRNA
ENSG0000(	PLCXD1	2043.653	-0.22463	0.010508	0.033409	protein_coding
ENSG0000(	ENSG0000(	109.7714	0.232804	0.010511	0.033416	lncRNA
ENSG0000(	RNU6-5P	1077.397	0.363064	0.010513	0.033419	snRNA
ENSG0000(	EDN2	60.32939	-0.90068	0.010517	0.033427	protein_coding
ENSG0000(	ENSG0000(	44.55786	0.328344	0.010523	0.033442	lncRNA
ENSG0000(	ENSG0000(	76.77195	-0.55641	0.010528	0.033457	lncRNA
ENSG0000(	KCTD1	312.9699	0.182764	0.010533	0.03347	protein_coding
ENSG0000(	MRPS2	251.6329	-0.20834	0.010542	0.033494	protein_coding
ENSG0000(	ZSCAN31	226.8738	-0.41439	0.010545	0.0335	protein_coding
ENSG0000(	OLFML3	145.5423	0.384237	0.010558	0.033539	protein_coding
ENSG0000(	CAPZA1	3525.048	0.149802	0.010582	0.03361	protein_coding
ENSG0000(	ARHGAP11	805.6932	-0.29642	0.010599	0.033655	protein_coding
ENSG0000(	ARMC6	363.1481	-0.17891	0.010598	0.033655	protein_coding
ENSG0000(	TBC1D26	40.25661	0.547894	0.010599	0.033655	protein_coding
ENSG0000(	ICAM5	38.25543	0.496997	0.010602	0.033662	protein_coding
ENSG0000(	CLDN23	833.5222	-0.5606	0.010613	0.033691	protein_coding
ENSG0000(	CBWD5	2262.86	0.144469	0.010615	0.033694	protein_coding
ENSG0000(	MIA2	3106.41	-0.1721	0.010616	0.033694	protein_coding
ENSG0000(	C1orf141	56.61085	-0.34663	0.01062	0.033705	protein_coding
ENSG0000(	AGAP1	3110.885	-0.24459	0.010636	0.033751	protein_coding
ENSG0000(	ENSG0000(	124.0242	-0.25133	0.010642	0.033767	lncRNA
ENSG0000(	L3HYPDH	568.8998	0.161157	0.010645	0.033773	protein_coding
ENSG0000(	FGF13-AS1	46.40376	0.252636	0.010658	0.033807	lncRNA
ENSG0000(	RNU6-1176	32.38795	0.432763	0.010658	0.033807	snRNA
ENSG0000(	ENSG0000(	47.49637	-0.36427	0.010671	0.033846	lncRNA
ENSG0000(	ENSG0000(	97.89535	-0.27243	0.010672	0.033846	TEC
ENSG0000(	ARHGEF2-A	142.1438	0.222648	0.010705	0.033938	lncRNA
ENSG0000(	ENSG0000(	936.4501	-0.46122	0.010705	0.033938	lncRNA
ENSG0000(	ENSG0000(	2194.169	0.113924	0.010703	0.033938	TEC
ENSG0000(	RAI1	1402.757	0.184798	0.010707	0.033942	protein_coding
ENSG0000(	SDCBP2	1459.456	-0.45444	0.010709	0.033946	protein_coding
ENSG0000(	CEP295NL	179.4083	0.292104	0.010718	0.033971	protein_coding
ENSG0000(	TFG	3927.388	0.127967	0.01072	0.033972	protein_coding
ENSG0000(	ENSG0000(	17.87378	0.485526	0.010722	0.033976	lncRNA

ENSG0000(	SELENOT	1062.925	-0.19369	0.010727	0.033988	protein_coding
ENSG0000(	EIPR1	454.5989	-0.16178	0.010735	0.03401	protein_coding
ENSG0000(	JDP2-AS1	64.18861	0.279655	0.010736	0.034012	lncRNA
ENSG0000(	CSPG4P11	414.6719	0.258238	0.010739	0.034017	transcribed_unprocessed_pseudo
ENSG0000(	CPEB1-AS1	807.9139	-0.12163	0.010755	0.034063	lncRNA
ENSG0000(	HLA-F-AS1	186.5706	0.270478	0.010756	0.034064	lncRNA
ENSG0000(	BBS7-DT	1095.013	0.156922	0.010765	0.034083	lncRNA
ENSG0000(	LUCAT1	2066.39	0.34146	0.010764	0.034083	lncRNA
ENSG0000(	ENY2	888.4555	-0.1158	0.010767	0.034087	protein_coding
ENSG0000(	ZNF655	6201.721	0.086169	0.010778	0.03412	protein_coding
ENSG0000(	TRPC3	147.4996	0.380619	0.010782	0.03413	protein_coding
ENSG0000(	ZNF30	126.2063	-0.26945	0.010785	0.034135	protein_coding
ENSG0000(	SMAD7	788.4698	-0.25647	0.010795	0.034162	protein_coding
ENSG0000(	HDC	305.8097	0.516272	0.010806	0.034194	protein_coding
ENSG0000(	GNA11	4214.441	-0.32245	0.010808	0.034197	protein_coding
ENSG0000(	ENSG0000(	206.2432	-0.23158	0.01082	0.034233	lncRNA
ENSG0000(	ENSG0000(	149.3795	-0.20326	0.01083	0.034259	processed_pseudogene
ENSG0000(	ENSG0000(	227.9582	-0.46068	0.010831	0.03426	lncRNA
ENSG0000(	MIR4728	12.61899	-0.60741	0.010834	0.034266	miRNA
ENSG0000(	PTCHD3	61.64464	-0.25725	0.010841	0.034285	polymorphic_pseudogene
ENSG0000(	ENSG0000(	47.37294	-0.3015	0.010849	0.034306	lncRNA
ENSG0000(	MRPL40	155.4106	-0.18295	0.01085	0.034306	protein_coding
ENSG0000(	GGA3	1610.815	-0.11277	0.010854	0.034315	protein_coding
ENSG0000(	ERN2	3565.299	-0.34	0.010864	0.034344	protein_coding
ENSG0000(	ANKRD44	3398.802	0.350529	0.010868	0.034353	protein_coding
ENSG0000(	ENSG0000(	35.13532	0.349741	0.010875	0.034369	lncRNA
ENSG0000(	LRP11	574.0276	-0.22584	0.010877	0.034373	protein_coding
ENSG0000(	ENSG0000(	82.45827	0.244713	0.010879	0.034376	lncRNA
ENSG0000(	TRIM16	724.0834	-0.17439	0.010888	0.034396	protein_coding
ENSG0000(	ZNF782	991.0758	-0.13831	0.010887	0.034396	protein_coding
ENSG0000(	ENSG0000(	26.35527	-0.45705	0.010891	0.034404	lncRNA
ENSG0000(	ENSG0000(	965.3579	-0.14112	0.010894	0.03441	transcribed_unprocessed_pseudo
ENSG0000(	OIP5-AS1	6607.809	-0.07939	0.010911	0.034459	lncRNA
ENSG0000(	SYN1	12.96701	0.793971	0.010917	0.034474	protein_coding
ENSG0000(	ENSG0000(	234.3778	0.155841	0.010926	0.034499	lncRNA
ENSG0000(	ENSG0000(	50.67694	0.27434	0.010933	0.034519	TEC
ENSG0000(	LINC01104	105.3523	0.222943	0.010936	0.034523	lncRNA
ENSG0000(	CDH10	19.61722	-0.42938	0.010953	0.034571	protein_coding
ENSG0000(	NUDT16	2037.029	0.200634	0.010952	0.034571	protein_coding
ENSG0000(	ENSG0000(	47.07204	0.467067	0.010955	0.034574	lncRNA
ENSG0000(	MTRNR2L1	55.67559	-0.69164	0.010956	0.034574	protein_coding
ENSG0000(	MIR663AH(	61466.83	-0.52056	0.010962	0.034588	lncRNA
ENSG0000(	TNFRSF14	3300.363	-0.12072	0.010971	0.034615	protein_coding
ENSG0000(	CFDP1	835.3265	-0.12182	0.010977	0.034629	protein_coding
ENSG0000(	IGLC1	3578.191	0.659149	0.01098	0.034634	IG_C_gene
ENSG0000(	DEPDC1B	166.253	-0.25027	0.010986	0.034652	protein_coding
ENSG0000(	MSL3P1	37.06422	-0.74383	0.01099	0.03466	transcribed_processed_pseudoge

ENSG0000(	RNF34	1265.182	0.093579	0.010994	0.034669	protein_coding
ENSG0000(	TAMM41	1043.535	-0.12985	0.010998	0.034677	protein_coding
ENSG0000(	RN7SL558F	143.4846	0.211147	0.011003	0.034691	misc_RNA
ENSG0000(	ALG1	1856.446	-0.06793	0.011011	0.034713	protein_coding
ENSG0000(	ARMC3	49.52649	0.373645	0.011019	0.034733	protein_coding
ENSG0000(	SAP18	867.9548	-0.17487	0.011022	0.034741	protein_coding
ENSG0000(	ENSG0000(	74.82921	0.358424	0.011028	0.034755	lncRNA
ENSG0000(	SYNE2	17892.17	-0.13552	0.011034	0.034769	protein_coding
ENSG0000(	PER3	1211.009	-0.43307	0.011036	0.034774	protein_coding
ENSG0000(	ENSG0000(	20.81773	0.741818	0.011044	0.034793	lncRNA
ENSG0000(	ENSG0000(	192.4062	-1.36206	0.011047	0.034793	lncRNA
ENSG0000(	EVI2A	388.6494	0.366355	0.011045	0.034793	protein_coding
ENSG0000(	MRPL34	383.7142	-0.16676	0.011046	0.034793	protein_coding
ENSG0000(	SYNPR	25.51463	0.494519	0.011048	0.034793	protein_coding
ENSG0000(	NTNG1	43.95091	0.693338	0.011056	0.034816	protein_coding
ENSG0000(	LINC00957	839.1029	0.136692	0.01106	0.034825	lncRNA
ENSG0000(	AGAP5	758.1192	0.138925	0.011063	0.034826	protein_coding
ENSG0000(	ZCCHC8	1865.56	-0.08208	0.011063	0.034826	protein_coding
ENSG0000(	ENSG0000(	662.0987	0.203664	0.011066	0.034834	lncRNA
ENSG0000(	POLG-DT	11.62482	-0.55972	0.011068	0.034836	lncRNA
ENSG0000(	AMN	1702.294	-0.45213	0.011072	0.034844	protein_coding
ENSG0000(	CELF2	2780.32	0.234376	0.011075	0.034844	protein_coding
ENSG0000(	ENSG0000(	50.10058	0.279981	0.011073	0.034844	lncRNA
ENSG0000(	NCAPG2	709.1968	-0.28229	0.011075	0.034844	protein_coding
ENSG0000(	RNU6-979F	17.37887	0.573728	0.011077	0.034847	snRNA
ENSG0000(	LINC02660	41.67361	-0.3004	0.011102	0.034917	lncRNA
ENSG0000(	TMSB15B-/	159.0381	0.249601	0.011101	0.034917	lncRNA
ENSG0000(	SYTL1	920.0312	0.233097	0.01111	0.034939	protein_coding
ENSG0000(	ENSG0000(	442.8335	-0.13961	0.011113	0.034943	lncRNA
ENSG0000(	LINC02345	30.84464	0.461461	0.011113	0.034943	lncRNA
ENSG0000(	OR2A1	19.49695	0.876933	0.011115	0.034944	protein_coding
ENSG0000(	ENSG0000(	112.0843	0.296453	0.011122	0.034963	lncRNA
ENSG0000(	RGS17P1	111.0256	-0.23675	0.011128	0.034978	processed_pseudogene
ENSG0000(	MRPS18C	833.7416	-0.1197	0.011132	0.034988	protein_coding
ENSG0000(	TNRC6A	5271.107	0.086231	0.011141	0.035012	protein_coding
ENSG0000(	LGI1	410.4465	0.251718	0.011157	0.035058	protein_coding
ENSG0000(	TMEM94	1854.571	-0.22837	0.011158	0.035058	protein_coding
ENSG0000(	ENSG0000(	69.77124	0.244199	0.011159	0.035058	lncRNA
ENSG0000(	UBC	5597.465	-0.2641	0.011183	0.035131	protein_coding
ENSG0000(	ENSG0000(	1232.247	0.171872	0.011185	0.035134	lncRNA
ENSG0000(	CAHM	17.1503	0.477495	0.01119	0.035145	lncRNA
ENSG0000(	DHX9	2012.224	-0.15835	0.011191	0.035145	protein_coding
ENSG0000(	XRCC3	539.622	-0.21551	0.011199	0.035166	protein_coding
ENSG0000(	FARP1	4729.691	0.107484	0.01121	0.035196	protein_coding
ENSG0000(	GNLY	215.1372	0.420164	0.011212	0.035196	protein_coding
ENSG0000(	UAP1L1	638.2415	0.171868	0.011211	0.035196	protein_coding
ENSG0000(	CEP89	1532.371	0.138829	0.011213	0.035197	protein_coding

ENSG0000(	TGFBR2	3673.319	0.219613	0.01124	0.035279	protein_coding
ENSG0000(	ENSG0000(	43.95033	0.322366	0.011244	0.035288	lncRNA
ENSG0000(	CRIP1	128.9143	-0.41609	0.011247	0.035292	protein_coding
ENSG0000(	DYNLL2	2191.912	-0.13198	0.011253	0.035304	protein_coding
ENSG0000(	RPL10P3	17.40872	0.495195	0.011252	0.035304	processed_pseudogene
ENSG0000(	MX1	3100.531	0.378812	0.011259	0.03532	protein_coding
ENSG0000(	SLAMF6	470.8914	0.359864	0.011261	0.035321	protein_coding
ENSG0000(	ZNF407	2831.364	0.137737	0.011269	0.035343	protein_coding
ENSG0000(	ZNF69	990.208	-0.22499	0.011273	0.035352	protein_coding
ENSG0000(	FAM166C	16.6322	0.740607	0.011277	0.035361	protein_coding
ENSG0000(	SRI	3795.149	-0.44466	0.011293	0.03541	protein_coding
ENSG0000(	CCDC134	1408.137	0.114803	0.011301	0.035429	protein_coding
ENSG0000(	LINC01807	80.72281	-0.30321	0.011309	0.03545	lncRNA
ENSG0000(	HLX	276.4359	0.332939	0.011311	0.03545	protein_coding
ENSG0000(	SLC25A24	1719.722	-0.24051	0.011311	0.03545	protein_coding
ENSG0000(	ENSG0000(	44.09648	0.335011	0.011317	0.035466	lncRNA
ENSG0000(	ENSG0000(	4125.237	0.41654	0.011328	0.035498	lncRNA
ENSG0000(	FLAD1	881.5868	-0.10478	0.011331	0.035503	protein_coding
ENSG0000(	NFATC1	516.6388	0.452163	0.011332	0.035503	protein_coding
ENSG0000(	C19orf48	574.9601	-0.17683	0.011343	0.035534	transcribed_unitary_pseudogene
ENSG0000(	ENSG0000(	418.6858	0.092738	0.011345	0.035536	lncRNA
ENSG0000(	WDR11	2782.762	0.086847	0.011346	0.035536	protein_coding
ENSG0000(	ENSG0000(	22.67398	0.469877	0.011353	0.035555	lncRNA
ENSG0000(	DDB2	981.8454	0.132356	0.01136	0.03557	protein_coding
ENSG0000(	ZBTB2P1	10.18463	-0.51721	0.011359	0.03557	processed_pseudogene
ENSG0000(	FADS6	340.6102	0.185098	0.011367	0.035589	protein_coding
ENSG0000(	PNPO	966.9792	-0.14145	0.011403	0.035696	protein_coding
ENSG0000(	SLC35F3	10.8566	0.726223	0.011405	0.0357	protein_coding
ENSG0000(	EIF2B5	1238.31	-0.1246	0.011407	0.035701	protein_coding
ENSG0000(	KNSTRN	315.3701	-0.16448	0.01141	0.035702	protein_coding
ENSG0000(	RAB20	298.1114	-0.24949	0.011408	0.035702	protein_coding
ENSG0000(	GUSBP11	2831.331	-0.14081	0.011415	0.035715	lncRNA
ENSG0000(	LINC01480	137.924	0.302804	0.011421	0.035733	lncRNA
ENSG0000(	ONECUT3	338.618	0.150358	0.011425	0.035739	protein_coding
ENSG0000(	ENSG0000(	220.2966	-0.18037	0.01144	0.035782	lncRNA
ENSG0000(	ZBTB42	223.7423	-0.17816	0.011441	0.035782	protein_coding
ENSG0000(	ENSG0000(	77.06893	0.319722	0.011444	0.035783	lncRNA
ENSG0000(	ENSG0000(	32.22933	0.310629	0.011443	0.035783	TEC
ENSG0000(	SNRNP200	4606.666	-0.0879	0.011444	0.035783	protein_coding
ENSG0000(	CCDC136	163.8167	0.322281	0.011451	0.035799	protein_coding
ENSG0000(	GNB1L	206.7411	-0.34322	0.011454	0.035806	protein_coding
ENSG0000(	ANKRD52	2704.388	-0.1005	0.011464	0.035826	protein_coding
ENSG0000(	PRMT6	279.1703	-0.19292	0.011463	0.035826	protein_coding
ENSG0000(	RBM14	4053.092	-0.10474	0.011463	0.035826	protein_coding
ENSG0000(	ENSG0000(	173.0565	-0.17173	0.011471	0.035845	processed_pseudogene
ENSG0000(	ENSG0000(	177.2759	-0.147	0.011473	0.035848	lncRNA
ENSG0000(	ENSG0000(	100.2264	0.283578	0.011476	0.035853	lncRNA

ENSG0000(	FLT3	121.8659	0.605473	0.011482	0.035864	protein_coding
ENSG0000(	UNKL	983.7733	0.216281	0.011481	0.035864	protein_coding
ENSG0000(	RNU7-96P	14.38753	0.520992	0.011488	0.03588	snRNA
ENSG0000(	ENSG0000(	12.37718	0.955045	0.011498	0.035906	lncRNA
ENSG0000(	ZNF280D	2229.201	0.123179	0.011503	0.035919	protein_coding
ENSG0000(	ENSG0000(	549.9682	0.11824	0.01151	0.035938	TEC
ENSG0000(	OR8J3	44.17442	-0.40822	0.011512	0.035941	protein_coding
ENSG0000(	SMIM14-D`	847.6191	-0.11459	0.011515	0.035947	lncRNA
ENSG0000(	CKS1B	291.4067	-0.1771	0.011524	0.035971	protein_coding
ENSG0000(	LANCL3	576.3664	-0.39409	0.011528	0.03598	protein_coding
ENSG0000(	IGHV3-20	183.7039	0.676946	0.01153	0.035983	IG_V_gene
ENSG0000(	LINC02343	38.41082	0.439892	0.011532	0.035983	lncRNA
ENSG0000(	ENSG0000(	782.026	-0.31104	0.011534	0.035986	lncRNA
ENSG0000(	MLLT10	1862.429	-0.13453	0.011535	0.035986	protein_coding
ENSG0000(	PIP4K2A	1251.249	0.268669	0.011537	0.035989	protein_coding
ENSG0000(	ENSG0000(	418.6847	-0.19826	0.011541	0.035995	lncRNA
ENSG0000(	ZNF846	710.2152	-0.16269	0.011541	0.035995	protein_coding
ENSG0000(	CMTM3	235.4389	0.315988	0.011544	0.035997	protein_coding
ENSG0000(	ENSG0000(	109.4249	0.405838	0.011544	0.035997	protein_coding
ENSG0000(	ENSG0000(	91.72246	0.251859	0.011549	0.035999	lncRNA
ENSG0000(	HAUS4	374.5434	-0.19715	0.011549	0.035999	protein_coding
ENSG0000(	PEX3	187.6984	-0.33496	0.011546	0.035999	protein_coding
ENSG0000(	RMND5A	2564.896	-0.26206	0.011548	0.035999	protein_coding
ENSG0000(	CNDP1	257.1534	-0.17702	0.011555	0.036013	protein_coding
ENSG0000(	ENSG0000(	31.36368	0.317851	0.011564	0.036037	lncRNA
ENSG0000(	PTCHD1	513.2815	-0.17114	0.011573	0.036061	protein_coding
ENSG0000(	CCNL2	5793.39	0.086784	0.011577	0.036071	protein_coding
ENSG0000(	ZNF563	425.1903	-0.1368	0.011579	0.036073	protein_coding
ENSG0000(	PNKP	1157.015	-0.13502	0.011581	0.036075	protein_coding
ENSG0000(	MARK4	578.7397	0.119909	0.011588	0.036096	protein_coding
ENSG0000(	SPG7	14334.16	0.149358	0.01159	0.036097	protein_coding
ENSG0000(	BLNK	1987.079	0.193209	0.011591	0.036097	protein_coding
ENSG0000(	ENSG0000(	44.10012	0.306612	0.011595	0.036104	lncRNA
ENSG0000(	UBE2R2-AS	75.17554	0.374195	0.011596	0.036105	lncRNA
ENSG0000(	B4GALT1	4390.525	-0.39821	0.011599	0.03611	protein_coding
ENSG0000(	NCKAP1L	2463.762	0.259152	0.011601	0.036114	protein_coding
ENSG0000(	ENSG0000(	310.5168	-0.16679	0.011605	0.036124	lncRNA
ENSG0000(	ENSG0000(	24.78583	0.399483	0.011608	0.03613	misc_RNA
ENSG0000(	ENSG0000(	11.09235	0.589322	0.011617	0.036141	processed_pseudogene
ENSG0000(	ENSG0000(	41.7582	0.222237	0.011616	0.036141	lncRNA
ENSG0000(	LINC01567	205.5994	-0.1648	0.011618	0.036141	lncRNA
ENSG0000(	PKHD1L1	777.784	0.426208	0.011615	0.036141	protein_coding
ENSG0000(	YBX1P4	9.161852	-0.57149	0.011614	0.036141	processed_pseudogene
ENSG0000(	ENSG0000(	231.6632	-0.16988	0.011623	0.036142	lncRNA
ENSG0000(	FA2H	559.5957	-0.37276	0.011621	0.036142	protein_coding
ENSG0000(	INSYN1	224.7731	-0.14152	0.011623	0.036142	protein_coding
ENSG0000(	TMEM64	557.013	0.20177	0.011622	0.036142	protein_coding

ENSG0000(	LARP1	5856.209	-0.12756	0.011629	0.036158	protein_coding
ENSG0000(	LINC00844	332.9384	0.127618	0.011638	0.036181	lncRNA
ENSG0000(	CDKN1C	49.71274	0.3935	0.011642	0.036186	protein_coding
ENSG0000(	PCAT6	19.84032	0.66217	0.011642	0.036186	lncRNA
ENSG0000(	ENSG0000(	436.6679	-0.19936	0.011644	0.036191	TEC
ENSG0000(	ENSG0000(	16.04848	0.622575	0.011649	0.036203	TEC
ENSG0000(	RICTOR	3873.224	0.091857	0.011652	0.036206	protein_coding
ENSG0000(	ENSG0000(	58.25901	0.294327	0.011655	0.036214	lncRNA
ENSG0000(	DECR1	1894.221	0.121113	0.011658	0.03622	protein_coding
ENSG0000(	OGA	8755.617	0.10907	0.01166	0.03622	protein_coding
ENSG0000(	ENSG0000(	27.3092	0.365787	0.011671	0.036251	TEC
ENSG0000(	ENSG0000(	151.7887	0.192839	0.011674	0.036258	lncRNA
ENSG0000(	CCDC85C	1791.856	-0.28415	0.011692	0.03631	protein_coding
ENSG0000(	RN7SL525F	183.2032	-0.19766	0.011693	0.03631	misc_RNA
ENSG0000(	ABCB6	196.2583	-0.32779	0.011703	0.036337	protein_coding
ENSG0000(	DENR	1216.652	-0.08615	0.011706	0.036341	protein_coding
ENSG0000(	TYW5	2370.797	0.087862	0.011707	0.036341	protein_coding
ENSG0000(	MYL6B-AS1	15.59421	0.52663	0.011723	0.036387	lncRNA
ENSG0000(	CNRIP1	228.975	0.278333	0.011725	0.036393	protein_coding
ENSG0000(	NAB1	1590.823	0.134778	0.01174	0.036434	protein_coding
ENSG0000(	VN1R1	70.2349	0.264996	0.011741	0.036434	protein_coding
ENSG0000(	VAMP8	681.5659	-0.32055	0.011749	0.036453	protein_coding
ENSG0000(	ENSG0000(	8.841741	0.678813	0.011758	0.036475	lncRNA
ENSG0000(	ENSG0000(	17.01519	-0.51121	0.011757	0.036475	lncRNA
ENSG0000(	ABCF2	828.587	-0.185	0.011759	0.036476	protein_coding
ENSG0000(	PABPC4	3548.087	-0.11302	0.011773	0.036515	protein_coding
ENSG0000(	RPL7P14	84.4708	-0.3297	0.011777	0.036525	processed_pseudogene
ENSG0000(	RWDD4	323.7358	-0.28484	0.011786	0.036547	protein_coding
ENSG0000(	ARFGAP3	774.6086	0.226827	0.01179	0.036553	protein_coding
ENSG0000(	TBC1D10A	1004.431	-0.14489	0.01179	0.036553	protein_coding
ENSG0000(	NPM1	3933.258	-0.19636	0.0118	0.036582	protein_coding
ENSG0000(	RN7SL213F	10.75188	-0.50187	0.011805	0.036592	misc_RNA
ENSG0000(	ENSG0000(	213.5441	-0.39283	0.011808	0.036599	protein_coding
ENSG0000(	ALDH1A2	100.7911	0.32665	0.011821	0.036633	protein_coding
ENSG0000(	ENSG0000(	9.938431	0.780452	0.011832	0.036664	lncRNA
ENSG0000(	CCDC61	170.4113	-0.30682	0.011841	0.036688	protein_coding
ENSG0000(	ENSG0000(	52.16598	0.33908	0.011846	0.036699	lncRNA
ENSG0000(	NIPSNAP3E	213.5828	0.231163	0.011847	0.036699	protein_coding
ENSG0000(	ENSG0000(	36.59987	0.262148	0.011856	0.036724	lncRNA
ENSG0000(	ENSG0000(	10.21245	-0.65753	0.011883	0.036806	lncRNA
ENSG0000(	SLC66A1	367.3323	0.131912	0.011887	0.03681	protein_coding
ENSG0000(	WWTR1-IT:	132.7906	0.161617	0.011887	0.03681	lncRNA
ENSG0000(	LINC02086	1655.444	-0.29301	0.011889	0.036813	lncRNA
ENSG0000(	MED11	407.0167	-0.12601	0.011893	0.03682	protein_coding
ENSG0000(	LIN28B-AS1	576.829	-0.13488	0.011903	0.036848	lncRNA
ENSG0000(	SMPD3	1639.651	-0.22494	0.011913	0.036877	protein_coding
ENSG0000(	ENSG0000(	740.8146	-0.22754	0.011916	0.03688	lncRNA

ENSG0000(	RBM12B	2482.565	0.171637	0.011921	0.036895	protein_coding
ENSG0000(	NFU1	178.9677	-0.19689	0.011924	0.036898	protein_coding
ENSG0000(	ZNF174	163.054	-0.17883	0.011926	0.036902	protein_coding
ENSG0000(	BRICD5	44.47679	-0.31257	0.01193	0.03691	protein_coding
ENSG0000(	ENSG0000(	237.7651	-0.17103	0.011934	0.036918	lncRNA
ENSG0000(	S1PR4	96.64028	0.570092	0.011942	0.03694	protein_coding
ENSG0000(	ENSG0000(	204.1448	-0.1713	0.011945	0.036945	lncRNA
ENSG0000(	ENSG0000(	89.50758	0.279533	0.011957	0.036975	lncRNA
ENSG0000(	ENSG0000(	202.9929	0.180674	0.011957	0.036975	TEC
ENSG0000(	MYO9A	4283.981	-0.17685	0.011966	0.037	protein_coding
ENSG0000(	FGD5-AS1	3051.659	-0.11678	0.011972	0.037015	lncRNA
ENSG0000(	ENSG0000(	285.4316	0.185758	0.011978	0.037028	lncRNA
ENSG0000(	KCNJ5	609.2088	0.152733	0.011979	0.037028	protein_coding
ENSG0000(	ENSG0000(	28.60638	0.448917	0.01199	0.03706	lncRNA
ENSG0000(	DBNDD2	612.0089	-0.25515	0.011998	0.037079	protein_coding
ENSG0000(	KIAA1328	3320.76	-0.1531	0.011999	0.037079	protein_coding
ENSG0000(	PRF1	113.8719	0.485244	0.012006	0.037097	protein_coding
ENSG0000(	ENSG0000(	28.6626	0.399854	0.012017	0.037123	lncRNA
ENSG0000(	LINC00327	137.9892	-0.20225	0.012016	0.037123	lncRNA
ENSG0000(	ENSG0000(	34.35598	0.378685	0.012019	0.037125	lncRNA
ENSG0000(	TMSB4X	12357.49	-0.23579	0.01202	0.037126	protein_coding
ENSG0000(	HSPA4L	1265.267	-0.17427	0.012021	0.037126	protein_coding
ENSG0000(	ENSG0000(	35.30838	0.303251	0.012031	0.03715	lncRNA
ENSG0000(	SNORD110	22.25036	0.577354	0.01203	0.03715	snoRNA
ENSG0000(	PDCD4-AS1	174.0746	-0.16024	0.012057	0.037226	lncRNA
ENSG0000(	CYRIB	1839.121	0.140932	0.012058	0.037227	protein_coding
ENSG0000(	ENSG0000(	180.6333	0.146852	0.012067	0.037249	TEC
ENSG0000(	YWHAZP10	32.18797	-0.43451	0.012069	0.037253	processed_pseudogene
ENSG0000(	ENSG0000(	24.48061	-0.40518	0.012092	0.03732	lncRNA
ENSG0000(	FANCF	453.3291	-0.12876	0.012098	0.037334	protein_coding
ENSG0000(	HOXD9	145.8643	0.347523	0.012102	0.037339	protein_coding
ENSG0000(	ZNF233	218.8846	0.167122	0.012102	0.037339	protein_coding
ENSG0000(	MAP2K4P1	43.87049	0.302986	0.012107	0.03735	transcribed_processed_pseudoge
ENSG0000(	POLR1F	288.843	-0.22225	0.01211	0.037357	protein_coding
ENSG0000(	ANKFY1	3240.446	0.126873	0.012121	0.03738	protein_coding
ENSG0000(	IGLV2-8	713.8405	0.708742	0.01212	0.03738	IG_V_gene
ENSG0000(	LINC01013	213.6991	-0.28006	0.012121	0.03738	lncRNA
ENSG0000(	IKZF3	4314.389	0.329515	0.012127	0.037393	protein_coding
ENSG0000(	CCL11	106.7847	0.677003	0.012137	0.037418	protein_coding
ENSG0000(	TTC32	204.6884	0.153578	0.012137	0.037418	protein_coding
ENSG0000(	ENSG0000(	120.9161	0.211059	0.01214	0.037423	lncRNA
ENSG0000(	TMEM92-A	13.97968	0.631652	0.012143	0.037427	lncRNA
ENSG0000(	SCARF1	799.2956	0.243301	0.012147	0.037437	protein_coding
ENSG0000(	IGKV1D-13	125.0183	1.052013	0.012174	0.037511	IG_V_gene
ENSG0000(	MIR4802	46.14678	-0.39105	0.012175	0.037511	miRNA
ENSG0000(	SEC22A	884.1459	-0.10202	0.012174	0.037511	protein_coding
ENSG0000(	ENSG0000(	9.727937	0.582429	0.012177	0.037514	lncRNA

ENSG0000(	ENSG0000(	42.59759	0.338891	0.012182	0.037525	lncRNA
ENSG0000(	ZFAND4	701.0637	-0.15879	0.012183	0.037525	protein_coding
ENSG0000(	ENSG0000(	30.43836	0.461717	0.012185	0.037527	lncRNA
ENSG0000(	LIAS	2395.938	-0.08515	0.012186	0.037527	protein_coding
ENSG0000(	ENSG0000(	332.4404	0.133977	0.012192	0.037537	lncRNA
ENSG0000(	IQCA1	31.61197	0.636025	0.012191	0.037537	protein_coding
ENSG0000(	ENSG0000(	191.7138	0.216498	0.012194	0.037542	lncRNA
ENSG0000(	RNF169	2282.136	0.099894	0.0122	0.037556	protein_coding
ENSG0000(	RNF121	662.5144	-0.12845	0.012206	0.03757	protein_coding
ENSG0000(	SFXN3	678.186	0.24088	0.012207	0.03757	protein_coding
ENSG0000(	ENSG0000(	90.91674	-0.40817	0.012212	0.037583	lncRNA
ENSG0000(	ACAP3	1542.478	-0.18351	0.012218	0.037597	protein_coding
ENSG0000(	CTU2	127.3872	-0.28081	0.012221	0.037597	protein_coding
ENSG0000(	ENSG0000(	84.30808	-0.77037	0.012221	0.037597	lncRNA
ENSG0000(	REXO1	1085.504	-0.18324	0.012223	0.037602	protein_coding
ENSG0000(	NR5A2	2094.097	-0.25299	0.012243	0.037659	protein_coding
ENSG0000(	RN7SL502P	31.729	0.428858	0.012245	0.037661	misc_RNA
ENSG0000(	DPP9	2524.014	-0.11544	0.012266	0.03772	protein_coding
ENSG0000(	ENSG0000(	32.34926	0.407011	0.012266	0.03772	snoRNA
ENSG0000(	ENSG0000(	64.90858	0.347078	0.012273	0.037732	lncRNA
ENSG0000(	GORAB	1677.579	0.106942	0.012272	0.037732	protein_coding
ENSG0000(	TYW1B	294.1837	-0.21416	0.012276	0.037735	protein_coding
ENSG0000(	ZNF570	698.3637	-0.16427	0.012276	0.037735	protein_coding
ENSG0000(	ZFX	2370.436	0.144979	0.01228	0.037742	protein_coding
ENSG0000(	LINC01209	224.3843	-0.22708	0.012285	0.037755	lncRNA
ENSG0000(	ENSG0000(	13.65166	0.567269	0.012291	0.037763	processed_pseudogene
ENSG0000(	EPHB2	2118.962	-0.31103	0.012289	0.037763	protein_coding
ENSG0000(	GRAMD2B	1346.352	-0.25616	0.012291	0.037763	protein_coding
ENSG0000(	ENSG0000(	90.57813	0.215082	0.012296	0.037769	lncRNA
ENSG0000(	SSBP3-AS1	18.87635	0.510028	0.012295	0.037769	lncRNA
ENSG0000(	ENSG0000(	16.65439	-0.74932	0.012297	0.03777	processed_pseudogene
ENSG0000(	IRF3	2305.601	-0.10475	0.012301	0.037778	protein_coding
ENSG0000(	ENSG0000(	19.50528	0.537088	0.012306	0.037789	transcribed_processed_pseudoge
ENSG0000(	MCPH1	3744.538	0.07429	0.012307	0.037789	protein_coding
ENSG0000(	ENSG0000(	236.422	0.217029	0.012316	0.037812	lncRNA
ENSG0000(	ENSG0000(	54.23558	0.254636	0.012321	0.037826	lncRNA
ENSG0000(	HMGN5	69.34605	-0.39996	0.012326	0.037835	protein_coding
ENSG0000(	ENSG0000(	34.22791	0.375593	0.012334	0.037856	lncRNA
ENSG0000(	OST4	458.9459	-0.25311	0.012343	0.037881	protein_coding
ENSG0000(	GPC5	21.09182	0.826052	0.012352	0.037906	protein_coding
ENSG0000(	MAS1L	17.397	-0.78097	0.012369	0.037953	protein_coding
ENSG0000(	BARX2	432.6419	-0.34152	0.012375	0.037969	protein_coding
ENSG0000(	ENSG0000(	110.5463	0.186179	0.012387	0.037998	lncRNA
ENSG0000(	IGHV3OR1(	13.4394	0.827277	0.012386	0.037998	protein_coding
ENSG0000(	ENSG0000(	70.9848	0.216023	0.012391	0.038005	lncRNA
ENSG0000(	NATD1	335.179	0.173586	0.012392	0.038005	protein_coding
ENSG0000(	ENSG0000(	80.5463	0.312239	0.012398	0.038021	lncRNA

ENSG0000(	MYL12-AS1	13.67918	0.474929	0.012403	0.038031	lncRNA
ENSG0000(	ENSG0000(	61.44058	0.271341	0.012414	0.038056	lncRNA
ENSG0000(	TRIM40	451.6658	0.776478	0.012413	0.038056	protein_coding
ENSG0000(	AGPAT1	584.8086	-0.16315	0.012417	0.038064	protein_coding
ENSG0000(	LINC01411	141.3079	0.263979	0.012425	0.038079	lncRNA
ENSG0000(	USP38-DT	100.7838	0.334693	0.012424	0.038079	lncRNA
ENSG0000(	ENSG0000(	49.99893	0.29072	0.012432	0.038092	lncRNA
ENSG0000(	IL11RA	536.8973	-0.20199	0.012432	0.038092	protein_coding
ENSG0000(	SEC14L4	211.8571	0.155413	0.012431	0.038092	protein_coding
ENSG0000(	NUMA1	9048.714	-0.0962	0.012434	0.038093	protein_coding
ENSG0000(	ZNF77	157.4496	-0.23561	0.012448	0.038133	protein_coding
ENSG0000(	NUDT14	253.6309	-0.22567	0.012464	0.038178	protein_coding
ENSG0000(	HOXB9	2140.52	-0.33733	0.012471	0.038189	protein_coding
ENSG0000(	P4HTM	1306.795	-0.14293	0.01247	0.038189	protein_coding
ENSG0000(	TRGV10	23.68971	-0.50145	0.012471	0.038189	TR_V_gene
ENSG0000(	ENSG0000(	207.5755	0.254934	0.012473	0.038189	lncRNA
ENSG0000(	ENSG0000(	98.21969	0.199033	0.01248	0.038205	lncRNA
ENSG0000(	RASGRF1	160.744	0.389162	0.01248	0.038205	protein_coding
ENSG0000(	ZNF704	2821.303	-0.37728	0.012483	0.038209	protein_coding
ENSG0000(	LPCAT4	1748.869	-0.21873	0.012494	0.038239	protein_coding
ENSG0000(	LIG3	2140.072	-0.12868	0.012503	0.038262	protein_coding
ENSG0000(	LINC02511	154.8363	0.168195	0.012505	0.038266	lncRNA
ENSG0000(	IGSF9B	665.8581	-0.44882	0.012513	0.038286	protein_coding
ENSG0000(	LRCH2	96.61695	0.379771	0.012517	0.038294	protein_coding
ENSG0000(	LINC01770	12.59274	0.633653	0.012519	0.038297	lncRNA
ENSG0000(	TOX	485.2552	-0.22341	0.012526	0.038314	protein_coding
ENSG0000(	TTC21B	4908.252	0.092273	0.012527	0.038314	protein_coding
ENSG0000(	AP1S3	1246.513	0.121378	0.012533	0.038328	protein_coding
ENSG0000(	ENSG0000(	132.9723	0.267366	0.012538	0.038339	lncRNA
ENSG0000(	ETAA1	587.8999	0.152725	0.012541	0.038342	protein_coding
ENSG0000(	ZNF146	1604.148	-0.12781	0.012541	0.038342	protein_coding
ENSG0000(	CENPL	751.6695	-0.11922	0.012551	0.038368	protein_coding
ENSG0000(	ENSG0000(	24.84843	0.42516	0.012569	0.03842	lncRNA
ENSG0000(	ENSG0000(	178.046	0.260387	0.012591	0.03848	lncRNA
ENSG0000(	ITCH-AS1	26.19117	0.348259	0.012591	0.03848	lncRNA
ENSG0000(	PRH1	489.873	0.209554	0.0126	0.038502	protein_coding
ENSG0000(	ENSG0000(	147.8227	-0.60466	0.012605	0.038514	protein_coding
ENSG0000(	RN7SL648F	372.9574	0.14832	0.012609	0.038522	misc_RNA
ENSG0000(	COL6A4P2	48.26404	0.603998	0.012621	0.038555	transcribed_unitary_pseudogene
ENSG0000(	CNPY1	47.35107	0.225658	0.012625	0.038565	protein_coding
ENSG0000(	ENSG0000(	366.0459	0.240586	0.012629	0.038572	TEC
ENSG0000(	LINC00587	14.6221	0.507706	0.012633	0.03858	lncRNA
ENSG0000(	LINC02909	224.7614	0.159271	0.012638	0.038593	lncRNA
ENSG0000(	ENSG0000(	83.19093	0.279469	0.012648	0.03862	lncRNA
ENSG0000(	ABL1	3107.049	-0.16145	0.01265	0.038622	protein_coding
ENSG0000(	WDR18	159.5941	-0.2863	0.01266	0.038647	protein_coding
ENSG0000(	ENSG0000(	169.5069	0.332708	0.012661	0.038647	lncRNA

ENSG0000(	ENSG0000(	806.2356	-0.36258	0.012674	0.03868	lncRNA
ENSG0000(	FIGNL2	46.71433	0.392959	0.012674	0.03868	protein_coding
ENSG0000(	LINC02302	191.1453	0.171882	0.012675	0.03868	lncRNA
ENSG0000(	ENSG0000(	175.8469	0.13787	0.012681	0.038695	lncRNA
ENSG0000(	KCNQ1OT1	22717.07	0.113158	0.012691	0.038721	lncRNA
ENSG0000(	B3GLCT	206.6778	-0.19629	0.012701	0.038746	protein_coding
ENSG0000(	ENSG0000(	61.35824	0.282798	0.012703	0.038746	lncRNA
ENSG0000(	LAMTOR2	146.3653	-0.22339	0.012703	0.038746	protein_coding
ENSG0000(	GEMIN2	293.5352	-0.17313	0.01271	0.038763	protein_coding
ENSG0000(	CUTALP	1964.304	0.345296	0.012712	0.038763	transcribed_unitary_pseudogene
ENSG0000(	SLC8A1-AS:	319.6002	0.192409	0.012712	0.038763	lncRNA
ENSG0000(	ATPAF1	928.2915	-0.15851	0.012717	0.038773	protein_coding
ENSG0000(	SNORD118	922.4855	0.393317	0.012728	0.038803	snoRNA
ENSG0000(	NSRP1P1	52.01071	-0.42184	0.01273	0.038805	processed_pseudogene
ENSG0000(	MED31	114.0431	-0.19966	0.012738	0.038825	protein_coding
ENSG0000(	CPSF2	2894.617	-0.16441	0.012743	0.038839	protein_coding
ENSG0000(	CERCAM	517.4498	0.177825	0.012748	0.038848	protein_coding
ENSG0000(	ENSG0000(	75.96439	0.303392	0.012751	0.038854	lncRNA
ENSG0000(	ENSG0000(	325.3061	-0.12816	0.012758	0.038872	lncRNA
ENSG0000(	TBC1D24	984.041	-0.0864	0.012766	0.038892	protein_coding
ENSG0000(	ENSG0000(	386.3665	0.154979	0.012799	0.038985	TEC
ENSG0000(	VSTM5	70.27674	0.431404	0.012799	0.038985	protein_coding
ENSG0000(	ENSG0000(	836.7326	-0.20985	0.012846	0.039123	lncRNA
ENSG0000(	CNKSR1	655.1165	-0.24413	0.012854	0.039133	protein_coding
ENSG0000(	DNAJC9	583.3654	-0.18663	0.012851	0.039133	protein_coding
ENSG0000(	KLF2	490.1091	0.445085	0.012853	0.039133	protein_coding
ENSG0000(	TXNDC16	371.2755	-0.23019	0.012854	0.039133	protein_coding
ENSG0000(	TMEM183/	1274.943	-0.14085	0.012874	0.03919	protein_coding
ENSG0000(	ENSG0000(	13.40026	-0.70725	0.012898	0.039261	TEC
ENSG0000(	ENSG0000(	47.45437	0.308996	0.012907	0.039279	lncRNA
ENSG0000(	ZNF479	157.2636	-0.18056	0.012905	0.039279	protein_coding
ENSG0000(	COA6-AS1	25.9774	-0.37883	0.01291	0.039282	lncRNA
ENSG0000(	RNU6-407F	20.41162	0.45103	0.01291	0.039282	snRNA
ENSG0000(	MARCKS	4834.902	-0.22621	0.012914	0.03929	protein_coding
ENSG0000(	NUDT11	19.05948	0.484725	0.012919	0.0393	protein_coding
ENSG0000(	ENSG0000(	520.8209	0.101195	0.012922	0.039307	lncRNA
ENSG0000(	BCAN-AS1	307.3178	0.230234	0.012924	0.039309	lncRNA
ENSG0000(	DNAJC14	934.6901	-0.10225	0.012925	0.039309	protein_coding
ENSG0000(	SLC35E3	6127.464	0.104073	0.012941	0.039353	protein_coding
ENSG0000(	CNST	2231.454	-0.21717	0.012948	0.039363	protein_coding
ENSG0000(	PKD2L2	236.2213	-0.20807	0.012947	0.039363	protein_coding
ENSG0000(	PSMD10	265.5229	-0.16137	0.012947	0.039363	protein_coding
ENSG0000(	ENSG0000(	81.15537	0.291279	0.01295	0.039366	TEC
ENSG0000(	ENSG0000(	41.9418	0.287453	0.012953	0.03937	lncRNA
ENSG0000(	SIAH3	111.5821	0.195686	0.012954	0.03937	protein_coding
ENSG0000(	CLIC4	1388.174	0.305289	0.012959	0.039378	protein_coding
ENSG0000(	SLC12A3	55.58278	0.470241	0.012959	0.039378	protein_coding

ENSG0000(	LINC00345	388.7223	0.151829	0.012963	0.039385	lncRNA
ENSG0000(	OR2A25	64.35324	-0.26456	0.012969	0.039398	protein_coding
ENSG0000(	ARNTL	828.9061	0.289116	0.012985	0.039436	protein_coding
ENSG0000(	ENSG0000(	345.8689	-0.27412	0.012985	0.039436	lncRNA
ENSG0000(	NPIPB5	15468.58	0.127982	0.012984	0.039436	protein_coding
ENSG0000(	ENSG0000(	72.41786	0.274056	0.012997	0.039465	lncRNA
ENSG0000(	OR2T10	15.22846	1.130782	0.012997	0.039465	protein_coding
ENSG0000(	PDGFA	3297.158	0.314307	0.013007	0.039491	protein_coding
ENSG0000(	ENSG0000(	269.7983	0.126158	0.013018	0.039522	lncRNA
ENSG0000(	IRX2	20.4694	0.46694	0.013028	0.039547	protein_coding
ENSG0000(	LINC01355	1575.991	0.145995	0.013041	0.039584	lncRNA
ENSG0000(	ENSG0000(	914.2362	0.142594	0.01305	0.039603	lncRNA
ENSG0000(	FUT11	720.4708	0.121291	0.013049	0.039603	protein_coding
ENSG0000(	ENSG0000(	231.6295	-0.1767	0.013054	0.039608	TEC
ENSG0000(	HAPLN1	241.2703	-0.54062	0.013054	0.039608	protein_coding
ENSG0000(	ENSG0000(	184.2691	-0.16047	0.013081	0.039685	lncRNA
ENSG0000(	RSU1P3	221.364	0.275606	0.013086	0.039696	processed_pseudogene
ENSG0000(	ZNF250	836.5655	-0.14806	0.013088	0.039699	protein_coding
ENSG0000(	CASKIN1	20.48599	-0.76786	0.013096	0.03972	protein_coding
ENSG0000(	UGT2B10	11.42074	-0.70955	0.013097	0.03972	protein_coding
ENSG0000(	CPVL	210.6275	0.354918	0.013113	0.039759	protein_coding
ENSG0000(	TLK2	3087.42	0.082059	0.013113	0.039759	protein_coding
ENSG0000(	LYRM1	424.059	0.143568	0.013114	0.039759	protein_coding
ENSG0000(	ENSG0000(	24.71123	-0.42792	0.013122	0.039781	lncRNA
ENSG0000(	ENSG0000(	449.7243	0.274169	0.01313	0.039799	lncRNA
ENSG0000(	TNFRSF4	55.03871	0.527906	0.013134	0.039809	protein_coding
ENSG0000(	MTCO1P25	204.6836	-0.204	0.013145	0.039838	unprocessed_pseudogene
ENSG0000(	IRF5	364.3101	0.206355	0.013157	0.03987	protein_coding
ENSG0000(	ENSG0000(	38.93039	0.367841	0.013159	0.039873	lncRNA
ENSG0000(	TBC1D22B	549.4608	-0.14809	0.013162	0.039878	protein_coding
ENSG0000(	ENSG0000(	16.75118	0.518882	0.013164	0.039879	lncRNA
ENSG0000(	ENSG0000(	620.0752	-0.11421	0.013168	0.039889	lncRNA
ENSG0000(	ENSG0000(	508.0108	-0.0961	0.013172	0.039893	lncRNA
ENSG0000(	LINC00526	73.23847	-0.35105	0.013171	0.039893	lncRNA
ENSG0000(	ZNF626	619.5562	-0.22156	0.01319	0.039942	protein_coding
ENSG0000(	IL23A	13.16512	0.662394	0.013193	0.039946	protein_coding
ENSG0000(	RAMP2	47.34784	0.380668	0.013194	0.039946	protein_coding
ENSG0000(	RN7SL750F	55.24554	-0.30246	0.013203	0.039971	misc_RNA
ENSG0000(	SART1	673.0642	-0.18809	0.013214	0.039999	protein_coding
ENSG0000(	RNU6-1025	18.54987	0.470819	0.013222	0.040021	snRNA
ENSG0000(	BET1	712.8813	0.146172	0.013232	0.040044	protein_coding
ENSG0000(	ENSG0000(	12.47584	0.626652	0.013232	0.040044	snoRNA
ENSG0000(	TOR1A	747.8625	-0.11072	0.013243	0.040071	protein_coding
ENSG0000(	CLDN18	92.53104	0.369469	0.013248	0.040084	protein_coding
ENSG0000(	CPM	2300.748	-0.13607	0.013249	0.040084	protein_coding
ENSG0000(	ENSG0000(	17.1687	0.396586	0.013253	0.04009	lncRNA
ENSG0000(	LINC00161	17.53719	0.42702	0.013265	0.040124	lncRNA

ENSG0000(	FUCA1	1540.256	-0.35118	0.013273	0.040144	protein_coding
ENSG0000(	PCF11-AS1	197.6307	0.186221	0.013278	0.040154	lncRNA
ENSG0000(	ENSG0000(	143.7064	0.155835	0.01328	0.040157	lncRNA
ENSG0000(	CTNNA1	5476.032	-0.16821	0.013292	0.040191	protein_coding
ENSG0000(	SNORA78	15.88263	-0.67741	0.013307	0.040231	snoRNA
ENSG0000(	ENSG0000(	126.8395	0.320702	0.013317	0.040258	transcribed_processed_pseudoge
ENSG0000(	DOCK6	1963.005	-0.18014	0.013327	0.040285	protein_coding
ENSG0000(	MFF	1389.34	-0.10437	0.013333	0.040299	protein_coding
ENSG0000(	YIF1A	296.8044	-0.20125	0.013341	0.040318	protein_coding
ENSG0000(	ENSG0000(	405.8474	0.110204	0.013346	0.040328	lncRNA
ENSG0000(	GPRIN3	3053.473	0.220768	0.013351	0.04034	protein_coding
ENSG0000(	LNCOG	44.30084	0.386774	0.013356	0.040348	lncRNA
ENSG0000(	PRODH	221.6293	-0.36526	0.013356	0.040348	protein_coding
ENSG0000(	ENSG0000(	40.00426	0.267943	0.013361	0.040358	TEC
ENSG0000(	ENSG0000(	601.8161	-0.17038	0.013365	0.040368	lncRNA
ENSG0000(	ENSG0000(	502.5371	0.178533	0.013368	0.040373	lncRNA
ENSG0000(	ENSG0000(	174.407	-0.17554	0.013375	0.040391	lncRNA
ENSG0000(	ENSG0000(	2481.247	0.100009	0.013393	0.040441	lncRNA
ENSG0000(	DAB2IP	2988.356	-0.25021	0.01342	0.040513	protein_coding
ENSG0000(	TMEM221	49.11875	0.294196	0.013418	0.040513	protein_coding
ENSG0000(	GRB7	353.4506	-0.37972	0.013425	0.040524	protein_coding
ENSG0000(	LINC01579	1449.47	0.113475	0.01343	0.040538	lncRNA
ENSG0000(	BPIFB1	112.1266	0.355482	0.013435	0.040548	protein_coding
ENSG0000(	ENSG0000(	64.89925	-0.39456	0.013437	0.04055	lncRNA
ENSG0000(	ENSG0000(	89.7867	0.618303	0.013445	0.040571	unprocessed_pseudogene
ENSG0000(	CCAR2	1262.761	-0.1618	0.013454	0.040591	protein_coding
ENSG0000(	CHCHD6	86.97056	-0.25914	0.013454	0.040591	protein_coding
ENSG0000(	ISCA2	591.0888	-0.10707	0.013459	0.040601	protein_coding
ENSG0000(	CENPNP1	275.4944	0.146142	0.013466	0.040619	processed_pseudogene
ENSG0000(	ARMC5	286.696	0.252009	0.013471	0.040624	protein_coding
ENSG0000(	MBTPS1	3238.293	0.106428	0.01347	0.040624	protein_coding
ENSG0000(	DLGAP1-AS	93.94841	0.217376	0.013485	0.040664	lncRNA
ENSG0000(	ENSG0000(	258.3608	-0.13943	0.013499	0.040701	lncRNA
ENSG0000(	ENSG0000(	377.2023	-0.21246	0.013511	0.040734	TEC
ENSG0000(	ENSG0000(	139.2963	-0.19709	0.013516	0.040745	lncRNA
ENSG0000(	LINC02503	4406.588	-0.22502	0.01352	0.040754	lncRNA
ENSG0000(	SYNJ2-IT1	69.22028	-0.22374	0.013529	0.040776	lncRNA
ENSG0000(	DDX3ILA1	402.9954	-0.38091	0.013532	0.04078	lncRNA
ENSG0000(	ENSG0000(	10.83496	0.614331	0.013533	0.040781	lncRNA
ENSG0000(	PAQR9-AS1	443.4441	-0.13083	0.013539	0.040795	lncRNA
ENSG0000(	RPL3P7	10.12455	-0.83054	0.013548	0.040817	processed_pseudogene
ENSG0000(	PPIP5K1	2911.682	-0.17915	0.013551	0.040824	protein_coding
ENSG0000(	ENSG0000(	121.2174	-0.24793	0.013555	0.040832	lncRNA
ENSG0000(	MORN1	789.2188	-0.12776	0.013561	0.040844	protein_coding
ENSG0000(	RBM48	940.5507	-0.10408	0.013565	0.040852	protein_coding
ENSG0000(	ENSG0000(	518.3469	0.132439	0.013573	0.040872	TEC
ENSG0000(	ENSG0000(	683.1124	0.12768	0.013581	0.040892	TEC

ENSG0000(	ENSG0000(	107.929	-0.16236	0.013583	0.040894	lncRNA
ENSG0000(	AFF4	5920.31	0.10784	0.013584	0.040896	protein_coding
ENSG0000(	ENSG0000(	22.47018	0.460445	0.013591	0.040912	lncRNA
ENSG0000(	NAV3	1007.185	-0.229	0.013594	0.040916	protein_coding
ENSG0000(	PCGEM1	9.485862	-0.65089	0.013598	0.040924	lncRNA
ENSG0000(	GNPNAT1	943.4089	-0.22611	0.013602	0.040933	protein_coding
ENSG0000(	BCL2L12	90.46022	-0.39061	0.013603	0.040933	protein_coding
ENSG0000(	GIMAP5	1195.086	0.360618	0.01361	0.040949	protein_coding
ENSG0000(	PCDH7	946.1424	0.314498	0.013621	0.040977	protein_coding
ENSG0000(	GSN-AS1	238.1945	0.158762	0.013624	0.040985	lncRNA
ENSG0000(	ENSG0000(	13.9049	-0.56362	0.013633	0.041006	lncRNA
ENSG0000(	PDC-AS1	666.0454	0.113382	0.013635	0.041007	lncRNA
ENSG0000(	MAST4-IT1	48.64579	-0.31931	0.013637	0.041008	lncRNA
ENSG0000(	NFE2L3P1	58.4374	-0.41987	0.013636	0.041008	processed_pseudogene
ENSG0000(	MRPL2	354.0716	-0.15061	0.013643	0.041021	protein_coding
ENSG0000(	NXPE4	2384.231	-0.54514	0.013651	0.04104	protein_coding
ENSG0000(	ENSG0000(	161.1493	-0.41519	0.013653	0.041044	lncRNA
ENSG0000(	DCTN2	3074.54	-0.1315	0.013655	0.041044	protein_coding
ENSG0000(	ZNF888	1134.35	-0.20242	0.013665	0.041071	protein_coding
ENSG0000(	ENSG0000(	116.9373	-0.17231	0.013673	0.041091	processed_pseudogene
ENSG0000(	ENSG0000(	509.8895	-0.13765	0.013678	0.041104	protein_coding
ENSG0000(	ARHGAP11	15.55342	0.921846	0.013695	0.041151	protein_coding
ENSG0000(	MAFK	1147.722	-0.18227	0.013704	0.041171	protein_coding
ENSG0000(	SEPHS1	613.1116	-0.17716	0.013705	0.041171	protein_coding
ENSG0000(	USP6NL	1470.379	0.108164	0.013711	0.041187	protein_coding
ENSG0000(	MOK	1266.488	0.110329	0.01372	0.04121	protein_coding
ENSG0000(	TIMM29	299.0574	-0.13612	0.013731	0.041238	protein_coding
ENSG0000(	C1orf226	547.8151	-0.27084	0.01374	0.04126	protein_coding
ENSG0000(	BDKRB2	1127.513	0.307721	0.013743	0.041265	protein_coding
ENSG0000(	ENSG0000(	29.169	-0.34563	0.013749	0.041281	lncRNA
ENSG0000(	TUBB	3321.223	-0.25571	0.013754	0.041291	protein_coding
ENSG0000(	LONP2	4872.613	-0.10738	0.013758	0.041298	protein_coding
ENSG0000(	VAV3	2179.634	-0.24681	0.01376	0.041302	protein_coding
ENSG0000(	PHLPP2	5559.302	-0.45445	0.013764	0.041309	protein_coding
ENSG0000(	ENSG0000(	66.77439	0.261088	0.013768	0.041313	lncRNA
ENSG0000(	PICALM	3750.049	0.163684	0.013767	0.041313	protein_coding
ENSG0000(	FHDC1	1082.129	-0.26831	0.013774	0.041328	protein_coding
ENSG0000(	SLC39A6	324.1852	0.259981	0.013789	0.041369	protein_coding
ENSG0000(	ENSG0000(	243.5389	0.368823	0.013794	0.041378	lncRNA
ENSG0000(	ENSG0000(	13.20605	0.532636	0.013804	0.041406	lncRNA
ENSG0000(	ENSG0000(	90.53608	0.232666	0.013807	0.041411	lncRNA
ENSG0000(	PM20D1	16.25828	0.715062	0.013827	0.041467	protein_coding
ENSG0000(	ENSG0000(	80.93959	0.289068	0.013837	0.041493	lncRNA
ENSG0000(	ATAD1	1582.32	-0.12797	0.013841	0.041501	protein_coding
ENSG0000(	ENSG0000(	36.42011	0.513135	0.013847	0.041513	lncRNA
ENSG0000(	ENSG0000(	506.8115	0.190635	0.013851	0.041522	processed_pseudogene
ENSG0000(	LIPG	2607.344	0.273287	0.013856	0.041532	protein_coding

ENSG0000(	PPP1R3C	88.3158	-0.40883	0.013863	0.041551	protein_coding
ENSG0000(	ZNF629	823.293	-0.1639	0.013866	0.041556	protein_coding
ENSG0000(	PSMD13	966.2769	-0.13178	0.013871	0.041566	protein_coding
ENSG0000(	ENSG0000(	16.46091	0.472265	0.013872	0.041567	processed_pseudogene
ENSG0000(	ENSG0000(	204.6976	0.160197	0.013878	0.041581	lncRNA
ENSG0000(	IGHJ1	18.40418	0.6774	0.01388	0.041582	IG_J_gene
ENSG0000(	BEND3	406.3453	-0.23475	0.013892	0.041612	protein_coding
ENSG0000(	ENSG0000(	26.34495	-0.42809	0.0139	0.041626	transcribed_processed_pseudoge
ENSG0000(	PLGLA	75.73012	-0.29052	0.013898	0.041626	transcribed_unprocessed_pseudo
ENSG0000(	PUS3	158.026	-0.23407	0.0139	0.041626	protein_coding
ENSG0000(	ALOX12-AS	922.2599	0.106534	0.013915	0.041665	lncRNA
ENSG0000(	STRADB	830.556	-0.28116	0.013916	0.041665	protein_coding
ENSG0000(	ENSG0000(	933.3786	-0.19646	0.013926	0.041691	lncRNA
ENSG0000(	RN7SL552F	12.50276	0.452002	0.013928	0.041694	misc_RNA
ENSG0000(	MTFR1L	1528.59	-0.19791	0.013931	0.041699	protein_coding
ENSG0000(	OAS3	3626.611	0.313378	0.013939	0.041719	protein_coding
ENSG0000(	ENSG0000(	20.42928	0.509681	0.01395	0.041743	lncRNA
ENSG0000(	ENSG0000(	12.38675	0.546523	0.01395	0.041743	lncRNA
ENSG0000(	PFKM	864.4615	-0.11365	0.013952	0.041745	protein_coding
ENSG0000(	RARRES2	398.7183	0.395149	0.013956	0.041752	protein_coding
ENSG0000(	ACAT1	984.3963	-0.13884	0.013963	0.04177	protein_coding
ENSG0000(	ACVR1B	1736.203	-0.23711	0.013971	0.041789	protein_coding
ENSG0000(	ENSG0000(	223.1182	0.134605	0.013977	0.041803	lncRNA
ENSG0000(	RNU6-322F	25.10793	0.62904	0.013979	0.041807	snRNA
ENSG0000(	ENSG0000(	295.2462	-0.38492	0.013987	0.041826	lncRNA
ENSG0000(	LINC01033	44.31418	0.423568	0.013989	0.041827	lncRNA
ENSG0000(	ENSG0000(	176.8234	0.223557	0.013998	0.041852	lncRNA
ENSG0000(	KMT2C	19882.7	0.06978	0.014003	0.041861	protein_coding
ENSG0000(	MRPL55	181.425	-0.22988	0.014005	0.041861	protein_coding
ENSG0000(	TMEM139	596.9962	-0.37551	0.014005	0.041861	protein_coding
ENSG0000(	C9orf64	472.9008	-0.12839	0.01402	0.0419	protein_coding
ENSG0000(	ENSG0000(	37.9172	0.390671	0.014024	0.041907	lncRNA
ENSG0000(	NOTO	113.5778	-0.22101	0.014028	0.041913	protein_coding
ENSG0000(	TMEM30BF	85.76651	-0.28278	0.014028	0.041913	processed_pseudogene
ENSG0000(	RN7SL277F	32.7557	0.522539	0.014036	0.04193	misc_RNA
ENSG0000(	TRMO	415.3214	0.192812	0.014036	0.04193	protein_coding
ENSG0000(	TPTEP1	1282.725	0.124434	0.014045	0.04195	transcribed_unprocessed_pseudo
ENSG0000(	ENSG0000(	287.2606	0.123571	0.014047	0.041954	lncRNA
ENSG0000(	ZDHHC23	2267.204	-0.30781	0.014066	0.042005	protein_coding
ENSG0000(	RNU4-53P	13.16807	0.577915	0.014071	0.042016	snRNA
ENSG0000(	REV1	1908.192	-0.1286	0.014079	0.042036	protein_coding
ENSG0000(	NHLRC1	112.3584	-0.18713	0.01409	0.042066	protein_coding
ENSG0000(	ENSG0000(	115.2566	0.212975	0.014095	0.042068	processed_pseudogene
ENSG0000(	ENSG0000(	19.5069	0.615244	0.014095	0.042068	lncRNA
ENSG0000(	TSKU	1549.529	-0.20338	0.014093	0.042068	protein_coding
ENSG0000(	DDX46	1714.967	-0.10617	0.014098	0.04207	protein_coding
ENSG0000(	ENSG0000(	323.0181	0.182757	0.014099	0.04207	lncRNA

ENSG0000(	NBEAL1	10610.55	-0.17741	0.0141	0.04207	protein_coding
ENSG0000(	SDF4	1776.634	-0.15827	0.014109	0.042094	protein_coding
ENSG0000(	CLIP2	2675.548	-0.19948	0.014116	0.042111	protein_coding
ENSG0000(	IGKV1-12	359.0545	0.650039	0.014133	0.042159	IG_V_gene
ENSG0000(	ENSG0000(	552.2724	-0.09209	0.014142	0.042181	lncRNA
ENSG0000(	ENSG0000(	3799.167	-0.13057	0.014143	0.042181	lncRNA
ENSG0000(	ASB4	309.3078	0.169243	0.014158	0.042217	protein_coding
ENSG0000(	ENSG0000(	82.48547	0.269534	0.01416	0.042217	processed_pseudogene
ENSG0000(	PPP1R3E	1188.828	0.168535	0.014159	0.042217	protein_coding
ENSG0000(	ENSG0000(	103.629	0.210728	0.014173	0.042253	lncRNA
ENSG0000(	DENND2A	872.3094	0.120375	0.014178	0.04226	protein_coding
ENSG0000(	UHRF1	584.9993	-0.18402	0.014178	0.04226	protein_coding
ENSG0000(	SMOX	164.3657	0.388693	0.01419	0.042291	protein_coding
ENSG0000(	ENSG0000(	735.7035	-0.10404	0.014198	0.04231	lncRNA
ENSG0000(	H3P31	83.5576	-0.25649	0.014208	0.042336	processed_pseudogene
ENSG0000(	UGT1A2P	140.5523	-0.57718	0.014229	0.042394	unprocessed_pseudogene
ENSG0000(	BICD1-AS1	41.068	-0.31753	0.014236	0.042413	lncRNA
ENSG0000(	ENSG0000(	25.35836	-0.5454	0.014238	0.042413	processed_pseudogene
ENSG0000(	ENSG0000(	45.70028	0.281518	0.01424	0.042415	lncRNA
ENSG0000(	ENSG0000(	173.8951	-0.42894	0.014249	0.042438	processed_pseudogene
ENSG0000(	LINC02515	360.1618	0.130186	0.014251	0.04244	lncRNA
ENSG0000(	SLC25A45	1389.663	0.132573	0.014252	0.04244	protein_coding
ENSG0000(	CFHR1	92.70484	-0.31479	0.014254	0.042441	protein_coding
ENSG0000(	THAP11	221.894	-0.20111	0.014262	0.042462	protein_coding
ENSG0000(	NLGN3	618.3426	0.114617	0.014264	0.042464	protein_coding
ENSG0000(	RBM17	1676.788	-0.08753	0.014267	0.042468	protein_coding
ENSG0000(	ENSG0000(	39.69382	-0.28918	0.014282	0.04251	lncRNA
ENSG0000(	ENSG0000(	9.888232	-0.67211	0.014286	0.042517	lncRNA
ENSG0000(	ENSG0000(	17.88857	0.507192	0.014289	0.042521	unprocessed_pseudogene
ENSG0000(	LMBRD2	987.1354	-0.17397	0.014293	0.042531	protein_coding
ENSG0000(	SNORA81	1176.081	-0.30089	0.014311	0.04258	snoRNA
ENSG0000(	JADE2	1863.794	0.175461	0.014315	0.042586	protein_coding
ENSG0000(	CADM3-AS	167.4494	0.44873	0.014323	0.042595	lncRNA
ENSG0000(	ENSG0000(	139.0017	-0.15972	0.014323	0.042595	lncRNA
ENSG0000(	LINC02827	14.30199	0.73717	0.01432	0.042595	lncRNA
ENSG0000(	PTGER2	332.4317	-0.25237	0.014321	0.042595	protein_coding
ENSG0000(	GLA	888.1206	-0.12253	0.014353	0.04268	protein_coding
ENSG0000(	ENSG0000(	16.61574	-0.60699	0.014366	0.042715	lncRNA
ENSG0000(	RNF220	1219.911	-0.07776	0.014374	0.042733	protein_coding
ENSG0000(	MIR4473	14.58667	0.492193	0.014377	0.042739	miRNA
ENSG0000(	ENSG0000(	10.98437	-0.9083	0.01439	0.042772	protein_coding
ENSG0000(	ESPNP	25.91483	-0.71459	0.014395	0.042784	transcribed_unprocessed_pseudo
ENSG0000(	ENSG0000(	251.45	-0.24054	0.014407	0.042803	lncRNA
ENSG0000(	FFAR1	114.0743	0.191703	0.014404	0.042803	protein_coding
ENSG0000(	SAMD5	1373.96	-0.2322	0.014406	0.042803	protein_coding
ENSG0000(	SIGLEC16	599.4157	0.098984	0.014406	0.042803	polymorphic_pseudogene
ENSG0000(	TAF2	5955.688	-0.10467	0.014415	0.042823	protein_coding

ENSG0000(	ENSG0000(	534.8875	0.135249	0.014416	0.042823	lncRNA
ENSG0000(	LINC02795	85.98446	0.289925	0.014419	0.042826	lncRNA
ENSG0000(	TANGO6	972.0825	-0.14373	0.014423	0.042836	protein_coding
ENSG0000(	EXOC3L4	622.3752	0.232086	0.014425	0.042838	protein_coding
ENSG0000(	ENSG0000(	41.71225	0.293422	0.014438	0.042872	lncRNA
ENSG0000(	ENSG0000(	83.39029	0.222687	0.014444	0.042882	lncRNA
ENSG0000(	ENSG0000(	17.80829	0.709132	0.014446	0.042882	lncRNA
ENSG0000(	YY1	2204.064	-0.13057	0.014446	0.042882	protein_coding
ENSG0000(	LINC01098	28.75985	0.367414	0.014457	0.042912	lncRNA
ENSG0000(	WASHC5	957.9715	-0.16001	0.014473	0.042954	protein_coding
ENSG0000(	ATP23	682.7978	-0.17105	0.014474	0.042955	protein_coding
ENSG0000(	ISLR	103.2388	0.47507	0.014484	0.04298	protein_coding
ENSG0000(	ENSG0000(	9.772458	-0.58749	0.014488	0.042984	lncRNA
ENSG0000(	FERMT2	828.0198	0.253044	0.014488	0.042984	protein_coding
ENSG0000(	EXO5	181.3068	-0.22623	0.014496	0.043002	protein_coding
ENSG0000(	PEPD	361.3616	-0.28483	0.014499	0.043007	protein_coding
ENSG0000(	LINC01531	455.949	-0.15669	0.014503	0.043015	lncRNA
ENSG0000(	ENSG0000(	144.6762	0.202393	0.014504	0.043016	lncRNA
ENSG0000(	CDH6	418.9727	0.266284	0.014514	0.04304	protein_coding
ENSG0000(	ENSG0000(	33.13892	0.403939	0.014521	0.043056	lncRNA
ENSG0000(	LINC01290	31.16204	-0.39555	0.014528	0.043072	lncRNA
ENSG0000(	CTDNEP1	1006.665	-0.15546	0.014532	0.043081	protein_coding
ENSG0000(	MYO7B	5958.254	-0.28904	0.014535	0.043081	protein_coding
ENSG0000(	SLC37A2	7340.579	-0.87436	0.014534	0.043081	protein_coding
ENSG0000(	DOK5	12.97681	0.829635	0.014538	0.043083	protein_coding
ENSG0000(	ENSG0000(	261.7767	0.193807	0.014537	0.043083	lncRNA
ENSG0000(	RAMP1	13.38934	0.704128	0.01454	0.043083	protein_coding
ENSG0000(	CYB561A3	3102.3	-0.09516	0.014544	0.043091	protein_coding
ENSG0000(	ENSG0000(	120.4106	0.184303	0.014547	0.043096	lncRNA
ENSG0000(	CFAP206	45.57003	-0.30888	0.014551	0.0431	protein_coding
ENSG0000(	ENSG0000(	27.58736	0.368922	0.014551	0.0431	lncRNA
ENSG0000(	ENSG0000(	179.5718	-0.22356	0.014552	0.0431	lncRNA
ENSG0000(	ARL5A	1684.485	0.136942	0.014557	0.043109	protein_coding
ENSG0000(	C7orf50	518.0553	-0.14964	0.01456	0.043114	protein_coding
ENSG0000(	PHTF1	783.846	0.166734	0.014587	0.04319	protein_coding
ENSG0000(	CAMTA2	1365.939	-0.13256	0.014597	0.043216	protein_coding
ENSG0000(	MGAM	442.9956	0.739649	0.014599	0.043218	protein_coding
ENSG0000(	GCSAML	180.6016	0.161139	0.01461	0.043248	protein_coding
ENSG0000(	ZBTB40	2387.533	0.117141	0.01462	0.043273	protein_coding
ENSG0000(	ENSG0000(	167.0207	0.182448	0.014635	0.04331	lncRNA
ENSG0000(	RGPD4-AS1	576.2751	-0.27587	0.014636	0.04331	lncRNA
ENSG0000(	RNF25	148.2926	-0.26698	0.014639	0.043316	protein_coding
ENSG0000(	ATM	14479.49	0.20703	0.014648	0.043333	protein_coding
ENSG0000(	IGHV4-34	342.449	0.784778	0.014647	0.043333	IG_V_gene
ENSG0000(	ENSG0000(	283.0756	0.169972	0.014658	0.043359	lncRNA
ENSG0000(	ENSG0000(	125.0583	-0.30654	0.014662	0.043367	lncRNA
ENSG0000(	AEBP2	1393.576	-0.11422	0.014664	0.04337	protein_coding

ENSG0000(	ENSG0000(	99.18	0.209205	0.014674	0.043395	lncRNA
ENSG0000(	ENSG0000(	765.1369	-0.20182	0.014676	0.043397	lncRNA
ENSG0000(	MAP2	88.28142	0.469125	0.014683	0.043412	protein_coding
ENSG0000(	ENSG0000(	128.8295	0.177182	0.014693	0.04344	lncRNA
ENSG0000(	ENSG0000(	464.8136	0.222844	0.014709	0.043483	lncRNA
ENSG0000(	ENSG0000(	80.68391	-1.04746	0.014735	0.043549	protein_coding
ENSG0000(	MFHAS1	1458.89	-0.16619	0.014734	0.043549	protein_coding
ENSG0000(	ENSG0000(	78.4408	0.222358	0.014739	0.043559	lncRNA
ENSG0000(	NR2F1	226.0931	0.341492	0.014751	0.043589	protein_coding
ENSG0000(	CDYL2	519.2798	0.303849	0.014753	0.04359	protein_coding
ENSG0000(	RRN3P1	1890.7	-0.11852	0.014762	0.043615	transcribed_unprocessed_pseudo
ENSG0000(	TAOK2	2107.507	-0.09472	0.014775	0.043641	protein_coding
ENSG0000(	TMEM250	629.733	-0.18468	0.014773	0.043641	protein_coding
ENSG0000(	ZBTB46-AS	65.54536	0.3281	0.014775	0.043641	lncRNA
ENSG0000(	PABPC4L	29.73835	0.406445	0.014779	0.043646	protein_coding
ENSG0000(	SPPL2A	5219.745	-0.17645	0.014795	0.043691	protein_coding
ENSG0000(	KANSL1-AS	309.4292	0.178334	0.014802	0.043708	lncRNA
ENSG0000(	GNAI3	2599.582	-0.091	0.014807	0.043719	protein_coding
ENSG0000(	IRGQ	2371.773	-0.09431	0.014811	0.043722	protein_coding
ENSG0000(	RBM7	1117.12	-0.16221	0.014811	0.043722	protein_coding
ENSG0000(	ENSG0000(	659.2151	0.086069	0.014825	0.04376	lncRNA
ENSG0000(	FKBP11	1493.229	0.181309	0.014827	0.04376	protein_coding
ENSG0000(	CHUK	692.2162	-0.15297	0.014846	0.043814	protein_coding
ENSG0000(	ENSG0000(	293.3638	-0.19796	0.014868	0.043874	transcribed_unprocessed_pseudo
ENSG0000(	PMEL	31.6812	0.475639	0.014875	0.043889	protein_coding
ENSG0000(	EVPL	737.7451	-0.20187	0.014892	0.043935	protein_coding
ENSG0000(	ENSG0000(	47.92988	-0.40375	0.014901	0.043958	lncRNA
ENSG0000(	RNA5SP527	11.59631	-0.51985	0.014919	0.044006	rRNA
ENSG0000(	SEPTIN6	2010.923	0.35959	0.014924	0.044018	protein_coding
ENSG0000(	ENSG0000(	94.7821	-0.19825	0.014944	0.044067	lncRNA
ENSG0000(	LINC01755	23.16076	-0.4836	0.014943	0.044067	lncRNA
ENSG0000(	MIR429	40.84085	-0.49856	0.014948	0.044076	miRNA
ENSG0000(	LINC01592	11.77157	0.710531	0.014951	0.04408	lncRNA
ENSG0000(	MFAP5	184.9728	0.265615	0.014966	0.04412	protein_coding
ENSG0000(	ENSG0000(	16.07956	0.495008	0.014968	0.044123	processed_pseudogene
ENSG0000(	SETP6	12.42382	-0.59047	0.014973	0.04413	processed_pseudogene
ENSG0000(	TMEM135	1548.378	-0.134	0.014973	0.04413	protein_coding
ENSG0000(	ADAMTSL3	1691.746	-0.20411	0.014982	0.044146	protein_coding
ENSG0000(	CGN	5623.852	-0.39956	0.014981	0.044146	protein_coding
ENSG0000(	ENSG0000(	137.8055	-0.21587	0.014994	0.044173	lncRNA
ENSG0000(	ENSG0000(	70.29851	0.23606	0.014993	0.044173	lncRNA
ENSG0000(	ENSG0000(	701.8245	0.298517	0.014999	0.044185	transcribed_unprocessed_pseudo
ENSG0000(	ENSG0000(	40.15066	0.385787	0.015004	0.044188	lncRNA
ENSG0000(	FAM171B	152.8151	0.307355	0.015004	0.044188	protein_coding
ENSG0000(	SNAP25	1455.115	0.194715	0.015002	0.044188	protein_coding
ENSG0000(	MIR1-1HG	38.09663	0.518796	0.015013	0.04421	lncRNA
ENSG0000(	ENSG0000(	66.12567	0.419012	0.015028	0.044246	lncRNA

ENSG0000(	RAB38	41.76569	0.319551	0.015028	0.044246	protein_coding
ENSG0000(	CDHR2	3042.473	-0.4927	0.015033	0.044255	protein_coding
ENSG0000(	ENSG0000(	91.07874	-0.18257	0.015034	0.044255	lncRNA
ENSG0000(	LINC02242	324.2444	0.143149	0.015039	0.044264	lncRNA
ENSG0000(	ANKRD33B	360.5952	0.410098	0.015044	0.044276	protein_coding
ENSG0000(	ENSG0000(	72.16412	-0.38776	0.015051	0.044293	unprocessed_pseudogene
ENSG0000(	KIF9-AS1	940.036	-0.08504	0.015063	0.044323	lncRNA
ENSG0000(	ENSG0000(	39.66319	0.327655	0.01508	0.044362	processed_pseudogene
ENSG0000(	ENSG0000(	152.7737	-0.85582	0.01508	0.044362	protein_coding
ENSG0000(	RNU6-1136	17.10748	0.483003	0.015079	0.044362	snRNA
ENSG0000(	HEBP2	1930.277	-0.22189	0.015089	0.04438	protein_coding
ENSG0000(	MIRLET7A1	2196.01	0.106499	0.01509	0.04438	lncRNA
ENSG0000(	LINC01115	142.1743	0.175283	0.015093	0.044381	lncRNA
ENSG0000(	RPL10P19	35.00391	0.36807	0.015092	0.044381	transcribed_processed_pseudoge
ENSG0000(	LINC02185	419.2021	0.108382	0.0151	0.0444	lncRNA
ENSG0000(	SAMD13	248.751	-0.33377	0.015121	0.044456	protein_coding
ENSG0000(	MTCO1P30	12.33856	0.569083	0.015131	0.044482	processed_pseudogene
ENSG0000(	FHIP1B	3891.435	-0.23885	0.01514	0.044504	protein_coding
ENSG0000(	COL5A3	636.9118	0.352722	0.015151	0.044532	protein_coding
ENSG0000(	PPP3R2	14.83561	0.815004	0.015155	0.044538	protein_coding
ENSG0000(	AKIRIN2	720.5028	0.168816	0.015157	0.04454	protein_coding
ENSG0000(	DDX19A-DT	163.5328	-0.17629	0.015175	0.044588	lncRNA
ENSG0000(	SLC26A7	41.02207	0.546187	0.015179	0.044599	protein_coding
ENSG0000(	SNORD14D	19.98425	0.910289	0.015188	0.04462	snoRNA
ENSG0000(	ENSG0000(	20.23699	0.448222	0.015199	0.044649	lncRNA
ENSG0000(	HES1	646.21	-0.27148	0.015214	0.044689	protein_coding
ENSG0000(	EIF3J	1051.241	-0.16105	0.015218	0.044695	protein_coding
ENSG0000(	FCRL2	1220.524	0.382259	0.01522	0.044695	protein_coding
ENSG0000(	CYP51A1	1233.338	-0.348	0.015231	0.044725	protein_coding
ENSG0000(	MROH1	3454.855	-0.15777	0.015236	0.044736	protein_coding
ENSG0000(	CUL3	2368.049	-0.10788	0.015239	0.04474	protein_coding
ENSG0000(	MAP3K6	387.3036	0.211592	0.015243	0.044744	protein_coding
ENSG0000(	MT-TV	185.7714	0.48769	0.015242	0.044744	Mt_tRNA
ENSG0000(	ENSG0000(	535.1964	0.123057	0.015252	0.044766	lncRNA
ENSG0000(	ENSG0000(	14.25712	0.596158	0.015259	0.044781	lncRNA
ENSG0000(	DKK3	523.1653	0.331382	0.015261	0.044783	protein_coding
ENSG0000(	ABO	4142.685	-0.20404	0.015263	0.044786	protein_coding
ENSG0000(	C10orf99	1311.994	-0.42708	0.015278	0.044826	protein_coding
ENSG0000(	ENSG0000(	108.004	0.247345	0.015283	0.04483	lncRNA
ENSG0000(	ENSG0000(	114.4589	-0.22243	0.015284	0.04483	lncRNA
ENSG0000(	H6PD	3480.148	0.153746	0.015282	0.04483	protein_coding
ENSG0000(	ADCY1	301.8789	0.242533	0.015286	0.044831	protein_coding
ENSG0000(	ADH1A	16.98744	-0.55649	0.015289	0.044837	protein_coding
ENSG0000(	DCTN1	1808.45	-0.11252	0.015295	0.044845	protein_coding
ENSG0000(	ENSG0000(	19.59719	0.418136	0.015294	0.044845	lncRNA
ENSG0000(	RAET1G	9.807072	0.532964	0.015299	0.044852	protein_coding
ENSG0000(	CSKMT	358.8218	-0.25747	0.015312	0.044878	protein_coding

ENSG0000(	ENSG0000(	143.9945	0.283833	0.015313	0.044878	lncRNA
ENSG0000(	HDGFL2	645.8612	-0.09864	0.015312	0.044878	protein_coding
ENSG0000(	PSG9	605.4893	0.371622	0.015312	0.044878	protein_coding
ENSG0000(	ENSG0000(	73.60737	0.249654	0.015321	0.044898	lncRNA
ENSG0000(	USF1	1117.662	0.175452	0.015327	0.044909	protein_coding
ENSG0000(	FXYD3	4410.26	-0.50742	0.015353	0.044982	protein_coding
ENSG0000(	CACNB3	1218.494	-0.21311	0.015356	0.044986	protein_coding
ENSG0000(	ENSG0000(	208.1616	-0.19307	0.015363	0.045002	lncRNA
ENSG0000(	FAM3D-AS:	388.0182	0.153023	0.015368	0.045012	lncRNA
ENSG0000(	SNORA23	326.01	-0.2613	0.015374	0.045027	snoRNA
ENSG0000(	ERC2-IT1	103.3012	0.15226	0.015383	0.045049	lncRNA
ENSG0000(	ISOC1	291.6015	-0.20258	0.015385	0.045049	protein_coding
ENSG0000(	LONP1	768.7587	-0.11037	0.015395	0.045075	protein_coding
ENSG0000(	GIPC1	1545.544	-0.30003	0.015401	0.045089	protein_coding
ENSG0000(	ENSG0000(	162.3988	0.185924	0.015407	0.045103	lncRNA
ENSG0000(	MIR3677H(	280.948	-0.13496	0.015431	0.045167	lncRNA
ENSG0000(	RTBDN	184.3257	-0.16449	0.01544	0.045191	protein_coding
ENSG0000(	RNF138P1	203.0416	-0.30666	0.01545	0.045211	processed_pseudogene
ENSG0000(	USF3	3850.873	-0.10566	0.015449	0.045211	protein_coding
ENSG0000(	GIMAP1	597.0621	0.262048	0.015456	0.045219	protein_coding
ENSG0000(	HMGA2	1477.623	-0.26882	0.015455	0.045219	protein_coding
ENSG0000(	NOC3L	479.5511	-0.2353	0.015462	0.045234	protein_coding
ENSG0000(	LINC02643	16.5302	-0.48857	0.015473	0.04526	lncRNA
ENSG0000(	ENSG0000(	13.39046	0.801124	0.015475	0.045262	TEC
ENSG0000(	ENSG0000(	199.868	-0.52216	0.015479	0.045265	unprocessed_pseudogene
ENSG0000(	TTL	2750.413	0.160016	0.015478	0.045265	protein_coding
ENSG0000(	NYNRIN	926.2342	-0.28647	0.015484	0.045276	protein_coding
ENSG0000(	LINC01128	3029.66	0.09712	0.015492	0.045291	lncRNA
ENSG0000(	POLR2K	254.9192	-0.1904	0.015491	0.045291	protein_coding
ENSG0000(	KRT42P	124.0747	-0.24109	0.015494	0.045294	transcribed_unprocessed_pseudo
ENSG0000(	ENSG0000(	12.61952	0.533069	0.015496	0.045294	lncRNA
ENSG0000(	CCNG1	1163.696	-0.24316	0.015503	0.04531	protein_coding
ENSG0000(	ENSG0000(	353.4245	0.116537	0.015504	0.04531	lncRNA
ENSG0000(	MTHFD1L	360.0203	0.324112	0.015507	0.045313	protein_coding
ENSG0000(	ENSG0000(	21.78965	0.327428	0.01551	0.045318	lncRNA
ENSG0000(	ENSG0000(	1242.176	0.294676	0.015521	0.045343	lncRNA
ENSG0000(	GUCY1A2	223.6873	0.413973	0.015521	0.045343	protein_coding
ENSG0000(	ENSG0000(	18.73944	0.475155	0.015523	0.045345	lncRNA
ENSG0000(	ENSG0000(	27.19941	0.286208	0.01553	0.045361	transcribed_unitary_pseudogene
ENSG0000(	RNU6-957F	20.4693	0.479784	0.015533	0.045364	snRNA
ENSG0000(	ENSG0000(	16.72865	-0.49092	0.015536	0.045369	lncRNA
ENSG0000(	CNIH1	997.4988	-0.19822	0.015549	0.045396	protein_coding
ENSG0000(	MSI1	188.0773	-0.28003	0.015547	0.045396	protein_coding
ENSG0000(	TDRD9	64.0311	0.626349	0.015549	0.045396	protein_coding
ENSG0000(	SH3PXD2A-	48.18268	0.335475	0.015558	0.045417	lncRNA
ENSG0000(	LAMTOR5-/	896.0523	-0.1012	0.015567	0.045437	lncRNA
ENSG0000(	ENSG0000(	75.11208	0.239166	0.01557	0.045442	lncRNA

ENSG0000(	DPY19L2P3	168.278	0.205768	0.015574	0.045445	transcribed_unprocessed_pseudo
ENSG0000(	UGT1A1	852.4491	-0.23626	0.015573	0.045445	protein_coding
ENSG0000(	GM2A	1375.108	0.293292	0.015582	0.045466	protein_coding
ENSG0000(	PCDHB5	24.18064	-0.4888	0.01559	0.045481	protein_coding
ENSG0000(	POP7	94.97267	-0.22339	0.01559	0.045481	protein_coding
ENSG0000(	YWHABP2	30.71352	-0.48003	0.015604	0.045516	processed_pseudogene
ENSG0000(	LINC02574	20.44505	0.776886	0.01561	0.045531	lncRNA
ENSG0000(	CREBZF	3555.402	0.10801	0.015633	0.045588	protein_coding
ENSG0000(	ENSG0000(	192.7484	0.199637	0.015632	0.045588	lncRNA
ENSG0000(	ATXN7L3	1422.981	-0.15658	0.015638	0.045598	protein_coding
ENSG0000(	SNORA79B	285.672	-0.25488	0.015667	0.045678	snoRNA
ENSG0000(	MAP3K5	1381.163	0.164575	0.015674	0.045695	protein_coding
ENSG0000(	TOP3B	848.1329	-0.12316	0.015677	0.045699	protein_coding
ENSG0000(	ENSG0000(	50.19418	-0.31932	0.015681	0.045707	lncRNA
ENSG0000(	FICD	273.2033	0.244199	0.015686	0.045712	protein_coding
ENSG0000(	PTPN18	4109.986	-0.18137	0.015685	0.045712	protein_coding
ENSG0000(	ENSG0000(	16.86284	0.623907	0.015691	0.045722	TEC
ENSG0000(	ENSG0000(	57.18952	0.383963	0.015696	0.045734	transcribed_processed_pseudoge
ENSG0000(	ENSG0000(	759.2427	0.248972	0.0157	0.045742	lncRNA
ENSG0000(	IGHV3-21	667.7331	0.57611	0.015712	0.045771	IG_V_gene
ENSG0000(	DIO2-AS1	25.78088	-0.32347	0.015745	0.045863	lncRNA
ENSG0000(	LDAF1	487.6778	-0.17639	0.015769	0.045928	protein_coding
ENSG0000(	CSTF3-DT	345.843	-0.13742	0.01578	0.045957	lncRNA
ENSG0000(	PCBP1	1476.595	-0.18076	0.015802	0.046018	protein_coding
ENSG0000(	KDELR3	170.8313	0.398166	0.01581	0.046036	protein_coding
ENSG0000(	GCSIR	93.12959	0.330531	0.015816	0.046051	lncRNA
ENSG0000(	NFX1	3473.289	0.077427	0.015829	0.046082	protein_coding
ENSG0000(	SETMAR	337.9702	-0.18373	0.015832	0.046087	protein_coding
ENSG0000(	ENSG0000(	645.6333	-0.15661	0.015839	0.046103	lncRNA
ENSG0000(	REEP5	1215.158	-0.13665	0.015861	0.046164	protein_coding
ENSG0000(	CHIAP2	79.93115	0.252067	0.015888	0.046236	transcribed_unprocessed_pseudo
ENSG0000(	LINC00485	127.0689	-0.20043	0.015889	0.046236	lncRNA
ENSG0000(	ENSG0000(	140.9181	0.200709	0.015892	0.046239	TEC
ENSG0000(	FAM107A	191.4186	0.392391	0.015893	0.046239	protein_coding
ENSG0000(	ENSG0000(	190.8409	-0.22063	0.015897	0.046246	lncRNA
ENSG0000(	ENSG0000(	1726.482	-0.22092	0.015914	0.046293	lncRNA
ENSG0000(	ENSG0000(	117.7998	0.26085	0.015917	0.046297	lncRNA
ENSG0000(	ENSG0000(	141.6413	0.185726	0.015921	0.046303	lncRNA
ENSG0000(	ENSG0000(	15.50505	-0.67966	0.01593	0.046325	processed_pseudogene
ENSG0000(	ENSG0000(	21.7708	0.503162	0.015933	0.046331	lncRNA
ENSG0000(	ASNSD1	407.1321	-0.18423	0.015942	0.046347	protein_coding
ENSG0000(	GAS2L3	1388.322	-0.20804	0.015942	0.046347	protein_coding
ENSG0000(	ENSG0000(	22.39904	0.419017	0.015945	0.046352	processed_pseudogene
ENSG0000(	ENSG0000(	732.9586	0.091008	0.015949	0.046358	lncRNA
ENSG0000(	STXBP6	580.9881	-0.26934	0.015951	0.046359	protein_coding
ENSG0000(	MAP3K3	910.1375	0.175206	0.015956	0.04637	protein_coding
ENSG0000(	NOS1	773.6296	0.10204	0.015959	0.046371	protein_coding

ENSG0000(	SLC35A3	9137.901	-0.20163	0.015959	0.046371	protein_coding
ENSG0000(	TSPAN14	4254.577	-0.11743	0.015961	0.046371	protein_coding
ENSG0000(	DYNLT2	231.992	0.176943	0.016	0.046481	protein_coding
ENSG0000(	RNU2-17P	14.16218	-0.7222	0.016009	0.046502	snRNA
ENSG0000(	EEA1	1728.927	-0.19173	0.016026	0.046548	protein_coding
ENSG0000(	TCTN1	1669.235	0.096654	0.01603	0.046556	protein_coding
ENSG0000(	CCNB1IP1	670.2349	-0.12201	0.016037	0.04657	protein_coding
ENSG0000(	IGLV9-49	21.14168	0.866659	0.01604	0.046574	IG_V_gene
ENSG0000(	NPFF	71.04511	-0.21636	0.016042	0.046574	protein_coding
ENSG0000(	SLC13A3	109.3672	0.213539	0.016042	0.046574	protein_coding
ENSG0000(	GLUD1P2	63.36609	0.271496	0.016044	0.046575	transcribed_unprocessed_pseudo
ENSG0000(	LINC00575	16.90575	0.490609	0.016055	0.046601	lncRNA
ENSG0000(	ENSG0000(	98.4175	0.176843	0.016061	0.046614	transcribed_unprocessed_pseudo
ENSG0000(	DRAP1	159.3565	-0.23976	0.016066	0.046626	protein_coding
ENSG0000(	RN7SL452F	14.07967	0.436533	0.016072	0.046639	misc_RNA
ENSG0000(	VPS26C	1868.486	-0.09605	0.016077	0.046649	protein_coding
ENSG0000(	RN7SKP95	123.008	0.218898	0.016085	0.046667	misc_RNA
ENSG0000(	CAPN15	3417.419	-0.19542	0.016092	0.046683	protein_coding
ENSG0000(	ENSG0000(	30.79752	0.402066	0.016096	0.046691	TEC
ENSG0000(	SDCBP2-AS	443.8564	0.199066	0.016109	0.046723	lncRNA
ENSG0000(	MBD1	2064.205	-0.1034	0.016128	0.046773	protein_coding
ENSG0000(	SDSL	171.4773	-0.23377	0.016131	0.046779	protein_coding
ENSG0000(	ENSG0000(	92.78334	-0.26009	0.016137	0.046792	lncRNA
ENSG0000(	MEA1	267.5031	-0.19211	0.016148	0.046821	protein_coding
ENSG0000(	MED28-DT	235.2703	-0.11733	0.016155	0.046833	lncRNA
ENSG0000(	SMYD3	529.3494	0.156767	0.016156	0.046833	protein_coding
ENSG0000(	ENSG0000(	198.8528	0.19755	0.016159	0.046839	protein_coding
ENSG0000(	ENSG0000(	170.0367	-0.15062	0.016171	0.046868	lncRNA
ENSG0000(	ENSG0000(	235.3128	0.142966	0.016175	0.046873	lncRNA
ENSG0000(	ENSG0000(	45.4102	0.322728	0.016175	0.046873	lncRNA
ENSG0000(	B4GAT1	102.2847	-0.29872	0.016197	0.046926	protein_coding
ENSG0000(	ENSG0000(	26.4296	0.520204	0.016197	0.046926	lncRNA
ENSG0000(	SPATA2L	134.2967	-0.25439	0.016198	0.046926	protein_coding
ENSG0000(	ZNF669	508.1865	-0.12862	0.0162	0.046928	protein_coding
ENSG0000(	KPNA6	2673.159	-0.09778	0.016202	0.046929	protein_coding
ENSG0000(	RSC1A1	180.6786	-0.39798	0.016209	0.046943	protein_coding
ENSG0000(	AFG1L	436.3783	-0.15588	0.016213	0.046951	protein_coding
ENSG0000(	RCN3	750.2618	-0.38577	0.016221	0.04697	protein_coding
ENSG0000(	ENSG0000(	26.05576	0.35017	0.016227	0.046984	lncRNA
ENSG0000(	OGDHL	53.41179	-0.59217	0.016234	0.046999	protein_coding
ENSG0000(	ENSG0000(	1016.569	0.108323	0.016238	0.047001	lncRNA
ENSG0000(	ENSG0000(	54.42976	0.237187	0.016238	0.047001	lncRNA
ENSG0000(	GULP1	887.0455	-0.25136	0.016241	0.047003	protein_coding
ENSG0000(	VASH2	320.1024	0.160126	0.016241	0.047003	protein_coding
ENSG0000(	LINC02671	128.3277	-0.22425	0.016243	0.047005	lncRNA
ENSG0000(	USP42	742.3369	-0.1058	0.016261	0.047052	protein_coding
ENSG0000(	GJC2	22.3028	0.441056	0.016277	0.047093	protein_coding

ENSG0000(	CRLF2	40.6508	0.388033	0.016279	0.047094	protein_coding
ENSG0000(	ENSG0000(	105.3041	0.177939	0.016283	0.047103	lncRNA
ENSG0000(	ZSCAN20	623.2252	0.128573	0.016286	0.047105	protein_coding
ENSG0000(	ENSG0000(	72.10781	0.241675	0.0163	0.047124	lncRNA
ENSG0000(	ENSG0000(	194.214	-0.14597	0.0163	0.047124	lncRNA
ENSG0000(	ENSG0000(	980.5209	-0.35157	0.016296	0.047124	lncRNA
ENSG0000(	TMPO	2537.543	-0.16278	0.016298	0.047124	protein_coding
ENSG0000(	USP2	587.2557	-0.62857	0.016298	0.047124	protein_coding
ENSG0000(	AKAP10	1499.979	-0.10656	0.016313	0.047158	protein_coding
ENSG0000(	ENSG0000(	98.75407	-0.23363	0.016318	0.047167	TEC
ENSG0000(	GJB6	27.26551	0.395494	0.01632	0.04717	protein_coding
ENSG0000(	ENSG0000(	88.32704	-0.21072	0.016331	0.047197	lncRNA
ENSG0000(	PCID2	1164.677	-0.13233	0.016334	0.0472	protein_coding
ENSG0000(	ENSG0000(	137.333	0.214054	0.016369	0.047297	lncRNA
ENSG0000(	MED1	1866.567	-0.11758	0.016387	0.047345	protein_coding
ENSG0000(	RAMP2-AS1	613.114	0.116772	0.016396	0.047369	lncRNA
ENSG0000(	FAM151B-I	629.149	-0.11745	0.016414	0.047416	lncRNA
ENSG0000(	TMEM218	872.145	0.149455	0.016416	0.047417	protein_coding
ENSG0000(	CYP19A1	21.09374	1.073902	0.016418	0.047418	protein_coding
ENSG0000(	ATG14	1326.243	0.099125	0.016429	0.047432	protein_coding
ENSG0000(	ENSG0000(	714.7215	0.135801	0.016431	0.047432	protein_coding
ENSG0000(	MRFAP1	1226.982	-0.18272	0.016431	0.047432	protein_coding
ENSG0000(	PLOD3	1026.851	-0.11142	0.016428	0.047432	protein_coding
ENSG0000(	WHRN	427.2655	0.294543	0.016429	0.047432	protein_coding
ENSG0000(	ARMC8	1873.038	-0.10083	0.016439	0.047445	protein_coding
ENSG0000(	C4orf36	72.85823	-0.25818	0.01644	0.047445	protein_coding
ENSG0000(	ENSG0000(	25.20532	-0.33999	0.016439	0.047445	lncRNA
ENSG0000(	MFSD5	214.7655	-0.31165	0.016456	0.047489	protein_coding
ENSG0000(	LINC01951	72.81009	0.263413	0.016459	0.047489	lncRNA
ENSG0000(	MIR581	25.02393	0.39304	0.016458	0.047489	miRNA
ENSG0000(	ENSG0000(	124.1723	0.354265	0.016464	0.047499	lncRNA
ENSG0000(	ARMCX2	234.4994	0.32921	0.016479	0.047538	protein_coding
ENSG0000(	PHF7	353.4907	-0.20093	0.016482	0.047542	protein_coding
ENSG0000(	TLCD4	1911.821	-0.44721	0.016494	0.047571	protein_coding
ENSG0000(	ATP9A	3791.59	-0.29104	0.016507	0.047596	protein_coding
ENSG0000(	ENSG0000(	122.9859	-0.20982	0.016505	0.047596	lncRNA
ENSG0000(	LINC02471	82.0105	0.287532	0.016505	0.047596	lncRNA
ENSG0000(	TWF2	298.2767	-0.2311	0.016508	0.047596	protein_coding
ENSG0000(	RN7SKP104	46.23379	-0.6588	0.016527	0.047645	misc_RNA
ENSG0000(	LPL	109.0875	0.615457	0.016543	0.047682	protein_coding
ENSG0000(	RPL23P2	13.32839	0.447645	0.016542	0.047682	processed_pseudogene
ENSG0000(	ENSG0000(	106.0451	-0.28373	0.016552	0.047698	lncRNA
ENSG0000(	PAQR7	410.8475	-0.14244	0.016552	0.047698	protein_coding
ENSG0000(	PLEKHH1	4687.116	-0.24722	0.016553	0.047698	protein_coding
ENSG0000(	ENSG0000(	37.82721	0.311822	0.016558	0.047707	lncRNA
ENSG0000(	EPHX2	1659.272	-0.20423	0.016575	0.047747	protein_coding
ENSG0000(	PRRG1	495.6589	0.149885	0.016576	0.047747	protein_coding

ENSG0000(	SETX	9037.922	0.145379	0.016576	0.047747	protein_coding
ENSG0000(	IRAK4	1660.397	0.109946	0.016584	0.047766	protein_coding
ENSG0000(	TSPAN8	4378.487	-0.3072	0.016594	0.04779	protein_coding
ENSG0000(	LINC02465	97.40876	-0.25262	0.0166	0.047796	lncRNA
ENSG0000(	RAB40A	200.682	0.191155	0.0166	0.047796	protein_coding
ENSG0000(	SKA3	137.7494	-0.33757	0.016601	0.047796	protein_coding
ENSG0000(	ADAMTS5	399.8964	0.386985	0.016614	0.047831	protein_coding
ENSG0000(	ADAMTS9-/	780.3196	0.209398	0.016624	0.047853	lncRNA
ENSG0000(	MRPL50	415.8747	-0.14056	0.016629	0.047863	protein_coding
ENSG0000(	C1orf43	1556.471	-0.12147	0.016635	0.047876	protein_coding
ENSG0000(	ENSG0000(	39.43208	-0.96516	0.016645	0.047901	lncRNA
ENSG0000(	MTND3P22	12.11564	0.663258	0.016672	0.047975	processed_pseudogene
ENSG0000(	ABCA2	1599.76	-0.27136	0.016679	0.047985	protein_coding
ENSG0000(	GGT8P	29.78458	-0.73581	0.016679	0.047985	unprocessed_pseudogene
ENSG0000(	ENSG0000(	84.56438	0.296759	0.01668	0.047985	lncRNA
ENSG0000(	RN7SL833F	13.41357	-0.50984	0.016687	0.048001	misc_RNA
ENSG0000(	CUL1	1229.02	-0.12078	0.016691	0.048008	protein_coding
ENSG0000(	RPL23AP82	998.3161	0.129388	0.016702	0.048035	transcribed_unprocessed_pseudo
ENSG0000(	TMEM39B	174.87	-0.17638	0.016704	0.048035	protein_coding
ENSG0000(	EPS8L3	3484.257	-0.30759	0.016716	0.048066	protein_coding
ENSG0000(	ENSG0000(	40.44193	-0.42684	0.01672	0.048073	lncRNA
ENSG0000(	ENSG0000(	158.3789	-0.20064	0.016726	0.048086	lncRNA
ENSG0000(	ENSG0000(	9.896448	0.563239	0.016747	0.048141	transcribed_unprocessed_pseudo
ENSG0000(	ENSG0000(	328.3132	0.188628	0.016754	0.048156	lncRNA
ENSG0000(	PODXL2	137.1502	-0.356	0.016765	0.048185	protein_coding
ENSG0000(	POM121	2373.989	-0.08479	0.016777	0.048215	protein_coding
ENSG0000(	ENSG0000(	9.77182	0.47998	0.016783	0.048227	lncRNA
ENSG0000(	ENSG0000(	55.11918	0.477384	0.016793	0.048253	lncRNA
ENSG0000(	ENSG0000(	83.86579	-0.36438	0.01682	0.048325	protein_coding
ENSG0000(	PSTK	287.639	-0.15723	0.016823	0.048329	protein_coding
ENSG0000(	ENSG0000(	999.6485	0.116706	0.016828	0.048339	lncRNA
ENSG0000(	GAN	3063.844	-0.24554	0.016856	0.048416	protein_coding
ENSG0000(	CREB3L1	1086.401	0.226481	0.01686	0.048418	protein_coding
ENSG0000(	ENSG0000(	109.0574	0.261671	0.01686	0.048418	lncRNA
ENSG0000(	ENSG0000(	411.3981	0.203075	0.016874	0.04845	transcribed_unprocessed_pseudo
ENSG0000(	TMEM214	1391.457	-0.15685	0.016874	0.04845	protein_coding
ENSG0000(	DHX29	837.1591	-0.15466	0.016933	0.048614	protein_coding
ENSG0000(	ENSG0000(	252.9992	0.168528	0.016938	0.048623	TEC
ENSG0000(	PLA2G10	178.4325	-0.38659	0.016947	0.048644	protein_coding
ENSG0000(	RNU6-853F	17.30379	0.466743	0.016951	0.048653	snRNA
ENSG0000(	STK26	340.9253	-0.20088	0.016956	0.048662	protein_coding
ENSG0000(	ENSG0000(	13.71226	0.460849	0.016966	0.048686	lncRNA
ENSG0000(	PARVG	1413.34	0.360587	0.016975	0.048708	protein_coding
ENSG0000(	ENSG0000(	13.93089	0.510666	0.016987	0.048737	unprocessed_pseudogene
ENSG0000(	SH3RF1	2295.363	-0.29559	0.017001	0.048772	protein_coding
ENSG0000(	ENSG0000(	11.57538	0.48426	0.017011	0.048798	lncRNA
ENSG0000(	LINC02504	76.7803	-0.31854	0.017016	0.048807	lncRNA

ENSG0000(	ENSG0000(	104.891	-0.2131	0.017038	0.048861	TEC
ENSG0000(	PRR33	10.8417	0.920742	0.017037	0.048861	protein_coding
ENSG0000(	ENSG0000(	185.5548	-0.19096	0.017046	0.048879	lncRNA
ENSG0000(	CAMK2G	2413.735	-0.16229	0.017049	0.04888	protein_coding
ENSG0000(	ENSG0000(	133.6472	0.28706	0.017048	0.04888	lncRNA
ENSG0000(	ANKS6	712.7002	-0.17687	0.017062	0.048913	protein_coding
ENSG0000(	BRCA1	1291.987	-0.1236	0.017065	0.048917	protein_coding
ENSG0000(	ENSG0000(	84.02881	-0.24208	0.017069	0.048922	lncRNA
ENSG0000(	KRT8P15	15.73705	-0.52793	0.017077	0.048937	processed_pseudogene
ENSG0000(	SHPK	1190.036	-0.17472	0.017076	0.048937	protein_coding
ENSG0000(	ENSG0000(	30.67153	0.367729	0.017096	0.048986	lncRNA
ENSG0000(	ENSG0000(	12.21783	-0.49835	0.017102	0.048996	lncRNA
ENSG0000(	NCF1C	123.1601	0.501514	0.017102	0.048996	unprocessed_pseudogene
ENSG0000(	SNORA3A	210.0285	0.253865	0.017104	0.048997	snoRNA
ENSG0000(	ENSG0000(	563.8068	0.11929	0.017113	0.049019	TEC
ENSG0000(	PDE4C	823.4584	-0.17829	0.01712	0.049032	protein_coding
ENSG0000(	DSCC1	67.02667	-0.41531	0.017125	0.049042	protein_coding
ENSG0000(	ZNF37A	3324.531	0.110731	0.017127	0.049046	protein_coding
ENSG0000(	ENSG0000(	9.614646	1.024052	0.017136	0.049064	lncRNA
ENSG0000(	TMC4	1177.822	-0.35282	0.017141	0.049076	protein_coding
ENSG0000(	DPY19L3	1859.736	0.091168	0.017163	0.049133	protein_coding
ENSG0000(	NBEAL2	4621.691	-0.21806	0.017177	0.049166	protein_coding
ENSG0000(	ST6GALNA(	1950.372	-0.3774	0.017176	0.049166	protein_coding
ENSG0000(	PCNT	2575.157	-0.15106	0.017209	0.049253	protein_coding
ENSG0000(	AQP4-AS1	355.2048	0.141172	0.017223	0.04927	lncRNA
ENSG0000(	ENSG0000(	30.64435	-0.58084	0.01722	0.04927	transcribed_unprocessed_pseudo
ENSG0000(	NBPF19	6019.144	-0.26534	0.017221	0.04927	protein_coding
ENSG0000(	RNU5A-1	3487.119	0.307546	0.017222	0.04927	snRNA
ENSG0000(	ZBTB8B	1619.523	-0.10043	0.017222	0.04927	protein_coding
ENSG0000(	RRAS2	647.7462	-0.15932	0.017228	0.049279	protein_coding
ENSG0000(	ENSG0000(	49.06285	0.233016	0.01723	0.049281	lncRNA
ENSG0000(	DGKH	4923.744	0.137799	0.017247	0.049325	protein_coding
ENSG0000(	ENSG0000(	17.7937	0.432463	0.017257	0.049349	lncRNA
ENSG0000(	GTF3A	575.8515	-0.20462	0.017276	0.049394	protein_coding
ENSG0000(	LINC01748	756.4719	0.192981	0.017275	0.049394	lncRNA
ENSG0000(	TOB2P1	14.91557	-0.44017	0.017279	0.049398	processed_pseudogene
ENSG0000(	BECN1	2165.431	-0.10803	0.017293	0.049435	protein_coding
ENSG0000(	ENSG0000(	78.0239	-0.1966	0.017295	0.049436	lncRNA
ENSG0000(	ENSG0000(	662.6172	0.121837	0.0173	0.049444	lncRNA
ENSG0000(	RNU6-177F	10.27888	0.606273	0.017366	0.049624	snRNA
ENSG0000(	ZNF777	173.5508	-0.20528	0.017365	0.049624	protein_coding
ENSG0000(	CD300LD-A	166.2692	0.158959	0.01738	0.049659	lncRNA
ENSG0000(	RPN2	1494.563	-0.22573	0.017393	0.049692	protein_coding
ENSG0000(	ENSG0000(	21.42569	0.456676	0.017399	0.049705	lncRNA
ENSG0000(	MARCHF11	66.63861	0.243606	0.017403	0.049712	lncRNA
ENSG0000(	CFAP97D1	553.0859	-0.10919	0.017413	0.049731	protein_coding
ENSG0000(	LYST	5451.04	0.189903	0.017413	0.049731	protein_coding

ENSG0000(	ENSG0000(	18.15455	0.521266	0.017432	0.049777	lncRNA
ENSG0000(	SIN3A	2302.95	-0.10149	0.017431	0.049777	protein_coding
ENSG0000(	LINC01798	80.85535	0.255786	0.017444	0.049802	lncRNA
ENSG0000(	MARK2	3335.518	-0.13838	0.017444	0.049802	protein_coding
ENSG0000(	PDLIM2	1419.798	-0.22626	0.017449	0.049812	protein_coding
ENSG0000(	PECR	403.5391	-0.15697	0.01746	0.049837	protein_coding
ENSG0000(	ENSG0000(	12.43696	1.021496	0.017469	0.04985	lncRNA
ENSG0000(	ENSG0000(	98.87701	-0.30541	0.017469	0.04985	lncRNA
ENSG0000(	ENSG0000(	35.59929	0.311405	0.017466	0.04985	TEC
ENSG0000(	ENSG0000(	25.31761	0.322492	0.017476	0.049862	lncRNA
ENSG0000(	TMX1	1028.009	-0.10647	0.017476	0.049862	protein_coding
ENSG0000(	STK11IP	489.9605	-0.16098	0.017486	0.049885	protein_coding
ENSG0000(	LINC02540	203.3451	-0.18141	0.01749	0.049891	lncRNA
ENSG0000(	ENSG0000(	79.79921	0.239329	0.017502	0.049918	lncRNA
ENSG0000(	SNORA70D	40.71659	-0.59642	0.017502	0.049918	snoRNA
ENSG0000(	LINC02327	300.0151	-0.17357	0.017514	0.049948	lncRNA
ENSG0000(	METTL23	254.5875	-0.15416	0.017517	0.04995	protein_coding
ENSG0000(	MCMBP	1056.109	-0.11635	0.017521	0.049952	protein_coding
ENSG0000(	RNH1	1187.198	-0.12138	0.017522	0.049952	protein_coding
ENSG0000(	STX18-AS1	4486.474	0.082377	0.017522	0.049952	lncRNA
ENSG0000(	ENSG0000(	14.1888	0.431758	0.017524	0.049952	unprocessed_pseudogene
ENSG0000(	FANCI	1767.761	-0.1828	0.017536	0.049982	protein_coding
ENSG0000(	MIR3648-1	3174.392	-0.52874	0.017538	0.049983	miRNA
ENSG0000(	ENSG0000(	137.0186	0.191584	0.017556	0.050031	lncRNA
ENSG0000(	ENSG0000(	55.85583	0.253005	0.017558	0.050031	lncRNA
ENSG0000(	NT5DC3	1187.69	-0.15987	0.017559	0.050031	protein_coding
ENSG0000(	ENSG0000(	18.75453	0.503379	0.017566	0.050044	lncRNA
ENSG0000(	MTND5P2	88.52131	0.244757	0.017573	0.05006	processed_pseudogene
ENSG0000(	TUT7	2791.031	0.134773	0.017575	0.050062	protein_coding
ENSG0000(	ENSG0000(	126.6005	0.225894	0.017592	0.050101	lncRNA
ENSG0000(	GJC1	1195.442	0.153829	0.017592	0.050101	protein_coding
ENSG0000(	ABCA5	5542.425	0.242985	0.017605	0.050132	protein_coding
ENSG0000(	ENSG0000(	148.9993	0.210338	0.01761	0.050142	lncRNA
ENSG0000(	PIP4P2	94.38164	0.425893	0.017612	0.050144	protein_coding
ENSG0000(	CCDC137	301.871	-0.22302	0.017618	0.050155	protein_coding
ENSG0000(	ENSG0000(	36.54512	-0.32267	0.017629	0.050182	lncRNA
ENSG0000(	RPL7AP52	31.57239	-0.59711	0.01763	0.050182	processed_pseudogene
ENSG0000(	ENSG0000(	11.81752	0.598747	0.017649	0.050225	lncRNA
ENSG0000(	SPAG5-AS1	147.4124	0.182858	0.017648	0.050225	lncRNA
ENSG0000(	PYGM	315.8991	-0.1598	0.017674	0.050292	protein_coding
ENSG0000(	CCL15	239.1773	-0.35507	0.017679	0.050297	protein_coding
ENSG0000(	ENSG0000(	116.5155	-0.20029	0.017679	0.050297	lncRNA
ENSG0000(	MAOA	4835.875	-0.40832	0.017684	0.050307	protein_coding
ENSG0000(	RN7SL328F	87.35196	0.255242	0.017687	0.05031	misc_RNA
ENSG0000(	ENSG0000(	138.6771	-0.15854	0.017691	0.050319	lncRNA
ENSG0000(	VN1R96P	9.818139	0.692413	0.017693	0.050321	unitary_pseudogene
ENSG0000(	KIAA0087	85.05084	-0.21948	0.017711	0.050367	lncRNA

ENSG0000(	ENSG0000(	135.6934	0.283625	0.017716	0.050371	TEC
ENSG0000(	SERINC5	6819.29	-0.18011	0.017715	0.050371	protein_coding
ENSG0000(	KMT2B	2608.713	-0.1527	0.017726	0.050394	protein_coding
ENSG0000(	HAR1B	138.6406	-0.16251	0.01773	0.050398	lncRNA
ENSG0000(	YJU2B	796.7998	-0.09788	0.01773	0.050398	protein_coding
ENSG0000(	IFNAR1	2462.001	0.120811	0.017734	0.050405	protein_coding
ENSG0000(	OTUB1	1484.56	-0.07903	0.017738	0.050408	protein_coding
ENSG0000(	SMG1P3	13958.14	0.199093	0.017738	0.050408	transcribed_unprocessed_pseudo
ENSG0000(	KRR1	1596.052	-0.13513	0.017742	0.050412	protein_coding
ENSG0000(	RGS3	1888.569	-0.18307	0.017748	0.050426	protein_coding
ENSG0000(	ENSG0000(	66.30726	0.203862	0.017758	0.050448	processed_pseudogene
ENSG0000(	NAP1L2	20.70094	-0.55214	0.01776	0.05045	protein_coding
ENSG0000(	AVPI1	269.9128	-0.37171	0.017773	0.050477	protein_coding
ENSG0000(	ENSG0000(	12.91295	0.430065	0.017772	0.050477	lncRNA
ENSG0000(	ENSG0000(	141.6637	0.157038	0.017774	0.050477	lncRNA
ENSG0000(	PTS	500.5528	0.144022	0.01779	0.050517	protein_coding
ENSG0000(	NSD3	5243.671	0.093104	0.017806	0.050557	protein_coding
ENSG0000(	ENSG0000(	17.2485	0.737246	0.01782	0.050588	lncRNA
ENSG0000(	XRN1	5634.271	0.162169	0.017819	0.050588	protein_coding
ENSG0000(	MIR593	9.079252	0.484078	0.017823	0.050592	miRNA
ENSG0000(	KIF14	469.9991	-0.23648	0.017826	0.050597	protein_coding
ENSG0000(	ARHGAP15	27.59301	-0.62865	0.017835	0.050617	lncRNA
ENSG0000(	NUTM2A	48.58886	0.795166	0.017839	0.05062	protein_coding
ENSG0000(	RXRA	2856.749	-0.20676	0.017838	0.05062	protein_coding
ENSG0000(	CEP164P1	209.0266	0.138435	0.017847	0.050638	transcribed_processed_pseudoge
ENSG0000(	TTPA	148.6551	-0.28056	0.017886	0.050744	protein_coding
ENSG0000(	FAR1	3387.982	-0.12823	0.017895	0.050766	protein_coding
ENSG0000(	B3GAT3	397.0553	-0.14151	0.017921	0.050829	protein_coding
ENSG0000(	RN7SL14P	8.415287	0.598614	0.017921	0.050829	misc_RNA
ENSG0000(	SLC44A3-A:	1221.852	-0.19827	0.017946	0.050892	transcribed_processed_pseudoge
ENSG0000(	ZYG11A	302.52	0.143263	0.017947	0.050892	protein_coding
ENSG0000(	TBXA2R	324.7403	-0.14757	0.017952	0.050903	protein_coding
ENSG0000(	ENSG0000(	241.033	0.156494	0.017996	0.051022	TEC
ENSG0000(	MPLKIP	2094.418	-0.07586	0.017997	0.051022	protein_coding
ENSG0000(	ENSG0000(	21.69369	-0.34771	0.018016	0.051071	lncRNA
ENSG0000(	NRAS	1782.671	-0.1699	0.018022	0.051083	protein_coding
ENSG0000(	ADAMTS13	230.9657	-0.30267	0.018045	0.051139	protein_coding
ENSG0000(	LRRC70	77.08316	0.304585	0.018045	0.051139	protein_coding
ENSG0000(	MTCO1P24	22.87782	0.824491	0.018055	0.051163	processed_pseudogene
ENSG0000(	CCDC138	266.9663	-0.18966	0.018059	0.05117	protein_coding
ENSG0000(	UPK1A-AS1	71.18208	0.23551	0.018065	0.051181	lncRNA
ENSG0000(	ENSG0000(	170.2473	-0.15485	0.018068	0.051187	lncRNA
ENSG0000(	GPBP1	3035.266	0.142859	0.018071	0.051188	protein_coding
ENSG0000(	BNIP3P28	278.9066	-0.14038	0.018076	0.051199	processed_pseudogene
ENSG0000(	CELSR1	608.3524	0.344809	0.018078	0.051201	protein_coding
ENSG0000(	ENSG0000(	108.8446	0.165049	0.018086	0.051217	lncRNA
ENSG0000(	SPG11	7638.177	0.074812	0.018099	0.05125	protein_coding

ENSG0000(	SOHLH2	36.7769	0.288399	0.018113	0.051285	protein_coding
ENSG0000(	ENSG0000(	95.29698	0.210851	0.018119	0.051298	lncRNA
ENSG0000(	OTULIN-DT	129.9155	0.25828	0.018123	0.051301	lncRNA
ENSG0000(	RHOT2	1537.643	-0.18971	0.018123	0.051301	protein_coding
ENSG0000(	SLC6A1	646.4147	0.114726	0.018135	0.051329	protein_coding
ENSG0000(	ENSG0000(	23.96417	0.440206	0.018143	0.051346	lncRNA
ENSG0000(	ENSG0000(	177.3371	0.151004	0.018148	0.051356	lncRNA
ENSG0000(	ENSG0000(	239.2075	0.16193	0.018154	0.051368	lncRNA
ENSG0000(	PRR16	212.1386	0.319228	0.018163	0.051389	protein_coding
ENSG0000(	MYO1D	7894.946	-0.27144	0.018165	0.05139	protein_coding
ENSG0000(	GCNT3	10512.26	-0.41012	0.01817	0.051401	protein_coding
ENSG0000(	FBXL5	1817.417	0.20875	0.018176	0.051409	protein_coding
ENSG0000(	SNAPIN	212.0966	-0.15541	0.018175	0.051409	protein_coding
ENSG0000(	DPYSL2	1626.585	0.171361	0.018207	0.051489	protein_coding
ENSG0000(	ENSG0000(	71.26561	0.271099	0.018208	0.051489	lncRNA
ENSG0000(	SNX30	2935.442	-0.24072	0.01821	0.051489	protein_coding
ENSG0000(	NEK10	292.5291	0.182959	0.018219	0.051511	protein_coding
ENSG0000(	IKBIP	353.3358	0.219022	0.018221	0.051513	protein_coding
ENSG0000(	LINC01843	233.7494	-0.28201	0.018228	0.051527	lncRNA
ENSG0000(	ENSG0000(	43.09706	0.305781	0.01823	0.051528	lncRNA
ENSG0000(	ENSG0000(	260.3729	0.165964	0.018246	0.051568	lncRNA
ENSG0000(	RN7SL342F	43.47237	0.297998	0.018247	0.051568	misc_RNA
ENSG0000(	ENSG0000(	108.6774	-0.23553	0.018258	0.051594	lncRNA
ENSG0000(	PURA	3163.48	0.141844	0.018262	0.051601	protein_coding
ENSG0000(	ENSG0000(	13.69735	0.487629	0.018266	0.051607	lncRNA
ENSG0000(	WDR11-AS	358.0372	-0.23842	0.018275	0.051627	lncRNA
ENSG0000(	PPP4R1L	801.207	0.147445	0.018288	0.051659	transcribed_unprocessed_pseudo
ENSG0000(	D2HGDH	1774.788	-0.1804	0.018314	0.051728	protein_coding
ENSG0000(	FBXO2	74.95593	0.266804	0.018316	0.05173	protein_coding
ENSG0000(	RNU6-118F	34.2464	0.321614	0.018333	0.051773	snRNA
ENSG0000(	DPM3	72.79428	-0.25373	0.018344	0.051791	protein_coding
ENSG0000(	NFYB	459.8111	-0.13041	0.018341	0.051791	protein_coding
ENSG0000(	WASL	2456.702	-0.25924	0.018344	0.051791	protein_coding
ENSG0000(	ENSG0000(	100.6907	0.28407	0.01836	0.051831	transcribed_processed_pseudoge
ENSG0000(	MALSU1	265.3344	-0.14364	0.018373	0.051862	protein_coding
ENSG0000(	ENSG0000(	81.55881	0.223611	0.018391	0.051907	lncRNA
ENSG0000(	ENSG0000(	111.8934	0.156607	0.018398	0.051918	lncRNA
ENSG0000(	ENSG0000(	80.70825	0.20827	0.018398	0.051918	lncRNA
ENSG0000(	DYNC1H1	16467.34	-0.05678	0.01841	0.051949	protein_coding
ENSG0000(	ENSG0000(	294.1808	-0.20953	0.018446	0.052044	lncRNA
ENSG0000(	RHOU	1088.5	-0.40331	0.018466	0.052097	protein_coding
ENSG0000(	ENSG0000(	101.0544	0.309152	0.018472	0.052105	lncRNA
ENSG0000(	RNU6-107F	40.85065	0.299533	0.018472	0.052105	snRNA
ENSG0000(	IL12RB1	693.6371	0.166687	0.01848	0.052123	protein_coding
ENSG0000(	ENSG0000(	17.5869	-0.35135	0.018486	0.052131	lncRNA
ENSG0000(	FLJ40194	312.9295	0.217403	0.018485	0.052131	lncRNA
ENSG0000(	VGLL4	1722.329	-0.0937	0.018492	0.052143	protein_coding

ENSG0000(	ENSG0000(	1028.442	-0.12582	0.018499	0.052157	lncRNA
ENSG0000(	CACTIN	771.8028	-0.11381	0.018511	0.052186	protein_coding
ENSG0000(	RPL4P7	59.89341	0.415058	0.018516	0.052194	processed_pseudogene
ENSG0000(	CNTNAP2	719.6738	-0.35764	0.018531	0.052221	protein_coding
ENSG0000(	MON1B	2622.452	-0.1178	0.018531	0.052221	protein_coding
ENSG0000(	RAC2	774.958	0.391594	0.018532	0.052221	protein_coding
ENSG0000(	YWHAB	5662.106	-0.17805	0.018532	0.052221	protein_coding
ENSG0000(	TMEM9B	635.0977	-0.20933	0.018534	0.052223	protein_coding
ENSG0000(	CASP3	968.943	0.160128	0.018554	0.052273	protein_coding
ENSG0000(	AGAP2-AS1	23.26603	0.477953	0.018557	0.052277	lncRNA
ENSG0000(	CCT6P3	563.755	-0.15789	0.01856	0.05228	transcribed_unprocessed_pseudo
ENSG0000(	ENSG0000(	16.48691	0.492486	0.018569	0.052297	lncRNA
ENSG0000(	ENSG0000(	23.30784	-0.53638	0.018568	0.052297	lncRNA
ENSG0000(	P2RY12	20.12288	0.672372	0.018571	0.052298	protein_coding
ENSG0000(	ZNF544	1971.355	-0.11407	0.018579	0.052316	protein_coding
ENSG0000(	RNU2-65P	52.21967	-0.26932	0.018586	0.052331	snRNA
ENSG0000(	NUP188	1201.019	-0.16824	0.018591	0.052342	protein_coding
ENSG0000(	CNTN1	153.4704	-0.20524	0.018594	0.052343	protein_coding
ENSG0000(	RN7SL93P	29.93377	0.561525	0.018597	0.052347	misc_RNA
ENSG0000(	GUSBP9	780.7398	-0.43828	0.018606	0.052369	unprocessed_pseudogene
ENSG0000(	TSPYL5	213.0325	0.252976	0.018608	0.05237	protein_coding
ENSG0000(	PRICKLE2	2107.158	0.166407	0.018615	0.052384	protein_coding
ENSG0000(	LRRC32	398.6184	0.302938	0.018624	0.052406	protein_coding
ENSG0000(	ENSG0000(	119.8085	0.314205	0.018627	0.052407	lncRNA
ENSG0000(	MGAT4EP	237.7619	0.110329	0.018629	0.05241	transcribed_unitary_pseudogene
ENSG0000(	ENSG0000(	36.77158	0.383782	0.018633	0.052415	lncRNA
ENSG0000(	ENSG0000(	204.2007	-0.2464	0.018634	0.052415	lncRNA
ENSG0000(	DDHD1-DT	20.44536	0.457869	0.018638	0.052418	lncRNA
ENSG0000(	PAK3	297.5716	0.231452	0.018639	0.052418	protein_coding
ENSG0000(	ENSG0000(	20.3707	0.443669	0.018656	0.052462	lncRNA
ENSG0000(	ENSG0000(	120.6167	0.225693	0.018664	0.052481	lncRNA
ENSG0000(	ENSG0000(	34.32923	0.268552	0.018668	0.052486	lncRNA
ENSG0000(	ENSG0000(	44.14585	0.316903	0.018673	0.052496	lncRNA
ENSG0000(	RN7SL8P	13.94627	0.410081	0.018689	0.052536	misc_RNA
ENSG0000(	ENSG0000(	130.6012	0.227266	0.018705	0.052565	lncRNA
ENSG0000(	OACYLP	28.94269	0.765579	0.018702	0.052565	transcribed_unitary_pseudogene
ENSG0000(	ZSWIM5	573.613	-0.23155	0.018703	0.052565	protein_coding
ENSG0000(	SPATA13	4009.605	0.137617	0.018715	0.05259	protein_coding
ENSG0000(	ENSG0000(	72.77082	-0.23267	0.018717	0.052591	lncRNA
ENSG0000(	ZNF321P	966.78	-0.15276	0.018734	0.052635	transcribed_processed_pseudoge
ENSG0000(	RNA5S9	14.219	0.621831	0.018738	0.05264	rRNA
ENSG0000(	C20orf96	140.6969	-0.24977	0.018741	0.052645	protein_coding
ENSG0000(	ENSG0000(	30.68699	0.352539	0.01875	0.052664	lncRNA
ENSG0000(	LINC01553	248.135	-0.1817	0.018755	0.052671	lncRNA
ENSG0000(	PDCL3P6	21.23839	-0.52846	0.018756	0.052671	processed_pseudogene
ENSG0000(	SEPTIN8	1947.407	-0.14543	0.018771	0.052709	protein_coding
ENSG0000(	ARL9	117.0064	0.236993	0.018795	0.052763	protein_coding

ENSG0000(	CISH	792.6953	0.172936	0.018794	0.052763	protein_coding
ENSG0000(	ENSG0000(	39.50269	0.293333	0.018795	0.052763	lncRNA
ENSG0000(	PANCR	49.35953	0.462667	0.018807	0.05279	lncRNA
ENSG0000(	REM2	21.25925	0.439386	0.018808	0.05279	protein_coding
ENSG0000(	MAGEH1	95.30757	0.348129	0.018834	0.052858	protein_coding
ENSG0000(	AKAP17A	1888.059	0.116924	0.018843	0.052866	protein_coding
ENSG0000(	CEP63	2694.256	0.068751	0.018844	0.052866	protein_coding
ENSG0000(	ENSG0000(	89.23521	0.290316	0.018843	0.052866	processed_pseudogene
ENSG0000(	HP1BP3	8143.091	-0.11899	0.018841	0.052866	protein_coding
ENSG0000(	RCHY1	913.8006	-0.08896	0.018861	0.05291	protein_coding
ENSG0000(	CYB5RL	2565.469	0.10838	0.018876	0.052943	protein_coding
ENSG0000(	ENSG0000(	380.4101	-0.16117	0.018875	0.052943	lncRNA
ENSG0000(	STIMATE-N	307.5406	0.376981	0.018881	0.052952	protein_coding
ENSG0000(	ENSG0000(	137.8235	-0.21442	0.018889	0.05297	lncRNA
ENSG0000(	SCARNA7	3861.121	-0.19614	0.018918	0.053046	scaRNA
ENSG0000(	LINC01602	93.08458	0.199248	0.018936	0.053091	lncRNA
ENSG0000(	ENSG0000(	8685.967	-0.20363	0.018947	0.053118	lncRNA
ENSG0000(	PARD6B	997.4112	-0.22319	0.018949	0.053118	protein_coding
ENSG0000(	LCMT1	391.973	-0.17349	0.018967	0.053162	protein_coding
ENSG0000(	SFT2D1	817.1056	-0.12632	0.018968	0.053162	protein_coding
ENSG0000(	ENSG0000(	3646.451	-0.15394	0.01897	0.053163	lncRNA
ENSG0000(	ENSG0000(	817.6013	0.234721	0.018979	0.053184	lncRNA
ENSG0000(	ENSG0000(	792.4021	0.210179	0.018982	0.053186	lncRNA
ENSG0000(	CLEC20A	21.10968	0.348431	0.019006	0.053246	protein_coding
ENSG0000(	PLPPR2	204.8722	0.220758	0.019006	0.053246	protein_coding
ENSG0000(	GATA6-AS1	131.1053	-0.46466	0.019017	0.05327	lncRNA
ENSG0000(	LINC01609	2191.885	-0.21512	0.019025	0.053283	lncRNA
ENSG0000(	OPRD1	734.3911	0.096321	0.019025	0.053283	protein_coding
ENSG0000(	ENSG0000(	282.9458	-0.15996	0.019041	0.053325	lncRNA
ENSG0000(	PFDN4	98.88069	-0.25208	0.019061	0.053376	protein_coding
ENSG0000(	KLHL21	1118.188	0.118517	0.019076	0.053406	protein_coding
ENSG0000(	TERF2IP	718.7733	0.128109	0.019075	0.053406	protein_coding
ENSG0000(	MTND4P3(	177.469	-0.14671	0.019079	0.053412	unprocessed_pseudogene
ENSG0000(	ENSG0000(	328.9919	0.111912	0.019087	0.05343	lncRNA
ENSG0000(	ENSG0000(	51.10638	0.299194	0.019094	0.053442	lncRNA
ENSG0000(	SERPINB8	341.9449	0.307042	0.019111	0.053485	protein_coding
ENSG0000(	LINC02562	26.70682	-0.51449	0.019113	0.053486	lncRNA
ENSG0000(	ALG1L9P	942.4046	0.111205	0.019117	0.053491	lncRNA
ENSG0000(	GOLGA2P1	166.3366	-0.43086	0.019118	0.053491	transcribed_unprocessed_pseudo
ENSG0000(	NPY4R	181.5171	0.665179	0.019156	0.053592	protein_coding
ENSG0000(	IRF8	1380.979	0.267051	0.019179	0.053653	protein_coding
ENSG0000(	IGLV8-61	148.3359	0.892279	0.019183	0.053659	IG_V_gene
ENSG0000(	ENSG0000(	33.79636	-0.78025	0.019186	0.053663	lncRNA
ENSG0000(	LDHD	298.6387	-0.55917	0.019228	0.053774	protein_coding
ENSG0000(	ENSG0000(	10.25328	-0.52207	0.01923	0.053776	TEC
ENSG0000(	EIF2S2P1	140.7217	-0.28751	0.019233	0.053781	processed_pseudogene
ENSG0000(	GALNT12	1171.487	-0.3392	0.019239	0.053791	protein_coding

ENSG0000(	TAS2R64P	438.2114	0.271672	0.019247	0.053808	transcribed_unprocessed_pseudo
ENSG0000(	SYNCRIP	4783.88	-0.10328	0.019264	0.053853	protein_coding
ENSG0000(	LINC02624	116.3683	-0.19686	0.019277	0.053878	lncRNA
ENSG0000(	SNORA5A	98.28306	-0.30399	0.019275	0.053878	snoRNA
ENSG0000(	HIPK1-AS1	382.0794	0.11493	0.019282	0.053889	lncRNA
ENSG0000(	SNORA24	181.858	-0.27252	0.019299	0.053931	snoRNA
ENSG0000(	HDX	188.8804	0.245822	0.019303	0.053937	protein_coding
ENSG0000(	LINC02835	17.39585	0.374498	0.019305	0.053937	lncRNA
ENSG0000(	ENSG0000(	11.90412	0.42513	0.019318	0.053966	TEC
ENSG0000(	ZNF253	701.3461	-0.20231	0.019318	0.053966	protein_coding
ENSG0000(	HCG15	167.2555	-0.17401	0.019323	0.053974	lncRNA
ENSG0000(	PGS1	1849.806	0.149103	0.019339	0.054011	protein_coding
ENSG0000(	RN7SL647F	20.48117	0.406804	0.01934	0.054011	misc_RNA
ENSG0000(	ENSG0000(	29.71411	-0.36228	0.019352	0.054039	lncRNA
ENSG0000(	ENSG0000(	119.4627	-0.51215	0.019357	0.054045	transcribed_processed_pseudoge
ENSG0000(	ENSG0000(	22.22544	-0.41751	0.019357	0.054045	lncRNA
ENSG0000(	ENSG0000(	13.87935	0.508158	0.019359	0.054045	lncRNA
ENSG0000(	C16orf89	105.1251	-0.18959	0.019368	0.054052	protein_coding
ENSG0000(	ENSG0000(	124.2856	-0.20323	0.019365	0.054052	lncRNA
ENSG0000(	ENSG0000(	296.7485	0.228399	0.019368	0.054052	lncRNA
ENSG0000(	ZNF189	1223.772	0.124396	0.019365	0.054052	protein_coding
ENSG0000(	UNK	1304.107	0.098843	0.019371	0.054055	protein_coding
ENSG0000(	KLLN	305.1554	0.257087	0.01939	0.054101	protein_coding
ENSG0000(	ENSG0000(	315.8755	0.113144	0.019408	0.054149	lncRNA
ENSG0000(	ENSG0000(	23.64259	0.738841	0.01941	0.054149	lncRNA
ENSG0000(	ENSG0000(	73.98646	0.703916	0.019433	0.054209	lncRNA
ENSG0000(	LNX1-AS2	241.3037	0.165952	0.01944	0.054223	lncRNA
ENSG0000(	RNU2-68P	11.13405	0.503625	0.019457	0.054264	snRNA
ENSG0000(	ENSG0000(	75.60976	0.262533	0.019468	0.054285	lncRNA
ENSG0000(	GNB5	1164.55	0.1675	0.019469	0.054285	protein_coding
ENSG0000(	ZBBX	45.37794	-0.53444	0.019466	0.054285	protein_coding
ENSG0000(	RNU1-83P	93.36392	0.503732	0.019473	0.05429	snRNA
ENSG0000(	RN7SL822F	11.83557	0.46722	0.019475	0.054291	misc_RNA
ENSG0000(	CHAF1A	578.2687	-0.20913	0.019487	0.054319	protein_coding
ENSG0000(	CPSF1	2198.172	-0.24085	0.019489	0.054319	protein_coding
ENSG0000(	ENSG0000(	47.96123	-0.31755	0.0195	0.054346	lncRNA
ENSG0000(	AHCYL1	1987.942	-0.12513	0.019504	0.054354	protein_coding
ENSG0000(	ENSG0000(	89.40405	0.338578	0.019537	0.054434	processed_pseudogene
ENSG0000(	HNF4G	1799.878	-0.29568	0.019536	0.054434	protein_coding
ENSG0000(	LGALS9	2681.128	-0.18954	0.019541	0.054442	protein_coding
ENSG0000(	PHF24	40.49673	0.771162	0.019548	0.054455	protein_coding
ENSG0000(	ENSG0000(	27.42308	0.345386	0.019551	0.05446	lncRNA
ENSG0000(	ENSG0000(	272.9523	0.129191	0.019558	0.054475	processed_pseudogene
ENSG0000(	ENSG0000(	285.2323	-0.19368	0.019561	0.054477	TEC
ENSG0000(	FGB	79.47329	0.522825	0.019563	0.054477	protein_coding
ENSG0000(	IQCB2P	24.91189	0.430754	0.019577	0.054512	processed_pseudogene
ENSG0000(	ENSG0000(	84.44448	0.342833	0.019583	0.054525	lncRNA

ENSG0000(	DUOXA1	38.99615	0.590432	0.019601	0.054571	protein_coding
ENSG0000(	CEP83	1025.602	-0.14936	0.01962	0.054619	protein_coding
ENSG0000(	ENSG0000(	36.51309	-0.49855	0.01963	0.054636	lncRNA
ENSG0000(	MALL	5075.28	-0.41257	0.01963	0.054636	protein_coding
ENSG0000(	ENSG0000(	20.68082	-1.3425	0.019635	0.054644	protein_coding
ENSG0000(	ENSG0000(	132.3468	-0.26829	0.019645	0.054662	transcribed_unprocessed_pseudo
ENSG0000(	SMARCA1	282.4217	0.280683	0.019645	0.054662	protein_coding
ENSG0000(	ENSG0000(	169.951	-0.2234	0.019669	0.054726	lncRNA
ENSG0000(	ZNF445	5395.631	0.090563	0.019683	0.054758	protein_coding
ENSG0000(	ENSG0000(	193.5371	0.200039	0.01969	0.054768	lncRNA
ENSG0000(	RHOXF1P1	38.33893	0.316602	0.019688	0.054768	transcribed_unprocessed_pseudo
ENSG0000(	PIH1D2	74.45127	-0.24609	0.019697	0.054782	protein_coding
ENSG0000(	SESN2	523.3955	-0.24703	0.019701	0.05479	protein_coding
ENSG0000(	ENSG0000(	17.45223	0.466943	0.019704	0.054792	lncRNA
ENSG0000(	ZBTB39	515.2158	0.132692	0.019705	0.054792	protein_coding
ENSG0000(	GAPDHP43	97.10215	-0.267	0.019709	0.054796	processed_pseudogene
ENSG0000(	BRD8	1407.011	-0.08996	0.019724	0.054834	protein_coding
ENSG0000(	ENSG0000(	185.2594	-0.18934	0.019733	0.054855	lncRNA
ENSG0000(	ENSG0000(	22.23975	0.701353	0.019747	0.054882	lncRNA
ENSG0000(	MOB2	762.8326	-0.16562	0.019746	0.054882	protein_coding
ENSG0000(	DHX15	5113.852	-0.10611	0.01975	0.054888	protein_coding
ENSG0000(	ENSG0000(	43.27278	-0.35936	0.01977	0.054938	lncRNA
ENSG0000(	HAGH	981.0189	-0.10329	0.019773	0.054942	protein_coding
ENSG0000(	HSD17B7	817.9947	0.166673	0.019778	0.05495	protein_coding
ENSG0000(	PHC1	916.3901	0.236694	0.019782	0.054957	protein_coding
ENSG0000(	TTLL7-IT1	689.2415	0.148702	0.019784	0.054957	lncRNA
ENSG0000(	TSC22D1-A	299.8312	0.178139	0.019799	0.054994	lncRNA
ENSG0000(	MAP2K4	1037.769	-0.12197	0.019801	0.054996	protein_coding
ENSG0000(	DHRS7	585.321	-0.1207	0.019813	0.055018	protein_coding
ENSG0000(	PKP1	24.05499	0.692301	0.019813	0.055018	protein_coding
ENSG0000(	ENSG0000(	59.50032	0.295435	0.019817	0.055024	lncRNA
ENSG0000(	EGF	184.9017	-0.54897	0.019823	0.055027	protein_coding
ENSG0000(	SASS6	218.3483	-0.25348	0.019822	0.055027	protein_coding
ENSG0000(	SMOC1	139.2684	-0.24496	0.019821	0.055027	protein_coding
ENSG0000(	ENSG0000(	25.36722	0.39289	0.019827	0.055031	TEC
ENSG0000(	AK4P1	8.890399	-1.05962	0.019831	0.055039	processed_pseudogene
ENSG0000(	ENSG0000(	12.86915	-0.46413	0.019837	0.05505	lncRNA
ENSG0000(	ARAP2	2822.26	0.183856	0.019854	0.055092	protein_coding
ENSG0000(	DBH-AS1	78.16324	0.539295	0.01986	0.055102	lncRNA
ENSG0000(	LINC00462	13.44912	0.413465	0.019861	0.055102	lncRNA
ENSG0000(	ENSG0000(	618.3382	0.111309	0.019864	0.055103	lncRNA
ENSG0000(	LINC02127	19.47686	0.394949	0.019865	0.055103	lncRNA
ENSG0000(	BRF2	213.4181	-0.16996	0.019886	0.055152	protein_coding
ENSG0000(	TAF8	2832.16	0.070324	0.019885	0.055152	protein_coding
ENSG0000(	CTBS	605.6208	0.171457	0.019898	0.055166	protein_coding
ENSG0000(	ENSG0000(	107.538	0.187307	0.019898	0.055166	lncRNA
ENSG0000(	MRPL22	358.2677	-0.14814	0.019896	0.055166	protein_coding

ENSG0000(	TJP3	2579.869	-0.35617	0.019897	0.055166	protein_coding
ENSG0000(	ENSG0000(	24.94242	0.382005	0.019902	0.05517	lncRNA
ENSG0000(	EYA1	93.93303	-0.26471	0.019904	0.05517	protein_coding
ENSG0000(	INMT	455.6841	-0.15601	0.019905	0.05517	protein_coding
ENSG0000(	GLYCTK-AS	21.99175	0.774832	0.019909	0.055176	lncRNA
ENSG0000(	EARS2	1240.079	-0.10398	0.019914	0.05518	protein_coding
ENSG0000(	RN7SKP213	26.12269	-0.36254	0.019912	0.05518	misc_RNA
ENSG0000(	ENSG0000(	480.2301	-0.20263	0.019927	0.055213	TEC
ENSG0000(	DROSHA	1556.429	-0.11914	0.01994	0.055238	protein_coding
ENSG0000(	LINC00337	247.2198	-0.14684	0.01994	0.055238	lncRNA
ENSG0000(	ENSG0000(	22.65008	0.374209	0.019946	0.055244	lncRNA
ENSG0000(	LINC00556	84.14943	0.245132	0.019945	0.055244	lncRNA
ENSG0000(	C8orf86	321.256	-0.11491	0.01997	0.055307	lncRNA
ENSG0000(	ENSG0000(	24.67474	-0.99545	0.019995	0.055371	unprocessed_pseudogene
ENSG0000(	RNPEPL1	1430.364	-0.20153	0.020021	0.055437	protein_coding
ENSG0000(	PNO1	437.0728	-0.13327	0.020042	0.055491	protein_coding
ENSG0000(	CIAO1	2308.342	-0.06574	0.020055	0.055512	protein_coding
ENSG0000(	ENSG0000(	11.55773	0.595613	0.020055	0.055512	lncRNA
ENSG0000(	ENSG0000(	44.84132	0.315607	0.020051	0.055512	TEC
ENSG0000(	TLR10	745.2132	0.444435	0.020057	0.055512	protein_coding
ENSG0000(	LAMP5	25.60179	0.669911	0.020075	0.055558	protein_coding
ENSG0000(	NRSN2-AS1	1874.916	-0.12053	0.020077	0.055558	lncRNA
ENSG0000(	ENSG0000(	9.841246	0.645683	0.020103	0.055626	processed_pseudogene
ENSG0000(	ANAPC10	317.6749	0.127805	0.020111	0.055644	protein_coding
ENSG0000(	STAG3	1270.033	0.16544	0.020126	0.05568	protein_coding
ENSG0000(	SEMA3D	272.2711	-0.15776	0.020131	0.055687	protein_coding
ENSG0000(	CBWD3	1635.657	0.153406	0.02014	0.055709	protein_coding
ENSG0000(	BSN	123.1548	0.354364	0.020145	0.055717	protein_coding
ENSG0000(	NR1D2	1177.266	-0.28363	0.020167	0.055774	protein_coding
ENSG0000(	HGS	2246.523	-0.22612	0.020171	0.05578	protein_coding
ENSG0000(	HJURP	513.436	-0.21508	0.020177	0.055791	protein_coding
ENSG0000(	ENSG0000(	260.8136	-0.13504	0.020188	0.055817	lncRNA
ENSG0000(	ENSG0000(	16.15665	-0.5837	0.020196	0.055823	processed_pseudogene
ENSG0000(	ENSG0000(	156.4445	-0.17047	0.020196	0.055823	transcribed_processed_pseudoge
ENSG0000(	KRAS	2575.675	-0.14345	0.020198	0.055823	protein_coding
ENSG0000(	MSANTD3	227.7464	0.269616	0.020195	0.055823	protein_coding
ENSG0000(	ENSG0000(	17.10101	0.330699	0.020233	0.055916	lncRNA
ENSG0000(	ENSG0000(	116.4359	0.205121	0.020269	0.05601	lncRNA
ENSG0000(	TUG1	10166	0.053958	0.02027	0.05601	protein_coding
ENSG0000(	RNU6-593F	15.46904	0.422633	0.020278	0.056025	snRNA
ENSG0000(	ENSG0000(	233.4744	-0.1464	0.020289	0.056052	lncRNA
ENSG0000(	GKAP1	132.6791	-0.2715	0.020294	0.05606	protein_coding
ENSG0000(	KYAT3	389.3852	-0.16021	0.020298	0.056065	protein_coding
ENSG0000(	ENSG0000(	1289.982	0.07701	0.020302	0.056067	lncRNA
ENSG0000(	PRKCD	1239.23	-0.21424	0.020302	0.056067	protein_coding
ENSG0000(	ENSG0000(	167.8139	0.182565	0.020309	0.056081	lncRNA
ENSG0000(	CDK5RAP3	3498.189	0.13784	0.020316	0.056095	protein_coding

ENSG0000(	ENSG0000(	68.24919	0.239922	0.020317	0.056095	lncRNA
ENSG0000(	TAF9	1760.728	-0.10881	0.020324	0.056109	protein_coding
ENSG0000(	ENSG0000(	17.21587	0.465439	0.020335	0.056131	lncRNA
ENSG0000(	LINC00888	136.83	0.207331	0.020336	0.056131	transcribed_unprocessed_pseudo
ENSG0000(	RNF213-AS	370.2154	-0.13993	0.020339	0.056135	lncRNA
ENSG0000(	SLC15A1	520.346	0.34022	0.02035	0.056161	protein_coding
ENSG0000(	ARHGEF11	3147.49	-0.16538	0.020353	0.056162	protein_coding
ENSG0000(	HNF1B	1302.035	-0.34249	0.020361	0.056165	protein_coding
ENSG0000(	HS2ST1	795.4528	0.133952	0.02036	0.056165	protein_coding
ENSG0000(	RN7SL677F	43.17763	0.275467	0.020359	0.056165	misc_RNA
ENSG0000(	SERPINB1	3046.964	-0.22902	0.02036	0.056165	protein_coding
ENSG0000(	NGEF	818.9048	-0.26039	0.020373	0.056194	protein_coding
ENSG0000(	TNFRSF11A	1783.448	-0.30725	0.020376	0.056196	protein_coding
ENSG0000(	ENSG0000(	63.70577	0.231106	0.020407	0.056277	TEC
ENSG0000(	MYH11	4834.753	-0.27037	0.02041	0.05628	protein_coding
ENSG0000(	MPEG1	2679.783	0.278198	0.020451	0.05639	protein_coding
ENSG0000(	ENSG0000(	25.73092	-0.40734	0.020479	0.056462	lncRNA
ENSG0000(	RN7SL708F	10.68823	0.49878	0.020492	0.056492	misc_RNA
ENSG0000(	HSF2	172.9396	-0.23736	0.020523	0.056566	protein_coding
ENSG0000(	SPCS1	1231.838	-0.22217	0.020522	0.056566	protein_coding
ENSG0000(	ENSG0000(	52.38907	-0.21282	0.020532	0.056587	pseudogene
ENSG0000(	GATAD2B	2654.916	0.22675	0.020544	0.056617	protein_coding
ENSG0000(	TMEM204	158.4476	0.277361	0.020551	0.056631	protein_coding
ENSG0000(	SPRED2	1692.284	-0.24553	0.020562	0.056654	protein_coding
ENSG0000(	ENSG0000(	249.0319	0.409726	0.02057	0.056673	lncRNA
ENSG0000(	ENSG0000(	41.89686	0.302165	0.020573	0.056674	lncRNA
ENSG0000(	ACOX2	312.8799	-0.30821	0.020587	0.056699	protein_coding
ENSG0000(	CD2BP2-DT	53.74099	-0.32558	0.020583	0.056699	lncRNA
ENSG0000(	ENSG0000(	134.3559	0.151424	0.020586	0.056699	lncRNA
ENSG0000(	LRRIQ3	39.53578	0.509982	0.020597	0.056721	protein_coding
ENSG0000(	SLC5A12	288.5051	0.179107	0.020607	0.056744	protein_coding
ENSG0000(	ENSG0000(	30.48239	0.406485	0.020611	0.056745	lncRNA
ENSG0000(	ENSG0000(	121.2869	0.232475	0.020611	0.056745	lncRNA
ENSG0000(	FOXA3	356.7404	-0.32603	0.020631	0.056796	protein_coding
ENSG0000(	KIF12	243.2034	-0.47861	0.020649	0.05684	protein_coding
ENSG0000(	ENSG0000(	22.18378	0.605296	0.020659	0.056858	lncRNA
ENSG0000(	NUP155	1634.595	-0.15072	0.020658	0.056858	protein_coding
ENSG0000(	MAP1A	270.9955	0.344485	0.020674	0.056893	protein_coding
ENSG0000(	SMOC2	348.5163	0.465698	0.020686	0.056921	protein_coding
ENSG0000(	PRRC2A	3918.7	-0.08658	0.020694	0.05694	protein_coding
ENSG0000(	RTL5	86.54136	0.230508	0.020698	0.056944	protein_coding
ENSG0000(	IGHJ2	37.39158	0.609768	0.020701	0.056949	IG_J_gene
ENSG0000(	ENSG0000(	828.3327	0.214517	0.020711	0.05697	TEC
ENSG0000(	RN7SL531F	191.6636	-0.14037	0.020729	0.057015	misc_RNA
ENSG0000(	EMP1	5491.828	-0.47207	0.020735	0.057017	protein_coding
ENSG0000(	MGP	319.7086	0.329933	0.020734	0.057017	protein_coding
ENSG0000(	TBX6	248.4634	0.141488	0.020732	0.057017	protein_coding

ENSG0000(	MTCO1P31	13.5895	0.504341	0.020744	0.057036	processed_pseudogene
ENSG0000(	MAEA	2360.755	-0.09947	0.020755	0.05706	protein_coding
ENSG0000(	YRDC	148.1036	-0.21135	0.020766	0.057086	protein_coding
ENSG0000(	RAB40B	740.7387	-0.25936	0.02078	0.05712	protein_coding
ENSG0000(	ENSG0000(	16.1634	-0.49382	0.020825	0.057239	misc_RNA
ENSG0000(	CLEC14A	110.5149	0.340996	0.020828	0.057241	protein_coding
ENSG0000(	NALT1	24.74181	-0.43979	0.020829	0.057241	lncRNA
ENSG0000(	DSCAML1	99.0961	-0.53217	0.020843	0.057263	protein_coding
ENSG0000(	HIVEP1	3580.175	0.113277	0.020844	0.057263	protein_coding
ENSG0000(	LINC00365	35.85135	0.417475	0.020845	0.057263	lncRNA
ENSG0000(	MED22	1200.316	-0.11395	0.020841	0.057263	protein_coding
ENSG0000(	ZNRF1	803.6612	-0.13541	0.020872	0.057333	protein_coding
ENSG0000(	ENSG0000(	313.2774	0.126163	0.020913	0.057431	lncRNA
ENSG0000(	PLA2G12B	50.02458	-0.82893	0.020912	0.057431	protein_coding
ENSG0000(	SMG8	258.3374	-0.18564	0.02091	0.057431	protein_coding
ENSG0000(	FAM215A	73.60014	-0.27593	0.020918	0.057434	lncRNA
ENSG0000(	IGKV1D-8	24.06232	0.773064	0.020918	0.057434	IG_V_gene
ENSG0000(	BDNF-AS	714.7036	0.117533	0.020929	0.057457	lncRNA
ENSG0000(	ENSG0000(	17.84229	-0.46457	0.020934	0.057469	lncRNA
ENSG0000(	STK4	4291.489	0.169452	0.020942	0.057485	protein_coding
ENSG0000(	MTRNR2L1	9.51748	0.588908	0.020951	0.057503	protein_coding
ENSG0000(	LINC01715	65.11234	0.231929	0.020962	0.05753	lncRNA
ENSG0000(	TFPI2	50.98288	0.750806	0.020968	0.057541	protein_coding
ENSG0000(	ATP2B2	13.15626	-0.78542	0.021003	0.057631	protein_coding
ENSG0000(	LINC00523	15.82135	0.534391	0.021019	0.057668	lncRNA
ENSG0000(	MADCAM1	148.7895	-0.1772	0.02102	0.057668	lncRNA
ENSG0000(	CCSER2	2165.24	0.132089	0.021029	0.057687	protein_coding
ENSG0000(	TMPRSS4	7265.038	-0.30085	0.021042	0.057718	protein_coding
ENSG0000(	MAGT1	2056.158	-0.11748	0.021046	0.057724	protein_coding
ENSG0000(	CCNI2	258.6356	-0.31218	0.021055	0.057744	protein_coding
ENSG0000(	NDUFA4	722.9635	-0.1507	0.021059	0.05775	protein_coding
ENSG0000(	LINC01082	53.89713	-0.44109	0.021074	0.057785	lncRNA
ENSG0000(	SUN2	2518.998	-0.16758	0.021078	0.057791	protein_coding
ENSG0000(	RSPH4A	24.85936	0.444133	0.021105	0.057861	protein_coding
ENSG0000(	LUNAR1	66.9674	0.224308	0.021116	0.057885	lncRNA
ENSG0000(	LINC02365	14.50022	0.461831	0.021123	0.057899	lncRNA
ENSG0000(	POLE4	241.0261	-0.17567	0.021143	0.05795	protein_coding
ENSG0000(	CCNB3	26.18926	-0.67793	0.021146	0.057954	protein_coding
ENSG0000(	ENSG0000(	16.02292	0.460497	0.02115	0.05796	lncRNA
ENSG0000(	TARS2	675.2418	-0.10368	0.021156	0.057969	protein_coding
ENSG0000(	ENSG0000(	12.2284	0.735177	0.021176	0.058019	protein_coding
ENSG0000(	LRRC59	1872.359	-0.22298	0.021182	0.05803	protein_coding
ENSG0000(	GTF2A2	669.3473	-0.18421	0.021187	0.058039	protein_coding
ENSG0000(	ENSG0000(	383.7077	0.127393	0.02119	0.058042	lncRNA
ENSG0000(	ENSG0000(	116.2023	-0.17659	0.021202	0.05806	lncRNA
ENSG0000(	LINC01091	542.3287	-0.13167	0.021201	0.05806	lncRNA
ENSG0000(	SYNJ1	1049.832	0.135578	0.021201	0.05806	protein_coding

ENSG0000(	KLHL31	73.14873	-0.31854	0.021204	0.058061	protein_coding
ENSG0000(	COMMD8	88.54227	-0.27672	0.021222	0.058098	protein_coding
ENSG0000(	ENSG0000(	276.4618	0.111803	0.021223	0.058098	TEC
ENSG0000(	HMGB1P5	91.36047	-0.41157	0.021223	0.058098	transcribed_processed_pseudoge
ENSG0000(	LINC01259	51.84582	-0.32655	0.02124	0.05814	lncRNA
ENSG0000(	RDH16	836.3143	0.105572	0.021243	0.058142	protein_coding
ENSG0000(	PSMD7	591.0895	-0.15047	0.021246	0.058144	protein_coding
ENSG0000(	ENSG0000(	13.67539	0.391889	0.021249	0.058145	lncRNA
ENSG0000(	RASL11A	176.6676	-0.36713	0.021249	0.058145	protein_coding
ENSG0000(	SLC9A7	1417.176	0.192367	0.02126	0.058166	protein_coding
ENSG0000(	TCEAL7	13.54374	0.50099	0.021261	0.058166	protein_coding
ENSG0000(	ENSG0000(	72.58765	-0.21582	0.021277	0.058199	lncRNA
ENSG0000(	NANOS1	224.1976	-0.14377	0.021275	0.058199	protein_coding
ENSG0000(	NECAB1	48.09541	0.387789	0.021317	0.058305	protein_coding
ENSG0000(	ENSG0000(	255.2922	-0.13335	0.021328	0.058326	lncRNA
ENSG0000(	KRT8P3	16.95821	-0.60423	0.021329	0.058326	processed_pseudogene
ENSG0000(	ARHGEF18	2548.579	-0.21792	0.021341	0.058355	protein_coding
ENSG0000(	ENSG0000(	11.76476	0.477465	0.021349	0.058359	lncRNA
ENSG0000(	JUP	3452.265	-0.28465	0.021349	0.058359	protein_coding
ENSG0000(	PROX1-AS1	740.8298	0.182879	0.021347	0.058359	lncRNA
ENSG0000(	ZNF211	665.3009	0.133715	0.02135	0.058359	protein_coding
ENSG0000(	CPLANE2	59.64367	-0.31302	0.021371	0.058412	protein_coding
ENSG0000(	UBXN6	677.2216	-0.18702	0.021383	0.058438	protein_coding
ENSG0000(	CBX3P4	19.09003	0.374735	0.021386	0.058442	transcribed_processed_pseudoge
ENSG0000(	LINC02454	40.23075	0.329446	0.021394	0.058458	lncRNA
ENSG0000(	ENSG0000(	22.67188	0.551791	0.021398	0.05846	lncRNA
ENSG0000(	SORD2P	82.57139	0.682437	0.021399	0.05846	transcribed_unprocessed_pseudo
ENSG0000(	MIR4502	12.44724	0.499454	0.02141	0.058487	miRNA
ENSG0000(	MSTO1	614.4305	-0.19827	0.021416	0.058498	protein_coding
ENSG0000(	ENSG0000(	573.8307	0.102597	0.021426	0.058521	lncRNA
ENSG0000(	DYRK1A	7027.948	-0.07286	0.021435	0.058539	protein_coding
ENSG0000(	LPGAT1	2662.489	0.193122	0.021437	0.058539	protein_coding
ENSG0000(	RDM1P5	317.9482	0.121536	0.021442	0.058548	transcribed_processed_pseudoge
ENSG0000(	OOSP1	38.43314	0.266092	0.021449	0.058562	protein_coding
ENSG0000(	ERAP1	2901.893	-0.12757	0.021454	0.05857	protein_coding
ENSG0000(	KLF10	750.2762	-0.25099	0.021468	0.058601	protein_coding
ENSG0000(	MAN2C1	3377.642	-0.13552	0.021469	0.058601	protein_coding
ENSG0000(	ENSG0000(	69.34882	-0.30604	0.021479	0.058623	lncRNA
ENSG0000(	C2orf81	65.03339	0.27423	0.021486	0.058636	protein_coding
ENSG0000(	ENSG0000(	55.48869	0.249131	0.021487	0.058636	lncRNA
ENSG0000(	APOM	19.86311	-0.40632	0.021498	0.05866	protein_coding
ENSG0000(	ENSG0000(	32.56817	0.361072	0.0215	0.05866	processed_pseudogene
ENSG0000(	CIDEC	42.90418	-0.68089	0.021513	0.058691	protein_coding
ENSG0000(	RC3H1-IT1	224.0278	-0.13646	0.02152	0.058704	lncRNA
ENSG0000(	ENSG0000(	26.19405	-0.4117	0.021546	0.058772	lncRNA
ENSG0000(	LINC01667	65.59823	-0.31712	0.021551	0.05878	transcribed_processed_pseudoge
ENSG0000(	ME1	272.3572	-0.40825	0.021561	0.058799	protein_coding

ENSG0000(	ZNF611	1494.951	-0.10655	0.021562	0.058799	protein_coding
ENSG0000(	TGFB2	68.53432	0.487858	0.021569	0.058813	protein_coding
ENSG0000(	ENSG0000(	235.9441	0.162025	0.021574	0.058821	lncRNA
ENSG0000(	NAP1L4	2266.155	-0.09896	0.02158	0.058832	protein_coding
ENSG0000(	TSPAN6	1392.381	-0.19885	0.021597	0.058875	protein_coding
ENSG0000(	ENSG0000(	24.24194	-0.39926	0.021605	0.058889	lncRNA
ENSG0000(	LRRC37A16	37.91435	0.585603	0.021606	0.058889	transcribed_unprocessed_pseudo
ENSG0000(	KANSL1	9953.319	0.16092	0.021619	0.058918	protein_coding
ENSG0000(	OR7E91P	15.95238	-0.47333	0.021625	0.058929	transcribed_unprocessed_pseudo
ENSG0000(	ENSG0000(	166.8261	0.310278	0.021631	0.058941	lncRNA
ENSG0000(	TMEM52	13.64295	-0.52951	0.021639	0.058956	protein_coding
ENSG0000(	F3	2783.47	0.341904	0.021657	0.059002	protein_coding
ENSG0000(	PARM1-AS1	16.62228	0.888327	0.021663	0.059014	lncRNA
ENSG0000(	RN7SL555P	47.59434	-0.37827	0.021679	0.059053	misc_RNA
ENSG0000(	ENSG0000(	391.0776	-0.10372	0.021691	0.059075	lncRNA
ENSG0000(	PCMT1	381.7523	-0.18221	0.02169	0.059075	protein_coding
ENSG0000(	ZNF613	296.614	-0.16259	0.02172	0.059149	protein_coding
ENSG0000(	PPIAP41	176.5923	-0.14735	0.021732	0.059175	processed_pseudogene
ENSG0000(	HOXB8	173.0974	-0.29947	0.021751	0.059221	protein_coding
ENSG0000(	CFAP54	2284.198	-0.17426	0.021753	0.059222	protein_coding
ENSG0000(	ENSG0000(	36.95978	0.36452	0.021762	0.05924	lncRNA
ENSG0000(	IMMP2L	300.0965	-0.20094	0.021763	0.05924	protein_coding
ENSG0000(	ENSG0000(	13.20601	0.52524	0.021771	0.059254	lncRNA
ENSG0000(	ENSG0000(	220.2858	0.143509	0.021791	0.059304	TEC
ENSG0000(	KCNH5	48.80912	-0.24779	0.021796	0.059312	protein_coding
ENSG0000(	DUSP14	43.32911	0.50689	0.021807	0.059338	protein_coding
ENSG0000(	LINC01516	29.16176	-0.33081	0.02182	0.059367	lncRNA
ENSG0000(	RN7SL812P	25.0761	0.330974	0.021824	0.059374	misc_RNA
ENSG0000(	RN7SL461P	67.85385	0.22109	0.021829	0.059384	misc_RNA
ENSG0000(	SNORA60	47.37545	0.370238	0.021834	0.059391	snoRNA
ENSG0000(	ENSG0000(	10.26662	-0.7107	0.021838	0.059397	processed_pseudogene
ENSG0000(	MIR553	14.83429	0.42889	0.021857	0.059444	miRNA
ENSG0000(	LINC02451	41.92573	-0.26508	0.021871	0.059475	lncRNA
ENSG0000(	PPP1CB	4459.964	-0.20311	0.021873	0.059477	protein_coding
ENSG0000(	ENSG0000(	15.5333	0.497144	0.021891	0.059521	lncRNA
ENSG0000(	ZNF592	2272.42	-0.10889	0.021902	0.059545	protein_coding
ENSG0000(	WFIKKN1	120.4207	-0.51577	0.021908	0.059557	protein_coding
ENSG0000(	ZNF208	587.1482	-0.15993	0.021915	0.059571	protein_coding
ENSG0000(	RASAL2-AS	3511.249	0.118717	0.021929	0.059602	lncRNA
ENSG0000(	MIR548Z	51.92828	0.258156	0.021941	0.059626	miRNA
ENSG0000(	RUBCN	2398.199	0.128559	0.02194	0.059626	protein_coding
ENSG0000(	LINC00566	32.56291	0.283468	0.021953	0.059652	lncRNA
ENSG0000(	ELOA-AS1	349.0444	-0.12805	0.021957	0.059659	lncRNA
ENSG0000(	ENSG0000(	158.4522	0.40013	0.021975	0.059702	lncRNA
ENSG0000(	LCMT1-AS2	136.4724	0.158849	0.021977	0.059702	lncRNA
ENSG0000(	SNX14	6703.185	-0.06074	0.022013	0.059794	protein_coding
ENSG0000(	ENSG0000(	255.1197	0.208037	0.022018	0.059802	lncRNA

ENSG0000(	GLUD2	13.40584	0.720487	0.022034	0.059841	protein_coding
ENSG0000(	EFEMP1	179.399	0.418756	0.022036	0.059842	protein_coding
ENSG0000(	FAM209B	11.92887	0.619754	0.022046	0.059852	protein_coding
ENSG0000(	NUF2	105.0793	-0.37301	0.022042	0.059852	protein_coding
ENSG0000(	PIR	150.443	-0.20617	0.022049	0.059852	protein_coding
ENSG0000(	POLA2	821.9942	-0.13468	0.022051	0.059852	protein_coding
ENSG0000(	PTPRS	1225.32	0.207924	0.02205	0.059852	protein_coding
ENSG0000(	RUVBL1	466.1095	-0.18614	0.022047	0.059852	protein_coding
ENSG0000(	ENSG0000(	18.65742	-0.4437	0.022068	0.059891	lncRNA
ENSG0000(	SLC22A9	115.4995	0.188536	0.022088	0.05994	protein_coding
ENSG0000(	ENSG0000(	53.54805	-0.4664	0.022096	0.059952	lncRNA
ENSG0000(	SVBP	79.53734	-0.25777	0.022095	0.059952	protein_coding
ENSG0000(	DEGS2	318.8093	-0.35863	0.022103	0.059967	protein_coding
ENSG0000(	CNTNAP3P	8.776295	0.802609	0.022108	0.059974	unprocessed_pseudogene
ENSG0000(	CHRM3	839.1132	0.300132	0.022116	0.059991	protein_coding
ENSG0000(	PRR4	101.1759	0.292326	0.022121	0.06	protein_coding
ENSG0000(	COL4A6	227.8584	-0.41387	0.022132	0.060025	protein_coding
ENSG0000(	CLSTN1	3185.68	-0.2075	0.022139	0.060037	protein_coding
ENSG0000(	MYCNOS	214.0582	0.13027	0.022141	0.060037	lncRNA
ENSG0000(	HIGD1AP1(	42.88303	0.330475	0.022148	0.060048	processed_pseudogene
ENSG0000(	TRMT2B	1756.541	-0.09149	0.022147	0.060048	protein_coding
ENSG0000(	GET4	1067.383	-0.18162	0.022167	0.060092	protein_coding
ENSG0000(	CAMK2N1	1987.34	-0.36987	0.022169	0.060094	protein_coding
ENSG0000(	LINC02802	128.121	-0.3123	0.022187	0.060136	lncRNA
ENSG0000(	LSM8	3427.109	0.075376	0.022224	0.060233	protein_coding
ENSG0000(	SNHG3	2405.938	-0.21125	0.022239	0.060268	lncRNA
ENSG0000(	BMERB1	177.7742	0.302684	0.022247	0.060282	protein_coding
ENSG0000(	MAPKBP1	1178.919	0.132944	0.022257	0.060306	protein_coding
ENSG0000(	H2AL1MP	35.33116	-0.44759	0.02229	0.060388	unprocessed_pseudogene
ENSG0000(	ENSG0000(	40.85334	0.33064	0.02231	0.060438	lncRNA
ENSG0000(	LINC02723	148.6865	0.169273	0.022323	0.060469	lncRNA
ENSG0000(	FOXP2	999.6705	-0.26807	0.022326	0.06047	protein_coding
ENSG0000(	GIPR	808.1404	0.18184	0.022331	0.060479	protein_coding
ENSG0000(	ZBTB11	1534.975	-0.1134	0.022339	0.060495	protein_coding
ENSG0000(	ENSG0000(	132.4645	0.160709	0.022344	0.060499	lncRNA
ENSG0000(	ENSG0000(	17.5457	0.562508	0.022343	0.060499	lncRNA
ENSG0000(	LINC00279	53.16523	-0.5258	0.022351	0.060513	lncRNA
ENSG0000(	STARD8	410.9347	0.169104	0.022369	0.060557	protein_coding
ENSG0000(	HIP1R	1887.921	-0.15814	0.022385	0.060594	protein_coding
ENSG0000(	ENSG0000(	1054.902	0.114985	0.022396	0.060613	TEC
ENSG0000(	ENSG0000(	58.96739	0.245067	0.022398	0.060613	lncRNA
ENSG0000(	KTN1	7806.841	-0.15151	0.022398	0.060613	protein_coding
ENSG0000(	PSMD11	1550.58	-0.11526	0.02241	0.06064	protein_coding
ENSG0000(	KIAA0930	1267.778	0.210772	0.022416	0.060653	protein_coding
ENSG0000(	ENSG0000(	32.00054	0.284961	0.022424	0.060668	lncRNA
ENSG0000(	ENSG0000(	409.5514	-0.1458	0.022431	0.060683	transcribed_processed_pseudoge
ENSG0000(	CALCOCO1	2194.839	0.149725	0.022447	0.060714	protein_coding

ENSG0000(	CTSE	365.1144	0.412733	0.022446	0.060714	protein_coding
ENSG0000(	TYROBP	129.1426	0.432738	0.022449	0.060715	protein_coding
ENSG0000(	XPNPEP1	1770.904	-0.16035	0.022451	0.060715	protein_coding
ENSG0000(	ENSG0000(	89.59412	0.224769	0.022459	0.060728	lncRNA
ENSG0000(	PPP1R14D	422.0961	-0.35192	0.022458	0.060728	protein_coding
ENSG0000(	PSPC1	1510.795	0.105932	0.022482	0.060782	protein_coding
ENSG0000(	ANKRD36B	37.33098	0.309711	0.02251	0.060839	transcribed_processed_pseudoge
ENSG0000(	ENSG0000(	24.66599	0.41311	0.022509	0.060839	lncRNA
ENSG0000(	LINC02436	112.1218	0.191285	0.022507	0.060839	lncRNA
ENSG0000(	SERPINB10	205.2578	0.155713	0.022511	0.060839	protein_coding
ENSG0000(	FCSK	1101.042	-0.13393	0.022527	0.060879	protein_coding
ENSG0000(	RRM2B	586.8859	-0.19673	0.02253	0.060881	protein_coding
ENSG0000(	ST3GAL3-A	52.6769	0.255429	0.02255	0.060929	lncRNA
ENSG0000(	RNU6-790F	9.130876	-0.47767	0.022552	0.06093	snRNA
ENSG0000(	HYMAI	325.4968	0.204917	0.022555	0.060934	lncRNA
ENSG0000(	ENSG0000(	15.12415	0.422865	0.022591	0.061019	lncRNA
ENSG0000(	SPSB1	480.5374	0.177758	0.022589	0.061019	protein_coding
ENSG0000(	CA5B	1579.867	0.132165	0.022614	0.061076	protein_coding
ENSG0000(	AAGAB	1162.924	-0.16352	0.022632	0.06112	protein_coding
ENSG0000(	LINC02316	26.90284	0.352147	0.022636	0.061127	lncRNA
ENSG0000(	ENSG0000(	117.4345	0.187863	0.022649	0.061156	lncRNA
ENSG0000(	KRT18P4	68.6683	-0.29498	0.022655	0.061166	processed_pseudogene
ENSG0000(	PDZD9	231.3308	0.156457	0.022658	0.06117	protein_coding
ENSG0000(	GRAMD1C	612.0208	-0.20567	0.022664	0.061174	protein_coding
ENSG0000(	NUDT9	935.0765	-0.1068	0.022663	0.061174	protein_coding
ENSG0000(	SHMT1	937.6892	-0.21932	0.022668	0.061181	protein_coding
ENSG0000(	ATN1	2473.041	-0.14703	0.022697	0.061242	protein_coding
ENSG0000(	CIBAR2	58.28207	0.490342	0.022694	0.061242	protein_coding
ENSG0000(	RPEL1	20.44641	0.431193	0.022695	0.061242	protein_coding
ENSG0000(	ENSG0000(	256.5937	0.307309	0.022713	0.061281	processed_pseudogene
ENSG0000(	SNRPEP10	74.73478	-0.60522	0.022731	0.061322	processed_pseudogene
ENSG0000(	TH2LCRR	11.41719	0.457725	0.022732	0.061322	lncRNA
ENSG0000(	ENSG0000(	11.01801	-0.54912	0.022739	0.061328	lncRNA
ENSG0000(	FOXM1	926.9152	-0.12615	0.022737	0.061328	protein_coding
ENSG0000(	ENSG0000(	7658.884	0.181801	0.022754	0.061364	lncRNA
ENSG0000(	FAM124B	106.249	0.208031	0.022756	0.061364	protein_coding
ENSG0000(	C9orf116	9.210115	0.62871	0.022762	0.061372	protein_coding
ENSG0000(	IER2	1369.558	0.195381	0.022761	0.061372	protein_coding
ENSG0000(	DANT2	2312.851	-0.24597	0.022791	0.061444	lncRNA
ENSG0000(	SURF4	2486.174	-0.17856	0.022812	0.061496	protein_coding
ENSG0000(	ENSG0000(	170.8539	0.140764	0.022836	0.061554	lncRNA
ENSG0000(	AFAP1L2	616.0432	-0.22779	0.02284	0.061561	protein_coding
ENSG0000(	ALAS1	899.3284	-0.25458	0.022843	0.061562	protein_coding
ENSG0000(	ZFYVE16	3927.591	0.106962	0.022855	0.06159	protein_coding
ENSG0000(	IRF2	845.4135	0.149576	0.022865	0.061612	protein_coding
ENSG0000(	ENSG0000(	11.64861	0.505044	0.022873	0.061626	TEC
ENSG0000(	ENSG0000(	124.7949	-0.19091	0.022876	0.06163	lncRNA

ENSG0000(	ENSG0000(	42.19478	0.26444	0.022896	0.06168	lncRNA
ENSG0000(	INO80C	1404.796	0.105394	0.02291	0.061712	protein_coding
ENSG0000(	GSN	4234.78	-0.15262	0.022919	0.061729	protein_coding
ENSG0000(	ENSG0000(	9.329604	0.827734	0.022928	0.061748	lncRNA
ENSG0000(	PTPRG-AS1	322.3117	-0.15424	0.02293	0.061748	lncRNA
ENSG0000(	ENSG0000(	40.8616	0.281197	0.022953	0.061804	unprocessed_pseudogene
ENSG0000(	KLHL8	1060.377	-0.10813	0.022954	0.061804	protein_coding
ENSG0000(	LINC00276	507.9053	0.117264	0.022999	0.061918	lncRNA
ENSG0000(	ENSG0000(	14.75492	0.523519	0.023012	0.061949	lncRNA
ENSG0000(	PPIAP40	9.998015	0.669625	0.023015	0.06195	processed_pseudogene
ENSG0000(	PORCN	123.8608	0.285267	0.023025	0.061974	protein_coding
ENSG0000(	CTSA	4194.665	-0.2901	0.023047	0.062028	protein_coding
ENSG0000(	C19orf38	52.69014	0.352416	0.023069	0.062081	protein_coding
ENSG0000(	SNRPGP9	18.87904	0.575978	0.023079	0.062101	processed_pseudogene
ENSG0000(	ENSG0000(	32.97255	0.333129	0.023094	0.062136	lncRNA
ENSG0000(	LINC01864	115.0203	0.229501	0.023108	0.06217	lncRNA
ENSG0000(	DNM1P47	751.7524	0.348206	0.023113	0.062175	transcribed_unprocessed_pseudo
ENSG0000(	ENSG0000(	3294.413	0.178138	0.023114	0.062175	lncRNA
ENSG0000(	ZGPAT	629.308	-0.17448	0.023117	0.062177	protein_coding
ENSG0000(	RBFOX1	945.7025	-0.22425	0.02312	0.06218	protein_coding
ENSG0000(	CRYZ	230.1957	-0.26037	0.023122	0.062181	protein_coding
ENSG0000(	VN1R108P	15.27694	0.495163	0.023128	0.06219	unprocessed_pseudogene
ENSG0000(	DNM1P46	173.4957	0.176675	0.023131	0.062193	transcribed_unprocessed_pseudo
ENSG0000(	UNC13D	990.4649	0.25423	0.023136	0.062201	protein_coding
ENSG0000(	PRICKLE2-A	179.5664	-0.16207	0.02314	0.062206	lncRNA
ENSG0000(	DCAF8L2	9.586469	0.754328	0.023144	0.062214	protein_coding
ENSG0000(	ENSG0000(	48.46485	-0.2729	0.023149	0.062217	lncRNA
ENSG0000(	ENSG0000(	30.02431	0.262186	0.02315	0.062217	lncRNA
ENSG0000(	ENSG0000(	198.5424	-0.72769	0.023167	0.062259	unprocessed_pseudogene
ENSG0000(	A1CF	2468.241	-0.37133	0.023172	0.062266	protein_coding
ENSG0000(	ENSG0000(	199.1705	0.226603	0.023193	0.062317	lncRNA
ENSG0000(	IFT46	228.5046	-0.17464	0.023196	0.06232	protein_coding
ENSG0000(	ENSG0000(	893.963	0.187257	0.023233	0.062414	lncRNA
ENSG0000(	KRBOX4	695.9558	0.117723	0.023236	0.062417	protein_coding
ENSG0000(	ENSG0000(	18.54706	0.448635	0.023241	0.062426	lncRNA
ENSG0000(	ENSG0000(	14.43727	-0.67239	0.023272	0.062502	lncRNA
ENSG0000(	ENSG0000(	2784.682	-0.16821	0.02328	0.062518	lncRNA
ENSG0000(	AKR7L	1090.262	-0.15233	0.023295	0.062528	polymorphic_pseudogene
ENSG0000(	MTMR3	3435.202	-0.14271	0.023291	0.062528	protein_coding
ENSG0000(	PDE4D	2717.169	0.152759	0.023289	0.062528	protein_coding
ENSG0000(	PRPSAP2	618.9234	-0.10952	0.023292	0.062528	protein_coding
ENSG0000(	SLC30A6-D	228.2856	-0.13354	0.023288	0.062528	lncRNA
ENSG0000(	STOML1	781.9629	0.147969	0.023295	0.062528	protein_coding
ENSG0000(	CENPU	259.4563	-0.25722	0.023321	0.062592	protein_coding
ENSG0000(	ENSG0000(	929.5427	0.161583	0.023327	0.062602	lncRNA
ENSG0000(	PI4KAP2	2219.37	-0.16992	0.023329	0.062603	transcribed_unitary_pseudogene
ENSG0000(	ENSG0000(	16.16595	0.442395	0.023331	0.062603	lncRNA

ENSG0000(	SHISA2	119.2551	-0.17492	0.023335	0.062607	protein_coding
ENSG0000(	ENSG0000(	25.4582	0.340959	0.02335	0.062637	lncRNA
ENSG0000(	ENSG0000(	77.79726	-0.21043	0.02335	0.062637	lncRNA
ENSG0000(	ENSG0000(	531.6256	0.14114	0.02338	0.062712	lncRNA
ENSG0000(	GPX8	303.9302	0.276029	0.023384	0.062715	protein_coding
ENSG0000(	KPNA1	1757.066	-0.10196	0.023385	0.062715	protein_coding
ENSG0000(	FRG1-DT	2250.788	0.199501	0.023398	0.062745	lncRNA
ENSG0000(	RNU6-668F	13.65704	0.605377	0.023414	0.062782	snRNA
ENSG0000(	EFNB2	2757.808	-0.28454	0.023447	0.062866	protein_coding
ENSG0000(	ENSG0000(	29.82787	0.397882	0.02345	0.062867	lncRNA
ENSG0000(	PLEKHA4	206.9073	0.260663	0.023465	0.062902	protein_coding
ENSG0000(	ENSG0000(	22.15075	-0.40471	0.02351	0.063017	unprocessed_pseudogene
ENSG0000(	ENSG0000(	106.06	0.15806	0.02356	0.063141	TEC
ENSG0000(	RPTOR	1746.998	-0.09966	0.02356	0.063141	protein_coding
ENSG0000(	ENSG0000(	165.5759	-0.3475	0.023575	0.063176	processed_pseudogene
ENSG0000(	ZNF562	5631.887	-0.12206	0.023584	0.063193	protein_coding
ENSG0000(	IGKV1D-37	14.15465	1.270582	0.023595	0.063215	IG_V_gene
ENSG0000(	SCARA5	775.2901	-0.28609	0.023596	0.063215	protein_coding
ENSG0000(	ENSG0000(	194.1813	-0.21282	0.023601	0.063223	lncRNA
ENSG0000(	ENSG0000(	10.97103	-1.38544	0.023616	0.063259	protein_coding
ENSG0000(	RNU4-23P	16.478	-0.50595	0.023619	0.063262	snRNA
ENSG0000(	CDC42-IT1	180.4006	-0.183	0.023638	0.063307	lncRNA
ENSG0000(	ENSG0000(	66.52141	0.248087	0.023645	0.063319	lncRNA
ENSG0000(	IGLV7-43	102.3124	0.620146	0.023653	0.063335	IG_V_gene
ENSG0000(	LINC02531	37.83363	0.288771	0.023656	0.063338	lncRNA
ENSG0000(	ADGRD1	475.8943	0.280731	0.023675	0.063384	protein_coding
ENSG0000(	FOXD4	10.63011	-0.6948	0.023697	0.063438	protein_coding
ENSG0000(	RPA3	124.5876	-0.27353	0.023711	0.06347	protein_coding
ENSG0000(	ENSG0000(	58.12876	0.229836	0.023714	0.063471	lncRNA
ENSG0000(	ENSG0000(	443.7816	0.118849	0.023717	0.063474	protein_coding
ENSG0000(	SYNDIG1	50.52091	0.418384	0.023725	0.063485	protein_coding
ENSG0000(	TSPAN9	399.349	0.317244	0.023725	0.063485	protein_coding
ENSG0000(	ESAM-AS1	187.7969	-0.22921	0.023758	0.063567	lncRNA
ENSG0000(	SLC35E1P1	54.31468	-0.36267	0.023762	0.063574	processed_pseudogene
ENSG0000(	LINC02629	17.52006	-0.4578	0.023785	0.063629	lncRNA
ENSG0000(	SAFB	2015.618	-0.09753	0.023793	0.063646	protein_coding
ENSG0000(	COQ8A	656.7717	-0.22708	0.023799	0.063656	protein_coding
ENSG0000(	ENSG0000(	29.26006	0.3222	0.023826	0.063723	lncRNA
ENSG0000(	ENSG0000(	12.77346	0.495848	0.023847	0.063773	processed_pseudogene
ENSG0000(	CFAP298-T(	982.4825	0.112983	0.023863	0.063805	protein_coding
ENSG0000(	ENSG0000(	46.31212	1.534894	0.023862	0.063805	lncRNA
ENSG0000(	RPL7P41	66.9258	0.208729	0.02388	0.063847	processed_pseudogene
ENSG0000(	ENSG0000(	14.67991	0.607826	0.023887	0.06386	TEC
ENSG0000(	ENSG0000(	15.914	0.501186	0.023897	0.063881	lncRNA
ENSG0000(	DCAF13P3	68.53244	-0.27557	0.023914	0.063921	processed_pseudogene
ENSG0000(	LDB3	502.364	-0.25558	0.023922	0.063937	protein_coding
ENSG0000(	ENSG0000(	47.06448	0.289799	0.023937	0.06397	lncRNA

ENSG0000(	ENSG0000(	302.5585	0.148648	0.023959	0.064018	TEC
ENSG0000(	LINC01998	31.28054	0.298617	0.023957	0.064018	lncRNA
ENSG0000(	MIR196A1	113.3244	-0.36813	0.023972	0.064049	miRNA
ENSG0000(	RNU6-1079	18.17442	0.363877	0.023975	0.064051	snRNA
ENSG0000(	SLC10A7	1135.671	0.156383	0.024001	0.064113	protein_coding
ENSG0000(	ENSG0000(	916.2229	-0.11742	0.024004	0.064117	protein_coding
ENSG0000(	FAM168A	1333.183	0.173948	0.024023	0.064147	protein_coding
ENSG0000(	MIR326	14.61772	-0.5386	0.024023	0.064147	miRNA
ENSG0000(	SMG1	30713.13	0.078175	0.024024	0.064147	protein_coding
ENSG0000(	UBE2B	868.7665	0.134281	0.024023	0.064147	protein_coding
ENSG0000(	TTC1	1693.094	-0.06785	0.024028	0.064152	protein_coding
ENSG0000(	ENSG0000(	188.3929	0.131841	0.024031	0.064156	transcribed_unitary_pseudogene
ENSG0000(	BLM	1129.204	0.161824	0.024047	0.064192	protein_coding
ENSG0000(	PIN1	954.9786	-0.09532	0.024089	0.0643	protein_coding
ENSG0000(	RXRB	1087.521	-0.09382	0.024095	0.064311	protein_coding
ENSG0000(	ENPP7	21.84657	0.792872	0.024105	0.064332	protein_coding
ENSG0000(	CYB5AP2	220.0363	-0.14603	0.02411	0.064339	processed_pseudogene
ENSG0000(	OR52H1	16.27309	0.515372	0.024115	0.064347	protein_coding
ENSG0000(	SPATA17	455.6538	-0.155	0.024126	0.064372	protein_coding
ENSG0000(	CHST5	745.4868	-0.43893	0.024162	0.064454	protein_coding
ENSG0000(	CNTLN	756.7175	0.212764	0.02416	0.064454	protein_coding
ENSG0000(	GADL1	792.4317	0.126671	0.024163	0.064454	protein_coding
ENSG0000(	NOL9	2723.05	-0.0925	0.024183	0.064501	protein_coding
ENSG0000(	DLGAP4-AS	114.9044	0.212757	0.024193	0.064521	lncRNA
ENSG0000(	ENSG0000(	63.7281	-0.2852	0.024198	0.064531	lncRNA
ENSG0000(	LINC02873	18.63217	0.426491	0.024213	0.06456	lncRNA
ENSG0000(	PRKXP1	359.9777	0.405394	0.024212	0.06456	processed_pseudogene
ENSG0000(	CYTH1	4197.401	0.12134	0.024228	0.064594	protein_coding
ENSG0000(	ENSG0000(	290.0839	-0.12675	0.024235	0.064606	lncRNA
ENSG0000(	SETP9	10.4626	0.722695	0.024242	0.064621	transcribed_processed_pseudoge
ENSG0000(	EMSY-DT	188.0923	-0.14858	0.024264	0.064673	lncRNA
ENSG0000(	OAF	1486.654	-0.19901	0.024267	0.064675	protein_coding
ENSG0000(	SUGP1	595.7099	-0.11478	0.024277	0.064698	protein_coding
ENSG0000(	FAM186B	27.43232	0.405856	0.024282	0.064705	protein_coding
ENSG0000(	ENSG0000(	14.34645	0.508869	0.024301	0.064748	processed_pseudogene
ENSG0000(	RAB39A	95.46739	0.240859	0.024303	0.064748	protein_coding
ENSG0000(	BMS1P8	86.33941	0.310497	0.024317	0.064772	transcribed_unprocessed_pseudo
ENSG0000(	NDUFAF5	873.2115	-0.15283	0.024318	0.064772	protein_coding
ENSG0000(	NPNT	1839.104	-0.2762	0.024315	0.064772	protein_coding
ENSG0000(	LINC02388	555.5905	0.122355	0.024337	0.064818	lncRNA
ENSG0000(	HECW1	175.2581	-0.19329	0.024373	0.064909	protein_coding
ENSG0000(	FPR2	124.0424	0.388574	0.024397	0.064967	protein_coding
ENSG0000(	ENSG0000(	8.609389	-0.62412	0.024401	0.064971	processed_pseudogene
ENSG0000(	ENSG0000(	38.6621	0.380247	0.024409	0.064987	lncRNA
ENSG0000(	PISD	1064.616	-0.1155	0.024433	0.065045	protein_coding
ENSG0000(	BCL7B	569.074	-0.1487	0.024442	0.065065	protein_coding
ENSG0000(	ASPM	1559.255	-0.2439	0.024451	0.065083	protein_coding

ENSG0000(	KBTBD6	1718.371	0.207813	0.024466	0.065118	protein_coding
ENSG0000(	ENSG0000(	62.12448	-0.42889	0.024469	0.065118	lncRNA
ENSG0000(	IGHV2-26	58.44464	0.639714	0.02448	0.065142	IG_V_gene
ENSG0000(	LYL1	273.3289	0.311587	0.024486	0.065154	protein_coding
ENSG0000(	ENSG0000(	11.22848	0.572012	0.02449	0.065157	lncRNA
ENSG0000(	ENSG0000(	215.6217	0.235259	0.024498	0.065176	lncRNA
ENSG0000(	IFT20	798.2484	-0.08983	0.024544	0.065292	protein_coding
ENSG0000(	ACACA	2299.044	-0.15343	0.024557	0.065321	protein_coding
ENSG0000(	EFHC1	2268.451	0.174418	0.024561	0.065325	protein_coding
ENSG0000(	ENSG0000(	145.9198	-0.20005	0.024563	0.065325	lncRNA
ENSG0000(	ETS2	4345.658	0.208142	0.024566	0.065329	protein_coding
ENSG0000(	IGKV1D-43	16.89054	0.860805	0.024581	0.065357	IG_V_gene
ENSG0000(	RHBG	263.7683	-0.14143	0.024581	0.065357	protein_coding
ENSG0000(	ENSG0000(	78.18623	-0.2649	0.024586	0.065363	TEC
ENSG0000(	CDIP1	346.7869	0.174697	0.024622	0.065449	protein_coding
ENSG0000(	JMJD1C-AS	11.46382	0.555798	0.024622	0.065449	lncRNA
ENSG0000(	LINC00954	715.8511	0.174463	0.024625	0.065451	lncRNA
ENSG0000(	WDR83OS	169.9687	-0.51086	0.024628	0.065454	protein_coding
ENSG0000(	S1PR5	45.47411	0.302996	0.024638	0.065475	protein_coding
ENSG0000(	ENSG0000(	77.09727	0.220039	0.024649	0.065499	lncRNA
ENSG0000(	ENSG0000(	91.6341	0.17914	0.024655	0.065508	lncRNA
ENSG0000(	PDCD6IP-D	469.7563	0.282078	0.024671	0.065546	lncRNA
ENSG0000(	DUX4L50	11.39829	0.561252	0.024682	0.065569	unprocessed_pseudogene
ENSG0000(	CAPN14	43.06037	0.491416	0.024694	0.06559	protein_coding
ENSG0000(	CPE	138.952	-0.37004	0.024699	0.06559	protein_coding
ENSG0000(	ENSG0000(	267.3188	-0.28021	0.024699	0.06559	lncRNA
ENSG0000(	EXOSC5	390.6669	-0.13581	0.0247	0.06559	protein_coding
ENSG0000(	PIK3IP1	1101.317	0.30458	0.024698	0.06559	protein_coding
ENSG0000(	IQCG	494.0737	0.107345	0.02472	0.065637	protein_coding
ENSG0000(	EIF2AK2	3896.368	0.22242	0.024734	0.065669	protein_coding
ENSG0000(	TASP1	432.7111	-0.16697	0.024772	0.065763	protein_coding
ENSG0000(	ANKRD65	76.94306	0.31001	0.024792	0.065806	protein_coding
ENSG0000(	RNU1-19P	99.35417	0.284074	0.024792	0.065806	snRNA
ENSG0000(	MARCHF7	3291.477	-0.1162	0.024796	0.065811	protein_coding
ENSG0000(	RNF208	31.1986	-0.35106	0.02481	0.065843	protein_coding
ENSG0000(	RPL36AP45	14.61875	0.470582	0.024818	0.065857	processed_pseudogene
ENSG0000(	ANKRD45	339.837	-0.15156	0.024826	0.065874	protein_coding
ENSG0000(	DDX31	416.8911	-0.16818	0.024834	0.065884	protein_coding
ENSG0000(	IGKV2-28	786.5119	0.636809	0.024834	0.065884	IG_V_gene
ENSG0000(	ENSG0000(	11.67586	0.494916	0.024856	0.065935	lncRNA
ENSG0000(	SMIM10L2I	32.72432	0.410579	0.02486	0.06594	lncRNA
ENSG0000(	AKAP8	1442.593	-0.07638	0.02487	0.065963	protein_coding
ENSG0000(	TMEM254-	166.1795	0.189879	0.024877	0.065976	lncRNA
ENSG0000(	FECH	1262.833	0.096501	0.02488	0.065978	protein_coding
ENSG0000(	ENSG0000(	175.2567	-0.13992	0.024906	0.06603	TEC
ENSG0000(	ENSG0000(	236.0715	0.119714	0.024903	0.06603	lncRNA
ENSG0000(	ENSG0000(	283.0837	-0.13332	0.024906	0.06603	lncRNA

ENSG0000(	GPATCH3	192.7653	-0.19191	0.024926	0.066077	protein_coding
ENSG0000(	DMGDH	113.55	-0.18297	0.024944	0.066118	protein_coding
ENSG0000(	ZNF649	525.9729	0.130577	0.024967	0.066175	protein_coding
ENSG0000(	UGCG	1795.422	0.206542	0.024972	0.066183	protein_coding
ENSG0000(	ENSG0000(	20.18988	0.439479	0.024982	0.066201	lncRNA
ENSG0000(	TAB1	532.2046	-0.16647	0.024983	0.066201	protein_coding
ENSG0000(	ENSG0000(	275.2449	-0.13513	0.024988	0.066208	lncRNA
ENSG0000(	SLC37A3	1598.947	-0.08359	0.025001	0.066237	protein_coding
ENSG0000(	ENSG0000(	355.3851	-0.28699	0.025023	0.066269	processed_pseudogene
ENSG0000(	RGS12	1697.14	0.120606	0.025024	0.066269	protein_coding
ENSG0000(	RN7SL811F	232.375	-0.18725	0.025021	0.066269	misc_RNA
ENSG0000(	SMIM10L2I	31.14567	0.282203	0.025021	0.066269	protein_coding
ENSG0000(	TADA1	275.0846	-0.14135	0.025019	0.066269	protein_coding
ENSG0000(	PRDX1	1002.165	-0.27129	0.02503	0.066279	protein_coding
ENSG0000(	GATB	555.7878	-0.15367	0.02506	0.066349	protein_coding
ENSG0000(	IGHV6-1	131.613	0.724464	0.02506	0.066349	IG_V_gene
ENSG0000(	C4orf19	1809.984	-0.16575	0.025077	0.066381	protein_coding
ENSG0000(	LINC02576	290.9156	0.163818	0.025075	0.066381	lncRNA
ENSG0000(	ENSG0000(	98.89848	0.186807	0.025092	0.066417	lncRNA
ENSG0000(	RIOX1	247.5537	-0.16859	0.025102	0.066436	protein_coding
ENSG0000(	ESRRB	12.17641	0.712339	0.025113	0.066453	protein_coding
ENSG0000(	MLLT11	124.289	-0.21292	0.025113	0.066453	protein_coding
ENSG0000(	PHRF1	1720.245	-0.10535	0.025114	0.066453	protein_coding
ENSG0000(	ENSG0000(	42.79234	0.384669	0.025119	0.066458	lncRNA
ENSG0000(	ESYT3	349.0835	-0.16949	0.025132	0.066488	protein_coding
ENSG0000(	C1orf109	344.5267	-0.13313	0.02514	0.066503	protein_coding
ENSG0000(	IL12A	21.20175	0.438132	0.025181	0.066607	protein_coding
ENSG0000(	ENSG0000(	50.69307	-0.24312	0.025202	0.066656	lncRNA
ENSG0000(	ENSG0000(	116.9691	-0.20968	0.025205	0.066659	lncRNA
ENSG0000(	ENSG0000(	57.15704	0.246959	0.02523	0.066718	lncRNA
ENSG0000(	RN7SL470F	150.603	0.158799	0.025237	0.066733	misc_RNA
ENSG0000(	ENSG0000(	113.7691	0.201093	0.025245	0.066747	TEC
ENSG0000(	TAF1C	1460.629	-0.11865	0.025255	0.066768	protein_coding
ENSG0000(	ENSG0000(	363.1355	-0.12946	0.025265	0.066789	lncRNA
ENSG0000(	ADGRF1	1439.117	0.355582	0.025275	0.06681	protein_coding
ENSG0000(	TEPP	138.1463	-0.24521	0.025289	0.066842	protein_coding
ENSG0000(	ENSG0000(	42.65027	-0.32501	0.025297	0.066857	processed_pseudogene
ENSG0000(	ENSG0000(	184.0768	0.175654	0.025299	0.066857	lncRNA
ENSG0000(	GPR35	3101.867	-0.25752	0.025313	0.066888	protein_coding
ENSG0000(	ENSG0000(	125.3175	-0.27242	0.025321	0.066903	lncRNA
ENSG0000(	CTBP1-AS	108.5012	0.191021	0.025324	0.066906	lncRNA
ENSG0000(	PLCG2	2361.666	0.297184	0.025331	0.066912	protein_coding
ENSG0000(	URI1	1286.14	-0.11048	0.025329	0.066912	protein_coding
ENSG0000(	IGLV2-23	722.7935	0.560015	0.025336	0.06692	IG_V_gene
ENSG0000(	ENSG0000(	428.2841	-0.14361	0.025345	0.066939	lncRNA
ENSG0000(	MYBL2	269.2883	-0.33097	0.025351	0.066949	protein_coding
ENSG0000(	ENSG0000(	94.85779	-0.22897	0.025368	0.066981	lncRNA

ENSG0000(	PTGDS	484.0226	0.506583	0.025366	0.066981	protein_coding
ENSG0000(	RN7SL621F	23.65723	0.384481	0.025373	0.06699	misc_RNA
ENSG0000(	ENSG0000(	533.54	0.209889	0.025381	0.067004	lncRNA
ENSG0000(	C15orf62	21.80129	0.381097	0.025402	0.067054	protein_coding
ENSG0000(	CLTB	985.9611	-0.27778	0.025404	0.067054	protein_coding
ENSG0000(	TPTE	23.93901	0.421194	0.025409	0.067062	protein_coding
ENSG0000(	MIR576	10.77523	0.537118	0.025413	0.067068	miRNA
ENSG0000(	CD177	3188.405	-0.73311	0.025443	0.067135	protein_coding
ENSG0000(	ITGB2-AS1	303.4054	0.460403	0.025442	0.067135	lncRNA
ENSG0000(	SIRPB3P	21.18945	0.667332	0.025467	0.067192	unprocessed_pseudogene
ENSG0000(	LINC02323	42.5363	-0.57444	0.025473	0.067202	lncRNA
ENSG0000(	ENSG0000(	733.4507	0.099573	0.025482	0.067221	protein_coding
ENSG0000(	RNF217	1045.808	0.117552	0.025487	0.067228	protein_coding
ENSG0000(	FMO6P	13.72023	0.557242	0.025504	0.067262	transcribed_unitary_pseudogene
ENSG0000(	PMP22	1290.552	0.224057	0.025504	0.067262	protein_coding
ENSG0000(	FASTKD2	1624.25	-0.11164	0.025558	0.067395	protein_coding
ENSG0000(	VAC14-AS1	322.2254	0.127894	0.025559	0.067395	lncRNA
ENSG0000(	PIWIL2	314.5674	-0.15616	0.025569	0.067416	protein_coding
ENSG0000(	DLG1	6412.631	-0.12934	0.025573	0.067416	protein_coding
ENSG0000(	OTUB2	62.61516	0.335111	0.025573	0.067416	protein_coding
ENSG0000(	ENSG0000(	41.41256	0.285145	0.025583	0.067437	lncRNA
ENSG0000(	ATP1B2	235.9189	-0.13613	0.025593	0.067456	protein_coding
ENSG0000(	IQSEC3	59.88735	0.70781	0.025598	0.067461	protein_coding
ENSG0000(	NKX3-1	32.44126	0.413418	0.025599	0.067461	protein_coding
ENSG0000(	CNTF	192.4863	-0.14622	0.025603	0.067466	protein_coding
ENSG0000(	LINC01451	45.75013	-0.44019	0.025617	0.067498	lncRNA
ENSG0000(	ENSG0000(	129.2138	0.209761	0.025629	0.067524	lncRNA
ENSG0000(	ENSG0000(	13.90101	0.423337	0.025633	0.067528	lncRNA
ENSG0000(	LINC02056	413.8372	0.187205	0.02564	0.067541	lncRNA
ENSG0000(	LINC02391	11.19282	0.708625	0.025645	0.067548	lncRNA
ENSG0000(	ENSG0000(	186.4487	0.164046	0.025655	0.067569	lncRNA
ENSG0000(	ENSG0000(	95.06157	-0.16009	0.025662	0.067578	lncRNA
ENSG0000(	ENSG0000(	16.68455	1.07645	0.025663	0.067578	TEC
ENSG0000(	LRRC24	25.1485	-0.39761	0.025667	0.067582	protein_coding
ENSG0000(	ENSG0000(	175.1962	0.25787	0.025675	0.067592	processed_pseudogene
ENSG0000(	LINC01902	960.425	-0.1095	0.025675	0.067592	lncRNA
ENSG0000(	NMRAL1	501.5254	0.111746	0.02569	0.067625	protein_coding
ENSG0000(	SP1	6200.619	0.147643	0.025691	0.067625	protein_coding
ENSG0000(	HAS2	371.1392	0.288826	0.025707	0.067659	protein_coding
ENSG0000(	FKBP3	487.2587	-0.16711	0.025709	0.06766	protein_coding
ENSG0000(	ZADH2	2181.864	-0.13283	0.025719	0.06768	protein_coding
ENSG0000(	ENSG0000(	14.73814	0.411875	0.025729	0.067696	lncRNA
ENSG0000(	RNU2-70P	25.02406	-0.41444	0.025729	0.067696	snRNA
ENSG0000(	MRPL57	714.3608	-0.08066	0.025732	0.067696	protein_coding
ENSG0000(	PAQR8	3121.654	-0.32343	0.025734	0.067696	protein_coding
ENSG0000(	C2CD5	2837.178	-0.17346	0.025736	0.067697	protein_coding
ENSG0000(	ENSG0000(	160.9703	0.153003	0.025744	0.06771	processed_pseudogene

ENSG0000(	SRSF4	5705.558	-0.06201	0.025746	0.06771	protein_coding
ENSG0000(	ENSG0000(	37.40983	-0.2854	0.025757	0.067735	lncRNA
ENSG0000(	RPL23AP34	27.4432	-0.39356	0.025761	0.067739	processed_pseudogene
ENSG0000(	ENSG0000(	196.5234	0.227004	0.025766	0.067744	lncRNA
ENSG0000(	PYROXD1	874.0081	-0.16853	0.025767	0.067744	protein_coding
ENSG0000(	MTCO3P39	52.13291	0.467602	0.02577	0.067747	processed_pseudogene
ENSG0000(	C1QTNF3	345.8212	0.204121	0.02578	0.067768	protein_coding
ENSG0000(	ENSG0000(	49.90204	0.305529	0.025801	0.067817	lncRNA
ENSG0000(	DMTN	305.3973	-0.26742	0.025818	0.067849	protein_coding
ENSG0000(	SEZ6L	38.28172	-0.58994	0.025818	0.067849	protein_coding
ENSG0000(	TBP	441.1481	-0.10141	0.025872	0.067986	protein_coding
ENSG0000(	ENSG0000(	128.8506	0.263775	0.025877	0.067994	lncRNA
ENSG0000(	PLEKHA8	1106.331	0.131449	0.025907	0.068066	protein_coding
ENSG0000(	ENSG0000(	258.6369	-0.14199	0.025909	0.068066	lncRNA
ENSG0000(	ENSG0000(	118.0185	0.232827	0.025919	0.068085	lncRNA
ENSG0000(	AGO4	1606.635	0.192694	0.025923	0.068091	protein_coding
ENSG0000(	RNU6-1111	21.2128	0.410133	0.025929	0.068101	snRNA
ENSG0000(	TRPV3	1088.001	-0.34356	0.025934	0.068109	protein_coding
ENSG0000(	ENSG0000(	77.99577	0.259088	0.025959	0.068168	lncRNA
ENSG0000(	ENSG0000(	20.0368	0.441495	0.025974	0.068201	lncRNA
ENSG0000(	EXPH5	3719.441	-0.3361	0.025982	0.068217	protein_coding
ENSG0000(	C21orf62-A	1770.185	0.073537	0.025988	0.068227	lncRNA
ENSG0000(	ODCP	176.4007	0.150429	0.026022	0.068311	processed_pseudogene
ENSG0000(	ENSG0000(	71.08302	-0.62852	0.026045	0.068365	lncRNA
ENSG0000(	CHST9	151.3356	-0.18085	0.026048	0.068367	protein_coding
ENSG0000(	RPL12P20	26.57073	0.361357	0.026061	0.068395	processed_pseudogene
ENSG0000(	C9orf78	1162.332	-0.07041	0.026081	0.068442	protein_coding
ENSG0000(	FBXO42	1411.89	0.075503	0.026095	0.068474	protein_coding
ENSG0000(	GLT8D2	144.7489	0.273407	0.026099	0.068477	protein_coding
ENSG0000(	GALNT7	2368.943	-0.21069	0.026105	0.068489	protein_coding
ENSG0000(	ENSG0000(	12.65911	1.110735	0.026113	0.068499	lncRNA
ENSG0000(	PPP1R26P1	9.158174	0.517629	0.026112	0.068499	processed_pseudogene
ENSG0000(	CENPP	409.6287	-0.27673	0.026124	0.068515	protein_coding
ENSG0000(	ENSG0000(	205.6242	0.137379	0.026122	0.068515	lncRNA
ENSG0000(	CORO2A	2616.515	-0.24178	0.026149	0.068575	protein_coding
ENSG0000(	HEATR3	1450.911	-0.0976	0.026155	0.068586	protein_coding
ENSG0000(	ENSG0000(	281.4108	0.132877	0.02617	0.068617	TEC
ENSG0000(	HSPE1P25	23.35818	0.358946	0.026171	0.068617	processed_pseudogene
ENSG0000(	ENSG0000(	261.8026	-0.1295	0.02618	0.068622	lncRNA
ENSG0000(	LINC02188	164.1265	-0.14843	0.026182	0.068622	lncRNA
ENSG0000(	LINC02254	3056.676	0.086465	0.026181	0.068622	lncRNA
ENSG0000(	TMEM256	113.2296	-0.25773	0.026178	0.068622	protein_coding
ENSG0000(	KCTD6	456.7737	-0.1431	0.026189	0.068634	protein_coding
ENSG0000(	ZNF841	2052.411	0.138231	0.026207	0.068675	protein_coding
ENSG0000(	ENSG0000(	63.40439	0.211072	0.026216	0.068693	lncRNA
ENSG0000(	ENSG0000(	457.5523	0.130052	0.026238	0.068745	lncRNA
ENSG0000(	ENSG0000(	78.66828	-0.18936	0.02626	0.068797	lncRNA

ENSG0000(	APLNR	256.3537	0.408805	0.026274	0.068827	protein_coding
ENSG0000(	OSBPL6	419.9787	-0.22079	0.026277	0.068831	protein_coding
ENSG0000(	FAM227B	261.4602	0.137715	0.026294	0.06887	protein_coding
ENSG0000(	GCATP1	48.29642	-0.37836	0.026326	0.068941	processed_pseudogene
ENSG0000(	MT2A	436.7522	0.509833	0.026326	0.068941	protein_coding
ENSG0000(	MINDY2	1890.204	0.083916	0.026334	0.068945	protein_coding
ENSG0000(	PCMTD2	1518.204	0.153862	0.026334	0.068945	protein_coding
ENSG0000(	SSPN	349.5491	0.301104	0.026331	0.068945	protein_coding
ENSG0000(	LINC01612	299.8464	-0.38399	0.026339	0.068952	lncRNA
ENSG0000(	NANOGP1	214.0195	0.142958	0.026367	0.069015	transcribed_unprocessed_pseudo
ENSG0000(	WASH3P	838.5768	-0.16169	0.026367	0.069015	transcribed_unprocessed_pseudo
ENSG0000(	ENSG0000(	19.94262	0.416492	0.026373	0.069023	lncRNA
ENSG0000(	OR10A2	233.2154	-0.17927	0.026377	0.069023	protein_coding
ENSG0000(	ZCWPW1	123.3381	0.206211	0.026377	0.069023	protein_coding
ENSG0000(	LINC01873	1863.987	-0.18086	0.026386	0.069041	lncRNA
ENSG0000(	ENSG0000(	95.61234	-0.1985	0.026392	0.069048	lncRNA
ENSG0000(	LINC02200	152.3034	0.199308	0.026394	0.069048	lncRNA
ENSG0000(	RAPGEF1	3393.567	0.144532	0.026411	0.069087	protein_coding
ENSG0000(	HENMT1	240.5836	-0.1749	0.026431	0.069135	protein_coding
ENSG0000(	CEACAM20	16.84847	0.799533	0.026439	0.069149	protein_coding
ENSG0000(	CCDC83	15.77614	0.458867	0.026477	0.069237	protein_coding
ENSG0000(	ENSG0000(	20.77998	-0.27476	0.026475	0.069237	lncRNA
ENSG0000(	GTF2IP14	23.24149	-0.36752	0.026484	0.069249	unprocessed_pseudogene
ENSG0000(	TDG	975.5488	-0.08457	0.026486	0.069249	protein_coding
ENSG0000(	SOD1	776.6589	-0.21854	0.026508	0.069301	protein_coding
ENSG0000(	SAT2	278.8477	-0.20647	0.026517	0.069319	protein_coding
ENSG0000(	MTCO3P17	14.06368	0.485797	0.026531	0.06935	unprocessed_pseudogene
ENSG0000(	DNMT1	3041.839	-0.13911	0.026577	0.069465	protein_coding
ENSG0000(	ENSG0000(	15.64661	0.733052	0.026595	0.069507	lncRNA
ENSG0000(	PI4KAP1	921.5571	-0.42571	0.026608	0.069534	transcribed_unprocessed_pseudo
ENSG0000(	ENSG0000(	59.75595	0.248174	0.02661	0.069534	lncRNA
ENSG0000(	ENSG0000(	16.87298	0.402499	0.026613	0.069535	lncRNA
ENSG0000(	ENSG0000(	581.7306	0.126444	0.026616	0.069537	lncRNA
ENSG0000(	ENSG0000(	247.8846	0.127953	0.026651	0.069624	lncRNA
ENSG0000(	AMD1P4	181.6716	-0.14261	0.026659	0.069629	processed_pseudogene
ENSG0000(	ENSG0000(	14.76097	0.383418	0.02666	0.069629	lncRNA
ENSG0000(	WDR5B-DT	466.1354	0.096431	0.026656	0.069629	lncRNA
ENSG0000(	BDH2	632.6288	-0.15784	0.026678	0.069671	protein_coding
ENSG0000(	ENSG0000(	277.1797	-0.12274	0.026682	0.069675	lncRNA
ENSG0000(	UMAD1	519.141	0.202949	0.026691	0.069692	protein_coding
ENSG0000(	ENSG0000(	306.37	-0.13672	0.026712	0.069741	TEC
ENSG0000(	ENSG0000(	121.1912	-0.16537	0.026732	0.069778	lncRNA
ENSG0000(	ENSG0000(	11.31033	0.576513	0.026733	0.069778	lncRNA
ENSG0000(	KLHL14	74.32807	0.566825	0.02673	0.069778	protein_coding
ENSG0000(	ENSG0000(	17.40453	0.741495	0.026735	0.069779	lncRNA
ENSG0000(	ENSG0000(	411.915	0.111396	0.02675	0.069811	lncRNA
ENSG0000(	DRD4	33.62516	0.351384	0.026758	0.069825	protein_coding

ENSG0000(	ENSG0000(	43.7347	0.361102	0.02676	0.069826	lncRNA
ENSG0000(	FUOM	109.9937	-0.25675	0.026764	0.069831	protein_coding
ENSG0000(	ENSG0000(	49.82698	0.371266	0.026779	0.069864	lncRNA
ENSG0000(	IGLV4-60	36.13133	0.894263	0.026786	0.069878	IG_V_gene
ENSG0000(	FAM151B	203.8616	0.209408	0.026789	0.069878	protein_coding
ENSG0000(	ENSG0000(	74.78772	0.198966	0.026796	0.06989	lncRNA
ENSG0000(	RNU6-1266	11.99074	0.516641	0.026803	0.069903	snRNA
ENSG0000(	TRMT11	812.424	-0.11041	0.026806	0.069904	protein_coding
ENSG0000(	ENSG0000(	10.33672	-0.60863	0.026828	0.06995	processed_pseudogene
ENSG0000(	SATL1	753.6038	-0.18633	0.026826	0.06995	protein_coding
ENSG0000(	TRIM5	893.6611	0.152201	0.026844	0.069986	protein_coding
ENSG0000(	ITLN1	929.594	-0.59796	0.026853	0.070006	protein_coding
ENSG0000(	SMC4	2348.951	-0.14857	0.026856	0.070006	protein_coding
ENSG0000(	ENSG0000(	13.21612	0.596215	0.026876	0.070054	lncRNA
ENSG0000(	ENSG0000(	630.6862	-0.2072	0.026892	0.070089	protein_coding
ENSG0000(	TBR1	36.27814	0.334858	0.026917	0.070148	protein_coding
ENSG0000(	MTND2P2	39.44206	0.303431	0.026924	0.070159	processed_pseudogene
ENSG0000(	LINC00896	66.69871	0.272127	0.026926	0.070161	lncRNA
ENSG0000(	POLH-AS1	197.2632	0.163149	0.026948	0.070211	lncRNA
ENSG0000(	FAM74A6	208.5183	-0.16637	0.026951	0.070212	transcribed_processed_pseudoge
ENSG0000(	MTERF4	1908.521	0.125027	0.02697	0.070256	protein_coding
ENSG0000(	ENSG0000(	82.27996	0.592096	0.026978	0.070269	lncRNA
ENSG0000(	PSG4	73.37982	0.439424	0.026979	0.070269	protein_coding
ENSG0000(	RNF19B	1148.231	0.242391	0.026997	0.070311	protein_coding
ENSG0000(	ENSG0000(	59.36538	0.302061	0.027072	0.070498	lncRNA
ENSG0000(	TCTN2	675.9883	-0.10136	0.027089	0.070537	protein_coding
ENSG0000(	RNASET2	5543.836	0.136539	0.027117	0.070605	protein_coding
ENSG0000(	ENSG0000(	14.28251	0.440505	0.027127	0.070624	lncRNA
ENSG0000(	ENSG0000(	716.4963	0.124624	0.027148	0.070674	lncRNA
ENSG0000(	ENSG0000(	90.6419	0.221102	0.02716	0.070698	lncRNA
ENSG0000(	CRYBB3	77.50019	-0.45579	0.027164	0.070704	protein_coding
ENSG0000(	ENSG0000(	52.09666	0.235642	0.027168	0.070708	lncRNA
ENSG0000(	RPS4XP7	51.14189	-0.58028	0.027176	0.070724	processed_pseudogene
ENSG0000(	ENSG0000(	28.88906	0.419836	0.027258	0.070924	snoRNA
ENSG0000(	TRRAP	6054.072	-0.07417	0.027257	0.070924	protein_coding
ENSG0000(	ALDH1A3-A	562.5626	-0.17633	0.027263	0.070926	lncRNA
ENSG0000(	ENSG0000(	63.53006	-0.27001	0.027261	0.070926	lncRNA
ENSG0000(	ENSG0000(	1237.593	0.242167	0.027272	0.070938	lncRNA
ENSG0000(	ZNF184	268.3326	-0.15447	0.027272	0.070938	protein_coding
ENSG0000(	TMSB15B	495.1929	0.132738	0.02728	0.070951	protein_coding
ENSG0000(	GSDMB	4763.161	0.209509	0.027283	0.070954	protein_coding
ENSG0000(	STMP1	1211.545	-0.08839	0.027285	0.070954	protein_coding
ENSG0000(	F11-AS1	209.3359	0.221013	0.027291	0.070958	lncRNA
ENSG0000(	STYX	884.2001	-0.1686	0.027291	0.070958	protein_coding
ENSG0000(	RAB35	843.3245	-0.12202	0.027295	0.070961	protein_coding
ENSG0000(	TMEM101	253.4032	-0.14849	0.027316	0.07101	protein_coding
ENSG0000(	RN7SL824P	12.2948	0.511329	0.027319	0.071013	misc_RNA

ENSG0000(	TMEM51-A	941.0035	0.139792	0.02734	0.071061	lncRNA
ENSG0000(	ENSG0000(	138.2846	0.173311	0.027345	0.071068	lncRNA
ENSG0000(	LINC01940	63.44787	-0.22418	0.027354	0.071085	lncRNA
ENSG0000(	FEM1A	2730.143	-0.05561	0.027361	0.071098	protein_coding
ENSG0000(	AK1	733.9385	-0.34112	0.027418	0.071239	protein_coding
ENSG0000(	ENSG0000(	471.7046	0.12153	0.027424	0.071247	lncRNA
ENSG0000(	PDE9A-AS1	56.26326	0.271045	0.027425	0.071247	lncRNA
ENSG0000(	WDR70	944.6913	-0.1734	0.027452	0.07131	protein_coding
ENSG0000(	RNU6-323F	8.74012	0.54984	0.027459	0.071323	snRNA
ENSG0000(	ENSG0000(	28.64898	-0.37088	0.027471	0.071348	lncRNA
ENSG0000(	GNG5	1591.168	-0.21824	0.027478	0.071359	protein_coding
ENSG0000(	ENSG0000(	39.48865	0.265006	0.027501	0.071413	lncRNA
ENSG0000(	ENSG0000(	20.1759	0.386892	0.027509	0.071422	lncRNA
ENSG0000(	ENSG0000(	51.27542	0.327505	0.027507	0.071422	lncRNA
ENSG0000(	SNAI2	356.7665	0.291121	0.027511	0.071422	protein_coding
ENSG0000(	ENSG0000(	146.9136	0.202554	0.027519	0.071436	lncRNA
ENSG0000(	COL25A1	35.49322	-0.66452	0.027538	0.071472	protein_coding
ENSG0000(	ENSG0000(	165.8458	-0.17333	0.027537	0.071472	processed_pseudogene
ENSG0000(	ENSG0000(	60.3918	0.441429	0.027539	0.071472	lncRNA
ENSG0000(	ARHGEF12	8566.758	-0.07901	0.027544	0.071476	protein_coding
ENSG0000(	ENSG0000(	29.80315	0.288682	0.027545	0.071476	lncRNA
ENSG0000(	C16orf54	379.7738	0.265573	0.027551	0.071478	protein_coding
ENSG0000(	KCNJ11	241.9173	-0.1638	0.027549	0.071478	protein_coding
ENSG0000(	ENSG0000(	484.0037	0.150798	0.02756	0.071496	lncRNA
ENSG0000(	ENSG0000(	558.7585	0.177778	0.027578	0.071536	lncRNA
ENSG0000(	HEPACAM	25.29941	0.459809	0.02758	0.071536	protein_coding
ENSG0000(	DDX28	109.9037	-0.18952	0.02759	0.071555	protein_coding
ENSG0000(	CHMP3	2053.319	-0.12591	0.027611	0.071595	protein_coding
ENSG0000(	LINC01476	463.714	-0.11419	0.027612	0.071595	lncRNA
ENSG0000(	TUBGCP5	899.2469	-0.12621	0.027611	0.071595	protein_coding
ENSG0000(	ENSG0000(	65.16806	-0.21642	0.027624	0.071615	lncRNA
ENSG0000(	SCARNA9	3046.325	-0.14022	0.027624	0.071615	lncRNA
ENSG0000(	ATP6V1B2	1955.461	0.163444	0.027656	0.071692	protein_coding
ENSG0000(	LINC02341	12.90823	0.561872	0.02768	0.071747	lncRNA
ENSG0000(	ENSG0000(	51.80863	-0.30913	0.027707	0.071811	lncRNA
ENSG0000(	DGUOK-AS	76.23725	-0.31132	0.02771	0.071814	lncRNA
ENSG0000(	BRCA2	1202.894	0.121281	0.027739	0.071884	protein_coding
ENSG0000(	MBD2	1900.516	-0.13592	0.027759	0.071928	protein_coding
ENSG0000(	ADAMTS18	51.81347	0.621303	0.027782	0.071977	protein_coding
ENSG0000(	ZNF561	1335.136	-0.10066	0.027782	0.071977	protein_coding
ENSG0000(	SLC2A4RG	354.1088	-0.22344	0.027786	0.071982	protein_coding
ENSG0000(	OR6J1	281.0262	-0.11559	0.027809	0.072034	protein_coding
ENSG0000(	SNX13	4660.588	-0.15922	0.027816	0.072048	protein_coding
ENSG0000(	MMAB	758.9754	-0.10627	0.027828	0.072071	protein_coding
ENSG0000(	MINAR2	401.4723	0.154189	0.027846	0.072113	protein_coding
ENSG0000(	PDIA3	4826.46	-0.15605	0.027853	0.072125	protein_coding
ENSG0000(	CHCHD3P2	14.56007	-0.64543	0.027856	0.072127	processed_pseudogene

ENSG0000(	POTEI	90.43402	0.279241	0.027867	0.07215	protein_coding
ENSG0000(	SCARNA13	1923.647	-0.19193	0.027879	0.072174	scaRNA
ENSG0000(	STAM	489.0351	-0.17195	0.02789	0.072197	protein_coding
ENSG0000(	ENSG0000(	110.9354	0.182184	0.027892	0.072197	lncRNA
ENSG0000(	FAM83G	1572.538	-0.23624	0.027949	0.072324	protein_coding
ENSG0000(	STMN2	157.3368	-0.43791	0.027947	0.072324	protein_coding
ENSG0000(	ZNF414	472.0043	0.23058	0.027947	0.072324	protein_coding
ENSG0000(	DOK4	1339.803	-0.26972	0.027962	0.072347	protein_coding
ENSG0000(	IGHV2-70	69.66531	0.896132	0.027962	0.072347	IG_V_gene
ENSG0000(	ENSG0000(	164.7493	0.24547	0.027971	0.072364	TEC
ENSG0000(	CHORDC1	2419.037	-0.12271	0.027977	0.072373	protein_coding
ENSG0000(	CNTFR	131.5963	-0.71856	0.027985	0.072388	protein_coding
ENSG0000(	CXXC4-AS1	560.9707	-0.13447	0.027988	0.072389	lncRNA
ENSG0000(	HDHD2	829.451	-0.11326	0.028006	0.07243	protein_coding
ENSG0000(	LINC00461	455.2416	0.157345	0.028017	0.072451	lncRNA
ENSG0000(	LINC02078	665.1313	0.105721	0.028019	0.072451	lncRNA
ENSG0000(	RN7SKP273	22.84974	0.368002	0.028023	0.072458	misc_RNA
ENSG0000(	ENSG0000(	103.7591	-0.22097	0.028028	0.072463	lncRNA
ENSG0000(	ASB16	76.02878	0.271189	0.028034	0.072473	protein_coding
ENSG0000(	RGMB-AS1	51.95167	0.463034	0.028063	0.072543	lncRNA
ENSG0000(	DNMBP-AS	350.0054	0.100778	0.028107	0.072651	lncRNA
ENSG0000(	SZT2-AS1	29.73515	-0.33605	0.02811	0.072651	lncRNA
ENSG0000(	ENSG0000(	273.2135	0.126545	0.028128	0.072692	lncRNA
ENSG0000(	IGKV2D-40	20.2396	1.475026	0.028152	0.072748	IG_V_gene
ENSG0000(	RAB22A	2106.84	-0.12099	0.028155	0.07275	protein_coding
ENSG0000(	DRICH1	59.24885	-0.37814	0.028175	0.072795	protein_coding
ENSG0000(	CHST7	46.16503	0.326457	0.02819	0.072815	protein_coding
ENSG0000(	IGLV2-14	1471.54	0.533514	0.028189	0.072815	IG_V_gene
ENSG0000(	LIN37	244.7294	-0.17864	0.028185	0.072815	protein_coding
ENSG0000(	ENSG0000(	26.57118	0.382116	0.028201	0.072839	TEC
ENSG0000(	LYPLA1	980.3246	-0.11221	0.028205	0.072839	protein_coding
ENSG0000(	NUDT9P1	14.63587	-0.37462	0.028206	0.072839	processed_pseudogene
ENSG0000(	KRTAP5-7	16.4284	0.490726	0.02821	0.072843	protein_coding
ENSG0000(	ENSG0000(	134.6932	0.142167	0.028225	0.072877	lncRNA
ENSG0000(	ENSG0000(	196.1946	-0.14547	0.028236	0.07289	lncRNA
ENSG0000(	LINC02030	22.6204	0.36803	0.028235	0.07289	lncRNA
ENSG0000(	PPM1M	302.0789	0.299239	0.028237	0.07289	protein_coding
ENSG0000(	ENSG0000(	32.32971	0.348336	0.028261	0.072946	lncRNA
ENSG0000(	HM13	3318.392	0.137612	0.028275	0.072975	protein_coding
ENSG0000(	C1D	144.0692	-0.20644	0.0283	0.073033	protein_coding
ENSG0000(	WDR26	4681.527	0.078694	0.028355	0.07317	protein_coding
ENSG0000(	ZNF582	73.57148	-0.28908	0.028369	0.073201	protein_coding
ENSG0000(	ANAPC2	1322.381	-0.1027	0.028379	0.073219	protein_coding
ENSG0000(	CPS1	895.778	0.175126	0.028383	0.073224	protein_coding
ENSG0000(	ECD	878.6738	-0.11719	0.02839	0.073235	protein_coding
ENSG0000(	ENSG0000(	101.5431	0.308076	0.028395	0.073243	lncRNA
ENSG0000(	TRIB1	1894.765	0.229958	0.028402	0.073255	protein_coding

ENSG0000(	ENSG0000(	156.9399	0.296448	0.028412	0.073276	lncRNA
ENSG0000(	BNIPL	257.0527	-0.13622	0.028422	0.073294	protein_coding
ENSG0000(	TTYH2	290.0026	0.288944	0.028431	0.07331	protein_coding
ENSG0000(	ENSG0000(	381.3912	-0.18271	0.02845	0.073356	lncRNA
ENSG0000(	ENSG0000(	22.76814	-0.51583	0.028456	0.073363	lncRNA
ENSG0000(	ENSG0000(	101.4365	-0.19083	0.028469	0.073391	lncRNA
ENSG0000(	FRG1JP	37.518	-0.39268	0.028476	0.073404	unprocessed_pseudogene
ENSG0000(	GPRASP2	146.6168	0.212518	0.028483	0.073416	protein_coding
ENSG0000(	ENSG0000(	243.5967	-0.20256	0.028486	0.073417	lncRNA
ENSG0000(	RHOT1P1	49.8235	0.472803	0.028497	0.07344	processed_pseudogene
ENSG0000(	ENSG0000(	97.48771	0.198197	0.02851	0.073466	lncRNA
ENSG0000(	A1BG-AS1	276.7634	0.150276	0.028521	0.07349	lncRNA
ENSG0000(	DMXL2	6820.227	0.146462	0.028527	0.073499	protein_coding
ENSG0000(	RPP25L	117.6841	-0.26781	0.028537	0.073518	protein_coding
ENSG0000(	ENSG0000(	31.51177	0.340309	0.028561	0.073573	lncRNA
ENSG0000(	CSDC2	39.63448	0.492664	0.028565	0.073579	protein_coding
ENSG0000(	ACTR2	5052.79	-0.17666	0.028576	0.073602	protein_coding
ENSG0000(	MIR378E	13.9891	0.540335	0.028581	0.073607	miRNA
ENSG0000(	ANO5	653.9925	-0.19251	0.028604	0.073652	protein_coding
ENSG0000(	ENSG0000(	576.3457	-0.10776	0.028606	0.073652	lncRNA
ENSG0000(	LINC02405	414.0156	-0.21208	0.028602	0.073652	lncRNA
ENSG0000(	ENSG0000(	331.7799	0.114627	0.028618	0.073678	lncRNA
ENSG0000(	FOXI3	60.03872	-0.26797	0.028639	0.073726	protein_coding
ENSG0000(	MAD2L1	392.298	-0.24747	0.028655	0.073755	protein_coding
ENSG0000(	UBE2R2	1305.858	-0.13095	0.028654	0.073755	protein_coding
ENSG0000(	ENSG0000(	110.1562	0.200065	0.028658	0.073758	lncRNA
ENSG0000(	ENSG0000(	63.62585	0.468934	0.028661	0.07376	lncRNA
ENSG0000(	TXNRD1	1577.654	-0.26059	0.028673	0.073784	protein_coding
ENSG0000(	CD5L	10.83145	0.726918	0.028743	0.073956	protein_coding
ENSG0000(	NSUN4	1256.05	0.136914	0.028745	0.073956	protein_coding
ENSG0000(	GPR19	26.60407	0.480788	0.028759	0.073987	protein_coding
ENSG0000(	UPF3AP2	13.86629	-0.57953	0.028761	0.073987	transcribed_processed_pseudoge
ENSG0000(	MAGI3	3234.7	-0.24969	0.028804	0.074089	protein_coding
ENSG0000(	SND1	2727.689	0.157241	0.028807	0.074092	protein_coding
ENSG0000(	ARID2	3576.189	0.072043	0.028811	0.074095	protein_coding
ENSG0000(	ENSG0000(	301.2207	-0.10973	0.028827	0.07413	TEC
ENSG0000(	ENSG0000(	75.85951	-0.24047	0.028849	0.074181	lncRNA
ENSG0000(	FAM185A	250.7328	-0.16018	0.028866	0.074218	protein_coding
ENSG0000(	ARF3	3875.622	-0.18162	0.028891	0.074278	protein_coding
ENSG0000(	ETV5	243.4666	0.241262	0.028903	0.074302	protein_coding
ENSG0000(	ZNF462	2911.3	-0.1832	0.028906	0.074302	protein_coding
ENSG0000(	DHRS7B	618.7181	-0.09178	0.028952	0.074415	protein_coding
ENSG0000(	AMMECR1-	85.49849	-0.20532	0.028964	0.074441	lncRNA
ENSG0000(	ENSG0000(	274.7274	0.153517	0.028975	0.074452	lncRNA
ENSG0000(	ENSG0000(	393.2181	0.107487	0.028976	0.074452	lncRNA
ENSG0000(	HEXB	848.7297	-0.14133	0.028974	0.074452	protein_coding
ENSG0000(	ITPKA	169.6643	-0.44841	0.02899	0.074483	protein_coding

ENSG0000(	TSPAN18	175.3657	0.340083	0.028994	0.074488	protein_coding
ENSG0000(	ABHD14A-/	123.7237	-0.26618	0.029006	0.074511	protein_coding
ENSG0000(	ENSG0000(	18.92644	0.361972	0.029028	0.074562	lncRNA
ENSG0000(	ENSG0000(	45.28099	0.282593	0.029046	0.074601	miRNA
ENSG0000(	ENSG0000(	45.10602	-0.25475	0.029061	0.074629	lncRNA
ENSG0000(	LINC00596	105.4852	-0.19873	0.029061	0.074629	lncRNA
ENSG0000(	STAG1	2106.932	0.10592	0.029067	0.074638	protein_coding
ENSG0000(	ENSG0000(	18.50682	-0.45029	0.02907	0.074639	lncRNA
ENSG0000(	ZNF793-AS	22.58832	-0.41964	0.029072	0.074639	lncRNA
ENSG0000(	ZNF726	804.6531	-0.14279	0.02908	0.074653	protein_coding
ENSG0000(	GORAB-AS:	181.9346	0.163267	0.029084	0.074656	lncRNA
ENSG0000(	C3orf38	522.6236	0.140574	0.029103	0.074699	protein_coding
ENSG0000(	RUFY1	897.1296	-0.09379	0.029107	0.074703	protein_coding
ENSG0000(	CERS3	16.01433	0.764262	0.029175	0.074873	protein_coding
ENSG0000(	RNU1-20P	9.287889	-0.52834	0.029186	0.074895	snRNA
ENSG0000(	CDC23	542.9243	-0.13544	0.029205	0.074936	protein_coding
ENSG0000(	ENSG0000(	194.8655	-0.14956	0.029232	0.075	lncRNA
ENSG0000(	GTF2H4	166.8535	-0.18744	0.029237	0.075006	protein_coding
ENSG0000(	FAM24B	93.46713	-0.2703	0.029252	0.075038	protein_coding
ENSG0000(	CEP295	1793.732	-0.11649	0.029275	0.075086	protein_coding
ENSG0000(	SREBF2	3881.433	-0.15119	0.029275	0.075086	protein_coding
ENSG0000(	LINC00869	718.7936	0.163258	0.029286	0.075108	transcribed_unprocessed_pseudo
ENSG0000(	CCNY	1211.689	-0.12692	0.029291	0.075111	protein_coding
ENSG0000(	ENSG0000(	33.35461	-0.35177	0.029294	0.075111	lncRNA
ENSG0000(	LINC01882	22.21299	0.4129	0.029293	0.075111	lncRNA
ENSG0000(	LINC00504	9867.54	-0.14204	0.029311	0.075148	lncRNA
ENSG0000(	HSPA12B	88.14593	0.327693	0.029347	0.075233	protein_coding
ENSG0000(	SNORD15B	450.3649	-0.25981	0.029349	0.075233	snoRNA
ENSG0000(	UBE2D4	582.5206	0.145887	0.029371	0.075283	protein_coding
ENSG0000(	ENSG0000(	98.39395	0.162022	0.029382	0.075305	lncRNA
ENSG0000(	COG5	2499.169	-0.12178	0.029388	0.075313	protein_coding
ENSG0000(	CWC15	316.2189	-0.17347	0.02939	0.075313	protein_coding
ENSG0000(	ENSG0000(	109.8876	0.185628	0.029416	0.075374	lncRNA
ENSG0000(	METTL7A	2558.86	-0.19355	0.029439	0.075425	protein_coding
ENSG0000(	SLC1A1	522.5547	-0.49289	0.029445	0.075435	protein_coding
ENSG0000(	PCTP	507.1412	-0.17049	0.029449	0.075438	protein_coding
ENSG0000(	ENSG0000(	29.94221	-0.32539	0.029456	0.07545	lncRNA
ENSG0000(	TUBB8P7	11.73566	0.820086	0.029479	0.075504	transcribed_unprocessed_pseudo
ENSG0000(	MIR548AQ	13.02612	0.542183	0.029483	0.075508	miRNA
ENSG0000(	LINC02328	212.8285	0.133151	0.029553	0.07568	lncRNA
ENSG0000(	FZD5	6180.228	-0.3123	0.029556	0.075683	protein_coding
ENSG0000(	MUCL1	110.6227	0.273158	0.029565	0.0757	protein_coding
ENSG0000(	CAVIN2-AS	68.1317	0.238336	0.029576	0.075721	lncRNA
ENSG0000(	LINC00624	41.36213	0.299033	0.029587	0.075744	lncRNA
ENSG0000(	ENSG0000(	31.58364	0.332085	0.029598	0.075752	lncRNA
ENSG0000(	HDGFL3P1	121.7988	-0.18794	0.029597	0.075752	processed_pseudogene
ENSG0000(	RN7SL284F	64.11418	0.266528	0.0296	0.075752	misc_RNA

ENSG0000(	SKOR1	29.99408	0.385552	0.0296	0.075752	protein_coding
ENSG0000(	VEGFC	47.21069	0.575753	0.029606	0.075761	protein_coding
ENSG0000(	CCDC78	25.15308	0.599693	0.029626	0.075805	protein_coding
ENSG0000(	MOB4	886.7627	-0.11304	0.029629	0.075807	protein_coding
ENSG0000(	BORCS6	117.6577	-0.18091	0.02964	0.075825	protein_coding
ENSG0000(	CCDC175	37.75532	-0.6442	0.029641	0.075825	protein_coding
ENSG0000(	HSPB8	103.0646	0.37461	0.029655	0.075854	protein_coding
ENSG0000(	RN7SL181F	26.4937	0.26493	0.029667	0.075879	misc_RNA
ENSG0000(	IGFBP3	969.6934	-0.338	0.029692	0.075937	protein_coding
ENSG0000(	ENSG0000(	119.0912	-0.16624	0.029709	0.075974	TEC
ENSG0000(	ENSG0000(	15.58422	0.465824	0.02973	0.076023	TEC
ENSG0000(	CBLN2	67.90806	-0.49567	0.029743	0.076046	protein_coding
ENSG0000(	ENSG0000(	10.79589	-0.42858	0.029744	0.076046	processed_pseudogene
ENSG0000(	ENSG0000(	27.02968	0.645193	0.029748	0.076049	lncRNA
ENSG0000(	ENSG0000(	237.9396	0.147894	0.029751	0.07605	lncRNA
ENSG0000(	PINX1	129.9205	-0.24368	0.029754	0.076051	protein_coding
ENSG0000(	DHDDS-AS1	92.78721	0.235523	0.029756	0.076051	lncRNA
ENSG0000(	FBXW4P1	115.3793	-0.19121	0.029771	0.076083	processed_pseudogene
ENSG0000(	PDE8A	3674.432	-0.21175	0.029778	0.076094	protein_coding
ENSG0000(	ENSG0000(	20.52161	0.368101	0.02978	0.076094	TEC
ENSG0000(	NRSN1	55.98747	-0.27225	0.029791	0.076115	protein_coding
ENSG0000(	SLC25A35	255.2175	-0.17454	0.029796	0.076122	protein_coding
ENSG0000(	ENSG0000(	182.4817	0.154475	0.02984	0.076229	lncRNA
ENSG0000(	ANKRD20A	123.2055	0.320259	0.029852	0.076254	transcribed_unprocessed_pseudo
ENSG0000(	KPNB1	4638.781	-0.11869	0.029903	0.076377	protein_coding
ENSG0000(	PGAP2	512.564	-0.17717	0.029908	0.076382	protein_coding
ENSG0000(	CLDND2	26.65579	-0.4337	0.02991	0.076383	protein_coding
ENSG0000(	RNU6-1255	10.38905	0.547887	0.029917	0.076393	snRNA
ENSG0000(	ENSG0000(	103.9794	-0.19262	0.02992	0.076396	lncRNA
ENSG0000(	GADD45G	62.72352	-0.33566	0.029934	0.076418	protein_coding
ENSG0000(	TSPAN1	5657.902	-0.33784	0.029932	0.076418	protein_coding
ENSG0000(	CFAP20DC-	26.21456	0.355567	0.029943	0.076422	lncRNA
ENSG0000(	CLECL1	81.68233	0.414752	0.029943	0.076422	transcribed_unitary_pseudogene
ENSG0000(	TAS2R1	56.05228	0.398332	0.029939	0.076422	protein_coding
ENSG0000(	ENSG0000(	174.6775	0.23799	0.029991	0.07654	lncRNA
ENSG0000(	PLD2	540.2685	-0.16249	0.030005	0.076568	protein_coding
ENSG0000(	FMO4	241.1283	-0.29727	0.030016	0.076588	protein_coding
ENSG0000(	OR9G1	14.11053	0.464655	0.030017	0.076588	protein_coding
ENSG0000(	MST1P2	88.29879	-0.51836	0.03004	0.076639	unprocessed_pseudogene
ENSG0000(	SIRPG-AS1	150.3759	-0.19973	0.030053	0.076667	lncRNA
ENSG0000(	ADM-DT	18.63182	0.394488	0.030057	0.076669	lncRNA
ENSG0000(	ENSG0000(	93.71167	0.186372	0.030068	0.076691	lncRNA
ENSG0000(	ENSG0000(	11.06606	0.848295	0.030072	0.076697	protein_coding
ENSG0000(	PRPF39	963.3209	-0.12937	0.030083	0.076719	protein_coding
ENSG0000(	COA4	500.5566	-0.10732	0.030109	0.076778	protein_coding
ENSG0000(	CTDSP1	2234.493	-0.1258	0.030133	0.076832	protein_coding
ENSG0000(	TEX45	125.8966	0.216757	0.030142	0.076849	protein_coding

ENSG0000(	LINC01394	15.98706	0.450583	0.03015	0.076864	lncRNA
ENSG0000(	ENSG0000(	26.85106	-0.29878	0.030165	0.076874	lncRNA
ENSG0000(	ENSG0000(	112.1334	0.164276	0.030167	0.076874	protein_coding
ENSG0000(	LINC00399	16.86743	0.381673	0.030167	0.076874	lncRNA
ENSG0000(	MICB	414.8197	0.127029	0.030166	0.076874	protein_coding
ENSG0000(	SECISBP2	4521.495	0.073319	0.030164	0.076874	protein_coding
ENSG0000(	IGKV1-33	601.8492	0.630559	0.030173	0.076878	IG_V_gene
ENSG0000(	ZNF805	1533.434	-0.09749	0.030172	0.076878	protein_coding
ENSG0000(	KLHL12	908.1997	-0.11937	0.030197	0.076933	protein_coding
ENSG0000(	ENSG0000(	106.2384	-0.25796	0.030221	0.076986	lncRNA
ENSG0000(	MET	1529.718	-0.24467	0.030247	0.077047	protein_coding
ENSG0000(	ANKRD20A	497.0774	0.23596	0.030288	0.077134	transcribed_unprocessed_pseudo
ENSG0000(	ENSG0000(	14.03869	0.475543	0.030285	0.077134	lncRNA
ENSG0000(	NAALADL1	212.4295	-0.54777	0.030287	0.077134	protein_coding
ENSG0000(	ISG15	81.79876	0.544239	0.030291	0.077135	protein_coding
ENSG0000(	TSC1	3716.316	0.085496	0.030303	0.077159	protein_coding
ENSG0000(	LINC01871	70.92345	0.368863	0.030317	0.077189	lncRNA
ENSG0000(	ARHGAP22	573.5661	0.148868	0.030329	0.077213	protein_coding
ENSG0000(	UTP11	768.4977	-0.15961	0.030337	0.077226	protein_coding
ENSG0000(	ENSG0000(	163.9623	0.193774	0.030345	0.077241	lncRNA
ENSG0000(	ENSG0000(	19.52009	0.390704	0.030357	0.077264	lncRNA
ENSG0000(	MIR548K	18.39149	0.408961	0.030363	0.077274	miRNA
ENSG0000(	ECE1	3280.948	-0.19054	0.030386	0.077326	protein_coding
ENSG0000(	ACSS1	1644.229	0.109489	0.030396	0.077346	protein_coding
ENSG0000(	PRRT2	226.9779	0.340345	0.030415	0.077388	protein_coding
ENSG0000(	LINC01923	445.9856	0.161506	0.030452	0.077475	lncRNA
ENSG0000(	LINC02902	67.58482	0.201088	0.030455	0.077475	lncRNA
ENSG0000(	NPR3	82.5706	0.280336	0.030458	0.077479	protein_coding
ENSG0000(	SORBS1	1564.162	-0.29038	0.030461	0.07748	protein_coding
ENSG0000(	PNPLA6	1068.907	-0.1398	0.030473	0.077503	protein_coding
ENSG0000(	CTTN	4309.612	-0.14124	0.030496	0.077545	protein_coding
ENSG0000(	ENSG0000(	21559.67	0.587783	0.030493	0.077545	lncRNA
ENSG0000(	SCN3A	278.5009	0.270662	0.030497	0.077545	protein_coding
ENSG0000(	CTSLP3	76.61653	0.22287	0.030507	0.07756	transcribed_unprocessed_pseudo
ENSG0000(	ENSG0000(	63.89835	0.289773	0.030508	0.07756	lncRNA
ENSG0000(	ENSG0000(	75.20639	0.194937	0.030538	0.077631	lncRNA
ENSG0000(	CLEC2D	2442.78	0.318036	0.030547	0.077647	protein_coding
ENSG0000(	ENSG0000(	214.6338	0.181223	0.030585	0.077737	lncRNA
ENSG0000(	CHMP4A	603.8095	-0.09231	0.030592	0.077744	protein_coding
ENSG0000(	ENSG0000(	85.25737	0.274525	0.030592	0.077744	TEC
ENSG0000(	CCDC82	3142.084	0.12146	0.0306	0.077756	protein_coding
ENSG0000(	ENSG0000(	20.24365	-0.50529	0.030608	0.077771	lncRNA
ENSG0000(	HMBOX1	2229.745	0.077123	0.030614	0.077774	protein_coding
ENSG0000(	SIGLEC7	38.57488	0.441549	0.030614	0.077774	protein_coding
ENSG0000(	MC1R	678.0632	0.184021	0.030625	0.077794	protein_coding
ENSG0000(	CYCSP24	21.63402	-0.32571	0.030647	0.077844	processed_pseudogene
ENSG0000(	ZNF213-AS	1881.39	-0.08611	0.030657	0.077864	lncRNA

ENSG0000(	CDK5R1	160.648	0.413783	0.030667	0.077882	protein_coding
ENSG0000(	SLC52A3	777.3197	-0.29389	0.03067	0.077885	protein_coding
ENSG0000(	ENSG0000(	73.68268	0.203462	0.030687	0.07792	lncRNA
ENSG0000(	ENSG0000(	110.5784	0.18713	0.030692	0.077928	lncRNA
ENSG0000(	ENSG0000(	16.89256	0.508691	0.030719	0.077991	lncRNA
ENSG0000(	ENSG0000(	869.0757	-0.07366	0.030727	0.078003	lncRNA
ENSG0000(	FBXO15	2486.477	0.141726	0.030755	0.078067	protein_coding
ENSG0000(	IGHV3-72	241.653	0.560991	0.030764	0.078085	IG_V_gene
ENSG0000(	LOXL1-AS1	522.9123	-0.11953	0.030766	0.078085	lncRNA
ENSG0000(	IL4R	3953.071	0.154303	0.030778	0.078107	protein_coding
ENSG0000(	HSP90AB7I	140.0774	-0.15414	0.030816	0.078197	processed_pseudogene
ENSG0000(	ENSG0000(	32.2302	0.672013	0.030822	0.078207	lncRNA
ENSG0000(	ENSG0000(	85.8426	0.186084	0.030828	0.078217	lncRNA
ENSG0000(	ENSG0000(	10.45171	0.533885	0.030858	0.078275	processed_pseudogene
ENSG0000(	ENSG0000(	36.64355	-0.35031	0.030859	0.078275	lncRNA
ENSG0000(	FAM91A1	1746.341	0.094969	0.030857	0.078275	protein_coding
ENSG0000(	VPS35L	1840.403	0.076673	0.030865	0.078284	protein_coding
ENSG0000(	ZBTB16	1887.77	0.497454	0.030868	0.078284	protein_coding
ENSG0000(	NUBPL	1078.651	-0.09902	0.030874	0.078293	protein_coding
ENSG0000(	SCG3	123.8163	0.217767	0.030897	0.078347	protein_coding
ENSG0000(	GFRA3	17.65661	-0.49373	0.030905	0.078359	protein_coding
ENSG0000(	RGS10	212.05	-0.21646	0.030908	0.078361	protein_coding
ENSG0000(	HSD17B13	409.6365	-0.15335	0.03092	0.078385	protein_coding
ENSG0000(	ENSG0000(	110.7868	0.369154	0.03093	0.078405	lncRNA
ENSG0000(	RN7SK	413920	-0.11977	0.03095	0.07845	snRNA
ENSG0000(	RNU5B-1	2423.688	0.318809	0.030976	0.078508	snRNA
ENSG0000(	ENSG0000(	180.5722	0.128616	0.03098	0.078513	lncRNA
ENSG0000(	PIWIL1	111.7154	-0.25307	0.030994	0.078534	protein_coding
ENSG0000(	ZNF347	1867.139	-0.10363	0.030991	0.078534	protein_coding
ENSG0000(	MTND5P5	46.67349	0.828456	0.031004	0.078546	processed_pseudogene
ENSG0000(	SPTSSA	768.468	-0.15431	0.031006	0.078546	protein_coding
ENSG0000(	TMEM185/	495.7782	-0.12638	0.031003	0.078546	protein_coding
ENSG0000(	C10orf53	552.8509	0.225013	0.031052	0.07865	protein_coding
ENSG0000(	RNASE10	96.66077	-0.25711	0.031051	0.07865	protein_coding
ENSG0000(	ENSG0000(	105.1617	-0.16043	0.03106	0.078663	lncRNA
ENSG0000(	ENSG0000(	114.8224	-0.21928	0.031073	0.078691	lncRNA
ENSG0000(	ENSG0000(	25.12205	-0.39496	0.031088	0.078723	processed_pseudogene
ENSG0000(	ENSG0000(	62.37477	-0.22003	0.031095	0.078732	lncRNA
ENSG0000(	CDKN1B	1747.02	0.117545	0.031105	0.078746	protein_coding
ENSG0000(	ENSG0000(	36.24049	-0.32303	0.031105	0.078746	lncRNA
ENSG0000(	LINC01549	148.5221	-0.28875	0.03111	0.078752	lncRNA
ENSG0000(	IGHV3-64	98.41449	0.876514	0.031123	0.078779	IG_V_gene
ENSG0000(	ENSG0000(	17.48602	0.420544	0.031151	0.078843	lncRNA
ENSG0000(	RNF20	703.138	-0.13078	0.031158	0.078854	protein_coding
ENSG0000(	JUN	1713.692	0.285717	0.031162	0.078857	protein_coding
ENSG0000(	TAS2R31	216.3402	0.182568	0.03117	0.078873	protein_coding
ENSG0000(	ENSG0000(	493.0583	0.205142	0.031192	0.078922	lncRNA

ENSG0000(	RN7SL381F	181.5003	0.168119	0.031216	0.078977	misc_RNA
ENSG0000(	PDE4A	415.9635	0.168426	0.031221	0.078982	protein_coding
ENSG0000(	MDC1	1390.689	-0.15323	0.031226	0.078987	protein_coding
ENSG0000(	MTCYBP3	113.4421	0.208844	0.031232	0.078998	processed_pseudogene
ENSG0000(	POP1	264.2367	-0.2905	0.031246	0.079025	protein_coding
ENSG0000(	MIPOL1	5079.656	-0.12643	0.031255	0.079041	protein_coding
ENSG0000(	C1orf115	1042.763	-0.38771	0.031276	0.079086	protein_coding
ENSG0000(	ENSG0000(	12.22939	0.417704	0.031277	0.079086	TEC
ENSG0000(	WDR41	941.1123	0.107535	0.031328	0.079208	protein_coding
ENSG0000(	ENSG0000(	335.1857	-0.22223	0.031338	0.079226	lncRNA
ENSG0000(	LRRC39	23.14554	0.38043	0.031341	0.079229	protein_coding
ENSG0000(	NFE2L2	2436.1	-0.13462	0.031346	0.079235	protein_coding
ENSG0000(	LINC00486	389.0623	0.101004	0.031352	0.079244	lncRNA
ENSG0000(	ENSG0000(	154.0924	0.130175	0.031355	0.079244	lncRNA
ENSG0000(	WDR75	990.7794	-0.15197	0.031366	0.079266	protein_coding
ENSG0000(	RNF4	1987.505	-0.07206	0.031369	0.079266	protein_coding
ENSG0000(	MRPL42	3566.216	-0.14122	0.031372	0.079269	protein_coding
ENSG0000(	L3MBTL2	964.0032	-0.10932	0.031379	0.07928	protein_coding
ENSG0000(	IGLC2	7981.564	0.514227	0.031383	0.079283	IG_C_gene
ENSG0000(	GUF1	1169.405	-0.11856	0.031389	0.079293	protein_coding
ENSG0000(	LINC01858	245.9564	-0.15315	0.031399	0.07931	lncRNA
ENSG0000(	UHRF1BP1I	43.4019	-0.25857	0.031405	0.079319	lncRNA
ENSG0000(	BAK1	1042.622	-0.20513	0.031415	0.079331	protein_coding
ENSG0000(	ENSG0000(	608.1265	-0.11996	0.031414	0.079331	lncRNA
ENSG0000(	ENSG0000(	33.17747	-0.28219	0.031418	0.079333	lncRNA
ENSG0000(	SLC37A1	1991.912	0.220944	0.031421	0.079335	protein_coding
ENSG0000(	CFD	197.2234	-0.21783	0.031429	0.079347	protein_coding
ENSG0000(	CA15P1	26.71217	-0.38639	0.031448	0.079388	unitary_pseudogene
ENSG0000(	PPP2R5C	4359.985	0.106534	0.031454	0.079398	protein_coding
ENSG0000(	IL17D	10.08841	-0.59844	0.031458	0.0794	protein_coding
ENSG0000(	NSUN5	400.3891	-0.13977	0.031482	0.079457	protein_coding
ENSG0000(	ENSG0000(	94.32236	0.186732	0.031515	0.079533	lncRNA
ENSG0000(	ENSG0000(	118.6493	0.172479	0.031523	0.079546	protein_coding
ENSG0000(	AJUBA	176.29	0.248739	0.031528	0.079553	protein_coding
ENSG0000(	ENSG0000(	467.1906	0.121759	0.031533	0.079559	lncRNA
ENSG0000(	ACOXL-AS1	33.5208	0.401462	0.031546	0.07958	lncRNA
ENSG0000(	KLB	361.2108	-0.14699	0.031544	0.07958	protein_coding
ENSG0000(	ENSG0000(	69.85317	0.227209	0.031567	0.079625	lncRNA
ENSG0000(	IFITM1	579.9856	0.338219	0.031574	0.079637	protein_coding
ENSG0000(	CLIP3	238.5976	0.237886	0.031592	0.079655	protein_coding
ENSG0000(	ENSG0000(	250.4368	-0.31273	0.03159	0.079655	lncRNA
ENSG0000(	ENSG0000(	14.50826	-0.44926	0.031592	0.079655	lncRNA
ENSG0000(	ZNF32	162.8421	-0.18213	0.031586	0.079655	protein_coding
ENSG0000(	ERMP1	3193.348	-0.11465	0.031601	0.079672	protein_coding
ENSG0000(	FOXK1	3787.695	0.08603	0.031607	0.079681	protein_coding
ENSG0000(	CD248	95.29079	0.466002	0.031612	0.079683	protein_coding
ENSG0000(	MAP7D1	700.5282	0.162945	0.031613	0.079683	protein_coding

ENSG0000(	MEMO1	1042.072	-0.12971	0.031615	0.079683	protein_coding
ENSG0000(	RPL21P7	18.18363	0.66477	0.03162	0.079689	processed_pseudogene
ENSG0000(	BAG1	2144.636	-0.17482	0.031635	0.07972	protein_coding
ENSG0000(	ENSG0000(	114.4451	-0.33622	0.031654	0.079761	transcribed_unprocessed_pseudo
ENSG0000(	NHLH1	16.34186	0.455212	0.031695	0.079857	protein_coding
ENSG0000(	CADM2-AS	143.9803	0.130749	0.031706	0.079878	lncRNA
ENSG0000(	BCHE	25.92299	-0.63908	0.031717	0.079892	protein_coding
ENSG0000(	BLOC1S6P1	412.6509	0.136481	0.031718	0.079892	processed_pseudogene
ENSG0000(	ENSG0000(	1049.9	-0.11272	0.031719	0.079892	lncRNA
ENSG0000(	ENSG0000(	14.68423	0.508186	0.031721	0.079892	TEC
ENSG0000(	ENSG0000(	9.732887	0.578374	0.031729	0.079906	lncRNA
ENSG0000(	ENSG0000(	327.3087	0.133904	0.031736	0.079916	TEC
ENSG0000(	OVAAL	16.18647	-0.42554	0.031752	0.07995	lncRNA
ENSG0000(	LINC02672	18.69763	0.42954	0.031778	0.08001	lncRNA
ENSG0000(	PIGH	518.4171	-0.10221	0.031794	0.080044	protein_coding
ENSG0000(	SCYL2	2135.372	-0.1266	0.031804	0.080061	protein_coding
ENSG0000(	FUT3	3465.151	-0.24399	0.031806	0.080061	protein_coding
ENSG0000(	ENSG0000(	21.72446	-0.33092	0.031819	0.080081	lncRNA
ENSG0000(	MAMDC2	171.0407	-0.42778	0.031819	0.080081	protein_coding
ENSG0000(	ENSG0000(	18.43523	0.45363	0.031837	0.08012	processed_pseudogene
ENSG0000(	UNC45A	789.3382	-0.07643	0.031856	0.080161	protein_coding
ENSG0000(	TXNDC12	428.7692	-0.25189	0.031859	0.080162	protein_coding
ENSG0000(	ENSG0000(	47.31562	0.243319	0.031866	0.080172	lncRNA
ENSG0000(	CD83	469.9947	0.523936	0.031872	0.08018	protein_coding
ENSG0000(	BBS7	338.9079	-0.33677	0.031882	0.080194	protein_coding
ENSG0000(	ENSG0000(	177.4597	-0.16612	0.03188	0.080194	lncRNA
ENSG0000(	ENSG0000(	179.6183	-0.13498	0.031903	0.080241	TEC
ENSG0000(	ENSG0000(	407.0915	-0.31048	0.031921	0.08028	lncRNA
ENSG0000(	NUFIP1	121.1084	-0.21695	0.03193	0.080289	protein_coding
ENSG0000(	UCKL1-AS1	337.42	0.132466	0.031928	0.080289	lncRNA
ENSG0000(	CREG1	983.9752	0.207887	0.031936	0.080298	protein_coding
ENSG0000(	ATG10-IT1	173.7272	-0.1711	0.031944	0.080301	lncRNA
ENSG0000(	ENSG0000(	170.8082	0.238625	0.031944	0.080301	lncRNA
ENSG0000(	ENSG0000(	1228.608	0.194768	0.031945	0.080301	lncRNA
ENSG0000(	ENSG0000(	54.19243	-0.29675	0.031958	0.080325	lncRNA
ENSG0000(	RPL21P99	19.60151	0.464248	0.03196	0.080325	processed_pseudogene
ENSG0000(	ORC6	10924.22	0.131111	0.031967	0.080337	protein_coding
ENSG0000(	MDP1	30.83769	-0.41533	0.031972	0.080343	protein_coding
ENSG0000(	NME7	495.2291	0.13902	0.031975	0.080345	protein_coding
ENSG0000(	THOC3-AS1	18.36588	0.367758	0.031993	0.080381	lncRNA
ENSG0000(	CACNA2D1	284.7226	0.31987	0.03201	0.080418	protein_coding
ENSG0000(	ITSN2	3184.654	0.114474	0.032013	0.080419	protein_coding
ENSG0000(	ZNF274	1786.834	0.111698	0.032025	0.080443	protein_coding
ENSG0000(	KAZN-AS1	27.79066	0.340584	0.032047	0.08048	lncRNA
ENSG0000(	PPA2	1156.413	-0.11838	0.032043	0.08048	protein_coding
ENSG0000(	SIPA1L1-AS	16.27286	-0.38966	0.032046	0.08048	lncRNA
ENSG0000(	ENSG0000(	18.07796	-0.54447	0.032099	0.0806	lncRNA

ENSG0000(	LRMDA	506.0329	0.163679	0.0321	0.0806	protein_coding
ENSG0000(	OSTCP5	10.94474	-0.48275	0.032103	0.0806	processed_pseudogene
ENSG0000(	MAP2K5-D	107.8514	-0.21648	0.03211	0.080604	lncRNA
ENSG0000(	MKRN1	3626.15	-0.17986	0.032107	0.080604	protein_coding
ENSG0000(	TPT1	17051.31	-0.1896	0.032122	0.080629	protein_coding
ENSG0000(	ENSG0000(	11.96288	0.47183	0.032128	0.08063	lncRNA
ENSG0000(	LIF	449.0851	0.229237	0.032127	0.08063	protein_coding
ENSG0000(	LINC01973	30.17182	0.368	0.032133	0.080638	lncRNA
ENSG0000(	QSOX1	2798.592	0.252904	0.032142	0.080654	protein_coding
ENSG0000(	DIRAS1	37.53825	-0.46156	0.032154	0.080677	protein_coding
ENSG0000(	ENSG0000(	28.8955	0.593155	0.03216	0.080682	lncRNA
ENSG0000(	FN1	2971.381	0.48588	0.032161	0.080682	protein_coding
ENSG0000(	RADIL	96.85608	-0.24597	0.032184	0.080731	protein_coding
ENSG0000(	OSGEPL1	130.4086	-0.19896	0.032191	0.080745	protein_coding
ENSG0000(	BLACE	19.66102	-0.30642	0.032201	0.080763	lncRNA
ENSG0000(	SPTY2D1OS	96.71476	-0.2235	0.032229	0.080827	protein_coding
ENSG0000(	LGR4	2565.929	-0.2202	0.032248	0.080866	protein_coding
ENSG0000(	ENSG0000(	40.91106	-0.28617	0.032255	0.080879	lncRNA
ENSG0000(	LSM5	608.7678	-0.15097	0.032259	0.080883	protein_coding
ENSG0000(	ENSG0000(	1388.876	-0.0727	0.032268	0.080896	lncRNA
ENSG0000(	HMGB1P31	28.12139	0.298269	0.03227	0.080896	processed_pseudogene
ENSG0000(	SLC26A4-A:	3508.509	-0.15194	0.032284	0.080924	lncRNA
ENSG0000(	ENSG0000(	159.3095	-0.1529	0.032287	0.080927	lncRNA
ENSG0000(	MYCBPAP	678.0684	0.132605	0.032319	0.080999	protein_coding
ENSG0000(	MT-TM	172.8398	0.384707	0.032329	0.081016	Mt_tRNA
ENSG0000(	NOL3	488.7786	0.181379	0.032331	0.081016	protein_coding
ENSG0000(	IGKV1-27	265.9248	0.543221	0.032361	0.08108	IG_V_gene
ENSG0000(	PPP2CB	1475.454	-0.19932	0.03236	0.08108	protein_coding
ENSG0000(	IGHV3-48	648.0358	0.538731	0.032369	0.081091	IG_V_gene
ENSG0000(	H2AZ1-DT	243.1223	-0.12144	0.032376	0.081104	lncRNA
ENSG0000(	ATP6V1E1	686.8308	-0.13327	0.032382	0.081107	protein_coding
ENSG0000(	ATR	5202.007	0.085579	0.03238	0.081107	protein_coding
ENSG0000(	ENSG0000(	34.76906	-0.29702	0.032418	0.08119	lncRNA
ENSG0000(	LCAT	253.0276	0.190454	0.032432	0.081218	protein_coding
ENSG0000(	ENSG0000(	312.0971	-0.13387	0.032443	0.081239	lncRNA
ENSG0000(	SNORA70	383.7255	-0.23259	0.032447	0.081243	snoRNA
ENSG0000(	ITGA1	3882.933	0.136146	0.032489	0.081341	protein_coding
ENSG0000(	MORF4	9.852067	-0.5937	0.032492	0.081341	processed_pseudogene
ENSG0000(	GPR141BP	11.35177	0.621808	0.032495	0.081343	unitary_pseudogene
ENSG0000(	HAS2-AS1	83.79087	-0.21417	0.032511	0.081377	lncRNA
ENSG0000(	CHRFAM7A	102.2596	0.284726	0.032536	0.081433	protein_coding
ENSG0000(	CIAPIN1	494.7608	-0.12332	0.032539	0.081434	protein_coding
ENSG0000(	ST20-MTHI	39.07991	-0.4969	0.032545	0.081442	protein_coding
ENSG0000(	PHF3	10219.98	-0.11107	0.03255	0.081447	protein_coding
ENSG0000(	PUDP	417.191	-0.13332	0.03256	0.081466	protein_coding
ENSG0000(	SEC62-AS1	141.116	-0.17874	0.032576	0.081499	lncRNA
ENSG0000(	ENSG0000(	77.13047	0.211981	0.03259	0.081528	lncRNA

ENSG0000(	ENSG0000(	53.15716	0.246228	0.032594	0.081533	TEC
ENSG0000(	DMPK	1379.288	0.0976	0.032604	0.081551	protein_coding
ENSG0000(	CDCA7L	704.4036	-0.20347	0.032625	0.08159	protein_coding
ENSG0000(	ENSG0000(	81.12778	0.517535	0.032623	0.08159	transcribed_unprocessed_pseudo
ENSG0000(	CNTNAP3B	544.3997	0.181312	0.032644	0.081632	protein_coding
ENSG0000(	ASS1	2238.4	0.380905	0.03266	0.081666	protein_coding
ENSG0000(	DHX40	671.2975	-0.11438	0.032681	0.081708	protein_coding
ENSG0000(	KIF1B	5993.697	-0.18674	0.032683	0.081708	protein_coding
ENSG0000(	ENSG0000(	38.90638	0.227904	0.032701	0.081748	lncRNA
ENSG0000(	ENSG0000(	336.5785	-0.16081	0.032715	0.081776	lncRNA
ENSG0000(	IGHV1-2	480.1633	0.666841	0.032719	0.081778	IG_V_gene
ENSG0000(	ENSG0000(	9.384832	0.576796	0.032722	0.081781	lncRNA
ENSG0000(	TES	1632.648	-0.09801	0.032729	0.081792	protein_coding
ENSG0000(	ZNF526	741.1339	-0.0958	0.032741	0.081814	protein_coding
ENSG0000(	LINC01095	21.77348	-0.43792	0.032764	0.081865	lncRNA
ENSG0000(	ABCG8	771.635	0.091864	0.032788	0.081899	protein_coding
ENSG0000(	MAP1LC3B	1208.782	0.183604	0.032785	0.081899	protein_coding
ENSG0000(	PRKAR2A-A	973.3762	-0.08499	0.032781	0.081899	lncRNA
ENSG0000(	TVP23B	1328.93	0.112915	0.032786	0.081899	protein_coding
ENSG0000(	WDR53	118.6517	-0.21855	0.032797	0.081916	protein_coding
ENSG0000(	GOLPH3L	1314.581	-0.16619	0.032806	0.081923	protein_coding
ENSG0000(	SULT1A3	821.1399	-0.17396	0.032805	0.081923	protein_coding
ENSG0000(	ALG3	285.2879	-0.18399	0.032846	0.082014	protein_coding
ENSG0000(	CDR2L	87.66706	0.351465	0.032847	0.082014	protein_coding
ENSG0000(	NKAPP1	490.8109	0.143156	0.032859	0.082038	transcribed_processed_pseudoge
ENSG0000(	NUP153	2141.403	-0.09293	0.032874	0.082067	protein_coding
ENSG0000(	EGFL7	341.1298	0.296604	0.032883	0.082085	protein_coding
ENSG0000(	NUDT12	316.4764	-0.30193	0.032892	0.082101	protein_coding
ENSG0000(	MAP3K13	3122.898	-0.14925	0.032901	0.082116	protein_coding
ENSG0000(	ENSG0000(	408.6654	0.148069	0.032913	0.082139	lncRNA
ENSG0000(	LINC00269	235.093	-0.14905	0.032916	0.082139	lncRNA
ENSG0000(	ENSG0000(	9.473037	0.671226	0.032953	0.082222	lncRNA
ENSG0000(	MYO15B	11571.14	-0.21746	0.032954	0.082222	protein_coding
ENSG0000(	ENSG0000(	113.1896	0.239151	0.032975	0.082268	TEC
ENSG0000(	ENSG0000(	342.7696	0.207235	0.032992	0.082304	lncRNA
ENSG0000(	ENSG0000(	188.7634	-0.15105	0.033001	0.082319	lncRNA
ENSG0000(	LYVE1	75.33439	0.470219	0.033016	0.082349	protein_coding
ENSG0000(	DBN1	224.3931	0.312295	0.033036	0.082393	protein_coding
ENSG0000(	ENSG0000(	30.36955	0.313388	0.033044	0.0824	lncRNA
ENSG0000(	FGD6	2722.133	0.177964	0.033043	0.0824	protein_coding
ENSG0000(	ENSG0000(	9.694531	0.487561	0.033055	0.082422	lncRNA
ENSG0000(	RN7SL204F	103.0208	-0.14774	0.033059	0.082423	misc_RNA
ENSG0000(	SNX2	1452.053	-0.15772	0.033061	0.082423	protein_coding
ENSG0000(	ARFIP2	1255.11	-0.10774	0.033069	0.082436	protein_coding
ENSG0000(	ENSG0000(	19.31393	0.421416	0.033076	0.082446	lncRNA
ENSG0000(	ENSG0000(	38.37205	0.283447	0.033078	0.082446	lncRNA
ENSG0000(	ENSG0000(	1616.634	-0.16427	0.033163	0.082652	lncRNA

ENSG0000(	PTPRT	169.1137	-0.54833	0.033176	0.082676	protein_coding
ENSG0000(	ENSG0000(	8.619213	-0.53152	0.033193	0.082713	lncRNA
ENSG0000(	LINC01676	27.28113	-0.34625	0.033213	0.08275	lncRNA
ENSG0000(	RAD54B	138.7539	-0.28114	0.033211	0.08275	protein_coding
ENSG0000(	IFT52	216.8	-0.22874	0.033219	0.082757	protein_coding
ENSG0000(	ABHD17C	2421.102	-0.24679	0.033227	0.082771	protein_coding
ENSG0000(	THSD4-AS1	86.20019	0.225033	0.033237	0.082788	lncRNA
ENSG0000(	SMARCAD1	1093.233	-0.13571	0.03325	0.082816	protein_coding
ENSG0000(	ASXL2	3550.634	-0.13616	0.033257	0.082826	protein_coding
ENSG0000(	ENSG0000(	27.58343	0.322284	0.033261	0.082831	lncRNA
ENSG0000(	ENSG0000(	30.58985	0.280729	0.033298	0.082916	TEC
ENSG0000(	ENSG0000(	77.66005	0.421667	0.033303	0.082921	TEC
ENSG0000(	ENSG0000(	20.45551	-0.37834	0.033327	0.082974	lncRNA
ENSG0000(	ENSG0000(	202.2678	0.211567	0.033337	0.082993	lncRNA
ENSG0000(	ITM2C	6678.532	-0.29078	0.033376	0.083084	protein_coding
ENSG0000(	TMEM178E	247.481	0.492243	0.033388	0.083106	protein_coding
ENSG0000(	STX10	412.7909	-0.15652	0.033402	0.083134	protein_coding
ENSG0000(	ENSG0000(	24.06265	0.381871	0.03341	0.083148	lncRNA
ENSG0000(	SELENOO	415.4437	-0.13029	0.033433	0.083198	protein_coding
ENSG0000(	PID1	654.4409	0.23953	0.033453	0.083234	protein_coding
ENSG0000(	PRR34-AS1	369.8917	0.21085	0.033451	0.083234	lncRNA
ENSG0000(	DNASE2	353.8816	0.149448	0.033468	0.083265	protein_coding
ENSG0000(	LINC01250	12.97289	0.4915	0.033508	0.083358	lncRNA
ENSG0000(	CYB5B	3343.442	-0.13608	0.033526	0.083397	protein_coding
ENSG0000(	HRG	156.3453	-0.26156	0.033537	0.08341	protein_coding
ENSG0000(	VAMP3	1332.717	-0.1414	0.033535	0.08341	protein_coding
ENSG0000(	CCDC30	3018.987	-0.06031	0.033553	0.083438	protein_coding
ENSG0000(	CCR6	472.5197	0.473104	0.033554	0.083438	protein_coding
ENSG0000(	ENSG0000(	113.652	-0.20605	0.033564	0.083459	lncRNA
ENSG0000(	SQSTM1	3837.833	-0.20438	0.03357	0.083467	protein_coding
ENSG0000(	ENSG0000(	179.0036	-0.23688	0.033602	0.083538	lncRNA
ENSG0000(	CHRNA5	280.6093	-0.14518	0.033608	0.083548	protein_coding
ENSG0000(	SEPHS2	1069.97	-0.22345	0.033619	0.083569	protein_coding
ENSG0000(	ENSG0000(	157.4344	0.201909	0.033628	0.083585	lncRNA
ENSG0000(	MNX1	450.5703	-0.38528	0.033657	0.083642	protein_coding
ENSG0000(	ZNF473	281.7496	-0.15644	0.033655	0.083642	protein_coding
ENSG0000(	LINC02693	707.5869	0.155767	0.033676	0.083676	lncRNA
ENSG0000(	NOL4	38.27877	-0.58733	0.033675	0.083676	protein_coding
ENSG0000(	ENSG0000(	27.09313	-0.33094	0.033689	0.083694	lncRNA
ENSG0000(	KANK3	98.73127	0.247859	0.033688	0.083694	protein_coding
ENSG0000(	ENSG0000(	1275.185	0.090062	0.033694	0.083701	TEC
ENSG0000(	ENSG0000(	30.28829	0.305401	0.033718	0.083755	lncRNA
ENSG0000(	TMBIM1	3437.626	-0.18327	0.033722	0.083758	protein_coding
ENSG0000(	SP6	129.2678	0.408719	0.033743	0.083802	protein_coding
ENSG0000(	FCAR	636.311	0.145946	0.033748	0.083809	protein_coding
ENSG0000(	ENSG0000(	407.7403	0.094139	0.033797	0.083924	lncRNA
ENSG0000(	RMDN3	664.4416	-0.11855	0.033814	0.083959	protein_coding

ENSG0000(	RN7SL356F	54.62684	-0.22062	0.033825	0.083979	misc_RNA
ENSG0000(	ZCCHC14-D	34.39574	-0.36907	0.033829	0.083983	lncRNA
ENSG0000(	HHIP	1226.201	-0.34248	0.03391	0.084178	protein_coding
ENSG0000(	PDZRN4	20.79652	0.636559	0.033922	0.084199	protein_coding
ENSG0000(	RBM23	3440.061	-0.08222	0.033946	0.084252	protein_coding
ENSG0000(	IGHV1-18	563.9967	0.588998	0.033986	0.084346	IG_V_gene
ENSG0000(	ORMDL3	1122.579	0.182971	0.033997	0.084361	protein_coding
ENSG0000(	RNU6-883F	25.46507	0.420382	0.033995	0.084361	snRNA
ENSG0000(	PSPH	457.7379	-0.1202	0.034032	0.084441	protein_coding
ENSG0000(	PTGR1	1631.133	-0.1597	0.034037	0.084446	protein_coding
ENSG0000(	ABL2	3506.947	0.1315	0.034051	0.084467	protein_coding
ENSG0000(	AS3MT	478.9557	-0.10248	0.03405	0.084467	protein_coding
ENSG0000(	WNT9B	216.2004	-0.14007	0.034067	0.0845	protein_coding
ENSG0000(	PPP2R2B	331.024	0.220265	0.034103	0.084583	protein_coding
ENSG0000(	MIR3671	14.04297	0.417248	0.034123	0.084624	miRNA
ENSG0000(	RN7SL337F	19.68056	0.488683	0.034148	0.084682	misc_RNA
ENSG0000(	SRP54	1575.462	0.105961	0.034154	0.08469	protein_coding
ENSG0000(	FRK	10386.84	-0.12202	0.034167	0.084715	protein_coding
ENSG0000(	FEZ2	1989.894	-0.11664	0.034179	0.084737	protein_coding
ENSG0000(	ENSG0000(	522.5584	-0.14154	0.034185	0.084746	processed_pseudogene
ENSG0000(	GGA2	3595.427	0.17904	0.0342	0.084776	protein_coding
ENSG0000(	TUBA4A	358.7739	-0.23076	0.034222	0.084825	protein_coding
ENSG0000(	RFT1	951.8898	-0.13431	0.034227	0.084829	protein_coding
ENSG0000(	MECR	572.5535	-0.14125	0.034249	0.084876	protein_coding
ENSG0000(	IRAG1	1101.105	0.217976	0.034262	0.084903	protein_coding
ENSG0000(	ENSG0000(	47.55997	-0.26183	0.034269	0.084914	processed_pseudogene
ENSG0000(	NIPAL2	2578.765	-0.18703	0.034327	0.08505	protein_coding
ENSG0000(	P3H3	255.0871	0.179682	0.034337	0.085068	protein_coding
ENSG0000(	TLCD3A	161.5599	-0.28481	0.034342	0.085075	protein_coding
ENSG0000(	ATP6V1C1	1253.37	0.135444	0.034357	0.085105	protein_coding
ENSG0000(	ENSG0000(	98.47188	0.198225	0.03437	0.085131	lncRNA
ENSG0000(	SUSD4	103.3388	0.390692	0.034375	0.085136	protein_coding
ENSG0000(	ENSG0000(	224.2719	-0.13493	0.034385	0.085154	lncRNA
ENSG0000(	CTU1	85.11772	-0.17792	0.034389	0.085155	protein_coding
ENSG0000(	NOMO3	2699.374	-0.09584	0.034391	0.085155	protein_coding
ENSG0000(	ENSG0000(	3058.373	0.158102	0.034407	0.085188	transcribed_unprocessed_pseudo
ENSG0000(	ARHGEF40	2114.967	-0.2351	0.034428	0.085233	protein_coding
ENSG0000(	TSFM	341.8433	-0.13609	0.034458	0.085295	protein_coding
ENSG0000(	ZNF8-DT	209.4626	0.244927	0.034457	0.085295	lncRNA
ENSG0000(	ENSG0000(	34.88339	-0.5674	0.03448	0.085328	lncRNA
ENSG0000(	KLHL33	956.2638	0.126416	0.034476	0.085328	protein_coding
ENSG0000(	TBC1D29P	152.1998	-0.20488	0.03448	0.085328	transcribed_unprocessed_pseudo
ENSG0000(	RBBP6	2962.977	0.096223	0.034524	0.08543	protein_coding
ENSG0000(	ENSG0000(	21.12852	0.38824	0.034548	0.085482	lncRNA
ENSG0000(	ENSG0000(	9.042843	-0.5234	0.034557	0.085494	processed_pseudogene
ENSG0000(	ENSG0000(	18.56019	0.394545	0.034558	0.085494	lncRNA
ENSG0000(	SNORA52	277.9267	-0.2815	0.034561	0.085494	snoRNA

ENSG0000(	ENSG0000(	150.9279	0.179526	0.034585	0.085547	lncRNA
ENSG0000(	SIGLEC15	195.062	0.151733	0.034588	0.085547	protein_coding
ENSG0000(	RAB11FIP1	5006.203	-0.16923	0.034591	0.085548	protein_coding
ENSG0000(	ENSG0000(	41.85704	-0.22632	0.034611	0.085585	lncRNA
ENSG0000(	ENSG0000(	13.04843	0.997451	0.034609	0.085585	processed_pseudogene
ENSG0000(	SLC9A7P1	54.68912	0.216136	0.034632	0.085629	transcribed_processed_pseudoge
ENSG0000(	ENSG0000(	137.6174	0.207336	0.034637	0.085635	lncRNA
ENSG0000(	CACNB1	442.9121	0.244538	0.03465	0.085647	protein_coding
ENSG0000(	ENSG0000(	1151.653	0.283009	0.034651	0.085647	lncRNA
ENSG0000(	SNHG14	15753.97	0.093628	0.034652	0.085647	lncRNA
ENSG0000(	SNORA80A	56.24672	-0.29084	0.03465	0.085647	snoRNA
ENSG0000(	CHMP1B	1434.656	-0.1596	0.034672	0.085687	protein_coding
ENSG0000(	EIF4BP6	20.12372	-0.41147	0.034686	0.085716	processed_pseudogene
ENSG0000(	DACT1	322.1529	-0.23446	0.034698	0.085738	protein_coding
ENSG0000(	CEP72	461.3808	-0.27308	0.034711	0.085763	protein_coding
ENSG0000(	RNU2-3P	13.80715	-0.46628	0.034713	0.085763	snRNA
ENSG0000(	ENTPD2	160.729	-0.3903	0.03475	0.085846	protein_coding
ENSG0000(	ZDHHC9	726.5001	-0.1671	0.034754	0.085849	protein_coding
ENSG0000(	GNL1	798.9177	0.132677	0.034766	0.085872	protein_coding
ENSG0000(	MIR133A1I	22.61759	0.446776	0.034772	0.085881	lncRNA
ENSG0000(	PPT1	740.2553	0.258312	0.034782	0.085898	protein_coding
ENSG0000(	GOT2P7	58.74861	0.2769	0.034794	0.085917	processed_pseudogene
ENSG0000(	RAP1B	6044.544	0.115119	0.034795	0.085917	protein_coding
ENSG0000(	TRMT12	355.9427	-0.15838	0.034811	0.08595	protein_coding
ENSG0000(	ENSG0000(	10.04371	0.700774	0.034825	0.085977	lncRNA
ENSG0000(	LYPLAL1-AS	358.5934	0.187217	0.034829	0.085981	lncRNA
ENSG0000(	AKNA	5105.535	0.207027	0.034854	0.086028	protein_coding
ENSG0000(	TCEA1P2	81.41839	-0.40306	0.034853	0.086028	processed_pseudogene
ENSG0000(	DNAL1	1312.121	-0.11942	0.034868	0.086056	protein_coding
ENSG0000(	SLC22A16	36.99416	0.297977	0.034887	0.086098	protein_coding
ENSG0000(	GRIP1	211.3673	-0.31176	0.034917	0.086162	protein_coding
ENSG0000(	LINC01605	65.16362	-0.44983	0.034919	0.086162	lncRNA
ENSG0000(	ENSG0000(	12.52106	0.819502	0.03493	0.086183	lncRNA
ENSG0000(	SATB2	8835.013	-0.33132	0.034936	0.086191	protein_coding
ENSG0000(	SLC30A8	187.2882	-0.19434	0.034946	0.086209	protein_coding
ENSG0000(	ENSG0000(	14.22042	-0.42621	0.034955	0.086224	lncRNA
ENSG0000(	FCGBP	77876.59	-0.33964	0.034958	0.086224	protein_coding
ENSG0000(	MRM2	324.211	-0.13859	0.034995	0.086309	protein_coding
ENSG0000(	ENSG0000(	47.32358	0.259619	0.035019	0.086361	processed_pseudogene
ENSG0000(	NUDT16-DT	1127.625	0.157204	0.035048	0.086425	lncRNA
ENSG0000(	ENSG0000(	16.06699	0.817696	0.035053	0.086432	lncRNA
ENSG0000(	ENSG0000(	20.16647	0.453458	0.035067	0.086454	lncRNA
ENSG0000(	ZNF776	1455.923	-0.13591	0.035067	0.086454	protein_coding
ENSG0000(	ZNF692	754.9808	-0.13488	0.035081	0.086479	protein_coding
ENSG0000(	ZC3H4	1449.805	-0.10852	0.035089	0.086493	protein_coding
ENSG0000(	ZNF485	199.9542	-0.19959	0.035095	0.086501	protein_coding
ENSG0000(	TEX30	76.66621	-0.27231	0.035102	0.086512	protein_coding

ENSG0000(	ENSG0000(	90.59029	-0.23727	0.03512	0.08655	lncRNA
ENSG0000(	ENSG0000(	15.72279	-0.4906	0.035131	0.086563	lncRNA
ENSG0000(	TUB	166.5626	0.244276	0.035131	0.086563	protein_coding
ENSG0000(	RTCB	851.3802	-0.1341	0.035137	0.08657	protein_coding
ENSG0000(	C4orf3	779.8097	0.171991	0.035181	0.086672	protein_coding
ENSG0000(	ENSG0000(	30.67698	0.31578	0.035184	0.086672	lncRNA
ENSG0000(	TRAF2	575.9141	-0.14868	0.035201	0.086708	protein_coding
ENSG0000(	OTULIN	2877.723	0.098318	0.035217	0.086739	protein_coding
ENSG0000(	QDPR	211.5916	-0.18902	0.035224	0.08675	protein_coding
ENSG0000(	SLC25A18	14.54962	0.710338	0.03524	0.086782	protein_coding
ENSG0000(	DRC7	12.71965	0.628282	0.035251	0.086804	protein_coding
ENSG0000(	PCLAF	785.4991	-0.143	0.035264	0.086828	protein_coding
ENSG0000(	CAST	9513.734	-0.17375	0.035273	0.086837	protein_coding
ENSG0000(	CTNNBL1	405.1125	-0.13031	0.035276	0.086837	protein_coding
ENSG0000(	RTN3	2100.615	-0.16659	0.035274	0.086837	protein_coding
ENSG0000(	ENSG0000(	23.04004	0.406164	0.03528	0.08684	lncRNA
ENSG0000(	CCDC191	427.1713	0.143176	0.035291	0.08686	protein_coding
ENSG0000(	ENSG0000(	37.97355	0.27198	0.035298	0.086872	lncRNA
ENSG0000(	ENSG0000(	83.02936	0.18329	0.035322	0.086923	lncRNA
ENSG0000(	ENSG0000(	413.1044	0.08684	0.035327	0.086926	lncRNA
ENSG0000(	ENSG0000(	116.698	-0.17849	0.035328	0.086926	lncRNA
ENSG0000(	ENSG0000(	48.13912	-0.23005	0.035333	0.086931	lncRNA
ENSG0000(	ENSG0000(	26.02757	0.338776	0.03534	0.08694	lncRNA
ENSG0000(	ENSG0000(	2695.66	0.123328	0.035352	0.086962	TEC
ENSG0000(	UNC5D	78.49032	-0.44995	0.035357	0.086968	protein_coding
ENSG0000(	MKX	12.88341	0.790624	0.035359	0.086968	protein_coding
ENSG0000(	MEP1A	1671.538	-0.47941	0.035364	0.086971	protein_coding
ENSG0000(	SFXN2	1513.746	-0.13174	0.035372	0.086985	protein_coding
ENSG0000(	ENSG0000(	60.02466	-0.20831	0.035377	0.08699	processed_pseudogene
ENSG0000(	ENSG0000(	164.0914	-0.19225	0.03538	0.08699	lncRNA
ENSG0000(	AGO1	2558.439	-0.08955	0.035393	0.086995	protein_coding
ENSG0000(	BMPR1A	1916.942	-0.13491	0.035391	0.086995	protein_coding
ENSG0000(	ENSG0000(	856.8686	0.086275	0.035391	0.086995	lncRNA
ENSG0000(	H3P37	262.3134	0.120861	0.035387	0.086995	transcribed_unprocessed_pseudo
ENSG0000(	ENSG0000(	188.1164	0.13334	0.035402	0.08701	lncRNA
ENSG0000(	ENSG0000(	174.1751	0.154288	0.035416	0.087039	lncRNA
ENSG0000(	RDH12	15.91704	1.143972	0.035426	0.087056	protein_coding
ENSG0000(	ENSG0000(	764.849	-0.10261	0.035434	0.08707	lncRNA
ENSG0000(	DES	270.9717	-0.66928	0.035486	0.08719	protein_coding
ENSG0000(	ZNF814	3657.268	-0.12773	0.035505	0.087229	protein_coding
ENSG0000(	WDR49	23.07683	0.56391	0.03554	0.08731	protein_coding
ENSG0000(	CLPB	2245.423	0.109284	0.03555	0.087326	protein_coding
ENSG0000(	LAPTM4B	735.9582	-0.18857	0.035558	0.08734	protein_coding
ENSG0000(	C2orf83	177.0206	-0.14712	0.035571	0.087344	transcribed_unprocessed_pseudo
ENSG0000(	CNP	953.1391	0.109948	0.035571	0.087344	protein_coding
ENSG0000(	COL11A1	29.60486	0.549045	0.035567	0.087344	protein_coding
ENSG0000(	RN7SL743F	57.67193	0.247471	0.035565	0.087344	misc_RNA

ENSG0000(	PTGES3L-A	36.84905	-0.65636	0.035586	0.087369	protein_coding
ENSG0000(	SPIN2B	122.6773	-0.22912	0.03559	0.087369	protein_coding
ENSG0000(	TTC36-AS1	15.27717	0.58795	0.03559	0.087369	lncRNA
ENSG0000(	ENSG0000(	96.0951	-0.15646	0.035598	0.087382	lncRNA
ENSG0000(	LINC01271	16.72853	0.572675	0.035607	0.087399	lncRNA
ENSG0000(	GPSM3	343.4722	0.343576	0.03562	0.087423	protein_coding
ENSG0000(	IQGAP3	925.8696	-0.28179	0.035636	0.087455	protein_coding
ENSG0000(	ENSG0000(	14.10485	0.427855	0.035652	0.087487	TEC
ENSG0000(	YWHAEP7	60.897	0.414972	0.035661	0.087503	transcribed_unprocessed_pseudo
ENSG0000(	ENSG0000(	169.5609	-0.12631	0.035673	0.087525	lncRNA
ENSG0000(	ENSG0000(	72.73922	-0.20832	0.035675	0.087525	lncRNA
ENSG0000(	ENSG0000(	630.8724	0.112865	0.035683	0.087538	lncRNA
ENSG0000(	CLASP1	2474.444	0.09749	0.035723	0.0876	protein_coding
ENSG0000(	COX19	1323.354	-0.09831	0.03572	0.0876	protein_coding
ENSG0000(	ENSG0000(	14.53547	-0.83708	0.035715	0.0876	unprocessed_pseudogene
ENSG0000(	FGF7P3	45.93949	-0.40491	0.035721	0.0876	transcribed_unprocessed_pseudo
ENSG0000(	HELZ	5051.305	0.085481	0.035713	0.0876	protein_coding
ENSG0000(	ELK4	4651.932	0.118161	0.035726	0.087601	protein_coding
ENSG0000(	DNAAF4	176.8508	-0.14112	0.035758	0.087661	protein_coding
ENSG0000(	MFSD4B	7107.994	-0.09044	0.035756	0.087661	protein_coding
ENSG0000(	RN7SL434P	25.06781	0.291897	0.035759	0.087661	misc_RNA
ENSG0000(	ENSG0000(	16.32389	0.407427	0.035765	0.087669	lncRNA
ENSG0000(	ENSG0000(	251.0655	0.211769	0.03578	0.087699	lncRNA
ENSG0000(	MXD1	5375.025	0.326693	0.035784	0.087703	protein_coding
ENSG0000(	DDX6	6412.113	-0.0757	0.03579	0.08771	protein_coding
ENSG0000(	CCDC124	256.2103	-0.14995	0.035795	0.087716	protein_coding
ENSG0000(	CATSPER2	1774.86	-0.1699	0.035806	0.087736	protein_coding
ENSG0000(	ENSG0000(	15.21665	0.454721	0.035818	0.087757	lncRNA
ENSG0000(	ENSG0000(	534.8509	-0.13956	0.035835	0.087792	lncRNA
ENSG0000(	DLG5	2183.022	-0.13513	0.035843	0.087806	protein_coding
ENSG0000(	ARF1	3320.122	-0.1447	0.03585	0.087813	protein_coding
ENSG0000(	ENSG0000(	793.1187	-0.10682	0.035852	0.087813	lncRNA
ENSG0000(	LINC02691	94.65427	-0.31589	0.035855	0.087813	lncRNA
ENSG0000(	ARHGEF37	788.4603	-0.27932	0.03587	0.087842	protein_coding
ENSG0000(	STRIP1	1142.438	-0.08511	0.035872	0.087842	protein_coding
ENSG0000(	UPF3B	512.5803	-0.09806	0.035887	0.087871	protein_coding
ENSG0000(	RNU6ATAC	29.28291	0.333621	0.035929	0.087968	snRNA
ENSG0000(	TTC12	488.0085	-0.1578	0.03594	0.087987	protein_coding
ENSG0000(	EDDM13	10.23493	0.785351	0.035947	0.08799	protein_coding
ENSG0000(	MAP3K20	1669.259	0.15505	0.035945	0.08799	protein_coding
ENSG0000(	ARPC1B	1488.1	0.128836	0.035996	0.088097	protein_coding
ENSG0000(	MSH5-SAP(	232.6079	-0.29218	0.035994	0.088097	protein_coding
ENSG0000(	BRCC3P1	52.66788	-0.27052	0.036005	0.088112	processed_pseudogene
ENSG0000(	RN7SL346P	31.21572	0.517359	0.036029	0.088164	misc_RNA
ENSG0000(	ENSG0000(	299.9302	-0.11961	0.036042	0.088188	lncRNA
ENSG0000(	RPSAP16	82.39635	-0.19923	0.036087	0.088291	processed_pseudogene
ENSG0000(	ENSG0000(	18.36255	0.400363	0.036094	0.088303	TEC

ENSG0000(	TPT1P8	169.8995	-0.18619	0.0361	0.088309	transcribed_processed_pseudoge
ENSG0000(	CIC	2108.134	0.148407	0.036119	0.088321	protein_coding
ENSG0000(	ENSG0000(	179.5577	-0.19984	0.036115	0.088321	lncRNA
ENSG0000(	ENSG0000(	193.7009	0.178768	0.036114	0.088321	lncRNA
ENSG0000(	ENSG0000(	795.4634	0.100575	0.036121	0.088321	TEC
ENSG0000(	MED29	817.1792	-0.09858	0.036117	0.088321	protein_coding
ENSG0000(	PLAC9	15.61379	-0.50367	0.036107	0.088321	protein_coding
ENSG0000(	ENSG0000(	175.6351	-0.46133	0.036126	0.088324	TEC
ENSG0000(	RNU6-238F	9.13605	-0.515	0.036162	0.088405	snRNA
ENSG0000(	ENSG0000(	79.31612	-0.24293	0.036194	0.088477	lncRNA
ENSG0000(	LRRC40	276.471	-0.17816	0.0362	0.088486	protein_coding
ENSG0000(	TEX14	62.27288	0.266997	0.03622	0.088527	protein_coding
ENSG0000(	ENSG0000(	19.22993	0.322368	0.036223	0.088528	lncRNA
ENSG0000(	ENSG0000(	11.54481	0.398651	0.03626	0.088611	lncRNA
ENSG0000(	C16orf72	10325.44	0.086417	0.036272	0.088633	protein_coding
ENSG0000(	ACSL6	430.9312	-0.13957	0.036284	0.088643	protein_coding
ENSG0000(	ENSG0000(	17.85866	0.357829	0.036287	0.088643	lncRNA
ENSG0000(	FPGS	555.248	-0.13106	0.036282	0.088643	protein_coding
ENSG0000(	LINC01782	44.13935	-0.23832	0.036282	0.088643	lncRNA
ENSG0000(	PFKFB2	4667.42	-0.19998	0.036316	0.088706	protein_coding
ENSG0000(	ENSG0000(	29.55699	-0.28131	0.036329	0.088732	lncRNA
ENSG0000(	ATP2B1-AS	133.1265	0.186638	0.03636	0.088801	lncRNA
ENSG0000(	ENSG0000(	49.34775	0.294758	0.036471	0.089065	lncRNA
ENSG0000(	ENSG0000(	512.2767	0.122142	0.036506	0.089143	TEC
ENSG0000(	ENSG0000(	25.85184	0.414073	0.036541	0.08922	lncRNA
ENSG0000(	ENSG0000(	106.6024	-0.15841	0.036553	0.089245	transcribed_unitary_pseudogene
ENSG0000(	DNASE2B	13.06731	-0.57302	0.036559	0.089251	protein_coding
ENSG0000(	MAGI2	1784.97	0.242701	0.036571	0.089274	protein_coding
ENSG0000(	SEMA4F	493.5873	0.147322	0.036574	0.089274	protein_coding
ENSG0000(	ENSG0000(	31.80692	0.391087	0.036583	0.089288	lncRNA
ENSG0000(	KIAA0232	3547.276	-0.21425	0.036617	0.089361	protein_coding
ENSG0000(	RN7SL430F	55.61743	0.246446	0.036618	0.089361	misc_RNA
ENSG0000(	ENPP7P14	14.79412	-0.49447	0.036635	0.089396	unprocessed_pseudogene
ENSG0000(	ENSG0000(	53.13446	0.203583	0.03664	0.0894	lncRNA
ENSG0000(	KIF20B	629.9929	-0.23318	0.036683	0.089492	protein_coding
ENSG0000(	RNU1-30P	21.67123	-0.35738	0.036681	0.089492	snRNA
ENSG0000(	PLEKHA8P1	203.7023	0.135355	0.036689	0.0895	transcribed_processed_pseudoge
ENSG0000(	ENSG0000(	23.73988	0.365919	0.036737	0.08961	snoRNA
ENSG0000(	ENSG0000(	1103.521	0.095395	0.036748	0.089615	lncRNA
ENSG0000(	PHF1	639.7708	0.151081	0.036751	0.089615	protein_coding
ENSG0000(	SPIN3	2368.616	0.078561	0.036747	0.089615	protein_coding
ENSG0000(	TPM1	11331.85	0.173118	0.036744	0.089615	protein_coding
ENSG0000(	ENSG0000(	238.9954	0.145433	0.036758	0.089622	lncRNA
ENSG0000(	SFXN1	2410.793	-0.15551	0.036762	0.089622	protein_coding
ENSG0000(	ZNF75A	1632.498	0.10038	0.036761	0.089622	protein_coding
ENSG0000(	LINC00320	671.0812	0.0988	0.036766	0.089623	lncRNA
ENSG0000(	AURKB	245.7678	-0.23448	0.03678	0.089637	protein_coding

ENSG0000(	ENSG0000(	33.39786	-0.77218	0.03678	0.089637	lncRNA
ENSG0000(	TMEM61	31.41293	-0.42446	0.036778	0.089637	protein_coding
ENSG0000(	LSP1P4	942.5066	-0.23799	0.03679	0.089655	transcribed_unprocessed_pseudo
ENSG0000(	RNF31	798.1066	-0.14015	0.036812	0.089702	protein_coding
ENSG0000(	ENSG0000(	54.19932	-0.42324	0.036818	0.089709	lncRNA
ENSG0000(	ENSG0000(	231.7935	0.127832	0.036838	0.089744	lncRNA
ENSG0000(	ZNF878	23.09903	-0.39663	0.036838	0.089744	protein_coding
ENSG0000(	EEF1E1	195.6233	-0.19088	0.036842	0.089746	protein_coding
ENSG0000(	SLC12A1	68.37891	0.221713	0.036875	0.08982	protein_coding
ENSG0000(	SF3B1	11337.88	0.078766	0.036913	0.089906	protein_coding
ENSG0000(	ENSG0000(	49.48152	0.265911	0.036918	0.089909	lncRNA
ENSG0000(	CDYL	897.3336	-0.1243	0.036925	0.089921	protein_coding
ENSG0000(	GASK1B-AS	318.1037	0.280438	0.036944	0.089959	lncRNA
ENSG0000(	ENSG0000(	1043.863	-0.10443	0.036949	0.089966	protein_coding
ENSG0000(	SFTPA2	21.42576	0.661545	0.036957	0.089976	protein_coding
ENSG0000(	ENSG0000(	301.4172	0.401674	0.036971	0.090004	lncRNA
ENSG0000(	ADAMTSL2	171.5845	-0.17728	0.036978	0.090014	protein_coding
ENSG0000(	EDNRB-AS1	232.2248	-0.17486	0.036988	0.090033	lncRNA
ENSG0000(	ZNF710-AS	346.1325	0.137496	0.037016	0.090092	lncRNA
ENSG0000(	HEATR5A-D	249.5008	-0.15444	0.037058	0.090189	lncRNA
ENSG0000(	ENSG0000(	35.58468	0.262824	0.037062	0.090191	lncRNA
ENSG0000(	ENSG0000(	12.6068	-0.59924	0.037067	0.090196	lncRNA
ENSG0000(	BCL2L15	4579.417	-0.20284	0.037078	0.090216	protein_coding
ENSG0000(	ENSG0000(	53.95064	0.31494	0.037086	0.090227	lncRNA
ENSG0000(	ENSG0000(	95.43864	0.184513	0.037092	0.090235	lncRNA
ENSG0000(	GPR171	105.3724	0.447765	0.037105	0.090253	protein_coding
ENSG0000(	NTN4	550.6685	-0.26062	0.037104	0.090253	protein_coding
ENSG0000(	ARHGDIB	1015.196	0.355622	0.037109	0.090255	protein_coding
ENSG0000(	ENSG0000(	439.5477	0.114681	0.037126	0.090289	lncRNA
ENSG0000(	ENSG0000(	56.55066	0.323128	0.037132	0.090299	lncRNA
ENSG0000(	MYADM	1095.453	0.164219	0.03715	0.090335	protein_coding
ENSG0000(	PAX3	60.14318	0.269033	0.037161	0.090355	protein_coding
ENSG0000(	ENSG0000(	204.1642	0.387277	0.037191	0.09042	protein_coding
ENSG0000(	LMBRD1	1447.458	-0.10202	0.037225	0.090483	protein_coding
ENSG0000(	RAB7B	109.158	0.33474	0.037226	0.090483	protein_coding
ENSG0000(	RNU1-88P	232.2486	0.413487	0.037221	0.090483	snRNA
ENSG0000(	SLC20A1	11855.77	-0.4339	0.037229	0.090483	protein_coding
ENSG0000(	TSPAN5	440.4299	0.224854	0.037249	0.090527	protein_coding
ENSG0000(	POFUT1	1471.25	-0.13138	0.037255	0.090533	protein_coding
ENSG0000(	CYCSP28	9.549176	0.496168	0.037278	0.090581	processed_pseudogene
ENSG0000(	HIP1	1248.14	0.215629	0.037293	0.090611	protein_coding
ENSG0000(	C17orf107	70.37591	0.33056	0.037311	0.090648	protein_coding
ENSG0000(	ENSG0000(	130.3375	0.244035	0.037349	0.090731	lncRNA
ENSG0000(	RN7SL850F	104.3582	-0.21764	0.037351	0.090731	misc_RNA
ENSG0000(	ENSG0000(	31.73631	0.244133	0.037366	0.090755	lncRNA
ENSG0000(	PPP1R12B	6527.28	0.133604	0.037364	0.090755	protein_coding
ENSG0000(	ECPAS	4210.06	-0.10241	0.03738	0.090774	protein_coding

ENSG0000(	ENSG0000(	25.28812	-0.33131	0.037379	0.090774	lncRNA
ENSG0000(	ENSG0000(	95.77475	0.210314	0.037387	0.090785	lncRNA
ENSG0000(	LINC01859	93.57705	-0.18489	0.037398	0.090803	lncRNA
ENSG0000(	PCOLCE-AS	40.58531	0.259668	0.037402	0.090806	lncRNA
ENSG0000(	ENSG0000(	86.51541	0.217566	0.03741	0.090818	processed_pseudogene
ENSG0000(	ENSG0000(	1708.003	0.145632	0.037448	0.090903	lncRNA
ENSG0000(	ANKRD34A	28.34565	0.423401	0.037467	0.090935	protein_coding
ENSG0000(	HERC2P2	5466.81	0.126899	0.037466	0.090935	transcribed_unprocessed_pseudo
ENSG0000(	TDRP	205.0431	0.271867	0.037475	0.090947	protein_coding
ENSG0000(	ENSG0000(	11.51869	-0.49863	0.037502	0.090999	processed_pseudogene
ENSG0000(	POGK	1991.654	-0.0902	0.0375	0.090999	protein_coding
ENSG0000(	LINC02552	595.6572	0.099835	0.037521	0.091038	lncRNA
ENSG0000(	KDM8	210.6867	0.172994	0.037526	0.091044	protein_coding
ENSG0000(	CA1	1761.264	-0.70686	0.037549	0.091092	protein_coding
ENSG0000(	ENSG0000(	23.53561	0.46403	0.037589	0.091182	lncRNA
ENSG0000(	ENSG0000(	22.32614	0.294101	0.0376	0.091201	lncRNA
ENSG0000(	GNE	2030.701	-0.18324	0.037686	0.091403	protein_coding
ENSG0000(	ENSG0000(	69.4095	0.194561	0.0377	0.091429	lncRNA
ENSG0000(	PDE6B-AS1	12.78369	0.78338	0.037706	0.091432	lncRNA
ENSG0000(	SPSB2	163.3916	-0.19369	0.037709	0.091432	protein_coding
ENSG0000(	ZNF90P1	66.66328	-0.25856	0.037709	0.091432	processed_pseudogene
ENSG0000(	BTG1-DT	227.1257	0.3227	0.037722	0.091454	lncRNA
ENSG0000(	ENSG0000(	70.87192	0.335841	0.037741	0.091487	transcribed_processed_pseudoge
ENSG0000(	NCKAP1	4203.332	-0.16821	0.037739	0.091487	protein_coding
ENSG0000(	AHCTF1	2427.09	-0.12834	0.037756	0.091512	protein_coding
ENSG0000(	CCDC150	788.9435	-0.13908	0.037757	0.091512	protein_coding
ENSG0000(	MATN2	1287.725	-0.23464	0.037849	0.091728	protein_coding
ENSG0000(	EMD	247.2565	-0.16348	0.03787	0.091771	protein_coding
ENSG0000(	ENSG0000(	142.4535	-0.19793	0.037898	0.091831	processed_pseudogene
ENSG0000(	PDSS2	636.0535	-0.13562	0.037921	0.091881	protein_coding
ENSG0000(	ENSG0000(	246.9228	0.22261	0.037943	0.091922	lncRNA
ENSG0000(	UTP23	707.0229	-0.10373	0.037944	0.091922	protein_coding
ENSG0000(	CETN4P	145.4298	-0.34107	0.037953	0.091938	transcribed_unitary_pseudogene
ENSG0000(	EMID1	376.9691	0.2385	0.037965	0.091959	protein_coding
ENSG0000(	TOX2	98.73352	0.323841	0.037977	0.091982	protein_coding
ENSG0000(	CPEB1	246.9937	0.225144	0.03799	0.092006	protein_coding
ENSG0000(	PROSER2	146.0069	-0.29025	0.038004	0.092033	protein_coding
ENSG0000(	ENSG0000(	45.58595	0.286804	0.038015	0.092051	lncRNA
ENSG0000(	RBBP8	557.9914	-0.18987	0.038021	0.092058	protein_coding
ENSG0000(	GATA3	92.1592	0.340671	0.038034	0.092084	protein_coding
ENSG0000(	ENSG0000(	16.8284	-0.44054	0.038038	0.092086	lncRNA
ENSG0000(	IL17REL	128.8801	0.669377	0.038071	0.092157	protein_coding
ENSG0000(	HSPA8P4	19.69538	0.423646	0.038109	0.092235	processed_pseudogene
ENSG0000(	SEC22C	1162.065	0.116934	0.038108	0.092235	protein_coding
ENSG0000(	ARL4AP5	14.94025	0.536442	0.03812	0.092248	processed_pseudogene
ENSG0000(	DUTP6	9.465773	0.507869	0.038117	0.092248	processed_pseudogene
ENSG0000(	CYP51A1P3	51.86959	0.272839	0.038128	0.092262	processed_pseudogene

ENSG0000(	NIN	3088.387	0.172433	0.038144	0.092293	protein_coding
ENSG0000(	LINC00663	442.4832	0.147897	0.038215	0.092456	lncRNA
ENSG0000(	PPP6R2	1981.232	-0.07196	0.038228	0.092481	protein_coding
ENSG0000(	OTUD6B	466.4844	-0.1347	0.038242	0.092509	protein_coding
ENSG0000(	BRPF1	721.3963	-0.11101	0.038248	0.092515	protein_coding
ENSG0000(	LINC00857	146.0308	0.174367	0.038264	0.092547	lncRNA
ENSG0000(	CERS2	1173.302	-0.14088	0.03827	0.092553	protein_coding
ENSG0000(	EMG1	912.1413	-0.11765	0.038276	0.092563	protein_coding
ENSG0000(	RNU6-202F	29.12871	-0.27011	0.038291	0.092591	snRNA
ENSG0000(	BNIP3P4	210.2026	0.512659	0.038296	0.092596	processed_pseudogene
ENSG0000(	ZNF506	3485.117	-0.08361	0.038332	0.092676	protein_coding
ENSG0000(	SS18L1	940.5399	-0.11385	0.038352	0.092717	protein_coding
ENSG0000(	HEXIM1	928.3301	-0.17321	0.038392	0.092805	protein_coding
ENSG0000(	RN7SL559F	109.1428	0.186361	0.038406	0.092834	misc_RNA
ENSG0000(	LINC02549	54.23635	-0.25221	0.03843	0.092885	lncRNA
ENSG0000(	GINS1	514.467	-0.14277	0.038439	0.092897	protein_coding
ENSG0000(	ENSG0000(	53.54521	0.293512	0.038468	0.092961	lncRNA
ENSG0000(	PIAS2	2831.885	0.124285	0.038497	0.093024	protein_coding
ENSG0000(	MEGF11	28.93781	0.54443	0.038501	0.093026	protein_coding
ENSG0000(	ZDHHC4	521.1887	-0.11207	0.038504	0.093027	protein_coding
ENSG0000(	ENSG0000(	127.7231	-0.18558	0.038522	0.093059	lncRNA
ENSG0000(	ENSG0000(	52.25503	-0.19623	0.038524	0.093059	lncRNA
ENSG0000(	DAB1	46.31145	0.612365	0.038563	0.093149	protein_coding
ENSG0000(	E2F7	191.4529	-0.30796	0.038577	0.093174	protein_coding
ENSG0000(	RN7SL778F	36.39189	0.27508	0.038587	0.093191	misc_RNA
ENSG0000(	LINC01235	157.2934	0.207643	0.038601	0.093218	lncRNA
ENSG0000(	RAD51B	575.1272	0.151514	0.03865	0.093329	protein_coding
ENSG0000(	RPSAP11	19.56457	-0.69089	0.038653	0.093329	processed_pseudogene
ENSG0000(	CCDC39	476.6522	0.10789	0.038674	0.093372	protein_coding
ENSG0000(	ENSG0000(	24.72694	0.323726	0.038681	0.093381	lncRNA
ENSG0000(	ENSG0000(	47.60076	0.205119	0.038683	0.093381	TEC
ENSG0000(	ENSG0000(	24.47455	0.262432	0.038695	0.093395	lncRNA
ENSG0000(	RNU6-1152	12.78813	0.605655	0.038693	0.093395	snRNA
ENSG0000(	RDM1P4	55.23369	0.380813	0.038703	0.093405	transcribed_processed_pseudoge
ENSG0000(	MAP3K1	1622.827	0.183262	0.038706	0.093407	protein_coding
ENSG0000(	ENSG0000(	134.2465	-0.14607	0.038742	0.093476	lncRNA
ENSG0000(	ENSG0000(	1127.69	0.101301	0.038742	0.093476	lncRNA
ENSG0000(	PCDH12	120.4242	0.392576	0.038744	0.093476	protein_coding
ENSG0000(	ENSG0000(	21.53767	0.330149	0.038782	0.093561	TEC
ENSG0000(	ADAMTSL1	951.2347	-0.28142	0.038796	0.093585	protein_coding
ENSG0000(	DOCK10	2962.323	0.242727	0.038799	0.093585	protein_coding
ENSG0000(	MTCO1P53	46.67168	-0.78344	0.038801	0.093585	processed_pseudogene
ENSG0000(	ZNF252P	1553.132	-0.11339	0.038818	0.093619	transcribed_unprocessed_pseudo
ENSG0000(	ENSG0000(	2547.539	-0.15213	0.038824	0.093626	lncRNA
ENSG0000(	RN7SKP29(	21.31546	0.451523	0.038855	0.093694	misc_RNA
ENSG0000(	CLCF1	129.2072	0.325788	0.03887	0.093723	protein_coding
ENSG0000(	DEFB124	10.20974	0.560808	0.03889	0.093763	protein_coding

ENSG0000(	ENSG0000(	125.572	-0.15882	0.038899	0.093778	lncRNA
ENSG0000(	ENSG0000(	12.31501	-0.41372	0.038907	0.09379	lncRNA
ENSG0000(	UCHL5	951.9662	-0.10843	0.038913	0.093797	protein_coding
ENSG0000(	PIWIL3	131.5538	-0.1577	0.03892	0.093807	protein_coding
ENSG0000(	ENSG0000(	3293.965	-0.06078	0.038933	0.093826	lncRNA
ENSG0000(	ENSG0000(	48.42129	0.268709	0.038934	0.093826	lncRNA
ENSG0000(	ENSG0000(	16.18961	0.439673	0.038962	0.093886	lncRNA
ENSG0000(	RN7SKP13(	12.21066	-0.62907	0.038988	0.093936	misc_RNA
ENSG0000(	ZBTB9	146.9368	-0.21386	0.038989	0.093936	protein_coding
ENSG0000(	OR6C6	56.26643	-0.18175	0.038992	0.093938	protein_coding
ENSG0000(	CPLANE1	4269.453	0.067908	0.039004	0.093958	protein_coding
ENSG0000(	ENSG0000(	32.11699	0.385758	0.039018	0.093986	lncRNA
ENSG0000(	ENSG0000(	15.75601	0.505857	0.039023	0.093991	lncRNA
ENSG0000(	GXYLT1P3	13.99	1.095003	0.039047	0.094041	processed_pseudogene
ENSG0000(	C11orf52	61.552	-0.35111	0.039052	0.094045	protein_coding
ENSG0000(	AP3D1	2845.575	-0.12945	0.039067	0.094074	protein_coding
ENSG0000(	GPR37	18.68432	0.523281	0.039081	0.094079	protein_coding
ENSG0000(	SLC29A1	255.0879	0.22552	0.039077	0.094079	protein_coding
ENSG0000(	STEAP1	188.5332	-0.24197	0.03908	0.094079	protein_coding
ENSG0000(	TBC1D8B	6164.543	-0.13943	0.039077	0.094079	protein_coding
ENSG0000(	ENSG0000(	25.62623	0.323234	0.039091	0.094095	transcribed_unitary_pseudogene
ENSG0000(	DAB2	2312.682	0.182549	0.039108	0.094129	protein_coding
ENSG0000(	ENSG0000(	28.91102	-0.31771	0.039111	0.094129	lncRNA
ENSG0000(	CPEB2-DT	86.16175	0.235775	0.039118	0.094139	lncRNA
ENSG0000(	ENSG0000(	167.6559	0.136403	0.039127	0.094154	lncRNA
ENSG0000(	CCT8P1	141.3553	-0.38924	0.039147	0.094194	processed_pseudogene
ENSG0000(	ENSG0000(	52.16629	0.270507	0.039161	0.094221	lncRNA
ENSG0000(	FTH1P23	17.1266	-0.45393	0.039165	0.094223	processed_pseudogene
ENSG0000(	CA3	85.37072	0.35035	0.039176	0.094241	protein_coding
ENSG0000(	SLC23A2	1564.929	0.181391	0.039226	0.094355	protein_coding
ENSG0000(	ENSG0000(	52.47167	0.219594	0.03924	0.094367	lncRNA
ENSG0000(	LINC02211	50.69371	-0.28493	0.039238	0.094367	lncRNA
ENSG0000(	ZNF709	278.9973	0.250286	0.039237	0.094367	protein_coding
ENSG0000(	ENSG0000(	56.57502	0.242182	0.039255	0.094395	lncRNA
ENSG0000(	BBS1	885.0046	-0.08492	0.039271	0.094428	protein_coding
ENSG0000(	LINC02458	11.17773	0.536351	0.039279	0.094438	lncRNA
ENSG0000(	ST3GAL6-A	422.8586	0.137463	0.039305	0.094495	lncRNA
ENSG0000(	GASAL1	115.1553	0.181007	0.039312	0.094505	lncRNA
ENSG0000(	TNFAIP8	1690.681	0.186859	0.039347	0.094581	protein_coding
ENSG0000(	LUZP2	177.4123	-0.2183	0.039358	0.0946	protein_coding
ENSG0000(	ENSG0000(	133.9663	0.216545	0.039366	0.094612	lncRNA
ENSG0000(	ENSG0000(	11.65551	0.429139	0.039391	0.094665	lncRNA
ENSG0000(	LCN8	11.44045	1.243617	0.039429	0.094749	protein_coding
ENSG0000(	KIR3DX1	812.6647	0.068525	0.039441	0.094765	transcribed_unprocessed_pseudo
ENSG0000(	PKD1P6-NF	76.53587	0.408567	0.039442	0.094765	lncRNA
ENSG0000(	ENSG0000(	110.8897	-0.16442	0.039467	0.094818	lncRNA
ENSG0000(	ENSG0000(	203.2454	-0.14953	0.039473	0.094825	lncRNA

ENSG0000(	NXPE2	765.5702	-0.36487	0.039481	0.094836	protein_coding
ENSG0000(	ENSG0000(	131.1574	0.187015	0.039484	0.094837	lncRNA
ENSG0000(	RN7SL455F	247.4712	-0.12375	0.039488	0.094839	misc_RNA
ENSG0000(	ENSG0000(	111.9709	0.249786	0.039503	0.094868	TEC
ENSG0000(	ENSG0000(	16.79915	0.427444	0.039506	0.094869	processed_pseudogene
ENSG0000(	YBEY	227.6333	-0.21148	0.03952	0.094894	protein_coding
ENSG0000(	VWFP1	12.2278	0.710852	0.039527	0.094905	transcribed_unprocessed_pseudo
ENSG0000(	LSS	1593.329	0.186404	0.039538	0.094923	protein_coding
ENSG0000(	ENSG0000(	84.50095	0.215892	0.039563	0.094975	lncRNA
ENSG0000(	MYH9	19090.1	-0.0916	0.039573	0.094994	protein_coding
ENSG0000(	RMND1	1115.14	-0.07515	0.039586	0.095009	protein_coding
ENSG0000(	ZNF710	1088.025	0.143388	0.039585	0.095009	protein_coding
ENSG0000(	ENSG0000(	209.6055	0.128468	0.03961	0.09506	lncRNA
ENSG0000(	HEXD-IT1	952.9384	0.075653	0.039619	0.09506	lncRNA
ENSG0000(	NHS	550.9995	0.227209	0.039617	0.09506	protein_coding
ENSG0000(	SMG6-IT1	86.93318	0.198537	0.039617	0.09506	TEC
ENSG0000(	DCP1A	1597.251	-0.07977	0.039639	0.0951	protein_coding
ENSG0000(	ENSG0000(	374.863	-0.11847	0.039643	0.095103	lncRNA
ENSG0000(	SEPTIN3	136.0256	0.224626	0.039647	0.095105	protein_coding
ENSG0000(	ENSG0000(	10.87676	0.506601	0.039652	0.09511	lncRNA
ENSG0000(	RAB40AL	17.29334	-0.42742	0.039656	0.095112	protein_coding
ENSG0000(	ENSG0000(	201.722	0.208005	0.039669	0.095136	TEC
ENSG0000(	P2RX6	57.99873	-0.237	0.039672	0.095136	protein_coding
ENSG0000(	ENSG0000(	173.6069	0.53064	0.03968	0.095147	protein_coding
ENSG0000(	LINC02412	60.38719	-0.21626	0.03969	0.095163	lncRNA
ENSG0000(	ENSG0000(	172.275	-0.16436	0.039714	0.095214	TEC
ENSG0000(	SNORA17B	228.7827	-0.28815	0.039722	0.095226	snoRNA
ENSG0000(	DNM2	15058.33	-0.16569	0.039732	0.095243	protein_coding
ENSG0000(	LINC02757	17.67537	0.432442	0.039768	0.095323	lncRNA
ENSG0000(	ENSG0000(	43.27404	0.278038	0.0398	0.095384	lncRNA
ENSG0000(	IGKV6-21	57.33679	0.541955	0.0398	0.095384	IG_V_gene
ENSG0000(	RGCC	96.92943	0.330059	0.039804	0.095386	protein_coding
ENSG0000(	ZYG11B	2220.088	-0.10976	0.03982	0.095418	protein_coding
ENSG0000(	CCDC9B	1910.185	-0.29815	0.039827	0.095426	protein_coding
ENSG0000(	RNU6-789F	16.55816	0.434564	0.03983	0.095427	snRNA
ENSG0000(	ENSG0000(	14.93188	-0.48079	0.039844	0.095447	lncRNA
ENSG0000(	L3MBTL2-A	693.6098	0.104306	0.039845	0.095447	lncRNA
ENSG0000(	ENSG0000(	178.07	0.158119	0.039872	0.095505	lncRNA
ENSG0000(	FARS2	404.3378	-0.15911	0.039876	0.095508	protein_coding
ENSG0000(	SDC2	299.1869	0.262804	0.039912	0.095587	protein_coding
ENSG0000(	RSF1	3023.949	-0.09298	0.039963	0.095702	protein_coding
ENSG0000(	ENSG0000(	23.58332	0.327131	0.039975	0.095723	lncRNA
ENSG0000(	AP1G2	2211.539	-0.12325	0.039987	0.095738	protein_coding
ENSG0000(	HMSD	21.04622	0.636262	0.039991	0.095738	protein_coding
ENSG0000(	TEKT5	15.27932	-0.55102	0.039989	0.095738	protein_coding
ENSG0000(	ENSG0000(	198.3155	-0.14992	0.039994	0.095739	lncRNA
ENSG0000(	ENSG0000(	47.2493	-0.25608	0.040005	0.095758	lncRNA

ENSG0000(	ENSG0000(	768.8634	-0.13297	0.040014	0.095761	lncRNA
ENSG0000(	ENSG0000(	11.78135	-0.43352	0.040013	0.095761	lncRNA
ENSG0000(	NEIL1	1167.771	-0.14043	0.040016	0.095761	protein_coding
ENSG0000(	SMARCA2	5548.797	0.150965	0.040028	0.095783	protein_coding
ENSG0000(	CARM1P1	23.05736	-0.39087	0.040038	0.095799	transcribed_unprocessed_pseudo
ENSG0000(	ENSG0000(	30.03291	0.437125	0.040046	0.095811	processed_pseudogene
ENSG0000(	SFRP1	92.02778	-0.48242	0.040052	0.095818	protein_coding
ENSG0000(	MANCR	217.3279	-0.16949	0.040056	0.095821	lncRNA
ENSG0000(	CENPQ	56.13481	-0.30179	0.040064	0.095834	protein_coding
ENSG0000(	IGKV1-9	418.8522	0.544236	0.040069	0.095838	IG_V_gene
ENSG0000(	TNFSF13	567.5315	0.233806	0.040087	0.095873	protein_coding
ENSG0000(	ENSG0000(	14.2088	0.374176	0.040117	0.095929	lncRNA
ENSG0000(	GARRE1	2177.937	0.103385	0.040118	0.095929	protein_coding
ENSG0000(	LINC00904	199.4755	0.098585	0.04012	0.095929	lncRNA
ENSG0000(	ENSG0000(	77.04382	0.184185	0.040137	0.095963	lncRNA
ENSG0000(	CAVIN1	727.0766	0.289448	0.040155	0.096	protein_coding
ENSG0000(	ENSG0000(	595.7471	-0.10339	0.04017	0.096029	TEC
ENSG0000(	COQ8B	2014.353	0.071915	0.040189	0.096066	protein_coding
ENSG0000(	GNG2	845.7607	0.21671	0.040215	0.096121	protein_coding
ENSG0000(	SAP30	69.89317	-0.28906	0.040222	0.09613	protein_coding
ENSG0000(	ENSG0000(	30.23767	0.2734	0.040239	0.096157	processed_pseudogene
ENSG0000(	MIR762HG	93.47918	0.164805	0.040239	0.096157	lncRNA
ENSG0000(	HMGB1P19	11.85539	-0.41286	0.040257	0.096191	processed_pseudogene
ENSG0000(	OR2AT4	1557.57	0.09207	0.040294	0.096272	protein_coding
ENSG0000(	ENSG0000(	236.1253	-0.11226	0.040348	0.096395	lncRNA
ENSG0000(	ENSG0000(	267.7942	0.127911	0.04036	0.096416	protein_coding
ENSG0000(	SLC26A3	18098.8	-0.54487	0.040364	0.096419	protein_coding
ENSG0000(	LINC01964	58.31671	0.244624	0.040383	0.096457	lncRNA
ENSG0000(	P2RX4	2794.289	-0.16326	0.040395	0.096476	protein_coding
ENSG0000(	GABRA4	39.93876	0.391105	0.040403	0.096489	protein_coding
ENSG0000(	ZFP91P1	8.785447	-0.48715	0.040408	0.096495	processed_pseudogene
ENSG0000(	ANKRD12	6268.705	0.107488	0.040429	0.096532	protein_coding
ENSG0000(	SCD	1617.832	0.3563	0.04043	0.096532	protein_coding
ENSG0000(	LAIR2	11.39039	0.719186	0.040438	0.096544	protein_coding
ENSG0000(	DNAJC25	252.3151	-0.18775	0.040451	0.096559	protein_coding
ENSG0000(	SKIDA1	208.097	-0.189	0.040451	0.096559	protein_coding
ENSG0000(	ENSG0000(	107.4072	-0.19423	0.040462	0.096578	lncRNA
ENSG0000(	C1orf116	1107.489	-0.31878	0.040483	0.096622	protein_coding
ENSG0000(	TIGD6	169.9507	0.222781	0.040511	0.09668	protein_coding
ENSG0000(	ENSG0000(	34.29232	0.309091	0.040529	0.096716	lncRNA
ENSG0000(	ENSG0000(	361.9543	-0.13216	0.040542	0.096741	lncRNA
ENSG0000(	PRTG	188.3198	0.312661	0.040557	0.096768	protein_coding
ENSG0000(	ARFIP1	1137.445	-0.13988	0.040569	0.096789	protein_coding
ENSG0000(	NDUFB8P2	15.0001	0.441802	0.040576	0.096797	processed_pseudogene
ENSG0000(	ENSG0000(	144.4283	0.15868	0.040589	0.096822	lncRNA
ENSG0000(	ENSG0000(	36.0457	0.233391	0.040601	0.096836	lncRNA
ENSG0000(	PRDM6	142.0989	0.478251	0.040601	0.096836	protein_coding

ENSG0000(	IGFBP6	31.03151	-0.42087	0.040607	0.096842	protein_coding
ENSG0000(	BTNL8	1129.783	-0.37679	0.040624	0.096877	protein_coding
ENSG0000(	PIK3R2	609.9264	-0.1348	0.040648	0.096927	protein_coding
ENSG0000(	DRD5	76.16681	-0.713	0.040664	0.096958	protein_coding
ENSG0000(	ENSG0000(	4105.704	0.066253	0.040675	0.096977	lncRNA
ENSG0000(	ENSG0000(	28.78588	-0.35059	0.040684	0.096991	lncRNA
ENSG0000(	ENSG0000(	79.97961	0.176403	0.040688	0.096991	lncRNA
ENSG0000(	KRT18P15	75.46833	-0.2952	0.040703	0.097019	processed_pseudogene
ENSG0000(	EPB41L5	1371.848	-0.24398	0.040718	0.09705	protein_coding
ENSG0000(	DDIAS	180.3889	-0.19094	0.040727	0.097063	protein_coding
ENSG0000(	ENSG0000(	2052.52	-0.14667	0.040736	0.097076	lncRNA
ENSG0000(	ENSG0000(	34.18829	0.387424	0.040764	0.097137	lncRNA
ENSG0000(	CALML6	15.68375	0.499621	0.040777	0.097153	protein_coding
ENSG0000(	CEP55	190.7272	-0.2273	0.04078	0.097153	protein_coding
ENSG0000(	LCA10	210.1131	0.136245	0.04078	0.097153	lncRNA
ENSG0000(	ENSG0000(	740.6827	0.101618	0.040789	0.097154	lncRNA
ENSG0000(	ENSG0000(	390.8383	0.095072	0.040793	0.097154	lncRNA
ENSG0000(	ENSG0000(	90.93367	0.24449	0.040793	0.097154	lncRNA
ENSG0000(	ZNF622	270.3448	-0.17419	0.04079	0.097154	protein_coding
ENSG0000(	CDK5RAP1	717.6537	-0.08984	0.040802	0.097168	protein_coding
ENSG0000(	IGF2BP3	391.3602	0.234295	0.040807	0.097171	protein_coding
ENSG0000(	SLC4A4	5875.481	-0.39551	0.040855	0.097278	protein_coding
ENSG0000(	FAM178B	108.8609	-0.17719	0.040875	0.097319	protein_coding
ENSG0000(	RPL21P31	9.632799	0.45325	0.040923	0.097425	processed_pseudogene
ENSG0000(	ENSG0000(	67.61817	-0.2264	0.040932	0.097433	lncRNA
ENSG0000(	ENSG0000(	302.7281	-0.14909	0.040931	0.097433	TEC
ENSG0000(	ENSG0000(	70.21052	-0.18159	0.040936	0.097436	lncRNA
ENSG0000(	CTSL	701.2	0.230831	0.040952	0.097465	protein_coding
ENSG0000(	FBXO9	1661.047	-0.09498	0.04096	0.097477	protein_coding
ENSG0000(	UHRF2	2773.287	0.105804	0.040975	0.097505	protein_coding
ENSG0000(	ENSG0000(	14.80241	-0.48333	0.040991	0.097536	lncRNA
ENSG0000(	ENSG0000(	80.889	-0.60343	0.041018	0.097593	lncRNA
ENSG0000(	TRIM47	253.0651	0.257421	0.041022	0.097594	protein_coding
ENSG0000(	ENSG0000(	597.5421	0.071482	0.04104	0.09763	lncRNA
ENSG0000(	STK32C	99.09727	-0.18094	0.041045	0.097635	protein_coding
ENSG0000(	SNORA64	164.5432	-0.29806	0.041051	0.097641	snoRNA
ENSG0000(	S1PR1	410.3146	0.336277	0.041059	0.097652	protein_coding
ENSG0000(	MMGT1	723.5432	-0.14054	0.041063	0.097656	protein_coding
ENSG0000(	DUSP6	1447.806	0.258818	0.041067	0.097657	protein_coding
ENSG0000(	LINC00381	59.86629	-0.2131	0.04107	0.097658	lncRNA
ENSG0000(	SLC16A1-A:	2954.721	0.126851	0.041084	0.097677	lncRNA
ENSG0000(	SYS1	1051.52	0.122567	0.041085	0.097677	protein_coding
ENSG0000(	RN7SKP103	70.02905	-0.33317	0.041096	0.097697	misc_RNA
ENSG0000(	RPS10P7	823.2304	0.077381	0.041106	0.097712	transcribed_processed_pseudoge
ENSG0000(	SIRT2	1069.403	0.078137	0.041109	0.097712	protein_coding
ENSG0000(	RAVER2	3285.065	-0.28897	0.041116	0.097722	protein_coding
ENSG0000(	PTPN12	2222.941	0.116145	0.041123	0.097732	protein_coding

ENSG0000(	MCM5	1258.587	-0.17437	0.041143	0.097765	protein_coding
ENSG0000(	PRR19	332.4402	-0.11727	0.041144	0.097765	protein_coding
ENSG0000(	MIR3681H(	24.59532	0.571392	0.041147	0.097766	lncRNA
ENSG0000(	CCND2-AS1	84.77443	0.181075	0.041157	0.097774	lncRNA
ENSG0000(	RAB3GAP1	4768.205	0.053787	0.041157	0.097774	protein_coding
ENSG0000(	5S_rRNA	9.604416	0.499528	0.041183	0.097829	rRNA_pseudogene
ENSG0000(	RRAGC	921.2564	-0.1435	0.041187	0.097831	protein_coding
ENSG0000(	OAZ3	223.6523	0.151902	0.041192	0.097836	protein_coding
ENSG0000(	MAP3K14-/	403.1214	0.117294	0.041199	0.097843	lncRNA
ENSG0000(	TMEM35A	52.51477	-0.27778	0.041229	0.097907	protein_coding
ENSG0000(	ENSG0000(	69.42031	-0.18925	0.041255	0.097956	lncRNA
ENSG0000(	ENSG0000(	1099.783	0.1186	0.041255	0.097956	lncRNA
ENSG0000(	ENSG0000(	27.67544	0.293943	0.041266	0.097965	transcribed_unitary_pseudogene
ENSG0000(	SCAMP3	335.3953	-0.15653	0.041265	0.097965	protein_coding
ENSG0000(	LINC02857	72.37416	0.21931	0.041273	0.097976	lncRNA
ENSG0000(	HSD3B7	340.8503	0.148619	0.04131	0.098055	protein_coding
ENSG0000(	IQCF1	51.52652	0.236867	0.041345	0.098124	protein_coding
ENSG0000(	SCRN2	394.2677	-0.15217	0.041344	0.098124	protein_coding
ENSG0000(	ITGAD	84.4485	-0.28213	0.04137	0.098168	protein_coding
ENSG0000(	STIL	1038.266	-0.1332	0.041369	0.098168	protein_coding
ENSG0000(	GTPBP8	396.7152	-0.0987	0.041375	0.098173	protein_coding
ENSG0000(	KATNB1	224.2952	-0.2193	0.041404	0.098235	protein_coding
ENSG0000(	SNUPN	545.9565	-0.08418	0.041433	0.098296	protein_coding
ENSG0000(	ENSG0000(	19.88733	-0.31509	0.041507	0.098464	lncRNA
ENSG0000(	ZNF436-AS	167.1167	0.158312	0.041512	0.098469	lncRNA
ENSG0000(	ZNF33B	2481.336	-0.14272	0.041521	0.098481	protein_coding
ENSG0000(	EFNA4	139.6811	-0.23492	0.041528	0.098492	protein_coding
ENSG0000(	WASH9P	147.0412	-0.34673	0.041537	0.098505	unprocessed_pseudogene
ENSG0000(	ENSG0000(	24.53149	-0.42476	0.041541	0.098506	lncRNA
ENSG0000(	ENSG0000(	106.3847	0.214897	0.04155	0.098521	lncRNA
ENSG0000(	ENSG0000(	129.7096	0.149938	0.041566	0.098552	TEC
ENSG0000(	PCYOX1L	489.2912	0.199052	0.041585	0.098588	protein_coding
ENSG0000(	RN7SKP26ɛ	33.81598	0.391602	0.041616	0.098654	misc_RNA
ENSG0000(	LINC02064	147.1482	0.182278	0.041629	0.09868	lncRNA
ENSG0000(	ENSG0000(	107.7631	-0.16824	0.041661	0.098748	lncRNA
ENSG0000(	AXL	875.2526	0.176068	0.041676	0.098775	protein_coding
ENSG0000(	BCL2	3886.806	0.16795	0.041686	0.098792	protein_coding
ENSG0000(	NACA3P	12.13134	-0.67442	0.041707	0.098834	processed_pseudogene
ENSG0000(	ENSG0000(	68.20783	0.368941	0.041723	0.098863	TEC
ENSG0000(	ENSG0000(	9.354114	0.622202	0.041749	0.098917	lncRNA
ENSG0000(	ENSG0000(	27.81402	0.290134	0.041757	0.098928	lncRNA
ENSG0000(	LINC00167	87.59331	-0.17293	0.04176	0.098928	lncRNA
ENSG0000(	ENSG0000(	45.63965	-0.39406	0.041789	0.098989	lncRNA
ENSG0000(	HHAT	304.2739	0.176026	0.0418	0.099009	protein_coding
ENSG0000(	ENSG0000(	62.88955	-0.21506	0.041806	0.099009	lncRNA
ENSG0000(	ENSG0000(	28.44826	0.27418	0.041804	0.099009	lncRNA
ENSG0000(	DCLK1	340.2499	0.192965	0.041819	0.09903	protein_coding

ENSG0000(	ENSG0000(	288.3709	0.157693	0.041841	0.099076	TEC
ENSG0000(	HRAT92	152.1967	0.213521	0.041864	0.099123	lncRNA
ENSG0000(	LDLR	3059.196	0.195434	0.041882	0.099157	protein_coding
ENSG0000(	CLCA4-AS1	157.2707	0.211387	0.041896	0.099182	lncRNA
ENSG0000(	BIRC6-AS2	25.13093	0.270698	0.041927	0.09925	transcribed_unprocessed_pseudo
ENSG0000(	LRRC51	621.6058	-0.13977	0.041954	0.099305	protein_coding
ENSG0000(	PIGR	124995.2	-0.31345	0.041968	0.099331	protein_coding
ENSG0000(	LINC01186	29.63865	0.379285	0.041995	0.099388	lncRNA
ENSG0000(	ENSG0000(	18.14647	0.367629	0.042012	0.099421	lncRNA
ENSG0000(	MLXIP	13365.51	-0.15389	0.042034	0.099465	protein_coding
ENSG0000(	POU2F2	959.2717	0.37511	0.042044	0.099481	protein_coding
ENSG0000(	KCTD2	587.2139	-0.10379	0.042051	0.09949	protein_coding
ENSG0000(	ENSG0000(	57.10192	-0.20387	0.042062	0.099509	lncRNA
ENSG0000(	NUDT19P5	20.67329	0.454031	0.042071	0.099523	processed_pseudogene
ENSG0000(	CALM3	2410.265	-0.15789	0.042086	0.099542	protein_coding
ENSG0000(	ENSG0000(	28.92444	0.387019	0.042086	0.099542	lncRNA
ENSG0000(	ACTRT3	20.27049	-0.39359	0.042111	0.099595	protein_coding
ENSG0000(	DECR2	700.833	-0.16293	0.042119	0.099606	protein_coding
ENSG0000(	C16orf96	13.7141	0.467713	0.042124	0.09961	protein_coding
ENSG0000(	ENSG0000(	43.77404	0.240571	0.042159	0.099677	lncRNA
ENSG0000(	ZNF80	71.05378	-0.31526	0.042158	0.099677	protein_coding
ENSG0000(	ENSG0000(	275.7701	0.12987	0.04217	0.099697	lncRNA
ENSG0000(	ENSG0000(	22.50347	0.333675	0.042174	0.099698	lncRNA
ENSG0000(	ENSG0000(	59.77582	0.25618	0.042189	0.099722	lncRNA
ENSG0000(	SCLT1	1158.683	0.093326	0.04219	0.099722	protein_coding
ENSG0000(	ZNF160	2643.054	0.07799	0.042211	0.099762	protein_coding
ENSG0000(	SREBF1	1517.29	-0.16613	0.04222	0.099777	protein_coding
ENSG0000(	PAICSP1	9.852879	0.488539	0.042225	0.099781	processed_pseudogene
ENSG0000(	LINC02809	251.2867	0.128649	0.042235	0.099797	lncRNA
ENSG0000(	C21orf58	621.3354	-0.14288	0.042281	0.099898	protein_coding
ENSG0000(	CDC20B	351.9963	0.096149	0.04236	0.100077	protein_coding
ENSG0000(	ENSG0000(	37.2544	0.281681	0.042377	0.100111	lncRNA
ENSG0000(	ENSG0000(	39.38157	-0.24529	0.042385	0.10012	lncRNA
ENSG0000(	CRYAB	88.84088	-0.3352	0.042445	0.100255	protein_coding
ENSG0000(	PPP2R5CP	21.41595	0.352655	0.042458	0.100278	processed_pseudogene
ENSG0000(	ENSG0000(	20.05111	0.284838	0.042478	0.100311	lncRNA
ENSG0000(	LGALS12	20.02527	0.440393	0.042477	0.100311	protein_coding
ENSG0000(	IGHV1-67	9.505884	0.751159	0.042498	0.10035	IG_V_pseudogene
ENSG0000(	AUH	469.0324	-0.12082	0.042506	0.100351	protein_coding
ENSG0000(	ENSG0000(	155.7961	-0.17102	0.042506	0.100351	TEC
ENSG0000(	ENSG0000(	115.4058	0.185557	0.042508	0.100351	lncRNA
ENSG0000(	NPAP1P6	16.31312	0.686847	0.042522	0.100376	processed_pseudogene
ENSG0000(	FIG4	3764.532	0.07115	0.042536	0.100401	protein_coding
ENSG0000(	ENSG0000(	41.42661	-0.25426	0.042549	0.100425	lncRNA
ENSG0000(	NME1	344.8142	-0.19548	0.042565	0.100456	protein_coding
ENSG0000(	ENSG0000(	226.0526	0.222499	0.042574	0.100461	lncRNA
ENSG0000(	PINLYP	316.7728	0.129336	0.042572	0.100461	protein_coding

ENSG0000(	TRBV29-1	45.53764	-0.30278	0.042583	0.100476	TR_V_gene
ENSG0000(	CORIN	498.9897	0.100786	0.042625	0.100544	protein_coding
ENSG0000(	CYP7A1	13.58872	0.39986	0.04262	0.100544	protein_coding
ENSG0000(	ENSG0000(	35.79561	0.321983	0.042624	0.100544	lncRNA
ENSG0000(	SPDYE16	58.59646	0.330998	0.042619	0.100544	protein_coding
ENSG0000(	CCNO	13.20268	0.658893	0.04264	0.100572	protein_coding
ENSG0000(	ZFAS1	1663.351	-0.10621	0.042651	0.100591	lncRNA
ENSG0000(	TTK	202.3667	-0.2866	0.04267	0.100626	protein_coding
ENSG0000(	RN7SL448F	9.848131	0.417251	0.04268	0.100643	misc_RNA
ENSG0000(	MYB	1775.512	-0.2549	0.042705	0.100689	protein_coding
ENSG0000(	PKD2	776.6808	0.181874	0.042706	0.100689	protein_coding
ENSG0000(	ELOCP19	158.7524	0.192136	0.042746	0.100761	processed_pseudogene
ENSG0000(	ENSG0000(	30.85586	-0.36715	0.042743	0.100761	lncRNA
ENSG0000(	LAMA2	1310.232	0.163534	0.042746	0.100761	protein_coding
ENSG0000(	VCP	4455.821	-0.11791	0.042759	0.100785	protein_coding
ENSG0000(	DLK2	58.93048	-0.18405	0.042787	0.10084	protein_coding
ENSG0000(	ENSG0000(	61.19865	-0.21571	0.042789	0.10084	lncRNA
ENSG0000(	RN7SKP205	9.444286	0.424297	0.042836	0.100942	misc_RNA
ENSG0000(	SELENOI	2191.811	-0.12251	0.042896	0.101077	protein_coding
ENSG0000(	AKTIP	783.578	-0.11466	0.042917	0.101118	protein_coding
ENSG0000(	PENK-AS1	2156.828	-0.15982	0.042922	0.101122	lncRNA
ENSG0000(	ZP2	62.73534	0.234114	0.042925	0.101122	protein_coding
ENSG0000(	RN7SL170F	8.744317	-0.48208	0.042939	0.101147	misc_RNA
ENSG0000(	AQP11	74.14793	-0.38107	0.042968	0.101208	protein_coding
ENSG0000(	CDHR5	5492.484	-0.38128	0.042975	0.101216	protein_coding
ENSG0000(	ENSG0000(	165.3754	0.167841	0.042984	0.101222	lncRNA
ENSG0000(	ENSG0000(	230.8666	0.246447	0.042981	0.101222	lncRNA
ENSG0000(	ENSG0000(	39.54067	0.248525	0.042991	0.101231	lncRNA
ENSG0000(	HPN-AS1	158.634	0.169568	0.043007	0.101262	lncRNA
ENSG0000(	BMP4	522.6497	-0.26558	0.043032	0.101314	protein_coding
ENSG0000(	POP4	329.8868	-0.11661	0.043037	0.101317	protein_coding
ENSG0000(	LINC02249	215.0449	0.12633	0.043042	0.10132	lncRNA
ENSG0000(	IBA57	1807.312	0.077461	0.043057	0.101349	protein_coding
ENSG0000(	KMT5A	1002.403	-0.10438	0.043064	0.101353	protein_coding
ENSG0000(	RNU4ATAC	136.1524	0.249	0.043065	0.101353	snRNA
ENSG0000(	ENSG0000(	592.8653	0.117616	0.043103	0.101434	transcribed_unprocessed_pseudo
ENSG0000(	ENSG0000(	31.57873	0.520399	0.043157	0.101555	lncRNA
ENSG0000(	MYCN	29.29007	-0.39904	0.043266	0.101803	protein_coding
ENSG0000(	DOCK1	5447.935	-0.22099	0.043277	0.101821	protein_coding
ENSG0000(	TM2D1	1965.312	-0.09071	0.043292	0.101849	protein_coding
ENSG0000(	RYR2	338.9074	0.509556	0.043297	0.101853	protein_coding
ENSG0000(	ENSG0000(	14.86881	0.481618	0.043336	0.101937	lncRNA
ENSG0000(	FKBP1B	88.0927	0.286646	0.04334	0.101937	protein_coding
ENSG0000(	ENSG0000(	64.46725	0.245756	0.043358	0.101972	lncRNA
ENSG0000(	MUC17	3039.084	0.447805	0.043373	0.102002	protein_coding
ENSG0000(	NECAP1	1883.944	-0.12031	0.043387	0.102027	protein_coding
ENSG0000(	SLC49A3	193.5412	0.209958	0.043456	0.10218	protein_coding

ENSG0000(	MAF	1564.595	0.167935	0.043487	0.102247	protein_coding
ENSG0000(	LINC02773	13.05456	0.449668	0.043495	0.102257	lncRNA
ENSG0000(	ENSG0000(	17.31343	0.44767	0.043522	0.102307	TEC
ENSG0000(	MIR194-2H	5093.554	-0.25659	0.043523	0.102307	lncRNA
ENSG0000(	LUADT1	80.58305	-0.2057	0.043531	0.102318	lncRNA
ENSG0000(	RN7SL344F	55.03801	0.224244	0.043567	0.102395	misc_RNA
ENSG0000(	ENSG0000(	20.89107	0.45321	0.043578	0.102413	lncRNA
ENSG0000(	ENSG0000(	9.995327	0.527966	0.043601	0.10246	lncRNA
ENSG0000(	ENSG0000(	27.31817	-0.43796	0.043618	0.102493	lncRNA
ENSG0000(	ENSG0000(	58.54134	0.296791	0.043682	0.102634	lncRNA
ENSG0000(	ENSG0000(	14.42374	0.631817	0.043685	0.102634	lncRNA
ENSG0000(	RPL36AP16	60.78187	0.265436	0.0437	0.102661	processed_pseudogene
ENSG0000(	ENSG0000(	298.2259	-0.1499	0.043705	0.102665	lncRNA
ENSG0000(	LINC01732	23.69189	0.257516	0.04375	0.102764	lncRNA
ENSG0000(	TRIM44	3653.355	-0.10003	0.043766	0.102794	protein_coding
ENSG0000(	ENSG0000(	44.78877	-0.344	0.043774	0.102804	lncRNA
ENSG0000(	ISL2	26.36842	0.368977	0.043818	0.102901	protein_coding
ENSG0000(	ENSG0000(	87.98915	0.215662	0.043836	0.102934	lncRNA
ENSG0000(	MOXD1	106.7357	0.337811	0.043864	0.102992	protein_coding
ENSG0000(	PP2672	85.52341	-0.20512	0.04387	0.102999	lncRNA
ENSG0000(	ENSG0000(	339.7162	0.148623	0.043881	0.10301	lncRNA
ENSG0000(	ENSG0000(	84.32633	0.255335	0.043882	0.10301	transcribed_unprocessed_pseudo
ENSG0000(	THOC1	1143.075	-0.11722	0.043884	0.10301	protein_coding
ENSG0000(	ERICH1	2542.167	0.167663	0.04389	0.103016	protein_coding
ENSG0000(	PIGCP1	8.658661	0.62101	0.043894	0.103016	processed_pseudogene
ENSG0000(	CRYBB2	11.10615	-0.76187	0.043902	0.103029	protein_coding
ENSG0000(	MRTFA	1262.268	0.129332	0.043906	0.10303	protein_coding
ENSG0000(	SRXN1	321.4774	-0.26073	0.04391	0.103031	protein_coding
ENSG0000(	ENSG0000(	479.2643	0.108549	0.043916	0.103038	lncRNA
ENSG0000(	RN7SL865F	14.67031	0.361308	0.043934	0.103071	misc_RNA
ENSG0000(	LINC00449	99.69396	-0.1932	0.043951	0.103104	lncRNA
ENSG0000(	BSCL2	850.0483	-0.10024	0.043966	0.103131	protein_coding
ENSG0000(	LARS2	1254.581	-0.10522	0.04399	0.103171	protein_coding
ENSG0000(	MIR200B	38.79292	-0.40954	0.04399	0.103171	miRNA
ENSG0000(	RB1CC1	2113.378	0.083292	0.044006	0.103201	protein_coding
ENSG0000(	ENSG0000(	204.5996	-0.14496	0.044019	0.103217	TEC
ENSG0000(	ENSG0000(	56.55169	0.218432	0.044018	0.103217	TEC
ENSG0000(	CDR2	580.4124	-0.1243	0.04403	0.103228	protein_coding
ENSG0000(	FAM86C1P	509.5155	0.106086	0.04403	0.103228	transcribed_unprocessed_pseudo
ENSG0000(	IMPDH1P8	29.76964	-0.30675	0.044036	0.103233	processed_pseudogene
ENSG0000(	RNU4-86P	58.25774	-0.23084	0.044048	0.103246	snRNA
ENSG0000(	UGT2B4	54.89731	-0.22438	0.044045	0.103246	protein_coding
ENSG0000(	TSGA13	158.3059	-0.15525	0.044057	0.103259	protein_coding
ENSG0000(	MTG2	1504.884	0.062814	0.044064	0.103268	protein_coding
ENSG0000(	ROGDI	271.2239	-0.20902	0.04407	0.103275	protein_coding
ENSG0000(	SNORD116	11.97076	0.516456	0.044088	0.103309	snoRNA
ENSG0000(	ENSG0000(	253.7847	0.133499	0.044122	0.103381	TEC

ENSG0000(	FOXL1	243.2492	-0.29437	0.044196	0.103546	protein_coding
ENSG0000(	CDC73	2135.434	-0.0566	0.044221	0.103584	protein_coding
ENSG0000(	GRHL3	25.59541	0.70531	0.044222	0.103584	protein_coding
ENSG0000(	ST8SIA1	1185.3	0.148717	0.044215	0.103584	protein_coding
ENSG0000(	KLKP1	25.40704	0.289861	0.044265	0.103675	transcribed_unprocessed_pseudo
ENSG0000(	PTPRD-AS1	470.3333	-0.10992	0.044267	0.103675	lncRNA
ENSG0000(	CCND3	1120.773	0.35211	0.044278	0.103692	protein_coding
ENSG0000(	SNORA73B	6892.73	-0.22745	0.044285	0.1037	snoRNA
ENSG0000(	ENSG0000(	76.31624	-0.1894	0.044301	0.10373	lncRNA
ENSG0000(	RN7SL91P	26.83964	0.288296	0.044311	0.103747	misc_RNA
ENSG0000(	STK19	378.4228	0.111749	0.044333	0.10379	protein_coding
ENSG0000(	RASSF1	753.1211	0.136006	0.04435	0.103823	protein_coding
ENSG0000(	ENSG0000(	85.0341	-0.16333	0.044358	0.103833	lncRNA
ENSG0000(	CDH8-AS1	114.5707	-0.17518	0.044363	0.103838	lncRNA
ENSG0000(	DACH2	31.80051	-0.29562	0.044397	0.103908	protein_coding
ENSG0000(	DUS2	281.2769	-0.14606	0.044427	0.103955	protein_coding
ENSG0000(	ENSG0000(	54.88495	-0.19989	0.044424	0.103955	lncRNA
ENSG0000(	ENSG0000(	71.64316	0.199469	0.044427	0.103955	lncRNA
ENSG0000(	ENSG0000(	535.614	-0.08693	0.044441	0.10398	lncRNA
ENSG0000(	ENSG0000(	217.9075	0.161845	0.044451	0.103988	lncRNA
ENSG0000(	NFIA-AS2	719.0132	0.110184	0.04445	0.103988	lncRNA
ENSG0000(	TCIM	816.6638	0.370568	0.044459	0.104	protein_coding
ENSG0000(	LAMB2	1358.787	0.228881	0.044472	0.104021	protein_coding
ENSG0000(	RNASE6	112.7842	0.350068	0.044489	0.104054	protein_coding
ENSG0000(	ENSG0000(	28.36306	0.277087	0.044507	0.104088	lncRNA
ENSG0000(	YES1P1	100.6874	0.351294	0.044522	0.104115	processed_pseudogene
ENSG0000(	SGTB	630.994	0.151527	0.044529	0.104117	protein_coding
ENSG0000(	TNFSF9	27.21957	0.259312	0.044528	0.104117	protein_coding
ENSG0000(	SCML2	137.7662	-0.29239	0.044549	0.104156	protein_coding
ENSG0000(	CELF3	59.26374	-0.30309	0.044591	0.104229	protein_coding
ENSG0000(	ENSG0000(	16.6194	0.356234	0.044588	0.104229	lncRNA
ENSG0000(	STOX2	854.8073	-0.28666	0.044587	0.104229	protein_coding
ENSG0000(	ENSG0000(	204.6915	-0.14776	0.044609	0.104251	lncRNA
ENSG0000(	ENSG0000(	13.97185	0.355267	0.044606	0.104251	lncRNA
ENSG0000(	SH3TC2-DT	36.26001	0.554488	0.04461	0.104251	lncRNA
ENSG0000(	LINC00571	102.6881	0.18431	0.044635	0.104302	lncRNA
ENSG0000(	FRG1HP	1392.272	0.120741	0.044639	0.104303	transcribed_unprocessed_pseudo
ENSG0000(	ENSG0000(	395.6896	0.123326	0.044671	0.104371	TEC
ENSG0000(	PDPK1	3201.759	-0.08238	0.044705	0.104441	protein_coding
ENSG0000(	MOSMO	568.571	0.114206	0.044709	0.104442	protein_coding
ENSG0000(	C2orf49	941.3044	-0.07573	0.044752	0.104528	protein_coding
ENSG0000(	ENSG0000(	258.3391	0.147255	0.044752	0.104528	lncRNA
ENSG0000(	NBPF10	1784.689	-0.21816	0.044763	0.104547	protein_coding
ENSG0000(	RNU6-39P	12.23874	0.438082	0.044801	0.104626	snRNA
ENSG0000(	OPCML	18.332	0.971632	0.044824	0.10467	protein_coding
ENSG0000(	RNU6-1237	12.93105	0.382255	0.044826	0.10467	snRNA
ENSG0000(	DNM1P51	640.7528	0.168913	0.044861	0.104743	transcribed_unprocessed_pseudo

ENSG0000(	MEOX2	26.66273	0.627074	0.044873	0.104764	protein_coding
ENSG0000(	ZNF962P	14.69367	-0.53044	0.04488	0.104773	unprocessed_pseudogene
ENSG0000(	SENP8	463.2564	-0.12514	0.044893	0.104795	protein_coding
ENSG0000(	L3MBTL1	1697.62	0.098503	0.04491	0.104828	protein_coding
ENSG0000(	LINC01180	189.1763	-0.12947	0.044924	0.104853	lncRNA
ENSG0000(	ENSG0000(	77.01363	-0.19294	0.044944	0.104883	lncRNA
ENSG0000(	LINC02476	157.6877	-0.17694	0.044944	0.104883	lncRNA
ENSG0000(	ENSG0000(	23.53806	0.310029	0.04495	0.104889	lncRNA
ENSG0000(	MFSD6L	81.84897	-0.24848	0.044959	0.104901	protein_coding
ENSG0000(	ENSG0000(	337.9094	0.09496	0.04499	0.104968	lncRNA
ENSG0000(	ENSG0000(	10.11279	-0.67288	0.044997	0.104975	processed_pseudogene
ENSG0000(	CBR3	14.90317	0.498162	0.045008	0.104979	protein_coding
ENSG0000(	ENSG0000(	217.3809	0.112851	0.045005	0.104979	lncRNA
ENSG0000(	UXT-AS1	747.267	0.070667	0.045009	0.104979	lncRNA
ENSG0000(	ZSWIM3	177.884	-0.20022	0.045017	0.10499	protein_coding
ENSG0000(	ENSG0000(	144.0276	0.19955	0.045022	0.104994	lncRNA
ENSG0000(	TLCD1	96.75888	-0.18778	0.045025	0.104995	protein_coding
ENSG0000(	CIAO2A	585.1784	-0.18504	0.045042	0.10502	protein_coding
ENSG0000(	PPP1R14A	34.46239	-0.37062	0.045043	0.10502	protein_coding
ENSG0000(	HLA-A	9244.623	0.240532	0.045065	0.105063	protein_coding
ENSG0000(	FEM1C	1906.715	-0.17544	0.045069	0.105064	protein_coding
ENSG0000(	ENSG0000(	707.2036	-0.09047	0.045085	0.105094	lncRNA
ENSG0000(	ENSG0000(	13.0115	-0.53926	0.045093	0.105105	unprocessed_pseudogene
ENSG0000(	ANO1	357.8751	-0.25371	0.045127	0.105176	protein_coding
ENSG0000(	KCTD11	257.3548	0.17858	0.045131	0.105179	protein_coding
ENSG0000(	IGHV1-3	314.5845	0.699464	0.045164	0.105248	IG_V_gene
ENSG0000(	ENSG0000(	383.2175	0.187991	0.045177	0.105263	lncRNA
ENSG0000(	POU6F2	103.221	0.253028	0.045178	0.105263	protein_coding
ENSG0000(	PPIAP53	10.87604	0.470696	0.045181	0.105263	processed_pseudogene
ENSG0000(	MEPCE	661.111	-0.11713	0.045213	0.105321	protein_coding
ENSG0000(	TMEM150E	237.3453	-0.20987	0.04521	0.105321	protein_coding
ENSG0000(	PRAF2	81.16957	0.298385	0.045227	0.105346	protein_coding
ENSG0000(	RN7SL113F	57.4846	0.241819	0.045245	0.10538	misc_RNA
ENSG0000(	ENSG0000(	118.1756	-0.3919	0.045282	0.105451	lncRNA
ENSG0000(	ENSG0000(	33.78324	-0.38811	0.045281	0.105451	lncRNA
ENSG0000(	CD14	241.4533	0.318241	0.045292	0.105467	protein_coding
ENSG0000(	ENSG0000(	1541.298	0.14031	0.045312	0.105505	protein_coding
ENSG0000(	ABCA9	903.3376	0.132783	0.045357	0.105585	protein_coding
ENSG0000(	ADAM15	1118.846	-0.17365	0.045356	0.105585	protein_coding
ENSG0000(	MND1	22.76066	-0.45681	0.045361	0.105585	protein_coding
ENSG0000(	RCBTB2	466.7491	0.173438	0.045361	0.105585	protein_coding
ENSG0000(	SLC9A8	1459.887	0.135042	0.045363	0.105585	protein_coding
ENSG0000(	LINC01797	19.29355	0.486748	0.045367	0.105586	lncRNA
ENSG0000(	LRIG3	1588.61	-0.21772	0.045373	0.105593	protein_coding
ENSG0000(	KLHDC2	1211.575	-0.15312	0.045389	0.105622	protein_coding
ENSG0000(	TPCN1	4185.451	-0.14311	0.045419	0.105683	protein_coding
ENSG0000(	MTIF3	468.5656	-0.10223	0.045454	0.105756	protein_coding

ENSG0000(	HEPH	7843.78	-0.25204	0.045479	0.105808	protein_coding
ENSG0000(	PPIH	1287.589	-0.0859	0.045489	0.105824	protein_coding
ENSG0000(	BRINP3	346.3183	-0.35618	0.045502	0.105845	protein_coding
ENSG0000(	OR6A2	93.08012	0.351111	0.045578	0.106015	protein_coding
ENSG0000(	TRPM8	211.7312	-0.20043	0.045582	0.106016	protein_coding
ENSG0000(	MICAL3	7464.314	-0.16318	0.045592	0.10603	protein_coding
ENSG0000(	CHERP	770.8115	-0.10185	0.045596	0.106032	protein_coding
ENSG0000(	LINC02059	21.09351	0.501474	0.045603	0.106042	lncRNA
ENSG0000(	CRTC2	751.1266	-0.12688	0.045629	0.106086	protein_coding
ENSG0000(	SCFD2	618.1499	-0.20663	0.045629	0.106086	protein_coding
ENSG0000(	LIG1	699.7098	-0.21279	0.045646	0.106116	protein_coding
ENSG0000(	ALS2	3214.707	0.07028	0.045659	0.106131	protein_coding
ENSG0000(	SNN	278.5661	0.320412	0.045657	0.106131	protein_coding
ENSG0000(	UTP14A	322.8439	-0.14696	0.045668	0.106145	protein_coding
ENSG0000(	EIF1B-AS1	3513.148	0.078777	0.045673	0.106147	lncRNA
ENSG0000(	HEPACAM2	817.7049	-0.42141	0.045676	0.106147	protein_coding
ENSG0000(	LINC01031	33.92037	0.243017	0.045723	0.106248	lncRNA
ENSG0000(	RN7SKP16(	26.31809	0.351697	0.045727	0.10625	misc_RNA
ENSG0000(	STAU1	1719.137	-0.11214	0.045744	0.106281	protein_coding
ENSG0000(	ENSG0000(	99.2925	0.172406	0.04576	0.106306	lncRNA
ENSG0000(	LINC01228	76.55323	0.184071	0.045761	0.106306	lncRNA
ENSG0000(	FAM66B	282.7536	0.200162	0.045768	0.106315	lncRNA
ENSG0000(	ENSG0000(	25.98749	-0.3294	0.045821	0.106423	TEC
ENSG0000(	HSPA13	381.8096	0.20799	0.045822	0.106423	protein_coding
ENSG0000(	ENSG0000(	140.1687	0.145805	0.045848	0.106475	lncRNA
ENSG0000(	MIR3936H(	2327.329	-0.19061	0.04587	0.106518	lncRNA
ENSG0000(	CEBPZOS	1290.398	-0.09284	0.045876	0.106526	protein_coding
ENSG0000(	LRRC77P	107.8665	0.284765	0.045898	0.10653	transcribed_unitary_pseudogene
ENSG0000(	PXK	972.3778	0.178441	0.045893	0.10653	protein_coding
ENSG0000(	SPCS3	3158.724	0.133257	0.045897	0.10653	protein_coding
ENSG0000(	SUZ12P1	1420.201	-0.07561	0.045899	0.10653	transcribed_unprocessed_pseudo
ENSG0000(	TAS2R13	214.1674	0.249639	0.045893	0.10653	protein_coding
ENSG0000(	TMEM150(	301.411	-0.20471	0.045894	0.10653	protein_coding
ENSG0000(	PGLS-DT	446.1535	-0.08542	0.045913	0.106556	lncRNA
ENSG0000(	PER1	1165.733	0.334798	0.045918	0.10656	protein_coding
ENSG0000(	PPM1J	33.05712	0.411732	0.045948	0.106622	protein_coding
ENSG0000(	RUNDC3A-/	890.2634	-0.06474	0.046	0.106733	lncRNA
ENSG0000(	CASTOR2	760.224	0.100741	0.046063	0.106873	protein_coding
ENSG0000(	APBA3	794.1783	-0.10979	0.046079	0.106888	protein_coding
ENSG0000(	ENSG0000(	246.154	-0.11971	0.04608	0.106888	lncRNA
ENSG0000(	SCARNA5	2349.18	-0.16105	0.046079	0.106888	scaRNA
ENSG0000(	ENSG0000(	185.043	0.466917	0.046097	0.106918	unprocessed_pseudogene
ENSG0000(	UPK1A	9.354333	0.454085	0.046104	0.106927	protein_coding
ENSG0000(	ENSG0000(	58.29188	-0.2215	0.046124	0.106965	pseudogene
ENSG0000(	BCORP1	152.5449	0.199897	0.046131	0.106974	transcribed_unprocessed_pseudo
ENSG0000(	ENSG0000(	67.66005	0.240349	0.046138	0.106983	lncRNA
ENSG0000(	ENSG0000(	75.21049	-0.17093	0.046161	0.107012	lncRNA

ENSG0000(	KAT6A	5402.897	0.076117	0.046161	0.107012	protein_coding
ENSG0000(	TNRC18P1	143.1562	0.213326	0.046154	0.107012	processed_pseudogene
ENSG0000(	SOX30	14.07635	0.393559	0.046169	0.107022	protein_coding
ENSG0000(	PTGFRN	1708.354	-0.22007	0.04618	0.10704	protein_coding
ENSG0000(	ENSG0000(	58.05299	-0.63316	0.046193	0.107062	lncRNA
ENSG0000(	GREP1	319.9357	0.142472	0.046209	0.107085	protein_coding
ENSG0000(	TMED5	2069.567	-0.12418	0.046209	0.107085	protein_coding
ENSG0000(	ENSG0000(	44.58228	-0.25942	0.046242	0.107153	transcribed_unprocessed_pseudo
ENSG0000(	CSNK1G1	2004.682	0.115075	0.046246	0.107154	protein_coding
ENSG0000(	CACNG2	19.47456	0.539157	0.046288	0.107242	protein_coding
ENSG0000(	LRP2	87.4652	0.386933	0.046342	0.10736	protein_coding
ENSG0000(	CACNA1D	1908.713	-0.19756	0.04638	0.10744	protein_coding
ENSG0000(	DOC2GP	40.80142	-0.33017	0.046387	0.107449	transcribed_unitary_pseudogene
ENSG0000(	LINC02057	160.3617	0.147207	0.046393	0.107455	lncRNA
ENSG0000(	FAM87A	27.36671	0.400002	0.0464	0.107462	lncRNA
ENSG0000(	URAD	202.2087	-0.36868	0.046428	0.10752	protein_coding
ENSG0000(	ANGPTL6	31.96307	0.421961	0.046437	0.107533	protein_coding
ENSG0000(	B4GALNT3	2650.069	-0.27902	0.04645	0.107539	protein_coding
ENSG0000(	TPR	4864.652	-0.09405	0.046447	0.107539	protein_coding
ENSG0000(	TRUB1	278.0236	-0.20561	0.046449	0.107539	protein_coding
ENSG0000(	OR11A1	564.5261	0.188237	0.046489	0.10762	protein_coding
ENSG0000(	FUT1	505.1399	0.138696	0.046499	0.107637	protein_coding
ENSG0000(	ZNF132-DT	968.674	0.087365	0.046556	0.10776	lncRNA
ENSG0000(	RECQL4	248.1494	-0.29095	0.046561	0.107763	protein_coding
ENSG0000(	DCHS1	1329.969	0.220681	0.046578	0.107795	protein_coding
ENSG0000(	ENSG0000(	100.1897	-0.29951	0.046597	0.10783	lncRNA
ENSG0000(	TPM3	8992.195	-0.12496	0.046646	0.107936	protein_coding
ENSG0000(	TCP10L3	196.6188	0.234739	0.046668	0.107979	transcribed_unprocessed_pseudo
ENSG0000(	AIDA	1152.38	0.135812	0.046682	0.108003	protein_coding
ENSG0000(	LMAN1L	15.0482	0.65814	0.046696	0.108028	protein_coding
ENSG0000(	UBR4	12376.56	-0.07157	0.046728	0.108093	protein_coding
ENSG0000(	DPY19L3-D	48.92823	0.211508	0.046744	0.108122	lncRNA
ENSG0000(	SLC5A7	8.966601	-0.58026	0.046764	0.108162	protein_coding
ENSG0000(	DEPDC7	148.475	-0.2988	0.046773	0.108174	protein_coding
ENSG0000(	HOXB-AS4	12.60893	0.604291	0.046804	0.108239	lncRNA
ENSG0000(	ENSG0000(	323.1411	-0.10701	0.046866	0.108372	TEC
ENSG0000(	PPARG	5337.45	-0.29269	0.046902	0.108448	protein_coding
ENSG0000(	BCAM	539.597	-0.11383	0.046909	0.108456	protein_coding
ENSG0000(	CR1L	31.34722	0.595816	0.046927	0.108481	protein_coding
ENSG0000(	MS4A2	610.4702	0.229194	0.046926	0.108481	protein_coding
ENSG0000(	RPS2P36	31.88332	0.472913	0.046947	0.10852	processed_pseudogene
ENSG0000(	CEP131	205.4909	-0.21799	0.046972	0.108571	protein_coding
ENSG0000(	ENSG0000(	18.61406	-0.30932	0.047011	0.108652	lncRNA
ENSG0000(	ENSG0000(	67.83912	0.216793	0.047033	0.108696	TEC
ENSG0000(	WARS2-AS1	5228.713	0.112064	0.047061	0.108746	lncRNA
ENSG0000(	ZNF749	770.2029	-0.20287	0.047062	0.108746	protein_coding
ENSG0000(	ENSG0000(	188.0447	0.177081	0.047077	0.108773	lncRNA

ENSG0000(	GPC6	422.0402	0.279024	0.04711	0.108841	protein_coding
ENSG0000(	SYTL4	2299.827	-0.19337	0.047127	0.108871	protein_coding
ENSG0000(	ENSG0000(	26.93913	0.243209	0.047136	0.108884	lncRNA
ENSG0000(	ENSG0000(	112.7929	0.218049	0.047154	0.108918	lncRNA
ENSG0000(	ALDH5A1	1016.91	-0.12925	0.047165	0.108936	protein_coding
ENSG0000(	CERKL	130.8053	-0.18527	0.047177	0.108955	protein_coding
ENSG0000(	PLBD2	668.6282	0.163526	0.047193	0.108983	protein_coding
ENSG0000(	IGKV1-16	246.446	0.519889	0.047221	0.109041	IG_V_gene
ENSG0000(	ENSG0000(	17.59453	0.376986	0.047247	0.109093	lncRNA
ENSG0000(	LIMS3	89.21148	0.408353	0.047262	0.10912	protein_coding
ENSG0000(	LINC01179	157.7721	-0.18768	0.047277	0.109146	lncRNA
ENSG0000(	SPOP	1647.131	0.05722	0.047281	0.109148	protein_coding
ENSG0000(	SCAMP1-A!	622.2354	0.110814	0.047288	0.109155	lncRNA
ENSG0000(	MAP3K9	1136.238	-0.13855	0.047295	0.109163	protein_coding
ENSG0000(	NSMAF	1107.279	-0.11273	0.047347	0.109276	protein_coding
ENSG0000(	TRIM14	2750.021	0.131837	0.047355	0.109286	protein_coding
ENSG0000(	RPN1	1811.13	-0.12853	0.047363	0.109295	protein_coding
ENSG0000(	ENSG0000(	307.6757	0.102035	0.047388	0.109337	protein_coding
ENSG0000(	PATJ	6604.078	-0.18021	0.047388	0.109337	protein_coding
ENSG0000(	MIER2	383.5211	0.124367	0.047405	0.10937	protein_coding
ENSG0000(	ANXA13	548.1838	-0.15144	0.047419	0.109393	protein_coding
ENSG0000(	ENSG0000(	213.6414	-0.17173	0.047435	0.109422	lncRNA
ENSG0000(	DNAJB6P7	182.8608	0.151839	0.047447	0.109442	processed_pseudogene
ENSG0000(	TP63	106.4196	-0.17865	0.047461	0.109465	protein_coding
ENSG0000(	EIF1B	241.4445	-0.15663	0.047469	0.109474	protein_coding
ENSG0000(	MAP2K6	5573.634	0.232898	0.047472	0.109474	protein_coding
ENSG0000(	TNKS1BP1	2936.775	0.17036	0.047519	0.109574	protein_coding
ENSG0000(	ZNF614	326.8661	-0.14094	0.047576	0.1097	protein_coding
ENSG0000(	CEP97	1140.997	0.149206	0.047583	0.109706	protein_coding
ENSG0000(	SHROOM4	1470.352	0.149292	0.047592	0.109719	protein_coding
ENSG0000(	SSH2	4225.317	0.101672	0.047597	0.109722	protein_coding
ENSG0000(	CDPF1	84.08639	-0.21424	0.047616	0.109758	protein_coding
ENSG0000(	SHROOM1	2004.67	-0.25595	0.047643	0.109812	protein_coding
ENSG0000(	EMC7	245.0896	-0.17256	0.047665	0.109852	protein_coding
ENSG0000(	ENSG0000(	46.48282	-0.2341	0.047667	0.109852	lncRNA
ENSG0000(	PGAM1P6	47.44051	-0.21875	0.04767	0.109852	processed_pseudogene
ENSG0000(	UACA	2558.352	0.156216	0.047676	0.109857	protein_coding
ENSG0000(	IGLV1-40	381.0645	0.56392	0.047686	0.109866	IG_V_gene
ENSG0000(	LINC02374	23.1359	-0.3041	0.047687	0.109866	lncRNA
ENSG0000(	ENSG0000(	10.93754	-0.39126	0.047694	0.10987	lncRNA
ENSG0000(	ENSG0000(	9.457572	0.522428	0.047696	0.10987	lncRNA
ENSG0000(	ENSG0000(	44.17751	-0.23758	0.047726	0.109932	lncRNA
ENSG0000(	SPNS1	901.6455	-0.07661	0.04776	0.110002	protein_coding
ENSG0000(	PPRC1	1250.5	-0.19299	0.04778	0.110039	protein_coding
ENSG0000(	ZNF117	1518.844	0.121509	0.047808	0.110095	protein_coding
ENSG0000(	ENSG0000(	137.2927	-0.16506	0.047827	0.110125	lncRNA
ENSG0000(	PMF1	488.5897	-0.09071	0.047828	0.110125	protein_coding

ENSG0000(	HAND2	43.00054	0.442535	0.047832	0.110127	protein_coding
ENSG0000(	ZNF470	1235.22	-0.0668	0.047842	0.110141	protein_coding
ENSG0000(	DHRS13	194.8088	0.207519	0.047847	0.110144	protein_coding
ENSG0000(	ILRUN-AS1	17.39441	-0.39769	0.04786	0.110167	lncRNA
ENSG0000(	KCNT2	179.8899	0.310701	0.047892	0.110233	protein_coding
ENSG0000(	PARP6	2544.117	0.076443	0.047908	0.11026	protein_coding
ENSG0000(	ENSG0000(	120.0332	0.293436	0.047923	0.110271	lncRNA
ENSG0000(	EPHB3	643.187	-0.33176	0.047917	0.110271	protein_coding
ENSG0000(	SOSTDC1	120.4792	-0.4438	0.04792	0.110271	protein_coding
ENSG0000(	ENSG0000(	252.399	0.112012	0.04794	0.110301	lncRNA
ENSG0000(	RNU4ATAC	43.63257	0.307778	0.047944	0.110302	snRNA
ENSG0000(	IGLV1-41	24.6231	0.904129	0.047956	0.110316	IG_V_pseudogene
ENSG0000(	PTOV1-AS2	221.5137	-0.13967	0.047953	0.110316	lncRNA
ENSG0000(	MBLAC1	39.12149	-0.30602	0.047963	0.110323	protein_coding
ENSG0000(	DPRXP2	46.20438	-0.25842	0.047975	0.110342	transcribed_processed_pseudoge
ENSG0000(	TMEM170E	327.063	0.15801	0.048029	0.110459	protein_coding
ENSG0000(	ENSG0000(	67.96234	-0.25493	0.048067	0.110536	lncRNA
ENSG0000(	MCPH1-AS	1379.759	0.08292	0.048077	0.110536	lncRNA
ENSG0000(	SI	1132.607	-0.43788	0.048077	0.110536	protein_coding
ENSG0000(	UFSP2	258.3447	-0.19796	0.048071	0.110536	protein_coding
ENSG0000(	RN7SL690F	258.45	0.128136	0.048081	0.110537	misc_RNA
ENSG0000(	ENSG0000(	112.7061	-0.24355	0.048085	0.110538	transcribed_unprocessed_pseudo
ENSG0000(	ENSG0000(	226.591	0.152473	0.048091	0.110545	TEC
ENSG0000(	ENSG0000(	34.073	-0.40376	0.048099	0.110554	lncRNA
ENSG0000(	HNRNPA1P	57.46431	0.366711	0.048113	0.110578	protein_coding
ENSG0000(	TNFRSF13E	189.8804	0.338749	0.048141	0.110634	protein_coding
ENSG0000(	ZBTB4	1560.396	0.131166	0.048154	0.110657	protein_coding
ENSG0000(	SLC16A12	233.3466	-0.11515	0.0482	0.110754	protein_coding
ENSG0000(	ARNT2	501.4311	0.131378	0.048212	0.110773	protein_coding
ENSG0000(	TYRO3	1439.817	-0.08185	0.048225	0.110795	protein_coding
ENSG0000(	GALNTL5	23.57353	0.313344	0.048239	0.110811	protein_coding
ENSG0000(	PLEKHA2	2589.131	0.163904	0.048238	0.110811	protein_coding
ENSG0000(	EDEM2	401.901	-0.13274	0.048253	0.110835	protein_coding
ENSG0000(	ENSG0000(	58.81173	0.260929	0.048256	0.110835	processed_pseudogene
ENSG0000(	GIHCG	159.6789	0.145634	0.048313	0.110953	lncRNA
ENSG0000(	RIT2	30.32168	0.275085	0.048315	0.110953	protein_coding
ENSG0000(	PCED1A	664.5851	0.176356	0.048328	0.110969	protein_coding
ENSG0000(	RSPH10B	326.0102	-0.09689	0.048329	0.110969	protein_coding
ENSG0000(	PSEN2	3234.302	-0.11339	0.048335	0.110974	protein_coding
ENSG0000(	CDHR3	853.2809	-0.16448	0.048348	0.110997	protein_coding
ENSG0000(	ENSG0000(	13.74024	-0.4293	0.048366	0.111028	lncRNA
ENSG0000(	ADNP-AS1	53.63297	-0.20387	0.048377	0.111047	lncRNA
ENSG0000(	ENSG0000(	49.55408	0.218407	0.048387	0.111062	lncRNA
ENSG0000(	DFFA	2627.017	-0.06128	0.048399	0.111079	protein_coding
ENSG0000(	ENSG0000(	19.53571	0.340622	0.048402	0.111079	lncRNA
ENSG0000(	ENSG0000(	194.0795	0.21219	0.048416	0.111102	lncRNA
ENSG0000(	ENSG0000(	342.8319	0.104626	0.048419	0.111103	lncRNA

ENSG0000(	PTK6	2186.271	-0.24413	0.048469	0.111208	protein_coding
ENSG0000(	C20orf204	122.8337	-0.19537	0.048493	0.111255	protein_coding
ENSG0000(	KLK3	143.118	-0.44389	0.048529	0.111331	protein_coding
ENSG0000(	HIGD1AP14	24.76957	0.491155	0.048576	0.111429	processed_pseudogene
ENSG0000(	ENSG0000(	238.8755	-0.16166	0.048581	0.111434	lncRNA
ENSG0000(	CACNA1F	154.3049	-0.16617	0.04859	0.111434	protein_coding
ENSG0000(	ENSG0000(	18.58727	0.334623	0.048592	0.111434	TEC
ENSG0000(	PYCARD	150.099	-0.26959	0.048592	0.111434	protein_coding
ENSG0000(	ZNF671	314.4152	0.212698	0.048611	0.111469	protein_coding
ENSG0000(	EI24	1132.287	-0.0923	0.048637	0.111519	protein_coding
ENSG0000(	RPS2P46	10.31242	-0.83393	0.048664	0.111574	processed_pseudogene
ENSG0000(	ENSG0000(	27.159	-0.39277	0.048669	0.111578	lncRNA
ENSG0000(	SIX5	195.8131	0.197281	0.048689	0.111616	protein_coding
ENSG0000(	HLA-DRB6	289.2505	0.444111	0.048714	0.111656	transcribed_unprocessed_pseudo
ENSG0000(	NOL6	782.8113	-0.13446	0.048714	0.111656	protein_coding
ENSG0000(	SWAP70	1565.137	0.188944	0.048743	0.111714	protein_coding
ENSG0000(	LRRC29	80.75358	0.221442	0.048775	0.111779	transcribed_unitary_pseudogene
ENSG0000(	GABPB2	3816.992	-0.06081	0.048859	0.111954	protein_coding
ENSG0000(	JRK	1786.039	0.112911	0.048857	0.111954	protein_coding
ENSG0000(	LINC01564	10.48618	-0.80021	0.048862	0.111954	lncRNA
ENSG0000(	ENSG0000(	187.5231	-0.18014	0.048892	0.112007	lncRNA
ENSG0000(	ENSG0000(	163.5015	0.122367	0.04889	0.112007	lncRNA
ENSG0000(	SH2D4A	589.3777	-0.21233	0.0489	0.112017	protein_coding
ENSG0000(	HDAC10	608.668	-0.12868	0.048915	0.112042	protein_coding
ENSG0000(	ENSG0000(	107.6018	-0.14589	0.048944	0.1121	lncRNA
ENSG0000(	LHFPL2	2540.458	-0.15902	0.048986	0.112189	protein_coding
ENSG0000(	LRRFIP1P1	265.7086	0.170758	0.049022	0.112264	processed_pseudogene
ENSG0000(	OR52V1P	11.18365	0.483866	0.049082	0.112391	unprocessed_pseudogene
ENSG0000(	ENSG0000(	35.10621	0.352219	0.049113	0.112454	lncRNA
ENSG0000(	LSM3	414.8808	-0.10722	0.049129	0.112485	protein_coding
ENSG0000(	CTSW	148.7551	-0.16355	0.049139	0.11249	protein_coding
ENSG0000(	RORB-AS1	60.26209	-0.20252	0.049136	0.11249	lncRNA
ENSG0000(	ENSG0000(	80.50172	-0.21823	0.049146	0.112498	lncRNA
ENSG0000(	CEP70	796.7523	-0.19583	0.049208	0.112625	protein_coding
ENSG0000(	LINC01771	13.93184	0.400563	0.049209	0.112625	lncRNA
ENSG0000(	ENSG0000(	62.0105	-1.21359	0.049252	0.112715	protein_coding
ENSG0000(	ENSG0000(	537.2818	-0.09776	0.049268	0.112743	lncRNA
ENSG0000(	PAFAH1B2	2058.406	-0.08216	0.049302	0.112813	protein_coding
ENSG0000(	ZNF587	6549.751	-0.1006	0.049309	0.11282	protein_coding
ENSG0000(	ENSG0000(	63.4725	0.207334	0.04934	0.11287	lncRNA
ENSG0000(	ENSG0000(	54.45862	0.24701	0.049341	0.11287	lncRNA
ENSG0000(	RN7SL644P	183.27	-0.14975	0.049336	0.11287	misc_RNA
ENSG0000(	FNBP1L	2087.628	-0.16569	0.049349	0.112881	protein_coding
ENSG0000(	RAI2	100.5361	0.293634	0.049353	0.112881	protein_coding
ENSG0000(	ENSG0000(	16.33651	0.438654	0.049363	0.112896	snoRNA
ENSG0000(	SENP6	4029.31	-0.07863	0.049368	0.112899	protein_coding
ENSG0000(	GPR107	2575.966	0.14198	0.049389	0.112939	protein_coding

ENSG0000(	ENSG0000(	10.45674	0.444099	0.049408	0.112973	lncRNA
ENSG0000(	C3orf18	165.0691	0.170193	0.049412	0.112974	protein_coding
ENSG0000(	FMR1-AS1	25.22713	0.309699	0.049433	0.113015	lncRNA
ENSG0000(	ENSG0000(	10.65127	0.555725	0.049447	0.113039	lncRNA
ENSG0000(	DYNLT4	17.52547	0.384783	0.049476	0.113096	protein_coding
ENSG0000(	EMX1	19.52246	-0.63109	0.049487	0.113109	protein_coding
ENSG0000(	ENSG0000(	18.2184	-0.46045	0.049489	0.113109	TEC
ENSG0000(	ENSG0000(	288.5263	0.123975	0.04952	0.113171	lncRNA
ENSG0000(	C1orf220	723.6549	-0.15785	0.049569	0.113274	lncRNA
ENSG0000(	PRR5-ARH(	153.7666	-0.2423	0.04959	0.113316	protein_coding
ENSG0000(	CEACAM19	625.7186	-0.12627	0.049598	0.113322	protein_coding
ENSG0000(	RETREG1	1633.948	0.169474	0.0496	0.113322	protein_coding
ENSG0000(	NDUFB5	1283.066	-0.15241	0.049642	0.113409	protein_coding
ENSG0000(	METTL21C	186.9637	0.220252	0.049689	0.113509	protein_coding
ENSG0000(	AMPD3	1148.213	0.156457	0.04972	0.113547	protein_coding
ENSG0000(	ENSG0000(	16.25041	0.587234	0.049719	0.113547	processed_pseudogene
ENSG0000(	ENSG0000(	217.9322	0.109738	0.049717	0.113547	TEC
ENSG0000(	PLPP1	892.7492	-0.2323	0.049721	0.113547	protein_coding
ENSG0000(	FAM13A	2629.665	-0.2171	0.049734	0.113568	protein_coding
ENSG0000(	DIPK2A	447.3439	0.186255	0.049745	0.113585	protein_coding
ENSG0000(	RCOR3	2151.596	0.088615	0.049758	0.113608	protein_coding
ENSG0000(	NPR1	89.39038	0.421973	0.049766	0.113617	protein_coding
ENSG0000(	ENSG0000(	105.8371	0.468864	0.04978	0.11364	lncRNA
ENSG0000(	MBL1P	51.89922	0.317694	0.049783	0.11364	transcribed_unitary_pseudogene
ENSG0000(	CTRL	22.25904	0.382233	0.049811	0.113693	protein_coding
ENSG0000(	ENSG0000(	65.13528	0.206667	0.049813	0.113693	lncRNA
ENSG0000(	ENSG0000(	542.7462	-0.07731	0.049818	0.113694	lncRNA
ENSG0000(	BOK-AS1	49.69057	0.247967	0.049856	0.113774	lncRNA
ENSG0000(	ENSG0000(	14.6104	0.50494	0.049864	0.113783	lncRNA
ENSG0000(	ENSG0000(	16.05951	0.427573	0.049872	0.113785	lncRNA
ENSG0000(	IGHV3OR1(	16.91355	0.683767	0.049869	0.113785	IG_V_gene
ENSG0000(	ATP1A1	9936.928	-0.2551	0.049903	0.113846	protein_coding
ENSG0000(	ZNF460	2303.268	0.095067	0.049906	0.113846	protein_coding
ENSG0000(	IGHV3-38	12.89029	0.638285	0.049939	0.113913	IG_V_gene
ENSG0000(	OR4K16P	79.60944	0.157299	0.049952	0.113934	transcribed_unprocessed_pseudo
ENSG0000(	ENSG0000(	91.2549	0.173733	0.049995	0.114023	lncRNA
ENSG0000(	CFAP97	1296.577	0.09057	0.050015	0.114053	protein_coding
ENSG0000(	R3HDM2	2315.492	-0.08543	0.050012	0.114053	protein_coding
ENSG0000(	LINC01410	349.7142	0.191007	0.050034	0.114087	lncRNA
ENSG0000(	UBP1	3445.55	-0.10711	0.050072	0.114149	protein_coding
ENSG0000(	ZBED6	6115.042	0.087079	0.05007	0.114149	protein_coding
ENSG0000(	ZNF599	390.2223	-0.08768	0.050065	0.114149	protein_coding
ENSG0000(	LINC02675	109.0639	0.181709	0.050078	0.114156	lncRNA
ENSG0000(	GSG1L2	266.0715	0.099861	0.050109	0.114201	protein_coding
ENSG0000(	ICAM2	338.9413	0.164884	0.050102	0.114201	protein_coding
ENSG0000(	LINC00520	266.7768	-0.13118	0.050107	0.114201	lncRNA
ENSG0000(	RNU7-124F	19.39338	0.419252	0.050122	0.114221	snRNA

ENSG0000(	COL16A1	1260.162	0.230347	0.050131	0.114235	protein_coding
ENSG0000(	ENSG0000(	132.9915	-0.14254	0.05014	0.114246	lncRNA
ENSG0000(	KCMF1	1638.914	-0.1381	0.050157	0.114276	protein_coding
ENSG0000(	LINC00273	71.01975	0.624665	0.050184	0.114331	lncRNA
ENSG0000(	ENSG0000(	12.31674	-0.58251	0.05019	0.114335	processed_pseudogene
ENSG0000(	CCDC169	862.3336	0.105169	0.050201	0.114352	protein_coding
ENSG0000(	SCARNA21I	9.468825	0.456695	0.050252	0.114459	scaRNA
ENSG0000(	FXYD7	8.396354	0.628428	0.050277	0.114508	protein_coding
ENSG0000(	ACTR3C	2074.811	-0.14983	0.050286	0.114521	protein_coding
ENSG0000(	KCTD15	482.9807	-0.17762	0.050311	0.11457	protein_coding
ENSG0000(	ENSG0000(	219.6851	-0.17055	0.050331	0.114603	lncRNA
ENSG0000(	SDHA	5155.814	-0.11817	0.050333	0.114603	protein_coding
ENSG0000(	ENSG0000(	95.64887	-0.18817	0.050349	0.114631	lncRNA
ENSG0000(	BRPF3-AS1	200.3801	0.116015	0.050387	0.114692	lncRNA
ENSG0000(	ENSG0000(	110.2344	-0.13054	0.050382	0.114692	lncRNA
ENSG0000(	ENSG0000(	142.0926	-0.13779	0.050387	0.114692	lncRNA
ENSG0000(	CIP2A	325.7203	-0.24635	0.050407	0.114728	protein_coding
ENSG0000(	TMEM26	131.3657	0.272466	0.050425	0.114762	protein_coding
ENSG0000(	ENSG0000(	141.8796	-0.16048	0.050429	0.114763	lncRNA
ENSG0000(	CNPY2	1305.238	0.081528	0.050445	0.114792	protein_coding
ENSG0000(	ENSG0000(	20.0581	-0.35482	0.050487	0.114874	lncRNA
ENSG0000(	ZNF664	3877.751	-0.12498	0.050489	0.114874	protein_coding
ENSG0000(	PPP1R7	396.983	-0.12935	0.050514	0.114921	protein_coding
ENSG0000(	ENSG0000(	299.0748	0.155488	0.05053	0.114943	TEC
ENSG0000(	ZNF112	264.7252	-0.17415	0.05053	0.114943	protein_coding
ENSG0000(	ENSG0000(	191.6353	-0.12502	0.050558	0.114997	lncRNA
ENSG0000(	FOXK2	1675.386	-0.10238	0.05058	0.115038	protein_coding
ENSG0000(	LINC02253	367.9981	0.142523	0.050583	0.115038	lncRNA
ENSG0000(	DMXL1-DT	118.0131	-0.18269	0.050594	0.11504	lncRNA
ENSG0000(	GPS1	624.3663	-0.14186	0.050595	0.11504	protein_coding
ENSG0000(	MTRF1L	721.9136	-0.15635	0.050591	0.11504	protein_coding
ENSG0000(	ENSG0000(	486.4173	0.185428	0.050612	0.115071	lncRNA
ENSG0000(	HAX1	398.9975	-0.15999	0.050648	0.115144	protein_coding
ENSG0000(	RSF1-IT2	86.55373	0.147988	0.050676	0.115199	lncRNA
ENSG0000(	CANX	8355.439	-0.17871	0.050686	0.115213	protein_coding
ENSG0000(	C12orf76	457.4273	-0.12219	0.05071	0.115259	protein_coding
ENSG0000(	SPAST	1415.743	-0.07709	0.050714	0.11526	protein_coding
ENSG0000(	RNA5SP47:	29.76518	0.199905	0.050775	0.115391	rRNA_pseudogene
ENSG0000(	ENSG0000(	124.3735	0.230153	0.05079	0.115416	lncRNA
ENSG0000(	RPEP3	41.28292	0.281146	0.050798	0.115426	processed_pseudogene
ENSG0000(	LINC02176	103.3657	-0.17552	0.050842	0.115509	lncRNA
ENSG0000(	ZNF586	2219.214	0.096772	0.050838	0.115509	protein_coding
ENSG0000(	EEF2KMT	140.4392	-0.17511	0.050847	0.115513	protein_coding
ENSG0000(	ENSG0000(	30.64806	0.353867	0.050857	0.115527	lncRNA
ENSG0000(	RTN4IP1	378.9225	-0.23396	0.050901	0.115618	protein_coding
ENSG0000(	LINC01975	12.17057	-0.60629	0.050971	0.115769	lncRNA
ENSG0000(	TMEM160	26.18846	-0.30182	0.051017	0.115865	protein_coding

ENSG0000(	ENSG0000(	14.86017	0.426102	0.051029	0.115884	lncRNA
ENSG0000(	ENSG0000(	12.76646	0.435237	0.051057	0.115908	snoRNA
ENSG0000(	ING2	140.7929	-0.21192	0.051061	0.115908	protein_coding
ENSG0000(	LINC00378	40.55223	-0.30922	0.051048	0.115908	lncRNA
ENSG0000(	NANOGNB	28.59951	-0.30963	0.051053	0.115908	protein_coding
ENSG0000(	RAB28	386.8381	-0.18631	0.051052	0.115908	protein_coding
ENSG0000(	ZNF43	1927.9	-0.08814	0.051062	0.115908	protein_coding
ENSG0000(	ACKR3	159.2089	0.28524	0.05107	0.115919	protein_coding
ENSG0000(	DUX4	47.28873	0.386323	0.051088	0.115951	protein_coding
ENSG0000(	RSL24D1	847.1011	-0.10605	0.051101	0.115962	protein_coding
ENSG0000(	ZSCAN30	2000.827	0.093664	0.051101	0.115962	protein_coding
ENSG0000(	ENSG0000(	44.11517	0.231738	0.051136	0.11603	lncRNA
ENSG0000(	TET2-AS1	25.86772	-0.30322	0.051138	0.11603	lncRNA
ENSG0000(	ANKRD18B	76.1157	-0.34606	0.051159	0.116069	protein_coding
ENSG0000(	ENSG0000(	47.75915	0.286857	0.051191	0.116133	lncRNA
ENSG0000(	UBE2E1	821.2059	-0.13448	0.051199	0.116142	protein_coding
ENSG0000(	GON4L	3563.246	0.071066	0.051206	0.116143	protein_coding
ENSG0000(	MYRF	858.2557	0.268115	0.051206	0.116143	protein_coding
ENSG0000(	ENSG0000(	155.3393	0.117414	0.051218	0.11616	lncRNA
ENSG0000(	PLXNA2	4450.401	-0.26057	0.051229	0.116177	protein_coding
ENSG0000(	SRRM4	220.1156	0.224551	0.051233	0.116179	protein_coding
ENSG0000(	DGCR11	447.4236	0.114066	0.05125	0.116207	lncRNA
ENSG0000(	ADCY6-DT	12.90804	-0.46694	0.051264	0.116223	lncRNA
ENSG0000(	BANK1	812.3297	0.603629	0.051264	0.116223	protein_coding
ENSG0000(	ENSG0000(	489.5996	0.123871	0.051295	0.116277	lncRNA
ENSG0000(	PAQR3	677.7415	0.157644	0.051295	0.116277	protein_coding
ENSG0000(	ADAM28	4255.12	0.184338	0.051333	0.116354	protein_coding
ENSG0000(	LAMC3	554.746	0.195908	0.051346	0.116376	protein_coding
ENSG0000(	EFCAB6-DT	51.59894	0.206159	0.051351	0.116379	lncRNA
ENSG0000(	PCSK6	2902.369	-0.21806	0.051372	0.116417	protein_coding
ENSG0000(	VSIR	1245.458	0.186267	0.051393	0.116455	protein_coding
ENSG0000(	CYP1B1-AS	2284.864	0.146796	0.051418	0.116497	lncRNA
ENSG0000(	UNGP1	107.3668	-0.1564	0.051416	0.116497	processed_pseudogene
ENSG0000(	TRIM8-DT	23.2129	0.267568	0.051435	0.116525	lncRNA
ENSG0000(	VTRNA2-1	12.61456	-0.9496	0.051451	0.116554	vault_RNA
ENSG0000(	CHCHD7	833.4743	-0.09805	0.051455	0.116555	protein_coding
ENSG0000(	ENSG0000(	76.88731	0.185819	0.051479	0.116601	lncRNA
ENSG0000(	ENSG0000(	302.4327	0.216861	0.05157	0.116798	lncRNA
ENSG0000(	ENSG0000(	154.0215	0.280797	0.05158	0.116813	lncRNA
ENSG0000(	RN7SL715F	22.9694	0.360589	0.051588	0.116813	misc_RNA
ENSG0000(	UROD	268.4647	-0.14375	0.051585	0.116813	protein_coding
ENSG0000(	SOCS7	1230.676	-0.09496	0.0516	0.116833	protein_coding
ENSG0000(	SMIM31	926.4322	-0.15157	0.051629	0.116889	protein_coding
ENSG0000(	ENSG0000(	49.52065	-0.24832	0.051661	0.116954	lncRNA
ENSG0000(	RN7SL255F	24.57992	0.365832	0.05168	0.116989	misc_RNA
ENSG0000(	RNF216P1	1229.067	0.090713	0.051724	0.117079	lncRNA
ENSG0000(	ENSG0000(	45.43313	0.226878	0.05175	0.11713	lncRNA

ENSG0000(	HROB	74.76935	-0.26	0.051816	0.11727	protein_coding
ENSG0000(	SOGA1	2338.277	0.115769	0.05183	0.117293	protein_coding
ENSG0000(	RAB5A	1203.443	0.120833	0.051846	0.117322	protein_coding
ENSG0000(	AZIN2	1366.558	0.261969	0.051908	0.117408	protein_coding
ENSG0000(	CMC4	48.14731	-0.54125	0.051893	0.117408	protein_coding
ENSG0000(	ENSG0000(	10.24572	0.48773	0.051897	0.117408	processed_pseudogene
ENSG0000(	LYRM9	224.1519	0.194142	0.051905	0.117408	protein_coding
ENSG0000(	SNHG17	2436.624	-0.0919	0.05189	0.117408	lncRNA
ENSG0000(	TMEM63C	292.885	-0.46297	0.051911	0.117408	protein_coding
ENSG0000(	ZNF358	219.6761	-0.20407	0.051902	0.117408	protein_coding
ENSG0000(	RADX	139.4432	0.178815	0.051916	0.117411	protein_coding
ENSG0000(	ENSG0000(	43.25074	0.243876	0.051939	0.117455	lncRNA
ENSG0000(	MYBPC1	38.69041	-0.83246	0.051959	0.117492	protein_coding
ENSG0000(	DBP	705.731	-0.14266	0.052039	0.117653	protein_coding
ENSG0000(	ENSG0000(	79.8843	0.311103	0.052045	0.117653	TEC
ENSG0000(	GHDC	318.1622	-0.13292	0.052035	0.117653	protein_coding
ENSG0000(	PRRT3	255.8942	0.117704	0.052042	0.117653	protein_coding
ENSG0000(	WFDC8	104.2779	-0.15976	0.052074	0.117709	protein_coding
ENSG0000(	SRP54-AS1	372.0335	-0.12023	0.052082	0.11772	transcribed_unitary_pseudogene
ENSG0000(	ENSG0000(	1078.52	0.088217	0.052087	0.117721	lncRNA
ENSG0000(	PJA2	3287.3	0.078918	0.052094	0.117728	protein_coding
ENSG0000(	GNA14	279.9286	0.210782	0.052132	0.117805	protein_coding
ENSG0000(	GOLGA8S	11.03515	0.540845	0.052135	0.117805	protein_coding
ENSG0000(	DDN	10.21494	0.562258	0.052185	0.11791	protein_coding
ENSG0000(	ENSG0000(	13.00694	0.414258	0.052198	0.117927	lncRNA
ENSG0000(	ENSG0000(	16.09246	-0.50479	0.052204	0.117927	lncRNA
ENSG0000(	MMRN1	133.7073	0.554541	0.052204	0.117927	protein_coding
ENSG0000(	ADAM33	582.3646	0.260966	0.052221	0.117956	protein_coding
ENSG0000(	RN7SL757F	11.59974	0.361156	0.052288	0.118098	misc_RNA
ENSG0000(	CDC25B	1139.831	0.227354	0.052294	0.118105	protein_coding
ENSG0000(	SP2	540.4268	0.138362	0.052309	0.118129	protein_coding
ENSG0000(	ENSG0000(	169.6598	-0.15612	0.052354	0.118211	processed_pseudogene
ENSG0000(	ENSG0000(	29.08574	0.282859	0.052361	0.118211	lncRNA
ENSG0000(	ENSG0000(	22.79873	0.366897	0.052368	0.118211	lncRNA
ENSG0000(	ENSG0000(	171.605	-0.16232	0.05236	0.118211	processed_pseudogene
ENSG0000(	ENSG0000(	502.9917	-0.1252	0.052366	0.118211	lncRNA
ENSG0000(	TENM2	68.01225	-0.58746	0.05236	0.118211	protein_coding
ENSG0000(	CREB3L4	226.8994	-0.14365	0.052382	0.118236	protein_coding
ENSG0000(	CASC9	527.3478	-0.20303	0.052417	0.118304	lncRNA
ENSG0000(	ENSG0000(	315.3927	0.318537	0.052434	0.118336	TEC
ENSG0000(	HUS1	1414.338	-0.12672	0.052443	0.118338	protein_coding
ENSG0000(	NRG4	214.0838	-0.25695	0.05244	0.118338	protein_coding
ENSG0000(	ENSG0000(	34.65534	0.360949	0.052459	0.118348	lncRNA
ENSG0000(	IGLV2-5	26.77688	0.540013	0.052453	0.118348	IG_V_pseudogene
ENSG0000(	LYSMD3	1071.098	-0.12138	0.052457	0.118348	protein_coding
ENSG0000(	ERVMER34	86.77586	-0.17398	0.052529	0.118498	protein_coding
ENSG0000(	ENSG0000(	250.8386	0.123808	0.052547	0.118531	lncRNA

ENSG0000(	LINC02478	17.78992	0.409076	0.052559	0.118532	lncRNA
ENSG0000(	SPATA5	1822.683	-0.12543	0.052559	0.118532	protein_coding
ENSG0000(	UBE3A	4725.555	0.06443	0.052559	0.118532	protein_coding
ENSG0000(	TNPO1	4090.011	-0.0896	0.052583	0.118577	protein_coding
ENSG0000(	AP1M1	3597.126	0.104136	0.052595	0.118592	protein_coding
ENSG0000(	TFRC	5782.315	-0.13257	0.052597	0.118592	protein_coding
ENSG0000(	TAS2R50	84.4608	0.263027	0.052617	0.118628	protein_coding
ENSG0000(	PITPNC1	948.0841	0.233952	0.052632	0.118654	protein_coding
ENSG0000(	LIPH	2330.473	-0.2921	0.052638	0.118657	protein_coding
ENSG0000(	ZNF271P	1109.917	0.072752	0.052647	0.118669	transcribed_unitary_pseudogene
ENSG0000(	SNORC	470.1033	-0.21989	0.052667	0.118707	protein_coding
ENSG0000(	ENSG0000(	23.51327	0.454203	0.052679	0.118725	lncRNA
ENSG0000(	KIF27	554.9124	-0.1241	0.052702	0.118759	protein_coding
ENSG0000(	KIF3B	2312.016	-0.13054	0.052703	0.118759	protein_coding
ENSG0000(	SLC4A1AP	1097.479	-0.07191	0.052705	0.118759	protein_coding
ENSG0000(	ENSG0000(	21.25888	0.410108	0.052718	0.11878	lncRNA
ENSG0000(	ENSG0000(	123.738	0.127403	0.052742	0.118815	lncRNA
ENSG0000(	PPM1A	3449.263	-0.15466	0.052738	0.118815	protein_coding
ENSG0000(	ITGB5	821.9705	-0.1748	0.052753	0.118832	protein_coding
ENSG0000(	LMX1B	251.8875	0.158397	0.052774	0.118867	protein_coding
ENSG0000(	TNFAIP8L1	1106.781	0.075022	0.052776	0.118867	protein_coding
ENSG0000(	B3GNT4	52.56404	0.2531	0.052801	0.118914	protein_coding
ENSG0000(	RAB42	274.982	0.11888	0.052815	0.118937	protein_coding
ENSG0000(	KLHL4	50.39189	0.25989	0.052872	0.119041	protein_coding
ENSG0000(	RNU2-6P	45.74044	0.274516	0.052871	0.119041	snRNA
ENSG0000(	RNU6-196F	10.53157	0.40667	0.052872	0.119041	snRNA
ENSG0000(	CARM1	851.9818	-0.11177	0.052893	0.119067	protein_coding
ENSG0000(	CBARP	14.97786	0.50935	0.052899	0.119067	protein_coding
ENSG0000(	NIPAL1	1522.045	-0.24118	0.052889	0.119067	protein_coding
ENSG0000(	POMZP3	221.8484	-0.1465	0.052896	0.119067	protein_coding
ENSG0000(	SIRT7	2058.225	-0.16222	0.052905	0.119071	protein_coding
ENSG0000(	PTPRR	801.2277	-0.45463	0.052914	0.119082	protein_coding
ENSG0000(	ENSG0000(	29.01724	0.251805	0.052934	0.119111	lncRNA
ENSG0000(	RN7SL127F	73.58628	0.214085	0.052933	0.119111	misc_RNA
ENSG0000(	FNDC11	11.98676	0.477218	0.052954	0.119148	protein_coding
ENSG0000(	ENSG0000(	116.6521	0.15234	0.05301	0.119266	lncRNA
ENSG0000(	BBIP1	1153.684	0.104455	0.053032	0.119307	protein_coding
ENSG0000(	ZFYVE26	3080.656	0.071668	0.053038	0.11931	protein_coding
ENSG0000(	HNMT	924.0555	-0.11321	0.05305	0.119325	protein_coding
ENSG0000(	RNU2-4P	9.919363	-1.68375	0.053052	0.119325	snRNA
ENSG0000(	PDE11A	668.4085	0.170678	0.053059	0.119332	protein_coding
ENSG0000(	GGT6	1895.251	-0.25256	0.053068	0.119343	protein_coding
ENSG0000(	GTF2F2P1	11.37376	0.593356	0.053087	0.119377	processed_pseudogene
ENSG0000(	M1AP	70.37897	0.18328	0.053095	0.119387	protein_coding
ENSG0000(	PTENP1	25.82061	0.352031	0.053099	0.119388	transcribed_processed_pseudoge
ENSG0000(	G3BP1P1	32.19874	-0.29541	0.05311	0.119403	processed_pseudogene
ENSG0000(	ENSG0000(	174.8585	0.258344	0.053114	0.119404	TEC

ENSG0000(	RN7SKP134	27.80327	-0.29984	0.053127	0.119424	misc_RNA
ENSG0000(	RIC8A	1123.325	-0.09964	0.053237	0.119663	protein_coding
ENSG0000(	NHEJ1	1436.89	-0.22346	0.053246	0.119676	protein_coding
ENSG0000(	CCDC116	21.7887	-0.39724	0.053303	0.119795	protein_coding
ENSG0000(	IST1	3617.864	-0.08708	0.053313	0.119808	protein_coding
ENSG0000(	BOK	202.7407	-0.26536	0.053322	0.11982	protein_coding
ENSG0000(	ENSG0000(	847.9034	0.254174	0.053338	0.119848	lncRNA
ENSG0000(	PDE1C	1002.684	0.187411	0.053345	0.119856	protein_coding
ENSG0000(	CCNQ	122.2816	-0.17109	0.053352	0.119861	protein_coding
ENSG0000(	CRY2	1298.551	-0.1036	0.053356	0.119863	protein_coding
ENSG0000(	ENSG0000(	10.70763	0.488767	0.053373	0.119877	lncRNA
ENSG0000(	ENSG0000(	15.17227	-0.53829	0.053374	0.119877	miRNA
ENSG0000(	METTL14	1254.694	-0.09595	0.053373	0.119877	protein_coding
ENSG0000(	EML5	373.1935	-0.15569	0.053384	0.119891	protein_coding
ENSG0000(	ENSG0000(	491.6577	-0.09785	0.053412	0.119946	TEC
ENSG0000(	ENSG0000(	64.16253	0.171034	0.053435	0.119988	lncRNA
ENSG0000(	SCN4B	133.5962	-0.28277	0.053461	0.120037	protein_coding
ENSG0000(	ARHGAP18	1226.716	-0.16516	0.053465	0.120038	protein_coding
ENSG0000(	DPY19L2	151.0618	0.295912	0.053499	0.120106	protein_coding
ENSG0000(	ENSG0000(	56.05307	0.205465	0.05352	0.120135	lncRNA
ENSG0000(	ZNF730	54.10343	-0.35901	0.053518	0.120135	protein_coding
ENSG0000(	IDH3A	2071.637	-0.1347	0.053524	0.120136	protein_coding
ENSG0000(	LILRA4	16.9555	0.629366	0.053614	0.12033	protein_coding
ENSG0000(	ZNF808	2552.014	-0.13407	0.053639	0.120376	protein_coding
ENSG0000(	FABP3	47.25845	-0.56959	0.05367	0.120437	protein_coding
ENSG0000(	BMI1	675.5267	-0.16522	0.053682	0.120457	protein_coding
ENSG0000(	ATP10B	10760.25	0.239278	0.053717	0.120527	protein_coding
ENSG0000(	RAB6A	2151.706	-0.11084	0.053723	0.120531	protein_coding
ENSG0000(	ENSG0000(	676.2542	0.079343	0.05373	0.120538	lncRNA
ENSG0000(	MED23	1578.512	-0.08429	0.053834	0.120762	protein_coding
ENSG0000(	SLC39A13	390.5184	0.175936	0.05384	0.120766	protein_coding
ENSG0000(	ENSG0000(	174.195	0.148933	0.053885	0.120859	lncRNA
ENSG0000(	EPS15	2991.161	0.089957	0.05391	0.120906	protein_coding
ENSG0000(	NRIP3-DT	217.1433	0.142445	0.053918	0.120916	lncRNA
ENSG0000(	ENSG0000(	11.32876	-0.55182	0.053937	0.120944	transcribed_unprocessed_pseudo
ENSG0000(	LINC02125	27.00714	0.303189	0.053938	0.120944	lncRNA
ENSG0000(	ENSG0000(	15.63799	0.366904	0.053973	0.121006	lncRNA
ENSG0000(	SIM2	289.8067	0.139248	0.053971	0.121006	protein_coding
ENSG0000(	ENSG0000(	273.5697	-0.24829	0.054019	0.1211	lncRNA
ENSG0000(	ENSG0000(	12.28127	0.43691	0.054025	0.121104	lncRNA
ENSG0000(	GJB7	33.73292	0.410824	0.054036	0.121119	protein_coding
ENSG0000(	ENSG0000(	14.92721	-0.34967	0.054054	0.121152	lncRNA
ENSG0000(	ENSG0000(	33.91979	-0.2394	0.054153	0.121364	lncRNA
ENSG0000(	ENSG0000(	135.8379	-0.1733	0.054235	0.121539	TEC
ENSG0000(	SLAIN1	70.09486	0.354135	0.054244	0.121552	protein_coding
ENSG0000(	ERICD	16.93846	0.372011	0.054251	0.121553	lncRNA
ENSG0000(	RN7SL151F	482.4154	0.328876	0.054253	0.121553	misc_RNA

ENSG0000(	ENSG0000(	43.13745	-0.19958	0.05428	0.121605	lncRNA
ENSG0000(	BTNL3	1486.542	-0.3991	0.054295	0.121632	protein_coding
ENSG0000(	HSPE1P26	98.75338	0.209126	0.054302	0.121637	processed_pseudogene
ENSG0000(	B3GALT1-A	13.19584	0.424859	0.054321	0.121672	lncRNA
ENSG0000(	MFN1	2566.788	-0.11428	0.054326	0.121674	protein_coding
ENSG0000(	GDF11	444.0863	0.170386	0.054451	0.121945	protein_coding
ENSG0000(	PITX2	1009.43	-0.48346	0.054493	0.12203	protein_coding
ENSG0000(	TUBD1	620.9754	-0.10952	0.05452	0.122081	protein_coding
ENSG0000(	ENSG0000(	28.15222	0.395164	0.054528	0.122093	lncRNA
ENSG0000(	ENSG0000(	26.57763	-0.23819	0.054535	0.122099	lncRNA
ENSG0000(	ENSG0000(	54.30401	0.236337	0.054542	0.1221	lncRNA
ENSG0000(	UGP2	3445.333	-0.21014	0.054543	0.1221	protein_coding
ENSG0000(	RAB3A	36.80734	-0.2999	0.054587	0.122189	protein_coding
ENSG0000(	CD72	252.6089	0.433662	0.054598	0.122196	protein_coding
ENSG0000(	ENSG0000(	21.73413	-0.28868	0.054598	0.122196	lncRNA
ENSG0000(	LINC01952	37.40153	0.234522	0.054603	0.122199	lncRNA
ENSG0000(	RIMS4	19.11916	-0.42742	0.054627	0.122235	protein_coding
ENSG0000(	RNU5D-1	20.12524	0.50057	0.054626	0.122235	snRNA
ENSG0000(	ENSG0000(	17.16821	0.411915	0.054656	0.122272	processed_pseudogene
ENSG0000(	ENSG0000(	32.7122	0.257838	0.054651	0.122272	lncRNA
ENSG0000(	RAB19	775.8612	-0.1021	0.054655	0.122272	protein_coding
ENSG0000(	RER1	2840.234	-0.08425	0.054672	0.1223	protein_coding
ENSG0000(	ATP6V0A1	3644.794	-0.09163	0.05469	0.122332	protein_coding
ENSG0000(	ENSG0000(	22.74475	-0.47961	0.054702	0.12235	lncRNA
ENSG0000(	MED10	255.2513	0.102689	0.054719	0.122379	protein_coding
ENSG0000(	ENSG0000(	592.4524	0.076589	0.054728	0.122381	lncRNA
ENSG0000(	ENSG0000(	66.54753	-0.17465	0.054724	0.122381	lncRNA
ENSG0000(	PSIP1	1007.235	0.120838	0.054738	0.122395	protein_coding
ENSG0000(	ENSG0000(	12.77572	0.448762	0.054743	0.122398	lncRNA
ENSG0000(	TMEM63A	5905.177	-0.13995	0.054765	0.122438	protein_coding
ENSG0000(	DNM3	240.6481	0.161945	0.054807	0.122504	protein_coding
ENSG0000(	ENSG0000(	19.70342	-0.35763	0.054809	0.122504	lncRNA
ENSG0000(	RASA2	1767.597	0.116373	0.054804	0.122504	protein_coding
ENSG0000(	SERTM2	11.40637	0.71216	0.05481	0.122504	protein_coding
ENSG0000(	PPP2R2C	616.3091	0.331244	0.054815	0.122505	protein_coding
ENSG0000(	SPACA5	17.54778	0.369979	0.054822	0.122512	protein_coding
ENSG0000(	COX11	1647.116	-0.14817	0.05484	0.122539	protein_coding
ENSG0000(	PUS10	905.4773	-0.12681	0.054841	0.122539	protein_coding
ENSG0000(	TPD52L1	943.5481	0.09061	0.054847	0.122542	protein_coding
ENSG0000(	PEX7	420.1445	-0.11061	0.054859	0.122562	protein_coding
ENSG0000(	PLEKHB2	3074.182	-0.13168	0.054866	0.122564	protein_coding
ENSG0000(	TSGA10	492.1687	-0.14329	0.054868	0.122564	protein_coding
ENSG0000(	KIAA0319L	2916.669	0.08319	0.054882	0.122586	protein_coding
ENSG0000(	ENSG0000(	101.612	0.15213	0.054928	0.122679	lncRNA
ENSG0000(	LINC00880	54.56797	0.173669	0.054974	0.122768	lncRNA
ENSG0000(	NEK1	1529.562	0.075931	0.054991	0.122768	protein_coding
ENSG0000(	NME9	101.3122	0.149027	0.054987	0.122768	protein_coding

ENSG0000(	PCDHB12	26.00664	-0.32846	0.054988	0.122768	protein_coding
ENSG0000(	RINL	506.2359	0.120708	0.054986	0.122768	protein_coding
ENSG0000(	TAS2R14	260.6583	0.185514	0.054982	0.122768	protein_coding
ENSG0000(	LINC01846	12.99498	0.385406	0.055012	0.122806	lncRNA
ENSG0000(	PDLIM1	2060.295	-0.17631	0.055016	0.122806	protein_coding
ENSG0000(	SLC25A19	532.2169	-0.13199	0.055052	0.122878	protein_coding
ENSG0000(	EIF3I	2754.681	-0.07876	0.055057	0.12288	protein_coding
ENSG0000(	NFAT5	9279.079	0.091254	0.055079	0.122921	protein_coding
ENSG0000(	ENSG0000(	4316.3	-0.1634	0.0551	0.12296	TEC
ENSG0000(	OR10AE1P	22.43991	0.312947	0.055126	0.123008	unprocessed_pseudogene
ENSG0000(	C2orf49-DT	217.7392	0.130766	0.055258	0.123294	lncRNA
ENSG0000(	KCTD9P1	49.61748	0.278952	0.05528	0.123323	transcribed_processed_pseudoge
ENSG0000(	MYADM-AS	18.85166	-0.39559	0.055287	0.123323	lncRNA
ENSG0000(	TBC1D2	557.5972	-0.16898	0.055279	0.123323	protein_coding
ENSG0000(	YIPF7	82.65769	-0.24251	0.055284	0.123323	protein_coding
ENSG0000(	TMEM86B	106.9047	-0.20384	0.055296	0.123334	protein_coding
ENSG0000(	LINC01422	1341.154	0.089786	0.055336	0.123415	lncRNA
ENSG0000(	ENSG0000(	252.6881	-0.11373	0.055365	0.12347	lncRNA
ENSG0000(	FCRL6	62.09585	-0.38298	0.055385	0.123499	protein_coding
ENSG0000(	RREB1	8669.474	-0.10505	0.055385	0.123499	protein_coding
ENSG0000(	ELOA	1359.2	-0.08282	0.055406	0.123535	protein_coding
ENSG0000(	XPR1	1274.922	-0.12575	0.055436	0.123593	protein_coding
ENSG0000(	LINC01544	295.8546	0.134642	0.055451	0.123618	lncRNA
ENSG0000(	FOXRED2	877.7257	0.082272	0.055456	0.123621	protein_coding
ENSG0000(	ENSG0000(	84.34886	0.157988	0.055485	0.123658	lncRNA
ENSG0000(	IGHV1-69D	349.9846	0.629298	0.055483	0.123658	IG_V_gene
ENSG0000(	IL6ST	7875.775	0.179848	0.055479	0.123658	protein_coding
ENSG0000(	MTG1	1275.39	-0.09529	0.055492	0.123665	protein_coding
ENSG0000(	ENSG0000(	13.45698	0.38892	0.055498	0.123671	processed_pseudogene
ENSG0000(	CECR3	91.93454	0.361834	0.055519	0.123708	lncRNA
ENSG0000(	IGLV5-45	71.40214	0.500458	0.055551	0.12377	IG_V_gene
ENSG0000(	GABPB1-AS	6723.184	0.151239	0.055561	0.123785	lncRNA
ENSG0000(	ADAT1	2427.168	0.06768	0.055596	0.123854	protein_coding
ENSG0000(	COG8	1477.868	-0.07226	0.055616	0.123871	protein_coding
ENSG0000(	ENSG0000(	15.68043	0.438794	0.055614	0.123871	scaRNA
ENSG0000(	HBG2	11.29348	0.530652	0.055608	0.123871	protein_coding
ENSG0000(	TBL2	910.2073	-0.08344	0.05564	0.123915	protein_coding
ENSG0000(	ENSG0000(	402.094	0.124366	0.055667	0.123968	lncRNA
ENSG0000(	DAOA-AS1	10.08734	0.495748	0.055693	0.124016	lncRNA
ENSG0000(	ENSG0000(	30.63065	0.3168	0.055709	0.124035	processed_pseudogene
ENSG0000(	PHF5CP	28.17027	0.306706	0.055707	0.124035	processed_pseudogene
ENSG0000(	CACNA1G	116.6955	0.161448	0.055785	0.124177	protein_coding
ENSG0000(	FBXW11	1340.313	0.108889	0.055777	0.124177	protein_coding
ENSG0000(	KCNIP1-OT	72.95663	0.161845	0.055782	0.124177	lncRNA
ENSG0000(	LINC02731	161.6165	-0.17864	0.055801	0.124205	lncRNA
ENSG0000(	ADGRB1	105.1839	-0.27808	0.055828	0.124255	protein_coding
ENSG0000(	EXOC6B	2012.62	-0.20274	0.055862	0.124322	protein_coding

ENSG0000(	DIP2A-IT1	63.69429	0.1865	0.055874	0.124339	lncRNA
ENSG0000(	DOCK11	1135.449	0.288756	0.055881	0.124339	protein_coding
ENSG0000(	JMJD4	398.5029	-0.14145	0.055881	0.124339	protein_coding
ENSG0000(	DMBX1	106.3792	0.157897	0.055902	0.124376	protein_coding
ENSG0000(	WWP2	2502.255	-0.09267	0.055925	0.124417	protein_coding
ENSG0000(	ENSG0000(	80.07533	0.263663	0.055941	0.124446	unprocessed_pseudogene
ENSG0000(	RELL1	1611.482	-0.20896	0.055946	0.124447	protein_coding
ENSG0000(	LRFN3	301.3963	-0.22588	0.055964	0.124479	protein_coding
ENSG0000(	NFKBIE	264.3845	0.2684	0.055971	0.124485	protein_coding
ENSG0000(	MTCO2P12	67.94999	-0.43532	0.055994	0.124526	unprocessed_pseudogene
ENSG0000(	ENSG0000(	201.2552	-0.13093	0.056004	0.12454	lncRNA
ENSG0000(	MAPT	321.0147	-0.28793	0.056037	0.124606	protein_coding
ENSG0000(	SENP7	1809.732	-0.09299	0.056042	0.124607	protein_coding
ENSG0000(	ENSG0000(	43.36368	0.232281	0.056063	0.124644	lncRNA
ENSG0000(	HIC2	683.0702	-0.10061	0.05608	0.124674	protein_coding
ENSG0000(	ENSG0000(	22.0618	0.310279	0.056087	0.124681	lncRNA
ENSG0000(	MARS1	1546.948	0.150171	0.056092	0.124681	protein_coding
ENSG0000(	SOWAHA	336.223	-0.39921	0.056095	0.124681	protein_coding
ENSG0000(	DPCD	43.88749	-0.24672	0.056108	0.124701	protein_coding
ENSG0000(	ENSG0000(	239.0539	-0.11834	0.056115	0.124708	processed_pseudogene
ENSG0000(	ENSG0000(	300.2803	-0.34445	0.056132	0.124736	processed_pseudogene
ENSG0000(	CXCL13	453.7129	0.967219	0.056208	0.124897	protein_coding
ENSG0000(	ENSG0000(	38.24094	0.225087	0.05622	0.124914	lncRNA
ENSG0000(	CCDC92	737.9618	0.11025	0.056246	0.124963	protein_coding
ENSG0000(	MIR151A	7.580457	0.475032	0.056255	0.124975	miRNA
ENSG0000(	IQCM	587.119	-0.18607	0.056278	0.125016	protein_coding
ENSG0000(	FBXO32	1574.208	0.277859	0.056303	0.125063	protein_coding
ENSG0000(	HMGB1P39	124.8815	-0.13351	0.056335	0.125126	processed_pseudogene
ENSG0000(	NHLRC2	3141.933	-0.07542	0.05637	0.125195	protein_coding
ENSG0000(	ENSG0000(	12.21036	-0.49052	0.056385	0.125202	lncRNA
ENSG0000(	ENSG0000(	492.18	0.136422	0.05638	0.125202	lncRNA
ENSG0000(	LINC01220	206.9254	0.122398	0.056383	0.125202	lncRNA
ENSG0000(	COL6A2	4066.46	0.23277	0.056402	0.125229	protein_coding
ENSG0000(	ENSG0000(	30.30882	0.407219	0.056436	0.125297	lncRNA
ENSG0000(	DEPDC4	545.9706	-0.11098	0.056441	0.125299	protein_coding
ENSG0000(	SLC9B1	94.7534	0.165895	0.056448	0.125306	protein_coding
ENSG0000(	MUS81	829.7703	-0.08902	0.05648	0.125368	protein_coding
ENSG0000(	ENSG0000(	10.65037	0.53261	0.056489	0.12537	lncRNA
ENSG0000(	ENSG0000(	272.6829	0.172413	0.056488	0.12537	lncRNA
ENSG0000(	G6PD	274.7552	-0.19106	0.056549	0.125494	protein_coding
ENSG0000(	KLRC3	59.09631	-0.45292	0.056555	0.125497	protein_coding
ENSG0000(	ENSG0000(	10053.62	-0.13098	0.0566	0.125588	lncRNA
ENSG0000(	SNORD116	21.61464	0.42499	0.056626	0.125637	snoRNA
ENSG0000(	USP18	106.9766	0.421351	0.05663	0.125637	protein_coding
ENSG0000(	EEF1A1P14	17.47058	0.442033	0.056644	0.125661	processed_pseudogene
ENSG0000(	ENSG0000(	70.37374	-0.20221	0.056696	0.125765	lncRNA
ENSG0000(	CCDC102B	540.7153	0.201778	0.056707	0.125781	protein_coding

ENSG0000(	RN7SL753F	16.11999	0.299342	0.05671	0.125781	misc_RNA
ENSG0000(	COLCA2	246.3131	-0.32419	0.056722	0.125798	protein_coding
ENSG0000(	ENSG0000(	242.5349	-0.24781	0.056761	0.125865	lncRNA
ENSG0000(	JAZF1-AS1	97.77399	-0.1498	0.056758	0.125865	lncRNA
ENSG0000(	CNGB1	125.9785	0.178558	0.05677	0.125877	protein_coding
ENSG0000(	RN7SKP50	43.77349	0.308846	0.05678	0.125891	misc_RNA
ENSG0000(	ENSG0000(	29.04291	0.631994	0.05685	0.126035	protein_coding
ENSG0000(	OR6K3	124.3648	-0.16752	0.05686	0.126049	protein_coding
ENSG0000(	RNU6-1226	20.63477	0.394848	0.056871	0.126066	snRNA
ENSG0000(	ENSG0000(	292.6217	0.131414	0.056888	0.126095	lncRNA
ENSG0000(	LINGO4	18.49165	-0.46915	0.05691	0.126124	protein_coding
ENSG0000(	P4HA1	603.908	0.205723	0.056906	0.126124	protein_coding
ENSG0000(	NEXN	327.3641	0.166977	0.056952	0.12621	protein_coding
ENSG0000(	PTPRQ	25.74146	0.79375	0.056971	0.126242	protein_coding
ENSG0000(	GPN1	355.6203	-0.1506	0.057026	0.126355	protein_coding
ENSG0000(	LGALS2	59.73391	-0.37555	0.057038	0.126372	protein_coding
ENSG0000(	MUC4	41438.44	0.281451	0.057066	0.126426	protein_coding
ENSG0000(	ENSG0000(	16.84771	0.372935	0.057081	0.126451	lncRNA
ENSG0000(	ENSG0000(	135.4865	0.149826	0.057088	0.126455	lncRNA
ENSG0000(	SSTR3	36.36827	0.441765	0.057122	0.126523	protein_coding
ENSG0000(	MXD3	265.0382	0.180436	0.057147	0.12657	protein_coding
ENSG0000(	ENSG0000(	33.53576	-0.22328	0.057179	0.126611	snoRNA
ENSG0000(	ENSG0000(	61.07921	-0.21167	0.057182	0.126611	lncRNA
ENSG0000(	ENSG0000(	343.0594	0.165568	0.05718	0.126611	TEC
ENSG0000(	RN7SL541F	74.94317	-0.31966	0.057181	0.126611	misc_RNA
ENSG0000(	GRAP2	621.6918	0.28613	0.057203	0.126648	protein_coding
ENSG0000(	CD28	690.865	0.342323	0.057237	0.12671	protein_coding
ENSG0000(	DXO	183.9685	-0.14772	0.057243	0.12671	protein_coding
ENSG0000(	TOGARAM1	2002.311	-0.12481	0.05724	0.12671	protein_coding
ENSG0000(	ENSG0000(	819.3317	-0.08746	0.057256	0.126729	lncRNA
ENSG0000(	ASTN2	5533.88	-0.08511	0.057277	0.126767	protein_coding
ENSG0000(	ENSG0000(	237.3107	0.18662	0.057341	0.126883	lncRNA
ENSG0000(	MPPED1	15.29618	-0.65443	0.057341	0.126883	protein_coding
ENSG0000(	ZIM3	172.3387	-0.12906	0.05734	0.126883	protein_coding
ENSG0000(	CNR2	1122.859	0.141346	0.057368	0.126914	protein_coding
ENSG0000(	MMP24OS	1027.637	-0.1081	0.057366	0.126914	protein_coding
ENSG0000(	VIPAS39	508.5891	-0.10947	0.057363	0.126914	protein_coding
ENSG0000(	TP53BP1	2718.119	0.112382	0.057389	0.126952	protein_coding
ENSG0000(	LINC00642	144.6956	0.151321	0.057405	0.12698	lncRNA
ENSG0000(	CUZD1	46.55183	-0.2713	0.057413	0.126987	protein_coding
ENSG0000(	RGMB	2585.887	-0.30354	0.057421	0.126996	protein_coding
ENSG0000(	OR10K1	1734.265	0.108599	0.057432	0.127013	protein_coding
ENSG0000(	ENSG0000(	1022.656	0.111421	0.057449	0.12704	lncRNA
ENSG0000(	ENSG0000(	370.2405	0.239977	0.057489	0.12712	lncRNA
ENSG0000(	SPINK5	115.2276	-0.43473	0.057524	0.127188	protein_coding
ENSG0000(	ENSG0000(	680.2539	0.289538	0.057534	0.127202	transcribed_unprocessed_pseudo
ENSG0000(	ENSG0000(	70.42099	0.207938	0.057544	0.127215	lncRNA

ENSG0000(	ENSG0000(	88.49037	0.200955	0.05759	0.127298	lncRNA
ENSG0000(	SCUBE3	189.8009	0.107218	0.05759	0.127298	protein_coding
ENSG0000(	ENSG0000(	19.5159	0.305164	0.057625	0.127365	lncRNA
ENSG0000(	ENSG0000(	63.77916	-0.23176	0.05765	0.12741	processed_pseudogene
ENSG0000(	GMPR2	659.5463	-0.11864	0.057653	0.12741	protein_coding
ENSG0000(	IRAK1	1255.603	-0.12499	0.057723	0.127556	protein_coding
ENSG0000(	CFAP91	136.8559	0.231574	0.057732	0.127567	protein_coding
ENSG0000(	ENSG0000(	17.46032	-0.29653	0.057736	0.127568	lncRNA
ENSG0000(	HCFC2	605.7648	0.150606	0.05775	0.127589	protein_coding
ENSG0000(	ARHGAP42	1649.459	-0.22454	0.057765	0.127612	protein_coding
ENSG0000(	ALG9-IT1	38.43914	0.27539	0.057789	0.127656	lncRNA
ENSG0000(	SEPTIN7-D'	210.4421	0.150064	0.057799	0.127671	lncRNA
ENSG0000(	IKZF4	353.1941	0.162118	0.057808	0.127671	protein_coding
ENSG0000(	MRPL36	88.5679	-0.22914	0.057806	0.127671	protein_coding
ENSG0000(	ENSG0000(	21.63473	-0.40384	0.057915	0.127899	processed_pseudogene
ENSG0000(	ENSG0000(	20.26173	0.312639	0.05793	0.127923	lncRNA
ENSG0000(	TTC28-AS1	1562.963	-0.07285	0.057946	0.127948	lncRNA
ENSG0000(	FMN1	5317.696	-0.19872	0.058003	0.128057	protein_coding
ENSG0000(	ZFPL1	572.2082	-0.11204	0.057999	0.128057	protein_coding
ENSG0000(	ENSG0000(	186.5834	0.143525	0.058022	0.12809	transcribed_processed_pseudoge
ENSG0000(	RNU2-36P	22.65314	-0.40171	0.058033	0.128105	snRNA
ENSG0000(	LZTR1	1848.845	-0.09549	0.058049	0.128132	protein_coding
ENSG0000(	ENSG0000(	536.2059	0.093794	0.058069	0.128162	lncRNA
ENSG0000(	ENSG0000(	69.84549	-0.18142	0.058071	0.128162	lncRNA
ENSG0000(	ENSG0000(	676.7004	0.246966	0.058081	0.128165	transcribed_processed_pseudoge
ENSG0000(	ZSWIM1	653.9426	-0.09934	0.058077	0.128165	protein_coding
ENSG0000(	ENSG0000(	273.8651	-0.14187	0.058111	0.128223	lncRNA
ENSG0000(	ENSG0000(	41.48611	0.253875	0.058146	0.128291	lncRNA
ENSG0000(	ENSG0000(	192.7306	-0.1534	0.058156	0.128304	lncRNA
ENSG0000(	ENSG0000(	299.7054	-0.14452	0.058161	0.128306	lncRNA
ENSG0000(	CLK4	2099.862	0.074615	0.058169	0.128307	protein_coding
ENSG0000(	LINC01788	26.2587	-0.28043	0.05817	0.128307	lncRNA
ENSG0000(	LINC00328-	44.85336	0.389737	0.058192	0.128348	processed_pseudogene
ENSG0000(	ARRB1	2790.285	-0.13049	0.058201	0.128358	protein_coding
ENSG0000(	PWWP2A	1708.975	-0.12451	0.058213	0.128375	protein_coding
ENSG0000(	ENPP3	397.1361	-0.25796	0.058219	0.128381	protein_coding
ENSG0000(	EIF2AK3-D1	129.3802	0.151849	0.058229	0.128393	lncRNA
ENSG0000(	ZNF234	997.6971	-0.07162	0.058242	0.128412	protein_coding
ENSG0000(	SLC38A7	800.8924	0.101851	0.058288	0.128506	protein_coding
ENSG0000(	H4-16	748.9061	-0.15104	0.05831	0.128545	protein_coding
ENSG0000(	ENSG0000(	3076.804	-0.27351	0.058339	0.128572	miRNA
ENSG0000(	ENSG0000(	10.34502	0.596947	0.058336	0.128572	lncRNA
ENSG0000(	SLC48A1	665.3098	0.137998	0.058335	0.128572	protein_coding
ENSG0000(	TKFC	695.212	-0.13859	0.058329	0.128572	protein_coding
ENSG0000(	ZNF44	1836.263	-0.13565	0.058359	0.128607	protein_coding
ENSG0000(	RRP36	652.7222	-0.08583	0.058381	0.128646	protein_coding
ENSG0000(	PLD5	119.3827	0.210834	0.058438	0.128763	protein_coding

ENSG0000(	DUSP5-DT	143.0447	0.149385	0.058453	0.128786	lncRNA
ENSG0000(	ENSG0000(	204.9093	0.128165	0.058484	0.128847	lncRNA
ENSG0000(	KCNG1	458.8212	-0.15417	0.058494	0.128859	protein_coding
ENSG0000(	SLC22A2	94.25164	0.189941	0.05851	0.128886	protein_coding
ENSG0000(	TNS4	612.51	-0.32542	0.058522	0.128903	protein_coding
ENSG0000(	GRIK3	163.4244	-0.32629	0.058533	0.128917	protein_coding
ENSG0000(	IDI1	773.7693	0.15609	0.05856	0.128968	protein_coding
ENSG0000(	C17orf100	97.67411	0.180998	0.058566	0.128973	protein_coding
ENSG0000(	CYP21A1P	70.6891	0.22753	0.058576	0.128979	transcribed_unprocessed_pseudo
ENSG0000(	ENSG0000(	280.7175	0.235759	0.058582	0.128979	lncRNA
ENSG0000(	FAT3	276.6678	-0.23294	0.058579	0.128979	protein_coding
ENSG0000(	TMEM40	186.1086	-0.1059	0.058599	0.129009	protein_coding
ENSG0000(	ENSG0000(	10.09609	0.45546	0.058669	0.129154	processed_pseudogene
ENSG0000(	TMEM205	200.1057	-0.15284	0.05868	0.129169	protein_coding
ENSG0000(	ZNF71	416.9162	0.145879	0.058749	0.129312	protein_coding
ENSG0000(	METTL2A	2178.428	-0.08875	0.058755	0.129316	protein_coding
ENSG0000(	EEF1A1P10	47.46388	0.32487	0.058788	0.129379	processed_pseudogene
ENSG0000(	IFNLR1	817.6677	-0.25092	0.058817	0.129433	protein_coding
ENSG0000(	AK4P4	13.50657	-0.38771	0.05883	0.129435	processed_pseudogene
ENSG0000(	ETNK1-DT	20.64752	-0.41678	0.058828	0.129435	lncRNA
ENSG0000(	TMEM126E	433.0336	-0.10355	0.058826	0.129435	protein_coding
ENSG0000(	MIX23	689.4009	-0.20879	0.058871	0.129516	protein_coding
ENSG0000(	ENSG0000(	47.96625	-0.22473	0.058884	0.129535	processed_pseudogene
ENSG0000(	HDAC4-AS1	16.69678	0.324739	0.058901	0.129564	lncRNA
ENSG0000(	ENSG0000(	227.9796	-0.26015	0.058907	0.129569	lncRNA
ENSG0000(	ENSG0000(	412.8925	-0.10462	0.058917	0.129582	lncRNA
ENSG0000(	TEX29	13.49596	-0.39548	0.058978	0.129706	protein_coding
ENSG0000(	ARF4-AS1	54.44387	0.224134	0.059001	0.12973	lncRNA
ENSG0000(	LRRC37A2	1644.107	0.201993	0.058998	0.12973	protein_coding
ENSG0000(	SLC47A1	475.7886	0.10615	0.058995	0.12973	protein_coding
ENSG0000(	ARL17A	2806.241	0.263905	0.059015	0.129752	protein_coding
ENSG0000(	RNU4ATAC	22.81894	-0.29416	0.059059	0.129833	snRNA
ENSG0000(	XRCC2	1136.729	-0.07636	0.059061	0.129833	protein_coding
ENSG0000(	GCLM	982.51	-0.12151	0.059082	0.12987	protein_coding
ENSG0000(	ENSG0000(	140.8527	0.148447	0.059099	0.129898	lncRNA
ENSG0000(	MATN3	13.53669	-0.48899	0.059117	0.12993	protein_coding
ENSG0000(	CNTNAP2-/	175.8321	0.135292	0.059164	0.130018	lncRNA
ENSG0000(	RAP2C	772.6158	0.129913	0.059166	0.130018	protein_coding
ENSG0000(	DONSON	372.1307	-0.17086	0.059195	0.130068	protein_coding
ENSG0000(	MRPL41	223.5563	-0.21484	0.059197	0.130068	protein_coding
ENSG0000(	ENSG0000(	40.61136	0.256443	0.059301	0.130281	lncRNA
ENSG0000(	ENSG0000(	269.1981	0.150499	0.059302	0.130281	lncRNA
ENSG0000(	TTC5	819.9738	0.101867	0.059317	0.130306	protein_coding
ENSG0000(	TCEA2	128.1875	0.238655	0.059323	0.130309	protein_coding
ENSG0000(	MS4A4E	1451.441	0.097023	0.059331	0.130318	protein_coding
ENSG0000(	COL8A2	91.12151	0.268386	0.059351	0.130334	protein_coding
ENSG0000(	HDAC4	1666.585	0.099341	0.059347	0.130334	protein_coding

ENSG0000(	NUDT8	50.41092	-0.33936	0.059344	0.130334	protein_coding
ENSG0000(	ENSG0000(	474.4047	0.108179	0.059366	0.130358	lncRNA
ENSG0000(	ENSG0000(	1840.148	0.083168	0.05939	0.130401	lncRNA
ENSG0000(	VWA5B1	272.9657	-0.149	0.059411	0.130438	protein_coding
ENSG0000(	RBM27	1102.607	-0.16315	0.059425	0.130459	protein_coding
ENSG0000(	ENSG0000(	38.8208	0.247416	0.059446	0.130496	lncRNA
ENSG0000(	LINC02871	179.4124	0.145391	0.059471	0.130543	lncRNA
ENSG0000(	MAPKAPK3	488.5548	-0.14931	0.059497	0.13059	protein_coding
ENSG0000(	ENSG0000(	620.215	0.103679	0.059525	0.130624	lncRNA
ENSG0000(	ENSG0000(	204.5446	-0.12911	0.059524	0.130624	lncRNA
ENSG0000(	TRIM4	1439.422	-0.06391	0.059522	0.130624	protein_coding
ENSG0000(	FCER1A	11.96369	-0.55346	0.059548	0.130666	protein_coding
ENSG0000(	ENSG0000(	276.1423	-0.14829	0.059567	0.130697	lncRNA
ENSG0000(	IQCN	1601.088	0.155237	0.059573	0.130702	protein_coding
ENSG0000(	ENSG0000(	47.11986	0.235575	0.059598	0.130742	lncRNA
ENSG0000(	OR7E25P	706.6876	-0.08155	0.0596	0.130742	transcribed_unprocessed_pseudo
ENSG0000(	NUTM2B-A	6012.387	0.108929	0.059606	0.130747	lncRNA
ENSG0000(	FBXL3	1509.603	-0.13733	0.05962	0.130769	protein_coding
ENSG0000(	CAMTA1-D	11.82953	-0.40705	0.05964	0.130801	lncRNA
ENSG0000(	LINC01585	81.24687	0.256409	0.059643	0.130801	lncRNA
ENSG0000(	ENSG0000(	23.44471	0.3289	0.059656	0.13081	lncRNA
ENSG0000(	LINC01819	97.61955	0.34678	0.059652	0.13081	lncRNA
ENSG0000(	RN7SL732P	23.89702	0.280857	0.059694	0.130884	misc_RNA
ENSG0000(	ZNF474	11.44944	0.470813	0.059701	0.130892	protein_coding
ENSG0000(	XKR9	1146.33	0.154412	0.059715	0.130913	protein_coding
ENSG0000(	MKS1	1186.338	0.082437	0.05973	0.130936	protein_coding
ENSG0000(	C21orf62	708.3202	-0.10887	0.059765	0.131004	protein_coding
ENSG0000(	ENSG0000(	409.4047	0.110808	0.059809	0.131091	lncRNA
ENSG0000(	CD58	539.5589	-0.14613	0.059854	0.131182	protein_coding
ENSG0000(	ENSG0000(	67.96811	-0.19586	0.059883	0.131235	lncRNA
ENSG0000(	RN7SL396P	152.0538	0.306107	0.059911	0.131286	misc_RNA
ENSG0000(	ANKLE1	1342.806	0.283598	0.059941	0.131322	protein_coding
ENSG0000(	ENSG0000(	184.3772	0.134997	0.059943	0.131322	lncRNA
ENSG0000(	ENSG0000(	185.5754	0.150809	0.059936	0.131322	lncRNA
ENSG0000(	ENSG0000(	212.4608	0.142503	0.059944	0.131322	TEC
ENSG0000(	ENSG0000(	24.11154	0.294688	0.059956	0.131334	lncRNA
ENSG0000(	SCGB2B2	375.8876	0.181432	0.059957	0.131334	protein_coding
ENSG0000(	SENP3	615.784	-0.15986	0.059969	0.13135	protein_coding
ENSG0000(	CLPX	1242.725	-0.09723	0.060022	0.131456	protein_coding
ENSG0000(	ARMCX7P	27.41055	1.024014	0.06003	0.131464	transcribed_unprocessed_pseudo
ENSG0000(	ENSG0000(	95.79345	0.237082	0.06011	0.131631	TEC
ENSG0000(	AADACL2	23.4378	0.35764	0.060115	0.131632	protein_coding
ENSG0000(	ZNF630	493.0254	-0.12395	0.06019	0.131787	protein_coding
ENSG0000(	CD59	5763.377	-0.22418	0.060202	0.131804	protein_coding
ENSG0000(	ENSG0000(	80.64645	0.175839	0.060244	0.131886	lncRNA
ENSG0000(	GFRA2	130.4814	-0.32539	0.060271	0.131929	protein_coding
ENSG0000(	RN7SL798P	20.53649	0.326842	0.060268	0.131929	misc_RNA

ENSG0000(	OTOP2	175.4363	-0.66335	0.060288	0.131956	protein_coding
ENSG0000(	ENSG0000(	214.1441	0.12624	0.06031	0.131994	lncRNA
ENSG0000(	ENSG0000(	121.6472	-0.15977	0.06033	0.132023	lncRNA
ENSG0000(	PLEKHF1	99.58363	0.275179	0.060332	0.132023	protein_coding
ENSG0000(	RBX1	508.4483	-0.10962	0.060336	0.132023	protein_coding
ENSG0000(	ENSG0000(	23.18777	0.489241	0.060348	0.132042	lncRNA
ENSG0000(	AQP3	161.5267	0.284554	0.060359	0.132056	protein_coding
ENSG0000(	YTHDC2	2127.949	-0.1026	0.060377	0.132085	protein_coding
ENSG0000(	ENSG0000(	15.38959	-0.42286	0.060398	0.132104	lncRNA
ENSG0000(	ENSG0000(	1442.398	0.085339	0.060396	0.132104	lncRNA
ENSG0000(	HPF1	90.53525	-0.2272	0.060398	0.132104	protein_coding
ENSG0000(	ENSG0000(	163.1811	-0.15435	0.060435	0.132167	lncRNA
ENSG0000(	TM6SF2	102.9148	-0.50611	0.060435	0.132167	protein_coding
ENSG0000(	KCTD13	1322.962	-0.10006	0.060448	0.132185	protein_coding
ENSG0000(	TMEM128	229.942	-0.15478	0.060459	0.1322	protein_coding
ENSG0000(	ENSG0000(	17.23038	0.340668	0.060468	0.132211	lncRNA
ENSG0000(	ENSG0000(	77.96047	-0.31543	0.060495	0.132262	lncRNA
ENSG0000(	MISP3	291.0246	-0.26462	0.06051	0.132284	protein_coding
ENSG0000(	DCAF13	1247.193	-0.09639	0.060519	0.132292	protein_coding
ENSG0000(	ZNF536	314.1066	0.218502	0.060522	0.132292	protein_coding
ENSG0000(	HCFC1	2450.613	-0.09622	0.060533	0.132307	protein_coding
ENSG0000(	PSMD1	3746.107	-0.13265	0.060539	0.13231	protein_coding
ENSG0000(	TLCD4-RWI	164.9541	-0.2308	0.060563	0.132354	protein_coding
ENSG0000(	LINC01707	135.0935	0.14046	0.060571	0.132361	lncRNA
ENSG0000(	PCGF6	337.9267	-0.14828	0.060575	0.132361	protein_coding
ENSG0000(	EFTUD2	1545.118	-0.08156	0.060585	0.132365	protein_coding
ENSG0000(	SYCP1	243.5746	-0.12077	0.060585	0.132365	protein_coding
ENSG0000(	MTCO1P1	22.45189	0.414877	0.060603	0.132396	processed_pseudogene
ENSG0000(	ENSG0000(	15.75396	0.457118	0.060611	0.132404	lncRNA
ENSG0000(	ENSG0000(	156.6509	0.20341	0.060663	0.132507	lncRNA
ENSG0000(	SNX18P26	34.46856	0.390542	0.060699	0.132577	processed_pseudogene
ENSG0000(	SRSF10	5459.467	-0.06301	0.060704	0.132579	protein_coding
ENSG0000(	C1QC	608.5302	0.316306	0.060714	0.132592	protein_coding
ENSG0000(	ENSG0000(	181.3831	-0.12377	0.060727	0.13261	lncRNA
ENSG0000(	ENSG0000(	123.4377	0.221263	0.060738	0.132626	lncRNA
ENSG0000(	LINC00683	534.2094	0.155608	0.060748	0.132638	lncRNA
ENSG0000(	ENSG0000(	87.82741	0.179338	0.06077	0.132677	lncRNA
ENSG0000(	LOH12CR2	247.4441	-0.14167	0.060778	0.132685	lncRNA
ENSG0000(	LINC01315	13.81738	0.464903	0.060792	0.132702	lncRNA
ENSG0000(	RN7SL679F	27.32271	0.242196	0.060794	0.132702	misc_RNA
ENSG0000(	ZNF2	333.4936	-0.12431	0.060835	0.132782	protein_coding
ENSG0000(	SINHCAFP3	427.1443	0.204888	0.060867	0.132843	processed_pseudogene
ENSG0000(	HELLS	1777.419	-0.19077	0.060898	0.132899	protein_coding
ENSG0000(	IZUMO1	255.2923	0.095392	0.060903	0.132901	protein_coding
ENSG0000(	PLXNA4	257.5016	0.243114	0.060925	0.132942	protein_coding
ENSG0000(	SIGLEC22P	12.38699	0.486678	0.06094	0.132965	transcribed_unprocessed_pseudo
ENSG0000(	ENSG0000(	75.8111	0.127542	0.060951	0.132979	lncRNA

ENSG0000(	ENSG0000(	32.81584	0.344441	0.060958	0.132986	lncRNA
ENSG0000(	ENSG0000(	236.1463	0.142774	0.060968	0.132998	lncRNA
ENSG0000(	PMFBP1	259.01	-0.29734	0.060973	0.132999	protein_coding
ENSG0000(	GPR63	639.8842	0.188871	0.061002	0.133026	protein_coding
ENSG0000(	ID1	1018.001	-0.28654	0.061	0.133026	protein_coding
ENSG0000(	LINC01565	31.05695	-0.26596	0.060999	0.133026	lncRNA
ENSG0000(	ZNF688	347.0279	0.116084	0.060995	0.133026	protein_coding
ENSG0000(	ENSG0000(	9.303214	0.593125	0.061031	0.133078	processed_pseudogene
ENSG0000(	PRAMEF9	42.20472	0.237367	0.061054	0.133121	protein_coding
ENSG0000(	VSX1	28.339	0.243406	0.061148	0.133315	protein_coding
ENSG0000(	LINC02298	381.1327	-0.1681	0.061156	0.133324	lncRNA
ENSG0000(	RN7SL564F	112.3111	-0.41386	0.06117	0.133346	misc_RNA
ENSG0000(	IL7	225.1229	0.178323	0.06119	0.13338	protein_coding
ENSG0000(	PRKD2	1755.433	0.096574	0.061197	0.133385	protein_coding
ENSG0000(	UTP3	460.8347	-0.10626	0.061213	0.133412	protein_coding
ENSG0000(	ENSG0000(	10.50159	0.323677	0.061234	0.133439	lncRNA
ENSG0000(	ENSG0000(	198.4394	0.290698	0.061231	0.133439	TEC
ENSG0000(	GUSBP5	28.59394	0.378541	0.061272	0.133511	transcribed_processed_pseudoge
ENSG0000(	ENSG0000(	28.04586	-0.33612	0.061284	0.133527	lncRNA
ENSG0000(	FBXO8	235.325	-0.14381	0.061288	0.133527	protein_coding
ENSG0000(	CHMP1B2F	268.7703	-0.24632	0.061301	0.133546	transcribed_unitary_pseudogene
ENSG0000(	PDE4DIPP2	1853.953	0.145845	0.061305	0.133547	transcribed_unprocessed_pseudo
ENSG0000(	DGKA	4101.177	-0.14511	0.061313	0.133555	protein_coding
ENSG0000(	ENSG0000(	13.50614	0.410566	0.061345	0.133615	lncRNA
ENSG0000(	ISM1-AS1	9.128287	0.451485	0.061362	0.133643	lncRNA
ENSG0000(	ENSG0000(	298.1714	-0.31589	0.06137	0.133651	lncRNA
ENSG0000(	GZF1	373.0901	0.159039	0.06138	0.133652	protein_coding
ENSG0000(	LEFTY1	315.0868	-0.39412	0.061383	0.133652	protein_coding
ENSG0000(	TBC1D22A-	181.5621	0.115171	0.061378	0.133652	lncRNA
ENSG0000(	GOLT1B	508.0474	0.179782	0.061414	0.13371	protein_coding
ENSG0000(	BOLA3P3	18.70119	-0.46294	0.061419	0.133711	processed_pseudogene
ENSG0000(	CCDC196	18.57365	-0.34056	0.061428	0.133722	protein_coding
ENSG0000(	ETNK1	17344.82	-0.33507	0.06147	0.133794	protein_coding
ENSG0000(	ZSCAN29	1567.8	-0.07172	0.06147	0.133794	protein_coding
ENSG0000(	CD164	8125.629	-0.13444	0.061568	0.133999	protein_coding
ENSG0000(	ENSG0000(	24.46268	-0.57292	0.061582	0.13402	lncRNA
ENSG0000(	OVCH1	28.57706	0.591509	0.061617	0.134085	protein_coding
ENSG0000(	SBF2-AS1	1681.502	0.077354	0.061642	0.134132	lncRNA
ENSG0000(	ENSG0000(	18.47605	0.290501	0.061709	0.134259	processed_pseudogene
ENSG0000(	ENSG0000(	79.98906	-0.22967	0.061706	0.134259	lncRNA
ENSG0000(	GLIS3	1194.308	0.329459	0.061727	0.134288	protein_coding
ENSG0000(	BCAN	11.50736	0.691341	0.061812	0.134464	protein_coding
ENSG0000(	SRF	766.676	0.118177	0.061821	0.134474	protein_coding
ENSG0000(	LIMCH1	537.5669	0.185378	0.0619	0.134626	protein_coding
ENSG0000(	RHOA-IT1	113.2973	0.151745	0.061895	0.134626	lncRNA
ENSG0000(	AFAP1L1	466.269	0.238046	0.061922	0.134665	protein_coding
ENSG0000(	LINC01996	50.65327	0.185026	0.061928	0.13467	lncRNA

ENSG0000(	ENSG0000(	77.78461	0.164827	0.061966	0.134733	lncRNA
ENSG0000(	LGALSL	803.6172	-0.259	0.061964	0.134733	protein_coding
ENSG0000(	MGC4859	27.70894	0.313045	0.061971	0.134733	lncRNA
ENSG0000(	LINC02593	235.7786	0.263413	0.061981	0.134748	lncRNA
ENSG0000(	LINC02822	2575.632	-0.17866	0.061991	0.134759	lncRNA
ENSG0000(	STPG4	97.36399	0.224997	0.061999	0.134768	protein_coding
ENSG0000(	CLEC9A	31.23479	0.435488	0.062039	0.134841	protein_coding
ENSG0000(	RN7SL254F	25.2189	0.336052	0.062041	0.134841	misc_RNA
ENSG0000(	DENND4A	2582.382	0.16136	0.062075	0.134905	protein_coding
ENSG0000(	ENSG0000(	84.58169	0.168864	0.062089	0.134925	lncRNA
ENSG0000(	COPG1	2125.477	-0.10931	0.062116	0.134975	protein_coding
ENSG0000(	COMT	960.3973	0.100459	0.062173	0.135081	protein_coding
ENSG0000(	ENSG0000(	10.6234	-0.50499	0.062174	0.135081	processed_pseudogene
ENSG0000(	ENSG0000(	252.0493	-0.16967	0.062189	0.135104	TEC
ENSG0000(	CLLU1	212.1564	-0.14289	0.062216	0.135125	lncRNA
ENSG0000(	ENSG0000(	86.15005	-0.18002	0.062206	0.135125	lncRNA
ENSG0000(	LINC01566	14.93349	0.461227	0.062214	0.135125	lncRNA
ENSG0000(	TNIP1	2610.79	0.139926	0.062212	0.135125	protein_coding
ENSG0000(	FLT4	266.5977	0.291484	0.06224	0.13517	protein_coding
ENSG0000(	IGIP	614.6647	0.090081	0.062268	0.135221	protein_coding
ENSG0000(	NR1I2	2358.096	-0.26488	0.062312	0.135306	protein_coding
ENSG0000(	SIPA1L1	3338.35	0.095878	0.062321	0.135317	protein_coding
ENSG0000(	TUT4	3164.242	0.147606	0.062329	0.135325	protein_coding
ENSG0000(	ENSG0000(	24.44573	0.361377	0.062336	0.13533	TEC
ENSG0000(	MAST2	4365.292	-0.26473	0.062352	0.135356	protein_coding
ENSG0000(	RNU6-946F	28.6624	0.263421	0.062377	0.135401	snRNA
ENSG0000(	UCP2	790.9651	0.279798	0.062382	0.135402	protein_coding
ENSG0000(	EEF1A1P30	94.1048	0.191221	0.062389	0.135409	processed_pseudogene
ENSG0000(	APBA1	501.8106	-0.14532	0.062428	0.135483	protein_coding
ENSG0000(	MIR7-3HG	129.4908	0.1776	0.062462	0.135547	lncRNA
ENSG0000(	ENSG0000(	21.82875	0.43756	0.062494	0.135607	processed_pseudogene
ENSG0000(	ENSG0000(	203.0751	0.192319	0.062507	0.135627	lncRNA
ENSG0000(	NECAP2	1238.431	0.114186	0.062518	0.135641	protein_coding
ENSG0000(	ENSG0000(	74.33742	0.155626	0.062523	0.135642	lncRNA
ENSG0000(	GSTM3	699.5375	-0.09821	0.062534	0.135657	protein_coding
ENSG0000(	PDCD4	5713.497	-0.23422	0.062588	0.135765	protein_coding
ENSG0000(	PDE7A	2949.476	0.242873	0.062608	0.135798	protein_coding
ENSG0000(	ARHGAP30	1246.105	0.289087	0.062643	0.135865	protein_coding
ENSG0000(	ENSG0000(	126.0707	0.207955	0.062648	0.135867	lncRNA
ENSG0000(	ENSG0000(	79.25538	-0.15128	0.062768	0.136116	processed_pseudogene
ENSG0000(	FAM76B	614.0853	-0.14636	0.062772	0.136116	protein_coding
ENSG0000(	NUAK2	997.2333	-0.14121	0.06279	0.136147	protein_coding
ENSG0000(	CHD6	3001.517	0.119511	0.062818	0.136196	protein_coding
ENSG0000(	SHROOM2	483.8353	-0.22843	0.062848	0.136253	protein_coding
ENSG0000(	EXD3	996.7669	-0.16112	0.062871	0.136283	protein_coding
ENSG0000(	PPP3CA	1182.202	0.13466	0.062867	0.136283	protein_coding
ENSG0000(	ZNF277	627.3445	-0.08659	0.062898	0.136332	protein_coding

ENSG0000(	ENSG0000(	261.2305	0.151973	0.062907	0.136344	protein_coding
ENSG0000(	CBFB	1075.357	-0.08481	0.062916	0.136353	protein_coding
ENSG0000(	ENSG0000(	215.79	0.165794	0.062932	0.136369	lncRNA
ENSG0000(	ZNF789	1732.702	-0.08273	0.062931	0.136369	protein_coding
ENSG0000(	TSC22D4	829.5902	-0.11865	0.062945	0.136387	protein_coding
ENSG0000(	OR52B2	10.14184	-0.53078	0.062954	0.136398	protein_coding
ENSG0000(	ENSG0000(	58.64522	-0.16875	0.062969	0.136414	lncRNA
ENSG0000(	ENSG0000(	29.96485	0.300538	0.06297	0.136414	lncRNA
ENSG0000(	HIF1A-AS3	4012.786	-0.11676	0.06298	0.136416	lncRNA
ENSG0000(	NIPSNAP3A	272.4198	-0.19794	0.062976	0.136416	protein_coding
ENSG0000(	ENSG0000(	22.09993	-0.38837	0.062988	0.136423	lncRNA
ENSG0000(	RN7SL334P	24.24154	0.352124	0.062997	0.136433	misc_RNA
ENSG0000(	ENSG0000(	254.733	-0.14639	0.06306	0.136561	lncRNA
ENSG0000(	CDH18	195.5902	-0.14467	0.063072	0.136577	protein_coding
ENSG0000(	ENSG0000(	1686.79	-0.16499	0.063128	0.13669	lncRNA
ENSG0000(	ENSG0000(	529.5362	0.146954	0.063166	0.136748	processed_pseudogene
ENSG0000(	LINC02627	76.9259	-0.20034	0.063168	0.136748	lncRNA
ENSG0000(	MRPL30	1103.158	-0.10752	0.063167	0.136748	protein_coding
ENSG0000(	RILPL1	965.8648	0.065714	0.06319	0.136785	protein_coding
ENSG0000(	GLUD1P3	238.5282	0.155088	0.063194	0.136785	transcribed_unprocessed_pseudo
ENSG0000(	CENPO	576.984	-0.12243	0.063213	0.136817	protein_coding
ENSG0000(	ENSG0000(	373.4414	-0.11966	0.063241	0.136867	protein_coding
ENSG0000(	XPO6	2843.347	0.135045	0.063253	0.136883	protein_coding
ENSG0000(	LINC00638	102.7699	0.194455	0.063273	0.136918	lncRNA
ENSG0000(	ENSG0000(	85.93943	0.184725	0.063319	0.137008	lncRNA
ENSG0000(	CBFA2T3	480.2336	0.247876	0.063331	0.137025	protein_coding
ENSG0000(	PKD1P1	1337.102	-0.45429	0.063361	0.13708	unprocessed_pseudogene
ENSG0000(	NIFKP4	462.0681	0.102493	0.063404	0.137162	processed_pseudogene
ENSG0000(	ATP13A1	1355.222	-0.10483	0.063429	0.137198	protein_coding
ENSG0000(	SERHL2	61.75486	-0.19475	0.063428	0.137198	protein_coding
ENSG0000(	PLCB2	1324.113	0.186247	0.063449	0.137231	protein_coding
ENSG0000(	ENSG0000(	190.4267	-0.18611	0.063454	0.137233	protein_coding
ENSG0000(	MTRNR2L5	123.7635	-0.17186	0.063465	0.137248	protein_coding
ENSG0000(	ZC3H12D	1560.89	0.188651	0.06347	0.137249	protein_coding
ENSG0000(	RPL13AP12	52.86758	0.204444	0.063505	0.137316	processed_pseudogene
ENSG0000(	BCDIN3D	609.6519	0.110948	0.063516	0.137329	protein_coding
ENSG0000(	ENSG0000(	11.35836	0.389239	0.063526	0.137343	lncRNA
ENSG0000(	ENSG0000(	19.0301	-0.3875	0.063547	0.137358	lncRNA
ENSG0000(	SLC19A2	370.5684	0.16149	0.063539	0.137358	protein_coding
ENSG0000(	TOP1P1	29.02265	-0.36191	0.063546	0.137358	processed_pseudogene
ENSG0000(	CCDC28B	341.6315	0.095322	0.06359	0.137434	protein_coding
ENSG0000(	ENSG0000(	22.43381	-0.36543	0.063587	0.137434	processed_pseudogene
ENSG0000(	H2BC21	354.9614	-0.19993	0.063601	0.137447	protein_coding
ENSG0000(	ATP5MGP6	15.5982	-0.50587	0.063618	0.137474	processed_pseudogene
ENSG0000(	ELOVL2-AS	218.2752	0.164716	0.063646	0.137526	lncRNA
ENSG0000(	ENSG0000(	214.0925	0.15146	0.063656	0.137536	lncRNA
ENSG0000(	UBA6	2610.929	-0.09809	0.063668	0.137554	protein_coding

ENSG0000(	ENSG0000(	160.1293	0.167297	0.063675	0.137559	lncRNA
ENSG0000(	H3-3A	4611.436	-0.16695	0.063735	0.13768	protein_coding
ENSG0000(	LINC02047	15.87707	0.329411	0.063744	0.13769	lncRNA
ENSG0000(	ZNF286A-T	246.1952	0.271242	0.063777	0.13775	protein_coding
ENSG0000(	FLOT1	1029.685	-0.11463	0.063823	0.137841	protein_coding
ENSG0000(	ENSG0000(	20.23974	-0.38218	0.063832	0.13785	lncRNA
ENSG0000(	LINC00649	1755.812	0.102032	0.063847	0.137864	lncRNA
ENSG0000(	MTND4P32	46.97033	0.223766	0.063847	0.137864	processed_pseudogene
ENSG0000(	RNU6-377F	36.02591	-0.36639	0.063864	0.137891	snRNA
ENSG0000(	EXTL3	1422.352	-0.09802	0.063883	0.137923	protein_coding
ENSG0000(	COQ7	655.8114	-0.11332	0.063901	0.137936	protein_coding
ENSG0000(	ENSG0000(	61.68823	0.17889	0.063911	0.137936	lncRNA
ENSG0000(	ENSG0000(	16.14245	0.349407	0.063899	0.137936	lncRNA
ENSG0000(	FBLN2	195.2072	0.48421	0.063907	0.137936	protein_coding
ENSG0000(	TXNIP	12971.98	-0.16668	0.06391	0.137936	protein_coding
ENSG0000(	PRSS8	2248.631	-0.25645	0.063956	0.138024	protein_coding
ENSG0000(	ENSG0000(	164.8717	-0.24547	0.063987	0.138072	lncRNA
ENSG0000(	WNT9A	104.5145	-0.24351	0.063985	0.138072	protein_coding
ENSG0000(	ENSG0000(	116.0801	0.170429	0.063996	0.138082	lncRNA
ENSG0000(	TRIM63	12.39702	0.501282	0.064002	0.138085	protein_coding
ENSG0000(	LINC00943	143.8244	-0.20069	0.064102	0.13829	lncRNA
ENSG0000(	SAPCD1-AS	12.89591	-0.48967	0.064115	0.13831	lncRNA
ENSG0000(	BORCS7	632.7068	-0.09728	0.064122	0.138314	protein_coding
ENSG0000(	INPP5F	1065.96	0.147124	0.064197	0.138467	protein_coding
ENSG0000(	ENSG0000(	134.9133	0.283215	0.064202	0.138468	lncRNA
ENSG0000(	SLC35B4	884.0441	0.128849	0.064219	0.138496	protein_coding
ENSG0000(	TREH	53.68029	-0.40011	0.064273	0.138603	protein_coding
ENSG0000(	PLEKHA3	1851.769	0.103603	0.064278	0.138603	protein_coding
ENSG0000(	CTBP2P8	91.20694	-0.24454	0.064287	0.138613	processed_pseudogene
ENSG0000(	DNAJC5	3028.332	-0.13304	0.064294	0.138618	protein_coding
ENSG0000(	ENSG0000(	205.7373	0.27165	0.064305	0.138633	lncRNA
ENSG0000(	KCP	463.7021	0.113578	0.064312	0.138637	protein_coding
ENSG0000(	HLF	186.0481	-0.30156	0.064325	0.138658	protein_coding
ENSG0000(	ACTG1P25	120.3124	-0.16412	0.064369	0.138742	transcribed_processed_pseudoge
ENSG0000(	SPATA20	1199.12	-0.18319	0.064375	0.138745	protein_coding
ENSG0000(	LINC02864	258.1265	0.122913	0.064383	0.138753	lncRNA
ENSG0000(	CDIPT	534.3212	-0.13313	0.064408	0.138797	protein_coding
ENSG0000(	BDP1	3980.775	-0.10322	0.064438	0.138853	protein_coding
ENSG0000(	ENSG0000(	20.28965	0.313577	0.064448	0.138857	lncRNA
ENSG0000(	ENSG0000(	18.95385	0.285366	0.064449	0.138857	lncRNA
ENSG0000(	KYAT1	108.0944	0.219617	0.064455	0.13886	protein_coding
ENSG0000(	MRGPRF	106.5094	0.367402	0.064459	0.13886	protein_coding
ENSG0000(	ENSG0000(	59.83616	0.159952	0.064475	0.138875	lncRNA
ENSG0000(	ENSG0000(	41.08306	0.255368	0.064475	0.138875	TEC
ENSG0000(	SUDS3P1	9.530674	-0.48895	0.064491	0.1389	processed_pseudogene
ENSG0000(	FER1L5	225.1122	0.102447	0.064509	0.138929	protein_coding
ENSG0000(	ENSG0000(	27.83831	0.360755	0.064523	0.138951	lncRNA

ENSG0000(	C3orf70	835.7856	-0.14058	0.064545	0.138988	protein_coding
ENSG0000(	ENSG0000(	637.0946	0.189404	0.064576	0.139045	lncRNA
ENSG0000(	ENSG0000(	64.78194	0.1553	0.064582	0.139048	lncRNA
ENSG0000(	PCED1B-AS	774.4768	0.164616	0.064587	0.139048	lncRNA
ENSG0000(	RRP1	534.7307	-0.0898	0.064608	0.139084	protein_coding
ENSG0000(	FABP5P9	97.29734	-0.24269	0.064619	0.139099	transcribed_processed_pseudoge
ENSG0000(	ADAM32	5317.489	0.081814	0.064639	0.139133	protein_coding
ENSG0000(	LINC02682	48.48149	0.372249	0.064656	0.139151	lncRNA
ENSG0000(	NTRK3	750.2311	-0.16527	0.064657	0.139151	protein_coding
ENSG0000(	C5orf52	25.07903	-0.37877	0.064687	0.139208	protein_coding
ENSG0000(	DNAAF1	594.3233	0.096492	0.064711	0.13924	protein_coding
ENSG0000(	RFWD3	1192.933	-0.09961	0.064707	0.13924	protein_coding
ENSG0000(	ENSG0000(	115.8087	-0.18668	0.064721	0.139251	lncRNA
ENSG0000(	ENSG0000(	33.9009	-0.4422	0.064725	0.139251	misc_RNA
ENSG0000(	CFAP36	385.2053	0.114468	0.064762	0.139315	protein_coding
ENSG0000(	ENSG0000(	129.4947	0.156206	0.064766	0.139315	lncRNA
ENSG0000(	YPEL4	59.55228	-0.35678	0.064768	0.139315	protein_coding
ENSG0000(	ENSG0000(	422.8564	-0.09516	0.064777	0.139325	TEC
ENSG0000(	DNAJC22	1186.655	-0.19991	0.064787	0.139336	protein_coding
ENSG0000(	ELF2	2195.935	0.066855	0.064838	0.139436	protein_coding
ENSG0000(	RRS1-DT	283.2665	0.164421	0.064843	0.139436	lncRNA
ENSG0000(	PGR-AS1	39.93396	0.381762	0.064877	0.139501	lncRNA
ENSG0000(	EGLN1	1483.953	-0.13665	0.064919	0.139582	protein_coding
ENSG0000(	CXorf65	25.67102	0.602035	0.064927	0.139589	protein_coding
ENSG0000(	LGALS14	52.51926	0.217216	0.064979	0.139672	protein_coding
ENSG0000(	NBEA	2178.003	0.148403	0.064974	0.139672	protein_coding
ENSG0000(	PTPN21	1328.827	-0.20331	0.064979	0.139672	protein_coding
ENSG0000(	ENSG0000(	30.5444	0.297136	0.065003	0.139714	TEC
ENSG0000(	NKD2	72.22247	-0.3238	0.065016	0.139732	protein_coding
ENSG0000(	LINC00412	61.44051	0.246543	0.065027	0.139746	lncRNA
ENSG0000(	ENSG0000(	44.82044	-0.49668	0.065046	0.139758	lncRNA
ENSG0000(	SEPTIN14P	63.47384	0.282862	0.065042	0.139758	transcribed_unprocessed_pseudo
ENSG0000(	TXNL4A	1300.941	-0.08895	0.065042	0.139758	protein_coding
ENSG0000(	FAM78A	650.9757	0.236229	0.065083	0.139828	protein_coding
ENSG0000(	PARP10	1063.935	0.161676	0.065106	0.139868	protein_coding
ENSG0000(	KLF17P1	17.76727	-0.29997	0.065122	0.139893	processed_pseudogene
ENSG0000(	COL4A3	659.732	0.206246	0.065162	0.139962	protein_coding
ENSG0000(	OAT	526.9202	-0.15673	0.065168	0.139962	protein_coding
ENSG0000(	PLAAT2	47.86244	-0.51978	0.065165	0.139962	protein_coding
ENSG0000(	DERA	578.4697	-0.13142	0.065202	0.139998	protein_coding
ENSG0000(	ENSG0000(	84.17185	-0.22375	0.0652	0.139998	lncRNA
ENSG0000(	MIR6891	10.74836	0.540734	0.065198	0.139998	miRNA
ENSG0000(	OR2C3	77.20438	0.277374	0.0652	0.139998	protein_coding
ENSG0000(	LINC02429	53.69879	0.166867	0.065232	0.140053	lncRNA
ENSG0000(	ENSG0000(	265.0604	0.119785	0.065242	0.140054	TEC
ENSG0000(	HM13-IT1	104.8865	0.15435	0.06524	0.140054	lncRNA
ENSG0000(	NKX2-3	160.5579	-0.21028	0.065258	0.140079	protein_coding

ENSG0000(	AP5Z1	1152.168	-0.10237	0.065265	0.140085	protein_coding
ENSG0000(	LINC01354	34.81818	0.331106	0.065285	0.140117	lncRNA
ENSG0000(	UBE2QL1	36.23641	0.341098	0.065293	0.140125	protein_coding
ENSG0000(	RN7SKP154	33.27134	-0.35846	0.065308	0.140148	misc_RNA
ENSG0000(	ALG8	2106.949	0.063322	0.065326	0.140178	protein_coding
ENSG0000(	TMEM233	93.25804	0.149614	0.065343	0.140204	protein_coding
ENSG0000(	DHRSX	773.1303	-0.08994	0.065395	0.140306	protein_coding
ENSG0000(	ITIH1	35.76224	0.261367	0.065424	0.140358	protein_coding
ENSG0000(	PDCL3P5	31.7147	-0.34425	0.065435	0.140372	processed_pseudogene
ENSG0000(	REG4	657.9884	0.63009	0.065468	0.140433	protein_coding
ENSG0000(	CCDC144N	1002.127	0.094737	0.06548	0.140449	lncRNA
ENSG0000(	ENSG0000(	34.63126	0.394966	0.065549	0.140579	lncRNA
ENSG0000(	ENSG0000(	355.6927	-0.13795	0.065548	0.140579	lncRNA
ENSG0000(	IGHV3-33	739.483	0.500389	0.065573	0.14062	IG_V_gene
ENSG0000(	ITPRID1	276.4061	0.40934	0.0656	0.140659	protein_coding
ENSG0000(	TRPV6	60.52598	0.559391	0.065598	0.140659	protein_coding
ENSG0000(	ENSG0000(	98.74156	0.153869	0.065703	0.14087	lncRNA
ENSG0000(	DNAAF11	120.2807	-0.28439	0.065729	0.140915	protein_coding
ENSG0000(	MIB1	2209.454	0.082233	0.06583	0.141123	protein_coding
ENSG0000(	MPV17	1042.614	0.097589	0.065838	0.14113	protein_coding
ENSG0000(	ENSG0000(	328.6866	0.268889	0.065883	0.141217	lncRNA
ENSG0000(	EHD4	1861.138	0.162028	0.065901	0.141246	protein_coding
ENSG0000(	IPCEF1	610.6921	0.313565	0.065942	0.141325	protein_coding
ENSG0000(	SNORA70B	19.45371	0.30281	0.065963	0.141361	snoRNA
ENSG0000(	ENSG0000(	11.04773	-0.43514	0.065973	0.141371	lncRNA
ENSG0000(	ASB6	824.5195	-0.06729	0.065983	0.141377	protein_coding
ENSG0000(	LINC02100	8.766745	0.455951	0.065989	0.141377	lncRNA
ENSG0000(	SYT12	415.3111	0.121798	0.065988	0.141377	protein_coding
ENSG0000(	TMEM186	204.2778	-0.11762	0.066014	0.14142	protein_coding
ENSG0000(	TDRD5	83.67662	-0.33073	0.066034	0.141448	protein_coding
ENSG0000(	ZBED1	1382.431	-0.12159	0.066036	0.141448	protein_coding
ENSG0000(	BPTFP1	223.5752	-0.28106	0.066061	0.141492	unprocessed_pseudogene
ENSG0000(	CFL2	450.3971	0.155752	0.06607	0.141502	protein_coding
ENSG0000(	ENSG0000(	66.58726	-0.17267	0.066084	0.141511	lncRNA
ENSG0000(	LEAP2	133.184	-0.1512	0.06608	0.141511	protein_coding
ENSG0000(	MTND5P11	46.71953	-0.50498	0.066116	0.141571	processed_pseudogene
ENSG0000(	ENSG0000(	307.6892	0.155362	0.066135	0.141601	lncRNA
ENSG0000(	ACE	960.8454	-0.19473	0.066196	0.141723	protein_coding
ENSG0000(	CYP2U1	246.4986	0.166672	0.066211	0.141745	protein_coding
ENSG0000(	ENSG0000(	98.8487	0.222372	0.066215	0.141745	lncRNA
ENSG0000(	RMI1	381.4201	-0.11522	0.066226	0.141758	protein_coding
ENSG0000(	ENSG0000(	745.125	0.152402	0.066241	0.141766	transcribed_processed_pseudoge
ENSG0000(	ENSG0000(	80.65134	0.16271	0.066243	0.141766	TEC
ENSG0000(	ENSG0000(	37.20569	0.217148	0.066236	0.141766	lncRNA
ENSG0000(	C3orf85	413.9925	-0.34043	0.06631	0.141879	protein_coding
ENSG0000(	ENSG0000(	31.19647	0.284131	0.066305	0.141879	lncRNA
ENSG0000(	PCNX4	4565.858	0.075111	0.066306	0.141879	protein_coding

ENSG0000(	KCNMB4	168.4274	-0.17722	0.066344	0.141933	protein_coding
ENSG0000(	MTND5P15	33.79966	0.298528	0.06634	0.141933	processed_pseudogene
ENSG0000(	RNF11	932.15	-0.17749	0.066355	0.141946	protein_coding
ENSG0000(	ACSS2	4696.056	-0.23156	0.066362	0.141951	protein_coding
ENSG0000(	ENSG0000(	16.94863	-0.32073	0.066366	0.141951	lncRNA
ENSG0000(	ENSG0000(	108.2511	-0.16794	0.066394	0.142	lncRNA
ENSG0000(	ENSG0000(	12.35471	0.394001	0.066404	0.142012	lncRNA
ENSG0000(	ZNF442	680.5216	-0.11458	0.066443	0.142086	protein_coding
ENSG0000(	ENSG0000(	44.39221	0.707966	0.066473	0.142141	transcribed_unprocessed_pseudo
ENSG0000(	ENSG0000(	141.6311	-0.11633	0.066487	0.142141	lncRNA
ENSG0000(	ENSG0000(	111.2564	0.258706	0.066479	0.142141	lncRNA
ENSG0000(	HES2	829.5704	-0.11017	0.066485	0.142141	protein_coding
ENSG0000(	CD8B2	98.98905	0.203767	0.066491	0.142141	protein_coding
ENSG0000(	ACOT8	643.5528	-0.16469	0.066504	0.142159	protein_coding
ENSG0000(	BICD2	998.4481	0.118086	0.066515	0.142172	protein_coding
ENSG0000(	ABHD17B	544.7207	-0.14835	0.066537	0.142187	protein_coding
ENSG0000(	FAM72A	109.9153	0.272323	0.06654	0.142187	protein_coding
ENSG0000(	SYT17	495.4605	-0.17073	0.066536	0.142187	protein_coding
ENSG0000(	ZBTB38	5459.838	-0.09343	0.06653	0.142187	protein_coding
ENSG0000(	ARHGEF19	164.8836	-0.19109	0.06655	0.142199	protein_coding
ENSG0000(	PRCP	2801.79	-0.12052	0.06657	0.142232	protein_coding
ENSG0000(	CBWD1	3717.189	0.103128	0.066588	0.142261	protein_coding
ENSG0000(	IGHJ3	114.006	0.416071	0.066604	0.142285	IG_J_gene
ENSG0000(	ENSG0000(	117.8856	-0.14448	0.066633	0.142338	lncRNA
ENSG0000(	HAUS3	1225.873	-0.07024	0.066658	0.142381	protein_coding
ENSG0000(	SPDYE3	35.68684	0.308912	0.066668	0.142392	protein_coding
ENSG0000(	TSPAN7	785.6065	-0.31706	0.066696	0.142442	protein_coding
ENSG0000(	SIK2	2161.016	-0.0792	0.066703	0.142447	protein_coding
ENSG0000(	PAK6	151.8162	-0.18673	0.066715	0.142464	protein_coding
ENSG0000(	NR1H4	555.6426	-0.38283	0.066723	0.142471	protein_coding
ENSG0000(	ENSG0000(	24.06	0.280604	0.066751	0.142522	lncRNA
ENSG0000(	RNU4ATAC	30.8702	0.284417	0.066756	0.142522	snRNA
ENSG0000(	LINC02816	19.41759	0.298188	0.066763	0.142526	lncRNA
ENSG0000(	CPD	3339.779	0.091331	0.066773	0.142539	protein_coding
ENSG0000(	ELAPOR1	2162.905	-0.26849	0.066779	0.142543	protein_coding
ENSG0000(	FKBP8	745.6174	-0.1359	0.066791	0.142558	protein_coding
ENSG0000(	EBF4	177.8401	0.234688	0.066838	0.142648	protein_coding
ENSG0000(	ADGRG5	385.6464	0.297194	0.06685	0.142663	protein_coding
ENSG0000(	ENSG0000(	40.84833	0.225788	0.06698	0.142933	lncRNA
ENSG0000(	CYP4F26P	154.2345	0.144845	0.066993	0.14294	lncRNA
ENSG0000(	ENSG0000(	49.2371	-0.208	0.066993	0.14294	lncRNA
ENSG0000(	ENSG0000(	302.9917	0.202207	0.067008	0.142962	transcribed_unprocessed_pseudo
ENSG0000(	JOSD1	2568.106	-0.20135	0.06702	0.142977	protein_coding
ENSG0000(	ENSG0000(	55.45563	0.296541	0.067035	0.142999	lncRNA
ENSG0000(	ENSG0000(	31.62335	0.22607	0.067072	0.143069	lncRNA
ENSG0000(	RBM25	6573.275	0.068323	0.06708	0.143076	protein_coding
ENSG0000(	ENSG0000(	43.85421	0.203898	0.067098	0.143105	lncRNA

ENSG0000(	LINC01907	366.3668	-0.10178	0.067149	0.143204	lncRNA
ENSG0000(	HNRNPL	3589.289	-0.10047	0.067165	0.14323	protein_coding
ENSG0000(	PRDM2	3372.312	0.099805	0.067232	0.143363	protein_coding
ENSG0000(	ENSG0000(	10.36774	0.495451	0.067282	0.14345	lncRNA
ENSG0000(	LONRF1	535.3646	0.146301	0.067283	0.14345	protein_coding
ENSG0000(	PIK3C2B	4488.376	-0.10537	0.067305	0.143489	protein_coding
ENSG0000(	ENSG0000(	13.81287	-0.3481	0.067329	0.14353	TEC
ENSG0000(	ENSG0000(	66.36944	-0.21464	0.067378	0.143614	lncRNA
ENSG0000(	IGLC6	14.02758	0.55372	0.067374	0.143614	IG_C_pseudogene
ENSG0000(	KLF2-DT	26.80812	0.284894	0.06742	0.143695	lncRNA
ENSG0000(	ENSG0000(	17.60136	0.36718	0.067428	0.143703	lncRNA
ENSG0000(	ENSG0000(	13.39516	0.406915	0.067448	0.143722	lncRNA
ENSG0000(	IFNAR2	1585.873	0.159991	0.067454	0.143722	protein_coding
ENSG0000(	RN7SKP292	38.55027	0.22548	0.067453	0.143722	misc_RNA
ENSG0000(	RN7SL774P	241.4202	0.129281	0.067456	0.143722	misc_RNA
ENSG0000(	LINC00879	12.33431	0.386209	0.067472	0.143747	lncRNA
ENSG0000(	ENSG0000(	55.04187	0.198717	0.067484	0.143762	lncRNA
ENSG0000(	MAP4K1	669.5228	-0.14424	0.067496	0.143779	protein_coding
ENSG0000(	GCAT	152.252	-0.15137	0.067552	0.143886	protein_coding
ENSG0000(	RASA1	1411.419	-0.06006	0.067604	0.143988	protein_coding
ENSG0000(	SLC7A2	276.1009	0.266825	0.067615	0.144002	protein_coding
ENSG0000(	LINC01020	90.81809	0.14516	0.067668	0.144103	lncRNA
ENSG0000(	MCUR1	1521.697	-0.10398	0.067672	0.144103	protein_coding
ENSG0000(	HES5	23.45896	-0.49039	0.067694	0.14414	protein_coding
ENSG0000(	ENSG0000(	61.02489	-0.19049	0.067701	0.144147	lncRNA
ENSG0000(	ENSG0000(	15.50349	0.393191	0.067746	0.144231	lncRNA
ENSG0000(	CCL21	510.2597	-0.7812	0.067773	0.144279	protein_coding
ENSG0000(	ENSG0000(	35.36171	0.255945	0.067787	0.1443	lncRNA
ENSG0000(	BAMBI	86.50046	-0.37518	0.06782	0.144341	protein_coding
ENSG0000(	BEAN1	142.7269	0.203823	0.067817	0.144341	protein_coding
ENSG0000(	KLF4	4355.426	-0.28164	0.067812	0.144341	protein_coding
ENSG0000(	GLRX	1392.859	0.193485	0.06786	0.144416	protein_coding
ENSG0000(	TRPC4	95.65181	0.41064	0.067872	0.14443	protein_coding
ENSG0000(	RN7SL173P	127.2549	0.14062	0.067882	0.144442	misc_RNA
ENSG0000(	ENSG0000(	14.37968	0.349054	0.067912	0.144496	TEC
ENSG0000(	LRRTM4	363.5733	0.123347	0.068013	0.144692	protein_coding
ENSG0000(	RPL4P6	146.4148	0.165165	0.068012	0.144692	processed_pseudogene
ENSG0000(	AGPAT3	3265.999	-0.09333	0.068077	0.144818	protein_coding
ENSG0000(	RN7SL494P	17.10773	0.33998	0.068102	0.144858	misc_RNA
ENSG0000(	SLC18A1	76.65259	0.258174	0.068105	0.144858	protein_coding
ENSG0000(	ENSG0000(	15.0907	-0.36314	0.068157	0.14494	lncRNA
ENSG0000(	ODC1	909.4951	-0.21783	0.068157	0.14494	protein_coding
ENSG0000(	RNU4-2	29703.33	-0.15952	0.068148	0.14494	snRNA
ENSG0000(	GSTO1	193.2279	-0.19806	0.068168	0.144953	protein_coding
ENSG0000(	BDNF	34.119	0.427963	0.0682	0.145002	protein_coding
ENSG0000(	ENSG0000(	30.07652	0.230119	0.0682	0.145002	lncRNA
ENSG0000(	PPP1R13B	2176.245	-0.13643	0.068231	0.145057	protein_coding

ENSG0000(	PRKAG2	2605.976	-0.09547	0.068247	0.145082	protein_coding
ENSG0000(	NENFP1	23.2451	0.269215	0.068264	0.145108	processed_pseudogene
ENSG0000(	ENSG0000(	59.57392	0.182404	0.068286	0.145144	lncRNA
ENSG0000(	PPIB	2273.397	-0.18686	0.068292	0.145147	protein_coding
ENSG0000(	HES6	111.7207	-0.29064	0.06832	0.145198	protein_coding
ENSG0000(	LARP4	2389.194	-0.09853	0.068327	0.145202	protein_coding
ENSG0000(	CHRNA7	754.477	-0.13389	0.068334	0.145207	protein_coding
ENSG0000(	ENSG0000(	32.26208	0.276038	0.068381	0.145286	lncRNA
ENSG0000(	RN7SL146F	29.46806	0.249448	0.068379	0.145286	misc_RNA
ENSG0000(	DNLZ	52.17773	-0.31573	0.068387	0.14529	protein_coding
ENSG0000(	SLC25A51P	369.4699	0.079908	0.068446	0.145406	transcribed_unprocessed_pseudo
ENSG0000(	ICAM4	130.1129	0.145599	0.068524	0.14556	protein_coding
ENSG0000(	ENSG0000(	71.10879	0.155093	0.068532	0.145565	TEC
ENSG0000(	PROX1	1047.296	0.307562	0.068535	0.145565	protein_coding
ENSG0000(	AGAP7P	55.87811	0.664677	0.068556	0.145589	unprocessed_pseudogene
ENSG0000(	TUBE1	861.8913	0.080154	0.068553	0.145589	protein_coding
ENSG0000(	ENSG0000(	27.94804	0.263103	0.068561	0.145591	processed_pseudogene
ENSG0000(	GATC	1251.986	-0.08353	0.06857	0.1456	protein_coding
ENSG0000(	PGAP6	1253.423	-0.20533	0.068623	0.145702	protein_coding
ENSG0000(	SOX12	182.5266	0.183637	0.068682	0.145817	protein_coding
ENSG0000(	ENSG0000(	25.54409	-0.32754	0.068692	0.14582	lncRNA
ENSG0000(	RPL36AP39	10.83697	0.380216	0.06869	0.14582	processed_pseudogene
ENSG0000(	PCDHGB7	164.358	-0.17659	0.06876	0.145952	protein_coding
ENSG0000(	ENSG0000(	36.49717	-0.2056	0.068782	0.14599	lncRNA
ENSG0000(	ST8SIA3	31.20792	0.529955	0.068824	0.146069	protein_coding
ENSG0000(	CSPG4P13	38.3001	0.484975	0.068851	0.146108	transcribed_unprocessed_pseudo
ENSG0000(	LINC00454	39.83927	0.320521	0.068848	0.146108	lncRNA
ENSG0000(	SESTD1	1326.557	0.212638	0.06887	0.146138	protein_coding
ENSG0000(	ARHGAP35	4430.722	-0.14549	0.06888	0.146148	protein_coding
ENSG0000(	ENSG0000(	10.03644	0.703118	0.068921	0.146225	lncRNA
ENSG0000(	CHD2	9565.341	0.107173	0.068939	0.146254	protein_coding
ENSG0000(	ENSG0000(	119.891	0.373385	0.068955	0.146279	lncRNA
ENSG0000(	RAB7A	2821.75	-0.12141	0.068963	0.146285	protein_coding
ENSG0000(	FOSL1	82.51392	0.283715	0.068996	0.146344	protein_coding
ENSG0000(	RBMXP2	7.852026	0.815341	0.069031	0.146409	processed_pseudogene
ENSG0000(	MIR3975	29.97082	0.323254	0.069045	0.146429	miRNA
ENSG0000(	ENSG0000(	365.3428	0.144914	0.069051	0.146433	transcribed_unprocessed_pseudo
ENSG0000(	ENSG0000(	365.4754	0.078687	0.069083	0.14649	lncRNA
ENSG0000(	ENSG0000(	10.92133	0.629064	0.069089	0.146492	lncRNA
ENSG0000(	HOXA9	788.0386	-0.22042	0.069094	0.146493	protein_coding
ENSG0000(	ENSG0000(	55.47942	-0.24132	0.069109	0.146505	transcribed_processed_pseudoge
ENSG0000(	ENSG0000(	171.3946	-0.17134	0.069105	0.146505	protein_coding
ENSG0000(	SLC25A23	1544.552	-0.28223	0.069127	0.146534	protein_coding
ENSG0000(	MIR3183	20.49867	-0.33879	0.069145	0.146561	miRNA
ENSG0000(	ENSG0000(	194.5512	0.177061	0.069167	0.146598	lncRNA
ENSG0000(	ARMCX4	1314.103	0.137294	0.069202	0.146632	protein_coding
ENSG0000(	FRG1	380.4906	-0.15964	0.069202	0.146632	protein_coding

ENSG0000(	IGKV1D-39	1391.775	0.502575	0.069195	0.146632	IG_V_gene
ENSG0000(	LINC00944	146.2984	0.155958	0.06919	0.146632	lncRNA
ENSG0000(	USP9X	6881.999	-0.10464	0.069251	0.146727	protein_coding
ENSG0000(	ENSG0000(	10.01825	-0.75304	0.06929	0.146792	lncRNA
ENSG0000(	ENSG0000(	194.2364	0.342287	0.069291	0.146792	lncRNA
ENSG0000(	LINC00974	11.96983	-0.47315	0.069344	0.146885	lncRNA
ENSG0000(	STX4	875.9801	-0.07378	0.069342	0.146885	protein_coding
ENSG0000(	TENM3-AS1	216.1823	0.10493	0.069384	0.146958	lncRNA
ENSG0000(	SH3PXD2A	6686.952	-0.13043	0.069414	0.147012	protein_coding
ENSG0000(	CAND2	81.93837	0.359425	0.069439	0.147055	protein_coding
ENSG0000(	OR2T8	15.47501	0.441372	0.069477	0.147127	protein_coding
ENSG0000(	FTSJ1	329.1605	-0.15509	0.069482	0.147127	protein_coding
ENSG0000(	DDX20	444.9894	-0.13091	0.069515	0.147185	protein_coding
ENSG0000(	ENSG0000(	67.72636	0.496891	0.069596	0.147348	unprocessed_pseudogene
ENSG0000(	SCAF8	2875.183	-0.06477	0.069612	0.147373	protein_coding
ENSG0000(	ENSG0000(	40.2794	-0.26798	0.069624	0.147388	lncRNA
ENSG0000(	ENSG0000(	193.001	-0.10892	0.069638	0.147408	lncRNA
ENSG0000(	RGL4	61.69512	0.432849	0.069674	0.147474	protein_coding
ENSG0000(	VTRNA1-1	66.91675	0.345723	0.0697	0.147519	misc_RNA
ENSG0000(	C6	27.27303	-0.57615	0.069728	0.147567	protein_coding
ENSG0000(	VCAM1	526.0499	0.307925	0.069753	0.14761	protein_coding
ENSG0000(	ENSG0000(	247.1019	-0.12654	0.069774	0.147645	lncRNA
ENSG0000(	ENSG0000(	877.9173	0.096512	0.0698	0.14769	lncRNA
ENSG0000(	STX3	2993.252	-0.19514	0.069811	0.147704	protein_coding
ENSG0000(	ENSG0000(	115.7307	-0.16252	0.069848	0.147771	lncRNA
ENSG0000(	POLG2	773.6693	0.120976	0.069912	0.147897	protein_coding
ENSG0000(	FITM1	50.53879	-0.2213	0.069941	0.147949	protein_coding
ENSG0000(	PSME2	1080.835	0.164395	0.069953	0.147963	protein_coding
ENSG0000(	ENSG0000(	115.2654	0.192015	0.069999	0.14805	lncRNA
ENSG0000(	ENSG0000(	17.09591	0.356242	0.070038	0.148106	lncRNA
ENSG0000(	LINC02587	33.3453	0.26437	0.07003	0.148106	lncRNA
ENSG0000(	LRP10	4623.164	-0.18675	0.07004	0.148106	protein_coding
ENSG0000(	KLHL6-AS1	206.5766	0.150532	0.070074	0.148169	lncRNA
ENSG0000(	RIMS2	515.0903	-0.13612	0.070098	0.148209	protein_coding
ENSG0000(	ENSG0000(	14.75394	-0.33249	0.070141	0.148292	lncRNA
ENSG0000(	ENSG0000(	42.00438	0.235578	0.070155	0.148301	lncRNA
ENSG0000(	FAHD2A	672.3794	-0.1094	0.070154	0.148301	protein_coding
ENSG0000(	UBE3B	2314.367	-0.07963	0.070179	0.14834	protein_coding
ENSG0000(	NIPA2P4	16.0766	0.550472	0.070188	0.148351	processed_pseudogene
ENSG0000(	CD300LG	102.8638	-0.21616	0.070195	0.148355	protein_coding
ENSG0000(	THADA	3123.965	0.11294	0.070208	0.148372	protein_coding
ENSG0000(	BMS1P23	2337.444	0.125915	0.070218	0.148383	transcribed_unprocessed_pseudo
ENSG0000(	ASRGL1	569.6936	0.164992	0.070248	0.148437	protein_coding
ENSG0000(	RPS6KC1	917.9104	0.117594	0.070255	0.148442	protein_coding
ENSG0000(	HMGN1P24	9.646746	-0.44102	0.070304	0.148534	processed_pseudogene
ENSG0000(	SLF1	450.9976	-0.14478	0.070337	0.148595	protein_coding
ENSG0000(	PNLIPP1	46.54727	-0.19078	0.070359	0.148631	unprocessed_pseudogene

ENSG0000(	ENSG0000(	12.72273	-0.45693	0.070397	0.14869	protein_coding
ENSG0000(	REG3A	330.9218	2.446832	0.070401	0.14869	protein_coding
ENSG0000(	TNFRSF19	137.0457	0.174489	0.070395	0.14869	protein_coding
ENSG0000(	ENSG0000(	48.64172	0.290342	0.070423	0.148724	lncRNA
ENSG0000(	ENSG0000(	117.1835	0.157605	0.070427	0.148724	lncRNA
ENSG0000(	PDZD4	96.71325	-0.23584	0.070434	0.148728	protein_coding
ENSG0000(	TTTY14	1835.746	0.093154	0.070483	0.148822	lncRNA
ENSG0000(	CORO1A-A:	15.052	0.492668	0.070506	0.14886	lncRNA
ENSG0000(	PHBP8	44.34657	-0.32446	0.070513	0.148867	processed_pseudogene
ENSG0000(	ENSG0000(	594.1231	0.100689	0.070553	0.14892	lncRNA
ENSG0000(	MOB3B	2077.716	-0.10906	0.07055	0.14892	protein_coding
ENSG0000(	RIC3-DT	14.35059	0.381943	0.070549	0.14892	lncRNA
ENSG0000(	VTRNA1-2	29.9512	0.409767	0.070603	0.149015	misc_RNA
ENSG0000(	TMEM107	528.9102	0.091392	0.070634	0.149072	protein_coding
ENSG0000(	ENSG0000(	25.87668	0.327379	0.07064	0.149073	TEC
ENSG0000(	APPBP2	1668.073	-0.06459	0.070652	0.149088	protein_coding
ENSG0000(	ZMYM1	757.2127	-0.13777	0.070767	0.149321	protein_coding
ENSG0000(	SHLD3	111.5035	0.171349	0.070775	0.149328	protein_coding
ENSG0000(	SERINC4	18.67443	0.467489	0.070784	0.149337	protein_coding
ENSG0000(	PRRC2C	12207.14	-0.0827	0.070789	0.149337	protein_coding
ENSG0000(	ENSG0000(	9.813676	0.583175	0.070857	0.14946	lncRNA
ENSG0000(	SLC13A2	70.47921	-0.63709	0.070856	0.14946	protein_coding
ENSG0000(	ENSG0000(	20.34936	0.357863	0.070909	0.149561	lncRNA
ENSG0000(	ENSG0000(	188.3296	0.11528	0.070961	0.149643	lncRNA
ENSG0000(	ENSG0000(	70.25049	0.189083	0.070962	0.149643	TEC
ENSG0000(	P4HA3-AS1	166.213	-0.11648	0.070962	0.149643	lncRNA
ENSG0000(	ENSG0000(	19.21068	0.322882	0.070974	0.149657	lncRNA
ENSG0000(	GORASP1	1858.779	-0.10999	0.070989	0.149669	protein_coding
ENSG0000(	XXYLT1-AS:	12.49412	0.474132	0.070987	0.149669	lncRNA
ENSG0000(	LINC00393	13.2153	-0.56285	0.071001	0.149683	lncRNA
ENSG0000(	ADA	110.4614	0.295283	0.071067	0.149814	protein_coding
ENSG0000(	LINC00933	151.6193	0.120924	0.071074	0.149817	transcribed_unprocessed_pseudo
ENSG0000(	KNL1	1150.779	-0.17941	0.071111	0.149886	protein_coding
ENSG0000(	FLG2	149.6625	-0.2138	0.071124	0.149894	protein_coding
ENSG0000(	OR10Z1	95.79875	0.151162	0.071121	0.149894	protein_coding
ENSG0000(	CIB1	828.765	-0.19939	0.071158	0.149944	protein_coding
ENSG0000(	TTC9-DT	25.76963	0.523732	0.071155	0.149944	lncRNA
ENSG0000(	RPS26P47	9.751325	0.685269	0.071163	0.149945	processed_pseudogene
ENSG0000(	LETR1	579.1616	0.125419	0.071175	0.149959	lncRNA
ENSG0000(	PCDH10	30.48944	-0.30234	0.071191	0.149983	protein_coding
ENSG0000(	INTS13	345.0925	-0.19392	0.071247	0.150072	protein_coding
ENSG0000(	KCNIP2	269.9161	-0.1288	0.071248	0.150072	protein_coding
ENSG0000(	MINDY1	800.352	-0.12777	0.071252	0.150072	protein_coding
ENSG0000(	RNU6-936F	9.918084	0.417179	0.071241	0.150072	snRNA
ENSG0000(	ENSG0000(	63.64534	0.201598	0.071282	0.150124	processed_pseudogene
ENSG0000(	C1QTNF1-A	142.5245	0.211861	0.07129	0.150132	lncRNA
ENSG0000(	DIMT1	1485.533	-0.0598	0.071324	0.150194	protein_coding

ENSG0000(	SEC24AP1	111.3783	0.241273	0.071358	0.150255	processed_pseudogene
ENSG0000(	HSFY3P	16.32373	0.320782	0.071398	0.150329	transcribed_unprocessed_pseudo
ENSG0000(	ENSG0000(	111.0746	-0.22674	0.071438	0.150402	lncRNA
ENSG0000(	ENSG0000(	33.77917	-0.56421	0.071448	0.150411	lncRNA
ENSG0000(	IGKV1D-16	52.12864	0.577502	0.071456	0.150411	IG_V_gene
ENSG0000(	RN7SL660F	39.89899	0.228271	0.071456	0.150411	misc_RNA
ENSG0000(	PTGER4P2-	177.6472	0.167343	0.071486	0.150463	lncRNA
ENSG0000(	TAF15	5890.954	0.074248	0.071508	0.1505	protein_coding
ENSG0000(	RHOC	2039.487	-0.23236	0.071529	0.150534	protein_coding
ENSG0000(	RN7SL48P	346.7367	0.09893	0.071534	0.150535	misc_RNA
ENSG0000(	ZCCHC18	63.77449	0.317793	0.071579	0.150617	protein_coding
ENSG0000(	MALLP2	26.3231	0.416826	0.071592	0.150635	transcribed_unprocessed_pseudo
ENSG0000(	RNU2-18P	11.96012	0.304649	0.071732	0.150921	snRNA
ENSG0000(	ENSG0000(	663.673	0.113947	0.071761	0.150971	lncRNA
ENSG0000(	ENSG0000(	64.4327	0.189815	0.071796	0.151035	lncRNA
ENSG0000(	LINC01949	1126.709	-0.10845	0.071846	0.15113	lncRNA
ENSG0000(	SELENOF	870.4358	-0.12898	0.071853	0.151134	protein_coding
ENSG0000(	ENSG0000(	155.5225	-0.20661	0.071904	0.151232	lncRNA
ENSG0000(	PRDM11	609.4219	-0.12058	0.071924	0.151263	protein_coding
ENSG0000(	ENSG0000(	74.56557	-0.15055	0.072046	0.151505	lncRNA
ENSG0000(	RHEB	631.3183	-0.12022	0.072049	0.151505	protein_coding
ENSG0000(	ENSG0000(	59.3046	-0.14844	0.072083	0.151557	lncRNA
ENSG0000(	SAMMSON	509.6567	-0.08186	0.072083	0.151557	lncRNA
ENSG0000(	AGXT	234.9082	-0.13168	0.072117	0.151617	protein_coding
ENSG0000(	CLDN9	9.849394	-0.42915	0.072125	0.151625	protein_coding
ENSG0000(	CKAP2L	442.0636	-0.16298	0.072136	0.151637	protein_coding
ENSG0000(	LINC00899	316.714	-0.12927	0.072152	0.15166	lncRNA
ENSG0000(	MARCHF1C	52.86846	0.182964	0.072156	0.15166	lncRNA
ENSG0000(	ADCY7	2252.519	0.136942	0.072186	0.151701	protein_coding
ENSG0000(	SPTBN5	281.0395	0.435117	0.072183	0.151701	protein_coding
ENSG0000(	LINC01301	334.3361	0.092887	0.072195	0.151707	lncRNA
ENSG0000(	LINC01582	17.75589	-0.39083	0.072198	0.151707	lncRNA
ENSG0000(	ZSCAN5A	752.9007	0.081382	0.072206	0.151714	protein_coding
ENSG0000(	C6orf52	9.848046	-0.48217	0.072229	0.151751	protein_coding
ENSG0000(	ENSG0000(	327.4152	0.094506	0.072249	0.151773	lncRNA
ENSG0000(	SMYD4	1798.648	0.070936	0.072248	0.151773	protein_coding
ENSG0000(	ENSG0000(	11.91927	0.364856	0.072265	0.151796	lncRNA
ENSG0000(	CFL1P5	44.68463	-0.30554	0.072279	0.1518	processed_pseudogene
ENSG0000(	GABARAP	854.4652	-0.18859	0.072281	0.1518	protein_coding
ENSG0000(	SIGLEC11	239.3261	0.125673	0.072274	0.1518	protein_coding
ENSG0000(	SLC25A42	646.575	-0.12359	0.072319	0.151869	protein_coding
ENSG0000(	MELTF	756.4141	0.158068	0.072329	0.151879	protein_coding
ENSG0000(	ENSG0000(	199.9895	0.164124	0.072378	0.151971	lncRNA
ENSG0000(	GPM6A	49.24637	0.300561	0.07239	0.151987	protein_coding
ENSG0000(	EIF1	3459.26	-0.12273	0.072405	0.152003	protein_coding
ENSG0000(	POGLUT2P	202.4299	-0.26803	0.072407	0.152003	processed_pseudogene
ENSG0000(	CAPN5	4477.066	-0.22226	0.072424	0.152029	protein_coding

ENSG0000(	ENSG0000(	77.90885	0.176475	0.072433	0.152031	unprocessed_pseudogene
ENSG0000(	VAPB	2161.085	-0.06864	0.072435	0.152031	protein_coding
ENSG0000(	ZNF155	248.1453	-0.18891	0.072471	0.152097	protein_coding
ENSG0000(	LRRC1	1791.801	-0.23543	0.07248	0.152105	protein_coding
ENSG0000(	APPL2	3129.712	-0.22132	0.072494	0.152108	protein_coding
ENSG0000(	LINC00863	727.8835	0.110321	0.072496	0.152108	lncRNA
ENSG0000(	LINC02530	41.60594	-0.2214	0.072492	0.152108	lncRNA
ENSG0000(	ALPK3	214.3785	0.29662	0.072565	0.152222	protein_coding
ENSG0000(	CTDSP2	6147.68	-0.14301	0.07257	0.152222	protein_coding
ENSG0000(	ENSG0000(	38.8337	0.340502	0.072567	0.152222	lncRNA
ENSG0000(	NHLRC3	1803.145	0.106354	0.072559	0.152222	protein_coding
ENSG0000(	EFHD2	1592.391	-0.13535	0.072638	0.152355	protein_coding
ENSG0000(	PIP4P1	737.0073	-0.11275	0.07265	0.15236	protein_coding
ENSG0000(	SCIN	2490.531	-0.30029	0.072647	0.15236	protein_coding
ENSG0000(	EPB41L4A	1024.875	0.115731	0.07267	0.152391	protein_coding
ENSG0000(	VPS4A	1481.982	0.080815	0.072678	0.152399	protein_coding
ENSG0000(	C5AR1	497.3415	-0.12978	0.072717	0.152469	protein_coding
ENSG0000(	MON2	6302.334	0.074937	0.07273	0.152487	protein_coding
ENSG0000(	ARHGEF38-	64.47608	0.26441	0.072808	0.15264	lncRNA
ENSG0000(	ENSG0000(	9.092673	0.385146	0.072823	0.152661	lncRNA
ENSG0000(	CSPG4P12	1548.471	0.115909	0.07284	0.152676	transcribed_unprocessed_pseudo
ENSG0000(	PAPPA-AS1	73.82823	0.199459	0.072836	0.152676	lncRNA
ENSG0000(	RN7SL520F	17.0016	0.358017	0.072893	0.152778	misc_RNA
ENSG0000(	SLC26A2	37326.16	-0.45196	0.072912	0.152806	protein_coding
ENSG0000(	TCEAL3	62.70531	0.22422	0.072918	0.152809	protein_coding
ENSG0000(	KCTD7	903.4875	0.101985	0.072934	0.152822	protein_coding
ENSG0000(	MATK	176.1502	-0.12093	0.072931	0.152822	protein_coding
ENSG0000(	ANKRD30B	235.8565	0.246724	0.072944	0.152829	transcribed_unprocessed_pseudo
ENSG0000(	COPB1	1692.825	0.087996	0.072947	0.152829	protein_coding
ENSG0000(	MAD2L2	79.76379	0.235093	0.073006	0.152942	protein_coding
ENSG0000(	ENSG0000(	10.20109	0.459146	0.073027	0.152975	lncRNA
ENSG0000(	ENSG0000(	199.9184	-0.11166	0.073035	0.152982	lncRNA
ENSG0000(	MITD1	1223.781	-0.08856	0.073081	0.153068	protein_coding
ENSG0000(	IGLV1-50	12.56276	0.452132	0.073088	0.153073	IG_V_gene
ENSG0000(	SSR4P1	29.87766	0.312605	0.07311	0.153109	transcribed_processed_pseudoge
ENSG0000(	SPATA31C1	133.4529	-0.11351	0.073148	0.153177	transcribed_unprocessed_pseudo
ENSG0000(	ELP2	2209.352	-0.11502	0.073226	0.153322	protein_coding
ENSG0000(	TMEM243	426.5706	0.185202	0.073226	0.153322	protein_coding
ENSG0000(	SNORD17	2004.756	-0.27145	0.073275	0.153413	snoRNA
ENSG0000(	ENSG0000(	17.0356	0.350805	0.073326	0.153478	lncRNA
ENSG0000(	INTS7	849.9795	-0.1386	0.073319	0.153478	protein_coding
ENSG0000(	MORF4L1P	12.65071	-0.53582	0.073315	0.153478	processed_pseudogene
ENSG0000(	ZNF417	2055.044	0.110826	0.073321	0.153478	protein_coding
ENSG0000(	ACOT4	20.15107	-0.69707	0.073335	0.153487	protein_coding
ENSG0000(	ENSG0000(	18.80739	0.647442	0.073392	0.153597	lncRNA
ENSG0000(	ENSG0000(	2567.655	0.071545	0.073413	0.15362	lncRNA
ENSG0000(	ENSG0000(	17.3256	-0.27389	0.073411	0.15362	lncRNA

ENSG0000(	OR4E2	42.94947	-0.20785	0.07342	0.153624	protein_coding
ENSG0000(	ENSG0000(	19.39647	-0.34719	0.073451	0.153671	transcribed_processed_pseudoge
ENSG0000(	S100A4	121.2237	0.345764	0.073452	0.153671	protein_coding
ENSG0000(	GADD45A	282.6258	-0.22651	0.073474	0.153706	protein_coding
ENSG0000(	IGKV1-8	38.63368	0.610382	0.0735	0.153741	IG_V_gene
ENSG0000(	PGGHG	2046.146	0.259134	0.073496	0.153741	protein_coding
ENSG0000(	IGLV2-18	106.3105	0.526159	0.073508	0.153745	IG_V_gene
ENSG0000(	AKR1C1	325.3748	-0.20064	0.073524	0.15377	protein_coding
ENSG0000(	LINC02274	71.29563	0.167435	0.073572	0.153859	lncRNA
ENSG0000(	ANK2-AS1	64.971	0.159144	0.073591	0.153888	lncRNA
ENSG0000(	ENSG0000(	23.48723	0.306485	0.073597	0.153892	lncRNA
ENSG0000(	ENSG0000(	23.34394	-0.29539	0.073604	0.153896	lncRNA
ENSG0000(	EPHX3	12.30398	0.588001	0.073617	0.153913	protein_coding
ENSG0000(	STX2	462.0344	0.166542	0.073622	0.153913	protein_coding
ENSG0000(	PGBD1	107.5268	0.141228	0.073632	0.153924	protein_coding
ENSG0000(	LRRC75A	1566.447	-0.2926	0.073643	0.153936	protein_coding
ENSG0000(	NPM1P9	9.153147	-0.46201	0.07371	0.154065	processed_pseudogene
ENSG0000(	ENSG0000(	36.2105	-0.3676	0.073741	0.154121	lncRNA
ENSG0000(	NARS2	383.294	-0.18764	0.073774	0.154179	protein_coding
ENSG0000(	ENSG0000(	46.25877	0.212858	0.073795	0.154212	lncRNA
ENSG0000(	LINC01152	101.3556	-0.11708	0.073815	0.154245	lncRNA
ENSG0000(	ENSG0000(	61.08989	0.202028	0.073845	0.154296	lncRNA
ENSG0000(	ENSG0000(	69.34409	0.232635	0.073866	0.15433	lncRNA
ENSG0000(	TIMD4	51.47067	-0.42162	0.073901	0.154393	protein_coding
ENSG0000(	ENSG0000(	84.15625	0.387302	0.073915	0.154412	lncRNA
ENSG0000(	ENSG0000(	14.08854	0.439135	0.073964	0.154504	processed_pseudogene
ENSG0000(	ENSG0000(	15.96772	0.423868	0.07403	0.154621	lncRNA
ENSG0000(	ENSG0000(	168.8718	0.183309	0.074029	0.154621	lncRNA
ENSG0000(	ENSG0000(	44.94996	-0.2368	0.074049	0.15465	lncRNA
ENSG0000(	MEX3D	165.134	-0.16991	0.074055	0.154652	protein_coding
ENSG0000(	NCR3LG1	1372.575	-0.20807	0.074094	0.154715	protein_coding
ENSG0000(	TMEM143	244.6008	-0.13673	0.074095	0.154715	protein_coding
ENSG0000(	TFR2	271.6482	0.110827	0.074142	0.154793	protein_coding
ENSG0000(	WNK2	4771.056	-0.28436	0.074138	0.154793	protein_coding
ENSG0000(	BEX3	144.3686	-0.20608	0.074161	0.154815	protein_coding
ENSG0000(	LINC01366	137.1908	0.13535	0.074162	0.154815	lncRNA
ENSG0000(	LINC00997	216.5785	0.16404	0.074245	0.154978	lncRNA
ENSG0000(	ITGA7	15109.59	0.122399	0.074257	0.154991	protein_coding
ENSG0000(	SNORD8	39.84461	0.259504	0.074272	0.155012	snoRNA
ENSG0000(	DIPK1A	299.5866	0.189066	0.074368	0.155203	protein_coding
ENSG0000(	WDR19	923.8921	0.115561	0.074407	0.155274	protein_coding
ENSG0000(	HACE1	926.3202	-0.0889	0.074446	0.155345	protein_coding
ENSG0000(	PTPN22	678.7842	0.150256	0.074459	0.155352	protein_coding
ENSG0000(	RN7SL98P	11.6745	0.330325	0.074456	0.155352	misc_RNA
ENSG0000(	FBXO25	1298.635	-0.16931	0.074471	0.155367	protein_coding
ENSG0000(	ZNF513	498.4053	-0.12778	0.074477	0.155367	protein_coding
ENSG0000(	CYFIP2	1413.167	0.265025	0.074528	0.155453	protein_coding

ENSG0000(	ENSG0000(	130.7631	-0.1447	0.074527	0.155453	lncRNA
ENSG0000(	LINC01764	453.8231	0.097863	0.074566	0.155523	lncRNA
ENSG0000(	ROBO4	333.1297	0.22755	0.074605	0.155592	protein_coding
ENSG0000(	ENSG0000(	61.34587	0.175418	0.074623	0.155621	lncRNA
ENSG0000(	CT69	153.8694	0.144234	0.074643	0.155652	lncRNA
ENSG0000(	DCLRE1A	313.6887	-0.19364	0.074654	0.155654	protein_coding
ENSG0000(	RN7SL138F	49.53504	0.245249	0.074651	0.155654	misc_RNA
ENSG0000(	ENSG0000(	21.53511	0.350723	0.074674	0.155686	lncRNA
ENSG0000(	ENSG0000(	41.6496	0.332692	0.074681	0.155691	lncRNA
ENSG0000(	TMEM8B	1678.53	-0.14892	0.074732	0.155786	protein_coding
ENSG0000(	SCUBE1-AS	200.779	-0.11463	0.074765	0.155844	lncRNA
ENSG0000(	ENPP2	854.3171	0.236649	0.074773	0.155849	protein_coding
ENSG0000(	TOP1MT	181.6017	-0.19809	0.074783	0.155857	protein_coding
ENSG0000(	VENTXP4	126.2737	0.131474	0.074786	0.155857	processed_pseudogene
ENSG0000(	RAB6C-AS1	50.95181	0.216512	0.0748	0.155874	transcribed_unprocessed_pseudo
ENSG0000(	ENSG0000(	238.1688	-0.10265	0.074813	0.155893	lncRNA
ENSG0000(	KDM4A-AS	429.5529	-0.13532	0.074836	0.15593	lncRNA
ENSG0000(	SPIRE1	363.9409	0.185812	0.074847	0.155943	protein_coding
ENSG0000(	ENSG0000(	11.60313	-0.40502	0.074864	0.155966	lncRNA
ENSG0000(	ENSG0000(	16.53581	0.372456	0.074893	0.156017	lncRNA
ENSG0000(	ENSG0000(	1541.432	0.087157	0.074913	0.156039	lncRNA
ENSG0000(	PALB2	529.642	-0.12223	0.074913	0.156039	protein_coding
ENSG0000(	ENSG0000(	44.92631	0.204459	0.074944	0.156091	lncRNA
ENSG0000(	PRND	211.8648	-0.10158	0.07498	0.156156	protein_coding
ENSG0000(	ADH1B	2286.509	-0.23605	0.075047	0.156286	protein_coding
ENSG0000(	RPSAP18	9.905116	0.377807	0.075196	0.156586	processed_pseudogene
ENSG0000(	BRCA1P1	12.47561	0.391899	0.075247	0.156666	unprocessed_pseudogene
ENSG0000(	CASC17	141.4035	-0.1737	0.07525	0.156666	lncRNA
ENSG0000(	MIR4320	31.24647	-0.38707	0.075247	0.156666	miRNA
ENSG0000(	ETS1	4533.605	0.172758	0.075287	0.156734	protein_coding
ENSG0000(	ENSG0000(	12.40492	0.398219	0.075317	0.156785	processed_pseudogene
ENSG0000(	LINC02337	36.13063	0.23323	0.075365	0.156874	lncRNA
ENSG0000(	ENSG0000(	449.3146	-0.08612	0.075409	0.156956	lncRNA
ENSG0000(	CD180	608.9936	0.354049	0.075431	0.156992	protein_coding
ENSG0000(	TSPAN31	1116.857	-0.07806	0.075466	0.157054	protein_coding
ENSG0000(	ENSG0000(	5513.726	0.067558	0.075592	0.157307	lncRNA
ENSG0000(	ENSG0000(	44.13786	-0.28379	0.075615	0.157342	protein_coding
ENSG0000(	ENSG0000(	13.84891	-0.42725	0.075629	0.15735	TEC
ENSG0000(	XPO1	10100.59	-0.0642	0.075625	0.15735	protein_coding
ENSG0000(	RNU6-90P	35.93305	0.254648	0.075663	0.157411	snRNA
ENSG0000(	KNOP1	1449.435	-0.09289	0.075688	0.157453	protein_coding
ENSG0000(	PPP2CA	1950.449	-0.097	0.075708	0.157483	protein_coding
ENSG0000(	IGHV4-59	901.5431	0.476751	0.07573	0.157509	IG_V_gene
ENSG0000(	LINC01821	2153.012	-0.1781	0.075727	0.157509	lncRNA
ENSG0000(	CCDC122	2642.263	-0.08798	0.075761	0.157553	protein_coding
ENSG0000(	SYNE4	157.9952	0.133449	0.075756	0.157553	protein_coding
ENSG0000(	PRSS55	33.32102	0.24567	0.075769	0.157558	protein_coding

ENSG0000(	ENSG0000(	10.76586	0.394569	0.075808	0.157629	lncRNA
ENSG0000(	BTG3-AS1	36.99968	-0.33158	0.075815	0.157634	lncRNA
ENSG0000(	GRAMD2A	165.2299	-0.19247	0.075831	0.157655	protein_coding
ENSG0000(	ENSG0000(	19.5569	0.352108	0.075891	0.157771	lncRNA
ENSG0000(	ENSG0000(	238.5389	0.100413	0.075932	0.157846	TEC
ENSG0000(	SH3GLB2	3404.071	-0.15757	0.075939	0.157848	protein_coding
ENSG0000(	ENSG0000(	62.65299	0.1838	0.075945	0.157851	lncRNA
ENSG0000(	ENSG0000(	786.8942	-0.28475	0.075969	0.15789	lncRNA
ENSG0000(	NRL	135.5696	0.188796	0.076	0.157944	protein_coding
ENSG0000(	ENSG0000(	24.86714	-0.32382	0.076016	0.157968	lncRNA
ENSG0000(	RAP1A	1479.651	-0.12398	0.076028	0.157981	protein_coding
ENSG0000(	DAP	1346.248	0.117106	0.076086	0.158091	protein_coding
ENSG0000(	ENSG0000(	43.94984	0.397816	0.076143	0.1582	processed_pseudogene
ENSG0000(	ENSG0000(	55.23364	-0.45204	0.07616	0.158225	lncRNA
ENSG0000(	MIR648	36.52499	-0.3316	0.076167	0.158228	miRNA
ENSG0000(	ENSG0000(	134.6482	0.11927	0.07621	0.158308	lncRNA
ENSG0000(	TCEAL8	206.1458	-0.18812	0.076224	0.158325	protein_coding
ENSG0000(	RPS18P9	224.2652	-0.1101	0.076327	0.158528	transcribed_processed_pseudoge
ENSG0000(	GTF2IRD1P	328.3794	0.085593	0.07637	0.158607	transcribed_unprocessed_pseudo
ENSG0000(	NUDCD3	1899.081	-0.0949	0.076381	0.158619	protein_coding
ENSG0000(	ENSG0000(	41.88098	0.252037	0.076434	0.15872	lncRNA
ENSG0000(	SEC61A2	282.324	0.144472	0.076462	0.158757	protein_coding
ENSG0000(	TBKBP1	202.6114	0.216114	0.076457	0.158757	protein_coding
ENSG0000(	LINC01645	194.3176	-0.60642	0.076476	0.158776	lncRNA
ENSG0000(	HK1	1621.715	-0.12186	0.076498	0.158811	protein_coding
ENSG0000(	C14orf180	18.07873	-0.62685	0.076514	0.158823	protein_coding
ENSG0000(	ZNF600	1313.478	-0.1211	0.076514	0.158823	protein_coding
ENSG0000(	ENSG0000(	78.35907	0.163156	0.076551	0.15889	lncRNA
ENSG0000(	GNB3	67.9082	0.274149	0.076647	0.159077	protein_coding
ENSG0000(	NDUFAF4P	20.52049	0.287459	0.07666	0.159095	processed_pseudogene
ENSG0000(	ITGA2-AS1	58.6355	0.212328	0.07669	0.159144	lncRNA
ENSG0000(	ZFP28	254.384	0.193215	0.076694	0.159144	protein_coding
ENSG0000(	ANK3	4606.608	-0.21283	0.076716	0.159159	protein_coding
ENSG0000(	IGHV1-46	270.753	0.440067	0.076717	0.159159	IG_V_gene
ENSG0000(	ZNF217	4604.758	-0.16783	0.076713	0.159159	protein_coding
ENSG0000(	ENSG0000(	47.77185	0.238608	0.076723	0.159162	lncRNA
ENSG0000(	OR4E1	32.05145	-0.25395	0.076745	0.159198	protein_coding
ENSG0000(	WDR87BP	26.64266	-0.33095	0.076797	0.159294	transcribed_unprocessed_pseudo
ENSG0000(	EXOC3-AS1	200.3905	-0.12542	0.076828	0.159347	lncRNA
ENSG0000(	LINC02142	347.993	0.093626	0.076848	0.159379	lncRNA
ENSG0000(	ENSG0000(	30.65419	-0.30332	0.076872	0.159418	lncRNA
ENSG0000(	ARGFX	1206.043	-0.06905	0.076877	0.159418	protein_coding
ENSG0000(	HPS3	897.4744	0.126989	0.076902	0.159448	protein_coding
ENSG0000(	MANSC4	102.5103	0.156424	0.076899	0.159448	protein_coding
ENSG0000(	RN7SL519F	11.57761	0.376465	0.076938	0.159502	misc_RNA
ENSG0000(	URB1-AS1	25.67073	-0.30297	0.076935	0.159502	lncRNA
ENSG0000(	ENSG0000(	97.85408	0.134781	0.076975	0.159556	lncRNA

ENSG0000(	PTCD2	1100.146	-0.08228	0.076972	0.159556	protein_coding
ENSG0000(	ENSG0000(	768.5444	-0.18157	0.077032	0.159652	lncRNA
ENSG0000(	NAPEPLD	2318.299	-0.17655	0.077039	0.159652	protein_coding
ENSG0000(	TFEB	810.5434	0.119842	0.077039	0.159652	protein_coding
ENSG0000(	USP35	353.1336	0.164499	0.077041	0.159652	protein_coding
ENSG0000(	FGF10-AS1	19.1272	0.485118	0.077079	0.159718	lncRNA
ENSG0000(	NR2C1	1070.42	-0.08596	0.077083	0.159718	protein_coding
ENSG0000(	PCDHGA10	334.2067	0.136188	0.077092	0.159725	protein_coding
ENSG0000(	ENSG0000(	99.0741	-0.2719	0.077109	0.159751	processed_pseudogene
ENSG0000(	TMEM71	169.5902	0.350798	0.077118	0.159759	protein_coding
ENSG0000(	ENSG0000(	193.1918	0.135748	0.077144	0.159791	lncRNA
ENSG0000(	ZKSCAN2	237.6551	0.133115	0.077144	0.159791	protein_coding
ENSG0000(	CDK19	1339.169	0.123164	0.077149	0.159792	protein_coding
ENSG0000(	IFT22	1638.232	0.056861	0.077187	0.159858	protein_coding
ENSG0000(	ENSG0000(	45.19351	0.234332	0.077213	0.159892	processed_pseudogene
ENSG0000(	TMEM209	882.3987	-0.10603	0.077211	0.159892	protein_coding
ENSG0000(	STS	1347.079	0.124643	0.077219	0.159892	protein_coding
ENSG0000(	ENSG0000(	30.94945	-0.27927	0.077274	0.159986	lncRNA
ENSG0000(	NCOA3	3971.871	0.109688	0.077274	0.159986	protein_coding
ENSG0000(	FBRSL1	1554.037	-0.09505	0.077283	0.159993	protein_coding
ENSG0000(	XAGE5	20.89917	-0.34266	0.077288	0.159993	protein_coding
ENSG0000(	EOLA2	296.2158	-0.11626	0.077334	0.160079	protein_coding
ENSG0000(	RN7SKP193	21.20116	0.281298	0.077429	0.160264	misc_RNA
ENSG0000(	KCTD21-AS	559.2736	-0.09641	0.077449	0.160295	lncRNA
ENSG0000(	ENSG0000(	67.69551	0.173416	0.077468	0.160319	transcribed_processed_pseudoge
ENSG0000(	NCEH1	767.4734	-0.20832	0.077476	0.160319	protein_coding
ENSG0000(	PUSL1	107.0257	-0.24069	0.077474	0.160319	protein_coding
ENSG0000(	GPD2	2109.585	-0.17954	0.07751	0.16038	protein_coding
ENSG0000(	BST1	285.0329	0.134491	0.077531	0.160389	protein_coding
ENSG0000(	LINC01087	358.0976	0.094873	0.077521	0.160389	lncRNA
ENSG0000(	OR4K13	127.493	-0.17044	0.077535	0.160389	protein_coding
ENSG0000(	TATDN2	1936.163	-0.10775	0.077532	0.160389	protein_coding
ENSG0000(	DNAJB5	149.7993	0.227316	0.077565	0.160429	protein_coding
ENSG0000(	SST	19.23884	-0.5518	0.077561	0.160429	protein_coding
ENSG0000(	TRIM80P	10.1124	-0.48262	0.077605	0.1605	unitary_pseudogene
ENSG0000(	PLBD1-AS1	264.3407	-0.19621	0.077655	0.160595	lncRNA
ENSG0000(	ADAM11	21.8449	0.390601	0.077672	0.160618	protein_coding
ENSG0000(	QRFPR	143.5971	-0.13878	0.07769	0.160645	protein_coding
ENSG0000(	ENSG0000(	27.52665	0.278256	0.077701	0.160657	lncRNA
ENSG0000(	TRIM6	134.212	-0.1544	0.07773	0.160706	protein_coding
ENSG0000(	RBM5-AS1	114.6861	-0.1736	0.077765	0.160769	lncRNA
ENSG0000(	BMS1P1	3181.16	0.105627	0.077807	0.160845	transcribed_unprocessed_pseudo
ENSG0000(	KIF13B	6147.49	-0.16428	0.077837	0.160896	protein_coding
ENSG0000(	ENSG0000(	110.7951	0.190994	0.077895	0.161005	lncRNA
ENSG0000(	NUMB	4127.283	0.068018	0.077903	0.16101	protein_coding
ENSG0000(	GPC4	488.1724	-0.18756	0.077923	0.161042	protein_coding
ENSG0000(	ULK3	1403.875	-0.18231	0.077947	0.161081	protein_coding

ENSG0000(	ENSG0000(	94.67562	0.248272	0.077981	0.161134	lncRNA
ENSG0000(	KLF2P2	31.14473	0.292889	0.077983	0.161134	processed_pseudogene
ENSG0000(	EXT1	4080.115	-0.10968	0.078034	0.16123	protein_coding
ENSG0000(	FBN2	444.4583	-0.24913	0.07805	0.161249	protein_coding
ENSG0000(	SFT2D3	568.5625	-0.06224	0.078054	0.161249	protein_coding
ENSG0000(	ENSG0000(	16.47554	-0.42228	0.078082	0.161297	lncRNA
ENSG0000(	INHBE	81.95009	-0.13811	0.078102	0.161324	protein_coding
ENSG0000(	LINC01776	503.6959	0.09439	0.078106	0.161324	lncRNA
ENSG0000(	ENSG0000(	13.91716	0.427355	0.078155	0.161416	lncRNA
ENSG0000(	SH2D3C	329.7767	0.275283	0.078162	0.161419	protein_coding
ENSG0000(	ACTR5	243.7502	-0.08847	0.078192	0.161459	protein_coding
ENSG0000(	MIR548AJ2	27.86378	0.281413	0.078192	0.161459	miRNA
ENSG0000(	LYSMD1	211.4069	-0.11131	0.078236	0.161539	protein_coding
ENSG0000(	ENSG0000(	463.0228	0.11432	0.078248	0.161554	lncRNA
ENSG0000(	LINC01569	899.4185	0.066035	0.078278	0.161604	lncRNA
ENSG0000(	NAA30	1010.374	-0.10543	0.078283	0.161604	protein_coding
ENSG0000(	LINC01415	14.18605	0.449653	0.078359	0.161751	lncRNA
ENSG0000(	CTSD	5069.547	-0.20444	0.078372	0.161756	protein_coding
ENSG0000(	LTO1	1356.924	0.069761	0.07837	0.161756	protein_coding
ENSG0000(	CSNK1E	2722.489	0.086701	0.078406	0.161816	protein_coding
ENSG0000(	ENSG0000(	67.35436	0.212173	0.078415	0.161816	lncRNA
ENSG0000(	RN7SL278F	55.95122	0.213564	0.078416	0.161816	misc_RNA
ENSG0000(	BSDC1	2569.602	0.098544	0.078505	0.161957	protein_coding
ENSG0000(	ENSG0000(	86.94037	0.188031	0.078502	0.161957	processed_pseudogene
ENSG0000(	ENSG0000(	44.67399	0.246551	0.078506	0.161957	lncRNA
ENSG0000(	SHANK2	1202.96	-0.20623	0.078494	0.161957	protein_coding
ENSG0000(	PXDN	1297.257	0.218469	0.078525	0.161986	protein_coding
ENSG0000(	FAM229B	154.0449	0.106617	0.078544	0.162015	protein_coding
ENSG0000(	RN7SKP102	70.22494	-0.34685	0.078578	0.162074	misc_RNA
ENSG0000(	ENSG0000(	14.85987	0.469244	0.0786	0.162098	transcribed_processed_pseudoge
ENSG0000(	ENSG0000(	10.14111	0.354121	0.078604	0.162098	lncRNA
ENSG0000(	RBM22P2	645.5481	0.096769	0.078605	0.162098	processed_pseudogene
ENSG0000(	ENSG0000(	45.84068	0.195475	0.078617	0.162113	processed_pseudogene
ENSG0000(	STAC	22.02165	0.56357	0.07866	0.162191	protein_coding
ENSG0000(	ENSG0000(	126	0.136373	0.078667	0.162193	lncRNA
ENSG0000(	TMEM230	1054.29	-0.09925	0.078712	0.162276	protein_coding
ENSG0000(	CRTC1	626.0846	-0.11782	0.078721	0.162284	protein_coding
ENSG0000(	RFESD	55.70229	0.289969	0.078734	0.162299	protein_coding
ENSG0000(	DPRXP3	74.81128	0.172429	0.078748	0.162317	processed_pseudogene
ENSG0000(	AOAH	1331.253	-0.17888	0.078775	0.162349	protein_coding
ENSG0000(	EID3	145.8004	-0.22684	0.078774	0.162349	protein_coding
ENSG0000(	PHC1P1	54.36966	-0.44532	0.078779	0.162349	processed_pseudogene
ENSG0000(	ENSG0000(	306.0408	0.125244	0.078796	0.162375	lncRNA
ENSG0000(	ENSG0000(	177.3746	-0.13966	0.078882	0.16254	lncRNA
ENSG0000(	LINC02556	31.41903	-0.26719	0.078895	0.162556	lncRNA
ENSG0000(	IGKV3D-15	67.65105	0.589935	0.07891	0.162577	IG_V_gene
ENSG0000(	TAF7	994.2505	-0.09739	0.078952	0.162653	protein_coding

ENSG0000(	MYO6	4983.623	-0.13783	0.07896	0.162658	protein_coding
ENSG0000(	ENSG0000(	21.43619	0.289811	0.07906	0.162824	lncRNA
ENSG0000(	ENSG0000(	36.71505	0.250103	0.079061	0.162824	lncRNA
ENSG0000(	EPS8	3806.776	-0.17348	0.079066	0.162824	protein_coding
ENSG0000(	ITGB4	3208.391	-0.17588	0.079047	0.162824	protein_coding
ENSG0000(	MIR210HG	420.3623	0.298253	0.079065	0.162824	lncRNA
ENSG0000(	RPRD1A	1235.836	0.069025	0.079078	0.162838	protein_coding
ENSG0000(	ATP6V1G1	811.9424	0.099415	0.079095	0.162861	protein_coding
ENSG0000(	IQCB1	832.7505	0.09216	0.07912	0.162902	protein_coding
ENSG0000(	ENSG0000(	52.78158	-0.2177	0.07913	0.162912	lncRNA
ENSG0000(	ENSG0000(	24.61982	0.356732	0.079174	0.162992	lncRNA
ENSG0000(	TNNI3K	13.3465	-0.51302	0.079223	0.163081	protein_coding
ENSG0000(	ENSG0000(	64.04599	-0.44183	0.07924	0.163087	TEC
ENSG0000(	MAPRE1	1256.518	-0.11092	0.079232	0.163087	protein_coding
ENSG0000(	MRPS30-DT	357.0479	0.107573	0.079241	0.163087	lncRNA
ENSG0000(	IQUB	19.41039	0.2893	0.079254	0.163104	protein_coding
ENSG0000(	AHR	2446.578	0.161359	0.079282	0.16315	protein_coding
ENSG0000(	ENSG0000(	39.64125	0.248554	0.079325	0.163228	lncRNA
ENSG0000(	ALG11	4079.107	0.055601	0.079346	0.16326	protein_coding
ENSG0000(	COA1	2654.541	0.098251	0.079372	0.163303	protein_coding
ENSG0000(	RPL23AP35	68.47148	-0.20721	0.079425	0.163391	processed_pseudogene
ENSG0000(	SRRT	1574.478	-0.09583	0.079423	0.163391	protein_coding
ENSG0000(	ENSG0000(	88.89237	-0.18164	0.079434	0.163398	lncRNA
ENSG0000(	H3-3A-DT	79.17132	0.190306	0.079445	0.1634	lncRNA
ENSG0000(	RNU1-103F	11.50016	-0.38202	0.079442	0.1634	snRNA
ENSG0000(	ENSG0000(	78.02847	0.154167	0.079478	0.163456	lncRNA
ENSG0000(	TMEM216	134.7257	-0.15204	0.079505	0.163502	protein_coding
ENSG0000(	RN7SL242F	12.65946	0.383892	0.079531	0.163544	misc_RNA
ENSG0000(	TTTY9B	11.14394	0.393732	0.07961	0.163696	lncRNA
ENSG0000(	ATF4	2829.582	-0.09232	0.079638	0.163726	protein_coding
ENSG0000(	ENSG0000(	50.69385	-0.19501	0.079639	0.163726	lncRNA
ENSG0000(	SRRM1	3394.723	-0.04918	0.07964	0.163726	protein_coding
ENSG0000(	RPL23AP32	24.16326	-0.33764	0.079733	0.163905	processed_pseudogene
ENSG0000(	AGK	1682.981	0.070112	0.079796	0.164025	protein_coding
ENSG0000(	EXD2	585.6413	-0.12466	0.079819	0.164051	protein_coding
ENSG0000(	ZNF181	728.4099	-0.10259	0.079816	0.164051	protein_coding
ENSG0000(	ENSG0000(	287.2443	-0.15441	0.079828	0.164058	lncRNA
ENSG0000(	TMEM72	166.7808	-0.51658	0.079923	0.164242	protein_coding
ENSG0000(	ENSG0000(	292.482	-0.10853	0.079945	0.164277	lncRNA
ENSG0000(	PIK3R6	189.8377	0.170706	0.079978	0.164333	protein_coding
ENSG0000(	CCT7P1	9.491595	-0.58849	0.080014	0.164382	processed_pseudogene
ENSG0000(	FBXO38-DT	16113.84	-0.12549	0.080018	0.164382	lncRNA
ENSG0000(	LINC01322	811.9543	0.096406	0.080012	0.164382	lncRNA
ENSG0000(	CLCNKB	113.7843	0.127938	0.080023	0.164383	protein_coding
ENSG0000(	ENSG0000(	227.0784	0.125346	0.080032	0.164387	lncRNA
ENSG0000(	SLC5A9	70.52611	0.469808	0.080036	0.164387	protein_coding
ENSG0000(	MIR1256	15.4469	0.350208	0.080127	0.164563	miRNA

ENSG0000(	ENSG0000(	78.8796	-0.22499	0.080178	0.164635	TEC
ENSG0000(	ENSG0000(	46.79629	0.250015	0.080172	0.164635	lncRNA
ENSG0000(	PLAAT3	282.6979	-0.16231	0.080175	0.164635	protein_coding
ENSG0000(	DDX42	2897.788	-0.06045	0.080197	0.164654	protein_coding
ENSG0000(	IGHV3-52	10.04146	0.58724	0.080196	0.164654	IG_V_pseudogene
ENSG0000(	RPS12P26	136.6913	-0.22674	0.080217	0.164684	transcribed_processed_pseudoge
ENSG0000(	FKBP7	397.8264	0.089847	0.080233	0.164701	protein_coding
ENSG0000(	USP33	3084.445	-0.077	0.080236	0.164701	protein_coding
ENSG0000(	ZNF543	588.2054	-0.10538	0.08026	0.16474	protein_coding
ENSG0000(	ENSG0000(	134.6267	0.157503	0.080267	0.164743	lncRNA
ENSG0000(	TRIB3	61.56064	0.361686	0.080305	0.164811	protein_coding
ENSG0000(	FAT2	23.21039	0.436036	0.080313	0.164816	protein_coding
ENSG0000(	RNF157-AS	246.0215	-0.11139	0.080325	0.164829	lncRNA
ENSG0000(	ENSG0000(	506.8353	-0.09491	0.080339	0.164839	TEC
ENSG0000(	LINC00685	346.9951	0.134632	0.08034	0.164839	lncRNA
ENSG0000(	VPS9D1	533.5482	0.164509	0.080365	0.164879	protein_coding
ENSG0000(	LINC01946	14.48358	-0.34983	0.080382	0.164904	lncRNA
ENSG0000(	P2RY1	1462.864	-0.23491	0.08039	0.16491	protein_coding
ENSG0000(	NSF	1526.065	-0.15722	0.0804	0.164919	protein_coding
ENSG0000(	ENSG0000(	179.4491	0.142247	0.080422	0.164943	lncRNA
ENSG0000(	LINC01257	107.6259	0.135323	0.080422	0.164943	lncRNA
ENSG0000(	CLDN11	136.2423	0.17549	0.080437	0.164963	protein_coding
ENSG0000(	PDX1	14.25027	0.545298	0.080448	0.164974	protein_coding
ENSG0000(	HERC6	620.9582	0.275621	0.080462	0.164991	protein_coding
ENSG0000(	ENSG0000(	246.7045	-0.09233	0.080517	0.165094	lncRNA
ENSG0000(	C2CD3	5133.533	-0.05356	0.080531	0.165107	protein_coding
ENSG0000(	RPL32P13	20.25093	0.3713	0.080534	0.165107	processed_pseudogene
ENSG0000(	FAM86C2P	148.7465	-0.17845	0.080571	0.165173	transcribed_unprocessed_pseudo
ENSG0000(	ENSG0000(	159.0134	0.256309	0.080664	0.165348	lncRNA
ENSG0000(	ENSG0000(	53.97189	0.152109	0.080668	0.165348	lncRNA
ENSG0000(	PIAS3	602.6505	0.119073	0.080675	0.165354	protein_coding
ENSG0000(	CEP19	264.1742	-0.13299	0.080711	0.165415	protein_coding
ENSG0000(	B3GAT2	41.77176	0.170222	0.080724	0.165431	protein_coding
ENSG0000(	ARPC3	1139.07	-0.14373	0.080734	0.16544	protein_coding
ENSG0000(	ENSG0000(	24.54736	0.296121	0.080748	0.165458	lncRNA
ENSG0000(	RUFY3	3036.807	0.082828	0.08079	0.165534	protein_coding
ENSG0000(	ENSG0000(	20.86698	0.309919	0.0808	0.165543	lncRNA
ENSG0000(	C2CD2L	1436.073	-0.14193	0.080872	0.165681	protein_coding
ENSG0000(	GAS1RR	117.6943	-0.17085	0.080895	0.165716	lncRNA
ENSG0000(	ARPP19	2808.284	0.104039	0.080946	0.165801	protein_coding
ENSG0000(	LINC01342	171.7096	-0.28742	0.080947	0.165801	lncRNA
ENSG0000(	ANAPC1P2	417.8507	-0.14452	0.080965	0.165806	lncRNA
ENSG0000(	DCAF6	2073.469	-0.07873	0.080955	0.165806	protein_coding
ENSG0000(	MMP17	90.90217	0.256165	0.080962	0.165806	protein_coding
ENSG0000(	ENSG0000(	440.1437	0.087356	0.08098	0.165814	processed_pseudogene
ENSG0000(	ENSG0000(	30.3777	-0.31915	0.080978	0.165814	lncRNA
ENSG0000(	ENSG0000(	15.06828	0.378778	0.080995	0.165814	lncRNA

ENSG0000(	ENSG0000(	867.0249	-0.15416	0.080994	0.165814	protein_coding
ENSG0000(	PRR29-AS1	300.7029	0.11627	0.080996	0.165814	lncRNA
ENSG0000(	SEPTIN9-D	20.70548	-0.47181	0.081031	0.165876	lncRNA
ENSG0000(	AR	166.2938	0.237966	0.081042	0.165886	protein_coding
ENSG0000(	NGDN	435.329	-0.10336	0.081057	0.165886	protein_coding
ENSG0000(	RNF148	40.09655	-0.26918	0.081052	0.165886	protein_coding
ENSG0000(	USB1	904.3764	0.124067	0.081052	0.165886	protein_coding
ENSG0000(	ENSG0000(	413.2948	0.181665	0.081074	0.165888	lncRNA
ENSG0000(	ENSG0000(	802.9361	0.066056	0.081074	0.165888	TEC
ENSG0000(	PSD	81.62655	-0.20754	0.081071	0.165888	protein_coding
ENSG0000(	RNU4-40P	32.26804	0.31883	0.081113	0.165957	snRNA
ENSG0000(	DHRS2	174.1649	0.13891	0.08112	0.165961	protein_coding
ENSG0000(	ENSG0000(	49.97651	-0.17626	0.081131	0.165971	lncRNA
ENSG0000(	LINC00900	429.3364	0.135675	0.081143	0.165985	lncRNA
ENSG0000(	CAP2P1	163.3466	-0.21656	0.081149	0.165987	transcribed_processed_pseudoge
ENSG0000(	ENSG0000(	9.391306	-0.41734	0.081155	0.165988	misc_RNA
ENSG0000(	ENSG0000(	10.86566	0.370059	0.081172	0.165993	lncRNA
ENSG0000(	TAS2R63P	106.0978	0.22568	0.081173	0.165993	unprocessed_pseudogene
ENSG0000(	TCEANC	424.9116	-0.08995	0.081163	0.165993	protein_coding
ENSG0000(	ENSG0000(	334.5144	-0.12509	0.081188	0.166001	lncRNA
ENSG0000(	FAM214B	866.5363	-0.22114	0.081185	0.166001	protein_coding
ENSG0000(	SESN3	2196.139	0.245902	0.081237	0.166079	protein_coding
ENSG0000(	SLC11A2	2231.831	0.110958	0.081237	0.166079	protein_coding
ENSG0000(	ENSG0000(	13.47935	-0.37506	0.081282	0.166161	lncRNA
ENSG0000(	CTRC	366.4622	-0.10052	0.081311	0.166209	protein_coding
ENSG0000(	EID2B	227.6974	0.215129	0.081342	0.166261	protein_coding
ENSG0000(	ENSG0000(	109.6161	0.185492	0.081368	0.166305	lncRNA
ENSG0000(	PRIMA1	177.4141	-0.24258	0.081426	0.166402	protein_coding
ENSG0000(	ZNF836	663.0602	-0.1028	0.081423	0.166402	protein_coding
ENSG0000(	HSPE1P10	101.0022	-0.13321	0.081433	0.166405	processed_pseudogene
ENSG0000(	ABHD13	839.9524	0.078866	0.081462	0.166453	protein_coding
ENSG0000(	COX6CP1	66.32895	-0.18172	0.081549	0.166619	processed_pseudogene
ENSG0000(	OR6B1	113.4638	0.12536	0.081578	0.166669	protein_coding
ENSG0000(	C5AR2	1482.714	0.080192	0.081608	0.166718	protein_coding
ENSG0000(	ENSG0000(	82.756	-0.22309	0.081621	0.166733	lncRNA
ENSG0000(	CERS4	420.3163	0.221002	0.081628	0.166737	protein_coding
ENSG0000(	SGSM2	1791.304	0.114569	0.081638	0.166746	protein_coding
ENSG0000(	NADSYN1	5336.898	-0.10709	0.081661	0.166783	protein_coding
ENSG0000(	HUS1B	22.75191	-0.31498	0.081678	0.166808	protein_coding
ENSG0000(	LINC02416	15.28448	0.339776	0.081702	0.166824	lncRNA
ENSG0000(	LRRC37B	969.5462	0.112961	0.081697	0.166824	protein_coding
ENSG0000(	RN7SKP255	411.1718	0.263755	0.081694	0.166824	misc_RNA
ENSG0000(	SNED1	1443.205	0.171007	0.081751	0.166913	protein_coding
ENSG0000(	NIBAN3	1847.929	0.177616	0.08178	0.166961	protein_coding
ENSG0000(	EEF1A1P19	41.78917	-0.25103	0.081814	0.167013	processed_pseudogene
ENSG0000(	ENSG0000(	17.84254	0.489241	0.081817	0.167013	transcribed_unprocessed_pseudo
ENSG0000(	LINC01534	730.4975	-0.08226	0.081822	0.167013	lncRNA

ENSG0000(	ENSG0000(	435.0139	0.064974	0.081861	0.16706	lncRNA
ENSG0000(	ENSG0000(	264.1221	0.109961	0.081854	0.16706	lncRNA
ENSG0000(	LINC00113	52.48288	0.164405	0.081859	0.16706	lncRNA
ENSG0000(	OTX2-AS1	321.1724	-0.18769	0.081917	0.167163	lncRNA
ENSG0000(	KATNBL1	1611.864	-0.05377	0.081945	0.167211	protein_coding
ENSG0000(	ENSG0000(	10.46479	0.628187	0.082002	0.167316	TEC
ENSG0000(	RFX6	29.91516	-0.46185	0.082033	0.167369	protein_coding
ENSG0000(	ENSG0000(	29.26965	0.284952	0.082056	0.167405	lncRNA
ENSG0000(	TCTA	919.0056	-0.07924	0.082065	0.167412	protein_coding
ENSG0000(	SCARNA18I	10.35241	0.375197	0.082077	0.167424	snoRNA
ENSG0000(	FAM90A1	14.23749	0.534864	0.082134	0.167531	protein_coding
ENSG0000(	OBSCN-AS1	67.22074	0.284503	0.082198	0.16763	lncRNA
ENSG0000(	RFX2	696.3591	-0.15342	0.082199	0.16763	protein_coding
ENSG0000(	RIBC1	29.4161	0.3293	0.082194	0.16763	protein_coding
ENSG0000(	FCHSD1	954.9655	0.10431	0.082209	0.167639	protein_coding
ENSG0000(	LMTK2	3295.609	-0.18338	0.082245	0.167703	protein_coding
ENSG0000(	GUSBP1	2307.342	0.118142	0.082261	0.167724	transcribed_unprocessed_pseudo
ENSG0000(	IGLV2-11	608.0196	0.436969	0.082289	0.167769	IG_V_gene
ENSG0000(	SMAD2	13593.08	-0.06327	0.082294	0.167769	protein_coding
ENSG0000(	ENSG0000(	684.6164	-0.20799	0.082308	0.167787	transcribed_unprocessed_pseudo
ENSG0000(	ENSG0000(	252.7192	0.185506	0.082319	0.167787	lncRNA
ENSG0000(	ENSG0000(	117.0431	0.1748	0.082315	0.167787	lncRNA
ENSG0000(	ENSG0000(	207.4194	-0.14369	0.082337	0.167794	lncRNA
ENSG0000(	LINC00929	121.5011	-0.12952	0.082338	0.167794	lncRNA
ENSG0000(	PRPH2	107.4323	0.226614	0.082336	0.167794	protein_coding
ENSG0000(	MDS2	126.244	-0.18435	0.082379	0.167867	lncRNA
ENSG0000(	ENSG0000(	31.23392	-0.3793	0.082433	0.167965	processed_pseudogene
ENSG0000(	URB1	1589.859	-0.1392	0.082447	0.167983	protein_coding
ENSG0000(	RAD54L	257.4264	-0.14502	0.082464	0.168007	protein_coding
ENSG0000(	APIP	644.3822	-0.1762	0.08249	0.168037	protein_coding
ENSG0000(	KRT18P61	26.07495	-0.29563	0.08249	0.168037	processed_pseudogene
ENSG0000(	ENSG0000(	10.50936	0.370525	0.082499	0.168045	lncRNA
ENSG0000(	WBP2NL	484.6748	0.104413	0.082523	0.168083	protein_coding
ENSG0000(	LINC02271	357.8822	0.088407	0.08253	0.168085	lncRNA
ENSG0000(	LRRIQ1	106.9303	-0.16094	0.082541	0.168097	protein_coding
ENSG0000(	TBC1D32	1224.051	-0.11301	0.082559	0.168123	protein_coding
ENSG0000(	CLOCK	1858.305	0.114031	0.082565	0.168124	protein_coding
ENSG0000(	ENSG0000(	25.78961	-0.27303	0.082583	0.16815	TEC
ENSG0000(	CHGA	708.8504	-0.371	0.082594	0.168161	protein_coding
ENSG0000(	FCRLB	17.50993	0.504865	0.0826	0.168163	protein_coding
ENSG0000(	SPRY2	908.6316	-0.17331	0.082632	0.168216	protein_coding
ENSG0000(	MALINC1	681.3765	0.077067	0.082649	0.168241	lncRNA
ENSG0000(	BCL3	1120.788	0.157908	0.08272	0.168342	protein_coding
ENSG0000(	CERS6	3378.464	-0.1623	0.082719	0.168342	protein_coding
ENSG0000(	EID2	92.48156	-0.17322	0.082705	0.168342	protein_coding
ENSG0000(	PLG	118.2786	0.250169	0.082716	0.168342	protein_coding
ENSG0000(	DDX11-AS1	108.9599	-0.21026	0.082757	0.168406	lncRNA

ENSG0000(	ENSG0000(	480.7211	0.094062	0.082777	0.168436	lncRNA
ENSG0000(	LINC01473	550.5814	0.141048	0.082789	0.168448	lncRNA
ENSG0000(	SMIM12	2060.739	-0.06527	0.082802	0.168465	protein_coding
ENSG0000(	FAM89B	254.326	0.119103	0.082818	0.168485	protein_coding
ENSG0000(	ALKBH6	589.9575	-0.08987	0.082847	0.168521	protein_coding
ENSG0000(	LIPJ	16.89321	-0.3912	0.082852	0.168521	protein_coding
ENSG0000(	ZHX2	840.2477	0.177244	0.082843	0.168521	protein_coding
ENSG0000(	ENSG0000(	14.91555	-0.317	0.082858	0.168524	lncRNA
ENSG0000(	MCEE	250.3361	0.143606	0.082884	0.168565	protein_coding
ENSG0000(	ENSG0000(	33.91827	-0.77257	0.082945	0.168679	lncRNA
ENSG0000(	LINC02303	61.16143	0.202534	0.083002	0.168784	lncRNA
ENSG0000(	BHLHE41	478.1563	0.272992	0.083022	0.168813	protein_coding
ENSG0000(	SPTLC1P1	20.35872	0.447143	0.083042	0.168842	transcribed_processed_pseudoge
ENSG0000(	VIP	36.32398	0.707333	0.083129	0.169009	protein_coding
ENSG0000(	ENSG0000(	53.43116	0.201295	0.083137	0.169014	lncRNA
ENSG0000(	SLC9A3-AS:	1705.89	-0.30082	0.083148	0.169025	lncRNA
ENSG0000(	KSR2	406.143	-0.22539	0.083204	0.169129	protein_coding
ENSG0000(	ENSG0000(	236.6986	0.265551	0.083215	0.169139	TEC
ENSG0000(	DELEC1	699.8584	0.086126	0.083238	0.169175	lncRNA
ENSG0000(	DEDD	657.9987	0.102424	0.083273	0.169236	protein_coding
ENSG0000(	PSG8-AS1	37.73587	-0.70231	0.083333	0.169346	lncRNA
ENSG0000(	FAM83F	3637.872	-0.20926	0.08336	0.16938	protein_coding
ENSG0000(	TGM1	63.32152	-0.31405	0.083359	0.16938	protein_coding
ENSG0000(	ALG9	1155.795	0.073936	0.083389	0.169419	protein_coding
ENSG0000(	KDR	487.9732	0.267175	0.08339	0.169419	protein_coding
ENSG0000(	NRG3	36.87839	0.21657	0.083398	0.169424	protein_coding
ENSG0000(	RABAC1	261.0175	-0.16954	0.083412	0.16944	protein_coding
ENSG0000(	SNORA58	23.77286	0.245125	0.08342	0.169446	snoRNA
ENSG0000(	RN7SL861F	17.65403	0.377632	0.083455	0.169506	misc_RNA
ENSG0000(	ENSG0000(	46.99627	0.246929	0.083478	0.169543	lncRNA
ENSG0000(	TUFT1	841.0014	-0.17207	0.083493	0.169561	protein_coding
ENSG0000(	ENSG0000(	25.06724	0.316326	0.083505	0.169575	lncRNA
ENSG0000(	ENSG0000(	94.01925	0.199963	0.083516	0.169585	lncRNA
ENSG0000(	METTL25B	503.4762	-0.10361	0.083549	0.169643	protein_coding
ENSG0000(	ATG9A	858.1157	-0.11945	0.0836	0.169695	protein_coding
ENSG0000(	CEL	52.66055	-0.25408	0.083582	0.169695	protein_coding
ENSG0000(	ENSG0000(	589.1305	0.271987	0.083593	0.169695	lncRNA
ENSG0000(	ENSG0000(	87.76511	-0.15743	0.083602	0.169695	lncRNA
ENSG0000(	PRSS2	40.12961	0.579727	0.083594	0.169695	protein_coding
ENSG0000(	PI4K2A	381.5427	0.122896	0.08361	0.1697	protein_coding
ENSG0000(	ZNF764	409.4965	-0.13276	0.083629	0.169728	protein_coding
ENSG0000(	KRT8P12	105.0237	-0.15072	0.083663	0.169783	transcribed_processed_pseudoge
ENSG0000(	RFNG	333.0934	-0.13526	0.083667	0.169783	protein_coding
ENSG0000(	ENSG0000(	36.74542	-0.30348	0.083719	0.169878	TEC
ENSG0000(	ENSG0000(	2670.368	-0.1014	0.08374	0.169898	lncRNA
ENSG0000(	SCARA3	288.9154	0.220441	0.083737	0.169898	protein_coding
ENSG0000(	DERL1	2078.77	0.088947	0.083761	0.16993	protein_coding

ENSG0000(	KASH5	60.03692	0.262047	0.083783	0.169963	protein_coding
ENSG0000(	CCDC28A-A	66.11139	-0.25088	0.083791	0.169968	lncRNA
ENSG0000(	ENSG0000(	96.70799	0.142758	0.083807	0.16999	lncRNA
ENSG0000(	RSAD1	818.4971	-0.12069	0.083842	0.170049	protein_coding
ENSG0000(	ENSG0000(	10.3968	0.415588	0.08387	0.170096	processed_pseudogene
ENSG0000(	DIP2B	2800.461	-0.08187	0.083902	0.170149	protein_coding
ENSG0000(	CITED2	575.1214	0.174159	0.083946	0.170227	protein_coding
ENSG0000(	DNAJC8	708.7877	-0.06958	0.083969	0.170242	protein_coding
ENSG0000(	ENSG0000(	16.79071	-0.29857	0.083975	0.170242	lncRNA
ENSG0000(	ENSG0000(	76.05717	-0.15278	0.083975	0.170242	TEC
ENSG0000(	ENSG0000(	1030.278	0.068093	0.083972	0.170242	TEC
ENSG0000(	ENSG0000(	417.6845	0.097012	0.084003	0.170288	lncRNA
ENSG0000(	SLC12A2	6896.493	-0.21527	0.084009	0.170288	protein_coding
ENSG0000(	PGM2L1	1265.432	0.095214	0.084017	0.170294	protein_coding
ENSG0000(	ENSG0000(	21.17413	-0.26864	0.084054	0.170358	lncRNA
ENSG0000(	RN7SL592F	128.1921	0.14635	0.084068	0.170376	misc_RNA
ENSG0000(	PFN2	417.9646	-0.17133	0.084106	0.170441	protein_coding
ENSG0000(	ZNF707	438.9858	-0.10963	0.08414	0.170499	protein_coding
ENSG0000(	HMCN2	3024.191	0.362506	0.084153	0.170515	protein_coding
ENSG0000(	GGNBP1	107.4726	0.170104	0.084187	0.170573	transcribed_unitary_pseudogene
ENSG0000(	SPRED1	2425.59	-0.1826	0.084193	0.170573	protein_coding
ENSG0000(	LSINCT5	175.9303	-0.14743	0.084231	0.170638	lncRNA
ENSG0000(	RPL10L	66.31425	-0.3841	0.084256	0.170679	protein_coding
ENSG0000(	DNAI4	940.918	-0.20728	0.084271	0.170686	protein_coding
ENSG0000(	HIPK3	3136.632	0.087713	0.084265	0.170686	protein_coding
ENSG0000(	NEB	692.2688	0.13871	0.084418	0.170974	protein_coding
ENSG0000(	H4C15	416.4601	0.307955	0.084425	0.170977	protein_coding
ENSG0000(	LINP1	76.79894	-0.15281	0.084474	0.171064	lncRNA
ENSG0000(	CLMN	8282.667	-0.17714	0.084524	0.171156	protein_coding
ENSG0000(	ENSG0000(	31.95309	-0.2374	0.084536	0.171167	lncRNA
ENSG0000(	ENSG0000(	178.9475	0.145759	0.084559	0.171194	TEC
ENSG0000(	HEYL	201.382	0.210147	0.08456	0.171194	protein_coding
ENSG0000(	MED15P9	301.6013	0.112478	0.084573	0.17121	transcribed_unprocessed_pseudo
ENSG0000(	ENSG0000(	181.5724	0.151163	0.08458	0.171213	lncRNA
ENSG0000(	LINC02767	89.30082	0.200853	0.084611	0.171254	lncRNA
ENSG0000(	RNU6ATAC	58.28098	0.210746	0.084611	0.171254	snRNA
ENSG0000(	HSPD1P11	315.2503	0.172415	0.084645	0.171311	processed_pseudogene
ENSG0000(	UGT2B29P	17.36886	0.664619	0.084654	0.171318	unprocessed_pseudogene
ENSG0000(	GBA3	169.3603	-0.38702	0.084686	0.171361	polymorphic_pseudogene
ENSG0000(	TMIGD1	487.4878	-0.51963	0.084683	0.171361	protein_coding
ENSG0000(	ENSG0000(	8.052219	-0.56443	0.084743	0.171466	lncRNA
ENSG0000(	UBIAD1	584.7374	-0.09873	0.084808	0.171586	protein_coding
ENSG0000(	ENSG0000(	33.00656	-0.2666	0.084861	0.171681	lncRNA
ENSG0000(	SMPD4	2312.909	-0.085	0.084885	0.171718	protein_coding
ENSG0000(	HIVEP2	2066.246	0.193686	0.084902	0.171742	protein_coding
ENSG0000(	PSMG3-AS:	313.3602	-0.15358	0.084914	0.171756	lncRNA
ENSG0000(	ENSG0000(	1656.395	-0.0852	0.084927	0.17176	transcribed_processed_pseudoge

ENSG0000(	FAM238A	15.23918	0.387943	0.084922	0.17176	transcribed_unprocessed_pseudo
ENSG0000(	TMEM105	242.7645	0.163777	0.084946	0.171786	lncRNA
ENSG0000(	GRIK1-AS1	399.8642	-0.09523	0.084961	0.171807	lncRNA
ENSG0000(	GPR89B	910.7667	0.091568	0.084997	0.171868	protein_coding
ENSG0000(	HAUS2	804.9163	-0.10006	0.085003	0.171869	protein_coding
ENSG0000(	ENSG0000(	37.34026	-0.2864	0.085016	0.171884	processed_pseudogene
ENSG0000(	PPP1R35	121.1518	-0.14677	0.085034	0.17191	protein_coding
ENSG0000(	CMPK1	2607.849	-0.14623	0.085058	0.171947	protein_coding
ENSG0000(	KIAA0040	1033.333	0.174328	0.085084	0.171978	protein_coding
ENSG0000(	PHF13	286.6773	0.170464	0.085084	0.171978	protein_coding
ENSG0000(	LINC01901	106.6126	0.147893	0.085098	0.171995	lncRNA
ENSG0000(	OTULINL	2004.426	-0.16088	0.085119	0.172014	protein_coding
ENSG0000(	SMURF2	1624.612	0.108239	0.085115	0.172014	protein_coding
ENSG0000(	IL10RB	1131.778	-0.14019	0.085144	0.172055	protein_coding
ENSG0000(	RNU2-52P	12.42804	-0.67751	0.085164	0.172084	snRNA
ENSG0000(	SMAP1	1067.692	-0.14135	0.085179	0.172103	protein_coding
ENSG0000(	DNAJC1	417.2103	0.140188	0.085209	0.172153	protein_coding
ENSG0000(	ATG4A	322.9735	-0.17176	0.085246	0.172216	protein_coding
ENSG0000(	ENSG0000(	22.69733	-1.04195	0.085257	0.172227	protein_coding
ENSG0000(	ENSG0000(	1205.311	0.129955	0.085286	0.172275	lncRNA
ENSG0000(	ZNF66	2334.916	-0.11125	0.085303	0.172297	protein_coding
ENSG0000(	ZKSCAN7-A	11.55722	0.434037	0.08531	0.172301	lncRNA
ENSG0000(	ADAM22	389.7878	-0.22161	0.08533	0.172331	protein_coding
ENSG0000(	BMPER	198.4188	0.163281	0.085399	0.172446	protein_coding
ENSG0000(	KRTAP5-9	17.05626	0.414353	0.085396	0.172446	protein_coding
ENSG0000(	ENSG0000(	28.25442	0.237299	0.085429	0.17249	lncRNA
ENSG0000(	ENSG0000(	157.3682	0.158481	0.085432	0.17249	lncRNA
ENSG0000(	ENSG0000(	92.16314	0.149253	0.085441	0.172498	lncRNA
ENSG0000(	ENSG0000(	20.84918	0.263351	0.085458	0.172517	lncRNA
ENSG0000(	PDIA4	1698.245	-0.19498	0.085461	0.172517	protein_coding
ENSG0000(	ENSG0000(	602.762	0.116752	0.085508	0.172601	lncRNA
ENSG0000(	EFCAB14	9459.306	0.072178	0.085573	0.17271	protein_coding
ENSG0000(	ENSG0000(	46.16254	0.375386	0.085574	0.17271	lncRNA
ENSG0000(	OLA1P3	1095.002	0.064225	0.085598	0.172737	processed_pseudogene
ENSG0000(	TUBBP10	31.96995	-0.64902	0.085597	0.172737	processed_pseudogene
ENSG0000(	PPP4R3A	2778.76	-0.07424	0.085651	0.172834	protein_coding
ENSG0000(	B3GALT6	222.2446	-0.13646	0.085662	0.172835	protein_coding
ENSG0000(	CADPS	537.6404	0.193212	0.085663	0.172835	protein_coding
ENSG0000(	C5orf60	118.9166	-0.17214	0.085713	0.172914	lncRNA
ENSG0000(	ENSG0000(	399.6186	0.106041	0.085709	0.172914	lncRNA
ENSG0000(	MTHFSD	666.336	0.078995	0.08572	0.172917	protein_coding
ENSG0000(	KIAA0895L	1054.56	-0.14	0.085743	0.172952	protein_coding
ENSG0000(	ENSG0000(	39.7257	0.182217	0.085768	0.172991	lncRNA
ENSG0000(	ENSG0000(	105.9193	-0.15032	0.085778	0.173001	lncRNA
ENSG0000(	NPTX1	57.59032	-0.32558	0.08581	0.173054	protein_coding
ENSG0000(	ZNF235	761.3275	-0.10653	0.085865	0.173152	protein_coding
ENSG0000(	LINC02166	19.07968	0.363051	0.08587	0.173153	lncRNA

ENSG0000(	MYL6P4	22.73659	0.319128	0.085927	0.173255	processed_pseudogene
ENSG0000(	RNU6-97P	17.64188	0.3613	0.085942	0.173274	snRNA
ENSG0000(	LINC00589	17.22054	-0.37181	0.085959	0.173296	lncRNA
ENSG0000(	NOC2LP1	33.52341	-0.41896	0.085964	0.173296	transcribed_processed_pseudoge
ENSG0000(	IL10RB-DT	135.0203	0.115416	0.085974	0.173305	lncRNA
ENSG0000(	RNU6-761F	21.21054	0.548441	0.08605	0.173447	snRNA
ENSG0000(	LINC00616	26.60974	0.271303	0.086058	0.173452	lncRNA
ENSG0000(	LINC02821	70.13458	-0.30269	0.086067	0.17346	lncRNA
ENSG0000(	LINC01099	96.91215	-0.1638	0.086096	0.173507	lncRNA
ENSG0000(	KDM5C	3648.005	-0.05918	0.086117	0.173538	protein_coding
ENSG0000(	SPNS2	565.7105	0.312459	0.086156	0.173606	protein_coding
ENSG0000(	PRKG1-AS1	9636.459	-0.07031	0.086199	0.17368	lncRNA
ENSG0000(	TSC22D1	5105.256	-0.14733	0.086306	0.173886	protein_coding
ENSG0000(	ZNF714	1775.359	-0.08117	0.08633	0.173923	protein_coding
ENSG0000(	ENSG0000(	110.4103	-0.13392	0.086375	0.174001	lncRNA
ENSG0000(	DET1	1051.617	0.181529	0.08645	0.174126	protein_coding
ENSG0000(	ENSG0000(	113.8733	-0.53044	0.086449	0.174126	lncRNA
ENSG0000(	ENSG0000(	17.85484	0.298001	0.086454	0.174126	lncRNA
ENSG0000(	ENSG0000(	19.21036	0.38723	0.08649	0.174179	lncRNA
ENSG0000(	ENSG0000(	15.70899	0.297307	0.086497	0.174179	lncRNA
ENSG0000(	SH2D1A	147.2887	0.339731	0.086495	0.174179	protein_coding
ENSG0000(	ARID1A	5742.216	0.048649	0.086514	0.174204	protein_coding
ENSG0000(	EPHA7	2320.258	0.104369	0.086541	0.174246	protein_coding
ENSG0000(	BFSP2-AS1	43.52545	0.26063	0.086599	0.174343	lncRNA
ENSG0000(	ENSG0000(	239.125	0.474909	0.0866	0.174343	lncRNA
ENSG0000(	TGIF2P1	36.46326	0.238681	0.086611	0.174354	processed_pseudogene
ENSG0000(	RN7SL663F	37.32148	0.233303	0.086637	0.174393	misc_RNA
ENSG0000(	STK40	1170.149	-0.14611	0.086642	0.174393	protein_coding
ENSG0000(	ACSM3	651.6951	-0.13031	0.086655	0.174403	protein_coding
ENSG0000(	HMBOX1-IT	56.9795	0.146598	0.086661	0.174403	lncRNA
ENSG0000(	MFGE8	1378.203	0.20256	0.086669	0.174403	protein_coding
ENSG0000(	ZNF540	383.7319	-0.13594	0.086669	0.174403	protein_coding
ENSG0000(	ENSG0000(	141.7439	0.199775	0.08668	0.174414	lncRNA
ENSG0000(	DCDC1	247.6282	0.111488	0.086713	0.17446	protein_coding
ENSG0000(	LINC01068	16.33061	-0.51748	0.086714	0.17446	lncRNA
ENSG0000(	SSX2IP	404.8308	-0.16119	0.08673	0.174481	protein_coding
ENSG0000(	ENSG0000(	301.8838	-0.19077	0.086762	0.174522	lncRNA
ENSG0000(	FAM192BP	23.4206	-0.3304	0.086759	0.174522	processed_pseudogene
ENSG0000(	NASP	2897.229	-0.07645	0.086771	0.174529	protein_coding
ENSG0000(	ENSG0000(	27.63899	-0.27152	0.086805	0.174588	lncRNA
ENSG0000(	CAPN13	476.9315	-0.37759	0.086834	0.174632	protein_coding
ENSG0000(	RCN1	997.2112	0.148759	0.086838	0.174632	protein_coding
ENSG0000(	ENSG0000(	208.4253	-0.09726	0.086864	0.174672	lncRNA
ENSG0000(	LINC00595	71.78919	0.212887	0.086898	0.174729	lncRNA
ENSG0000(	LINC00298	37.27735	0.264536	0.086905	0.174731	lncRNA
ENSG0000(	ENSG0000(	167.6479	-0.16752	0.086923	0.174757	lncRNA
ENSG0000(	OR5AS1	3177.76	-0.1248	0.086934	0.174769	protein_coding

ENSG0000(	C1orf112	452.2231	-0.16991	0.086943	0.174775	protein_coding
ENSG0000(	ENSG0000(	25.48354	0.240528	0.086979	0.174832	lncRNA
ENSG0000(	PRORY	65.02052	0.239688	0.086983	0.174832	lncRNA
ENSG0000(	SDHAF3	85.5235	-0.20508	0.086993	0.174841	protein_coding
ENSG0000(	TRAM1L1	13.66206	-0.43151	0.087063	0.174971	protein_coding
ENSG0000(	FCHO1	513.9383	0.111745	0.087072	0.174977	protein_coding
ENSG0000(	RPL7P23	10.41088	0.634252	0.087082	0.174986	processed_pseudogene
ENSG0000(	NUP58	1646.731	0.113195	0.087101	0.175014	protein_coding
ENSG0000(	LMO7	5075.269	-0.22869	0.087129	0.175059	protein_coding
ENSG0000(	CAP1	3620.836	-0.16622	0.087142	0.175073	protein_coding
ENSG0000(	ABHD5	1655.068	-0.20275	0.087299	0.175377	protein_coding
ENSG0000(	CENPF	1878.16	-0.10954	0.087338	0.175434	protein_coding
ENSG0000(	RN7SKP245	23.19751	0.417901	0.087333	0.175434	misc_RNA
ENSG0000(	COG3	2111.908	-0.08519	0.08736	0.175466	protein_coding
ENSG0000(	COPZ1	1005.843	-0.11309	0.087376	0.175475	protein_coding
ENSG0000(	KCNC1	73.49189	-0.187	0.087375	0.175475	protein_coding
ENSG0000(	AIG1	817.0141	-0.07782	0.087396	0.175506	protein_coding
ENSG0000(	RN7SL460F	337.4031	0.108388	0.087436	0.175574	misc_RNA
ENSG0000(	ILDR1	334.6936	-0.18381	0.087463	0.175617	protein_coding
ENSG0000(	ENSG0000(	12.62096	0.401654	0.08748	0.175639	lncRNA
ENSG0000(	TMEM220	485.4947	-0.17292	0.08757	0.175809	protein_coding
ENSG0000(	ENSG0000(	114.372	-0.14257	0.087585	0.175828	lncRNA
ENSG0000(	TBX2	974.8955	-0.18581	0.087617	0.17588	protein_coding
ENSG0000(	GEMIN8P4	9.498947	-0.49864	0.087632	0.175899	processed_pseudogene
ENSG0000(	ENSG0000(	15.17638	0.300836	0.087707	0.176039	TEC
ENSG0000(	METTL21EF	142.0309	0.146866	0.08772	0.176053	transcribed_unprocessed_pseudo
ENSG0000(	LINC02232	19.00985	0.440396	0.087775	0.176153	lncRNA
ENSG0000(	EBI3	40.71201	0.394055	0.087787	0.176165	protein_coding
ENSG0000(	TRPV2	336.1376	0.215793	0.087846	0.176272	protein_coding
ENSG0000(	AGRN	1475.975	0.126579	0.087864	0.176286	protein_coding
ENSG0000(	SOX9-AS1	1300.871	-0.09988	0.087864	0.176286	lncRNA
ENSG0000(	SNORA22C	19.04725	-0.31184	0.087874	0.176296	snoRNA
ENSG0000(	SCAND2P	2205.728	0.076923	0.087889	0.176313	transcribed_unprocessed_pseudo
ENSG0000(	GOLGA8B	3728.585	0.224921	0.087907	0.176338	protein_coding
ENSG0000(	TMED9	1083.156	-0.10818	0.087913	0.176339	protein_coding
ENSG0000(	PDE8B	119.4591	0.224062	0.087939	0.176381	protein_coding
ENSG0000(	ENSG0000(	116.4529	-0.12509	0.088	0.176492	lncRNA
ENSG0000(	JAM3	383.7749	0.193337	0.088017	0.176514	protein_coding
ENSG0000(	ENSG0000(	26.71561	0.260055	0.088042	0.176552	lncRNA
ENSG0000(	COLEC10	25.25252	0.423052	0.088084	0.176626	protein_coding
ENSG0000(	ENSG0000(	109.2166	-0.17995	0.088176	0.176799	lncRNA
ENSG0000(	ENSG0000(	29.34527	-0.25032	0.088188	0.176812	protein_coding
ENSG0000(	CLRN1	42.1924	0.214207	0.088222	0.176868	protein_coding
ENSG0000(	MSANTD3-	17.0022	0.754784	0.08824	0.176893	protein_coding
ENSG0000(	ENSG0000(	129.0624	0.135954	0.08827	0.17693	lncRNA
ENSG0000(	PRKAR2B-A	212.9983	0.246299	0.088269	0.17693	lncRNA
ENSG0000(	GPANK1	382.3419	-0.09403	0.088279	0.176937	protein_coding

ENSG0000(	LINC01811	102.5933	0.250867	0.08831	0.176989	lncRNA
ENSG0000(	S100A13	227.3843	-0.20978	0.088365	0.177086	protein_coding
ENSG0000(	ENSG0000(	2097.465	0.064318	0.088438	0.177203	lncRNA
ENSG0000(	ENSG0000(	77.33458	-0.39354	0.088434	0.177203	protein_coding
ENSG0000(	MYDGF	285.8118	-0.1869	0.08844	0.177203	protein_coding
ENSG0000(	PCSK7	13325.39	-0.07893	0.088484	0.177279	protein_coding
ENSG0000(	ENSG0000(	21.77258	0.268597	0.088497	0.177294	lncRNA
ENSG0000(	ENSG0000(	19.6005	0.276948	0.088516	0.17732	lncRNA
ENSG0000(	ENSG0000(	47.9597	0.709237	0.088532	0.177342	unprocessed_pseudogene
ENSG0000(	SH3D19	4345.633	-0.16835	0.088563	0.177393	protein_coding
ENSG0000(	ENSG0000(	15.87662	0.514272	0.088598	0.177451	lncRNA
ENSG0000(	MTCO1P4(	173.7062	-0.84054	0.088615	0.177474	processed_pseudogene
ENSG0000(	PIK3C3	2860.633	0.060891	0.088632	0.177497	protein_coding
ENSG0000(	RHPN2P1	76.17262	0.165792	0.088639	0.1775	unprocessed_pseudogene
ENSG0000(	ENSG0000(	869.0244	0.091038	0.088664	0.177538	lncRNA
ENSG0000(	ENSG0000(	19.8509	0.393991	0.088682	0.177551	transcribed_processed_pseudoge
ENSG0000(	SELENOP	4825.447	0.291943	0.088682	0.177551	protein_coding
ENSG0000(	RBP4	11.40951	0.563723	0.088713	0.177602	protein_coding
ENSG0000(	ARHGEF4	346.3964	0.130882	0.088744	0.177642	protein_coding
ENSG0000(	SPIN2A	118.7103	0.171125	0.08874	0.177642	protein_coding
ENSG0000(	FZR1	1252.729	-0.11205	0.088757	0.177656	protein_coding
ENSG0000(	ENSG0000(	41.78302	0.276591	0.088771	0.177673	lncRNA
ENSG0000(	PKD1L1	482.1058	-0.09732	0.088777	0.177674	protein_coding
ENSG0000(	MYRF-AS1	13.67892	0.486651	0.088836	0.177769	lncRNA
ENSG0000(	NBPF26	1056.108	0.318574	0.088831	0.177769	protein_coding
ENSG0000(	ZNF767P	1457.113	0.083052	0.088929	0.177943	transcribed_unprocessed_pseudo
ENSG0000(	ENSG0000(	1770.319	0.082826	0.088997	0.178068	lncRNA
ENSG0000(	ELF4	2579.409	-0.14539	0.08905	0.178163	protein_coding
ENSG0000(	AKR1B15	511.3176	0.097129	0.08906	0.178171	protein_coding
ENSG0000(	MACROD2-	8.727852	0.458523	0.08908	0.178189	lncRNA
ENSG0000(	RBM26	3377.071	0.069983	0.089078	0.178189	protein_coding
ENSG0000(	CYREN	675.898	-0.19902	0.089142	0.178302	protein_coding
ENSG0000(	GATAD1	2848.286	0.055284	0.089149	0.178303	protein_coding
ENSG0000(	ENSG0000(	15.40039	0.332782	0.089164	0.178311	lncRNA
ENSG0000(	GABRB2	398.5042	-0.12333	0.08917	0.178311	protein_coding
ENSG0000(	SMTN	2012.01	-0.16156	0.089167	0.178311	protein_coding
ENSG0000(	TRAM1	1468.731	0.184368	0.089213	0.178375	protein_coding
ENSG0000(	TRMT61B	223.4188	-0.14466	0.089213	0.178375	protein_coding
ENSG0000(	SENP2	1571.32	-0.06388	0.089314	0.178565	protein_coding
ENSG0000(	MRGBP	205.056	-0.13678	0.089339	0.178604	protein_coding
ENSG0000(	MS4A8	469.5731	0.360068	0.08935	0.178615	protein_coding
ENSG0000(	TAX1BP3	953.7021	-0.27161	0.0894	0.178704	protein_coding
ENSG0000(	HYLS1	245.2367	-0.09085	0.089409	0.178709	protein_coding
ENSG0000(	ITSN1	2170.573	-0.10258	0.089426	0.178733	protein_coding
ENSG0000(	ENKD1	147.5036	-0.16365	0.089467	0.178802	protein_coding
ENSG0000(	TMTC4	568.6022	-0.13663	0.089493	0.178844	protein_coding
ENSG0000(	RPL17P50	44.93733	0.233608	0.089555	0.178955	processed_pseudogene

ENSG0000(	TRIM54	10.39895	-0.47103	0.089583	0.178999	protein_coding
ENSG0000(	ENSG0000(	132.2349	0.277236	0.089626	0.179075	protein_coding
ENSG0000(	ENSG0000(	178.2132	0.129414	0.089651	0.179113	lncRNA
ENSG0000(	HIF3A	845.3536	0.117186	0.089667	0.179134	protein_coding
ENSG0000(	SNORD116	13.2266	0.884452	0.089694	0.179176	snoRNA
ENSG0000(	PCDHB16	146.4583	0.120082	0.089717	0.179211	protein_coding
ENSG0000(	MIR548V	22.30824	0.310753	0.089735	0.179235	miRNA
ENSG0000(	HHATL	12.61168	-0.72196	0.08976	0.179273	protein_coding
ENSG0000(	ENSG0000(	16.38533	0.425708	0.089766	0.179274	lncRNA
ENSG0000(	ENSG0000(	225.7404	-0.12769	0.089839	0.179409	protein_coding
ENSG0000(	SCHLAP1	149.6168	0.123612	0.089846	0.179412	lncRNA
ENSG0000(	ZNF300	321.9636	-0.13652	0.089878	0.179463	protein_coding
ENSG0000(	ENSG0000(	22.60396	0.344015	0.089917	0.179522	lncRNA
ENSG0000(	PRPS1	244.9985	0.171727	0.089919	0.179522	protein_coding
ENSG0000(	FAM151A	14.44903	-0.53273	0.089925	0.179522	protein_coding
ENSG0000(	CMTM8	46.12035	-0.18992	0.08995	0.179561	protein_coding
ENSG0000(	ENSG0000(	5602.014	-0.10948	0.089988	0.179614	lncRNA
ENSG0000(	TTC39A	1211.104	-0.23515	0.089983	0.179614	protein_coding
ENSG0000(	LIG4	992.8065	0.085893	0.090003	0.179632	protein_coding
ENSG0000(	ENSG0000(	91.66808	0.160162	0.090017	0.17965	lncRNA
ENSG0000(	AATF	1086.469	-0.0726	0.090065	0.179723	protein_coding
ENSG0000(	UOX	16.24579	0.54485	0.090066	0.179723	transcribed_unitary_pseudogene
ENSG0000(	DYNLT3	390.1885	-0.13367	0.090086	0.179753	protein_coding
ENSG0000(	ENSG0000(	13.34722	-0.29364	0.090092	0.179753	lncRNA
ENSG0000(	UBE2Q2	472.433	0.162194	0.090142	0.179841	protein_coding
ENSG0000(	ZNF695	97.14296	-0.25462	0.090151	0.179848	protein_coding
ENSG0000(	ENSG0000(	345.1811	0.116177	0.090189	0.179901	TEC
ENSG0000(	ENSG0000(	169.5827	0.110056	0.090188	0.179901	lncRNA
ENSG0000(	ARHGEF17-	10.89362	0.3966	0.090207	0.179921	lncRNA
ENSG0000(	ENSG0000(	387.3923	-0.13577	0.090211	0.179921	transcribed_processed_pseudoge
ENSG0000(	RN7SL215F	8.932396	0.436209	0.090252	0.179985	misc_RNA
ENSG0000(	RPL23AP63	26.91393	-0.90065	0.090254	0.179985	processed_pseudogene
ENSG0000(	ENSG0000(	87.55015	-0.1625	0.090276	0.179988	lncRNA
ENSG0000(	ENSG0000(	47.21471	0.311069	0.090285	0.179988	TEC
ENSG0000(	ENSG0000(	40.57663	0.199389	0.090282	0.179988	lncRNA
ENSG0000(	PPP5D1P	622.3454	0.085593	0.090278	0.179988	transcribed_unprocessed_pseudo
ENSG0000(	RIF1	4454.84	0.09197	0.090274	0.179988	protein_coding
ENSG0000(	ENSG0000(	16.32513	0.371195	0.090322	0.180052	lncRNA
ENSG0000(	ARMCX1	120.9836	0.167835	0.090442	0.180263	protein_coding
ENSG0000(	IGLV1-44	333.3426	0.498266	0.090446	0.180263	IG_V_gene
ENSG0000(	WDR62	678.1643	-0.09089	0.090443	0.180263	protein_coding
ENSG0000(	CTSZ	5390.863	-0.13693	0.090477	0.180313	protein_coding
ENSG0000(	ENSG0000(	158.1695	-0.29722	0.090524	0.180396	lncRNA
ENSG0000(	GJB3	49.69741	-0.49998	0.090542	0.180421	protein_coding
ENSG0000(	ENSG0000(	71.90842	0.178281	0.090559	0.180443	lncRNA
ENSG0000(	RABGGTB	2505.128	-0.08361	0.09057	0.180453	protein_coding
ENSG0000(	ENSG0000(	25.01467	-0.33808	0.090585	0.180472	lncRNA

ENSG0000(	CTAGE4	571.7662	-0.55488	0.090601	0.18048	protein_coding
ENSG0000(	LINC00987	93.97334	0.257696	0.090607	0.18048	lncRNA
ENSG0000(	OCEL1	241.17	-0.1901	0.090603	0.18048	protein_coding
ENSG0000(	F2RL3	602.1182	0.117431	0.090627	0.180509	protein_coding
ENSG0000(	ENSG0000(	51.89851	0.186927	0.090641	0.180526	lncRNA
ENSG0000(	ENSG0000(	11.06174	0.402229	0.090651	0.180535	lncRNA
ENSG0000(	ENSG0000(	59.54173	-0.1494	0.090738	0.180696	lncRNA
ENSG0000(	ENSG0000(	14.96142	-0.75083	0.090745	0.180698	processed_pseudogene
ENSG0000(	NBPF20	281.3796	-0.15923	0.090752	0.180702	protein_coding
ENSG0000(	WWP1	2472.293	-0.10639	0.090793	0.180771	protein_coding
ENSG0000(	XACT	125558	0.118462	0.090856	0.180885	lncRNA
ENSG0000(	SSH3	748.8652	-0.18729	0.090876	0.180914	protein_coding
ENSG0000(	FANCC	1110.28	-0.09402	0.090888	0.180915	protein_coding
ENSG0000(	LTK	222.2021	-0.31474	0.090889	0.180915	protein_coding
ENSG0000(	HACD3	2240.116	0.07595	0.090896	0.180918	protein_coding
ENSG0000(	ENSG0000(	70.89054	0.154052	0.090922	0.180937	lncRNA
ENSG0000(	MIR548AA:	59.86983	0.194715	0.090919	0.180937	miRNA
ENSG0000(	ZNF594	784.1211	-0.12564	0.090922	0.180937	protein_coding
ENSG0000(	CYP20A1	3975.363	-0.05508	0.090958	0.180986	protein_coding
ENSG0000(	GALNT17	83.09549	-0.23185	0.090959	0.180986	protein_coding
ENSG0000(	ENSG0000(	20.25211	0.299595	0.09099	0.181024	lncRNA
ENSG0000(	MIR645	12.16287	0.398421	0.090985	0.181024	miRNA
ENSG0000(	PCSK4	70.00614	-0.32071	0.091015	0.181064	protein_coding
ENSG0000(	GIMAP6	488.3358	0.19938	0.091049	0.18111	protein_coding
ENSG0000(	RCOR2	26.89857	-0.33573	0.09105	0.18111	protein_coding
ENSG0000(	VASH1-DT	10.26653	0.413994	0.091123	0.181244	lncRNA
ENSG0000(	TPP2	2961.258	-0.09056	0.09113	0.181245	protein_coding
ENSG0000(	ENSG0000(	40.38121	0.34484	0.09115	0.181274	lncRNA
ENSG0000(	ENSG0000(	19.44159	-0.31727	0.091173	0.181297	lncRNA
ENSG0000(	TNS1-AS1	96.55834	-0.23076	0.091173	0.181297	lncRNA
ENSG0000(	ZCCHC4	629.9745	-0.08089	0.09118	0.1813	protein_coding
ENSG0000(	TMEM240	16.91877	-0.29764	0.091243	0.181413	protein_coding
ENSG0000(	ENSG0000(	309.1434	-0.0981	0.091309	0.181532	lncRNA
ENSG0000(	PALS1	3066.163	-0.12636	0.091321	0.181545	protein_coding
ENSG0000(	FRMD5	125.7106	0.283894	0.091339	0.181568	protein_coding
ENSG0000(	ENSG0000(	115.9843	0.121832	0.091358	0.181595	lncRNA
ENSG0000(	GRIA4	314.7064	0.211103	0.091446	0.181736	protein_coding
ENSG0000(	SEMA6D	2622.542	-0.20024	0.091446	0.181736	protein_coding
ENSG0000(	TPRG1	132.4382	0.176581	0.091444	0.181736	protein_coding
ENSG0000(	ENSG0000(	10.23395	0.423	0.091497	0.181815	lncRNA
ENSG0000(	STMN4	47.44078	0.206968	0.091496	0.181815	protein_coding
ENSG0000(	ENSG0000(	20.32088	0.30032	0.091515	0.181837	lncRNA
ENSG0000(	ENSG0000(	75.0781	-0.18623	0.091541	0.181878	lncRNA
ENSG0000(	BAALC-AS1	460.2785	-0.09221	0.091553	0.18189	lncRNA
ENSG0000(	NDUFA9	4370.327	-0.08213	0.091566	0.181906	protein_coding
ENSG0000(	FABP2	449.7301	-0.27395	0.091575	0.181911	protein_coding
ENSG0000(	PILRA	119.2997	0.301059	0.0916	0.18195	protein_coding

ENSG0000(	APOC4	28.81712	0.319057	0.091692	0.182121	protein_coding
ENSG0000(	ENSG0000(	651.8841	0.086387	0.091714	0.182133	lncRNA
ENSG0000(	ENSG0000(	23.46432	-0.3342	0.091716	0.182133	transcribed_unprocessed_pseudo
ENSG0000(	RARB	515.5508	0.157785	0.09171	0.182133	protein_coding
ENSG0000(	SNORA3B	132.8657	0.165857	0.091739	0.182167	snoRNA
ENSG0000(	FTLP6	162.5819	-0.11413	0.09176	0.182198	processed_pseudogene
ENSG0000(	HERC2P7	807.7189	0.141747	0.091801	0.182267	unprocessed_pseudogene
ENSG0000(	PDCD6	2934.26	-0.06665	0.091878	0.182409	protein_coding
ENSG0000(	OR4F15	108.6585	-0.15115	0.091888	0.182418	protein_coding
ENSG0000(	ENSG0000(	216.8852	-0.09664	0.091904	0.182436	lncRNA
ENSG0000(	SNX9	3571.271	-0.1172	0.091914	0.182445	protein_coding
ENSG0000(	LINC00598	660.0991	-0.12205	0.091931	0.182468	lncRNA
ENSG0000(	ZNF781	155.2772	0.179587	0.091969	0.182531	protein_coding
ENSG0000(	OR8D1	117.3684	0.143487	0.091977	0.182536	protein_coding
ENSG0000(	ENSG0000(	168.8776	-0.11889	0.091992	0.182543	lncRNA
ENSG0000(	PELI3	160.3594	0.144533	0.091988	0.182543	protein_coding
ENSG0000(	GSPT2	209.5272	-0.14728	0.092	0.182546	protein_coding
ENSG0000(	MED16	667.5299	-0.12711	0.092016	0.182567	protein_coding
ENSG0000(	EYA3	2118.966	-0.06664	0.09205	0.182623	protein_coding
ENSG0000(	ENSG0000(	61.46703	0.16111	0.092081	0.182673	lncRNA
ENSG0000(	ENSG0000(	143.5153	0.148647	0.092094	0.182688	TEC
ENSG0000(	FAM110D	230.1834	0.157292	0.09212	0.182728	protein_coding
ENSG0000(	LINC01488	12.71222	0.368823	0.092142	0.182759	lncRNA
ENSG0000(	ENSG0000(	61.34179	0.169541	0.092153	0.182769	lncRNA
ENSG0000(	PCDHB4	33.7558	0.270447	0.092198	0.182847	protein_coding
ENSG0000(	ENSG0000(	417.1095	0.091031	0.092219	0.182877	lncRNA
ENSG0000(	ENSG0000(	27.22657	0.344148	0.092255	0.182935	processed_pseudogene
ENSG0000(	ENSG0000(	23.32092	-0.24482	0.09226	0.182935	lncRNA
ENSG0000(	MLF1	130.2752	-0.14419	0.092293	0.182989	protein_coding
ENSG0000(	NRG2	32.79941	-0.36675	0.092303	0.182996	protein_coding
ENSG0000(	TRAM2-AS:	548.98	0.067214	0.092311	0.183001	lncRNA
ENSG0000(	ENSG0000(	106.4486	0.113043	0.092431	0.183228	lncRNA
ENSG0000(	CEACAM8	218.4136	-0.16094	0.092476	0.183305	protein_coding
ENSG0000(	RBM38-AS:	59.34937	0.175481	0.09249	0.18331	lncRNA
ENSG0000(	RPL41P2	16.67817	0.380281	0.092488	0.18331	processed_pseudogene
ENSG0000(	ENSG0000(	191.318	0.191748	0.092532	0.183381	lncRNA
ENSG0000(	GCHFR	47.70767	-0.29006	0.092588	0.18348	protein_coding
ENSG0000(	ENSG0000(	155.3703	-0.14619	0.092599	0.183491	lncRNA
ENSG0000(	ENSG0000(	310.9988	-0.18994	0.092611	0.183503	unprocessed_pseudogene
ENSG0000(	LRRC46	11.56418	0.496953	0.092686	0.18364	protein_coding
ENSG0000(	ENSG0000(	188.5583	-0.19834	0.092743	0.183742	lncRNA
ENSG0000(	RASGEF1C	36.46781	-0.52016	0.092761	0.183755	protein_coding
ENSG0000(	SPTBN2	566.3904	0.160273	0.092762	0.183755	protein_coding
ENSG0000(	NDUFA12	2838.772	-0.08508	0.092773	0.183765	protein_coding
ENSG0000(	ALMS1	5039.736	0.066881	0.092802	0.183812	protein_coding
ENSG0000(	PLB1	172.8254	0.307613	0.092849	0.183893	protein_coding
ENSG0000(	DNAH14	683.9848	-0.16886	0.092864	0.183912	protein_coding

ENSG0000(	C19orf47	489.6862	-0.10503	0.092882	0.183923	protein_coding
ENSG0000(	CCDC173	12.07418	0.526791	0.092881	0.183923	protein_coding
ENSG0000(	ENSG0000(	11.16083	-0.47124	0.092919	0.183986	protein_coding
ENSG0000(	NBAS	2357.929	0.101607	0.09295	0.184034	protein_coding
ENSG0000(	PROSER2-A	78.23349	-0.14071	0.092978	0.184078	lncRNA
ENSG0000(	LINC01436	31.30036	0.293497	0.093007	0.184124	lncRNA
ENSG0000(	MMADHC	563.5811	-0.13235	0.093069	0.184234	protein_coding
ENSG0000(	BRWD3	2758.948	0.119856	0.093117	0.1843	protein_coding
ENSG0000(	ENSG0000(	1004.117	-0.0875	0.093118	0.1843	lncRNA
ENSG0000(	FANCD2	1521.09	-0.16307	0.093119	0.1843	protein_coding
ENSG0000(	COX15	1289.606	-0.09378	0.093139	0.184328	protein_coding
ENSG0000(	ENSG0000(	45.57961	0.239591	0.093173	0.184382	TEC
ENSG0000(	ANKRD11	6977.681	0.05776	0.093204	0.184433	protein_coding
ENSG0000(	GCGR	10.12654	0.531136	0.093263	0.184526	protein_coding
ENSG0000(	KRT8P50	25.13877	-0.28854	0.09326	0.184526	processed_pseudogene
ENSG0000(	ENSG0000(	40.96473	-1.56399	0.093312	0.184611	transcribed_unprocessed_pseudo
ENSG0000(	PDP2	1473.112	-0.09308	0.093341	0.184657	protein_coding
ENSG0000(	ARFGAP1	1835.002	0.086598	0.093395	0.18474	protein_coding
ENSG0000(	ENSG0000(	111.0461	-0.145	0.09339	0.18474	lncRNA
ENSG0000(	ENSG0000(	1211.753	0.181591	0.09341	0.184747	TEC
ENSG0000(	FUNDC1	72.37111	-0.16433	0.093409	0.184747	protein_coding
ENSG0000(	TICRR	444.3136	-0.19636	0.093467	0.184848	protein_coding
ENSG0000(	ENSG0000(	74.11762	0.158621	0.093476	0.184853	lncRNA
ENSG0000(	CDC6	473.0883	-0.18664	0.093592	0.185014	protein_coding
ENSG0000(	CPSF3	459.7947	-0.12416	0.093577	0.185014	protein_coding
ENSG0000(	DCAF4	523.6974	-0.1263	0.093571	0.185014	protein_coding
ENSG0000(	IGHV3-30	1121.431	0.381927	0.093587	0.185014	IG_V_gene
ENSG0000(	PLEKHG4B	382.6431	0.129809	0.093572	0.185014	protein_coding
ENSG0000(	SCYGR4	125.5445	0.101556	0.093592	0.185014	protein_coding
ENSG0000(	BAAT	300.2895	0.12868	0.093612	0.185041	protein_coding
ENSG0000(	ZNF558	1334.093	-0.11155	0.093618	0.185042	protein_coding
ENSG0000(	SOX10	80.86172	-0.29955	0.093724	0.185239	protein_coding
ENSG0000(	ENSG0000(	298.7007	-0.10623	0.093736	0.18525	lncRNA
ENSG0000(	ENSG0000(	45.48458	0.183505	0.093748	0.185263	lncRNA
ENSG0000(	ENSG0000(	298.6178	-0.12363	0.093762	0.185267	lncRNA
ENSG0000(	PRPF4	577.7837	-0.09355	0.09376	0.185267	protein_coding
ENSG0000(	KCTD14	86.6337	-0.27412	0.093775	0.185282	protein_coding
ENSG0000(	LHX9	18.65179	-0.48702	0.093849	0.185404	protein_coding
ENSG0000(	RHOD	36.23303	-0.4475	0.093848	0.185404	protein_coding
ENSG0000(	HFE	732.0268	-0.08518	0.093881	0.185456	protein_coding
ENSG0000(	ENSG0000(	326.0437	0.156686	0.093922	0.185525	lncRNA
ENSG0000(	ENSG0000(	35.04575	0.279047	0.09396	0.185587	processed_pseudogene
ENSG0000(	ENSG0000(	309.3479	-0.07767	0.094007	0.185669	lncRNA
ENSG0000(	ACAP1	1381.24	0.178968	0.094023	0.185688	protein_coding
ENSG0000(	ENSG0000(	161.0841	-0.20294	0.094049	0.185729	lncRNA
ENSG0000(	LINC02554	18.39539	0.350232	0.094059	0.185737	lncRNA
ENSG0000(	ENSG0000(	113.0525	0.150709	0.094066	0.185739	lncRNA

ENSG0000(	ENSG0000(	451.8016	0.148611	0.094132	0.185858	lncRNA
ENSG0000(	ENSG0000(	302.7768	0.079632	0.094188	0.185956	lncRNA
ENSG0000(	ENSG0000(	27.73265	-0.22305	0.094233	0.186033	lncRNA
ENSG0000(	TMEM19	1396.58	-0.08066	0.094301	0.186155	protein_coding
ENSG0000(	NAIF1	419.6305	-0.07907	0.094317	0.186175	protein_coding
ENSG0000(	ENSG0000(	115.8606	-0.16987	0.094328	0.186185	lncRNA
ENSG0000(	ENSG0000(	117.847	-0.18301	0.094343	0.186197	protein_coding
ENSG0000(	SRSF6P2	9.270413	0.354483	0.094346	0.186197	processed_pseudogene
ENSG0000(	FLJ20021	28.58498	0.278589	0.094369	0.186231	lncRNA
ENSG0000(	EFCAB15P	24.48588	-0.29709	0.094378	0.186237	transcribed_unitary_pseudogene
ENSG0000(	USP21	409.4356	-0.10171	0.094403	0.186275	protein_coding
ENSG0000(	PMCHL2	86.28653	-0.19931	0.094431	0.186319	transcribed_unprocessed_pseudo
ENSG0000(	ZNRD1ASP	2232.709	0.095417	0.094462	0.186367	transcribed_unitary_pseudogene
ENSG0000(	C9orf153	81.33068	-0.15487	0.094485	0.1864	protein_coding
ENSG0000(	TTC4	292.4506	-0.13017	0.094566	0.186537	protein_coding
ENSG0000(	ZNF839	819.7501	-0.0752	0.094564	0.186537	protein_coding
ENSG0000(	ENSG0000(	248.6221	-0.12063	0.094621	0.186635	lncRNA
ENSG0000(	DHX34	616.8081	-0.12211	0.094696	0.18677	protein_coding
ENSG0000(	TECTA	71.17674	0.292462	0.09474	0.186845	protein_coding
ENSG0000(	FGD3	903.1509	0.273072	0.094746	0.186846	protein_coding
ENSG0000(	THBS3	567.7524	0.12922	0.094768	0.186877	protein_coding
ENSG0000(	PARK7P1	54.80624	0.184541	0.094804	0.186935	processed_pseudogene
ENSG0000(	ENSG0000(	93.06128	-0.34624	0.094811	0.186935	lncRNA
ENSG0000(	ENSG0000(	117.5397	0.143285	0.094816	0.186935	lncRNA
ENSG0000(	SMIM38	65.37275	0.379955	0.094882	0.187054	protein_coding
ENSG0000(	LINC01266	303.7041	0.123647	0.094895	0.187068	lncRNA
ENSG0000(	CEMP1	23.25668	0.406827	0.094905	0.187076	protein_coding
ENSG0000(	CGA	38.21702	-0.19925	0.095	0.187248	protein_coding
ENSG0000(	LARGE-IT1	57.38686	-0.20499	0.095004	0.187248	lncRNA
ENSG0000(	SIAH1	941.2331	-0.08482	0.095032	0.18729	protein_coding
ENSG0000(	TCAM1P	222.294	0.133453	0.095045	0.187305	transcribed_unitary_pseudogene
ENSG0000(	RRAGD	177.3035	0.244836	0.095083	0.187369	protein_coding
ENSG0000(	ERCC6L2	4751.79	0.053114	0.095124	0.187437	protein_coding
ENSG0000(	LRRC58	1811.06	-0.08319	0.095138	0.187453	protein_coding
ENSG0000(	APOF	70.2134	0.145227	0.095159	0.187482	protein_coding
ENSG0000(	ENSG0000(	293.0482	0.110994	0.095195	0.187541	lncRNA
ENSG0000(	MAP9	216.0446	0.291467	0.095287	0.187712	protein_coding
ENSG0000(	TTC30A	226.4694	-0.15637	0.095295	0.187714	protein_coding
ENSG0000(	VEGFD	15.36422	0.426978	0.095322	0.187756	protein_coding
ENSG0000(	CNN2	1887.406	0.123702	0.09541	0.187918	protein_coding
ENSG0000(	RNF182	20.85983	0.332643	0.095447	0.187979	protein_coding
ENSG0000(	ENSG0000(	552.6067	-0.1295	0.095484	0.188016	lncRNA
ENSG0000(	ENSG0000(	1315.73	-0.0707	0.095482	0.188016	processed_pseudogene
ENSG0000(	GALC	1184.712	0.077838	0.095479	0.188016	protein_coding
ENSG0000(	ENSG0000(	26.2125	0.297213	0.095523	0.188081	transcribed_processed_pseudoge
ENSG0000(	HARS1	3436.556	-0.04835	0.095532	0.188086	protein_coding
ENSG0000(	GATD3A	418.8504	-0.23446	0.095558	0.188127	protein_coding

ENSG0000(	FAM50B	46.79074	0.218252	0.095606	0.188208	protein_coding
ENSG0000(	MLPH	2332.259	0.193186	0.095616	0.188217	protein_coding
ENSG0000(	MPV17L	1663.073	-0.10096	0.095634	0.18824	protein_coding
ENSG0000(	NT5DC2	248.1409	0.215203	0.095651	0.188261	protein_coding
ENSG0000(	FBLN1	1837.082	0.143007	0.095777	0.188498	protein_coding
ENSG0000(	ENSG0000(	34.01496	0.28605	0.095812	0.188555	lncRNA
ENSG0000(	LINC01028	9.547607	0.390389	0.095859	0.188636	lncRNA
ENSG0000(	NPIPB14P	258.9069	-0.30918	0.095869	0.188643	transcribed_unprocessed_pseudo
ENSG0000(	GOLGA6L9	1166.628	0.145998	0.095961	0.188813	protein_coding
ENSG0000(	ENSG0000(	63.08972	0.167804	0.095991	0.188861	lncRNA
ENSG0000(	RNU6-653F	25.07683	0.285466	0.096022	0.188909	snRNA
ENSG0000(	RBMS3-AS3	807.994	-0.12971	0.096029	0.18891	lncRNA
ENSG0000(	ENSG0000(	39.18778	0.223299	0.096044	0.188928	lncRNA
ENSG0000(	ENSG0000(	20.10648	0.257321	0.096153	0.189131	lncRNA
ENSG0000(	RN7SL160F	12.67571	0.372054	0.096161	0.189136	misc_RNA
ENSG0000(	SLITRK5	333.08	-0.10935	0.096172	0.189145	protein_coding
ENSG0000(	PKHD1	308.1864	-0.313	0.096194	0.189175	protein_coding
ENSG0000(	ENSG0000(	52.08034	0.171862	0.096213	0.189202	lncRNA
ENSG0000(	KIF3A	625.9126	0.114639	0.09622	0.189204	protein_coding
ENSG0000(	RIDA	116.6611	-0.19087	0.096251	0.189253	protein_coding
ENSG0000(	FOXP1	10011.45	0.090199	0.096259	0.189256	protein_coding
ENSG0000(	ENSG0000(	226.4029	0.14708	0.096282	0.189277	lncRNA
ENSG0000(	GP2	414.8073	0.087563	0.096282	0.189277	protein_coding
ENSG0000(	ENSG0000(	58.71718	-0.28262	0.096292	0.189286	lncRNA
ENSG0000(	LMF2	829.5305	0.10692	0.096309	0.189308	protein_coding
ENSG0000(	CTIF	584.875	0.145755	0.096326	0.189322	protein_coding
ENSG0000(	PRSS3	593.4629	-0.27316	0.096335	0.189322	protein_coding
ENSG0000(	SEPTIN7	4583.727	0.066899	0.096334	0.189322	protein_coding
ENSG0000(	ENSG0000(	82.83334	0.189046	0.096354	0.189325	TEC
ENSG0000(	ENSG0000(	176.909	0.118073	0.096353	0.189325	lncRNA
ENSG0000(	ENSG0000(	173.5298	-0.36675	0.096355	0.189325	lncRNA
ENSG0000(	SMURF1	1913.747	0.101832	0.096374	0.189352	protein_coding
ENSG0000(	SAMD3	229.7776	0.181288	0.096397	0.189385	protein_coding
ENSG0000(	SLC9C1	39.03777	0.464432	0.096425	0.189428	protein_coding
ENSG0000(	CCDC86	455.0854	-0.0942	0.096438	0.18943	protein_coding
ENSG0000(	MZT1	97.75502	-0.19339	0.096435	0.18943	protein_coding
ENSG0000(	GAS8	452.5114	0.157837	0.096452	0.189434	protein_coding
ENSG0000(	RNF216	1849.067	0.100741	0.09645	0.189434	protein_coding
ENSG0000(	ENSG0000(	515.3308	0.090519	0.096473	0.189463	TEC
ENSG0000(	ABCD2	197.0025	0.276689	0.096507	0.189506	protein_coding
ENSG0000(	DLC1	1210.821	0.147692	0.096502	0.189506	protein_coding
ENSG0000(	ZNF79	208.6179	-0.11937	0.096553	0.189583	protein_coding
ENSG0000(	RNF214	604.2122	-0.08213	0.096568	0.189602	protein_coding
ENSG0000(	C3	2577.423	0.299374	0.096577	0.189608	protein_coding
ENSG0000(	CH25H	86.3059	0.288834	0.09662	0.189668	protein_coding
ENSG0000(	ENSG0000(	32.7962	0.23943	0.096618	0.189668	lncRNA
ENSG0000(	CDKAL1	1067.067	-0.07412	0.096643	0.189694	protein_coding

ENSG0000(	SNORD32A	68.39495	0.431288	0.096645	0.189694	snoRNA
ENSG0000(	GNGT2	31.84059	0.307664	0.096672	0.189735	protein_coding
ENSG0000(	RN7SL326F	34.96118	0.190746	0.09671	0.189796	misc_RNA
ENSG0000(	UBN1	2092.504	-0.09181	0.096721	0.189806	protein_coding
ENSG0000(	SKINT1L	865.475	0.140431	0.096744	0.18984	transcribed_unprocessed_pseudo
ENSG0000(	PHEX	102.0865	0.275213	0.096773	0.189885	protein_coding
ENSG0000(	RNU6-469F	9.146727	0.417921	0.0968	0.189927	snRNA
ENSG0000(	ZNF383	2077.442	-0.07502	0.096812	0.189937	protein_coding
ENSG0000(	BICDL2	2525.223	-0.23459	0.096824	0.18995	protein_coding
ENSG0000(	PNCK	30.5626	0.357186	0.096904	0.190094	protein_coding
ENSG0000(	KRT23	14.60325	0.511909	0.09692	0.190113	protein_coding
ENSG0000(	CEROX1	521.5502	0.188785	0.096973	0.190205	lncRNA
ENSG0000(	ENSG0000(	420.4583	0.129556	0.097041	0.190327	lncRNA
ENSG0000(	ENSG0000(	12.41236	0.351476	0.097061	0.19035	lncRNA
ENSG0000(	HSD17B6	49.36032	-0.34387	0.097065	0.19035	protein_coding
ENSG0000(	ENSG0000(	18.77438	-0.4335	0.097084	0.190367	lncRNA
ENSG0000(	POLR2J2	1274.576	0.16115	0.097086	0.190367	protein_coding
ENSG0000(	ITGAV	2399.529	0.148779	0.097098	0.19038	protein_coding
ENSG0000(	RHOBTB2	435.2888	0.233582	0.097225	0.190617	protein_coding
ENSG0000(	ENSG0000(	72.8366	0.147901	0.097238	0.19063	lncRNA
ENSG0000(	ESPN	297.8425	-0.31017	0.097287	0.190714	protein_coding
ENSG0000(	GALNS	1705.197	-0.08132	0.097379	0.190882	protein_coding
ENSG0000(	IGKV1-17	306.6914	0.435535	0.097393	0.190898	IG_V_gene
ENSG0000(	ENSG0000(	15.41616	0.329294	0.097423	0.190944	lncRNA
ENSG0000(	PLD1	3379.116	-0.14468	0.097442	0.190966	protein_coding
ENSG0000(	RAB36	200.3239	0.152646	0.097446	0.190966	protein_coding
ENSG0000(	FAM174B	514.1037	-0.10911	0.097476	0.191012	protein_coding
ENSG0000(	PCDHB15	76.6941	0.251255	0.097538	0.191121	protein_coding
ENSG0000(	GAREM1	1265.244	-0.20616	0.097547	0.191127	protein_coding
ENSG0000(	METRN	151.0059	-0.27011	0.097563	0.191148	protein_coding
ENSG0000(	SASH3	641.0611	0.284581	0.097595	0.191187	protein_coding
ENSG0000(	STARD5	1229.871	0.136996	0.097595	0.191187	protein_coding
ENSG0000(	HEIH	283.0393	0.102751	0.097648	0.191277	lncRNA
ENSG0000(	C1QTNF2	118.8713	-0.13883	0.09767	0.191308	protein_coding
ENSG0000(	LINC01787	101.5028	0.169352	0.09769	0.191335	lncRNA
ENSG0000(	MID2	227.98	0.232191	0.09771	0.191364	protein_coding
ENSG0000(	TRMT13	656.351	-0.10465	0.09772	0.19137	protein_coding
ENSG0000(	RNU2-63P	14.82319	0.378734	0.097737	0.191392	snRNA
ENSG0000(	CHL1-AS2	14.9468	0.469498	0.09775	0.191403	lncRNA
ENSG0000(	ENSG0000(	1863.388	0.116802	0.097755	0.191403	lncRNA
ENSG0000(	KCNT1	223.0832	-0.41026	0.097773	0.191426	protein_coding
ENSG0000(	ENSG0000(	31.82386	-0.2272	0.097855	0.191576	processed_pseudogene
ENSG0000(	CELA2A	95.35055	0.133151	0.097872	0.191585	protein_coding
ENSG0000(	TPH2	946.4758	0.07531	0.097872	0.191585	protein_coding
ENSG0000(	ENSG0000(	506.0063	0.094922	0.097908	0.191642	lncRNA
ENSG0000(	GPRIN1	41.57725	0.250651	0.097914	0.191643	protein_coding
ENSG0000(	ACOT9	1712.857	0.078288	0.097924	0.191649	protein_coding

ENSG0000(	TMOD1	94.11044	0.26285	0.097936	0.191661	protein_coding
ENSG0000(	ENSG0000(	22.85117	0.343392	0.097961	0.191698	lncRNA
ENSG0000(	ENSG0000(	13.84198	0.461923	0.098044	0.19185	processed_pseudogene
ENSG0000(	OR4D5	15.18541	-0.60905	0.09807	0.191888	protein_coding
ENSG0000(	DEGS1	474.7722	0.14919	0.098119	0.191959	protein_coding
ENSG0000(	PDE6D	269.3875	0.084467	0.098113	0.191959	protein_coding
ENSG0000(	MIR4538	18.62009	0.485236	0.098148	0.192004	miRNA
ENSG0000(	CEP170P1	77.01194	0.196894	0.098164	0.192023	transcribed_unprocessed_pseudo
ENSG0000(	RASA4	1095.635	0.207448	0.098211	0.192103	protein_coding
ENSG0000(	AGAP9	831.8992	0.266464	0.098221	0.192111	protein_coding
ENSG0000(	CRCP	1197.952	-0.08519	0.09825	0.192156	protein_coding
ENSG0000(	MGAT3	283.8735	0.208763	0.098273	0.192189	protein_coding
ENSG0000(	ENSG0000(	13.33241	-0.32339	0.098301	0.19222	processed_pseudogene
ENSG0000(	ENSG0000(	44.32488	0.209757	0.098299	0.19222	protein_coding
ENSG0000(	VKORC1L1	1432.285	-0.0823	0.098319	0.192243	protein_coding
ENSG0000(	SPATA21	141.5937	0.143254	0.098365	0.19232	protein_coding
ENSG0000(	LINC00628	72.1393	-0.24042	0.098374	0.192326	lncRNA
ENSG0000(	FAM186A	205.5507	0.068085	0.098395	0.192355	protein_coding
ENSG0000(	ENSG0000(	21.59875	0.349551	0.098437	0.192412	lncRNA
ENSG0000(	OXA1L	2119.506	-0.06786	0.098433	0.192412	protein_coding
ENSG0000(	B3GALNT2	1911.266	0.072412	0.098458	0.192442	protein_coding
ENSG0000(	HECTD2	638.7797	0.088765	0.098469	0.192452	protein_coding
ENSG0000(	SLC2A14	79.42064	0.177017	0.098523	0.192545	protein_coding
ENSG0000(	LINC02551	329.4352	0.090413	0.098535	0.192557	lncRNA
ENSG0000(	ITGAL	2866.187	0.185381	0.098569	0.192599	protein_coding
ENSG0000(	LEPR	314.0342	0.161928	0.098567	0.192599	protein_coding
ENSG0000(	LINC01559	1934.409	-0.42862	0.098622	0.192691	lncRNA
ENSG0000(	HMGB1P24	30.54583	-0.26253	0.098654	0.192741	transcribed_processed_pseudoge
ENSG0000(	DEFB1	19.81347	-0.53396	0.098696	0.192811	protein_coding
ENSG0000(	ANGPT1	206.2603	0.225909	0.098724	0.192853	protein_coding
ENSG0000(	NELL1	40.02973	0.354826	0.098732	0.192856	protein_coding
ENSG0000(	ZNF528	1484.452	0.093525	0.098764	0.192907	protein_coding
ENSG0000(	ENSG0000(	374.177	-0.09464	0.098794	0.192954	lncRNA
ENSG0000(	ENSG0000(	20.54587	0.346037	0.098851	0.193033	lncRNA
ENSG0000(	ENSG0000(	36.66419	0.253475	0.098853	0.193033	lncRNA
ENSG0000(	MOV10L1	134.6953	0.220827	0.09885	0.193033	protein_coding
ENSG0000(	U2	15988.58	-0.75936	0.098861	0.193036	snRNA
ENSG0000(	LANCL1-AS	35.03497	0.203297	0.098909	0.193119	lncRNA
ENSG0000(	UNC13A	173.9053	0.25478	0.098973	0.193232	protein_coding
ENSG0000(	FLG	57.92168	-0.90347	0.099	0.193251	protein_coding
ENSG0000(	LINC00460	23.28194	0.310074	0.099001	0.193251	lncRNA
ENSG0000(	SNORA74B	332.9074	-0.26104	0.098995	0.193251	snoRNA
ENSG0000(	PIF1	671.6336	0.084635	0.099106	0.193444	protein_coding
ENSG0000(	ENSG0000(	86.86083	0.17884	0.099114	0.193447	lncRNA
ENSG0000(	ENSG0000(	74.34974	-0.16812	0.099158	0.19352	lncRNA
ENSG0000(	ENSG0000(	17.35461	-0.28779	0.099205	0.193593	lncRNA
ENSG0000(	TMPRSS15	39.32941	-0.79304	0.099208	0.193593	protein_coding

ENSG0000(	MCUB	188.5665	0.275142	0.099216	0.193597	protein_coding
ENSG0000(	ENSG0000(	14.06901	0.402039	0.099293	0.193735	processed_pseudogene
ENSG0000(	FAM86EP	184.1267	-0.16225	0.09937	0.193861	transcribed_unprocessed_pseudo
ENSG0000(	RNF185	819.3876	-0.11434	0.099368	0.193861	protein_coding
ENSG0000(	ENSG0000(	52.45265	-0.16522	0.099391	0.193876	lncRNA
ENSG0000(	FAM222A-/	17.62448	0.283965	0.099392	0.193876	lncRNA
ENSG0000(	TMEM200E	113.1526	0.242355	0.099396	0.193876	protein_coding
ENSG0000(	ENSG0000(	97.25831	0.168261	0.099408	0.193888	lncRNA
ENSG0000(	SH3YL1	2281.695	-0.11653	0.0995	0.194054	protein_coding
ENSG0000(	DARS1	797.4484	-0.10042	0.099515	0.194072	protein_coding
ENSG0000(	COL20A1	48.42495	-0.38652	0.099526	0.194081	protein_coding
ENSG0000(	ST6GALNA(	1378.144	-0.21519	0.09955	0.194117	protein_coding
ENSG0000(	SMARCE1	2616.912	0.064278	0.099565	0.194132	protein_coding
ENSG0000(	KLC3	332.859	0.120175	0.099601	0.194191	protein_coding
ENSG0000(	ILRUN	1812.202	-0.11053	0.099635	0.194245	protein_coding
ENSG0000(	LINC02607	78.90101	0.170195	0.09968	0.194309	lncRNA
ENSG0000(	NCSTN	1057.576	-0.07063	0.099678	0.194309	protein_coding
ENSG0000(	SDHAF2	469.2713	-0.08014	0.099688	0.194312	protein_coding
ENSG0000(	SH3RF2	3592.726	-0.2037	0.099712	0.194347	protein_coding
ENSG0000(	SMARCA4	3974.362	-0.05475	0.099736	0.194381	protein_coding
ENSG0000(	SHC1P2	182.628	0.111276	0.099744	0.194385	processed_pseudogene
ENSG0000(	COBL	1792.756	-0.25092	0.099814	0.194485	protein_coding
ENSG0000(	NAF1	409.0272	-0.09758	0.099809	0.194485	protein_coding
ENSG0000(	RNU6-479F	11.17607	0.36676	0.099804	0.194485	snRNA
ENSG0000(	TIMM50	1684.772	-0.07008	0.09982	0.194485	protein_coding
ENSG0000(	NPY5R	12.52162	-0.58773	0.099894	0.194591	protein_coding
ENSG0000(	PGPEP1	3253.522	-0.11463	0.0999	0.194591	protein_coding
ENSG0000(	PRSS12	2210.5	-0.2474	0.09989	0.194591	protein_coding
ENSG0000(	SMARCAL1	584.1513	-0.08743	0.099885	0.194591	protein_coding
ENSG0000(	ZNF107	2627.341	0.135182	0.099909	0.194598	protein_coding
ENSG0000(	ENSG0000(	12.6463	0.454785	0.099917	0.1946	lncRNA
ENSG0000(	ENSG0000(	110.7027	0.143096	0.099934	0.194622	lncRNA
ENSG0000(	ENSG0000(	81.69553	0.139365	0.099993	0.194725	lncRNA
ENSG0000(	CST7	36.45929	0.395109	0.100031	0.194773	protein_coding
ENSG0000(	LINC01285	1301.886	0.055467	0.10003	0.194773	lncRNA
ENSG0000(	ENSG0000(	40.01613	0.281238	0.100063	0.1948	lncRNA
ENSG0000(	ENSG0000(	77.65418	0.135804	0.100062	0.1948	TEC
ENSG0000(	RIOK3	3343.365	-0.17842	0.100053	0.1948	protein_coding
ENSG0000(	C1QTNF7	99.25884	-0.36429	0.100076	0.194813	protein_coding
ENSG0000(	ENSG0000(	24.57424	0.246748	0.100126	0.194887	lncRNA
ENSG0000(	INCA1	44.03671	-0.1955	0.100122	0.194887	protein_coding
ENSG0000(	CAMKMT	367.2577	-0.09629	0.100133	0.194887	protein_coding
ENSG0000(	DNALI1	156.3127	0.164307	0.1002	0.195007	protein_coding
ENSG0000(	LINC00836	622.5434	0.078373	0.10022	0.195033	lncRNA
ENSG0000(	ENSG0000(	211.9509	0.109896	0.100247	0.195074	lncRNA
ENSG0000(	CFAP92	1711.785	0.098106	0.100326	0.195206	protein_coding
ENSG0000(	ZDHHC18	1211.034	-0.13872	0.100328	0.195206	protein_coding

ENSG0000(	LINC00471	234.6097	0.143952	0.100344	0.195226	lncRNA
ENSG0000(	ENSG0000(	627.5511	-0.10539	0.10039	0.195304	lncRNA
ENSG0000(	GCNT2P1	12.16301	-0.37317	0.100423	0.195355	processed_pseudogene
ENSG0000(	ENSG0000(	301.1866	0.138243	0.10043	0.195357	lncRNA
ENSG0000(	KLF5	8014.732	-0.28123	0.100475	0.195431	protein_coding
ENSG0000(	ZNF813	796.3637	-0.07215	0.100482	0.195433	protein_coding
ENSG0000(	SEMA6C	248.2004	-0.21018	0.100494	0.195445	protein_coding
ENSG0000(	ENSG0000(	122.4036	0.152121	0.100559	0.195547	lncRNA
ENSG0000(	RNVU1-31	1710.53	0.184222	0.100557	0.195547	snRNA
ENSG0000(	CNOT7	1635.544	-0.08468	0.100598	0.195598	protein_coding
ENSG0000(	ENSG0000(	17.52546	0.313155	0.100598	0.195598	lncRNA
ENSG0000(	TAF10	889.0549	-0.15581	0.100622	0.195634	protein_coding
ENSG0000(	RN7SKP178	16.37095	-0.35132	0.100647	0.195669	misc_RNA
ENSG0000(	CEACAM22	400.8841	0.11701	0.100666	0.195675	transcribed_unprocessed_pseudo
ENSG0000(	ENSG0000(	34.09209	0.205049	0.100657	0.195675	lncRNA
ENSG0000(	ENSG0000(	45.57806	0.165755	0.100669	0.195675	lncRNA
ENSG0000(	SLC4A5	532.7966	0.085314	0.100702	0.195727	protein_coding
ENSG0000(	ENSG0000(	1076.592	0.066207	0.100773	0.19584	transcribed_processed_pseudoge
ENSG0000(	FAM216B	216.0608	0.177836	0.100766	0.19584	protein_coding
ENSG0000(	RNU6-490F	10.24107	0.427549	0.100807	0.195894	snRNA
ENSG0000(	ITPR1-DT	43.14256	0.236467	0.10082	0.195908	lncRNA
ENSG0000(	DESI1	1502.285	0.093711	0.100835	0.195924	protein_coding
ENSG0000(	ENSG0000(	63.87551	0.152062	0.100847	0.195937	lncRNA
ENSG0000(	UBTD1	23.9841	0.399825	0.100869	0.195966	protein_coding
ENSG0000(	FABP5P3	36.74397	-0.23342	0.100892	0.196	transcribed_processed_pseudoge
ENSG0000(	ENSG0000(	42.55177	0.224466	0.100985	0.196156	lncRNA
ENSG0000(	FBXW10	157.3896	0.108346	0.100981	0.196156	protein_coding
ENSG0000(	SNORA40	21.89391	0.759804	0.101033	0.196237	snoRNA
ENSG0000(	PTAR1	3907.531	0.075053	0.101102	0.196359	protein_coding
ENSG0000(	PTOV1-AS1	171.1655	0.098153	0.101117	0.196375	lncRNA
ENSG0000(	LINC02024	27.87815	-0.44908	0.101133	0.196385	lncRNA
ENSG0000(	RNU7-73P	11.56156	0.468722	0.101134	0.196385	snRNA
ENSG0000(	ENSG0000(	89.01446	0.133409	0.101145	0.196393	lncRNA
ENSG0000(	WFDC2	65.60492	-0.48636	0.101152	0.196394	protein_coding
ENSG0000(	ENSG0000(	49.38694	-0.17933	0.101161	0.196399	transcribed_processed_pseudoge
ENSG0000(	PSMC6	1684.199	-0.05129	0.101284	0.196627	protein_coding
ENSG0000(	GPR83	235.1563	0.099692	0.101352	0.196747	protein_coding
ENSG0000(	CC2D2A	1133.786	-0.07063	0.101359	0.196748	protein_coding
ENSG0000(	LINC01036	213.1452	0.145948	0.101368	0.196753	lncRNA
ENSG0000(	C21orf91-C	11.0636	0.358992	0.101418	0.196838	lncRNA
ENSG0000(	ENSG0000(	134.0658	-0.12775	0.101432	0.196841	processed_pseudogene
ENSG0000(	ENSG0000(	69.60749	-0.1739	0.101433	0.196841	lncRNA
ENSG0000(	MGLL	13704.59	-0.26477	0.101438	0.196841	protein_coding
ENSG0000(	TFAP4	1164.231	0.16442	0.101521	0.196989	protein_coding
ENSG0000(	MICAL2	8288.311	-0.19485	0.101549	0.19703	protein_coding
ENSG0000(	USP12	2059.835	-0.09281	0.101557	0.197035	protein_coding
ENSG0000(	CCDC187	30.40139	0.389168	0.101595	0.197096	protein_coding

ENSG0000(	TCN2	190.4244	0.240462	0.101614	0.19712	protein_coding
ENSG0000(	ZNF70	1599.632	-0.05071	0.101622	0.197123	protein_coding
ENSG0000(	ENSG0000(	142.2174	-0.22924	0.101641	0.197148	lncRNA
ENSG0000(	ENSG0000(	8.996693	0.501656	0.101665	0.197183	lncRNA
ENSG0000(	ZNF330	424.7526	-0.08861	0.101677	0.197194	protein_coding
ENSG0000(	KAT7	4041.348	-0.05736	0.101747	0.197316	protein_coding
ENSG0000(	GOLPH3	1486.249	-0.1128	0.10181	0.197426	protein_coding
ENSG0000(	MIF	201.9571	-0.21015	0.101895	0.19758	protein_coding
ENSG0000(	EIF3CL	647.4036	-0.30214	0.101966	0.197705	protein_coding
ENSG0000(	RN7SL597F	47.3595	0.174607	0.102052	0.197859	misc_RNA
ENSG0000(	ENSG0000(	70.88495	-0.17563	0.102066	0.197874	lncRNA
ENSG0000(	ACBD7	993.2253	-0.05463	0.102076	0.197881	protein_coding
ENSG0000(	ENSG0000(	120.6645	0.140755	0.102094	0.197904	processed_pseudogene
ENSG0000(	ZNF236-DT	80.98474	-0.12968	0.102232	0.198159	lncRNA
ENSG0000(	PCDHB7	16.59111	-0.3541	0.102239	0.198161	protein_coding
ENSG0000(	GSTCD	760.3259	-0.10934	0.102316	0.198298	protein_coding
ENSG0000(	RNU4-82P	14.11509	0.294957	0.102336	0.198325	snRNA
ENSG0000(	ATP6V1G2	24.03381	0.302379	0.102465	0.198562	protein_coding
ENSG0000(	ENSG0000(	100.9973	0.188843	0.102512	0.198641	lncRNA
ENSG0000(	ZNF423	388.1104	-0.20449	0.102527	0.198657	protein_coding
ENSG0000(	TEDDM1	61.42914	0.158811	0.102555	0.198699	protein_coding
ENSG0000(	ENSG0000(	83.83631	-0.1635	0.102588	0.198738	TEC
ENSG0000(	ENSG0000(	279.1287	0.106584	0.102587	0.198738	lncRNA
ENSG0000(	SPTY2D1	876.6997	-0.08926	0.102618	0.198784	protein_coding
ENSG0000(	COL11A2	83.79553	0.285817	0.102628	0.198791	protein_coding
ENSG0000(	KLHL7	988.3914	0.096535	0.102635	0.198793	protein_coding
ENSG0000(	KCNK6	3211.941	0.064876	0.10265	0.198801	protein_coding
ENSG0000(	OR7D4	438.5717	-0.07828	0.102652	0.198801	protein_coding
ENSG0000(	RHOV	27.67238	-0.33437	0.102705	0.19889	protein_coding
ENSG0000(	DLL1	564.5399	0.116778	0.102728	0.198923	protein_coding
ENSG0000(	ENSG0000(	124.169	0.288482	0.102735	0.198924	lncRNA
ENSG0000(	RNA5-8SN2	1135.905	-0.34643	0.102764	0.198969	rRNA
ENSG0000(	ABCB10	581.5446	-0.12008	0.102822	0.199068	protein_coding
ENSG0000(	RNU6-574F	12.90382	-0.41506	0.102837	0.199085	snRNA
ENSG0000(	ARHGEF10I	3298.81	-0.15649	0.102851	0.199088	protein_coding
ENSG0000(	ENSG0000(	1913.591	-0.05486	0.102846	0.199088	protein_coding
ENSG0000(	ADAMTS3	61.34573	0.341862	0.102867	0.199106	protein_coding
ENSG0000(	ZFAND2A-I	1045.039	0.080101	0.10292	0.199196	lncRNA
ENSG0000(	CASQ1	38.33659	0.286816	0.102929	0.199202	protein_coding
ENSG0000(	OR10G3	983.5542	0.067232	0.102942	0.199203	protein_coding
ENSG0000(	PPM1L-DT	64.87868	0.183493	0.102949	0.199203	lncRNA
ENSG0000(	ZKSCAN8P1	75.15975	0.219498	0.102944	0.199203	protein_coding
ENSG0000(	CD3D	107.9512	-0.37126	0.102955	0.199203	protein_coding
ENSG0000(	A2MP1	26.56815	0.619951	0.103007	0.19929	transcribed_unprocessed_pseudo
ENSG0000(	SLC7A6	2959.3	-0.10514	0.103018	0.1993	protein_coding
ENSG0000(	ENSG0000(	597.5624	-0.0924	0.103049	0.199348	lncRNA
ENSG0000(	ENSG0000(	181.4059	0.13612	0.103072	0.199379	lncRNA

ENSG0000(	ENSG0000(	122.4501	0.185918	0.103137	0.199493	lncRNA
ENSG0000(	ENSG0000(	9.257878	0.398574	0.103151	0.199507	lncRNA
ENSG0000(	ENSG0000(	103.1012	0.180401	0.103175	0.199542	lncRNA
ENSG0000(	PNLDC1	19.04138	-0.43368	0.103237	0.199649	protein_coding
ENSG0000(	ENSG0000(	211.0368	-0.11163	0.103255	0.199669	lncRNA
ENSG0000(	ENSG0000(	22.46991	-0.2556	0.10326	0.199669	lncRNA
ENSG0000(	ENSG0000(	125.3415	-0.15019	0.103291	0.199704	processed_pseudogene
ENSG0000(	TNFSF4	142.296	0.217899	0.103289	0.199704	protein_coding
ENSG0000(	ENSG0000(	27.75149	0.238651	0.103327	0.199761	processed_pseudogene
ENSG0000(	PPCDC	567.3741	0.126075	0.103365	0.199823	protein_coding
ENSG0000(	ZNF286A	775.1297	0.135965	0.103381	0.199841	protein_coding
ENSG0000(	ENSG0000(	36.59322	0.243953	0.1034	0.199865	lncRNA
ENSG0000(	ENSG0000(	206.4683	0.097289	0.103524	0.200094	lncRNA
ENSG0000(	DDB1	3205.004	-0.04812	0.103698	0.200405	protein_coding
ENSG0000(	PPM1N	843.9981	0.278272	0.103698	0.200405	protein_coding
ENSG0000(	ENSG0000(	18.96816	0.451602	0.103719	0.200433	lncRNA
ENSG0000(	LIPC	127.5424	-0.14105	0.103765	0.200502	protein_coding
ENSG0000(	SLCO5A1-A	45.61674	0.236985	0.103768	0.200502	lncRNA
ENSG0000(	SLX1A	621.3114	-0.14243	0.103883	0.200713	protein_coding
ENSG0000(	LRRC56	255.5469	-0.19326	0.10395	0.200805	protein_coding
ENSG0000(	MIR137HG	89.13093	0.144083	0.103947	0.200805	lncRNA
ENSG0000(	TEAD2	360.182	0.161675	0.10395	0.200805	protein_coding
ENSG0000(	ENSG0000(	25.97697	0.287248	0.103983	0.200855	lncRNA
ENSG0000(	RN7SL180F	70.15367	0.165062	0.104013	0.200902	misc_RNA
ENSG0000(	B3GALT1	704.1471	-0.25012	0.104024	0.20091	protein_coding
ENSG0000(	CRAT	1292.697	-0.13254	0.10409	0.201025	protein_coding
ENSG0000(	FAM106A	1569.921	0.126849	0.104118	0.201067	lncRNA
ENSG0000(	ZNRF2P2	43.60345	-0.21623	0.104161	0.201137	transcribed_processed_pseudoge
ENSG0000(	LINC01281	28.2978	0.509118	0.104174	0.20115	lncRNA
ENSG0000(	CD68	585.8854	0.257812	0.104282	0.201307	protein_coding
ENSG0000(	DTNBP1	363.5019	0.095296	0.104283	0.201307	protein_coding
ENSG0000(	ENSG0000(	143.5729	-0.13446	0.104274	0.201307	lncRNA
ENSG0000(	ENSG0000(	20.77128	-0.34536	0.104276	0.201307	lncRNA
ENSG0000(	SPAG9	5137.247	-0.09865	0.104287	0.201307	protein_coding
ENSG0000(	ENSG0000(	241.9182	-0.12003	0.104356	0.201406	lncRNA
ENSG0000(	ENSG0000(	80.71189	-0.14998	0.104358	0.201406	lncRNA
ENSG0000(	NRAD1	18.88845	-0.52142	0.104346	0.201406	lncRNA
ENSG0000(	SMIM2-AS1	851.0678	-0.28329	0.104368	0.201406	lncRNA
ENSG0000(	TOX4	2005.507	0.071543	0.104371	0.201406	protein_coding
ENSG0000(	HSPD1P13	32.00368	0.333445	0.104386	0.201423	processed_pseudogene
ENSG0000(	E2F3P2	325.5434	-0.12799	0.1044	0.201437	processed_pseudogene
ENSG0000(	SNORA73	54.89195	0.163424	0.10441	0.201444	snoRNA
ENSG0000(	ENSG0000(	588.8255	-0.05922	0.104477	0.201561	lncRNA
ENSG0000(	ENSG0000(	113.307	0.133667	0.104579	0.201747	lncRNA
ENSG0000(	AKNAD1	37.41398	-0.25052	0.104623	0.201819	protein_coding
ENSG0000(	CD99L2	607.2462	0.082615	0.104703	0.201959	protein_coding
ENSG0000(	DDX3Y	944.5404	0.540744	0.104738	0.202015	protein_coding

ENSG0000(	NDRG4	154.5263	0.194329	0.104749	0.202023	protein_coding
ENSG0000(	DTX3	399.2597	0.130579	0.104759	0.20203	protein_coding
ENSG0000(	ENSG0000(	20.56427	-0.32894	0.104785	0.202057	lncRNA
ENSG0000(	MTCO1P19	18.65115	0.30994	0.104779	0.202057	processed_pseudogene
ENSG0000(	RN7SL785F	79.33093	0.140232	0.104851	0.20217	misc_RNA
ENSG0000(	AMY2B	1202.62	0.126024	0.104894	0.202242	protein_coding
ENSG0000(	ENSG0000(	25.53786	-0.27253	0.104938	0.202314	lncRNA
ENSG0000(	ZHX3	1557.464	0.141332	0.104988	0.202397	protein_coding
ENSG0000(	OR7A2P	12.92888	0.368092	0.105001	0.20241	transcribed_unprocessed_pseudo
ENSG0000(	SLCO4A1	223.2296	0.19946	0.105024	0.202441	protein_coding
ENSG0000(	ZNF324	597.1251	-0.09797	0.105066	0.202511	protein_coding
ENSG0000(	CEP57L1	2736.964	0.054998	0.105166	0.20269	protein_coding
ENSG0000(	CNTN2	410.0743	-0.09171	0.105196	0.202736	protein_coding
ENSG0000(	PRDM16-D	35.74825	-0.39816	0.10539	0.203096	lncRNA
ENSG0000(	ZNRF2P1	126.6696	-0.18903	0.105434	0.203169	transcribed_processed_pseudoge
ENSG0000(	ENSG0000(	178.5622	0.116348	0.105465	0.203218	lncRNA
ENSG0000(	CTSS	4034.685	0.102791	0.105475	0.203224	protein_coding
ENSG0000(	ANKMY1	1585.867	-0.10236	0.105558	0.203371	protein_coding
ENSG0000(	CAB39	2968.724	-0.13478	0.10558	0.2034	protein_coding
ENSG0000(	CRHBP	26.01641	-0.38033	0.105616	0.203433	protein_coding
ENSG0000(	RN7SL838F	53.03436	-0.21456	0.105616	0.203433	misc_RNA
ENSG0000(	VDAC1P13	35.89844	-0.34638	0.105605	0.203433	processed_pseudogene
ENSG0000(	ENSG0000(	124.055	-0.12209	0.105679	0.203542	lncRNA
ENSG0000(	CTBP1-DT	928.3433	0.07736	0.105721	0.203598	lncRNA
ENSG0000(	SNRPCP19	14.82698	0.384622	0.105715	0.203598	processed_pseudogene
ENSG0000(	RN7SL269F	49.07718	0.176056	0.105728	0.203599	misc_RNA
ENSG0000(	MIR505	10.96304	0.326672	0.105745	0.203619	miRNA
ENSG0000(	ENSG0000(	132.3429	-0.13247	0.105778	0.203669	unprocessed_pseudogene
ENSG0000(	LINC01795	106.8374	0.163814	0.105822	0.203742	lncRNA
ENSG0000(	ANKRD24	81.92464	0.304068	0.105915	0.203908	protein_coding
ENSG0000(	ZNF503	144.0376	0.204657	0.105949	0.203961	protein_coding
ENSG0000(	TRAV13-1	9.460556	-0.44727	0.105962	0.203973	TR_V_gene
ENSG0000(	SNORA71B	142.4915	0.157545	0.106033	0.204097	snoRNA
ENSG0000(	MIR6793	8.704227	-0.41064	0.106081	0.204166	miRNA
ENSG0000(	SPDYA	278.3835	0.154812	0.106078	0.204166	protein_coding
ENSG0000(	ENPP7P11	109.5612	-0.29083	0.106112	0.204213	processed_pseudogene
ENSG0000(	ENSG0000(	1385.816	0.105144	0.106135	0.204244	TEC
ENSG0000(	ENSG0000(	29.8208	-0.4842	0.106292	0.204533	processed_pseudogene
ENSG0000(	ENSG0000(	23.83142	0.228939	0.106337	0.204608	miRNA
ENSG0000(	PAIP2	822.0571	-0.09008	0.106349	0.204617	protein_coding
ENSG0000(	ENSG0000(	160.916	-0.23747	0.106391	0.204674	lncRNA
ENSG0000(	NDE1P2	104.3581	0.13855	0.106391	0.204674	processed_pseudogene
ENSG0000(	ENSG0000(	413.1133	0.118973	0.106416	0.204709	lncRNA
ENSG0000(	RALGPS2-A	34.48494	0.287698	0.106477	0.204814	lncRNA
ENSG0000(	MYT1L	2156.372	0.039644	0.106488	0.204823	protein_coding
ENSG0000(	ENSG0000(	92.59891	-0.17649	0.106497	0.204826	lncRNA
ENSG0000(	ENSG0000(	17.08106	-0.55454	0.106518	0.204854	transcribed_processed_pseudoge

ENSG0000(	TOR1AIP1	1029.13	0.084516	0.106584	0.204968	protein_coding
ENSG0000(	ENSG0000(	67.28606	0.165927	0.106658	0.205072	lncRNA
ENSG0000(	ENSG0000(	1366.619	0.312998	0.106647	0.205072	lncRNA
ENSG0000(	TSSC4	220.7958	-0.12104	0.106655	0.205072	protein_coding
ENSG0000(	IDI2-AS1	99.93269	-0.17462	0.106782	0.205274	lncRNA
ENSG0000(	ISX	885.4328	-0.34015	0.106777	0.205274	protein_coding
ENSG0000(	RAD51AP1	145.8913	-0.2331	0.106774	0.205274	protein_coding
ENSG0000(	SLC25A26	2896.896	0.073023	0.10684	0.205373	protein_coding
ENSG0000(	ENSG0000(	134.0955	-0.11578	0.106869	0.205416	TEC
ENSG0000(	BANP	1047.41	0.076884	0.106886	0.205437	protein_coding
ENSG0000(	NKAPD1	1144.403	-0.06327	0.106917	0.205472	protein_coding
ENSG0000(	RN7SL769F	18.84931	0.267516	0.106918	0.205472	misc_RNA
ENSG0000(	RAB8A	1729.779	-0.09142	0.106926	0.205474	protein_coding
ENSG0000(	ARPC4	1542.01	-0.08779	0.106957	0.20552	protein_coding
ENSG0000(	ENSG0000(	30.38755	0.251095	0.106963	0.20552	lncRNA
ENSG0000(	ENSG0000(	20.79946	0.366319	0.106985	0.205551	lncRNA
ENSG0000(	SLC35D2	2019.676	-0.14183	0.106999	0.205564	protein_coding
ENSG0000(	WDR24	191.5709	-0.1087	0.107101	0.205749	protein_coding
ENSG0000(	ENSG0000(	136.0716	-0.17166	0.107125	0.205781	transcribed_processed_pseudoge
ENSG0000(	ENSG0000(	13.04368	-0.31249	0.107165	0.205832	lncRNA
ENSG0000(	FOXP4-AS1	18.13559	-0.43986	0.107158	0.205832	lncRNA
ENSG0000(	A2M-AS1	215.5615	0.301543	0.107196	0.20588	lncRNA
ENSG0000(	ENSG0000(	132.9346	-0.13309	0.107209	0.205892	lncRNA
ENSG0000(	HNRNPR	3828.797	-0.097	0.107251	0.205961	protein_coding
ENSG0000(	ATF7IP2	1043.461	-0.10629	0.107266	0.205966	protein_coding
ENSG0000(	RASL10A	48.88306	-0.2552	0.107274	0.205966	protein_coding
ENSG0000(	RPL9P33	9.107341	0.511228	0.107269	0.205966	processed_pseudogene
ENSG0000(	LINC00501	291.9125	-0.10376	0.107302	0.206008	lncRNA
ENSG0000(	GMFG	342.2999	0.17215	0.107385	0.206141	protein_coding
ENSG0000(	RAB33B	528.9495	-0.0781	0.10738	0.206141	protein_coding
ENSG0000(	ENSG0000(	88.7439	0.144384	0.107444	0.206243	lncRNA
ENSG0000(	TNNT2	197.2277	-0.17403	0.107451	0.206243	protein_coding
ENSG0000(	STX17	2966.713	0.066125	0.107469	0.206265	protein_coding
ENSG0000(	ENSG0000(	104.9109	-0.13336	0.107487	0.206286	lncRNA
ENSG0000(	ENSG0000(	28.23482	0.224552	0.107494	0.206288	lncRNA
ENSG0000(	ENSG0000(	78.05449	-0.25511	0.107509	0.206305	lncRNA
ENSG0000(	PNLIPRP2	22.03987	-0.50677	0.107583	0.20643	polymorphic_pseudogene
ENSG0000(	ZNF831	714.2676	0.402691	0.107588	0.20643	protein_coding
ENSG0000(	BHMT	48.49026	0.274685	0.107595	0.206432	protein_coding
ENSG0000(	YWHAH-AS	668.9466	0.062882	0.107611	0.206449	lncRNA
ENSG0000(	ENPP5	132.8232	-0.29376	0.107651	0.206513	protein_coding
ENSG0000(	AKAP11	4194.002	0.063614	0.107713	0.206618	protein_coding
ENSG0000(	GTF2IP1	1430.037	0.140877	0.107722	0.206624	transcribed_unprocessed_pseudo
ENSG0000(	RRP1B	751.5764	-0.10126	0.107822	0.206803	protein_coding
ENSG0000(	ENSG0000(	12.75719	0.347546	0.107864	0.206858	lncRNA
ENSG0000(	MOBP	462.7647	-0.16679	0.107863	0.206858	protein_coding
ENSG0000(	RPP40	40.38491	-0.26239	0.107875	0.206866	protein_coding

ENSG0000(	ENSG0000(	9.297646	0.318362	0.10789	0.206882	lncRNA
ENSG0000(	ENSG0000(	71.2279	0.432626	0.107927	0.206941	lncRNA
ENSG0000(	NDUFV2-A!	51.11553	0.208831	0.108037	0.207139	lncRNA
ENSG0000(	TBX18-AS1	16.84452	-0.34525	0.108044	0.207139	lncRNA
ENSG0000(	ATG12P1	10.83572	-0.37728	0.108093	0.20722	processed_pseudogene
ENSG0000(	DNMBP	3233.088	-0.15269	0.108119	0.207248	protein_coding
ENSG0000(	ENSG0000(	148.8372	-0.1191	0.10812	0.207248	lncRNA
ENSG0000(	RPL29P24	36.44634	0.207222	0.108133	0.20726	processed_pseudogene
ENSG0000(	STPG1	854.6535	-0.13308	0.108149	0.207277	protein_coding
ENSG0000(	CNPY4	288.1996	-0.09508	0.108187	0.20731	protein_coding
ENSG0000(	ENSG0000(	34.21933	0.268407	0.108177	0.20731	lncRNA
ENSG0000(	F13A1	501.9189	-0.34119	0.108192	0.20731	protein_coding
ENSG0000(	WDR48	1222.486	-0.10948	0.10818	0.20731	protein_coding
ENSG0000(	SEMA3A	418.9532	-0.25921	0.108239	0.207379	protein_coding
ENSG0000(	SLC4A10	239.0666	-0.27943	0.108242	0.207379	protein_coding
ENSG0000(	PRKAR1AP:	39.25884	0.279217	0.108248	0.207379	processed_pseudogene
ENSG0000(	LIMA1	5835.835	-0.12868	0.108301	0.207467	protein_coding
ENSG0000(	RPL23AP74	11.44627	-0.44475	0.108403	0.207649	processed_pseudogene
ENSG0000(	LEMD1-AS:	270.2893	-0.09423	0.108427	0.207683	lncRNA
ENSG0000(	LINC00440	58.27303	-0.1979	0.108449	0.207713	lncRNA
ENSG0000(	FHL1	979.0077	0.174277	0.108477	0.207753	protein_coding
ENSG0000(	DNM1P35	299.6041	0.114866	0.108488	0.207761	lncRNA
ENSG0000(	ZMAT1	1055.761	0.152027	0.108568	0.207902	protein_coding
ENSG0000(	LMNTD2-A	111.6561	0.375656	0.108612	0.207974	lncRNA
ENSG0000(	ENSG0000(	103.1292	0.335987	0.108621	0.207978	lncRNA
ENSG0000(	FXYD5	315.723	0.235361	0.108635	0.207991	protein_coding
ENSG0000(	ATP1A1-AS	907.4276	0.120618	0.108667	0.20803	lncRNA
ENSG0000(	ENSG0000(	63.93625	0.163005	0.108673	0.20803	TEC
ENSG0000(	MACIR	950.8051	-0.23917	0.108675	0.20803	protein_coding
ENSG0000(	PCDHGB3	134.3483	-0.19154	0.108732	0.208127	protein_coding
ENSG0000(	ENSG0000(	54.22798	0.245684	0.108801	0.208246	transcribed_processed_pseudoge
ENSG0000(	ENSG0000(	88.53315	-0.33862	0.10882	0.208269	lncRNA
ENSG0000(	NTAQ1	523.6005	-0.10456	0.108842	0.2083	protein_coding
ENSG0000(	ENSG0000(	13.52803	0.296225	0.10889	0.208365	lncRNA
ENSG0000(	FUT4	2112.806	-0.11994	0.108887	0.208365	protein_coding
ENSG0000(	GALNT18	125.6362	0.206958	0.108944	0.208456	protein_coding
ENSG0000(	ENSG0000(	265.5329	-0.18849	0.108975	0.208502	lncRNA
ENSG0000(	ENSG0000(	12.35624	0.410701	0.109001	0.208539	processed_pseudogene
ENSG0000(	OR2L5	19.42115	0.376347	0.10902	0.208563	protein_coding
ENSG0000(	LINC01637	142.2132	-0.27857	0.109045	0.208597	lncRNA
ENSG0000(	C20orf144	97.21054	0.133408	0.109092	0.208662	protein_coding
ENSG0000(	LINC02020	76.96138	0.157369	0.109086	0.208662	lncRNA
ENSG0000(	RAB3IL1	94.82024	-0.24181	0.10911	0.208683	protein_coding
ENSG0000(	IGLJ3	25.71395	0.42513	0.109131	0.208711	IG_J_gene
ENSG0000(	RRP7A	2224.976	-0.14422	0.109146	0.208728	protein_coding
ENSG0000(	ENSG0000(	40.13982	0.219058	0.109168	0.208757	lncRNA
ENSG0000(	IGLV1-47	241.6066	0.409864	0.109239	0.208879	IG_V_gene

ENSG0000(	ENSG0000(	288.3435	0.098969	0.10929	0.208952	TEC
ENSG0000(	UBE2F-SCL'	20.31384	0.350879	0.109285	0.208952	protein_coding
ENSG0000(	ENSG0000(	59.04989	-0.4107	0.109334	0.208997	lncRNA
ENSG0000(	ENSG0000(	76.1636	0.176098	0.109331	0.208997	processed_pseudogene
ENSG0000(	ENSG0000(	81.88524	0.154945	0.109333	0.208997	lncRNA
ENSG0000(	LINC02288	231.5631	0.147324	0.109361	0.209037	lncRNA
ENSG0000(	ENSG0000(	63.22757	0.1985	0.109431	0.209157	processed_pseudogene
ENSG0000(	SNTG2	53.96247	0.293809	0.109447	0.209175	protein_coding
ENSG0000(	LMO7-AS1	191.4258	0.121758	0.109466	0.209199	lncRNA
ENSG0000(	NOL4L	3649.686	-0.12018	0.109485	0.209222	protein_coding
ENSG0000(	NPHP3-AS1	32.27298	0.251202	0.109524	0.209284	lncRNA
ENSG0000(	FCRL1	910.0525	0.508214	0.109544	0.209309	protein_coding
ENSG0000(	ENSG0000(	106.475	0.139573	0.10957	0.209346	protein_coding
ENSG0000(	ENSG0000(	18.54906	-0.34613	0.109597	0.209372	lncRNA
ENSG0000(	ENSG0000(	560.9232	-0.0693	0.10959	0.209372	lncRNA
ENSG0000(	RNU6-786F	11.93604	0.381425	0.109616	0.209396	snRNA
ENSG0000(	VAX1	27.80585	-0.21615	0.109648	0.209445	protein_coding
ENSG0000(	ENSG0000(	60.75444	0.199891	0.109671	0.209475	TEC
ENSG0000(	ELMOD3	1653.988	-0.06312	0.109702	0.209523	protein_coding
ENSG0000(	EIF5A2	211.954	-0.14986	0.109749	0.209599	protein_coding
ENSG0000(	TNFRSF13C	1223.689	0.281662	0.109758	0.209602	protein_coding
ENSG0000(	ZNF432	909.2512	0.10191	0.109774	0.209621	protein_coding
ENSG0000(	ENSG0000(	124.1044	-0.11923	0.109788	0.209635	lncRNA
ENSG0000(	ENSG0000(	151.4987	0.10769	0.109805	0.209642	lncRNA
ENSG0000(	MUC5B-AS	15.54675	0.468168	0.109804	0.209642	lncRNA
ENSG0000(	BABAM2	594.7281	0.090111	0.109846	0.209695	protein_coding
ENSG0000(	UGT2A1	28.75974	-0.29425	0.109846	0.209695	protein_coding
ENSG0000(	PARP11	940.1017	0.113418	0.109863	0.209714	protein_coding
ENSG0000(	ABCA1	4061.603	0.142452	0.109964	0.209894	protein_coding
ENSG0000(	YIF1B	449.5188	-0.09164	0.109995	0.209941	protein_coding
ENSG0000(	PIKFYVE	3108.515	0.094196	0.110016	0.209968	protein_coding
ENSG0000(	CRB2	181.2271	-0.23033	0.110078	0.210023	protein_coding
ENSG0000(	ENSG0000(	569.6244	0.130195	0.110072	0.210023	lncRNA
ENSG0000(	GRAPL	304.7008	0.375008	0.110073	0.210023	protein_coding
ENSG0000(	MMP11	281.5601	0.115143	0.110075	0.210023	protein_coding
ENSG0000(	TTC23L-AS:	17.6742	0.293121	0.110074	0.210023	lncRNA
ENSG0000(	ENSG0000(	110.2567	0.289124	0.110097	0.210046	TEC
ENSG0000(	SEMA7A	235.3621	0.328788	0.110121	0.21007	protein_coding
ENSG0000(	TLR7	432.5959	0.171644	0.110124	0.21007	protein_coding
ENSG0000(	LINC01670	156.7481	-0.25568	0.110137	0.210083	lncRNA
ENSG0000(	ENSG0000(	423.5898	-0.10889	0.110146	0.210087	lncRNA
ENSG0000(	TRIM45	236.3897	-0.11377	0.110201	0.210179	protein_coding
ENSG0000(	ENSG0000(	351.0491	-0.4587	0.110214	0.210192	protein_coding
ENSG0000(	RRAS	175.449	-0.17975	0.110269	0.210283	protein_coding
ENSG0000(	ABCA3	181.2885	-0.17561	0.110284	0.2103	protein_coding
ENSG0000(	GMCL1	606.0107	-0.10016	0.110303	0.210309	protein_coding
ENSG0000(	PRR15L	2216.991	-0.23498	0.110302	0.210309	protein_coding

ENSG0000(	ADRB3	72.52133	0.257107	0.11033	0.210322	protein_coding
ENSG0000(	LINC00391	30.58186	0.220233	0.110327	0.210322	lncRNA
ENSG0000(	USP32P1	927.4576	0.444689	0.110318	0.210322	transcribed_unprocessed_pseudo
ENSG0000(	RN7SL583F	9.601081	0.426402	0.110347	0.210342	misc_RNA
ENSG0000(	POLR3G	128.5743	0.148968	0.110373	0.21038	protein_coding
ENSG0000(	BUD23	995.5828	-0.09095	0.110442	0.210484	protein_coding
ENSG0000(	MCU	1609.15	-0.13101	0.110441	0.210484	protein_coding
ENSG0000(	ENSG0000(	1142.642	-0.06833	0.110454	0.210496	protein_coding
ENSG0000(	ERCC2	486.7828	-0.1066	0.11049	0.210551	protein_coding
ENSG0000(	MYRIP	306.7757	-0.21245	0.110497	0.210551	protein_coding
ENSG0000(	ENSG0000(	55.24666	-0.22722	0.110504	0.210552	unprocessed_pseudogene
ENSG0000(	ENSG0000(	13.92037	0.315965	0.110543	0.210613	lncRNA
ENSG0000(	ENSG0000(	1037.24	0.10463	0.110566	0.210645	lncRNA
ENSG0000(	EHBP1	1359.543	-0.09105	0.110578	0.210655	protein_coding
ENSG0000(	HLA-DQB2	158.94	0.24907	0.110629	0.210739	protein_coding
ENSG0000(	POC1B	2334.744	0.053607	0.110649	0.210764	protein_coding
ENSG0000(	CREBBP	5596.894	0.145235	0.110666	0.210784	protein_coding
ENSG0000(	C4orf48	40.49178	-0.3504	0.110694	0.210824	protein_coding
ENSG0000(	ENSG0000(	69.49781	-0.14566	0.110701	0.210824	lncRNA
ENSG0000(	GOLGA8DP	12.3964	0.596764	0.110766	0.210936	transcribed_unprocessed_pseudo
ENSG0000(	RN7SL149F	29.40913	0.271153	0.110808	0.211003	misc_RNA
ENSG0000(	ENSG0000(	104.7661	-0.25536	0.110837	0.211045	lncRNA
ENSG0000(	SPIDR	1907.662	0.074974	0.110847	0.211052	protein_coding
ENSG0000(	GEMIN8	688.8084	0.080688	0.110869	0.211063	protein_coding
ENSG0000(	UVSSA	3637.203	0.103766	0.110873	0.211063	protein_coding
ENSG0000(	ZBTB40-IT1	35.85401	0.185958	0.110863	0.211063	lncRNA
ENSG0000(	D21S2088E	72.75358	0.177849	0.110906	0.2111	lncRNA
ENSG0000(	ENSG0000(	61.35514	-0.20197	0.110905	0.2111	lncRNA
ENSG0000(	CYP8B1	209.6732	0.092652	0.110971	0.211197	protein_coding
ENSG0000(	ENSG0000(	1170.347	0.132734	0.11097	0.211197	lncRNA
ENSG0000(	NPIPA1	6529.8	0.08302	0.111043	0.211309	protein_coding
ENSG0000(	PNMA8A	109.9254	0.126249	0.111037	0.211309	protein_coding
ENSG0000(	FCGR3B	186.2935	2.081781	0.11107	0.211347	protein_coding
ENSG0000(	TM9SF1	550.609	-0.08966	0.111076	0.211347	protein_coding
ENSG0000(	ENSG0000(	156.0972	0.128415	0.111144	0.211462	lncRNA
ENSG0000(	DACT3	1029.753	0.062622	0.111184	0.211525	protein_coding
ENSG0000(	TVP23A	175.3164	0.203551	0.111209	0.211561	protein_coding
ENSG0000(	SLC50A1	880.8797	-0.08082	0.111217	0.211562	protein_coding
ENSG0000(	NIPBL-DT	1330.447	-0.0702	0.111239	0.211592	lncRNA
ENSG0000(	ARL15	555.6473	-0.15189	0.11125	0.2116	protein_coding
ENSG0000(	SRBD1	783.1907	0.117033	0.111299	0.21168	protein_coding
ENSG0000(	ENSG0000(	82.54281	0.199064	0.111322	0.211712	lncRNA
ENSG0000(	MFSD14C	7170.154	0.052351	0.111413	0.211872	transcribed_unprocessed_pseudo
ENSG0000(	ENSG0000(	1042.813	-0.1053	0.111476	0.211939	lncRNA
ENSG0000(	ENSG0000(	477.7574	-0.09301	0.111458	0.211939	lncRNA
ENSG0000(	ENSG0000(	59.9722	0.15605	0.111475	0.211939	lncRNA
ENSG0000(	SUCLA2-AS	9.437768	0.3654	0.111473	0.211939	lncRNA

ENSG0000(	CCNT2-AS1	294.6985	0.104953	0.111513	0.21196	lncRNA
ENSG0000(	ENSG0000(	219.293	0.413236	0.111496	0.21196	lncRNA
ENSG0000(	ENSG0000(	169.1769	-0.09047	0.111511	0.21196	lncRNA
ENSG0000(	ZIK1P1	280.1662	0.102204	0.111514	0.21196	processed_pseudogene
ENSG0000(	ENSG0000(	62.85795	0.122832	0.111524	0.211967	lncRNA
ENSG0000(	SLC17A4	5038.059	-0.35847	0.11157	0.212041	protein_coding
ENSG0000(	ENSG0000(	354.701	-0.12675	0.11158	0.212047	lncRNA
ENSG0000(	PYHIN1	450.3626	0.164534	0.111601	0.212074	protein_coding
ENSG0000(	ENSG0000(	86.01732	-0.16095	0.111688	0.2122	lncRNA
ENSG0000(	LINC02280	892.1905	0.080221	0.111688	0.2122	lncRNA
ENSG0000(	PEBP1P2	26.48057	-0.32123	0.111675	0.2122	processed_pseudogene
ENSG0000(	ENSG0000(	101.6719	-0.288	0.111758	0.21232	lncRNA
ENSG0000(	USP13	1754.271	-0.23374	0.111785	0.212359	protein_coding
ENSG0000(	ARHGAP8	1388.523	-0.18148	0.111875	0.212497	protein_coding
ENSG0000(	CYP39A1	67.28974	0.294503	0.111892	0.212497	protein_coding
ENSG0000(	ENSG0000(	28.89099	-0.57883	0.111867	0.212497	lncRNA
ENSG0000(	STON1-GTF	63.84211	0.659288	0.111883	0.212497	protein_coding
ENSG0000(	TOR1B	766.451	-0.13831	0.111889	0.212497	protein_coding
ENSG0000(	SPOCK2	993.6004	0.340175	0.111907	0.212514	protein_coding
ENSG0000(	C5orf34	80.50991	-0.22106	0.112055	0.212782	protein_coding
ENSG0000(	ZZEF1	11917.22	-0.14254	0.112067	0.212791	protein_coding
ENSG0000(	ENSG0000(	31.88333	0.266025	0.112113	0.212866	lncRNA
ENSG0000(	ENSG0000(	16.37134	0.394633	0.112129	0.212883	lncRNA
ENSG0000(	RN7SL591F	32.23676	-0.28161	0.112173	0.212954	misc_RNA
ENSG0000(	KCTD3	1174.29	-0.09891	0.112258	0.213101	protein_coding
ENSG0000(	DAPK1	1480.687	0.138056	0.112298	0.213166	protein_coding
ENSG0000(	ENSG0000(	228.6754	-0.26146	0.112305	0.213166	lncRNA
ENSG0000(	GGA1	2976.457	-0.05092	0.112342	0.213223	protein_coding
ENSG0000(	SUGT1P1	35.32414	0.296442	0.112406	0.213331	unprocessed_pseudogene
ENSG0000(	ENSG0000(	117.1625	0.140504	0.112422	0.213337	lncRNA
ENSG0000(	LAP3P1	15.3115	0.288174	0.112421	0.213337	processed_pseudogene
ENSG0000(	PCBD2	2050.752	-0.10271	0.112458	0.213391	protein_coding
ENSG0000(	OR7A17	514.2118	-0.09045	0.112479	0.213418	protein_coding
ENSG0000(	SEC13	1176.39	0.09267	0.112581	0.213598	protein_coding
ENSG0000(	LINC00620	67.14579	0.16678	0.112646	0.213708	lncRNA
ENSG0000(	ENSG0000(	171.3842	-0.12338	0.112696	0.213791	lncRNA
ENSG0000(	C1orf198	433.7684	0.20946	0.112708	0.213801	protein_coding
ENSG0000(	RN7SL566F	56.6188	0.130034	0.112747	0.213862	misc_RNA
ENSG0000(	RPAP1	759.207	-0.07936	0.112769	0.21389	protein_coding
ENSG0000(	LINC00612	46.87361	0.187817	0.112779	0.213896	lncRNA
ENSG0000(	ADAMTS16	704.8667	0.115621	0.112805	0.213906	lncRNA
ENSG0000(	CRY1	344.3352	-0.16365	0.112825	0.213906	protein_coding
ENSG0000(	EGOT	11.42552	0.524017	0.112809	0.213906	lncRNA
ENSG0000(	ENSG0000(	16.7301	-0.33333	0.112816	0.213906	lncRNA
ENSG0000(	ENSG0000(	47.23067	0.187456	0.112818	0.213906	lncRNA
ENSG0000(	LINC01881	648.0156	0.085677	0.11282	0.213906	transcribed_unprocessed_pseudo
ENSG0000(	MIOS-DT	107.2895	-0.22093	0.112957	0.214143	lncRNA

ENSG0000(	CES4A	512.8235	0.103463	0.112999	0.214209	protein_coding
ENSG0000(	FLJ30679	53.67704	0.171531	0.113043	0.21428	TEC
ENSG0000(	RNU1-82P	12.66278	-0.46053	0.11307	0.214319	snRNA
ENSG0000(	ENSG0000(	185.3741	0.118688	0.113093	0.214348	processed_pseudogene
ENSG0000(	ENSG0000(	73.12173	0.17012	0.113226	0.214588	processed_pseudogene
ENSG0000(	MIR569	20.13601	0.277844	0.113235	0.214592	miRNA
ENSG0000(	LBH	694.0111	0.232631	0.113282	0.214668	protein_coding
ENSG0000(	LRRC23	69.99591	-0.19568	0.113301	0.214679	protein_coding
ENSG0000(	PDCL	427.1131	-0.09537	0.113301	0.214679	protein_coding
ENSG0000(	PCAT5	113.0944	0.146084	0.113339	0.214738	lncRNA
ENSG0000(	H1-0	1886.788	-0.20171	0.113409	0.214857	protein_coding
ENSG0000(	TRAJ35	13.86029	-0.43929	0.113416	0.214857	TR_J_gene
ENSG0000(	ENSG0000(	68.65344	0.143169	0.113426	0.214864	lncRNA
ENSG0000(	ENSG0000(	54.31958	0.188771	0.113534	0.215055	lncRNA
ENSG0000(	GSTM4	760.0853	-0.1822	0.113566	0.215075	protein_coding
ENSG0000(	IL1RAPL1	55.51371	-0.24734	0.113561	0.215075	protein_coding
ENSG0000(	TSNAX	1172.017	-0.0678	0.113559	0.215075	protein_coding
ENSG0000(	GTF3C2	1533.689	-0.05719	0.113608	0.21513	protein_coding
ENSG0000(	RRP15	1091.582	0.134623	0.113607	0.21513	protein_coding
ENSG0000(	ENSG0000(	18.69444	-0.29702	0.113691	0.215274	processed_pseudogene
ENSG0000(	C7orf61	17.37627	-0.31265	0.113707	0.215291	protein_coding
ENSG0000(	RBBP8NL	115.6625	-0.32075	0.113738	0.215337	protein_coding
ENSG0000(	ENSG0000(	458.8832	-0.20113	0.113769	0.215356	protein_coding
ENSG0000(	OR2F1	174.0152	0.124774	0.113768	0.215356	protein_coding
ENSG0000(	RNU6-100F	25.25305	0.246931	0.113757	0.215356	snRNA
ENSG0000(	ABTB1	828.3992	0.132611	0.113839	0.215476	protein_coding
ENSG0000(	SNORA11	83.65608	0.189919	0.113902	0.215582	snoRNA
ENSG0000(	CCR10	17.43664	0.315785	0.113928	0.215618	protein_coding
ENSG0000(	DLGAP1-AS	171.7125	-0.11239	0.114021	0.215768	lncRNA
ENSG0000(	TMEM91	280.6013	-0.08881	0.114015	0.215768	protein_coding
ENSG0000(	AHNAK2	378.5178	0.333986	0.114073	0.215852	protein_coding
ENSG0000(	CD6	704.6249	0.202605	0.114101	0.215887	protein_coding
ENSG0000(	ENSG0000(	496.1364	0.145168	0.114105	0.215887	TEC
ENSG0000(	TM4SF18	124.7194	0.20362	0.114173	0.215991	protein_coding
ENSG0000(	TSPAN4	314.3306	0.139473	0.114172	0.215991	protein_coding
ENSG0000(	RELL2	107.5342	0.176254	0.11421	0.216047	protein_coding
ENSG0000(	ENSG0000(	17.30399	-0.31111	0.114232	0.216076	protein_coding
ENSG0000(	LINC01692	24.26643	-0.2465	0.114239	0.216077	lncRNA
ENSG0000(	ENSG0000(	128.9017	-0.11431	0.114269	0.216119	lncRNA
ENSG0000(	SH3BP2	5579.857	0.109647	0.114336	0.216234	protein_coding
ENSG0000(	ENSG0000(	95.32027	0.127591	0.114362	0.216268	lncRNA
ENSG0000(	ENSG0000(	18.75734	0.709116	0.114371	0.216273	lncRNA
ENSG0000(	GABRA2	525.4515	0.230382	0.114406	0.216313	protein_coding
ENSG0000(	NACAD	64.36335	-0.38475	0.114406	0.216313	protein_coding
ENSG0000(	ENSG0000(	542.1946	0.098252	0.114414	0.216316	lncRNA
ENSG0000(	ENSG0000(	273.2522	-0.08364	0.114454	0.216376	lncRNA
ENSG0000(	IRF2BP2	3335.149	-0.11002	0.11446	0.216376	protein_coding

ENSG0000(	MIR608	21.73438	-0.3712	0.114511	0.216459	miRNA
ENSG0000(	MANEALP1	13.51483	-0.32222	0.114543	0.216507	processed_pseudogene
ENSG0000(	BBOF1	155.2603	-0.19322	0.114667	0.216729	protein_coding
ENSG0000(	ENSG0000(	210.4083	0.133401	0.114704	0.216784	TEC
ENSG0000(	FAM111B	712.5267	-0.15569	0.11473	0.216821	protein_coding
ENSG0000(	TUBA1A	277.9171	0.235319	0.114758	0.21686	protein_coding
ENSG0000(	STK10	2215.641	0.113192	0.114812	0.216949	protein_coding
ENSG0000(	RN7SL88P	13.08392	0.339701	0.114847	0.217002	misc_RNA
ENSG0000(	ENSG0000(	16.51926	0.412928	0.114889	0.217068	lncRNA
ENSG0000(	ARHGAP11	513.8905	-0.17242	0.114937	0.217136	protein_coding
ENSG0000(	LINC01312	15.95667	0.35478	0.114938	0.217136	lncRNA
ENSG0000(	ENSG0000(	415.8004	-0.20139	0.115127	0.217478	lncRNA
ENSG0000(	ENSG0000(	8.687965	0.448466	0.115161	0.217531	snoRNA
ENSG0000(	ENSG0000(	8.614732	-0.44381	0.115221	0.21763	lncRNA
ENSG0000(	TMEM25	211.226	-0.16717	0.115312	0.217789	protein_coding
ENSG0000(	CIDEB	140.9439	0.159289	0.115325	0.217791	protein_coding
ENSG0000(	TF	2753.408	0.108606	0.115327	0.217791	protein_coding
ENSG0000(	C12orf60	313.7892	0.085383	0.115386	0.217889	protein_coding
ENSG0000(	ENSG0000(	10.96182	0.340843	0.115396	0.217896	lncRNA
ENSG0000(	U2AF1L4	565.6396	-0.08209	0.115441	0.217966	protein_coding
ENSG0000(	ENSG0000(	33.44501	-0.2688	0.115456	0.217982	lncRNA
ENSG0000(	DNAJA1	1097.598	-0.12603	0.11548	0.218002	protein_coding
ENSG0000(	ENSG0000(	57.54637	0.167911	0.115475	0.218002	lncRNA
ENSG0000(	ENSG0000(	479.1238	-0.09265	0.115489	0.218004	lncRNA
ENSG0000(	ERMAP	910.7118	0.130536	0.115585	0.218172	protein_coding
ENSG0000(	C18orf32	1120.753	-0.0805	0.115622	0.218224	protein_coding
ENSG0000(	DCAF17	932.6266	-0.07553	0.115626	0.218224	protein_coding
ENSG0000(	SIAH2	467.2718	-0.10251	0.115703	0.218356	protein_coding
ENSG0000(	ENSG0000(	18.81687	-0.35002	0.115714	0.218364	lncRNA
ENSG0000(	ENSG0000(	144.7579	0.123918	0.115723	0.218368	lncRNA
ENSG0000(	YEATS2	2011.395	0.096838	0.115743	0.218392	protein_coding
ENSG0000(	ENSG0000(	9.893408	0.383069	0.115759	0.218409	lncRNA
ENSG0000(	ENSG0000(	117.4854	-0.12409	0.115783	0.218441	processed_pseudogene
ENSG0000(	ZNF646	1292.714	-0.12469	0.11579	0.218441	protein_coding
ENSG0000(	ENSG0000(	121.9373	-0.14872	0.115857	0.218554	protein_coding
ENSG0000(	LINC02533	165.8895	0.096241	0.115871	0.218568	lncRNA
ENSG0000(	ENSG0000(	13.73491	-0.40678	0.115905	0.218618	protein_coding
ENSG0000(	SRSF11	5750.602	0.082158	0.115917	0.218628	protein_coding
ENSG0000(	ENSG0000(	45.66198	0.234915	0.115928	0.218636	lncRNA
ENSG0000(	ENSG0000(	60.39251	0.199327	0.115985	0.218729	lncRNA
ENSG0000(	NTN1	223.3997	0.322528	0.11602	0.218782	protein_coding
ENSG0000(	COX4I1P2	18.08669	-0.29954	0.116075	0.218862	processed_pseudogene
ENSG0000(	ENSG0000(	161.9387	-0.12004	0.116076	0.218862	lncRNA
ENSG0000(	PMP2	94.52275	0.194392	0.116111	0.218914	protein_coding
ENSG0000(	MMP21	19.29129	0.285554	0.116169	0.219011	protein_coding
ENSG0000(	ENSG0000(	13.49624	-0.3619	0.116203	0.219062	lncRNA
ENSG0000(	CCL5	980.2616	-0.12832	0.116223	0.21908	protein_coding

ENSG0000(	RYBP	1658.171	-0.09619	0.116227	0.21908	protein_coding
ENSG0000(	ENSG0000(	258.6035	0.087346	0.116238	0.219089	lncRNA
ENSG0000(	UCK2	1070.847	-0.12817	0.116321	0.219231	protein_coding
ENSG0000(	ENSG0000(	248.7777	0.117007	0.116336	0.219247	lncRNA
ENSG0000(	DPEP2	269.3595	0.233457	0.116346	0.219251	protein_coding
ENSG0000(	ENSG0000(	25.98923	0.25641	0.116362	0.219269	lncRNA
ENSG0000(	ANKLE2	5591.563	0.071567	0.116372	0.219274	protein_coding
ENSG0000(	DACH1	508.1601	-0.15583	0.116404	0.219307	protein_coding
ENSG0000(	RPUSD3	899.5469	-0.06893	0.116405	0.219307	protein_coding
ENSG0000(	TNFSF18	75.29068	-0.22105	0.11641	0.219307	protein_coding
ENSG0000(	LINC01060	308.536	-0.12138	0.116422	0.219315	lncRNA
ENSG0000(	YME1L1	2672.524	-0.07205	0.116463	0.219379	protein_coding
ENSG0000(	HSBP1L1	787.3909	-0.17635	0.116507	0.219449	protein_coding
ENSG0000(	METTL5	359.5929	-0.07696	0.116541	0.219501	protein_coding
ENSG0000(	FKBP5	3583.508	0.268655	0.116558	0.219519	protein_coding
ENSG0000(	GBP3	1777.466	0.172597	0.116585	0.219557	protein_coding
ENSG0000(	PPP1R26-A	727.358	-0.14638	0.116598	0.219567	lncRNA
ENSG0000(	ENSG0000(	389.8669	-0.09081	0.116612	0.21958	lncRNA
ENSG0000(	ENSG0000(	197.8201	-0.10587	0.116625	0.21958	lncRNA
ENSG0000(	FABP7	55.70026	0.160818	0.11662	0.21958	protein_coding
ENSG0000(	IL2RB	521.7354	0.173764	0.116708	0.219723	protein_coding
ENSG0000(	PTGR2	1244.654	-0.10165	0.116721	0.219733	protein_coding
ENSG0000(	LRSAM1	1414.782	0.066526	0.116783	0.219838	protein_coding
ENSG0000(	RPS26	1347.007	-0.25441	0.116808	0.219872	protein_coding
ENSG0000(	LINC00536	39.16099	0.185364	0.116868	0.21997	lncRNA
ENSG0000(	ENSG0000(	380.0768	-0.07906	0.116896	0.220011	lncRNA
ENSG0000(	POLRMTP1	30.64893	-0.44979	0.116914	0.22003	processed_pseudogene
ENSG0000(	LINC01809	12.01333	0.328306	0.116939	0.220065	lncRNA
ENSG0000(	ENSG0000(	32.37454	0.270904	0.116974	0.220117	lncRNA
ENSG0000(	HS1BP3	1186.985	0.185705	0.116989	0.22012	protein_coding
ENSG0000(	NPC1L1	40.8132	-0.2314	0.116987	0.22012	protein_coding
ENSG0000(	ENSG0000(	15.87072	0.271786	0.116997	0.220121	lncRNA
ENSG0000(	CPNE3	1580.186	-0.12286	0.117087	0.220251	protein_coding
ENSG0000(	ENSG0000(	35.15935	-0.38727	0.117094	0.220251	lncRNA
ENSG0000(	HOXC6	52.3638	0.441873	0.117088	0.220251	protein_coding
ENSG0000(	LINC01939	18.07563	-0.31485	0.117076	0.220251	lncRNA
ENSG0000(	ENSG0000(	28.44859	0.249644	0.117147	0.220336	processed_pseudogene
ENSG0000(	ENSG0000(	2186.852	-0.1159	0.117156	0.22034	lncRNA
ENSG0000(	C9orf135-D	14.34059	-0.30232	0.117182	0.220377	lncRNA
ENSG0000(	SLC16A13	116.106	0.141958	0.117191	0.220379	protein_coding
ENSG0000(	ENSG0000(	18.0987	0.22498	0.117201	0.220386	lncRNA
ENSG0000(	MIR4733H(	61.39798	0.156157	0.117248	0.220461	lncRNA
ENSG0000(	PABPC5	26.83672	0.290161	0.117377	0.22069	protein_coding
ENSG0000(	DYNC2H1	1243.259	0.195789	0.117385	0.220692	protein_coding
ENSG0000(	SPACA3	9.799376	-0.34464	0.117455	0.22081	protein_coding
ENSG0000(	ENSG0000(	27.31148	0.227896	0.117466	0.220811	lncRNA
ENSG0000(	PURG	25.77961	0.26451	0.117473	0.220811	protein_coding

ENSG0000(	SUMF1	889.8673	-0.08704	0.117477	0.220811	protein_coding
ENSG0000(	NUTM2A-A	6791.486	0.086546	0.117504	0.220848	lncRNA
ENSG0000(	TAF11	2202.091	-0.05481	0.117564	0.220948	protein_coding
ENSG0000(	SORBS2	1447.878	-0.15478	0.117583	0.220971	protein_coding
ENSG0000(	MEN1	480.645	-0.08045	0.117616	0.221019	protein_coding
ENSG0000(	ENSG0000(	236.9959	-0.10148	0.117629	0.221031	lncRNA
ENSG0000(	MRPL23	257.6769	-0.14541	0.117651	0.221058	protein_coding
ENSG0000(	AKAP12	827.0713	0.165227	0.117776	0.221254	protein_coding
ENSG0000(	ENSG0000(	102.6983	0.141436	0.117776	0.221254	lncRNA
ENSG0000(	MST1L	714.0576	-0.45812	0.117771	0.221254	transcribed_unprocessed_pseudo
ENSG0000(	ENSG0000(	344.3379	-0.15087	0.117798	0.221269	lncRNA
ENSG0000(	SPESP1	13.81572	-0.52209	0.117798	0.221269	protein_coding
ENSG0000(	ENSA	2927.694	-0.0578	0.117825	0.221292	protein_coding
ENSG0000(	MAU2	2754.241	-0.05076	0.117824	0.221292	protein_coding
ENSG0000(	KAZN	738.1107	0.146754	0.117848	0.221323	protein_coding
ENSG0000(	LRP1B	63.01513	0.547252	0.117885	0.221379	protein_coding
ENSG0000(	THRAP3P3	23.98763	0.31471	0.117909	0.22141	processed_pseudogene
ENSG0000(	SRSF9	1216.814	-0.08145	0.117938	0.221438	protein_coding
ENSG0000(	TRGV2	11.64801	-0.47368	0.117937	0.221438	TR_V_gene
ENSG0000(	CACNA2D4	198.4477	0.158795	0.118019	0.221576	protein_coding
ENSG0000(	RPE	1367.872	-0.07302	0.118042	0.221606	protein_coding
ENSG0000(	MVK	352.0646	-0.16638	0.11805	0.22161	protein_coding
ENSG0000(	ENSG0000(	9.461539	0.555914	0.118074	0.221641	unprocessed_pseudogene
ENSG0000(	SCX	55.11115	-0.25783	0.118136	0.221744	protein_coding
ENSG0000(	FAM204A	1501.522	-0.10889	0.11818	0.2218	protein_coding
ENSG0000(	TLN1	5245.597	-0.10686	0.118175	0.2218	protein_coding
ENSG0000(	CTDP1	890.978	0.100241	0.118216	0.221853	protein_coding
ENSG0000(	FAM230C	112.9135	0.283879	0.11824	0.221868	lncRNA
ENSG0000(	MTMR6	608.9005	0.128281	0.118234	0.221868	protein_coding
ENSG0000(	TAS2R4	791.4645	0.094266	0.118245	0.221868	protein_coding
ENSG0000(	VRTN	72.51822	0.154292	0.118278	0.221916	protein_coding
ENSG0000(	TAC4	584.1421	0.179685	0.118285	0.221917	protein_coding
ENSG0000(	ENSG0000(	55.12379	0.218074	0.118377	0.222035	lncRNA
ENSG0000(	ENSG0000(	194.2994	0.160105	0.118367	0.222035	lncRNA
ENSG0000(	EPS15-AS1	98.57285	-0.25786	0.118373	0.222035	lncRNA
ENSG0000(	WASHC3	359.3277	0.108896	0.11837	0.222035	protein_coding
ENSG0000(	MMP26	32.99378	-0.22944	0.118387	0.222042	protein_coding
ENSG0000(	ENSG0000(	163.1419	0.099239	0.118407	0.222059	lncRNA
ENSG0000(	RN7SKP275	16.25661	-0.44938	0.118414	0.222059	misc_RNA
ENSG0000(	TRAJ43	11.27831	0.472484	0.118418	0.222059	TR_J_gene
ENSG0000(	CDC42SE1	3746.393	0.10592	0.118448	0.222102	protein_coding
ENSG0000(	ENSG0000(	9.633814	0.489775	0.118555	0.222289	lncRNA
ENSG0000(	TGDS	239.8007	-0.12299	0.118632	0.222421	protein_coding
ENSG0000(	LDHBP1	158.6971	-0.11318	0.118654	0.222448	processed_pseudogene
ENSG0000(	CWC27	7012.572	0.037504	0.11867	0.222465	protein_coding
ENSG0000(	AP4E1	1162.621	-0.1503	0.118734	0.222573	protein_coding
ENSG0000(	ENSG0000(	56.30054	0.170457	0.118744	0.222577	lncRNA

ENSG0000(	ENSG0000(	9.688221	0.377506	0.118757	0.222588	lncRNA
ENSG0000(	ENSG0000(	59.19312	0.127811	0.118794	0.222645	lncRNA
ENSG0000(	TRAJ34	16.11161	-0.40214	0.118809	0.22266	TR_J_gene
ENSG0000(	ENSG0000(	17.12849	0.31063	0.118842	0.222708	lncRNA
ENSG0000(	MMS22L	2614.122	-0.08759	0.118861	0.222729	protein_coding
ENSG0000(	ENSG0000(	87.7553	0.291177	0.118907	0.222798	protein_coding
ENSG0000(	ENSG0000(	205.9267	-0.09841	0.118911	0.222798	lncRNA
ENSG0000(	FRMD8	976.0466	-0.11798	0.118922	0.222805	protein_coding
ENSG0000(	MTRNR2L8	63.57447	-0.75658	0.118959	0.22286	protein_coding
ENSG0000(	MTRNR2L1	11.59883	0.342521	0.118988	0.222888	protein_coding
ENSG0000(	OR2T11	47.8745	0.312978	0.118983	0.222888	protein_coding
ENSG0000(	ATP2A1	74.73952	-0.32288	0.119023	0.222913	protein_coding
ENSG0000(	ENSG0000(	541.1372	0.075588	0.119022	0.222913	lncRNA
ENSG0000(	SUGT1P2	94.6546	-0.12997	0.119022	0.222913	processed_pseudogene
ENSG0000(	ENSG0000(	322.6212	0.080886	0.119044	0.222927	TEC
ENSG0000(	FLNB-AS1	14.34937	0.626436	0.119051	0.222927	lncRNA
ENSG0000(	SENP5	3210.4	-0.06262	0.119051	0.222927	protein_coding
ENSG0000(	FLRT1	28.77505	-0.29035	0.119065	0.222939	protein_coding
ENSG0000(	ENSG0000(	13.35434	0.270933	0.119104	0.222998	lncRNA
ENSG0000(	FAM181A-/	399.219	-0.06837	0.119126	0.223013	lncRNA
ENSG0000(	RN7SL623F	32.99329	0.227463	0.119123	0.223013	misc_RNA
ENSG0000(	C1QTNF9	48.52912	0.22995	0.119177	0.223095	protein_coding
ENSG0000(	IFNGR1	1359.74	0.140273	0.119224	0.22317	protein_coding
ENSG0000(	ENSG0000(	24.74491	0.251133	0.119234	0.223175	lncRNA
ENSG0000(	MSLN	14.51774	0.664123	0.119303	0.22329	protein_coding
ENSG0000(	ENSG0000(	25.62196	0.285622	0.119389	0.223439	lncRNA
ENSG0000(	ENSG0000(	38.32602	-0.23201	0.119432	0.223478	lncRNA
ENSG0000(	HSP90B1	7763.855	-0.14948	0.119427	0.223478	protein_coding
ENSG0000(	THSD4	1961.355	-0.22285	0.119424	0.223478	protein_coding
ENSG0000(	DBIL5P2	76.02978	0.16011	0.119443	0.223487	transcribed_processed_pseudoge
ENSG0000(	SH3BGRL3	1149.496	-0.1846	0.119508	0.22358	protein_coding
ENSG0000(	STMN3	203.3735	0.254599	0.119506	0.22358	protein_coding
ENSG0000(	ABHD3	2028.268	-0.16264	0.119573	0.223689	protein_coding
ENSG0000(	ENSG0000(	19.29292	0.266806	0.119651	0.223821	lncRNA
ENSG0000(	HMCN1	497.2254	-0.29308	0.11968	0.223849	protein_coding
ENSG0000(	RNF41	1779.122	-0.06569	0.119676	0.223849	protein_coding
ENSG0000(	ENSG0000(	202.332	0.094207	0.119717	0.223905	lncRNA
ENSG0000(	UBL3	1027.701	-0.13324	0.119751	0.223955	protein_coding
ENSG0000(	ENSG0000(	16.10295	-0.38291	0.119805	0.224043	unitary_pseudogene
ENSG0000(	FAM180A	28.07346	0.278602	0.119828	0.224072	protein_coding
ENSG0000(	TSHZ3-AS1	10.14618	0.427998	0.119864	0.224125	lncRNA
ENSG0000(	LINC00102	76.23612	-0.13628	0.11991	0.224199	lncRNA
ENSG0000(	ENSG0000(	152.3984	0.12875	0.119928	0.224219	lncRNA
ENSG0000(	MUC3A	6588.288	-0.19367	0.119987	0.224316	protein_coding
ENSG0000(	ENSG0000(	59.05945	0.254339	0.120021	0.224366	processed_pseudogene
ENSG0000(	FAM131B	26.94903	0.347748	0.12003	0.224368	protein_coding
ENSG0000(	ENSG0000(	124.9164	0.135795	0.12004	0.224374	lncRNA

ENSG0000(	ENSG0000(	27.63511	0.315218	0.120058	0.224395	lncRNA
ENSG0000(	ENSG0000(	22.87279	0.294298	0.120067	0.224399	snoRNA
ENSG0000(	TCF3P1	79.39669	0.242451	0.120084	0.224417	processed_pseudogene
ENSG0000(	ENSG0000(	111.6663	-0.13406	0.120123	0.224476	lncRNA
ENSG0000(	LIPN	21.48595	1.428763	0.120143	0.2245	protein_coding
ENSG0000(	RNY1P12	9.583769	0.332843	0.120177	0.22455	misc_RNA
ENSG0000(	FMO5	1095.148	-0.18783	0.120195	0.224571	protein_coding
ENSG0000(	ENSG0000(	27.79065	-0.26776	0.120244	0.224648	transcribed_processed_pseudoge
ENSG0000(	ANKRD16	507.2026	0.087665	0.120274	0.224689	protein_coding
ENSG0000(	ENSG0000(	13.67372	0.26655	0.12028	0.224689	lncRNA
ENSG0000(	ENSG0000(	19.53233	0.322588	0.120442	0.224966	processed_pseudogene
ENSG0000(	LINC00563	10.9996	0.356758	0.120443	0.224966	lncRNA
ENSG0000(	TFAP2A-AS	16.65247	0.242315	0.120493	0.225047	lncRNA
ENSG0000(	ENSG0000(	83.52058	-0.13306	0.120508	0.225047	lncRNA
ENSG0000(	RNASEH2A	293.7976	-0.08104	0.120502	0.225047	protein_coding
ENSG0000(	CENPN	995.4449	-0.06395	0.120515	0.225048	protein_coding
ENSG0000(	ENSG0000(	52.08963	-0.18804	0.120565	0.225126	lncRNA
ENSG0000(	ENSG0000(	47.78888	0.163644	0.120602	0.225183	lncRNA
ENSG0000(	ENSG0000(	585.894	0.105012	0.120617	0.225198	lncRNA
ENSG0000(	PCM1	5556.547	-0.06718	0.120636	0.22522	protein_coding
ENSG0000(	VIPR2	150.7054	0.154372	0.120653	0.225238	protein_coding
ENSG0000(	ENSG0000(	121.079	0.134174	0.120668	0.225252	processed_pseudogene
ENSG0000(	PPEF2	241.7792	-0.20554	0.120691	0.225282	protein_coding
ENSG0000(	ENSG0000(	35.16768	0.221426	0.120705	0.225295	lncRNA
ENSG0000(	TBC1D17	1016.208	-0.06944	0.120727	0.225322	protein_coding
ENSG0000(	AADAC	12.93307	-0.63805	0.120739	0.225331	protein_coding
ENSG0000(	ENSG0000(	729.8192	-0.12091	0.12077	0.225375	lncRNA
ENSG0000(	BHLHE40-A	650.3362	-0.06657	0.120781	0.225382	lncRNA
ENSG0000(	ENSG0000(	130.0696	-0.12257	0.120916	0.225621	lncRNA
ENSG0000(	ENSG0000(	12.59624	1.351234	0.120935	0.225629	lncRNA
ENSG0000(	TCP11L2	933.1229	0.135904	0.120931	0.225629	protein_coding
ENSG0000(	ENSG0000(	82.5878	0.147576	0.120959	0.22566	lncRNA
ENSG0000(	PCAT7	390.7507	-0.13097	0.120979	0.225685	lncRNA
ENSG0000(	RAD21L1	37.89806	-0.26858	0.121002	0.225714	protein_coding
ENSG0000(	AURKC	23.15308	0.313902	0.121017	0.225728	protein_coding
ENSG0000(	ENSG0000(	150.5575	-0.15304	0.121067	0.225809	unprocessed_pseudogene
ENSG0000(	ENSG0000(	8.944493	0.429759	0.121089	0.225835	lncRNA
ENSG0000(	CDRT15P4	19.05353	0.340154	0.12112	0.225877	transcribed_unprocessed_pseudo
ENSG0000(	ENSG0000(	177.6335	-0.09751	0.121125	0.225877	lncRNA
ENSG0000(	TBCB	515.9253	-0.0744	0.121153	0.225901	protein_coding
ENSG0000(	TIAM1	600.2423	0.140459	0.121148	0.225901	protein_coding
ENSG0000(	CNTRL	3485.168	0.154249	0.121161	0.225902	protein_coding
ENSG0000(	RN7SL630F	30.20181	0.249381	0.121183	0.22593	misc_RNA
ENSG0000(	ENSG0000(	27.49522	0.195281	0.121203	0.225954	lncRNA
ENSG0000(	BRWD1-AS	18.98349	0.314093	0.121233	0.225969	lncRNA
ENSG0000(	POPDC2	93.53507	0.226023	0.121231	0.225969	protein_coding
ENSG0000(	USP10	1540.799	-0.07331	0.121225	0.225969	protein_coding

ENSG0000(	TULP3	430.143	0.10016	0.121254	0.225995	protein_coding
ENSG0000(	DHX30	1349.831	-0.07576	0.121347	0.226155	protein_coding
ENSG0000(	ENSG0000(	25.16996	-0.47325	0.121354	0.226155	transcribed_unprocessed_pseudo
ENSG0000(	MRGPRF-A	13.20987	0.356224	0.121367	0.226166	lncRNA
ENSG0000(	LINC01193	52.71811	0.209167	0.12138	0.226176	lncRNA
ENSG0000(	PLEKHF2	810.3947	-0.1207	0.121391	0.226184	protein_coding
ENSG0000(	LINC01358	1042.022	0.083972	0.121488	0.22635	lncRNA
ENSG0000(	TAS2R10	41.43506	0.262482	0.121497	0.226354	protein_coding
ENSG0000(	ARMC1	584.185	-0.08472	0.121549	0.226436	protein_coding
ENSG0000(	ENSG0000(	18.56051	0.309154	0.121571	0.226466	lncRNA
ENSG0000(	RARA	639.3722	-0.13163	0.121591	0.226489	protein_coding
ENSG0000(	ENSG0000(	38.46117	-0.35569	0.12164	0.226567	lncRNA
ENSG0000(	ENSG0000(	11.42919	0.426266	0.121649	0.226569	processed_pseudogene
ENSG0000(	ENSG0000(	726.3207	0.077992	0.121676	0.226606	lncRNA
ENSG0000(	ENSG0000(	2724.051	-0.11378	0.121683	0.226606	lncRNA
ENSG0000(	EWSR1	7746.177	0.047186	0.121705	0.226634	protein_coding
ENSG0000(	ENSG0000(	22.19482	-0.33937	0.121727	0.226662	transcribed_unprocessed_pseudo
ENSG0000(	ENSG0000(	278.1895	0.083187	0.121745	0.226682	lncRNA
ENSG0000(	CATSPERD	26.52852	0.201199	0.121769	0.226712	protein_coding
ENSG0000(	ENSG0000(	42.4046	0.222335	0.121835	0.226821	lncRNA
ENSG0000(	ENSG0000(	93.79529	0.14447	0.12188	0.226891	lncRNA
ENSG0000(	SRCIN1	233.6877	-0.18018	0.121941	0.226992	protein_coding
ENSG0000(	MYC	656.8638	-0.16752	0.121961	0.227016	protein_coding
ENSG0000(	ENSG0000(	31.35199	-0.20649	0.122054	0.227175	lncRNA
ENSG0000(	PATL1	1315.943	0.08996	0.122068	0.227188	protein_coding
ENSG0000(	ZC3HAV1	3667.388	0.095267	0.122112	0.227257	protein_coding
ENSG0000(	ENSG0000(	35.09508	0.199592	0.122207	0.22742	lncRNA
ENSG0000(	LINC02488	38.80823	0.187327	0.122265	0.227513	lncRNA
ENSG0000(	TMEM176/	869.5816	-0.15424	0.122279	0.227525	protein_coding
ENSG0000(	ENSG0000(	73.65152	0.135567	0.122296	0.227532	lncRNA
ENSG0000(	LDHAL6FP	43.65402	-0.23069	0.122297	0.227532	processed_pseudogene
ENSG0000(	ENSG0000(	286.9475	-0.07741	0.122349	0.227604	lncRNA
ENSG0000(	LGR5	336.1221	-0.3567	0.12235	0.227604	protein_coding
ENSG0000(	MVB12A	386.1085	-0.08462	0.122359	0.227607	protein_coding
ENSG0000(	SOD2-OT1	68.49655	0.1344	0.122412	0.227691	lncRNA
ENSG0000(	SELENOH	481.6516	-0.08718	0.122422	0.227698	protein_coding
ENSG0000(	ENSG0000(	9.458167	0.357974	0.122519	0.227864	lncRNA
ENSG0000(	CRAMP1	1363.717	-0.09899	0.122559	0.227925	protein_coding
ENSG0000(	LILRB5	699.3932	-0.1715	0.122606	0.227999	protein_coding
ENSG0000(	ENSG0000(	46.76184	0.790873	0.122653	0.228074	TEC
ENSG0000(	CKMT2-AS:	985.9792	0.072437	0.122715	0.228147	lncRNA
ENSG0000(	GRAMD4P:	713.0689	0.166361	0.12271	0.228147	processed_pseudogene
ENSG0000(	ICE1	2396.77	0.07913	0.122702	0.228147	protein_coding
ENSG0000(	RN7SL336F	32.36806	0.188806	0.122756	0.22821	misc_RNA
ENSG0000(	ENSG0000(	329.2323	-0.05879	0.122776	0.228233	lncRNA
ENSG0000(	ENSG0000(	216.9617	-0.10796	0.122853	0.228364	transcribed_unprocessed_pseudo
ENSG0000(	CASC19	920.2707	0.138693	0.122914	0.228463	lncRNA

ENSG0000(	ENSG0000(	19.02078	0.269528	0.122987	0.228585	lncRNA
ENSG0000(	RAD17P1	170.3194	-0.2644	0.123018	0.228629	processed_pseudogene
ENSG0000(	ZZZ3	2095.887	-0.07322	0.123034	0.228646	protein_coding
ENSG0000(	LINC01638	137.7216	-0.13555	0.123064	0.228687	lncRNA
ENSG0000(	ENSG0000(	39.18494	-0.22486	0.123133	0.228802	lncRNA
ENSG0000(	ENSG0000(	252.2133	-0.12849	0.12315	0.22882	lncRNA
ENSG0000(	GIMAP4	445.4106	0.254908	0.123185	0.228871	protein_coding
ENSG0000(	GPBAR1	39.73309	0.183189	0.123203	0.228891	protein_coding
ENSG0000(	ABHD18	1601.094	-0.06224	0.123297	0.229053	protein_coding
ENSG0000(	USF2	1396.478	-0.07814	0.12332	0.229081	protein_coding
ENSG0000(	ENSG0000(	314.9812	-0.11496	0.123372	0.229164	lncRNA
ENSG0000(	ENSG0000(	25.32043	-0.18709	0.123394	0.229179	lncRNA
ENSG0000(	SMAD4	4779.322	0.048033	0.123393	0.229179	protein_coding
ENSG0000(	ENSG0000(	126.6549	-0.27236	0.123439	0.229247	protein_coding
ENSG0000(	FAM215B	786.1326	0.166729	0.12346	0.229274	lncRNA
ENSG0000(	ATP1B1	13702.04	-0.20369	0.123497	0.229329	protein_coding
ENSG0000(	BTG1	3254.458	0.132821	0.123507	0.229334	protein_coding
ENSG0000(	FAM197Y6	14.02358	-0.87996	0.123548	0.229395	lncRNA
ENSG0000(	ENSG0000(	44.56376	0.202765	0.123679	0.229612	lncRNA
ENSG0000(	SLC6A17	15.1251	0.393506	0.123676	0.229612	protein_coding
ENSG0000(	CARD10	1114.61	-0.18879	0.123732	0.229696	protein_coding
ENSG0000(	POMGNT2	156.9926	-0.13573	0.123753	0.229722	protein_coding
ENSG0000(	ENSG0000(	103.1459	-0.46948	0.123775	0.229749	processed_pseudogene
ENSG0000(	RPS10-NUD	348.357	-0.25397	0.123809	0.229785	protein_coding
ENSG0000(	ZNF142	1673.955	-0.08892	0.123802	0.229785	protein_coding
ENSG0000(	RPL9P3	21.20113	0.267054	0.123821	0.229794	processed_pseudogene
ENSG0000(	CLEC12A-A	2769.39	-0.13917	0.12385	0.229834	lncRNA
ENSG0000(	ENSG0000(	294.2379	0.168132	0.123858	0.229835	lncRNA
ENSG0000(	MAN2B1	1411.091	0.108318	0.123884	0.229869	protein_coding
ENSG0000(	ABCC6P1	348.5367	0.265498	0.123969	0.229948	transcribed_unprocessed_pseudo
ENSG0000(	BCLAF1	5262.334	0.070694	0.123958	0.229948	protein_coding
ENSG0000(	ENSG0000(	263.1616	0.093366	0.12397	0.229948	lncRNA
ENSG0000(	ENSG0000(	808.3322	-0.05155	0.123947	0.229948	TEC
ENSG0000(	ENSG0000(	126.5037	0.129279	0.123961	0.229948	lncRNA
ENSG0000(	RPL7L1P10	29.95937	0.244093	0.12397	0.229948	processed_pseudogene
ENSG0000(	ENSG0000(	20.13572	0.242229	0.123995	0.22998	snoRNA
ENSG0000(	KLHL25	583.2127	-0.12769	0.124005	0.229985	protein_coding
ENSG0000(	ZNF385B	57.76907	-0.40462	0.124051	0.230057	protein_coding
ENSG0000(	EPS8L1	595.34	-0.29929	0.12408	0.230097	protein_coding
ENSG0000(	ENSG0000(	22.59958	0.268862	0.124108	0.230107	lncRNA
ENSG0000(	MMEL1	45.16651	-0.34346	0.124101	0.230107	protein_coding
ENSG0000(	SNX25P1	21.9493	0.313317	0.124106	0.230107	unprocessed_pseudogene
ENSG0000(	RNU6-1275	17.05276	0.319263	0.124213	0.230288	snRNA
ENSG0000(	FGD1	130.8034	0.180142	0.124237	0.23032	protein_coding
ENSG0000(	ZNF391	153.2405	0.263808	0.124248	0.230326	protein_coding
ENSG0000(	CXCR1	48.98679	2.49126	0.124277	0.230366	protein_coding
ENSG0000(	ZNF573	500.6347	-0.12048	0.124291	0.230378	protein_coding

ENSG0000(	ENSG0000(	27.68164	-0.31813	0.124314	0.230408	lncRNA
ENSG0000(	ENSG0000(	15.89648	0.421704	0.124371	0.230499	TEC
ENSG0000(	ENSG0000(	368.479	-0.08975	0.124453	0.230639	lncRNA
ENSG0000(	ENSG0000(	76.14373	-0.25061	0.124463	0.230643	protein_coding
ENSG0000(	NOSTRIN	1227.392	-0.18469	0.124498	0.230694	protein_coding
ENSG0000(	ENSG0000(	191.6551	0.109979	0.124513	0.230702	lncRNA
ENSG0000(	ENSG0000(	54.49453	0.285011	0.124517	0.230702	lncRNA
ENSG0000(	ACTG2	504.9846	0.248783	0.124553	0.230741	protein_coding
ENSG0000(	LINC01816	8.61801	0.374198	0.124552	0.230741	lncRNA
ENSG0000(	HDHD5-AS:	15.85417	-0.32642	0.124561	0.230743	lncRNA
ENSG0000(	ENSG0000(	60.15632	-0.19602	0.124607	0.230814	transcribed_unprocessed_pseudo
ENSG0000(	ENSG0000(	12.2043	0.36659	0.124624	0.230831	lncRNA
ENSG0000(	ENSG0000(	62.77374	0.26081	0.124636	0.230841	lncRNA
ENSG0000(	PPP2CA-DT	33.9376	0.208253	0.124718	0.230979	lncRNA
ENSG0000(	ENSG0000(	82.77394	0.304519	0.12474	0.230991	lncRNA
ENSG0000(	MXRA5Y	9.99906	0.585902	0.124737	0.230991	unprocessed_pseudogene
ENSG0000(	IMPDH1P5	63.28761	-0.24931	0.124781	0.231054	processed_pseudogene
ENSG0000(	CYP4F30P	54.46089	-0.31783	0.124833	0.231137	transcribed_unprocessed_pseudo
ENSG0000(	RNU6-681F	34.18617	0.235549	0.124883	0.231216	snRNA
ENSG0000(	ENSG0000(	12.27075	-0.51262	0.124924	0.231278	protein_coding
ENSG0000(	SLC16A10	961.7911	0.115698	0.124952	0.231317	protein_coding
ENSG0000(	ENSG0000(	982.8106	0.088849	0.125048	0.231481	TEC
ENSG0000(	ZBTB18	949.8767	0.089817	0.125056	0.231481	protein_coding
ENSG0000(	ENSG0000(	4840.045	0.09998	0.125067	0.231489	lncRNA
ENSG0000(	ENSG0000(	27.22236	0.211966	0.125109	0.23155	processed_pseudogene
ENSG0000(	ENSG0000(	28.59542	-0.39368	0.125115	0.23155	lncRNA
ENSG0000(	UBQLN4	633.8279	-0.12326	0.125161	0.231621	protein_coding
ENSG0000(	ENSG0000(	11.45498	0.313433	0.125189	0.231659	lncRNA
ENSG0000(	RMDN2	913.6495	-0.17643	0.125235	0.23173	protein_coding
ENSG0000(	HNRNPU	20743.21	-0.05297	0.125253	0.23175	protein_coding
ENSG0000(	NENF	186.4421	-0.1622	0.125267	0.231762	protein_coding
ENSG0000(	RSPH9	33.20824	0.288382	0.125315	0.231836	protein_coding
ENSG0000(	KLHL20	600.1473	-0.08782	0.125388	0.231958	protein_coding
ENSG0000(	ENSG0000(	247.4752	0.096854	0.125409	0.231984	lncRNA
ENSG0000(	RN7SL618F	17.62326	0.248756	0.125429	0.232008	misc_RNA
ENSG0000(	USP3	2512.559	0.048383	0.125461	0.232053	protein_coding
ENSG0000(	MAGED2	409.4984	-0.16948	0.125628	0.232348	protein_coding
ENSG0000(	TNFRSF11E	126.0912	0.201926	0.125646	0.232368	protein_coding
ENSG0000(	ENSG0000(	40.20322	-0.16285	0.125699	0.232441	processed_pseudogene
ENSG0000(	MIR4644	16.52658	0.312857	0.125709	0.232441	miRNA
ENSG0000(	SARS1	1085.123	-0.07435	0.125704	0.232441	protein_coding
ENSG0000(	TMEM177	313.8835	-0.13638	0.12572	0.232441	protein_coding
ENSG0000(	USP32P3	2197.682	0.093472	0.125723	0.232441	transcribed_unprocessed_pseudo
ENSG0000(	SPATA1	79.76408	0.195583	0.125833	0.23263	protein_coding
ENSG0000(	ENSG0000(	1282.077	-0.10253	0.125865	0.232636	lncRNA
ENSG0000(	PPM1D	1885.819	-0.04815	0.125866	0.232636	protein_coding
ENSG0000(	SARDH	174.0195	0.170327	0.12585	0.232636	protein_coding

ENSG0000(	TBCC	365.2892	-0.1178	0.125854	0.232636	protein_coding
ENSG0000(	RNASEK	494.33	-0.1362	0.125928	0.232738	protein_coding
ENSG0000(	LRRC3B	51.23288	-0.5048	0.125963	0.232786	protein_coding
ENSG0000(	MIR663A	4131.088	-0.27483	0.125977	0.232786	miRNA
ENSG0000(	NECTIN4	221.2344	0.257442	0.125972	0.232786	protein_coding
ENSG0000(	ATP11C	1104.223	0.101851	0.126	0.232816	protein_coding
ENSG0000(	ENSG0000(	144.6371	0.1279	0.126063	0.232918	lncRNA
ENSG0000(	PCDHGC4	72.75503	-0.18484	0.126117	0.233004	protein_coding
ENSG0000(	RAD21-AS1	202.4686	0.119782	0.126171	0.233085	lncRNA
ENSG0000(	SUGCT-AS1	187.1416	-0.11263	0.126176	0.233085	lncRNA
ENSG0000(	EDAR	100.7064	-0.2044	0.1262	0.233117	protein_coding
ENSG0000(	ENSG0000(	53.5936	0.196768	0.12623	0.233129	lncRNA
ENSG0000(	ENSG0000(	13.12919	0.331268	0.126223	0.233129	lncRNA
ENSG0000(	PTPRK	3895.871	-0.10309	0.126222	0.233129	protein_coding
ENSG0000(	ENSG0000(	16.70914	0.537026	0.126273	0.233195	lncRNA
ENSG0000(	RAB26	125.2724	0.2898	0.126298	0.233228	protein_coding
ENSG0000(	IGFLR1	281.6207	0.123353	0.126318	0.23325	protein_coding
ENSG0000(	ETF1	1656.735	-0.08501	0.126328	0.233256	protein_coding
ENSG0000(	ENSG0000(	48.05905	0.147799	0.12636	0.233302	lncRNA
ENSG0000(	COTL1	1830.61	0.135006	0.12644	0.233435	protein_coding
ENSG0000(	BRWD1	9108.394	-0.05857	0.126472	0.233467	protein_coding
ENSG0000(	DCTN6-DT	26.8666	0.202798	0.126465	0.233467	lncRNA
ENSG0000(	LHX4	52.43913	-0.36931	0.12656	0.233615	protein_coding
ENSG0000(	EML4	4691.669	-0.07546	0.126619	0.233711	protein_coding
ENSG0000(	GPR75	200.8907	-0.15403	0.126642	0.233739	protein_coding
ENSG0000(	DNHD1	1861.76	0.086454	0.126697	0.233827	protein_coding
ENSG0000(	CPLX1	33.95959	0.258693	0.126715	0.233832	protein_coding
ENSG0000(	ENSG0000(	99.5141	0.157413	0.126708	0.233832	processed_pseudogene
ENSG0000(	INTS9-AS1	121.8353	-0.1573	0.12674	0.233864	lncRNA
ENSG0000(	NHEG1	55.86566	0.154959	0.126762	0.233891	lncRNA
ENSG0000(	CDKN1A	1942.384	0.209762	0.126782	0.233915	protein_coding
ENSG0000(	KRTAP9-6	26.27182	-0.21355	0.126808	0.233948	protein_coding
ENSG0000(	LINC00608	509.5548	-0.09625	0.126821	0.233958	lncRNA
ENSG0000(	ENSG0000(	30.63002	0.245986	0.12687	0.234022	lncRNA
ENSG0000(	SNORD9	41.91178	0.174132	0.126868	0.234022	snoRNA
ENSG0000(	SEC62	1930.894	0.084289	0.127004	0.234254	protein_coding
ENSG0000(	CCL13	68.59727	0.415291	0.127022	0.234275	protein_coding
ENSG0000(	ENSG0000(	85.59155	-0.12223	0.127048	0.234309	TEC
ENSG0000(	HNRNPA1P	16.21626	0.361697	0.12706	0.234317	processed_pseudogene
ENSG0000(	THAP12	1561.271	-0.08157	0.127121	0.234415	protein_coding
ENSG0000(	ENSG0000(	138.7423	0.17504	0.127234	0.234611	lncRNA
ENSG0000(	ENSG0000(	20.40829	0.309401	0.127258	0.23464	lncRNA
ENSG0000(	ARHGEF9	2342.777	-0.16623	0.127269	0.234647	protein_coding
ENSG0000(	LEMD1-DT	372.2922	-0.07814	0.127297	0.234684	lncRNA
ENSG0000(	ZMYM2	5396.411	0.052869	0.127315	0.234705	protein_coding
ENSG0000(	MADD	2543.217	0.084547	0.127399	0.234845	protein_coding
ENSG0000(	CHKB-CPT1	218.0222	-0.21334	0.127474	0.234969	protein_coding

ENSG0000(	ENSG0000(	139.2995	-0.11455	0.127485	0.234975	lncRNA
ENSG0000(	ENSG0000(	23.37614	0.419343	0.127508	0.234991	processed_pseudogene
ENSG0000(	SKAP1-AS1	309.5487	-0.10296	0.127503	0.234991	lncRNA
ENSG0000(	NELL2	252.7669	-0.36495	0.127621	0.235185	protein_coding
ENSG0000(	LINC01719	801.0919	-0.09142	0.127641	0.235208	lncRNA
ENSG0000(	ATP13A3	5005.758	0.130754	0.12771	0.235321	protein_coding
ENSG0000(	ENSG0000(	101.882	0.198043	0.127723	0.235331	lncRNA
ENSG0000(	ENSG0000(	19.04992	0.278099	0.127773	0.235402	lncRNA
ENSG0000(	GFRA1	470.6327	-0.18441	0.127791	0.235402	protein_coding
ENSG0000(	LINC02520	16.47193	0.659636	0.12778	0.235402	lncRNA
ENSG0000(	NUCB1	1345.896	-0.1133	0.127791	0.235402	protein_coding
ENSG0000(	MFF-DT	1272.594	0.054544	0.127807	0.235403	lncRNA
ENSG0000(	RN7SL121F	479.5081	0.107609	0.1278	0.235403	misc_RNA
ENSG0000(	TSEN2	3334.664	-0.12117	0.127817	0.235408	protein_coding
ENSG0000(	CEP250	2355.128	-0.1177	0.127851	0.235432	protein_coding
ENSG0000(	CTXN2-AS1	30.15045	0.258361	0.12784	0.235432	lncRNA
ENSG0000(	OCIAD2	641.8466	-0.21299	0.127853	0.235432	protein_coding
ENSG0000(	FHIP1A-DT	54.52929	-0.27767	0.127891	0.235461	lncRNA
ENSG0000(	TXNRD2	837.9438	-0.06012	0.127891	0.235461	protein_coding
ENSG0000(	ZNF367	180.1708	-0.21007	0.127879	0.235461	protein_coding
ENSG0000(	RN7SL239F	94.5224	0.149737	0.127903	0.23547	misc_RNA
ENSG0000(	ANO8	431.0462	0.083553	0.127976	0.23559	protein_coding
ENSG0000(	ENSG0000(	382.261	-0.15534	0.128022	0.235648	protein_coding
ENSG0000(	ENSG0000(	18.955	0.369008	0.12803	0.235648	lncRNA
ENSG0000(	ENSG0000(	51.55211	0.194841	0.128026	0.235648	lncRNA
ENSG0000(	SAMD11	199.8058	-0.25195	0.128053	0.23567	protein_coding
ENSG0000(	SLC2A12	40.58626	0.315677	0.128057	0.23567	protein_coding
ENSG0000(	ENSG0000(	215.5502	-0.09263	0.12809	0.235715	lncRNA
ENSG0000(	ENSG0000(	9.427207	0.349405	0.128135	0.235785	lncRNA
ENSG0000(	IGKV6D-21	24.49882	0.649743	0.12815	0.235786	IG_V_gene
ENSG0000(	WDR5B	457.4406	0.10134	0.128143	0.235786	protein_coding
ENSG0000(	ALKBH4	346.2055	-0.07479	0.128166	0.2358	protein_coding
ENSG0000(	TP53I3	1000.149	-0.17833	0.128192	0.235835	protein_coding
ENSG0000(	BFAR	1200.364	-0.07094	0.128223	0.235878	protein_coding
ENSG0000(	ENSG0000(	49.27268	0.18333	0.128339	0.236056	lncRNA
ENSG0000(	RBM11	24.08458	0.352392	0.128332	0.236056	protein_coding
ENSG0000(	RFFL	3627.913	-0.11449	0.128343	0.236056	protein_coding
ENSG0000(	ZNF419	1150.101	0.073038	0.128362	0.236077	protein_coding
ENSG0000(	SUGCT	235.5289	-0.23678	0.128396	0.236126	protein_coding
ENSG0000(	ENSG0000(	70.05929	0.166539	0.128418	0.236153	lncRNA
ENSG0000(	TCTE1	39.56234	0.213644	0.128491	0.236274	protein_coding
ENSG0000(	DCST1	33.21481	-0.29271	0.128526	0.236313	protein_coding
ENSG0000(	KPNA2P3	270.4914	-0.25692	0.128543	0.236313	transcribed_unprocessed_pseudo
ENSG0000(	MAPK7	752.0239	-0.14938	0.128534	0.236313	protein_coding
ENSG0000(	RNF138	1145.305	-0.13055	0.128543	0.236313	protein_coding
ENSG0000(	CNPY2-AS1	220.5668	-0.12311	0.128637	0.236375	lncRNA
ENSG0000(	ENSG0000(	19.6804	0.242983	0.128633	0.236375	lncRNA

ENSG0000(	KRT18P5	79.27508	-0.20713	0.128623	0.236375	processed_pseudogene
ENSG0000(	MAP4K2	835.4486	-0.16427	0.128589	0.236375	protein_coding
ENSG0000(	SAMD8	1837.817	0.094688	0.128611	0.236375	protein_coding
ENSG0000(	SLC6A12	43.70679	0.315276	0.128625	0.236375	protein_coding
ENSG0000(	TMEM258	380.9754	-0.1254	0.128601	0.236375	protein_coding
ENSG0000(	ZNF812P	20.18996	0.386115	0.128617	0.236375	transcribed_unprocessed_pseudo
ENSG0000(	FBXO3	1728.261	-0.056	0.128668	0.236404	protein_coding
ENSG0000(	SWT1	383.9231	0.130231	0.128668	0.236404	protein_coding
ENSG0000(	ENSG0000(	42.48578	0.210235	0.128726	0.236497	TEC
ENSG0000(	ENSG0000(	13.53197	0.28948	0.128735	0.2365	lncRNA
ENSG0000(	CDK4P1	90.01525	0.154753	0.128776	0.236561	processed_pseudogene
ENSG0000(	CDK1	280.1516	-0.17956	0.12879	0.236572	protein_coding
ENSG0000(	CHD9	7474.412	0.070879	0.128824	0.236621	protein_coding
ENSG0000(	PYGO1	377.6986	0.102076	0.128831	0.236621	protein_coding
ENSG0000(	RN7SL38P	50.85711	0.182972	0.128872	0.236681	misc_RNA
ENSG0000(	STARD3	918.1437	-0.0949	0.128903	0.236726	protein_coding
ENSG0000(	ENSG0000(	23.72402	0.290954	0.128929	0.236759	lncRNA
ENSG0000(	LCORL	2187.662	0.058578	0.128947	0.236763	protein_coding
ENSG0000(	NLRP8	94.56822	0.125476	0.128946	0.236763	protein_coding
ENSG0000(	NOMO2	651.9568	-0.16317	0.128965	0.236783	protein_coding
ENSG0000(	B4GALT1-A	292.7639	0.10984	0.129006	0.236834	lncRNA
ENSG0000(	ENSG0000(	73.87093	-0.16341	0.129008	0.236834	lncRNA
ENSG0000(	OR8B9P	607.2972	-0.08577	0.129029	0.236844	transcribed_unprocessed_pseudo
ENSG0000(	RBFADN	1041.146	0.060899	0.129022	0.236844	lncRNA
ENSG0000(	ENSG0000(	201.3565	0.154019	0.129106	0.236971	TEC
ENSG0000(	SYT16	255.7445	-0.1745	0.129113	0.236971	protein_coding
ENSG0000(	KCNMB2	22.31857	-0.33959	0.129121	0.236971	protein_coding
ENSG0000(	ENSG0000(	234.9961	-0.09	0.129153	0.237016	lncRNA
ENSG0000(	ALOX12P1	19.15289	-0.27144	0.129198	0.237085	unprocessed_pseudogene
ENSG0000(	CCNE1	102.7811	-0.17939	0.129227	0.237099	protein_coding
ENSG0000(	NDFIP1	1463.238	0.096749	0.129217	0.237099	protein_coding
ENSG0000(	SH2D4B	12.63187	0.369876	0.129236	0.237099	protein_coding
ENSG0000(	ZNF92	389.2404	0.132915	0.129232	0.237099	protein_coding
ENSG0000(	HNRNPA3	8148.925	-0.05731	0.129261	0.237131	protein_coding
ENSG0000(	GPR156	13.17393	-0.41514	0.12928	0.237152	protein_coding
ENSG0000(	CSPG4P10	276.1918	0.207639	0.129353	0.237258	transcribed_unprocessed_pseudo
ENSG0000(	ENSG0000(	176.8492	0.116618	0.129346	0.237258	lncRNA
ENSG0000(	P2RX5	533.3622	0.411707	0.129384	0.237301	protein_coding
ENSG0000(	ENSG0000(	26.83336	-0.23377	0.129409	0.23732	lncRNA
ENSG0000(	IGF2R	9939.807	0.080196	0.129402	0.23732	protein_coding
ENSG0000(	MNT	1854.21	0.083046	0.12956	0.237583	protein_coding
ENSG0000(	ENSG0000(	231.8862	-0.10532	0.129593	0.237614	lncRNA
ENSG0000(	MIGA1	1653.397	-0.04728	0.129591	0.237614	protein_coding
ENSG0000(	CHEK2	734.511	-0.06799	0.129612	0.237627	protein_coding
ENSG0000(	KBTBD11-A	220.9894	-0.12614	0.129615	0.237627	lncRNA
ENSG0000(	RN7SL441F	61.6242	0.178193	0.129632	0.237645	misc_RNA
ENSG0000(	ENSG0000(	43.21967	-0.17654	0.129659	0.23768	lncRNA

ENSG0000(	UPRT	373.8749	-0.08554	0.129708	0.237756	protein_coding
ENSG0000(	MIR3663H(	11.52939	0.485269	0.129716	0.237757	lncRNA
ENSG0000(	SDHCP3	44.1033	0.163163	0.129794	0.237886	processed_pseudogene
ENSG0000(	WIPF2	3205.31	-0.11205	0.129848	0.237971	protein_coding
ENSG0000(	PRR7-AS1	104.226	-0.22616	0.129862	0.237983	lncRNA
ENSG0000(	CYP2B7P	625.3461	-0.26554	0.129888	0.238017	transcribed_unprocessed_pseudo
ENSG0000(	ENSG0000(	27.07175	0.245845	0.129934	0.238086	lncRNA
ENSG0000(	LRRC52-AS	22.45569	0.447879	0.129959	0.238118	lncRNA
ENSG0000(	LINC00687	167.2086	0.102103	0.130005	0.238189	lncRNA
ENSG0000(	ENSG0000(	1099.23	-0.09271	0.130017	0.238195	TEC
ENSG0000(	MFSD12	776.5702	0.104423	0.130033	0.238195	protein_coding
ENSG0000(	PPP3R1	1114.81	-0.07386	0.130027	0.238195	protein_coding
ENSG0000(	PSKH1	934.8277	-0.14097	0.130039	0.238195	protein_coding
ENSG0000(	ENSG0000(	21.91193	0.247517	0.130058	0.238216	lncRNA
ENSG0000(	VPS13B-DT	135.7968	0.102064	0.130121	0.238317	lncRNA
ENSG0000(	ARL13B	423.8767	0.102816	0.130131	0.238322	protein_coding
ENSG0000(	H1-3	289.2886	-0.22377	0.130148	0.238339	protein_coding
ENSG0000(	CD101	184.4823	-0.20374	0.130269	0.238546	protein_coding
ENSG0000(	FASTKD3	267.6512	-0.14885	0.130297	0.238572	protein_coding
ENSG0000(	TMEM50A	984.1638	-0.12424	0.130298	0.238572	protein_coding
ENSG0000(	BOD1	440.3125	-0.0911	0.130403	0.238749	protein_coding
ENSG0000(	SOX2-OT	1398.869	0.044088	0.13041	0.238749	lncRNA
ENSG0000(	CHADL	18.86363	-0.39098	0.130511	0.23891	protein_coding
ENSG0000(	LINC00665	2513.191	-0.0639	0.130515	0.23891	lncRNA
ENSG0000(	TAF1D	3429.539	-0.08192	0.130521	0.23891	protein_coding
ENSG0000(	DAZAP2	2756.527	-0.13219	0.130559	0.238938	protein_coding
ENSG0000(	ENSG0000(	28.48469	0.229513	0.130553	0.238938	lncRNA
ENSG0000(	XPO5	1154.928	-0.09634	0.130549	0.238938	protein_coding
ENSG0000(	MALRD1	146.8761	0.515557	0.130567	0.238939	protein_coding
ENSG0000(	IGLV3-19	311.7232	0.453429	0.130609	0.238994	IG_V_gene
ENSG0000(	LRRC37A	1196.294	0.202895	0.130612	0.238994	protein_coding
ENSG0000(	LNCAROD	22.13667	0.35411	0.130624	0.239	lncRNA
ENSG0000(	ENSG0000(	280.4425	0.082228	0.130652	0.239038	lncRNA
ENSG0000(	ENSG0000(	29.09218	0.225765	0.130664	0.239046	TEC
ENSG0000(	ENSG0000(	17.24657	0.307913	0.130707	0.239111	lncRNA
ENSG0000(	RETNLB	358.4183	-0.41205	0.130801	0.239269	protein_coding
ENSG0000(	ENSG0000(	1919.473	-0.52969	0.13084	0.239322	transcribed_processed_pseudoge
ENSG0000(	ENSG0000(	39.97147	0.318552	0.130845	0.239322	lncRNA
ENSG0000(	ATG4C	229.3069	-0.1671	0.130855	0.239326	protein_coding
ENSG0000(	POU5F1B	260.5965	-0.09116	0.130882	0.239361	protein_coding
ENSG0000(	ENSG0000(	189.4397	0.11576	0.130893	0.239367	lncRNA
ENSG0000(	OR10K2	71.73773	0.155832	0.130985	0.239521	protein_coding
ENSG0000(	ENSG0000(	127.1786	0.151631	0.13103	0.239589	transcribed_unprocessed_pseudo
ENSG0000(	GIPC3	89.80467	0.20816	0.131039	0.239591	protein_coding
ENSG0000(	LY6E-DT	176.3281	0.119612	0.131052	0.239602	lncRNA
ENSG0000(	COL17A1	2909.763	-0.24571	0.131133	0.239735	protein_coding
ENSG0000(	PRKCI	1278.567	-0.09023	0.131162	0.239774	protein_coding

ENSG0000(	NUDT1	125.725	0.146472	0.131237	0.239898	protein_coding
ENSG0000(	CHAC2	36.18158	-0.25855	0.131272	0.239924	protein_coding
ENSG0000(	CLCN3	2925.068	-0.10397	0.131281	0.239924	protein_coding
ENSG0000(	ENSG0000(	46.45274	0.164556	0.131282	0.239924	transcribed_unprocessed_pseudo
ENSG0000(	RN7SKP13(	17.14195	0.39509	0.131279	0.239924	misc_RNA
ENSG0000(	TMEM192	3200.137	-0.03951	0.131318	0.239976	protein_coding
ENSG0000(	FAM180B	98.34528	0.246522	0.131333	0.239988	protein_coding
ENSG0000(	COL14A1	1362.075	0.244526	0.13135	0.239999	protein_coding
ENSG0000(	ENSG0000(	60.88211	0.220496	0.131354	0.239999	lncRNA
ENSG0000(	DYNLRB1	547.6087	-0.10852	0.131408	0.24006	protein_coding
ENSG0000(	POGLUT3	895.3304	-0.09093	0.13141	0.24006	protein_coding
ENSG0000(	PPAN-P2RY	13.10232	-0.93205	0.131395	0.24006	protein_coding
ENSG0000(	PGD	643.9314	0.142266	0.131475	0.240164	protein_coding
ENSG0000(	ENSG0000(	27.3853	-0.24677	0.131505	0.240206	lncRNA
ENSG0000(	HSBP1P1	11.50498	0.353813	0.131532	0.240218	processed_pseudogene
ENSG0000(	SPATA22	10.0555	0.588583	0.131535	0.240218	protein_coding
ENSG0000(	WHSC1L2P	220.8509	-0.09926	0.131531	0.240218	processed_pseudogene
ENSG0000(	USP37	1350.384	-0.09233	0.131603	0.240328	protein_coding
ENSG0000(	ENSG0000(	73.31457	0.183945	0.131649	0.240398	lncRNA
ENSG0000(	FOXF2	209.1855	-0.18252	0.131776	0.240616	protein_coding
ENSG0000(	ENSG0000(	246.2546	0.11248	0.131865	0.240765	lncRNA
ENSG0000(	ENSG0000(	60.01863	0.198415	0.131891	0.240798	lncRNA
ENSG0000(	SLC17A5	714.2953	-0.14578	0.13197	0.240929	protein_coding
ENSG0000(	ZNF41	1020.075	-0.08055	0.132107	0.241164	protein_coding
ENSG0000(	ENSG0000(	33.64249	-0.26707	0.132121	0.241175	lncRNA
ENSG0000(	ENSG0000(	47.5834	-0.18393	0.132145	0.241206	lncRNA
ENSG0000(	SH2B2	90.85124	0.266208	0.132164	0.241227	protein_coding
ENSG0000(	IGLON5	56.93631	0.203711	0.132182	0.241231	protein_coding
ENSG0000(	TOR2A	244.3377	-0.13995	0.132182	0.241231	protein_coding
ENSG0000(	ENSG0000(	14.52865	0.313464	0.132194	0.241238	lncRNA
ENSG0000(	ENSG0000(	30.21351	0.178457	0.132221	0.241274	lncRNA
ENSG0000(	ZNF404	73.1304	-0.22862	0.132232	0.24128	protein_coding
ENSG0000(	ENSG0000(	1554.774	-0.08111	0.132262	0.241306	lncRNA
ENSG0000(	ERLEC1	2704.09	0.064388	0.132259	0.241306	protein_coding
ENSG0000(	PTPRJ	3565.016	0.117258	0.132285	0.241335	protein_coding
ENSG0000(	ENSG0000(	11.73535	-0.36047	0.1323	0.241348	lncRNA
ENSG0000(	LETM2	418.8483	0.071626	0.132428	0.24156	protein_coding
ENSG0000(	TEFM	629.5718	-0.06197	0.132432	0.24156	protein_coding
ENSG0000(	IGHV4-39	850.6509	0.405741	0.13245	0.24158	IG_V_gene
ENSG0000(	RASEF	2485.218	-0.15581	0.132497	0.24165	protein_coding
ENSG0000(	SNORA77B	45.18712	-0.27382	0.132567	0.241763	snoRNA
ENSG0000(	ENSG0000(	134.6825	0.145689	0.132586	0.241785	lncRNA
ENSG0000(	ENSG0000(	11.67897	0.384454	0.132634	0.241839	lncRNA
ENSG0000(	ENSG0000(	9.606083	0.653451	0.132647	0.241839	lncRNA
ENSG0000(	GNG11	128.2483	0.225895	0.132644	0.241839	protein_coding
ENSG0000(	SH3D21	1182.473	-0.12491	0.132642	0.241839	protein_coding
ENSG0000(	TIMM23B	1240.605	-0.07422	0.132672	0.24187	protein_coding

ENSG0000(	ENSG0000(	348.1031	0.114996	0.132741	0.241982	TEC
ENSG0000(	NINL	325.4611	-0.15162	0.132749	0.241983	protein_coding
ENSG0000(	CFAP65	31.99034	0.360227	0.132762	0.241992	protein_coding
ENSG0000(	LINC02569	84.58415	-0.15309	0.132791	0.242031	lncRNA
ENSG0000(	KANSL3	3684.672	-0.05184	0.132814	0.242058	protein_coding
ENSG0000(	ENSG0000(	102.158	0.309262	0.132859	0.242126	lncRNA
ENSG0000(	ENSG0000(	12.84517	0.324382	0.132892	0.242173	lncRNA
ENSG0000(	SC5D	1222.461	0.088707	0.132948	0.242261	protein_coding
ENSG0000(	ENSG0000(	1079.851	0.100766	0.132965	0.242278	TEC
ENSG0000(	LRATD1	3867.159	-0.18098	0.132981	0.242293	protein_coding
ENSG0000(	POLL	1085.555	-0.07322	0.133013	0.242337	protein_coding
ENSG0000(	ENSG0000(	320.7175	0.081703	0.133029	0.242347	lncRNA
ENSG0000(	RIT1	1031.422	-0.09273	0.133034	0.242347	protein_coding
ENSG0000(	ACVR2A	803.2692	-0.14517	0.133046	0.242355	protein_coding
ENSG0000(	SLC2A9	307.8925	0.114206	0.133067	0.242379	protein_coding
ENSG0000(	ENSG0000(	221.229	-0.08506	0.133145	0.242503	lncRNA
ENSG0000(	TPSB2	342.9102	-0.20406	0.133151	0.242503	protein_coding
ENSG0000(	STON2	2170.822	-0.12345	0.133211	0.242599	protein_coding
ENSG0000(	DCAF10	1993.774	-0.06219	0.13325	0.242641	protein_coding
ENSG0000(	MANEAL	115.8392	-0.19431	0.133244	0.242641	protein_coding
ENSG0000(	SUMO1	724.3064	-0.107	0.133262	0.24265	protein_coding
ENSG0000(	TSTD2	931.9446	0.084444	0.133296	0.242697	protein_coding
ENSG0000(	MICOS10P:	13.81663	0.248849	0.133317	0.242707	processed_pseudogene
ENSG0000(	RN7SL246F	53.52878	0.17298	0.133312	0.242707	misc_RNA
ENSG0000(	ENSG0000(	9245.867	-0.05731	0.133334	0.242725	lncRNA
ENSG0000(	VPS37A	1045.564	0.072864	0.133356	0.242749	protein_coding
ENSG0000(	B3GALT5	3285.059	0.381678	0.133437	0.242867	protein_coding
ENSG0000(	ENSG0000(	422.1349	-0.07532	0.133459	0.242867	lncRNA
ENSG0000(	ENSG0000(	32.32638	0.242493	0.133439	0.242867	lncRNA
ENSG0000(	MROCKI	395.9703	-0.14267	0.133455	0.242867	lncRNA
ENSG0000(	RFLNB	510.2349	0.16638	0.133449	0.242867	protein_coding
ENSG0000(	C5orf34-AS	55.15176	0.187567	0.133471	0.242875	lncRNA
ENSG0000(	ENSG0000(	628.5291	0.260459	0.133529	0.242966	lncRNA
ENSG0000(	MAGED1	1641.217	0.100405	0.133552	0.242993	protein_coding
ENSG0000(	CYHR1	1607.137	-0.08018	0.133577	0.243024	protein_coding
ENSG0000(	RNU1-52P	21.06877	-0.32262	0.133642	0.243128	snRNA
ENSG0000(	ENSG0000(	26.30392	-0.32161	0.13374	0.243293	TEC
ENSG0000(	ENSG0000(	36.69882	-0.19141	0.133759	0.243314	lncRNA
ENSG0000(	TNFSF12	149.1333	0.146432	0.133768	0.243315	protein_coding
ENSG0000(	MKRN3	212.5507	-0.16048	0.133807	0.243373	protein_coding
ENSG0000(	ENSG0000(	10.19381	0.358122	0.133863	0.24346	lncRNA
ENSG0000(	ENSG0000(	38.45446	-0.16095	0.133921	0.243538	TEC
ENSG0000(	ENSG0000(	398.2971	0.318604	0.133916	0.243538	lncRNA
ENSG0000(	ASH1L	9962.03	0.046213	0.133996	0.24366	protein_coding
ENSG0000(	ENSG0000(	76.59003	-0.19242	0.134022	0.243683	lncRNA
ENSG0000(	MIF4GD-DT	125.5962	-0.1169	0.134025	0.243683	lncRNA
ENSG0000(	GRM8	206.2403	-0.34204	0.134075	0.243761	protein_coding

ENSG0000(	ARFGEF3	2298.811	-0.1621	0.134107	0.243801	protein_coding
ENSG0000(	MRPS11	841.614	-0.08365	0.134121	0.243801	protein_coding
ENSG0000(	OPN4	25.1518	-0.40729	0.134117	0.243801	protein_coding
ENSG0000(	CSNK1G3	1413.012	-0.11358	0.13418	0.243895	protein_coding
ENSG0000(	ENSG0000(	13.9269	0.281999	0.134198	0.243914	lncRNA
ENSG0000(	ENSG0000(	71.32795	-0.14883	0.134207	0.243916	lncRNA
ENSG0000(	TXK	528.4818	0.147587	0.134259	0.243996	protein_coding
ENSG0000(	ENSG0000(	54.55294	-0.20221	0.134332	0.244025	lncRNA
ENSG0000(	ENSG0000(	259.1663	-0.13711	0.134324	0.244025	transcribed_unprocessed_pseudo
ENSG0000(	ENSG0000(	65.12791	-0.20772	0.134321	0.244025	protein_coding
ENSG0000(	HOXA1	53.30173	0.208656	0.134323	0.244025	protein_coding
ENSG0000(	LINC01695	884.8316	0.079842	0.134311	0.244025	lncRNA
ENSG0000(	PRDM5	629.194	-0.07205	0.134337	0.244025	protein_coding
ENSG0000(	RNY4P34	18.404	0.290473	0.134335	0.244025	misc_RNA
ENSG0000(	TNKS2	2204.17	0.059544	0.134296	0.244025	protein_coding
ENSG0000(	IGLV10-54	123.7912	0.643499	0.13438	0.244088	IG_V_gene
ENSG0000(	ENSG0000(	143.3494	0.126155	0.13441	0.244128	lncRNA
ENSG0000(	ENSG0000(	79.53738	0.170383	0.134438	0.244165	TEC
ENSG0000(	ENSG0000(	84.21595	0.142939	0.134458	0.244188	lncRNA
ENSG0000(	LYNX1	88.03302	0.297343	0.134493	0.244236	protein_coding
ENSG0000(	ENSG0000(	20.48835	-0.22873	0.134535	0.244299	lncRNA
ENSG0000(	ANKRD20A	8.708672	-0.35742	0.13458	0.244353	unprocessed_pseudogene
ENSG0000(	DDX60	5307.543	0.260241	0.134577	0.244353	protein_coding
ENSG0000(	EIF4EBP3	84.82963	-0.2166	0.134592	0.24436	protein_coding
ENSG0000(	TIMM22	222.4714	-0.12806	0.134647	0.244445	protein_coding
ENSG0000(	LINC01551	1242.937	-0.17241	0.134689	0.244508	lncRNA
ENSG0000(	PHC2-AS1	10.28134	0.410127	0.134732	0.244571	lncRNA
ENSG0000(	NBEAP1	43.81735	0.230198	0.134785	0.244653	transcribed_unprocessed_pseudo
ENSG0000(	NANOGP11	11.04331	0.442686	0.134797	0.24466	lncRNA
ENSG0000(	ENTPD4-DT	46.71299	0.155171	0.134805	0.244661	lncRNA
ENSG0000(	ENSG0000(	131.8824	0.122737	0.134819	0.244673	lncRNA
ENSG0000(	ENSG0000(	305.1252	-0.09506	0.13484	0.244683	transcribed_processed_pseudoge
ENSG0000(	RNU6-444F	62.36372	-0.22369	0.134838	0.244683	snRNA
ENSG0000(	ENSG0000(	11.42832	-0.39822	0.134849	0.244683	lncRNA
ENSG0000(	ENSG0000(	71.93861	0.163539	0.134889	0.244728	lncRNA
ENSG0000(	UGGT1	4542.466	-0.0851	0.134887	0.244728	protein_coding
ENSG0000(	KCTD10	2113.024	0.08525	0.134912	0.244757	protein_coding
ENSG0000(	ENSG0000(	14.58531	0.347852	0.134944	0.2448	lncRNA
ENSG0000(	RPL13P5	89.39176	-0.15476	0.134957	0.244809	transcribed_processed_pseudoge
ENSG0000(	ZNF260	1055.707	-0.12041	0.134968	0.244814	protein_coding
ENSG0000(	IGHD	174.0019	0.555318	0.135002	0.244862	IG_C_gene
ENSG0000(	PSMD10P1	10.24982	0.449287	0.135071	0.244973	processed_pseudogene
ENSG0000(	WASH6P	2763.873	-0.09278	0.135097	0.245007	protein_coding
ENSG0000(	DESI2	1086.435	-0.07444	0.135166	0.245118	protein_coding
ENSG0000(	VSIG10L-AS	11.15423	0.304703	0.135193	0.245151	lncRNA
ENSG0000(	SGK3	1516.403	0.050923	0.135223	0.245192	protein_coding
ENSG0000(	VAV2	982.3361	-0.1563	0.135288	0.245295	protein_coding

ENSG0000(	ENSG0000(	17.1735	0.254683	0.135334	0.245365	transcribed_unprocessed_pseudo
ENSG0000(	PBOV1	99.98935	-0.14549	0.135387	0.245446	protein_coding
ENSG0000(	SLC30A6	1211.273	-0.09079	0.135406	0.245467	protein_coding
ENSG0000(	ENSG0000(	284.7392	0.10627	0.135417	0.245473	lncRNA
ENSG0000(	ENSG0000(	55.89868	-0.17421	0.13544	0.245501	lncRNA
ENSG0000(	TTLL5	1571.127	-0.06886	0.1355	0.245596	protein_coding
ENSG0000(	LPXN	711.7825	0.135841	0.13564	0.245833	protein_coding
ENSG0000(	HVCN1	465.4982	0.205734	0.135747	0.246013	protein_coding
ENSG0000(	GNPDA2	283.1001	-0.09852	0.135756	0.246016	protein_coding
ENSG0000(	ZXDB	345.7771	0.094766	0.135824	0.246125	protein_coding
ENSG0000(	LINC02854	57.56266	0.322846	0.135836	0.246133	lncRNA
ENSG0000(	ENSG0000(	60.04626	0.171405	0.135921	0.246272	lncRNA
ENSG0000(	PCNX2	2126.401	0.061037	0.135938	0.246282	protein_coding
ENSG0000(	YBX3	2554.142	0.096552	0.135942	0.246282	protein_coding
ENSG0000(	ENSG0000(	32.2562	0.183922	0.135982	0.24634	lncRNA
ENSG0000(	ENSG0000(	148.7261	-0.1146	0.136025	0.246361	lncRNA
ENSG0000(	FANCG	289.1223	-0.13891	0.136011	0.246361	protein_coding
ENSG0000(	SLFN14	423.8263	0.081455	0.136018	0.246361	protein_coding
ENSG0000(	WDR31	996.8265	-0.05758	0.136004	0.246361	protein_coding
ENSG0000(	ENSG0000(	36.23792	0.208209	0.136107	0.246489	lncRNA
ENSG0000(	ERVH-1	55.97097	0.24641	0.136139	0.246489	lncRNA
ENSG0000(	MEIS3P1	87.18266	-0.17862	0.136143	0.246489	processed_pseudogene
ENSG0000(	MUTYH	966.2262	-0.06495	0.136122	0.246489	protein_coding
ENSG0000(	NXT2	107.0861	-0.21529	0.136142	0.246489	protein_coding
ENSG0000(	USP39	598.86	-0.07084	0.136115	0.246489	protein_coding
ENSG0000(	PPP1R9B	1058.491	0.134005	0.136235	0.246641	protein_coding
ENSG0000(	PSORS1C3	118.0414	0.327405	0.136284	0.246715	lncRNA
ENSG0000(	ENSG0000(	1891.204	0.09838	0.136391	0.246895	lncRNA
ENSG0000(	RHOG	432.0267	0.149488	0.136482	0.247046	protein_coding
ENSG0000(	ENSG0000(	204.6531	-0.12148	0.136507	0.247077	lncRNA
ENSG0000(	CCDC181	11.11064	0.516424	0.136552	0.247144	protein_coding
ENSG0000(	NUP107-DT	45.20527	0.205749	0.136564	0.24715	lncRNA
ENSG0000(	USP27X	73.13073	-0.15121	0.136616	0.24723	protein_coding
ENSG0000(	LINC02313	14.06468	-0.35501	0.136657	0.24729	lncRNA
ENSG0000(	ENSG0000(	11.37911	0.439013	0.13668	0.247317	lncRNA
ENSG0000(	ZNF496	1889.488	0.068618	0.136707	0.247352	protein_coding
ENSG0000(	RN7SL718P	1389.742	-0.12048	0.136715	0.247352	misc_RNA
ENSG0000(	C11orf71	297.8128	0.097123	0.136746	0.247375	protein_coding
ENSG0000(	ENSG0000(	46.05698	0.175639	0.136737	0.247375	lncRNA
ENSG0000(	MYO1H	12.70475	0.546553	0.136751	0.247375	protein_coding
ENSG0000(	ENSG0000(	9.97911	-0.35081	0.136814	0.247447	processed_pseudogene
ENSG0000(	ENSG0000(	37.89556	0.19204	0.1368	0.247447	TEC
ENSG0000(	ENSG0000(	847.3628	0.076577	0.136808	0.247447	lncRNA
ENSG0000(	DCX	64.38635	-0.16887	0.136876	0.247515	protein_coding
ENSG0000(	FAM3B	576.8422	-0.37337	0.136868	0.247515	protein_coding
ENSG0000(	RPGRIP1L	701.95	-0.07384	0.136869	0.247515	protein_coding
ENSG0000(	ANKRD18A	572.8561	-0.22067	0.136912	0.247551	protein_coding

ENSG0000(	RABEPK	596.2705	-0.06542	0.136907	0.247551	protein_coding
ENSG0000(	ENSG0000(	11.29753	0.537181	0.136942	0.247592	lncRNA
ENSG0000(	STIM2	1357.138	0.085877	0.136985	0.247655	protein_coding
ENSG0000(	ENSG0000(	16.32687	0.273059	0.137015	0.247695	lncRNA
ENSG0000(	RN7SL122F	42.89026	0.190285	0.137036	0.247718	misc_RNA
ENSG0000(	SELENBP1	6904.144	-0.31978	0.137058	0.247744	protein_coding
ENSG0000(	OR2M4	376.4815	-0.07135	0.137079	0.247767	protein_coding
ENSG0000(	ENSG0000(	9.940568	-0.34033	0.137117	0.247794	lncRNA
ENSG0000(	GOLGA8O	281.0327	0.384024	0.137113	0.247794	protein_coding
ENSG0000(	HSD17B12	1765.567	-0.096	0.137115	0.247794	protein_coding
ENSG0000(	ENSG0000(	22.91633	-0.24627	0.137211	0.247945	lncRNA
ENSG0000(	ENSG0000(	105.4253	0.113725	0.137217	0.247945	lncRNA
ENSG0000(	RRH	111.0847	0.147306	0.137354	0.248178	protein_coding
ENSG0000(	HRAT17	429.1168	0.134256	0.137435	0.24831	lncRNA
ENSG0000(	RNU6ATAC	19.34341	0.277442	0.137577	0.248552	snRNA
ENSG0000(	ENSG0000(	901.973	-0.08167	0.13764	0.248652	TEC
ENSG0000(	ZNF564	908.4615	-0.10771	0.137662	0.248677	protein_coding
ENSG0000(	CSTL1	100.2586	-0.14233	0.137677	0.248684	protein_coding
ENSG0000(	HCN1	180.8798	-0.45526	0.137682	0.248684	protein_coding
ENSG0000(	BMP2K-DT	74.52129	0.127129	0.137704	0.24871	lncRNA
ENSG0000(	ENSG0000(	63.0442	0.147902	0.137716	0.248717	lncRNA
ENSG0000(	ENSG0000(	145.378	-0.11664	0.137733	0.248734	lncRNA
ENSG0000(	SCOCP1	12.59766	0.397978	0.137792	0.248827	processed_pseudogene
ENSG0000(	RN7SKP204	49.76584	-0.25928	0.137816	0.248855	misc_RNA
ENSG0000(	EIF2S3B	49.87072	-0.19832	0.137826	0.24886	protein_coding
ENSG0000(	ENSG0000(	185.3622	-0.11246	0.137874	0.24893	lncRNA
ENSG0000(	ENSG0000(	238.9926	0.087638	0.137934	0.249011	TEC
ENSG0000(	RBBP9	475.4296	-0.07767	0.137932	0.249011	protein_coding
ENSG0000(	EMILIN3	33.36521	0.294177	0.137968	0.249052	protein_coding
ENSG0000(	ENSG0000(	11.71935	0.392411	0.137973	0.249052	lncRNA
ENSG0000(	ENSG0000(	68.89355	-0.23468	0.137997	0.249082	lncRNA
ENSG0000(	DENND2C	811.1307	0.066074	0.138017	0.249089	protein_coding
ENSG0000(	ZNF81	1579.233	-0.0876	0.138016	0.249089	protein_coding
ENSG0000(	ENSG0000(	160.7959	-0.09731	0.13815	0.249294	lncRNA
ENSG0000(	ENSG0000(	13.65065	-0.44853	0.138171	0.249294	lncRNA
ENSG0000(	ENSG0000(	146.8926	0.148851	0.138167	0.249294	lncRNA
ENSG0000(	RNVU1-17	18.73803	-0.33153	0.138149	0.249294	snRNA
ENSG0000(	ZNF546	953.0964	-0.07822	0.138164	0.249294	protein_coding
ENSG0000(	C7orf26	441.2421	-0.07383	0.138246	0.249416	protein_coding
ENSG0000(	FAM81A	151.8059	-0.26016	0.138322	0.249539	protein_coding
ENSG0000(	CCBE1	583.3387	0.125142	0.138468	0.249787	protein_coding
ENSG0000(	ENSG0000(	35.15787	-0.18867	0.138483	0.2498	lncRNA
ENSG0000(	SNORA16B	24.85895	0.263927	0.138504	0.249823	snoRNA
ENSG0000(	GSKIP	1396.932	-0.18341	0.138577	0.249941	protein_coding
ENSG0000(	CA10	14.45106	0.560015	0.138645	0.250048	protein_coding
ENSG0000(	SNORA2C	250.6569	-0.17487	0.13868	0.250098	snoRNA
ENSG0000(	RC3H2	3083.286	0.06311	0.13869	0.2501	protein_coding

ENSG0000(	YIPF3	679.9573	-0.07133	0.138703	0.250111	protein_coding
ENSG0000(	ENSG0000(	58.51318	-0.18785	0.138724	0.250126	lncRNA
ENSG0000(	ENSG0000(	83.6978	-0.1685	0.138728	0.250126	lncRNA
ENSG0000(	KCTD12	1597.342	0.171499	0.138833	0.250302	protein_coding
ENSG0000(	SUCLG2P4	70.94742	0.144147	0.138878	0.250368	processed_pseudogene
ENSG0000(	ENSG0000(	72.3716	-0.15846	0.138931	0.250419	lncRNA
ENSG0000(	PIEZO2	1396.039	0.07698	0.13893	0.250419	protein_coding
ENSG0000(	SPATC1L	46.87734	-0.3686	0.13893	0.250419	protein_coding
ENSG0000(	CRADD	350.1224	0.105048	0.138967	0.250442	protein_coding
ENSG0000(	ENSG0000(	620.1437	-0.06207	0.138963	0.250442	lncRNA
ENSG0000(	ZER1	1046.76	-0.14868	0.138965	0.250442	protein_coding
ENSG0000(	H2BE1	9.997718	-0.36234	0.139023	0.250528	protein_coding
ENSG0000(	ENSG0000(	27.53502	-0.24313	0.139066	0.250591	lncRNA
ENSG0000(	NUTM2B	31.83308	0.435711	0.139263	0.250933	protein_coding
ENSG0000(	ENSG0000(	29.45938	-0.20093	0.13934	0.251055	processed_pseudogene
ENSG0000(	LGALS8	2623.372	-0.08443	0.139381	0.251101	protein_coding
ENSG0000(	LINC01854	11.41312	-0.39192	0.13938	0.251101	lncRNA
ENSG0000(	REPS1	1643.071	-0.07066	0.139401	0.251108	protein_coding
ENSG0000(	VAMP7	459.5626	-0.12281	0.139399	0.251108	protein_coding
ENSG0000(	ENSG0000(	15.34991	0.379709	0.139418	0.251124	processed_pseudogene
ENSG0000(	CLCN5	1156.615	-0.09742	0.13945	0.251152	protein_coding
ENSG0000(	TRIM73	992.2719	-0.07991	0.139447	0.251152	protein_coding
ENSG0000(	ENSG0000(	14.58431	-0.34485	0.139473	0.251179	lncRNA
ENSG0000(	DPP8	2496.651	-0.04888	0.139534	0.251274	protein_coding
ENSG0000(	PTPA	1152.424	-0.06763	0.139568	0.251308	protein_coding
ENSG0000(	TMCO1-AS	442.4245	-0.07203	0.139565	0.251308	lncRNA
ENSG0000(	ARHGAP32	5504.619	-0.14257	0.13959	0.251333	protein_coding
ENSG0000(	ENSG0000(	172.7319	-0.09868	0.139605	0.251345	lncRNA
ENSG0000(	LINC01653	168.7598	-0.10313	0.139638	0.251386	lncRNA
ENSG0000(	SNRPCP9	26.14496	0.362703	0.139643	0.251386	processed_pseudogene
ENSG0000(	BOLA2P2	28.40839	0.183165	0.139653	0.251388	transcribed_processed_pseudoge
ENSG0000(	RNF8	504.1719	-0.09446	0.139663	0.251391	protein_coding
ENSG0000(	PAIP2B	532.7161	-0.13637	0.139744	0.251523	protein_coding
ENSG0000(	ENSG0000(	256.7368	0.088971	0.139761	0.25154	lncRNA
ENSG0000(	CD1A	12.58881	0.58915	0.139772	0.251544	protein_coding
ENSG0000(	ENSG0000(	519.2315	0.090965	0.13979	0.251548	lncRNA
ENSG0000(	TDRD3	1207.571	-0.06864	0.13979	0.251548	protein_coding
ENSG0000(	LINC00630	12391.57	0.066717	0.139921	0.251769	lncRNA
ENSG0000(	ENSG0000(	29.11801	0.279536	0.140025	0.251913	lncRNA
ENSG0000(	KLF3P1	78.88671	-0.16443	0.140024	0.251913	processed_pseudogene
ENSG0000(	LOX	173.2667	0.270057	0.140019	0.251913	protein_coding
ENSG0000(	LRAT	242.3662	0.088317	0.140061	0.251963	protein_coding
ENSG0000(	METTL25	374.4689	-0.11267	0.14013	0.252058	protein_coding
ENSG0000(	RNF10	3031.036	-0.07369	0.140127	0.252058	protein_coding
ENSG0000(	ENPP7P8	160.3099	-0.27739	0.140188	0.252148	transcribed_unprocessed_pseudo
ENSG0000(	ENSG0000(	31.75398	0.279395	0.140246	0.252239	lncRNA
ENSG0000(	EIF2B2	543.1621	-0.08617	0.140293	0.252307	protein_coding

ENSG0000(	ENSG0000(	22.50942	0.21946	0.14032	0.252343	lncRNA
ENSG0000(	PIPOX	267.8396	0.096694	0.140366	0.252411	protein_coding
ENSG0000(	ENSG0000(	10.87447	0.312345	0.140401	0.252459	lncRNA
ENSG0000(	ENSG0000(	28.31538	0.251977	0.140424	0.252485	lncRNA
ENSG0000(	UVRAG	1511.275	0.081646	0.140452	0.252522	protein_coding
ENSG0000(	UBA7	1913.677	0.116911	0.140477	0.252552	protein_coding
ENSG0000(	MBOAT7	1361.936	-0.10889	0.140503	0.252585	protein_coding
ENSG0000(	ENSG0000(	12.12364	-0.37786	0.140525	0.252609	lncRNA
ENSG0000(	RN7SL656F	65.17167	0.396485	0.140533	0.25261	misc_RNA
ENSG0000(	RN7SKP8	260.0244	0.213823	0.140548	0.252622	misc_RNA
ENSG0000(	PRR7	20.01153	0.350777	0.14056	0.252629	protein_coding
ENSG0000(	REPS2	1354.609	-0.10737	0.140592	0.252656	protein_coding
ENSG0000(	SLC24A3	223.8799	0.158299	0.140587	0.252656	protein_coding
ENSG0000(	ENHO	46.26473	-0.22268	0.140727	0.252884	protein_coding
ENSG0000(	ENSG0000(	37.13016	0.196484	0.140759	0.252928	lncRNA
ENSG0000(	FUT2	2416.637	-0.16016	0.140776	0.252944	protein_coding
ENSG0000(	MEX3B	50.31029	0.24064	0.140834	0.253033	protein_coding
ENSG0000(	ENSG0000(	526.6873	-0.10342	0.140851	0.253035	lncRNA
ENSG0000(	ENSG0000(	15.12389	0.383263	0.14085	0.253035	lncRNA
ENSG0000(	KIAA2026	3784.205	0.070432	0.14087	0.253054	protein_coding
ENSG0000(	IFT122	2190.018	-0.07678	0.14093	0.253134	protein_coding
ENSG0000(	NPDC1	1338.331	0.166006	0.140929	0.253134	protein_coding
ENSG0000(	ENSG0000(	10.1053	0.321193	0.140944	0.253136	snoRNA
ENSG0000(	IMPG2	168.0795	0.122099	0.140948	0.253136	protein_coding
ENSG0000(	CHRM3-AS	597.7893	0.329096	0.140968	0.25315	lncRNA
ENSG0000(	LINC02052	35.78507	0.164983	0.140976	0.25315	lncRNA
ENSG0000(	UBQLN3	22.52176	0.301491	0.14098	0.25315	protein_coding
ENSG0000(	SCARNA1	123.9121	-0.17256	0.141005	0.253181	scaRNA
ENSG0000(	CYB561	1046.899	-0.11147	0.141032	0.253215	protein_coding
ENSG0000(	ENSG0000(	10.85763	0.355028	0.141051	0.253235	processed_pseudogene
ENSG0000(	AGAP2	381.1068	0.236538	0.141085	0.253282	protein_coding
ENSG0000(	RPL12P25	24.90974	-0.31401	0.14111	0.253296	transcribed_processed_pseudoge
ENSG0000(	SPRYD3	489.6996	-0.08016	0.141106	0.253296	protein_coding
ENSG0000(	ABHD14A	172.1668	-0.09895	0.141154	0.253361	protein_coding
ENSG0000(	ENSG0000(	120.8242	-0.21725	0.141263	0.253543	lncRNA
ENSG0000(	ENSG0000(	764.1632	0.078327	0.141302	0.253597	lncRNA
ENSG0000(	ENSG0000(	10.36013	0.315861	0.141334	0.253641	lncRNA
ENSG0000(	TANC2	2073.128	0.133409	0.141417	0.253774	protein_coding
ENSG0000(	ENSG0000(	557.1553	0.106482	0.141456	0.253831	lncRNA
ENSG0000(	CRIP2	212.6138	0.192958	0.14148	0.253859	protein_coding
ENSG0000(	ENSG0000(	119.777	0.095642	0.141495	0.253872	lncRNA
ENSG0000(	ENSG0000(	9.719444	0.440858	0.14151	0.253883	lncRNA
ENSG0000(	ENSG0000(	14.12246	-0.35625	0.14153	0.253889	lncRNA
ENSG0000(	ENSG0000(	1128.699	-0.40179	0.14153	0.253889	miRNA
ENSG0000(	LDHAL6A	56.24064	-0.14645	0.141538	0.253889	protein_coding
ENSG0000(	TUBA8	571.1905	0.10238	0.141565	0.253922	protein_coding
ENSG0000(	VWC2	101.0805	-0.19183	0.141572	0.253922	protein_coding

ENSG0000(	G6PC1	220.3257	-0.16552	0.141581	0.253923	protein_coding
ENSG0000(	ZSWIM8	2315.694	0.088632	0.141615	0.25397	protein_coding
ENSG0000(	ENSG0000(	10.97855	0.429183	0.141632	0.253986	lncRNA
ENSG0000(	PCDH20	233.6238	0.463471	0.141647	0.253999	protein_coding
ENSG0000(	DYRK2	5625.293	-0.14336	0.141688	0.254057	protein_coding
ENSG0000(	TRADD	420.3274	0.078739	0.141738	0.254131	protein_coding
ENSG0000(	ENSG0000(	11.23475	0.300121	0.141848	0.254314	lncRNA
ENSG0000(	ZNF433	222.5028	0.137902	0.141856	0.254315	protein_coding
ENSG0000(	ENSG0000(	34.7191	-0.21159	0.141869	0.254323	lncRNA
ENSG0000(	ENSG0000(	9.761112	0.365219	0.141997	0.254538	lncRNA
ENSG0000(	ENSG0000(	103.7786	0.111427	0.142116	0.254736	TEC
ENSG0000(	TRAJ39	23.43899	0.353766	0.142124	0.254736	TR_J_gene
ENSG0000(	TPPP3	43.09708	0.272603	0.142193	0.254845	protein_coding
ENSG0000(	LINC01148	80.1286	-0.16056	0.142238	0.254912	lncRNA
ENSG0000(	ENSG0000(	12.53772	0.315352	0.142254	0.254927	lncRNA
ENSG0000(	LINC01700	18.93605	0.285424	0.142282	0.254961	lncRNA
ENSG0000(	ENSG0000(	24.239	0.261079	0.142301	0.25498	lncRNA
ENSG0000(	ENTPD3-AS	624.0246	-0.07927	0.142328	0.255014	lncRNA
ENSG0000(	ENSG0000(	84.44579	0.124895	0.142336	0.255014	lncRNA
ENSG0000(	GPRACR	171.878	0.100882	0.142357	0.255037	lncRNA
ENSG0000(	BIRC5	604.5732	-0.08413	0.1424	0.255059	protein_coding
ENSG0000(	ENSG0000(	41.88617	0.234101	0.142403	0.255059	snoRNA
ENSG0000(	LINC00574	206.1999	-0.07433	0.142392	0.255059	lncRNA
ENSG0000(	SLC30A4	1580.793	0.062912	0.142409	0.255059	protein_coding
ENSG0000(	SMIM11B	268.9579	-0.16643	0.14241	0.255059	protein_coding
ENSG0000(	MIR3978	9.263637	-0.34837	0.142432	0.255085	miRNA
ENSG0000(	MNX1-AS1	10.50141	-0.45972	0.142475	0.255146	lncRNA
ENSG0000(	IVD	2095.937	-0.08924	0.142511	0.255184	protein_coding
ENSG0000(	MINCR	99.95678	-0.19497	0.142512	0.255184	lncRNA
ENSG0000(	ENSG0000(	169.8232	0.097453	0.142534	0.255208	TEC
ENSG0000(	ENSG0000(	15.14584	0.734936	0.142546	0.255211	protein_coding
ENSG0000(	RGS6	176.4188	0.192865	0.142552	0.255211	protein_coding
ENSG0000(	ENSG0000(	34.36289	0.259393	0.142577	0.255241	lncRNA
ENSG0000(	ENSG0000(	78.73015	0.131413	0.142619	0.255287	lncRNA
ENSG0000(	RPS3AP34	103.4522	-0.31485	0.14261	0.255287	processed_pseudogene
ENSG0000(	ENSG0000(	214.4501	-0.07991	0.142653	0.255334	lncRNA
ENSG0000(	RN7SL329F	78.08262	0.146957	0.14272	0.255439	misc_RNA
ENSG0000(	ENSG0000(	14.91096	0.32366	0.142793	0.255556	lncRNA
ENSG0000(	ENSG0000(	14.69408	0.513727	0.142953	0.255812	processed_pseudogene
ENSG0000(	USP9Y	1535.394	0.100601	0.142953	0.255812	protein_coding
ENSG0000(	ANKRD29	319.5709	0.227686	0.142977	0.25584	protein_coding
ENSG0000(	FRMPD1	261.8919	-0.09241	0.142991	0.255851	protein_coding
ENSG0000(	DMRTC1B	36.25101	-0.27755	0.143039	0.255877	protein_coding
ENSG0000(	ENSG0000(	98.5148	-0.25441	0.143036	0.255877	transcribed_unprocessed_pseudo
ENSG0000(	ENSG0000(	347.1595	0.073657	0.143018	0.255877	lncRNA
ENSG0000(	FAT1	14049.05	-0.12051	0.143023	0.255877	protein_coding
ENSG0000(	RN7SL748F	9.415868	0.277732	0.14308	0.255937	misc_RNA

ENSG0000(	ENSG0000(	14.22544	0.299651	0.143091	0.255941	lncRNA
ENSG0000(	ENSG0000(	17.74845	0.243793	0.143127	0.255991	lncRNA
ENSG0000(	ENSG0000(	226.2898	0.131586	0.143204	0.256114	lncRNA
ENSG0000(	RSPRY1	1900.098	-0.07128	0.143389	0.25643	protein_coding
ENSG0000(	ITPKB-IT1	95.44063	0.244312	0.1434	0.256435	lncRNA
ENSG0000(	SF1-DT	20.21098	0.208746	0.143444	0.256484	lncRNA
ENSG0000(	TYR	32.47286	0.177934	0.143441	0.256484	protein_coding
ENSG0000(	PPP1R2	1146.445	0.073996	0.143459	0.256497	protein_coding
ENSG0000(	TTLL1	54.56005	-0.15455	0.143482	0.256523	protein_coding
ENSG0000(	OTOGL	117.3066	-0.35739	0.143504	0.256548	protein_coding
ENSG0000(	AFTPH	2166.893	-0.0536	0.143534	0.256588	protein_coding
ENSG0000(	ENSG0000(	241.1579	0.102734	0.143567	0.256631	TEC
ENSG0000(	ENSG0000(	100.5294	-0.12068	0.143595	0.256652	lncRNA
ENSG0000(	RNU11-6P	12.89299	-0.37917	0.143595	0.256652	snRNA
ENSG0000(	ENSG0000(	167.5828	0.263294	0.143632	0.256704	lncRNA
ENSG0000(	ENSG0000(	17.79788	0.309152	0.143666	0.25675	lncRNA
ENSG0000(	ACTR3-AS1	131.2325	0.162258	0.143744	0.256874	lncRNA
ENSG0000(	RASL12	130.7058	0.20555	0.143762	0.256892	protein_coding
ENSG0000(	TBCD	2878.939	0.071422	0.143785	0.256919	protein_coding
ENSG0000(	LINC01442	35.41048	0.203735	0.143804	0.256939	lncRNA
ENSG0000(	PXYLP1	1028.338	-0.12796	0.143826	0.256949	protein_coding
ENSG0000(	RNU7-80P	47.22152	0.269752	0.143822	0.256949	snRNA
ENSG0000(	ENSG0000(	127.8102	0.108198	0.143838	0.256955	lncRNA
ENSG0000(	MLYCD	2771.919	0.069107	0.143887	0.257027	protein_coding
ENSG0000(	RNU4-78P	33.82867	0.217725	0.144055	0.257314	snRNA
ENSG0000(	WDFY1	2798.544	0.091531	0.144067	0.257319	protein_coding
ENSG0000(	SNORA20	163.4659	-0.16789	0.144104	0.257372	snoRNA
ENSG0000(	ADORA2A-/	20.19503	0.22096	0.144144	0.257427	lncRNA
ENSG0000(	OR6C4	165.819	-0.09789	0.144191	0.257497	protein_coding
ENSG0000(	ENSG0000(	14.63125	-0.33949	0.144325	0.257707	lncRNA
ENSG0000(	LRRC42	133.3423	-0.16497	0.144325	0.257707	protein_coding
ENSG0000(	ENSG0000(	54.42127	0.201276	0.144371	0.257759	lncRNA
ENSG0000(	FBXO46	818.9286	0.113187	0.144363	0.257759	protein_coding
ENSG0000(	GOT2P6	64.26469	-0.21395	0.144395	0.257787	processed_pseudogene
ENSG0000(	NKAIN3	7665.016	0.058171	0.144472	0.257911	protein_coding
ENSG0000(	EIF4ENIF1	1227.038	0.055765	0.144484	0.257918	protein_coding
ENSG0000(	ENSG0000(	113.256	-0.12676	0.144539	0.257986	lncRNA
ENSG0000(	NFIL3	363.1844	0.181198	0.144532	0.257986	protein_coding
ENSG0000(	TMEM138	2141.644	0.045182	0.144619	0.258114	protein_coding
ENSG0000(	LINC01255	62.52183	-0.12966	0.144657	0.258167	lncRNA
ENSG0000(	ENSG0000(	45.74186	0.187479	0.144728	0.258279	unprocessed_pseudogene
ENSG0000(	ENSG0000(	81.85854	-0.22433	0.14479	0.258375	lncRNA
ENSG0000(	OR6C74	1338.819	0.089162	0.144823	0.258418	protein_coding
ENSG0000(	ENSG0000(	93.88949	-0.1419	0.144843	0.25844	protein_coding
ENSG0000(	RN7SL60P	18.71063	0.251514	0.144881	0.258493	misc_RNA
ENSG0000(	SMARCB1	2474.879	-0.04393	0.144897	0.258508	protein_coding
ENSG0000(	BLOC1S5	479.7657	-0.0716	0.14492	0.258533	protein_coding

ENSG0000(	TAS2R5	105.2854	0.146909	0.144943	0.258559	protein_coding
ENSG0000(	EME2	1664.887	-0.08764	0.144954	0.258564	protein_coding
ENSG0000(	ENSG0000(	197.6916	0.094427	0.144962	0.258564	lncRNA
ENSG0000(	SANBR	1194.78	-0.07487	0.145123	0.258836	protein_coding
ENSG0000(	LINC02135	18.66267	0.299296	0.145154	0.258862	lncRNA
ENSG0000(	MT-ND6	1899.267	-0.33402	0.145146	0.258862	protein_coding
ENSG0000(	ENSG0000(	134.1532	0.118045	0.145163	0.258864	lncRNA
ENSG0000(	ODAD3	87.06507	0.151118	0.145227	0.258962	protein_coding
ENSG0000(	MAFG	1067.092	-0.11872	0.145268	0.25902	protein_coding
ENSG0000(	CDC37L1	289.06	0.100581	0.145296	0.259056	protein_coding
ENSG0000(	ENSG0000(	24.94548	0.355937	0.145305	0.259057	lncRNA
ENSG0000(	RPS2P45	185.9698	0.101938	0.145324	0.259077	transcribed_processed_pseudoge
ENSG0000(	LAMTOR1	723.5543	-0.07809	0.145335	0.259082	protein_coding
ENSG0000(	ENSG0000(	11.80386	0.351816	0.145399	0.259181	lncRNA
ENSG0000(	ENSG0000(	28.27012	0.263434	0.145529	0.259388	lncRNA
ENSG0000(	RPS3AP22	8.349743	0.380576	0.145532	0.259388	processed_pseudogene
ENSG0000(	ENSG0000(	195.134	-0.10865	0.145544	0.259395	lncRNA
ENSG0000(	ENSG0000(	34.43236	-0.26981	0.145554	0.259398	lncRNA
ENSG0000(	ENSG0000(	176.0294	0.100162	0.145591	0.259423	lncRNA
ENSG0000(	LINC01513	318.1946	0.094182	0.145583	0.259423	lncRNA
ENSG0000(	MTCO1P12	1894.637	-0.79226	0.145609	0.259423	unprocessed_pseudogene
ENSG0000(	RPL32P2	13.73685	-0.22032	0.145609	0.259423	processed_pseudogene
ENSG0000(	TMEM225E	37.46469	0.222742	0.14561	0.259423	protein_coding
ENSG0000(	TMEM37	922.037	-0.27291	0.145624	0.259434	protein_coding
ENSG0000(	ENSG0000(	694.3481	0.093816	0.145654	0.259473	transcribed_unprocessed_pseudo
ENSG0000(	ZNF428	147.4449	-0.11378	0.145675	0.259494	protein_coding
ENSG0000(	VEPH1	44.4731	0.278783	0.145691	0.25951	protein_coding
ENSG0000(	ENSG0000(	167.8022	0.109834	0.145717	0.259532	lncRNA
ENSG0000(	ENSG0000(	18.48091	-0.64736	0.145721	0.259532	lncRNA
ENSG0000(	TOP2B	2585.834	-0.08177	0.145745	0.25956	protein_coding
ENSG0000(	TEDC2-AS1	484.9971	0.077595	0.145756	0.259565	lncRNA
ENSG0000(	B3GALT9	646.4185	-0.06479	0.145808	0.25962	protein_coding
ENSG0000(	ENSG0000(	332.0404	-0.08332	0.145808	0.25962	lncRNA
ENSG0000(	PIP5K1A	1886.501	0.052952	0.145812	0.25962	protein_coding
ENSG0000(	NBR1	4025.696	-0.09275	0.145825	0.25963	protein_coding
ENSG0000(	DCTN5	3855.11	-0.0768	0.145853	0.259665	protein_coding
ENSG0000(	EIF2S2P3	12.86053	0.412396	0.145917	0.259761	processed_pseudogene
ENSG0000(	ENSG0000(	136.5434	-0.11615	0.145924	0.259761	lncRNA
ENSG0000(	ENSG0000(	201.1628	-0.09358	0.145933	0.259761	lncRNA
ENSG0000(	RTCA	908.6552	-0.06782	0.145952	0.259782	protein_coding
ENSG0000(	FBXO48	647.1244	-0.07287	0.145977	0.259796	protein_coding
ENSG0000(	STX16-NPE	249.6235	0.159505	0.145975	0.259796	protein_coding
ENSG0000(	C1orf122	127.6713	-0.18496	0.14602	0.259858	protein_coding
ENSG0000(	ENSG0000(	11.10272	-0.36613	0.146041	0.259881	processed_pseudogene
ENSG0000(	INO80E	2858.582	-0.08258	0.146082	0.259939	protein_coding
ENSG0000(	LINC02175	810.012	0.083773	0.1461	0.259956	lncRNA
ENSG0000(	C9orf43	24.28605	0.273756	0.146124	0.259984	protein_coding

ENSG0000(	ENSG0000(	333.582	0.136176	0.14617	0.260051	processed_pseudogene
ENSG0000(	DDX39B-AS	106.9499	-0.12831	0.146185	0.260063	lncRNA
ENSG0000(	MTND4LP1	12.27687	-0.47871	0.146256	0.260174	processed_pseudogene
ENSG0000(	ENSG0000(	74.51202	0.152216	0.146298	0.260234	processed_pseudogene
ENSG0000(	TTLL11	370.5411	-0.11298	0.146379	0.260363	protein_coding
ENSG0000(	PCDHGC3	279.6261	0.152889	0.146431	0.26043	protein_coding
ENSG0000(	SLC3A1	943.7563	-0.1684	0.146433	0.26043	protein_coding
ENSG0000(	HOXD10	311.4136	0.22238	0.146452	0.26045	protein_coding
ENSG0000(	CCDC197	12.89398	0.283721	0.146521	0.260542	protein_coding
ENSG0000(	CSAD	1323.457	0.066007	0.146513	0.260542	protein_coding
ENSG0000(	LRP1	11960.39	-0.10814	0.146532	0.260547	protein_coding
ENSG0000(	ITPKC	629.0877	-0.14951	0.146579	0.260615	protein_coding
ENSG0000(	SHISA6	18.22347	0.564096	0.146643	0.260714	protein_coding
ENSG0000(	ENSG0000(	454.011	-0.05846	0.146697	0.260795	lncRNA
ENSG0000(	FCF1	1710.271	-0.05953	0.146736	0.26085	protein_coding
ENSG0000(	RNU1-18P	27.18421	0.317517	0.146797	0.260944	snRNA
ENSG0000(	LIPA	812.0922	0.202889	0.146835	0.260997	protein_coding
ENSG0000(	RPL5P18	18.50029	-0.27269	0.146844	0.260998	processed_pseudogene
ENSG0000(	ENSG0000(	25.81464	-0.24708	0.14689	0.26106	lncRNA
ENSG0000(	MMAA	563.5686	0.097387	0.146896	0.26106	protein_coding
ENSG0000(	CHKB-DT	366.6135	0.082671	0.14694	0.261123	lncRNA
ENSG0000(	FRG1FP	13.35466	0.422409	0.14696	0.261145	unprocessed_pseudogene
ENSG0000(	FOXO3B	234.4839	-0.19268	0.147023	0.261242	protein_coding
ENSG0000(	ZNF135	389.6309	-0.09739	0.147085	0.261337	protein_coding
ENSG0000(	CFAP251	1483.639	0.041185	0.147159	0.261453	protein_coding
ENSG0000(	ENSG0000(	235.4691	0.126496	0.147172	0.261462	lncRNA
ENSG0000(	LINC02367	64.40995	0.23618	0.147206	0.261508	lncRNA
ENSG0000(	MCCD1P2	10.2805	-0.33479	0.147244	0.261561	unprocessed_pseudogene
ENSG0000(	BTBD6P1	65.02894	0.435953	0.14726	0.261574	processed_pseudogene
ENSG0000(	SRP14-DT	538.4861	0.10389	0.147278	0.26159	lncRNA
ENSG0000(	EP400P1	1986.962	0.098484	0.147299	0.261606	transcribed_unprocessed_pseudo
ENSG0000(	POLR2M	767.5839	-0.07911	0.147303	0.261606	protein_coding
ENSG0000(	ENSG0000(	189.435	0.084272	0.147317	0.261615	lncRNA
ENSG0000(	PPIAP46	440.1738	0.094254	0.147401	0.26175	transcribed_processed_pseudoge
ENSG0000(	MIR200A	22.13239	-0.34173	0.147432	0.261789	miRNA
ENSG0000(	FAM171A2	16.13991	0.393318	0.147461	0.261812	protein_coding
ENSG0000(	ORAI1	399.9855	-0.08341	0.147454	0.261812	protein_coding
ENSG0000(	ENSG0000(	9.901383	-0.5078	0.147523	0.261907	lncRNA
ENSG0000(	ESPL1	606.6586	-0.12602	0.147709	0.262222	protein_coding
ENSG0000(	ENSG0000(	252.1569	0.075291	0.147727	0.262239	lncRNA
ENSG0000(	IGF1R	2700.892	0.115004	0.147806	0.262365	protein_coding
ENSG0000(	PPIAP50	11.48083	0.374471	0.14783	0.262392	processed_pseudogene
ENSG0000(	H4C11	144.7451	-0.45881	0.147871	0.262451	protein_coding
ENSG0000(	ENSG0000(	58.6126	0.178169	0.147948	0.262557	processed_pseudogene
ENSG0000(	ENSG0000(	67.69001	0.120058	0.147942	0.262557	lncRNA
ENSG0000(	RN7SL322P	25.6904	0.234679	0.148034	0.262694	misc_RNA
ENSG0000(	USP34	10445.59	0.051267	0.148074	0.26275	protein_coding

ENSG0000(	RDH10	780.3953	-0.12367	0.148117	0.262813	protein_coding
ENSG0000(	CCR9	25.76764	-0.43091	0.148141	0.26284	protein_coding
ENSG0000(	ENSG0000(	205.1604	0.146613	0.148166	0.262869	lncRNA
ENSG0000(	SNORA63D	32.06963	0.242879	0.148223	0.262955	snoRNA
ENSG0000(	ENSG0000(	348.0473	0.078785	0.148243	0.262976	lncRNA
ENSG0000(	BEND7	801.7297	-0.11454	0.148369	0.263184	protein_coding
ENSG0000(	ENSG0000(	50.44793	-0.16811	0.148398	0.263222	lncRNA
ENSG0000(	FAHD2CP	133.4444	0.161241	0.148437	0.263276	transcribed_unprocessed_pseudo
ENSG0000(	TAS2R41	10.3929	0.440186	0.148522	0.263411	protein_coding
ENSG0000(	CARD11	481.9188	0.299804	0.148617	0.263555	protein_coding
ENSG0000(	FAM184A	95.81438	0.263784	0.14862	0.263555	protein_coding
ENSG0000(	ANKRD46	504.0128	-0.06976	0.14865	0.263594	protein_coding
ENSG0000(	MAP4	5043.745	-0.06588	0.148679	0.263627	protein_coding
ENSG0000(	SNX19	5639.107	0.096048	0.148686	0.263627	protein_coding
ENSG0000(	PRPS2	424.0299	-0.15202	0.148703	0.263643	protein_coding
ENSG0000(	TM7SF2	200.9609	-0.1664	0.148724	0.263664	protein_coding
ENSG0000(	EGLN3	1419.375	0.272522	0.148745	0.263688	protein_coding
ENSG0000(	ST6GALNA(	333.3019	-0.19581	0.148763	0.263703	protein_coding
ENSG0000(	RAB2A	1198.296	-0.07658	0.148812	0.263776	protein_coding
ENSG0000(	CBS	99.95583	0.316673	0.148849	0.263827	protein_coding
ENSG0000(	HRCT1	173.615	-0.39199	0.148897	0.263896	protein_coding
ENSG0000(	SRRM2	52867.91	0.072756	0.148961	0.263994	protein_coding
ENSG0000(	ATP6V0E1	741.397	-0.14603	0.148969	0.263995	protein_coding
ENSG0000(	MED6	718.1453	0.064605	0.149053	0.264128	protein_coding
ENSG0000(	C16orf46	12.48537	-0.40798	0.149129	0.264249	protein_coding
ENSG0000(	ENSG0000(	134.0354	0.097813	0.149208	0.264373	lncRNA
ENSG0000(	LINC01570	55.29371	0.168539	0.149229	0.264396	lncRNA
ENSG0000(	TM2D3	1337.775	0.057405	0.149257	0.264429	protein_coding
ENSG0000(	ENSG0000(	37.62583	-0.19086	0.149355	0.264588	protein_coding
ENSG0000(	HCG9	61.98264	0.211602	0.149364	0.26459	lncRNA
ENSG0000(	ENSG0000(	176.9162	0.132164	0.149423	0.264679	lncRNA
ENSG0000(	AJAP1	182.4303	0.307061	0.149485	0.264761	protein_coding
ENSG0000(	LINC01030	9.59803	0.363755	0.149493	0.264761	lncRNA
ENSG0000(	MRPS10P2	32.85133	0.219052	0.149495	0.264761	processed_pseudogene
ENSG0000(	CFAP53	16.91845	-0.35031	0.149509	0.264771	protein_coding
ENSG0000(	SNORA80E	162.0215	0.157411	0.149584	0.26489	snoRNA
ENSG0000(	ENSG0000(	95.54842	-0.17296	0.149613	0.264911	lncRNA
ENSG0000(	TSHZ1	914.3644	-0.11908	0.149613	0.264911	protein_coding
ENSG0000(	TAF5L	804.9982	0.074057	0.149658	0.264975	protein_coding
ENSG0000(	CERNA1	29.84315	0.245847	0.149687	0.265011	lncRNA
ENSG0000(	UCHL1-DT	29.65473	0.191504	0.149704	0.265026	lncRNA
ENSG0000(	ENSG0000(	103.4226	0.124515	0.149771	0.26513	lncRNA
ENSG0000(	ARPC5	3352.106	-0.11972	0.149857	0.265268	protein_coding
ENSG0000(	ENSG0000(	307.7157	-0.08819	0.149885	0.265302	lncRNA
ENSG0000(	BRWD1-AS	40.67029	0.187821	0.149944	0.265392	lncRNA
ENSG0000(	NMNAT2	59.25621	0.232458	0.150046	0.265557	protein_coding
ENSG0000(	RAB23	272.0249	0.132337	0.15012	0.265673	protein_coding

ENSG0000(	ENSG0000(	35.44442	0.202807	0.150145	0.265702	lncRNA
ENSG0000(	FLVCR1-DT	512.0776	-0.07639	0.150225	0.265829	lncRNA
ENSG0000(	BCL2L14	1097.212	-0.15256	0.150247	0.265842	protein_coding
ENSG0000(	ENSG0000(	96.24072	0.138657	0.150255	0.265842	lncRNA
ENSG0000(	ENSG0000(	18.25526	0.313722	0.150258	0.265842	lncRNA
ENSG0000(	MST1	319.3422	-0.20514	0.150274	0.265854	protein_coding
ENSG0000(	NUDT5	923.2335	-0.07919	0.150295	0.265862	protein_coding
ENSG0000(	VRK1	17809.92	0.054458	0.150291	0.265862	protein_coding
ENSG0000(	ENSG0000(	260.3811	0.093284	0.150312	0.265877	lncRNA
ENSG0000(	CCDC43	356.8556	-0.0891	0.15036	0.265933	protein_coding
ENSG0000(	RPL7L1P2	58.3589	-0.27318	0.150357	0.265933	processed_pseudogene
ENSG0000(	GABBR2	66.13491	0.372054	0.150417	0.266002	protein_coding
ENSG0000(	LINC02446	68.07676	-0.35182	0.150413	0.266002	lncRNA
ENSG0000(	ENSG0000(	10.36404	-0.36693	0.150463	0.266038	processed_pseudogene
ENSG0000(	ING3	405.9519	-0.09588	0.150451	0.266038	protein_coding
ENSG0000(	SEMA3F-AS	60.32903	0.149505	0.150459	0.266038	lncRNA
ENSG0000(	ENSG0000(	12.84524	-0.28191	0.15057	0.266212	lncRNA
ENSG0000(	ENSG0000(	252.488	-0.12683	0.150586	0.266226	lncRNA
ENSG0000(	PPA1	1362.817	0.104002	0.150631	0.266291	protein_coding
ENSG0000(	NCAPD3	1624.848	-0.08587	0.150797	0.266568	protein_coding
ENSG0000(	ENSG0000(	115.2299	-0.13816	0.150844	0.266637	lncRNA
ENSG0000(	ART4	193.0929	-0.12448	0.150861	0.266652	protein_coding
ENSG0000(	ENSG0000(	77.12595	0.154323	0.150869	0.266652	lncRNA
ENSG0000(	HOGA1	199.4951	0.115975	0.15091	0.266709	protein_coding
ENSG0000(	OR2G6	1257.218	0.056933	0.150922	0.266715	protein_coding
ENSG0000(	RFPL1S	511.5263	0.085454	0.150943	0.266737	lncRNA
ENSG0000(	ENSG0000(	116.4534	-0.25204	0.15097	0.266754	lncRNA
ENSG0000(	RBFOX3	364.9487	0.094575	0.15097	0.266754	protein_coding
ENSG0000(	ABCB4	207.6662	0.319921	0.151003	0.266798	protein_coding
ENSG0000(	ENSG0000(	36.33438	-0.22618	0.151018	0.266808	lncRNA
ENSG0000(	ENSG0000(	46.39958	-0.20764	0.15105	0.266836	lncRNA
ENSG0000(	ENSG0000(	300.5326	-0.10732	0.15105	0.266836	lncRNA
ENSG0000(	RN7SL144F	64.64321	0.180396	0.151087	0.266886	misc_RNA
ENSG0000(	ENSG0000(	612.2506	-0.07203	0.151163	0.267005	lncRNA
ENSG0000(	TRIM8	1419.993	0.100316	0.151287	0.267209	protein_coding
ENSG0000(	MTSS2	599.3126	-0.13584	0.151333	0.267275	protein_coding
ENSG0000(	ENSG0000(	70.23838	0.159082	0.151399	0.267377	lncRNA
ENSG0000(	ENSG0000(	97.97546	0.149252	0.151429	0.267415	TEC
ENSG0000(	ENSG0000(	272.0039	-0.07884	0.151457	0.267449	lncRNA
ENSG0000(	ENSG0000(	126.3726	0.147384	0.151548	0.267594	lncRNA
ENSG0000(	PPIEL	964.3362	0.072207	0.151562	0.267605	transcribed_unprocessed_pseudo
ENSG0000(	IRF7	449.0576	0.209169	0.151589	0.267631	protein_coding
ENSG0000(	OTUD7A	699.1856	-0.14344	0.151594	0.267631	protein_coding
ENSG0000(	ENSG0000(	97.80591	0.778535	0.151622	0.267665	lncRNA
ENSG0000(	ZSCAN22	481.1447	-0.06712	0.151758	0.267889	protein_coding
ENSG0000(	C8orf58	173.3306	0.117649	0.151788	0.267904	protein_coding
ENSG0000(	ENSG0000(	227.8702	0.078631	0.151792	0.267904	lncRNA

ENSG0000(	MIR571	69.85362	-0.16241	0.151792	0.267904	miRNA
ENSG0000(	ENSG0000(	95.948	-0.18885	0.15181	0.267921	lncRNA
ENSG0000(	TM4SF1-AS	13.582	-0.44451	0.151826	0.267934	lncRNA
ENSG0000(	ARLNC1	656.3762	-0.07579	0.151933	0.268107	lncRNA
ENSG0000(	CSNK1G2	1033.604	0.078919	0.15204	0.268281	protein_coding
ENSG0000(	EP400	4163.864	0.048437	0.152057	0.268281	protein_coding
ENSG0000(	RPL7AP60	63.90305	-0.17841	0.152057	0.268281	processed_pseudogene
ENSG0000(	ENSG0000(	23.17895	-0.27734	0.152141	0.2684	lncRNA
ENSG0000(	NAIPP2	418.6742	0.267856	0.152142	0.2684	unprocessed_pseudogene
ENSG0000(	ZNF503-AS	179.4185	0.284646	0.15215	0.2684	lncRNA
ENSG0000(	WDTC1	1390.218	-0.06976	0.152198	0.268469	protein_coding
ENSG0000(	NUP210L	25.72277	0.224417	0.152264	0.26857	protein_coding
ENSG0000(	SNORA50A	100.3496	-0.13012	0.152281	0.268585	snoRNA
ENSG0000(	ENSG0000(	867.9825	-0.08619	0.152298	0.268589	lncRNA
ENSG0000(	LINC02340	88.43919	0.122527	0.152309	0.268589	lncRNA
ENSG0000(	TMLHE-AS1	47.65752	0.310529	0.152301	0.268589	lncRNA
ENSG0000(	ENSG0000(	86.17848	0.140761	0.152346	0.26864	lncRNA
ENSG0000(	RGS20	15.10205	0.280884	0.152374	0.268673	protein_coding
ENSG0000(	ENSG0000(	25.79609	-0.21167	0.152406	0.268714	lncRNA
ENSG0000(	OSGEPL1-A	198.8366	-0.07741	0.152482	0.268834	lncRNA
ENSG0000(	IGSF1	57.23821	-0.29989	0.152536	0.268902	protein_coding
ENSG0000(	XPO7	1753.237	-0.05719	0.152538	0.268902	protein_coding
ENSG0000(	GSE1	4299.645	-0.07424	0.152627	0.269044	protein_coding
ENSG0000(	RNU6-10P	11.04741	0.380401	0.152668	0.269101	snRNA
ENSG0000(	ADGRG1	1143.119	-0.11321	0.152703	0.269133	protein_coding
ENSG0000(	ZNF114	170.751	-0.117	0.1527	0.269133	protein_coding
ENSG0000(	ENSG0000(	208.6743	-0.19755	0.152724	0.269153	lncRNA
ENSG0000(	TRANK1	7864.077	0.167384	0.152753	0.269183	protein_coding
ENSG0000(	ZNF236	1424.476	-0.10857	0.152758	0.269183	protein_coding
ENSG0000(	DDX60L	3871.748	0.182508	0.152801	0.269244	protein_coding
ENSG0000(	SLC35E4	319.8728	-0.11041	0.15281	0.269244	protein_coding
ENSG0000(	CXADR	1841.669	-0.20551	0.152938	0.269455	protein_coding
ENSG0000(	OR8H1	135.5053	-0.12907	0.152976	0.269506	protein_coding
ENSG0000(	NBPF9	11424.18	-0.08745	0.152991	0.269518	protein_coding
ENSG0000(	CCDC8	276.446	0.11161	0.153027	0.269566	protein_coding
ENSG0000(	MRPL20-DT	362.3005	0.077867	0.153088	0.269658	lncRNA
ENSG0000(	ENSG0000(	129.7686	0.123942	0.153189	0.269822	TEC
ENSG0000(	ENSG0000(	114.4893	0.0941	0.153327	0.27005	lncRNA
ENSG0000(	HCG24	66.39721	0.136535	0.15334	0.270056	lncRNA
ENSG0000(	LINC01672	520.7803	0.084012	0.153385	0.270122	lncRNA
ENSG0000(	RN7SKP217	31.59282	-0.28764	0.153426	0.270176	misc_RNA
ENSG0000(	UBE2D3-AS	45.0653	-0.22767	0.153434	0.270176	lncRNA
ENSG0000(	ENSG0000(	146.7176	0.124959	0.153559	0.270381	lncRNA
ENSG0000(	LRRN2	403.5561	-0.3593	0.153572	0.27039	protein_coding
ENSG0000(	AFMID	1301.229	0.064856	0.153624	0.27046	protein_coding
ENSG0000(	RPS19P3	84.27183	0.133355	0.15363	0.27046	processed_pseudogene
ENSG0000(	XKR4	981.2443	-0.08987	0.153721	0.270606	protein_coding

ENSG0000(	ZNF706	2110.698	0.060959	0.153735	0.270616	protein_coding
ENSG0000(	C9orf50	135.4888	-0.12502	0.153748	0.270622	protein_coding
ENSG0000(	IKZF5	601.9372	0.078938	0.153861	0.270806	protein_coding
ENSG0000(	ENSG0000(	556.6491	-0.06239	0.15394	0.270931	lncRNA
ENSG0000(	ENSG0000(	246.5002	-0.08742	0.153965	0.27096	lncRNA
ENSG0000(	KDM6A	9236.91	0.057716	0.154025	0.271034	protein_coding
ENSG0000(	PRKD3	1911.916	0.11001	0.154024	0.271034	protein_coding
ENSG0000(	THBS1-AS1	23.14348	-0.44221	0.154035	0.271037	lncRNA
ENSG0000(	PART1	411.0072	0.065388	0.154055	0.271058	lncRNA
ENSG0000(	CCNB3P1	191.3636	0.09677	0.154076	0.271078	processed_pseudogene
ENSG0000(	WBP11	859.4572	-0.07948	0.154115	0.271132	protein_coding
ENSG0000(	ENSG0000(	136.3806	0.128933	0.154143	0.271157	lncRNA
ENSG0000(	ENSG0000(	136.7249	-0.11112	0.154154	0.271157	lncRNA
ENSG0000(	SLC10A3	203.2877	-0.1141	0.154155	0.271157	protein_coding
ENSG0000(	RRAGAP1-/	83.48886	0.133376	0.154189	0.271201	lncRNA
ENSG0000(	OR2AG2	197.0908	0.119139	0.15421	0.271207	protein_coding
ENSG0000(	TEKT4P2	874.3386	0.287809	0.154209	0.271207	transcribed_unprocessed_pseudo
ENSG0000(	MCTS1	2701.536	-0.06455	0.154239	0.271242	protein_coding
ENSG0000(	ENSG0000(	52.77808	0.186526	0.154259	0.271248	lncRNA
ENSG0000(	ENSG0000(	55.50103	0.137248	0.154256	0.271248	lncRNA
ENSG0000(	CENPC	2145.385	0.078084	0.154312	0.271326	protein_coding
ENSG0000(	ENSG0000(	25.33906	-0.25794	0.154384	0.271429	lncRNA
ENSG0000(	MAP2K5	783.8621	0.089043	0.154388	0.271429	protein_coding
ENSG0000(	ENSG0000(	23.0342	0.484408	0.154409	0.271451	lncRNA
ENSG0000(	MYLK-AS1	500.614	-0.07014	0.154434	0.271478	lncRNA
ENSG0000(	ENSG0000(	407.9568	0.062986	0.154447	0.271487	lncRNA
ENSG0000(	IZUMO4	39.60231	0.309395	0.154482	0.271533	protein_coding
ENSG0000(	ENSG0000(	55.67444	0.180447	0.154509	0.271552	lncRNA
ENSG0000(	NUP205	1620.887	-0.09384	0.15451	0.271552	protein_coding
ENSG0000(	ENSG0000(	265.8	0.115905	0.154562	0.271628	lncRNA
ENSG0000(	ENSG0000(	14.76418	0.317809	0.154611	0.271698	processed_pseudogene
ENSG0000(	DDX43	65.72948	-0.20354	0.154621	0.271701	protein_coding
ENSG0000(	DUSP7	353.9683	0.122088	0.154669	0.271764	protein_coding
ENSG0000(	LINC00484	163.1348	-0.16191	0.154674	0.271764	lncRNA
ENSG0000(	SNRNP40	588.3089	-0.09741	0.1547	0.271794	protein_coding
ENSG0000(	EEF1A1P11	11.92379	-0.31234	0.154833	0.271998	processed_pseudogene
ENSG0000(	MIR3139	20.44227	0.285856	0.154834	0.271998	miRNA
ENSG0000(	ENSG0000(	13.57708	0.258542	0.154845	0.272004	lncRNA
ENSG0000(	CTPS2	1004.474	-0.08655	0.154881	0.272042	protein_coding
ENSG0000(	ENSG0000(	65.40532	0.154232	0.154885	0.272042	lncRNA
ENSG0000(	ENSG0000(	246.9465	0.073077	0.154954	0.272149	protein_coding
ENSG0000(	ENSG0000(	1945.858	0.080764	0.154974	0.272156	TEC
ENSG0000(	RN7SL538F	42.23641	0.174382	0.154976	0.272156	misc_RNA
ENSG0000(	EXOSC3P1	39.57796	0.183324	0.155011	0.272197	processed_pseudogene
ENSG0000(	MIR9-3HG	198.6682	0.182498	0.155016	0.272197	lncRNA
ENSG0000(	LAMA4-AS:	35.31958	0.213415	0.155038	0.27221	lncRNA
ENSG0000(	PAPSS2	3362.955	-0.19437	0.155041	0.27221	protein_coding

ENSG0000(	MRPL3	1478.48	0.06259	0.155091	0.272282	protein_coding
ENSG0000(	ABCF1	1514.095	-0.05791	0.155119	0.272305	protein_coding
ENSG0000(	DLGAP1-AS	716.8651	-0.07272	0.155129	0.272305	lncRNA
ENSG0000(	ENSG0000(	18.93163	0.205352	0.15513	0.272305	lncRNA
ENSG0000(	ENSG0000(	126.4165	0.115138	0.155247	0.272494	lncRNA
ENSG0000(	ENSG0000(	370.773	-0.09578	0.155295	0.272562	lncRNA
ENSG0000(	PPFIBP2	1883.36	-0.08793	0.155317	0.272562	protein_coding
ENSG0000(	RANBP17	753.1071	0.118888	0.155315	0.272562	protein_coding
ENSG0000(	SLC12A4	1040.394	0.126451	0.15532	0.272562	protein_coding
ENSG0000(	OR10G4	356.5942	-0.10555	0.15533	0.272564	protein_coding
ENSG0000(	ANKRD20A	777.2056	0.188227	0.155369	0.272616	transcribed_unprocessed_pseudo
ENSG0000(	LINC02775	35.67591	-0.1995	0.15538	0.272621	lncRNA
ENSG0000(	LINC01483	233.5251	0.097101	0.155398	0.272637	lncRNA
ENSG0000(	ENSG0000(	52.09859	0.190828	0.15545	0.272713	lncRNA
ENSG0000(	ENSG0000(	24.25094	0.234144	0.155535	0.272848	processed_pseudogene
ENSG0000(	ENSG0000(	100.5595	0.143263	0.155556	0.272869	lncRNA
ENSG0000(	LINC01555	33.47619	0.175299	0.15557	0.272877	lncRNA
ENSG0000(	PPP1R21	887.5623	-0.07907	0.155578	0.272877	protein_coding
ENSG0000(	TMEM187	59.59308	-0.23105	0.155602	0.272888	protein_coding
ENSG0000(	UBE2H	2312.012	0.077674	0.155601	0.272888	protein_coding
ENSG0000(	ENSG0000(	37.27681	0.169898	0.15562	0.272891	lncRNA
ENSG0000(	FEZF2	34.58149	0.65172	0.155621	0.272891	protein_coding
ENSG0000(	NOP10	257.1713	-0.16994	0.155654	0.272934	protein_coding
ENSG0000(	IL18BP	813.1437	0.09362	0.155673	0.272936	protein_coding
ENSG0000(	MKNK1	2756.388	0.05324	0.155664	0.272936	protein_coding
ENSG0000(	DNAJC24	911.0561	0.071632	0.155701	0.272971	protein_coding
ENSG0000(	ADAM20P1	130.1514	0.151217	0.155811	0.273148	transcribed_unprocessed_pseudo
ENSG0000(	ENSG0000(	14.23234	0.309825	0.15582	0.273148	snoRNA
ENSG0000(	ENSG0000(	48.53168	-0.19593	0.155851	0.273188	lncRNA
ENSG0000(	ENSG0000(	92.70239	-0.14179	0.155893	0.27323	lncRNA
ENSG0000(	RBM28	3675.689	-0.08166	0.15589	0.27323	protein_coding
ENSG0000(	MYOM2	46.5807	-0.324	0.155923	0.273267	protein_coding
ENSG0000(	SLC39A10	800.4028	0.132112	0.155973	0.27334	protein_coding
ENSG0000(	ENSG0000(	21.23005	0.258491	0.156029	0.273423	lncRNA
ENSG0000(	ENSG0000(	50.76215	0.149712	0.156048	0.273441	lncRNA
ENSG0000(	ENSG0000(	71.41148	-0.19658	0.15617	0.273639	lncRNA
ENSG0000(	KBTBD11-C	131.4773	-0.27038	0.156183	0.273646	protein_coding
ENSG0000(	LINC00467	1360.534	0.057031	0.156284	0.273808	lncRNA
ENSG0000(	ENSG0000(	141.1818	-0.09598	0.156366	0.273936	lncRNA
ENSG0000(	CACNA1B	53.39645	0.239492	0.156394	0.273969	protein_coding
ENSG0000(	ENSG0000(	19.8822	0.250317	0.156516	0.274168	processed_pseudogene
ENSG0000(	LINC00680	556.4668	-0.16242	0.156552	0.274201	transcribed_unprocessed_pseudo
ENSG0000(	NR1I3	125.9604	-0.1028	0.156561	0.274201	protein_coding
ENSG0000(	ZNF256	278.9399	0.117993	0.15656	0.274201	protein_coding
ENSG0000(	ENSG0000(	42.39069	-0.17593	0.156592	0.27424	lncRNA
ENSG0000(	POLH	2303.115	-0.06256	0.156627	0.274271	protein_coding
ENSG0000(	SEMA4C	1286.176	-0.07389	0.156624	0.274271	protein_coding

ENSG0000(	PPHLN1	3194.75	0.057472	0.15673	0.274435	protein_coding
ENSG0000(	OR2M3	3358.958	0.082266	0.156764	0.27448	protein_coding
ENSG0000(	GAPDHP62	38.0089	-0.24496	0.156877	0.274662	processed_pseudogene
ENSG0000(	RN7SKP9	1379.82	0.195962	0.156891	0.274671	misc_RNA
ENSG0000(	FOXO1	2125.405	0.136176	0.156961	0.274735	protein_coding
ENSG0000(	PLK2	554.0701	-0.18505	0.15696	0.274735	protein_coding
ENSG0000(	SIRT1	679.4207	-0.10729	0.156962	0.274735	protein_coding
ENSG0000(	SORCS3	15.9084	0.519522	0.156961	0.274735	protein_coding
ENSG0000(	ATP6V1FNI	33.76365	0.200455	0.157105	0.274969	protein_coding
ENSG0000(	ENSG0000(	99.12086	0.745712	0.157121	0.274981	protein_coding
ENSG0000(	AP5B1	1563.001	0.0886	0.157194	0.275095	protein_coding
ENSG0000(	ARHGAP28	1395.633	0.10918	0.157206	0.275099	protein_coding
ENSG0000(	ENSG0000(	136.3674	0.176955	0.157249	0.275159	lncRNA
ENSG0000(	ENSG0000(	379.7554	0.068375	0.157267	0.275176	lncRNA
ENSG0000(	ENSG0000(	179.2658	0.107372	0.157315	0.275245	TEC
ENSG0000(	TDRD10	66.57558	-0.21093	0.157344	0.275279	protein_coding
ENSG0000(	USP8	7020.994	-0.05796	0.157354	0.275281	protein_coding
ENSG0000(	ENSG0000(	3123.161	0.070405	0.157369	0.275293	lncRNA
ENSG0000(	BMP3	642.8873	-0.31485	0.157435	0.275393	protein_coding
ENSG0000(	DOCK9-DT	19.20485	0.240459	0.157453	0.275408	lncRNA
ENSG0000(	ENSG0000(	18.77557	0.296203	0.157506	0.275485	lncRNA
ENSG0000(	IGLL5	1496.928	0.281314	0.157548	0.275543	protein_coding
ENSG0000(	MAML2	2766.315	-0.07576	0.157591	0.275603	protein_coding
ENSG0000(	ENSG0000(	49.82655	0.156459	0.157613	0.275621	processed_pseudogene
ENSG0000(	ZNF701	1402.98	-0.09564	0.157619	0.275621	protein_coding
ENSG0000(	WIPI2	1195.896	0.057681	0.157676	0.275705	protein_coding
ENSG0000(	ENSG0000(	46.6432	0.184613	0.157697	0.275727	miRNA
ENSG0000(	MIR1267	33.27716	0.21657	0.15772	0.275752	miRNA
ENSG0000(	ENSG0000(	127.7232	0.143654	0.15777	0.275824	lncRNA
ENSG0000(	BEND4	192.7677	0.262298	0.15785	0.275949	protein_coding
ENSG0000(	CHMP1B-A	12.11243	0.571248	0.1579	0.276005	lncRNA
ENSG0000(	EEF1A1P3	271.2771	-0.11346	0.157896	0.276005	processed_pseudogene
ENSG0000(	ENSG0000(	43.33072	0.209168	0.157936	0.276012	lncRNA
ENSG0000(	ENSG0000(	18.5033	0.232923	0.157932	0.276012	lncRNA
ENSG0000(	TMEM262	11.16944	0.478993	0.157939	0.276012	protein_coding
ENSG0000(	VPS54	1661.182	0.039734	0.157916	0.276012	protein_coding
ENSG0000(	ZBED3	353.8159	-0.19746	0.15803	0.276155	protein_coding
ENSG0000(	ENSG0000(	42.97857	0.152337	0.158348	0.27664	lncRNA
ENSG0000(	ENSG0000(	81.49043	-0.33123	0.158346	0.27664	lncRNA
ENSG0000(	LINC01978	219.2906	-0.08242	0.158326	0.27664	lncRNA
ENSG0000(	RN7SKP185	20.45106	0.238124	0.158352	0.27664	misc_RNA
ENSG0000(	TMEM14C	414.843	-0.13897	0.158352	0.27664	protein_coding
ENSG0000(	ENSG0000(	37.11406	0.193056	0.158391	0.276694	lncRNA
ENSG0000(	PRKCE	1280.653	0.088663	0.158446	0.276773	protein_coding
ENSG0000(	RPL31P52	11.81154	0.304279	0.158495	0.276845	processed_pseudogene
ENSG0000(	ENSG0000(	458.5604	-0.12507	0.158518	0.276868	lncRNA
ENSG0000(	ENSG0000(	203.297	0.182381	0.158652	0.277087	lncRNA

ENSG0000(	ENSG0000(	76.81053	-0.24514	0.158681	0.277122	processed_pseudogene
ENSG0000(	RAD17	750.596	-0.09374	0.158719	0.277173	protein_coding
ENSG0000(	ARG1	13.48032	0.300023	0.158745	0.277203	protein_coding
ENSG0000(	ARHGEF18-	45.80735	0.263214	0.158889	0.277434	lncRNA
ENSG0000(	NEDD4L	8959.62	-0.16394	0.158895	0.277434	protein_coding
ENSG0000(	CCDC144A	2982.77	0.163664	0.158959	0.277515	protein_coding
ENSG0000(	SCARNA23	10.25993	0.370467	0.158954	0.277515	scaRNA
ENSG0000(	ENSG0000(	306.1943	0.096853	0.158972	0.277522	lncRNA
ENSG0000(	ENSG0000(	136.3833	-0.15444	0.158998	0.277552	lncRNA
ENSG0000(	ENSG0000(	49.05558	-0.15332	0.159029	0.277591	lncRNA
ENSG0000(	ELP1	2978.462	0.035234	0.159066	0.277639	protein_coding
ENSG0000(	ECT2L	61.54097	-0.22183	0.159101	0.277685	protein_coding
ENSG0000(	LRRC37A6F	31.43202	-0.31715	0.15912	0.277704	transcribed_processed_pseudoge
ENSG0000(	LINC01572	607.7209	-0.10824	0.159169	0.277772	lncRNA
ENSG0000(	LINC01132	19.21168	0.301549	0.159212	0.277832	lncRNA
ENSG0000(	ENSG0000(	114.4807	-0.10146	0.159232	0.277852	lncRNA
ENSG0000(	TBPL2	27.07378	-0.28035	0.15925	0.277867	protein_coding
ENSG0000(	ZNF879	220.9089	-0.12294	0.159278	0.277901	protein_coding
ENSG0000(	ZNF766	1894.767	-0.06407	0.15932	0.27796	protein_coding
ENSG0000(	ENSG0000(	211.7783	-0.06991	0.159332	0.277964	transcribed_unprocessed_pseudo
ENSG0000(	CYP2W1	38.58819	-0.24596	0.159358	0.277994	protein_coding
ENSG0000(	HOMER2	468.4002	0.111741	0.159385	0.278026	protein_coding
ENSG0000(	SEPTIN14P	11.0997	-0.39143	0.159411	0.278055	processed_pseudogene
ENSG0000(	GRM4	117.4646	0.136552	0.159421	0.278058	protein_coding
ENSG0000(	ENSG0000(	35.87162	0.225696	0.159459	0.278109	lncRNA
ENSG0000(	ENSG0000(	740.1315	0.05658	0.159479	0.278128	lncRNA
ENSG0000(	C10orf88	267.4237	-0.13753	0.159503	0.278154	protein_coding
ENSG0000(	RN7SL397F	32.63944	0.188106	0.159512	0.278154	misc_RNA
ENSG0000(	E2F3	749.8425	0.081984	0.159533	0.278175	protein_coding
ENSG0000(	ZNF784	125.7631	-0.13324	0.159548	0.278186	protein_coding
ENSG0000(	ENSG0000(	99.56877	-0.22039	0.15962	0.278296	TEC
ENSG0000(	SEC31B	1207.679	-0.10734	0.159692	0.278406	protein_coding
ENSG0000(	RASSF9	199.1851	-0.15292	0.159705	0.278413	protein_coding
ENSG0000(	CATSPERG	736.4974	-0.11576	0.15983	0.278616	protein_coding
ENSG0000(	DICER1	5220.507	0.050891	0.15994	0.278777	protein_coding
ENSG0000(	SYBU	435.2288	-0.22124	0.159939	0.278777	protein_coding
ENSG0000(	ENSG0000(	26.28864	0.216251	0.16003	0.278917	lncRNA
ENSG0000(	CABP4	880.4836	0.088392	0.160075	0.278934	protein_coding
ENSG0000(	ENSG0000(	75.07638	-0.15435	0.160071	0.278934	transcribed_unprocessed_pseudo
ENSG0000(	RAP1GDS1	888.1724	-0.07038	0.160071	0.278934	protein_coding
ENSG0000(	RELCH	3893.692	-0.07477	0.160053	0.278934	protein_coding
ENSG0000(	MEIS3P2	26.17716	-0.3237	0.160089	0.278942	processed_pseudogene
ENSG0000(	FAM135A	3865.803	-0.10434	0.160133	0.279003	protein_coding
ENSG0000(	ABCC13	1035.171	0.258244	0.16015	0.279018	transcribed_unprocessed_pseudo
ENSG0000(	LINC02428	239.6119	-0.09142	0.16018	0.279054	lncRNA
ENSG0000(	TTC33	518.0728	-0.07194	0.16024	0.279144	protein_coding
ENSG0000(	CCDC102A	49.64279	0.233433	0.160282	0.279201	protein_coding

ENSG0000(	HSPE1P21	11.5048	0.34702	0.160353	0.279309	processed_pseudogene
ENSG0000(	ENSG0000(	239.1883	-0.11731	0.160368	0.27932	lncRNA
ENSG0000(	ENSG0000(	32.99984	0.451781	0.160406	0.27937	lncRNA
ENSG0000(	ENSG0000(	92.65692	-0.11906	0.160438	0.279411	lncRNA
ENSG0000(	PAX8	152.4293	-0.35037	0.160482	0.27947	protein_coding
ENSG0000(	RAB11FIP2	803.4312	0.095537	0.16049	0.27947	protein_coding
ENSG0000(	CERT1	2571.98	-0.06493	0.160546	0.279552	protein_coding
ENSG0000(	ASB1	1090.825	-0.10324	0.160656	0.279729	protein_coding
ENSG0000(	LINC02642	313.0349	0.074564	0.160686	0.279765	lncRNA
ENSG0000(	ENSG0000(	131.8263	0.102021	0.160736	0.279837	lncRNA
ENSG0000(	TMEM255/	47.73815	0.334347	0.160764	0.279854	protein_coding
ENSG0000(	ZG16B	158.0914	0.280094	0.160756	0.279854	protein_coding
ENSG0000(	LMNTD2	100.9272	-0.16314	0.160782	0.279869	protein_coding
ENSG0000(	ENSG0000(	9.826283	0.354858	0.160809	0.279902	lncRNA
ENSG0000(	AREG	248.7036	0.348349	0.160855	0.279965	protein_coding
ENSG0000(	C18orf21	126.5248	-0.12814	0.160905	0.280037	protein_coding
ENSG0000(	ENSG0000(	24.19832	-0.21513	0.160945	0.28006	lncRNA
ENSG0000(	ENSG0000(	328.3314	0.224801	0.160945	0.28006	protein_coding
ENSG0000(	SNX19P2	15.72108	-0.3471	0.160938	0.28006	processed_pseudogene
ENSG0000(	KCNA7	493.7718	0.065889	0.160997	0.280134	protein_coding
ENSG0000(	TRGV5	8.067832	-0.43172	0.161035	0.280185	TR_V_gene
ENSG0000(	ENSG0000(	258.2607	0.095744	0.16109	0.280265	lncRNA
ENSG0000(	BTBD11	189.9817	0.156744	0.161163	0.280362	protein_coding
ENSG0000(	CRNKL1	629.3803	-0.10057	0.161163	0.280362	protein_coding
ENSG0000(	CDHR1	2553.805	-0.33258	0.161201	0.280371	protein_coding
ENSG0000(	FLNC	289.4861	-0.32574	0.161196	0.280371	protein_coding
ENSG0000(	PCDHGB8P	262.6388	-0.08003	0.161197	0.280371	transcribed_unitary_pseudogene
ENSG0000(	PDCD2	1257.57	-0.0648	0.161205	0.280371	protein_coding
ENSG0000(	OPHN1	7701.482	-0.08969	0.161401	0.280697	protein_coding
ENSG0000(	NANOGP5	94.26724	-0.16059	0.161458	0.28078	transcribed_processed_pseudoge
ENSG0000(	HTR3B	764.0867	0.054775	0.161579	0.280976	protein_coding
ENSG0000(	ENSG0000(	16.60159	0.294433	0.161596	0.28099	processed_pseudogene
ENSG0000(	CAPZA2	2109.717	0.119472	0.161617	0.28101	protein_coding
ENSG0000(	ENSG0000(	17.50169	0.552279	0.161635	0.281027	lncRNA
ENSG0000(	FAM245A	111.1468	0.107388	0.161702	0.281111	lncRNA
ENSG0000(	PHBP2	73.44737	-0.25883	0.161697	0.281111	processed_pseudogene
ENSG0000(	NKG7	43.15494	0.291066	0.161725	0.281136	protein_coding
ENSG0000(	ENSG0000(	10.61397	-0.30118	0.161752	0.281166	lncRNA
ENSG0000(	THAP5	1157.559	-0.06566	0.161762	0.281168	protein_coding
ENSG0000(	IGLV3-9	60.8026	0.507024	0.161783	0.28119	IG_V_gene
ENSG0000(	BOLA1	80.03037	-0.16439	0.161795	0.281194	protein_coding
ENSG0000(	IGF2BP2	1712.781	-0.1897	0.161828	0.281237	protein_coding
ENSG0000(	LINC02338	248.0918	0.08971	0.161877	0.281306	lncRNA
ENSG0000(	IQCJ-SCHIP	51.11746	0.239126	0.161888	0.281309	protein_coding
ENSG0000(	GOLGA6L7	168.8086	0.130824	0.161933	0.281371	protein_coding
ENSG0000(	SPTB	262.8283	0.091188	0.161954	0.281392	protein_coding
ENSG0000(	ENSG0000(	458.9551	-0.12409	0.161979	0.281403	lncRNA

ENSG0000(	LIN28B	14.67021	0.292088	0.161987	0.281403	protein_coding
ENSG0000(	RN7SL776F	13.93829	0.261666	0.161971	0.281403	misc_RNA
ENSG0000(	ENSG0000(	71.86517	0.486909	0.162099	0.281567	lncRNA
ENSG0000(	RNU6-611F	39.163	0.168315	0.162094	0.281567	snRNA
ENSG0000(	DMKN	263.3769	0.144528	0.162109	0.281567	protein_coding
ENSG0000(	TAF1A-AS1	41.03396	-0.15976	0.162117	0.281567	lncRNA
ENSG0000(	DEFA6	98.9588	1.892137	0.162131	0.281569	protein_coding
ENSG0000(	FCRL4	86.71508	0.471311	0.162136	0.281569	protein_coding
ENSG0000(	SKIV2L	1138.83	-0.08633	0.162177	0.281623	protein_coding
ENSG0000(	CADPS2	757.1053	0.117042	0.162288	0.281798	protein_coding
ENSG0000(	ENSG0000(	46.17093	0.238722	0.162295	0.281798	lncRNA
ENSG0000(	LINC02811	26.45026	-0.21964	0.16236	0.281895	lncRNA
ENSG0000(	HTR1D	111.8139	-0.1395	0.162385	0.281921	protein_coding
ENSG0000(	MYH3	454.7078	-0.08643	0.162393	0.281921	protein_coding
ENSG0000(	ZNF677	859.7231	-0.05813	0.16241	0.281935	protein_coding
ENSG0000(	ENSG0000(	291.1081	0.076401	0.162438	0.281948	lncRNA
ENSG0000(	MAST1	313.5075	0.089619	0.162442	0.281948	protein_coding
ENSG0000(	PI4KA	3665.233	-0.0627	0.162445	0.281948	protein_coding
ENSG0000(	AMIGO1	174.4371	-0.18523	0.162513	0.282051	protein_coding
ENSG0000(	CMTM6	1547.579	0.129037	0.162587	0.282163	protein_coding
ENSG0000(	LINC01198	450.6458	0.086012	0.162619	0.282202	lncRNA
ENSG0000(	ENSG0000(	10.48476	0.377998	0.162642	0.282212	lncRNA
ENSG0000(	RN7SL711F	49.60225	0.141157	0.162641	0.282212	misc_RNA
ENSG0000(	FSCN2	42.96708	-0.22699	0.16275	0.282383	protein_coding
ENSG0000(	ENSG0000(	272.8757	-0.2333	0.16277	0.282387	lncRNA
ENSG0000(	YWHAZP4	15.3368	-0.28402	0.162767	0.282387	processed_pseudogene
ENSG0000(	LRRC37A5F	134.0677	-0.08827	0.162808	0.282438	transcribed_unprocessed_pseudo
ENSG0000(	ACTG1P14	162.1474	-0.13103	0.162846	0.282487	processed_pseudogene
ENSG0000(	CNNM2	2202.843	-0.15057	0.162856	0.282489	protein_coding
ENSG0000(	FAM110C	931.4465	-0.18072	0.162916	0.282577	protein_coding
ENSG0000(	ENSG0000(	30.38684	-0.19634	0.162957	0.282633	lncRNA
ENSG0000(	TTN	6763.166	0.302153	0.163037	0.282757	protein_coding
ENSG0000(	ENSG0000(	78.70933	0.144218	0.163069	0.282796	lncRNA
ENSG0000(	HERC3	1750.007	0.078071	0.16309	0.282817	protein_coding
ENSG0000(	RIPK4	271.6978	-0.23726	0.163128	0.282866	protein_coding
ENSG0000(	RNF216-IT1	46.64232	0.216092	0.163217	0.282989	lncRNA
ENSG0000(	SAMD15	235.9027	-0.07754	0.163217	0.282989	protein_coding
ENSG0000(	ENSG0000(	61.82001	0.151962	0.163232	0.283001	processed_pseudogene
ENSG0000(	ENSG0000(	14.61035	0.299248	0.163251	0.283018	processed_pseudogene
ENSG0000(	GLT8D1	510.6444	-0.07572	0.163265	0.283026	protein_coding
ENSG0000(	ENSG0000(	27.65343	0.177768	0.163281	0.283031	lncRNA
ENSG0000(	RP9P	209.4006	-0.09998	0.163286	0.283031	transcribed_unprocessed_pseudo
ENSG0000(	ENSG0000(	25.58525	-0.19888	0.163298	0.283036	lncRNA
ENSG0000(	ENSG0000(	38.80104	-0.17299	0.163323	0.283055	lncRNA
ENSG0000(	ENSG0000(	12.42153	0.2449	0.163336	0.283055	lncRNA
ENSG0000(	RSRC1	2284.836	0.046218	0.16333	0.283055	protein_coding
ENSG0000(	NKILA	42.82381	0.268376	0.1634	0.28315	lncRNA

ENSG0000(	ENSG0000(	221.1842	-0.1102	0.163417	0.283163	lncRNA
ENSG0000(	JADE3	285.7966	0.123245	0.163443	0.283194	protein_coding
ENSG0000(	ENSG0000(	1499.127	0.078171	0.163483	0.283242	lncRNA
ENSG0000(	ENSG0000(	267.8046	-0.08361	0.163489	0.283242	TEC
ENSG0000(	CCNO-DT	19.6142	-0.23053	0.163566	0.283357	lncRNA
ENSG0000(	MAN2B2	1437.159	-0.06254	0.163574	0.283357	protein_coding
ENSG0000(	WIZ	940.2133	-0.08292	0.163598	0.283383	protein_coding
ENSG0000(	RNA5SP33	12.53175	0.346304	0.163619	0.283403	rRNA_pseudogene
ENSG0000(	ENSG0000(	345.9596	0.065549	0.163733	0.283586	lncRNA
ENSG0000(	SNORA11E	16.85642	0.380688	0.163853	0.283778	snoRNA
ENSG0000(	ENSG0000(	52.34291	0.148465	0.163963	0.283942	lncRNA
ENSG0000(	ENSG0000(	14.20372	0.288097	0.163966	0.283942	TEC
ENSG0000(	COX6CP18	10.81341	0.343043	0.163976	0.283944	processed_pseudogene
ENSG0000(	ENSG0000(	57.47086	0.187287	0.163992	0.283956	processed_pseudogene
ENSG0000(	ALMS1P1	431.5949	0.097861	0.164017	0.283983	transcribed_unprocessed_pseudo
ENSG0000(	PTK2	3657.3	-0.04202	0.164035	0.283999	protein_coding
ENSG0000(	ANKZF1	1582.707	-0.06174	0.164074	0.284035	protein_coding
ENSG0000(	ARSK	305.6504	-0.09463	0.164068	0.284035	protein_coding
ENSG0000(	ENSG0000(	157.0911	-0.14389	0.164117	0.284094	processed_pseudogene
ENSG0000(	ENSG0000(	102.5893	-0.18114	0.164193	0.284209	lncRNA
ENSG0000(	ENSG0000(	14.16088	0.289512	0.164203	0.28421	lncRNA
ENSG0000(	GNS	2066.998	0.1283	0.164251	0.284262	protein_coding
ENSG0000(	LINC01393	47.96736	0.155708	0.16425	0.284262	lncRNA
ENSG0000(	CD44-AS1	150.6588	0.139336	0.164285	0.28429	lncRNA
ENSG0000(	ENSG0000(	97.00482	0.149073	0.164277	0.28429	TEC
ENSG0000(	IGSF21	15.84001	-0.41232	0.164347	0.284381	protein_coding
ENSG0000(	ENSG0000(	308.7916	-0.06993	0.164372	0.284409	lncRNA
ENSG0000(	OSR2	57.66294	0.349671	0.1644	0.284442	protein_coding
ENSG0000(	ENSG0000(	308.7989	0.083654	0.164427	0.284472	lncRNA
ENSG0000(	CA14	248.8191	-0.09611	0.164482	0.284553	protein_coding
ENSG0000(	ACP1	1141.746	-0.06901	0.164538	0.284629	protein_coding
ENSG0000(	ENSG0000(	23.26866	-0.41496	0.164545	0.284629	lncRNA
ENSG0000(	ZNF569	308.7416	0.069129	0.164698	0.284878	protein_coding
ENSG0000(	ENSG0000(	716.6375	0.181259	0.164734	0.284924	protein_coding
ENSG0000(	CCL22	551.5043	0.164188	0.1648	0.285024	protein_coding
ENSG0000(	ENSG0000(	721.6168	0.069774	0.164849	0.285093	TEC
ENSG0000(	HMG20B	950.2624	-0.07	0.164888	0.285143	protein_coding
ENSG0000(	RN7SL728F	32.46438	0.283906	0.164919	0.285181	misc_RNA
ENSG0000(	CBX5	6157.235	-0.07523	0.164952	0.285222	protein_coding
ENSG0000(	ENSG0000(	22.66977	0.234504	0.164971	0.28524	lncRNA
ENSG0000(	MXD4	1655.268	-0.07706	0.164996	0.285267	protein_coding
ENSG0000(	PCDHA10	510.1937	-0.0838	0.165017	0.285274	protein_coding
ENSG0000(	RD3	155.3515	-0.09849	0.165018	0.285274	protein_coding
ENSG0000(	MIR562	12.60568	0.295345	0.165116	0.285428	miRNA
ENSG0000(	SNORD88A	13.08121	-0.30364	0.165158	0.285484	snoRNA
ENSG0000(	TSPAN12	295.2057	-0.17265	0.165208	0.285556	protein_coding
ENSG0000(	PRDM16	157.876	-0.12963	0.165232	0.28558	protein_coding

ENSG0000(	ENSG0000(	88.33332	-0.13381	0.165273	0.285637	lncRNA
ENSG0000(	ENSG0000(	18.59306	0.25207	0.165307	0.28568	lncRNA
ENSG0000(	RNY1P11	18.35634	0.30824	0.165333	0.285707	misc_RNA
ENSG0000(	LINC01446	417.4262	0.067186	0.165384	0.285781	lncRNA
ENSG0000(	ENSG0000(	540.5788	0.099636	0.165432	0.285848	TEC
ENSG0000(	PINK1	877.3342	-0.1296	0.165498	0.285946	protein_coding
ENSG0000(	HACD1	302.015	-0.07733	0.165515	0.285959	protein_coding
ENSG0000(	ENSG0000(	834.98	-0.0519	0.165558	0.28601	lncRNA
ENSG0000(	ENSG0000(	988.974	0.054764	0.165563	0.28601	TEC
ENSG0000(	ENSG0000(	100.1807	0.122513	0.165591	0.286043	lncRNA
ENSG0000(	ENSG0000(	30.19528	0.260697	0.165677	0.286176	lncRNA
ENSG0000(	ENSG0000(	98.63122	0.127791	0.165711	0.286219	lncRNA
ENSG0000(	ENSG0000(	10.13607	-0.36163	0.165768	0.286301	processed_pseudogene
ENSG0000(	RNU6-25P	19.23868	0.866078	0.165829	0.286392	snRNA
ENSG0000(	CTD-2194D	50.06158	0.166491	0.165894	0.286488	lncRNA
ENSG0000(	LINC01230	38.34375	-0.18427	0.165976	0.286613	lncRNA
ENSG0000(	ENSG0000(	18.98857	-0.31945	0.166051	0.286699	misc_RNA
ENSG0000(	FAT4	2695.156	0.11092	0.166037	0.286699	protein_coding
ENSG0000(	GS1-24F4.2	448.003	0.123086	0.166053	0.286699	lncRNA
ENSG0000(	FBXL8	406.4946	-0.08946	0.166138	0.286829	protein_coding
ENSG0000(	MAP1B	772.8969	0.206917	0.166147	0.286829	protein_coding
ENSG0000(	EML4-AS1	25.49549	0.240884	0.166168	0.286834	lncRNA
ENSG0000(	SLC2A1	1018.896	-0.16809	0.166164	0.286834	protein_coding
ENSG0000(	ENSG0000(	91.71507	0.140365	0.166188	0.286853	lncRNA
ENSG0000(	SCARNA21	742.864	-0.13156	0.166226	0.286903	scaRNA
ENSG0000(	ENSG0000(	11.22002	-0.32832	0.166238	0.286907	lncRNA
ENSG0000(	RBP5	254.9126	0.165544	0.166303	0.287003	protein_coding
ENSG0000(	CEPT1	1822.998	0.075991	0.166361	0.28705	protein_coding
ENSG0000(	ENSG0000(	58.53853	-0.1444	0.166355	0.28705	lncRNA
ENSG0000(	RPGR	1036.387	-0.07527	0.166366	0.28705	protein_coding
ENSG0000(	UBE2Z	2473.79	0.062944	0.166351	0.28705	protein_coding
ENSG0000(	GPR139	20.30988	0.31153	0.166394	0.287082	protein_coding
ENSG0000(	GOLGA7B	70.14329	0.226722	0.166453	0.287151	protein_coding
ENSG0000(	TEX26-AS1	222.1962	-0.0869	0.166452	0.287151	lncRNA
ENSG0000(	ENSG0000(	254.1934	0.117402	0.166486	0.287192	lncRNA
ENSG0000(	ENSG0000(	575.6621	0.070423	0.166581	0.28734	TEC
ENSG0000(	RN7SL392F	11.98981	0.273023	0.166637	0.287421	misc_RNA
ENSG0000(	ENSG0000(	13.61148	0.368242	0.166698	0.287511	unprocessed_pseudogene
ENSG0000(	BOD1L1	5056.085	0.072631	0.166739	0.287545	protein_coding
ENSG0000(	CASTOR3	537.6218	0.097114	0.166746	0.287545	transcribed_unprocessed_pseudo
ENSG0000(	CCDC7	729.1931	-0.10685	0.166736	0.287545	protein_coding
ENSG0000(	NBEAP2	24.88528	0.331456	0.166801	0.287625	transcribed_unprocessed_pseudo
ENSG0000(	RN7SL561F	28.69607	0.227671	0.166815	0.287633	misc_RNA
ENSG0000(	RGPD2	623.0711	0.547624	0.166834	0.287651	protein_coding
ENSG0000(	ATF2	1334.848	0.061509	0.167001	0.287891	protein_coding
ENSG0000(	EMBP1	603.8926	0.095627	0.166988	0.287891	transcribed_unprocessed_pseudo
ENSG0000(	ZNF284	427.3851	-0.05439	0.166998	0.287891	protein_coding

ENSG0000(	LINC00993	9.079528	-0.34819	0.167027	0.28792	transcribed_unprocessed_pseudo
ENSG0000(	SNORA51	23.43634	-0.23017	0.16704	0.287925	snoRNA
ENSG0000(	RASSF3	1682.894	-0.11096	0.167077	0.287975	protein_coding
ENSG0000(	CD163	1305.583	-0.11144	0.167234	0.288181	protein_coding
ENSG0000(	ENSG0000(	125.1401	-0.10486	0.167221	0.288181	lncRNA
ENSG0000(	MICU2	698.2292	-0.07078	0.167221	0.288181	protein_coding
ENSG0000(	PRKCG	70.28918	0.184197	0.167233	0.288181	protein_coding
ENSG0000(	RC3H1	4440.439	-0.07718	0.167274	0.288234	protein_coding
ENSG0000(	PIGA	849.991	0.070085	0.167293	0.288252	protein_coding
ENSG0000(	TPCN2	990.3314	0.080274	0.167311	0.288266	protein_coding
ENSG0000(	GLS2	20.57988	0.32685	0.167363	0.288339	protein_coding
ENSG0000(	ADD1	6665.102	-0.05108	0.167373	0.288341	protein_coding
ENSG0000(	NMRAL2P	513.1012	-0.24261	0.167418	0.288403	transcribed_unprocessed_pseudo
ENSG0000(	TP53TG5	17.68119	0.291236	0.167572	0.288646	protein_coding
ENSG0000(	ZFP69	116.0707	0.136657	0.167577	0.288646	protein_coding
ENSG0000(	RN7SL202F	66.5723	-0.15358	0.167604	0.288676	misc_RNA
ENSG0000(	ASB7	834.9345	-0.11456	0.16767	0.288774	protein_coding
ENSG0000(	RN7SKP62	48.31249	0.27623	0.167702	0.288812	misc_RNA
ENSG0000(	NMD3	784.5259	-0.07907	0.167755	0.288888	protein_coding
ENSG0000(	MYCBP2-AS	38.53285	0.193678	0.167767	0.288893	lncRNA
ENSG0000(	LLPH	1735.456	-0.04865	0.167789	0.288915	protein_coding
ENSG0000(	CEACAMP1	22.34865	0.238406	0.167832	0.288928	transcribed_processed_pseudoge
ENSG0000(	ENSG0000(	138.8016	-0.22884	0.167834	0.288928	protein_coding
ENSG0000(	ENSG0000(	203.7974	0.131709	0.167816	0.288928	lncRNA
ENSG0000(	SECTM1	669.4239	0.278035	0.167808	0.288928	protein_coding
ENSG0000(	AVPR1A	140.5291	0.197837	0.167903	0.288972	protein_coding
ENSG0000(	C22orf46	505.924	-0.07651	0.167904	0.288972	transcribed_unitary_pseudogene
ENSG0000(	ENSG0000(	24.81723	0.206577	0.167906	0.288972	lncRNA
ENSG0000(	SKAP2	1511.305	-0.06176	0.167901	0.288972	protein_coding
ENSG0000(	UXS1	452.8055	-0.1233	0.167903	0.288972	protein_coding
ENSG0000(	CDC40	980.651	-0.09702	0.167926	0.288991	protein_coding
ENSG0000(	CCDC167	39.29473	-0.20933	0.167971	0.289039	protein_coding
ENSG0000(	FGF1	40.50423	0.232826	0.167972	0.289039	protein_coding
ENSG0000(	IL21-AS1	10.93257	0.571731	0.167982	0.28904	lncRNA
ENSG0000(	RIC3	521.1468	0.124725	0.168053	0.28914	protein_coding
ENSG0000(	SIRT3	707.348	-0.09403	0.168058	0.28914	protein_coding
ENSG0000(	JADRR	407.185	-0.08448	0.168185	0.289342	lncRNA
ENSG0000(	ENSG0000(	37.95267	-0.1762	0.168353	0.289614	processed_pseudogene
ENSG0000(	OGFOD1	789.2195	-0.08518	0.168388	0.289658	protein_coding
ENSG0000(	ENSG0000(	279.8798	-0.13864	0.16843	0.289715	lncRNA
ENSG0000(	SMARCA5	1879.26	-0.10004	0.16847	0.289753	protein_coding
ENSG0000(	VMA21	1103.281	-0.08005	0.168471	0.289753	protein_coding
ENSG0000(	TADA2B	980.5336	-0.04499	0.1685	0.289788	protein_coding
ENSG0000(	ZNF133	731.5542	0.071563	0.168528	0.289819	protein_coding
ENSG0000(	RPL39L	38.26423	-0.2428	0.168639	0.289995	protein_coding
ENSG0000(	TBC1D23	852.8705	0.093842	0.168696	0.290076	protein_coding
ENSG0000(	GPCPD1	4065.394	0.0953	0.168717	0.290098	protein_coding

ENSG0000(	CDS2	2932.169	0.054493	0.168799	0.290222	protein_coding
ENSG0000(	SNORA19	9.983345	0.4029	0.168881	0.290347	snoRNA
ENSG0000(	ENSG0000(	231.0616	-0.11971	0.169032	0.290591	lncRNA
ENSG0000(	RN7SL503F	22.43987	0.230486	0.169054	0.290612	misc_RNA
ENSG0000(	LINC01726	46.83457	-0.16958	0.169127	0.290711	lncRNA
ENSG0000(	PDPK2P	453.6309	0.089446	0.16913	0.290711	transcribed_unprocessed_pseudo
ENSG0000(	LINC00114	114.1476	0.104475	0.169183	0.290786	lncRNA
ENSG0000(	TAF4B	447.6012	0.139709	0.169198	0.290796	protein_coding
ENSG0000(	ARHGEF26	381.9371	-0.10334	0.169228	0.290819	protein_coding
ENSG0000(	TRAPPC13	652.9225	-0.0893	0.16923	0.290819	protein_coding
ENSG0000(	ENSG0000(	13.82194	-0.55885	0.169254	0.290845	unprocessed_pseudogene
ENSG0000(	DHH	90.54785	0.145494	0.169322	0.290921	protein_coding
ENSG0000(	ENSG0000(	171.0749	0.119194	0.169336	0.290921	lncRNA
ENSG0000(	ENSG0000(	57.76728	-0.18904	0.169313	0.290921	lncRNA
ENSG0000(	KLF11	402.4836	-0.14275	0.169327	0.290921	protein_coding
ENSG0000(	NUMBL	623.596	0.090296	0.169351	0.290932	protein_coding
ENSG0000(	ENSG0000(	210.0324	-0.09819	0.169362	0.290934	lncRNA
ENSG0000(	STRIT1	78.98039	0.15546	0.169406	0.290993	protein_coding
ENSG0000(	ENSG0000(	115.6567	0.203697	0.169428	0.291011	lncRNA
ENSG0000(	FBXW11P1	24.10817	0.304676	0.169444	0.291011	processed_pseudogene
ENSG0000(	RN7SKP48	55.39063	-0.26327	0.16944	0.291011	misc_RNA
ENSG0000(	MIR4292	15.93583	-0.32435	0.169483	0.291062	miRNA
ENSG0000(	SHANK2-AS	211.5184	0.093454	0.169492	0.291062	lncRNA
ENSG0000(	HOXA11	86.50216	-0.22517	0.169559	0.291161	protein_coding
ENSG0000(	ENSG0000(	252.9657	-0.08394	0.169611	0.291226	lncRNA
ENSG0000(	ENSG0000(	247.4005	-0.08685	0.169616	0.291226	lncRNA
ENSG0000(	SNORD3A	63366.75	-0.2414	0.169644	0.291258	snoRNA
ENSG0000(	ENSG0000(	67.9586	-0.22484	0.169675	0.291297	lncRNA
ENSG0000(	ENSG0000(	75.72532	0.134806	0.169821	0.291531	TEC
ENSG0000(	ACOT1	25.45852	-0.41897	0.169865	0.291574	protein_coding
ENSG0000(	LINC01498	10.42948	0.476362	0.169862	0.291574	lncRNA
ENSG0000(	CATSPER2F	314.2446	-0.08609	0.169892	0.291605	transcribed_unprocessed_pseudo
ENSG0000(	ST6GALNA(	125.0699	-0.18589	0.169987	0.291751	protein_coding
ENSG0000(	RN7SL220F	154.5431	-0.14099	0.17002	0.291792	misc_RNA
ENSG0000(	CDKN2D	61.98605	-0.16836	0.170163	0.291928	protein_coding
ENSG0000(	ENSG0000(	13.34761	0.260219	0.170132	0.291928	lncRNA
ENSG0000(	ENSG0000(	21.6796	0.700974	0.170131	0.291928	transcribed_unprocessed_pseudo
ENSG0000(	ENSG0000(	217.4569	0.117455	0.170162	0.291928	protein_coding
ENSG0000(	MIR4440	29.53663	0.224795	0.170162	0.291928	miRNA
ENSG0000(	PTH2R	27.54918	0.289659	0.170111	0.291928	protein_coding
ENSG0000(	SRGAP1	3813.51	-0.11925	0.170165	0.291928	protein_coding
ENSG0000(	ZNF609	4036.865	0.049033	0.17024	0.292041	protein_coding
ENSG0000(	ENSG0000(	53.72551	-0.14692	0.170329	0.292165	lncRNA
ENSG0000(	HOPX	407.7982	-0.08629	0.170331	0.292165	protein_coding
ENSG0000(	ENSG0000(	31.44025	-0.22294	0.170386	0.292243	processed_pseudogene
ENSG0000(	LINC00658	36.04097	0.199594	0.170409	0.292267	lncRNA
ENSG0000(	IGBP1-AS1	165.7237	-0.11256	0.170445	0.292313	lncRNA

ENSG0000(	ATP6V0CP4	52.90078	0.202638	0.170459	0.292322	processed_pseudogene
ENSG0000(	REEP6	29.43147	-0.28077	0.170504	0.292382	protein_coding
ENSG0000(	LINC00051	12.21708	0.263227	0.170521	0.292396	lncRNA
ENSG0000(	ENSG0000(	212.0634	-0.10264	0.170537	0.292407	TEC
ENSG0000(	ANTXR2	2403.458	0.10054	0.17055	0.292409	protein_coding
ENSG0000(	VPS13B	8008.87	0.05867	0.170557	0.292409	protein_coding
ENSG0000(	AP2M1	1812.1	-0.10583	0.170587	0.292445	protein_coding
ENSG0000(	MIR6832	9.285672	-0.31355	0.170624	0.292492	miRNA
ENSG0000(	MYOCOS	232.1731	0.106194	0.170692	0.292584	protein_coding
ENSG0000(	RUSF1	1099.444	-0.088	0.170697	0.292584	protein_coding
ENSG0000(	TPBG	427.4183	0.123554	0.17071	0.292591	protein_coding
ENSG0000(	BTF3-DT	12.70017	0.345904	0.170727	0.292604	lncRNA
ENSG0000(	RENO1	400.3611	-0.08143	0.170756	0.292638	lncRNA
ENSG0000(	TXLNGY	3821.193	0.196336	0.170771	0.292647	transcribed_unprocessed_pseudo
ENSG0000(	STARD7-AS	505.484	0.075278	0.170796	0.292675	lncRNA
ENSG0000(	ENSG0000(	65.68008	-0.13586	0.17087	0.292778	lncRNA
ENSG0000(	MIR8086	91.65599	-0.11344	0.170885	0.292778	miRNA
ENSG0000(	NPIPP1	234.8529	-0.12124	0.170884	0.292778	transcribed_unprocessed_pseudo
ENSG0000(	RNU6-32P	15.81576	0.368314	0.170918	0.29282	snRNA
ENSG0000(	DTX2P1-UF	4649.731	-0.10227	0.170964	0.292878	lncRNA
ENSG0000(	ENSG0000(	71.2448	-0.22931	0.170971	0.292878	processed_pseudogene
ENSG0000(	BRMS1L	102.8446	-0.19013	0.170993	0.292883	protein_coding
ENSG0000(	SRP72	1311.405	-0.09545	0.170988	0.292883	protein_coding
ENSG0000(	ENSG0000(	45.36396	0.440198	0.171037	0.29294	lncRNA
ENSG0000(	HTR7P1	209.0306	0.092835	0.171045	0.29294	transcribed_processed_pseudoge
ENSG0000(	ENSG0000(	117.8106	0.146171	0.171062	0.292953	lncRNA
ENSG0000(	BORCS8-M	42.00738	-0.27677	0.171108	0.292996	protein_coding
ENSG0000(	SLC22A11	110.0214	0.131963	0.171107	0.292996	protein_coding
ENSG0000(	TBC1D3B	278.6131	-0.12963	0.171115	0.292996	protein_coding
ENSG0000(	ALKBH2	100.2697	-0.12173	0.171135	0.293015	protein_coding
ENSG0000(	BACH1-AS1	302.0527	-0.08989	0.171205	0.293118	lncRNA
ENSG0000(	RABEP2	939.2729	0.081699	0.171303	0.293269	protein_coding
ENSG0000(	ENSG0000(	131.6787	0.286561	0.171345	0.293326	lncRNA
ENSG0000(	ENSG0000(	387.0925	-0.08206	0.171416	0.293406	lncRNA
ENSG0000(	RHBDL1	23.83371	-0.2254	0.171417	0.293406	protein_coding
ENSG0000(	RN7SL118F	78.22178	-0.15051	0.17142	0.293406	misc_RNA
ENSG0000(	RN7SL128F	135.9679	-0.11139	0.171431	0.293408	misc_RNA
ENSG0000(	MXI1	1699.176	-0.25008	0.171446	0.293419	protein_coding
ENSG0000(	CDCA4	224.2569	-0.10827	0.171482	0.29345	protein_coding
ENSG0000(	KAZALD1	207.3456	-0.19729	0.171484	0.29345	protein_coding
ENSG0000(	ENSG0000(	70.31432	0.146904	0.171619	0.293667	lncRNA
ENSG0000(	GEMIN4	596.9194	-0.10486	0.171636	0.293679	protein_coding
ENSG0000(	TEX21P	515.9657	-0.06693	0.171708	0.293786	transcribed_unitary_pseudogene
ENSG0000(	ENSG0000(	12.97353	-0.3527	0.171825	0.293938	unprocessed_pseudogene
ENSG0000(	ENSG0000(	281.7886	0.18285	0.171822	0.293938	lncRNA
ENSG0000(	RALGAPA2	5725.335	-0.08618	0.171823	0.293938	protein_coding
ENSG0000(	ENSG0000(	12.05162	0.341486	0.171868	0.293996	lncRNA

ENSG0000(	PLOD2	2199.714	-0.19265	0.171888	0.293998	protein_coding
ENSG0000(	SERF1B	541.6284	-0.14054	0.171884	0.293998	protein_coding
ENSG0000(	TMPRSS5	60.476	0.244881	0.171913	0.294024	protein_coding
ENSG0000(	LPGAT1-AS	15.42281	0.24219	0.171937	0.29405	lncRNA
ENSG0000(	ENSG0000(	14.45341	-0.33292	0.171968	0.294071	processed_pseudogene
ENSG0000(	MAN1B1	1579.216	-0.067	0.17196	0.294071	protein_coding
ENSG0000(	CUEDC1	529.8567	0.093366	0.17199	0.294091	protein_coding
ENSG0000(	ENSG0000(	274.4084	0.147411	0.172065	0.294204	lncRNA
ENSG0000(	ENSG0000(	255.9599	-0.12609	0.172113	0.294271	lncRNA
ENSG0000(	NIFK-AS1	581.7163	0.064231	0.172287	0.294552	lncRNA
ENSG0000(	LINC02800	79.68356	0.220806	0.172309	0.294573	lncRNA
ENSG0000(	ASXL3	244.4263	-0.14079	0.172339	0.294607	protein_coding
ENSG0000(	ENSG0000(	1729.079	-0.06363	0.172415	0.294705	lncRNA
ENSG0000(	ZNF835	292.8072	-0.08087	0.172406	0.294705	protein_coding
ENSG0000(	ASTE1	368.394	-0.11669	0.172458	0.294763	protein_coding
ENSG0000(	BLK	402.532	0.362973	0.172506	0.294829	protein_coding
ENSG0000(	ADAMTS1C	513.8886	0.179222	0.172548	0.294835	protein_coding
ENSG0000(	ENSG0000(	11.5057	0.355278	0.172539	0.294835	lncRNA
ENSG0000(	ENSG0000(	135.9972	-0.1168	0.172528	0.294835	lncRNA
ENSG0000(	IGHA1	24081.86	0.302343	0.172557	0.294835	IG_C_gene
ENSG0000(	TRIM3	1388.587	-0.17429	0.172533	0.294835	protein_coding
ENSG0000(	ENSG0000(	255.1529	-0.09526	0.172697	0.295037	lncRNA
ENSG0000(	EXOSC8	1174.681	-0.06759	0.172706	0.295037	protein_coding
ENSG0000(	MYLK3	2197.598	0.118751	0.17269	0.295037	protein_coding
ENSG0000(	SLC29A3	177.4445	0.131821	0.172712	0.295037	protein_coding
ENSG0000(	ENSG0000(	21.59449	-0.21019	0.172784	0.295127	transcribed_processed_pseudoge
ENSG0000(	ENSG0000(	459.5441	-0.06643	0.17278	0.295127	lncRNA
ENSG0000(	FHOD3	190.2318	0.203654	0.172824	0.295179	protein_coding
ENSG0000(	WBP1LP2	18.06919	-0.22342	0.172899	0.295291	processed_pseudogene
ENSG0000(	MRPL35P3	34.60349	0.239937	0.172923	0.295315	processed_pseudogene
ENSG0000(	SPTLC3	1452.893	-0.11115	0.172946	0.295339	protein_coding
ENSG0000(	MAML3	1140.037	-0.12967	0.173026	0.295459	protein_coding
ENSG0000(	ABCB8	1096.393	-0.06774	0.173053	0.29547	protein_coding
ENSG0000(	ENSG0000(	18.17993	-0.24302	0.17306	0.29547	lncRNA
ENSG0000(	ENSG0000(	10.14765	0.310172	0.173051	0.29547	lncRNA
ENSG0000(	HOXA2	166.3274	-0.1847	0.17318	0.295657	protein_coding
ENSG0000(	ENSG0000(	26.48995	0.244884	0.173227	0.295691	transcribed_unprocessed_pseudo
ENSG0000(	ENSG0000(	70.47387	0.162663	0.173214	0.295691	lncRNA
ENSG0000(	PCDHA6	18.63772	0.334171	0.173228	0.295691	protein_coding
ENSG0000(	SNORA8	380.8592	-0.19276	0.173275	0.295755	snoRNA
ENSG0000(	ENSG0000(	126.4876	0.277185	0.173316	0.295808	lncRNA
ENSG0000(	THUMPD1	2028.535	-0.06224	0.17336	0.295868	protein_coding
ENSG0000(	ARHGAP21	4683.555	-0.08249	0.173395	0.295912	protein_coding
ENSG0000(	TASOR2	4371.467	-0.08153	0.173426	0.295948	protein_coding
ENSG0000(	ENSG0000(	121.8353	0.111766	0.173555	0.296153	lncRNA
ENSG0000(	ST8SIA5	291.1333	0.110419	0.173633	0.296269	protein_coding
ENSG0000(	FBN3	18.6851	-0.65729	0.173685	0.296341	protein_coding

ENSG0000(	ARMC12	11.01672	0.327374	0.17371	0.296369	protein_coding
ENSG0000(	FOXP1-IT1	861.9954	0.101263	0.173743	0.296408	lncRNA
ENSG0000(	ENSG0000(	13.29379	0.274275	0.173788	0.296453	lncRNA
ENSG0000(	RN7SL333F	19.80407	0.214447	0.173788	0.296453	misc_RNA
ENSG0000(	C1orf146	63.64041	-0.12268	0.173803	0.296462	protein_coding
ENSG0000(	ENSG0000(	307.9475	-0.1677	0.173823	0.29648	TEC
ENSG0000(	KLHDC4	1765.702	-0.11191	0.173833	0.296482	protein_coding
ENSG0000(	ANKRD49	611.0887	0.065685	0.173885	0.296538	protein_coding
ENSG0000(	PNPLA3	280.8979	0.133893	0.173878	0.296538	protein_coding
ENSG0000(	IPO8	1807.727	-0.07541	0.173917	0.296577	protein_coding
ENSG0000(	ENSG0000(	35.7999	0.160011	0.17395	0.296616	lncRNA
ENSG0000(	HEATR5A	2432.647	-0.08994	0.174034	0.296723	protein_coding
ENSG0000(	OR2J3	19.3774	-0.21821	0.174041	0.296723	protein_coding
ENSG0000(	PADI1	10.6767	-0.53829	0.174037	0.296723	protein_coding
ENSG0000(	BCKDK	362.366	-0.11122	0.174051	0.296723	protein_coding
ENSG0000(	ITM2A	90.6353	-0.26317	0.174078	0.296754	protein_coding
ENSG0000(	MXRA8	343.1049	0.155708	0.174096	0.296768	protein_coding
ENSG0000(	ENSG0000(	19.60597	0.395911	0.174148	0.296823	transcribed_unprocessed_pseudo
ENSG0000(	RAD1	970.0211	-0.08315	0.174147	0.296823	protein_coding
ENSG0000(	ENSG0000(	19.40661	0.283043	0.174186	0.296873	lncRNA
ENSG0000(	CBY1	344.7407	0.073881	0.174205	0.296888	protein_coding
ENSG0000(	ENSG0000(	21.67034	-0.21803	0.174221	0.2969	lncRNA
ENSG0000(	RRP12	748.4857	-0.06534	0.174253	0.296939	protein_coding
ENSG0000(	GNPTG	1065.367	-0.06493	0.174288	0.296982	protein_coding
ENSG0000(	ENSG0000(	9.737596	-0.35815	0.174324	0.297027	processed_pseudogene
ENSG0000(	IFITM10	126.2161	0.196086	0.174376	0.297099	protein_coding
ENSG0000(	MS4A1	2144.717	0.523134	0.174412	0.297144	protein_coding
ENSG0000(	ENSG0000(	327.1346	0.069468	0.17445	0.297189	lncRNA
ENSG0000(	LINC02082	387.9419	-0.09152	0.174457	0.297189	lncRNA
ENSG0000(	ENSG0000(	455.5973	0.13156	0.174477	0.297206	TEC
ENSG0000(	ZNF200	1091.64	0.059386	0.174578	0.297362	protein_coding
ENSG0000(	MIR2052H(	50.67694	0.169986	0.174616	0.297394	lncRNA
ENSG0000(	TRBC1	268.4168	-0.2839	0.174609	0.297394	TR_C_gene
ENSG0000(	SLCO2B1	4301.901	0.10224	0.174638	0.297416	protein_coding
ENSG0000(	TPTE2	35.67949	0.239156	0.174674	0.297462	protein_coding
ENSG0000(	LRRC7	1572.922	-0.1063	0.174718	0.297513	protein_coding
ENSG0000(	MT-TR	66.58048	0.615322	0.174733	0.297513	Mt_tRNA
ENSG0000(	TENM1	155.8881	-0.24891	0.174728	0.297513	protein_coding
ENSG0000(	RNU1-11P	442.6704	-0.24637	0.174779	0.297576	snRNA
ENSG0000(	ENSG0000(	16.16102	0.249347	0.174866	0.297681	lncRNA
ENSG0000(	ENSG0000(	10.08331	0.310468	0.17487	0.297681	TEC
ENSG0000(	MBTPS2	484.0264	0.069229	0.174856	0.297681	protein_coding
ENSG0000(	ENSG0000(	57.04932	0.139444	0.174902	0.297704	processed_pseudogene
ENSG0000(	HSDL1	444.3108	0.095994	0.174901	0.297704	protein_coding
ENSG0000(	ODF2	922.3298	-0.07879	0.174966	0.29778	protein_coding
ENSG0000(	TNFAIP8L3	302.7225	0.083562	0.174963	0.29778	protein_coding
ENSG0000(	ENSG0000(	224.4813	0.111018	0.174983	0.297794	TEC

ENSG0000(	FDXACB1	207.2187	-0.09893	0.175026	0.297851	protein_coding
ENSG0000(	ENSG0000(	135.5357	0.119678	0.175053	0.297867	lncRNA
ENSG0000(	ENSG0000(	23.4478	0.189701	0.175055	0.297867	lncRNA
ENSG0000(	EIF2B5-DT	10.23631	-0.3399	0.175128	0.297974	lncRNA
ENSG0000(	PFN4	15.47094	-0.39508	0.175176	0.29804	protein_coding
ENSG0000(	HK2-DT	192.7837	0.091691	0.175203	0.298071	lncRNA
ENSG0000(	ENSG0000(	189.284	-0.37541	0.175222	0.298087	lncRNA
ENSG0000(	PRKAA1	2874.98	-0.05316	0.175284	0.298175	protein_coding
ENSG0000(	HRH4	190.2755	-0.11308	0.175362	0.298292	protein_coding
ENSG0000(	EXT2	3269.255	0.065758	0.175432	0.298395	protein_coding
ENSG0000(	ENSG0000(	252.8888	-0.0937	0.175452	0.298413	lncRNA
ENSG0000(	ENSG0000(	120.097	-0.19008	0.17549	0.298433	transcribed_unprocessed_pseudo
ENSG0000(	ERVFRD-1	33.01505	0.208318	0.175474	0.298433	protein_coding
ENSG0000(	RECQL5	1149.517	0.069814	0.175493	0.298433	protein_coding
ENSG0000(	ENSG0000(	116.3148	-0.10659	0.175539	0.298496	lncRNA
ENSG0000(	ENSG0000(	283.3039	-0.11736	0.175577	0.298544	lncRNA
ENSG0000(	PHF19	502.4199	-0.14558	0.175623	0.298596	protein_coding
ENSG0000(	ZFY	261.1221	0.259711	0.175627	0.298596	protein_coding
ENSG0000(	SPDYE18	52.78297	0.197507	0.175639	0.298601	protein_coding
ENSG0000(	LINC02664	328.4256	-0.08757	0.175679	0.298647	lncRNA
ENSG0000(	NEGR1	2940.457	0.078576	0.175685	0.298647	protein_coding
ENSG0000(	LINC02463	15.76005	0.285462	0.175765	0.298766	lncRNA
ENSG0000(	LINC01055	72.82407	-0.1358	0.175803	0.298814	lncRNA
ENSG0000(	MARVELD3	2165.608	-0.16347	0.175883	0.298935	protein_coding
ENSG0000(	NPHP4	605.0893	0.098438	0.17601	0.299133	protein_coding
ENSG0000(	ZNF319	228.0909	0.166386	0.176028	0.299148	protein_coding
ENSG0000(	SLC23A1	135.4228	-0.22325	0.176049	0.299167	protein_coding
ENSG0000(	ENSG0000(	102.7162	0.126874	0.176112	0.299259	lncRNA
ENSG0000(	ENSG0000(	53.25254	0.14789	0.176219	0.299424	lncRNA
ENSG0000(	ENSG0000(	553.4218	-0.1411	0.176326	0.29959	lncRNA
ENSG0000(	ENSG0000(	15.7049	0.264899	0.176412	0.299717	lncRNA
ENSG0000(	ENSG0000(	28.73619	-0.26237	0.17642	0.299717	lncRNA
ENSG0000(	ENSG0000(	10.72969	-0.27776	0.176449	0.299733	processed_pseudogene
ENSG0000(	MARCHF2	382.7167	0.082923	0.176449	0.299733	protein_coding
ENSG0000(	ENSG0000(	22.16325	-0.23666	0.17648	0.299769	lncRNA
ENSG0000(	ENSG0000(	13.24534	0.299196	0.17651	0.299788	miRNA
ENSG0000(	TAB2	2946.803	0.056913	0.176507	0.299788	protein_coding
ENSG0000(	CDH13	534.1258	0.137142	0.176522	0.29979	protein_coding
ENSG0000(	GLYCTK	1976.03	-0.13571	0.176533	0.29979	protein_coding
ENSG0000(	HSPA8P3	174.3191	-0.16117	0.17654	0.29979	processed_pseudogene
ENSG0000(	ENSG0000(	13.59461	-0.30094	0.176593	0.299864	lncRNA
ENSG0000(	FLVCR1	1391.421	-0.09767	0.176618	0.29989	protein_coding
ENSG0000(	IL27RA	324.125	0.176179	0.176643	0.2999	protein_coding
ENSG0000(	RTN4R	24.61877	-0.28287	0.176636	0.2999	protein_coding
ENSG0000(	KDM4B	2447	0.055833	0.176667	0.299924	protein_coding
ENSG0000(	ENSG0000(	53.52619	0.158631	0.176697	0.299959	lncRNA
ENSG0000(	ENSG0000(	20.23071	0.180361	0.176709	0.299964	lncRNA

ENSG0000(	DOCK9	2345.055	0.085855	0.176723	0.29997	protein_coding
ENSG0000(	CPA6	97.0033	-0.29432	0.176757	0.300012	protein_coding
ENSG0000(	USP3-AS1	184.0193	0.138506	0.176787	0.300031	lncRNA
ENSG0000(	ZNF491	302.184	0.115504	0.176784	0.300031	protein_coding
ENSG0000(	CYP17A1	210.7557	-0.07265	0.176845	0.300113	protein_coding
ENSG0000(	ENSG0000(	11.4242	-0.30524	0.176902	0.300192	processed_pseudogene
ENSG0000(	ENSG0000(	13.93424	0.326203	0.176923	0.300212	lncRNA
ENSG0000(	ENSG0000(	17.46203	-0.30384	0.176998	0.300324	lncRNA
ENSG0000(	ENSG0000(	46.61611	-0.18777	0.177034	0.300369	lncRNA
ENSG0000(	SPP2	130.2178	-0.17408	0.177045	0.300371	protein_coding
ENSG0000(	ENSG0000(	88.64899	0.117288	0.177067	0.300392	lncRNA
ENSG0000(	ENSG0000(	60.18038	-0.13845	0.177161	0.300519	lncRNA
ENSG0000(	LEO1	654.256	-0.08027	0.177161	0.300519	protein_coding
ENSG0000(	KRT17P5	29.26251	-0.66977	0.1772	0.300557	transcribed_unprocessed_pseudo
ENSG0000(	MAGOHB	533.9568	0.098705	0.177203	0.300557	protein_coding
ENSG0000(	TMEM219	847.1963	-0.09814	0.177228	0.300583	protein_coding
ENSG0000(	NUP153-AS	19.4068	0.2794	0.177286	0.300664	lncRNA
ENSG0000(	ATAD2	860.7158	-0.10433	0.17738	0.300808	protein_coding
ENSG0000(	LINC02812	86.39267	0.136318	0.177409	0.300825	lncRNA
ENSG0000(	SULT1C4	262.064	-0.08247	0.177406	0.300825	protein_coding
ENSG0000(	ENSG0000(	34.4039	-0.18089	0.177503	0.300936	processed_pseudogene
ENSG0000(	ENSG0000(	8091.252	0.086046	0.1775	0.300936	lncRNA
ENSG0000(	LINC00463	44.37609	0.169279	0.177499	0.300936	lncRNA
ENSG0000(	ENSG0000(	764.044	-0.11993	0.177659	0.301154	lncRNA
ENSG0000(	IGKV1D-17	26.23951	0.703938	0.177651	0.301154	IG_V_gene
ENSG0000(	MYD88	1382.273	0.112165	0.177661	0.301154	protein_coding
ENSG0000(	ENSG0000(	71.88705	0.143778	0.177692	0.30119	lncRNA
ENSG0000(	LINC02167	18.66849	0.302222	0.1778	0.301358	lncRNA
ENSG0000(	ELF3	6875.77	-0.17757	0.177832	0.301394	protein_coding
ENSG0000(	TTC6	251.0275	-0.12016	0.177983	0.301635	protein_coding
ENSG0000(	ENSG0000(	48.9359	-0.21789	0.178185	0.301952	lncRNA
ENSG0000(	ENSG0000(	9.609661	0.376682	0.178189	0.301952	processed_pseudogene
ENSG0000(	CSF1	1007.285	0.118117	0.178281	0.302091	protein_coding
ENSG0000(	ACVRL1	1918.686	0.143213	0.17831	0.302123	protein_coding
ENSG0000(	BTF3P3	23.05741	-0.25943	0.17836	0.302191	processed_pseudogene
ENSG0000(	SLC7A7	643.0123	0.135648	0.178444	0.302317	protein_coding
ENSG0000(	MSTO2P	49.0848	-0.20864	0.178464	0.302318	unprocessed_pseudogene
ENSG0000(	TTC8	479.5742	0.095415	0.178459	0.302318	protein_coding
ENSG0000(	ERBB4	106.2201	-0.57455	0.178476	0.302323	protein_coding
ENSG0000(	BGLAP	29.41451	-0.26787	0.178569	0.302464	protein_coding
ENSG0000(	ENSG0000(	77.20301	-0.14053	0.178651	0.302579	lncRNA
ENSG0000(	LINC00487	16.38868	-0.44328	0.178656	0.302579	lncRNA
ENSG0000(	DUXA	35.39269	0.157569	0.178699	0.302629	protein_coding
ENSG0000(	ENSG0000(	69.47738	0.121811	0.178705	0.302629	lncRNA
ENSG0000(	PKDREJ	84.13803	-0.29235	0.178725	0.302646	protein_coding
ENSG0000(	IGHJ6	253.6858	0.243204	0.178819	0.302789	IG_J_gene
ENSG0000(	ENSG0000(	187.9702	-0.09515	0.178831	0.302793	lncRNA

ENSG0000(	ENSG0000(	31.53207	0.215414	0.178851	0.30281	lncRNA
ENSG0000(	IQGAP1	7848.869	-0.05894	0.178961	0.302981	protein_coding
ENSG0000(	HPS5	1142.382	0.05863	0.178994	0.303003	protein_coding
ENSG0000(	NAP1L3	16.41684	0.361067	0.178987	0.303003	protein_coding
ENSG0000(	RAD54L2	2395.486	0.081318	0.179011	0.303016	protein_coding
ENSG0000(	ZNF723	17.83081	-0.23715	0.179105	0.303159	protein_coding
ENSG0000(	UBE2FP1	87.83942	-0.16677	0.179162	0.303238	processed_pseudogene
ENSG0000(	ENSG0000(	20.82694	-0.21165	0.179228	0.303334	lncRNA
ENSG0000(	ENSG0000(	112.7249	0.141734	0.179269	0.303387	lncRNA
ENSG0000(	GNL3L	3902.222	-0.05909	0.179335	0.303481	protein_coding
ENSG0000(	F10	11.9119	-0.40219	0.179451	0.303662	protein_coding
ENSG0000(	ENSG0000(	101.1189	0.100896	0.179509	0.303743	lncRNA
ENSG0000(	ENSG0000(	96.24396	0.1111	0.17954	0.303746	processed_pseudogene
ENSG0000(	ENSG0000(	201.6677	-0.26797	0.179535	0.303746	processed_pseudogene
ENSG0000(	GRIN2B	1119.554	0.519286	0.179536	0.303746	protein_coding
ENSG0000(	ENSG0000(	433.8944	-0.08404	0.179626	0.303876	lncRNA
ENSG0000(	ANKUB1	143.3249	0.089406	0.179683	0.303955	protein_coding
ENSG0000(	SLC1A6	509.2744	0.054919	0.179692	0.303955	protein_coding
ENSG0000(	HERC2P3	1365.905	-0.14534	0.179744	0.304026	transcribed_unprocessed_pseudo
ENSG0000(	LINC01515	1862.58	-0.12462	0.179777	0.304065	lncRNA
ENSG0000(	ENSG0000(	13.22967	0.384274	0.179787	0.304066	unprocessed_pseudogene
ENSG0000(	DSG3	133.005	0.192591	0.179892	0.304227	protein_coding
ENSG0000(	PAK2	2729.376	-0.0869	0.179931	0.304277	protein_coding
ENSG0000(	BCAT1	497.1597	0.131936	0.179953	0.304297	protein_coding
ENSG0000(	ENSG0000(	128.0569	-0.12037	0.179972	0.304312	lncRNA
ENSG0000(	YEATS2-AS:	48.19605	-0.15975	0.180025	0.304386	lncRNA
ENSG0000(	ALOX12P2	241.582	0.139839	0.180079	0.304461	transcribed_unprocessed_pseudo
ENSG0000(	RNF207	1433.198	-0.1251	0.180132	0.304534	protein_coding
ENSG0000(	USP46-DT	103.6181	0.114164	0.180151	0.30455	lncRNA
ENSG0000(	ENSG0000(	18.38262	-0.24015	0.18017	0.304565	lncRNA
ENSG0000(	ENSG0000(	17.86169	0.347833	0.180208	0.304597	unprocessed_pseudogene
ENSG0000(	ENSG0000(	68.52188	-0.14751	0.1802	0.304597	TEC
ENSG0000(	ENSG0000(	9.539453	0.336611	0.180231	0.304619	lncRNA
ENSG0000(	SNORD99	33.95786	0.176752	0.180257	0.304647	snoRNA
ENSG0000(	ENSG0000(	158.6908	-0.1963	0.180327	0.304748	TEC
ENSG0000(	MTND4P35	23.42469	-0.37528	0.180351	0.304773	processed_pseudogene
ENSG0000(	IFT43	274.966	0.078858	0.180424	0.304879	protein_coding
ENSG0000(	ENSG0000(	52.63992	0.156748	0.180545	0.305068	lncRNA
ENSG0000(	CYP2R1	744.7913	-0.09577	0.180575	0.305101	protein_coding
ENSG0000(	ANXA2	8425.962	-0.14	0.180618	0.305158	protein_coding
ENSG0000(	CDRT15P6	41.65582	0.213801	0.180645	0.305187	transcribed_unprocessed_pseudo
ENSG0000(	LHX5-AS1	12.27917	-0.37827	0.180657	0.30519	lncRNA
ENSG0000(	ENSG0000(	32.00388	0.200424	0.180683	0.305219	lncRNA
ENSG0000(	GOLGA2	3123.77	0.051168	0.180696	0.305224	protein_coding
ENSG0000(	IGKV5-2	23.96438	0.373091	0.180734	0.305272	IG_V_gene
ENSG0000(	ENSG0000(	108.1899	-0.10966	0.180763	0.305303	lncRNA
ENSG0000(	ARL6IP5	1126.866	-0.11975	0.180778	0.305312	protein_coding

ENSG0000(	GFOD2	2540.293	-0.0579	0.180827	0.305372	protein_coding
ENSG0000(	KATNAL2	838.0615	0.062451	0.180833	0.305372	protein_coding
ENSG0000(	ENSG0000(	77.83102	0.385418	0.180892	0.305455	lncRNA
ENSG0000(	ENSG0000(	16.08578	-0.22035	0.180918	0.305484	lncRNA
ENSG0000(	RXYLT1	928.5644	0.047894	0.180972	0.305541	protein_coding
ENSG0000(	TAT-AS1	146.5173	-0.09806	0.180967	0.305541	lncRNA
ENSG0000(	SLC10A1	11.13496	0.479583	0.181052	0.305659	protein_coding
ENSG0000(	ENSG0000(	71.8673	-0.24167	0.181247	0.305972	lncRNA
ENSG0000(	RPS29P14	26.35647	0.250547	0.181291	0.306031	processed_pseudogene
ENSG0000(	DLL4	328.0602	0.152582	0.181335	0.306055	protein_coding
ENSG0000(	ENSG0000(	13.65595	-0.27297	0.181325	0.306055	lncRNA
ENSG0000(	ENSG0000(	15.9491	0.472597	0.181325	0.306055	protein_coding
ENSG0000(	PRELID1P1	11.26782	-0.3863	0.181361	0.306082	transcribed_processed_pseudoge
ENSG0000(	PAK1	2051.397	-0.07098	0.181415	0.306157	protein_coding
ENSG0000(	FAIM	295.904	-0.10067	0.181436	0.306164	protein_coding
ENSG0000(	TENM3	132.0668	0.392055	0.181439	0.306164	protein_coding
ENSG0000(	ARNT	2036.097	-0.05967	0.181454	0.306173	protein_coding
ENSG0000(	RYR3	635.3324	-0.28868	0.18158	0.30637	protein_coding
ENSG0000(	FRAT1	158.646	0.130082	0.181653	0.306476	protein_coding
ENSG0000(	JCADP1	15.94536	-0.30614	0.181688	0.306519	processed_pseudogene
ENSG0000(	METTL21A	5333.185	-0.06734	0.181708	0.306536	protein_coding
ENSG0000(	FLJ43315	51.67466	-0.29224	0.181723	0.306544	transcribed_unprocessed_pseudo
ENSG0000(	LINC01909	202.7218	-0.10678	0.181737	0.306551	lncRNA
ENSG0000(	ITCH-IT1	57.41198	0.162865	0.181834	0.306698	lncRNA
ENSG0000(	ENSG0000(	23.60443	0.190449	0.181861	0.30671	lncRNA
ENSG0000(	LMOD1	459.2893	-0.19371	0.181854	0.30671	protein_coding
ENSG0000(	BEND7P1	14.54496	0.247496	0.181905	0.306769	processed_pseudogene
ENSG0000(	ENSG0000(	39.85255	0.142807	0.181937	0.306803	lncRNA
ENSG0000(	SNTA1	61.16802	0.149146	0.181945	0.306803	protein_coding
ENSG0000(	ARHGEF28	1029.702	0.149334	0.181961	0.306812	protein_coding
ENSG0000(	ZNF93	430.8957	0.092236	0.181988	0.306842	protein_coding
ENSG0000(	ENSG0000(	440.6688	-0.11787	0.182022	0.306882	lncRNA
ENSG0000(	ENSG0000(	95.06756	-0.10065	0.18204	0.306898	TEC
ENSG0000(	ENSG0000(	49.59506	0.147112	0.182095	0.306973	lncRNA
ENSG0000(	TONSL-AS1	16.81242	-0.276	0.182136	0.307025	lncRNA
ENSG0000(	SNORA31	106.1806	0.145841	0.182178	0.307079	snoRNA
ENSG0000(	U2SURP	3520.829	0.054325	0.182189	0.307082	protein_coding
ENSG0000(	ENSG0000(	169.8201	-0.09615	0.182236	0.307144	lncRNA
ENSG0000(	ENSG0000(	147.1074	-0.08413	0.182257	0.307164	lncRNA
ENSG0000(	ENSG0000(	44.51214	0.172313	0.182296	0.307213	processed_pseudogene
ENSG0000(	ENSG0000(	14.32771	0.393743	0.182306	0.307214	lncRNA
ENSG0000(	BSPRY	396.9878	-0.1245	0.182336	0.307246	protein_coding
ENSG0000(	BMP6	189.486	0.190499	0.182385	0.307296	protein_coding
ENSG0000(	LCK	441.2116	-0.15939	0.182378	0.307296	protein_coding
ENSG0000(	ENSG0000(	256.5573	0.077781	0.182463	0.307411	lncRNA
ENSG0000(	LINC02266	20.19029	0.209022	0.182522	0.307478	lncRNA
ENSG0000(	SEC1P	110.0788	0.124218	0.182516	0.307478	transcribed_unitary_pseudogene

ENSG0000(	ARHGAP26	8194.379	-0.08909	0.182562	0.307528	protein_coding
ENSG0000(	CLIC2	225.0035	0.146759	0.182585	0.30755	protein_coding
ENSG0000(	MTMR14	1191.967	0.069242	0.182601	0.307561	protein_coding
ENSG0000(	ADH7	20.81269	0.407752	0.182636	0.307603	protein_coding
ENSG0000(	DPP7	428.0066	0.120682	0.1827	0.307694	protein_coding
ENSG0000(	ENSG0000(	96.87175	-0.12011	0.182745	0.307754	lncRNA
ENSG0000(	GAPDHP47	21.18371	0.260397	0.182797	0.307825	processed_pseudogene
ENSG0000(	LINC01606	99.13659	-0.15649	0.182848	0.307893	lncRNA
ENSG0000(	DHRS3	776.1634	-0.12142	0.182864	0.307905	protein_coding
ENSG0000(	ENSG0000(	87.60627	0.123489	0.182885	0.307922	TEC
ENSG0000(	ENSG0000(	78.6927	0.180864	0.182921	0.307967	lncRNA
ENSG0000(	ENSG0000(	109.1927	0.25646	0.182951	0.307986	lncRNA
ENSG0000(	NPLOC4	5015.435	-0.07347	0.182952	0.307986	protein_coding
ENSG0000(	PAX5	1018.46	0.282593	0.183003	0.308055	protein_coding
ENSG0000(	ARHGEF5	2978.202	-0.21666	0.183053	0.308124	protein_coding
ENSG0000(	ENSG0000(	24.06573	0.284247	0.183158	0.308283	misc_RNA
ENSG0000(	PLS1	4358.03	-0.19062	0.183191	0.308322	protein_coding
ENSG0000(	ADAMTS7	304.4364	0.187135	0.183256	0.308346	protein_coding
ENSG0000(	ART3	221.6404	0.099195	0.18325	0.308346	protein_coding
ENSG0000(	ASH1L-AS1	216.2481	0.117324	0.183232	0.308346	lncRNA
ENSG0000(	ENSG0000(	153.1572	-0.15194	0.183233	0.308346	TEC
ENSG0000(	ENSG0000(	74.65083	-0.1714	0.183264	0.308346	lncRNA
ENSG0000(	RN7SL378F	66.10837	0.138137	0.183263	0.308346	misc_RNA
ENSG0000(	ENSG0000(	179.5055	0.078623	0.183314	0.308379	lncRNA
ENSG0000(	ENSG0000(	72.46369	0.158899	0.183295	0.308379	lncRNA
ENSG0000(	IL20RA	289.2863	-0.24226	0.183311	0.308379	protein_coding
ENSG0000(	RNU1-43P	26.72139	0.332291	0.183328	0.308387	snRNA
ENSG0000(	FOXD3	8.52423	-0.36173	0.183359	0.308406	protein_coding
ENSG0000(	GATA2-AS1	42.75573	0.217804	0.183355	0.308406	lncRNA
ENSG0000(	ENSG0000(	180.4785	-0.09986	0.183403	0.308462	TEC
ENSG0000(	ENSG0000(	436.0796	0.142324	0.183444	0.308515	lncRNA
ENSG0000(	ENSG0000(	36.47108	0.166213	0.183467	0.308521	lncRNA
ENSG0000(	NTF3	36.39952	0.246782	0.183466	0.308521	protein_coding
ENSG0000(	ENSG0000(	21.20095	-0.26369	0.183524	0.308601	lncRNA
ENSG0000(	MAP3K20-/	30.8684	0.335155	0.183535	0.308602	lncRNA
ENSG0000(	ENSG0000(	242.1499	0.073264	0.18355	0.308611	lncRNA
ENSG0000(	ENSG0000(	99.46586	-0.10864	0.183671	0.308798	lncRNA
ENSG0000(	ENSG0000(	441.5894	-0.08528	0.183709	0.308829	lncRNA
ENSG0000(	PEAR1	340.1678	0.143325	0.183705	0.308829	protein_coding
ENSG0000(	ENSG0000(	300.8663	0.07048	0.183779	0.308929	lncRNA
ENSG0000(	PTGER1	10.40078	0.346044	0.183789	0.308929	protein_coding
ENSG0000(	KRT18P52	9.989916	0.360002	0.183839	0.308996	processed_pseudogene
ENSG0000(	ENSG0000(	21.08788	0.228806	0.183857	0.309011	lncRNA
ENSG0000(	ENSG0000(	136.6958	-0.09718	0.183923	0.309105	lncRNA
ENSG0000(	ENSG0000(	12.13699	0.446934	0.183938	0.309114	lncRNA
ENSG0000(	ENSG0000(	9.465478	0.3542	0.184048	0.309283	lncRNA
ENSG0000(	RAB5B	3487.139	-0.06468	0.184142	0.309423	protein_coding

ENSG0000(	FAM66E	44.94454	0.159297	0.184244	0.309578	lncRNA
ENSG0000(	ENSG0000(	19.23436	0.267551	0.184293	0.309644	processed_pseudogene
ENSG0000(	VPS26A	829.3105	-0.13645	0.184363	0.309745	protein_coding
ENSG0000(	LINC01554	67.14961	-0.15714	0.184539	0.310024	lncRNA
ENSG0000(	HP	138.3799	-0.12923	0.184575	0.310068	protein_coding
ENSG0000(	ENSG0000(	43.67343	-0.2245	0.184606	0.310103	lncRNA
ENSG0000(	PRDM14	79.53537	0.142733	0.184688	0.310202	protein_coding
ENSG0000(	SLC5A2	21.5436	0.372206	0.184687	0.310202	protein_coding
ENSG0000(	USP21P1	216.0961	-0.08868	0.184695	0.310202	processed_pseudogene
ENSG0000(	CBX8	117.4008	0.165785	0.184722	0.310231	protein_coding
ENSG0000(	RPL34-DT	246.1054	0.103014	0.184738	0.310236	lncRNA
ENSG0000(	SYNPO	3559.634	-0.15178	0.184745	0.310236	protein_coding
ENSG0000(	KIF24	288.4274	-0.16015	0.184818	0.310342	protein_coding
ENSG0000(	GRK6	932.0198	-0.1252	0.184887	0.310442	protein_coding
ENSG0000(	ENSG0000(	47.97292	0.210699	0.184918	0.310474	lncRNA
ENSG0000(	PLEKHG3	3456.469	-0.12355	0.184926	0.310474	protein_coding
ENSG0000(	IKZF2	1212.834	0.117379	0.184952	0.3105	protein_coding
ENSG0000(	TCERG1L	27.75799	-0.25799	0.184983	0.310536	protein_coding
ENSG0000(	ENSG0000(	489.8459	0.0706	0.184997	0.310542	TEC
ENSG0000(	SCYL2P1	238.7802	-0.10045	0.185031	0.310569	processed_pseudogene
ENSG0000(	SLC22A17	151.8395	0.160605	0.185033	0.310569	protein_coding
ENSG0000(	SERINC1	2215.52	-0.08099	0.185044	0.310571	protein_coding
ENSG0000(	C5orf63	780.8519	-0.11842	0.185076	0.310596	protein_coding
ENSG0000(	DBET	112.4316	-0.4517	0.185078	0.310596	lncRNA
ENSG0000(	APLP1	167.0096	0.107976	0.185149	0.31068	protein_coding
ENSG0000(	GLMP	443.544	-0.09101	0.185139	0.31068	protein_coding
ENSG0000(	ENSG0000(	5919.811	-0.09005	0.185165	0.310691	lncRNA
ENSG0000(	LINC02251	61.99654	-0.33468	0.185175	0.310692	lncRNA
ENSG0000(	ENSG0000(	10.86456	-0.35787	0.185206	0.310728	lncRNA
ENSG0000(	SCML4	1611.779	0.154749	0.185294	0.310857	protein_coding
ENSG0000(	ENSG0000(	167.1585	-0.10806	0.185311	0.310869	lncRNA
ENSG0000(	DEPTOR	268.6641	-0.2148	0.185357	0.310914	protein_coding
ENSG0000(	SLC39A9	2171.532	-0.0708	0.185356	0.310914	protein_coding
ENSG0000(	RN7SL262F	10.014	0.280813	0.185383	0.31094	misc_RNA
ENSG0000(	CTCF-DT	73.99861	0.189248	0.185407	0.310946	lncRNA
ENSG0000(	RBM4	3112.843	0.042239	0.185416	0.310946	protein_coding
ENSG0000(	SEMA3F	228.7152	-0.21727	0.185407	0.310946	protein_coding
ENSG0000(	ENSG0000(	132.3488	-0.08422	0.185433	0.310958	lncRNA
ENSG0000(	ENSG0000(	14.98653	-0.29312	0.18547	0.311003	TEC
ENSG0000(	ENSG0000(	15.24688	0.26011	0.1855	0.311037	lncRNA
ENSG0000(	VHL	2620.053	-0.04624	0.185532	0.311073	protein_coding
ENSG0000(	HBS1L	1508.285	-0.06923	0.185586	0.311147	protein_coding
ENSG0000(	DDN-AS1	40.50238	0.24084	0.185665	0.311264	lncRNA
ENSG0000(	MIR548F2	12.11593	0.280585	0.185689	0.311287	miRNA
ENSG0000(	BSN-AS1	103.3758	-0.10037	0.185719	0.311306	lncRNA
ENSG0000(	ENSG0000(	53.78071	0.175366	0.18574	0.311306	lncRNA
ENSG0000(	GVQW3	2429.486	-0.04852	0.185732	0.311306	protein_coding

ENSG0000(	WHAMMP3	1290.179	0.05516	0.185724	0.311306	transcribed_unprocessed_pseudo
ENSG0000(	CA5BP1	246.7576	-0.07729	0.185781	0.311309	transcribed_unprocessed_pseudo
ENSG0000(	CCDC14	7818.513	-0.10718	0.185777	0.311309	protein_coding
ENSG0000(	ENSG0000(	42.29429	-0.22359	0.185782	0.311309	processed_pseudogene
ENSG0000(	ENSG0000(	19.26954	-0.25779	0.185782	0.311309	lncRNA
ENSG0000(	REELD1	45.97086	0.210336	0.185816	0.311349	protein_coding
ENSG0000(	PCK1	3955.476	-0.38391	0.185828	0.311353	protein_coding
ENSG0000(	FBXL19-AS1	659.356	0.0621	0.185853	0.311378	lncRNA
ENSG0000(	ENSG0000(	24.75212	-0.19148	0.185869	0.311388	lncRNA
ENSG0000(	EXOC3	1787.089	-0.07789	0.185907	0.311436	protein_coding
ENSG0000(	ZNF101	2152.013	0.075775	0.185938	0.31147	protein_coding
ENSG0000(	ENSG0000(	10.95131	0.446839	0.185952	0.311478	lncRNA
ENSG0000(	NEK6	1820.347	-0.08479	0.185991	0.311525	protein_coding
ENSG0000(	ENSG0000(	38.64129	-0.16109	0.186079	0.311656	lncRNA
ENSG0000(	LINC02433	36.42536	0.166524	0.186139	0.311741	lncRNA
ENSG0000(	HOXD8	92.72606	-0.20221	0.186231	0.311878	protein_coding
ENSG0000(	ENSG0000(	396.9	0.075149	0.186256	0.311902	lncRNA
ENSG0000(	ENSG0000(	79.34777	-0.22751	0.186274	0.311916	lncRNA
ENSG0000(	TMEM62	858.6962	0.055483	0.186318	0.311973	protein_coding
ENSG0000(	ENSG0000(	367.6551	0.070569	0.18635	0.312011	processed_pseudogene
ENSG0000(	ENSG0000(	22.93108	0.204715	0.186446	0.312153	lncRNA
ENSG0000(	ENSG0000(	67.24873	0.15845	0.186484	0.312184	lncRNA
ENSG0000(	TUBA5P	268.2464	-0.11511	0.186483	0.312184	transcribed_unprocessed_pseudo
ENSG0000(	MINDY4	357.5288	0.092497	0.186619	0.312393	protein_coding
ENSG0000(	ENSG0000(	194.3306	-0.08332	0.18675	0.312596	lncRNA
ENSG0000(	U4	19.99368	0.193547	0.186926	0.312875	snRNA
ENSG0000(	TRAPPC3	746.5525	-0.05861	0.186941	0.312882	protein_coding
ENSG0000(	IGHV2-5	342.6822	0.334191	0.186988	0.312945	IG_V_gene
ENSG0000(	PSD4	4196.742	0.099823	0.187014	0.312971	protein_coding
ENSG0000(	TRBJ1-2	11.14643	0.511839	0.187041	0.313	TR_J_gene
ENSG0000(	ENSG0000(	1813.612	0.100331	0.187066	0.313025	lncRNA
ENSG0000(	ENSG0000(	133.913	0.147526	0.187115	0.313089	lncRNA
ENSG0000(	GNG12	3187.167	-0.17402	0.187127	0.313093	protein_coding
ENSG0000(	C16orf87	616.8985	-0.07372	0.187151	0.313099	protein_coding
ENSG0000(	LINC02210	2017.959	-0.06609	0.187144	0.313099	transcribed_unitary_pseudogene
ENSG0000(	ENSG0000(	317.4082	0.086946	0.187179	0.31313	lncRNA
ENSG0000(	ENSG0000(	466.7642	-0.44299	0.187208	0.313146	lncRNA
ENSG0000(	IL17RC	402.179	-0.09565	0.187204	0.313146	protein_coding
ENSG0000(	ENSG0000(	41.6634	0.226533	0.187224	0.313155	TEC
ENSG0000(	ENSG0000(	49.6946	-0.34448	0.187248	0.313162	TEC
ENSG0000(	PPIL6	325.7835	-0.07706	0.187241	0.313162	protein_coding
ENSG0000(	GABRB3	385.6028	-0.34453	0.187364	0.313339	protein_coding
ENSG0000(	ENSG0000(	12.64282	0.275135	0.187378	0.313346	lncRNA
ENSG0000(	TPD52	3955.256	-0.09207	0.1874	0.313366	protein_coding
ENSG0000(	P4HB	6643.906	-0.11345	0.187436	0.313409	protein_coding
ENSG0000(	ZCCHC7	1047.773	0.079173	0.187504	0.313506	protein_coding
ENSG0000(	PSMB8	775.8121	0.12169	0.187549	0.313564	protein_coding

ENSG0000(	ENSG0000(	15.03965	-0.34032	0.187582	0.313569	lncRNA
ENSG0000(	ENSG0000(	235.5327	-0.07725	0.187575	0.313569	TEC
ENSG0000(	ILDR2	253.9279	0.220504	0.187563	0.313569	protein_coding
ENSG0000(	ENSG0000(	59.51652	0.158653	0.187627	0.313628	processed_pseudogene
ENSG0000(	ITIH3	26.83401	0.464934	0.187791	0.313879	protein_coding
ENSG0000(	NEU1	970.3835	-0.10258	0.187797	0.313879	protein_coding
ENSG0000(	FHIP2B	1665.922	-0.04625	0.18785	0.313933	protein_coding
ENSG0000(	IKBKE	672.473	0.094069	0.18784	0.313933	protein_coding
ENSG0000(	RAPH1	3419.054	0.101396	0.187867	0.313945	protein_coding
ENSG0000(	EVL	4412.184	0.191063	0.187973	0.314104	protein_coding
ENSG0000(	ENSG0000(	123.9001	-0.14772	0.188003	0.314125	lncRNA
ENSG0000(	OR6D1P	815.3895	-0.09442	0.188005	0.314125	transcribed_unitary_pseudogene
ENSG0000(	EMB	936.2677	0.240583	0.188063	0.314205	protein_coding
ENSG0000(	RN7SL846F	16.08207	0.224077	0.188085	0.314226	misc_RNA
ENSG0000(	IFI35	388.6309	0.093068	0.188112	0.314254	protein_coding
ENSG0000(	ANXA2R	95.76285	0.229723	0.188141	0.314274	protein_coding
ENSG0000(	ENSG0000(	105.1663	-0.22581	0.188144	0.314274	processed_pseudogene
ENSG0000(	OBSCN	4553.636	-0.10797	0.188207	0.314362	protein_coding
ENSG0000(	ENSG0000(	20.23206	-0.25405	0.188227	0.314379	lncRNA
ENSG0000(	GTF3C2-AS	228.888	0.075677	0.188246	0.314393	lncRNA
ENSG0000(	ENSG0000(	9.83711	-0.34235	0.188273	0.314421	processed_pseudogene
ENSG0000(	PCDHB2	94.92135	-0.18008	0.18831	0.314467	protein_coding
ENSG0000(	RN7SL191F	17.27495	0.244338	0.188461	0.314702	misc_RNA
ENSG0000(	LINC01806	126.092	0.148586	0.188579	0.314883	lncRNA
ENSG0000(	ENSG0000(	419.4014	0.067248	0.188647	0.314979	lncRNA
ENSG0000(	MUC19	62.42591	-0.14458	0.188729	0.315098	polymorphic_pseudogene
ENSG0000(	FZD6	423.3346	-0.09573	0.188747	0.315109	protein_coding
ENSG0000(	SPINK1	440.6189	0.258759	0.188755	0.315109	protein_coding
ENSG0000(	ZNF252P-A	266.9354	-0.08947	0.188863	0.315273	lncRNA
ENSG0000(	DGLUCY	3468.144	0.080247	0.188879	0.315282	protein_coding
ENSG0000(	ENSG0000(	14.10051	0.223435	0.188891	0.315286	lncRNA
ENSG0000(	GPR135	288.0875	0.137982	0.188923	0.315322	protein_coding
ENSG0000(	PGAM1	1376.532	-0.11809	0.189025	0.315476	protein_coding
ENSG0000(	USP29	35.82445	0.174901	0.189038	0.31548	protein_coding
ENSG0000(	ENSG0000(	13.83423	0.22542	0.189077	0.315528	lncRNA
ENSG0000(	REXO1L1P	354.5893	-0.33255	0.189121	0.315585	transcribed_processed_pseudoge
ENSG0000(	LCTL	312.9535	0.071217	0.189143	0.315605	protein_coding
ENSG0000(	KCNAB1	23.1517	0.392531	0.189173	0.315639	protein_coding
ENSG0000(	ENSG0000(	162.0764	0.115927	0.189247	0.315745	lncRNA
ENSG0000(	ADORA3	85.24292	-0.23954	0.189276	0.315762	protein_coding
ENSG0000(	MRFAP1L1	796.428	-0.05956	0.189278	0.315762	protein_coding
ENSG0000(	CCNJ	390.0653	-0.07398	0.189357	0.315878	protein_coding
ENSG0000(	ENSG0000(	341.4263	0.071184	0.189437	0.315995	TEC
ENSG0000(	ZNF101P2	59.94327	-0.18579	0.18949	0.316067	processed_pseudogene
ENSG0000(	ANKHD1	7572.94	-0.04854	0.189511	0.316085	protein_coding
ENSG0000(	ABCA6	1837.296	0.111264	0.189537	0.31611	protein_coding
ENSG0000(	WNT7B	180.6612	0.097621	0.189569	0.316146	protein_coding

ENSG0000(	ENSG0000(	464.0216	0.076561	0.189592	0.316169	lncRNA
ENSG0000(	ENSG0000(	100.6304	0.238175	0.189621	0.3162	lncRNA
ENSG0000(	KLHL32	59.4865	0.226475	0.189662	0.31624	protein_coding
ENSG0000(	MAP1LC3C	42.27963	-0.22387	0.189665	0.31624	protein_coding
ENSG0000(	ENSG0000(	18.02217	0.250259	0.189711	0.316299	lncRNA
ENSG0000(	ENSG0000(	195.6334	0.141794	0.189851	0.316516	lncRNA
ENSG0000(	SNTG1	121.6436	-0.12733	0.189927	0.316625	protein_coding
ENSG0000(	SNORD6	11.72407	-0.25502	0.189949	0.316646	snoRNA
ENSG0000(	INO80D	5061.936	-0.0484	0.18996	0.316647	protein_coding
ENSG0000(	RAB11FIP3	1024.972	-0.088	0.189984	0.316671	protein_coding
ENSG0000(	ENSG0000(	26.85302	0.208598	0.19011	0.316863	lncRNA
ENSG0000(	GART	1258.952	-0.05932	0.190145	0.316905	protein_coding
ENSG0000(	HCG18	7871.216	0.051229	0.190175	0.316925	lncRNA
ENSG0000(	ULK4P1	12.02229	0.522447	0.190177	0.316925	transcribed_unprocessed_pseudo
ENSG0000(	ENSG0000(	96.11495	-0.12193	0.190231	0.316997	lncRNA
ENSG0000(	AGBL2	259.6578	-0.10071	0.190252	0.317015	protein_coding
ENSG0000(	RN7SL178F	35.46803	0.145317	0.190274	0.317036	misc_RNA
ENSG0000(	ENSG0000(	152.2555	-0.0843	0.190345	0.317117	lncRNA
ENSG0000(	ENSG0000(	1958.044	-0.20212	0.190353	0.317117	lncRNA
ENSG0000(	ENSG0000(	496.625	0.056011	0.190351	0.317117	TEC
ENSG0000(	PCLO	2198.612	-0.18746	0.190418	0.317207	protein_coding
ENSG0000(	DOCK7	4638.116	-0.11037	0.190499	0.317292	protein_coding
ENSG0000(	ERBB3	7843.182	-0.13568	0.190483	0.317292	protein_coding
ENSG0000(	LINC01633	9.870317	0.326437	0.190496	0.317292	lncRNA
ENSG0000(	LDHC	30.80094	-0.24391	0.19053	0.317314	protein_coding
ENSG0000(	TBC1D4	1807.79	-0.15016	0.190533	0.317314	protein_coding
ENSG0000(	SAXO1	114.5176	0.184553	0.190831	0.317794	protein_coding
ENSG0000(	ENSG0000(	30.53108	0.159688	0.190855	0.317817	lncRNA
ENSG0000(	ARHGAP19	12.46178	0.385027	0.190951	0.317946	protein_coding
ENSG0000(	POLR2D	1644.105	-0.05111	0.190953	0.317946	protein_coding
ENSG0000(	ENSG0000(	79.03107	-0.13803	0.190993	0.317996	lncRNA
ENSG0000(	ENSG0000(	25.85855	0.44247	0.19105	0.318075	unprocessed_pseudogene
ENSG0000(	ENSG0000(	211.3751	0.089801	0.191073	0.318086	lncRNA
ENSG0000(	LINC02832	646.5096	-0.06938	0.191078	0.318086	lncRNA
ENSG0000(	AFG3L1P	1584.221	-0.06147	0.191094	0.318096	transcribed_unitary_pseudogene
ENSG0000(	OS9	2737.7	-0.06567	0.191108	0.318103	protein_coding
ENSG0000(	ISL1	30.70777	0.280947	0.191148	0.318153	protein_coding
ENSG0000(	ENSG0000(	196.1238	-0.25251	0.19117	0.318172	lncRNA
ENSG0000(	RIOK2	580.996	-0.0838	0.191186	0.318182	protein_coding
ENSG0000(	ENSG0000(	51.05782	0.191467	0.191207	0.3182	TEC
ENSG0000(	ARMCX5	558.6302	0.066715	0.191234	0.318229	protein_coding
ENSG0000(	LTF	130.9338	0.401941	0.191337	0.318383	protein_coding
ENSG0000(	FAM219B	2192.89	-0.07355	0.19139	0.318454	protein_coding
ENSG0000(	RASSF6	2009.554	-0.10287	0.19146	0.318553	protein_coding
ENSG0000(	PHLDA3	299.1648	0.104287	0.191484	0.318576	protein_coding
ENSG0000(	ENSG0000(	12.7133	0.371477	0.191524	0.318626	protein_coding
ENSG0000(	ENSG0000(	18.20678	-0.30632	0.191539	0.318634	lncRNA

ENSG0000(	ENSG0000(	120.7746	-0.13048	0.191629	0.318732	lncRNA
ENSG0000(	PCBP1-AS1	6137.602	0.041171	0.191621	0.318732	lncRNA
ENSG0000(	PDE11A-AS	35.9644	0.32789	0.19162	0.318732	lncRNA
ENSG0000(	SEC23B	864.3796	-0.09323	0.191639	0.318732	protein_coding
ENSG0000(	CELF4	99.22036	0.246835	0.191668	0.318765	protein_coding
ENSG0000(	ENSG0000(	587.5256	0.076985	0.191679	0.318765	protein_coding
ENSG0000(	LRRC4C	34.21712	0.264979	0.191735	0.318842	protein_coding
ENSG0000(	HNRNPC	6390.646	0.06888	0.191747	0.318844	protein_coding
ENSG0000(	ENSG0000(	16.97774	0.278851	0.191785	0.318891	lncRNA
ENSG0000(	CTNS	522.0787	0.079438	0.192071	0.319335	protein_coding
ENSG0000(	ENSG0000(	21.39604	0.218227	0.192073	0.319335	lncRNA
ENSG0000(	ENSG0000(	8.690554	0.309242	0.192172	0.319482	scaRNA
ENSG0000(	IL32	1062.441	-0.19412	0.192211	0.319513	protein_coding
ENSG0000(	ZKSCAN1	8381.106	-0.05991	0.192208	0.319513	protein_coding
ENSG0000(	ZNF18	387.7992	-0.1071	0.192247	0.319556	protein_coding
ENSG0000(	ENSG0000(	14.03484	0.241075	0.192526	0.320003	lncRNA
ENSG0000(	ZMAT5	373.8728	0.068275	0.192537	0.320005	protein_coding
ENSG0000(	ENSG0000(	16.78614	0.19995	0.192594	0.320031	lncRNA
ENSG0000(	HGD	168.7599	0.200701	0.192592	0.320031	protein_coding
ENSG0000(	IQSEC2	1252.569	0.081888	0.192574	0.320031	protein_coding
ENSG0000(	ZNF672	735.9048	0.047336	0.19258	0.320031	protein_coding
ENSG0000(	TPGS2	1935.436	0.073477	0.192607	0.320036	protein_coding
ENSG0000(	IAPP	223.9412	-0.11327	0.192624	0.320048	protein_coding
ENSG0000(	GPX6	11.33683	-0.30003	0.192641	0.320058	protein_coding
ENSG0000(	AQP12B	93.97256	-0.42162	0.192727	0.320168	protein_coding
ENSG0000(	ENSG0000(	23.62593	0.221917	0.19272	0.320168	lncRNA
ENSG0000(	ENSG0000(	22.07752	0.204442	0.192781	0.32024	lncRNA
ENSG0000(	KLHDC9	38.80676	-0.25794	0.192861	0.32035	protein_coding
ENSG0000(	NCS1	134.7809	0.332604	0.192868	0.32035	protein_coding
ENSG0000(	IGHV3-41	10.11884	0.426701	0.192881	0.320356	IG_V_pseudogene
ENSG0000(	DUS4L-BCA	402.8649	0.080277	0.193067	0.320646	protein_coding
ENSG0000(	CHORDC1P	62.04744	-0.10066	0.193104	0.320674	processed_pseudogene
ENSG0000(	ENSG0000(	47.52172	0.18762	0.193099	0.320674	lncRNA
ENSG0000(	BRD1	1621.319	-0.06205	0.193135	0.320708	protein_coding
ENSG0000(	RN7SKP89	15.20277	-0.34212	0.193195	0.320792	misc_RNA
ENSG0000(	ADAL	509.2293	-0.0827	0.193283	0.32092	protein_coding
ENSG0000(	RN7SKP184	40.76679	0.238644	0.193304	0.320938	misc_RNA
ENSG0000(	UNC5C-AS1	32.40898	0.181395	0.193325	0.320956	lncRNA
ENSG0000(	ENSG0000(	24.52476	-0.4105	0.193354	0.320987	lncRNA
ENSG0000(	ENSG0000(	63.62509	0.133463	0.193408	0.321031	lncRNA
ENSG0000(	ENSG0000(	225.9587	0.085604	0.193411	0.321031	lncRNA
ENSG0000(	ERCC1	974.2287	-0.06357	0.1934	0.321031	protein_coding
ENSG0000(	ADGRL2	1425.061	0.119185	0.193462	0.321099	protein_coding
ENSG0000(	ENSG0000(	26.5505	0.183476	0.193535	0.321186	lncRNA
ENSG0000(	RPS3P1	19.77761	-0.25271	0.193532	0.321186	processed_pseudogene
ENSG0000(	SHB	663.2338	-0.12945	0.193599	0.321274	protein_coding
ENSG0000(	ENSG0000(	67.30684	0.14179	0.193638	0.321289	lncRNA

ENSG0000(	ENSG0000(	19.63271	0.344455	0.193629	0.321289	lncRNA
ENSG0000(	PRELID2	4681.889	-0.15595	0.193627	0.321289	protein_coding
ENSG0000(	CCDC168	36.04108	0.234201	0.193689	0.321346	protein_coding
ENSG0000(	ENSG0000(	10.91616	-0.39597	0.193697	0.321346	processed_pseudogene
ENSG0000(	HOXC4	41.58786	0.276013	0.193704	0.321346	protein_coding
ENSG0000(	RN7SKP119	13.95602	-0.27406	0.193728	0.321369	misc_RNA
ENSG0000(	C10orf67	90.64438	0.107509	0.193749	0.321387	protein_coding
ENSG0000(	IMMP1L	719.6829	-0.13725	0.193772	0.321409	protein_coding
ENSG0000(	ENSG0000(	554.0762	0.105914	0.193805	0.321445	lncRNA
ENSG0000(	NUDCP1	59.35409	-0.15482	0.193825	0.321462	processed_pseudogene
ENSG0000(	HMGCR	2324.622	-0.10543	0.193964	0.321676	protein_coding
ENSG0000(	FEM1B	2264.249	-0.06177	0.194043	0.321789	protein_coding
ENSG0000(	TMEM131L	1019.404	0.226505	0.194055	0.321792	protein_coding
ENSG0000(	IGHV3-13	103.0589	0.349687	0.194083	0.321807	IG_V_gene
ENSG0000(	STAU2-AS1	119.9461	-0.10125	0.194085	0.321807	lncRNA
ENSG0000(	PARM1	4522.904	0.157875	0.194101	0.321818	protein_coding
ENSG0000(	VPS26B	1296.132	-0.05054	0.194131	0.32185	protein_coding
ENSG0000(	ARHGAP19	587.88	-0.0914	0.194168	0.321878	protein_coding
ENSG0000(	TOR1AIP2	7317.843	-0.06217	0.194161	0.321878	protein_coding
ENSG0000(	SRRM2-AS1	327.5616	-0.06316	0.194189	0.321896	lncRNA
ENSG0000(	ENSG0000(	3750.795	-0.05984	0.194241	0.321964	lncRNA
ENSG0000(	TRIM10	184.7184	-0.20336	0.194282	0.322015	protein_coding
ENSG0000(	ENSG0000(	29.56697	0.216199	0.194347	0.322089	lncRNA
ENSG0000(	LINC02885	32.01299	0.224882	0.194337	0.322089	lncRNA
ENSG0000(	OAZ2	2083.96	0.10893	0.194402	0.322163	protein_coding
ENSG0000(	ENSG0000(	9.032821	0.385546	0.194432	0.322196	lncRNA
ENSG0000(	C4orf46	140.8976	-0.17277	0.194472	0.322244	protein_coding
ENSG0000(	MAD2L1P1	23.97564	0.237628	0.194487	0.322248	processed_pseudogene
ENSG0000(	PRPH	15.49969	0.382538	0.194495	0.322248	protein_coding
ENSG0000(	ENSG0000(	267.9656	-0.07848	0.194529	0.322287	lncRNA
ENSG0000(	ZBTB25	2845.104	0.078194	0.194579	0.322353	protein_coding
ENSG0000(	LINC02014	27.34696	-0.18303	0.194637	0.322433	lncRNA
ENSG0000(	ENSG0000(	120.52	-0.09198	0.194652	0.322441	lncRNA
ENSG0000(	AVIL	1387.713	-0.18025	0.194685	0.322445	protein_coding
ENSG0000(	ENSG0000(	9.801958	0.311841	0.194674	0.322445	TEC
ENSG0000(	ZNF529	1089.507	0.064008	0.194682	0.322445	protein_coding
ENSG0000(	CPAMD8	515.7777	-0.0756	0.194796	0.322594	protein_coding
ENSG0000(	FANCB	136.9757	-0.19496	0.19479	0.322594	protein_coding
ENSG0000(	ALK	40.12093	-0.40783	0.194947	0.322827	protein_coding
ENSG0000(	ENSG0000(	469.2766	-0.08352	0.194974	0.322854	lncRNA
ENSG0000(	MIR5689H(	258.2813	0.074934	0.19499	0.322863	lncRNA
ENSG0000(	ENSG0000(	59.91282	-0.13493	0.195074	0.322985	lncRNA
ENSG0000(	ENSG0000(	127.7939	-0.09165	0.195091	0.322996	lncRNA
ENSG0000(	FKTN	1974.818	0.064979	0.195108	0.323007	protein_coding
ENSG0000(	ENSG0000(	15.41386	0.333564	0.195144	0.323042	lncRNA
ENSG0000(	STAC2	194.147	0.093977	0.195149	0.323042	protein_coding
ENSG0000(	PHF2P2	10.36644	0.415212	0.19528	0.323241	transcribed_unprocessed_pseudo

ENSG0000(	OVCA2	36.96192	-0.36495	0.195354	0.323346	protein_coding
ENSG0000(	THUMPD2	454.3704	-0.11698	0.195386	0.323382	protein_coding
ENSG0000(	ENSG0000(	75.58802	-0.12005	0.195487	0.323515	lncRNA
ENSG0000(	ENSG0000(	19.55279	0.211932	0.195477	0.323515	lncRNA
ENSG0000(	EIF2B3	400.2328	-0.08955	0.195578	0.323627	protein_coding
ENSG0000(	MSANTD2	811.7026	-0.07029	0.195586	0.323627	protein_coding
ENSG0000(	PABPC1L	817.9584	0.09881	0.195565	0.323627	protein_coding
ENSG0000(	KIF13A	2161.162	0.108084	0.195602	0.323637	protein_coding
ENSG0000(	ENSG0000(	192.7002	-0.10483	0.195618	0.323647	lncRNA
ENSG0000(	CPNE1	1162.06	-0.06724	0.195707	0.323741	protein_coding
ENSG0000(	CYP27A1	294.9525	-0.1775	0.195694	0.323741	protein_coding
ENSG0000(	ENSG0000(	184.3233	0.087644	0.195697	0.323741	lncRNA
ENSG0000(	DRC3	205.2214	0.084219	0.195737	0.323757	protein_coding
ENSG0000(	RNU6-126F	23.75801	0.247494	0.195728	0.323757	snRNA
ENSG0000(	HNRNPA1P	27.26344	-0.37114	0.195777	0.323806	processed_pseudogene
ENSG0000(	NFKBIB	696.5928	0.065036	0.195903	0.323997	protein_coding
ENSG0000(	ENSG0000(	105.0203	-0.10471	0.195951	0.32406	lncRNA
ENSG0000(	ENSG0000(	18.5892	-0.29654	0.195966	0.324068	lncRNA
ENSG0000(	ENSG0000(	231.6599	-0.10673	0.19606	0.324207	lncRNA
ENSG0000(	ABCG1	1225.758	0.105364	0.196183	0.32439	protein_coding
ENSG0000(	ERBIN	7893.804	-0.1094	0.196192	0.32439	protein_coding
ENSG0000(	GRIK5	49.48873	-0.24794	0.19637	0.324667	protein_coding
ENSG0000(	ASCL2	70.73714	-0.26734	0.196399	0.324698	protein_coding
ENSG0000(	CWC25	1073.26	-0.06002	0.196539	0.324912	protein_coding
ENSG0000(	RN7SL745F	17.70059	0.207813	0.196614	0.325019	misc_RNA
ENSG0000(	ENSG0000(	165.2632	0.123563	0.196687	0.325121	lncRNA
ENSG0000(	ITPRID2-DT	189.9768	-0.10153	0.196696	0.325121	lncRNA
ENSG0000(	ENSG0000(	68.90357	0.144546	0.196741	0.325151	lncRNA
ENSG0000(	PON2	1632.695	-0.10435	0.196746	0.325151	protein_coding
ENSG0000(	RNU6-316F	11.21728	0.362945	0.196726	0.325151	snRNA
ENSG0000(	CCDC17	452.4133	-0.14681	0.196779	0.325188	protein_coding
ENSG0000(	ENSG0000(	54.44868	-0.14125	0.196829	0.325254	lncRNA
ENSG0000(	GLE1	2150.798	-0.02802	0.196858	0.325284	protein_coding
ENSG0000(	HFM1	41.65121	0.341806	0.196872	0.32529	protein_coding
ENSG0000(	ABCG4	26.37541	0.22072	0.196887	0.325298	protein_coding
ENSG0000(	ENSG0000(	12.93631	0.264738	0.196931	0.325354	lncRNA
ENSG0000(	CADM4	42.25333	0.25917	0.197076	0.325544	protein_coding
ENSG0000(	ENSG0000(	35.97855	0.248687	0.197077	0.325544	processed_pseudogene
ENSG0000(	RPL13AP2C	16.21628	-0.24799	0.197069	0.325544	processed_pseudogene
ENSG0000(	PTDSS2	439.3527	-0.08359	0.197105	0.325572	protein_coding
ENSG0000(	DDX11	1991.225	-0.09405	0.197121	0.325582	protein_coding
ENSG0000(	INGX	197.029	-0.09114	0.197137	0.325591	transcribed_processed_pseudoge
ENSG0000(	TMEM33	2661.492	-0.05607	0.197166	0.325621	protein_coding
ENSG0000(	NLK	1077.132	0.070905	0.197178	0.325624	protein_coding
ENSG0000(	TERB2	191.6237	-0.08462	0.197247	0.325721	protein_coding
ENSG0000(	RPS4XP16	38.67167	-0.1986	0.197262	0.325729	transcribed_processed_pseudoge
ENSG0000(	ENSG0000(	91.90445	-0.11474	0.197275	0.325732	lncRNA

ENSG0000(	ZNF226	1265.718	0.077244	0.197377	0.325884	protein_coding
ENSG0000(	TRIM71	52.0529	0.222063	0.197408	0.325918	protein_coding
ENSG0000(	ALG13-AS1	20.77926	-0.22247	0.197472	0.325989	lncRNA
ENSG0000(	MPZ	91.36754	0.148228	0.197467	0.325989	protein_coding
ENSG0000(	LINC01716	34.11009	0.179969	0.197504	0.326025	lncRNA
ENSG0000(	PRIM2	711.6151	-0.06551	0.197611	0.326185	protein_coding
ENSG0000(	TRAF3IP2-/	1126.285	0.062811	0.197643	0.32622	lncRNA
ENSG0000(	ADCY10P1	562.3133	-0.09337	0.197726	0.326322	transcribed_unprocessed_pseudo
ENSG0000(	NFATC4	602.0407	0.176237	0.197715	0.326322	protein_coding
ENSG0000(	CBWD2	966.989	-0.07481	0.197759	0.326342	protein_coding
ENSG0000(	ENSG0000(	302.2627	0.181563	0.197756	0.326342	lncRNA
ENSG0000(	ENSG0000(	167.0273	-2.22372	0.197778	0.326357	protein_coding
ENSG0000(	C1orf127	68.73266	0.119286	0.197917	0.326569	protein_coding
ENSG0000(	RN7SL492F	15.47461	0.230635	0.197963	0.326627	misc_RNA
ENSG0000(	RNU6-125F	30.84716	-0.16455	0.198045	0.326745	snRNA
ENSG0000(	MAPK8IP3	4143.199	-0.06651	0.198056	0.326747	protein_coding
ENSG0000(	RNU1-63P	13.93197	0.282949	0.198077	0.326763	snRNA
ENSG0000(	TMEM234	676.5297	-0.07182	0.198097	0.326779	protein_coding
ENSG0000(	MAP4K3	969.8177	-0.08737	0.198155	0.326858	protein_coding
ENSG0000(	RMC1	543.3786	-0.07409	0.198208	0.326928	protein_coding
ENSG0000(	PCSK9	253.8222	0.330043	0.198241	0.326966	protein_coding
ENSG0000(	ENSG0000(	457.1372	-0.08631	0.198269	0.326978	lncRNA
ENSG0000(	ENSG0000(	71.01461	0.165361	0.198265	0.326978	lncRNA
ENSG0000(	ANXA3	597.1735	0.142715	0.198295	0.327002	protein_coding
ENSG0000(	ATP11B	3927.281	0.06324	0.198313	0.327014	protein_coding
ENSG0000(	ENSG0000(	722.8172	0.068114	0.198358	0.327053	lncRNA
ENSG0000(	MPHOSPH(	368.8662	-0.09235	0.198367	0.327053	protein_coding
ENSG0000(	RN7SL615F	71.23324	0.12362	0.198352	0.327053	misc_RNA
ENSG0000(	MOAP1	480.2401	-0.05384	0.198395	0.327081	protein_coding
ENSG0000(	SLC27A6	17.51319	-0.36293	0.198425	0.327113	protein_coding
ENSG0000(	ENSG0000(	10.79648	0.303811	0.1985	0.327219	lncRNA
ENSG0000(	RFX8	20.9828	1.029892	0.198528	0.327248	protein_coding
ENSG0000(	RNFT1	633.4392	-0.07828	0.198584	0.327324	protein_coding
ENSG0000(	SCML2P2	50.04583	0.172627	0.198611	0.327351	processed_pseudogene
ENSG0000(	SNORA5C	96.09807	-0.13066	0.198636	0.327375	snoRNA
ENSG0000(	IQCH	90.21842	0.146237	0.198708	0.327459	protein_coding
ENSG0000(	KDM4D	13.3685	0.250618	0.198718	0.327459	protein_coding
ENSG0000(	MMP23B	50.4555	-0.20661	0.198699	0.327459	protein_coding
ENSG0000(	ZNF800	1525.305	-0.06032	0.198737	0.327472	protein_coding
ENSG0000(	ENSG0000(	40.50952	0.215398	0.198795	0.327551	lncRNA
ENSG0000(	ENSG0000(	22.99719	-0.25225	0.19884	0.327608	lncRNA
ENSG0000(	YY1AP1	2023.839	-0.03669	0.198874	0.327646	protein_coding
ENSG0000(	NNT-AS1	2393.341	0.039857	0.198918	0.327702	lncRNA
ENSG0000(	ENSG0000(	66.45314	0.132767	0.198977	0.327782	lncRNA
ENSG0000(	PIK3CD	1232.339	0.229563	0.199016	0.327829	protein_coding
ENSG0000(	IFRD1	2263.472	0.126494	0.199048	0.327864	protein_coding
ENSG0000(	BIVM	1788.574	-0.05414	0.199124	0.327966	protein_coding

ENSG0000(	SLC24A4	789.252	0.088385	0.199131	0.327966	protein_coding
ENSG0000(	KRT17P7	16.95045	1.251806	0.199162	0.328	transcribed_unprocessed_pseudo
ENSG0000(	SLC22A10	162.4553	-0.09267	0.199177	0.328008	protein_coding
ENSG0000(	YWHAQ	1904.031	-0.10909	0.199299	0.328191	protein_coding
ENSG0000(	TRAT1	80.38456	0.31225	0.199321	0.32821	protein_coding
ENSG0000(	ENSG0000(	16.014	0.223736	0.199608	0.328666	lncRNA
ENSG0000(	ITFG2-AS1	26.4319	0.189261	0.199659	0.328733	lncRNA
ENSG0000(	TMEM263-	22.41348	0.285639	0.199735	0.328839	lncRNA
ENSG0000(	ENSG0000(	31.09434	-0.20304	0.199766	0.328873	lncRNA
ENSG0000(	ENSG0000(	64.90428	0.168618	0.199838	0.328944	lncRNA
ENSG0000(	ENSG0000(	233.7022	0.120133	0.19984	0.328944	lncRNA
ENSG0000(	LINC00355	376.6217	-0.07024	0.199837	0.328944	lncRNA
ENSG0000(	GSDMA	297.8727	0.069174	0.199891	0.32901	protein_coding
ENSG0000(	LINC02680	536.6027	0.058059	0.199927	0.329052	lncRNA
ENSG0000(	ENSG0000(	61.04244	0.305313	0.199971	0.329107	transcribed_unprocessed_pseudo
ENSG0000(	ENSG0000(	13.0047	-0.25156	0.200023	0.329176	lncRNA
ENSG0000(	SLC30A2	118.6959	0.091526	0.200048	0.329198	protein_coding
ENSG0000(	ENSG0000(	34.98087	0.282508	0.200076	0.3292	lncRNA
ENSG0000(	LINC01117	24.91518	0.219576	0.200071	0.3292	lncRNA
ENSG0000(	SNORA57	532.9637	-0.16103	0.20008	0.3292	snoRNA
ENSG0000(	ENSG0000(	17.08302	-0.41674	0.200109	0.32923	lncRNA
ENSG0000(	TBX3-AS1	58.40114	-0.119	0.200138	0.32926	lncRNA
ENSG0000(	GRM2	77.01748	0.211146	0.200168	0.329293	protein_coding
ENSG0000(	CCDC65	12.13065	0.348912	0.200196	0.329311	protein_coding
ENSG0000(	FAM230A	104.4499	-0.12427	0.2002	0.329311	lncRNA
ENSG0000(	ENSG0000(	14.29247	0.242696	0.200345	0.329532	lncRNA
ENSG0000(	SH3GL3	62.60271	-0.14533	0.200386	0.329582	protein_coding
ENSG0000(	RN7SL65P	25.92471	0.184941	0.200403	0.329593	misc_RNA
ENSG0000(	GNRH2	26.72442	-0.24255	0.200435	0.329628	protein_coding
ENSG0000(	ENSG0000(	17.38722	0.214308	0.200453	0.329641	snoRNA
ENSG0000(	ENSG0000(	132.3661	0.229287	0.200464	0.329642	TEC
ENSG0000(	BLOC1S3	375.5672	-0.06518	0.200491	0.329668	protein_coding
ENSG0000(	ENSG0000(	72.84961	-0.10565	0.200524	0.329704	TEC
ENSG0000(	ENSG0000(	16.91625	-0.26992	0.200538	0.329708	lncRNA
ENSG0000(	ENSG0000(	9.855464	0.508343	0.200547	0.329708	IG_V_pseudogene
ENSG0000(	ENOX1-AS1	87.02026	-0.10643	0.200645	0.329834	lncRNA
ENSG0000(	ENSG0000(	9.648286	-0.41495	0.200637	0.329834	lncRNA
ENSG0000(	ACD	123.7758	-0.15201	0.200668	0.329856	protein_coding
ENSG0000(	CCDC144CI	480.4898	0.087194	0.200782	0.330011	transcribed_processed_pseudoge
ENSG0000(	ENSG0000(	374.4959	0.089544	0.20079	0.330011	lncRNA
ENSG0000(	SRP68	1140.925	0.051904	0.200795	0.330011	protein_coding
ENSG0000(	HNRNPUL2	163.1876	-2.06997	0.200808	0.330015	protein_coding
ENSG0000(	ENSG0000(	857.534	-0.07333	0.200832	0.330038	lncRNA
ENSG0000(	AAR2	783.3587	-0.05383	0.200871	0.330067	protein_coding
ENSG0000(	CHKA	1782.884	-0.16162	0.200871	0.330067	protein_coding
ENSG0000(	FBXO36	299.2827	-0.08571	0.200888	0.330078	protein_coding
ENSG0000(	H4C10P	8.868663	-0.42807	0.201047	0.330321	processed_pseudogene

ENSG0000(	CLDND1	1273.875	0.077731	0.201115	0.330416	protein_coding
ENSG0000(	LHPP	262.4971	0.111545	0.201152	0.330459	protein_coding
ENSG0000(	ENSG0000(	436.6879	-0.09468	0.201206	0.330513	protein_coding
ENSG0000(	FGFBP3	68.9906	0.152867	0.201197	0.330513	protein_coding
ENSG0000(	ACADL	518.4078	-0.07003	0.201277	0.330578	protein_coding
ENSG0000(	ENSG0000(	18.85672	0.267029	0.201256	0.330578	lncRNA
ENSG0000(	LINC01494	11.2414	0.303098	0.201269	0.330578	lncRNA
ENSG0000(	TUSC1	135.5231	-0.1328	0.201383	0.330735	protein_coding
ENSG0000(	ENSG0000(	57.234	-0.15137	0.201471	0.330863	lncRNA
ENSG0000(	MED17	3146.288	-0.03324	0.201511	0.330909	protein_coding
ENSG0000(	NSG1	255.9356	0.13692	0.201521	0.330909	protein_coding
ENSG0000(	PIGG	2299.183	-0.04406	0.201553	0.330945	protein_coding
ENSG0000(	MAP3K11	2820.647	-0.06101	0.201625	0.331045	protein_coding
ENSG0000(	CCDC97	748.7067	-0.06369	0.201665	0.331093	protein_coding
ENSG0000(	H2AW	30.36746	-0.33775	0.201687	0.331112	protein_coding
ENSG0000(	ENSG0000(	17.71653	0.230949	0.201698	0.331113	lncRNA
ENSG0000(	DZIP3	1117.311	0.071741	0.201923	0.33143	protein_coding
ENSG0000(	ELOF1	450.4718	-0.07962	0.20191	0.33143	protein_coding
ENSG0000(	ENSG0000(	25.94672	-0.28664	0.201914	0.33143	lncRNA
ENSG0000(	ENSG0000(	12.12457	0.303387	0.201955	0.331448	lncRNA
ENSG0000(	UHRF1BP1	1773.488	0.055805	0.201946	0.331448	protein_coding
ENSG0000(	ENSG0000(	601.8589	0.052547	0.20199	0.331487	lncRNA
ENSG0000(	TRAF5	2976.353	0.103496	0.202002	0.33149	protein_coding
ENSG0000(	AAAS	537.3868	-0.06576	0.202055	0.331507	protein_coding
ENSG0000(	ENSG0000(	88.64048	-0.11166	0.202045	0.331507	lncRNA
ENSG0000(	SLC25A16	2682.507	-0.06209	0.202054	0.331507	protein_coding
ENSG0000(	ZC3H3	863.4164	0.136426	0.202025	0.331507	protein_coding
ENSG0000(	ULK1	939.9016	0.08571	0.202069	0.331513	protein_coding
ENSG0000(	ZNF14	371.3231	-0.08678	0.202099	0.331546	protein_coding
ENSG0000(	PTPRD	1971.007	-0.1719	0.202151	0.331613	protein_coding
ENSG0000(	ABCC11	119.492	-0.11495	0.202248	0.331744	protein_coding
ENSG0000(	TRIM59	880.0152	-0.06986	0.202252	0.331744	protein_coding
ENSG0000(	DHRS4-AS1	2245.245	0.036366	0.202318	0.331799	lncRNA
ENSG0000(	ENSG0000(	22.76607	0.269269	0.202317	0.331799	lncRNA
ENSG0000(	ENSG0000(	13.6623	-0.26897	0.202315	0.331799	lncRNA
ENSG0000(	APTR	1248.425	-0.0594	0.202367	0.331863	lncRNA
ENSG0000(	COPS8	626.2812	0.07641	0.202426	0.331917	protein_coding
ENSG0000(	OR5AN1	223.7089	0.099162	0.202431	0.331917	protein_coding
ENSG0000(	TEX2	1362.058	-0.07545	0.202432	0.331917	protein_coding
ENSG0000(	ENSG0000(	13.07157	0.356983	0.202476	0.331972	processed_pseudogene
ENSG0000(	ENSG0000(	29.55532	0.167285	0.202504	0.331984	lncRNA
ENSG0000(	RN7SL851P	10.86882	0.295516	0.202502	0.331984	misc_RNA
ENSG0000(	TTC31	917.6143	-0.0619	0.202624	0.332162	protein_coding
ENSG0000(	ADAMTS7P	105.2303	-0.16099	0.202658	0.332201	transcribed_unprocessed_pseudo
ENSG0000(	ZC3HC1	227.3901	-0.09562	0.202704	0.332259	protein_coding
ENSG0000(	ENSG0000(	36.27528	0.164184	0.202809	0.332414	lncRNA
ENSG0000(	FAM219A	500.085	0.107656	0.202931	0.332567	protein_coding

ENSG0000(	PITPNA-AS:	28.62244	-0.19658	0.202922	0.332567	lncRNA
ENSG0000(	TMEM267	321.3186	-0.08621	0.202935	0.332567	protein_coding
ENSG0000(	LMNTD1	159.8059	-0.10917	0.20296	0.332591	protein_coding
ENSG0000(	BAK1P1	13.85631	-0.28542	0.202989	0.332621	processed_pseudogene
ENSG0000(	C11orf24	699.5269	-0.09472	0.203032	0.332657	protein_coding
ENSG0000(	FHIT	757.6422	-0.08054	0.203031	0.332657	protein_coding
ENSG0000(	ENSG0000(	73.23288	0.181241	0.203058	0.332665	lncRNA
ENSG0000(	ZFP69B	92.8964	0.147237	0.203052	0.332665	protein_coding
ENSG0000(	Vault	49.77456	0.197128	0.203072	0.332671	misc_RNA
ENSG0000(	COPE	679.5552	-0.10666	0.203116	0.332707	protein_coding
ENSG0000(	TAF1A	264.2704	-0.09192	0.203113	0.332707	protein_coding
ENSG0000(	ENSG0000(	23.55323	0.201489	0.203143	0.332734	transcribed_unprocessed_pseudo
ENSG0000(	LINC02666	22.45443	0.219923	0.203159	0.332743	lncRNA
ENSG0000(	PPIAP2	35.2113	-0.14868	0.203275	0.332916	processed_pseudogene
ENSG0000(	DCTN4	1929.016	0.052253	0.203302	0.332942	protein_coding
ENSG0000(	ENSG0000(	341.987	-0.06433	0.203339	0.332972	TEC
ENSG0000(	METTL27	83.33338	-0.23507	0.203342	0.332972	protein_coding
ENSG0000(	NFRKB	1056.192	0.075379	0.203369	0.332999	protein_coding
ENSG0000(	ACSM5	33.05644	-0.25536	0.203424	0.333073	protein_coding
ENSG0000(	ENSG0000(	18.27427	-0.2295	0.203446	0.33309	lncRNA
ENSG0000(	ENSG0000(	42.00419	-0.155	0.203456	0.33309	lncRNA
ENSG0000(	ACRP1	13.08869	-0.36339	0.203504	0.333151	processed_pseudogene
ENSG0000(	ENSG0000(	12.02699	-0.3447	0.20352	0.33316	lncRNA
ENSG0000(	SNORA31B	103.8016	-0.19284	0.203616	0.3333	snoRNA
ENSG0000(	MED14	2538.627	-0.06396	0.203655	0.333346	protein_coding
ENSG0000(	ENSG0000(	161.2346	-0.09668	0.203802	0.333569	lncRNA
ENSG0000(	CTTN-DT	140.45	-0.13182	0.203883	0.333632	lncRNA
ENSG0000(	ENSG0000(	49.55914	-0.26431	0.203855	0.333632	TEC
ENSG0000(	PIN4	368.2308	-0.05896	0.203873	0.333632	protein_coding
ENSG0000(	RN7SL717F	9.658205	-0.26365	0.203864	0.333632	misc_RNA
ENSG0000(	ENSG0000(	8.884147	0.415331	0.203921	0.333676	lncRNA
ENSG0000(	AP1M2P1	16.44122	-0.26227	0.203981	0.333722	processed_pseudogene
ENSG0000(	FARP2	5587.751	-0.09765	0.20397	0.333722	protein_coding
ENSG0000(	RABGAP1L-	2651.907	0.073116	0.203972	0.333722	lncRNA
ENSG0000(	RALGDS	2538.062	0.094801	0.204052	0.33382	protein_coding
ENSG0000(	GTF2A1	1015.705	0.075037	0.204081	0.33385	protein_coding
ENSG0000(	ENSG0000(	123.9556	-0.09861	0.204134	0.333891	processed_pseudogene
ENSG0000(	GUSBP4	161.3352	-0.14536	0.204118	0.333891	transcribed_unprocessed_pseudo
ENSG0000(	NTM	104.4153	0.210511	0.204148	0.333891	protein_coding
ENSG0000(	STRBP	2166.887	-0.0829	0.204159	0.333891	protein_coding
ENSG0000(	USH2A	40.01312	0.525119	0.204156	0.333891	protein_coding
ENSG0000(	LGALS9B	223.1618	-0.26287	0.204177	0.333896	protein_coding
ENSG0000(	SAPCD1	80.39608	-0.18173	0.204184	0.333896	protein_coding
ENSG0000(	ELOC	2605.896	0.036077	0.204247	0.333967	protein_coding
ENSG0000(	KRT17P4	12.33987	-1.30568	0.204253	0.333967	transcribed_unprocessed_pseudo
ENSG0000(	MIR548XH(	112.0111	0.108479	0.204259	0.333967	lncRNA
ENSG0000(	CYP2G1P	80.8967	0.112102	0.204334	0.334072	transcribed_unprocessed_pseudo

ENSG0000(	ST20	186.2808	-0.10442	0.204393	0.334151	protein_coding
ENSG0000(	ENSG0000(	136.2161	2.242631	0.204442	0.334214	lncRNA
ENSG0000(	ENSG0000(	161.0143	-0.09409	0.204457	0.334221	TEC
ENSG0000(	CRTC3-AS1	77.00354	-0.12751	0.204567	0.334366	lncRNA
ENSG0000(	WBP11P1	13.56386	0.231354	0.204566	0.334366	transcribed_processed_pseudoge
ENSG0000(	ITGB3BP	1570.684	0.288156	0.204578	0.334367	protein_coding
ENSG0000(	ENSG0000(	198.9589	0.201546	0.204694	0.334538	TEC
ENSG0000(	PRLHR	125.6063	-0.09045	0.204759	0.334628	protein_coding
ENSG0000(	ENSG0000(	181.0261	0.088252	0.204774	0.334635	lncRNA
ENSG0000(	ZNF530	523.5979	0.08042	0.204814	0.334683	protein_coding
ENSG0000(	ACP7	358.982	0.086896	0.204842	0.334687	protein_coding
ENSG0000(	CRYZL2P	2990.464	-0.09753	0.204849	0.334687	transcribed_unitary_pseudogene
ENSG0000(	SNHG11	264.5801	-0.12785	0.204842	0.334687	lncRNA
ENSG0000(	CELSR3	224.7423	0.177621	0.20487	0.334697	protein_coding
ENSG0000(	TOX3	1058.146	-0.15401	0.204877	0.334697	protein_coding
ENSG0000(	ENSG0000(	39.74432	0.152741	0.204934	0.334757	lncRNA
ENSG0000(	TTBK1	108.8492	0.144842	0.20493	0.334757	protein_coding
ENSG0000(	RNU6-1201	12.99427	0.270148	0.204952	0.334768	snRNA
ENSG0000(	PFKP	1821.261	-0.11052	0.204995	0.334804	protein_coding
ENSG0000(	TRGV3	15.20175	-0.29559	0.204985	0.334804	TR_V_gene
ENSG0000(	ENSG0000(	12.76875	0.28549	0.205048	0.334871	lncRNA
ENSG0000(	BLOC1S2	335.4524	-0.10696	0.205093	0.334911	protein_coding
ENSG0000(	ENSG0000(	837.7712	-0.39557	0.205083	0.334911	protein_coding
ENSG0000(	ENSG0000(	154.1185	-0.09628	0.20523	0.335118	lncRNA
ENSG0000(	SLC27A5	2331.74	0.056471	0.205265	0.335139	protein_coding
ENSG0000(	ZNF444	1205.915	-0.08791	0.205256	0.335139	protein_coding
ENSG0000(	HPCAL1	1555.609	0.090728	0.205306	0.335188	protein_coding
ENSG0000(	ANKRD18D	29.65684	0.343415	0.205328	0.335208	transcribed_unprocessed_pseudo
ENSG0000(	ANGPT4	110.5298	0.109727	0.20538	0.335264	protein_coding
ENSG0000(	ENSG0000(	13.92874	-0.23994	0.205384	0.335264	lncRNA
ENSG0000(	CASQ2	27.79051	0.279247	0.205514	0.335458	protein_coding
ENSG0000(	BCAR3-AS1	395.6861	-0.20058	0.205629	0.335498	lncRNA
ENSG0000(	ENSG0000(	600.3959	0.092951	0.205622	0.335498	lncRNA
ENSG0000(	ENSG0000(	85.5376	0.100534	0.205569	0.335498	lncRNA
ENSG0000(	ENSG0000(	27.80893	-0.21002	0.205634	0.335498	lncRNA
ENSG0000(	LINC02025	61.4101	-0.14064	0.205612	0.335498	lncRNA
ENSG0000(	RSF1-IT1	67.82244	0.093123	0.205605	0.335498	lncRNA
ENSG0000(	RSU1P2	109.0932	0.102372	0.205592	0.335498	transcribed_unprocessed_pseudo
ENSG0000(	SQLE	331.5131	0.184518	0.20559	0.335498	protein_coding
ENSG0000(	TMIGD2	129.9045	0.113355	0.205635	0.335498	protein_coding
ENSG0000(	SMYD3-IT1	122.861	0.109164	0.205786	0.335727	lncRNA
ENSG0000(	SLC35C2	1476.795	-0.0539	0.205929	0.335942	protein_coding
ENSG0000(	VPS37B	2549.874	-0.09182	0.20599	0.336025	protein_coding
ENSG0000(	EPHB6	146.3956	0.295036	0.206038	0.336062	protein_coding
ENSG0000(	THA1P	10.10806	0.324978	0.206035	0.336062	unitary_pseudogene
ENSG0000(	UBA2	1270.778	-0.11141	0.206045	0.336062	protein_coding
ENSG0000(	DPH3	429.9077	0.102934	0.206067	0.336074	protein_coding

ENSG0000(	RN7SKP47	11.16343	-0.40192	0.206074	0.336074	misc_RNA
ENSG0000(	ENSG0000(	29.29295	0.213822	0.206147	0.336176	lncRNA
ENSG0000(	ENSG0000(	13.47021	0.253073	0.206185	0.33622	lncRNA
ENSG0000(	SMAD1	1118.004	0.075188	0.206196	0.336221	protein_coding
ENSG0000(	F8A1	294.378	0.25342	0.206473	0.336654	protein_coding
ENSG0000(	UGDH-AS1	9264.165	-0.07828	0.206504	0.336687	lncRNA
ENSG0000(	PLEKHD1	427.314	0.061879	0.206538	0.336725	protein_coding
ENSG0000(	SNIP1	1157.692	0.044988	0.206552	0.33673	protein_coding
ENSG0000(	SSBP3	1452.766	-0.07458	0.20675	0.337036	protein_coding
ENSG0000(	ENSG0000(	110.7224	-0.09519	0.206775	0.337041	processed_pseudogene
ENSG0000(	JMY	1143.988	0.090799	0.20677	0.337041	protein_coding
ENSG0000(	ENSG0000(	92.32941	-0.1041	0.206795	0.337056	lncRNA
ENSG0000(	ENSG0000(	9.622742	0.328978	0.206864	0.337151	lncRNA
ENSG0000(	ENSG0000(	53.21752	-0.15668	0.206891	0.337178	lncRNA
ENSG0000(	KMT2A	10843.2	0.05459	0.206922	0.337211	protein_coding
ENSG0000(	F12	27.73272	-0.33793	0.207006	0.337312	protein_coding
ENSG0000(	GNAQ	3587.788	-0.08352	0.207	0.337312	protein_coding
ENSG0000(	KIAA0753	1004.003	-0.08476	0.207024	0.337324	protein_coding
ENSG0000(	NAPSB	205.5595	0.294451	0.207053	0.337354	transcribed_unprocessed_pseudo
ENSG0000(	PCDHGA1	42.20208	0.176174	0.207093	0.337402	protein_coding
ENSG0000(	ENSG0000(	41.06897	-0.15597	0.207116	0.337403	lncRNA
ENSG0000(	ID2	628.0047	-0.14553	0.207112	0.337403	protein_coding
ENSG0000(	PLXNB2	5199.098	-0.11184	0.207218	0.337552	protein_coding
ENSG0000(	ZNF876P	196.1312	-0.09135	0.207283	0.337641	transcribed_unprocessed_pseudo
ENSG0000(	ENSG0000(	271.4848	-0.05918	0.207306	0.33766	lncRNA
ENSG0000(	ENSG0000(	35.65905	0.178206	0.207325	0.337664	lncRNA
ENSG0000(	LINC00886	261.5521	0.118043	0.20733	0.337664	lncRNA
ENSG0000(	ENSG0000(	29.72283	-0.18724	0.207341	0.337664	lncRNA
ENSG0000(	ENSG0000(	75.03954	0.147199	0.207388	0.337724	lncRNA
ENSG0000(	ISM1	8.906856	0.378453	0.207407	0.337737	protein_coding
ENSG0000(	TRIM32	454.4609	-0.08697	0.207683	0.338169	protein_coding
ENSG0000(	BEGAIN	108.3378	0.216427	0.2077	0.338171	protein_coding
ENSG0000(	RGS7BP	56.62685	0.222403	0.207706	0.338171	protein_coding
ENSG0000(	RNU6-998F	28.12145	0.22962	0.207746	0.338219	snRNA
ENSG0000(	TOX4P1	34.68638	0.192475	0.207768	0.338236	processed_pseudogene
ENSG0000(	ENSG0000(	22.6681	-0.24666	0.207793	0.338259	TEC
ENSG0000(	BEAN1-AS1	24.7147	0.209664	0.207859	0.338334	lncRNA
ENSG0000(	HRH1	702.3182	0.161157	0.207871	0.338334	protein_coding
ENSG0000(	ZNF264	3241.76	0.055272	0.207867	0.338334	protein_coding
ENSG0000(	BBS4	428.9766	0.08305	0.207906	0.338374	protein_coding
ENSG0000(	ENSG0000(	144.0098	-0.18026	0.208031	0.338543	lncRNA
ENSG0000(	GJA9	57.07813	0.169022	0.208032	0.338543	protein_coding
ENSG0000(	ENSG0000(	18.42935	0.261383	0.208048	0.338552	lncRNA
ENSG0000(	PLEKHA7	3625.628	-0.15183	0.208119	0.338649	protein_coding
ENSG0000(	ENSG0000(	73.89432	0.159244	0.208141	0.33865	lncRNA
ENSG0000(	ENSG0000(	75.20873	0.140688	0.208138	0.33865	lncRNA
ENSG0000(	SCARF2	95.87643	0.151336	0.208166	0.338674	protein_coding

ENSG0000(	ENSG0000(	152.6524	-0.52616	0.208204	0.338699	lncRNA
ENSG0000(	ENSG0000(	58.94071	0.15856	0.208197	0.338699	lncRNA
ENSG0000(	ENSG0000(	28.63922	-0.22625	0.208348	0.338916	protein_coding
ENSG0000(	LINC01778	28.03979	-0.18648	0.208423	0.33902	lncRNA
ENSG0000(	ENSG0000(	212.7538	-0.08835	0.208442	0.339034	lncRNA
ENSG0000(	FLT1	1223.258	0.103973	0.208602	0.339276	protein_coding
ENSG0000(	MED9	1765.986	0.069278	0.20863	0.339304	protein_coding
ENSG0000(	ENSG0000(	203.3802	-0.0803	0.208709	0.339416	lncRNA
ENSG0000(	ABHD11-AS	109.8743	-0.25998	0.20879	0.339493	transcribed_unitary_pseudogene
ENSG0000(	ENSG0000(	74.31733	0.152925	0.20879	0.339493	lncRNA
ENSG0000(	ENSG0000(	22.45482	0.251347	0.2088	0.339493	lncRNA
ENSG0000(	ZSCAN12P1	58.22515	-0.23828	0.208791	0.339493	transcribed_unprocessed_pseudo
ENSG0000(	ESPNL	26.20973	0.39437	0.208839	0.339539	protein_coding
ENSG0000(	COX20	1339.032	-0.0563	0.20888	0.339588	protein_coding
ENSG0000(	ITGB1-DT	155.1199	-0.0991	0.208905	0.33961	lncRNA
ENSG0000(	LINC01524	182.0052	0.092913	0.208936	0.339644	lncRNA
ENSG0000(	PDCD6IP	4611.965	-0.08252	0.208966	0.339668	protein_coding
ENSG0000(	RN7SL464F	17.83296	0.218989	0.208973	0.339668	misc_RNA
ENSG0000(	BAZ2A	6063.987	-0.05957	0.208995	0.339671	protein_coding
ENSG0000(	NAT16	101.9627	0.144986	0.208997	0.339671	protein_coding
ENSG0000(	TNNT3	23.96943	-0.38026	0.20902	0.339691	protein_coding
ENSG0000(	APOO	165.6209	-0.13294	0.209059	0.339718	protein_coding
ENSG0000(	ARHGAP29	60.44653	0.160143	0.209069	0.339718	lncRNA
ENSG0000(	CD36	1123.891	-0.12521	0.209061	0.339718	protein_coding
ENSG0000(	APPL1	1676.222	-0.0709	0.209163	0.339853	protein_coding
ENSG0000(	TLNRD1	698.0844	0.076788	0.2092	0.339896	protein_coding
ENSG0000(	FPGT	693.0626	-0.09417	0.209239	0.339941	protein_coding
ENSG0000(	ENSG0000(	12.63807	0.226323	0.209293	0.340007	lncRNA
ENSG0000(	LINC00294	1213.869	-0.09055	0.209301	0.340007	lncRNA
ENSG0000(	TMEM80	905.9012	-0.0925	0.209348	0.340065	protein_coding
ENSG0000(	SORT1	3188.479	-0.0963	0.209368	0.34008	protein_coding
ENSG0000(	ZNF790	385.606	-0.09529	0.209501	0.340279	protein_coding
ENSG0000(	LTB4R2	249.4092	-0.09485	0.209582	0.340384	protein_coding
ENSG0000(	NCOA6	3946.578	-0.06685	0.209588	0.340384	protein_coding
ENSG0000(	ENSG0000(	30.36084	0.17716	0.209614	0.340392	lncRNA
ENSG0000(	ENSG0000(	76.17919	-0.195	0.209609	0.340392	lncRNA
ENSG0000(	CD8A	194.5527	-0.26561	0.20966	0.340448	protein_coding
ENSG0000(	ENSG0000(	8.421401	-0.33713	0.2097	0.340496	transcribed_processed_pseudoge
ENSG0000(	ENSG0000(	32.37977	0.188407	0.209788	0.340586	processed_pseudogene
ENSG0000(	ENSG0000(	11030.64	0.078525	0.209789	0.340586	TEC
ENSG0000(	ENSG0000(	288.6706	0.063987	0.209786	0.340586	lncRNA
ENSG0000(	ENSG0000(	490.1041	0.081178	0.209801	0.340589	lncRNA
ENSG0000(	ENSG0000(	31.08117	-0.2075	0.209877	0.340694	TEC
ENSG0000(	ENSG0000(	51.60551	-0.18975	0.209949	0.340794	lncRNA
ENSG0000(	ENSG0000(	39.78988	0.143786	0.210104	0.341015	lncRNA
ENSG0000(	GAS5-AS1	48.8594	0.120846	0.210107	0.341015	lncRNA
ENSG0000(	ENSG0000(	98.93959	-0.1476	0.210158	0.341081	lncRNA

ENSG0000(	TBC1D22A-	33.63055	-0.15596	0.210217	0.341157	lncRNA
ENSG0000(	ENSG0000(	318.8467	0.089275	0.210456	0.341528	lncRNA
ENSG0000(	EHD1	1248.938	0.095829	0.210474	0.341539	protein_coding
ENSG0000(	ZMAT4	17.35957	0.275581	0.210506	0.341574	protein_coding
ENSG0000(	TRGV4	19.81947	-0.37336	0.210655	0.341798	TR_V_gene
ENSG0000(	ENSG0000(	18.34386	0.224862	0.210737	0.341895	lncRNA
ENSG0000(	SUSD3	104.5828	0.207219	0.210734	0.341895	protein_coding
ENSG0000(	CARHSP1	1078.068	0.079476	0.210768	0.341927	protein_coding
ENSG0000(	ENSG0000(	125.0869	0.106753	0.21078	0.341929	lncRNA
ENSG0000(	DDX50P1	370.5368	-0.08234	0.210794	0.341935	processed_pseudogene
ENSG0000(	SNHG22	2528.803	-0.09678	0.210929	0.342137	lncRNA
ENSG0000(	ENSG0000(	21.65702	0.22512	0.210949	0.342142	lncRNA
ENSG0000(	FAM71E1	12.24711	-0.26141	0.210955	0.342142	protein_coding
ENSG0000(	ENSG0000(	72.38052	-0.12567	0.211002	0.342202	unprocessed_pseudogene
ENSG0000(	FAM149A	737.985	-0.10926	0.211063	0.342266	protein_coding
ENSG0000(	MICU3	103.8332	0.22633	0.211064	0.342266	protein_coding
ENSG0000(	DAAM1	1498.84	-0.0614	0.211194	0.34246	protein_coding
ENSG0000(	ENSG0000(	2794.95	-0.07503	0.211226	0.342493	lncRNA
ENSG0000(	ENSG0000(	10.44377	-0.45301	0.211259	0.342529	protein_coding
ENSG0000(	ROR1	188.7186	0.159174	0.211288	0.342558	protein_coding
ENSG0000(	ENSG0000(	506.3739	0.070318	0.21135	0.342641	lncRNA
ENSG0000(	ENSG0000(	96.67083	0.109534	0.211361	0.342642	lncRNA
ENSG0000(	ENSG0000(	12.60487	0.242173	0.211401	0.342674	lncRNA
ENSG0000(	UGT2B7	24.22091	-0.31567	0.211403	0.342674	protein_coding
ENSG0000(	FKBP6	205.7925	0.079609	0.211444	0.342694	protein_coding
ENSG0000(	PSTPIP1	417.3505	0.157169	0.211449	0.342694	protein_coding
ENSG0000(	SYT14	454.2754	-0.17455	0.211443	0.342694	protein_coding
ENSG0000(	MIR8065	14.93587	-0.33079	0.211467	0.342707	miRNA
ENSG0000(	ENSG0000(	67.10768	0.194776	0.211511	0.34276	protein_coding
ENSG0000(	IRAK1BP1	469.7112	0.094369	0.211539	0.342788	protein_coding
ENSG0000(	ENSG0000(	107.4492	0.109376	0.211602	0.342872	lncRNA
ENSG0000(	MRPL33	218.9267	-0.11081	0.211629	0.342898	protein_coding
ENSG0000(	GSG1L	33.79323	-0.21124	0.21167	0.342947	protein_coding
ENSG0000(	TOMM20P.	48.74405	0.250622	0.211692	0.342964	processed_pseudogene
ENSG0000(	ENSG0000(	48.45532	-0.16136	0.211753	0.343046	lncRNA
ENSG0000(	ENSG0000(	19.9692	0.246964	0.211767	0.343051	transcribed_processed_pseudoge
ENSG0000(	ZNF300P1	147.3527	-0.26499	0.211821	0.34312	transcribed_unprocessed_pseudo
ENSG0000(	ENSG0000(	11.85021	-0.26092	0.211839	0.343132	lncRNA
ENSG0000(	BMP5	811.5048	-0.196	0.211905	0.343203	protein_coding
ENSG0000(	PITPNA	1843.109	0.073625	0.211904	0.343203	protein_coding
ENSG0000(	ENSG0000(	241.271	0.124045	0.212014	0.343348	lncRNA
ENSG0000(	LHFPL3	12.09025	0.335485	0.212017	0.343348	protein_coding
ENSG0000(	AFDN	7533.546	-0.08513	0.212054	0.34339	protein_coding
ENSG0000(	APCDD1L-D	160.7531	0.09206	0.21211	0.343446	lncRNA
ENSG0000(	DYNC1LI2-I	226.4335	-0.08349	0.212104	0.343446	lncRNA
ENSG0000(	ENSG0000(	16.74937	-0.2804	0.212134	0.343455	lncRNA
ENSG0000(	ENSG0000(	219.8014	0.074941	0.212141	0.343455	lncRNA

ENSG0000(	RHAG	82.12277	-0.10932	0.212148	0.343455	protein_coding
ENSG0000(	ENSG0000(	447.1637	0.054806	0.212194	0.34351	lncRNA
ENSG0000(	WASHC2C	2654.169	-0.1213	0.212221	0.343536	protein_coding
ENSG0000(	ENSG0000(	288.0162	0.218883	0.212265	0.34359	TEC
ENSG0000(	KCNRG	91.96442	0.126437	0.212333	0.343682	protein_coding
ENSG0000(	LINC01703	115.7884	-0.08988	0.212411	0.343778	lncRNA
ENSG0000(	MAP3K7CL	352.4256	0.081668	0.212416	0.343778	protein_coding
ENSG0000(	SSR2	2248.729	-0.08711	0.212425	0.343778	protein_coding
ENSG0000(	MGME1	530.6661	-0.07061	0.212457	0.343789	protein_coding
ENSG0000(	RAD9A	1084.433	-0.05421	0.212465	0.343789	protein_coding
ENSG0000(	RN7SL403F	85.6098	0.098319	0.212457	0.343789	misc_RNA
ENSG0000(	TM2D2	417.2752	-0.08986	0.212482	0.343799	protein_coding
ENSG0000(	ARSD	2046.925	-0.07375	0.212548	0.343871	protein_coding
ENSG0000(	ENSG0000(	1466.413	-0.09511	0.212547	0.343871	lncRNA
ENSG0000(	EEF1AKMT:	447.9887	0.088387	0.212563	0.343875	protein_coding
ENSG0000(	MTND6P4	21.05148	-0.31854	0.212573	0.343875	processed_pseudogene
ENSG0000(	ENSG0000(	224.2865	0.144152	0.21263	0.34395	lncRNA
ENSG0000(	FSD1	245.4369	0.079217	0.21267	0.343996	protein_coding
ENSG0000(	ENSG0000(	467.8132	-0.14573	0.212726	0.344069	lncRNA
ENSG0000(	ENSG0000(	10.53645	-0.35895	0.212856	0.344262	lncRNA
ENSG0000(	ENSG0000(	42.22739	-0.14741	0.212883	0.344287	processed_pseudogene
ENSG0000(	USP41	24.69117	-0.47376	0.212896	0.344291	protein_coding
ENSG0000(	ENSG0000(	47.00513	0.126515	0.212968	0.344389	lncRNA
ENSG0000(	ENSG0000(	18.07619	-1.32084	0.213	0.344423	protein_coding
ENSG0000(	ENSG0000(	63.31637	-0.23139	0.213092	0.344554	lncRNA
ENSG0000(	EMC3-AS1	463.072	-0.0871	0.213177	0.344674	transcribed_unprocessed_pseudo
ENSG0000(	ENSG0000(	9.954124	-0.44425	0.213268	0.344803	lncRNA
ENSG0000(	LINC00377	68.22788	0.118234	0.213303	0.344842	lncRNA
ENSG0000(	ENSG0000(	886.5284	0.145098	0.213356	0.344887	protein_coding
ENSG0000(	KIAA1143	1247.588	-0.04975	0.213364	0.344887	protein_coding
ENSG0000(	ZNF420	268.5297	0.104069	0.213354	0.344887	protein_coding
ENSG0000(	POLR2A	3356.353	-0.06546	0.213376	0.344888	protein_coding
ENSG0000(	ZNF35	222.1626	0.097228	0.213399	0.344908	protein_coding
ENSG0000(	SNAPC2	76.7577	-0.17841	0.213419	0.344923	protein_coding
ENSG0000(	SCN4A	50.38231	-0.40352	0.213486	0.345013	protein_coding
ENSG0000(	OTOF	14.8682	0.425672	0.213533	0.345071	protein_coding
ENSG0000(	GOLM2P1	12.94669	0.327831	0.213609	0.345152	processed_pseudogene
ENSG0000(	RSBN1L	1839.418	-0.04521	0.213604	0.345152	protein_coding
ENSG0000(	ZBTB24	913.3094	0.058745	0.213616	0.345152	protein_coding
ENSG0000(	ADTRP	2594.702	-0.16085	0.213639	0.345152	protein_coding
ENSG0000(	SNORD105	13.74263	0.262477	0.213636	0.345152	snoRNA
ENSG0000(	SNORD22	580.3954	0.140861	0.213649	0.345152	snoRNA
ENSG0000(	CDKN2B	1853.775	-0.28182	0.213661	0.345154	protein_coding
ENSG0000(	ENSG0000(	9.912134	-0.27623	0.2137	0.345194	lncRNA
ENSG0000(	LIN52	815.4237	0.060432	0.213708	0.345194	protein_coding
ENSG0000(	ENSG0000(	155.2474	-0.08819	0.213737	0.345222	lncRNA
ENSG0000(	RNF13	866.9512	0.071662	0.213794	0.345296	protein_coding

ENSG0000(	SLC6A11	153.2344	0.076921	0.213827	0.345332	protein_coding
ENSG0000(	CCDC159	404.8221	-0.06088	0.213888	0.345378	protein_coding
ENSG0000(	ENSG0000(	33.81297	0.408324	0.213889	0.345378	lncRNA
ENSG0000(	MCF2	23.47339	0.333555	0.213869	0.345378	protein_coding
ENSG0000(	TTC39C	919.232	0.113463	0.214005	0.345549	protein_coding
ENSG0000(	SLITRK3	40.66255	-0.16599	0.214031	0.345573	protein_coding
ENSG0000(	C8orf88	23.44239	0.229428	0.214106	0.34564	protein_coding
ENSG0000(	HECA	2465.544	0.071508	0.214099	0.34564	protein_coding
ENSG0000(	TPT1-AS1	4011.84	0.063944	0.214094	0.34564	lncRNA
ENSG0000(	PMM2	5146.67	-0.06528	0.214118	0.345641	protein_coding
ENSG0000(	GALNT11	752.8584	-0.06775	0.214154	0.345682	protein_coding
ENSG0000(	RPL34P6	15.76342	0.269144	0.2142	0.345738	processed_pseudogene
ENSG0000(	INKA2	401.7772	0.106606	0.214221	0.345755	protein_coding
ENSG0000(	RASA3	985.2043	0.128955	0.214239	0.345765	protein_coding
ENSG0000(	BMS1P2	385.4202	0.162318	0.214278	0.345793	transcribed_unprocessed_pseudo
ENSG0000(	MYO3B-AS	611.3213	0.043555	0.214276	0.345793	lncRNA
ENSG0000(	ENSG0000(	82.83052	-0.14369	0.214341	0.345876	lncRNA
ENSG0000(	ENSG0000(	66.21672	-0.45533	0.214377	0.345917	lncRNA
ENSG0000(	ENSG0000(	16.10954	0.355061	0.214391	0.345922	processed_pseudogene
ENSG0000(	ENSG0000(	15.8529	-0.25788	0.214428	0.345963	lncRNA
ENSG0000(	RAD50	2612.066	-0.06833	0.214468	0.34601	protein_coding
ENSG0000(	CFAP410	602.6122	-0.11991	0.21451	0.346052	protein_coding
ENSG0000(	ENSG0000(	24.85138	0.246203	0.214516	0.346052	processed_pseudogene
ENSG0000(	ENSG0000(	23.67651	-0.20438	0.214529	0.346055	lncRNA
ENSG0000(	DENND4B	1909.413	-0.08384	0.214575	0.346111	protein_coding
ENSG0000(	ENSG0000(	10.74472	-0.29769	0.214667	0.346241	lncRNA
ENSG0000(	LINC02027	329.0033	-0.11466	0.214678	0.346241	lncRNA
ENSG0000(	TEKT2	47.90947	-0.17312	0.214757	0.346351	protein_coding
ENSG0000(	FAM118A	3478.373	0.201916	0.2148	0.346403	protein_coding
ENSG0000(	PCDHA8	12.77346	-0.38748	0.214941	0.346612	protein_coding
ENSG0000(	ENSG0000(	52.17145	-0.13789	0.214953	0.346613	processed_pseudogene
ENSG0000(	LINC02154	38.82291	0.137752	0.215025	0.346693	lncRNA
ENSG0000(	MRPL20-AS	308.2054	-0.09432	0.215022	0.346693	lncRNA
ENSG0000(	ENSG0000(	103.3434	0.163206	0.215063	0.346734	lncRNA
ENSG0000(	LIME1	230.2547	-0.13437	0.215072	0.346734	protein_coding
ENSG0000(	EMC1-AS1	38.81008	0.209336	0.215096	0.346755	lncRNA
ENSG0000(	SEPTIN7P1	19.78379	0.247142	0.215162	0.346842	transcribed_unprocessed_pseudo
ENSG0000(	TMEM70	179.0623	-0.13961	0.215172	0.346842	protein_coding
ENSG0000(	HNRNPLP2	12.1811	0.334504	0.215184	0.346843	unprocessed_pseudogene
ENSG0000(	ENSG0000(	218.4387	0.099779	0.215268	0.346947	lncRNA
ENSG0000(	ENSG0000(	35.48121	-0.16424	0.215281	0.346947	lncRNA
ENSG0000(	ENSG0000(	243.695	-0.14976	0.215273	0.346947	lncRNA
ENSG0000(	ENSG0000(	493.8556	0.107284	0.215303	0.346964	protein_coding
ENSG0000(	ENSG0000(	81.69342	0.116125	0.215338	0.346985	lncRNA
ENSG0000(	ENSG0000(	66.53328	-0.25556	0.215328	0.346985	TEC
ENSG0000(	LRRC2-AS1	13.46317	0.298322	0.215479	0.347194	lncRNA
ENSG0000(	ANKRA2	334.2701	-0.10047	0.215556	0.347299	protein_coding

ENSG0000(	STMN1P1	15.29054	-0.31584	0.215571	0.347305	processed_pseudogene
ENSG0000(	ENSG0000(	18.26271	-0.29578	0.215658	0.347428	processed_pseudogene
ENSG0000(	B3GNT5	2241.837	-0.14799	0.215689	0.347442	protein_coding
ENSG0000(	ENSG0000(	35.37669	-0.31246	0.215687	0.347442	lncRNA
ENSG0000(	PPM1F	1394.625	0.066524	0.215766	0.347531	protein_coding
ENSG0000(	SSR3	2880.666	0.084243	0.215766	0.347531	protein_coding
ENSG0000(	ASPA	1253.552	0.051018	0.215793	0.347556	protein_coding
ENSG0000(	RNU6-540F	9.463708	0.276624	0.215824	0.347589	snRNA
ENSG0000(	ENSG0000(	38.56753	0.18317	0.215854	0.3476	processed_pseudogene
ENSG0000(	TESPA1	633.9714	0.169647	0.215845	0.3476	protein_coding
ENSG0000(	ENSG0000(	18.74835	0.226183	0.215891	0.347642	lncRNA
ENSG0000(	ZNF528-AS	676.6972	0.05144	0.215908	0.347651	lncRNA
ENSG0000(	LINC02269	84.26463	0.1036	0.216038	0.347843	lncRNA
ENSG0000(	YIPF5	679.3864	0.085348	0.21606	0.347861	protein_coding
ENSG0000(	POLR2C	743.8098	-0.06106	0.216173	0.348024	protein_coding
ENSG0000(	PPP1R14BF	31.00458	-0.21685	0.2162	0.348051	processed_pseudogene
ENSG0000(	SNAI3	40.38761	0.198904	0.216217	0.34806	protein_coding
ENSG0000(	ENSG0000(	44.27222	-0.2897	0.216297	0.348171	processed_pseudogene
ENSG0000(	MORN4	322.8892	-0.06063	0.21641	0.348334	protein_coding
ENSG0000(	HINFP	2755.803	-0.03766	0.216421	0.348335	protein_coding
ENSG0000(	VPS13D	10831.63	-0.05767	0.216516	0.348469	protein_coding
ENSG0000(	ENSG0000(	12.97766	0.296081	0.216598	0.348584	lncRNA
ENSG0000(	PDE1A	220.7817	0.114085	0.216624	0.348607	protein_coding
ENSG0000(	ENSG0000(	794.3807	-0.10478	0.216637	0.348609	lncRNA
ENSG0000(	ENSG0000(	51.1775	0.191765	0.216667	0.348641	lncRNA
ENSG0000(	SCNN1D	122.1457	-0.17333	0.216728	0.34872	protein_coding
ENSG0000(	NAP1L6P	162.5036	0.089761	0.216901	0.348981	transcribed_processed_pseudoge
ENSG0000(	MORC3	1757.641	0.054851	0.217002	0.349126	protein_coding
ENSG0000(	LINC02586	10.46943	0.28954	0.217108	0.349278	lncRNA
ENSG0000(	ENSG0000(	478.2716	0.065175	0.217188	0.349389	lncRNA
ENSG0000(	JARID2	1193.583	0.064181	0.217236	0.349445	protein_coding
ENSG0000(	SLCO2A1	1573.227	0.171176	0.217245	0.349445	protein_coding
ENSG0000(	SEMA3E	312.2697	0.19181	0.217259	0.34945	protein_coding
ENSG0000(	GARNL3	408.0668	-0.11473	0.21741	0.349674	protein_coding
ENSG0000(	CCHCR1	689.6312	-0.08713	0.217448	0.3497	protein_coding
ENSG0000(	ENSG0000(	21.68518	-0.21453	0.217448	0.3497	lncRNA
ENSG0000(	ENSG0000(	10.63847	0.303262	0.217586	0.349903	lncRNA
ENSG0000(	SAYSD1	403.5516	-0.06471	0.217659	0.350003	protein_coding
ENSG0000(	ENSG0000(	33.88821	0.174222	0.217674	0.350008	protein_coding
ENSG0000(	ENSG0000(	54.11765	0.187256	0.217795	0.350167	lncRNA
ENSG0000(	OR2T2	13.17424	0.613789	0.217784	0.350167	protein_coding
ENSG0000(	SFTA1P	175.5307	0.139735	0.217828	0.350203	lncRNA
ENSG0000(	HDAC5	781.8728	0.100254	0.217887	0.350279	protein_coding
ENSG0000(	ENSG0000(	39.4626	-0.1712	0.217927	0.35029	lncRNA
ENSG0000(	RASIP1	80.45517	0.181651	0.217908	0.35029	protein_coding
ENSG0000(	ZFP64	667.0106	-0.05759	0.217924	0.35029	protein_coding
ENSG0000(	FAM27E3	316.215	-0.14379	0.217955	0.350304	lncRNA

ENSG0000(	RPL7L1P1	19.37682	-0.20806	0.217958	0.350304	processed_pseudogene
ENSG0000(	RPL18P10	13.65252	-0.2335	0.217991	0.350338	processed_pseudogene
ENSG0000(	ENSG0000(	280.082	-0.06237	0.218009	0.350349	lncRNA
ENSG0000(	SPTBN1	19798.39	-0.06618	0.218132	0.350528	protein_coding
ENSG0000(	ENSG0000(	112.0007	0.185889	0.218216	0.350628	lncRNA
ENSG0000(	PRMT2	2015.742	0.07631	0.218216	0.350628	protein_coding
ENSG0000(	FAM193A	1975.739	-0.046	0.218387	0.350884	protein_coding
ENSG0000(	ENSG0000(	90.86723	-0.11892	0.218483	0.351007	lncRNA
ENSG0000(	RAB29	1046.822	0.070963	0.218486	0.351007	protein_coding
ENSG0000(	ENSG0000(	52.27878	0.137668	0.218502	0.351015	lncRNA
ENSG0000(	ENSG0000(	11.26689	-0.24574	0.218608	0.351167	lncRNA
ENSG0000(	ENSG0000(	90.30067	0.244808	0.218639	0.351181	TEC
ENSG0000(	MEF2D	2161.989	0.084074	0.218635	0.351181	protein_coding
ENSG0000(	CNDP2	3045.589	0.056865	0.218699	0.35126	protein_coding
ENSG0000(	ZNF134	747.1941	-0.08153	0.218739	0.351306	protein_coding
ENSG0000(	OR52T1P	14.8099	0.261209	0.218791	0.351358	unprocessed_pseudogene
ENSG0000(	PGAP1	1377.874	-0.13568	0.218794	0.351358	protein_coding
ENSG0000(	GNG10	263.9902	0.196903	0.218818	0.351378	protein_coding
ENSG0000(	ENSG0000(	75.16278	-0.14141	0.218843	0.3514	lncRNA
ENSG0000(	ENSG0000(	688.762	0.181777	0.218883	0.351447	transcribed_unprocessed_pseudo
ENSG0000(	COX7A1	13.09604	-0.27872	0.218973	0.351484	protein_coding
ENSG0000(	ENSG0000(	149.6183	-0.34712	0.218939	0.351484	protein_coding
ENSG0000(	MSTN	10.05251	0.26079	0.218965	0.351484	protein_coding
ENSG0000(	PHF5GP	83.13691	-0.15179	0.218933	0.351484	processed_pseudogene
ENSG0000(	POLR2KP1	23.5894	0.195387	0.218973	0.351484	processed_pseudogene
ENSG0000(	QRSL1	1183.942	-0.06273	0.218941	0.351484	protein_coding
ENSG0000(	ENSG0000(	410.4483	0.057576	0.21906	0.351573	lncRNA
ENSG0000(	ENSG0000(	435.7515	0.063724	0.219061	0.351573	lncRNA
ENSG0000(	PLEKHM1	2662.886	-0.05577	0.219063	0.351573	protein_coding
ENSG0000(	GDPGP1	1163.48	-0.05047	0.21911	0.351631	protein_coding
ENSG0000(	ZNF551	931.7731	-0.0817	0.219158	0.351689	protein_coding
ENSG0000(	SELE	79.48217	1.355889	0.21922	0.351771	protein_coding
ENSG0000(	GLUD1	4810.944	-0.05988	0.219406	0.352051	protein_coding
ENSG0000(	SYNJ2BP	3278.465	-0.07136	0.219552	0.352268	protein_coding
ENSG0000(	ENSG0000(	12.27688	-0.28655	0.219584	0.352301	lncRNA
ENSG0000(	EFCAB3	304.0415	-0.1254	0.219598	0.352305	protein_coding
ENSG0000(	GOLGA2P8	14.27325	0.294688	0.219623	0.352328	transcribed_unprocessed_pseudo
ENSG0000(	ENSG0000(	57.07209	-0.17924	0.219651	0.352353	processed_pseudogene
ENSG0000(	ST7	345.9551	0.10038	0.219738	0.352475	protein_coding
ENSG0000(	ENSG0000(	11.81304	0.282867	0.21977	0.352509	lncRNA
ENSG0000(	ENSG0000(	58.73831	0.126562	0.219815	0.352546	lncRNA
ENSG0000(	ENSG0000(	151.7268	0.091969	0.219816	0.352546	lncRNA
ENSG0000(	A2ML1-AS2	15.652	-0.24298	0.219854	0.352589	lncRNA
ENSG0000(	LINC02202	376.0672	-0.09296	0.219894	0.352636	lncRNA
ENSG0000(	LPAL2	363.1628	0.116904	0.219968	0.352718	transcribed_unprocessed_pseudo
ENSG0000(	PEX10	316.2575	-0.1068	0.219965	0.352718	protein_coding
ENSG0000(	ENSG0000(	26.40412	0.199915	0.219981	0.352721	lncRNA

ENSG0000(	ATP6V1C2	37.75169	-0.33415	0.220028	0.352749	protein_coding
ENSG0000(	CHRNA3	77.24414	-0.17283	0.220033	0.352749	protein_coding
ENSG0000(	TIAL1	3055.982	-0.0738	0.220029	0.352749	protein_coding
ENSG0000(	CNKSR3	4366.498	-0.13084	0.220048	0.352755	protein_coding
ENSG0000(	CD96	800.2734	-0.1695	0.220128	0.352834	protein_coding
ENSG0000(	LINC02235	131.746	-0.1349	0.22012	0.352834	lncRNA
ENSG0000(	LRP2BP	292.6102	0.090936	0.220131	0.352834	protein_coding
ENSG0000(	VRK2	445.6916	0.091307	0.2202	0.352927	protein_coding
ENSG0000(	CDRT1	240.575	-0.18321	0.220226	0.352933	protein_coding
ENSG0000(	STAC3	108.7799	-0.11236	0.220218	0.352933	protein_coding
ENSG0000(	ENSG0000(	141.9162	0.081978	0.220291	0.353019	lncRNA
ENSG0000(	HOXB3	2668.163	-0.13004	0.220427	0.353218	protein_coding
ENSG0000(	MTND2P16	10.87139	0.340907	0.22056	0.353396	processed_pseudogene
ENSG0000(	ZNF718	806.7405	-0.07134	0.220561	0.353396	protein_coding
ENSG0000(	ENSG0000(	68.31312	0.113441	0.2207	0.353601	lncRNA
ENSG0000(	IFT81	846.9494	-0.08146	0.220746	0.353657	protein_coding
ENSG0000(	ENSG0000(	19.36839	0.304932	0.220784	0.3537	TEC
ENSG0000(	NPEPPSP1	1033.912	-0.08505	0.220891	0.353853	unprocessed_pseudogene
ENSG0000(	GTF2IRD1	793.8283	-0.11722	0.220964	0.353934	protein_coding
ENSG0000(	SLC29A4	830.6635	-0.06262	0.220955	0.353934	protein_coding
ENSG0000(	C1GALT1P2	11.39343	-0.29875	0.22111	0.35415	processed_pseudogene
ENSG0000(	TMEM175	563.8872	-0.09275	0.221372	0.354551	protein_coding
ENSG0000(	ENSG0000(	826.2611	0.061308	0.221421	0.354612	lncRNA
ENSG0000(	CRTAP	1268.064	-0.1015	0.221435	0.354616	protein_coding
ENSG0000(	ENSG0000(	663.9236	0.069388	0.22151	0.354711	lncRNA
ENSG0000(	ENSG0000(	24.71972	-0.26307	0.221517	0.354711	lncRNA
ENSG0000(	ENSG0000(	46.79017	0.201855	0.221562	0.354765	lncRNA
ENSG0000(	MIR222HG	802.0809	0.2124	0.221575	0.354767	lncRNA
ENSG0000(	EGLN2	2656.677	-0.06532	0.221685	0.35489	protein_coding
ENSG0000(	ENOX2	272.2538	-0.11941	0.221677	0.35489	protein_coding
ENSG0000(	PBRM1	3279.247	-0.04816	0.221666	0.35489	protein_coding
ENSG0000(	TOLLIP	829.8183	-0.06717	0.221725	0.354935	protein_coding
ENSG0000(	ENSG0000(	172.035	0.11553	0.221839	0.355063	lncRNA
ENSG0000(	OR8A1	584.7684	0.061674	0.221823	0.355063	protein_coding
ENSG0000(	ZNF12	1333.28	-0.07511	0.221833	0.355063	protein_coding
ENSG0000(	ENSG0000(	39.0646	0.1757	0.221852	0.355065	lncRNA
ENSG0000(	CTNND2	71.51356	0.278219	0.221912	0.355143	protein_coding
ENSG0000(	TPD52L2	1573.127	-0.07333	0.221955	0.355194	protein_coding
ENSG0000(	ENSG0000(	11.08614	-0.31645	0.22203	0.355296	lncRNA
ENSG0000(	ATXN3	2431.156	-0.03391	0.222054	0.355298	protein_coding
ENSG0000(	ENSG0000(	21.62786	0.227638	0.222047	0.355298	lncRNA
ENSG0000(	CD19	297.174	0.396916	0.222094	0.355343	protein_coding
ENSG0000(	MTARC2	367.5953	-0.13791	0.222163	0.355435	protein_coding
ENSG0000(	ENSG0000(	42.50844	0.202945	0.222217	0.355504	lncRNA
ENSG0000(	EFCAB7	224.9173	-0.13412	0.222268	0.355568	protein_coding
ENSG0000(	SAAL1	206.5312	-0.10479	0.222324	0.355638	protein_coding
ENSG0000(	RN7SL635F	12.16236	0.268298	0.222344	0.355653	misc_RNA

ENSG0000(	ENSG0000(	23.78586	0.263418	0.222372	0.355679	lncRNA
ENSG0000(	TSPYL2	606.9865	0.13294	0.222503	0.355871	protein_coding
ENSG0000(	ADARB1	1054.613	0.163108	0.222564	0.35595	protein_coding
ENSG0000(	SDHAP3	2363.001	0.085061	0.222579	0.355955	transcribed_unprocessed_pseudo
ENSG0000(	ENSG0000(	151.7713	0.100224	0.222611	0.355988	lncRNA
ENSG0000(	ENSG0000(	163.0828	0.111471	0.222634	0.356007	transcribed_unprocessed_pseudo
ENSG0000(	ENSG0000(	24.03602	-0.29194	0.222651	0.356016	lncRNA
ENSG0000(	ENSG0000(	31.083	0.155972	0.222673	0.356033	lncRNA
ENSG0000(	DCST1-AS1	27.70103	-0.23529	0.222711	0.356076	lncRNA
ENSG0000(	MIR3909	9.911397	0.322302	0.22276	0.356135	miRNA
ENSG0000(	ENSG0000(	21.66741	0.213478	0.222803	0.356186	lncRNA
ENSG0000(	ENSG0000(	36.137	0.160925	0.222842	0.356231	lncRNA
ENSG0000(	TECPR2	997.9551	0.116929	0.222857	0.356236	protein_coding
ENSG0000(	ZNF969P	545.4051	-0.08398	0.222937	0.356345	processed_pseudogene
ENSG0000(	SH3KBP1	1500.756	-0.08175	0.222961	0.356365	protein_coding
ENSG0000(	C1QL1	9.603482	0.268155	0.222996	0.356404	protein_coding
ENSG0000(	FAM181B	126.8721	0.090466	0.223141	0.356618	protein_coding
ENSG0000(	TAFAZZIN	1039.008	0.049585	0.223175	0.356653	protein_coding
ENSG0000(	ENSG0000(	12.18405	-0.26591	0.223205	0.356683	lncRNA
ENSG0000(	ENSG0000(	59.49911	0.152248	0.223356	0.356906	lncRNA
ENSG0000(	BTG1P1	15.6298	-0.31255	0.223476	0.357061	processed_pseudogene
ENSG0000(	ENSG0000(	89.86535	-0.09437	0.223473	0.357061	lncRNA
ENSG0000(	NCMAP	516.8251	0.058942	0.223649	0.35732	protein_coding
ENSG0000(	ENSG0000(	33.52701	0.149243	0.2237	0.357383	snoRNA
ENSG0000(	INTS2	913.653	-0.06831	0.223801	0.357526	protein_coding
ENSG0000(	ENSG0000(	30.79927	0.225958	0.223887	0.35761	lncRNA
ENSG0000(	ENSG0000(	70.89239	-0.12259	0.223888	0.35761	lncRNA
ENSG0000(	PBXIP1	1228.324	0.110702	0.223875	0.35761	protein_coding
ENSG0000(	CA11	34.16934	0.258828	0.223953	0.357695	protein_coding
ENSG0000(	LINC02823	32.85702	0.146752	0.223965	0.357696	lncRNA
ENSG0000(	ENSG0000(	495.9783	-0.05403	0.224028	0.357779	lncRNA
ENSG0000(	AGBL1	278.1763	0.125979	0.22407	0.357809	protein_coding
ENSG0000(	ANKRD62	215.8062	-0.10293	0.224064	0.357809	protein_coding
ENSG0000(	ASIC3	33.16312	-0.21683	0.224295	0.358132	protein_coding
ENSG0000(	ENSG0000(	22.85624	0.325049	0.224288	0.358132	lncRNA
ENSG0000(	ENSG0000(	108.9836	-0.09075	0.224317	0.35815	lncRNA
ENSG0000(	NFKBIL1	117.6272	-0.12361	0.224473	0.35838	protein_coding
ENSG0000(	IGHV1-69-2	25.51814	0.792611	0.224535	0.358461	IG_V_gene
ENSG0000(	ACTG1P22	10.06118	-0.32929	0.224599	0.358507	transcribed_processed_pseudoge
ENSG0000(	MTFR1	588.8405	-0.08108	0.224585	0.358507	protein_coding
ENSG0000(	PRSS53	222.1258	-0.08394	0.224589	0.358507	protein_coding
ENSG0000(	CLDN5	79.48914	-0.17099	0.224648	0.358555	protein_coding
ENSG0000(	INHBA-AS1	170.0397	-0.10394	0.224651	0.358555	lncRNA
ENSG0000(	ENSG0000(	45.89987	0.19181	0.224699	0.358612	lncRNA
ENSG0000(	SCARNA10	6542.303	-0.09854	0.22471	0.358612	snoRNA
ENSG0000(	MIS12	705.0534	-0.10287	0.224752	0.35866	protein_coding
ENSG0000(	ENSG0000(	56.60635	-0.18348	0.224862	0.358817	TEC

ENSG0000(	C20orf27	132.2734	-0.13508	0.224888	0.35884	protein_coding
ENSG0000(	IGLV3-16	13.59876	0.410155	0.224919	0.358871	IG_V_gene
ENSG0000(	ENSG0000(	35.91069	-0.2375	0.224932	0.358874	protein_coding
ENSG0000(	ZNF487	403.5211	0.111829	0.224986	0.358942	protein_coding
ENSG0000(	ENSG0000(	121.8543	-0.17905	0.225017	0.358951	lncRNA
ENSG0000(	NDNF	110.1818	-0.18713	0.225026	0.358951	protein_coding
ENSG0000(	SETD1B	2207.397	-0.05471	0.22501	0.358951	protein_coding
ENSG0000(	DAAM2-AS	195.4961	-0.31611	0.225132	0.359101	lncRNA
ENSG0000(	HIPK1	3411.037	0.060133	0.225152	0.359116	protein_coding
ENSG0000(	ENSG0000(	5801.03	0.076551	0.225172	0.359129	lncRNA
ENSG0000(	ENSG0000(	108.9088	-0.09209	0.225207	0.359167	TEC
ENSG0000(	ATCAY	681.3262	0.058287	0.22522	0.359169	protein_coding
ENSG0000(	ENSG0000(	74.15947	-0.12219	0.225236	0.359176	lncRNA
ENSG0000(	ENSG0000(	18.2402	0.220819	0.225264	0.359203	TEC
ENSG0000(	ENSG0000(	214.6134	0.0721	0.225362	0.359315	lncRNA
ENSG0000(	ENSG0000(	216.8405	-0.09013	0.225392	0.359315	lncRNA
ENSG0000(	ENSG0000(	189.1076	0.083527	0.225392	0.359315	processed_pseudogene
ENSG0000(	LAMP1	2298.489	-0.1025	0.225388	0.359315	protein_coding
ENSG0000(	NSUN5P1	4654.891	-0.06423	0.225403	0.359315	transcribed_unprocessed_pseudo
ENSG0000(	RPS7P3	10.08248	-0.31394	0.225376	0.359315	processed_pseudogene
ENSG0000(	ENSG0000(	94.73347	-0.10689	0.225436	0.359348	lncRNA
ENSG0000(	FGF14-AS2	9.612239	-0.26017	0.225503	0.359437	lncRNA
ENSG0000(	ENSG0000(	56.98867	0.128068	0.225569	0.359524	lncRNA
ENSG0000(	MEGF6	479.3649	-0.17287	0.225605	0.359564	protein_coding
ENSG0000(	IFT80	1332.17	-0.05944	0.225675	0.359656	protein_coding
ENSG0000(	KCNIP4-IT1	206.6346	0.104684	0.22569	0.359662	lncRNA
ENSG0000(	TMEM50B	1577.181	0.050479	0.225716	0.359685	protein_coding
ENSG0000(	AGAP4	731.9186	-0.07896	0.225927	0.360002	protein_coding
ENSG0000(	AKIRIN1	1231.35	-0.08412	0.22615	0.360326	protein_coding
ENSG0000(	ENSG0000(	1272.764	0.102467	0.226153	0.360326	lncRNA
ENSG0000(	LINC01625	147.4105	0.092082	0.226249	0.360456	lncRNA
ENSG0000(	POLR3F	212.7243	-0.09338	0.226258	0.360456	protein_coding
ENSG0000(	ENSG0000(	10.93168	0.241589	0.226369	0.360564	lncRNA
ENSG0000(	ENSG0000(	58.97752	-0.22644	0.226359	0.360564	lncRNA
ENSG0000(	GSTM5	179.2233	0.201259	0.22635	0.360564	protein_coding
ENSG0000(	RNU6-1053	32.40663	0.165969	0.226371	0.360564	snRNA
ENSG0000(	ENSG0000(	194.3871	0.13777	0.226443	0.36066	lncRNA
ENSG0000(	ENSG0000(	281.3238	-0.0704	0.226481	0.360701	lncRNA
ENSG0000(	PARN	1760.513	-0.04329	0.226528	0.36075	protein_coding
ENSG0000(	RN7SKP6	9.749067	-0.36417	0.226534	0.36075	misc_RNA
ENSG0000(	AGER	181.9412	-0.16415	0.226571	0.360771	protein_coding
ENSG0000(	ENSG0000(	250.13	0.152933	0.226565	0.360771	TEC
ENSG0000(	RNF145	1646.06	0.09857	0.226632	0.36085	protein_coding
ENSG0000(	LINC00892	27.00753	0.295226	0.226658	0.360873	lncRNA
ENSG0000(	ENSG0000(	29.25093	-0.23642	0.226708	0.360935	lncRNA
ENSG0000(	ARHGEF26-	1295.61	0.04724	0.226757	0.360979	lncRNA
ENSG0000(	IGLV7-46	134.3936	0.37554	0.226759	0.360979	IG_V_gene

ENSG0000(	ENSG0000(	525.7728	0.062827	0.226786	0.360985	lncRNA
ENSG0000(	TMLHE	836.5509	-0.05286	0.226778	0.360985	protein_coding
ENSG0000(	ENSG0000(	12.93591	0.240688	0.226934	0.361185	lncRNA
ENSG0000(	ZCCHC10	512.3231	-0.06858	0.226931	0.361185	protein_coding
ENSG0000(	ENSG0000(	206.0216	0.147228	0.226979	0.361237	TEC
ENSG0000(	STAT5B	2087.559	0.073217	0.226994	0.361243	protein_coding
ENSG0000(	ENSG0000(	1127.738	-0.17537	0.227098	0.36139	lncRNA
ENSG0000(	CDV3	3708.214	0.079169	0.227131	0.361424	protein_coding
ENSG0000(	GFUS	484.0929	-0.12299	0.227492	0.361981	protein_coding
ENSG0000(	LINC00852	295.443	0.10831	0.227512	0.361989	lncRNA
ENSG0000(	ZSCAN5A-A	427.1052	-0.09271	0.227521	0.361989	lncRNA
ENSG0000(	IRS4	67.21366	0.153236	0.227696	0.362231	protein_coding
ENSG0000(	VIRMA	2041.783	-0.04178	0.227686	0.362231	protein_coding
ENSG0000(	ENSG0000(	200.4128	-0.10881	0.227775	0.362328	lncRNA
ENSG0000(	ZNF337	1030.071	0.067626	0.22778	0.362328	protein_coding
ENSG0000(	AASDHPPT	570.1473	-0.06853	0.227846	0.362396	protein_coding
ENSG0000(	ENSG0000(	138.7723	-0.07976	0.227839	0.362396	lncRNA
ENSG0000(	MGC32805	251.0229	-0.27424	0.227869	0.362414	lncRNA
ENSG0000(	ENSG0000(	45.15303	0.139531	0.227937	0.362502	lncRNA
ENSG0000(	ENSG0000(	340.8151	-0.04857	0.227948	0.362502	lncRNA
ENSG0000(	ENSG0000(	23.82356	0.214293	0.227986	0.362545	lncRNA
ENSG0000(	ENSG0000(	72.00841	-0.24403	0.228057	0.362639	lncRNA
ENSG0000(	TVP23CP2	71.49245	0.117486	0.228087	0.362669	processed_pseudogene
ENSG0000(	ENSG0000(	63.09891	0.129662	0.228107	0.362682	lncRNA
ENSG0000(	ENSG0000(	76.94149	0.100426	0.228165	0.362753	lncRNA
ENSG0000(	OR8D4	216.6378	-0.14172	0.228175	0.362753	protein_coding
ENSG0000(	FAM185BP	119.5089	-0.11296	0.228193	0.362764	transcribed_unprocessed_pseudo
ENSG0000(	TINF2	703.908	-0.07562	0.228205	0.362764	protein_coding
ENSG0000(	SPAG8	40.01383	0.216688	0.228226	0.362779	protein_coding
ENSG0000(	ENSG0000(	17.21022	0.329955	0.228248	0.362795	processed_pseudogene
ENSG0000(	ENSG0000(	10.84976	0.30749	0.228457	0.363067	lncRNA
ENSG0000(	ENSG0000(	84.01885	-0.11463	0.228463	0.363067	lncRNA
ENSG0000(	MYO16	106.27	0.205207	0.228465	0.363067	protein_coding
ENSG0000(	PATZ1	564.7457	-0.08274	0.228432	0.363067	protein_coding
ENSG0000(	ENSG0000(	81.46363	0.129358	0.228477	0.363067	transcribed_processed_pseudoge
ENSG0000(	MIR548T	11.45086	0.286552	0.2285	0.363084	miRNA
ENSG0000(	PARGP1	1087.346	0.057555	0.228609	0.36324	transcribed_unprocessed_pseudo
ENSG0000(	ENSG0000(	112.6747	0.122253	0.228644	0.363259	lncRNA
ENSG0000(	NPM3	47.18038	-0.17881	0.228639	0.363259	protein_coding
ENSG0000(	SPDYE2B	17.88888	0.49156	0.228719	0.36336	protein_coding
ENSG0000(	SLC13A4	155.8868	0.099833	0.228732	0.363361	protein_coding
ENSG0000(	ENSG0000(	27.76771	0.172352	0.228814	0.363473	lncRNA
ENSG0000(	RN7SL706F	27.88118	0.334641	0.228866	0.363537	misc_RNA
ENSG0000(	TNFRSF10A	20.26426	-0.20994	0.228879	0.36354	lncRNA
ENSG0000(	RN7SKP127	42.70218	-0.40521	0.228989	0.363696	misc_RNA
ENSG0000(	PJA1	140.9376	0.155613	0.229007	0.363707	protein_coding
ENSG0000(	ENSG0000(	391.1357	0.053565	0.22917	0.363927	lncRNA

ENSG0000(	RNU6-476F	21.26308	0.213319	0.229168	0.363927	snRNA
ENSG0000(	LINC02321	99.68832	-0.11825	0.229211	0.363975	lncRNA
ENSG0000(	ENSG0000(	12.26434	0.219654	0.229224	0.363976	lncRNA
ENSG0000(	GLIDR	573.113	-0.0606	0.229243	0.363988	lncRNA
ENSG0000(	AJUBA-DT	12.9958	0.24782	0.229349	0.364117	lncRNA
ENSG0000(	KCNC2	57.08892	-0.19919	0.229359	0.364117	protein_coding
ENSG0000(	SNORD3D	26628.16	-0.2677	0.229347	0.364117	snoRNA
ENSG0000(	NONO	4035.652	0.068675	0.229415	0.364188	protein_coding
ENSG0000(	OR2V2	319.6874	0.061823	0.229509	0.364318	protein_coding
ENSG0000(	ADIRF-AS1	421.12	-0.0984	0.229563	0.364385	lncRNA
ENSG0000(	ZBTB3	750.1482	-0.06496	0.229642	0.364492	protein_coding
ENSG0000(	ENSG0000(	88.25952	0.111495	0.229712	0.364575	lncRNA
ENSG0000(	MPP2	52.42398	-0.2254	0.229717	0.364575	protein_coding
ENSG0000(	RN7SKP269	110.8269	-0.15343	0.22978	0.364637	misc_RNA
ENSG0000(	TPM2	486.407	0.178413	0.229769	0.364637	protein_coding
ENSG0000(	UBR1	2257.51	-0.07045	0.229829	0.364697	protein_coding
ENSG0000(	OLFML1	147.1256	0.147134	0.229844	0.364702	protein_coding
ENSG0000(	WDR45B	1359.522	-0.05435	0.229878	0.364737	protein_coding
ENSG0000(	CD2	282.4926	0.214131	0.230046	0.364938	protein_coding
ENSG0000(	ENSG0000(	58.67066	-0.12579	0.230066	0.364938	lncRNA
ENSG0000(	ENSG0000(	12.62975	0.3492	0.230074	0.364938	processed_pseudogene
ENSG0000(	ENSG0000(	40.99018	0.154665	0.230075	0.364938	lncRNA
ENSG0000(	ENSG0000(	55.07115	-0.24532	0.230057	0.364938	lncRNA
ENSG0000(	TRBV28	10.35225	-0.50912	0.23004	0.364938	TR_V_gene
ENSG0000(	ENSG0000(	318.6702	0.056105	0.230106	0.364969	lncRNA
ENSG0000(	UBE2W	1589.804	0.065836	0.230247	0.365175	protein_coding
ENSG0000(	CFAP74	702.0175	0.063385	0.230292	0.365228	protein_coding
ENSG0000(	ANAPC15	847.5828	0.053103	0.230309	0.365233	protein_coding
ENSG0000(	RPS3AP13	16.29984	-0.20739	0.230319	0.365233	processed_pseudogene
ENSG0000(	MTMR7	20.20791	0.418302	0.230379	0.365307	protein_coding
ENSG0000(	SH3GL1P2	44.9285	-0.5294	0.230389	0.365307	processed_pseudogene
ENSG0000(	TRIM36-IT1	70.47366	-0.25617	0.230458	0.365398	lncRNA
ENSG0000(	PYROXD2	313.4769	-0.10055	0.230508	0.36546	protein_coding
ENSG0000(	TJP1	3915.227	-0.12311	0.230528	0.365473	protein_coding
ENSG0000(	LINC01913	121.7007	-0.10106	0.230628	0.365612	lncRNA
ENSG0000(	TRIM52	2081.697	0.048951	0.230644	0.365619	protein_coding
ENSG0000(	EMCN	479.8171	0.088304	0.230666	0.365636	protein_coding
ENSG0000(	ETFBKMT	611.6412	-0.07653	0.230687	0.365649	protein_coding
ENSG0000(	SLC16A11	15.2294	-0.26525	0.230721	0.365685	protein_coding
ENSG0000(	RN7SL870F	14.88896	0.265281	0.230755	0.36572	misc_RNA
ENSG0000(	ZNF90P3	93.74138	-0.12194	0.230831	0.365822	processed_pseudogene
ENSG0000(	DNAJC12	64.77099	-0.14589	0.230878	0.365879	protein_coding
ENSG0000(	LINC02444	2320.356	-0.0893	0.230923	0.365931	lncRNA
ENSG0000(	PGBD5	334.5679	-0.13354	0.230941	0.365942	protein_coding
ENSG0000(	METTL8	1726.262	0.05848	0.230979	0.365982	protein_coding
ENSG0000(	ENSG0000(	8.802426	0.399845	0.231033	0.366049	lncRNA
ENSG0000(	ENSG0000(	98.81131	-0.11478	0.231091	0.366104	lncRNA

ENSG0000(	LINC00967	159.5014	-0.191	0.231087	0.366104	lncRNA
ENSG0000(	ZNF502	262.0663	-0.10985	0.23115	0.36618	protein_coding
ENSG0000(	ZNF138	435.0426	-0.08908	0.231215	0.366264	protein_coding
ENSG0000(	ACTBP7	10.30042	-0.32403	0.231235	0.366277	processed_pseudogene
ENSG0000(	GPR12	96.91664	-0.10159	0.231281	0.366293	protein_coding
ENSG0000(	HNRNPA1P	58.45142	-0.16868	0.231267	0.366293	processed_pseudogene
ENSG0000(	NFIA	18445.61	-0.0829	0.231292	0.366293	protein_coding
ENSG0000(	SLC30A1	1648.206	-0.12423	0.231291	0.366293	protein_coding
ENSG0000(	GRM7	159.1231	-0.1703	0.231413	0.366457	protein_coding
ENSG0000(	HMGB1P6	30.63605	-0.69006	0.231419	0.366457	processed_pseudogene
ENSG0000(	AFF1	7739.552	0.062788	0.231475	0.366527	protein_coding
ENSG0000(	HOXB2	107.544	0.164361	0.231533	0.3666	protein_coding
ENSG0000(	KHDRBS3	231.1338	-0.1266	0.231664	0.36679	protein_coding
ENSG0000(	CREBL2	967.1893	0.086585	0.231699	0.366816	protein_coding
ENSG0000(	PINCR	18.71276	0.218069	0.231704	0.366816	lncRNA
ENSG0000(	KLK13	11.10979	-0.35717	0.231758	0.366856	protein_coding
ENSG0000(	MBOAT2	1934.811	-0.11732	0.231765	0.366856	protein_coding
ENSG0000(	ZNF585B	2244.139	-0.10399	0.231748	0.366856	protein_coding
ENSG0000(	MAST3	2014.294	-0.06879	0.231839	0.366955	protein_coding
ENSG0000(	LRWD1	407.1855	-0.09046	0.231889	0.367016	protein_coding
ENSG0000(	ENSG0000(	21.0581	0.195378	0.231965	0.367098	lncRNA
ENSG0000(	LINC01885	43.05037	0.193372	0.231954	0.367098	lncRNA
ENSG0000(	SLU7	833.2793	-0.07428	0.231987	0.367115	protein_coding
ENSG0000(	ENSG0000(	74.37732	-0.19153	0.232029	0.367162	lncRNA
ENSG0000(	IGKV1-6	177.072	0.305873	0.23204	0.367162	IG_V_gene
ENSG0000(	C17orf75	1180.88	-0.05202	0.232086	0.367209	protein_coding
ENSG0000(	LRRC4B	40.52955	-0.23461	0.232093	0.367209	protein_coding
ENSG0000(	ENSG0000(	186.8465	-0.1041	0.232122	0.367218	unprocessed_pseudogene
ENSG0000(	LINC01361	375.7877	0.086654	0.232113	0.367218	lncRNA
ENSG0000(	CCDC184	13.56479	0.300503	0.232171	0.367233	protein_coding
ENSG0000(	GTF2IP23	29.31629	0.199096	0.232154	0.367233	unprocessed_pseudogene
ENSG0000(	MMP20	13.08847	-0.31471	0.232179	0.367233	protein_coding
ENSG0000(	RPL34P20	11.46554	0.304587	0.232168	0.367233	processed_pseudogene
ENSG0000(	ENSG0000(	990.1597	0.054724	0.232192	0.367234	TEC
ENSG0000(	RPS3AP14	44.80657	-0.34936	0.23221	0.367245	processed_pseudogene
ENSG0000(	ENSG0000(	2095.466	0.055542	0.232308	0.367372	protein_coding
ENSG0000(	MYO3B	276.7647	0.063443	0.232321	0.367372	protein_coding
ENSG0000(	ODR4	920.9262	-0.06967	0.232326	0.367372	protein_coding
ENSG0000(	ENSG0000(	67.17125	-0.12881	0.232367	0.367401	lncRNA
ENSG0000(	FMN2	79.58803	-0.19631	0.232358	0.367401	protein_coding
ENSG0000(	LINC00907	589.3744	0.064067	0.232622	0.367785	lncRNA
ENSG0000(	ENSG0000(	731.8108	-0.04901	0.232655	0.367819	TEC
ENSG0000(	SV2B	232.4987	-0.15801	0.232687	0.36785	protein_coding
ENSG0000(	BAG2	425.1899	0.099682	0.232758	0.367919	protein_coding
ENSG0000(	ENSG0000(	193.2281	-0.08785	0.232757	0.367919	lncRNA
ENSG0000(	PEF1	556.2875	-0.07106	0.232765	0.367919	protein_coding
ENSG0000(	PPIL2	2946.834	-0.07075	0.232798	0.367952	protein_coding

ENSG0000(	RCN1P2	19.492	-0.32763	0.232934	0.368148	processed_pseudogene
ENSG0000(	ANKEF1	624.165	0.082547	0.233022	0.368268	protein_coding
ENSG0000(	RN7SKP74	96.0796	0.202446	0.233033	0.368268	misc_RNA
ENSG0000(	SLC26A5	11.30691	-0.33662	0.233086	0.368332	protein_coding
ENSG0000(	ENSG0000(	11.0677	0.30074	0.233112	0.368336	lncRNA
ENSG0000(	WAC-AS1	974.4004	-0.06534	0.233102	0.368336	lncRNA
ENSG0000(	CAMK4	995.2054	-0.17987	0.233134	0.368352	protein_coding
ENSG0000(	ITGB1BP2	74.48176	-0.13308	0.233156	0.368369	protein_coding
ENSG0000(	E2F5	557.7142	0.120525	0.233174	0.368379	protein_coding
ENSG0000(	NAIPP1	138.7382	-0.13002	0.233302	0.368562	transcribed_unprocessed_pseudo
ENSG0000(	KNDC1	293.9627	0.111522	0.233338	0.368563	protein_coding
ENSG0000(	RDH11	842.863	0.089017	0.233315	0.368563	protein_coding
ENSG0000(	RPL7P46	40.51454	-0.14867	0.233327	0.368563	processed_pseudogene
ENSG0000(	SDR16C5	80.24794	-0.28186	0.233452	0.368725	protein_coding
ENSG0000(	ATP1B3	1606.362	-0.16111	0.233484	0.368756	protein_coding
ENSG0000(	FSIP2-AS1	83.72703	0.124729	0.233562	0.368806	lncRNA
ENSG0000(	JAM2	668.097	0.06785	0.233551	0.368806	protein_coding
ENSG0000(	SALL4	205.7539	0.165985	0.233553	0.368806	protein_coding
ENSG0000(	SLC25A5-A:	57.93376	0.1592	0.233545	0.368806	lncRNA
ENSG0000(	GNB1	3864.851	-0.06409	0.233609	0.368861	protein_coding
ENSG0000(	ODAD1	379.9912	0.069889	0.233728	0.369029	protein_coding
ENSG0000(	ENSG0000(	18.62026	-0.36568	0.233806	0.369134	processed_pseudogene
ENSG0000(	ENSG0000(	11.50245	0.245853	0.233824	0.369144	lncRNA
ENSG0000(	CD160	158.8501	-0.17341	0.233991	0.369389	protein_coding
ENSG0000(	OR10A6	269.2886	0.106674	0.234022	0.36942	protein_coding
ENSG0000(	NSL1	1595.071	0.083209	0.234065	0.369469	protein_coding
ENSG0000(	CALHM1	66.44331	0.109269	0.234187	0.369606	protein_coding
ENSG0000(	KLK7	68.73078	0.187588	0.234174	0.369606	protein_coding
ENSG0000(	SNORD38C	10.4006	0.263053	0.234188	0.369606	snoRNA
ENSG0000(	RN7SL390F	26.36797	0.204987	0.234202	0.369611	misc_RNA
ENSG0000(	ENSG0000(	98.28131	0.186764	0.234363	0.369829	lncRNA
ENSG0000(	ENSG0000(	285.2818	0.102817	0.234364	0.369829	lncRNA
ENSG0000(	RPS4XP5	205.8337	0.099855	0.234406	0.369877	transcribed_processed_pseudoge
ENSG0000(	CD109	440.143	0.12041	0.234434	0.369901	protein_coding
ENSG0000(	RN7SL569F	17.89794	0.218424	0.234484	0.369953	misc_RNA
ENSG0000(	SMIM5	2169.65	-0.21718	0.23449	0.369953	protein_coding
ENSG0000(	ADSS1	329.6751	-0.08749	0.234537	0.370009	protein_coding
ENSG0000(	CX3CR1	43.60587	0.225983	0.23459	0.370073	protein_coding
ENSG0000(	ENSG0000(	221.186	-0.07721	0.234626	0.370111	lncRNA
ENSG0000(	FLCN	2662.604	0.083914	0.234739	0.370271	protein_coding
ENSG0000(	ENSG0000(	13.62564	0.293912	0.234798	0.370346	lncRNA
ENSG0000(	ENSG0000(	10.65574	0.258613	0.234846	0.370402	lncRNA
ENSG0000(	MPPE1	1697.447	0.063873	0.234871	0.370423	protein_coding
ENSG0000(	ENSG0000(	17.0144	-0.24675	0.234891	0.370435	transcribed_processed_pseudoge
ENSG0000(	ENSG0000(	476.0514	-0.07213	0.234918	0.370459	lncRNA
ENSG0000(	ALKBH5	1033.512	-0.0804	0.234942	0.370479	protein_coding
ENSG0000(	LINC02613	298.3457	0.074691	0.235043	0.370603	lncRNA

ENSG0000(	RPL39P36	17.10981	-0.18789	0.235044	0.370603	processed_pseudogene
ENSG0000(	NEPNP	10.95611	-0.26246	0.235059	0.370607	unitary_pseudogene
ENSG0000(	HMOX1	498.0183	-0.21301	0.235098	0.370651	protein_coding
ENSG0000(	LGI2	99.62522	0.298062	0.235226	0.370833	protein_coding
ENSG0000(	SLC2A13	1472.131	0.096258	0.235244	0.370843	protein_coding
ENSG0000(	ENSG0000(	42.45302	0.211281	0.235355	0.370999	lncRNA
ENSG0000(	ENSG0000(	70.97488	-0.1128	0.235616	0.371392	lncRNA
ENSG0000(	ENSG0000(	354.3676	0.053404	0.235746	0.371578	lncRNA
ENSG0000(	ECM1	195.0171	-0.15548	0.235834	0.371699	protein_coding
ENSG0000(	ENSG0000(	52.98208	-0.14727	0.235852	0.371708	lncRNA
ENSG0000(	ENSG0000(	19.30332	-0.21307	0.236023	0.371922	lncRNA
ENSG0000(	SOX7	187.6633	-0.08128	0.236013	0.371922	protein_coding
ENSG0000(	STK24-AS1	56.96396	0.116747	0.236019	0.371922	lncRNA
ENSG0000(	ENSG0000(	78.60912	0.098567	0.23608	0.371992	lncRNA
ENSG0000(	MFSD2A	472.3294	-0.20737	0.236106	0.372015	protein_coding
ENSG0000(	ENSG0000(	14.57512	-0.28131	0.23614	0.372048	lncRNA
ENSG0000(	SMIM6	50.75176	-0.26565	0.236151	0.372048	protein_coding
ENSG0000(	SPOPL	1351.404	-0.06407	0.236243	0.372174	protein_coding
ENSG0000(	ENSG0000(	33.76302	0.164632	0.236312	0.372245	lncRNA
ENSG0000(	ZDHHC8P1	231.9178	-0.10556	0.236301	0.372245	transcribed_unprocessed_pseudo
ENSG0000(	ENSG0000(	126.5663	0.12402	0.236324	0.372246	lncRNA
ENSG0000(	ENSG0000(	10.23797	-0.37706	0.236387	0.372307	lncRNA
ENSG0000(	TUSC3	178.5063	0.147875	0.236375	0.372307	protein_coding
ENSG0000(	ENSG0000(	156.609	-0.09654	0.236573	0.372581	lncRNA
ENSG0000(	ENSG0000(	22.71464	-0.21967	0.236586	0.372583	lncRNA
ENSG0000(	RNF139-DT	548.3412	0.068972	0.236855	0.372988	lncRNA
ENSG0000(	PTPN6	1135.07	0.123418	0.23692	0.373072	protein_coding
ENSG0000(	BTD	2064.884	-0.04301	0.236949	0.373098	protein_coding
ENSG0000(	CDH15	122.7087	0.101237	0.236963	0.373102	protein_coding
ENSG0000(	WT1	115.3123	0.130019	0.236986	0.373119	protein_coding
ENSG0000(	INSYN2A	17.5469	0.310584	0.236998	0.37312	protein_coding
ENSG0000(	DCST2	78.20144	-0.19574	0.237078	0.373226	protein_coding
ENSG0000(	DENND10	1187.248	-0.07389	0.237111	0.37326	protein_coding
ENSG0000(	ASMT	51.53462	-0.17258	0.237165	0.373326	protein_coding
ENSG0000(	HLA-J	272.7182	0.181944	0.23721	0.373378	transcribed_unprocessed_pseudo
ENSG0000(	CALB2	11.34073	-0.43187	0.237267	0.373448	protein_coding
ENSG0000(	ENSG0000(	68.17607	-0.14169	0.237343	0.373549	processed_pseudogene
ENSG0000(	ENSG0000(	116.7265	-0.11722	0.237411	0.373637	lncRNA
ENSG0000(	DOC2A	399.1332	-0.11757	0.237482	0.373731	protein_coding
ENSG0000(	ENSG0000(	18.50024	0.218952	0.237522	0.373775	lncRNA
ENSG0000(	PRORP	1724.695	-0.06599	0.237608	0.373891	protein_coding
ENSG0000(	OR2L3	52.04097	0.166548	0.237684	0.373992	protein_coding
ENSG0000(	ENSG0000(	9.831489	-0.26837	0.237725	0.374038	processed_pseudogene
ENSG0000(	NEK8	580.1531	0.060667	0.237845	0.374208	protein_coding
ENSG0000(	DDX11L10	10.12566	-0.53115	0.237864	0.374219	transcribed_unprocessed_pseudo
ENSG0000(	CLEC17A	379.5217	0.1864	0.237914	0.374259	protein_coding
ENSG0000(	TRAJ37	27.02588	-0.26324	0.237914	0.374259	TR_J_gene

ENSG0000(	ENSG0000(	134.2878	-0.1377	0.238143	0.374601	lncRNA
ENSG0000(	LRRC34	269.0256	0.086556	0.238156	0.374602	protein_coding
ENSG0000(	FHL2	2525.926	0.128327	0.238234	0.374707	protein_coding
ENSG0000(	CBX5P1	23.52998	0.222113	0.238367	0.374896	processed_pseudogene
ENSG0000(	ZNF542P	587.9587	-0.06093	0.23849	0.375072	transcribed_unprocessed_pseudo
ENSG0000(	TEC	413.7356	-0.11899	0.238519	0.375099	protein_coding
ENSG0000(	CCZ1	1692.832	-0.06645	0.238569	0.375121	protein_coding
ENSG0000(	FBXO11	4187.015	0.037895	0.238553	0.375121	protein_coding
ENSG0000(	KARS1	942.8561	-0.07411	0.238566	0.375121	protein_coding
ENSG0000(	RAB13	601.7019	0.103024	0.238589	0.375133	protein_coding
ENSG0000(	LINC02891	56.49648	-0.15574	0.238698	0.375286	lncRNA
ENSG0000(	HOMEZ	959.0016	-0.07761	0.238928	0.375621	protein_coding
ENSG0000(	SIM1	42.01392	0.152279	0.238936	0.375621	protein_coding
ENSG0000(	ENSG0000(	157.1942	-0.13257	0.238975	0.375665	processed_pseudogene
ENSG0000(	CYP3A4	84.35928	-0.26935	0.239154	0.375927	protein_coding
ENSG0000(	OR7E122P	885.958	-0.06033	0.239277	0.376101	transcribed_unprocessed_pseudo
ENSG0000(	ENSG0000(	35.30749	-0.1653	0.23931	0.376135	lncRNA
ENSG0000(	ENSG0000(	137.2122	0.084688	0.239365	0.376201	lncRNA
ENSG0000(	ENSG0000(	62.22628	0.194026	0.23947	0.376347	lncRNA
ENSG0000(	ENSG0000(	15.3188	0.323174	0.239514	0.376379	lncRNA
ENSG0000(	NAB2	187.6589	-0.16338	0.239514	0.376379	protein_coding
ENSG0000(	ZNF17	564.423	0.064358	0.239533	0.37639	protein_coding
ENSG0000(	ENSG0000(	54.53503	-0.11292	0.239555	0.376406	lncRNA
ENSG0000(	ENSG0000(	44.18726	0.156895	0.239584	0.376433	lncRNA
ENSG0000(	ZNF280C	330.2912	-0.08161	0.239603	0.376443	protein_coding
ENSG0000(	RELA-DT	687.1112	-0.0463	0.239747	0.37665	lncRNA
ENSG0000(	KTI12	130.3322	-0.13099	0.239793	0.376704	protein_coding
ENSG0000(	GYPB	28.75822	0.191952	0.239874	0.376812	protein_coding
ENSG0000(	DNAH17-A!	277.8904	-0.08586	0.239904	0.37684	lncRNA
ENSG0000(	ENSG0000(	283.5339	0.092249	0.239965	0.376879	TEC
ENSG0000(	LINC00840	90.77155	0.112127	0.239957	0.376879	lncRNA
ENSG0000(	LINC02700	39.98206	-0.2182	0.239944	0.376879	lncRNA
ENSG0000(	SNORA26	116.6618	0.149001	0.240021	0.376948	snoRNA
ENSG0000(	SNORA49	232.8157	-0.12715	0.240062	0.376994	snoRNA
ENSG0000(	ENSG0000(	19.37935	0.215706	0.24021	0.377208	lncRNA
ENSG0000(	ENSG0000(	450.1012	0.148104	0.240232	0.377222	protein_coding
ENSG0000(	PNPLA2	2193.238	-0.1544	0.240279	0.377277	protein_coding
ENSG0000(	C1orf167	238.675	0.08575	0.240311	0.377307	protein_coding
ENSG0000(	FAM224B	52.51013	-3.43415	0.240333	0.377307	lncRNA
ENSG0000(	PPIL4	593.6988	-0.05203	0.240328	0.377307	protein_coding
ENSG0000(	WFDC10B	7.58939	0.440862	0.240357	0.377325	protein_coding
ENSG0000(	CBX3	1723.009	-0.0569	0.240389	0.377357	protein_coding
ENSG0000(	ENSG0000(	671.02	0.058965	0.240407	0.377366	lncRNA
ENSG0000(	ENSG0000(	11.22589	-0.25912	0.24062	0.377681	lncRNA
ENSG0000(	RPL13AP5	21.60303	0.600119	0.240641	0.377694	processed_pseudogene
ENSG0000(	WDPCP	2877.951	0.063599	0.24071	0.377784	protein_coding
ENSG0000(	DM1-AS	22.55476	-0.23062	0.240771	0.377804	lncRNA

ENSG0000(	ISLR2	129.6595	0.133211	0.24075	0.377804	protein_coding
ENSG0000(	VAV3-AS1	12.28206	0.486821	0.24076	0.377804	lncRNA
ENSG0000(	ZNF8-ERVK	198.6533	0.080007	0.240755	0.377804	lncRNA
ENSG0000(	PBX2	1620.842	0.085429	0.240818	0.377859	protein_coding
ENSG0000(	ENSG0000(	9.256367	-0.25039	0.24085	0.37789	lncRNA
ENSG0000(	STX17-DT	3382.796	-0.05389	0.240917	0.377976	lncRNA
ENSG0000(	BET1-AS1	14.29895	-0.19613	0.240975	0.378048	lncRNA
ENSG0000(	EDNRB	613.4245	-0.12075	0.241052	0.378131	protein_coding
ENSG0000(	RN7SL553P	27.13178	0.164663	0.241044	0.378131	misc_RNA
ENSG0000(	ENSG0000(	97.1382	-0.13165	0.241087	0.378167	TEC
ENSG0000(	NKD1	710.0498	0.177648	0.241103	0.378173	protein_coding
ENSG0000(	ENSG0000(	14.34	0.299591	0.241146	0.378202	lncRNA
ENSG0000(	ENSG0000(	43.65562	-0.16344	0.241134	0.378202	lncRNA
ENSG0000(	AP1B1P1	20.5048	0.21418	0.241167	0.378217	transcribed_unprocessed_pseudo
ENSG0000(	ENSG0000(	309.3503	0.063745	0.241228	0.378293	lncRNA
ENSG0000(	CNPY3	398.2433	-0.10979	0.24129	0.378371	protein_coding
ENSG0000(	ZNF239	144.354	-0.15077	0.241308	0.37838	protein_coding
ENSG0000(	ENSG0000(	104.5852	0.117184	0.241434	0.37856	lncRNA
ENSG0000(	OR2A9P	597.0482	0.13709	0.241501	0.378646	transcribed_unprocessed_pseudo
ENSG0000(	TMEM217	41.57878	-0.19392	0.24166	0.378876	protein_coding
ENSG0000(	LINC01048	11.51763	0.233103	0.241689	0.378901	lncRNA
ENSG0000(	NEIL2	193.985	0.111777	0.241778	0.379023	protein_coding
ENSG0000(	ERC2	45.00146	0.214513	0.241982	0.379304	protein_coding
ENSG0000(	TRPC4AP	1860.863	-0.05245	0.241981	0.379304	protein_coding
ENSG0000(	MTUS1	6281.06	-0.09076	0.242023	0.37935	protein_coding
ENSG0000(	ZBTB20-AS	10.97306	-0.37885	0.242037	0.379352	lncRNA
ENSG0000(	ENSG0000(	67.32983	0.093009	0.242124	0.379471	lncRNA
ENSG0000(	DPY19L2P1	400.0247	0.07852	0.242143	0.37948	transcribed_unprocessed_pseudo
ENSG0000(	ENSG0000(	8.862661	-0.48846	0.242157	0.379484	lncRNA
ENSG0000(	AIRE	179.7185	0.107617	0.242239	0.379593	protein_coding
ENSG0000(	CCDC190	13.84288	0.278088	0.242349	0.379719	protein_coding
ENSG0000(	SNHG31	705.1341	-0.09468	0.242333	0.379719	lncRNA
ENSG0000(	TRIM68	668.8118	-0.07244	0.242356	0.379719	protein_coding
ENSG0000(	ADGRD2	439.3304	-0.05756	0.242388	0.37975	protein_coding
ENSG0000(	ENSG0000(	765.0255	0.145271	0.242429	0.379796	lncRNA
ENSG0000(	ENSG0000(	10.07263	0.427485	0.242449	0.379809	lncRNA
ENSG0000(	ENSG0000(	31.51113	-0.18559	0.242526	0.37991	lncRNA
ENSG0000(	ADRA2B	31.32175	-0.2476	0.242573	0.379959	protein_coding
ENSG0000(	HSPE1P19	15.30639	0.255545	0.242582	0.379959	processed_pseudogene
ENSG0000(	KY	38.46528	0.198079	0.242643	0.380036	protein_coding
ENSG0000(	ENSG0000(	143.5982	-0.09634	0.242687	0.380086	TEC
ENSG0000(	SH3TC2	626.1022	-0.09028	0.242741	0.380151	protein_coding
ENSG0000(	ENSG0000(	150.9881	0.077861	0.242768	0.380174	lncRNA
ENSG0000(	TMEM272	146.3888	0.2574	0.242795	0.380198	protein_coding
ENSG0000(	ENSG0000(	88.46505	-0.10793	0.242854	0.380271	lncRNA
ENSG0000(	RPL36AP15	40.82969	0.14621	0.242888	0.380306	processed_pseudogene
ENSG0000(	ENSG0000(	169.8484	0.104348	0.24296	0.3804	protein_coding

ENSG0000(	ENSG0000(	21.98916	0.287198	0.243146	0.38067	processed_pseudogene
ENSG0000(	ENSG0000(	20.25815	0.214371	0.243195	0.380672	lncRNA
ENSG0000(	ENSG0000(	167.0243	0.097332	0.243187	0.380672	unprocessed_pseudogene
ENSG0000(	ENSG0000(	234.2606	0.07481	0.243186	0.380672	lncRNA
ENSG0000(	HSF2BP	19.32111	0.239736	0.243176	0.380672	protein_coding
ENSG0000(	COX7A2L	1005.287	-0.06983	0.243307	0.380794	protein_coding
ENSG0000(	ENSG0000(	1081.65	-0.0579	0.24331	0.380794	lncRNA
ENSG0000(	PIGQ	862.2545	-0.10652	0.243303	0.380794	protein_coding
ENSG0000(	PLGLB2	240.5086	0.324394	0.243327	0.380801	protein_coding
ENSG0000(	LINC01992	239.08	-0.06505	0.243372	0.380854	lncRNA
ENSG0000(	ENSG0000(	14.57461	-0.25449	0.243409	0.380855	lncRNA
ENSG0000(	ENSG0000(	70.87107	0.146514	0.243402	0.380855	lncRNA
ENSG0000(	EXOSC9	511.9158	-0.08247	0.243388	0.380855	protein_coding
ENSG0000(	NANOGNBI	9.793147	-0.27201	0.243445	0.380892	processed_pseudogene
ENSG0000(	ZNF341	510.5391	-0.07977	0.2435	0.380958	protein_coding
ENSG0000(	SIAH2-AS1	98.92423	-0.18721	0.24363	0.381143	lncRNA
ENSG0000(	ASCC2	1637.656	-0.06367	0.243691	0.381159	protein_coding
ENSG0000(	CRAT37	430.871	-0.08556	0.243678	0.381159	lncRNA
ENSG0000(	CYGB	319.6258	-0.14561	0.243686	0.381159	protein_coding
ENSG0000(	ENSG0000(	9.112034	0.30151	0.243671	0.381159	lncRNA
ENSG0000(	WNT11	21.11717	-0.38878	0.243701	0.381159	protein_coding
ENSG0000(	ACVR1C	654.1521	-0.20222	0.243788	0.381276	protein_coding
ENSG0000(	LINC01378	116.2009	0.146113	0.24388	0.3814	lncRNA
ENSG0000(	ENSG0000(	46.67169	0.170991	0.243945	0.381483	TEC
ENSG0000(	NPIPA3	41.03927	-0.29537	0.243985	0.381526	protein_coding
ENSG0000(	CAP2	59.17902	-0.19211	0.244008	0.381543	protein_coding
ENSG0000(	ANKRD53	19.83624	0.317828	0.244025	0.381551	protein_coding
ENSG0000(	WDR73	2724.675	0.060671	0.244066	0.381595	protein_coding
ENSG0000(	ZNF19	921.869	-0.09914	0.244131	0.381679	protein_coding
ENSG0000(	DNAJA4-DT	21.73869	-0.18376	0.244196	0.381703	lncRNA
ENSG0000(	ENSG0000(	94.75164	-0.16304	0.244191	0.381703	lncRNA
ENSG0000(	ENSG0000(	10.95241	0.300386	0.244173	0.381703	lncRNA
ENSG0000(	RORC	764.9768	-0.11616	0.244188	0.381703	protein_coding
ENSG0000(	TUBB8	13.71525	0.475613	0.244253	0.381773	protein_coding
ENSG0000(	ENSG0000(	193.1245	0.081485	0.244267	0.381777	lncRNA
ENSG0000(	ENSG0000(	39.05446	0.172836	0.244314	0.381831	lncRNA
ENSG0000(	STX18	899.5265	-0.06035	0.244396	0.381935	protein_coding
ENSG0000(	TUBAP11	29.56891	0.165145	0.244405	0.381935	processed_pseudogene
ENSG0000(	ENSG0000(	14.83697	0.210401	0.244473	0.381986	lncRNA
ENSG0000(	ENSG0000(	114.3642	0.125983	0.244457	0.381986	lncRNA
ENSG0000(	IL2RG	866.2634	-0.11743	0.244486	0.381986	protein_coding
ENSG0000(	LINC01521	589.8955	0.061542	0.244476	0.381986	lncRNA
ENSG0000(	C1orf56	40.98192	-0.218	0.244617	0.38217	protein_coding
ENSG0000(	CCDC9	690.0629	0.06633	0.244678	0.382209	protein_coding
ENSG0000(	CHURC1	1972.504	0.056861	0.244668	0.382209	protein_coding
ENSG0000(	FMO3	135.5636	-0.10806	0.244655	0.382209	protein_coding
ENSG0000(	ENSG0000(	292.6776	-0.06786	0.244692	0.382212	lncRNA

ENSG0000(	OR8B8	84.92611	-0.09423	0.244799	0.38236	protein_coding
ENSG0000(	ENSG0000(	233.5984	-0.07882	0.244831	0.382391	protein_coding
ENSG0000(	CES1P1	27.32998	0.211545	0.244853	0.382406	transcribed_unprocessed_pseudo
ENSG0000(	CCDC183	192.4772	-0.30894	0.244991	0.382525	protein_coding
ENSG0000(	ENSG0000(	65.00905	0.138437	0.244958	0.382525	lncRNA
ENSG0000(	ENSG0000(	421.1773	0.104595	0.244982	0.382525	transcribed_processed_pseudoge
ENSG0000(	ENSG0000(	16.85914	0.202887	0.244982	0.382525	lncRNA
ENSG0000(	ENSG0000(	15.79594	-0.24235	0.245003	0.382525	lncRNA
ENSG0000(	OR6C75	505.74	0.072323	0.244943	0.382525	protein_coding
ENSG0000(	HIF1AN	3803.582	-0.05105	0.245018	0.38253	protein_coding
ENSG0000(	SRC	2133.6	-0.08157	0.245078	0.382605	protein_coding
ENSG0000(	ADAMTSL5	152.2699	0.178092	0.245113	0.382639	protein_coding
ENSG0000(	ISCU	1133.616	0.0804	0.245302	0.38291	protein_coding
ENSG0000(	RN7SL789F	31.40165	0.177615	0.245311	0.38291	misc_RNA
ENSG0000(	ENSG0000(	39.63856	0.179143	0.245356	0.382933	lncRNA
ENSG0000(	MYO18B	35.65428	-0.42638	0.245362	0.382933	protein_coding
ENSG0000(	RRAGA	366.7085	-0.0841	0.245362	0.382933	protein_coding
ENSG0000(	MED30	78.5969	-0.14081	0.245542	0.383195	protein_coding
ENSG0000(	ENSG0000(	147.9214	0.178894	0.245562	0.383206	lncRNA
ENSG0000(	KLK14	60.84958	-0.20258	0.245687	0.383382	protein_coding
ENSG0000(	CDADC1	217.5823	-0.09555	0.245737	0.383442	protein_coding
ENSG0000(	CCDC90B	3098.347	-0.04936	0.245773	0.383479	protein_coding
ENSG0000(	SPAG7	640.5223	-0.04738	0.245815	0.383509	protein_coding
ENSG0000(	WBP2	1095.287	0.05831	0.245817	0.383509	protein_coding
ENSG0000(	ENSG0000(	84.12048	0.101373	0.245851	0.383543	lncRNA
ENSG0000(	OPCML-IT1	17.86622	0.19761	0.246137	0.38397	lncRNA
ENSG0000(	ENSG0000(	19.97239	0.215476	0.246178	0.384014	lncRNA
ENSG0000(	ENSG0000(	2870.641	-0.08782	0.246318	0.384213	lncRNA
ENSG0000(	VWDE	100.9897	-0.31304	0.246411	0.38434	protein_coding
ENSG0000(	MIR378J	11.90295	-0.2467	0.246455	0.384389	miRNA
ENSG0000(	KBTBD8	275.8506	-0.16917	0.246497	0.384417	protein_coding
ENSG0000(	RRN3P2	353.9042	0.108665	0.246496	0.384417	lncRNA
ENSG0000(	GPR85	3219.953	0.190053	0.246514	0.384424	protein_coding
ENSG0000(	NAXD	878.4433	-0.05901	0.246784	0.384826	protein_coding
ENSG0000(	ZNF490	1130.146	0.073219	0.246801	0.384832	protein_coding
ENSG0000(	C12orf42	64.10563	0.363515	0.246919	0.384958	protein_coding
ENSG0000(	ENSG0000(	43.98356	0.132772	0.246943	0.384958	lncRNA
ENSG0000(	RN7SKP55	259.963	-0.24298	0.246937	0.384958	misc_RNA
ENSG0000(	SDC1	2052.475	-0.11904	0.246924	0.384958	protein_coding
ENSG0000(	ZNF334	498.1121	0.172637	0.246912	0.384958	protein_coding
ENSG0000(	MIR554	15.03193	-0.25814	0.247048	0.385103	miRNA
ENSG0000(	LINC01018	162.8249	0.07337	0.247064	0.385109	lncRNA
ENSG0000(	RNU6-140F	29.949	-0.32824	0.247138	0.385205	snRNA
ENSG0000(	ENSG0000(	340.8249	-0.06155	0.247201	0.385277	lncRNA
ENSG0000(	ENSG0000(	35.35096	0.141238	0.247209	0.385277	lncRNA
ENSG0000(	A2M	4805.087	-0.12848	0.247331	0.385448	protein_coding
ENSG0000(	RINT1	636.8389	-0.06839	0.247435	0.385591	protein_coding

ENSG0000(	ARL3	111.4746	0.135737	0.247453	0.3856	protein_coding
ENSG0000(	ENSG0000(	46.92386	-0.14413	0.247488	0.385632	lncRNA
ENSG0000(	ENSG0000(	28.54817	0.159798	0.247498	0.385632	lncRNA
ENSG0000(	PTGER4	1126.172	0.091022	0.247605	0.385779	protein_coding
ENSG0000(	NPM1P37	96.36678	-0.10345	0.247625	0.385791	processed_pseudogene
ENSG0000(	ANAPC10P	29.34776	0.159028	0.247653	0.385815	processed_pseudogene
ENSG0000(	DAZL	18.54683	1.050687	0.247862	0.386121	protein_coding
ENSG0000(	AQP12A	12.62219	-0.54433	0.248001	0.3863	protein_coding
ENSG0000(	JKAMP	878.4339	-0.06003	0.248	0.3863	protein_coding
ENSG0000(	LINC02620	23.88184	0.175198	0.248103	0.38644	lncRNA
ENSG0000(	DGKG	170.9073	-0.18083	0.248167	0.386516	protein_coding
ENSG0000(	LINC00824	194.9797	-0.08497	0.248177	0.386516	lncRNA
ENSG0000(	EPHA1-AS1	495.2608	0.104064	0.2482	0.386533	lncRNA
ENSG0000(	OMD	94.05459	-0.10159	0.248228	0.386537	protein_coding
ENSG0000(	TMEM30A	2302.213	0.093853	0.248216	0.386537	protein_coding
ENSG0000(	MIRLET7BH	2270.954	0.066966	0.24843	0.386833	lncRNA
ENSG0000(	PTPRO	1251.483	-0.15975	0.248468	0.386873	protein_coding
ENSG0000(	ENSG0000(	16.83696	-0.44207	0.248494	0.386895	lncRNA
ENSG0000(	ENSG0000(	71.27788	0.099099	0.248509	0.386899	lncRNA
ENSG0000(	TPSD1	29.31333	-0.47573	0.248566	0.386967	protein_coding
ENSG0000(	AGBL4	234.1105	0.108754	0.248593	0.38699	protein_coding
ENSG0000(	GDF15	95.87719	0.236827	0.248606	0.386991	protein_coding
ENSG0000(	ELAC1	418.2408	0.058482	0.248634	0.386997	protein_coding
ENSG0000(	NECTIN1	756.9658	-0.13385	0.248624	0.386997	protein_coding
ENSG0000(	TNR	183.4014	0.131921	0.248647	0.386997	protein_coding
ENSG0000(	ADORA1	48.1777	0.246986	0.248684	0.387036	protein_coding
ENSG0000(	LCA5L	131.2485	-0.09203	0.248768	0.387148	protein_coding
ENSG0000(	ENSG0000(	29.8769	0.191026	0.248933	0.387385	lncRNA
ENSG0000(	ZNF311	124.0858	-0.15065	0.248994	0.38746	protein_coding
ENSG0000(	ENSG0000(	68.32909	-0.1126	0.24915	0.387684	lncRNA
ENSG0000(	ENSG0000(	16.19211	0.264226	0.249237	0.387771	lncRNA
ENSG0000(	GTSE1	461.4838	-0.08031	0.249231	0.387771	protein_coding
ENSG0000(	LINC02619	29.84451	0.162631	0.249243	0.387771	lncRNA
ENSG0000(	MZF1-AS1	442.6988	-0.07002	0.249275	0.387801	lncRNA
ENSG0000(	NUTM2G	12.03563	0.495892	0.249332	0.387871	protein_coding
ENSG0000(	ARPC5L	527.8211	-0.09386	0.249378	0.387904	protein_coding
ENSG0000(	CMAHP	1511.453	0.156022	0.249381	0.387904	transcribed_unitary_pseudogene
ENSG0000(	PNPLA8	951.1369	-0.08125	0.249391	0.387904	protein_coding
ENSG0000(	ENSG0000(	14.55637	0.277061	0.249418	0.387927	lncRNA
ENSG0000(	FTH1P10	10.78788	-0.30131	0.249439	0.38794	transcribed_processed_pseudoge
ENSG0000(	RN7SL797F	20.05518	0.185526	0.249463	0.387959	misc_RNA
ENSG0000(	DCUN1D4	1317.639	-0.06723	0.249484	0.38796	protein_coding
ENSG0000(	ENSG0000(	461.1491	-0.08283	0.249489	0.38796	lncRNA
ENSG0000(	ARL4A	1386.211	-0.13345	0.249588	0.388094	protein_coding
ENSG0000(	MIR4432H(	262.5927	0.151935	0.249695	0.388242	lncRNA
ENSG0000(	ENSG0000(	27.74805	0.271915	0.249782	0.388358	lncRNA
ENSG0000(	C1orf54	77.23212	-0.15882	0.249849	0.388442	protein_coding

ENSG0000(	LINC02301	1319.886	0.081576	0.249875	0.388464	lncRNA
ENSG0000(	ADIPOR2	2284.075	-0.07852	0.249918	0.388511	protein_coding
ENSG0000(	SOCS2	465.1533	-0.11696	0.25	0.38862	protein_coding
ENSG0000(	NOS1AP	617.5366	0.12292	0.250024	0.388637	protein_coding
ENSG0000(	CTC1	2032.213	-0.0873	0.25012	0.388748	protein_coding
ENSG0000(	ENSG0000(	136.0913	-0.10677	0.250114	0.388748	lncRNA
ENSG0000(	ENSG0000(	79.10311	0.137791	0.250225	0.388873	lncRNA
ENSG0000(	GPAA1P2	88.32479	0.098197	0.25022	0.388873	transcribed_processed_pseudoge
ENSG0000(	ENSG0000(	230.5513	0.083271	0.250295	0.388962	lncRNA
ENSG0000(	TSSK3	53.90365	0.141329	0.25034	0.389012	protein_coding
ENSG0000(	CREB3	238.0608	-0.09152	0.250412	0.389105	protein_coding
ENSG0000(	HPX	146.4213	-0.21578	0.250447	0.38914	protein_coding
ENSG0000(	PRLR	1014.024	-0.1767	0.250473	0.389162	protein_coding
ENSG0000(	SMPDL3A	616.2185	-0.14356	0.250519	0.389214	protein_coding
ENSG0000(	RAP1GAP2	1416.527	-0.15703	0.250637	0.389346	protein_coding
ENSG0000(	VAPA	3608.53	-0.0772	0.250637	0.389346	protein_coding
ENSG0000(	WASL-DT	213.5713	0.11118	0.250641	0.389346	lncRNA
ENSG0000(	LINC00511	8645.805	-0.17309	0.250691	0.389404	lncRNA
ENSG0000(	ENSG0000(	46.93069	0.137836	0.250816	0.389578	lncRNA
ENSG0000(	ENSG0000(	40.86491	0.156119	0.250909	0.389691	lncRNA
ENSG0000(	ZNF559-ZN	170.7712	-0.13935	0.250914	0.389691	protein_coding
ENSG0000(	ENSG0000(	42.38372	-0.15394	0.250951	0.389692	lncRNA
ENSG0000(	ITGB6	1108.229	0.153133	0.250943	0.389692	protein_coding
ENSG0000(	NRTN	17.54262	0.337223	0.250947	0.389692	protein_coding
ENSG0000(	RN7SL45P	109.7217	0.095156	0.250965	0.389694	misc_RNA
ENSG0000(	ENSG0000(	98.87596	0.109253	0.25112	0.389915	lncRNA
ENSG0000(	ENSG0000(	4666.669	-0.0489	0.25116	0.389945	lncRNA
ENSG0000(	NREP	1039.811	0.105401	0.251164	0.389945	protein_coding
ENSG0000(	ENSG0000(	205.5105	-0.08306	0.251274	0.390096	lncRNA
ENSG0000(	ENSG0000(	18.41216	0.223795	0.251368	0.390223	lncRNA
ENSG0000(	ENSG0000(	1185.801	0.148535	0.251461	0.390345	lncRNA
ENSG0000(	RN7SL810F	8.118477	0.288486	0.251472	0.390345	misc_RNA
ENSG0000(	CENPM	173.1981	0.113297	0.251497	0.390365	protein_coding
ENSG0000(	ROS1	298.3301	-0.11694	0.251576	0.390468	protein_coding
ENSG0000(	LINC00698	31.94126	0.185377	0.25162	0.390498	lncRNA
ENSG0000(	TRIM34	349.5129	0.077921	0.251612	0.390498	protein_coding
ENSG0000(	ENSG0000(	29.76801	0.210257	0.251648	0.390513	transcribed_unitary_pseudogene
ENSG0000(	NAAA	1402.471	-0.13785	0.251655	0.390513	protein_coding
ENSG0000(	GBA	555.0404	-0.0804	0.251692	0.390551	protein_coding
ENSG0000(	RN7SL200F	58.28428	0.143589	0.251734	0.390597	misc_RNA
ENSG0000(	ENSG0000(	295.06	-0.08074	0.25182	0.390692	lncRNA
ENSG0000(	ZC3H6	1557.288	0.086845	0.251818	0.390692	protein_coding
ENSG0000(	ENSG0000(	45.43121	0.214104	0.251941	0.39086	TEC
ENSG0000(	ENSG0000(	117.5219	-0.08946	0.252005	0.390939	lncRNA
ENSG0000(	PNLIPRP1	243.3919	-0.06724	0.252039	0.390961	protein_coding
ENSG0000(	PPP6R1	1747.667	-0.05925	0.252044	0.390961	protein_coding
ENSG0000(	RAB15	743.3797	-0.13398	0.252113	0.391049	protein_coding

ENSG0000(	ENSG0000(	156.8902	-0.08594	0.252165	0.39111	lncRNA
ENSG0000(	TESC	64.84969	-0.14538	0.252193	0.391134	protein_coding
ENSG0000(	C2orf72	973.1105	-0.17014	0.252223	0.391161	protein_coding
ENSG0000(	ENSG0000(	14.1592	-0.24338	0.252294	0.391252	lncRNA
ENSG0000(	COL2A1	35.09617	-0.38695	0.252344	0.391303	protein_coding
ENSG0000(	WDR37	1367.995	0.044089	0.252352	0.391303	protein_coding
ENSG0000(	TTLL7	915.5264	-0.07087	0.252419	0.391388	protein_coding
ENSG0000(	CLSPN	1340.149	-0.0616	0.252477	0.391438	protein_coding
ENSG0000(	ENSG0000(	11.0643	0.272696	0.252471	0.391438	lncRNA
ENSG0000(	ENSG0000(	9.487703	0.327594	0.252515	0.39147	lncRNA
ENSG0000(	ENSG0000(	23.32176	0.235603	0.252534	0.39147	lncRNA
ENSG0000(	INAFM1	74.39834	-0.10499	0.25253	0.39147	protein_coding
ENSG0000(	ENSG0000(	86.38514	0.086791	0.252558	0.391487	processed_pseudogene
ENSG0000(	ENSG0000(	177.1643	-0.06663	0.252578	0.391498	lncRNA
ENSG0000(	C14orf93	341.4817	-0.09227	0.252661	0.391607	protein_coding
ENSG0000(	SOAT1	1302.513	0.060585	0.252729	0.391694	protein_coding
ENSG0000(	GOSR2-DT	18.47661	0.230636	0.252759	0.39172	lncRNA
ENSG0000(	THAP2	182.1292	-0.091	0.252832	0.391815	protein_coding
ENSG0000(	CHRDL1	189.2715	-0.20894	0.252861	0.39184	protein_coding
ENSG0000(	SPICE1	1532.424	-0.06427	0.252964	0.391981	protein_coding
ENSG0000(	TRNT1	1053.882	-0.05311	0.252991	0.391983	protein_coding
ENSG0000(	ZNF304	375.9776	0.075702	0.252982	0.391983	protein_coding
ENSG0000(	SEPSECS	1351.931	0.071594	0.253011	0.391994	protein_coding
ENSG0000(	SACS	2831.406	0.068252	0.253092	0.3921	protein_coding
ENSG0000(	SNORA59A	23.07998	-0.29375	0.253151	0.392173	snoRNA
ENSG0000(	ENSG0000(	21.59836	0.212406	0.253196	0.392223	lncRNA
ENSG0000(	AKAP13	15800.51	0.06392	0.253256	0.392296	protein_coding
ENSG0000(	ENSG0000(	11.32973	0.245216	0.253308	0.392338	lncRNA
ENSG0000(	RNU4-12P	20.16234	0.246118	0.253299	0.392338	snRNA
ENSG0000(	RBM12	3686.794	0.05928	0.253368	0.392412	protein_coding
ENSG0000(	MIR5009	12.92434	0.266407	0.253427	0.392484	miRNA
ENSG0000(	P3H2-AS1	355.0998	0.056898	0.253447	0.392495	lncRNA
ENSG0000(	CDO1	13.56328	0.370355	0.253484	0.392532	protein_coding
ENSG0000(	MAST4-AS1	16.43834	-0.26553	0.2535	0.392538	lncRNA
ENSG0000(	MICU1	882.0088	-0.05526	0.253601	0.392675	protein_coding
ENSG0000(	ENSG0000(	50.4818	0.23231	0.253621	0.392687	lncRNA
ENSG0000(	SPATA24	35.21753	0.167132	0.253652	0.392715	protein_coding
ENSG0000(	ENSG0000(	128.0008	-0.09195	0.253751	0.392831	lncRNA
ENSG0000(	PHETA2	242.3536	0.104073	0.253752	0.392831	protein_coding
ENSG0000(	RPL24P8	10.09204	-0.32377	0.253769	0.392838	processed_pseudogene
ENSG0000(	LINC02037	156.9359	0.088981	0.253827	0.392909	lncRNA
ENSG0000(	TIGD7	279.2193	0.098265	0.253858	0.392937	protein_coding
ENSG0000(	PACRGL	1114.466	0.100008	0.253887	0.392962	protein_coding
ENSG0000(	DEFA5	368.8514	1.71278	0.254043	0.393184	protein_coding
ENSG0000(	AMMECR1	668.6757	0.074397	0.25406	0.393192	protein_coding
ENSG0000(	ENSG0000(	94.61885	-0.17283	0.25412	0.393265	lncRNA
ENSG0000(	ADPGK-AS1	188.6742	0.073203	0.254184	0.393267	lncRNA

ENSG0000(	ENSG0000(	1114.927	-0.06945	0.254171	0.393267	lncRNA
ENSG0000(	ENSG0000(	52.03594	-0.11795	0.254179	0.393267	lncRNA
ENSG0000(	INHCAP	31.90136	0.196786	0.254146	0.393267	transcribed_unprocessed_pseudo
ENSG0000(	LINC01419	18.46156	-0.32959	0.254151	0.393267	lncRNA
ENSG0000(	ENSG0000(	185.7954	-0.11148	0.254231	0.39328	lncRNA
ENSG0000(	FAM187A	17.91211	0.404145	0.254229	0.39328	protein_coding
ENSG0000(	RB1-DT	45.60253	0.12562	0.254226	0.39328	lncRNA
ENSG0000(	LINC01911	21.1568	-0.24163	0.254449	0.393598	lncRNA
ENSG0000(	NLRP10	314.7402	0.054255	0.254684	0.393943	protein_coding
ENSG0000(	ENSG0000(	119.5283	-0.20431	0.254706	0.393957	lncRNA
ENSG0000(	ENSG0000(	19.96455	0.210579	0.254775	0.394031	lncRNA
ENSG0000(	ENSG0000(	74.57534	0.15794	0.254786	0.394031	lncRNA
ENSG0000(	SNURF	62.14404	0.187138	0.254791	0.394031	protein_coding
ENSG0000(	FGF5	380.7363	-0.06071	0.254901	0.394181	protein_coding
ENSG0000(	C11orf49	981.7829	-0.05575	0.255011	0.394312	protein_coding
ENSG0000(	ENSG0000(	22.2714	0.174457	0.255006	0.394312	lncRNA
ENSG0000(	ENSG0000(	21.37325	-0.23939	0.255072	0.394368	lncRNA
ENSG0000(	SV2A	185.5025	0.101603	0.255067	0.394368	protein_coding
ENSG0000(	GFOD1	1667.76	0.089227	0.255117	0.394397	protein_coding
ENSG0000(	SMCO4	248.0511	0.098662	0.255114	0.394397	protein_coding
ENSG0000(	ENSG0000(	228.648	-0.09228	0.25515	0.39443	lncRNA
ENSG0000(	ENSG0000(	65.31129	-0.38455	0.255163	0.39443	protein_coding
ENSG0000(	ENSG0000(	244.2903	-0.2806	0.255215	0.39449	lncRNA
ENSG0000(	ENSG0000(	850.5326	0.076659	0.255242	0.394514	protein_coding
ENSG0000(	RN7SL482F	44.45972	0.137756	0.255277	0.394548	misc_RNA
ENSG0000(	ENSG0000(	10.0859	0.365404	0.255315	0.394583	lncRNA
ENSG0000(	ENSG0000(	680.548	0.04644	0.255325	0.394583	transcribed_processed_pseudoge
ENSG0000(	ENSG0000(	141.8348	-0.07466	0.255377	0.394643	lncRNA
ENSG0000(	ENSG0000(	39.51085	-0.25916	0.255405	0.394667	lncRNA
ENSG0000(	LCN12	119.9979	-0.09311	0.255456	0.394727	protein_coding
ENSG0000(	ATRX	6136.821	0.095357	0.255512	0.394794	protein_coding
ENSG0000(	ENSG0000(	14.58236	0.287911	0.255585	0.394887	lncRNA
ENSG0000(	LINC02516	51.97982	0.271158	0.255602	0.394894	lncRNA
ENSG0000(	BEND5	39.95512	0.207402	0.255689	0.395009	protein_coding
ENSG0000(	CCDC172	12.28737	0.234388	0.255768	0.395111	protein_coding
ENSG0000(	AP3M1	1593.904	-0.0491	0.255815	0.395126	protein_coding
ENSG0000(	CD200	329.2208	0.127414	0.25581	0.395126	protein_coding
ENSG0000(	SCARNA20	26.54582	0.151625	0.255799	0.395126	scaRNA
ENSG0000(	CEP85	743.2056	-0.07613	0.255829	0.395127	protein_coding
ENSG0000(	GRAMD4P7	19.03015	0.23826	0.255863	0.39516	processed_pseudogene
ENSG0000(	ENSG0000(	86.73989	0.106126	0.255914	0.395219	lncRNA
ENSG0000(	NOX5	383.1281	0.077209	0.25595	0.395255	protein_coding
ENSG0000(	TADA2A	898.2394	-0.06568	0.255991	0.395299	protein_coding
ENSG0000(	LEMD2	1441.729	0.040806	0.256043	0.39536	protein_coding
ENSG0000(	ENSG0000(	28.76035	-0.21183	0.256101	0.395366	lncRNA
ENSG0000(	FLJ12825	333.5769	0.090111	0.256074	0.395366	lncRNA
ENSG0000(	FNIP2	3538.989	-0.11297	0.256092	0.395366	protein_coding

ENSG0000(	LINC02268	1072.422	-0.10072	0.256089	0.395366	lncRNA
ENSG0000(	PRSS40A	257.4914	0.072711	0.25611	0.395366	transcribed_unprocessed_pseudo
ENSG0000(	SMG1P7	1208.215	-0.06431	0.256149	0.395406	transcribed_unprocessed_pseudo
ENSG0000(	PARP3	388.5539	0.086189	0.256269	0.395567	protein_coding
ENSG0000(	ZNF415	268.9219	-0.09597	0.256278	0.395567	protein_coding
ENSG0000(	ENSG0000(	54.03347	0.145814	0.256295	0.395574	lncRNA
ENSG0000(	BCAP29	567.7642	-0.0749	0.256327	0.395604	protein_coding
ENSG0000(	CYB5D2	259.3947	0.080809	0.256431	0.395728	protein_coding
ENSG0000(	ENSG0000(	127.5108	-0.09915	0.256436	0.395728	lncRNA
ENSG0000(	MTERF3	218.176	-0.1006	0.256445	0.395728	protein_coding
ENSG0000(	DYNLL2-DT	196.3407	0.066704	0.256472	0.395749	lncRNA
ENSG0000(	LINC02636	18.21213	0.234133	0.256548	0.395847	lncRNA
ENSG0000(	KITLG	1320.475	-0.10571	0.256773	0.396174	protein_coding
ENSG0000(	PTPRZ1	168.2835	-0.16338	0.256805	0.396204	protein_coding
ENSG0000(	ENSG0000(	178.567	-0.09965	0.25683	0.396224	processed_pseudogene
ENSG0000(	LINC02209	20.49292	0.257728	0.256866	0.39626	lncRNA
ENSG0000(	CCDC201	21.25171	0.196048	0.256901	0.396274	protein_coding
ENSG0000(	LZTFL1	528.2415	0.075423	0.2569	0.396274	protein_coding
ENSG0000(	ENSG0000(	21.70815	0.195777	0.256921	0.396286	lncRNA
ENSG0000(	ENSG0000(	89.1753	0.100852	0.256937	0.396291	lncRNA
ENSG0000(	ENSG0000(	291.0737	-0.10653	0.257014	0.396391	lncRNA
ENSG0000(	ENSG0000(	313.045	0.076111	0.257058	0.396438	lncRNA
ENSG0000(	ENSG0000(	38.59089	0.149719	0.257112	0.396443	lncRNA
ENSG0000(	ENSG0000(	108.4039	0.121467	0.257107	0.396443	TEC
ENSG0000(	RCC2	2120.075	-0.06907	0.257085	0.396443	protein_coding
ENSG0000(	SNORD14A	32.19494	0.181805	0.257092	0.396443	snoRNA
ENSG0000(	PASK	1179.74	-0.09213	0.257246	0.396611	protein_coding
ENSG0000(	YAP1	1922.098	-0.06531	0.257235	0.396611	protein_coding
ENSG0000(	ENSG0000(	487.287	0.060371	0.257284	0.39665	lncRNA
ENSG0000(	RPS3AP37	21.73144	0.218261	0.257297	0.396651	processed_pseudogene
ENSG0000(	ENSG0000(	315.7308	-0.07228	0.257355	0.396701	lncRNA
ENSG0000(	GALNT14	38.19281	0.343438	0.257351	0.396701	protein_coding
ENSG0000(	TP53AIP1	72.59071	0.130808	0.257382	0.396723	protein_coding
ENSG0000(	ENSG0000(	24.56342	-0.18501	0.257419	0.39676	lncRNA
ENSG0000(	KCNK12	174.2808	0.190695	0.257642	0.397085	protein_coding
ENSG0000(	ENSG0000(	27.93089	0.17645	0.257742	0.397218	lncRNA
ENSG0000(	ENSG0000(	18.74485	0.216878	0.257775	0.39725	lncRNA
ENSG0000(	ENSG0000(	21.63098	0.171215	0.257793	0.397258	lncRNA
ENSG0000(	ALPI	87.05624	-0.36956	0.257822	0.39727	protein_coding
ENSG0000(	AOX2P	95.64219	0.085983	0.257826	0.39727	transcribed_unprocessed_pseudo
ENSG0000(	OR9H1P	1352.067	-0.05173	0.257872	0.397322	polymorphic_pseudogene
ENSG0000(	PAEP	72.53981	-0.11882	0.257994	0.39749	protein_coding
ENSG0000(	ENSG0000(	27.25775	-0.18681	0.258045	0.397529	lncRNA
ENSG0000(	ZKSCAN7	330.0263	-0.07473	0.258037	0.397529	protein_coding
ENSG0000(	TFPI2-DT	27.88432	0.154638	0.258128	0.397637	lncRNA
ENSG0000(	TSPOAP1	873.4812	0.11814	0.258154	0.397658	protein_coding
ENSG0000(	ATXN7L3-A	114.406	0.14683	0.258215	0.397713	lncRNA

ENSG0000(	KCNH4	13.1149	0.320982	0.258214	0.397713	protein_coding
ENSG0000(	LINC02019	76.48495	0.108469	0.258298	0.397822	lncRNA
ENSG0000(	KIFBP	611.7073	0.05141	0.25839	0.397943	protein_coding
ENSG0000(	TRAJ8	12.96299	0.353216	0.258462	0.398035	TR_J_pseudogene
ENSG0000(	CDK15	91.34519	0.190685	0.258538	0.398106	protein_coding
ENSG0000(	CIB2	64.07009	-0.21714	0.258547	0.398106	protein_coding
ENSG0000(	MTCP1	91.95802	-0.11737	0.258537	0.398106	protein_coding
ENSG0000(	PROCR	354.1172	0.067908	0.258608	0.39818	protein_coding
ENSG0000(	HMGA2-AS	187.5938	-0.08317	0.258748	0.398376	lncRNA
ENSG0000(	ENSG0000(	26.46224	-0.13907	0.258832	0.398467	lncRNA
ENSG0000(	LETMD1	3206.985	-0.04595	0.258826	0.398467	protein_coding
ENSG0000(	ENSG0000(	816.9156	-0.05483	0.258888	0.398525	lncRNA
ENSG0000(	ZNF497-AS	60.7574	-0.16176	0.258895	0.398525	lncRNA
ENSG0000(	ZNF516-DT	12.2648	0.266915	0.258946	0.398584	lncRNA
ENSG0000(	KNG1	84.37113	-0.14229	0.259	0.398648	protein_coding
ENSG0000(	TERC	92.15739	-0.238	0.259081	0.398752	lncRNA
ENSG0000(	ZNF624	385.9552	-0.08511	0.259097	0.398757	protein_coding
ENSG0000(	ENSG0000(	54.66041	0.145267	0.259118	0.398759	lncRNA
ENSG0000(	ENSG0000(	21.30207	0.195151	0.259126	0.398759	TEC
ENSG0000(	LINC02028	249.5972	0.090423	0.259136	0.398759	lncRNA
ENSG0000(	ENSG0000(	677.387	0.059839	0.259232	0.398887	lncRNA
ENSG0000(	C8orf34	510.6054	0.060285	0.259374	0.399016	protein_coding
ENSG0000(	CRNDE	309.0233	0.063081	0.259368	0.399016	lncRNA
ENSG0000(	ENSG0000(	287.7273	-0.13401	0.259373	0.399016	lncRNA
ENSG0000(	KIAA1109	16204.69	-0.04804	0.25938	0.399016	protein_coding
ENSG0000(	ZDHHC13	1480.729	-0.08018	0.25936	0.399016	protein_coding
ENSG0000(	ENSG0000(	232.7843	0.059205	0.259427	0.399029	lncRNA
ENSG0000(	ENSG0000(	124.9436	0.079086	0.259418	0.399029	transcribed_unprocessed_pseudo
ENSG0000(	ZNF26	4967.128	0.042869	0.259425	0.399029	protein_coding
ENSG0000(	AFDN-DT	593.8857	-0.07551	0.259442	0.399032	lncRNA
ENSG0000(	FZD1	448.3795	-0.10539	0.259468	0.399053	protein_coding
ENSG0000(	ZNF454	172.123	-0.08825	0.25951	0.399098	protein_coding
ENSG0000(	SALL2	95.90556	0.165986	0.259606	0.399226	protein_coding
ENSG0000(	MUC15	39.36537	0.176934	0.259709	0.399365	protein_coding
ENSG0000(	ENSG0000(	49.89531	-0.11938	0.25974	0.399393	lncRNA
ENSG0000(	ZNF727	425.1254	-0.1219	0.259791	0.399451	protein_coding
ENSG0000(	ENSG0000(	14.02209	0.223365	0.26001	0.399756	lncRNA
ENSG0000(	LINC00158	14.54371	0.329655	0.260022	0.399756	lncRNA
ENSG0000(	RNU6-942F	89.98862	0.145967	0.260027	0.399756	snRNA
ENSG0000(	ADAMTS8	23.78256	0.245334	0.260047	0.399767	protein_coding
ENSG0000(	ENSG0000(	68.0462	0.105183	0.260091	0.399815	processed_pseudogene
ENSG0000(	ENSG0000(	17.05645	-0.19887	0.260121	0.399841	lncRNA
ENSG0000(	AK4P3	79.00341	0.163571	0.260225	0.399961	protein_coding
ENSG0000(	ENSG0000(	13.39336	-0.33909	0.260213	0.399961	lncRNA
ENSG0000(	GAS2	53.71505	0.182213	0.260245	0.399973	protein_coding
ENSG0000(	NUDT6	519.1446	-0.07532	0.260283	0.400011	protein_coding
ENSG0000(	SFTPD-AS1	952.9073	0.034967	0.260379	0.400125	lncRNA

ENSG0000(	ZNF610	202.964	-0.09262	0.260383	0.400125	protein_coding
ENSG0000(	CCDC73	12.44924	0.304422	0.260432	0.400142	protein_coding
ENSG0000(	ENSG0000(	15.42474	0.266894	0.260432	0.400142	lncRNA
ENSG0000(	FLNA	9215.553	0.144608	0.260428	0.400142	protein_coding
ENSG0000(	CMTM1	69.04463	0.150362	0.260464	0.400152	protein_coding
ENSG0000(	ERF	577.0384	0.087114	0.260461	0.400152	protein_coding
ENSG0000(	ENSG0000(	59.63619	-0.10529	0.260482	0.400159	lncRNA
ENSG0000(	NFKB2	954.41	0.126855	0.260589	0.400305	protein_coding
ENSG0000(	ENSG0000(	141.1504	0.137845	0.260627	0.400343	lncRNA
ENSG0000(	ENSG0000(	166.4723	0.0693	0.26064	0.400344	TEC
ENSG0000(	ENSG0000(	95.89717	-0.09523	0.260682	0.400388	lncRNA
ENSG0000(	GRAP	237.5022	0.247421	0.260807	0.400561	protein_coding
ENSG0000(	GPRC5C	794.6694	-0.15249	0.260961	0.400718	protein_coding
ENSG0000(	ITIH4	125.8788	0.158192	0.260927	0.400718	protein_coding
ENSG0000(	ODAD2	38.39266	0.186468	0.26095	0.400718	protein_coding
ENSG0000(	SMCHD1	13355.17	0.079398	0.260957	0.400718	protein_coding
ENSG0000(	PACSIN3	48.90637	0.216563	0.261055	0.400844	protein_coding
ENSG0000(	ENSG0000(	273.2489	-0.37416	0.261159	0.400984	lncRNA
ENSG0000(	ENSG0000(	32.46655	0.172052	0.261184	0.401002	lncRNA
ENSG0000(	C2orf16	14.70966	0.742883	0.261204	0.401006	protein_coding
ENSG0000(	DPYSL4	16.8316	0.468874	0.261212	0.401006	protein_coding
ENSG0000(	LCT	28.4182	-0.36565	0.261239	0.401027	protein_coding
ENSG0000(	LLPH-DT	22.08564	-0.21566	0.261324	0.401139	lncRNA
ENSG0000(	RPS29P16	17.93195	0.184712	0.261356	0.401167	processed_pseudogene
ENSG0000(	GUCY2GP	36.61467	0.255715	0.261369	0.401167	transcribed_unitary_pseudogene
ENSG0000(	ENSG0000(	12.77619	0.220018	0.261449	0.401271	lncRNA
ENSG0000(	NEURL1B	2294.277	-0.18074	0.261476	0.401293	protein_coding
ENSG0000(	NOP2	481.3081	-0.09361	0.261512	0.401328	protein_coding
ENSG0000(	PLEKHM1P	5291.689	-0.0531	0.261563	0.401386	transcribed_unprocessed_pseudo
ENSG0000(	ENSG0000(	96.65295	0.109059	0.261641	0.401428	lncRNA
ENSG0000(	ENSG0000(	10.10766	0.230742	0.261634	0.401428	lncRNA
ENSG0000(	JMJD7	98.83006	-0.13357	0.261614	0.401428	protein_coding
ENSG0000(	TAF6L	201.7022	-0.07827	0.261618	0.401428	protein_coding
ENSG0000(	LINC01058	17.55965	0.282821	0.261657	0.401432	lncRNA
ENSG0000(	MIR3908	15.54114	-0.22872	0.261782	0.401605	miRNA
ENSG0000(	AMACR	766.5063	-0.19857	0.26183	0.401627	protein_coding
ENSG0000(	ENSG0000(	22.81037	-0.19186	0.261835	0.401627	lncRNA
ENSG0000(	ZRANB1	1192.491	0.062335	0.261822	0.401627	protein_coding
ENSG0000(	GAPDHP76	25.29097	0.187804	0.261869	0.401659	unprocessed_pseudogene
ENSG0000(	ENSG0000(	492.3939	0.050209	0.261941	0.40175	lncRNA
ENSG0000(	ENSG0000(	46.82761	-0.15889	0.261979	0.401789	lncRNA
ENSG0000(	SFPQ	10596.93	0.037022	0.262004	0.401807	protein_coding
ENSG0000(	LINC01649	15.08951	0.223646	0.262109	0.401948	lncRNA
ENSG0000(	DSEL	835.5632	0.085401	0.262162	0.401988	protein_coding
ENSG0000(	ENSG0000(	593.7933	-0.05109	0.262173	0.401988	lncRNA
ENSG0000(	KCNS1	28.80949	0.197497	0.26216	0.401988	protein_coding
ENSG0000(	ACAT2	290.6872	-0.14547	0.262199	0.401988	protein_coding

ENSG0000(	ENSG0000(	43.2733	0.168767	0.262187	0.401988	lncRNA
ENSG0000(	KLC2	381.3654	0.104763	0.262229	0.402014	protein_coding
ENSG0000(	CAVIN3	50.50199	0.209613	0.262439	0.402316	protein_coding
ENSG0000(	ENPP7P1	22.19089	-0.22161	0.262469	0.402342	transcribed_unprocessed_pseudo
ENSG0000(	ENSG0000(	158.4048	-0.09516	0.262482	0.402342	lncRNA
ENSG0000(	STEAP2-AS	9.980513	0.329902	0.262587	0.402483	lncRNA
ENSG0000(	SNORA80B	76.3959	0.143965	0.262662	0.402579	snoRNA
ENSG0000(	ENSG0000(	30.75759	0.295072	0.262764	0.402677	lncRNA
ENSG0000(	MT-TK	80.00307	0.347055	0.262761	0.402677	Mt_tRNA
ENSG0000(	PTOV1	1132.408	-0.05576	0.262764	0.402677	protein_coding
ENSG0000(	BMP1	1381.669	0.090275	0.26278	0.402682	protein_coding
ENSG0000(	IFIT1P1	16.56235	0.216895	0.262823	0.402727	processed_pseudogene
ENSG0000(	DIO3	9.84363	-0.4053	0.262905	0.402825	protein_coding
ENSG0000(	ENSG0000(	10.12035	0.396068	0.262926	0.402825	lncRNA
ENSG0000(	TSPEAR-AS	12.72263	0.407999	0.262917	0.402825	lncRNA
ENSG0000(	ILF3-DT	835.5842	0.038526	0.262942	0.402831	lncRNA
ENSG0000(	ZNF197-AS	45.48702	0.171465	0.263003	0.402904	lncRNA
ENSG0000(	ADRA1B	12.98389	0.366333	0.263053	0.402958	protein_coding
ENSG0000(	SNORA1	61.43762	-0.15136	0.263064	0.402958	snoRNA
ENSG0000(	ENSG0000(	330.1497	0.111633	0.26315	0.403071	lncRNA
ENSG0000(	ENSG0000(	10.63552	0.212303	0.263251	0.403171	lncRNA
ENSG0000(	MGMT	481.1415	0.074581	0.263254	0.403171	protein_coding
ENSG0000(	SNORD3B-:	167.7378	-0.26915	0.263253	0.403171	snoRNA
ENSG0000(	MCF2L	2942.561	0.10129	0.263347	0.403293	protein_coding
ENSG0000(	PAN3	2711.699	-0.04806	0.263375	0.403317	protein_coding
ENSG0000(	ENSG0000(	9.91366	0.30814	0.263451	0.403413	transcribed_unprocessed_pseudo
ENSG0000(	CBLL1-AS1	42.11193	-0.21048	0.263505	0.403476	lncRNA
ENSG0000(	NECTIN2	992.8925	0.100663	0.263541	0.403511	protein_coding
ENSG0000(	ENSG0000(	39.28315	0.177442	0.263707	0.403745	TEC
ENSG0000(	PSMC2	879.9392	-0.06916	0.263758	0.403805	protein_coding
ENSG0000(	ATF7	2492.909	0.051785	0.263852	0.403908	protein_coding
ENSG0000(	LIFR	1857.551	0.156677	0.263844	0.403908	protein_coding
ENSG0000(	ENSG0000(	51.0716	-0.1564	0.263869	0.403914	processed_pseudogene
ENSG0000(	ENSG0000(	43.69353	0.129752	0.263913	0.403943	lncRNA
ENSG0000(	TMTC2	1067.43	0.079894	0.263908	0.403943	protein_coding
ENSG0000(	CENPN-AS1	390.435	0.056351	0.264201	0.404232	lncRNA
ENSG0000(	ENSG0000(	105.4959	0.126719	0.264145	0.404232	TEC
ENSG0000(	FAM102B	1836.408	-0.06406	0.264206	0.404232	protein_coding
ENSG0000(	OR10V3P	21.08619	0.211886	0.264184	0.404232	unprocessed_pseudogene
ENSG0000(	PPP2R5A	1285.133	-0.0762	0.264194	0.404232	protein_coding
ENSG0000(	RN7SL190F	145.2966	-0.07874	0.264138	0.404232	misc_RNA
ENSG0000(	SMIM8	1062.582	-0.07302	0.264171	0.404232	protein_coding
ENSG0000(	SRPRA	1573.078	0.068031	0.264127	0.404232	protein_coding
ENSG0000(	AKAP8L	1986.698	-0.05119	0.264267	0.404305	protein_coding
ENSG0000(	UPK3B	121.4766	-0.14639	0.264389	0.404472	protein_coding
ENSG0000(	KDM5C-IT1	139.8767	-0.07922	0.264508	0.404635	lncRNA
ENSG0000(	GPR143	19.42672	-0.26253	0.264637	0.404813	protein_coding

ENSG0000(	ENSG0000(	19.66222	-0.22281	0.264769	0.404995	transcribed_unprocessed_pseudo
ENSG0000(	ENSG0000(	26.45412	0.184788	0.264786	0.405002	lncRNA
ENSG0000(	IYD	1224.703	0.099156	0.264859	0.405082	protein_coding
ENSG0000(	RN7SKP38	16.58898	0.196733	0.264865	0.405082	misc_RNA
ENSG0000(	TNK2-AS1	123.0693	0.099064	0.264994	0.40526	lncRNA
ENSG0000(	MAGOH2P	44.34138	-0.20821	0.265024	0.405285	transcribed_processed_pseudoge
ENSG0000(	GP6	267.4232	0.08102	0.265133	0.405433	protein_coding
ENSG0000(	TRIM39	489.8346	0.072918	0.265158	0.40545	protein_coding
ENSG0000(	CDC7	295.7507	-0.15883	0.265187	0.405476	protein_coding
ENSG0000(	FBXL4	1755.456	-0.05051	0.265226	0.405516	protein_coding
ENSG0000(	ENSG0000(	13.75087	0.23115	0.265281	0.40558	processed_pseudogene
ENSG0000(	ENSG0000(	10.89455	0.229649	0.265338	0.405647	lncRNA
ENSG0000(	ENSG0000(	168.5671	0.074516	0.265392	0.405709	lncRNA
ENSG0000(	ENSG0000(	22.7466	-0.21364	0.26541	0.405718	lncRNA
ENSG0000(	PLSCR3	197.9455	0.132165	0.265426	0.405721	protein_coding
ENSG0000(	CPB1	43.29525	0.214006	0.265443	0.405729	protein_coding
ENSG0000(	KLHDC7A	48.35637	-0.31384	0.265557	0.405883	protein_coding
ENSG0000(	ENSG0000(	49.43369	-0.12355	0.265587	0.405908	lncRNA
ENSG0000(	ENSG0000(	33.68946	0.147642	0.265628	0.405952	lncRNA
ENSG0000(	ENSG0000(	20.31651	-0.24027	0.265709	0.406036	lncRNA
ENSG0000(	GREB1L	40.43931	-0.32716	0.265698	0.406036	protein_coding
ENSG0000(	ENSG0000(	113.686	0.096458	0.265821	0.406186	lncRNA
ENSG0000(	GPER1	54.1015	0.198835	0.265866	0.406236	protein_coding
ENSG0000(	TEPSIN	882.4612	-0.06301	0.265936	0.406323	protein_coding
ENSG0000(	GLIS1	46.82832	-0.22635	0.266009	0.406414	protein_coding
ENSG0000(	ENSG0000(	13.41537	0.226373	0.266023	0.406416	unprocessed_pseudogene
ENSG0000(	NARF-AS2	130.0022	0.122127	0.266056	0.406447	lncRNA
ENSG0000(	TMEM252	17.254	-0.33124	0.266143	0.40656	protein_coding
ENSG0000(	ENSG0000(	49.10376	-0.09474	0.266163	0.40657	lncRNA
ENSG0000(	UNC79	193.0613	-0.07224	0.266229	0.406651	protein_coding
ENSG0000(	MYG1-AS1	114.8058	-0.09075	0.266274	0.406699	lncRNA
ENSG0000(	HOXB-AS2	8.222301	-0.41994	0.266327	0.406762	lncRNA
ENSG0000(	ENSG0000(	28.187	0.172855	0.266409	0.406867	lncRNA
ENSG0000(	ENSG0000(	30.42032	0.169194	0.266492	0.406973	lncRNA
ENSG0000(	PLCXD2	770.794	0.083542	0.266517	0.406992	protein_coding
ENSG0000(	NPAS4	13.06024	0.430128	0.266564	0.407043	protein_coding
ENSG0000(	ITFG1	1225.782	-0.0575	0.266627	0.40712	protein_coding
ENSG0000(	ENSG0000(	213.2662	-0.06597	0.266751	0.407255	lncRNA
ENSG0000(	ENSG0000(	264.7474	-0.08634	0.266767	0.407255	lncRNA
ENSG0000(	ENSG0000(	117.8274	-0.08568	0.266734	0.407255	IG_V_pseudogene
ENSG0000(	TFAP2E-AS	19.17381	-0.28714	0.266755	0.407255	lncRNA
ENSG0000(	CCDC188	13.07991	0.266242	0.266888	0.407418	protein_coding
ENSG0000(	SHISA4	28.71675	0.196064	0.2669	0.407418	protein_coding
ENSG0000(	ENSG0000(	148.9429	0.091082	0.266965	0.407477	lncRNA
ENSG0000(	ENSG0000(	48.95607	-0.12783	0.266956	0.407477	lncRNA
ENSG0000(	HMGB1P8	152.5056	-0.08024	0.266994	0.407502	processed_pseudogene
ENSG0000(	H3P13	166.2196	0.078638	0.267047	0.407563	processed_pseudogene

ENSG0000(	SH3BP4	843.328	0.12882	0.267088	0.407604	protein_coding
ENSG0000(	LINC00894	2059.301	-0.0769	0.26711	0.407618	lncRNA
ENSG0000(	TTI2	254.7613	-0.07633	0.267143	0.407649	protein_coding
ENSG0000(	LINC02798	142.8948	-0.09919	0.267187	0.407697	lncRNA
ENSG0000(	LRRC37A17	119.9555	0.294514	0.267209	0.407711	transcribed_unprocessed_pseudo
ENSG0000(	CTC-338M1	423.9	-0.0895	0.26723	0.407711	lncRNA
ENSG0000(	ENSG0000(	119.3169	0.109483	0.267248	0.407711	lncRNA
ENSG0000(	LINC01681	14.77856	-0.21651	0.26724	0.407711	lncRNA
ENSG0000(	SPTA1	54.37805	0.279701	0.267291	0.407756	protein_coding
ENSG0000(	ENSG0000(	700.8713	0.057824	0.267446	0.407972	lncRNA
ENSG0000(	TTLL13P	25.9623	0.286877	0.267508	0.408047	protein_coding
ENSG0000(	SNORD67	67.15316	0.137971	0.26754	0.408076	snoRNA
ENSG0000(	S100B	53.07819	0.14845	0.267665	0.408226	protein_coding
ENSG0000(	TPI1P2	24.25659	0.210744	0.267664	0.408226	transcribed_processed_pseudoge
ENSG0000(	SIPA1L2	3847.203	-0.11503	0.267728	0.408302	protein_coding
ENSG0000(	ENSG0000(	215.3382	-0.07733	0.267741	0.408303	lncRNA
ENSG0000(	HOXD11	144.9519	0.174636	0.267782	0.408326	protein_coding
ENSG0000(	PIGV	845.3164	-0.04788	0.267774	0.408326	protein_coding
ENSG0000(	ENSG0000(	13.41959	0.226754	0.267822	0.408366	lncRNA
ENSG0000(	SORD	1041.364	0.112291	0.267871	0.408401	protein_coding
ENSG0000(	TTC39B	3923.971	0.081406	0.267861	0.408401	protein_coding
ENSG0000(	SLC39A14	3195.169	-0.10925	0.267949	0.4085	protein_coding
ENSG0000(	ARHGAP26	115.9416	0.125827	0.268028	0.408581	lncRNA
ENSG0000(	ORC2	1563.057	-0.04231	0.268024	0.408581	protein_coding
ENSG0000(	SCOC-AS1	259.1606	0.109063	0.268041	0.408581	lncRNA
ENSG0000(	ENSG0000(	743.4948	0.059521	0.268079	0.408599	TEC
ENSG0000(	ENSG0000(	11.94294	-0.23591	0.268066	0.408599	lncRNA
ENSG0000(	ENSG0000(	36.97094	-0.34547	0.268102	0.408614	processed_pseudogene
ENSG0000(	RN7SL12P	18.2928	0.180183	0.268146	0.408662	misc_RNA
ENSG0000(	ENSG0000(	31.5839	0.1956	0.268162	0.408666	lncRNA
ENSG0000(	ENSG0000(	40.35951	0.147075	0.268197	0.408696	lncRNA
ENSG0000(	ESAM	251.4387	0.144966	0.268208	0.408696	protein_coding
ENSG0000(	ENSG0000(	15.53142	0.278087	0.268318	0.408824	lncRNA
ENSG0000(	NUDT2	145.4034	-0.07767	0.268313	0.408824	protein_coding
ENSG0000(	ENSG0000(	15.20274	0.206487	0.268345	0.408845	lncRNA
ENSG0000(	ENSG0000(	113.2597	-0.22905	0.26836	0.408848	lncRNA
ENSG0000(	RMI2	210.4773	-0.12072	0.268448	0.40894	protein_coding
ENSG0000(	SEPTIN7P2	1078.559	0.052932	0.268459	0.40894	transcribed_unprocessed_pseudo
ENSG0000(	ZRANB3	702.6577	-0.08389	0.268445	0.40894	protein_coding
ENSG0000(	ZNF407-AS	813.028	-0.06052	0.268484	0.408957	lncRNA
ENSG0000(	LINC02292	10.31332	0.275503	0.268525	0.40899	lncRNA
ENSG0000(	RN7SL279F	50.37013	-0.10629	0.268532	0.40899	misc_RNA
ENSG0000(	MED20	465.6781	-0.07069	0.268602	0.409077	protein_coding
ENSG0000(	ENSG0000(	11.00014	-0.40044	0.268657	0.409141	lncRNA
ENSG0000(	ENSG0000(	35.10848	0.186496	0.268696	0.409181	lncRNA
ENSG0000(	ENSG0000(	34.81095	0.133027	0.268737	0.409222	lncRNA
ENSG0000(	LINC01796	91.1346	0.092543	0.268762	0.409222	lncRNA

ENSG0000(	RN7SL835F	89.98302	0.093365	0.26875	0.409222	misc_RNA
ENSG0000(	SMIM4	264.6771	-0.09291	0.268789	0.409242	protein_coding
ENSG0000(	RPS4XP22	25.71549	-0.42167	0.268846	0.40929	processed_pseudogene
ENSG0000(	TNXB	1341.677	0.101068	0.268835	0.40929	protein_coding
ENSG0000(	ENSG0000(	90.05828	0.114289	0.26898	0.409473	TEC
ENSG0000(	RNU6-122F	40.94444	0.194091	0.269026	0.409524	snRNA
ENSG0000(	MT-TS2	81.37966	0.313969	0.269126	0.409656	Mt_tRNA
ENSG0000(	PTCSC2	239.1397	-0.08681	0.269172	0.409706	lncRNA
ENSG0000(	ENSG0000(	113.4985	-0.09444	0.269215	0.409752	lncRNA
ENSG0000(	NOVA1	572.4168	-0.12325	0.269236	0.409763	protein_coding
ENSG0000(	TPM3P6	22.93935	-0.20828	0.269347	0.409913	processed_pseudogene
ENSG0000(	TMED10	4227.116	-0.08803	0.269425	0.410011	protein_coding
ENSG0000(	CHGB	261.5311	-0.0839	0.269443	0.410019	protein_coding
ENSG0000(	SSX5	20.28435	0.26191	0.269496	0.41008	protein_coding
ENSG0000(	MIR7161	13.6315	0.236216	0.269512	0.410083	miRNA
ENSG0000(	ENSG0000(	71.1833	0.191984	0.269535	0.410099	lncRNA
ENSG0000(	GDNF	262.3946	0.139905	0.26987	0.410578	protein_coding
ENSG0000(	KDM1A	3269.233	-0.03468	0.269876	0.410578	protein_coding
ENSG0000(	MDGA2	42.24029	-0.2051	0.269936	0.410649	protein_coding
ENSG0000(	RPUSD4	868.2483	-0.03871	0.269958	0.410662	protein_coding
ENSG0000(	CHRNG	214.6358	0.075119	0.269983	0.410681	protein_coding
ENSG0000(	STAP1	120.4232	0.308166	0.270064	0.410784	protein_coding
ENSG0000(	LINC02245	530.3664	0.073887	0.270083	0.410792	lncRNA
ENSG0000(	LRRC41	2138.388	0.034331	0.270103	0.410802	protein_coding
ENSG0000(	ENSG0000(	63.69073	0.184988	0.270142	0.410822	lncRNA
ENSG0000(	HEATR9	12.62953	-0.33225	0.270132	0.410822	protein_coding
ENSG0000(	ZNNT1	415.2247	0.085426	0.27018	0.41086	lncRNA
ENSG0000(	RPS12P27	63.00528	0.141348	0.270197	0.410865	processed_pseudogene
ENSG0000(	ENSG0000(	79.90999	0.124007	0.27021	0.410866	lncRNA
ENSG0000(	ENSG0000(	22.80547	-0.20418	0.270251	0.410909	lncRNA
ENSG0000(	ENSG0000(	287.4301	-0.06746	0.270278	0.410929	lncRNA
ENSG0000(	BCAR3	714.8811	-0.08037	0.270292	0.41093	protein_coding
ENSG0000(	OLMALINC	22143.64	-0.06075	0.270304	0.41093	lncRNA
ENSG0000(	UPF3A	2011.553	-0.04094	0.270331	0.41095	protein_coding
ENSG0000(	ENSG0000(	26.49252	0.191222	0.270381	0.411007	lncRNA
ENSG0000(	RNU6-255F	10.10638	0.285798	0.270458	0.411103	snRNA
ENSG0000(	PIGT	3736.213	-0.03462	0.270521	0.411178	protein_coding
ENSG0000(	LINC02046	63.51222	-0.1394	0.27054	0.411188	lncRNA
ENSG0000(	PRSS16	249.5014	-0.14811	0.270655	0.411343	protein_coding
ENSG0000(	NAMPTP1	47.21394	0.486295	0.270716	0.411411	processed_pseudogene
ENSG0000(	PDCD6IPP1	176.5675	0.149819	0.270726	0.411411	transcribed_unprocessed_pseudo
ENSG0000(	SAMD4B	2900.522	-0.04056	0.270774	0.411463	protein_coding
ENSG0000(	UBXN8	402.2101	-0.06728	0.2708	0.411483	protein_coding
ENSG0000(	WASF1	75.02097	0.187774	0.270821	0.411495	protein_coding
ENSG0000(	CENPBD1	318.8197	-0.05763	0.270907	0.411602	transcribed_unitary_pseudogene
ENSG0000(	TLCD5	355.431	0.073741	0.270918	0.411602	protein_coding
ENSG0000(	HSPA1L	40.88478	-0.13572	0.270998	0.411704	protein_coding

ENSG0000(	ENSG0000(	22.15059	-0.18624	0.271037	0.411743	lncRNA
ENSG0000(	ETNK2	46.305	0.153148	0.271066	0.411747	protein_coding
ENSG0000(	SLC13A1	37.18928	-0.27604	0.271053	0.411747	protein_coding
ENSG0000(	PACSIN2	2268.203	-0.08349	0.271208	0.411943	protein_coding
ENSG0000(	ENSG0000(	1490.365	0.058646	0.27123	0.411956	lncRNA
ENSG0000(	ENSG0000(	104.6603	0.104662	0.271343	0.412108	lncRNA
ENSG0000(	LINC02915	31.21693	0.179264	0.271433	0.412224	lncRNA
ENSG0000(	PPIAP67	45.56624	-0.15324	0.271516	0.412331	processed_pseudogene
ENSG0000(	TCF7L2	4690.03	-0.13387	0.271543	0.412351	protein_coding
ENSG0000(	LINC02216	54.08159	-0.10672	0.271584	0.412394	lncRNA
ENSG0000(	ENSG0000(	25.61039	0.17849	0.271612	0.412416	lncRNA
ENSG0000(	AHSA2P	5312.756	-0.04727	0.271832	0.412662	transcribed_unitary_pseudogene
ENSG0000(	ENSG0000(	8.607732	-0.27755	0.27182	0.412662	lncRNA
ENSG0000(	ENSG0000(	127.6675	0.080518	0.271841	0.412662	transcribed_unprocessed_pseudo
ENSG0000(	KLHL24	2690.743	-0.05895	0.27184	0.412662	protein_coding
ENSG0000(	SFT2D2	9766.189	0.047577	0.271805	0.412662	protein_coding
ENSG0000(	ZNF678	863.2559	-0.08321	0.271853	0.412662	protein_coding
ENSG0000(	BBS2	2014.953	-0.05041	0.27193	0.412759	protein_coding
ENSG0000(	ARL2BP	425.7053	-0.08285	0.271988	0.412827	protein_coding
ENSG0000(	MIR124-2H	11.97035	0.28903	0.272082	0.412949	lncRNA
ENSG0000(	CDK5RAP2	3453.035	0.055319	0.272109	0.41295	protein_coding
ENSG0000(	ENSG0000(	9.357616	0.365156	0.272106	0.41295	lncRNA
ENSG0000(	PPARGC1B	4470.01	-0.11554	0.272122	0.41295	protein_coding
ENSG0000(	R3HCC1L	1263.892	0.048366	0.272206	0.413057	protein_coding
ENSG0000(	CLPTM1L	1956.863	0.111693	0.272323	0.413215	protein_coding
ENSG0000(	ALG5	1591.612	0.051921	0.272347	0.413231	protein_coding
ENSG0000(	ENSG0000(	65.88865	0.128082	0.272447	0.413363	lncRNA
ENSG0000(	LY9	863.2377	0.153611	0.272487	0.413403	protein_coding
ENSG0000(	ZNF83	3023.571	0.070998	0.272542	0.413467	protein_coding
ENSG0000(	HSD11B1L	242.9407	0.087596	0.272565	0.413482	protein_coding
ENSG0000(	GABARAPL:	18.53927	0.24791	0.272697	0.413662	TEC
ENSG0000(	ENSG0000(	231.8901	-0.14846	0.272718	0.413673	transcribed_unprocessed_pseudo
ENSG0000(	RAB9A	341.3791	-0.1062	0.272786	0.413736	protein_coding
ENSG0000(	TLE1P1	16.01172	-0.21363	0.272785	0.413736	processed_pseudogene
ENSG0000(	JRKL-AS1	11.60829	-0.26445	0.272968	0.413992	lncRNA
ENSG0000(	IL6R-AS1	203.6114	-0.07384	0.272994	0.414003	lncRNA
ENSG0000(	RN7SL125F	39.86594	-0.13092	0.273001	0.414003	misc_RNA
ENSG0000(	RNF215	479.375	0.094553	0.273016	0.414004	protein_coding
ENSG0000(	B3GNTL1P1	12.80853	0.203478	0.273033	0.414012	processed_pseudogene
ENSG0000(	ENSG0000(	131.37	0.079774	0.273068	0.414044	lncRNA
ENSG0000(	PYCARD-AS	26.30117	0.152724	0.273089	0.414055	lncRNA
ENSG0000(	WDR44	605.2788	-0.06603	0.273108	0.414064	protein_coding
ENSG0000(	DPY19L4	1127.271	-0.04918	0.273294	0.414326	protein_coding
ENSG0000(	ENSG0000(	9.541696	-0.28604	0.273355	0.414398	lncRNA
ENSG0000(	SYNM	400.2764	-0.1258	0.273454	0.414529	protein_coding
ENSG0000(	POLB	455.5357	0.064824	0.273501	0.414564	protein_coding
ENSG0000(	UTP25	1060.801	-0.05567	0.273504	0.414564	protein_coding

ENSG0000(	SPACA7	36.24937	0.129364	0.273622	0.414724	protein_coding
ENSG0000(	C3orf52	1879.152	-0.13641	0.273697	0.414806	protein_coding
ENSG0000(	EPPK1	3581.338	-0.14387	0.273703	0.414806	protein_coding
ENSG0000(	ENSG0000(	50.45403	0.300891	0.273746	0.414851	protein_coding
ENSG0000(	XPO4	1890.227	-0.08392	0.273777	0.414878	protein_coding
ENSG0000(	ENSG0000(	30.42736	-0.20269	0.273797	0.414885	transcribed_unprocessed_pseudo
ENSG0000(	GALNT8	350.3176	-0.23285	0.273822	0.414885	protein_coding
ENSG0000(	VGLL3	571.9486	0.11469	0.273812	0.414885	protein_coding
ENSG0000(	ENSG0000(	512.0066	0.05006	0.274004	0.415141	lncRNA
ENSG0000(	ADGRL3	1483.621	-0.15668	0.27402	0.415145	protein_coding
ENSG0000(	B4GALT7	478.4818	0.066179	0.274151	0.415323	protein_coding
ENSG0000(	ELF3-AS1	1391.408	-0.08178	0.27435	0.415584	lncRNA
ENSG0000(	NICN1	488.0872	0.110915	0.274343	0.415584	protein_coding
ENSG0000(	ENSG0000(	266.5448	0.092768	0.274419	0.415667	lncRNA
ENSG0000(	ENSG0000(	21.38714	0.230838	0.274431	0.415667	lncRNA
ENSG0000(	ARL2	697.1519	-0.04501	0.274452	0.415679	protein_coding
ENSG0000(	ENSG0000(	86.64963	-0.12201	0.274498	0.415728	lncRNA
ENSG0000(	DIS3L	1226.061	-0.04952	0.274564	0.415773	protein_coding
ENSG0000(	DUS4L	246.4857	-0.10989	0.274588	0.415773	protein_coding
ENSG0000(	ENSG0000(	149.1117	-0.15124	0.274578	0.415773	processed_pseudogene
ENSG0000(	ENTPD3	56.16094	0.198097	0.274594	0.415773	protein_coding
ENSG0000(	MRPL17	287.3836	-0.09575	0.274552	0.415773	protein_coding
ENSG0000(	ENSG0000(	39.78362	0.157806	0.274684	0.41589	lncRNA
ENSG0000(	ENSG0000(	13.16438	0.180366	0.274758	0.415982	lncRNA
ENSG0000(	ENSG0000(	15.29513	0.217128	0.274803	0.416029	lncRNA
ENSG0000(	ENSG0000(	300.0409	0.084307	0.274837	0.41606	lncRNA
ENSG0000(	WT1-AS	109.9799	-0.1076	0.274901	0.416138	lncRNA
ENSG0000(	MYL6B	243.8743	0.06563	0.274928	0.416157	protein_coding
ENSG0000(	ENSG0000(	285.0557	0.091446	0.274942	0.416158	lncRNA
ENSG0000(	CTNND1	22327.7	-0.09726	0.274992	0.416174	protein_coding
ENSG0000(	MYEF2	946.0485	0.067149	0.274983	0.416174	protein_coding
ENSG0000(	PUM1	2936.11	-0.03518	0.274969	0.416174	protein_coding
ENSG0000(	GPR173	252.3525	-0.08257	0.275044	0.416213	protein_coding
ENSG0000(	MSMO1	648.5416	-0.11859	0.275039	0.416213	protein_coding
ENSG0000(	ENSG0000(	87.34631	0.117198	0.275125	0.416315	lncRNA
ENSG0000(	ENSG0000(	82.8416	0.116545	0.275181	0.41634	lncRNA
ENSG0000(	ENSG0000(	31.77791	0.167102	0.275172	0.41634	TEC
ENSG0000(	ZNF736	1618.535	-0.07692	0.275176	0.41634	protein_coding
ENSG0000(	CALHM2	160.7248	0.150545	0.275221	0.416359	protein_coding
ENSG0000(	MAP3K2-D	531.3342	0.048065	0.275216	0.416359	lncRNA
ENSG0000(	ENSG0000(	25.01555	-0.31372	0.275332	0.416507	lncRNA
ENSG0000(	ENSG0000(	57.72919	0.117789	0.275384	0.416565	lncRNA
ENSG0000(	LINC00559	61.81608	0.108987	0.275454	0.416651	lncRNA
ENSG0000(	ENSG0000(	59.81572	-0.10937	0.275778	0.417122	lncRNA
ENSG0000(	ONECUT1	21.04122	0.236104	0.275798	0.417132	protein_coding
ENSG0000(	ABITRAM	298.7449	-0.07717	0.275867	0.417216	protein_coding
ENSG0000(	ENSG0000(	123.0852	0.088483	0.27594	0.417286	lncRNA

ENSG0000(	RNF141	1127.067	-0.06061	0.275929	0.417286	protein_coding
ENSG0000(	HMGN2P17	31.93866	-0.19464	0.275968	0.417307	processed_pseudogene
ENSG0000(	ENSG0000(	302.4247	-0.05247	0.276082	0.41746	TEC
ENSG0000(	RN7SKP137	16.32551	-0.25127	0.276096	0.417461	misc_RNA
ENSG0000(	PJVK	241.0727	0.078762	0.276129	0.417492	protein_coding
ENSG0000(	ENSG0000(	13.16281	0.234274	0.276219	0.417566	lncRNA
ENSG0000(	LMLN2	18.48996	0.193785	0.276208	0.417566	protein_coding
ENSG0000(	MTRNR2L4	313.5001	-0.06876	0.276196	0.417566	protein_coding
ENSG0000(	ENSG0000(	197.656	0.115524	0.276244	0.417584	lncRNA
ENSG0000(	OCLNP1	31.7822	0.538131	0.276308	0.417661	unprocessed_pseudogene
ENSG0000(	DNAJB6	3378.831	0.057478	0.276322	0.417661	protein_coding
ENSG0000(	HPGD	2085.792	-0.20918	0.276353	0.417688	protein_coding
ENSG0000(	KCNJ2	813.7284	0.132467	0.27639	0.417724	protein_coding
ENSG0000(	CCDC28A	170.0337	-0.11828	0.276478	0.417827	protein_coding
ENSG0000(	INPPL1	2203.805	-0.06926	0.276485	0.417827	protein_coding
ENSG0000(	NAA16	859.8769	-0.07883	0.276606	0.41799	protein_coding
ENSG0000(	ENSG0000(	140.7155	0.131192	0.276648	0.418026	lncRNA
ENSG0000(	ENSG0000(	26.82133	-0.15744	0.276657	0.418026	lncRNA
ENSG0000(	GGACT	167.2233	0.145435	0.276728	0.418114	protein_coding
ENSG0000(	FAM138A	177.7418	-0.16472	0.276789	0.418185	lncRNA
ENSG0000(	CD244	94.79566	-0.16786	0.276834	0.418234	protein_coding
ENSG0000(	NCOA4P4	11.90961	0.260445	0.276909	0.418323	processed_pseudogene
ENSG0000(	SPATA32	39.0641	-0.1683	0.27692	0.418323	protein_coding
ENSG0000(	EXTL2	303.1323	0.08913	0.276946	0.418343	protein_coding
ENSG0000(	ENSG0000(	49.30864	0.107652	0.277	0.418403	lncRNA
ENSG0000(	FAM3C	610.442	0.10786	0.277056	0.418467	protein_coding
ENSG0000(	ENSG0000(	230.17	-0.06604	0.277091	0.418489	lncRNA
ENSG0000(	ENSG0000(	187.4109	-0.08956	0.277097	0.418489	lncRNA
ENSG0000(	SNX22	578.3928	0.126435	0.27713	0.418499	protein_coding
ENSG0000(	VANGL2	289.485	0.140255	0.277118	0.418499	protein_coding
ENSG0000(	PHTF2	1181.095	0.067439	0.277181	0.418555	protein_coding
ENSG0000(	FKBP1A	3441.413	0.080976	0.277344	0.418781	protein_coding
ENSG0000(	NUDT13	140.7855	-0.0987	0.277377	0.418797	protein_coding
ENSG0000(	SNX17	814.6024	-0.05099	0.277381	0.418797	protein_coding
ENSG0000(	IGLJ1	20.77316	0.292633	0.277405	0.418812	IG_J_gene
ENSG0000(	ENSG0000(	115.0882	0.108982	0.277469	0.41887	lncRNA
ENSG0000(	ENSG0000(	79.31885	0.105569	0.277497	0.41887	lncRNA
ENSG0000(	NEURL1	757.9693	-0.10975	0.277494	0.41887	protein_coding
ENSG0000(	SNORA6	151.1931	-0.15366	0.27748	0.41887	snoRNA
ENSG0000(	RARS2	4027.252	-0.02581	0.277595	0.418998	protein_coding
ENSG0000(	CRYM	54.16384	-0.21803	0.277676	0.4191	protein_coding
ENSG0000(	COX17	249.9401	-0.08113	0.27772	0.419147	protein_coding
ENSG0000(	ZFP37	163.6232	-0.08829	0.277742	0.41916	protein_coding
ENSG0000(	SNX18P8	75.85976	0.328594	0.277816	0.419251	unprocessed_pseudogene
ENSG0000(	ENSG0000(	133.7389	0.08792	0.277991	0.419496	lncRNA
ENSG0000(	C9orf57	18.84345	-0.16641	0.278013	0.419501	protein_coding
ENSG0000(	ENSG0000(	4745.505	-0.02601	0.278022	0.419501	lncRNA

ENSG0000(	RN7SL665F	32.80155	0.207914	0.278074	0.419539	misc_RNA
ENSG0000(	SRRD	594.261	-0.07145	0.278071	0.419539	protein_coding
ENSG0000(	ENSG0000(	11.92775	0.265034	0.278106	0.419567	lncRNA
ENSG0000(	OR4P4	119.1533	-0.10007	0.278123	0.419572	protein_coding
ENSG0000(	ENSG0000(	57.78326	-0.12278	0.278161	0.419603	lncRNA
ENSG0000(	MIR1284	11.1126	0.251376	0.27817	0.419603	miRNA
ENSG0000(	DMD	4800.24	-0.05663	0.278201	0.41963	protein_coding
ENSG0000(	RN7SKP229	10.17339	-0.31205	0.278221	0.419639	misc_RNA
ENSG0000(	ENSG0000(	20.26468	0.193347	0.27834	0.419784	lncRNA
ENSG0000(	SERPINI1	46.74517	-0.1823	0.278344	0.419784	protein_coding
ENSG0000(	FAM223A	48.01815	-0.10669	0.27837	0.419804	lncRNA
ENSG0000(	UHMK1	3744.88	-0.06164	0.278408	0.419841	protein_coding
ENSG0000(	PTMAP5	14.06288	0.252625	0.278438	0.419866	transcribed_processed_pseudoge
ENSG0000(	ENSG0000(	404.2247	-0.0517	0.278505	0.419935	lncRNA
ENSG0000(	ENSG0000(	860.0972	0.084751	0.278511	0.419935	lncRNA
ENSG0000(	GTF3C3	1118.375	-0.04923	0.27856	0.419989	protein_coding
ENSG0000(	SYNGR3	31.25746	0.252709	0.27864	0.420089	protein_coding
ENSG0000(	ENSG0000(	15.20565	-0.26399	0.278769	0.420239	lncRNA
ENSG0000(	ENSG0000(	18.30277	0.212004	0.27878	0.420239	lncRNA
ENSG0000(	LINC00667	2464.151	-0.05804	0.278757	0.420239	lncRNA
ENSG0000(	KIN	1299.423	-0.05519	0.278806	0.420259	protein_coding
ENSG0000(	RPH3AL-AS	16.84236	0.290985	0.278855	0.420312	lncRNA
ENSG0000(	CAPN11	24.54443	0.274102	0.278981	0.420462	protein_coding
ENSG0000(	H2AZ2P1	182.8936	-0.09445	0.278977	0.420462	transcribed_processed_pseudoge
ENSG0000(	CSRNP3	363.7546	-0.15224	0.279029	0.420468	protein_coding
ENSG0000(	ENSG0000(	17.3293	-0.30669	0.279026	0.420468	lncRNA
ENSG0000(	METTL1	139.1285	-0.13622	0.279039	0.420468	protein_coding
ENSG0000(	SCAMP1	906.9522	-0.06818	0.279	0.420468	protein_coding
ENSG0000(	LRRC37A15	64.63519	0.181226	0.279057	0.42047	processed_pseudogene
ENSG0000(	TRMT9B	1965.009	-0.08005	0.279067	0.42047	protein_coding
ENSG0000(	ENSG0000(	13.46605	-0.21557	0.279086	0.420478	processed_pseudogene
ENSG0000(	CDH4	1088.393	0.181246	0.279254	0.420711	protein_coding
ENSG0000(	RPL7AP66	13.03209	-0.30187	0.279326	0.420799	processed_pseudogene
ENSG0000(	CNTD1	21.60415	0.271407	0.279343	0.420804	protein_coding
ENSG0000(	FKBP9	1316.056	-0.06751	0.279408	0.420882	protein_coding
ENSG0000(	ENSG0000(	75.82504	0.123749	0.279437	0.420905	lncRNA
ENSG0000(	HIBADH	384.2587	-0.08357	0.279452	0.420908	protein_coding
ENSG0000(	MIA2-AS1	16.33892	0.240783	0.279502	0.420962	lncRNA
ENSG0000(	ENSG0000(	16.85726	-0.20611	0.279528	0.420982	lncRNA
ENSG0000(	ENSG0000(	117.4538	0.083929	0.279625	0.421107	lncRNA
ENSG0000(	SMN1	770.2743	0.103635	0.279671	0.421156	protein_coding
ENSG0000(	MTPN	2091.601	-0.102	0.279696	0.421174	protein_coding
ENSG0000(	GINS3	366.3896	-0.10265	0.279711	0.421177	protein_coding
ENSG0000(	FRMD4A	1521.878	0.101439	0.279752	0.421218	protein_coding
ENSG0000(	NSMCE2	619.2157	0.038496	0.279781	0.42124	protein_coding
ENSG0000(	ENSG0000(	2937.74	0.066333	0.279863	0.421345	lncRNA
ENSG0000(	ENSG0000(	36.62478	0.133314	0.279917	0.421404	lncRNA

ENSG0000(	ENSG0000(	87.30005	-0.1285	0.280015	0.421532	transcribed_processed_pseudoge
ENSG0000(	SLC43A1	194.914	0.120593	0.280085	0.421617	protein_coding
ENSG0000(	SMUG1	887.4392	0.053012	0.280101	0.421621	protein_coding
ENSG0000(	FAM120B	2348.486	0.039676	0.280233	0.421799	protein_coding
ENSG0000(	ENSG0000(	20.29736	0.176141	0.280307	0.42189	TEC
ENSG0000(	AMFR	1181.554	-0.06268	0.280366	0.421952	protein_coding
ENSG0000(	ENSG0000(	31.92241	0.139017	0.280387	0.421952	lncRNA
ENSG0000(	LINC00326	14.03429	-0.2363	0.280389	0.421952	lncRNA
ENSG0000(	ENSG0000(	231.7777	0.08238	0.280468	0.422052	TEC
ENSG0000(	VIPR1	3626.47	-0.19251	0.280524	0.422115	protein_coding
ENSG0000(	EHMT2	1318.89	-0.0432	0.280729	0.422384	protein_coding
ENSG0000(	ENSG0000(	79.8745	-0.093	0.280723	0.422384	lncRNA
ENSG0000(	IGHV4-28	151.3905	0.291138	0.280826	0.422508	IG_V_gene
ENSG0000(	ENSG0000(	37.25282	0.271709	0.280878	0.422566	lncRNA
ENSG0000(	ENSG0000(	64.16271	-0.13138	0.280935	0.422612	processed_pseudogene
ENSG0000(	VKORC1	165.867	-0.10171	0.280927	0.422612	protein_coding
ENSG0000(	SMYD2	831.4147	-0.07701	0.281069	0.422793	protein_coding
ENSG0000(	ENSG0000(	144.8621	0.08371	0.281338	0.423158	lncRNA
ENSG0000(	ZNF568	923.8862	-0.05919	0.281332	0.423158	protein_coding
ENSG0000(	ZNF565	155.2612	-0.098	0.281355	0.423162	protein_coding
ENSG0000(	ENSG0000(	30.2801	0.17196	0.281392	0.423198	lncRNA
ENSG0000(	ENSG0000(	99.8899	0.087895	0.281421	0.423222	lncRNA
ENSG0000(	ENSG0000(	376.2645	-0.0579	0.28146	0.423239	TEC
ENSG0000(	TRAJ17	25.49145	0.288798	0.281456	0.423239	TR_J_gene
ENSG0000(	DHX32	983.8972	-0.10582	0.281519	0.423307	protein_coding
ENSG0000(	REM1	31.46337	0.193493	0.281557	0.423345	protein_coding
ENSG0000(	DPM2	448.769	-0.08287	0.281702	0.423512	protein_coding
ENSG0000(	RPH3AL	731.8051	-0.10632	0.281711	0.423512	protein_coding
ENSG0000(	TCF21	735.4422	-0.16386	0.281723	0.423512	protein_coding
ENSG0000(	ZNF623	1653.292	-0.04138	0.281717	0.423512	protein_coding
ENSG0000(	LINC02315	156.1375	-0.1229	0.281971	0.423865	lncRNA
ENSG0000(	ENSG0000(	91.05693	0.101633	0.281989	0.423872	lncRNA
ENSG0000(	TLL1	176.4112	0.184768	0.282079	0.423986	protein_coding
ENSG0000(	VWCE	121.0019	-0.09091	0.282148	0.424069	protein_coding
ENSG0000(	NRIP2	81.65874	0.18346	0.282206	0.424136	protein_coding
ENSG0000(	LINC02570	15.54821	-0.18141	0.28226	0.424198	lncRNA
ENSG0000(	ITGB1BP1	623.4392	-0.07017	0.282304	0.424222	protein_coding
ENSG0000(	ZFR2	126.448	0.144965	0.2823	0.424222	protein_coding
ENSG0000(	CCR7	522.2373	0.387151	0.282429	0.424389	protein_coding
ENSG0000(	C4orf33	444.2641	0.079468	0.282575	0.424548	protein_coding
ENSG0000(	DRG2	927.9882	-0.05626	0.282558	0.424548	protein_coding
ENSG0000(	ENSG0000(	186.7409	0.074739	0.282572	0.424548	lncRNA
ENSG0000(	PTP4A2P1	16.4847	0.216651	0.282769	0.42482	processed_pseudogene
ENSG0000(	ENSG0000(	340.3433	-0.05993	0.283043	0.425197	lncRNA
ENSG0000(	SLIT2-IT1	19.0744	0.234516	0.283047	0.425197	lncRNA
ENSG0000(	RIMBP2	171.6179	0.176706	0.283065	0.425202	protein_coding
ENSG0000(	PRR13P2	35.04281	-0.14792	0.283111	0.425251	processed_pseudogene

ENSG0000(	DLEC1	262.5079	0.111489	0.283213	0.425363	protein_coding
ENSG0000(	TMEM156	436.744	0.103464	0.283201	0.425363	protein_coding
ENSG0000(	KBTBD2	1722.533	0.051556	0.283265	0.425422	protein_coding
ENSG0000(	LINC00304	18.1955	-0.3007	0.283407	0.425593	lncRNA
ENSG0000(	NPAS2	1705.018	0.129227	0.283402	0.425593	protein_coding
ENSG0000(	PRSS21	9.241636	0.324616	0.283515	0.425735	protein_coding
ENSG0000(	IQANK1	637.6812	-0.06999	0.283547	0.425763	protein_coding
ENSG0000(	ENSG0000(	109.0743	-0.09894	0.283595	0.425815	lncRNA
ENSG0000(	AP3S1	644.7799	0.082232	0.283631	0.425848	protein_coding
ENSG0000(	LPAR4	50.97893	0.155376	0.283662	0.425875	protein_coding
ENSG0000(	CYP4F24P	25.72709	-0.52383	0.283689	0.425878	unprocessed_pseudogene
ENSG0000(	PLXNB3	104.4435	-0.23129	0.283691	0.425878	protein_coding
ENSG0000(	ENSG0000(	19.94656	0.185283	0.283742	0.425933	lncRNA
ENSG0000(	PSME1	689.3414	-0.10559	0.283842	0.426063	protein_coding
ENSG0000(	DYNC1LI2	7325.349	-0.07245	0.283896	0.426117	protein_coding
ENSG0000(	SKIL	3764.814	0.098837	0.283906	0.426117	protein_coding
ENSG0000(	CYP4V2	2938.051	-0.06959	0.283946	0.426158	protein_coding
ENSG0000(	DEXI	914.1931	-0.0636	0.283973	0.426177	protein_coding
ENSG0000(	LRRC31	825.4516	-0.2024	0.284021	0.426229	protein_coding
ENSG0000(	GAPT	195.1176	0.216319	0.284158	0.426394	protein_coding
ENSG0000(	LINC02101	31.06946	0.231125	0.284155	0.426394	lncRNA
ENSG0000(	GIGYF1	2491.352	-0.06371	0.284225	0.426474	protein_coding
ENSG0000(	ENSG0000(	32.47696	0.20472	0.284429	0.42676	lncRNA
ENSG0000(	SUMF2	1560.507	-0.04508	0.284506	0.426855	protein_coding
ENSG0000(	KLC1	5548.535	0.045868	0.284536	0.426879	protein_coding
ENSG0000(	ENSG0000(	28.34781	-0.38605	0.284625	0.426992	processed_pseudogene
ENSG0000(	ENSG0000(	108.1105	0.095845	0.284715	0.427046	lncRNA
ENSG0000(	ENSG0000(	95.70796	0.090516	0.284692	0.427046	lncRNA
ENSG0000(	NEK9	3910.349	-0.03433	0.284705	0.427046	protein_coding
ENSG0000(	RPS29P12	27.10624	-0.25442	0.284692	0.427046	processed_pseudogene
ENSG0000(	ENSG0000(	14.76379	-0.2544	0.284732	0.427051	lncRNA
ENSG0000(	CARD8-AS1	229.1603	0.086432	0.284747	0.427052	lncRNA
ENSG0000(	MECP2	3701.699	0.086097	0.284781	0.427082	protein_coding
ENSG0000(	MARCHF8	1846.406	-0.0654	0.284861	0.427183	protein_coding
ENSG0000(	NKIRAS2	1133.757	-0.0498	0.284912	0.427239	protein_coding
ENSG0000(	CD81	1686.915	0.103621	0.284974	0.42731	protein_coding
ENSG0000(	IARS1	4768.856	0.061951	0.284988	0.427311	protein_coding
ENSG0000(	ADCY5	456.6765	-0.15367	0.285018	0.427316	protein_coding
ENSG0000(	CCDC13	343.3839	0.119611	0.285005	0.427316	protein_coding
ENSG0000(	SNORD21	16.19621	0.281837	0.285057	0.427353	snoRNA
ENSG0000(	UGT2B17	3465.48	-0.37882	0.285184	0.427523	protein_coding
ENSG0000(	DNAH17	107.0955	0.119863	0.285198	0.427523	protein_coding
ENSG0000(	ENSG0000(	54.72792	-0.13382	0.28527	0.427611	lncRNA
ENSG0000(	CALCB	38.54071	0.116781	0.285362	0.427729	protein_coding
ENSG0000(	RUNDC1	2047.045	-0.04872	0.285456	0.42785	protein_coding
ENSG0000(	ENSG0000(	42.26853	0.146322	0.285482	0.427867	lncRNA
ENSG0000(	POU2F2-AS	21.62894	0.337551	0.285539	0.427932	lncRNA

ENSG0000(	ENSG0000(	61.11556	0.11833	0.285566	0.427953	lncRNA
ENSG0000(	KATNAL1	687.4935	0.079348	0.285661	0.428072	protein_coding
ENSG0000(	SCGN	25.05058	-0.24247	0.285673	0.428072	protein_coding
ENSG0000(	ENSG0000(	10.57662	0.253332	0.285714	0.428091	lncRNA
ENSG0000(	ENSG0000(	56.6648	0.120628	0.285708	0.428091	lncRNA
ENSG0000(	AEN	651.4209	0.136567	0.285746	0.428119	protein_coding
ENSG0000(	ENSG0000(	13.59746	0.236337	0.285761	0.428122	lncRNA
ENSG0000(	ENSG0000(	71.88408	-0.28809	0.285789	0.428123	lncRNA
ENSG0000(	TMEM53	478.8817	-0.08919	0.285779	0.428123	protein_coding
ENSG0000(	ZNF705E	28.69611	0.178112	0.285858	0.428205	protein_coding
ENSG0000(	ENSG0000(	20.65921	-0.17624	0.285875	0.42821	lncRNA
ENSG0000(	ENSG0000(	187.1451	0.131874	0.285907	0.428237	lncRNA
ENSG0000(	ENSG0000(	134.1195	0.068342	0.285976	0.428321	lncRNA
ENSG0000(	ENSG0000(	40.48338	0.152115	0.286075	0.428448	lncRNA
ENSG0000(	MPRIP-AS1	35.55942	-0.11827	0.286243	0.428679	lncRNA
ENSG0000(	PELO	516.6034	0.085291	0.286288	0.428726	protein_coding
ENSG0000(	WDR20	1362.891	-0.04639	0.286343	0.428788	protein_coding
ENSG0000(	AP3B2	405.7614	0.088245	0.286407	0.428802	protein_coding
ENSG0000(	ARRDC2	1034.365	0.092177	0.286395	0.428802	protein_coding
ENSG0000(	B4GALNT4	53.0884	-0.2711	0.286402	0.428802	protein_coding
ENSG0000(	SNORD13	853.2215	0.135932	0.286381	0.428802	snoRNA
ENSG0000(	ENSG0000(	78.91376	-0.25767	0.286441	0.428818	processed_pseudogene
ENSG0000(	TMEM266	382.2543	0.055527	0.286445	0.428818	protein_coding
ENSG0000(	EMX2OS	849.0078	0.05227	0.286489	0.428862	lncRNA
ENSG0000(	CPA4	388.8509	-0.08845	0.286551	0.428911	protein_coding
ENSG0000(	ENSG0000(	1191.683	-0.06799	0.286558	0.428911	lncRNA
ENSG0000(	ERVH48-1	36.06434	0.167634	0.286575	0.428911	protein_coding
ENSG0000(	PTCH1	4837.545	-0.12125	0.286576	0.428911	protein_coding
ENSG0000(	ENSG0000(	47.18066	0.133961	0.286591	0.428912	lncRNA
ENSG0000(	NABP2	492.153	-0.05267	0.286787	0.429186	protein_coding
ENSG0000(	ENSG0000(	819.0536	-0.08094	0.286805	0.429192	lncRNA
ENSG0000(	ENSG0000(	261.6249	-0.12129	0.286844	0.42921	lncRNA
ENSG0000(	PCDHGA12	137.1191	-0.12713	0.286843	0.42921	protein_coding
ENSG0000(	BNIP1	113.5559	-0.10761	0.286864	0.429218	protein_coding
ENSG0000(	SLC4A3	39.81053	0.262433	0.286929	0.429295	protein_coding
ENSG0000(	ENSG0000(	112.4888	-0.08325	0.286978	0.429348	lncRNA
ENSG0000(	MXRA7	1342.879	0.08839	0.287096	0.429483	protein_coding
ENSG0000(	TTF2	2171.182	-0.07332	0.287094	0.429483	protein_coding
ENSG0000(	MFAP1P1	15.06563	-0.23036	0.287141	0.429489	processed_pseudogene
ENSG0000(	PNPLA4	595.1555	0.076445	0.287136	0.429489	protein_coding
ENSG0000(	STAB1	6266.31	-0.11741	0.287115	0.429489	protein_coding
ENSG0000(	ENSG0000(	54.36764	0.128373	0.287168	0.429509	lncRNA
ENSG0000(	MIF4GD	267.6892	-0.06444	0.287204	0.429524	protein_coding
ENSG0000(	NUDT17	43.1648	-0.14769	0.287205	0.429524	protein_coding
ENSG0000(	SNORD16	25.62044	0.280915	0.287268	0.429597	snoRNA
ENSG0000(	PRR23C	134.9235	-0.07617	0.287282	0.429597	protein_coding
ENSG0000(	ZNF454-DT	40.24668	-0.11858	0.287363	0.429698	lncRNA

ENSG0000(	PPOX	251.5952	0.078161	0.287388	0.429715	protein_coding
ENSG0000(	LINC02281	104.5511	-0.09758	0.287494	0.429838	lncRNA
ENSG0000(	SPARCL1	922.3699	0.146425	0.287498	0.429838	protein_coding
ENSG0000(	MGAT4C	727.0964	-0.08767	0.287522	0.429853	protein_coding
ENSG0000(	DBT	1866.017	-0.04392	0.28784	0.430288	protein_coding
ENSG0000(	INO80B	221.2908	-0.09311	0.287827	0.430288	protein_coding
ENSG0000(	ENSG0000(	72.62536	0.177668	0.287898	0.430353	lncRNA
ENSG0000(	IL5RA	76.01863	0.207088	0.287953	0.430416	protein_coding
ENSG0000(	ENSG0000(	15.28153	0.271616	0.287968	0.430417	processed_pseudogene
ENSG0000(	IFT140	518.5047	0.082686	0.288011	0.430461	protein_coding
ENSG0000(	ENSG0000(	369.9136	-0.09696	0.288103	0.430557	lncRNA
ENSG0000(	PROS1	452.7096	-0.08143	0.288091	0.430557	protein_coding
ENSG0000(	ENSG0000(	177.2804	0.086325	0.28813	0.430578	lncRNA
ENSG0000(	ALDH4A1	105.3284	-0.12804	0.288187	0.430589	protein_coding
ENSG0000(	CAMKK1	481.5085	-0.06285	0.288166	0.430589	protein_coding
ENSG0000(	PIPSL	200.1992	0.23666	0.288184	0.430589	transcribed_processed_pseudoge
ENSG0000(	RN7SL580F	26.90866	0.171255	0.288206	0.430589	misc_RNA
ENSG0000(	TCP10L2	10.0748	0.291312	0.288205	0.430589	protein_coding
ENSG0000(	ENSG0000(	20.5856	0.205641	0.288264	0.430654	lncRNA
ENSG0000(	DRAIC	1165.027	-0.07262	0.288318	0.430714	lncRNA
ENSG0000(	ENSG0000(	44.67076	0.145882	0.288358	0.430753	lncRNA
ENSG0000(	ENSG0000(	15.18428	-0.2131	0.288408	0.430807	processed_pseudogene
ENSG0000(	ENSG0000(	124.5428	0.074779	0.288512	0.430942	lncRNA
ENSG0000(	BRDT	108.5624	-0.10009	0.288584	0.431029	protein_coding
ENSG0000(	ENSG0000(	152.7764	0.064927	0.288668	0.431135	lncRNA
ENSG0000(	PLEKHA5	3758.55	0.063165	0.288739	0.431219	protein_coding
ENSG0000(	ENSG0000(	11.00939	0.23833	0.288826	0.431329	lncRNA
ENSG0000(	ENSG0000(	317.235	-0.07781	0.28884	0.43133	lncRNA
ENSG0000(	IGHV3-6	13.83341	0.296826	0.288954	0.431478	IG_V_pseudogene
ENSG0000(	FSIP1	145.8954	-0.32608	0.289117	0.431701	protein_coding
ENSG0000(	SYPL2	48.26491	0.204127	0.289135	0.431707	protein_coding
ENSG0000(	ANKRD50	1917.716	0.076609	0.289387	0.432064	protein_coding
ENSG0000(	ACTG1P9	288.0769	0.053873	0.289514	0.432233	processed_pseudogene
ENSG0000(	ENSG0000(	22.95758	0.150016	0.2897	0.43249	lncRNA
ENSG0000(	ENSG0000(	56.50335	0.111749	0.289814	0.432639	lncRNA
ENSG0000(	ZNF148	2155.985	-0.04158	0.289865	0.432694	protein_coding
ENSG0000(	ENSG0000(	47.89241	-0.1409	0.289927	0.432767	lncRNA
ENSG0000(	IBA57-DT	42.45047	-0.13445	0.289966	0.432804	lncRNA
ENSG0000(	VRK3	1794.365	-0.05001	0.290118	0.43301	protein_coding
ENSG0000(	TSLP	27.42885	-0.22737	0.290225	0.433149	protein_coding
ENSG0000(	TMEM79	214.2578	-0.06604	0.290275	0.433203	protein_coding
ENSG0000(	TIGD4	24.39871	-0.18135	0.290314	0.43322	protein_coding
ENSG0000(	ZNF180	475.8296	-0.06504	0.290306	0.43322	protein_coding
ENSG0000(	ENSG0000(	273.1106	0.128603	0.290358	0.433244	lncRNA
ENSG0000(	S100Z	20.23032	0.235497	0.290348	0.433244	protein_coding
ENSG0000(	RN7SL775F	68.7705	-0.10649	0.290493	0.433425	misc_RNA
ENSG0000(	ENSG0000(	235.5584	0.075539	0.290598	0.433542	lncRNA

ENSG0000(	ENSG0000(	235.9321	0.056369	0.290599	0.433542	lncRNA
ENSG0000(	ENSG0000(	661.7334	0.033556	0.290723	0.433706	lncRNA
ENSG0000(	ENSG0000(	18.65201	0.174014	0.290777	0.433746	lncRNA
ENSG0000(	ENSG0000(	17.23226	0.214528	0.290768	0.433746	lncRNA
ENSG0000(	ENSG0000(	9.012121	0.208576	0.290887	0.433889	lncRNA
ENSG0000(	ENSG0000(	37.91413	0.246529	0.291012	0.434054	protein_coding
ENSG0000(	ENSG0000(	235.5863	-0.0851	0.291049	0.434082	lncRNA
ENSG0000(	ENSG0000(	133.389	-0.13793	0.291058	0.434082	protein_coding
ENSG0000(	ADK	659.8607	-0.07028	0.291144	0.434153	protein_coding
ENSG0000(	ENSG0000(	11.04473	0.388668	0.291139	0.434153	lncRNA
ENSG0000(	RIMS1	148.3909	-0.08077	0.291161	0.434153	protein_coding
ENSG0000(	TMEM88B	314.7681	0.062059	0.291153	0.434153	protein_coding
ENSG0000(	ENSG0000(	20.03111	0.223898	0.291199	0.434188	TEC
ENSG0000(	MARCHF9	392.7503	-0.06844	0.291261	0.434261	protein_coding
ENSG0000(	ZNF516-AS	15.59793	0.235048	0.291311	0.434314	lncRNA
ENSG0000(	EHMT1	4899.101	-0.0652	0.291683	0.434847	protein_coding
ENSG0000(	EXOSC1	690.2239	-0.04143	0.291741	0.434914	protein_coding
ENSG0000(	ENSG0000(	55.35785	0.100276	0.291813	0.43498	lncRNA
ENSG0000(	TP73-AS1	1474.5	0.047154	0.291801	0.43498	transcribed_unitary_pseudogene
ENSG0000(	TRPC6	228.8479	-0.12386	0.291828	0.434981	protein_coding
ENSG0000(	FXYD6	276.4454	-0.1481	0.291908	0.43508	protein_coding
ENSG0000(	ENSG0000(	29.59494	-0.16922	0.292292	0.43558	lncRNA
ENSG0000(	ENSG0000(	79.77893	0.127078	0.292287	0.43558	lncRNA
ENSG0000(	PNMA3	84.8973	0.248115	0.292263	0.43558	protein_coding
ENSG0000(	RNF152	2549.025	0.163288	0.2923	0.43558	protein_coding
ENSG0000(	ENSG0000(	178.5849	0.071414	0.292359	0.435647	transcribed_processed_pseudoge
ENSG0000(	FAM133CP	19.42639	0.251923	0.292415	0.43571	processed_pseudogene
ENSG0000(	ENSG0000(	127.4011	-0.06146	0.292478	0.435784	lncRNA
ENSG0000(	CFLAR-AS1	441.3352	-0.07184	0.292542	0.435817	lncRNA
ENSG0000(	LINC02727	23.53044	-0.17352	0.292528	0.435817	lncRNA
ENSG0000(	TK2	1313.02	0.081491	0.292521	0.435817	protein_coding
ENSG0000(	PSMA5	875.6646	-0.07574	0.292591	0.435869	protein_coding
ENSG0000(	FCF1P1	34.62356	0.135744	0.292608	0.435874	processed_pseudogene
ENSG0000(	GNAI1	1516.448	0.075011	0.292625	0.435878	protein_coding
ENSG0000(	ENSG0000(	22.3302	-0.16535	0.292661	0.435911	lncRNA
ENSG0000(	ENSG0000(	141.8286	-0.06788	0.292804	0.436103	lncRNA
ENSG0000(	SLC8A3	57.84525	-0.17859	0.292818	0.436104	protein_coding
ENSG0000(	MERTK	683.5902	0.121806	0.292918	0.436232	protein_coding
ENSG0000(	CELSR2	319.7566	-0.13893	0.292972	0.436242	protein_coding
ENSG0000(	ENSG0000(	3438.738	-0.03643	0.292956	0.436242	lncRNA
ENSG0000(	LINC01847	32.2068	0.157294	0.293009	0.436242	lncRNA
ENSG0000(	RN7SL652F	27.55711	0.174214	0.293001	0.436242	misc_RNA
ENSG0000(	SNORA66	132.282	-0.1272	0.292962	0.436242	snoRNA
ENSG0000(	TPSAB1	190.3132	-0.22418	0.293023	0.436242	protein_coding
ENSG0000(	ZNF131	1511.397	0.034986	0.293018	0.436242	protein_coding
ENSG0000(	ARHGEF34I	2234.461	-0.20588	0.293054	0.436268	unprocessed_pseudogene
ENSG0000(	ENO1P3	71.44024	0.115693	0.293147	0.436343	processed_pseudogene

ENSG0000(	ENSG0000(	15.072	-0.18004	0.29316	0.436343	lncRNA
ENSG0000(	ILKAP	1011.222	-0.04771	0.293129	0.436343	protein_coding
ENSG0000(	PLA2G2C	108.9575	0.124106	0.293149	0.436343	protein_coding
ENSG0000(	ENSG0000(	17.79157	-0.2082	0.29321	0.436397	lncRNA
ENSG0000(	FBXL19	650.6093	-0.06599	0.293304	0.436515	protein_coding
ENSG0000(	KTN1-AS1	705.838	0.04088	0.293422	0.436669	lncRNA
ENSG0000(	FAM117A	434.6839	-0.06906	0.293451	0.436672	protein_coding
ENSG0000(	ZNF596	333.7462	-0.07415	0.293445	0.436672	protein_coding
ENSG0000(	ENSG0000(	20.25907	0.182832	0.293568	0.436801	lncRNA
ENSG0000(	FAM230J	53.4071	0.245641	0.29358	0.436801	lncRNA
ENSG0000(	ZBTB11-AS	409.5356	0.0446	0.293577	0.436801	lncRNA
ENSG0000(	CDCA3	425.2276	-0.07218	0.293647	0.436881	protein_coding
ENSG0000(	ENSG0000(	53.28801	-0.11735	0.293736	0.436992	lncRNA
ENSG0000(	LTB4R	458.8973	-0.11278	0.293762	0.43701	protein_coding
ENSG0000(	ENSG0000(	20.2243	-0.19338	0.294022	0.437376	lncRNA
ENSG0000(	KLK10	282.0866	0.092135	0.294238	0.437677	protein_coding
ENSG0000(	ENSG0000(	40.44291	0.134793	0.29428	0.437718	lncRNA
ENSG0000(	ENSG0000(	65.17517	0.12056	0.294331	0.437773	lncRNA
ENSG0000(	ENSG0000(	28.57219	0.155256	0.294486	0.437982	lncRNA
ENSG0000(	ENSG0000(	141.2628	-0.07805	0.294574	0.438093	TEC
ENSG0000(	ZNF663P	18.14086	-0.19293	0.294713	0.438278	transcribed_unitary_pseudogene
ENSG0000(	ENSG0000(	20.48452	-0.20214	0.29479	0.438372	lncRNA
ENSG0000(	FAM72D	49.12988	0.919938	0.294826	0.438404	protein_coding
ENSG0000(	TUBB2B	27.78839	-0.25678	0.294856	0.438428	protein_coding
ENSG0000(	ENSG0000(	29.34234	-0.17591	0.294923	0.438507	processed_pseudogene
ENSG0000(	SCNN1A	3862.032	-0.13029	0.294944	0.438518	protein_coding
ENSG0000(	CKMT2	13.75867	-0.28068	0.294972	0.438538	protein_coding
ENSG0000(	CHRM1	24.0691	0.309789	0.295007	0.438549	protein_coding
ENSG0000(	ENSG0000(	108.8709	0.140901	0.294994	0.438549	lncRNA
ENSG0000(	CCDC74B	46.20739	0.197235	0.295046	0.438586	protein_coding
ENSG0000(	ENSG0000(	707.336	0.168447	0.295091	0.438612	TEC
ENSG0000(	ENSG0000(	226.1782	0.076421	0.295082	0.438612	lncRNA
ENSG0000(	AGO3	5825.75	0.040216	0.295199	0.43875	protein_coding
ENSG0000(	ROR1-AS1	2239.167	0.045093	0.295322	0.438913	lncRNA
ENSG0000(	ENSG0000(	103.4892	0.21287	0.295489	0.439098	lncRNA
ENSG0000(	LINC02506	34.21706	-0.15357	0.295482	0.439098	lncRNA
ENSG0000(	UBALD1	387.3675	0.083433	0.29547	0.439098	protein_coding
ENSG0000(	PATE2	36.09524	0.150729	0.29552	0.439124	protein_coding
ENSG0000(	ENSG0000(	9.743694	-0.29031	0.295543	0.439128	lncRNA
ENSG0000(	ENSG0000(	350.0148	-0.05568	0.295551	0.439128	lncRNA
ENSG0000(	RDH14	214.5991	-0.06228	0.295875	0.439589	protein_coding
ENSG0000(	ENSG0000(	53.73535	0.140925	0.295947	0.439675	lncRNA
ENSG0000(	TRMT10A	133.4465	-0.1368	0.29599	0.439718	protein_coding
ENSG0000(	ENSG0000(	16.05526	-0.21958	0.296037	0.439746	lncRNA
ENSG0000(	ENSG0000(	110.0311	-0.09094	0.296033	0.439746	lncRNA
ENSG0000(	ATP6V0C	604.5839	-0.09538	0.296101	0.43982	protein_coding
ENSG0000(	AOAH-IT1	148.834	0.143021	0.296252	0.440023	lncRNA

ENSG0000(	ENSG0000(	129.076	-0.09453	0.296313	0.440093	lncRNA
ENSG0000(	ENSG0000(	67.91766	0.116811	0.296378	0.440127	lncRNA
ENSG0000(	MTF1	1606.939	-0.07733	0.29636	0.440127	protein_coding
ENSG0000(	RN7SL610F	12.4327	0.266726	0.296374	0.440127	misc_RNA
ENSG0000(	GSTT4	38.95928	0.135873	0.296488	0.440268	protein_coding
ENSG0000(	RNU6-341F	42.60058	0.127689	0.296501	0.440268	snRNA
ENSG0000(	ATP1B1P1	78.58305	-0.08875	0.296641	0.440423	unprocessed_pseudogene
ENSG0000(	RALGAPA1I	358.5565	-0.11677	0.296648	0.440423	processed_pseudogene
ENSG0000(	ZNF852	667.9993	0.043676	0.296637	0.440423	protein_coding
ENSG0000(	ANTXRLP1	82.32524	0.251141	0.296724	0.440456	unprocessed_pseudogene
ENSG0000(	LINC01562	972.7173	-0.03533	0.296723	0.440456	lncRNA
ENSG0000(	RNY1P16	72.27665	-0.18814	0.296726	0.440456	misc_RNA
ENSG0000(	TRAJ6	19.59632	0.264988	0.29672	0.440456	TR_J_gene
ENSG0000(	SCNM1	761.0809	-0.03974	0.296794	0.440535	protein_coding
ENSG0000(	CYP26B1	183.5388	0.285604	0.296845	0.440591	protein_coding
ENSG0000(	ANKRD42	518.7507	-0.06133	0.296968	0.440689	protein_coding
ENSG0000(	ENSG0000(	93.73919	0.144852	0.296964	0.440689	lncRNA
ENSG0000(	ENSG0000(	15.54153	-0.23511	0.296938	0.440689	lncRNA
ENSG0000(	SMAD6	1106.932	-0.09368	0.296959	0.440689	protein_coding
ENSG0000(	PARP4P2	20.98834	-0.22985	0.297006	0.440724	unprocessed_pseudogene
ENSG0000(	ERO1A	2064.278	0.098979	0.297138	0.440899	protein_coding
ENSG0000(	ENSG0000(	14.19147	0.15693	0.297193	0.440924	lncRNA
ENSG0000(	ENSG0000(	190.8464	-0.06744	0.297197	0.440924	lncRNA
ENSG0000(	TTC30B	163.8097	0.092343	0.297189	0.440924	protein_coding
ENSG0000(	PROM1	4170.136	-0.18898	0.297278	0.441024	protein_coding
ENSG0000(	ASIC2	27.51358	0.35812	0.297305	0.44104	protein_coding
ENSG0000(	ENDOV	732.6832	0.058391	0.297317	0.44104	protein_coding
ENSG0000(	C6orf226	18.1814	-0.21393	0.297399	0.44114	protein_coding
ENSG0000(	GLP1R	24.02808	-0.21283	0.297415	0.441142	protein_coding
ENSG0000(	EXOC7	3911.081	0.04988	0.297441	0.44116	protein_coding
ENSG0000(	DCXR-DT	45.48082	0.137686	0.297492	0.441194	lncRNA
ENSG0000(	ENSG0000(	72.45033	-0.09127	0.297488	0.441194	lncRNA
ENSG0000(	ACTL10	24.33754	-0.19643	0.297548	0.441256	protein_coding
ENSG0000(	BANF1	282.0416	-0.10481	0.297596	0.441307	protein_coding
ENSG0000(	ZBTB43	1952.931	0.045905	0.297712	0.441458	protein_coding
ENSG0000(	DSTNP2	158.9611	-0.06713	0.297728	0.441461	transcribed_unprocessed_pseudo
ENSG0000(	ZC3H11A	3845.3	0.058326	0.297766	0.441496	protein_coding
ENSG0000(	RN7SL380F	13.3984	0.228847	0.297829	0.441569	misc_RNA
ENSG0000(	ZNF778	1172.165	-0.08837	0.297934	0.441703	protein_coding
ENSG0000(	ENSG0000(	1959.868	-0.07571	0.298083	0.441903	TEC
ENSG0000(	KICS2	426.229	0.075556	0.298126	0.441946	protein_coding
ENSG0000(	RN7SKP248	11.40076	0.2345	0.298176	0.441999	misc_RNA
ENSG0000(	ENSG0000(	263.1751	-0.09587	0.298357	0.442226	lncRNA
ENSG0000(	ENSG0000(	210.4931	-0.06899	0.298353	0.442226	lncRNA
ENSG0000(	ENSG0000(	13.11207	0.231958	0.298396	0.442263	lncRNA
ENSG0000(	SNTB1	640.6663	-0.08737	0.298517	0.442421	protein_coding
ENSG0000(	JAG2	232.6874	0.140745	0.298536	0.442429	protein_coding

ENSG0000(	DBF4B	695.61	0.051794	0.298617	0.442527	protein_coding
ENSG0000(	SPAG16	370.2011	-0.0768	0.298694	0.44262	protein_coding
ENSG0000(	CPSF4	495.4437	-0.05592	0.298717	0.442633	protein_coding
ENSG0000(	ENSG0000(	343.9222	0.050644	0.298759	0.442674	lncRNA
ENSG0000(	C1orf159	602.2191	-0.06462	0.298825	0.442751	protein_coding
ENSG0000(	SGPP2	1829.778	-0.11472	0.298913	0.44286	protein_coding
ENSG0000(	ARHGAP45	1650.459	0.136805	0.298932	0.442867	protein_coding
ENSG0000(	ENSG0000(	106.9172	0.105873	0.299224	0.44328	lncRNA
ENSG0000(	PGAM1P5	101.78	0.101644	0.29926	0.443311	transcribed_processed_pseudoge
ENSG0000(	ZDHHC24	1184.689	0.045947	0.299349	0.443423	protein_coding
ENSG0000(	COQ6	1233.956	-0.03393	0.299402	0.44348	protein_coding
ENSG0000(	ZNF862	1159.885	-0.07283	0.299491	0.44359	protein_coding
ENSG0000(	FAM241A	29947.59	-0.0406	0.299555	0.443661	protein_coding
ENSG0000(	LACTB	530.5029	0.076445	0.299566	0.443661	protein_coding
ENSG0000(	BNIP2	2544.234	0.051029	0.299632	0.443738	protein_coding
ENSG0000(	DNAJC9-AS	271.303	-0.06419	0.299813	0.443983	lncRNA
ENSG0000(	ENSG0000(	18.15753	0.252024	0.299966	0.44419	lncRNA
ENSG0000(	LINC01849	41.31598	-0.13826	0.300189	0.444499	lncRNA
ENSG0000(	MTRNR2L6	19.87651	0.232429	0.300211	0.44451	protein_coding
ENSG0000(	FAM104B	207.8921	-0.11621	0.30023	0.444517	protein_coding
ENSG0000(	MIR1915H(	104.5697	0.09068	0.300318	0.444627	lncRNA
ENSG0000(	NCR3	14.38794	-0.317	0.300354	0.444659	protein_coding
ENSG0000(	ENSG0000(	267.294	0.052215	0.300446	0.444773	lncRNA
ENSG0000(	ENSG0000(	640.6699	0.049923	0.300474	0.444791	lncRNA
ENSG0000(	ZMIZ2	3202.72	0.048316	0.300486	0.444791	protein_coding
ENSG0000(	GOT2P3	12.29949	-0.33944	0.300511	0.444807	transcribed_processed_pseudoge
ENSG0000(	ENSG0000(	52.98222	-0.13548	0.300557	0.444832	TEC
ENSG0000(	ENSG0000(	457.0563	-0.11873	0.300547	0.444832	lncRNA
ENSG0000(	RN7SL323F	9.204658	0.211001	0.300582	0.444849	misc_RNA
ENSG0000(	SULT2B1	119.1961	-0.11719	0.300602	0.444857	protein_coding
ENSG0000(	ENSG0000(	31.76491	0.217525	0.30062	0.444863	lncRNA
ENSG0000(	ATP10A	808.5945	0.120909	0.300648	0.444883	protein_coding
ENSG0000(	WASH7P	99.90284	-0.28228	0.300681	0.444912	unprocessed_pseudogene
ENSG0000(	C11orf94	17.00078	-0.22873	0.300704	0.444924	protein_coding
ENSG0000(	KIAA2012	399.2728	-0.04521	0.300923	0.445227	protein_coding
ENSG0000(	PHF21B	19.95444	0.314589	0.301101	0.445451	protein_coding
ENSG0000(	RBMX2P3	16.63702	-0.17533	0.301117	0.445451	processed_pseudogene
ENSG0000(	TMEM47	225.0166	-0.12503	0.301112	0.445451	protein_coding
ENSG0000(	LDHB	684.8162	-0.12279	0.301168	0.445505	protein_coding
ENSG0000(	ENSG0000(	172.6238	0.08461	0.301195	0.445525	TEC
ENSG0000(	ENSG0000(	223.3946	0.058467	0.301268	0.445611	lncRNA
ENSG0000(	RBM14-RBI	672.9108	0.062513	0.301315	0.445659	protein_coding
ENSG0000(	SNORA58B	40.46891	0.173122	0.301354	0.445696	snoRNA
ENSG0000(	ENSG0000(	57.49928	0.1249	0.301447	0.445786	lncRNA
ENSG0000(	LINC01460	162.4007	0.060094	0.301457	0.445786	lncRNA
ENSG0000(	SLC16A6	121.2556	0.152957	0.301435	0.445786	protein_coding
ENSG0000(	AGKP1	11.12605	0.2627	0.301525	0.445854	processed_pseudogene

ENSG0000(	CIDECP1	482.3413	-0.03985	0.301561	0.445854	transcribed_unprocessed_pseudo
ENSG0000(	PCDHAC2	38.89824	-0.3491	0.301537	0.445854	protein_coding
ENSG0000(	SATB1	2306.168	0.110221	0.301557	0.445854	protein_coding
ENSG0000(	NUDT18	171.2424	0.080916	0.301581	0.445863	protein_coding
ENSG0000(	ZNF670	157.2819	-0.10514	0.3016	0.44587	protein_coding
ENSG0000(	ENSG0000(	232.2672	0.067039	0.301669	0.445952	lncRNA
ENSG0000(	ENSG0000(	12.23286	-0.23815	0.30175	0.44605	lncRNA
ENSG0000(	MPV17L2	155.9134	-0.09607	0.301829	0.446146	protein_coding
ENSG0000(	ENSG0000(	97.681	0.162358	0.301899	0.446227	lncRNA
ENSG0000(	ENSG0000(	16.25021	0.223625	0.301961	0.446299	lncRNA
ENSG0000(	RN7SKP203	321.6932	0.169308	0.301993	0.446325	misc_RNA
ENSG0000(	ENSG0000(	281.1183	0.136743	0.30215	0.446536	TEC
ENSG0000(	CCDC85A	25.26014	-0.2504	0.302171	0.446542	protein_coding
ENSG0000(	ENSG0000(	12.60078	0.194427	0.302183	0.446542	lncRNA
ENSG0000(	ENSG0000(	245.2198	0.073932	0.302198	0.446544	lncRNA
ENSG0000(	ENSG0000(	66.01905	-0.10994	0.302239	0.446582	lncRNA
ENSG0000(	WASH4P	16.32354	-1.89655	0.30232	0.446681	unprocessed_pseudogene
ENSG0000(	CBX3P2	43.81029	-0.12331	0.302347	0.446681	transcribed_processed_pseudoge
ENSG0000(	ENSG0000(	127.2216	0.191185	0.302349	0.446681	lncRNA
ENSG0000(	NINJ2	26.16874	0.186776	0.302365	0.446685	protein_coding
ENSG0000(	DIAPH2-AS	64.39483	0.117945	0.302424	0.44675	lncRNA
ENSG0000(	ENSG0000(	100.0528	0.089427	0.302438	0.446751	lncRNA
ENSG0000(	ENSG0000(	74.58648	0.192332	0.302491	0.446806	lncRNA
ENSG0000(	BMS1P22	24.80318	0.370722	0.302629	0.446989	transcribed_unprocessed_pseudo
ENSG0000(	ITGB8-AS1	16.93272	-0.22855	0.302751	0.447149	lncRNA
ENSG0000(	ENSG0000(	24.65445	0.192406	0.302776	0.447165	lncRNA
ENSG0000(	BCDIN3D-A	407.2759	-0.04627	0.302882	0.447238	lncRNA
ENSG0000(	CTDSPL2P1	177.2233	0.086991	0.302883	0.447238	processed_pseudogene
ENSG0000(	ENSG0000(	1066.593	-0.07666	0.302865	0.447238	lncRNA
ENSG0000(	KIR2DL4	14.04975	-0.29514	0.302881	0.447238	protein_coding
ENSG0000(	ADAM1B	86.68415	0.094149	0.302993	0.44738	unitary_pseudogene
ENSG0000(	ENSG0000(	12.09072	0.300205	0.303267	0.447763	lncRNA
ENSG0000(	ENSG0000(	95.50108	-0.11999	0.303293	0.447781	TEC
ENSG0000(	ENSG0000(	52.82255	0.153619	0.30332	0.4478	lncRNA
ENSG0000(	ENSG0000(	20.54415	-0.18527	0.30349	0.448008	lncRNA
ENSG0000(	IGKV3-7	29.63298	0.330758	0.303485	0.448008	IG_V_gene
ENSG0000(	ENSG0000(	12.95769	0.264641	0.303526	0.448022	lncRNA
ENSG0000(	ENSG0000(	18.96401	-0.222	0.303528	0.448022	lncRNA
ENSG0000(	ENSG0000(	241.7767	-0.05994	0.303575	0.44807	lncRNA
ENSG0000(	ENSG0000(	85.26131	-0.12916	0.303691	0.44822	protein_coding
ENSG0000(	KEL	63.20225	0.210046	0.303724	0.448248	protein_coding
ENSG0000(	RN7SL836P	27.79142	-0.17609	0.303739	0.448248	misc_RNA
ENSG0000(	ENSG0000(	43.89909	0.13849	0.303758	0.448255	lncRNA
ENSG0000(	LINC02669	186.8772	0.073029	0.303812	0.448314	lncRNA
ENSG0000(	LINC01435	177.2462	0.073333	0.303862	0.448345	lncRNA
ENSG0000(	ZNF581	115.6068	-0.11014	0.303851	0.448345	protein_coding
ENSG0000(	CRYZL1	1586.114	0.032408	0.303951	0.448456	protein_coding

ENSG0000(	GAS2L1	270.0189	-0.1094	0.304083	0.448628	protein_coding
ENSG0000(	EFHB	74.02028	0.163464	0.304105	0.44864	protein_coding
ENSG0000(	CATIP	292.2421	-0.05525	0.304261	0.44885	protein_coding
ENSG0000(	UFL1	822.2278	0.081764	0.304309	0.448894	protein_coding
ENSG0000(	ZNF772	640.1836	-0.07097	0.304326	0.448894	protein_coding
ENSG0000(	ZNF821	137.6378	-0.13126	0.304334	0.448894	protein_coding
ENSG0000(	INPP1	636.209	-0.12183	0.304351	0.448897	protein_coding
ENSG0000(	ARPIN-AP3	198.2976	-1.13266	0.304485	0.449074	protein_coding
ENSG0000(	ENSG0000(	19.59976	0.219367	0.304617	0.449248	lncRNA
ENSG0000(	ENSG0000(	31.56477	-0.18001	0.304701	0.44935	TEC
ENSG0000(	OR5AU1	451.3591	-0.0605	0.304721	0.449359	protein_coding
ENSG0000(	MIR2117H(	15.31824	-0.22896	0.304759	0.449393	lncRNA
ENSG0000(	ENSG0000(	101.6334	0.12794	0.304813	0.449452	TEC
ENSG0000(	ENSG0000(	137.7053	-0.06829	0.304828	0.449452	lncRNA
ENSG0000(	U2AF1	2528.454	0.289658	0.304849	0.449463	protein_coding
ENSG0000(	ENSG0000(	26.41757	0.181347	0.304916	0.449523	lncRNA
ENSG0000(	NMUR1	225.8008	-0.0679	0.304919	0.449523	protein_coding
ENSG0000(	FRMD1	2274.012	-0.22466	0.305013	0.44964	protein_coding
ENSG0000(	IGHV4-55	89.22644	0.268257	0.305035	0.449652	IG_V_pseudogene
ENSG0000(	C1orf50	830.4172	-0.05091	0.305154	0.449764	protein_coding
ENSG0000(	ENSG0000(	186.658	0.073577	0.30514	0.449764	lncRNA
ENSG0000(	RANBP6	883.8286	0.046832	0.305154	0.449764	protein_coding
ENSG0000(	CXXC5	641.2522	0.060193	0.305222	0.449843	protein_coding
ENSG0000(	ADRA1A	305.8377	-0.08018	0.305407	0.450094	protein_coding
ENSG0000(	CDKL3	338.0004	0.070771	0.305459	0.45015	protein_coding
ENSG0000(	ENSG0000(	29.47406	0.142597	0.305494	0.450164	lncRNA
ENSG0000(	ENSG0000(	151.2064	-0.06509	0.305541	0.450164	lncRNA
ENSG0000(	ENSG0000(	54.86261	-0.11777	0.305534	0.450164	lncRNA
ENSG0000(	PRR12	1544.432	-0.07726	0.305541	0.450164	protein_coding
ENSG0000(	SAMD10	135.1875	0.154825	0.305538	0.450164	protein_coding
ENSG0000(	GXYLT1P4	17.35045	0.291797	0.305571	0.450188	transcribed_processed_pseudoge
ENSG0000(	CIBAR1-DT	54.83582	-0.22553	0.305588	0.450191	lncRNA
ENSG0000(	PRNP	606.5975	0.130318	0.305618	0.450214	protein_coding
ENSG0000(	HAAO	278.1949	0.09754	0.305638	0.450222	protein_coding
ENSG0000(	CELF1	5731.675	0.036997	0.305765	0.450353	protein_coding
ENSG0000(	RASAL2	2114.189	0.060564	0.305777	0.450353	protein_coding
ENSG0000(	SREBF2-AS:	54.03806	0.111342	0.305751	0.450353	lncRNA
ENSG0000(	TMEM212	1913.971	-0.06913	0.305784	0.450353	protein_coding
ENSG0000(	KCNJ6	987.3296	0.09303	0.30584	0.450413	protein_coding
ENSG0000(	ENSG0000(	161.5498	0.216076	0.30587	0.450436	protein_coding
ENSG0000(	C2CD4D-AS	31.57295	0.169436	0.305905	0.450456	lncRNA
ENSG0000(	LINC02069	160.4447	0.073509	0.305912	0.450456	lncRNA
ENSG0000(	ENSG0000(	743.3	-0.0692	0.305976	0.450529	lncRNA
ENSG0000(	NECTIN3-A	39.29588	0.19322	0.306038	0.450599	lncRNA
ENSG0000(	ENSG0000(	21.9144	-0.1744	0.306087	0.450651	processed_pseudogene
ENSG0000(	LINC02361	27.63151	-0.19862	0.3062	0.450796	lncRNA
ENSG0000(	ENSG0000(	14.01323	0.283257	0.306234	0.450824	lncRNA

ENSG0000(	PSORS1C1	354.2146	0.291109	0.306309	0.450913	protein_coding
ENSG0000(	FAM66A	21.30328	0.248527	0.306333	0.450928	lncRNA
ENSG0000(	RN7SL820F	32.3076	0.150227	0.306483	0.451114	misc_RNA
ENSG0000(	TMEM132I	20.71806	0.250895	0.306488	0.451114	protein_coding
ENSG0000(	MAPKAP1	1394.016	0.037206	0.30653	0.45115	protein_coding
ENSG0000(	PPP1R27	39.06032	0.120852	0.306542	0.45115	protein_coding
ENSG0000(	SMARCE1P	40.84432	-0.12538	0.306591	0.451201	processed_pseudogene
ENSG0000(	ENSG0000(	127.5409	0.078276	0.306633	0.451242	lncRNA
ENSG0000(	COLGALT2	44.68108	-0.19648	0.306693	0.451308	protein_coding
ENSG0000(	NRIR	219.9636	0.100677	0.306722	0.451331	lncRNA
ENSG0000(	METRNL	749.5474	-0.1239	0.306746	0.451344	protein_coding
ENSG0000(	INTU	5068.339	0.045911	0.306782	0.451375	protein_coding
ENSG0000(	ENSG0000(	47.83657	0.158515	0.306816	0.451383	TEC
ENSG0000(	RP1	127.5013	-0.084	0.306807	0.451383	protein_coding
ENSG0000(	ASAP3	866.3219	-0.07111	0.306855	0.451396	protein_coding
ENSG0000(	KIAA1217	3257.679	0.086138	0.30684	0.451396	protein_coding
ENSG0000(	KRT15	452.7526	-0.14854	0.306868	0.451396	protein_coding
ENSG0000(	PTCD1	662.5526	-0.05721	0.306907	0.451432	protein_coding
ENSG0000(	EEF1DP2	21.75583	0.215837	0.306935	0.451452	processed_pseudogene
ENSG0000(	ENSG0000(	28.21192	0.163333	0.307069	0.45162	lncRNA
ENSG0000(	PRRT1	96.63109	-0.16109	0.307078	0.45162	protein_coding
ENSG0000(	RNU2-24P	39.95195	0.158413	0.307205	0.451786	snRNA
ENSG0000(	ENSG0000(	111.7511	0.085374	0.307282	0.451878	lncRNA
ENSG0000(	ENSG0000(	28.07457	-0.14072	0.307298	0.451879	lncRNA
ENSG0000(	ENSG0000(	26.24807	0.153929	0.307318	0.451889	lncRNA
ENSG0000(	ENSG0000(	79.4335	0.153932	0.307361	0.45193	lncRNA
ENSG0000(	ENSG0000(	269.6564	0.062975	0.307456	0.452049	lncRNA
ENSG0000(	ENSG0000(	50.67384	0.13783	0.307576	0.452182	TEC
ENSG0000(	RGPD1	246.1325	0.385746	0.307572	0.452182	protein_coding
ENSG0000(	TAX1BP1	2266.295	0.048128	0.307612	0.452214	protein_coding
ENSG0000(	CABLES2	313.9189	-0.08426	0.307651	0.45223	protein_coding
ENSG0000(	RN7SL131F	247.5517	-0.0607	0.307648	0.45223	misc_RNA
ENSG0000(	RAB17	728.7035	-0.1729	0.307727	0.45232	protein_coding
ENSG0000(	SNAI3-AS1	384.4386	0.087278	0.307744	0.452323	lncRNA
ENSG0000(	PI4KB	1650.554	-0.04491	0.3078	0.452384	protein_coding
ENSG0000(	ENSG0000(	98.1868	-0.08789	0.307872	0.45246	lncRNA
ENSG0000(	FASN	2041.981	0.186247	0.30788	0.45246	protein_coding
ENSG0000(	SLC24A1	589.9227	0.080979	0.307964	0.452561	protein_coding
ENSG0000(	PDXP-DT	11.04728	0.289517	0.308058	0.452679	lncRNA
ENSG0000(	HTR3A	16.92841	0.412597	0.30817	0.452822	protein_coding
ENSG0000(	GJD2-DT	221.6634	0.049387	0.308217	0.452869	lncRNA
ENSG0000(	CCDC89	136.5882	-0.17269	0.308253	0.452901	protein_coding
ENSG0000(	FAR2P3	137.562	0.112732	0.308325	0.452985	transcribed_unprocessed_pseudo
ENSG0000(	CALN1	213.1325	0.076824	0.308418	0.453101	protein_coding
ENSG0000(	SNORD71	21.48878	0.169055	0.308489	0.453184	snoRNA
ENSG0000(	ENSG0000(	6327.869	0.055325	0.308582	0.453299	lncRNA
ENSG0000(	SF1	4985.083	0.046745	0.308597	0.453299	protein_coding

ENSG0000(	LINC00992	49.81858	-0.19533	0.308665	0.453378	lncRNA
ENSG0000(	PAPPA2	123.6291	0.331786	0.308691	0.453395	protein_coding
ENSG0000(	GFAP	355.3973	0.082511	0.30878	0.453505	protein_coding
ENSG0000(	PDK4	1693.862	-0.21255	0.308847	0.453582	protein_coding
ENSG0000(	APOBEC3D	629.3276	0.0585	0.308869	0.453592	protein_coding
ENSG0000(	TRAF3IP3	1329.829	0.183962	0.308884	0.453594	protein_coding
ENSG0000(	ENSG0000(	191.1673	0.080325	0.309124	0.45392	lncRNA
ENSG0000(	SCUBE1	814.653	-0.1067	0.309136	0.45392	protein_coding
ENSG0000(	ENSG0000(	349.9275	0.048283	0.309226	0.454032	lncRNA
ENSG0000(	DCT	15.53239	0.317616	0.309299	0.454096	protein_coding
ENSG0000(	SYNPO2	2921.953	0.10775	0.309289	0.454096	protein_coding
ENSG0000(	ENSG0000(	27.69508	0.153083	0.309365	0.454172	lncRNA
ENSG0000(	FBXO4	230.7483	0.085739	0.30959	0.454438	protein_coding
ENSG0000(	MTO1	3118.248	-0.03427	0.309581	0.454438	protein_coding
ENSG0000(	TPT1P5	15.25187	0.242513	0.30959	0.454438	processed_pseudogene
ENSG0000(	OR5H14	271.4957	0.063012	0.309744	0.454643	protein_coding
ENSG0000(	RBM5	8144.052	-0.02424	0.309833	0.454752	protein_coding
ENSG0000(	TBPL1	419.9612	-0.10016	0.309874	0.454791	protein_coding
ENSG0000(	ENSG0000(	219.3666	0.068945	0.30994	0.454866	lncRNA
ENSG0000(	OPN3	609.3865	-0.16381	0.309959	0.454874	protein_coding
ENSG0000(	PCOTH	12.76777	-0.25461	0.309981	0.454884	lncRNA
ENSG0000(	ENSG0000(	9.926223	0.269953	0.310111	0.455054	lncRNA
ENSG0000(	TBC1D16	1427.289	0.098715	0.310148	0.455086	protein_coding
ENSG0000(	ENSG0000(	749.5049	-0.044	0.310217	0.455166	lncRNA
ENSG0000(	ENSG0000(	936.5862	0.054151	0.310346	0.455313	lncRNA
ENSG0000(	TIPARP-AS1	17.43531	-0.17783	0.310339	0.455313	lncRNA
ENSG0000(	FAM98B	1174.921	0.044038	0.310378	0.455339	protein_coding
ENSG0000(	ENSG0000(	21.71493	0.175621	0.310401	0.455351	lncRNA
ENSG0000(	IGLJ2	18.60877	0.296963	0.310541	0.455535	IG_J_gene
ENSG0000(	ZBED3-AS1	4348.076	-0.04887	0.310634	0.45565	lncRNA
ENSG0000(	ENSG0000(	8.543314	-0.45883	0.3107	0.455725	protein_coding
ENSG0000(	LINC01007	21.35878	0.263409	0.310733	0.455752	lncRNA
ENSG0000(	RTF1	1130.422	-0.0663	0.31079	0.455815	protein_coding
ENSG0000(	ELN	628.3584	0.078446	0.310817	0.455833	protein_coding
ENSG0000(	ENSG0000(	37.79655	-0.15022	0.310937	0.455988	transcribed_processed_pseudoge
ENSG0000(	ERCC6	1972.537	-0.05369	0.310984	0.456035	protein_coding
ENSG0000(	CHFR	3344.034	0.040416	0.31101	0.456051	protein_coding
ENSG0000(	SSBP4	350.5254	-0.09987	0.311044	0.45608	protein_coding
ENSG0000(	ENSG0000(	400.001	0.050116	0.311075	0.456081	lncRNA
ENSG0000(	FIZ1	284.1898	0.089039	0.311065	0.456081	protein_coding
ENSG0000(	GOLGA5	781.8535	-0.06231	0.311088	0.456081	protein_coding
ENSG0000(	ENSG0000(	11.40741	-0.25312	0.311131	0.456123	unprocessed_pseudogene
ENSG0000(	CARS2	1596.802	-0.04274	0.311181	0.456153	protein_coding
ENSG0000(	MT-TH	125.8801	0.309071	0.311167	0.456153	Mt_tRNA
ENSG0000(	CCDC38	105.0426	-0.11468	0.311227	0.456199	protein_coding
ENSG0000(	ARSI	42.16743	0.194921	0.311269	0.456217	protein_coding
ENSG0000(	MTCO3P44	16.21488	0.21145	0.311257	0.456217	unprocessed_pseudogene

ENSG0000(	KCNJ12	87.68222	-0.13439	0.311315	0.456264	protein_coding
ENSG0000(	STAR	290.3961	0.054537	0.311339	0.456277	protein_coding
ENSG0000(	ANKRD34C	288.595	0.086827	0.311377	0.456299	lncRNA
ENSG0000(	PRO1804	160.3007	0.155669	0.311383	0.456299	TEC
ENSG0000(	LINC02145	189.9759	-0.10936	0.311434	0.456352	lncRNA
ENSG0000(	ENSG0000(	161.8568	-0.07673	0.311483	0.456403	lncRNA
ENSG0000(	MYOT	51.02778	0.192033	0.311659	0.45664	protein_coding
ENSG0000(	ENSG0000(	592.729	-0.05848	0.311682	0.456651	lncRNA
ENSG0000(	ENSG0000(	27.8008	-0.1865	0.311718	0.456684	lncRNA
ENSG0000(	ENSG0000(	55.44281	-0.09734	0.311772	0.456741	lncRNA
ENSG0000(	TTC39A-AS	60.98179	-0.19485	0.311802	0.456763	lncRNA
ENSG0000(	ENSG0000(	22.4623	0.25004	0.312016	0.457055	unprocessed_pseudogene
ENSG0000(	ENSG0000(	190.6748	-0.104	0.312123	0.457138	lncRNA
ENSG0000(	ENSG0000(	181.9609	-0.06732	0.312131	0.457138	TEC
ENSG0000(	LINC01895	38.75295	0.122591	0.312111	0.457138	lncRNA
ENSG0000(	MAPK8IP1	121.1033	0.290579	0.312105	0.457138	protein_coding
ENSG0000(	SNX3	711.5831	-0.10922	0.312163	0.457142	protein_coding
ENSG0000(	ST8SIA6-AS	267.6654	-0.07915	0.312151	0.457142	lncRNA
ENSG0000(	DNASE1L1	417.0411	0.056268	0.31222	0.457204	protein_coding
ENSG0000(	ENSG0000(	9.702977	-0.23501	0.3123	0.457299	lncRNA
ENSG0000(	PMS1	776.6719	-0.05836	0.312573	0.457678	protein_coding
ENSG0000(	SLC10A5P1	30.91551	0.276755	0.312669	0.457776	unprocessed_pseudogene
ENSG0000(	TOB1-AS1	123.7188	-0.09982	0.312662	0.457776	lncRNA
ENSG0000(	ENSG0000(	24.20546	-0.12545	0.312811	0.457923	lncRNA
ENSG0000(	ENSG0000(	8.420714	0.266548	0.31279	0.457923	lncRNA
ENSG0000(	SLC12A9	2104.977	0.051805	0.312813	0.457923	protein_coding
ENSG0000(	LINC02752	14.30378	0.241901	0.312945	0.458083	lncRNA
ENSG0000(	RNF135	509.7232	-0.08842	0.312952	0.458083	protein_coding
ENSG0000(	FBXO24	220.6184	0.063694	0.312967	0.458083	protein_coding
ENSG0000(	USP38	1904.427	0.058204	0.313005	0.458118	protein_coding
ENSG0000(	C2orf69	484.5096	-0.05962	0.313033	0.458137	protein_coding
ENSG0000(	ENSG0000(	85.31289	0.070783	0.313207	0.45836	lncRNA
ENSG0000(	RGPD8	1049.735	0.148129	0.313214	0.45836	protein_coding
ENSG0000(	NAT9	575.3048	-0.05559	0.313385	0.458567	protein_coding
ENSG0000(	TCEAL1	70.82987	-0.11943	0.313376	0.458567	protein_coding
ENSG0000(	ENPP6	238.872	-0.08728	0.31341	0.458583	protein_coding
ENSG0000(	ENSG0000(	115.0821	-0.14272	0.313486	0.458671	lncRNA
ENSG0000(	NEXN-AS1	139.7599	-0.15678	0.313523	0.458705	lncRNA
ENSG0000(	ZNF230	411.2577	-0.04516	0.313622	0.458828	protein_coding
ENSG0000(	TLX1	10.98891	0.404438	0.313669	0.458875	protein_coding
ENSG0000(	ENSG0000(	19.54243	0.197816	0.31371	0.458913	TEC
ENSG0000(	COL13A1	336.7526	-0.14718	0.31384	0.459082	protein_coding
ENSG0000(	FAR1-IT1	76.57192	-0.12907	0.313886	0.459128	lncRNA
ENSG0000(	CLEC16A	2478.666	0.048513	0.313932	0.459158	protein_coding
ENSG0000(	ENSG0000(	28.50005	0.155835	0.313936	0.459158	lncRNA
ENSG0000(	ENSG0000(	141.1482	0.100447	0.313983	0.459188	lncRNA
ENSG0000(	MTARC1	1916.055	-0.12621	0.313986	0.459188	protein_coding

ENSG0000(	CAMSAP2	869.0234	0.105965	0.314043	0.45925	protein_coding
ENSG0000(	KIF3C	118.8858	0.151239	0.314068	0.459265	protein_coding
ENSG0000(	MTMR1	953.7146	0.058226	0.314104	0.459297	protein_coding
ENSG0000(	ENSG0000(	21.63407	0.170253	0.314186	0.459309	lncRNA
ENSG0000(	ENSG0000(	149.4771	-0.09354	0.31414	0.459309	processed_pseudogene
ENSG0000(	ENSG0000(	129.2446	0.086903	0.314179	0.459309	lncRNA
ENSG0000(	FOXD4L4	10.5821	-0.3509	0.314146	0.459309	protein_coding
ENSG0000(	MICAL1	2651.151	-0.0716	0.314163	0.459309	protein_coding
ENSG0000(	ENSG0000(	90.01318	-0.42516	0.314247	0.459355	lncRNA
ENSG0000(	LINC02623	10.76702	0.27708	0.314246	0.459355	lncRNA
ENSG0000(	ENSG0000(	51.11166	0.246874	0.314361	0.459501	unprocessed_pseudogene
ENSG0000(	CLUAP1	964.9692	0.045744	0.314383	0.459509	protein_coding
ENSG0000(	FGF10	127.0005	0.148359	0.314396	0.459509	protein_coding
ENSG0000(	ENSG0000(	13.73638	-0.17152	0.314451	0.459568	lncRNA
ENSG0000(	ENSG0000(	33.15053	-0.11494	0.314611	0.459763	lncRNA
ENSG0000(	SEC24B-AS	378.2083	-0.0574	0.314614	0.459763	lncRNA
ENSG0000(	NOX1	542.4723	-0.23952	0.314658	0.459805	protein_coding
ENSG0000(	ENSG0000(	203.8336	0.056678	0.314757	0.459929	lncRNA
ENSG0000(	ANO7L1	69.43143	0.16638	0.314926	0.460155	transcribed_unprocessed_pseudo
ENSG0000(	SVIL	4413.877	0.068929	0.31499	0.460226	protein_coding
ENSG0000(	GEMIN7	222.2318	-0.08638	0.315207	0.460522	protein_coding
ENSG0000(	ENSG0000(	14.75955	0.280044	0.315295	0.460629	protein_coding
ENSG0000(	ENSG0000(	680.8438	-0.04236	0.315434	0.460811	lncRNA
ENSG0000(	ENSG0000(	11.14626	-0.32409	0.315503	0.46089	lncRNA
ENSG0000(	ENSG0000(	21.73837	-0.15788	0.315608	0.461009	lncRNA
ENSG0000(	TRAJ41	10.12613	0.280604	0.315614	0.461009	TR_J_gene
ENSG0000(	MIR7854	11.90796	0.24704	0.315649	0.461038	miRNA
ENSG0000(	AFF3	1573.163	0.132486	0.315724	0.461126	protein_coding
ENSG0000(	ENSG0000(	198.7117	0.224377	0.315756	0.46115	lncRNA
ENSG0000(	PCAT19	611.814	0.067764	0.315769	0.46115	lncRNA
ENSG0000(	ENSG0000(	90.64034	-0.10617	0.315844	0.461166	lncRNA
ENSG0000(	ENSG0000(	489.7508	0.041932	0.315813	0.461166	TEC
ENSG0000(	ENSG0000(	371.8643	-0.07051	0.315845	0.461166	lncRNA
ENSG0000(	LINC00692	28.13728	-0.16008	0.31582	0.461166	lncRNA
ENSG0000(	ZNF615	450.0523	-0.0738	0.315854	0.461166	protein_coding
ENSG0000(	ENSG0000(	47.09969	-0.10784	0.315906	0.461198	lncRNA
ENSG0000(	ENSG0000(	48.57625	-0.17496	0.315893	0.461198	lncRNA
ENSG0000(	NUDT19-D1	10.64182	0.229872	0.315959	0.461254	lncRNA
ENSG0000(	RN7SL49P	38.27152	0.146755	0.31599	0.461277	misc_RNA
ENSG0000(	RNU2-66P	10.85252	-0.23391	0.316004	0.461277	snRNA
ENSG0000(	LSP1	1222.876	-0.08864	0.316031	0.461294	protein_coding
ENSG0000(	MAGI1	2385.69	-0.12659	0.316049	0.4613	protein_coding
ENSG0000(	ENSG0000(	324.1509	-0.05375	0.316069	0.461307	lncRNA
ENSG0000(	ENSG0000(	25.34536	0.156302	0.316129	0.461373	lncRNA
ENSG0000(	FOXO6	8.951637	-0.26739	0.31628	0.461528	protein_coding
ENSG0000(	TMEM88	42.06645	0.167719	0.316257	0.461528	protein_coding
ENSG0000(	ZSWIM9	215.7991	-0.11045	0.31628	0.461528	protein_coding

ENSG0000(	FRAS1	287.9979	-0.18295	0.316323	0.461548	protein_coding
ENSG0000(	LINC02568	167.9762	-0.12878	0.31631	0.461548	lncRNA
ENSG0000(	DPEP1	63.81836	0.304172	0.31643	0.461683	protein_coding
ENSG0000(	ENSG0000(	25.40778	-0.15989	0.316493	0.461754	processed_pseudogene
ENSG0000(	TRAJ26	33.16045	0.252201	0.316553	0.46182	TR_J_gene
ENSG0000(	R3HDM1	1587.408	-0.03774	0.316583	0.461841	protein_coding
ENSG0000(	RN7SL546F	39.74478	0.120083	0.316599	0.461843	misc_RNA
ENSG0000(	NME4	315.5094	-0.08664	0.31664	0.461881	protein_coding
ENSG0000(	OSGIN1	314.1147	0.107757	0.316665	0.461897	protein_coding
ENSG0000(	LINC01704	35.27828	0.160031	0.316832	0.462108	lncRNA
ENSG0000(	VWA5B2	47.42117	-0.21785	0.31684	0.462108	protein_coding
ENSG0000(	ENSG0000(	73.45728	-0.10694	0.31694	0.462224	lncRNA
ENSG0000(	SNORA61	277.6588	0.114172	0.316948	0.462224	snoRNA
ENSG0000(	ENSG0000(	21.54794	-0.26538	0.317013	0.462296	lncRNA
ENSG0000(	IGHV3-43	206.0453	0.253071	0.317074	0.462354	IG_V_gene
ENSG0000(	LINC00499	585.9988	0.048791	0.317082	0.462354	lncRNA
ENSG0000(	ENSG0000(	187.383	-0.11589	0.317228	0.462545	lncRNA
ENSG0000(	ENSG0000(	100.4119	-0.23121	0.31731	0.462644	lncRNA
ENSG0000(	SEC14L1P1	137.1573	-0.09598	0.317328	0.462648	processed_pseudogene
ENSG0000(	PXDNL	32.34176	0.195054	0.317474	0.462817	protein_coding
ENSG0000(	RSL1D1-DT	21.97333	0.146096	0.317468	0.462817	lncRNA
ENSG0000(	BCO2	447.6989	0.064694	0.317492	0.462823	protein_coding
ENSG0000(	ARHGAP42	139.5806	0.085137	0.317514	0.462832	processed_pseudogene
ENSG0000(	ENSG0000(	196.0972	-0.06083	0.317541	0.462851	lncRNA
ENSG0000(	ZNF350-AS	290.7318	0.1002	0.31757	0.462871	lncRNA
ENSG0000(	SLC22A20P	311.5262	0.090629	0.317796	0.463179	transcribed_unitary_pseudogene
ENSG0000(	EGFR	3524.926	-0.09524	0.317875	0.463256	protein_coding
ENSG0000(	METTL6	998.2821	0.038201	0.317878	0.463256	protein_coding
ENSG0000(	C11orf91	20.33129	0.393641	0.317896	0.46326	protein_coding
ENSG0000(	ZNF583	561.3257	-0.08651	0.317945	0.46331	protein_coding
ENSG0000(	ENSG0000(	246.777	-0.05373	0.318053	0.463446	lncRNA
ENSG0000(	ENSG0000(	284.1843	0.088306	0.318119	0.463521	lncRNA
ENSG0000(	CDC42SE2	3721.337	-0.0874	0.318231	0.463619	protein_coding
ENSG0000(	ENSG0000(	72.0044	0.084732	0.318226	0.463619	lncRNA
ENSG0000(	RRN3P3	1377.155	-0.05836	0.318212	0.463619	transcribed_unprocessed_pseudo
ENSG0000(	MTNR1A	46.90396	0.22852	0.318329	0.46374	protein_coding
ENSG0000(	AKR1A1	1303.76	-0.04445	0.318413	0.463841	protein_coding
ENSG0000(	ENSG0000(	81.81052	-0.12107	0.318483	0.463921	lncRNA
ENSG0000(	TAF7L	21.23792	-0.17784	0.318583	0.464045	protein_coding
ENSG0000(	FAM13B-AS	10.02102	0.235246	0.318702	0.464196	lncRNA
ENSG0000(	FAM86JP	76.99884	0.131056	0.318753	0.464249	transcribed_unprocessed_pseudo
ENSG0000(	GOLGA8N	593.2024	0.101184	0.318934	0.46446	protein_coding
ENSG0000(	ITGA9	762.4699	-0.09348	0.318951	0.46446	protein_coding
ENSG0000(	LINC02137	55.25251	-0.1057	0.318932	0.46446	lncRNA
ENSG0000(	LIPC-AS1	107.3393	-0.09848	0.318957	0.46446	lncRNA
ENSG0000(	ASB18	440.5596	-0.0447	0.319111	0.464654	protein_coding
ENSG0000(	ZNF394	2577.772	0.030094	0.31912	0.464654	protein_coding

ENSG0000(	ENSG0000(	10.51099	0.255505	0.319145	0.464669	lncRNA
ENSG0000(	LINC02846	16.87929	0.184386	0.319209	0.46474	lncRNA
ENSG0000(	LPAR1	934.4828	-0.06184	0.319226	0.464743	protein_coding
ENSG0000(	ENSG0000(	24.30063	0.144836	0.319412	0.464992	lncRNA
ENSG0000(	KLRA1P	150.1322	0.118871	0.319569	0.465199	transcribed_unprocessed_pseudo
ENSG0000(	SETDB1	1817.815	-0.03201	0.319636	0.465276	protein_coding
ENSG0000(	ENSG0000(	124.2061	0.065685	0.319759	0.465432	lncRNA
ENSG0000(	ENSG0000(	18.71925	0.460143	0.319782	0.465444	lncRNA
ENSG0000(	ABLIM1	6488.785	-0.05544	0.319829	0.46547	protein_coding
ENSG0000(	PARP1	2223.052	0.070454	0.319826	0.46547	protein_coding
ENSG0000(	ACE2	741.6611	-0.16484	0.320165	0.465915	protein_coding
ENSG0000(	ENSG0000(	15.15253	-0.18737	0.320151	0.465915	lncRNA
ENSG0000(	C12orf43	754.6882	-0.0383	0.320181	0.465916	protein_coding
ENSG0000(	CDC37L1-D	26.15652	-0.17117	0.320217	0.465948	lncRNA
ENSG0000(	ENSG0000(	240.582	0.057532	0.320278	0.466007	lncRNA
ENSG0000(	ENSG0000(	495.914	0.045547	0.320302	0.466007	lncRNA
ENSG0000(	ENSG0000(	112.4479	0.210686	0.320293	0.466007	lncRNA
ENSG0000(	STUM	420.5608	-0.23851	0.320388	0.466088	protein_coding
ENSG0000(	TARBP2	308.9075	-0.06218	0.320385	0.466088	protein_coding
ENSG0000(	BCYRN1	523.1512	-0.09238	0.320423	0.466117	scRNA
ENSG0000(	LINC02016	110.706	0.10898	0.320452	0.466137	lncRNA
ENSG0000(	ENSG0000(	12.94099	0.223078	0.320487	0.466167	lncRNA
ENSG0000(	TMEM42	446.3699	0.052919	0.320533	0.466212	protein_coding
ENSG0000(	ALG14	1791.08	-0.10657	0.320614	0.466309	protein_coding
ENSG0000(	ENSG0000(	189.4208	0.098449	0.320643	0.466328	TEC
ENSG0000(	FHL5	78.33254	0.1959	0.320749	0.466461	protein_coding
ENSG0000(	LINC02472	29.00548	0.13794	0.320784	0.466491	lncRNA
ENSG0000(	ENSG0000(	74.79444	-0.11193	0.320972	0.466737	transcribed_unprocessed_pseudo
ENSG0000(	LINC01977	236.9013	0.063877	0.320983	0.466737	lncRNA
ENSG0000(	ENSG0000(	58.1998	-0.09438	0.321001	0.466741	lncRNA
ENSG0000(	ENSG0000(	22.79687	-0.16037	0.321059	0.466804	lncRNA
ENSG0000(	LINC02102	25.86223	0.211692	0.321104	0.466848	lncRNA
ENSG0000(	ENSG0000(	23.02664	-0.16961	0.321178	0.466934	snoRNA
ENSG0000(	RN7SKP16(	36.23375	0.134621	0.321386	0.467191	misc_RNA
ENSG0000(	SRD5A3-AS	645.4976	-0.03742	0.321388	0.467191	lncRNA
ENSG0000(	ZEB1-AS1	210.069	0.090254	0.3214	0.467191	lncRNA
ENSG0000(	ITGBL1	80.4608	0.143317	0.321474	0.467277	protein_coding
ENSG0000(	PIK3IP1-DT	17.2918	0.191177	0.321566	0.467388	lncRNA
ENSG0000(	ENSG0000(	88.00363	0.167699	0.321607	0.467426	lncRNA
ENSG0000(	ENSG0000(	60.89611	0.138117	0.321675	0.467503	lncRNA
ENSG0000(	ASPHD1	22.58042	0.264788	0.321691	0.467506	protein_coding
ENSG0000(	ENSG0000(	59.98038	0.14238	0.321751	0.467551	protein_coding
ENSG0000(	FUCA2	975.9147	-0.04289	0.321752	0.467551	protein_coding
ENSG0000(	EML2-AS1	26.3612	0.180497	0.321911	0.46765	lncRNA
ENSG0000(	ENSG0000(	9.077385	-0.2473	0.321847	0.46765	processed_pseudogene
ENSG0000(	ENSG0000(	112.9593	-0.12036	0.3219	0.46765	lncRNA
ENSG0000(	ENSG0000(	176.0987	-0.07254	0.321904	0.46765	lncRNA

ENSG0000(	PMS2P8	26.01607	-0.34503	0.321897	0.46765	unprocessed_pseudogene
ENSG0000(	RN7SL535F	93.81801	0.103877	0.321859	0.46765	misc_RNA
ENSG0000(	ENSG0000(	62.32564	0.132959	0.322022	0.467791	lncRNA
ENSG0000(	HPYR1	32.73322	0.150304	0.322053	0.467814	lncRNA
ENSG0000(	ENSG0000(	105.1672	-0.09554	0.32218	0.467977	lncRNA
ENSG0000(	LINC01044	52.99184	-0.11155	0.322242	0.468045	lncRNA
ENSG0000(	IFI27L2	72.40796	0.1676	0.322278	0.468076	protein_coding
ENSG0000(	CHST14	87.30555	0.097922	0.322319	0.468097	protein_coding
ENSG0000(	DCLRE1C	1853.032	0.048682	0.322323	0.468097	protein_coding
ENSG0000(	POT1	512.8313	0.07521	0.322359	0.468127	protein_coding
ENSG0000(	AASDH	565.4403	-0.08611	0.322498	0.468308	protein_coding
ENSG0000(	ENSG0000(	235.6947	-0.06799	0.322514	0.468309	lncRNA
ENSG0000(	SNORA71A	138.0033	-0.17316	0.322545	0.468333	snoRNA
ENSG0000(	CTRB1	30.18431	0.161007	0.322625	0.468415	protein_coding
ENSG0000(	RIBC2	38.59719	-0.23041	0.322632	0.468415	protein_coding
ENSG0000(	INTS3	3782.113	0.035377	0.322844	0.46868	protein_coding
ENSG0000(	LINC02746	116.8655	-0.15235	0.32284	0.46868	lncRNA
ENSG0000(	ENSG0000(	124.8595	0.077914	0.322891	0.468726	lncRNA
ENSG0000(	MTCYBP14	13.22837	0.218151	0.322909	0.46873	processed_pseudogene
ENSG0000(	ENSG0000(	417.2758	-0.07792	0.322945	0.468761	lncRNA
ENSG0000(	FHOD1	604.6187	-0.08372	0.322979	0.468789	protein_coding
ENSG0000(	CACNB2	1708.624	-0.07266	0.32302	0.468805	protein_coding
ENSG0000(	ENSG0000(	58.29604	-0.10336	0.323014	0.468805	lncRNA
ENSG0000(	GLRA1	67.54551	0.101432	0.32309	0.468885	protein_coding
ENSG0000(	APOBR	2062.227	-0.08921	0.323281	0.46914	protein_coding
ENSG0000(	BET1L	1065.243	0.048891	0.323361	0.469191	protein_coding
ENSG0000(	GALK2	1342.858	-0.05013	0.32334	0.469191	protein_coding
ENSG0000(	RTTN	3957.644	0.039781	0.323359	0.469191	protein_coding
ENSG0000(	RPL23AP49	23.96429	0.172212	0.323533	0.469418	transcribed_processed_pseudoge
ENSG0000(	HERC1	7368.085	-0.04009	0.323717	0.469663	protein_coding
ENSG0000(	IFIT1	332.5878	0.207123	0.323737	0.469672	protein_coding
ENSG0000(	ENSG0000(	601.4058	0.043616	0.323853	0.469802	lncRNA
ENSG0000(	ENSG0000(	352.5195	-0.04869	0.323903	0.469802	lncRNA
ENSG0000(	KCNQ5	254.0432	0.189082	0.323874	0.469802	protein_coding
ENSG0000(	MSH3	1863.684	-0.04036	0.323865	0.469802	protein_coding
ENSG0000(	RNU6-701F	13.39788	-0.1959	0.323893	0.469802	snRNA
ENSG0000(	BEX4	157.9605	-0.08935	0.324067	0.470003	protein_coding
ENSG0000(	LINC01979	17.70555	0.166384	0.324071	0.470003	lncRNA
ENSG0000(	MEIS2	826.2278	0.182291	0.324197	0.470163	protein_coding
ENSG0000(	IGHV1OR1!	12.30123	0.371036	0.324263	0.470237	IG_V_pseudogene
ENSG0000(	ENSG0000(	505.6609	-0.04285	0.324278	0.470238	lncRNA
ENSG0000(	GPAT4-AS1	37.15393	0.112245	0.324372	0.47033	lncRNA
ENSG0000(	LINGO3	10.36416	0.287574	0.32436	0.47033	protein_coding
ENSG0000(	CYP2C8	30.35054	0.200222	0.324432	0.470396	protein_coding
ENSG0000(	OR2T33	344.7423	0.062499	0.324458	0.470411	protein_coding
ENSG0000(	GRIK4	54.39354	0.269751	0.324533	0.470499	protein_coding
ENSG0000(	ENSG0000(	16.64891	-0.18153	0.3246	0.470564	lncRNA

ENSG0000(	FER	1817.395	-0.04831	0.324608	0.470564	protein_coding
ENSG0000(	ZRANB2-AS	343.0142	-0.09854	0.324724	0.47071	lncRNA
ENSG0000(	XKR5	137.4246	0.060826	0.324869	0.470898	protein_coding
ENSG0000(	ENSG0000(	177.0615	0.068334	0.324938	0.470962	lncRNA
ENSG0000(	TMEM212-	153.032	0.077822	0.324943	0.470962	lncRNA
ENSG0000(	ENSG0000(	27.30918	0.139422	0.32496	0.470965	lncRNA
ENSG0000(	ENO1-AS1	11.60627	0.229505	0.325074	0.471108	lncRNA
ENSG0000(	ENSG0000(	298.42	0.085529	0.325144	0.471187	lncRNA
ENSG0000(	POTEC	100.047	-0.15698	0.325226	0.471285	protein_coding
ENSG0000(	ENSG0000(	11.70102	0.202068	0.325424	0.47155	lncRNA
ENSG0000(	LINC00410	92.41639	-0.08615	0.325623	0.471817	lncRNA
ENSG0000(	CUL4B	12452.92	0.028664	0.325655	0.47184	protein_coding
ENSG0000(	GDF5-AS1	104.5643	0.079069	0.325678	0.471852	lncRNA
ENSG0000(	ARPIN	3497.077	-0.06276	0.325734	0.471894	protein_coding
ENSG0000(	ENSG0000(	31.52133	0.153485	0.325737	0.471894	transcribed_unprocessed_pseudo
ENSG0000(	ENSG0000(	114.4007	-0.08182	0.325787	0.471944	lncRNA
ENSG0000(	AHI1	5496.045	0.053881	0.325813	0.47196	protein_coding
ENSG0000(	ENSG0000(	40.17975	0.134685	0.325856	0.472001	TEC
ENSG0000(	ENSG0000(	150.4617	0.080189	0.325906	0.472052	lncRNA
ENSG0000(	ENSG0000(	56.57278	-0.23541	0.326085	0.472288	lncRNA
ENSG0000(	PAPOLG	1217.945	-0.06393	0.326141	0.472347	protein_coding
ENSG0000(	ENSG0000(	9.516457	0.257559	0.326187	0.472393	lncRNA
ENSG0000(	CAMK1D	2573.675	-0.05499	0.326231	0.472434	protein_coding
ENSG0000(	ENSG0000(	33.81361	-0.16902	0.326259	0.472453	processed_pseudogene
ENSG0000(	MYRFL	66.96484	-0.28511	0.326337	0.472522	protein_coding
ENSG0000(	ZNF354C	301.637	0.07726	0.326323	0.472522	protein_coding
ENSG0000(	AANAT	10.88825	0.279219	0.326359	0.472533	protein_coding
ENSG0000(	CHD7	4805.429	0.059426	0.32643	0.472613	protein_coding
ENSG0000(	ARVCF	265.2398	-0.14566	0.326472	0.472652	protein_coding
ENSG0000(	CCDC171	718.6603	-0.08157	0.32653	0.472715	protein_coding
ENSG0000(	ENSG0000(	779.1625	0.086321	0.326604	0.472755	lncRNA
ENSG0000(	ENSG0000(	15.12776	0.180557	0.326591	0.472755	lncRNA
ENSG0000(	PROSER1	1295.329	0.067343	0.326588	0.472755	protein_coding
ENSG0000(	ENSG0000(	47.6617	-0.12127	0.326632	0.472774	lncRNA
ENSG0000(	IGKV3D-11	28.63918	0.34648	0.326752	0.472926	IG_V_gene
ENSG0000(	ENSG0000(	268.8088	0.046561	0.326937	0.473152	lncRNA
ENSG0000(	STX18-IT1	56.66572	0.098212	0.326938	0.473152	lncRNA
ENSG0000(	ENSG0000(	143.4938	0.085523	0.326995	0.47319	lncRNA
ENSG0000(	SYT15	573.4385	0.075592	0.32699	0.47319	protein_coding
ENSG0000(	EXTL1	22.58338	-0.25891	0.327052	0.473241	protein_coding
ENSG0000(	TATDN3	644.4448	-0.05578	0.32706	0.473241	protein_coding
ENSG0000(	C2orf42	304.915	0.059993	0.327225	0.473435	protein_coding
ENSG0000(	PGM5P4-A	112.8091	0.085476	0.327211	0.473435	lncRNA
ENSG0000(	POLK	1613.57	0.053048	0.327304	0.473528	protein_coding
ENSG0000(	SERTAD4	212.1057	-0.15697	0.327328	0.473541	protein_coding
ENSG0000(	ENSG0000(	75.78609	-0.10032	0.32754	0.473825	lncRNA
ENSG0000(	ENSG0000(	47.19407	0.227784	0.327711	0.47403	processed_pseudogene

ENSG0000(	ENSG0000(	179.7772	0.059738	0.327698	0.47403	lncRNA
ENSG0000(	ENSG0000(	14.4807	0.195826	0.327789	0.474119	lncRNA
ENSG0000(	ENSG0000(	18.53395	0.173414	0.327863	0.474205	lncRNA
ENSG0000(	RND3	1426.309	0.106935	0.327883	0.474211	protein_coding
ENSG0000(	ENSG0000(	147.2221	-0.20979	0.327946	0.474272	lncRNA
ENSG0000(	MED12	3633.706	-0.02974	0.327955	0.474272	protein_coding
ENSG0000(	ENSG0000(	108.3316	-0.11899	0.32803	0.474358	lncRNA
ENSG0000(	HERC2P4	269.8056	0.1029	0.328163	0.474529	transcribed_unprocessed_pseudo
ENSG0000(	RBCK1	1694.901	0.0525	0.328272	0.474665	protein_coding
ENSG0000(	CDCP1	3070.31	-0.09687	0.328303	0.474687	protein_coding
ENSG0000(	NAV2	1559.486	-0.08481	0.328326	0.474699	protein_coding
ENSG0000(	CLEC18A	306.0668	-0.12278	0.328367	0.474737	protein_coding
ENSG0000(	RHBDD2	464.2589	0.080989	0.328446	0.474828	protein_coding
ENSG0000(	ENSG0000(	522.765	0.07991	0.328479	0.474855	lncRNA
ENSG0000(	ENSG0000(	14.0069	0.226767	0.328549	0.474934	lncRNA
ENSG0000(	ENSG0000(	16.69507	-0.25621	0.328651	0.475046	lncRNA
ENSG0000(	RN7SL388F	34.61281	0.132773	0.328657	0.475046	misc_RNA
ENSG0000(	ENSG0000(	232.3518	-0.10613	0.328778	0.475176	lncRNA
ENSG0000(	ENSG0000(	48.61561	0.114159	0.328773	0.475176	lncRNA
ENSG0000(	C1orf158	357.9168	0.047785	0.328803	0.47519	protein_coding
ENSG0000(	CNOT6L	2559.863	0.065299	0.328835	0.475194	protein_coding
ENSG0000(	OSTCP1	12.41415	0.213542	0.328835	0.475194	transcribed_processed_pseudoge
ENSG0000(	CSNK1G2P1	30.99694	0.178124	0.328859	0.475206	processed_pseudogene
ENSG0000(	CCDC40	403.7847	0.064604	0.328898	0.475241	protein_coding
ENSG0000(	ENSG0000(	12.5346	0.188501	0.328957	0.475303	lncRNA
ENSG0000(	CD27-AS1	826.5287	0.057294	0.329033	0.475385	lncRNA
ENSG0000(	FOXN3-AS1	18.67762	-0.16773	0.329044	0.475385	lncRNA
ENSG0000(	SAMD1	515.0964	-0.06424	0.329083	0.47542	protein_coding
ENSG0000(	ASS1P2	24.04361	-0.1599	0.329134	0.475472	processed_pseudogene
ENSG0000(	UCN	8.199995	0.25838	0.329153	0.475477	protein_coding
ENSG0000(	DERPC	459.6364	-0.14668	0.329299	0.475666	protein_coding
ENSG0000(	KL	62.42912	0.153523	0.329336	0.475697	protein_coding
ENSG0000(	CD46	6827.723	-0.04764	0.329356	0.475704	protein_coding
ENSG0000(	RN7SL790F	32.90051	0.159955	0.329407	0.475756	misc_RNA
ENSG0000(	ENSG0000(	41.5769	0.108256	0.329462	0.475814	lncRNA
ENSG0000(	PIK3CB	2069.068	0.057329	0.329667	0.476088	protein_coding
ENSG0000(	MIR3659H(	36.00396	-0.1509	0.329773	0.476218	lncRNA
ENSG0000(	LINC01990	23.36931	-0.15783	0.329811	0.476252	lncRNA
ENSG0000(	ENSG0000(	14.63104	0.200081	0.329828	0.476254	lncRNA
ENSG0000(	IL21	22.71631	0.362422	0.329989	0.476465	protein_coding
ENSG0000(	ENSG0000(	18.10496	0.1703	0.330037	0.476482	lncRNA
ENSG0000(	LINC02307	13.20587	0.267842	0.330062	0.476482	lncRNA
ENSG0000(	RNY4P37	13.09558	0.200226	0.330028	0.476482	misc_RNA
ENSG0000(	TMEM18	922.3257	0.077638	0.330053	0.476482	protein_coding
ENSG0000(	ENSG0000(	85.69458	0.093188	0.330341	0.476855	lncRNA
ENSG0000(	OR2T6	40.58861	0.109912	0.330351	0.476855	protein_coding
ENSG0000(	PRICKLE4	42.64963	-0.21365	0.3305	0.477035	protein_coding

ENSG0000(	SLIT3	552.9495	0.107526	0.330506	0.477035	protein_coding
ENSG0000(	LINC00623	159.745	0.120652	0.330533	0.477052	lncRNA
ENSG0000(	WDR1	5147.21	-0.06356	0.330685	0.477249	protein_coding
ENSG0000(	ENSG0000(	9.505906	0.318085	0.330787	0.477352	lncRNA
ENSG0000(	RN7SKP36	73.62304	-0.17738	0.330781	0.477352	misc_RNA
ENSG0000(	SMIM13	306.1055	-0.07535	0.330824	0.477384	protein_coding
ENSG0000(	ZNF316	1196.233	-0.06084	0.330841	0.477386	protein_coding
ENSG0000(	SAXO2	127.9444	-0.24896	0.330974	0.477556	protein_coding
ENSG0000(	RN7SL716F	131.2164	-0.08242	0.331026	0.477609	misc_RNA
ENSG0000(	KCNH3	29.3847	0.320731	0.331057	0.477632	protein_coding
ENSG0000(	CLCA2	44.18019	0.254639	0.331153	0.477726	protein_coding
ENSG0000(	ZNF653	136.0521	-0.08333	0.331139	0.477726	protein_coding
ENSG0000(	CMSS1	1680.235	0.037504	0.331196	0.477734	protein_coding
ENSG0000(	ENSG0000(	12.69686	-0.23209	0.331239	0.477734	lncRNA
ENSG0000(	ENSG0000(	31.21705	-0.13536	0.331296	0.477734	lncRNA
ENSG0000(	ENSG0000(	81.43306	0.142992	0.331214	0.477734	lncRNA
ENSG0000(	ENSG0000(	843.4921	0.054133	0.331265	0.477734	protein_coding
ENSG0000(	FAAH2	228.2274	-0.11077	0.331249	0.477734	protein_coding
ENSG0000(	OR10AC1	34.86234	-0.13434	0.331278	0.477734	polymorphic_pseudogene
ENSG0000(	RPL26P6	52.35945	0.095894	0.331176	0.477734	processed_pseudogene
ENSG0000(	WASF3-AS1	297.8886	0.051803	0.33129	0.477734	lncRNA
ENSG0000(	RBM47	7220.724	-0.08352	0.33138	0.477833	protein_coding
ENSG0000(	ENSG0000(	274.9045	0.08911	0.3314	0.47784	lncRNA
ENSG0000(	ENSG0000(	68.06139	-0.11946	0.331499	0.477961	lncRNA
ENSG0000(	RYR3-DT	77.22322	-0.14974	0.331551	0.478014	lncRNA
ENSG0000(	CRLF3	1128.334	0.047993	0.331597	0.478036	protein_coding
ENSG0000(	ENSG0000(	11.18367	0.199252	0.33159	0.478036	lncRNA
ENSG0000(	PKIA-AS1	126.8716	0.084318	0.331651	0.478092	lncRNA
ENSG0000(	GACAT3	214.5832	-0.05955	0.331707	0.47815	lncRNA
ENSG0000(	LRTM1	232.9587	0.164282	0.331744	0.478181	protein_coding
ENSG0000(	PNMA8C	305.1708	-0.04943	0.331759	0.478181	protein_coding
ENSG0000(	ENSG0000(	28.61142	-0.14223	0.3318	0.478218	lncRNA
ENSG0000(	CCDC24	395.1722	-0.07395	0.331854	0.478275	protein_coding
ENSG0000(	HMGN3	280.8703	-0.09275	0.331957	0.4784	protein_coding
ENSG0000(	BBLN	244.1855	-0.09783	0.332052	0.478451	protein_coding
ENSG0000(	EIPR1-IT1	62.13363	-0.11947	0.332069	0.478451	lncRNA
ENSG0000(	ENSG0000(	88.17617	-0.1082	0.332036	0.478451	lncRNA
ENSG0000(	ITLN2	15.2858	0.426585	0.332061	0.478451	protein_coding
ENSG0000(	LINC00640	10.02398	0.269508	0.332017	0.478451	lncRNA
ENSG0000(	ENSG0000(	208.2749	-0.07784	0.332183	0.478583	protein_coding
ENSG0000(	LINC02156	55.32317	0.134339	0.332191	0.478583	lncRNA
ENSG0000(	ENSG0000(	128.9178	-0.09566	0.332302	0.478651	lncRNA
ENSG0000(	PARP2	342.1402	0.054741	0.332289	0.478651	protein_coding
ENSG0000(	RN7SL418F	20.31368	0.164658	0.33226	0.478651	misc_RNA
ENSG0000(	RNA5SP104	14.58423	0.191695	0.332315	0.478651	rRNA_pseudogene
ENSG0000(	VEGFA	6119.389	0.097892	0.332298	0.478651	protein_coding
ENSG0000(	MT-TQ	152.1741	0.348068	0.332392	0.478741	Mt_tRNA

ENSG0000(	LY86	49.12815	0.240099	0.332448	0.4788	protein_coding
ENSG0000(	ENSG0000(	15.49824	-0.8165	0.332473	0.478813	protein_coding
ENSG0000(	AGMO	165.7365	-0.13905	0.332616	0.478975	protein_coding
ENSG0000(	LINC01275	66.69517	0.099104	0.332616	0.478975	lncRNA
ENSG0000(	ZNF446	223.7203	-0.07283	0.332634	0.478978	protein_coding
ENSG0000(	FAM118B	790.5779	-0.08224	0.332674	0.479015	protein_coding
ENSG0000(	LINC02076	55.3775	-0.09533	0.332695	0.479022	lncRNA
ENSG0000(	ZNF525	810.8542	-0.06363	0.332779	0.479122	protein_coding
ENSG0000(	ENSG0000(	68.13431	0.093711	0.332873	0.479212	lncRNA
ENSG0000(	MAP1LC3B	54.34875	0.109763	0.332859	0.479212	protein_coding
ENSG0000(	KIAA1614-/	71.39076	0.090099	0.332953	0.479305	lncRNA
ENSG0000(	ENSG0000(	114.2352	-0.12005	0.333025	0.479388	lncRNA
ENSG0000(	MROH2A	94.74904	-0.4259	0.333075	0.479437	protein_coding
ENSG0000(	ENSG0000(	320.6898	-0.0655	0.333125	0.479488	lncRNA
ENSG0000(	DNAJC3	2653.223	-0.08038	0.33324	0.479631	protein_coding
ENSG0000(	ENSG0000(	16.96773	-0.17726	0.333327	0.479734	TEC
ENSG0000(	SLC20A2	2207.21	-0.09592	0.333347	0.479741	protein_coding
ENSG0000(	ICA1L	761.3337	-0.06624	0.333399	0.479793	protein_coding
ENSG0000(	SUGT1P4-S	105.0697	-0.18152	0.333463	0.479863	lncRNA
ENSG0000(	COPS7B	1387.685	-0.04717	0.333501	0.479895	protein_coding
ENSG0000(	ENSG0000(	165.9613	0.066004	0.333519	0.479899	lncRNA
ENSG0000(	ENSG0000(	149.2443	-0.06216	0.33354	0.479907	lncRNA
ENSG0000(	KC6	39.44398	0.165311	0.333563	0.479919	lncRNA
ENSG0000(	CYB561D2	247.1046	-0.07066	0.333604	0.479955	protein_coding
ENSG0000(	ENSG0000(	78.58455	-0.08964	0.333646	0.479976	lncRNA
ENSG0000(	PPP1R3B	1052.133	0.081474	0.333649	0.479976	protein_coding
ENSG0000(	ENSG0000(	23.82778	-0.17572	0.333675	0.479992	lncRNA
ENSG0000(	TMPRSS2	7335.636	-0.15273	0.333861	0.480237	protein_coding
ENSG0000(	CHML	2390.677	0.100136	0.333889	0.480256	protein_coding
ENSG0000(	CHKB	1459.434	-0.05349	0.333967	0.480329	protein_coding
ENSG0000(	OSTN-AS1	94.66372	-0.07234	0.333971	0.480329	lncRNA
ENSG0000(	NLRC3	1196.025	0.117893	0.334023	0.480382	protein_coding
ENSG0000(	WASHC4	2750.664	0.067712	0.334038	0.480382	protein_coding
ENSG0000(	EFCAB11	938.7944	-0.045	0.334062	0.480394	protein_coding
ENSG0000(	ENSG0000(	207.4893	0.06025	0.334103	0.480414	lncRNA
ENSG0000(	ENSG0000(	54.63215	0.115097	0.334107	0.480414	lncRNA
ENSG0000(	EIF5AP3	31.67843	-0.11924	0.334146	0.480448	processed_pseudogene
ENSG0000(	UPP2	28.61476	0.159011	0.334279	0.480618	protein_coding
ENSG0000(	RPS20P22	252.5271	0.071057	0.334309	0.480639	transcribed_processed_pseudoge
ENSG0000(	CFAP418	241.2348	-0.07131	0.334347	0.480671	protein_coding
ENSG0000(	KIAA1586	246.6619	0.086794	0.33438	0.480696	protein_coding
ENSG0000(	ENSG0000(	65.2444	0.097662	0.334452	0.480755	lncRNA
ENSG0000(	RN7SL625F	85.23873	0.084482	0.334446	0.480755	misc_RNA
ENSG0000(	ENSG0000(	17.30965	-0.15595	0.334506	0.480811	lncRNA
ENSG0000(	ENSG0000(	74.41604	0.117631	0.334639	0.48098	lncRNA
ENSG0000(	SUCLG2-AS	1932.631	-0.0446	0.334741	0.481104	lncRNA
ENSG0000(	RHEBL1	16.50991	0.228297	0.334763	0.481114	protein_coding

ENSG0000(	MYPOP	166.1347	0.072292	0.334921	0.481319	protein_coding
ENSG0000(	ENSG0000(	311.3151	-0.07607	0.335099	0.481553	lncRNA
ENSG0000(	OR13I1P	53.74588	-0.09756	0.33517	0.481633	unprocessed_pseudogene
ENSG0000(	ENSG0000(	28.51033	-0.40055	0.335221	0.481683	transcribed_processed_pseudoge
ENSG0000(	ENSG0000(	303.4038	0.046199	0.335271	0.481733	TEC
ENSG0000(	NEFM	13.25279	0.364951	0.335317	0.481778	protein_coding
ENSG0000(	ENSG0000(	22.623	-0.1633	0.335401	0.481876	lncRNA
ENSG0000(	SLC6A19	228.5592	0.449254	0.335423	0.481886	protein_coding
ENSG0000(	ENSG0000(	14.39933	0.225506	0.335453	0.481906	lncRNA
ENSG0000(	IFITM4P	12.38245	0.270495	0.335472	0.481911	processed_pseudogene
ENSG0000(	ENSG0000(	41.67077	-0.1264	0.335581	0.482045	lncRNA
ENSG0000(	ENSG0000(	51.34407	-0.11865	0.335633	0.482076	lncRNA
ENSG0000(	MIR9-1HG	639.788	-0.05308	0.335619	0.482076	lncRNA
ENSG0000(	TBC1D3P5	13.40024	-0.22116	0.335758	0.482234	transcribed_unprocessed_pseudo
ENSG0000(	ENSG0000(	13.34405	1.063038	0.335911	0.482409	lncRNA
ENSG0000(	TAS2R6P	64.15831	0.113187	0.335896	0.482409	processed_pseudogene
ENSG0000(	MYO1B	2362.524	0.061341	0.33599	0.4825	protein_coding
ENSG0000(	AMOTL1	821.6644	0.124044	0.336044	0.482555	protein_coding
ENSG0000(	TIMP4	13.51621	-0.24352	0.336109	0.482627	protein_coding
ENSG0000(	RXFP1	41.70248	-0.17553	0.336185	0.482714	protein_coding
ENSG0000(	ENSG0000(	26.4854	0.135692	0.336341	0.482898	lncRNA
ENSG0000(	ENSG0000(	17.73307	-0.17133	0.336375	0.482898	lncRNA
ENSG0000(	MEGF10	59.26725	0.274872	0.336369	0.482898	protein_coding
ENSG0000(	NME6	1711.888	-0.0368	0.336372	0.482898	protein_coding
ENSG0000(	ENSG0000(	37.664	0.142425	0.336437	0.48296	lncRNA
ENSG0000(	ERFL	31.99973	-0.16605	0.336449	0.48296	protein_coding
ENSG0000(	CEACAM16	592.0788	0.103562	0.33647	0.482969	lncRNA
ENSG0000(	DLG4	926.7073	0.074881	0.336495	0.482981	protein_coding
ENSG0000(	TRHDE	483.2306	-0.18762	0.336528	0.483007	protein_coding
ENSG0000(	PFKFB1	21.70572	0.153186	0.336594	0.483058	protein_coding
ENSG0000(	SLC14A1	322.5301	0.073379	0.336588	0.483058	protein_coding
ENSG0000(	ENSG0000(	170.3393	0.082094	0.336616	0.483065	lncRNA
ENSG0000(	TMEM161E	11153.07	0.054484	0.33663	0.483065	lncRNA
ENSG0000(	TRIM7	230.3844	-0.10749	0.336767	0.48324	protein_coding
ENSG0000(	ENSG0000(	62.78236	-0.09741	0.336788	0.483247	transcribed_processed_pseudoge
ENSG0000(	RN7SL58P	46.39031	0.124255	0.336836	0.483271	misc_RNA
ENSG0000(	TP53INP2	3230.796	-0.18415	0.336826	0.483271	protein_coding
ENSG0000(	EIF3EP2	130.8088	-0.08246	0.336928	0.483381	processed_pseudogene
ENSG0000(	HSPE1P28	50.32952	0.114038	0.336983	0.483393	processed_pseudogene
ENSG0000(	RN7SL423F	13.77828	0.195135	0.336956	0.483393	misc_RNA
ENSG0000(	ULK4P3	12.56217	-0.36347	0.336972	0.483393	transcribed_unprocessed_pseudo
ENSG0000(	NDUFAF6	632.7724	0.053716	0.337111	0.483556	protein_coding
ENSG0000(	FFAR4	572.1675	0.087131	0.337166	0.483613	protein_coding
ENSG0000(	BRD9	1578.027	-0.04496	0.337186	0.483618	protein_coding
ENSG0000(	JCHAIN	17047.96	0.206204	0.337243	0.483678	protein_coding
ENSG0000(	ENSG0000(	8.357538	0.342136	0.337291	0.483725	lncRNA
ENSG0000(	ENSG0000(	29.14192	0.123855	0.337394	0.48385	lncRNA

ENSG0000(	ENPP4	668.7569	-0.07194	0.337608	0.484112	protein_coding
ENSG0000(	ENSG0000(	21.11191	0.185456	0.337601	0.484112	lncRNA
ENSG0000(	ENSG0000(	25.04572	0.149839	0.337791	0.484353	lncRNA
ENSG0000(	CTD-3080P	73.78464	0.114953	0.338095	0.484767	lncRNA
ENSG0000(	AKAP9	13283.29	0.050701	0.338185	0.484874	protein_coding
ENSG0000(	SERGEF	390.9874	0.049885	0.338265	0.484966	protein_coding
ENSG0000(	PEX11G	37.22802	-0.14774	0.338346	0.48506	protein_coding
ENSG0000(	LAMTOR5P	28.40899	-0.16766	0.338445	0.485174	processed_pseudogene
ENSG0000(	RN7SL30P	36.77464	0.123479	0.338457	0.485174	misc_RNA
ENSG0000(	RUSC1-AS1	341.9634	0.071318	0.338499	0.485212	lncRNA
ENSG0000(	RN7SL589F	53.75936	0.094312	0.338536	0.485233	misc_RNA
ENSG0000(	TRAJ25	14.35481	0.245688	0.338544	0.485233	TR_J_gene
ENSG0000(	NEFH	17.01738	0.230245	0.338566	0.485241	protein_coding
ENSG0000(	SUPT3H	258.5074	0.067337	0.338636	0.48532	protein_coding
ENSG0000(	RN7SL612F	25.94183	0.137782	0.33877	0.48549	misc_RNA
ENSG0000(	ENSG0000(	39.3971	-0.14401	0.338918	0.485679	lncRNA
ENSG0000(	GNAZ	35.66834	-0.23275	0.338974	0.485715	protein_coding
ENSG0000(	TAF3	1622.423	0.042473	0.338961	0.485715	protein_coding
ENSG0000(	VWA2	596.7351	0.074675	0.339033	0.485778	protein_coding
ENSG0000(	IGFBP4	1787.429	0.132933	0.339108	0.485863	protein_coding
ENSG0000(	MAB21L3	14523.67	-0.05859	0.339126	0.485866	protein_coding
ENSG0000(	ENSG0000(	8.695928	0.230026	0.339188	0.485909	lncRNA
ENSG0000(	ENSG0000(	25.64123	0.264817	0.339176	0.485909	lncRNA
ENSG0000(	KIF1A	164.4504	-0.26808	0.339218	0.485909	protein_coding
ENSG0000(	PRR34	84.37514	0.087323	0.339206	0.485909	lncRNA
ENSG0000(	ADAD2	46.19425	-0.11484	0.339243	0.485922	protein_coding
ENSG0000(	ENSG0000(	585.0688	0.049299	0.339282	0.485934	lncRNA
ENSG0000(	WDR97	324.6009	-0.06703	0.339277	0.485934	protein_coding
ENSG0000(	ENSG0000(	113.1122	-0.07453	0.339429	0.486121	lncRNA
ENSG0000(	SREK1IP1	947.7462	0.041806	0.339543	0.486264	protein_coding
ENSG0000(	C17orf113	380.2089	-0.05444	0.339585	0.4863	protein_coding
ENSG0000(	NLGN1	51.43181	-0.19414	0.339658	0.486384	protein_coding
ENSG0000(	HID1	1021.555	0.073382	0.339684	0.486398	protein_coding
ENSG0000(	LINC02899	210.5493	0.125902	0.339708	0.48641	lncRNA
ENSG0000(	B9D1	254.6791	0.082008	0.339764	0.486468	protein_coding
ENSG0000(	IGHV5-51	477.6277	0.223079	0.340046	0.48685	IG_V_gene
ENSG0000(	ENSG0000(	10.28588	0.323814	0.340064	0.486853	lncRNA
ENSG0000(	ENSG0000(	11.56573	0.240595	0.340146	0.486949	lncRNA
ENSG0000(	ENSG0000(	25.34015	-0.15132	0.340222	0.487013	lncRNA
ENSG0000(	RHEX	62.86888	0.150672	0.340211	0.487013	protein_coding
ENSG0000(	GPR82	905.7278	0.060925	0.340277	0.487046	protein_coding
ENSG0000(	NGF	10.45841	0.337958	0.340272	0.487046	protein_coding
ENSG0000(	ENSG0000(	90.0229	0.075018	0.340336	0.487108	lncRNA
ENSG0000(	TNIP2	521.1014	0.058397	0.340394	0.487169	protein_coding
ENSG0000(	ENSG0000(	83.94317	0.080207	0.340473	0.48726	lncRNA
ENSG0000(	GJA1	423.7938	0.152699	0.340535	0.487327	protein_coding
ENSG0000(	TBC1D27P	463.6627	0.081561	0.340561	0.487342	transcribed_unprocessed_pseudo

ENSG0000(	ENSG0000(	224.3323	0.064445	0.340581	0.487347	lncRNA
ENSG0000(	MAGIX	326.7857	-0.07378	0.340622	0.487385	protein_coding
ENSG0000(	SNORA21	227.5527	-0.11402	0.340725	0.487509	snoRNA
ENSG0000(	ARHGEF7	3035.776	0.037856	0.340804	0.487556	protein_coding
ENSG0000(	ENSG0000(	18.16231	0.159407	0.34079	0.487556	lncRNA
ENSG0000(	RN7SL586F	9.280216	-0.23759	0.340803	0.487556	misc_RNA
ENSG0000(	SNORA63	1855.444	-0.1226	0.34087	0.487627	snoRNA
ENSG0000(	KCNA6	84.76746	0.16296	0.340904	0.487654	protein_coding
ENSG0000(	LINC01997	352.5141	0.069589	0.340957	0.487707	lncRNA
ENSG0000(	ENSG0000(	178.794	0.060464	0.341273	0.488137	lncRNA
ENSG0000(	FAM228B	2179.302	-0.06288	0.341356	0.488182	protein_coding
ENSG0000(	HOXA11-AS	159.1958	-0.35771	0.341367	0.488182	lncRNA
ENSG0000(	KCNK17	22.285	0.168995	0.341344	0.488182	protein_coding
ENSG0000(	LINC02756	27.62915	0.17601	0.341353	0.488182	lncRNA
ENSG0000(	IQCE	1348.476	-0.06491	0.341419	0.488235	protein_coding
ENSG0000(	TIGAR	1256.992	0.084709	0.341554	0.488405	protein_coding
ENSG0000(	RAD51AP2	14.14422	-0.21602	0.341661	0.488536	protein_coding
ENSG0000(	IGHV5-10-1	124.0459	0.522374	0.341827	0.488729	IG_V_gene
ENSG0000(	RN7SL4P	110.4039	0.160242	0.341825	0.488729	misc_RNA
ENSG0000(	ATAD2B	3511.887	-0.03737	0.341894	0.488735	protein_coding
ENSG0000(	ENSG0000(	569.1017	-0.077	0.341851	0.488735	transcribed_unprocessed_pseudo
ENSG0000(	ENSG0000(	19.14406	0.642469	0.341881	0.488735	processed_pseudogene
ENSG0000(	MRAP2	219.5642	0.104348	0.341874	0.488735	protein_coding
ENSG0000(	ARFGEF2	5035.214	-0.04191	0.341987	0.488818	protein_coding
ENSG0000(	CD22	1530.532	0.254915	0.341999	0.488818	protein_coding
ENSG0000(	HOXB-AS1	50.72241	0.157468	0.341985	0.488818	lncRNA
ENSG0000(	CIART	275.8546	-0.06544	0.342056	0.488842	protein_coding
ENSG0000(	GPR119	243.7869	-0.05405	0.342063	0.488842	protein_coding
ENSG0000(	RN7SKP175	17.2751	0.992074	0.342039	0.488842	misc_RNA
ENSG0000(	GDPD4	17.85799	0.222475	0.342138	0.488927	protein_coding
ENSG0000(	ENSG0000(	61.54481	0.167083	0.342248	0.488995	lncRNA
ENSG0000(	FER1L6	1227.023	-0.16667	0.342219	0.488995	protein_coding
ENSG0000(	HCST	27.11297	-0.22432	0.342218	0.488995	protein_coding
ENSG0000(	ZNF667-AS	942.088	-0.03571	0.342244	0.488995	lncRNA
ENSG0000(	LINC01374	9.001785	0.271865	0.34234	0.489105	lncRNA
ENSG0000(	ENSG0000(	8.900844	-0.19946	0.342426	0.489183	snoRNA
ENSG0000(	UBE2Q2P1	419.3057	0.055349	0.342417	0.489183	transcribed_unprocessed_pseudo
ENSG0000(	ZNF222-DT	12.28254	0.198472	0.342562	0.489354	lncRNA
ENSG0000(	ENSG0000(	29.24664	0.218501	0.342581	0.489359	lncRNA
ENSG0000(	ENSG0000(	97.87915	-0.1873	0.34262	0.489393	lncRNA
ENSG0000(	PLCL2	1372.007	0.075034	0.342653	0.489417	protein_coding
ENSG0000(	ENSG0000(	129.1397	0.093237	0.34267	0.489419	lncRNA
ENSG0000(	TRIM31-AS	10.73305	0.225386	0.34269	0.489425	lncRNA
ENSG0000(	RN7SL693F	253.8475	-0.0736	0.342757	0.489499	misc_RNA
ENSG0000(	COL6A6	40.10547	0.840567	0.3429	0.489666	protein_coding
ENSG0000(	GPR176-DT	185.7568	0.063677	0.342937	0.489666	lncRNA
ENSG0000(	IGHV4-61	468.769	0.240139	0.342914	0.489666	IG_V_gene

ENSG0000(	LCMT1-AS1	294.9149	-0.0646	0.342924	0.489666	lncRNA
ENSG0000(	ENSG0000(	120.8378	-0.07567	0.343035	0.489717	lncRNA
ENSG0000(	ENSG0000(	128.3707	-0.07353	0.343025	0.489717	TEC
ENSG0000(	SHISA3	14.44618	-0.35881	0.343026	0.489717	protein_coding
ENSG0000(	ZNF658	308.9288	-0.09353	0.343	0.489717	protein_coding
ENSG0000(	CCR4	704.1155	0.049459	0.343101	0.489733	protein_coding
ENSG0000(	GTF2IP5	21.85396	-0.17572	0.343109	0.489733	unprocessed_pseudogene
ENSG0000(	LINC01933	106.3891	0.105167	0.343093	0.489733	lncRNA
ENSG0000(	PYGL	810.9441	0.080319	0.343101	0.489733	protein_coding
ENSG0000(	ENSG0000(	15.93344	-0.21354	0.34319	0.489803	lncRNA
ENSG0000(	SOX4	1252.487	0.090313	0.343182	0.489803	protein_coding
ENSG0000(	ENSG0000(	393.8316	0.05537	0.343236	0.489846	protein_coding
ENSG0000(	ENSG0000(	579.647	-0.05265	0.343251	0.489846	lncRNA
ENSG0000(	KANK1	5566.226	-0.07942	0.343308	0.489861	protein_coding
ENSG0000(	PGM5P2	516.9139	-0.06747	0.343296	0.489861	transcribed_unprocessed_pseudo
ENSG0000(	SEPTIN5	51.6228	-0.1851	0.343295	0.489861	protein_coding
ENSG0000(	MUC5B	9877.634	-0.20043	0.343394	0.489961	protein_coding
ENSG0000(	TCEAL4	218.6058	0.092599	0.343451	0.49002	protein_coding
ENSG0000(	LINC02580	223.3362	0.120974	0.343477	0.490035	lncRNA
ENSG0000(	LBHD1	659.0648	0.039863	0.343564	0.490136	protein_coding
ENSG0000(	PMS2P4	2052.011	0.033899	0.343594	0.490157	transcribed_unprocessed_pseudo
ENSG0000(	ENSG0000(	12.15687	-0.91121	0.343766	0.490379	lncRNA
ENSG0000(	TMBIM4	1597.724	-0.05285	0.343791	0.490393	protein_coding
ENSG0000(	ENSG0000(	28.61838	0.206685	0.343981	0.490619	lncRNA
ENSG0000(	ZC3H12C	2284.385	0.085901	0.343979	0.490619	protein_coding
ENSG0000(	MT-TG	51.74328	0.42494	0.344046	0.49069	Mt_tRNA
ENSG0000(	ENSG0000(	16.89808	0.190176	0.344108	0.490756	lncRNA
ENSG0000(	ENSG0000(	50.83621	0.102907	0.344129	0.490764	lncRNA
ENSG0000(	CLTRN	36.93663	-0.11497	0.34419	0.490827	protein_coding
ENSG0000(	NXPE1	8703.892	-0.18791	0.344244	0.490882	protein_coding
ENSG0000(	PITX1	266.9618	-0.11582	0.344305	0.490946	protein_coding
ENSG0000(	SEMA6A	5333.918	-0.12299	0.344321	0.490947	protein_coding
ENSG0000(	SERTAD2	1206.058	-0.08652	0.344366	0.490967	protein_coding
ENSG0000(	SNTN	232.9615	-0.06878	0.344355	0.490967	protein_coding
ENSG0000(	ENSG0000(	31.86318	0.241919	0.344395	0.490985	unprocessed_pseudogene
ENSG0000(	HERPUD2	897.9981	0.060938	0.344441	0.491029	protein_coding
ENSG0000(	HSF5	37.35401	0.24024	0.344537	0.491144	protein_coding
ENSG0000(	ENSG0000(	69.87291	0.098416	0.344602	0.491147	lncRNA
ENSG0000(	LINC01311	67.87938	0.112488	0.344584	0.491147	lncRNA
ENSG0000(	SHISA8	9.271627	0.379884	0.344589	0.491147	protein_coding
ENSG0000(	ZRSR2	525.3224	-0.06755	0.344584	0.491147	protein_coding
ENSG0000(	ENSG0000(	518.1165	0.067864	0.344643	0.491183	lncRNA
ENSG0000(	RHD	285.9929	-0.05462	0.344666	0.491193	protein_coding
ENSG0000(	PHKA2	1596.974	0.067588	0.34479	0.491347	protein_coding
ENSG0000(	MALAT1	1526934	0.073191	0.344821	0.49137	lncRNA
ENSG0000(	ENSG0000(	177.5488	-0.06347	0.344913	0.491478	TEC
ENSG0000(	DDX25	426.1801	-0.04691	0.345072	0.491658	protein_coding

ENSG0000(	FZD2	132.4638	0.105325	0.345079	0.491658	protein_coding
ENSG0000(	LIPE	148.6486	-0.097	0.345087	0.491658	protein_coding
ENSG0000(	ENSG0000(	384.8958	-0.04284	0.345148	0.491723	lncRNA
ENSG0000(	ENSG0000(	192.3425	-0.06372	0.345173	0.491736	lncRNA
ENSG0000(	STT3A	1794.994	-0.05669	0.345196	0.491747	protein_coding
ENSG0000(	ENSG0000(	37.9676	0.101096	0.345217	0.491755	lncRNA
ENSG0000(	LPAR2	370.9551	0.104779	0.345305	0.491857	protein_coding
ENSG0000(	FERMT3	468.6547	0.10387	0.345488	0.492095	protein_coding
ENSG0000(	ENSG0000(	19.14815	0.250334	0.345562	0.492133	lncRNA
ENSG0000(	ENSG0000(	10.64557	0.247775	0.345531	0.492133	processed_pseudogene
ENSG0000(	ENSG0000(	9.256036	-0.23423	0.345552	0.492133	lncRNA
ENSG0000(	NDUFV2P1	10.94208	-0.28134	0.345648	0.49222	processed_pseudogene
ENSG0000(	PSG10P	40.66352	0.243173	0.345654	0.49222	transcribed_unprocessed_pseudo
ENSG0000(	PRKAA2	281.02	-0.11935	0.345795	0.492398	protein_coding
ENSG0000(	ENSG0000(	17.16945	0.134305	0.345839	0.492438	lncRNA
ENSG0000(	DYNLRB2-A	122.4072	0.120709	0.345891	0.492445	lncRNA
ENSG0000(	RNU2-48P	14.87688	-0.24538	0.345871	0.492445	snRNA
ENSG0000(	TXLNG	630.5753	-0.04707	0.345887	0.492445	protein_coding
ENSG0000(	GANC	1839.912	0.064322	0.345908	0.492447	protein_coding
ENSG0000(	EIF2B4	746.7739	-0.05012	0.345994	0.492529	protein_coding
ENSG0000(	PALD1	278.5284	0.098236	0.345997	0.492529	protein_coding
ENSG0000(	ENSG0000(	35.60538	0.146329	0.346125	0.492689	lncRNA
ENSG0000(	ENSG0000(	18.11276	0.197207	0.346171	0.492732	lncRNA
ENSG0000(	ENSG0000(	28.20931	-0.11686	0.346305	0.492899	lncRNA
ENSG0000(	ENSG0000(	98.79332	-0.08044	0.346361	0.492957	lncRNA
ENSG0000(	ENSG0000(	17.4494	0.224297	0.346693	0.493408	lncRNA
ENSG0000(	MT1E	468.3539	-0.21822	0.346729	0.493437	protein_coding
ENSG0000(	LINC01968	168.3624	-0.07079	0.346848	0.493583	lncRNA
ENSG0000(	CETP	104.4253	0.096168	0.346917	0.493658	protein_coding
ENSG0000(	OR2T12	1444.679	-0.0455	0.346942	0.493672	protein_coding
ENSG0000(	ENSG0000(	25.25617	-0.25742	0.347011	0.493675	lncRNA
ENSG0000(	ENSG0000(	196.1061	-0.07824	0.347017	0.493675	lncRNA
ENSG0000(	EYA2	484.0502	-0.21516	0.347015	0.493675	protein_coding
ENSG0000(	PCSK6-AS1	64.98799	-0.12039	0.347023	0.493675	lncRNA
ENSG0000(	RNF40	1450.481	-0.05755	0.346974	0.493675	protein_coding
ENSG0000(	PEG13	388.9974	-0.05676	0.347078	0.493731	lncRNA
ENSG0000(	TRIM61	274.2649	-0.06722	0.347111	0.493755	protein_coding
ENSG0000(	DLEU1	1516.222	-0.08618	0.347189	0.493843	lncRNA
ENSG0000(	C16orf95-D	18.18823	-0.19645	0.347258	0.493905	lncRNA
ENSG0000(	PODXL	983.9119	0.094191	0.347264	0.493905	protein_coding
ENSG0000(	PIEZO1	3404.883	0.068801	0.347359	0.494018	protein_coding
ENSG0000(	ENSG0000(	28.80598	0.151657	0.347379	0.494024	lncRNA
ENSG0000(	SLC6A16	249.1689	-0.09013	0.347406	0.49404	protein_coding
ENSG0000(	MIR205HG	103.1005	-0.0767	0.347468	0.494105	lncRNA
ENSG0000(	ARFGEF1	5630.36	-0.04158	0.347619	0.494297	protein_coding
ENSG0000(	ENSG0000(	130.6502	-0.13126	0.347667	0.494344	lncRNA
ENSG0000(	ENSG0000(	334.124	0.064829	0.347686	0.494348	lncRNA

ENSG0000(	ANKRD20A	285.5534	0.223035	0.347717	0.494369	protein_coding
ENSG0000(	ENSG0000(	5175.575	-0.02265	0.34774	0.49438	lncRNA
ENSG0000(	KCNH2	120.2716	-0.13467	0.347952	0.494658	protein_coding
ENSG0000(	ENSG0000(	233.115	-0.07566	0.348005	0.494689	lncRNA
ENSG0000(	ENSG0000(	13.40332	-0.23398	0.347989	0.494689	lncRNA
ENSG0000(	ENSG0000(	28.66137	0.152028	0.34802	0.494689	lncRNA
ENSG0000(	LENG9	188.6081	-0.09045	0.348207	0.494931	protein_coding
ENSG0000(	ENSG0000(	31.06595	-0.12719	0.348294	0.495032	lncRNA
ENSG0000(	ENSG0000(	10.43243	0.289175	0.348392	0.495149	transcribed_processed_pseudoge
ENSG0000(	HEATR3-AS	10.19201	0.219805	0.348604	0.495428	lncRNA
ENSG0000(	ENSG0000(	96.51788	-0.09146	0.348642	0.495447	lncRNA
ENSG0000(	ENSG0000(	60.92159	0.102	0.348681	0.495447	lncRNA
ENSG0000(	ENSG0000(	146.759	0.054738	0.348666	0.495447	lncRNA
ENSG0000(	TMEM231F	190.8657	-0.14959	0.348653	0.495447	transcribed_unprocessed_pseudo
ENSG0000(	STK31	208.2689	0.083212	0.348719	0.49548	protein_coding
ENSG0000(	ENSG0000(	72.41597	0.121295	0.34882	0.495588	transcribed_unprocessed_pseudo
ENSG0000(	KCNJ13	13.65782	0.416503	0.348828	0.495588	protein_coding
ENSG0000(	RN7SL274F	257.5963	0.065383	0.348848	0.495595	misc_RNA
ENSG0000(	ENSG0000(	256.9152	0.058088	0.348961	0.495732	lncRNA
ENSG0000(	SNORD116	12.99949	0.309694	0.349052	0.49584	snoRNA
ENSG0000(	DUSP28	490.0338	-0.0455	0.349158	0.495968	protein_coding
ENSG0000(	KCTD19	319.6405	0.076262	0.3492	0.495982	protein_coding
ENSG0000(	ZFAND2A	270.7315	-0.09406	0.349197	0.495982	protein_coding
ENSG0000(	MEX3A	78.28996	-0.14226	0.349309	0.496092	protein_coding
ENSG0000(	TCAP	20.52344	-0.20316	0.349302	0.496092	protein_coding
ENSG0000(	ALG1L13P	369.112	0.059909	0.349354	0.496132	transcribed_unprocessed_pseudo
ENSG0000(	NKX6-3	12.23338	-0.33423	0.349536	0.49637	protein_coding
ENSG0000(	SCAMP5	341.8962	0.146007	0.349567	0.49639	protein_coding
ENSG0000(	ENSG0000(	387.2594	-0.04718	0.349664	0.496483	lncRNA
ENSG0000(	OFD1	3840.6	-0.0453	0.349655	0.496483	protein_coding
ENSG0000(	IBTK	2618.737	-0.05693	0.349734	0.49656	protein_coding
ENSG0000(	ERP27	80.3216	0.178094	0.349833	0.496677	protein_coding
ENSG0000(	LCT-AS1	15.92396	0.178288	0.349884	0.496727	lncRNA
ENSG0000(	ENSG0000(	852.7419	0.059579	0.349979	0.49684	lncRNA
ENSG0000(	CCDC69	1427.539	0.079311	0.350076	0.496955	protein_coding
ENSG0000(	ENSG0000(	547.471	-0.12664	0.350115	0.496988	processed_pseudogene
ENSG0000(	DNAJC7	3766.038	0.029772	0.350251	0.497158	protein_coding
ENSG0000(	DEF8	1082.506	0.081957	0.35032	0.497201	protein_coding
ENSG0000(	ENSG0000(	9.933213	0.466824	0.350338	0.497201	lncRNA
ENSG0000(	ENSG0000(	152.9309	-0.17113	0.350314	0.497201	protein_coding
ENSG0000(	RNU2-55P	42.01797	0.139652	0.350344	0.497201	snRNA
ENSG0000(	ENSG0000(	50.30454	0.103818	0.350444	0.49732	lncRNA
ENSG0000(	CTTNBP2NI	3665.927	0.08536	0.350681	0.497611	protein_coding
ENSG0000(	ENSG0000(	59.4069	0.089834	0.350674	0.497611	lncRNA
ENSG0000(	ENSG0000(	14.92464	0.205431	0.350755	0.49767	lncRNA
ENSG0000(	ENSG0000(	139.7161	0.067408	0.350746	0.49767	lncRNA
ENSG0000(	DCHS2	283.3729	0.118473	0.350865	0.497804	protein_coding

ENSG0000(	CUX1	3886.596	-0.03782	0.350933	0.497878	protein_coding
ENSG0000(	LIF-AS1	30.65824	0.107202	0.350972	0.497889	lncRNA
ENSG0000(	SSTR5-AS1	74.29127	-0.14372	0.350964	0.497889	lncRNA
ENSG0000(	GRIN1	93.89909	0.218618	0.351036	0.497957	protein_coding
ENSG0000(	RN7SL271F	12.07002	-0.16388	0.35121	0.49818	misc_RNA
ENSG0000(	ANKRD36B	2462.115	0.061215	0.351267	0.498227	protein_coding
ENSG0000(	LINC00540	112.2072	-0.22377	0.351275	0.498227	lncRNA
ENSG0000(	KDM4C	3405.104	0.039055	0.351329	0.498281	protein_coding
ENSG0000(	ATP6V1H	538.1115	0.044952	0.351397	0.498356	protein_coding
ENSG0000(	CNIH2	24.28626	0.189808	0.351413	0.498356	protein_coding
ENSG0000(	PSME3IP1	2250.464	0.047977	0.351535	0.498506	protein_coding
ENSG0000(	CYP11A1	44.48074	0.135148	0.351588	0.498515	protein_coding
ENSG0000(	ENSG0000(	19.35941	0.131695	0.351647	0.498515	lncRNA
ENSG0000(	ENSG0000(	246.2143	-0.05444	0.351662	0.498515	transcribed_processed_pseudoge
ENSG0000(	ENSG0000(	79.22049	-0.12051	0.351675	0.498515	lncRNA
ENSG0000(	OR2J2	50.33741	-0.10633	0.351642	0.498515	protein_coding
ENSG0000(	RANBP3L	57.75548	-0.1288	0.351649	0.498515	protein_coding
ENSG0000(	RPEP4	66.90643	0.098346	0.351685	0.498515	processed_pseudogene
ENSG0000(	RPL7L1P12	21.67392	0.128929	0.351683	0.498515	processed_pseudogene
ENSG0000(	SNRPCP5	13.66595	0.304678	0.351618	0.498515	processed_pseudogene
ENSG0000(	S100A2	11.28631	-0.25352	0.351728	0.498553	protein_coding
ENSG0000(	NPIPB8	40.10748	0.27498	0.351749	0.498561	protein_coding
ENSG0000(	ENSG0000(	42.91332	-0.1492	0.351777	0.498577	lncRNA
ENSG0000(	TTYH1	260.4003	0.067228	0.351823	0.498621	protein_coding
ENSG0000(	ENSG0000(	107.5531	0.075781	0.351972	0.498809	lncRNA
ENSG0000(	LINC01119	65.85539	0.147135	0.352015	0.498847	lncRNA
ENSG0000(	ARHGAP12	2175.094	-0.04377	0.352142	0.498967	protein_coding
ENSG0000(	RP9	803.2402	-0.03251	0.352137	0.498967	protein_coding
ENSG0000(	TMCC3	2032.044	-0.13094	0.352147	0.498967	protein_coding
ENSG0000(	SORL1	7535.956	0.092528	0.352229	0.49906	protein_coding
ENSG0000(	AKAP1-DT	324.5713	0.04816	0.352278	0.499085	lncRNA
ENSG0000(	ENSG0000(	9.761151	0.431493	0.352267	0.499085	lncRNA
ENSG0000(	ENSG0000(	142.2852	0.180742	0.352379	0.499206	lncRNA
ENSG0000(	ENSG0000(	48.63408	-0.12722	0.352411	0.499228	lncRNA
ENSG0000(	KLF17	72.23332	0.085102	0.352456	0.499252	protein_coding
ENSG0000(	PSMD5	798.2745	-0.05206	0.35246	0.499252	protein_coding
ENSG0000(	ATP8A2	796.6073	0.040326	0.35256	0.499347	protein_coding
ENSG0000(	FAM241B	21.64064	-0.16698	0.352586	0.499347	protein_coding
ENSG0000(	MEAF6	1301.081	0.043409	0.352547	0.499347	protein_coding
ENSG0000(	SMG1P1	15908.97	-0.05725	0.352591	0.499347	transcribed_unprocessed_pseudo
ENSG0000(	RN7SL391F	90.78441	-0.09763	0.352613	0.499356	misc_RNA
ENSG0000(	LINC00243	297.7103	0.069685	0.352672	0.499417	lncRNA
ENSG0000(	ENSG0000(	19.44667	0.169959	0.352718	0.499436	lncRNA
ENSG0000(	TARS1	1003.091	0.068232	0.352706	0.499436	protein_coding
ENSG0000(	AIPL1	221.013	0.0578	0.352808	0.499519	protein_coding
ENSG0000(	ENSG0000(	72.30525	-0.07948	0.352807	0.499519	lncRNA
ENSG0000(	LINC01090	57.71693	-0.0906	0.352835	0.499534	lncRNA

ENSG0000(	ENSG0000(	22.58914	0.129172	0.352855	0.49954	lncRNA
ENSG0000(	CARD18	77.90975	-0.09815	0.352921	0.499611	protein_coding
ENSG0000(	DLGAP2	42.85427	0.296526	0.352975	0.499665	protein_coding
ENSG0000(	ENSG0000(	18.48729	-0.22516	0.353048	0.499745	lncRNA
ENSG0000(	BORCS5	1316.219	0.054499	0.353066	0.499748	protein_coding
ENSG0000(	PEX5L	203.8068	-0.08226	0.353093	0.499764	protein_coding
ENSG0000(	PHF10	1518.995	-0.06252	0.353147	0.499817	protein_coding
ENSG0000(	ACRBP	56.20877	-0.13939	0.353217	0.499894	protein_coding
ENSG0000(	PLCG1-AS1	612.9288	-0.03832	0.35325	0.499918	lncRNA
ENSG0000(	KCTD4	14.52735	-0.31734	0.353302	0.499969	protein_coding
ENSG0000(	PKD1L1-AS	18.26701	0.14747	0.353318	0.499969	lncRNA
ENSG0000(	FSIP2-AS2	10.71016	-0.2802	0.353347	0.499988	lncRNA
ENSG0000(	OR5B2	52.11893	0.119384	0.353457	0.50012	protein_coding
ENSG0000(	CASC2	655.4163	0.048297	0.353501	0.500137	lncRNA
ENSG0000(	ENSG0000(	30.53662	-0.14292	0.353488	0.500137	lncRNA
ENSG0000(	PPTC7	1585.753	-0.05089	0.353531	0.500158	protein_coding
ENSG0000(	GRK1	11.76465	-0.26596	0.35356	0.500176	protein_coding
ENSG0000(	ADAM10	4466.894	-0.03973	0.353599	0.500208	protein_coding
ENSG0000(	CYBA	1367.717	0.080835	0.353659	0.500271	protein_coding
ENSG0000(	RN7SL36P	15.44957	0.162715	0.353716	0.500329	misc_RNA
ENSG0000(	GPAT2	80.52129	0.209938	0.353747	0.500349	protein_coding
ENSG0000(	MUC16	132.1323	0.320395	0.35385	0.500472	protein_coding
ENSG0000(	GULOP	53.17833	-0.20323	0.354002	0.500665	unitary_pseudogene
ENSG0000(	ARL6	198.3725	-0.06539	0.354048	0.500694	protein_coding
ENSG0000(	RNASE4	279.4822	0.164958	0.354054	0.500694	protein_coding
ENSG0000(	RNU12	692.1839	0.104959	0.354094	0.500727	snRNA
ENSG0000(	LONRF2	368.0144	-0.06793	0.35413	0.500756	protein_coding
ENSG0000(	ENSG0000(	18.53674	-0.19124	0.354187	0.500814	lncRNA
ENSG0000(	BVES-AS1	102.0115	0.100379	0.35424	0.50083	lncRNA
ENSG0000(	C12orf56	24.70459	-0.24553	0.354218	0.50083	protein_coding
ENSG0000(	LINC00324	110.3687	0.075889	0.354247	0.50083	lncRNA
ENSG0000(	LINC02348	239.2791	0.054686	0.3543	0.500883	lncRNA
ENSG0000(	GABRG3	305.3615	-0.11028	0.354345	0.500924	protein_coding
ENSG0000(	HDAC6	2167.626	-0.03414	0.35441	0.500976	protein_coding
ENSG0000(	TMEM178/	81.21585	0.12713	0.354414	0.500976	protein_coding
ENSG0000(	LINC00240	68.17869	-0.14461	0.354492	0.501064	lncRNA
ENSG0000(	ENSG0000(	222.5369	-0.06876	0.354529	0.501093	lncRNA
ENSG0000(	LRTOMT	12.56326	-0.2467	0.354655	0.501248	protein_coding
ENSG0000(	ENSG0000(	32.61317	-0.11064	0.354735	0.501339	lncRNA
ENSG0000(	MCOLN1	546.53	-0.04605	0.354795	0.501401	protein_coding
ENSG0000(	ENSG0000(	21.94721	-0.14016	0.354909	0.501497	lncRNA
ENSG0000(	ENSG0000(	63.41965	-0.13738	0.354911	0.501497	lncRNA
ENSG0000(	TPRX1	13.29804	0.209218	0.354893	0.501497	protein_coding
ENSG0000(	FOXF2-DT	44.97493	0.125294	0.354961	0.501545	lncRNA
ENSG0000(	ENSG0000(	74.91107	-0.22197	0.355104	0.501725	TEC
ENSG0000(	THOC1-DT	230.3351	0.050951	0.355125	0.501732	lncRNA
ENSG0000(	ENSG0000(	31.78278	-0.1186	0.355293	0.501947	TEC

ENSG0000(	DOLK	105.5183	-0.09497	0.355373	0.501992	protein_coding
ENSG0000(	INSM1	28.60656	-0.19718	0.355361	0.501992	protein_coding
ENSG0000(	ZNF548	1105.107	0.055929	0.355357	0.501992	protein_coding
ENSG0000(	CASP12	21.30692	0.225123	0.355405	0.502009	polymorphic_pseudogene
ENSG0000(	ENSG0000(	182.5638	0.063153	0.355418	0.502009	lncRNA
ENSG0000(	JPH4	23.87845	-0.18851	0.355436	0.502012	protein_coding
ENSG0000(	ENSG0000(	107.4959	-0.07117	0.355543	0.50214	lncRNA
ENSG0000(	ENSG0000(	41.35342	-0.10464	0.355644	0.50226	lncRNA
ENSG0000(	LINC01539	16.07825	0.238691	0.355736	0.502368	lncRNA
ENSG0000(	SAR1A	2452.179	0.048626	0.355771	0.502394	protein_coding
ENSG0000(	ENSG0000(	211.4479	-0.06103	0.355837	0.502443	lncRNA
ENSG0000(	IAH1	1155.791	-0.04077	0.355834	0.502443	protein_coding
ENSG0000(	RAPSN	35.85796	0.165739	0.355937	0.50256	protein_coding
ENSG0000(	ARIH2OS	181.0332	0.047096	0.356001	0.502629	lncRNA
ENSG0000(	ENSG0000(	600.109	-0.07165	0.356055	0.502637	lncRNA
ENSG0000(	ENSG0000(	164.67	0.06282	0.356041	0.502637	lncRNA
ENSG0000(	TTC9	175.4157	0.210252	0.356045	0.502637	protein_coding
ENSG0000(	LRP4-AS1	66.797	0.107963	0.356111	0.502684	lncRNA
ENSG0000(	RANBP2	6237.123	-0.05227	0.356121	0.502684	protein_coding
ENSG0000(	ENSG0000(	67.21049	-0.14585	0.356178	0.502743	processed_pseudogene
ENSG0000(	MID1	2584.882	0.046979	0.356311	0.502908	protein_coding
ENSG0000(	DUSP8P5	121.7309	-0.10097	0.356471	0.50311	processed_pseudogene
ENSG0000(	DYNC1LI1	652.0022	-0.05909	0.356565	0.503197	protein_coding
ENSG0000(	ENSG0000(	16.53793	-0.14478	0.356554	0.503197	processed_pseudogene
ENSG0000(	ENSG0000(	20.0736	-0.14617	0.356667	0.503318	lncRNA
ENSG0000(	ANAPC13	302.8282	-0.08418	0.356774	0.503447	protein_coding
ENSG0000(	ENSG0000(	17.44661	0.174401	0.356885	0.503543	lncRNA
ENSG0000(	ENSG0000(	23.94336	0.177694	0.35689	0.503543	lncRNA
ENSG0000(	FBXL14	299.3242	-0.09408	0.356865	0.503543	protein_coding
ENSG0000(	PCDHB3	32.65428	0.138369	0.356942	0.503594	protein_coding
ENSG0000(	ENSG0000(	17.84299	0.197079	0.356964	0.503597	processed_pseudogene
ENSG0000(	OR10AH1P	316.719	0.044777	0.356977	0.503597	transcribed_unprocessed_pseudo
ENSG0000(	LINC02217	214.8237	0.067557	0.356998	0.503604	lncRNA
ENSG0000(	ENSG0000(	15.52185	-0.25679	0.357065	0.503652	lncRNA
ENSG0000(	TMEM132E	40.47075	0.132082	0.357064	0.503652	lncRNA
ENSG0000(	ERVE-1	290.6147	-0.08414	0.357147	0.503747	lncRNA
ENSG0000(	RN7SL636F	11.77987	0.22228	0.357226	0.503835	misc_RNA
ENSG0000(	KCNQ4	81.11968	-0.1315	0.357257	0.503856	protein_coding
ENSG0000(	AP5S1	1367.045	-0.03642	0.357348	0.503962	protein_coding
ENSG0000(	GTSF1	94.55235	0.094658	0.357397	0.504008	protein_coding
ENSG0000(	DENND5B-/	19.3349	0.200037	0.357424	0.504024	lncRNA
ENSG0000(	TAS2R43	138.2406	-0.14568	0.357448	0.504035	protein_coding
ENSG0000(	TSTD3	1505.397	0.038107	0.357485	0.504064	protein_coding
ENSG0000(	GLIPR1L2	758.4507	0.047501	0.35763	0.5042	protein_coding
ENSG0000(	LINC01743	18.23783	0.208228	0.357602	0.5042	lncRNA
ENSG0000(	SMO	218.2584	0.099362	0.357622	0.5042	protein_coding
ENSG0000(	ENSG0000(	110.6059	-0.07524	0.357742	0.504336	lncRNA

ENSG0000(	ENSG0000(	65.8319	0.115898	0.357781	0.504345	transcribed_processed_pseudoge
ENSG0000(	NPIPB3	11547.56	0.056835	0.357768	0.504345	protein_coding
ENSG0000(	GTF2IRD2P	19.82993	-0.32958	0.357864	0.504439	transcribed_unprocessed_pseudo
ENSG0000(	PCDHGA8	561.4844	-0.04264	0.357913	0.504485	protein_coding
ENSG0000(	GPRIN2	2515.235	0.112389	0.357937	0.504496	protein_coding
ENSG0000(	TTC23L	24.30947	0.28421	0.357974	0.504525	protein_coding
ENSG0000(	EXOC3L1	41.11156	0.17408	0.358077	0.504648	protein_coding
ENSG0000(	ENSG0000(	172.2681	-0.11558	0.358124	0.504669	lncRNA
ENSG0000(	PHKA1	257.2218	-0.09324	0.358113	0.504669	protein_coding
ENSG0000(	SNORA50C	56.11742	-0.18458	0.358232	0.504799	snoRNA
ENSG0000(	DPRXP4	100.2009	0.092203	0.358274	0.504811	processed_pseudogene
ENSG0000(	NCDN	327.7254	0.071873	0.358266	0.504811	protein_coding
ENSG0000(	ENSG0000(	170.2382	0.086901	0.358326	0.504863	lncRNA
ENSG0000(	RNF212	237.5791	-0.07964	0.358357	0.504883	protein_coding
ENSG0000(	LINC02087	45.15863	-0.13916	0.358374	0.504885	lncRNA
ENSG0000(	ENSG0000(	48.73112	0.107002	0.35857	0.505138	protein_coding
ENSG0000(	ENSG0000(	15.32419	-0.35443	0.358747	0.505365	processed_pseudogene
ENSG0000(	ENSG0000(	11.75058	-0.22025	0.358764	0.505366	lncRNA
ENSG0000(	ENSG0000(	254.2602	0.046806	0.35884	0.505443	TEC
ENSG0000(	HSPD1P4	12.72436	-0.26377	0.358851	0.505443	processed_pseudogene
ENSG0000(	SLC35A5	927.0783	0.056851	0.358906	0.505497	protein_coding
ENSG0000(	ZNF266	2256.153	0.071065	0.358943	0.505526	protein_coding
ENSG0000(	BCAR4	9.845721	-0.25993	0.358988	0.505567	lncRNA
ENSG0000(	ENSG0000(	128.4355	-0.11262	0.359059	0.505645	protein_coding
ENSG0000(	UGGT2	1592.565	0.041599	0.359079	0.50565	protein_coding
ENSG0000(	LINC01822	10.1196	0.26042	0.359207	0.505808	lncRNA
ENSG0000(	HYPK	2166.453	-0.03433	0.359333	0.505962	protein_coding
ENSG0000(	SPRY4	1571.866	0.108748	0.359466	0.506126	protein_coding
ENSG0000(	ENSG0000(	81.16452	-0.08989	0.359507	0.506162	lncRNA
ENSG0000(	ENSG0000(	16.16847	0.287611	0.359549	0.506198	lncRNA
ENSG0000(	AP4S1	1664.92	-0.02833	0.359607	0.506257	protein_coding
ENSG0000(	TMPRSS11I	108.4101	0.088703	0.359681	0.506338	transcribed_unprocessed_pseudo
ENSG0000(	ENSG0000(	27.23924	0.134611	0.359833	0.506529	processed_pseudogene
ENSG0000(	USP24	4078.355	-0.04071	0.360008	0.506752	protein_coding
ENSG0000(	LINC01429	110.1918	-0.08648	0.360162	0.506919	lncRNA
ENSG0000(	PPIAL4C	15.10983	-0.4941	0.360209	0.506919	protein_coding
ENSG0000(	PTPDC1	624.1685	-0.05117	0.360199	0.506919	protein_coding
ENSG0000(	RN7SL426P	11.5854	-0.18291	0.360216	0.506919	misc_RNA
ENSG0000(	TIMM10B	1380.881	-0.037	0.360216	0.506919	protein_coding
ENSG0000(	TMIGD3	25.27554	0.281055	0.360223	0.506919	protein_coding
ENSG0000(	ENSG0000(	101.7234	0.073271	0.36047	0.507223	lncRNA
ENSG0000(	ENSG0000(	26.22822	0.135192	0.360472	0.507223	lncRNA
ENSG0000(	ANKRD35	15.94067	0.172268	0.360536	0.507234	protein_coding
ENSG0000(	ENSG0000(	137.3044	0.084913	0.360504	0.507234	lncRNA
ENSG0000(	ENSG0000(	12.21756	0.184644	0.360562	0.507234	lncRNA
ENSG0000(	GIMAP2	646.9513	0.087885	0.360571	0.507234	protein_coding
ENSG0000(	MIR4664	14.9116	-0.19127	0.360577	0.507234	miRNA

ENSG0000(	STK38L	1518.025	-0.04649	0.360529	0.507234	protein_coding
ENSG0000(	DCDC2C	85.84421	-0.08182	0.360779	0.507495	protein_coding
ENSG0000(	LINC00865	210.0136	-0.0963	0.360954	0.507719	lncRNA
ENSG0000(	ENSG0000(	36.59653	-0.12912	0.360979	0.507731	processed_pseudogene
ENSG0000(	ENSG0000(	514.8811	-0.04684	0.361033	0.507785	lncRNA
ENSG0000(	EPS15P1	71.91129	-0.09682	0.361141	0.507913	transcribed_processed_pseudoge
ENSG0000(	USP43	442.0137	-0.09835	0.361246	0.508038	protein_coding
ENSG0000(	ENSG0000(	10.81047	-0.18643	0.361301	0.508069	lncRNA
ENSG0000(	NBPF4	39.60617	0.272958	0.361287	0.508069	protein_coding
ENSG0000(	ENSG0000(	295.0165	-0.04497	0.361378	0.508155	lncRNA
ENSG0000(	MRE11	1861.271	-0.05887	0.361443	0.508223	protein_coding
ENSG0000(	ZFPM2-AS1	102.8842	-0.08707	0.361558	0.508362	lncRNA
ENSG0000(	ENSG0000(	66.80017	0.185793	0.3618	0.508662	lncRNA
ENSG0000(	NUDT7	129.6536	-0.08454	0.361803	0.508662	protein_coding
ENSG0000(	ENSG0000(	37.63033	0.134879	0.361872	0.508695	lncRNA
ENSG0000(	ENSG0000(	39.19035	0.123541	0.361907	0.508695	processed_pseudogene
ENSG0000(	NLGN4Y-AS	19.6093	0.149976	0.361863	0.508695	lncRNA
ENSG0000(	PCDHGB4	113.4185	-0.12248	0.361888	0.508695	protein_coding
ENSG0000(	RERG	209.0524	-0.0791	0.361908	0.508695	protein_coding
ENSG0000(	MAPK12	309.925	0.088005	0.361935	0.508709	protein_coding
ENSG0000(	ENSG0000(	34.65554	-0.12946	0.361968	0.508711	TEC
ENSG0000(	GZMM	12.71194	0.285775	0.361956	0.508711	protein_coding
ENSG0000(	ENSG0000(	34.10677	-0.20315	0.3621	0.50885	lncRNA
ENSG0000(	IFI27L1	229.4013	0.079534	0.362097	0.50885	protein_coding
ENSG0000(	LINC01509	10.5877	0.223524	0.362198	0.508964	lncRNA
ENSG0000(	ENSG0000(	91.7087	-0.08481	0.362264	0.509035	processed_pseudogene
ENSG0000(	ANKRD13B	79.9946	-0.13722	0.362317	0.509064	protein_coding
ENSG0000(	TRBJ2-7	13.86965	0.300921	0.362304	0.509064	TR_J_gene
ENSG0000(	ENSG0000(	23.55488	-0.18117	0.362466	0.50925	lncRNA
ENSG0000(	KRT10-AS1	288.1975	-0.05751	0.362511	0.50929	lncRNA
ENSG0000(	APOA2	41.61777	0.168202	0.362556	0.509307	protein_coding
ENSG0000(	ENSG0000(	59.39139	0.117651	0.36254	0.509307	TEC
ENSG0000(	CCM2L	60.99024	0.131233	0.362577	0.509314	protein_coding
ENSG0000(	DDX5	19105.14	0.06537	0.362615	0.509322	protein_coding
ENSG0000(	LINC00702	617.7608	-0.05087	0.362615	0.509322	lncRNA
ENSG0000(	CNOT6	1385.818	-0.06599	0.362642	0.509337	protein_coding
ENSG0000(	ATP13A2	709.803	-0.06789	0.362909	0.50969	protein_coding
ENSG0000(	ENSG0000(	98.2712	-0.07936	0.363051	0.509866	lncRNA
ENSG0000(	ZNF397	3864.378	0.030135	0.363216	0.510074	protein_coding
ENSG0000(	CCDC57	2634.796	0.041722	0.363296	0.510165	protein_coding
ENSG0000(	ENSG0000(	13.94916	-0.17318	0.363361	0.510232	lncRNA
ENSG0000(	ENSG0000(	58.14812	0.106544	0.363385	0.510243	lncRNA
ENSG0000(	MAP3K7	1398.646	0.03908	0.363436	0.510292	protein_coding
ENSG0000(	ENSG0000(	18.30983	-0.15901	0.36354	0.510415	processed_pseudogene
ENSG0000(	AMBRA1	2178.792	0.055813	0.363643	0.51049	protein_coding
ENSG0000(	LINC02474	36.43058	-0.17134	0.363635	0.51049	lncRNA
ENSG0000(	PCDHGA9	204.1503	-0.07862	0.363622	0.51049	protein_coding

ENSG0000(	ENSG0000(	14.11565	-0.26983	0.363686	0.510528	lncRNA
ENSG0000(	ENSG0000(	35.88923	-0.11648	0.363747	0.510535	lncRNA
ENSG0000(	ENSG0000(	31.79796	0.223064	0.363719	0.510535	lncRNA
ENSG0000(	ENSG0000(	355.2076	-0.04236	0.363729	0.510535	TEC
ENSG0000(	PARPBP	354.0538	-0.06324	0.363756	0.510535	protein_coding
ENSG0000(	CD1C	104.5981	0.247309	0.363873	0.510676	protein_coding
ENSG0000(	RN7SL37P	42.91354	0.097926	0.363996	0.510827	misc_RNA
ENSG0000(	LAMB2P1	41.67083	0.116675	0.364109	0.510962	unprocessed_pseudogene
ENSG0000(	ENSG0000(	22.41147	-0.12371	0.364209	0.51102	lncRNA
ENSG0000(	FZD4-DT	25.58755	0.18597	0.364222	0.51102	lncRNA
ENSG0000(	LPP-AS2	177.4406	0.057856	0.364174	0.51102	lncRNA
ENSG0000(	PCDHA11	13.94613	0.219255	0.364232	0.51102	protein_coding
ENSG0000(	RECK	254.2943	0.079078	0.364186	0.51102	protein_coding
ENSG0000(	GPR174	1414.831	0.156253	0.364342	0.511151	protein_coding
ENSG0000(	ZNF215	573.6025	0.088913	0.364365	0.51116	protein_coding
ENSG0000(	RPSAP70	15.18469	-0.21491	0.364508	0.511321	processed_pseudogene
ENSG0000(	SYNJ2BP-C(	10.27457	-0.37382	0.364512	0.511321	protein_coding
ENSG0000(	PPFIA1	2682.364	-0.04399	0.364552	0.511354	protein_coding
ENSG0000(	KRT8P31	13.40755	-0.19312	0.364629	0.511438	processed_pseudogene
ENSG0000(	RNU1-16P	24.59222	-0.15293	0.364697	0.511511	snRNA
ENSG0000(	CCDC96	19.91118	-0.17409	0.36487	0.511731	protein_coding
ENSG0000(	GTF2H2C	1966.516	0.08891	0.364917	0.511758	protein_coding
ENSG0000(	RNU6-767F	27.90155	0.176988	0.364922	0.511758	snRNA
ENSG0000(	ENSG0000(	17.46409	0.4235	0.364957	0.511784	lncRNA
ENSG0000(	ENSG0000(	13.63443	-0.18819	0.365046	0.511886	TEC
ENSG0000(	FXNP2	18.70487	0.198244	0.36512	0.511945	processed_pseudogene
ENSG0000(	RUNDC3A	226.1837	0.063828	0.365112	0.511945	protein_coding
ENSG0000(	SLC10A6	28.34563	0.18097	0.365157	0.511973	protein_coding
ENSG0000(	ENSG0000(	151.8292	0.073848	0.365222	0.512037	lncRNA
ENSG0000(	MIR17HG	489.8591	-0.09857	0.365236	0.512037	lncRNA
ENSG0000(	OR14J1	3735.614	0.043098	0.365316	0.512127	protein_coding
ENSG0000(	STAMBPL1	1402.758	-0.06223	0.365373	0.512184	protein_coding
ENSG0000(	TMC7	851.4305	-0.06612	0.365449	0.512267	protein_coding
ENSG0000(	ENSG0000(	140.3347	-0.11698	0.365473	0.512278	lncRNA
ENSG0000(	ENSG0000(	16.9961	0.207065	0.365566	0.512362	transcribed_processed_pseudoge
ENSG0000(	MRAP-AS1	45.23916	-0.13513	0.365552	0.512362	lncRNA
ENSG0000(	C6orf62	2658.475	0.054186	0.365607	0.512388	protein_coding
ENSG0000(	ENSG0000(	67.51618	-0.11252	0.365617	0.512388	lncRNA
ENSG0000(	ENSG0000(	69.50746	0.08585	0.365703	0.512485	lncRNA
ENSG0000(	FAM217B	730.878	-0.04282	0.365984	0.512847	protein_coding
ENSG0000(	RN7SKP133	40.45671	-0.19303	0.365993	0.512847	misc_RNA
ENSG0000(	FN3K	624.5876	-0.06709	0.366084	0.51295	protein_coding
ENSG0000(	ENSG0000(	135.4758	0.0794	0.366233	0.513078	lncRNA
ENSG0000(	PARK7P2	44.11327	0.101857	0.36624	0.513078	processed_pseudogene
ENSG0000(	SOWAHD	26.16298	-0.14444	0.36621	0.513078	protein_coding
ENSG0000(	TNKS	2831.005	0.031044	0.366223	0.513078	protein_coding
ENSG0000(	RN7SKP72	10.56801	-0.24586	0.366282	0.513112	misc_RNA

ENSG0000(	ENSG0000(	60.82642	0.089843	0.366366	0.513208	lncRNA
ENSG0000(	TMEM161/	226.351	-0.08516	0.366411	0.513247	protein_coding
ENSG0000(	CLEC4OP	8.460987	0.382212	0.366472	0.513311	transcribed_unprocessed_pseudo
ENSG0000(	MCHR2	151.4322	-0.07013	0.366507	0.513336	protein_coding
ENSG0000(	ZNF837	22.7766	-0.13639	0.366589	0.513429	protein_coding
ENSG0000(	ENSG0000(	48.59265	0.093926	0.366703	0.513565	lncRNA
ENSG0000(	HEXIM2	43.88223	0.092681	0.366826	0.513713	protein_coding
ENSG0000(	RNU6-1061	12.21588	0.247382	0.36687	0.513753	snRNA
ENSG0000(	C22orf39	1271.994	-0.03107	0.367018	0.513923	protein_coding
ENSG0000(	ENSG0000(	47.2425	0.10829	0.367041	0.513923	lncRNA
ENSG0000(	ENSG0000(	11.44663	0.221869	0.367035	0.513923	lncRNA
ENSG0000(	NRF1	636.7847	-0.04933	0.367109	0.513995	protein_coding
ENSG0000(	PPP4R2	1608.158	-0.04291	0.367186	0.51408	protein_coding
ENSG0000(	ABI3BP	1618.422	0.094686	0.367214	0.514097	protein_coding
ENSG0000(	ENSG0000(	49.34988	0.099854	0.367254	0.514129	lncRNA
ENSG0000(	ENSG0000(	12.00198	0.199529	0.367344	0.514233	lncRNA
ENSG0000(	ENSG0000(	67.45178	-0.12336	0.36738	0.51426	lncRNA
ENSG0000(	ERI1	598.7989	0.074405	0.367397	0.514261	protein_coding
ENSG0000(	SLC26A11	551.2921	0.059848	0.367459	0.514301	protein_coding
ENSG0000(	TPTE2P1	434.2206	-0.05159	0.36745	0.514301	transcribed_unprocessed_pseudo
ENSG0000(	ENSG0000(	178.3482	0.069387	0.367503	0.514331	lncRNA
ENSG0000(	XXYLT1	278.3405	0.076505	0.367513	0.514331	protein_coding
ENSG0000(	ADSS2	823.4455	-0.03604	0.367576	0.514396	protein_coding
ENSG0000(	CEMIP2	5629.952	-0.06433	0.367739	0.5146	protein_coding
ENSG0000(	ENSG0000(	375.8206	0.055896	0.36783	0.514705	lncRNA
ENSG0000(	ENSG0000(	1021.848	-0.08753	0.3679	0.514757	lncRNA
ENSG0000(	MAT1A	25.5687	0.348863	0.367898	0.514757	protein_coding
ENSG0000(	TMC6	3280.085	0.080941	0.367926	0.51477	protein_coding
ENSG0000(	ENSG0000(	67.47369	-0.3128	0.368075	0.514936	protein_coding
ENSG0000(	SMIM43	14.89911	0.203248	0.368077	0.514936	protein_coding
ENSG0000(	LINC01134	477.4791	-0.07982	0.368136	0.514995	lncRNA
ENSG0000(	SEC11A	4483.579	-0.02801	0.368174	0.515025	protein_coding
ENSG0000(	MT1M	71.5331	0.276204	0.368269	0.515113	protein_coding
ENSG0000(	XPOT	1610.471	0.05729	0.368262	0.515113	protein_coding
ENSG0000(	MIR181A2I	28.49429	0.142265	0.368348	0.515199	lncRNA
ENSG0000(	LINC02402	10.77302	0.224287	0.36839	0.515236	lncRNA
ENSG0000(	ENSG0000(	222.4032	-0.06649	0.368493	0.515334	lncRNA
ENSG0000(	ENSG0000(	251.345	0.071302	0.368481	0.515334	TEC
ENSG0000(	CHD1-DT	1124.492	0.04446	0.368746	0.515664	lncRNA
ENSG0000(	PCDHB1	800.4795	-0.04109	0.368778	0.515686	protein_coding
ENSG0000(	ENSG0000(	606.5978	0.060241	0.368871	0.51576	lncRNA
ENSG0000(	RN7SL416P	17.81895	0.148027	0.368881	0.51576	misc_RNA
ENSG0000(	UBE2Q2P2	494.9364	-0.05207	0.368871	0.51576	transcribed_unprocessed_pseudo
ENSG0000(	CYCSP34	110.5597	0.1057	0.369054	0.515956	processed_pseudogene
ENSG0000(	ENSG0000(	94.33343	0.078584	0.369049	0.515956	lncRNA
ENSG0000(	TMEM220-	173.8408	-0.11817	0.369077	0.515965	lncRNA
ENSG0000(	ENSG0000(	275.2709	0.04769	0.369208	0.516126	lncRNA

ENSG0000(	ENSG0000(	10.83778	0.191618	0.369316	0.516253	processed_pseudogene
ENSG0000(	OSER1	546.1443	-0.05099	0.369464	0.516438	protein_coding
ENSG0000(	DCP2	2890.789	0.059219	0.369506	0.516472	protein_coding
ENSG0000(	ENSG0000(	315.7923	0.044519	0.369741	0.516762	lncRNA
ENSG0000(	GLCE	1124.277	-0.06905	0.369746	0.516762	protein_coding
ENSG0000(	ENSG0000(	23.09373	0.16519	0.369825	0.51685	lncRNA
ENSG0000(	ENSG0000(	375.8915	-0.04123	0.369891	0.516918	lncRNA
ENSG0000(	ENSG0000(	74.13173	-0.09387	0.369926	0.516944	lncRNA
ENSG0000(	CBSL	45.40669	0.517763	0.370091	0.517152	protein_coding
ENSG0000(	ENSG0000(	47.30323	0.107631	0.370121	0.517157	lncRNA
ENSG0000(	SNX33	2355.446	-0.05892	0.370128	0.517157	protein_coding
ENSG0000(	CLU	898.081	0.190188	0.370175	0.517191	protein_coding
ENSG0000(	SNORA22	47.9777	0.13508	0.370186	0.517191	snoRNA
ENSG0000(	RN7SL784F	235.9237	-0.05504	0.370246	0.517252	misc_RNA
ENSG0000(	LINC01747	27.03941	0.149223	0.370263	0.517253	lncRNA
ENSG0000(	AP1AR-DT	30.95424	0.116981	0.370293	0.517257	lncRNA
ENSG0000(	SNHG10	421.5796	-0.05176	0.370298	0.517257	lncRNA
ENSG0000(	ENSG0000(	64.13041	0.150965	0.370316	0.517258	lncRNA
ENSG0000(	ENSG0000(	298.0907	0.070096	0.370385	0.517331	lncRNA
ENSG0000(	RN7SKP189	22.8566	-0.22149	0.370401	0.517331	misc_RNA
ENSG0000(	ENSG0000(	263.1604	-0.04634	0.370456	0.517385	lncRNA
ENSG0000(	CAPRIN2	1450.192	0.051019	0.370578	0.517508	protein_coding
ENSG0000(	LINC00926	892.8258	0.157621	0.370566	0.517508	lncRNA
ENSG0000(	ENSG0000(	199.2333	0.052095	0.370646	0.51758	lncRNA
ENSG0000(	ENSG0000(	61.90316	-0.12435	0.37075	0.517687	TEC
ENSG0000(	FAM189B	602.9135	0.042113	0.370756	0.517687	protein_coding
ENSG0000(	PLS3-AS1	150.5928	0.077094	0.370875	0.51783	lncRNA
ENSG0000(	UTP14C	153.2847	0.308523	0.37091	0.517856	protein_coding
ENSG0000(	APP-DT	311.1951	-0.04849	0.37098	0.517931	lncRNA
ENSG0000(	PCA3	671.0409	-0.05459	0.371055	0.518013	lncRNA
ENSG0000(	SLC45A4	1053.639	0.081925	0.371108	0.518064	protein_coding
ENSG0000(	TRAJ21	17.05973	0.28511	0.37115	0.5181	TR_J_gene
ENSG0000(	HOXB-AS3	31.06467	-0.16783	0.371235	0.518171	lncRNA
ENSG0000(	NAA20	361.1586	-0.06114	0.37123	0.518171	protein_coding
ENSG0000(	NAT8L	19.25845	-0.22001	0.371259	0.518182	protein_coding
ENSG0000(	ACER1	89.35131	0.080878	0.371315	0.518238	protein_coding
ENSG0000(	ENSG0000(	15.21938	0.160984	0.371376	0.518298	lncRNA
ENSG0000(	MTHFD2P1	200.1673	-0.06914	0.371419	0.518336	transcribed_processed_pseudoge
ENSG0000(	ENSG0000(	141.307	-0.10543	0.371438	0.518339	transcribed_processed_pseudoge
ENSG0000(	EDEM3	5108.89	-0.04324	0.371532	0.518447	protein_coding
ENSG0000(	LGALS1	236.1002	0.194721	0.371715	0.51868	protein_coding
ENSG0000(	RASA4CP	370.4541	-0.08368	0.37178	0.518747	transcribed_unprocessed_pseudo
ENSG0000(	FGD5	910.4517	0.066503	0.371862	0.518777	protein_coding
ENSG0000(	PRKCA	3593.26	0.044424	0.371839	0.518777	protein_coding
ENSG0000(	RN7SL19P	13.93931	0.173498	0.37183	0.518777	misc_RNA
ENSG0000(	RNU6-101F	8.61585	0.231227	0.371868	0.518777	snRNA
ENSG0000(	H2BU1	525.6053	-0.12229	0.371887	0.51878	protein_coding

ENSG0000(	ATF6	2609.007	0.034876	0.371966	0.518867	protein_coding
ENSG0000(	KIF5A	405.9537	-0.12181	0.372088	0.519015	protein_coding
ENSG0000(	HTR4	615.0458	0.142241	0.372323	0.519319	protein_coding
ENSG0000(	ENSG0000(	17.71216	0.168318	0.372464	0.519492	misc_RNA
ENSG0000(	ENSG0000(	281.5344	-0.07605	0.372556	0.519599	lncRNA
ENSG0000(	PIK3C2A	3174.404	0.047888	0.372684	0.519754	protein_coding
ENSG0000(	RN7SKP93	30.0706	0.179676	0.372754	0.519828	misc_RNA
ENSG0000(	ENSG0000(	336.4392	0.062919	0.372929	0.520025	lncRNA
ENSG0000(	TBC1D22A	1722.683	-0.04038	0.372945	0.520025	protein_coding
ENSG0000(	VAMP2	1230.995	0.041944	0.372945	0.520025	protein_coding
ENSG0000(	ENSG0000(	9.339832	0.299713	0.373002	0.52008	lncRNA
ENSG0000(	THPO	15.51886	-0.2962	0.373029	0.520095	protein_coding
ENSG0000(	LINC02250	27.8176	0.119993	0.373071	0.520131	lncRNA
ENSG0000(	PIGX	774.2372	0.04115	0.373102	0.520151	protein_coding
ENSG0000(	ZNF518A	5145.346	-0.05667	0.373144	0.520186	protein_coding
ENSG0000(	LINC01563	359.5646	-0.04717	0.37321	0.520255	lncRNA
ENSG0000(	ENSG0000(	105.6228	0.078602	0.373266	0.520309	lncRNA
ENSG0000(	ENSG0000(	192.918	0.06613	0.373439	0.520527	lncRNA
ENSG0000(	GARS1	3172.161	0.053122	0.373477	0.520558	protein_coding
ENSG0000(	GMPPB	1440.112	-0.05864	0.373567	0.520657	protein_coding
ENSG0000(	LINC01135	106.1402	0.101281	0.373582	0.520657	lncRNA
ENSG0000(	MRGPRX3	18.67854	-0.1555	0.373622	0.520668	protein_coding
ENSG0000(	TBC1D31	763.5072	-0.04885	0.373623	0.520668	protein_coding
ENSG0000(	BICDL1	572.743	-0.08803	0.373735	0.520777	protein_coding
ENSG0000(	KCNQ5-DT	11.47265	0.167155	0.373731	0.520777	lncRNA
ENSG0000(	APOC1	91.50775	0.156974	0.373902	0.520824	protein_coding
ENSG0000(	CSMD3	494.0471	-0.07388	0.373826	0.520824	protein_coding
ENSG0000(	ENSG0000(	64.66419	0.11157	0.373843	0.520824	lncRNA
ENSG0000(	ENSG0000(	26.47793	0.150395	0.373901	0.520824	TEC
ENSG0000(	ENSG0000(	593.7041	-0.05795	0.373861	0.520824	unprocessed_pseudogene
ENSG0000(	ENSG0000(	9.336714	0.215774	0.373894	0.520824	lncRNA
ENSG0000(	MFSD4A	515.4012	-0.13202	0.373875	0.520824	protein_coding
ENSG0000(	SRD5A1	2263.213	0.039099	0.373832	0.520824	protein_coding
ENSG0000(	PZP	35.53359	0.152871	0.373945	0.520862	protein_coding
ENSG0000(	MOB3A	1479.273	0.059572	0.374207	0.521203	protein_coding
ENSG0000(	INMT-MINI	22.08343	0.376821	0.374344	0.521359	protein_coding
ENSG0000(	LINC00173	300.5081	0.062347	0.374352	0.521359	lncRNA
ENSG0000(	ENSG0000(	101.1651	0.269295	0.374369	0.52136	TEC
ENSG0000(	PSMA6	1856.096	-0.05857	0.374499	0.521517	protein_coding
ENSG0000(	ENSG0000(	12.77442	0.280756	0.374575	0.52153	processed_pseudogene
ENSG0000(	ENSG0000(	543.7203	-0.0455	0.374543	0.52153	lncRNA
ENSG0000(	FTL	8132.18	-0.10878	0.374562	0.52153	protein_coding
ENSG0000(	KBTBD3	145.2174	-0.07958	0.374553	0.52153	protein_coding
ENSG0000(	ENSG0000(	27.33951	0.131823	0.374821	0.521849	lncRNA
ENSG0000(	TRIL	170.6119	-0.12687	0.374844	0.521858	protein_coding
ENSG0000(	LAG3	59.41373	0.168201	0.374867	0.521866	protein_coding
ENSG0000(	RERE-AS1	27.74114	0.156955	0.374884	0.521867	lncRNA

ENSG0000(	ENSG0000(	19.15235	0.172675	0.375015	0.522003	lncRNA
ENSG0000(	ENSG0000(	35.50708	0.13447	0.374998	0.522003	lncRNA
ENSG0000(	ENSG0000(	12.39511	0.251675	0.375096	0.522092	lncRNA
ENSG0000(	ZUP1	271.2465	-0.07437	0.375235	0.522263	protein_coding
ENSG0000(	ENSG0000(	3439.473	0.065834	0.375318	0.522309	transcribed_unprocessed_pseudo
ENSG0000(	RSPH10B2	80.73169	0.300649	0.375297	0.522309	protein_coding
ENSG0000(	ZFAND5	4044.617	0.065302	0.375308	0.522309	protein_coding
ENSG0000(	NFKBID	453.9652	0.071443	0.375543	0.522598	protein_coding
ENSG0000(	ENSG0000(	8.694169	-0.26114	0.375578	0.522604	lncRNA
ENSG0000(	ENSG0000(	42.77546	-0.10993	0.375597	0.522604	lncRNA
ENSG0000(	ZCCHC2	1366.148	-0.07086	0.375592	0.522604	protein_coding
ENSG0000(	TMEM104	473.7996	-0.08331	0.375655	0.522662	protein_coding
ENSG0000(	ENSG0000(	30.34253	-0.13749	0.375675	0.522667	lncRNA
ENSG0000(	ACER3	7754.618	0.05317	0.375723	0.522675	protein_coding
ENSG0000(	CCDC81	194.6756	-0.05021	0.375732	0.522675	protein_coding
ENSG0000(	MIR6769A	9.98061	-0.21371	0.375704	0.522675	miRNA
ENSG0000(	RAF1	5803.432	0.020396	0.375759	0.52269	protein_coding
ENSG0000(	ENSG0000(	42.82984	-0.2253	0.375806	0.522728	processed_pseudogene
ENSG0000(	ENSG0000(	117.441	0.073191	0.37582	0.522728	lncRNA
ENSG0000(	FRYL	17982.37	-0.0769	0.375863	0.522765	protein_coding
ENSG0000(	CCDC92B	304.3559	-0.06644	0.375881	0.522766	protein_coding
ENSG0000(	IRF6	1796.93	-0.12094	0.375955	0.522846	protein_coding
ENSG0000(	PDCD1	56.71869	0.233443	0.376072	0.522986	protein_coding
ENSG0000(	ENSG0000(	93.41085	-0.06806	0.376291	0.523267	lncRNA
ENSG0000(	ENSG0000(	276.3399	-0.05441	0.376473	0.523497	lncRNA
ENSG0000(	CEACAM1	16787.98	-0.17467	0.376508	0.523521	protein_coding
ENSG0000(	TUBGCP2	2648.93	-0.02301	0.376524	0.523521	protein_coding
ENSG0000(	CEP250-AS	147.4843	0.074284	0.376633	0.523649	lncRNA
ENSG0000(	HOXA-AS3	131.853	-0.16317	0.376768	0.52379	lncRNA
ENSG0000(	PDXDC1	6042.713	0.063258	0.376753	0.52379	protein_coding
ENSG0000(	CARS1	2457.416	0.044574	0.376825	0.523846	protein_coding
ENSG0000(	FAM197Y3	10.79561	0.431002	0.377065	0.524156	transcribed_unprocessed_pseudo
ENSG0000(	IGKV1-37	9.771086	-0.59365	0.377121	0.524212	IG_V_gene
ENSG0000(	ZNF843	324.7782	0.041403	0.377176	0.524265	protein_coding
ENSG0000(	CEP78	1584.196	-0.05846	0.377285	0.524381	protein_coding
ENSG0000(	PNN	4570.992	-0.05697	0.377293	0.524381	protein_coding
ENSG0000(	ENSG0000(	13.55111	0.199786	0.377471	0.5246	lncRNA
ENSG0000(	ENSG0000(	12.13035	-0.60233	0.377484	0.5246	protein_coding
ENSG0000(	RABL2B	1570.899	-0.04256	0.377531	0.524641	protein_coding
ENSG0000(	ENSG0000(	14.15507	0.191907	0.377568	0.52467	lncRNA
ENSG0000(	ENSG0000(	26.58366	0.142612	0.377612	0.524707	processed_pseudogene
ENSG0000(	ZNF696	664.1328	0.062241	0.377648	0.524734	protein_coding
ENSG0000(	ENSG0000(	113.3654	-0.08369	0.377699	0.524781	lncRNA
ENSG0000(	ENSG0000(	224.3069	-0.06825	0.377755	0.524826	lncRNA
ENSG0000(	TEX15	34.10549	-0.13722	0.377764	0.524826	protein_coding
ENSG0000(	SIN3B	2179.061	0.029496	0.377822	0.524882	protein_coding
ENSG0000(	TSACC	16.17633	-0.16153	0.377919	0.524994	protein_coding

ENSG0000(	ENSG0000(	10.32917	0.207331	0.377973	0.525045	lncRNA
ENSG0000(	PPP1R11	955.0837	-0.07733	0.378011	0.525075	protein_coding
ENSG0000(	ENSG0000(	66.28447	-0.10842	0.378098	0.52515	lncRNA
ENSG0000(	ST3GAL3	2469.58	0.029965	0.378095	0.52515	protein_coding
ENSG0000(	COL6A4P1	26.83515	0.242329	0.378218	0.525293	transcribed_unprocessed_pseudo
ENSG0000(	ENSG0000(	13.47374	0.178195	0.378236	0.525295	lncRNA
ENSG0000(	ENSG0000(	169.7533	-0.05923	0.378295	0.52533	lncRNA
ENSG0000(	SNRPCP13	13.26094	0.285097	0.378291	0.52533	processed_pseudogene
ENSG0000(	BANCR	27.19697	0.153495	0.378423	0.525484	lncRNA
ENSG0000(	GABRR3	73.89584	0.093717	0.378484	0.525492	protein_coding
ENSG0000(	PRKAB2	1329.009	-0.06258	0.378487	0.525492	protein_coding
ENSG0000(	SEMA6B	375.3484	0.058647	0.378486	0.525492	protein_coding
ENSG0000(	TMEM119	595.2944	0.082515	0.378496	0.525492	protein_coding
ENSG0000(	ENSG0000(	11.63448	-0.20197	0.378524	0.525507	lncRNA
ENSG0000(	LINC00313	15.70203	0.212084	0.378609	0.525602	lncRNA
ENSG0000(	PRH2	68.82318	0.116444	0.378673	0.525668	protein_coding
ENSG0000(	CFAP46	13.53024	0.329815	0.378722	0.525713	protein_coding
ENSG0000(	ENSG0000(	91.44163	0.12504	0.378764	0.525724	lncRNA
ENSG0000(	RPL21P10	53.35774	0.10124	0.378761	0.525724	processed_pseudogene
ENSG0000(	SLC25A6P3	25.9521	0.148745	0.37879	0.525736	processed_pseudogene
ENSG0000(	KPNA4	4168.217	-0.03615	0.378815	0.525748	protein_coding
ENSG0000(	MIX23P1	31.94023	-0.24832	0.378856	0.525781	processed_pseudogene
ENSG0000(	INTS9	297.3985	-0.06087	0.378914	0.525839	protein_coding
ENSG0000(	ENSG0000(	1689.667	0.037438	0.378982	0.52591	lncRNA
ENSG0000(	PRR5L	4810.931	-0.12933	0.379174	0.526153	protein_coding
ENSG0000(	LINC00659	13.49753	0.234705	0.379219	0.526192	lncRNA
ENSG0000(	ENSG0000(	68.15842	0.100728	0.379295	0.526273	TEC
ENSG0000(	ENSG0000(	16.75108	0.159368	0.379503	0.52654	lncRNA
ENSG0000(	WNK3	190.0012	-0.10115	0.379571	0.52661	protein_coding
ENSG0000(	ENSG0000(	15.33002	0.161848	0.3796	0.526628	lncRNA
ENSG0000(	LINC02291	57.30446	0.109586	0.379645	0.526666	lncRNA
ENSG0000(	ENSG0000(	25.17741	-0.16096	0.379724	0.526705	lncRNA
ENSG0000(	ENSG0000(	207.1288	0.066135	0.379709	0.526705	lncRNA
ENSG0000(	ZNF680	635.7736	0.057618	0.379699	0.526705	protein_coding
ENSG0000(	ENSG0000(	14.68316	-0.29914	0.37982	0.526793	processed_pseudogene
ENSG0000(	ENSG0000(	229.7818	-0.16617	0.37982	0.526793	protein_coding
ENSG0000(	ZNF331	1189.762	-0.05294	0.379872	0.526841	protein_coding
ENSG0000(	BTAF1	4336.016	0.052594	0.380086	0.527092	protein_coding
ENSG0000(	CELF6	142.7701	-0.09527	0.380079	0.527092	protein_coding
ENSG0000(	DGCR6	131.5303	0.107546	0.38025	0.527225	protein_coding
ENSG0000(	ENOSF1	5361.257	-0.05911	0.380236	0.527225	protein_coding
ENSG0000(	ENSG0000(	77.29249	-0.09323	0.380207	0.527225	lncRNA
ENSG0000(	NDUFA2	315.4805	-0.0651	0.380224	0.527225	protein_coding
ENSG0000(	ENSG0000(	206.5297	-0.07467	0.380339	0.527279	lncRNA
ENSG0000(	PIM1	849.4668	0.097942	0.380306	0.527279	protein_coding
ENSG0000(	SNTB2	2578.094	0.045809	0.380331	0.527279	protein_coding
ENSG0000(	MCOLN2	877.6782	-0.15747	0.3806	0.527617	protein_coding

ENSG0000(	ENSG0000(	18.79742	-0.11648	0.380735	0.527695	lncRNA
ENSG0000(	GRAMD1A-	206.6721	0.058906	0.380694	0.527695	lncRNA
ENSG0000(	NIT2	1310.427	0.039516	0.380725	0.527695	protein_coding
ENSG0000(	TCEA1	1438.266	-0.04954	0.380699	0.527695	protein_coding
ENSG0000(	ZNRF3-IT1	74.68167	-0.16749	0.380741	0.527695	lncRNA
ENSG0000(	SMCO1	16.80955	0.162297	0.380864	0.527843	protein_coding
ENSG0000(	NDUFB2-A!	83.01376	-0.05733	0.38093	0.52791	lncRNA
ENSG0000(	CHMP5P1	10.02401	0.292177	0.381017	0.527973	processed_pseudogene
ENSG0000(	ENSG0000(	632.0347	0.105309	0.381026	0.527973	TEC
ENSG0000(	MYOZ3	141.0694	-0.10835	0.381017	0.527973	protein_coding
ENSG0000(	CASR	1371.741	0.050168	0.381237	0.528032	protein_coding
ENSG0000(	ENSG0000(	257.4777	0.078315	0.381237	0.528032	protein_coding
ENSG0000(	ENSG0000(	77.51948	0.11207	0.38122	0.528032	lncRNA
ENSG0000(	GUSBP6	17.41063	-0.18988	0.381209	0.528032	transcribed_unprocessed_pseudo
ENSG0000(	LINC02158	25.72292	-0.15954	0.381231	0.528032	lncRNA
ENSG0000(	RN7SL219F	11.77369	0.183306	0.381219	0.528032	misc_RNA
ENSG0000(	RNY3P11	14.86962	0.247314	0.381148	0.528032	misc_RNA
ENSG0000(	SLC22A1	94.98968	-0.1254	0.381121	0.528032	protein_coding
ENSG0000(	TRIM49D1	385.9881	0.063348	0.381206	0.528032	protein_coding
ENSG0000(	TRPM3	285.2057	0.044896	0.381152	0.528032	protein_coding
ENSG0000(	PIMREGP2	8.769378	-0.23191	0.381278	0.528066	processed_pseudogene
ENSG0000(	P2RX3	56.86269	-0.10555	0.381328	0.528111	protein_coding
ENSG0000(	RNU2-33P	28.5505	0.179093	0.381363	0.528135	snRNA
ENSG0000(	HASPIN	106.2974	-0.10343	0.381448	0.52823	protein_coding
ENSG0000(	CHD5	24.25398	-0.17923	0.381597	0.528414	protein_coding
ENSG0000(	DLGAP1-AS	189.7368	0.044968	0.381761	0.528593	lncRNA
ENSG0000(	ZNF451-AS	223.5403	-0.05737	0.38175	0.528593	lncRNA
ENSG0000(	ENSG0000(	11.51736	-0.2597	0.381836	0.528627	lncRNA
ENSG0000(	HHLA3	91.99779	0.064519	0.381828	0.528627	lncRNA
ENSG0000(	PRPF40B	384.5974	0.067456	0.381836	0.528627	protein_coding
ENSG0000(	CSRP1	3200.893	-0.06515	0.381914	0.528712	protein_coding
ENSG0000(	LINC01683	127.4203	0.072676	0.382005	0.528791	lncRNA
ENSG0000(	UQCRFS1P:	11.33157	-0.46342	0.382005	0.528791	processed_pseudogene
ENSG0000(	ENSG0000(	47.27626	-0.10398	0.382083	0.528875	lncRNA
ENSG0000(	ENSG0000(	67.9996	-0.07871	0.382257	0.529093	lncRNA
ENSG0000(	MIR600HG	1346.699	0.063865	0.382359	0.529211	lncRNA
ENSG0000(	SOX6	1855.831	0.108239	0.382486	0.529363	protein_coding
ENSG0000(	ENSG0000(	247.4661	0.055554	0.382619	0.529524	lncRNA
ENSG0000(	ENSG0000(	21.78424	-0.14306	0.38264	0.52953	lncRNA
ENSG0000(	SEC16A	4810.778	0.039275	0.382657	0.52953	protein_coding
ENSG0000(	GUCA1B	173.1621	0.062306	0.382746	0.529629	protein_coding
ENSG0000(	GFI1B	509.1	-0.04233	0.382888	0.529802	protein_coding
ENSG0000(	ZIC1	10.75656	-0.31542	0.382935	0.529844	protein_coding
ENSG0000(	ENSG0000(	200.9129	0.101731	0.383003	0.529914	lncRNA
ENSG0000(	ENSG0000(	466.1096	-0.0741	0.383035	0.529935	protein_coding
ENSG0000(	ENSG0000(	115.0225	0.066856	0.383079	0.529973	lncRNA
ENSG0000(	C1GALT1C1	261.9245	-0.07098	0.3832	0.530097	protein_coding

ENSG0000(	SOX8	84.59911	-0.20717	0.383203	0.530097	protein_coding
ENSG0000(	CBLN1	10.04895	-0.2478	0.383318	0.530233	protein_coding
ENSG0000(	C1orf21	2814.967	-0.08648	0.383567	0.530554	protein_coding
ENSG0000(	SNX29P1	191.5724	-0.10514	0.383743	0.530773	unprocessed_pseudogene
ENSG0000(	ENSG0000(	71.46829	0.095443	0.38382	0.530857	lncRNA
ENSG0000(	CAPNS2	19.56671	0.17285	0.383856	0.530884	protein_coding
ENSG0000(	ENSG0000(	10.97663	0.200258	0.383876	0.530888	lncRNA
ENSG0000(	KPNA7	26.44976	-0.22361	0.383961	0.530961	protein_coding
ENSG0000(	OTOA	263.9442	0.066605	0.38398	0.530961	protein_coding
ENSG0000(	RN7SL376F	116.877	0.076282	0.383974	0.530961	misc_RNA
ENSG0000(	ENSG0000(	515.032	0.043882	0.384057	0.530973	lncRNA
ENSG0000(	ENSG0000(	69.97164	0.092267	0.384055	0.530973	lncRNA
ENSG0000(	RN7SL210F	19.61186	0.162872	0.384041	0.530973	misc_RNA
ENSG0000(	SON	15734.84	0.029619	0.38405	0.530973	protein_coding
ENSG0000(	LINC01176	926.0526	-0.07043	0.384078	0.530979	lncRNA
ENSG0000(	ENSG0000(	402.0077	-0.07813	0.384106	0.530994	lncRNA
ENSG0000(	F7	11.12476	0.29093	0.384179	0.531072	protein_coding
ENSG0000(	ENSG0000(	916.9258	-0.02915	0.384241	0.53111	lncRNA
ENSG0000(	ITGA2	1828.952	0.145352	0.384275	0.53111	protein_coding
ENSG0000(	UCHL1	24.1438	0.242309	0.384275	0.53111	protein_coding
ENSG0000(	ZNF22-AS1	67.52194	0.094682	0.384261	0.53111	lncRNA
ENSG0000(	ENSG0000(	10.66349	0.180876	0.384342	0.531168	lncRNA
ENSG0000(	LINC01136	294.569	0.059217	0.384351	0.531168	lncRNA
ENSG0000(	LINC00507	947.0785	0.045489	0.38438	0.531186	lncRNA
ENSG0000(	ENSG0000(	10.35132	0.209641	0.384476	0.531218	lncRNA
ENSG0000(	ENSG0000(	211.3049	-0.10807	0.384457	0.531218	TEC
ENSG0000(	LRRTM2	81.09085	0.174833	0.384431	0.531218	protein_coding
ENSG0000(	PLA2G2F	13.65849	0.293294	0.384489	0.531218	protein_coding
ENSG0000(	SREK1	5517.972	0.037874	0.384464	0.531218	protein_coding
ENSG0000(	C2orf15	266.0654	-0.053	0.384613	0.531366	protein_coding
ENSG0000(	ADAT2	1268.961	0.07855	0.38469	0.531449	protein_coding
ENSG0000(	SELENOV	15.10163	0.177894	0.384709	0.531452	protein_coding
ENSG0000(	FAM86MP	15.19364	0.206004	0.384844	0.531615	unprocessed_pseudogene
ENSG0000(	NAPSA	71.58245	-0.16051	0.384867	0.531623	protein_coding
ENSG0000(	ENSG0000(	47.67169	0.111969	0.384919	0.531648	transcribed_unprocessed_pseudo
ENSG0000(	MCTS2P	28.12613	-0.42788	0.384919	0.531648	protein_coding
ENSG0000(	ENSG0000(	13.47027	0.167473	0.385085	0.531854	processed_pseudogene
ENSG0000(	CES3	3174.181	-0.1282	0.385122	0.531882	protein_coding
ENSG0000(	GPAT4	1835.247	0.038893	0.385197	0.531961	protein_coding
ENSG0000(	SNORA59B	67.88165	-0.0837	0.38522	0.531969	snoRNA
ENSG0000(	FSD2	223.2152	-0.09066	0.38526	0.532001	protein_coding
ENSG0000(	ENSG0000(	264.1751	-0.04906	0.38531	0.532047	lncRNA
ENSG0000(	ENSG0000(	45.7443	-0.11341	0.385372	0.532108	lncRNA
ENSG0000(	ENSG0000(	86.47467	0.083506	0.385397	0.53212	lncRNA
ENSG0000(	NRIP1	4416.627	0.068692	0.385492	0.532228	protein_coding
ENSG0000(	UBR2	4375.682	0.041996	0.385544	0.532276	protein_coding
ENSG0000(	ENSG0000(	15.93882	0.182401	0.38562	0.53233	lncRNA

ENSG0000(	LINC01424	84.15183	0.079316	0.385631	0.53233	lncRNA
ENSG0000(	LINC02257	21.38935	0.158608	0.385634	0.53233	lncRNA
ENSG0000(	DNAAF3	9.824409	0.247247	0.385661	0.532344	protein_coding
ENSG0000(	ENSG0000(	32.79848	0.125086	0.385701	0.532376	lncRNA
ENSG0000(	PARP4	5093.642	-0.05767	0.385743	0.53241	protein_coding
ENSG0000(	LMNA	2541.23	0.103074	0.386046	0.532805	protein_coding
ENSG0000(	TMEM44	1124.657	-0.07457	0.386075	0.532821	protein_coding
ENSG0000(	DCP1B	367.0741	0.048332	0.386104	0.532838	protein_coding
ENSG0000(	ENSG0000(	12.78881	-0.16712	0.38618	0.532919	lncRNA
ENSG0000(	ENSG0000(	25.17578	0.12508	0.386256	0.532977	lncRNA
ENSG0000(	MTCYBP18	38.9745	-0.40375	0.386241	0.532977	processed_pseudogene
ENSG0000(	RNY3	1943.395	-0.10646	0.386291	0.533001	misc_RNA
ENSG0000(	EMC2	1005.502	-0.03701	0.38631	0.533004	protein_coding
ENSG0000(	CEP68	1311.671	0.045378	0.38638	0.533077	protein_coding
ENSG0000(	CFAP44	1624.528	0.070175	0.386491	0.533206	protein_coding
ENSG0000(	SLC6A9	683.7023	-0.13742	0.38673	0.533513	protein_coding
ENSG0000(	CACNB4	1175.873	0.071138	0.386835	0.533611	protein_coding
ENSG0000(	METTL18	100.6321	-0.09573	0.386825	0.533611	protein_coding
ENSG0000(	ZNF212	384.2909	-0.05882	0.386856	0.533616	protein_coding
ENSG0000(	HNRNPA1P	48.41274	-0.12252	0.386918	0.533655	processed_pseudogene
ENSG0000(	R3HDM2-D	50.00635	-0.1036	0.386905	0.533655	lncRNA
ENSG0000(	UBE2Q2P6	15.48473	0.248685	0.387103	0.533887	transcribed_unprocessed_pseudo
ENSG0000(	ENSG0000(	55.67865	0.132898	0.387156	0.533914	protein_coding
ENSG0000(	RN7SL815F	24.24579	0.131697	0.387157	0.533914	misc_RNA
ENSG0000(	ENSG0000(	32.48398	-0.26487	0.387187	0.53393	lncRNA
ENSG0000(	NAIPP3	409.0368	0.10604	0.387215	0.53393	transcribed_unprocessed_pseudo
ENSG0000(	SNORA55	94.45421	0.094938	0.38722	0.53393	snoRNA
ENSG0000(	ALG1L	86.04348	-0.09437	0.387332	0.534061	transcribed_unprocessed_pseudo
ENSG0000(	ZNF579	86.32194	-0.11429	0.387407	0.53414	protein_coding
ENSG0000(	ARG2	55.27085	-0.22892	0.387531	0.534265	protein_coding
ENSG0000(	ENSG0000(	833.946	0.052078	0.387527	0.534265	lncRNA
ENSG0000(	RN7SL478F	24.0468	-0.13367	0.387573	0.534299	misc_RNA
ENSG0000(	PARG	999.7103	0.043175	0.38759	0.534299	protein_coding
ENSG0000(	ENSG0000(	18.82779	0.151488	0.387639	0.534342	lncRNA
ENSG0000(	ENSG0000(	353.0879	-0.03846	0.387692	0.534393	TEC
ENSG0000(	MYLK	6211.084	0.065108	0.387737	0.534431	protein_coding
ENSG0000(	ZNF471	1989.925	-0.06522	0.387817	0.534518	protein_coding
ENSG0000(	ENSG0000(	92.19266	-0.09891	0.387891	0.534597	lncRNA
ENSG0000(	USP25	2046.562	0.044085	0.387909	0.534597	protein_coding
ENSG0000(	TCERG1	2854.35	-0.04415	0.38795	0.53463	protein_coding
ENSG0000(	ELL2P1	10.92069	0.226788	0.38804	0.53473	processed_pseudogene
ENSG0000(	LYG1	41.06179	0.128321	0.388057	0.53473	protein_coding
ENSG0000(	KCTD20	2075.306	0.047189	0.388219	0.534929	protein_coding
ENSG0000(	RPL10P9	65.24811	-0.39231	0.38831	0.535032	processed_pseudogene
ENSG0000(	ATP8B4	808.742	0.07051	0.388366	0.535083	protein_coding
ENSG0000(	CHRM5	68.27289	-0.09942	0.388381	0.535083	protein_coding
ENSG0000(	ENSG0000(	186.088	0.056871	0.388535	0.535271	transcribed_unprocessed_pseudo

ENSG0000(	NTMT1	287.9287	-0.05458	0.388573	0.535276	protein_coding
ENSG0000(	XRCC4	136.2802	0.081587	0.388565	0.535276	protein_coding
ENSG0000(	ENSG0000(	22.42258	-0.19419	0.388599	0.535288	lncRNA
ENSG0000(	CD37	1080.881	0.204264	0.388642	0.535312	protein_coding
ENSG0000(	RAB44	64.77277	0.157046	0.388651	0.535312	protein_coding
ENSG0000(	NUP160	1693.434	-0.03993	0.388805	0.535501	protein_coding
ENSG0000(	ENSG0000(	26.40073	0.128809	0.38893	0.53565	processed_pseudogene
ENSG0000(	TMED7	1476.885	-0.07606	0.389163	0.535948	protein_coding
ENSG0000(	ANK2	3689.55	0.048751	0.389206	0.535948	protein_coding
ENSG0000(	RMDN1	1167.503	-0.0354	0.389215	0.535948	protein_coding
ENSG0000(	TRIM26	1487.65	-0.04777	0.389187	0.535948	protein_coding
ENSG0000(	EIF4G3	6377.107	-0.06115	0.389253	0.535963	protein_coding
ENSG0000(	MTND4P12	325.9547	-0.31201	0.389275	0.535963	processed_pseudogene
ENSG0000(	TMEM254	358.9998	-0.07861	0.389277	0.535963	protein_coding
ENSG0000(	RBIS	501.0198	0.056006	0.389307	0.535981	protein_coding
ENSG0000(	LRRC61	62.63155	-0.10495	0.389364	0.536035	protein_coding
ENSG0000(	ENSG0000(	576.8559	0.048575	0.389489	0.536184	lncRNA
ENSG0000(	ENSG0000(	11.73753	0.212852	0.389525	0.536187	lncRNA
ENSG0000(	KATNBL1P2	61.21353	0.136455	0.38951	0.536187	processed_pseudogene
ENSG0000(	RN7SL740F	96.26117	0.083976	0.389584	0.536244	misc_RNA
ENSG0000(	ENSG0000(	140.8817	0.06467	0.389636	0.536292	TEC
ENSG0000(	ENSG0000(	29.90194	0.119759	0.389653	0.536292	processed_pseudogene
ENSG0000(	MMP3	723.2534	2.331298	0.389706	0.536318	protein_coding
ENSG0000(	RN7SKP3	15.85807	-0.1419	0.389701	0.536318	misc_RNA
ENSG0000(	CPVL-AS1	8.921703	0.221734	0.38988	0.53651	lncRNA
ENSG0000(	ENSG0000(	273.9314	0.052741	0.38988	0.53651	lncRNA
ENSG0000(	ENSG0000(	52.88966	-0.10393	0.389973	0.536614	lncRNA
ENSG0000(	RN7SL619F	245.8298	-0.04314	0.390035	0.536675	misc_RNA
ENSG0000(	ENSG0000(	261.0277	-0.04794	0.390143	0.536801	lncRNA
ENSG0000(	ENSG0000(	33.19139	-0.17748	0.390201	0.536833	lncRNA
ENSG0000(	LINC02564	30.31418	0.13509	0.390187	0.536833	lncRNA
ENSG0000(	ARPC2	5422.134	0.055401	0.390221	0.536838	protein_coding
ENSG0000(	PTPMT1	327.2735	-0.07502	0.390396	0.537054	protein_coding
ENSG0000(	SNORA71C	33.14052	-0.15036	0.390453	0.537109	snoRNA
ENSG0000(	BTBD3	2032.837	-0.10279	0.390485	0.53713	protein_coding
ENSG0000(	LINC00940	25.14108	-0.20086	0.390513	0.537143	lncRNA
ENSG0000(	TTC24	226.8326	0.136442	0.390529	0.537143	protein_coding
ENSG0000(	ENSG0000(	85.247	-0.06549	0.390645	0.537207	lncRNA
ENSG0000(	ENSG0000(	159.8827	-0.05157	0.390637	0.537207	lncRNA
ENSG0000(	LURAP1	186.8709	0.058289	0.390624	0.537207	protein_coding
ENSG0000(	MACROH2/	1744.089	0.035122	0.390632	0.537207	protein_coding
ENSG0000(	GADD45B	219.5126	0.123304	0.390768	0.537353	protein_coding
ENSG0000(	DUSP18	645.2517	0.061645	0.390814	0.537393	protein_coding
ENSG0000(	NPAP1P2	40.33702	-0.14969	0.390946	0.53755	processed_pseudogene
ENSG0000(	HCN3	460.0674	-0.11947	0.391001	0.537579	protein_coding
ENSG0000(	HMGN4	986.5849	0.108446	0.390992	0.537579	protein_coding
ENSG0000(	ACKR1	86.3161	0.183365	0.391079	0.537638	protein_coding

ENSG0000(	ENSG0000(	22.69119	0.144756	0.391077	0.537638	lncRNA
ENSG0000(	ENSG0000(	105.8836	0.100202	0.391211	0.537797	lncRNA
ENSG0000(	ENSG0000(	194.6886	0.058762	0.391269	0.537854	lncRNA
ENSG0000(	CLEC18C	72.02492	0.175918	0.391318	0.537878	protein_coding
ENSG0000(	FBRS	2086.485	-0.04388	0.391326	0.537878	protein_coding
ENSG0000(	UBA1	3961.423	-0.03032	0.391339	0.537878	protein_coding
ENSG0000(	ENSG0000(	1011.188	-0.02899	0.391478	0.538045	lncRNA
ENSG0000(	NDE1	1691.332	-0.05689	0.391523	0.538083	protein_coding
ENSG0000(	ENSG0000(	58.47421	-0.08675	0.391555	0.538104	TEC
ENSG0000(	ENSG0000(	128.6743	0.085566	0.391635	0.53819	TEC
ENSG0000(	KIF26A	122.6314	-0.13646	0.391717	0.538268	protein_coding
ENSG0000(	TLL2	293.681	-0.04569	0.391726	0.538268	protein_coding
ENSG0000(	TTC22	1489.907	-0.09731	0.391757	0.538288	protein_coding
ENSG0000(	ENSG0000(	20.49352	-0.17535	0.391849	0.538389	lncRNA
ENSG0000(	NAP1L1	3614.595	0.075076	0.392068	0.538646	protein_coding
ENSG0000(	RPL17P15	14.66966	0.19685	0.39207	0.538646	processed_pseudogene
ENSG0000(	CARMIL3	74.86713	0.155662	0.392172	0.538763	protein_coding
ENSG0000(	ENSG0000(	2745.872	0.024818	0.392217	0.538794	lncRNA
ENSG0000(	ENSG0000(	123.0032	-0.08406	0.392229	0.538794	lncRNA
ENSG0000(	ENSG0000(	36.46383	0.128803	0.392284	0.538846	lncRNA
ENSG0000(	RN7SL488F	114.4875	-0.07048	0.392349	0.538911	misc_RNA
ENSG0000(	ENSG0000(	111.4591	-0.0648	0.392375	0.538923	lncRNA
ENSG0000(	ENSG0000(	212.8965	-0.06469	0.392396	0.538928	lncRNA
ENSG0000(	GBAP1	244.8954	-0.13857	0.392518	0.539073	transcribed_unprocessed_pseudo
ENSG0000(	ENSG0000(	36.28886	0.127848	0.392571	0.539098	protein_coding
ENSG0000(	SPIC	10.84156	-0.27257	0.392565	0.539098	protein_coding
ENSG0000(	ADH4	218.5374	0.059282	0.392687	0.539228	protein_coding
ENSG0000(	SEMA5B	286.3885	0.072948	0.392701	0.539228	protein_coding
ENSG0000(	VPS45	1024.832	-0.03745	0.392828	0.539379	protein_coding
ENSG0000(	ENSG0000(	46.61308	-0.13621	0.392903	0.539459	lncRNA
ENSG0000(	ASIC1	77.90781	-0.14479	0.392921	0.53946	protein_coding
ENSG0000(	CADM2	54.6164	-0.20567	0.392976	0.539512	protein_coding
ENSG0000(	CCNC	1193.81	0.040973	0.393047	0.539515	protein_coding
ENSG0000(	HAPLN4	113.7015	0.595391	0.393024	0.539515	protein_coding
ENSG0000(	LINC00550	107.985	-0.12497	0.393041	0.539515	lncRNA
ENSG0000(	TMEM87A	1829.155	-0.04607	0.393046	0.539515	protein_coding
ENSG0000(	IDO2	477.8454	0.057233	0.393091	0.539552	protein_coding
ENSG0000(	ANKRD13D	1037.222	0.057228	0.393209	0.539571	protein_coding
ENSG0000(	BTG3	201.008	-0.07509	0.393196	0.539571	protein_coding
ENSG0000(	ENSG0000(	12.16424	0.199446	0.393181	0.539571	lncRNA
ENSG0000(	ENSG0000(	19.79469	0.154678	0.393156	0.539571	lncRNA
ENSG0000(	PLAT	1793.756	-0.0885	0.393199	0.539571	protein_coding
ENSG0000(	PURPL	424.5586	-0.05024	0.393141	0.539571	lncRNA
ENSG0000(	ENSG0000(	381.8248	-0.07388	0.393239	0.539588	lncRNA
ENSG0000(	ENSG0000(	82.12671	-0.08786	0.393311	0.539663	lncRNA
ENSG0000(	STIM1	1526.897	0.038934	0.393455	0.539838	protein_coding
ENSG0000(	ENSG0000(	582.4654	0.134693	0.393489	0.53986	lncRNA

ENSG0000(	ENSG0000(	18.16417	-0.14748	0.393628	0.540027	lncRNA
ENSG0000(	ENSG0000(	11.37272	-0.22662	0.393926	0.540413	lncRNA
ENSG0000(	COLEC11	109.7989	0.088331	0.393944	0.540413	protein_coding
ENSG0000(	ENSG0000(	19.06399	-0.15599	0.394048	0.540533	lncRNA
ENSG0000(	ENSG0000(	51.56714	-0.08689	0.394188	0.540701	lncRNA
ENSG0000(	ENSG0000(	127.2301	-0.14158	0.394225	0.540728	lncRNA
ENSG0000(	PDGFRL	13.56344	0.211539	0.394289	0.540792	protein_coding
ENSG0000(	ZAP70	668.2825	-0.16497	0.39435	0.540852	protein_coding
ENSG0000(	CFAP300	123.5389	-0.09907	0.394422	0.540928	protein_coding
ENSG0000(	ENSG0000(	78.37692	-0.08311	0.394442	0.540931	lncRNA
ENSG0000(	SECISBP2L	2668.943	-0.0341	0.394532	0.541031	protein_coding
ENSG0000(	RN7SKP96	22.99293	0.160788	0.394581	0.541073	misc_RNA
ENSG0000(	ADARB2	136.2606	0.159013	0.394735	0.541238	protein_coding
ENSG0000(	ENSG0000(	29.01712	-0.1171	0.394719	0.541238	processed_pseudogene
ENSG0000(	ENSG0000(	293.2133	0.095212	0.394846	0.541367	lncRNA
ENSG0000(	PLCD4	172.6803	0.08288	0.394939	0.54147	protein_coding
ENSG0000(	ENSG0000(	110.779	0.066146	0.394967	0.541484	lncRNA
ENSG0000(	TMUB2	1358.079	-0.03408	0.395144	0.541704	protein_coding
ENSG0000(	TRAJ19	23.59503	0.213295	0.395219	0.541783	TR_J_gene
ENSG0000(	TRIO	8740.866	0.041647	0.395279	0.54184	protein_coding
ENSG0000(	ZNF550	575.9835	-0.05959	0.3953	0.541846	protein_coding
ENSG0000(	CCDC74A	70.9309	0.091086	0.39544	0.541966	protein_coding
ENSG0000(	DRD1	12.20141	-0.25664	0.395452	0.541966	protein_coding
ENSG0000(	ENSG0000(	31.6693	0.149911	0.395468	0.541966	lncRNA
ENSG0000(	RN7SL498F	45.00693	0.105546	0.395474	0.541966	misc_RNA
ENSG0000(	TMED6	84.18158	-0.12152	0.395405	0.541966	protein_coding
ENSG0000(	PLA2G15	460.596	0.05087	0.395534	0.542	protein_coding
ENSG0000(	RN7SL248F	11.584	0.20786	0.395525	0.542	misc_RNA
ENSG0000(	NSFP1	372.7266	-0.5843	0.395574	0.542032	unprocessed_pseudogene
ENSG0000(	GPR157	894.18	0.048743	0.395682	0.542156	protein_coding
ENSG0000(	AARD	315.9983	0.048519	0.395792	0.542259	protein_coding
ENSG0000(	ENSG0000(	21.32273	0.161051	0.395779	0.542259	lncRNA
ENSG0000(	NLGN4Y	202.4268	0.122162	0.396029	0.542536	protein_coding
ENSG0000(	ZBTB21	756.4127	0.06783	0.396029	0.542536	protein_coding
ENSG0000(	TCF7	1540.933	-0.1984	0.396075	0.542576	protein_coding
ENSG0000(	MYCL	299.0617	-0.07428	0.396109	0.542598	protein_coding
ENSG0000(	ENSG0000(	203.431	0.061717	0.396138	0.542615	lncRNA
ENSG0000(	ENSG0000(	101.261	0.060411	0.39617	0.542625	lncRNA
ENSG0000(	SRR	127.8195	0.088386	0.396181	0.542625	protein_coding
ENSG0000(	MARCHF5	646.5179	-0.04557	0.396292	0.542753	protein_coding
ENSG0000(	RNU5E-6P	99.66511	0.192483	0.396331	0.542776	snRNA
ENSG0000(	ZNF804B	9.909508	-0.38028	0.396343	0.542776	protein_coding
ENSG0000(	CP	468.9905	0.098796	0.39638	0.542803	protein_coding
ENSG0000(	SCARNA3	81.36602	0.079334	0.396473	0.542907	scaRNA
ENSG0000(	CCDC162P	699.1888	-0.11935	0.396552	0.542991	transcribed_unitary_pseudogene
ENSG0000(	C13orf42	199.8324	0.06419	0.396611	0.543048	protein_coding
ENSG0000(	KLHDC1	195.5235	0.065792	0.396672	0.543108	protein_coding

ENSG0000(	TRAJ36	26.99291	0.198282	0.396702	0.543125	TR_J_gene
ENSG0000(	ENSG0000(	133.0222	-0.07373	0.396871	0.543333	lncRNA
ENSG0000(	ZNF207	12860.17	-0.02815	0.396925	0.543383	protein_coding
ENSG0000(	ENSG0000(	12.98685	-0.29594	0.396998	0.543435	processed_pseudogene
ENSG0000(	UTY	2470.76	0.066184	0.396981	0.543435	protein_coding
ENSG0000(	ENSG0000(	23.1784	-0.16014	0.397099	0.543525	lncRNA
ENSG0000(	PCDHGA3	37.16691	-0.15714	0.397087	0.543525	protein_coding
ENSG0000(	MIR4434	8.993792	-0.22593	0.397273	0.543717	miRNA
ENSG0000(	UBR3	2384.955	0.044754	0.397262	0.543717	protein_coding
ENSG0000(	FAM95B1	1015.8	0.182748	0.397328	0.543744	transcribed_unprocessed_pseudo
ENSG0000(	NIT1	1339.461	-0.05063	0.397328	0.543744	protein_coding
ENSG0000(	ASB8	837.3736	-0.04078	0.397514	0.543927	protein_coding
ENSG0000(	LRRC15	99.25521	0.311315	0.397493	0.543927	protein_coding
ENSG0000(	PMS2CL	722.4013	-0.0427	0.397503	0.543927	transcribed_unprocessed_pseudo
ENSG0000(	ENSG0000(	61.3397	-0.10724	0.397561	0.543968	processed_pseudogene
ENSG0000(	DKFZP434A	49.31241	-0.1397	0.397678	0.544068	lncRNA
ENSG0000(	ENSG0000(	231.2409	-0.14727	0.397704	0.544068	lncRNA
ENSG0000(	GCLC	1747.288	-0.07406	0.397672	0.544068	protein_coding
ENSG0000(	RN7SL713F	9.343427	0.200279	0.397689	0.544068	misc_RNA
ENSG0000(	PCDHB11	144.0115	-0.05658	0.397733	0.544084	protein_coding
ENSG0000(	HERC2P9	4052.986	0.034991	0.39796	0.544347	transcribed_unprocessed_pseudo
ENSG0000(	TBC1D3L	1430.646	0.080704	0.397944	0.544347	protein_coding
ENSG0000(	ENSG0000(	109.3437	-0.07519	0.397992	0.544348	lncRNA
ENSG0000(	ENSG0000(	68.80305	0.075152	0.397995	0.544348	lncRNA
ENSG0000(	CERS6-AS1	101.8321	-0.07296	0.398094	0.544459	lncRNA
ENSG0000(	ENSG0000(	46.82202	-0.08981	0.398118	0.544468	lncRNA
ENSG0000(	RNF103	3369.799	0.080592	0.398159	0.544501	protein_coding
ENSG0000(	AMER2	103.5854	0.062588	0.398334	0.544714	protein_coding
ENSG0000(	ENSG0000(	479.1594	0.077016	0.398403	0.544714	lncRNA
ENSG0000(	SCN11A	347.0221	0.076662	0.398396	0.544714	protein_coding
ENSG0000(	VPS36	3073.144	-0.03213	0.398365	0.544714	protein_coding
ENSG0000(	ZNF713	1322.675	-0.02689	0.398401	0.544714	protein_coding
ENSG0000(	CARF	1059.769	0.045366	0.398462	0.544772	protein_coding
ENSG0000(	LRRIQ4	57.13051	0.099822	0.398507	0.544809	protein_coding
ENSG0000(	ENSG0000(	25.7289	-0.34912	0.398567	0.544855	lncRNA
ENSG0000(	MFSD2B	70.57361	-0.08055	0.398575	0.544855	protein_coding
ENSG0000(	RLF	1239.678	-0.04184	0.398667	0.544956	protein_coding
ENSG0000(	KREMEN1	2260.37	-0.04191	0.398772	0.545076	protein_coding
ENSG0000(	LINC02181	76.11464	0.075526	0.398798	0.545087	lncRNA
ENSG0000(	SERPINB6	7039.186	0.071209	0.398835	0.545115	protein_coding
ENSG0000(	LINC00861	866.0435	0.196179	0.398862	0.545128	lncRNA
ENSG0000(	ENSG0000(	9.206993	0.205615	0.39891	0.545169	lncRNA
ENSG0000(	ENSG0000(	11.74766	-0.34143	0.398928	0.545171	processed_pseudogene
ENSG0000(	CHTF8	526.9829	-0.10255	0.398963	0.545195	protein_coding
ENSG0000(	CYCSP38	12.83493	0.177246	0.399124	0.54539	transcribed_processed_pseudoge
ENSG0000(	BUB1	407.667	-0.09705	0.399176	0.545438	protein_coding
ENSG0000(	ENSG0000(	69.65775	-0.0964	0.399259	0.545511	lncRNA

ENSG0000(	MIR941-2	26.37048	-0.22151	0.399265	0.545511	miRNA
ENSG0000(	ENSG0000(	189.9225	0.145247	0.39929	0.545522	lncRNA
ENSG0000(	OLFML2A	697.2792	-0.06984	0.399323	0.545543	protein_coding
ENSG0000(	ARL8A	212.9099	-0.08619	0.399355	0.545556	protein_coding
ENSG0000(	CCNYL6	51.34451	-0.08973	0.399372	0.545556	transcribed_processed_pseudoge
ENSG0000(	ENSG0000(	15.98984	0.193782	0.399385	0.545556	lncRNA
ENSG0000(	MIDEAS	6042.234	-0.06935	0.399441	0.545609	protein_coding
ENSG0000(	ENSG0000(	10.98938	0.292149	0.399501	0.545666	misc_RNA
ENSG0000(	ENSG0000(	11.30935	-0.16392	0.399587	0.545761	lncRNA
ENSG0000(	DLG5-AS1	237.0527	-0.05313	0.399787	0.54601	lncRNA
ENSG0000(	IFT27	992.7366	-0.05509	0.399819	0.54603	protein_coding
ENSG0000(	MEG8	2394.456	0.064975	0.399912	0.546133	lncRNA
ENSG0000(	ENSG0000(	33.74116	-0.16251	0.399981	0.54618	lncRNA
ENSG0000(	PCDHGA11	148.4376	-0.07903	0.399972	0.54618	protein_coding
ENSG0000(	HMGB1P1	55.82101	-0.0885	0.400009	0.546194	protein_coding
ENSG0000(	EPIST	16.27118	-0.16745	0.40005	0.546226	lncRNA
ENSG0000(	ATPSCKMT	567.4337	-0.05405	0.400122	0.5463	protein_coding
ENSG0000(	Metazoa_S	22281.63	0.026008	0.400295	0.546513	misc_RNA
ENSG0000(	RN7SL424F	24.59341	0.136876	0.400374	0.546574	misc_RNA
ENSG0000(	SERP1	2079.973	0.05096	0.400366	0.546574	protein_coding
ENSG0000(	ROCK1P1	935.9865	-0.25849	0.400491	0.546709	transcribed_unprocessed_pseudo
ENSG0000(	FAM53B	2038.438	0.049068	0.400549	0.546765	protein_coding
ENSG0000(	ENSG0000(	290.0684	0.044152	0.400653	0.546882	lncRNA
ENSG0000(	ENSG0000(	8.215567	-0.25729	0.400737	0.546971	processed_pseudogene
ENSG0000(	NLRP6	63.30244	0.204291	0.400753	0.546971	protein_coding
ENSG0000(	ENSG0000(	83.55342	-0.07793	0.40086	0.54707	lncRNA
ENSG0000(	RNU6-171F	62.74045	0.116598	0.400845	0.54707	snRNA
ENSG0000(	ENSG0000(	201.643	-0.0562	0.400902	0.547103	transcribed_processed_pseudoge
ENSG0000(	ENSG0000(	25.89555	0.12994	0.401128	0.547366	lncRNA
ENSG0000(	HPS4	2165.973	0.032032	0.40113	0.547366	protein_coding
ENSG0000(	TARS1-DT	103.3625	-0.13862	0.401226	0.547473	lncRNA
ENSG0000(	EOMES	60.02007	0.190284	0.401294	0.547542	protein_coding
ENSG0000(	CHST1	241.1719	0.061632	0.401353	0.547598	protein_coding
ENSG0000(	AQP7	781.8884	0.131633	0.401435	0.547619	protein_coding
ENSG0000(	EIF4BP7	19.14139	-0.1779	0.401426	0.547619	processed_pseudogene
ENSG0000(	ENSG0000(	39.40051	-0.09896	0.401438	0.547619	lncRNA
ENSG0000(	ZNF788P	1122.214	-0.04617	0.401413	0.547619	transcribed_unprocessed_pseudo
ENSG0000(	PDZK1	89.64609	-0.13071	0.401459	0.547624	protein_coding
ENSG0000(	ENSG0000(	19.91022	0.15707	0.401525	0.547666	lncRNA
ENSG0000(	RNU6-1316	16.41791	0.198224	0.401521	0.547666	snRNA
ENSG0000(	LACTB2	122.2243	-0.10121	0.401556	0.547685	protein_coding
ENSG0000(	STAG1-DT	122.9413	-0.05767	0.401617	0.547744	lncRNA
ENSG0000(	ENSG0000(	27.4774	-0.1068	0.401635	0.547744	lncRNA
ENSG0000(	DYNC1I1	52.75723	0.105054	0.401755	0.547799	protein_coding
ENSG0000(	ENSG0000(	175.2904	0.120698	0.40176	0.547799	lncRNA
ENSG0000(	ENSG0000(	22.00681	0.14345	0.401721	0.547799	lncRNA
ENSG0000(	ENSG0000(	93.1528	-0.07691	0.401763	0.547799	lncRNA

ENSG0000(	JRKL	429.4345	-0.05732	0.401736	0.547799	protein_coding
ENSG0000(	PRKCQ-AS1	559.2132	0.08901	0.401848	0.547892	lncRNA
ENSG0000(	MSH4	41.27751	-0.15626	0.401989	0.54806	protein_coding
ENSG0000(	ENSG0000(	8.212303	0.252128	0.402036	0.548076	lncRNA
ENSG0000(	ENSG0000(	25.77124	-0.1303	0.402025	0.548076	lncRNA
ENSG0000(	NFXL1	630.7478	-0.06383	0.402237	0.548326	protein_coding
ENSG0000(	ENSG0000(	173.0417	0.055978	0.402264	0.548339	lncRNA
ENSG0000(	ENSG0000(	17.29476	0.547068	0.402304	0.54837	lncRNA
ENSG0000(	ENSG0000(	69.76209	0.094009	0.402498	0.548611	lncRNA
ENSG0000(	ENSG0000(	111.0319	-0.06328	0.402521	0.548618	lncRNA
ENSG0000(	IER3IP1	531.4711	-0.04587	0.402815	0.548994	protein_coding
ENSG0000(	ENSG0000(	52.88665	0.078635	0.402981	0.549174	lncRNA
ENSG0000(	LINC01121	220.7565	0.059016	0.402973	0.549174	lncRNA
ENSG0000(	CASC20	32.5272	0.111163	0.403112	0.549232	lncRNA
ENSG0000(	ENSG0000(	24.94027	0.115389	0.403061	0.549232	lncRNA
ENSG0000(	FMR1-IT1	154.9848	-0.07794	0.403083	0.549232	lncRNA
ENSG0000(	KIAA1210	67.64799	-0.13901	0.403106	0.549232	protein_coding
ENSG0000(	RPS20P9	23.46819	0.143663	0.403067	0.549232	processed_pseudogene
ENSG0000(	LINC02297	13.50516	0.376419	0.403158	0.549271	lncRNA
ENSG0000(	ENSG0000(	76.08918	0.15573	0.403246	0.549366	lncRNA
ENSG0000(	ENSG0000(	120.1454	-0.09424	0.403271	0.549377	lncRNA
ENSG0000(	LIMS4	222.3823	0.233373	0.403424	0.549539	protein_coding
ENSG0000(	OR1AB1P	62.82647	0.107577	0.403426	0.549539	unprocessed_pseudogene
ENSG0000(	ENSG0000(	3298.557	-0.06811	0.403462	0.549565	transcribed_unprocessed_pseudo
ENSG0000(	CALML3-AS	257.607	-0.04954	0.40348	0.549566	lncRNA
ENSG0000(	ENSG0000(	130.1581	-0.10283	0.403553	0.549641	lncRNA
ENSG0000(	RN7SL687F	10.67328	0.208938	0.403609	0.549694	misc_RNA
ENSG0000(	DNAJC19P5	97.46226	0.069645	0.403896	0.55006	processed_pseudogene
ENSG0000(	RN7SL864F	10.69335	0.215331	0.403913	0.55006	misc_RNA
ENSG0000(	METTL9	1252.883	0.058366	0.403969	0.550112	protein_coding
ENSG0000(	ENSG0000(	574.7434	0.034568	0.404145	0.550328	transcribed_processed_pseudoge
ENSG0000(	SEPTIN11	4035.999	-0.04549	0.404166	0.550332	protein_coding
ENSG0000(	NHLRC4	23.8727	0.14873	0.404267	0.550446	protein_coding
ENSG0000(	RARA-AS1	51.47531	0.127774	0.404303	0.55047	lncRNA
ENSG0000(	ENSG0000(	20.30854	0.133043	0.404367	0.550534	lncRNA
ENSG0000(	ENSG0000(	24.80343	0.14302	0.404417	0.550554	lncRNA
ENSG0000(	FAM43A	358.7349	0.154663	0.404405	0.550554	protein_coding
ENSG0000(	CCND1	1268.603	-0.08332	0.404505	0.550647	protein_coding
ENSG0000(	MYCT1	122.1251	0.121612	0.40452	0.550647	protein_coding
ENSG0000(	ENSG0000(	19.66459	-0.17607	0.404544	0.550655	protein_coding
ENSG0000(	ENSG0000(	76.64394	-0.1777	0.404799	0.550979	lncRNA
ENSG0000(	UBXN10	163.4426	0.11834	0.404885	0.551072	protein_coding
ENSG0000(	LANCL1	816.4467	-0.05921	0.404928	0.551106	protein_coding
ENSG0000(	PTK2B	3065.049	0.053244	0.404975	0.551145	protein_coding
ENSG0000(	CD300LD	71.57647	0.0718	0.405073	0.551231	protein_coding
ENSG0000(	SUPT16HP1	9.119061	-0.19811	0.405065	0.551231	processed_pseudogene
ENSG0000(	CCNY-AS1	532.5355	0.035328	0.4051	0.551244	lncRNA

ENSG0000(	FAAP20	1049.854	-0.04931	0.40513	0.551261	protein_coding
ENSG0000(	RAD23BP1	9.425298	-0.24999	0.40517	0.551267	processed_pseudogene
ENSG0000(	UFD1	2042.242	0.0359	0.405155	0.551267	protein_coding
ENSG0000(	ENSG0000(	111.9973	-0.09523	0.405212	0.551277	protein_coding
ENSG0000(	LINC02068	199.9301	0.074834	0.405207	0.551277	lncRNA
ENSG0000(	DHX37	680.3293	-0.0397	0.405243	0.551294	protein_coding
ENSG0000(	ENSG0000(	126.3701	-0.21652	0.405334	0.551394	lncRNA
ENSG0000(	GDAP1L1	18.86718	0.145435	0.405369	0.551394	protein_coding
ENSG0000(	MFSD14A	486.5646	0.104995	0.405366	0.551394	protein_coding
ENSG0000(	PROKR2	257.8095	0.043803	0.405514	0.551567	protein_coding
ENSG0000(	AZGP1P1	14.84949	-0.21485	0.405556	0.5516	transcribed_unprocessed_pseudo
ENSG0000(	ELAPOR2	378.2148	-0.13317	0.405577	0.551605	protein_coding
ENSG0000(	MUC13	7200.128	0.130332	0.405689	0.551734	protein_coding
ENSG0000(	AVPR1B	28.25246	-0.20832	0.405776	0.551828	protein_coding
ENSG0000(	ADAMTS15	194.4126	0.132125	0.405818	0.551861	protein_coding
ENSG0000(	ALOX12B	8.540815	-0.29944	0.405855	0.551887	protein_coding
ENSG0000(	RAG1	92.07969	0.175795	0.405952	0.551995	protein_coding
ENSG0000(	ENSG0000(	12.66844	0.169283	0.406069	0.552131	lncRNA
ENSG0000(	EIF2A	1392.699	0.044398	0.406136	0.552177	protein_coding
ENSG0000(	ENSG0000(	407.7698	0.051584	0.406139	0.552177	lncRNA
ENSG0000(	ENSG0000(	32.4004	-0.22681	0.4062	0.552212	protein_coding
ENSG0000(	OR1L8	17.90356	-0.1196	0.406186	0.552212	protein_coding
ENSG0000(	ENSG0000(	9.754622	-0.15566	0.40625	0.552257	lncRNA
ENSG0000(	DMWD	1301.448	-0.02808	0.406282	0.552276	protein_coding
ENSG0000(	R3HCC1	212.2947	-0.06708	0.406331	0.552318	protein_coding
ENSG0000(	ALKBH1	270.2849	0.061885	0.406457	0.552441	protein_coding
ENSG0000(	PRR29	73.93524	0.091979	0.406453	0.552441	protein_coding
ENSG0000(	CLUHP3	2020.712	-0.03917	0.40651	0.55249	transcribed_unprocessed_pseudo
ENSG0000(	NSD1	7066.818	0.029799	0.406565	0.55254	protein_coding
ENSG0000(	SNRNP27	263.7326	-0.07177	0.406583	0.55254	protein_coding
ENSG0000(	GPR21	187.1231	0.055538	0.406621	0.552569	protein_coding
ENSG0000(	XKR8	585.8369	0.045216	0.406809	0.5528	protein_coding
ENSG0000(	SLC25A41	53.1082	-0.10173	0.40684	0.552818	protein_coding
ENSG0000(	DDTL	148.8024	-0.20035	0.40691	0.552889	protein_coding
ENSG0000(	WLS	1070.887	-0.07777	0.406966	0.552941	protein_coding
ENSG0000(	ENSG0000(	956.6867	-0.05341	0.407032	0.552983	lncRNA
ENSG0000(	SMIM27	280.4325	0.062892	0.407024	0.552983	protein_coding
ENSG0000(	ENSG0000(	33.29048	-0.145	0.407082	0.553021	lncRNA
ENSG0000(	SP3	4848.714	0.041517	0.407096	0.553021	protein_coding
ENSG0000(	MEAK7	1573.937	-0.05378	0.407142	0.55306	protein_coding
ENSG0000(	DNAJB6P8	11.66596	0.205875	0.40751	0.553536	transcribed_processed_pseudoge
ENSG0000(	HRK	233.0397	-0.09512	0.407659	0.55369	protein_coding
ENSG0000(	PCBP2-OT1	76.69276	-0.0824	0.407654	0.55369	lncRNA
ENSG0000(	PAX4	11.15343	0.230979	0.407741	0.553778	protein_coding
ENSG0000(	SUSD2	41.84387	-0.15244	0.4078	0.553834	protein_coding
ENSG0000(	APOC1P1	66.29133	-0.11318	0.40782	0.553835	transcribed_unprocessed_pseudo
ENSG0000(	C10orf95	23.15262	0.123389	0.407872	0.553835	protein_coding

ENSG0000(	SLC9A5	99.28954	0.105834	0.407854	0.553835	protein_coding
ENSG0000(	TRIM2	6920.894	-0.08184	0.407858	0.553835	protein_coding
ENSG0000(	ENSG0000(	11.11885	0.213984	0.408032	0.554028	lncRNA
ENSG0000(	OTUD5	872.8015	-0.04318	0.40805	0.554029	protein_coding
ENSG0000(	C1orf21-DT	11.13994	-0.16037	0.408115	0.554093	lncRNA
ENSG0000(	WRAP53	129.789	-0.08499	0.408174	0.554149	protein_coding
ENSG0000(	KLF9	1315.381	0.077795	0.408232	0.554204	protein_coding
ENSG0000(	ENSG0000(	180.5166	0.045487	0.408305	0.554237	processed_pseudogene
ENSG0000(	ENSG0000(	29.83572	-0.20296	0.408327	0.554237	lncRNA
ENSG0000(	GID4	772.8487	0.037157	0.408292	0.554237	protein_coding
ENSG0000(	MYORG	3375.371	-0.07278	0.408318	0.554237	protein_coding
ENSG0000(	C3orf20	31.66548	0.163563	0.408441	0.554368	protein_coding
ENSG0000(	ENSG0000(	38.03555	0.272306	0.408461	0.554371	lncRNA
ENSG0000(	FOXO6-AS1	30.30814	0.112759	0.408576	0.554502	lncRNA
ENSG0000(	SIDT1	1762.771	0.06489	0.408626	0.554546	protein_coding
ENSG0000(	ENSG0000(	13.63526	0.192597	0.408678	0.55459	lncRNA
ENSG0000(	ENSG0000(	599.118	0.037911	0.408709	0.55459	lncRNA
ENSG0000(	ENSG0000(	35.40335	-0.12887	0.408711	0.55459	lncRNA
ENSG0000(	ENSG0000(	55.36349	-0.08254	0.408746	0.554613	lncRNA
ENSG0000(	HCN2	9.920505	0.247366	0.408844	0.554703	protein_coding
ENSG0000(	IGKV1OR-2	7.530712	0.452357	0.408857	0.554703	IG_V_pseudogene
ENSG0000(	NOP14-AS1	560.9381	0.046269	0.408865	0.554703	lncRNA
ENSG0000(	NBR2	833.681	-0.04293	0.408896	0.55472	lncRNA
ENSG0000(	ARSD-AS1	35.74527	-0.22286	0.40902	0.554845	lncRNA
ENSG0000(	ENSG0000(	12.89465	0.175814	0.409024	0.554845	lncRNA
ENSG0000(	ZC3H10	671.4341	0.049546	0.4091	0.554924	protein_coding
ENSG0000(	ENSG0000(	47.08968	0.130159	0.409163	0.554975	lncRNA
ENSG0000(	ZNF507	2280.247	-0.03258	0.409173	0.554975	protein_coding
ENSG0000(	PCK2	942.7047	-0.08599	0.409272	0.555086	protein_coding
ENSG0000(	ENSG0000(	17.61121	-0.15759	0.409296	0.555094	lncRNA
ENSG0000(	PARD6G	308.7449	-0.04563	0.409347	0.555139	protein_coding
ENSG0000(	DENND1C	501.7119	0.135993	0.40942	0.555189	protein_coding
ENSG0000(	ENSG0000(	189.5884	0.046817	0.409405	0.555189	lncRNA
ENSG0000(	LINC02261	320.7833	-0.04299	0.409674	0.55551	lncRNA
ENSG0000(	RN7SL331P	11.91316	0.187647	0.40973	0.555563	misc_RNA
ENSG0000(	AOC4P	78.12967	-0.08533	0.40976	0.555579	transcribed_unprocessed_pseudo
ENSG0000(	FZD8	147.4175	-0.09897	0.409805	0.555615	protein_coding
ENSG0000(	OLR1	226.0698	-0.06549	0.409964	0.555783	protein_coding
ENSG0000(	SOX9	806.3682	-0.11803	0.409956	0.555783	protein_coding
ENSG0000(	ITM2B	3391.433	-0.10017	0.409998	0.555793	protein_coding
ENSG0000(	SYVN1	2023.352	-0.07366	0.410007	0.555793	protein_coding
ENSG0000(	HMGN1P1(	29.26291	-0.11882	0.410157	0.555972	processed_pseudogene
ENSG0000(	ATP6V1G2-	11.27365	-0.63897	0.410239	0.556059	protein_coding
ENSG0000(	MTND4P9	30.90002	0.145161	0.410357	0.556195	unprocessed_pseudogene
ENSG0000(	CEP350	5242.491	-0.04895	0.410454	0.55628	protein_coding
ENSG0000(	MEIG1	33.37763	-0.1466	0.410455	0.55628	protein_coding
ENSG0000(	PCDHB13	284.5912	0.041735	0.410542	0.556373	protein_coding

ENSG0000(	RBM4B	438.5927	-0.04501	0.410575	0.556394	protein_coding
ENSG0000(	ENSG0000(	1670.444	-0.05733	0.410634	0.556427	lncRNA
ENSG0000(	MC5R	15.43293	0.155352	0.410629	0.556427	protein_coding
ENSG0000(	ENSG0000(	175.1443	-0.05269	0.410784	0.556582	TEC
ENSG0000(	LINC02575	124.48	0.055388	0.41078	0.556582	lncRNA
ENSG0000(	APBB2	1595.186	-0.05365	0.410828	0.556617	protein_coding
ENSG0000(	GRIN2C	37.22986	0.108438	0.410856	0.55663	protein_coding
ENSG0000(	GGNBP2	2796.867	-0.03607	0.410903	0.55667	protein_coding
ENSG0000(	KLK2	125.9165	0.069213	0.410992	0.556767	protein_coding
ENSG0000(	VEGFB	319.7527	-0.07554	0.411258	0.557103	protein_coding
ENSG0000(	ACP6	1234.273	-0.06411	0.411303	0.557139	protein_coding
ENSG0000(	ENSG0000(	11.74337	0.190578	0.41133	0.557151	lncRNA
ENSG0000(	PLAAT5	11.5247	-0.24883	0.411349	0.557154	protein_coding
ENSG0000(	IKBKG	856.3295	0.046613	0.411374	0.557163	protein_coding
ENSG0000(	ADAM23	92.20828	0.128077	0.411424	0.557207	protein_coding
ENSG0000(	ENSG0000(	32.44614	-0.12782	0.411526	0.55732	lncRNA
ENSG0000(	ABCC6	831.1264	0.133557	0.411634	0.557419	protein_coding
ENSG0000(	C17orf99	276.2642	0.078985	0.411618	0.557419	protein_coding
ENSG0000(	CHN2	1132.7	-0.0584	0.411706	0.557492	protein_coding
ENSG0000(	ENSG0000(	133.4132	-0.05701	0.411734	0.557506	transcribed_processed_pseudoge
ENSG0000(	BBOX1-AS1	67.98743	0.094419	0.411801	0.557549	lncRNA
ENSG0000(	KLHL15	333.7353	0.057364	0.411789	0.557549	protein_coding
ENSG0000(	CYP4F62P	21.48319	0.453771	0.411898	0.557568	transcribed_unprocessed_pseudo
ENSG0000(	ENSG0000(	19.56824	-0.15264	0.411839	0.557568	lncRNA
ENSG0000(	ENSG0000(	177.6139	0.053132	0.411921	0.557568	lncRNA
ENSG0000(	ENSG0000(	23.67477	-0.12807	0.411922	0.557568	lncRNA
ENSG0000(	ENSG0000(	10.14822	0.187517	0.411891	0.557568	lncRNA
ENSG0000(	UBE2L5	113.6483	0.071344	0.411898	0.557568	protein_coding
ENSG0000(	ENSG0000(	22.61393	0.14791	0.411981	0.557624	lncRNA
ENSG0000(	REEP1	251.3832	-0.06044	0.41204	0.557679	protein_coding
ENSG0000(	DPF2	1798.337	-0.02558	0.412134	0.557782	protein_coding
ENSG0000(	LINC02688	65.03154	0.128893	0.412189	0.557832	lncRNA
ENSG0000(	ENSG0000(	8.902735	0.205462	0.412336	0.558007	lncRNA
ENSG0000(	PIGP	493.5025	-0.05706	0.412542	0.558261	protein_coding
ENSG0000(	ENSG0000(	395.8609	-0.04708	0.412599	0.558315	lncRNA
ENSG0000(	DBNL	5164.539	0.049832	0.412698	0.558425	protein_coding
ENSG0000(	ZBTB6	368.8526	-0.0439	0.412814	0.558557	protein_coding
ENSG0000(	SLCO1C1	78.68629	-0.15561	0.412938	0.5587	protein_coding
ENSG0000(	ENSG0000(	10.93484	0.19695	0.41316	0.558977	lncRNA
ENSG0000(	ZNF702P	532.1795	-0.05194	0.413213	0.559025	transcribed_processed_pseudoge
ENSG0000(	ASPSCR1	1273.87	0.031061	0.413279	0.559066	protein_coding
ENSG0000(	KRBA1	158.9596	0.104677	0.413278	0.559066	protein_coding
ENSG0000(	ENSG0000(	245.5532	0.049232	0.413436	0.559253	lncRNA
ENSG0000(	FHAD1-AS1	84.24158	-0.06808	0.413467	0.559272	lncRNA
ENSG0000(	SNORD13D	14.68628	-0.25137	0.413529	0.559331	snoRNA
ENSG0000(	FAM149B1	750.376	0.04473	0.413649	0.559464	protein_coding
ENSG0000(	ZNF251	839.9267	-0.04226	0.413663	0.559464	protein_coding

ENSG0000(	ENSG0000(	11.89481	-0.19641	0.413717	0.55949	processed_pseudogene
ENSG0000(	HARBI1	266.2454	-0.04994	0.413718	0.55949	protein_coding
ENSG0000(	EVA1C	421.6442	0.072881	0.41387	0.559648	protein_coding
ENSG0000(	LINC01502	122.6655	0.07021	0.413858	0.559648	lncRNA
ENSG0000(	BAIAP2L1	2474.433	0.096807	0.413913	0.55968	protein_coding
ENSG0000(	OFCC1	14.02803	0.181616	0.41415	0.559977	transcribed_unitary_pseudogene
ENSG0000(	ENSG0000(	92.92853	-0.0624	0.4142	0.560021	lncRNA
ENSG0000(	MLXIPL	500.2472	0.066311	0.414377	0.560236	protein_coding
ENSG0000(	DNAJB2	811.7214	0.040898	0.414496	0.560373	protein_coding
ENSG0000(	LINC01583	16.51014	0.141304	0.414522	0.560384	lncRNA
ENSG0000(	KHNYN	5098.206	-0.04961	0.414594	0.560446	protein_coding
ENSG0000(	SYNGR2	1947.989	-0.05609	0.414604	0.560446	protein_coding
ENSG0000(	RN7SL680F	12.09317	0.17237	0.414638	0.560468	misc_RNA
ENSG0000(	ACAP2-IT1	115.7089	0.120183	0.414677	0.560496	lncRNA
ENSG0000(	CNGA3	9.782698	-0.29046	0.414706	0.560511	protein_coding
ENSG0000(	ENSG0000(	65.86276	-0.08694	0.414744	0.560537	lncRNA
ENSG0000(	ANKHD1-EI	2090.471	-0.05253	0.414853	0.560661	protein_coding
ENSG0000(	ENSG0000(	16.43655	-0.19156	0.414916	0.560722	lncRNA
ENSG0000(	WARS2	379.6754	-0.06664	0.415075	0.560913	protein_coding
ENSG0000(	ENSG0000(	47.29812	-0.09488	0.415229	0.561073	lncRNA
ENSG0000(	ENSG0000(	10.8667	-0.18781	0.41523	0.561073	lncRNA
ENSG0000(	TLE1	980.5379	0.068081	0.415498	0.561412	protein_coding
ENSG0000(	C12orf50	2374.564	-0.0448	0.41558	0.561497	protein_coding
ENSG0000(	ENSG0000(	24.73154	0.134385	0.415604	0.561506	lncRNA
ENSG0000(	ENSG0000(	10.42823	0.187792	0.415624	0.561509	lncRNA
ENSG0000(	ENSG0000(	20.10432	0.559904	0.415674	0.561552	lncRNA
ENSG0000(	ENSG0000(	44.21439	-0.09405	0.415755	0.561564	lncRNA
ENSG0000(	ENSG0000(	17.75261	0.17536	0.415735	0.561564	lncRNA
ENSG0000(	ENSG0000(	230.946	0.070802	0.415706	0.561564	TEC
ENSG0000(	NUS1	1348.914	0.052859	0.415743	0.561564	protein_coding
ENSG0000(	FBXO7	2258.215	0.030635	0.415776	0.561569	protein_coding
ENSG0000(	HRNR	20.3728	0.819377	0.415935	0.561759	protein_coding
ENSG0000(	LINC00682	61.70957	-0.07224	0.416009	0.561822	lncRNA
ENSG0000(	PRSS42P	20.50933	0.183571	0.416017	0.561822	transcribed_unitary_pseudogene
ENSG0000(	DENND6B	374.3472	0.059716	0.416052	0.561844	protein_coding
ENSG0000(	BCL2L2	1944.03	-0.04688	0.416098	0.561849	protein_coding
ENSG0000(	ENSG0000(	49.34744	0.105101	0.416077	0.561849	lncRNA
ENSG0000(	HOXD4	156.6287	-0.13262	0.416109	0.561849	protein_coding
ENSG0000(	C19orf73	11.95749	-0.19213	0.416245	0.562008	protein_coding
ENSG0000(	RNU6-233F	29.24304	0.097028	0.416343	0.562116	snRNA
ENSG0000(	LACC1	773.324	0.074754	0.416412	0.562186	protein_coding
ENSG0000(	ADAMTS19	38.26103	-0.26972	0.416498	0.562252	protein_coding
ENSG0000(	NCAM2	75.67565	-0.19232	0.416493	0.562252	protein_coding
ENSG0000(	KIAA2012-/	237.2482	0.046171	0.416565	0.562319	lncRNA
ENSG0000(	LINC02246	254.2383	0.06673	0.416593	0.562332	lncRNA
ENSG0000(	ENSG0000(	4593.358	0.034095	0.416806	0.562595	lncRNA
ENSG0000(	AMOT	465.034	-0.11803	0.41684	0.562601	protein_coding

ENSG0000(	HTT-AS	100.4162	-0.06252	0.416846	0.562601	lncRNA
ENSG0000(	ENSG0000(	31.84958	0.132062	0.416892	0.562614	lncRNA
ENSG0000(	ENSG0000(	291.2345	-0.14374	0.416879	0.562614	lncRNA
ENSG0000(	FGF11	197.2024	-0.05971	0.416939	0.562653	protein_coding
ENSG0000(	LINC00384	24.17651	-0.19094	0.41703	0.562727	lncRNA
ENSG0000(	NIPAL4	19.6629	0.19168	0.417028	0.562727	protein_coding
ENSG0000(	SALL1	527.7837	0.120075	0.417054	0.562735	protein_coding
ENSG0000(	ENSG0000(	164.1469	-0.06811	0.417094	0.562765	lncRNA
ENSG0000(	ENSG0000(	72.71051	0.081497	0.41712	0.562776	lncRNA
ENSG0000(	MYNN	578.1181	-0.04327	0.417157	0.562802	protein_coding
ENSG0000(	FAM226B	65.02823	0.104547	0.417251	0.562904	lncRNA
ENSG0000(	RPL5P4	112.4094	0.078037	0.417308	0.562957	processed_pseudogene
ENSG0000(	TYW3	621.8677	-0.04257	0.417438	0.563103	protein_coding
ENSG0000(	ZNRD2-DT	295.5236	-0.0415	0.417452	0.563103	lncRNA
ENSG0000(	ITGAE	713.5892	-0.04316	0.41766	0.56336	protein_coding
ENSG0000(	IGHV4-4	282.1532	0.280664	0.417761	0.563447	IG_V_gene
ENSG0000(	SNORD101	24.18769	0.14202	0.417745	0.563447	snoRNA
ENSG0000(	LINC02635	85.17214	0.089544	0.417795	0.563468	lncRNA
ENSG0000(	ENSG0000(	60.61654	0.1086	0.417949	0.563651	lncRNA
ENSG0000(	APBB3	650.1418	0.047458	0.418067	0.563749	protein_coding
ENSG0000(	CRMP1	52.34659	0.149295	0.418093	0.563749	protein_coding
ENSG0000(	ENSG0000(	10.76664	0.167334	0.418081	0.563749	lncRNA
ENSG0000(	ENSG0000(	51.47012	-0.09206	0.418075	0.563749	lncRNA
ENSG0000(	ENSG0000(	31.58838	-0.1002	0.418151	0.563803	lncRNA
ENSG0000(	ENSG0000(	85.8778	-0.08046	0.418211	0.563836	lncRNA
ENSG0000(	KRBOX1	18.12711	-0.24479	0.418212	0.563836	protein_coding
ENSG0000(	TGFBR3	683.7504	-0.09372	0.418375	0.564031	protein_coding
ENSG0000(	CMPK2	384.0491	0.187911	0.418501	0.564177	protein_coding
ENSG0000(	ENSG0000(	32.91171	-0.14609	0.418682	0.564396	lncRNA
ENSG0000(	MIR5190	15.27849	0.166899	0.41871	0.564411	miRNA
ENSG0000(	SPX	25.25219	0.155985	0.418862	0.564591	protein_coding
ENSG0000(	ENSG0000(	36.41196	0.117248	0.418942	0.564675	lncRNA
ENSG0000(	RN7SKP25:	37.73764	-0.12926	0.418993	0.564718	misc_RNA
ENSG0000(	ENSG0000(	246.7749	-0.04678	0.41927	0.564994	lncRNA
ENSG0000(	ENSG0000(	24.44201	0.148893	0.41925	0.564994	lncRNA
ENSG0000(	FAM199X	1479.075	-0.04989	0.419267	0.564994	protein_coding
ENSG0000(	HPCA	12.68288	-0.23546	0.419218	0.564994	protein_coding
ENSG0000(	ENSG0000(	98.18372	-0.0666	0.41933	0.565051	lncRNA
ENSG0000(	IDUA	370.3266	0.075357	0.419529	0.565295	protein_coding
ENSG0000(	ZNF654	930.0642	-0.04749	0.419552	0.565301	protein_coding
ENSG0000(	DUSP3	1534.798	-0.04667	0.419586	0.565323	protein_coding
ENSG0000(	PEAK3	114.6157	0.082713	0.419627	0.565354	protein_coding
ENSG0000(	ENSG0000(	191.0166	-0.34813	0.419787	0.565545	transcribed_processed_pseudoge
ENSG0000(	RBM12B-D	26.09058	0.135731	0.420009	0.565819	lncRNA
ENSG0000(	ENSG0000(	317.3011	0.073444	0.420065	0.565871	lncRNA
ENSG0000(	ENSG0000(	59.97497	-0.09152	0.420157	0.56597	transcribed_unprocessed_pseudo
ENSG0000(	HOXC8	15.2929	0.245419	0.420175	0.56597	protein_coding

ENSG0000(	CDH2	69.93947	0.095795	0.420257	0.565983	protein_coding
ENSG0000(	ENSG0000(	1113.055	-0.05661	0.420219	0.565983	lncRNA
ENSG0000(	OR2S1P	147.467	0.05683	0.420244	0.565983	transcribed_unprocessed_pseudo
ENSG0000(	OSTCP8	81.3108	0.085448	0.420246	0.565983	processed_pseudogene
ENSG0000(	ANKRD23	122.1914	-0.07913	0.420297	0.565997	protein_coding
ENSG0000(	TRPC1	176.0093	0.100785	0.420304	0.565997	protein_coding
ENSG0000(	ENSG0000(	422.3174	0.074951	0.420463	0.566188	processed_pseudogene
ENSG0000(	ENSG0000(	10.619	0.221924	0.420542	0.566222	processed_pseudogene
ENSG0000(	ENSG0000(	515.4651	-0.05838	0.420543	0.566222	protein_coding
ENSG0000(	ZNF576	487.9638	-0.03592	0.420522	0.566222	protein_coding
ENSG0000(	MIR5010	13.64882	-0.16697	0.420565	0.566228	miRNA
ENSG0000(	RAB3B	1164.545	0.059049	0.42065	0.566317	protein_coding
ENSG0000(	ENSG0000(	18.54794	0.12808	0.420756	0.566419	lncRNA
ENSG0000(	ENSG0000(	129.0641	-0.0866	0.420777	0.566419	lncRNA
ENSG0000(	LGALS8-AS:	14.84269	-0.15077	0.420786	0.566419	lncRNA
ENSG0000(	LYRM4-AS1	173.3559	0.05197	0.420798	0.566419	lncRNA
ENSG0000(	ENSG0000(	44.02148	-0.16576	0.420914	0.56655	processed_pseudogene
ENSG0000(	ENSG0000(	197.0843	-0.05183	0.420969	0.566576	lncRNA
ENSG0000(	ENSG0000(	29.70296	0.120775	0.420952	0.566576	snoRNA
ENSG0000(	ENSG0000(	51.02506	0.170398	0.421083	0.566677	lncRNA
ENSG0000(	FGF9	311.3903	0.089569	0.421098	0.566677	protein_coding
ENSG0000(	NCOA2	3318.414	0.038321	0.42107	0.566677	protein_coding
ENSG0000(	ENSG0000(	62.14814	-0.07961	0.421509	0.567188	lncRNA
ENSG0000(	NKIRAS1	218.6454	0.058118	0.421514	0.567188	protein_coding
ENSG0000(	HLA-W	20.73286	0.18556	0.421548	0.567209	unprocessed_pseudogene
ENSG0000(	SGSH	1171.762	0.054431	0.421643	0.567298	protein_coding
ENSG0000(	WDR6	3100.911	-0.05247	0.421651	0.567298	protein_coding
ENSG0000(	SNORA38B	63.53539	-0.11124	0.421706	0.567349	snoRNA
ENSG0000(	ENSG0000(	461.7537	0.044929	0.421736	0.567365	lncRNA
ENSG0000(	ENSG0000(	13.77624	-0.17575	0.421763	0.567376	lncRNA
ENSG0000(	ENSG0000(	33.42122	0.206559	0.421832	0.567443	transcribed_unprocessed_pseudo
ENSG0000(	PWRN2	32.45512	0.375271	0.421849	0.567443	lncRNA
ENSG0000(	LINC01363	27.08364	0.147999	0.421885	0.567467	lncRNA
ENSG0000(	ENSG0000(	47.1254	-0.09563	0.421943	0.567521	lncRNA
ENSG0000(	ENSG0000(	132.8502	-0.06174	0.422059	0.567627	lncRNA
ENSG0000(	NPIPA7	257.9936	-0.08458	0.422042	0.567627	protein_coding
ENSG0000(	ENSG0000(	211.9851	0.0426	0.422095	0.567652	lncRNA
ENSG0000(	API5P2	31.41488	0.180837	0.422122	0.567664	processed_pseudogene
ENSG0000(	LINC01304	235.9676	-0.05486	0.422161	0.567692	lncRNA
ENSG0000(	SNORA75	43.7278	0.121508	0.422202	0.567722	snoRNA
ENSG0000(	AP3S2	2914.249	-0.06226	0.422265	0.567782	protein_coding
ENSG0000(	LINC02080	1247.284	0.027288	0.422343	0.567864	lncRNA
ENSG0000(	OIT3	32.94294	-0.13228	0.422439	0.567968	protein_coding
ENSG0000(	SART3	1401.889	0.030828	0.422738	0.568346	protein_coding
ENSG0000(	GAPLINC	447.1542	0.041069	0.422851	0.568473	lncRNA
ENSG0000(	METTL14-D	323.766	-0.05115	0.42288	0.568487	lncRNA
ENSG0000(	CENPT	717.8375	-0.07592	0.423067	0.568704	protein_coding

ENSG0000(	RSAD2	865.9257	0.184429	0.423077	0.568704	protein_coding
ENSG0000(	ENSG0000(	54.09959	-0.09234	0.423202	0.568847	lncRNA
ENSG0000(	SNORA74	13.96517	-0.15771	0.42323	0.56886	snoRNA
ENSG0000(	SPNS3	30.29737	-0.19153	0.423263	0.568881	protein_coding
ENSG0000(	GZMA	52.43227	0.19126	0.423352	0.56895	protein_coding
ENSG0000(	MIR3976H(	137.8928	0.087112	0.423342	0.56895	lncRNA
ENSG0000(	ENSG0000(	26.24764	0.163642	0.423404	0.568972	lncRNA
ENSG0000(	TTLL2	23.6992	-0.14463	0.4234	0.568972	protein_coding
ENSG0000(	ADGRB3	141.6027	-0.11876	0.423592	0.569089	protein_coding
ENSG0000(	CCDC33	12.21211	-0.25429	0.423594	0.569089	protein_coding
ENSG0000(	ENSG0000(	9.277789	-0.1913	0.423571	0.569089	snoRNA
ENSG0000(	ENSG0000(	72.82317	0.167484	0.423548	0.569089	lncRNA
ENSG0000(	LINC00265	1337.504	-0.02892	0.423532	0.569089	lncRNA
ENSG0000(	MRPS6	702.0088	-0.05356	0.4236	0.569089	protein_coding
ENSG0000(	ANKHD1-D'	58.00914	-0.07968	0.423635	0.569111	lncRNA
ENSG0000(	AMDHD1	78.98559	-0.15413	0.423692	0.569114	protein_coding
ENSG0000(	MIR4472-2	17.66244	0.176889	0.423676	0.569114	miRNA
ENSG0000(	SNORD53B	12.40391	0.159629	0.42366	0.569114	snoRNA
ENSG0000(	HYDIN	332.3107	-0.0817	0.423775	0.569202	protein_coding
ENSG0000(	ENSG0000(	75.91086	-0.10185	0.423917	0.569269	lncRNA
ENSG0000(	KCNH8	158.3645	-0.1229	0.423887	0.569269	protein_coding
ENSG0000(	MIR4774	9.037546	0.213658	0.423867	0.569269	miRNA
ENSG0000(	PEX12	86.65914	-0.07859	0.42391	0.569269	protein_coding
ENSG0000(	SLCO4C1	147.3631	0.111058	0.423907	0.569269	protein_coding
ENSG0000(	ABCA10	659.4254	0.113436	0.424043	0.569384	protein_coding
ENSG0000(	CYP3A43	187.7934	-0.06012	0.424075	0.569384	protein_coding
ENSG0000(	ENSG0000(	90.8856	-0.06338	0.424061	0.569384	lncRNA
ENSG0000(	ENSG0000(	23.36361	-0.16956	0.424036	0.569384	lncRNA
ENSG0000(	ENSG0000(	645.2858	0.043093	0.424249	0.569573	TEC
ENSG0000(	MIR4714	11.73264	0.215015	0.424252	0.569573	miRNA
ENSG0000(	DYDC2	130.6254	0.063019	0.424272	0.569575	protein_coding
ENSG0000(	CATSPER3	35.46657	-0.15772	0.424507	0.569866	protein_coding
ENSG0000(	LRRC2	752.9977	0.035537	0.42461	0.56998	protein_coding
ENSG0000(	TRGV8	13.06694	-0.18561	0.424674	0.570042	TR_V_gene
ENSG0000(	ENSG0000(	67.22871	-0.08495	0.424736	0.570101	lncRNA
ENSG0000(	MAPRE2	983.1309	-0.04344	0.42488	0.57027	protein_coding
ENSG0000(	DCTN1-AS1	98.14618	-0.06097	0.42495	0.570315	lncRNA
ENSG0000(	ENSG0000(	18.23451	0.132584	0.42495	0.570315	lncRNA
ENSG0000(	AKR1C7P	9.964138	-0.22202	0.425051	0.570401	transcribed_unprocessed_pseudo
ENSG0000(	SLC27A3	347.9303	-0.08726	0.425038	0.570401	protein_coding
ENSG0000(	PLEKHH2	2403.987	-0.05753	0.425081	0.570416	protein_coding
ENSG0000(	KCNS3	241.9104	-0.14588	0.42511	0.570431	protein_coding
ENSG0000(	ENSG0000(	15.55034	-0.22264	0.425238	0.570578	lncRNA
ENSG0000(	ENSG0000(	19.32988	0.136509	0.425289	0.570623	lncRNA
ENSG0000(	ENSG0000(	29.74613	-0.1056	0.425416	0.570745	lncRNA
ENSG0000(	ENSG0000(	31.49408	0.210476	0.425435	0.570745	lncRNA
ENSG0000(	PPP2R2A	1614.481	0.036863	0.425428	0.570745	protein_coding

ENSG0000(	MIR544B	12.48755	0.201514	0.42546	0.570754	miRNA
ENSG0000(	ENSG0000(	27.10649	-0.10686	0.425483	0.57076	processed_pseudogene
ENSG0000(	CSTF3	582.3128	-0.0498	0.425631	0.570935	protein_coding
ENSG0000(	C10orf95-A	2170.875	0.020392	0.425906	0.571253	lncRNA
ENSG0000(	ENSG0000(	314.0627	-0.11528	0.425889	0.571253	lncRNA
ENSG0000(	ENSG0000(	14.53269	-0.28666	0.42596	0.571253	lncRNA
ENSG0000(	OSR1	145.6799	-0.13437	0.42594	0.571253	protein_coding
ENSG0000(	ZNF143	472.6175	0.059985	0.425957	0.571253	protein_coding
ENSG0000(	ENSG0000(	11.62859	-0.17195	0.425998	0.571279	processed_pseudogene
ENSG0000(	LINC02006	248.9933	-0.05221	0.426023	0.571288	lncRNA
ENSG0000(	IPO9	3029.429	0.053842	0.426053	0.571305	protein_coding
ENSG0000(	RNU6-251F	45.71874	0.151391	0.426086	0.571324	snRNA
ENSG0000(	HSPB6	29.48541	-0.22229	0.426148	0.571382	protein_coding
ENSG0000(	ENSG0000(	85.43314	0.104935	0.426173	0.571388	lncRNA
ENSG0000(	RN7SL11P	17.73818	0.138358	0.426188	0.571388	misc_RNA
ENSG0000(	CNOT8	1294.762	-0.03294	0.426256	0.571453	protein_coding
ENSG0000(	DAO	16.5268	-0.3352	0.426351	0.571537	protein_coding
ENSG0000(	MT-TS1	137.1707	0.247878	0.426414	0.571537	Mt_tRNA
ENSG0000(	NDUFS5P1	52.28829	-0.114	0.426407	0.571537	processed_pseudogene
ENSG0000(	PP7080	6002.884	-0.21082	0.426446	0.571537	lncRNA
ENSG0000(	RPL13AP3	11.35491	-0.19014	0.426446	0.571537	transcribed_processed_pseudoge
ENSG0000(	SIDT1-AS1	22.1664	-0.1426	0.42644	0.571537	lncRNA
ENSG0000(	WDR47	323.0285	-0.07222	0.42643	0.571537	protein_coding
ENSG0000(	SNORD15A	105.4455	-0.10408	0.426557	0.571661	snoRNA
ENSG0000(	ENSG0000(	73.03687	1.147661	0.426576	0.571663	protein_coding
ENSG0000(	ENSG0000(	17.68703	0.15778	0.426693	0.571794	unprocessed_pseudogene
ENSG0000(	CCDC174	660.6443	-0.04372	0.426795	0.571893	protein_coding
ENSG0000(	MVD	496.233	-0.08289	0.426803	0.571893	protein_coding
ENSG0000(	ENSG0000(	21.45875	-0.12951	0.426865	0.571903	lncRNA
ENSG0000(	NRXN3	670.9735	0.10408	0.426854	0.571903	protein_coding
ENSG0000(	PTCHD4	151.6124	-0.0801	0.426832	0.571903	protein_coding
ENSG0000(	LINC01793	15.52455	0.166445	0.427002	0.572062	lncRNA
ENSG0000(	MAPK4	17.67013	-0.27769	0.42703	0.572074	protein_coding
ENSG0000(	ENSG0000(	271.9413	0.055453	0.427152	0.572115	lncRNA
ENSG0000(	ENSG0000(	19.93355	-0.5054	0.427126	0.572115	unprocessed_pseudogene
ENSG0000(	LSM1P1	10.22787	0.199142	0.427082	0.572115	processed_pseudogene
ENSG0000(	MAP2K7	1102.292	-0.04041	0.427135	0.572115	protein_coding
ENSG0000(	SMN2	465.9471	-0.15558	0.427152	0.572115	protein_coding
ENSG0000(	ENSG0000(	320.6551	-0.04273	0.427277	0.572259	lncRNA
ENSG0000(	ENSG0000(	41.68428	-0.22113	0.427324	0.572297	lncRNA
ENSG0000(	CAND1	3712.468	-0.03665	0.427362	0.572324	protein_coding
ENSG0000(	ACP2	601.3239	0.057563	0.427403	0.572329	protein_coding
ENSG0000(	CABYR	83.62318	-0.06891	0.42739	0.572329	protein_coding
ENSG0000(	AK9	741.8455	-0.0418	0.427588	0.57243	protein_coding
ENSG0000(	ENSG0000(	96.51574	-0.07744	0.427553	0.57243	lncRNA
ENSG0000(	ENSG0000(	252.0301	-0.08301	0.427505	0.57243	lncRNA
ENSG0000(	ENSG0000(	111.8122	0.106924	0.427575	0.57243	lncRNA

ENSG0000(	ENSG0000(	132.1592	0.065329	0.427534	0.57243	lncRNA
ENSG0000(	GOLGA6C	37.95119	0.149043	0.427572	0.57243	protein_coding
ENSG0000(	LINC02058	30.06378	0.092955	0.427717	0.572578	lncRNA
ENSG0000(	ENSG0000(	39.66334	0.130206	0.427816	0.572686	unprocessed_pseudogene
ENSG0000(	ENSG0000(	11.65245	-0.17576	0.427936	0.572822	lncRNA
ENSG0000(	ENSG0000(	428.2096	0.039392	0.428032	0.572854	processed_pseudogene
ENSG0000(	ENSG0000(	31.67014	0.128717	0.428033	0.572854	lncRNA
ENSG0000(	SPC25	604.8818	-0.05128	0.428008	0.572854	protein_coding
ENSG0000(	ZNF354A	383.2323	0.06632	0.42802	0.572854	protein_coding
ENSG0000(	ENSG0000(	239.5255	0.088372	0.428173	0.572993	transcribed_unprocessed_pseudo
ENSG0000(	STX1A	80.31096	0.109814	0.428168	0.572993	protein_coding
ENSG0000(	ENSG0000(	13.45079	0.197847	0.428286	0.573118	lncRNA
ENSG0000(	GATA2	205.1079	0.12524	0.428373	0.573186	protein_coding
ENSG0000(	LINC02772	16.89986	0.201427	0.428372	0.573186	lncRNA
ENSG0000(	STX19	226.489	-0.12991	0.428398	0.573195	protein_coding
ENSG0000(	ENSG0000(	346.4227	0.077703	0.428432	0.573215	lncRNA
ENSG0000(	USHBP1	621.9599	-0.05008	0.428498	0.573281	protein_coding
ENSG0000(	ENSG0000(	19.95559	0.143993	0.42854	0.573312	lncRNA
ENSG0000(	TLE6	23.77409	0.15499	0.428596	0.573363	protein_coding
ENSG0000(	URB2	478.1919	-0.07746	0.428768	0.573567	protein_coding
ENSG0000(	WDR27	3022.409	0.042225	0.42894	0.573773	protein_coding
ENSG0000(	DNAH9	174.8587	0.075455	0.428964	0.57378	protein_coding
ENSG0000(	ENSG0000(	7.475772	-0.40731	0.429015	0.573824	protein_coding
ENSG0000(	BMS1P10	92.02722	0.138357	0.429285	0.57416	transcribed_unprocessed_pseudo
ENSG0000(	LINC00551	58.99611	0.101042	0.429322	0.574185	lncRNA
ENSG0000(	ENSG0000(	10.88952	-0.19602	0.429435	0.574312	lncRNA
ENSG0000(	TBC1D3I	145.756	0.120543	0.429455	0.574315	protein_coding
ENSG0000(	ARHGEF38	975.0386	-0.06827	0.429574	0.57444	protein_coding
ENSG0000(	GATM	486.1914	0.089478	0.429586	0.57444	protein_coding
ENSG0000(	ENSG0000(	33.88712	0.132542	0.429692	0.57451	lncRNA
ENSG0000(	ENSG0000(	26.68472	0.105379	0.429693	0.57451	lncRNA
ENSG0000(	KRT73	12.83314	0.265407	0.429683	0.57451	protein_coding
ENSG0000(	ENSG0000(	17.08669	0.159299	0.429761	0.574577	lncRNA
ENSG0000(	GTF2I	5626.62	0.03299	0.429849	0.574645	protein_coding
ENSG0000(	ZNF232-AS	127.2271	-0.07675	0.429835	0.574645	lncRNA
ENSG0000(	ALDH8A1	197.1234	0.056688	0.429923	0.57472	protein_coding
ENSG0000(	ENSG0000(	79.76675	0.087498	0.430113	0.574949	TEC
ENSG0000(	MIR3150BH	103.4651	0.205655	0.430274	0.575137	lncRNA
ENSG0000(	PDCL3P4	1597.634	-0.02419	0.43029	0.575137	transcribed_processed_pseudoge
ENSG0000(	ENSG0000(	10.25197	0.217109	0.430316	0.575146	lncRNA
ENSG0000(	ENSG0000(	46.91442	0.114308	0.430376	0.575202	lncRNA
ENSG0000(	ENSG0000(	11.1264	0.192167	0.430478	0.575289	lncRNA
ENSG0000(	PGAM4	37.3821	0.125706	0.430461	0.575289	protein_coding
ENSG0000(	IL31RA	412.8448	-0.04797	0.430513	0.575311	protein_coding
ENSG0000(	PROSER3	1133.828	0.027147	0.430555	0.575343	protein_coding
ENSG0000(	SNORD14E	21.33925	-0.1256	0.4306	0.575378	snoRNA
ENSG0000(	EIF5AP2	13.69709	0.188555	0.430698	0.575476	processed_pseudogene

ENSG0000(	RNFT1-DT	34.76043	0.175886	0.43071	0.575476	lncRNA
ENSG0000(	SNHG27	149.7864	0.087409	0.430928	0.575743	lncRNA
ENSG0000(	CCNYL1	1089.833	-0.08681	0.431203	0.576075	protein_coding
ENSG0000(	ERCC4	1786.824	0.040157	0.431213	0.576075	protein_coding
ENSG0000(	PRDX6-AS1	791.9921	0.071768	0.431276	0.576133	lncRNA
ENSG0000(	APOE	584.2575	-0.0968	0.431312	0.576135	protein_coding
ENSG0000(	ENSG0000(	134.6442	-0.10778	0.431314	0.576135	lncRNA
ENSG0000(	ENSG0000(	13.91499	-0.1854	0.431337	0.576141	processed_pseudogene
ENSG0000(	FOXD4L1	21.5413	0.137033	0.431403	0.576205	protein_coding
ENSG0000(	ENSG0000(	23.69505	0.14525	0.431462	0.576235	processed_pseudogene
ENSG0000(	SNORD70	9.645953	0.206614	0.431462	0.576235	snoRNA
ENSG0000(	SMC5	2805.509	-0.03729	0.431574	0.57636	protein_coding
ENSG0000(	PSMG4	1453.385	-0.02997	0.431673	0.576468	protein_coding
ENSG0000(	OR2A7	1263.928	0.152178	0.431712	0.576495	protein_coding
ENSG0000(	ENSG0000(	16.84695	0.182238	0.431815	0.576582	transcribed_unprocessed_pseudo
ENSG0000(	SNORD123	11.33942	-0.20719	0.431801	0.576582	snoRNA
ENSG0000(	AMPD1	171.4767	-0.14556	0.431879	0.576644	protein_coding
ENSG0000(	NAP1L5	30.32493	0.141248	0.432047	0.576843	protein_coding
ENSG0000(	LTBP3	2081.446	0.060553	0.43207	0.576849	protein_coding
ENSG0000(	ORC3	574.9984	0.042947	0.432103	0.576868	protein_coding
ENSG0000(	WDR33	3830.586	0.027436	0.432121	0.576869	protein_coding
ENSG0000(	DCAF7	3990.301	-0.02066	0.432261	0.577006	protein_coding
ENSG0000(	RPL7P49	56.76128	-0.09767	0.432249	0.577006	processed_pseudogene
ENSG0000(	ENSG0000(	17.26996	0.246466	0.432329	0.577073	transcribed_unprocessed_pseudo
ENSG0000(	ENSG0000(	10.42355	0.204271	0.432613	0.5774	lncRNA
ENSG0000(	SYS1-DBND	26.52784	0.1606	0.432593	0.5774	protein_coding
ENSG0000(	VCPIP1	2660.962	0.02767	0.43263	0.5774	protein_coding
ENSG0000(	LTC4S	21.54086	-0.1358	0.432693	0.57746	protein_coding
ENSG0000(	ENSG0000(	99.29513	0.055865	0.432752	0.577498	lncRNA
ENSG0000(	GRID2IP	78.91654	0.093061	0.432777	0.577498	protein_coding
ENSG0000(	LINC00672	864.5117	0.053214	0.432773	0.577498	protein_coding
ENSG0000(	PLA2R1	2288.301	-0.11514	0.432809	0.577515	protein_coding
ENSG0000(	LINC02897	202.9776	-0.05982	0.43284	0.577532	lncRNA
ENSG0000(	FRS2	1023.444	-0.05248	0.432861	0.577536	protein_coding
ENSG0000(	MROH8	139.8446	0.066537	0.432919	0.577588	protein_coding
ENSG0000(	ENSG0000(	222.0487	0.06422	0.432973	0.577612	lncRNA
ENSG0000(	THAP1	123.5453	0.071715	0.432974	0.577612	protein_coding
ENSG0000(	ENSG0000(	22.86622	-0.12854	0.433162	0.57774	snoRNA
ENSG0000(	ENSG0000(	153.4271	-0.13035	0.433142	0.57774	lncRNA
ENSG0000(	ENSG0000(	3798.324	-0.0179	0.433109	0.57774	lncRNA
ENSG0000(	OTUD3	1400.93	0.067793	0.433134	0.57774	protein_coding
ENSG0000(	SEPTIN9	5305.169	-0.0465	0.433151	0.57774	protein_coding
ENSG0000(	RN7SKP35	16.42212	-0.17443	0.433203	0.57777	misc_RNA
ENSG0000(	KHDC1-AS1	1478.988	0.03545	0.433241	0.577797	lncRNA
ENSG0000(	SNX5	3614.339	-0.03644	0.433329	0.57789	protein_coding
ENSG0000(	ZSCAN9	531.1275	0.044046	0.433419	0.577984	protein_coding
ENSG0000(	FBLN7	272.5713	0.068948	0.433526	0.578103	protein_coding

ENSG0000(	ANGPTL1	51.1339	0.168262	0.433619	0.578202	protein_coding
ENSG0000(	ZKSCAN3	1279.251	-0.02131	0.433776	0.578387	protein_coding
ENSG0000(	ENSG0000(	33.72777	-0.86041	0.433858	0.578425	protein_coding
ENSG0000(	EXOC2	948.0079	0.044196	0.433836	0.578425	protein_coding
ENSG0000(	LINC01054	21.87734	-0.14505	0.433873	0.578425	lncRNA
ENSG0000(	MAD1L1	562.5509	-0.04561	0.433879	0.578425	protein_coding
ENSG0000(	MTAP	2552.13	0.047112	0.433913	0.578446	protein_coding
ENSG0000(	ENSG0000(	14.62707	0.173272	0.434106	0.578679	lncRNA
ENSG0000(	ENSG0000(	293.7161	-2.15316	0.434193	0.578769	lncRNA
ENSG0000(	GCNA	399.1364	0.045999	0.434255	0.578828	protein_coding
ENSG0000(	STYK1	668.8052	0.103692	0.434376	0.578965	protein_coding
ENSG0000(	MEIOC	340.5115	-0.04505	0.434486	0.579087	protein_coding
ENSG0000(	ENSG0000(	18.77821	-0.13028	0.434699	0.579345	lncRNA
ENSG0000(	UBAC2-AS1	225.6801	0.045802	0.434732	0.579365	lncRNA
ENSG0000(	CNTNAP1	355.6534	0.056697	0.434764	0.579383	protein_coding
ENSG0000(	ERICH6B	19.87024	0.124572	0.434851	0.579474	protein_coding
ENSG0000(	PALM	281.5426	0.052134	0.434891	0.579503	protein_coding
ENSG0000(	C2orf80	38.45173	0.10269	0.43504	0.579676	protein_coding
ENSG0000(	CEACAM7	20754.94	-0.19619	0.43508	0.579705	protein_coding
ENSG0000(	ENSG0000(	90.85981	-0.08093	0.435112	0.579723	lncRNA
ENSG0000(	TLCD3B	309.622	-0.03883	0.435248	0.579879	protein_coding
ENSG0000(	ENO2	423.1844	0.068574	0.435401	0.580058	protein_coding
ENSG0000(	FTO	4450.729	0.025833	0.435581	0.580274	protein_coding
ENSG0000(	RN7SL602F	566.1249	-0.06367	0.435623	0.580305	misc_RNA
ENSG0000(	LINC01785	17.12905	0.161024	0.435715	0.580399	lncRNA
ENSG0000(	MIR548D1	19.39839	0.177414	0.435749	0.580399	miRNA
ENSG0000(	MPZL1	2526.675	-0.04326	0.435736	0.580399	protein_coding
ENSG0000(	EXTL3-AS1	759.9337	0.051526	0.435831	0.580483	lncRNA
ENSG0000(	CA7	279.1874	-0.24396	0.435871	0.580512	protein_coding
ENSG0000(	FKBP14-AS	13.32747	0.216089	0.43607	0.580728	lncRNA
ENSG0000(	KCTD21	383.3911	0.040794	0.436054	0.580728	protein_coding
ENSG0000(	SLC35F5	6298.167	-0.0372	0.436133	0.580787	protein_coding
ENSG0000(	PPP1R9A	1614.182	-0.10667	0.436193	0.580841	protein_coding
ENSG0000(	GJD4	26.7534	0.120672	0.436218	0.58085	protein_coding
ENSG0000(	CORO7	1492.048	-0.03492	0.436333	0.580978	protein_coding
ENSG0000(	IWS1	1248.193	0.03616	0.436381	0.581018	protein_coding
ENSG0000(	KANSL2	608.7752	-0.04312	0.436492	0.581141	protein_coding
ENSG0000(	ENSG0000(	209.721	0.041767	0.436707	0.581393	lncRNA
ENSG0000(	FAM162B	10.77223	0.21684	0.436756	0.581393	protein_coding
ENSG0000(	HIC1	716.7394	0.076561	0.436728	0.581393	protein_coding
ENSG0000(	RBSN	1704.728	-0.03447	0.436742	0.581393	protein_coding
ENSG0000(	ENSG0000(	186.5814	0.057509	0.436786	0.581408	lncRNA
ENSG0000(	ENSG0000(	57.3706	-0.29486	0.436868	0.581472	lncRNA
ENSG0000(	PIRT	10.29875	0.27446	0.436871	0.581472	protein_coding
ENSG0000(	ENSG0000(	10.06198	0.17582	0.436984	0.581572	processed_pseudogene
ENSG0000(	PIANP	27.9483	-0.17063	0.436977	0.581572	protein_coding
ENSG0000(	LINC02535	130.9704	-0.21691	0.437038	0.58162	lncRNA

ENSG0000(	AGGF1P2	27.96919	0.179078	0.4371	0.581678	processed_pseudogene
ENSG0000(	ENSG0000(	49.23508	0.121085	0.437169	0.581745	lncRNA
ENSG0000(	MROH7	2215.621	-0.11314	0.437195	0.581755	protein_coding
ENSG0000(	RYK	1374.247	-0.03977	0.437267	0.581813	protein_coding
ENSG0000(	SMG1P4	1086.789	0.177034	0.437276	0.581813	transcribed_unprocessed_pseudo
ENSG0000(	ZNF699	1052.918	-0.03469	0.437339	0.581872	protein_coding
ENSG0000(	DNAAF6	115.3591	-0.08994	0.437368	0.581885	protein_coding
ENSG0000(	ENSG0000(	11.03498	0.162462	0.437566	0.582125	lncRNA
ENSG0000(	ENSG0000(	11.65507	0.232885	0.437662	0.582153	lncRNA
ENSG0000(	IFTAP	2769.381	-0.03577	0.437648	0.582153	protein_coding
ENSG0000(	PABPC1P11	51.04797	-0.08148	0.437659	0.582153	processed_pseudogene
ENSG0000(	ZNF10	550.0008	0.060095	0.437619	0.582153	protein_coding
ENSG0000(	ERO1B	2001.269	0.049529	0.437688	0.582162	protein_coding
ENSG0000(	ENSG0000(	73.3058	-0.07487	0.437804	0.582292	lncRNA
ENSG0000(	GMPR	102.3856	0.073943	0.437887	0.582378	protein_coding
ENSG0000(	CTSF	260.2451	0.058159	0.437957	0.582397	protein_coding
ENSG0000(	RPP30	502.7964	-0.0432	0.437956	0.582397	protein_coding
ENSG0000(	SEC61B	352.4855	-0.07861	0.437928	0.582397	protein_coding
ENSG0000(	EPC1	3228.736	-0.03237	0.438054	0.582479	protein_coding
ENSG0000(	SLC66A2	886.5175	-0.058	0.438056	0.582479	protein_coding
ENSG0000(	ENSG0000(	86.01534	-0.10568	0.438144	0.582571	lncRNA
ENSG0000(	ENSG0000(	874.4351	-0.03053	0.438164	0.582573	lncRNA
ENSG0000(	MATR3	6339.22	-0.03521	0.438358	0.582807	protein_coding
ENSG0000(	FAXC	290.3221	0.053651	0.438411	0.582852	protein_coding
ENSG0000(	ENSG0000(	257.2959	0.055727	0.438466	0.5829	lncRNA
ENSG0000(	ENSG0000(	298.7714	0.047437	0.438512	0.582936	lncRNA
ENSG0000(	CCDC200	238.4563	0.055362	0.438555	0.582969	protein_coding
ENSG0000(	PRSS23	1723.111	0.099547	0.438619	0.583029	protein_coding
ENSG0000(	GET1	574.9432	-0.0591	0.43865	0.583046	protein_coding
ENSG0000(	PEG3	251.4161	0.187504	0.438683	0.583065	protein_coding
ENSG0000(	C8orf17	96.06144	0.059458	0.438802	0.583173	TEC
ENSG0000(	ENSG0000(	51.87842	0.099982	0.438794	0.583173	lncRNA
ENSG0000(	ENSG0000(	102.3011	-0.06123	0.438907	0.583288	TEC
ENSG0000(	ZNF561-AS	1777.287	0.027194	0.439085	0.583501	lncRNA
ENSG0000(	B3GALT4	288.4704	-0.05528	0.439125	0.583529	protein_coding
ENSG0000(	ENSG0000(	9485.786	0.061131	0.439317	0.583734	lncRNA
ENSG0000(	NME3	162.1063	-0.06928	0.439316	0.583734	protein_coding
ENSG0000(	OPRK1	112.7348	-0.06965	0.439372	0.583783	protein_coding
ENSG0000(	FAM189A2	163.3046	0.108832	0.439531	0.583968	protein_coding
ENSG0000(	ENSG0000(	9.574511	0.156351	0.439554	0.583974	lncRNA
ENSG0000(	RNF130	1089.179	0.07056	0.439653	0.584081	protein_coding
ENSG0000(	ENSG0000(	46.02033	-0.0988	0.439704	0.584124	transcribed_processed_pseudoge
ENSG0000(	LINC02732	264.9669	0.044557	0.439751	0.584133	lncRNA
ENSG0000(	TUBB3	867.1219	0.048239	0.439767	0.584133	protein_coding
ENSG0000(	ZNF597	239.3927	0.05173	0.439745	0.584133	protein_coding
ENSG0000(	MUC20-OT	15084.13	-0.0741	0.439788	0.584136	lncRNA
ENSG0000(	TUT1	324.0397	0.063645	0.439814	0.584146	protein_coding

ENSG0000(	TM4SF19-A	197.9972	-0.07046	0.439949	0.584301	lncRNA
ENSG0000(	UNC93B1	541.6083	0.067593	0.439973	0.584308	protein_coding
ENSG0000(	DPY19L2P2	1152.246	0.046109	0.44	0.584318	transcribed_unprocessed_pseudo
ENSG0000(	ENSG0000(	36.84253	-0.14962	0.440155	0.5845	lncRNA
ENSG0000(	ENSG0000(	25.05598	0.130239	0.44026	0.58459	misc_RNA
ENSG0000(	PLA2G4E	77.73529	0.114869	0.440252	0.58459	protein_coding
ENSG0000(	IFT172	2356.868	0.03034	0.440306	0.5846	protein_coding
ENSG0000(	RN7SL28P	9.174218	0.16979	0.440291	0.5846	misc_RNA
ENSG0000(	LINC01530	15.33533	0.152621	0.440391	0.584689	lncRNA
ENSG0000(	ANKRD37	99.01206	0.127633	0.440493	0.584702	protein_coding
ENSG0000(	ENSG0000(	447.3092	-0.04445	0.440513	0.584702	TEC
ENSG0000(	ENSG0000(	19.82318	0.160736	0.440484	0.584702	lncRNA
ENSG0000(	LBX2-AS1	197.8218	-0.09874	0.44048	0.584702	lncRNA
ENSG0000(	PKD1P3	1449.549	-0.11973	0.440478	0.584702	transcribed_unprocessed_pseudo
ENSG0000(	SMIM29	74.19802	-0.09479	0.440508	0.584702	protein_coding
ENSG0000(	ENSG0000(	312.2824	0.05179	0.4406	0.584792	lncRNA
ENSG0000(	LRRC17	44.62353	0.123972	0.440624	0.584799	protein_coding
ENSG0000(	ENSG0000(	55.02303	-0.07278	0.44066	0.584822	lncRNA
ENSG0000(	FENDRR	3079.676	0.073709	0.440691	0.584839	lncRNA
ENSG0000(	KIFC2	1338.574	-0.0859	0.440773	0.584914	protein_coding
ENSG0000(	LINC01360	73.79435	-0.08896	0.440785	0.584914	lncRNA
ENSG0000(	LYPD6	103.1479	-0.12189	0.440907	0.585051	protein_coding
ENSG0000(	PHYKPL	7578.82	-0.05097	0.440985	0.58513	protein_coding
ENSG0000(	DNAJC28	118.803	-0.05921	0.441201	0.585392	protein_coding
ENSG0000(	ENSG0000(	314.4557	0.04121	0.441267	0.585429	lncRNA
ENSG0000(	TDRD1	108.7997	0.076527	0.441265	0.585429	protein_coding
ENSG0000(	TPBGL-AS1	39.18228	-0.117	0.441353	0.585519	lncRNA
ENSG0000(	MTDH	2814.85	-0.06326	0.441449	0.585621	protein_coding
ENSG0000(	PACC1	152.3734	0.122192	0.441542	0.58572	protein_coding
ENSG0000(	ENSG0000(	17.34764	0.145552	0.441574	0.585731	processed_pseudogene
ENSG0000(	PRAMENP	21.17403	0.190242	0.441588	0.585731	transcribed_unprocessed_pseudo
ENSG0000(	OR52E4	160.8567	-0.06355	0.441608	0.585733	protein_coding
ENSG0000(	ATXN2L	3244.145	-0.03886	0.441727	0.585866	protein_coding
ENSG0000(	ENSG0000(	38.72599	-0.1148	0.441789	0.585901	lncRNA
ENSG0000(	ENSG0000(	16.44139	0.154798	0.441791	0.585901	lncRNA
ENSG0000(	ENSG0000(	248.3654	0.050685	0.441947	0.586064	lncRNA
ENSG0000(	FAM138C	19.5835	-0.878	0.441951	0.586064	lncRNA
ENSG0000(	ENSG0000(	37.65287	0.132947	0.442063	0.586187	lncRNA
ENSG0000(	ABHD12	913.3179	-0.04785	0.442125	0.586245	protein_coding
ENSG0000(	ENSG0000(	17.26805	-0.16499	0.442257	0.586385	lncRNA
ENSG0000(	MSRB3	658.0735	0.07828	0.442269	0.586385	protein_coding
ENSG0000(	CIRBP-AS1	19.59543	-0.15582	0.442312	0.586393	lncRNA
ENSG0000(	MT-TP	119.1297	0.27941	0.442311	0.586393	Mt_tRNA
ENSG0000(	ACAD8	988.0613	-0.03611	0.442372	0.586423	protein_coding
ENSG0000(	ENSG0000(	19.07044	0.162949	0.442358	0.586423	lncRNA
ENSG0000(	ENSG0000(	61.32619	0.084094	0.442478	0.586539	lncRNA
ENSG0000(	ENSG0000(	67.33403	-0.12589	0.4425	0.586542	lncRNA

ENSG0000(	TAFA1	12.32347	0.260569	0.442636	0.586698	protein_coding
ENSG0000(	ENSG0000(	155.6736	-0.058	0.442655	0.586699	lncRNA
ENSG0000(	THSD7B	18.47656	0.286112	0.442734	0.586779	protein_coding
ENSG0000(	ENSG0000(	23.47017	0.123399	0.442824	0.586873	lncRNA
ENSG0000(	LYPD9P	20.85988	0.142235	0.442845	0.586876	transcribed_unitary_pseudogene
ENSG0000(	RNA5S1	719.4771	0.140654	0.442906	0.586932	rRNA
ENSG0000(	TNRC6B	9722.85	0.036114	0.443383	0.587539	protein_coding
ENSG0000(	ENSG0000(	16.05089	-0.12882	0.443403	0.587541	processed_pseudogene
ENSG0000(	DUX4L35	9.957588	-0.2653	0.443455	0.587584	unprocessed_pseudogene
ENSG0000(	ENSG0000(	29.17282	0.127064	0.443528	0.587657	lncRNA
ENSG0000(	MIR3939	16.24627	-0.15209	0.443582	0.587703	miRNA
ENSG0000(	ERVK13-1	5996.264	-0.04077	0.443738	0.587885	lncRNA
ENSG0000(	ENSG0000(	43.1886	-0.08968	0.443865	0.588003	lncRNA
ENSG0000(	MYLIP	795.0456	-0.06281	0.443861	0.588003	protein_coding
ENSG0000(	ENSG0000(	11.20642	-0.18463	0.443917	0.588047	lncRNA
ENSG0000(	SPACA9	282.9419	-0.04878	0.443936	0.588047	protein_coding
ENSG0000(	TMX3	1914.671	0.031548	0.443984	0.588086	protein_coding
ENSG0000(	LEFTY2	19.4899	-0.3177	0.444143	0.588221	protein_coding
ENSG0000(	NUAK1	360.4264	0.090646	0.444128	0.588221	protein_coding
ENSG0000(	RN7SL143F	10.90794	0.197084	0.44414	0.588221	misc_RNA
ENSG0000(	ENSG0000(	49.24512	0.105811	0.444263	0.588355	TEC
ENSG0000(	MUC6	227.8588	1.373516	0.444401	0.588513	protein_coding
ENSG0000(	ST7L	887.9013	-0.03317	0.444508	0.588631	protein_coding
ENSG0000(	ENSG0000(	38.76672	-0.1246	0.444544	0.588636	TEC
ENSG0000(	ENSG0000(	89.21596	0.06354	0.44455	0.588636	lncRNA
ENSG0000(	PPARGC1A	2669.902	-0.13858	0.444577	0.588648	protein_coding
ENSG0000(	FAM230G	183.7301	-0.31019	0.444622	0.588682	lncRNA
ENSG0000(	LINC00554	24.52432	-0.11188	0.444644	0.588686	lncRNA
ENSG0000(	NKPD1	87.42818	0.06064	0.44497	0.589093	protein_coding
ENSG0000(	ENSG0000(	18.54506	0.125107	0.445094	0.589232	TEC
ENSG0000(	PLEKHB1	110.6929	-0.11752	0.445146	0.589275	protein_coding
ENSG0000(	WWTR1	734.8402	0.0788	0.445219	0.589347	protein_coding
ENSG0000(	INSL6	103.6859	-0.10988	0.445281	0.589405	protein_coding
ENSG0000(	SUN3	31.47529	-0.12309	0.445374	0.589503	protein_coding
ENSG0000(	ITIH5	1772.029	0.065296	0.445447	0.589573	protein_coding
ENSG0000(	UBTD2	605.8737	-0.05035	0.445465	0.589573	protein_coding
ENSG0000(	ENSG0000(	36.58947	-0.09295	0.445515	0.589614	lncRNA
ENSG0000(	NT5M	35.56379	-0.11368	0.445537	0.589619	protein_coding
ENSG0000(	ENSG0000(	88.05291	0.102651	0.445732	0.589852	lncRNA
ENSG0000(	PRMT7	1465.061	-0.05061	0.445762	0.589866	protein_coding
ENSG0000(	LINC01684	1129.695	0.053082	0.445827	0.589878	lncRNA
ENSG0000(	P2RX1	707.1748	0.064076	0.44581	0.589878	protein_coding
ENSG0000(	ZNF204P	348.8313	-0.09198	0.445819	0.589878	transcribed_unprocessed_pseudo
ENSG0000(	ENSG0000(	22.0817	-0.12707	0.445945	0.589959	lncRNA
ENSG0000(	GOSR1	2973.799	-0.02057	0.445919	0.589959	protein_coding
ENSG0000(	RIPK1	3073.392	0.042964	0.445928	0.589959	protein_coding
ENSG0000(	ENSG0000(	10.57498	0.166592	0.446016	0.590028	lncRNA

ENSG0000(	MIR6081	22.38221	-0.15388	0.446129	0.590153	miRNA
ENSG0000(	CLDN16	164.594	-0.06357	0.446292	0.590318	protein_coding
ENSG0000(	TRAJ46	14.20364	-0.21652	0.446283	0.590318	TR_J_gene
ENSG0000(	PPP1R11P2	17.85862	0.130027	0.446371	0.590397	processed_pseudogene
ENSG0000(	ENSG0000(	11.09066	0.181077	0.446469	0.590502	lncRNA
ENSG0000(	SDC4	1331.059	-0.08097	0.446524	0.59055	protein_coding
ENSG0000(	CIRBP	5427.835	-0.03497	0.446602	0.590628	protein_coding
ENSG0000(	ITPRID2	8139.521	-0.08558	0.446723	0.590763	protein_coding
ENSG0000(	KDM2B	1504.234	0.069365	0.446783	0.590775	protein_coding
ENSG0000(	LINC01532	26.25143	0.119993	0.446753	0.590775	lncRNA
ENSG0000(	TRA2A	2247.252	-0.03072	0.446788	0.590775	protein_coding
ENSG0000(	ABCG5	83.13089	0.071836	0.446917	0.590919	protein_coding
ENSG0000(	FRY-AS1	96.43934	0.071363	0.446967	0.590961	lncRNA
ENSG0000(	ENSG0000(	22.38934	0.150102	0.447106	0.591119	processed_pseudogene
ENSG0000(	ENSG0000(	49.47289	0.138932	0.447224	0.591194	lncRNA
ENSG0000(	FAM182B	70.12659	0.100257	0.447214	0.591194	lncRNA
ENSG0000(	GUCY1B2	49.07312	0.323363	0.447265	0.591194	transcribed_unitary_pseudogene
ENSG0000(	HS3ST1	373.5496	0.107794	0.447196	0.591194	protein_coding
ENSG0000(	SFR1P1	22.76292	-0.14041	0.447251	0.591194	processed_pseudogene
ENSG0000(	TM4SF1	547.8506	-0.15653	0.447276	0.591194	protein_coding
ENSG0000(	SCAT8	120.0827	0.053385	0.447318	0.591225	lncRNA
ENSG0000(	ENSG0000(	39.18675	-0.10699	0.447411	0.591323	processed_pseudogene
ENSG0000(	ENSG0000(	12.50468	-0.22096	0.447521	0.591368	lncRNA
ENSG0000(	ENSG0000(	23.63253	0.136692	0.447515	0.591368	lncRNA
ENSG0000(	ENSG0000(	32.52108	0.094215	0.44749	0.591368	lncRNA
ENSG0000(	RNF111	1340.58	0.043274	0.447465	0.591368	protein_coding
ENSG0000(	ZNF815P	112.6847	0.105663	0.44759	0.591434	transcribed_unprocessed_pseudo
ENSG0000(	GLIS2	136.5013	0.117313	0.447667	0.591457	protein_coding
ENSG0000(	KLHL23	449.9804	-0.07574	0.447631	0.591457	protein_coding
ENSG0000(	SEC23A-AS	19.43277	-0.12828	0.447682	0.591457	lncRNA
ENSG0000(	SGMS2	991.1865	-0.07439	0.447656	0.591457	protein_coding
ENSG0000(	ENSG0000(	43.20275	0.093378	0.447791	0.591529	lncRNA
ENSG0000(	SFSWAP	2950.194	0.035148	0.447794	0.591529	protein_coding
ENSG0000(	TMEM169	125.3266	-0.0626	0.447772	0.591529	protein_coding
ENSG0000(	FAM201A	101.9329	-0.08633	0.447836	0.59156	lncRNA
ENSG0000(	SYT7	449.4776	-0.11133	0.447901	0.59162	protein_coding
ENSG0000(	C3orf14	62.28301	0.122734	0.447993	0.591704	protein_coding
ENSG0000(	ENSG0000(	16.65622	0.136462	0.448002	0.591704	lncRNA
ENSG0000(	ENSG0000(	83.42622	0.083815	0.448063	0.59176	lncRNA
ENSG0000(	RDH8	15.24527	0.139524	0.448178	0.591887	protein_coding
ENSG0000(	SLC17A6-D	133.1007	-0.17488	0.448259	0.591969	lncRNA
ENSG0000(	ENSG0000(	24.85983	0.632212	0.448483	0.592164	protein_coding
ENSG0000(	ENSG0000(	51.92305	-0.09148	0.448476	0.592164	lncRNA
ENSG0000(	KLHL17	211.456	0.069688	0.44844	0.592164	protein_coding
ENSG0000(	THAP12P7	150.9231	0.094485	0.448465	0.592164	processed_pseudogene
ENSG0000(	DMXL1	4597.403	-0.0489	0.448811	0.592557	protein_coding
ENSG0000(	OR2AP1	99.53415	0.071387	0.448818	0.592557	protein_coding

ENSG0000(	CPA3	197.5869	-0.14238	0.448848	0.592572	protein_coding
ENSG0000(	SSTR2	1405.283	-0.0379	0.448916	0.592636	protein_coding
ENSG0000(	ENSG0000(	22.68251	-0.12392	0.449065	0.592807	lncRNA
ENSG0000(	ENSG0000(	88.73259	-0.08743	0.449146	0.592853	lncRNA
ENSG0000(	MAP3K14	1000.745	-0.04176	0.449149	0.592853	protein_coding
ENSG0000(	SNORA18	85.20407	0.090683	0.449157	0.592853	snoRNA
ENSG0000(	PDGFRA	3904.44	0.071283	0.449181	0.59286	protein_coding
ENSG0000(	LINC01673	24.99812	0.120785	0.449381	0.5931	lncRNA
ENSG0000(	LINC00331	138.6402	0.073655	0.449401	0.5931	lncRNA
ENSG0000(	MRPS31P5	868.6901	0.058471	0.449498	0.593203	transcribed_unprocessed_pseudo
ENSG0000(	ZNF582-DT	222.1849	0.05414	0.449651	0.593381	lncRNA
ENSG0000(	COX7A2P1	19.1215	-0.15663	0.449689	0.593406	processed_pseudogene
ENSG0000(	CETN2	98.90693	-0.10773	0.449792	0.593466	protein_coding
ENSG0000(	FCHO2-DT	10.02913	0.155877	0.449765	0.593466	lncRNA
ENSG0000(	OARD1	830.136	0.040092	0.449775	0.593466	protein_coding
ENSG0000(	ENSG0000(	14.77994	0.166566	0.449821	0.59348	lncRNA
ENSG0000(	RNF32	85.38047	0.11319	0.449842	0.593482	protein_coding
ENSG0000(	ZDHHC20	1975.096	-0.04906	0.449885	0.593514	protein_coding
ENSG0000(	ENSG0000(	25.38845	0.123557	0.450186	0.593881	lncRNA
ENSG0000(	LINC01527	9.793483	0.223134	0.450201	0.593881	lncRNA
ENSG0000(	ENSG0000(	288.9492	0.042173	0.450327	0.594016	unprocessed_pseudogene
ENSG0000(	FLJ36000	60.96354	-0.124	0.45036	0.594016	lncRNA
ENSG0000(	LRIF1	438.3129	0.050067	0.450347	0.594016	protein_coding
ENSG0000(	SCG5	31.35122	0.174322	0.45038	0.594017	protein_coding
ENSG0000(	CNOT10	1324.95	-0.02952	0.450417	0.59404	protein_coding
ENSG0000(	YPEL1	434.0143	-0.05675	0.450441	0.594047	protein_coding
ENSG0000(	OSBPL9P4	12.63821	-0.15948	0.450621	0.59426	processed_pseudogene
ENSG0000(	ENSG0000(	33.19273	-0.10043	0.450742	0.594394	lncRNA
ENSG0000(	RSPH1	112.6861	-0.09592	0.450783	0.594423	protein_coding
ENSG0000(	AADACL2-A	23.33929	0.143057	0.450854	0.594457	lncRNA
ENSG0000(	ATG12	2000.233	0.025223	0.450866	0.594457	protein_coding
ENSG0000(	LINC02177	237.726	-0.07767	0.450829	0.594457	lncRNA
ENSG0000(	ENSG0000(	97.607	-0.0947	0.450885	0.594458	lncRNA
ENSG0000(	CASZ1	2044.103	-0.08215	0.450956	0.594526	protein_coding
ENSG0000(	ENSG0000(	802.7304	0.041397	0.451083	0.594668	lncRNA
ENSG0000(	OR7E100P	26.80144	0.113376	0.451136	0.594713	unprocessed_pseudogene
ENSG0000(	IGHV2-70D	55.64971	0.317303	0.451338	0.594944	IG_V_gene
ENSG0000(	LGR4-AS1	33.62124	-0.10028	0.45135	0.594944	lncRNA
ENSG0000(	HAUS8	614.5461	-0.04013	0.451461	0.595066	protein_coding
ENSG0000(	RN7SL608P	84.06926	0.064834	0.451524	0.595124	misc_RNA
ENSG0000(	NRG1	3272.11	-0.14061	0.45158	0.595172	protein_coding
ENSG0000(	ENSG0000(	20.58059	0.14932	0.451771	0.5954	lncRNA
ENSG0000(	IDH2-DT	76.71023	-0.0725	0.451954	0.595591	lncRNA
ENSG0000(	TTBK2	1552.768	0.053035	0.451954	0.595591	protein_coding
ENSG0000(	ENSG0000(	32.45917	-0.14905	0.452047	0.595663	processed_pseudogene
ENSG0000(	IGFN1	97.03652	-0.2344	0.452042	0.595663	protein_coding
ENSG0000(	PRKY	1553.104	0.066401	0.452157	0.595783	transcribed_unitary_pseudogene

ENSG0000(	ENSG0000(	22.03034	-0.1319	0.452185	0.595795	lncRNA
ENSG0000(	ENSG0000(	386.9366	0.073956	0.452207	0.595799	lncRNA
ENSG0000(	TP53TG1	572.2925	-0.0485	0.452286	0.595878	lncRNA
ENSG0000(	RN7SL162F	18.20235	0.133675	0.452358	0.595926	misc_RNA
ENSG0000(	SENP3-EIF4	75.24765	-0.19984	0.452361	0.595926	protein_coding
ENSG0000(	LINC00710	321.0704	-0.06123	0.452392	0.595942	lncRNA
ENSG0000(	MIPEPP3	96.34426	0.075875	0.452481	0.596033	transcribed_unprocessed_pseudo
ENSG0000(	ENSG0000(	13.86844	-0.19229	0.452553	0.596104	lncRNA
ENSG0000(	ENSG0000(	9.633004	-0.22134	0.452588	0.596124	lncRNA
ENSG0000(	ENSG0000(	36.25301	0.152366	0.452671	0.596208	lncRNA
ENSG0000(	HLA-T	17.44105	0.212573	0.452814	0.596368	unprocessed_pseudogene
ENSG0000(	LINC02204	33.99197	0.096393	0.45283	0.596368	lncRNA
ENSG0000(	RPS15AP6	109.0723	0.070334	0.452869	0.596394	processed_pseudogene
ENSG0000(	IGLV3-1	423.5597	0.215896	0.452991	0.596529	IG_V_gene
ENSG0000(	ENSG0000(	11.16219	0.165073	0.453032	0.596559	lncRNA
ENSG0000(	LINC00293	20.6033	0.218598	0.453053	0.596561	lncRNA
ENSG0000(	HAUS7	203.1334	-0.08734	0.453074	0.596564	protein_coding
ENSG0000(	ENSG0000(	9.656088	-0.24125	0.45322	0.59673	lncRNA
ENSG0000(	RN7SL32P	204.6985	0.059878	0.453348	0.596874	misc_RNA
ENSG0000(	LINC02430	35.95299	-0.19303	0.453538	0.597073	lncRNA
ENSG0000(	RPS6P25	42.14929	-0.09553	0.453532	0.597073	processed_pseudogene
ENSG0000(	RN7SL198F	88.05776	0.07394	0.453648	0.597194	misc_RNA
ENSG0000(	LINC01892	195.7635	-0.05895	0.453747	0.597299	lncRNA
ENSG0000(	ENSG0000(	23.8285	0.194921	0.453815	0.597364	lncRNA
ENSG0000(	EDN3	545.0849	-0.15222	0.453928	0.597486	protein_coding
ENSG0000(	ENSG0000(	25.40621	0.147145	0.453969	0.597515	lncRNA
ENSG0000(	CCL24	543.522	0.098394	0.453998	0.597528	protein_coding
ENSG0000(	ENSG0000(	12.38925	0.176852	0.454112	0.597653	lncRNA
ENSG0000(	ABCC9	4819.644	0.030267	0.454199	0.597696	protein_coding
ENSG0000(	ENSG0000(	206.707	-1.12853	0.454202	0.597696	protein_coding
ENSG0000(	RNU6-866F	22.29449	0.113062	0.454194	0.597696	snRNA
ENSG0000(	ENSG0000(	50.06199	-0.84529	0.454247	0.597731	processed_pseudogene
ENSG0000(	ENSG0000(	11.03518	0.164976	0.454297	0.597772	lncRNA
ENSG0000(	RN7SL698F	46.6324	-0.09297	0.454325	0.597783	misc_RNA
ENSG0000(	SMAD5	1958.271	-0.04672	0.454427	0.597892	protein_coding
ENSG0000(	CLINT1	2984.554	0.041507	0.454569	0.598054	protein_coding
ENSG0000(	FTH1P20	10.75125	0.247962	0.454592	0.598059	processed_pseudogene
ENSG0000(	ENSG0000(	2448.926	-0.04004	0.454647	0.598106	TEC
ENSG0000(	LINC01533	65.84472	-0.11453	0.454679	0.598123	lncRNA
ENSG0000(	NARF	1602.675	0.03249	0.454749	0.598189	protein_coding
ENSG0000(	ENSG0000(	9.350811	0.214121	0.454904	0.598318	lncRNA
ENSG0000(	HNRNPD-D	16.98481	0.13252	0.454868	0.598318	lncRNA
ENSG0000(	RBM43	643.4153	0.048576	0.454893	0.598318	protein_coding
ENSG0000(	ENSG0000(	137.3414	-0.06935	0.454952	0.598356	lncRNA
ENSG0000(	ENSG0000(	34.21299	-0.1323	0.455011	0.598408	lncRNA
ENSG0000(	ENSG0000(	25.80037	0.123373	0.455034	0.598414	processed_pseudogene
ENSG0000(	AGAP12P	123.8424	-0.21989	0.455104	0.598455	transcribed_unprocessed_pseudo

ENSG0000(	RN7SL405F	239.2246	0.050706	0.455102	0.598455	misc_RNA
ENSG0000(	ENSG0000(	8.520357	0.461832	0.455294	0.598675	misc_RNA
ENSG0000(	ENSG0000(	53.90104	-0.09029	0.455328	0.598675	lncRNA
ENSG0000(	EPHA3	349.9692	0.074092	0.45531	0.598675	protein_coding
ENSG0000(	PRRT1B	32.78535	-0.20023	0.455354	0.598683	protein_coding
ENSG0000(	USP4	2801.814	-0.02996	0.455381	0.598694	protein_coding
ENSG0000(	POTEH	29.67099	0.52161	0.455428	0.598716	protein_coding
ENSG0000(	RNU4-1	8145.575	-0.07971	0.455436	0.598716	snRNA
ENSG0000(	ENSG0000(	14.78164	-0.14839	0.455458	0.598719	lncRNA
ENSG0000(	RPL7P18	19.65955	0.178644	0.455552	0.598818	processed_pseudogene
ENSG0000(	SETP12	35.27559	0.115067	0.455627	0.598891	processed_pseudogene
ENSG0000(	MMRN2	337.2107	0.075697	0.455698	0.59896	protein_coding
ENSG0000(	ENSG0000(	19.64478	-0.12296	0.455842	0.599123	lncRNA
ENSG0000(	HAL	96.58395	0.124231	0.455974	0.599272	protein_coding
ENSG0000(	ENSG0000(	181.8605	-0.13078	0.456128	0.599424	processed_pseudogene
ENSG0000(	PDCD7	767.8242	0.046736	0.45612	0.599424	protein_coding
ENSG0000(	CASC6	148.4061	0.048948	0.456499	0.59981	lncRNA
ENSG0000(	CFH	1472.71	0.096838	0.456556	0.59981	protein_coding
ENSG0000(	ENSG0000(	30.99148	-0.38899	0.456535	0.59981	protein_coding
ENSG0000(	MEF2A	2221.847	-0.0394	0.456515	0.59981	protein_coding
ENSG0000(	PHBP19	323.7352	-0.04992	0.456497	0.59981	transcribed_processed_pseudoge
ENSG0000(	PIGZ	1800.081	-0.15819	0.456551	0.59981	protein_coding
ENSG0000(	RGPD6	242.3499	-0.13479	0.456552	0.59981	protein_coding
ENSG0000(	ZC3H11B	20.25764	0.158114	0.456632	0.599884	protein_coding
ENSG0000(	FAM133B	1667.332	-0.02701	0.456736	0.599996	protein_coding
ENSG0000(	ENSG0000(	44.36692	-1.33944	0.456773	0.600019	protein_coding
ENSG0000(	ENSG0000(	23.16634	-0.12123	0.456794	0.600021	lncRNA
ENSG0000(	COCH	32.79162	0.194681	0.456902	0.600139	protein_coding
ENSG0000(	SIGLEC1	1050.049	0.105446	0.456994	0.600235	protein_coding
ENSG0000(	ENSG0000(	26.18974	0.113881	0.457048	0.600255	lncRNA
ENSG0000(	LINC02805	169.9482	-0.05393	0.457043	0.600255	lncRNA
ENSG0000(	OR56A4	680.0709	0.038007	0.457069	0.600257	protein_coding
ENSG0000(	TFAP2A	37.21965	-0.13965	0.457112	0.600289	protein_coding
ENSG0000(	MAPK6	2542.79	-0.05428	0.457148	0.600291	protein_coding
ENSG0000(	TCAIM	1023.437	-0.03387	0.457152	0.600291	protein_coding
ENSG0000(	TMEM39A	1154.763	0.055972	0.457177	0.600299	protein_coding
ENSG0000(	ZNF322	948.0329	-0.06818	0.45738	0.600539	protein_coding
ENSG0000(	ZNF510	863.4947	0.048964	0.45746	0.600619	protein_coding
ENSG0000(	BRI3BPP1	15.1105	0.15772	0.457504	0.600626	processed_pseudogene
ENSG0000(	IDH2	1141.458	-0.05877	0.457504	0.600626	protein_coding
ENSG0000(	ENSG0000(	381.7158	0.04311	0.457554	0.600667	transcribed_unprocessed_pseudo
ENSG0000(	ENSG0000(	20.369	-0.15559	0.45758	0.600676	lncRNA
ENSG0000(	CCNL2P1	216.8062	0.081201	0.457699	0.600807	processed_pseudogene
ENSG0000(	AGAP14P	19.00285	0.38891	0.457762	0.600826	unprocessed_pseudogene
ENSG0000(	FAM72B	73.49316	0.143932	0.457771	0.600826	protein_coding
ENSG0000(	SGK1	3417.726	0.160947	0.457741	0.600826	protein_coding
ENSG0000(	PDE7B	565.2255	-0.07497	0.458155	0.601305	protein_coding

ENSG0000(	ENSG0000(	37.43945	-0.08461	0.458265	0.601424	lncRNA
ENSG0000(	ENSG0000(	120.6393	-0.06743	0.458287	0.601427	lncRNA
ENSG0000(	TIRAP	622.0886	-0.03794	0.458397	0.601546	protein_coding
ENSG0000(	ENSG0000(	326.4406	0.050301	0.458465	0.60161	lncRNA
ENSG0000(	TMEM132[	46.28397	0.135104	0.458507	0.60164	lncRNA
ENSG0000(	CLEC3A	147.6326	-0.04641	0.458644	0.601647	protein_coding
ENSG0000(	ENSG0000(	81.79962	-0.0605	0.458598	0.601647	lncRNA
ENSG0000(	ENSG0000(	201.1049	0.048964	0.458637	0.601647	lncRNA
ENSG0000(	ENSG0000(	14.1714	-0.17715	0.458647	0.601647	TEC
ENSG0000(	HNRNPA1P	106.3593	-0.17986	0.458647	0.601647	processed_pseudogene
ENSG0000(	NIM1K	169.5777	-0.04898	0.458637	0.601647	protein_coding
ENSG0000(	RHOBTB3	2304.182	-0.06138	0.458547	0.601647	protein_coding
ENSG0000(	LINC01963	277.544	0.073015	0.458681	0.601666	lncRNA
ENSG0000(	RN7SL505F	26.43431	0.119425	0.458747	0.601728	misc_RNA
ENSG0000(	SCARB2	7672.509	0.036023	0.458893	0.601894	protein_coding
ENSG0000(	ENSG0000(	11.69287	-0.17728	0.459012	0.601924	lncRNA
ENSG0000(	ENSG0000(	55.56204	0.101368	0.458943	0.601924	lncRNA
ENSG0000(	LINC00882	1860.286	-0.03661	0.458994	0.601924	lncRNA
ENSG0000(	LINC02449	458.31	0.046263	0.458982	0.601924	lncRNA
ENSG0000(	ZNF572	145.1405	0.057409	0.458973	0.601924	protein_coding
ENSG0000(	ENSG0000(	51.87016	-0.07817	0.459108	0.601953	lncRNA
ENSG0000(	OR5H1	117.8935	0.067314	0.45909	0.601953	protein_coding
ENSG0000(	OXCT2	62.56955	0.128129	0.4591	0.601953	protein_coding
ENSG0000(	PGK1	2710.046	-0.06787	0.459111	0.601953	protein_coding
ENSG0000(	TRAJ38	23.07793	-0.18869	0.459137	0.601961	TR_J_gene
ENSG0000(	LINC01644	19.05581	0.181431	0.459194	0.601986	lncRNA
ENSG0000(	SLC22A14	17.33442	0.19564	0.459179	0.601986	protein_coding
ENSG0000(	ENSG0000(	437.5281	0.044339	0.459367	0.602163	lncRNA
ENSG0000(	PRORSD1P	316.0025	-0.05364	0.459359	0.602163	transcribed_unitary_pseudogene
ENSG0000(	MIR3127	16.2211	-0.19462	0.45939	0.602168	miRNA
ENSG0000(	ATP8B3	228.5763	0.050194	0.459429	0.60219	protein_coding
ENSG0000(	MBTD1	1486.575	-0.03764	0.459462	0.60219	protein_coding
ENSG0000(	SLC2A8	310.331	-0.05942	0.459465	0.60219	protein_coding
ENSG0000(	ENSG0000(	149.1963	0.055874	0.459553	0.60228	lncRNA
ENSG0000(	ENSG0000(	15.78107	0.176498	0.459582	0.602293	lncRNA
ENSG0000(	ENSG0000(	260.9089	-0.07753	0.459642	0.602346	lncRNA
ENSG0000(	ENSG0000(	456.7871	0.0333	0.45975	0.602463	lncRNA
ENSG0000(	SAP30BP	1424.489	-0.03341	0.459774	0.602469	protein_coding
ENSG0000(	HDDC3	323.8293	-0.04694	0.459811	0.60247	protein_coding
ENSG0000(	HIPK2	5130.961	0.039126	0.459813	0.60247	protein_coding
ENSG0000(	C1QTNF12	63.38221	-0.14014	0.45991	0.602521	protein_coding
ENSG0000(	ENSG0000(	19.11381	0.174218	0.459886	0.602521	lncRNA
ENSG0000(	ZNF56P	136.3977	-0.05283	0.459909	0.602521	transcribed_unprocessed_pseudo
ENSG0000(	PNMA8B	34.22344	-0.13945	0.460149	0.602808	protein_coding
ENSG0000(	PCDH11X	418.6843	-0.09121	0.460284	0.60296	protein_coding
ENSG0000(	OSBP2	274.9377	0.056314	0.460312	0.602971	protein_coding
ENSG0000(	ENSG0000(	372.4472	0.038737	0.460511	0.603207	lncRNA

ENSG0000(	FSBP	675.3666	-0.05031	0.460532	0.603209	protein_coding
ENSG0000(	YBX2P2	21.03315	0.200152	0.460555	0.603214	transcribed_processed_pseudoge
ENSG0000(	ENSG0000(	17.69742	0.162798	0.460647	0.603309	lncRNA
ENSG0000(	ENSG0000(	14.5226	-0.14443	0.460771	0.603416	lncRNA
ENSG0000(	ENSG0000(	440.9381	-0.05212	0.460806	0.603416	lncRNA
ENSG0000(	FASLG	69.29018	0.097046	0.460777	0.603416	protein_coding
ENSG0000(	LINC02031	58.91758	-0.06727	0.460802	0.603416	lncRNA
ENSG0000(	ENSG0000(	101.4987	-0.10863	0.460917	0.603512	lncRNA
ENSG0000(	SALRNA2	21.00642	0.103678	0.460907	0.603512	lncRNA
ENSG0000(	TMEM72-A	342.2956	-0.04127	0.461036	0.603642	lncRNA
ENSG0000(	ENSG0000(	65.08181	-0.07432	0.461076	0.603669	lncRNA
ENSG0000(	ENSG0000(	122.1972	0.059778	0.461109	0.603687	lncRNA
ENSG0000(	RN7SL363F	12.81798	-0.13926	0.461322	0.60394	misc_RNA
ENSG0000(	FBXO21	907.7311	-0.04277	0.461342	0.603941	protein_coding
ENSG0000(	ENSG0000(	27.44684	0.125664	0.461369	0.603951	lncRNA
ENSG0000(	ENSG0000(	29.14764	0.097928	0.461463	0.604049	lncRNA
ENSG0000(	CASTOR1	63.76579	0.116924	0.461524	0.604078	protein_coding
ENSG0000(	SNAP23	1586.311	0.035141	0.46151	0.604078	protein_coding
ENSG0000(	CFAP20DC	235.1314	0.067124	0.461556	0.604095	protein_coding
ENSG0000(	TPTE2P5	337.4404	-0.08306	0.461622	0.604156	transcribed_unprocessed_pseudo
ENSG0000(	TFF1	154.9386	0.244016	0.461673	0.604197	protein_coding
ENSG0000(	RNU1-93P	10.16151	0.151104	0.461693	0.604198	snRNA
ENSG0000(	ENSG0000(	72.23009	0.07745	0.461717	0.604204	lncRNA
ENSG0000(	ENSG0000(	77.89559	-0.08376	0.461775	0.604255	lncRNA
ENSG0000(	BORCS8	2138.898	0.047896	0.461826	0.604255	protein_coding
ENSG0000(	ENSG0000(	186.2044	-0.09162	0.461825	0.604255	lncRNA
ENSG0000(	SMIM10	26.32084	0.124259	0.461833	0.604255	protein_coding
ENSG0000(	KLK4	116.9908	-0.12798	0.461886	0.604299	protein_coding
ENSG0000(	PPP1R32	97.84271	0.087763	0.461938	0.604342	protein_coding
ENSG0000(	ENSG0000(	46.35475	-0.10033	0.461979	0.60437	lncRNA
ENSG0000(	TRIM72	1381.155	0.027168	0.462001	0.604373	protein_coding
ENSG0000(	ENSG0000(	70.31061	-0.08014	0.462033	0.604391	lncRNA
ENSG0000(	ENSG0000(	134.5565	-0.08784	0.462178	0.604554	lncRNA
ENSG0000(	ENSG0000(	27.10804	-0.10629	0.462237	0.604594	lncRNA
ENSG0000(	LINC01118	125.0673	0.052445	0.462247	0.604594	lncRNA
ENSG0000(	CACUL1	3195.306	-0.03636	0.462475	0.604867	protein_coding
ENSG0000(	ENSG0000(	9.166504	0.189278	0.462622	0.605034	lncRNA
ENSG0000(	ENSG0000(	36.6542	0.136322	0.46269	0.605098	lncRNA
ENSG0000(	MIR924HG	556.0553	-0.08307	0.462759	0.605162	lncRNA
ENSG0000(	ENSG0000(	181.0733	-0.05581	0.462802	0.605194	lncRNA
ENSG0000(	SSC5D	285.6805	0.089159	0.462839	0.605217	protein_coding
ENSG0000(	INAVA	3942.81	-0.08875	0.462888	0.605255	protein_coding
ENSG0000(	TMEM38A	328.3089	-0.0505	0.463076	0.605476	protein_coding
ENSG0000(	SNORA30	25.48807	-0.14498	0.463114	0.6055	snoRNA
ENSG0000(	ENSG0000(	14.41861	0.168011	0.463141	0.60551	lncRNA
ENSG0000(	GOLGA7	610.763	-0.04829	0.463212	0.605557	protein_coding
ENSG0000(	VSIG1	427.8254	-0.05545	0.463216	0.605557	protein_coding

ENSG0000(	SCN2A	550.9112	0.048474	0.463314	0.60566	protein_coding
ENSG0000(	ENSG0000(	86.89227	0.067793	0.463414	0.605766	lncRNA
ENSG0000(	ARGFXP2	79.48349	-0.05953	0.463536	0.605899	processed_pseudogene
ENSG0000(	CARD14	580.2612	0.042363	0.463555	0.605899	protein_coding
ENSG0000(	B3GNTL1	332.8507	0.065849	0.463623	0.605913	protein_coding
ENSG0000(	ENSG0000(	25.26665	-0.13481	0.463588	0.605913	lncRNA
ENSG0000(	STAG3L4	640.5193	-0.04812	0.463618	0.605913	transcribed_unprocessed_pseudo
ENSG0000(	ENSG0000(	314.7577	-0.05949	0.463733	0.606031	lncRNA
ENSG0000(	SMG9	1398.322	0.038015	0.463759	0.606039	protein_coding
ENSG0000(	ENSG0000(	44.51957	-0.10537	0.463887	0.606131	lncRNA
ENSG0000(	ENSG0000(	32.61016	-1.11721	0.463886	0.606131	protein_coding
ENSG0000(	OR1C1	51.07598	0.104559	0.463872	0.606131	protein_coding
ENSG0000(	ENSG0000(	1603.945	0.030219	0.463989	0.606239	lncRNA
ENSG0000(	COPA	3762.625	0.036908	0.464057	0.606303	protein_coding
ENSG0000(	ENSG0000(	14.86717	0.151629	0.464107	0.606343	lncRNA
ENSG0000(	ENSG0000(	134.1568	0.152702	0.464174	0.606359	transcribed_unprocessed_pseudo
ENSG0000(	POLD3	908.6034	0.051826	0.464167	0.606359	protein_coding
ENSG0000(	SDHAF4	401.1396	-0.04137	0.464178	0.606359	protein_coding
ENSG0000(	SNORA33	131.2728	0.061214	0.464276	0.606462	snoRNA
ENSG0000(	SNORA27	66.42898	-0.10117	0.464363	0.60655	snoRNA
ENSG0000(	PRR22	98.21786	-0.08406	0.464528	0.60674	protein_coding
ENSG0000(	ZNF484	681.7818	0.048654	0.464607	0.606818	protein_coding
ENSG0000(	KLHL9	1927.583	-0.02524	0.464648	0.606847	protein_coding
ENSG0000(	LINC02870	10.96678	-0.25204	0.464678	0.60686	lncRNA
ENSG0000(	ENSG0000(	1038.561	0.072248	0.464728	0.6069	lncRNA
ENSG0000(	IGHV3-71	10.40897	0.279253	0.464773	0.606933	IG_V_pseudogene
ENSG0000(	ENSG0000(	15.19038	0.158718	0.464923	0.607105	processed_pseudogene
ENSG0000(	NOS2P3	138.9309	0.048012	0.465142	0.607339	transcribed_unprocessed_pseudo
ENSG0000(	THRB	562.9499	-0.19231	0.465128	0.607339	protein_coding
ENSG0000(	ENSG0000(	44.24392	0.068418	0.465239	0.60744	lncRNA
ENSG0000(	LSM6	333.608	0.055073	0.465296	0.60744	protein_coding
ENSG0000(	MIR6724-1	11.39934	-0.21647	0.46527	0.60744	miRNA
ENSG0000(	WNK1	6478.18	0.03695	0.465292	0.60744	protein_coding
ENSG0000(	ENSG0000(	37.79986	0.087123	0.465414	0.607513	lncRNA
ENSG0000(	ENSG0000(	118.8437	0.058039	0.465414	0.607513	lncRNA
ENSG0000(	OR7E162P	14.62866	0.180949	0.465385	0.607513	unprocessed_pseudogene
ENSG0000(	ZNF625	168.9984	0.050652	0.465431	0.607513	protein_coding
ENSG0000(	ENSG0000(	13.06512	0.162144	0.465535	0.607625	lncRNA
ENSG0000(	ARMH4	167.7143	0.098507	0.465833	0.607937	protein_coding
ENSG0000(	MUC2	45001.26	-0.16468	0.465801	0.607937	protein_coding
ENSG0000(	WDCP	240.1452	0.070475	0.465831	0.607937	protein_coding
ENSG0000(	TLCD2	3297.89	-0.0652	0.465895	0.607992	protein_coding
ENSG0000(	ENSG0000(	156.9153	-0.04782	0.466069	0.608189	lncRNA
ENSG0000(	MILR1	386.4765	0.053628	0.466084	0.608189	protein_coding
ENSG0000(	ENSG0000(	11.62024	0.151751	0.466167	0.608248	lncRNA
ENSG0000(	SYCE2	24.56695	-0.12711	0.466168	0.608248	protein_coding
ENSG0000(	ENSG0000(	82.84761	-0.14543	0.466231	0.608304	lncRNA

ENSG0000(	DENND2D	3119.573	-0.05748	0.466487	0.608613	protein_coding
ENSG0000(	TRAPPC2B	193.9659	0.05026	0.466575	0.608702	protein_coding
ENSG0000(	BOLA3-AS1	169.1675	0.051732	0.466809	0.608957	lncRNA
ENSG0000(	MIR1-1HG-	152.9525	-0.06888	0.466791	0.608957	lncRNA
ENSG0000(	MAP1S	595.96	-0.04043	0.466891	0.609039	protein_coding
ENSG0000(	GTF2E1	401.8601	-0.04732	0.467168	0.609374	protein_coding
ENSG0000(	ENSG0000(	31.06901	-0.1462	0.467367	0.609608	lncRNA
ENSG0000(	RN7SL832F	283.9845	0.039465	0.467457	0.6097	lncRNA
ENSG0000(	ENSG0000(	15.93424	-0.1451	0.467522	0.609727	lncRNA
ENSG0000(	GLI1	249.0035	-0.10095	0.467527	0.609727	protein_coding
ENSG0000(	ZNF248	1226.059	0.039647	0.467536	0.609727	protein_coding
ENSG0000(	LINC01448	51.878	-0.10222	0.467611	0.609799	lncRNA
ENSG0000(	ENSG0000(	20.58454	0.165008	0.467668	0.609848	lncRNA
ENSG0000(	RN7SL285F	9.697839	0.182751	0.467694	0.609857	misc_RNA
ENSG0000(	ACTA2-AS1	528.4977	0.067807	0.46774	0.609891	lncRNA
ENSG0000(	ENSG0000(	46.72409	0.118192	0.467881	0.610028	lncRNA
ENSG0000(	PMF1-BGL/	11.40306	-0.55068	0.467884	0.610028	protein_coding
ENSG0000(	CNEP1R1	656.694	-0.0405	0.46794	0.61005	protein_coding
ENSG0000(	ZNF451	3700.595	0.027613	0.46794	0.61005	protein_coding
ENSG0000(	ESCO1	1537.798	-0.05445	0.46809	0.610191	protein_coding
ENSG0000(	FNBP4	4577.697	0.030501	0.468126	0.610191	protein_coding
ENSG0000(	LINC02740	29.59862	-0.09517	0.468071	0.610191	lncRNA
ENSG0000(	TACC1	5586.403	0.035668	0.468117	0.610191	protein_coding
ENSG0000(	ETF1P2	61.58358	-0.07424	0.46816	0.61021	processed_pseudogene
ENSG0000(	TRAPPC12	2403.127	-0.02477	0.468227	0.610272	protein_coding
ENSG0000(	ENSG0000(	37.45203	-0.1538	0.468303	0.61032	lncRNA
ENSG0000(	ZDHHC11B	438.495	-0.12846	0.468293	0.61032	protein_coding
ENSG0000(	ZBTB44	2086.846	0.044902	0.468331	0.610332	protein_coding
ENSG0000(	EPC2	990.3596	-0.03701	0.46837	0.610357	protein_coding
ENSG0000(	ASTL	14.49749	0.214453	0.468439	0.610421	protein_coding
ENSG0000(	ENSG0000(	13.03415	0.148525	0.468523	0.610469	lncRNA
ENSG0000(	FAM21FP	42.11969	0.133266	0.468532	0.610469	transcribed_unprocessed_pseudo
ENSG0000(	GNAS	9113.816	-0.04336	0.468534	0.610469	protein_coding
ENSG0000(	LINC02359	502.1231	0.038194	0.46857	0.610489	lncRNA
ENSG0000(	FGF14	87.0035	-0.16317	0.468622	0.610507	protein_coding
ENSG0000(	SLC38A3	31.78993	-0.15623	0.468603	0.610507	protein_coding
ENSG0000(	ENSG0000(	50.1266	-0.0719	0.468703	0.610587	lncRNA
ENSG0000(	MMP25-AS	900.2121	0.046795	0.468729	0.610596	lncRNA
ENSG0000(	ATP2C2-AS	157.4882	-0.08169	0.468749	0.610596	lncRNA
ENSG0000(	ABCA17P	37.74242	-0.12745	0.468878	0.610713	transcribed_unitary_pseudogene
ENSG0000(	ENSG0000(	15.88321	0.235376	0.46886	0.610713	lncRNA
ENSG0000(	ENSG0000(	12.45086	0.169151	0.468938	0.610766	lncRNA
ENSG0000(	USP53	8532.317	0.08961	0.468968	0.610779	protein_coding
ENSG0000(	PAAF1	1422.967	0.023821	0.469026	0.61083	protein_coding
ENSG0000(	DTD1	378.4026	0.03145	0.469125	0.610933	protein_coding
ENSG0000(	EMILIN1	818.3938	0.082755	0.469182	0.610982	protein_coding
ENSG0000(	DGKB	265.8863	-0.09479	0.469308	0.611044	protein_coding

ENSG0000(	GABRG2	2190.3	0.041944	0.469289	0.611044	protein_coding
ENSG0000(	IGHA2	14829.35	-0.1692	0.469278	0.611044	IG_C_gene
ENSG0000(	NALF1	114.2779	0.086859	0.469253	0.611044	protein_coding
ENSG0000(	LINC01381	22.78799	0.102606	0.469344	0.611065	lncRNA
ENSG0000(	NDUFAF7	670.6462	-0.0385	0.469392	0.611103	protein_coding
ENSG0000(	ENSG0000(	61.30281	0.082546	0.469428	0.611124	lncRNA
ENSG0000(	ENSG0000(	202.309	-0.06498	0.469522	0.611221	lncRNA
ENSG0000(	VIPR1-AS1	225.5747	0.052558	0.46967	0.611388	lncRNA
ENSG0000(	ENSG0000(	74.64009	-0.09238	0.469706	0.61141	TEC
ENSG0000(	ENSG0000(	9.438674	0.152554	0.46978	0.611455	lncRNA
ENSG0000(	FTH1P16	52.81946	0.099569	0.469767	0.611455	processed_pseudogene
[truncated: 607,372 more chars]
