# Supplementary material for: Linking gene expression to clinical outcomes in pediatric Crohn’s disease using machine learning
Source: Sci Rep. 2024 Feb 1;14:2667. doi: 10.1038/s41598-024-52678-0 (PMC10834600; doi:10.1038/s41598-024-52678-0)
Supplement: Supplementary file 2 — Supplementary Table 2. [file 41598_2024_52678_MOESM2_ESM.pdf]

ensembl	gene	baseMean	log2FoldCh	pvalue	padj	biotype
ENSG0000(	HCAR3	340.5366	6.085196	1.93E-37	6.09E-33	protein_coding
ENSG0000(	AQP9	272.7622	6.091092	9.19E-33	1.45E-28	protein_coding
ENSG0000(	CXCL8	326.329	4.998463	2.87E-32	2.48E-28	protein_coding
ENSG0000(	MUC1	740.3332	2.762535	3.14E-32	2.48E-28	protein_coding
ENSG0000(	MMP3	600.9684	6.544629	3.43E-31	2.16E-27	protein_coding
ENSG0000(	FCGR3B	213.4524	4.777364	1.49E-30	7.82E-27	protein_coding
ENSG0000(	FCGR1B	219.3068	3.625016	3.66E-29	1.65E-25	protein_coding
ENSG0000(	UCN2	87.72115	5.588192	7.56E-29	2.98E-25	protein_coding
ENSG0000(	FCGR3A	391.7728	3.383717	2.31E-28	7.40E-25	protein_coding
ENSG0000(	IL1B	849.9423	4.506426	2.35E-28	7.40E-25	protein_coding
ENSG0000(	ENSG0000(	235.9858	3.245858	4.28E-28	1.12E-24	lncRNA
ENSG0000(	HCAR2	214.1298	4.484333	3.95E-28	1.12E-24	protein_coding
ENSG0000(	CXCL5	644.4911	4.968794	7.49E-28	1.82E-24	protein_coding
ENSG0000(	ENSG0000(	201648.1	2.482848	2.60E-25	5.86E-22	processed_pseudogene
ENSG0000(	TREM1	181.786	4.246371	2.97E-25	6.25E-22	protein_coding
ENSG0000(	ENSG0000(	279421.9	2.943847	3.49E-25	6.86E-22	lncRNA
ENSG0000(	ENSG0000(	45.39616	6.002564	8.81E-25	1.63E-21	lncRNA
ENSG0000(	FCGR1A	121.5791	3.491098	3.46E-24	6.05E-21	protein_coding
ENSG0000(	MTND5P32	60.59462	5.311702	4.27E-24	7.07E-21	processed_pseudogene
ENSG0000(	S100A8	113.1881	5.207857	1.97E-23	3.03E-20	protein_coding
ENSG0000(	TNFAIP6	39.95912	4.422471	2.02E-23	3.03E-20	protein_coding
ENSG0000(	COL7A1	2908.197	3.143018	3.14E-23	4.50E-20	protein_coding
ENSG0000(	CHI3L1	768.54	3.610401	4.42E-23	6.05E-20	protein_coding
ENSG0000(	IL1RN	359.5353	3.62913	2.14E-22	2.81E-19	protein_coding
ENSG0000(	S100A9	132.8324	4.420347	2.99E-22	3.77E-19	protein_coding
ENSG0000(	INHBA	584.462	3.097246	6.73E-22	8.16E-19	protein_coding
ENSG0000(	WNT5A	1106.404	1.789702	1.02E-21	1.19E-18	protein_coding
ENSG0000(	DUOX2	4873.272	3.828457	2.03E-21	2.29E-18	protein_coding
ENSG0000(	TFAP2C	33.06438	4.021198	7.53E-21	8.18E-18	protein_coding
ENSG0000(	KCNJ2	438.9938	1.588926	2.84E-20	2.99E-17	protein_coding
ENSG0000(	DUOXA2	173.4221	4.4146	4.39E-20	4.46E-17	protein_coding
ENSG0000(	GLT1D1	54.7637	3.305805	5.28E-20	5.20E-17	protein_coding
ENSG0000(	IL1A	60.58407	4.094031	2.96E-19	2.82E-16	protein_coding
ENSG0000(	MAP3K20-/	74.00621	2.735851	4.20E-19	3.89E-16	lncRNA
ENSG0000(	CXCL1	411.0148	2.102948	4.38E-19	3.94E-16	protein_coding
ENSG0000(	ENSG0000(	35.51638	4.402618	5.01E-19	4.38E-16	processed_pseudogene
ENSG0000(	SRPX2	517.3388	1.673861	5.54E-19	4.72E-16	protein_coding
ENSG0000(	CSF3R	1497.417	1.990146	6.15E-19	5.10E-16	protein_coding
ENSG0000(	PAQR9	327.8063	2.237571	2.94E-18	2.37E-15	protein_coding
ENSG0000(	GBP5	3569.736	2.275454	4.20E-18	3.31E-15	protein_coding
ENSG0000(	ADAMTSL4	453.9456	1.114692	5.26E-18	4.04E-15	lncRNA
ENSG0000(	ENSG0000(	4124.928	2.773178	6.18E-18	4.64E-15	processed_pseudogene
ENSG0000(	GBP1P1	160.9459	1.870945	8.21E-18	6.02E-15	transcribed_unprocessed_pseudo
ENSG0000(	SLED1	69.08558	2.598432	8.60E-18	6.16E-15	transcribed_processed_pseudoge
ENSG0000(	MIRLET7IH	928.5151	0.746561	1.29E-17	9.05E-15	lncRNA
ENSG0000(	ENSG0000(	48.18034	1.564595	1.40E-17	9.61E-15	unprocessed_pseudogene

ENSG0000(	IL1R2	142.3494	2.191807	1.46E-17	9.79E-15	protein_coding
ENSG0000(	SUPT20HL2	1621.104	1.591582	5.62E-17	3.69E-14	protein_coding
ENSG0000(	PRRG3	5907.249	1.928834	7.02E-17	4.51E-14	protein_coding
ENSG0000(	LILRB2	515.0746	1.588026	7.71E-17	4.86E-14	protein_coding
ENSG0000(	ANKRD22	402.3476	1.867162	8.56E-17	5.29E-14	protein_coding
ENSG0000(	HGF	261.5077	1.412088	9.64E-17	5.84E-14	protein_coding
ENSG0000(	PRDM1	1540.233	1.294363	1.04E-16	6.16E-14	protein_coding
ENSG0000(	GBP1	2671.608	1.757887	1.10E-16	6.44E-14	protein_coding
ENSG0000(	CCL3	98.25904	2.488732	1.23E-16	7.07E-14	protein_coding
ENSG0000(	ENSG0000(	36.44522	2.635091	1.26E-16	7.09E-14	lncRNA
ENSG0000(	ZNF449	323.9054	0.540647	1.49E-16	8.24E-14	protein_coding
ENSG0000(	CLEC4D	45.09036	3.175755	1.65E-16	8.79E-14	protein_coding
ENSG0000(	FPR1	169.423	2.158354	1.62E-16	8.79E-14	protein_coding
ENSG0000(	USP7	3656.613	-0.26355	1.69E-16	8.90E-14	protein_coding
ENSG0000(	CD300E	532.9219	1.340527	1.80E-16	9.28E-14	protein_coding
ENSG0000(	HSPA6	184.3271	2.025406	2.30E-16	1.17E-13	protein_coding
ENSG0000(	ACSL1	1275.864	2.012918	3.30E-16	1.65E-13	protein_coding
ENSG0000(	FAP	160.464	2.577874	4.38E-16	2.15E-13	protein_coding
ENSG0000(	CREB5	883.9801	2.190718	5.12E-16	2.48E-13	protein_coding
ENSG0000(	KIF26B	266.1816	1.708668	5.27E-16	2.51E-13	protein_coding
ENSG0000(	DPPA4	1904.825	1.330646	5.69E-16	2.62E-13	protein_coding
ENSG0000(	ENSG0000(	40.80376	2.433312	5.67E-16	2.62E-13	lncRNA
ENSG0000(	PIK3R3	1281.662	0.393786	5.74E-16	2.62E-13	protein_coding
ENSG0000(	ENSG0000(	3833.137	2.028106	9.66E-16	4.35E-13	lncRNA
ENSG0000(	TRPC4	54.7169	1.768931	1.22E-15	5.40E-13	protein_coding
ENSG0000(	LILRB3	526.4211	1.629317	1.81E-15	7.93E-13	protein_coding
ENSG0000(	SGIP1	870.6589	1.371218	1.88E-15	8.11E-13	protein_coding
ENSG0000(	SLC39A8	932.6363	1.102905	2.38E-15	1.01E-12	protein_coding
ENSG0000(	TLR2	1025.344	1.323645	2.81E-15	1.18E-12	protein_coding
ENSG0000(	FCGR2A	900.5275	1.940936	2.95E-15	1.21E-12	protein_coding
ENSG0000(	N4BP1	1915.914	0.467643	2.95E-15	1.21E-12	protein_coding
ENSG0000(	ENSG0000(	696.7652	0.287524	3.20E-15	1.28E-12	protein_coding
ENSG0000(	RN7SL141F	69.92578	0.982344	3.20E-15	1.28E-12	misc_RNA
ENSG0000(	MIR34AHG	599.3445	1.186268	3.92E-15	1.53E-12	lncRNA
ENSG0000(	RIPOR3	778.6798	1.01092	3.89E-15	1.53E-12	protein_coding
ENSG0000(	NAMPT	10182.13	1.728047	4.31E-15	1.64E-12	protein_coding
ENSG0000(	TSHZ2	1154.745	0.851328	4.28E-15	1.64E-12	protein_coding
ENSG0000(	APOBEC3A	255.5102	1.689125	4.70E-15	1.76E-12	protein_coding
ENSG0000(	FAM20A	463.8404	1.55185	5.11E-15	1.89E-12	protein_coding
ENSG0000(	CXCL6	112.5842	2.268924	5.81E-15	2.13E-12	protein_coding
ENSG0000(	TM4SF4	183.6062	-2.45718	6.53E-15	2.36E-12	protein_coding
ENSG0000(	FOLH1	549.0842	2.171636	7.19E-15	2.57E-12	protein_coding
ENSG0000(	DCUN1D3	818.7378	0.914739	7.29E-15	2.58E-12	protein_coding
ENSG0000(	GPR84	38.54549	2.663818	9.24E-15	3.23E-12	protein_coding
ENSG0000(	ADGRG3	174.5263	1.748192	1.05E-14	3.62E-12	protein_coding
ENSG0000(	ENSG0000(	57.77524	1.335698	1.12E-14	3.82E-12	TEC
ENSG0000(	PLAU	491.1129	1.707118	1.14E-14	3.88E-12	protein_coding

ENSG0000(	MYOF	1512.187	0.825125	1.25E-14	4.18E-12	protein_coding
ENSG0000(	ENSG0000(	12.43022	2.11772	1.31E-14	4.35E-12	lncRNA
ENSG0000(	ENSG0000(	18416.8	2.010733	1.36E-14	4.46E-12	lncRNA
ENSG0000(	NCF2	352.5195	1.679901	1.47E-14	4.78E-12	protein_coding
ENSG0000(	ENSG0000(	50.33163	2.530911	1.65E-14	5.29E-12	lncRNA
ENSG0000(	RNF182	28.6247	1.576127	1.68E-14	5.34E-12	protein_coding
ENSG0000(	ENSG0000(	280.0725	1.709617	1.75E-14	5.47E-12	lncRNA
ENSG0000(	ENSG0000(	46.0641	1.489414	1.75E-14	5.47E-12	TEC
ENSG0000(	IGHV3-15	516.6651	2.263514	1.81E-14	5.58E-12	IG_V_gene
ENSG0000(	PDPN	310.4703	1.315622	1.87E-14	5.72E-12	protein_coding
ENSG0000(	LRRC69	1778.045	0.999297	2.20E-14	6.67E-12	protein_coding
ENSG0000(	LINC02352	229.6628	0.39073	2.60E-14	7.79E-12	lncRNA
ENSG0000(	MYEOV	76.15448	2.039135	2.67E-14	7.92E-12	protein_coding
ENSG0000(	CXCR2	206.2205	1.438957	2.78E-14	8.20E-12	protein_coding
ENSG0000(	ENSG0000(	22.08429	2.112765	2.84E-14	8.29E-12	lncRNA
ENSG0000(	GAS1	126.7621	2.702854	2.96E-14	8.56E-12	protein_coding
ENSG0000(	ENSG0000(	84.1816	1.96358	3.29E-14	9.43E-12	lncRNA
ENSG0000(	C6orf223	102.3749	1.213246	4.22E-14	1.20E-11	lncRNA
ENSG0000(	IL6	54.31116	3.002696	4.56E-14	1.28E-11	protein_coding
ENSG0000(	ENSG0000(	390.5957	0.49269	4.96E-14	1.37E-11	lncRNA
ENSG0000(	IL24	29.91577	4.148598	4.92E-14	1.37E-11	protein_coding
ENSG0000(	ENSG0000(	34.93249	1.150136	5.10E-14	1.40E-11	lncRNA
ENSG0000(	FNDC3B	4035.557	0.829872	5.98E-14	1.63E-11	protein_coding
ENSG0000(	SLC1A3	882.3394	1.772227	6.31E-14	1.70E-11	protein_coding
ENSG0000(	LILRA6	130.455	1.593474	6.53E-14	1.74E-11	protein_coding
ENSG0000(	PSG6	45.62206	1.469753	6.65E-14	1.76E-11	protein_coding
ENSG0000(	ZBP1	741.7001	0.826911	6.73E-14	1.77E-11	protein_coding
ENSG0000(	LCN2	702.6326	2.612751	7.16E-14	1.86E-11	protein_coding
ENSG0000(	CEMIP	563.8315	2.728803	7.64E-14	1.97E-11	protein_coding
ENSG0000(	SLC7A11	824.4104	0.792022	8.20E-14	2.10E-11	protein_coding
ENSG0000(	NFE2	26.65493	2.717336	8.69E-14	2.21E-11	protein_coding
ENSG0000(	CELF5	2573.073	1.255924	9.24E-14	2.31E-11	protein_coding
ENSG0000(	VMP1	11463.54	0.817807	9.17E-14	2.31E-11	protein_coding
ENSG0000(	LMO3	117.2914	1.293744	9.67E-14	2.40E-11	protein_coding
ENSG0000(	KCNJ2-AS1	38.20979	1.345312	1.07E-13	2.64E-11	lncRNA
ENSG0000(	CCN4	638.3944	1.759767	1.22E-13	2.99E-11	protein_coding
ENSG0000(	LIPN	18.68652	2.538408	1.27E-13	3.08E-11	protein_coding
ENSG0000(	WSB1	7455.325	0.400296	1.30E-13	3.12E-11	protein_coding
ENSG0000(	CTSK	1549.419	0.637814	1.49E-13	3.56E-11	protein_coding
ENSG0000(	COL6A3	9842.015	1.135654	1.50E-13	3.56E-11	protein_coding
ENSG0000(	NEUROD2	3233.577	1.499526	1.78E-13	4.18E-11	protein_coding
ENSG0000(	ADGRE2	1356.519	0.904835	1.88E-13	4.36E-11	protein_coding
ENSG0000(	CD274	519.6723	1.483923	1.87E-13	4.36E-11	protein_coding
ENSG0000(	RAB1AP1	305.1296	1.35132	2.81E-13	6.46E-11	processed_pseudogene
ENSG0000(	XBP1	4056.916	0.986857	2.95E-13	6.74E-11	protein_coding
ENSG0000(	TMEM158	64.41356	2.284556	3.02E-13	6.85E-11	protein_coding
ENSG0000(	DUSP4	327.7102	1.022878	4.28E-13	9.63E-11	protein_coding

ENSG0000(	ENSG0000(	286.6162	1.195614	4.33E-13	9.67E-11	TEC
ENSG0000(	UQCRB	1274.714	-0.51389	4.82E-13	1.07E-10	protein_coding
ENSG0000(	ENSG0000(	15.94169	2.061752	5.53E-13	1.22E-10	lncRNA
ENSG0000(	ADGRE1	99.07287	0.999968	5.72E-13	1.24E-10	protein_coding
ENSG0000(	RPSAP48	154.2505	1.40628	5.71E-13	1.24E-10	processed_pseudogene
ENSG0000(	GLUL	4052.93	1.089695	5.94E-13	1.27E-10	protein_coding
ENSG0000(	MUC4	6097.861	2.13218	5.94E-13	1.27E-10	protein_coding
ENSG0000(	ENSG0000(	29.16299	2.04611	6.99E-13	1.49E-10	lncRNA
ENSG0000(	ENSG0000(	146.8206	0.675851	7.57E-13	1.60E-10	TEC
ENSG0000(	IGHG2	4212.122	2.956513	7.61E-13	1.60E-10	IG_C_gene
ENSG0000(	PELATON	267.6213	0.966057	7.88E-13	1.64E-10	lncRNA
ENSG0000(	CLEC12A	132.8816	1.107953	8.21E-13	1.70E-10	protein_coding
ENSG0000(	HK3	211.8207	1.619959	8.55E-13	1.76E-10	protein_coding
ENSG0000(	PAPPA	489.2326	1.191192	8.93E-13	1.83E-10	protein_coding
ENSG0000(	CHAC1	29.61032	1.556439	9.07E-13	1.84E-10	protein_coding
ENSG0000(	THBS1-IT1	154.0575	1.170844	9.30E-13	1.88E-10	lncRNA
ENSG0000(	RUNX1	3139.504	0.643193	1.18E-12	2.37E-10	protein_coding
ENSG0000(	TSHZ3	343.859	0.909508	1.20E-12	2.39E-10	protein_coding
ENSG0000(	OSCAR	64.30021	1.441173	1.27E-12	2.52E-10	protein_coding
ENSG0000(	ENSG0000(	60.24037	2.854627	1.28E-12	2.53E-10	lncRNA
ENSG0000(	PSTPIP2	591.0409	1.293995	1.31E-12	2.56E-10	protein_coding
ENSG0000(	COL6A6	41.30759	2.044222	1.44E-12	2.80E-10	protein_coding
ENSG0000(	RNF24	945.9192	0.890577	1.79E-12	3.47E-10	protein_coding
ENSG0000(	FAM157A	652.7008	1.541899	1.83E-12	3.51E-10	transcribed_unprocessed_pseudo
ENSG0000(	ENSG0000(	454.8026	0.440504	1.88E-12	3.60E-10	lncRNA
ENSG0000(	BTN2A3P	633.3943	0.422512	1.97E-12	3.71E-10	transcribed_unprocessed_pseudo
ENSG0000(	CTHRC1	35.01888	2.026514	1.97E-12	3.71E-10	protein_coding
ENSG0000(	SAA1	29.26127	3.075969	1.99E-12	3.73E-10	protein_coding
ENSG0000(	ADAMTS2	691.9996	1.199104	2.19E-12	4.09E-10	protein_coding
ENSG0000(	CLEC6A	23.79053	2.968864	2.20E-12	4.09E-10	protein_coding
ENSG0000(	CEBPB	307.9053	1.202819	2.34E-12	4.30E-10	protein_coding
ENSG0000(	IDO1	917.5169	1.561966	2.46E-12	4.50E-10	protein_coding
ENSG0000(	FAM184B	450.4977	0.819692	2.50E-12	4.55E-10	protein_coding
ENSG0000(	ENSG0000(	408.6147	0.544962	2.60E-12	4.70E-10	TEC
ENSG0000(	ENSG0000(	93.34866	1.25854	2.74E-12	4.94E-10	lncRNA
ENSG0000(	ADAMTSL4	83.12661	0.916497	2.81E-12	5.03E-10	lncRNA
ENSG0000(	RFX8	24.74456	2.297775	2.87E-12	5.10E-10	protein_coding
ENSG0000(	LILRA1	68.17518	1.5768	3.12E-12	5.51E-10	protein_coding
ENSG0000(	ENSG0000(	51.21015	1.398357	3.25E-12	5.72E-10	lncRNA
ENSG0000(	ENSG0000(	147.5929	0.613862	3.52E-12	6.11E-10	lncRNA
ENSG0000(	GCNT1P3	16.9436	1.356057	3.52E-12	6.11E-10	processed_pseudogene
ENSG0000(	IRAK3	1633.752	1.081334	3.53E-12	6.11E-10	protein_coding
ENSG0000(	MSX1	38.28204	1.136471	3.74E-12	6.40E-10	protein_coding
ENSG0000(	THBS2	323.0756	2.300409	3.73E-12	6.40E-10	protein_coding
ENSG0000(	MINAR1	71.7715	1.0082	3.89E-12	6.59E-10	protein_coding
ENSG0000(	MMP10	27.43098	3.759655	3.89E-12	6.59E-10	protein_coding
ENSG0000(	LINC01303	70.12886	1.573446	4.02E-12	6.75E-10	lncRNA

ENSG0000(	SERPINE1	334.3795	1.669916	4.03E-12	6.75E-10	protein_coding
ENSG0000(	ENSG0000(	623.4541	0.564572	4.06E-12	6.76E-10	lncRNA
ENSG0000(	GBP2	2647.083	0.767639	4.17E-12	6.92E-10	protein_coding
ENSG0000(	TXNDC5	3734.442	1.01004	4.21E-12	6.95E-10	protein_coding
ENSG0000(	LINC01679	24.80432	1.698098	4.37E-12	7.18E-10	lncRNA
ENSG0000(	AJAP1	108.2774	1.677089	4.42E-12	7.22E-10	protein_coding
ENSG0000(	ENSG0000(	35.72021	1.815014	4.84E-12	7.87E-10	lncRNA
ENSG0000(	LILRA5	196.6411	0.90864	4.97E-12	8.03E-10	protein_coding
ENSG0000(	USP3-AS1	137.7252	0.611516	5.19E-12	8.34E-10	lncRNA
ENSG0000(	APOL4	666.6599	0.75734	5.35E-12	8.56E-10	protein_coding
ENSG0000(	ARSA	4462.971	0.946314	5.42E-12	8.63E-10	protein_coding
ENSG0000(	ROR2	232.5951	1.139996	5.67E-12	8.97E-10	protein_coding
ENSG0000(	H2AC6	763.6477	-0.56089	5.70E-12	8.97E-10	protein_coding
ENSG0000(	CLEC5A	62.00187	1.950341	6.53E-12	1.02E-09	protein_coding
ENSG0000(	ENSG0000(	886.3982	0.331087	6.55E-12	1.02E-09	lncRNA
ENSG0000(	ALPK1	3127.04	0.436626	6.93E-12	1.08E-09	protein_coding
ENSG0000(	BACE2-IT1	50.28801	0.787963	7.03E-12	1.09E-09	lncRNA
ENSG0000(	CEACAM4	43.25756	1.871562	7.26E-12	1.12E-09	protein_coding
ENSG0000(	ENSG0000(	195.818	0.367429	7.45E-12	1.14E-09	lncRNA
ENSG0000(	NR2F1-AS1	2807.816	0.253526	7.52E-12	1.14E-09	lncRNA
ENSG0000(	FOXQ1	31.89365	1.794604	7.62E-12	1.15E-09	protein_coding
ENSG0000(	ENSG0000(	921.5266	0.833749	7.74E-12	1.17E-09	lncRNA
ENSG0000(	MIR4435-2	3850.544	1.085957	7.77E-12	1.17E-09	lncRNA
ENSG0000(	ENSG0000(	32.83704	1.675998	7.83E-12	1.17E-09	lncRNA
ENSG0000(	PTGS2	230.9119	2.007607	8.76E-12	1.30E-09	protein_coding
ENSG0000(	BTN3A1	4342.079	0.541215	9.78E-12	1.45E-09	protein_coding
ENSG0000(	IGHV3-73	103.0871	2.026711	9.86E-12	1.45E-09	IG_V_gene
ENSG0000(	IGHV3-7	748.5127	1.884357	1.09E-11	1.59E-09	IG_V_gene
ENSG0000(	MTCO1P11	317.6925	0.692937	1.12E-11	1.64E-09	unprocessed_pseudogene
ENSG0000(	DYSF	799.8509	1.290074	1.17E-11	1.70E-09	protein_coding
ENSG0000(	STEAP4	1412.345	1.171302	1.22E-11	1.76E-09	protein_coding
ENSG0000(	IRF1	7448.782	0.701438	1.29E-11	1.85E-09	protein_coding
ENSG0000(	HNRNPA1P	3081.021	0.226584	1.35E-11	1.94E-09	processed_pseudogene
ENSG0000(	FER1L4	1019.956	0.808495	1.41E-11	2.01E-09	transcribed_unitary_pseudogene
ENSG0000(	TRIM9	105.8858	1.486097	1.48E-11	2.09E-09	protein_coding
ENSG0000(	CXCR2P1	266.2525	1.404344	1.52E-11	2.14E-09	transcribed_unprocessed_pseudo
ENSG0000(	LINC01001	1303.054	0.707019	1.53E-11	2.15E-09	lncRNA
ENSG0000(	CEACAM3	123.016	1.252334	1.62E-11	2.27E-09	protein_coding
ENSG0000(	ENSG0000(	49.35675	1.328485	1.68E-11	2.34E-09	lncRNA
ENSG0000(	CCN1	291.2872	1.657319	1.81E-11	2.51E-09	protein_coding
ENSG0000(	LINC01094	443.4309	0.343157	1.84E-11	2.54E-09	lncRNA
ENSG0000(	ENSG0000(	28.00974	-2.03324	1.95E-11	2.68E-09	processed_pseudogene
ENSG0000(	MEDAG	24.78049	2.014006	2.10E-11	2.87E-09	protein_coding
ENSG0000(	ENSG0000(	184.67	0.877271	2.21E-11	3.01E-09	lncRNA
ENSG0000(	IER3	232.8175	1.386062	2.25E-11	3.05E-09	protein_coding
ENSG0000(	MTND5P14	124.1095	0.759938	2.29E-11	3.10E-09	unprocessed_pseudogene
ENSG0000(	DOCK4	1152.363	0.979162	2.38E-11	3.20E-09	protein_coding

ENSG0000(	C2	1194.438	0.50793	2.47E-11	3.31E-09	protein_coding
ENSG0000(	CFI	863.0628	0.711373	2.52E-11	3.34E-09	protein_coding
ENSG0000(	COL1A1	5641.554	1.406061	2.53E-11	3.34E-09	protein_coding
ENSG0000(	PCDH17	267.9567	1.300335	2.52E-11	3.34E-09	protein_coding
ENSG0000(	WARS1	3152.512	1.255135	3.01E-11	3.96E-09	protein_coding
ENSG0000(	ENSG0000(	17.25199	2.552283	3.10E-11	4.07E-09	processed_pseudogene
ENSG0000(	ENSG0000(	4067.879	0.259659	3.23E-11	4.22E-09	lncRNA
ENSG0000(	ENSG0000(	371.8174	0.813307	3.40E-11	4.43E-09	protein_coding
ENSG0000(	LINC01278	1659.569	0.25008	3.51E-11	4.55E-09	lncRNA
ENSG0000(	SNORD68	48.14964	1.768153	3.65E-11	4.71E-09	snoRNA
ENSG0000(	HACD2	1823.746	-0.40801	3.77E-11	4.84E-09	protein_coding
ENSG0000(	ENSG0000(	369.2835	0.436116	3.79E-11	4.85E-09	lncRNA
ENSG0000(	MSC-AS1	717.3655	0.699613	3.80E-11	4.85E-09	lncRNA
ENSG0000(	PSG11	142.3451	1.435458	3.97E-11	5.00E-09	protein_coding
ENSG0000(	PSG7	22.81349	1.893799	3.96E-11	5.00E-09	protein_coding
ENSG0000(	STAT1	8315.436	1.132048	3.96E-11	5.00E-09	protein_coding
ENSG0000(	VCAN	2067.866	1.23958	3.98E-11	5.00E-09	protein_coding
ENSG0000(	SAMD9L	4290.427	1.004467	4.11E-11	5.14E-09	protein_coding
ENSG0000(	CXCL3	188.583	2.081465	4.20E-11	5.23E-09	protein_coding
ENSG0000(	CLK1	3737.579	-0.25341	4.24E-11	5.26E-09	protein_coding
ENSG0000(	TLR8	178.85	1.436566	4.44E-11	5.48E-09	protein_coding
ENSG0000(	SNORD66	15.97095	1.319569	4.65E-11	5.72E-09	snoRNA
ENSG0000(	IL11	227.8521	1.333105	4.95E-11	6.07E-09	protein_coding
ENSG0000(	BLACAT1	139.672	0.946532	5.29E-11	6.46E-09	protein_coding
ENSG0000(	NOS2	2489.939	2.035542	5.37E-11	6.54E-09	protein_coding
ENSG0000(	PSG2	90.36188	0.991916	5.66E-11	6.86E-09	protein_coding
ENSG0000(	LINC01127	88.75539	0.663867	5.68E-11	6.86E-09	lncRNA
ENSG0000(	TNFRSF10C	131.1398	1.343421	5.83E-11	7.01E-09	protein_coding
ENSG0000(	SELE	73.11909	3.068994	6.05E-11	7.22E-09	protein_coding
ENSG0000(	ZNF618	656.9061	0.724728	6.04E-11	7.22E-09	protein_coding
ENSG0000(	ENSG0000(	190.3045	1.926408	6.19E-11	7.33E-09	lncRNA
ENSG0000(	SLAMF7	1614.259	1.028746	6.18E-11	7.33E-09	protein_coding
ENSG0000(	DENND2B	2236.54	0.565928	6.33E-11	7.41E-09	protein_coding
ENSG0000(	ENSG0000(	48.34375	1.254132	6.31E-11	7.41E-09	lncRNA
ENSG0000(	FFAR2	403.3589	0.80816	6.29E-11	7.41E-09	protein_coding
ENSG0000(	LINC02362	1029.987	0.786755	6.70E-11	7.82E-09	lncRNA
ENSG0000(	CXCL2	152.0624	1.795273	6.78E-11	7.88E-09	protein_coding
ENSG0000(	KIF23-AS1	129.9901	0.459411	6.80E-11	7.88E-09	lncRNA
ENSG0000(	PTPN13	566.3356	0.751304	6.83E-11	7.88E-09	protein_coding
ENSG0000(	PHKA1	169.9759	0.655447	7.08E-11	8.14E-09	protein_coding
ENSG0000(	SHC3	121.8992	2.121273	7.81E-11	8.94E-09	protein_coding
ENSG0000(	LMOD3	395.6842	0.414894	7.86E-11	8.97E-09	protein_coding
ENSG0000(	ENSG0000(	466.7513	0.640473	8.37E-11	9.51E-09	lncRNA
ENSG0000(	CYP24A1	25.04132	2.397624	8.40E-11	9.52E-09	protein_coding
ENSG0000(	KIAA0825	1304.666	0.33871	8.70E-11	9.82E-09	protein_coding
ENSG0000(	CYTOR	1839.925	1.082204	9.11E-11	1.02E-08	lncRNA
ENSG0000(	SLC7A5	347.0864	1.223943	9.17E-11	1.03E-08	protein_coding

ENSG0000(	ENSG0000(	311.7077	0.437889	9.71E-11	1.08E-08	lncRNA
ENSG0000(	FCN1	770.6536	0.586326	9.77E-11	1.09E-08	protein_coding
ENSG0000(	ACSL4	1646.82	1.155875	9.96E-11	1.10E-08	protein_coding
ENSG0000(	PRR16	162.977	1.145308	1.02E-10	1.13E-08	protein_coding
ENSG0000(	PTGES3P1	83.79377	0.957447	1.03E-10	1.14E-08	processed_pseudogene
ENSG0000(	ENSG0000(	38.42922	1.304657	1.04E-10	1.15E-08	lncRNA
ENSG0000(	ENSG0000(	77.75154	0.7028	1.05E-10	1.15E-08	processed_pseudogene
ENSG0000(	ADM	220.5769	1.351604	1.08E-10	1.18E-08	protein_coding
ENSG0000(	ECH1	642.8541	-0.40342	1.09E-10	1.18E-08	protein_coding
ENSG0000(	WDR86	58.13457	1.460452	1.10E-10	1.20E-08	protein_coding
ENSG0000(	FOSL1	52.30691	0.941835	1.16E-10	1.25E-08	protein_coding
ENSG0000(	AATBC	40.57741	1.51058	1.18E-10	1.27E-08	lncRNA
ENSG0000(	RN7SL473F	116.2804	0.832826	1.25E-10	1.34E-08	misc_RNA
ENSG0000(	OR1I1	2500.374	0.820637	1.29E-10	1.38E-08	protein_coding
ENSG0000(	SIGLEC9	113.6999	0.915586	1.29E-10	1.38E-08	protein_coding
ENSG0000(	ENSG0000(	1873.767	0.422253	1.35E-10	1.43E-08	lncRNA
ENSG0000(	ENSG0000(	170.3979	0.373697	1.46E-10	1.54E-08	lncRNA
ENSG0000(	MNDA	125.7712	1.684336	1.51E-10	1.59E-08	protein_coding
ENSG0000(	PML	3104.518	0.568794	1.51E-10	1.59E-08	protein_coding
ENSG0000(	LINC01465	16.30789	1.270694	1.57E-10	1.64E-08	lncRNA
ENSG0000(	ENSG0000(	28.10392	1.544453	1.63E-10	1.70E-08	lncRNA
ENSG0000(	ENSG0000(	1427.77	0.270422	1.69E-10	1.75E-08	lncRNA
ENSG0000(	NR4A3	147.8457	1.499218	1.72E-10	1.78E-08	protein_coding
ENSG0000(	ENSG0000(	4832.739	0.96732	1.78E-10	1.84E-08	lncRNA
ENSG0000(	ALDH1L2	514.2655	0.866742	1.89E-10	1.94E-08	protein_coding
ENSG0000(	ENSG0000(	80.01328	0.722677	1.91E-10	1.95E-08	lncRNA
ENSG0000(	ENSG0000(	167.4779	0.785207	1.91E-10	1.95E-08	lncRNA
ENSG0000(	ZNF469	343.9919	1.242004	1.91E-10	1.95E-08	protein_coding
ENSG0000(	ENSG0000(	508.3715	0.34583	1.94E-10	1.97E-08	lncRNA
ENSG0000(	KCND3	540.1035	0.704242	1.94E-10	1.97E-08	protein_coding
ENSG0000(	TMEM132/	431.9239	1.281729	2.04E-10	2.06E-08	protein_coding
ENSG0000(	ENSG0000(	62.28285	0.647518	2.12E-10	2.13E-08	lncRNA
ENSG0000(	IGHJ4	376.3492	1.843911	2.12E-10	2.13E-08	IG_J_gene
ENSG0000(	TWIST1	23.02574	2.287798	2.14E-10	2.14E-08	protein_coding
ENSG0000(	ENSG0000(	58.23738	0.831149	2.15E-10	2.14E-08	processed_pseudogene
ENSG0000(	LINC02432	428.7187	0.26907	2.21E-10	2.20E-08	lncRNA
ENSG0000(	SLC6A14	257.9745	1.318574	2.24E-10	2.22E-08	protein_coding
ENSG0000(	ALPL	132.3456	1.688577	2.44E-10	2.41E-08	protein_coding
ENSG0000(	NACA	3732.489	-0.42983	2.60E-10	2.56E-08	protein_coding
ENSG0000(	RN7SL600F	173.2463	0.790777	2.61E-10	2.56E-08	misc_RNA
ENSG0000(	APPL1	2238.793	-0.36701	2.64E-10	2.58E-08	protein_coding
ENSG0000(	C9orf72	1159.593	0.463075	2.69E-10	2.63E-08	protein_coding
ENSG0000(	ADAMTS19	17.93197	1.803111	2.73E-10	2.64E-08	lncRNA
ENSG0000(	ATG7	1652.196	0.313935	2.73E-10	2.64E-08	protein_coding
ENSG0000(	BHLHA15	131.6128	1.317583	2.73E-10	2.64E-08	protein_coding
ENSG0000(	ZBED9-AS1	19.70194	1.284067	2.74E-10	2.64E-08	lncRNA
ENSG0000(	NFATC2IP	3068.151	0.241946	2.75E-10	2.64E-08	protein_coding

ENSG0000(	IGKV2-24	99.93808	1.877048	2.80E-10	2.69E-08	IG_V_gene
ENSG0000(	COL4A1	3059.822	1.134868	3.05E-10	2.91E-08	protein_coding
ENSG0000(	DPYD	2153.512	0.39694	3.26E-10	3.10E-08	protein_coding
ENSG0000(	TMEM121E	18.43563	1.526716	3.31E-10	3.14E-08	protein_coding
ENSG0000(	PHLDA1	1049.916	0.85032	3.48E-10	3.29E-08	protein_coding
ENSG0000(	PDGFRB	1876.135	0.812421	3.53E-10	3.33E-08	protein_coding
ENSG0000(	PDE11A-AS	14.96938	1.398291	3.59E-10	3.38E-08	lncRNA
ENSG0000(	LITAF	2093.977	0.988015	3.63E-10	3.40E-08	protein_coding
ENSG0000(	PLAUR	592.1772	0.850517	3.91E-10	3.65E-08	protein_coding
ENSG0000(	BLZF2P	71.75755	0.755568	4.11E-10	3.83E-08	unprocessed_pseudogene
ENSG0000(	LINC02225	253.4451	0.430377	4.32E-10	4.01E-08	lncRNA
ENSG0000(	MITF	418.9706	0.509359	4.36E-10	4.04E-08	protein_coding
ENSG0000(	SOCS3	864.8064	1.824597	4.43E-10	4.09E-08	protein_coding
ENSG0000(	MLKL	1403.334	0.570779	4.45E-10	4.10E-08	protein_coding
ENSG0000(	ENSG0000(	250.9464	0.559352	4.48E-10	4.12E-08	lncRNA
ENSG0000(	CXCL10	390.4252	2.396721	4.64E-10	4.25E-08	protein_coding
ENSG0000(	CYP27B1	51.05009	1.548303	4.67E-10	4.26E-08	protein_coding
ENSG0000(	ABALON	20.77004	1.253859	4.75E-10	4.32E-08	lncRNA
ENSG0000(	ENSG0000(	824.7925	0.225566	4.81E-10	4.37E-08	processed_pseudogene
ENSG0000(	ADAMTS12	321.9882	0.959261	4.89E-10	4.43E-08	protein_coding
ENSG0000(	SAA2	223.5445	1.166251	4.92E-10	4.44E-08	protein_coding
ENSG0000(	ZNF407	2931.065	0.247697	5.06E-10	4.55E-08	protein_coding
ENSG0000(	MUC5AC	367.8638	3.319409	5.10E-10	4.58E-08	protein_coding
ENSG0000(	PDZK1IP1	216.6311	1.57298	5.14E-10	4.60E-08	protein_coding
ENSG0000(	STON1	565.3493	0.544144	5.18E-10	4.63E-08	protein_coding
ENSG0000(	ENSG0000(	28.05293	1.092532	5.34E-10	4.75E-08	lncRNA
ENSG0000(	E2F3	634.5477	0.464546	5.44E-10	4.82E-08	protein_coding
ENSG0000(	CCL4	130.4459	1.689741	5.55E-10	4.92E-08	protein_coding
ENSG0000(	MIR503HG	67.3318	0.961803	5.70E-10	5.03E-08	lncRNA
ENSG0000(	PLEK	1755.704	1.746272	5.80E-10	5.11E-08	protein_coding
ENSG0000(	DLGAP1-AS	104.3078	0.505679	5.86E-10	5.14E-08	lncRNA
ENSG0000(	MECOM	1639.591	0.695852	5.91E-10	5.17E-08	protein_coding
ENSG0000(	EDNRA	167.5527	1.182505	5.98E-10	5.22E-08	protein_coding
ENSG0000(	MYH13	13.20984	1.6797	6.02E-10	5.24E-08	protein_coding
ENSG0000(	PTK7	748.8203	0.466223	6.06E-10	5.26E-08	protein_coding
ENSG0000(	BMPR1B	32.56871	1.118019	6.10E-10	5.28E-08	protein_coding
ENSG0000(	ENSG0000(	2239.731	0.433	6.11E-10	5.28E-08	transcribed_unprocessed_pseudo
ENSG0000(	TFPI2	25.40294	1.596672	6.24E-10	5.37E-08	protein_coding
ENSG0000(	GORAB	1188.109	0.305054	6.33E-10	5.43E-08	protein_coding
ENSG0000(	ENSG0000(	45.93082	0.808044	6.66E-10	5.70E-08	lncRNA
ENSG0000(	ENSG0000(	139.5444	0.696692	6.71E-10	5.73E-08	lncRNA
ENSG0000(	STC2	48.47282	2.010337	6.78E-10	5.77E-08	protein_coding
ENSG0000(	IL12RB2	370.0336	0.702656	6.99E-10	5.93E-08	protein_coding
ENSG0000(	RAB3B	975.3623	0.326107	7.72E-10	6.54E-08	protein_coding
ENSG0000(	PPIAP10	18.83477	1.345892	7.78E-10	6.57E-08	processed_pseudogene
ENSG0000(	MMP2	1425.324	1.274857	8.16E-10	6.87E-08	protein_coding
ENSG0000(	PRSS22	35.62466	1.539877	8.27E-10	6.95E-08	protein_coding

ENSG0000(	CAPS2	677.5812	0.278862	8.43E-10	7.06E-08	protein_coding
ENSG0000(	SOCS1	176.3998	1.26479	8.93E-10	7.46E-08	protein_coding
ENSG0000(	STEAP2	1732.337	0.826102	9.01E-10	7.51E-08	protein_coding
ENSG0000(	RNF183	17.41561	1.644016	9.31E-10	7.74E-08	protein_coding
ENSG0000(	COL18A1	2062.568	0.897732	9.35E-10	7.76E-08	protein_coding
ENSG0000(	SNORD95	37.49292	1.332336	9.42E-10	7.79E-08	snoRNA
ENSG0000(	ENSG0000(	68.70494	0.865066	9.72E-10	8.02E-08	lncRNA
ENSG0000(	HSD11B1	12.69813	2.303751	9.84E-10	8.09E-08	protein_coding
ENSG0000(	ETV6	2954.423	0.377745	1.02E-09	8.35E-08	protein_coding
ENSG0000(	PLSCR2	89.68712	0.798168	1.04E-09	8.51E-08	protein_coding
ENSG0000(	SOD2	43662.44	0.825757	1.05E-09	8.58E-08	protein_coding
ENSG0000(	SPHK1	154.963	1.187788	1.06E-09	8.67E-08	protein_coding
ENSG0000(	NOS2P1	284.2654	1.110481	1.09E-09	8.84E-08	transcribed_unprocessed_pseudo
ENSG0000(	PITPNM3	298.5531	0.588332	1.17E-09	9.44E-08	protein_coding
ENSG0000(	WDR64	20.79005	1.677122	1.21E-09	9.74E-08	protein_coding
ENSG0000(	ENSG0000(	2450.962	0.356847	1.26E-09	1.01E-07	lncRNA
ENSG0000(	KCNE3	1286.67	0.797929	1.25E-09	1.01E-07	protein_coding
ENSG0000(	LSM14A	2314.81	-0.20614	1.25E-09	1.01E-07	protein_coding
ENSG0000(	UCKL1	780.7476	-0.43097	1.27E-09	1.02E-07	protein_coding
ENSG0000(	ENSG0000(	468.1849	0.246654	1.28E-09	1.02E-07	lncRNA
ENSG0000(	LIPG	2035.946	0.563948	1.28E-09	1.02E-07	protein_coding
ENSG0000(	CXCL9	1533.246	2.415155	1.33E-09	1.06E-07	protein_coding
ENSG0000(	CHPF2	1927.363	0.25078	1.36E-09	1.07E-07	protein_coding
ENSG0000(	ENSG0000(	13.35071	1.540061	1.36E-09	1.08E-07	protein_coding
ENSG0000(	ENSG0000(	72.54167	0.674154	1.38E-09	1.08E-07	lncRNA
ENSG0000(	GPX8	307.3145	0.644695	1.40E-09	1.10E-07	protein_coding
ENSG0000(	OCIAD1	1600.649	-0.17919	1.44E-09	1.13E-07	protein_coding
ENSG0000(	MTND4P14	153.2948	0.70635	1.44E-09	1.13E-07	unprocessed_pseudogene
ENSG0000(	ENSG0000(	183.0167	0.336352	1.46E-09	1.13E-07	lncRNA
ENSG0000(	VWF	1844.465	1.027355	1.51E-09	1.17E-07	protein_coding
ENSG0000(	KCNJ15	2077.016	0.83406	1.53E-09	1.19E-07	protein_coding
ENSG0000(	PSAT1	70.75298	1.057538	1.53E-09	1.19E-07	protein_coding
ENSG0000(	CRISPLD2	1480.283	0.711082	1.54E-09	1.19E-07	protein_coding
ENSG0000(	ENSG0000(	90.59597	0.62439	1.65E-09	1.26E-07	processed_pseudogene
ENSG0000(	LINC01761	1098.056	0.259326	1.64E-09	1.26E-07	lncRNA
ENSG0000(	TUNAR	146.4668	0.374858	1.65E-09	1.26E-07	protein_coding
ENSG0000(	WIPI1	847.2926	0.35994	1.65E-09	1.26E-07	protein_coding
ENSG0000(	CXCL11	106.6754	2.509007	1.66E-09	1.27E-07	protein_coding
ENSG0000(	LINC02043	468.6694	0.417027	1.67E-09	1.27E-07	lncRNA
ENSG0000(	ENSG0000(	35.75985	1.311293	1.72E-09	1.31E-07	lncRNA
ENSG0000(	ENSG0000(	124.3787	0.649675	1.72E-09	1.31E-07	TEC
ENSG0000(	OR2I1P	120.5953	0.596108	1.75E-09	1.32E-07	protein_coding
ENSG0000(	TMEM165	2014.747	0.461278	1.76E-09	1.33E-07	protein_coding
ENSG0000(	ENSG0000(	687.9345	0.271412	1.85E-09	1.39E-07	lncRNA
ENSG0000(	IMMP2L	368.8125	-0.33865	1.84E-09	1.39E-07	protein_coding
ENSG0000(	FAM111A	4537.098	0.227729	1.87E-09	1.40E-07	protein_coding
ENSG0000(	HSF1	938.4191	-0.27988	1.89E-09	1.41E-07	protein_coding

ENSG0000(	ENSG0000(	1786.547	0.207206	1.90E-09	1.42E-07	lncRNA
ENSG0000(	BATF2	338.5718	1.099975	1.91E-09	1.42E-07	protein_coding
ENSG0000(	COL3A1	8789.323	1.178174	1.92E-09	1.42E-07	protein_coding
ENSG0000(	STX11	425.671	0.642017	1.92E-09	1.42E-07	protein_coding
ENSG0000(	ENSG0000(	54.30364	0.945284	1.93E-09	1.42E-07	protein_coding
ENSG0000(	APOL2	2036.947	0.630822	1.93E-09	1.42E-07	protein_coding
ENSG0000(	TPBG	306.7329	0.638712	1.95E-09	1.43E-07	protein_coding
ENSG0000(	ZNF527	1075.341	-0.24718	1.96E-09	1.43E-07	protein_coding
ENSG0000(	ENSG0000(	44.39518	1.398041	1.96E-09	1.44E-07	lncRNA
ENSG0000(	RN7SL398F	83.04267	0.533993	2.09E-09	1.53E-07	misc_RNA
ENSG0000(	GOLGA4-AS	487.011	0.279344	2.11E-09	1.53E-07	lncRNA
ENSG0000(	TRPV4	34.47182	1.288297	2.11E-09	1.53E-07	protein_coding
ENSG0000(	IGLV7-46	78.719	2.353866	2.13E-09	1.54E-07	IG_V_gene
ENSG0000(	TMCC1	1008.719	0.243847	2.16E-09	1.56E-07	protein_coding
ENSG0000(	ENSG0000(	94.40186	1.317204	2.21E-09	1.59E-07	lncRNA
ENSG0000(	ENSG0000(	27.66367	0.767835	2.23E-09	1.60E-07	lncRNA
ENSG0000(	C8orf82	315.8043	-0.51204	2.25E-09	1.62E-07	protein_coding
ENSG0000(	CLIC6	185.452	0.850663	2.28E-09	1.64E-07	protein_coding
ENSG0000(	MIR1302-9	45.77809	1.292443	2.32E-09	1.66E-07	lncRNA
ENSG0000(	NDUFA13	434.2689	-0.40392	2.33E-09	1.66E-07	protein_coding
ENSG0000(	MUC5B	918.9159	2.683366	2.39E-09	1.70E-07	protein_coding
ENSG0000(	ENSG0000(	448.6075	0.379118	2.45E-09	1.74E-07	lncRNA
ENSG0000(	TNFAIP2	3794.794	0.9763	2.47E-09	1.75E-07	protein_coding
ENSG0000(	ENSG0000(	98.14907	-0.56731	2.49E-09	1.76E-07	lncRNA
ENSG0000(	C19orf38	37.62626	1.076354	2.55E-09	1.79E-07	protein_coding
ENSG0000(	ELL3	509.2641	-0.81796	2.55E-09	1.80E-07	protein_coding
ENSG0000(	ENSG0000(	31.4367	1.651765	2.65E-09	1.86E-07	TEC
ENSG0000(	ENSG0000(	14.45271	1.189942	2.69E-09	1.89E-07	TEC
ENSG0000(	LIMK2	2680.69	0.690821	2.73E-09	1.91E-07	protein_coding
ENSG0000(	ENSG0000(	56.93369	0.822773	2.75E-09	1.91E-07	processed_pseudogene
ENSG0000(	ATP5MG	1685.307	-0.35297	2.78E-09	1.94E-07	protein_coding
ENSG0000(	ENSG0000(	25.29615	1.425994	2.82E-09	1.95E-07	lncRNA
ENSG0000(	EMP2	3419.325	0.23094	2.91E-09	2.02E-07	protein_coding
ENSG0000(	PDK1	3537.068	0.744615	3.03E-09	2.10E-07	protein_coding
ENSG0000(	PNRC2	2435.495	-0.28362	3.08E-09	2.13E-07	protein_coding
ENSG0000(	CLEC4E	351.6095	0.728801	3.14E-09	2.16E-07	protein_coding
ENSG0000(	ARHGAP29	1493.326	0.313133	3.16E-09	2.17E-07	protein_coding
ENSG0000(	LINC01270	108.4978	0.909164	3.21E-09	2.20E-07	lncRNA
ENSG0000(	FAM225B	128.3566	0.740699	3.34E-09	2.28E-07	lncRNA
ENSG0000(	LINC00467	1293.204	0.208781	3.34E-09	2.28E-07	lncRNA
ENSG0000(	SPATS2	2147.78	0.203991	3.36E-09	2.29E-07	protein_coding
ENSG0000(	MBNL3	2001.405	-0.38138	3.38E-09	2.30E-07	protein_coding
ENSG0000(	BACE2	1236.296	0.729406	3.42E-09	2.31E-07	protein_coding
ENSG0000(	DCLK3	21.91976	1.736195	3.42E-09	2.31E-07	protein_coding
ENSG0000(	ENSG0000(	27.45432	1.169301	3.43E-09	2.32E-07	TEC
ENSG0000(	CSRNP2	947.374	0.345858	3.47E-09	2.34E-07	protein_coding
ENSG0000(	NECTIN4	113.5163	0.620187	3.51E-09	2.35E-07	protein_coding

ENSG0000(	BACE1	681.4899	0.298758	3.63E-09	2.43E-07	protein_coding
ENSG0000(	SNAI1	24.03477	1.283491	3.63E-09	2.43E-07	protein_coding
ENSG0000(	FGF10	157.624	0.688497	3.72E-09	2.48E-07	protein_coding
ENSG0000(	TPRKB	271.8259	-0.31409	3.72E-09	2.48E-07	protein_coding
ENSG0000(	ENSG0000(	134.1285	0.43099	3.78E-09	2.51E-07	TEC
ENSG0000(	ENSG0000(	135.4873	0.560548	3.79E-09	2.51E-07	lncRNA
ENSG0000(	HCK	531.3601	0.870616	3.80E-09	2.51E-07	protein_coding
ENSG0000(	MTMR8	182.6353	-0.49245	3.89E-09	2.57E-07	protein_coding
ENSG0000(	LPAR6	752.3279	0.447505	3.95E-09	2.61E-07	protein_coding
ENSG0000(	BTN2A1	1269.935	0.291219	4.03E-09	2.65E-07	protein_coding
ENSG0000(	LINC01923	478.3724	0.304882	4.08E-09	2.68E-07	lncRNA
ENSG0000(	RAB11FIP5	262.613	0.641505	4.13E-09	2.70E-07	protein_coding
ENSG0000(	TMC3-AS1	939.9942	0.531646	4.38E-09	2.86E-07	lncRNA
ENSG0000(	P2RX7	888.5955	0.552058	4.44E-09	2.89E-07	protein_coding
ENSG0000(	RAB12	765.4339	0.624612	4.44E-09	2.89E-07	protein_coding
ENSG0000(	MPP3	90.92934	0.932739	4.49E-09	2.92E-07	protein_coding
ENSG0000(	ALOX15B	51.11078	1.236876	4.55E-09	2.95E-07	protein_coding
ENSG0000(	STC1	68.1924	1.610785	4.61E-09	2.98E-07	protein_coding
ENSG0000(	GPR137B	370.2754	0.479716	4.65E-09	3.00E-07	protein_coding
ENSG0000(	LINC02631	54.21576	0.52706	4.89E-09	3.15E-07	lncRNA
ENSG0000(	DCC	142.0075	1.103924	4.91E-09	3.16E-07	protein_coding
ENSG0000(	NOD2	332.7459	1.108899	4.93E-09	3.16E-07	protein_coding
ENSG0000(	ENSG0000(	51.94117	1.458566	4.98E-09	3.19E-07	processed_pseudogene
ENSG0000(	ACP3	160.8598	0.689993	5.05E-09	3.22E-07	protein_coding
ENSG0000(	ENSG0000(	522.3192	0.426974	5.05E-09	3.22E-07	TEC
ENSG0000(	ENSG0000(	41.02822	0.674503	5.08E-09	3.23E-07	lncRNA
ENSG0000(	ENSG0000(	55.91948	0.805837	5.09E-09	3.23E-07	lncRNA
ENSG0000(	RAMACL	157.5139	0.630535	5.25E-09	3.33E-07	protein_coding
ENSG0000(	FOSB	182.6978	1.082589	5.26E-09	3.33E-07	protein_coding
ENSG0000(	BHLHB9P1	18.20706	1.478676	5.49E-09	3.47E-07	processed_pseudogene
ENSG0000(	LILRB4	488.3052	1.197491	5.51E-09	3.47E-07	protein_coding
ENSG0000(	TMSB4XP4	41.61934	-0.66998	5.55E-09	3.49E-07	processed_pseudogene
ENSG0000(	CD276	457.8461	0.609461	5.64E-09	3.53E-07	protein_coding
ENSG0000(	LINC02887	441.8543	0.756601	5.63E-09	3.53E-07	lncRNA
ENSG0000(	ZMIZ1-AS1	86.29484	0.662192	5.71E-09	3.57E-07	lncRNA
ENSG0000(	ENSG0000(	68.39075	1.033774	5.80E-09	3.62E-07	lncRNA
ENSG0000(	THEMIS2	1100.13	1.04498	5.84E-09	3.64E-07	protein_coding
ENSG0000(	PPP6R3	4103.356	-0.18011	5.86E-09	3.64E-07	protein_coding
ENSG0000(	KIAA0513	1325.4	0.355423	5.89E-09	3.65E-07	protein_coding
ENSG0000(	L1TD1	294.2025	1.135189	5.89E-09	3.65E-07	protein_coding
ENSG0000(	GUCY1A2	191.1567	0.916837	5.93E-09	3.66E-07	protein_coding
ENSG0000(	TNPO3	1481.479	-0.1934	6.03E-09	3.72E-07	protein_coding
ENSG0000(	LINC00635	267.894	0.280081	6.26E-09	3.85E-07	lncRNA
ENSG0000(	ENSG0000(	1278.135	0.545994	6.47E-09	3.97E-07	lncRNA
ENSG0000(	NNMT	396.4243	1.054357	6.47E-09	3.97E-07	protein_coding
ENSG0000(	PAQR5	1265.936	-1.01485	6.52E-09	3.99E-07	protein_coding
ENSG0000(	ENSG0000(	61.78533	1.23786	6.65E-09	4.06E-07	processed_pseudogene

ENSG0000(	STK32B	35.83888	1.052212	6.66E-09	4.06E-07	protein_coding
ENSG0000(	ADAMTS4	1220.283	0.878068	6.70E-09	4.08E-07	protein_coding
ENSG0000(	DDAH2	288.8131	-0.50159	6.99E-09	4.24E-07	protein_coding
ENSG0000(	F11-AS1	159.2196	0.338309	7.01E-09	4.25E-07	lncRNA
ENSG0000(	HK2	1571.722	1.122853	7.06E-09	4.27E-07	protein_coding
ENSG0000(	ENSG0000(	20.48266	1.077761	7.15E-09	4.32E-07	lncRNA
ENSG0000(	CSMD2	275.6817	0.737791	7.20E-09	4.33E-07	protein_coding
ENSG0000(	RAPGEF4	227.478	0.85917	7.20E-09	4.33E-07	protein_coding
ENSG0000(	CLEC7A	929.018	0.634005	7.37E-09	4.42E-07	protein_coding
ENSG0000(	SLC27A1	1661.243	0.266539	7.38E-09	4.42E-07	protein_coding
ENSG0000(	PLPP7	130.5406	0.503258	7.41E-09	4.43E-07	protein_coding
ENSG0000(	ENSG0000(	148.3183	0.779627	7.44E-09	4.44E-07	lncRNA
ENSG0000(	LINC00884	3380.771	0.171845	7.49E-09	4.46E-07	lncRNA
ENSG0000(	COL5A1	2572.396	0.901852	7.88E-09	4.69E-07	protein_coding
ENSG0000(	GLIS3	782.6457	0.865271	8.37E-09	4.97E-07	protein_coding
ENSG0000(	C3orf36	31.96119	1.107611	8.45E-09	5.01E-07	lncRNA
ENSG0000(	IL18RAP	195.768	0.9665	8.51E-09	5.03E-07	protein_coding
ENSG0000(	HTRA3	128.7782	1.145889	8.80E-09	5.19E-07	protein_coding
ENSG0000(	ABL2	3332.358	0.277102	9.06E-09	5.32E-07	protein_coding
ENSG0000(	FJX1	22.8832	1.286774	9.04E-09	5.32E-07	protein_coding
ENSG0000(	HNRNPK	5005.607	-0.16589	9.05E-09	5.32E-07	protein_coding
ENSG0000(	PTAFR	759.2298	0.738279	9.59E-09	5.61E-07	protein_coding
ENSG0000(	CCR1	227.676	1.076585	9.72E-09	5.68E-07	protein_coding
ENSG0000(	MIR3677H(	289.8959	-0.3041	9.82E-09	5.73E-07	lncRNA
ENSG0000(	BEST3	367.9138	0.338727	9.98E-09	5.81E-07	protein_coding
ENSG0000(	FBXL3	1546.456	-0.31126	1.02E-08	5.94E-07	protein_coding
ENSG0000(	GBP4	3432.348	0.842087	1.02E-08	5.94E-07	protein_coding
ENSG0000(	IGHV3-23	1012.732	1.464592	1.05E-08	6.08E-07	IG_V_gene
ENSG0000(	MIR3945H(	155.8012	0.889204	1.05E-08	6.09E-07	lncRNA
ENSG0000(	ENSG0000(	425.9268	0.652035	1.07E-08	6.13E-07	lncRNA
ENSG0000(	ENSG0000(	460.7574	0.391471	1.06E-08	6.13E-07	lncRNA
ENSG0000(	C2CD4A	221.9336	0.512827	1.07E-08	6.16E-07	protein_coding
ENSG0000(	ICAM1	1133.516	1.070757	1.07E-08	6.16E-07	protein_coding
ENSG0000(	BDNF	16.2059	1.209271	1.10E-08	6.25E-07	protein_coding
ENSG0000(	FGF7	379.4897	1.165727	1.10E-08	6.25E-07	protein_coding
ENSG0000(	KIRREL1-IT1	38.17023	0.88238	1.09E-08	6.25E-07	lncRNA
ENSG0000(	TAP1	2871.945	0.649491	1.10E-08	6.25E-07	protein_coding
ENSG0000(	EIF2AK3	3714.636	0.173493	1.10E-08	6.27E-07	protein_coding
ENSG0000(	DAW1	120.3748	0.71601	1.11E-08	6.29E-07	protein_coding
ENSG0000(	COL1A2	6048.422	1.050923	1.12E-08	6.34E-07	protein_coding
ENSG0000(	JAK2	2213.826	0.592276	1.12E-08	6.34E-07	protein_coding
ENSG0000(	ENSG0000(	24.11691	1.450409	1.13E-08	6.40E-07	lncRNA
ENSG0000(	SLAMF8	515.0204	1.042223	1.14E-08	6.44E-07	protein_coding
ENSG0000(	ENC1	2672.932	0.509323	1.15E-08	6.48E-07	protein_coding
ENSG0000(	HNRNPA1P	10.75627	1.654369	1.17E-08	6.54E-07	processed_pseudogene
ENSG0000(	PIM3	841.9098	0.53601	1.19E-08	6.65E-07	protein_coding
ENSG0000(	TGFBI	1666.402	0.668675	1.19E-08	6.68E-07	protein_coding

ENSG0000(	UBTD1	22.71328	1.156683	1.20E-08	6.70E-07	protein_coding
ENSG0000(	ENSG0000(	11.07618	2.269793	1.21E-08	6.75E-07	lncRNA
ENSG0000(	ERN1	3713.887	0.412569	1.22E-08	6.80E-07	protein_coding
ENSG0000(	P2RY13	376.0973	0.848243	1.23E-08	6.85E-07	protein_coding
ENSG0000(	ACRBP	94.43364	-0.64642	1.27E-08	7.02E-07	protein_coding
ENSG0000(	SOX11	91.30422	0.753546	1.27E-08	7.02E-07	protein_coding
ENSG0000(	ENSG0000(	621.04	-0.29102	1.28E-08	7.05E-07	lncRNA
ENSG0000(	LINC02863	18.23841	1.100533	1.28E-08	7.05E-07	lncRNA
ENSG0000(	SRSF3	5032.012	-0.16743	1.31E-08	7.19E-07	protein_coding
ENSG0000(	LRRTM2	65.98831	0.73987	1.31E-08	7.22E-07	protein_coding
ENSG0000(	ACAA1	2191.198	-0.61786	1.33E-08	7.27E-07	protein_coding
ENSG0000(	ATAD3C	432.6852	0.588709	1.33E-08	7.27E-07	protein_coding
ENSG0000(	ENSG0000(	18.30138	1.26603	1.33E-08	7.27E-07	lncRNA
ENSG0000(	PLA2G4C	1209.247	-0.44395	1.33E-08	7.27E-07	protein_coding
ENSG0000(	RPL36AP33	37.95707	0.735133	1.33E-08	7.27E-07	processed_pseudogene
ENSG0000(	CEP128	662.6194	0.936992	1.36E-08	7.39E-07	protein_coding
ENSG0000(	SPAG16-DT	984.5937	0.232979	1.37E-08	7.42E-07	lncRNA
ENSG0000(	ST7-AS1	25.33739	0.760165	1.37E-08	7.45E-07	lncRNA
ENSG0000(	RASSF8	494.1577	0.648131	1.41E-08	7.61E-07	protein_coding
ENSG0000(	KCNK3	429.5587	0.25802	1.41E-08	7.63E-07	protein_coding
ENSG0000(	ENSG0000(	143.9826	0.597173	1.44E-08	7.76E-07	lncRNA
ENSG0000(	IDH3B	527.556	-0.38138	1.46E-08	7.87E-07	protein_coding
ENSG0000(	RARRES1	414.7136	1.119096	1.46E-08	7.87E-07	protein_coding
ENSG0000(	LINC00273	57.08338	1.659557	1.47E-08	7.89E-07	lncRNA
ENSG0000(	ENSG0000(	248.5308	0.513408	1.47E-08	7.89E-07	lncRNA
ENSG0000(	IGHV3-74	341.0265	1.440784	1.48E-08	7.90E-07	IG_V_gene
ENSG0000(	ENSG0000(	56.40436	0.568828	1.48E-08	7.91E-07	lncRNA
ENSG0000(	IGKJ4	79.77362	1.539915	1.50E-08	7.99E-07	IG_J_gene
ENSG0000(	PAIP2	819.0515	-0.24488	1.50E-08	7.99E-07	protein_coding
ENSG0000(	TNIP3	257.0735	0.795339	1.51E-08	8.00E-07	protein_coding
ENSG0000(	MTND1P11	134.1027	0.707348	1.51E-08	8.00E-07	unprocessed_pseudogene
ENSG0000(	MMP13	19.11011	1.530561	1.54E-08	8.16E-07	protein_coding
ENSG0000(	MTCO3P11	144.656	0.601361	1.54E-08	8.16E-07	unprocessed_pseudogene
ENSG0000(	CLIP1	4595.567	0.144686	1.55E-08	8.19E-07	protein_coding
ENSG0000(	NXF1	2668.925	0.26656	1.56E-08	8.20E-07	protein_coding
ENSG0000(	IGFBP5	4740.342	1.126092	1.58E-08	8.30E-07	protein_coding
ENSG0000(	ENSG0000(	1039.969	0.273692	1.66E-08	8.70E-07	lncRNA
ENSG0000(	ENSG0000(	28.20668	0.72194	1.67E-08	8.77E-07	lncRNA
ENSG0000(	ANKRD36B	3108.197	0.71772	1.72E-08	8.98E-07	transcribed_unprocessed_pseudo
ENSG0000(	CCL2	312.578	1.030217	1.74E-08	9.07E-07	protein_coding
ENSG0000(	PROB1	56.81911	0.712689	1.77E-08	9.21E-07	protein_coding
ENSG0000(	COL4A2	3906.464	0.717952	1.78E-08	9.26E-07	protein_coding
ENSG0000(	ENSG0000(	417.7256	0.36468	1.79E-08	9.32E-07	lncRNA
ENSG0000(	ENSG0000(	94.7807	0.833584	1.81E-08	9.38E-07	lncRNA
ENSG0000(	PCDH9	2172.419	0.804458	1.82E-08	9.42E-07	protein_coding
ENSG0000(	TBK1	2864.131	0.202148	1.82E-08	9.44E-07	protein_coding
ENSG0000(	NLRC4	111.1428	0.872381	1.84E-08	9.52E-07	protein_coding

ENSG0000(	NAA50	2084.351	-0.22829	1.85E-08	9.54E-07	protein_coding
ENSG0000(	PIM2	1888.932	1.153291	1.87E-08	9.60E-07	protein_coding
ENSG0000(	ENSG0000(	3081.018	0.360015	1.88E-08	9.67E-07	transcribed_unprocessed_pseudo
ENSG0000(	SIKE1	1343.831	-0.24377	1.89E-08	9.70E-07	protein_coding
ENSG0000(	PFN1P2	100.8094	0.616524	1.90E-08	9.74E-07	transcribed_processed_pseudoge
ENSG0000(	RRAGAP1-/	72.80816	0.475838	1.91E-08	9.78E-07	lncRNA
ENSG0000(	SLC25A2	44.10327	0.667907	1.92E-08	9.82E-07	protein_coding
ENSG0000(	ENSG0000(	106.9048	0.428255	1.93E-08	9.83E-07	lncRNA
ENSG0000(	CCR8	13.63624	1.654215	1.95E-08	9.91E-07	protein_coding
ENSG0000(	TENT5C	2228.436	0.814479	1.99E-08	1.01E-06	protein_coding
ENSG0000(	ADAM12	331.7528	1.332017	1.99E-08	1.01E-06	protein_coding
ENSG0000(	SLC9A8	1216.289	0.302439	1.99E-08	1.01E-06	protein_coding
ENSG0000(	ENSG0000(	152.3273	0.326791	2.02E-08	1.02E-06	lncRNA
ENSG0000(	LINC01232	2780.534	0.272612	2.04E-08	1.03E-06	lncRNA
ENSG0000(	LINC00652	864.5481	0.225594	2.05E-08	1.03E-06	lncRNA
ENSG0000(	SPON2	914.9308	0.585829	2.05E-08	1.03E-06	protein_coding
ENSG0000(	ENSG0000(	20.25746	1.298111	2.06E-08	1.03E-06	lncRNA
ENSG0000(	MRPL3	1376.376	0.294879	2.07E-08	1.03E-06	protein_coding
ENSG0000(	STEAP3-AS	19.11099	0.770376	2.06E-08	1.03E-06	lncRNA
ENSG0000(	AOPEP	2599.968	0.289409	2.10E-08	1.05E-06	protein_coding
ENSG0000(	TRAFD1	1087.253	0.466165	2.19E-08	1.09E-06	protein_coding
ENSG0000(	LINC01146	326.6239	0.478662	2.21E-08	1.10E-06	lncRNA
ENSG0000(	ENSG0000(	2494.749	0.205439	2.25E-08	1.12E-06	lncRNA
ENSG0000(	APOL1	1587.423	0.572359	2.27E-08	1.13E-06	protein_coding
ENSG0000(	ENSG0000(	37.9537	0.639365	2.35E-08	1.17E-06	lncRNA
ENSG0000(	SLC31A2	364.6185	0.673974	2.35E-08	1.17E-06	protein_coding
ENSG0000(	ESRRAP2	113.4347	0.461047	2.37E-08	1.17E-06	transcribed_processed_pseudoge
ENSG0000(	FIBIN	85.76852	1.376851	2.37E-08	1.17E-06	protein_coding
ENSG0000(	ENSG0000(	10.7626	1.565166	2.38E-08	1.17E-06	lncRNA
ENSG0000(	ENSG0000(	14.3988	1.440245	2.45E-08	1.21E-06	lncRNA
ENSG0000(	ENSG0000(	286.4	0.391815	2.48E-08	1.22E-06	lncRNA
ENSG0000(	COX7C	1112.768	-0.5851	2.51E-08	1.23E-06	protein_coding
ENSG0000(	GALT	977.9487	-0.33534	2.53E-08	1.24E-06	protein_coding
ENSG0000(	ENSG0000(	23.4385	1.462289	2.56E-08	1.25E-06	lncRNA
ENSG0000(	ZNF7	1876.361	0.15104	2.56E-08	1.25E-06	protein_coding
ENSG0000(	PDE4B	1098.325	1.214001	2.59E-08	1.26E-06	protein_coding
ENSG0000(	ENSG0000(	108.9786	1.839848	2.65E-08	1.29E-06	lncRNA
ENSG0000(	SDC3	1412.744	0.602356	2.66E-08	1.29E-06	protein_coding
ENSG0000(	ENSG0000(	189.2559	0.659106	2.67E-08	1.30E-06	lncRNA
ENSG0000(	ENSG0000(	28.32843	0.905057	2.70E-08	1.31E-06	lncRNA
ENSG0000(	PHETA2	189.0253	0.507545	2.75E-08	1.33E-06	protein_coding
ENSG0000(	ENSG0000(	1760.369	0.595221	2.76E-08	1.33E-06	lncRNA
ENSG0000(	FGF10-AS1	22.58152	1.219477	2.77E-08	1.33E-06	lncRNA
ENSG0000(	OR4K17	1189.805	0.308908	2.77E-08	1.33E-06	protein_coding
ENSG0000(	ASCC3	3165.245	0.273525	2.82E-08	1.35E-06	protein_coding
ENSG0000(	LINC01484	624.3678	0.485083	2.84E-08	1.36E-06	lncRNA
ENSG0000(	PRECSIT	864.5805	0.278344	2.84E-08	1.36E-06	lncRNA

ENSG0000(	PDCD1LG2	110.2756	1.142452	2.92E-08	1.40E-06	protein_coding
ENSG0000(	DERL3	721.0069	1.061008	2.97E-08	1.42E-06	protein_coding
ENSG0000(	EMX2	429.01	0.808019	2.99E-08	1.43E-06	protein_coding
ENSG0000(	KSR1	2166.307	0.384508	3.01E-08	1.44E-06	protein_coding
ENSG0000(	ENSG0000(	45.25564	0.907802	3.02E-08	1.44E-06	lncRNA
ENSG0000(	IRS3P	129.0902	0.51856	3.05E-08	1.45E-06	processed_pseudogene
ENSG0000(	CRABP2	13.17826	1.336323	3.08E-08	1.46E-06	protein_coding
ENSG0000(	GABRR3	62.47509	0.539344	3.08E-08	1.46E-06	protein_coding
ENSG0000(	LCMT1-AS2	130.3626	0.357658	3.10E-08	1.47E-06	lncRNA
ENSG0000(	ENSG0000(	110.5358	0.726824	3.11E-08	1.47E-06	lncRNA
ENSG0000(	IL15RA	439.8905	0.508422	3.12E-08	1.47E-06	protein_coding
ENSG0000(	ENSG0000(	179.4861	0.646727	3.13E-08	1.47E-06	lncRNA
ENSG0000(	LINC02528	11.89383	1.777301	3.13E-08	1.47E-06	lncRNA
ENSG0000(	IGKV2-30	211.4414	1.596167	3.18E-08	1.49E-06	IG_V_gene
ENSG0000(	ZNF521	570.4506	0.74085	3.19E-08	1.50E-06	protein_coding
ENSG0000(	SBK1	577.944	0.911398	3.26E-08	1.53E-06	protein_coding
ENSG0000(	ENSG0000(	1007.454	0.434851	3.28E-08	1.53E-06	lncRNA
ENSG0000(	GABRR2	459.657	0.375535	3.33E-08	1.55E-06	protein_coding
ENSG0000(	ENSG0000(	643.3229	0.235531	3.34E-08	1.56E-06	processed_pseudogene
ENSG0000(	DNAJC7	3573.254	0.16453	3.37E-08	1.57E-06	protein_coding
ENSG0000(	TWIST2	44.04152	1.33124	3.42E-08	1.59E-06	protein_coding
ENSG0000(	ZNF841	1926.921	0.234344	3.42E-08	1.59E-06	protein_coding
ENSG0000(	RNF149	2493.147	0.405547	3.45E-08	1.60E-06	protein_coding
ENSG0000(	ENSG0000(	861.477	0.220116	3.50E-08	1.62E-06	lncRNA
ENSG0000(	TCEANC2	2042.289	-0.20544	3.50E-08	1.62E-06	protein_coding
ENSG0000(	RAB31	1028.079	0.977168	3.53E-08	1.63E-06	protein_coding
ENSG0000(	ST13	1686.563	-0.35967	3.53E-08	1.63E-06	protein_coding
ENSG0000(	COL5A2	1685.486	0.829562	3.54E-08	1.63E-06	protein_coding
ENSG0000(	ENSG0000(	44.4738	0.717733	3.61E-08	1.66E-06	lncRNA
ENSG0000(	IGHV2-70	39.5959	2.100639	3.62E-08	1.66E-06	IG_V_gene
ENSG0000(	DGKD	3506.345	-0.31767	3.65E-08	1.67E-06	protein_coding
ENSG0000(	ENSG0000(	102.9176	-0.45759	3.66E-08	1.67E-06	TEC
ENSG0000(	NOL3	391.586	0.465136	3.66E-08	1.67E-06	protein_coding
ENSG0000(	SERINC4	13.20867	1.291121	3.68E-08	1.68E-06	protein_coding
ENSG0000(	CA9	26.42653	2.092354	3.71E-08	1.69E-06	protein_coding
ENSG0000(	CALU	2193.198	0.409836	3.72E-08	1.69E-06	protein_coding
ENSG0000(	ZNF302	2213.181	0.581033	3.72E-08	1.69E-06	protein_coding
ENSG0000(	ENSG0000(	15.10589	1.4183	3.74E-08	1.69E-06	lncRNA
ENSG0000(	ENSG0000(	257.8688	0.431472	3.75E-08	1.70E-06	lncRNA
ENSG0000(	ENSG0000(	13.99694	1.582512	3.79E-08	1.71E-06	unprocessed_pseudogene
ENSG0000(	CCSAP	529.5739	0.366326	3.80E-08	1.72E-06	protein_coding
ENSG0000(	DGKH	3786.563	0.269245	3.81E-08	1.72E-06	protein_coding
ENSG0000(	ENSG0000(	296.7434	0.285228	3.83E-08	1.72E-06	lncRNA
ENSG0000(	ENSG0000(	546.5342	0.270596	3.83E-08	1.72E-06	TEC
ENSG0000(	ENSG0000(	354.9942	0.288755	3.86E-08	1.73E-06	lncRNA
ENSG0000(	KCNIP4	41.85311	1.399979	3.86E-08	1.73E-06	protein_coding
ENSG0000(	ANGEL1	1104.356	0.32639	3.97E-08	1.78E-06	protein_coding

ENSG0000(	ADAMTS6	295.0088	0.846443	4.05E-08	1.81E-06	protein_coding
ENSG0000(	PLIN5	383.6307	0.26887	4.11E-08	1.84E-06	protein_coding
ENSG0000(	LINC01671	134.2515	0.440203	4.12E-08	1.84E-06	lncRNA
ENSG0000(	TBCEL	909.4506	0.360854	4.12E-08	1.84E-06	protein_coding
ENSG0000(	OTUB2	38.73417	0.9119	4.14E-08	1.84E-06	protein_coding
ENSG0000(	CD300C	32.97648	0.992821	4.20E-08	1.86E-06	protein_coding
ENSG0000(	KLRB1	156.7243	-0.8209	4.21E-08	1.86E-06	protein_coding
ENSG0000(	OR52K3P	145.9236	0.556721	4.27E-08	1.89E-06	transcribed_unprocessed_pseudo
ENSG0000(	LGALS17A	37.30825	1.397522	4.28E-08	1.89E-06	transcribed_unprocessed_pseudo
ENSG0000(	ENSG0000(	245.0908	0.296017	4.30E-08	1.90E-06	TEC
ENSG0000(	ENSG0000(	1195.825	0.234604	4.31E-08	1.90E-06	lncRNA
ENSG0000(	LINC01191	66.15788	0.98782	4.33E-08	1.91E-06	lncRNA
ENSG0000(	HNRNPUL2	2876.434	-0.23009	4.45E-08	1.95E-06	protein_coding
ENSG0000(	MEPCE	671.7304	-0.25268	4.46E-08	1.96E-06	protein_coding
ENSG0000(	CSNK2B	979.1324	-0.31699	4.48E-08	1.96E-06	protein_coding
ENSG0000(	ALDH9A1	724.2874	-0.41491	4.60E-08	2.01E-06	protein_coding
ENSG0000(	CFB	1505.036	0.896186	4.61E-08	2.02E-06	protein_coding
ENSG0000(	RNF167	721.8973	-0.33238	4.71E-08	2.06E-06	protein_coding
ENSG0000(	SH3PXD2B	885.9531	0.893439	4.72E-08	2.06E-06	protein_coding
ENSG0000(	ENSG0000(	146.3606	0.590784	4.78E-08	2.08E-06	lncRNA
ENSG0000(	ARMC9	2130.218	0.264947	4.82E-08	2.09E-06	protein_coding
ENSG0000(	IGHEP2	79.37791	0.778867	4.82E-08	2.09E-06	IG_C_pseudogene
ENSG0000(	LINC02871	170.3833	0.327952	4.86E-08	2.11E-06	lncRNA
ENSG0000(	R3HDM2	2367.803	-0.20432	4.90E-08	2.12E-06	protein_coding
ENSG0000(	PCMTD1	2984.935	0.18078	4.94E-08	2.14E-06	protein_coding
ENSG0000(	NOL12	604.0789	-0.24033	4.96E-08	2.14E-06	protein_coding
ENSG0000(	EEF1B2	1720.156	-0.45644	4.98E-08	2.15E-06	protein_coding
ENSG0000(	ZNF438	323.8772	0.439458	5.01E-08	2.16E-06	protein_coding
ENSG0000(	EVA1A-AS	275.669	0.272192	5.04E-08	2.17E-06	lncRNA
ENSG0000(	SNORA53	588.7204	-0.8293	5.06E-08	2.17E-06	snoRNA
ENSG0000(	ENSG0000(	757.8234	0.260861	5.08E-08	2.18E-06	TEC
ENSG0000(	C4orf50	106.3698	0.60226	5.09E-08	2.18E-06	protein_coding
ENSG0000(	IGHV3-49	132.1202	1.749521	5.11E-08	2.18E-06	IG_V_gene
ENSG0000(	MMP25	439.9662	1.296878	5.14E-08	2.20E-06	protein_coding
ENSG0000(	ENSG0000(	27.54529	0.922822	5.20E-08	2.22E-06	TEC
ENSG0000(	DVL2	631.2122	-0.21989	5.21E-08	2.22E-06	protein_coding
ENSG0000(	ENSG0000(	47.28019	0.643133	5.22E-08	2.22E-06	TEC
ENSG0000(	ENSG0000(	829.4393	0.237719	5.25E-08	2.23E-06	lncRNA
ENSG0000(	ENSG0000(	189.8344	0.382002	5.27E-08	2.24E-06	lncRNA
ENSG0000(	ENSG0000(	188.6116	0.821104	5.32E-08	2.25E-06	lncRNA
ENSG0000(	MAN1A2	2760.646	0.211545	5.35E-08	2.26E-06	protein_coding
ENSG0000(	MCTP2	3029.1	0.389095	5.40E-08	2.28E-06	protein_coding
ENSG0000(	COL5A3	512.5748	0.847137	5.43E-08	2.29E-06	protein_coding
ENSG0000(	RN7SL124F	14.66427	1.494347	5.45E-08	2.30E-06	misc_RNA
ENSG0000(	CFLAR	24378.67	0.278562	5.52E-08	2.32E-06	protein_coding
ENSG0000(	IGHV3-72	117.6509	1.551191	5.53E-08	2.32E-06	IG_V_gene
ENSG0000(	RBM8A	1466.913	-0.23873	5.58E-08	2.34E-06	protein_coding

ENSG0000(	CBY1	301.3243	0.242325	5.67E-08	2.37E-06	protein_coding
ENSG0000(	GOLGA8K	133.3589	0.615323	5.67E-08	2.37E-06	protein_coding
ENSG0000(	ENSG0000(	9.696526	1.923647	5.70E-08	2.38E-06	lncRNA
ENSG0000(	PDE10A	933.8765	0.576367	5.70E-08	2.38E-06	protein_coding
ENSG0000(	UNC80	1389.565	0.246124	5.71E-08	2.38E-06	protein_coding
ENSG0000(	CDK12	4713.679	0.187408	5.81E-08	2.42E-06	protein_coding
ENSG0000(	WAC	4003.43	-0.14233	5.80E-08	2.42E-06	protein_coding
ENSG0000(	PYGO2	871.7128	-0.20371	5.88E-08	2.44E-06	protein_coding
ENSG0000(	ACER3	6290.341	0.1768	5.97E-08	2.47E-06	protein_coding
ENSG0000(	ENSG0000(	505.611	0.259821	5.98E-08	2.47E-06	lncRNA
ENSG0000(	H4C5	419.6768	-0.54221	6.00E-08	2.48E-06	protein_coding
ENSG0000(	DGKI	70.77777	1.429186	6.03E-08	2.49E-06	protein_coding
ENSG0000(	C3AR1	216.0082	0.790598	6.05E-08	2.49E-06	protein_coding
ENSG0000(	MXRA5	2041.927	0.877411	6.07E-08	2.50E-06	protein_coding
ENSG0000(	ZFP30	840.797	-0.26579	6.09E-08	2.50E-06	protein_coding
ENSG0000(	LPCAT2	608.5428	0.574247	6.10E-08	2.51E-06	protein_coding
ENSG0000(	ZC3H13	2133.049	-0.20258	6.19E-08	2.54E-06	protein_coding
ENSG0000(	TMEM92-A	10.65478	1.12547	6.26E-08	2.56E-06	lncRNA
ENSG0000(	IRF1-AS1	5167.129	0.351648	6.28E-08	2.57E-06	lncRNA
ENSG0000(	NUTM2B	27.33712	1.239374	6.30E-08	2.58E-06	protein_coding
ENSG0000(	ENSG0000(	582.6284	0.269887	6.31E-08	2.58E-06	processed_pseudogene
ENSG0000(	FDPSP3	102.5794	0.700917	6.35E-08	2.59E-06	transcribed_processed_pseudoge
ENSG0000(	APOL3	1845.927	0.683438	6.37E-08	2.59E-06	protein_coding
ENSG0000(	IGFBP3	869.5877	-0.63212	6.56E-08	2.67E-06	protein_coding
ENSG0000(	NAV1	3189.436	0.289763	6.61E-08	2.68E-06	protein_coding
ENSG0000(	MMP19	460.0358	0.888158	6.65E-08	2.70E-06	protein_coding
ENSG0000(	ENSG0000(	56.96583	0.695979	6.85E-08	2.77E-06	processed_pseudogene
ENSG0000(	ENSG0000(	353.6546	-0.34372	6.87E-08	2.78E-06	lncRNA
ENSG0000(	CSNK1E	2224.27	0.259531	6.88E-08	2.78E-06	protein_coding
ENSG0000(	DNM3OS	354.532	0.543212	6.91E-08	2.79E-06	lncRNA
ENSG0000(	AUP1	1411.267	-0.28297	6.94E-08	2.80E-06	protein_coding
ENSG0000(	CCT8	900.2809	-0.23384	6.96E-08	2.80E-06	protein_coding
ENSG0000(	ENSG0000(	16.96385	1.1666	6.98E-08	2.80E-06	misc_RNA
ENSG0000(	ENSG0000(	252.1084	0.5882	7.10E-08	2.85E-06	lncRNA
ENSG0000(	ENSG0000(	47.17865	0.565526	7.13E-08	2.85E-06	lncRNA
ENSG0000(	PHOSPHO1	19.51965	1.634689	7.13E-08	2.85E-06	protein_coding
ENSG0000(	PRSS23	1061.775	0.45879	7.15E-08	2.86E-06	protein_coding
ENSG0000(	ENSG0000(	216.6016	0.406907	7.23E-08	2.89E-06	lncRNA
ENSG0000(	VCL	2925.142	0.450599	7.25E-08	2.89E-06	protein_coding
ENSG0000(	ENSG0000(	812.3477	0.242524	7.26E-08	2.89E-06	lncRNA
ENSG0000(	PSMD8	681.4544	-0.28928	7.39E-08	2.94E-06	protein_coding
ENSG0000(	FKBP4	719.2432	-0.37443	7.41E-08	2.94E-06	protein_coding
ENSG0000(	IGKC	12362.5	1.38355	7.52E-08	2.98E-06	IG_C_gene
ENSG0000(	ENSG0000(	908.0288	0.244157	7.58E-08	3.00E-06	lncRNA
ENSG0000(	TPM4	6799.844	0.400601	7.57E-08	3.00E-06	protein_coding
ENSG0000(	AKR1B10	1215.116	-1.37469	7.60E-08	3.00E-06	protein_coding
ENSG0000(	RTEL1P1	262.4112	0.432626	7.61E-08	3.00E-06	transcribed_processed_pseudoge

ENSG0000(	ITGA5	1126.923	0.905518	7.63E-08	3.01E-06	protein_coding
ENSG0000(	CLEC12B	23.31773	1.14163	7.68E-08	3.03E-06	protein_coding
ENSG0000(	IGKV3-20	1007.533	1.459393	7.75E-08	3.05E-06	IG_V_gene
ENSG0000(	LINC02701	38.59234	0.680354	7.79E-08	3.06E-06	lncRNA
ENSG0000(	ENSG0000(	22.8666	0.965212	7.88E-08	3.09E-06	lncRNA
ENSG0000(	CEP104	2257.702	-0.21918	7.94E-08	3.11E-06	protein_coding
ENSG0000(	NPIPB11	618.1731	0.475578	7.99E-08	3.13E-06	protein_coding
ENSG0000(	ERVFRD-3	41.80042	0.709749	8.04E-08	3.14E-06	transcribed_processed_pseudoge
ENSG0000(	LAMA4	2691.332	0.506048	8.07E-08	3.15E-06	protein_coding
ENSG0000(	SULF2	1096.222	0.682303	8.07E-08	3.15E-06	protein_coding
ENSG0000(	NIPAL4-DT	11.89987	1.353692	8.12E-08	3.16E-06	lncRNA
ENSG0000(	RN7SL368F	35.89953	1.441152	8.21E-08	3.19E-06	misc_RNA
ENSG0000(	TSPAN9	361.4326	0.597408	8.22E-08	3.19E-06	protein_coding
ENSG0000(	CICP14	23.30153	2.098568	8.40E-08	3.26E-06	processed_pseudogene
ENSG0000(	ELL2	1494.188	0.660052	8.42E-08	3.26E-06	protein_coding
ENSG0000(	HM13	2777.639	0.356621	8.49E-08	3.29E-06	protein_coding
ENSG0000(	MZB1	553.3857	0.913636	8.55E-08	3.30E-06	protein_coding
ENSG0000(	SND1-IT1	652.7804	0.362605	8.56E-08	3.30E-06	lncRNA
ENSG0000(	ENSG0000(	92.86328	0.418937	8.61E-08	3.32E-06	lncRNA
ENSG0000(	TAAR3P	15.56101	1.360076	8.74E-08	3.37E-06	transcribed_unprocessed_pseudo
ENSG0000(	GAPDHP14	12.92789	1.389179	8.83E-08	3.39E-06	processed_pseudogene
ENSG0000(	NRP2	1262.564	0.56886	8.83E-08	3.39E-06	protein_coding
ENSG0000(	ADAMTSL4	382.8666	0.702311	8.87E-08	3.40E-06	protein_coding
ENSG0000(	PFKFB3	1705.451	0.742381	8.87E-08	3.40E-06	protein_coding
ENSG0000(	ANGPTL2	389.6846	1.071921	8.90E-08	3.41E-06	protein_coding
ENSG0000(	EPHB1	122.506	0.795367	9.02E-08	3.45E-06	protein_coding
ENSG0000(	GON4L	3583.455	0.176079	9.02E-08	3.45E-06	protein_coding
ENSG0000(	WFDC2	19.66992	1.153446	9.46E-08	3.61E-06	protein_coding
ENSG0000(	PDCD6-DT	25.64754	0.756992	9.54E-08	3.64E-06	lncRNA
ENSG0000(	ENSG0000(	2060.903	0.404004	9.60E-08	3.65E-06	transcribed_unprocessed_pseudo
ENSG0000(	SEC16A	4700.241	0.206337	9.64E-08	3.66E-06	protein_coding
ENSG0000(	KLF7-IT1	150.3218	0.503999	9.68E-08	3.68E-06	lncRNA
ENSG0000(	EMILIN2	588.0911	0.688676	9.70E-08	3.68E-06	protein_coding
ENSG0000(	CHST13	12.81133	-1.48237	9.87E-08	3.74E-06	protein_coding
ENSG0000(	PYGL	569.1173	0.417871	1.00E-07	3.78E-06	protein_coding
ENSG0000(	FCER1G	290.176	0.791434	1.01E-07	3.81E-06	protein_coding
ENSG0000(	ELAVL3	90.95291	0.895488	1.01E-07	3.82E-06	protein_coding
ENSG0000(	FKBP10	588.2374	0.480711	1.04E-07	3.92E-06	protein_coding
ENSG0000(	SEMA5A	2630.54	-0.5381	1.06E-07	4.00E-06	protein_coding
ENSG0000(	PPP6C	912.0014	-0.28298	1.07E-07	4.04E-06	protein_coding
ENSG0000(	MKNK2	3163.332	-0.46664	1.08E-07	4.04E-06	protein_coding
ENSG0000(	ENSG0000(	30.69926	0.589268	1.09E-07	4.08E-06	lncRNA
ENSG0000(	TRPM7	5491.091	-0.1831	1.09E-07	4.08E-06	protein_coding
ENSG0000(	ENSG0000(	383.9973	0.227401	1.11E-07	4.14E-06	lncRNA
ENSG0000(	SLC17A9	535.341	0.996506	1.11E-07	4.15E-06	protein_coding
ENSG0000(	RPS6KA2	753.8979	0.468248	1.11E-07	4.15E-06	protein_coding
ENSG0000(	SPAG4	65.96215	1.02311	1.11E-07	4.15E-06	protein_coding

ENSG0000(	OIP5-AS1	6846.913	-0.16523	1.12E-07	4.16E-06	lncRNA
ENSG0000(	RNF227	782.5535	-0.22398	1.12E-07	4.17E-06	protein_coding
ENSG0000(	CKAP4	655.1662	0.535003	1.15E-07	4.26E-06	protein_coding
ENSG0000(	ENSG0000(	81.26926	0.6396	1.15E-07	4.27E-06	lncRNA
ENSG0000(	CIBAR1	230.3528	0.503974	1.15E-07	4.28E-06	protein_coding
ENSG0000(	IGF2R	7820.303	0.313845	1.16E-07	4.30E-06	protein_coding
ENSG0000(	SPATS1	415.3394	0.404321	1.19E-07	4.40E-06	protein_coding
ENSG0000(	FZD7	371.9082	-0.63986	1.20E-07	4.44E-06	protein_coding
ENSG0000(	FBN1	2094.88	0.602834	1.21E-07	4.46E-06	protein_coding
ENSG0000(	WARS2-IT1	1398.996	0.233085	1.21E-07	4.46E-06	lncRNA
ENSG0000(	ZNF385D	293.0845	1.001705	1.21E-07	4.46E-06	protein_coding
ENSG0000(	SH3RF3	385.8729	0.679698	1.22E-07	4.47E-06	protein_coding
ENSG0000(	CCN2	421.5046	0.899548	1.24E-07	4.54E-06	protein_coding
ENSG0000(	COMMD6	232.8122	-0.51279	1.25E-07	4.59E-06	protein_coding
ENSG0000(	ENSG0000(	125.7192	0.435945	1.26E-07	4.61E-06	lncRNA
ENSG0000(	ZNF710	775.3698	0.402545	1.26E-07	4.61E-06	protein_coding
ENSG0000(	KCNJ8	33.73925	0.921875	1.27E-07	4.64E-06	protein_coding
ENSG0000(	CEACAM5	4113.409	1.375182	1.30E-07	4.74E-06	protein_coding
ENSG0000(	ENSG0000(	496.0821	0.201964	1.35E-07	4.91E-06	lncRNA
ENSG0000(	ENSG0000(	92.48206	0.483015	1.35E-07	4.93E-06	lncRNA
ENSG0000(	ENSG0000(	370.7159	0.447372	1.36E-07	4.94E-06	lncRNA
ENSG0000(	CARMN	13182.37	0.549115	1.39E-07	5.06E-06	lncRNA
ENSG0000(	SCO2	240.8021	0.872696	1.41E-07	5.11E-06	protein_coding
ENSG0000(	ORMDL1	1329.371	0.21317	1.42E-07	5.16E-06	protein_coding
ENSG0000(	ZNF75D	1228.765	0.14879	1.42E-07	5.16E-06	protein_coding
ENSG0000(	BCL6	1388.863	1.10879	1.44E-07	5.23E-06	protein_coding
ENSG0000(	ENSG0000(	885.04	0.195434	1.49E-07	5.37E-06	lncRNA
ENSG0000(	IL1R1	1969.794	0.681565	1.53E-07	5.52E-06	protein_coding
ENSG0000(	PPP1CC	4095.748	-0.3881	1.54E-07	5.55E-06	protein_coding
ENSG0000(	TRIM69	1647.704	0.369336	1.54E-07	5.55E-06	protein_coding
ENSG0000(	SDK1	853.0253	0.80697	1.56E-07	5.60E-06	protein_coding
ENSG0000(	ENSG0000(	58.65863	0.432639	1.57E-07	5.65E-06	lncRNA
ENSG0000(	ENSG0000(	307.1946	0.389151	1.57E-07	5.65E-06	lncRNA
ENSG0000(	LINC00159	180.2268	0.455975	1.59E-07	5.69E-06	lncRNA
ENSG0000(	PI15	476.2993	2.018747	1.59E-07	5.70E-06	protein_coding
ENSG0000(	ENSG0000(	368.8568	0.298508	1.62E-07	5.79E-06	lncRNA
ENSG0000(	VNN3	434.0804	0.457639	1.64E-07	5.85E-06	protein_coding
ENSG0000(	ERRFI1	1433.37	1.016832	1.65E-07	5.88E-06	protein_coding
ENSG0000(	ENSG0000(	545.0896	0.337849	1.66E-07	5.92E-06	lncRNA
ENSG0000(	LPL	234.2659	1.024786	1.66E-07	5.92E-06	protein_coding
ENSG0000(	U2AF2	1704.151	-0.20634	1.67E-07	5.92E-06	protein_coding
ENSG0000(	CD14	160.0783	1.092235	1.67E-07	5.94E-06	protein_coding
ENSG0000(	LAP3	1113.614	0.74481	1.68E-07	5.95E-06	protein_coding
ENSG0000(	VASH1-AS1	472.736	0.241067	1.68E-07	5.97E-06	lncRNA
ENSG0000(	FSTL1	1828.588	0.728737	1.70E-07	6.01E-06	protein_coding
ENSG0000(	GYG1	998.0459	0.259884	1.70E-07	6.02E-06	protein_coding
ENSG0000(	ENSG0000(	28.48097	1.765038	1.71E-07	6.03E-06	lncRNA

ENSG0000(	C4BPB	733.1847	0.362575	1.72E-07	6.07E-06	protein_coding
ENSG0000(	ASPHD2	145.5781	0.732903	1.74E-07	6.11E-06	protein_coding
ENSG0000(	SUPT7L	1938.972	0.166342	1.74E-07	6.11E-06	protein_coding
ENSG0000(	SNORD33	229.9016	0.799789	1.74E-07	6.11E-06	snoRNA
ENSG0000(	ENSG0000(	19.64082	1.611716	1.78E-07	6.27E-06	lncRNA
ENSG0000(	IQCH	69.86324	0.605556	1.82E-07	6.38E-06	protein_coding
ENSG0000(	ENSG0000(	109.9942	0.624758	1.82E-07	6.39E-06	lncRNA
ENSG0000(	NR1D2	1377.623	-0.53361	1.84E-07	6.42E-06	protein_coding
ENSG0000(	ENSG0000(	528.1995	0.202098	1.84E-07	6.43E-06	transcribed_processed_pseudoge
ENSG0000(	NFAM1	572.2043	0.611959	1.84E-07	6.43E-06	protein_coding
ENSG0000(	PDHX	586.3854	-0.28957	1.84E-07	6.43E-06	protein_coding
ENSG0000(	PLEKHM1P	4353.037	0.150374	1.85E-07	6.44E-06	transcribed_unprocessed_pseudo
ENSG0000(	CT55	9.808591	1.204689	1.85E-07	6.44E-06	protein_coding
ENSG0000(	SBF2	5228.956	0.178859	1.87E-07	6.50E-06	protein_coding
ENSG0000(	ST20-AS1	818.8019	0.2836	1.87E-07	6.50E-06	lncRNA
ENSG0000(	SULF1	1452.132	0.569678	1.89E-07	6.57E-06	protein_coding
ENSG0000(	ENSG0000(	99.0494	0.447329	1.91E-07	6.63E-06	lncRNA
ENSG0000(	ENSG0000(	154.9235	0.37907	1.92E-07	6.65E-06	lncRNA
ENSG0000(	CTSB	6981.101	0.529052	1.93E-07	6.66E-06	protein_coding
ENSG0000(	ENSG0000(	41.49094	0.67143	1.95E-07	6.70E-06	lncRNA
ENSG0000(	ENSG0000(	20.54919	0.933476	1.95E-07	6.70E-06	lncRNA
ENSG0000(	ENSG0000(	265.1425	0.510774	1.95E-07	6.70E-06	lncRNA
ENSG0000(	MYOSLID	155.7923	0.417914	1.95E-07	6.70E-06	lncRNA
ENSG0000(	SLC6A12	54.48086	1.125428	1.95E-07	6.70E-06	protein_coding
ENSG0000(	H4C8	446.9751	-0.57282	1.99E-07	6.84E-06	protein_coding
ENSG0000(	CBR3	17.27948	0.892287	2.02E-07	6.93E-06	protein_coding
ENSG0000(	SUCNR1	55.94747	1.082274	2.04E-07	6.99E-06	protein_coding
ENSG0000(	MOB3C	1087.671	0.32327	2.05E-07	7.01E-06	protein_coding
ENSG0000(	ENSG0000(	384.2243	-0.52863	2.06E-07	7.05E-06	lncRNA
ENSG0000(	LINC02013	747.2256	0.271714	2.06E-07	7.05E-06	lncRNA
ENSG0000(	MFAP2	75.73965	0.937561	2.08E-07	7.09E-06	protein_coding
ENSG0000(	ENSG0000(	12.55958	1.335773	2.08E-07	7.09E-06	lncRNA
ENSG0000(	ENSG0000(	1120.427	0.252915	2.08E-07	7.09E-06	TEC
ENSG0000(	BICC1	426.1458	0.797266	2.09E-07	7.12E-06	protein_coding
ENSG0000(	OR6B1	108.4079	0.411032	2.09E-07	7.12E-06	protein_coding
ENSG0000(	RPS26P21	58.10627	0.791825	2.12E-07	7.18E-06	processed_pseudogene
ENSG0000(	ZNF415P1	40.20116	1.041405	2.12E-07	7.18E-06	processed_pseudogene
ENSG0000(	ENSG0000(	46.42558	0.516019	2.14E-07	7.24E-06	lncRNA
ENSG0000(	SNORD83B	26.44975	1.192568	2.16E-07	7.30E-06	snoRNA
ENSG0000(	WNT9B	213.777	-0.2793	2.20E-07	7.43E-06	protein_coding
ENSG0000(	DCUN1D1	1442.504	-0.26246	2.21E-07	7.47E-06	protein_coding
ENSG0000(	ADAMDEC:	3489.7	-0.78393	2.24E-07	7.54E-06	protein_coding
ENSG0000(	GFPT2	116.5604	1.374937	2.24E-07	7.55E-06	protein_coding
ENSG0000(	NMNAT1	1563.012	-0.19777	2.26E-07	7.61E-06	protein_coding
ENSG0000(	GRIN2D	30.60153	0.89729	2.28E-07	7.65E-06	protein_coding
ENSG0000(	ENSG0000(	15.41017	1.231694	2.29E-07	7.69E-06	lncRNA
ENSG0000(	PPP1R9A-A	152.1107	0.372002	2.29E-07	7.70E-06	lncRNA

ENSG0000(	SPDYE3	30.55064	0.810963	2.30E-07	7.70E-06	protein_coding
ENSG0000(	CLPTM1	805.1085	-0.26254	2.35E-07	7.87E-06	protein_coding
ENSG0000(	VPS26BP1	128.6854	0.407654	2.37E-07	7.94E-06	processed_pseudogene
ENSG0000(	ENSG0000(	87.68324	0.642175	2.39E-07	7.96E-06	lncRNA
ENSG0000(	KRT42P	111.3356	-0.41489	2.39E-07	7.96E-06	transcribed_unprocessed_pseudo
ENSG0000(	SYPL1P2	35.49965	0.609058	2.39E-07	7.98E-06	processed_pseudogene
ENSG0000(	TMPRSS3	172.0633	0.751319	2.41E-07	8.04E-06	protein_coding
ENSG0000(	TRAM2	2211.739	0.37183	2.45E-07	8.16E-06	protein_coding
ENSG0000(	ADAMTS14	724.8129	0.715585	2.46E-07	8.19E-06	protein_coding
ENSG0000(	ENSG0000(	333.6947	0.244317	2.47E-07	8.19E-06	lncRNA
ENSG0000(	ENSG0000(	72.69102	0.708202	2.48E-07	8.22E-06	TEC
ENSG0000(	LINC01002	1545.236	0.672348	2.50E-07	8.29E-06	lncRNA
ENSG0000(	EVI5L	373.559	0.266062	2.53E-07	8.38E-06	protein_coding
ENSG0000(	SRSF7	2279.467	-0.17381	2.54E-07	8.39E-06	protein_coding
ENSG0000(	BAG3	195.6554	0.464075	2.55E-07	8.42E-06	protein_coding
ENSG0000(	FABP1	4687.256	-1.27972	2.55E-07	8.42E-06	protein_coding
ENSG0000(	ENSG0000(	1283.376	0.222419	2.57E-07	8.46E-06	lncRNA
ENSG0000(	ENSG0000(	91.54512	0.511544	2.57E-07	8.46E-06	lncRNA
ENSG0000(	MEG3	3951.179	0.663173	2.57E-07	8.46E-06	lncRNA
ENSG0000(	ENSG0000(	161.836	-0.38644	2.59E-07	8.52E-06	protein_coding
ENSG0000(	GALNTL6	48.90864	1.104736	2.61E-07	8.56E-06	protein_coding
ENSG0000(	CYP26B1	172.1989	1.035495	2.61E-07	8.56E-06	protein_coding
ENSG0000(	DBP	1239.648	-0.4491	2.61E-07	8.56E-06	protein_coding
ENSG0000(	NLRP14	48.42025	1.09149	2.62E-07	8.57E-06	protein_coding
ENSG0000(	ENSG0000(	255.1488	0.349488	2.62E-07	8.57E-06	transcribed_processed_pseudoge
ENSG0000(	LARP6	194.6162	0.508256	2.63E-07	8.60E-06	protein_coding
ENSG0000(	SYNPO2L-A	186.3494	0.480549	2.65E-07	8.64E-06	lncRNA
ENSG0000(	SSH1	3514.406	0.335355	2.66E-07	8.67E-06	protein_coding
ENSG0000(	COL27A1	2236.107	0.485615	2.69E-07	8.77E-06	protein_coding
ENSG0000(	RGS13	439.9219	-1.13374	2.73E-07	8.87E-06	protein_coding
ENSG0000(	GTF3C2	1494.63	-0.14327	2.75E-07	8.91E-06	protein_coding
ENSG0000(	IGKV1-5	652.4985	1.359416	2.75E-07	8.91E-06	IG_V_gene
ENSG0000(	IFNG	14.77088	2.027318	2.77E-07	8.97E-06	protein_coding
ENSG0000(	EIF2AK4	1480.095	0.197165	2.82E-07	9.14E-06	protein_coding
ENSG0000(	AASS	1867.594	0.280615	2.83E-07	9.15E-06	protein_coding
ENSG0000(	C13orf42	171.2675	0.327995	2.84E-07	9.16E-06	protein_coding
ENSG0000(	RPS28P7	208.6426	-0.63949	2.84E-07	9.16E-06	processed_pseudogene
ENSG0000(	CDK20	422.3442	-0.82553	2.87E-07	9.25E-06	protein_coding
ENSG0000(	IL20RA	41.28214	1.775051	2.88E-07	9.26E-06	protein_coding
ENSG0000(	TYMP	1605.596	0.814129	2.90E-07	9.33E-06	protein_coding
ENSG0000(	ENSG0000(	323.7057	0.229054	2.93E-07	9.42E-06	lncRNA
ENSG0000(	FLG-AS1	534.7219	0.213212	2.96E-07	9.49E-06	lncRNA
ENSG0000(	FRZB	151.7564	-0.61482	2.96E-07	9.50E-06	protein_coding
ENSG0000(	PRPF8	6077.556	-0.19031	2.97E-07	9.50E-06	protein_coding
ENSG0000(	COX6C	682.2737	-0.41374	2.98E-07	9.54E-06	protein_coding
ENSG0000(	CASC15	2879.023	0.221557	3.01E-07	9.60E-06	lncRNA
ENSG0000(	ENSG0000(	27.5483	0.71405	3.00E-07	9.60E-06	lncRNA

ENSG0000(	ENSG0000(	10.91819	1.733669	3.01E-07	9.60E-06	lncRNA
ENSG0000(	ENSG0000(	43.99913	0.62743	3.01E-07	9.61E-06	lncRNA
ENSG0000(	LINC02913	246.4234	0.253711	3.06E-07	9.73E-06	lncRNA
ENSG0000(	AK7	289.1276	-0.35001	3.06E-07	9.73E-06	protein_coding
ENSG0000(	GRHL3	15.92465	1.205782	3.08E-07	9.79E-06	protein_coding
ENSG0000(	IGHJ5	266.1619	1.271058	3.09E-07	9.82E-06	IG_J_gene
ENSG0000(	ENSG0000(	32.18199	0.713138	3.10E-07	9.82E-06	lncRNA
ENSG0000(	PSG9	102.5324	1.100606	3.10E-07	9.82E-06	protein_coding
ENSG0000(	FXR2	707.666	-0.20399	3.14E-07	9.93E-06	protein_coding
ENSG0000(	PPAT	304.283	0.302314	3.14E-07	9.93E-06	protein_coding
ENSG0000(	ENSG0000(	22.45627	1.123794	3.18E-07	1.01E-05	lncRNA
ENSG0000(	ENSG0000(	623.7133	0.300554	3.19E-07	1.01E-05	lncRNA
ENSG0000(	YARS1	1838.749	0.223126	3.19E-07	1.01E-05	protein_coding
ENSG0000(	TIMM21	505.9416	-0.24661	3.22E-07	1.01E-05	protein_coding
ENSG0000(	ENSG0000(	301.8918	0.216103	3.30E-07	1.04E-05	lncRNA
ENSG0000(	AMPH	57.43477	0.950694	3.32E-07	1.04E-05	protein_coding
ENSG0000(	EXOC4	2824.683	0.171396	3.32E-07	1.04E-05	protein_coding
ENSG0000(	ZNF621	3036.904	0.148077	3.36E-07	1.05E-05	protein_coding
ENSG0000(	IGHV3-21	305.3188	1.438612	3.37E-07	1.06E-05	IG_V_gene
ENSG0000(	FKBP14	1482.024	0.195676	3.37E-07	1.06E-05	protein_coding
ENSG0000(	CLSTN2	437.3372	-0.52101	3.40E-07	1.06E-05	protein_coding
ENSG0000(	ENSG0000(	244.9021	0.749932	3.41E-07	1.07E-05	lncRNA
ENSG0000(	ENSG0000(	74.91117	0.590861	3.46E-07	1.08E-05	lncRNA
ENSG0000(	ENSG0000(	93.43857	0.310269	3.46E-07	1.08E-05	lncRNA
ENSG0000(	CCL4L2	48.7633	1.599669	3.55E-07	1.11E-05	protein_coding
ENSG0000(	H2BC4	309.8542	-0.49587	3.55E-07	1.11E-05	protein_coding
ENSG0000(	RPS20P20	15.72409	1.239758	3.60E-07	1.12E-05	processed_pseudogene
ENSG0000(	MIR5690	40.38566	1.569844	3.62E-07	1.12E-05	miRNA
ENSG0000(	CCL16	300.3635	-0.2062	3.62E-07	1.13E-05	protein_coding
ENSG0000(	MCTP1	700.3763	0.717669	3.63E-07	1.13E-05	protein_coding
ENSG0000(	ENSG0000(	395.1368	0.355911	3.71E-07	1.15E-05	lncRNA
ENSG0000(	AGTRAP	200.1048	0.605561	3.76E-07	1.16E-05	protein_coding
ENSG0000(	ENSG0000(	261.1861	0.282198	3.79E-07	1.17E-05	lncRNA
ENSG0000(	KRT17	16.29333	1.790263	3.81E-07	1.18E-05	protein_coding
ENSG0000(	ENSG0000(	39.8533	1.042395	3.84E-07	1.19E-05	lncRNA
ENSG0000(	ADORA2B	143.5605	0.509858	3.85E-07	1.19E-05	protein_coding
ENSG0000(	RPP21	159.0822	-0.38864	3.86E-07	1.19E-05	protein_coding
ENSG0000(	CPXM1	74.75601	1.217187	3.90E-07	1.20E-05	protein_coding
ENSG0000(	ENSG0000(	616.7326	0.570106	3.91E-07	1.20E-05	lncRNA
ENSG0000(	ENSG0000(	377.3683	0.391578	3.93E-07	1.21E-05	lncRNA
ENSG0000(	ENSG0000(	124.2289	0.323726	3.93E-07	1.21E-05	lncRNA
ENSG0000(	GNG4	402.4661	-0.46074	3.95E-07	1.21E-05	protein_coding
ENSG0000(	ENSG0000(	197.9039	0.278294	3.96E-07	1.21E-05	lncRNA
ENSG0000(	KIF19	211.0607	0.437277	3.96E-07	1.21E-05	protein_coding
ENSG0000(	ZNF77	110.3552	0.361814	3.96E-07	1.21E-05	protein_coding
ENSG0000(	EIF4A2	7016.179	-0.17732	4.02E-07	1.23E-05	protein_coding
ENSG0000(	AAMDC	447.0367	-0.25883	4.03E-07	1.23E-05	protein_coding

ENSG0000(	NFKBIZ	2685.86	0.62272	4.04E-07	1.23E-05	protein_coding
ENSG0000(	GRIN3A	70.15704	1.343625	4.07E-07	1.24E-05	protein_coding
ENSG0000(	BTN3A3	3258.241	0.438848	4.10E-07	1.25E-05	protein_coding
ENSG0000(	GLDN	84.46252	0.656612	4.11E-07	1.25E-05	protein_coding
ENSG0000(	PTPRE	2209.729	0.343558	4.19E-07	1.27E-05	protein_coding
ENSG0000(	C3orf86	40.73782	0.898864	4.24E-07	1.29E-05	transcribed_unitary_pseudogene
ENSG0000(	ENSG0000(	44.59052	0.591945	4.29E-07	1.30E-05	TEC
ENSG0000(	PSG3	23.20561	0.936601	4.29E-07	1.30E-05	protein_coding
ENSG0000(	ENSG0000(	74.78889	0.582693	4.31E-07	1.30E-05	lncRNA
ENSG0000(	FGR	422.1775	1.089097	4.34E-07	1.31E-05	protein_coding
ENSG0000(	PTGES	330.9576	0.543871	4.37E-07	1.32E-05	protein_coding
ENSG0000(	SRSF1	5720.126	-0.19695	4.37E-07	1.32E-05	protein_coding
ENSG0000(	CDH11	1271.999	0.649197	4.40E-07	1.33E-05	protein_coding
ENSG0000(	ENSG0000(	13.54315	1.022087	4.41E-07	1.33E-05	lncRNA
ENSG0000(	RIPK2	209.9682	0.753138	4.45E-07	1.34E-05	protein_coding
ENSG0000(	KLHDC7B-D	8.935598	1.530209	4.47E-07	1.34E-05	lncRNA
ENSG0000(	LGI1	352.6687	0.422978	4.48E-07	1.34E-05	protein_coding
ENSG0000(	GALNT2	1131.743	0.273888	4.51E-07	1.35E-05	protein_coding
ENSG0000(	IGKJ1	61.95251	1.397465	4.50E-07	1.35E-05	IG_J_gene
ENSG0000(	LMBRD1	1688.62	-0.24784	4.51E-07	1.35E-05	protein_coding
ENSG0000(	PKIA-AS1	123.2876	0.359387	4.51E-07	1.35E-05	lncRNA
ENSG0000(	TBX18	42.4198	1.609257	4.55E-07	1.36E-05	protein_coding
ENSG0000(	MTATP6P1	111.8855	0.64131	4.55E-07	1.36E-05	unprocessed_pseudogene
ENSG0000(	VPS52	1186.874	-0.21907	4.61E-07	1.37E-05	protein_coding
ENSG0000(	IGHV2-26	36.77145	1.602066	4.66E-07	1.39E-05	IG_V_gene
ENSG0000(	IMMT	1200.823	-0.17592	4.66E-07	1.39E-05	protein_coding
ENSG0000(	SNORA71B	112.186	0.459631	4.72E-07	1.40E-05	snoRNA
ENSG0000(	ATP5F1C	1270.347	-0.28865	4.73E-07	1.41E-05	protein_coding
ENSG0000(	IGKV2D-29	48.06014	1.760313	4.75E-07	1.41E-05	IG_V_gene
ENSG0000(	ENSG0000(	99.53378	0.89483	4.78E-07	1.42E-05	lncRNA
ENSG0000(	ENSG0000(	63.55146	1.096032	4.80E-07	1.42E-05	unprocessed_pseudogene
ENSG0000(	SIRPB2	502.9851	0.665156	4.81E-07	1.42E-05	protein_coding
ENSG0000(	FLII	1754.972	-0.28969	4.82E-07	1.42E-05	protein_coding
ENSG0000(	NALF1-IT1	30.36543	1.182624	4.85E-07	1.43E-05	lncRNA
ENSG0000(	ZNF33BP1	90.33172	0.505702	4.90E-07	1.45E-05	processed_pseudogene
ENSG0000(	MIR762HG	88.81841	0.415324	4.92E-07	1.45E-05	lncRNA
ENSG0000(	SCFD1	5409.806	0.15456	4.95E-07	1.46E-05	protein_coding
ENSG0000(	MTCO2P11	127.1723	0.615151	5.05E-07	1.49E-05	unprocessed_pseudogene
ENSG0000(	CSNK1G2P1	34.29274	0.755996	5.11E-07	1.50E-05	processed_pseudogene
ENSG0000(	GSEC	270.8778	0.426134	5.11E-07	1.50E-05	lncRNA
ENSG0000(	LINC00703	28.1286	0.735202	5.11E-07	1.50E-05	lncRNA
ENSG0000(	PARP14	14951.19	0.612992	5.12E-07	1.50E-05	protein_coding
ENSG0000(	ANXA13	835.4646	-0.60904	5.14E-07	1.50E-05	protein_coding
ENSG0000(	ENSG0000(	117.7674	-0.31284	5.18E-07	1.52E-05	unprocessed_pseudogene
ENSG0000(	SLC25A37	5432.778	0.525698	5.19E-07	1.52E-05	protein_coding
ENSG0000(	ENSG0000(	180.3204	0.248611	5.25E-07	1.53E-05	lncRNA
ENSG0000(	SNORD14C	80.48118	0.838763	5.26E-07	1.53E-05	snoRNA

ENSG0000(	INTS11	1905.412	-0.27764	5.30E-07	1.55E-05	protein_coding
ENSG0000(	LRRC39	20.771	0.75526	5.32E-07	1.55E-05	protein_coding
ENSG0000(	LAMC3	409.5876	0.417595	5.38E-07	1.56E-05	protein_coding
ENSG0000(	PGF	499.4537	0.534971	5.39E-07	1.57E-05	protein_coding
ENSG0000(	RGS4	39.02129	0.956753	5.42E-07	1.57E-05	protein_coding
ENSG0000(	ENSG0000(	311.6844	0.297237	5.46E-07	1.58E-05	lncRNA
ENSG0000(	TNFSF13	339.4803	0.617564	5.53E-07	1.60E-05	protein_coding
ENSG0000(	LINC01919	119.2642	0.332399	5.54E-07	1.60E-05	lncRNA
ENSG0000(	TCEA1	1627.391	-0.209	5.64E-07	1.63E-05	protein_coding
ENSG0000(	ENSG0000(	35.38247	0.654138	5.66E-07	1.64E-05	lncRNA
ENSG0000(	RPS6KA2-IT	32.21247	0.664082	5.67E-07	1.64E-05	lncRNA
ENSG0000(	ENSG0000(	12.8085	1.443591	5.69E-07	1.64E-05	lncRNA
ENSG0000(	SERINC1	2634.158	-0.31907	5.71E-07	1.65E-05	protein_coding
ENSG0000(	LINC01016	907.706	0.35375	5.76E-07	1.66E-05	lncRNA
ENSG0000(	IGSF6	853.8868	0.500592	5.78E-07	1.66E-05	protein_coding
ENSG0000(	LINC00174	1939.494	0.249491	5.78E-07	1.66E-05	lncRNA
ENSG0000(	EVA1A	58.41417	0.978323	5.81E-07	1.67E-05	protein_coding
ENSG0000(	PALS2	1506.918	-0.69885	5.81E-07	1.67E-05	protein_coding
ENSG0000(	ENSG0000(	1154.061	0.17418	5.84E-07	1.67E-05	lncRNA
ENSG0000(	ENSG0000(	485.3959	0.47927	5.86E-07	1.68E-05	TEC
ENSG0000(	H2BC9	170.6275	-0.60073	5.88E-07	1.68E-05	protein_coding
ENSG0000(	SLC38A4	52.62242	-1.29918	5.88E-07	1.68E-05	protein_coding
ENSG0000(	ERVW-1	1740.874	0.304868	5.90E-07	1.68E-05	protein_coding
ENSG0000(	ADGRE3	70.91195	1.064372	5.93E-07	1.69E-05	protein_coding
ENSG0000(	ENSG0000(	20.21385	1.447433	5.92E-07	1.69E-05	lncRNA
ENSG0000(	SNX10	330.6963	1.031642	5.99E-07	1.71E-05	protein_coding
ENSG0000(	LPAR5	466.9459	-0.35914	6.01E-07	1.71E-05	protein_coding
ENSG0000(	CCT3	1079.096	-0.22394	6.07E-07	1.73E-05	protein_coding
ENSG0000(	SLC22A1	59.64953	0.631755	6.08E-07	1.73E-05	protein_coding
ENSG0000(	ENSG0000(	31.43279	1.986871	6.10E-07	1.73E-05	lncRNA
ENSG0000(	ANXA3	447.5458	0.457667	6.12E-07	1.73E-05	protein_coding
ENSG0000(	DENND5A	3118.72	0.527739	6.14E-07	1.74E-05	protein_coding
ENSG0000(	ENSG0000(	59.04489	-1.23552	6.14E-07	1.74E-05	processed_pseudogene
ENSG0000(	TGM2	2798.956	0.588755	6.16E-07	1.74E-05	protein_coding
ENSG0000(	FPR3	453.2556	0.530238	6.20E-07	1.75E-05	protein_coding
ENSG0000(	CCL3L3	45.15519	1.796803	6.27E-07	1.77E-05	protein_coding
ENSG0000(	CRIP1	492.2019	-1.12209	6.33E-07	1.78E-05	protein_coding
ENSG0000(	ENSG0000(	482.8011	0.228443	6.32E-07	1.78E-05	lncRNA
ENSG0000(	TIMP1	301.5868	1.155833	6.33E-07	1.78E-05	protein_coding
ENSG0000(	ENSG0000(	365.8387	0.351102	6.37E-07	1.79E-05	lncRNA
ENSG0000(	ENSG0000(	106.2043	0.759935	6.43E-07	1.81E-05	lncRNA
ENSG0000(	ZNF445	5092.993	0.174821	6.48E-07	1.82E-05	protein_coding
ENSG0000(	SLC35F2	543.4179	-0.43959	6.53E-07	1.83E-05	protein_coding
ENSG0000(	LINC02384	534.7043	0.630399	6.53E-07	1.83E-05	lncRNA
ENSG0000(	ENSG0000(	125.7162	0.514275	6.55E-07	1.84E-05	lncRNA
ENSG0000(	SH2D7	35.19211	-1.2139	6.58E-07	1.84E-05	protein_coding
ENSG0000(	GJD3	135.3111	0.687109	6.61E-07	1.85E-05	protein_coding

ENSG0000(	ODF3B	351.3203	0.642449	6.61E-07	1.85E-05	protein_coding
ENSG0000(	PHF21A	5201.062	0.301603	6.63E-07	1.85E-05	protein_coding
ENSG0000(	ENSG0000(	263.8218	0.301909	6.73E-07	1.88E-05	lncRNA
ENSG0000(	ENSG0000(	229.6992	0.227821	6.74E-07	1.88E-05	lncRNA
ENSG0000(	ST3GAL4	447.9502	0.618966	6.80E-07	1.89E-05	protein_coding
ENSG0000(	ENSG0000(	197.1998	0.344894	6.83E-07	1.90E-05	lncRNA
ENSG0000(	ENSG0000(	121.5952	0.922981	6.89E-07	1.91E-05	transcribed_processed_pseudoge
ENSG0000(	ENSG0000(	134.2039	0.407876	6.89E-07	1.91E-05	TEC
ENSG0000(	KIRREL1	988.7214	0.550285	6.92E-07	1.92E-05	protein_coding
ENSG0000(	SHANK2-AS	576.0287	0.193564	7.00E-07	1.94E-05	lncRNA
ENSG0000(	HSP90AB1	3062.687	-0.34826	7.02E-07	1.94E-05	protein_coding
ENSG0000(	ENSG0000(	450.7099	0.258685	7.03E-07	1.94E-05	lncRNA
ENSG0000(	MSANTD3	204.5691	0.638507	7.08E-07	1.96E-05	protein_coding
ENSG0000(	ADM-DT	10.82595	1.247785	7.16E-07	1.98E-05	lncRNA
ENSG0000(	BID	682.0289	0.356197	7.17E-07	1.98E-05	protein_coding
ENSG0000(	PLRG1	724.9577	-0.30757	7.18E-07	1.98E-05	protein_coding
ENSG0000(	ENSG0000(	105.0201	0.40576	7.22E-07	1.99E-05	lncRNA
ENSG0000(	ENSG0000(	163.3592	-0.31721	7.26E-07	2.00E-05	TEC
ENSG0000(	EDF1	1115.187	-0.52318	7.33E-07	2.01E-05	protein_coding
ENSG0000(	ENSG0000(	281.4989	0.310627	7.33E-07	2.01E-05	lncRNA
ENSG0000(	LINC00838	76.30569	0.40824	7.32E-07	2.01E-05	lncRNA
ENSG0000(	UBE2D2	1259.617	-0.24693	7.33E-07	2.01E-05	protein_coding
ENSG0000(	LATS2	371.3201	0.59237	7.38E-07	2.02E-05	protein_coding
ENSG0000(	LINC00691	279.7818	0.295529	7.37E-07	2.02E-05	lncRNA
ENSG0000(	PYCR1	119.8681	0.81173	7.36E-07	2.02E-05	protein_coding
ENSG0000(	STAT2	4969.97	0.40373	7.41E-07	2.03E-05	protein_coding
ENSG0000(	SOCS5	1066.539	0.183608	7.53E-07	2.06E-05	protein_coding
ENSG0000(	SMARCC1	2598.724	-0.23495	7.66E-07	2.09E-05	protein_coding
ENSG0000(	TRPS1	1431.334	0.725941	7.71E-07	2.10E-05	protein_coding
ENSG0000(	MTCH2	1086.38	-0.46812	7.84E-07	2.13E-05	protein_coding
ENSG0000(	TEF	596.807	-0.77927	7.84E-07	2.13E-05	protein_coding
ENSG0000(	PCSK9	129.5732	1.250264	7.86E-07	2.14E-05	protein_coding
ENSG0000(	QSOX1	2450.205	0.529522	7.89E-07	2.14E-05	protein_coding
ENSG0000(	ENSG0000(	91.96695	0.431888	7.91E-07	2.15E-05	lncRNA
ENSG0000(	ENSG0000(	15.43397	1.074731	7.95E-07	2.16E-05	lncRNA
ENSG0000(	ENSG0000(	28.54447	0.863956	7.98E-07	2.16E-05	lncRNA
ENSG0000(	SRD5A3	504.1075	0.347569	8.08E-07	2.19E-05	protein_coding
ENSG0000(	ENSG0000(	89.01512	0.512175	8.10E-07	2.19E-05	lncRNA
ENSG0000(	LZTS1	158.7255	0.549112	8.12E-07	2.19E-05	protein_coding
ENSG0000(	LINC00486	367.3264	0.24906	8.15E-07	2.20E-05	lncRNA
ENSG0000(	ENSG0000(	70.28131	0.660752	8.24E-07	2.22E-05	processed_pseudogene
ENSG0000(	MICALL1	1036.935	0.244001	8.27E-07	2.23E-05	protein_coding
ENSG0000(	ALDH1L1	567.4373	0.599304	8.29E-07	2.23E-05	protein_coding
ENSG0000(	LINC01050	341.5701	0.324252	8.29E-07	2.23E-05	lncRNA
ENSG0000(	PLAC4	109.5974	0.501822	8.35E-07	2.24E-05	lncRNA
ENSG0000(	LINC02890	74.89696	-0.46494	8.41E-07	2.26E-05	lncRNA
ENSG0000(	ERVH48-1	32.42041	0.587165	8.51E-07	2.28E-05	protein_coding

ENSG0000(	GDI2	3131.213	-0.30924	8.52E-07	2.29E-05	protein_coding
ENSG0000(	NHS	575.0902	0.390308	8.54E-07	2.29E-05	protein_coding
ENSG0000(	UBB	1793.374	-0.60957	8.57E-07	2.30E-05	protein_coding
ENSG0000(	NFU1	173.5592	-0.34873	8.60E-07	2.30E-05	protein_coding
ENSG0000(	CAMLG	1464.103	-0.20233	8.62E-07	2.30E-05	protein_coding
ENSG0000(	LINC01783	58.00366	0.89897	8.69E-07	2.32E-05	lncRNA
ENSG0000(	ENSG0000(	11.81977	0.826667	8.72E-07	2.32E-05	lncRNA
ENSG0000(	SRA1	423.4209	0.313681	8.72E-07	2.32E-05	protein_coding
ENSG0000(	ABCF3	820.3317	-0.24871	8.76E-07	2.33E-05	protein_coding
ENSG0000(	HAPLN3	534.7488	1.379095	8.77E-07	2.33E-05	protein_coding
ENSG0000(	SLC2A3P2	99.55021	0.540107	8.86E-07	2.36E-05	processed_pseudogene
ENSG0000(	SLC28A2	5744.146	-1.15064	8.92E-07	2.37E-05	protein_coding
ENSG0000(	ENSG0000(	312.7446	0.308129	8.93E-07	2.37E-05	lncRNA
ENSG0000(	PLA2G7	653.9085	0.673003	8.94E-07	2.37E-05	protein_coding
ENSG0000(	RPL21P44	102.9612	0.438381	8.96E-07	2.37E-05	processed_pseudogene
ENSG0000(	MAP3K8	549.4845	0.592842	8.98E-07	2.38E-05	protein_coding
ENSG0000(	STAG3L3	2259.952	-0.20714	8.99E-07	2.38E-05	transcribed_unprocessed_pseudo
ENSG0000(	PRPSAP2	659.724	-0.22231	9.09E-07	2.40E-05	protein_coding
ENSG0000(	CHST15	1665.778	0.809415	9.24E-07	2.44E-05	protein_coding
ENSG0000(	KYNU	6415.94	0.462116	9.29E-07	2.45E-05	protein_coding
ENSG0000(	MT-RNR2	46169.92	-0.81398	9.46E-07	2.49E-05	Mt_rRNA
ENSG0000(	CAPNS2	15.33999	0.887716	9.79E-07	2.58E-05	protein_coding
ENSG0000(	CD80	402.6478	1.152668	9.93E-07	2.61E-05	protein_coding
ENSG0000(	ENSG0000(	40.33061	0.616303	9.92E-07	2.61E-05	lncRNA
ENSG0000(	NPM1P40	70.3003	0.63156	9.97E-07	2.62E-05	processed_pseudogene
ENSG0000(	SMIM36	220.6767	0.30354	9.98E-07	2.62E-05	protein_coding
ENSG0000(	C3orf80	14.92354	1.006053	1.00E-06	2.63E-05	protein_coding
ENSG0000(	SLC14A2	293.9969	-0.54361	1.00E-06	2.63E-05	protein_coding
ENSG0000(	RETREG2	1391.625	-0.28506	1.00E-06	2.63E-05	protein_coding
ENSG0000(	ENSG0000(	42.99674	0.568227	1.01E-06	2.64E-05	lncRNA
ENSG0000(	SF3A2	572.6206	-0.19091	1.01E-06	2.64E-05	protein_coding
ENSG0000(	ENSG0000(	25.10403	0.626225	1.02E-06	2.66E-05	lncRNA
ENSG0000(	NPHP1	768.6009	0.19899	1.02E-06	2.67E-05	protein_coding
ENSG0000(	LINC02289	48.00376	0.857352	1.03E-06	2.68E-05	lncRNA
ENSG0000(	ENSG0000(	1029.935	0.489075	1.03E-06	2.70E-05	lncRNA
ENSG0000(	DIPK2B	657.5757	0.499432	1.04E-06	2.70E-05	protein_coding
ENSG0000(	ENSG0000(	116.6093	0.321468	1.04E-06	2.71E-05	processed_pseudogene
ENSG0000(	TLR4	1866.446	0.802505	1.04E-06	2.71E-05	protein_coding
ENSG0000(	RBM4B	490.4646	-0.24765	1.06E-06	2.75E-05	protein_coding
ENSG0000(	ENSG0000(	246.4015	0.328929	1.08E-06	2.79E-05	lncRNA
ENSG0000(	DYNLL1	847.2224	-0.38161	1.09E-06	2.84E-05	protein_coding
ENSG0000(	ENSG0000(	3032.16	0.282596	1.10E-06	2.85E-05	lncRNA
ENSG0000(	C1QTNF6	1053.509	0.247925	1.10E-06	2.85E-05	protein_coding
ENSG0000(	ABCA1	2771.991	0.558654	1.13E-06	2.92E-05	protein_coding
ENSG0000(	CYP2E1	87.93418	0.708215	1.13E-06	2.92E-05	protein_coding
ENSG0000(	NUTM2G	10.09152	1.936128	1.15E-06	2.96E-05	protein_coding
ENSG0000(	GJA9	19.62964	0.664341	1.15E-06	2.96E-05	protein_coding

ENSG0000(	PHB2	1093.342	-0.26977	1.15E-06	2.97E-05	protein_coding
ENSG0000(	UPP1	757.7028	0.523454	1.15E-06	2.98E-05	protein_coding
ENSG0000(	ENSG0000(	18.40533	0.755052	1.16E-06	2.98E-05	lncRNA
ENSG0000(	ENSG0000(	1875.226	0.193696	1.16E-06	2.98E-05	lncRNA
ENSG0000(	CDRT4	763.0687	0.36342	1.17E-06	2.99E-05	protein_coding
ENSG0000(	WTAP	2377.686	0.289335	1.17E-06	3.00E-05	protein_coding
ENSG0000(	LDLRAD2	60.87309	0.925548	1.18E-06	3.03E-05	protein_coding
ENSG0000(	DNAJA3	1540.254	-0.20485	1.19E-06	3.05E-05	protein_coding
ENSG0000(	USF2	1609.492	-0.20947	1.20E-06	3.07E-05	protein_coding
ENSG0000(	HLA-L	1394.261	0.241088	1.21E-06	3.10E-05	transcribed_unprocessed_pseudo
ENSG0000(	TBXAS1	542.9597	0.441893	1.23E-06	3.14E-05	protein_coding
ENSG0000(	ENSG0000(	65.1468	0.540064	1.24E-06	3.17E-05	lncRNA
ENSG0000(	TMEM245	2797.077	-0.27915	1.24E-06	3.17E-05	protein_coding
ENSG0000(	ATP5IF1	946.7895	-0.38796	1.24E-06	3.17E-05	protein_coding
ENSG0000(	DENND3-A:	344.3939	0.280518	1.25E-06	3.18E-05	lncRNA
ENSG0000(	PTCSC3	503.1057	0.369396	1.26E-06	3.21E-05	lncRNA
ENSG0000(	HMGB1P21	70.02278	-0.60073	1.27E-06	3.23E-05	processed_pseudogene
ENSG0000(	LOXL3	187.0593	0.497508	1.27E-06	3.23E-05	protein_coding
ENSG0000(	LINC01271	19.24391	1.068642	1.27E-06	3.23E-05	lncRNA
ENSG0000(	SBDSP1	303.5808	-0.24826	1.28E-06	3.25E-05	transcribed_unprocessed_pseudo
ENSG0000(	ENSG0000(	77.02772	0.576574	1.29E-06	3.27E-05	lncRNA
ENSG0000(	FABP2	3825.554	-1.10735	1.29E-06	3.27E-05	protein_coding
ENSG0000(	ITM2B	4677.483	-0.50771	1.29E-06	3.27E-05	protein_coding
ENSG0000(	LINC02035	532.8306	0.224533	1.30E-06	3.28E-05	lncRNA
ENSG0000(	OLFML2B	133.0099	1.034028	1.30E-06	3.28E-05	protein_coding
ENSG0000(	SLC26A7	28.11167	0.999607	1.30E-06	3.29E-05	protein_coding
ENSG0000(	FRMD5	62.27941	0.929805	1.30E-06	3.29E-05	protein_coding
ENSG0000(	ENSG0000(	46.52591	0.76014	1.31E-06	3.29E-05	lncRNA
ENSG0000(	HCP5	5696.718	0.535078	1.32E-06	3.32E-05	lncRNA
ENSG0000(	ENSG0000(	11.08398	1.343154	1.33E-06	3.34E-05	lncRNA
ENSG0000(	MMP14	1509.626	0.581213	1.33E-06	3.34E-05	protein_coding
ENSG0000(	SMCO2	15.41592	1.095303	1.33E-06	3.34E-05	protein_coding
ENSG0000(	TACO1	313.9392	-0.37429	1.33E-06	3.34E-05	protein_coding
ENSG0000(	NRP1	1868.12	0.3968	1.34E-06	3.36E-05	protein_coding
ENSG0000(	ENSG0000(	24.12252	0.655878	1.34E-06	3.37E-05	lncRNA
ENSG0000(	GPATCH8	2994.888	-0.16754	1.37E-06	3.43E-05	protein_coding
ENSG0000(	MRGPRF-A	14.84144	1.241211	1.37E-06	3.44E-05	lncRNA
ENSG0000(	NHSL2	916.8013	0.491019	1.38E-06	3.46E-05	protein_coding
ENSG0000(	MPC2	867.2052	-0.49956	1.39E-06	3.47E-05	protein_coding
ENSG0000(	PPCDC	454.921	0.34199	1.39E-06	3.47E-05	protein_coding
ENSG0000(	ENSG0000(	96.68513	0.410784	1.39E-06	3.47E-05	lncRNA
ENSG0000(	RPL12	3192.639	-0.40249	1.39E-06	3.47E-05	protein_coding
ENSG0000(	CPNE4	29.64715	1.333551	1.39E-06	3.48E-05	protein_coding
ENSG0000(	CAT	1147.301	-0.34182	1.40E-06	3.49E-05	protein_coding
ENSG0000(	MED23	1646.775	-0.18754	1.40E-06	3.50E-05	protein_coding
ENSG0000(	LINC00641	1905.825	0.26611	1.41E-06	3.50E-05	lncRNA
ENSG0000(	ENSG0000(	11.77179	1.28716	1.41E-06	3.51E-05	lncRNA

ENSG0000(	LINC01182	704.5556	0.239556	1.43E-06	3.54E-05	lncRNA
ENSG0000(	NOX4	316.7014	0.309385	1.43E-06	3.55E-05	protein_coding
ENSG0000(	ENSG0000(	1860.425	-0.20938	1.43E-06	3.55E-05	lncRNA
ENSG0000(	CLMP	1072.206	0.432693	1.44E-06	3.56E-05	protein_coding
ENSG0000(	JAKMIP3	83.02847	0.612729	1.44E-06	3.56E-05	protein_coding
ENSG0000(	BATF3	123.5139	0.710336	1.45E-06	3.59E-05	protein_coding
ENSG0000(	CICP27	134.1029	0.72443	1.45E-06	3.59E-05	processed_pseudogene
ENSG0000(	DIAPH2	1676.505	0.265464	1.46E-06	3.60E-05	protein_coding
ENSG0000(	SYPL1	836.2749	-0.36016	1.47E-06	3.62E-05	protein_coding
ENSG0000(	KLF10	758.3418	-0.53034	1.48E-06	3.64E-05	protein_coding
ENSG0000(	HPGDS	102.3023	-0.77157	1.48E-06	3.65E-05	protein_coding
ENSG0000(	STEAP1	61.9246	1.065733	1.50E-06	3.69E-05	protein_coding
ENSG0000(	ENSG0000(	28.24893	0.923603	1.50E-06	3.70E-05	lncRNA
ENSG0000(	HIF1A	2678.94	0.477778	1.50E-06	3.70E-05	protein_coding
ENSG0000(	ENSG0000(	122.3965	0.367238	1.51E-06	3.71E-05	lncRNA
ENSG0000(	ABCB1	3580.944	-1.00419	1.52E-06	3.72E-05	protein_coding
ENSG0000(	TRDC	65.78324	-0.89046	1.52E-06	3.72E-05	TR_C_gene
ENSG0000(	H2BC5	305.3051	-0.50132	1.54E-06	3.77E-05	protein_coding
ENSG0000(	ENSG0000(	271.5704	0.239845	1.54E-06	3.77E-05	lncRNA
ENSG0000(	ENSG0000(	828.0131	0.184104	1.54E-06	3.77E-05	lncRNA
ENSG0000(	ENSG0000(	18.29586	0.632554	1.54E-06	3.78E-05	lncRNA
ENSG0000(	VPS72	220.7334	-0.24973	1.55E-06	3.79E-05	protein_coding
ENSG0000(	HTR1F	10.16302	1.529738	1.56E-06	3.80E-05	protein_coding
ENSG0000(	ENSG0000(	338.108	0.244504	1.56E-06	3.81E-05	lncRNA
ENSG0000(	LYPD1	67.21376	1.312271	1.57E-06	3.81E-05	protein_coding
ENSG0000(	ENSG0000(	447.0792	0.269264	1.57E-06	3.83E-05	TEC
ENSG0000(	ENSG0000(	166.1414	0.418117	1.58E-06	3.85E-05	lncRNA
ENSG0000(	AGMAT	1017.411	-0.22754	1.59E-06	3.87E-05	protein_coding
ENSG0000(	ZNF432	872.2135	0.257633	1.60E-06	3.88E-05	protein_coding
ENSG0000(	POM121L7	140.5032	0.614898	1.60E-06	3.89E-05	processed_pseudogene
ENSG0000(	FCGR2C	569.8336	1.204099	1.62E-06	3.92E-05	polymorphic_pseudogene
ENSG0000(	ENSG0000(	15.13916	1.086295	1.63E-06	3.94E-05	lncRNA
ENSG0000(	ENSG0000(	194.0629	0.526369	1.63E-06	3.94E-05	TEC
ENSG0000(	ENSG0000(	327.6076	0.369799	1.64E-06	3.97E-05	lncRNA
ENSG0000(	ASPH	3998.891	0.338806	1.65E-06	3.98E-05	protein_coding
ENSG0000(	PSMB9	1478.569	0.366154	1.65E-06	3.98E-05	protein_coding
ENSG0000(	ENSG0000(	172.6205	0.24977	1.66E-06	4.00E-05	lncRNA
ENSG0000(	PRPF38A	1702.678	-0.16689	1.66E-06	4.00E-05	protein_coding
ENSG0000(	YPEL4	44.14641	0.763789	1.66E-06	4.00E-05	protein_coding
ENSG0000(	ENSG0000(	167.7803	0.423709	1.66E-06	4.01E-05	TEC
ENSG0000(	RAB4A	506.1307	-0.35046	1.66E-06	4.01E-05	protein_coding
ENSG0000(	ENSG0000(	272.9727	0.194712	1.68E-06	4.03E-05	TEC
ENSG0000(	GNA15	58.41257	1.056735	1.68E-06	4.03E-05	protein_coding
ENSG0000(	HINT3	733.1873	-0.17405	1.68E-06	4.03E-05	protein_coding
ENSG0000(	ANGPT1	218.0116	0.517006	1.69E-06	4.04E-05	protein_coding
ENSG0000(	ENSG0000(	10.13773	1.13785	1.71E-06	4.10E-05	lncRNA
ENSG0000(	TNFRSF18	47.27374	0.820678	1.72E-06	4.13E-05	protein_coding

ENSG0000(	INSYN2B	17.99609	1.633397	1.73E-06	4.15E-05	protein_coding
ENSG0000(	LRG1	437.1978	0.354056	1.74E-06	4.17E-05	protein_coding
ENSG0000(	CHCHD10	379.1271	-0.5186	1.75E-06	4.17E-05	protein_coding
ENSG0000(	PCAT14	878.2125	0.309033	1.75E-06	4.17E-05	lncRNA
ENSG0000(	RND1	334.6648	0.392094	1.75E-06	4.17E-05	protein_coding
ENSG0000(	LINC01623	246.6982	0.353413	1.75E-06	4.18E-05	lncRNA
ENSG0000(	CALHM5	168.9887	0.454299	1.77E-06	4.21E-05	protein_coding
ENSG0000(	MTHFD2L	630.3875	0.270644	1.80E-06	4.27E-05	protein_coding
ENSG0000(	RN7SKP16	70.64103	0.527323	1.80E-06	4.28E-05	misc_RNA
ENSG0000(	ENSG0000(	153.1805	-0.41485	1.81E-06	4.30E-05	unprocessed_pseudogene
ENSG0000(	NRBP2	845.5654	0.303159	1.81E-06	4.30E-05	protein_coding
ENSG0000(	ADCYAP1	28.90969	1.909184	1.81E-06	4.30E-05	protein_coding
ENSG0000(	ENSG0000(	493.4889	0.61193	1.84E-06	4.36E-05	lncRNA
ENSG0000(	PALMD	225.7148	0.606368	1.85E-06	4.38E-05	protein_coding
ENSG0000(	ENSG0000(	41.84652	0.90779	1.85E-06	4.38E-05	lncRNA
ENSG0000(	IL1RAP	1100.489	0.303915	1.87E-06	4.44E-05	protein_coding
ENSG0000(	SMC2-DT	673.4312	0.282385	1.88E-06	4.44E-05	lncRNA
ENSG0000(	CCL28	1123.948	0.540378	1.89E-06	4.47E-05	protein_coding
ENSG0000(	ENSG0000(	97.67223	0.389832	1.89E-06	4.47E-05	lncRNA
ENSG0000(	GPX4	745.4325	-0.55428	1.89E-06	4.47E-05	protein_coding
ENSG0000(	PRRX1	527.6335	1.578687	1.89E-06	4.47E-05	protein_coding
ENSG0000(	ENSG0000(	36.24303	0.527473	1.91E-06	4.50E-05	lncRNA
ENSG0000(	IRF2-DT	684.1254	0.244618	1.91E-06	4.51E-05	lncRNA
ENSG0000(	ENSG0000(	191.1015	0.293622	1.93E-06	4.53E-05	lncRNA
ENSG0000(	ENSG0000(	60.44953	0.993979	1.94E-06	4.56E-05	lncRNA
ENSG0000(	ENSG0000(	227.6192	0.254741	1.96E-06	4.60E-05	lncRNA
ENSG0000(	ENSG0000(	544.3433	0.526302	1.98E-06	4.64E-05	processed_pseudogene
ENSG0000(	AKAP17A	1782.963	0.221479	1.98E-06	4.64E-05	protein_coding
ENSG0000(	ENSG0000(	11.01524	1.849241	2.02E-06	4.74E-05	lncRNA
ENSG0000(	ID2	643.1166	-0.5482	2.02E-06	4.74E-05	protein_coding
ENSG0000(	BACH1-IT1	231.5502	0.427215	2.04E-06	4.77E-05	lncRNA
ENSG0000(	CNTN4	473.6601	-0.63901	2.06E-06	4.82E-05	protein_coding
ENSG0000(	IGKJ3	39.97795	1.35877	2.07E-06	4.85E-05	IG_J_gene
ENSG0000(	BBS5	388.992	0.327507	2.08E-06	4.87E-05	protein_coding
ENSG0000(	LINC00310	295.6109	0.321337	2.09E-06	4.88E-05	lncRNA
ENSG0000(	ENSG0000(	65.46207	-0.43982	2.11E-06	4.93E-05	pseudogene
ENSG0000(	PBDC1	176.4812	-0.4563	2.13E-06	4.96E-05	protein_coding
ENSG0000(	MTMR9LP	410.1119	0.399647	2.17E-06	5.05E-05	transcribed_unprocessed_pseudo
ENSG0000(	JSRP1	12.87716	1.243213	2.17E-06	5.05E-05	protein_coding
ENSG0000(	SH3RF3-AS	27.69376	0.75309	2.18E-06	5.06E-05	lncRNA
ENSG0000(	ENSG0000(	8.340932	1.366678	2.18E-06	5.06E-05	lncRNA
ENSG0000(	FAS	3994.717	0.380895	2.18E-06	5.07E-05	protein_coding
ENSG0000(	SNORD35A	24.18492	1.227761	2.18E-06	5.07E-05	snoRNA
ENSG0000(	RPL9P16	62.49282	0.589851	2.20E-06	5.10E-05	processed_pseudogene
ENSG0000(	CIPC	1131.735	-0.38899	2.20E-06	5.10E-05	protein_coding
ENSG0000(	MEOX1	94.39994	0.916226	2.21E-06	5.12E-05	protein_coding
ENSG0000(	SHISAL1	284.9359	0.524907	2.23E-06	5.15E-05	protein_coding

ENSG0000(	PRPH2	99.34871	0.597349	2.24E-06	5.18E-05	protein_coding
ENSG0000(	ENSG0000(	364.9226	-0.73521	2.25E-06	5.20E-05	transcribed_unprocessed_pseudo
ENSG0000(	SHC4	284.204	0.378445	2.28E-06	5.25E-05	protein_coding
ENSG0000(	ENSG0000(	30.78574	0.712034	2.28E-06	5.26E-05	lncRNA
ENSG0000(	LINC01359	1156.449	0.243351	2.31E-06	5.32E-05	lncRNA
ENSG0000(	ENSG0000(	711.4983	0.425001	2.33E-06	5.36E-05	lncRNA
ENSG0000(	ENSG0000(	82.09562	0.418592	2.33E-06	5.36E-05	lncRNA
ENSG0000(	BCL2A1	140.6608	1.245835	2.33E-06	5.37E-05	protein_coding
ENSG0000(	ITGA11	697.9502	0.47224	2.34E-06	5.38E-05	protein_coding
ENSG0000(	ACTN1	3506.716	0.452014	2.34E-06	5.38E-05	protein_coding
ENSG0000(	ENSG0000(	162.4635	-0.38013	2.35E-06	5.40E-05	lncRNA
ENSG0000(	NMT2	1587.872	-0.26909	2.35E-06	5.40E-05	protein_coding
ENSG0000(	BACH1-IT3	77.20631	0.53247	2.37E-06	5.42E-05	lncRNA
ENSG0000(	MIR223HG	1176.561	0.826934	2.37E-06	5.42E-05	lncRNA
ENSG0000(	ENSG0000(	291.9742	0.21971	2.38E-06	5.45E-05	transcribed_unprocessed_pseudo
ENSG0000(	ENSG0000(	283.8716	0.209773	2.38E-06	5.45E-05	lncRNA
ENSG0000(	CACNA1E	216.47	1.155584	2.39E-06	5.45E-05	protein_coding
ENSG0000(	ENSG0000(	73.95591	0.497921	2.41E-06	5.50E-05	lncRNA
ENSG0000(	SERBP1	3257.822	-0.20779	2.41E-06	5.50E-05	protein_coding
ENSG0000(	SLC35E4	186.8838	0.254468	2.41E-06	5.50E-05	protein_coding
ENSG0000(	ITIH3	65.40289	-1.28123	2.44E-06	5.55E-05	protein_coding
ENSG0000(	IGHV4-28	64.44416	1.373746	2.49E-06	5.66E-05	IG_V_gene
ENSG0000(	PAICS	1584.274	-0.21147	2.49E-06	5.66E-05	protein_coding
ENSG0000(	TRAPPC2	1576.13	-0.18389	2.49E-06	5.66E-05	protein_coding
ENSG0000(	ENSG0000(	559.0939	0.275651	2.50E-06	5.68E-05	lncRNA
ENSG0000(	LINC00578	538.2743	0.295554	2.50E-06	5.68E-05	lncRNA
ENSG0000(	HNRNPH3	2331.262	-0.19077	2.54E-06	5.76E-05	protein_coding
ENSG0000(	ARSJ	134.1588	0.530147	2.55E-06	5.79E-05	protein_coding
ENSG0000(	PRTFDC1	189.1412	-0.61254	2.56E-06	5.80E-05	protein_coding
ENSG0000(	MCL1	10481.53	0.347572	2.56E-06	5.80E-05	protein_coding
ENSG0000(	ENSG0000(	45.71745	0.980284	2.57E-06	5.80E-05	processed_pseudogene
ENSG0000(	PTPN14	2248.064	0.226706	2.57E-06	5.81E-05	protein_coding
ENSG0000(	SFMBT2	1908.95	0.497121	2.58E-06	5.83E-05	protein_coding
ENSG0000(	ENSG0000(	233.7231	0.314352	2.59E-06	5.84E-05	lncRNA
ENSG0000(	ENSG0000(	59.14127	0.575754	2.61E-06	5.89E-05	lncRNA
ENSG0000(	TENT4B	1165.559	-0.51984	2.62E-06	5.92E-05	protein_coding
ENSG0000(	ENSG0000(	75.13074	0.487272	2.63E-06	5.93E-05	TEC
ENSG0000(	CYYR1-AS1	658.7253	0.188987	2.65E-06	5.96E-05	lncRNA
ENSG0000(	APOBR	856.7273	0.361316	2.65E-06	5.97E-05	protein_coding
ENSG0000(	IGKJ5	52.05364	1.255649	2.66E-06	5.98E-05	IG_J_gene
ENSG0000(	ENSG0000(	15.22999	0.779286	2.69E-06	6.04E-05	TEC
ENSG0000(	ENSG0000(	244.7608	0.542746	2.71E-06	6.08E-05	lncRNA
ENSG0000(	MRPL43	375.2371	-0.26551	2.75E-06	6.17E-05	protein_coding
ENSG0000(	LUCAT1	1921.23	0.475101	2.75E-06	6.17E-05	lncRNA
ENSG0000(	ENSG0000(	32.99972	0.61669	2.76E-06	6.18E-05	TEC
ENSG0000(	ENSG0000(	360.0896	0.510891	2.78E-06	6.22E-05	transcribed_unprocessed_pseudo
ENSG0000(	SPTLC2	3340.399	-0.17972	2.78E-06	6.22E-05	protein_coding

ENSG0000(	ENSG0000(	129.0709	0.322141	2.79E-06	6.23E-05	lncRNA
ENSG0000(	WWTR1-IT:	131.14	0.266975	2.79E-06	6.24E-05	lncRNA
ENSG0000(	RGS16	118.5536	1.197517	2.81E-06	6.27E-05	protein_coding
ENSG0000(	CRLS1	1133.964	-0.38263	2.81E-06	6.27E-05	protein_coding
ENSG0000(	ENSG0000(	18.46548	0.985417	2.83E-06	6.30E-05	lncRNA
ENSG0000(	AKTIP	1011.593	-0.3057	2.85E-06	6.35E-05	protein_coding
ENSG0000(	ADD3	4112.576	-0.34297	2.88E-06	6.41E-05	protein_coding
ENSG0000(	CGRRF1	206.4584	-0.37704	2.88E-06	6.41E-05	protein_coding
ENSG0000(	H2AC8	160.0503	-0.58411	2.89E-06	6.41E-05	protein_coding
ENSG0000(	KCNMA1	1775.87	0.399483	2.89E-06	6.42E-05	protein_coding
ENSG0000(	NEBL	580.1597	0.645981	2.90E-06	6.44E-05	protein_coding
ENSG0000(	TFAP2A-AS	13.47729	0.821284	2.91E-06	6.44E-05	lncRNA
ENSG0000(	ENSG0000(	576.4444	0.22203	2.91E-06	6.45E-05	lncRNA
ENSG0000(	HSPA7	244.0871	1.157873	2.92E-06	6.46E-05	unprocessed_pseudogene
ENSG0000(	LINC02344	99.32184	0.564836	2.93E-06	6.47E-05	lncRNA
ENSG0000(	CRY2	1394.851	-0.31	2.93E-06	6.47E-05	protein_coding
ENSG0000(	IGKV3D-11	13.15076	1.930199	2.93E-06	6.48E-05	IG_V_gene
ENSG0000(	LINC00507	878.8553	0.248247	2.94E-06	6.48E-05	lncRNA
ENSG0000(	ENSG0000(	10.8998	1.004032	2.95E-06	6.51E-05	lncRNA
ENSG0000(	ENSG0000(	19.48592	0.582956	2.98E-06	6.56E-05	lncRNA
ENSG0000(	HIP1	1227.013	0.328438	2.98E-06	6.56E-05	protein_coding
ENSG0000(	ODF2L	4068.634	0.237004	2.98E-06	6.57E-05	protein_coding
ENSG0000(	CCDC198	177.5458	-0.32944	3.00E-06	6.60E-05	protein_coding
ENSG0000(	ACVRL1	833.9189	0.457717	3.00E-06	6.61E-05	protein_coding
ENSG0000(	ENSG0000(	597.2705	0.4484	3.03E-06	6.66E-05	lncRNA
ENSG0000(	POLR2I	60.08597	-0.55911	3.04E-06	6.68E-05	protein_coding
ENSG0000(	IGLV10-54	85.35146	2.140182	3.06E-06	6.72E-05	IG_V_gene
ENSG0000(	CNBP	3016.598	-0.19322	3.08E-06	6.74E-05	protein_coding
ENSG0000(	ENSG0000(	1865.578	0.268739	3.09E-06	6.77E-05	lncRNA
ENSG0000(	ENSG0000(	63.32103	0.472807	3.10E-06	6.78E-05	lncRNA
ENSG0000(	PEX19	744.2556	-0.25614	3.11E-06	6.80E-05	protein_coding
ENSG0000(	ENSG0000(	107.8604	0.504826	3.12E-06	6.83E-05	lncRNA
ENSG0000(	HSPA1B	824.329	-0.61953	3.13E-06	6.84E-05	protein_coding
ENSG0000(	ENSG0000(	343.1862	0.283663	3.17E-06	6.92E-05	lncRNA
ENSG0000(	CEP95	1864.058	-0.19384	3.21E-06	7.01E-05	protein_coding
ENSG0000(	PMF1	500.1726	-0.18628	3.22E-06	7.02E-05	protein_coding
ENSG0000(	SMCR5	106.9316	0.37526	3.24E-06	7.05E-05	lncRNA
ENSG0000(	CD55	1417.657	0.747128	3.26E-06	7.09E-05	protein_coding
ENSG0000(	ENSG0000(	3392.171	0.209641	3.26E-06	7.09E-05	lncRNA
ENSG0000(	STX16	5391.459	0.147908	3.27E-06	7.10E-05	protein_coding
ENSG0000(	MOSPD2	735.0569	0.537285	3.27E-06	7.11E-05	protein_coding
ENSG0000(	AKAP6	928.7239	-0.3449	3.28E-06	7.11E-05	protein_coding
ENSG0000(	SLC25A42	650.9937	-0.245	3.28E-06	7.12E-05	protein_coding
ENSG0000(	RLIMP2	11.20271	1.262302	3.29E-06	7.13E-05	processed_pseudogene
ENSG0000(	IGHV4-55	33.48083	1.421907	3.30E-06	7.15E-05	IG_V_pseudogene
ENSG0000(	LINC02412	63.13267	-0.38848	3.30E-06	7.15E-05	lncRNA
ENSG0000(	ZNF738	1163.915	-0.18919	3.30E-06	7.15E-05	protein_coding

ENSG0000(	ENSG0000(	411.5969	0.336353	3.31E-06	7.15E-05	lncRNA
ENSG0000(	ACTR6	327.0604	-0.26741	3.32E-06	7.17E-05	protein_coding
ENSG0000(	AOX1	96.02205	1.210632	3.32E-06	7.17E-05	protein_coding
ENSG0000(	BAIAP3	221.5524	-0.80387	3.33E-06	7.19E-05	protein_coding
ENSG0000(	CICP18	958.1342	0.329637	3.35E-06	7.21E-05	transcribed_processed_pseudoge
ENSG0000(	RPL37	5452.07	-0.43074	3.35E-06	7.21E-05	protein_coding
ENSG0000(	LMCD1	623.1339	0.561719	3.36E-06	7.24E-05	protein_coding
ENSG0000(	MIR378D2I	16.25635	0.775799	3.37E-06	7.26E-05	lncRNA
ENSG0000(	RN7SL559F	107.6949	0.334699	3.38E-06	7.26E-05	misc_RNA
ENSG0000(	SELENOF	864.2945	-0.29206	3.38E-06	7.26E-05	protein_coding
ENSG0000(	LINC00211	70.06961	0.354322	3.39E-06	7.28E-05	lncRNA
ENSG0000(	PI3	38.25558	1.571476	3.40E-06	7.29E-05	protein_coding
ENSG0000(	HSPE1	339.596	-0.3686	3.42E-06	7.33E-05	protein_coding
ENSG0000(	IGHJ3P	268.9925	1.028204	3.42E-06	7.33E-05	IG_J_pseudogene
ENSG0000(	FAM110B	112.7875	0.731883	3.43E-06	7.34E-05	protein_coding
ENSG0000(	MMGT1	622.8134	-0.33178	3.43E-06	7.35E-05	protein_coding
ENSG0000(	IGHV3-64D	62.72111	1.952718	3.44E-06	7.36E-05	IG_V_gene
ENSG0000(	PITX1-AS1	115.2862	0.551643	3.45E-06	7.38E-05	lncRNA
ENSG0000(	IGKV1-17	148.6519	1.432243	3.47E-06	7.41E-05	IG_V_gene
ENSG0000(	ENSG0000(	295.4258	0.259448	3.54E-06	7.55E-05	TEC
ENSG0000(	LINC01537	802.3323	0.239558	3.54E-06	7.55E-05	lncRNA
ENSG0000(	RN7SL683F	97.87622	0.417807	3.54E-06	7.55E-05	misc_RNA
ENSG0000(	CASS4	268.9004	0.450848	3.55E-06	7.56E-05	protein_coding
ENSG0000(	SPRN	11.04742	1.313926	3.58E-06	7.62E-05	protein_coding
ENSG0000(	IFI30	1172.067	0.834108	3.58E-06	7.62E-05	protein_coding
ENSG0000(	IGHV3-62	12.85057	1.572969	3.60E-06	7.65E-05	IG_V_pseudogene
ENSG0000(	ENSG0000(	57.99005	0.430681	3.61E-06	7.66E-05	lncRNA
ENSG0000(	NLRP3	307.3391	0.616971	3.60E-06	7.66E-05	protein_coding
ENSG0000(	SPI1	383.5372	0.768721	3.62E-06	7.68E-05	protein_coding
ENSG0000(	PRR33	13.68373	1.928233	3.66E-06	7.76E-05	protein_coding
ENSG0000(	ADAMTS1	680.1436	0.571821	3.70E-06	7.83E-05	protein_coding
ENSG0000(	BHLHE22	62.90863	1.12836	3.70E-06	7.83E-05	protein_coding
ENSG0000(	GSN-AS1	242.5474	0.271183	3.71E-06	7.86E-05	lncRNA
ENSG0000(	ENSG0000(	608.4713	0.702808	3.73E-06	7.89E-05	lncRNA
ENSG0000(	FAM168B	2131.847	-0.23403	3.76E-06	7.94E-05	protein_coding
ENSG0000(	TCP1	1292.579	-0.22122	3.76E-06	7.95E-05	protein_coding
ENSG0000(	EIF1B	261.1121	-0.28492	3.78E-06	7.98E-05	protein_coding
ENSG0000(	SFXN1	2301.848	-0.28853	3.83E-06	8.07E-05	protein_coding
ENSG0000(	ENSG0000(	1560.081	0.553965	3.83E-06	8.07E-05	TEC
ENSG0000(	RAP2C-AS1	336.2417	0.206103	3.84E-06	8.08E-05	lncRNA
ENSG0000(	ENSG0000(	1599.108	0.213918	3.85E-06	8.09E-05	lncRNA
ENSG0000(	IGHJ3	60.17962	1.298417	3.86E-06	8.12E-05	IG_J_gene
ENSG0000(	SLC28A3	280.7309	0.808417	3.87E-06	8.12E-05	protein_coding
ENSG0000(	ENSG0000(	14.40274	1.147717	3.88E-06	8.15E-05	lncRNA
ENSG0000(	KLLN	261.3734	0.406882	3.88E-06	8.15E-05	protein_coding
ENSG0000(	ADAR	10592.28	0.222093	3.94E-06	8.27E-05	protein_coding
ENSG0000(	GSTA1	1231.822	-1.39884	3.95E-06	8.27E-05	protein_coding

ENSG0000(	THY1	561.6716	0.846562	3.96E-06	8.29E-05	protein_coding
ENSG0000(	ENSG0000(	14.86749	0.91224	3.98E-06	8.33E-05	lncRNA
ENSG0000(	ZNFX1	3556.003	0.279344	3.98E-06	8.34E-05	protein_coding
ENSG0000(	SLC7A11-A:	150.9777	0.398451	4.01E-06	8.39E-05	lncRNA
ENSG0000(	TP53INP1	3218.628	0.420171	4.02E-06	8.41E-05	protein_coding
ENSG0000(	RPS19P3	66.79025	0.365428	4.03E-06	8.42E-05	processed_pseudogene
ENSG0000(	SKP1	4039.484	-0.26942	4.05E-06	8.45E-05	protein_coding
ENSG0000(	ZYX	1353.416	0.401548	4.05E-06	8.45E-05	protein_coding
ENSG0000(	GLB1L3	705.841	0.292684	4.06E-06	8.46E-05	protein_coding
ENSG0000(	TTLL13P	22.65725	1.061845	4.06E-06	8.46E-05	protein_coding
ENSG0000(	WDR59	2152.962	-0.18652	4.08E-06	8.48E-05	protein_coding
ENSG0000(	THYN1	236.8719	-0.26979	4.11E-06	8.54E-05	protein_coding
ENSG0000(	ACBD4	752.7942	-0.44018	4.13E-06	8.59E-05	protein_coding
ENSG0000(	ELMO2	836.7059	0.310542	4.16E-06	8.65E-05	protein_coding
ENSG0000(	ENSG0000(	8.592447	1.110846	4.17E-06	8.65E-05	lncRNA
ENSG0000(	ENSG0000(	16.62188	1.002584	4.18E-06	8.66E-05	lncRNA
ENSG0000(	LINC02649	479.2051	0.345853	4.18E-06	8.67E-05	lncRNA
ENSG0000(	LINC02605	2299.39	0.536962	4.19E-06	8.68E-05	lncRNA
ENSG0000(	ZFYVE19	651.2753	-0.41433	4.24E-06	8.77E-05	protein_coding
ENSG0000(	ENSG0000(	310.8432	0.237942	4.27E-06	8.84E-05	lncRNA
ENSG0000(	KCND3-IT1	14.25159	1.135118	4.31E-06	8.91E-05	lncRNA
ENSG0000(	OPA1	2215.526	-0.21618	4.32E-06	8.93E-05	protein_coding
ENSG0000(	ENSG0000(	11.49853	2.194471	4.33E-06	8.95E-05	lncRNA
ENSG0000(	ENSG0000(	92.58757	0.441704	4.35E-06	8.97E-05	lncRNA
ENSG0000(	SND1	2459.139	0.245942	4.40E-06	9.06E-05	protein_coding
ENSG0000(	ENSG0000(	235.409	0.360436	4.41E-06	9.08E-05	lncRNA
ENSG0000(	HSPE1P18	123.5361	0.47356	4.48E-06	9.22E-05	processed_pseudogene
ENSG0000(	ENSG0000(	737.8385	0.266903	4.51E-06	9.29E-05	lncRNA
ENSG0000(	MEIS1	1113.574	0.596114	4.52E-06	9.30E-05	protein_coding
ENSG0000(	COL15A1	834.8737	0.605152	4.55E-06	9.33E-05	protein_coding
ENSG0000(	ENSG0000(	41.08337	-0.5476	4.54E-06	9.33E-05	transcribed_processed_pseudoge
ENSG0000(	IGKV3-15	405.9429	1.345711	4.54E-06	9.33E-05	IG_V_gene
ENSG0000(	ENSG0000(	1034.145	0.264184	4.56E-06	9.34E-05	lncRNA
ENSG0000(	ATF1	292.0483	-0.22219	4.58E-06	9.38E-05	protein_coding
ENSG0000(	MT-CO3	27470.89	-0.81013	4.58E-06	9.38E-05	protein_coding
ENSG0000(	SRRM2	49182.15	0.185415	4.58E-06	9.38E-05	protein_coding
ENSG0000(	ENSG0000(	15.97833	1.39618	4.59E-06	9.40E-05	unprocessed_pseudogene
ENSG0000(	USP32	1608.243	0.239386	4.60E-06	9.41E-05	protein_coding
ENSG0000(	CES2	6522.941	-0.80214	4.65E-06	9.49E-05	protein_coding
ENSG0000(	TMLHE-AS1	39.33863	0.768407	4.65E-06	9.50E-05	lncRNA
ENSG0000(	PANX2	10.56391	1.466016	4.67E-06	9.53E-05	protein_coding
ENSG0000(	GNA14	205.8215	0.550625	4.69E-06	9.57E-05	protein_coding
ENSG0000(	THBS1	2626.75	0.731148	4.70E-06	9.58E-05	protein_coding
ENSG0000(	PIGS	1177.187	-0.29579	4.73E-06	9.64E-05	protein_coding
ENSG0000(	NHLH1	17.79475	0.698312	4.77E-06	9.71E-05	protein_coding
ENSG0000(	FBXO6	238.9867	0.339071	4.78E-06	9.73E-05	protein_coding
ENSG0000(	USP35	359.1357	0.40415	4.81E-06	9.78E-05	protein_coding

ENSG0000(	B3GNT4	44.73932	0.49603	4.83E-06	9.80E-05	protein_coding
ENSG0000(	ENSG0000(	14.53829	1.595521	4.83E-06	9.81E-05	lncRNA
ENSG0000(	EGR1	592.6951	0.85941	4.85E-06	9.83E-05	protein_coding
ENSG0000(	PARP9	2764.474	0.531092	4.86E-06	9.85E-05	protein_coding
ENSG0000(	RUSC2	406.8988	0.518195	4.86E-06	9.85E-05	protein_coding
ENSG0000(	SFRP2	42.17809	2.257622	4.86E-06	9.85E-05	protein_coding
ENSG0000(	ARHGEF17	1246.176	0.431222	4.87E-06	9.85E-05	protein_coding
ENSG0000(	SIPA1L1	2984.816	0.285076	4.87E-06	9.85E-05	protein_coding
ENSG0000(	KDM3AP1	19.75823	0.865629	4.92E-06	9.95E-05	processed_pseudogene
ENSG0000(	ENSG0000(	19.01882	1.210532	4.95E-06	9.99E-05	miRNA
ENSG0000(	CNIH1	958.791	-0.38278	4.95E-06	1.00E-04	protein_coding
ENSG0000(	GALNT10	2195.557	0.180165	4.96E-06	1.00E-04	protein_coding
ENSG0000(	ENSG0000(	486.7492	0.196978	4.97E-06	0.0001	TEC
ENSG0000(	DUSP6	909.4152	0.546702	4.99E-06	0.000101	protein_coding
ENSG0000(	FICD	215.031	0.470246	5.01E-06	0.000101	protein_coding
ENSG0000(	ZEB2-AS1	66.99549	1.420967	5.01E-06	0.000101	lncRNA
ENSG0000(	ENSG0000(	12.45858	0.901873	5.07E-06	0.000102	processed_pseudogene
ENSG0000(	TMEM185E	1089.465	0.379533	5.07E-06	0.000102	protein_coding
ENSG0000(	CCRL2	214.3386	0.606171	5.08E-06	0.000102	protein_coding
ENSG0000(	ENSG0000(	18.38714	1.3348	5.08E-06	0.000102	unprocessed_pseudogene
ENSG0000(	FABP6	3086.871	-1.70453	5.09E-06	0.000102	protein_coding
ENSG0000(	CCR3	66.60784	0.597125	5.11E-06	0.000102	protein_coding
ENSG0000(	CYP7B1	740.1834	0.714463	5.11E-06	0.000102	protein_coding
ENSG0000(	ENSG0000(	284.5751	0.224368	5.10E-06	0.000102	TEC
ENSG0000(	ZNF639	1567.551	-0.17501	5.11E-06	0.000102	protein_coding
ENSG0000(	PPP4R3B	3494.231	-0.2124	5.13E-06	0.000103	protein_coding
ENSG0000(	ENSG0000(	113.9123	0.350707	5.15E-06	0.000103	lncRNA
ENSG0000(	ENSG0000(	205.9067	-0.29381	5.19E-06	0.000104	lncRNA
ENSG0000(	ERC2-IT1	97.75672	0.305837	5.19E-06	0.000104	lncRNA
ENSG0000(	SLC48A1	579.0822	0.296202	5.20E-06	0.000104	protein_coding
ENSG0000(	ENSG0000(	53.21945	0.42437	5.21E-06	0.000104	lncRNA
ENSG0000(	ENSG0000(	564.0224	0.231206	5.22E-06	0.000104	lncRNA
ENSG0000(	CTSL	603.0547	0.584745	5.25E-06	0.000104	protein_coding
ENSG0000(	LEFTY1	18.85248	1.537398	5.28E-06	0.000105	protein_coding
ENSG0000(	CCNY	1340.103	-0.28657	5.28E-06	0.000105	protein_coding
ENSG0000(	ENSG0000(	15.1224	1.183007	5.30E-06	0.000105	lncRNA
ENSG0000(	IGKV3-11	492.2767	1.315956	5.35E-06	0.000106	IG_V_gene
ENSG0000(	ZNF267	1236.978	0.508649	5.37E-06	0.000106	protein_coding
ENSG0000(	ENSG0000(	156.5916	0.356062	5.41E-06	0.000107	lncRNA
ENSG0000(	NOTCH4	1168.433	0.325235	5.41E-06	0.000107	protein_coding
ENSG0000(	RGS17	411.523	0.22298	5.44E-06	0.000108	protein_coding
ENSG0000(	MEG9	41.30249	0.857344	5.47E-06	0.000108	lncRNA
ENSG0000(	ENSG0000(	93.94801	0.334063	5.48E-06	0.000108	lncRNA
ENSG0000(	PGA3	77.49202	-0.92019	5.48E-06	0.000108	protein_coding
ENSG0000(	CRLF2	49.90197	0.806309	5.49E-06	0.000108	protein_coding
ENSG0000(	CYP1B1	117.0058	1.102604	5.50E-06	0.000109	protein_coding
ENSG0000(	NACC2	930.995	0.337378	5.51E-06	0.000109	protein_coding

ENSG0000(	ENSG0000(	104.5731	-0.34426	5.52E-06	0.000109	lncRNA
ENSG0000(	RAB9A	263.0359	-0.34042	5.53E-06	0.000109	protein_coding
ENSG0000(	SNORA80B	54.02447	0.557758	5.54E-06	0.000109	snoRNA
ENSG0000(	DKK3	510.4155	0.573292	5.57E-06	0.00011	protein_coding
ENSG0000(	GSTA2	202.7217	-1.54255	5.58E-06	0.00011	protein_coding
ENSG0000(	ENSG0000(	203.6272	0.399215	5.58E-06	0.00011	lncRNA
ENSG0000(	APCDD1	470.0994	0.46682	5.66E-06	0.000111	protein_coding
ENSG0000(	ATP5MK	277.0123	-0.55835	5.66E-06	0.000111	protein_coding
ENSG0000(	ENSG0000(	18.96831	0.832511	5.65E-06	0.000111	lncRNA
ENSG0000(	LINC00706	139.5698	0.313116	5.68E-06	0.000111	lncRNA
ENSG0000(	IGLV9-49	15.25321	1.668817	5.70E-06	0.000112	IG_V_gene
ENSG0000(	ENSG0000(	30.30027	1.559051	5.75E-06	0.000113	lncRNA
ENSG0000(	AKT1S1	855.1705	0.44757	5.78E-06	0.000113	protein_coding
ENSG0000(	CASP5	376.0877	1.171683	5.79E-06	0.000113	protein_coding
ENSG0000(	IGLV1-47	133.6316	1.265073	5.83E-06	0.000114	IG_V_gene
ENSG0000(	ENSG0000(	35.19411	0.753591	5.84E-06	0.000114	protein_coding
ENSG0000(	TMCO1	1584.402	-0.43757	5.86E-06	0.000114	protein_coding
ENSG0000(	RUNX3-AS1	10.70479	1.161269	5.87E-06	0.000114	lncRNA
ENSG0000(	EMB	3201.684	-0.38534	5.87E-06	0.000115	protein_coding
ENSG0000(	RPL23	3310.86	-0.395	5.89E-06	0.000115	protein_coding
ENSG0000(	ENSG0000(	78.45525	0.359235	5.93E-06	0.000115	TEC
ENSG0000(	PRAM1	317.8562	0.30307	5.93E-06	0.000115	protein_coding
ENSG0000(	APEH	738.2424	-0.28402	5.96E-06	0.000116	protein_coding
ENSG0000(	CHSY1	988.1061	0.558568	5.96E-06	0.000116	protein_coding
ENSG0000(	GATD1	2647.661	-0.13461	5.98E-06	0.000116	protein_coding
ENSG0000(	SLITRK1	11.34557	1.147454	6.00E-06	0.000116	protein_coding
ENSG0000(	ENSG0000(	46.38815	0.628479	6.04E-06	0.000117	lncRNA
ENSG0000(	MARCHF5	712.8403	-0.19811	6.04E-06	0.000117	protein_coding
ENSG0000(	ENSG0000(	12.29498	1.23851	6.06E-06	0.000117	protein_coding
ENSG0000(	ATL2	1705.574	-0.22935	6.10E-06	0.000118	protein_coding
ENSG0000(	MIR124-2H	10.33792	0.893325	6.10E-06	0.000118	lncRNA
ENSG0000(	NDUFA3	545.8652	-0.23394	6.12E-06	0.000118	protein_coding
ENSG0000(	ENSG0000(	96.24512	0.332869	6.17E-06	0.000119	lncRNA
ENSG0000(	ENSG0000(	245.565	0.26277	6.17E-06	0.000119	TEC
ENSG0000(	PPIF	786.0184	0.628998	6.17E-06	0.000119	protein_coding
ENSG0000(	DGLUCY	2925.36	0.163963	6.21E-06	0.00012	protein_coding
ENSG0000(	LURAP1L	313.5913	0.361684	6.21E-06	0.00012	protein_coding
ENSG0000(	MIR181A1H	533.0096	0.566929	6.21E-06	0.00012	lncRNA
ENSG0000(	CDK5RAP3	3500.956	0.206456	6.33E-06	0.000122	protein_coding
ENSG0000(	DZIP1L	290.1345	0.509617	6.32E-06	0.000122	protein_coding
ENSG0000(	ENSG0000(	1023.101	0.238511	6.33E-06	0.000122	lncRNA
ENSG0000(	CGB7	9.755837	1.176188	6.33E-06	0.000122	protein_coding
ENSG0000(	ENSG0000(	512.5639	0.535828	6.34E-06	0.000122	lncRNA
ENSG0000(	SPOCD1	232.0284	0.379439	6.34E-06	0.000122	protein_coding
ENSG0000(	GRM2	76.58145	0.707298	6.36E-06	0.000122	protein_coding
ENSG0000(	IL18R1	522.5482	0.562123	6.37E-06	0.000122	protein_coding
ENSG0000(	FRS2	1085.759	-0.23948	6.39E-06	0.000123	protein_coding

ENSG0000(	ANO9	4656.945	-0.55049	6.44E-06	0.000123	protein_coding
ENSG0000(	ENSG0000(	1685.536	0.191504	6.46E-06	0.000123	lncRNA
ENSG0000(	ENSG0000(	12.50093	1.099868	6.45E-06	0.000123	lncRNA
ENSG0000(	H3-2	458.0721	0.382049	6.45E-06	0.000123	protein_coding
ENSG0000(	EBF3	38.57663	1.04279	6.47E-06	0.000124	protein_coding
ENSG0000(	TMEM179E	328.5535	-0.32231	6.48E-06	0.000124	protein_coding
ENSG0000(	CCDC115	406.8828	-0.23259	6.49E-06	0.000124	protein_coding
ENSG0000(	ENSG0000(	17.10381	1.437134	6.51E-06	0.000124	lncRNA
ENSG0000(	ENSG0000(	42.2537	0.689169	6.51E-06	0.000124	lncRNA
ENSG0000(	IGHV3-13	48.6882	1.337302	6.55E-06	0.000125	IG_V_gene
ENSG0000(	SBNO2	2146.225	0.42787	6.58E-06	0.000125	protein_coding
ENSG0000(	HRH2	273.035	0.947177	6.61E-06	0.000126	protein_coding
ENSG0000(	RPRD2	2214.96	-0.19971	6.61E-06	0.000126	protein_coding
ENSG0000(	TEX261	983.0319	-0.29375	6.61E-06	0.000126	protein_coding
ENSG0000(	ENSG0000(	787.2694	0.233581	6.65E-06	0.000126	processed_pseudogene
ENSG0000(	IGLV2-8	296.1829	1.402476	6.68E-06	0.000127	IG_V_gene
ENSG0000(	OACYLP	15.44005	1.435891	6.68E-06	0.000127	transcribed_unitary_pseudogene
ENSG0000(	ENSG0000(	108.6311	0.317143	6.70E-06	0.000127	lncRNA
ENSG0000(	TPPP	574.0278	-0.99542	6.70E-06	0.000127	protein_coding
ENSG0000(	RNF213	37212.01	0.375875	6.77E-06	0.000128	protein_coding
ENSG0000(	CHD5	25.32446	0.900706	6.79E-06	0.000128	protein_coding
ENSG0000(	ENSG0000(	16.61898	0.879703	6.79E-06	0.000128	lncRNA
ENSG0000(	MASP1	451.9478	0.896749	6.80E-06	0.000129	protein_coding
ENSG0000(	ENSG0000(	160.0979	0.216755	6.81E-06	0.000129	lncRNA
ENSG0000(	MAFF	922.06	0.426245	6.81E-06	0.000129	protein_coding
ENSG0000(	RPS3AP32	16.87953	1.087529	6.82E-06	0.000129	processed_pseudogene
ENSG0000(	LRRK2	2308.643	0.887405	6.85E-06	0.000129	protein_coding
ENSG0000(	GDPD3	234.0183	-0.53355	6.88E-06	0.00013	protein_coding
ENSG0000(	ADIRF	785.242	-1.12828	6.90E-06	0.00013	protein_coding
ENSG0000(	ENSG0000(	44.30963	-1.38342	6.89E-06	0.00013	lncRNA
ENSG0000(	ENSG0000(	25.59172	0.927796	6.94E-06	0.00013	lncRNA
ENSG0000(	IGLC1	1759.481	1.258866	6.94E-06	0.00013	IG_C_gene
ENSG0000(	LIPE	296.9451	-0.47405	6.94E-06	0.00013	protein_coding
ENSG0000(	CATSPERB	567.8593	0.488774	6.98E-06	0.000131	protein_coding
ENSG0000(	UBL7-DT	2896.999	0.199051	6.98E-06	0.000131	lncRNA
ENSG0000(	IGKV3D-20	36.14749	1.544478	7.00E-06	0.000131	IG_V_gene
ENSG0000(	SPTBN5	180.0246	0.870747	7.00E-06	0.000131	protein_coding
ENSG0000(	TMEM14C	375.7068	-0.36163	7.11E-06	0.000133	protein_coding
ENSG0000(	TMED3	1543.222	0.350772	7.12E-06	0.000133	protein_coding
ENSG0000(	LINC02603	38.12759	0.586693	7.14E-06	0.000134	lncRNA
ENSG0000(	RCBTB1	584.0029	0.336836	7.14E-06	0.000134	protein_coding
ENSG0000(	ENSG0000(	84.41984	0.613696	7.15E-06	0.000134	lncRNA
ENSG0000(	ANKRD27	1107.559	-0.26316	7.15E-06	0.000134	protein_coding
ENSG0000(	ENSG0000(	628.2786	-0.17005	7.17E-06	0.000134	lncRNA
ENSG0000(	ZMYND15	109.4475	0.606186	7.17E-06	0.000134	protein_coding
ENSG0000(	ENSG0000(	27.07033	0.965138	7.19E-06	0.000134	processed_pseudogene
ENSG0000(	ENSG0000(	61.29493	0.485441	7.19E-06	0.000134	lncRNA

ENSG0000(	TMEM18-D	383.385	0.260998	7.19E-06	0.000134	lncRNA
ENSG0000(	ERI1	616.6169	0.316381	7.20E-06	0.000134	protein_coding
ENSG0000(	OFD1	4387.55	-0.20187	7.22E-06	0.000134	protein_coding
ENSG0000(	ZNF697	386.3878	0.476297	7.25E-06	0.000135	protein_coding
ENSG0000(	ENSG0000(	11.20496	1.262338	7.26E-06	0.000135	lncRNA
ENSG0000(	HMGCS2	332.2799	-1.78656	7.27E-06	0.000135	protein_coding
ENSG0000(	PRICKLE2	1878.412	0.275399	7.33E-06	0.000136	protein_coding
ENSG0000(	NRCAM	215.3786	1.169855	7.34E-06	0.000136	protein_coding
ENSG0000(	TRIO	5821.92	0.216904	7.38E-06	0.000137	protein_coding
ENSG0000(	YY1	2279.897	-0.15907	7.42E-06	0.000138	protein_coding
ENSG0000(	IGKV1D-16	23.45677	1.645389	7.43E-06	0.000138	IG_V_gene
ENSG0000(	ECI1	361.3693	-0.40042	7.47E-06	0.000138	protein_coding
ENSG0000(	ATG4B	1685.587	-0.26437	7.49E-06	0.000139	protein_coding
ENSG0000(	MIR1-1HG	33.87168	0.866437	7.55E-06	0.00014	lncRNA
ENSG0000(	ENSG0000(	753.2936	0.201371	7.58E-06	0.00014	lncRNA
ENSG0000(	ENSG0000(	32.70634	0.913916	7.59E-06	0.00014	processed_pseudogene
ENSG0000(	ANO2	117.7697	0.840604	7.62E-06	0.000141	protein_coding
ENSG0000(	TRIM39	511.1412	0.224539	7.62E-06	0.000141	protein_coding
ENSG0000(	GCA	406.7751	0.690525	7.65E-06	0.000141	protein_coding
ENSG0000(	HPSE	527.9312	0.206671	7.67E-06	0.000141	protein_coding
ENSG0000(	RMND5A	2023.162	-0.4367	7.69E-06	0.000142	protein_coding
ENSG0000(	TMEM126E	422.6141	-0.24952	7.71E-06	0.000142	protein_coding
ENSG0000(	ERBIN	9726.905	-0.42415	7.72E-06	0.000142	protein_coding
ENSG0000(	ENSG0000(	46.38146	1.250374	7.79E-06	0.000143	unprocessed_pseudogene
ENSG0000(	RN7SL130F	46.41352	0.347314	7.79E-06	0.000143	misc_RNA
ENSG0000(	ATP2B1	3088.525	-0.26142	7.85E-06	0.000144	protein_coding
ENSG0000(	PRRT3-AS1	37.24732	-0.48109	7.87E-06	0.000144	lncRNA
ENSG0000(	GTPBP1	3098.206	0.302837	7.95E-06	0.000146	protein_coding
ENSG0000(	LINC02833	52.43161	0.472664	7.94E-06	0.000146	lncRNA
ENSG0000(	EOGT	603.27	0.331314	7.99E-06	0.000146	protein_coding
ENSG0000(	MAGED1	1326.933	0.268054	8.01E-06	0.000146	protein_coding
ENSG0000(	NUDT16L1	240.0442	-0.32676	8.01E-06	0.000146	protein_coding
ENSG0000(	LNCTAM34	22.64535	0.983159	8.10E-06	0.000148	lncRNA
ENSG0000(	ENSG0000(	95.02817	0.393891	8.12E-06	0.000148	lncRNA
ENSG0000(	PER3	1536.208	-0.83951	8.14E-06	0.000148	protein_coding
ENSG0000(	SMC3	2903.276	-0.22314	8.14E-06	0.000148	protein_coding
ENSG0000(	TMEM14EF	20.97742	1.38867	8.14E-06	0.000148	transcribed_processed_pseudoge
ENSG0000(	SPDYE10P	282.1238	0.325074	8.20E-06	0.000149	protein_coding
ENSG0000(	EIF1	3148.266	-0.28686	8.20E-06	0.000149	protein_coding
ENSG0000(	ENSG0000(	26.35258	0.571025	8.24E-06	0.00015	lncRNA
ENSG0000(	ZNF658B	98.17729	0.594512	8.26E-06	0.00015	transcribed_unprocessed_pseudo
ENSG0000(	HARBI1	241.4197	0.241322	8.32E-06	0.000151	protein_coding
ENSG0000(	DDR2	1962.014	0.496506	8.34E-06	0.000151	protein_coding
ENSG0000(	ENSG0000(	183.4732	0.715785	8.35E-06	0.000152	lncRNA
ENSG0000(	DLX6-AS1	491.4488	-0.22047	8.36E-06	0.000152	lncRNA
ENSG0000(	TECR	1267.828	-0.23064	8.38E-06	0.000152	protein_coding
ENSG0000(	CHRAC1	299.1216	-0.2791	8.40E-06	0.000152	protein_coding

ENSG0000(	ENSG0000(	467.8642	0.23786	8.40E-06	0.000152	TEC
ENSG0000(	ENSG0000(	679.6612	0.19095	8.52E-06	0.000154	lncRNA
ENSG0000(	YPEL3-DT	22.09936	0.64626	8.53E-06	0.000154	lncRNA
ENSG0000(	MS4A4A	621.6415	0.340579	8.60E-06	0.000156	protein_coding
ENSG0000(	ENSG0000(	20.5272	0.756696	8.65E-06	0.000156	lncRNA
ENSG0000(	UBE2Q1	827.0122	-0.35375	8.66E-06	0.000156	protein_coding
ENSG0000(	ST6GALNA(	106.5859	0.974234	8.70E-06	0.000157	protein_coding
ENSG0000(	FAM124A	288.9614	0.404501	8.71E-06	0.000157	protein_coding
ENSG0000(	ENSG0000(	48.47354	0.426094	8.72E-06	0.000157	lncRNA
ENSG0000(	LRRC40	375.1279	-0.35629	8.73E-06	0.000157	protein_coding
ENSG0000(	CASP1P2	43.95489	0.900082	8.79E-06	0.000158	unprocessed_pseudogene
ENSG0000(	BRAP	686.2732	-0.17709	8.80E-06	0.000158	protein_coding
ENSG0000(	ENSG0000(	61.43093	0.474603	8.82E-06	0.000159	lncRNA
ENSG0000(	WDR82	2756.14	-0.13245	8.84E-06	0.000159	protein_coding
ENSG0000(	DDX10P1	74.12528	0.559532	8.86E-06	0.000159	processed_pseudogene
ENSG0000(	HAR1B	135.3849	-0.26347	8.88E-06	0.00016	lncRNA
ENSG0000(	POM121C	2073.294	-0.18674	8.96E-06	0.000161	protein_coding
ENSG0000(	ENSG0000(	76.94812	0.524339	9.01E-06	0.000162	lncRNA
ENSG0000(	KRT23	10.25606	1.328933	9.04E-06	0.000162	protein_coding
ENSG0000(	PILRA	120.9376	0.773079	9.04E-06	0.000162	protein_coding
ENSG0000(	IGLV8-61	77.23727	1.698934	9.06E-06	0.000162	IG_V_gene
ENSG0000(	GTF2IP20	1049.084	0.187566	9.08E-06	0.000163	transcribed_unprocessed_pseudo
ENSG0000(	ENSG0000(	120.1671	0.327255	9.11E-06	0.000163	lncRNA
ENSG0000(	NDUFS4	228.6978	-0.33288	9.14E-06	0.000163	protein_coding
ENSG0000(	ANXA5	815.1233	0.555859	9.19E-06	0.000164	protein_coding
ENSG0000(	SAFB2	2595.184	-0.14227	9.23E-06	0.000165	protein_coding
ENSG0000(	PRKCSH	1586.546	-0.22685	9.24E-06	0.000165	protein_coding
ENSG0000(	PHLDB3	394.8323	-0.24774	9.26E-06	0.000165	protein_coding
ENSG0000(	PLBD1-AS1	164.8076	0.392287	9.27E-06	0.000165	lncRNA
ENSG0000(	ENSG0000(	96.14403	0.44909	9.31E-06	0.000166	lncRNA
ENSG0000(	ENSG0000(	1095.002	0.202809	9.34E-06	0.000166	lncRNA
ENSG0000(	TCAF2	1306.667	0.298161	9.36E-06	0.000167	protein_coding
ENSG0000(	LINC02693	700.9542	0.274812	9.39E-06	0.000167	lncRNA
ENSG0000(	ADAMTS16	612.3596	0.306013	9.43E-06	0.000167	lncRNA
ENSG0000(	ENSG0000(	80.35992	0.51927	9.42E-06	0.000167	lncRNA
ENSG0000(	YPEL2	2104.547	0.208583	9.42E-06	0.000167	protein_coding
ENSG0000(	EAPP	331.8024	-0.37599	9.45E-06	0.000168	protein_coding
ENSG0000(	PCYT2	730.7598	-0.60962	9.45E-06	0.000168	protein_coding
ENSG0000(	CCDC113	309.2323	-0.24401	9.49E-06	0.000168	protein_coding
ENSG0000(	PTGIR	475.4866	0.412837	9.51E-06	0.000168	protein_coding
ENSG0000(	DHRS7	980.9639	-0.50232	9.53E-06	0.000169	protein_coding
ENSG0000(	LGALS2	268.8733	-0.84785	9.55E-06	0.000169	protein_coding
ENSG0000(	GOLGA8G	41.1557	0.732417	9.60E-06	0.00017	protein_coding
ENSG0000(	ENSG0000(	72.25139	0.528627	9.62E-06	0.00017	lncRNA
ENSG0000(	TCERG1	2360.303	0.188054	9.64E-06	0.00017	protein_coding
ENSG0000(	GLIS1	35.10652	0.823816	9.66E-06	0.000171	protein_coding
ENSG0000(	ENSG0000(	170.326	0.241489	9.71E-06	0.000171	lncRNA

ENSG0000(	ENSG0000(	79.06527	0.269303	9.75E-06	0.000172	TEC
ENSG0000(	EIF5	4277.558	-0.15854	9.78E-06	0.000173	protein_coding
ENSG0000(	ENSG0000(	71.29984	0.33492	9.84E-06	0.000173	lncRNA
ENSG0000(	ENSG0000(	204.8306	0.997989	9.85E-06	0.000173	processed_pseudogene
ENSG0000(	PA2G4	825.0336	-0.24068	9.85E-06	0.000173	protein_coding
ENSG0000(	TRAF4	1532.532	-0.4604	9.86E-06	0.000173	protein_coding
ENSG0000(	TRIM71	45.14895	0.544198	9.84E-06	0.000173	protein_coding
ENSG0000(	ENSG0000(	16.8081	0.936546	1.00E-05	0.000176	TEC
ENSG0000(	CDYLP1	59.51322	0.402586	1.00E-05	0.000176	unprocessed_pseudogene
ENSG0000(	ASPHD1	19.50816	1.095925	1.01E-05	0.000177	protein_coding
ENSG0000(	LINC02511	148.4987	0.337314	1.01E-05	0.000177	lncRNA
ENSG0000(	ENSG0000(	238.1788	0.21158	1.01E-05	0.000177	lncRNA
ENSG0000(	CDKL1	1783.007	0.693495	1.01E-05	0.000177	protein_coding
ENSG0000(	TCIRG1	2344.19	0.355962	1.01E-05	0.000178	protein_coding
ENSG0000(	SLC52A3	968.7984	-0.81345	1.02E-05	0.000179	protein_coding
ENSG0000(	RNU6-433F	22.89324	0.640247	1.02E-05	0.000179	snRNA
ENSG0000(	TLNRD1	546.5442	0.261693	1.02E-05	0.000179	protein_coding
ENSG0000(	TCF3P1	85.61516	0.604485	1.03E-05	0.00018	processed_pseudogene
ENSG0000(	FBXO42	1460.376	0.11686	1.03E-05	0.00018	protein_coding
ENSG0000(	SPARC	2799.126	0.78107	1.03E-05	0.00018	protein_coding
ENSG0000(	DSE	1688.58	0.457429	1.03E-05	0.000181	protein_coding
ENSG0000(	YJEFN3	298.766	-0.41822	1.04E-05	0.000181	protein_coding
ENSG0000(	KIAA1191	1370.92	-0.19255	1.05E-05	0.000182	protein_coding
ENSG0000(	SHROOM3-	22.21654	1.004896	1.05E-05	0.000182	lncRNA
ENSG0000(	TMEM59	2554.447	-0.48073	1.06E-05	0.000185	protein_coding
ENSG0000(	MT-CO1	97012.85	-0.81032	1.06E-05	0.000185	protein_coding
ENSG0000(	ATP5ME	257.6002	-0.54341	1.07E-05	0.000186	protein_coding
ENSG0000(	RIC1	2546.614	0.154239	1.07E-05	0.000186	protein_coding
ENSG0000(	PIWIL2	339.976	-0.27548	1.08E-05	0.000187	protein_coding
ENSG0000(	ENSG0000(	154.7128	0.258328	1.08E-05	0.000187	lncRNA
ENSG0000(	STARD13	889.8439	0.421218	1.09E-05	0.000189	protein_coding
ENSG0000(	APOC2	259.0371	-0.64436	1.09E-05	0.000189	protein_coding
ENSG0000(	LINC01632	30.32228	1.024843	1.09E-05	0.000189	lncRNA
ENSG0000(	ENSG0000(	265.6025	0.227006	1.10E-05	0.00019	lncRNA
ENSG0000(	PRR23C	126.3658	-0.32333	1.10E-05	0.00019	protein_coding
ENSG0000(	LINC00547	882.2045	0.226667	1.10E-05	0.00019	lncRNA
ENSG0000(	NPL	498.7808	0.609307	1.11E-05	0.000191	protein_coding
ENSG0000(	TRAPPC10	2830.358	-0.17206	1.11E-05	0.000191	protein_coding
ENSG0000(	GPLD1	591.9923	0.207385	1.12E-05	0.000192	protein_coding
ENSG0000(	PPEF2	251.2559	-0.50391	1.11E-05	0.000192	protein_coding
ENSG0000(	SYT16	209.7231	0.44853	1.11E-05	0.000192	protein_coding
ENSG0000(	ENSG0000(	10.72832	0.768893	1.12E-05	0.000193	lncRNA
ENSG0000(	HTR1D	442.5575	-0.71844	1.12E-05	0.000193	protein_coding
ENSG0000(	SCAF4	1489.312	-0.15449	1.13E-05	0.000194	protein_coding
ENSG0000(	FOXJ3	2442.102	-0.21173	1.13E-05	0.000194	protein_coding
ENSG0000(	RPL11	3388.499	-0.37474	1.13E-05	0.000194	protein_coding
ENSG0000(	CALCOCO2	3805.351	-0.1618	1.14E-05	0.000195	protein_coding

ENSG0000(	GPR85	2670.945	0.638713	1.14E-05	0.000196	protein_coding
ENSG0000(	ENSG0000(	61.07449	0.935307	1.14E-05	0.000196	lncRNA
ENSG0000(	SLC2A3	1241.212	0.726159	1.14E-05	0.000196	protein_coding
ENSG0000(	CCT5	943.6998	-0.23368	1.15E-05	0.000197	protein_coding
ENSG0000(	GEMIN8	633.5019	0.193869	1.15E-05	0.000197	protein_coding
ENSG0000(	RN7SL98P	11.20513	0.763788	1.15E-05	0.000197	misc_RNA
ENSG0000(	POSTN	369.0045	1.115754	1.16E-05	0.000198	protein_coding
ENSG0000(	VWA3B	96.21459	0.426652	1.16E-05	0.000198	protein_coding
ENSG0000(	PTGES2-AS	600.527	0.22213	1.17E-05	0.000199	lncRNA
ENSG0000(	DNM1P34	39.91337	0.711272	1.17E-05	0.0002	transcribed_unprocessed_pseudo
ENSG0000(	RPS23	2711.014	-0.54007	1.17E-05	0.0002	protein_coding
ENSG0000(	SIGMAR1	750.6635	-0.27081	1.18E-05	0.000201	protein_coding
ENSG0000(	ADAMTS7F	125.1483	-0.46927	1.18E-05	0.000202	transcribed_unprocessed_pseudo
ENSG0000(	ENSG0000(	142.0353	-0.32952	1.19E-05	0.000203	lncRNA
ENSG0000(	REG3G	103.0757	-1.73599	1.19E-05	0.000203	protein_coding
ENSG0000(	VTA1	862.8532	-0.37255	1.19E-05	0.000203	protein_coding
ENSG0000(	BRI3BP	2565.524	-0.27089	1.19E-05	0.000203	protein_coding
ENSG0000(	GCH1	630.8699	0.475061	1.20E-05	0.000204	protein_coding
ENSG0000(	CPZ	67.92486	1.084049	1.20E-05	0.000204	protein_coding
ENSG0000(	TGFB3	277.7687	0.685268	1.20E-05	0.000205	protein_coding
ENSG0000(	RPS14P4	40.54302	1.017762	1.21E-05	0.000205	processed_pseudogene
ENSG0000(	CYBRD1	5816.394	-0.708	1.21E-05	0.000205	protein_coding
ENSG0000(	ENSG0000(	42.1286	0.614277	1.21E-05	0.000206	lncRNA
ENSG0000(	IGKV1-33	243.4534	1.254755	1.22E-05	0.000206	IG_V_gene
ENSG0000(	EIF3F	3145.332	-0.30702	1.23E-05	0.000208	protein_coding
ENSG0000(	AHI1	4225.359	0.246817	1.23E-05	0.000208	protein_coding
ENSG0000(	GNAI1	1184.64	0.287616	1.23E-05	0.000209	protein_coding
ENSG0000(	KCNE1	390.0543	0.51572	1.23E-05	0.000209	protein_coding
ENSG0000(	B3GNT6	522.2213	0.804803	1.23E-05	0.000209	protein_coding
ENSG0000(	SIRT1	699.3788	-0.3212	1.24E-05	0.000209	protein_coding
ENSG0000(	DEAF1	990.917	-0.20496	1.24E-05	0.00021	protein_coding
ENSG0000(	ACVR1	999.4754	0.229312	1.25E-05	0.000212	protein_coding
ENSG0000(	FBXW4	1193.87	-0.18201	1.26E-05	0.000212	protein_coding
ENSG0000(	C3orf33	204.2735	-0.31152	1.26E-05	0.000213	protein_coding
ENSG0000(	ENSG0000(	388.8458	0.274832	1.26E-05	0.000213	TEC
ENSG0000(	COL8A1	961.3882	0.351635	1.26E-05	0.000213	protein_coding
ENSG0000(	ENSG0000(	17.47073	0.858102	1.27E-05	0.000213	processed_pseudogene
ENSG0000(	ENSG0000(	13.29124	1.442787	1.28E-05	0.000215	lncRNA
ENSG0000(	FAU	744.9561	-0.36122	1.28E-05	0.000215	protein_coding
ENSG0000(	PGBD1	108.798	0.336087	1.28E-05	0.000215	protein_coding
ENSG0000(	ENSG0000(	665.9952	0.183958	1.29E-05	0.000217	lncRNA
ENSG0000(	NRN1	61.28258	0.597935	1.29E-05	0.000217	protein_coding
ENSG0000(	LINC00513	420.2405	0.226773	1.30E-05	0.000218	lncRNA
ENSG0000(	VPS9D1	516.4865	0.381354	1.30E-05	0.000218	protein_coding
ENSG0000(	RNU2-52P	12.96854	-1.35428	1.30E-05	0.000218	snRNA
ENSG0000(	ATAD1	1287.838	-0.13672	1.30E-05	0.000219	protein_coding
ENSG0000(	SLC25A6	2208.697	-0.2924	1.31E-05	0.00022	protein_coding

ENSG0000(	RASSF8-AS:	733.3036	0.246666	1.32E-05	0.000221	lncRNA
ENSG0000(	FUOM	233.6239	-0.52383	1.32E-05	0.000221	protein_coding
ENSG0000(	CACYBP	507.1289	-0.25319	1.33E-05	0.000222	protein_coding
ENSG0000(	RN7SL81P	64.45723	0.457217	1.33E-05	0.000222	misc_RNA
ENSG0000(	ID3	467.9376	-0.43739	1.33E-05	0.000222	protein_coding
ENSG0000(	GAD1	27.78502	1.493323	1.33E-05	0.000223	protein_coding
ENSG0000(	ENSG0000(	75.9078	0.602347	1.34E-05	0.000223	lncRNA
ENSG0000(	LINC02402	10.39089	0.830041	1.34E-05	0.000224	lncRNA
ENSG0000(	ENSG0000(	470.6342	0.191722	1.34E-05	0.000224	lncRNA
ENSG0000(	AMDHD2	851.7995	-0.29119	1.35E-05	0.000224	protein_coding
ENSG0000(	BHMT2	467.5552	0.312112	1.35E-05	0.000224	protein_coding
ENSG0000(	SLTM	2793.901	-0.14656	1.35E-05	0.000224	protein_coding
ENSG0000(	SDHB	418.089	-0.34979	1.35E-05	0.000225	protein_coding
ENSG0000(	PXMP2	243.4982	-0.71261	1.36E-05	0.000226	protein_coding
ENSG0000(	ENSG0000(	254.7718	0.76975	1.36E-05	0.000227	lncRNA
ENSG0000(	UBE2L6	785.9752	0.541887	1.36E-05	0.000227	protein_coding
ENSG0000(	STAP2	958.5487	-0.4157	1.37E-05	0.000227	protein_coding
ENSG0000(	IL2RA	529.7322	0.756423	1.37E-05	0.000228	protein_coding
ENSG0000(	ZKSCAN2	223.4679	0.307178	1.37E-05	0.000228	protein_coding
ENSG0000(	RN7SL274F	260.2461	0.234313	1.39E-05	0.00023	misc_RNA
ENSG0000(	DHFR	1244.583	-0.27008	1.40E-05	0.000232	protein_coding
ENSG0000(	ENSG0000(	310.2292	0.386086	1.41E-05	0.000233	lncRNA
ENSG0000(	MRPL45P2	815.5254	-0.17701	1.42E-05	0.000234	transcribed_unprocessed_pseudo
ENSG0000(	NDUFB1	598.2783	-0.33661	1.42E-05	0.000234	protein_coding
ENSG0000(	SEPTIN14	481.4538	0.273162	1.42E-05	0.000234	protein_coding
ENSG0000(	TAP2	3270.393	0.418581	1.42E-05	0.000234	protein_coding
ENSG0000(	TGFB2	60.62315	0.650696	1.42E-05	0.000234	protein_coding
ENSG0000(	TMEM237	278.42	0.478045	1.42E-05	0.000234	protein_coding
ENSG0000(	ENSG0000(	16.39475	0.661686	1.42E-05	0.000235	lncRNA
ENSG0000(	LINC00337	248.3123	-0.20158	1.44E-05	0.000238	lncRNA
ENSG0000(	ENSG0000(	25.39629	0.647399	1.45E-05	0.000239	lncRNA
ENSG0000(	TTC19	1960.748	-0.23928	1.45E-05	0.000239	protein_coding
ENSG0000(	HMGN1P8	166.844	0.327958	1.46E-05	0.00024	processed_pseudogene
ENSG0000(	IGHV3-20	84.00767	1.2358	1.46E-05	0.00024	IG_V_gene
ENSG0000(	ENSG0000(	29.14798	0.675642	1.46E-05	0.00024	lncRNA
ENSG0000(	ENSG0000(	159.7734	0.514139	1.46E-05	0.000241	lncRNA
ENSG0000(	LCP2	2584.462	0.595044	1.47E-05	0.000242	protein_coding
ENSG0000(	H19	78.29131	1.260069	1.48E-05	0.000243	lncRNA
ENSG0000(	OR2V2	310.449	0.196406	1.48E-05	0.000243	protein_coding
ENSG0000(	SETP12	21.09185	0.811772	1.48E-05	0.000243	processed_pseudogene
ENSG0000(	ABHD14B	599.4652	-0.38335	1.49E-05	0.000245	protein_coding
ENSG0000(	RHOD	40.78358	-0.94622	1.49E-05	0.000245	protein_coding
ENSG0000(	CCDC57	2606.494	0.148268	1.49E-05	0.000245	protein_coding
ENSG0000(	SASH1	1239.208	0.299732	1.49E-05	0.000245	protein_coding
ENSG0000(	ENSG0000(	49.4867	-0.93389	1.50E-05	0.000245	lncRNA
ENSG0000(	ENSG0000(	13.67418	1.140088	1.50E-05	0.000245	lncRNA
ENSG0000(	RHOJ	448.9312	0.413089	1.50E-05	0.000246	protein_coding

ENSG0000(	ZNF726	684.5035	-0.22849	1.51E-05	0.000246	protein_coding
ENSG0000(	IGLV1-51	220.3368	1.337133	1.51E-05	0.000247	IG_V_gene
ENSG0000(	LCAL1	80.59037	0.507983	1.51E-05	0.000247	lncRNA
ENSG0000(	NSMCE1	311.7185	-0.28609	1.51E-05	0.000247	protein_coding
ENSG0000(	NUP88	1392.986	-0.22794	1.52E-05	0.000247	protein_coding
ENSG0000(	ENSG0000(	599.6311	0.187631	1.52E-05	0.000247	lncRNA
ENSG0000(	LUZP2	132.4942	-0.47429	1.52E-05	0.000247	protein_coding
ENSG0000(	PTPRO	818.5493	0.301727	1.54E-05	0.00025	protein_coding
ENSG0000(	USP1	768.2462	-0.1957	1.54E-05	0.00025	protein_coding
ENSG0000(	ENSG0000(	289.845	0.248469	1.54E-05	0.000251	TEC
ENSG0000(	ENSG0000(	163.8944	0.259739	1.54E-05	0.000251	TEC
ENSG0000(	LINC00161	16.93798	0.736185	1.54E-05	0.000251	lncRNA
ENSG0000(	HDLBP	6631.987	0.248037	1.55E-05	0.000252	protein_coding
ENSG0000(	EPHA7	1829.082	0.227798	1.55E-05	0.000252	protein_coding
ENSG0000(	UGT1A10	455.6926	-1.11283	1.55E-05	0.000252	protein_coding
ENSG0000(	ENSG0000(	260.4878	0.332892	1.56E-05	0.000252	TEC
ENSG0000(	ENSG0000(	20.15299	0.599799	1.56E-05	0.000253	lncRNA
ENSG0000(	TEC	403.0351	-0.36595	1.56E-05	0.000253	protein_coding
ENSG0000(	STIP1	773.2813	-0.20707	1.56E-05	0.000254	protein_coding
ENSG0000(	ATPAF1	794.2682	-0.19894	1.57E-05	0.000255	protein_coding
ENSG0000(	CREB3L3	3435.343	-1.27957	1.57E-05	0.000255	protein_coding
ENSG0000(	TRIM56	7780.914	0.208007	1.58E-05	0.000255	protein_coding
ENSG0000(	TLCD3B	329.3863	-0.17789	1.59E-05	0.000256	protein_coding
ENSG0000(	C9orf40	233.3632	-0.60001	1.59E-05	0.000257	protein_coding
ENSG0000(	CD93	1450.192	0.583256	1.59E-05	0.000257	protein_coding
ENSG0000(	FNTA	4040.705	-0.11071	1.59E-05	0.000257	protein_coding
ENSG0000(	IGHV1-18	231.3378	1.136092	1.59E-05	0.000257	IG_V_gene
ENSG0000(	MAFB	505.0483	0.617169	1.59E-05	0.000257	protein_coding
ENSG0000(	SLC6A2	47.61842	0.466062	1.59E-05	0.000257	protein_coding
ENSG0000(	FAM157B	148.2667	1.330597	1.60E-05	0.000257	transcribed_unprocessed_pseudo
ENSG0000(	ENSG0000(	68.38433	0.48718	1.60E-05	0.000258	lncRNA
ENSG0000(	YWHAE	2030.901	-0.20583	1.60E-05	0.000258	protein_coding
ENSG0000(	RPS25	1992.833	-0.39621	1.60E-05	0.000258	protein_coding
ENSG0000(	SUGT1	3645.192	-0.17027	1.62E-05	0.00026	protein_coding
ENSG0000(	KAT14	544.582	-0.32077	1.62E-05	0.00026	protein_coding
ENSG0000(	LINC02700	30.57573	0.667859	1.62E-05	0.00026	lncRNA
ENSG0000(	IGHJ2	17.79194	1.385188	1.62E-05	0.00026	IG_J_gene
ENSG0000(	COX7B	925.9752	-0.40851	1.62E-05	0.000261	protein_coding
ENSG0000(	FAF1	1350.337	-0.23846	1.65E-05	0.000264	protein_coding
ENSG0000(	ENSG0000(	310.7393	0.247839	1.65E-05	0.000265	lncRNA
ENSG0000(	ZNF280B	167.8452	0.462542	1.65E-05	0.000265	protein_coding
ENSG0000(	SBDS	580.614	-0.45149	1.66E-05	0.000265	protein_coding
ENSG0000(	ONECUT2	83.51883	1.227699	1.66E-05	0.000265	protein_coding
ENSG0000(	ENSG0000(	92.15188	0.353625	1.66E-05	0.000266	lncRNA
ENSG0000(	RNF128	465.6805	-0.89819	1.67E-05	0.000267	protein_coding
ENSG0000(	SNORA71D	92.52962	0.5948	1.67E-05	0.000267	snoRNA
ENSG0000(	IWS1	1286.492	0.160379	1.70E-05	0.000271	protein_coding

ENSG0000(	NXN	612.5819	0.435474	1.70E-05	0.000271	protein_coding
ENSG0000(	DDX1	1983.827	-0.16038	1.71E-05	0.000272	protein_coding
ENSG0000(	RN7SL751F	69.27191	0.58571	1.71E-05	0.000272	misc_RNA
ENSG0000(	ENSG0000(	707.3885	0.347011	1.71E-05	0.000273	TEC
ENSG0000(	PPP2CA	1880.685	-0.16484	1.71E-05	0.000273	protein_coding
ENSG0000(	POM121L1	11.43575	1.099423	1.71E-05	0.000273	transcribed_unprocessed_pseudo
ENSG0000(	TPM3P7	35.93747	-0.44103	1.73E-05	0.000276	processed_pseudogene
ENSG0000(	API5	1549.72	-0.17443	1.74E-05	0.000276	protein_coding
ENSG0000(	REPS1	1749.006	-0.21743	1.74E-05	0.000277	protein_coding
ENSG0000(	PDE4A	279.5368	0.468485	1.74E-05	0.000277	protein_coding
ENSG0000(	RPL30	2827.872	-0.36027	1.75E-05	0.000278	protein_coding
ENSG0000(	SDHCP1	10.57343	1.068408	1.76E-05	0.000279	processed_pseudogene
ENSG0000(	BACH1-IT2	259.4758	0.409744	1.77E-05	0.00028	lncRNA
ENSG0000(	CDKN2AIP	519.928	-0.28711	1.77E-05	0.00028	protein_coding
ENSG0000(	CPEB4	2558.214	0.42814	1.77E-05	0.00028	protein_coding
ENSG0000(	ENSG0000(	1003.457	0.35624	1.77E-05	0.00028	TEC
ENSG0000(	GYG2	56.1053	0.608735	1.77E-05	0.00028	protein_coding
ENSG0000(	RDX	704.3947	0.463335	1.76E-05	0.00028	protein_coding
ENSG0000(	ENSG0000(	632.1657	0.503935	1.78E-05	0.000281	lncRNA
ENSG0000(	ENSG0000(	73.11739	0.429092	1.78E-05	0.000281	transcribed_processed_pseudoge
ENSG0000(	VGLL4	1705.451	-0.17647	1.78E-05	0.000282	protein_coding
ENSG0000(	LIN7C	1397.662	-0.27831	1.79E-05	0.000282	protein_coding
ENSG0000(	CTD-3080P	198.4321	-0.86498	1.79E-05	0.000282	lncRNA
ENSG0000(	PECAM1	2411.97	0.577847	1.80E-05	0.000283	protein_coding
ENSG0000(	MS4A10	2220.005	-1.10995	1.80E-05	0.000284	protein_coding
ENSG0000(	FAM157C	1249.55	0.523615	1.80E-05	0.000284	lncRNA
ENSG0000(	EDA2R	51.92545	0.650045	1.80E-05	0.000284	protein_coding
ENSG0000(	RPS10P7	757.712	0.149016	1.80E-05	0.000284	transcribed_processed_pseudoge
ENSG0000(	AIFM1	2218.61	-0.12908	1.82E-05	0.000286	protein_coding
ENSG0000(	GGT5	1116.266	0.382964	1.82E-05	0.000286	protein_coding
ENSG0000(	ENSG0000(	21.13368	-0.74616	1.82E-05	0.000287	lncRNA
ENSG0000(	VSTM2B-D1	211.9653	0.286798	1.83E-05	0.000288	lncRNA
ENSG0000(	RAD51-AS1	1688.54	0.170247	1.83E-05	0.000288	lncRNA
ENSG0000(	CHD9	6079.102	0.136919	1.84E-05	0.000288	protein_coding
ENSG0000(	FCER1A	13.46137	-1.25593	1.84E-05	0.000288	protein_coding
ENSG0000(	RTRAF	1094.974	-0.26643	1.84E-05	0.000288	protein_coding
ENSG0000(	ENSG0000(	12.44359	1.102642	1.84E-05	0.000288	lncRNA
ENSG0000(	PRAP1	1473.801	-1.10692	1.84E-05	0.000289	protein_coding
ENSG0000(	ENSG0000(	300.3141	0.438105	1.84E-05	0.000289	lncRNA
ENSG0000(	VPS51	1098.222	-0.20835	1.84E-05	0.000289	protein_coding
ENSG0000(	ENSG0000(	274.736	0.326287	1.85E-05	0.000289	lncRNA
ENSG0000(	HIPK1-AS1	410.1821	0.204891	1.85E-05	0.000289	lncRNA
ENSG0000(	ENSG0000(	26.31785	0.574205	1.86E-05	0.000291	lncRNA
ENSG0000(	ZDBF2	755.8175	0.343222	1.86E-05	0.000291	protein_coding
ENSG0000(	ZNF8-ERVK	186.327	0.339333	1.86E-05	0.000291	lncRNA
ENSG0000(	CYP4F11	594.467	-1.17047	1.87E-05	0.000292	protein_coding
ENSG0000(	NPFFR1	1273.551	0.20653	1.87E-05	0.000292	protein_coding

ENSG0000(	ENSG0000(	106.6504	-0.32292	1.89E-05	0.000294	lncRNA
ENSG0000(	WNT5B	185.1904	0.474284	1.89E-05	0.000295	protein_coding
ENSG0000(	CLBA1	259.7286	0.427287	1.90E-05	0.000295	protein_coding
ENSG0000(	DNAJA2-DT	33.11394	0.574095	1.90E-05	0.000296	lncRNA
ENSG0000(	EIF4B	6463.284	-0.18582	1.90E-05	0.000296	protein_coding
ENSG0000(	DYNAP	235.5079	0.260503	1.91E-05	0.000297	protein_coding
ENSG0000(	ENSG0000(	11.761	0.972569	1.91E-05	0.000297	lncRNA
ENSG0000(	TMEM86B	134.7137	-0.33188	1.92E-05	0.000298	protein_coding
ENSG0000(	SETD6	1156.293	-0.15943	1.92E-05	0.000298	protein_coding
ENSG0000(	FXR1	2138.225	-0.16057	1.92E-05	0.000298	protein_coding
ENSG0000(	POM121L9	154.6758	0.777582	1.92E-05	0.000298	transcribed_unprocessed_pseudo
ENSG0000(	TBC1D2B	3401.163	0.177996	1.93E-05	0.0003	protein_coding
ENSG0000(	LHX6	73.3459	0.694203	1.94E-05	0.0003	protein_coding
ENSG0000(	SLC35G2	188.7268	0.346327	1.94E-05	0.000301	protein_coding
ENSG0000(	ARHGEF4	356.0597	0.222922	1.94E-05	0.000301	protein_coding
ENSG0000(	ZC3H15	837.2541	-0.20407	1.94E-05	0.000301	protein_coding
ENSG0000(	TNNC2	18.97037	-1.0764	1.96E-05	0.000303	protein_coding
ENSG0000(	LINC02604	309.1317	0.388325	1.96E-05	0.000303	lncRNA
ENSG0000(	ENSG0000(	883.4716	0.292983	1.98E-05	0.000305	processed_pseudogene
ENSG0000(	C10orf99	229.5142	-1.38093	1.98E-05	0.000306	protein_coding
ENSG0000(	IGDCC4	505.792	0.917615	1.99E-05	0.000306	protein_coding
ENSG0000(	ENSG0000(	278.0748	0.268174	2.00E-05	0.000308	lncRNA
ENSG0000(	GRM1	64.2978	0.785975	2.01E-05	0.000309	protein_coding
ENSG0000(	ENSG0000(	95.98406	0.37802	2.01E-05	0.00031	lncRNA
ENSG0000(	CCDC32	1654.466	0.270607	2.01E-05	0.00031	protein_coding
ENSG0000(	PDLIM7	629.3626	0.513122	2.01E-05	0.00031	protein_coding
ENSG0000(	LINC02864	257.3681	0.270506	2.02E-05	0.000311	lncRNA
ENSG0000(	ENSG0000(	576.6824	0.221985	2.02E-05	0.000311	protein_coding
ENSG0000(	ENSG0000(	176.3447	-0.29598	2.03E-05	0.000313	lncRNA
ENSG0000(	NPFFR2	59.40268	-0.40934	2.03E-05	0.000313	protein_coding
ENSG0000(	PHC2	983.3538	0.377833	2.04E-05	0.000313	protein_coding
ENSG0000(	BAZ1A	2445.724	0.24037	2.04E-05	0.000313	protein_coding
ENSG0000(	SLC5A6	583.833	-0.25036	2.04E-05	0.000313	protein_coding
ENSG0000(	ITGAX	3097.065	0.735921	2.04E-05	0.000313	protein_coding
ENSG0000(	OR2A42	121.172	0.280691	2.04E-05	0.000313	protein_coding
ENSG0000(	RPS27A	2256.555	-0.39471	2.04E-05	0.000313	protein_coding
ENSG0000(	ENSG0000(	293.8134	0.234102	2.05E-05	0.000313	TEC
ENSG0000(	BTN3A2	4847.716	0.395062	2.05E-05	0.000314	protein_coding
ENSG0000(	BLOC1S1	396.4339	-0.41463	2.07E-05	0.000317	protein_coding
ENSG0000(	APOA4	5559.92	-1.59375	2.07E-05	0.000317	protein_coding
ENSG0000(	ENSG0000(	13.6741	0.846961	2.08E-05	0.000317	TEC
ENSG0000(	PTPRM	1928.056	0.275808	2.08E-05	0.000318	protein_coding
ENSG0000(	ENSG0000(	29.38419	0.76302	2.08E-05	0.000318	lncRNA
ENSG0000(	KMT5B	2466.953	-0.22286	2.09E-05	0.000318	protein_coding
ENSG0000(	ENSG0000(	102.9043	0.49114	2.09E-05	0.000318	lncRNA
ENSG0000(	PEX16	349.4504	-0.2527	2.09E-05	0.000318	protein_coding
ENSG0000(	ENSG0000(	909.3218	0.292129	2.09E-05	0.000319	lncRNA

ENSG0000(	BMERB1	163.5056	0.512152	2.11E-05	0.000321	protein_coding
ENSG0000(	AREG	48.24693	1.104717	2.11E-05	0.000322	protein_coding
ENSG0000(	LNP1	51.64125	0.51011	2.12E-05	0.000322	protein_coding
ENSG0000(	LZIC	1317.647	-0.24759	2.12E-05	0.000323	protein_coding
ENSG0000(	ENDOD1	916.3501	-0.33528	2.13E-05	0.000323	protein_coding
ENSG0000(	RN7SL4P	100.898	0.6117	2.13E-05	0.000324	misc_RNA
ENSG0000(	SNRPA	494.3539	-0.22741	2.13E-05	0.000324	protein_coding
ENSG0000(	ENSG0000(	15.08025	1.204105	2.13E-05	0.000324	lncRNA
ENSG0000(	SLFN12	328.9798	0.49896	2.14E-05	0.000324	protein_coding
ENSG0000(	SNRPD3	1522.414	-0.15655	2.14E-05	0.000324	protein_coding
ENSG0000(	ENSG0000(	11.79192	1.052768	2.14E-05	0.000324	lncRNA
ENSG0000(	NAB2	292.7661	-0.36974	2.15E-05	0.000325	protein_coding
ENSG0000(	RAB44	57.91268	0.689097	2.15E-05	0.000326	protein_coding
ENSG0000(	LINC02773	15.90958	0.802953	2.16E-05	0.000328	lncRNA
ENSG0000(	ZNF90	688.9822	-0.24662	2.17E-05	0.000328	protein_coding
ENSG0000(	SNORD118	722.615	0.652605	2.18E-05	0.000329	snoRNA
ENSG0000(	PLA1A	44.72315	1.123224	2.18E-05	0.000329	protein_coding
ENSG0000(	IGHJ6	147.4435	0.981444	2.19E-05	0.000331	IG_J_gene
ENSG0000(	WTIP	3081.828	0.188801	2.22E-05	0.000335	protein_coding
ENSG0000(	IGLV2-11	294.8944	1.247944	2.22E-05	0.000336	IG_V_gene
ENSG0000(	ENTPD1-AS	3337.434	0.122936	2.24E-05	0.000338	lncRNA
ENSG0000(	IGHG3	1807.29	1.510799	2.24E-05	0.000338	IG_C_gene
ENSG0000(	ENSG0000(	15.77282	1.351941	2.24E-05	0.000338	lncRNA
ENSG0000(	OGDH	3102.83	-0.49504	2.25E-05	0.000338	protein_coding
ENSG0000(	SPG11	7450.556	0.094467	2.26E-05	0.000341	protein_coding
ENSG0000(	CNNM3	1501.167	-0.27785	2.27E-05	0.000341	protein_coding
ENSG0000(	BAG6	2451.941	-0.22796	2.30E-05	0.000345	protein_coding
ENSG0000(	ENSG0000(	21.15142	0.901424	2.30E-05	0.000345	lncRNA
ENSG0000(	ZG16B	83.86428	0.875523	2.31E-05	0.000347	protein_coding
ENSG0000(	ATP5PF	301.9178	-0.50176	2.31E-05	0.000347	protein_coding
ENSG0000(	LINC02471	82.54782	0.496142	2.32E-05	0.000348	lncRNA
ENSG0000(	COL28A1	610.8034	0.32673	2.32E-05	0.000348	protein_coding
ENSG0000(	IGHV1-58	14.07209	1.572321	2.32E-05	0.000348	IG_V_gene
ENSG0000(	IRS1	883.7837	0.460723	2.32E-05	0.000348	protein_coding
ENSG0000(	PRXL2B	619.2607	-0.45995	2.32E-05	0.000348	protein_coding
ENSG0000(	MYO1B	1431.764	0.410655	2.32E-05	0.000348	protein_coding
ENSG0000(	ENSG0000(	21.00045	0.803857	2.34E-05	0.000349	lncRNA
ENSG0000(	ENSG0000(	51.81054	0.507226	2.33E-05	0.000349	processed_pseudogene
ENSG0000(	ENSG0000(	232.8377	0.251945	2.33E-05	0.000349	TEC
ENSG0000(	CSF2RA	1046.492	0.355422	2.35E-05	0.000352	protein_coding
ENSG0000(	B9D1	194.5317	0.404713	2.36E-05	0.000352	protein_coding
ENSG0000(	SPTLC1P1	21.9003	0.799026	2.36E-05	0.000352	transcribed_processed_pseudoge
ENSG0000(	APOBEC3B-	16.25847	0.704086	2.37E-05	0.000353	lncRNA
ENSG0000(	ENSG0000(	78.09694	0.405622	2.37E-05	0.000353	lncRNA
ENSG0000(	ENSG0000(	286.9253	-0.22524	2.37E-05	0.000354	transcribed_unprocessed_pseudo
ENSG0000(	IFT20	855.0925	-0.1864	2.37E-05	0.000354	protein_coding
ENSG0000(	SYDE1	186.9163	0.55771	2.38E-05	0.000355	protein_coding

ENSG0000(	UHMK1	4196.337	-0.25222	2.38E-05	0.000355	protein_coding
ENSG0000(	CIAO2B	189.509	-0.30351	2.39E-05	0.000356	protein_coding
ENSG0000(	KIAA0100	5305.642	-0.164	2.40E-05	0.000357	protein_coding
ENSG0000(	FUT7	21.37872	1.217612	2.40E-05	0.000357	protein_coding
ENSG0000(	NDUFA6	334.7362	-0.44058	2.41E-05	0.000358	protein_coding
ENSG0000(	MARK3	8452.785	0.104024	2.41E-05	0.000358	protein_coding
ENSG0000(	SLCO3A1	904.5063	0.487347	2.41E-05	0.000359	protein_coding
ENSG0000(	ATXN1-AS1	88.77683	0.333749	2.42E-05	0.000359	lncRNA
ENSG0000(	CLDN1	59.38126	1.131291	2.42E-05	0.000359	protein_coding
ENSG0000(	RPL23AP35	64.24446	-0.42922	2.42E-05	0.000359	processed_pseudogene
ENSG0000(	SMIM20	153.0716	-0.41256	2.42E-05	0.000359	protein_coding
ENSG0000(	NDUFB6	150.9918	-0.36226	2.45E-05	0.000362	protein_coding
ENSG0000(	IGLC2	4046.884	1.14236	2.46E-05	0.000364	IG_C_gene
ENSG0000(	EIF3L	1918.121	-0.37201	2.47E-05	0.000365	protein_coding
ENSG0000(	SLC13A3	117.3627	0.341965	2.47E-05	0.000366	protein_coding
ENSG0000(	ENSG0000(	17.66409	1.786027	2.48E-05	0.000367	TEC
ENSG0000(	TRAPPC4	927.4394	-0.1985	2.51E-05	0.000371	protein_coding
ENSG0000(	PLK5	499.8462	0.214212	2.52E-05	0.000372	protein_coding
ENSG0000(	LINC01356	771.8203	0.260089	2.53E-05	0.000373	lncRNA
ENSG0000(	SMYD3-IT1	124.4696	0.282289	2.53E-05	0.000373	lncRNA
ENSG0000(	AHSA1	604.9731	-0.21176	2.53E-05	0.000374	protein_coding
ENSG0000(	CADM2-AS	91.86146	0.319864	2.54E-05	0.000375	lncRNA
ENSG0000(	HOXC6	229.8871	0.457885	2.56E-05	0.000378	protein_coding
ENSG0000(	PANK1	446.0236	-0.67132	2.57E-05	0.000379	protein_coding
ENSG0000(	NFE4	13.03407	0.736467	2.60E-05	0.000382	lncRNA
ENSG0000(	RAB24	467.2304	0.349548	2.60E-05	0.000382	protein_coding
ENSG0000(	ATP5MGL	203.6522	0.221157	2.60E-05	0.000383	protein_coding
ENSG0000(	ENSG0000(	472.205	-0.20779	2.60E-05	0.000383	protein_coding
ENSG0000(	PKD1L3	143.6661	0.82625	2.60E-05	0.000383	protein_coding
ENSG0000(	GSK3A	1066.038	-0.21333	2.61E-05	0.000384	protein_coding
ENSG0000(	KPNA2P3	155.3466	0.543076	2.62E-05	0.000384	transcribed_unprocessed_pseudo
ENSG0000(	ENSG0000(	77.83151	0.429363	2.64E-05	0.000387	lncRNA
ENSG0000(	ENSG0000(	82.83278	0.389201	2.66E-05	0.00039	TEC
ENSG0000(	ENSG0000(	125.616	0.283096	2.67E-05	0.000391	lncRNA
ENSG0000(	SLC5A8	114.1332	1.017235	2.67E-05	0.000391	protein_coding
ENSG0000(	CHN1	454.5239	0.413271	2.68E-05	0.000393	protein_coding
ENSG0000(	SMIM26	162.586	-0.33768	2.69E-05	0.000393	protein_coding
ENSG0000(	ENSG0000(	60.62475	0.388245	2.69E-05	0.000393	lncRNA
ENSG0000(	ENSG0000(	87.61506	0.453725	2.69E-05	0.000393	lncRNA
ENSG0000(	PDGFRL	10.67046	1.087431	2.69E-05	0.000394	protein_coding
ENSG0000(	ENSG0000(	68.49304	0.394243	2.69E-05	0.000394	lncRNA
ENSG0000(	ENSG0000(	240.8659	0.270237	2.71E-05	0.000396	lncRNA
ENSG0000(	RBPMS	2103.244	0.433625	2.71E-05	0.000396	protein_coding
ENSG0000(	SLC7A1	1408.472	0.362639	2.71E-05	0.000396	protein_coding
ENSG0000(	PDZRN3	444.2374	0.548438	2.72E-05	0.000397	protein_coding
ENSG0000(	RSBN1	1156.569	-0.25516	2.73E-05	0.000399	protein_coding
ENSG0000(	NLGN2	577.156	0.509851	2.74E-05	0.000399	protein_coding

ENSG0000(	ENSG0000(	133.0315	0.277004	2.74E-05	0.000399	lncRNA
ENSG0000(	NLRC5	6802.693	0.575532	2.74E-05	0.000399	protein_coding
ENSG0000(	LINC01705	31.62475	0.604662	2.74E-05	0.000399	lncRNA
ENSG0000(	ZNF821	177.1002	-0.39947	2.77E-05	0.000403	protein_coding
ENSG0000(	B4GALNT3	433.9323	0.810826	2.78E-05	0.000404	protein_coding
ENSG0000(	YDJC	181.0344	-0.25528	2.78E-05	0.000404	protein_coding
ENSG0000(	UQCRC2	2486.796	-0.27292	2.78E-05	0.000404	protein_coding
ENSG0000(	LINC01750	31.88827	0.905918	2.78E-05	0.000404	lncRNA
ENSG0000(	NDUFA9	3398.864	0.1333	2.79E-05	0.000405	protein_coding
ENSG0000(	ENSG0000(	119.2562	0.287799	2.80E-05	0.000406	lncRNA
ENSG0000(	CCDC148	98.71981	0.318923	2.80E-05	0.000406	protein_coding
ENSG0000(	ADGRF1	1145.007	0.541312	2.80E-05	0.000406	protein_coding
ENSG0000(	ENSG0000(	12.36902	2.031818	2.81E-05	0.000407	processed_pseudogene
ENSG0000(	LINC02656	259.4842	0.526573	2.81E-05	0.000407	lncRNA
ENSG0000(	ZDHHC11	509.8356	-0.90021	2.82E-05	0.000408	protein_coding
ENSG0000(	ENSG0000(	79.77577	0.523682	2.82E-05	0.000408	lncRNA
ENSG0000(	FAAP24	735.263	0.153914	2.84E-05	0.00041	protein_coding
ENSG0000(	IGLC3	1749.85	1.262685	2.83E-05	0.00041	IG_C_gene
ENSG0000(	OR5A2	618.8869	0.198589	2.84E-05	0.00041	protein_coding
ENSG0000(	GALK1	141.3728	-0.35025	2.85E-05	0.000411	protein_coding
ENSG0000(	SMIM11B	321.4147	-0.37913	2.85E-05	0.000412	protein_coding
ENSG0000(	WNT10B	66.8811	0.674511	2.86E-05	0.000413	protein_coding
ENSG0000(	FLJ46284	176.734	0.343952	2.87E-05	0.000414	lncRNA
ENSG0000(	ENSG0000(	290.2841	0.198921	2.87E-05	0.000414	lncRNA
ENSG0000(	VPS9D1-AS	71.99421	0.607965	2.87E-05	0.000414	lncRNA
ENSG0000(	UGT1A1	818.029	-0.55965	2.88E-05	0.000415	protein_coding
ENSG0000(	HHIPL1	361.4464	0.279044	2.88E-05	0.000415	protein_coding
ENSG0000(	TRIM4	1513.437	-0.14528	2.89E-05	0.000416	protein_coding
ENSG0000(	TMTC1	952.327	0.334169	2.89E-05	0.000416	protein_coding
ENSG0000(	GPRIN1	36.91869	0.575022	2.90E-05	0.000417	protein_coding
ENSG0000(	IGHV4-39	365.2988	1.206484	2.92E-05	0.00042	IG_V_gene
ENSG0000(	KANSL1L	1230.586	0.1784	2.92E-05	0.00042	protein_coding
ENSG0000(	ARNTL2	952.5693	0.477917	2.93E-05	0.00042	protein_coding
ENSG0000(	ENSG0000(	63.05506	0.48698	2.93E-05	0.000421	lncRNA
ENSG0000(	ENSG0000(	96.73538	-0.50216	2.95E-05	0.000423	lncRNA
ENSG0000(	SCARF1	774.8102	0.377224	2.95E-05	0.000423	protein_coding
ENSG0000(	IGHV3-69-1	43.37075	1.474249	2.96E-05	0.000424	IG_V_pseudogene
ENSG0000(	TEAD1	1845.708	0.298546	2.96E-05	0.000424	protein_coding
ENSG0000(	NUDCP1	43.16284	0.678524	2.97E-05	0.000425	processed_pseudogene
ENSG0000(	RN7SKP184	53.94359	0.579912	2.98E-05	0.000426	misc_RNA
ENSG0000(	ENSG0000(	119.5341	0.280377	2.99E-05	0.000427	lncRNA
ENSG0000(	ENSG0000(	111.4768	0.271982	2.99E-05	0.000427	lncRNA
ENSG0000(	ENSG0000(	11.42861	0.954146	2.99E-05	0.000427	lncRNA
ENSG0000(	TRMT61B	303.2165	-0.35807	2.99E-05	0.000427	protein_coding
ENSG0000(	GJA6P	23.75503	0.531925	2.99E-05	0.000427	processed_pseudogene
ENSG0000(	ERVK3-1	2275.128	0.165584	2.99E-05	0.000427	protein_coding
ENSG0000(	PRR7-AS1	72.02322	0.396589	3.00E-05	0.000428	lncRNA

ENSG0000(	IRF5	530.5938	-0.25341	3.00E-05	0.000428	protein_coding
ENSG0000(	IGHA2	4040.602	1.180081	3.00E-05	0.000428	IG_C_gene
ENSG0000(	NAV2-AS5	34.00179	0.602496	3.03E-05	0.000431	lncRNA
ENSG0000(	ENSG0000(	182.5833	-0.60377	3.03E-05	0.000432	lncRNA
ENSG0000(	ENSG0000(	57.98572	0.375124	3.03E-05	0.000432	lncRNA
ENSG0000(	RAB22A	2198.651	-0.21801	3.04E-05	0.000432	protein_coding
ENSG0000(	RETSAT	2545.187	-0.71732	3.04E-05	0.000432	protein_coding
ENSG0000(	SPDYE16	55.49286	0.637274	3.04E-05	0.000432	protein_coding
ENSG0000(	ENSG0000(	78.39424	0.359912	3.06E-05	0.000435	lncRNA
ENSG0000(	SNHG6	357.6246	-0.36094	3.07E-05	0.000436	lncRNA
ENSG0000(	AGPAT4	654.2104	0.507197	3.07E-05	0.000436	protein_coding
ENSG0000(	ENSG0000(	100.0074	0.344921	3.08E-05	0.000437	lncRNA
ENSG0000(	LINC02580	254.2807	0.453168	3.08E-05	0.000437	lncRNA
ENSG0000(	SNHG7	876.5989	-0.22942	3.08E-05	0.000437	lncRNA
ENSG0000(	PLCB1	596.6368	0.549175	3.09E-05	0.000438	protein_coding
ENSG0000(	FN1	2579.635	0.964901	3.10E-05	0.000439	protein_coding
ENSG0000(	RWDD2A	243.3107	0.262626	3.10E-05	0.000439	protein_coding
ENSG0000(	GOLGA6L5I	235.3596	0.429388	3.10E-05	0.000439	transcribed_unprocessed_pseudo
ENSG0000(	FTX	21704.3	0.114622	3.11E-05	0.00044	lncRNA
ENSG0000(	LINC01856	292.386	0.304453	3.12E-05	0.000441	lncRNA
ENSG0000(	VN1R2	56.07628	0.394327	3.12E-05	0.000442	protein_coding
ENSG0000(	COL22A1	152.8107	1.002487	3.13E-05	0.000442	protein_coding
ENSG0000(	DLEU2	3359.591	0.217696	3.14E-05	0.000443	lncRNA
ENSG0000(	FMNL2	1254.432	0.422199	3.14E-05	0.000443	protein_coding
ENSG0000(	GLS	7551.697	-0.39703	3.15E-05	0.000445	protein_coding
ENSG0000(	MIR133A1I	18.11686	0.792781	3.16E-05	0.000446	lncRNA
ENSG0000(	ENSG0000(	177.6756	0.408503	3.17E-05	0.000447	lncRNA
ENSG0000(	DNASE1	5871.574	-0.59385	3.18E-05	0.000448	protein_coding
ENSG0000(	FMN1	2204.626	0.437831	3.18E-05	0.000448	protein_coding
ENSG0000(	THEM6	206.634	-0.39215	3.19E-05	0.000449	protein_coding
ENSG0000(	SH3BP4	600.2588	0.472006	3.20E-05	0.00045	protein_coding
ENSG0000(	ENSG0000(	333.7486	-0.23013	3.21E-05	0.000452	lncRNA
ENSG0000(	ENSG0000(	11.77844	0.694324	3.24E-05	0.000456	lncRNA
ENSG0000(	SLC27A4	679.4441	-0.63642	3.25E-05	0.000456	protein_coding
ENSG0000(	RSPH10B	309.9933	-0.20456	3.25E-05	0.000457	protein_coding
ENSG0000(	DHDH	98.36135	-1.26162	3.27E-05	0.000459	protein_coding
ENSG0000(	ENSG0000(	15.20808	0.752049	3.27E-05	0.000459	processed_pseudogene
ENSG0000(	MACF1	33144.8	0.196925	3.27E-05	0.000459	protein_coding
ENSG0000(	TUBB4B	1117.347	-0.46767	3.27E-05	0.000459	protein_coding
ENSG0000(	ENSG0000(	1030.315	0.28471	3.28E-05	0.00046	TEC
ENSG0000(	TTLL3	3743.003	0.184586	3.28E-05	0.00046	protein_coding
ENSG0000(	NECAB1	42.79419	0.675859	3.29E-05	0.000461	protein_coding
ENSG0000(	ENSG0000(	60.20768	0.466248	3.30E-05	0.000462	lncRNA
ENSG0000(	ENO3	252.5855	-0.25579	3.30E-05	0.000462	protein_coding
ENSG0000(	ENSG0000(	323.8766	0.223037	3.30E-05	0.000462	lncRNA
ENSG0000(	ENSG0000(	413.1844	-0.22894	3.32E-05	0.000464	lncRNA
ENSG0000(	KL	101.1998	-0.54204	3.32E-05	0.000464	protein_coding

ENSG0000(	NOD1	847.7875	0.415896	3.32E-05	0.000464	protein_coding
ENSG0000(	RN7SL610F	12.56201	0.658284	3.34E-05	0.000466	misc_RNA
ENSG0000(	SRP14	860.5434	-0.24724	3.34E-05	0.000466	protein_coding
ENSG0000(	LDLRAD3	306.4722	0.480155	3.34E-05	0.000466	protein_coding
ENSG0000(	ENSG0000(	1353.032	0.26362	3.36E-05	0.000468	lncRNA
ENSG0000(	STOML2	280.5436	-0.34258	3.37E-05	0.00047	protein_coding
ENSG0000(	ENSG0000(	54.25571	0.568495	3.40E-05	0.000473	lncRNA
ENSG0000(	SERPING1	1751.993	0.439183	3.41E-05	0.000474	protein_coding
ENSG0000(	BCAM	518.575	-0.20663	3.42E-05	0.000476	protein_coding
ENSG0000(	ENSG0000(	136.4042	0.214701	3.43E-05	0.000477	lncRNA
ENSG0000(	ENSG0000(	902.1125	0.214332	3.44E-05	0.000478	TEC
ENSG0000(	FAM151A	415.7426	-1.56569	3.44E-05	0.000478	protein_coding
ENSG0000(	ENSG0000(	17.92135	0.690671	3.46E-05	0.00048	lncRNA
ENSG0000(	ING1	883.8649	-0.15115	3.46E-05	0.00048	protein_coding
ENSG0000(	ENSG0000(	104.0498	0.338466	3.48E-05	0.000483	lncRNA
ENSG0000(	SEC14L1	2160.774	0.449904	3.48E-05	0.000483	protein_coding
ENSG0000(	HLA-B	16561.06	0.413885	3.50E-05	0.000486	protein_coding
ENSG0000(	ENSG0000(	136.4368	0.434598	3.51E-05	0.000486	lncRNA
ENSG0000(	LRRC27	1011.904	0.258488	3.53E-05	0.000488	protein_coding
ENSG0000(	ENSG0000(	876.7867	0.236855	3.53E-05	0.000489	protein_coding
ENSG0000(	ENSG0000(	61.12892	-0.44084	3.54E-05	0.00049	pseudogene
ENSG0000(	CUL3	2273.136	-0.14709	3.55E-05	0.000491	protein_coding
ENSG0000(	PRKD1	273.9646	0.363069	3.55E-05	0.000491	protein_coding
ENSG0000(	ENSG0000(	101.5809	0.367562	3.58E-05	0.000495	lncRNA
ENSG0000(	RNU1-100F	32.26239	0.571491	3.59E-05	0.000495	snRNA
ENSG0000(	PLEKHJ1	1109.53	-0.35997	3.59E-05	0.000495	protein_coding
ENSG0000(	RN7SL127F	82.60465	0.344558	3.60E-05	0.000497	misc_RNA
ENSG0000(	ENSG0000(	68.64731	0.329349	3.61E-05	0.000499	lncRNA
ENSG0000(	PSMD3	1023.452	-0.18267	3.62E-05	0.000499	protein_coding
ENSG0000(	SURF6	970.754	-0.19294	3.62E-05	0.000499	protein_coding
ENSG0000(	WEE1	1500.462	-0.27341	3.62E-05	0.000499	protein_coding
ENSG0000(	ENSG0000(	13.45412	0.786875	3.63E-05	0.000499	lncRNA
ENSG0000(	IGKV1D-13	53.10049	1.81082	3.63E-05	0.000499	IG_V_gene
ENSG0000(	VKORC1	323.814	-0.59018	3.63E-05	0.000499	protein_coding
ENSG0000(	ENSG0000(	556.8447	0.245465	3.64E-05	0.000501	lncRNA
ENSG0000(	TRIB2	1350.89	0.631995	3.64E-05	0.000501	protein_coding
ENSG0000(	CARNMT1	728.0142	-0.29721	3.65E-05	0.000501	protein_coding
ENSG0000(	ENSG0000(	34.20262	0.42467	3.65E-05	0.000502	lncRNA
ENSG0000(	ENSG0000(	269.6439	0.220406	3.66E-05	0.000503	lncRNA
ENSG0000(	RNF144B	641.8457	0.823976	3.68E-05	0.000505	protein_coding
ENSG0000(	FFAR4	373.681	0.206303	3.68E-05	0.000505	protein_coding
ENSG0000(	MALSU1	259.8898	-0.23434	3.69E-05	0.000507	protein_coding
ENSG0000(	H2AZ2	3314.32	-0.1982	3.70E-05	0.000507	protein_coding
ENSG0000(	ENSG0000(	108.0138	0.323307	3.70E-05	0.000507	lncRNA
ENSG0000(	SLC9A5	86.76707	0.540656	3.71E-05	0.000508	protein_coding
ENSG0000(	EXOG	918.3359	0.257342	3.73E-05	0.00051	protein_coding
ENSG0000(	RPS7	2745.707	-0.36092	3.73E-05	0.000511	protein_coding

ENSG0000(	QKI	2353.858	0.438713	3.74E-05	0.000511	protein_coding
ENSG0000(	CDC42EP4	1366.051	-0.41719	3.75E-05	0.000512	protein_coding
ENSG0000(	MTHFD2	2410.631	0.25441	3.78E-05	0.000516	protein_coding
ENSG0000(	RPS15A	3172.961	-0.40776	3.78E-05	0.000516	protein_coding
ENSG0000(	SCN4B	158.8917	-0.701	3.77E-05	0.000516	protein_coding
ENSG0000(	ZFP91	2745.495	-0.24954	3.78E-05	0.000516	protein_coding
ENSG0000(	CTSZ	6103.639	-0.28184	3.78E-05	0.000516	protein_coding
ENSG0000(	DAD1	367.1831	-0.40286	3.79E-05	0.000517	protein_coding
ENSG0000(	GPAA1	524.143	-0.3067	3.81E-05	0.00052	protein_coding
ENSG0000(	BEST1	973.7221	0.413493	3.82E-05	0.000521	protein_coding
ENSG0000(	HNRNPF	2152.716	-0.23832	3.84E-05	0.000522	protein_coding
ENSG0000(	ENSG0000(	42.98616	0.653531	3.84E-05	0.000523	TEC
ENSG0000(	COX5A	689.1901	-0.51933	3.85E-05	0.000523	protein_coding
ENSG0000(	ENSG0000(	89.56103	0.527801	3.85E-05	0.000523	lncRNA
ENSG0000(	APOL6	7272.658	0.317303	3.86E-05	0.000525	protein_coding
ENSG0000(	C2orf16	9.77551	1.298395	3.86E-05	0.000525	protein_coding
ENSG0000(	ENSG0000(	11.69321	1.451604	3.86E-05	0.000525	lncRNA
ENSG0000(	PRPF39	1259.582	-0.27796	3.86E-05	0.000525	protein_coding
ENSG0000(	REEP5	1199.541	-0.18432	3.86E-05	0.000525	protein_coding
ENSG0000(	GPBP1L1	4550.304	-0.24496	3.87E-05	0.000526	protein_coding
ENSG0000(	IGLV1-44	164.8577	1.128204	3.88E-05	0.000526	IG_V_gene
ENSG0000(	LINC02074	58.57182	0.374826	3.89E-05	0.000527	lncRNA
ENSG0000(	PRXL2A	1531.402	-0.5395	3.90E-05	0.000529	protein_coding
ENSG0000(	MRPL49	474.4243	-0.21137	3.91E-05	0.00053	protein_coding
ENSG0000(	SLC25A29	1487.201	0.3946	3.93E-05	0.000532	protein_coding
ENSG0000(	SSR4P1	33.08143	0.595809	3.94E-05	0.000533	transcribed_processed_pseudoge
ENSG0000(	TMEM69	266.7134	-0.19985	3.95E-05	0.000534	protein_coding
ENSG0000(	UPK1A-AS1	69.79507	0.326404	3.95E-05	0.000534	lncRNA
ENSG0000(	ENSG0000(	10.36003	0.927408	3.96E-05	0.000535	processed_pseudogene
ENSG0000(	ST7-OT4	318.3194	0.286317	3.96E-05	0.000535	lncRNA
ENSG0000(	TAS1R3	55.03752	0.7101	3.96E-05	0.000535	protein_coding
ENSG0000(	ENSG0000(	70.14561	0.60595	3.96E-05	0.000535	lncRNA
ENSG0000(	IGHV3-48	309.0517	1.070478	3.99E-05	0.000538	IG_V_gene
ENSG0000(	ADCY1	305.1899	0.372494	4.00E-05	0.000539	protein_coding
ENSG0000(	SLFN13	1357.914	0.522622	4.00E-05	0.00054	protein_coding
ENSG0000(	ENSG0000(	360.3674	-0.27035	4.01E-05	0.000541	lncRNA
ENSG0000(	ENSG0000(	21.11621	0.659434	4.03E-05	0.000543	lncRNA
ENSG0000(	HSPE1P11	172.569	0.314066	4.04E-05	0.000544	processed_pseudogene
ENSG0000(	TMEM170/	1798.512	-0.22274	4.04E-05	0.000544	protein_coding
ENSG0000(	CUEDC1	363.3935	0.296376	4.05E-05	0.000545	protein_coding
ENSG0000(	FPR2	137.0543	0.841296	4.05E-05	0.000545	protein_coding
ENSG0000(	BTF3	1824.919	-0.29933	4.06E-05	0.000545	protein_coding
ENSG0000(	LST1	585.0293	0.412726	4.06E-05	0.000546	protein_coding
ENSG0000(	DUSP26	16.97522	0.915239	4.07E-05	0.000546	protein_coding
ENSG0000(	G0S2	205.4377	1.226361	4.07E-05	0.000546	protein_coding
ENSG0000(	KLF6	4217.269	0.406683	4.07E-05	0.000546	protein_coding
ENSG0000(	OSBPL7	1272.246	-0.45618	4.07E-05	0.000546	protein_coding

ENSG0000(	TUT7	2790.923	0.215968	4.07E-05	0.000546	protein_coding
ENSG0000(	IGLV4-69	51.55581	1.112788	4.08E-05	0.000546	IG_V_gene
ENSG0000(	UGT3A1	104.1121	-0.55788	4.08E-05	0.000546	protein_coding
ENSG0000(	WDTC1	1535.29	-0.18433	4.09E-05	0.000547	protein_coding
ENSG0000(	CALHM6	93.66101	1.118178	4.10E-05	0.000549	protein_coding
ENSG0000(	MROH7-TT	332.7706	-0.52429	4.10E-05	0.000549	protein_coding
ENSG0000(	RPL36AL	586.7131	-0.30935	4.12E-05	0.00055	protein_coding
ENSG0000(	SLC25A51	788.7334	-0.23771	4.11E-05	0.00055	protein_coding
ENSG0000(	COL12A1	3862.037	0.695415	4.12E-05	0.00055	protein_coding
ENSG0000(	ENSG0000(	50.48481	0.433428	4.12E-05	0.00055	lncRNA
ENSG0000(	RAB11B	709.0219	-0.18996	4.13E-05	0.000551	protein_coding
ENSG0000(	SEC24B	1584.832	-0.16864	4.15E-05	0.000554	protein_coding
ENSG0000(	RAPGEF3	853.1224	0.397774	4.16E-05	0.000555	protein_coding
ENSG0000(	WIPF3	320.5834	-0.40762	4.17E-05	0.000556	protein_coding
ENSG0000(	ENSG0000(	44.18108	-1.17007	4.20E-05	0.00056	lncRNA
ENSG0000(	ENSG0000(	112.173	0.354296	4.22E-05	0.000562	lncRNA
ENSG0000(	CHRNA6	9.677959	1.326323	4.23E-05	0.000563	protein_coding
ENSG0000(	PRKAR2B-A	191.7228	0.45191	4.23E-05	0.000563	lncRNA
ENSG0000(	ENSG0000(	10.69757	0.751688	4.24E-05	0.000564	lncRNA
ENSG0000(	HSPD1P11	251.3593	0.447999	4.24E-05	0.000564	processed_pseudogene
ENSG0000(	RGMB-AS1	39.0196	0.745499	4.25E-05	0.000566	lncRNA
ENSG0000(	GPS2	415.8264	-0.26377	4.27E-05	0.000567	protein_coding
ENSG0000(	MT-CYB	20559.68	-0.70833	4.27E-05	0.000568	protein_coding
ENSG0000(	AP1AR	797.5072	-0.46804	4.30E-05	0.000571	protein_coding
ENSG0000(	RBM44	124.9517	0.599462	4.31E-05	0.000572	protein_coding
ENSG0000(	F3	1770.764	0.481453	4.31E-05	0.000572	protein_coding
ENSG0000(	IRF2BPL	533.5521	0.265405	4.34E-05	0.000575	protein_coding
ENSG0000(	NELFE	311.9051	-0.28803	4.34E-05	0.000576	protein_coding
ENSG0000(	RECQL5	1061.488	0.171693	4.34E-05	0.000576	protein_coding
ENSG0000(	BCDIN3D	633.8127	0.229203	4.35E-05	0.000577	protein_coding
ENSG0000(	CYP4F2	1597.503	-1.23753	4.38E-05	0.000579	protein_coding
ENSG0000(	IGKV1-16	119.2612	1.143331	4.38E-05	0.000579	IG_V_gene
ENSG0000(	SULT2B1	179.4091	-0.51872	4.39E-05	0.000581	protein_coding
ENSG0000(	ARSB	785.5264	0.283887	4.40E-05	0.000582	protein_coding
ENSG0000(	C9orf24	43.95694	-1.24427	4.40E-05	0.000582	protein_coding
ENSG0000(	NFATC4	553.8024	0.495738	4.41E-05	0.000583	protein_coding
ENSG0000(	RPL17	2673.509	-0.30825	4.42E-05	0.000583	protein_coding
ENSG0000(	ZNF23	595.2772	0.248488	4.42E-05	0.000584	protein_coding
ENSG0000(	CLDN15	2045.268	-0.62516	4.43E-05	0.000584	protein_coding
ENSG0000(	ATP5F1A	5315.029	-0.27389	4.44E-05	0.000586	protein_coding
ENSG0000(	MRPL35	788.9751	-0.26637	4.44E-05	0.000586	protein_coding
ENSG0000(	RN7SL749F	103.1734	0.379164	4.45E-05	0.000587	misc_RNA
ENSG0000(	DSEL	688.4246	0.260098	4.46E-05	0.000588	protein_coding
ENSG0000(	SNX27	3903.753	0.222887	4.46E-05	0.000588	protein_coding
ENSG0000(	TNPO1	4200.968	-0.14823	4.47E-05	0.000588	protein_coding
ENSG0000(	IARS2	954.439	-0.26059	4.47E-05	0.000588	protein_coding
ENSG0000(	PIGH	564.9935	-0.20112	4.47E-05	0.000588	protein_coding

ENSG0000(	ENSG0000(	30.07058	0.526374	4.48E-05	0.000589	lncRNA
ENSG0000(	ENSG0000(	14.9508	1.132383	4.48E-05	0.000589	lncRNA
ENSG0000(	SNX3	767.164	-0.31924	4.48E-05	0.000589	protein_coding
ENSG0000(	SETD7	1766.514	0.280786	4.49E-05	0.000589	protein_coding
ENSG0000(	SHROOM2	134.4482	0.460264	4.50E-05	0.000591	protein_coding
ENSG0000(	GLIPR2	355.2866	0.503566	4.52E-05	0.000592	protein_coding
ENSG0000(	SLC11A2	2396.93	0.159057	4.52E-05	0.000592	protein_coding
ENSG0000(	RN7SK	405737.4	-0.2632	4.53E-05	0.000594	snRNA
ENSG0000(	LINC00707	202.5157	0.253201	4.54E-05	0.000594	lncRNA
ENSG0000(	WBP4	274.234	-0.22669	4.55E-05	0.000596	protein_coding
ENSG0000(	C1QTNF1	248.5627	0.445005	4.56E-05	0.000597	protein_coding
ENSG0000(	GRIP2	185.8527	0.65044	4.59E-05	0.0006	protein_coding
ENSG0000(	MAP1A	328.3949	0.5313	4.64E-05	0.000607	protein_coding
ENSG0000(	GNPAT	877.7302	-0.22237	4.65E-05	0.000609	protein_coding
ENSG0000(	PTP4A3	295.0437	0.594587	4.66E-05	0.000609	protein_coding
ENSG0000(	MIR3976H(	136.5924	0.39035	4.68E-05	0.000611	lncRNA
ENSG0000(	TP53I13	681.3314	-0.41509	4.68E-05	0.000611	protein_coding
ENSG0000(	H2BC12	276.3493	-0.4472	4.69E-05	0.000612	protein_coding
ENSG0000(	LINC00575	15.05783	0.646277	4.69E-05	0.000612	lncRNA
ENSG0000(	RHOB	549.5917	0.481226	4.70E-05	0.000612	protein_coding
ENSG0000(	FKBP15	2929.69	0.141463	4.70E-05	0.000613	protein_coding
ENSG0000(	FLVCR1	3951.267	-0.74632	4.72E-05	0.000614	protein_coding
ENSG0000(	LINC01675	48.39509	0.485327	4.74E-05	0.000617	lncRNA
ENSG0000(	ENSG0000(	42.96823	0.570345	4.75E-05	0.000618	lncRNA
ENSG0000(	TUBG2	215.8681	0.404144	4.75E-05	0.000618	protein_coding
ENSG0000(	MBTPS1	3048.187	0.140319	4.76E-05	0.000619	protein_coding
ENSG0000(	RGS18	95.62809	0.632056	4.76E-05	0.000619	protein_coding
ENSG0000(	ENSG0000(	64.05279	0.438934	4.76E-05	0.000619	lncRNA
ENSG0000(	SLC22A25	2830.279	0.152675	4.77E-05	0.000619	protein_coding
ENSG0000(	SUGP2	5159.402	0.266842	4.78E-05	0.00062	protein_coding
ENSG0000(	TSNARE1	611.2685	-0.40595	4.77E-05	0.00062	protein_coding
ENSG0000(	C12orf75	164.6436	-0.46533	4.78E-05	0.000621	protein_coding
ENSG0000(	ENSG0000(	95.78342	0.446688	4.79E-05	0.000621	lncRNA
ENSG0000(	TXNL1	1010.886	-0.21047	4.81E-05	0.000623	protein_coding
ENSG0000(	LINC01996	53.62834	0.362932	4.82E-05	0.000625	lncRNA
ENSG0000(	TAGAP	753.8662	0.885023	4.83E-05	0.000626	protein_coding
ENSG0000(	FADS1	845.3459	0.402481	4.86E-05	0.000629	protein_coding
ENSG0000(	RPL21P123	246.3715	0.439345	4.86E-05	0.000629	transcribed_processed_pseudoge
ENSG0000(	NR1D1	255.6531	-0.80569	4.87E-05	0.00063	protein_coding
ENSG0000(	APLP2	4043.631	-0.24459	4.88E-05	0.000631	protein_coding
ENSG0000(	ENSG0000(	183.5071	0.195688	4.89E-05	0.000632	lncRNA
ENSG0000(	HS3ST3B1	671.1833	0.475236	4.89E-05	0.000632	protein_coding
ENSG0000(	USP30-AS1	52.4885	0.489895	4.90E-05	0.000633	lncRNA
ENSG0000(	ENSG0000(	555.9976	0.262984	4.90E-05	0.000633	TEC
ENSG0000(	RN7SL69P	11.07901	0.870917	4.90E-05	0.000633	misc_RNA
ENSG0000(	RBM25	5991.432	0.119756	4.91E-05	0.000633	protein_coding
ENSG0000(	KLHDC7B	64.18131	0.950633	4.92E-05	0.000634	protein_coding

ENSG0000(	TMPRSS5	49.05651	0.570385	4.92E-05	0.000634	protein_coding
ENSG0000(	HSP90B2P	197.9925	-0.62269	4.93E-05	0.000636	processed_pseudogene
ENSG0000(	IGHV6-1	73.01402	1.353225	4.95E-05	0.000637	IG_V_gene
ENSG0000(	REG1CP	11.97292	1.385187	4.96E-05	0.000639	transcribed_unprocessed_pseudo
ENSG0000(	DDX60L	3609.163	0.473671	4.97E-05	0.000639	protein_coding
ENSG0000(	KCNMB1	780.2549	0.204997	4.97E-05	0.000639	protein_coding
ENSG0000(	SPATA9	26.21503	0.661444	4.98E-05	0.00064	protein_coding
ENSG0000(	ENSG0000(	155.5775	-0.29519	4.98E-05	0.00064	lncRNA
ENSG0000(	ENSG0000(	283.4552	-0.19859	4.98E-05	0.00064	lncRNA
ENSG0000(	SIGLEC1	956.3263	0.486544	5.01E-05	0.000643	protein_coding
ENSG0000(	ENSG0000(	2425.172	0.283029	5.01E-05	0.000644	lncRNA
ENSG0000(	MGAT4EP	244.0979	0.200243	5.04E-05	0.000647	transcribed_unitary_pseudogene
ENSG0000(	PGS1	1799.584	0.232611	5.07E-05	0.00065	protein_coding
ENSG0000(	ENSG0000(	143.9654	-0.26999	5.07E-05	0.00065	lncRNA
ENSG0000(	RBMS3	1391.963	0.251589	5.08E-05	0.000651	protein_coding
ENSG0000(	C1orf52	498.5501	-0.23308	5.10E-05	0.000653	protein_coding
ENSG0000(	NME8	37.99746	0.744551	5.10E-05	0.000653	protein_coding
ENSG0000(	CXCR6	183.8224	0.597567	5.12E-05	0.000655	protein_coding
ENSG0000(	MIR8485	51.26714	-0.60726	5.20E-05	0.000665	miRNA
ENSG0000(	COX4I1	1898.466	-0.43905	5.20E-05	0.000665	protein_coding
ENSG0000(	ENSG0000(	603.9384	0.259569	5.21E-05	0.000666	lncRNA
ENSG0000(	RTEL1-TNFI	721.2623	0.265355	5.23E-05	0.000668	protein_coding
ENSG0000(	UMPS	1165.509	-0.20538	5.23E-05	0.000668	protein_coding
ENSG0000(	PLIN1	48.33479	-0.6112	5.23E-05	0.000668	protein_coding
ENSG0000(	FRMD4A	1529.216	0.413383	5.24E-05	0.000668	protein_coding
ENSG0000(	NRDC	2839.153	0.133757	5.24E-05	0.000668	protein_coding
ENSG0000(	FBH1	2200.734	0.154014	5.24E-05	0.000669	protein_coding
ENSG0000(	IGHV3OR1(	17.00876	1.311287	5.25E-05	0.000669	IG_V_gene
ENSG0000(	THSD1	96.89863	0.455576	5.26E-05	0.000671	protein_coding
ENSG0000(	DPEP1	3261.623	-1.05038	5.31E-05	0.000676	protein_coding
ENSG0000(	IL1RL2	20.29095	0.939631	5.31E-05	0.000676	protein_coding
ENSG0000(	ENSG0000(	190.003	0.237036	5.34E-05	0.000679	lncRNA
ENSG0000(	GADL1	722.6671	0.213551	5.34E-05	0.000679	protein_coding
ENSG0000(	GOLGA8A	7265.813	0.364681	5.34E-05	0.000679	protein_coding
ENSG0000(	HAUS4	375.4613	-0.24425	5.34E-05	0.000679	protein_coding
ENSG0000(	MAD2L1-D	223.025	0.307257	5.36E-05	0.000681	lncRNA
ENSG0000(	ENSG0000(	44.73533	0.329871	5.37E-05	0.000682	lncRNA
ENSG0000(	GCK	108.2584	0.283832	5.37E-05	0.000682	protein_coding
ENSG0000(	PHKA2	1713.776	-0.20586	5.38E-05	0.000682	protein_coding
ENSG0000(	TIGD1	5626.027	0.143132	5.38E-05	0.000682	protein_coding
ENSG0000(	XRCC6	1234.346	-0.17289	5.38E-05	0.000682	protein_coding
ENSG0000(	LINC01366	148.9944	0.217421	5.39E-05	0.000684	lncRNA
ENSG0000(	ENSG0000(	303.2208	-0.21311	5.41E-05	0.000686	TEC
ENSG0000(	ENSG0000(	30.27456	0.632901	5.42E-05	0.000687	processed_pseudogene
ENSG0000(	ADAM10	4577.642	-0.14444	5.42E-05	0.000687	protein_coding
ENSG0000(	ENSG0000(	1653.06	0.213017	5.44E-05	0.000688	lncRNA
ENSG0000(	YAP1P1	9.49385	0.963325	5.44E-05	0.000688	processed_pseudogene

ENSG0000(	ZNF706	2373.145	-0.19642	5.44E-05	0.000688	protein_coding
ENSG0000(	POLD4	1506.731	-0.27098	5.46E-05	0.00069	protein_coding
ENSG0000(	SHANK3	2032.049	0.347363	5.47E-05	0.000691	protein_coding
ENSG0000(	ENSG0000(	95.4992	0.361157	5.47E-05	0.000691	lncRNA
ENSG0000(	SLX4IP	1021.211	0.171177	5.47E-05	0.000691	protein_coding
ENSG0000(	ENSG0000(	48.22616	0.835502	5.48E-05	0.000691	lncRNA
ENSG0000(	IRAG1	937.8811	0.374467	5.48E-05	0.000691	protein_coding
ENSG0000(	SAT1	7645.409	0.444356	5.49E-05	0.000692	protein_coding
ENSG0000(	MT-ND4	30015.55	-0.64067	5.49E-05	0.000692	protein_coding
ENSG0000(	ENSG0000(	57.69269	0.41505	5.50E-05	0.000693	lncRNA
ENSG0000(	METAP2	722.5063	-0.25543	5.51E-05	0.000694	protein_coding
ENSG0000(	NTRK1	132.8373	0.308664	5.52E-05	0.000695	protein_coding
ENSG0000(	MAIP1	127.2206	-0.43343	5.54E-05	0.000698	protein_coding
ENSG0000(	ENSG0000(	437.141	0.23556	5.56E-05	0.0007	lncRNA
ENSG0000(	FERMT2	786.4388	0.361318	5.56E-05	0.0007	protein_coding
ENSG0000(	MATN1	28.05841	0.610903	5.59E-05	0.000702	protein_coding
ENSG0000(	ENSG0000(	133.3365	-0.34473	5.60E-05	0.000704	lncRNA
ENSG0000(	LIFR-AS1	716.6679	0.216682	5.60E-05	0.000704	lncRNA
ENSG0000(	PELI1	1683.6	0.318648	5.61E-05	0.000705	protein_coding
ENSG0000(	IGFBP2	352.6155	0.611885	5.63E-05	0.000707	protein_coding
ENSG0000(	RPL7	3778.818	-0.33355	5.63E-05	0.000707	protein_coding
ENSG0000(	IGHJ1	10.55353	1.456377	5.64E-05	0.000707	IG_J_gene
ENSG0000(	CD300LB	21.97656	0.804078	5.65E-05	0.000707	protein_coding
ENSG0000(	CD86	245.3804	0.698133	5.65E-05	0.000707	protein_coding
ENSG0000(	ENSG0000(	18.26128	0.780321	5.65E-05	0.000707	lncRNA
ENSG0000(	TTC31	1047.799	-0.19015	5.65E-05	0.000707	protein_coding
ENSG0000(	ZNF571-AS	344.2123	-0.2509	5.65E-05	0.000707	lncRNA
ENSG0000(	GASK1B	929.3811	0.426872	5.65E-05	0.000707	protein_coding
ENSG0000(	SPINK8	37.75586	0.495526	5.67E-05	0.000709	protein_coding
ENSG0000(	ASIC2	40.07644	0.860522	5.67E-05	0.000709	protein_coding
ENSG0000(	AADAC	444.8279	-1.30555	5.68E-05	0.00071	protein_coding
ENSG0000(	HIRIP3	131.3222	-0.36149	5.68E-05	0.00071	protein_coding
ENSG0000(	ZNF566	849.1214	-0.19708	5.69E-05	0.000711	protein_coding
ENSG0000(	NDUFA5	1828.536	-0.31474	5.73E-05	0.000715	protein_coding
ENSG0000(	ENSG0000(	99.63908	0.479484	5.73E-05	0.000715	lncRNA
ENSG0000(	ENSG0000(	317.6381	0.701395	5.74E-05	0.000715	processed_pseudogene
ENSG0000(	TTC8	379.1709	0.283908	5.74E-05	0.000715	protein_coding
ENSG0000(	NSUN5P2	341.1136	-0.29817	5.74E-05	0.000716	transcribed_unprocessed_pseudo
ENSG0000(	ENSG0000(	79.78335	0.401834	5.74E-05	0.000716	lncRNA
ENSG0000(	VASH1	1151.104	0.435409	5.76E-05	0.000717	protein_coding
ENSG0000(	ATP5F1AP3	11.34098	1.064835	5.77E-05	0.000718	processed_pseudogene
ENSG0000(	LINC01580	88.24844	0.298565	5.77E-05	0.000718	lncRNA
ENSG0000(	SHISA4	25.45717	0.705452	5.77E-05	0.000718	protein_coding
ENSG0000(	ARNILA	244.1247	0.339598	5.78E-05	0.000718	lncRNA
ENSG0000(	ENSG0000(	232.4977	0.206395	5.78E-05	0.000718	lncRNA
ENSG0000(	PSPN	466.0591	-0.18147	5.78E-05	0.000718	protein_coding
ENSG0000(	FBXL22	77.38486	0.49433	5.80E-05	0.00072	protein_coding

ENSG0000(	ENSG0000(	328.8027	0.279209	5.80E-05	0.00072	protein_coding
ENSG0000(	LINC02680	527.2911	0.167375	5.81E-05	0.000721	lncRNA
ENSG0000(	SEM1	863.6791	-0.17405	5.82E-05	0.000722	protein_coding
ENSG0000(	ENSG0000(	37.41947	0.401708	5.82E-05	0.000722	lncRNA
ENSG0000(	ENSG0000(	203.0995	0.222422	5.83E-05	0.000722	TEC
ENSG0000(	GPR27	13.07731	0.993499	5.83E-05	0.000723	protein_coding
ENSG0000(	ENSG0000(	384.0783	-0.4555	5.84E-05	0.000723	protein_coding
ENSG0000(	LRFN5	62.83954	1.066818	5.84E-05	0.000723	protein_coding
ENSG0000(	CALM2	7781.36	-0.34827	5.85E-05	0.000724	protein_coding
ENSG0000(	ENSG0000(	393.3256	0.239532	5.86E-05	0.000725	lncRNA
ENSG0000(	PLPBP	856.1837	-0.26064	5.87E-05	0.000726	protein_coding
ENSG0000(	RN7SL172F	63.5297	0.494117	5.90E-05	0.00073	misc_RNA
ENSG0000(	GARS1-DT	11321.32	0.14471	5.92E-05	0.000731	lncRNA
ENSG0000(	CSDC2	41.43107	0.679745	5.94E-05	0.000734	protein_coding
ENSG0000(	EIF1AX	1112.634	-0.29581	5.96E-05	0.000736	protein_coding
ENSG0000(	PAPPA2	44.23819	1.262167	5.98E-05	0.000738	protein_coding
ENSG0000(	ALG12	678.0804	-0.27091	5.99E-05	0.000738	protein_coding
ENSG0000(	ENSG0000(	86.38004	0.403375	5.99E-05	0.000738	lncRNA
ENSG0000(	SUN2	3148.912	-0.29831	6.00E-05	0.00074	protein_coding
ENSG0000(	ENSG0000(	20.61572	0.579946	6.01E-05	0.000741	lncRNA
ENSG0000(	RPL26	2489.394	-0.3291	6.03E-05	0.000743	protein_coding
ENSG0000(	UXT-AS1	725.1218	0.160325	6.04E-05	0.000744	lncRNA
ENSG0000(	CHRD	345.7806	0.325041	6.06E-05	0.000746	protein_coding
ENSG0000(	MCPH1-DT	498.7298	0.28346	6.06E-05	0.000746	lncRNA
ENSG0000(	MRPS18B	375.7313	-0.24385	6.08E-05	0.000748	protein_coding
ENSG0000(	ANGPT2	507.5023	0.220896	6.13E-05	0.000754	protein_coding
ENSG0000(	TOMM5	569.0031	-0.27813	6.14E-05	0.000754	protein_coding
ENSG0000(	ENSG0000(	16.78899	0.561799	6.17E-05	0.000758	lncRNA
ENSG0000(	LINC00621	2475.123	0.138823	6.18E-05	0.000758	lncRNA
ENSG0000(	ALG1L2	222.7005	0.329357	6.19E-05	0.000759	protein_coding
ENSG0000(	LINC00368	50.10285	0.364521	6.21E-05	0.000762	lncRNA
ENSG0000(	SLC51A	1040.283	-0.7494	6.22E-05	0.000763	protein_coding
ENSG0000(	PDLIM2	2346.226	-0.3914	6.23E-05	0.000763	protein_coding
ENSG0000(	RAB13	567.5146	0.302784	6.23E-05	0.000763	protein_coding
ENSG0000(	ZBTB11-AS	416.5849	0.14933	6.23E-05	0.000763	lncRNA
ENSG0000(	IGLV2-14	651.3926	1.108911	6.26E-05	0.000767	IG_V_gene
ENSG0000(	KHDRBS2	93.82122	-0.78204	6.27E-05	0.000767	protein_coding
ENSG0000(	AHNAK	48522.04	-0.45182	6.27E-05	0.000767	protein_coding
ENSG0000(	C1RL-AS1	2065.59	0.269458	6.28E-05	0.000768	lncRNA
ENSG0000(	GRAMD1A	1464.547	0.430431	6.31E-05	0.000771	protein_coding
ENSG0000(	ENSG0000(	173.4045	0.236698	6.31E-05	0.000772	lncRNA
ENSG0000(	GP2	661.5983	-0.40611	6.32E-05	0.000772	protein_coding
ENSG0000(	ENSG0000(	378.1294	-0.27326	6.33E-05	0.000772	lncRNA
ENSG0000(	ZNF182	468.436	0.328647	6.33E-05	0.000773	protein_coding
ENSG0000(	ENSG0000(	179.4085	0.210411	6.33E-05	0.000773	TEC
ENSG0000(	ITPRIPL2	1995.371	0.315105	6.34E-05	0.000773	protein_coding
ENSG0000(	CNGB3	35.59539	0.766856	6.35E-05	0.000774	protein_coding

ENSG0000(	OSMR-DT	223.9269	0.278312	6.38E-05	0.000778	lncRNA
ENSG0000(	RN7SKP97	48.04771	0.39354	6.39E-05	0.000778	misc_RNA
ENSG0000(	ENSG0000(	167.1763	0.25372	6.40E-05	0.000779	lncRNA
ENSG0000(	C1QA	564.0152	0.592375	6.40E-05	0.00078	protein_coding
ENSG0000(	LMBRD2	1045.874	-0.25799	6.42E-05	0.000781	protein_coding
ENSG0000(	MIA	52.49663	-0.42683	6.42E-05	0.000781	protein_coding
ENSG0000(	ENSG0000(	562.3109	0.172684	6.44E-05	0.000783	lncRNA
ENSG0000(	NME5	15.42498	0.752674	6.45E-05	0.000785	protein_coding
ENSG0000(	RN7SL713F	9.311892	0.762356	6.48E-05	0.000787	misc_RNA
ENSG0000(	CAMK1G	11.74822	1.268928	6.50E-05	0.000789	protein_coding
ENSG0000(	ROBO4	332.062	0.494542	6.50E-05	0.000789	protein_coding
ENSG0000(	ENSG0000(	9.796993	1.037216	6.51E-05	0.000791	TEC
ENSG0000(	HERPUD2-/	784.3088	0.128612	6.54E-05	0.000794	lncRNA
ENSG0000(	ENSG0000(	52.97369	0.399444	6.57E-05	0.000796	lncRNA
ENSG0000(	NAXE	547.0292	-0.2594	6.58E-05	0.000798	protein_coding
ENSG0000(	ANO8	396.9603	0.245828	6.59E-05	0.000798	protein_coding
ENSG0000(	ENSG0000(	274.047	0.549469	6.62E-05	0.000802	processed_pseudogene
ENSG0000(	LINC01132	17.0048	0.811274	6.63E-05	0.000803	lncRNA
ENSG0000(	SLC5A4-AS:	242.1312	-0.29898	6.64E-05	0.000803	lncRNA
ENSG0000(	CNPPD1	866.639	-0.3345	6.66E-05	0.000805	protein_coding
ENSG0000(	GDAP1L1	18.38238	0.598392	6.67E-05	0.000807	protein_coding
ENSG0000(	ENSG0000(	1107.546	0.225014	6.68E-05	0.000807	TEC
ENSG0000(	ENSG0000(	113.1326	-0.27856	6.69E-05	0.000809	lncRNA
ENSG0000(	CCDC13	284.2722	0.317738	6.71E-05	0.000811	protein_coding
ENSG0000(	H4C2	161.952	-0.40252	6.73E-05	0.000812	protein_coding
ENSG0000(	CCR2	230.0852	0.778175	6.75E-05	0.000815	protein_coding
ENSG0000(	IGLV2-23	308.1086	1.117892	6.77E-05	0.000816	IG_V_gene
ENSG0000(	CMKLR1	693.8531	0.508374	6.79E-05	0.000818	protein_coding
ENSG0000(	PDHB	1115.191	-0.16796	6.79E-05	0.000818	protein_coding
ENSG0000(	CCDC71L	639.4043	0.395143	6.79E-05	0.000818	protein_coding
ENSG0000(	LINC01737	8.364365	0.763051	6.80E-05	0.000818	lncRNA
ENSG0000(	LINC01913	148.6455	0.472198	6.79E-05	0.000818	lncRNA
ENSG0000(	OTOP2	43.27045	-1.35082	6.80E-05	0.000818	protein_coding
ENSG0000(	PTCSC1	41.91089	0.718784	6.80E-05	0.000818	lncRNA
ENSG0000(	ENSG0000(	59.60158	0.632559	6.81E-05	0.000819	lncRNA
ENSG0000(	ENSG0000(	12.40362	0.826282	6.82E-05	0.00082	lncRNA
ENSG0000(	RBM20	225.5656	0.446791	6.83E-05	0.000821	protein_coding
ENSG0000(	ENSG0000(	49.49638	0.465465	6.84E-05	0.000821	lncRNA
ENSG0000(	CTSE	491.9478	-0.6378	6.84E-05	0.000822	protein_coding
ENSG0000(	MIR635	8.643051	0.894466	6.85E-05	0.000822	miRNA
ENSG0000(	NDUFA7	306.3076	-0.17832	6.85E-05	0.000822	protein_coding
ENSG0000(	LINC00299	1078.166	0.229303	6.86E-05	0.000823	lncRNA
ENSG0000(	TBC1D16	1286.573	0.326134	6.87E-05	0.000824	protein_coding
ENSG0000(	ENSG0000(	100.8222	-0.27943	6.89E-05	0.000826	lncRNA
ENSG0000(	TATDN2P2	13.06127	0.901524	6.89E-05	0.000826	processed_pseudogene
ENSG0000(	MUC12-AS	66.97517	0.427896	6.90E-05	0.000827	lncRNA
ENSG0000(	CPEB1-AS1	829.1115	-0.17122	6.92E-05	0.000828	lncRNA

ENSG0000(	ENSG0000(	19.73141	0.68432	6.93E-05	0.000829	lncRNA
ENSG0000(	MIR3142H(	321.2486	0.681352	6.93E-05	0.000829	lncRNA
ENSG0000(	PRPF18	509.04	-0.23124	6.94E-05	0.00083	protein_coding
ENSG0000(	TRIM21	377.9911	0.349845	6.94E-05	0.00083	protein_coding
ENSG0000(	VPS4B	1530.268	-0.22788	6.94E-05	0.00083	protein_coding
ENSG0000(	RNU5E-6P	77.61072	1.088243	6.96E-05	0.000832	snRNA
ENSG0000(	LINC00663	441.5507	0.241958	6.96E-05	0.000832	lncRNA
ENSG0000(	ENSG0000(	177.2515	0.261157	6.97E-05	0.000832	processed_pseudogene
ENSG0000(	ACOX1	6191.452	-0.656	6.99E-05	0.000834	protein_coding
ENSG0000(	PLXNA4	304.6534	0.451428	7.00E-05	0.000835	protein_coding
ENSG0000(	PDGFB	208.802	0.546202	7.01E-05	0.000835	protein_coding
ENSG0000(	POLE	2953.648	-0.15711	7.02E-05	0.000837	protein_coding
ENSG0000(	LYPD6B	43.9175	0.958858	7.03E-05	0.000838	protein_coding
ENSG0000(	COPS5	2208.263	-0.14839	7.04E-05	0.000839	protein_coding
ENSG0000(	MAST4	2136.619	0.204626	7.05E-05	0.00084	protein_coding
ENSG0000(	SERPINH1	1066.035	0.466489	7.07E-05	0.000842	protein_coding
ENSG0000(	SLC39A4	791.6152	-0.63659	7.07E-05	0.000842	protein_coding
ENSG0000(	CYLD-AS1	224.8885	0.498728	7.08E-05	0.000842	lncRNA
ENSG0000(	ENSG0000(	10.80023	0.804548	7.10E-05	0.000844	lncRNA
ENSG0000(	PCBP2	8363.568	-0.16566	7.11E-05	0.000845	protein_coding
ENSG0000(	ENSG0000(	13.74285	0.901208	7.11E-05	0.000845	lncRNA
ENSG0000(	DISC1	1032.58	0.424207	7.12E-05	0.000845	protein_coding
ENSG0000(	ENSG0000(	57.63234	-1.40156	7.14E-05	0.000848	lncRNA
ENSG0000(	ENSG0000(	2141.344	0.228983	7.15E-05	0.000848	lncRNA
ENSG0000(	ZNF275	907.4549	0.395469	7.19E-05	0.000853	protein_coding
ENSG0000(	NT5C	278.0506	-0.28856	7.20E-05	0.000853	protein_coding
ENSG0000(	NAE1	847.9797	-0.12367	7.24E-05	0.000858	protein_coding
ENSG0000(	ENSG0000(	25.08815	0.524748	7.25E-05	0.000859	lncRNA
ENSG0000(	LAX1	662.7151	0.594366	7.26E-05	0.00086	protein_coding
ENSG0000(	NBN	1213.751	0.377129	7.26E-05	0.00086	protein_coding
ENSG0000(	PPFIA4	106.4179	0.624377	7.28E-05	0.000861	protein_coding
ENSG0000(	ENSG0000(	797.7967	0.19443	7.31E-05	0.000865	lncRNA
ENSG0000(	F2RL2	275.8357	0.366794	7.32E-05	0.000866	protein_coding
ENSG0000(	LYPLAL1	390.6087	-0.22129	7.33E-05	0.000866	protein_coding
ENSG0000(	SIX5	159.0863	0.445473	7.33E-05	0.000866	protein_coding
ENSG0000(	CBARP	10.82746	0.919082	7.34E-05	0.000867	protein_coding
ENSG0000(	POLE4	210.2285	-0.31982	7.34E-05	0.000867	protein_coding
ENSG0000(	RPS6P20	177.143	-0.25375	7.37E-05	0.00087	unprocessed_pseudogene
ENSG0000(	GPM6B	90.71496	0.799794	7.38E-05	0.000871	protein_coding
ENSG0000(	ENSG0000(	64.81768	0.436817	7.41E-05	0.000874	lncRNA
ENSG0000(	DPF1	168.391	0.249068	7.43E-05	0.000876	protein_coding
ENSG0000(	MYBPC3	37.2598	0.727278	7.44E-05	0.000876	protein_coding
ENSG0000(	P2RX6	51.74923	-0.38003	7.43E-05	0.000876	protein_coding
ENSG0000(	SNPH	184.8232	0.517808	7.44E-05	0.000876	protein_coding
ENSG0000(	COPZ2	62.156	0.584792	7.47E-05	0.000879	protein_coding
ENSG0000(	LRRTM4	328.8714	0.236798	7.48E-05	0.00088	protein_coding
ENSG0000(	VAC14	1475.769	0.281491	7.48E-05	0.00088	protein_coding

ENSG0000(	ENSG0000(	14.78725	0.762865	7.49E-05	0.00088	lncRNA
ENSG0000(	ENSG0000(	61.05447	0.432843	7.51E-05	0.000883	processed_pseudogene
ENSG0000(	ENSG0000(	324.4147	0.21729	7.51E-05	0.000883	lncRNA
ENSG0000(	GDF15	55.37695	0.696413	7.51E-05	0.000883	protein_coding
ENSG0000(	OR7E100P	29.1285	0.445849	7.54E-05	0.000885	unprocessed_pseudogene
ENSG0000(	ENSG0000(	109.5987	0.299667	7.59E-05	0.000891	lncRNA
ENSG0000(	SCG5	31.76147	0.558237	7.60E-05	0.000892	protein_coding
ENSG0000(	WDFY3	3838.05	0.302407	7.63E-05	0.000895	protein_coding
ENSG0000(	ASRGL1	440.8512	0.248814	7.65E-05	0.000897	protein_coding
ENSG0000(	PALLD	1833.374	0.24434	7.65E-05	0.000897	protein_coding
ENSG0000(	ENSG0000(	63.50443	0.522503	7.66E-05	0.000897	transcribed_processed_pseudoge
ENSG0000(	BCAR3	672.2765	-0.28069	7.68E-05	0.0009	protein_coding
ENSG0000(	ENSG0000(	31.15947	0.449752	7.70E-05	0.000901	lncRNA
ENSG0000(	MMP26	32.86522	-0.4214	7.70E-05	0.000901	protein_coding
ENSG0000(	ENSG0000(	446.6283	0.193141	7.71E-05	0.000902	lncRNA
ENSG0000(	CTR9	793.8276	-0.22198	7.73E-05	0.000904	protein_coding
ENSG0000(	NBPF25P	921.2885	0.278501	7.74E-05	0.000904	transcribed_unprocessed_pseudo
ENSG0000(	RTL8C	239.4539	0.342229	7.74E-05	0.000905	protein_coding
ENSG0000(	CNOT1	8052.751	-0.15722	7.75E-05	0.000905	protein_coding
ENSG0000(	RPS14	3410.783	-0.38842	7.75E-05	0.000905	protein_coding
ENSG0000(	SLC30A7	2428.122	0.185714	7.75E-05	0.000905	protein_coding
ENSG0000(	MNT	1762.69	0.162978	7.78E-05	0.000907	protein_coding
ENSG0000(	ZDHHC14	1234.476	-0.25473	7.79E-05	0.000908	protein_coding
ENSG0000(	NKX1-2	17.28062	-1.40153	7.79E-05	0.000908	protein_coding
ENSG0000(	VPS35	3225.21	-0.372	7.79E-05	0.000908	protein_coding
ENSG0000(	TMEM116	1637.979	-0.19052	7.80E-05	0.000908	protein_coding
ENSG0000(	IGKV1-27	120.2843	1.151658	7.81E-05	0.00091	IG_V_gene
ENSG0000(	ENSG0000(	29.41242	0.481007	7.82E-05	0.000911	lncRNA
ENSG0000(	SLC25A3	4211.31	-0.28421	7.83E-05	0.000912	protein_coding
ENSG0000(	TMEM263	1087.619	0.206225	7.83E-05	0.000912	protein_coding
ENSG0000(	MIR4500H(	488.0296	0.262663	7.85E-05	0.000912	lncRNA
ENSG0000(	IGKJ2	14.23868	1.250621	7.85E-05	0.000913	IG_J_gene
ENSG0000(	LENG8-AS1	908.8226	-0.1738	7.85E-05	0.000913	lncRNA
ENSG0000(	PRDM10	717.4327	-0.28417	7.86E-05	0.000913	protein_coding
ENSG0000(	ENSG0000(	705.156	-0.14685	7.93E-05	0.000921	lncRNA
ENSG0000(	PDE6A	1064.362	0.184863	7.99E-05	0.000927	protein_coding
ENSG0000(	CNIH3	178.3065	0.213592	7.99E-05	0.000928	protein_coding
ENSG0000(	EZR	6225.038	-0.56955	8.00E-05	0.000928	protein_coding
ENSG0000(	ENSG0000(	9.388695	0.883235	8.05E-05	0.000933	lncRNA
ENSG0000(	LINC01291	20.79067	1.548222	8.06E-05	0.000934	lncRNA
ENSG0000(	SMIM33	46.86808	0.805176	8.06E-05	0.000934	protein_coding
ENSG0000(	EPSTI1	1825.899	0.464813	8.15E-05	0.000944	protein_coding
ENSG0000(	ENSG0000(	542.1098	0.152804	8.15E-05	0.000944	lncRNA
ENSG0000(	CD300LG	91.87993	-0.41564	8.17E-05	0.000946	protein_coding
ENSG0000(	SSR3	2305.795	0.268486	8.21E-05	0.000949	protein_coding
ENSG0000(	DIRC3	343.8319	0.460792	8.22E-05	0.00095	lncRNA
ENSG0000(	MGAT3	952.3201	-0.59969	8.23E-05	0.000952	protein_coding

ENSG0000(	SLC10A7	1116.205	0.19796	8.24E-05	0.000952	protein_coding
ENSG0000(	KCNJ6	977.4529	0.278558	8.24E-05	0.000952	protein_coding
ENSG0000(	UBTF	1831.919	-0.20718	8.25E-05	0.000953	protein_coding
ENSG0000(	MRPL48	719.838	-0.18444	8.27E-05	0.000955	protein_coding
ENSG0000(	PGM2L1	899.9665	0.283164	8.29E-05	0.000956	protein_coding
ENSG0000(	SIPA1L3	4512.551	-0.24145	8.30E-05	0.000957	protein_coding
ENSG0000(	GARS1	2792.397	0.171429	8.31E-05	0.000959	protein_coding
ENSG0000(	ENSG0000(	8.172987	0.987414	8.36E-05	0.000964	lncRNA
ENSG0000(	RN7SL752F	187.8139	0.505467	8.37E-05	0.000965	misc_RNA
ENSG0000(	CSNK1G3	1470.241	-0.27895	8.38E-05	0.000965	protein_coding
ENSG0000(	SH3BP1	994.9767	-0.2896	8.38E-05	0.000965	protein_coding
ENSG0000(	ENSG0000(	10.12474	1.958387	8.39E-05	0.000966	lncRNA
ENSG0000(	DSC2	3831.278	-0.6983	8.41E-05	0.000968	protein_coding
ENSG0000(	PAM	960.7043	0.370236	8.43E-05	0.000969	protein_coding
ENSG0000(	ENSG0000(	34.09755	0.927498	8.44E-05	0.00097	lncRNA
ENSG0000(	ENSG0000(	39.0182	0.456397	8.46E-05	0.000973	lncRNA
ENSG0000(	APOA1	3095.199	-1.6579	8.47E-05	0.000973	protein_coding
ENSG0000(	PKP4-AS1	11.43957	0.876132	8.48E-05	0.000974	lncRNA
ENSG0000(	ENSG0000(	53.47256	0.358446	8.49E-05	0.000975	lncRNA
ENSG0000(	CD99	1512.793	-0.19446	8.59E-05	0.000985	protein_coding
ENSG0000(	SRSF5	5644.156	-0.18705	8.59E-05	0.000985	protein_coding
ENSG0000(	CRYZ	435.9885	-0.45915	8.61E-05	0.000987	protein_coding
ENSG0000(	CLDND1	1503.831	-0.20063	8.62E-05	0.000988	protein_coding
ENSG0000(	ZNF674-AS	337.717	-0.2933	8.64E-05	0.00099	lncRNA
ENSG0000(	SLC11A1	741.0588	0.519489	8.66E-05	0.000992	protein_coding
ENSG0000(	SLC16A2	206.3693	0.368928	8.66E-05	0.000992	protein_coding
ENSG0000(	UQCRH	741.0839	-0.2892	8.66E-05	0.000992	protein_coding
ENSG0000(	AEBP1	1155.76	0.640717	8.68E-05	0.000993	protein_coding
ENSG0000(	PHF10	1780.491	-0.17909	8.69E-05	0.000994	protein_coding
ENSG0000(	MAP1LC3A	236.8229	-0.38348	8.73E-05	0.000999	protein_coding
ENSG0000(	SINHCAFP3	390.4493	0.445422	8.74E-05	0.000999	processed_pseudogene
ENSG0000(	ASIC3	37.21565	-0.5225	8.78E-05	0.001002	protein_coding
ENSG0000(	ENSG0000(	199.6573	0.245726	8.77E-05	0.001002	lncRNA
ENSG0000(	LRRC41	1958.275	0.120735	8.77E-05	0.001002	protein_coding
ENSG0000(	ZFP62	1510.756	-0.15214	8.78E-05	0.001002	protein_coding
ENSG0000(	GJB7	27.90126	0.573814	8.80E-05	0.001004	protein_coding
ENSG0000(	TRAK2	1899.617	-0.24178	8.82E-05	0.001007	protein_coding
ENSG0000(	ENSG0000(	784.4876	0.179892	8.83E-05	0.001007	lncRNA
ENSG0000(	CCDC28A	217.5583	-0.38481	8.84E-05	0.001007	protein_coding
ENSG0000(	TMEM184E	1175.304	0.300901	8.84E-05	0.001007	protein_coding
ENSG0000(	SAT2	717.5877	-0.67858	8.84E-05	0.001007	protein_coding
ENSG0000(	ENSG0000(	352.8106	0.159472	8.86E-05	0.001009	unprocessed_pseudogene
ENSG0000(	CHST12	3848.284	0.171325	8.87E-05	0.00101	protein_coding
ENSG0000(	UBQLN1	2532.156	-0.14511	8.87E-05	0.00101	protein_coding
ENSG0000(	P2RY14	211.5148	0.592253	8.89E-05	0.001011	protein_coding
ENSG0000(	PGBD5	233.3062	0.223873	8.89E-05	0.001011	protein_coding
ENSG0000(	C17orf107	61.96056	0.578123	8.90E-05	0.001011	protein_coding

ENSG0000(	PLIN2	1519.675	-0.67542	8.91E-05	0.001012	protein_coding
ENSG0000(	IGHG1	6802.478	1.418869	8.92E-05	0.001013	IG_C_gene
ENSG0000(	RAI1	1418.018	0.225545	8.92E-05	0.001013	protein_coding
ENSG0000(	LINC01012	656.46	-0.14698	9.00E-05	0.001022	lncRNA
ENSG0000(	ENOX1	241.2527	0.492522	9.02E-05	0.001023	protein_coding
ENSG0000(	ENSG0000(	22.72647	0.75301	9.02E-05	0.001023	TEC
ENSG0000(	MEIS3	171.8513	0.433902	9.08E-05	0.00103	protein_coding
ENSG0000(	TTTY8B	11.62224	0.810109	9.11E-05	0.001033	lncRNA
ENSG0000(	APC2	29.30182	0.573813	9.12E-05	0.001033	protein_coding
ENSG0000(	ENSG0000(	36.77088	0.443928	9.13E-05	0.001034	lncRNA
ENSG0000(	NFE2L1	4431.64	-0.34586	9.14E-05	0.001035	protein_coding
ENSG0000(	LINC01320	450.0414	0.213303	9.17E-05	0.001038	lncRNA
ENSG0000(	MRC2	721.7726	0.562455	9.21E-05	0.001042	protein_coding
ENSG0000(	KCNS2	24.51279	0.947111	9.23E-05	0.001044	protein_coding
ENSG0000(	ENSG0000(	99.74336	0.513499	9.25E-05	0.001046	unprocessed_pseudogene
ENSG0000(	IGSF3	1252.779	-0.79837	9.27E-05	0.001048	protein_coding
ENSG0000(	ENSG0000(	48.57622	0.350428	9.33E-05	0.001054	lncRNA
ENSG0000(	CLASRP	939.3572	-0.15998	9.34E-05	0.001055	protein_coding
ENSG0000(	ENSG0000(	26.81305	-1.20868	9.35E-05	0.001055	transcribed_unprocessed_pseudo
ENSG0000(	GNA15-DT	12.81219	0.89792	9.35E-05	0.001055	lncRNA
ENSG0000(	TMEM131	4324.978	-0.18082	9.36E-05	0.001056	protein_coding
ENSG0000(	EEA1	1484.832	-0.27108	9.37E-05	0.001056	protein_coding
ENSG0000(	ENSG0000(	17.52879	1.105865	9.37E-05	0.001056	processed_pseudogene
ENSG0000(	LINC01643	22.30229	0.664222	9.39E-05	0.001058	lncRNA
ENSG0000(	NR4A2	180.5686	0.358082	9.40E-05	0.001059	protein_coding
ENSG0000(	C1S	2845.77	0.452451	9.41E-05	0.001059	protein_coding
ENSG0000(	ENSG0000(	10.32869	0.922842	9.44E-05	0.001062	lncRNA
ENSG0000(	RNF139	571.3603	-0.2256	9.49E-05	0.001067	protein_coding
ENSG0000(	S100A11	240.9907	0.637013	9.51E-05	0.00107	protein_coding
ENSG0000(	TESK1	495.9132	-0.22396	9.52E-05	0.00107	protein_coding
ENSG0000(	PTENP1-AS	127.9255	0.294041	9.53E-05	0.001072	lncRNA
ENSG0000(	ENSG0000(	50.62669	0.600101	9.57E-05	0.001075	lncRNA
ENSG0000(	KLF3-AS1	1265.246	0.171343	9.57E-05	0.001075	lncRNA
ENSG0000(	ZNF562	4026.78	0.15991	9.57E-05	0.001075	protein_coding
ENSG0000(	ENSG0000(	18.65945	0.64166	9.58E-05	0.001076	lncRNA
ENSG0000(	MTG2	1467.426	0.127651	9.59E-05	0.001076	protein_coding
ENSG0000(	SGF29	204.2663	-0.26509	9.60E-05	0.001077	protein_coding
ENSG0000(	ENSG0000(	381.5834	0.379487	9.62E-05	0.001078	lncRNA
ENSG0000(	ENSG0000(	250.2152	0.405744	9.62E-05	0.001079	lncRNA
ENSG0000(	SPRYD4	2146.893	0.114141	9.67E-05	0.001083	protein_coding
ENSG0000(	EVC	709.5129	0.432747	9.68E-05	0.001084	protein_coding
ENSG0000(	ENSG0000(	631.6963	0.383298	9.70E-05	0.001086	protein_coding
ENSG0000(	ATRN	2408.796	-0.41114	9.71E-05	0.001087	protein_coding
ENSG0000(	ENSG0000(	22.86741	0.617748	9.71E-05	0.001087	lncRNA
ENSG0000(	ARMCX3	900.3625	0.230234	9.75E-05	0.00109	protein_coding
ENSG0000(	SLC25A22	413.2331	-0.27703	9.75E-05	0.00109	protein_coding
ENSG0000(	SEZ6	26.68062	0.818855	9.75E-05	0.00109	protein_coding

ENSG0000(	SNRNP48	737.0237	-0.19798	9.76E-05	0.00109	protein_coding
ENSG0000(	ENSG0000(	16.82431	0.617563	9.76E-05	0.00109	lncRNA
ENSG0000(	RDH5	426.0616	-0.28103	9.77E-05	0.001091	protein_coding
ENSG0000(	CLVS1	145.6285	-0.26685	9.78E-05	0.001091	protein_coding
ENSG0000(	SLFN11	1304.956	0.590086	9.79E-05	0.001092	protein_coding
ENSG0000(	ENSG0000(	509.26	0.235634	9.81E-05	0.001094	lncRNA
ENSG0000(	ENSG0000(	76.06668	0.672445	9.81E-05	0.001094	TEC
ENSG0000(	ENSG0000(	187.0883	0.378962	9.85E-05	0.001098	lncRNA
ENSG0000(	ALAD	1158.865	-0.31833	9.86E-05	0.001099	protein_coding
ENSG0000(	SMARCD2	892.9319	-0.17321	9.88E-05	0.0011	protein_coding
ENSG0000(	ENSG0000(	159.371	0.232868	9.89E-05	0.001101	processed_pseudogene
ENSG0000(	ENSG0000(	61.12495	0.351654	9.90E-05	0.001102	lncRNA
ENSG0000(	C1RL	1444.055	0.244946	9.92E-05	0.001104	protein_coding
ENSG0000(	ENSG0000(	154.5804	0.27644	9.92E-05	0.001104	TEC
ENSG0000(	N4BP2	1980.342	0.228202	9.96E-05	0.001108	protein_coding
ENSG0000(	ENSG0000(	12.87255	1.00601	9.98E-05	0.001109	TEC
ENSG0000(	ENSG0000(	89.05568	-0.37301	0.0001	0.001112	lncRNA
ENSG0000(	HSD17B11	2232.936	-0.64467	0.0001	0.001112	protein_coding
ENSG0000(	PROX1-AS1	668.6959	0.275742	0.0001	0.001112	lncRNA
ENSG0000(	SNTG2-AS1	16.06049	-1.06152	0.0001	0.001112	lncRNA
ENSG0000(	TLE3	2993.451	-0.21767	0.0001	0.001112	protein_coding
ENSG0000(	MAP3K2	5346.767	-0.27471	0.0001	0.001113	protein_coding
ENSG0000(	GIN1	391.6435	-0.21631	0.000101	0.001115	protein_coding
ENSG0000(	SCP2	2404.726	-0.60366	0.000101	0.001118	protein_coding
ENSG0000(	HSPA9	2428.253	-0.25218	0.000101	0.001118	protein_coding
ENSG0000(	ENSG0000(	73.3084	0.523569	0.000101	0.001121	lncRNA
ENSG0000(	ENSG0000(	139.996	0.256338	0.000101	0.001121	lncRNA
ENSG0000(	NDUFA8	111.6584	-0.4434	0.000101	0.001121	protein_coding
ENSG0000(	AKIRIN2	639.8529	0.294539	0.000101	0.001123	protein_coding
ENSG0000(	ACSF2	1334.088	-0.68575	0.000102	0.001124	protein_coding
ENSG0000(	IGKV1-8	17.7802	1.341159	0.000102	0.001124	IG_V_gene
ENSG0000(	STT3B	3327.394	-0.1422	0.000102	0.001124	protein_coding
ENSG0000(	RPL37A	2955.403	-0.38603	0.000102	0.001125	protein_coding
ENSG0000(	TOR3A	864.1126	0.192953	0.000102	0.001125	protein_coding
ENSG0000(	SRGAP2	2710.916	0.301776	0.000102	0.001127	protein_coding
ENSG0000(	CCL11	162.2207	0.859534	0.000102	0.001127	protein_coding
ENSG0000(	MS4A4E	1363.081	0.203882	0.000103	0.001133	protein_coding
ENSG0000(	ENSG0000(	468.834	0.267802	0.000103	0.001133	lncRNA
ENSG0000(	ENSG0000(	3710.576	0.635226	0.000103	0.001135	lncRNA
ENSG0000(	MYL3	215.6759	0.20651	0.000103	0.001135	protein_coding
ENSG0000(	ENSG0000(	154.0544	0.239922	0.000103	0.001137	lncRNA
ENSG0000(	HOXC11	20.6511	-1.32084	0.000103	0.001137	protein_coding
ENSG0000(	SNORD110	23.62265	0.77463	0.000103	0.001137	snoRNA
ENSG0000(	ENSG0000(	100.7823	0.998071	0.000104	0.001139	protein_coding
ENSG0000(	VPS28	998.4049	-0.24396	0.000104	0.00114	protein_coding
ENSG0000(	TMEM98	1403.498	-0.4066	0.000104	0.001142	protein_coding
ENSG0000(	ENSG0000(	58.59128	0.35735	0.000104	0.001147	TEC

ENSG0000(	RPS6KB2	987.9282	-0.19935	0.000105	0.001149	protein_coding
ENSG0000(	NCK2	1036.978	-0.23761	0.000105	0.00115	protein_coding
ENSG0000(	LINC01150	48.10165	0.423539	0.000105	0.001155	lncRNA
ENSG0000(	TSNAXIP1	22.69231	0.689835	0.000106	0.001164	protein_coding
ENSG0000(	ST8SIA5-DT	196.0294	-0.24771	0.000106	0.001166	lncRNA
ENSG0000(	SSBP3-AS1	18.24402	0.665086	0.000107	0.001172	lncRNA
ENSG0000(	REM2	25.19697	0.642211	0.000107	0.001172	protein_coding
ENSG0000(	LIPA	1883.119	-0.54834	0.000107	0.001176	protein_coding
ENSG0000(	ENSG0000(	47.21015	0.430647	0.000107	0.001178	lncRNA
ENSG0000(	ENSG0000(	21.88899	0.543683	0.000108	0.001178	TEC
ENSG0000(	ATG4D	551.312	-0.5285	0.000108	0.00118	protein_coding
ENSG0000(	ENSG0000(	42.67609	-1.53491	0.000108	0.00118	lncRNA
ENSG0000(	BMP2K-DT	69.70834	0.350414	0.000108	0.00118	lncRNA
ENSG0000(	MEFV	727.1895	0.406838	0.000108	0.001181	protein_coding
ENSG0000(	ENSG0000(	57.34042	0.32209	0.000109	0.001187	lncRNA
ENSG0000(	IGHV3-43	101.543	1.141324	0.000109	0.00119	IG_V_gene
ENSG0000(	ENSG0000(	229.9314	-0.3345	0.000109	0.001191	lncRNA
ENSG0000(	DDX27	1234.344	-0.21682	0.00011	0.001196	protein_coding
ENSG0000(	ENSG0000(	35.62675	0.401325	0.00011	0.001205	processed_pseudogene
ENSG0000(	C11orf45	31.76281	0.462944	0.00011	0.001205	protein_coding
ENSG0000(	TERF1	2646.143	0.128588	0.00011	0.001205	protein_coding
ENSG0000(	ENSG0000(	37.65299	0.764158	0.000111	0.001208	lncRNA
ENSG0000(	SCARNA3	68.87287	0.339843	0.000111	0.001211	scaRNA
ENSG0000(	ENSG0000(	12.5545	0.863338	0.000111	0.001212	lncRNA
ENSG0000(	MRPL37	350.3972	-0.24533	0.000112	0.001216	protein_coding
ENSG0000(	ENSG0000(	28.56121	0.652537	0.000112	0.001219	lncRNA
ENSG0000(	NINJ2-AS1	321.5189	0.360439	0.000112	0.001219	lncRNA
ENSG0000(	CFAP298	524.5097	-0.17727	0.000112	0.001219	protein_coding
ENSG0000(	CTCFL	585.4267	0.142275	0.000112	0.001219	protein_coding
ENSG0000(	FAN1	3290.093	0.163264	0.000112	0.001219	protein_coding
ENSG0000(	FRMD6	363.655	0.454837	0.000112	0.001219	protein_coding
ENSG0000(	NDEL1	2096.572	0.178176	0.000112	0.001219	protein_coding
ENSG0000(	TSHR	790.3865	0.184827	0.000112	0.001219	protein_coding
ENSG0000(	TSPAN9-IT1	18.08899	0.6775	0.000112	0.001219	lncRNA
ENSG0000(	ENSG0000(	13.90873	0.692774	0.000113	0.001223	TEC
ENSG0000(	SNORD89	345.2486	0.409621	0.000113	0.001228	snoRNA
ENSG0000(	SLC25A21	223.8648	-0.19839	0.000113	0.001231	protein_coding
ENSG0000(	HSP90AA1	4349.217	-0.28682	0.000114	0.001234	protein_coding
ENSG0000(	ACBD5	2697.668	-0.19345	0.000114	0.001235	protein_coding
ENSG0000(	ELL	794.2626	0.270596	0.000114	0.001235	protein_coding
ENSG0000(	CHMP4B	944.8038	-0.46355	0.000114	0.001236	protein_coding
ENSG0000(	FZD10-AS1	343.773	0.385633	0.000114	0.001236	lncRNA
ENSG0000(	HYI	678.2808	-0.46734	0.000114	0.001236	protein_coding
ENSG0000(	TMEM19	1675.786	-0.1665	0.000114	0.001239	protein_coding
ENSG0000(	SNORA12	252.3225	-0.38027	0.000115	0.001243	snoRNA
ENSG0000(	ENSG0000(	253.0228	0.168792	0.000115	0.001243	TEC
ENSG0000(	TLL1	394.0764	-0.53543	0.000115	0.001246	protein_coding

ENSG0000(	BFSP1	16.50523	0.706247	0.000115	0.001247	protein_coding
ENSG0000(	SCN1B	608.669	0.336387	0.000116	0.001249	protein_coding
ENSG0000(	CMAHP	948.726	0.382103	0.000116	0.001249	transcribed_unitary_pseudogene
ENSG0000(	FRMD6-AS:	269.7612	0.1895	0.000116	0.001249	lncRNA
ENSG0000(	IGLV1-40	175.612	1.135811	0.000116	0.00125	IG_V_gene
ENSG0000(	ENSG0000(	136.0252	0.308636	0.000116	0.00125	lncRNA
ENSG0000(	CAVIN4	75.42664	0.344695	0.000116	0.001251	protein_coding
ENSG0000(	SYNJ2	2370.279	-0.13551	0.000116	0.001251	protein_coding
ENSG0000(	POLR2J	114.4715	-0.4183	0.000116	0.001252	protein_coding
ENSG0000(	LDB2	466.5566	0.425595	0.000116	0.001252	protein_coding
ENSG0000(	C1QB	493.6021	0.598174	0.000116	0.001252	protein_coding
ENSG0000(	MCFD2	4410.921	0.104301	0.000116	0.001252	protein_coding
ENSG0000(	SPART	597.8477	0.415405	0.000118	0.001266	protein_coding
ENSG0000(	COMMD1	222.8587	-0.27534	0.000118	0.001269	protein_coding
ENSG0000(	MUCL1	113.0059	0.283646	0.000118	0.001272	protein_coding
ENSG0000(	RNF181	207.8574	-0.29677	0.000118	0.001272	protein_coding
ENSG0000(	RRBP1	7673.022	0.352901	0.000118	0.001273	protein_coding
ENSG0000(	IL15	1174.641	0.215453	0.000119	0.001276	protein_coding
ENSG0000(	PARVA	2338.626	0.210481	0.000119	0.001276	protein_coding
ENSG0000(	RPS21	805.3936	-0.41129	0.000119	0.001276	protein_coding
ENSG0000(	SRGN	681.9425	0.841023	0.000119	0.001276	protein_coding
ENSG0000(	NAMA	12.00698	0.889653	0.000119	0.001277	lncRNA
ENSG0000(	SNX12	783.7359	-0.3922	0.000119	0.001277	protein_coding
ENSG0000(	MTRNR2L1	12.24283	0.817954	0.000119	0.001278	protein_coding
ENSG0000(	ENSG0000(	290.4036	0.314331	0.00012	0.001285	processed_pseudogene
ENSG0000(	ACSM1	19.24828	0.692255	0.00012	0.001286	protein_coding
ENSG0000(	ENSG0000(	550.8049	-0.19308	0.00012	0.001289	lncRNA
ENSG0000(	NPTX2	22.35368	1.110719	0.00012	0.001289	protein_coding
ENSG0000(	ENSG0000(	336.1323	-0.16907	0.000121	0.001293	lncRNA
ENSG0000(	PATL2	601.0087	0.443752	0.000121	0.001293	protein_coding
ENSG0000(	IGLV7-43	50.3803	1.353563	0.000121	0.001293	IG_V_gene
ENSG0000(	TAFA2	252.3815	0.392733	0.000121	0.001293	protein_coding
ENSG0000(	TBX19	261.4393	0.289363	0.000121	0.001294	protein_coding
ENSG0000(	FBXL8	294.6645	0.207348	0.000121	0.001294	protein_coding
ENSG0000(	DMBT1	19328.07	1.193519	0.000122	0.001302	protein_coding
ENSG0000(	ENSG0000(	63.14505	1.272486	0.000122	0.001303	lncRNA
ENSG0000(	TMEM50A	918.8018	-0.28159	0.000122	0.001305	protein_coding
ENSG0000(	ENSG0000(	13.70712	0.766789	0.000123	0.00131	lncRNA
ENSG0000(	IGKV4-1	635.1189	1.065225	0.000123	0.001311	IG_V_gene
ENSG0000(	IGKV1-9	177.4575	1.212318	0.000123	0.001312	IG_V_gene
ENSG0000(	MTCL1	506.4899	0.367746	0.000123	0.001312	protein_coding
ENSG0000(	RNU6-5P	1102.832	0.430056	0.000123	0.001312	snRNA
ENSG0000(	SPOCK1	25.2217	1.282798	0.000123	0.001312	protein_coding
ENSG0000(	CYP4X1	28.03568	0.808094	0.000124	0.001316	protein_coding
ENSG0000(	CDR2	664.8999	-0.30796	0.000124	0.001317	protein_coding
ENSG0000(	ENSG0000(	96.5758	0.399774	0.000124	0.00132	lncRNA
ENSG0000(	SREBF1	1746.21	-0.39771	0.000124	0.00132	protein_coding

ENSG0000(	MSX2	28.92528	1.022044	0.000124	0.00132	protein_coding
ENSG0000(	COL11A2	72.08222	0.711552	0.000124	0.001322	protein_coding
ENSG0000(	ENSG0000(	129.9338	0.26862	0.000125	0.001333	TEC
ENSG0000(	ENSG0000(	700.3852	0.312229	0.000126	0.001335	transcribed_unprocessed_pseudo
ENSG0000(	ENSG0000(	12.92167	0.774766	0.000126	0.001336	TEC
ENSG0000(	ZC3H14	3432.268	-0.20049	0.000126	0.001338	protein_coding
ENSG0000(	ENSG0000(	10.94131	0.907711	0.000126	0.00134	lncRNA
ENSG0000(	IGHV4-34	167.9613	1.049803	0.000127	0.001346	IG_V_gene
ENSG0000(	NFAT5	9414.604	0.11895	0.000127	0.001346	protein_coding
ENSG0000(	PCED1A	645.4839	0.324574	0.000127	0.001346	protein_coding
ENSG0000(	RN7SKP118	110.674	-0.71961	0.000127	0.001346	misc_RNA
ENSG0000(	DUOXA1	32.15022	0.821097	0.000127	0.001348	protein_coding
ENSG0000(	HSPD1	2524.687	-0.27501	0.000127	0.001348	protein_coding
ENSG0000(	ENSG0000(	134.0968	0.238767	0.000127	0.001348	TEC
ENSG0000(	KLHDC3	493.5303	-0.25414	0.000128	0.001355	protein_coding
ENSG0000(	SNTB2	2323.642	0.163335	0.000128	0.001355	protein_coding
ENSG0000(	CCNYL6	45.09193	0.372719	0.000128	0.001355	transcribed_processed_pseudoge
ENSG0000(	ENSG0000(	265.1714	-0.26705	0.000128	0.001355	lncRNA
ENSG0000(	ZEB2	8957.628	0.430863	0.000128	0.001357	protein_coding
ENSG0000(	ANKRD36C	5377.328	0.280364	0.000129	0.001362	protein_coding
ENSG0000(	ENSG0000(	180.3511	0.238997	0.000129	0.001366	lncRNA
ENSG0000(	ENSG0000(	15.73873	0.793449	0.000129	0.001366	TEC
ENSG0000(	ZFR	1903.459	-0.148	0.000129	0.001366	protein_coding
ENSG0000(	ZSWIM7	486.173	-0.18271	0.000129	0.001366	protein_coding
ENSG0000(	GOLIM4	2688.628	-0.58651	0.00013	0.001367	protein_coding
ENSG0000(	ENSG0000(	60.93716	0.302535	0.00013	0.001368	processed_pseudogene
ENSG0000(	KHSRP	1645.691	-0.24106	0.00013	0.001374	protein_coding
ENSG0000(	TOX4P1	32.56792	0.554347	0.00013	0.001374	processed_pseudogene
ENSG0000(	EMILIN3	26.04916	0.668717	0.00013	0.001375	protein_coding
ENSG0000(	ENSG0000(	54.50538	0.370813	0.000131	0.001377	lncRNA
ENSG0000(	SLC51B	276.1723	-0.92752	0.000131	0.001377	protein_coding
ENSG0000(	DDIT4	659.5896	0.615814	0.000131	0.001382	protein_coding
ENSG0000(	WDFY1	2331.269	0.295253	0.000131	0.001382	protein_coding
ENSG0000(	FAM102A	3762.875	-0.34805	0.000131	0.001383	protein_coding
ENSG0000(	SDR42E1	1402.921	-0.43244	0.000132	0.001385	protein_coding
ENSG0000(	CACNA1C	1260.778	0.47953	0.000132	0.001388	protein_coding
ENSG0000(	ENSG0000(	85.34988	0.357116	0.000132	0.001388	lncRNA
ENSG0000(	NT5DC2	162.5942	0.629079	0.000132	0.00139	protein_coding
ENSG0000(	DHPS	605.5652	-0.22785	0.000132	0.00139	protein_coding
ENSG0000(	FUCA1	999.4495	-0.36774	0.000132	0.00139	protein_coding
ENSG0000(	SNW1	591.3896	-0.25536	0.000132	0.00139	protein_coding
ENSG0000(	CSF2RB	1522.697	0.871254	0.000133	0.001395	protein_coding
ENSG0000(	ENSG0000(	131.4082	-0.33863	0.000133	0.001395	pseudogene
ENSG0000(	CDH13	400.9413	0.293425	0.000133	0.001398	protein_coding
ENSG0000(	ENSG0000(	185.1163	-0.23514	0.000133	0.001398	lncRNA
ENSG0000(	HOXC-AS2	10.48822	0.984512	0.000133	0.001398	lncRNA
ENSG0000(	MIR302CH(	13.31448	1.011054	0.000133	0.001398	lncRNA

ENSG0000(	NORAD	7296.242	-0.16297	0.000133	0.001398	lncRNA
ENSG0000(	PEBP1	2383.403	-0.56221	0.000134	0.001399	protein_coding
ENSG0000(	PDGFRA	3273.475	0.405043	0.000134	0.0014	protein_coding
ENSG0000(	ARHGEF17-	11.4658	0.80298	0.000134	0.001405	lncRNA
ENSG0000(	CBR1	1191.565	-0.8023	0.000134	0.001407	protein_coding
ENSG0000(	ENSG0000(	256.5729	-0.19524	0.000135	0.001407	lncRNA
ENSG0000(	GHITM	1963.64	-0.38932	0.000135	0.001407	protein_coding
ENSG0000(	ENSG0000(	27.66348	0.553	0.000135	0.001411	lncRNA
ENSG0000(	UXT	195.4501	-0.30274	0.000135	0.001415	protein_coding
ENSG0000(	ERAP2	3198.612	0.347946	0.000136	0.001416	protein_coding
ENSG0000(	FA2H	277.8359	-0.48896	0.000136	0.001417	protein_coding
ENSG0000(	RNY4P18	8.899714	0.991928	0.000136	0.001418	misc_RNA
ENSG0000(	GLUD1	4974.718	-0.2358	0.000136	0.001418	protein_coding
ENSG0000(	BGLAP	30.83694	-0.69401	0.000136	0.001418	protein_coding
ENSG0000(	FAM3A	1033.268	-0.31143	0.000136	0.001418	protein_coding
ENSG0000(	HSDL2	751.0029	-0.37948	0.000136	0.001419	protein_coding
ENSG0000(	FAM3C	1724.008	-0.45917	0.000136	0.001419	protein_coding
ENSG0000(	ENSG0000(	803.3205	0.168984	0.000137	0.001424	lncRNA
ENSG0000(	PPIAP86	11.2109	0.765807	0.000137	0.001424	processed_pseudogene
ENSG0000(	ENSG0000(	16.94529	0.594601	0.000137	0.001424	snoRNA
ENSG0000(	CARD6	530.2833	0.254167	0.000137	0.001426	protein_coding
ENSG0000(	HMGB3P9	152.1196	0.307615	0.000138	0.001434	processed_pseudogene
ENSG0000(	LINC01235	150.1766	0.365647	0.000138	0.001439	lncRNA
ENSG0000(	FARSA	280.766	-0.30657	0.000139	0.001443	protein_coding
ENSG0000(	LINC01943	54.3921	1.012604	0.000139	0.001443	lncRNA
ENSG0000(	ENSG0000(	109.7672	0.267146	0.000139	0.001445	lncRNA
ENSG0000(	ENSG0000(	21.59948	0.505327	0.000139	0.001447	lncRNA
ENSG0000(	RAI14	939.7661	0.476006	0.000139	0.001448	protein_coding
ENSG0000(	CD33	319.4351	0.366888	0.00014	0.00145	protein_coding
ENSG0000(	RPS13	1602.653	-0.32578	0.00014	0.001453	protein_coding
ENSG0000(	CYS1	64.8855	-0.64345	0.00014	0.001454	protein_coding
ENSG0000(	ECSCR	57.39662	0.656498	0.00014	0.001455	protein_coding
ENSG0000(	LINC02688	61.62822	0.668489	0.00014	0.001456	lncRNA
ENSG0000(	REX1BD	784.0858	-0.15236	0.000141	0.001456	protein_coding
ENSG0000(	SNHG12	877.4965	-0.16527	0.000141	0.001456	lncRNA
ENSG0000(	HAS2	398.5478	0.408561	0.000141	0.00146	protein_coding
ENSG0000(	BTBD19	766.9121	0.358691	0.000141	0.001461	protein_coding
ENSG0000(	IQCK	331.39	0.186133	0.000141	0.001461	protein_coding
ENSG0000(	CDHR1	2863.153	-1.09355	0.000141	0.001463	protein_coding
ENSG0000(	LINC00648	1509.175	0.326073	0.000141	0.001463	lncRNA
ENSG0000(	ENSG0000(	48.45335	0.392894	0.000142	0.001464	lncRNA
ENSG0000(	FAM234A	1651.954	-0.37827	0.000142	0.001464	protein_coding
ENSG0000(	IGHG4	1297.413	1.521191	0.000142	0.001464	IG_C_gene
ENSG0000(	MBTD1	1614.687	-0.13514	0.000142	0.001464	protein_coding
ENSG0000(	TAFA1	14.26903	1.15152	0.000142	0.001465	protein_coding
ENSG0000(	ENSG0000(	253.8561	0.182488	0.000142	0.001466	protein_coding
ENSG0000(	LINC02293	41.9859	-1.41912	0.000142	0.001466	lncRNA

ENSG0000(	TPRG1L	872.1129	-0.46078	0.000142	0.001469	protein_coding
ENSG0000(	IGHV3OR1(	10.74673	1.345805	0.000143	0.001469	IG_V_gene
ENSG0000(	ENSG0000(	116.1236	0.329838	0.000143	0.001471	TEC
ENSG0000(	MAPK3	1488.758	-0.30354	0.000143	0.001476	protein_coding
ENSG0000(	TECPR2	871.0361	0.324912	0.000143	0.001477	protein_coding
ENSG0000(	DYRK1A	7138.293	-0.10361	0.000144	0.00148	protein_coding
ENSG0000(	MIR5689H(	245.0212	0.185731	0.000144	0.00148	lncRNA
ENSG0000(	TRHDE-AS1	657.893	-0.931	0.000144	0.00148	lncRNA
ENSG0000(	TNS1	4344.178	0.362451	0.000144	0.00148	protein_coding
ENSG0000(	PAICSP1	11.36603	0.674168	0.000144	0.001483	processed_pseudogene
ENSG0000(	RPA1	865.6699	-0.23042	0.000145	0.001487	protein_coding
ENSG0000(	SPEN	6116.503	-0.10949	0.000145	0.001493	protein_coding
ENSG0000(	HADHA	3318.566	-0.36903	0.000146	0.001498	protein_coding
ENSG0000(	NELFA	711.706	-0.17436	0.000146	0.001498	protein_coding
ENSG0000(	ENSG0000(	48.42158	0.381239	0.000146	0.001502	lncRNA
ENSG0000(	ENSG0000(	24.36444	0.686799	0.000147	0.001506	lncRNA
ENSG0000(	ENSG0000(	18.02102	0.742299	0.000147	0.001506	processed_pseudogene
ENSG0000(	OR7G2	258.7092	-0.17802	0.000148	0.001514	protein_coding
ENSG0000(	DENND5B-/	23.92557	0.571484	0.000148	0.001514	lncRNA
ENSG0000(	ENSG0000(	2846.262	0.176257	0.000148	0.001514	lncRNA
ENSG0000(	TNC	3287.666	0.804178	0.000148	0.001515	protein_coding
ENSG0000(	CTDSPL2	2175.01	-0.19035	0.000148	0.00152	protein_coding
ENSG0000(	FRA10AC1	1213.532	0.208008	0.000148	0.00152	protein_coding
ENSG0000(	ICAM4	119.1167	0.243745	0.000149	0.001521	protein_coding
ENSG0000(	MOCS1	545.9085	-0.46519	0.00015	0.001533	protein_coding
ENSG0000(	ZNF22-AS1	60.64268	0.370425	0.00015	0.001537	lncRNA
ENSG0000(	ENSG0000(	247.1722	-0.20785	0.00015	0.001538	lncRNA
ENSG0000(	INF2	3908.17	-0.34498	0.00015	0.001538	protein_coding
ENSG0000(	ADIPOQ-AS	256.6782	0.201394	0.000151	0.001539	lncRNA
ENSG0000(	P3H1	1691.589	0.149185	0.000151	0.001539	protein_coding
ENSG0000(	ZNF395	1704.619	-0.29095	0.000151	0.001539	protein_coding
ENSG0000(	CCDC50	3391.033	-0.1866	0.000152	0.001547	protein_coding
ENSG0000(	OTOGL	64.09617	0.853934	0.000152	0.001547	protein_coding
ENSG0000(	H2AC21	64.57199	-0.6057	0.000152	0.001547	protein_coding
ENSG0000(	IGHV3-66	67.85672	1.122006	0.000152	0.001548	IG_V_gene
ENSG0000(	ESRRB	10.26989	1.059199	0.000152	0.001548	protein_coding
ENSG0000(	CNTFR	159.9483	-1.46451	0.000152	0.001553	protein_coding
ENSG0000(	PGAM5	965.6029	-0.14126	0.000152	0.001554	protein_coding
ENSG0000(	ENSG0000(	38.57548	0.476769	0.000153	0.001559	lncRNA
ENSG0000(	ENSG0000(	142.8323	0.227754	0.000153	0.001562	lncRNA
ENSG0000(	ENSG0000(	323.5332	0.23734	0.000154	0.001564	lncRNA
ENSG0000(	SNORA3A	184.4279	0.439823	0.000154	0.001564	snoRNA
ENSG0000(	LINC00311	513.9308	0.184656	0.000154	0.001564	lncRNA
ENSG0000(	TMEM213	617.6988	0.135218	0.000154	0.001564	protein_coding
ENSG0000(	PIGR	30134.77	0.873026	0.000154	0.001571	protein_coding
ENSG0000(	TBCA	332.1299	-0.33946	0.000155	0.001575	protein_coding
ENSG0000(	ENSG0000(	69.14791	0.386316	0.000155	0.001576	lncRNA

ENSG0000(	BCKDHA	226.3947	-0.28098	0.000155	0.001576	protein_coding
ENSG0000(	FHL3	136.3166	0.560249	0.000155	0.001577	protein_coding
ENSG0000(	SCART1	808.7254	0.27042	0.000156	0.001579	protein_coding
ENSG0000(	MAP3K20	1881.553	0.212898	0.000156	0.001585	protein_coding
ENSG0000(	ETS2	4482.308	0.399326	0.000156	0.001586	protein_coding
ENSG0000(	PNLIPRP2	326.3208	-1.0206	0.000156	0.001586	polymorphic_pseudogene
ENSG0000(	ENSG0000(	652.7063	0.240684	0.000157	0.001591	lncRNA
ENSG0000(	WDR12	1407.554	-0.22664	0.000157	0.001592	protein_coding
ENSG0000(	UHRF1BP1	1605.406	0.172459	0.000157	0.001594	protein_coding
ENSG0000(	D21S2088E	67.32309	0.323601	0.000157	0.001594	lncRNA
ENSG0000(	ZC3H12A	841.544	0.536972	0.000158	0.001597	protein_coding
ENSG0000(	ENSG0000(	22.34114	0.51311	0.000158	0.0016	lncRNA
ENSG0000(	ENSG0000(	844.0804	0.174841	0.000158	0.0016	lncRNA
ENSG0000(	ENSG0000(	16.76748	0.616753	0.000158	0.0016	lncRNA
ENSG0000(	ENSG0000(	388.4123	0.778459	0.000159	0.001604	lncRNA
ENSG0000(	LINC02672	17.55313	0.575286	0.000159	0.001607	lncRNA
ENSG0000(	PRELID3B	1178.021	-0.48167	0.00016	0.001612	protein_coding
ENSG0000(	MATN2	2307.167	-0.63518	0.00016	0.001615	protein_coding
ENSG0000(	ENSG0000(	145.0702	0.271108	0.00016	0.001618	TEC
ENSG0000(	LINC01961	52.25379	-0.37252	0.00016	0.001618	lncRNA
ENSG0000(	SNRNP27	280.4839	-0.24554	0.000161	0.001625	protein_coding
ENSG0000(	RASSF4	3060.78	0.343491	0.000161	0.001628	protein_coding
ENSG0000(	ENSG0000(	149.3748	0.282133	0.000162	0.001631	lncRNA
ENSG0000(	ENSG0000(	103.0828	0.271195	0.000162	0.001631	lncRNA
ENSG0000(	MIR4307H(	359.2601	-0.75644	0.000162	0.001631	lncRNA
ENSG0000(	NUDT14	259.3291	-0.3199	0.000162	0.001631	protein_coding
ENSG0000(	PDXP	504.8452	-0.64562	0.000162	0.001631	protein_coding
ENSG0000(	RAB27A	1571.536	0.264822	0.000162	0.001631	protein_coding
ENSG0000(	TCIM	612.5511	0.465624	0.000162	0.001631	protein_coding
ENSG0000(	UHRF1BP1I	37.58335	-0.4543	0.000162	0.001631	lncRNA
ENSG0000(	SSNA1	149.7281	-0.35549	0.000163	0.001634	protein_coding
ENSG0000(	ZSWIM4	230.7101	0.52817	0.000162	0.001634	protein_coding
ENSG0000(	RPS3P7	68.60372	0.407287	0.000163	0.001643	processed_pseudogene
ENSG0000(	ENSG0000(	1151.3	0.306991	0.000164	0.001648	lncRNA
ENSG0000(	RPL38	1804.068	-0.33512	0.000164	0.001651	protein_coding
ENSG0000(	MRPL18	212.6102	-0.30457	0.000165	0.001653	protein_coding
ENSG0000(	RDH16	840.4646	0.138808	0.000165	0.001653	protein_coding
ENSG0000(	VSIG4	130.1877	0.782033	0.000165	0.001655	protein_coding
ENSG0000(	ENSG0000(	15.70624	0.915041	0.000165	0.001659	transcribed_unprocessed_pseudo
ENSG0000(	ENSG0000(	10.69773	1.037044	0.000166	0.001665	snoRNA
ENSG0000(	ENSG0000(	74.69978	0.375812	0.000166	0.001665	TEC
ENSG0000(	ENSG0000(	206.1275	-0.23568	0.000166	0.001665	lncRNA
ENSG0000(	PRDX2	466.6596	-0.35754	0.000166	0.001665	protein_coding
ENSG0000(	RITA1	244.4669	-0.34666	0.000166	0.001666	protein_coding
ENSG0000(	ENSG0000(	421.099	0.25236	0.000166	0.001667	lncRNA
ENSG0000(	ENSG0000(	439.3654	0.152263	0.000167	0.00167	lncRNA
ENSG0000(	RNF207	1282.449	-0.34216	0.000167	0.001672	protein_coding

ENSG0000(	C5orf34-AS	52.5006	0.411385	0.000168	0.001676	lncRNA
ENSG0000(	SLC35B1	940.5343	-0.27139	0.000168	0.001679	protein_coding
ENSG0000(	NHLRC2	3298.961	-0.12669	0.000168	0.001682	protein_coding
ENSG0000(	ENSG0000(	36.23564	0.465974	0.000168	0.001684	lncRNA
ENSG0000(	PMVK	148.2793	-0.39743	0.000168	0.001684	protein_coding
ENSG0000(	MIR378G	26.52917	1.447557	0.000169	0.001689	miRNA
ENSG0000(	SNORD16	20.22993	0.818321	0.00017	0.001693	snoRNA
ENSG0000(	ENSG0000(	289.936	0.187289	0.00017	0.001699	lncRNA
ENSG0000(	ECM1	131.4743	0.492847	0.000171	0.001701	protein_coding
ENSG0000(	ENSG0000(	259.2937	0.2419	0.000171	0.001701	processed_pseudogene
ENSG0000(	ENSG0000(	150.7935	-0.24215	0.00017	0.001701	TEC
ENSG0000(	ENSG0000(	266.6929	-0.52737	0.000171	0.001704	lncRNA
ENSG0000(	MED13L	9513.995	0.135165	0.000171	0.001707	protein_coding
ENSG0000(	ENSG0000(	295.1508	-0.28512	0.000172	0.001709	lncRNA
ENSG0000(	MT-ATP6	12451.33	-0.60032	0.000172	0.00171	protein_coding
ENSG0000(	CALD1	2821.735	0.445853	0.000172	0.001714	protein_coding
ENSG0000(	KDM2A	5148.505	0.124304	0.000172	0.001715	protein_coding
ENSG0000(	HSPA8	6660.065	-0.27632	0.000173	0.001717	protein_coding
ENSG0000(	HCCS	199.8827	-0.35138	0.000173	0.001717	protein_coding
ENSG0000(	PNPLA6	1315.376	-0.2549	0.000173	0.001717	protein_coding
ENSG0000(	MS4A6E	35.51704	0.668338	0.000174	0.001725	protein_coding
ENSG0000(	MSC	89.03118	0.868188	0.000174	0.001725	protein_coding
ENSG0000(	ZNF587B	2576.564	0.264717	0.000174	0.001725	protein_coding
ENSG0000(	ENSG0000(	153.7491	0.570257	0.000174	0.001728	lncRNA
ENSG0000(	DLEC1	200.621	0.360086	0.000174	0.001729	protein_coding
ENSG0000(	ZRANB2-AS	300.5528	-0.3424	0.000175	0.001736	lncRNA
ENSG0000(	PCOLCE	246.8565	0.678361	0.000175	0.001737	protein_coding
ENSG0000(	CHMP2A	786.0186	-0.32083	0.000175	0.001738	protein_coding
ENSG0000(	TIFA	491.3482	0.702916	0.000175	0.001738	protein_coding
ENSG0000(	TM7SF3	1577.3	-0.16299	0.000175	0.001738	protein_coding
ENSG0000(	DNAJC19	835.6841	-0.19486	0.000177	0.001751	protein_coding
ENSG0000(	JPH1	103.6099	0.629715	0.000177	0.001751	protein_coding
ENSG0000(	CUL2	2275.376	-0.1442	0.000177	0.001752	protein_coding
ENSG0000(	TNFRSF8	40.45055	0.834567	0.000177	0.001752	protein_coding
ENSG0000(	ENSG0000(	58.6223	-0.39157	0.000177	0.001753	transcribed_unprocessed_pseudo
ENSG0000(	ARHGAP28	1182.709	0.258378	0.000177	0.001755	protein_coding
ENSG0000(	ENSG0000(	80.72029	0.425267	0.000177	0.001755	protein_coding
ENSG0000(	ENSG0000(	333.7898	0.476235	0.000178	0.001755	lncRNA
ENSG0000(	TMEM102	119.929	-0.29678	0.000178	0.001755	protein_coding
ENSG0000(	MACO1	756.1668	-0.21166	0.000178	0.001757	protein_coding
ENSG0000(	OR7M1P	15.51433	0.728392	0.000178	0.001757	unprocessed_pseudogene
ENSG0000(	TPRKBP2	46.61866	0.446756	0.000178	0.001757	processed_pseudogene
ENSG0000(	ENSG0000(	28.11918	-0.5001	0.000178	0.001758	lncRNA
ENSG0000(	USP15	4761.66	0.235045	0.000178	0.00176	protein_coding
ENSG0000(	ENSG0000(	7.528699	1.099002	0.000179	0.001761	TEC
ENSG0000(	MLLT10	1851.011	-0.15246	0.000179	0.001763	protein_coding
ENSG0000(	PAF1	303.5785	-0.27329	0.00018	0.001776	protein_coding

ENSG0000(	RTN4	3673.472	-0.38013	0.00018	0.001776	protein_coding
ENSG0000(	ZNF121	3141.417	0.170858	0.00018	0.001778	protein_coding
ENSG0000(	ABCC1	2534.319	0.326983	0.000181	0.001784	protein_coding
ENSG0000(	MORC4	6661.248	-0.20686	0.000181	0.001787	protein_coding
ENSG0000(	SLC5A4	154.9853	-0.87142	0.000182	0.001788	protein_coding
ENSG0000(	ERG	323.0706	0.424188	0.000182	0.001789	protein_coding
ENSG0000(	H4C3	207.7699	-0.50759	0.000182	0.001793	protein_coding
ENSG0000(	SEMA3E	247.5844	0.588048	0.000182	0.001793	protein_coding
ENSG0000(	ZNF710-AS	387.6703	0.271646	0.000182	0.001793	lncRNA
ENSG0000(	SORCS2	200.0066	0.565666	0.000183	0.001796	protein_coding
ENSG0000(	TSEN54	708.0296	-0.1822	0.000183	0.001797	protein_coding
ENSG0000(	EIF1AD	654.5202	-0.15372	0.000183	0.001799	protein_coding
ENSG0000(	PCID2	1151.089	-0.15257	0.000183	0.001799	protein_coding
ENSG0000(	HACE1	1013.188	-0.15216	0.000183	0.001801	protein_coding
ENSG0000(	LGALS3	3278.804	-0.68586	0.000183	0.001801	protein_coding
ENSG0000(	SAMD10	153.3382	-0.42215	0.000184	0.001803	protein_coding
ENSG0000(	GAS2L1	321.7263	-0.37943	0.000184	0.001807	protein_coding
ENSG0000(	RPL34	2062.487	-0.33255	0.000184	0.001807	protein_coding
ENSG0000(	DND1	49.79692	0.464244	0.000184	0.001808	protein_coding
ENSG0000(	ENSG0000(	469.5884	-0.2942	0.000185	0.001811	protein_coding
ENSG0000(	ENSG0000(	159.5309	-0.3635	0.000185	0.001814	lncRNA
ENSG0000(	POLI	1230.087	0.199532	0.000185	0.001814	protein_coding
ENSG0000(	SEPTIN7P1:	978.5144	0.193257	0.000185	0.001815	transcribed_unprocessed_pseudo
ENSG0000(	OR52N5	26.37613	0.70333	0.000186	0.001817	protein_coding
ENSG0000(	FBXL13	56.03269	0.624399	0.000187	0.00183	protein_coding
ENSG0000(	GPAT3	516.5585	-0.74108	0.000187	0.00183	protein_coding
ENSG0000(	ANKRD36	5641.664	0.293483	0.000187	0.00183	protein_coding
ENSG0000(	ENSG0000(	57.82129	0.313688	0.000187	0.00183	lncRNA
ENSG0000(	CCR9	161.0695	-0.79012	0.000188	0.001833	protein_coding
ENSG0000(	WDR19	932.68	0.284596	0.000188	0.001835	protein_coding
ENSG0000(	ZNF844	1878.818	-0.1131	0.000188	0.001836	protein_coding
ENSG0000(	ADD2	425.4704	0.502995	0.000188	0.001837	protein_coding
ENSG0000(	MRPL19	914.8922	-0.21327	0.000189	0.001842	protein_coding
ENSG0000(	ENSG0000(	164.8459	0.277572	0.000189	0.001842	lncRNA
ENSG0000(	CYP19A1	12.15733	1.232462	0.000189	0.001848	protein_coding
ENSG0000(	CFAP20	148.5559	-0.29475	0.00019	0.001848	protein_coding
ENSG0000(	SPTA1	49.97554	0.871739	0.00019	0.001848	protein_coding
ENSG0000(	SMPD5	11.99671	1.16975	0.00019	0.001854	transcribed_unitary_pseudogene
ENSG0000(	ENSG0000(	117.7396	-0.3222	0.00019	0.001855	TEC
ENSG0000(	TRUB2	1792.109	-0.08158	0.000191	0.001855	protein_coding
ENSG0000(	ENSG0000(	17.71642	-0.59634	0.000191	0.001859	lncRNA
ENSG0000(	ENSG0000(	368.1941	0.181515	0.000191	0.00186	protein_coding
ENSG0000(	PRR19	330.9175	-0.22945	0.000191	0.00186	protein_coding
ENSG0000(	VASN	97.82268	0.687071	0.000191	0.001861	protein_coding
ENSG0000(	STK36	705.6986	0.267034	0.000192	0.001863	protein_coding
ENSG0000(	LINC01762	157.0207	-0.25594	0.000192	0.001865	lncRNA
ENSG0000(	AMFR	1363.68	-0.18313	0.000192	0.001866	protein_coding

ENSG0000(	ENSG0000(	133.4174	0.406085	0.000192	0.001866	lncRNA
ENSG0000(	GPR78	154.3176	0.353799	0.000192	0.001866	protein_coding
ENSG0000(	GRIK4	39.10945	0.894175	0.000193	0.001872	protein_coding
ENSG0000(	BLVRB	277.5746	-0.48091	0.000194	0.00188	protein_coding
ENSG0000(	MIR4472-2	13.35431	0.802876	0.000194	0.00188	miRNA
ENSG0000(	ENSG0000(	150.3939	0.261707	0.000194	0.001881	lncRNA
ENSG0000(	RNU2-23P	13.63273	-0.76673	0.000195	0.001892	snRNA
ENSG0000(	TSC22D3	1194.765	0.81658	0.000195	0.001894	protein_coding
ENSG0000(	SLC23A1	1072.101	-0.89043	0.000196	0.001899	protein_coding
ENSG0000(	ENSG0000(	63.5225	0.527351	0.000196	0.001902	lncRNA
ENSG0000(	ENSG0000(	17.26214	1.189167	0.000196	0.001902	lncRNA
ENSG0000(	TEAD4	62.92817	0.665297	0.000196	0.001902	protein_coding
ENSG0000(	TMCO5B	189.8953	1.11024	0.000197	0.001905	transcribed_unitary_pseudogene
ENSG0000(	ARAP3	349.6834	0.368537	0.000197	0.001907	protein_coding
ENSG0000(	ENSG0000(	204.3228	0.280206	0.000197	0.001907	lncRNA
ENSG0000(	LINC01648	34.98321	0.949835	0.000197	0.001907	lncRNA
ENSG0000(	ZBTB44	2480.045	-0.17658	0.000197	0.001907	protein_coding
ENSG0000(	MTND6P5	58.56763	0.506571	0.000198	0.001909	unprocessed_pseudogene
ENSG0000(	SNX2	1849.84	-0.28656	0.000198	0.001912	protein_coding
ENSG0000(	LY86-AS1	934.7154	0.230375	0.000198	0.001917	lncRNA
ENSG0000(	BORCS8	1971.934	0.203625	0.000199	0.001923	protein_coding
ENSG0000(	ENSG0000(	27.2958	0.434151	0.000199	0.001925	lncRNA
ENSG0000(	P2RY6	2269.964	0.239928	0.0002	0.001935	protein_coding
ENSG0000(	TRIM38	5018.062	0.151396	0.000201	0.001935	protein_coding
ENSG0000(	TSTD1	268.535	-0.3846	0.000201	0.001936	protein_coding
ENSG0000(	KLHDC7A	18.52547	-0.76399	0.000201	0.001938	protein_coding
ENSG0000(	C1orf198	430.8606	0.382821	0.000202	0.001947	protein_coding
ENSG0000(	ENSG0000(	53.70259	0.576967	0.000202	0.001948	lncRNA
ENSG0000(	ROGDI	314.1509	-0.35008	0.000203	0.001951	protein_coding
ENSG0000(	TPT1	17425.54	-0.28964	0.000203	0.001951	protein_coding
ENSG0000(	CHST3	349.7887	0.303745	0.000203	0.001953	protein_coding
ENSG0000(	RIT2	27.95843	0.580825	0.000204	0.001959	protein_coding
ENSG0000(	ENSG0000(	150.0914	0.30819	0.000204	0.00196	processed_pseudogene
ENSG0000(	ENSG0000(	116.2503	0.367176	0.000204	0.00196	lncRNA
ENSG0000(	LINC02146	12.30976	0.688107	0.000204	0.00196	lncRNA
ENSG0000(	RPL7P14	82.49456	-0.51286	0.000204	0.00196	processed_pseudogene
ENSG0000(	DNAJC21	1432.336	-0.14419	0.000204	0.001964	protein_coding
ENSG0000(	PLXND1	1971.139	0.474707	0.000205	0.001965	protein_coding
ENSG0000(	TPK1	994.8944	0.22461	0.000205	0.001967	protein_coding
ENSG0000(	ALDH7A1	2367.808	-0.11122	0.000205	0.001968	protein_coding
ENSG0000(	DYNLT4	15.70194	0.704292	0.000205	0.001968	protein_coding
ENSG0000(	LINC02675	95.54135	0.309903	0.000205	0.001968	lncRNA
ENSG0000(	PDS5A	4099.182	-0.14219	0.000206	0.001975	protein_coding
ENSG0000(	ENSG0000(	27.00376	1.136618	0.000206	0.001976	lncRNA
ENSG0000(	RPS12	2089.614	-0.37897	0.000207	0.00198	protein_coding
ENSG0000(	TMX2	826.2358	-0.14661	0.000207	0.00198	protein_coding
ENSG0000(	COL2A1	50.21164	-1.06954	0.000207	0.001982	protein_coding

ENSG0000(	PXDN	1108.721	0.430331	0.000207	0.001982	protein_coding
ENSG0000(	STARD13-IT	45.57	0.475196	0.000207	0.001983	lncRNA
ENSG0000(	PRICKLE2-A	58.74656	0.316529	0.000207	0.001984	lncRNA
ENSG0000(	PTPN2	2740.25	0.212172	0.000207	0.001984	protein_coding
ENSG0000(	TNS4	213.5738	0.537005	0.000208	0.001985	protein_coding
ENSG0000(	ENSG0000(	16.78958	0.70422	0.000208	0.001986	lncRNA
ENSG0000(	MIR7848	17.17782	0.911288	0.000208	0.00199	miRNA
ENSG0000(	VAV3	1415.352	-0.22395	0.000208	0.001991	protein_coding
ENSG0000(	ENSG0000(	60.34532	0.433159	0.000208	0.001991	lncRNA
ENSG0000(	ENSG0000(	189.2632	0.337909	0.000209	0.001992	lncRNA
ENSG0000(	LPP-AS2	161.4261	0.24018	0.00021	0.002001	lncRNA
ENSG0000(	ENSG0000(	68.26652	-0.4385	0.00021	0.002004	lncRNA
ENSG0000(	C1GALT1P1	54.74145	0.386141	0.000211	0.002011	processed_pseudogene
ENSG0000(	ENSG0000(	66.27349	0.364496	0.000211	0.002012	lncRNA
ENSG0000(	ENSG0000(	30.67238	0.665448	0.000212	0.002016	lncRNA
ENSG0000(	GUCA2B	354.0585	-1.47538	0.000212	0.002016	protein_coding
ENSG0000(	SLC38A5	107.2647	0.578128	0.000212	0.002016	protein_coding
ENSG0000(	ABCB7	1026.485	-0.19149	0.000212	0.00202	protein_coding
ENSG0000(	RPP25L	122.0858	-0.41728	0.000212	0.002022	protein_coding
ENSG0000(	ENSG0000(	88.54985	0.640206	0.000213	0.002023	lncRNA
ENSG0000(	ENSG0000(	213.6386	0.238144	0.000213	0.002023	lncRNA
ENSG0000(	ASAP1-IT2	398.0934	0.207316	0.000214	0.00203	lncRNA
ENSG0000(	ENSG0000(	25.21272	0.472308	0.000213	0.00203	lncRNA
ENSG0000(	ENSG0000(	38.33715	0.416	0.000214	0.00203	lncRNA
ENSG0000(	ENSG0000(	88.59834	-0.31071	0.000213	0.00203	TEC
ENSG0000(	ENSG0000(	121.3269	0.328579	0.000215	0.002045	lncRNA
ENSG0000(	GPR68	115.4967	0.616005	0.000215	0.002046	protein_coding
ENSG0000(	CHCHD3P2	28.63523	-1.05537	0.000216	0.002048	processed_pseudogene
ENSG0000(	FH	939.6016	-0.2436	0.000216	0.002048	protein_coding
ENSG0000(	RAP2B	1556.332	0.152205	0.000216	0.002048	protein_coding
ENSG0000(	SIGLEC6	617.9676	-0.35788	0.000216	0.002048	protein_coding
ENSG0000(	SLC38A6	630.6266	0.267805	0.000216	0.002049	protein_coding
ENSG0000(	C9orf78	1197.328	-0.13318	0.000216	0.002053	protein_coding
ENSG0000(	PTP4A2	4281.393	-0.18254	0.000217	0.002053	protein_coding
ENSG0000(	ENSG0000(	111.2142	0.328366	0.000217	0.002053	lncRNA
ENSG0000(	ENSG0000(	54.05664	-0.33233	0.000217	0.002057	lncRNA
ENSG0000(	IL17RB	473.6255	-0.49776	0.000217	0.002057	protein_coding
ENSG0000(	MEA1	263.3244	-0.22253	0.000217	0.002057	protein_coding
ENSG0000(	SEC23A	2917.827	-0.20635	0.000217	0.002057	protein_coding
ENSG0000(	FAM98C	1949.483	-0.14691	0.000217	0.002057	protein_coding
ENSG0000(	RBBP9	531.3582	-0.17345	0.000217	0.002057	protein_coding
ENSG0000(	ENSG0000(	78.73262	0.312101	0.000218	0.002059	lncRNA
ENSG0000(	TTL	2639.376	0.264875	0.000218	0.002064	protein_coding
ENSG0000(	GABRP	861.9962	0.254109	0.000218	0.002065	protein_coding
ENSG0000(	ENSG0000(	19.90311	0.47055	0.000219	0.002067	TEC
ENSG0000(	LZTFL1	800.9624	-0.36919	0.00022	0.002081	protein_coding
ENSG0000(	COX5B	583.884	-0.31016	0.00022	0.002082	protein_coding

ENSG0000(	LINC02334	684.3994	0.175186	0.000221	0.002087	lncRNA
ENSG0000(	PRDX5	394.3795	-0.43344	0.000221	0.002087	protein_coding
ENSG0000(	IPO7	2150.566	-0.23153	0.000221	0.002088	protein_coding
ENSG0000(	KY	40.67683	0.502484	0.000221	0.002088	protein_coding
ENSG0000(	ENSG0000(	392.4657	0.196924	0.000222	0.002089	lncRNA
ENSG0000(	ENSG0000(	203.5886	-0.28084	0.000222	0.002089	lncRNA
ENSG0000(	ENSG0000(	554.0639	0.171253	0.000222	0.002091	lncRNA
ENSG0000(	LINC02447	343.0777	-0.15954	0.000222	0.002092	lncRNA
ENSG0000(	ATP13A3	4185.337	0.276724	0.000223	0.002101	protein_coding
ENSG0000(	CUTA	236.3539	-0.34189	0.000223	0.002102	protein_coding
ENSG0000(	MT-ND5	19026.62	-0.55003	0.000224	0.002105	protein_coding
ENSG0000(	SRRM4	211.7201	0.395851	0.000224	0.002105	protein_coding
ENSG0000(	CBLL1	766.1533	-0.15232	0.000224	0.002106	protein_coding
ENSG0000(	GCOM1	638.648	-0.7017	0.000224	0.002106	protein_coding
ENSG0000(	MMP7	27.0457	0.824646	0.000225	0.002115	protein_coding
ENSG0000(	TRAF7	1058.343	-0.33506	0.000225	0.002116	protein_coding
ENSG0000(	MRAP2	134.9973	0.474443	0.000226	0.002121	protein_coding
ENSG0000(	MRAS	343.4715	0.512609	0.000226	0.002121	protein_coding
ENSG0000(	H2BC8	185.8119	-0.43959	0.000226	0.002122	protein_coding
ENSG0000(	ITGB3	1067.778	0.334893	0.000226	0.002124	protein_coding
ENSG0000(	KIAA0930	1332.693	0.278201	0.000226	0.002125	protein_coding
ENSG0000(	ENSG0000(	143.1663	0.301558	0.000227	0.00213	lncRNA
ENSG0000(	PCNX1	6167.366	0.235482	0.000227	0.00213	protein_coding
ENSG0000(	TRIB1	1275.746	0.361871	0.000228	0.002135	protein_coding
ENSG0000(	TMC3	111.6309	0.24681	0.000228	0.002135	protein_coding
ENSG0000(	LTBP4	2586.452	-0.28128	0.000228	0.002136	protein_coding
ENSG0000(	SELENOT	1055.244	-0.22518	0.000228	0.002136	protein_coding
ENSG0000(	UGT1A5	178.97	-1.02963	0.000228	0.002138	protein_coding
ENSG0000(	MIR3690	15.63694	0.85104	0.000229	0.002145	miRNA
ENSG0000(	H4C9	58.12291	-0.53596	0.000229	0.002146	protein_coding
ENSG0000(	PRSS51	14.61807	0.935727	0.00023	0.002152	protein_coding
ENSG0000(	ENSG0000(	139.3623	0.219666	0.00023	0.002152	lncRNA
ENSG0000(	C15orf65	10.4084	0.750124	0.00023	0.002153	protein_coding
ENSG0000(	ENSG0000(	19.23608	-0.61127	0.00023	0.002154	lncRNA
ENSG0000(	RPS15	1119.26	-0.31588	0.000231	0.002154	protein_coding
ENSG0000(	GOLGA3	5560.356	0.151093	0.000231	0.002155	protein_coding
ENSG0000(	LINC02228	94.9215	0.31816	0.000231	0.002155	lncRNA
ENSG0000(	NCKIPSD	385.7014	-0.30262	0.000231	0.002156	protein_coding
ENSG0000(	KLHL13	120.5236	-0.55103	0.000231	0.002159	protein_coding
ENSG0000(	TRGV9	21.43575	-0.50667	0.000232	0.002161	TR_V_gene
ENSG0000(	OR8D1	120.6033	0.302928	0.000232	0.002163	protein_coding
ENSG0000(	CDK16	956.4731	-0.18357	0.000233	0.002169	protein_coding
ENSG0000(	DLD	1081.073	-0.20265	0.000233	0.002172	protein_coding
ENSG0000(	LOXL2	628.1494	0.286916	0.000233	0.002174	protein_coding
ENSG0000(	ENSG0000(	195.1881	0.233888	0.000234	0.002177	lncRNA
ENSG0000(	ENSG0000(	347.889	0.172372	0.000234	0.002177	lncRNA
ENSG0000(	ENSG0000(	226.9335	0.30349	0.000234	0.002178	transcribed_unprocessed_pseudo

ENSG0000(	SLAMF1	518.8128	0.698623	0.000234	0.002179	protein_coding
ENSG0000(	ELF1	1997.737	-0.17731	0.000234	0.00218	protein_coding
ENSG0000(	ENSG0000(	1902.05	0.181396	0.000235	0.002186	lncRNA
ENSG0000(	LINC00881	54.72613	0.382764	0.000235	0.002186	lncRNA
ENSG0000(	RBP2	943.8105	-0.98407	0.000235	0.002186	protein_coding
ENSG0000(	CORO6	82.67789	0.642408	0.000235	0.002187	protein_coding
ENSG0000(	GUCY2D	11.2607	1.187841	0.000236	0.00219	protein_coding
ENSG0000(	RRP15	918.8251	0.375226	0.000236	0.00219	protein_coding
ENSG0000(	MARS1	1347.939	0.263338	0.000236	0.00219	protein_coding
ENSG0000(	PLEKHN1	46.55076	0.731006	0.000236	0.00219	protein_coding
ENSG0000(	USP31	1132.005	0.191504	0.000236	0.002191	protein_coding
ENSG0000(	KREMEN1	1746.943	0.167551	0.000236	0.002192	protein_coding
ENSG0000(	AGAP13P	13.1407	-1.03236	0.000237	0.002195	unprocessed_pseudogene
ENSG0000(	FAM86JP	48.66394	0.417715	0.000237	0.002195	transcribed_unprocessed_pseudo
ENSG0000(	PSG8	50.35685	0.906422	0.000237	0.002195	protein_coding
ENSG0000(	TRIM28	1345.974	-0.26271	0.000237	0.002195	protein_coding
ENSG0000(	TSEN15	1660.647	0.189212	0.000237	0.002195	protein_coding
ENSG0000(	ORC4	10574.68	-0.13617	0.000237	0.002195	protein_coding
ENSG0000(	PCSK6-AS1	64.14354	-0.35143	0.000237	0.002195	lncRNA
ENSG0000(	TMEM14A	68.57945	-0.5354	0.000238	0.002206	protein_coding
ENSG0000(	ENSG0000(	83.00222	0.266483	0.000239	0.002211	lncRNA
ENSG0000(	STK11	1376.295	-0.15056	0.000239	0.002213	protein_coding
ENSG0000(	ENSG0000(	61.02823	-0.52106	0.00024	0.002216	miRNA
ENSG0000(	ENSG0000(	67.55439	0.292985	0.00024	0.002216	lncRNA
ENSG0000(	ATP10D	1238.538	0.206003	0.00024	0.002218	protein_coding
ENSG0000(	SPATA4	48.59807	0.425518	0.00024	0.002218	protein_coding
ENSG0000(	ENSG0000(	143.1054	-0.2467	0.00024	0.002219	lncRNA
ENSG0000(	EIF2B1	766.8184	-0.12506	0.000241	0.002228	protein_coding
ENSG0000(	ENSG0000(	35.56288	-0.5092	0.000241	0.002229	lncRNA
ENSG0000(	COX7A2	544.0323	-0.31175	0.000242	0.002231	protein_coding
ENSG0000(	ENSG0000(	123.1552	0.273261	0.000242	0.002232	lncRNA
ENSG0000(	PPP2R1A	2067.335	-0.17029	0.000242	0.002236	protein_coding
ENSG0000(	SMIM24	942.2713	-0.97564	0.000242	0.002236	protein_coding
ENSG0000(	DLST	2205.78	-0.14957	0.000243	0.002238	protein_coding
ENSG0000(	ENSG0000(	430.4005	0.29172	0.000243	0.002238	TEC
ENSG0000(	ENSG0000(	58.05427	-0.34807	0.000243	0.002239	lncRNA
ENSG0000(	WASF3	122.3812	0.4236	0.000243	0.002239	protein_coding
ENSG0000(	PTCD3	3380.536	-0.14585	0.000243	0.002242	protein_coding
ENSG0000(	SLC30A1	2254.507	-0.5192	0.000244	0.002247	protein_coding
ENSG0000(	GUCA2A	1749.76	-1.07381	0.000244	0.002249	protein_coding
ENSG0000(	TMEM256	200.1795	-0.58962	0.000244	0.002249	protein_coding
ENSG0000(	RNF32-AS1	129.9789	0.447894	0.000245	0.002254	lncRNA
ENSG0000(	DCT	14.03432	0.908446	0.000246	0.002257	protein_coding
ENSG0000(	ENSG0000(	247.8618	0.333768	0.000246	0.002257	TEC
ENSG0000(	LINC00472	1491.726	0.251613	0.000246	0.002257	lncRNA
ENSG0000(	FBXL5	2036.333	0.257861	0.000246	0.002258	protein_coding
ENSG0000(	ENSG0000(	199.2415	0.210708	0.000246	0.002263	lncRNA

ENSG0000(	VPS50	934.4289	-0.20167	0.000247	0.002266	protein_coding
ENSG0000(	ENSG0000(	12.55968	0.755498	0.000247	0.002269	lncRNA
ENSG0000(	ENSG0000(	21.13956	0.965039	0.000248	0.002273	processed_pseudogene
ENSG0000(	KIF25	90.4266	0.404365	0.000248	0.002273	protein_coding
ENSG0000(	IFIT2	761.0043	0.669148	0.000248	0.002277	protein_coding
ENSG0000(	ENSG0000(	94.75792	0.353542	0.00025	0.002294	lncRNA
ENSG0000(	ACADM	3184.173	-0.2681	0.00025	0.002295	protein_coding
ENSG0000(	E2F3-IT1	93.19863	0.420009	0.000251	0.002296	lncRNA
ENSG0000(	ENSG0000(	238.8049	0.203683	0.000251	0.002296	lncRNA
ENSG0000(	PYURF	406.1139	-0.25911	0.000251	0.002298	protein_coding
ENSG0000(	B3GLCT	211.1342	-0.25107	0.000251	0.002299	protein_coding
ENSG0000(	GRHL1	315.8212	0.266176	0.000251	0.0023	protein_coding
ENSG0000(	AGPAT2	1231.69	-0.75558	0.000251	0.002301	protein_coding
ENSG0000(	DYRK3	470.4323	0.288788	0.000252	0.002302	protein_coding
ENSG0000(	ATP5PO	788.3864	-0.37124	0.000253	0.002314	protein_coding
ENSG0000(	UGGT2	1402.19	0.1408	0.000253	0.002314	protein_coding
ENSG0000(	ENSG0000(	105.9031	0.333247	0.000253	0.002314	lncRNA
ENSG0000(	HINT2P1	24.90938	-0.62437	0.000253	0.002314	processed_pseudogene
ENSG0000(	AK1	354.7285	0.308885	0.000254	0.002318	protein_coding
ENSG0000(	ENSG0000(	2274.692	0.186679	0.000255	0.002326	lncRNA
ENSG0000(	MYL12B	1568.793	-0.44167	0.000255	0.002326	protein_coding
ENSG0000(	STRIP2	500.8232	0.415879	0.000255	0.002326	protein_coding
ENSG0000(	CARD16	314.8766	0.43408	0.000255	0.002327	protein_coding
ENSG0000(	CBS	189.2012	-0.696	0.000255	0.002327	protein_coding
ENSG0000(	ENSG0000(	275.3129	0.218414	0.000255	0.002327	lncRNA
ENSG0000(	SLC36A4	460.6968	0.367185	0.000255	0.002327	protein_coding
ENSG0000(	HOMER3-A	40.03311	0.361255	0.000255	0.002328	lncRNA
ENSG0000(	ENSG0000(	667.3202	-0.22096	0.000256	0.002328	lncRNA
ENSG0000(	GAS7	1426.614	0.390153	0.000256	0.00233	protein_coding
ENSG0000(	ENSG0000(	462.9262	0.276031	0.000256	0.002332	lncRNA
ENSG0000(	ENSG0000(	216.0992	0.254826	0.000256	0.002333	lncRNA
ENSG0000(	ENSG0000(	156.9711	-0.2683	0.000257	0.002335	lncRNA
ENSG0000(	LINC00456	238.5537	0.237554	0.000257	0.002335	lncRNA
ENSG0000(	LINC01160	281.8463	0.18229	0.000257	0.002335	lncRNA
ENSG0000(	EEF2	11595.98	-0.22061	0.000257	0.002336	protein_coding
ENSG0000(	LINC02346	344.7913	0.234335	0.000258	0.002344	lncRNA
ENSG0000(	ACOT12	52.29484	-1.1652	0.000258	0.002345	protein_coding
ENSG0000(	GPR176	185.3951	0.796808	0.000258	0.002347	protein_coding
ENSG0000(	IGHV3-11	168.0547	0.97705	0.000259	0.002351	IG_V_gene
ENSG0000(	RARS2	4593.181	-0.16869	0.000259	0.002354	protein_coding
ENSG0000(	ENSG0000(	8.072319	-0.82871	0.00026	0.002362	processed_pseudogene
ENSG0000(	GOT1	457.9939	-0.43035	0.00026	0.002364	protein_coding
ENSG0000(	ENSG0000(	26.95437	0.495981	0.000261	0.002369	lncRNA
ENSG0000(	VWA1	271.8806	0.506355	0.000261	0.002371	protein_coding
ENSG0000(	IGKV1-12	155.106	1.106488	0.000262	0.002372	IG_V_gene
ENSG0000(	IGLV5-45	50.21587	1.216935	0.000262	0.002374	IG_V_gene
ENSG0000(	MMP12	198.3763	1.158747	0.000262	0.002374	protein_coding

ENSG0000(	IFIT1P1	13.72676	0.749654	0.000263	0.002379	processed_pseudogene
ENSG0000(	CD58	624.6102	-0.28435	0.000263	0.002382	protein_coding
ENSG0000(	ENSG0000(	45.64131	0.416572	0.000263	0.002382	lncRNA
ENSG0000(	GABARAPL:	688.0589	-0.22859	0.000264	0.002385	protein_coding
ENSG0000(	VTI1B	1537.307	-0.24901	0.000264	0.002387	protein_coding
ENSG0000(	AGAP2-AS1	22.66119	0.651289	0.000264	0.002387	lncRNA
ENSG0000(	ENSG0000(	29.0247	0.439906	0.000264	0.002387	lncRNA
ENSG0000(	DACT1	216.6372	0.434602	0.000264	0.00239	protein_coding
ENSG0000(	TYROBP	149.3926	0.589316	0.000264	0.00239	protein_coding
ENSG0000(	OSBPL3	1584.204	0.284228	0.000265	0.002393	protein_coding
ENSG0000(	TPST2	888.401	0.28723	0.000265	0.002393	protein_coding
ENSG0000(	ENSG0000(	219.7493	0.278163	0.000265	0.002397	lncRNA
ENSG0000(	ENSG0000(	126.5466	0.301372	0.000266	0.002399	lncRNA
ENSG0000(	TRPM6	3658.519	-0.7324	0.000266	0.002399	protein_coding
ENSG0000(	TLE1	475.7503	0.34545	0.000266	0.002404	protein_coding
ENSG0000(	PET100	319.9343	-0.31182	0.000267	0.002412	protein_coding
ENSG0000(	NDUFA1	235.7072	-0.4718	0.000268	0.002412	protein_coding
ENSG0000(	SLC16A5	591.7651	-0.45151	0.000268	0.002413	protein_coding
ENSG0000(	SNORA48	2081.222	0.363776	0.000268	0.002419	snoRNA
ENSG0000(	LINC00519	263.3681	0.209668	0.000269	0.002425	lncRNA
ENSG0000(	PLEKHM1	2069.63	0.139154	0.00027	0.002429	protein_coding
ENSG0000(	IL4I1	204.5693	0.853572	0.00027	0.002431	protein_coding
ENSG0000(	IL32	2205	-0.67452	0.000271	0.002441	protein_coding
ENSG0000(	PHGR1	3519.632	-0.87627	0.000272	0.002446	protein_coding
ENSG0000(	ENSG0000(	48.29852	0.333183	0.000272	0.002449	TEC
ENSG0000(	OXGR1	9.993342	0.869969	0.000272	0.00245	protein_coding
ENSG0000(	ENSG0000(	16.65716	0.580887	0.000273	0.002454	snoRNA
ENSG0000(	ENSG0000(	14.46386	0.816979	0.000273	0.002457	lncRNA
ENSG0000(	ENSG0000(	294.7896	0.18418	0.000273	0.002457	lncRNA
ENSG0000(	KBTBD6	1523.108	0.315758	0.000273	0.002457	protein_coding
ENSG0000(	PFN2	608.145	-0.51541	0.000274	0.002461	protein_coding
ENSG0000(	ENSG0000(	605.2315	0.140111	0.000275	0.002467	lncRNA
ENSG0000(	BGN	589.4943	0.679113	0.000275	0.002468	protein_coding
ENSG0000(	H4C4	166.9911	-0.48522	0.000275	0.00247	protein_coding
ENSG0000(	ENSG0000(	214.3118	-0.21214	0.000275	0.002471	lncRNA
ENSG0000(	ENSG0000(	215.957	0.212374	0.000276	0.002476	lncRNA
ENSG0000(	ENSG0000(	404.1009	0.18238	0.000276	0.002476	lncRNA
ENSG0000(	NT5C1A	461.6139	0.263762	0.000276	0.002476	protein_coding
ENSG0000(	LINC00865	194.4623	0.383114	0.000277	0.002479	lncRNA
ENSG0000(	GFM2	472.7797	-0.20811	0.000277	0.002482	protein_coding
ENSG0000(	LGMN	1147.875	-0.25782	0.000277	0.002482	protein_coding
ENSG0000(	HSD3B1	229.0159	-1.07782	0.000277	0.002484	protein_coding
ENSG0000(	ADAMTS18	36.74675	1.121892	0.000278	0.002487	protein_coding
ENSG0000(	PRSS42P	19.28455	0.777326	0.000279	0.002492	transcribed_unitary_pseudogene
ENSG0000(	DGKA	5693.507	-0.24883	0.00028	0.002502	protein_coding
ENSG0000(	ENSG0000(	78.8566	0.399334	0.00028	0.002502	processed_pseudogene
ENSG0000(	LPCAT3	1349.29	-0.45737	0.00028	0.002502	protein_coding

ENSG0000(	POLN	537.7241	0.212627	0.00028	0.002502	protein_coding
ENSG0000(	ACIN1	6572.248	0.073412	0.00028	0.002504	protein_coding
ENSG0000(	CCND1	1046.164	0.423614	0.00028	0.002504	protein_coding
ENSG0000(	KIF5C	2263.197	0.141015	0.00028	0.002504	protein_coding
ENSG0000(	LYPD4	11.24396	0.703346	0.00028	0.002504	protein_coding
ENSG0000(	RN7SL552F	12.57508	0.634009	0.000281	0.00251	misc_RNA
ENSG0000(	ENSG0000(	133.2846	0.257081	0.000282	0.002513	lncRNA
ENSG0000(	ITPRIP	747.6369	0.607359	0.000282	0.002513	protein_coding
ENSG0000(	ZFAND1	634.6683	-0.20591	0.000282	0.002513	protein_coding
ENSG0000(	BPNT1	1103.829	-0.32498	0.000282	0.002514	protein_coding
ENSG0000(	H2BC18	233.165	-0.54783	0.000282	0.002514	protein_coding
ENSG0000(	TBC1D17	1145.184	-0.14759	0.000282	0.002515	protein_coding
ENSG0000(	AMD1P4	173.9871	-0.17992	0.000283	0.002518	processed_pseudogene
ENSG0000(	DLGAP4	1801.741	0.142529	0.000283	0.002519	protein_coding
ENSG0000(	LINC02549	51.03731	-0.34011	0.000283	0.002519	lncRNA
ENSG0000(	PYROXD1	927.2427	-0.27216	0.000283	0.002519	protein_coding
ENSG0000(	GMCL1	701.9821	-0.22251	0.000283	0.00252	protein_coding
ENSG0000(	SLAIN2	1349.522	-0.23374	0.000283	0.00252	protein_coding
ENSG0000(	TINAG	200.7863	-0.78481	0.000283	0.00252	protein_coding
ENSG0000(	LINC02365	14.37792	0.645664	0.000284	0.002521	lncRNA
ENSG0000(	PEPD	1424.636	-0.69872	0.000284	0.002523	protein_coding
ENSG0000(	ENSG0000(	71.84222	0.325676	0.000284	0.002523	TEC
ENSG0000(	RPS19BP1	598.2924	-0.16786	0.000285	0.002532	protein_coding
ENSG0000(	MOCS2	435.7025	-0.23597	0.000285	0.002534	protein_coding
ENSG0000(	QARS1	1524.59	-0.22241	0.000286	0.002537	protein_coding
ENSG0000(	SNAI2	261.1103	0.483919	0.000286	0.002542	protein_coding
ENSG0000(	ENSG0000(	364.836	0.221613	0.000287	0.002547	lncRNA
ENSG0000(	ENSG0000(	58.17732	0.613051	0.000288	0.002551	lncRNA
ENSG0000(	TEAD2	324.0653	0.387643	0.000288	0.002553	protein_coding
ENSG0000(	CERS2	1716.058	-0.41335	0.000288	0.002553	protein_coding
ENSG0000(	TMEM262	10.42774	1.212289	0.000288	0.002555	protein_coding
ENSG0000(	Telomerase	166.5936	0.472613	0.000288	0.002555	misc_RNA
ENSG0000(	ENSG0000(	45.63829	0.367232	0.000288	0.002555	lncRNA
ENSG0000(	ENSG0000(	13.45933	0.645716	0.00029	0.002564	lncRNA
ENSG0000(	TSR2	562.1842	-0.13843	0.00029	0.002571	protein_coding
ENSG0000(	DHDDS	1132.428	0.196654	0.000291	0.002578	protein_coding
ENSG0000(	LINC00471	221.6568	0.268153	0.000292	0.002581	lncRNA
ENSG0000(	LYN	2328.198	0.429364	0.000292	0.002586	protein_coding
ENSG0000(	ENSG0000(	554.1703	-0.22189	0.000293	0.002593	lncRNA
ENSG0000(	ENSG0000(	114.2792	0.88183	0.000293	0.002593	lncRNA
ENSG0000(	CDH8-AS1	104.8126	-0.24267	0.000294	0.0026	lncRNA
ENSG0000(	DPY19L1	1580.657	0.248773	0.000296	0.002615	protein_coding
ENSG0000(	MIR6753	16.27236	0.692291	0.000296	0.002615	miRNA
ENSG0000(	SLC27A2	345.2385	-0.7412	0.000296	0.002615	protein_coding
ENSG0000(	KLHL30-AS:	152.4898	0.280596	0.000297	0.00262	lncRNA
ENSG0000(	NELL2	501.8159	-0.5755	0.000298	0.002628	protein_coding
ENSG0000(	LINC01409	1322.073	0.211327	0.000298	0.00263	lncRNA

ENSG0000(	MRI1	1145.673	-0.18172	0.000298	0.00263	protein_coding
ENSG0000(	ENSG0000(	7.961025	1.072415	0.000299	0.002633	lncRNA
ENSG0000(	SPACA6P-A	369.9613	0.167615	0.000299	0.002633	lncRNA
ENSG0000(	VSTM5	47.42973	0.605572	0.000299	0.002635	protein_coding
ENSG0000(	SNCAIP	280.2352	0.450147	0.000299	0.002636	protein_coding
ENSG0000(	MT-ND3	2675.341	-0.64874	0.000299	0.002636	protein_coding
ENSG0000(	PDE6C	41.60325	0.366328	0.000299	0.002637	protein_coding
ENSG0000(	ENSG0000(	248.141	0.16526	0.0003	0.002637	lncRNA
ENSG0000(	STX18-AS1	4098.177	0.127686	0.0003	0.00264	lncRNA
ENSG0000(	ENG	792.0666	0.432233	0.000301	0.002645	protein_coding
ENSG0000(	NDUFC2	585.4122	-0.49063	0.000301	0.002647	protein_coding
ENSG0000(	ALPI	2140.909	-0.97896	0.000302	0.002655	protein_coding
ENSG0000(	TECTA	62.09256	0.570961	0.000302	0.002657	protein_coding
ENSG0000(	PIP4P1	773.2256	-0.17604	0.000303	0.00266	protein_coding
ENSG0000(	ENSG0000(	11.61795	0.734501	0.000303	0.002663	lncRNA
ENSG0000(	MT-ND2	12749.3	-0.64174	0.000303	0.002663	protein_coding
ENSG0000(	ENSG0000(	190.2136	-0.20561	0.000304	0.002666	unprocessed_pseudogene
ENSG0000(	ENSG0000(	353.0178	0.228814	0.000304	0.00267	lncRNA
ENSG0000(	COL9A1	363.8794	-0.20973	0.000305	0.002677	protein_coding
ENSG0000(	RPL41	4771.672	-0.28981	0.000305	0.002677	protein_coding
ENSG0000(	RN7SL442F	10.72734	0.618843	0.000305	0.002677	misc_RNA
ENSG0000(	ENSG0000(	834.3486	0.170053	0.000306	0.002685	TEC
ENSG0000(	MTA1	1329.01	-0.14707	0.000306	0.002685	protein_coding
ENSG0000(	ENSG0000(	14.30036	1.998735	0.000307	0.002686	unprocessed_pseudogene
ENSG0000(	ENSG0000(	20.72784	0.581663	0.000306	0.002686	lncRNA
ENSG0000(	RGS1	483.1223	0.844798	0.000307	0.00269	protein_coding
ENSG0000(	NFS1	807.1837	-0.15604	0.000308	0.002695	protein_coding
ENSG0000(	ENSG0000(	65.35118	0.301244	0.000308	0.0027	lncRNA
ENSG0000(	ZCCHC24	494.1617	0.43719	0.000309	0.002701	protein_coding
ENSG0000(	NANOGP7	18.00549	0.888	0.000309	0.002706	transcribed_processed_pseudoge
ENSG0000(	ENSG0000(	551.2676	0.234127	0.00031	0.00271	TEC
ENSG0000(	ASGR1	236.0832	0.300561	0.00031	0.002711	protein_coding
ENSG0000(	AZIN1	2501.962	-0.27493	0.00031	0.002711	protein_coding
ENSG0000(	TANC1	2801.258	0.255417	0.00031	0.002711	protein_coding
ENSG0000(	TMA7	523.629	-0.24273	0.00031	0.002712	protein_coding
ENSG0000(	IGHV1-46	113.4681	1.023943	0.000311	0.002714	IG_V_gene
ENSG0000(	SRRM3	40.99506	0.465356	0.000311	0.002719	protein_coding
ENSG0000(	ENSG0000(	637.0693	0.217927	0.000312	0.002726	lncRNA
ENSG0000(	ENSG0000(	93.2209	0.342254	0.000313	0.002729	lncRNA
ENSG0000(	MTHFSD	603.4905	0.165335	0.000315	0.002748	protein_coding
ENSG0000(	EVA1B	45.45198	0.48279	0.000315	0.002749	protein_coding
ENSG0000(	ENSG0000(	15.32771	0.633953	0.000316	0.002753	lncRNA
ENSG0000(	ENSG0000(	62.75569	-0.7129	0.000316	0.002753	lncRNA
ENSG0000(	ENSG0000(	38.46156	-0.36344	0.000316	0.002756	lncRNA
ENSG0000(	GEMIN2	284.569	-0.20598	0.000317	0.002764	protein_coding
ENSG0000(	ING4	425.1311	-0.21868	0.000317	0.002764	protein_coding
ENSG0000(	TMEM200E	107.5965	0.383636	0.000317	0.002764	protein_coding

ENSG0000(	DIRAS2	183.1613	-0.2896	0.000318	0.002766	protein_coding
ENSG0000(	ENSG0000(	23.38147	0.547887	0.000318	0.002769	lncRNA
ENSG0000(	UBE2M	219.3784	-0.24322	0.000318	0.002769	protein_coding
ENSG0000(	TIGD6	166.14	0.308652	0.000319	0.002771	protein_coding
ENSG0000(	FBXW7	1803.871	0.235353	0.000319	0.002773	protein_coding
ENSG0000(	U3	187.267	0.282706	0.000319	0.002774	snoRNA
ENSG0000(	ENSG0000(	150.663	0.318724	0.000319	0.002774	lncRNA
ENSG0000(	MRGPRF	112.2817	0.614803	0.000319	0.002774	protein_coding
ENSG0000(	PGRMC1	1232.487	-0.60093	0.000319	0.002775	protein_coding
ENSG0000(	YWHAZ	8671.153	-0.19021	0.00032	0.002776	protein_coding
ENSG0000(	ABCG2	3171.603	-1.0626	0.00032	0.002777	protein_coding
ENSG0000(	TDO2	292.8632	0.917852	0.00032	0.002779	protein_coding
ENSG0000(	ENPP7P3	12.97709	-1.21681	0.00032	0.002779	processed_pseudogene
ENSG0000(	UGT2A3	1307.666	-0.90778	0.00032	0.00278	protein_coding
ENSG0000(	ANKRD28	1879.623	0.235439	0.000321	0.002785	protein_coding
ENSG0000(	ENSG0000(	120.882	0.642605	0.000321	0.002785	protein_coding
ENSG0000(	ENSG0000(	162.2448	0.197563	0.000322	0.002785	TEC
ENSG0000(	ENSG0000(	56.87363	0.307353	0.000322	0.002785	lncRNA
ENSG0000(	RIPOR3-AS:	11.16568	0.852894	0.000321	0.002785	lncRNA
ENSG0000(	ENSG0000(	351.5811	0.180241	0.000323	0.002793	lncRNA
ENSG0000(	NUP50-DT	853.4913	0.157276	0.000323	0.002793	lncRNA
ENSG0000(	SNORA81	1234.744	-0.44662	0.000322	0.002793	snoRNA
ENSG0000(	UGT1A9	242.5378	-0.8971	0.000323	0.002793	protein_coding
ENSG0000(	NOP53	1761.181	-0.21002	0.000324	0.002801	protein_coding
ENSG0000(	TUSC8	138.1838	0.441197	0.000324	0.002801	lncRNA
ENSG0000(	PMP22	5469.796	-0.67633	0.000324	0.002804	protein_coding
ENSG0000(	UBL4A	277.2293	-0.29367	0.000324	0.002804	protein_coding
ENSG0000(	LRRC57	2021.297	-0.12054	0.000325	0.002805	protein_coding
ENSG0000(	RPL11P3	321.9413	0.205815	0.000325	0.002805	processed_pseudogene
ENSG0000(	SEPTIN9	6410.653	-0.16109	0.000325	0.002807	protein_coding
ENSG0000(	ENSG0000(	277.9977	-0.20113	0.000325	0.002809	lncRNA
ENSG0000(	ENSG0000(	387.4544	-0.68798	0.000325	0.002809	processed_pseudogene
ENSG0000(	ENSG0000(	62.46514	0.391201	0.000326	0.00281	lncRNA
ENSG0000(	FAM20B	1434.638	-0.12922	0.000326	0.00281	protein_coding
ENSG0000(	TMEM255E	321.8196	0.517544	0.000326	0.002813	protein_coding
ENSG0000(	SMDT1	371.3145	-0.2913	0.000326	0.002815	protein_coding
ENSG0000(	EIF4G3	3697.656	0.165693	0.000327	0.002821	protein_coding
ENSG0000(	NAALADL1	2096.145	-1.00199	0.000328	0.002822	protein_coding
ENSG0000(	CD47	9350.2	0.384523	0.000328	0.002823	protein_coding
ENSG0000(	ENSG0000(	335.6848	0.198768	0.000328	0.002825	lncRNA
ENSG0000(	ENSG0000(	179.2343	0.210122	0.000328	0.002825	TEC
ENSG0000(	GNA13	2611.45	-0.19404	0.000328	0.002827	protein_coding
ENSG0000(	SNORD55	26.42909	0.772883	0.000329	0.002828	snoRNA
ENSG0000(	METTL16	1777.724	-0.12344	0.000329	0.002831	protein_coding
ENSG0000(	SAMHD1	4422.63	0.280821	0.00033	0.002839	protein_coding
ENSG0000(	ENSG0000(	117.4116	0.294314	0.00033	0.00284	lncRNA
ENSG0000(	TICAM2	230.2373	0.618895	0.00033	0.00284	protein_coding

ENSG0000(	MAP3K12	999.3471	0.268944	0.00033	0.00284	protein_coding
ENSG0000(	DNASE1L3	722.5941	-0.44572	0.000331	0.002841	protein_coding
ENSG0000(	DGKQ	1304.578	-0.39264	0.000331	0.002842	protein_coding
ENSG0000(	GLT8D2	130.4218	0.446204	0.000331	0.002843	protein_coding
ENSG0000(	RPL17P10	41.89789	0.527509	0.000333	0.002861	processed_pseudogene
ENSG0000(	SULT1C2P1	121.9753	0.266925	0.000333	0.002861	transcribed_unprocessed_pseudo
ENSG0000(	MFSD9	871.939	-0.35525	0.000334	0.002862	protein_coding
ENSG0000(	LINC01715	64.933	0.322445	0.000334	0.002866	lncRNA
ENSG0000(	IGKV3-7	12.43407	1.282674	0.000335	0.002873	IG_V_gene
ENSG0000(	INHBB	25.00233	0.794406	0.000335	0.002874	protein_coding
ENSG0000(	KHDRBS1	1729.022	-0.13437	0.000336	0.002878	protein_coding
ENSG0000(	RCHY1	849.4331	-0.10964	0.000336	0.002881	protein_coding
ENSG0000(	ENSG0000(	496.9663	0.299128	0.000337	0.002888	TEC
ENSG0000(	CACNA1A	3859.604	0.095741	0.000337	0.002889	protein_coding
ENSG0000(	EIF4EBP2	5692.065	-0.44096	0.000337	0.002889	protein_coding
ENSG0000(	GLTP	1272.069	-0.22346	0.000338	0.002891	protein_coding
ENSG0000(	CYP51A1P3	48.5277	0.33955	0.000339	0.002901	processed_pseudogene
ENSG0000(	ENSG0000(	66.11883	0.310926	0.000339	0.002901	lncRNA
ENSG0000(	TMEM252	212.1558	-1.30122	0.000339	0.002901	protein_coding
ENSG0000(	ENSG0000(	86.72259	0.331381	0.00034	0.002905	lncRNA
ENSG0000(	MAST3	2226.868	-0.14879	0.00034	0.002905	protein_coding
ENSG0000(	STAG2	5174.373	0.2766	0.00034	0.002905	protein_coding
ENSG0000(	SLC16A3	1453.665	0.452404	0.00034	0.002906	protein_coding
ENSG0000(	SVIL	3163.774	0.318549	0.00034	0.002906	protein_coding
ENSG0000(	NAGS	384.356	-0.73387	0.000341	0.002911	protein_coding
ENSG0000(	MYO7A	487.5828	0.311665	0.000342	0.002921	protein_coding
ENSG0000(	TH2LCRR	11.98061	0.680751	0.000343	0.002928	lncRNA
ENSG0000(	IGBP1	538.5735	-0.31282	0.000343	0.002928	protein_coding
ENSG0000(	PGRMC2	1730.828	-0.45346	0.000343	0.002928	protein_coding
ENSG0000(	ZNF746	1581.677	0.139994	0.000344	0.002934	protein_coding
ENSG0000(	ALG8	2024.833	0.142051	0.000344	0.002935	protein_coding
ENSG0000(	ENSG0000(	1059.523	0.173715	0.000346	0.002949	TEC
ENSG0000(	BCL7B	515.0655	-0.20987	0.000347	0.002953	protein_coding
ENSG0000(	PSMA7	521.8582	-0.23668	0.000347	0.002953	protein_coding
ENSG0000(	ENSG0000(	68.80836	0.297735	0.000348	0.002963	lncRNA
ENSG0000(	ENSG0000(	70.81642	0.27212	0.000348	0.002966	lncRNA
ENSG0000(	LINC02394	91.47951	0.323508	0.000349	0.002968	lncRNA
ENSG0000(	TRIM22	4131.284	0.457966	0.00035	0.002982	protein_coding
ENSG0000(	ENSG0000(	14.87553	0.651567	0.000352	0.002994	lncRNA
ENSG0000(	ENSG0000(	128.9342	0.35346	0.000353	0.002999	lncRNA
ENSG0000(	RAD21	3548.535	-0.1527	0.000353	0.002999	protein_coding
ENSG0000(	MEIS3P1	45.84581	0.547699	0.000353	0.002999	processed_pseudogene
ENSG0000(	CDC42EP3	1238.787	0.188919	0.000353	0.002999	protein_coding
ENSG0000(	ENSG0000(	92.4678	0.309416	0.000353	0.002999	lncRNA
ENSG0000(	ZNF91	8398.948	-0.37324	0.000353	0.003	protein_coding
ENSG0000(	GPD1L	1121.671	-0.38639	0.000354	0.003005	protein_coding
ENSG0000(	SMIM19	380.51	-0.36478	0.000354	0.003006	protein_coding

ENSG0000(	TRPV3	1469.866	-0.71125	0.000354	0.003007	protein_coding
ENSG0000(	ENSG0000(	23.22794	0.502036	0.000355	0.003013	lncRNA
ENSG0000(	BNIP3P4	60.66418	0.983385	0.000355	0.003015	processed_pseudogene
ENSG0000(	CYP51A1	1006.965	-0.46154	0.000357	0.003024	protein_coding
ENSG0000(	KCNA2	311.7376	0.533541	0.000357	0.003026	protein_coding
ENSG0000(	ENSG0000(	131.1656	0.279205	0.000358	0.003031	lncRNA
ENSG0000(	LINC01707	134.7551	0.218301	0.000358	0.003035	lncRNA
ENSG0000(	IGF1R	2435.683	0.238058	0.000358	0.003035	protein_coding
ENSG0000(	ENSG0000(	196.8879	-0.30534	0.000359	0.003037	lncRNA
ENSG0000(	OR56B1	21.39837	0.968704	0.000359	0.003037	protein_coding
ENSG0000(	PKD1L2	964.3721	0.170322	0.000359	0.003037	polymorphic_pseudogene
ENSG0000(	CEPT1	2514.832	-0.16715	0.000359	0.003041	protein_coding
ENSG0000(	ATG12	2238.918	-0.12626	0.00036	0.003043	protein_coding
ENSG0000(	ENSG0000(	56.86511	0.339812	0.00036	0.003045	lncRNA
ENSG0000(	ENSG0000(	22.34686	-1.10841	0.000361	0.003053	lncRNA
ENSG0000(	TMEM134	939.8851	-0.17826	0.000361	0.003055	protein_coding
ENSG0000(	ARRDC3	1566.968	0.298855	0.000362	0.003055	protein_coding
ENSG0000(	SF3B2	2234.56	-0.12054	0.000362	0.003055	protein_coding
ENSG0000(	SLC25A38	467.5009	-0.2217	0.000362	0.003055	protein_coding
ENSG0000(	SUCLG2	1272.95	-0.31371	0.000362	0.003055	protein_coding
ENSG0000(	FOXC1	23.12269	0.758626	0.000362	0.003059	protein_coding
ENSG0000(	APLN	15.20258	1.165216	0.000363	0.003064	protein_coding
ENSG0000(	OR52K1	424.6294	0.2068	0.000363	0.003064	protein_coding
ENSG0000(	ENSG0000(	48.53915	0.322357	0.000364	0.003067	lncRNA
ENSG0000(	IL3RA	91.08173	0.760681	0.000364	0.003067	protein_coding
ENSG0000(	CHID1	530.5588	-0.23344	0.000365	0.003072	protein_coding
ENSG0000(	LAMTOR4	438.2447	-0.19009	0.000364	0.003072	protein_coding
ENSG0000(	ENSG0000(	48.54914	0.499876	0.000366	0.003085	TEC
ENSG0000(	TREH	494.8702	-0.94147	0.000367	0.003088	protein_coding
ENSG0000(	DNAI3	17.3117	1.083791	0.000367	0.003089	protein_coding
ENSG0000(	ENSG0000(	21.10171	0.581176	0.000367	0.003093	lncRNA
ENSG0000(	IGHV3-33	358.0762	0.968984	0.000368	0.003095	IG_V_gene
ENSG0000(	PWP2	158.5403	-0.63366	0.000368	0.003095	protein_coding
ENSG0000(	TSHZ1	1266.577	-0.41681	0.000368	0.003098	protein_coding
ENSG0000(	ZNF770	1267.669	-0.14261	0.000369	0.003102	protein_coding
ENSG0000(	CYP2D8P	59.97489	-0.37778	0.000369	0.003105	unprocessed_pseudogene
ENSG0000(	TONSL	908.1316	-0.12495	0.00037	0.003113	protein_coding
ENSG0000(	ZNF616	607.0029	-0.20982	0.000371	0.003114	protein_coding
ENSG0000(	MRPL37P1	123.519	0.340484	0.000373	0.003135	processed_pseudogene
ENSG0000(	ENSG0000(	1498.042	0.1916	0.000373	0.003136	lncRNA
ENSG0000(	OR10G3	935.0096	0.12967	0.000374	0.003136	protein_coding
ENSG0000(	ENSG0000(	16.78148	1.188894	0.000374	0.003143	lncRNA
ENSG0000(	SLC43A3	449.2981	0.466329	0.000375	0.003145	protein_coding
ENSG0000(	FGF2	282.2246	0.352318	0.000375	0.003148	protein_coding
ENSG0000(	SFI1	3507.717	-0.15692	0.000376	0.00315	protein_coding
ENSG0000(	CDC25B	1300.832	0.39812	0.000376	0.003152	protein_coding
ENSG0000(	DNAJB5	168.3558	0.338339	0.000376	0.003154	protein_coding

ENSG0000(	DGAT1	4085.533	-0.75175	0.000377	0.003155	protein_coding
ENSG0000(	SNRPGP9	16.23906	0.666679	0.000377	0.003157	processed_pseudogene
ENSG0000(	NDFIP2	1190.605	-0.59564	0.000377	0.003161	protein_coding
ENSG0000(	LSM14B	747.7731	-0.14759	0.000378	0.003162	protein_coding
ENSG0000(	EMC10	2601.196	-0.16613	0.000378	0.003163	protein_coding
ENSG0000(	ENSG0000(	59.60869	0.323536	0.000378	0.003166	lncRNA
ENSG0000(	SERPINB9P	1045.185	0.284723	0.000379	0.003168	lncRNA
ENSG0000(	TUFM	729.909	-0.26071	0.000379	0.003168	protein_coding
ENSG0000(	IQSEC1	2528.271	0.471971	0.000379	0.003172	protein_coding
ENSG0000(	GPR155	1755.781	0.292936	0.000379	0.003172	protein_coding
ENSG0000(	ZNF341	400.0702	0.145342	0.00038	0.003173	protein_coding
ENSG0000(	LMCD1-AS:	794.8034	0.165279	0.00038	0.003175	lncRNA
ENSG0000(	IGKV1-6	80.38595	1.015083	0.00038	0.003176	IG_V_gene
ENSG0000(	RPL29	1937.126	-0.24596	0.000381	0.003177	protein_coding
ENSG0000(	EFNA2	799.797	0.634823	0.000381	0.00318	protein_coding
ENSG0000(	SRGAP2B	876.2918	0.293505	0.000381	0.003182	protein_coding
ENSG0000(	SCMH1	1109.548	-0.18904	0.000382	0.003185	protein_coding
ENSG0000(	TAGAP-AS1	1531.521	0.15503	0.000382	0.003188	lncRNA
ENSG0000(	ADGRF5	617.6707	0.469193	0.000382	0.003189	protein_coding
ENSG0000(	ENSG0000(	40.58494	0.558454	0.000383	0.003193	transcribed_processed_pseudoge
ENSG0000(	ENSG0000(	59.05698	0.350283	0.000383	0.003194	lncRNA
ENSG0000(	GALNT1	1467.444	-0.27117	0.000383	0.003194	protein_coding
ENSG0000(	PLAAT2	75.68019	-0.89225	0.000384	0.003199	protein_coding
ENSG0000(	ANKLE2	5685.358	0.16922	0.000384	0.0032	protein_coding
ENSG0000(	RPS12P3	63.6167	-0.53262	0.000385	0.003202	processed_pseudogene
ENSG0000(	ENSG0000(	23.47006	-0.98719	0.000385	0.003203	protein_coding
ENSG0000(	ENSG0000(	474.2633	-0.25257	0.000386	0.003212	transcribed_unprocessed_pseudo
ENSG0000(	SP2-AS1	160.7365	0.255362	0.000386	0.003214	lncRNA
ENSG0000(	ENSG0000(	26.78214	0.533076	0.000387	0.003214	lncRNA
ENSG0000(	NAA11	25.53665	0.603654	0.000386	0.003214	protein_coding
ENSG0000(	ENSG0000(	16.74902	0.690012	0.000387	0.003218	lncRNA
ENSG0000(	FBXO3	1897.976	-0.14378	0.000388	0.003223	protein_coding
ENSG0000(	CXCL14	3809.558	-0.3786	0.000388	0.003224	protein_coding
ENSG0000(	CYCSP28	10.36964	0.704973	0.000388	0.003226	processed_pseudogene
ENSG0000(	EIF4H	3270.395	-0.14747	0.000388	0.003226	protein_coding
ENSG0000(	IL12B	33.60831	0.44082	0.000389	0.003229	protein_coding
ENSG0000(	ENSG0000(	23.87361	0.876845	0.000391	0.003244	lncRNA
ENSG0000(	AP1B1	1997.251	-0.19893	0.000392	0.00325	protein_coding
ENSG0000(	AOAH	1005.635	0.337148	0.000393	0.003255	protein_coding
ENSG0000(	DDHD2	2610.36	-0.20958	0.000393	0.003255	protein_coding
ENSG0000(	ENSG0000(	34.34258	-0.59274	0.000393	0.003255	processed_pseudogene
ENSG0000(	ENSG0000(	193.4685	0.308046	0.000393	0.003255	protein_coding
ENSG0000(	GOLGA6L9	912.0888	0.223513	0.000393	0.003257	protein_coding
ENSG0000(	ENSG0000(	90.4652	0.468243	0.000394	0.003266	lncRNA
ENSG0000(	SLC46A3	1916.331	-0.60187	0.000395	0.00327	protein_coding
ENSG0000(	DACT3-AS1	18.7567	0.699817	0.000395	0.003272	lncRNA
ENSG0000(	PPP1CB	3334.073	-0.25292	0.000396	0.003274	protein_coding

ENSG0000(	ENSG0000(	246.6096	0.180362	0.000396	0.003274	lncRNA
ENSG0000(	ZNF137P	365.2828	-0.30472	0.000396	0.003276	transcribed_unprocessed_pseudo
ENSG0000(	ATP11A	2282.715	0.183959	0.000396	0.003277	protein_coding
ENSG0000(	ENSG0000(	75.36874	-0.28455	0.000396	0.003277	lncRNA
ENSG0000(	GPR180	555.2962	0.263608	0.000396	0.003277	protein_coding
ENSG0000(	ENSG0000(	169.9779	0.242909	0.000397	0.003279	lncRNA
ENSG0000(	FN3KRP	447.318	-0.23016	0.000397	0.003279	protein_coding
ENSG0000(	ENSG0000(	169.4244	0.246131	0.000397	0.00328	TEC
ENSG0000(	HLX	287.3541	0.490144	0.000397	0.00328	protein_coding
ENSG0000(	SNORA55	68.16739	0.375957	0.000397	0.00328	snoRNA
ENSG0000(	ENSG0000(	15.86079	0.516203	0.000398	0.003284	lncRNA
ENSG0000(	ENSG0000(	20.91126	0.466862	0.000398	0.003284	lncRNA
ENSG0000(	SNORD53B	10.25682	0.686454	0.000399	0.003289	snoRNA
ENSG0000(	LRRC25	226.1033	0.311436	0.0004	0.003299	protein_coding
ENSG0000(	ZNF133	654.5974	0.142522	0.000402	0.003316	protein_coding
ENSG0000(	HEBP1	972.2611	-0.58682	0.000403	0.003319	protein_coding
ENSG0000(	ENSG0000(	122.1405	0.240431	0.000403	0.003319	lncRNA
ENSG0000(	SF3B6	222.3511	-0.3186	0.000403	0.00332	protein_coding
ENSG0000(	SSC5D	259.8183	0.422961	0.000403	0.00332	protein_coding
ENSG0000(	HMGXB4	738.8392	0.157909	0.000403	0.003321	protein_coding
ENSG0000(	H2BC6	203.5163	-0.59525	0.000404	0.003322	protein_coding
ENSG0000(	ANKRD6	464.0846	0.208657	0.000404	0.003328	protein_coding
ENSG0000(	LINC02055	709.4154	0.211357	0.000405	0.003331	lncRNA
ENSG0000(	IK	1185.012	-0.12605	0.000405	0.003333	protein_coding
ENSG0000(	NUAK2	1122.438	-0.29048	0.000405	0.003333	protein_coding
ENSG0000(	SUDS3	2084.998	-0.14808	0.000405	0.003333	protein_coding
ENSG0000(	C5AR1	455.5943	-0.22773	0.000406	0.003337	protein_coding
ENSG0000(	DDT	663.5133	-0.29643	0.000406	0.003337	protein_coding
ENSG0000(	NLRP12	343.7365	0.185454	0.000406	0.003337	protein_coding
ENSG0000(	RNY3P2	14.69887	0.603925	0.000406	0.003337	misc_RNA
ENSG0000(	ENSG0000(	702.6337	-0.33509	0.000407	0.003338	protein_coding
ENSG0000(	ENSG0000(	61.32237	0.285773	0.000408	0.00335	lncRNA
ENSG0000(	PADI2	961.024	-0.73161	0.000408	0.00335	protein_coding
ENSG0000(	ENSG0000(	12.76608	0.869309	0.000408	0.003351	lncRNA
ENSG0000(	RNU7-119F	11.14123	0.897983	0.000409	0.003351	snRNA
ENSG0000(	E2F7	141.4145	0.597684	0.000409	0.003353	protein_coding
ENSG0000(	MYO15B	14810.65	-0.44034	0.000409	0.003353	protein_coding
ENSG0000(	DRAM1	968.9603	0.281955	0.000409	0.003356	protein_coding
ENSG0000(	ENSG0000(	594.361	0.23845	0.00041	0.003356	lncRNA
ENSG0000(	THAP3	194.5901	-0.20271	0.00041	0.003361	protein_coding
ENSG0000(	RCN1	740.6296	0.387585	0.00041	0.003362	protein_coding
ENSG0000(	ENSG0000(	158.2505	0.298742	0.000412	0.003377	lncRNA
ENSG0000(	BPIFB1	133.8517	0.495489	0.000413	0.003383	protein_coding
ENSG0000(	MOB2	701.5506	-0.19232	0.000413	0.003384	protein_coding
ENSG0000(	AIMP2	212.5785	-0.22853	0.000414	0.003387	protein_coding
ENSG0000(	LINC01776	493.813	0.152843	0.000415	0.003393	lncRNA
ENSG0000(	AOAH-IT1	108.6161	0.476779	0.000415	0.003394	lncRNA

ENSG0000(	FAM117B	1368.284	-0.23385	0.000415	0.003395	protein_coding
ENSG0000(	SRP9	1638.475	-0.16143	0.000415	0.003395	protein_coding
ENSG0000(	ADAMTS9	711.0552	0.460598	0.000417	0.00341	protein_coding
ENSG0000(	ENSG0000(	490.5471	0.170116	0.000417	0.00341	lncRNA
ENSG0000(	IRF4	1686.843	0.756561	0.000418	0.00341	protein_coding
ENSG0000(	MR1	1658.247	0.230355	0.000418	0.003416	protein_coding
ENSG0000(	ENSG0000(	435.6928	0.214959	0.000419	0.003417	processed_pseudogene
ENSG0000(	MED31	128.4307	-0.29343	0.00042	0.003431	protein_coding
ENSG0000(	ENSG0000(	100.6845	0.25973	0.000421	0.003436	processed_pseudogene
ENSG0000(	ENSG0000(	155.1761	0.241715	0.000421	0.003436	lncRNA
ENSG0000(	GCNT2	1460.029	-0.25309	0.000422	0.003442	protein_coding
ENSG0000(	MIR4635	24.99517	-0.72345	0.000422	0.003442	miRNA
ENSG0000(	VIL1	7019.496	-0.78199	0.000422	0.003442	protein_coding
ENSG0000(	MIR548O2	16.30763	0.578168	0.000423	0.003443	miRNA
ENSG0000(	C9orf153	87.9907	-0.34667	0.000423	0.003444	protein_coding
ENSG0000(	MEP1B	3162.665	-1.06753	0.000424	0.003451	protein_coding
ENSG0000(	NFILZ	323.1721	0.200369	0.000424	0.003453	protein_coding
ENSG0000(	LINC02196	79.3776	0.322543	0.000424	0.003455	lncRNA
ENSG0000(	CPB2-AS1	879.1251	0.201199	0.000425	0.00346	lncRNA
ENSG0000(	ENSG0000(	215.7094	0.38007	0.000426	0.003469	TEC
ENSG0000(	PSMD2	974.6944	-0.2831	0.000427	0.00347	protein_coding
ENSG0000(	ENSG0000(	862.3229	0.515213	0.000427	0.003472	lncRNA
ENSG0000(	PDK2	911.7601	-0.55799	0.000429	0.003487	protein_coding
ENSG0000(	ENSG0000(	13.76385	0.559575	0.000429	0.003489	lncRNA
ENSG0000(	ENSG0000(	58.26743	0.378687	0.000431	0.003498	TEC
ENSG0000(	ENSG0000(	391.2696	0.21896	0.000431	0.003498	lncRNA
ENSG0000(	TRIM7-AS1	278.5931	-0.25149	0.000431	0.0035	lncRNA
ENSG0000(	GAL3ST1	255.8664	-0.89205	0.000433	0.003511	protein_coding
ENSG0000(	ENSG0000(	1044.443	-0.19488	0.000433	0.003514	transcribed_unprocessed_pseudo
ENSG0000(	HAUS1	275.8128	-0.22908	0.000434	0.003522	protein_coding
ENSG0000(	NDUFS1	4565.23	-0.23129	0.000434	0.003524	protein_coding
ENSG0000(	DUSP10	230.3524	0.483184	0.000435	0.003528	protein_coding
ENSG0000(	C4BPA	50.65287	0.44568	0.000435	0.003529	protein_coding
ENSG0000(	ECHS1	564.2639	-0.47407	0.000435	0.003529	protein_coding
ENSG0000(	IGKV2-28	354.9885	1.429924	0.000436	0.003533	IG_V_gene
ENSG0000(	DDX46	1860.311	-0.1363	0.000437	0.003537	protein_coding
ENSG0000(	FKBP7	395.3273	0.180931	0.000437	0.003539	protein_coding
ENSG0000(	ENSG0000(	9.679802	0.618714	0.000438	0.003544	processed_pseudogene
ENSG0000(	ENSG0000(	541.6709	-0.13597	0.000438	0.003544	lncRNA
ENSG0000(	RORA	2152.372	0.267189	0.000438	0.003544	protein_coding
ENSG0000(	ENSG0000(	91.50335	0.30292	0.000438	0.003546	lncRNA
ENSG0000(	CYP4B1	72.85668	-0.3237	0.000438	0.003546	protein_coding
ENSG0000(	AKR1C1	358.1335	0.372269	0.000439	0.00355	protein_coding
ENSG0000(	VWFP1	11.12961	1.337978	0.000439	0.00355	transcribed_unprocessed_pseudo
ENSG0000(	BUD31	449.9471	-0.16341	0.00044	0.00356	protein_coding
ENSG0000(	CCDC170	223.1027	0.213927	0.000442	0.003572	protein_coding
ENSG0000(	ZNF281	830.7911	0.300605	0.000443	0.003582	protein_coding

ENSG0000(	MAF1	508.6765	-0.2378	0.000444	0.003582	protein_coding
ENSG0000(	PFDN5	1329.83	-0.25411	0.000444	0.003583	protein_coding
ENSG0000(	MID1	2216.907	0.150294	0.000444	0.003588	protein_coding
ENSG0000(	FKBP11	1146.706	0.303148	0.000445	0.003588	protein_coding
ENSG0000(	PRSS53	457.0268	-0.50352	0.000445	0.003589	protein_coding
ENSG0000(	BICRAL	1617.229	-0.13283	0.000446	0.003594	protein_coding
ENSG0000(	ENSG0000(	98.73672	0.265502	0.000447	0.003603	lncRNA
ENSG0000(	ENSG0000(	874.8888	-0.16296	0.000447	0.003603	TEC
ENSG0000(	KLHL2	437.8503	0.278481	0.000447	0.003603	protein_coding
ENSG0000(	FER1L5	225.5507	0.168814	0.000447	0.003604	protein_coding
ENSG0000(	SNORD13	650.746	0.492508	0.000448	0.003607	snoRNA
ENSG0000(	LINC01139	260.3529	0.327497	0.00045	0.00362	lncRNA
ENSG0000(	SNORD105	15.01467	0.83526	0.00045	0.00362	snoRNA
ENSG0000(	ENSG0000(	356.7102	0.172743	0.00045	0.003625	TEC
ENSG0000(	ITGA3	2872.421	-0.39733	0.000451	0.003627	protein_coding
ENSG0000(	NPY	17.23454	-1.26141	0.000453	0.003642	protein_coding
ENSG0000(	ENSG0000(	88.84106	0.311541	0.000453	0.003647	lncRNA
ENSG0000(	ENSG0000(	34.72941	0.444957	0.000454	0.003648	lncRNA
ENSG0000(	CBLB	3299.351	0.142117	0.000454	0.003648	protein_coding
ENSG0000(	RNU7-124F	16.21887	0.709894	0.000454	0.003648	snRNA
ENSG0000(	SDHD	1453.141	-0.26007	0.000454	0.00365	protein_coding
ENSG0000(	RPS20	3698.227	-0.34264	0.000455	0.003656	protein_coding
ENSG0000(	ENSG0000(	218.0872	0.327204	0.000456	0.003666	lncRNA
ENSG0000(	CYP2U1	486.1553	-0.26939	0.000458	0.003676	protein_coding
ENSG0000(	GFER	424.3291	-0.48831	0.000458	0.003676	protein_coding
ENSG0000(	SERPINA3	82.37509	0.972884	0.000458	0.003676	protein_coding
ENSG0000(	YBX1	2884.844	-0.27694	0.000459	0.003679	protein_coding
ENSG0000(	DUSP14	44.40891	0.610269	0.000459	0.00368	protein_coding
ENSG0000(	RPS28	1048.447	-0.34258	0.000459	0.00368	protein_coding
ENSG0000(	CASP1	1685.435	0.376228	0.000461	0.003693	protein_coding
ENSG0000(	ARNTL	844.332	0.306173	0.000461	0.003695	protein_coding
ENSG0000(	NUP54	533.2073	-0.14614	0.000461	0.003695	protein_coding
ENSG0000(	C2CD5	2075.976	-0.18315	0.000462	0.003699	protein_coding
ENSG0000(	INPP5B	2624.475	-0.15005	0.000462	0.003699	protein_coding
ENSG0000(	ENSG0000(	33.51271	0.36043	0.000463	0.003709	lncRNA
ENSG0000(	FGD1	112.1293	0.413476	0.000464	0.00371	protein_coding
ENSG0000(	LAMB3	1348.504	-0.68279	0.000464	0.00371	protein_coding
ENSG0000(	ANK1	178.1337	0.561945	0.000465	0.003718	protein_coding
ENSG0000(	TRHDE	872.6192	-0.75297	0.000467	0.003736	protein_coding
ENSG0000(	ENSG0000(	51.16032	0.376196	0.000468	0.003744	lncRNA
ENSG0000(	MCRIP2	1025.24	-0.39469	0.000468	0.003744	protein_coding
ENSG0000(	ENSG0000(	10.50233	0.764489	0.00047	0.003755	lncRNA
ENSG0000(	SRSF8	1023.872	-0.20717	0.00047	0.003755	protein_coding
ENSG0000(	LINC01238	678.5216	0.251033	0.00047	0.003758	lncRNA
ENSG0000(	SF3A1	1561.459	-0.18899	0.000471	0.003758	protein_coding
ENSG0000(	LINC00565	423.3785	0.186218	0.000471	0.00376	lncRNA
ENSG0000(	TMEM72	15.2884	-1.00689	0.000471	0.003761	protein_coding

ENSG0000(	ENSG0000(	142.7691	0.407609	0.000472	0.003763	lncRNA
ENSG0000(	ENSG0000(	84.06224	0.400031	0.000472	0.003765	TEC
ENSG0000(	FBXL19-AS:	625.8836	0.127095	0.000472	0.003765	lncRNA
ENSG0000(	TMIGD1	1327.32	-0.96136	0.000472	0.003765	protein_coding
ENSG0000(	GREB1	665.8493	0.177612	0.000474	0.003776	protein_coding
ENSG0000(	TGOLN2	10319.61	-0.18276	0.000474	0.003778	protein_coding
ENSG0000(	ENSG0000(	412.263	0.191858	0.000474	0.003779	lncRNA
ENSG0000(	OSBPL10-A	70.24477	-0.37628	0.000475	0.003781	lncRNA
ENSG0000(	ENSG0000(	56.8101	0.539294	0.000476	0.003786	lncRNA
ENSG0000(	LINC00963	2556.797	0.151134	0.000476	0.003786	lncRNA
ENSG0000(	MRO	440.8646	-0.47052	0.000476	0.003787	protein_coding
ENSG0000(	PRODH	4570.941	-0.8756	0.000477	0.003796	protein_coding
ENSG0000(	MS4A14	223.4766	0.555107	0.000477	0.003798	protein_coding
ENSG0000(	EHF	3041.322	0.5263	0.000478	0.0038	protein_coding
ENSG0000(	SLC19A3	1675.069	-0.44074	0.000478	0.0038	protein_coding
ENSG0000(	OOSP1	37.06347	0.394542	0.000478	0.003802	protein_coding
ENSG0000(	MCF2L2	1373.483	0.178038	0.000479	0.003805	protein_coding
ENSG0000(	TMEM161E	1870.571	-0.12444	0.000479	0.00381	protein_coding
ENSG0000(	EGFLAM	145.8743	0.345743	0.00048	0.003813	protein_coding
ENSG0000(	ARMC12	11.49451	0.881958	0.00048	0.003815	protein_coding
ENSG0000(	FUNDC2	621.5204	-0.17467	0.00048	0.003815	protein_coding
ENSG0000(	LINC00844	312.2005	0.140714	0.000481	0.003818	lncRNA
ENSG0000(	GNB1	4773.91	-0.22207	0.000481	0.003821	protein_coding
ENSG0000(	ENSG0000(	27.02695	0.464467	0.000482	0.003824	lncRNA
ENSG0000(	ENSG0000(	63.18614	0.281593	0.000482	0.003824	lncRNA
ENSG0000(	FOXK1	3578.866	0.148209	0.000482	0.003824	protein_coding
ENSG0000(	HRNR	16.17846	1.353499	0.000482	0.003824	protein_coding
ENSG0000(	OR2L2	732.204	-0.20624	0.000482	0.003824	protein_coding
ENSG0000(	ACAN	184.113	0.212203	0.000483	0.003826	protein_coding
ENSG0000(	PANK3	8007.741	-0.41702	0.000483	0.003826	protein_coding
ENSG0000(	HCG4B	37.89409	0.512745	0.000484	0.003834	unprocessed_pseudogene
ENSG0000(	ENSG0000(	214.4427	0.268316	0.000484	0.003834	lncRNA
ENSG0000(	S100A4	132.9701	0.645688	0.000485	0.003838	protein_coding
ENSG0000(	EHBP1	1365.461	-0.15432	0.000485	0.003839	protein_coding
ENSG0000(	CST3	1536.929	-0.40635	0.000486	0.003841	protein_coding
ENSG0000(	ENSG0000(	1882.349	-0.11125	0.000487	0.003849	protein_coding
ENSG0000(	TMEM41B	1627.576	-0.21338	0.000487	0.003849	protein_coding
ENSG0000(	ENSG0000(	519.128	0.194758	0.000488	0.003858	TEC
ENSG0000(	ZNF737	2420.706	-0.20885	0.000488	0.003858	protein_coding
ENSG0000(	SHROOM4	1405.778	0.21994	0.000488	0.003859	protein_coding
ENSG0000(	MTCO1P25	201.0823	-0.22667	0.000489	0.003859	unprocessed_pseudogene
ENSG0000(	CLEC2B	465.9569	0.430887	0.000489	0.003861	protein_coding
ENSG0000(	PSMC1	960.5377	-0.26615	0.000489	0.003864	protein_coding
ENSG0000(	LRMDA	455.5149	0.293715	0.00049	0.003865	protein_coding
ENSG0000(	TENT4A	1201.22	0.149236	0.00049	0.003866	protein_coding
ENSG0000(	TOE1	215.1123	-0.21891	0.00049	0.003866	protein_coding
ENSG0000(	TRAF6	1926.304	-0.20808	0.00049	0.003869	protein_coding

ENSG0000(	ENSG0000(	16.746	0.532478	0.000493	0.00389	lncRNA
ENSG0000(	CORO1C	1544.88	0.225062	0.000494	0.003891	protein_coding
ENSG0000(	DUSP11	699.4115	-0.14716	0.000494	0.003893	protein_coding
ENSG0000(	ZNF691	237.9054	0.237991	0.000494	0.003893	protein_coding
ENSG0000(	RBMS1	2089.183	0.338232	0.000495	0.003896	protein_coding
ENSG0000(	SEMA3G	609.2069	-0.80655	0.000496	0.003908	protein_coding
ENSG0000(	AK3	2418.285	-0.30163	0.000497	0.003913	protein_coding
ENSG0000(	ENSG0000(	58.25101	0.338211	0.000497	0.003916	lncRNA
ENSG0000(	ENSG0000(	10.44554	0.784408	0.000498	0.003921	lncRNA
ENSG0000(	SPINT2	2090.416	-0.50026	0.0005	0.003931	protein_coding
ENSG0000(	ENSG0000(	45.09509	0.334042	0.0005	0.003932	lncRNA
ENSG0000(	ENSG0000(	203.4677	0.224324	0.000501	0.003936	lncRNA
ENSG0000(	H3C7	109.4953	-0.53754	0.000501	0.003936	protein_coding
ENSG0000(	F5	384.7446	0.469005	0.000502	0.003944	protein_coding
ENSG0000(	PLEKHG6	1378.231	-0.72877	0.000502	0.003948	protein_coding
ENSG0000(	HTT	5245.226	-0.13125	0.000503	0.003951	protein_coding
ENSG0000(	FAM89A	70.67374	0.602888	0.000503	0.003953	protein_coding
ENSG0000(	ENSG0000(	45.31999	-0.86926	0.000503	0.003954	unprocessed_pseudogene
ENSG0000(	ENSG0000(	25.9141	0.525297	0.000504	0.003956	lncRNA
ENSG0000(	SNORA7A	431.3529	0.380892	0.000504	0.003956	snoRNA
ENSG0000(	ERCC4	1799.437	0.154862	0.000505	0.003958	protein_coding
ENSG0000(	IGHV1-2	210.0413	1.104676	0.000504	0.003958	IG_V_gene
ENSG0000(	SLC4A11	34.37536	0.773122	0.000505	0.003959	protein_coding
ENSG0000(	SLFN14	428.7903	0.13624	0.000505	0.003961	protein_coding
ENSG0000(	NAGLU	441.7844	0.201283	0.000506	0.003965	protein_coding
ENSG0000(	TMEM218	801.7681	0.205288	0.000507	0.003973	protein_coding
ENSG0000(	FLACC1	177.6098	-0.27265	0.000509	0.003989	protein_coding
ENSG0000(	LINC02336	225.2224	0.239864	0.000509	0.003989	lncRNA
ENSG0000(	RN7SL449F	23.40191	0.521785	0.000509	0.003989	misc_RNA
ENSG0000(	LYPD9P	15.32812	0.580812	0.00051	0.003991	transcribed_unitary_pseudogene
ENSG0000(	MXD3	240.1056	0.341269	0.00051	0.003992	protein_coding
ENSG0000(	PINLYP	335.9105	0.145801	0.00051	0.003992	protein_coding
ENSG0000(	ENSG0000(	186.5084	0.171709	0.000511	0.003997	lncRNA
ENSG0000(	TMBIM6	9598.353	-0.34211	0.000511	0.003997	protein_coding
ENSG0000(	ENSG0000(	11.3934	0.893895	0.000512	0.004003	lncRNA
ENSG0000(	ENSG0000(	11.99804	0.549604	0.000513	0.004007	lncRNA
ENSG0000(	ENSG0000(	300.2391	-0.16994	0.000513	0.004008	transcribed_unprocessed_pseudo
ENSG0000(	ENSG0000(	1075.441	0.132752	0.000513	0.004008	lncRNA
ENSG0000(	CREM	307.7497	0.240488	0.000513	0.00401	protein_coding
ENSG0000(	ENSG0000(	95.12258	0.24098	0.000514	0.004014	lncRNA
ENSG0000(	ENSG0000(	49.09762	0.371863	0.000514	0.004016	lncRNA
ENSG0000(	RN7SL275F	582.1252	0.195593	0.000515	0.004023	misc_RNA
ENSG0000(	IGFBP6	34.25895	-0.53284	0.000516	0.004023	protein_coding
ENSG0000(	ELK1	345.5329	0.240182	0.000516	0.004028	protein_coding
ENSG0000(	GPR17	13.57276	0.843356	0.000517	0.00403	protein_coding
ENSG0000(	ENSG0000(	61.6108	0.580442	0.000518	0.004039	TEC
ENSG0000(	RNU7-40P	22.18154	0.63599	0.00052	0.004053	snRNA

ENSG0000(	ESR1	133.5502	0.837034	0.000521	0.004061	protein_coding
ENSG0000(	SCGB2B2	376.6846	0.296906	0.000522	0.004067	protein_coding
ENSG0000(	SELP	137.644	0.722504	0.000523	0.004072	protein_coding
ENSG0000(	ATP13A4-A	59.84946	0.378312	0.000523	0.004077	lncRNA
ENSG0000(	SNRPD2	287.6783	-0.31008	0.000524	0.004077	protein_coding
ENSG0000(	ENSG0000(	2394.947	0.508456	0.000526	0.004091	processed_pseudogene
ENSG0000(	PSME3	1051.82	-0.1913	0.000526	0.004091	protein_coding
ENSG0000(	ENSG0000(	296.832	0.214684	0.000526	0.004091	lncRNA
ENSG0000(	ENSG0000(	225.7696	0.22384	0.000526	0.004092	lncRNA
ENSG0000(	LSM6	364.2926	-0.18968	0.000526	0.004092	protein_coding
ENSG0000(	PGAP2	475.5652	-0.21548	0.000527	0.004096	protein_coding
ENSG0000(	SEC22B	3888.673	0.140796	0.000527	0.0041	protein_coding
ENSG0000(	HLA-F	3268.64	0.338267	0.000531	0.004123	protein_coding
ENSG0000(	DIABLO	1035.727	-0.11586	0.000531	0.004125	protein_coding
ENSG0000(	BTN2A2	1359.589	0.491555	0.000532	0.004128	protein_coding
ENSG0000(	CYP2J2	1139.126	-0.92739	0.000532	0.004128	protein_coding
ENSG0000(	ENSG0000(	53.34074	0.271273	0.000532	0.004128	lncRNA
ENSG0000(	ENSG0000(	15.95005	0.819053	0.000532	0.004128	lncRNA
ENSG0000(	MINAR2	407.0087	0.216586	0.000532	0.004128	protein_coding
ENSG0000(	SLC39A13	412.1379	0.306576	0.000532	0.004128	protein_coding
ENSG0000(	ENSG0000(	98.48452	0.319504	0.000532	0.004128	lncRNA
ENSG0000(	ENSG0000(	544.7756	0.123496	0.000533	0.004135	transcribed_processed_pseudoge
ENSG0000(	EIF3K	1022.547	-0.16363	0.000534	0.004139	protein_coding
ENSG0000(	ABO	4194.736	-0.352	0.000536	0.004148	protein_coding
ENSG0000(	ENSG0000(	224.41	0.236533	0.000535	0.004148	lncRNA
ENSG0000(	IFITM3	787.0436	0.304486	0.000536	0.004148	protein_coding
ENSG0000(	MIR4453H(	474.4299	0.252145	0.000535	0.004148	lncRNA
ENSG0000(	LATS1	2875.898	0.079308	0.000536	0.004149	protein_coding
ENSG0000(	TRIM11	1694.34	0.108972	0.000536	0.004151	protein_coding
ENSG0000(	C1orf159	417.5909	0.202848	0.000537	0.004157	protein_coding
ENSG0000(	TSIX	9013.265	0.205739	0.000538	0.004161	lncRNA
ENSG0000(	SAMSN1	498.6747	0.704089	0.000538	0.004162	protein_coding
ENSG0000(	ENSG0000(	10.00537	0.66244	0.000538	0.004162	lncRNA
ENSG0000(	ENSG0000(	59.69985	0.29799	0.000539	0.004167	lncRNA
ENSG0000(	IL6-AS1	25.06603	1.258603	0.000539	0.004167	lncRNA
ENSG0000(	ZNF627	520.9366	0.151215	0.000539	0.004167	protein_coding
ENSG0000(	EDIL3	737.9721	-0.40682	0.000541	0.00418	protein_coding
ENSG0000(	HTR2B	9.314118	0.801709	0.000541	0.00418	protein_coding
ENSG0000(	LINC01327	31.91217	0.401523	0.000541	0.00418	lncRNA
ENSG0000(	ENSG0000(	119.5805	0.344633	0.000542	0.004183	lncRNA
ENSG0000(	GOLGA2P1	299.5461	0.199429	0.000542	0.004186	transcribed_unprocessed_pseudo
ENSG0000(	OSTF1	434.9551	-0.22069	0.000542	0.004186	protein_coding
ENSG0000(	ENSG0000(	172.8717	-0.21318	0.000543	0.004186	lncRNA
ENSG0000(	RPL14	3032.12	-0.27812	0.000543	0.004189	protein_coding
ENSG0000(	TMX3	2293.081	-0.169	0.000543	0.004189	protein_coding
ENSG0000(	POLR3E	1403.045	-0.14982	0.000544	0.004193	protein_coding
ENSG0000(	SH2D6	127.35	-0.85083	0.000544	0.004193	protein_coding

ENSG0000(	ENSG0000(	20.26459	0.58421	0.000545	0.0042	lncRNA
ENSG0000(	RN7SL448F	11.6264	0.562148	0.000545	0.0042	misc_RNA
ENSG0000(	NAA30	1108.189	-0.20083	0.000547	0.004216	protein_coding
ENSG0000(	ENSG0000(	24.27586	0.583719	0.00055	0.004234	lncRNA
ENSG0000(	ENSG0000(	151.7837	0.582615	0.00055	0.004236	lncRNA
ENSG0000(	STPG4	88.15427	0.350884	0.00055	0.004236	protein_coding
ENSG0000(	HSPE1P26	91.03612	0.287349	0.000551	0.004236	processed_pseudogene
ENSG0000(	ENSG0000(	31.2931	0.54674	0.000552	0.004244	lncRNA
ENSG0000(	NRG1	1551.332	0.435402	0.000552	0.004244	protein_coding
ENSG0000(	RUFY1	939.9638	-0.13978	0.000553	0.004255	protein_coding
ENSG0000(	HIGD1A	1355.148	-0.40268	0.000554	0.004257	protein_coding
ENSG0000(	GABPB1-IT:	1539.634	0.233299	0.000555	0.004266	lncRNA
ENSG0000(	PPFIA2	90.91923	0.803421	0.000556	0.004268	protein_coding
ENSG0000(	YAE1	366.8957	-0.22443	0.000557	0.00428	protein_coding
ENSG0000(	CRBN	1777.855	-0.16152	0.000557	0.00428	protein_coding
ENSG0000(	ENSG0000(	36.91862	0.347294	0.000558	0.004281	lncRNA
ENSG0000(	ENSG0000(	13.8176	-0.74012	0.000558	0.004284	lncRNA
ENSG0000(	ENSG0000(	230.798	0.204458	0.000561	0.004307	TEC
ENSG0000(	ENSG0000(	12.59166	0.871936	0.000562	0.004307	lncRNA
ENSG0000(	ARL6IP1	1756.077	-0.22129	0.000563	0.004317	protein_coding
ENSG0000(	CA7	75.13373	-1.10318	0.000563	0.004317	protein_coding
ENSG0000(	FOXO4	523.5639	-0.37478	0.000563	0.004317	protein_coding
ENSG0000(	ENSG0000(	131.2195	0.216365	0.000564	0.004318	lncRNA
ENSG0000(	ENSG0000(	31.43401	0.457636	0.000564	0.004318	lncRNA
ENSG0000(	PTPN23-DT	187.0296	0.208914	0.000564	0.004318	lncRNA
ENSG0000(	CNTLN	922.5459	0.208504	0.000564	0.004322	protein_coding
ENSG0000(	CAMTA1	470.6415	-0.1988	0.000565	0.004323	protein_coding
ENSG0000(	RPL23A	2240.305	-0.25215	0.000565	0.004323	protein_coding
ENSG0000(	ATP6V0D2	251.9575	-0.2041	0.000567	0.004334	protein_coding
ENSG0000(	OAZ1	2624.946	-0.2463	0.000566	0.004334	protein_coding
ENSG0000(	RN7SKP48	50.63705	-0.56096	0.000567	0.004335	misc_RNA
ENSG0000(	ZPR1	1417.232	0.280223	0.000567	0.004335	protein_coding
ENSG0000(	CCNG1	1772.172	-0.40214	0.000567	0.004336	protein_coding
ENSG0000(	TEPSIN	959.0568	-0.19639	0.000567	0.004337	protein_coding
ENSG0000(	ST3GAL1-D	29.45563	0.753621	0.000568	0.004337	lncRNA
ENSG0000(	RPS18P9	240.8835	-0.20229	0.000568	0.004338	transcribed_processed_pseudoge
ENSG0000(	SPIB	1519.851	-0.58916	0.000568	0.004338	protein_coding
ENSG0000(	SLC25A23	1139.738	-0.50387	0.000569	0.004342	protein_coding
ENSG0000(	CD99L2	560.5091	0.196918	0.000569	0.004342	protein_coding
ENSG0000(	UIMC1	1231.959	-0.14809	0.000569	0.004342	protein_coding
ENSG0000(	HEATR6-DT	257.9645	0.164654	0.00057	0.00435	lncRNA
ENSG0000(	PLEKHO2	768.5501	0.490759	0.00057	0.00435	protein_coding
ENSG0000(	REC8	394.2811	0.378585	0.00057	0.00435	protein_coding
ENSG0000(	CCT8P1	347.7603	-0.77057	0.000571	0.004356	processed_pseudogene
ENSG0000(	TNFAIP3	3005.12	0.461278	0.000572	0.004356	protein_coding
ENSG0000(	YBX3	1850.164	0.263844	0.000571	0.004356	protein_coding
ENSG0000(	ENSG0000(	14.24686	0.706341	0.000573	0.004364	lncRNA

ENSG0000(	UBE2H	2276.752	0.133677	0.000573	0.004364	protein_coding
ENSG0000(	CAPZB	5849.909	0.12635	0.000574	0.004371	protein_coding
ENSG0000(	KIAA1614	443.4604	0.321774	0.000574	0.004373	protein_coding
ENSG0000(	NPTN-IT1	664.2931	0.212556	0.000574	0.004373	lncRNA
ENSG0000(	IGHV3-64	40.18656	1.178616	0.000575	0.004373	IG_V_gene
ENSG0000(	ENSG0000(	15.25839	0.825707	0.000576	0.004379	lncRNA
ENSG0000(	PFDN6	100.7585	-0.2918	0.000576	0.004384	protein_coding
ENSG0000(	ZDHHC21	4126.233	-0.19424	0.000577	0.004385	protein_coding
ENSG0000(	LINC00221	212.1292	0.328013	0.000577	0.004386	lncRNA
ENSG0000(	RRAS2	929.5822	-0.36486	0.000578	0.004394	protein_coding
ENSG0000(	ERAL1	246.6981	-0.2219	0.000579	0.004396	protein_coding
ENSG0000(	SLC25A34	814.517	-0.45679	0.000579	0.004396	protein_coding
ENSG0000(	STXBP1	750.1126	0.262874	0.000579	0.004396	protein_coding
ENSG0000(	CHROMR	1285.31	0.20506	0.00058	0.004403	lncRNA
ENSG0000(	ENSG0000(	48.14004	0.291665	0.00058	0.004404	lncRNA
ENSG0000(	ENSG0000(	38.78101	-0.58672	0.000581	0.004408	processed_pseudogene
ENSG0000(	GSAP	1738.04	0.463033	0.000581	0.004408	protein_coding
ENSG0000(	POGLUT3	797.4442	0.149675	0.000581	0.00441	protein_coding
ENSG0000(	YEATS4	112.0016	-0.305	0.000582	0.00441	protein_coding
ENSG0000(	MLF2	712.8798	-0.2364	0.000585	0.004435	protein_coding
ENSG0000(	ENSG0000(	155.5379	0.281238	0.000585	0.004436	lncRNA
ENSG0000(	MKS1	1139.433	0.150233	0.000585	0.004436	protein_coding
ENSG0000(	PZP	33.36505	0.568116	0.000585	0.004436	protein_coding
ENSG0000(	ISCA1	585.9416	-0.18924	0.000586	0.004436	protein_coding
ENSG0000(	ENSG0000(	28.72186	0.73554	0.000586	0.004439	lncRNA
ENSG0000(	ESYT2	2889.645	-0.15204	0.000587	0.004441	protein_coding
ENSG0000(	SLC2A6	350.7248	0.643932	0.000588	0.004453	protein_coding
ENSG0000(	DANCR	1286.115	0.220608	0.000589	0.004456	lncRNA
ENSG0000(	GLOD4	1060.028	-0.18642	0.000589	0.004456	protein_coding
ENSG0000(	TANC2	1436.536	0.301941	0.000589	0.004456	protein_coding
ENSG0000(	LINC02803	84.925	0.258094	0.000589	0.004458	lncRNA
ENSG0000(	USP2	382.465	-0.97551	0.00059	0.004464	protein_coding
ENSG0000(	FBXO39	18.84716	0.726289	0.000591	0.004466	protein_coding
ENSG0000(	HNRNPD	3506.449	-0.14788	0.000592	0.004471	protein_coding
ENSG0000(	XPR1	1585.511	-0.21662	0.000592	0.004472	protein_coding
ENSG0000(	HOXC5	61.49466	0.470668	0.000592	0.004474	protein_coding
ENSG0000(	ZNF792	462.7801	0.202841	0.000593	0.004474	protein_coding
ENSG0000(	ENSG0000(	537.7126	0.194117	0.000593	0.004476	lncRNA
ENSG0000(	C1R	1588.359	0.465983	0.000593	0.004477	protein_coding
ENSG0000(	PMCHL2	93.68202	-0.32603	0.000593	0.004477	transcribed_unprocessed_pseudo
ENSG0000(	RPL27A	3760.415	-0.30178	0.000595	0.004489	protein_coding
ENSG0000(	ENSG0000(	28.13909	0.520692	0.000597	0.004498	lncRNA
ENSG0000(	ENSG0000(	96.48278	0.243761	0.000597	0.004498	lncRNA
ENSG0000(	LINC01901	93.53859	0.29588	0.000597	0.004498	lncRNA
ENSG0000(	MAZ	1269.267	-0.18505	0.000597	0.004498	protein_coding
ENSG0000(	METTL21C	165.7525	0.389405	0.000597	0.004498	protein_coding
ENSG0000(	SLC22A5	5087.75	-0.64314	0.000597	0.004498	protein_coding

ENSG0000(	VIP	29.70975	1.026135	0.000597	0.004498	protein_coding
ENSG0000(	GCFC2	541.2295	-0.22477	0.000598	0.0045	protein_coding
ENSG0000(	ARRDC3-AS	787.4654	0.165905	0.000598	0.004503	lncRNA
ENSG0000(	TMEM167E	882.4557	0.138288	0.000599	0.004505	protein_coding
ENSG0000(	MT-ND1	11675.6	-0.5258	0.0006	0.004511	protein_coding
ENSG0000(	NOA1	322.1145	-0.19505	0.0006	0.004511	protein_coding
ENSG0000(	SOX4	945.3665	0.300454	0.0006	0.004511	protein_coding
ENSG0000(	GJC1	1266.393	0.207417	0.000601	0.004516	protein_coding
ENSG0000(	ATP5MC3	1114.567	-0.39272	0.000602	0.004528	protein_coding
ENSG0000(	ENSG0000(	267.3101	0.159337	0.000603	0.004531	lncRNA
ENSG0000(	LINC00106	212.6459	-0.24948	0.000603	0.004533	lncRNA
ENSG0000(	RNU6-43P	23.8489	0.52428	0.000604	0.004536	snRNA
ENSG0000(	RPS6KB1	1643.113	-0.13545	0.000605	0.004545	protein_coding
ENSG0000(	TICAM2-AS	67.46979	0.691975	0.000605	0.004545	lncRNA
ENSG0000(	ENSG0000(	86.85126	0.263976	0.000606	0.004548	lncRNA
ENSG0000(	ENSG0000(	367.7235	0.141022	0.000606	0.004548	lncRNA
ENSG0000(	THSD7A	220.1251	0.536688	0.000606	0.004548	protein_coding
ENSG0000(	HPRT1	165.4656	-0.34458	0.000608	0.004561	protein_coding
ENSG0000(	ENSG0000(	221.8018	0.271019	0.000608	0.004562	TEC
ENSG0000(	POU6F2	93.37575	0.32883	0.000609	0.004567	protein_coding
ENSG0000(	ATXN1	4668.304	0.169739	0.00061	0.004574	protein_coding
ENSG0000(	GTF2IP7	452.323	0.157007	0.00061	0.004574	transcribed_unprocessed_pseudo
ENSG0000(	ELP3	682.3175	-0.18214	0.000612	0.004586	protein_coding
ENSG0000(	ZNF780A	2361.243	0.122491	0.000612	0.004586	protein_coding
ENSG0000(	MPZL3	818.3314	-0.25339	0.000613	0.004594	protein_coding
ENSG0000(	ENSG0000(	11.62944	0.809009	0.000615	0.004601	TEC
ENSG0000(	LINC02275	803.7166	0.172705	0.000615	0.004601	lncRNA
ENSG0000(	MAS1	364.8598	0.181493	0.000615	0.004601	protein_coding
ENSG0000(	SNORD14D	21.99393	1.21069	0.000615	0.004601	snoRNA
ENSG0000(	EXOC3L1	41.21405	0.623212	0.000617	0.004613	protein_coding
ENSG0000(	PSPC1	1636.327	0.195743	0.000617	0.004613	protein_coding
ENSG0000(	SLC4A1	304.8447	0.231003	0.000617	0.004613	protein_coding
ENSG0000(	SPDYE2	16.77818	1.948499	0.000617	0.004614	protein_coding
ENSG0000(	BCAS2	176.6064	-0.24289	0.000618	0.004617	protein_coding
ENSG0000(	FOXP3	92.04664	0.840387	0.000619	0.004628	protein_coding
ENSG0000(	CCDC102B	433.8855	0.431402	0.00062	0.004629	protein_coding
ENSG0000(	SLC10A2	3406.428	-0.99147	0.00062	0.004631	protein_coding
ENSG0000(	CCDC188	13.73189	0.591869	0.00062	0.004631	protein_coding
ENSG0000(	ENSG0000(	11537.5	-0.58137	0.000621	0.004637	lncRNA
ENSG0000(	UTS2R	683.7358	0.319912	0.000622	0.004641	protein_coding
ENSG0000(	ENSG0000(	323.1796	0.225077	0.000623	0.004648	lncRNA
ENSG0000(	FAM78B-AS	109.245	-0.26113	0.000623	0.00465	lncRNA
ENSG0000(	C15orf39	1093.453	0.314359	0.000624	0.00465	protein_coding
ENSG0000(	ENSG0000(	120.6518	0.234197	0.000625	0.004659	lncRNA
ENSG0000(	ENSG0000(	236.5438	-0.17812	0.000625	0.004661	lncRNA
ENSG0000(	LINC01558	311.9853	-0.37572	0.000626	0.004661	lncRNA
ENSG0000(	KCTD10	1405.014	0.203563	0.000626	0.004664	protein_coding

ENSG0000(	ENSG0000(	671.8639	0.18205	0.000626	0.004665	lncRNA
ENSG0000(	ICA1	982.3304	0.279337	0.000627	0.004671	protein_coding
ENSG0000(	KCNS1	20.9282	0.542462	0.000628	0.004671	protein_coding
ENSG0000(	SLC25A33	450.2455	-0.15337	0.000628	0.004671	protein_coding
ENSG0000(	LAMP1	2413.281	-0.24818	0.000628	0.004673	protein_coding
ENSG0000(	SURF1	256.3498	-0.2281	0.000629	0.004676	protein_coding
ENSG0000(	FLJ36000	62.80229	0.423878	0.000629	0.004679	lncRNA
ENSG0000(	CYRIA	699.8825	0.567051	0.000631	0.004693	protein_coding
ENSG0000(	DNAH2	303.6947	0.156044	0.000631	0.004693	protein_coding
ENSG0000(	ENSG0000(	9.923506	0.820347	0.000631	0.004693	lncRNA
ENSG0000(	ENSG0000(	11.47748	0.792517	0.000632	0.004693	lncRNA
ENSG0000(	AK2	4637.378	-0.26439	0.000633	0.0047	protein_coding
ENSG0000(	LINC01770	11.11226	0.85313	0.000633	0.0047	lncRNA
ENSG0000(	ENSG0000(	16.70566	0.569328	0.000633	0.004702	lncRNA
ENSG0000(	GRAMD1C	1204.799	-0.55981	0.000634	0.004709	protein_coding
ENSG0000(	DAZAP2	2603.865	-0.22363	0.000635	0.004716	protein_coding
ENSG0000(	ENSG0000(	277.4455	0.188965	0.000636	0.004716	lncRNA
ENSG0000(	AKAP3	45.13907	0.485544	0.000636	0.004719	protein_coding
ENSG0000(	ENSG0000(	264.533	0.210205	0.000637	0.004721	lncRNA
ENSG0000(	ENSG0000(	123.9699	0.292798	0.000638	0.004727	lncRNA
ENSG0000(	B3GNT9	151.871	0.375428	0.000638	0.004729	protein_coding
ENSG0000(	ENSG0000(	120.0238	-0.25001	0.000638	0.004729	lncRNA
ENSG0000(	ARL15	519.8452	-0.21984	0.000638	0.004731	protein_coding
ENSG0000(	TFCP2L1	336.7344	0.580763	0.00064	0.004743	protein_coding
ENSG0000(	STRN	2993.941	-0.19859	0.000641	0.004747	protein_coding
ENSG0000(	RPL28	2272.314	-0.30964	0.000641	0.004748	protein_coding
ENSG0000(	SOCAR	16.24922	0.921677	0.000642	0.00475	lncRNA
ENSG0000(	NDOR1	664.2959	-0.23374	0.000642	0.004751	protein_coding
ENSG0000(	JDP2	1052.488	-0.32094	0.000644	0.004766	protein_coding
ENSG0000(	SLC25A1	725.2821	-0.49308	0.000644	0.004768	protein_coding
ENSG0000(	THRAP3	2636.574	-0.13523	0.000645	0.004771	protein_coding
ENSG0000(	ATP5PD	875.9496	-0.26351	0.000646	0.004773	protein_coding
ENSG0000(	EPCAM	3985.196	-0.66009	0.000646	0.004777	protein_coding
ENSG0000(	ENSG0000(	22.55978	0.757135	0.000647	0.004778	lncRNA
ENSG0000(	HDAC4	1662.777	0.185924	0.000647	0.00478	protein_coding
ENSG0000(	ENSG0000(	124.9258	0.371373	0.000647	0.00478	lncRNA
ENSG0000(	METAP1	783.7201	-0.16485	0.000647	0.00478	protein_coding
ENSG0000(	TBXA2R	323.4773	-0.19231	0.000647	0.00478	protein_coding
ENSG0000(	LINC02861	406.819	-0.17358	0.000648	0.004784	lncRNA
ENSG0000(	ENSG0000(	1077.496	-0.26541	0.000651	0.004801	lncRNA
ENSG0000(	ENSG0000(	257.1999	0.183206	0.000651	0.004801	TEC
ENSG0000(	PC	612.9285	-0.64965	0.000651	0.004801	protein_coding
ENSG0000(	ENSG0000(	174.4259	-0.25029	0.000651	0.004803	lncRNA
ENSG0000(	FNDC4	19.95624	0.727951	0.000653	0.004813	protein_coding
ENSG0000(	MIR23AHG	4277.018	0.222361	0.000653	0.004816	lncRNA
ENSG0000(	ENSG0000(	9.942449	0.708549	0.000654	0.004818	processed_pseudogene
ENSG0000(	SLC36A3	113.8368	-0.24778	0.000655	0.00483	protein_coding

ENSG0000(	HMGB3P4	204.1506	0.273737	0.000656	0.00483	processed_pseudogene
ENSG0000(	DUBR	412.7076	0.2279	0.000657	0.004835	lncRNA
ENSG0000(	KRCC1	478.7922	-0.20236	0.000658	0.004848	protein_coding
ENSG0000(	ENSG0000(	517.3374	0.202827	0.00066	0.004857	lncRNA
ENSG0000(	COL6A1	3888.308	0.40385	0.00066	0.004858	protein_coding
ENSG0000(	ENSG0000(	149.4709	-0.27835	0.000661	0.004861	lncRNA
ENSG0000(	ENSG0000(	120.7009	-0.34615	0.000661	0.004862	lncRNA
ENSG0000(	ENSG0000(	23.71279	0.821983	0.000661	0.004862	lncRNA
ENSG0000(	ENSG0000(	14.70201	0.778565	0.000661	0.004862	lncRNA
ENSG0000(	ENSG0000(	12.7888	0.6991	0.000662	0.004868	lncRNA
ENSG0000(	UNC5A	9.771707	0.886699	0.000662	0.004868	protein_coding
ENSG0000(	SACM1L	1736.833	-0.10511	0.000663	0.004872	protein_coding
ENSG0000(	PCDH12	100.4432	0.572511	0.000664	0.004878	protein_coding
ENSG0000(	HINT1	2589.066	-0.24863	0.000664	0.00488	protein_coding
ENSG0000(	ELOVL1	573.4019	-0.24592	0.000665	0.004881	protein_coding
ENSG0000(	RAB21	3391.345	-0.19007	0.000665	0.004884	protein_coding
ENSG0000(	BMPR1A	1952.955	-0.27714	0.000667	0.00489	protein_coding
ENSG0000(	DUSP12	163.6878	-0.26269	0.000666	0.00489	protein_coding
ENSG0000(	ENSG0000(	98.53114	0.416126	0.000667	0.00489	lncRNA
ENSG0000(	PHGDH	361.9516	0.200299	0.000667	0.00489	protein_coding
ENSG0000(	TSNAX-DIS(	26.42547	0.716881	0.000667	0.00489	protein_coding
ENSG0000(	ENSG0000(	114.9403	-0.30782	0.000667	0.004891	lncRNA
ENSG0000(	HMGCL	992.9991	0.126476	0.000668	0.004897	protein_coding
ENSG0000(	ENSG0000(	38.59118	0.381127	0.000669	0.0049	lncRNA
ENSG0000(	IGLV2-18	50.10905	1.198926	0.000669	0.004901	IG_V_gene
ENSG0000(	LRRN2	135.5487	0.273102	0.000669	0.004901	protein_coding
ENSG0000(	DNAJA2	1116.868	-0.11063	0.000669	0.004901	protein_coding
ENSG0000(	ARPIN-AP3	97.44236	-1.55828	0.000671	0.00491	protein_coding
ENSG0000(	ENSG0000(	10.82068	0.710402	0.000673	0.004924	processed_pseudogene
ENSG0000(	ENSG0000(	251.8252	-0.57288	0.000674	0.004932	lncRNA
ENSG0000(	LINC02624	111.6033	-0.2352	0.000675	0.004937	lncRNA
ENSG0000(	NMT1	1950.03	-0.13742	0.000675	0.004937	protein_coding
ENSG0000(	ATP8A1	5941.726	-0.3214	0.000675	0.004939	protein_coding
ENSG0000(	KRT18P31	255.3911	-0.23342	0.000678	0.004955	processed_pseudogene
ENSG0000(	SLC38A9	1176.825	0.146365	0.000679	0.004965	protein_coding
ENSG0000(	GIGYF2	3738.807	-0.11649	0.00068	0.004967	protein_coding
ENSG0000(	HMX3	138.8848	-0.24147	0.00068	0.004967	protein_coding
ENSG0000(	RBBP4P1	116.2631	-0.38707	0.00068	0.004967	processed_pseudogene
ENSG0000(	P4HA3	115.878	0.348973	0.000681	0.00497	protein_coding
ENSG0000(	SOWAHB	408.2521	-0.67524	0.000681	0.00497	protein_coding
ENSG0000(	HRAT92	127.7879	0.32326	0.000684	0.00499	lncRNA
ENSG0000(	PARTICL	63.73385	0.33006	0.000685	0.004995	lncRNA
ENSG0000(	CDC42EP1	511.7695	0.272746	0.000685	0.004997	protein_coding
ENSG0000(	UBE2K	1806.211	-0.16423	0.000686	0.005006	protein_coding
ENSG0000(	PDLIM4	94.81609	0.471138	0.000687	0.00501	protein_coding
ENSG0000(	ENSG0000(	460.1641	0.142093	0.000688	0.005017	lncRNA
ENSG0000(	COX11	1500.889	-0.17267	0.000689	0.005023	protein_coding

ENSG0000(	ENSG0000(	162.8978	-0.29376	0.00069	0.00503	lncRNA
ENSG0000(	FES	325.2446	0.329311	0.000691	0.005031	protein_coding
ENSG0000(	PERP	1129.729	-0.57548	0.000692	0.00504	protein_coding
ENSG0000(	SLC16A1-A:	2898.369	0.222711	0.000692	0.005041	lncRNA
ENSG0000(	HNRNPA1P	22.1191	-1.11158	0.000693	0.005047	processed_pseudogene
ENSG0000(	ZDHHC20	2077.767	-0.20329	0.000694	0.005049	protein_coding
ENSG0000(	CHMP4C	445.1356	-0.39831	0.000694	0.005051	protein_coding
ENSG0000(	ENSG0000(	24.26412	0.459721	0.000695	0.005052	lncRNA
ENSG0000(	HYAL2	328.0917	0.31821	0.000695	0.005052	protein_coding
ENSG0000(	LINC02728	147.5633	0.268135	0.000697	0.005066	lncRNA
ENSG0000(	SHISA2	133.6359	-0.22204	0.000697	0.00507	protein_coding
ENSG0000(	PPID	467.5622	-0.21914	0.000698	0.005073	protein_coding
ENSG0000(	KCNK5	1752.966	-0.65284	0.000698	0.005075	protein_coding
ENSG0000(	LINC02363	259.7647	0.216583	0.000701	0.00509	lncRNA
ENSG0000(	LRRC2	724.8924	0.127544	0.000702	0.005095	protein_coding
ENSG0000(	UGT1A3	184.2199	-0.94046	0.000702	0.005095	protein_coding
ENSG0000(	IGHV3-30	550.7042	0.952457	0.000704	0.005111	IG_V_gene
ENSG0000(	CUTC	244.0887	-0.20939	0.000707	0.005127	protein_coding
ENSG0000(	ENSG0000(	21.34812	0.660209	0.000707	0.005127	TEC
ENSG0000(	HNRNPA1P	54.33007	0.364217	0.000707	0.005127	processed_pseudogene
ENSG0000(	ENSG0000(	17.09942	0.499838	0.000707	0.005128	lncRNA
ENSG0000(	ENSG0000(	38.64067	0.39567	0.000708	0.005132	TEC
ENSG0000(	ATXN7	5576.592	0.105577	0.000708	0.005135	protein_coding
ENSG0000(	GIPR	597.0259	0.132975	0.000711	0.005151	protein_coding
ENSG0000(	PKLR	126.9912	-0.98483	0.000711	0.005152	protein_coding
ENSG0000(	ZDHHC11B	1396.533	-0.81612	0.000711	0.005154	protein_coding
ENSG0000(	GPR141	1005.548	0.193794	0.000712	0.005155	protein_coding
ENSG0000(	MRS2	1147.063	-0.10179	0.000712	0.005155	protein_coding
ENSG0000(	LINC01726	46.31657	-0.33684	0.000713	0.005161	lncRNA
ENSG0000(	MRPL50	419.0619	-0.17035	0.000713	0.005161	protein_coding
ENSG0000(	ADD1	6739.187	-0.10873	0.000713	0.005162	protein_coding
ENSG0000(	ENSG0000(	84.42264	0.345062	0.000714	0.005163	lncRNA
ENSG0000(	DCAF4L1	102.4867	0.249776	0.000715	0.00517	protein_coding
ENSG0000(	ENSG0000(	631.9357	0.369875	0.000715	0.00517	lncRNA
ENSG0000(	MT-RNR1	8865.29	-0.55982	0.000715	0.00517	Mt_rRNA
ENSG0000(	ENSG0000(	286.2402	0.210469	0.000716	0.005174	lncRNA
ENSG0000(	DCN	1824.367	0.404612	0.000718	0.005189	protein_coding
ENSG0000(	HTT-AS	102.0966	-0.22141	0.00072	0.005205	lncRNA
ENSG0000(	ZNF793	2127.621	-0.11825	0.000721	0.005206	protein_coding
ENSG0000(	COLQ	1178.348	0.283752	0.000722	0.005211	protein_coding
ENSG0000(	SPINT1-AS1	138.1155	-0.53403	0.000722	0.005215	lncRNA
ENSG0000(	PDE4DIP	6763.193	0.146765	0.000723	0.005218	protein_coding
ENSG0000(	HMOX2	583.3972	-0.14475	0.000724	0.005221	protein_coding
ENSG0000(	BCAP31	605.7917	-0.23846	0.000726	0.00524	protein_coding
ENSG0000(	C11orf53	15.18274	-0.71187	0.000727	0.005243	protein_coding
ENSG0000(	ENSG0000(	283.5233	-0.19604	0.000727	0.005243	TEC
ENSG0000(	MAP2K4	979.2025	-0.14356	0.000727	0.005243	protein_coding

ENSG0000(	TRIM65	878.3827	-0.16133	0.000727	0.005243	protein_coding
ENSG0000(	ZEB1	1727.161	0.389994	0.000728	0.005249	protein_coding
ENSG0000(	GHDC	389.4123	-0.20629	0.000732	0.005273	protein_coding
ENSG0000(	ENSG0000(	1674.663	0.129058	0.000733	0.005278	lncRNA
ENSG0000(	HLA-C	8961.562	0.340938	0.000733	0.005278	protein_coding
ENSG0000(	NRIP3-DT	197.7779	0.209522	0.000735	0.005291	lncRNA
ENSG0000(	CLCN2	1073.664	-0.42785	0.000735	0.005292	protein_coding
ENSG0000(	KDR	422.3325	0.430559	0.000735	0.005292	protein_coding
ENSG0000(	SETD9	84.98294	-0.50991	0.000736	0.005292	protein_coding
ENSG0000(	ENSG0000(	4914.371	0.259452	0.000736	0.005292	lncRNA
ENSG0000(	ADGRL4	172.8818	0.458922	0.000736	0.005294	protein_coding
ENSG0000(	CRTC3	1893.082	0.290074	0.000737	0.005299	protein_coding
ENSG0000(	ENSG0000(	453.0777	-0.12709	0.000738	0.005304	lncRNA
ENSG0000(	KCNN3	553.4671	0.374567	0.000738	0.005306	protein_coding
ENSG0000(	LINC02577	242.3647	-0.29647	0.000739	0.005309	lncRNA
ENSG0000(	JAML	2639.071	0.255545	0.000739	0.005312	protein_coding
ENSG0000(	RN7SL750F	60.85438	-0.33002	0.00074	0.005316	misc_RNA
ENSG0000(	RPL29P2	14.90764	0.523851	0.00074	0.005316	transcribed_processed_pseudoge
ENSG0000(	PRUNE1	456.1975	-0.22927	0.000742	0.005326	protein_coding
ENSG0000(	BIRC3	11719.61	0.46766	0.000742	0.005328	protein_coding
ENSG0000(	C2orf81	65.82446	0.396839	0.000743	0.005328	protein_coding
ENSG0000(	GP6-AS1	1245.607	0.204935	0.000743	0.005333	lncRNA
ENSG0000(	LINC01505	141.9916	0.273715	0.000744	0.005333	lncRNA
ENSG0000(	LINC01411	169.0394	0.421196	0.000744	0.005334	lncRNA
ENSG0000(	GMPPB	1092.393	0.191311	0.000744	0.005335	protein_coding
ENSG0000(	IGLL5	763.0543	0.78833	0.000745	0.005341	protein_coding
ENSG0000(	WASH5P	15104.1	0.216659	0.000746	0.005342	transcribed_processed_pseudoge
ENSG0000(	LINC01093	140.6245	0.308332	0.000746	0.005345	lncRNA
ENSG0000(	ALG11	3778.814	0.101742	0.000746	0.005346	protein_coding
ENSG0000(	ASF1A	253.4044	-0.24136	0.000748	0.005354	protein_coding
ENSG0000(	SI	19070.97	-0.91855	0.000749	0.00536	protein_coding
ENSG0000(	PFN1	2267.035	-0.24407	0.00075	0.005366	protein_coding
ENSG0000(	ENSG0000(	195.5416	0.397696	0.00075	0.005367	lncRNA
ENSG0000(	PARVB	1292.739	0.263709	0.000751	0.005369	protein_coding
ENSG0000(	TULP3	389.4245	0.225685	0.000752	0.005381	protein_coding
ENSG0000(	ACOX2	748.6084	-0.63983	0.000753	0.005382	protein_coding
ENSG0000(	NOTCH2NL	8451.158	0.321187	0.000753	0.005382	protein_coding
ENSG0000(	TARBP1	2543.466	0.279433	0.000753	0.005383	protein_coding
ENSG0000(	ENSG0000(	192.9698	0.410457	0.000755	0.005394	lncRNA
ENSG0000(	MGAT4B	3375.387	-0.47008	0.000756	0.005398	protein_coding
ENSG0000(	STRADA	1957.088	0.1773	0.000757	0.00541	protein_coding
ENSG0000(	ENSG0000(	25.95673	0.470733	0.000759	0.005418	lncRNA
ENSG0000(	ENSG0000(	107.1153	0.439323	0.000759	0.00542	lncRNA
ENSG0000(	CDH1	5941.471	-0.49116	0.000761	0.005434	protein_coding
ENSG0000(	ENSG0000(	94.52207	0.280168	0.000762	0.005434	lncRNA
ENSG0000(	RRAS	187.3076	-0.26349	0.000762	0.005434	protein_coding
ENSG0000(	PLSCR1	1532.865	0.339857	0.000762	0.005435	protein_coding

ENSG0000(	ENSG0000(	224.7573	-0.23798	0.000763	0.005444	lncRNA
ENSG0000(	FHAD1	309.4311	0.325375	0.000764	0.005447	protein_coding
ENSG0000(	SBF1	3801.257	-0.19001	0.000765	0.005451	protein_coding
ENSG0000(	DEDD2	537.8322	-0.22835	0.000767	0.005466	protein_coding
ENSG0000(	ENSG0000(	17.37294	0.566049	0.000767	0.005466	lncRNA
ENSG0000(	LINC02207	434.0356	0.168019	0.000767	0.005466	lncRNA
ENSG0000(	PLA2G4D	184.4654	0.228289	0.000768	0.005467	protein_coding
ENSG0000(	THBS3-AS1	30.29572	0.504121	0.000768	0.005471	lncRNA
ENSG0000(	ENSG0000(	26.21249	0.487644	0.00077	0.005478	TEC
ENSG0000(	RCE1	250.3723	-0.25579	0.00077	0.005478	protein_coding
ENSG0000(	FAM41C	86.1519	0.666977	0.00077	0.005479	lncRNA
ENSG0000(	TIMM13	307.2193	-0.36873	0.000771	0.005488	protein_coding
ENSG0000(	LRRC4	59.8633	0.823012	0.000772	0.005493	protein_coding
ENSG0000(	RHOQ	1494.403	0.536865	0.000774	0.005504	protein_coding
ENSG0000(	IL19	306.1879	0.185957	0.000774	0.005505	protein_coding
ENSG0000(	CLCF1	104.0068	0.524205	0.000775	0.005506	protein_coding
ENSG0000(	PCM1	6455.553	-0.10655	0.000775	0.005506	protein_coding
ENSG0000(	ANKRD20A	503.5691	0.317489	0.000775	0.005507	transcribed_unprocessed_pseudo
ENSG0000(	ART3	199.393	0.220823	0.000777	0.005519	protein_coding
ENSG0000(	ENSG0000(	118.4925	0.38615	0.000778	0.005524	lncRNA
ENSG0000(	STK32A	87.96282	0.466948	0.000778	0.005524	protein_coding
ENSG0000(	DHRSX	725.6935	-0.13953	0.000781	0.005546	protein_coding
ENSG0000(	PAN2	2015.401	-0.13987	0.000782	0.005547	protein_coding
ENSG0000(	EEF1A1	55624.28	-0.2536	0.000783	0.005553	protein_coding
ENSG0000(	EBPL	489.1922	-0.25121	0.000786	0.005574	protein_coding
ENSG0000(	SVEP1	786.7687	0.485676	0.000787	0.005579	protein_coding
ENSG0000(	PHOSPHO2	239.3439	-0.31057	0.000787	0.005581	protein_coding
ENSG0000(	LINC00391	30.57116	0.365448	0.00079	0.005598	lncRNA
ENSG0000(	ENSG0000(	54.04469	-0.61276	0.00079	0.005599	lncRNA
ENSG0000(	MYO18A	5172.845	-0.15807	0.000791	0.005605	protein_coding
ENSG0000(	ENSG0000(	20.67937	0.479015	0.000794	0.005622	lncRNA
ENSG0000(	PEX6	584.3267	-0.28588	0.000794	0.005625	protein_coding
ENSG0000(	GPR89A	1216.493	-0.28228	0.000795	0.005629	protein_coding
ENSG0000(	ENSG0000(	96.31968	0.232174	0.000797	0.005644	lncRNA
ENSG0000(	LINC02747	236.3819	-0.70748	0.000799	0.005652	lncRNA
ENSG0000(	PRSS3	465.4333	-0.69797	0.0008	0.005661	protein_coding
ENSG0000(	RWDD1	812.5755	-0.21677	0.0008	0.005661	protein_coding
ENSG0000(	ENSG0000(	64.1672	0.35911	0.000801	0.005663	lncRNA
ENSG0000(	ETV1	129.5155	0.470408	0.000803	0.005678	protein_coding
ENSG0000(	LSM7	470.9325	-0.20453	0.000804	0.005682	protein_coding
ENSG0000(	VSIG10L	78.85734	-0.40909	0.000807	0.005702	protein_coding
ENSG0000(	IFIT3	1273.894	0.527854	0.000807	0.005703	protein_coding
ENSG0000(	C11orf80	971.7064	-0.28591	0.000807	0.005703	protein_coding
ENSG0000(	LINC01010	114.3484	0.21332	0.000809	0.005715	lncRNA
ENSG0000(	SHC2	233.785	0.385899	0.000809	0.005715	protein_coding
ENSG0000(	GTF2E2	584.6356	-0.21234	0.00081	0.005716	protein_coding
ENSG0000(	MAPKAPK2	1592.284	0.261253	0.000811	0.005723	protein_coding

ENSG0000(	CHRM3	739.8199	0.38047	0.000811	0.005724	protein_coding
ENSG0000(	LAMA2	941.3492	0.286403	0.000814	0.005739	protein_coding
ENSG0000(	DLAT	807.8314	-0.16893	0.000814	0.00574	protein_coding
ENSG0000(	APBB3	548.0481	0.212832	0.000816	0.005753	protein_coding
ENSG0000(	ZFPL1	548.3616	-0.16022	0.000816	0.005753	protein_coding
ENSG0000(	ENSG0000(	98.88004	-0.66724	0.000816	0.005753	TEC
ENSG0000(	HELB	1007.442	0.288417	0.000818	0.005762	protein_coding
ENSG0000(	ENSG0000(	48.04134	0.470315	0.000819	0.005769	lncRNA
ENSG0000(	DENND3	3519.137	0.467803	0.00082	0.005774	protein_coding
ENSG0000(	MYO1C	3049.452	-0.29614	0.000823	0.005795	protein_coding
ENSG0000(	ENSG0000(	43.14012	0.358546	0.000824	0.005802	lncRNA
ENSG0000(	ENSG0000(	13.47758	0.780152	0.000825	0.005808	lncRNA
ENSG0000(	ENSG0000(	201.672	0.8664	0.000825	0.005808	lncRNA
ENSG0000(	B4GALT1	1539.576	0.336841	0.000828	0.005825	protein_coding
ENSG0000(	ENSG0000(	394.4034	0.19723	0.000828	0.005825	lncRNA
ENSG0000(	SLC25A20	527.5186	-0.46113	0.000828	0.005825	protein_coding
ENSG0000(	ENSG0000(	276.0688	0.404473	0.00083	0.005835	lncRNA
ENSG0000(	NRXN2	239.0437	0.40911	0.00083	0.005836	protein_coding
ENSG0000(	TMEM44	560.1719	0.230612	0.000831	0.005839	protein_coding
ENSG0000(	ELFN1	41.81661	0.537485	0.000832	0.005844	protein_coding
ENSG0000(	LINC02733	74.62416	0.346986	0.000832	0.005844	lncRNA
ENSG0000(	RPL21	3000.394	-0.30575	0.000832	0.005844	protein_coding
ENSG0000(	RPL5	3359.878	-0.22645	0.000832	0.005844	protein_coding
ENSG0000(	ZDHHC1	89.39801	0.353377	0.000833	0.005845	protein_coding
ENSG0000(	KIAA1549	562.3445	0.167296	0.000835	0.005863	protein_coding
ENSG0000(	PEX1	1344.787	-0.15502	0.000836	0.005865	protein_coding
ENSG0000(	EMSLR	1086.001	-0.11861	0.000837	0.005875	lncRNA
ENSG0000(	RHBDD3	377.1106	0.179791	0.00084	0.005895	protein_coding
ENSG0000(	ANTXR1	2139.747	0.418887	0.000842	0.005904	protein_coding
ENSG0000(	ENSG0000(	713.5596	0.260099	0.000842	0.005905	lncRNA
ENSG0000(	PTX3	9.08524	0.795029	0.000845	0.005919	protein_coding
ENSG0000(	NTS	248.1889	-1.19778	0.000846	0.005928	protein_coding
ENSG0000(	TBC1D8	1378.003	0.172918	0.000846	0.005928	protein_coding
ENSG0000(	ANO1	133.6247	0.652288	0.000848	0.005943	protein_coding
ENSG0000(	PDCD5	351.4288	-0.2365	0.000849	0.005947	protein_coding
ENSG0000(	LIPC	123.7731	-0.25407	0.00085	0.005948	protein_coding
ENSG0000(	VAMP3	1255.383	-0.23462	0.00085	0.005948	protein_coding
ENSG0000(	IGSF9	1152.209	-0.96196	0.000851	0.005957	protein_coding
ENSG0000(	DAAM1	1406.455	-0.2397	0.000851	0.005957	protein_coding
ENSG0000(	AIF1	155.92	0.514998	0.000852	0.00596	protein_coding
ENSG0000(	GUSB	990.8098	-0.13536	0.000852	0.00596	protein_coding
ENSG0000(	CRKL	2042.408	-0.15466	0.000853	0.005962	protein_coding
ENSG0000(	ENSG0000(	221.2319	0.204781	0.000855	0.005979	lncRNA
ENSG0000(	ENSG0000(	9.994183	0.680233	0.000857	0.005987	lncRNA
ENSG0000(	ENSG0000(	21.96942	0.589019	0.000858	0.005991	lncRNA
ENSG0000(	GZMB	33.4144	0.988433	0.000857	0.005991	protein_coding
ENSG0000(	LRP1B	88.91274	0.91994	0.000858	0.005991	protein_coding

ENSG0000(	COL9A2	257.3879	0.504283	0.000861	0.006012	protein_coding
ENSG0000(	MTREX	1787.943	-0.12967	0.000861	0.006012	protein_coding
ENSG0000(	NCF4	341.3963	0.458437	0.000861	0.006012	protein_coding
ENSG0000(	ENSG0000(	22.71747	0.605462	0.000863	0.006019	lncRNA
ENSG0000(	LINC01104	113.418	0.250508	0.000863	0.006019	lncRNA
ENSG0000(	OSMR	1063.964	0.446225	0.000865	0.006032	protein_coding
ENSG0000(	ENSG0000(	119.8801	0.247494	0.000866	0.00604	TEC
ENSG0000(	DHX57	890.9033	0.203697	0.00087	0.006064	protein_coding
ENSG0000(	HNRNPH2	827.1149	-0.21218	0.000871	0.00607	protein_coding
ENSG0000(	MT-TG	41.61416	1.567637	0.000871	0.006072	Mt_tRNA
ENSG0000(	ENSG0000(	194.8438	0.280212	0.000871	0.006072	lncRNA
ENSG0000(	PCNPP1	38.54349	-0.37905	0.000872	0.006078	processed_pseudogene
ENSG0000(	ARHGAP17	3929.647	-0.13576	0.000874	0.006084	protein_coding
ENSG0000(	PAXX	209.2258	-0.28497	0.000874	0.006085	protein_coding
ENSG0000(	CIDEB	532.7948	-0.5394	0.000874	0.006086	protein_coding
ENSG0000(	ENSG0000(	3706.773	-0.1548	0.000875	0.006092	lncRNA
ENSG0000(	RASSF6	1670.957	-0.25598	0.000877	0.006099	protein_coding
ENSG0000(	ENSG0000(	351.9697	-0.1671	0.000877	0.006103	lncRNA
ENSG0000(	LINC02125	26.07464	0.424452	0.000878	0.006105	lncRNA
ENSG0000(	CPT1B	631.4747	-0.29173	0.00088	0.006113	protein_coding
ENSG0000(	ENSG0000(	212.513	0.25269	0.000879	0.006113	lncRNA
ENSG0000(	IGHV1-3	145.2415	1.232519	0.000879	0.006113	IG_V_gene
ENSG0000(	IGKV2-29	19.40395	1.594737	0.000881	0.006121	IG_V_pseudogene
ENSG0000(	ENSG0000(	54.09929	0.391018	0.000882	0.006125	lncRNA
ENSG0000(	CPNE2	977.0194	-0.41246	0.000883	0.006132	protein_coding
ENSG0000(	ENSG0000(	57.43509	-0.70256	0.000886	0.00615	processed_pseudogene
ENSG0000(	ING5	2056.661	-0.15314	0.000887	0.006158	protein_coding
ENSG0000(	RNU6-98P	26.16963	0.72601	0.00089	0.006174	snRNA
ENSG0000(	OAT	5200.67	-0.77167	0.000891	0.006186	protein_coding
ENSG0000(	ENSG0000(	35.7557	0.394852	0.000892	0.006189	lncRNA
ENSG0000(	SNAPIN	193.9841	-0.16521	0.000894	0.006203	protein_coding
ENSG0000(	CT75	136.2425	0.254783	0.000895	0.006209	transcribed_unprocessed_pseudo
ENSG0000(	ENSG0000(	30.37831	0.39129	0.000895	0.006209	lncRNA
ENSG0000(	ENSG0000(	275.991	-0.71427	0.000897	0.006216	lncRNA
ENSG0000(	ENSG0000(	473.0009	0.12545	0.000897	0.006217	TEC
ENSG0000(	RPLP0P2	484.6581	0.247223	0.000898	0.006222	transcribed_processed_pseudoge
ENSG0000(	SLC35F5	6652.46	-0.21938	0.000899	0.006229	protein_coding
ENSG0000(	ENSG0000(	21.0555	1.034283	0.000901	0.006241	lncRNA
ENSG0000(	ZBTB26	624.4858	0.16084	0.000902	0.006243	protein_coding
ENSG0000(	CPT1A	4243.495	-0.40117	0.000902	0.006246	protein_coding
ENSG0000(	ENSG0000(	80.46457	0.344186	0.000903	0.006252	lncRNA
ENSG0000(	GSPT1	2080.013	-0.15265	0.000904	0.006253	protein_coding
ENSG0000(	ENSG0000(	89.72443	0.286607	0.000906	0.006271	lncRNA
ENSG0000(	NPTN	1264.597	-0.22104	0.000909	0.006287	protein_coding
ENSG0000(	TNFRSF11B	130.7163	0.338925	0.00091	0.006295	protein_coding
ENSG0000(	OR51L1	140.1392	0.31051	0.000911	0.006296	protein_coding
ENSG0000(	LIMK1	1115.283	0.260438	0.000912	0.006302	protein_coding

ENSG0000(	AIP	243.6963	-0.28698	0.000914	0.006315	protein_coding
ENSG0000(	SEMA3C	707.4027	0.269912	0.000914	0.006315	protein_coding
ENSG0000(	ENSG0000(	39.58638	0.409915	0.000916	0.006326	lncRNA
ENSG0000(	RPS17	2429.917	-0.3024	0.000916	0.006326	protein_coding
ENSG0000(	SNORA35B	16.72956	0.592937	0.000917	0.006329	snoRNA
ENSG0000(	STBD1	513.2532	-0.98332	0.000918	0.006335	protein_coding
ENSG0000(	MED1	1831.938	-0.15627	0.000919	0.006341	protein_coding
ENSG0000(	PSENEN	222.015	0.271849	0.000921	0.006353	protein_coding
ENSG0000(	ADGRG1	691.4226	0.253108	0.000921	0.006354	protein_coding
ENSG0000(	LIG4	986.5775	0.161782	0.000921	0.006354	protein_coding
ENSG0000(	NSMAF	1079.371	-0.16829	0.000922	0.006359	protein_coding
ENSG0000(	NAIP	4029.725	0.278902	0.000923	0.00636	protein_coding
ENSG0000(	SPATA5L1	257.8184	-0.35064	0.000923	0.006361	protein_coding
ENSG0000(	REEP6	606.7863	-0.92587	0.000924	0.006367	protein_coding
ENSG0000(	ENSG0000(	9.392387	0.593945	0.000925	0.006371	lncRNA
ENSG0000(	CYB5R4	3087.971	0.122479	0.000925	0.006371	protein_coding
ENSG0000(	STX6	1265.163	0.149097	0.000925	0.006372	protein_coding
ENSG0000(	PODNL1	234.5188	0.204196	0.000926	0.006375	protein_coding
ENSG0000(	CYP4F3	1706.345	-0.60124	0.000927	0.00638	protein_coding
ENSG0000(	NBPF15	1898.283	0.33403	0.000928	0.006385	protein_coding
ENSG0000(	SLC18B1	317.6937	0.236441	0.000928	0.006385	protein_coding
ENSG0000(	JUND	1392.629	-0.37454	0.000929	0.006387	protein_coding
ENSG0000(	NEU1	937.7297	-0.29219	0.000929	0.006387	protein_coding
ENSG0000(	ENSG0000(	50.57233	2.314809	0.000929	0.00639	lncRNA
ENSG0000(	ENSG0000(	1093.151	0.656404	0.00093	0.006393	lncRNA
ENSG0000(	LINC02574	26.43815	0.850695	0.00093	0.006393	lncRNA
ENSG0000(	DDX23	1506.675	-0.12073	0.000932	0.006399	protein_coding
ENSG0000(	LINC01501	49.0676	0.364133	0.000932	0.006399	lncRNA
ENSG0000(	URGCP	2368.821	-0.3081	0.000931	0.006399	protein_coding
ENSG0000(	ENSG0000(	40.18606	0.399724	0.000932	0.006401	lncRNA
ENSG0000(	TG	140.9873	0.611384	0.000933	0.006404	protein_coding
ENSG0000(	ENSG0000(	119.2236	0.202918	0.000933	0.006405	lncRNA
ENSG0000(	VSIG10	3104.461	-0.42885	0.000934	0.006409	protein_coding
ENSG0000(	ZNF208	584.3482	-0.19136	0.000941	0.006455	protein_coding
ENSG0000(	FZD4	1037.211	0.26355	0.000942	0.006462	protein_coding
ENSG0000(	ERVH-1	36.64807	0.544752	0.000943	0.006466	lncRNA
ENSG0000(	VDAC1	2053.065	-0.28383	0.000944	0.006475	protein_coding
ENSG0000(	S1PR5	43.97314	0.423886	0.000946	0.006482	protein_coding
ENSG0000(	UCA1	204.9156	0.277368	0.000947	0.00649	lncRNA
ENSG0000(	KATNAL2	758.195	0.138666	0.000948	0.006496	protein_coding
ENSG0000(	C15orf40	4362.993	-0.11142	0.00095	0.006511	protein_coding
ENSG0000(	CHP2	2826.411	-0.60401	0.000953	0.00653	protein_coding
ENSG0000(	IGHV1-69D	179.3801	1.081675	0.000954	0.006533	IG_V_gene
ENSG0000(	RNF175	22.9317	0.771375	0.000955	0.006537	protein_coding
ENSG0000(	ENSG0000(	36.11867	0.647444	0.000956	0.006541	lncRNA
ENSG0000(	RCBTB2	389.8027	0.283524	0.000956	0.006542	protein_coding
ENSG0000(	H3C4	152.2792	-0.56741	0.000956	0.006542	protein_coding

ENSG0000(	OR10A3	134.4409	-0.27379	0.000956	0.006542	protein_coding
ENSG0000(	TPMT	894.8961	-0.26644	0.000957	0.006544	protein_coding
ENSG0000(	LINC01702	88.66675	-0.30001	0.000957	0.006544	lncRNA
ENSG0000(	NPHP4	527.8692	0.237385	0.000957	0.006544	protein_coding
ENSG0000(	N4BP2L2	16799.44	0.131316	0.000958	0.006546	protein_coding
ENSG0000(	ENSG0000(	73.38209	0.477773	0.000958	0.006549	lncRNA
ENSG0000(	LRRC8B	1109.516	0.188673	0.000963	0.006576	protein_coding
ENSG0000(	SLA	2076.456	0.653133	0.000963	0.006576	protein_coding
ENSG0000(	HECTD3	1516.147	-0.3984	0.000963	0.006577	protein_coding
ENSG0000(	SLC7A14-A:	643.1894	0.15043	0.000964	0.006579	lncRNA
ENSG0000(	LETM1	1947.087	-0.2024	0.000967	0.006598	protein_coding
ENSG0000(	MTA3	1587.353	-0.14356	0.000967	0.006598	protein_coding
ENSG0000(	MIRLET7A1	1996.227	0.120435	0.000967	0.0066	lncRNA
ENSG0000(	ENSG0000(	139.1845	-0.31294	0.000968	0.006603	lncRNA
ENSG0000(	RETREG3	2022.616	-0.16976	0.000968	0.006603	protein_coding
ENSG0000(	BRCA2	1008.077	0.229216	0.000969	0.006607	protein_coding
ENSG0000(	ENSG0000(	678.3496	-0.16831	0.00097	0.006609	lncRNA
ENSG0000(	FITM1	80.04893	-0.44028	0.000974	0.006639	protein_coding
ENSG0000(	CCDC47	1341.641	-0.22722	0.000975	0.006642	protein_coding
ENSG0000(	CC2D1B	1635.4	-0.26499	0.000977	0.006646	protein_coding
ENSG0000(	CFAP418-A	111.5991	0.349121	0.000976	0.006646	lncRNA
ENSG0000(	ENSG0000(	1168.715	0.401559	0.000976	0.006646	lncRNA
ENSG0000(	OR2A1-AS1	336.2592	-0.30448	0.000976	0.006646	lncRNA
ENSG0000(	PWWP3A	2076.089	-0.13757	0.000977	0.006646	protein_coding
ENSG0000(	ST7	409.0972	0.217431	0.000976	0.006646	protein_coding
ENSG0000(	CRYBG3	4229.749	0.228875	0.000977	0.006649	protein_coding
ENSG0000(	FANCL	465.0582	-0.20508	0.000978	0.006652	protein_coding
ENSG0000(	ENSG0000(	183.5574	0.235912	0.000979	0.006659	lncRNA
ENSG0000(	RNU6-196F	11.57024	0.666094	0.00098	0.006663	snRNA
ENSG0000(	HNRNPH1F	162.136	0.302713	0.00098	0.006663	processed_pseudogene
ENSG0000(	ELOVL2	27.34913	0.689723	0.000981	0.006666	protein_coding
ENSG0000(	LINC01470	62.26117	-0.44025	0.000982	0.00667	lncRNA
ENSG0000(	ENSG0000(	50.95647	-0.36871	0.000982	0.00667	lncRNA
ENSG0000(	LINC02709	66.56801	0.257631	0.000983	0.006674	lncRNA
ENSG0000(	RIF1	4270.992	0.159586	0.000985	0.006687	protein_coding
ENSG0000(	ENSG0000(	61.32672	-0.41382	0.000986	0.006693	lncRNA
ENSG0000(	ENSG0000(	293.7901	0.184599	0.000987	0.0067	TEC
ENSG0000(	ENSG0000(	139.5166	-0.25887	0.000987	0.0067	lncRNA
ENSG0000(	PHF5GP	52.21819	0.363885	0.000988	0.0067	processed_pseudogene
ENSG0000(	ENSG0000(	16.97535	0.520093	0.000988	0.006701	lncRNA
ENSG0000(	ENSG0000(	805.1338	0.161525	0.000988	0.006701	lncRNA
ENSG0000(	RPEP4	69.00333	0.289998	0.00099	0.006709	processed_pseudogene
ENSG0000(	MPZ	104.486	0.378827	0.00099	0.006709	protein_coding
ENSG0000(	LRRC32	330.9681	0.49343	0.000992	0.00672	protein_coding
ENSG0000(	ENSG0000(	10.67978	0.952785	0.000992	0.006722	lncRNA
ENSG0000(	LINC00601	11.77215	0.615074	0.000992	0.006722	lncRNA
ENSG0000(	SIDT2	2804.006	-0.2791	0.000993	0.006723	protein_coding

ENSG0000(	KLHDC2	1367.691	-0.30475	0.000993	0.006726	protein_coding
ENSG0000(	ENSG0000(	102.5271	0.260654	0.000997	0.006748	TEC
ENSG0000(	ENSG0000(	25.11395	0.408732	0.000998	0.006752	lncRNA
ENSG0000(	PA2G4P4	179.4571	-0.37059	0.000998	0.006756	processed_pseudogene
ENSG0000(	LINC00266-	41.39476	0.420267	0.000999	0.006758	lncRNA
ENSG0000(	ADAM9	2209.641	0.240766	0.001	0.006761	protein_coding
ENSG0000(	ENSG0000(	22.11225	0.472975	0.001	0.006761	lncRNA
ENSG0000(	ENSG0000(	399.9345	0.216835	0.001	0.006761	lncRNA
ENSG0000(	CASP10	2958.074	0.258621	0.001001	0.006764	protein_coding
ENSG0000(	LINC02086	544.6429	0.425053	0.001002	0.006768	lncRNA
ENSG0000(	SUMO3	706.969	-0.17569	0.001002	0.006768	protein_coding
ENSG0000(	ENSG0000(	632.9082	0.400644	0.001003	0.006776	lncRNA
ENSG0000(	ZFYVE16	3499.461	0.149287	0.001004	0.00678	protein_coding
ENSG0000(	ENSG0000(	117.6209	0.294202	0.001005	0.006783	lncRNA
ENSG0000(	UGT1A7	695.0999	-0.62149	0.001005	0.006783	protein_coding
ENSG0000(	ENSG0000(	202.0431	0.1986	0.001006	0.006788	lncRNA
ENSG0000(	HMGN1P1	76.88763	0.275549	0.001006	0.006791	processed_pseudogene
ENSG0000(	ENSG0000(	80.85514	-0.52139	0.001007	0.006793	processed_pseudogene
ENSG0000(	MIR4300H(	170.3599	-0.23368	0.001007	0.006793	lncRNA
ENSG0000(	CLTCL1	210.2699	0.503687	0.001008	0.006796	protein_coding
ENSG0000(	FBXL2	461.7563	0.200372	0.00101	0.006807	protein_coding
ENSG0000(	ZP3	243.931	-0.23129	0.00101	0.006807	protein_coding
ENSG0000(	ENSG0000(	81.99558	-0.64756	0.00101	0.006809	lncRNA
ENSG0000(	SMARCD3	213.4128	0.343561	0.001011	0.006812	protein_coding
ENSG0000(	ZNF414	429.8789	0.247669	0.001012	0.00682	protein_coding
ENSG0000(	HLA-E	9870.567	0.306146	0.001014	0.006826	protein_coding
ENSG0000(	MRFAP1L1	826.5163	-0.11941	0.001014	0.006826	protein_coding
ENSG0000(	FECH	1174.982	0.130307	0.001017	0.006843	protein_coding
ENSG0000(	PROC	17.54199	-0.78008	0.001017	0.006843	protein_coding
ENSG0000(	RPS15AP6	70.37313	0.292303	0.001017	0.006845	processed_pseudogene
ENSG0000(	ENSG0000(	21.84283	0.453995	0.001018	0.006852	lncRNA
ENSG0000(	SLC9A3R1	2342.684	-0.58148	0.00102	0.006859	protein_coding
ENSG0000(	MTFMT	873.8066	-0.14038	0.001022	0.006871	protein_coding
ENSG0000(	GPAT4	1645.503	0.198795	0.001022	0.006872	protein_coding
ENSG0000(	SPINK4	781.301	0.86414	0.001023	0.006874	protein_coding
ENSG0000(	ENSG0000(	466.3422	0.14453	0.001024	0.006879	lncRNA
ENSG0000(	ENSG0000(	9.645564	0.634911	0.001024	0.006881	lncRNA
ENSG0000(	C11orf1	773.2897	0.130435	0.001027	0.006901	protein_coding
ENSG0000(	IGLV3-19	131.9391	0.958447	0.001028	0.006902	IG_V_gene
ENSG0000(	WDR48	1240.535	-0.1866	0.001028	0.006902	protein_coding
ENSG0000(	ENSG0000(	215.5786	0.205742	0.001029	0.006907	lncRNA
ENSG0000(	ITGA6-AS1	24.1725	0.611304	0.001029	0.006907	lncRNA
ENSG0000(	PLTP	226.7562	0.549211	0.00103	0.006909	protein_coding
ENSG0000(	AGO1	3406.224	-0.20418	0.001031	0.006915	protein_coding
ENSG0000(	HOXA6	300.7395	-0.4267	0.001031	0.006918	protein_coding
ENSG0000(	ENSG0000(	445.3397	0.231183	0.001032	0.00692	lncRNA
ENSG0000(	GPAM	519.4855	-0.21844	0.001033	0.006924	protein_coding

ENSG0000(	ANKIB1	2007.726	-0.12357	0.001033	0.006926	protein_coding
ENSG0000(	METTL17	1069.825	-0.15522	0.001033	0.006926	protein_coding
ENSG0000(	CCNL2	5944.565	0.100314	0.001035	0.006931	protein_coding
ENSG0000(	PMM1	661.8621	-0.41559	0.001035	0.006931	protein_coding
ENSG0000(	RNF138	925.2408	-0.19817	0.001034	0.006931	protein_coding
ENSG0000(	ENSG0000(	29.49571	0.381697	0.001035	0.006932	TEC
ENSG0000(	ENSG0000(	26.93205	0.347737	0.001039	0.006954	lncRNA
ENSG0000(	METTL25B	511.1205	-0.15136	0.001039	0.006954	protein_coding
ENSG0000(	ZSWIM5	717.8521	-0.41603	0.001039	0.006954	protein_coding
ENSG0000(	TTN-AS1	2397.35	0.154386	0.00104	0.006956	lncRNA
ENSG0000(	ENSG0000(	36.70887	0.398994	0.001042	0.00697	lncRNA
ENSG0000(	ATP5F1E	1060.444	-0.16655	0.001042	0.00697	protein_coding
ENSG0000(	OXSR1	1432.533	-0.21085	0.001043	0.006972	protein_coding
ENSG0000(	ENSG0000(	148.5511	0.437421	0.001044	0.006977	TEC
ENSG0000(	IL11RA	657.9403	-0.24907	0.001048	0.007001	protein_coding
ENSG0000(	NDUFC1	306.901	-0.38077	0.001048	0.007001	protein_coding
ENSG0000(	TRDV1	11.85985	-0.85873	0.001048	0.007002	TR_V_gene
ENSG0000(	SLBP	278.372	-0.22627	0.001049	0.007008	protein_coding
ENSG0000(	LINC01067	31.14133	0.404434	0.00105	0.007009	lncRNA
ENSG0000(	MIR4644	16.80682	0.501683	0.00105	0.007009	miRNA
ENSG0000(	ALYREF	183.1376	-0.29437	0.001051	0.007013	protein_coding
ENSG0000(	ENSG0000(	254.3585	0.171539	0.001051	0.007013	lncRNA
ENSG0000(	PRKAG1	767.6598	0.094693	0.001051	0.007014	protein_coding
ENSG0000(	ENSG0000(	21.61595	0.653435	0.001052	0.007015	lncRNA
ENSG0000(	IL1RL1	528.0366	0.4895	0.001052	0.007019	protein_coding
ENSG0000(	PRNCR1	1170.864	0.155752	0.001054	0.007025	lncRNA
ENSG0000(	LILRB1	1256.836	0.552952	0.001054	0.007027	protein_coding
ENSG0000(	SPDYE2B	20.29272	1.45333	0.001055	0.007032	protein_coding
ENSG0000(	MFAP1	228.9723	-0.22685	0.001057	0.00704	protein_coding
ENSG0000(	HEPACAM	22.09379	0.670662	0.001057	0.007044	protein_coding
ENSG0000(	TBC1D14	1994.605	0.159823	0.001058	0.007045	protein_coding
ENSG0000(	CLCA4-AS1	117.8857	0.312654	0.001058	0.007047	lncRNA
ENSG0000(	ENSG0000(	181.8176	0.198008	0.001059	0.007053	lncRNA
ENSG0000(	CAPN10-DT	538.6863	0.142419	0.001062	0.007067	lncRNA
ENSG0000(	RIOK2	536.8878	-0.14949	0.001062	0.00707	protein_coding
ENSG0000(	KCNH6	392.8959	-0.43458	0.001063	0.007074	protein_coding
ENSG0000(	PTBP3	4637.712	-0.17224	0.001063	0.007074	protein_coding
ENSG0000(	FMO4	418.0657	-0.57163	0.001064	0.007076	protein_coding
ENSG0000(	BISPR	683.8378	0.328196	0.001066	0.007089	lncRNA
ENSG0000(	ENSG0000(	911.1382	-0.14453	0.001066	0.00709	lncRNA
ENSG0000(	HLA-V	2032.755	0.504793	0.001068	0.007101	transcribed_unprocessed_pseudo
ENSG0000(	MARCHF2	340.7441	0.20237	0.001069	0.007107	protein_coding
ENSG0000(	OSGEPL1-A	201.4335	-0.16736	0.00107	0.007107	lncRNA
ENSG0000(	CTSS	3605.872	0.239233	0.001071	0.007109	protein_coding
ENSG0000(	RN7SKP6	10.09856	-0.83507	0.00107	0.007109	misc_RNA
ENSG0000(	SCLT1	1166.475	0.164798	0.00107	0.007109	protein_coding
ENSG0000(	LINC02389	103.2992	0.271171	0.001071	0.00711	lncRNA

ENSG0000(	ARHGAP18	1410.733	-0.3764	0.001075	0.007133	protein_coding
ENSG0000(	ENSG0000(	84.67551	0.265861	0.001076	0.00714	lncRNA
ENSG0000(	MT-ND4L	4822.263	-0.53208	0.001077	0.007147	protein_coding
ENSG0000(	CSNK1G2-A	20.84683	0.468649	0.001078	0.007148	lncRNA
ENSG0000(	LINC01806	129.1229	0.335022	0.001078	0.007148	lncRNA
ENSG0000(	VCPIP1	2694.238	0.106763	0.001079	0.007155	protein_coding
ENSG0000(	IGHA1	7831.63	0.833651	0.00108	0.007157	IG_C_gene
ENSG0000(	CDK2	492.309	0.26725	0.001083	0.007176	protein_coding
ENSG0000(	SMIM3	159.5389	0.399522	0.001086	0.007194	protein_coding
ENSG0000(	RB1CC1	2508.056	-0.17225	0.001087	0.007199	protein_coding
ENSG0000(	ENSG0000(	388.1653	0.196837	0.001087	0.007202	lncRNA
ENSG0000(	NAPRT	712.5615	-0.44083	0.001088	0.007203	protein_coding
ENSG0000(	MANEA	635.6725	0.237314	0.001089	0.00721	protein_coding
ENSG0000(	SVIP	779.6973	-0.20552	0.001089	0.00721	protein_coding
ENSG0000(	ENSG0000(	34.45977	0.526854	0.00109	0.007213	lncRNA
ENSG0000(	MGC16275	80.74668	0.284353	0.001091	0.007221	lncRNA
ENSG0000(	SLC2A4RG	346.1881	-0.28108	0.001092	0.007222	protein_coding
ENSG0000(	CERNA1	35.89028	0.599206	0.001095	0.007238	lncRNA
ENSG0000(	ENSG0000(	238.2802	0.233707	0.001098	0.007257	lncRNA
ENSG0000(	RASA3-IT1	38.72184	0.699278	0.001098	0.007257	lncRNA
ENSG0000(	HAND2	30.4293	0.630417	0.001098	0.00726	protein_coding
ENSG0000(	LINC00298	36.31298	0.423429	0.0011	0.00727	lncRNA
ENSG0000(	CHMP2B	1012.121	-0.25261	0.001101	0.007273	protein_coding
ENSG0000(	ANAPC15	759.7253	0.123541	0.001102	0.007278	protein_coding
ENSG0000(	TCTN3	1727.921	-0.16351	0.001102	0.007279	protein_coding
ENSG0000(	MAPK9	1277.678	-0.13358	0.001103	0.007282	protein_coding
ENSG0000(	ENSG0000(	23.71189	0.426835	0.001104	0.007285	lncRNA
ENSG0000(	ENSG0000(	88.33711	0.214634	0.001106	0.007296	lncRNA
ENSG0000(	ENSG0000(	221.452	0.249486	0.001106	0.007296	lncRNA
ENSG0000(	PQBP1	442.4272	-0.20405	0.001106	0.007296	protein_coding
ENSG0000(	TOMM20	1564.366	-0.15037	0.001109	0.007316	protein_coding
ENSG0000(	MSANTD4	489.2534	0.246939	0.001109	0.007316	protein_coding
ENSG0000(	GABARAPL	25.67456	0.61875	0.001112	0.007334	TEC
ENSG0000(	ENSG0000(	15.02094	0.522096	0.001113	0.007338	processed_pseudogene
ENSG0000(	ENSG0000(	324.9431	0.148674	0.001117	0.007358	lncRNA
ENSG0000(	ENSG0000(	65.87515	-0.58967	0.001117	0.007361	lncRNA
ENSG0000(	CHORDC1P	340.4571	0.468573	0.00112	0.007377	transcribed_processed_pseudoge
ENSG0000(	MTRNR2L7	46.62107	0.384468	0.001121	0.007384	protein_coding
ENSG0000(	MAT2A	6760.244	-0.15744	0.001124	0.007399	protein_coding
ENSG0000(	FLJ40194	317.007	0.211371	0.001125	0.007408	lncRNA
ENSG0000(	AFG1L	488.6447	-0.19696	0.001127	0.007418	protein_coding
ENSG0000(	STRIT1	70.19066	0.323201	0.001127	0.007418	protein_coding
ENSG0000(	STRADB	1109.87	-0.52828	0.001128	0.00742	protein_coding
ENSG0000(	HDGFL3	1400.095	0.25936	0.001129	0.007427	protein_coding
ENSG0000(	YJU2	158.9742	-0.19987	0.00113	0.007432	protein_coding
ENSG0000(	ENSG0000(	672.5542	0.173638	0.001132	0.007443	lncRNA
ENSG0000(	ENSG0000(	159.5698	0.536424	0.001134	0.007452	lncRNA

ENSG0000(	MT-TS1	96.93725	1.072353	0.001134	0.007453	Mt_tRNA
ENSG0000(	DMAC2	611.5644	-0.26717	0.001134	0.007453	protein_coding
ENSG0000(	ENSG0000(	9.375129	0.74688	0.001135	0.007455	processed_pseudogene
ENSG0000(	WEE2-AS1	180.2076	0.311006	0.001136	0.007459	lncRNA
ENSG0000(	MINDY3	645.4964	-0.16945	0.001137	0.007467	protein_coding
ENSG0000(	SMOX	160.3753	0.355479	0.001137	0.007467	protein_coding
ENSG0000(	SLC6A20	2350.021	-0.59265	0.00114	0.007483	protein_coding
ENSG0000(	SUCLG2P4	68.50545	0.320559	0.00114	0.007483	processed_pseudogene
ENSG0000(	SLC23A3	1130.625	-0.72575	0.001142	0.007493	protein_coding
ENSG0000(	ENSG0000(	12.26742	0.824532	0.001144	0.007501	processed_pseudogene
ENSG0000(	ENSG0000(	190.5713	0.22687	0.001144	0.007501	lncRNA
ENSG0000(	ENSG0000(	17.26976	0.49367	0.001144	0.007501	lncRNA
ENSG0000(	PDGFD	187.6577	-0.37849	0.001146	0.007514	protein_coding
ENSG0000(	AGR3	336.3857	-0.70879	0.001148	0.007517	protein_coding
ENSG0000(	ALKBH5	1037.695	-0.1863	0.001148	0.007517	protein_coding
ENSG0000(	CCDC12	530.5336	-0.10949	0.001148	0.007517	protein_coding
ENSG0000(	EFR3B	359.492	-0.13932	0.001148	0.007517	protein_coding
ENSG0000(	SAP30L	1194.793	-0.25394	0.001148	0.007517	protein_coding
ENSG0000(	UQCR10	319.384	-0.29299	0.001148	0.007518	protein_coding
ENSG0000(	RPL35	1108.877	-0.29115	0.001149	0.007521	protein_coding
ENSG0000(	MRC1	626.9166	0.477362	0.00115	0.007526	protein_coding
ENSG0000(	MIR99AHG	2231.764	0.1257	0.001151	0.007532	lncRNA
ENSG0000(	SUGP1	645.1033	-0.12593	0.001153	0.007541	protein_coding
ENSG0000(	JAKMIP1	38.09543	0.717744	0.001153	0.007544	protein_coding
ENSG0000(	VINAC1P	57.98465	-0.3701	0.001155	0.007551	transcribed_unitary_pseudogene
ENSG0000(	DNPEP	1903.51	-0.27815	0.001158	0.007569	protein_coding
ENSG0000(	CIDEC	343.4462	-0.82747	0.001159	0.007573	protein_coding
ENSG0000(	CLRN1	41.47955	0.392976	0.001163	0.007599	protein_coding
ENSG0000(	ENSG0000(	88.53981	0.446032	0.001163	0.007599	lncRNA
ENSG0000(	FAR1-IT1	51.0811	0.31315	0.001165	0.00761	lncRNA
ENSG0000(	VPS13C	13324.08	0.20942	0.001169	0.007636	protein_coding
ENSG0000(	LIMS1	2269.475	0.165428	0.00117	0.007639	protein_coding
ENSG0000(	RAD52	657.3312	-0.20677	0.00117	0.007639	protein_coding
ENSG0000(	SLCO5A1	567.4129	0.427776	0.001172	0.007645	protein_coding
ENSG0000(	TSPAN2	125.4676	0.481642	0.001171	0.007645	protein_coding
ENSG0000(	KLRC4	83.77174	-0.51737	0.001175	0.007667	protein_coding
ENSG0000(	ENSG0000(	124.2618	-0.31584	0.001176	0.007672	lncRNA
ENSG0000(	ZMYM2	5285.894	0.084577	0.001176	0.007672	protein_coding
ENSG0000(	MEGF11	28.43095	0.792484	0.001177	0.007676	protein_coding
ENSG0000(	OR6D1P	662.2354	0.180818	0.001179	0.007684	transcribed_unitary_pseudogene
ENSG0000(	HOXC4	137.2893	0.361719	0.001179	0.007687	protein_coding
ENSG0000(	RN7SL273F	9.48775	0.558932	0.00118	0.00769	misc_RNA
ENSG0000(	CYP4A22-A	425.8948	0.152919	0.001182	0.007702	lncRNA
ENSG0000(	SGK2	1241.399	-0.56687	0.001186	0.007725	protein_coding
ENSG0000(	OST4	434.7587	-0.29849	0.001188	0.007735	protein_coding
ENSG0000(	ZBTB2	354.0508	-0.13802	0.001188	0.007735	protein_coding
ENSG0000(	ABHD6	799.7382	-0.48716	0.001191	0.00775	protein_coding

ENSG0000(	ZNHIT3	1121.38	-0.17236	0.001193	0.007761	protein_coding
ENSG0000(	SLC39A12-/	323.7823	0.201635	0.001194	0.007767	lncRNA
ENSG0000(	ENSG0000(	70.84893	0.273284	0.001195	0.007776	lncRNA
ENSG0000(	ENSG0000(	242.7405	0.135465	0.001196	0.007779	lncRNA
ENSG0000(	RNU6-1005	27.14824	0.459943	0.001196	0.007779	snRNA
ENSG0000(	LINC02848	592.3268	0.139396	0.001198	0.007789	lncRNA
ENSG0000(	ENSG0000(	41.1375	-0.64776	0.001199	0.007794	lncRNA
ENSG0000(	AFMID	1176.026	0.135161	0.001203	0.007815	protein_coding
ENSG0000(	BRWD1	9018.734	-0.12173	0.001203	0.007815	protein_coding
ENSG0000(	DFFB	833.98	-0.12631	0.001204	0.007819	protein_coding
ENSG0000(	EVC2	222.9368	0.413031	0.001204	0.007819	protein_coding
ENSG0000(	FAM13C	288.496	0.335401	0.001204	0.007819	protein_coding
ENSG0000(	FGF13-AS1	54.19407	0.294114	0.001204	0.007819	lncRNA
ENSG0000(	RPS26P28	29.81397	0.48562	0.001205	0.007819	processed_pseudogene
ENSG0000(	SERINC3	2394.062	-0.14614	0.001205	0.007819	protein_coding
ENSG0000(	ENSG0000(	309.8144	0.137602	0.001205	0.007819	lncRNA
ENSG0000(	NANOG	797.8542	-0.11925	0.001205	0.007819	protein_coding
ENSG0000(	SPP1	331.6256	0.333955	0.001206	0.00782	protein_coding
ENSG0000(	ENSG0000(	236.7727	0.276813	0.001207	0.007825	lncRNA
ENSG0000(	DAPK1-IT1	148.4387	0.249827	0.001208	0.007826	lncRNA
ENSG0000(	ENSG0000(	462.5497	0.214387	0.001208	0.007826	lncRNA
ENSG0000(	LINC00989	159.7762	0.257213	0.001208	0.007826	lncRNA
ENSG0000(	OR2A12	113.0766	0.234726	0.001208	0.007826	protein_coding
ENSG0000(	LINC01013	207.0586	-0.32295	0.00121	0.007835	lncRNA
ENSG0000(	SLC25A28	687.9883	0.233204	0.00121	0.007835	protein_coding
ENSG0000(	C1orf210	413.9363	-0.50651	0.00121	0.007836	protein_coding
ENSG0000(	CEACAM6	1466.596	0.862531	0.001213	0.007851	protein_coding
ENSG0000(	HDDC3	314.7108	-0.16582	0.001213	0.007851	protein_coding
ENSG0000(	RPS6KA1	2603.443	-0.36408	0.001214	0.007853	protein_coding
ENSG0000(	CYP4V2	4705.736	-0.36807	0.001214	0.007857	protein_coding
ENSG0000(	KMT2E	5805.406	-0.10677	0.001217	0.007871	protein_coding
ENSG0000(	RPL23AP4S	22.49039	0.559036	0.001218	0.007875	transcribed_processed_pseudoge
ENSG0000(	DHRS11	4326.412	-0.64089	0.001219	0.00788	protein_coding
ENSG0000(	HGSNAT	1949.237	0.118798	0.00122	0.007884	protein_coding
ENSG0000(	SLITRK2	107.4308	-0.30082	0.00122	0.007884	protein_coding
ENSG0000(	NDUFA6-D	614.0019	-0.31024	0.001222	0.007897	lncRNA
ENSG0000(	LINC01140	178.9274	0.250345	0.001222	0.007897	lncRNA
ENSG0000(	ATF3	285.2194	0.645421	0.001223	0.007898	protein_coding
ENSG0000(	C2CD2L	2022.072	-0.37025	0.001223	0.007898	protein_coding
ENSG0000(	CCDC74B	40.13214	0.519286	0.001224	0.007903	protein_coding
ENSG0000(	GAS5	1724.27	-0.2065	0.001224	0.007903	lncRNA
ENSG0000(	IGHV4-4	128.6785	1.14477	0.001227	0.007919	IG_V_gene
ENSG0000(	ASGR2	18.6074	0.731868	0.001228	0.00792	protein_coding
ENSG0000(	ENSG0000(	499.3492	0.185215	0.001228	0.00792	lncRNA
ENSG0000(	SH3GL1	969.7071	-0.15923	0.001229	0.007926	protein_coding
ENSG0000(	ACTG1P3	70.26088	-0.28991	0.00123	0.00793	processed_pseudogene
ENSG0000(	BRSK1	134.6989	0.369139	0.001232	0.007941	protein_coding

ENSG0000(	SEPHS2	1326.233	-0.46592	0.001232	0.007941	protein_coding
ENSG0000(	ENSG0000(	17.2952	0.561983	0.001233	0.007946	lncRNA
ENSG0000(	CFDP1	801.3201	-0.18835	0.001235	0.007951	protein_coding
ENSG0000(	ENSG0000(	593.518	0.193017	0.001234	0.007951	lncRNA
ENSG0000(	ENSG0000(	262.1071	0.497898	0.001235	0.007951	lncRNA
ENSG0000(	ENSG0000(	72.29577	0.256545	0.001235	0.007951	lncRNA
ENSG0000(	LINC02551	319.5882	0.140149	0.001235	0.007951	lncRNA
ENSG0000(	SYTL3	254.0943	0.347644	0.001235	0.007951	protein_coding
ENSG0000(	ZNF283	1475.513	-0.14998	0.001236	0.007951	protein_coding
ENSG0000(	KLHL41	24.62601	0.561404	0.001236	0.007952	protein_coding
ENSG0000(	SNAPC5	334.0958	-0.14512	0.001237	0.007955	protein_coding
ENSG0000(	RANBP9	817.3535	-0.16014	0.001238	0.007961	protein_coding
ENSG0000(	SPHK2	1000.999	-0.24019	0.001238	0.007962	protein_coding
ENSG0000(	LINC02395	537.1259	-0.15865	0.001239	0.007965	lncRNA
ENSG0000(	CELA2A	95.54472	0.217615	0.00124	0.007972	protein_coding
ENSG0000(	FOXRED1	1142.269	0.179577	0.001241	0.007974	protein_coding
ENSG0000(	DOLPP1	384.5103	-0.46098	0.001242	0.007976	protein_coding
ENSG0000(	RAB14	1837.911	-0.14904	0.001244	0.007988	protein_coding
ENSG0000(	TRIR	618.4897	-0.13702	0.001244	0.007988	protein_coding
ENSG0000(	ENSG0000(	89.24878	-0.33317	0.001247	0.008004	transcribed_processed_pseudoge
ENSG0000(	HMGXB3	1281.357	0.239963	0.001248	0.008011	protein_coding
ENSG0000(	ENSG0000(	1080.717	-0.21724	0.001249	0.008016	lncRNA
ENSG0000(	SLC1A1	1490.045	-0.75468	0.00125	0.008019	protein_coding
ENSG0000(	MRTFA-AS1	332.1534	-0.16114	0.001251	0.008023	lncRNA
ENSG0000(	ENSG0000(	289.4964	-0.22591	0.001252	0.008032	lncRNA
ENSG0000(	ENSG0000(	1269.007	0.075891	0.001254	0.008042	lncRNA
ENSG0000(	ATG13	1469.458	0.120225	0.001256	0.008053	protein_coding
ENSG0000(	BCCIP	443.4031	-0.17163	0.001259	0.008067	protein_coding
ENSG0000(	DCAF11	2492.851	-0.29027	0.001258	0.008067	protein_coding
ENSG0000(	PPIAP53	13.85787	0.573693	0.00126	0.008072	processed_pseudogene
ENSG0000(	GPR4	137.1314	0.376636	0.001262	0.008083	protein_coding
ENSG0000(	EGFLAM-AS	26.92079	0.481181	0.001265	0.008099	lncRNA
ENSG0000(	MGAT5	4320.595	-0.2174	0.001265	0.008099	protein_coding
ENSG0000(	CNST	1920.206	-0.2418	0.001266	0.008107	protein_coding
ENSG0000(	KDM7A-DT	711.1472	0.191825	0.001266	0.008107	lncRNA
ENSG0000(	ZBTB40	2669.057	0.153685	0.001267	0.008109	protein_coding
ENSG0000(	MTRF1	495.5194	-0.17638	0.001268	0.008115	protein_coding
ENSG0000(	BAIAP2-DT	609.378	0.118556	0.001269	0.00812	lncRNA
ENSG0000(	ENSG0000(	65.5033	0.383003	0.001272	0.008135	lncRNA
ENSG0000(	HADHB	2405.346	-0.33839	0.001273	0.008142	protein_coding
ENSG0000(	ENSG0000(	13.66294	0.764586	0.001275	0.008148	lncRNA
ENSG0000(	ADCY9	1645.269	-0.40356	0.001275	0.008149	protein_coding
ENSG0000(	ACOT7	382.5964	-0.35775	0.001275	0.008149	protein_coding
ENSG0000(	ENSG0000(	165.8475	0.237082	0.001279	0.008171	lncRNA
ENSG0000(	ENSG0000(	12.66849	0.721514	0.00128	0.008174	lncRNA
ENSG0000(	RAMP3	186.3296	0.554548	0.00128	0.008174	protein_coding
ENSG0000(	SYNPR	26.14192	0.448819	0.001281	0.008181	protein_coding

ENSG0000(	CHST5	2217.902	-0.68582	0.001282	0.008183	protein_coding
ENSG0000(	F2R	803.3505	0.241087	0.001282	0.008183	protein_coding
ENSG0000(	ENSG0000(	35.18573	0.485425	0.001284	0.008192	lncRNA
ENSG0000(	LRRC4B	47.40962	-0.55913	0.001284	0.008193	protein_coding
ENSG0000(	ENSG0000(	9.120692	0.77282	0.001285	0.008195	TEC
ENSG0000(	ENSG0000(	423.3683	-0.12294	0.001286	0.008201	processed_pseudogene
ENSG0000(	MRM1	271.5578	-0.22587	0.001286	0.008201	protein_coding
ENSG0000(	ZNF528-AS	670.7649	0.133516	0.001286	0.008201	lncRNA
ENSG0000(	ENSG0000(	85.18785	0.273643	0.001287	0.008203	lncRNA
ENSG0000(	ENSG0000(	408.4156	0.212109	0.001288	0.008208	lncRNA
ENSG0000(	ENSG0000(	455.7005	-0.24242	0.001291	0.008225	lncRNA
ENSG0000(	GPD1	1157.924	-0.75158	0.001291	0.008225	protein_coding
ENSG0000(	INSIG1-DT	23.92294	0.451469	0.001293	0.008233	lncRNA
ENSG0000(	ENSG0000(	58.64957	0.305871	0.001295	0.008242	lncRNA
ENSG0000(	ENSG0000(	266.3837	0.349204	0.001295	0.008242	TEC
ENSG0000(	ENSG0000(	487.4788	0.197674	0.001296	0.008248	protein_coding
ENSG0000(	GRPEL1	298.7402	-0.19693	0.001297	0.008256	protein_coding
ENSG0000(	OR52E4	143.3049	0.240004	0.001299	0.008265	protein_coding
ENSG0000(	DNAJC3-DT	340.7028	0.262476	0.0013	0.00827	lncRNA
ENSG0000(	LINC02478	20.95312	0.587984	0.001301	0.008275	lncRNA
ENSG0000(	RPS29	2943.763	-0.28868	0.001305	0.008301	protein_coding
ENSG0000(	IGHV5-51	226.0538	0.828625	0.001306	0.008304	IG_V_gene
ENSG0000(	ENSG0000(	35.72733	0.345362	0.001307	0.008308	lncRNA
ENSG0000(	EIF3D	1165.671	-0.15977	0.001308	0.008314	protein_coding
ENSG0000(	ENSG0000(	191.2042	-0.23564	0.001311	0.00833	lncRNA
ENSG0000(	ENSG0000(	732.493	0.161888	0.001312	0.00833	lncRNA
ENSG0000(	RHOBTB3	1732.792	0.31047	0.001312	0.00833	protein_coding
ENSG0000(	IGSF8	370.7333	-0.3167	0.001314	0.008342	protein_coding
ENSG0000(	ADORA1	42.13044	0.657247	0.001314	0.008342	protein_coding
ENSG0000(	RUNX1T1	815.5131	0.306481	0.001315	0.008342	protein_coding
ENSG0000(	VAV2	1669.069	-0.32191	0.001314	0.008342	protein_coding
ENSG0000(	EIF2AK1	1542.22	-0.18897	0.001315	0.008343	protein_coding
ENSG0000(	CRNDE	283.5102	0.173425	0.001316	0.008348	lncRNA
ENSG0000(	ENSG0000(	49.1391	0.400151	0.001317	0.008353	lncRNA
ENSG0000(	GCNT4	2447.557	-0.6932	0.001318	0.008357	protein_coding
ENSG0000(	ENSG0000(	160.2587	0.275881	0.001319	0.008362	lncRNA
ENSG0000(	AP5S1	1188.775	0.100925	0.001321	0.008373	protein_coding
ENSG0000(	ATP5PBP1	50.54009	0.442641	0.001322	0.008375	processed_pseudogene
ENSG0000(	TST	507.5325	-0.5805	0.001323	0.008385	protein_coding
ENSG0000(	FRG1BP	277.6849	-0.4276	0.001325	0.008391	unprocessed_pseudogene
ENSG0000(	ENSG0000(	531.6419	0.229533	0.001325	0.008394	lncRNA
ENSG0000(	ENSG0000(	22.93914	0.465657	0.001326	0.008397	transcribed_processed_pseudoge
ENSG0000(	PHYH	351.7005	-0.5067	0.001329	0.008411	protein_coding
ENSG0000(	LINC01555	42.39491	0.440211	0.00133	0.008419	lncRNA
ENSG0000(	ALDH2	4532.938	-0.27247	0.001334	0.00844	protein_coding
ENSG0000(	NOL10	564.0598	-0.19667	0.001334	0.00844	protein_coding
ENSG0000(	ZNF57	289.0535	-0.52811	0.001335	0.008442	protein_coding

ENSG0000(	ENSG0000(	70.07792	0.292264	0.001338	0.00846	lncRNA
ENSG0000(	ECI2	873.7383	-0.33634	0.001341	0.008478	protein_coding
ENSG0000(	ENSG0000(	11.87601	0.751112	0.001342	0.008485	TEC
ENSG0000(	MLXIPL	2481.083	-0.55059	0.001343	0.008489	protein_coding
ENSG0000(	PPM1H	265.6179	0.419488	0.001344	0.008491	protein_coding
ENSG0000(	ENSG0000(	43.1384	0.382414	0.001344	0.008492	TEC
ENSG0000(	INTS4P1	95.67947	0.343836	0.001344	0.008492	transcribed_unprocessed_pseudo
ENSG0000(	MTX2	392.1061	-0.21834	0.001345	0.008494	protein_coding
ENSG0000(	STARD13-A	121.8377	0.315999	0.001345	0.008494	lncRNA
ENSG0000(	ZC3H18	1279.083	-0.11614	0.001346	0.008494	protein_coding
ENSG0000(	VGLL3	577.7063	0.190818	0.001347	0.008502	protein_coding
ENSG0000(	THOC1-DT	222.1134	0.172318	0.001348	0.008503	lncRNA
ENSG0000(	SLC25A5	2680.127	-0.55635	0.001348	0.008505	protein_coding
ENSG0000(	ZNF433	197.4766	0.2935	0.001348	0.008505	protein_coding
ENSG0000(	SGMS1-AS1	1005.233	0.175035	0.001349	0.008508	lncRNA
ENSG0000(	RN7SL715F	21.98418	0.643281	0.00135	0.008513	misc_RNA
ENSG0000(	JCAD	403.4403	0.408483	0.001351	0.008517	protein_coding
ENSG0000(	ENSG0000(	15.60088	-0.5815	0.001356	0.008543	lncRNA
ENSG0000(	HOXA4	140.3482	-0.38861	0.001356	0.008547	protein_coding
ENSG0000(	DYNLL2	2289.072	-0.21702	0.001358	0.008553	protein_coding
ENSG0000(	RUNX2	816.809	0.42721	0.001358	0.008553	protein_coding
ENSG0000(	KLRK1-AS1	591.4357	0.183305	0.00136	0.008562	lncRNA
ENSG0000(	ARHGAP8	604.9625	0.281221	0.001363	0.008579	protein_coding
ENSG0000(	ENSG0000(	239.3881	0.151466	0.001363	0.008579	lncRNA
ENSG0000(	ANO10	1501.232	-0.36436	0.001366	0.008596	protein_coding
ENSG0000(	STARD3	931.4454	-0.16769	0.001366	0.008596	protein_coding
ENSG0000(	ALDH3A2	3715.831	-0.21192	0.001368	0.008609	protein_coding
ENSG0000(	CD8B	307.1186	-0.50436	0.001371	0.008622	protein_coding
ENSG0000(	GYS1	773.2226	-0.17652	0.001371	0.008622	protein_coding
ENSG0000(	RN7SL525F	190.9077	-0.22954	0.001372	0.008629	misc_RNA
ENSG0000(	ENSG0000(	62.85516	-0.26742	0.001373	0.00863	lncRNA
ENSG0000(	LRRC19	1682.211	-0.62089	0.001373	0.00863	protein_coding
ENSG0000(	BNC2	1259.397	0.301248	0.001376	0.008641	protein_coding
ENSG0000(	DLG1-AS1	11.15601	0.638258	0.001376	0.008641	lncRNA
ENSG0000(	ENSG0000(	14.08792	-0.59067	0.001376	0.008641	lncRNA
ENSG0000(	ENSG0000(	383.1032	0.517118	0.001376	0.008641	lncRNA
ENSG0000(	RALYL	89.1478	-1.19552	0.001381	0.008669	protein_coding
ENSG0000(	AVPR1A	130.6987	0.405655	0.001382	0.008674	protein_coding
ENSG0000(	ENSG0000(	118.6871	0.220461	0.001388	0.008711	lncRNA
ENSG0000(	MARCKSL1	53.62404	0.340896	0.001389	0.008713	processed_pseudogene
ENSG0000(	RPL21P135	458.8259	0.172921	0.001392	0.008734	processed_pseudogene
ENSG0000(	ENSG0000(	77.05857	0.320022	0.001393	0.008735	lncRNA
ENSG0000(	XPNPEP2	4326.728	-0.85224	0.001394	0.00874	protein_coding
ENSG0000(	IGKV1D-17	12.13778	1.512486	0.001396	0.008753	IG_V_gene
ENSG0000(	FBXW8	633.2941	0.253165	0.001397	0.008756	protein_coding
ENSG0000(	FGD5-AS1	2944.313	-0.16239	0.001398	0.00876	lncRNA
ENSG0000(	NPIPB10P	56.92567	0.475755	0.001398	0.00876	unprocessed_pseudogene

ENSG0000(	BAHD1	815.1211	-0.19317	0.001399	0.008765	protein_coding
ENSG0000(	GGT1	1251.069	-0.56745	0.001399	0.008765	protein_coding
ENSG0000(	SIRT5	1930.491	-0.12157	0.0014	0.008765	protein_coding
ENSG0000(	PPIP5K1	3031.866	-0.25837	0.001401	0.008768	protein_coding
ENSG0000(	RSPRY1	2082.665	-0.17629	0.001401	0.008768	protein_coding
ENSG0000(	PMPCB	1320.026	-0.12051	0.001403	0.008781	protein_coding
ENSG0000(	ABCC2	1538.681	-0.53787	0.001404	0.008786	protein_coding
ENSG0000(	EFCAB5	245.5379	0.196495	0.001405	0.008787	protein_coding
ENSG0000(	TMEM94	1976.792	-0.29428	0.001405	0.008787	protein_coding
ENSG0000(	IATPR	147.353	0.216194	0.001405	0.008787	lncRNA
ENSG0000(	WRAP73	826.5561	-0.21279	0.001405	0.008787	protein_coding
ENSG0000(	COL17A1	2643.798	-0.68991	0.001407	0.008792	protein_coding
ENSG0000(	DYNC2LI1	770.6877	-0.16802	0.001408	0.008798	protein_coding
ENSG0000(	RPS10	1491.732	-0.31571	0.001409	0.008805	protein_coding
ENSG0000(	TMEM150/	149.4357	-0.26863	0.001413	0.008828	protein_coding
ENSG0000(	ENSG0000(	27.97789	-0.38508	0.001414	0.00883	lncRNA
ENSG0000(	ARHGEF3	637.1939	0.378995	0.001414	0.00883	protein_coding
ENSG0000(	SEMA3D	381.8085	-0.18511	0.001414	0.008831	protein_coding
ENSG0000(	CD163L1	1312.253	-0.45777	0.001418	0.008847	protein_coding
ENSG0000(	ENSG0000(	20.45939	0.967875	0.001418	0.008847	protein_coding
ENSG0000(	INPP5K	822.5792	-0.14691	0.001417	0.008847	protein_coding
ENSG0000(	PLPP2	1472.135	-0.3822	0.001418	0.008847	protein_coding
ENSG0000(	LINC01833	23.07345	0.463429	0.00142	0.008856	lncRNA
ENSG0000(	ENSG0000(	111.2429	0.288881	0.001421	0.008862	lncRNA
ENSG0000(	POLR2H	1188.028	-0.25801	0.001422	0.008862	protein_coding
ENSG0000(	SPATA7	204.5347	0.267524	0.001422	0.008862	protein_coding
ENSG0000(	ENSG0000(	14.84789	0.590007	0.001422	0.008864	lncRNA
ENSG0000(	MIEN1	216.3499	-0.32098	0.001422	0.008864	protein_coding
ENSG0000(	PAIP1	516.3543	-0.26735	0.001424	0.008871	protein_coding
ENSG0000(	ENSG0000(	79.94781	0.225159	0.001425	0.008875	TEC
ENSG0000(	ENSG0000(	22.28762	0.475665	0.001426	0.008881	lncRNA
ENSG0000(	SFRP1	149.4958	0.750078	0.001428	0.008894	protein_coding
ENSG0000(	IFNGR2	1081.537	-0.24044	0.00143	0.008904	protein_coding
ENSG0000(	USP12	1895.907	-0.21162	0.001432	0.008911	protein_coding
ENSG0000(	ENSG0000(	118.6651	0.1965	0.001433	0.00892	lncRNA
ENSG0000(	ENSG0000(	88.20126	-0.43469	0.001436	0.008933	lncRNA
ENSG0000(	PITPNM1	1505.797	-0.22996	0.001436	0.008933	protein_coding
ENSG0000(	PIK3CD-AS:	9.816996	0.931632	0.001437	0.008939	lncRNA
ENSG0000(	CLUAP1	928.096	0.124039	0.001439	0.008949	protein_coding
ENSG0000(	NPEPL1	629.8977	0.217962	0.001441	0.00896	protein_coding
ENSG0000(	ZMYND19	169.9151	0.252581	0.001442	0.00896	protein_coding
ENSG0000(	MARVELD1	343.0026	0.294133	0.001442	0.008963	protein_coding
ENSG0000(	RNU1-140F	20.91816	-0.76828	0.001445	0.00898	snRNA
ENSG0000(	ENSG0000(	935.9146	0.243327	0.001446	0.008982	transcribed_unprocessed_pseudo
ENSG0000(	H2BC3	47.13466	-0.61115	0.001448	0.008992	protein_coding
ENSG0000(	ENSG0000(	485.9418	0.189285	0.001448	0.008992	lncRNA
ENSG0000(	APOB	82861.74	-0.97268	0.001452	0.009015	protein_coding

ENSG0000(	TAF5	498.0298	-0.11859	0.001452	0.009015	protein_coding
ENSG0000(	GLYR1	1843.797	-0.10565	0.001454	0.009025	protein_coding
ENSG0000(	LRRK2-DT	64.16362	0.609821	0.001456	0.009035	lncRNA
ENSG0000(	LAMB1	7432.651	0.345941	0.001457	0.009036	protein_coding
ENSG0000(	LINC01948	360.4015	0.298545	0.001458	0.009044	lncRNA
ENSG0000(	SMIM38	69.26439	0.784692	0.001458	0.009044	protein_coding
ENSG0000(	BRSK2	19.7084	0.74062	0.001465	0.00908	protein_coding
ENSG0000(	KRT20	4763.178	-0.67708	0.001465	0.009082	protein_coding
ENSG0000(	ENSG0000(	11.56559	0.730598	0.001466	0.009083	lncRNA
ENSG0000(	ENSG0000(	13.2546	0.846558	0.001467	0.009088	lncRNA
ENSG0000(	NTN1	271.8792	0.556075	0.001467	0.009088	protein_coding
ENSG0000(	UBE2B	942.1977	-0.18564	0.001467	0.009088	protein_coding
ENSG0000(	LINC01997	311.4787	0.203278	0.001472	0.009112	lncRNA
ENSG0000(	EMC4	1135.465	-0.09428	0.001472	0.009112	protein_coding
ENSG0000(	ENSG0000(	25.72163	0.370055	0.001473	0.009115	lncRNA
ENSG0000(	RPL39	1898.028	-0.35952	0.001473	0.009116	protein_coding
ENSG0000(	ENTPD5	3477.187	-0.46457	0.001474	0.009121	protein_coding
ENSG0000(	LINC00923	2175.267	0.120251	0.001474	0.009121	lncRNA
ENSG0000(	ENSG0000(	115.2665	0.185678	0.001475	0.009122	lncRNA
ENSG0000(	HELZ2	2180.288	0.357346	0.001475	0.009122	protein_coding
ENSG0000(	SPINT1	2533.088	-0.63595	0.001475	0.009122	protein_coding
ENSG0000(	ASTL	15.41169	1.0926	0.001478	0.009135	protein_coding
ENSG0000(	IQCN	1463.228	0.195034	0.001479	0.00914	protein_coding
ENSG0000(	ENSG0000(	133.6227	0.246454	0.00148	0.009145	lncRNA
ENSG0000(	GATAD2A	2089.867	-0.14052	0.001484	0.009166	protein_coding
ENSG0000(	CTSW	152.4368	-0.22567	0.001485	0.009169	protein_coding
ENSG0000(	FAM200B	699.575	-0.19641	0.001486	0.009176	protein_coding
ENSG0000(	RGS9	516.0603	-0.16177	0.001486	0.009176	protein_coding
ENSG0000(	COX6A1	708.0715	-0.37891	0.001489	0.00919	protein_coding
ENSG0000(	CXCL16	626.645	0.275904	0.00149	0.009194	protein_coding
ENSG0000(	HMCES	383.3435	-0.28401	0.00149	0.009194	protein_coding
ENSG0000(	ARFGEF1-D	37.38908	0.400163	0.001491	0.009201	lncRNA
ENSG0000(	HPS4	2139.946	0.112118	0.001495	0.009219	protein_coding
ENSG0000(	LINC01973	37.75576	0.51628	0.001496	0.009227	lncRNA
ENSG0000(	RIC3-DT	12.17845	0.509423	0.001497	0.009227	lncRNA
ENSG0000(	KLF7P1	24.14757	0.474364	0.001499	0.009239	processed_pseudogene
ENSG0000(	LINC01797	16.4714	0.828958	0.0015	0.009245	lncRNA
ENSG0000(	ADORA2A	121.1782	0.439337	0.001503	0.00926	protein_coding
ENSG0000(	ENSG0000(	250.512	-0.20301	0.001507	0.009284	lncRNA
ENSG0000(	HERPUD1	2891.75	0.235751	0.00151	0.009304	protein_coding
ENSG0000(	EEF1G	6324.816	-0.2655	0.001513	0.009315	protein_coding
ENSG0000(	SOX8	97.71653	-0.81252	0.001515	0.009327	protein_coding
ENSG0000(	PDGFA	2560.797	0.390835	0.001516	0.00933	protein_coding
ENSG0000(	ABCG4	19.09777	0.533604	0.001517	0.009339	protein_coding
ENSG0000(	ENSG0000(	30.54598	0.430739	0.001518	0.009339	lncRNA
ENSG0000(	ENSG0000(	79.86407	0.262072	0.001519	0.009342	lncRNA
ENSG0000(	ENSG0000(	678.2265	-0.12707	0.001519	0.009342	lncRNA

ENSG0000(	FCGR2B	1307.549	0.643958	0.001519	0.009342	protein_coding
ENSG0000(	PNPLA3	246.1786	0.183777	0.00152	0.009344	protein_coding
ENSG0000(	ZRANB2	2393.929	-0.13547	0.00152	0.009344	protein_coding
ENSG0000(	TOMM7	647.8759	-0.23145	0.00152	0.009345	protein_coding
ENSG0000(	GRK5-IT1	190.5097	0.253615	0.001526	0.009378	lncRNA
ENSG0000(	ENSG0000(	115.668	0.489377	0.001527	0.009382	transcribed_unprocessed_pseudo
ENSG0000(	SLC30A8	202.6187	-0.26319	0.001528	0.009387	protein_coding
ENSG0000(	ENSG0000(	37.69214	-0.33074	0.001529	0.009388	lncRNA
ENSG0000(	LINC02785	114.5326	0.537278	0.00153	0.009393	lncRNA
ENSG0000(	HSD17B10	397.8298	-0.20147	0.001531	0.009398	protein_coding
ENSG0000(	CKLF	230.9225	0.24488	0.001532	0.009401	protein_coding
ENSG0000(	ENSG0000(	737.1362	0.142099	0.001534	0.009413	lncRNA
ENSG0000(	LINC01151	671.1076	0.106484	0.001534	0.009413	lncRNA
ENSG0000(	ENSG0000(	82.70891	0.240337	0.001536	0.009418	lncRNA
ENSG0000(	ENSG0000(	48.90486	0.439526	0.001536	0.009418	lncRNA
ENSG0000(	LINC00836	562.6571	0.115467	0.001536	0.009418	lncRNA
ENSG0000(	ADCY4	779.6309	0.234886	0.001538	0.009429	protein_coding
ENSG0000(	BLVRA	123.7672	0.418004	0.001539	0.009432	protein_coding
ENSG0000(	DBI	590.7712	-0.28263	0.001539	0.009432	protein_coding
ENSG0000(	RPL24	1486.145	-0.26424	0.001539	0.009432	protein_coding
ENSG0000(	NEXN	356.3731	0.19854	0.00154	0.009433	protein_coding
ENSG0000(	KLC4	1435.332	-0.31918	0.001542	0.009444	protein_coding
ENSG0000(	ADGRA2	852.3362	0.371653	0.001545	0.009463	protein_coding
ENSG0000(	NCKAP5L	746.8554	0.245079	0.001547	0.009472	protein_coding
ENSG0000(	GEMIN7-AS	177.7337	0.205957	0.001547	0.009472	lncRNA
ENSG0000(	DEPDC5	3071.444	-0.10726	0.001548	0.009472	protein_coding
ENSG0000(	ENSG0000(	58.85473	0.854417	0.001553	0.0095	unprocessed_pseudogene
ENSG0000(	KRT8	8051.114	-0.47647	0.001556	0.009516	protein_coding
ENSG0000(	HDAC8	3698.902	0.169689	0.001557	0.009522	protein_coding
ENSG0000(	ENSG0000(	27.77521	-0.39971	0.001559	0.009535	lncRNA
ENSG0000(	MAPK11	138.1527	0.482863	0.00156	0.009535	protein_coding
ENSG0000(	ATP5F1B	2983.425	-0.30507	0.00156	0.009536	protein_coding
ENSG0000(	PABPC1	10767.07	-0.17728	0.00156	0.009536	protein_coding
ENSG0000(	SLC25A4	273.0493	-0.34081	0.001563	0.009554	protein_coding
ENSG0000(	MPND	211.4235	-0.35883	0.001565	0.009559	protein_coding
ENSG0000(	ERV3-1	1472.449	0.242222	0.001565	0.009561	protein_coding
ENSG0000(	DCAF17	953.7431	-0.13514	0.001566	0.009564	protein_coding
ENSG0000(	RPL3L	309.6306	0.245277	0.001568	0.009571	protein_coding
ENSG0000(	SULT1A2	1173.125	-0.69511	0.001568	0.009571	protein_coding
ENSG0000(	RASL12	115.0431	0.420463	0.001569	0.009575	protein_coding
ENSG0000(	ENSG0000(	90.7182	-0.42463	0.00157	0.009583	lncRNA
ENSG0000(	ESD	486.8515	-0.24392	0.001571	0.009584	protein_coding
ENSG0000(	GNAS-AS1	165.3411	0.228733	0.001572	0.009587	lncRNA
ENSG0000(	NID1	1768.492	0.33363	0.001576	0.009607	protein_coding
ENSG0000(	NUAK1	261.8889	0.415161	0.001575	0.009607	protein_coding
ENSG0000(	GPC6	437.6492	0.418477	0.001576	0.009611	protein_coding
ENSG0000(	CSGALNAC	444.4049	0.589163	0.001578	0.009617	protein_coding

ENSG0000(	ENSG0000(	154.6736	0.225194	0.001579	0.009624	lncRNA
ENSG0000(	GABBR1	1536.45	0.360905	0.00158	0.009624	protein_coding
ENSG0000(	ENSG0000(	64.44393	0.33577	0.00158	0.009628	lncRNA
ENSG0000(	CTBP1-AS	101.8754	0.222699	0.001583	0.009644	lncRNA
ENSG0000(	DHX33	670.1593	0.140772	0.001584	0.009645	protein_coding
ENSG0000(	CEP83-DT	24.67591	0.411123	0.001585	0.009647	lncRNA
ENSG0000(	ESPNP	119.5931	-1.02318	0.001586	0.009654	transcribed_unprocessed_pseudo
ENSG0000(	CD164	8433.505	-0.33875	0.001587	0.009659	protein_coding
ENSG0000(	RIMKLA	276.5151	0.177173	0.001588	0.009661	protein_coding
ENSG0000(	ENSG0000(	137.5921	0.229034	0.001589	0.009666	lncRNA
ENSG0000(	HDHD2	790.4451	-0.13495	0.001589	0.009666	protein_coding
ENSG0000(	CPO	589.0758	-1.10995	0.00159	0.009667	protein_coding
ENSG0000(	TMEM9B	527.0194	-0.25518	0.001594	0.009692	protein_coding
ENSG0000(	USP46	902.5765	0.175422	0.001595	0.009695	protein_coding
ENSG0000(	SPOPL	1363.859	-0.14838	0.001596	0.009701	protein_coding
ENSG0000(	CDC14A	1585.891	-0.42406	0.001598	0.00971	protein_coding
ENSG0000(	ENSG0000(	744.4474	0.172247	0.001599	0.009717	lncRNA
ENSG0000(	B3GNT8	305.703	-0.6485	0.001602	0.009728	protein_coding
ENSG0000(	NSMF	859.8462	-0.36158	0.001602	0.009728	protein_coding
ENSG0000(	S100P	40.81313	0.944764	0.001603	0.009728	protein_coding
ENSG0000(	SAFB	2084.075	-0.12576	0.001602	0.009728	protein_coding
ENSG0000(	HMGN1	3586.975	-0.16164	0.001604	0.009736	protein_coding
ENSG0000(	ARHGAP5	6003.888	-0.32491	0.001607	0.009754	protein_coding
ENSG0000(	TIMM44	464.4012	-0.18576	0.001608	0.009758	protein_coding
ENSG0000(	ENSG0000(	139.9711	-0.18074	0.00161	0.009764	lncRNA
ENSG0000(	ENSG0000(	7517.546	0.15509	0.001611	0.009772	TEC
ENSG0000(	CYP4F12	5018.12	-0.67398	0.001612	0.009775	protein_coding
ENSG0000(	ERCC6	2038.596	-0.1536	0.001613	0.009778	protein_coding
ENSG0000(	FAM9B	176.6206	0.178021	0.001613	0.009778	protein_coding
ENSG0000(	ARL5AP2	52.7417	0.578665	0.001614	0.009779	processed_pseudogene
ENSG0000(	ARFGAP1	1774.942	0.160908	0.001619	0.009812	protein_coding
ENSG0000(	TERT	188.2849	-0.28275	0.00162	0.009815	protein_coding
ENSG0000(	C16orf89	125.1546	-0.28608	0.001621	0.009818	protein_coding
ENSG0000(	PJA2	3273.034	-0.09644	0.001622	0.00982	protein_coding
ENSG0000(	ENSG0000(	111.9534	0.419535	0.001622	0.009823	lncRNA
ENSG0000(	ENSG0000(	26.5516	0.434786	0.001624	0.009831	lncRNA
ENSG0000(	CWC22	489.2321	-0.1664	0.001625	0.009831	protein_coding
ENSG0000(	ENSG0000(	328.7288	0.17436	0.001625	0.009831	TEC
ENSG0000(	ENSG0000(	51.63803	0.332217	0.001625	0.009831	TEC
ENSG0000(	FBXO8	250.6651	-0.22505	0.001626	0.009836	protein_coding
ENSG0000(	TNF	158.7618	0.65982	0.001628	0.009847	protein_coding
ENSG0000(	MIER3	1600.817	-0.52442	0.00163	0.009854	protein_coding
ENSG0000(	CROT	690.2456	-0.35213	0.00163	0.009854	protein_coding
ENSG0000(	ENSG0000(	62.18892	-0.33719	0.001632	0.009866	pseudogene
ENSG0000(	MYBL1	416.7432	-0.60033	0.001634	0.009871	protein_coding
ENSG0000(	CISD1	448.0579	-0.44415	0.001636	0.00988	protein_coding
ENSG0000(	CRHBP	28.16971	-0.67906	0.001636	0.00988	protein_coding

ENSG0000(	ENSG0000(	111.4962	0.234662	0.001636	0.00988	TEC
ENSG0000(	SIRPA	1115.825	0.338202	0.001636	0.00988	protein_coding
ENSG0000(	MT-CO2	16025.1	-0.53093	0.001637	0.009881	protein_coding
ENSG0000(	ENSG0000(	71.13888	0.265628	0.001637	0.009881	lncRNA
ENSG0000(	ENSG0000(	116.3278	-0.20985	0.001638	0.009886	processed_pseudogene
ENSG0000(	SLC35E3	6121.636	0.12398	0.001639	0.009889	protein_coding
ENSG0000(	IGHV3-53	97.34788	0.861719	0.00164	0.00989	IG_V_gene
ENSG0000(	UMODL1	54.3244	-0.7032	0.001641	0.009897	protein_coding
ENSG0000(	CCDC25	779.5813	-0.27226	0.001643	0.009907	protein_coding
ENSG0000(	IGKV5-2	11.36205	0.925918	0.001644	0.009911	IG_V_gene
ENSG0000(	TUG1	9846.104	0.072391	0.001645	0.009914	protein_coding
ENSG0000(	EME1	61.22232	0.433487	0.001646	0.009921	protein_coding
ENSG0000(	ENSG0000(	18.94053	0.81881	0.001648	0.009927	lncRNA
ENSG0000(	HS1BP3-IT1	18.32536	0.46311	0.001648	0.009927	lncRNA
ENSG0000(	PEX13	1603.091	-0.17324	0.001648	0.009927	protein_coding
ENSG0000(	MAGOH	165.7347	-0.18192	0.00165	0.009936	protein_coding
ENSG0000(	ENSG0000(	368.8771	0.220996	0.001651	0.009939	transcribed_unprocessed_pseudo
ENSG0000(	THOC2	3081.693	-0.14932	0.001652	0.009943	protein_coding
ENSG0000(	NFYA	1634.602	-0.1386	0.001656	0.009966	protein_coding
ENSG0000(	ENSG0000(	28.47414	0.439887	0.001657	0.009972	lncRNA
ENSG0000(	MAPK1	2718.873	-0.12141	0.00166	0.009986	protein_coding
ENSG0000(	EHBP1L1	6784.563	0.184795	0.001661	0.009993	protein_coding
ENSG0000(	EPC2	1050.211	-0.15756	0.001664	0.010006	protein_coding
ENSG0000(	LGALS4	4342.657	-0.55825	0.001664	0.010006	protein_coding
ENSG0000(	CYP2B6	824.9011	-0.81655	0.001668	0.010025	protein_coding
ENSG0000(	BCL6B	152.17	0.437156	0.001671	0.01004	protein_coding
ENSG0000(	FAM53A	44.4853	0.593931	0.001675	0.010063	protein_coding
ENSG0000(	ING3	393.4633	-0.18725	0.001676	0.010068	protein_coding
ENSG0000(	KCCAT198	46.55115	0.492589	0.001677	0.010075	lncRNA
ENSG0000(	PPP1R7	392.6095	-0.1902	0.001678	0.010078	protein_coding
ENSG0000(	CLSTN3	1362.514	0.327419	0.00168	0.010089	protein_coding
ENSG0000(	MRPL55	205.0942	-0.22554	0.001681	0.010093	protein_coding
ENSG0000(	ENSG0000(	58.52213	0.36148	0.001682	0.010095	lncRNA
ENSG0000(	ENSG0000(	25.17506	0.432412	0.001682	0.010096	lncRNA
ENSG0000(	ZNF213-AS	1935.163	-0.09382	0.001683	0.010098	lncRNA
ENSG0000(	CNTNAP5	47.43098	0.728949	0.001685	0.010102	protein_coding
ENSG0000(	DPP4	5504.926	-0.6484	0.001685	0.010102	protein_coding
ENSG0000(	MRPS36	147.1606	-0.39037	0.001685	0.010102	protein_coding
ENSG0000(	MSR1	305.2968	0.282879	0.001685	0.010102	protein_coding
ENSG0000(	MTRNR2L1	46.54033	-0.84199	0.001684	0.010102	protein_coding
ENSG0000(	GINM1	709.5009	-0.16455	0.001687	0.010105	protein_coding
ENSG0000(	NDUFAF4P	21.73765	0.448452	0.001686	0.010105	processed_pseudogene
ENSG0000(	TAGLN2	1575.52	-0.25702	0.001687	0.010105	protein_coding
ENSG0000(	ENSG0000(	195.1421	0.136903	0.001687	0.010106	TEC
ENSG0000(	ZDHHC6	2316.696	-0.14223	0.001688	0.010109	protein_coding
ENSG0000(	ENSG0000(	217.2128	0.193307	0.001695	0.01015	lncRNA
ENSG0000(	LINC02232	41.59431	-1.12	0.001696	0.01015	lncRNA

ENSG0000(	MLANA	348.8529	-0.21249	0.001696	0.01015	protein_coding
ENSG0000(	ALG1L9P	965.2556	0.147788	0.001702	0.010187	lncRNA
ENSG0000(	ENSG0000(	1107.78	0.099617	0.001704	0.010193	lncRNA
ENSG0000(	ENSG0000(	21.58574	-0.42757	0.001705	0.010201	lncRNA
ENSG0000(	LINC02416	17.71277	0.586782	0.001706	0.010201	lncRNA
ENSG0000(	ENSG0000(	63.14172	0.526749	0.001707	0.010205	lncRNA
ENSG0000(	HOOK2	1940.454	-0.31909	0.001707	0.010205	protein_coding
ENSG0000(	LINC01184	3077.271	0.212464	0.001707	0.010205	lncRNA
ENSG0000(	KCTD9P1	60.07027	0.300756	0.001711	0.010227	transcribed_processed_pseudoge
ENSG0000(	ENSG0000(	12.06364	0.522439	0.001714	0.01024	TEC
ENSG0000(	ENSG0000(	253.8307	0.151589	0.001714	0.01024	lncRNA
ENSG0000(	MT-TR	51.78658	1.594924	0.001716	0.010248	Mt_tRNA
ENSG0000(	RN7SL483F	38.93282	0.330622	0.001718	0.010257	misc_RNA
ENSG0000(	IGHGP	233.053	1.22189	0.001719	0.010261	IG_C_pseudogene
ENSG0000(	RHOF	1164.443	-0.22189	0.001723	0.010285	protein_coding
ENSG0000(	RNU5F-1	13.9872	1.131505	0.001723	0.010285	snRNA
ENSG0000(	SLIRP	426.8093	-0.17785	0.001726	0.0103	protein_coding
ENSG0000(	SMPD3	3400.28	-0.56256	0.001726	0.0103	protein_coding
ENSG0000(	ENSG0000(	65.35053	0.264931	0.001727	0.0103	TEC
ENSG0000(	FADD	204.3635	-0.19138	0.001727	0.0103	protein_coding
ENSG0000(	RHOQ-AS1	11.31434	0.669531	0.001727	0.0103	lncRNA
ENSG0000(	HROB	53.83623	0.49158	0.001729	0.010307	protein_coding
ENSG0000(	ENSG0000(	203.8794	0.175667	0.001731	0.01032	lncRNA
ENSG0000(	ENSG0000(	1559.328	0.302659	0.001733	0.010327	lncRNA
ENSG0000(	FAM21FP	43.43693	0.434226	0.001735	0.010335	transcribed_unprocessed_pseudo
ENSG0000(	ENSG0000(	140.5315	-0.18857	0.001736	0.010342	lncRNA
ENSG0000(	FAM228A	17.82741	0.67868	0.001737	0.010342	protein_coding
ENSG0000(	RBPJ	3312.184	0.12269	0.001737	0.010342	protein_coding
ENSG0000(	ANKRA2	365.9412	-0.17097	0.001741	0.010364	protein_coding
ENSG0000(	C1orf220	753.7969	-0.21249	0.001746	0.010394	lncRNA
ENSG0000(	SLX1B-SUL	1314.155	-0.24662	0.001747	0.010394	lncRNA
ENSG0000(	NDUFB11	212.7253	-0.3269	0.001748	0.010403	protein_coding
ENSG0000(	ENSG0000(	104.9455	0.232486	0.00175	0.010409	lncRNA
ENSG0000(	SBNO1	3411.488	-0.09582	0.001751	0.010417	protein_coding
ENSG0000(	CCDC201	22.12728	0.502286	0.001755	0.010428	protein_coding
ENSG0000(	ENSG0000(	121.1864	-0.22248	0.001755	0.010428	processed_pseudogene
ENSG0000(	LEAP2	414.5305	-0.5509	0.001754	0.010428	protein_coding
ENSG0000(	OLA1	810.8816	-0.16709	0.001755	0.010428	protein_coding
ENSG0000(	SIK1B	362.7833	0.646318	0.001754	0.010428	protein_coding
ENSG0000(	SIN3A	2262.014	-0.13643	0.001757	0.010436	protein_coding
ENSG0000(	H2BC13	70.44717	-0.54664	0.001757	0.010437	protein_coding
ENSG0000(	PPIL6	306.2899	-0.14288	0.001759	0.010445	protein_coding
ENSG0000(	TSPYL1	2050.985	-0.12527	0.001762	0.010465	protein_coding
ENSG0000(	VAMP7	496.1903	-0.26555	0.001764	0.010473	protein_coding
ENSG0000(	GOLGA6L7	155.362	0.268971	0.001768	0.010494	protein_coding
ENSG0000(	UNC50	493.5358	-0.18767	0.00177	0.010501	protein_coding
ENSG0000(	ZACN	18.44208	0.979889	0.001771	0.010508	protein_coding

ENSG0000(	ENSG0000(	112.3411	-0.2544	0.001772	0.010511	lncRNA
ENSG0000(	KLKP1	25.02862	0.342037	0.001773	0.010515	transcribed_unprocessed_pseudo
ENSG0000(	FOXA2	86.2198	0.562563	0.001775	0.010528	protein_coding
ENSG0000(	RAD54L2	2327.702	0.15544	0.001779	0.010547	protein_coding
ENSG0000(	PCBD1	253.5143	-0.32025	0.001783	0.010567	protein_coding
ENSG0000(	EDC4	1179.416	-0.18868	0.001784	0.010575	protein_coding
ENSG0000(	ENSG0000(	887.868	0.549489	0.001786	0.010585	unprocessed_pseudogene
ENSG0000(	KIAA0895L	708.4703	0.23593	0.001788	0.010594	protein_coding
ENSG0000(	KLK11	190.6405	0.202197	0.001789	0.010597	protein_coding
ENSG0000(	RPPH1	24181.79	-0.40593	0.00179	0.0106	ribozyme
ENSG0000(	ENSG0000(	10.78516	0.568285	0.00179	0.010601	lncRNA
ENSG0000(	ENSG0000(	1751.965	0.15937	0.001791	0.010602	protein_coding
ENSG0000(	ETFRF1	259.2631	-0.17848	0.001792	0.010602	protein_coding
ENSG0000(	LAMP3	647.7369	0.529479	0.001791	0.010602	protein_coding
ENSG0000(	IL2RG	1089.709	-0.34352	0.001792	0.010603	protein_coding
ENSG0000(	ENSG0000(	39.35102	0.440098	0.001793	0.010608	lncRNA
ENSG0000(	MTND2P13	13.47227	0.58795	0.001795	0.010616	unprocessed_pseudogene
ENSG0000(	ENSG0000(	28.10348	0.497928	0.001798	0.010627	transcribed_processed_pseudoge
ENSG0000(	ENSG0000(	50.40484	0.3023	0.001798	0.010627	lncRNA
ENSG0000(	L3HYPDH	608.244	0.181578	0.001798	0.010627	protein_coding
ENSG0000(	CCDC73	10.57257	0.843404	0.001799	0.010628	protein_coding
ENSG0000(	ENSG0000(	54.46525	0.693861	0.001799	0.010628	TEC
ENSG0000(	LRRC8C	1048.118	0.405416	0.0018	0.010631	protein_coding
ENSG0000(	PLXDC1	1266.934	0.294759	0.001801	0.01064	protein_coding
ENSG0000(	SMAD7	824.9802	-0.39316	0.001802	0.010641	protein_coding
ENSG0000(	NOL11	651.1479	-0.20431	0.001804	0.010654	protein_coding
ENSG0000(	PRUNE2	3426.871	0.428633	0.001805	0.010656	protein_coding
ENSG0000(	ENSG0000(	158.6094	0.198861	0.001806	0.010656	lncRNA
ENSG0000(	ENSG0000(	98.66442	0.211758	0.001807	0.010662	lncRNA
ENSG0000(	SH3BGRL3	626.0373	0.298102	0.001809	0.010673	protein_coding
ENSG0000(	SLC26A2	4560.68	-0.55343	0.001812	0.010689	protein_coding
ENSG0000(	PIH1D1	576.6285	-0.14403	0.001814	0.010702	protein_coding
ENSG0000(	ENSG0000(	10.64373	1.03003	0.001816	0.01071	lncRNA
ENSG0000(	TUBB2B	37.51253	-0.62317	0.001817	0.010712	protein_coding
ENSG0000(	RFX4	135.101	0.298352	0.001818	0.010715	protein_coding
ENSG0000(	TNFRSF14	3829.779	-0.16615	0.00182	0.010724	protein_coding
ENSG0000(	SPIN1	2138.055	-0.21595	0.001821	0.010732	protein_coding
ENSG0000(	ENSG0000(	119.4653	0.221566	0.001823	0.010742	lncRNA
ENSG0000(	ENSG0000(	123.8468	0.200206	0.001824	0.010744	lncRNA
ENSG0000(	RUFY4	109.8591	0.639779	0.001824	0.010744	protein_coding
ENSG0000(	CLUH	1523.457	-0.32499	0.001826	0.010748	protein_coding
ENSG0000(	ENSG0000(	50.88688	-0.50311	0.001826	0.010748	processed_pseudogene
ENSG0000(	TCF12	3395.238	-0.12478	0.001826	0.010748	protein_coding
ENSG0000(	ENSG0000(	12.48131	0.652125	0.001827	0.010749	lncRNA
ENSG0000(	TAT	170.4232	-0.32805	0.001828	0.010755	protein_coding
ENSG0000(	ENSG0000(	4502.326	0.224267	0.00183	0.010762	lncRNA
ENSG0000(	EPN1	8102.266	-0.32696	0.00183	0.010765	protein_coding

ENSG0000(	RNF115	3698.99	0.121495	0.001834	0.010783	protein_coding
ENSG0000(	SIGLEC16	584.1116	0.123881	0.001835	0.010785	polymorphic_pseudogene
ENSG0000(	ENSG0000(	15.24588	0.592923	0.001837	0.010796	lncRNA
ENSG0000(	ENSG0000(	65.56508	0.301666	0.001837	0.010796	lncRNA
ENSG0000(	ENSG0000(	164.6008	0.198268	0.00184	0.010811	TEC
ENSG0000(	ENSG0000(	19.11047	0.576558	0.001841	0.010817	lncRNA
ENSG0000(	CFAP161	18.91212	0.524177	0.001842	0.01082	protein_coding
ENSG0000(	ENSG0000(	15.56934	0.568268	0.001843	0.010821	lncRNA
ENSG0000(	ENSG0000(	37.73804	0.324847	0.001844	0.010826	lncRNA
ENSG0000(	ENSG0000(	98.94396	0.238854	0.001846	0.010837	lncRNA
ENSG0000(	IP6K2	2163.609	-0.21709	0.001847	0.010838	protein_coding
ENSG0000(	ILRUN	2001.758	-0.19461	0.001849	0.010846	protein_coding
ENSG0000(	LMO7-AS1	209.5039	0.219038	0.001849	0.010846	lncRNA
ENSG0000(	ENSG0000(	11.91612	0.835755	0.001849	0.010848	unitary_pseudogene
ENSG0000(	THADA	3223.71	0.230183	0.001852	0.010861	protein_coding
ENSG0000(	ENSG0000(	14.59282	-0.47382	0.001853	0.010865	lncRNA
ENSG0000(	IKZF5	678.4086	-0.13508	0.001856	0.010881	protein_coding
ENSG0000(	OCLN	3389.185	-0.30252	0.001856	0.010881	protein_coding
ENSG0000(	MRPL40	150.2501	-0.21264	0.001858	0.010887	protein_coding
ENSG0000(	PTPN1	1535.857	0.351307	0.001858	0.010887	protein_coding
ENSG0000(	IGLV2-5	12.86125	0.83956	0.00186	0.010896	IG_V_pseudogene
ENSG0000(	DEPP1	415.5438	0.470084	0.001861	0.0109	protein_coding
ENSG0000(	POLR2C	732.876	-0.13942	0.001864	0.010914	protein_coding
ENSG0000(	DDN	9.770044	0.732311	0.001865	0.010918	protein_coding
ENSG0000(	ZNF721	1935.862	-0.20435	0.001865	0.010919	protein_coding
ENSG0000(	CYB5AP2	231.7253	-0.17045	0.001867	0.010928	processed_pseudogene
ENSG0000(	ENSG0000(	21.50122	0.492518	0.00187	0.010944	lncRNA
ENSG0000(	LINC02485	17.28922	-0.45335	0.001872	0.010951	lncRNA
ENSG0000(	HINT2	139.8458	-0.26819	0.001872	0.010952	protein_coding
ENSG0000(	PHLPP2	5973.296	-0.58536	0.001874	0.010962	protein_coding
ENSG0000(	ENSG0000(	12.28479	0.559669	0.001875	0.010965	lncRNA
ENSG0000(	KRT26	47.67863	0.534117	0.001876	0.010965	protein_coding
ENSG0000(	B4GALT6	342.942	-0.54962	0.001887	0.01103	protein_coding
ENSG0000(	POLR3D	881.2265	0.158275	0.001888	0.011035	protein_coding
ENSG0000(	ENSG0000(	31.55219	0.331916	0.00189	0.011044	lncRNA
ENSG0000(	ENSG0000(	333.0994	0.179396	0.001892	0.011054	lncRNA
ENSG0000(	ARHGAP1	1835.657	0.115805	0.001893	0.011058	protein_coding
ENSG0000(	DDX49	212.9492	-0.27923	0.001894	0.01106	protein_coding
ENSG0000(	KYAT3	378.8566	-0.17373	0.001894	0.01106	protein_coding
ENSG0000(	PLEKHH2	2253.313	0.256237	0.001896	0.011066	protein_coding
ENSG0000(	RHEBL1	17.54803	0.810558	0.001896	0.011066	protein_coding
ENSG0000(	SP6	202.1405	0.492965	0.001897	0.01107	protein_coding
ENSG0000(	CHD2	9691.034	0.152993	0.0019	0.011083	protein_coding
ENSG0000(	ENSG0000(	29.36095	0.353322	0.0019	0.011083	lncRNA
ENSG0000(	ANK2-AS1	67.03027	0.232398	0.001901	0.011088	lncRNA
ENSG0000(	CCDC152	217.3341	0.55722	0.001902	0.011091	protein_coding
ENSG0000(	ENSG0000(	105.6093	0.371837	0.001903	0.011092	protein_coding

ENSG0000(	BACH1	3622.936	0.255854	0.001903	0.011093	protein_coding
ENSG0000(	NUDT11	18.18943	0.536592	0.001904	0.011094	protein_coding
ENSG0000(	ENSG0000(	152.3292	0.239529	0.001905	0.011099	lncRNA
ENSG0000(	AEN	552.2768	0.431878	0.001907	0.011111	protein_coding
ENSG0000(	PTPRN	37.92485	0.977754	0.001908	0.011116	protein_coding
ENSG0000(	CRB1	49.88343	0.464971	0.001909	0.011118	protein_coding
ENSG0000(	GGA3	1604.74	-0.13548	0.00191	0.011121	protein_coding
ENSG0000(	ENSG0000(	955.5415	0.188985	0.001911	0.011123	lncRNA
ENSG0000(	ENSG0000(	40.92667	0.309133	0.001912	0.011123	lncRNA
ENSG0000(	PARP16	269.9505	-0.19488	0.001911	0.011123	protein_coding
ENSG0000(	PCAT5	103.1754	0.244719	0.001911	0.011123	lncRNA
ENSG0000(	ENSG0000(	17.11328	0.47481	0.001915	0.011139	lncRNA
ENSG0000(	ENSG0000(	103.3407	-0.24398	0.001917	0.011146	lncRNA
ENSG0000(	ENSG0000(	65.05395	0.402068	0.001918	0.011146	lncRNA
ENSG0000(	GPR171	145.6758	0.516098	0.001917	0.011146	protein_coding
ENSG0000(	RNF6	1029.805	-0.17715	0.001917	0.011146	protein_coding
ENSG0000(	CYB5R2	226.0734	0.268455	0.001919	0.011153	protein_coding
ENSG0000(	H4C14	349.7212	-0.55229	0.001919	0.011153	protein_coding
ENSG0000(	PHLDA1-DT	19.31426	0.524428	0.001926	0.011189	lncRNA
ENSG0000(	RPS4XP16	50.79546	-0.31781	0.001926	0.011189	transcribed_processed_pseudoge
ENSG0000(	CLCA4	717.4009	1.521051	0.001934	0.011233	protein_coding
ENSG0000(	FNDC3A	3564.277	0.143101	0.001935	0.011233	protein_coding
ENSG0000(	ENSG0000(	139.1404	-0.24536	0.001941	0.011271	lncRNA
ENSG0000(	ABHD12	1020.333	-0.23035	0.001942	0.011272	protein_coding
ENSG0000(	KDM4A	1814.803	-0.1737	0.001942	0.011272	protein_coding
ENSG0000(	ENSG0000(	365.0278	0.197619	0.001944	0.011275	lncRNA
ENSG0000(	NCOA4	3903.204	-0.32568	0.001944	0.011275	protein_coding
ENSG0000(	HMGN2P4(	347.2776	0.218199	0.001946	0.011285	transcribed_processed_pseudoge
ENSG0000(	GUCD1	2414.843	-0.33609	0.001946	0.011287	protein_coding
ENSG0000(	ENSG0000(	38.11211	-0.43772	0.001947	0.01129	lncRNA
ENSG0000(	FAM133CP	17.52973	0.798614	0.001948	0.01129	processed_pseudogene
ENSG0000(	STRA6	295.7856	0.39806	0.001952	0.011315	protein_coding
ENSG0000(	ENSG0000(	152.7407	0.188426	0.001954	0.011324	lncRNA
ENSG0000(	LGALS9	1404.042	0.275827	0.001955	0.011325	protein_coding
ENSG0000(	RAP2C	798.1165	0.172882	0.001957	0.011336	protein_coding
ENSG0000(	LINC00566	28.7384	0.369345	0.001958	0.011341	lncRNA
ENSG0000(	TRIM35	617.8158	-0.20344	0.001959	0.011343	protein_coding
ENSG0000(	TFAM	698.5449	-0.17621	0.001959	0.011344	protein_coding
ENSG0000(	ERH	379.1206	-0.24779	0.00196	0.011347	protein_coding
ENSG0000(	CDKN2AIPN	328.274	-0.17341	0.001961	0.011349	protein_coding
ENSG0000(	RN7SL785P	77.90131	0.212449	0.001962	0.011354	misc_RNA
ENSG0000(	PPM1N	765.317	0.524862	0.001963	0.011358	protein_coding
ENSG0000(	ENSG0000(	25.27823	-0.64989	0.001964	0.011361	lncRNA
ENSG0000(	APOC4	27.07867	0.546843	0.001965	0.011364	protein_coding
ENSG0000(	CHMP1A	1669.293	-0.2403	0.001965	0.011364	protein_coding
ENSG0000(	ENSG0000(	30.79341	0.405161	0.001967	0.011371	lncRNA
ENSG0000(	PAFAH1B1	7633.288	-0.12385	0.001967	0.011371	protein_coding

ENSG0000(	ENSG0000(	9.242611	0.589921	0.001969	0.011378	lncRNA
ENSG0000(	COX17	250.6533	-0.20793	0.001972	0.01139	protein_coding
ENSG0000(	DGKZ	2806.121	-0.20397	0.001972	0.01139	protein_coding
ENSG0000(	ENSG0000(	254.9234	0.159234	0.001971	0.01139	lncRNA
ENSG0000(	ENSG0000(	52.19394	0.359676	0.001973	0.011394	lncRNA
ENSG0000(	USF3	3753.386	-0.1192	0.001974	0.011399	protein_coding
ENSG0000(	CTNNA3	179.3717	0.189718	0.001975	0.011402	protein_coding
ENSG0000(	ENSG0000(	348.9161	-0.25885	0.001976	0.011402	lncRNA
ENSG0000(	ENSG0000(	339.6466	0.235464	0.001976	0.011402	lncRNA
ENSG0000(	CAMKK1	421.7646	0.194909	0.001979	0.011415	protein_coding
ENSG0000(	ENSG0000(	183.5606	-0.19828	0.001979	0.011415	lncRNA
ENSG0000(	HSPH1	1306.907	-0.29346	0.001979	0.011415	protein_coding
ENSG0000(	ENSG0000(	11.13638	0.554768	0.00198	0.011417	lncRNA
ENSG0000(	SH3TC1	852.8517	0.543242	0.001981	0.011418	protein_coding
ENSG0000(	MT-TV	132.4409	0.681826	0.001981	0.011419	Mt_tRNA
ENSG0000(	MMADHC	596.2146	-0.20711	0.001983	0.011429	protein_coding
ENSG0000(	STAT3	7315.889	0.231825	0.001987	0.011448	protein_coding
ENSG0000(	LINC02669	178.5317	0.211386	0.001988	0.01145	lncRNA
ENSG0000(	NFATC3	3493.887	-0.15309	0.001989	0.011454	protein_coding
ENSG0000(	EEF1AKMT:	969.1583	-0.14552	0.001991	0.011466	protein_coding
ENSG0000(	BATF	65.19444	0.647504	0.001993	0.011474	protein_coding
ENSG0000(	TANK	1514.183	0.206886	0.001995	0.011483	protein_coding
ENSG0000(	ENSG0000(	107.0092	-0.25267	0.001996	0.011488	lncRNA
ENSG0000(	GIMAP8	970.0759	0.328197	0.001996	0.011488	protein_coding
ENSG0000(	ASH1L	9965.561	0.087712	0.001998	0.011494	protein_coding
ENSG0000(	BEST4	106.4345	-0.79895	0.001998	0.011494	protein_coding
ENSG0000(	PCYOX1	1538.462	-0.205	0.001999	0.011494	protein_coding
ENSG0000(	NFE2L3P1	67.93514	-0.52016	0.002002	0.011513	processed_pseudogene
ENSG0000(	LINC00607	235.8896	-0.17813	0.002007	0.01154	lncRNA
ENSG0000(	WASF1	82.39005	0.438067	0.00201	0.011555	protein_coding
ENSG0000(	RBM34	720.0681	-0.1465	0.002011	0.011556	protein_coding
ENSG0000(	ENSG0000(	39.578	0.407044	0.002011	0.011558	miRNA
ENSG0000(	ENSG0000(	476.761	-0.15208	0.002013	0.011565	lncRNA
ENSG0000(	ENSG0000(	49.78103	0.596665	0.002017	0.011586	lncRNA
ENSG0000(	LINC00653	92.78734	0.272081	0.002017	0.011586	lncRNA
ENSG0000(	ENSG0000(	229.8262	-0.207	0.002021	0.011606	lncRNA
ENSG0000(	ENSG0000(	18.8709	0.610527	0.002023	0.011607	lncRNA
ENSG0000(	LSR	620.5866	-0.36616	0.002022	0.011607	protein_coding
ENSG0000(	NEK4	1258.715	-0.15676	0.002022	0.011607	protein_coding
ENSG0000(	CHMP5	539.1518	-0.30273	0.002023	0.011609	protein_coding
ENSG0000(	LASP1	7483.076	-0.2916	0.002024	0.011609	protein_coding
ENSG0000(	CRNKL1	659.6897	-0.156	0.002025	0.011616	protein_coding
ENSG0000(	ENSG0000(	90.63086	-0.45927	0.002026	0.011616	protein_coding
ENSG0000(	ENSG0000(	293.2358	0.233609	0.002029	0.011634	TEC
ENSG0000(	ENSG0000(	16.14177	0.454291	0.002031	0.011641	lncRNA
ENSG0000(	PCDHGC4	59.66416	0.381648	0.002031	0.011641	protein_coding
ENSG0000(	ENSG0000(	203.2635	-0.18167	0.002033	0.01165	TEC

ENSG0000(	COX6CP17	31.818	0.440926	0.002038	0.011675	processed_pseudogene
ENSG0000(	SERPINB9	1789.954	0.495846	0.002039	0.01168	protein_coding
ENSG0000(	ENSG0000(	157.8545	0.280081	0.002041	0.011685	transcribed_unprocessed_pseudo
ENSG0000(	ENSG0000(	83.66595	0.207646	0.00204	0.011685	lncRNA
ENSG0000(	UBC	5004.391	-0.36132	0.002042	0.011693	protein_coding
ENSG0000(	RPS24	5136.98	-0.25175	0.002043	0.011693	protein_coding
ENSG0000(	ACY1	1452.451	-0.49058	0.002044	0.011701	protein_coding
ENSG0000(	AKAP13	13319.36	0.143451	0.002046	0.011705	protein_coding
ENSG0000(	LINC01193	45.16279	0.414968	0.002047	0.011705	lncRNA
ENSG0000(	MMP28	188.2375	-0.67483	0.002047	0.011705	protein_coding
ENSG0000(	TRMT1L	520.3812	-0.19755	0.002046	0.011705	protein_coding
ENSG0000(	RRAGD	150.0484	0.414907	0.002048	0.011712	protein_coding
ENSG0000(	ZNF460-AS	313.462	0.17236	0.00205	0.011722	lncRNA
ENSG0000(	ZW10	331.3534	-0.19698	0.002054	0.011738	protein_coding
ENSG0000(	UBE2F	782.4641	0.130085	0.002055	0.011744	protein_coding
ENSG0000(	ENSG0000(	9.412725	0.778447	0.002056	0.011747	processed_pseudogene
ENSG0000(	UGT2B7	159.0669	-0.80211	0.002057	0.011752	protein_coding
ENSG0000(	ENSG0000(	52.31012	0.311117	0.002059	0.011757	lncRNA
ENSG0000(	NAPSA	101.2967	-0.36667	0.002059	0.011757	protein_coding
ENSG0000(	RN7SL832F	298.3485	0.158707	0.00206	0.011764	lncRNA
ENSG0000(	IGKV3D-15	27.93342	1.354776	0.002065	0.011786	IG_V_gene
ENSG0000(	ENSG0000(	41.57952	0.342977	0.002066	0.011787	lncRNA
ENSG0000(	LINC01764	408.0653	0.140751	0.002065	0.011787	lncRNA
ENSG0000(	RPP38	262.0901	-0.16522	0.002066	0.011787	protein_coding
ENSG0000(	HECW2	872.7157	0.257057	0.002066	0.011788	protein_coding
ENSG0000(	RGL1	907.0276	0.258564	0.002069	0.011798	protein_coding
ENSG0000(	H3-3A	3763.639	-0.20739	0.002072	0.011817	protein_coding
ENSG0000(	ENSG0000(	20.14857	0.435293	0.002074	0.011824	lncRNA
ENSG0000(	ENSG0000(	140.4809	0.181079	0.002075	0.011828	lncRNA
ENSG0000(	ENSG0000(	27.75514	-0.45507	0.002076	0.011829	lncRNA
ENSG0000(	ENSG0000(	38.60532	0.407213	0.002076	0.01183	lncRNA
ENSG0000(	ENSG0000(	9.494839	0.822938	0.002077	0.011833	unprocessed_pseudogene
ENSG0000(	SF3B3	3368.32	-0.16896	0.002078	0.011837	protein_coding
ENSG0000(	HS3ST1	356.8407	0.382897	0.002079	0.011839	protein_coding
ENSG0000(	RNU6-998F	29.84412	0.386232	0.002081	0.011852	snRNA
ENSG0000(	PRR5	1175.228	-0.24679	0.002084	0.011865	protein_coding
ENSG0000(	ENSG0000(	10.48976	0.674148	0.002087	0.011881	lncRNA
ENSG0000(	RCC2P6	23.94055	0.514951	0.002088	0.011881	processed_pseudogene
ENSG0000(	PDHA1	1325.517	-0.23838	0.002088	0.011881	protein_coding
ENSG0000(	GGACT	143.1365	0.359165	0.002089	0.011886	protein_coding
ENSG0000(	GOLT1A	177.5919	-0.725	0.002092	0.011897	protein_coding
ENSG0000(	NAT16	88.8741	0.266668	0.002098	0.011931	protein_coding
ENSG0000(	SYNPO	4216.656	-0.38735	0.0021	0.01194	protein_coding
ENSG0000(	RFK	701.2246	-0.31775	0.002102	0.01195	protein_coding
ENSG0000(	USB1	832.2862	0.151282	0.002106	0.011968	protein_coding
ENSG0000(	TEX14	68.25317	0.364517	0.002108	0.011981	protein_coding
ENSG0000(	NBPF8	1205.414	0.445621	0.002111	0.011996	transcribed_unprocessed_pseudo

ENSG0000(	RPL10A	1943.198	-0.32709	0.002113	0.012001	protein_coding
ENSG0000(	ENSG0000(	216.5736	-0.30527	0.002116	0.01202	processed_pseudogene
ENSG0000(	LPAR1	1036.486	-0.22488	0.002118	0.012026	protein_coding
ENSG0000(	EPS8L2	4094.826	-0.62624	0.002122	0.012048	protein_coding
ENSG0000(	ST20-MTHI	42.69778	-0.54526	0.002122	0.012048	protein_coding
ENSG0000(	BNIP3	351.7659	0.27656	0.002123	0.012048	protein_coding
ENSG0000(	PRAG1	1693.552	-0.4011	0.002123	0.012048	protein_coding
ENSG0000(	SLC2A11	1128.596	0.142605	0.002123	0.012048	protein_coding
ENSG0000(	MEIS2	634.1873	0.442552	0.002124	0.01205	protein_coding
ENSG0000(	ENSG0000(	37.0041	-0.44773	0.002126	0.012051	processed_pseudogene
ENSG0000(	MAT2B	1335.473	-0.22123	0.002125	0.012051	protein_coding
ENSG0000(	UBE2G1	1133.324	-0.21197	0.002126	0.012051	protein_coding
ENSG0000(	ZNF347	1877.714	-0.16115	0.002126	0.012051	protein_coding
ENSG0000(	GLDC	309.7458	0.238442	0.002128	0.012063	protein_coding
ENSG0000(	PEAR1	340.6331	0.353746	0.002132	0.012081	protein_coding
ENSG0000(	ENSG0000(	318.7329	-0.17733	0.002137	0.012107	lncRNA
ENSG0000(	DDX17	45153.01	0.079956	0.00214	0.012119	protein_coding
ENSG0000(	SH2D4B	9.71021	0.822601	0.002141	0.012124	protein_coding
ENSG0000(	ENSG0000(	29.52583	0.419247	0.002141	0.012126	lncRNA
ENSG0000(	CIBAR1-DT	37.45694	0.570594	0.002142	0.012126	lncRNA
ENSG0000(	ENSG0000(	17.60691	0.708445	0.002144	0.012133	unprocessed_pseudogene
ENSG0000(	MAD2L2	91.34566	0.394519	0.002145	0.012141	protein_coding
ENSG0000(	NUP62	1726.482	0.147134	0.002148	0.01215	protein_coding
ENSG0000(	PDE1B	730.2735	0.173299	0.002148	0.01215	protein_coding
ENSG0000(	FRMD1	1035.191	-0.80799	0.002153	0.012179	protein_coding
ENSG0000(	HCFC2	644.9701	0.164053	0.002154	0.012181	protein_coding
ENSG0000(	C2orf49-DT	202.5901	0.208948	0.002158	0.012203	lncRNA
ENSG0000(	NCBP1	1778.358	-0.11262	0.002161	0.012217	protein_coding
ENSG0000(	AP3S1	968.2802	-0.31299	0.002162	0.012219	protein_coding
ENSG0000(	SCN8A	506.3977	0.407848	0.002162	0.012219	protein_coding
ENSG0000(	ANKZF1	1810.273	-0.11877	0.002165	0.012235	protein_coding
ENSG0000(	LAMTOR3	617.1959	-0.25942	0.002167	0.012241	protein_coding
ENSG0000(	PRR13P2	40.6326	-0.31551	0.002167	0.012242	processed_pseudogene
ENSG0000(	LHFPL2	2476.8	-0.27956	0.002169	0.012246	protein_coding
ENSG0000(	ENSG0000(	50.93283	0.45428	0.00217	0.012254	lncRNA
ENSG0000(	ENSG0000(	694.2652	-0.20327	0.002174	0.01227	protein_coding
ENSG0000(	ENSG0000(	40.56653	-0.36246	0.002176	0.01228	lncRNA
ENSG0000(	ENSG0000(	158.7039	0.253613	0.002178	0.012293	TEC
ENSG0000(	CCDC172	9.387372	0.727565	0.002179	0.012294	protein_coding
ENSG0000(	ENSG0000(	170.5452	-0.16941	0.002179	0.012294	processed_pseudogene
ENSG0000(	CBLC	538.9619	-0.54194	0.002181	0.012301	protein_coding
ENSG0000(	ZNF773	1127.363	0.157229	0.002181	0.012301	protein_coding
ENSG0000(	RN7SL734F	23.96282	0.347901	0.002185	0.012321	misc_RNA
ENSG0000(	ARHGEF10I	1827.229	0.349135	0.002187	0.012326	protein_coding
ENSG0000(	RPS3A	4480.558	-0.29352	0.002187	0.012326	protein_coding
ENSG0000(	ENSG0000(	12.11244	0.519825	0.002187	0.012326	lncRNA
ENSG0000(	RNU4-23P	16.93218	-0.55369	0.002188	0.012327	snRNA

ENSG0000(	ACSM2A	406.3386	0.209828	0.00219	0.012335	protein_coding
ENSG0000(	ENSG0000(	9.599938	0.727799	0.002191	0.01234	lncRNA
ENSG0000(	ABCA8	1029.452	-0.60102	0.002194	0.012349	protein_coding
ENSG0000(	ELF2	2309.299	0.083577	0.002195	0.012349	protein_coding
ENSG0000(	ENSG0000(	490.9831	0.113097	0.002194	0.012349	TEC
ENSG0000(	ENSG0000(	231.8523	-0.20736	0.002193	0.012349	lncRNA
ENSG0000(	HRAT17	402.71	0.26627	0.002195	0.012349	lncRNA
ENSG0000(	OR9M1P	52.58653	0.354675	0.002195	0.012349	transcribed_unprocessed_pseudo
ENSG0000(	TGIF1	940.6002	0.203524	0.002196	0.012354	protein_coding
ENSG0000(	NID2	425.7795	0.353095	0.002199	0.012366	protein_coding
ENSG0000(	ARHGAP10	708.0098	0.277776	0.002201	0.012375	protein_coding
ENSG0000(	GSR	1745.967	-0.37599	0.002201	0.012375	protein_coding
ENSG0000(	SMARCA5	2038.655	-0.18215	0.002202	0.012378	protein_coding
ENSG0000(	OR51I2	13.06212	0.648536	0.002203	0.01238	protein_coding
ENSG0000(	EGR2	109.3788	0.683565	0.002203	0.012381	protein_coding
ENSG0000(	ENSG0000(	228.0513	0.193037	0.002204	0.012381	lncRNA
ENSG0000(	ZNF124	1428.196	-0.15485	0.002205	0.012383	protein_coding
ENSG0000(	TRPV6	70.08299	0.853005	0.002209	0.012408	protein_coding
ENSG0000(	ACADS	746.6207	-0.5126	0.002212	0.012415	protein_coding
ENSG0000(	EGR3	294.9941	0.488068	0.002212	0.012415	protein_coding
ENSG0000(	PPP2R5D	944.98	-0.36658	0.002212	0.012415	protein_coding
ENSG0000(	SAP30-DT	1195.318	-0.21418	0.002213	0.01242	lncRNA
ENSG0000(	EPS15-AS1	181.1099	-0.6125	0.002214	0.01242	lncRNA
ENSG0000(	SNORA60	46.57946	0.443239	0.002215	0.012428	snoRNA
ENSG0000(	NAGK	3087.301	0.252601	0.002217	0.012436	protein_coding
ENSG0000(	ENSG0000(	60.14461	0.320973	0.00222	0.01245	lncRNA
ENSG0000(	TTC28	1815.329	0.258465	0.002223	0.012465	protein_coding
ENSG0000(	TET2	6195.014	0.145416	0.002224	0.012467	protein_coding
ENSG0000(	NINJ1	442.5098	0.36177	0.002225	0.012472	protein_coding
ENSG0000(	ENSG0000(	25.0476	0.448104	0.002226	0.012477	processed_pseudogene
ENSG0000(	ARHGAP26	6920.545	0.185273	0.002231	0.012503	protein_coding
ENSG0000(	CDK2AP1	1119.075	0.197815	0.002232	0.012505	protein_coding
ENSG0000(	HORMAD1	41.61146	0.604609	0.002234	0.012515	protein_coding
ENSG0000(	ETFA	3164.629	-0.16049	0.002237	0.012523	protein_coding
ENSG0000(	MEP1A	6351.779	-0.78205	0.002237	0.012523	protein_coding
ENSG0000(	PRR7	24.31781	0.537862	0.002237	0.012523	protein_coding
ENSG0000(	SPRED2	1314.242	-0.32721	0.002237	0.012523	protein_coding
ENSG0000(	CWC15	327.4096	-0.1788	0.002242	0.012541	protein_coding
ENSG0000(	ST3GAL1	2178.012	0.462811	0.002241	0.012541	protein_coding
ENSG0000(	UGT1A13P	124.4252	-0.77705	0.002242	0.012541	unprocessed_pseudogene
ENSG0000(	CISH	693.9188	0.242722	0.002246	0.012559	protein_coding
ENSG0000(	PECR	597.0681	-0.27732	0.002246	0.012559	protein_coding
ENSG0000(	ENSG0000(	44.52781	0.337419	0.002246	0.012559	lncRNA
ENSG0000(	SNX5	3720.62	-0.11918	0.00225	0.012577	protein_coding
ENSG0000(	MSH2	948.6567	-0.22283	0.002256	0.01261	protein_coding
ENSG0000(	MARCHF3	484.979	0.220133	0.002257	0.012613	protein_coding
ENSG0000(	ENSG0000(	29.21102	0.371988	0.002258	0.012614	lncRNA

ENSG0000(	ENSG0000(	12.42417	0.692333	0.002262	0.012633	lncRNA
ENSG0000(	SLC35G1	977.7096	-0.57267	0.002262	0.012633	protein_coding
ENSG0000(	CATSPERG	681.6013	-0.21701	0.002263	0.012635	protein_coding
ENSG0000(	MARK4	578.0411	0.15143	0.002264	0.012639	protein_coding
ENSG0000(	DALRD3	170.4619	-0.29021	0.002271	0.012675	protein_coding
ENSG0000(	ATP6V1E2	106.2334	0.504829	0.002271	0.012675	protein_coding
ENSG0000(	GTSE1-DT	41.5738	0.356427	0.002273	0.012684	lncRNA
ENSG0000(	VN1R108P	18.43251	0.582067	0.002276	0.012696	unprocessed_pseudogene
ENSG0000(	MTERF1	1042.928	0.151036	0.002276	0.012698	protein_coding
ENSG0000(	ZKSCAN5	734.082	0.10196	0.00228	0.012718	protein_coding
ENSG0000(	CD2AP	4516.155	-0.48398	0.002281	0.01272	protein_coding
ENSG0000(	TMEM223	125.9554	-0.27756	0.002283	0.012727	protein_coding
ENSG0000(	ENSG0000(	18.83297	0.554441	0.002284	0.012733	lncRNA
ENSG0000(	DENND4C	4364.88	-0.26381	0.002286	0.012739	protein_coding
ENSG0000(	ENSG0000(	21.48284	0.547735	0.002287	0.012745	lncRNA
ENSG0000(	TPST1	288.7926	0.498253	0.002291	0.012762	protein_coding
ENSG0000(	GTPBP6	405.2206	-0.21562	0.002292	0.012765	protein_coding
ENSG0000(	HOXB-AS1	46.01114	0.497767	0.002293	0.012773	lncRNA
ENSG0000(	IGHM	7171.988	0.715289	0.002294	0.012773	IG_C_gene
ENSG0000(	HINFP	2681.494	-0.09043	0.002294	0.012774	protein_coding
ENSG0000(	SLC2A5	1720.994	-0.39444	0.002297	0.012787	protein_coding
ENSG0000(	DGUOK	303.4485	-0.14409	0.002301	0.012804	protein_coding
ENSG0000(	GRM4	110.3934	0.27853	0.002303	0.012817	protein_coding
ENSG0000(	RDM1P5	308.4543	0.171848	0.002304	0.012821	transcribed_processed_pseudoge
ENSG0000(	FAM83A	82.07696	0.423689	0.002306	0.012823	protein_coding
ENSG0000(	TOPBP1	1485.052	-0.1959	0.002305	0.012823	protein_coding
ENSG0000(	CCDC120	760.5783	-0.34144	0.002307	0.012827	protein_coding
ENSG0000(	PLA2G4C-A	34.25284	0.317021	0.002308	0.012834	lncRNA
ENSG0000(	OR2V1	54.10619	0.30729	0.002313	0.012855	protein_coding
ENSG0000(	CRAT	1750.055	-0.35739	0.002315	0.012862	protein_coding
ENSG0000(	SSU72	2485.725	-0.10432	0.002315	0.012862	protein_coding
ENSG0000(	DNAJB1	1007.634	-0.21568	0.002315	0.012862	protein_coding
ENSG0000(	B3GALT2	114.4718	-0.45651	0.002316	0.012867	protein_coding
ENSG0000(	RNU1-52P	23.75372	-0.65255	0.002317	0.012869	snRNA
ENSG0000(	ENSG0000(	80.01279	0.24789	0.002318	0.012871	lncRNA
ENSG0000(	ENSG0000(	24.93837	0.377222	0.002321	0.012884	lncRNA
ENSG0000(	SCHLAP1	127.6577	0.211407	0.002321	0.012884	lncRNA
ENSG0000(	ENSG0000(	65.70916	0.471402	0.002323	0.012891	lncRNA
ENSG0000(	PIK3R4	1003.49	-0.19802	0.002325	0.012902	protein_coding
ENSG0000(	SELENON	1147.514	0.202299	0.002328	0.012913	protein_coding
ENSG0000(	GAN	2266.16	-0.22936	0.002328	0.012913	protein_coding
ENSG0000(	TMEM115	346.4685	-0.25799	0.00233	0.012923	protein_coding
ENSG0000(	TUBAL3	508.7194	-0.76953	0.002331	0.012926	protein_coding
ENSG0000(	DIP2B	2364.503	0.113506	0.002334	0.01294	protein_coding
ENSG0000(	NRADDP	20.85001	0.438366	0.002334	0.01294	transcribed_unitary_pseudogene
ENSG0000(	UQCR11	408.4329	-0.29585	0.002336	0.012947	protein_coding
ENSG0000(	EIF4G2	10692.21	-0.13331	0.002337	0.012949	protein_coding

ENSG0000(	FBXW10	155.8658	0.171349	0.002338	0.012955	protein_coding
ENSG0000(	ENSG0000(	10.60965	0.781387	0.00234	0.012959	lncRNA
ENSG0000(	WWC2-AS2	11.74148	0.601521	0.002342	0.012968	lncRNA
ENSG0000(	DLEU2L	151.0263	0.282493	0.002345	0.012986	lncRNA
ENSG0000(	IDH1	1379.533	-0.33587	0.002346	0.012988	protein_coding
ENSG0000(	MS4A18	44.59028	1.39177	0.002347	0.01299	protein_coding
ENSG0000(	MT-TP	87.49068	1.178537	0.002348	0.012996	Mt_tRNA
ENSG0000(	ENSG0000(	61.49078	-0.37874	0.002351	0.013006	lncRNA
ENSG0000(	HNRNPUL1	3150.681	-0.07477	0.002355	0.013028	protein_coding
ENSG0000(	ENSG0000(	550.6542	0.165703	0.002356	0.013031	lncRNA
ENSG0000(	MOK	1219.993	0.126149	0.002356	0.013031	protein_coding
ENSG0000(	C1orf50	839.3954	-0.13393	0.002357	0.013034	protein_coding
ENSG0000(	ENSG0000(	119.3282	-0.29057	0.002361	0.013049	lncRNA
ENSG0000(	ENSG0000(	17.59787	1.01517	0.002361	0.013049	lncRNA
ENSG0000(	MESD	1645.813	0.114195	0.002361	0.013049	protein_coding
ENSG0000(	RPL8	2665.615	-0.26136	0.002365	0.013067	protein_coding
ENSG0000(	NOL4L	3758.334	-0.2189	0.002366	0.013073	protein_coding
ENSG0000(	MT-TM	129.0817	0.648613	0.00237	0.013089	Mt_tRNA
ENSG0000(	ENSG0000(	162.577	-0.18654	0.002375	0.013115	processed_pseudogene
ENSG0000(	ARHGEF2-A	182.1306	0.222868	0.002377	0.013118	lncRNA
ENSG0000(	CMTM1	76.50361	0.318559	0.002377	0.013118	protein_coding
ENSG0000(	FAM72A	117.0643	0.46581	0.002377	0.013118	protein_coding
ENSG0000(	MYO9A	3186.939	-0.1267	0.002377	0.013118	protein_coding
ENSG0000(	ZNF845	729.2359	-0.14201	0.002376	0.013118	protein_coding
ENSG0000(	KLF7	1964.13	0.194827	0.002379	0.013122	protein_coding
ENSG0000(	ENSG0000(	351.2998	0.244471	0.002384	0.013147	lncRNA
ENSG0000(	UBXN1	374.6546	-0.23169	0.002384	0.013147	protein_coding
ENSG0000(	ENSG0000(	81.94884	0.20126	0.002385	0.013148	lncRNA
ENSG0000(	CLCC1	5181.104	0.086368	0.002385	0.01315	protein_coding
ENSG0000(	MXD4	1853.079	-0.15152	0.002386	0.013152	protein_coding
ENSG0000(	RN7SL838F	67.05725	-0.36739	0.002387	0.013156	misc_RNA
ENSG0000(	ENSG0000(	273.877	0.165385	0.002389	0.013159	TEC
ENSG0000(	NUDT19	644.6306	-0.15099	0.002388	0.013159	protein_coding
ENSG0000(	ATP6V1B1	13.96768	0.690028	0.002393	0.013173	protein_coding
ENSG0000(	ENSG0000(	206.139	0.436223	0.002393	0.013173	lncRNA
ENSG0000(	ENSG0000(	35.39309	0.329225	0.002392	0.013173	lncRNA
ENSG0000(	ENSG0000(	422.1445	-0.81916	0.002393	0.013173	lncRNA
ENSG0000(	RN7SL520F	16.93375	0.374953	0.002393	0.013173	misc_RNA
ENSG0000(	ENSG0000(	258.4567	0.164033	0.002398	0.013194	lncRNA
ENSG0000(	MIR3605	13.36822	0.533025	0.002399	0.013196	miRNA
ENSG0000(	NR1H2	603.9278	-0.1787	0.002399	0.013196	protein_coding
ENSG0000(	LAMTOR5	659.2501	-0.25055	0.002399	0.013197	protein_coding
ENSG0000(	SNHG29	2166.097	-0.27117	0.002402	0.013208	lncRNA
ENSG0000(	HTATSF1	377.2119	-0.22603	0.002406	0.013228	protein_coding
ENSG0000(	SNRPN	614.2856	-0.26049	0.002406	0.013228	protein_coding
ENSG0000(	SELENOI	3368.541	-0.32982	0.002409	0.013238	protein_coding
ENSG0000(	ZNF252P	1427.429	-0.13706	0.002411	0.013251	transcribed_unprocessed_pseudo

ENSG0000(	MLLT1	848.116	0.220771	0.002413	0.013261	protein_coding
ENSG0000(	ZNF571	644.779	0.159037	0.002415	0.013267	protein_coding
ENSG0000(	LINC02886	203.425	-0.31386	0.002415	0.013267	lncRNA
ENSG0000(	ENSG0000(	75.07556	0.321528	0.002419	0.013284	lncRNA
ENSG0000(	OTULIN-DT	150.7911	0.307055	0.002419	0.013284	lncRNA
ENSG0000(	ENSG0000(	112.8502	-0.32358	0.002422	0.013294	processed_pseudogene
ENSG0000(	ABHD15	827.8864	-0.20005	0.002424	0.013306	protein_coding
ENSG0000(	ACLY	1882.34	-0.24433	0.002425	0.013306	protein_coding
ENSG0000(	IGSF23	133.3914	-0.92124	0.002426	0.013309	protein_coding
ENSG0000(	RALY-AS1	1276.873	0.093966	0.00243	0.013331	lncRNA
ENSG0000(	VPS53	7495.329	0.069142	0.002432	0.013339	protein_coding
ENSG0000(	ADCY2	1020.944	0.167292	0.002434	0.013344	protein_coding
ENSG0000(	C1orf116	1434.096	-0.5935	0.002434	0.013344	protein_coding
ENSG0000(	OR6C66P	148.8924	-0.24799	0.002433	0.013344	transcribed_unprocessed_pseudo
ENSG0000(	ENSG0000(	2895.596	0.105495	0.002435	0.013346	lncRNA
ENSG0000(	LINC00528	130.7548	0.433445	0.002442	0.013382	lncRNA
ENSG0000(	SNHG21	26.86351	-0.42346	0.002442	0.013382	lncRNA
ENSG0000(	ENSG0000(	358.4131	-0.16817	0.002448	0.01341	lncRNA
ENSG0000(	ADGRE5	1641.679	0.241272	0.002449	0.013412	protein_coding
ENSG0000(	GCAWKR	162.4284	0.269661	0.002451	0.013425	lncRNA
ENSG0000(	MRPL2	315.4089	-0.18585	0.002454	0.013436	protein_coding
ENSG0000(	ACTA2-AS1	453.7906	0.367622	0.002455	0.013437	lncRNA
ENSG0000(	ENSG0000(	67.1108	0.271529	0.002455	0.013439	lncRNA
ENSG0000(	TRPC3	126.2281	0.341835	0.002456	0.013439	protein_coding
ENSG0000(	BMF	1832.931	-0.28634	0.002458	0.013451	protein_coding
ENSG0000(	OR8H2	20.09272	-0.5639	0.002459	0.013453	protein_coding
ENSG0000(	SMIM6	25.27963	-0.71717	0.00246	0.013453	protein_coding
ENSG0000(	ENSG0000(	44.90667	0.296962	0.002466	0.013487	lncRNA
ENSG0000(	GJB1	748.306	-0.37212	0.002469	0.013498	protein_coding
ENSG0000(	MT-TC	72.0579	0.89744	0.002475	0.013531	Mt_tRNA
ENSG0000(	LINC02167	17.8322	0.479479	0.002476	0.013536	lncRNA
ENSG0000(	PIPOX	283.8213	0.167414	0.002478	0.013538	protein_coding
ENSG0000(	RMDN3	1187.539	-0.41669	0.002478	0.013538	protein_coding
ENSG0000(	SMIM17	251.6907	-0.21865	0.002479	0.013541	protein_coding
ENSG0000(	TRPV1	1385.533	-0.34124	0.002479	0.013543	protein_coding
ENSG0000(	THBD	189.4676	0.436585	0.002481	0.01355	protein_coding
ENSG0000(	ENSG0000(	14.04198	0.488808	0.002482	0.013551	lncRNA
ENSG0000(	TMEM107	473.5597	0.162208	0.002485	0.013566	protein_coding
ENSG0000(	PKIB	636.3134	-0.35203	0.002486	0.013568	protein_coding
ENSG0000(	PPP4R4	20.27194	0.824434	0.002486	0.013568	protein_coding
ENSG0000(	VEGFD	25.6401	-0.67362	0.002487	0.013572	protein_coding
ENSG0000(	ADAMTS3	51.75983	0.599695	0.00249	0.013579	protein_coding
ENSG0000(	ATP10B	3855.678	0.473701	0.00249	0.013579	protein_coding
ENSG0000(	CHFR	3164.132	0.103626	0.00249	0.013579	protein_coding
ENSG0000(	ENSG0000(	231.2314	0.169525	0.002491	0.013579	lncRNA
ENSG0000(	GPR37	23.69435	0.551075	0.00249	0.013579	protein_coding
ENSG0000(	ENSG0000(	26.75741	0.336832	0.002491	0.01358	lncRNA

ENSG0000(	AANAT	9.543844	0.718843	0.002492	0.013583	protein_coding
ENSG0000(	DCTN1	1698.406	-0.12099	0.002494	0.013588	protein_coding
ENSG0000(	ENSG0000(	21.62749	0.465061	0.002495	0.01359	snoRNA
ENSG0000(	ENSG0000(	476.1378	0.155026	0.002496	0.013596	TEC
ENSG0000(	RN7SKP227	429.7322	-0.40721	0.002498	0.013605	misc_RNA
ENSG0000(	DDRGK1	384.6101	-0.15225	0.002501	0.01362	protein_coding
ENSG0000(	ENSG0000(	212.0025	0.183558	0.002502	0.013622	lncRNA
ENSG0000(	CAPN7	2140.015	-0.11204	0.002503	0.013624	protein_coding
ENSG0000(	LINC00189	49.62547	0.399469	0.002505	0.013634	lncRNA
ENSG0000(	RNF186	118.3383	0.681478	0.002506	0.013635	protein_coding
ENSG0000(	ENSG0000(	127.5759	0.405543	0.002513	0.013669	lncRNA
ENSG0000(	BRK1	490.4754	-0.20748	0.00252	0.013703	protein_coding
ENSG0000(	CLDN12	1147.691	0.28043	0.00252	0.013703	protein_coding
ENSG0000(	SNCA	78.94123	0.284458	0.002519	0.013703	protein_coding
ENSG0000(	SUB1	1995.751	0.148796	0.002521	0.013707	protein_coding
ENSG0000(	SSBP2	2891.065	0.124066	0.00253	0.01375	protein_coding
ENSG0000(	OR4K2	145.3719	0.267617	0.002531	0.013756	protein_coding
ENSG0000(	C4orf3	1233.784	-0.17626	0.002533	0.01376	protein_coding
ENSG0000(	ENSG0000(	70.17638	0.287245	0.002533	0.01376	lncRNA
ENSG0000(	ABCG5	402.9066	-0.58258	0.002538	0.013785	protein_coding
ENSG0000(	MRPL39	122.9221	-0.2492	0.002539	0.013789	protein_coding
ENSG0000(	OR2AG2	178.1062	0.19964	0.002547	0.013827	protein_coding
ENSG0000(	RN7SL385F	23.51789	0.496977	0.002547	0.013827	misc_RNA
ENSG0000(	TVP23CP1	37.94862	0.65777	0.002548	0.013832	processed_pseudogene
ENSG0000(	ENSG0000(	9.024682	0.787332	0.002549	0.013832	lncRNA
ENSG0000(	ENSG0000(	105.3479	0.260099	0.002551	0.013844	lncRNA
ENSG0000(	ENSG0000(	50.7566	0.308774	0.002554	0.013855	lncRNA
ENSG0000(	MOB4	818.3044	-0.12983	0.002556	0.013864	protein_coding
ENSG0000(	ENSG0000(	2213.816	0.252201	0.002558	0.013871	lncRNA
ENSG0000(	ENSG0000(	110.5112	-0.70814	0.002558	0.013871	lncRNA
ENSG0000(	FOXD4L5	119.4366	0.285101	0.00256	0.013878	protein_coding
ENSG0000(	POLR2L	157.3657	-0.25234	0.002561	0.013883	protein_coding
ENSG0000(	ENSG0000(	17.02039	0.496541	0.002564	0.013893	lncRNA
ENSG0000(	MIR29B2CH	7063.008	0.199794	0.002564	0.013893	lncRNA
ENSG0000(	DLGAP1-AS	168.1345	-0.17949	0.002565	0.013895	lncRNA
ENSG0000(	SNRPEP10	11.48594	1.018807	0.002566	0.013901	processed_pseudogene
ENSG0000(	FAM245A	101.3201	0.238856	0.002568	0.013908	lncRNA
ENSG0000(	LINC02800	54.83305	0.352584	0.002572	0.013929	lncRNA
ENSG0000(	CCDC150	728.3045	0.155205	0.002573	0.013931	protein_coding
ENSG0000(	ENSG0000(	99.11333	0.57308	0.002574	0.013931	lncRNA
ENSG0000(	TLCD1	78.17379	-0.22696	0.002573	0.013931	protein_coding
ENSG0000(	RALBP1	1678.599	-0.11446	0.002579	0.013958	protein_coding
ENSG0000(	LRRN1	139.0415	-0.47993	0.00258	0.01396	protein_coding
ENSG0000(	NDC80	379.6257	-0.14753	0.002581	0.01396	protein_coding
ENSG0000(	RERG-IT1	8.670734	0.815723	0.002583	0.013968	lncRNA
ENSG0000(	UBFD1	1159.749	0.145029	0.002584	0.013975	protein_coding
ENSG0000(	FAH	480.7009	-0.22108	0.002585	0.013975	protein_coding

ENSG0000(	CHCHD2	714.4739	-0.27088	0.002587	0.013987	protein_coding
ENSG0000(	HOXD3	176.5671	0.306547	0.002591	0.014005	protein_coding
ENSG0000(	RTL5	86.84636	0.325092	0.002592	0.014006	protein_coding
ENSG0000(	SNORD22	606.927	0.430806	0.002593	0.014011	snoRNA
ENSG0000(	ENSG0000(	58.33447	0.409703	0.002595	0.014021	lncRNA
ENSG0000(	H2AC13	137.4997	-0.54749	0.002596	0.014024	protein_coding
ENSG0000(	SNORA26	90.89403	0.396088	0.002598	0.014028	snoRNA
ENSG0000(	ENSG0000(	126.7941	-0.26343	0.002603	0.014052	pseudogene
ENSG0000(	KCNG3	43.703	0.581734	0.002603	0.014052	protein_coding
ENSG0000(	TP53BP1	2410.962	0.216549	0.002604	0.014055	protein_coding
ENSG0000(	CCDC59	403.1949	-0.17524	0.002605	0.014059	protein_coding
ENSG0000(	DELEC1	690.2225	0.118014	0.002606	0.014064	lncRNA
ENSG0000(	TAL1	212.4268	0.327	0.002611	0.014086	protein_coding
ENSG0000(	MX2	3028.041	0.362477	0.002612	0.014086	protein_coding
ENSG0000(	HTR3B	758.8252	0.095219	0.002616	0.014107	protein_coding
ENSG0000(	IREB2	3505.034	-0.1209	0.002616	0.014107	protein_coding
ENSG0000(	CPT1C	527.2857	0.130175	0.002618	0.014111	protein_coding
ENSG0000(	RLF	1358.877	-0.1179	0.002618	0.014111	protein_coding
ENSG0000(	TMEM30A-	370.1383	-0.17949	0.002618	0.014111	lncRNA
ENSG0000(	ICAM5	38.46198	0.54142	0.002622	0.014128	protein_coding
ENSG0000(	TMCC1-DT	1965.2	0.161658	0.002625	0.014145	lncRNA
ENSG0000(	ENSG0000(	17.65295	0.494872	0.002631	0.014173	lncRNA
ENSG0000(	BLCAP	3091.87	-0.20089	0.002632	0.014173	protein_coding
ENSG0000(	TMEM230	976.6948	-0.13366	0.002632	0.014173	protein_coding
ENSG0000(	VRK2	368.738	0.275619	0.002633	0.014177	protein_coding
ENSG0000(	ENSG0000(	250.1796	0.124322	0.002636	0.014185	lncRNA
ENSG0000(	RSPH4A	21.19799	0.486511	0.002635	0.014185	protein_coding
ENSG0000(	CADM3-AS	194.9756	0.589651	0.002638	0.014194	lncRNA
ENSG0000(	ENSG0000(	20.94855	0.405279	0.002638	0.014194	lncRNA
ENSG0000(	AQP11	277.9936	-0.75192	0.002641	0.014207	protein_coding
ENSG0000(	BTG3	151.794	0.311205	0.002643	0.014213	protein_coding
ENSG0000(	ENSG0000(	114.663	0.299306	0.002644	0.014218	lncRNA
ENSG0000(	ENSG0000(	308.3549	0.119184	0.002646	0.014219	lncRNA
ENSG0000(	POP5	384.5521	-0.15596	0.002646	0.014219	protein_coding
ENSG0000(	UBAC2	1424.974	-0.11583	0.002645	0.014219	protein_coding
ENSG0000(	ENSG0000(	616.2688	-0.15818	0.002646	0.01422	lncRNA
ENSG0000(	PANX1	291.5171	0.369475	0.002647	0.014223	protein_coding
ENSG0000(	PTCHD1	464.0634	-0.15321	0.002649	0.01423	protein_coding
ENSG0000(	CYBB	2915.152	0.602466	0.002651	0.014235	protein_coding
ENSG0000(	ENSG0000(	26.46729	-0.4724	0.002651	0.014235	TEC
ENSG0000(	ENSG0000(	16.0946	-0.64066	0.002653	0.014244	lncRNA
ENSG0000(	MKKS	766.7645	-0.31662	0.002653	0.014244	protein_coding
ENSG0000(	BAG1	1588.55	-0.1681	0.002655	0.01425	protein_coding
ENSG0000(	HDC	406.2252	0.494899	0.002656	0.014254	protein_coding
ENSG0000(	DAPL1	23.28276	-0.44723	0.002663	0.014287	protein_coding
ENSG0000(	SEMA3B	2462.679	-0.62541	0.002663	0.014288	protein_coding
ENSG0000(	APBA3	951.0806	-0.14822	0.002665	0.014296	protein_coding

ENSG0000(	LINC01506	166.8311	-0.19604	0.002668	0.014308	lncRNA
ENSG0000(	CCL8	57.29082	0.753971	0.00267	0.014317	protein_coding
ENSG0000(	ENSG0000(	54.8377	0.355502	0.002671	0.014319	lncRNA
ENSG0000(	ENSG0000(	10.91179	0.612773	0.002672	0.014319	lncRNA
ENSG0000(	FAM87A	28.49464	0.587758	0.002672	0.01432	lncRNA
ENSG0000(	SPTBN2	522.2189	0.246221	0.002677	0.014344	protein_coding
ENSG0000(	EFCAB6	259.719	0.223889	0.002682	0.014368	protein_coding
ENSG0000(	EPHX2	2502.764	-0.37652	0.002683	0.014368	protein_coding
ENSG0000(	NDUFS5	232.9336	-0.28103	0.002683	0.014368	protein_coding
ENSG0000(	ENSG0000(	39.88023	0.506075	0.002684	0.014373	lncRNA
ENSG0000(	HEXIM1	934.7039	-0.22886	0.002685	0.014377	protein_coding
ENSG0000(	ENSG0000(	102.7659	0.244207	0.002687	0.014383	lncRNA
ENSG0000(	UBALD1	357.5311	0.222611	0.002688	0.014384	protein_coding
ENSG0000(	MAPK10	3110.216	0.18225	0.002689	0.014391	protein_coding
ENSG0000(	HOXB-AS3	13.73821	0.583745	0.002691	0.014397	lncRNA
ENSG0000(	STAG3L5P-	691.2253	-0.40961	0.002692	0.014401	lncRNA
ENSG0000(	ENSG0000(	38.81049	0.450409	0.002693	0.014405	lncRNA
ENSG0000(	ENSG0000(	345.4011	0.520195	0.002694	0.014406	lncRNA
ENSG0000(	AOC1	2322.162	-0.67427	0.002696	0.014412	protein_coding
ENSG0000(	PMS2CL	722.9809	0.152652	0.0027	0.014431	transcribed_unprocessed_pseudo
ENSG0000(	MASP2	57.52651	0.269927	0.002701	0.014438	protein_coding
ENSG0000(	PPP2R5CP	23.41967	0.399828	0.002702	0.014438	processed_pseudogene
ENSG0000(	CYSLTR2	377.8908	0.300783	0.002703	0.01444	protein_coding
ENSG0000(	ENSG0000(	184.3801	-0.19029	0.002703	0.01444	lncRNA
ENSG0000(	SNORA80E	152.7565	0.315637	0.002707	0.014454	snoRNA
ENSG0000(	ZNF185	90.60533	0.352534	0.002706	0.014454	protein_coding
ENSG0000(	ZIK1P1	281.5585	0.155522	0.002708	0.014457	processed_pseudogene
ENSG0000(	CKAP2LP1	12.69528	0.543245	0.002712	0.014478	processed_pseudogene
ENSG0000(	ENSG0000(	59.87197	0.281704	0.002717	0.014499	lncRNA
ENSG0000(	ACSF3	3999.466	-0.14942	0.002719	0.014508	protein_coding
ENSG0000(	ENSG0000(	119.9044	-0.19773	0.002719	0.014508	lncRNA
ENSG0000(	ENSG0000(	169.7692	0.268194	0.00272	0.014511	lncRNA
ENSG0000(	EXTL2	267.1614	0.203789	0.002721	0.014511	protein_coding
ENSG0000(	MRTFA	1275.469	0.179538	0.002721	0.014511	protein_coding
ENSG0000(	ROBO1	1829.863	0.331953	0.002722	0.014515	protein_coding
ENSG0000(	EIF4E	1126.373	-0.10625	0.002724	0.014521	protein_coding
ENSG0000(	PINCR	17.29555	0.49019	0.002731	0.014556	lncRNA
ENSG0000(	ALDH1A1	1201.007	-0.48525	0.002733	0.014563	protein_coding
ENSG0000(	FAM13A-A:	2381.452	0.146708	0.002735	0.01457	lncRNA
ENSG0000(	LINC02390	46.70939	0.426188	0.002735	0.01457	lncRNA
ENSG0000(	ISX	1048.879	-0.6202	0.002737	0.014577	protein_coding
ENSG0000(	ENSG0000(	11.64109	0.630884	0.00274	0.014593	lncRNA
ENSG0000(	LINC01579	1410.064	0.128548	0.002741	0.014595	lncRNA
ENSG0000(	CHST2	392.0337	0.450318	0.002742	0.014597	protein_coding
ENSG0000(	ENSG0000(	23.61507	-0.76646	0.002743	0.014601	transcribed_processed_pseudoge
ENSG0000(	ENSG0000(	18.80331	0.404025	0.002744	0.014601	lncRNA
ENSG0000(	DTWD1	1648.275	0.141982	0.002748	0.014614	protein_coding

ENSG0000(	ENSG0000(	190.6885	-0.19304	0.002747	0.014614	lncRNA
ENSG0000(	REEP3	1612.025	-0.32808	0.002747	0.014614	protein_coding
ENSG0000(	RTCB	1033.621	-0.23385	0.002748	0.014614	protein_coding
ENSG0000(	AKR1A1	1782.808	-0.22305	0.002756	0.014653	protein_coding
ENSG0000(	RN7SKP136	12.32634	-0.72375	0.002758	0.014663	misc_RNA
ENSG0000(	SLC26A5	8.953602	0.884801	0.002758	0.014663	protein_coding
ENSG0000(	ENSG0000(	12.20772	0.587135	0.00276	0.014664	lncRNA
ENSG0000(	LINC01709	52.32042	0.898531	0.002759	0.014664	lncRNA
ENSG0000(	BTBD2	844.0383	-0.14071	0.002764	0.014678	protein_coding
ENSG0000(	BZW2	484.0852	-0.16123	0.002766	0.014678	protein_coding
ENSG0000(	CASC19	898.0832	0.173485	0.002764	0.014678	lncRNA
ENSG0000(	ENSG0000(	13.68344	-0.71208	0.002765	0.014678	lncRNA
ENSG0000(	ENSG0000(	18.62084	0.40835	0.002766	0.014678	lncRNA
ENSG0000(	KLHL24	3193.445	-0.18234	0.002765	0.014678	protein_coding
ENSG0000(	MIB1	2013.916	-0.10048	0.002765	0.014678	protein_coding
ENSG0000(	THAP8	51.03329	-0.32826	0.00277	0.014698	protein_coding
ENSG0000(	SVIL2P	64.19959	0.460533	0.002773	0.014711	transcribed_unprocessed_pseudo
ENSG0000(	TRIM32	609.0692	-0.21972	0.002774	0.014716	protein_coding
ENSG0000(	TMEM78	210.0934	0.20365	0.002776	0.014725	lncRNA
ENSG0000(	RMC1	668.8393	-0.15559	0.002777	0.014729	protein_coding
ENSG0000(	ENSG0000(	112.648	0.181904	0.00278	0.014738	lncRNA
ENSG0000(	SOBP	214.9355	0.310564	0.002781	0.014744	protein_coding
ENSG0000(	PRRG2	107.5463	-0.51314	0.002786	0.014769	protein_coding
ENSG0000(	TMEM253	446.6217	-0.63509	0.002788	0.014773	protein_coding
ENSG0000(	TMEM167A	1898.788	0.123815	0.002788	0.014773	protein_coding
ENSG0000(	TTC38	1709.952	-0.35718	0.00279	0.014779	protein_coding
ENSG0000(	XRCC3	406.4864	0.225602	0.00279	0.014781	protein_coding
ENSG0000(	ENSG0000(	390.12	0.303895	0.002794	0.014795	lncRNA
ENSG0000(	SPIN4	151.6053	0.216224	0.002795	0.014799	protein_coding
ENSG0000(	COL6A2	3770.114	0.362227	0.002799	0.014817	protein_coding
ENSG0000(	NIP7	460.0192	-0.13147	0.002799	0.014817	protein_coding
ENSG0000(	ENSG0000(	42.88735	0.41749	0.002801	0.014821	processed_pseudogene
ENSG0000(	HOMER3	196.9997	0.23088	0.002801	0.014821	protein_coding
ENSG0000(	TMEM79	206.1528	-0.16636	0.002802	0.014826	protein_coding
ENSG0000(	CPTP	313.4092	-0.28284	0.002805	0.014836	protein_coding
ENSG0000(	MARCHF11	65.99764	0.250881	0.002805	0.014836	lncRNA
ENSG0000(	ENSG0000(	1958.817	0.634285	0.002807	0.014841	lncRNA
ENSG0000(	C3orf18	165.9446	0.206928	0.002808	0.014841	protein_coding
ENSG0000(	ENSG0000(	182.1014	0.233393	0.002809	0.014841	lncRNA
ENSG0000(	ENSG0000(	501.9054	0.168925	0.002808	0.014841	lncRNA
ENSG0000(	NRG3	37.23756	-0.32836	0.002809	0.014841	protein_coding
ENSG0000(	SYNC	225.6346	0.227927	0.002808	0.014841	protein_coding
ENSG0000(	PIPSL	195.3473	0.540736	0.002811	0.014851	transcribed_processed_pseudoge
ENSG0000(	HMGCLL1	46.5924	-0.46887	0.002812	0.014852	protein_coding
ENSG0000(	ACYP1	313.197	-0.17616	0.002814	0.014862	protein_coding
ENSG0000(	TBC1D3I	145.2531	0.42312	0.002815	0.014866	protein_coding
ENSG0000(	ENSG0000(	13.12172	0.52388	0.002819	0.014884	lncRNA

ENSG0000(	MADD	2528.872	0.135373	0.002821	0.014888	protein_coding
ENSG0000(	ADRA1B	27.79051	-0.69119	0.002827	0.014921	protein_coding
ENSG0000(	LYZ	7030.69	0.478474	0.002829	0.014928	protein_coding
ENSG0000(	IQGAP3	626.4727	0.436376	0.002835	0.014956	protein_coding
ENSG0000(	ENSG0000(	382.7361	0.211393	0.002837	0.014967	lncRNA
ENSG0000(	ENSG0000(	207.4751	-0.19905	0.002838	0.014968	lncRNA
ENSG0000(	RPL19	4092.21	-0.26616	0.002839	0.014969	protein_coding
ENSG0000(	UQCRQ	719.6518	-0.41913	0.002839	0.014969	protein_coding
ENSG0000(	PDE11A	701.5838	0.273064	0.002843	0.014985	protein_coding
ENSG0000(	SMYD2	841.5664	-0.16481	0.002847	0.015007	protein_coding
ENSG0000(	MACC1	4007.854	-0.45577	0.002851	0.015024	protein_coding
ENSG0000(	ENSG0000(	22.87886	0.4848	0.00286	0.015062	lncRNA
ENSG0000(	ENSG0000(	192.9017	0.150646	0.00286	0.015062	lncRNA
ENSG0000(	RNU2-36P	24.73623	-0.52083	0.002859	0.015062	snRNA
ENSG0000(	LINC00534	1036.187	0.104611	0.00287	0.015112	lncRNA
ENSG0000(	FAM135B	285.859	-0.33748	0.002871	0.015117	protein_coding
ENSG0000(	ENSG0000(	17.04103	0.572169	0.002873	0.015125	lncRNA
ENSG0000(	FZD10	11.90679	0.626709	0.002874	0.015126	protein_coding
ENSG0000(	CLDND2	32.04927	-0.5176	0.002875	0.015132	protein_coding
ENSG0000(	GPANK1	522.795	-0.22561	0.002877	0.015135	protein_coding
ENSG0000(	ARF5	444.6149	-0.25562	0.002879	0.015142	protein_coding
ENSG0000(	ENSG0000(	257.0382	0.181691	0.002879	0.015142	lncRNA
ENSG0000(	TMEM248	2012.959	-0.11606	0.002879	0.015142	protein_coding
ENSG0000(	TSPAN7	393.9822	-0.36778	0.002881	0.015146	protein_coding
ENSG0000(	ENSG0000(	10.44047	0.586785	0.002883	0.015152	lncRNA
ENSG0000(	LINC01859	89.80212	-0.1623	0.002882	0.015152	lncRNA
ENSG0000(	GET3	143.7175	-0.2941	0.002883	0.015154	protein_coding
ENSG0000(	PPP2R2A	1761.456	-0.13852	0.002885	0.015159	protein_coding
ENSG0000(	ENSG0000(	136.4913	0.230325	0.002886	0.01516	lncRNA
ENSG0000(	HTRA1	169.6715	0.458284	0.002887	0.015164	protein_coding
ENSG0000(	ENSG0000(	102.9902	0.533311	0.002888	0.015168	lncRNA
ENSG0000(	ENSG0000(	129.1689	-0.34802	0.00289	0.015173	lncRNA
ENSG0000(	ESAM	233.0518	0.314493	0.00289	0.015173	protein_coding
ENSG0000(	IFFO1	651.7756	0.304301	0.002891	0.015175	protein_coding
ENSG0000(	SH2D3A	1979.834	-0.16784	0.002891	0.015175	protein_coding
ENSG0000(	ENSG0000(	75.64476	0.245929	0.002893	0.015179	processed_pseudogene
ENSG0000(	ENSG0000(	412.7942	-0.31	0.002894	0.015183	lncRNA
ENSG0000(	ENSG0000(	88.133	0.186421	0.002894	0.015183	lncRNA
ENSG0000(	IFITM2	361.5756	0.444685	0.002896	0.015188	protein_coding
ENSG0000(	BAG4	823.4418	-0.17997	0.002897	0.01519	protein_coding
ENSG0000(	LINC02455	25.91854	-0.40807	0.002901	0.015206	lncRNA
ENSG0000(	TMEM14B	971.7101	-0.09713	0.0029	0.015206	protein_coding
ENSG0000(	WFIKKN1	98.41019	-0.62028	0.002901	0.015206	protein_coding
ENSG0000(	RN7SL380F	11.68511	0.510661	0.002903	0.015212	misc_RNA
ENSG0000(	ENSG0000(	126.4809	0.207386	0.002906	0.015225	lncRNA
ENSG0000(	CTSC	8174.196	0.241983	0.002912	0.015255	protein_coding
ENSG0000(	SLC25A53	615.6393	-0.15294	0.002912	0.015255	protein_coding

ENSG0000(	ENSG0000(	206.9167	0.18342	0.002914	0.015256	TEC
ENSG0000(	ENSG0000(	53.21988	0.311385	0.002913	0.015256	TEC
ENSG0000(	SETD3	1036.504	-0.19697	0.002914	0.015256	protein_coding
ENSG0000(	ENSG0000(	538.5213	0.201183	0.002916	0.015265	lncRNA
ENSG0000(	ENSG0000(	9.856125	0.556062	0.002921	0.015284	lncRNA
ENSG0000(	PNMA8B	42.69117	0.472693	0.002921	0.015284	protein_coding
ENSG0000(	DNM3	216.0367	0.310847	0.002924	0.015297	protein_coding
ENSG0000(	NDUFS7	1225.498	-0.1419	0.002925	0.015298	protein_coding
ENSG0000(	ENSG0000(	26.15694	0.457044	0.002929	0.015319	lncRNA
ENSG0000(	ENSG0000(	331.7426	0.287581	0.002929	0.015319	lncRNA
ENSG0000(	RNF222	232.7634	0.165765	0.00293	0.015319	protein_coding
ENSG0000(	ENSG0000(	10.13908	0.633734	0.002935	0.015343	processed_pseudogene
ENSG0000(	NAPB	1016.862	0.116087	0.002938	0.015354	protein_coding
ENSG0000(	ACBD7	971.2726	-0.08778	0.00294	0.015362	protein_coding
ENSG0000(	ENSG0000(	80.60731	0.217739	0.002942	0.015372	lncRNA
ENSG0000(	NFE2L3	514.1766	0.247717	0.002944	0.015381	protein_coding
ENSG0000(	ENSG0000(	397.697	0.300839	0.002949	0.015394	lncRNA
ENSG0000(	ENSG0000(	431.0342	0.124826	0.002948	0.015394	TEC
ENSG0000(	GAPVD1	3097.048	0.104473	0.002949	0.015394	protein_coding
ENSG0000(	GPCPD1	4358.695	0.184923	0.002949	0.015394	protein_coding
ENSG0000(	PTMS	286.1553	-0.27452	0.00295	0.015394	protein_coding
ENSG0000(	ZNF724	524.3654	-0.13223	0.002949	0.015394	protein_coding
ENSG0000(	ENSG0000(	31.44191	0.323472	0.002952	0.015402	lncRNA
ENSG0000(	MIR4728	12.37323	-0.74689	0.002953	0.015405	miRNA
ENSG0000(	MDGA1	473.7762	0.633734	0.002956	0.015417	protein_coding
ENSG0000(	ENSG0000(	174.749	0.169372	0.002964	0.01546	lncRNA
ENSG0000(	CSGALNAC	1004.176	0.36401	0.002965	0.015463	protein_coding
ENSG0000(	TMEM205	212.8308	-0.20919	0.002969	0.01548	protein_coding
ENSG0000(	ANAPC11	223.5443	-0.21414	0.002973	0.015496	protein_coding
ENSG0000(	CEBPA	737.9812	-0.42334	0.002973	0.015496	protein_coding
ENSG0000(	MDP1	35.7677	-0.34219	0.002976	0.015508	protein_coding
ENSG0000(	CSF1R	1094.618	0.25095	0.002977	0.015512	protein_coding
ENSG0000(	CD300A	342.7325	0.384598	0.002981	0.015529	protein_coding
ENSG0000(	IGBP1-AS2	17.05313	-0.50549	0.002983	0.015535	lncRNA
ENSG0000(	NALCN	454.6824	-0.14899	0.002983	0.015535	protein_coding
ENSG0000(	NMD3	795.0227	-0.12984	0.002986	0.015545	protein_coding
ENSG0000(	PRPF40A	2445.036	-0.1485	0.002988	0.015556	protein_coding
ENSG0000(	DYRK1B	292.0511	-0.23572	0.002989	0.015558	protein_coding
ENSG0000(	ENSG0000(	175.3013	0.251367	0.002993	0.015578	lncRNA
ENSG0000(	ENSG0000(	1325.639	0.101243	0.002994	0.015578	lncRNA
ENSG0000(	ENSG0000(	17.0347	-0.77113	0.002996	0.015586	lncRNA
ENSG0000(	NRROS	166.4401	0.513439	0.002996	0.015586	protein_coding
ENSG0000(	CNPY2-AS1	192.9043	-0.2124	0.002997	0.015587	lncRNA
ENSG0000(	ADNP	3871.028	-0.11748	0.003003	0.01561	protein_coding
ENSG0000(	PRDM8-AS	8.308183	0.961157	0.003003	0.01561	lncRNA
ENSG0000(	RMDN2-AS	47.85098	0.4011	0.003002	0.01561	lncRNA
ENSG0000(	NOL7	299.863	-0.18173	0.003004	0.015614	protein_coding

ENSG0000(	DGCR5	668.9943	0.156331	0.003006	0.015621	lncRNA
ENSG0000(	NEU3	1268.707	0.245405	0.003007	0.015621	protein_coding
ENSG0000(	SEC24D	2408.315	0.228644	0.003007	0.015621	protein_coding
ENSG0000(	SMIM15	712.365	-0.24083	0.00301	0.015636	protein_coding
ENSG0000(	ACTG1P9	274.6814	0.138947	0.003016	0.015658	processed_pseudogene
ENSG0000(	ENSG0000(	140.8874	-0.21092	0.003016	0.015658	lncRNA
ENSG0000(	HDAC10	602.5423	-0.18836	0.003017	0.015662	protein_coding
ENSG0000(	IGHV4-61	175.9165	0.90664	0.003019	0.015672	IG_V_gene
ENSG0000(	APPL2	2597.342	-0.3108	0.003023	0.015687	protein_coding
ENSG0000(	SOAT2	144.043	-0.98841	0.003026	0.0157	protein_coding
ENSG0000(	ENSG0000(	14.05897	0.403352	0.003028	0.015709	lncRNA
ENSG0000(	ENSG0000(	35.83043	0.307889	0.003031	0.015721	TEC
ENSG0000(	NIBAN1	1063.562	0.244466	0.003031	0.015721	protein_coding
ENSG0000(	CUBN	23268.03	-0.98455	0.003033	0.015723	protein_coding
ENSG0000(	ENSG0000(	1244.537	0.189441	0.003033	0.015723	transcribed_processed_pseudoge
ENSG0000(	RYR1	254.2958	-0.3499	0.003033	0.015723	protein_coding
ENSG0000(	ENSG0000(	15.54519	0.568055	0.003042	0.015766	lncRNA
ENSG0000(	JMJD8	344.2073	-0.19841	0.003044	0.015772	protein_coding
ENSG0000(	ENSG0000(	193.391	0.195831	0.003045	0.015775	TEC
ENSG0000(	CASC2	584.7874	0.111452	0.003047	0.015786	lncRNA
ENSG0000(	ENSG0000(	144.7611	0.239194	0.003049	0.015789	lncRNA
ENSG0000(	MCM10	176.6113	0.389593	0.003049	0.015789	protein_coding
ENSG0000(	SHLD2	1281.441	-0.22873	0.00305	0.015794	protein_coding
ENSG0000(	LINC02064	148.5005	0.239621	0.003052	0.015801	lncRNA
ENSG0000(	ENSG0000(	32.66787	0.363699	0.003054	0.015809	lncRNA
ENSG0000(	ABCD4	2022.518	-0.09865	0.003055	0.015811	protein_coding
ENSG0000(	H2BC15	460.2325	-0.26907	0.003056	0.015812	protein_coding
ENSG0000(	ACE	8256.852	-0.72025	0.003063	0.015845	protein_coding
ENSG0000(	CTNNBL1	429.262	-0.17874	0.003063	0.015845	protein_coding
ENSG0000(	TOB2	1460.507	-0.28195	0.003065	0.015851	protein_coding
ENSG0000(	ENSG0000(	21.81487	0.388999	0.003067	0.015858	lncRNA
ENSG0000(	DIO2	202.6286	0.651078	0.00307	0.015873	protein_coding
ENSG0000(	ENSG0000(	715.8164	-0.16634	0.003072	0.015882	lncRNA
ENSG0000(	NUP153-AS	19.67743	0.628343	0.003075	0.015893	lncRNA
ENSG0000(	FAM185BP	135.2432	-0.27233	0.003077	0.015903	transcribed_unprocessed_pseudo
ENSG0000(	ZNF24	2650.98	-0.08711	0.003078	0.015905	protein_coding
ENSG0000(	ENSG0000(	74.44848	-0.88313	0.003079	0.015906	lncRNA
ENSG0000(	SAMD9	2295.892	0.302497	0.00308	0.015908	protein_coding
ENSG0000(	ENSG0000(	487.8302	0.182397	0.003082	0.015913	lncRNA
ENSG0000(	NT5DC1	1008.263	-0.27602	0.003082	0.015913	protein_coding
ENSG0000(	GTPBP2	1768.172	0.110955	0.003083	0.015918	protein_coding
ENSG0000(	C8orf33	585.1845	0.133661	0.003086	0.015921	protein_coding
ENSG0000(	ENSG0000(	9.854161	0.570725	0.003086	0.015921	lncRNA
ENSG0000(	ENSG0000(	14.2036	0.636875	0.003086	0.015921	lncRNA
ENSG0000(	ENSG0000(	77.97641	0.287398	0.003085	0.015921	lncRNA
ENSG0000(	TPM1-AS	575.5036	0.663046	0.003087	0.015922	lncRNA
ENSG0000(	CYP2S1	799.3442	-0.6567	0.003092	0.015941	protein_coding

ENSG0000(	ENKD1	120.7242	0.227595	0.003092	0.015941	protein_coding
ENSG0000(	NDUFV1-D'	243.3747	0.286378	0.003091	0.015941	lncRNA
ENSG0000(	SULT1B1	19436.37	-0.25898	0.003092	0.015941	protein_coding
ENSG0000(	ENSG0000(	22.25053	0.438227	0.003093	0.015941	lncRNA
ENSG0000(	DYDC1	76.9051	-0.28031	0.003094	0.015944	protein_coding
ENSG0000(	CORIN	473.1099	0.133847	0.003095	0.015945	protein_coding
ENSG0000(	CCDC88A	5215.717	0.211541	0.003096	0.015949	protein_coding
ENSG0000(	LINC01906	15.51877	0.686011	0.003097	0.015953	lncRNA
ENSG0000(	PHIP	5312.987	0.090112	0.003101	0.015971	protein_coding
ENSG0000(	ENSG0000(	339.161	0.123703	0.003103	0.015976	lncRNA
ENSG0000(	ENSG0000(	126.2332	0.17703	0.003107	0.015996	lncRNA
ENSG0000(	ATXN7L3-A	45.68204	0.328102	0.00311	0.016007	lncRNA
ENSG0000(	SRPK2	1143.196	0.149233	0.003111	0.016009	protein_coding
ENSG0000(	ZNF230-DT	49.54781	0.271351	0.003111	0.016009	lncRNA
ENSG0000(	RN7SL192F	41.88976	0.338866	0.003112	0.016012	misc_RNA
ENSG0000(	ENSG0000(	32.14789	0.387911	0.003113	0.016013	lncRNA
ENSG0000(	HPN	163.1004	0.256576	0.003117	0.01603	protein_coding
ENSG0000(	TARS3	711.2333	-0.17882	0.003118	0.016034	protein_coding
ENSG0000(	ENSG0000(	186.4896	0.204813	0.003119	0.016037	lncRNA
ENSG0000(	ENSG0000(	362.7239	0.172682	0.003122	0.016048	lncRNA
ENSG0000(	ENSG0000(	41.30812	0.448958	0.003123	0.01605	processed_pseudogene
ENSG0000(	LINC01519	72.21827	0.234994	0.003124	0.01605	lncRNA
ENSG0000(	NRSN2	92.88031	0.376311	0.003124	0.01605	protein_coding
ENSG0000(	MAP3K3	1020.283	0.198535	0.003125	0.016052	protein_coding
ENSG0000(	MIR137HG	93.50204	0.224468	0.003126	0.016058	lncRNA
ENSG0000(	LYST	5403.711	0.217107	0.003127	0.016059	protein_coding
ENSG0000(	LINC01721	405.4994	0.202979	0.003132	0.01608	lncRNA
ENSG0000(	OGG1	2111.198	-0.19089	0.003132	0.016081	protein_coding
ENSG0000(	SNORD3D	17839.29	0.485233	0.003133	0.016083	snoRNA
ENSG0000(	ENSG0000(	89.4954	0.229471	0.003139	0.016109	processed_pseudogene
ENSG0000(	TCHP	965.6451	0.257525	0.003142	0.016123	protein_coding
ENSG0000(	CA4	101.1761	1.027395	0.003143	0.016126	protein_coding
ENSG0000(	GFRAL	72.04309	-0.27852	0.003144	0.016128	protein_coding
ENSG0000(	ENSG0000(	39.67498	0.329352	0.003146	0.016136	lncRNA
ENSG0000(	ENSG0000(	105.8847	-0.2837	0.003147	0.016139	pseudogene
ENSG0000(	COLEC10	22.58672	0.659494	0.00315	0.016149	protein_coding
ENSG0000(	LGALS1	209.9203	0.52613	0.003152	0.016156	protein_coding
ENSG0000(	CYYR1	189.3878	0.3648	0.003153	0.016161	protein_coding
ENSG0000(	IPMK	1075.373	-0.43072	0.003154	0.016161	protein_coding
ENSG0000(	RPLP2	1849.864	-0.29211	0.003156	0.01617	protein_coding
ENSG0000(	ENSG0000(	344.0848	-0.18089	0.003176	0.016269	TEC
ENSG0000(	ENSG0000(	220.1265	-0.62867	0.003177	0.016272	lncRNA
ENSG0000(	MAMDC2	189.9707	-0.68276	0.003179	0.016281	protein_coding
ENSG0000(	MON1A	324.903	-0.28411	0.00318	0.016281	protein_coding
ENSG0000(	GOLGA7B	137.0204	-0.36251	0.00318	0.016282	protein_coding
ENSG0000(	ENSG0000(	35.64645	0.417749	0.003181	0.016285	lncRNA
ENSG0000(	MTHFS	265.4287	-0.30128	0.003183	0.016293	protein_coding

ENSG0000(	ENSG0000(	93.21981	0.267675	0.003187	0.016311	lncRNA
ENSG0000(	ENSG0000(	119.8213	0.218366	0.00319	0.016319	lncRNA
ENSG0000(	CCNO	16.45354	1.101398	0.003191	0.016323	protein_coding
ENSG0000(	ENSG0000(	11.94605	0.606747	0.003206	0.016399	processed_pseudogene
ENSG0000(	MT-TH	90.20982	0.921238	0.003213	0.016433	Mt_tRNA
ENSG0000(	H2BC10	101.0125	-0.4575	0.003217	0.016445	protein_coding
ENSG0000(	INTS3	3803.72	0.111196	0.003217	0.016445	protein_coding
ENSG0000(	SNX15	756.1961	-0.17034	0.00322	0.016457	protein_coding
ENSG0000(	TDGF1P3	72.12238	-0.242	0.003223	0.016472	protein_coding
ENSG0000(	SPATA2L	136.0189	-0.37064	0.003225	0.016477	protein_coding
ENSG0000(	MIR100HG	3256.761	0.428753	0.003226	0.016481	lncRNA
ENSG0000(	ENSG0000(	218.1885	0.186623	0.003228	0.016488	lncRNA
ENSG0000(	GRID2	13.83069	1.037892	0.003228	0.016488	protein_coding
ENSG0000(	DNAH11	334.826	0.179062	0.003238	0.016537	protein_coding
ENSG0000(	IGLV6-57	56.73713	0.91206	0.003239	0.016539	IG_V_gene
ENSG0000(	ENSG0000(	10.73719	0.508084	0.003245	0.016565	lncRNA
ENSG0000(	NCL	3340.377	-0.14593	0.003248	0.016576	protein_coding
ENSG0000(	SMAD5	1669.246	0.130524	0.003248	0.016576	protein_coding
ENSG0000(	CCL5	1132.441	-0.17923	0.003251	0.016582	protein_coding
ENSG0000(	ENSG0000(	33.17704	0.473341	0.00325	0.016582	TEC
ENSG0000(	LILRA2	451.7693	0.171332	0.003251	0.016582	protein_coding
ENSG0000(	ENSG0000(	192.2995	0.22227	0.003254	0.016597	lncRNA
ENSG0000(	ATRAID	389.9138	-0.17798	0.003256	0.016602	protein_coding
ENSG0000(	CYB5A	3550.93	-0.44644	0.003258	0.016604	protein_coding
ENSG0000(	ENSG0000(	9.874871	0.54229	0.003257	0.016604	lncRNA
ENSG0000(	ENSG0000(	513.6235	0.123122	0.003258	0.016604	lncRNA
ENSG0000(	GFRA1	532.0302	-0.43058	0.003258	0.016604	protein_coding
ENSG0000(	SOSTDC1	68.23073	-0.65446	0.003259	0.016605	protein_coding
ENSG0000(	RPL13A	5626.747	-0.27008	0.003261	0.016609	protein_coding
ENSG0000(	EFL1	951.5572	-0.30847	0.003264	0.016622	protein_coding
ENSG0000(	AKR7A3	586.8912	-0.62232	0.003267	0.016637	protein_coding
ENSG0000(	SELENOW	2441.148	-0.22369	0.00327	0.016647	protein_coding
ENSG0000(	TREML2	300.3623	0.469884	0.00327	0.016647	protein_coding
ENSG0000(	TMED9	819.0424	0.180237	0.003274	0.016665	protein_coding
ENSG0000(	ETV7	275.1247	0.369291	0.003277	0.016674	protein_coding
ENSG0000(	RFX2	661.1515	-0.30667	0.003281	0.016693	protein_coding
ENSG0000(	LINC00342	6385.74	0.201535	0.003281	0.016694	lncRNA
ENSG0000(	ENSG0000(	84.16029	0.319846	0.003289	0.016728	lncRNA
ENSG0000(	ENSG0000(	808.2389	0.10132	0.00329	0.016728	lncRNA
ENSG0000(	GCHFR	133.6414	-0.53613	0.003289	0.016728	protein_coding
ENSG0000(	ENSG0000(	208.8563	0.162249	0.003296	0.016755	lncRNA
ENSG0000(	HPS1	1610.293	-0.1011	0.003296	0.016755	protein_coding
ENSG0000(	LINC02031	57.90001	-0.27292	0.003297	0.016755	lncRNA
ENSG0000(	TGFA	454.1216	-0.43241	0.003297	0.016755	protein_coding
ENSG0000(	RORB-AS1	61.49059	-0.34156	0.0033	0.016764	lncRNA
ENSG0000(	TCN2	617.9274	-0.47574	0.0033	0.016764	protein_coding
ENSG0000(	ENSG0000(	290.6351	0.210396	0.003305	0.016787	lncRNA

ENSG0000(	ENSG0000(	586.1005	0.122951	0.003306	0.016788	lncRNA
ENSG0000(	ENSG0000(	95.89958	-0.9051	0.003307	0.016789	unprocessed_pseudogene
ENSG0000(	ERLIN2	1183.361	-0.13493	0.003308	0.016789	protein_coding
ENSG0000(	KRT10	71.84443	-0.30027	0.003307	0.016789	protein_coding
ENSG0000(	PALM2AKA	2824.212	0.425731	0.003308	0.016789	protein_coding
ENSG0000(	TMEM120/	577.4631	-0.49325	0.003311	0.016802	protein_coding
ENSG0000(	CDC20B	332.1325	0.122489	0.003316	0.016822	protein_coding
ENSG0000(	MRPS33	1063.343	-0.10385	0.003323	0.016857	protein_coding
ENSG0000(	ENSG0000(	176.1804	-0.23887	0.003328	0.016879	lncRNA
ENSG0000(	N4BP2L1	1613.295	0.145208	0.00333	0.016885	protein_coding
ENSG0000(	MRM3	158.1177	-0.23938	0.003331	0.016891	protein_coding
ENSG0000(	ANGPTL4	172.0987	-0.40639	0.003333	0.016897	protein_coding
ENSG0000(	ASAP1	1873.714	0.239908	0.003336	0.016906	protein_coding
ENSG0000(	RAD51C	863.6653	-0.13294	0.003336	0.016906	protein_coding
ENSG0000(	LINC02908	337.3514	0.318476	0.003337	0.016908	lncRNA
ENSG0000(	ANKRD46	559.8603	-0.17156	0.003338	0.016909	protein_coding
ENSG0000(	MTCO1P2	15.35843	-1.08008	0.003338	0.01691	unprocessed_pseudogene
ENSG0000(	SLC13A1	1242.144	-0.88993	0.003342	0.016926	protein_coding
ENSG0000(	ANO4	11.62503	0.877931	0.003344	0.016928	protein_coding
ENSG0000(	ENSG0000(	55.59781	0.426464	0.003343	0.016928	TEC
ENSG0000(	TOB1	1071.353	-0.45112	0.003346	0.016936	protein_coding
ENSG0000(	CA6	256.3922	-0.14807	0.003349	0.016946	protein_coding
ENSG0000(	RPS3	3613.117	-0.25109	0.003348	0.016946	protein_coding
ENSG0000(	FBP1	1238.71	-0.66385	0.003353	0.016967	protein_coding
ENSG0000(	ZNF619	897.2585	0.1116	0.003355	0.016973	protein_coding
ENSG0000(	ENSG0000(	32.30736	0.52693	0.003358	0.016984	lncRNA
ENSG0000(	HTD2	301.8757	-0.29621	0.003361	0.016996	protein_coding
ENSG0000(	RAB3IP	2791.752	-0.28054	0.003362	0.016999	protein_coding
ENSG0000(	ARRDC2	981.8602	0.265107	0.003364	0.017004	protein_coding
ENSG0000(	TNKS1BP1	2221.488	0.316099	0.003367	0.017017	protein_coding
ENSG0000(	HRG	154.5153	-0.34064	0.003369	0.017026	protein_coding
ENSG0000(	FASTKD1	1326.648	-0.21269	0.003371	0.017035	protein_coding
ENSG0000(	GNAT3	14.04949	-0.86834	0.003372	0.017036	protein_coding
ENSG0000(	ENSG0000(	60.14962	0.272203	0.003373	0.017039	lncRNA
ENSG0000(	RN7SL205F	13.73103	0.532897	0.003374	0.017039	misc_RNA
ENSG0000(	HOOK1	2038.684	-0.47247	0.003382	0.017081	protein_coding
ENSG0000(	ISG20	2455.316	0.467719	0.003383	0.017081	protein_coding
ENSG0000(	ENSG0000(	9.136008	0.642133	0.003385	0.017085	lncRNA
ENSG0000(	SCAMP1-A!	642.0719	0.16085	0.003385	0.017085	lncRNA
ENSG0000(	TLK2P2	110.6523	0.24754	0.003385	0.017085	processed_pseudogene
ENSG0000(	RBM17	1664.9	-0.08103	0.003387	0.017091	protein_coding
ENSG0000(	ENSG0000(	15.93748	0.556376	0.003394	0.01712	unprocessed_pseudogene
ENSG0000(	TPD52L1	842.6807	0.124971	0.003394	0.017122	protein_coding
ENSG0000(	VPS26B	1419.84	-0.16775	0.003397	0.017132	protein_coding
ENSG0000(	PAAF1	1460.909	0.082211	0.003398	0.017133	protein_coding
ENSG0000(	RNF121	894.0219	-0.23159	0.0034	0.01714	protein_coding
ENSG0000(	ERFE	67.86225	0.361716	0.003405	0.017163	protein_coding

ENSG0000(	ENSG0000(	73.78201	-0.25702	0.003407	0.01717	transcribed_processed_pseudoge
ENSG0000(	C1orf131	765.7102	-0.14909	0.003407	0.017171	protein_coding
ENSG0000(	LINC00491	291.8917	0.219111	0.003409	0.017176	lncRNA
ENSG0000(	SOD2-OT1	66.46663	0.227998	0.003409	0.017176	lncRNA
ENSG0000(	ENSG0000(	340.2035	-0.11805	0.003418	0.017216	lncRNA
ENSG0000(	HRK	217.4529	-0.38713	0.003419	0.017217	protein_coding
ENSG0000(	TRA2A	2265.722	-0.10802	0.003419	0.017217	protein_coding
ENSG0000(	CREG2	323.23	-0.13334	0.003423	0.017235	protein_coding
ENSG0000(	ARID5A	592.2499	0.505799	0.003425	0.017242	protein_coding
ENSG0000(	ENSG0000(	210.955	-0.1668	0.003428	0.017247	lncRNA
ENSG0000(	HK1	957.063	0.220311	0.003428	0.017247	protein_coding
ENSG0000(	KANSL1L-A	642.0277	0.135812	0.003428	0.017247	lncRNA
ENSG0000(	ENSG0000(	9.054582	0.639144	0.003432	0.017266	processed_pseudogene
ENSG0000(	ARF6	2586.998	-0.15068	0.003436	0.017282	protein_coding
ENSG0000(	CERK	2546.497	-0.20925	0.003437	0.017282	protein_coding
ENSG0000(	NOSIP	626.874	-0.19245	0.00344	0.017296	protein_coding
ENSG0000(	ZNF131	1474.644	0.11499	0.00344	0.017296	protein_coding
ENSG0000(	FOXF2	190.1371	-0.28792	0.003443	0.017309	protein_coding
ENSG0000(	RPSAP21	32.44299	-0.3924	0.003445	0.017313	processed_pseudogene
ENSG0000(	ARHGAP23	469.0955	0.374989	0.003448	0.017326	protein_coding
ENSG0000(	FGF7P6	62.32683	0.449665	0.003452	0.017342	transcribed_unprocessed_pseudo
ENSG0000(	ATF6B	1866.651	-0.07529	0.003453	0.017342	protein_coding
ENSG0000(	ENSG0000(	119.5441	0.210867	0.003454	0.017342	lncRNA
ENSG0000(	NDUFB10	223.7348	-0.33641	0.003453	0.017342	protein_coding
ENSG0000(	NRGN	15.71053	0.5637	0.003454	0.017342	protein_coding
ENSG0000(	WDR37	1370.397	0.104199	0.003453	0.017342	protein_coding
ENSG0000(	ENSG0000(	3766.6	-0.07993	0.003458	0.017356	transcribed_unprocessed_pseudo
ENSG0000(	ENSG0000(	570.1748	0.139295	0.003465	0.017388	lncRNA
ENSG0000(	CD207	43.11647	-0.59458	0.003466	0.01739	protein_coding
ENSG0000(	ENSG0000(	16.77673	0.548726	0.003466	0.01739	lncRNA
ENSG0000(	LINC02732	594.7008	-0.48747	0.003468	0.017398	lncRNA
ENSG0000(	ENSG0000(	33.62102	0.534029	0.003471	0.017406	lncRNA
ENSG0000(	GDNF-AS1	413.1505	0.123156	0.003473	0.017413	lncRNA
ENSG0000(	TMEM106(	1304.846	-0.36644	0.003473	0.017413	protein_coding
ENSG0000(	TMEM128	226.2387	-0.20697	0.003474	0.017413	protein_coding
ENSG0000(	SCARNA4	150.1799	0.277302	0.003477	0.017428	scaRNA
ENSG0000(	ENSG0000(	68.51779	0.320873	0.003481	0.017444	lncRNA
ENSG0000(	BCYRN1	440.3719	0.213573	0.003484	0.017457	scRNA
ENSG0000(	ENSG0000(	108.2095	0.242693	0.003487	0.01747	lncRNA
ENSG0000(	TSC2	3686.816	-0.12182	0.003488	0.017471	protein_coding
ENSG0000(	AMPD2	1001.149	0.213966	0.003493	0.017492	protein_coding
ENSG0000(	S100A14	767.5469	-0.55449	0.003493	0.017492	protein_coding
ENSG0000(	TUBB2A	199.2779	-0.502	0.003497	0.017508	protein_coding
ENSG0000(	DDX28	97.71105	0.30992	0.00351	0.017569	protein_coding
ENSG0000(	PEX3	231.2986	-0.29341	0.003511	0.017575	protein_coding
ENSG0000(	ENSG0000(	35.91984	0.482672	0.003516	0.017596	misc_RNA
ENSG0000(	PPP2R3C	429.3666	-0.14302	0.003521	0.017619	protein_coding

ENSG0000(	SIPA1L2	1631.905	0.298494	0.003523	0.017624	protein_coding
ENSG0000(	ENSG0000(	18.82043	-0.7819	0.003525	0.017631	lncRNA
ENSG0000(	SERPINB5	266.9694	-0.37711	0.003529	0.017646	protein_coding
ENSG0000(	CH25H	79.90215	0.491523	0.003529	0.017646	protein_coding
ENSG0000(	WT1	98.48678	0.288438	0.003535	0.017674	protein_coding
ENSG0000(	S100A10	1035.045	-0.38234	0.003538	0.017684	protein_coding
ENSG0000(	MS4A7	1098.109	0.279329	0.003541	0.017699	protein_coding
ENSG0000(	ATP2B4	3305.754	0.226895	0.003543	0.017701	protein_coding
ENSG0000(	EFNA1	1149.658	-0.68356	0.003543	0.017701	protein_coding
ENSG0000(	SAMD4A	1908.799	0.209385	0.003544	0.017702	protein_coding
ENSG0000(	ENSG0000(	86.01558	0.282096	0.003554	0.01775	lncRNA
ENSG0000(	MIA2-AS1	15.54019	0.46233	0.003557	0.017762	lncRNA
ENSG0000(	KCND1	133.7504	0.35759	0.003558	0.017764	protein_coding
ENSG0000(	TMEM140	733.1039	0.244499	0.003563	0.017788	protein_coding
ENSG0000(	DEGS2	654.3298	-0.55271	0.003567	0.017802	protein_coding
ENSG0000(	RAB43	947.8739	0.202716	0.003569	0.017811	protein_coding
ENSG0000(	AFAP1-AS1	145.8681	0.577809	0.00357	0.017812	lncRNA
ENSG0000(	ENPP3	583.8665	-0.67777	0.003571	0.017816	protein_coding
ENSG0000(	RASA2-IT1	108.921	0.281774	0.003572	0.017817	lncRNA
ENSG0000(	MAP3K10	434.5637	0.153457	0.003573	0.017821	protein_coding
ENSG0000(	RN7SKP55	251.0449	-0.51624	0.003576	0.017835	misc_RNA
ENSG0000(	ENSG0000(	276.2055	-0.15345	0.00358	0.017846	lncRNA
ENSG0000(	GPT2	409.7342	0.16409	0.00358	0.017846	protein_coding
ENSG0000(	RN7SKP151	39.45479	0.357759	0.003582	0.017852	misc_RNA
ENSG0000(	SIL1	476.0963	0.163577	0.003583	0.017852	protein_coding
ENSG0000(	SNORA38	77.33621	0.380973	0.003583	0.017852	snoRNA
ENSG0000(	ENSG0000(	25.38701	0.400902	0.003587	0.017867	processed_pseudogene
ENSG0000(	ENSG0000(	12.51406	0.696493	0.003586	0.017867	lncRNA
ENSG0000(	ACOXL	236.7333	0.232037	0.003592	0.017884	protein_coding
ENSG0000(	ENSG0000(	186.1945	-0.27563	0.003591	0.017884	lncRNA
ENSG0000(	ENSG0000(	92.73993	0.235126	0.003592	0.017884	lncRNA
ENSG0000(	HSD17B2	2496.644	-0.57365	0.003593	0.017886	protein_coding
ENSG0000(	BAIAP2L2	2746.378	-0.49854	0.003596	0.01789	protein_coding
ENSG0000(	DAB2	2239.797	0.224509	0.003596	0.01789	protein_coding
ENSG0000(	DPP4-DT	137.3369	0.285652	0.003595	0.01789	lncRNA
ENSG0000(	PDX1	14.03803	0.725103	0.003595	0.01789	protein_coding
ENSG0000(	CLEC3B	52.43364	-0.44412	0.003597	0.017892	protein_coding
ENSG0000(	ENSG0000(	10.73884	0.62963	0.0036	0.017906	lncRNA
ENSG0000(	ENSG0000(	23.6515	0.384087	0.003602	0.017912	lncRNA
ENSG0000(	JDP2-AS1	78.63734	0.22573	0.003607	0.017931	lncRNA
ENSG0000(	UGT1A4	195.1882	-0.78465	0.003612	0.017955	protein_coding
ENSG0000(	ENSG0000(	23.9482	0.427134	0.003615	0.017966	lncRNA
ENSG0000(	CCZ1	1611.464	-0.13539	0.003617	0.017976	protein_coding
ENSG0000(	MTPN	2133.306	-0.18355	0.003624	0.018004	protein_coding
ENSG0000(	ENSG0000(	78.41331	0.222946	0.003629	0.018023	lncRNA
ENSG0000(	RN7SL173F	146.3679	0.202233	0.003629	0.018023	misc_RNA
ENSG0000(	ISL2	29.05334	0.516282	0.00363	0.018025	protein_coding

ENSG0000(	ENSG0000(	32.38031	0.363741	0.003631	0.018029	lncRNA
ENSG0000(	PRDX3	936.29	-0.21384	0.003632	0.018029	protein_coding
ENSG0000(	NTNG2	138.3147	0.470169	0.003633	0.018032	protein_coding
ENSG0000(	SLC7A9	2161.557	-0.66567	0.003635	0.018039	protein_coding
ENSG0000(	QPCT	36.91487	0.680482	0.003636	0.018042	protein_coding
ENSG0000(	ENSG0000(	117.3969	-0.20711	0.003641	0.018062	lncRNA
ENSG0000(	ENSG0000(	33.22018	0.366259	0.003641	0.018062	lncRNA
ENSG0000(	STX2	515.425	0.270539	0.003642	0.018062	protein_coding
ENSG0000(	TMEM141	314.5896	-0.34729	0.003643	0.018066	protein_coding
ENSG0000(	RAB6A	2257.588	-0.22671	0.003644	0.018071	protein_coding
ENSG0000(	ENSG0000(	9.021426	0.498133	0.00365	0.018094	lncRNA
ENSG0000(	HOXB8	90.97933	0.469865	0.003652	0.018103	protein_coding
ENSG0000(	CIAPIN1	394.3008	0.124902	0.003656	0.018117	protein_coding
ENSG0000(	CPB1	37.9791	0.464564	0.003657	0.018123	protein_coding
ENSG0000(	ENSG0000(	132.9307	-0.17764	0.003659	0.018131	TEC
ENSG0000(	ARRB2	1384.54	0.204714	0.003661	0.018133	protein_coding
ENSG0000(	HNRNPM	1801.447	-0.10692	0.003661	0.018133	protein_coding
ENSG0000(	UGT1A8	85.47403	-0.80131	0.003662	0.018133	protein_coding
ENSG0000(	ANKRD34C	13.51384	0.455924	0.003662	0.018134	protein_coding
ENSG0000(	ENSG0000(	225.242	0.185713	0.00367	0.018167	lncRNA
ENSG0000(	ENSG0000(	346.8804	-0.36302	0.003671	0.018167	lncRNA
ENSG0000(	RN7SL138F	44.75354	0.355659	0.003672	0.018167	misc_RNA
ENSG0000(	RTF2	672.4576	-0.15129	0.003672	0.018167	protein_coding
ENSG0000(	XACT	81555.5	0.100095	0.003671	0.018167	lncRNA
ENSG0000(	RPL9	2661.968	-0.39286	0.003678	0.018192	protein_coding
ENSG0000(	TRAPPC8	2331.138	-0.14166	0.003678	0.018192	protein_coding
ENSG0000(	TERF2	1220.319	-0.13836	0.003682	0.018208	protein_coding
ENSG0000(	HSD17B7P2	170.6319	0.185266	0.003688	0.018231	transcribed_unprocessed_pseudo
ENSG0000(	HSD17B8	99.08378	-0.28275	0.003688	0.018231	protein_coding
ENSG0000(	CHKB-DT	375.3283	0.181719	0.003689	0.018236	lncRNA
ENSG0000(	DNAJC16	1382.654	-0.14709	0.003691	0.018237	protein_coding
ENSG0000(	LINC01600	13.78457	0.693306	0.00369	0.018237	lncRNA
ENSG0000(	ENSG0000(	23.71257	0.538589	0.003694	0.018251	lncRNA
ENSG0000(	IGHV1-24	42.40962	0.972182	0.003695	0.018251	IG_V_gene
ENSG0000(	ENSG0000(	33.65069	0.355142	0.003698	0.018261	TEC
ENSG0000(	LRRC8C-DT	539.8744	0.3445	0.003698	0.018261	lncRNA
ENSG0000(	C5orf64	1456.482	0.117871	0.0037	0.018269	lncRNA
ENSG0000(	SLC35D1	2341.547	-0.30644	0.003705	0.018291	protein_coding
ENSG0000(	ENSG0000(	49.66563	0.308926	0.003707	0.018293	processed_pseudogene
ENSG0000(	NREP	862.3466	0.265156	0.003707	0.018293	protein_coding
ENSG0000(	ENSG0000(	30.65532	0.295631	0.003708	0.018295	lncRNA
ENSG0000(	TMEM130	338.8952	0.197223	0.003708	0.018295	protein_coding
ENSG0000(	FAM229A	512.4702	-0.25039	0.00371	0.0183	protein_coding
ENSG0000(	LINC02182	21.96206	0.44608	0.003711	0.0183	lncRNA
ENSG0000(	RNU6-1226	18.48977	0.482622	0.00371	0.0183	snRNA
ENSG0000(	SCRG1	803.0327	0.170634	0.003712	0.018303	protein_coding
ENSG0000(	ENSG0000(	10.61387	0.908009	0.003719	0.018334	processed_pseudogene

ENSG0000(	PACERR	40.67895	0.443184	0.00372	0.018334	lncRNA
ENSG0000(	LL22NC03-(	548.3236	0.132655	0.003722	0.018341	lncRNA
ENSG0000(	ENSG0000(	272.2857	0.193688	0.003724	0.018349	protein_coding
ENSG0000(	ITPK1-AS1	61.97893	0.267865	0.003725	0.018352	lncRNA
ENSG0000(	ENSG0000(	79.62323	0.228018	0.003727	0.018352	lncRNA
ENSG0000(	H2AC17	109.3542	-0.40989	0.003726	0.018352	protein_coding
ENSG0000(	UBA2	1330.358	-0.22987	0.003727	0.018352	protein_coding
ENSG0000(	RN7SL105F	61.34428	0.423412	0.003728	0.018354	misc_RNA
ENSG0000(	SMARCA1	296.541	0.271893	0.00373	0.018362	protein_coding
ENSG0000(	ENSG0000(	15.12733	0.461804	0.003733	0.018373	TEC
ENSG0000(	KLHL8	1069.766	-0.14067	0.003735	0.01838	protein_coding
ENSG0000(	KATNBL1P2	32.84163	0.515236	0.003737	0.018388	processed_pseudogene
ENSG0000(	MAP4K5	1557.619	-0.12013	0.003738	0.01839	protein_coding
ENSG0000(	RACK1	4436.348	-0.1963	0.003741	0.0184	protein_coding
ENSG0000(	SLC7A6OS	1030.847	0.145133	0.003741	0.0184	protein_coding
ENSG0000(	BTG2	2579.616	0.329221	0.003742	0.018403	protein_coding
ENSG0000(	KLHL42	1331.108	0.169834	0.003745	0.018413	protein_coding
ENSG0000(	ENSG0000(	233.3007	0.223991	0.003749	0.018429	lncRNA
ENSG0000(	LINC02085	15.34691	2.337202	0.003751	0.018436	lncRNA
ENSG0000(	MYO18B-A	10.47308	0.649756	0.003751	0.018436	lncRNA
ENSG0000(	PHLPP1	1072.86	-0.22243	0.003752	0.018436	protein_coding
ENSG0000(	ENSG0000(	586.6932	0.113846	0.003755	0.018444	lncRNA
ENSG0000(	MLXIP	7751.237	0.128005	0.003755	0.018444	protein_coding
ENSG0000(	UTP6	1630.179	-0.12191	0.003755	0.018444	protein_coding
ENSG0000(	SAP18	778.9691	-0.18225	0.003758	0.018452	protein_coding
ENSG0000(	FAM222A	78.22228	-0.36979	0.003761	0.018464	protein_coding
ENSG0000(	ANKMY2	574.9487	-0.28698	0.003763	0.018471	protein_coding
ENSG0000(	PRRT2	176.6817	0.464074	0.003766	0.018482	protein_coding
ENSG0000(	PLPPR2	223.9809	0.229304	0.003769	0.018496	protein_coding
ENSG0000(	PPFIA1	2177.226	0.103676	0.003771	0.018503	protein_coding
ENSG0000(	CYP26C1	15.02068	0.506268	0.003772	0.018505	protein_coding
ENSG0000(	TACR1	57.24168	0.634355	0.003773	0.018507	protein_coding
ENSG0000(	ENSG0000(	55.00282	0.425225	0.003774	0.018507	TEC
ENSG0000(	TKFC	2483.88	-0.49164	0.003774	0.018507	protein_coding
ENSG0000(	ENSG0000(	221.2622	0.176778	0.003782	0.018538	lncRNA
ENSG0000(	ENSG0000(	44.87857	0.353161	0.003782	0.018538	lncRNA
ENSG0000(	TTC39A	624.369	0.389705	0.003782	0.018538	protein_coding
ENSG0000(	ENSG0000(	100.9175	0.205779	0.003792	0.01858	lncRNA
ENSG0000(	MALL	5815.223	-0.61864	0.003792	0.01858	protein_coding
ENSG0000(	ENSG0000(	11.96334	0.472938	0.003793	0.018581	lncRNA
ENSG0000(	THRA	1206.035	-0.29683	0.0038	0.018612	protein_coding
ENSG0000(	PPP1R42	26.96115	0.34956	0.003805	0.018634	protein_coding
ENSG0000(	RPA4	68.10646	0.246661	0.003808	0.018649	protein_coding
ENSG0000(	PPP2R2B	333.9031	0.254424	0.003813	0.01867	protein_coding
ENSG0000(	ENSG0000(	71.71304	0.258223	0.003814	0.018671	lncRNA
ENSG0000(	NOB1	224.6186	-0.28236	0.003815	0.018674	protein_coding
ENSG0000(	CCL13	48.12874	0.88657	0.003819	0.01869	protein_coding

ENSG0000(	ENSG0000(	201.6957	0.172841	0.00382	0.018691	lncRNA
ENSG0000(	CAPN2	3281.661	0.217578	0.003824	0.018699	protein_coding
ENSG0000(	ENSG0000(	81.12948	0.242413	0.003823	0.018699	lncRNA
ENSG0000(	ENSG0000(	653.4675	0.213672	0.003824	0.018699	lncRNA
ENSG0000(	NPAS2	1384.887	0.386705	0.003822	0.018699	protein_coding
ENSG0000(	ENSG0000(	30.00123	0.385289	0.003828	0.01871	transcribed_processed_pseudoge
ENSG0000(	LCMT2	662.0564	0.159062	0.003827	0.01871	protein_coding
ENSG0000(	MAGI2-AS3	1294.942	0.217113	0.003828	0.01871	lncRNA
ENSG0000(	ENSG0000(	203.1363	-0.18631	0.003834	0.018735	lncRNA
ENSG0000(	ENSG0000(	49.39803	-0.31488	0.003838	0.018755	lncRNA
ENSG0000(	BCO2	580.8423	-0.27104	0.00384	0.01876	protein_coding
ENSG0000(	LINC00271	1069.085	0.145323	0.003842	0.018765	lncRNA
ENSG0000(	RNU6-46P	9.816908	-0.76291	0.003853	0.018819	snRNA
ENSG0000(	DICER1	4627.034	0.113533	0.003854	0.018821	protein_coding
ENSG0000(	HCG24	61.04008	0.224644	0.003855	0.018824	lncRNA
ENSG0000(	ELMO3	400.3841	-0.43781	0.003858	0.018835	protein_coding
ENSG0000(	ABHD11-AS	95.66799	-0.61351	0.003861	0.018843	transcribed_unitary_pseudogene
ENSG0000(	ZFHX4	149.4562	0.306689	0.003862	0.018845	protein_coding
ENSG0000(	MGAT4A	7546.162	-0.2717	0.003871	0.018886	protein_coding
ENSG0000(	OTC	333.3396	-0.63553	0.003872	0.018892	protein_coding
ENSG0000(	ENSG0000(	130.594	-0.15779	0.003875	0.018901	lncRNA
ENSG0000(	ISL1-DT	34.93418	0.531106	0.003877	0.018907	lncRNA
ENSG0000(	LINC02019	87.95429	-0.32094	0.003877	0.018907	lncRNA
ENSG0000(	KHK	1591.951	-0.76677	0.00388	0.018915	protein_coding
ENSG0000(	PCBP1	1474.83	-0.18604	0.00388	0.018915	protein_coding
ENSG0000(	ENSG0000(	144.1974	0.176623	0.003881	0.018918	lncRNA
ENSG0000(	ENSG0000(	238.6779	-0.11769	0.003882	0.018921	lncRNA
ENSG0000(	ZNF700	1744.414	0.100278	0.003884	0.018924	protein_coding
ENSG0000(	ENSG0000(	17.62223	-0.52059	0.003891	0.018955	transcribed_processed_pseudoge
ENSG0000(	CDCA4P4	47.2615	-0.33406	0.003893	0.018961	unprocessed_pseudogene
ENSG0000(	SS18L2	316.4572	-0.19731	0.003896	0.018972	protein_coding
ENSG0000(	CHRDL1	279.938	-0.4422	0.003897	0.018976	protein_coding
ENSG0000(	ENSG0000(	26.00724	0.457954	0.003898	0.018977	lncRNA
ENSG0000(	CADM1	479.4906	0.329724	0.003899	0.018982	protein_coding
ENSG0000(	MT-TQ	110.5053	1.083293	0.003905	0.019009	Mt_tRNA
ENSG0000(	TMEM108	167.0707	0.41629	0.003911	0.019033	protein_coding
ENSG0000(	PEDS1	1343.53	0.114478	0.003918	0.019063	protein_coding
ENSG0000(	TMEM25	957.0673	-0.49995	0.003918	0.019063	protein_coding
ENSG0000(	ENSG0000(	124.777	0.279266	0.003921	0.019064	lncRNA
ENSG0000(	ENSG0000(	423.3032	0.168911	0.00392	0.019064	lncRNA
ENSG0000(	ENSG0000(	16.63982	0.487258	0.00392	0.019064	lncRNA
ENSG0000(	TSPAN11	699.4401	0.520785	0.003921	0.019064	protein_coding
ENSG0000(	VIM-AS1	145.657	0.451479	0.00392	0.019064	lncRNA
ENSG0000(	ENSG0000(	127.4217	-0.27123	0.003924	0.01907	transcribed_unprocessed_pseudo
ENSG0000(	NCSTN	1242.041	-0.16439	0.003924	0.01907	protein_coding
ENSG0000(	BMP1	1142.664	0.238373	0.003926	0.019073	protein_coding
ENSG0000(	ENTPD1	6680.972	0.296521	0.003926	0.019073	protein_coding

ENSG0000(	PUS10	1211.687	-0.22307	0.003933	0.019103	protein_coding
ENSG0000(	ACOT11	5007.03	-0.38777	0.003936	0.019114	protein_coding
ENSG0000(	COPS7A	520.8721	0.121716	0.003947	0.019165	protein_coding
ENSG0000(	ENSG0000(	39.92735	-0.26598	0.003948	0.019168	lncRNA
ENSG0000(	RASL11A	136.0505	-0.43119	0.003948	0.019168	protein_coding
ENSG0000(	ADAT1	2483.886	0.100138	0.00395	0.019174	protein_coding
ENSG0000(	MIB2	1072.184	-0.22331	0.003951	0.019175	protein_coding
ENSG0000(	ARMC7	271.5032	-0.19948	0.003954	0.019186	protein_coding
ENSG0000(	ENSG0000(	55.88352	0.263718	0.003955	0.019186	lncRNA
ENSG0000(	FOXN3	4548.291	-0.14361	0.003962	0.019219	protein_coding
ENSG0000(	STAT5A	1040.119	0.379058	0.003964	0.019223	protein_coding
ENSG0000(	TLDC2	764.0825	-0.58538	0.003964	0.019224	protein_coding
ENSG0000(	ZNF626	692.4477	-0.26069	0.003967	0.019235	protein_coding
ENSG0000(	ENSG0000(	54.46024	0.567381	0.00397	0.019245	lncRNA
ENSG0000(	ENSG0000(	240.0966	0.185103	0.003972	0.019253	lncRNA
ENSG0000(	MDH2	830.9216	-0.22376	0.003976	0.019269	protein_coding
ENSG0000(	DHRS4L2	238.9013	-0.28933	0.003981	0.019288	protein_coding
ENSG0000(	BRCC3	772.0327	-0.13128	0.003984	0.019295	protein_coding
ENSG0000(	LINC01187	8.313395	0.690132	0.003984	0.019295	lncRNA
ENSG0000(	TUSC2	231.6628	-0.17964	0.003983	0.019295	protein_coding
ENSG0000(	DCBLD1	1274.782	0.290237	0.003992	0.019332	protein_coding
ENSG0000(	CPEB1	207.9412	0.294191	0.003994	0.01934	protein_coding
ENSG0000(	DIO3OS	248.0869	-0.40193	0.004005	0.019384	lncRNA
ENSG0000(	DOC2A	243.5241	0.237127	0.004005	0.019384	protein_coding
ENSG0000(	MAOA	7172.11	-0.62542	0.004004	0.019384	protein_coding
ENSG0000(	SOWAHA	778.5288	-0.69768	0.004007	0.019391	protein_coding
ENSG0000(	CCBE1	629.5868	0.194897	0.004019	0.019443	protein_coding
ENSG0000(	ENSG0000(	186.6492	0.225424	0.00402	0.019443	lncRNA
ENSG0000(	OSCP1	32.96339	0.468469	0.00402	0.019443	protein_coding
ENSG0000(	ENSG0000(	230.1808	0.216697	0.004021	0.019445	TEC
ENSG0000(	XXYLT1-AS:	26.10957	0.547923	0.004021	0.019445	lncRNA
ENSG0000(	SORBS3	672.9455	-0.26543	0.004027	0.019463	protein_coding
ENSG0000(	STX7	6900.516	-0.18167	0.004026	0.019463	protein_coding
ENSG0000(	DNAH17	121.2562	0.353785	0.00403	0.019476	protein_coding
ENSG0000(	ENSG0000(	288.8252	0.168478	0.004035	0.019496	lncRNA
ENSG0000(	PLEKHB2	2803.447	-0.19193	0.004037	0.019504	protein_coding
ENSG0000(	CHP1	3642.398	-0.34891	0.004042	0.019522	protein_coding
ENSG0000(	GLI3	597.9034	0.457771	0.004042	0.019522	protein_coding
ENSG0000(	ENSG0000(	28.63834	0.874137	0.004043	0.019525	lncRNA
ENSG0000(	ENSG0000(	46.9852	0.251165	0.004044	0.019528	lncRNA
ENSG0000(	LINC00470	472.1739	0.172921	0.004048	0.019544	lncRNA
ENSG0000(	MTTP	4837.966	-0.70432	0.00405	0.019548	protein_coding
ENSG0000(	ATP5PB	978.2451	-0.274	0.004053	0.019554	protein_coding
ENSG0000(	ENSG0000(	25.75439	-0.43557	0.004053	0.019554	lncRNA
ENSG0000(	ZSCAN16	163.3353	-0.19639	0.004052	0.019554	protein_coding
ENSG0000(	FAM178B	105.948	-0.20644	0.004054	0.019557	protein_coding
ENSG0000(	MS4A6A	1739.941	0.280653	0.004055	0.019558	protein_coding

ENSG0000(	MARVELD2	1521.029	-0.31469	0.004057	0.019564	protein_coding
ENSG0000(	IKZF4	346.9894	0.191445	0.004059	0.01957	protein_coding
ENSG0000(	LRP8	1193.072	0.229198	0.00406	0.019571	protein_coding
ENSG0000(	RNF126	488.9046	-0.15154	0.004061	0.019575	protein_coding
ENSG0000(	TM9SF2	2436.962	-0.21008	0.004061	0.019575	protein_coding
ENSG0000(	CLCN4	157.6543	0.527975	0.004064	0.019585	protein_coding
ENSG0000(	ACVR2A	1020.646	-0.38601	0.004067	0.019597	protein_coding
ENSG0000(	CNGB1	124.3906	0.242073	0.00407	0.019603	protein_coding
ENSG0000(	WLS	680.9511	0.242778	0.004069	0.019603	protein_coding
ENSG0000(	ENSG0000(	75.14836	0.221776	0.004072	0.019609	lncRNA
ENSG0000(	ENSG0000(	48.32465	0.466171	0.004074	0.019616	processed_pseudogene
ENSG0000(	ATP5MF	442.3099	-0.27965	0.004076	0.019622	protein_coding
ENSG0000(	POLRMT	708.7521	-0.24037	0.004077	0.019622	protein_coding
ENSG0000(	RN7SL824F	12.60811	0.522087	0.004078	0.019622	misc_RNA
ENSG0000(	RPL36AP16	56.18818	0.315628	0.004076	0.019622	processed_pseudogene
ENSG0000(	PTGES3	2487.575	-0.16214	0.004079	0.019625	protein_coding
ENSG0000(	LINC01284	422.3406	0.142139	0.004081	0.01963	lncRNA
ENSG0000(	YWHABP2	37.11717	-0.34376	0.004081	0.01963	processed_pseudogene
ENSG0000(	CASP16P	658.9248	-0.4509	0.004083	0.019632	transcribed_unitary_pseudogene
ENSG0000(	ENSG0000(	46.68155	0.374655	0.004083	0.019632	lncRNA
ENSG0000(	LINC00622	64.70805	0.339479	0.004083	0.019632	lncRNA
ENSG0000(	PRADC1	100.7029	-0.38877	0.004086	0.01964	protein_coding
ENSG0000(	DUX4L26	21.591	0.940647	0.004088	0.01965	processed_pseudogene
ENSG0000(	FABP5	292.9318	-0.23895	0.004095	0.019677	protein_coding
ENSG0000(	ENSG0000(	77.26483	0.329528	0.004096	0.019679	transcribed_unprocessed_pseudo
ENSG0000(	ENSG0000(	99.53395	0.301698	0.004098	0.019683	lncRNA
ENSG0000(	RIOK3	3414.874	-0.35537	0.004098	0.019683	protein_coding
ENSG0000(	ENSG0000(	80.84878	0.254018	0.004099	0.019684	lncRNA
ENSG0000(	ENSG0000(	8.5439	-0.69534	0.004104	0.019709	lncRNA
ENSG0000(	ENSG0000(	28.66467	0.347728	0.004108	0.019723	lncRNA
ENSG0000(	ENSG0000(	75.31064	0.394467	0.004112	0.019741	lncRNA
ENSG0000(	SNURF	81.61405	-0.42328	0.004116	0.019755	protein_coding
ENSG0000(	CSRNP1	424.4662	0.298857	0.00412	0.019772	protein_coding
ENSG0000(	TIFAB	409.2736	0.481545	0.004121	0.019772	protein_coding
ENSG0000(	ENSG0000(	8.835047	0.83489	0.004122	0.019775	lncRNA
ENSG0000(	ENSG0000(	366.1797	0.220045	0.004123	0.019777	transcribed_unprocessed_pseudo
ENSG0000(	PSMD6	1155.38	-0.0974	0.004125	0.019786	protein_coding
ENSG0000(	ACOT9	1709.589	0.132623	0.004132	0.019816	protein_coding
ENSG0000(	NDUFS3	692.5893	-0.22516	0.004134	0.019819	protein_coding
ENSG0000(	ENSG0000(	34.71347	0.342967	0.004138	0.019829	lncRNA
ENSG0000(	ENSG0000(	158.9693	0.165329	0.004137	0.019829	lncRNA
ENSG0000(	ENSG0000(	167.5348	0.191001	0.004138	0.019829	lncRNA
ENSG0000(	METTL7B	311.2445	-0.64245	0.004137	0.019829	protein_coding
ENSG0000(	ENSG0000(	132.9095	0.163512	0.004154	0.019899	lncRNA
ENSG0000(	ZNF300	301.5927	0.212327	0.004154	0.019899	protein_coding
ENSG0000(	ENSG0000(	152.6664	-0.81439	0.004168	0.019964	unprocessed_pseudogene
ENSG0000(	H2BC7	130.9764	-0.37454	0.00417	0.019968	protein_coding

ENSG0000(	POMT2	1077.353	-0.10436	0.00417	0.019968	protein_coding
ENSG0000(	ENSG0000(	12.17452	0.492977	0.004172	0.019969	lncRNA
ENSG0000(	FAM189A2	176.7475	0.342659	0.004172	0.019969	protein_coding
ENSG0000(	CAPN8	890.9865	0.54071	0.004179	0.020002	protein_coding
ENSG0000(	LINC02393	37.01759	0.430889	0.004182	0.020007	lncRNA
ENSG0000(	LRRC36	301.9426	-0.17448	0.004181	0.020007	protein_coding
ENSG0000(	TDRKH-AS1	186.1842	-0.166	0.004182	0.020007	lncRNA
ENSG0000(	RPS5	1206.251	-0.29457	0.004187	0.020027	protein_coding
ENSG0000(	AK6	204.1184	-0.20092	0.004188	0.020028	protein_coding
ENSG0000(	AHR	2435.536	0.227014	0.004189	0.020032	protein_coding
ENSG0000(	LACTB2	172.0013	-0.2941	0.004191	0.020037	protein_coding
ENSG0000(	ENSG0000(	108.3582	0.26264	0.004193	0.020044	lncRNA
ENSG0000(	ENSG0000(	13.74162	0.51668	0.0042	0.020075	lncRNA
ENSG0000(	ANO6	2146.661	-0.2152	0.004202	0.020079	protein_coding
ENSG0000(	ENSG0000(	221.3596	-0.42609	0.004202	0.020079	lncRNA
ENSG0000(	PDIA4	1225.506	0.293843	0.004209	0.020106	protein_coding
ENSG0000(	ENSG0000(	151.7417	-0.18581	0.004213	0.020122	lncRNA
ENSG0000(	MRPL10	411.7363	-0.15638	0.004221	0.020156	protein_coding
ENSG0000(	ARHGEF40	974.9521	0.290552	0.004222	0.020162	protein_coding
ENSG0000(	ENSG0000(	235.7024	-0.15349	0.004223	0.020162	lncRNA
ENSG0000(	NAV2	1241.21	0.241217	0.004224	0.020162	protein_coding
ENSG0000(	PEX5	506.6111	-0.20038	0.004224	0.020162	protein_coding
ENSG0000(	BSN-DT	405.6336	0.12425	0.004225	0.020165	lncRNA
ENSG0000(	PLD3	1446.795	0.096993	0.004228	0.020175	protein_coding
ENSG0000(	DNAH10OS	298.0539	0.134135	0.00423	0.020181	lncRNA
ENSG0000(	ENSG0000(	106.5973	0.233513	0.004231	0.020181	lncRNA
ENSG0000(	ENSG0000(	40.7153	0.968108	0.004237	0.020207	unprocessed_pseudogene
ENSG0000(	DSP	6259.87	-0.42939	0.00425	0.020264	protein_coding
ENSG0000(	ENSG0000(	35.31795	-0.34734	0.00425	0.020264	lncRNA
ENSG0000(	DZIP1	672.6082	0.301602	0.004253	0.020273	protein_coding
ENSG0000(	CXCR1	53.67418	2.993132	0.004254	0.020275	protein_coding
ENSG0000(	RPL32	3322.158	-0.26437	0.004258	0.020291	protein_coding
ENSG0000(	ENSG0000(	10.62696	0.696239	0.004263	0.020313	lncRNA
ENSG0000(	CACNA1C-I	63.94039	0.522119	0.004267	0.020328	lncRNA
ENSG0000(	NINJ2	22.09073	0.542503	0.004269	0.020337	protein_coding
ENSG0000(	SPATC1	29.54137	0.770639	0.00427	0.020337	protein_coding
ENSG0000(	ENSG0000(	134.2781	0.210078	0.004271	0.020341	lncRNA
ENSG0000(	L3MBTL2-A	686.5641	0.110001	0.004274	0.020351	lncRNA
ENSG0000(	LINC02150	38.86807	0.400537	0.004276	0.020355	lncRNA
ENSG0000(	KIFAP3	601.1577	-0.22067	0.004278	0.020365	protein_coding
ENSG0000(	ERICH6-AS1	915.1471	0.148408	0.004283	0.020382	lncRNA
ENSG0000(	RHBDF1	365.5337	0.29168	0.004292	0.020422	protein_coding
ENSG0000(	ENSG0000(	254.446	0.172313	0.004294	0.020428	lncRNA
ENSG0000(	GPT	416.5057	-0.62672	0.004297	0.020438	protein_coding
ENSG0000(	HFE	693.1813	0.11038	0.004297	0.020438	protein_coding
ENSG0000(	CNN2P9	19.60769	0.416662	0.004301	0.020455	processed_pseudogene
ENSG0000(	ENSG0000(	164.3445	-0.34279	0.004305	0.020465	protein_coding

ENSG0000(	IQCB2P	21.98341	0.46896	0.004304	0.020465	processed_pseudogene
ENSG0000(	FYB1	3568.055	0.448258	0.004306	0.020469	protein_coding
ENSG0000(	AQP3	1536.01	-0.68596	0.00431	0.020481	protein_coding
ENSG0000(	HMGN2	3250.497	-0.18285	0.00431	0.020481	protein_coding
ENSG0000(	PHETA1	820.2056	-0.51962	0.004311	0.020481	protein_coding
ENSG0000(	ENSG0000(	18.65503	0.391564	0.004312	0.020483	lncRNA
ENSG0000(	HNRNPA1	9056.099	-0.14601	0.004312	0.020483	protein_coding
ENSG0000(	SYNE4	160.4821	-0.19312	0.004322	0.020526	protein_coding
ENSG0000(	CEACAM18	219.8029	-0.89857	0.004324	0.020532	protein_coding
ENSG0000(	ENSG0000(	481.7433	-0.15066	0.004325	0.020533	lncRNA
ENSG0000(	ENSG0000(	12.14726	0.755653	0.004325	0.020533	transcribed_unitary_pseudogene
ENSG0000(	ZFPM2	416.4134	0.203864	0.004326	0.020533	protein_coding
ENSG0000(	ENSG0000(	138.1885	0.20214	0.00433	0.020547	lncRNA
ENSG0000(	ADAP2	632.5054	0.191657	0.004333	0.02056	protein_coding
ENSG0000(	KRT12	75.30022	-0.63441	0.004335	0.020567	protein_coding
ENSG0000(	ENSG0000(	106.1669	0.209131	0.004341	0.020587	lncRNA
ENSG0000(	TM4SF5	165.2997	-0.85644	0.004341	0.020587	protein_coding
ENSG0000(	CNIH3-AS2	1087.198	0.170245	0.004346	0.020608	lncRNA
ENSG0000(	SENP7	1919.79	-0.13572	0.004347	0.020608	protein_coding
ENSG0000(	ENSG0000(	16.14288	0.51019	0.004353	0.020636	snoRNA
ENSG0000(	HMGB1	3980.164	-0.1484	0.004366	0.020696	protein_coding
ENSG0000(	DDX5	19196.73	0.171248	0.004369	0.0207	protein_coding
ENSG0000(	ENSG0000(	99.46251	0.241601	0.004369	0.0207	lncRNA
ENSG0000(	GRM8	29.59512	0.535476	0.004369	0.0207	protein_coding
ENSG0000(	DAPP1	1034.994	0.597921	0.004372	0.020707	protein_coding
ENSG0000(	PMEPA1	717.1135	0.303638	0.004371	0.020707	protein_coding
ENSG0000(	SNRPF	172.4481	-0.19476	0.004378	0.020734	protein_coding
ENSG0000(	SLC12A7	3586.224	-0.31757	0.004383	0.020753	protein_coding
ENSG0000(	ENSG0000(	113.1207	0.209784	0.004385	0.020761	lncRNA
ENSG0000(	FAM13B	2078.271	-0.18026	0.004388	0.020769	protein_coding
ENSG0000(	RPL18	1929.31	-0.23857	0.004391	0.020782	protein_coding
ENSG0000(	RNU4-82P	15.27026	0.446644	0.004397	0.020808	snRNA
ENSG0000(	ENSG0000(	46.20315	0.291844	0.004399	0.020814	lncRNA
ENSG0000(	TIAM2	1509.214	-0.30016	0.0044	0.020816	protein_coding
ENSG0000(	KCNJ13	1548.159	-0.81938	0.004405	0.020836	protein_coding
ENSG0000(	ENSG0000(	20.54509	0.555471	0.004407	0.020841	processed_pseudogene
ENSG0000(	ENSG0000(	29.08872	0.5141	0.004408	0.020843	processed_pseudogene
ENSG0000(	SENP2	1716.184	-0.15518	0.004409	0.020845	protein_coding
ENSG0000(	RN7SL408F	37.15726	0.317525	0.004415	0.020869	misc_RNA
ENSG0000(	C1GALT1	2010.65	0.191575	0.004416	0.020874	protein_coding
ENSG0000(	LRRFIP1P1	224.7804	0.26832	0.004419	0.020877	processed_pseudogene
ENSG0000(	PARK7	654.8442	-0.13668	0.004418	0.020877	protein_coding
ENSG0000(	LINC00868	194.9966	0.273838	0.004423	0.020894	lncRNA
ENSG0000(	RN7SL870F	13.2477	0.513923	0.004426	0.020907	misc_RNA
ENSG0000(	PIDD1	574.5881	-0.21658	0.004431	0.020928	protein_coding
ENSG0000(	PRR15	636.5582	-0.62415	0.004437	0.020954	protein_coding
ENSG0000(	RNF217-AS	535.0396	0.11125	0.004438	0.020955	lncRNA

ENSG0000(	GEMIN6	1286.115	-0.08449	0.004442	0.020968	protein_coding
ENSG0000(	ASPSCR1	1280.524	0.100866	0.004442	0.020968	protein_coding
ENSG0000(	PIGBOS1	250.9193	-0.14975	0.004452	0.021006	protein_coding
ENSG0000(	RPS27L	2563.168	-0.19308	0.004452	0.021006	protein_coding
ENSG0000(	SLFN5	4712.533	0.377327	0.004452	0.021006	protein_coding
ENSG0000(	EIF6	498.896	-0.32394	0.004454	0.021009	protein_coding
ENSG0000(	MAP7D2	89.97926	-0.69293	0.004455	0.021011	protein_coding
ENSG0000(	MIR5194	44.66251	0.436203	0.004456	0.021011	miRNA
ENSG0000(	ZNF625	151.3192	0.195736	0.004456	0.021011	protein_coding
ENSG0000(	LTO1	1302.174	0.10865	0.00447	0.021073	protein_coding
ENSG0000(	MOV10L1	151.8631	0.312192	0.004474	0.021088	protein_coding
ENSG0000(	ENSG0000(	42.85783	0.53575	0.004475	0.02109	lncRNA
ENSG0000(	ENSG0000(	34.60587	-0.56524	0.004478	0.0211	lncRNA
ENSG0000(	TOMM6	260.6196	-0.21055	0.004478	0.0211	protein_coding
ENSG0000(	GNPTG	1004.295	-0.12757	0.00448	0.021106	protein_coding
ENSG0000(	GBAP1	191.027	0.434567	0.004484	0.021118	transcribed_unprocessed_pseudo
ENSG0000(	SLC16A9	128.693	-0.80146	0.004484	0.021118	protein_coding
ENSG0000(	ENSG0000(	453.6538	0.113009	0.004491	0.021148	protein_coding
ENSG0000(	OTOF	22.02438	0.770647	0.004496	0.021167	protein_coding
ENSG0000(	BZW1	2460.966	-0.22054	0.0045	0.021173	protein_coding
ENSG0000(	CLK2	1420.491	-0.0936	0.004498	0.021173	protein_coding
ENSG0000(	ENSG0000(	111.108	0.18927	0.004499	0.021173	lncRNA
ENSG0000(	KLHL11	828.9184	-0.12773	0.004501	0.021173	protein_coding
ENSG0000(	OTOP3	25.59879	-1.34155	0.004499	0.021173	protein_coding
ENSG0000(	SRCIN1	220.1168	-0.27535	0.004502	0.021173	protein_coding
ENSG0000(	TNFSF8	362.1759	0.679569	0.004502	0.021173	protein_coding
ENSG0000(	C1orf35	177.7425	-0.21953	0.004503	0.021174	protein_coding
ENSG0000(	CDC5L	1272.602	-0.11061	0.004504	0.021174	protein_coding
ENSG0000(	SFRP5	263.8544	-0.92225	0.004506	0.021181	protein_coding
ENSG0000(	USP45	922.2776	-0.15748	0.004507	0.021185	protein_coding
ENSG0000(	TFE3	1018.284	0.153347	0.00451	0.021195	protein_coding
ENSG0000(	MBIP	386.2166	-0.20965	0.004514	0.02121	protein_coding
ENSG0000(	B3GAT1	162.8826	0.278424	0.004517	0.02122	protein_coding
ENSG0000(	HOXD1	78.51457	-0.4871	0.004517	0.02122	protein_coding
ENSG0000(	SCTR	79.67379	0.203158	0.004518	0.02122	protein_coding
ENSG0000(	FLJ31104	374.0725	0.181701	0.004521	0.021228	lncRNA
ENSG0000(	PEX11B	256.2393	-0.15733	0.004521	0.021228	protein_coding
ENSG0000(	CDX1	895.6113	-0.4982	0.004522	0.021228	protein_coding
ENSG0000(	ENSG0000(	26.43215	0.399482	0.004525	0.021235	lncRNA
ENSG0000(	NSUN5P1	5002.932	-0.13144	0.004526	0.021235	transcribed_unprocessed_pseudo
ENSG0000(	RPS15AP1(	153.3887	-0.24162	0.004524	0.021235	processed_pseudogene
ENSG0000(	SRGAP2C	741.2445	0.268689	0.004526	0.021235	protein_coding
ENSG0000(	GHR	541.0357	-0.12519	0.00453	0.021249	protein_coding
ENSG0000(	ENSG0000(	91.63041	0.351741	0.004531	0.021253	lncRNA
ENSG0000(	ENSG0000(	152.8969	0.200681	0.004536	0.021272	lncRNA
ENSG0000(	NUP210L	22.79578	0.509441	0.004542	0.021298	protein_coding
ENSG0000(	MT-TL2	50.47466	1.022663	0.004545	0.021306	Mt_tRNA

ENSG0000(	STK4-AS1	56.78171	0.27024	0.004545	0.021306	lncRNA
ENSG0000(	ENSG0000(	8.819748	0.552096	0.004546	0.021307	lncRNA
ENSG0000(	FLJ42393	652.3643	0.195867	0.004552	0.021333	TEC
ENSG0000(	ENSG0000(	254.5081	-0.14756	0.004554	0.021334	lncRNA
ENSG0000(	PRPF6	947.5477	-0.15734	0.004553	0.021334	protein_coding
ENSG0000(	LINC02558	857.4903	0.135444	0.004556	0.02134	lncRNA
ENSG0000(	PRDM4	1115.283	-0.17725	0.004556	0.02134	protein_coding
ENSG0000(	CTDNEP1	1024.097	-0.15146	0.00456	0.021356	protein_coding
ENSG0000(	PLA2G12B	431.772	-0.65784	0.004561	0.021356	protein_coding
ENSG0000(	TSHZ3-AS1	9.909095	0.557624	0.004569	0.021389	lncRNA
ENSG0000(	DGCR2	1705.247	-0.13185	0.004572	0.021396	protein_coding
ENSG0000(	ENSG0000(	80.19734	0.237409	0.004572	0.021396	lncRNA
ENSG0000(	PTGR1	4020.046	-0.4759	0.004571	0.021396	protein_coding
ENSG0000(	CMTR1	1483.372	0.162009	0.00458	0.021427	protein_coding
ENSG0000(	TMEM70	158.9183	-0.3174	0.004584	0.021442	protein_coding
ENSG0000(	GEM	424.5704	0.411079	0.004586	0.02145	protein_coding
ENSG0000(	LINC00887	251.7219	-0.20442	0.004591	0.021468	lncRNA
ENSG0000(	PHKB	3837.54	0.080149	0.004592	0.021471	protein_coding
ENSG0000(	HIVEP3	1117.105	0.256065	0.004594	0.021478	protein_coding
ENSG0000(	ADGRG2	596.4781	-0.21846	0.004596	0.021482	protein_coding
ENSG0000(	ALOX5AP	129.9036	0.50409	0.004597	0.021486	protein_coding
ENSG0000(	FENDRR	2192.922	0.246949	0.004604	0.021511	lncRNA
ENSG0000(	FLVCR1-DT	640.4209	-0.19917	0.004603	0.021511	lncRNA
ENSG0000(	LAMTOR2	131.8498	-0.25337	0.004604	0.021511	protein_coding
ENSG0000(	TMED4	3039.405	-0.23977	0.004624	0.021597	protein_coding
ENSG0000(	CLDN4	1721.934	-0.47014	0.004625	0.021601	protein_coding
ENSG0000(	NDUFB4	352.5853	-0.23178	0.004627	0.021606	protein_coding
ENSG0000(	UGCG	2246.187	-0.20545	0.004634	0.021636	protein_coding
ENSG0000(	EPM2A	1052.198	0.179093	0.004641	0.021666	protein_coding
ENSG0000(	ENSG0000(	68.38663	0.238912	0.004643	0.021668	lncRNA
ENSG0000(	RPL21P99	21.18353	0.493209	0.004642	0.021668	processed_pseudogene
ENSG0000(	IGLV4-60	17.50424	1.099078	0.004646	0.021675	IG_V_gene
ENSG0000(	TXNDC12	381.8956	-0.22672	0.004646	0.021675	protein_coding
ENSG0000(	SSB	578.0607	-0.16944	0.004647	0.021679	protein_coding
ENSG0000(	LINC01625	144.3852	0.175177	0.004652	0.021693	lncRNA
ENSG0000(	USP9X	7260.767	-0.17308	0.004652	0.021693	protein_coding
ENSG0000(	ODCP	179.3237	0.144607	0.004658	0.021721	processed_pseudogene
ENSG0000(	ENSG0000(	115.6695	0.245723	0.004665	0.021747	lncRNA
ENSG0000(	ENSG0000(	76.45083	-0.3609	0.004673	0.021777	lncRNA
ENSG0000(	LINC01572	492.2139	0.149196	0.004673	0.021777	lncRNA
ENSG0000(	ZFYVE21	1102.792	-0.15434	0.004672	0.021777	protein_coding
ENSG0000(	KATNA1	209.0208	-0.14909	0.004682	0.021815	protein_coding
ENSG0000(	PM20D2	833.2707	-0.15073	0.004683	0.021816	protein_coding
ENSG0000(	SNORD83A	23.69043	0.442923	0.004688	0.021836	snoRNA
ENSG0000(	TSR3	136.1541	-0.2703	0.004692	0.021853	protein_coding
ENSG0000(	ZFP3	913.4024	-0.15294	0.004694	0.021856	protein_coding
ENSG0000(	MCCC1	744.4604	-0.21519	0.0047	0.021879	protein_coding

ENSG0000(	UROD	285.0566	-0.17416	0.004699	0.021879	protein_coding
ENSG0000(	KRT7	15.72374	0.864175	0.004701	0.021881	protein_coding
ENSG0000(	RASGRP4	258.1069	0.220873	0.004708	0.021909	protein_coding
ENSG0000(	MT-TS2	58.99464	0.959612	0.004712	0.021926	Mt_tRNA
ENSG0000(	USH1G	45.78504	-0.5624	0.004714	0.021931	protein_coding
ENSG0000(	MFN2	2970.755	-0.25535	0.004724	0.021973	protein_coding
ENSG0000(	ENSG0000(	14.45782	0.796696	0.004726	0.02198	protein_coding
ENSG0000(	AAMP	568.602	-0.23513	0.004727	0.021983	protein_coding
ENSG0000(	C10orf55	19.95501	0.328687	0.00473	0.021994	lncRNA
ENSG0000(	RPP14	1964.261	-0.1139	0.004735	0.022012	protein_coding
ENSG0000(	ENSG0000(	129.3038	0.29035	0.004736	0.022015	transcribed_unprocessed_pseudo
ENSG0000(	LNX2	1372.042	-0.3505	0.00474	0.022028	protein_coding
ENSG0000(	ENSG0000(	19.00996	0.436514	0.004744	0.022034	lncRNA
ENSG0000(	ENSG0000(	11.24952	0.530754	0.004743	0.022034	lncRNA
ENSG0000(	ENSG0000(	80.84786	0.201449	0.004742	0.022034	lncRNA
ENSG0000(	RAB3D	694.9857	0.197376	0.004744	0.022034	protein_coding
ENSG0000(	ZNF883	130.2695	0.217643	0.004744	0.022034	transcribed_unprocessed_pseudo
ENSG0000(	RNASEH1	1166.237	0.109189	0.004746	0.022038	protein_coding
ENSG0000(	ENSG0000(	148.2353	-0.69652	0.004748	0.022039	transcribed_unprocessed_pseudo
ENSG0000(	HOXA5	486.9253	-0.37933	0.004748	0.022039	protein_coding
ENSG0000(	P3H3	257.6828	0.266586	0.004748	0.022039	protein_coding
ENSG0000(	PDLIM1P4	37.22734	0.421132	0.004749	0.022039	processed_pseudogene
ENSG0000(	SULT1A1	3326.888	-0.41126	0.00475	0.022042	protein_coding
ENSG0000(	ENSG0000(	124.4973	0.189115	0.004755	0.022055	lncRNA
ENSG0000(	ENSG0000(	13.41887	0.633099	0.004754	0.022055	lncRNA
ENSG0000(	RFX1	466.7916	-0.17002	0.004755	0.022055	protein_coding
ENSG0000(	ENSG0000(	41.69616	0.309239	0.004758	0.022063	lncRNA
ENSG0000(	PCGF5	2164.382	-0.19058	0.004758	0.022063	protein_coding
ENSG0000(	NUP50	2533.214	0.120577	0.00476	0.022065	protein_coding
ENSG0000(	VILL	2212.998	-0.46539	0.004767	0.022098	protein_coding
ENSG0000(	ENSG0000(	130.8973	0.205069	0.004769	0.022103	lncRNA
ENSG0000(	SPRY3	503.0911	0.200383	0.004772	0.022113	protein_coding
ENSG0000(	HOXB7	186.4848	-0.27878	0.004773	0.022114	protein_coding
ENSG0000(	ENSG0000(	919.9786	-0.16037	0.004776	0.022124	TEC
ENSG0000(	ITPK1	2076.378	-0.32911	0.004791	0.022192	protein_coding
ENSG0000(	BMP6	83.70293	0.543253	0.004795	0.022209	protein_coding
ENSG0000(	TNK1	264.359	-0.29652	0.004799	0.022223	protein_coding
ENSG0000(	ENSG0000(	293.2286	-0.12208	0.004805	0.022245	lncRNA
ENSG0000(	LYPLAL1-AS	331.2545	0.188172	0.004806	0.022247	lncRNA
ENSG0000(	NPY4R2	104.1885	-0.8227	0.004808	0.022252	protein_coding
ENSG0000(	ACSM3	488.9272	0.158443	0.00481	0.022258	protein_coding
ENSG0000(	AMMECR1-	95.29765	-0.29637	0.004812	0.022262	lncRNA
ENSG0000(	HOMER2	483.9806	0.255194	0.004812	0.022262	protein_coding
ENSG0000(	ENSG0000(	1037.081	0.126668	0.004817	0.02228	lncRNA
ENSG0000(	LINC01926	20.75995	0.328294	0.00482	0.022293	lncRNA
ENSG0000(	IGLC7	33.86146	0.982383	0.004824	0.02231	IG_C_gene
ENSG0000(	MYL6B	246.6017	0.173246	0.004826	0.022313	protein_coding

ENSG0000(	PRORP	1472.456	0.14263	0.004827	0.022317	protein_coding
ENSG0000(	IFT22	1617.746	0.07704	0.00483	0.022322	protein_coding
ENSG0000(	ZNF330	394.2887	-0.14176	0.00483	0.022322	protein_coding
ENSG0000(	PSMD7-DT	578.1965	0.098294	0.004836	0.022349	lncRNA
ENSG0000(	PPP1CA	932.4756	-0.19386	0.004838	0.022355	protein_coding
ENSG0000(	MCUR1	1679.865	-0.19935	0.004843	0.022374	protein_coding
ENSG0000(	ENSG0000(	1862.642	0.122782	0.004848	0.022392	lncRNA
ENSG0000(	INTS6-AS1	916.6277	0.134251	0.004849	0.022392	lncRNA
ENSG0000(	CFAP99	17.98845	0.671751	0.004854	0.022413	protein_coding
ENSG0000(	OR2A1	17.36092	0.735171	0.004854	0.022413	protein_coding
ENSG0000(	HOMER1	150.8727	0.304658	0.004857	0.022423	protein_coding
ENSG0000(	ENSG0000(	34.2001	0.349868	0.004859	0.022429	transcribed_unitary_pseudogene
ENSG0000(	ENSG0000(	75.82219	0.326172	0.004863	0.022442	lncRNA
ENSG0000(	PIWIL4	483.6142	-0.22837	0.004864	0.022443	protein_coding
ENSG0000(	PI4KA	3756.774	-0.11491	0.004867	0.022454	protein_coding
ENSG0000(	NDN	91.61667	-0.33182	0.004869	0.02246	protein_coding
ENSG0000(	ENSG0000(	95.26775	0.22188	0.004872	0.022469	lncRNA
ENSG0000(	LINC02615	412.0819	-0.21799	0.004875	0.022479	lncRNA
ENSG0000(	CCDC125	4486.536	0.081363	0.004876	0.022485	protein_coding
ENSG0000(	ENSG0000(	14.10368	0.499682	0.004878	0.022488	lncRNA
ENSG0000(	ENSG0000(	725.6797	-0.1188	0.004896	0.022569	lncRNA
ENSG0000(	H2AC4	40.57511	-0.43823	0.004897	0.022572	protein_coding
ENSG0000(	ACTG1P25	190.4042	-0.33141	0.0049	0.022581	transcribed_processed_pseudoge
ENSG0000(	ENSG0000(	827.0825	0.472398	0.004903	0.022588	lncRNA
ENSG0000(	COQ4	2095.125	-0.13868	0.00491	0.022615	protein_coding
ENSG0000(	FAM86DP	399.1605	0.176393	0.00491	0.022615	transcribed_unprocessed_pseudo
ENSG0000(	LINC01301	297.554	0.153974	0.004911	0.022616	lncRNA
ENSG0000(	RMDN2	904.5557	-0.40592	0.004914	0.022627	protein_coding
ENSG0000(	PLA2R1	4234.134	-0.39277	0.004915	0.022628	protein_coding
ENSG0000(	CLRN3	606.3221	-0.58717	0.00492	0.022648	protein_coding
ENSG0000(	ENSG0000(	82.09286	0.314355	0.004924	0.022664	lncRNA
ENSG0000(	TARDBP	3464.128	-0.1004	0.004928	0.022679	protein_coding
ENSG0000(	ENSG0000(	123.9745	0.219329	0.00493	0.022686	lncRNA
ENSG0000(	MYO1F	1556.437	0.270897	0.004932	0.022689	protein_coding
ENSG0000(	MIS18BP1	1882.206	0.155419	0.004933	0.02269	protein_coding
ENSG0000(	ENSG0000(	160.9642	-0.16689	0.004935	0.022696	lncRNA
ENSG0000(	ENSG0000(	199.2174	-0.1562	0.004946	0.022746	lncRNA
ENSG0000(	FASTK	981.5904	-0.1641	0.004952	0.022768	protein_coding
ENSG0000(	AMD1P1	11.91872	0.606688	0.004954	0.022775	processed_pseudogene
ENSG0000(	ENSG0000(	65.58265	0.275514	0.004955	0.022775	lncRNA
ENSG0000(	CDK11A	989.5662	-0.26464	0.004955	0.022776	protein_coding
ENSG0000(	CCNT2-AS1	604.3842	-0.41481	0.004957	0.02278	lncRNA
ENSG0000(	ITGA9	1076.495	-0.27923	0.004958	0.02278	protein_coding
ENSG0000(	ENSG0000(	15.45467	-0.67105	0.004962	0.022784	lncRNA
ENSG0000(	ENSG0000(	201.9048	0.163469	0.004963	0.022784	lncRNA
ENSG0000(	ENSG0000(	120.1612	0.273149	0.004962	0.022784	lncRNA
ENSG0000(	PCBP3	334.5902	0.393608	0.004962	0.022784	protein_coding

ENSG0000(	RAB11FIP4	2506.538	-0.15925	0.004962	0.022784	protein_coding
ENSG0000(	TMUB1	329.1615	-0.22469	0.004963	0.022784	protein_coding
ENSG0000(	ENSG0000(	70.1099	0.272111	0.004971	0.022819	lncRNA
ENSG0000(	OBI1-AS1	15.72174	0.830359	0.004977	0.02284	lncRNA
ENSG0000(	PSMB1	941.066	-0.17903	0.004977	0.02284	protein_coding
ENSG0000(	RPS3AP12	44.3054	0.275394	0.004981	0.022853	processed_pseudogene
ENSG0000(	ZNF887P	32.82553	0.484342	0.004983	0.022861	transcribed_unprocessed_pseudo
ENSG0000(	TEPP	121.4018	-0.24155	0.004986	0.022868	protein_coding
ENSG0000(	RTL8B	42.56516	0.372127	0.004994	0.022901	protein_coding
ENSG0000(	TMEM39A	1053.876	0.174389	0.005004	0.022947	protein_coding
ENSG0000(	C11orf96	160.4779	0.35129	0.005007	0.022952	protein_coding
ENSG0000(	ENSG0000(	474.0978	0.161135	0.005007	0.022952	lncRNA
ENSG0000(	MYZAP	321.0751	-0.59759	0.005008	0.022952	protein_coding
ENSG0000(	TLR1	854.3358	0.50568	0.005008	0.022952	protein_coding
ENSG0000(	MICU2	703.3934	-0.11549	0.005011	0.022958	protein_coding
ENSG0000(	RN7SL364F	61.70039	0.257642	0.005011	0.022958	misc_RNA
ENSG0000(	AURKC	19.99347	0.547302	0.005018	0.022984	protein_coding
ENSG0000(	RBM15-AS:	228.7102	-0.30256	0.005021	0.022994	lncRNA
ENSG0000(	CENPV	811.7865	-0.34679	0.005026	0.023013	protein_coding
ENSG0000(	LY96	37.69767	0.634045	0.005026	0.023013	protein_coding
ENSG0000(	ENSG0000(	98.92316	0.205126	0.005036	0.023056	lncRNA
ENSG0000(	ENSG0000(	1618.435	0.147065	0.005039	0.023067	TEC
ENSG0000(	TMPO	2852.877	-0.15653	0.005042	0.023077	protein_coding
ENSG0000(	ENSG0000(	198.6131	-0.19237	0.005044	0.023079	lncRNA
ENSG0000(	PPM1G	761.9666	-0.14572	0.005044	0.023079	protein_coding
ENSG0000(	PDCD2L	36.68661	-0.43728	0.005053	0.023117	protein_coding
ENSG0000(	ENSG0000(	45.6882	0.383158	0.005054	0.023119	lncRNA
ENSG0000(	ENSG0000(	59.90873	-0.42271	0.005061	0.023147	TEC
ENSG0000(	RN7SL48P	357.9248	0.128168	0.005064	0.023157	misc_RNA
ENSG0000(	B3GAT2	35.50622	0.286433	0.005066	0.023161	protein_coding
ENSG0000(	ENSG0000(	60.77325	0.234482	0.005066	0.023161	lncRNA
ENSG0000(	ENSG0000(	14.42773	0.497795	0.005075	0.023192	lncRNA
ENSG0000(	LRFN4	101.0751	0.42126	0.005074	0.023192	protein_coding
ENSG0000(	PTPRA	1034.718	-0.1626	0.005076	0.023192	protein_coding
ENSG0000(	CYP27A1	788.5631	-0.39764	0.005082	0.02322	protein_coding
ENSG0000(	ENSG0000(	58.13732	0.291528	0.005084	0.023226	processed_pseudogene
ENSG0000(	ENSG0000(	114.283	0.284722	0.005089	0.02324	lncRNA
ENSG0000(	TBCEL-TEC	25.40037	0.442803	0.005089	0.02324	protein_coding
ENSG0000(	PAFAH2	1073.42	-0.211	0.005092	0.023247	protein_coding
ENSG0000(	VPS4A	1407.121	0.115803	0.005092	0.023247	protein_coding
ENSG0000(	ENSG0000(	543.0866	0.113831	0.005093	0.02325	lncRNA
ENSG0000(	CXXC4	280.2843	-0.51122	0.005098	0.023266	protein_coding
ENSG0000(	SCPEP1	620.2614	0.187567	0.005102	0.023282	protein_coding
ENSG0000(	RPL22	2330.709	-0.26313	0.005104	0.023286	protein_coding
ENSG0000(	ARFGEF3	1752.254	0.375248	0.005105	0.023288	protein_coding
ENSG0000(	RLIM	2924.793	0.124006	0.005105	0.023288	protein_coding
ENSG0000(	UTRN	11728.84	0.226925	0.005107	0.023291	protein_coding

ENSG0000(	ENSG0000(	68.77899	0.357232	0.005111	0.023307	lncRNA
ENSG0000(	TNFRSF4	63.0043	0.634045	0.005117	0.023331	protein_coding
ENSG0000(	ENSG0000(	52.10158	0.249429	0.005121	0.023344	lncRNA
ENSG0000(	ENSG0000(	653.1491	0.13312	0.005127	0.023367	lncRNA
ENSG0000(	CCNB3P1	196.6455	0.149745	0.005133	0.023395	processed_pseudogene
ENSG0000(	CCDC90B-A	341.692	0.211125	0.005135	0.023398	lncRNA
ENSG0000(	ENSG0000(	19.27967	0.464722	0.005135	0.023398	lncRNA
ENSG0000(	COQ7-DT	95.60161	-0.55826	0.005138	0.023406	lncRNA
ENSG0000(	ARAF	867.1791	0.138863	0.005144	0.023428	protein_coding
ENSG0000(	ENSG0000(	12.49244	0.525133	0.005154	0.023464	lncRNA
ENSG0000(	STMN2	43.8454	-0.49452	0.005153	0.023464	protein_coding
ENSG0000(	ZNF595	786.0048	-0.24539	0.005154	0.023464	protein_coding
ENSG0000(	PLCB3	3945.46	-0.50535	0.005157	0.023477	protein_coding
ENSG0000(	EEPD1	627.497	-0.50728	0.005165	0.023507	protein_coding
ENSG0000(	SARNP	619.0525	-0.13625	0.005165	0.023507	protein_coding
ENSG0000(	FAM167A-/	166.8547	0.255921	0.005168	0.023514	lncRNA
ENSG0000(	RPS27	3097.154	-0.30639	0.005168	0.023514	protein_coding
ENSG0000(	NLGN3	579.9326	0.107285	0.005174	0.023532	protein_coding
ENSG0000(	OR6A2	62.49307	0.510964	0.005173	0.023532	protein_coding
ENSG0000(	RN7SL3	3885.944	0.454967	0.005177	0.023541	misc_RNA
ENSG0000(	ALDOC	383.0255	-0.62088	0.005178	0.023544	protein_coding
ENSG0000(	ENSG0000(	13.08189	0.671699	0.005179	0.023544	lncRNA
ENSG0000(	ENSG0000(	141.0842	-0.24387	0.005185	0.023571	lncRNA
ENSG0000(	LRP11	354.8329	0.282496	0.005187	0.023575	protein_coding
ENSG0000(	CDK13-DT	105.023	0.183939	0.005189	0.023577	lncRNA
ENSG0000(	PBXIP1	1983.015	-0.21092	0.005189	0.023577	protein_coding
ENSG0000(	ZBTB47	297.2083	0.298158	0.005194	0.023597	protein_coding
ENSG0000(	ENSG0000(	149.5044	0.272611	0.005196	0.023601	lncRNA
ENSG0000(	ENSG0000(	24.74793	-0.40506	0.005198	0.023609	lncRNA
ENSG0000(	ENSG0000(	17.25108	0.392494	0.005201	0.023617	lncRNA
ENSG0000(	RNU6-343F	13.37369	0.497557	0.005202	0.023619	snRNA
ENSG0000(	ENSG0000(	45.54556	-0.31832	0.005204	0.023624	lncRNA
ENSG0000(	FCRL4	228.7942	-0.96997	0.005205	0.023627	protein_coding
ENSG0000(	WSCD1	453.1614	-0.59639	0.005207	0.023633	protein_coding
ENSG0000(	ASZ1	172.3966	0.224957	0.005213	0.023654	protein_coding
ENSG0000(	SNORD100	15.55935	0.467614	0.00522	0.023686	snoRNA
ENSG0000(	TIGD5	280.0831	-0.13911	0.005227	0.023713	protein_coding
ENSG0000(	PARP8	2741.54	0.235814	0.005228	0.023715	protein_coding
ENSG0000(	ARL4AP4	10.94915	0.49953	0.005238	0.023755	processed_pseudogene
ENSG0000(	ENSG0000(	9.125255	0.462292	0.005244	0.023777	TEC
ENSG0000(	ZBTB7B	3343.791	-0.42881	0.005247	0.023789	protein_coding
ENSG0000(	CD44-AS1	166.5932	0.231377	0.005253	0.023808	lncRNA
ENSG0000(	ENSG0000(	257.8907	0.171633	0.005252	0.023808	lncRNA
ENSG0000(	YY2	123.2782	0.190741	0.005253	0.023808	protein_coding
ENSG0000(	USP21P1	216.6588	-0.16014	0.005263	0.023848	processed_pseudogene
ENSG0000(	PRKCZ	1557.066	-0.35954	0.005265	0.023855	protein_coding
ENSG0000(	RPL36	1145.732	-0.24021	0.005267	0.023859	protein_coding

ENSG0000(	ZMYND11	1271.989	-0.12891	0.005272	0.023879	protein_coding
ENSG0000(	ENSG0000(	320.6665	0.175422	0.005275	0.023887	lncRNA
ENSG0000(	ENSG0000(	185.7298	0.167117	0.005276	0.023891	lncRNA
ENSG0000(	RNU5E-4P	8.982409	0.714718	0.00528	0.023905	snRNA
ENSG0000(	ZBTB46-AS	62.44864	0.282765	0.005286	0.023929	lncRNA
ENSG0000(	ENSG0000(	19.02559	0.512526	0.005293	0.023954	lncRNA
ENSG0000(	ENSG0000(	15.17677	0.652862	0.005294	0.023957	lncRNA
ENSG0000(	LINC01876	232.6197	0.413897	0.005295	0.023957	lncRNA
ENSG0000(	ODC1-DT	661.6651	0.151654	0.005295	0.023957	lncRNA
ENSG0000(	SLC22A15	222.366	0.364511	0.005299	0.023972	protein_coding
ENSG0000(	CD160	211.837	-0.39019	0.005308	0.024009	protein_coding
ENSG0000(	MRPL45	208.4248	-0.19122	0.005318	0.024047	protein_coding
ENSG0000(	PLEKHA1	3600.151	-0.25252	0.005319	0.02405	protein_coding
ENSG0000(	ALDH1A3	923.0583	-0.3591	0.005322	0.024056	protein_coding
ENSG0000(	ENSG0000(	11.59959	0.939663	0.005323	0.024056	lncRNA
ENSG0000(	HMGA1	1145.211	-0.21118	0.005322	0.024056	protein_coding
ENSG0000(	APH1B	461.8933	0.309231	0.005325	0.024062	protein_coding
ENSG0000(	ENSG0000(	159.2126	0.300933	0.005328	0.024075	lncRNA
ENSG0000(	PRDX1	1002.166	-0.33274	0.005337	0.024109	protein_coding
ENSG0000(	GXYLT1	1103.014	-0.1298	0.005342	0.024128	protein_coding
ENSG0000(	CBLL1P1	164.1253	0.168106	0.005343	0.024131	processed_pseudogene
ENSG0000(	ENSG0000(	37.46117	0.325184	0.005345	0.024135	lncRNA
ENSG0000(	WWTR1	672.1687	0.238674	0.005356	0.024184	protein_coding
ENSG0000(	STIL	888.6972	0.143395	0.005358	0.02419	protein_coding
ENSG0000(	ENSG0000(	31.58172	0.370258	0.00536	0.024195	lncRNA
ENSG0000(	BMP5	796.7991	-0.39112	0.005367	0.024222	protein_coding
ENSG0000(	H4-16	797.0144	-0.23125	0.005382	0.024286	protein_coding
ENSG0000(	CC2D1A	2367.751	-0.15405	0.005383	0.024288	protein_coding
ENSG0000(	SNHG18	388.0785	-0.40967	0.005387	0.024303	lncRNA
ENSG0000(	ARIH2	2846.63	-0.09501	0.005389	0.024305	protein_coding
ENSG0000(	ENSG0000(	133.6404	0.212848	0.00539	0.024305	lncRNA
ENSG0000(	TPTE2	31.10667	0.421277	0.00539	0.024305	protein_coding
ENSG0000(	ENSG0000(	75.94323	0.341472	0.005392	0.024309	lncRNA
ENSG0000(	EP400	4253.195	0.11759	0.005392	0.024309	protein_coding
ENSG0000(	PTN	22.28823	-0.59689	0.005396	0.024323	protein_coding
ENSG0000(	ENSG0000(	44.64465	0.321617	0.005403	0.024348	lncRNA
ENSG0000(	RPL7L1P11	10.67768	0.781702	0.005406	0.024359	processed_pseudogene
ENSG0000(	SPR	135.7745	-0.45309	0.005411	0.024378	protein_coding
ENSG0000(	FMO1	641.5011	-0.89378	0.005413	0.024383	protein_coding
ENSG0000(	CDKL3	330.0731	0.195638	0.005417	0.024394	protein_coding
ENSG0000(	FIBP	438.6205	-0.21448	0.005417	0.024394	protein_coding
ENSG0000(	TMEM106/	1512.461	0.137948	0.005416	0.024394	protein_coding
ENSG0000(	ENSG0000(	19.30944	0.523289	0.005422	0.024411	lncRNA
ENSG0000(	ENSG0000(	50.10662	0.319683	0.005425	0.02442	lncRNA
ENSG0000(	ARL6IP4	374.7879	-0.26974	0.005429	0.024434	protein_coding
ENSG0000(	ENSG0000(	195.4274	0.170342	0.00543	0.024436	lncRNA
ENSG0000(	FGFR3	1073.655	-0.60089	0.005441	0.024481	protein_coding

ENSG0000(	ENSG0000(	27.40711	0.406103	0.005444	0.024489	lncRNA
ENSG0000(	LDHAP7	12.64554	-0.66471	0.005444	0.024489	processed_pseudogene
ENSG0000(	ANKRD30B	139.3198	0.318465	0.005448	0.024505	transcribed_unprocessed_pseudo
ENSG0000(	RTKN	698.1454	-0.35852	0.00545	0.024509	protein_coding
ENSG0000(	DYNC2I2	311.0904	-0.21746	0.005456	0.024527	protein_coding
ENSG0000(	PANK2	2356.343	0.12474	0.005456	0.024527	protein_coding
ENSG0000(	SHH	180.2936	0.506281	0.005455	0.024527	protein_coding
ENSG0000(	ENSG0000(	33.75298	0.327241	0.005459	0.024535	TEC
ENSG0000(	ENSG0000(	215.2884	0.139537	0.005464	0.024552	lncRNA
ENSG0000(	KCNB1	2181.657	0.13434	0.005465	0.024554	protein_coding
ENSG0000(	CHAC2	28.51261	0.390964	0.005469	0.024567	protein_coding
ENSG0000(	DCTN6	339.1214	-0.12088	0.005468	0.024567	protein_coding
ENSG0000(	ENSG0000(	334.5536	0.15581	0.005471	0.024569	lncRNA
ENSG0000(	MYRF-AS1	36.01651	0.606711	0.005471	0.024569	lncRNA
ENSG0000(	NEDD4	8071.751	-0.21082	0.005475	0.024581	protein_coding
ENSG0000(	ENSG0000(	43.82157	-0.30692	0.005478	0.024594	lncRNA
ENSG0000(	MYOT	67.62123	-0.47874	0.005483	0.024611	protein_coding
ENSG0000(	RBM14	4147.997	-0.12196	0.005487	0.024621	protein_coding
ENSG0000(	TXNDC15	1706.768	0.162918	0.005486	0.024621	protein_coding
ENSG0000(	LINC02801	125.9541	0.177487	0.00549	0.024632	lncRNA
ENSG0000(	RPS8	3579.235	-0.28328	0.005498	0.024665	protein_coding
ENSG0000(	PSMB4	600.3154	-0.21016	0.005508	0.024705	protein_coding
ENSG0000(	PTPN12	2058.984	0.143063	0.005525	0.024777	protein_coding
ENSG0000(	ENSG0000(	148.6457	0.180375	0.005527	0.024786	lncRNA
ENSG0000(	RN7SL597F	44.9809	0.286043	0.005532	0.024801	misc_RNA
ENSG0000(	FRAS1	526.8974	-0.58669	0.005542	0.024846	protein_coding
ENSG0000(	PPP1R35	124.0469	-0.18416	0.005547	0.024862	protein_coding
ENSG0000(	PDE3B	1812.907	0.327703	0.005548	0.024864	protein_coding
ENSG0000(	GOLGA6L4	227.7628	0.270263	0.005557	0.024903	protein_coding
ENSG0000(	ENSG0000(	17.07003	0.347405	0.005559	0.024905	lncRNA
ENSG0000(	CTTNBP2	239.0343	-0.36582	0.005561	0.024909	protein_coding
ENSG0000(	ENSG0000(	153.6353	0.225375	0.005561	0.024909	lncRNA
ENSG0000(	RN7SL265F	30.50677	0.292802	0.005562	0.024909	misc_RNA
ENSG0000(	ENSG0000(	227.1063	-1.00705	0.005574	0.024962	lncRNA
ENSG0000(	RN7SL757F	12.67643	0.5393	0.005578	0.024976	misc_RNA
ENSG0000(	ENSG0000(	38.7662	0.305653	0.005584	0.024992	lncRNA
ENSG0000(	SIGLEC12	55.54109	0.440804	0.005584	0.024992	protein_coding
ENSG0000(	MIGA2	1618.774	-0.31388	0.005586	0.024998	protein_coding
ENSG0000(	ADAMTS7	343.3557	0.322916	0.005589	0.025005	protein_coding
ENSG0000(	MPZL2	365.6217	0.319187	0.005589	0.025005	protein_coding
ENSG0000(	TRBV29-1	41.46689	-0.43383	0.005592	0.025015	TR_V_gene
ENSG0000(	CCNI	4038.421	-0.12646	0.0056	0.025046	protein_coding
ENSG0000(	LINC00616	23.60812	0.412155	0.0056	0.025046	lncRNA
ENSG0000(	ENTPD7	2996.976	-0.42611	0.005603	0.02505	protein_coding
ENSG0000(	LNPK	1202.741	-0.19005	0.005603	0.02505	protein_coding
ENSG0000(	CDIPT	587.1436	-0.2291	0.005609	0.025075	protein_coding
ENSG0000(	GALNT18	117.2252	0.322596	0.005611	0.025077	protein_coding

ENSG0000(	ENSG0000(	126.3252	0.203318	0.005615	0.025091	TEC
ENSG0000(	FAM192BP	21.29777	-0.46173	0.005617	0.0251	processed_pseudogene
ENSG0000(	CCDC88B	2384.92	0.30603	0.005621	0.025115	protein_coding
ENSG0000(	DMBX1	118.0126	0.211904	0.005623	0.02512	protein_coding
ENSG0000(	RPL21P65	37.95189	0.328612	0.005625	0.025123	processed_pseudogene
ENSG0000(	STOML1	832.905	0.162177	0.005631	0.025145	protein_coding
ENSG0000(	HNF1A-AS1	3190.396	-0.4115	0.005646	0.025204	lncRNA
ENSG0000(	HP	147.7521	0.34114	0.005645	0.025204	protein_coding
ENSG0000(	USP42	727.6731	-0.14263	0.005646	0.025204	protein_coding
ENSG0000(	ENSG0000(	45.98311	0.307582	0.005653	0.025229	lncRNA
ENSG0000(	XPO6	2863.66	0.223028	0.005653	0.025229	protein_coding
ENSG0000(	ENSG0000(	17.77845	0.369871	0.005657	0.02524	lncRNA
ENSG0000(	PDE4DIPP8	20.70175	-0.91812	0.005662	0.02526	unprocessed_pseudogene
ENSG0000(	HPGD	1971.368	-0.54364	0.005665	0.025261	protein_coding
ENSG0000(	RNU5D-1	16.81917	0.796193	0.005665	0.025261	snRNA
ENSG0000(	SELENOM	500.8783	0.332515	0.005663	0.025261	protein_coding
ENSG0000(	USF1P1	49.4871	0.266794	0.005667	0.025269	processed_pseudogene
ENSG0000(	MPI	1320.375	-0.23564	0.005669	0.025275	protein_coding
ENSG0000(	ENSG0000(	172.3275	0.160041	0.005671	0.025278	protein_coding
ENSG0000(	ENSG0000(	43.96192	-0.32934	0.005673	0.025283	lncRNA
ENSG0000(	LARGE-AS1	9.034681	0.648374	0.005674	0.025283	lncRNA
ENSG0000(	ENSG0000(	174.4899	-0.23687	0.005676	0.025291	lncRNA
ENSG0000(	TECRL	132.2993	0.195612	0.005682	0.025313	protein_coding
ENSG0000(	NSUN6	853.4433	-0.12988	0.005683	0.025316	protein_coding
ENSG0000(	EHD4-AS1	70.17004	0.237236	0.005686	0.025323	lncRNA
ENSG0000(	POMP	297.9198	-0.28475	0.005686	0.025323	protein_coding
ENSG0000(	VPS16	619.6597	-0.13927	0.005688	0.025325	protein_coding
ENSG0000(	ENSG0000(	88.06979	0.222231	0.005695	0.025356	lncRNA
ENSG0000(	DAPK2	3686.353	-0.268	0.005697	0.025359	protein_coding
ENSG0000(	CISD3	635.7903	-0.38231	0.005699	0.025359	protein_coding
ENSG0000(	EZH1	2838.939	-0.10832	0.005699	0.025359	protein_coding
ENSG0000(	FIGNL2	52.18729	0.373233	0.0057	0.025359	protein_coding
ENSG0000(	ENSG0000(	22.52549	0.448151	0.005703	0.025372	lncRNA
ENSG0000(	MIR325HG	260.5101	0.173179	0.005709	0.025393	lncRNA
ENSG0000(	PPP1R14B	356.6322	-0.20003	0.005713	0.025407	protein_coding
ENSG0000(	CBX6	1721.222	0.354337	0.005716	0.025416	protein_coding
ENSG0000(	EBF4	155.3785	0.3531	0.005726	0.025461	protein_coding
ENSG0000(	CASP4	2306.144	0.198395	0.005729	0.025467	protein_coding
ENSG0000(	ENSG0000(	127.8883	0.225014	0.005732	0.025479	lncRNA
ENSG0000(	RAB17	1848.214	-0.6401	0.005741	0.025513	protein_coding
ENSG0000(	ENSG0000(	97.77973	-0.2209	0.005744	0.025525	lncRNA
ENSG0000(	POLR2B	1823.15	-0.1312	0.005747	0.025533	protein_coding
ENSG0000(	MT-TN	91.20018	0.974025	0.005753	0.025559	Mt_tRNA
ENSG0000(	APOBEC1	256.8623	-0.69201	0.005759	0.02558	protein_coding
ENSG0000(	BCAT1	531.763	0.250697	0.005764	0.0256	protein_coding
ENSG0000(	C11orf86	278.2504	-0.67689	0.005768	0.025612	protein_coding
ENSG0000(	ENSG0000(	37.13257	0.531778	0.005776	0.025643	lncRNA

ENSG0000(	ENSG0000(	105.9343	-0.20725	0.005778	0.025648	lncRNA
ENSG0000(	P2RY2	1310.435	-0.43332	0.005778	0.025648	protein_coding
ENSG0000(	ENSG0000(	260.3318	0.160003	0.005781	0.025657	lncRNA
ENSG0000(	ENSG0000(	69.16771	-0.20578	0.005782	0.025658	lncRNA
ENSG0000(	ENSG0000(	149.566	0.211503	0.005783	0.025659	lncRNA
ENSG0000(	ENSG0000(	86.54416	0.237598	0.005796	0.025714	lncRNA
ENSG0000(	AKAP5	971.6453	-0.1571	0.005799	0.025717	protein_coding
ENSG0000(	ENSG0000(	21.22712	0.572846	0.005798	0.025717	lncRNA
ENSG0000(	SLC6A4	2861.308	-0.76862	0.005801	0.025726	protein_coding
ENSG0000(	RFLNA	204.8713	-0.44972	0.005806	0.025743	protein_coding
ENSG0000(	PARPBP	315.5021	0.183275	0.005809	0.025754	protein_coding
ENSG0000(	PTP4A1	2537.738	-0.412	0.005811	0.025755	protein_coding
ENSG0000(	PGLYRP3	660.805	0.493811	0.005813	0.02576	protein_coding
ENSG0000(	ENSG0000(	1830.607	-0.12407	0.005827	0.025818	lncRNA
ENSG0000(	ETFB	2485.122	-0.13372	0.005827	0.025818	protein_coding
ENSG0000(	ELOCP33	210.4654	-0.16231	0.005833	0.025837	processed_pseudogene
ENSG0000(	ZNRF2P1	118.2711	0.231551	0.005833	0.025837	transcribed_processed_pseudoge
ENSG0000(	UBE2D1	232.8389	0.289306	0.005834	0.025837	protein_coding
ENSG0000(	LRP2	55.45777	0.410234	0.005835	0.025837	protein_coding
ENSG0000(	LINC02804	68.96306	0.283138	0.005842	0.025864	lncRNA
ENSG0000(	HDAC3	685.1182	-0.11965	0.005844	0.025869	protein_coding
ENSG0000(	FCHO2	1093.643	-0.21844	0.005846	0.025874	protein_coding
ENSG0000(	RPS6	5415.374	-0.2543	0.005846	0.025874	protein_coding
ENSG0000(	JPT2	900.3257	-0.15029	0.005852	0.025895	protein_coding
ENSG0000(	MIR4729	13.62281	0.51728	0.005855	0.025907	miRNA
ENSG0000(	ENSG0000(	136.6701	-0.16858	0.005859	0.025919	TEC
ENSG0000(	HDDC2	467.4581	-0.17264	0.005861	0.025922	protein_coding
ENSG0000(	NUDT9	998.1079	-0.14303	0.005862	0.025924	protein_coding
ENSG0000(	STK19	332.6605	0.158517	0.005863	0.025927	protein_coding
ENSG0000(	CRB3	229.2761	-0.53758	0.005864	0.025928	protein_coding
ENSG0000(	ZBTB43	1704.164	0.091759	0.005871	0.025953	protein_coding
ENSG0000(	PLAAT3	323.3551	-0.23932	0.005872	0.025954	protein_coding
ENSG0000(	ENSG0000(	90.23316	0.204076	0.005879	0.025981	lncRNA
ENSG0000(	ENSG0000(	10.7677	0.553745	0.00588	0.025984	lncRNA
ENSG0000(	RSBN1L	1823.448	-0.08852	0.005885	0.025998	protein_coding
ENSG0000(	SGSM3-AS1	100.7553	0.205475	0.005885	0.025998	lncRNA
ENSG0000(	ENSG0000(	169.185	0.247767	0.005888	0.026007	lncRNA
ENSG0000(	ENSG0000(	12.42415	0.769192	0.005889	0.026007	lncRNA
ENSG0000(	GET4	1014.142	-0.17759	0.00589	0.026007	protein_coding
ENSG0000(	APBA2	660.292	0.344613	0.005894	0.026014	protein_coding
ENSG0000(	BMP3	558.9808	-0.5209	0.005895	0.026014	protein_coding
ENSG0000(	TM6SF2	1624.172	-0.6657	0.005894	0.026014	protein_coding
ENSG0000(	ZNF540	510.5262	-0.20424	0.005893	0.026014	protein_coding
ENSG0000(	RBMS3-AS2	146.8549	-0.19634	0.005902	0.026041	lncRNA
ENSG0000(	ENSG0000(	204.658	0.181708	0.005903	0.026044	lncRNA
ENSG0000(	ENSG0000(	49.74498	-0.25525	0.005905	0.026048	lncRNA
ENSG0000(	AURKAP1	69.66941	-0.35277	0.005907	0.026052	transcribed_processed_pseudoge

ENSG0000(	ENSG0000(	648.663	0.178929	0.005907	0.026052	lncRNA
ENSG0000(	ENSG0000(	35.66766	0.407466	0.005917	0.026092	lncRNA
ENSG0000(	SLC28A1	1482.348	-0.82701	0.005918	0.026092	protein_coding
ENSG0000(	CA12	1221.883	0.58422	0.005922	0.026108	protein_coding
ENSG0000(	LENG1	96.12799	-0.20421	0.005935	0.026158	protein_coding
ENSG0000(	ZNF175	893.5272	-0.15814	0.005936	0.026158	protein_coding
ENSG0000(	ENSG0000(	163.0686	-0.21187	0.005944	0.02619	lncRNA
ENSG0000(	STAM-DT	130.3532	0.19919	0.005949	0.026211	lncRNA
ENSG0000(	C10orf95	17.31377	0.363882	0.00595	0.026213	protein_coding
ENSG0000(	LIMD1	3298.197	0.143557	0.005952	0.026217	protein_coding
ENSG0000(	OVOL1	420.4696	-0.54444	0.005954	0.026221	protein_coding
ENSG0000(	UGT1A6	507.738	-0.73027	0.005955	0.026223	protein_coding
ENSG0000(	DIRC3-AS1	21.87791	-0.62831	0.00596	0.026241	lncRNA
ENSG0000(	ELP5	261.2115	-0.18147	0.005964	0.026254	protein_coding
ENSG0000(	RNU5E-1	320.1935	0.605774	0.005968	0.02627	snRNA
ENSG0000(	ENSG0000(	19.08902	0.400869	0.00597	0.026271	lncRNA
ENSG0000(	LINC00839	402.3846	0.156163	0.005973	0.026281	lncRNA
ENSG0000(	WASL	1675.929	-0.3426	0.005973	0.026281	protein_coding
ENSG0000(	PHLDA3	294.0345	0.189257	0.005985	0.026318	protein_coding
ENSG0000(	RNF11	948.9913	-0.29381	0.005983	0.026318	protein_coding
ENSG0000(	SUSD2	901.8989	-0.81551	0.005985	0.026318	protein_coding
ENSG0000(	USP49	1089.403	0.107385	0.005983	0.026318	protein_coding
ENSG0000(	GSTM4	1179.758	-0.39459	0.005987	0.026325	protein_coding
ENSG0000(	SOX17	31.61901	0.502899	0.005993	0.026347	protein_coding
ENSG0000(	ROBO2	451.9133	-0.34294	0.005998	0.026365	protein_coding
ENSG0000(	IARS1	4081.089	0.184918	0.006006	0.026396	protein_coding
ENSG0000(	ENSG0000(	8.639103	0.631063	0.006009	0.026406	transcribed_unprocessed_pseudo
ENSG0000(	RNU6-1231	9.616581	0.524287	0.00601	0.026406	snRNA
ENSG0000(	SLC30A4	2076.738	-0.21544	0.006012	0.026409	protein_coding
ENSG0000(	SLCO1B3	11.34983	0.71384	0.006014	0.026414	protein_coding
ENSG0000(	ENSG0000(	185.3257	0.160261	0.006025	0.026461	lncRNA
ENSG0000(	STPG2-AS1	523.5052	0.163589	0.00603	0.026477	lncRNA
ENSG0000(	SAMD14	127.4253	0.406706	0.006032	0.026485	protein_coding
ENSG0000(	ENSG0000(	179.1032	0.180846	0.006035	0.026492	TEC
ENSG0000(	ENSG0000(	55.58115	0.319165	0.006042	0.02652	lncRNA
ENSG0000(	APOC3	1002.568	-1.06798	0.006061	0.026597	protein_coding
ENSG0000(	RBM39	11541.42	-0.0704	0.006061	0.026597	protein_coding
ENSG0000(	SDHDP4	22.62303	-0.5596	0.006065	0.02661	processed_pseudogene
ENSG0000(	ABCA3	215.0268	-0.28967	0.006068	0.026616	protein_coding
ENSG0000(	ENSG0000(	17.8196	0.635171	0.006067	0.026616	processed_pseudogene
ENSG0000(	ENSG0000(	111.946	0.186627	0.006069	0.026616	lncRNA
ENSG0000(	STRN4	1164.13	-0.11542	0.00607	0.026617	protein_coding
ENSG0000(	CILK1	1033.194	-0.19024	0.006071	0.02662	protein_coding
ENSG0000(	C6orf136	352.3565	-0.38637	0.006077	0.026639	protein_coding
ENSG0000(	SPON1	864.2689	-0.31956	0.006086	0.026675	protein_coding
ENSG0000(	CRTAC1	10.95143	0.885187	0.006088	0.026681	protein_coding
ENSG0000(	PPP2R2C	565.3213	0.407252	0.00609	0.026686	protein_coding

ENSG0000(	ENSG0000(	24.62499	0.453499	0.006094	0.026698	processed_pseudogene
ENSG0000(	ST3GAL6	365.2086	0.276253	0.006098	0.026712	protein_coding
ENSG0000(	ENSG0000(	52.99357	0.292291	0.006107	0.026746	lncRNA
ENSG0000(	ENSG0000(	22.94991	0.351054	0.006108	0.026746	lncRNA
ENSG0000(	RHOU	1512.292	-0.54419	0.006108	0.026746	protein_coding
ENSG0000(	ENSG0000(	767.0207	0.125374	0.006109	0.026747	TEC
ENSG0000(	IARS2P1	148.7805	0.267605	0.006121	0.026794	processed_pseudogene
ENSG0000(	C3orf85	2427.787	-0.72487	0.006122	0.026798	protein_coding
ENSG0000(	ENSG0000(	45.46849	0.354725	0.006123	0.026798	lncRNA
ENSG0000(	ENSG0000(	19.23797	0.438549	0.006126	0.026805	TEC
ENSG0000(	NALF1	134.4008	0.290649	0.006127	0.026807	protein_coding
ENSG0000(	ENSG0000(	284.6848	-0.23125	0.006131	0.026823	protein_coding
ENSG0000(	ZNF85	558.3554	-0.16683	0.006139	0.026853	protein_coding
ENSG0000(	MTRF1L	675.3946	-0.19972	0.006155	0.026919	protein_coding
ENSG0000(	ENSG0000(	49.59786	-0.29023	0.006164	0.026944	lncRNA
ENSG0000(	ENSG0000(	235.8527	0.146467	0.006164	0.026944	lncRNA
ENSG0000(	HIBADH	382.0913	-0.19618	0.006162	0.026944	protein_coding
ENSG0000(	PRICKLE3	281.352	-0.1864	0.006164	0.026944	protein_coding
ENSG0000(	SEPTIN3	110.9795	0.314772	0.006167	0.026952	protein_coding
ENSG0000(	ENSG0000(	58.14827	0.277453	0.006174	0.026978	lncRNA
ENSG0000(	ZNF566-AS	96.86017	0.242745	0.006185	0.027021	lncRNA
ENSG0000(	ENSG0000(	37.27465	0.395583	0.00619	0.02704	lncRNA
ENSG0000(	LIPT1	346.3446	-0.13991	0.0062	0.027082	protein_coding
ENSG0000(	PRKAR1A	3101.761	0.165798	0.006203	0.02709	protein_coding
ENSG0000(	TCEAL8	216.4022	-0.22728	0.006205	0.027094	protein_coding
ENSG0000(	IPO5P1	678.0354	0.300672	0.006206	0.027098	transcribed_processed_pseudoge
ENSG0000(	ID1	708.7246	-0.46816	0.006211	0.027113	protein_coding
ENSG0000(	NLRX1	272.5616	0.20281	0.006216	0.027132	protein_coding
ENSG0000(	EFCAB10	35.72489	0.340962	0.00622	0.027141	protein_coding
ENSG0000(	ENSG0000(	410.5429	0.251056	0.00622	0.027141	lncRNA
ENSG0000(	RAI2	135.8892	-0.31261	0.006221	0.027141	protein_coding
ENSG0000(	SLC8A2	10.52553	0.935619	0.006222	0.027141	protein_coding
ENSG0000(	ENSG0000(	84.2656	0.230834	0.006227	0.02716	lncRNA
ENSG0000(	AFF4	5117.476	0.088264	0.00623	0.02717	protein_coding
ENSG0000(	ENSG0000(	201.2541	-0.25777	0.006233	0.027179	lncRNA
ENSG0000(	ENSG0000(	45.95052	0.316019	0.006238	0.027198	lncRNA
ENSG0000(	FMO5	2047.507	-0.50103	0.006242	0.027212	protein_coding
ENSG0000(	KDM8	520.7527	-0.42028	0.006245	0.02722	protein_coding
ENSG0000(	SDCBP2	1123.614	-0.58734	0.006252	0.027247	protein_coding
ENSG0000(	NSRP1P1	73.18771	-0.29849	0.006254	0.027253	processed_pseudogene
ENSG0000(	ARV1	247.9783	-0.15084	0.00626	0.027277	protein_coding
ENSG0000(	CTC-338M1	392.8127	0.17274	0.006269	0.027308	lncRNA
ENSG0000(	ENSG0000(	165.9927	-0.24354	0.006269	0.027308	lncRNA
ENSG0000(	CYP1A2	465.6568	-0.10215	0.006272	0.027316	protein_coding
ENSG0000(	ELP2	2503.517	-0.15782	0.006277	0.027333	protein_coding
ENSG0000(	ENSG0000(	11.06944	0.389281	0.006283	0.027351	lncRNA
ENSG0000(	ENSG0000(	70.33043	-0.22521	0.006282	0.027351	lncRNA

ENSG0000(	GET1	711.8131	-0.15969	0.006283	0.027351	protein_coding
ENSG0000(	MSI2	4226.832	0.255019	0.006285	0.027352	protein_coding
ENSG0000(	CHDH	2824.4	-0.45758	0.006286	0.027355	protein_coding
ENSG0000(	FAT4	2588.482	0.174897	0.006289	0.027363	protein_coding
ENSG0000(	ACE2	8338.438	-0.75673	0.006294	0.02738	protein_coding
ENSG0000(	ARHGEF9	1991.877	0.21189	0.006295	0.027382	protein_coding
ENSG0000(	CDK9	1107.895	-0.16558	0.006297	0.027387	protein_coding
ENSG0000(	ENSG0000(	62.07397	0.214783	0.006299	0.027392	lncRNA
ENSG0000(	MAOB	978.2149	-0.66052	0.006302	0.027398	protein_coding
ENSG0000(	MMP24	46.43935	-0.65047	0.006302	0.027398	protein_coding
ENSG0000(	SPCS1	1140.289	-0.20251	0.006304	0.027402	protein_coding
ENSG0000(	ENSG0000(	45.95949	0.211614	0.006306	0.027408	lncRNA
ENSG0000(	ENSG0000(	15.87651	0.522385	0.006309	0.027416	lncRNA
ENSG0000(	TFF1	175.2619	0.875563	0.00631	0.027418	protein_coding
ENSG0000(	DDC	1013.581	-0.62683	0.006313	0.027429	protein_coding
ENSG0000(	ENSG0000(	265.5182	0.184395	0.006316	0.027437	lncRNA
ENSG0000(	ENSG0000(	22.24434	0.446548	0.006318	0.02744	lncRNA
ENSG0000(	ENSG0000(	187.7159	0.191574	0.006319	0.027441	lncRNA
ENSG0000(	ENSG0000(	80.16975	-0.26123	0.00632	0.027444	lncRNA
ENSG0000(	ENSG0000(	13.89889	0.475502	0.006323	0.027453	lncRNA
ENSG0000(	CYTH4	1625.625	0.376473	0.006327	0.027464	protein_coding
ENSG0000(	CHMP4BP1	72.11935	-0.33447	0.006328	0.027464	processed_pseudogene
ENSG0000(	ENSG0000(	21.92043	0.466546	0.006334	0.027489	lncRNA
ENSG0000(	TICAM1	379.854	-0.29483	0.006337	0.027495	protein_coding
ENSG0000(	KLF17P1	17.14409	0.430548	0.00634	0.027507	processed_pseudogene
ENSG0000(	ENSG0000(	42.61509	0.340881	0.006342	0.027509	lncRNA
ENSG0000(	ENSG0000(	67.95939	-0.25158	0.006348	0.027531	lncRNA
ENSG0000(	ENSG0000(	855.7752	0.239786	0.006353	0.027549	TEC
ENSG0000(	RNU6-113(	15.6031	0.472213	0.006357	0.027564	snRNA
ENSG0000(	SMG1P7	1195.669	-0.15294	0.006358	0.027564	transcribed_unprocessed_pseudo
ENSG0000(	RPL21P5	55.64444	0.238764	0.00636	0.027566	processed_pseudogene
ENSG0000(	TERB1	218.8984	-0.22333	0.00636	0.027566	protein_coding
ENSG0000(	TMED5	1919.493	-0.15951	0.006365	0.027583	protein_coding
ENSG0000(	GP5	53.05428	0.333325	0.006369	0.027597	protein_coding
ENSG0000(	CDHR5	10811.7	-0.66132	0.006373	0.027609	protein_coding
ENSG0000(	AKR1D1	145.2857	0.206448	0.006376	0.027619	protein_coding
ENSG0000(	TTLL9	121.7793	0.213804	0.006381	0.027637	protein_coding
ENSG0000(	ENSG0000(	229.341	-0.14387	0.006384	0.027643	lncRNA
ENSG0000(	ZYG11B	1773.049	0.078794	0.006384	0.027643	protein_coding
ENSG0000(	ZNF263	784.2086	0.108783	0.006391	0.027672	protein_coding
ENSG0000(	POLR2E	781.7651	-0.16045	0.006394	0.027679	protein_coding
ENSG0000(	ENSG0000(	86.43546	0.321384	0.006395	0.027681	lncRNA
ENSG0000(	ENSG0000(	225.8652	0.219421	0.006399	0.027692	lncRNA
ENSG0000(	ENSG0000(	147.2522	0.27428	0.00641	0.027732	lncRNA
ENSG0000(	ZNF663P	13.92255	0.572085	0.00641	0.027732	transcribed_unitary_pseudogene
ENSG0000(	N4BP2L2-IT	1704.199	0.137079	0.006411	0.027734	lncRNA
ENSG0000(	AQP7	1267.354	-0.52213	0.006417	0.027753	protein_coding

ENSG0000(	C18orf21	115.5713	-0.21672	0.006418	0.027753	protein_coding
ENSG0000(	MBL1P	54.05071	0.373922	0.006418	0.027753	transcribed_unitary_pseudogene
ENSG0000(	BCR	1420.797	0.20304	0.006422	0.027766	protein_coding
ENSG0000(	ENSG0000(	1671.432	0.128987	0.00643	0.027797	TEC
ENSG0000(	ENSG0000(	17.28866	0.716008	0.006432	0.027801	lncRNA
ENSG0000(	ENSG0000(	37.36364	0.456391	0.006434	0.027808	unprocessed_pseudogene
ENSG0000(	NECAB3	404.9771	-0.26152	0.006449	0.027866	protein_coding
ENSG0000(	LINC00487	21.98631	-0.87893	0.006451	0.027872	lncRNA
ENSG0000(	TYW1	684.162	-0.15688	0.006456	0.027889	protein_coding
ENSG0000(	PRELID1	753.4032	-0.30083	0.006458	0.027893	protein_coding
ENSG0000(	IL12A	18.088	0.535071	0.006461	0.027902	protein_coding
ENSG0000(	ABCB10	594.0936	-0.15556	0.00647	0.027941	protein_coding
ENSG0000(	MADCAM1	142.4886	-0.16797	0.006476	0.027962	lncRNA
ENSG0000(	PCBP2P1	37.7726	0.299737	0.00648	0.027973	processed_pseudogene
ENSG0000(	ZNF132-DT	994.4355	0.102243	0.006481	0.027973	lncRNA
ENSG0000(	FHIP2A	1161.7	-0.22183	0.006486	0.027989	protein_coding
ENSG0000(	GPA33	2445.089	-0.57686	0.006487	0.027989	protein_coding
ENSG0000(	POLR1E	223.6477	-0.23714	0.006487	0.027989	protein_coding
ENSG0000(	ENSG0000(	207.2504	0.172749	0.006492	0.028002	protein_coding
ENSG0000(	PDP2	1482.53	-0.12349	0.006491	0.028002	protein_coding
ENSG0000(	RBM43	734.5362	0.150802	0.006498	0.028026	protein_coding
ENSG0000(	NR1I3	179.4033	-0.34564	0.006503	0.028043	protein_coding
ENSG0000(	ENSG0000(	897.4405	-0.21982	0.006505	0.02805	lncRNA
ENSG0000(	COX6B1	537.0835	-0.30614	0.00651	0.028064	protein_coding
ENSG0000(	ZNF695	78.98028	0.47302	0.00651	0.028064	protein_coding
ENSG0000(	ENSG0000(	52.00086	0.249412	0.006516	0.028084	lncRNA
ENSG0000(	TMEM82	105.3695	-0.62984	0.00652	0.028097	protein_coding
ENSG0000(	NEDD8	1075.055	-0.11154	0.006522	0.028104	protein_coding
ENSG0000(	RNU6-476F	23.46172	-0.39872	0.006524	0.028106	snRNA
ENSG0000(	MT-TE	110.2286	0.834838	0.00653	0.028131	Mt_tRNA
ENSG0000(	SRL	120.2276	-0.24423	0.006535	0.028146	protein_coding
ENSG0000(	ENSG0000(	21.52331	0.406114	0.006539	0.02816	lncRNA
ENSG0000(	API5P2	36.21928	0.589086	0.006542	0.028168	processed_pseudogene
ENSG0000(	ENSG0000(	36.72174	0.310994	0.006546	0.028179	lncRNA
ENSG0000(	ENSG0000(	131.4424	-0.20852	0.006546	0.028179	processed_pseudogene
ENSG0000(	ENSG0000(	257.2402	0.16377	0.006547	0.028179	TEC
ENSG0000(	CDC42-IT1	176.2671	-0.1699	0.00655	0.02819	lncRNA
ENSG0000(	NCDN	338.7037	0.314612	0.006559	0.028222	protein_coding
ENSG0000(	NME6	1588.27	0.103705	0.00656	0.028222	protein_coding
ENSG0000(	MYO3A	64.50752	-0.69165	0.006567	0.028248	protein_coding
ENSG0000(	TLCD4	795.0833	-0.51648	0.006571	0.028263	protein_coding
ENSG0000(	LINC00970	393.1446	-0.14049	0.006572	0.028263	lncRNA
ENSG0000(	RN7SKP24(	9.638889	0.538357	0.006583	0.028305	misc_RNA
ENSG0000(	UBR7	453.5648	-0.15823	0.006587	0.028318	protein_coding
ENSG0000(	ENSG0000(	480.4771	0.219897	0.006594	0.028348	lncRNA
ENSG0000(	ENSG0000(	34.38946	0.311277	0.006598	0.02835	lncRNA
ENSG0000(	ME2	3835.724	-0.13291	0.006597	0.02835	protein_coding

ENSG0000(	SDR16C5	66.16461	-0.7419	0.006596	0.02835	protein_coding
ENSG0000(	ENSG0000(	17.81863	0.585257	0.006599	0.028352	lncRNA
ENSG0000(	DNM1P46	185.3696	0.198748	0.006602	0.028358	transcribed_unprocessed_pseudo
ENSG0000(	RCAN2	317.3741	-0.35548	0.006602	0.028358	protein_coding
ENSG0000(	HOXC8	87.79626	0.33963	0.006604	0.028361	protein_coding
ENSG0000(	LRRC77P	94.18364	0.356976	0.006605	0.028361	transcribed_unitary_pseudogene
ENSG0000(	MINK1	2823.774	-0.21055	0.00661	0.02838	protein_coding
ENSG0000(	CACHD1	308.6835	0.260132	0.006612	0.028386	protein_coding
ENSG0000(	CCN5	323.3147	0.19991	0.006619	0.028411	protein_coding
ENSG0000(	RNF5	366.8397	-0.35985	0.006635	0.028475	protein_coding
ENSG0000(	SPATA18	173.0006	0.437028	0.006636	0.028475	protein_coding
ENSG0000(	TRAF1	1452.862	0.466258	0.006641	0.028496	protein_coding
ENSG0000(	ENSG0000(	14.53769	0.696445	0.006654	0.028544	processed_pseudogene
ENSG0000(	PCGF1	156.3638	-0.21652	0.00666	0.02857	protein_coding
ENSG0000(	CHMP1B	1325.448	-0.18996	0.00667	0.028608	protein_coding
ENSG0000(	ZNF580	1098.773	0.195917	0.006674	0.028618	protein_coding
ENSG0000(	KAZALD1	288.3659	-0.52472	0.006679	0.028634	protein_coding
ENSG0000(	TMEM254-	162.2635	0.203489	0.006679	0.028634	lncRNA
ENSG0000(	CNOT7	1766.451	-0.11463	0.006698	0.028702	protein_coding
ENSG0000(	ENSG0000(	29.90153	0.398299	0.006698	0.028702	lncRNA
ENSG0000(	ENSG0000(	63.85216	0.259738	0.006697	0.028702	lncRNA
ENSG0000(	ATP5F1D	495.0892	-0.211	0.006707	0.028733	protein_coding
ENSG0000(	ENSG0000(	307.4118	-0.20795	0.006706	0.028733	lncRNA
ENSG0000(	MGAM	14107.48	-0.69123	0.00671	0.028743	protein_coding
ENSG0000(	RPL5P13	62.80208	0.28659	0.006712	0.028749	processed_pseudogene
ENSG0000(	MPST	3095.68	-0.33547	0.006716	0.02876	protein_coding
ENSG0000(	CIRBP-AS1	14.15731	0.423452	0.006725	0.028789	lncRNA
ENSG0000(	ENSG0000(	15.75837	0.552259	0.006725	0.028789	lncRNA
ENSG0000(	SCIN	7737.138	-0.6298	0.006725	0.028789	protein_coding
ENSG0000(	ENSG0000(	127.029	0.2359	0.006726	0.02879	lncRNA
ENSG0000(	LINC01163	10.42862	0.917894	0.006734	0.028817	lncRNA
ENSG0000(	CLEC4A	93.93166	0.446142	0.006742	0.028847	protein_coding
ENSG0000(	PLEKHO1	1886.827	0.40907	0.006746	0.028859	protein_coding
ENSG0000(	STYXL1	293.6449	-0.18789	0.006746	0.028859	protein_coding
ENSG0000(	PLEKHM3	2016.104	0.084005	0.006749	0.028865	protein_coding
ENSG0000(	PPL	402.2008	-0.45954	0.006752	0.028875	protein_coding
ENSG0000(	TMEM150E	556.6679	-0.4529	0.006753	0.028876	protein_coding
ENSG0000(	FAM126A	3117.234	0.171769	0.006754	0.028878	protein_coding
ENSG0000(	TAFA5	32.08255	0.410504	0.006755	0.028878	protein_coding
ENSG0000(	SNX25P1	22.29345	0.452841	0.006758	0.028884	unprocessed_pseudogene
ENSG0000(	SETX	10208.16	0.152336	0.00677	0.028934	protein_coding
ENSG0000(	MTCO1P53	27.42466	-0.86247	0.006776	0.028957	processed_pseudogene
ENSG0000(	ENSG0000(	433.1413	-0.13327	0.006779	0.028965	lncRNA
ENSG0000(	ENSG0000(	65.77265	0.24029	0.006781	0.028966	lncRNA
ENSG0000(	ENSG0000(	882.5309	0.097834	0.006789	0.028999	lncRNA
ENSG0000(	CLHC1	610.3029	0.233604	0.006798	0.029028	protein_coding
ENSG0000(	ENSG0000(	10.32317	0.795312	0.006799	0.029028	lncRNA

ENSG0000(	NUP133	1039.386	0.115303	0.006797	0.029028	protein_coding
ENSG0000(	ENSG0000(	69.63412	0.33778	0.0068	0.02903	processed_pseudogene
ENSG0000(	ENSG0000(	44.69095	0.463583	0.006804	0.029039	lncRNA
ENSG0000(	ICMT	1368.892	-0.23701	0.006804	0.029039	protein_coding
ENSG0000(	ENSG0000(	16.17305	0.711191	0.006806	0.029043	processed_pseudogene
ENSG0000(	MDFI	38.79821	0.563672	0.006807	0.029045	protein_coding
ENSG0000(	MAF	7244.581	-0.49295	0.00681	0.029054	protein_coding
ENSG0000(	RGS5	2270.561	0.222503	0.006814	0.029058	protein_coding
ENSG0000(	STXBP3	1432.101	-0.10776	0.006814	0.029058	protein_coding
ENSG0000(	TRPM5	133.3503	-0.51112	0.006814	0.029058	protein_coding
ENSG0000(	ADAM33	615.0555	0.397911	0.006826	0.029105	protein_coding
ENSG0000(	TMSB4X	10150.37	-0.19691	0.006827	0.029105	protein_coding
ENSG0000(	DAND5	321.3597	-0.15667	0.006828	0.029107	protein_coding
ENSG0000(	TOP2B	2807.483	-0.1556	0.006831	0.029115	protein_coding
ENSG0000(	ACTR10	657.4106	-0.14043	0.006833	0.029119	protein_coding
ENSG0000(	ENSG0000(	102.3191	0.197004	0.006835	0.02912	TEC
ENSG0000(	PARD6B	1058.086	-0.32943	0.006835	0.02912	protein_coding
ENSG0000(	CSNK1D	4954.146	-0.20552	0.006847	0.029151	protein_coding
ENSG0000(	ENSG0000(	213.9989	-0.12912	0.006848	0.029151	lncRNA
ENSG0000(	ENSG0000(	37.368	0.396839	0.006846	0.029151	lncRNA
ENSG0000(	ENSG0000(	1052.585	0.155714	0.006845	0.029151	lncRNA
ENSG0000(	ENSG0000(	678.1843	0.093144	0.006846	0.029151	TEC
ENSG0000(	H6PD	3515.077	0.12019	0.006847	0.029151	protein_coding
ENSG0000(	ZNF34	381.3147	-0.12972	0.006852	0.029163	protein_coding
ENSG0000(	PPIAP46	367.6841	0.161094	0.006855	0.029172	transcribed_processed_pseudoge
ENSG0000(	ENSG0000(	336.0903	0.12207	0.00687	0.029233	lncRNA
ENSG0000(	H2AC11	159.1753	-0.44815	0.006883	0.02928	protein_coding
ENSG0000(	ZNF664	4556.844	-0.30175	0.006883	0.02928	protein_coding
ENSG0000(	NKAP	1637.85	-0.12262	0.006887	0.029295	protein_coding
ENSG0000(	ENSG0000(	10.33305	0.522108	0.006893	0.029317	lncRNA
ENSG0000(	HDGF	2151.039	-0.24492	0.006895	0.02932	protein_coding
ENSG0000(	EMC6	61.87608	-0.30575	0.006901	0.029342	protein_coding
ENSG0000(	ENTHD1	13.02505	0.866906	0.006902	0.029342	protein_coding
ENSG0000(	FRMD8	1070.374	-0.21297	0.006903	0.029343	protein_coding
ENSG0000(	PIN4	375.0941	-0.10786	0.00691	0.029367	protein_coding
ENSG0000(	ENSG0000(	308.0082	-0.31619	0.006925	0.02942	lncRNA
ENSG0000(	ENSG0000(	113.3762	0.265273	0.006925	0.02942	lncRNA
ENSG0000(	UBXN11	475.0019	0.289397	0.006923	0.02942	protein_coding
ENSG0000(	TRIB3	67.01738	0.400782	0.006926	0.029421	protein_coding
ENSG0000(	COA4	551.6238	-0.16602	0.006928	0.029427	protein_coding
ENSG0000(	LINC01533	61.08545	0.424955	0.006936	0.029453	lncRNA
ENSG0000(	TLN2	1766.357	-0.32714	0.006953	0.029525	protein_coding
ENSG0000(	ENSG0000(	268.6628	0.137849	0.006959	0.029545	lncRNA
ENSG0000(	PAPSS1	360.9758	0.252803	0.00698	0.029629	protein_coding
ENSG0000(	LIX1L-AS1	212.4383	-0.1702	0.006985	0.029647	lncRNA
ENSG0000(	LINC02185	402.5068	0.132911	0.006987	0.029653	lncRNA
ENSG0000(	ENSG0000(	14.75334	0.468613	0.006995	0.029681	lncRNA

ENSG0000(	ENSG0000(	169.3687	0.184822	0.006998	0.029689	lncRNA
ENSG0000(	ENSG0000(	38.76418	0.363095	0.007002	0.029698	processed_pseudogene
ENSG0000(	GLYCTK	2767.756	-0.32358	0.007003	0.029698	protein_coding
ENSG0000(	HAVCR2	578.5568	0.244134	0.007003	0.029698	protein_coding
ENSG0000(	ENSG0000(	13.15612	-0.49133	0.007015	0.029745	lncRNA
ENSG0000(	ENSG0000(	33.95986	0.514149	0.007021	0.02977	lncRNA
ENSG0000(	NOTCH2NL	3270.522	0.230538	0.007023	0.02977	protein_coding
ENSG0000(	ENSG0000(	123.1261	-0.36558	0.007028	0.029772	lncRNA
ENSG0000(	ENSG0000(	193.3891	0.245555	0.007026	0.029772	lncRNA
ENSG0000(	EPB41	5717.646	-0.1229	0.007028	0.029772	protein_coding
ENSG0000(	LAMC1	1938.172	0.275224	0.007026	0.029772	protein_coding
ENSG0000(	NMRAL1	466.3922	0.138484	0.007026	0.029772	protein_coding
ENSG0000(	RN7SL531F	193.2238	-0.1533	0.007035	0.029801	misc_RNA
ENSG0000(	ENSG0000(	70.29415	-0.80786	0.007041	0.02982	lncRNA
ENSG0000(	ENSG0000(	71.3558	0.225912	0.007042	0.02982	lncRNA
ENSG0000(	SLC1A7	283.2892	-0.85008	0.007046	0.029835	protein_coding
ENSG0000(	IGLV3-21	138.6531	0.885952	0.00705	0.029848	IG_V_gene
ENSG0000(	ENSG0000(	361.8637	0.141826	0.007052	0.029849	lncRNA
ENSG0000(	ENSG0000(	93.74986	-0.65641	0.007056	0.029862	unprocessed_pseudogene
ENSG0000(	HAAO	523.2234	-0.36596	0.007057	0.029863	protein_coding
ENSG0000(	NABP1	12000.22	0.266276	0.007058	0.029863	protein_coding
ENSG0000(	CYC1	510.9164	-0.29181	0.007065	0.029888	protein_coding
ENSG0000(	SEL1L	3483.58	0.184222	0.00707	0.029908	protein_coding
ENSG0000(	ARL14	359.9444	-0.55625	0.007072	0.02991	protein_coding
ENSG0000(	ZNF816	334.5801	-0.18298	0.007075	0.029919	protein_coding
ENSG0000(	RILPL1	959.6412	0.091017	0.007076	0.02992	protein_coding
ENSG0000(	ENSG0000(	7198.377	0.183603	0.007078	0.029924	lncRNA
ENSG0000(	RBM22	809.0559	-0.09021	0.007082	0.029937	protein_coding
ENSG0000(	GNPDA2	301.8185	-0.13373	0.007086	0.029949	protein_coding
ENSG0000(	ADAMTS16	38.26339	0.430399	0.007091	0.029967	protein_coding
ENSG0000(	ENSG0000(	85.58737	0.308371	0.007094	0.029978	lncRNA
ENSG0000(	ENSG0000(	19.49288	0.338061	0.007103	0.030007	TEC
ENSG0000(	ITIH5	2159.492	-0.2208	0.007103	0.030007	protein_coding
ENSG0000(	LINC01497	18.13936	0.395493	0.007109	0.030028	lncRNA
ENSG0000(	PPM1D	1911.225	-0.0702	0.007112	0.030036	protein_coding
ENSG0000(	GLB1L2	301.0384	-0.26232	0.007113	0.030038	protein_coding
ENSG0000(	ENSG0000(	47.73059	0.249713	0.007119	0.030047	lncRNA
ENSG0000(	IGHV4-59	302.2461	0.704999	0.007119	0.030047	IG_V_gene
ENSG0000(	MIR4507	10.41278	0.886775	0.007119	0.030047	miRNA
ENSG0000(	ZNF573	549.7757	-0.20507	0.007118	0.030047	protein_coding
ENSG0000(	ENSG0000(	70.2948	-0.21942	0.007122	0.030055	lncRNA
ENSG0000(	ENSG0000(	62.99381	0.277359	0.007128	0.030076	lncRNA
ENSG0000(	ENSG0000(	15.31345	0.460058	0.007129	0.030076	lncRNA
ENSG0000(	CLCN5	1680.187	-0.28347	0.007133	0.030088	protein_coding
ENSG0000(	MIR3659H(	39.65387	-0.34916	0.007136	0.030097	lncRNA
ENSG0000(	ENSG0000(	9.960969	0.641361	0.00714	0.030104	lncRNA
ENSG0000(	ENSG0000(	270.0185	0.168997	0.00714	0.030104	lncRNA

ENSG0000(	HSPE1P19	15.50227	0.481049	0.00714	0.030104	processed_pseudogene
ENSG0000(	ENSG0000(	182.5092	0.246555	0.007142	0.030106	lncRNA
ENSG0000(	CLDN23	256.4531	-0.60328	0.007144	0.030111	protein_coding
ENSG0000(	ENSG0000(	255.9926	-0.19789	0.007148	0.030122	lncRNA
ENSG0000(	MBOAT2	1149.993	0.223253	0.007152	0.03013	protein_coding
ENSG0000(	PPIL3	705.3883	-0.10373	0.007152	0.03013	protein_coding
ENSG0000(	XPA	385.6779	-0.15827	0.007152	0.03013	protein_coding
ENSG0000(	ENSG0000(	165.5998	0.169285	0.007157	0.030144	lncRNA
ENSG0000(	ENSG0000(	15.84736	-0.40602	0.007163	0.030161	lncRNA
ENSG0000(	TRO	174.1283	0.332018	0.007162	0.030161	protein_coding
ENSG0000(	IGSF5	293.2319	0.22079	0.007165	0.030169	protein_coding
ENSG0000(	LINC00642	140.224	0.163098	0.007169	0.030179	lncRNA
ENSG0000(	ENSG0000(	185.2631	0.208775	0.007172	0.030187	lncRNA
ENSG0000(	ZNF467	106.9731	0.338559	0.007173	0.030187	protein_coding
ENSG0000(	ABCD3	2262.199	-0.35978	0.007184	0.03023	protein_coding
ENSG0000(	CACNG2	16.82497	0.611419	0.007188	0.030242	protein_coding
ENSG0000(	ZNF224	2816.62	0.088505	0.00719	0.030247	protein_coding
ENSG0000(	ENSG0000(	38.61724	0.277987	0.007192	0.030254	lncRNA
ENSG0000(	SCNN1B	30.35825	0.726803	0.007199	0.030278	protein_coding
ENSG0000(	STON1-GTF	58.53005	0.97751	0.007206	0.030302	protein_coding
ENSG0000(	ENSG0000(	153.6135	0.17525	0.007209	0.030306	lncRNA
ENSG0000(	LINC02197	745.7314	0.373924	0.007208	0.030306	lncRNA
ENSG0000(	URI1	1188.021	-0.14812	0.007213	0.03032	protein_coding
ENSG0000(	ENSG0000(	45.26007	0.431459	0.007215	0.030325	lncRNA
ENSG0000(	MIR210HG	320.574	0.443452	0.007219	0.030336	lncRNA
ENSG0000(	RPL13AP12	55.24309	0.275898	0.007232	0.030388	processed_pseudogene
ENSG0000(	LINC02837	218.4663	-0.15618	0.007242	0.030428	lncRNA
ENSG0000(	MYO1E	3662.553	-0.2976	0.007246	0.030432	protein_coding
ENSG0000(	SAP30	77.83795	0.315812	0.007245	0.030432	protein_coding
ENSG0000(	SUCLG1	1468.36	-0.34422	0.007247	0.030432	protein_coding
ENSG0000(	TRMT10B	623.8117	-0.11043	0.007246	0.030432	protein_coding
ENSG0000(	TPT1P8	169.0434	-0.18858	0.007251	0.030443	transcribed_processed_pseudoge
ENSG0000(	IL17RA	2195.055	0.211888	0.007254	0.030452	protein_coding
ENSG0000(	ENSG0000(	91.21381	0.284124	0.007257	0.030461	lncRNA
ENSG0000(	IGKV6-21	22.13855	0.980048	0.007268	0.0305	IG_V_gene
ENSG0000(	KLF8	1700.858	0.114187	0.007268	0.0305	protein_coding
ENSG0000(	C2CD4B	42.10661	0.603816	0.007275	0.030521	protein_coding
ENSG0000(	CRYBG2	554.2247	-0.66208	0.007275	0.030521	protein_coding
ENSG0000(	ENSG0000(	33.93211	0.329912	0.007276	0.030522	lncRNA
ENSG0000(	GOLGA8J	121.1913	0.20561	0.00728	0.030535	protein_coding
ENSG0000(	ENSG0000(	12.27814	0.633262	0.007289	0.030567	lncRNA
ENSG0000(	FAM72D	55.2717	0.682454	0.007296	0.030591	protein_coding
ENSG0000(	ENSG0000(	37.45497	0.341624	0.007298	0.030598	lncRNA
ENSG0000(	PRG4	11.29994	0.947747	0.007301	0.030606	protein_coding
ENSG0000(	CCDC3	117.5194	0.551134	0.007304	0.030607	protein_coding
ENSG0000(	CXXC1	693.1735	-0.17013	0.007304	0.030607	protein_coding
ENSG0000(	SLC3A1	2330.929	-0.4973	0.007303	0.030607	protein_coding

ENSG0000(	CS	3082.86	-0.10109	0.007307	0.030613	protein_coding
ENSG0000(	KNOP1	1251.578	0.133777	0.007308	0.030613	protein_coding
ENSG0000(	ENSG0000(	97.31074	0.208341	0.007311	0.030624	lncRNA
ENSG0000(	ENSG0000(	27.71407	0.344759	0.007318	0.030631	lncRNA
ENSG0000(	FGFR1	2018.807	0.19673	0.007316	0.030631	protein_coding
ENSG0000(	MT-TK	55.7406	0.917284	0.007318	0.030631	Mt_tRNA
ENSG0000(	RPLP1	2495.534	-0.25326	0.007315	0.030631	protein_coding
ENSG0000(	ZNF117	1528.133	0.202764	0.007316	0.030631	protein_coding
ENSG0000(	DTX3L	3387.986	0.191057	0.007323	0.03065	protein_coding
ENSG0000(	UQCRC1	799.9846	-0.31049	0.007329	0.030668	protein_coding
ENSG0000(	ENSG0000(	84.72154	-0.21086	0.00733	0.030671	lncRNA
ENSG0000(	NAIPP2	405.9558	0.426012	0.007333	0.030677	unprocessed_pseudogene
ENSG0000(	ENSG0000(	453.3667	-0.10909	0.007335	0.030682	lncRNA
ENSG0000(	ENSG0000(	24.34673	0.369817	0.007338	0.030685	lncRNA
ENSG0000(	MIR4502	12.83593	0.456737	0.007337	0.030685	miRNA
ENSG0000(	P2RX3	55.43472	-0.29901	0.007352	0.03074	protein_coding
ENSG0000(	PTPN23	1262.903	-0.16614	0.007355	0.030751	protein_coding
ENSG0000(	ENSG0000(	149.3467	0.198934	0.00736	0.030767	lncRNA
ENSG0000(	ZNF474	9.068617	0.70895	0.00737	0.030804	protein_coding
ENSG0000(	CCDC80	1650.712	0.380011	0.007379	0.030838	protein_coding
ENSG0000(	ENSG0000(	159.5085	-0.17566	0.007382	0.030847	lncRNA
ENSG0000(	CENPNP1	268.0474	0.133625	0.007388	0.030852	processed_pseudogene
ENSG0000(	CLEC11A	49.99579	0.374323	0.007387	0.030852	protein_coding
ENSG0000(	DHRS4	358.2726	-0.37122	0.007388	0.030852	protein_coding
ENSG0000(	H2AC20	194.877	-0.27055	0.007386	0.030852	protein_coding
ENSG0000(	ST3GAL5	2897.573	0.354375	0.007385	0.030852	protein_coding
ENSG0000(	CPT2	1218.879	-0.12858	0.007391	0.030853	protein_coding
ENSG0000(	ZNF333	1763.971	0.090325	0.00739	0.030853	protein_coding
ENSG0000(	ENSG0000(	352.8345	0.231774	0.007396	0.030869	lncRNA
ENSG0000(	SNORD104	42.20895	0.433056	0.007396	0.030869	snoRNA
ENSG0000(	RAB26	127.843	0.464769	0.007398	0.03087	protein_coding
ENSG0000(	MIR648	29.14977	-0.3963	0.007406	0.030899	miRNA
ENSG0000(	WSB2	1381.622	-0.21628	0.007412	0.030921	protein_coding
ENSG0000(	ENSG0000(	254.6368	0.204951	0.007415	0.030932	lncRNA
ENSG0000(	DPY30	221.3419	-0.16001	0.007417	0.030933	protein_coding
ENSG0000(	PLPP1	646.5314	-0.22938	0.007428	0.030978	protein_coding
ENSG0000(	ENSG0000(	70.68156	0.230702	0.00743	0.030981	lncRNA
ENSG0000(	DNAJA4	1075.49	-0.237	0.007455	0.031081	protein_coding
ENSG0000(	MTMR11	3510.522	-0.47865	0.007456	0.031081	protein_coding
ENSG0000(	PLEKHG3	1859.182	0.244806	0.007461	0.031098	protein_coding
ENSG0000(	LINC02904	332.085	0.174454	0.007476	0.031154	lncRNA
ENSG0000(	ENSG0000(	61.52127	0.231409	0.007479	0.031163	lncRNA
ENSG0000(	ENSG0000(	358.9083	0.106055	0.007481	0.031168	lncRNA
ENSG0000(	ENSG0000(	595.103	0.094865	0.007488	0.031196	lncRNA
ENSG0000(	MANEA-DT	104.112	0.293697	0.007494	0.031213	lncRNA
ENSG0000(	FADS6	562.8449	-0.33124	0.007499	0.031229	protein_coding
ENSG0000(	FAM32A	686.2311	-0.17475	0.0075	0.031229	protein_coding

ENSG0000(	METTL7A	2008.463	-0.1709	0.007502	0.031229	protein_coding
ENSG0000(	TTC34	680.0607	0.208374	0.007501	0.031229	protein_coding
ENSG0000(	ABRACL	236.2548	-0.25165	0.007503	0.031233	protein_coding
ENSG0000(	CHIC2	275.5583	-0.20533	0.00751	0.031254	protein_coding
ENSG0000(	ZBED4	1039.502	0.105107	0.007512	0.031259	protein_coding
ENSG0000(	C18orf15	440.6142	0.208182	0.007514	0.031264	TEC
ENSG0000(	ENSG0000(	18.23872	0.363538	0.00754	0.031364	lncRNA
ENSG0000(	ENSG0000(	22.17482	0.343347	0.00754	0.031364	lncRNA
ENSG0000(	AMOTL2	558.2403	0.211019	0.007544	0.031366	protein_coding
ENSG0000(	ENSG0000(	1769.252	0.112287	0.007543	0.031366	lncRNA
ENSG0000(	FARP1	3146.565	0.126692	0.007543	0.031366	protein_coding
ENSG0000(	FBXW11	1384.841	0.110831	0.007544	0.031366	protein_coding
ENSG0000(	KIFBP	570.6103	0.114651	0.007548	0.031376	protein_coding
ENSG0000(	SNORA62	184.1024	0.319164	0.00755	0.031382	snoRNA
ENSG0000(	BMT2	298.7609	-0.19658	0.007553	0.031384	protein_coding
ENSG0000(	ENSG0000(	23.66615	0.339904	0.007553	0.031384	lncRNA
ENSG0000(	LINC02327	310.2807	-0.15149	0.007554	0.031384	lncRNA
ENSG0000(	COX10	930.7865	-0.21676	0.007555	0.031385	protein_coding
ENSG0000(	H1-2	551.4814	-0.28554	0.007556	0.031385	protein_coding
ENSG0000(	INPP5J	1212.952	-0.64684	0.007574	0.031456	protein_coding
ENSG0000(	ENSG0000(	569.7096	-0.09247	0.007575	0.031457	lncRNA
ENSG0000(	RPL17P50	55.98375	0.312074	0.007577	0.03146	processed_pseudogene
ENSG0000(	NFX1	3355.205	0.073444	0.007583	0.031478	protein_coding
ENSG0000(	OVOL2	240.696	-0.5176	0.007582	0.031478	protein_coding
ENSG0000(	ENSG0000(	9.92162	0.550521	0.007586	0.031484	lncRNA
ENSG0000(	ENSG0000(	44.32553	-0.26526	0.007588	0.03149	lncRNA
ENSG0000(	ENSG0000(	38.72462	0.311441	0.00759	0.031494	lncRNA
ENSG0000(	CEP44	929.6929	-0.13872	0.007594	0.031508	protein_coding
ENSG0000(	STX8	593.0056	-0.10169	0.007601	0.031529	protein_coding
ENSG0000(	ENSG0000(	41.58633	0.292413	0.007614	0.03158	lncRNA
ENSG0000(	ENSG0000(	87.21934	-0.25304	0.007617	0.031587	lncRNA
ENSG0000(	PDZK1	1877.762	-0.69072	0.007619	0.031591	protein_coding
ENSG0000(	ENSG0000(	286.5427	-0.14967	0.007624	0.031606	TEC
ENSG0000(	SELENOP	24233.54	-0.6591	0.007624	0.031606	protein_coding
ENSG0000(	IMPDH1	384.5552	0.294457	0.007627	0.031614	protein_coding
ENSG0000(	RN7SL43P	37.09861	0.291693	0.007631	0.031628	misc_RNA
ENSG0000(	ENSG0000(	59.88729	-0.30206	0.007633	0.031631	lncRNA
ENSG0000(	AUH	537.3869	-0.19241	0.007641	0.031645	protein_coding
ENSG0000(	KLF2-DT	28.79784	0.338116	0.007644	0.031645	lncRNA
ENSG0000(	LINC02436	96.59659	0.217919	0.007641	0.031645	lncRNA
ENSG0000(	LINC02723	148.779	0.141329	0.007643	0.031645	lncRNA
ENSG0000(	RN7SL461F	69.12725	0.234494	0.007639	0.031645	misc_RNA
ENSG0000(	RPGR	888.7678	0.142572	0.00764	0.031645	protein_coding
ENSG0000(	STARD9	3706.89	0.13281	0.007643	0.031645	protein_coding
ENSG0000(	ENSG0000(	10.11188	0.533681	0.007646	0.031652	lncRNA
ENSG0000(	ENSG0000(	199.7927	0.169805	0.007649	0.031659	lncRNA
ENSG0000(	GIPC2	1461.974	-0.5419	0.007658	0.031692	protein_coding

ENSG0000(	XAF1	8455.231	0.40432	0.00767	0.031736	protein_coding
ENSG0000(	ENSG0000(	693.6409	0.179341	0.007671	0.031736	lncRNA
ENSG0000(	LRRC18	49.19694	0.285301	0.007672	0.031736	protein_coding
ENSG0000(	C9orf50	140.8484	-0.1786	0.007673	0.031738	protein_coding
ENSG0000(	ENSG0000(	24.4867	0.344593	0.00768	0.031763	lncRNA
ENSG0000(	ENSG0000(	110.2523	-0.21734	0.007684	0.031773	lncRNA
ENSG0000(	DACT2	42.42515	-0.65932	0.007698	0.031824	protein_coding
ENSG0000(	NNT-AS1	2308.564	0.07995	0.007698	0.031824	lncRNA
ENSG0000(	GDF6	66.91445	0.519259	0.007701	0.031832	protein_coding
ENSG0000(	ENSG0000(	11.6271	0.62985	0.007704	0.031837	lncRNA
ENSG0000(	ENSG0000(	262.222	-0.15991	0.007704	0.031837	TEC
ENSG0000(	ENSG0000(	75.70276	0.195907	0.00771	0.031859	lncRNA
ENSG0000(	SLC5A10	31.67205	0.432291	0.007713	0.031861	protein_coding
ENSG0000(	ZFTA	482.2992	0.236784	0.007713	0.031861	protein_coding
ENSG0000(	ENSG0000(	551.5625	0.308096	0.007716	0.031871	lncRNA
ENSG0000(	ANKRD40C	1555.933	-0.34343	0.007728	0.031908	protein_coding
ENSG0000(	ENSG0000(	61.49772	-0.33305	0.007728	0.031908	lncRNA
ENSG0000(	RPS11	3497.571	-0.23655	0.007728	0.031908	protein_coding
ENSG0000(	GJA5	50.02313	0.598678	0.00773	0.03191	protein_coding
ENSG0000(	ENSG0000(	17.24043	0.535178	0.007737	0.031935	lncRNA
ENSG0000(	RAPGEFL1	2416.965	-0.44055	0.007747	0.031973	protein_coding
ENSG0000(	GTPBP8	393.7616	-0.13481	0.007749	0.031975	protein_coding
ENSG0000(	USP22	3310.64	-0.09279	0.007751	0.03198	protein_coding
ENSG0000(	ENSG0000(	19.92987	0.407654	0.007755	0.031986	TEC
ENSG0000(	PLXNC1	1566.552	0.458486	0.007755	0.031986	protein_coding
ENSG0000(	RN7SKP71	11.43926	-0.5466	0.007756	0.031986	misc_RNA
ENSG0000(	RPL35A	1480.211	-0.22841	0.007755	0.031986	protein_coding
ENSG0000(	RNU6-1316	19.98702	-0.51269	0.007764	0.032011	snRNA
ENSG0000(	UAP1L1	554.1251	0.281213	0.007765	0.032011	protein_coding
ENSG0000(	ENSG0000(	628.0827	0.149135	0.007771	0.032033	lncRNA
ENSG0000(	RPEL1	20.56244	0.458754	0.007782	0.032074	protein_coding
ENSG0000(	CELSR3	197.4421	0.2991	0.007783	0.032075	protein_coding
ENSG0000(	NDUFB7	132.5236	-0.3337	0.007787	0.032084	protein_coding
ENSG0000(	VSIG10L-AS	11.56622	-0.43821	0.007787	0.032084	lncRNA
ENSG0000(	TULP4	5502.946	-0.25304	0.007795	0.032113	protein_coding
ENSG0000(	LINC00399	10.46146	0.622992	0.007802	0.032137	lncRNA
ENSG0000(	ENSG0000(	149.9244	-0.46165	0.007812	0.032173	TEC
ENSG0000(	CEP152	1444.736	0.119664	0.007814	0.032176	protein_coding
ENSG0000(	CCDC110	25.6138	0.555526	0.007823	0.03221	protein_coding
ENSG0000(	ENSG0000(	552.0166	-0.1166	0.007827	0.032222	TEC
ENSG0000(	FAM218A	45.21198	0.324356	0.007832	0.032234	lncRNA
ENSG0000(	ZFAND2B	473.8783	-0.20457	0.007831	0.032234	protein_coding
ENSG0000(	ENSG0000(	357.2751	-0.10902	0.00784	0.032262	lncRNA
ENSG0000(	CYP2AB1P	21.71212	0.432929	0.007847	0.032288	transcribed_unitary_pseudogene
ENSG0000(	CFAP54	2346.381	-0.16276	0.007859	0.032329	protein_coding
ENSG0000(	KAT2B	1942.641	-0.13269	0.007858	0.032329	protein_coding
ENSG0000(	ENSG0000(	165.7236	0.197344	0.007863	0.03234	lncRNA

ENSG0000(	LDAF1	374.8029	-0.15886	0.007868	0.032355	protein_coding
ENSG0000(	RN7SL566F	56.71778	0.207408	0.007868	0.032355	misc_RNA
ENSG0000(	ENSG0000(	7.959189	0.704418	0.007871	0.032359	lncRNA
ENSG0000(	FAM200A	158.385	-0.18459	0.007871	0.032359	protein_coding
ENSG0000(	GTF2IP12	3008.075	0.13657	0.007877	0.032377	transcribed_unprocessed_pseudo
ENSG0000(	FBXL7	277.0388	0.255885	0.007881	0.032389	protein_coding
ENSG0000(	PRR3	1055.823	0.145083	0.007893	0.032436	protein_coding
ENSG0000(	TACR2	93.2811	0.264855	0.007903	0.03247	protein_coding
ENSG0000(	ZFHX2-AS1	700.0218	0.163106	0.007906	0.03248	lncRNA
ENSG0000(	ERMAP	707.9506	0.144385	0.007912	0.032499	protein_coding
ENSG0000(	ENSG0000(	164.0922	0.160115	0.007913	0.032499	lncRNA
ENSG0000(	EIF3H	1362.565	-0.15187	0.007914	0.032501	protein_coding
ENSG0000(	GPR199P	121.8367	0.308074	0.007916	0.032502	transcribed_unitary_pseudogene
ENSG0000(	ADPGK	3161.867	0.203853	0.007917	0.032505	protein_coding
ENSG0000(	C19orf33	174.4783	-0.61349	0.007925	0.032531	protein_coding
ENSG0000(	ZNF417	1968.635	0.128376	0.007927	0.032537	protein_coding
ENSG0000(	ENSG0000(	85.89714	0.30823	0.007935	0.032565	lncRNA
ENSG0000(	IER5	624.0951	0.211442	0.00794	0.032583	protein_coding
ENSG0000(	DPY19L2	162.451	0.360953	0.007944	0.032588	protein_coding
ENSG0000(	ENSG0000(	122.1841	0.221818	0.007945	0.032588	lncRNA
ENSG0000(	SLITRK6	414.7605	-0.66615	0.007943	0.032588	protein_coding
ENSG0000(	PROKR2	257.505	0.120879	0.007959	0.032637	protein_coding
ENSG0000(	RNU1-21P	21.64656	-0.70056	0.007959	0.032637	snRNA
ENSG0000(	LINC02005	23.3545	0.334324	0.007961	0.032644	lncRNA
ENSG0000(	NDRG1	11272.3	-0.48315	0.007965	0.032654	protein_coding
ENSG0000(	ODAD2	42.48221	0.374579	0.007972	0.032677	protein_coding
ENSG0000(	ENSG0000(	142.2795	0.152354	0.007981	0.032712	lncRNA
ENSG0000(	RASAL2	1627.529	0.166988	0.007984	0.032719	protein_coding
ENSG0000(	ENSG0000(	328.4755	0.230912	0.007992	0.032745	TEC
ENSG0000(	TMEM184/	3168.916	-0.5169	0.007992	0.032745	protein_coding
ENSG0000(	TMCO4	492.1696	0.160265	0.008001	0.032777	protein_coding
ENSG0000(	ENSG0000(	301.8332	0.269889	0.008008	0.03279	lncRNA
ENSG0000(	LINC02119	67.71236	0.270674	0.008006	0.03279	lncRNA
ENSG0000(	NAPG	1478.24	0.12289	0.008008	0.03279	protein_coding
ENSG0000(	GABRB2	386.1122	-0.12748	0.00801	0.032794	protein_coding
ENSG0000(	ENSG0000(	260.8537	-0.14282	0.008012	0.032799	lncRNA
ENSG0000(	PTMAP5	9.597531	0.560865	0.008022	0.032835	transcribed_processed_pseudoge
ENSG0000(	ENSG0000(	36.59948	0.31606	0.008024	0.032841	transcribed_processed_pseudoge
ENSG0000(	ENSG0000(	62.53112	0.294316	0.008027	0.03285	lncRNA
ENSG0000(	SMIM2-AS:	884.6165	-0.56465	0.008029	0.032852	lncRNA
ENSG0000(	DPF2	1920.047	-0.07797	0.008038	0.032885	protein_coding
ENSG0000(	TMTC3	648.6918	-0.16875	0.008039	0.032885	protein_coding
ENSG0000(	ENSG0000(	310.3789	0.151249	0.008043	0.032896	lncRNA
ENSG0000(	PPARA	6398.431	-0.29941	0.008044	0.032898	protein_coding
ENSG0000(	ENSG0000(	32.23967	0.501881	0.008052	0.032925	lncRNA
ENSG0000(	SLC22A3	221.9492	0.376229	0.008053	0.032925	protein_coding
ENSG0000(	IGLV3-1	141.1047	0.667358	0.00807	0.032989	IG_V_gene

ENSG0000(	ENSG0000(	3710.697	-0.10763	0.008075	0.033006	lncRNA
ENSG0000(	ENSG0000(	17.76882	0.381411	0.008077	0.033007	lncRNA
ENSG0000(	LINC00167	89.30323	-0.1622	0.008078	0.033007	lncRNA
ENSG0000(	SMC1A	2452.379	-0.16342	0.008078	0.033007	protein_coding
ENSG0000(	ENSG0000(	24.40059	0.321422	0.008089	0.033028	lncRNA
ENSG0000(	ENSG0000(	38.36066	0.300568	0.00809	0.033028	lncRNA
ENSG0000(	ENSG0000(	308.7589	0.151971	0.00809	0.033028	lncRNA
ENSG0000(	RN7SL36P	16.02587	0.377691	0.008088	0.033028	misc_RNA
ENSG0000(	RN7SL569F	22.16726	0.436505	0.008088	0.033028	misc_RNA
ENSG0000(	TMEM44-A	189.0598	0.198343	0.008086	0.033028	lncRNA
ENSG0000(	SLC25A18	11.88894	0.715356	0.008097	0.033052	protein_coding
ENSG0000(	LINC01011	265.8853	0.184046	0.008099	0.033055	lncRNA
ENSG0000(	ENSG0000(	233.0427	0.124197	0.008102	0.033063	lncRNA
ENSG0000(	NUDT19P5	26.66348	0.446414	0.008111	0.033098	processed_pseudogene
ENSG0000(	SNX4	490.699	-0.19334	0.008112	0.033098	protein_coding
ENSG0000(	ENSG0000(	10.06607	0.587539	0.008116	0.033109	lncRNA
ENSG0000(	ZMAT4	13.06785	0.602141	0.00812	0.03312	protein_coding
ENSG0000(	IL4R	3505.567	0.234261	0.00813	0.033156	protein_coding
ENSG0000(	GLO1	634.6024	-0.29745	0.008136	0.03318	protein_coding
ENSG0000(	ENSG0000(	140.1659	-0.1417	0.008148	0.033218	lncRNA
ENSG0000(	UBTFL11	62.90846	0.303894	0.008148	0.033218	processed_pseudogene
ENSG0000(	ENSG0000(	443.5665	0.166939	0.008151	0.033228	lncRNA
ENSG0000(	ENSG0000(	12.50864	0.535538	0.008163	0.033271	processed_pseudogene
ENSG0000(	LINC02112	57.34875	0.244539	0.008169	0.03329	lncRNA
ENSG0000(	ARPC1A	780.7097	-0.22441	0.008175	0.033311	protein_coding
ENSG0000(	RN7SL657F	28.79063	1.09667	0.008176	0.033311	misc_RNA
ENSG0000(	ENSG0000(	12.36145	0.525689	0.00818	0.033321	lncRNA
ENSG0000(	GZF1	311.1727	0.187935	0.008185	0.033337	protein_coding
ENSG0000(	CCL15	211.8414	-0.42527	0.008196	0.033377	protein_coding
ENSG0000(	KCNMB3	403.573	0.198518	0.008197	0.033377	protein_coding
ENSG0000(	TMEM238	49.89876	-0.44883	0.008201	0.033389	protein_coding
ENSG0000(	TRIM50	34.88083	0.647839	0.008206	0.033408	protein_coding
ENSG0000(	ENSG0000(	314.7372	-0.15725	0.008215	0.033441	lncRNA
ENSG0000(	ENSG0000(	1528.064	-0.42633	0.008217	0.033442	lncRNA
ENSG0000(	SLC30A6	1168.82	-0.14034	0.008218	0.033442	protein_coding
ENSG0000(	ENSG0000(	25.60363	0.381719	0.008223	0.033458	lncRNA
ENSG0000(	SGSM2	2090.381	0.148259	0.008224	0.033458	protein_coding
ENSG0000(	PARL	405.9545	-0.12349	0.008226	0.033464	protein_coding
ENSG0000(	ENSG0000(	167.1578	-0.20886	0.008228	0.033468	lncRNA
ENSG0000(	CCDC93	2966.733	-0.17585	0.008232	0.033478	protein_coding
ENSG0000(	LINC02376	170.9044	0.355936	0.008233	0.033479	lncRNA
ENSG0000(	ENSG0000(	35.23502	0.323938	0.008236	0.033486	lncRNA
ENSG0000(	NOTCH3	1039.897	0.18197	0.008237	0.033486	protein_coding
ENSG0000(	ENSG0000(	879.9922	0.113175	0.008241	0.033496	lncRNA
ENSG0000(	SNHG17	1962.029	0.099392	0.008247	0.033519	lncRNA
ENSG0000(	ENSG0000(	114.0375	0.226853	0.008257	0.033552	lncRNA
ENSG0000(	RN7SL516F	9.571915	0.501133	0.008257	0.033552	misc_RNA

ENSG0000(	ENSG0000(	57.62042	0.307054	0.008261	0.033563	lncRNA
ENSG0000(	MFF-DT	1264.964	0.093607	0.008262	0.033563	lncRNA
ENSG0000(	LINC02609	445.4353	0.106539	0.008271	0.033592	lncRNA
ENSG0000(	ENSG0000(	52.25411	0.354295	0.008285	0.033648	lncRNA
ENSG0000(	ENSG0000(	51.54889	-0.24522	0.008289	0.033657	processed_pseudogene
ENSG0000(	LINC01602	90.91747	0.211099	0.008292	0.033668	lncRNA
ENSG0000(	ABCA4	86.29994	0.548308	0.008297	0.033675	protein_coding
ENSG0000(	DNAJC25-G	144.3841	0.457693	0.008296	0.033675	protein_coding
ENSG0000(	IFI44L	2397.649	0.759405	0.008297	0.033675	protein_coding
ENSG0000(	EMC9	133.8577	-0.28541	0.0083	0.033678	protein_coding
ENSG0000(	EVPL	772.3178	-0.32118	0.0083	0.033678	protein_coding
ENSG0000(	LRCH2	113.0063	0.281544	0.008319	0.033748	protein_coding
ENSG0000(	ENSG0000(	83.05443	0.285082	0.008324	0.033765	lncRNA
ENSG0000(	PEX11A	564.6907	-0.15968	0.00833	0.033786	protein_coding
ENSG0000(	PHF21B	19.33465	0.631071	0.008339	0.033817	protein_coding
ENSG0000(	KLHL5	2546.988	0.27608	0.008341	0.03382	protein_coding
ENSG0000(	NCKAP1	4511.873	-0.30207	0.008342	0.033821	protein_coding
ENSG0000(	MCC	916.2363	0.290341	0.008348	0.033839	protein_coding
ENSG0000(	ATG9A	933.353	-0.20832	0.008351	0.033846	protein_coding
ENSG0000(	ENSG0000(	353.3347	0.130057	0.008351	0.033846	lncRNA
ENSG0000(	IRF9	2234.576	0.183098	0.008354	0.033848	protein_coding
ENSG0000(	RIPK2-DT	14471.3	0.162737	0.008353	0.033848	lncRNA
ENSG0000(	ENSG0000(	45.03765	0.270485	0.008356	0.033852	lncRNA
ENSG0000(	TAFAZZIN	1027.63	0.122788	0.008358	0.033856	protein_coding
ENSG0000(	ENSG0000(	25.80288	0.340138	0.008372	0.033909	lncRNA
ENSG0000(	KCNJ11	231.609	-0.14341	0.008375	0.033916	protein_coding
ENSG0000(	RN7SL378F	69.75351	0.183992	0.008382	0.033939	misc_RNA
ENSG0000(	PDZD3	1527.203	-0.57277	0.008392	0.033976	protein_coding
ENSG0000(	ENSG0000(	81.72556	-0.69239	0.008409	0.034041	lncRNA
ENSG0000(	ASB2	385.2146	-0.31631	0.008412	0.034049	protein_coding
ENSG0000(	PABIR3	1451.79	-0.0884	0.008418	0.034068	protein_coding
ENSG0000(	ENSG0000(	28.68679	0.300019	0.008433	0.034117	processed_pseudogene
ENSG0000(	VWA8	1444.781	-0.16147	0.008433	0.034117	protein_coding
ENSG0000(	ZFYVE1	784.6515	-0.21921	0.008432	0.034117	protein_coding
ENSG0000(	RRN3	758.0731	-0.15979	0.008452	0.034187	protein_coding
ENSG0000(	ZNF383	1992.745	-0.09879	0.008455	0.034197	protein_coding
ENSG0000(	ZNF480	1204.562	-0.15145	0.008462	0.034219	protein_coding
ENSG0000(	PANTR1	243.3235	0.14399	0.008474	0.034265	lncRNA
ENSG0000(	RNF217	1019.499	0.150229	0.008476	0.034265	protein_coding
ENSG0000(	SPRTN	370.8784	0.119889	0.008477	0.034265	protein_coding
ENSG0000(	ZBED8	139.9571	0.203133	0.008478	0.034265	protein_coding
ENSG0000(	ENSG0000(	12.94233	-0.50959	0.008488	0.034299	lncRNA
ENSG0000(	KCNA3	448.6025	0.497641	0.008488	0.034299	protein_coding
ENSG0000(	ENSG0000(	10.61362	0.648192	0.00849	0.034302	lncRNA
ENSG0000(	RPL23AP82	1012.081	0.134061	0.008491	0.034302	transcribed_unprocessed_pseudo
ENSG0000(	ENSG0000(	12.66399	0.612623	0.0085	0.034331	processed_pseudogene
ENSG0000(	ENSG0000(	1251.807	0.085615	0.0085	0.034331	lncRNA

ENSG0000(	THAP12	1460.446	-0.13127	0.008506	0.03435	protein_coding
ENSG0000(	POU2F3	266.0821	-0.17703	0.008512	0.034368	protein_coding
ENSG0000(	SGSH	1147.305	0.167729	0.008514	0.034373	protein_coding
ENSG0000(	KLHL21	1086.013	0.155288	0.008519	0.03439	protein_coding
ENSG0000(	CREBZF	3637.479	0.092585	0.008522	0.034393	protein_coding
ENSG0000(	DRGX	16.64303	-1.08933	0.008522	0.034393	protein_coding
ENSG0000(	C9orf57	39.85108	-0.42795	0.008534	0.03443	protein_coding
ENSG0000(	XYLB	943.8476	-0.30103	0.008533	0.03443	protein_coding
ENSG0000(	CTSD	4936.971	-0.27693	0.008536	0.034434	protein_coding
ENSG0000(	C6orf132	1543.293	-0.31188	0.008537	0.034436	protein_coding
ENSG0000(	EML2	830.2533	0.137591	0.008541	0.03444	protein_coding
ENSG0000(	ENSG0000(	127.687	-0.18309	0.008539	0.03444	pseudogene
ENSG0000(	RPL7P18	21.51441	0.504891	0.008542	0.034442	processed_pseudogene
ENSG0000(	SNORA2A	42.4174	0.251405	0.008548	0.03446	snoRNA
ENSG0000(	TRDN-AS1	1115.574	0.170731	0.008557	0.034495	lncRNA
ENSG0000(	CAMK2N1	1873.171	-0.52482	0.008568	0.034533	protein_coding
ENSG0000(	ENSG0000(	849.8772	0.111369	0.008569	0.034534	lncRNA
ENSG0000(	PBX3	631.3413	0.289215	0.008579	0.034569	protein_coding
ENSG0000(	AKAP8	1366.467	0.088162	0.008581	0.034573	protein_coding
ENSG0000(	AGPAT1	715.6663	-0.22588	0.00859	0.034604	protein_coding
ENSG0000(	RAB9B	78.76131	0.26787	0.008607	0.034667	protein_coding
ENSG0000(	ENSG0000(	302.2408	-0.13005	0.008608	0.034669	lncRNA
ENSG0000(	ENSG0000(	8.659031	0.573523	0.008617	0.0347	TEC
ENSG0000(	PON1	30.5893	-0.36858	0.00862	0.034707	protein_coding
ENSG0000(	ENSG0000(	110.1091	0.187062	0.008626	0.034726	lncRNA
ENSG0000(	ENSG0000(	18.23621	0.454101	0.00863	0.034738	lncRNA
ENSG0000(	HNF4G	4939.819	-0.54064	0.008633	0.034745	protein_coding
ENSG0000(	PCP2	14.79597	-0.46325	0.008648	0.034801	protein_coding
ENSG0000(	KIF25-AS1	69.90154	-0.20554	0.008652	0.034815	lncRNA
ENSG0000(	CPSF1	2067.751	-0.26681	0.008653	0.034815	protein_coding
ENSG0000(	TMEM147	148.0488	-0.29093	0.008656	0.034821	protein_coding
ENSG0000(	ENSG0000(	762.2072	0.202848	0.008659	0.034824	lncRNA
ENSG0000(	ENSG0000(	8.200583	0.657453	0.008659	0.034824	lncRNA
ENSG0000(	DNAJC1	380.0253	0.182244	0.008665	0.034842	protein_coding
ENSG0000(	HEMK1	3094.346	0.107293	0.008666	0.034845	protein_coding
ENSG0000(	TDRP	240.1519	0.316279	0.008671	0.03486	protein_coding
ENSG0000(	NUTM2E	90.10996	0.314915	0.008676	0.034873	protein_coding
ENSG0000(	MED14	2473.325	-0.13359	0.008686	0.034912	protein_coding
ENSG0000(	ENSG0000(	70.046	0.469884	0.008689	0.034919	lncRNA
ENSG0000(	RPS20P9	23.89797	0.301252	0.008692	0.034927	processed_pseudogene
ENSG0000(	ENSG0000(	74.49535	-0.23435	0.0087	0.034953	lncRNA
ENSG0000(	ENSG0000(	185.4318	-0.17556	0.008714	0.035001	lncRNA
ENSG0000(	ENSG0000(	61.32951	0.323124	0.008715	0.035001	lncRNA
ENSG0000(	RTN1	130.8796	0.370083	0.008715	0.035001	protein_coding
ENSG0000(	DYRK2	5999.571	-0.26748	0.008728	0.035042	protein_coding
ENSG0000(	ENSG0000(	121.0353	0.163044	0.008727	0.035042	lncRNA
ENSG0000(	CNGA1	219.6993	-0.51168	0.008732	0.035055	protein_coding

ENSG0000(	DUTP6	9.861892	0.529516	0.008747	0.035112	processed_pseudogene
ENSG0000(	SSUH2	300.7685	-0.46321	0.008755	0.035137	protein_coding
ENSG0000(	CEACAM7	512.6785	1.703477	0.008762	0.035152	protein_coding
ENSG0000(	ENSG0000(	15.51522	0.38781	0.008761	0.035152	lncRNA
ENSG0000(	RRM2B	585.5941	-0.2075	0.00876	0.035152	protein_coding
ENSG0000(	PSME4	2904.509	0.177703	0.008766	0.035164	protein_coding
ENSG0000(	ENSG0000(	160.1328	0.149384	0.00877	0.035175	lncRNA
ENSG0000(	GP6	239.0952	0.148442	0.008771	0.035177	protein_coding
ENSG0000(	MFSD4B	9509.078	-0.21864	0.008774	0.035185	protein_coding
ENSG0000(	LUC7L	2962.344	-0.09808	0.00878	0.035203	protein_coding
ENSG0000(	TPD52	3277.995	-0.15507	0.008786	0.035221	protein_coding
ENSG0000(	ENSG0000(	39.31168	0.289918	0.008794	0.035252	lncRNA
ENSG0000(	ENSG0000(	34.77369	-0.38578	0.008797	0.035256	lncRNA
ENSG0000(	NOVA1-DT	56.26453	-0.55825	0.008802	0.035267	lncRNA
ENSG0000(	RMRP	24652.3	-0.33146	0.008802	0.035267	lncRNA
ENSG0000(	ENSG0000(	102.2092	0.190344	0.008803	0.03527	lncRNA
ENSG0000(	ENSG0000(	77.45468	-0.23834	0.008805	0.03527	lncRNA
ENSG0000(	OSGIN1	505.5033	-0.38742	0.008807	0.035273	protein_coding
ENSG0000(	PLEKHA8P1	250.8741	0.151851	0.008809	0.03528	transcribed_processed_pseudoge
ENSG0000(	ENSG0000(	126.8729	-0.16108	0.008819	0.035314	lncRNA
ENSG0000(	ENSG0000(	1405.741	0.13363	0.008829	0.035343	lncRNA
ENSG0000(	FKBP3	502.0411	-0.12634	0.008828	0.035343	protein_coding
ENSG0000(	RPAP3-DT	312.9778	0.199042	0.008841	0.03539	lncRNA
ENSG0000(	ATP6V0E1	706.1903	-0.20334	0.008843	0.035394	protein_coding
ENSG0000(	C1orf141	55.99399	-0.34323	0.008846	0.035399	protein_coding
ENSG0000(	GNL1	790.5663	0.150914	0.008858	0.035443	protein_coding
ENSG0000(	RBM5	8426.205	-0.05068	0.008865	0.035465	protein_coding
ENSG0000(	SEC23IP	1882.476	0.143644	0.008867	0.03547	protein_coding
ENSG0000(	SECTM1	1134.114	-0.53665	0.008872	0.035486	protein_coding
ENSG0000(	YWHAH	771.2278	-0.16674	0.008874	0.03549	protein_coding
ENSG0000(	SCGB3A2	436.756	0.242394	0.008878	0.035499	protein_coding
ENSG0000(	OR7E7P	77.97653	-0.29003	0.008889	0.03554	unprocessed_pseudogene
ENSG0000(	SLC26A3	3310.01	-0.76778	0.008893	0.035552	protein_coding
ENSG0000(	PROK2	33.42081	2.683462	0.008899	0.035571	protein_coding
ENSG0000(	ENSG0000(	90.54188	0.31206	0.008905	0.035588	lncRNA
ENSG0000(	GZMK	49.40704	-0.77802	0.008905	0.035588	protein_coding
ENSG0000(	LINC02316	23.30273	0.399544	0.008914	0.035618	lncRNA
ENSG0000(	CD2BP2	851.872	-0.11595	0.008919	0.035631	protein_coding
ENSG0000(	ENSG0000(	19.6088	-0.56954	0.00892	0.035631	processed_pseudogene
ENSG0000(	TTC32-DT	8.585505	0.542283	0.008924	0.035644	lncRNA
ENSG0000(	ENSG0000(	112.2543	0.169022	0.00893	0.035664	lncRNA
ENSG0000(	CNOT6	1398.84	-0.1559	0.008933	0.035671	protein_coding
ENSG0000(	GGT8P	121.2208	-0.73935	0.008938	0.035687	unprocessed_pseudogene
ENSG0000(	LIG1	783.2696	-0.21608	0.008954	0.035745	protein_coding
ENSG0000(	MRPS18C	846.4403	-0.10016	0.008962	0.035772	protein_coding
ENSG0000(	CEBPD	348.5601	0.34687	0.008965	0.035779	protein_coding
ENSG0000(	AGXT	217.568	-0.16221	0.008966	0.03578	protein_coding

ENSG0000(	RBM27	1191.555	-0.15693	0.008971	0.035795	protein_coding
ENSG0000(	BNIP5	1095.299	-0.64462	0.008974	0.035805	protein_coding
ENSG0000(	ENSG0000(	78.38839	0.317225	0.008983	0.035835	protein_coding
ENSG0000(	DHX9	2049.724	-0.13248	0.008989	0.035854	protein_coding
ENSG0000(	ENSG0000(	14.95867	0.517507	0.008994	0.03587	lncRNA
ENSG0000(	RN7SL703F	11.36425	0.438947	0.009003	0.0359	misc_RNA
ENSG0000(	ENSG0000(	255.0898	-0.13434	0.009017	0.035951	processed_pseudogene
ENSG0000(	ENSG0000(	62.24105	0.281182	0.009024	0.035961	transcribed_processed_pseudoge
ENSG0000(	ENSG0000(	10.31896	0.642478	0.009023	0.035961	lncRNA
ENSG0000(	PIK3R5	935.9493	0.402694	0.009021	0.035961	protein_coding
ENSG0000(	RELL1	1148.827	-0.21453	0.009023	0.035961	protein_coding
ENSG0000(	PCNA	208.9787	-0.2998	0.009027	0.035967	protein_coding
ENSG0000(	PDE1A	219.9482	0.256586	0.009028	0.035967	protein_coding
ENSG0000(	FGF1	42.75308	0.346015	0.009037	0.035998	protein_coding
ENSG0000(	TOR4A	550.1363	0.189282	0.009038	0.036	protein_coding
ENSG0000(	ADAT2	1010.735	0.201648	0.009044	0.036003	protein_coding
ENSG0000(	AGPAT3	3773.118	-0.17078	0.009043	0.036003	protein_coding
ENSG0000(	ENSG0000(	154.3105	0.326056	0.009043	0.036003	lncRNA
ENSG0000(	GNAL	484.6712	-0.25028	0.009043	0.036003	protein_coding
ENSG0000(	ENSG0000(	209.4964	0.215792	0.009052	0.036023	lncRNA
ENSG0000(	FILIP1	347.7943	0.188437	0.009052	0.036023	protein_coding
ENSG0000(	OR52P1P	11.54289	0.784235	0.009052	0.036023	polymorphic_pseudogene
ENSG0000(	OR6K3	122.46	-0.19081	0.009053	0.036023	protein_coding
ENSG0000(	ENSG0000(	579.4651	0.160863	0.009062	0.036048	lncRNA
ENSG0000(	ENSG0000(	127.6775	0.220972	0.009061	0.036048	protein_coding
ENSG0000(	AMPD1	59.90793	0.660211	0.009067	0.036065	protein_coding
ENSG0000(	BTNL3	3015.886	-0.57698	0.009072	0.03608	protein_coding
ENSG0000(	ENSG0000(	347.0149	0.144182	0.009078	0.0361	lncRNA
ENSG0000(	ENSG0000(	26.76372	0.39672	0.00908	0.036103	lncRNA
ENSG0000(	ENSG0000(	104.0236	0.277048	0.009086	0.036124	processed_pseudogene
ENSG0000(	BDNF-AS	711.4952	0.128332	0.009102	0.036159	lncRNA
ENSG0000(	ENSG0000(	21.80619	0.37647	0.009102	0.036159	lncRNA
ENSG0000(	ENSG0000(	511.7477	0.112644	0.009102	0.036159	TEC
ENSG0000(	GPR65	519.5606	0.364467	0.009099	0.036159	protein_coding
ENSG0000(	SULT1A4	168.2612	-0.576	0.009097	0.036159	protein_coding
ENSG0000(	ZNF843	343.0588	-0.10547	0.009101	0.036159	protein_coding
ENSG0000(	CPLX1	30.69348	0.459961	0.009118	0.036217	protein_coding
ENSG0000(	LSS	1258.976	0.219131	0.00912	0.036221	protein_coding
ENSG0000(	IDH2	1113.206	0.236343	0.009123	0.036228	protein_coding
ENSG0000(	NKX2-3	187.4773	-0.30113	0.009133	0.036261	protein_coding
ENSG0000(	ENSG0000(	11.14309	-0.45386	0.009137	0.036271	lncRNA
ENSG0000(	ENSG0000(	241.5229	0.133618	0.009137	0.036271	lncRNA
ENSG0000(	OAS2	1896.206	0.31466	0.009147	0.036304	protein_coding
ENSG0000(	MNS1	114.5457	-0.25841	0.009149	0.036307	protein_coding
ENSG0000(	CEP112	857.3401	0.103804	0.009151	0.036311	protein_coding
ENSG0000(	RPA2	236.6345	-0.24285	0.009157	0.03633	protein_coding
ENSG0000(	TEN1-CDK3	644.3607	-0.16898	0.009167	0.036366	protein_coding

ENSG0000(	RN7SL487F	23.95354	0.369246	0.00918	0.036414	misc_RNA
ENSG0000(	ERMARD	824.6089	0.124705	0.009192	0.036444	protein_coding
ENSG0000(	LINC00620	53.2458	0.252772	0.009192	0.036444	lncRNA
ENSG0000(	MRPS24	253.6839	-0.24797	0.009193	0.036444	protein_coding
ENSG0000(	RBM26-AS:	578.7709	0.1402	0.009192	0.036444	lncRNA
ENSG0000(	FAM86HP	327.6857	0.226678	0.009196	0.036453	transcribed_unprocessed_pseudo
ENSG0000(	OR1D2	427.878	0.122651	0.009197	0.036453	protein_coding
ENSG0000(	GPKOW	168.1234	-0.19604	0.009201	0.036464	protein_coding
ENSG0000(	PRRC2C	13118.34	-0.1061	0.009206	0.036481	protein_coding
ENSG0000(	ENSG0000(	33.16183	0.291102	0.009222	0.036535	lncRNA
ENSG0000(	TCEANC	460.9399	-0.1344	0.009223	0.036535	protein_coding
ENSG0000(	MIR5699	9.175459	0.537796	0.009224	0.036538	miRNA
ENSG0000(	RN7SL530F	20.25076	0.400401	0.009244	0.036612	misc_RNA
ENSG0000(	LRRC37A16	34.38661	0.615999	0.009253	0.036642	transcribed_unprocessed_pseudo
ENSG0000(	GEN1	1924.296	0.233557	0.009258	0.036658	protein_coding
ENSG0000(	LINC02857	68.42739	0.197469	0.009272	0.036709	lncRNA
ENSG0000(	LINC00216	137.2178	0.283047	0.009275	0.036715	lncRNA
ENSG0000(	ABRAXAS2	399.7518	-0.11215	0.009286	0.036755	protein_coding
ENSG0000(	TPCN1	3783.621	-0.17528	0.009287	0.036755	protein_coding
ENSG0000(	MRPL11	142.8881	-0.27311	0.00929	0.036759	protein_coding
ENSG0000(	ITGA9-AS1	1343.174	0.098063	0.009292	0.036765	lncRNA
ENSG0000(	CDS1	1065.799	-0.50274	0.009294	0.036768	protein_coding
ENSG0000(	ENSG0000(	429.845	0.232926	0.009299	0.036781	lncRNA
ENSG0000(	RASAL1	181.4341	0.2789	0.009309	0.036818	protein_coding
ENSG0000(	ENSG0000(	38.8323	0.410951	0.009324	0.036872	lncRNA
ENSG0000(	ENSG0000(	22.51416	0.329454	0.009328	0.03688	lncRNA
ENSG0000(	MARCHF8	2138.869	-0.2088	0.009327	0.03688	protein_coding
ENSG0000(	ENSG0000(	126.1143	0.246876	0.009337	0.03691	TEC
ENSG0000(	RABGAP1L-	1292.249	-0.10363	0.009339	0.036915	lncRNA
ENSG0000(	SLC25A11	470.8583	-0.14788	0.009353	0.036962	protein_coding
ENSG0000(	ENSG0000(	14.04354	0.571455	0.009356	0.036972	lncRNA
ENSG0000(	FBXL17	880.8074	-0.13483	0.009368	0.037012	protein_coding
ENSG0000(	EXO1	140.9341	-0.29547	0.009376	0.037037	protein_coding
ENSG0000(	FAHD2A	870.8445	-0.21804	0.009376	0.037037	protein_coding
ENSG0000(	ENSG0000(	670.4012	-0.13186	0.009387	0.037075	lncRNA
ENSG0000(	ENSG0000(	194.5681	-0.17125	0.00939	0.037082	lncRNA
ENSG0000(	ENSG0000(	24.40711	0.419241	0.009391	0.037082	TEC
ENSG0000(	PANK4	489.7116	-0.15196	0.0094	0.037112	protein_coding
ENSG0000(	CCNDBP1	1772.699	-0.11763	0.009408	0.037129	protein_coding
ENSG0000(	CIRBP	5559.064	-0.10666	0.009414	0.037129	protein_coding
ENSG0000(	KIAA2013	1341.327	-0.27654	0.009408	0.037129	protein_coding
ENSG0000(	LINC01424	79.36273	0.20936	0.00941	0.037129	lncRNA
ENSG0000(	MPDZ	753.4559	0.32402	0.009413	0.037129	protein_coding
ENSG0000(	RNF145	1230.941	0.276548	0.009407	0.037129	protein_coding
ENSG0000(	RNU2-3P	12.28346	-0.55703	0.009413	0.037129	snRNA
ENSG0000(	TNFAIP8L1	1182.365	0.112614	0.009412	0.037129	protein_coding
ENSG0000(	GALNT14	222.586	-0.42089	0.009426	0.037173	protein_coding

ENSG0000(	COX6CP18	15.58868	0.470482	0.009429	0.037179	processed_pseudogene
ENSG0000(	H2BC20P	1469.563	0.149489	0.009438	0.037209	transcribed_unprocessed_pseudo
ENSG0000(	LRRC8E	189.886	-0.16262	0.009439	0.037212	protein_coding
ENSG0000(	DMAC1	289.3057	-0.26176	0.009443	0.037223	protein_coding
ENSG0000(	ENSG0000(	420.862	0.090489	0.009451	0.037247	lncRNA
ENSG0000(	ENSG0000(	11.4388	0.492266	0.009452	0.037247	lncRNA
ENSG0000(	XIAPP2	291.2295	-0.19313	0.009454	0.037251	processed_pseudogene
ENSG0000(	ENSG0000(	147.0546	0.148362	0.009457	0.037253	lncRNA
ENSG0000(	ENSG0000(	373.0038	-0.1796	0.009456	0.037253	lncRNA
ENSG0000(	ENSG0000(	45.04035	0.26931	0.009459	0.037253	lncRNA
ENSG0000(	NDUFA11	754.5763	-0.12398	0.009459	0.037253	protein_coding
ENSG0000(	ENSG0000(	24.40426	0.372089	0.00947	0.037281	lncRNA
ENSG0000(	PLCB1-IT1	39.2295	0.335895	0.00947	0.037281	lncRNA
ENSG0000(	RN7SL717F	8.630291	0.490079	0.009469	0.037281	misc_RNA
ENSG0000(	ENSG0000(	37.75919	0.343479	0.009474	0.037286	TEC
ENSG0000(	ENSG0000(	22.46629	0.439883	0.009473	0.037286	lncRNA
ENSG0000(	RRH	105.3252	0.234079	0.009478	0.0373	protein_coding
ENSG0000(	ENSG0000(	15.57898	0.489472	0.009484	0.037317	TEC
ENSG0000(	CHCHD4	125.9285	-0.22116	0.009493	0.03735	protein_coding
ENSG0000(	ENSG0000(	549.1087	0.213903	0.009498	0.037359	lncRNA
ENSG0000(	ENSG0000(	21.81443	0.618128	0.009498	0.037359	lncRNA
ENSG0000(	ARMH3	1337.061	-0.1004	0.009504	0.037373	protein_coding
ENSG0000(	IDS	3298.376	-0.12984	0.009504	0.037373	protein_coding
ENSG0000(	OR10AC1	28.77288	0.321758	0.009512	0.037401	polymorphic_pseudogene
ENSG0000(	SLC9A2	322.0415	-0.63052	0.009519	0.037425	protein_coding
ENSG0000(	SNRPCP5	14.75266	0.600547	0.009529	0.037456	processed_pseudogene
ENSG0000(	ENSG0000(	29.08769	0.363402	0.009536	0.037477	lncRNA
ENSG0000(	NAT14	52.68336	0.407522	0.009536	0.037477	protein_coding
ENSG0000(	OSBPL6	566.5197	-0.26257	0.009539	0.037478	protein_coding
ENSG0000(	PPP1R12C	2061.119	-0.11505	0.009539	0.037478	protein_coding
ENSG0000(	ENSG0000(	238.3825	0.153608	0.009541	0.037482	lncRNA
ENSG0000(	ENSG0000(	12.92764	0.390598	0.009554	0.037528	snoRNA
ENSG0000(	ENSG0000(	106.573	-0.17059	0.00956	0.037548	lncRNA
ENSG0000(	BICRA	969.2889	0.083774	0.009575	0.037595	protein_coding
ENSG0000(	ZFY-AS1	7.775223	-0.63324	0.009575	0.037595	lncRNA
ENSG0000(	ENSG0000(	175.3053	-0.1427	0.009577	0.037599	lncRNA
ENSG0000(	HDHD5-AS1	14.76571	0.446029	0.009582	0.037609	lncRNA
ENSG0000(	ZNF37BP	3547.233	0.141483	0.009581	0.037609	transcribed_processed_pseudoge
ENSG0000(	FBXO25	1202.237	-0.17771	0.009587	0.037625	protein_coding
ENSG0000(	ENSG0000(	107.0061	-0.6876	0.009591	0.037635	transcribed_unprocessed_pseudo
ENSG0000(	ENSG0000(	9.518785	0.508889	0.009606	0.037688	lncRNA
ENSG0000(	LINC00294	1545.038	-0.26838	0.009609	0.037693	lncRNA
ENSG0000(	NEU4	149.1847	-0.61464	0.009609	0.037693	protein_coding
ENSG0000(	SLC39A5	1962.497	-0.53675	0.009613	0.037702	protein_coding
ENSG0000(	ATP6V1FNI	35.74621	0.259204	0.00962	0.03772	protein_coding
ENSG0000(	ENSG0000(	13.95479	0.379786	0.00962	0.03772	processed_pseudogene
ENSG0000(	MRPL27	450.7739	-0.16018	0.009621	0.03772	protein_coding

ENSG0000(	CFAP20DC-	28.28743	0.359165	0.009625	0.037733	lncRNA
ENSG0000(	FAM240B	59.73787	-0.27036	0.009629	0.037743	protein_coding
ENSG0000(	LINC02133	105.6562	0.23032	0.00963	0.037743	lncRNA
ENSG0000(	ENSG0000(	264.6437	0.143273	0.009632	0.037745	processed_pseudogene
ENSG0000(	CLYBL	393.5285	-0.34639	0.009637	0.037748	protein_coding
ENSG0000(	ENSG0000(	306.6788	-0.14525	0.00964	0.037748	lncRNA
ENSG0000(	HSPA12B	99.8292	0.329914	0.009636	0.037748	protein_coding
ENSG0000(	MAP4K1	820.0269	-0.26838	0.009639	0.037748	protein_coding
ENSG0000(	PTP4A1P3	17.37938	0.667671	0.009636	0.037748	processed_pseudogene
ENSG0000(	SVOP	596.433	-0.12117	0.00964	0.037748	protein_coding
ENSG0000(	ENSG0000(	126.9377	0.172491	0.009642	0.037751	lncRNA
ENSG0000(	ADGRB3	171.6169	0.384455	0.009648	0.037771	protein_coding
ENSG0000(	HOXB9	780.0987	0.377669	0.009656	0.037797	protein_coding
ENSG0000(	ENSG0000(	318.4371	-0.12944	0.009659	0.037803	TEC
ENSG0000(	RPP40	28.89927	-0.51326	0.009661	0.037807	protein_coding
ENSG0000(	MRPL1	172.2139	-0.21805	0.009663	0.03781	protein_coding
ENSG0000(	PTS	516.9723	0.125476	0.009668	0.037825	protein_coding
ENSG0000(	LINC01124	11.29062	0.545588	0.009671	0.037832	lncRNA
ENSG0000(	LYRM9	266.4958	0.210037	0.00968	0.037861	protein_coding
ENSG0000(	ENSG0000(	86.26427	0.231177	0.009696	0.037914	lncRNA
ENSG0000(	ENSG0000(	80.46212	-0.23306	0.009695	0.037914	lncRNA
ENSG0000(	CTCF	2333.657	-0.08185	0.009704	0.037939	protein_coding
ENSG0000(	ENSG0000(	87.80079	0.214449	0.009704	0.037939	lncRNA
ENSG0000(	ENSG0000(	45.15117	0.270418	0.009709	0.037952	lncRNA
ENSG0000(	ST6GALNA(	25.25117	-0.37083	0.009713	0.037961	processed_pseudogene
ENSG0000(	KLHL12	770.2194	-0.12319	0.009717	0.037974	protein_coding
ENSG0000(	C9orf131	130.9716	0.205628	0.009719	0.037978	protein_coding
ENSG0000(	ENSG0000(	617.2113	0.130769	0.009744	0.038071	lncRNA
ENSG0000(	RASSF1-AS1	8.503612	0.581097	0.009747	0.038077	lncRNA
ENSG0000(	ENSG0000(	16.97504	0.358679	0.009756	0.038106	TEC
ENSG0000(	ENSG0000(	47.20882	0.26778	0.009761	0.038122	lncRNA
ENSG0000(	RNF38	1560.929	-0.09961	0.009766	0.038135	protein_coding
ENSG0000(	WAKMAR2	312.2004	0.227027	0.009772	0.038155	lncRNA
ENSG0000(	ADGRG4	499.2296	-0.49271	0.009778	0.038176	protein_coding
ENSG0000(	PRLR	2318.462	-0.48095	0.009787	0.038204	protein_coding
ENSG0000(	ROCK2	3748.541	-0.21502	0.009791	0.038215	protein_coding
ENSG0000(	ATF5	362.5188	0.215601	0.009794	0.038221	protein_coding
ENSG0000(	SHANK2-AS	203.8254	0.128703	0.0098	0.038239	lncRNA
ENSG0000(	ENSG0000(	289.8893	0.124361	0.009803	0.03825	lncRNA
ENSG0000(	GRB7	344.9683	-0.52146	0.009805	0.03825	protein_coding
ENSG0000(	SDAD1-AS1	284.7801	-0.15189	0.009806	0.03825	lncRNA
ENSG0000(	CPVL	269.7569	0.308367	0.00981	0.038261	protein_coding
ENSG0000(	ENSG0000(	870.116	0.126611	0.009811	0.038261	lncRNA
ENSG0000(	GLRX5	263.1019	-0.22911	0.009825	0.038311	protein_coding
ENSG0000(	ENSG0000(	186.1986	0.128708	0.009829	0.03832	lncRNA
ENSG0000(	MCPH1-AS	1278.982	0.082251	0.009833	0.038332	lncRNA
ENSG0000(	TCP11L2	1311.316	-0.20434	0.009836	0.038338	protein_coding

ENSG0000(	SUV39H2	321.6996	0.17801	0.009843	0.038362	protein_coding
ENSG0000(	RDH11	841.2182	0.145327	0.009849	0.038382	protein_coding
ENSG0000(	PTHLH	22.58944	0.843345	0.009854	0.038396	protein_coding
ENSG0000(	ACHE	402.8338	-0.44292	0.009869	0.038448	protein_coding
ENSG0000(	LINC01752	138.5253	0.273969	0.009874	0.038462	lncRNA
ENSG0000(	BEND7	1782.955	-0.47385	0.009878	0.038474	protein_coding
ENSG0000(	DENND6A-	192.2627	0.123063	0.009882	0.038486	lncRNA
ENSG0000(	RAB3A	34.19723	-0.37309	0.009886	0.038495	protein_coding
ENSG0000(	WDFY2	2263.377	0.20753	0.009887	0.038495	protein_coding
ENSG0000(	CHKB-CPT1	215.1132	-0.27227	0.009889	0.038496	protein_coding
ENSG0000(	ENSG0000(	15.0393	0.396865	0.009898	0.03853	lncRNA
ENSG0000(	ENSG0000(	15.46318	0.474654	0.009903	0.038542	lncRNA
ENSG0000(	AEBP2	1495.946	-0.10338	0.009909	0.038563	protein_coding
ENSG0000(	FZD5	3718.353	-0.43587	0.009915	0.038578	protein_coding
ENSG0000(	LMLN	583.949	0.140672	0.009931	0.038633	protein_coding
ENSG0000(	PLIN4	79.70371	0.583336	0.00993	0.038633	protein_coding
ENSG0000(	FOXD1	62.74681	-0.94128	0.009948	0.03869	protein_coding
ENSG0000(	SLC2A8	286.7438	-0.18384	0.009947	0.03869	protein_coding
ENSG0000(	ENSG0000(	146.3794	-0.19477	0.00995	0.038692	transcribed_unprocessed_pseudo
ENSG0000(	CDK3	81.86508	-0.33465	0.009959	0.038711	protein_coding
ENSG0000(	ENSG0000(	354.2862	0.19999	0.009961	0.038711	TEC
ENSG0000(	LMO4	1409.535	0.195734	0.00996	0.038711	protein_coding
ENSG0000(	OR2C3	85.54154	0.326452	0.00996	0.038711	protein_coding
ENSG0000(	REV1	1929.698	-0.15566	0.009957	0.038711	protein_coding
ENSG0000(	VAMP5	119.4004	0.292688	0.009974	0.038757	protein_coding
ENSG0000(	C1QTNF1-A	125.7709	0.236764	0.009982	0.038766	lncRNA
ENSG0000(	HDAC5	896.267	-0.17388	0.009981	0.038766	protein_coding
ENSG0000(	LINC02789	145.8623	0.14094	0.009982	0.038766	lncRNA
ENSG0000(	MISP	4112.912	-0.49746	0.009981	0.038766	protein_coding
ENSG0000(	MT-TD	73.4129	0.969477	0.009979	0.038766	Mt_tRNA
ENSG0000(	PMS2P10	17.04341	-1.14887	0.009989	0.038785	transcribed_unprocessed_pseudo
ENSG0000(	CIITA	16881.65	0.272154	0.009991	0.038788	protein_coding
ENSG0000(	HSPA4	1418.479	-0.13941	0.009993	0.038788	protein_coding
ENSG0000(	LINC02762	570.5148	0.191113	0.009993	0.038788	lncRNA
ENSG0000(	C14orf28	217.7939	-0.29251	0.009994	0.038788	protein_coding
ENSG0000(	SLC12A3	80.16361	0.530771	0.009996	0.038789	protein_coding
ENSG0000(	ENSG0000(	91.77123	0.184998	0.010007	0.038827	lncRNA
ENSG0000(	RPS4X	4101.82	-0.23532	0.010011	0.038839	protein_coding
ENSG0000(	TOR1A	698.4468	-0.11512	0.010013	0.038843	protein_coding
ENSG0000(	ENSG0000(	19.6992	-0.55338	0.010019	0.03886	lncRNA
ENSG0000(	PMM2	4337.026	0.108721	0.010026	0.038884	protein_coding
ENSG0000(	AGMO	613.2204	-0.46915	0.01003	0.038893	protein_coding
ENSG0000(	NDUFB3	231.0278	-0.2889	0.010039	0.038922	protein_coding
ENSG0000(	MAP3K4-A:	10.58179	0.618534	0.010055	0.038981	lncRNA
ENSG0000(	DNAAF2	161.824	-0.1877	0.010058	0.038987	protein_coding
ENSG0000(	ENSG0000(	42.35544	-0.31212	0.010066	0.039012	lncRNA
ENSG0000(	NT5E	697.321	-0.41269	0.010068	0.039012	protein_coding

ENSG0000(	SENP3-EIF4	49.79509	0.625191	0.010068	0.039012	protein_coding
ENSG0000(	ADCY6	3490.19	-0.34309	0.010075	0.039035	protein_coding
ENSG0000(	FLJ16779	9.653265	0.54402	0.010078	0.039041	lncRNA
ENSG0000(	CLPP	621.9312	-0.1275	0.010087	0.03907	protein_coding
ENSG0000(	SMG6	2445.768	0.09263	0.010091	0.03908	protein_coding
ENSG0000(	AGXT2	114.907	-0.8938	0.0101	0.039107	protein_coding
ENSG0000(	BPGM	158.1456	0.219956	0.0101	0.039107	protein_coding
ENSG0000(	RPL7L1	3361.295	-0.08494	0.010101	0.039107	protein_coding
ENSG0000(	ENSG0000(	951.4068	0.238635	0.01011	0.039136	lncRNA
ENSG0000(	TMEM259	2353.368	-0.13339	0.010112	0.039137	protein_coding
ENSG0000(	SLITRK4	198.4075	0.321437	0.010114	0.03914	protein_coding
ENSG0000(	ENSG0000(	106.8006	-0.38425	0.010118	0.039153	lncRNA
ENSG0000(	NELFB	509.8194	-0.1766	0.01012	0.039155	protein_coding
ENSG0000(	STN1	791.7293	-0.14925	0.010127	0.039176	protein_coding
ENSG0000(	FAM217B	898.8898	-0.1312	0.010137	0.03921	protein_coding
ENSG0000(	COL16A1	1529.358	0.284262	0.01014	0.039217	protein_coding
ENSG0000(	ENSG0000(	406.8614	-0.2224	0.010143	0.039222	lncRNA
ENSG0000(	LNCOC1	296.5599	0.153629	0.010143	0.039222	lncRNA
ENSG0000(	TFDP2	3773.994	-0.18158	0.010157	0.039268	protein_coding
ENSG0000(	ENSG0000(	83.35191	0.215149	0.010167	0.039304	TEC
ENSG0000(	MAMLD1	140.2868	0.357462	0.010176	0.039332	protein_coding
ENSG0000(	MIR205HG	95.35099	0.226178	0.010177	0.039334	lncRNA
ENSG0000(	SEPTIN7P9	449.7715	0.121726	0.010186	0.039361	transcribed_unprocessed_pseudo
ENSG0000(	ASNS	341.5953	0.212399	0.010199	0.039405	protein_coding
ENSG0000(	CDHR2	7164.109	-0.61203	0.0102	0.039405	protein_coding
ENSG0000(	LIPT2	96.88784	0.24581	0.010208	0.039433	protein_coding
ENSG0000(	KMT5AP3	86.71728	-0.51211	0.010221	0.039468	processed_pseudogene
ENSG0000(	LINC02087	40.79869	0.253125	0.01022	0.039468	lncRNA
ENSG0000(	PSMB8	847.2533	0.211027	0.01022	0.039468	protein_coding
ENSG0000(	RPL29P14	135.4975	0.183495	0.010222	0.039468	processed_pseudogene
ENSG0000(	SEC14L2	695.2127	-0.48094	0.010225	0.039473	protein_coding
ENSG0000(	CDC34	261.9149	-0.1872	0.01023	0.03949	protein_coding
ENSG0000(	ENSG0000(	31.45013	0.33349	0.010238	0.039504	lncRNA
ENSG0000(	ENSG0000(	1495.28	0.138832	0.010239	0.039504	lncRNA
ENSG0000(	LINC02515	389.1147	0.138117	0.010239	0.039504	lncRNA
ENSG0000(	LINGO1	18.88494	0.674742	0.010238	0.039504	protein_coding
ENSG0000(	C6orf47	720.2597	-0.1245	0.010247	0.039527	protein_coding
ENSG0000(	FAM156B	1581.616	0.455514	0.010247	0.039527	protein_coding
ENSG0000(	HSPB1	351.3514	-0.26834	0.010268	0.039601	protein_coding
ENSG0000(	ENSG0000(	33.95133	0.440931	0.01027	0.039605	lncRNA
ENSG0000(	ATP5MC2	1230.965	-0.17931	0.010275	0.039608	protein_coding
ENSG0000(	ENSG0000(	377.4096	-0.15192	0.010273	0.039608	lncRNA
ENSG0000(	ENSG0000(	263.5854	0.143738	0.010273	0.039608	lncRNA
ENSG0000(	ENSG0000(	29.32945	0.476817	0.010286	0.039637	processed_pseudogene
ENSG0000(	ENSG0000(	419.8214	-0.15272	0.010285	0.039637	lncRNA
ENSG0000(	ENSG0000(	10.08306	0.50096	0.010286	0.039637	lncRNA
ENSG0000(	MIR8061	16.66993	0.63072	0.010315	0.039743	miRNA

ENSG0000(	ENSG0000(	11.05919	-0.6446	0.010321	0.039762	unitary_pseudogene
ENSG0000(	GPR160	1754.954	-0.43162	0.010335	0.039813	protein_coding
ENSG0000(	ENSG0000(	1136.15	0.107722	0.010351	0.039863	transcribed_processed_pseudoge
ENSG0000(	KRTCAP3	143.5271	-0.31566	0.01035	0.039863	protein_coding
ENSG0000(	ENSG0000(	32.2369	0.360149	0.010353	0.039864	lncRNA
ENSG0000(	NPDC1	908.3926	0.324956	0.010357	0.039875	protein_coding
ENSG0000(	ENSG0000(	21.94871	0.372965	0.010366	0.039907	lncRNA
ENSG0000(	ENSG0000(	74.98659	-0.56253	0.010373	0.039927	lncRNA
ENSG0000(	FAM186B	22.82988	0.55117	0.010381	0.039955	protein_coding
ENSG0000(	FDXR	93.36017	0.263429	0.010386	0.039969	protein_coding
ENSG0000(	ZMYM1	712.8545	-0.15748	0.010395	0.039996	protein_coding
ENSG0000(	LRRC15	67.04207	0.954213	0.010403	0.040022	protein_coding
ENSG0000(	NHEJ1	1841.968	-0.4441	0.010404	0.040022	protein_coding
ENSG0000(	COX14	275.4842	-0.20937	0.010412	0.040049	protein_coding
ENSG0000(	TTC41P	88.94201	0.274136	0.010417	0.040064	transcribed_unitary_pseudogene
ENSG0000(	AKR7L	1771.86	-0.34473	0.010432	0.040115	polymorphic_pseudogene
ENSG0000(	DLC1	1267.335	0.20905	0.010436	0.040127	protein_coding
ENSG0000(	ENSG0000(	103.7785	0.246247	0.010438	0.040129	lncRNA
ENSG0000(	APAF1	1630.884	0.146206	0.010446	0.040151	protein_coding
ENSG0000(	GBA3	1144.392	-0.61441	0.010445	0.040151	polymorphic_pseudogene
ENSG0000(	CERCAM	493.2143	0.183526	0.010449	0.040153	protein_coding
ENSG0000(	ENSG0000(	89.37266	0.279246	0.010449	0.040153	lncRNA
ENSG0000(	ENSG0000(	15.78978	0.42223	0.010451	0.040155	lncRNA
ENSG0000(	EPAS1	3456.015	0.246014	0.010452	0.040155	protein_coding
ENSG0000(	ENSG0000(	50.18099	0.213689	0.010465	0.040199	lncRNA
ENSG0000(	ENSG0000(	11.8054	0.497003	0.010471	0.040216	lncRNA
ENSG0000(	ENSG0000(	29.70128	0.383284	0.010482	0.040255	lncRNA
ENSG0000(	ENSG0000(	22.56929	0.323975	0.010483	0.040255	lncRNA
ENSG0000(	AIPL1	216.6263	0.121982	0.010492	0.040281	protein_coding
ENSG0000(	DNAH14	337.8107	0.247931	0.010501	0.040296	protein_coding
ENSG0000(	EDN3	842.4287	-0.59484	0.0105	0.040296	protein_coding
ENSG0000(	SLC1A5	824.9676	0.172713	0.0105	0.040296	protein_coding
ENSG0000(	ZNF25	465.3733	0.162403	0.010497	0.040296	protein_coding
ENSG0000(	ENSG0000(	19.73476	0.472756	0.010504	0.040303	lncRNA
ENSG0000(	JMJD7	98.76017	-0.28123	0.010543	0.040449	protein_coding
ENSG0000(	MGP	364.3449	0.338696	0.010561	0.040514	protein_coding
ENSG0000(	SPATA25	85.14489	-0.40414	0.010563	0.040517	protein_coding
ENSG0000(	ENSG0000(	59.76086	0.246558	0.010568	0.04053	lncRNA
ENSG0000(	ENSG0000(	289.1242	0.117669	0.010573	0.040541	lncRNA
ENSG0000(	GTF2A2	516.939	-0.13519	0.010573	0.040541	protein_coding
ENSG0000(	ENSG0000(	11.94444	0.593914	0.010579	0.040556	snoRNA
ENSG0000(	MISP3	216.767	-0.41458	0.010582	0.040561	protein_coding
ENSG0000(	OBSL1	543.6925	0.224923	0.010582	0.040561	protein_coding
ENSG0000(	ENSG0000(	29.56373	0.307055	0.010584	0.040562	lncRNA
ENSG0000(	NAT8	51.38338	-1.00449	0.010587	0.040568	protein_coding
ENSG0000(	LARP7P1	10.15114	0.702822	0.010592	0.040579	processed_pseudogene
ENSG0000(	TMPRSS13	109.8538	0.276163	0.010591	0.040579	protein_coding

ENSG0000(	ENSG0000(	69.2881	0.270326	0.010599	0.040593	TEC
ENSG0000(	PTGER1	8.605321	0.788231	0.010599	0.040593	protein_coding
ENSG0000(	RTTN	3969.213	0.085316	0.010615	0.040652	protein_coding
ENSG0000(	C1D	148.1024	-0.19788	0.010626	0.040689	protein_coding
ENSG0000(	RBBP4	4765.182	-0.11082	0.01063	0.040699	protein_coding
ENSG0000(	TTC17	4452.578	-0.07868	0.010645	0.04075	protein_coding
ENSG0000(	ENSG0000(	147.5263	0.384625	0.010649	0.04076	lncRNA
ENSG0000(	CAP2P1	174.1752	-0.2494	0.01066	0.040789	transcribed_processed_pseudoge
ENSG0000(	MIR155HG	183.8306	0.603497	0.01066	0.040789	lncRNA
ENSG0000(	MMACHC	682.0453	0.116623	0.010659	0.040789	protein_coding
ENSG0000(	GTF2IP4	1789.023	-0.32703	0.010663	0.040795	transcribed_processed_pseudoge
ENSG0000(	ENSG0000(	130.3982	0.199637	0.010669	0.040802	lncRNA
ENSG0000(	GLP2R	339.9361	-0.54109	0.010668	0.040802	protein_coding
ENSG0000(	SCN3A	310.855	0.281733	0.010668	0.040802	protein_coding
ENSG0000(	ENSG0000(	18.0115	0.523551	0.010671	0.040806	lncRNA
ENSG0000(	NUDT16	2389.705	0.406995	0.010672	0.040807	protein_coding
ENSG0000(	VTRNA1-2	13.81725	0.811514	0.010678	0.040824	misc_RNA
ENSG0000(	MYLK3	2062.654	0.173068	0.010682	0.040833	protein_coding
ENSG0000(	ENSG0000(	27.65319	-0.66927	0.010688	0.040842	lncRNA
ENSG0000(	ENSG0000(	146.9878	0.254189	0.010688	0.040842	lncRNA
ENSG0000(	ENSG0000(	45.61277	-0.31189	0.010688	0.040842	protein_coding
ENSG0000(	LMAN1	2285.475	0.166677	0.010691	0.040848	protein_coding
ENSG0000(	ENSG0000(	1088.772	-0.1418	0.0107	0.040876	lncRNA
ENSG0000(	ENSG0000(	8.73876	0.567851	0.01071	0.040903	scaRNA
ENSG0000(	ENSG0000(	10.83445	0.648862	0.010711	0.040903	protein_coding
ENSG0000(	SNORA71A	80.72773	0.374417	0.010708	0.040903	snoRNA
ENSG0000(	ENSG0000(	21.55476	0.358583	0.010717	0.040922	lncRNA
ENSG0000(	PNCK	66.45192	0.707198	0.010721	0.040934	protein_coding
ENSG0000(	SENCR	9.288666	0.680825	0.010723	0.040936	lncRNA
ENSG0000(	MALINC1	673.7143	0.103042	0.010726	0.04094	lncRNA
ENSG0000(	ENSG0000(	102.4181	0.181802	0.010729	0.040948	processed_pseudogene
ENSG0000(	ENSG0000(	121.0121	0.204701	0.01073	0.040948	lncRNA
ENSG0000(	NEIL1	1079.704	-0.19163	0.010741	0.040975	protein_coding
ENSG0000(	PPP5D1P	572.6726	0.096016	0.010741	0.040975	transcribed_unprocessed_pseudo
ENSG0000(	SLC5A12	5412.384	-0.73033	0.010741	0.040975	protein_coding
ENSG0000(	CYP11A1	40.6592	0.256529	0.01075	0.041005	protein_coding
ENSG0000(	ACP1	1081.808	-0.08452	0.010762	0.04104	protein_coding
ENSG0000(	SMIM14-D'	848.457	-0.08515	0.010761	0.04104	lncRNA
ENSG0000(	ENSG0000(	152.6919	0.383942	0.010766	0.04105	TEC
ENSG0000(	SFXN5	1352.81	-0.10423	0.010769	0.041058	protein_coding
ENSG0000(	ZSWIM3	154.5548	-0.19719	0.010771	0.041058	protein_coding
ENSG0000(	KRT18	1656.296	-0.37074	0.010775	0.041069	protein_coding
ENSG0000(	ENSG0000(	49.24305	0.238921	0.010801	0.041163	lncRNA
ENSG0000(	LINS1	1562.626	0.170418	0.010803	0.041166	protein_coding
ENSG0000(	XPOT	1462.316	0.137592	0.010809	0.041183	protein_coding
ENSG0000(	ENSG0000(	142.9847	-0.18265	0.010812	0.041187	lncRNA
ENSG0000(	NLRP1	2716.012	0.377789	0.010812	0.041187	protein_coding

ENSG0000(	MT-TW	52.29223	1.135957	0.010814	0.041187	Mt_tRNA
ENSG0000(	ENSG0000(	58.7371	0.261878	0.010816	0.04119	lncRNA
ENSG0000(	ENSG0000(	8.882635	-0.60385	0.010822	0.04121	processed_pseudogene
ENSG0000(	PFKL	3497.544	-0.22161	0.010828	0.041224	protein_coding
ENSG0000(	ENSG0000(	338.9931	0.138719	0.010851	0.04131	lncRNA
ENSG0000(	ENSG0000(	11.60224	0.550599	0.010866	0.041356	lncRNA
ENSG0000(	GALNT15	582.2852	0.170297	0.010865	0.041356	protein_coding
ENSG0000(	IPPK	381.6142	0.156847	0.010878	0.041395	protein_coding
ENSG0000(	TMEM220	668.3741	-0.36691	0.010885	0.041418	protein_coding
ENSG0000(	ENSG0000(	140.7495	0.368563	0.010893	0.041443	TEC
ENSG0000(	ENSG0000(	1058.246	-0.2022	0.010904	0.041479	lncRNA
ENSG0000(	SLC36A1	4599.513	-0.40848	0.010919	0.041532	protein_coding
ENSG0000(	DPCD	27.91492	0.35915	0.010929	0.041565	protein_coding
ENSG0000(	HSPG2	6097.467	0.22331	0.010933	0.041576	protein_coding
ENSG0000(	EIF3G	762.4207	-0.12264	0.010936	0.041582	protein_coding
ENSG0000(	ENSG0000(	236.7152	0.142183	0.010939	0.041589	lncRNA
ENSG0000(	ENSG0000(	23.69961	0.376508	0.010954	0.04164	lncRNA
ENSG0000(	KIAA0319	63.99038	0.28886	0.010966	0.04168	protein_coding
ENSG0000(	ENSG0000(	343.9506	-0.12496	0.010972	0.041697	lncRNA
ENSG0000(	OR5T2	38.80193	0.260487	0.010977	0.041712	protein_coding
ENSG0000(	BAX	674.266	-0.13608	0.010985	0.041739	protein_coding
ENSG0000(	ENSG0000(	9.859783	0.577509	0.010994	0.041767	lncRNA
ENSG0000(	EPB41L4B	1637.219	-0.56852	0.011002	0.041794	protein_coding
ENSG0000(	ENSG0000(	213.6947	0.228587	0.011004	0.041794	lncRNA
ENSG0000(	ENSG0000(	94.08483	0.388619	0.011014	0.041827	processed_pseudogene
ENSG0000(	MMUT	465.2502	-0.23834	0.011023	0.041853	protein_coding
ENSG0000(	WFDC12	29.89412	0.350632	0.011023	0.041853	protein_coding
ENSG0000(	ZNF157	304.1899	-0.12046	0.011025	0.041856	protein_coding
ENSG0000(	MS4A2	695.6013	0.275657	0.011028	0.041857	protein_coding
ENSG0000(	POLR2K	208.6091	-0.16764	0.011028	0.041857	protein_coding
ENSG0000(	ENSG0000(	31.46954	0.340814	0.011049	0.04193	lncRNA
ENSG0000(	RN7SKP102	65.13692	-0.4285	0.011056	0.041954	misc_RNA
ENSG0000(	ENSG0000(	309.9603	0.301636	0.011059	0.041959	lncRNA
ENSG0000(	LRRC7	1376.227	0.1709	0.011073	0.042006	protein_coding
ENSG0000(	AMN	2574.789	-0.58306	0.011075	0.042011	protein_coding
ENSG0000(	ZFPM1-AS1	11.15071	0.522166	0.011077	0.042013	lncRNA
ENSG0000(	HECTD2	584.4938	0.127048	0.01108	0.042017	protein_coding
ENSG0000(	NINL	342.9279	0.261264	0.011084	0.042027	protein_coding
ENSG0000(	ADIPOQ	293.064	-0.14155	0.011089	0.042041	protein_coding
ENSG0000(	ENSG0000(	120.307	-0.21585	0.011096	0.042063	lncRNA
ENSG0000(	CUL7	910.3219	0.148809	0.01111	0.042112	protein_coding
ENSG0000(	ENSG0000(	30.03051	0.593514	0.011115	0.042126	lncRNA
ENSG0000(	PPP1R11	814.3778	-0.20352	0.011117	0.042127	protein_coding
ENSG0000(	TTYH1	267.2516	0.188569	0.011126	0.042156	protein_coding
ENSG0000(	LAMP5	25.41321	0.593624	0.011131	0.042173	protein_coding
ENSG0000(	F10	27.16435	-0.59062	0.011152	0.042245	protein_coding
ENSG0000(	NIFKP4	490.486	0.132447	0.011162	0.042279	processed_pseudogene

ENSG0000(	ENSG0000(	228.0617	0.135362	0.011165	0.042286	lncRNA
ENSG0000(	ENSG0000(	9.239921	0.679033	0.011167	0.042289	lncRNA
ENSG0000(	C20orf144	88.02322	0.216042	0.011185	0.042351	protein_coding
ENSG0000(	DDIT3	174.6262	-0.20668	0.011191	0.042369	protein_coding
ENSG0000(	CSDE1	7347.219	-0.11622	0.011193	0.04237	protein_coding
ENSG0000(	UPK3B	98.26739	-0.33453	0.011199	0.042384	protein_coding
ENSG0000(	WDR7	1833.821	0.117275	0.011199	0.042384	protein_coding
ENSG0000(	ELOC	2527.65	0.067605	0.011205	0.042398	protein_coding
ENSG0000(	PITHD1	460.5209	-0.11387	0.011212	0.04242	protein_coding
ENSG0000(	ENSG0000(	28.66248	0.317114	0.011219	0.042442	lncRNA
ENSG0000(	ENSG0000(	99.76989	0.253992	0.011224	0.042457	lncRNA
ENSG0000(	MTARC2	910.9753	-0.45139	0.011227	0.042462	protein_coding
ENSG0000(	ENSG0000(	79.36512	0.190336	0.011239	0.042503	lncRNA
ENSG0000(	PRSS36	51.77558	-0.43458	0.01124	0.042503	protein_coding
ENSG0000(	GOLGA8S	9.935903	0.704711	0.011247	0.042524	protein_coding
ENSG0000(	CINP	745.6864	-0.11059	0.011254	0.042544	protein_coding
ENSG0000(	RN7SL586F	9.144371	0.576229	0.011268	0.042593	misc_RNA
ENSG0000(	BRI3BPP1	12.03268	0.541838	0.011271	0.0426	processed_pseudogene
ENSG0000(	CCT4	669.5755	-0.20803	0.011285	0.042648	protein_coding
ENSG0000(	ENSG0000(	395.0276	-0.65698	0.011291	0.042658	lncRNA
ENSG0000(	ENSG0000(	32.99842	0.295929	0.011289	0.042658	lncRNA
ENSG0000(	SYF2	667.8827	-0.14812	0.011302	0.042695	protein_coding
ENSG0000(	TMEM150(	301.0262	-0.22064	0.011309	0.042716	protein_coding
ENSG0000(	ENSG0000(	20.81923	0.385486	0.011321	0.042758	lncRNA
ENSG0000(	NSMCE2	613.643	0.089599	0.011329	0.042782	protein_coding
ENSG0000(	ENSG0000(	29.24322	-0.77132	0.011335	0.042783	lncRNA
ENSG0000(	ENSG0000(	36.72006	0.339573	0.011334	0.042783	TEC
ENSG0000(	RGP1	2342.532	-0.15658	0.011334	0.042783	protein_coding
ENSG0000(	SMPD2	273.768	-0.17201	0.011333	0.042783	protein_coding
ENSG0000(	LINC02614	1949.865	0.130019	0.011346	0.042819	lncRNA
ENSG0000(	PYGO1	371.884	0.160645	0.011347	0.042819	protein_coding
ENSG0000(	ENSG0000(	127.1858	0.23399	0.011349	0.042821	lncRNA
ENSG0000(	ENSG0000(	70.60952	0.28871	0.011354	0.042834	lncRNA
ENSG0000(	KLF11	372.9553	-0.21103	0.011355	0.042834	protein_coding
ENSG0000(	RC3H2	3330.798	0.102847	0.011357	0.042837	protein_coding
ENSG0000(	SCNN1D	198.3525	-0.28736	0.01136	0.042845	protein_coding
ENSG0000(	METTL25	371.3206	-0.12721	0.011362	0.042846	protein_coding
ENSG0000(	ENSG0000(	267.728	0.126627	0.011364	0.042846	lncRNA
ENSG0000(	PAN3	2894.664	-0.07881	0.011379	0.042899	protein_coding
ENSG0000(	CHST11	1030.103	0.393978	0.011392	0.042944	protein_coding
ENSG0000(	ENSG0000(	111.2892	0.150991	0.011399	0.042962	lncRNA
ENSG0000(	SLC24A2	153.0849	0.147859	0.011406	0.042984	protein_coding
ENSG0000(	HOXD4	89.07394	0.308554	0.011409	0.042991	protein_coding
ENSG0000(	ENSG0000(	13.53689	0.395289	0.011417	0.043017	TEC
ENSG0000(	ENSG0000(	46.18693	0.333134	0.011423	0.043027	lncRNA
ENSG0000(	ZKSCAN8P1	90.2376	0.303218	0.011422	0.043027	protein_coding
ENSG0000(	ENSG0000(	127.6458	0.187643	0.011426	0.043034	lncRNA

ENSG0000(	RAPGEF2	2444.545	0.14622	0.011433	0.043053	protein_coding
ENSG0000(	ZFC3H1	4991.793	-0.1037	0.011434	0.043053	protein_coding
ENSG0000(	LINC01819	76.46326	0.378952	0.011463	0.043157	lncRNA
ENSG0000(	ACTR2	4312.414	-0.16051	0.011482	0.043219	protein_coding
ENSG0000(	ENSG0000(	247.3925	0.127371	0.011482	0.043219	transcribed_processed_pseudoge
ENSG0000(	ZIC1	12.50557	0.888231	0.011483	0.043219	protein_coding
ENSG0000(	PPFIBP2	2034.045	-0.17953	0.011485	0.043222	protein_coding
ENSG0000(	UQCRFS1-D	170.9688	0.132561	0.011487	0.043225	lncRNA
ENSG0000(	BBC3	155.1034	0.2995	0.011497	0.043246	protein_coding
ENSG0000(	MIR27B	17.2719	0.536442	0.011496	0.043246	miRNA
ENSG0000(	PLPP5	1700.789	0.118371	0.011495	0.043246	protein_coding
ENSG0000(	RARRES2	449.6907	0.360449	0.011507	0.043278	protein_coding
ENSG0000(	GS1-24F4.2	421.5042	0.235383	0.011517	0.043307	lncRNA
ENSG0000(	MRPS26	165.9802	-0.17162	0.011518	0.043307	protein_coding
ENSG0000(	CALM1	7440.896	-0.2314	0.011522	0.043318	protein_coding
ENSG0000(	C5orf24	1314.938	-0.0843	0.01153	0.043343	protein_coding
ENSG0000(	GPX7	111.1792	0.496541	0.011534	0.043355	protein_coding
ENSG0000(	ENSG0000(	18.52506	0.694912	0.011548	0.043395	lncRNA
ENSG0000(	HERC2P7	634.4918	0.173583	0.011547	0.043395	unprocessed_pseudogene
ENSG0000(	HAGLR	1059.171	-0.42288	0.011552	0.043404	lncRNA
ENSG0000(	TRIM6	148.6475	-0.21011	0.011556	0.043414	protein_coding
ENSG0000(	DLG5-AS1	238.4003	0.126628	0.01156	0.043424	lncRNA
ENSG0000(	LINC01257	101.9728	0.19473	0.011565	0.043431	lncRNA
ENSG0000(	NUFIP1	107.2575	-0.19282	0.011565	0.043431	protein_coding
ENSG0000(	PACSIN2	1516.549	0.103478	0.01157	0.043431	protein_coding
ENSG0000(	RN7SL519P	12.81982	0.471701	0.011569	0.043431	misc_RNA
ENSG0000(	RPGRIP1L	618.0145	0.108673	0.011568	0.043431	protein_coding
ENSG0000(	SLC25A35	198.676	0.198545	0.011567	0.043431	protein_coding
ENSG0000(	CDK14	534.7871	0.531336	0.011581	0.04347	protein_coding
ENSG0000(	RPL7A	3706.236	-0.22842	0.011585	0.043474	protein_coding
ENSG0000(	ST14	3366.703	-0.43728	0.011584	0.043474	protein_coding
ENSG0000(	DUSP5-DT	149.1478	0.185585	0.011593	0.043495	lncRNA
ENSG0000(	RNU6-123P	19.04407	0.385367	0.011595	0.043495	snRNA
ENSG0000(	SH3PXD2A	4066.424	0.196054	0.011594	0.043495	protein_coding
ENSG0000(	EPHX4	50.65406	0.211225	0.011597	0.043498	protein_coding
ENSG0000(	SLC37A4	1628.161	-0.39258	0.011612	0.043543	protein_coding
ENSG0000(	SMIM10L2I	26.25033	0.412401	0.011612	0.043543	lncRNA
ENSG0000(	NME3	174.4433	-0.1846	0.011614	0.043546	protein_coding
ENSG0000(	PLBD2	671.2058	0.192295	0.01163	0.043601	protein_coding
ENSG0000(	FCAR	621.9326	0.138873	0.011633	0.043606	protein_coding
ENSG0000(	IGKV1D-39	613.2366	0.678643	0.011644	0.043641	IG_V_gene
ENSG0000(	HLCS-IT1	55.65268	0.264445	0.011647	0.043647	lncRNA
ENSG0000(	EHD2	734.2095	0.224759	0.011649	0.043649	protein_coding
ENSG0000(	RALGAPA2	4300.181	0.160856	0.011663	0.043699	protein_coding
ENSG0000(	ETFDH	4486.868	-0.14778	0.011682	0.043762	protein_coding
ENSG0000(	KRT222	14.54576	-0.66667	0.011685	0.043768	protein_coding
ENSG0000(	MTHFR	3147.759	0.103038	0.011686	0.043769	protein_coding

ENSG0000(	JADE1	1694.125	0.151008	0.011692	0.043785	protein_coding
ENSG0000(	RN7SL553F	24.21627	0.276625	0.011697	0.043798	misc_RNA
ENSG0000(	ENSG0000(	20.89635	0.346182	0.011713	0.043818	lncRNA
ENSG0000(	ENSG0000(	107.973	0.224987	0.011705	0.043818	lncRNA
ENSG0000(	ENSG0000(	70.88995	-0.61567	0.011711	0.043818	processed_pseudogene
ENSG0000(	HEIH	311.6047	0.115505	0.011705	0.043818	lncRNA
ENSG0000(	LINC01102	219.2916	0.138905	0.011713	0.043818	lncRNA
ENSG0000(	NPM1P27	47.46288	-0.38092	0.011707	0.043818	processed_pseudogene
ENSG0000(	RGS17P1	114.5827	-0.22147	0.01171	0.043818	processed_pseudogene
ENSG0000(	TMEM176A	1239.397	-0.27044	0.011708	0.043818	protein_coding
ENSG0000(	ANKRD9	2613.47	-0.40557	0.011716	0.043824	protein_coding
ENSG0000(	C9orf163	17.93513	0.410712	0.011719	0.043831	lncRNA
ENSG0000(	ENSG0000(	11.35571	0.533685	0.011722	0.043835	lncRNA
ENSG0000(	ASLP1	116.5671	-0.16849	0.011735	0.043881	unprocessed_pseudogene
ENSG0000(	ERG28	259.8803	-0.18487	0.01176	0.043969	protein_coding
ENSG0000(	ZNF836	781.7763	-0.13564	0.011762	0.043971	protein_coding
ENSG0000(	DMPK	1319.951	0.132513	0.01177	0.04399	protein_coding
ENSG0000(	DNPH1	191.851	-0.27939	0.01177	0.04399	protein_coding
ENSG0000(	PHTF1	758.3247	0.149447	0.01179	0.044058	protein_coding
ENSG0000(	FAM27E3	402.5548	-0.30229	0.011795	0.044072	lncRNA
ENSG0000(	ZBED1	1290.112	-0.15165	0.011814	0.044137	protein_coding
ENSG0000(	ENSG0000(	130.121	0.168625	0.01182	0.044155	lncRNA
ENSG0000(	DUSP28	507.9996	-0.14605	0.011832	0.044194	protein_coding
ENSG0000(	ENSG0000(	52.20145	0.415966	0.011838	0.044211	lncRNA
ENSG0000(	SUFU	628.0028	0.108921	0.011841	0.044218	protein_coding
ENSG0000(	ENSG0000(	32.81754	-0.31183	0.011858	0.044275	lncRNA
ENSG0000(	IPO9-AS1	19.22161	0.343628	0.011866	0.044299	lncRNA
ENSG0000(	CNTNAP2	579.3855	0.195629	0.011868	0.0443	protein_coding
ENSG0000(	ENSG0000(	114.1156	0.180166	0.011869	0.0443	TEC
ENSG0000(	CDK15	90.77694	0.357089	0.011874	0.044313	protein_coding
ENSG0000(	ECD	906.2354	-0.10632	0.011876	0.044316	protein_coding
ENSG0000(	ZNF532	2588.144	0.133749	0.011883	0.044337	protein_coding
ENSG0000(	ENSG0000(	219.503	0.169003	0.011889	0.044353	lncRNA
ENSG0000(	BNIP3L	992.8982	0.149803	0.011899	0.044387	protein_coding
ENSG0000(	ENSG0000(	110.5176	0.219775	0.011907	0.044411	lncRNA
ENSG0000(	UTP14A	259.5252	0.190386	0.011908	0.044411	protein_coding
ENSG0000(	SUPT6H	3393.664	0.091327	0.01191	0.044413	protein_coding
ENSG0000(	SDHCP3	46.72143	0.296164	0.01192	0.044444	processed_pseudogene
ENSG0000(	TRIM16L	462.1846	-0.14986	0.011921	0.044444	transcribed_unprocessed_pseudo
ENSG0000(	DESI1	1526.39	0.129487	0.01193	0.044471	protein_coding
ENSG0000(	LINC02687	107.9637	-0.16261	0.011934	0.04448	lncRNA
ENSG0000(	LAYN	127.6792	0.272337	0.011935	0.04448	protein_coding
ENSG0000(	ENSG0000(	58.299	0.213063	0.011943	0.044502	lncRNA
ENSG0000(	MCCC2	3459.391	0.088993	0.011945	0.044506	protein_coding
ENSG0000(	SNORA61	262.8201	0.242988	0.011951	0.044521	snoRNA
ENSG0000(	ENSG0000(	209.8597	0.228851	0.011952	0.044522	lncRNA
ENSG0000(	CSE1L	726.5017	-0.17564	0.011963	0.044552	protein_coding

ENSG0000(	RALY	2181.497	-0.1219	0.011962	0.044552	protein_coding
ENSG0000(	RPL23AP32	26.67019	-0.4194	0.011966	0.044557	processed_pseudogene
ENSG0000(	NAA60	2172.404	-0.13332	0.011974	0.044583	protein_coding
ENSG0000(	ENSG0000(	86.45809	0.1994	0.011979	0.044596	lncRNA
ENSG0000(	ENSG0000(	43.4885	0.282473	0.011996	0.044644	TEC
ENSG0000(	PHYHIPL	408.8598	-0.5314	0.011996	0.044644	protein_coding
ENSG0000(	PSG1	20.77731	0.541471	0.011996	0.044644	protein_coding
ENSG0000(	PLCH2	473.2412	-0.35423	0.012008	0.044681	protein_coding
ENSG0000(	ENSG0000(	67.12748	-0.25902	0.012018	0.04471	lncRNA
ENSG0000(	TFAP2E	111.7461	0.289138	0.012018	0.04471	protein_coding
ENSG0000(	TMEM216	121.0064	-0.20356	0.01202	0.04471	protein_coding
ENSG0000(	EEF1D	3187.819	-0.12863	0.012029	0.04474	protein_coding
ENSG0000(	LINC01068	12.92571	-0.77936	0.012034	0.044753	lncRNA
ENSG0000(	DERA	694.826	-0.31956	0.01204	0.044771	protein_coding
ENSG0000(	UBE2V1P1	53.71531	0.27826	0.012048	0.044792	processed_pseudogene
ENSG0000(	LINC02869	78.01817	-0.30475	0.012064	0.04485	lncRNA
ENSG0000(	PRKG2	1165.175	-0.66252	0.012072	0.044873	protein_coding
ENSG0000(	FAM161B	443.6549	-0.19402	0.01208	0.044894	protein_coding
ENSG0000(	GID4	770.5025	-0.07473	0.012082	0.044894	protein_coding
ENSG0000(	MCAM	825.8051	0.306533	0.012082	0.044894	protein_coding
ENSG0000(	FAM210A	646.7716	-0.12737	0.012088	0.044899	protein_coding
ENSG0000(	NFYC-AS1	245.2341	0.229702	0.012088	0.044899	lncRNA
ENSG0000(	WASHC5	956.9671	-0.11804	0.012085	0.044899	protein_coding
ENSG0000(	ENSG0000(	174.0981	-0.15449	0.01209	0.044902	lncRNA
ENSG0000(	LINC01134	477.0146	-0.25794	0.012096	0.044919	lncRNA
ENSG0000(	C2orf27A	361.58	0.336224	0.012097	0.044919	lncRNA
ENSG0000(	MORF4L1P	10.58425	-0.6715	0.012105	0.044943	processed_pseudogene
ENSG0000(	AP1M2	328.7085	-0.36133	0.012113	0.044955	protein_coding
ENSG0000(	ENSG0000(	94.72226	0.307352	0.012112	0.044955	TEC
ENSG0000(	TMEM184(	1016.39	-0.12533	0.012113	0.044955	protein_coding
ENSG0000(	VPS26AP1	97.18384	-0.53753	0.01212	0.04497	processed_pseudogene
ENSG0000(	ZNF280D	2177.086	0.096038	0.012119	0.04497	protein_coding
ENSG0000(	CBLN3	259.7013	0.574348	0.012133	0.045009	protein_coding
ENSG0000(	LINC01775	15.02998	-0.44579	0.012132	0.045009	lncRNA
ENSG0000(	SNHG26	860.2501	0.228074	0.01214	0.045031	lncRNA
ENSG0000(	AMACR	366.4859	-0.3417	0.01215	0.045062	protein_coding
ENSG0000(	LINC01939	20.85139	-0.45026	0.012156	0.04508	lncRNA
ENSG0000(	OR10G4	353.4134	-0.18906	0.012158	0.04508	protein_coding
ENSG0000(	NCCRP1	221.2288	-0.15839	0.012167	0.045108	protein_coding
ENSG0000(	ENSG0000(	10.62641	0.539585	0.012176	0.045136	lncRNA
ENSG0000(	ENSG0000(	149.7382	0.203906	0.012179	0.045142	lncRNA
ENSG0000(	ENSG0000(	935.8259	0.211848	0.012182	0.045142	lncRNA
ENSG0000(	GTPBP10	1260.004	-0.12647	0.012181	0.045142	protein_coding
ENSG0000(	OR5AN1	231.9425	0.155556	0.012186	0.045154	protein_coding
ENSG0000(	GABRG3	317.8711	-0.23281	0.01219	0.045161	protein_coding
ENSG0000(	KCNE4	721.8924	0.121724	0.012195	0.045173	protein_coding
ENSG0000(	LAPTM4A	762.8902	-0.23893	0.012196	0.045173	protein_coding

ENSG0000(	ENSG0000(	20.62931	-0.37061	0.012211	0.045224	lncRNA
ENSG0000(	LINC01126	166.3486	0.256368	0.012215	0.045227	lncRNA
ENSG0000(	POLR1D	1520.788	-0.19316	0.012214	0.045227	protein_coding
ENSG0000(	KLHL22	734.6625	0.191762	0.01222	0.045243	protein_coding
ENSG0000(	FAM167B	22.72589	0.37456	0.01223	0.045273	protein_coding
ENSG0000(	BRICD5	43.75979	-0.32706	0.012244	0.045305	protein_coding
ENSG0000(	MYNN	598.5295	-0.10694	0.012244	0.045305	protein_coding
ENSG0000(	SLC38A7	741.6268	0.108486	0.012244	0.045305	protein_coding
ENSG0000(	ZNF211	706.2013	0.133732	0.012241	0.045305	protein_coding
ENSG0000(	MIR4306	12.69973	-0.48241	0.012248	0.045314	miRNA
ENSG0000(	LINC02823	30.04703	0.294401	0.01226	0.045353	lncRNA
ENSG0000(	ENSG0000(	362.6112	-0.15973	0.012262	0.045353	lncRNA
ENSG0000(	ENSG0000(	101.1596	0.167945	0.012264	0.045353	lncRNA
ENSG0000(	ENSG0000(	190.298	0.178184	0.012265	0.045353	lncRNA
ENSG0000(	ENSG0000(	21.44366	0.413471	0.012267	0.045358	lncRNA
ENSG0000(	ACOT4	16.97292	-1.13566	0.012275	0.04538	protein_coding
ENSG0000(	ENSG0000(	12.20184	0.424566	0.012279	0.045389	lncRNA
ENSG0000(	LINC01828	188.7429	0.180693	0.01228	0.04539	lncRNA
ENSG0000(	SLC37A2	1205.863	0.240508	0.012285	0.045402	protein_coding
ENSG0000(	ENSG0000(	10.66993	-0.52737	0.012302	0.045458	lncRNA
ENSG0000(	ABCG8	2147.637	-0.33995	0.012307	0.045472	protein_coding
ENSG0000(	LINC00685	339.8883	0.17332	0.012319	0.045513	lncRNA
ENSG0000(	SMOC2	293.0905	0.487497	0.012324	0.045525	protein_coding
ENSG0000(	IGKV3OR2-	15.64509	0.837289	0.012326	0.045525	IG_V_gene
ENSG0000(	SNRPEP9	30.9669	-0.33148	0.012334	0.04555	processed_pseudogene
ENSG0000(	STX1B	308.736	0.312693	0.012336	0.045552	protein_coding
ENSG0000(	LINC01547	219.9966	0.14131	0.01234	0.045564	lncRNA
ENSG0000(	ITGB1-DT	146.2724	0.156727	0.012362	0.04564	lncRNA
ENSG0000(	ENSG0000(	90.33688	0.193151	0.012365	0.045645	lncRNA
ENSG0000(	STRBP	2132.049	-0.18612	0.012368	0.045649	protein_coding
ENSG0000(	SYNE3	2556.68	0.215408	0.012381	0.045693	protein_coding
ENSG0000(	RCOR1	1749.383	-0.14432	0.012384	0.0457	protein_coding
ENSG0000(	FUBP1	4026.978	-0.14139	0.012386	0.045701	protein_coding
ENSG0000(	MAGI2	1695.945	0.242878	0.012394	0.045725	protein_coding
ENSG0000(	CLDN3	556.1458	-0.48436	0.012399	0.045732	protein_coding
ENSG0000(	ENSG0000(	1523.323	-0.48761	0.0124	0.045732	TEC
ENSG0000(	TMBIM1	3211.514	-0.25271	0.012399	0.045732	protein_coding
ENSG0000(	ENSG0000(	12.38925	0.505065	0.012412	0.045769	lncRNA
ENSG0000(	CEP170	1921.756	0.230465	0.012417	0.045781	protein_coding
ENSG0000(	LINC02694	842.8421	0.144919	0.01242	0.045781	lncRNA
ENSG0000(	SLC5A1	9057.176	-0.59878	0.012419	0.045781	protein_coding
ENSG0000(	ENSG0000(	20.26357	0.401998	0.012434	0.045823	lncRNA
ENSG0000(	SIGLEC22P	10.85942	0.572957	0.012433	0.045823	transcribed_unprocessed_pseudo
ENSG0000(	IDNK	336.7555	-0.20239	0.012437	0.045828	protein_coding
ENSG0000(	ACTN1-DT	19.17081	0.544207	0.012441	0.045838	lncRNA
ENSG0000(	PHPT1	438.4819	-0.25125	0.012443	0.045842	protein_coding
ENSG0000(	SPAG17	25.24487	1.002054	0.012449	0.045857	protein_coding

ENSG0000(	LINC02236	23.10789	0.396115	0.012451	0.045859	lncRNA
ENSG0000(	DCDC2	82.98394	-0.24937	0.012455	0.045869	protein_coding
ENSG0000(	LINC02452	255.475	0.126818	0.012458	0.045876	lncRNA
ENSG0000(	MIR215	22.89711	-0.69944	0.012472	0.04592	miRNA
ENSG0000(	ENSG0000(	1600.548	0.078981	0.012484	0.045954	TEC
ENSG0000(	NIPAL1	880.9394	-0.36984	0.012483	0.045954	protein_coding
ENSG0000(	TOMM70	1090.077	-0.13864	0.012515	0.046063	protein_coding
ENSG0000(	ACSL5	7989.865	-0.49891	0.012519	0.046074	protein_coding
ENSG0000(	ENSG0000(	121.0893	0.187558	0.012524	0.046085	lncRNA
ENSG0000(	SLC6A19	10091.28	-0.72526	0.012529	0.046098	protein_coding
ENSG0000(	CDHR4	373.092	-0.15031	0.012535	0.046114	protein_coding
ENSG0000(	LINC02145	201.8409	-0.27711	0.01254	0.046128	lncRNA
ENSG0000(	TMPRSS11I	283.5756	0.17726	0.012545	0.046142	protein_coding
ENSG0000(	ENSG0000(	1757.039	-0.09798	0.012563	0.046202	lncRNA
ENSG0000(	RANP3	15.46178	0.401548	0.012566	0.046208	processed_pseudogene
ENSG0000(	SDR39U1	636.8747	0.226655	0.012573	0.046227	protein_coding
ENSG0000(	ENSG0000(	101.4797	0.356893	0.012575	0.046231	lncRNA
ENSG0000(	ENSG0000(	10.22191	0.524761	0.012587	0.046269	lncRNA
ENSG0000(	TFPI2-DT	26.82418	0.291835	0.012606	0.046334	lncRNA
ENSG0000(	ENSG0000(	8.484292	0.546562	0.012614	0.046356	lncRNA
ENSG0000(	ENSG0000(	54.13852	0.198999	0.012626	0.046394	lncRNA
ENSG0000(	ENSG0000(	92.68662	-0.18086	0.012642	0.046432	lncRNA
ENSG0000(	REG1A	9026.485	0.816879	0.012642	0.046432	protein_coding
ENSG0000(	TPH1	1292.833	0.24731	0.012639	0.046432	protein_coding
ENSG0000(	ZNF609	4166.606	0.070912	0.012642	0.046432	protein_coding
ENSG0000(	TFIP11-DT	63.58215	0.243704	0.012653	0.046469	lncRNA
ENSG0000(	SLC13A4	158.499	0.162617	0.012665	0.046506	protein_coding
ENSG0000(	CCDC38	94.31973	0.20531	0.012685	0.046573	protein_coding
ENSG0000(	ENSG0000(	132.9325	0.211351	0.012686	0.046573	TEC
ENSG0000(	C2orf68	1961.439	-0.0816	0.012694	0.046592	protein_coding
ENSG0000(	CLEC4C	58.60136	0.321858	0.012696	0.046592	protein_coding
ENSG0000(	PTPRR	512.9286	-0.6112	0.012695	0.046592	protein_coding
ENSG0000(	ENSG0000(	270.2658	-0.13089	0.0127	0.046603	lncRNA
ENSG0000(	VAPA	3145.643	-0.14802	0.012704	0.04661	protein_coding
ENSG0000(	EXOC6B	3486.035	-0.42554	0.012705	0.046611	protein_coding
ENSG0000(	ENSG0000(	17.91472	0.496245	0.012716	0.046644	scaRNA
ENSG0000(	ZNF768	501.9219	-0.28643	0.012721	0.046657	protein_coding
ENSG0000(	GALNT6	1524.307	-0.32032	0.012725	0.046667	protein_coding
ENSG0000(	PROX1	1074.347	0.324968	0.012732	0.046685	protein_coding
ENSG0000(	LINC01618	116.5624	-0.1573	0.012739	0.046708	lncRNA
ENSG0000(	NR0B2	56.31952	-0.82126	0.012747	0.046731	protein_coding
ENSG0000(	ENSG0000(	37.61817	-0.32172	0.012758	0.046765	lncRNA
ENSG0000(	ENSG0000(	876.0291	-0.27561	0.012764	0.046776	lncRNA
ENSG0000(	ENSG0000(	32.73035	0.284921	0.012764	0.046776	lncRNA
ENSG0000(	ENSG0000(	98.65253	0.166048	0.01278	0.046832	lncRNA
ENSG0000(	ENSG0000(	104.4539	0.297553	0.0128	0.046899	lncRNA
ENSG0000(	ENSG0000(	12.44122	0.423677	0.012805	0.046908	lncRNA

ENSG0000(	SV2A	208.6693	0.157033	0.012806	0.046908	protein_coding
ENSG0000(	SMTNL2	12.77819	0.714465	0.012817	0.046942	protein_coding
ENSG0000(	TRAK1	5253.094	-0.2222	0.012818	0.046942	protein_coding
ENSG0000(	PLA2G12A	1669.962	-0.24144	0.012822	0.04695	protein_coding
ENSG0000(	C3orf62	984.8524	0.112188	0.012834	0.046985	protein_coding
ENSG0000(	MEGF9	872.6563	0.146317	0.012834	0.046985	protein_coding
ENSG0000(	CDC7	287.5917	0.335926	0.012836	0.046986	protein_coding
ENSG0000(	ENSG0000(	110.1667	0.182088	0.012839	0.046986	lncRNA
ENSG0000(	WDR76	605.9776	-0.14476	0.012839	0.046986	protein_coding
ENSG0000(	IRAK1	1163.533	0.154428	0.012851	0.047018	protein_coding
ENSG0000(	TMEM231I	676.7995	-0.59314	0.012849	0.047018	transcribed_unprocessed_pseudo
ENSG0000(	TMEM92	1254.131	-0.57673	0.012858	0.047039	protein_coding
ENSG0000(	ENSG0000(	254.4063	0.134965	0.01286	0.04704	lncRNA
ENSG0000(	GBA2	3316.714	-0.26912	0.012876	0.047093	protein_coding
ENSG0000(	TRIM67	9.541756	0.527177	0.012883	0.047114	protein_coding
ENSG0000(	SNORA11B	58.66651	0.222748	0.012887	0.047124	snoRNA
ENSG0000(	SP100	11293.05	0.211324	0.01289	0.047129	protein_coding
ENSG0000(	ENSG0000(	96.75493	-0.27846	0.012894	0.04714	lncRNA
ENSG0000(	ENSG0000(	152.623	0.144497	0.012903	0.047161	lncRNA
ENSG0000(	ENSG0000(	397.1089	0.099538	0.012903	0.047161	lncRNA
ENSG0000(	ENSG0000(	192.0048	0.235623	0.012914	0.047195	lncRNA
ENSG0000(	LINC01445	1991.641	0.175426	0.012916	0.047195	lncRNA
ENSG0000(	CEP164	1222.559	0.154584	0.012934	0.047255	protein_coding
ENSG0000(	LINC02337	34.70681	0.255075	0.012935	0.047255	lncRNA
ENSG0000(	HCG15	157.5696	-0.15719	0.01294	0.047266	lncRNA
ENSG0000(	ENSG0000(	47.23206	0.241978	0.012943	0.047275	TEC
ENSG0000(	RARA	800.2507	-0.19613	0.012947	0.047284	protein_coding
ENSG0000(	FCGRT	3172.679	-0.26771	0.012956	0.047311	protein_coding
ENSG0000(	GNE	1269.967	0.11299	0.012964	0.047334	protein_coding
ENSG0000(	ENSG0000(	26.98971	0.492625	0.012974	0.047366	lncRNA
ENSG0000(	AGTPBP1	722.9776	0.25081	0.012977	0.047371	protein_coding
ENSG0000(	SLC35B4	1146.946	-0.10868	0.012979	0.047373	protein_coding
ENSG0000(	ENSG0000(	22.04405	0.337202	0.012986	0.047389	lncRNA
ENSG0000(	RNVU1-15	361.1402	-0.31846	0.012987	0.047389	snRNA
ENSG0000(	SNORD93	10.23756	0.683434	0.012996	0.047416	snoRNA
ENSG0000(	DCHS2	245.6689	0.239073	0.01301	0.047446	protein_coding
ENSG0000(	ENSG0000(	621.611	-0.08692	0.01301	0.047446	lncRNA
ENSG0000(	EPRS1	1615.655	-0.1435	0.013011	0.047446	protein_coding
ENSG0000(	SMIM15-A!	116.4051	0.189417	0.013008	0.047446	lncRNA
ENSG0000(	SNHG8	736.453	-0.2732	0.013011	0.047446	lncRNA
ENSG0000(	ADA2	1891.971	0.190611	0.013019	0.047465	protein_coding
ENSG0000(	BLM	996.615	0.227995	0.013019	0.047465	protein_coding
ENSG0000(	PPM1B	2141.982	0.077422	0.013024	0.047471	protein_coding
ENSG0000(	RPS4XP2	26.91523	0.306144	0.013024	0.047471	transcribed_processed_pseudoge
ENSG0000(	GANAB	2855.443	-0.10199	0.01303	0.047476	protein_coding
ENSG0000(	HDAC1	1794.528	-0.10455	0.013028	0.047476	protein_coding
ENSG0000(	STEAP1B	145.4661	0.779314	0.013029	0.047476	protein_coding

ENSG0000(	ENSG0000(	91.92673	-0.64408	0.013032	0.047476	unprocessed_pseudogene
ENSG0000(	SLC39A11	816.3039	0.162291	0.013034	0.047476	protein_coding
ENSG0000(	SRP14P3	31.56966	0.289513	0.013035	0.047476	processed_pseudogene
ENSG0000(	AHNAK2	445.9094	0.411687	0.01304	0.047482	protein_coding
ENSG0000(	CIP2A	278.4269	0.290347	0.013041	0.047482	protein_coding
ENSG0000(	ENSG0000(	279.4858	0.259164	0.013039	0.047482	lncRNA
ENSG0000(	ENSG0000(	285.4397	0.179622	0.013044	0.047489	lncRNA
ENSG0000(	ENSG0000(	2438.678	0.136352	0.013048	0.047497	lncRNA
ENSG0000(	ENSG0000(	43.56649	0.313867	0.013051	0.047502	lncRNA
ENSG0000(	ISOC1	328.5756	-0.22434	0.01306	0.047526	protein_coding
ENSG0000(	RBBP5	1104.628	-0.08041	0.01306	0.047526	protein_coding
ENSG0000(	CHCHD1	187.758	-0.15376	0.013062	0.047528	protein_coding
ENSG0000(	RN7SL655F	70.94654	0.207986	0.013067	0.04754	misc_RNA
ENSG0000(	ENSG0000(	30.63914	-0.46883	0.013088	0.047609	lncRNA
ENSG0000(	FAM86C1P	445.4556	0.147516	0.013089	0.047609	transcribed_unprocessed_pseudo
ENSG0000(	CIZ1	1388.176	-0.09917	0.013095	0.047625	protein_coding
ENSG0000(	ENSG0000(	247.1998	0.199173	0.013107	0.047653	lncRNA
ENSG0000(	HTATIP2	540.9706	-0.33163	0.013106	0.047653	protein_coding
ENSG0000(	RNF185	838.5424	-0.16194	0.013107	0.047653	protein_coding
ENSG0000(	PACRG-AS1	189.2039	-0.13438	0.013115	0.047673	lncRNA
ENSG0000(	STARD4-AS	142.1906	0.189573	0.013124	0.047703	lncRNA
ENSG0000(	TBCD	2916.56	0.153513	0.01313	0.04772	protein_coding
ENSG0000(	ENSG0000(	498.3265	0.185739	0.013142	0.047758	lncRNA
ENSG0000(	OARD1	1139.451	-0.13198	0.013145	0.047761	protein_coding
ENSG0000(	CDK10	2648.122	-0.14644	0.013147	0.047765	protein_coding
ENSG0000(	RPL21P106	13.22984	0.880606	0.013149	0.047766	processed_pseudogene
ENSG0000(	DLGAP5	173.8318	0.388254	0.013155	0.047781	protein_coding
ENSG0000(	MIR583HG	15.62173	0.447704	0.013164	0.047805	lncRNA
ENSG0000(	MRPS17	586.9124	-0.09031	0.013164	0.047805	protein_coding
ENSG0000(	TEKT3	49.65286	-0.28739	0.013181	0.047853	protein_coding
ENSG0000(	UBE2Q2P1	397.4563	0.141619	0.01318	0.047853	transcribed_unprocessed_pseudo
ENSG0000(	ENSG0000(	131.1709	0.129464	0.013194	0.047896	lncRNA
ENSG0000(	ENSG0000(	50.04617	0.201807	0.013222	0.047991	lncRNA
ENSG0000(	PMAIP1	86.39492	0.366549	0.013223	0.047991	protein_coding
ENSG0000(	CATSPERD	24.25804	0.33579	0.01323	0.048008	protein_coding
ENSG0000(	ENSG0000(	38.00313	0.384825	0.013235	0.048012	lncRNA
ENSG0000(	KDM3B	3533.095	0.062376	0.013234	0.048012	protein_coding
ENSG0000(	XPC	1198.208	-0.13356	0.013235	0.048012	protein_coding
ENSG0000(	CCDC87	10.8338	0.451239	0.01324	0.048024	protein_coding
ENSG0000(	CLIC1	1184.202	-0.2537	0.013244	0.048034	protein_coding
ENSG0000(	MTOR	2938.649	0.107633	0.013246	0.048035	protein_coding
ENSG0000(	ENSG0000(	17.91572	0.451191	0.013248	0.048036	lncRNA
ENSG0000(	NTM	97.26672	0.400914	0.013253	0.048048	protein_coding
ENSG0000(	H3C10	174.3852	-0.38931	0.013269	0.048103	protein_coding
ENSG0000(	ENSG0000(	34.63343	0.218054	0.013277	0.048127	lncRNA
ENSG0000(	STAT6	8663.229	-0.14297	0.013293	0.048176	protein_coding
ENSG0000(	ATCAY	660.4202	0.105587	0.013304	0.048206	protein_coding

ENSG0000(	FAM209B	13.17729	0.534992	0.013303	0.048206	protein_coding
ENSG0000(	UBA6	2652.547	0.122702	0.013317	0.048248	protein_coding
ENSG0000(	GRM3	33.50874	0.397559	0.013321	0.048256	protein_coding
ENSG0000(	LDOC1	145.0483	0.140997	0.013322	0.048256	protein_coding
ENSG0000(	MOGAT3	1515.866	-0.39361	0.013327	0.048269	protein_coding
ENSG0000(	LINC02054	342.954	-0.12517	0.013331	0.048277	lncRNA
ENSG0000(	ENSG0000(	20.74495	0.302806	0.013347	0.048313	lncRNA
ENSG0000(	MAP1LC3B	47.92517	0.262205	0.013348	0.048313	protein_coding
ENSG0000(	ROMO1	81.3879	-0.30072	0.013345	0.048313	protein_coding
ENSG0000(	SAR1B	5893.472	-0.17575	0.013349	0.048313	protein_coding
ENSG0000(	SNORD111	15.86603	-0.36597	0.013344	0.048313	snoRNA
ENSG0000(	RNU11	90.53196	0.906898	0.013357	0.048336	snRNA
ENSG0000(	TRAPPC11	1374.352	-0.11911	0.013373	0.048389	protein_coding
ENSG0000(	ENSG0000(	31.828	-0.40312	0.013375	0.048392	lncRNA
ENSG0000(	ENSG0000(	30.99133	0.421357	0.013396	0.048446	lncRNA
ENSG0000(	ENSG0000(	186.4098	-0.15224	0.013393	0.048446	lncRNA
ENSG0000(	ENSG0000(	242.8692	-0.13003	0.013396	0.048446	lncRNA
ENSG0000(	LINC01355	1708.598	0.112303	0.013395	0.048446	lncRNA
ENSG0000(	RCN2	1802.782	-0.0867	0.0134	0.048453	protein_coding
ENSG0000(	IL12A-AS1	326.2525	0.119148	0.013404	0.048464	lncRNA
ENSG0000(	FBLN2	167.091	0.525603	0.013417	0.048504	protein_coding
ENSG0000(	CNRIP1	214.6375	0.215481	0.013423	0.048519	protein_coding
ENSG0000(	LINC02176	102.4574	-0.18583	0.013437	0.048564	lncRNA
ENSG0000(	ENSG0000(	31.98234	0.299164	0.013438	0.048565	lncRNA
ENSG0000(	SMCHD1	13593.98	0.131644	0.013442	0.048571	protein_coding
ENSG0000(	ENSG0000(	32.05436	0.433786	0.013451	0.048596	processed_pseudogene
ENSG0000(	XYLT1	1990.092	-0.17742	0.013452	0.048596	protein_coding
ENSG0000(	RHOC	1803.107	-0.38837	0.013456	0.048606	protein_coding
ENSG0000(	MTHFD1L	368.0685	0.300464	0.013467	0.04864	protein_coding
ENSG0000(	SPAG16	310.407	-0.14541	0.01348	0.048683	protein_coding
ENSG0000(	ENSG0000(	18.5326	0.385326	0.013489	0.04871	lncRNA
ENSG0000(	CAPN3	1623.713	0.124084	0.013507	0.048764	protein_coding
ENSG0000(	ENSG0000(	89.56746	0.170342	0.01351	0.048764	lncRNA
ENSG0000(	HSPBAP1	811.6321	0.115918	0.01351	0.048764	protein_coding
ENSG0000(	LINC00412	59.05356	0.322552	0.013507	0.048764	lncRNA
ENSG0000(	ENSG0000(	1326.818	0.201691	0.013528	0.048809	lncRNA
ENSG0000(	ENSG0000(	418.3033	-0.14154	0.013527	0.048809	lncRNA
ENSG0000(	WBP1	879.2271	-0.10931	0.013526	0.048809	protein_coding
ENSG0000(	HCLS1	2231.522	0.380048	0.013541	0.048852	protein_coding
ENSG0000(	LINC01348	204.6191	0.270625	0.013552	0.048887	lncRNA
ENSG0000(	ENSG0000(	112.5147	-0.16728	0.013558	0.0489	lncRNA
ENSG0000(	ZC3H10	707.555	0.131314	0.013563	0.048913	protein_coding
ENSG0000(	SSTR1	182.3327	-0.5811	0.013571	0.048939	protein_coding
ENSG0000(	EDEM3	4278.947	0.103563	0.013579	0.04896	protein_coding
ENSG0000(	ENSG0000(	30.27907	0.27736	0.013586	0.048969	lncRNA
ENSG0000(	SEC61G-DT	181.341	-0.14974	0.013584	0.048969	lncRNA
ENSG0000(	TRPC1	181.8915	0.271389	0.013588	0.048969	protein_coding

ENSG0000(	UTS2B	181.5458	0.243872	0.013586	0.048969	protein_coding
ENSG0000(	ZNRD2	60.64634	-0.24257	0.013596	0.048992	protein_coding
ENSG0000(	ENSG0000(	43.31822	0.29447	0.013604	0.049012	TEC
ENSG0000(	GPATCH2L	5450.766	0.090743	0.013604	0.049012	protein_coding
ENSG0000(	ENSG0000(	8.94667	0.535013	0.013606	0.049013	lncRNA
ENSG0000(	MMP15	1043.846	-0.44614	0.013611	0.049024	protein_coding
ENSG0000(	ENSG0000(	88.7438	0.168078	0.013618	0.049038	lncRNA
ENSG0000(	MPP7	675.5663	-0.20215	0.013617	0.049038	protein_coding
ENSG0000(	ENSG0000(	115.5114	-0.14785	0.013622	0.04905	lncRNA
ENSG0000(	ENSG0000(	51.00004	0.266368	0.013632	0.049079	lncRNA
ENSG0000(	LPIN1	3291.166	0.185803	0.013634	0.049079	protein_coding
ENSG0000(	CDH3	399.2007	-0.16095	0.013636	0.049083	protein_coding
ENSG0000(	NUP155	1558.244	-0.14308	0.01365	0.049128	protein_coding
ENSG0000(	DLGAP4-AS	122.1768	0.246103	0.013663	0.049167	lncRNA
ENSG0000(	SLX1A	831.4368	-0.23973	0.013677	0.049213	protein_coding
ENSG0000(	QTRT2	1668.801	0.109311	0.01368	0.049217	protein_coding
ENSG0000(	FGF13	2950.29	0.103496	0.01369	0.049249	protein_coding
ENSG0000(	ENSG0000(	39.3089	-0.32334	0.013708	0.049304	lncRNA
ENSG0000(	ENSG0000(	22.22436	0.493891	0.01371	0.049304	lncRNA
ENSG0000(	ENSG0000(	334.9139	0.177149	0.01371	0.049304	lncRNA
ENSG0000(	CDRT15P6	44.24329	0.366013	0.013713	0.049304	transcribed_unprocessed_pseudo
ENSG0000(	ENSG0000(	40.33564	-0.27275	0.013713	0.049304	lncRNA
ENSG0000(	LINC02548	831.2264	-0.11466	0.013724	0.049334	lncRNA
ENSG0000(	POLR2J2	1331.219	0.255346	0.013725	0.049334	protein_coding
ENSG0000(	ALOXE3P1	19.31387	0.506591	0.013727	0.049337	unprocessed_pseudogene
ENSG0000(	PDAP1	765.9637	-0.18066	0.013729	0.049337	protein_coding
ENSG0000(	GOLGA8H	66.12665	0.539598	0.01375	0.049408	protein_coding
ENSG0000(	RPL15	5045.634	-0.24249	0.013752	0.049409	protein_coding
ENSG0000(	MYO1G	1109.583	0.370115	0.013757	0.04942	protein_coding
ENSG0000(	FAAH	566.803	-0.36772	0.013762	0.049429	protein_coding
ENSG0000(	HDAC6	2001.996	-0.06218	0.013762	0.049429	protein_coding
ENSG0000(	OTUD5	1041.266	-0.11173	0.013766	0.049439	protein_coding
ENSG0000(	DMAP1	1479.231	-0.09431	0.013776	0.049466	protein_coding
ENSG0000(	CNTNAP3	799.6336	-0.15565	0.013781	0.049478	protein_coding
ENSG0000(	GLYCTK-AS	27.65043	0.793646	0.013785	0.049484	lncRNA
ENSG0000(	POMGNT1	1147.146	-0.12893	0.013784	0.049484	protein_coding
ENSG0000(	DNAJB13	43.62155	-0.83875	0.013789	0.049492	protein_coding
ENSG0000(	OR8A1	595.0531	0.128914	0.013792	0.049497	protein_coding
ENSG0000(	SNORD10	843.5013	0.343704	0.013794	0.049498	snoRNA
ENSG0000(	ZNF100	1016.006	-0.13251	0.013812	0.049556	protein_coding
ENSG0000(	EFR3A	1686.106	-0.11155	0.013827	0.049604	protein_coding
ENSG0000(	CADM2-AS	145.6017	0.142723	0.013833	0.049616	lncRNA
ENSG0000(	TIE1	501.8564	0.228791	0.013833	0.049616	protein_coding
ENSG0000(	CAPRIN1	4002.644	-0.08681	0.013836	0.049619	protein_coding
ENSG0000(	RING1	466.343	-0.12895	0.013844	0.049643	protein_coding
ENSG0000(	TMEM38B	262.4316	-0.15661	0.013847	0.049649	protein_coding
ENSG0000(	ARL8A	239.9519	-0.20246	0.013853	0.049663	protein_coding

ENSG0000(	MIR1200	13.26062	0.522793	0.013863	0.049694	miRNA
ENSG0000(	CDH12	365.8425	-0.16866	0.01387	0.049705	protein_coding
ENSG0000(	STAG3L5P	1710.716	-0.10347	0.013871	0.049705	transcribed_unprocessed_pseudo
ENSG0000(	VAT1	503.7325	-0.24459	0.013868	0.049705	protein_coding
ENSG0000(	CMTM6	1292.687	0.248338	0.013875	0.04971	protein_coding
ENSG0000(	RBM10	783.3221	-0.1343	0.013874	0.04971	protein_coding
ENSG0000(	ENSG0000(	22.49109	0.496364	0.013893	0.049768	lncRNA
ENSG0000(	ENSG0000(	62.37584	0.275215	0.013913	0.049836	lncRNA
ENSG0000(	GFAP	352.816	0.190064	0.013922	0.049861	protein_coding
ENSG0000(	CDH5	432.8091	0.321104	0.013938	0.049914	protein_coding
ENSG0000(	CEP350	7409.249	-0.15534	0.013942	0.049922	protein_coding
ENSG0000(	ENSG0000(	18.7494	0.342486	0.013966	0.050001	lncRNA
ENSG0000(	MRPL23	228.4281	-0.17239	0.013977	0.050036	protein_coding
ENSG0000(	MRPS30	1234.475	-0.10956	0.013982	0.050048	protein_coding
ENSG0000(	ADH6	348.9154	-0.47935	0.013987	0.050059	protein_coding
ENSG0000(	EGLN1	1350.613	-0.20397	0.014003	0.05011	protein_coding
ENSG0000(	ENSG0000(	123.2563	0.148106	0.014005	0.05011	lncRNA
ENSG0000(	LINC01531	466.6757	-0.15069	0.014006	0.05011	lncRNA
ENSG0000(	OVCH1	25.89269	0.49429	0.014018	0.050142	protein_coding
ENSG0000(	SZT2-AS1	32.68466	-0.42461	0.014018	0.050142	lncRNA
ENSG0000(	IFI16	2313.322	0.369465	0.014025	0.050144	protein_coding
ENSG0000(	LNCOG	35.83075	0.300515	0.014021	0.050144	lncRNA
ENSG0000(	SCARNA20	19.99817	0.351517	0.014025	0.050144	scaRNA
ENSG0000(	TAC3	49.76973	-0.4568	0.014023	0.050144	protein_coding
ENSG0000(	ENSG0000(	16.32927	0.428294	0.014038	0.050187	lncRNA
ENSG0000(	TRGC1	174.4894	-0.30381	0.014041	0.050189	TR_C_gene
ENSG0000(	CNKSR3	6933.084	-0.38878	0.014047	0.050206	protein_coding
ENSG0000(	PER2	1417.78	-0.25314	0.014049	0.050208	protein_coding
ENSG0000(	SEC11C	586.3358	0.175196	0.014054	0.050221	protein_coding
ENSG0000(	EDEM1	3441.34	0.215189	0.014072	0.050277	protein_coding
ENSG0000(	ELN-AS1	26.74461	0.346252	0.014092	0.050331	lncRNA
ENSG0000(	ENSG0000(	113.8607	0.24578	0.014089	0.050331	lncRNA
ENSG0000(	PPIL4	615.099	-0.11808	0.014091	0.050331	protein_coding
ENSG0000(	RFNG	293.57	-0.19451	0.014094	0.050333	protein_coding
ENSG0000(	PAUPAR	198.0627	0.153643	0.014096	0.050334	lncRNA
ENSG0000(	RPL4P6	136.9608	0.170813	0.0141	0.050345	processed_pseudogene
ENSG0000(	LDHD	36.76933	0.746769	0.014116	0.050396	protein_coding
ENSG0000(	LINC00842	104.4659	-0.38696	0.01412	0.050403	lncRNA
ENSG0000(	CCL15-CCL1	66.73994	-0.59631	0.014125	0.050414	protein_coding
ENSG0000(	GLUD1P3	228.2575	0.154747	0.014126	0.050415	transcribed_unprocessed_pseudo
ENSG0000(	PGM3	1576.08	0.225677	0.014133	0.050433	protein_coding
ENSG0000(	CDH7	231.5011	-0.16779	0.014144	0.050465	protein_coding
ENSG0000(	SNORD91B	10.91198	0.437755	0.014155	0.0505	snoRNA
ENSG0000(	VIPAS39	555.4367	-0.15166	0.014156	0.0505	protein_coding
ENSG0000(	KIAA1614-A	78.91256	0.222027	0.01416	0.050508	lncRNA
ENSG0000(	AKR7A2	378.9766	-0.25965	0.014173	0.050549	protein_coding
ENSG0000(	GATA6	1130.564	-0.46185	0.014179	0.050563	protein_coding

ENSG0000(	RN7SL820F	31.35272	0.269665	0.014198	0.050625	misc_RNA
ENSG0000(	EXOC3L4	878.3768	-0.25735	0.01421	0.050662	protein_coding
ENSG0000(	OR52U1P	22.33419	0.592969	0.014223	0.050698	unprocessed_pseudogene
ENSG0000(	UHRF2	2866.02	0.145448	0.014222	0.050698	protein_coding
ENSG0000(	SUCO	2148.042	-0.21581	0.014236	0.050736	protein_coding
ENSG0000(	INTS1	2267.678	-0.13883	0.014246	0.050768	protein_coding
ENSG0000(	LINC02454	37.76017	0.323103	0.014249	0.050772	lncRNA
ENSG0000(	MINDY4	336.5285	0.171401	0.014255	0.050787	protein_coding
ENSG0000(	ENSG0000(	27.42598	0.358849	0.014257	0.050791	lncRNA
ENSG0000(	ATP13A1	1402.934	-0.14851	0.014263	0.050804	protein_coding
ENSG0000(	BDH2	955.93	-0.26033	0.014265	0.050808	protein_coding
ENSG0000(	AKR1B15	499.2705	0.151648	0.014273	0.050828	protein_coding
ENSG0000(	TMEM236	3402.197	-0.39306	0.014285	0.050867	protein_coding
ENSG0000(	KCNIP1	8.499926	0.697832	0.014291	0.05088	protein_coding
ENSG0000(	ENSG0000(	491.8431	0.102559	0.014294	0.050886	TEC
ENSG0000(	ENSG0000(	72.36372	-0.64061	0.014312	0.050945	lncRNA
ENSG0000(	ENSG0000(	8.581053	0.556695	0.014315	0.050952	lncRNA
ENSG0000(	C1orf146	60.15247	0.270794	0.014318	0.050956	protein_coding
ENSG0000(	OAZ2	2352.981	0.166779	0.01432	0.050956	protein_coding
ENSG0000(	CATSPER2	2080.097	-0.2597	0.014324	0.050964	protein_coding
ENSG0000(	RPSAP6	30.77714	0.404516	0.014326	0.050966	processed_pseudogene
ENSG0000(	LINC02429	54.2505	0.199875	0.014336	0.050991	lncRNA
ENSG0000(	TOM1L1	1199.147	-0.33952	0.014335	0.050991	protein_coding
ENSG0000(	CD320	266.1192	-0.16104	0.014345	0.051017	protein_coding
ENSG0000(	SPIDR	1682.32	0.134708	0.014347	0.051018	protein_coding
ENSG0000(	ENSG0000(	20.94375	0.326551	0.014354	0.051038	lncRNA
ENSG0000(	LGALS12	20.54011	0.504019	0.014359	0.051049	protein_coding
ENSG0000(	ENSG0000(	424.1047	0.091006	0.014363	0.051056	lncRNA
ENSG0000(	NUCB1	1837.633	-0.21654	0.014374	0.051091	protein_coding
ENSG0000(	CERT1	2683.79	-0.09494	0.014396	0.051164	protein_coding
ENSG0000(	ENSG0000(	257.9351	0.116665	0.014399	0.051168	lncRNA
ENSG0000(	LL22NC01-;	106.8768	-0.20429	0.014404	0.051178	lncRNA
ENSG0000(	RNU2-65P	50.85331	-0.26743	0.01441	0.051195	snRNA
ENSG0000(	ENSG0000(	26.37595	0.412048	0.01442	0.051226	lncRNA
ENSG0000(	SCAMP1	855.9752	-0.11906	0.014427	0.051244	protein_coding
ENSG0000(	STARD7	2511.682	-0.11522	0.014434	0.051262	protein_coding
ENSG0000(	ENSG0000(	1009.936	0.176445	0.014446	0.051299	TEC
ENSG0000(	SH3BP2	4845.448	0.175721	0.01445	0.051307	protein_coding
ENSG0000(	VAT1L	171.7664	-0.4357	0.014451	0.051307	protein_coding
ENSG0000(	CHN2	1791.818	-0.26899	0.014453	0.051309	protein_coding
ENSG0000(	ENSG0000(	107.184	0.346302	0.014459	0.051325	lncRNA
ENSG0000(	C10orf105	29.07242	0.433257	0.014462	0.051328	protein_coding
ENSG0000(	MEIOC	331.1855	0.134394	0.01447	0.051352	protein_coding
ENSG0000(	PTGS1	658.7255	0.324966	0.014475	0.051361	protein_coding
ENSG0000(	TMEM80	906.9202	-0.14652	0.014477	0.051364	protein_coding
ENSG0000(	PRKAR1B	177.19	-0.22671	0.014481	0.051372	protein_coding
ENSG0000(	ENSG0000(	28.45472	-0.29057	0.014504	0.051447	lncRNA

ENSG0000(	POR	978.8052	-0.33199	0.014507	0.051453	protein_coding
ENSG0000(	NCALD	1066.808	0.166377	0.014518	0.051479	protein_coding
ENSG0000(	UNC5C-AS1	37.55578	0.306284	0.014517	0.051479	lncRNA
ENSG0000(	EBP	243.0666	-0.26751	0.01452	0.051483	protein_coding
ENSG0000(	BMP2	531.7402	-0.41347	0.014524	0.051491	protein_coding
ENSG0000(	ENSG0000(	15.13308	0.587187	0.014526	0.051492	protein_coding
ENSG0000(	ENSG0000(	222.1089	0.295978	0.014539	0.051533	lncRNA
ENSG0000(	AREL1	1944.425	-0.15212	0.014555	0.051583	protein_coding
ENSG0000(	ATXN8OS	329.3137	0.150781	0.014562	0.051596	lncRNA
ENSG0000(	C2orf88	1594.675	-0.40614	0.014561	0.051596	protein_coding
ENSG0000(	TTF2	1856.406	0.154696	0.01459	0.051688	protein_coding
ENSG0000(	SOD3	73.40704	0.40286	0.014592	0.05169	protein_coding
ENSG0000(	CCDC14	4945.916	0.127888	0.014594	0.051693	protein_coding
ENSG0000(	PRKXP1	255.1258	0.38326	0.014603	0.051717	processed_pseudogene
ENSG0000(	ENSG0000(	89.47296	0.200164	0.014615	0.051754	lncRNA
ENSG0000(	GNA11	5152.36	-0.3898	0.014617	0.051754	protein_coding
ENSG0000(	ENSG0000(	160.7991	0.169784	0.014619	0.051757	lncRNA
ENSG0000(	ENSG0000(	220.7608	-0.2441	0.014631	0.051794	lncRNA
ENSG0000(	LINC00955	1189.371	-0.76389	0.014633	0.051794	lncRNA
ENSG0000(	CHAD	238.1669	-0.68348	0.014637	0.051796	protein_coding
ENSG0000(	GRSF1	1884.142	-0.08185	0.01464	0.051796	protein_coding
ENSG0000(	PTGES3L	391.6993	-0.11921	0.014636	0.051796	protein_coding
ENSG0000(	PTPN4	1664.321	-0.1226	0.014639	0.051796	protein_coding
ENSG0000(	ACSS1	3177.869	-0.26484	0.014645	0.051808	protein_coding
ENSG0000(	MTA2	1091.38	-0.12726	0.014655	0.051838	protein_coding
ENSG0000(	RPL5P30	31.30793	-0.49136	0.014657	0.051838	processed_pseudogene
ENSG0000(	PACSIN3	45.37159	0.409772	0.014661	0.051848	protein_coding
ENSG0000(	EEF2K	2800.275	-0.19762	0.014668	0.05185	protein_coding
ENSG0000(	ENSG0000(	64.79754	0.395915	0.014668	0.05185	transcribed_unprocessed_pseudo
ENSG0000(	MME	6996.939	-0.62231	0.014669	0.05185	protein_coding
ENSG0000(	RGS20	9.036531	0.548927	0.01467	0.05185	protein_coding
ENSG0000(	SNF8P1	183.7125	0.148618	0.014669	0.05185	processed_pseudogene
ENSG0000(	GOLGA6C	39.35536	0.447744	0.014681	0.051883	protein_coding
ENSG0000(	CD248	96.60995	0.456999	0.014685	0.051891	protein_coding
ENSG0000(	ENSG0000(	210.456	0.211714	0.014692	0.05191	lncRNA
ENSG0000(	CADPS2	608.4548	0.256805	0.014696	0.05192	protein_coding
ENSG0000(	CDCP2	8.735086	0.613081	0.014703	0.051933	protein_coding
ENSG0000(	ENSG0000(	953.1097	-0.37496	0.014703	0.051933	lncRNA
ENSG0000(	DUXAP7	64.93736	0.1986	0.014709	0.05194	processed_pseudogene
ENSG0000(	SLC41A1	473.9329	0.348558	0.014708	0.05194	protein_coding
ENSG0000(	ZNF169	1178.797	-0.08542	0.014715	0.051955	protein_coding
ENSG0000(	RRAGB	316.7357	-0.14745	0.014722	0.051976	protein_coding
ENSG0000(	RFESD	57.1099	0.36948	0.014726	0.051985	protein_coding
ENSG0000(	ZNF329	904.445	-0.13015	0.014731	0.051995	protein_coding
ENSG0000(	NCF1	727.8434	0.377019	0.01474	0.05202	protein_coding
ENSG0000(	ANKRD20A	123.3727	0.258197	0.014756	0.052071	transcribed_unprocessed_pseudo
ENSG0000(	TNXB	2338.012	-0.27033	0.01476	0.052081	protein_coding

ENSG0000(	GPR141BP	13.44026	0.554261	0.014762	0.052082	unitary_pseudogene
ENSG0000(	PIGM	1099.507	0.102735	0.014773	0.052117	protein_coding
ENSG0000(	NR2F1	223.4055	0.290283	0.014776	0.05212	protein_coding
ENSG0000(	ENSG0000(	153.4627	0.218278	0.014783	0.052138	lncRNA
ENSG0000(	NAAA	1408.325	-0.29393	0.014788	0.052149	protein_coding
ENSG0000(	MIR5587	11.26124	-0.76049	0.014801	0.052192	miRNA
ENSG0000(	ENSG0000(	23.57425	0.415745	0.014805	0.052197	lncRNA
ENSG0000(	SDR42E2	465.0838	0.135889	0.014808	0.052204	protein_coding
ENSG0000(	ENSG0000(	38.69325	0.300639	0.014818	0.052234	unprocessed_pseudogene
ENSG0000(	EPS15L1	1352.071	-0.09262	0.014828	0.05226	protein_coding
ENSG0000(	LAD1	1141.273	-0.41746	0.014847	0.052324	protein_coding
ENSG0000(	ENSG0000(	272.4658	0.285677	0.01487	0.052397	protein_coding
ENSG0000(	ENSG0000(	12.26496	0.56839	0.014873	0.052405	processed_pseudogene
ENSG0000(	ENSG0000(	10.08903	0.465379	0.014876	0.052408	lncRNA
ENSG0000(	TPO	162.2675	-0.37701	0.014885	0.052433	protein_coding
ENSG0000(	AMZ1	224.8344	0.316246	0.014892	0.05245	protein_coding
ENSG0000(	ENSG0000(	31.72736	0.665438	0.014893	0.05245	unprocessed_pseudogene
ENSG0000(	IGF2BP2-A!	83.46478	0.193772	0.014897	0.05246	lncRNA
ENSG0000(	ENSG0000(	181.3612	0.116262	0.01491	0.0525	processed_pseudogene
ENSG0000(	ST6GALNA(	234.9598	0.318133	0.014914	0.052505	protein_coding
ENSG0000(	CTDSP1	2287.96	-0.15884	0.014916	0.052508	protein_coding
ENSG0000(	ENSG0000(	256.1188	0.158333	0.014918	0.052508	lncRNA
ENSG0000(	ALKBH7	128.3903	-0.35469	0.014929	0.052543	protein_coding
ENSG0000(	ENSG0000(	99.47833	0.145208	0.014933	0.052549	processed_pseudogene
ENSG0000(	ENSG0000(	41.16145	0.284031	0.01494	0.05257	lncRNA
ENSG0000(	ENSG0000(	243.1899	0.281463	0.014952	0.0526	TEC
ENSG0000(	KIAA0319L	3218.302	0.074953	0.014952	0.0526	protein_coding
ENSG0000(	ENSG0000(	27.19125	-0.45351	0.014972	0.052661	lncRNA
ENSG0000(	SRI	1689.354	-0.3679	0.014973	0.052661	protein_coding
ENSG0000(	ENSG0000(	249.6847	0.142991	0.014989	0.052705	TEC
ENSG0000(	SNHG10	406.7022	-0.10769	0.014988	0.052705	lncRNA
ENSG0000(	PHYKPL	6963.315	-0.1579	0.014994	0.052717	protein_coding
ENSG0000(	ENSG0000(	27.8656	0.575057	0.015001	0.052728	lncRNA
ENSG0000(	GRIN1	70.54563	0.420439	0.015002	0.052728	protein_coding
ENSG0000(	SPATA13	3860.519	0.153416	0.015002	0.052728	protein_coding
ENSG0000(	CIBAR2	51.74694	0.588909	0.015011	0.052749	protein_coding
ENSG0000(	LARP1	5742.037	-0.08539	0.015011	0.052749	protein_coding
ENSG0000(	ZNF232	180.1685	-0.21166	0.015032	0.052817	protein_coding
ENSG0000(	ABCC8	628.0949	0.103814	0.015034	0.052818	protein_coding
ENSG0000(	LUADT1	85.97607	-0.21832	0.015043	0.052842	lncRNA
ENSG0000(	KCTD13	1327.452	-0.11146	0.015048	0.052855	protein_coding
ENSG0000(	ACOT2	139.9706	-0.26315	0.015057	0.052861	protein_coding
ENSG0000(	BCAS3	1789.77	0.082271	0.01506	0.052861	protein_coding
ENSG0000(	LRP6	2185.937	-0.26579	0.015056	0.052861	protein_coding
ENSG0000(	NRSN2-AS1	1564.103	-0.08075	0.01506	0.052861	lncRNA
ENSG0000(	PITPNA-AS:	26.08415	-0.32802	0.015052	0.052861	lncRNA
ENSG0000(	PRDX6-AS1	582.7313	0.264934	0.015053	0.052861	lncRNA

ENSG0000(	ZNF507	2698.495	-0.11262	0.015065	0.052872	protein_coding
ENSG0000(	FABP6-AS1	1358.305	-0.40966	0.015081	0.052923	lncRNA
ENSG0000(	MTRNR2L5	121.11	0.188271	0.015092	0.052948	protein_coding
ENSG0000(	PET117	176.5558	-0.23701	0.015091	0.052948	protein_coding
ENSG0000(	GPR132	548.1241	0.413909	0.015106	0.052992	protein_coding
ENSG0000(	ANK3	4186.995	-0.24043	0.015122	0.053041	protein_coding
ENSG0000(	CCDC144N	243.0731	0.190584	0.015146	0.053122	transcribed_unprocessed_pseudo
ENSG0000(	MYO1A	10934.73	-0.55607	0.015152	0.053135	protein_coding
ENSG0000(	RN7SL375F	32.55595	0.264236	0.015161	0.053162	misc_RNA
ENSG0000(	RGS12	1704.673	0.145655	0.015171	0.053191	protein_coding
ENSG0000(	RPSAP18	8.865624	-0.54545	0.015173	0.053193	processed_pseudogene
ENSG0000(	WDR72	206.9503	0.162663	0.015175	0.053193	protein_coding
ENSG0000(	LINC01933	97.07517	0.266758	0.015195	0.053257	lncRNA
ENSG0000(	C4orf17	11.24515	0.501764	0.0152	0.053271	protein_coding
ENSG0000(	ARRB1	2751.468	-0.15746	0.015206	0.053277	protein_coding
ENSG0000(	KRT19	2720.685	-0.49355	0.015206	0.053277	protein_coding
ENSG0000(	CNTNAP3B	560.2954	0.191865	0.015211	0.053291	protein_coding
ENSG0000(	NIPA1	1589.748	-0.18177	0.015223	0.053325	protein_coding
ENSG0000(	PSMA4	953.3606	-0.17034	0.015247	0.053404	protein_coding
ENSG0000(	LINC02214	78.14843	-0.15825	0.015251	0.053411	lncRNA
ENSG0000(	WDR20	1422.542	-0.09497	0.015255	0.053421	protein_coding
ENSG0000(	JTB	665.1919	-0.1826	0.015257	0.053422	protein_coding
ENSG0000(	ZNF781	157.7726	0.234819	0.015296	0.053553	protein_coding
ENSG0000(	ENSG0000(	95.07928	0.243339	0.015307	0.053583	lncRNA
ENSG0000(	CARNS1	100.5947	0.352566	0.015309	0.053585	protein_coding
ENSG0000(	RN7SL258F	62.75345	0.200272	0.015313	0.053592	misc_RNA
ENSG0000(	TRBJ2-2P	12.79505	0.899739	0.015324	0.053626	TR_J_gene
ENSG0000(	CD300LD-A	172.6679	0.158606	0.015326	0.053629	lncRNA
ENSG0000(	ENSG0000(	355.8845	0.108365	0.015335	0.053654	lncRNA
ENSG0000(	ENSG0000(	210.2821	0.141067	0.015344	0.053678	lncRNA
ENSG0000(	CDK7	210.6086	-0.21883	0.015351	0.053692	protein_coding
ENSG0000(	ENSG0000(	396.3411	0.120601	0.015353	0.053692	TEC
ENSG0000(	UCK1	335.3377	-0.18634	0.015353	0.053692	protein_coding
ENSG0000(	RPL35AP9	37.1878	0.314426	0.015384	0.053795	processed_pseudogene
ENSG0000(	MIR6081	18.44765	0.452097	0.015401	0.053848	miRNA
ENSG0000(	TRAJ43	18.22571	-0.51174	0.015406	0.053858	TR_J_gene
ENSG0000(	ENSG0000(	171.5459	0.156365	0.015424	0.053915	lncRNA
ENSG0000(	ENSG0000(	14.46536	0.589998	0.015431	0.053935	lncRNA
ENSG0000(	CARD17	26.22655	0.650394	0.015433	0.053937	transcribed_unprocessed_pseudo
ENSG0000(	ZNF295-AS	226.3019	0.168966	0.015437	0.053945	lncRNA
ENSG0000(	HEBP2P1	93.32624	0.248636	0.015449	0.053981	processed_pseudogene
ENSG0000(	FBXO17	254.8097	0.160085	0.015463	0.054024	protein_coding
ENSG0000(	KCNJ3	1215.672	-0.40122	0.015465	0.054024	protein_coding
ENSG0000(	MTFR1	545.9776	-0.2025	0.015471	0.054039	protein_coding
ENSG0000(	ENPEP	9239.149	-0.59815	0.015482	0.054066	protein_coding
ENSG0000(	NBPF14	4088.76	0.251596	0.015482	0.054066	protein_coding
ENSG0000(	TMEM242	396.2267	-0.11681	0.015494	0.054102	protein_coding

ENSG0000(	ABL1	2225.992	0.156268	0.015498	0.054105	protein_coding
ENSG0000(	FMOD	436.401	0.276523	0.015499	0.054105	protein_coding
ENSG0000(	SNORA68	267.5594	0.25833	0.015501	0.054107	snoRNA
ENSG0000(	NFKBIL1	139.3611	-0.22005	0.015514	0.05414	protein_coding
ENSG0000(	TSTD2	765.4579	0.102027	0.015513	0.05414	protein_coding
ENSG0000(	SRP54-AS1	408.1869	-0.14237	0.015516	0.054142	transcribed_unitary_pseudogene
ENSG0000(	KCNH4	31.9043	-0.59144	0.015529	0.054182	protein_coding
ENSG0000(	STAG3L4	588.3173	-0.12797	0.015556	0.054271	transcribed_unprocessed_pseudo
ENSG0000(	LGALS14	44.15536	0.228628	0.015562	0.054285	protein_coding
ENSG0000(	HAL	112.711	0.314881	0.015571	0.054299	protein_coding
ENSG0000(	TRPC6	221.4958	0.231229	0.015569	0.054299	protein_coding
ENSG0000(	VPS13B-DT	152.5125	0.146247	0.015571	0.054299	lncRNA
ENSG0000(	MATK	189.3574	-0.173	0.015575	0.054305	protein_coding
ENSG0000(	MORC2	1476.182	0.099903	0.015588	0.054345	protein_coding
ENSG0000(	VIRMA	2090.136	-0.07199	0.015593	0.054356	protein_coding
ENSG0000(	LINC01204	239.8021	-0.16241	0.015602	0.054381	lncRNA
ENSG0000(	ENSG0000(	7.950955	0.742081	0.015625	0.054455	processed_pseudogene
ENSG0000(	WDCP	218.7346	0.227638	0.015635	0.054485	protein_coding
ENSG0000(	TRIM47	365.1046	-0.26275	0.015646	0.054517	protein_coding
ENSG0000(	ENSG0000(	151.2839	0.362804	0.015651	0.05453	protein_coding
ENSG0000(	ST18	262.9155	-0.1517	0.015661	0.054556	protein_coding
ENSG0000(	CLIP4	2395.33	0.256676	0.015681	0.054609	protein_coding
ENSG0000(	PES1	352.0976	-0.13059	0.015681	0.054609	protein_coding
ENSG0000(	RAB40A	166.8501	0.196333	0.015678	0.054609	protein_coding
ENSG0000(	ENSG0000(	21.68075	0.348191	0.015686	0.05462	lncRNA
ENSG0000(	CDH24	117.8077	0.262164	0.015694	0.05464	protein_coding
ENSG0000(	RPL7L1P12	26.90956	0.315545	0.015696	0.054643	processed_pseudogene
ENSG0000(	PLEKHF2	790.7545	-0.22648	0.015718	0.054713	protein_coding
ENSG0000(	FAM120B	2287.642	0.064003	0.015728	0.054743	protein_coding
ENSG0000(	SRCAP	5053.572	-0.09239	0.015749	0.054811	protein_coding
ENSG0000(	SEPTIN14P	64.48775	0.294404	0.015753	0.054819	transcribed_unprocessed_pseudo
ENSG0000(	F2RL3	535.279	0.12218	0.015762	0.054844	protein_coding
ENSG0000(	RPS4XP7	57.40845	-0.62851	0.015766	0.054851	processed_pseudogene
ENSG0000(	UBXN6	739.9975	-0.21516	0.015774	0.054872	protein_coding
ENSG0000(	ENSG0000(	784.5014	0.077833	0.01579	0.054923	TEC
ENSG0000(	ABHD17B	461.3511	-0.1789	0.015796	0.054936	protein_coding
ENSG0000(	LINC02193	758.216	0.21336	0.015799	0.054939	lncRNA
ENSG0000(	EEF1A1P9	34.05115	-0.34668	0.015822	0.055013	processed_pseudogene
ENSG0000(	PSRC1	76.95505	0.315681	0.015854	0.055122	protein_coding
ENSG0000(	HTR3A	26.57495	-0.81794	0.015859	0.055131	protein_coding
ENSG0000(	PSMC3	303.4737	-0.1838	0.015872	0.055172	protein_coding
ENSG0000(	RNU2-61P	10.44634	-0.52036	0.015885	0.055209	snRNA
ENSG0000(	ENSG0000(	563.764	-0.09222	0.015893	0.055231	lncRNA
ENSG0000(	ENSG0000(	169.9886	0.136012	0.015895	0.055233	lncRNA
ENSG0000(	PTGER4P2	18.21105	0.345894	0.015899	0.05524	transcribed_processed_pseudoge
ENSG0000(	ENSG0000(	120.2043	-1.27845	0.015903	0.055246	processed_pseudogene
ENSG0000(	ERICH4	255.8725	-0.62647	0.015909	0.055261	protein_coding

ENSG0000(	ENSG0000(	25.79877	0.325506	0.015915	0.055278	lncRNA
ENSG0000(	TMEM30B	1214.992	-0.3847	0.015917	0.055279	protein_coding
ENSG0000(	ENSG0000(	165.5606	0.195187	0.015923	0.055291	TEC
ENSG0000(	CDH17	6024.555	-0.44067	0.015927	0.055293	protein_coding
ENSG0000(	ENSG0000(	178.0635	-0.14738	0.015926	0.055293	lncRNA
ENSG0000(	TTTY2B	42.24599	0.757666	0.015941	0.055335	lncRNA
ENSG0000(	ENSG0000(	1105.151	-0.11311	0.015944	0.055343	protein_coding
ENSG0000(	ATXN7L3B	2007.476	-0.09915	0.015963	0.055401	protein_coding
ENSG0000(	TAMALIN	183.2671	0.292002	0.015976	0.055439	protein_coding
ENSG0000(	SMAD9	416.686	-0.15745	0.015982	0.055454	protein_coding
ENSG0000(	ENSG0000(	48.93246	0.237163	0.015986	0.055462	lncRNA
ENSG0000(	ENSG0000(	114.7371	0.164249	0.015993	0.05548	lncRNA
ENSG0000(	NOL6	692.0701	0.156433	0.016002	0.055506	protein_coding
ENSG0000(	ZNF765	1678.377	-0.08998	0.016015	0.055546	protein_coding
ENSG0000(	MFSD13A	428.0074	-0.14255	0.016022	0.055562	protein_coding
ENSG0000(	ENSG0000(	96.0173	-0.26538	0.016037	0.055605	lncRNA
ENSG0000(	P4HA1	666.8537	0.231066	0.016038	0.055605	protein_coding
ENSG0000(	TTC21B	4755.613	0.075046	0.016041	0.055609	protein_coding
ENSG0000(	LRR1	201.444	-0.19485	0.01605	0.055634	protein_coding
ENSG0000(	CAMK2G	2755.846	-0.20424	0.016054	0.055645	protein_coding
ENSG0000(	SF3B4	565.3663	-0.22651	0.01607	0.055694	protein_coding
ENSG0000(	CCDC194	68.15322	0.286056	0.016078	0.05571	protein_coding
ENSG0000(	ENSG0000(	2161.839	0.318337	0.016078	0.05571	lncRNA
ENSG0000(	NDUFAB1	274.8599	-0.22042	0.016086	0.055729	protein_coding
ENSG0000(	MFSD1	1125.318	0.145048	0.016093	0.055746	protein_coding
ENSG0000(	ENSG0000(	296.8097	0.152757	0.0161	0.055766	lncRNA
ENSG0000(	ULK2	729.2198	-0.17131	0.016105	0.055777	protein_coding
ENSG0000(	ENSG0000(	307.8916	-0.1944	0.016111	0.05579	TEC
ENSG0000(	ENSG0000(	17.21782	0.360761	0.016115	0.055801	lncRNA
ENSG0000(	HMGB1P39	118.5304	-0.16609	0.016121	0.055814	processed_pseudogene
ENSG0000(	AP2A2	2486.496	-0.10631	0.016125	0.055823	protein_coding
ENSG0000(	LINC02756	33.93358	0.364245	0.016132	0.05584	lncRNA
ENSG0000(	KIF12	486.9738	-0.50224	0.016145	0.055878	protein_coding
ENSG0000(	FBXO44	572.7255	0.188367	0.016157	0.055916	protein_coding
ENSG0000(	FAM168A	1717.429	0.126845	0.016171	0.055957	protein_coding
ENSG0000(	PIK3C2G	335.4396	-0.46986	0.016176	0.055967	protein_coding
ENSG0000(	ENSG0000(	32.08487	0.553363	0.016183	0.055987	processed_pseudogene
ENSG0000(	ANKRD10-I	517.3547	0.137046	0.016188	0.055998	lncRNA
ENSG0000(	ABCB11	423.0185	-0.20962	0.016191	0.056002	protein_coding
ENSG0000(	FAM222A-/	18.26742	0.416927	0.016197	0.056014	lncRNA
ENSG0000(	LINC02481	85.08386	0.244228	0.016199	0.056014	lncRNA
ENSG0000(	VTRNA1-1	62.77404	0.412314	0.0162	0.056014	misc_RNA
ENSG0000(	ENSG0000(	165.7255	0.150381	0.016204	0.056015	lncRNA
ENSG0000(	RPL3P13	8.251453	-0.68026	0.016203	0.056015	processed_pseudogene
ENSG0000(	LINC02345	26.81087	0.37904	0.016206	0.056016	lncRNA
ENSG0000(	ENSG0000(	199.5321	0.129323	0.016215	0.056039	lncRNA
ENSG0000(	ENSG0000(	133.2312	0.159758	0.016219	0.056047	lncRNA

ENSG0000(	HIPK2	4162.293	0.129647	0.01623	0.05608	protein_coding
ENSG0000(	NCAPH2	564.6865	-0.12858	0.016251	0.056147	protein_coding
ENSG0000(	ATP6V0A1	3446.821	-0.14946	0.016255	0.05615	protein_coding
ENSG0000(	ENSG0000(	11.51694	-0.91829	0.016255	0.05615	lncRNA
ENSG0000(	ENSG0000(	60.2237	0.37506	0.01626	0.056159	lncRNA
ENSG0000(	SRM	201.4908	0.244634	0.016262	0.056159	protein_coding
ENSG0000(	CREB3L2	5759.097	0.224855	0.016267	0.056173	protein_coding
ENSG0000(	ENSG0000(	9.165138	0.596095	0.016282	0.056217	processed_pseudogene
ENSG0000(	ENSG0000(	392.2753	0.158335	0.016297	0.056262	lncRNA
ENSG0000(	ENSG0000(	307.3096	0.20201	0.016304	0.05627	lncRNA
ENSG0000(	ENSG0000(	900.9924	-0.17665	0.016306	0.05627	lncRNA
ENSG0000(	ENSG0000(	85.74265	0.475418	0.016303	0.05627	lncRNA
ENSG0000(	PGAM1P8	325.5826	0.135836	0.016306	0.05627	transcribed_processed_pseudoge
ENSG0000(	NDRG3	628.1571	-0.11886	0.016311	0.056281	protein_coding
ENSG0000(	ENSG0000(	23.25296	0.403871	0.016313	0.056282	snoRNA
ENSG0000(	ENSG0000(	111.6338	0.179345	0.016321	0.056297	lncRNA
ENSG0000(	TSPAN14	3550.717	0.120442	0.016321	0.056297	protein_coding
ENSG0000(	ENSG0000(	144.5606	-0.20817	0.016366	0.056445	lncRNA
ENSG0000(	ARID5B	2813.177	0.289967	0.016371	0.056449	protein_coding
ENSG0000(	ENSG0000(	80.99293	0.308813	0.016373	0.056449	lncRNA
ENSG0000(	PYCARD	128.7521	-0.24725	0.016374	0.056449	protein_coding
ENSG0000(	RPL22L1	198.8121	-0.21217	0.016369	0.056449	protein_coding
ENSG0000(	WIPF2	2755.418	-0.16202	0.016379	0.056461	protein_coding
ENSG0000(	CTLA4	253.797	0.639942	0.016382	0.056463	protein_coding
ENSG0000(	ZNF804A	123.4645	-0.32486	0.016389	0.056481	protein_coding
ENSG0000(	TUBA1C	830.0794	-0.27388	0.016393	0.056489	protein_coding
ENSG0000(	FTSJ1	262.8054	0.20892	0.016396	0.056494	protein_coding
ENSG0000(	OR2T33	345.4904	0.156256	0.016399	0.056496	protein_coding
ENSG0000(	SLC9A4	308.9581	0.21085	0.016405	0.056511	protein_coding
ENSG0000(	ALOX12B	12.34693	-0.70761	0.016409	0.056521	protein_coding
ENSG0000(	DHRS2	178.7242	-0.15142	0.016413	0.056524	protein_coding
ENSG0000(	SCG2	34.63334	-0.53704	0.016414	0.056524	protein_coding
ENSG0000(	COQ2	296.0841	0.165541	0.016416	0.056526	protein_coding
ENSG0000(	ELF4	2232.212	-0.12781	0.016421	0.056534	protein_coding
ENSG0000(	TSEN2	1901.944	0.084388	0.016423	0.056536	protein_coding
ENSG0000(	LINC01777	16.56833	0.355114	0.016425	0.056537	lncRNA
ENSG0000(	DUSP3	1584.604	-0.1748	0.016433	0.056551	protein_coding
ENSG0000(	IGFL3	15.04975	0.434793	0.016431	0.056551	protein_coding
ENSG0000(	RN7SL271F	9.532323	0.417734	0.016437	0.05656	misc_RNA
ENSG0000(	MAN1A1	2470.916	-0.27439	0.016441	0.056567	protein_coding
ENSG0000(	ENSG0000(	622.5027	-0.08869	0.016447	0.056582	lncRNA
ENSG0000(	DDX6	6783.905	-0.09301	0.016464	0.056633	protein_coding
ENSG0000(	ENSG0000(	32.77025	0.350212	0.016467	0.056638	transcribed_unprocessed_pseudo
ENSG0000(	GATA5	321.9992	-0.50774	0.016476	0.056662	protein_coding
ENSG0000(	CHRNG	207.5334	0.149537	0.016491	0.056708	protein_coding
ENSG0000(	ENSG0000(	20.71979	0.422022	0.016507	0.056752	processed_pseudogene
ENSG0000(	NDUFB8	596.7483	-0.11824	0.016506	0.056752	protein_coding

ENSG0000(	ENSG0000(	134.1063	-0.18053	0.016513	0.056761	lncRNA
ENSG0000(	RTF1	1104.886	-0.13391	0.016513	0.056761	protein_coding
ENSG0000(	C2orf69	465.9239	-0.12061	0.016523	0.056786	protein_coding
ENSG0000(	CRCP	974.4959	-0.08613	0.016524	0.056786	protein_coding
ENSG0000(	MIF	163.9985	-0.31042	0.016527	0.056789	protein_coding
ENSG0000(	ENSG0000(	111.0397	0.16888	0.016529	0.056791	lncRNA
ENSG0000(	LCNL1	187.2847	0.627972	0.016532	0.056794	protein_coding
ENSG0000(	ENSG0000(	58.84881	0.3346	0.016535	0.056799	lncRNA
ENSG0000(	FAM221B	152.7491	0.147711	0.01654	0.05681	protein_coding
ENSG0000(	RILPL2	175.3814	0.361932	0.016551	0.056841	protein_coding
ENSG0000(	CLEC4F	164.8764	-0.43447	0.016555	0.056848	protein_coding
ENSG0000(	AQR	2494.582	-0.09268	0.016569	0.056884	protein_coding
ENSG0000(	ENSG0000(	272.2017	0.179434	0.016568	0.056884	protein_coding
ENSG0000(	ADGRF4	16.49546	0.703518	0.016574	0.056896	protein_coding
ENSG0000(	ENSG0000(	220.853	0.150884	0.01658	0.05691	lncRNA
ENSG0000(	LRAT	408.3989	-0.32174	0.016603	0.056981	protein_coding
ENSG0000(	KCTD18	1128.528	0.075264	0.016613	0.057003	protein_coding
ENSG0000(	SHCBP1L	53.73758	-0.26472	0.016612	0.057003	protein_coding
ENSG0000(	LINC02827	9.503822	0.601655	0.016621	0.057019	lncRNA
ENSG0000(	RPL7P24	9.287981	0.586823	0.016621	0.057019	processed_pseudogene
ENSG0000(	FST	86.4723	0.306114	0.016644	0.057092	protein_coding
ENSG0000(	TCTE1	41.9756	0.310084	0.016654	0.05712	protein_coding
ENSG0000(	FUT3	1656.706	0.37516	0.016658	0.057126	protein_coding
ENSG0000(	DSN1	364.9596	0.137246	0.016671	0.057165	protein_coding
ENSG0000(	ENSG0000(	87.87307	0.171481	0.01669	0.057225	lncRNA
ENSG0000(	ENSG0000(	854.2526	-0.06949	0.016701	0.057254	lncRNA
ENSG0000(	ENSG0000(	140.7042	-0.1638	0.016718	0.057306	lncRNA
ENSG0000(	PLEKHG2	2161.211	0.272151	0.01672	0.057309	protein_coding
ENSG0000(	STRIP1	1342.134	-0.14672	0.016728	0.057329	protein_coding
ENSG0000(	SYN1	11.0748	0.648616	0.01673	0.05733	protein_coding
ENSG0000(	ENSG0000(	13.49965	0.425923	0.01675	0.057386	processed_pseudogene
ENSG0000(	ENSG0000(	83.52363	0.232824	0.016749	0.057386	lncRNA
ENSG0000(	CD84	2867.125	0.38316	0.016759	0.057407	protein_coding
ENSG0000(	DUX4	47.02721	0.418356	0.01676	0.057407	protein_coding
ENSG0000(	ZNF487	475.3115	-0.14149	0.016774	0.057451	protein_coding
ENSG0000(	DLL1	521.9305	0.202409	0.016786	0.057483	protein_coding
ENSG0000(	ENSG0000(	67.75379	-0.18829	0.016799	0.057522	lncRNA
ENSG0000(	ENSG0000(	178.4823	-0.15175	0.016806	0.057541	lncRNA
ENSG0000(	SAP130	762.2583	-0.13232	0.01681	0.057548	protein_coding
ENSG0000(	ENSG0000(	17.53467	0.378808	0.016818	0.057558	lncRNA
ENSG0000(	RANBP3-D1	7.937267	-0.58547	0.01682	0.057558	lncRNA
ENSG0000(	STEEP1	786.2057	-0.06975	0.016819	0.057558	protein_coding
ENSG0000(	TOPORS	666.8025	-0.14128	0.016819	0.057558	protein_coding
ENSG0000(	ERCC5	3678.811	-0.10736	0.016822	0.057558	protein_coding
ENSG0000(	ENSG0000(	374.1471	0.154596	0.016828	0.057572	lncRNA
ENSG0000(	ENSG0000(	15.3357	0.489487	0.016831	0.057576	lncRNA
ENSG0000(	ZSCAN12P1	33.2685	0.428995	0.016833	0.057576	transcribed_unprocessed_pseudo

ENSG0000(	SMURF1	1444.965	0.115964	0.016841	0.057597	protein_coding
ENSG0000(	ENSG0000(	17.77679	0.372569	0.016855	0.057639	snoRNA
ENSG0000(	TDGF1	178.9441	-0.12989	0.016858	0.057644	protein_coding
ENSG0000(	PINX1	110.7714	-0.25352	0.016866	0.057664	protein_coding
ENSG0000(	ECHDC3	46.45396	0.386376	0.016874	0.057681	protein_coding
ENSG0000(	ENSG0000(	232.4136	-0.13921	0.016874	0.057681	lncRNA
ENSG0000(	ENSG0000(	756.8085	0.266553	0.016881	0.057697	lncRNA
ENSG0000(	ACSS3	454.0971	0.15626	0.016885	0.057706	protein_coding
ENSG0000(	ARL4AP5	16.16946	0.468861	0.016895	0.057724	processed_pseudogene
ENSG0000(	CD200R1L	9.036753	0.487553	0.016895	0.057724	protein_coding
ENSG0000(	ENSG0000(	328.5637	0.139755	0.016896	0.057724	lncRNA
ENSG0000(	ENSG0000(	40.14436	0.365769	0.0169	0.057732	unprocessed_pseudogene
ENSG0000(	SZRD1	1646.997	0.089463	0.016907	0.05775	protein_coding
ENSG0000(	ENSG0000(	407.0943	0.136758	0.016915	0.057768	lncRNA
ENSG0000(	ENSG0000(	376.0147	0.133475	0.016917	0.057768	TEC
ENSG0000(	DUSP22	1539.64	0.186341	0.016919	0.05777	protein_coding
ENSG0000(	NKAPL	11.58047	0.435618	0.016952	0.057877	protein_coding
ENSG0000(	CDC73	2232.424	-0.07051	0.016962	0.057902	protein_coding
ENSG0000(	CNOT8	1299.101	-0.08377	0.016965	0.057902	protein_coding
ENSG0000(	NUTF2	461.7512	-0.14859	0.016964	0.057902	protein_coding
ENSG0000(	RPL7P41	68.36355	0.166001	0.016969	0.057911	processed_pseudogene
ENSG0000(	NYAP2	148.8484	0.163096	0.016985	0.057958	protein_coding
ENSG0000(	FADS2	812.8297	0.286118	0.017008	0.058029	protein_coding
ENSG0000(	RN7SL50P	71.66582	0.206254	0.017016	0.058051	misc_RNA
ENSG0000(	ENSG0000(	17.54919	0.438223	0.017023	0.058069	lncRNA
ENSG0000(	ENSG0000(	284.8487	-0.17595	0.017028	0.058072	lncRNA
ENSG0000(	LRRC37A17	110.059	0.673602	0.017027	0.058072	transcribed_unprocessed_pseudo
ENSG0000(	LINC02008	109.5302	0.211269	0.017046	0.05813	lncRNA
ENSG0000(	MYCBP2-A!	59.91633	0.305019	0.017068	0.058189	lncRNA
ENSG0000(	MYOCOS	216.3075	0.153073	0.017069	0.058189	protein_coding
ENSG0000(	PHLDB2	1209.192	0.268272	0.017069	0.058189	protein_coding
ENSG0000(	H2BC21	404.3982	-0.21599	0.017072	0.058192	protein_coding
ENSG0000(	CREB3L1	926.4422	0.294214	0.017076	0.058195	protein_coding
ENSG0000(	TJP2	4088.56	-0.29853	0.017076	0.058195	protein_coding
ENSG0000(	ENSG0000(	29.62238	0.415046	0.017081	0.058205	lncRNA
ENSG0000(	PLS1	5087.551	-0.48705	0.017111	0.0583	protein_coding
ENSG0000(	CD34	984.0918	0.240729	0.017116	0.058312	protein_coding
ENSG0000(	APBB2	1211.645	0.179228	0.017123	0.058329	protein_coding
ENSG0000(	TMEM199	835.1628	0.096723	0.017131	0.05835	protein_coding
ENSG0000(	NSA2	578.5265	-0.2077	0.01714	0.058369	protein_coding
ENSG0000(	PROSER2	37.70569	0.443632	0.01714	0.058369	protein_coding
ENSG0000(	ENSG0000(	382.0198	-0.19654	0.01715	0.058393	lncRNA
ENSG0000(	ENSG0000(	142.5548	0.253752	0.017187	0.058509	lncRNA
ENSG0000(	RNU6-110(	11.76398	0.872559	0.017187	0.058509	snRNA
ENSG0000(	ENSG0000(	184.8322	-0.16519	0.017205	0.058563	lncRNA
ENSG0000(	ENSG0000(	276.211	0.100882	0.017207	0.058564	lncRNA
ENSG0000(	GMPS	1824.639	-0.12631	0.01722	0.058596	protein_coding

ENSG0000(	PLD5P1	514.6487	0.133429	0.01722	0.058596	protein_coding
ENSG0000(	ENSG0000(	254.8685	0.222188	0.017226	0.058611	lncRNA
ENSG0000(	RPL34P6	12.89664	0.45972	0.017233	0.058626	processed_pseudogene
ENSG0000(	ENSG0000(	467.1341	0.163255	0.017241	0.058646	processed_pseudogene
ENSG0000(	FAM180A	30.70705	0.291357	0.017242	0.058646	protein_coding
ENSG0000(	PPP3R1	1106.979	-0.08643	0.017246	0.058652	protein_coding
ENSG0000(	ENSG0000(	300.8052	0.124543	0.01725	0.058659	lncRNA
ENSG0000(	PICK1	477.0899	-0.12923	0.017253	0.058662	protein_coding
ENSG0000(	OAS3	3337.048	0.297181	0.017298	0.05881	protein_coding
ENSG0000(	ENSG0000(	182.3927	-0.10883	0.017305	0.058828	lncRNA
ENSG0000(	ENSG0000(	55.07045	0.18857	0.01731	0.058831	lncRNA
ENSG0000(	GNB4	1465.771	0.191096	0.01731	0.058831	protein_coding
ENSG0000(	CEBPZ	828.467	-0.15573	0.017313	0.058835	protein_coding
ENSG0000(	ENSG0000(	14.39822	0.429172	0.01732	0.058848	lncRNA
ENSG0000(	TRIM27	2412.358	0.112818	0.017319	0.058848	protein_coding
ENSG0000(	LINC02223	62.61699	0.318794	0.017327	0.058862	lncRNA
ENSG0000(	CYTH3	845.7056	-0.30895	0.017329	0.058865	protein_coding
ENSG0000(	LINC01811	131.6702	0.321321	0.017348	0.058911	lncRNA
ENSG0000(	RHBG	275.6913	-0.11963	0.017346	0.058911	protein_coding
ENSG0000(	SSX5	22.25888	-0.43318	0.017347	0.058911	protein_coding
ENSG0000(	ENSG0000(	30.93721	0.355294	0.017366	0.05895	lncRNA
ENSG0000(	ENSG0000(	305.9722	-0.15355	0.017363	0.05895	lncRNA
ENSG0000(	VCPKMT	458.6819	-0.15937	0.017364	0.05895	protein_coding
ENSG0000(	ENSG0000(	12.71277	0.475584	0.017373	0.058962	TEC
ENSG0000(	LINC01500	28.17428	0.300015	0.017371	0.058962	lncRNA
ENSG0000(	STXBP2	1173.829	-0.16389	0.017377	0.058972	protein_coding
ENSG0000(	ENSG0000(	15.45788	0.488774	0.017401	0.059039	snoRNA
ENSG0000(	EPG5	3229.816	0.099419	0.0174	0.059039	protein_coding
ENSG0000(	ENSG0000(	25.9176	0.296872	0.01741	0.059064	misc_RNA
ENSG0000(	TAS2R15P	203.6907	0.221631	0.017417	0.059081	unprocessed_pseudogene
ENSG0000(	WWC1	1329.091	-0.36081	0.017425	0.059103	protein_coding
ENSG0000(	LINC01877	154.4732	0.170636	0.017434	0.059125	lncRNA
ENSG0000(	SMS	469.3213	-0.19131	0.017447	0.059164	protein_coding
ENSG0000(	ZNF829	1227.599	-0.12461	0.017473	0.059246	protein_coding
ENSG0000(	APOOL	972.8178	-0.09468	0.017477	0.059252	protein_coding
ENSG0000(	CHPT1	2383.318	-0.26002	0.017488	0.059264	protein_coding
ENSG0000(	F2RL1	774.1182	-0.42316	0.017488	0.059264	protein_coding
ENSG0000(	RNU2-17P	11.69153	-0.56604	0.017486	0.059264	snRNA
ENSG0000(	UBE2F-SCL'	19.29349	0.46221	0.017483	0.059264	protein_coding
ENSG0000(	ENSG0000(	501.1988	-0.09276	0.017491	0.059268	lncRNA
ENSG0000(	ENSG0000(	28.75335	1.007358	0.017493	0.059269	lncRNA
ENSG0000(	ABCA2	1330.757	-0.23025	0.017499	0.059282	protein_coding
ENSG0000(	FIGN	320.7626	0.293124	0.017517	0.059336	protein_coding
ENSG0000(	TM7SF2	189.2296	-0.18171	0.017524	0.059353	protein_coding
ENSG0000(	ENSG0000(	19.82948	0.389326	0.017527	0.059358	lncRNA
ENSG0000(	TMCC3	1563.163	-0.258	0.017532	0.059368	protein_coding
ENSG0000(	COLEC12	1402.431	-0.13672	0.01754	0.059376	protein_coding

ENSG0000(	GOLGA8R	752.729	0.138469	0.01754	0.059376	protein_coding
ENSG0000(	LINC00615	14.98554	-0.5497	0.01754	0.059376	lncRNA
ENSG0000(	UBQLN4	544.9872	-0.12927	0.017555	0.059422	protein_coding
ENSG0000(	ENSG0000(	641.0706	0.100469	0.01757	0.059457	TEC
ENSG0000(	ENSG0000(	18.66699	0.527169	0.017571	0.059457	lncRNA
ENSG0000(	SEMA4A	411.0266	0.524874	0.017571	0.059457	protein_coding
ENSG0000(	LIME1	242.9968	-0.2925	0.017577	0.059464	protein_coding
ENSG0000(	RBX1	463.573	-0.12333	0.017577	0.059464	protein_coding
ENSG0000(	LAMA4-AS1	32.6855	0.234239	0.017582	0.059473	lncRNA
ENSG0000(	RSL1D1	1605.363	-0.14564	0.017586	0.059481	protein_coding
ENSG0000(	RPSAP36	249.0301	-0.12637	0.017592	0.059495	transcribed_processed_pseudoge
ENSG0000(	MAPKBP1	1810.08	-0.1774	0.017605	0.059532	protein_coding
ENSG0000(	MRPS35	296.9429	-0.21748	0.017611	0.059547	protein_coding
ENSG0000(	ANPEP	46886.61	-0.5712	0.017618	0.059565	protein_coding
ENSG0000(	RPS19	2245.584	-0.2547	0.017646	0.059653	protein_coding
ENSG0000(	VPS13B	8470.635	0.075572	0.017654	0.059671	protein_coding
ENSG0000(	GUSBP6	18.17645	-0.41037	0.017659	0.059682	transcribed_unprocessed_pseudo
ENSG0000(	POLR3G	108.4821	0.23507	0.017681	0.059753	protein_coding
ENSG0000(	RDH12	12.92633	1.181283	0.01769	0.05977	protein_coding
ENSG0000(	ZNF200	1099.908	0.120624	0.017689	0.05977	protein_coding
ENSG0000(	ENSG0000(	188.7056	-0.20638	0.017699	0.059781	lncRNA
ENSG0000(	ENSG0000(	219.019	0.111937	0.017701	0.059781	lncRNA
ENSG0000(	NAP1L6P	156.8115	0.134883	0.017699	0.059781	transcribed_processed_pseudoge
ENSG0000(	UBAP2L	2247.262	0.09052	0.017701	0.059781	protein_coding
ENSG0000(	GAPLINC	448.8112	0.123327	0.017705	0.059786	lncRNA
ENSG0000(	AATF	1208.34	-0.08228	0.017712	0.059804	protein_coding
ENSG0000(	ENSG0000(	28.10993	0.300391	0.01773	0.05986	lncRNA
ENSG0000(	CHKA	1204.4	-0.25578	0.017734	0.059865	protein_coding
ENSG0000(	ENSG0000(	37.68831	0.247864	0.017742	0.059887	lncRNA
ENSG0000(	C1orf43	1461.858	-0.13284	0.017754	0.059922	protein_coding
ENSG0000(	ENSG0000(	41.89021	0.340409	0.017779	0.059998	lncRNA
ENSG0000(	FUBP3	1246.248	-0.09537	0.01779	0.060028	protein_coding
ENSG0000(	LINC01684	1103.573	0.137852	0.017809	0.060087	lncRNA
ENSG0000(	KLHL35	329.4334	0.155868	0.017823	0.060116	protein_coding
ENSG0000(	MRPS35-DT	145.1183	0.207212	0.017821	0.060116	lncRNA
ENSG0000(	ZNF569	285.9681	0.091487	0.017823	0.060116	protein_coding
ENSG0000(	ENSG0000(	70.78019	0.34095	0.017828	0.060125	lncRNA
ENSG0000(	ENSG0000(	68.52612	0.226496	0.01784	0.060152	lncRNA
ENSG0000(	IFT172	2225.51	0.094125	0.017838	0.060152	protein_coding
ENSG0000(	ENSG0000(	45.8461	-0.59256	0.017842	0.060154	lncRNA
ENSG0000(	ENSG0000(	48.87902	-0.25689	0.01785	0.060174	lncRNA
ENSG0000(	FNBP1L	1999.16	-0.32531	0.017868	0.060228	protein_coding
ENSG0000(	ENSG0000(	693.1018	0.098736	0.01788	0.060256	TEC
ENSG0000(	LINC00939	1671.322	0.108567	0.01788	0.060256	lncRNA
ENSG0000(	EIF5A2P1	10.44444	-0.46535	0.017882	0.060256	processed_pseudogene
ENSG0000(	ENSG0000(	1444.742	0.092621	0.01789	0.060263	lncRNA
ENSG0000(	LDAH	597.828	-0.1652	0.017888	0.060263	protein_coding

ENSG0000(	SIK1	188.8356	0.583092	0.017886	0.060263	protein_coding
ENSG0000(	ENSG0000(	123.4842	0.162529	0.017892	0.060265	lncRNA
ENSG0000(	PLXNB1	2476.222	-0.26878	0.017897	0.060275	protein_coding
ENSG0000(	ENSG0000(	171.722	0.237712	0.017902	0.060283	lncRNA
ENSG0000(	ENSG0000(	11.89133	1.18036	0.01791	0.060285	unprocessed_pseudogene
ENSG0000(	ENSG0000(	54.41621	0.366644	0.017909	0.060285	lncRNA
ENSG0000(	ENSG0000(	164.4646	-0.20305	0.017914	0.060285	lncRNA
ENSG0000(	MXRA8	340.8238	0.269708	0.017913	0.060285	protein_coding
ENSG0000(	TMEM204	181.9258	0.261764	0.01791	0.060285	protein_coding
ENSG0000(	VAMP4	725.455	0.08566	0.017913	0.060285	protein_coding
ENSG0000(	EIF2S3	3554.491	-0.12966	0.017924	0.060314	protein_coding
ENSG0000(	DPF3	350.1186	-0.25363	0.017937	0.060344	protein_coding
ENSG0000(	ENSG0000(	19.60543	0.435137	0.017937	0.060344	lncRNA
ENSG0000(	RNU4-9P	9.186962	0.555548	0.017949	0.06038	snRNA
ENSG0000(	ENSG0000(	85.21798	0.437179	0.017957	0.060399	lncRNA
ENSG0000(	DNAJC8	666.6965	-0.08849	0.017966	0.060424	protein_coding
ENSG0000(	ENSG0000(	34.37694	-0.36918	0.017978	0.060456	lncRNA
ENSG0000(	ENSG0000(	181.1184	-0.14664	0.017994	0.060504	lncRNA
ENSG0000(	KBTBD11	1915.771	-0.36785	0.018029	0.060615	protein_coding
ENSG0000(	ENSG0000(	49.37631	0.375899	0.018036	0.060631	lncRNA
ENSG0000(	TMEM268	894.1037	0.240453	0.018038	0.060632	protein_coding
ENSG0000(	ENSG0000(	8.735824	0.515847	0.018057	0.060683	processed_pseudogene
ENSG0000(	ENSG0000(	105.3893	0.156945	0.018057	0.060683	lncRNA
ENSG0000(	ENSG0000(	41.91661	-0.55242	0.018063	0.060698	lncRNA
ENSG0000(	SH3BGRL2	1934.637	-0.37251	0.018095	0.060799	protein_coding
ENSG0000(	RPL7AP66	8.70826	0.74806	0.018108	0.060836	processed_pseudogene
ENSG0000(	ERBB4	31.50432	0.982675	0.018119	0.060865	protein_coding
ENSG0000(	JCHAIN	5744.507	0.521089	0.018146	0.06095	protein_coding
ENSG0000(	TFEC	1462.578	0.378651	0.018152	0.060962	protein_coding
ENSG0000(	PNPLA2	2079.483	-0.30419	0.018162	0.060988	protein_coding
ENSG0000(	ZFP41	854.6311	-0.12841	0.018163	0.060988	protein_coding
ENSG0000(	C1orf115	2448.889	-0.53568	0.018182	0.061009	protein_coding
ENSG0000(	ENSG0000(	99.78851	-0.16955	0.018181	0.061009	lncRNA
ENSG0000(	ENSG0000(	499.4262	-0.10957	0.018174	0.061009	TEC
ENSG0000(	GARNL3	473.9619	-0.20445	0.018179	0.061009	protein_coding
ENSG0000(	LINC02512	8.868831	0.481538	0.018185	0.061009	lncRNA
ENSG0000(	RN7SL698F	45.49498	0.249585	0.018176	0.061009	misc_RNA
ENSG0000(	SNORA9	56.57096	0.354147	0.01818	0.061009	snoRNA
ENSG0000(	TBX21	70.76858	0.373848	0.018184	0.061009	protein_coding
ENSG0000(	GSG1L2	280.679	0.099869	0.018188	0.061011	protein_coding
ENSG0000(	THAP11	208.2379	-0.18891	0.018211	0.061083	protein_coding
ENSG0000(	ENSG0000(	123.5303	-0.32499	0.018213	0.061084	lncRNA
ENSG0000(	LHFPL3-AS:	629.6394	-0.37396	0.018217	0.06109	lncRNA
ENSG0000(	ZNF311	80.18604	0.274196	0.018219	0.061091	protein_coding
ENSG0000(	SDCBP	1379.461	0.27195	0.018227	0.061109	protein_coding
ENSG0000(	ENSG0000(	35.82644	0.297105	0.018237	0.061137	lncRNA
ENSG0000(	SLC40A1	4291.741	0.288863	0.018241	0.061145	protein_coding

ENSG0000(	ENSG0000(	75.29982	0.164581	0.018247	0.061155	lncRNA
ENSG0000(	EXOC5	1584.996	-0.0933	0.01825	0.061155	protein_coding
ENSG0000(	ZNF780B	2837.641	-0.12093	0.018248	0.061155	protein_coding
ENSG0000(	ENSG0000(	58.571	-0.24675	0.018254	0.061157	TEC
ENSG0000(	KLF15	52.56194	0.376025	0.018254	0.061157	protein_coding
ENSG0000(	ZNF675	1294.802	-0.10904	0.01826	0.061168	protein_coding
ENSG0000(	CYP39A1	51.65758	0.418996	0.018264	0.061177	protein_coding
ENSG0000(	BCL7A	888.0973	-0.16544	0.018273	0.061196	protein_coding
ENSG0000(	PAIP1P1	150.7435	0.152594	0.018274	0.061196	processed_pseudogene
ENSG0000(	TDP2	3809.836	-0.47086	0.018278	0.061203	protein_coding
ENSG0000(	NEK8	566.8271	-0.1349	0.01828	0.061203	protein_coding
ENSG0000(	VEZF1	1454.745	-0.1178	0.018292	0.061239	protein_coding
ENSG0000(	LUM	877.5447	0.485201	0.018304	0.061271	protein_coding
ENSG0000(	SMAP2	1901.408	0.411287	0.018317	0.061307	protein_coding
ENSG0000(	USP29	37.8033	0.218813	0.01832	0.061311	protein_coding
ENSG0000(	ESR2	871.6115	0.233468	0.018328	0.061332	protein_coding
ENSG0000(	UGT1A2P	64.72897	-0.67055	0.018333	0.061343	unprocessed_pseudogene
ENSG0000(	MAP3K5	1373.404	0.166226	0.018337	0.061349	protein_coding
ENSG0000(	MAPT-AS1	399.2721	-0.10735	0.018359	0.061416	lncRNA
ENSG0000(	RPS25P9	21.1456	0.359876	0.018363	0.061423	processed_pseudogene
ENSG0000(	BAZ2B	8392.945	0.123277	0.018378	0.061464	protein_coding
ENSG0000(	ENSG0000(	54.18589	0.221005	0.018379	0.061464	lncRNA
ENSG0000(	LINC01785	16.96304	0.450116	0.018382	0.061467	lncRNA
ENSG0000(	CER1	14.2437	0.744207	0.018394	0.061493	protein_coding
ENSG0000(	DKC1	554.6208	-0.16802	0.018392	0.061493	protein_coding
ENSG0000(	TMEM65	1654.837	-0.1425	0.018397	0.061498	protein_coding
ENSG0000(	ENSG0000(	57.69647	0.277228	0.018413	0.061539	lncRNA
ENSG0000(	TLK1	2879.723	-0.08272	0.018413	0.061539	protein_coding
ENSG0000(	NOC2LP1	12.46353	0.585995	0.018422	0.061549	transcribed_processed_pseudoge
ENSG0000(	PHACTR3	279.1079	0.163384	0.018422	0.061549	protein_coding
ENSG0000(	SALL4	229.9115	0.274135	0.018422	0.061549	protein_coding
ENSG0000(	STAU1	1625.979	-0.12414	0.018427	0.061559	protein_coding
ENSG0000(	KCNN2	62.44086	0.196479	0.018435	0.06158	protein_coding
ENSG0000(	LINC01203	600.3604	0.08677	0.018437	0.061581	lncRNA
ENSG0000(	GJA1	406.0917	0.310809	0.018443	0.061591	protein_coding
ENSG0000(	ANKRD20A	644.7998	0.207332	0.018455	0.061627	transcribed_unprocessed_pseudo
ENSG0000(	RN7SL745F	14.76314	0.399684	0.018462	0.061643	misc_RNA
ENSG0000(	LINC02660	44.01005	-0.25676	0.018485	0.061713	lncRNA
ENSG0000(	GSDME	1212.566	-0.55003	0.018493	0.061733	protein_coding
ENSG0000(	LINC02324	1116.845	0.101099	0.018495	0.061734	lncRNA
ENSG0000(	CLEC20A	19.91544	0.41732	0.018499	0.061741	protein_coding
ENSG0000(	RN7SKP13:	39.83808	-0.41792	0.01852	0.061805	misc_RNA
ENSG0000(	WAPL	2510.111	-0.0851	0.018528	0.061824	protein_coding
ENSG0000(	RPL7AP15	15.87964	0.362309	0.01853	0.061825	processed_pseudogene
ENSG0000(	XRCC5	2927.467	-0.11708	0.018537	0.061841	protein_coding
ENSG0000(	ENSG0000(	74.41405	0.248759	0.018547	0.061868	lncRNA
ENSG0000(	ENSG0000(	130.3258	0.177309	0.01855	0.06187	lncRNA

ENSG0000(	ENSG0000(	41.30585	0.264424	0.018556	0.061883	TEC
ENSG0000(	JUNB	1024.954	0.307876	0.018569	0.06192	protein_coding
ENSG0000(	TBL1Y	11.09038	0.653082	0.018573	0.061927	protein_coding
ENSG0000(	ZNF496	1649.306	0.190552	0.018576	0.061931	protein_coding
ENSG0000(	KIAA2026	3539.863	0.080762	0.018584	0.061951	protein_coding
ENSG0000(	ENSG0000(	153.0652	0.130248	0.018608	0.062026	lncRNA
ENSG0000(	BHMT	46.35062	-0.27081	0.018615	0.062041	protein_coding
ENSG0000(	TM4SF19-A	195.9569	-0.18214	0.018626	0.062073	lncRNA
ENSG0000(	ENSG0000(	65.32061	0.27028	0.018633	0.062084	lncRNA
ENSG0000(	OR52N4	20.05496	0.663209	0.018633	0.062084	protein_coding
ENSG0000(	NOCT	424.5712	0.131291	0.018644	0.062112	protein_coding
ENSG0000(	ZNF628	108.9063	0.300012	0.018667	0.062183	protein_coding
ENSG0000(	TM4SF19	116.224	-0.17013	0.018671	0.06219	protein_coding
ENSG0000(	ENSG0000(	11.92695	0.47015	0.018685	0.062229	transcribed_unprocessed_pseudo
ENSG0000(	POLR2A	3155.365	-0.1044	0.018691	0.062242	protein_coding
ENSG0000(	ENSG0000(	34.82162	0.279194	0.018693	0.062242	lncRNA
ENSG0000(	SYT8	86.93584	-0.53349	0.018696	0.062247	protein_coding
ENSG0000(	DCAF8L2	10.6101	0.670235	0.0187	0.062252	protein_coding
ENSG0000(	CD1C	248.4447	-0.72204	0.018704	0.062259	protein_coding
ENSG0000(	FAM86B3P	347.0149	0.142673	0.018706	0.06226	transcribed_unprocessed_pseudo
ENSG0000(	ENSG0000(	1210.753	0.35286	0.018717	0.06229	TEC
ENSG0000(	ENSG0000(	32.48069	0.308215	0.018735	0.062329	lncRNA
ENSG0000(	GNG5	1097.852	-0.21668	0.018733	0.062329	protein_coding
ENSG0000(	PSEN2	3360.758	-0.1248	0.018731	0.062329	protein_coding
ENSG0000(	LINC00608	519.0103	-0.16394	0.018749	0.06237	lncRNA
ENSG0000(	ING2	261.5905	-0.35738	0.018756	0.062388	protein_coding
ENSG0000(	ENSG0000(	69.76449	0.214062	0.018759	0.06239	lncRNA
ENSG0000(	PRPF4	545.5593	-0.13113	0.018774	0.062432	protein_coding
ENSG0000(	ZNF366	562.7503	0.259397	0.018776	0.062435	protein_coding
ENSG0000(	ENSG0000(	78.90358	0.204766	0.018791	0.062478	lncRNA
ENSG0000(	ENSG0000(	299.8534	0.111178	0.018796	0.062488	lncRNA
ENSG0000(	STRC	393.6991	-0.15529	0.018818	0.062552	protein_coding
ENSG0000(	ENSG0000(	1040.641	0.320488	0.018828	0.06258	lncRNA
ENSG0000(	ANKS4B	2393.053	-0.38084	0.018841	0.062592	protein_coding
ENSG0000(	ENSG0000(	61.64222	0.230316	0.018836	0.062592	lncRNA
ENSG0000(	LIPE-AS1	112.6345	0.232381	0.018842	0.062592	lncRNA
ENSG0000(	LYRM1	442.208	0.141125	0.018836	0.062592	protein_coding
ENSG0000(	STRAP	496.1695	-0.19786	0.018838	0.062592	protein_coding
ENSG0000(	CEP295NL	192.2669	0.267032	0.018849	0.06261	protein_coding
ENSG0000(	ZXDA	384.016	0.160854	0.018859	0.062636	protein_coding
ENSG0000(	RPS2P45	196.7519	0.138886	0.018864	0.062647	transcribed_processed_pseudoge
ENSG0000(	ENSG0000(	23.52155	0.306008	0.018868	0.062654	TEC
ENSG0000(	CRYGS	212.0205	0.143578	0.01887	0.062654	protein_coding
ENSG0000(	DDB1	3369.084	-0.06378	0.018889	0.062711	protein_coding
ENSG0000(	LINC00343	2069.704	-0.18143	0.018893	0.062715	lncRNA
ENSG0000(	ENSG0000(	988.9325	0.125102	0.0189	0.062734	lncRNA
ENSG0000(	GDPD2	309.6431	-0.60478	0.018916	0.06278	protein_coding

ENSG0000(	SETD5	12267.73	0.061079	0.018919	0.062783	protein_coding
ENSG0000(	FAM182B	64.25591	0.223876	0.018932	0.062819	lncRNA
ENSG0000(	ENSG0000(	65.38128	0.26593	0.01894	0.06284	TEC
ENSG0000(	DNM1P41	194.0639	0.221254	0.018949	0.062855	TEC
ENSG0000(	MICOS13	351.3301	-0.10941	0.018948	0.062855	protein_coding
ENSG0000(	ENSG0000(	74.9309	0.191095	0.018966	0.062905	lncRNA
ENSG0000(	ENSG0000(	61.24646	-0.20001	0.018971	0.062914	lncRNA
ENSG0000(	DHRS13	176.9257	0.327449	0.018978	0.062931	protein_coding
ENSG0000(	REEP2	159.4092	-0.14146	0.018993	0.062977	protein_coding
ENSG0000(	RN7SL254F	28.88523	0.335743	0.019003	0.062997	misc_RNA
ENSG0000(	UCHL5	1010.867	-0.10422	0.019003	0.062997	protein_coding
ENSG0000(	ENSG0000(	28.67957	0.291301	0.019012	0.063018	lncRNA
ENSG0000(	PIM1	993.2458	0.211133	0.019021	0.063043	protein_coding
ENSG0000(	MTFR2	48.18334	0.343268	0.019024	0.063046	protein_coding
ENSG0000(	PKP4	1754.435	0.176866	0.019028	0.063051	protein_coding
ENSG0000(	IDH3A	1858.893	-0.1507	0.019031	0.063055	protein_coding
ENSG0000(	CCDC85C	1583.189	-0.35531	0.019047	0.063103	protein_coding
ENSG0000(	HERC2P2	4815.745	0.133541	0.01905	0.063105	transcribed_unprocessed_pseudo
ENSG0000(	FAM187A	16.77984	0.548282	0.019056	0.063117	protein_coding
ENSG0000(	ENSG0000(	48.4229	0.419542	0.019059	0.063121	lncRNA
ENSG0000(	SLIT1	72.53294	-0.2953	0.019066	0.063139	protein_coding
ENSG0000(	COMTD1	141.5242	-0.34414	0.019074	0.063144	protein_coding
ENSG0000(	ENSG0000(	88.38468	0.203182	0.019072	0.063144	lncRNA
ENSG0000(	RBFOX2	2684.796	0.164272	0.019072	0.063144	protein_coding
ENSG0000(	PEX26	7707.256	-0.20432	0.019088	0.063178	protein_coding
ENSG0000(	PIAS2	2469.256	0.125626	0.019087	0.063178	protein_coding
ENSG0000(	PRR13	3314.451	-0.27097	0.019101	0.063214	protein_coding
ENSG0000(	BOC	578.692	0.315376	0.019113	0.063228	protein_coding
ENSG0000(	C4A	1075.77	0.489269	0.019108	0.063228	protein_coding
ENSG0000(	ENSG0000(	38.36338	0.356817	0.019112	0.063228	lncRNA
ENSG0000(	TCF19	321.4624	0.251032	0.019113	0.063228	protein_coding
ENSG0000(	TNRC18	4276.115	0.106274	0.019125	0.063261	protein_coding
ENSG0000(	DHRS1	904.1959	-0.23265	0.019129	0.063266	protein_coding
ENSG0000(	OR7E11P	41.38053	0.394577	0.019132	0.06327	lncRNA
ENSG0000(	HUS1	1083.31	-0.09962	0.019139	0.06327	protein_coding
ENSG0000(	LINC01786	46.05154	-0.24593	0.01914	0.06327	lncRNA
ENSG0000(	MYH10	1944.667	0.132863	0.019138	0.06327	protein_coding
ENSG0000(	RNU6-130F	65.32969	0.283907	0.019134	0.06327	snRNA
ENSG0000(	TMEM138	2017.315	0.076178	0.019142	0.06327	protein_coding
ENSG0000(	ENSG0000(	23.56874	0.386519	0.019154	0.063302	TEC
ENSG0000(	FOXG1	11.39764	0.600132	0.019163	0.063327	protein_coding
ENSG0000(	ENSG0000(	31.79515	-0.56029	0.019194	0.063417	lncRNA
ENSG0000(	MRPL47	143.1838	-0.1551	0.019195	0.063417	protein_coding
ENSG0000(	SEPTIN4-AS	240.1411	0.187387	0.019199	0.063425	lncRNA
ENSG0000(	ENSG0000(	145.9602	0.348895	0.019204	0.063434	lncRNA
ENSG0000(	PHF6	2964.764	-0.10329	0.019226	0.063502	protein_coding
ENSG0000(	PPARG	1692.622	-0.32298	0.019236	0.063521	protein_coding

ENSG0000(	PTPRH	3600.586	-0.49412	0.019234	0.063521	protein_coding
ENSG0000(	TBX3	2621.8	-0.41853	0.019251	0.063564	protein_coding
ENSG0000(	TPI1	2104.483	-0.25408	0.019268	0.063611	protein_coding
ENSG0000(	OCEL1	252.3028	-0.22423	0.019284	0.06366	protein_coding
ENSG0000(	CCDC22	195.6718	-0.22567	0.019288	0.063667	protein_coding
ENSG0000(	LINC02541	92.15325	0.255651	0.019292	0.063673	lncRNA
ENSG0000(	LINC00971	99.33968	-0.22162	0.019296	0.063677	lncRNA
ENSG0000(	FDX1	587.8248	-0.16696	0.019308	0.063711	protein_coding
ENSG0000(	ENSG0000(	126.1766	0.150843	0.019318	0.063739	lncRNA
ENSG0000(	LINC01990	25.09391	-0.35194	0.019361	0.063875	lncRNA
ENSG0000(	BNIP3P41	132.8514	-0.96897	0.019369	0.063892	transcribed_processed_pseudoge
ENSG0000(	ENSG0000(	20.13147	0.374094	0.019373	0.063899	lncRNA
ENSG0000(	SALRNA3	199.5214	0.159488	0.019391	0.063953	lncRNA
ENSG0000(	FSCN1	321.1004	0.393931	0.019399	0.063972	protein_coding
ENSG0000(	ENSG0000(	185.2831	-0.16525	0.019407	0.063989	protein_coding
ENSG0000(	GPRASP2	149.7643	0.184094	0.019408	0.063989	protein_coding
ENSG0000(	BET1L	1054.139	0.11484	0.019415	0.064003	protein_coding
ENSG0000(	CDKN2A	100.4024	0.258172	0.019428	0.06404	protein_coding
ENSG0000(	MYCNOS	222.2645	0.127783	0.019431	0.064042	lncRNA
ENSG0000(	MPIG6B	162.2826	-0.18162	0.019444	0.06408	protein_coding
ENSG0000(	FTCDNL1	285.3483	0.171737	0.019476	0.064178	protein_coding
ENSG0000(	VEGFB	336.4086	-0.21066	0.019482	0.064193	protein_coding
ENSG0000(	ENSG0000(	139.3374	0.1722	0.019491	0.064214	TEC
ENSG0000(	ENSG0000(	17.02609	-0.62908	0.019498	0.06423	lncRNA
ENSG0000(	ENSG0000(	31.38001	0.248112	0.019512	0.064271	lncRNA
ENSG0000(	ENSG0000(	79.48177	0.458102	0.019528	0.064317	lncRNA
ENSG0000(	RAB11FIP1	5283.491	-0.25877	0.01953	0.064317	protein_coding
ENSG0000(	ENSG0000(	29.67184	0.350753	0.01954	0.064336	lncRNA
ENSG0000(	NR1H3	1274.822	0.206616	0.01954	0.064336	protein_coding
ENSG0000(	SPRY1	574.2607	0.178846	0.019545	0.064345	protein_coding
ENSG0000(	CELF4	103.8107	0.313829	0.019558	0.064376	protein_coding
ENSG0000(	ENSG0000(	26.70978	-0.32509	0.019556	0.064376	protein_coding
ENSG0000(	CD8A	349.0965	-0.34265	0.019588	0.064453	protein_coding
ENSG0000(	ITFG1	1181.709	-0.11936	0.019588	0.064453	protein_coding
ENSG0000(	NUDT1	125.844	0.187513	0.01959	0.064453	protein_coding
ENSG0000(	TRIM25	4086.076	0.174318	0.019588	0.064453	protein_coding
ENSG0000(	WDR25	200.9703	0.160443	0.019596	0.064465	protein_coding
ENSG0000(	ENSG0000(	65.14137	0.286592	0.019598	0.064467	lncRNA
ENSG0000(	JPH4	28.43959	0.506406	0.019629	0.06456	protein_coding
ENSG0000(	ENSG0000(	70.40539	0.210778	0.019656	0.064643	lncRNA
ENSG0000(	ENSG0000(	199.2484	0.132511	0.019661	0.064648	lncRNA
ENSG0000(	ZMYM3	804.3949	0.108969	0.019661	0.064648	protein_coding
ENSG0000(	BMS1P10	69.27611	0.325494	0.019671	0.064673	transcribed_unprocessed_pseudo
ENSG0000(	IKBIP	380.4602	0.186221	0.019687	0.064717	protein_coding
ENSG0000(	ACSBG1	375.6985	0.164851	0.0197	0.064734	protein_coding
ENSG0000(	CLEC1A	122.5592	0.222033	0.019697	0.064734	protein_coding
ENSG0000(	ENSG0000(	544.4564	0.163326	0.019696	0.064734	lncRNA

ENSG0000(	ENSG0000(	38.08058	-0.42382	0.019701	0.064734	lncRNA
ENSG0000(	LINC02391	11.65764	0.555631	0.019703	0.064734	lncRNA
ENSG0000(	TTC37	2675.489	-0.09059	0.019704	0.064734	protein_coding
ENSG0000(	RN7SL556F	25.08631	0.299464	0.01972	0.06478	misc_RNA
ENSG0000(	ENSG0000(	148.4184	0.194814	0.019723	0.064781	lncRNA
ENSG0000(	ZNF460	2091.429	0.102902	0.019737	0.064821	protein_coding
ENSG0000(	FOXM1	817.0755	0.119627	0.019769	0.064921	protein_coding
ENSG0000(	ENSG0000(	20.95865	0.336296	0.019789	0.064978	lncRNA
ENSG0000(	CACNA2D1	337.7481	0.330555	0.019805	0.065024	protein_coding
ENSG0000(	EPS8L3	4108.243	-0.49202	0.019828	0.065093	protein_coding
ENSG0000(	ENSG0000(	238.4774	0.223091	0.01984	0.065126	lncRNA
ENSG0000(	NUPR1	1910.528	0.138145	0.019849	0.06515	protein_coding
ENSG0000(	DPY19L2P4	10.9947	0.723083	0.019869	0.065207	transcribed_unprocessed_pseudo
ENSG0000(	RNU5B-1	2328.239	0.388718	0.019909	0.065331	snRNA
ENSG0000(	TPRA1	467.1845	-0.1354	0.019913	0.06534	protein_coding
ENSG0000(	ENSG0000(	492.4179	0.086487	0.019923	0.065363	lncRNA
ENSG0000(	EFL1P1	77.06106	0.235174	0.01993	0.065375	transcribed_unprocessed_pseudo
ENSG0000(	LINC02009	77.52244	0.281249	0.019929	0.065375	lncRNA
ENSG0000(	DCAF8	4608.687	0.074536	0.019935	0.065383	protein_coding
ENSG0000(	DYDC2	120.4148	0.178749	0.019951	0.06543	protein_coding
ENSG0000(	ENSG0000(	70.81149	0.194966	0.019963	0.065463	lncRNA
ENSG0000(	ENSG0000(	116.9602	0.185705	0.019976	0.065494	lncRNA
ENSG0000(	ENSG0000(	58.84863	0.208705	0.019979	0.065494	lncRNA
ENSG0000(	HSPA8P4	21.2121	0.366703	0.019978	0.065494	processed_pseudogene
ENSG0000(	RN7SL122F	43.92537	0.211811	0.019983	0.065501	misc_RNA
ENSG0000(	NOTCH2	4650.344	0.250785	0.019997	0.065539	protein_coding
ENSG0000(	RGSL1	79.49809	0.276681	0.020007	0.065563	protein_coding
ENSG0000(	RNU1-30P	14.53831	-0.42399	0.02001	0.065569	snRNA
ENSG0000(	RHOA	3491.975	-0.15473	0.020024	0.065607	protein_coding
ENSG0000(	PLK3	415.9536	0.218754	0.020027	0.065609	protein_coding
ENSG0000(	UBA52	1608.38	-0.21357	0.020048	0.065673	protein_coding
ENSG0000(	DPRXP2	41.29623	-0.32517	0.020059	0.065695	transcribed_processed_pseudoge
ENSG0000(	EIPR1-IT1	53.61656	0.243536	0.020059	0.065695	lncRNA
ENSG0000(	SH2B1	2799.058	-0.10198	0.020062	0.065698	protein_coding
ENSG0000(	RIC8A	1026.55	-0.1218	0.020078	0.065743	protein_coding
ENSG0000(	ARHGAP31	1660.932	0.254812	0.020083	0.065751	protein_coding
ENSG0000(	ENSG0000(	192.6382	0.239183	0.02009	0.06577	lncRNA
ENSG0000(	ENSG0000(	25.35081	0.45419	0.020096	0.065782	lncRNA
ENSG0000(	THRB	504.025	-0.31564	0.020099	0.065786	protein_coding
ENSG0000(	CEP89	1501.746	0.101517	0.020107	0.06579	protein_coding
ENSG0000(	RPL9P7	25.38872	0.654143	0.020105	0.06579	processed_pseudogene
ENSG0000(	TTC39A-AS	33.90293	0.449798	0.020104	0.06579	lncRNA
ENSG0000(	BSG	1155.513	-0.22505	0.020116	0.065813	protein_coding
ENSG0000(	FBXL14	210.1141	-0.22236	0.020121	0.065822	protein_coding
ENSG0000(	DOK4	1094.99	-0.41893	0.020137	0.065868	protein_coding
ENSG0000(	CYP2C8	63.2567	-0.59771	0.020142	0.065878	protein_coding
ENSG0000(	GPR142	11.41834	0.472414	0.020149	0.065887	protein_coding

ENSG0000(	IRF6	1250.079	-0.37494	0.020148	0.065887	protein_coding
ENSG0000(	ZDHHC3	3947.194	-0.24162	0.020154	0.065896	protein_coding
ENSG0000(	PDLIM1	1945.876	-0.27662	0.02017	0.065941	protein_coding
ENSG0000(	HLF	304.6688	-0.43419	0.020182	0.065967	protein_coding
ENSG0000(	LINC02893	395.3296	0.249142	0.02018	0.065967	lncRNA
ENSG0000(	C21orf91-C	11.38306	0.476357	0.020194	0.065999	lncRNA
ENSG0000(	ENSG0000(	13.44404	0.330846	0.020196	0.065999	lncRNA
ENSG0000(	FAM20C	820.5598	0.352646	0.020206	0.066019	protein_coding
ENSG0000(	MIR6774	19.83678	-0.42848	0.020206	0.066019	miRNA
ENSG0000(	SPATA1	64.35148	0.262625	0.020255	0.066171	protein_coding
ENSG0000(	FAR2P2	163.726	-0.24873	0.020261	0.066177	transcribed_unprocessed_pseudo
ENSG0000(	SERPINB8	293.6346	0.203611	0.020261	0.066177	protein_coding
ENSG0000(	ALOX12P2	391.7369	0.244453	0.020268	0.066187	transcribed_unprocessed_pseudo
ENSG0000(	LINC02301	1270.643	0.185512	0.020267	0.066187	lncRNA
ENSG0000(	CDYL2	1056.265	-0.1591	0.020276	0.066206	protein_coding
ENSG0000(	DPP10-AS1	28.72869	-0.33228	0.020281	0.066211	lncRNA
ENSG0000(	HMOX1	511.0269	-0.3508	0.020282	0.066211	protein_coding
ENSG0000(	RPL7AP22	71.46408	0.214397	0.020285	0.066215	processed_pseudogene
ENSG0000(	ZNF114-AS	11.87422	0.48047	0.020292	0.06623	lncRNA
ENSG0000(	UQCRFS1	666.8909	-0.17985	0.0203	0.06625	protein_coding
ENSG0000(	ENSG0000(	961.1211	-0.18665	0.020304	0.066257	protein_coding
ENSG0000(	ENSG0000(	248.9805	0.218729	0.020309	0.066264	lncRNA
ENSG0000(	RPL23AP42	532.3397	-0.48477	0.02032	0.066294	processed_pseudogene
ENSG0000(	ENSG0000(	11.35769	0.539379	0.020329	0.066317	lncRNA
ENSG0000(	PALM	298.4661	-0.14039	0.020331	0.066317	protein_coding
ENSG0000(	EIF2S1	752.6595	-0.16065	0.020353	0.066382	protein_coding
ENSG0000(	ENSG0000(	86.10087	0.159253	0.020371	0.066433	lncRNA
ENSG0000(	SKOR1	26.71164	0.330462	0.020379	0.066451	protein_coding
ENSG0000(	CLDN8	20.40674	-1.16177	0.020393	0.066489	protein_coding
ENSG0000(	RPL6	3548.579	-0.20267	0.020395	0.066489	protein_coding
ENSG0000(	HMGA1P4	119.8201	0.251887	0.020401	0.066497	lncRNA
ENSG0000(	PLEKHH3	574.3456	0.119001	0.0204	0.066497	protein_coding
ENSG0000(	ENSG0000(	959.7017	-0.29395	0.020418	0.066543	lncRNA
ENSG0000(	WDFY3-AS2	438.2418	0.10679	0.02042	0.066543	lncRNA
ENSG0000(	NUDT16-DT	1147.084	0.281241	0.02044	0.066603	lncRNA
ENSG0000(	ENSG0000(	244.8618	0.166049	0.020462	0.066648	lncRNA
ENSG0000(	ENSG0000(	22.8161	0.371553	0.020462	0.066648	lncRNA
ENSG0000(	PARD3B	1475.588	-0.23412	0.020465	0.066648	protein_coding
ENSG0000(	RAD50	2657.733	-0.09781	0.020461	0.066648	protein_coding
ENSG0000(	SOCS7	1186.833	-0.08635	0.020467	0.066648	protein_coding
ENSG0000(	SUPT20HL1	8.015551	0.626491	0.020464	0.066648	protein_coding
ENSG0000(	ENSG0000(	62.96665	-0.22459	0.020485	0.066701	lncRNA
ENSG0000(	ENSG0000(	1345.809	0.162272	0.020492	0.066718	protein_coding
ENSG0000(	ENSG0000(	86.1825	-0.173	0.020508	0.066764	lncRNA
ENSG0000(	CCL21	1629.918	-0.83559	0.020515	0.066777	protein_coding
ENSG0000(	ENSG0000(	15.0306	0.698473	0.020523	0.066798	processed_pseudogene
ENSG0000(	DLG4	896.4923	0.174452	0.020529	0.06681	protein_coding

ENSG0000(	DDR1-DT	1742.831	0.097575	0.020571	0.066941	lncRNA
ENSG0000(	ALDH18A1	3060.644	-0.39443	0.02058	0.066962	protein_coding
ENSG0000(	EFNA4	147.8417	-0.3398	0.020587	0.066977	protein_coding
ENSG0000(	ENSG0000(	196.1269	0.17339	0.020589	0.066977	lncRNA
ENSG0000(	ENSG0000(	17.36534	0.365532	0.020626	0.067085	lncRNA
ENSG0000(	RN7SL416F	17.64341	0.355308	0.020625	0.067085	misc_RNA
ENSG0000(	WWC2	464.5075	0.247428	0.020629	0.067087	protein_coding
ENSG0000(	ENSG0000(	109.2886	0.193291	0.020635	0.067094	lncRNA
ENSG0000(	PRPF31	305.0441	-0.14072	0.020635	0.067094	protein_coding
ENSG0000(	LINC01534	877.4563	-0.09919	0.020639	0.067097	lncRNA
ENSG0000(	PPT2-EGFL8	205.8674	-0.14113	0.020674	0.067206	protein_coding
ENSG0000(	FBXL12	607.8609	-0.13738	0.020681	0.06722	protein_coding
ENSG0000(	LINC00384	25.04881	0.572919	0.020684	0.067221	lncRNA
ENSG0000(	SNX18	940.8882	0.155646	0.020685	0.067221	protein_coding
ENSG0000(	DUXAP9	380.9297	0.263241	0.020688	0.067222	transcribed_processed_pseudoge
ENSG0000(	ENSG0000(	2951.174	0.174051	0.02069	0.067224	lncRNA
ENSG0000(	ENSG0000(	21.74106	0.376232	0.020705	0.067263	lncRNA
ENSG0000(	NXPE4	46.91236	0.77291	0.020711	0.067279	protein_coding
ENSG0000(	PCDHGC5	57.32805	0.33403	0.020734	0.067345	protein_coding
ENSG0000(	CHRNA3	71.92778	-0.31148	0.020744	0.06737	protein_coding
ENSG0000(	KCNJ10	277.1954	0.500838	0.020746	0.067371	protein_coding
ENSG0000(	CD151	1469.548	-0.19028	0.020755	0.067392	protein_coding
ENSG0000(	LINC01544	281.3536	0.134562	0.020764	0.067416	lncRNA
ENSG0000(	PCK2	2717.47	-0.44128	0.020777	0.067449	protein_coding
ENSG0000(	ENSG0000(	399.7503	0.098472	0.020787	0.067475	lncRNA
ENSG0000(	VPS54	1713.96	0.076142	0.020789	0.067475	protein_coding
ENSG0000(	ENSG0000(	1013.597	0.127088	0.020791	0.067475	lncRNA
ENSG0000(	ENSG0000(	315.0408	-0.17197	0.020799	0.067495	lncRNA
ENSG0000(	ARHGEF12	8790.827	-0.13344	0.020802	0.067495	protein_coding
ENSG0000(	ENSG0000(	28.81004	0.293813	0.020804	0.067495	lncRNA
ENSG0000(	ALPK3	216.5616	0.307437	0.020807	0.0675	protein_coding
ENSG0000(	SMURF2	2147.34	-0.13957	0.020813	0.067512	protein_coding
ENSG0000(	RBM23	3455.957	-0.07256	0.020831	0.067564	protein_coding
ENSG0000(	ENSG0000(	77.73518	-0.30219	0.020846	0.067599	lncRNA
ENSG0000(	SNRPE	250.6338	-0.25257	0.020847	0.067599	protein_coding
ENSG0000(	SSX2	39.6054	0.361807	0.020849	0.067599	protein_coding
ENSG0000(	ENSG0000(	116.3426	-0.18848	0.020852	0.067604	lncRNA
ENSG0000(	ENSG0000(	77.73015	0.177889	0.020861	0.067625	TEC
ENSG0000(	GRAMD2B	1253.877	-0.29833	0.020869	0.067641	protein_coding
ENSG0000(	INPP5A	530.0416	-0.1581	0.020872	0.067641	protein_coding
ENSG0000(	RRAGC	850.2806	-0.11533	0.020871	0.067641	protein_coding
ENSG0000(	ENSG0000(	130.7019	0.221116	0.020877	0.067645	lncRNA
ENSG0000(	TEX35	274.1335	0.201581	0.020878	0.067645	protein_coding
ENSG0000(	TGIF2P1	30.16855	0.27243	0.020881	0.067649	processed_pseudogene
ENSG0000(	ENSG0000(	269.9163	0.134655	0.020883	0.067649	lncRNA
ENSG0000(	LINC02132	34.76994	0.297643	0.020916	0.067737	lncRNA
ENSG0000(	LINC02795	84.39094	0.288758	0.020917	0.067737	lncRNA

ENSG0000(	TRABD2B	84.50911	0.383215	0.020914	0.067737	protein_coding
ENSG0000(	CNOT6L	3283.266	-0.10995	0.020935	0.067768	protein_coding
ENSG0000(	CRYL1	1361.645	-0.35553	0.020935	0.067768	protein_coding
ENSG0000(	ENSG0000(	140.9981	-0.13782	0.020929	0.067768	lncRNA
ENSG0000(	RPL31P52	14.2269	0.384645	0.020935	0.067768	processed_pseudogene
ENSG0000(	RN7SKP124	19.8779	-0.36862	0.020947	0.0678	misc_RNA
ENSG0000(	ENSG0000(	8.223956	0.575044	0.020991	0.067934	processed_pseudogene
ENSG0000(	PAPOLA	7211.296	-0.08959	0.021	0.067957	protein_coding
ENSG0000(	ENSG0000(	48.27245	0.250088	0.021031	0.06805	lncRNA
ENSG0000(	CNOT3	1190.712	-0.14579	0.021047	0.068096	protein_coding
ENSG0000(	LASTR	710.3678	0.363256	0.021061	0.068132	lncRNA
ENSG0000(	ZC3H12B	440.4141	-0.12269	0.021063	0.068132	protein_coding
ENSG0000(	KANTR	1961.178	0.079125	0.021079	0.068176	protein_coding
ENSG0000(	TET1	473.1711	0.182455	0.021083	0.068182	protein_coding
ENSG0000(	MKRN5P	145.9669	0.209127	0.021104	0.068245	processed_pseudogene
ENSG0000(	DIRAS3	21.2083	0.345287	0.021115	0.068273	protein_coding
ENSG0000(	MSL3	1729.338	0.209108	0.021123	0.068284	protein_coding
ENSG0000(	RN7SL145P	142.1404	0.157399	0.021123	0.068284	misc_RNA
ENSG0000(	TSPEAR-AS	18.76714	0.731239	0.021125	0.068284	lncRNA
ENSG0000(	TRIM34	402.3665	-0.14981	0.021133	0.068301	protein_coding
ENSG0000(	KCNJ14	536.2398	0.117648	0.021137	0.06831	protein_coding
ENSG0000(	SARAF	2266.158	-0.16891	0.021143	0.068321	protein_coding
ENSG0000(	CNTNAP3P	24.23333	0.585248	0.02116	0.06837	unprocessed_pseudogene
ENSG0000(	SLC22A18	1239.435	-0.38592	0.021189	0.068455	protein_coding
ENSG0000(	EFNB1	602.7395	-0.28728	0.021213	0.068527	protein_coding
ENSG0000(	IST1	3699.113	-0.1144	0.02122	0.068537	protein_coding
ENSG0000(	KMT2C	19546.78	0.058978	0.021221	0.068537	protein_coding
ENSG0000(	ENSG0000(	30.31903	-0.26963	0.021223	0.068539	lncRNA
ENSG0000(	ENSG0000(	8.337501	-0.47115	0.021226	0.068541	lncRNA
ENSG0000(	ENSG0000(	3024.865	-0.18619	0.021229	0.068544	lncRNA
ENSG0000(	H4C12	202.9756	-0.51983	0.021235	0.068555	protein_coding
ENSG0000(	PPM1F-AS1	1962.671	0.082255	0.021239	0.06856	lncRNA
ENSG0000(	BIRC6	15560.72	-0.09503	0.02125	0.068589	protein_coding
ENSG0000(	ACTR8	593.388	-0.14676	0.021252	0.06859	protein_coding
ENSG0000(	PEX14	276.1035	-0.17358	0.021269	0.068636	protein_coding
ENSG0000(	RN7SKP78	93.17253	0.244856	0.021277	0.068656	misc_RNA
ENSG0000(	ENSG0000(	539.0822	-0.08933	0.021281	0.06866	lncRNA
ENSG0000(	TRIM10	152.9471	-0.43103	0.021287	0.068675	protein_coding
ENSG0000(	KCTD11	242.5172	0.139973	0.02129	0.068677	protein_coding
ENSG0000(	CCDC126	176.3175	-0.1667	0.021296	0.068681	protein_coding
ENSG0000(	TMEM86A	208.6438	0.232304	0.021296	0.068681	protein_coding
ENSG0000(	TNFRSF1B	1702.917	0.236277	0.021303	0.068697	protein_coding
ENSG0000(	RB1-DT	48.47451	0.227244	0.021328	0.068771	lncRNA
ENSG0000(	ENSG0000(	70.0076	-0.20393	0.021333	0.068773	lncRNA
ENSG0000(	ENSG0000(	463.2916	-0.095	0.021332	0.068773	lncRNA
ENSG0000(	ENSG0000(	68.83651	0.242652	0.021352	0.068826	lncRNA
ENSG0000(	NAP1L4P1	137.9279	-0.24766	0.021356	0.068834	processed_pseudogene

ENSG0000(	BNIP1	93.21883	0.16991	0.021362	0.068847	protein_coding
ENSG0000(	LINC01787	101.3375	0.165795	0.021367	0.068854	lncRNA
ENSG0000(	ARPC5L	413.1155	0.162321	0.02139	0.06892	protein_coding
ENSG0000(	SNORD69	11.35626	0.450524	0.021392	0.06892	snoRNA
ENSG0000(	ENSG0000(	45.35453	0.248615	0.021407	0.068954	transcribed_processed_pseudoge
ENSG0000(	ENSG0000(	33.99754	0.325257	0.021407	0.068954	TEC
ENSG0000(	EMC7	222.8837	-0.1761	0.02141	0.068957	protein_coding
ENSG0000(	AMN1	1017.902	0.101069	0.021417	0.068973	protein_coding
ENSG0000(	ENSG0000(	111.6382	0.224165	0.021423	0.068984	lncRNA
ENSG0000(	RNU4-6P	29.75587	-0.39109	0.021433	0.069011	snRNA
ENSG0000(	CALN1	222.5224	0.130793	0.021437	0.069014	protein_coding
ENSG0000(	COX5BP6	22.03662	0.316472	0.021441	0.069014	processed_pseudogene
ENSG0000(	ENSG0000(	13.45148	0.324799	0.021441	0.069014	lncRNA
ENSG0000(	OGFRL1	1342.081	0.283048	0.021443	0.069014	protein_coding
ENSG0000(	THOC1	1141.04	-0.11577	0.021445	0.069015	protein_coding
ENSG0000(	COPS8	640.6965	0.101895	0.021468	0.069081	protein_coding
ENSG0000(	DVL1	716.2258	-0.21361	0.021477	0.069103	protein_coding
ENSG0000(	SNORA74D	54.61438	0.357353	0.021484	0.069117	snoRNA
ENSG0000(	CBX3P2	47.14131	0.202989	0.021498	0.069156	transcribed_processed_pseudoge
ENSG0000(	DTNB	346.6501	0.270959	0.0215	0.069156	protein_coding
ENSG0000(	IGHV1-69	88.85946	1.002836	0.021506	0.069168	IG_V_gene
ENSG0000(	TXLNA	1689.283	0.138652	0.02151	0.069175	protein_coding
ENSG0000(	ENSG0000(	99.06287	0.3463	0.021518	0.069192	lncRNA
ENSG0000(	ENSG0000(	80.8518	0.231698	0.021532	0.069232	lncRNA
ENSG0000(	H2BC19P	787.1645	0.16665	0.021552	0.069289	transcribed_unprocessed_pseudo
ENSG0000(	H2BP1	63.90802	0.319187	0.021561	0.06931	transcribed_unprocessed_pseudo
ENSG0000(	IBA57	1691.443	0.072848	0.02158	0.069353	protein_coding
ENSG0000(	MIR924HG	447.3336	0.223584	0.021578	0.069353	lncRNA
ENSG0000(	RN7SKP22(	9.401924	-0.59664	0.021581	0.069353	misc_RNA
ENSG0000(	SRSF6P2	10.52918	0.484974	0.021596	0.069394	processed_pseudogene
ENSG0000(	ENSG0000(	18.51311	0.373591	0.021601	0.069401	lncRNA
ENSG0000(	LINC00592	53.48656	0.253812	0.021607	0.069415	lncRNA
ENSG0000(	AKIRIN1	993.9335	-0.1177	0.021614	0.069417	protein_coding
ENSG0000(	ENSG0000(	458.2825	0.099239	0.021615	0.069417	lncRNA
ENSG0000(	ENSG0000(	233.0118	0.205502	0.021617	0.069417	lncRNA
ENSG0000(	MYCBP2-A!	35.60776	0.279683	0.021611	0.069417	lncRNA
ENSG0000(	ZBTB8OS	1131.447	-0.06884	0.021636	0.069474	protein_coding
ENSG0000(	AGGF1P2	33.58873	0.478557	0.021651	0.069514	processed_pseudogene
ENSG0000(	PPP1R26	464.9883	-0.22095	0.021671	0.06957	protein_coding
ENSG0000(	PPIG	2592.069	-0.06312	0.021674	0.069574	protein_coding
ENSG0000(	ENSG0000(	62.04554	-0.79954	0.021681	0.069588	lncRNA
ENSG0000(	NBL1	2490.412	-0.29595	0.021685	0.069596	protein_coding
ENSG0000(	CMYA5	265.162	0.267397	0.021697	0.069617	protein_coding
ENSG0000(	SNX19	5124.073	0.157752	0.021696	0.069617	protein_coding
ENSG0000(	DNM1P47	1000.61	0.382593	0.0217	0.069621	transcribed_unprocessed_pseudo
ENSG0000(	LINC02488	39.19715	0.240472	0.021702	0.069621	lncRNA
ENSG0000(	ENSG0000(	172.7223	0.127332	0.021708	0.069632	lncRNA

ENSG0000(	ELOB	675.3922	-0.1827	0.021736	0.069714	protein_coding
ENSG0000(	ENSG0000(	18.4099	0.434001	0.021742	0.069726	lncRNA
ENSG0000(	SNORA70D	48.04838	-0.54623	0.021745	0.069731	snoRNA
ENSG0000(	DUSP7	318.5689	0.192505	0.021749	0.069736	protein_coding
ENSG0000(	ENTPD8	593.7414	-0.51368	0.021751	0.069736	protein_coding
ENSG0000(	CMBL	2318.592	-0.49606	0.021772	0.069796	protein_coding
ENSG0000(	LINC00624	43.32454	0.249823	0.021791	0.06985	lncRNA
ENSG0000(	ENSG0000(	101.4886	0.146161	0.021799	0.06986	lncRNA
ENSG0000(	ENSG0000(	125.7685	0.263476	0.021798	0.06986	lncRNA
ENSG0000(	ENSG0000(	21.53193	0.359941	0.021802	0.069863	lncRNA
ENSG0000(	ENSG0000(	13.36436	0.390591	0.021822	0.06992	lncRNA
ENSG0000(	BST2	351.546	0.35521	0.021829	0.069936	protein_coding
ENSG0000(	GNLY	265.7775	0.375291	0.021837	0.069953	protein_coding
ENSG0000(	ENSG0000(	257.7129	0.106008	0.02184	0.069958	lncRNA
ENSG0000(	TFB1M	407.6724	-0.13183	0.021858	0.070008	protein_coding
ENSG0000(	MT-TF	70.15822	0.69233	0.021863	0.070016	Mt_tRNA
ENSG0000(	ENSG0000(	29.52374	-0.38806	0.021869	0.070027	transcribed_processed_pseudoge
ENSG0000(	RNF135	424.7427	-0.15623	0.021878	0.07005	protein_coding
ENSG0000(	ENSG0000(	156.5576	0.387459	0.021885	0.070066	lncRNA
ENSG0000(	RAB39A	111.8524	0.203176	0.02189	0.070073	protein_coding
ENSG0000(	OSBP	1728.953	-0.14899	0.021893	0.070075	protein_coding
ENSG0000(	OMP	47.41505	-0.47454	0.021896	0.070078	protein_coding
ENSG0000(	ULK4	664.9993	0.164485	0.021901	0.070089	protein_coding
ENSG0000(	RND2	150.2399	0.150677	0.021908	0.070104	protein_coding
ENSG0000(	LINC02433	38.60784	0.233408	0.021931	0.07017	lncRNA
ENSG0000(	ALDOB	36580.41	-0.57929	0.02194	0.070182	protein_coding
ENSG0000(	FCHO2-DT	10.15668	0.461145	0.021938	0.070182	lncRNA
ENSG0000(	ENSG0000(	82.1246	-0.1739	0.02197	0.070273	lncRNA
ENSG0000(	ENSG0000(	13.87507	0.352005	0.021975	0.070275	TEC
ENSG0000(	ENSG0000(	38.49268	0.383985	0.021973	0.070275	lncRNA
ENSG0000(	ZNF385A	313.4269	0.177791	0.021992	0.070322	protein_coding
ENSG0000(	TRMT13	701.1251	-0.13894	0.022	0.07034	protein_coding
ENSG0000(	ENSG0000(	268.4821	0.240251	0.022008	0.070358	lncRNA
ENSG0000(	ENSG0000(	68.24842	0.188677	0.022012	0.070365	lncRNA
ENSG0000(	OTUD7A	318.8044	0.150932	0.022017	0.070374	protein_coding
ENSG0000(	ENSG0000(	9.926521	0.521293	0.02202	0.070376	processed_pseudogene
ENSG0000(	SEMA6C	587.7796	-0.40892	0.022036	0.070407	protein_coding
ENSG0000(	SMCR8	2283.907	0.071879	0.022033	0.070407	protein_coding
ENSG0000(	ST3GAL2	2762.122	0.176452	0.022037	0.070407	protein_coding
ENSG0000(	ZFYVE26	3113.485	0.072993	0.022045	0.070426	protein_coding
ENSG0000(	SCARNA1	106.369	-0.22741	0.022055	0.070452	scaRNA
ENSG0000(	GNG12	3086.808	-0.39732	0.022095	0.070573	protein_coding
ENSG0000(	COL23A1	144.3509	0.396223	0.022116	0.070631	protein_coding
ENSG0000(	PLPP4	124.0522	-0.189	0.022131	0.070672	protein_coding
ENSG0000(	RNU7-45P	85.53385	0.286345	0.02214	0.07069	snRNA
ENSG0000(	TYK2	2827.585	0.097879	0.022141	0.07069	protein_coding
ENSG0000(	GP1BA	62.44456	-0.66888	0.022168	0.070763	protein_coding

ENSG0000(	KCNJ5	620.7188	0.1581	0.02217	0.070763	protein_coding
ENSG0000(	ZNF236-DT	81.60687	-0.17415	0.02217	0.070763	lncRNA
ENSG0000(	LIG3	1912.819	0.111275	0.022178	0.070779	protein_coding
ENSG0000(	RMDN1	1301.134	-0.11271	0.022181	0.070784	protein_coding
ENSG0000(	METTL3	1427.61	0.146486	0.022186	0.070791	protein_coding
ENSG0000(	UGT2B15	203.4405	-0.51158	0.022189	0.070794	protein_coding
ENSG0000(	ENSG0000(	16.82703	-0.72783	0.022215	0.070861	unprocessed_pseudogene
ENSG0000(	EP400P1	1846.046	0.165059	0.022214	0.070861	transcribed_unprocessed_pseudo
ENSG0000(	ADRB3	76.65469	0.29763	0.022217	0.070862	protein_coding
ENSG0000(	SLC43A1	175.0528	0.238113	0.022221	0.070867	protein_coding
ENSG0000(	ENSG0000(	15.92743	0.447199	0.022227	0.070874	lncRNA
ENSG0000(	RN7SL670F	421.9441	-0.11526	0.022228	0.070874	misc_RNA
ENSG0000(	DAPK1	2118.541	-0.15107	0.022239	0.070901	protein_coding
ENSG0000(	ENSG0000(	383.5567	0.21163	0.022245	0.070901	lncRNA
ENSG0000(	JCADP1	11.83313	0.513389	0.022245	0.070901	processed_pseudogene
ENSG0000(	MRPL15	241.0733	-0.20647	0.022244	0.070901	protein_coding
ENSG0000(	USP16	1824.379	0.055111	0.022253	0.07092	protein_coding
ENSG0000(	RN7SL834F	236.8434	0.220817	0.022265	0.070949	misc_RNA
ENSG0000(	RPS3AP39	71.8721	-0.51797	0.022272	0.070965	processed_pseudogene
ENSG0000(	CHRNA10	87.98439	0.199995	0.022286	0.071002	protein_coding
ENSG0000(	TWF1	1220.991	-0.21167	0.022335	0.07115	protein_coding
ENSG0000(	SLC27A6	30.2713	-0.59498	0.02234	0.071161	protein_coding
ENSG0000(	ZBTB39	514.4259	0.148336	0.022366	0.071236	protein_coding
ENSG0000(	SREBF2	3021.087	-0.12213	0.022373	0.071251	protein_coding
ENSG0000(	ATG16L1	1124.319	-0.08799	0.022383	0.071267	protein_coding
ENSG0000(	LINC01766	223.7284	-0.12179	0.022382	0.071267	lncRNA
ENSG0000(	ENSG0000(	638.7477	0.401932	0.022396	0.071302	lncRNA
ENSG0000(	CRELD2	631.0398	0.192521	0.022402	0.071315	protein_coding
ENSG0000(	TMEM260	1222.318	0.073222	0.022409	0.07133	protein_coding
ENSG0000(	SLC35G5	21.81644	0.419065	0.022419	0.071355	protein_coding
ENSG0000(	ZCCHC14	2452.94	-0.26791	0.022425	0.071365	protein_coding
ENSG0000(	PABPN1	2893.043	-0.0961	0.022447	0.071429	protein_coding
ENSG0000(	FAM215A	69.59233	-0.23589	0.022454	0.071443	lncRNA
ENSG0000(	UBA6-DT	1465.573	0.110178	0.022476	0.071505	lncRNA
ENSG0000(	LINC01269	40.51079	0.212915	0.022497	0.071566	lncRNA
ENSG0000(	EIF3A	3902.798	-0.0937	0.022501	0.071572	protein_coding
ENSG0000(	EIF4E3	2504.157	-0.23479	0.022504	0.071575	protein_coding
ENSG0000(	YTHDF1P1	16.78953	0.652756	0.022507	0.071576	processed_pseudogene
ENSG0000(	ENSG0000(	108.0858	-0.3515	0.022512	0.071584	lncRNA
ENSG0000(	NIT2	1206.582	0.121988	0.022519	0.071599	protein_coding
ENSG0000(	ENSG0000(	33.52728	0.317877	0.022522	0.0716	lncRNA
ENSG0000(	MIR643	10.33258	0.484843	0.022528	0.0716	miRNA
ENSG0000(	NPR3	81.72347	0.240992	0.022524	0.0716	protein_coding
ENSG0000(	TSPAN8	3033.336	-0.39579	0.022526	0.0716	protein_coding
ENSG0000(	OPRM1	1485.934	0.114059	0.022533	0.071609	protein_coding
ENSG0000(	DUOX1	413.0946	0.144879	0.022545	0.071638	protein_coding
ENSG0000(	ZNF419	1186.398	0.143956	0.022548	0.071641	protein_coding

ENSG0000(	MPHOSPH8	2538.898	-0.10007	0.022555	0.071648	protein_coding
ENSG0000(	TRAPPC5	1354.096	-0.12311	0.022554	0.071648	protein_coding
ENSG0000(	NHSL1	6020.812	-0.40673	0.022561	0.071654	protein_coding
ENSG0000(	PCGEM1	9.75016	-0.4582	0.022561	0.071654	lncRNA
ENSG0000(	ENSG0000(	114.6907	0.140956	0.02257	0.071669	lncRNA
ENSG0000(	HERC2P9	3557.375	0.075485	0.022569	0.071669	transcribed_unprocessed_pseudo
ENSG0000(	LINC01118	111.149	0.136214	0.022584	0.071706	lncRNA
ENSG0000(	LINC01727	698.8793	0.133433	0.022625	0.071827	lncRNA
ENSG0000(	ENSG0000(	620.1657	-0.11018	0.022631	0.071841	lncRNA
ENSG0000(	ENSG0000(	24.77108	0.340761	0.02264	0.071862	lncRNA
ENSG0000(	ELK3	850.2734	0.254566	0.022667	0.071941	protein_coding
ENSG0000(	FNTB	268.8924	0.195174	0.022702	0.072044	protein_coding
ENSG0000(	CHRNE	37.01067	0.371473	0.022712	0.072061	protein_coding
ENSG0000(	OSM	89.16406	2.332258	0.02271	0.072061	protein_coding
ENSG0000(	BAZ1A-AS1	56.99293	0.231955	0.022716	0.072067	lncRNA
ENSG0000(	ENSG0000(	11.45984	0.431572	0.022723	0.072083	lncRNA
ENSG0000(	UBE2E2	212.5747	0.391142	0.022744	0.072141	protein_coding
ENSG0000(	ANKRD35	18.44177	0.372597	0.022762	0.072191	protein_coding
ENSG0000(	FABP7P1	30.64373	0.367957	0.022785	0.072257	processed_pseudogene
ENSG0000(	CAVIN1	809.2311	0.304873	0.022835	0.072408	protein_coding
ENSG0000(	RANBP10	1092.73	-0.10841	0.022852	0.072454	protein_coding
ENSG0000(	ZSCAN9	480.7601	0.134114	0.022859	0.072469	protein_coding
ENSG0000(	ENSG0000(	122.4172	-0.61869	0.022894	0.072556	lncRNA
ENSG0000(	GCSAML	170.5559	0.130712	0.022891	0.072556	protein_coding
ENSG0000(	KLHL3	686.9925	0.245124	0.022896	0.072556	protein_coding
ENSG0000(	ZNF385C	328.0859	0.110931	0.02289	0.072556	protein_coding
ENSG0000(	ENSG0000(	105.8146	-0.66968	0.022901	0.072559	lncRNA
ENSG0000(	ENSG0000(	195.6785	-0.63054	0.0229	0.072559	lncRNA
ENSG0000(	ENSG0000(	65.92717	0.216608	0.022904	0.07256	lncRNA
ENSG0000(	DAGLB	775.4429	-0.11798	0.022908	0.072566	protein_coding
ENSG0000(	ENPP5	109.7891	-0.28031	0.022921	0.072599	protein_coding
ENSG0000(	ANKRD26	2659.005	0.069145	0.022926	0.072609	protein_coding
ENSG0000(	ENSG0000(	9.048221	0.450595	0.022932	0.072614	lncRNA
ENSG0000(	IGHV2-5	140.3211	0.612219	0.022932	0.072614	IG_V_gene
ENSG0000(	G3BP1	6529.016	0.129247	0.022938	0.072616	protein_coding
ENSG0000(	HEXD-IT1	950.8194	0.072408	0.02294	0.072616	lncRNA
ENSG0000(	PIP4K2B	1366.07	0.115946	0.022939	0.072616	protein_coding
ENSG0000(	CPNE5	530.9611	0.47742	0.022945	0.072616	protein_coding
ENSG0000(	ENSG0000(	27.35961	0.427885	0.022942	0.072616	lncRNA
ENSG0000(	ENSG0000(	62.97011	0.192409	0.022947	0.072616	lncRNA
ENSG0000(	NOXA1	206.2189	-0.30914	0.022956	0.072637	protein_coding
ENSG0000(	LINC00563	11.31211	0.57433	0.022968	0.072667	lncRNA
ENSG0000(	ENSG0000(	48.13212	-0.2303	0.022972	0.072671	lncRNA
ENSG0000(	RMND1	1335.7	-0.15056	0.022973	0.072671	protein_coding
ENSG0000(	ENSG0000(	401.6458	0.13865	0.022982	0.07269	lncRNA
ENSG0000(	SNORA8	359.3377	-0.28286	0.022991	0.072713	snoRNA
ENSG0000(	MIR4441	126.1813	0.211326	0.022995	0.072716	miRNA

ENSG0000(	CENPF	1664.031	0.140543	0.023006	0.072743	protein_coding
ENSG0000(	ADPRHL1	296.6526	-0.18425	0.023011	0.072747	protein_coding
ENSG0000(	PIP4K2C	536.1957	-0.1659	0.023009	0.072747	protein_coding
ENSG0000(	ENSG0000(	317.4236	-0.3326	0.023018	0.072759	lncRNA
ENSG0000(	LINC02641	110.5849	0.264361	0.023024	0.072772	lncRNA
ENSG0000(	LINC01644	12.15039	0.578367	0.023029	0.072782	lncRNA
ENSG0000(	CEP78	1451.959	0.176119	0.023048	0.072833	protein_coding
ENSG0000(	PAX4	11.29853	0.440503	0.023057	0.072855	protein_coding
ENSG0000(	NAXD	827.2603	0.103159	0.023066	0.072875	protein_coding
ENSG0000(	DDTL	155.8407	-0.52608	0.023071	0.072883	protein_coding
ENSG0000(	GDPD1	1040.419	-0.11493	0.023082	0.072912	protein_coding
ENSG0000(	LYPD6	105.5688	0.386163	0.023085	0.072912	protein_coding
ENSG0000(	ENSG0000(	25.80175	0.34917	0.023107	0.072969	lncRNA
ENSG0000(	UBE2V2	1026.98	-0.0855	0.023107	0.072969	protein_coding
ENSG0000(	ENSG0000(	211.7889	-0.27212	0.023112	0.072976	lncRNA
ENSG0000(	AP5B1	1625.77	0.118426	0.023123	0.072984	protein_coding
ENSG0000(	ENSG0000(	562.9597	0.131405	0.023117	0.072984	transcribed_processed_pseudoge
ENSG0000(	IMPDH1P5	58.88376	-0.32974	0.023123	0.072984	processed_pseudogene
ENSG0000(	STMN4	45.75499	0.213099	0.02312	0.072984	protein_coding
ENSG0000(	ASB4	321.5984	0.142578	0.023131	0.073001	protein_coding
ENSG0000(	ADIRF-AS1	450.4175	-0.17379	0.023145	0.073038	lncRNA
ENSG0000(	CDRT15P3	14.22217	0.652195	0.02315	0.073047	transcribed_unprocessed_pseudo
ENSG0000(	ENSG0000(	68.66825	-0.29069	0.023153	0.073048	lncRNA
ENSG0000(	KCNIP1-OT	73.54224	0.16221	0.023162	0.07307	lncRNA
ENSG0000(	ENSG0000(	106.8115	0.348237	0.023178	0.073105	TEC
ENSG0000(	GALNS	2047.92	-0.1256	0.023182	0.073105	protein_coding
ENSG0000(	LINC01634	96.21815	-0.19258	0.023182	0.073105	lncRNA
ENSG0000(	SLC27A3	366.6975	0.212047	0.023178	0.073105	protein_coding
ENSG0000(	DENND5B	3593.616	-0.23202	0.023187	0.07311	protein_coding
ENSG0000(	DUSP19	384.3359	-0.12076	0.023209	0.073155	protein_coding
ENSG0000(	ENSG0000(	547.305	0.09748	0.02321	0.073155	lncRNA
ENSG0000(	PLEKHA3	1927.43	-0.09634	0.023207	0.073155	protein_coding
ENSG0000(	SH3D21	1846.806	-0.27282	0.023206	0.073155	protein_coding
ENSG0000(	OLFM1	178.7638	0.40451	0.023238	0.073234	protein_coding
ENSG0000(	ENSG0000(	153.9127	0.20405	0.023255	0.073275	lncRNA
ENSG0000(	ENSG0000(	28.93806	-0.47651	0.023255	0.073275	lncRNA
ENSG0000(	ENSG0000(	34.88432	-0.36341	0.023258	0.073277	lncRNA
ENSG0000(	LZTS3	343.3596	0.267491	0.023269	0.073305	protein_coding
ENSG0000(	CHST7	43.60048	0.308586	0.023293	0.073374	protein_coding
ENSG0000(	MST1P2	120.0303	-0.46234	0.023317	0.073441	unprocessed_pseudogene
ENSG0000(	FLVCR2	1261.807	-0.37329	0.023326	0.073463	protein_coding
ENSG0000(	NRTN	17.45538	0.5996	0.023336	0.073484	protein_coding
ENSG0000(	ENSG0000(	299.7596	0.095534	0.023348	0.073512	lncRNA
ENSG0000(	NEK10	336.0042	0.145918	0.023349	0.073512	protein_coding
ENSG0000(	RBBP8NL	181.4485	-0.44251	0.023351	0.073512	protein_coding
ENSG0000(	ZZZ3	2043.834	-0.08292	0.023354	0.073513	protein_coding
ENSG0000(	RN7SL624F	73.13519	-0.16956	0.023357	0.073515	misc_RNA

ENSG0000(	ATG12P1	11.5134	0.391822	0.02336	0.073516	processed_pseudogene
ENSG0000(	CCR10	14.59395	0.483422	0.023362	0.073516	protein_coding
ENSG0000(	TRAV9-2	11.40448	-0.70727	0.023364	0.073516	TR_V_gene
ENSG0000(	MTCYBP14	14.95149	0.358068	0.023367	0.073518	processed_pseudogene
ENSG0000(	SIK2	2093.068	-0.08038	0.023406	0.073634	protein_coding
ENSG0000(	TOMM22	419.0837	-0.18849	0.023426	0.073689	protein_coding
ENSG0000(	OBI1	338.2073	-0.13946	0.023441	0.073728	protein_coding
ENSG0000(	ENSG0000(	1247.299	-0.15272	0.023446	0.073736	lncRNA
ENSG0000(	INO80C	1284.093	0.092301	0.023461	0.073768	protein_coding
ENSG0000(	KLF16	364.0824	-0.16698	0.023465	0.073768	protein_coding
ENSG0000(	LMNTD1	153.4451	0.138442	0.023465	0.073768	protein_coding
ENSG0000(	NKX2-2	24.44693	0.501886	0.023462	0.073768	protein_coding
ENSG0000(	ENSG0000(	38.9729	-0.45099	0.023484	0.073809	lncRNA
ENSG0000(	ENSG0000(	63.0327	0.222225	0.023486	0.073809	lncRNA
ENSG0000(	SLC25A10	245.9901	-0.33559	0.023485	0.073809	protein_coding
ENSG0000(	LINC01968	160.484	-0.13675	0.023494	0.073821	lncRNA
ENSG0000(	WASF3-AS1	293.1171	0.10969	0.023493	0.073821	lncRNA
ENSG0000(	GABPB1-AS	7066.59	0.19784	0.023497	0.073824	lncRNA
ENSG0000(	LPGAT1	6072.299	-0.29251	0.023505	0.073842	protein_coding
ENSG0000(	MAML1	2033.383	-0.07083	0.023509	0.073847	protein_coding
ENSG0000(	ENSG0000(	785.8406	0.147109	0.02352	0.073874	lncRNA
ENSG0000(	ZNF28	873.8483	-0.17995	0.023568	0.074018	protein_coding
ENSG0000(	OGN	86.82722	-0.5669	0.023589	0.074075	protein_coding
ENSG0000(	ENSG0000(	31.39517	-0.43271	0.023608	0.074128	processed_pseudogene
ENSG0000(	HEYL	129.391	0.289308	0.023612	0.074132	protein_coding
ENSG0000(	ENSG0000(	11.31299	0.399459	0.023617	0.07414	lncRNA
ENSG0000(	ENSG0000(	81.1406	0.293673	0.023628	0.07415	lncRNA
ENSG0000(	INTS8	1832.914	-0.1042	0.023629	0.07415	protein_coding
ENSG0000(	LRRC42	108.7375	-0.18957	0.023625	0.07415	protein_coding
ENSG0000(	MIAT	3682.457	0.263004	0.023625	0.07415	lncRNA
ENSG0000(	ENSG0000(	196.6184	-0.13075	0.023639	0.074174	TEC
ENSG0000(	RBP7	75.30555	-0.19498	0.023665	0.074247	protein_coding
ENSG0000(	LINC00261	617.2192	0.327376	0.02367	0.074255	lncRNA
ENSG0000(	BEND6	217.3415	0.153461	0.023695	0.074327	protein_coding
ENSG0000(	TIMP2	2280.898	0.217916	0.023698	0.074329	protein_coding
ENSG0000(	RPIA	249.2376	-0.13988	0.023709	0.074355	protein_coding
ENSG0000(	DBIL5P2	78.53328	0.187229	0.02374	0.074446	transcribed_processed_pseudoge
ENSG0000(	DNA2	484.6473	-0.17264	0.023747	0.074451	protein_coding
ENSG0000(	RN7SL735P	83.04326	-0.18033	0.023746	0.074451	misc_RNA
ENSG0000(	ENSG0000(	22.5311	-0.35875	0.023754	0.074466	lncRNA
ENSG0000(	CISD2	571.4186	-0.11209	0.023756	0.074467	protein_coding
ENSG0000(	ZSCAN5A	648.0508	0.095841	0.023774	0.074516	protein_coding
ENSG0000(	ENSG0000(	158.6112	0.134994	0.023782	0.074533	lncRNA
ENSG0000(	DNAJC17	888.1054	-0.13923	0.023791	0.074555	protein_coding
ENSG0000(	ENSG0000(	85.62388	0.198655	0.023833	0.074671	lncRNA
ENSG0000(	ENSG0000(	81.26652	-0.1908	0.023832	0.074671	lncRNA
ENSG0000(	RPS6KA5	6420.419	-0.1386	0.023837	0.074674	protein_coding

ENSG0000(	EFEMP2	325.0975	0.236841	0.02385	0.074708	protein_coding
ENSG0000(	MYORG	3225.995	-0.23897	0.023856	0.074713	protein_coding
ENSG0000(	SLC25A15	1206.116	-0.2693	0.023855	0.074713	protein_coding
ENSG0000(	CTDSP2	4909.976	-0.10743	0.023859	0.074716	protein_coding
ENSG0000(	NPY6R	746.3162	-0.37965	0.023864	0.074722	transcribed_unitary_pseudogene
ENSG0000(	S100Z	31.78288	-0.43311	0.023871	0.07473	protein_coding
ENSG0000(	TMEM229/	118.7689	-0.64708	0.023871	0.07473	protein_coding
ENSG0000(	ENSG0000(	41.72836	-0.44889	0.023885	0.074767	processed_pseudogene
ENSG0000(	XKR5	139.0427	0.139808	0.023924	0.07488	protein_coding
ENSG0000(	ATP1B1	9539.926	-0.38618	0.023931	0.074894	protein_coding
ENSG0000(	UBE2L3	832.0126	-0.14365	0.023933	0.074894	protein_coding
ENSG0000(	ENSG0000(	308.3173	0.167882	0.02394	0.07491	lncRNA
ENSG0000(	AFAP1L1	533.8885	0.221702	0.023967	0.074985	protein_coding
ENSG0000(	SUPT4H1	376.8256	-0.15535	0.023971	0.074988	protein_coding
ENSG0000(	SURF2	46.66494	-0.2777	0.023973	0.074988	protein_coding
ENSG0000(	NOC4L	116.3859	-0.18293	0.024	0.075068	protein_coding
ENSG0000(	ENSG0000(	505.0283	0.108858	0.024018	0.075115	lncRNA
ENSG0000(	MGST2	631.8273	-0.33081	0.024037	0.075168	protein_coding
ENSG0000(	IDI2-AS1	95.44843	-0.17328	0.024047	0.075191	lncRNA
ENSG0000(	MAP2K7	1115.978	-0.08781	0.024054	0.075205	protein_coding
ENSG0000(	CA3-AS1	10.95007	0.536679	0.024073	0.075246	lncRNA
ENSG0000(	CYLC2	27.80942	-0.43156	0.024074	0.075246	protein_coding
ENSG0000(	ENSG0000(	237.6976	-0.11841	0.024071	0.075246	lncRNA
ENSG0000(	ENSG0000(	37.27887	0.312904	0.024081	0.075259	lncRNA
ENSG0000(	ENSG0000(	144.159	0.151775	0.024099	0.075309	TEC
ENSG0000(	LINC02065	32.63643	0.265326	0.024111	0.075338	lncRNA
ENSG0000(	ENSG0000(	230.123	0.100951	0.024122	0.075366	lncRNA
ENSG0000(	SLC25A39	865.0266	-0.13297	0.024131	0.075385	protein_coding
ENSG0000(	ENSG0000(	4644.419	-0.10459	0.02414	0.075406	TEC
ENSG0000(	ENSG0000(	78.96331	-0.20103	0.024151	0.07543	lncRNA
ENSG0000(	RPSAP16	115.7206	-0.21574	0.024152	0.07543	processed_pseudogene
ENSG0000(	ENSG0000(	177.261	0.191552	0.024167	0.075468	lncRNA
ENSG0000(	LINC02750	26.151	0.272028	0.024171	0.075473	lncRNA
ENSG0000(	ABCD1	743.6756	-0.33649	0.024179	0.075491	protein_coding
ENSG0000(	ACTR1B	838.9792	-0.10405	0.02419	0.07551	protein_coding
ENSG0000(	ENSG0000(	336.1457	0.112864	0.024188	0.07551	lncRNA
ENSG0000(	SERPINB10	202.6424	0.161019	0.024234	0.075639	protein_coding
ENSG0000(	ENSG0000(	55.54598	0.250386	0.024236	0.07564	TEC
ENSG0000(	PUF60	631.2132	-0.12699	0.024245	0.07566	protein_coding
ENSG0000(	DBF4B	693.9396	0.12446	0.02425	0.075668	protein_coding
ENSG0000(	MAGOHB	473.1357	0.133326	0.024255	0.075676	protein_coding
ENSG0000(	ENSG0000(	223.476	0.19589	0.024268	0.075708	lncRNA
ENSG0000(	GREM1	7477.547	0.565583	0.024271	0.075712	protein_coding
ENSG0000(	ENSG0000(	167.2687	0.4361	0.024281	0.075736	lncRNA
ENSG0000(	GADD45G	68.18552	0.351614	0.024313	0.075818	protein_coding
ENSG0000(	L3MBTL1	1822.468	-0.0876	0.024312	0.075818	protein_coding
ENSG0000(	KRTAP5-11	64.50589	0.211821	0.024335	0.07588	protein_coding

ENSG0000(	CES4A	482.3456	0.130636	0.024339	0.075886	protein_coding
ENSG0000(	MYO15A	424.0185	0.094442	0.024342	0.075887	protein_coding
ENSG0000(	GRINA	1032.569	0.212535	0.024355	0.075918	protein_coding
ENSG0000(	RN7SKP104	44.66635	-0.53118	0.024357	0.075918	misc_RNA
ENSG0000(	CEP70	482.159	0.206537	0.024365	0.075937	protein_coding
ENSG0000(	GRK7	160.4144	0.129718	0.02438	0.075976	protein_coding
ENSG0000(	ARRDC5	292.2807	0.414727	0.024403	0.076033	protein_coding
ENSG0000(	ENSG0000(	13.66049	0.471172	0.024403	0.076033	lncRNA
ENSG0000(	RAB5C	943.548	-0.14613	0.024433	0.076119	protein_coding
ENSG0000(	ENSG0000(	20.84403	-0.33481	0.024445	0.076141	lncRNA
ENSG0000(	SNTG1	123.1039	-0.19534	0.024445	0.076141	protein_coding
ENSG0000(	DHX58	403.1492	0.258254	0.024452	0.076154	protein_coding
ENSG0000(	DPH6	790.1065	0.0913	0.024454	0.076154	protein_coding
ENSG0000(	ZNF607	424.4872	-0.19805	0.024463	0.076173	protein_coding
ENSG0000(	C12orf76	475.8726	-0.12247	0.024517	0.076334	protein_coding
ENSG0000(	ENSG0000(	135.4208	-0.15684	0.024528	0.076362	lncRNA
ENSG0000(	ENSG0000(	254.259	0.253816	0.024559	0.076451	lncRNA
ENSG0000(	SGPL1	3061.422	-0.17105	0.024571	0.076482	protein_coding
ENSG0000(	ENSG0000(	97.35557	0.241183	0.024575	0.076486	lncRNA
ENSG0000(	ENSG0000(	594.777	0.210257	0.024585	0.07651	protein_coding
ENSG0000(	BCS1L	462.0017	-0.12692	0.024615	0.076596	protein_coding
ENSG0000(	WFDC8	100.0936	-0.13366	0.024637	0.076656	protein_coding
ENSG0000(	ENSG0000(	22.81833	0.284661	0.024649	0.076684	lncRNA
ENSG0000(	CARS1	2251.172	0.102944	0.02466	0.076713	protein_coding
ENSG0000(	ENSG0000(	28.08868	0.258998	0.024666	0.076723	lncRNA
ENSG0000(	LRTM1	214.2402	0.366703	0.024668	0.076723	protein_coding
ENSG0000(	CHMP3	1784.01	-0.09887	0.024676	0.076731	protein_coding
ENSG0000(	NXPE3	1730.261	0.18449	0.024675	0.076731	protein_coding
ENSG0000(	MAJIN	13.22693	0.753205	0.024698	0.076791	protein_coding
ENSG0000(	LINC01091	533.9203	-0.12045	0.024712	0.076829	lncRNA
ENSG0000(	LINC02177	577.2766	-0.42592	0.02474	0.076908	lncRNA
ENSG0000(	JOSD1	1744.492	-0.22769	0.024747	0.07692	protein_coding
ENSG0000(	ENSG0000(	133.6173	0.231995	0.024772	0.076991	transcribed_unprocessed_pseudo
ENSG0000(	ENSG0000(	147.4487	0.408988	0.024774	0.076991	lncRNA
ENSG0000(	ENSG0000(	114.1991	0.178921	0.024784	0.077015	lncRNA
ENSG0000(	WDSUB1	204.6153	-0.15988	0.024797	0.077047	protein_coding
ENSG0000(	MCM8	1250.23	-0.10292	0.024801	0.077052	protein_coding
ENSG0000(	MAD2L1P1	22.73515	0.333347	0.024807	0.077063	processed_pseudogene
ENSG0000(	IMMTP1	17.03667	-0.55607	0.02481	0.077063	processed_pseudogene
ENSG0000(	H3C13	48.14166	-0.46679	0.024816	0.077067	protein_coding
ENSG0000(	RNU1-63P	15.64829	0.350996	0.024815	0.077067	snRNA
ENSG0000(	SUCLG2-AS	1737.132	0.079876	0.02482	0.077073	lncRNA
ENSG0000(	PPP1R16A	1479.791	-0.28136	0.024834	0.077109	protein_coding
ENSG0000(	ENSG0000(	97.4813	-0.17488	0.024839	0.077118	lncRNA
ENSG0000(	TNFSF13B	492.2414	0.337575	0.02485	0.077142	protein_coding
ENSG0000(	PSMD10P2	128.9303	-0.21104	0.024854	0.077148	transcribed_processed_pseudoge
ENSG0000(	ZNF878	12.84879	0.383443	0.02486	0.077158	protein_coding

ENSG0000(	CDK8	551.6058	-0.21121	0.024872	0.077182	protein_coding
ENSG0000(	COPS2	1149.128	-0.10834	0.024871	0.077182	protein_coding
ENSG0000(	BTNL8	2529.751	-0.50458	0.024905	0.077276	protein_coding
ENSG0000(	GRAMD2A	249.3015	0.256196	0.024931	0.077335	protein_coding
ENSG0000(	H3-3A-DT	96.5953	0.227158	0.024931	0.077335	lncRNA
ENSG0000(	TIMM29	288.9162	-0.11355	0.024932	0.077335	protein_coding
ENSG0000(	CD38	2150.985	0.411208	0.024945	0.077361	protein_coding
ENSG0000(	MFF	1227.237	-0.0815	0.024944	0.077361	protein_coding
ENSG0000(	TIAF1	1584.323	0.125345	0.024948	0.077364	protein_coding
ENSG0000(	C7orf50	402.6452	0.163035	0.024964	0.077404	protein_coding
ENSG0000(	ENSG0000(	103.5134	-0.2904	0.024973	0.077425	transcribed_unprocessed_pseudo
ENSG0000(	LINC02078	685.5206	0.111358	0.024975	0.077425	lncRNA
ENSG0000(	BNIPL	274.8572	-0.15486	0.02499	0.077465	protein_coding
ENSG0000(	CCZ1B	936.9321	-0.10955	0.025017	0.07754	protein_coding
ENSG0000(	CRX	520.6223	0.094566	0.025022	0.077547	protein_coding
ENSG0000(	ENSG0000(	30.94782	0.281413	0.025026	0.077552	lncRNA
ENSG0000(	ENSG0000(	46.46173	0.262957	0.025037	0.077578	TEC
ENSG0000(	ENSG0000(	412.2219	-0.08983	0.02504	0.07758	lncRNA
ENSG0000(	ANTXR2	2084.169	0.136941	0.025086	0.077715	protein_coding
ENSG0000(	RRM2	1252.438	0.183558	0.02511	0.077782	protein_coding
ENSG0000(	ENSG0000(	124.0907	0.15134	0.025115	0.077789	lncRNA
ENSG0000(	PLCG1	3150.645	-0.1643	0.025127	0.07782	protein_coding
ENSG0000(	ENSG0000(	12.99363	0.488875	0.025137	0.07784	lncRNA
ENSG0000(	PDE1C	799.7139	0.189973	0.025141	0.07784	protein_coding
ENSG0000(	TTYH3	2122.885	0.145713	0.02514	0.07784	protein_coding
ENSG0000(	IL1RAPL1	41.32415	-0.31096	0.025157	0.077882	protein_coding
ENSG0000(	ENSG0000(	24.79127	0.35252	0.025168	0.077901	lncRNA
ENSG0000(	LCT	607.2067	-0.98975	0.025166	0.077901	protein_coding
ENSG0000(	CDC42BPG	1634.759	-0.38754	0.025173	0.077907	protein_coding
ENSG0000(	ENSG0000(	367.2564	0.48238	0.025218	0.078039	lncRNA
ENSG0000(	CENATAC	1848.994	0.081167	0.025227	0.078052	protein_coding
ENSG0000(	UBE4A	3195.877	-0.0902	0.025227	0.078052	protein_coding
ENSG0000(	ACTG1P1	18.87529	0.344859	0.025238	0.078071	processed_pseudogene
ENSG0000(	NENFP1	21.57652	0.300435	0.025238	0.078071	processed_pseudogene
ENSG0000(	AACSP1	80.81496	0.227985	0.025241	0.078074	transcribed_unprocessed_pseudo
ENSG0000(	STK38	2760.025	-0.14263	0.02527	0.078154	protein_coding
ENSG0000(	SNORA40	14.59699	0.954773	0.025277	0.078167	snoRNA
ENSG0000(	DDX59-AS1	169.1808	-0.17956	0.025292	0.078193	lncRNA
ENSG0000(	EVI5	1836.728	-0.14557	0.025291	0.078193	protein_coding
ENSG0000(	SYT14	371.8178	0.205372	0.025293	0.078193	protein_coding
ENSG0000(	OCA2	18.92268	-0.51915	0.025308	0.078234	protein_coding
ENSG0000(	ENSG0000(	355.3911	-0.68758	0.025314	0.078245	lncRNA
ENSG0000(	CYP3A4	4337.389	-0.76338	0.025326	0.078267	protein_coding
ENSG0000(	RNF187	867.556	-0.10674	0.025326	0.078267	protein_coding
ENSG0000(	EIF5A2	232.3829	-0.21187	0.025353	0.078342	protein_coding
ENSG0000(	ASTN2	5521.763	-0.11692	0.02537	0.078386	protein_coding
ENSG0000(	TRAM2-AS:	525.2268	0.07873	0.025397	0.078461	lncRNA

ENSG0000(	CALM3	2627.543	-0.2115	0.025402	0.078461	protein_coding
ENSG0000(	ENSG0000(	18.76682	-0.31347	0.025401	0.078461	lncRNA
ENSG0000(	ENSG0000(	436.8786	0.19627	0.025432	0.078548	transcribed_unprocessed_pseudo
ENSG0000(	ENSG0000(	20.81569	-0.35991	0.02544	0.078565	processed_pseudogene
ENSG0000(	DYNLRB1	563.6731	-0.17405	0.025453	0.078595	protein_coding
ENSG0000(	ENSG0000(	50.47438	-0.21556	0.025463	0.07862	transcribed_unprocessed_pseudo
ENSG0000(	GPR160P1	111.1932	-0.22042	0.025473	0.078643	processed_pseudogene
ENSG0000(	RAB7B	114.1244	0.288425	0.025486	0.078675	protein_coding
ENSG0000(	SPAST	1566.934	-0.07966	0.025495	0.078696	protein_coding
ENSG0000(	CXADR	2515.844	-0.47332	0.025498	0.078697	protein_coding
ENSG0000(	ENSG0000(	118.6849	0.170449	0.025515	0.078734	lncRNA
ENSG0000(	SMURF2P1	126.1834	-0.1563	0.025514	0.078734	transcribed_unprocessed_pseudo
ENSG0000(	PREB	715.7969	0.177545	0.025521	0.078745	protein_coding
ENSG0000(	LIF	448.4528	0.221537	0.025525	0.078749	protein_coding
ENSG0000(	ENSG0000(	20.80926	0.392807	0.025545	0.078803	lncRNA
ENSG0000(	TVP23C	2685.624	0.103325	0.02555	0.078811	protein_coding
ENSG0000(	ARRDC4	1517.376	-0.27793	0.02556	0.078833	protein_coding
ENSG0000(	ADAM20P1	133.6246	0.261586	0.025567	0.078848	transcribed_unprocessed_pseudo
ENSG0000(	CAMSAP3	863.9173	-0.40122	0.025594	0.078922	protein_coding
ENSG0000(	MROH7	1920.624	-0.33469	0.025596	0.078922	protein_coding
ENSG0000(	GAPDHP62	40.88619	-0.35886	0.025607	0.078949	processed_pseudogene
ENSG0000(	ENSG0000(	151.4976	0.132671	0.025628	0.079005	lncRNA
ENSG0000(	ENSG0000(	65.03265	0.381093	0.025633	0.079009	TEC
ENSG0000(	SPECC1	3701.761	0.114973	0.025634	0.079009	protein_coding
ENSG0000(	SLC17A5	688.8409	-0.21154	0.02565	0.079049	protein_coding
ENSG0000(	GABPB1	424.9449	-0.12164	0.025657	0.079063	protein_coding
ENSG0000(	OLFML3	157.598	0.240141	0.025659	0.079063	protein_coding
ENSG0000(	AICDA	246.115	-0.21037	0.025676	0.079106	protein_coding
ENSG0000(	ENSG0000(	121.6203	0.178058	0.02569	0.079143	lncRNA
ENSG0000(	ARHGAP15	18.53086	-0.62507	0.025708	0.07919	lncRNA
ENSG0000(	LSM4	401.7831	-0.18066	0.025711	0.079193	protein_coding
ENSG0000(	RPL5P34	11.57659	0.501855	0.025723	0.07922	processed_pseudogene
ENSG0000(	ARHGAP11	430.9727	0.2641	0.025739	0.079261	protein_coding
ENSG0000(	GK-IT1	203.1971	0.353033	0.025751	0.07929	lncRNA
ENSG0000(	GCKR	46.62297	0.211275	0.025757	0.079304	protein_coding
ENSG0000(	ENSG0000(	85.88366	0.209121	0.025772	0.079342	lncRNA
ENSG0000(	GULP1	859.2908	-0.30322	0.025786	0.079375	protein_coding
ENSG0000(	IGFBPL1	171.9393	0.141764	0.02579	0.079381	protein_coding
ENSG0000(	FAM83G	1172.891	-0.2969	0.02581	0.079417	protein_coding
ENSG0000(	GNPTAB	2046.85	0.118913	0.025809	0.079417	protein_coding
ENSG0000(	PPIAL4G	22.54332	0.47483	0.025805	0.079417	protein_coding
ENSG0000(	EBF2	74.5288	0.238329	0.025824	0.079441	protein_coding
ENSG0000(	ENSG0000(	64.322	0.254472	0.02583	0.079441	lncRNA
ENSG0000(	ENSG0000(	110.3357	0.237789	0.025828	0.079441	lncRNA
ENSG0000(	ENSG0000(	130.4773	0.191652	0.02583	0.079441	lncRNA
ENSG0000(	USP30	586.5973	-0.20297	0.025824	0.079441	protein_coding
ENSG0000(	ARGFXP2	71.33284	-0.14933	0.025873	0.079559	processed_pseudogene

ENSG0000(	CENPO	454.8998	0.159607	0.025871	0.079559	protein_coding
ENSG0000(	EGFEM1P	97.26156	0.218217	0.025879	0.079568	transcribed_unitary_pseudogene
ENSG0000(	ACADVL	5832.245	-0.24515	0.025883	0.079573	protein_coding
ENSG0000(	FAM227A	973.3635	0.086201	0.025896	0.079607	protein_coding
ENSG0000(	FBXO45	616.6123	-0.10032	0.025912	0.079648	protein_coding
ENSG0000(	DNAJC4	438.7547	-0.10945	0.025948	0.079749	protein_coding
ENSG0000(	RYR3	572.2116	0.462088	0.025982	0.079845	protein_coding
ENSG0000(	ENSG0000(	100.3673	0.196058	0.025986	0.079849	lncRNA
ENSG0000(	MTATP6P1	1663.748	-0.40057	0.025988	0.079849	unprocessed_pseudogene
ENSG0000(	NSG1	237.0239	-0.24612	0.025993	0.079856	protein_coding
ENSG0000(	PRAMEF9	36.88083	0.250871	0.025998	0.079863	protein_coding
ENSG0000(	NIPAL2	1822.489	-0.19532	0.026002	0.079868	protein_coding
ENSG0000(	SNX20	689.2071	0.393095	0.02601	0.079885	protein_coding
ENSG0000(	TRIOBP	1627.994	-0.10236	0.026021	0.079911	protein_coding
ENSG0000(	FUNDC2P4	14.29602	0.57052	0.02603	0.079931	processed_pseudogene
ENSG0000(	ENSG0000(	18.3725	0.33581	0.02604	0.079956	lncRNA
ENSG0000(	ARFRP1	1879.661	-0.08994	0.026051	0.07997	protein_coding
ENSG0000(	ARMCX4	1356.598	-0.13048	0.026052	0.07997	protein_coding
ENSG0000(	THOC7	239.5659	-0.16354	0.026053	0.07997	protein_coding
ENSG0000(	ENSG0000(	27.02711	0.295567	0.02606	0.079978	lncRNA
ENSG0000(	ENSG0000(	220.3809	0.138095	0.026059	0.079978	lncRNA
ENSG0000(	ENSG0000(	119.799	0.130132	0.026087	0.080048	lncRNA
ENSG0000(	ZNF33B	2325.38	-0.14179	0.026088	0.080048	protein_coding
ENSG0000(	ENSG0000(	15.42629	0.643397	0.026099	0.080072	protein_coding
ENSG0000(	NKILA	34.49812	0.362516	0.026106	0.080087	lncRNA
ENSG0000(	CHMP6	314.8718	-0.12782	0.026125	0.080137	protein_coding
ENSG0000(	ENSG0000(	101.0017	0.164962	0.026136	0.080162	lncRNA
ENSG0000(	ANKRD34C	264.8093	0.156079	0.026139	0.080165	lncRNA
ENSG0000(	CPN2	103.888	0.253373	0.026151	0.080195	protein_coding
ENSG0000(	ENSG0000(	111.8505	0.188785	0.026174	0.080257	lncRNA
ENSG0000(	MLX	1483.422	-0.17247	0.026191	0.080286	protein_coding
ENSG0000(	POLD1	345.98	-0.21453	0.026189	0.080286	protein_coding
ENSG0000(	TAF6	970.842	-0.11351	0.026187	0.080286	protein_coding
ENSG0000(	ENSG0000(	64.90254	-0.18768	0.026203	0.080314	lncRNA
ENSG0000(	ENSG0000(	16.82501	-0.88209	0.02621	0.080329	lncRNA
ENSG0000(	UBR5	6009.087	0.072664	0.026215	0.080336	protein_coding
ENSG0000(	ENSG0000(	222.0642	0.142671	0.026232	0.080379	lncRNA
ENSG0000(	AKR1C3	1231.217	-0.41242	0.026235	0.080381	protein_coding
ENSG0000(	CMPK1	2422.643	-0.22986	0.026244	0.080394	protein_coding
ENSG0000(	FCRL6	76.58704	-0.36169	0.026243	0.080394	protein_coding
ENSG0000(	JPX	7099.248	0.111879	0.026252	0.080409	lncRNA
ENSG0000(	EHD3	279.101	0.321378	0.026259	0.080417	protein_coding
ENSG0000(	ENSG0000(	115.2527	-0.16143	0.026259	0.080417	IG_V_pseudogene
ENSG0000(	ERBB2	4430.67	-0.33565	0.02627	0.080442	protein_coding
ENSG0000(	FLNB	17929.25	-0.21668	0.026287	0.080485	protein_coding
ENSG0000(	ENSG0000(	121.2866	0.2209	0.026306	0.080536	lncRNA
ENSG0000(	ENSG0000(	78.81394	0.201092	0.026309	0.080536	lncRNA

ENSG0000(	ENSG0000(	41.72788	0.493796	0.026318	0.080556	lncRNA
ENSG0000(	ENSG0000(	380.8017	0.125443	0.02637	0.080705	lncRNA
ENSG0000(	SLC43A2	2176.041	0.225112	0.026372	0.080705	protein_coding
ENSG0000(	ENSG0000(	118.385	-0.19211	0.026377	0.080715	lncRNA
ENSG0000(	ENSG0000(	11.29294	0.395255	0.026394	0.080758	lncRNA
ENSG0000(	RNF169	2453.407	0.080639	0.026398	0.080762	protein_coding
ENSG0000(	ENSG0000(	186.1467	0.157544	0.026425	0.080838	lncRNA
ENSG0000(	PPDPF	666.9251	-0.30424	0.026442	0.080882	protein_coding
ENSG0000(	CSNK1G1	1648.238	0.106361	0.026449	0.080892	protein_coding
ENSG0000(	KLRA1P	145.187	0.162051	0.026451	0.080892	transcribed_unprocessed_pseudo
ENSG0000(	ENSG0000(	41.86372	0.280091	0.026469	0.080942	lncRNA
ENSG0000(	NGRN	800.7963	-0.12988	0.026473	0.080945	protein_coding
ENSG0000(	ABI1	1599.116	-0.09371	0.026487	0.080981	protein_coding
ENSG0000(	COASY	797.1889	-0.11415	0.026492	0.080988	protein_coding
ENSG0000(	MREG	734.5882	-0.16796	0.026497	0.080996	protein_coding
ENSG0000(	AURKB	206.0578	0.21058	0.0265	0.080996	protein_coding
ENSG0000(	ENSG0000(	94.51498	0.162773	0.026503	0.080998	lncRNA
ENSG0000(	SPRY4	1058.146	0.33095	0.026506	0.080998	protein_coding
ENSG0000(	LINC02323	105.6738	-0.43469	0.026512	0.081001	lncRNA
ENSG0000(	RAD23B	1784.947	-0.0984	0.026511	0.081001	protein_coding
ENSG0000(	ATP6V1G1	862.5692	-0.09706	0.02652	0.081012	protein_coding
ENSG0000(	NXNL1	18.36617	0.330785	0.026521	0.081012	protein_coding
ENSG0000(	BLOC1S6P1	396.9926	0.115282	0.026545	0.08108	processed_pseudogene
ENSG0000(	ENSG0000(	43.31638	0.253559	0.026552	0.081086	lncRNA
ENSG0000(	ENSG0000(	10.14353	0.431702	0.026552	0.081086	lncRNA
ENSG0000(	LINC02306	229.3508	0.228927	0.026575	0.081146	lncRNA
ENSG0000(	CD300LF	146.6427	0.366165	0.026598	0.081208	protein_coding
ENSG0000(	NUDT15	448.3129	-0.14572	0.026607	0.081227	protein_coding
ENSG0000(	AXIN2	571.9365	0.278422	0.026631	0.081293	protein_coding
ENSG0000(	ENSG0000(	42.97788	0.306854	0.026636	0.0813	lncRNA
ENSG0000(	INE1	534.9444	0.0972	0.026639	0.081303	lncRNA
ENSG0000(	CKS1B	260.798	-0.18526	0.026642	0.081305	protein_coding
ENSG0000(	FOXD2	17.55301	0.585605	0.02666	0.081335	protein_coding
ENSG0000(	MATN3	16.28085	-0.39043	0.026662	0.081335	protein_coding
ENSG0000(	POU2AF1	2550.323	0.449042	0.026658	0.081335	protein_coding
ENSG0000(	SHPK	1466.989	-0.2419	0.02666	0.081335	protein_coding
ENSG0000(	ENSG0000(	12.59503	-0.74165	0.026687	0.081394	unprocessed_pseudogene
ENSG0000(	LPIN3	2135.158	-0.32418	0.026686	0.081394	protein_coding
ENSG0000(	ENSG0000(	8.30015	0.562646	0.026711	0.081457	lncRNA
ENSG0000(	RPS15AP29	149.0442	0.181552	0.026713	0.081457	processed_pseudogene
ENSG0000(	ENSG0000(	130.0953	0.482269	0.026718	0.081464	protein_coding
ENSG0000(	PTPRG	2261.209	0.225272	0.026725	0.081479	protein_coding
ENSG0000(	ENSG0000(	17.64551	0.398984	0.026746	0.081522	lncRNA
ENSG0000(	PAMR1	91.03739	-0.38758	0.026742	0.081522	protein_coding
ENSG0000(	STIMATE-M	362.1245	0.30342	0.026747	0.081522	protein_coding
ENSG0000(	ABLIM3	435.9359	0.263686	0.026761	0.081558	protein_coding
ENSG0000(	CDC42	7079.287	-0.09655	0.026765	0.081561	protein_coding

ENSG0000(	ENSG0000(	89.99383	0.208555	0.02678	0.081592	lncRNA
ENSG0000(	ROCR	72.65525	0.307072	0.026779	0.081592	lncRNA
ENSG0000(	B4GALT7	401.2178	0.1398	0.026794	0.081613	protein_coding
ENSG0000(	ETAA1	638.3433	0.116005	0.026791	0.081613	protein_coding
ENSG0000(	WHAMMP2	1224.063	0.116797	0.026795	0.081613	transcribed_unprocessed_pseudo
ENSG0000(	EPHA6	619.4846	-0.13656	0.026799	0.081616	protein_coding
ENSG0000(	SLCO4A1	165.3843	0.278761	0.026809	0.08164	protein_coding
ENSG0000(	P2RY1	1154.87	-0.37037	0.026817	0.081655	protein_coding
ENSG0000(	ENSG0000(	48.92472	0.35101	0.026835	0.081703	lncRNA
ENSG0000(	ENSG0000(	227.6826	-0.1242	0.026867	0.081791	lncRNA
ENSG0000(	MTND4P35	16.40053	-0.57537	0.026881	0.081828	processed_pseudogene
ENSG0000(	ENSG0000(	60.49598	0.235719	0.026908	0.0819	transcribed_processed_pseudoge
ENSG0000(	MTIF2	717.3202	-0.09996	0.026941	0.081995	protein_coding
ENSG0000(	TMEM129	570.3848	-0.11939	0.026944	0.081996	protein_coding
ENSG0000(	STIMATE	640.1943	0.252254	0.026966	0.082054	protein_coding
ENSG0000(	RSAD1	664.5684	-0.11165	0.026995	0.082134	protein_coding
ENSG0000(	TTTY10	9624.989	-0.08972	0.027023	0.082213	lncRNA
ENSG0000(	CLK3	3123.744	-0.08762	0.027039	0.082247	protein_coding
ENSG0000(	PCAT19	637.3217	0.130724	0.027042	0.082247	lncRNA
ENSG0000(	SDHAP1	1427.535	-0.25777	0.027041	0.082247	transcribed_unprocessed_pseudo
ENSG0000(	DDI2	4053.412	-0.08361	0.027069	0.082321	protein_coding
ENSG0000(	SDS	254.9196	0.115996	0.027073	0.082325	protein_coding
ENSG0000(	RNF214	618.4476	-0.10429	0.027083	0.082342	protein_coding
ENSG0000(	RUSF1	1096.606	-0.11446	0.027084	0.082342	protein_coding
ENSG0000(	ENSG0000(	90.04324	0.178292	0.027113	0.082422	lncRNA
ENSG0000(	SLC12A9	2139.103	0.126557	0.027121	0.082434	protein_coding
ENSG0000(	TRIM17	42.64893	0.3358	0.027122	0.082434	protein_coding
ENSG0000(	GRTP1	784.4756	-0.3155	0.027138	0.082471	protein_coding
ENSG0000(	SLC16A14	120.0365	0.315433	0.027139	0.082471	protein_coding
ENSG0000(	CATIP-AS2	24.20185	-0.34109	0.027146	0.082481	lncRNA
ENSG0000(	ENSG0000(	449.7212	0.127727	0.027164	0.082528	lncRNA
ENSG0000(	LINC02263	237.896	0.143098	0.027187	0.082592	lncRNA
ENSG0000(	HLA-DPB2	120.4823	0.238882	0.027191	0.082595	transcribed_unprocessed_pseudo
ENSG0000(	RALGPS2-A	36.53884	0.363242	0.027217	0.082666	lncRNA
ENSG0000(	LSG1	1130.839	-0.12759	0.027231	0.082692	protein_coding
ENSG0000(	PLEC	15080.68	-0.21613	0.027231	0.082692	protein_coding
ENSG0000(	C1orf109	337.6771	-0.12406	0.027244	0.08272	protein_coding
ENSG0000(	IGHJ2P	9.537654	0.608383	0.027245	0.08272	IG_J_pseudogene
ENSG0000(	SNRPC	227.0403	-0.14402	0.027251	0.082731	protein_coding
ENSG0000(	SNHG32	472.2561	-0.19188	0.027255	0.082733	lncRNA
ENSG0000(	RANBP6	853.043	0.174749	0.027258	0.082735	protein_coding
ENSG0000(	OR6C74	1275.095	0.141188	0.027293	0.082832	protein_coding
ENSG0000(	PRPSAP1	1434.037	-0.08273	0.027306	0.082866	protein_coding
ENSG0000(	TRERF1	1067.822	0.178336	0.027315	0.082885	protein_coding
ENSG0000(	ENSG0000(	90.95932	0.182522	0.027335	0.082928	lncRNA
ENSG0000(	ENSG0000(	893.2974	-0.23975	0.027332	0.082928	lncRNA
ENSG0000(	GPR161	469.1687	0.263064	0.027348	0.082949	protein_coding

ENSG0000(	KCNMB4	189.7121	-0.18567	0.02735	0.082949	protein_coding
ENSG0000(	LINC00996	550.6695	-0.20372	0.027349	0.082949	lncRNA
ENSG0000(	CLVS2	12.82064	0.508684	0.027361	0.082975	protein_coding
ENSG0000(	TRAPPC2L	252.3776	-0.12982	0.027364	0.082977	protein_coding
ENSG0000(	TLE5	2188.271	-0.1251	0.027371	0.082989	protein_coding
ENSG0000(	LRCH4	2074.465	-0.1314	0.027379	0.083006	protein_coding
ENSG0000(	FAHD1	448.6224	-0.24933	0.027417	0.083112	protein_coding
ENSG0000(	RN7SKP189	20.14736	-0.38969	0.027423	0.083125	misc_RNA
ENSG0000(	ENSG0000(	115.5016	0.210775	0.027508	0.083371	protein_coding
ENSG0000(	ENSG0000(	800.9142	0.429886	0.02751	0.083371	lncRNA
ENSG0000(	SPDYE21	7.951169	0.723621	0.027514	0.083374	protein_coding
ENSG0000(	ENSG0000(	175.6944	-0.12603	0.027528	0.083409	TEC
ENSG0000(	EXD2	483.3798	0.130808	0.027539	0.083437	protein_coding
ENSG0000(	GMPR	87.1533	0.195294	0.027545	0.083444	protein_coding
ENSG0000(	IGHV1-69-2	21.37745	1.360456	0.027547	0.083444	IG_V_gene
ENSG0000(	LINC02291	59.93515	-0.22392	0.027555	0.083458	lncRNA
ENSG0000(	DTNBP1	422.8297	0.128726	0.027567	0.083487	protein_coding
ENSG0000(	PSMD12	1183.319	-0.11585	0.027576	0.083503	protein_coding
ENSG0000(	VASH2	334.0043	0.166076	0.027577	0.083503	protein_coding
ENSG0000(	ARPP21	15.80837	0.815293	0.027583	0.083511	protein_coding
ENSG0000(	ENSG0000(	78.84989	0.202956	0.027589	0.083521	lncRNA
ENSG0000(	ITGB8	2772.319	-0.18209	0.027603	0.083557	protein_coding
ENSG0000(	ENSG0000(	25.86862	0.295172	0.027609	0.083567	lncRNA
ENSG0000(	CYBC1	3901.506	0.209713	0.027617	0.083584	protein_coding
ENSG0000(	ENSG0000(	24.98755	0.316852	0.027629	0.083611	lncRNA
ENSG0000(	ENSG0000(	11.98456	-0.5485	0.027638	0.083632	processed_pseudogene
ENSG0000(	RPS6KC1	953.7759	0.102788	0.027642	0.083635	protein_coding
ENSG0000(	LGALSL	552.8481	-0.35808	0.027648	0.083641	protein_coding
ENSG0000(	TSPAN3	5934.624	-0.29541	0.027649	0.083641	protein_coding
ENSG0000(	GFRA2	113.5199	-0.32568	0.02766	0.083664	protein_coding
ENSG0000(	H1-10	440.4961	0.179946	0.02768	0.083718	protein_coding
ENSG0000(	RPL10	4918.9	-0.19373	0.027683	0.083718	protein_coding
ENSG0000(	ENSG0000(	195.4582	0.113529	0.027693	0.08374	lncRNA
ENSG0000(	BACE1-AS	698.6289	0.089461	0.027706	0.083764	lncRNA
ENSG0000(	TTI1	965.2183	0.116933	0.027704	0.083764	protein_coding
ENSG0000(	KIF14	380.3589	0.190495	0.027739	0.083856	protein_coding
ENSG0000(	ARGLU1	6051.874	0.078275	0.027753	0.083891	protein_coding
ENSG0000(	ENSG0000(	257.0839	0.11405	0.027786	0.083982	protein_coding
ENSG0000(	PHEX	203.5344	-0.3186	0.027802	0.084021	protein_coding
ENSG0000(	RHBDF2	1708.072	0.259653	0.027805	0.084022	protein_coding
ENSG0000(	PLD1	3593.441	-0.24401	0.027826	0.084078	protein_coding
ENSG0000(	PCDH7	1057.568	0.285012	0.027843	0.08412	protein_coding
ENSG0000(	ENSG0000(	77.43074	0.179705	0.027867	0.084185	lncRNA
ENSG0000(	NPB	30.57055	-0.41381	0.027875	0.084201	protein_coding
ENSG0000(	ENSG0000(	364.9389	0.109307	0.027892	0.084246	processed_pseudogene
ENSG0000(	ENSG0000(	23.13337	0.407598	0.027922	0.084329	TEC
ENSG0000(	BORCS5	1197.416	0.119336	0.02795	0.084404	protein_coding

ENSG0000(	ZNF296	51.87098	-0.23315	0.027979	0.084483	protein_coding
ENSG0000(	ENSG0000(	8.530626	0.538091	0.028006	0.084558	lncRNA
ENSG0000(	ENSG0000(	180.8269	-0.12047	0.028009	0.084558	lncRNA
ENSG0000(	TMEM41A	2481.774	-0.24576	0.028014	0.084567	protein_coding
ENSG0000(	LINC02798	170.529	-0.19351	0.028057	0.084686	lncRNA
ENSG0000(	NEK1	1466.714	0.115056	0.028076	0.084735	protein_coding
ENSG0000(	AJM1	282.7567	-0.23372	0.028089	0.084764	protein_coding
ENSG0000(	BIVM	1678.922	0.09873	0.02809	0.084764	protein_coding
ENSG0000(	ADH1A	8.88528	0.555919	0.028101	0.084786	protein_coding
ENSG0000(	ENSG0000(	23.93789	0.321878	0.028103	0.084786	lncRNA
ENSG0000(	UBR3	2345.405	-0.0972	0.028108	0.084794	protein_coding
ENSG0000(	UGDH	981.8072	-0.27195	0.028125	0.084836	protein_coding
ENSG0000(	NOVA2	220.4144	0.163578	0.028135	0.084856	protein_coding
ENSG0000(	ENSG0000(	50.78481	0.329798	0.028147	0.084884	lncRNA
ENSG0000(	SMOC1	133.6885	-0.15108	0.028153	0.084896	protein_coding
ENSG0000(	ENSG0000(	228.3461	0.209691	0.028159	0.084904	TEC
ENSG0000(	JAZF1-AS1	93.47324	-0.15158	0.028162	0.084905	lncRNA
ENSG0000(	ARHGDIG	164.5608	-0.66435	0.028168	0.084912	protein_coding
ENSG0000(	PPM1A	3174.221	-0.21354	0.028172	0.084912	protein_coding
ENSG0000(	PRKX	1776.703	0.174867	0.028172	0.084912	protein_coding
ENSG0000(	NLRP7	14.4384	0.675763	0.028177	0.084918	protein_coding
ENSG0000(	CBX4	341.2694	0.213953	0.028184	0.084929	protein_coding
ENSG0000(	RPS29P16	17.39293	0.359952	0.028186	0.084929	processed_pseudogene
ENSG0000(	ENSG0000(	34.01392	0.490985	0.02821	0.084995	lncRNA
ENSG0000(	ENSG0000(	20.69077	0.411734	0.028217	0.085008	processed_pseudogene
ENSG0000(	PRDX4	106.5422	0.311052	0.028223	0.085018	protein_coding
ENSG0000(	CACTIN-AS1	8.671883	0.467112	0.028254	0.085104	lncRNA
ENSG0000(	ENSG0000(	19.7016	0.404738	0.028258	0.085105	lncRNA
ENSG0000(	CEBPA-DT	183.3086	-0.32489	0.028288	0.085189	lncRNA
ENSG0000(	TSLP	21.90102	-0.43306	0.028326	0.085295	protein_coding
ENSG0000(	KHDC1	326.9529	0.109881	0.028358	0.085384	protein_coding
ENSG0000(	VDAC2	892.6911	-0.20884	0.028373	0.085419	protein_coding
ENSG0000(	SLC17A4	4297.689	-0.48574	0.028389	0.085461	protein_coding
ENSG0000(	C11orf68	267.6901	0.130841	0.02842	0.085545	protein_coding
ENSG0000(	ENSG0000(	9.044385	0.426444	0.028434	0.08557	processed_pseudogene
ENSG0000(	LINC01791	14.03387	0.440423	0.028434	0.08557	lncRNA
ENSG0000(	ENSG0000(	120.7312	0.184966	0.02846	0.085625	lncRNA
ENSG0000(	ENSG0000(	150.0553	0.150771	0.028457	0.085625	TEC
ENSG0000(	ENSG0000(	96.86434	0.301558	0.028458	0.085625	lncRNA
ENSG0000(	MSRA	735.5512	-0.25185	0.028494	0.085718	protein_coding
ENSG0000(	ENSG0000(	103.9035	0.51533	0.028505	0.085728	unprocessed_pseudogene
ENSG0000(	ENSG0000(	173.8239	-0.11865	0.028502	0.085728	lncRNA
ENSG0000(	SMARCD1	1196.281	-0.09483	0.028505	0.085728	protein_coding
ENSG0000(	FDX2	45.64771	-0.27458	0.02851	0.085735	protein_coding
ENSG0000(	RN7SL370P	14.67348	0.355768	0.028514	0.085739	misc_RNA
ENSG0000(	HERC2	4768.339	0.084304	0.02853	0.085778	protein_coding
ENSG0000(	ENSG0000(	365.2147	0.210333	0.028538	0.085792	TEC

ENSG0000(	ENSG0000(	8.772495	0.507099	0.028585	0.085922	TEC
ENSG0000(	ENSG0000(	11.26718	0.656819	0.028586	0.085922	protein_coding
ENSG0000(	PHF12	3345.435	0.065339	0.028596	0.085928	protein_coding
ENSG0000(	STARD10	650.3427	-0.217	0.028596	0.085928	protein_coding
ENSG0000(	TNFRSF17	83.80273	0.264104	0.028593	0.085928	protein_coding
ENSG0000(	SLC25A21-/	35.74405	0.33558	0.028607	0.085951	lncRNA
ENSG0000(	IMPDH2	584.7834	-0.21041	0.028618	0.085977	protein_coding
ENSG0000(	FAM219B	1776.695	-0.08761	0.028627	0.085993	protein_coding
ENSG0000(	LINC02026	28.23463	0.412743	0.028629	0.085993	lncRNA
ENSG0000(	LINC02451	43.77712	0.234096	0.028656	0.086066	lncRNA
ENSG0000(	MROH1	2662.59	-0.12579	0.028664	0.086082	protein_coding
ENSG0000(	ENSG0000(	46.42261	-0.19861	0.028671	0.086094	lncRNA
ENSG0000(	ATG5	844.6888	-0.1314	0.028685	0.086129	protein_coding
ENSG0000(	XKR8	535.3113	0.125536	0.028713	0.086205	protein_coding
ENSG0000(	ZNF728	25.9384	0.3365	0.028734	0.086258	protein_coding
ENSG0000(	ZBTB9	128.3482	0.227795	0.028759	0.086328	protein_coding
ENSG0000(	ENSG0000(	47.52313	0.339387	0.028774	0.086362	TEC
ENSG0000(	CSPP1	5119.192	0.080235	0.028791	0.086405	protein_coding
ENSG0000(	OR51E2	174.9434	-0.12112	0.028805	0.08644	protein_coding
ENSG0000(	LINC01133	2033.721	-0.39784	0.028826	0.086495	lncRNA
ENSG0000(	LRRC10	57.60305	-0.21094	0.028834	0.08651	protein_coding
ENSG0000(	RPL4P7	91.30524	0.39765	0.028853	0.08656	processed_pseudogene
ENSG0000(	IGHV2-70D	23.32271	1.03733	0.028864	0.086582	IG_V_gene
ENSG0000(	ENSG0000(	11.37311	-0.65572	0.028918	0.086738	transcribed_unprocessed_pseudo
ENSG0000(	DCUN1D2	758.7894	0.117974	0.028929	0.086761	protein_coding
ENSG0000(	BROX	1823.187	0.132348	0.028932	0.086762	protein_coding
ENSG0000(	ENSG0000(	117.3568	0.154101	0.02895	0.08681	TEC
ENSG0000(	ENSG0000(	53.41753	0.361018	0.028959	0.086827	lncRNA
ENSG0000(	ENSG0000(	64.21937	0.292683	0.028976	0.086862	lncRNA
ENSG0000(	SLC25A24	1323.5	-0.23842	0.028975	0.086862	protein_coding
ENSG0000(	POLD3	742.6081	0.176445	0.029	0.086926	protein_coding
ENSG0000(	EVPLL	14.58189	0.83938	0.029006	0.086934	protein_coding
ENSG0000(	ISY1	560.9442	-0.10102	0.029015	0.086955	protein_coding
ENSG0000(	PPFIBP1	2023.863	0.136752	0.02905	0.08705	protein_coding
ENSG0000(	ENSG0000(	19.24276	0.548852	0.029076	0.08712	lncRNA
ENSG0000(	RAVER1	808.0498	-0.15944	0.029116	0.087231	protein_coding
ENSG0000(	PKNOX2	799.9557	0.321976	0.029141	0.087299	protein_coding
ENSG0000(	SCN1A	279.4162	-0.15119	0.029155	0.087331	protein_coding
ENSG0000(	EEF1AKMT:	399.2858	0.127524	0.02918	0.087398	protein_coding
ENSG0000(	MS4A8	3131.144	-0.52286	0.029184	0.087398	protein_coding
ENSG0000(	TRIM36	1040.136	-0.38053	0.029185	0.087398	protein_coding
ENSG0000(	ENSG0000(	199.8409	-0.12125	0.0292	0.087432	lncRNA
ENSG0000(	LINC02896	88.45542	0.168671	0.029211	0.087457	lncRNA
ENSG0000(	ADCY5	404.8586	-0.33156	0.029224	0.087475	protein_coding
ENSG0000(	DBNDD1	57.45177	0.331432	0.029225	0.087475	protein_coding
ENSG0000(	FGFR4	612.8814	-0.2683	0.029224	0.087475	protein_coding
ENSG0000(	CSK	1454.677	-0.21911	0.029239	0.087507	protein_coding

ENSG0000(	CUL4A	1788.213	-0.0786	0.029244	0.087507	protein_coding
ENSG0000(	SLK	2420.545	-0.16527	0.029241	0.087507	protein_coding
ENSG0000(	UBE3C	3041.116	-0.07553	0.029274	0.087588	protein_coding
ENSG0000(	ENSG0000(	39.87802	0.26911	0.029289	0.087625	processed_pseudogene
ENSG0000(	ENSG0000(	107.1929	0.160985	0.029303	0.087659	lncRNA
ENSG0000(	RPL29P24	32.83331	0.201833	0.029313	0.087681	processed_pseudogene
ENSG0000(	CTSA	2591.681	-0.20489	0.029324	0.087705	protein_coding
ENSG0000(	ENSG0000(	14.7632	-0.48453	0.029335	0.087729	processed_pseudogene
ENSG0000(	HM13-IT1	96.59459	0.18906	0.029359	0.087792	lncRNA
ENSG0000(	AFG3L2	2217.01	-0.2034	0.029365	0.087803	protein_coding
ENSG0000(	RNU5A-1	3258.632	0.335488	0.029377	0.08783	snRNA
ENSG0000(	ALCAM	805.1754	0.172332	0.029417	0.087942	protein_coding
ENSG0000(	ENSG0000(	27.19858	-0.26008	0.029421	0.087943	processed_pseudogene
ENSG0000(	ENSG0000(	102.8713	0.138087	0.029427	0.087954	transcribed_unprocessed_pseudo
ENSG0000(	B3GALT5	9772.177	-0.45197	0.029437	0.087969	protein_coding
ENSG0000(	LPAR4	47.55083	0.326111	0.029435	0.087969	protein_coding
ENSG0000(	RNASE1	253.4309	-0.35213	0.02944	0.087969	protein_coding
ENSG0000(	ENSG0000(	279.3001	0.115028	0.029463	0.088011	processed_pseudogene
ENSG0000(	ENSG0000(	224.1938	-0.11806	0.029459	0.088011	lncRNA
ENSG0000(	LRRC63	41.25003	0.366312	0.029462	0.088011	protein_coding
ENSG0000(	NAT8B	106.0097	-0.7607	0.029489	0.088081	polymorphic_pseudogene
ENSG0000(	ENSG0000(	21.82068	0.359417	0.029494	0.088087	lncRNA
ENSG0000(	SHMT1	1043.522	-0.20916	0.029507	0.088117	protein_coding
ENSG0000(	MIGA1	1941.314	-0.09876	0.029514	0.08813	protein_coding
ENSG0000(	C11orf24	1253.225	-0.26519	0.029538	0.088195	protein_coding
ENSG0000(	OR7D2	546.382	-0.07916	0.029552	0.08822	protein_coding
ENSG0000(	USP14	1153.695	-0.13841	0.029552	0.08822	protein_coding
ENSG0000(	ENSG0000(	16.74319	1.105546	0.029561	0.088231	IG_V_gene
ENSG0000(	RN7SL11P	17.05838	0.370523	0.029561	0.088231	misc_RNA
ENSG0000(	AQP6	114.1527	-0.16424	0.029575	0.088262	protein_coding
ENSG0000(	ENSG0000(	196.8362	0.120966	0.029592	0.088306	lncRNA
ENSG0000(	ELP6	481.661	0.101461	0.029607	0.088341	protein_coding
ENSG0000(	GAS6-DT	277.3778	-0.1223	0.029626	0.088389	lncRNA
ENSG0000(	ENSG0000(	25.08878	-0.552	0.02963	0.088394	lncRNA
ENSG0000(	ENSG0000(	147.9098	0.17835	0.029643	0.088425	lncRNA
ENSG0000(	VPS35L	1715.258	0.069961	0.029654	0.088447	protein_coding
ENSG0000(	ENSG0000(	18.19235	0.350902	0.029668	0.088482	lncRNA
ENSG0000(	TNFRSF19	132.8865	0.193689	0.029673	0.088488	protein_coding
ENSG0000(	TSN	856.6174	-0.12095	0.029686	0.088518	protein_coding
ENSG0000(	ENSG0000(	15.65075	0.404256	0.029694	0.088535	lncRNA
ENSG0000(	MIR1267	36.30974	0.251015	0.029706	0.088561	miRNA
ENSG0000(	MRPL30	1071.08	-0.10334	0.029727	0.08861	protein_coding
ENSG0000(	RN7SL28P	9.432731	0.418195	0.029728	0.08861	misc_RNA
ENSG0000(	LRFN5-DT	194.3645	0.208768	0.029739	0.088635	lncRNA
ENSG0000(	GGH	204.6909	0.214354	0.029742	0.088636	protein_coding
ENSG0000(	ENSG0000(	17.84751	-0.32243	0.029753	0.08866	lncRNA
ENSG0000(	ANKH	2768.127	-0.1928	0.029788	0.088756	protein_coding

ENSG0000(	BCLAF3	1671.746	-0.11254	0.029798	0.088762	protein_coding
ENSG0000(	ENSG0000(	15.63429	0.519596	0.029799	0.088762	processed_pseudogene
ENSG0000(	FUS	11661.63	0.14591	0.029796	0.088762	protein_coding
ENSG0000(	EXOSC3	1217.979	-0.10042	0.029816	0.088805	protein_coding
ENSG0000(	ENSG0000(	159.9926	0.126584	0.029825	0.088824	lncRNA
ENSG0000(	SNORA80A	36.81131	0.285473	0.029841	0.088863	snoRNA
ENSG0000(	EFS	160.3967	0.228541	0.029846	0.088869	protein_coding
ENSG0000(	ENSG0000(	13.22457	0.451571	0.029855	0.088878	snoRNA
ENSG0000(	PTGFR	239.0814	0.442152	0.029853	0.088878	protein_coding
ENSG0000(	ENSG0000(	9.254312	0.44189	0.029864	0.088899	processed_pseudogene
ENSG0000(	FPGS	614.8728	-0.15176	0.029868	0.088902	protein_coding
ENSG0000(	EDN2	316.0583	-0.56558	0.029876	0.08891	protein_coding
ENSG0000(	SMPDL3A	763.9762	-0.41568	0.029874	0.08891	protein_coding
ENSG0000(	SMC5	2545.99	0.096738	0.02988	0.088912	protein_coding
ENSG0000(	CAVIN3	49.51126	0.398297	0.029908	0.088984	protein_coding
ENSG0000(	TAS2R3	177.5054	-0.1266	0.02991	0.088984	protein_coding
ENSG0000(	APOM	35.93333	-0.39912	0.029917	0.088998	protein_coding
ENSG0000(	PM20D1	22.45221	0.647175	0.029938	0.08905	protein_coding
ENSG0000(	HPYR1	30.00306	0.263678	0.029944	0.08906	lncRNA
ENSG0000(	ENSG0000(	125.4288	-0.13129	0.029966	0.089118	TEC
ENSG0000(	GOPC	1389.03	0.070935	0.029975	0.089135	protein_coding
ENSG0000(	ZMPSTE24	846.2591	-0.19898	0.029983	0.089151	protein_coding
ENSG0000(	GPR34	145.2322	-0.24021	0.02999	0.089155	protein_coding
ENSG0000(	RN7SL711F	50.73852	0.199702	0.02999	0.089155	misc_RNA
ENSG0000(	ATG14	1325.577	0.067388	0.030017	0.089226	protein_coding
ENSG0000(	LINC02531	37.25905	0.282913	0.030026	0.089245	lncRNA
ENSG0000(	MTCO1P22	19.95879	-0.66796	0.030031	0.089251	processed_pseudogene
ENSG0000(	LINC02877	145.0126	0.160803	0.03005	0.0893	lncRNA
ENSG0000(	CDHR3	1191.148	-0.24446	0.030061	0.089321	protein_coding
ENSG0000(	ENSG0000(	335.9503	0.104101	0.030066	0.089321	lncRNA
ENSG0000(	RN7SL47P	47.24331	0.210938	0.030066	0.089321	misc_RNA
ENSG0000(	KBTBD11-A	215.9054	-0.14757	0.030085	0.08937	lncRNA
ENSG0000(	ENSG0000(	15.53716	0.421712	0.030089	0.089374	TEC
ENSG0000(	NUDCD1	855.7265	0.14502	0.030104	0.089409	protein_coding
ENSG0000(	TGIF2	793.5937	0.301359	0.03011	0.089418	protein_coding
ENSG0000(	A2M	3735.867	0.249789	0.030122	0.089446	protein_coding
ENSG0000(	MIR765	14.46368	0.384823	0.030133	0.08947	miRNA
ENSG0000(	CHST6	1899.32	-0.2906	0.030142	0.089489	protein_coding
ENSG0000(	ELAC2	902.819	-0.12225	0.030147	0.089494	protein_coding
ENSG0000(	LINC02668	27.29062	-0.22458	0.030169	0.089553	lncRNA
ENSG0000(	SPTSSB	10.84003	0.690212	0.030177	0.089568	protein_coding
ENSG0000(	TCF7L1-IT1	25.91819	0.347077	0.030184	0.089581	lncRNA
ENSG0000(	ENPP7P1	13.80576	-0.51424	0.030206	0.089619	transcribed_unprocessed_pseudo
ENSG0000(	ENSG0000(	166.6511	-0.13695	0.030201	0.089619	TEC
ENSG0000(	RN7SL822F	13.84582	0.398164	0.030204	0.089619	misc_RNA
ENSG0000(	IQCJ-SCHIP	44.17578	0.324087	0.030225	0.089658	protein_coding
ENSG0000(	UBXN4	3699.833	0.077344	0.030224	0.089658	protein_coding

ENSG0000(	ENSG0000(	10.35451	0.439761	0.030252	0.08973	unprocessed_pseudogene
ENSG0000(	ENSG0000(	302.5427	0.132855	0.030259	0.089744	TEC
ENSG0000(	ENSG0000(	36.385	0.455427	0.030276	0.089778	lncRNA
ENSG0000(	PLA2G15	425.9345	-0.11227	0.030277	0.089778	protein_coding
ENSG0000(	REST	2258.25	-0.08369	0.030285	0.089795	protein_coding
ENSG0000(	ENSG0000(	11.27377	0.633997	0.030297	0.089821	unprocessed_pseudogene
ENSG0000(	HSDL2-AS1	416.4619	0.123287	0.030307	0.089843	lncRNA
ENSG0000(	SYN3	1318.901	0.16128	0.030345	0.089947	protein_coding
ENSG0000(	TRIM13	1573.331	-0.08181	0.030385	0.090057	protein_coding
ENSG0000(	ARHGAP26	102.966	0.215335	0.030403	0.090101	lncRNA
ENSG0000(	TRPA1	795.9389	0.311572	0.030406	0.090104	protein_coding
ENSG0000(	ENSG0000(	115.0229	0.150362	0.030413	0.090116	lncRNA
ENSG0000(	TUBA4A	404.4267	-0.19148	0.03043	0.090158	protein_coding
ENSG0000(	ANKRD54	655.4492	-0.07602	0.030441	0.09018	protein_coding
ENSG0000(	ENSG0000(	25.04925	0.28577	0.030446	0.090186	lncRNA
ENSG0000(	ENSG0000(	321.107	0.122443	0.03046	0.090212	lncRNA
ENSG0000(	SYT12	414.7643	0.104202	0.030459	0.090212	protein_coding
ENSG0000(	GPR143	9.86326	0.526366	0.030467	0.090224	protein_coding
ENSG0000(	MIR378J	13.07569	0.395387	0.03048	0.090253	miRNA
ENSG0000(	ENSG0000(	216.8824	0.111763	0.030494	0.09026	lncRNA
ENSG0000(	PRR11	1676.78	0.093151	0.030488	0.09026	protein_coding
ENSG0000(	TSSK4	53.83314	0.247896	0.030491	0.09026	protein_coding
ENSG0000(	UQCC1	1646.696	0.069684	0.030493	0.09026	protein_coding
ENSG0000(	DAXX	515.8768	-0.1601	0.030503	0.090279	protein_coding
ENSG0000(	ENSG0000(	33.40343	0.249287	0.030511	0.090294	processed_pseudogene
ENSG0000(	KEAP1	447.7271	-0.14643	0.030516	0.090302	protein_coding
ENSG0000(	ENSG0000(	287.8374	-0.1127	0.03054	0.090364	lncRNA
ENSG0000(	LINC01255	55.35854	0.173912	0.030543	0.090366	lncRNA
ENSG0000(	ISG20L2	1356.743	0.095933	0.030552	0.090381	protein_coding
ENSG0000(	SNORA21	176.6089	0.269297	0.030555	0.090382	snoRNA
ENSG0000(	GPR61	11.99688	0.701236	0.030575	0.090432	protein_coding
ENSG0000(	ENSG0000(	44.8342	0.302699	0.030581	0.090442	lncRNA
ENSG0000(	MPHOSPH:	495.9493	-0.10064	0.030588	0.090454	protein_coding
ENSG0000(	GPATCH11	584.5121	-0.13926	0.030597	0.090471	protein_coding
ENSG0000(	ENSG0000(	56.79536	-0.24234	0.030616	0.090518	lncRNA
ENSG0000(	ENSG0000(	118.1855	0.138217	0.030619	0.090518	lncRNA
ENSG0000(	FAR2P4	104.0395	0.263419	0.030621	0.090518	transcribed_unprocessed_pseudo
ENSG0000(	ENSG0000(	209.9662	0.158412	0.030625	0.090521	lncRNA
ENSG0000(	ALG5	1437.563	0.077909	0.030631	0.090523	protein_coding
ENSG0000(	OTUD6B-A:	893.2447	-0.12335	0.030629	0.090523	lncRNA
ENSG0000(	C1QTNF3	352.7731	0.166023	0.030642	0.090548	protein_coding
ENSG0000(	SLX1A-SUL	540.9841	-0.24818	0.03066	0.090592	lncRNA
ENSG0000(	ENSG0000(	107.4746	0.145906	0.0307	0.090689	TEC
ENSG0000(	ENSG0000(	596.7389	0.10926	0.030696	0.090689	lncRNA
ENSG0000(	TNFSF11	160.509	-0.64946	0.030702	0.090689	protein_coding
ENSG0000(	MYL12-AS1	16.7216	0.340258	0.030742	0.0908	lncRNA
ENSG0000(	PARP10	1292.945	0.174391	0.030761	0.090848	protein_coding

ENSG0000(	ENSG0000(	16.32309	0.345155	0.03077	0.090866	lncRNA
ENSG0000(	ENSG0000(	499.2655	0.110801	0.030782	0.09089	lncRNA
ENSG0000(	ENSG0000(	255.0592	0.174304	0.030784	0.09089	lncRNA
ENSG0000(	ENSG0000(	40.69837	-0.36257	0.030796	0.090917	lncRNA
ENSG0000(	FEM1B	2244.422	-0.09349	0.03081	0.090946	protein_coding
ENSG0000(	TOB2P1	12.39995	-0.43306	0.030812	0.090946	processed_pseudogene
ENSG0000(	C16orf91	51.2384	-0.23671	0.030823	0.090968	protein_coding
ENSG0000(	CPD	3586.609	0.133723	0.030828	0.090968	protein_coding
ENSG0000(	PRKAB2	2245.549	-0.31761	0.030825	0.090968	protein_coding
ENSG0000(	KCNIP3	63.33532	0.292125	0.030832	0.090972	protein_coding
ENSG0000(	DCLRE1B	123.8048	0.255081	0.030839	0.090985	protein_coding
ENSG0000(	POF1B	2931.603	-0.43573	0.030886	0.091113	protein_coding
ENSG0000(	RCAN1	1078.361	0.322471	0.030915	0.09119	protein_coding
ENSG0000(	LINC00960	814.3037	0.181544	0.030942	0.091263	lncRNA
ENSG0000(	SIGLEC5	70.58607	0.640701	0.030956	0.091295	protein_coding
ENSG0000(	ITGA8	627.6594	0.270632	0.030962	0.091296	protein_coding
ENSG0000(	SMG6-IT1	82.19332	0.173963	0.030961	0.091296	TEC
ENSG0000(	VIPR1-AS1	212.7935	0.156871	0.030969	0.091306	lncRNA
ENSG0000(	BAMBI	34.08558	-0.34362	0.030986	0.091323	protein_coding
ENSG0000(	ENSG0000(	25.89281	0.281204	0.030983	0.091323	lncRNA
ENSG0000(	ENSG0000(	23.14223	0.353688	0.030986	0.091323	lncRNA
ENSG0000(	MUC17	39334.83	-0.5062	0.030984	0.091323	protein_coding
ENSG0000(	ENSG0000(	144.5758	-0.74187	0.031001	0.091349	lncRNA
ENSG0000(	TRUB1	241.1905	-0.1895	0.030999	0.091349	protein_coding
ENSG0000(	ABCC5	2190.393	0.11751	0.031013	0.091372	protein_coding
ENSG0000(	ENSG0000(	106.9382	-0.31791	0.031015	0.091372	processed_pseudogene
ENSG0000(	SRRM5	24.5397	-0.22104	0.031017	0.091372	protein_coding
ENSG0000(	RPL30P13	30.50292	0.385578	0.031026	0.09139	processed_pseudogene
ENSG0000(	WHSC1L2P	216.7717	0.130037	0.031051	0.091455	processed_pseudogene
ENSG0000(	ENSG0000(	12.47252	0.396812	0.031061	0.091474	lncRNA
ENSG0000(	SFXN3	3074.211	-0.35832	0.031063	0.091474	protein_coding
ENSG0000(	LINC02519	11.31334	0.62197	0.031074	0.091498	lncRNA
ENSG0000(	ENSG0000(	31.83359	0.334155	0.031077	0.091499	lncRNA
ENSG0000(	ENSG0000(	52.55643	0.279522	0.031103	0.091564	lncRNA
ENSG0000(	RAB2A	1175.513	-0.11547	0.031116	0.091594	protein_coding
ENSG0000(	ENSG0000(	1016.736	-0.09329	0.031134	0.091639	TEC
ENSG0000(	ENSG0000(	113.3378	0.207188	0.031145	0.091662	TEC
ENSG0000(	ARL5A	2300.214	-0.141	0.031149	0.091665	protein_coding
ENSG0000(	LINC01892	230.7462	-0.2316	0.031169	0.091718	lncRNA
ENSG0000(	LINC02817	382.9548	0.115638	0.031173	0.091721	lncRNA
ENSG0000(	LINC02288	221.8807	0.153068	0.031185	0.091747	lncRNA
ENSG0000(	ENSG0000(	200.7034	-0.4456	0.031205	0.091797	transcribed_unprocessed_pseudo
ENSG0000(	MIXL1	119.1329	0.154702	0.031234	0.091873	protein_coding
ENSG0000(	CSKMT	310.5224	0.192609	0.031242	0.09188	protein_coding
ENSG0000(	ENSG0000(	75.0731	-0.19089	0.031241	0.09188	lncRNA
ENSG0000(	ZNF605	2185.874	0.089208	0.031249	0.091891	protein_coding
ENSG0000(	FUT10	951.2851	0.125767	0.031263	0.091924	protein_coding

ENSG0000(	FAIM2	106.7659	-0.51744	0.031275	0.091952	protein_coding
ENSG0000(	BEND2	148.7227	0.13494	0.031294	0.091983	protein_coding
ENSG0000(	ENSG0000(	11.85227	0.319675	0.031297	0.091983	lncRNA
ENSG0000(	ENSG0000(	41.48074	0.232624	0.031297	0.091983	lncRNA
ENSG0000(	LINC01914	14.25569	0.485219	0.031294	0.091983	lncRNA
ENSG0000(	ENTPD6	2378.757	-0.22597	0.031304	0.091995	protein_coding
ENSG0000(	HOXD-AS2	20.3358	0.318674	0.031314	0.092014	lncRNA
ENSG0000(	UGT2B4	53.42241	-0.23017	0.031317	0.092014	protein_coding
ENSG0000(	LINC02517	199.0862	-0.16423	0.031334	0.092056	lncRNA
ENSG0000(	COG3	1875.56	0.113674	0.031339	0.092063	protein_coding
ENSG0000(	ENSG0000(	101.7472	-0.47874	0.031376	0.092162	lncRNA
ENSG0000(	ANAPC5	5642.567	-0.06986	0.031382	0.092171	protein_coding
ENSG0000(	BCAN-AS1	352.8022	0.191258	0.031394	0.092197	lncRNA
ENSG0000(	PDE7B	384.0963	0.184437	0.031399	0.092204	protein_coding
ENSG0000(	APP-DT	294.5225	0.109898	0.031405	0.092214	lncRNA
ENSG0000(	ENSG0000(	340.848	-0.22392	0.031422	0.092255	lncRNA
ENSG0000(	ABLIM1	5921.817	-0.11816	0.031432	0.092275	protein_coding
ENSG0000(	ERICH5	11.64045	0.475175	0.031446	0.0923	protein_coding
ENSG0000(	SLC2A2	1055.229	-0.6321	0.031444	0.0923	protein_coding
ENSG0000(	ENSG0000(	9.92202	0.415462	0.03145	0.092302	lncRNA
ENSG0000(	ENSG0000(	774.1479	0.07537	0.031453	0.092304	TEC
ENSG0000(	PDCD6IP	4124.621	-0.12698	0.031471	0.092346	protein_coding
ENSG0000(	DNAAF11	98.19715	-0.29652	0.031492	0.092399	protein_coding
ENSG0000(	MT-ND6	1284.819	0.522158	0.0315	0.092416	protein_coding
ENSG0000(	ENSG0000(	25.41016	-0.66821	0.03151	0.092427	lncRNA
ENSG0000(	LINC02620	24.09955	0.298155	0.031509	0.092427	lncRNA
ENSG0000(	ENSG0000(	65.03549	0.177875	0.031544	0.092516	lncRNA
ENSG0000(	RBM48	961.836	-0.0803	0.031561	0.092558	protein_coding
ENSG0000(	ENSG0000(	13.85453	0.416831	0.031582	0.09261	lncRNA
ENSG0000(	ABCB5	240.9897	0.152932	0.031599	0.092652	protein_coding
ENSG0000(	ENSG0000(	106.3488	-0.20384	0.031624	0.092718	lncRNA
ENSG0000(	TLCD4-RWI	126.82	-0.25373	0.031634	0.092738	protein_coding
ENSG0000(	PKN2-AS1	333.7425	-0.10529	0.03167	0.092837	lncRNA
ENSG0000(	RPL35P3	28.40173	-0.32227	0.031719	0.092969	processed_pseudogene
ENSG0000(	ARID4A	1637.039	-0.09891	0.031745	0.09302	protein_coding
ENSG0000(	CEBPG	1795.026	-0.25392	0.03174	0.09302	protein_coding
ENSG0000(	N6AMT1	653.7325	-0.12718	0.031745	0.09302	protein_coding
ENSG0000(	DUSP23	76.44507	-0.24343	0.031754	0.093033	protein_coding
ENSG0000(	ENSG0000(	73.19622	-0.86946	0.031762	0.093033	lncRNA
ENSG0000(	GPRIN2	3411.441	-0.35755	0.031759	0.093033	protein_coding
ENSG0000(	H4C6	98.98118	-0.35099	0.031764	0.093033	protein_coding
ENSG0000(	MIR505	12.77671	0.353948	0.031758	0.093033	miRNA
ENSG0000(	COX7A1	12.957	0.397976	0.031769	0.093038	protein_coding
ENSG0000(	ASAH2B	618.6121	0.147733	0.031804	0.093123	protein_coding
ENSG0000(	ENSG0000(	278.3521	-0.12942	0.031807	0.093123	lncRNA
ENSG0000(	PABPC1P4	17.86627	0.422929	0.031801	0.093123	processed_pseudogene
ENSG0000(	MICAL1	3384.563	-0.11796	0.031822	0.093159	protein_coding

ENSG0000(	TSPAN10	829.7013	0.081585	0.031829	0.093172	protein_coding
ENSG0000(	HNRNPA1P	40.06082	-0.22447	0.031868	0.093267	processed_pseudogene
ENSG0000(	LGR5	414.1453	-0.48367	0.031867	0.093267	protein_coding
ENSG0000(	LINC01312	15.18441	0.376754	0.031885	0.093309	lncRNA
ENSG0000(	ENSG0000(	156.0055	-0.14994	0.031895	0.09333	lncRNA
ENSG0000(	ZNF789	1595.173	-0.0892	0.031919	0.093391	protein_coding
ENSG0000(	RPL31	3212.57	-0.21219	0.031948	0.093466	protein_coding
ENSG0000(	MRPL21	206.6758	-0.17328	0.031953	0.093473	protein_coding
ENSG0000(	FOXF2-DT	39.46048	0.303418	0.031966	0.093503	lncRNA
ENSG0000(	TRNP1	77.15846	0.340559	0.031977	0.093528	protein_coding
ENSG0000(	MAN1B1-D	712.6196	0.113812	0.031985	0.093541	lncRNA
ENSG0000(	ENSG0000(	336.6089	-0.08734	0.031995	0.093563	TEC
ENSG0000(	XXYLT1	243.6393	0.174324	0.032003	0.093578	protein_coding
ENSG0000(	NDRG2	1460.892	-0.14317	0.032016	0.093605	protein_coding
ENSG0000(	ANKRD39	393.2487	-0.14465	0.032024	0.093622	protein_coding
ENSG0000(	KRT18P59	34.10773	0.277792	0.03203	0.09363	transcribed_processed_pseudoge
ENSG0000(	FAT1	16208.47	-0.31528	0.03205	0.093675	protein_coding
ENSG0000(	RPSA	2762.856	-0.19694	0.032052	0.093675	protein_coding
ENSG0000(	ENSG0000(	55.39741	0.210239	0.032066	0.093702	TEC
ENSG0000(	HIPK3	3416.942	0.098058	0.032067	0.093702	protein_coding
ENSG0000(	RNU1-11P	386.989	-0.32761	0.032077	0.093723	snRNA
ENSG0000(	ENSG0000(	12.22603	0.476409	0.032091	0.093757	lncRNA
ENSG0000(	CREG1	951.6808	0.182885	0.032095	0.093757	protein_coding
ENSG0000(	SP2-DT	828.8697	0.136547	0.032104	0.093776	lncRNA
ENSG0000(	JPH2	610.6466	0.201366	0.032121	0.093817	protein_coding
ENSG0000(	ENSG0000(	74.46645	0.171379	0.032135	0.093849	lncRNA
ENSG0000(	ENSG0000(	302.2499	0.170183	0.032142	0.093862	lncRNA
ENSG0000(	HECW1	164.6862	-0.20091	0.032159	0.093901	protein_coding
ENSG0000(	ZNF524	151.0591	-0.14503	0.032172	0.09393	protein_coding
ENSG0000(	PARN	1772.898	-0.06954	0.032213	0.094043	protein_coding
ENSG0000(	LCORL	3266.084	-0.20497	0.032246	0.094131	protein_coding
ENSG0000(	ENSG0000(	268.9082	-0.11843	0.032257	0.094155	lncRNA
ENSG0000(	ITM2A	102.6587	-0.37441	0.032271	0.094185	protein_coding
ENSG0000(	GSTO1	274.2618	-0.25553	0.03228	0.094202	protein_coding
ENSG0000(	PLAAT5	12.42756	0.495526	0.032292	0.094226	protein_coding
ENSG0000(	VPS26C	1847.426	-0.07557	0.032294	0.094226	protein_coding
ENSG0000(	ERC1	3049.664	0.09398	0.032311	0.094267	protein_coding
ENSG0000(	KCNAB1	25.68659	0.566508	0.032325	0.0943	protein_coding
ENSG0000(	RN7SL743F	65.52686	0.206011	0.032338	0.094329	misc_RNA
ENSG0000(	HOXA-AS3	92.8708	0.364512	0.032352	0.09436	lncRNA
ENSG0000(	BCORP1	162.9833	0.220766	0.032355	0.094361	transcribed_unprocessed_pseudo
ENSG0000(	EFCAB15P	24.58677	-0.27549	0.032365	0.094372	transcribed_unitary_pseudogene
ENSG0000(	ENSG0000(	807.7558	-0.06341	0.032364	0.094372	TEC
ENSG0000(	PPIAP40	9.738315	0.459636	0.032385	0.094422	processed_pseudogene
ENSG0000(	ENSG0000(	548.8173	-0.07787	0.032389	0.094425	lncRNA
ENSG0000(	ANKRD20A	18.6316	0.772469	0.03241	0.09447	unprocessed_pseudogene
ENSG0000(	ENSG0000(	10.61252	0.762803	0.032411	0.09447	lncRNA

ENSG0000(	ENSG0000(	43.85968	0.24202	0.032413	0.09447	lncRNA
ENSG0000(	ENSG0000(	9.902656	0.428563	0.032427	0.094491	lncRNA
ENSG0000(	SPIN2A	99.69514	0.222779	0.032426	0.094491	protein_coding
ENSG0000(	PCSK4	60.70662	-0.32123	0.03243	0.094493	protein_coding
ENSG0000(	DOT1L	2519.174	-0.1212	0.032464	0.094583	protein_coding
ENSG0000(	ENSG0000(	30.75078	-0.47379	0.032494	0.094661	lncRNA
ENSG0000(	IDO2	533.5875	0.160281	0.032518	0.094721	protein_coding
ENSG0000(	CPLANE1	3955.131	0.077511	0.032541	0.094766	protein_coding
ENSG0000(	IGLV3-9	18.18853	0.714579	0.032538	0.094766	IG_V_gene
ENSG0000(	MT-TA	95.16417	0.791556	0.032542	0.094766	Mt_tRNA
ENSG0000(	YWHAZP10	31.90087	-0.27719	0.032559	0.094805	processed_pseudogene
ENSG0000(	ENSG0000(	138.2236	0.394327	0.032575	0.094843	processed_pseudogene
ENSG0000(	EPGN	126.3084	0.139371	0.032578	0.094845	protein_coding
ENSG0000(	ENSG0000(	145.039	-0.2399	0.032582	0.094847	transcribed_unprocessed_pseudo
ENSG0000(	RBM4	3143.105	0.079135	0.032588	0.094847	protein_coding
ENSG0000(	SMLR1	620.5802	-0.61858	0.032588	0.094847	protein_coding
ENSG0000(	CFHR1	95.05585	-0.22924	0.032621	0.094935	protein_coding
ENSG0000(	NBPF1	7120.773	-0.19988	0.032639	0.09497	protein_coding
ENSG0000(	TSG101	660.7884	-0.12465	0.032638	0.09497	protein_coding
ENSG0000(	ENSG0000(	12.3209	0.466863	0.03266	0.095016	lncRNA
ENSG0000(	GOLM1	1458.629	0.223287	0.032661	0.095016	protein_coding
ENSG0000(	ENSG0000(	38.26756	0.213485	0.032672	0.095021	lncRNA
ENSG0000(	ENSG0000(	256.8628	-0.1291	0.032671	0.095021	lncRNA
ENSG0000(	MIR4539	66.30042	0.608364	0.03267	0.095021	miRNA
ENSG0000(	ENSG0000(	22.68829	-0.28118	0.032691	0.095064	lncRNA
ENSG0000(	SRBD1	773.5565	0.128841	0.032693	0.095064	protein_coding
ENSG0000(	ENSG0000(	199.6701	0.125964	0.032702	0.095081	lncRNA
ENSG0000(	CALML6	13.07107	0.505111	0.032725	0.095142	protein_coding
ENSG0000(	GFPT1	4155.511	-0.18625	0.032763	0.095243	protein_coding
ENSG0000(	ENSG0000(	98.29839	0.218233	0.032776	0.095265	lncRNA
ENSG0000(	MDM4P1	8.501475	0.423848	0.032777	0.095265	processed_pseudogene
ENSG0000(	LINC01275	66.51101	-0.16369	0.032785	0.095279	lncRNA
ENSG0000(	SHB	506.2343	0.224413	0.032801	0.095316	protein_coding
ENSG0000(	COPS4	282.6368	-0.16287	0.032813	0.095317	protein_coding
ENSG0000(	ENSG0000(	22.79533	0.292333	0.032811	0.095317	processed_pseudogene
ENSG0000(	GNGT1	13.35105	0.480487	0.03281	0.095317	protein_coding
ENSG0000(	LINC00102	80.05292	0.172822	0.032812	0.095317	lncRNA
ENSG0000(	POLR1H	146.3069	-0.17198	0.032828	0.095351	protein_coding
ENSG0000(	MTCO3P39	45.50306	0.390394	0.032842	0.095383	processed_pseudogene
ENSG0000(	PRR29	69.99997	0.197623	0.032851	0.0954	protein_coding
ENSG0000(	ANKHD1-EI	1691.228	0.102625	0.032898	0.09553	protein_coding
ENSG0000(	SRGAP3	1087.033	0.101906	0.032908	0.095547	protein_coding
ENSG0000(	AGFG1	3390.538	-0.15679	0.03294	0.095634	protein_coding
ENSG0000(	VBP1	203.3982	-0.15723	0.032945	0.09564	protein_coding
ENSG0000(	ENSG0000(	28.92765	0.45483	0.032961	0.095659	lncRNA
ENSG0000(	LINC02627	81.31724	-0.17895	0.032961	0.095659	lncRNA
ENSG0000(	RBMS2	2379.32	0.13868	0.032957	0.095659	protein_coding

ENSG0000(	FRAT1	171.3597	0.131552	0.032987	0.095724	protein_coding
ENSG0000(	GUSBP11	2805.193	-0.08822	0.032999	0.09575	lncRNA
ENSG0000(	LYRM4-AS1	161.3412	0.14061	0.033014	0.095785	lncRNA
ENSG0000(	ENSG0000(	49.03803	0.23343	0.033044	0.095847	lncRNA
ENSG0000(	ENSG0000(	26.46422	0.410502	0.033041	0.095847	TEC
ENSG0000(	SLC38A3	30.06807	-0.40083	0.033042	0.095847	protein_coding
ENSG0000(	ATP2A1	63.48112	0.389848	0.03309	0.095971	protein_coding
ENSG0000(	RTCA	1003.26	-0.1308	0.033095	0.095977	protein_coding
ENSG0000(	ENSG0000(	189.067	0.1264	0.033108	0.096006	lncRNA
ENSG0000(	XBP1P1	65.87082	0.202735	0.033121	0.096035	processed_pseudogene
ENSG0000(	ENSG0000(	829.8882	-0.12368	0.033129	0.09605	protein_coding
ENSG0000(	METTL1	87.99172	0.289368	0.033134	0.096056	protein_coding
ENSG0000(	C7orf31	157.7135	-0.21412	0.033175	0.096166	protein_coding
ENSG0000(	ENSG0000(	13.92863	-0.53719	0.033197	0.096219	lncRNA
ENSG0000(	METTL26	139.6282	-0.20746	0.033224	0.096288	protein_coding
ENSG0000(	AZIN2	435.0922	0.254801	0.033243	0.096335	protein_coding
ENSG0000(	TUBAP11	32.75339	0.237208	0.033283	0.096442	processed_pseudogene
ENSG0000(	SCGN	28.28478	-0.54135	0.033302	0.09649	protein_coding
ENSG0000(	SDAD1	618.4162	-0.12246	0.033307	0.096494	protein_coding
ENSG0000(	ENSG0000(	10.5853	-0.83101	0.033338	0.096574	lncRNA
ENSG0000(	ENSG0000(	408.7201	0.099179	0.033353	0.09661	lncRNA
ENSG0000(	ENSG0000(	77.84748	-0.62414	0.033366	0.096631	lncRNA
ENSG0000(	RORC	1186.274	-0.35179	0.033367	0.096631	protein_coding
ENSG0000(	ENSG0000(	59.28205	0.333927	0.033382	0.096667	lncRNA
ENSG0000(	ENSG0000(	42.10264	0.208878	0.033388	0.096676	lncRNA
ENSG0000(	ENSG0000(	159.0316	0.19861	0.033406	0.09672	lncRNA
ENSG0000(	ANKRD40	1233.587	-0.07444	0.033422	0.096755	protein_coding
ENSG0000(	GCNT2P1	15.14373	-0.36275	0.033455	0.096844	processed_pseudogene
ENSG0000(	ITGB3BP	1291.539	0.456977	0.033476	0.096895	protein_coding
ENSG0000(	LINC01986	9.347079	0.488706	0.033521	0.097016	lncRNA
ENSG0000(	ENSG0000(	14.65781	0.376754	0.033537	0.097054	lncRNA
ENSG0000(	TCF23	665.8458	0.078105	0.033544	0.097066	protein_coding
ENSG0000(	GXYLT2	302.8028	0.158719	0.033559	0.097098	protein_coding
ENSG0000(	ATP6V1G1I	24.35591	-0.32897	0.033565	0.0971	processed_pseudogene
ENSG0000(	B3GNTL1	307.884	0.219105	0.033564	0.0971	protein_coding
ENSG0000(	TXN2P1	24.83235	0.412136	0.033579	0.097131	processed_pseudogene
ENSG0000(	ENSG0000(	146.462	0.17068	0.0336	0.097174	lncRNA
ENSG0000(	ENSG0000(	8.26628	0.572991	0.033599	0.097174	lncRNA
ENSG0000(	LINC01700	14.52965	0.416577	0.033607	0.097174	lncRNA
ENSG0000(	RCL1	564.1705	0.152752	0.033605	0.097174	protein_coding
ENSG0000(	JUP	2354.189	-0.33173	0.033627	0.097226	protein_coding
ENSG0000(	OR2J3	21.30113	0.27323	0.033637	0.097244	protein_coding
ENSG0000(	CDH18	204.2099	-0.15982	0.033662	0.097308	protein_coding
ENSG0000(	ENSG0000(	16.15754	0.473758	0.033695	0.09738	lncRNA
ENSG0000(	NFKB1	1452.082	0.186355	0.033696	0.09738	protein_coding
ENSG0000(	SOX5	413.711	0.225457	0.033695	0.09738	protein_coding
ENSG0000(	RAB1B	850.5225	-0.1662	0.033705	0.097396	protein_coding

ENSG0000(	ENSG0000(	253.4144	-0.37771	0.03372	0.09743	lncRNA
ENSG0000(	ENSG0000(	106.3407	-0.43184	0.03373	0.097441	processed_pseudogene
ENSG0000(	ENSG0000(	26.62648	0.281808	0.033735	0.097441	lncRNA
ENSG0000(	MYL5	526.6195	-0.20283	0.033734	0.097441	protein_coding
ENSG0000(	VWA7	231.0222	-0.24657	0.033736	0.097441	protein_coding
ENSG0000(	SORCS3	14.21476	-0.72137	0.033757	0.097492	protein_coding
ENSG0000(	PKM	3301.162	0.153968	0.033775	0.097536	protein_coding
ENSG0000(	SPINK9	99.44544	0.225672	0.03378	0.097541	protein_coding
ENSG0000(	GTF2IP1	2197.058	-0.21892	0.033794	0.097565	transcribed_unprocessed_pseudo
ENSG0000(	TRIP6	229.0661	0.202736	0.033794	0.097565	protein_coding
ENSG0000(	KIF26A	98.80096	0.328778	0.033803	0.097582	protein_coding
ENSG0000(	ENSG0000(	206.1996	-0.10214	0.033828	0.097645	TEC
ENSG0000(	RGS11	387.4049	-0.22633	0.033856	0.097703	protein_coding
ENSG0000(	RIMS3	238.5013	0.218172	0.033858	0.097703	protein_coding
ENSG0000(	RWDD3	353.013	-0.13421	0.033854	0.097703	protein_coding
ENSG0000(	ENSG0000(	181.5305	0.196045	0.033873	0.097738	lncRNA
ENSG0000(	ENSG0000(	82.92794	0.183801	0.033878	0.097745	processed_pseudogene
ENSG0000(	EPB41L5	1071.855	-0.24519	0.033884	0.097753	protein_coding
ENSG0000(	NUP107	1232.541	0.139008	0.033894	0.097771	protein_coding
ENSG0000(	LINC02809	274.0954	0.142162	0.033898	0.097773	lncRNA
ENSG0000(	SLC18A1	100.121	0.257283	0.033901	0.097773	protein_coding
ENSG0000(	ENSG0000(	99.0502	0.136927	0.033909	0.097788	lncRNA
ENSG0000(	TEX45	124.8835	0.193735	0.033917	0.097802	protein_coding
ENSG0000(	RPL23AP74	8.295121	0.496382	0.033921	0.097806	processed_pseudogene
ENSG0000(	SERPINF1	638.2435	0.209106	0.033926	0.09781	protein_coding
ENSG0000(	SNX24	391.3583	-0.24028	0.033932	0.097818	protein_coding
ENSG0000(	ZNF670-ZN	686.3568	0.078876	0.033946	0.09785	protein_coding
ENSG0000(	CCND2-AS1	85.05593	0.232925	0.033963	0.097874	lncRNA
ENSG0000(	FAM223A	49.16441	0.224441	0.03396	0.097874	lncRNA
ENSG0000(	FKBP2	193.5589	-0.30351	0.033963	0.097874	protein_coding
ENSG0000(	PDZRN4	37.04044	0.558	0.033967	0.097876	protein_coding
ENSG0000(	MPC1	330.9141	-0.2111	0.034015	0.098004	protein_coding
ENSG0000(	IGFBP7	819.2944	0.296566	0.034044	0.09808	protein_coding
ENSG0000(	RPL12P20	35.95508	0.299205	0.034054	0.098097	processed_pseudogene
ENSG0000(	CHRNA7	2219.285	-0.32892	0.034057	0.098099	protein_coding
ENSG0000(	CWC27	6841.574	0.06456	0.034063	0.098107	protein_coding
ENSG0000(	DDHD1	3996.11	0.172617	0.034092	0.098182	protein_coding
ENSG0000(	ENSG0000(	469.9605	-0.13717	0.034096	0.098183	lncRNA
ENSG0000(	OR2T7	60.99564	0.195479	0.034113	0.098224	polymorphic_pseudogene
ENSG0000(	KDM4B	2304.198	0.09619	0.034117	0.098226	protein_coding
ENSG0000(	FOCAD	1001.034	0.174012	0.034133	0.098263	protein_coding
ENSG0000(	SARM1	1407.672	-0.13729	0.034158	0.098326	protein_coding
ENSG0000(	ARL4A	1262.064	-0.30624	0.034184	0.098393	protein_coding
ENSG0000(	BICD1-AS1	40.98586	-0.22715	0.034239	0.098515	lncRNA
ENSG0000(	DDHD1-DT	21.13906	0.426219	0.034237	0.098515	lncRNA
ENSG0000(	DET1	1076.276	0.171896	0.034234	0.098515	protein_coding
ENSG0000(	SORD2P	42.17143	0.595985	0.034234	0.098515	transcribed_unprocessed_pseudo

ENSG0000(	ENSG0000(	73.92457	0.205259	0.034265	0.09858	lncRNA
ENSG0000(	LINC00893	361.2534	-0.13823	0.034297	0.098654	lncRNA
ENSG0000(	SLC25A25-/	3767.088	-0.1744	0.034295	0.098654	lncRNA
ENSG0000(	ENSG0000(	10.16685	0.436713	0.034322	0.0987	unprocessed_pseudogene
ENSG0000(	ENSG0000(	5029.551	0.133735	0.034318	0.0987	lncRNA
ENSG0000(	ZNF8-DT	228.748	0.208063	0.034322	0.0987	lncRNA
ENSG0000(	ENSG0000(	50.34121	0.209424	0.034342	0.098748	lncRNA
ENSG0000(	BAAT	273.0016	0.176268	0.034362	0.09877	protein_coding
ENSG0000(	C9orf85	776.8637	-0.0756	0.03436	0.09877	protein_coding
ENSG0000(	ENSG0000(	12.15076	0.476403	0.034365	0.09877	lncRNA
ENSG0000(	RNU1-20P	10.84057	-0.53341	0.034361	0.09877	snRNA
ENSG0000(	UNC5D	401.0902	-0.50225	0.034358	0.09877	protein_coding
ENSG0000(	LINC00328-	38.51489	0.376627	0.034378	0.098796	processed_pseudogene
ENSG0000(	MYOM3	892.6231	-0.2923	0.034395	0.098838	protein_coding
ENSG0000(	ARL11	729.0548	0.264127	0.034414	0.098884	protein_coding
ENSG0000(	GZMH	9.279756	0.484745	0.034418	0.098884	protein_coding
ENSG0000(	ENSG0000(	65.54874	0.235689	0.034474	0.099038	unprocessed_pseudogene
ENSG0000(	TRGC2	174.6067	-0.31124	0.034488	0.099067	TR_C_gene
ENSG0000(	UCN3	12.89985	-0.60462	0.034504	0.099104	protein_coding
ENSG0000(	CASTOR3	791.3191	-0.12518	0.034539	0.099146	transcribed_unprocessed_pseudo
ENSG0000(	ENSG0000(	874.8619	0.082546	0.03454	0.099146	lncRNA
ENSG0000(	ENSG0000(	197.0575	-0.32174	0.034535	0.099146	processed_pseudogene
ENSG0000(	ENSG0000(	30.60764	0.255719	0.034525	0.099146	lncRNA
ENSG0000(	P4HB	8526.706	-0.23862	0.03454	0.099146	protein_coding
ENSG0000(	PPP1R8	423.3712	-0.10353	0.034536	0.099146	protein_coding
ENSG0000(	SEMA4G	2024.938	-0.39136	0.034539	0.099146	protein_coding
ENSG0000(	FGF22	125.5738	-0.17325	0.034548	0.099151	protein_coding
ENSG0000(	LNPEP	7590.097	-0.20769	0.034548	0.099151	protein_coding
ENSG0000(	MAILR	933.1627	0.158729	0.034552	0.099153	lncRNA
ENSG0000(	MIER2	362.0462	0.121321	0.03456	0.099167	protein_coding
ENSG0000(	RNU2-2P	26893.46	-0.22888	0.034577	0.099206	snRNA
ENSG0000(	SNORD32A	65.41236	0.403781	0.034588	0.099229	snoRNA
ENSG0000(	NCOA5	651.6496	-0.08629	0.034623	0.099319	protein_coding
ENSG0000(	SLC6A15	374.2508	0.113747	0.034638	0.099355	protein_coding
ENSG0000(	GFUS	450.1354	0.203929	0.034652	0.099385	protein_coding
ENSG0000(	DLGAP1-AS	405.4224	0.165359	0.034671	0.099422	lncRNA
ENSG0000(	MADCAM1	74.07636	-0.43796	0.03468	0.099422	protein_coding
ENSG0000(	PYY	135.8955	-0.50188	0.034677	0.099422	protein_coding
ENSG0000(	RASGRP1	794.5746	0.344951	0.03468	0.099422	protein_coding
ENSG0000(	SLC17A8	455.2528	-0.60146	0.034675	0.099422	protein_coding
ENSG0000(	C2orf74	133.1888	-0.18353	0.034707	0.099463	protein_coding
ENSG0000(	ENSG0000(	16.76436	0.705755	0.034708	0.099463	processed_pseudogene
ENSG0000(	ENSG0000(	37.36038	0.255988	0.034707	0.099463	lncRNA
ENSG0000(	ENSG0000(	15.51551	0.347822	0.034707	0.099463	lncRNA
ENSG0000(	RHEBP2	10.41031	1.095168	0.034714	0.099473	processed_pseudogene
ENSG0000(	LINC00550	116.6127	-0.26052	0.034732	0.099515	lncRNA
ENSG0000(	CLEC19A	329.4167	0.09104	0.03474	0.099526	protein_coding

ENSG0000(	LCOR	9011.603	-0.1463	0.034742	0.099526	protein_coding
ENSG0000(	LINC01370	323.8235	0.1485	0.034755	0.099554	lncRNA
ENSG0000(	USH1C	2391.891	-0.37809	0.034758	0.099554	protein_coding
ENSG0000(	ENSG0000(	306.1752	0.122939	0.034844	0.09979	lncRNA
ENSG0000(	RN7SL237F	57.60344	0.218548	0.034847	0.09979	misc_RNA
ENSG0000(	DDX21	2063.12	0.143812	0.034857	0.099801	protein_coding
ENSG0000(	TXNDC11	1360.45	0.150832	0.034855	0.099801	protein_coding
ENSG0000(	ENSG0000(	13.54664	0.52306	0.034891	0.099888	processed_pseudogene
ENSG0000(	MTSS2	534.0659	0.238231	0.034898	0.0999	protein_coding
ENSG0000(	ANKS1A	1343.396	-0.11485	0.034903	0.099906	protein_coding
ENSG0000(	COL11A1	39.30796	0.511907	0.034911	0.09992	protein_coding
ENSG0000(	DNMT3B	70.02052	0.221708	0.034939	0.099989	protein_coding
ENSG0000(	ARHGAP22	19.23895	0.451325	0.034948	0.100006	lncRNA
ENSG0000(	ENDOV	722.0534	-0.10681	0.034959	0.100019	protein_coding
ENSG0000(	IQUB	19.43274	0.296862	0.034959	0.100019	protein_coding
ENSG0000(	ENSG0000(	29.96228	-0.28343	0.034974	0.100054	lncRNA
ENSG0000(	SNORA7B	155.9811	0.225744	0.034983	0.100069	snoRNA
ENSG0000(	HR	634.8881	-0.33727	0.034989	0.10007	protein_coding
ENSG0000(	MAFG-DT	110.9028	0.173147	0.034988	0.10007	lncRNA
ENSG0000(	KIF17	45.15211	0.288752	0.035023	0.100159	protein_coding
ENSG0000(	LY6K	102.2212	0.147499	0.035052	0.100231	protein_coding
ENSG0000(	KCP	769.6731	-0.22236	0.03506	0.100246	protein_coding
ENSG0000(	MAL2	885.3579	-0.36507	0.035074	0.100276	protein_coding
ENSG0000(	TTC23L-AS1	17.19912	0.373523	0.035077	0.100276	lncRNA
ENSG0000(	DCAF7	4245.289	-0.0492	0.035102	0.100339	protein_coding
ENSG0000(	RN7SL160F	9.518766	0.460291	0.035108	0.100345	misc_RNA
ENSG0000(	BBX	2289.627	0.159756	0.035113	0.10035	protein_coding
ENSG0000(	ENSG0000(	23.33716	0.301876	0.035119	0.10036	lncRNA
ENSG0000(	LRRC28	2097.108	-0.26554	0.035138	0.100405	protein_coding
ENSG0000(	RNU6-181F	15.26278	0.472411	0.03515	0.100429	snRNA
ENSG0000(	ENSG0000(	21.37908	0.827225	0.035154	0.100433	protein_coding
ENSG0000(	LINC01186	37.13833	0.265508	0.035172	0.100458	lncRNA
ENSG0000(	OLA1P3	1095.8	0.075354	0.035173	0.100458	processed_pseudogene
ENSG0000(	RPS3AP3	16.89133	0.407765	0.035172	0.100458	processed_pseudogene
ENSG0000(	BAP1	906.4606	0.10065	0.035189	0.100494	protein_coding
ENSG0000(	RPL13	3480.203	-0.21688	0.035192	0.100494	protein_coding
ENSG0000(	SUMO2	1593.946	-0.14925	0.035216	0.100553	protein_coding
ENSG0000(	RN7SL351F	66.92341	0.208765	0.03528	0.100727	misc_RNA
ENSG0000(	ENSG0000(	25.31928	0.334269	0.035293	0.100749	lncRNA
ENSG0000(	TSSC2	586.62	0.099831	0.035294	0.100749	transcribed_unprocessed_pseudo
ENSG0000(	SELPLG	396.0018	0.35558	0.0353	0.100756	protein_coding
ENSG0000(	GACAT3	221.8642	0.097193	0.035309	0.100773	lncRNA
ENSG0000(	PSMB8-AS1	631.9139	0.199316	0.035313	0.100777	lncRNA
ENSG0000(	RPL7P23	9.902471	-0.64636	0.035323	0.100795	processed_pseudogene
ENSG0000(	SNORA18	61.1675	0.276039	0.035328	0.1008	snoRNA
ENSG0000(	MIR186	13.68587	0.570797	0.035347	0.100846	miRNA
ENSG0000(	AMDHD1	73.46231	0.359953	0.035406	0.101004	protein_coding

ENSG0000(	XIRP1	25.1112	1.899072	0.035411	0.10101	protein_coding
ENSG0000(	ENSG0000(	222.4283	0.112234	0.035436	0.101073	lncRNA
ENSG0000(	MOGS	1512.386	-0.13437	0.035443	0.101082	protein_coding
ENSG0000(	MRPL51	202.5757	-0.21011	0.035461	0.101124	protein_coding
ENSG0000(	IQANK1	483.2235	0.119931	0.035464	0.101126	protein_coding
ENSG0000(	BAZ1B	2547.092	-0.06878	0.035469	0.10113	protein_coding
ENSG0000(	ENSG0000(	16.65862	0.327251	0.03549	0.101161	lncRNA
ENSG0000(	ENSG0000(	49.89495	-0.2035	0.035493	0.101161	lncRNA
ENSG0000(	MIR548D2	73.74324	0.267034	0.035491	0.101161	miRNA
ENSG0000(	ZNF30	117.8218	-0.19591	0.035485	0.101161	protein_coding
ENSG0000(	GMDS	1787.265	0.246296	0.035505	0.101186	protein_coding
ENSG0000(	PCDHB1	800.6675	0.086882	0.035527	0.10124	protein_coding
ENSG0000(	VLDLR-AS1	347.3114	0.079083	0.035544	0.10128	lncRNA
ENSG0000(	C2orf49	963.172	-0.07899	0.035576	0.101362	protein_coding
ENSG0000(	ENSG0000(	280.4596	0.244716	0.035597	0.101412	lncRNA
ENSG0000(	RIMBP3	57.27692	0.465451	0.035676	0.10163	protein_coding
ENSG0000(	NEGR1	2405.075	0.119271	0.035692	0.101665	protein_coding
ENSG0000(	DDX39B	9906.97	0.087587	0.035704	0.101681	protein_coding
ENSG0000(	SNHG28	156.9277	-0.20664	0.035703	0.101681	lncRNA
ENSG0000(	WDR45	1019.533	0.205942	0.035714	0.101701	protein_coding
ENSG0000(	ENSG0000(	96.45524	0.151153	0.035719	0.101706	lncRNA
ENSG0000(	NME7	510.0244	0.124865	0.035738	0.101749	protein_coding
ENSG0000(	TSPAN12	531.9695	-0.40112	0.035752	0.101779	protein_coding
ENSG0000(	ENSG0000(	19.84128	0.324245	0.035756	0.101782	lncRNA
ENSG0000(	ZDHHC8P1	253.8378	-0.22491	0.035764	0.101796	transcribed_unprocessed_pseudo
ENSG0000(	ENSG0000(	12.87246	0.39767	0.035776	0.101822	lncRNA
ENSG0000(	XAB2	507.5425	-0.11995	0.03579	0.101853	protein_coding
ENSG0000(	MSMO1	705.8635	-0.19315	0.035797	0.101863	protein_coding
ENSG0000(	PKP3	633.1998	-0.39775	0.0358	0.101863	protein_coding
ENSG0000(	SNHG30	400.0916	-0.09529	0.035837	0.101959	lncRNA
ENSG0000(	UFSP1	31.75152	0.260536	0.035862	0.102019	protein_coding
ENSG0000(	SNRPB	817.4291	-0.12981	0.035874	0.102045	protein_coding
ENSG0000(	ENSG0000(	32.96656	0.323986	0.035884	0.102065	lncRNA
ENSG0000(	MMAB	702.1965	-0.09969	0.035895	0.102087	protein_coding
ENSG0000(	ENSG0000(	154.4975	0.173393	0.035905	0.102106	processed_pseudogene
ENSG0000(	LHFPL4	13.93679	0.693197	0.035946	0.102211	protein_coding
ENSG0000(	TSGA10	473.3905	-0.16252	0.035956	0.102232	protein_coding
ENSG0000(	ACP7	319.7192	0.100611	0.035966	0.102241	protein_coding
ENSG0000(	ENSG0000(	760.0124	0.128012	0.035963	0.102241	protein_coding
ENSG0000(	LMO7	5581.063	-0.37847	0.035969	0.102241	protein_coding
ENSG0000(	CAPNS1	1444.852	-0.18331	0.035983	0.10227	protein_coding
ENSG0000(	ENSG0000(	54.97515	0.27471	0.036003	0.10232	lncRNA
ENSG0000(	GCLC	1565.332	-0.15674	0.036008	0.102324	protein_coding
ENSG0000(	C17orf78	1179.135	-0.53913	0.036027	0.102361	protein_coding
ENSG0000(	NANS	874.9229	0.124864	0.036027	0.102361	protein_coding
ENSG0000(	MCPH1	3771.458	0.067561	0.03604	0.102389	protein_coding
ENSG0000(	LINC01239	330.0295	-0.19529	0.036054	0.102419	lncRNA

ENSG0000(	CHIT1	147.1556	0.404453	0.036061	0.102429	protein_coding
ENSG0000(	ENSG0000(	42.70678	0.245333	0.03607	0.102445	processed_pseudogene
ENSG0000(	HSH2D	1439.013	0.177949	0.036085	0.102478	protein_coding
ENSG0000(	SNAP29	1092.774	-0.08788	0.03609	0.102483	protein_coding
ENSG0000(	ENSG0000(	1171.153	-0.10269	0.036111	0.102526	lncRNA
ENSG0000(	IGF2	672.4829	0.391337	0.036112	0.102526	protein_coding
ENSG0000(	HSPE1P27	33.82247	0.188272	0.036116	0.102529	processed_pseudogene
ENSG0000(	LINC00934	83.14802	-0.19239	0.036124	0.102542	lncRNA
ENSG0000(	GAS6	1353.995	0.206783	0.036132	0.102549	protein_coding
ENSG0000(	GLP1R	26.5957	0.316063	0.036133	0.102549	protein_coding
ENSG0000(	LINC01864	94.7071	0.198249	0.036136	0.10255	lncRNA
ENSG0000(	SLC34A3	64.83712	-0.65951	0.036153	0.102588	protein_coding
ENSG0000(	PLPP6	453.4751	-0.14092	0.036157	0.102591	protein_coding
ENSG0000(	ATP1B3	1112.098	-0.28417	0.036169	0.102616	protein_coding
ENSG0000(	SPPL3	1829.735	-0.08312	0.036173	0.102617	protein_coding
ENSG0000(	ENSG0000(	330.5286	0.161921	0.03618	0.102627	processed_pseudogene
ENSG0000(	RN7SL165F	8.823257	0.397496	0.036198	0.10266	misc_RNA
ENSG0000(	SLC9A3R2	433.1389	-0.20791	0.036196	0.10266	protein_coding
ENSG0000(	ENSG0000(	220.7918	0.146167	0.036229	0.10272	lncRNA
ENSG0000(	OXR1	1077.332	-0.10866	0.036226	0.10272	protein_coding
ENSG0000(	PTPRQ	20.8419	0.798358	0.036232	0.10272	protein_coding
ENSG0000(	RNU6-853F	14.27072	0.365223	0.036231	0.10272	snRNA
ENSG0000(	SHOC2	3411.433	-0.09463	0.036274	0.102828	protein_coding
ENSG0000(	RAB34	174.4864	0.282103	0.036282	0.102842	protein_coding
ENSG0000(	POC5	234.6878	-0.19669	0.036291	0.102859	protein_coding
ENSG0000(	ENSG0000(	227.1718	-0.15969	0.036311	0.102908	lncRNA
ENSG0000(	CD24	2818.399	0.345492	0.036322	0.102927	protein_coding
ENSG0000(	CYP4F8	543.7205	-0.07465	0.036325	0.102927	protein_coding
ENSG0000(	PEBP1P2	25.34194	-0.47435	0.03635	0.102989	processed_pseudogene
ENSG0000(	SLC22A23	3432.128	-0.29715	0.036359	0.103007	protein_coding
ENSG0000(	AP1G2	2230.322	-0.10776	0.036374	0.10304	protein_coding
ENSG0000(	ENSG0000(	148.213	-0.12815	0.036444	0.10322	lncRNA
ENSG0000(	NDUFAF4	265.0867	-0.15597	0.036444	0.10322	protein_coding
ENSG0000(	NAGPA	606.713	-0.1153	0.036477	0.103302	protein_coding
ENSG0000(	ENSG0000(	167.1382	-0.14002	0.03648	0.103303	lncRNA
ENSG0000(	YTHDF3	2275.695	-0.10666	0.036494	0.103332	protein_coding
ENSG0000(	LDHAL6FP	36.61221	-0.23416	0.036559	0.103507	processed_pseudogene
ENSG0000(	ENSG0000(	16.45742	0.398739	0.036572	0.103535	lncRNA
ENSG0000(	FAM171A1	621.0567	-0.17697	0.036576	0.103537	protein_coding
ENSG0000(	ENSG0000(	23.65956	0.398619	0.036586	0.103555	lncRNA
ENSG0000(	RN7SKP70	78.37206	-0.16817	0.036589	0.103555	misc_RNA
ENSG0000(	ITPKA	195.9912	-0.43397	0.036596	0.103566	protein_coding
ENSG0000(	ANAPC1P2	458.6677	-0.1499	0.036627	0.103633	lncRNA
ENSG0000(	LGALSL-DT	602.3414	-0.09825	0.03663	0.103633	lncRNA
ENSG0000(	SNORA31	85.43931	0.276245	0.03663	0.103633	snoRNA
ENSG0000(	OSR2	459.3783	-0.43148	0.036635	0.103638	protein_coding
ENSG0000(	CORO1A-A!	18.52359	0.421894	0.036652	0.103651	lncRNA

ENSG0000(	ENSG0000(	30.94166	0.301676	0.036655	0.103651	lncRNA
ENSG0000(	ENSG0000(	15.32196	0.527208	0.036653	0.103651	lncRNA
ENSG0000(	PPP1R26P1	10.70611	0.41948	0.036655	0.103651	processed_pseudogene
ENSG0000(	SCAMP2	1875	-0.16852	0.036656	0.103651	protein_coding
ENSG0000(	ENSG0000(	22.65271	0.33613	0.036663	0.103659	lncRNA
ENSG0000(	ENSG0000(	65.6598	0.182535	0.036665	0.103659	lncRNA
ENSG0000(	GLCCI1	1333.187	0.23413	0.036673	0.103661	protein_coding
ENSG0000(	TRAPPC1	212.7405	-0.16153	0.03667	0.103661	protein_coding
ENSG0000(	NFIA-AS2	713.1776	0.112925	0.036678	0.103667	lncRNA
ENSG0000(	VHL	2782.915	-0.08322	0.036682	0.103669	protein_coding
ENSG0000(	DHX36	2623.914	0.087933	0.036689	0.103679	protein_coding
ENSG0000(	ENSG0000(	88.44809	0.206355	0.036715	0.103736	processed_pseudogene
ENSG0000(	ENSG0000(	109.1771	-0.19151	0.036713	0.103736	lncRNA
ENSG0000(	YIPF5	778.2801	-0.17948	0.036733	0.103777	protein_coding
ENSG0000(	RAB15	670.1711	0.217492	0.036778	0.103884	protein_coding
ENSG0000(	SLC35A5	1055.044	-0.11598	0.036775	0.103884	protein_coding
ENSG0000(	HK2-DT	178.9827	0.142338	0.036797	0.103928	lncRNA
ENSG0000(	ANKRD13C	848.7083	-0.11654	0.036816	0.103973	protein_coding
ENSG0000(	OSTCP5	12.62727	-0.52255	0.036823	0.103985	processed_pseudogene
ENSG0000(	ENSG0000(	238.5389	-0.08988	0.036832	0.104001	processed_pseudogene
ENSG0000(	ENSG0000(	138.3463	0.393784	0.03686	0.104071	lncRNA
ENSG0000(	SPIRE1	298.3637	0.180013	0.036883	0.104124	protein_coding
ENSG0000(	EHD4	1390.947	0.184312	0.03692	0.104207	protein_coding
ENSG0000(	ENSG0000(	876.1998	0.166472	0.036919	0.104207	lncRNA
ENSG0000(	WDR11-AS	366.3876	-0.15435	0.036922	0.104207	lncRNA
ENSG0000(	LINC02473	229.0023	0.11864	0.036927	0.104212	lncRNA
ENSG0000(	KCNIP2	296.2812	-0.17384	0.036935	0.104224	protein_coding
ENSG0000(	AKAP9	14477.42	-0.12536	0.036938	0.104225	protein_coding
ENSG0000(	RP1L1	152.3732	0.117296	0.036946	0.104237	protein_coding
ENSG0000(	NAB1	1347.348	0.121395	0.036976	0.104314	protein_coding
ENSG0000(	ENSG0000(	107.6764	0.220829	0.036987	0.104335	lncRNA
ENSG0000(	ZWILCH	327.3172	-0.13781	0.037025	0.104434	protein_coding
ENSG0000(	FAM3D-AS:	360.0494	0.111769	0.037041	0.104468	lncRNA
ENSG0000(	HMGN2P47	13.99385	-0.70955	0.037055	0.104499	processed_pseudogene
ENSG0000(	MINDY2	1953.865	0.067708	0.037083	0.104567	protein_coding
ENSG0000(	PGD	613.3822	0.212037	0.037126	0.104679	protein_coding
ENSG0000(	RIPK4	453.863	-0.43755	0.037129	0.10468	protein_coding
ENSG0000(	H3C8	108.5166	-0.3631	0.037147	0.104718	protein_coding
ENSG0000(	SNORA2C	204.1815	0.225324	0.03715	0.104718	snoRNA
ENSG0000(	MLPH	2062.106	0.333641	0.037214	0.104892	protein_coding
ENSG0000(	ST8SIA6	298.7204	0.175168	0.037287	0.105086	protein_coding
ENSG0000(	CGGBP1	2680.397	-0.08292	0.037296	0.105096	protein_coding
ENSG0000(	RAB40C	719.935	-0.23581	0.037297	0.105096	protein_coding
ENSG0000(	ENSG0000(	279.5506	0.08451	0.037307	0.10511	lncRNA
ENSG0000(	ENSG0000(	115.0024	-0.14715	0.037309	0.10511	lncRNA
ENSG0000(	ADH4	230.5239	0.155839	0.037338	0.105173	protein_coding
ENSG0000(	LINC02878	253.0202	0.115855	0.037337	0.105173	lncRNA

ENSG0000(	ENSG0000(	181.0266	0.138118	0.037352	0.105204	lncRNA
ENSG0000(	ENSG0000(	57.58041	-0.30652	0.037372	0.105243	protein_coding
ENSG0000(	RN7SL517F	195.3991	0.132255	0.037372	0.105243	misc_RNA
ENSG0000(	C8G	75.88852	-0.49337	0.037408	0.105325	protein_coding
ENSG0000(	HPX	120.2532	-0.37824	0.037407	0.105325	protein_coding
ENSG0000(	TMEM171	191.1015	-0.42719	0.037442	0.105412	protein_coding
ENSG0000(	HIF1AN	3970.613	-0.06834	0.037476	0.105498	protein_coding
ENSG0000(	CLTA	785.3336	-0.12642	0.037486	0.105506	protein_coding
ENSG0000(	TFAP2A	23.73136	0.397828	0.037484	0.105506	protein_coding
ENSG0000(	ENSG0000(	148.8204	0.114585	0.03749	0.105507	lncRNA
ENSG0000(	ENSG0000(	16.28625	0.353132	0.037498	0.105514	TEC
ENSG0000(	SCYL3	1172.029	0.093921	0.037499	0.105514	protein_coding
ENSG0000(	CASP8	1961.302	0.122749	0.037521	0.105557	protein_coding
ENSG0000(	FMO6P	15.93519	0.329649	0.037521	0.105557	transcribed_unitary_pseudogene
ENSG0000(	ANO5	649.679	-0.21389	0.037548	0.105625	protein_coding
ENSG0000(	CASP6	494.9302	-0.2133	0.037571	0.105678	protein_coding
ENSG0000(	ENSG0000(	185.3142	0.355247	0.03758	0.105695	lncRNA
ENSG0000(	TMEM252-	10.09545	-0.50052	0.037585	0.105699	lncRNA
ENSG0000(	ENSG0000(	53.72734	0.335919	0.037602	0.105737	processed_pseudogene
ENSG0000(	BEGAIN	220.5497	-0.3144	0.037631	0.105805	protein_coding
ENSG0000(	ZNF485	157.2445	0.217997	0.037633	0.105805	protein_coding
ENSG0000(	ENSG0000(	113.1551	0.146123	0.037638	0.105812	lncRNA
ENSG0000(	CADM3	171.0631	-0.31991	0.037654	0.105847	protein_coding
ENSG0000(	DEPDC1B	177.0633	-0.18855	0.037667	0.105875	protein_coding
ENSG0000(	ZNF212	473.6796	-0.14438	0.037672	0.105879	protein_coding
ENSG0000(	PRKAR1B-A	29.60475	0.356884	0.03768	0.105891	lncRNA
ENSG0000(	ASB13	673.8361	-0.27113	0.037686	0.105899	protein_coding
ENSG0000(	ENSG0000(	33.59537	0.407408	0.037692	0.105906	lncRNA
ENSG0000(	ENSG0000(	78.97796	-0.19831	0.0377	0.105911	transcribed_processed_pseudoge
ENSG0000(	KLK10	275.773	-0.10169	0.037701	0.105911	protein_coding
ENSG0000(	CES1P1	30.04131	0.358044	0.037754	0.106034	transcribed_unprocessed_pseudo
ENSG0000(	DRG1	362.8176	-0.14274	0.037756	0.106034	protein_coding
ENSG0000(	ENSG0000(	76.18179	0.216988	0.037761	0.106034	lncRNA
ENSG0000(	ENSG0000(	569.0734	-0.09405	0.03775	0.106034	lncRNA
ENSG0000(	RTN4RL1	9.7618	-0.49673	0.037759	0.106034	protein_coding
ENSG0000(	ENSG0000(	36.29761	0.583008	0.037794	0.106108	lncRNA
ENSG0000(	PLS3	451.625	0.182047	0.037793	0.106108	protein_coding
ENSG0000(	DACH2	36.57282	-0.29591	0.037803	0.106124	protein_coding
ENSG0000(	SLC29A4	937.7325	-0.18943	0.03781	0.106134	protein_coding
ENSG0000(	ENSG0000(	41.18853	0.30172	0.037873	0.106283	TEC
ENSG0000(	ITGA1	4044.589	0.100032	0.037871	0.106283	protein_coding
ENSG0000(	LINC02343	36.54741	0.323372	0.037873	0.106283	lncRNA
ENSG0000(	ENSG0000(	50.68354	0.193562	0.037883	0.106291	lncRNA
ENSG0000(	SLC10A5	376.1537	-0.43324	0.03788	0.106291	protein_coding
ENSG0000(	MIR122HG	479.384	0.101049	0.037888	0.106297	lncRNA
ENSG0000(	ENSG0000(	30.53362	0.293941	0.037917	0.106368	lncRNA
ENSG0000(	ENSG0000(	48.0832	0.218091	0.037929	0.106392	unprocessed_pseudogene

ENSG0000(	ACTR1A	1353.157	-0.09757	0.037953	0.106449	protein_coding
ENSG0000(	CADM4	36.19218	0.382397	0.037957	0.106451	protein_coding
ENSG0000(	LRRC8D	1028.056	-0.16383	0.037965	0.106463	protein_coding
ENSG0000(	RNF148	33.75333	0.276599	0.03797	0.10647	protein_coding
ENSG0000(	LTV1	207.6708	-0.17573	0.038004	0.106554	protein_coding
ENSG0000(	G2E3	974.1428	-0.10196	0.038016	0.10658	protein_coding
ENSG0000(	ENSG0000(	20.9228	0.399265	0.038041	0.10664	lncRNA
ENSG0000(	ENSG0000(	152.3722	-0.14061	0.03805	0.106656	lncRNA
ENSG0000(	TAPT1	1033.07	-0.09873	0.038064	0.106685	protein_coding
ENSG0000(	LINC02347	89.38959	0.156754	0.038069	0.10669	lncRNA
ENSG0000(	CRYZL2P-SE	1770.479	-0.36159	0.038082	0.106705	lncRNA
ENSG0000(	WDR18	133.1754	-0.22897	0.038084	0.106705	protein_coding
ENSG0000(	ZNF134	759.7737	-0.13043	0.038085	0.106705	protein_coding
ENSG0000(	CD9	807.976	-0.23921	0.03809	0.106709	protein_coding
ENSG0000(	G6PC1	3797.092	-0.70859	0.038104	0.10672	protein_coding
ENSG0000(	RN7SL353P	183.3119	-0.14175	0.0381	0.10672	misc_RNA
ENSG0000(	ZCCHC10	438.8503	-0.12003	0.038103	0.10672	protein_coding
ENSG0000(	RAVER2	2092.501	-0.33108	0.038108	0.106723	protein_coding
ENSG0000(	RARS1	1344.119	-0.17539	0.038141	0.106804	protein_coding
ENSG0000(	PPP1R14C	53.30722	0.435456	0.038164	0.10686	protein_coding
ENSG0000(	RPL7AP26	12.01742	0.404678	0.038168	0.106862	processed_pseudogene
ENSG0000(	ENSG0000(	140.1855	0.139313	0.038194	0.106915	TEC
ENSG0000(	FBXW5	1161.353	-0.14758	0.038194	0.106915	protein_coding
ENSG0000(	PWP1	405.8799	-0.11021	0.038205	0.106937	protein_coding
ENSG0000(	ENSG0000(	151.2674	-0.1338	0.038231	0.106991	lncRNA
ENSG0000(	RFFL	2759.012	0.103615	0.038228	0.106991	protein_coding
ENSG0000(	ESPN	498.7171	-0.47994	0.038256	0.107042	protein_coding
ENSG0000(	HDAC4-AS1	18.18747	0.295291	0.038255	0.107042	lncRNA
ENSG0000(	ZNF592	2372.633	-0.09617	0.038263	0.107052	protein_coding
ENSG0000(	ENSG0000(	197.3384	0.144958	0.038289	0.107105	lncRNA
ENSG0000(	SMARCAD1	1040.983	-0.11747	0.038289	0.107105	protein_coding
ENSG0000(	OPRK1	107.0828	-0.1403	0.038294	0.107109	protein_coding
ENSG0000(	ALK	25.66794	0.545557	0.038345	0.107224	protein_coding
ENSG0000(	IL7R	4081.822	0.282113	0.038342	0.107224	protein_coding
ENSG0000(	PBLD	4457.286	-0.3932	0.038339	0.107224	protein_coding
ENSG0000(	ENSG0000(	57.78987	0.206378	0.038385	0.107328	lncRNA
ENSG0000(	ENSG0000(	18.56021	0.447832	0.038394	0.107337	lncRNA
ENSG0000(	UBXN10	92.49483	0.30821	0.038395	0.107337	protein_coding
ENSG0000(	RPL39P40	10.15638	-0.74614	0.038401	0.107342	processed_pseudogene
ENSG0000(	ENSG0000(	84.7398	0.162653	0.038414	0.107371	lncRNA
ENSG0000(	CHRFAM7A	104.1285	0.292545	0.038438	0.107427	protein_coding
ENSG0000(	ENSG0000(	11.79658	0.399164	0.038441	0.107427	lncRNA
ENSG0000(	ENSG0000(	283.2769	0.091025	0.038448	0.107437	lncRNA
ENSG0000(	FAM133B	1487.691	0.089671	0.03851	0.107571	protein_coding
ENSG0000(	KLF2	438.9324	0.394785	0.038509	0.107571	protein_coding
ENSG0000(	LIN37	248.3397	-0.14823	0.038509	0.107571	protein_coding
ENSG0000(	RPS18	3371.226	-0.21602	0.038507	0.107571	protein_coding

ENSG0000(	EPOP	51.89158	0.460819	0.038532	0.107623	protein_coding
ENSG0000(	NEURL4	819.7287	-0.15987	0.038544	0.107647	protein_coding
ENSG0000(	FDCSP	201.6214	-1.03802	0.038549	0.107651	protein_coding
ENSG0000(	TMEM222	572.4556	-0.0861	0.038552	0.107651	protein_coding
ENSG0000(	ANKRD17	5978.944	-0.07198	0.038575	0.107706	protein_coding
ENSG0000(	ENSG0000(	39.92004	0.221406	0.038584	0.107713	lncRNA
ENSG0000(	ENSG0000(	15.5819	0.657664	0.038585	0.107713	lncRNA
ENSG0000(	PTPRF	9964.286	-0.36221	0.038632	0.107827	protein_coding
ENSG0000(	ZBED6CL	508.5339	0.190994	0.038632	0.107827	protein_coding
ENSG0000(	ENSG0000(	407.8485	0.123449	0.038636	0.107829	lncRNA
ENSG0000(	RNU6-312F	16.71025	0.37935	0.03865	0.107856	snRNA
ENSG0000(	TMCO6	400.1403	-0.18126	0.038699	0.107984	protein_coding
ENSG0000(	NLRP6	2313.793	-0.38132	0.03871	0.108007	protein_coding
ENSG0000(	MED9	1592.061	0.11347	0.038722	0.108029	protein_coding
ENSG0000(	HPSE2	271.4736	0.42257	0.038732	0.108048	protein_coding
ENSG0000(	TENM4	463.7648	0.504189	0.038772	0.10815	protein_coding
ENSG0000(	ENSG0000(	211.0074	-0.14736	0.038784	0.108169	lncRNA
ENSG0000(	NPRL2	274.6036	-0.13484	0.038786	0.108169	protein_coding
ENSG0000(	CYP2D6	462.7365	-0.58723	0.038797	0.108191	protein_coding
ENSG0000(	COL21A1	50.28327	0.445715	0.03882	0.108244	protein_coding
ENSG0000(	PCOLCE-AS	50.3694	0.247711	0.038823	0.108244	lncRNA
ENSG0000(	NACA3P	11.62284	-0.62186	0.03883	0.108254	processed_pseudogene
ENSG0000(	ZDHHC12	197.4506	-0.21755	0.03884	0.108272	protein_coding
ENSG0000(	RPL17-C18(	50.69651	-1.29371	0.038848	0.108282	protein_coding
ENSG0000(	SLC2A7	13.84486	-0.65133	0.03885	0.108282	protein_coding
ENSG0000(	ARHGAP42	133.4425	0.130363	0.038874	0.108329	processed_pseudogene
ENSG0000(	WDR89	461.7824	0.149343	0.038871	0.108329	protein_coding
ENSG0000(	PRPF19	526.8855	-0.11952	0.038883	0.108334	protein_coding
ENSG0000(	YBEY	228.2471	-0.2115	0.038882	0.108334	protein_coding
ENSG0000(	ENSG0000(	20.94504	-0.37375	0.038902	0.108379	lncRNA
ENSG0000(	PIH1D2	78.42469	-0.23702	0.038906	0.10838	protein_coding
ENSG0000(	CHI3L2	221.1734	0.46458	0.038923	0.108417	protein_coding
ENSG0000(	MORF4L1	2473.943	-0.07039	0.038944	0.108466	protein_coding
ENSG0000(	SREBF2-AS1	53.01401	0.164894	0.038957	0.108494	lncRNA
ENSG0000(	ENSG0000(	186.0249	-0.12345	0.038968	0.108515	lncRNA
ENSG0000(	ENSG0000(	156.7026	-0.11514	0.038994	0.108569	processed_pseudogene
ENSG0000(	MELTF-AS1	20.33577	0.442542	0.038995	0.108569	lncRNA
ENSG0000(	NAT2	107.6894	-0.45722	0.039	0.108575	protein_coding
ENSG0000(	PPM1M	402.6678	0.279524	0.039023	0.108629	protein_coding
ENSG0000(	KCNK17	22.81597	-0.29767	0.039035	0.108651	protein_coding
ENSG0000(	LINC01443	287.0837	0.128687	0.03908	0.108769	lncRNA
ENSG0000(	MIR1256	9.633393	0.381188	0.039087	0.108777	miRNA
ENSG0000(	ENSG0000(	314.862	-0.11139	0.0391	0.108805	lncRNA
ENSG0000(	ENSG0000(	194.5062	0.130853	0.039116	0.108839	TEC
ENSG0000(	ENSG0000(	111.2372	-0.26504	0.039139	0.108893	lncRNA
ENSG0000(	ENSG0000(	21.36446	-0.63544	0.039153	0.108923	lncRNA
ENSG0000(	DYNC1I1	47.65271	0.21064	0.039169	0.108933	protein_coding

ENSG0000(	ENSG0000(	260.0729	0.176498	0.03917	0.108933	lncRNA
ENSG0000(	NUSAP1	451.1244	-0.19702	0.039165	0.108933	protein_coding
ENSG0000(	TMEM198I	1046.228	0.090666	0.039166	0.108933	transcribed_unitary_pseudogene
ENSG0000(	AOC4P	87.10101	0.175539	0.039201	0.109007	transcribed_unprocessed_pseudo
ENSG0000(	FAT3	249.2759	-0.24121	0.039204	0.109007	protein_coding
ENSG0000(	ENSG0000(	292.5082	0.101392	0.039214	0.109027	unprocessed_pseudogene
ENSG0000(	NDFIP1	1594.191	-0.09873	0.039232	0.109066	protein_coding
ENSG0000(	ENSG0000(	13.24087	0.357704	0.039295	0.109223	lncRNA
ENSG0000(	RBM25-AS1	12.19965	0.468017	0.039292	0.109223	lncRNA
ENSG0000(	SOGA3	16.32664	0.936369	0.039304	0.109238	protein_coding
ENSG0000(	LINC01494	11.74606	0.46538	0.039309	0.109241	lncRNA
ENSG0000(	DNASE1L2	28.65229	-0.26335	0.039341	0.109321	protein_coding
ENSG0000(	FAM171B	154.6659	0.2232	0.039391	0.109445	protein_coding
ENSG0000(	FIRRE	1078.216	0.098547	0.039393	0.109445	lncRNA
ENSG0000(	SLC6A6	2749.179	0.155829	0.039397	0.109448	protein_coding
ENSG0000(	ST3GAL6-A	371.9028	0.111699	0.039404	0.109458	lncRNA
ENSG0000(	KLHL20	617.6259	-0.1076	0.039413	0.109462	protein_coding
ENSG0000(	STK24-AS1	61.828	0.19769	0.03941	0.109462	lncRNA
ENSG0000(	ENSG0000(	174.5098	-0.15277	0.039432	0.109487	lncRNA
ENSG0000(	ENSG0000(	32.1246	0.265048	0.039426	0.109487	TEC
ENSG0000(	OR51E1	32.0622	0.317468	0.039429	0.109487	protein_coding
ENSG0000(	ALPK2	662.847	0.504059	0.039457	0.109547	protein_coding
ENSG0000(	LINC00562	352.095	0.108679	0.039465	0.10956	lncRNA
ENSG0000(	LBHD1	568.0723	0.130244	0.03948	0.109592	protein_coding
ENSG0000(	KIF5B	3535.157	-0.11541	0.039517	0.109684	protein_coding
ENSG0000(	ARL13B	430.3439	0.117165	0.039537	0.109728	protein_coding
ENSG0000(	INMT-MINI	21.3307	0.834817	0.03958	0.109841	protein_coding
ENSG0000(	TDRD9	60.5344	0.418142	0.039597	0.109876	protein_coding
ENSG0000(	ENSG0000(	302.4298	0.143119	0.039611	0.109907	transcribed_unprocessed_pseudo
ENSG0000(	ALG13-AS1	18.65808	-0.25928	0.039629	0.109928	lncRNA
ENSG0000(	CEP68	1210.257	0.160638	0.039635	0.109928	protein_coding
ENSG0000(	ENSG0000(	239.8192	-0.12121	0.039635	0.109928	TEC
ENSG0000(	SDHAF2	456.7517	-0.09006	0.039636	0.109928	protein_coding
ENSG0000(	SYNGAP1	1109.592	0.285597	0.039636	0.109928	protein_coding
ENSG0000(	ENSG0000(	47.31167	0.175047	0.039646	0.109945	lncRNA
ENSG0000(	ENSG0000(	22.30873	0.282313	0.03965	0.109946	lncRNA
ENSG0000(	FBXO2	91.49057	0.244348	0.039708	0.110097	protein_coding
ENSG0000(	CHRNA1	23.6459	0.295235	0.039714	0.110104	protein_coding
ENSG0000(	GPAT4-AS1	30.14114	0.210248	0.039724	0.110114	lncRNA
ENSG0000(	SULT2A1	681.5051	-0.49132	0.039724	0.110114	protein_coding
ENSG0000(	ENSG0000(	54.13344	-0.23044	0.039733	0.110128	lncRNA
ENSG0000(	RADIL	104.5993	-0.22612	0.039763	0.110201	protein_coding
ENSG0000(	ENSG0000(	101.3775	0.159899	0.039778	0.110235	TEC
ENSG0000(	FUT1	483.4263	0.148711	0.039789	0.110254	protein_coding
ENSG0000(	CCDC60	45.04538	0.27157	0.03982	0.110316	protein_coding
ENSG0000(	ENSG0000(	16.38662	-0.33255	0.039822	0.110316	lncRNA
ENSG0000(	PLCXD1	1512.404	-0.10863	0.03982	0.110316	protein_coding

ENSG0000(	ENSG0000(	27.01711	0.338914	0.039829	0.110326	lncRNA
ENSG0000(	ENSG0000(	478.6412	0.114267	0.039844	0.110359	lncRNA
ENSG0000(	TMED7	1368.262	-0.19174	0.039859	0.11039	protein_coding
ENSG0000(	SLC13A2	727.2314	-0.64667	0.039865	0.110396	protein_coding
ENSG0000(	RPL3	6096.306	-0.16719	0.039885	0.110442	protein_coding
ENSG0000(	ENSG0000(	789.9037	0.531795	0.039893	0.110455	miRNA
ENSG0000(	ARHGAP44	728.0712	0.222753	0.039901	0.110458	protein_coding
ENSG0000(	PPEF1	58.56038	0.266069	0.039901	0.110458	protein_coding
ENSG0000(	ENSG0000(	258.5106	0.237674	0.039918	0.110496	lncRNA
ENSG0000(	MTUS2	32.66394	0.606315	0.039932	0.110524	protein_coding
ENSG0000(	ENSG0000(	52.2432	0.206632	0.039952	0.11057	lncRNA
ENSG0000(	CYP2C18	2129.983	-0.45501	0.03999	0.110666	protein_coding
ENSG0000(	CCAR1	2633.149	-0.10006	0.040002	0.110688	protein_coding
ENSG0000(	CD8B2	112.3908	-0.21982	0.040008	0.110697	protein_coding
ENSG0000(	ENSG0000(	522.6232	-0.47212	0.040032	0.110751	lncRNA
ENSG0000(	BMS1P22	20.52585	0.582206	0.040048	0.110787	transcribed_unprocessed_pseudo
ENSG0000(	CNDP2	5534.215	-0.2312	0.040053	0.110792	protein_coding
ENSG0000(	ENSG0000(	180.4072	0.130283	0.04006	0.110801	lncRNA
ENSG0000(	GATA2	217.4108	0.283362	0.040065	0.110804	protein_coding
ENSG0000(	DCAF15	378.6181	-0.15864	0.040071	0.110812	protein_coding
ENSG0000(	C14orf119	556.8021	-0.12729	0.040086	0.110844	protein_coding
ENSG0000(	TMEM72-A	328.0079	0.100454	0.040094	0.110856	lncRNA
ENSG0000(	NT5C3B	424.844	0.102283	0.040149	0.110999	protein_coding
ENSG0000(	EIF3FP3	48.42861	-0.62833	0.040163	0.111028	processed_pseudogene
ENSG0000(	KIF5A	351.0452	0.175281	0.040169	0.111034	protein_coding
ENSG0000(	ENSG0000(	130.4722	0.127139	0.040198	0.111103	lncRNA
ENSG0000(	ENSG0000(	96.12205	0.14817	0.040231	0.111187	lncRNA
ENSG0000(	ENSG0000(	234.7694	-0.14283	0.040252	0.111235	TEC
ENSG0000(	HSPA14	226.0355	-0.12575	0.040259	0.111244	protein_coding
ENSG0000(	SPATA5	1620.017	0.127	0.040283	0.111301	protein_coding
ENSG0000(	LINC02757	17.88188	0.336871	0.040288	0.111305	lncRNA
ENSG0000(	MIR3939	19.98247	-0.40628	0.040304	0.111338	miRNA
ENSG0000(	SEPTIN11	3523.333	-0.13531	0.040319	0.111362	protein_coding
ENSG0000(	SNORA73	52.16959	0.203428	0.040317	0.111362	snoRNA
ENSG0000(	CD40	761.6077	0.356738	0.04033	0.11138	protein_coding
ENSG0000(	ENSG0000(	243.7883	0.120322	0.040357	0.111445	lncRNA
ENSG0000(	RHBDL1	34.09159	-0.40694	0.04037	0.111464	protein_coding
ENSG0000(	SMCO1	15.84958	0.28945	0.040371	0.111464	protein_coding
ENSG0000(	ENSG0000(	34.01817	0.229782	0.040398	0.111529	lncRNA
ENSG0000(	ENSG0000(	467.1171	0.147429	0.040407	0.111534	lncRNA
ENSG0000(	RBM12B-D	24.92794	0.268475	0.040404	0.111534	lncRNA
ENSG0000(	ENSG0000(	316.4078	-0.18564	0.040427	0.111581	lncRNA
ENSG0000(	SNRPA1	895.952	-0.09091	0.040442	0.111613	protein_coding
ENSG0000(	LINC02842	16.11062	0.466647	0.040458	0.111646	lncRNA
ENSG0000(	BMS1P14	97.12018	0.292398	0.040462	0.111647	lncRNA
ENSG0000(	ENSG0000(	15.3669	0.340326	0.040469	0.111657	lncRNA
ENSG0000(	INTS7	731.1738	-0.11744	0.040487	0.111696	protein_coding

ENSG0000(	HNF4A-AS1	437.0132	-0.59885	0.040494	0.111706	lncRNA
ENSG0000(	FLRT2	3447.807	0.36693	0.04051	0.111721	protein_coding
ENSG0000(	PYGM	325.7816	-0.12871	0.040505	0.111721	protein_coding
ENSG0000(	RBM38-AS1	55.61768	0.244599	0.040507	0.111721	lncRNA
ENSG0000(	ENSG0000(	74.21991	-0.36449	0.040515	0.111726	protein_coding
ENSG0000(	SAMD12	4899.666	-0.11027	0.040534	0.111767	protein_coding
ENSG0000(	SNORD3A	38588.57	0.315069	0.040537	0.111767	snoRNA
ENSG0000(	ENSG0000(	142.3563	-0.38091	0.040545	0.111779	lncRNA
ENSG0000(	CTIF	657.6222	0.157551	0.040551	0.111786	protein_coding
ENSG0000(	ENSG0000(	45.49743	0.251432	0.040586	0.111873	lncRNA
ENSG0000(	FAM180B	91.99208	0.257215	0.040616	0.111944	protein_coding
ENSG0000(	ENSG0000(	270.7039	0.153792	0.040627	0.111965	lncRNA
ENSG0000(	MRRF	1987.604	-0.10512	0.040631	0.111968	protein_coding
ENSG0000(	FSBP	603.2672	0.122079	0.040638	0.111978	protein_coding
ENSG0000(	LINC01524	174.1346	0.153904	0.040646	0.111988	lncRNA
ENSG0000(	KCTD3	1677.894	-0.21626	0.040684	0.112083	protein_coding
ENSG0000(	ENSG0000(	135.8978	-0.1487	0.040689	0.112088	lncRNA
ENSG0000(	ENSG0000(	26.67958	0.267455	0.040725	0.112176	lncRNA
ENSG0000(	ATP5MC1	457.3748	-0.25432	0.040732	0.112187	protein_coding
ENSG0000(	MIR5192	21.33843	0.307474	0.040755	0.112239	miRNA
ENSG0000(	FGF23	349.8086	0.319966	0.040782	0.112306	protein_coding
ENSG0000(	ENSG0000(	63.50669	0.20421	0.040789	0.112308	lncRNA
ENSG0000(	ENSG0000(	1386.023	0.105335	0.04079	0.112308	lncRNA
ENSG0000(	ENSG0000(	1008.872	0.093035	0.040814	0.112363	lncRNA
ENSG0000(	GPR52	598.2246	-0.18215	0.040832	0.112383	protein_coding
ENSG0000(	IKZF2	1183.33	0.248452	0.040825	0.112383	protein_coding
ENSG0000(	KPNB1P1	119.3626	0.138975	0.040835	0.112383	processed_pseudogene
ENSG0000(	NLK	917.1525	0.115185	0.040832	0.112383	protein_coding
ENSG0000(	CHRM5	68.16459	0.199209	0.040839	0.112384	protein_coding
ENSG0000(	EDNRB	740.439	-0.22466	0.040875	0.112463	protein_coding
ENSG0000(	ENSG0000(	108.8918	0.128745	0.040873	0.112463	lncRNA
ENSG0000(	CRMP1	64.68035	-0.26517	0.040889	0.112491	protein_coding
ENSG0000(	SMG1P4	1161.028	0.380843	0.0409	0.112511	transcribed_unprocessed_pseudo
ENSG0000(	MOGAT2	3676.777	-0.46566	0.040923	0.112565	protein_coding
ENSG0000(	CASKIN2	419.679	-0.28752	0.040936	0.112592	protein_coding
ENSG0000(	OR10H5	1040.562	0.062012	0.040957	0.112639	protein_coding
ENSG0000(	ENSG0000(	79.96647	-0.18066	0.040981	0.112676	lncRNA
ENSG0000(	MEIS3P2	16.32142	0.431144	0.040979	0.112676	processed_pseudogene
ENSG0000(	WNT7B	162.5686	0.144743	0.040976	0.112676	protein_coding
ENSG0000(	ENSG0000(	221.4195	-0.12737	0.040991	0.112692	lncRNA
ENSG0000(	ENSG0000(	28.30723	0.582729	0.041016	0.112741	unprocessed_pseudogene
ENSG0000(	PDK3	5355.703	-0.13294	0.041013	0.112741	protein_coding
ENSG0000(	ZSWIM6	1417.887	0.138208	0.041022	0.112749	protein_coding
ENSG0000(	ENSG0000(	51.52678	0.279568	0.041054	0.112828	lncRNA
ENSG0000(	DUS3L	369.9652	-0.19395	0.041066	0.112849	protein_coding
ENSG0000(	ENSG0000(	11.01988	0.417164	0.041083	0.112886	lncRNA
ENSG0000(	ENSG0000(	47.89039	0.168432	0.041103	0.112912	unprocessed_pseudogene

ENSG0000(	ENSG0000(	26.5547	-0.2691	0.041096	0.112912	lncRNA
ENSG0000(	FAM185A	239.0672	-0.13506	0.041101	0.112912	protein_coding
ENSG0000(	ENSG0000(	1700.863	-0.10227	0.041141	0.113004	transcribed_processed_pseudoge
ENSG0000(	TMEM217	41.31023	0.224261	0.041143	0.113004	protein_coding
ENSG0000(	MRPS31	410.5696	-0.09581	0.04116	0.11304	protein_coding
ENSG0000(	GSTA9P	12.23128	0.33736	0.04118	0.113083	transcribed_unprocessed_pseudo
ENSG0000(	ENSG0000(	11.42036	0.373666	0.041193	0.113107	lncRNA
ENSG0000(	NTAN1P2	94.84985	-0.14031	0.041195	0.113107	processed_pseudogene
ENSG0000(	CLDN7	2769.237	-0.37978	0.041204	0.113112	protein_coding
ENSG0000(	CRIP3	124.042	-0.33948	0.041201	0.113112	protein_coding
ENSG0000(	TMEM251	332.4181	-0.13534	0.04127	0.113281	protein_coding
ENSG0000(	MAP2K2	1821.052	-0.11354	0.041285	0.113312	protein_coding
ENSG0000(	ECSIT	984.2766	-0.08117	0.041292	0.113312	protein_coding
ENSG0000(	GASK1B-AS	388.1775	0.271854	0.04129	0.113312	lncRNA
ENSG0000(	ENSG0000(	88.37832	-0.17469	0.041298	0.113321	lncRNA
ENSG0000(	MDGA2	40.13774	0.396047	0.041306	0.113331	protein_coding
ENSG0000(	FBLIM1	3383.719	-0.26169	0.041324	0.113366	protein_coding
ENSG0000(	RASA3	1065.167	0.344374	0.041326	0.113366	protein_coding
ENSG0000(	ANP32B	695.2379	-0.12925	0.041375	0.113492	protein_coding
ENSG0000(	FOS	1319.163	0.438985	0.041387	0.113513	protein_coding
ENSG0000(	ENSG0000(	103.7762	0.353202	0.041415	0.113561	processed_pseudogene
ENSG0000(	GGNBP2	2864.065	-0.08218	0.041412	0.113561	protein_coding
ENSG0000(	ZNF827	3569.824	-0.11661	0.041411	0.113561	protein_coding
ENSG0000(	ARMC5	280.2246	0.167166	0.041441	0.113623	protein_coding
ENSG0000(	LINC02253	386.049	0.117353	0.041458	0.11366	lncRNA
ENSG0000(	C11orf54	1697.251	-0.15256	0.041477	0.113699	protein_coding
ENSG0000(	RN7SL399F	71.51142	0.190186	0.04148	0.113699	misc_RNA
ENSG0000(	ENSG0000(	27.737	0.229967	0.041529	0.113825	lncRNA
ENSG0000(	COPB2-DT	579.959	0.113393	0.041538	0.113829	lncRNA
ENSG0000(	ENSG0000(	20.44734	-0.31482	0.041535	0.113829	lncRNA
ENSG0000(	PKIA	96.98389	-0.4278	0.041584	0.113945	protein_coding
ENSG0000(	ELMOD2	957.2793	-0.15352	0.041592	0.113949	protein_coding
ENSG0000(	LHFPL6	372.8069	0.19138	0.041591	0.113949	protein_coding
ENSG0000(	NOTCH2NL	25.15574	0.797147	0.041612	0.113991	protein_coding
ENSG0000(	MIR2117H(	13.78637	0.426371	0.041616	0.113995	lncRNA
ENSG0000(	DEPTOR	314.7905	-0.27199	0.041643	0.114056	protein_coding
ENSG0000(	ENSG0000(	77.47914	0.24156	0.041653	0.114065	lncRNA
ENSG0000(	TAF4	949.3263	-0.15866	0.041651	0.114065	protein_coding
ENSG0000(	ENSG0000(	69.53482	0.184105	0.04168	0.114129	TEC
ENSG0000(	TUBA5P	362.3536	-0.27047	0.041691	0.114149	transcribed_unprocessed_pseudo
ENSG0000(	ENSG0000(	45.9074	0.233414	0.041764	0.114319	lncRNA
ENSG0000(	SNORA23	279.1644	-0.24138	0.041761	0.114319	snoRNA
ENSG0000(	ZNF567	515.9032	-0.12467	0.041758	0.114319	protein_coding
ENSG0000(	PHF3	9554.768	-0.08264	0.041797	0.114399	protein_coding
ENSG0000(	CCDC153	76.6815	-0.18707	0.041813	0.114434	protein_coding
ENSG0000(	CCR5	337.8989	0.245114	0.041817	0.114436	protein_coding
ENSG0000(	DNAH12	120.0017	0.1589	0.041828	0.114454	protein_coding

ENSG0000(	SCAT8	122.1034	-0.14043	0.04187	0.114559	lncRNA
ENSG0000(	ENSG0000(	284.91	0.090657	0.041902	0.114637	TEC
ENSG0000(	MARVELD3	2176.798	-0.30292	0.041906	0.114637	protein_coding
ENSG0000(	ENSG0000(	97.72484	0.43001	0.041932	0.1147	TEC
ENSG0000(	SAC3D1	39.18747	-0.2328	0.041949	0.114725	protein_coding
ENSG0000(	TAS2R20	306.5891	0.177487	0.041945	0.114725	protein_coding
ENSG0000(	ENSG0000(	173.4263	0.116452	0.041963	0.114756	lncRNA
ENSG0000(	TASL	240.7634	0.206935	0.041977	0.114783	protein_coding
ENSG0000(	ZNF677	858.076	-0.07662	0.041992	0.114815	protein_coding
ENSG0000(	LINC01980	81.82736	0.188043	0.041998	0.11482	lncRNA
ENSG0000(	FKRP	585.7538	-0.1007	0.042002	0.11482	protein_coding
ENSG0000(	ENSG0000(	66.80179	-0.18273	0.042028	0.114832	lncRNA
ENSG0000(	H2AC14	101.994	-0.35256	0.042024	0.114832	protein_coding
ENSG0000(	KTN1	5326.829	-0.06545	0.042022	0.114832	protein_coding
ENSG0000(	PKDCC	1138.791	-0.25067	0.042018	0.114832	protein_coding
ENSG0000(	RAB11A	1891.047	-0.14798	0.042021	0.114832	protein_coding
ENSG0000(	TUBA8	716.8415	-0.20196	0.04202	0.114832	protein_coding
ENSG0000(	MAGEB10	164.7098	-0.12192	0.042035	0.114842	protein_coding
ENSG0000(	ENSG0000(	18.9908	0.278331	0.042077	0.114946	lncRNA
ENSG0000(	TDH-AS1	304.857	0.197146	0.042081	0.114947	lncRNA
ENSG0000(	DEFB124	13.69725	0.485445	0.04209	0.114951	protein_coding
ENSG0000(	ENSG0000(	397.0437	-0.34529	0.042087	0.114951	protein_coding
ENSG0000(	CCL18	225.7607	0.799163	0.042106	0.114985	protein_coding
ENSG0000(	RN7SL388F	32.6797	0.246594	0.042146	0.115076	misc_RNA
ENSG0000(	RNU6-485F	10.37736	0.431009	0.042144	0.115076	snRNA
ENSG0000(	EML6	1251.805	-0.39734	0.0422	0.115184	protein_coding
ENSG0000(	KLF3	4417.347	-0.16491	0.042195	0.115184	protein_coding
ENSG0000(	LMBR1L	1743.536	-0.09725	0.042195	0.115184	protein_coding
ENSG0000(	SNRNP200	4846.053	-0.06628	0.042201	0.115184	protein_coding
ENSG0000(	PRAF2	90.03323	0.275129	0.04221	0.115199	protein_coding
ENSG0000(	COL13A1	221.4941	0.27511	0.042214	0.1152	protein_coding
ENSG0000(	ENSG0000(	38.67667	0.237917	0.04222	0.115201	lncRNA
ENSG0000(	NAPEPLD	2639.266	-0.24451	0.042221	0.115201	protein_coding
ENSG0000(	SCN7A	486.3078	0.134459	0.042236	0.115231	protein_coding
ENSG0000(	TSPAN31	1050.723	-0.07781	0.04224	0.115232	protein_coding
ENSG0000(	NR2F6	593.1802	-0.31239	0.042255	0.115262	protein_coding
ENSG0000(	ENSG0000(	55.22537	-0.21268	0.042261	0.115269	lncRNA
ENSG0000(	CYP8B1	212.5775	-0.1047	0.042304	0.115376	protein_coding
ENSG0000(	ARPP19	2744.609	-0.08115	0.042308	0.115379	protein_coding
ENSG0000(	TMEM39B	180.6664	-0.14466	0.042317	0.115392	protein_coding
ENSG0000(	CDKN2B-AS	3077.999	-0.35948	0.042341	0.115446	lncRNA
ENSG0000(	LINC01511	275.5864	0.135354	0.042348	0.115455	lncRNA
ENSG0000(	ENSG0000(	24.87695	-0.29332	0.042362	0.115484	lncRNA
ENSG0000(	AADACP1	345.2749	-0.52186	0.042444	0.115698	transcribed_unprocessed_pseudo
ENSG0000(	TATDN1	563.1324	-0.08293	0.042454	0.115714	protein_coding
ENSG0000(	LINC00667	2596.817	-0.1395	0.04246	0.115722	lncRNA
ENSG0000(	MTND3P22	12.03968	0.539281	0.042477	0.115759	processed_pseudogene

ENSG0000(	RANP8	25.63628	0.283352	0.042486	0.115772	processed_pseudogene
ENSG0000(	ANKRD37	76.26946	-0.26737	0.042494	0.115783	protein_coding
ENSG0000(	RNU2-48P	14.21458	-0.43631	0.042499	0.115788	snRNA
ENSG0000(	ENSG0000(	61.05458	0.245058	0.042519	0.115828	TEC
ENSG0000(	U2	14862.06	-0.89221	0.042521	0.115828	snRNA
ENSG0000(	C6orf226	17.20306	0.338687	0.042535	0.115855	protein_coding
ENSG0000(	AARS2	1112.582	-0.08825	0.042549	0.115884	protein_coding
ENSG0000(	SLC5A11	478.9454	-0.62744	0.042573	0.11594	protein_coding
ENSG0000(	FTLP14	73.3668	-0.38801	0.04258	0.11595	processed_pseudogene
ENSG0000(	ENSG0000(	359.4343	-0.23932	0.042586	0.115952	lncRNA
ENSG0000(	TNFRSF1A	2711.035	-0.25574	0.042588	0.115952	protein_coding
ENSG0000(	TNK2-AS1	128.0313	0.169601	0.042596	0.115963	lncRNA
ENSG0000(	C2orf66	13.20672	0.491767	0.042619	0.116014	protein_coding
ENSG0000(	ENSG0000(	33.60064	0.218848	0.042659	0.116113	processed_pseudogene
ENSG0000(	HSPA8P3	150.3077	0.219824	0.042697	0.116207	processed_pseudogene
ENSG0000(	ENSG0000(	15.75981	0.68874	0.04272	0.116258	lncRNA
ENSG0000(	MARF1	4209.267	-0.13914	0.042734	0.116288	protein_coding
ENSG0000(	ENSG0000(	30.69793	0.299827	0.042738	0.116289	lncRNA
ENSG0000(	ITGB2-AS1	482.2733	0.448284	0.042753	0.116318	lncRNA
ENSG0000(	ENSG0000(	68.65371	-0.33108	0.042773	0.116365	lncRNA
ENSG0000(	ENSG0000(	116.897	0.210491	0.042796	0.116395	lncRNA
ENSG0000(	ENSG0000(	9.237661	0.408443	0.04279	0.116395	TEC
ENSG0000(	ZG16	2017.45	-0.58027	0.042794	0.116395	protein_coding
ENSG0000(	ENSG0000(	209.0858	0.358136	0.042813	0.116417	TEC
ENSG0000(	HNRNPAB	1060.814	-0.13924	0.042811	0.116417	protein_coding
ENSG0000(	ZNF790-AS	1097.564	-0.08938	0.042815	0.116417	lncRNA
ENSG0000(	ENSG0000(	31.01857	0.223224	0.042821	0.116425	lncRNA
ENSG0000(	PALS1	3810.04	-0.26226	0.042836	0.116444	protein_coding
ENSG0000(	PLLP	2104.8	-0.26757	0.042836	0.116444	protein_coding
ENSG0000(	DM1-AS	18.64107	0.416176	0.042855	0.116467	lncRNA
ENSG0000(	ENSG0000(	71.12835	0.14226	0.042852	0.116467	lncRNA
ENSG0000(	LINC02318	141.9287	0.196927	0.042853	0.116467	lncRNA
ENSG0000(	F7	20.70212	-0.55052	0.042862	0.116467	protein_coding
ENSG0000(	LINC01318	47.97107	0.238691	0.042863	0.116467	lncRNA
ENSG0000(	AGGF1	1025.542	-0.09743	0.042906	0.116573	protein_coding
ENSG0000(	CD7	293.325	-0.28459	0.042912	0.116581	protein_coding
ENSG0000(	B3GALNT2	1951.683	0.101779	0.042927	0.1166	protein_coding
ENSG0000(	COPS3	332.0347	-0.14691	0.042926	0.1166	protein_coding
ENSG0000(	ZNF74	414.9516	0.096932	0.042933	0.116608	protein_coding
ENSG0000(	IFT140	411.9146	0.151464	0.04294	0.116618	protein_coding
ENSG0000(	SMU1	1545.662	-0.07584	0.042962	0.116666	protein_coding
ENSG0000(	ENSG0000(	395.1297	0.111012	0.04297	0.116678	transcribed_unprocessed_pseudo
ENSG0000(	RNF125	1898.813	-0.12952	0.042998	0.116745	protein_coding
ENSG0000(	BEX3	96.30521	0.227427	0.043003	0.116746	protein_coding
ENSG0000(	SLFN12L	5960.555	0.146099	0.043008	0.11675	protein_coding
ENSG0000(	CARD9	181.2077	0.228328	0.043017	0.116764	protein_coding
ENSG0000(	ENSG0000(	132.6506	0.152187	0.043047	0.116837	lncRNA

ENSG0000(	CHD4	4573.05	-0.08413	0.043057	0.116854	protein_coding
ENSG0000(	DNAJC22	1645.878	-0.32721	0.043084	0.116906	protein_coding
ENSG0000(	TRPM4	3013.939	-0.29598	0.043081	0.116906	protein_coding
ENSG0000(	PCF11-AS1	195.5839	0.11894	0.043116	0.116984	lncRNA
ENSG0000(	HIF1A-AS3	4233.539	-0.10954	0.043165	0.117105	lncRNA
ENSG0000(	LURAP1	181.1306	0.099981	0.04317	0.117111	protein_coding
ENSG0000(	C9orf43	25.65881	0.277677	0.043213	0.117215	protein_coding
ENSG0000(	ENSG0000(	149.5799	0.11626	0.043252	0.117312	TEC
ENSG0000(	ENSG0000(	1601.599	0.104934	0.043291	0.117397	lncRNA
ENSG0000(	GASAL1	149.4387	-0.16013	0.043295	0.117397	lncRNA
ENSG0000(	RPL32P3	1894.249	0.073945	0.043292	0.117397	transcribed_unprocessed_pseudo
ENSG0000(	CCNB1	165.7466	-0.28618	0.043311	0.117432	protein_coding
ENSG0000(	KLRD1	4286.837	0.081348	0.04332	0.117443	protein_coding
ENSG0000(	STAB1	4118.405	0.22409	0.043323	0.117443	protein_coding
ENSG0000(	CHAT	63.93673	-0.34259	0.043335	0.117466	protein_coding
ENSG0000(	CSPG4P11	422.0498	0.259776	0.043354	0.117498	transcribed_unprocessed_pseudo
ENSG0000(	LINC01202	167.1383	0.111737	0.043352	0.117498	lncRNA
ENSG0000(	EPPIN	471.4788	0.14585	0.043399	0.117598	protein_coding
ENSG0000(	LINC01772	641.6181	0.105234	0.043399	0.117598	lncRNA
ENSG0000(	CEMP1	25.33901	0.342736	0.043417	0.117638	protein_coding
ENSG0000(	OXSM	145.4562	-0.14952	0.043422	0.117642	protein_coding
ENSG0000(	LECT2	219.8795	0.217576	0.043438	0.117659	protein_coding
ENSG0000(	NBAS	3388.475	-0.16612	0.043442	0.117659	protein_coding
ENSG0000(	NR2C2	4007.412	-0.06653	0.043444	0.117659	protein_coding
ENSG0000(	TXNRD3	79.06249	0.249944	0.04344	0.117659	protein_coding
ENSG0000(	ENSG0000(	123.0899	0.133966	0.04349	0.117765	lncRNA
ENSG0000(	FBLN1	1687.044	0.188749	0.043487	0.117765	protein_coding
ENSG0000(	ENSG0000(	29.36334	0.291606	0.043498	0.117776	processed_pseudogene
ENSG0000(	SNAP23	1753.231	-0.11004	0.043523	0.117834	protein_coding
ENSG0000(	WHAMMP:	1428.769	0.086639	0.043535	0.117856	transcribed_unprocessed_pseudo
ENSG0000(	ENSG0000(	65.70794	0.184068	0.043554	0.117898	lncRNA
ENSG0000(	ENSG0000(	111.6316	0.226887	0.043565	0.117917	processed_pseudogene
ENSG0000(	RRS1-DT	217.4137	0.111083	0.043578	0.117944	lncRNA
ENSG0000(	SESN3	4214.21	-0.20333	0.043583	0.117946	protein_coding
ENSG0000(	GIT1	715.2813	-0.1056	0.043606	0.117999	protein_coding
ENSG0000(	ENTPD2	155.0812	-0.41782	0.043621	0.118025	protein_coding
ENSG0000(	ZNHIT1	1491.9	-0.12675	0.043623	0.118025	protein_coding
ENSG0000(	KPNA3	704.0857	-0.10507	0.043647	0.118078	protein_coding
ENSG0000(	ATR	4731.818	0.13206	0.043665	0.118116	protein_coding
ENSG0000(	CCDC200	247.4976	0.126889	0.043672	0.118117	protein_coding
ENSG0000(	ENSG0000(	29.52633	0.385074	0.043676	0.118117	lncRNA
ENSG0000(	INTS4	1221.028	-0.09449	0.043673	0.118117	protein_coding
ENSG0000(	ENSG0000(	224.8486	0.097901	0.043692	0.118151	lncRNA
ENSG0000(	IL6R	2866.938	0.178544	0.043717	0.118182	protein_coding
ENSG0000(	KIAA1522	3401.619	-0.34127	0.043719	0.118182	protein_coding
ENSG0000(	TENM1	234.6975	0.282557	0.043716	0.118182	protein_coding
ENSG0000(	TTC3P1	133.2718	-0.20433	0.043709	0.118182	processed_pseudogene

ENSG0000(	NMI	550.5572	0.142965	0.043724	0.118185	protein_coding
ENSG0000(	ENSG0000(	78.03408	-0.30842	0.043736	0.118208	TEC
ENSG0000(	ERAP1	3436.084	-0.12707	0.043766	0.118279	protein_coding
ENSG0000(	ENSG0000(	179.2271	0.129183	0.043796	0.118348	lncRNA
ENSG0000(	ENSG0000(	39.49926	0.337723	0.043814	0.118388	lncRNA
ENSG0000(	ENSG0000(	12.47157	-0.46888	0.043845	0.118461	lncRNA
ENSG0000(	ARHGAP23	8.77859	0.624516	0.043881	0.118547	transcribed_unprocessed_pseudo
ENSG0000(	CIAO2A	546.8118	-0.17878	0.043884	0.118547	protein_coding
ENSG0000(	DIXDC1	600.3163	0.132048	0.043916	0.118622	protein_coding
ENSG0000(	OR2A9P	466.1375	0.228338	0.043948	0.1187	transcribed_unprocessed_pseudo
ENSG0000(	POLR2D	1528.528	-0.06351	0.043959	0.118719	protein_coding
ENSG0000(	CRYBB3	57.01683	-0.40231	0.043988	0.11877	protein_coding
ENSG0000(	ENSG0000(	15.82572	0.454216	0.043986	0.11877	lncRNA
ENSG0000(	ENSG0000(	520.6833	0.190479	0.043989	0.11877	lncRNA
ENSG0000(	MIEF2	420.7089	-0.10887	0.043993	0.11877	protein_coding
ENSG0000(	ENSG0000(	138.4111	0.168365	0.044005	0.118782	lncRNA
ENSG0000(	LINC02303	55.69724	0.225346	0.044002	0.118782	lncRNA
ENSG0000(	COBL	3182.735	-0.43586	0.044029	0.118836	protein_coding
ENSG0000(	RN7SL65P	23.04637	0.240054	0.044055	0.118896	misc_RNA
ENSG0000(	ENSG0000(	501.5555	0.133133	0.044066	0.118915	lncRNA
ENSG0000(	TMEM202-	172.0173	0.158287	0.044102	0.119002	lncRNA
ENSG0000(	ACKR1	98.46507	0.464277	0.044122	0.119028	protein_coding
ENSG0000(	ENSG0000(	18.83541	-0.28897	0.044123	0.119028	lncRNA
ENSG0000(	RN7SKP25	203.8384	-0.2955	0.044116	0.119028	misc_RNA
ENSG0000(	CT69	159.7162	0.122239	0.044152	0.119088	lncRNA
ENSG0000(	ENSG0000(	14.51371	0.316306	0.044153	0.119088	lncRNA
ENSG0000(	RIPK3	670.2638	-0.19967	0.044156	0.119088	protein_coding
ENSG0000(	CLDN18	112.2002	0.262104	0.044182	0.119118	protein_coding
ENSG0000(	NSRP1	1929.951	-0.06578	0.044182	0.119118	protein_coding
ENSG0000(	PARP11	1107.712	0.18632	0.044177	0.119118	protein_coding
ENSG0000(	ZNF653	118.4684	-0.16734	0.044171	0.119118	protein_coding
ENSG0000(	ENSG0000(	221.6667	0.151254	0.044187	0.119121	lncRNA
ENSG0000(	LINC02056	377.7673	0.152808	0.044213	0.119173	lncRNA
ENSG0000(	ZNF736P9Y	28.55616	0.233139	0.044214	0.119173	transcribed_processed_pseudoge
ENSG0000(	SCAP	1958.951	-0.0963	0.044257	0.119279	protein_coding
ENSG0000(	INTU	4628.861	0.084526	0.044266	0.119291	protein_coding
ENSG0000(	ARHGEF18-	45.34554	-0.24365	0.044278	0.119314	lncRNA
ENSG0000(	MAP7	1102.646	-0.30996	0.04429	0.119336	protein_coding
ENSG0000(	SDE2	801.7666	-0.09102	0.044305	0.119366	protein_coding
ENSG0000(	TMEM63A	4813.864	0.159676	0.044309	0.119367	protein_coding
ENSG0000(	HRAS	248.6719	-0.2466	0.044318	0.119382	protein_coding
ENSG0000(	TMSB15B	531.7062	0.157782	0.044333	0.119411	protein_coding
ENSG0000(	SPATA31C1	135.9348	-0.12656	0.044356	0.119462	transcribed_unprocessed_pseudo
ENSG0000(	PDILT	21.21505	-0.29942	0.044362	0.119468	protein_coding
ENSG0000(	ENSG0000(	46.24904	0.209076	0.044372	0.119478	lncRNA
ENSG0000(	RN7SL329P	71.10612	0.161925	0.044373	0.119478	misc_RNA
ENSG0000(	CMC1	641.8535	-0.09873	0.044388	0.119499	protein_coding

ENSG0000(	ENSG0000(	287.541	0.104866	0.044391	0.119499	lncRNA
ENSG0000(	PALB2	543.2703	-0.10296	0.044392	0.119499	protein_coding
ENSG0000(	RN7SL644F	185.3552	-0.14074	0.044402	0.119505	misc_RNA
ENSG0000(	ZNF20	345.2083	-0.11123	0.0444	0.119505	protein_coding
ENSG0000(	RN7SKP292	34.05305	0.178828	0.044414	0.119527	misc_RNA
ENSG0000(	SPCS3	2889.033	0.132645	0.044423	0.119543	protein_coding
ENSG0000(	ENSG0000(	23.54689	-0.47698	0.044434	0.11955	lncRNA
ENSG0000(	POU5F2	1768.432	0.06581	0.044431	0.11955	protein_coding
ENSG0000(	ENSG0000(	21.9105	0.270833	0.044439	0.119553	lncRNA
ENSG0000(	ENSG0000(	62.17091	-0.19644	0.044461	0.119604	unprocessed_pseudogene
ENSG0000(	STAG3L1	549.3288	-0.10974	0.044469	0.119614	transcribed_unprocessed_pseudo
ENSG0000(	ENSG0000(	149.3666	-0.15868	0.044525	0.119754	lncRNA
ENSG0000(	GNAI3	2289.02	-0.05683	0.044573	0.119864	protein_coding
ENSG0000(	LINC02632	247.8454	0.1474	0.044572	0.119864	lncRNA
ENSG0000(	FAM238C	257.5029	0.221261	0.044579	0.119868	transcribed_unprocessed_pseudo
ENSG0000(	ENSG0000(	328.4415	0.091419	0.044586	0.119878	lncRNA
ENSG0000(	ENSG0000(	328.897	0.103155	0.044607	0.119923	lncRNA
ENSG0000(	ENSG0000(	62.35795	0.445456	0.044614	0.119932	lncRNA
ENSG0000(	ENSG0000(	9.951401	0.413778	0.044626	0.119954	lncRNA
ENSG0000(	AFTPH-DT	31.21791	0.435691	0.044632	0.119961	lncRNA
ENSG0000(	C19orf25	969.1854	-0.08288	0.044704	0.120145	protein_coding
ENSG0000(	ENSG0000(	324.0193	-0.11622	0.044736	0.12022	processed_pseudogene
ENSG0000(	SMAD9-IT1	10.75853	0.363381	0.044798	0.120375	lncRNA
ENSG0000(	ADAM22	255.2395	-0.31658	0.044827	0.120443	protein_coding
ENSG0000(	PYM1	382.5714	-0.09994	0.044836	0.120458	protein_coding
ENSG0000(	HID1-AS1	54.93339	0.161145	0.044846	0.120474	lncRNA
ENSG0000(	ENSG0000(	309.1476	-0.09642	0.04487	0.120529	lncRNA
ENSG0000(	ENSG0000(	60.46588	0.206129	0.044901	0.120601	lncRNA
ENSG0000(	ENSG0000(	561.7932	0.109231	0.044913	0.120622	transcribed_unprocessed_pseudo
ENSG0000(	XG	14.98896	0.608934	0.044935	0.120673	protein_coding
ENSG0000(	RN7SL582F	118.3261	-0.14242	0.044962	0.120734	misc_RNA
ENSG0000(	LINC00840	86.89667	0.159273	0.044973	0.120754	lncRNA
ENSG0000(	IGHV5-10-1	39.88373	0.938795	0.044982	0.120768	IG_V_gene
ENSG0000(	SNED1	1619.673	0.216332	0.044988	0.120774	protein_coding
ENSG0000(	ZSCAN23	105.2097	0.266228	0.044996	0.120785	protein_coding
ENSG0000(	AATK	3139.055	-0.4395	0.045004	0.120794	protein_coding
ENSG0000(	NFIC	2017.675	0.111631	0.045017	0.12082	protein_coding
ENSG0000(	ENSG0000(	34.83974	0.31512	0.045051	0.120888	lncRNA
ENSG0000(	HERC5	301.8611	0.327251	0.045049	0.120888	protein_coding
ENSG0000(	TIMM23B-/	847.8729	0.123943	0.045054	0.120888	protein_coding
ENSG0000(	THBS3	521.8395	0.161669	0.045061	0.120897	protein_coding
ENSG0000(	ENSG0000(	24.79516	-0.26855	0.045086	0.120952	lncRNA
ENSG0000(	CHD7	4292.324	0.098805	0.045096	0.120958	protein_coding
ENSG0000(	DENND2A	895.7984	0.085563	0.0451	0.120958	protein_coding
ENSG0000(	ENSG0000(	183.3459	-0.13173	0.045099	0.120958	lncRNA
ENSG0000(	ENSG0000(	178.6404	0.099018	0.045103	0.120958	TEC
ENSG0000(	ENSG0000(	9.502861	0.424801	0.045109	0.120963	lncRNA

ENSG0000(	C2CD6	32.24147	0.766708	0.045152	0.121055	protein_coding
ENSG0000(	ENSG0000(	25.03018	0.287815	0.045155	0.121055	unprocessed_pseudogene
ENSG0000(	NUDT22	949.6588	-0.09647	0.045155	0.121055	protein_coding
ENSG0000(	ABCA7	1163.013	0.248496	0.045175	0.121088	protein_coding
ENSG0000(	COL24A1	264.6457	0.418254	0.045172	0.121088	protein_coding
ENSG0000(	TMED2-DT	2675.212	-0.04877	0.045189	0.121117	lncRNA
ENSG0000(	TEX30	81.49659	-0.16543	0.045222	0.121195	protein_coding
ENSG0000(	ENSG0000(	1297.262	0.114085	0.045269	0.121311	TEC
ENSG0000(	PARK7P2	45.48812	0.19023	0.045281	0.121333	processed_pseudogene
ENSG0000(	ARHGEF16	1346.071	-0.37005	0.045316	0.121406	protein_coding
ENSG0000(	CMC4	44.48966	-0.45043	0.045313	0.121406	protein_coding
ENSG0000(	ENSG0000(	37.16022	0.205359	0.045322	0.121407	lncRNA
ENSG0000(	TSPYL4	430.4419	0.159943	0.045324	0.121407	protein_coding
ENSG0000(	ZDHHC23	1154.277	-0.22018	0.045341	0.121441	protein_coding
ENSG0000(	ENSG0000(	35.45742	-0.30328	0.045364	0.121493	lncRNA
ENSG0000(	ENSG0000(	26.37471	0.280294	0.04539	0.121542	lncRNA
ENSG0000(	HAPLN1	171.8242	-0.44476	0.045389	0.121542	protein_coding
ENSG0000(	FOXS1	13.21222	0.490668	0.045403	0.121565	protein_coding
ENSG0000(	ENSG0000(	57.06185	-0.62284	0.045414	0.121584	lncRNA
ENSG0000(	LACTB	553.146	0.132054	0.045448	0.121665	protein_coding
ENSG0000(	HECTD1	7730.473	-0.04878	0.045457	0.121679	protein_coding
ENSG0000(	ENSG0000(	10.54883	0.409666	0.045464	0.121688	lncRNA
ENSG0000(	MYRFL	1046.982	-0.43042	0.045526	0.121844	protein_coding
ENSG0000(	INSIG2	945.3196	-0.10895	0.045549	0.121896	protein_coding
ENSG0000(	ENSG0000(	310.8355	-0.1253	0.045559	0.121901	lncRNA
ENSG0000(	TSPAN5-DT	219.7889	0.167915	0.045557	0.121901	lncRNA
ENSG0000(	ENSG0000(	41.17985	0.195573	0.045588	0.121969	TEC
ENSG0000(	ARMC1	577.9607	-0.09234	0.045594	0.121974	protein_coding
ENSG0000(	ENSG0000(	22.79793	0.307746	0.045626	0.122039	lncRNA
ENSG0000(	ZNF304	350.7871	0.151242	0.045623	0.122039	protein_coding
ENSG0000(	GUCY2C	2023.147	-0.40227	0.045637	0.122057	protein_coding
ENSG0000(	XAGE5	21.97613	-0.30048	0.045648	0.122077	protein_coding
ENSG0000(	EML2-AS1	21.66586	0.344951	0.045653	0.122079	lncRNA
ENSG0000(	ENSG0000(	195.3779	0.174025	0.045694	0.122168	TEC
ENSG0000(	LINC02465	75.6977	-0.14221	0.045698	0.122168	lncRNA
ENSG0000(	OTUD4	2831.247	-0.08097	0.045705	0.122168	protein_coding
ENSG0000(	RHOT1P1	33.69145	0.358918	0.045702	0.122168	processed_pseudogene
ENSG0000(	UBL5	666.8944	-0.17022	0.045693	0.122168	protein_coding
ENSG0000(	USF1	1132.222	0.117765	0.045716	0.122185	protein_coding
ENSG0000(	ZC3H12C	1385.926	0.271475	0.04572	0.122187	protein_coding
ENSG0000(	NIPAL3	2325.838	-0.11495	0.045804	0.122402	protein_coding
ENSG0000(	PIGA	639.2598	0.099099	0.045817	0.122426	protein_coding
ENSG0000(	ENSG0000(	516.8203	0.071144	0.045828	0.122443	lncRNA
ENSG0000(	BBOX1-AS1	68.61604	0.188103	0.045836	0.122455	lncRNA
ENSG0000(	ENSG0000(	41.24815	-0.22809	0.045844	0.122465	lncRNA
ENSG0000(	ARMC2	180.3979	-0.16016	0.04585	0.122472	protein_coding
ENSG0000(	LINC02281	97.5727	0.154879	0.045869	0.122513	lncRNA

ENSG0000(	CCDC149	467.3333	0.212307	0.045911	0.122614	protein_coding
ENSG0000(	ENSG0000(	248.9775	-0.12674	0.04592	0.122617	lncRNA
ENSG0000(	TMEM178/	81.27704	0.277293	0.045917	0.122617	protein_coding
ENSG0000(	ENSG0000(	510.8091	0.091402	0.04595	0.122675	TEC
ENSG0000(	MT1F	386.547	-0.54081	0.045949	0.122675	protein_coding
ENSG0000(	ENSG0000(	19.36344	0.337404	0.045966	0.122707	lncRNA
ENSG0000(	ENSG0000(	52.32	-0.58648	0.045974	0.122721	TEC
ENSG0000(	NBEAL2	3786.581	0.165272	0.045995	0.122764	protein_coding
ENSG0000(	ENSG0000(	14.09293	0.281681	0.046006	0.122783	lncRNA
ENSG0000(	BCL2L13	1168.556	-0.09062	0.046015	0.122787	protein_coding
ENSG0000(	ENSG0000(	116.0366	-0.17323	0.046014	0.122787	lncRNA
ENSG0000(	NHLRC3	1723.846	0.086804	0.046036	0.122834	protein_coding
ENSG0000(	ENSG0000(	18.50763	-0.38514	0.046044	0.122845	lncRNA
ENSG0000(	OSBPL8	2312.873	-0.10084	0.04606	0.122875	protein_coding
ENSG0000(	RN7SKP47	10.67634	-0.49163	0.04614	0.123079	misc_RNA
ENSG0000(	ENSG0000(	10.75165	0.45249	0.046161	0.123125	lncRNA
ENSG0000(	BCHE	50.26857	-0.4462	0.046168	0.123134	protein_coding
ENSG0000(	ENSG0000(	444.688	-0.07386	0.046178	0.12315	lncRNA
ENSG0000(	ACR	10.95502	0.488884	0.046189	0.123168	protein_coding
ENSG0000(	RNU6-60P	32.94023	0.283473	0.046193	0.123169	snRNA
ENSG0000(	ENSG0000(	135.1037	-0.17735	0.046212	0.123199	lncRNA
ENSG0000(	ENSG0000(	76.47933	0.144581	0.046211	0.123199	lncRNA
ENSG0000(	AHCY	526.8747	-0.14327	0.04622	0.123206	protein_coding
ENSG0000(	ENSG0000(	41.56709	0.223009	0.046223	0.123206	protein_coding
ENSG0000(	SERHL	190.1545	0.129786	0.046234	0.123226	transcribed_unitary_pseudogene
ENSG0000(	ENSG0000(	261.0566	0.23927	0.046242	0.123236	TEC
ENSG0000(	MEN1	496.4249	-0.09576	0.046254	0.123258	protein_coding
ENSG0000(	ARL6IP5	1145.873	-0.14232	0.04626	0.123265	protein_coding
ENSG0000(	IAH1	1228.469	-0.06865	0.046287	0.123324	protein_coding
ENSG0000(	DHRS3	1023.954	-0.25408	0.046308	0.123362	protein_coding
ENSG0000(	ENSG0000(	51.6102	0.218734	0.046309	0.123362	lncRNA
ENSG0000(	ZNF334	486.5976	0.243215	0.046321	0.123385	protein_coding
ENSG0000(	ENSG0000(	93.30576	-0.38202	0.04633	0.123392	lncRNA
ENSG0000(	SCAMP5	517.2871	-0.35237	0.046332	0.123392	protein_coding
ENSG0000(	ENSG0000(	21.06438	0.376765	0.046371	0.123461	transcribed_unprocessed_pseudo
ENSG0000(	ENSG0000(	19.61804	0.591717	0.046367	0.123461	lncRNA
ENSG0000(	ENSG0000(	178.9246	-0.25502	0.046377	0.123461	lncRNA
ENSG0000(	GALM	729.4969	-0.13577	0.046374	0.123461	protein_coding
ENSG0000(	MIR194-2H	5351.329	-0.34655	0.046368	0.123461	lncRNA
ENSG0000(	ENSG0000(	207.2809	-0.14746	0.046402	0.123518	lncRNA
ENSG0000(	DIAPH2-AS	71.54437	0.165221	0.046414	0.123528	lncRNA
ENSG0000(	MSN	3092.883	0.232759	0.046413	0.123528	protein_coding
ENSG0000(	NR2F2-AS1	596.5932	0.213488	0.046427	0.12354	lncRNA
ENSG0000(	XCL2	8.848186	-0.51379	0.046424	0.12354	protein_coding
ENSG0000(	IL6R-AS1	198.3712	0.102727	0.046433	0.123546	lncRNA
ENSG0000(	ENSG0000(	30.45047	0.237628	0.046443	0.123552	lncRNA
ENSG0000(	ENSG0000(	22.96403	-0.29441	0.046442	0.123552	lncRNA

ENSG0000(	ENSG0000(	10.21879	0.383427	0.046456	0.123576	lncRNA
ENSG0000(	VEZT	2363.59	-0.06892	0.04646	0.123577	protein_coding
ENSG0000(	ENSG0000(	461.5522	0.181533	0.046466	0.123583	transcribed_processed_pseudoge
ENSG0000(	IL22RA1	1075.635	-0.44487	0.046476	0.123597	protein_coding
ENSG0000(	OR1J1	25.10655	0.272969	0.046481	0.1236	protein_coding
ENSG0000(	ENSG0000(	40.87639	0.212501	0.046486	0.123603	lncRNA
ENSG0000(	FKBP8	771.4499	-0.11463	0.04649	0.123604	protein_coding
ENSG0000(	ENSG0000(	76.115	-0.19674	0.046502	0.123627	lncRNA
ENSG0000(	RNU1-103F	10.50089	-0.42622	0.046527	0.123681	snRNA
ENSG0000(	HS3ST3A1	136.5505	0.437174	0.046535	0.123692	protein_coding
ENSG0000(	ENSG0000(	60.43194	0.217752	0.046559	0.123748	processed_pseudogene
ENSG0000(	ENSG0000(	43.21306	0.195392	0.046615	0.123883	lncRNA
ENSG0000(	USP38	1774.553	0.085042	0.046618	0.123883	protein_coding
ENSG0000(	CENATAC-D	444.9108	0.089034	0.046634	0.123908	lncRNA
ENSG0000(	ZNF506	3556.498	-0.11295	0.046636	0.123908	protein_coding
ENSG0000(	NPEPPSP1	1143.917	-0.16104	0.046644	0.123919	unprocessed_pseudogene
ENSG0000(	CATSPER3	34.94113	-0.32295	0.046648	0.12392	protein_coding
ENSG0000(	ADAM19	3042.516	0.347598	0.046672	0.123971	protein_coding
ENSG0000(	STAM	497.5011	-0.1826	0.046675	0.123971	protein_coding
ENSG0000(	CREBBP	6499.624	-0.12402	0.046687	0.123989	protein_coding
ENSG0000(	FBXO38	1788.579	0.058089	0.046698	0.123989	protein_coding
ENSG0000(	LINC00595	84.15334	0.226198	0.046692	0.123989	lncRNA
ENSG0000(	LINC01954	10.95417	-0.8561	0.046697	0.123989	lncRNA
ENSG0000(	CMTM4	2206.386	-0.29774	0.04674	0.12408	protein_coding
ENSG0000(	ZNF649	519.928	0.089817	0.046739	0.12408	protein_coding
ENSG0000(	TVP23CP2	64.33564	0.194376	0.046744	0.124081	processed_pseudogene
ENSG0000(	YWHAB	4517.32	-0.10811	0.046779	0.124164	protein_coding
ENSG0000(	ENSG0000(	142.3825	0.111621	0.046857	0.124339	lncRNA
ENSG0000(	GPR15	123.3212	-0.4486	0.046852	0.124339	protein_coding
ENSG0000(	MTRNR2L1	36.4827	0.491365	0.046856	0.124339	protein_coding
ENSG0000(	CLEC18A	274.3829	-0.22191	0.046863	0.124343	protein_coding
ENSG0000(	NFIL3	352.2225	0.285981	0.046868	0.124348	protein_coding
ENSG0000(	ZBTB18	1128.319	0.118663	0.046873	0.124349	protein_coding
ENSG0000(	ENSG0000(	252.8434	0.122401	0.046877	0.12435	TEC
ENSG0000(	SPG7	13004.46	0.09711	0.046886	0.124364	protein_coding
ENSG0000(	ENSG0000(	10.70169	-0.53308	0.046908	0.12441	lncRNA
ENSG0000(	ENSG0000(	20.61158	0.461104	0.046926	0.124449	transcribed_unprocessed_pseudo
ENSG0000(	ENSG0000(	23.66606	0.25816	0.046972	0.124538	lncRNA
ENSG0000(	TARBP2	270.9188	0.115093	0.046971	0.124538	protein_coding
ENSG0000(	ZNF222	86.55881	0.170617	0.04697	0.124538	protein_coding
ENSG0000(	RNU4ATAC	39.90832	0.233264	0.046976	0.124539	snRNA
ENSG0000(	ASB8	881.0332	-0.09286	0.046987	0.124557	protein_coding
ENSG0000(	IGLJ3	20.51442	0.584136	0.046995	0.124569	IG_J_gene
ENSG0000(	CDC42BPB	3014.132	-0.18076	0.047013	0.124605	protein_coding
ENSG0000(	LINC02154	35.22294	0.221369	0.047019	0.12461	lncRNA
ENSG0000(	SEPTIN12	22.6879	0.334274	0.047022	0.12461	protein_coding
ENSG0000(	PCDHGA1	37.66423	0.237896	0.047032	0.124625	protein_coding

ENSG0000(	ENSG0000(	117.2053	0.119467	0.047048	0.124645	lncRNA
ENSG0000(	MEG8	2334.496	0.111495	0.047051	0.124645	lncRNA
ENSG0000(	TES	1579.569	-0.09008	0.047047	0.124645	protein_coding
ENSG0000(	RORA-AS1	118.0045	-0.19213	0.047094	0.124746	lncRNA
ENSG0000(	ENSG0000(	145.8509	0.166885	0.047109	0.124777	lncRNA
ENSG0000(	IMPA1P1	51.30327	-0.41287	0.047142	0.124834	transcribed_unprocessed_pseudo
ENSG0000(	MIR3936H(	1860.223	-0.15097	0.04714	0.124834	lncRNA
ENSG0000(	RTKN2	649.755	0.155328	0.047143	0.124834	protein_coding
ENSG0000(	LINC01031	40.89797	0.211944	0.047149	0.12484	lncRNA
ENSG0000(	LYPLA2	432.4474	-0.15058	0.047168	0.124881	protein_coding
ENSG0000(	ENSG0000(	17.47285	0.299051	0.047222	0.125002	lncRNA
ENSG0000(	YY1AP1	2010.895	-0.04842	0.047222	0.125002	protein_coding
ENSG0000(	GRK5	925.5388	0.120931	0.047229	0.125009	protein_coding
ENSG0000(	NEO1	2428.817	-0.25488	0.047235	0.125016	protein_coding
ENSG0000(	TMLHE	841.1559	-0.09029	0.047251	0.125049	protein_coding
ENSG0000(	FHIP2B	1717.683	-0.07476	0.047274	0.125086	protein_coding
ENSG0000(	FZR1	1315.846	-0.13128	0.047271	0.125086	protein_coding
ENSG0000(	TESK2	569.3343	-0.28708	0.047279	0.125091	protein_coding
ENSG0000(	ADAM28	3842.948	0.311903	0.047347	0.125242	protein_coding
ENSG0000(	ENSG0000(	112.4168	-0.17752	0.047348	0.125242	lncRNA
ENSG0000(	ZNF620	485.8142	0.095191	0.047344	0.125242	protein_coding
ENSG0000(	ENSG0000(	111.4647	-0.19437	0.047354	0.125247	lncRNA
ENSG0000(	PLGLA	79.2906	-0.19337	0.047364	0.125262	transcribed_unprocessed_pseudo
ENSG0000(	CUL1	1213.483	0.089632	0.047388	0.125304	protein_coding
ENSG0000(	ENSG0000(	132.3268	0.29383	0.047386	0.125304	TEC
ENSG0000(	ENSG0000(	416.4106	0.166443	0.0474	0.125327	lncRNA
ENSG0000(	BRPF3-AS1	205.9981	0.123676	0.047424	0.125379	lncRNA
ENSG0000(	ENSG0000(	33.63821	-0.25859	0.047436	0.125393	lncRNA
ENSG0000(	TAF2	5872.156	-0.07324	0.047437	0.125393	protein_coding
ENSG0000(	SLC26A4-A:	3781.508	-0.1172	0.047449	0.125413	lncRNA
ENSG0000(	ITPR1	5969.159	0.266655	0.047487	0.125503	protein_coding
ENSG0000(	RNMT	1370.253	0.10757	0.047508	0.125548	protein_coding
ENSG0000(	CD36	1245.36	-0.23496	0.047526	0.125585	protein_coding
ENSG0000(	DPY19L2P1	434.9643	0.106252	0.047545	0.125626	transcribed_unprocessed_pseudo
ENSG0000(	ENSG0000(	206.2507	-0.10073	0.047577	0.125667	protein_coding
ENSG0000(	KMT2D	11552.01	0.04562	0.047577	0.125667	protein_coding
ENSG0000(	NBPF2P	102.3414	-0.20747	0.047565	0.125667	unprocessed_pseudogene
ENSG0000(	RS1	44.41421	0.242145	0.047571	0.125667	protein_coding
ENSG0000(	ENSG0000(	443.0606	0.078391	0.047589	0.125688	lncRNA
ENSG0000(	CEP43	2522.425	-0.1234	0.047594	0.12569	protein_coding
ENSG0000(	ENSG0000(	20.45438	0.306978	0.047609	0.125707	lncRNA
ENSG0000(	ENSG0000(	380.958	0.202866	0.047612	0.125707	protein_coding
ENSG0000(	SCCPDH	465.2715	-0.29197	0.047608	0.125707	protein_coding
ENSG0000(	RAP1A	1584.189	-0.0999	0.047646	0.125775	protein_coding
ENSG0000(	SCARNA16	356.6375	-0.16682	0.047645	0.125775	scaRNA
ENSG0000(	STRN3	1202.376	-0.0926	0.04766	0.125803	protein_coding
ENSG0000(	MAPK8	1641.092	-0.10827	0.047669	0.125815	protein_coding

ENSG0000(	ENSG0000(	275.5206	0.103931	0.047699	0.125883	lncRNA
ENSG0000(	ABHD16A	596.2204	-0.09652	0.047741	0.125985	protein_coding
ENSG0000(	RIMS1	169.4487	-0.12934	0.047755	0.126011	protein_coding
ENSG0000(	ATMIN	1153.433	-0.1132	0.047822	0.126176	protein_coding
ENSG0000(	PHRF1	1841.974	-0.10469	0.047831	0.126191	protein_coding
ENSG0000(	ZNF160	2755.121	0.068186	0.047843	0.126212	protein_coding
ENSG0000(	CX3CL1	646.181	0.128673	0.047854	0.126229	protein_coding
ENSG0000(	LDLRAD1	134.1429	0.11796	0.047862	0.126239	protein_coding
ENSG0000(	DDX19A-D⁻	153.3573	-0.11446	0.047935	0.126415	lncRNA
ENSG0000(	ENSG0000(	22.56035	0.261097	0.047936	0.126415	lncRNA
ENSG0000(	BCKDHB	919.3281	0.181411	0.047953	0.126449	protein_coding
ENSG0000(	ENSG0000(	20.63644	-0.48357	0.047959	0.126453	processed_pseudogene
ENSG0000(	RETREG1-A	98.13028	0.221182	0.047981	0.126502	lncRNA
ENSG0000(	CASKIN1	17.73058	0.611366	0.047989	0.126504	protein_coding
ENSG0000(	THNSL1	223.796	-0.13193	0.04799	0.126504	protein_coding
ENSG0000(	NCEH1	459.7063	-0.16909	0.047996	0.126509	protein_coding
ENSG0000(	NFE2L2	2197.293	-0.11573	0.048	0.126509	protein_coding
ENSG0000(	ARSK	327.3428	-0.09705	0.04803	0.126564	protein_coding
ENSG0000(	ENSG0000(	21.49045	-0.25499	0.048033	0.126564	lncRNA
ENSG0000(	ENSG0000(	330.8006	0.088178	0.048026	0.126564	lncRNA
ENSG0000(	MZF1	1654.886	-0.08972	0.048052	0.126602	protein_coding
ENSG0000(	ENSG0000(	114.4933	-0.15111	0.048074	0.126652	lncRNA
ENSG0000(	NPEPPS	6879.526	-0.08022	0.04808	0.126657	protein_coding
ENSG0000(	LINC02033	105.6596	0.214145	0.048103	0.126707	lncRNA
ENSG0000(	FKTN	1707.365	0.106481	0.048145	0.126794	protein_coding
ENSG0000(	SH2D2A	153.4515	0.26968	0.048141	0.126794	protein_coding
ENSG0000(	ALDH1A2	106.3854	0.258285	0.048154	0.126798	protein_coding
ENSG0000(	FAM193A	2017.68	-0.06451	0.048153	0.126798	protein_coding
ENSG0000(	TPRN	1410.475	-0.35011	0.048183	0.126864	protein_coding
ENSG0000(	SLC16A10	1173.326	0.130823	0.048187	0.126865	protein_coding
ENSG0000(	SGMS2	1173.904	-0.31971	0.048262	0.12705	protein_coding
ENSG0000(	ENSG0000(	17.55328	0.300103	0.048271	0.127065	lncRNA
ENSG0000(	ENSG0000(	32.01001	-0.37868	0.048301	0.12711	lncRNA
ENSG0000(	ENSG0000(	1128.512	0.073228	0.048301	0.12711	TEC
ENSG0000(	KCNH8	150.7817	0.283065	0.048305	0.12711	protein_coding
ENSG0000(	SCARA5	260.0318	-0.26748	0.048294	0.12711	protein_coding
ENSG0000(	LRRTM1	13.80627	0.589294	0.048331	0.127169	protein_coding
ENSG0000(	HABP2	13.17639	0.694097	0.048356	0.127223	protein_coding
ENSG0000(	ENSG0000(	120.1237	-0.1306	0.04836	0.127225	lncRNA
ENSG0000(	TUBD1	644.5818	-0.08847	0.048374	0.127249	protein_coding
ENSG0000(	ENSG0000(	724.7148	0.087882	0.048393	0.12729	lncRNA
ENSG0000(	SERPINB12	182.2297	0.111768	0.048401	0.1273	protein_coding
ENSG0000(	C16orf86	31.62784	0.304366	0.048406	0.127303	protein_coding
ENSG0000(	ATP8B2	1621.161	0.197049	0.048417	0.127308	protein_coding
ENSG0000(	ENSG0000(	11.84852	-0.48046	0.048426	0.127308	processed_pseudogene
ENSG0000(	EPB41L4A-/	502.4259	-0.12184	0.04843	0.127308	lncRNA
ENSG0000(	MIR6772	9.792305	-0.59589	0.048413	0.127308	miRNA

ENSG0000(	NPHS1	366.329	0.146296	0.048428	0.127308	protein_coding
ENSG0000(	SIGLEC7	45.86916	0.290416	0.048433	0.127308	protein_coding
ENSG0000(	PLIN3	1010.026	-0.2075	0.048438	0.127311	protein_coding
ENSG0000(	ENSG0000(	25.46366	0.291481	0.048443	0.127316	lncRNA
ENSG0000(	ENSG0000(	17.26373	-0.34222	0.04848	0.127395	lncRNA
ENSG0000(	MOB1A	3954.082	-0.10326	0.048484	0.127395	protein_coding
ENSG0000(	RNF112	147.0973	-0.26253	0.048486	0.127395	protein_coding
ENSG0000(	NUP85	1068.43	-0.10814	0.048505	0.127436	protein_coding
ENSG0000(	PRDM8	151.0844	0.383635	0.04851	0.127438	protein_coding
ENSG0000(	ENSG0000(	370.7742	0.100375	0.048528	0.127468	lncRNA
ENSG0000(	LINC00355	394.3192	-0.09683	0.04853	0.127468	lncRNA
ENSG0000(	SRSF12	359.2193	0.162674	0.048539	0.127481	protein_coding
ENSG0000(	RN7SKP1	14.73818	-0.41378	0.048618	0.127679	misc_RNA
ENSG0000(	ENSG0000(	17.78817	0.38552	0.048628	0.127694	lncRNA
ENSG0000(	OR9K1P	107.5945	-0.19732	0.048645	0.127729	transcribed_unitary_pseudogene
ENSG0000(	PLAT	1114.224	0.157016	0.048652	0.127737	protein_coding
ENSG0000(	YWHAQ	1470.22	-0.12617	0.048659	0.127743	protein_coding
ENSG0000(	C1QC	604.0023	0.266651	0.048677	0.127771	protein_coding
ENSG0000(	ENSG0000(	158.969	0.2224	0.048674	0.127771	lncRNA
ENSG0000(	KCNK12	242.6792	-0.47254	0.048699	0.127795	protein_coding
ENSG0000(	RUSC1-AS1	329.9264	0.156532	0.048696	0.127795	lncRNA
ENSG0000(	SPDYE18	64.23229	-0.224	0.048696	0.127795	protein_coding
ENSG0000(	CYCS	1839.747	-0.18393	0.048733	0.127873	protein_coding
ENSG0000(	DUXA	35.46418	0.216032	0.048737	0.127873	protein_coding
ENSG0000(	ENSG0000(	57.23998	-0.18342	0.048748	0.127893	lncRNA
ENSG0000(	ENSG0000(	628.3946	-0.07603	0.048764	0.127923	TEC
ENSG0000(	ENSG0000(	17.56557	0.288082	0.048771	0.127932	lncRNA
ENSG0000(	MCUR1P1	132.5698	0.120789	0.048794	0.127958	processed_pseudogene
ENSG0000(	PFDN2	78.03119	-0.18381	0.048787	0.127958	protein_coding
ENSG0000(	STX4	1032.997	-0.08627	0.048793	0.127958	protein_coding
ENSG0000(	SAV1	674.539	0.149463	0.04883	0.128042	protein_coding
ENSG0000(	GCC2	10843.72	0.141303	0.048838	0.128053	protein_coding
ENSG0000(	ENSG0000(	60.4221	-0.16598	0.048844	0.128058	lncRNA
ENSG0000(	CCDC162P	646.01	0.30897	0.048872	0.128121	transcribed_unitary_pseudogene
ENSG0000(	ENSG0000(	11.37358	0.572484	0.048878	0.128125	lncRNA
ENSG0000(	LINC01322	806.4134	0.108588	0.04891	0.128199	lncRNA
ENSG0000(	ENSG0000(	960.605	0.134038	0.048934	0.128252	lncRNA
ENSG0000(	SEPTIN7-D'	238.9754	0.161633	0.048943	0.128265	lncRNA
ENSG0000(	ADGRL1	733.3437	-0.1752	0.048972	0.128294	protein_coding
ENSG0000(	ENSG0000(	12.15859	0.355346	0.04897	0.128294	misc_RNA
ENSG0000(	GPX2	608.0568	0.368062	0.048979	0.128294	protein_coding
ENSG0000(	MTF1	1354.856	0.156281	0.048976	0.128294	protein_coding
ENSG0000(	PNOC	71.46161	0.489455	0.048978	0.128294	protein_coding
ENSG0000(	ZNF704	1981.338	-0.25975	0.048961	0.128294	protein_coding
ENSG0000(	ERLEC1	2616.355	0.063345	0.048991	0.128316	protein_coding
ENSG0000(	GRB10	1263.752	0.13644	0.048995	0.128316	protein_coding
ENSG0000(	GNRHR2	1699.621	-0.11534	0.049042	0.128429	transcribed_unitary_pseudogene

ENSG0000(	LRRC58	1834.037	-0.07255	0.049047	0.128431	protein_coding
ENSG0000(	IVNS1ABP	4989.397	0.16123	0.049052	0.128432	protein_coding
ENSG0000(	GPR108	582.754	-0.14584	0.049064	0.128435	protein_coding
ENSG0000(	GPR12	101.6352	-0.1187	0.049065	0.128435	protein_coding
ENSG0000(	MIR2909	9.222191	0.373711	0.049057	0.128435	miRNA
ENSG0000(	ENSG0000(	244.6267	-0.16092	0.049073	0.128446	lncRNA
ENSG0000(	SLC22A16	40.63224	0.229861	0.049121	0.128548	protein_coding
ENSG0000(	USP21	415.4205	-0.10415	0.049117	0.128548	protein_coding
ENSG0000(	RHOT1P2	9.682159	0.603668	0.049133	0.128569	processed_pseudogene
ENSG0000(	ENSG0000(	96.00161	0.233299	0.049148	0.1286	TEC
ENSG0000(	OMG	21.09511	0.418944	0.049152	0.1286	protein_coding
ENSG0000(	ZNF654	972.9009	-0.11397	0.049221	0.12877	protein_coding
ENSG0000(	DCUN1D4	1133.097	-0.10079	0.049233	0.128784	protein_coding
ENSG0000(	ENSG0000(	629.1136	-0.16136	0.049235	0.128784	lncRNA
ENSG0000(	LINC02679	23.80281	0.332414	0.049239	0.128784	lncRNA
ENSG0000(	ENSG0000(	10.53464	0.480387	0.049277	0.128858	lncRNA
ENSG0000(	ENSG0000(	163.1639	-0.16427	0.04928	0.128858	TEC
ENSG0000(	SYCP1	233.0154	-0.14	0.049278	0.128858	protein_coding
ENSG0000(	LINC01619	154.8796	0.353462	0.049296	0.128889	lncRNA
ENSG0000(	SLC15A2	1074.447	0.313028	0.049323	0.128949	protein_coding
ENSG0000(	RNU6-1327	47.48147	-0.29091	0.049357	0.12901	snRNA
ENSG0000(	SLIT3-AS2	514.5211	-0.22053	0.049358	0.12901	lncRNA
ENSG0000(	ZBTB21	637.1417	-0.13547	0.049353	0.12901	protein_coding
ENSG0000(	TRGV8	16.63435	-0.32293	0.049399	0.129105	TR_V_gene
ENSG0000(	DCTN4	1860.911	0.058379	0.049404	0.129107	protein_coding
ENSG0000(	DMXL2	7574.787	0.152219	0.04941	0.129113	protein_coding
ENSG0000(	SMG1P3	12779.26	0.160967	0.049421	0.129131	transcribed_unprocessed_pseudo
ENSG0000(	ANKK1	56.15614	0.205414	0.049433	0.129152	protein_coding
ENSG0000(	ENSG0000(	16.7018	0.340131	0.049475	0.129247	lncRNA
ENSG0000(	ENSG0000(	74.70111	0.127849	0.049478	0.129247	lncRNA
ENSG0000(	WASHC4	3506.774	-0.09757	0.0495	0.129295	protein_coding
ENSG0000(	ENSG0000(	157.197	-0.25792	0.049511	0.129312	protein_coding
ENSG0000(	ZFAS1	1471.009	-0.11969	0.049535	0.129364	lncRNA
ENSG0000(	ETHE1	659.121	-0.1846	0.049574	0.129456	protein_coding
ENSG0000(	KIAA0586	2283.29	-0.0826	0.049594	0.129496	protein_coding
ENSG0000(	ANAPC16	2416.96	-0.08709	0.049622	0.129539	protein_coding
ENSG0000(	MANBAL	359.3086	-0.11724	0.049623	0.129539	protein_coding
ENSG0000(	UBL3	896.4766	-0.15917	0.049618	0.129539	protein_coding
ENSG0000(	ENSG0000(	178.5002	0.156404	0.049639	0.129571	lncRNA
ENSG0000(	MAFTRR	186.2506	-0.24659	0.049649	0.129575	lncRNA
ENSG0000(	MIX23	371.7243	-0.13281	0.049646	0.129575	protein_coding
ENSG0000(	TAS2R31	225.8707	0.155932	0.049679	0.129642	protein_coding
ENSG0000(	ENSG0000(	31.5694	0.268181	0.049694	0.129662	lncRNA
ENSG0000(	KCNK6	3022.565	0.071138	0.049691	0.129662	protein_coding
ENSG0000(	ENSG0000(	77.98326	0.165899	0.049701	0.129669	lncRNA
ENSG0000(	RNVU1-2	2130.256	-0.21869	0.049716	0.129698	snRNA
ENSG0000(	ENSG0000(	40.59643	0.26542	0.049729	0.129721	lncRNA

ENSG0000(	ENSG0000(	110.3134	-0.16166	0.049742	0.129742	lncRNA
ENSG0000(	ENSG0000(	36.82896	0.302615	0.049761	0.129782	lncRNA
ENSG0000(	MTND2P28	881.8051	-0.45876	0.049765	0.129782	unprocessed_pseudogene
ENSG0000(	ENSG0000(	12.48748	0.718444	0.049781	0.129813	lncRNA
ENSG0000(	PMS2P8	22.17404	-0.65102	0.049788	0.12982	unprocessed_pseudogene
ENSG0000(	VRK3	1880.444	-0.09829	0.049826	0.129908	protein_coding
ENSG0000(	SPTLC1	2093.17	-0.06264	0.049858	0.129981	protein_coding
ENSG0000(	EMID1	337.3003	0.190211	0.049866	0.129991	protein_coding
ENSG0000(	TOP3B	798.7005	0.105646	0.049893	0.130051	protein_coding
ENSG0000(	RPL26P6	53.74189	0.215942	0.049902	0.130065	processed_pseudogene
ENSG0000(	TXNDC17	295.2764	-0.26258	0.049907	0.130065	protein_coding
ENSG0000(	ENSG0000(	87.74343	0.212781	0.049914	0.130073	lncRNA
ENSG0000(	ENPP4	557.6635	-0.17074	0.049936	0.130121	protein_coding
ENSG0000(	STX17-DT	3430.458	-0.08403	0.049952	0.130151	lncRNA
ENSG0000(	ORMDL2	501.9148	-0.08593	0.049974	0.130199	protein_coding
ENSG0000(	DDX55P1	15.61045	0.476677	0.049979	0.1302	processed_pseudogene
ENSG0000(	IGHV3-37	10.23028	0.513753	0.049997	0.130232	IG_V_pseudogene
ENSG0000(	KDSR	1339.24	-0.11487	0.05	0.130232	protein_coding
ENSG0000(	EIF4HP2	10.05739	0.404709	0.050014	0.130259	processed_pseudogene
ENSG0000(	USHBP1	669.3533	-0.10193	0.050037	0.130309	protein_coding
ENSG0000(	LINC01667	70.7052	-0.26104	0.050061	0.130361	transcribed_processed_pseudoge
ENSG0000(	AHCYL1	2127.094	-0.15489	0.050074	0.130383	protein_coding
ENSG0000(	ENSG0000(	73.97955	0.168765	0.050097	0.130432	TEC
ENSG0000(	ENSG0000(	272.745	0.122758	0.050107	0.130437	lncRNA
ENSG0000(	IGHV3OR16	9.502899	0.672455	0.050104	0.130437	IG_V_gene
ENSG0000(	CCDC157	418.4393	-0.10065	0.050134	0.130476	protein_coding
ENSG0000(	ENSG0000(	541.9816	0.216106	0.050135	0.130476	protein_coding
ENSG0000(	ENSG0000(	3649.898	-0.1008	0.050131	0.130476	lncRNA
ENSG0000(	DNAJB5-DT	34.79559	-0.58384	0.050147	0.130496	lncRNA
ENSG0000(	ENSG0000(	19.13164	0.278741	0.050155	0.130507	lncRNA
ENSG0000(	SRD5A1	2473.01	-0.10587	0.050161	0.130513	protein_coding
ENSG0000(	ENSG0000(	132.8052	0.132729	0.050176	0.13054	lncRNA
ENSG0000(	NSD2	4861.472	0.067833	0.050182	0.130546	protein_coding
ENSG0000(	LINC01030	9.487324	0.396457	0.050258	0.130732	lncRNA
ENSG0000(	ENSG0000(	49.235	-0.21329	0.050273	0.13076	lncRNA
ENSG0000(	HAS3	109.8127	0.46649	0.050299	0.130817	protein_coding
ENSG0000(	ENSG0000(	10.59847	0.519221	0.050321	0.130848	lncRNA
ENSG0000(	LINC01998	32.90977	0.248925	0.050323	0.130848	lncRNA
ENSG0000(	RD3	148.0851	-0.117	0.050323	0.130848	protein_coding
ENSG0000(	DNAJC25	202.1363	-0.16231	0.050331	0.130858	protein_coding
ENSG0000(	PRTG	169.7205	0.276372	0.050357	0.130915	protein_coding
ENSG0000(	ENSG0000(	63.54747	0.152321	0.050404	0.131025	lncRNA
ENSG0000(	ENSG0000(	146.0429	0.166168	0.050452	0.13114	TEC
ENSG0000(	ENSG0000(	24.27296	0.229005	0.05047	0.131175	lncRNA
ENSG0000(	MTMR4	3283.186	-0.15286	0.05049	0.131216	protein_coding
ENSG0000(	DNMBP-AS	352.6082	0.076926	0.050498	0.131226	lncRNA
ENSG0000(	CACNA1G	115.7768	0.152578	0.050506	0.131227	protein_coding

ENSG0000(	GEMIN4	514.7627	0.17425	0.050505	0.131227	protein_coding
ENSG0000(	AKR1C8P	13.59893	-0.46709	0.050526	0.131264	protein_coding
ENSG0000(	TMEM176I	1291.891	-0.18329	0.050529	0.131264	protein_coding
ENSG0000(	GPR139	15.57626	0.379055	0.050551	0.1313	protein_coding
ENSG0000(	LINC-PINT	4350.841	0.17738	0.050549	0.1313	lncRNA
ENSG0000(	LINC02071	227.6871	0.110103	0.050565	0.131315	lncRNA
ENSG0000(	PHF11	1299.793	0.110439	0.050561	0.131315	protein_coding
ENSG0000(	ENSG0000(	10.70828	0.517074	0.050575	0.13133	lncRNA
ENSG0000(	LEXM	88.50555	-0.45763	0.050617	0.131428	protein_coding
ENSG0000(	LLGL2	2988.338	-0.26856	0.050628	0.131444	protein_coding
ENSG0000(	HGS	2232.494	-0.16488	0.050639	0.131464	protein_coding
ENSG0000(	ANKRD49P	237.3111	0.128175	0.050644	0.131464	processed_pseudogene
ENSG0000(	FRY-AS1	95.20396	0.155909	0.050652	0.131474	lncRNA
ENSG0000(	RPS16	2069.411	-0.17315	0.050687	0.131554	protein_coding
ENSG0000(	TECPR1	1552.826	0.132246	0.050718	0.131626	protein_coding
ENSG0000(	ENSG0000(	656.8433	0.084008	0.050757	0.131715	protein_coding
ENSG0000(	GSTM5	214.2379	-0.28854	0.050811	0.13184	protein_coding
ENSG0000(	LINC00390	161.4246	0.185007	0.050814	0.13184	lncRNA
ENSG0000(	IGFL4	2672.47	-0.15807	0.050821	0.131848	protein_coding
ENSG0000(	CCNK	1475.628	-0.08787	0.050826	0.131851	protein_coding
ENSG0000(	NPIPB12	3001.458	0.187181	0.050832	0.131855	protein_coding
ENSG0000(	UPP2	25.13796	0.321128	0.050845	0.131877	protein_coding
ENSG0000(	C2CD4C	15.19387	0.389461	0.050876	0.131947	protein_coding
ENSG0000(	ENSG0000(	9.07443	0.392737	0.050888	0.131967	lncRNA
ENSG0000(	ENSG0000(	278.1749	0.123606	0.0509	0.131988	lncRNA
ENSG0000(	ZNF346-IT1	245.3614	0.102716	0.050927	0.132047	lncRNA
ENSG0000(	SLC24A1	583.4342	0.129538	0.050947	0.132089	protein_coding
ENSG0000(	DOK2	185.9493	0.269861	0.050963	0.132106	protein_coding
ENSG0000(	LINC02044	26.64727	0.260612	0.050967	0.132106	lncRNA
ENSG0000(	VWA5B1	242.9115	-0.11621	0.050961	0.132106	protein_coding
ENSG0000(	C5orf15	730.7539	-0.14263	0.050976	0.13212	protein_coding
ENSG0000(	SPSB4	24.64055	-0.39507	0.050982	0.132123	protein_coding
ENSG0000(	ENSG0000(	264.615	0.141865	0.050993	0.132124	lncRNA
ENSG0000(	FABP4	10.85125	-0.57869	0.050999	0.132124	protein_coding
ENSG0000(	HACL1	415.4341	-0.14639	0.050993	0.132124	protein_coding
ENSG0000(	KLK2	113.0316	0.17083	0.050998	0.132124	protein_coding
ENSG0000(	PAQR9-AS1	446.0022	-0.07753	0.051007	0.132134	lncRNA
ENSG0000(	RNU2-55P	40.03112	-0.2158	0.05103	0.132183	snRNA
ENSG0000(	RN7SL648F	401.3975	0.124529	0.051058	0.132246	misc_RNA
ENSG0000(	RNU4-2	29744.81	-0.16335	0.051067	0.132258	snRNA
ENSG0000(	ENSG0000(	32.68512	0.481386	0.051082	0.132284	lncRNA
ENSG0000(	ENSG0000(	42.51612	-0.50395	0.051086	0.132284	lncRNA
ENSG0000(	ENSG0000(	27.14136	-0.31948	0.051103	0.132308	lncRNA
ENSG0000(	RN7SKP89	12.68924	-0.40349	0.051102	0.132308	misc_RNA
ENSG0000(	GANC	1570.985	0.087691	0.051133	0.132375	protein_coding
ENSG0000(	CASTOR2	1166.593	-0.17198	0.05114	0.132375	protein_coding
ENSG0000(	NEURL3	135.9039	-0.4474	0.051142	0.132375	protein_coding

ENSG0000(	SCAPER	1699.908	-0.13823	0.051152	0.132392	protein_coding
ENSG0000(	APLNR	231.2757	0.343068	0.051199	0.13249	protein_coding
ENSG0000(	ENSG0000(	238.3091	-0.12398	0.051199	0.13249	lncRNA
ENSG0000(	PIK3AP1	1522.447	0.358772	0.051206	0.132498	protein_coding
ENSG0000(	FLT3	158.3814	0.420199	0.051215	0.132502	protein_coding
ENSG0000(	GCN1	2757.707	-0.12307	0.051216	0.132502	protein_coding
ENSG0000(	KCNG1	623.2295	-0.19135	0.051223	0.13251	protein_coding
ENSG0000(	C1orf162	382.2408	0.289871	0.051241	0.132534	protein_coding
ENSG0000(	PARM1-AS:	7.901576	0.768479	0.051237	0.132534	lncRNA
ENSG0000(	CPS1	3959.719	-0.26295	0.051249	0.132543	protein_coding
ENSG0000(	DEPDC1P2	59.71178	-0.23459	0.051265	0.132552	processed_pseudogene
ENSG0000(	IQCF1	48.56073	0.197542	0.05126	0.132552	protein_coding
ENSG0000(	TDP1	580.9039	0.1504	0.051265	0.132552	protein_coding
ENSG0000(	ENSG0000(	93.70407	0.13192	0.051271	0.132559	transcribed_unprocessed_pseudo
ENSG0000(	GRIA4	692.0532	-0.33739	0.051306	0.132636	protein_coding
ENSG0000(	ENSG0000(	186.5643	-0.11449	0.051351	0.132743	lncRNA
ENSG0000(	HAGH	1149.731	-0.12823	0.05137	0.13277	protein_coding
ENSG0000(	TMEM125	329.1288	-0.41458	0.051366	0.13277	protein_coding
ENSG0000(	POLR1G	370.5788	-0.08512	0.051376	0.132774	protein_coding
ENSG0000(	ENSG0000(	430.1678	0.072616	0.0514	0.132827	lncRNA
ENSG0000(	ENSG0000(	72.38577	0.215121	0.051425	0.132879	lncRNA
ENSG0000(	TRIM29	55.38329	0.311636	0.05143	0.13288	protein_coding
ENSG0000(	ABHD18	1616.714	-0.06538	0.051446	0.132911	protein_coding
ENSG0000(	ENSG0000(	25.87884	-0.24502	0.051466	0.132942	lncRNA
ENSG0000(	PABPC4L	35.46843	0.256384	0.051464	0.132942	protein_coding
ENSG0000(	ENSG0000(	1327.276	0.160222	0.051472	0.132947	lncRNA
ENSG0000(	ENSG0000(	380.5722	0.180115	0.051489	0.132979	TEC
ENSG0000(	PRKAR2B	298.903	0.218152	0.051509	0.13302	protein_coding
ENSG0000(	ATP9A	2839.425	-0.28011	0.051523	0.133024	protein_coding
ENSG0000(	ENSG0000(	26.25344	0.734454	0.051519	0.133024	TEC
ENSG0000(	SYNGR1	211.847	-0.26143	0.051523	0.133024	protein_coding
ENSG0000(	KRTAP5-7	12.9546	0.364695	0.05162	0.133262	protein_coding
ENSG0000(	TCAF2P1	67.95144	0.183784	0.051648	0.133324	transcribed_unprocessed_pseudo
ENSG0000(	ENSG0000(	278.7694	0.109776	0.051654	0.133329	lncRNA
ENSG0000(	ENSG0000(	19.23976	0.259166	0.051684	0.133396	lncRNA
ENSG0000(	ENSG0000(	181.1681	0.301621	0.051713	0.133438	lncRNA
ENSG0000(	FBL	482.303	-0.15485	0.051712	0.133438	protein_coding
ENSG0000(	GSTCD	654.806	0.108911	0.051709	0.133438	protein_coding
ENSG0000(	DDX43	55.73412	-0.20689	0.051735	0.133484	protein_coding
ENSG0000(	ENSG0000(	393.9123	0.090042	0.051764	0.133548	lncRNA
ENSG0000(	RNU4-62P	42.73365	0.41106	0.051774	0.133562	snRNA
ENSG0000(	MPG	137.1496	0.192525	0.051794	0.133601	protein_coding
ENSG0000(	NDUFA9P1	78.5174	0.270669	0.051809	0.133629	processed_pseudogene
ENSG0000(	AADAT	94.99275	0.201228	0.051814	0.133632	protein_coding
ENSG0000(	OR4K13	124.4765	-0.14107	0.051821	0.133636	protein_coding
ENSG0000(	TRIM15	438.4416	-0.41767	0.051824	0.133636	protein_coding
ENSG0000(	MT-TY	88.3262	0.66667	0.051842	0.133672	Mt_tRNA

ENSG0000(	ENSG0000(	18.26064	0.345035	0.051851	0.133685	lncRNA
ENSG0000(	ENSG0000(	31.2589	0.226706	0.051894	0.133772	lncRNA
ENSG0000(	ENSG0000(	58.89419	0.360468	0.051891	0.133772	transcribed_processed_pseudoge
ENSG0000(	ENSG0000(	33.14815	0.236931	0.05194	0.13388	lncRNA
ENSG0000(	ATP1A1-AS	1019.878	0.128035	0.051945	0.133883	lncRNA
ENSG0000(	AKAP1	2887.931	-0.2676	0.051972	0.133919	protein_coding
ENSG0000(	PLCD4	180.3954	0.215488	0.051963	0.133919	protein_coding
ENSG0000(	RPL29P25	52.07111	0.148709	0.051971	0.133919	processed_pseudogene
ENSG0000(	OR6U2P	269.6611	0.108051	0.051984	0.133939	transcribed_unprocessed_pseudo
ENSG0000(	FAM186A	198.5782	0.092505	0.051989	0.13394	protein_coding
ENSG0000(	ENSG0000(	42.26382	0.184065	0.051995	0.133945	lncRNA
ENSG0000(	FARSB	1105.812	0.105841	0.052013	0.133981	protein_coding
ENSG0000(	ENSG0000(	20.34614	0.374297	0.052022	0.133992	transcribed_processed_pseudoge
ENSG0000(	TCTN1	1669.366	0.103888	0.052033	0.134011	protein_coding
ENSG0000(	BUB1B	276.1186	-0.23112	0.052063	0.134066	protein_coding
ENSG0000(	SCFD2	553.7206	0.155145	0.05206	0.134066	protein_coding
ENSG0000(	ENSG0000(	25.91877	-0.4253	0.052091	0.134126	lncRNA
ENSG0000(	RN7SKP4	15.91451	0.329446	0.052123	0.134198	misc_RNA
ENSG0000(	ENSG0000(	20.5668	-0.30406	0.052148	0.13423	lncRNA
ENSG0000(	FBXL4	1844.699	-0.07125	0.052146	0.13423	protein_coding
ENSG0000(	ILVBL	696.1293	-0.20384	0.052146	0.13423	protein_coding
ENSG0000(	SSTR5-AS1	105.1668	-0.22543	0.052185	0.134314	lncRNA
ENSG0000(	ANKRD53	18.88981	0.372044	0.052213	0.134345	protein_coding
ENSG0000(	ENSG0000(	28.47404	0.325472	0.052212	0.134345	lncRNA
ENSG0000(	FBXO41	678.573	0.331765	0.052206	0.134345	protein_coding
ENSG0000(	LINC02450	51.43071	0.208785	0.052214	0.134345	lncRNA
ENSG0000(	ASMTL-AS1	435.8737	-0.12333	0.052236	0.134368	lncRNA
ENSG0000(	MICALL2	3777.882	-0.20694	0.052236	0.134368	protein_coding
ENSG0000(	TTC22	1563.509	-0.28801	0.052231	0.134368	protein_coding
ENSG0000(	THAP7	245.5836	-0.13306	0.052243	0.134375	protein_coding
ENSG0000(	FMNL1	1491.673	0.246297	0.052259	0.134407	protein_coding
ENSG0000(	ENSG0000(	15.60455	0.295715	0.052275	0.134433	processed_pseudogene
ENSG0000(	ENSG0000(	219.3713	0.122597	0.052278	0.134433	lncRNA
ENSG0000(	AK4P3	78.0279	-0.25093	0.052302	0.134452	protein_coding
ENSG0000(	ENSG0000(	309.2176	0.106252	0.052299	0.134452	lncRNA
ENSG0000(	GATC	1177.814	-0.06879	0.052303	0.134452	protein_coding
ENSG0000(	IPO9	2949.228	0.129394	0.052292	0.134452	protein_coding
ENSG0000(	PELI2	1014.629	-0.22527	0.052327	0.134505	protein_coding
ENSG0000(	ENSG0000(	27.67905	0.286398	0.052339	0.134523	lncRNA
ENSG0000(	LINC02311	450.4887	-0.15186	0.052363	0.134574	lncRNA
ENSG0000(	PNPLA4	593.0206	0.115467	0.052385	0.13462	protein_coding
ENSG0000(	ZNF354A	354.502	0.139108	0.052392	0.134627	protein_coding
ENSG0000(	AGFG2	1288.17	-0.14937	0.052414	0.134672	protein_coding
ENSG0000(	IFT122	1940.051	-0.08756	0.052419	0.134675	protein_coding
ENSG0000(	NR3C1	2841.766	0.106803	0.052508	0.134888	protein_coding
ENSG0000(	TFB2M	114.5671	-0.14925	0.052511	0.134888	protein_coding
ENSG0000(	ENSG0000(	133.2076	0.163685	0.052545	0.134965	lncRNA

ENSG0000(	PABPC1L2A	80.42242	0.221157	0.052586	0.13506	protein_coding
ENSG0000(	UQCC3	252.7175	-0.08799	0.052603	0.135093	protein_coding
ENSG0000(	ENSG0000(	285.5228	0.135727	0.052636	0.13515	lncRNA
ENSG0000(	ENSG0000(	23.44168	0.304017	0.052638	0.13515	lncRNA
ENSG0000(	PNMA6A	7.933279	0.580459	0.052635	0.13515	protein_coding
ENSG0000(	ENSG0000(	2072.227	-0.13739	0.052652	0.135164	transcribed_unprocessed_pseudo
ENSG0000(	ENSG0000(	18.10968	0.530267	0.052652	0.135164	processed_pseudogene
ENSG0000(	RN7SL326P	33.90694	0.295812	0.052673	0.135205	misc_RNA
ENSG0000(	CDH6	508.6904	0.170521	0.052694	0.135237	protein_coding
ENSG0000(	ENSG0000(	340.1108	0.290516	0.052692	0.135237	protein_coding
ENSG0000(	ENSG0000(	27.03061	0.322876	0.052708	0.135262	lncRNA
ENSG0000(	PRIM1	315.3145	-0.1529	0.052712	0.135262	protein_coding
ENSG0000(	LSAMP	694.3316	0.338776	0.05272	0.135272	protein_coding
ENSG0000(	ENSG0000(	73.97433	-0.13719	0.052735	0.135299	unprocessed_pseudogene
ENSG0000(	EMSY	1691.591	-0.08298	0.052773	0.135385	protein_coding
ENSG0000(	FMR1-AS1	22.85338	0.290277	0.052789	0.135416	lncRNA
ENSG0000(	REXO1	869.3603	-0.10638	0.052804	0.135442	protein_coding
ENSG0000(	CFAP206	40.51027	0.213573	0.052843	0.135503	protein_coding
ENSG0000(	ENSG0000(	25.18684	0.275348	0.052837	0.135503	lncRNA
ENSG0000(	MOB3B	2175.181	-0.12883	0.052845	0.135503	protein_coding
ENSG0000(	TP73	36.63362	0.471201	0.052842	0.135503	protein_coding
ENSG0000(	ENSG0000(	32.52733	-0.23521	0.052872	0.135562	lncRNA
ENSG0000(	SNX18P13	16.79361	0.459828	0.052883	0.135578	processed_pseudogene
ENSG0000(	ENSG0000(	9.060751	-0.405	0.052888	0.13558	lncRNA
ENSG0000(	CD44	7027.386	0.284664	0.052905	0.135613	protein_coding
ENSG0000(	ENSG0000(	38.77485	-0.1862	0.052917	0.135631	lncRNA
ENSG0000(	ENSG0000(	19.6923	0.379232	0.052921	0.135631	lncRNA
ENSG0000(	ENSG0000(	35.59447	0.186221	0.052949	0.135694	lncRNA
ENSG0000(	MICE	154.6002	0.176915	0.052962	0.135717	transcribed_unprocessed_pseudo
ENSG0000(	ENSG0000(	444.7733	0.097223	0.053027	0.13587	lncRNA
ENSG0000(	CDK2AP2	304.3816	-0.1568	0.053039	0.135882	protein_coding
ENSG0000(	ENSG0000(	17.97878	0.294014	0.05304	0.135882	processed_pseudogene
ENSG0000(	RBMX2P1	14.82519	-0.33117	0.053056	0.135913	processed_pseudogene
ENSG0000(	ENSG0000(	30.34213	0.253233	0.053079	0.13595	lncRNA
ENSG0000(	LINC00377	71.54228	0.143198	0.053079	0.13595	lncRNA
ENSG0000(	ENSG0000(	2117.182	0.087877	0.053097	0.135973	TEC
ENSG0000(	ENSG0000(	65.07398	0.234584	0.053096	0.135973	lncRNA
ENSG0000(	RNU6-548P	9.360562	0.459642	0.053113	0.136002	snRNA
ENSG0000(	ENSG0000(	63.91156	-0.17844	0.053128	0.136024	lncRNA
ENSG0000(	TMEM170E	346.0541	0.15918	0.05313	0.136024	protein_coding
ENSG0000(	ENSG0000(	9.779305	0.452294	0.053145	0.136051	lncRNA
ENSG0000(	INTS5	283.185	-0.12098	0.053151	0.136055	protein_coding
ENSG0000(	PIK3R2	644.2444	-0.12028	0.053179	0.136094	protein_coding
ENSG0000(	VN1R5	16.23184	0.250074	0.053179	0.136094	polymorphic_pseudogene
ENSG0000(	ZNF766	1942.477	-0.08515	0.053171	0.136094	protein_coding
ENSG0000(	TMEM263-	26.62731	0.229903	0.053204	0.136146	lncRNA
ENSG0000(	ARHGAP35	4593.544	-0.16867	0.053235	0.136203	protein_coding

ENSG0000(	ENSG0000(	15.57269	0.701756	0.053232	0.136203	lncRNA
ENSG0000(	ZNF812P	15.02856	0.406811	0.053241	0.13621	transcribed_unprocessed_pseudo
ENSG0000(	DBT	1828.421	-0.05394	0.053254	0.136231	protein_coding
ENSG0000(	CASR	1518.319	-0.13882	0.05328	0.136285	protein_coding
ENSG0000(	PINK1-AS	667.3523	0.089106	0.053288	0.136285	lncRNA
ENSG0000(	RPL23AP34	27.75131	-0.28033	0.053288	0.136285	processed_pseudogene
ENSG0000(	ENSG0000(	60.51412	0.198173	0.053325	0.136368	lncRNA
ENSG0000(	ENSG0000(	45.75983	-0.22483	0.053356	0.136437	lncRNA
ENSG0000(	TEX264	678.1333	-0.11764	0.053364	0.136445	protein_coding
ENSG0000(	EPHB3	373.9227	0.303699	0.053369	0.136447	protein_coding
ENSG0000(	ENSG0000(	141.4734	0.116564	0.053382	0.136469	lncRNA
ENSG0000(	DNASE2	339.7614	0.171302	0.053408	0.136503	protein_coding
ENSG0000(	RPP30	478.3601	-0.09707	0.053407	0.136503	protein_coding
ENSG0000(	SPDL1	217.7265	0.233146	0.053404	0.136503	protein_coding
ENSG0000(	MGC27382	364.749	0.113688	0.053464	0.136636	lncRNA
ENSG0000(	ENSG0000(	59.53388	0.231889	0.053479	0.136662	lncRNA
ENSG0000(	ENSG0000(	30.49265	0.234151	0.053505	0.136717	lncRNA
ENSG0000(	ENSG0000(	87.93359	-0.22636	0.053557	0.13684	lncRNA
ENSG0000(	CCL25	741.4897	-0.38109	0.053568	0.136857	protein_coding
ENSG0000(	TAS2R50	86.98595	0.204146	0.053594	0.13691	protein_coding
ENSG0000(	TCAIM	916.7103	0.083574	0.053598	0.13691	protein_coding
ENSG0000(	ENSG0000(	132.0711	0.157661	0.053639	0.137004	lncRNA
ENSG0000(	ADAM23	119.2278	-0.35812	0.053661	0.137049	protein_coding
ENSG0000(	ENSG0000(	91.16688	0.148807	0.05368	0.137088	lncRNA
ENSG0000(	ENSG0000(	13.75619	0.432736	0.053689	0.137099	lncRNA
ENSG0000(	ZKSCAN2-D	1126.417	0.090859	0.053735	0.137204	lncRNA
ENSG0000(	DBIL5P	13.76021	0.615752	0.053742	0.137212	transcribed_unitary_pseudogene
ENSG0000(	ENSG0000(	120.7184	-0.12602	0.053756	0.137238	lncRNA
ENSG0000(	ENSG0000(	94.38074	0.178543	0.053766	0.137251	lncRNA
ENSG0000(	ENSG0000(	19.12206	0.302283	0.053816	0.137368	lncRNA
ENSG0000(	ENSG0000(	14.06505	-0.87824	0.053826	0.137383	lncRNA
ENSG0000(	TMIGD3	18.02658	0.491424	0.053841	0.137408	protein_coding
ENSG0000(	SUMF1	785.3659	0.101295	0.053855	0.137435	protein_coding
ENSG0000(	GTF2F1	556.4533	-0.14058	0.053869	0.137458	protein_coding
ENSG0000(	ENSG0000(	268.8076	0.080271	0.053884	0.137485	lncRNA
ENSG0000(	LINC01080	15.40786	0.404726	0.053894	0.137499	lncRNA
ENSG0000(	PPP6R1	1929.211	-0.10219	0.053899	0.137502	protein_coding
ENSG0000(	ENSG0000(	140.2279	0.902715	0.053908	0.137508	lncRNA
ENSG0000(	FAM85B	90.14192	0.235562	0.05391	0.137508	lncRNA
ENSG0000(	ENSG0000(	94.49301	-0.12954	0.053933	0.137511	lncRNA
ENSG0000(	ENSG0000(	20.90241	-0.61521	0.053932	0.137511	lncRNA
ENSG0000(	ENSG0000(	14.44234	0.676064	0.053932	0.137511	lncRNA
ENSG0000(	MIR663AH(	44832.83	0.384324	0.053925	0.137511	lncRNA
ENSG0000(	RHPN1-AS1	74.7053	-0.14706	0.053926	0.137511	lncRNA
ENSG0000(	NCK1-DT	591.5795	0.096447	0.053943	0.137513	lncRNA
ENSG0000(	SEMA4F	527.5619	0.168455	0.053942	0.137513	protein_coding
ENSG0000(	ADAM11	22.36781	0.441173	0.053965	0.13752	protein_coding

ENSG0000(	DONSON	333.7995	0.110089	0.053958	0.13752	protein_coding
ENSG0000(	ENSG0000(	32.02668	-0.21027	0.053958	0.13752	lncRNA
ENSG0000(	INSYN2A	24.46003	0.39662	0.053967	0.13752	protein_coding
ENSG0000(	NSUN4	1139.945	0.106255	0.053962	0.13752	protein_coding
ENSG0000(	ENSG0000(	21.44957	0.244214	0.053997	0.137585	lncRNA
ENSG0000(	STAT4	475.0929	0.22283	0.054024	0.137641	protein_coding
ENSG0000(	RWDD4	257.7451	-0.18748	0.054045	0.137685	protein_coding
ENSG0000(	ENSG0000(	25.00804	-0.24237	0.054055	0.137698	lncRNA
ENSG0000(	RN7SKP203	304.425	-0.27129	0.054059	0.137698	misc_RNA
ENSG0000(	ENSG0000(	9.423308	0.448957	0.054083	0.137739	lncRNA
ENSG0000(	ENSG0000(	72.34318	0.170061	0.054084	0.137739	lncRNA
ENSG0000(	DCTN5	3509.607	0.099031	0.0541	0.137768	protein_coding
ENSG0000(	ENSG0000(	49.48351	-0.27841	0.054119	0.137795	lncRNA
ENSG0000(	NDUFA4L2	35.0417	0.320572	0.054117	0.137795	protein_coding
ENSG0000(	GS1-279B7	26.26289	0.229031	0.054128	0.137806	transcribed_processed_pseudoge
ENSG0000(	ENSG0000(	140.7643	0.118954	0.054134	0.13781	lncRNA
ENSG0000(	C3	3888.627	-0.37723	0.054155	0.137854	protein_coding
ENSG0000(	CD59	2795.068	0.219361	0.05421	0.13797	protein_coding
ENSG0000(	ENSG0000(	257.0395	-0.10125	0.054209	0.13797	lncRNA
ENSG0000(	ACTG1	11307.82	-0.15647	0.054233	0.138008	protein_coding
ENSG0000(	ENSG0000(	426.5319	0.098639	0.054235	0.138008	lncRNA
ENSG0000(	LINC02268	872.2946	-0.08847	0.054242	0.138008	lncRNA
ENSG0000(	NSUN7	337.1983	0.228934	0.054238	0.138008	protein_coding
ENSG0000(	LINC-ROR	230.3622	-0.12023	0.054248	0.138013	lncRNA
ENSG0000(	ENSG0000(	17.39977	-0.33702	0.054254	0.138016	lncRNA
ENSG0000(	ENSG0000(	512.692	-0.0817	0.054289	0.138093	lncRNA
ENSG0000(	CNNM4	1542.328	-0.24511	0.054317	0.138155	protein_coding
ENSG0000(	LINC02472	29.94595	0.221272	0.054335	0.138188	lncRNA
ENSG0000(	TRPM3	283.7736	0.095	0.054344	0.1382	protein_coding
ENSG0000(	FAM86EP	111.4901	0.170655	0.054369	0.138253	transcribed_unprocessed_pseudo
ENSG0000(	ZNF37A	3368.55	0.074651	0.054378	0.138263	protein_coding
ENSG0000(	KLK7	61.7874	-0.17518	0.054388	0.138279	protein_coding
ENSG0000(	DSG2	3598.093	-0.2774	0.054427	0.138361	protein_coding
ENSG0000(	ENSG0000(	9.721168	0.461545	0.054429	0.138361	lncRNA
ENSG0000(	PTPA	1053.722	0.09133	0.054545	0.138644	protein_coding
ENSG0000(	GAREM1	1075.666	-0.27325	0.054551	0.138649	protein_coding
ENSG0000(	ENSG0000(	492.8606	0.157209	0.054557	0.138653	lncRNA
ENSG0000(	ENSG0000(	683.0059	0.121597	0.054569	0.138673	TEC
ENSG0000(	RARG	240.1485	0.232028	0.054608	0.13876	protein_coding
ENSG0000(	CCDC159	465.5181	-0.07423	0.054623	0.138774	protein_coding
ENSG0000(	USP25	2181.377	-0.07528	0.054621	0.138774	protein_coding
ENSG0000(	ENSG0000(	155.2978	0.189612	0.054641	0.138805	processed_pseudogene
ENSG0000(	MGST3	3028.287	-0.1803	0.054644	0.138805	protein_coding
ENSG0000(	MIR4537	154.7276	0.497505	0.054666	0.138851	miRNA
ENSG0000(	KICS2	403.5165	0.173683	0.054673	0.138857	protein_coding
ENSG0000(	ENSG0000(	103.0867	0.138903	0.054679	0.138862	lncRNA
ENSG0000(	UBE2G2	3739.785	0.102703	0.054692	0.138884	protein_coding

ENSG0000(	LINC02483	19.19678	0.326849	0.054723	0.13895	lncRNA
ENSG0000(	TRIM33	2556.804	0.060399	0.054773	0.139068	protein_coding
ENSG0000(	ARID2	3789.889	-0.06416	0.054796	0.139114	protein_coding
ENSG0000(	BOLA2-SM(	1116.664	-0.12409	0.054839	0.139211	protein_coding
ENSG0000(	ENSG0000(	17.30521	0.381796	0.054858	0.139248	TEC
ENSG0000(	GOLGA6A	11.60297	0.469183	0.054867	0.139248	protein_coding
ENSG0000(	SDSL	130.888	0.206871	0.054863	0.139248	protein_coding
ENSG0000(	ENSG0000(	258.1579	0.219528	0.054877	0.139264	protein_coding
ENSG0000(	SMIM10	27.69503	0.271043	0.054884	0.13927	protein_coding
ENSG0000(	FRK	10473.64	-0.13723	0.054893	0.139281	protein_coding
ENSG0000(	COQ5	519.0968	-0.07221	0.054907	0.139305	protein_coding
ENSG0000(	UBAC2-AS1	226.0685	0.093785	0.054916	0.139317	lncRNA
ENSG0000(	ENSG0000(	200.9332	0.124947	0.054941	0.13937	lncRNA
ENSG0000(	ENSG0000(	15.00207	-0.27377	0.054952	0.139387	snoRNA
ENSG0000(	KRT80	45.17553	0.216369	0.055004	0.139507	protein_coding
ENSG0000(	ENSG0000(	73.00564	0.153149	0.055019	0.139534	lncRNA
ENSG0000(	ENSG0000(	59.16312	0.207039	0.055034	0.139562	lncRNA
ENSG0000(	CC2D2B	167.1666	0.313933	0.055071	0.139641	protein_coding
ENSG0000(	IFIH1	849.6008	0.212709	0.055078	0.139641	protein_coding
ENSG0000(	UNC13C	44.91913	-0.48018	0.055079	0.139641	protein_coding
ENSG0000(	MUC19	66.53217	0.17861	0.0551	0.139667	polymorphic_pseudogene
ENSG0000(	SNORA70	292.9166	0.183994	0.055099	0.139667	snoRNA
ENSG0000(	ZSCAN2	446.4194	-0.11321	0.055103	0.139667	protein_coding
ENSG0000(	SLC25A43	538.2241	-0.17722	0.05511	0.139675	protein_coding
ENSG0000(	ENSG0000(	84.80229	0.142601	0.05512	0.139689	lncRNA
ENSG0000(	MID1IP1	610.4547	-0.1157	0.055133	0.139709	protein_coding
ENSG0000(	ENSG0000(	64.02573	0.16374	0.055183	0.139796	lncRNA
ENSG0000(	FSIP2	10359.37	-0.10723	0.055177	0.139796	protein_coding
ENSG0000(	HDAC2-AS2	105.0609	0.13513	0.055193	0.139796	lncRNA
ENSG0000(	IQCB1	769.4838	0.13287	0.055192	0.139796	protein_coding
ENSG0000(	RNF146	873.7037	0.090326	0.055187	0.139796	protein_coding
ENSG0000(	URB2	359.745	0.232114	0.055175	0.139796	protein_coding
ENSG0000(	RN7SKP96	20.97886	0.30324	0.055215	0.139838	misc_RNA
ENSG0000(	KCNA1	12.40362	0.591344	0.055221	0.139843	protein_coding
ENSG0000(	ENSG0000(	49.30992	0.286842	0.055226	0.139845	transcribed_processed_pseudoge
ENSG0000(	LARP4B	5116.85	0.05339	0.055232	0.139848	protein_coding
ENSG0000(	RN7SL233P	36.23511	0.188202	0.055261	0.13991	misc_RNA
ENSG0000(	MRAP-AS1	33.94764	0.290218	0.05527	0.139923	lncRNA
ENSG0000(	ENSG0000(	9.879822	0.385435	0.05528	0.139927	processed_pseudogene
ENSG0000(	LINC02554	16.57917	0.366731	0.055281	0.139927	lncRNA
ENSG0000(	IMPG2	190.2424	0.131376	0.055291	0.139943	protein_coding
ENSG0000(	CARD10	1616.935	-0.35594	0.055298	0.139949	protein_coding
ENSG0000(	NBPF3	1230.125	0.107796	0.055311	0.139971	protein_coding
ENSG0000(	APP	3056.687	0.144956	0.055335	0.14001	protein_coding
ENSG0000(	TAT-AS1	132.3387	-0.12054	0.055335	0.14001	lncRNA
ENSG0000(	PRR5-ARH(	63.35626	0.253673	0.055341	0.140013	protein_coding
ENSG0000(	ENSG0000(	1498.54	0.069219	0.055382	0.140105	TEC

ENSG0000(	ENSG0000(	47.07663	0.228104	0.055404	0.14015	lncRNA
ENSG0000(	ENSG0000(	36.74571	-0.19373	0.055416	0.14015	lncRNA
ENSG0000(	HHLA2	3409.281	-0.4375	0.055418	0.14015	protein_coding
ENSG0000(	LINC01653	164.0227	0.113545	0.055411	0.14015	lncRNA
ENSG0000(	CEP20	506.6397	-0.1122	0.055428	0.140164	protein_coding
ENSG0000(	ENSG0000(	266.5367	0.387574	0.055444	0.140182	lncRNA
ENSG0000(	ENSG0000(	36.41045	0.445786	0.055439	0.140182	lncRNA
ENSG0000(	KIF21A	807.4178	-0.19431	0.055461	0.140214	protein_coding
ENSG0000(	IRF2BP2	3289.965	-0.11283	0.055491	0.140267	protein_coding
ENSG0000(	ZNF723	17.42318	0.302066	0.055489	0.140267	protein_coding
ENSG0000(	ADAM5	36.3298	-0.24058	0.055514	0.140315	transcribed_unitary_pseudogene
ENSG0000(	LINC02608	47.82895	0.25776	0.055528	0.140328	lncRNA
ENSG0000(	REXO4	339.9765	-0.10362	0.055528	0.140328	protein_coding
ENSG0000(	COG7	909.212	0.096707	0.055551	0.140374	protein_coding
ENSG0000(	FDPS	1253.087	-0.1039	0.05558	0.140437	protein_coding
ENSG0000(	SLC7A7	1622.918	-0.23913	0.055605	0.140489	protein_coding
ENSG0000(	ENSG0000(	12.22892	-0.36807	0.05562	0.140516	lncRNA
ENSG0000(	CCT6A	979.1553	-0.10909	0.055627	0.140521	protein_coding
ENSG0000(	TBC1D22A-	32.99328	-0.22818	0.055642	0.140548	lncRNA
ENSG0000(	TUBGCP4	1974.369	0.084556	0.055648	0.140553	protein_coding
ENSG0000(	ENSG0000(	13.02572	-0.50983	0.055709	0.140686	lncRNA
ENSG0000(	FBXL6	365.3349	-0.20998	0.05571	0.140686	protein_coding
ENSG0000(	ENSG0000(	1849.751	0.104565	0.055732	0.14073	lncRNA
ENSG0000(	LINC01559	85.79834	0.59428	0.055757	0.140772	lncRNA
ENSG0000(	LINC02561	14.48851	-0.38766	0.055753	0.140772	lncRNA
ENSG0000(	OVCH2	30.55618	0.22557	0.055762	0.140773	protein_coding
ENSG0000(	AQP1	1442.97	-0.33795	0.055798	0.140842	protein_coding
ENSG0000(	ENSG0000(	26.62175	0.296441	0.055798	0.140842	transcribed_processed_pseudoge
ENSG0000(	ARL2BP	418.9468	-0.12148	0.05581	0.140854	protein_coding
ENSG0000(	ENSG0000(	25.88373	0.276901	0.055812	0.140854	lncRNA
ENSG0000(	ILKAP	1011.106	-0.06947	0.055842	0.140917	protein_coding
ENSG0000(	ENSG0000(	27.72634	0.4024	0.055849	0.140925	processed_pseudogene
ENSG0000(	TFCP2	1117.951	-0.06784	0.055854	0.140925	protein_coding
ENSG0000(	ENSG0000(	293.7771	-0.13705	0.055868	0.140951	lncRNA
ENSG0000(	CTAGE6	33.57182	0.920572	0.055879	0.140966	protein_coding
ENSG0000(	POLR2F	487.1436	0.065685	0.055893	0.140989	protein_coding
ENSG0000(	ENSG0000(	50.0423	0.220732	0.055903	0.140997	lncRNA
ENSG0000(	ENSG0000(	43.79014	-0.18888	0.055905	0.140997	lncRNA
ENSG0000(	IYD	2399.661	-0.3119	0.055912	0.141004	protein_coding
ENSG0000(	ENSG0000(	23.65277	-0.50675	0.055921	0.141016	lncRNA
ENSG0000(	ENSG0000(	283.3276	0.118869	0.055951	0.141081	lncRNA
ENSG0000(	SVIL-AS1	1442.001	0.085929	0.055993	0.141173	transcribed_unprocessed_pseudo
ENSG0000(	RN7SL697F	12.11644	0.293626	0.056005	0.141193	misc_RNA
ENSG0000(	ACCS	1150.639	0.177079	0.056068	0.141341	protein_coding
ENSG0000(	ENSG0000(	28.57649	0.323589	0.05615	0.141524	lncRNA
ENSG0000(	ZNF683	34.18127	-0.31408	0.056149	0.141524	protein_coding
ENSG0000(	SMAD3	4502.254	-0.15047	0.056184	0.141591	protein_coding

ENSG0000(	THRB-IT1	337.2171	-0.16019	0.056185	0.141591	lncRNA
ENSG0000(	ENSG0000(	13.80076	0.264751	0.056203	0.141621	lncRNA
ENSG0000(	LSM12	802.2545	-0.09386	0.056206	0.141621	protein_coding
ENSG0000(	ENSG0000(	11.12888	0.276478	0.056226	0.14166	lncRNA
ENSG0000(	ENOX1-AS1	86.64537	0.150524	0.056318	0.141869	lncRNA
ENSG0000(	ENSG0000(	587.3366	0.10963	0.056316	0.141869	lncRNA
ENSG0000(	CAMKMT	338.2976	-0.12387	0.056344	0.141924	protein_coding
ENSG0000(	ENSG0000(	25.90926	0.258633	0.056378	0.141996	lncRNA
ENSG0000(	MTCYBP28	11.30906	0.40538	0.056394	0.142027	processed_pseudogene
ENSG0000(	SPATA41	99.02155	0.177876	0.056403	0.142038	lncRNA
ENSG0000(	ENSG0000(	16.38355	0.427979	0.056418	0.142065	lncRNA
ENSG0000(	ULBP3	483.2347	0.088206	0.056459	0.142155	protein_coding
ENSG0000(	RPS16P5	107.7801	0.314431	0.05648	0.142186	transcribed_processed_pseudoge
ENSG0000(	UNGP3	12.92824	-0.35114	0.056478	0.142186	processed_pseudogene
ENSG0000(	DPH3	538.3464	-0.22199	0.056486	0.14219	protein_coding
ENSG0000(	ZNF853	73.60074	-0.28589	0.056493	0.142196	protein_coding
ENSG0000(	CCDC68	1888.744	-0.31794	0.056553	0.142335	protein_coding
ENSG0000(	ERCC6L	85.59289	0.157862	0.056562	0.142347	protein_coding
ENSG0000(	ENSG0000(	246.2887	0.105238	0.056582	0.142387	lncRNA
ENSG0000(	CDO1	19.15843	-0.51407	0.056596	0.142411	protein_coding
ENSG0000(	ENSG0000(	10.42453	0.444796	0.05666	0.142559	lncRNA
ENSG0000(	RPS3AP40	30.28905	-0.47387	0.056672	0.142578	processed_pseudogene
ENSG0000(	ARL10	2005.78	0.177198	0.056694	0.142622	protein_coding
ENSG0000(	SSRP1	879.9531	-0.11345	0.056703	0.142633	protein_coding
ENSG0000(	ENSG0000(	117.7999	0.184516	0.056716	0.142654	lncRNA
ENSG0000(	FIG4	3659.498	0.071631	0.056829	0.142928	protein_coding
ENSG0000(	ENSG0000(	62.46072	-0.21228	0.056835	0.142931	lncRNA
ENSG0000(	PLCB4	1896.82	0.159182	0.056844	0.14294	protein_coding
ENSG0000(	SNU13	518.8982	-0.10576	0.056847	0.14294	protein_coding
ENSG0000(	LINC02241	528.7556	0.113645	0.056864	0.142969	lncRNA
ENSG0000(	ENSG0000(	9.080737	0.438801	0.056872	0.14298	lncRNA
ENSG0000(	ENSG0000(	55.57472	0.165792	0.056884	0.142998	lncRNA
ENSG0000(	ENSG0000(	13.22233	0.344134	0.0569	0.143026	lncRNA
ENSG0000(	SYS1-DBND	24.59103	0.270209	0.056922	0.143069	protein_coding
ENSG0000(	ENSG0000(	87.32625	-0.18049	0.056942	0.143108	lncRNA
ENSG0000(	ENSG0000(	12.32243	0.67002	0.056957	0.143135	lncRNA
ENSG0000(	ACAT1	766.3726	-0.08482	0.056981	0.143185	protein_coding
ENSG0000(	LINC01814	3329.639	-0.15887	0.057003	0.143228	lncRNA
ENSG0000(	NME4	252.8824	0.16156	0.057008	0.143229	protein_coding
ENSG0000(	ENSG0000(	467.8691	-0.16406	0.057032	0.143278	TEC
ENSG0000(	TMEM132E	247.6223	0.118787	0.057052	0.143317	protein_coding
ENSG0000(	DDX60	3167.309	0.222088	0.057068	0.143339	protein_coding
ENSG0000(	RBBP7	1780.515	-0.09658	0.057074	0.143339	protein_coding
ENSG0000(	RN7SKP90	49.93876	-0.31107	0.057071	0.143339	misc_RNA
ENSG0000(	METTL21EI	156.4395	0.131394	0.057125	0.143456	transcribed_unprocessed_pseudo
ENSG0000(	CNTF	197.6042	-0.13474	0.057163	0.143538	protein_coding
ENSG0000(	EPHB4	601.8352	0.127388	0.057179	0.143567	protein_coding

ENSG0000(	AURKAIP1	200.4215	-0.17478	0.0572	0.143609	protein_coding
ENSG0000(	SMPDL3B	116.5647	-0.27414	0.057218	0.143641	protein_coding
ENSG0000(	AIG1	1352.022	-0.17863	0.057248	0.143697	protein_coding
ENSG0000(	MSL1	3420.36	0.062002	0.057249	0.143697	protein_coding
ENSG0000(	VIPR1	2712.874	-0.36609	0.057266	0.143728	protein_coding
ENSG0000(	COMMD3	513.9142	-0.097	0.057304	0.143813	protein_coding
ENSG0000(	DICER1-AS:	1104.743	0.090757	0.057318	0.143837	lncRNA
ENSG0000(	PARP6	2874.605	-0.06471	0.057353	0.143912	protein_coding
ENSG0000(	ENSG0000(	8.449757	0.423551	0.057363	0.143925	lncRNA
ENSG0000(	SPPL2B	2952.115	-0.12572	0.057409	0.144029	protein_coding
ENSG0000(	OVCH1-AS:	493.8376	0.212499	0.057416	0.144037	lncRNA
ENSG0000(	ENSG0000(	197.1608	-0.11825	0.05746	0.144134	protein_coding
ENSG0000(	OR52N1	129.1655	0.207367	0.057466	0.144139	protein_coding
ENSG0000(	ENSG0000(	176.2375	-0.14441	0.057494	0.144186	lncRNA
ENSG0000(	LGSN	285.1589	0.1005	0.05749	0.144186	protein_coding
ENSG0000(	LIFR	2383.143	-0.22725	0.057504	0.14419	protein_coding
ENSG0000(	LINC01837	51.23925	0.249615	0.057505	0.14419	lncRNA
ENSG0000(	SCN5A	42.93157	0.444294	0.057518	0.144213	protein_coding
ENSG0000(	ENSG0000(	10.228	-0.37207	0.05753	0.144231	lncRNA
ENSG0000(	FSCN3	34.90003	0.315986	0.057551	0.144272	protein_coding
ENSG0000(	LCN15	682.8306	0.129845	0.057606	0.144397	protein_coding
ENSG0000(	C15orf61	233.4926	-0.13451	0.057612	0.144402	protein_coding
ENSG0000(	LINC00847	549.7596	0.1609	0.057653	0.144493	lncRNA
ENSG0000(	ENSG0000(	144.2736	0.216075	0.057662	0.144504	lncRNA
ENSG0000(	ASAH2	658.104	-0.38651	0.057695	0.144576	protein_coding
ENSG0000(	CLK4	2432.606	-0.06939	0.057729	0.144649	protein_coding
ENSG0000(	ENSG0000(	184.0442	0.107822	0.057743	0.144671	TEC
ENSG0000(	H2AC7	111.9743	-0.27812	0.057758	0.144698	protein_coding
ENSG0000(	ENSG0000(	39.03594	0.227199	0.057785	0.144754	lncRNA
ENSG0000(	CHRNB2	16.23001	0.530474	0.057796	0.14477	protein_coding
ENSG0000(	UBXN2A	1599.024	-0.07675	0.057812	0.144798	protein_coding
ENSG0000(	GPBAR1	29.12466	0.264261	0.057834	0.144818	protein_coding
ENSG0000(	MSH3	1831.893	0.077147	0.057833	0.144818	protein_coding
ENSG0000(	PFDN1	414.4726	-0.13471	0.057832	0.144818	protein_coding
ENSG0000(	PART1	406.0922	0.070135	0.057846	0.144838	lncRNA
ENSG0000(	A1CF	3671.014	-0.39126	0.057868	0.144869	protein_coding
ENSG0000(	PUDP	366.2507	-0.11929	0.057866	0.144869	protein_coding
ENSG0000(	IFNLR1	1041.629	-0.30935	0.057881	0.144873	protein_coding
ENSG0000(	RALGPS2	3488.018	-0.17575	0.057883	0.144873	protein_coding
ENSG0000(	SEZ6L	38.75384	-0.44192	0.057878	0.144873	protein_coding
ENSG0000(	MEF2B	136.256	-0.34621	0.05791	0.14493	protein_coding
ENSG0000(	ENSG0000(	54.56706	0.202946	0.05808	0.145332	lncRNA
ENSG0000(	SGPP1	459.0713	-0.23769	0.05808	0.145332	protein_coding
ENSG0000(	RAB6C-AS1	50.72372	0.219947	0.058092	0.145351	transcribed_unprocessed_pseudo
ENSG0000(	HBEGF	172.0635	0.383957	0.058128	0.145425	protein_coding
ENSG0000(	RNF213-AS	309.039	0.105715	0.058131	0.145425	lncRNA
ENSG0000(	UBE2J1	2297.581	0.121489	0.058145	0.145449	protein_coding

ENSG0000(	FAM83B	1747.103	-0.28845	0.058225	0.145637	protein_coding
ENSG0000(	PPP1R37	1029.504	-0.08717	0.058241	0.145666	protein_coding
ENSG0000(	DTNB-AS1	30.52413	0.468115	0.058288	0.145751	lncRNA
ENSG0000(	ENSG0000(	86.39055	0.142629	0.058289	0.145751	lncRNA
ENSG0000(	TANGO2	1419.402	0.154573	0.058283	0.145751	protein_coding
ENSG0000(	SNORA25	10.42054	0.800081	0.058319	0.145814	snoRNA
ENSG0000(	KRT73	21.86754	0.599325	0.058338	0.145849	protein_coding
ENSG0000(	DUSP5	893.2968	-0.29584	0.058375	0.145924	protein_coding
ENSG0000(	ENSG0000(	275.5207	0.135996	0.058377	0.145924	lncRNA
ENSG0000(	THA1P	11.68528	0.379023	0.058384	0.145929	unitary_pseudogene
ENSG0000(	ENSG0000(	8.778759	0.466167	0.058402	0.145952	lncRNA
ENSG0000(	MIR2467	8.769064	0.399922	0.058402	0.145952	miRNA
ENSG0000(	AHRR	251.2172	-0.31101	0.058407	0.145953	protein_coding
ENSG0000(	TLR3	993.2425	-0.22351	0.058434	0.146009	protein_coding
ENSG0000(	DGCR6L	131.4661	-0.22259	0.058465	0.146063	protein_coding
ENSG0000(	VAC14-AS1	341.4413	0.1063	0.058462	0.146063	lncRNA
ENSG0000(	ENSG0000(	74.00028	-0.14892	0.058481	0.146092	lncRNA
ENSG0000(	ENSG0000(	10.67733	0.407155	0.058487	0.146095	lncRNA
ENSG0000(	KRT8P50	27.78277	-0.2622	0.058493	0.146098	processed_pseudogene
ENSG0000(	LMBR1	1741.993	-0.10909	0.058534	0.146188	protein_coding
ENSG0000(	MIR9-1HG	630.7747	0.086556	0.058543	0.146189	lncRNA
ENSG0000(	MRPL28	241.2582	-0.10792	0.05854	0.146189	protein_coding
ENSG0000(	ENSG0000(	9.746709	0.403496	0.058575	0.146255	lncRNA
ENSG0000(	LINC01448	54.60947	0.271791	0.058579	0.146255	lncRNA
ENSG0000(	ENSG0000(	97.59303	-0.23031	0.058597	0.146289	TEC
ENSG0000(	ARHGAP25	1066.277	0.286955	0.058621	0.146335	protein_coding
ENSG0000(	ENSG0000(	240.8433	0.115575	0.058642	0.146376	lncRNA
ENSG0000(	DTWD2	881.1903	-0.0764	0.058654	0.146381	protein_coding
ENSG0000(	ENSG0000(	19.6598	0.270607	0.05865	0.146381	lncRNA
ENSG0000(	LINC00320	638.0806	0.105685	0.058657	0.146381	lncRNA
ENSG0000(	ENSG0000(	85.37315	-0.24195	0.058663	0.146382	lncRNA
ENSG0000(	ENSG0000(	352.966	0.098088	0.058667	0.146382	TEC
ENSG0000(	CP	587.4303	0.25618	0.058722	0.146489	protein_coding
ENSG0000(	ENSG0000(	353.5869	0.104285	0.058732	0.146489	transcribed_processed_pseudoge
ENSG0000(	ENSG0000(	365.1467	0.065755	0.058717	0.146489	TEC
ENSG0000(	JMJD1C-AS	10.82125	0.374052	0.058728	0.146489	lncRNA
ENSG0000(	PTPRN2	345.913	0.309028	0.058733	0.146489	protein_coding
ENSG0000(	ENSG0000(	125.0241	0.142534	0.05875	0.146511	lncRNA
ENSG0000(	SQSTM1	3017.844	-0.14671	0.058751	0.146511	protein_coding
ENSG0000(	ENSG0000(	9.222856	0.322184	0.058759	0.146519	lncRNA
ENSG0000(	ENSG0000(	40.00908	0.233628	0.058783	0.146567	lncRNA
ENSG0000(	SLC24A3	240.8605	0.175322	0.058796	0.146577	protein_coding
ENSG0000(	SNORD3B-:	71.90912	0.42434	0.058797	0.146577	snoRNA
ENSG0000(	RHOBTB2	1731.396	-0.33126	0.058813	0.146607	protein_coding
ENSG0000(	HACD3	2259.886	0.058283	0.058824	0.14662	protein_coding
ENSG0000(	TMEM43	1063.614	-0.0831	0.058828	0.14662	protein_coding
ENSG0000(	NANOGP4	99.78634	-0.25934	0.058842	0.146644	transcribed_processed_pseudoge

ENSG0000(	MRPL17	250.2422	0.147915	0.058859	0.146676	protein_coding
ENSG0000(	MGC32805	242.5947	-0.50968	0.058871	0.146694	lncRNA
ENSG0000(	SEMA3B-A!	20.98093	-0.48476	0.058881	0.146706	lncRNA
ENSG0000(	ENSG0000(	160.4705	0.126325	0.058904	0.146752	lncRNA
ENSG0000(	ENSG0000(	109.3291	0.130937	0.058916	0.146771	lncRNA
ENSG0000(	CASC3	1903.168	-0.06476	0.058927	0.146774	protein_coding
ENSG0000(	DCTD	569.8168	0.134524	0.058927	0.146774	protein_coding
ENSG0000(	GFI1B	488.8592	-0.0789	0.058931	0.146774	protein_coding
ENSG0000(	SMG7	2480.304	-0.08264	0.05894	0.146785	protein_coding
ENSG0000(	ACP6	1087.514	0.105157	0.058962	0.146828	protein_coding
ENSG0000(	ENSG0000(	127.3934	0.1225	0.058997	0.146901	transcribed_unprocessed_pseudo
ENSG0000(	UBOX5	726.906	0.09625	0.059001	0.146901	protein_coding
ENSG0000(	ENSG0000(	12.83517	0.379381	0.059025	0.146949	lncRNA
ENSG0000(	PCDH1	1860.173	-0.29562	0.059047	0.146991	protein_coding
ENSG0000(	MRPL52	759.1752	-0.09147	0.059071	0.147041	protein_coding
ENSG0000(	GORAB-AS:	152.406	0.104471	0.059115	0.147138	lncRNA
ENSG0000(	ENSG0000(	103.6157	0.145978	0.059135	0.147177	lncRNA
ENSG0000(	TENM3-AS:	226.5972	0.114516	0.059148	0.147198	lncRNA
ENSG0000(	IFRD2	311.7689	-0.14564	0.059189	0.147288	protein_coding
ENSG0000(	LINC01907	346.2379	-0.08444	0.059203	0.14731	lncRNA
ENSG0000(	ZNF251	806.2722	0.078278	0.059213	0.147323	protein_coding
ENSG0000(	ENSG0000(	114.2943	0.15191	0.059239	0.147378	lncRNA
ENSG0000(	ENSG0000(	503.9365	0.368799	0.059266	0.147421	lncRNA
ENSG0000(	OR13C1P	9.322164	0.586487	0.059266	0.147421	transcribed_unprocessed_pseudo
ENSG0000(	CLPX	1351.958	-0.07838	0.059318	0.147538	protein_coding
ENSG0000(	HIF3A	846.1155	0.14397	0.059328	0.147551	protein_coding
ENSG0000(	ENSG0000(	16.86552	0.322472	0.059383	0.147676	lncRNA
ENSG0000(	RNU1-138F	11.37983	-0.50847	0.059443	0.147814	snRNA
ENSG0000(	ENSG0000(	61.95108	0.23166	0.059456	0.147836	lncRNA
ENSG0000(	ENSG0000(	19.747	0.462847	0.059479	0.147854	transcribed_unprocessed_pseudo
ENSG0000(	GJD2-DT	216.2115	0.088251	0.059479	0.147854	lncRNA
ENSG0000(	PCDHGA5	243.1243	-0.11308	0.059474	0.147854	protein_coding
ENSG0000(	PVT1	3129.591	0.28125	0.059482	0.147854	lncRNA
ENSG0000(	ENSG0000(	94.79833	0.174286	0.05952	0.147937	lncRNA
ENSG0000(	ENSG0000(	134.1925	0.126933	0.059543	0.147981	lncRNA
ENSG0000(	LINC02607	63.49199	0.20913	0.059562	0.148017	lncRNA
ENSG0000(	H1-10-AS1	295.933	0.176468	0.059581	0.148053	lncRNA
ENSG0000(	ENSG0000(	275.6271	0.130912	0.059591	0.148067	protein_coding
ENSG0000(	ENSG0000(	157.163	0.16942	0.059635	0.148155	lncRNA
ENSG0000(	TMCO2	37.89525	-0.25058	0.059636	0.148155	protein_coding
ENSG0000(	ENSG0000(	28.18177	0.34348	0.059675	0.148239	lncRNA
ENSG0000(	ENSG0000(	110.4285	0.13471	0.059696	0.148279	lncRNA
ENSG0000(	TNKS2-AS1	79.57617	-0.14851	0.0597	0.148279	lncRNA
ENSG0000(	PLAA	614.4958	-0.12644	0.059775	0.148442	protein_coding
ENSG0000(	SET	2750.552	-0.11136	0.059772	0.148442	protein_coding
ENSG0000(	LANCL1	929.6004	-0.10731	0.059789	0.148465	protein_coding
ENSG0000(	ENSG0000(	346.9226	0.126675	0.059805	0.148492	lncRNA

ENSG0000(	LINC00880	51.95821	0.16216	0.05984	0.148557	lncRNA
ENSG0000(	LRCH3	4415.364	-0.09641	0.05984	0.148557	protein_coding
ENSG0000(	ENSG0000(	491.4108	-0.09799	0.05985	0.148568	lncRNA
ENSG0000(	APPBP2	1682.004	-0.06225	0.059883	0.148628	protein_coding
ENSG0000(	PPP2CB	1441.711	-0.18854	0.059881	0.148628	protein_coding
ENSG0000(	ENSG0000(	40.04392	0.884975	0.059898	0.148652	TEC
ENSG0000(	CCDC17	424.1792	-0.17617	0.059927	0.148654	protein_coding
ENSG0000(	ENSG0000(	19.47813	0.29972	0.059926	0.148654	TEC
ENSG0000(	LINC02854	42.19501	0.346305	0.059922	0.148654	lncRNA
ENSG0000(	POMC	13.45122	0.453869	0.059918	0.148654	protein_coding
ENSG0000(	PRC1-AS1	174.932	0.145585	0.059926	0.148654	lncRNA
ENSG0000(	ZNF462	1999.375	0.181391	0.059916	0.148654	protein_coding
ENSG0000(	PATJ	6067.949	-0.19141	0.059938	0.148661	protein_coding
ENSG0000(	TCEAL9	92.4312	0.212443	0.059944	0.148661	protein_coding
ENSG0000(	ZCRB1	824.3188	0.078243	0.05994	0.148661	protein_coding
ENSG0000(	SH3BGR	16.90624	0.388585	0.059949	0.148663	protein_coding
ENSG0000(	LINC01198	469.4994	0.138317	0.059958	0.148673	lncRNA
ENSG0000(	ENSG0000(	552.4602	0.321333	0.059978	0.148711	lncRNA
ENSG0000(	SLC25A48	312.8069	0.272748	0.060004	0.148764	protein_coding
ENSG0000(	ENSG0000(	40.42878	0.206851	0.060029	0.148813	lncRNA
ENSG0000(	ENSG0000(	51.18217	0.223826	0.060034	0.148814	processed_pseudogene
ENSG0000(	ENSG0000(	109.8977	0.127561	0.060047	0.148835	lncRNA
ENSG0000(	SCML1	783.5003	0.128098	0.060056	0.148846	protein_coding
ENSG0000(	CLSPN	1252.907	0.087099	0.060071	0.148871	protein_coding
ENSG0000(	LINC00910	1954.329	0.098783	0.060092	0.148912	lncRNA
ENSG0000(	C1QTNF12	97.97386	-0.43731	0.06012	0.148969	protein_coding
ENSG0000(	STAU2	2052.004	-0.16449	0.060142	0.149013	protein_coding
ENSG0000(	ENSG0000(	55.87198	0.221751	0.060193	0.149125	lncRNA
ENSG0000(	CPSF3	439.7793	-0.12678	0.060207	0.149137	protein_coding
ENSG0000(	ENSG0000(	25.09702	0.241067	0.060203	0.149137	lncRNA
ENSG0000(	OR9H1P	1310.683	-0.09454	0.060217	0.14915	polymorphic_pseudogene
ENSG0000(	ENSG0000(	18.3099	0.376621	0.060254	0.14922	transcribed_unprocessed_pseudo
ENSG0000(	TMEM26	128.218	0.240644	0.060252	0.14922	protein_coding
ENSG0000(	ENSG0000(	256.1034	0.091515	0.06026	0.149222	lncRNA
ENSG0000(	ZNF602P	108.6706	0.132797	0.060268	0.14923	transcribed_unprocessed_pseudo
ENSG0000(	C22orf46	463.3518	0.08543	0.060281	0.149251	transcribed_unitary_pseudogene
ENSG0000(	ENSG0000(	578.3133	0.109438	0.060297	0.149277	lncRNA
ENSG0000(	PCDHB11	131.8346	-0.13101	0.060304	0.149283	protein_coding
ENSG0000(	E2F6	369.6801	0.110505	0.060319	0.149285	protein_coding
ENSG0000(	LSM8	3494.136	0.078811	0.060313	0.149285	protein_coding
ENSG0000(	TRIQK	600.1589	-0.09762	0.060316	0.149285	protein_coding
ENSG0000(	ENSG0000(	27.74901	0.270562	0.060329	0.14929	lncRNA
ENSG0000(	ENSG0000(	479.112	-0.22704	0.06033	0.14929	lncRNA
ENSG0000(	ENSG0000(	47.2516	-0.29137	0.060388	0.149421	lncRNA
ENSG0000(	ENSG0000(	416.4358	0.246672	0.060417	0.149471	lncRNA
ENSG0000(	GPN3	112.3678	0.139361	0.060417	0.149471	protein_coding
ENSG0000(	SNRNP70	7010.952	-0.07467	0.060425	0.149478	protein_coding

ENSG0000(	TUBBP10	49.72834	-0.57882	0.060431	0.149481	processed_pseudogene
ENSG0000(	BRD9	1560.638	-0.08729	0.06048	0.149589	protein_coding
ENSG0000(	SIRPB1	614.496	0.291877	0.060484	0.149589	protein_coding
ENSG0000(	NPIPB15	7165.992	0.100899	0.060502	0.149621	protein_coding
ENSG0000(	EID2B	238.5911	0.196617	0.060544	0.149706	protein_coding
ENSG0000(	ENSG0000(	73.9391	0.147342	0.060546	0.149706	lncRNA
ENSG0000(	ILRUN-AS1	18.37284	-0.31504	0.060572	0.149712	lncRNA
ENSG0000(	RNU1-46P	27.8392	0.37992	0.060559	0.149712	snRNA
ENSG0000(	VNN2	239.9284	0.387381	0.060569	0.149712	protein_coding
ENSG0000(	XYLT2	762.4605	-0.09154	0.060565	0.149712	protein_coding
ENSG0000(	ZNF384	1337.715	-0.07761	0.060553	0.149712	protein_coding
ENSG0000(	ENSG0000(	238.0565	0.11062	0.060581	0.149721	lncRNA
ENSG0000(	ENSG0000(	55.96718	-0.21249	0.060678	0.149949	lncRNA
ENSG0000(	ENSG0000(	447.2589	0.079992	0.060715	0.150031	processed_pseudogene
ENSG0000(	PCSEAT	18.63481	0.413492	0.060721	0.150033	lncRNA
ENSG0000(	ENSG0000(	58.1226	-0.27447	0.060772	0.150145	lncRNA
ENSG0000(	SLC10A3	155.8015	-0.1325	0.060776	0.150145	protein_coding
ENSG0000(	POLQ	933.5041	0.087232	0.060784	0.150153	protein_coding
ENSG0000(	ENSG0000(	665.9207	0.156104	0.060802	0.150175	lncRNA
ENSG0000(	TUBB6	340.1263	0.243015	0.060799	0.150175	protein_coding
ENSG0000(	LINC01592	9.907357	0.494362	0.060821	0.150211	lncRNA
ENSG0000(	ACTRT3	14.32454	0.32606	0.06086	0.150295	protein_coding
ENSG0000(	LINC01436	28.22203	0.36484	0.060877	0.150317	lncRNA
ENSG0000(	MMP9	443.0824	0.617816	0.060879	0.150317	protein_coding
ENSG0000(	ENSG0000(	62.54363	-0.40562	0.060887	0.150326	lncRNA
ENSG0000(	ENSG0000(	168.773	0.120738	0.060909	0.150368	lncRNA
ENSG0000(	IHH	473.3538	-0.42091	0.060943	0.150441	protein_coding
ENSG0000(	ENSG0000(	17.50289	0.301263	0.06097	0.150495	lncRNA
ENSG0000(	TMSB15B-/	139.6109	0.254943	0.060989	0.150531	lncRNA
ENSG0000(	CANX	7506.329	-0.15331	0.060999	0.150543	protein_coding
ENSG0000(	ENSG0000(	20.49055	0.324042	0.061007	0.150552	lncRNA
ENSG0000(	HTATSF1P2	33.83095	0.406458	0.06103	0.150595	processed_pseudogene
ENSG0000(	ZNF10	467.0179	0.138377	0.061065	0.150672	protein_coding
ENSG0000(	SPTAN1	7252.912	-0.08526	0.061076	0.150687	protein_coding
ENSG0000(	ALG3	239.0408	-0.12728	0.061102	0.150738	protein_coding
ENSG0000(	POLB	460.6924	0.099708	0.061128	0.150791	protein_coding
ENSG0000(	ENSG0000(	76.7665	-0.19845	0.061168	0.150879	TEC
ENSG0000(	MICAL3	6123.42	-0.11406	0.061187	0.150912	protein_coding
ENSG0000(	EIF2S2P1	139.5956	-0.1863	0.06121	0.150945	processed_pseudogene
ENSG0000(	NDUFAF1	741.3316	-0.05429	0.061207	0.150945	protein_coding
ENSG0000(	CLUHP3	2503.1	-0.10886	0.061273	0.151078	transcribed_unprocessed_pseudo
ENSG0000(	COX19	1340.407	-0.08717	0.061278	0.151078	protein_coding
ENSG0000(	ESPL1	931.8887	-0.27665	0.061278	0.151078	protein_coding
ENSG0000(	EPHA4	290.7865	0.16489	0.061311	0.151148	protein_coding
ENSG0000(	TEX22	89.33278	-0.2315	0.061338	0.151203	protein_coding
ENSG0000(	ENSG0000(	77.62689	0.121742	0.061362	0.15125	unprocessed_pseudogene
ENSG0000(	CLEC3A	147.4882	-0.13538	0.061402	0.151336	protein_coding

ENSG0000(	UBE2A	846.3256	-0.09146	0.061412	0.15135	protein_coding
ENSG0000(	BICD1	1075.93	0.272487	0.061437	0.151351	protein_coding
ENSG0000(	ENSG0000(	9.515013	-0.30835	0.061431	0.151351	lncRNA
ENSG0000(	ENTPD4	4392.782	0.087791	0.061434	0.151351	protein_coding
ENSG0000(	H2AC12	99.03704	-0.30774	0.06142	0.151351	protein_coding
ENSG0000(	ZDHHC7	1647.135	-0.09608	0.061425	0.151351	protein_coding
ENSG0000(	ENSG0000(	1553.674	0.088219	0.061449	0.151358	lncRNA
ENSG0000(	PTPRJ	4345.865	-0.12273	0.061448	0.151358	protein_coding
ENSG0000(	LINC02910	29.0227	0.43174	0.061479	0.151418	lncRNA
ENSG0000(	RARB	438.4234	0.10998	0.0615	0.151448	protein_coding
ENSG0000(	SLF2	2763.655	0.082621	0.061499	0.151448	protein_coding
ENSG0000(	COA8	815.7392	-0.08331	0.061519	0.151471	protein_coding
ENSG0000(	RTN2	99.88218	0.153303	0.061516	0.151471	protein_coding
ENSG0000(	EID2	98.28673	-0.13091	0.061526	0.151476	protein_coding
ENSG0000(	TRAV21	9.550631	-0.61273	0.061531	0.151476	TR_V_gene
ENSG0000(	ENSG0000(	110.0911	-0.10878	0.061538	0.151481	lncRNA
ENSG0000(	H2BC14	81.71559	-0.38731	0.06158	0.151573	protein_coding
ENSG0000(	CYLD	4111.16	0.146549	0.061611	0.151638	protein_coding
ENSG0000(	ENSG0000(	45.8887	0.195821	0.061627	0.151665	lncRNA
ENSG0000(	RFX5	1874.151	0.170783	0.061652	0.151714	protein_coding
ENSG0000(	GPSM1	299.445	0.188621	0.061661	0.151725	protein_coding
ENSG0000(	SV2B	378.832	-0.27495	0.061691	0.151786	protein_coding
ENSG0000(	KLHL32	130.7384	0.263738	0.061698	0.151792	protein_coding
ENSG0000(	ALDH6A1	1930.325	-0.09846	0.061755	0.151921	protein_coding
ENSG0000(	ENSG0000(	14.26359	-0.58986	0.061818	0.152064	lncRNA
ENSG0000(	ENSG0000(	100.7036	-0.12837	0.061841	0.152109	lncRNA
ENSG0000(	CES5A	9.048209	0.44446	0.061858	0.15214	protein_coding
ENSG0000(	ENSG0000(	47.69954	0.219604	0.061874	0.152166	lncRNA
ENSG0000(	SLC50A1	871.4506	0.07222	0.061879	0.152166	protein_coding
ENSG0000(	ENSG0000(	49.45653	-0.2382	0.061895	0.152194	processed_pseudogene
ENSG0000(	ENSG0000(	33.31505	0.211953	0.061947	0.152305	lncRNA
ENSG0000(	PDIA5	467.435	0.147269	0.06195	0.152305	protein_coding
ENSG0000(	LINC02882	648.9108	0.101826	0.061995	0.152404	lncRNA
ENSG0000(	LINC02408	150.3103	0.185097	0.062074	0.152586	lncRNA
ENSG0000(	PKD1	4125.339	-0.11811	0.062091	0.152617	protein_coding
ENSG0000(	IL22RA2	40.52263	-0.66828	0.062109	0.152641	protein_coding
ENSG0000(	NXNL2	439.1407	0.130277	0.06211	0.152641	protein_coding
ENSG0000(	BTF3L4	1007.5	-0.08517	0.062118	0.152646	protein_coding
ENSG0000(	RN7SL815F	25.40632	0.19978	0.062152	0.15272	misc_RNA
ENSG0000(	ENSG0000(	410.5983	-0.08215	0.062184	0.152784	lncRNA
ENSG0000(	POLDIP2	564.1745	-0.1581	0.062218	0.152856	protein_coding
ENSG0000(	CA11	76.37376	-0.3026	0.062243	0.152908	protein_coding
ENSG0000(	HIRA	829.5554	-0.09262	0.062296	0.153026	protein_coding
ENSG0000(	CHCHD7	893.9572	-0.08617	0.062317	0.153052	protein_coding
ENSG0000(	ENSG0000(	69.92669	-0.18294	0.062313	0.153052	lncRNA
ENSG0000(	ADAMTS8	23.87369	0.342031	0.062371	0.15317	protein_coding
ENSG0000(	GUCY2GP	39.75835	0.538164	0.062374	0.15317	transcribed_unitary_pseudogene

ENSG0000(	ENSG0000(	359.8903	0.089562	0.062406	0.153235	lncRNA
ENSG0000(	ENSG0000(	9.662985	0.372736	0.062413	0.15324	lncRNA
ENSG0000(	ENSG0000(	56.74226	0.249817	0.062432	0.153264	lncRNA
ENSG0000(	TTC39B	3565.303	0.129882	0.06243	0.153264	protein_coding
ENSG0000(	CROCC2	33.50894	0.335851	0.062494	0.153401	protein_coding
ENSG0000(	RTP4	167.2797	0.20242	0.062498	0.153401	protein_coding
ENSG0000(	BLNK	3620.447	-0.17097	0.062555	0.153518	protein_coding
ENSG0000(	GGT2	117.2735	-0.51138	0.062553	0.153518	protein_coding
ENSG0000(	CFL1P1	731.9188	0.102251	0.062573	0.153549	transcribed_unprocessed_pseudo
ENSG0000(	ENSG0000(	613.311	0.09686	0.062583	0.153551	lncRNA
ENSG0000(	ENSG0000(	11.42191	0.321678	0.062581	0.153551	lncRNA
ENSG0000(	DNAAF9	945.8101	0.10059	0.062592	0.15356	protein_coding
ENSG0000(	ENSG0000(	364.3622	0.132481	0.062598	0.153563	lncRNA
ENSG0000(	ARL4AP2	16.00194	0.345137	0.062609	0.153565	processed_pseudogene
ENSG0000(	PAPLN-AS1	422.8939	0.081686	0.062607	0.153565	lncRNA
ENSG0000(	ANXA11	5682.417	-0.12603	0.062619	0.153578	protein_coding
ENSG0000(	CLN8	2188.475	0.110018	0.062641	0.15362	protein_coding
ENSG0000(	QPRT	953.2011	-0.10345	0.062673	0.153688	protein_coding
ENSG0000(	GATB	471.8788	0.114591	0.0627	0.153742	protein_coding
ENSG0000(	WTAPP1	147.5198	0.209893	0.062706	0.153744	transcribed_processed_pseudoge
ENSG0000(	PDCD4-AS1	169.6307	-0.09985	0.062717	0.153759	lncRNA
ENSG0000(	ITPRID1	158.68	-0.29876	0.062736	0.153794	protein_coding
ENSG0000(	TPRXL	189.3022	0.113027	0.062751	0.15382	transcribed_processed_pseudoge
ENSG0000(	IFNG-AS1	416.4618	0.389709	0.062757	0.153822	lncRNA
ENSG0000(	DROSHA	1385.561	-0.08811	0.062786	0.15388	protein_coding
ENSG0000(	ENSG0000(	86.85251	0.161645	0.062799	0.153901	lncRNA
ENSG0000(	DOCK8-AS1	223.557	-0.1112	0.062849	0.153987	lncRNA
ENSG0000(	GNS	2986.351	0.149127	0.06284	0.153987	protein_coding
ENSG0000(	RN7SL37P	35.90931	0.200638	0.062847	0.153987	misc_RNA
ENSG0000(	ENSG0000(	15.12567	0.441649	0.06288	0.154044	lncRNA
ENSG0000(	ENSG0000(	8.974926	-0.60581	0.062882	0.154044	transcribed_processed_pseudoge
ENSG0000(	MRPS14	513.2415	-0.08781	0.062889	0.15405	protein_coding
ENSG0000(	LINC02422	298.5265	0.467656	0.062918	0.154108	lncRNA
ENSG0000(	RN7SL333F	18.13226	0.252649	0.062929	0.154123	misc_RNA
ENSG0000(	FTL	11980.88	-0.26661	0.062947	0.154154	protein_coding
ENSG0000(	ENSG0000(	921.9628	0.292505	0.063017	0.154271	TEC
ENSG0000(	LINC01518	90.39433	-0.16482	0.063016	0.154271	lncRNA
ENSG0000(	METTL8	1659.751	0.093455	0.063019	0.154271	protein_coding
ENSG0000(	MT-TI	78.27394	0.605922	0.063012	0.154271	Mt_tRNA
ENSG0000(	RBP4	9.508779	0.570888	0.063014	0.154271	protein_coding
ENSG0000(	LINC01033	36.3529	0.265551	0.06303	0.154287	lncRNA
ENSG0000(	LY6E	360.042	0.315973	0.063073	0.15438	protein_coding
ENSG0000(	PAK1IP1	88.83348	-0.20904	0.063082	0.15439	protein_coding
ENSG0000(	RNU6-574F	11.59627	-0.38482	0.063129	0.154492	snRNA
ENSG0000(	ENSG0000(	54.84342	0.175269	0.063144	0.154518	lncRNA
ENSG0000(	ENSG0000(	273.6562	0.115285	0.063152	0.154525	lncRNA
ENSG0000(	SNORD15A	96.4593	0.217646	0.063169	0.154554	snoRNA

ENSG0000(	ATP6V1E1	603.6237	-0.08396	0.063177	0.154561	protein_coding
ENSG0000(	B4GALT1-A	257.7433	0.095207	0.063187	0.154575	lncRNA
ENSG0000(	IL17RE	1020.291	-0.29668	0.063195	0.154582	protein_coding
ENSG0000(	CLCNKA	9.755355	0.710354	0.063215	0.154607	protein_coding
ENSG0000(	RILP	213.6325	-0.30418	0.063212	0.154607	protein_coding
ENSG0000(	ENSG0000(	45.04894	0.317261	0.063239	0.154654	TEC
ENSG0000(	ENSG0000(	321.9748	0.174202	0.063255	0.154681	TEC
ENSG0000(	CLINT1	2610.842	0.093579	0.063273	0.15469	protein_coding
ENSG0000(	CNTN2	412.9926	-0.10292	0.06327	0.15469	protein_coding
ENSG0000(	ENSG0000(	284.2868	0.095672	0.063273	0.15469	lncRNA
ENSG0000(	DCST2	80.15186	-0.29922	0.063287	0.154712	protein_coding
ENSG0000(	NONOP2	161.2326	-0.24211	0.063301	0.154734	processed_pseudogene
ENSG0000(	ENSG0000(	53.39895	0.245975	0.063343	0.154823	snoRNA
ENSG0000(	ZWINT	172.9247	0.23984	0.063425	0.155013	protein_coding
ENSG0000(	TMEM87B	1906.635	-0.15988	0.063435	0.155024	protein_coding
ENSG0000(	BSN	125.0184	0.223236	0.063462	0.155066	protein_coding
ENSG0000(	ENSG0000(	9.536428	0.52789	0.063458	0.155066	lncRNA
ENSG0000(	GABRA1	54.07289	0.237603	0.063475	0.155086	protein_coding
ENSG0000(	ENSG0000(	36.01871	0.378271	0.063491	0.155108	lncRNA
ENSG0000(	ENSG0000(	82.3623	0.126465	0.063493	0.155108	lncRNA
ENSG0000(	DDN-AS1	39.84743	0.26793	0.063505	0.155123	lncRNA
ENSG0000(	ENSG0000(	111.9627	0.164359	0.063519	0.155134	processed_pseudogene
ENSG0000(	NEURL2	83.64454	-0.27816	0.063518	0.155134	protein_coding
ENSG0000(	LINC01414	151.4031	0.107248	0.063528	0.155142	lncRNA
ENSG0000(	PRKCD	1268.268	-0.17841	0.063532	0.155142	protein_coding
ENSG0000(	MYO5B	3659.354	-0.3563	0.063546	0.155165	protein_coding
ENSG0000(	PRDX2P3	67.96946	0.144581	0.063564	0.155195	processed_pseudogene
ENSG0000(	PKD2L1	10.79604	0.669392	0.063624	0.155331	protein_coding
ENSG0000(	SNX30	1789.57	-0.1119	0.063631	0.155335	protein_coding
ENSG0000(	LDC1P	28.84108	0.496292	0.063651	0.155372	transcribed_unitary_pseudogene
ENSG0000(	HSP90B1	6337.029	0.158895	0.063657	0.155376	protein_coding
ENSG0000(	ZNF516	1495.129	0.118396	0.063672	0.155399	protein_coding
ENSG0000(	MLH1	1367.478	0.088121	0.063685	0.155416	protein_coding
ENSG0000(	RGL4	75.54329	0.319771	0.063689	0.155416	protein_coding
ENSG0000(	APOBEC3F	1048.745	0.211307	0.063729	0.155491	protein_coding
ENSG0000(	NUDT12	290.7597	-0.26338	0.063727	0.155491	protein_coding
ENSG0000(	ZBED3	266.486	0.208176	0.06374	0.155504	protein_coding
ENSG0000(	ENSG0000(	248.3774	0.104559	0.063751	0.155519	lncRNA
ENSG0000(	ENSG0000(	72.9125	0.132345	0.063777	0.15556	lncRNA
ENSG0000(	SURF4	2795.406	-0.1758	0.063775	0.15556	protein_coding
ENSG0000(	P2RY4	168.8685	-0.41918	0.063791	0.155582	protein_coding
ENSG0000(	LINC02616	25.46838	0.363309	0.063806	0.155605	lncRNA
ENSG0000(	ENSG0000(	335.1695	0.09407	0.063815	0.155615	lncRNA
ENSG0000(	IFT74	676.4649	-0.1231	0.06384	0.155664	protein_coding
ENSG0000(	LINC00352	368.9849	-0.0875	0.063855	0.155688	lncRNA
ENSG0000(	PRR27	129.8323	-0.14089	0.063888	0.155758	protein_coding
ENSG0000(	SULT1A3	1064.616	-0.19583	0.06396	0.155921	protein_coding

ENSG0000(	ENSG0000(	38.02901	0.30118	0.063988	0.155977	misc_RNA
ENSG0000(	GUCY1A1	1208.243	0.359214	0.064059	0.156139	protein_coding
ENSG0000(	KRR1	1521.594	-0.11519	0.064094	0.156211	protein_coding
ENSG0000(	LFNG	645.8062	-0.14647	0.0641	0.156214	protein_coding
ENSG0000(	POGLUT1	1174.355	0.140593	0.064105	0.156215	protein_coding
ENSG0000(	KIF1C	3915.391	-0.18905	0.064122	0.156234	protein_coding
ENSG0000(	ZNF574	256.9944	-0.13248	0.064123	0.156234	protein_coding
ENSG0000(	ENSG0000(	32.04734	0.242841	0.064136	0.156254	processed_pseudogene
ENSG0000(	PPA2	1140.154	-0.07891	0.064156	0.15628	protein_coding
ENSG0000(	RSPH3	1614.044	-0.1408	0.064157	0.15628	protein_coding
ENSG0000(	ZNF488	574.1524	-0.52427	0.064163	0.156283	protein_coding
ENSG0000(	ATXN3	2383.215	-0.05881	0.064184	0.156299	protein_coding
ENSG0000(	ENSG0000(	194.3407	-0.12698	0.064194	0.156299	lncRNA
ENSG0000(	ESRRG	710.4555	-0.30866	0.064192	0.156299	protein_coding
ENSG0000(	PITRM1-AS	69.70334	0.267607	0.064177	0.156299	lncRNA
ENSG0000(	ST6GALNA(	37.5973	0.284501	0.064191	0.156299	protein_coding
ENSG0000(	ENSG0000(	131.1537	-0.27379	0.064207	0.156319	lncRNA
ENSG0000(	ENSG0000(	18.06284	0.269382	0.064261	0.156437	lncRNA
ENSG0000(	ENSG0000(	46.81603	0.19035	0.064276	0.156461	lncRNA
ENSG0000(	LINC02127	14.79936	0.288569	0.064303	0.156502	lncRNA
ENSG0000(	MDM2	3315.057	0.147949	0.064303	0.156502	protein_coding
ENSG0000(	TYRP1	24.74343	0.568055	0.06431	0.156509	protein_coding
ENSG0000(	GPER1	25.23159	0.34148	0.06432	0.15652	protein_coding
ENSG0000(	LINC02895	6631.814	-0.12754	0.064431	0.156778	lncRNA
ENSG0000(	EPDR1	218.0924	-0.27544	0.06447	0.156861	protein_coding
ENSG0000(	LINC00427	10.73735	0.495767	0.064498	0.156916	lncRNA
ENSG0000(	RSU1P2	80.93079	0.177028	0.064567	0.157072	transcribed_unprocessed_pseudo
ENSG0000(	COMMD2	720.2683	0.121276	0.064575	0.157079	protein_coding
ENSG0000(	COX6B1P4	9.058259	0.472308	0.064608	0.157148	processed_pseudogene
ENSG0000(	CDH16	10.11344	-0.66906	0.064621	0.157167	protein_coding
ENSG0000(	GOLGA5P1	125.0855	0.338679	0.064716	0.157385	processed_pseudogene
ENSG0000(	ENAH	1714.25	0.169284	0.064723	0.157392	protein_coding
ENSG0000(	TRAJ49	9.422088	-0.54377	0.064796	0.157556	TR_J_gene
ENSG0000(	CLEC14A	129.7607	0.242605	0.06482	0.157594	protein_coding
ENSG0000(	RABGAP1L	7164.305	-0.09346	0.064821	0.157594	protein_coding
ENSG0000(	ENSG0000(	149.9798	-0.1068	0.064839	0.157612	lncRNA
ENSG0000(	RNVU1-14	28.2114	-0.75408	0.064837	0.157612	snRNA
ENSG0000(	CBX5P1	21.68834	0.278332	0.06485	0.157616	processed_pseudogene
ENSG0000(	ERICH3-AS:	11.96639	0.383457	0.064849	0.157616	lncRNA
ENSG0000(	MARCHF9	440.5379	-0.11392	0.064863	0.157634	protein_coding
ENSG0000(	MED13	6383.755	-0.08431	0.064904	0.157721	protein_coding
ENSG0000(	AQP7P1	21.11009	0.609335	0.064911	0.157726	unprocessed_pseudogene
ENSG0000(	PAFAH1B2I	82.91414	-0.20039	0.064933	0.157767	transcribed_processed_pseudoge
ENSG0000(	TCEA1P4	61.8465	0.153837	0.064945	0.157785	processed_pseudogene
ENSG0000(	ENSG0000(	14.54364	0.371415	0.064958	0.157804	lncRNA
ENSG0000(	GOT2P6	54.09081	-0.20697	0.06498	0.157846	processed_pseudogene
ENSG0000(	SLC35A1	498.5022	-0.09012	0.065074	0.158062	protein_coding

ENSG0000(	IP6K3	32.55418	0.310545	0.065088	0.158083	protein_coding
ENSG0000(	LINC01681	11.82842	-0.34442	0.065095	0.158088	lncRNA
ENSG0000(	PATE4	299.9435	0.104617	0.065102	0.158094	protein_coding
ENSG0000(	AFDN-DT	684.7801	-0.13053	0.065129	0.158134	lncRNA
ENSG0000(	LTK	173.6598	-0.31262	0.065124	0.158134	protein_coding
ENSG0000(	ENSG0000(	98.50064	0.151507	0.065162	0.158203	lncRNA
ENSG0000(	WHAMM	1270.052	-0.08316	0.065186	0.158249	protein_coding
ENSG0000(	ENSG0000(	680.502	0.087332	0.065215	0.158306	lncRNA
ENSG0000(	ENSG0000(	64.14825	0.154084	0.065234	0.158341	TEC
ENSG0000(	NUDT6	407.8199	0.121282	0.065243	0.15835	protein_coding
ENSG0000(	CHMP4A	585.8412	-0.08614	0.065291	0.158455	protein_coding
ENSG0000(	PLCXD3	139.7004	0.267095	0.065303	0.158459	protein_coding
ENSG0000(	SNHG9	18.4708	-0.23758	0.065299	0.158459	lncRNA
ENSG0000(	ENSG0000(	12.40746	-0.34417	0.065309	0.158461	lncRNA
ENSG0000(	BORCS6	131.2838	-0.16201	0.065382	0.158625	protein_coding
ENSG0000(	SNX7	273.6462	-0.2908	0.065417	0.1587	protein_coding
ENSG0000(	CFAP47	10.39273	0.686705	0.065429	0.158716	protein_coding
ENSG0000(	CCT6P1	233.2861	0.140448	0.065445	0.158738	transcribed_unprocessed_pseudo
ENSG0000(	ENSG0000(	17.86697	0.300527	0.065448	0.158738	lncRNA
ENSG0000(	TBPL2	27.74345	0.292056	0.065471	0.158781	protein_coding
ENSG0000(	SMIM30	165.8911	0.158212	0.06548	0.158792	protein_coding
ENSG0000(	TJP3	1439.029	-0.35048	0.065546	0.158939	protein_coding
ENSG0000(	ENSG0000(	17.8721	0.274011	0.065566	0.158974	lncRNA
ENSG0000(	OFCC1	13.72521	0.328945	0.065607	0.159061	transcribed_unitary_pseudogene
ENSG0000(	MFAP4	941.3208	-0.26942	0.065617	0.159075	protein_coding
ENSG0000(	ENSG0000(	81.45114	0.124543	0.065623	0.159076	lncRNA
ENSG0000(	ENSG0000(	15.82772	0.289227	0.065638	0.15909	lncRNA
ENSG0000(	LINC00672	847.5431	0.115857	0.065639	0.15909	protein_coding
ENSG0000(	KIFC2	864.2782	-0.14984	0.06567	0.159154	protein_coding
ENSG0000(	ENSG0000(	72.47207	0.170764	0.065707	0.159232	lncRNA
ENSG0000(	YWHAZP5	8.474673	-0.61626	0.065729	0.159272	processed_pseudogene
ENSG0000(	ZNF415	248.3899	-0.11335	0.065738	0.159281	protein_coding
ENSG0000(	ENSG0000(	55.42001	0.242073	0.065778	0.159367	lncRNA
ENSG0000(	ENSG0000(	33.95795	0.273359	0.06581	0.159419	lncRNA
ENSG0000(	GKAP1	161.2944	-0.17526	0.065808	0.159419	protein_coding
ENSG0000(	LINC01048	8.676205	0.417853	0.065823	0.159439	lncRNA
ENSG0000(	ENSG0000(	25.06179	0.912312	0.065839	0.159457	protein_coding
ENSG0000(	GPALPP1	2559.816	0.072571	0.065841	0.159457	protein_coding
ENSG0000(	UBE2D3	6224.191	-0.06313	0.065848	0.159463	protein_coding
ENSG0000(	DOK5	14.0595	0.478635	0.065854	0.159464	protein_coding
ENSG0000(	TNK2	2339.811	0.233657	0.065904	0.159574	protein_coding
ENSG0000(	C1orf216	316.6229	-0.10555	0.065934	0.159621	protein_coding
ENSG0000(	ENSG0000(	129.3155	-0.11711	0.06593	0.159621	lncRNA
ENSG0000(	TMEM51-A	909.8654	0.153458	0.06596	0.159672	lncRNA
ENSG0000(	LINC01447	275.374	0.104776	0.065977	0.1597	lncRNA
ENSG0000(	ACO2	4302.218	-0.24726	0.065985	0.159708	protein_coding
ENSG0000(	ENSG0000(	129.1942	0.086543	0.066004	0.159731	processed_pseudogene

ENSG0000(	INKA1	22.40837	-0.37126	0.066002	0.159731	protein_coding
ENSG0000(	GOLPH3	1245.144	-0.13065	0.066038	0.159801	protein_coding
ENSG0000(	CNTNAP2-/	181.1937	0.106525	0.066056	0.159822	lncRNA
ENSG0000(	CPSF6	3650.073	-0.06656	0.066058	0.159822	protein_coding
ENSG0000(	HADH	2892.793	-0.22287	0.066122	0.159954	protein_coding
ENSG0000(	HMGN2P2ε	42.9265	-0.248	0.06612	0.159954	processed_pseudogene
ENSG0000(	ENSG0000(	120.2849	0.151399	0.06616	0.160034	lncRNA
ENSG0000(	ENSG0000(	86.20966	0.515185	0.066183	0.160077	lncRNA
ENSG0000(	ZBTB12	46.02889	0.186832	0.066264	0.160261	protein_coding
ENSG0000(	TRAF3IP2-/	1113.859	0.088604	0.066271	0.160266	lncRNA
ENSG0000(	LINC00336	127.1018	0.187594	0.066287	0.160268	lncRNA
ENSG0000(	LINC00504	9974.961	-0.1025	0.066283	0.160268	lncRNA
ENSG0000(	VOPP1	2989.93	0.151385	0.066284	0.160268	protein_coding
ENSG0000(	ENSG0000(	279.1201	0.116872	0.066302	0.16029	lncRNA
ENSG0000(	ENSG0000(	526.3746	0.385116	0.06633	0.160347	lncRNA
ENSG0000(	ENSG0000(	171.3495	0.173193	0.066346	0.160372	processed_pseudogene
ENSG0000(	THOC3	387.2733	-0.20109	0.066354	0.160379	protein_coding
ENSG0000(	ENSG0000(	66.32118	-0.2732	0.066365	0.160381	lncRNA
ENSG0000(	MED20	405.6279	0.100834	0.066363	0.160381	protein_coding
ENSG0000(	DBR1	397.9873	-0.10739	0.066377	0.160399	protein_coding
ENSG0000(	CACNG8	804.798	0.072697	0.066462	0.160585	protein_coding
ENSG0000(	PPP1R26-A	488.07	0.13139	0.066465	0.160585	lncRNA
ENSG0000(	INMT	466.988	-0.10604	0.066502	0.160652	protein_coding
ENSG0000(	LINC02782	37.74116	-0.26526	0.066502	0.160652	lncRNA
ENSG0000(	RNU6-20P	19.15167	-0.52765	0.066533	0.160714	snRNA
ENSG0000(	CLDN10	142.8966	0.133839	0.066541	0.16072	protein_coding
ENSG0000(	SNORD116	9.648765	0.549046	0.066593	0.160833	snoRNA
ENSG0000(	ENSG0000(	11.16925	0.380482	0.066599	0.160837	lncRNA
ENSG0000(	ENSG0000(	128.3552	-0.11599	0.066605	0.160839	lncRNA
ENSG0000(	EXOSC2	2532.304	-0.06404	0.066626	0.160876	protein_coding
ENSG0000(	GPR162	104.5894	0.210437	0.066639	0.160897	protein_coding
ENSG0000(	ATAT1	412.1155	-0.09817	0.066673	0.160956	protein_coding
ENSG0000(	STX18-IT1	59.81305	0.178527	0.066674	0.160956	lncRNA
ENSG0000(	ENSG0000(	246.4972	-0.15369	0.066692	0.160986	lncRNA
ENSG0000(	ENSG0000(	356.6496	-0.11316	0.066715	0.161018	lncRNA
ENSG0000(	ST6GAL1	4431.619	0.339701	0.066711	0.161018	protein_coding
ENSG0000(	ZNF181	781.1789	-0.11058	0.066758	0.161109	protein_coding
ENSG0000(	MYC	437.4744	0.298335	0.066788	0.161169	protein_coding
ENSG0000(	RPL7L1P9	23.61361	0.274418	0.066813	0.161216	processed_pseudogene
ENSG0000(	ENSG0000(	80.88441	-0.2091	0.066839	0.161268	TEC
ENSG0000(	BASP1-AS1	1104.17	-0.04823	0.066857	0.161298	lncRNA
ENSG0000(	P4HA3-AS1	157.7879	-0.10752	0.066867	0.161298	lncRNA
ENSG0000(	PI4KAP1	724.7051	-0.30605	0.066864	0.161298	transcribed_unprocessed_pseudo
ENSG0000(	DCTN6-DT	28.81505	0.196738	0.066895	0.161317	lncRNA
ENSG0000(	DEDD	643.9104	0.086247	0.066885	0.161317	protein_coding
ENSG0000(	ENSG0000(	355.1821	-0.08488	0.066894	0.161317	lncRNA
ENSG0000(	SNHG16	1627.051	0.105979	0.066894	0.161317	lncRNA

ENSG0000(	ZNF667	872.5671	0.093606	0.066905	0.161327	protein_coding
ENSG0000(	ENSG0000(	55.62166	-0.35184	0.066942	0.161404	lncRNA
ENSG0000(	APOBEC3H	17.37711	0.275542	0.067017	0.16155	protein_coding
ENSG0000(	ENSG0000(	13.12552	-0.45508	0.067014	0.16155	lncRNA
ENSG0000(	SEC24B-AS	314.1054	0.100078	0.067008	0.16155	lncRNA
ENSG0000(	OCIAD2	743.7412	-0.26121	0.06703	0.161567	protein_coding
ENSG0000(	ZNF491	281.6552	0.134281	0.067066	0.161642	protein_coding
ENSG0000(	NFATC2	1539.033	-0.1955	0.067072	0.161643	protein_coding
ENSG0000(	ENSG0000(	9.84755	0.369526	0.067109	0.161722	lncRNA
ENSG0000(	ENSG0000(	171.239	-0.10166	0.067159	0.161826	lncRNA
ENSG0000(	PKD1L1	433.1923	-0.09856	0.067163	0.161826	protein_coding
ENSG0000(	PGAP3	483.7843	-0.18345	0.067202	0.161908	protein_coding
ENSG0000(	OSER1	618.3943	-0.12496	0.067212	0.161919	protein_coding
ENSG0000(	ERMN	198.3008	0.300267	0.067234	0.161961	protein_coding
ENSG0000(	PSMG2	326.1169	-0.12693	0.067248	0.16197	protein_coding
ENSG0000(	SCARNA11	31.38686	0.238651	0.067249	0.16197	scaRNA
ENSG0000(	RPS2P44	87.35916	0.170375	0.067259	0.161983	processed_pseudogene
ENSG0000(	CCDC122	2606.041	-0.08863	0.067305	0.162079	protein_coding
ENSG0000(	MAP4	4805.812	0.077879	0.067312	0.162079	protein_coding
ENSG0000(	RALB	822.0562	0.170665	0.067314	0.162079	protein_coding
ENSG0000(	HAUS7	188.1513	0.19312	0.067336	0.162116	protein_coding
ENSG0000(	ILF3-DT	830.2866	0.065036	0.06734	0.162116	lncRNA
ENSG0000(	HLA-G	99.70267	0.321344	0.067349	0.162125	protein_coding
ENSG0000(	COLGALT1	1871.82	0.115078	0.067363	0.162146	protein_coding
ENSG0000(	CEP170P1	77.61322	0.202773	0.067408	0.162242	transcribed_unprocessed_pseudo
ENSG0000(	RNY4P37	11.8834	0.318104	0.067419	0.162256	misc_RNA
ENSG0000(	ENSG0000(	216.529	0.117155	0.067428	0.162267	lncRNA
ENSG0000(	ENSG0000(	10.3801	0.418543	0.067442	0.162288	lncRNA
ENSG0000(	ENSG0000(	752.6265	0.090949	0.067452	0.162288	lncRNA
ENSG0000(	RNF170	1207.235	0.064677	0.067452	0.162288	protein_coding
ENSG0000(	SGPP2	1488.29	-0.22495	0.067484	0.162351	protein_coding
ENSG0000(	ENSG0000(	541.5235	0.117255	0.0675	0.162365	lncRNA
ENSG0000(	ENSG0000(	52.9427	0.297783	0.067499	0.162365	lncRNA
ENSG0000(	ZNF784	135.9139	-0.17039	0.067506	0.162366	protein_coding
ENSG0000(	GDAP2	2791.844	-0.1241	0.067524	0.162398	protein_coding
ENSG0000(	ENSG0000(	585.354	-0.09473	0.067585	0.16253	lncRNA
ENSG0000(	PSME1	809.1509	-0.1569	0.067589	0.16253	protein_coding
ENSG0000(	AKR1B1	881.2399	0.114495	0.067599	0.162538	protein_coding
ENSG0000(	LOXL1	144.3545	0.346535	0.067603	0.162538	protein_coding
ENSG0000(	FBXO9	1772.261	-0.07356	0.067636	0.162583	protein_coding
ENSG0000(	LINC01685	27.44963	0.42112	0.067636	0.162583	lncRNA
ENSG0000(	RN7SKP269	88.04903	-0.21597	0.067637	0.162583	misc_RNA
ENSG0000(	FOXL1	194.6997	-0.30762	0.067663	0.162621	protein_coding
ENSG0000(	OR4D1	144.3232	-0.1455	0.067663	0.162621	protein_coding
ENSG0000(	ENSG0000(	674.3177	0.078407	0.067698	0.162693	lncRNA
ENSG0000(	MTND5P2	79.03303	0.17758	0.067724	0.162742	processed_pseudogene
ENSG0000(	ENSG0000(	16.94485	-0.26567	0.067747	0.162786	lncRNA

ENSG0000(	BTLA	684.5661	0.435259	0.067753	0.162786	protein_coding
ENSG0000(	CCDC154	68.74924	-0.38062	0.067758	0.162786	protein_coding
ENSG0000(	LINC01018	164.6838	0.102767	0.067773	0.162809	lncRNA
ENSG0000(	FGFBP3	69.10893	0.219298	0.067791	0.162835	protein_coding
ENSG0000(	PLXDC2	3336.375	0.19298	0.067794	0.162835	protein_coding
ENSG0000(	RSAD2	573.6432	0.369748	0.067812	0.162867	protein_coding
ENSG0000(	ENSG0000(	123.5815	0.126928	0.067829	0.162894	lncRNA
ENSG0000(	TCL6	610.3694	0.177622	0.067836	0.162899	lncRNA
ENSG0000(	ENSG0000(	143.6469	-0.17924	0.067883	0.163	lncRNA
ENSG0000(	ENSG0000(	10.7916	0.418645	0.067895	0.163015	lncRNA
ENSG0000(	PPIEL	836.2878	0.082538	0.067928	0.163084	transcribed_unprocessed_pseudo
ENSG0000(	ENSG0000(	14.05801	0.280836	0.067992	0.163215	lncRNA
ENSG0000(	PPIA	4733.32	-0.14047	0.067993	0.163215	protein_coding
ENSG0000(	RPL12P25	22.99438	-0.28562	0.068109	0.163468	transcribed_processed_pseudoge
ENSG0000(	ZNF430	1530.665	-0.10489	0.068105	0.163468	protein_coding
ENSG0000(	CNNM1	130.9154	-0.19724	0.068154	0.163564	protein_coding
ENSG0000(	ARHGAP20	234.2733	0.207105	0.068193	0.163646	protein_coding
ENSG0000(	SLC16A4	510.1184	-0.16829	0.068199	0.163647	protein_coding
ENSG0000(	ENSG0000(	10.10234	0.367099	0.068218	0.163679	lncRNA
ENSG0000(	RPS20P22	264.1463	0.105249	0.068246	0.163733	transcribed_processed_pseudoge
ENSG0000(	ENSG0000(	64.71298	0.220305	0.068306	0.163865	unprocessed_pseudogene
ENSG0000(	CCT2	808.4269	-0.1111	0.068325	0.163899	protein_coding
ENSG0000(	ENSG0000(	87.57806	-0.18419	0.068366	0.163984	lncRNA
ENSG0000(	MAPK8IP3	4368	-0.09565	0.068375	0.16399	protein_coding
ENSG0000(	SEMA6A	5155.253	-0.3423	0.068379	0.16399	protein_coding
ENSG0000(	ENSG0000(	46.53137	0.232177	0.068393	0.164012	lncRNA
ENSG0000(	ENSG0000(	22.54951	-0.28925	0.068427	0.16408	lncRNA
ENSG0000(	ACAD10	1756.993	-0.07935	0.068444	0.164101	protein_coding
ENSG0000(	ENSG0000(	34.95003	0.283989	0.068446	0.164101	lncRNA
ENSG0000(	HMGB1P31	30.29742	0.218389	0.068479	0.164168	processed_pseudogene
ENSG0000(	AMD1P3	24.98825	0.310749	0.068517	0.16421	processed_pseudogene
ENSG0000(	ENSG0000(	64.91125	-0.38192	0.068515	0.16421	processed_pseudogene
ENSG0000(	LHX9	15.43297	0.533474	0.068504	0.16421	protein_coding
ENSG0000(	ZNF716	403.2828	-0.11737	0.068514	0.16421	protein_coding
ENSG0000(	PDE4DIPP6	226.3521	0.171559	0.068581	0.164351	transcribed_unprocessed_pseudo
ENSG0000(	PAGR1	1566.577	0.114107	0.068626	0.164445	protein_coding
ENSG0000(	SEZ6L2	108.0756	0.270235	0.068641	0.16447	protein_coding
ENSG0000(	PAX8-AS1	4596.347	-0.33162	0.068716	0.164625	lncRNA
ENSG0000(	PTPN9	1475.176	-0.11884	0.068713	0.164625	protein_coding
ENSG0000(	SLC30A3	281.9196	0.101224	0.068768	0.164737	protein_coding
ENSG0000(	PDP1	706.1805	0.126725	0.068807	0.164816	protein_coding
ENSG0000(	ENSG0000(	189.3783	0.098456	0.06882	0.164828	lncRNA
ENSG0000(	SCOCP1	11.28074	0.340967	0.068822	0.164828	processed_pseudogene
ENSG0000(	ENSG0000(	17.97483	0.343099	0.068869	0.164915	TEC
ENSG0000(	LINC02582	56.01858	0.242244	0.068868	0.164915	lncRNA
ENSG0000(	SCRN2	484.7537	-0.16479	0.068885	0.16494	protein_coding
ENSG0000(	ENSG0000(	43.72466	0.257296	0.068938	0.165025	lncRNA

ENSG0000(	LINC01732	27.0562	0.245339	0.068936	0.165025	lncRNA
ENSG0000(	SRP19	2054.132	0.070192	0.068941	0.165025	protein_coding
ENSG0000(	XK	546.3883	-0.3512	0.06893	0.165025	protein_coding
ENSG0000(	CLDN2	503.6092	0.375819	0.06896	0.165033	protein_coding
ENSG0000(	ENSG0000(	12.58821	0.311167	0.068952	0.165033	lncRNA
ENSG0000(	FAM221A	2073.994	-0.07056	0.068965	0.165033	protein_coding
ENSG0000(	RNU6-125F	38.52531	-0.19836	0.068958	0.165033	snRNA
ENSG0000(	FCRL5	4591.577	0.381807	0.068995	0.165089	protein_coding
ENSG0000(	PKD1P2	293.4999	0.872396	0.068999	0.165089	unprocessed_pseudogene
ENSG0000(	GTF3C3	1114.998	-0.08728	0.069026	0.165129	protein_coding
ENSG0000(	KIF27	547.4741	-0.1009	0.069026	0.165129	protein_coding
ENSG0000(	TCP11L1	1498.827	-0.24472	0.069069	0.165218	protein_coding
ENSG0000(	ENSG0000(	14.10218	0.479106	0.069079	0.165229	lncRNA
ENSG0000(	ENSG0000(	23.63408	0.281369	0.069114	0.165301	lncRNA
ENSG0000(	GTF2I-AS1	193.0802	0.104538	0.069139	0.165349	lncRNA
ENSG0000(	ENSG0000(	91.89494	0.524906	0.069149	0.165359	lncRNA
ENSG0000(	ENSG0000(	9.328935	0.486979	0.069156	0.165365	TEC
ENSG0000(	BMS1P3	10.58147	-0.75214	0.06917	0.165373	transcribed_unprocessed_pseudo
ENSG0000(	RIBC2	30.62952	0.360128	0.069168	0.165373	protein_coding
ENSG0000(	RHBDL2	284.7664	0.094502	0.069189	0.165405	protein_coding
ENSG0000(	VPS13C-DT	13.93449	0.304199	0.069219	0.165465	lncRNA
ENSG0000(	ENSG0000(	9.606337	0.422629	0.069232	0.165471	lncRNA
ENSG0000(	PIAS3	643.2675	0.097614	0.06923	0.165471	protein_coding
ENSG0000(	SPRYD7	441.3655	-0.12307	0.069268	0.165543	protein_coding
ENSG0000(	ENSG0000(	242.8229	-0.10079	0.069326	0.165669	lncRNA
ENSG0000(	CYP21A1P	71.47344	0.190925	0.069355	0.165727	transcribed_unprocessed_pseudo
ENSG0000(	ATXN2L	3422.137	-0.06674	0.069371	0.165743	protein_coding
ENSG0000(	P2RY12	30.79156	0.390665	0.069373	0.165743	protein_coding
ENSG0000(	SOD1	722.6156	-0.18426	0.069382	0.165754	protein_coding
ENSG0000(	KIAA0895	144.965	0.152179	0.069409	0.16578	protein_coding
ENSG0000(	LRP4-AS1	55.82841	0.159319	0.0694	0.16578	lncRNA
ENSG0000(	SYNJ1	1086.423	0.112774	0.069405	0.16578	protein_coding
ENSG0000(	SNX18P26	23.63104	0.370747	0.069422	0.165798	processed_pseudogene
ENSG0000(	RSKR	1883.836	-0.06194	0.069433	0.165813	protein_coding
ENSG0000(	SF3A3	781.8354	-0.11256	0.069464	0.165872	protein_coding
ENSG0000(	ENSG0000(	10.01012	0.428741	0.069477	0.165884	TEC
ENSG0000(	SPATA6	562.107	0.146956	0.069479	0.165884	protein_coding
ENSG0000(	RN7SL786F	15.34588	0.317619	0.069495	0.165911	misc_RNA
ENSG0000(	AGPS	2017.246	-0.07733	0.069502	0.165914	protein_coding
ENSG0000(	ENSG0000(	14.04033	-0.53538	0.069513	0.165928	lncRNA
ENSG0000(	ENSG0000(	191.552	-0.08867	0.069541	0.165982	lncRNA
ENSG0000(	RPL7AP60	52.6922	-0.17983	0.069565	0.166027	processed_pseudogene
ENSG0000(	ENSG0000(	88.72112	0.134536	0.069594	0.166058	lncRNA
ENSG0000(	LINC01594	14.53048	-0.42322	0.069589	0.166058	lncRNA
ENSG0000(	SLC23A4P	32.82721	0.312731	0.069588	0.166058	transcribed_unprocessed_pseudo
ENSG0000(	RNU6-653F	18.0327	0.296346	0.069631	0.166122	snRNA
ENSG0000(	ZNF749	684.3819	0.18144	0.069628	0.166122	protein_coding

ENSG0000(	DOCK8	6678.852	0.230973	0.069652	0.166149	protein_coding
ENSG0000(	LETM2	423.4548	0.088251	0.069653	0.166149	protein_coding
ENSG0000(	IFTAP	2627.714	0.074185	0.069659	0.16615	protein_coding
ENSG0000(	EPHX1	206.2309	-0.21259	0.06967	0.166164	protein_coding
ENSG0000(	ALDH1B1	617.3812	-0.16642	0.069677	0.166167	protein_coding
ENSG0000(	MIR181A2I	28.06811	-0.26767	0.069716	0.166248	lncRNA
ENSG0000(	ENSG0000(	1291.609	0.078689	0.069774	0.166362	TEC
ENSG0000(	TC2N	3029.792	0.160114	0.06977	0.166362	protein_coding
ENSG0000(	FLJ30679	45.41139	0.195851	0.069816	0.166449	TEC
ENSG0000(	ENSG0000(	273.1779	0.090297	0.069858	0.166525	lncRNA
ENSG0000(	MIR548T	9.002601	0.421186	0.069858	0.166525	miRNA
ENSG0000(	ARPC3	1058.416	-0.10765	0.069873	0.166546	protein_coding
ENSG0000(	ENSG0000(	11.16072	0.353748	0.069902	0.166601	lncRNA
ENSG0000(	KRT17P8	31.40908	-0.41769	0.069906	0.166601	processed_pseudogene
ENSG0000(	CNIH2	19.01076	0.336756	0.069933	0.166652	protein_coding
ENSG0000(	PDCD4	4623.631	-0.19837	0.069962	0.16671	protein_coding
ENSG0000(	ENSG0000(	14.7603	0.243802	0.069988	0.166758	lncRNA
ENSG0000(	ZSCAN10	23.77401	-0.2468	0.069994	0.166759	protein_coding
ENSG0000(	ENSG0000(	25.78019	0.245361	0.070126	0.167061	lncRNA
ENSG0000(	TMEM177	363.9549	-0.16899	0.070159	0.167129	protein_coding
ENSG0000(	ENSG0000(	142.6741	0.11905	0.070167	0.167133	lncRNA
ENSG0000(	ENSG0000(	133.3843	0.206689	0.070223	0.167255	lncRNA
ENSG0000(	CNNM3-DT	14.45098	-0.28377	0.070278	0.167373	lncRNA
ENSG0000(	ENSG0000(	143.8765	0.126552	0.070289	0.167386	lncRNA
ENSG0000(	MAP10	170.2014	0.141596	0.070318	0.167443	protein_coding
ENSG0000(	NOC2L	1134.979	-0.08209	0.070328	0.167454	protein_coding
ENSG0000(	ENSG0000(	65.21279	-0.52337	0.070339	0.167468	lncRNA
ENSG0000(	ARHGAP27	4664.576	-0.1298	0.07036	0.167505	protein_coding
ENSG0000(	ENSG0000(	49.14707	0.16321	0.070435	0.167671	lncRNA
ENSG0000(	LINC00475	14.90375	0.456395	0.070494	0.167798	transcribed_unprocessed_pseudo
ENSG0000(	ENSG0000(	13.2975	0.330015	0.070509	0.167821	lncRNA
ENSG0000(	TUBB8P7	10.30076	0.80855	0.070521	0.167837	transcribed_unprocessed_pseudo
ENSG0000(	EEF1A1P30	114.4181	-0.12983	0.070529	0.167844	processed_pseudogene
ENSG0000(	C6orf120	623.4742	-0.07499	0.070556	0.167857	protein_coding
ENSG0000(	ERMP1	2666.472	-0.08481	0.070552	0.167857	protein_coding
ENSG0000(	ITPR1-DT	48.6039	0.204202	0.070556	0.167857	lncRNA
ENSG0000(	ZNF468	727.8279	-0.09651	0.070546	0.167857	protein_coding
ENSG0000(	CERS3	19.41119	0.541358	0.070561	0.167858	protein_coding
ENSG0000(	CASP8AP2	1668.308	-0.07357	0.070593	0.167903	protein_coding
ENSG0000(	CNTD1	16.34368	0.44837	0.070596	0.167903	protein_coding
ENSG0000(	PSD	72.03067	0.21862	0.070596	0.167903	protein_coding
ENSG0000(	CPEB3	771.6547	-0.16053	0.070621	0.167949	protein_coding
ENSG0000(	LINC01719	1040.278	-0.11873	0.070663	0.168035	lncRNA
ENSG0000(	SLC4A7	3122.372	-0.16066	0.070685	0.168076	protein_coding
ENSG0000(	APOC4-AP(	18.48774	-0.69707	0.070724	0.168156	protein_coding
ENSG0000(	NETO1	32.34655	0.594341	0.07074	0.168181	protein_coding
ENSG0000(	ZNF530	506.8258	0.111288	0.070755	0.168204	protein_coding

ENSG0000(	TLE4	1079.008	0.094684	0.070762	0.168207	protein_coding
ENSG0000(	FAM71F2	1453.437	0.096843	0.070779	0.168235	protein_coding
ENSG0000(	ENSG0000(	102.2937	0.155124	0.070796	0.168262	TEC
ENSG0000(	CDIP1	515.4922	-0.13044	0.070816	0.168263	protein_coding
ENSG0000(	MRPS12	166.6154	-0.10492	0.070808	0.168263	protein_coding
ENSG0000(	PPM1F	1385.358	0.091176	0.070817	0.168263	protein_coding
ENSG0000(	RNF216	2146.646	0.110715	0.070814	0.168263	protein_coding
ENSG0000(	SHLD2P1	55.34008	0.250626	0.070869	0.168373	transcribed_unprocessed_pseudo
ENSG0000(	CADPS	564.9391	0.218028	0.070876	0.168377	protein_coding
ENSG0000(	ENSG0000(	172.7741	-0.13108	0.070932	0.168496	processed_pseudogene
ENSG0000(	PRDX6	616.4255	-0.14651	0.070941	0.168496	protein_coding
ENSG0000(	PTPRG-AS1	304.1981	-0.12842	0.070942	0.168496	lncRNA
ENSG0000(	TBL3	420.6101	-0.13707	0.070952	0.168506	protein_coding
ENSG0000(	ENSG0000(	225.2252	0.084747	0.070999	0.168606	lncRNA
ENSG0000(	EMILIN1	761.7224	0.202472	0.07101	0.168614	protein_coding
ENSG0000(	MAPK14	2923.837	0.093937	0.071013	0.168614	protein_coding
ENSG0000(	RFX3-AS1	392.7618	0.108808	0.071087	0.168777	lncRNA
ENSG0000(	SDHAF1	155.6658	-0.12943	0.071094	0.16878	protein_coding
ENSG0000(	ENSG0000(	255.7904	-0.12604	0.071101	0.168784	lncRNA
ENSG0000(	ENSG0000(	15.52353	1.55488	0.071106	0.168784	lncRNA
ENSG0000(	ENSG0000(	704.3964	-0.09019	0.071117	0.168796	lncRNA
ENSG0000(	IMPACT	478.2337	-0.09595	0.071147	0.168855	protein_coding
ENSG0000(	PGLS	397.3003	-0.07551	0.071179	0.168919	protein_coding
ENSG0000(	RN7SL652F	26.02512	0.235507	0.071224	0.169013	misc_RNA
ENSG0000(	ZSCAN16-A	631.7154	0.120646	0.071256	0.169077	lncRNA
ENSG0000(	SPAG5-AS1	245.368	-0.18683	0.071274	0.169106	lncRNA
ENSG0000(	ARHGEF35-	2935.643	0.152614	0.071297	0.169147	lncRNA
ENSG0000(	ENSG0000(	11.50102	0.367876	0.071342	0.169241	processed_pseudogene
ENSG0000(	TPTE2P2	463.3871	0.083975	0.071358	0.169266	transcribed_unprocessed_pseudo
ENSG0000(	LINC01442	30.98975	0.237809	0.071388	0.169326	lncRNA
ENSG0000(	LINC02883	38.81661	-0.23405	0.071417	0.169382	lncRNA
ENSG0000(	ENDOG	34.90411	-0.28669	0.071436	0.169405	protein_coding
ENSG0000(	HSPE1P3	31.40432	0.225404	0.071443	0.169405	processed_pseudogene
ENSG0000(	PRELID3A	95.01703	-0.15239	0.071439	0.169405	protein_coding
ENSG0000(	B4GAT1	114.1752	-0.20195	0.07146	0.169432	protein_coding
ENSG0000(	APOA2	41.06392	0.283645	0.071502	0.169507	protein_coding
ENSG0000(	ENSG0000(	42.30907	0.196235	0.071502	0.169507	lncRNA
ENSG0000(	ENSG0000(	202.9945	-0.09805	0.071523	0.169543	lncRNA
ENSG0000(	ENSG0000(	33.49525	0.296606	0.071546	0.169577	lncRNA
ENSG0000(	OR2T29	19.82725	0.969385	0.071549	0.169577	protein_coding
ENSG0000(	OSTCP1	10.0837	0.372448	0.071553	0.169577	transcribed_processed_pseudoge
ENSG0000(	AGK	1638.319	0.077251	0.071611	0.169687	protein_coding
ENSG0000(	ENSG0000(	194.1391	0.175494	0.071608	0.169687	lncRNA
ENSG0000(	ENSG0000(	13.53358	0.534407	0.071641	0.169747	lncRNA
ENSG0000(	AAK1	7596.369	0.111575	0.071677	0.169818	protein_coding
ENSG0000(	SYNM	528.2373	-0.20502	0.071694	0.169847	protein_coding
ENSG0000(	ENSG0000(	229.7663	-0.1316	0.071724	0.169869	lncRNA

ENSG0000(	ENSG0000(	623.2655	0.175465	0.071725	0.169869	lncRNA
ENSG0000(	NSMCE4A	634.1784	-0.10544	0.071724	0.169869	protein_coding
ENSG0000(	OR5B2	56.60643	-0.21418	0.07172	0.169869	protein_coding
ENSG0000(	BMX	48.39493	-0.29977	0.071736	0.169883	protein_coding
ENSG0000(	ENSG0000(	68.16812	0.187365	0.071753	0.16991	lncRNA
ENSG0000(	ENSG0000(	213.0518	-0.42521	0.071772	0.169941	lncRNA
ENSG0000(	RB1	1145.697	0.126639	0.071792	0.169977	protein_coding
ENSG0000(	SIRT4	23.92917	-0.28889	0.071803	0.16999	protein_coding
ENSG0000(	CDKN2B	1756.91	-0.40879	0.071832	0.170045	protein_coding
ENSG0000(	SNRPG	257.1966	-0.11664	0.071842	0.170057	protein_coding
ENSG0000(	MFSD2B	73.04914	0.16628	0.071866	0.170101	protein_coding
ENSG0000(	NDUFS2	1785.963	-0.09218	0.071887	0.170138	protein_coding
ENSG0000(	FOXRED2	841.5987	0.075423	0.071934	0.170216	protein_coding
ENSG0000(	MAGED2	473.9158	-0.14662	0.071937	0.170216	protein_coding
ENSG0000(	ZNF84-DT	134.608	-0.13796	0.071928	0.170216	lncRNA
ENSG0000(	ENSG0000(	62.16296	0.204263	0.071945	0.170224	lncRNA
ENSG0000(	ASS1P2	20.57309	0.303454	0.071977	0.170274	processed_pseudogene
ENSG0000(	IL7	265.8031	-0.1514	0.071973	0.170274	protein_coding
ENSG0000(	EYA4	61.01563	0.234668	0.072018	0.170358	protein_coding
ENSG0000(	ENSG0000(	46.10437	0.173263	0.072033	0.170382	lncRNA
ENSG0000(	PNMA8A	114.9866	0.112134	0.072069	0.170454	protein_coding
ENSG0000(	CYP2B7P	354.1055	-0.1851	0.072084	0.170464	transcribed_unprocessed_pseudo
ENSG0000(	LSMEM2	10.33549	-0.43913	0.072084	0.170464	protein_coding
ENSG0000(	MAN2C1	3116.201	0.101585	0.07212	0.170523	protein_coding
ENSG0000(	PRKAR2A-A	963.2811	-0.07287	0.072117	0.170523	lncRNA
ENSG0000(	ANKRD20A	146.6864	0.405407	0.072189	0.170672	transcribed_unprocessed_pseudo
ENSG0000(	UFC1	560.4407	-0.09094	0.072218	0.170729	protein_coding
ENSG0000(	C12orf40	106.9079	0.132783	0.072231	0.170734	protein_coding
ENSG0000(	ENSG0000(	75.78833	0.168184	0.072236	0.170734	lncRNA
ENSG0000(	ENSG0000(	42.28714	0.23436	0.072242	0.170734	lncRNA
ENSG0000(	NCOA2	3587.869	-0.07815	0.072237	0.170734	protein_coding
ENSG0000(	TARS1	749.4767	0.101555	0.07228	0.17081	protein_coding
ENSG0000(	PPP4R2	1440.811	-0.09283	0.072308	0.170865	protein_coding
ENSG0000(	SLC9B2	309.2996	0.254471	0.072337	0.17092	protein_coding
ENSG0000(	MPC1-DT	401.8118	0.074156	0.072343	0.170921	lncRNA
ENSG0000(	CYP2D7	449.5777	-0.29999	0.072363	0.170956	polymorphic_pseudogene
ENSG0000(	GCGR	23.88667	0.336039	0.072374	0.170969	protein_coding
ENSG0000(	NAPA-AS1	70.03717	-0.19098	0.072393	0.171001	lncRNA
ENSG0000(	SCAND2P	2405.05	0.072785	0.072402	0.171009	transcribed_unprocessed_pseudo
ENSG0000(	YBX2P2	16.51432	0.4527	0.07242	0.171038	transcribed_processed_pseudoge
ENSG0000(	ENSG0000(	6558.143	0.149313	0.07246	0.171121	lncRNA
ENSG0000(	PMS2P4	2044.523	0.062814	0.072471	0.171134	transcribed_unprocessed_pseudo
ENSG0000(	ENSG0000(	87.58723	-0.53982	0.072491	0.171156	lncRNA
ENSG0000(	LINC01743	16.69858	0.355067	0.072489	0.171156	lncRNA
ENSG0000(	ENSG0000(	120.7289	0.226745	0.072507	0.171159	transcribed_processed_pseudoge
ENSG0000(	RELT	1052.536	0.337391	0.072509	0.171159	protein_coding
ENSG0000(	SUCLA2	799.417	-0.12295	0.072508	0.171159	protein_coding

ENSG0000(	SNX14	6780.681	-0.04762	0.072526	0.171188	protein_coding
ENSG0000(	ENSG0000(	83.44213	0.141084	0.072538	0.171204	lncRNA
ENSG0000(	LINC00640	10.49182	0.421823	0.07257	0.171266	lncRNA
ENSG0000(	TRIM58	316.6255	-0.11378	0.072582	0.17128	protein_coding
ENSG0000(	TMCO1-AS	458.0059	-0.08984	0.0726	0.171311	lncRNA
ENSG0000(	CEP55	166.978	0.154104	0.072617	0.171318	protein_coding
ENSG0000(	ENSG0000(	29.41707	-0.20296	0.07261	0.171318	lncRNA
ENSG0000(	LRRC37A4F	3951.672	0.359428	0.07262	0.171318	transcribed_unprocessed_pseudo
ENSG0000(	LINC02777	35.71638	-0.44671	0.072666	0.171402	lncRNA
ENSG0000(	PIP5K1A	2179.137	-0.06739	0.072666	0.171402	protein_coding
ENSG0000(	PCDHA5	61.01266	-0.15451	0.072762	0.171616	protein_coding
ENSG0000(	DDX19A	1629.1	-0.07295	0.072775	0.171633	protein_coding
ENSG0000(	LINC00680	541.971	-0.2076	0.072792	0.17166	transcribed_unprocessed_pseudo
ENSG0000(	NDC1	551.5739	-0.18491	0.072828	0.171733	protein_coding
ENSG0000(	ENSG0000(	322.1472	0.089077	0.072839	0.171746	lncRNA
ENSG0000(	HSD11B2	1927.455	-0.35959	0.072936	0.171961	protein_coding
ENSG0000(	STMN1	881.8606	-0.18472	0.072974	0.172038	protein_coding
ENSG0000(	ENSG0000(	73.9642	-0.15306	0.072983	0.172046	lncRNA
ENSG0000(	KBTBD8	364.3797	-0.33552	0.073005	0.172073	protein_coding
ENSG0000(	LINC00639	534.1824	0.095953	0.073	0.172073	lncRNA
ENSG0000(	RNF138P1	139.4288	-0.17041	0.073024	0.172105	processed_pseudogene
ENSG0000(	ENSG0000(	60.30022	0.169453	0.073037	0.172121	lncRNA
ENSG0000(	PGP	365.289	-0.10347	0.073042	0.172121	protein_coding
ENSG0000(	DUS1L	836.9028	0.118614	0.073062	0.17213	protein_coding
ENSG0000(	SLC9A3	4098.726	-0.40632	0.073054	0.17213	protein_coding
ENSG0000(	TBRG1	2384.374	0.070118	0.073059	0.17213	protein_coding
ENSG0000(	INSR	4174.892	-0.22551	0.073094	0.17218	protein_coding
ENSG0000(	VAPB	2084.148	-0.05586	0.073091	0.17218	protein_coding
ENSG0000(	ENSG0000(	70.16887	-0.13631	0.07314	0.172266	lncRNA
ENSG0000(	ENSG0000(	174.3092	0.182114	0.073142	0.172266	lncRNA
ENSG0000(	ENSG0000(	93.44369	0.138999	0.073158	0.172277	lncRNA
ENSG0000(	SCML2	136.5703	-0.27942	0.073159	0.172277	protein_coding
ENSG0000(	TLN1	5671.438	-0.11388	0.073163	0.172277	protein_coding
ENSG0000(	SNHG20	1370.599	-0.07982	0.073171	0.172283	lncRNA
ENSG0000(	VSIG10L2	29.9802	0.266728	0.073179	0.172291	protein_coding
ENSG0000(	CCM2L	60.74971	0.213432	0.073196	0.172318	protein_coding
ENSG0000(	CFAP45	13.61681	0.462835	0.07322	0.172361	protein_coding
ENSG0000(	LINC02284	134.1678	0.176389	0.073244	0.172403	lncRNA
ENSG0000(	GABPA	771.4775	-0.09543	0.073251	0.172407	protein_coding
ENSG0000(	SSPOP	330.3255	-0.25653	0.073261	0.172418	transcribed_unitary_pseudogene
ENSG0000(	GOSR2-DT	17.42042	0.261475	0.073276	0.172442	lncRNA
ENSG0000(	ENSG0000(	279.2929	-0.12384	0.073331	0.172556	lncRNA
ENSG0000(	BUB3	2121.228	-0.10412	0.073354	0.172591	protein_coding
ENSG0000(	NECAB2	9.771619	0.561298	0.073357	0.172591	protein_coding
ENSG0000(	METTL15P1	9.64638	0.564434	0.073395	0.172669	processed_pseudogene
ENSG0000(	SPIRE2	2088.912	-0.17791	0.073404	0.172677	protein_coding
ENSG0000(	TMEM105	267.5002	-0.16371	0.07341	0.172679	lncRNA

ENSG0000(	ENSG0000(	370.1513	-0.07654	0.073449	0.172756	TEC
ENSG0000(	MIR548I1	14.23008	0.38784	0.073477	0.17281	miRNA
ENSG0000(	PNPO	970.3107	-0.09338	0.073504	0.172847	protein_coding
ENSG0000(	RNU6-39P	12.03142	0.362189	0.073509	0.172847	snRNA
ENSG0000(	SLC16A7	1779.664	0.145921	0.073505	0.172847	protein_coding
ENSG0000(	NBEAP2	36.88793	0.34542	0.073523	0.172867	transcribed_unprocessed_pseudo
ENSG0000(	ZBTB46	441.0584	0.186503	0.073531	0.172874	protein_coding
ENSG0000(	KIR3DX1	790.1937	0.060534	0.073578	0.172969	transcribed_unprocessed_pseudo
ENSG0000(	ENSG0000(	402.7302	0.066981	0.073588	0.172977	lncRNA
ENSG0000(	RPL36AP45	15.2947	0.254586	0.073592	0.172977	processed_pseudogene
ENSG0000(	ENSG0000(	147.0971	0.13031	0.073663	0.173131	processed_pseudogene
ENSG0000(	ENPP1	928.8228	-0.16358	0.073692	0.173163	protein_coding
ENSG0000(	ENSG0000(	14.67391	0.311908	0.073686	0.173163	lncRNA
ENSG0000(	ENSG0000(	117.4466	0.119342	0.073693	0.173163	lncRNA
ENSG0000(	RNU6-1069	11.28746	0.47828	0.073702	0.173171	snRNA
ENSG0000(	KDM1B	974.3853	-0.07678	0.073741	0.173237	protein_coding
ENSG0000(	SSXP3	13.38571	-0.30372	0.073737	0.173237	unprocessed_pseudogene
ENSG0000(	ADAMTS9-/	739.3093	0.167368	0.073757	0.173263	lncRNA
ENSG0000(	PKN2	3482.503	-0.13269	0.073811	0.173377	protein_coding
ENSG0000(	ENSG0000(	42.87393	-0.19832	0.073846	0.173445	lncRNA
ENSG0000(	MNX1-AS1	9.792674	-0.41893	0.073871	0.173492	lncRNA
ENSG0000(	ENSG0000(	94.9899	0.143455	0.073878	0.173494	lncRNA
ENSG0000(	ENSG0000(	87.83075	0.160797	0.073898	0.173516	lncRNA
ENSG0000(	URM1	498.502	-0.1	0.073897	0.173516	protein_coding
ENSG0000(	ENSG0000(	60.2974	-0.17871	0.073931	0.173579	transcribed_processed_pseudoge
ENSG0000(	ENSG0000(	648.9592	0.080414	0.073985	0.173672	lncRNA
ENSG0000(	NARF-IT1	110.7084	0.134978	0.07398	0.173672	lncRNA
ENSG0000(	SLC12A5	65.15033	-0.34783	0.073987	0.173672	protein_coding
ENSG0000(	UTP14C	123.0685	0.574046	0.074	0.17369	protein_coding
ENSG0000(	ZNF382	1526.552	-0.14098	0.074025	0.173737	protein_coding
ENSG0000(	COG8	1518.056	-0.05969	0.07405	0.173777	protein_coding
ENSG0000(	ENSG0000(	41.22273	0.183869	0.074059	0.173777	lncRNA
ENSG0000(	PKIG	401.695	0.163251	0.074058	0.173777	protein_coding
ENSG0000(	VPS37D	15.87324	-0.35273	0.074108	0.173879	protein_coding
ENSG0000(	FAM110A	229.9902	-0.11131	0.074127	0.173898	protein_coding
ENSG0000(	GAREM2	287.4212	0.2023	0.074126	0.173898	protein_coding
ENSG0000(	RN7SL418F	20.3031	0.276417	0.074147	0.173931	misc_RNA
ENSG0000(	PNLIPP1	45.26737	-0.15355	0.074153	0.173933	unprocessed_pseudogene
ENSG0000(	SLC8A1	2724.28	0.204846	0.074167	0.173954	protein_coding
ENSG0000(	ENSG0000(	229.944	0.125713	0.074179	0.173967	lncRNA
ENSG0000(	AHCYL2	1885.574	-0.19456	0.074209	0.174024	protein_coding
ENSG0000(	TONSL-AS1	12.80453	0.333416	0.074214	0.174024	lncRNA
ENSG0000(	ENSG0000(	74.3492	-0.1504	0.074234	0.174057	lncRNA
ENSG0000(	ENSG0000(	57.34447	-0.26879	0.074252	0.174073	lncRNA
ENSG0000(	ENSG0000(	257.0642	0.08254	0.074246	0.174073	lncRNA
ENSG0000(	MMP24OS	1096.629	-0.11087	0.074266	0.174095	protein_coding
ENSG0000(	ABR	5525.104	-0.10045	0.074278	0.174109	protein_coding

ENSG0000(	YIPF1	320.0314	0.115465	0.074294	0.174133	protein_coding
ENSG0000(	ENSG0000(	22.14719	0.656244	0.07431	0.174159	lncRNA
ENSG0000(	BTF3-DT	20.91613	0.243042	0.074326	0.174171	lncRNA
ENSG0000(	GDF9	31.39403	0.189979	0.074322	0.174171	protein_coding
ENSG0000(	UBE2Q2	381.7132	0.186205	0.074369	0.174258	protein_coding
ENSG0000(	TRBV19	21.66042	-0.4561	0.07438	0.17427	TR_V_gene
ENSG0000(	ENSG0000(	20.60586	0.460434	0.074397	0.174297	TEC
ENSG0000(	ASS1P1	99.42425	-0.38081	0.074411	0.174317	processed_pseudogene
ENSG0000(	TMEM231	1215.005	-0.21879	0.074449	0.174394	protein_coding
ENSG0000(	DDX24	2339.562	0.097385	0.074464	0.174416	protein_coding
ENSG0000(	ENSG0000(	37.29626	0.632098	0.074482	0.174432	lncRNA
ENSG0000(	HOXA11-AS	9.019118	0.497849	0.07448	0.174432	lncRNA
ENSG0000(	LCLAT1	2731.37	-0.07587	0.07449	0.174436	protein_coding
ENSG0000(	ADAP1	936.9584	-0.22399	0.074514	0.174482	protein_coding
ENSG0000(	SNORA14B	63.08326	0.185488	0.074565	0.174587	snoRNA
ENSG0000(	ANXA1	492.5812	0.210504	0.074571	0.174589	protein_coding
ENSG0000(	PGAM1P7	15.96162	0.526715	0.07458	0.174597	processed_pseudogene
ENSG0000(	ENSG0000(	137.2883	0.172548	0.074588	0.174601	lncRNA
ENSG0000(	HCG11	1035.154	0.068965	0.0746	0.174617	lncRNA
ENSG0000(	RHEB	517.1918	-0.0916	0.07465	0.17472	protein_coding
ENSG0000(	ARF3	2229.788	0.102007	0.074679	0.174775	protein_coding
ENSG0000(	FGD5	1088.394	0.120188	0.074684	0.174775	protein_coding
ENSG0000(	ZMAT5	384.3557	0.072229	0.074706	0.174814	protein_coding
ENSG0000(	GATA6-AS1	324.3384	-0.40468	0.074717	0.174827	lncRNA
ENSG0000(	NEAT1	419443.4	0.255333	0.074742	0.174872	lncRNA
ENSG0000(	ENSG0000(	78.35726	-0.14497	0.074795	0.174984	lncRNA
ENSG0000(	ADAMTS13	204.1364	-0.20398	0.07482	0.175027	protein_coding
ENSG0000(	CDC42SE2	3184.102	-0.11208	0.074844	0.175044	protein_coding
ENSG0000(	LARP4P	52.7582	0.214946	0.074844	0.175044	transcribed_processed_pseudoge
ENSG0000(	PPCS	782.3136	-0.09071	0.074836	0.175044	protein_coding
ENSG0000(	ENSG0000(	11.60833	0.352233	0.074858	0.175066	lncRNA
ENSG0000(	ENSG0000(	121.8939	0.266297	0.07488	0.175103	lncRNA
ENSG0000(	ENSG0000(	32.64091	-0.30678	0.074906	0.175151	lncRNA
ENSG0000(	PCDH15	83.3184	0.328486	0.074913	0.175154	protein_coding
ENSG0000(	AP1S1	244.1664	-0.2272	0.074938	0.1752	protein_coding
ENSG0000(	ENSG0000(	261.2234	0.128862	0.074945	0.175204	lncRNA
ENSG0000(	PUM2	4673.867	-0.05037	0.075013	0.17535	protein_coding
ENSG0000(	NGEF	1075.372	-0.31862	0.075035	0.175388	protein_coding
ENSG0000(	ENSG0000(	14.56388	0.253866	0.075062	0.175437	lncRNA
ENSG0000(	ULK4P3	10.04252	0.585337	0.075072	0.175439	transcribed_unprocessed_pseudo
ENSG0000(	ZBTB3	743.9114	-0.07161	0.075077	0.175439	protein_coding
ENSG0000(	ZSWIM8-AS	40.32615	0.374214	0.075079	0.175439	lncRNA
ENSG0000(	PLXNA2	3161.019	-0.31411	0.075111	0.175501	protein_coding
ENSG0000(	CAV1	581.8861	0.250946	0.075146	0.175544	protein_coding
ENSG0000(	ENSG0000(	135.8504	0.1297	0.075143	0.175544	lncRNA
ENSG0000(	TP73-AS1	1488.516	0.093649	0.075138	0.175544	transcribed_unitary_pseudogene
ENSG0000(	MILR1	445.0285	-0.14193	0.075184	0.175618	protein_coding

ENSG0000(	CADM2	41.66528	0.375647	0.075216	0.17568	protein_coding
ENSG0000(	ENSG0000(	22.72626	0.274505	0.075251	0.175724	lncRNA
ENSG0000(	KRTAP5-AS	168.6091	-0.29426	0.075249	0.175724	lncRNA
ENSG0000(	RPL27	1199.443	-0.1725	0.075248	0.175724	protein_coding
ENSG0000(	TMEM135	1624.279	-0.1228	0.0753	0.175824	protein_coding
ENSG0000(	SCN11A	369.2084	0.134534	0.075325	0.175869	protein_coding
ENSG0000(	ENSG0000(	22.12477	-0.34441	0.075334	0.175878	lncRNA
ENSG0000(	RN7SL187F	14.35319	0.29519	0.075355	0.175914	misc_RNA
ENSG0000(	PODXL	1022.225	0.159703	0.075418	0.176047	protein_coding
ENSG0000(	MRPS9	196.1717	-0.1311	0.075428	0.176059	protein_coding
ENSG0000(	DNAL4	119.9373	0.127344	0.075466	0.176126	protein_coding
ENSG0000(	ENSG0000(	112.9686	0.131349	0.075468	0.176126	lncRNA
ENSG0000(	LDLR	2536.1	0.111612	0.075485	0.176153	protein_coding
ENSG0000(	ENSG0000(	18.81499	-0.2893	0.075567	0.176279	lncRNA
ENSG0000(	ENSG0000(	17.21534	0.403581	0.075552	0.176279	lncRNA
ENSG0000(	PLXNB3	105.4686	0.335983	0.075559	0.176279	protein_coding
ENSG0000(	POU2F2-AS	41.32343	-0.49597	0.075566	0.176279	lncRNA
ENSG0000(	RNU4-13P	21.25638	-0.31529	0.075546	0.176279	snRNA
ENSG0000(	MED4	672.551	-0.09525	0.075578	0.176291	protein_coding
ENSG0000(	TBC1D27P	605.4768	-0.24529	0.075588	0.176302	transcribed_unprocessed_pseudo
ENSG0000(	ENSG0000(	95.98198	-0.13703	0.075638	0.176391	lncRNA
ENSG0000(	ENSG0000(	7.4319	0.412362	0.075636	0.176391	lncRNA
ENSG0000(	ENSG0000(	264.2306	0.251165	0.07565	0.176406	lncRNA
ENSG0000(	TICRR	329.6657	0.190213	0.075657	0.17641	protein_coding
ENSG0000(	CBWD6	823.2834	-0.15603	0.075679	0.176436	protein_coding
ENSG0000(	FOXH1	17.31315	0.41693	0.075677	0.176436	protein_coding
ENSG0000(	ENSG0000(	51.28741	0.19908	0.075686	0.176438	lncRNA
ENSG0000(	MOAP1	507.239	-0.06673	0.075785	0.176656	protein_coding
ENSG0000(	ENSG0000(	75.92023	-0.14158	0.075802	0.176682	lncRNA
ENSG0000(	FAM219A	537.5233	0.11958	0.075818	0.176707	protein_coding
ENSG0000(	ROS1	310.7823	-0.14261	0.075836	0.176736	protein_coding
ENSG0000(	IRF3	2459.905	-0.06727	0.075855	0.176766	protein_coding
ENSG0000(	SYK	2543.081	-0.13203	0.07587	0.176789	protein_coding
ENSG0000(	RNU6-1294	10.31894	0.378565	0.075896	0.176836	snRNA
ENSG0000(	DNMBP	3791.223	-0.27431	0.075913	0.176836	protein_coding
ENSG0000(	KANK3	121.09	0.199981	0.075907	0.176836	protein_coding
ENSG0000(	MIR3150BH	53.55727	-0.40808	0.075908	0.176836	lncRNA
ENSG0000(	ENSG0000(	101.3376	-0.1077	0.075924	0.176849	lncRNA
ENSG0000(	DPY19L4	1087.222	-0.07581	0.075939	0.176858	protein_coding
ENSG0000(	NMNAT3	16233.94	-0.09191	0.075938	0.176858	protein_coding
ENSG0000(	ENSG0000(	47.81279	0.291385	0.075975	0.176929	lncRNA
ENSG0000(	WBP11P1	11.10159	0.331238	0.075987	0.176943	transcribed_processed_pseudoge
ENSG0000(	XPNPEP3	2476.679	0.06548	0.076132	0.177267	protein_coding
ENSG0000(	CFD	157.1811	-0.19666	0.076165	0.177307	protein_coding
ENSG0000(	ENSG0000(	16.2307	-0.44826	0.076161	0.177307	lncRNA
ENSG0000(	ENSG0000(	634.0954	0.063674	0.076156	0.177307	lncRNA
ENSG0000(	ZFP36	3232.768	0.211193	0.076174	0.177312	protein_coding

ENSG0000(	FAHD2CP	118.1287	0.18484	0.076207	0.177352	transcribed_unprocessed_pseudo
ENSG0000(	HERC2P10	60.11796	0.426122	0.076204	0.177352	unprocessed_pseudogene
ENSG0000(	LINC01378	106.5865	0.200644	0.076197	0.177352	lncRNA
ENSG0000(	GULOP	83.84574	-0.37441	0.076215	0.177358	unitary_pseudogene
ENSG0000(	CKLF-CMTN	58.99379	-0.26698	0.076232	0.177382	protein_coding
ENSG0000(	NDUFB5	1081.378	-0.14581	0.076238	0.177385	protein_coding
ENSG0000(	RPS2	3438.642	-0.21168	0.076252	0.177404	protein_coding
ENSG0000(	TWSG1	451.0227	0.125079	0.076261	0.17741	protein_coding
ENSG0000(	ENSG0000(	44.26893	0.199159	0.076294	0.177476	lncRNA
ENSG0000(	CKAP2L	350.6073	0.149761	0.076308	0.177494	protein_coding
ENSG0000(	ANKRD11	6922.599	0.06138	0.076366	0.177602	protein_coding
ENSG0000(	LINC00863	736.7121	0.118734	0.076364	0.177602	lncRNA
ENSG0000(	DHRSX-IT1	95.04055	0.142088	0.076447	0.177778	lncRNA
ENSG0000(	ENSG0000(	109.3	0.165446	0.07646	0.177795	lncRNA
ENSG0000(	TNR	238.7988	-0.2045	0.07652	0.177922	protein_coding
ENSG0000(	ENSG0000(	8.873526	-0.31705	0.07653	0.177932	unprocessed_pseudogene
ENSG0000(	DENND4B	2229.139	-0.14106	0.076548	0.177948	protein_coding
ENSG0000(	ENSG0000(	109.4461	0.109853	0.076543	0.177948	lncRNA
ENSG0000(	CMTR2	1649.69	-0.10737	0.076558	0.177957	protein_coding
ENSG0000(	AMOT	428.9509	-0.26399	0.076581	0.177999	protein_coding
ENSG0000(	ENSG0000(	214.9161	-0.13643	0.076596	0.17802	lncRNA
ENSG0000(	TRIM8-DT	28.75981	0.212681	0.076622	0.178067	lncRNA
ENSG0000(	ENSG0000(	78.75887	0.128051	0.076636	0.178087	lncRNA
ENSG0000(	MPPED1	10.66867	-0.53913	0.076659	0.178127	protein_coding
ENSG0000(	ENSG0000(	21.11517	-0.26069	0.07671	0.178223	lncRNA
ENSG0000(	OR2T35	14.26771	0.836862	0.076712	0.178223	protein_coding
ENSG0000(	LINC00205	795.5395	0.147141	0.076731	0.178247	lncRNA
ENSG0000(	RN7SKP87	15.24428	-0.41126	0.076733	0.178247	misc_RNA
ENSG0000(	ENSG0000(	9.709159	-0.3742	0.076765	0.178307	lncRNA
ENSG0000(	GYPE	84.96928	0.216696	0.076788	0.178334	protein_coding
ENSG0000(	YIPF6	2023.839	-0.04549	0.076786	0.178334	protein_coding
ENSG0000(	PPIAP71	23.81225	0.249616	0.076801	0.178351	processed_pseudogene
ENSG0000(	NDUFB2	1000.469	-0.06362	0.076811	0.17836	protein_coding
ENSG0000(	RAB32	74.15111	-0.21522	0.076817	0.178363	protein_coding
ENSG0000(	CDC26	142.9265	-0.18444	0.076838	0.178397	protein_coding
ENSG0000(	CKMT1A	990.6504	-0.3262	0.076846	0.178404	protein_coding
ENSG0000(	FBRS	2288.505	-0.06104	0.076859	0.178408	protein_coding
ENSG0000(	TAC1	11.148	0.680169	0.076856	0.178408	protein_coding
ENSG0000(	NPHP3-AS1	29.38706	0.204404	0.076867	0.178412	lncRNA
ENSG0000(	RPL7P7	79.72148	-0.15406	0.076884	0.17844	processed_pseudogene
ENSG0000(	PSMD10P1	13.71006	0.549079	0.076919	0.178506	processed_pseudogene
ENSG0000(	CLN6	1361.291	0.068669	0.076927	0.178513	protein_coding
ENSG0000(	ENSG0000(	292.6789	-0.0801	0.076961	0.178565	TEC
ENSG0000(	RSPO3	170.1902	0.44561	0.07696	0.178565	protein_coding
ENSG0000(	RN7SL126F	90.33986	0.136461	0.076987	0.178612	misc_RNA
ENSG0000(	ENSG0000(	21.47201	0.211246	0.077001	0.178618	lncRNA
ENSG0000(	NUDC	525.4244	-0.07866	0.076996	0.178618	protein_coding

ENSG0000(	ENSG0000(	31.97338	0.203014	0.077046	0.178697	lncRNA
ENSG0000(	RNU7-41P	14.07444	0.367301	0.077046	0.178697	snRNA
ENSG0000(	RIOX2	716.7621	0.17702	0.077069	0.178737	protein_coding
ENSG0000(	ENSG0000(	276.9523	0.183832	0.077076	0.178739	lncRNA
ENSG0000(	ADIPOR2	2086.852	-0.15771	0.077102	0.178761	protein_coding
ENSG0000(	ARHGAP5-/	46.66324	-0.2605	0.077094	0.178761	lncRNA
ENSG0000(	ENSG0000(	32.21071	0.224195	0.077099	0.178761	processed_pseudogene
ENSG0000(	ENSG0000(	92.39635	-0.34831	0.077132	0.178818	lncRNA
ENSG0000(	CDH23	394.5432	-0.17182	0.077143	0.178829	protein_coding
ENSG0000(	FER1L6-AS:	9.804471	0.33085	0.077177	0.178894	lncRNA
ENSG0000(	RN7SL288F	79.40924	0.249054	0.077218	0.178976	misc_RNA
ENSG0000(	COX6CP15	8.513573	0.420152	0.077225	0.17898	processed_pseudogene
ENSG0000(	CPSF7	2702.651	-0.05169	0.077251	0.179027	protein_coding
ENSG0000(	CYP21A2	28.85722	0.430368	0.077274	0.179045	protein_coding
ENSG0000(	ENSG0000(	20.95806	-0.25539	0.077279	0.179045	lncRNA
ENSG0000(	PMEL	33.06603	0.366504	0.077281	0.179045	protein_coding
ENSG0000(	VN2R10P	10.7125	-0.40128	0.077281	0.179045	unitary_pseudogene
ENSG0000(	OIT3	17.93343	0.339161	0.077343	0.179147	protein_coding
ENSG0000(	PPIH	1192.391	0.066821	0.077343	0.179147	protein_coding
ENSG0000(	VAMP2	1298.506	-0.07059	0.077332	0.179147	protein_coding
ENSG0000(	ENSG0000(	416.5962	0.331833	0.077361	0.179175	lncRNA
ENSG0000(	ENSG0000(	491.2958	0.115613	0.077387	0.179224	TEC
ENSG0000(	ENSG0000(	378.4685	-0.4927	0.077393	0.179225	lncRNA
ENSG0000(	ALDH5A1	1095.368	-0.11817	0.077443	0.179326	protein_coding
ENSG0000(	PARP2	324.5214	0.086127	0.077466	0.179368	protein_coding
ENSG0000(	UPF3B	522.9028	-0.08161	0.077474	0.179372	protein_coding
ENSG0000(	CFL1	4435.762	-0.17581	0.077486	0.179387	protein_coding
ENSG0000(	RN7SL269F	43.32541	0.161987	0.077492	0.179387	misc_RNA
ENSG0000(	ENSG0000(	27.07463	-0.23859	0.077526	0.179452	lncRNA
ENSG0000(	ENSG0000(	19.95617	0.254879	0.077539	0.17947	lncRNA
ENSG0000(	ENSG0000(	45.29729	0.35731	0.077591	0.179564	lncRNA
ENSG0000(	TRAM1L1	9.81893	0.363318	0.077586	0.179564	protein_coding
ENSG0000(	ENSG0000(	69.38481	0.14863	0.0776	0.17957	lncRNA
ENSG0000(	ENSG0000(	325.2827	0.165169	0.077644	0.179661	lncRNA
ENSG0000(	RIOK1	256.0612	-0.15019	0.07778	0.179962	protein_coding
ENSG0000(	PCSK7	10644.65	0.047168	0.077854	0.18012	protein_coding
ENSG0000(	FAM204BP	79.72255	-0.23041	0.077872	0.180148	processed_pseudogene
ENSG0000(	PTGDR2	86.17651	-0.3291	0.077881	0.180156	protein_coding
ENSG0000(	ENSG0000(	112.4966	0.192302	0.077891	0.180165	TEC
ENSG0000(	NAT10	805.3938	-0.12974	0.077898	0.180169	protein_coding
ENSG0000(	RN7SL804F	21.09062	0.219508	0.077933	0.180227	misc_RNA
ENSG0000(	ZNF271P	1125.159	0.050798	0.077935	0.180227	transcribed_unitary_pseudogene
ENSG0000(	EPM2AIP1	2596.635	0.089466	0.077986	0.180333	protein_coding
ENSG0000(	ENSG0000(	51.08295	0.186564	0.078	0.180351	lncRNA
ENSG0000(	EEF1AKMT	47.96572	0.175071	0.078038	0.180425	protein_coding
ENSG0000(	LINC01796	86.07768	0.138214	0.078053	0.180448	lncRNA
ENSG0000(	TOGARAM:	819.8602	0.078862	0.078059	0.180448	protein_coding

ENSG0000(	PHF7	329.7846	-0.12071	0.078105	0.180541	protein_coding
ENSG0000(	CFAP251	1509.818	0.053076	0.078114	0.18055	protein_coding
ENSG0000(	DAB1	1150.442	-0.42736	0.078173	0.180671	protein_coding
ENSG0000(	TRABD2A	2174.561	-0.16428	0.07819	0.180699	protein_coding
ENSG0000(	ENSG0000(	10.81496	-0.46665	0.078265	0.180857	processed_pseudogene
ENSG0000(	ADCK5	311.4735	-0.21547	0.078285	0.180878	protein_coding
ENSG0000(	SFRP4	13.5806	0.470031	0.078285	0.180878	protein_coding
ENSG0000(	ENSG0000(	105.39	0.174199	0.078303	0.180907	lncRNA
ENSG0000(	PEA15	956.9175	0.220612	0.078327	0.180949	protein_coding
ENSG0000(	DNAJC14	959.1373	-0.07254	0.078356	0.180993	protein_coding
ENSG0000(	ENSG0000(	405.314	0.070696	0.078369	0.180993	lncRNA
ENSG0000(	HLA-J	360.452	0.249188	0.078366	0.180993	transcribed_unprocessed_pseudo
ENSG0000(	TBPL1	447.5416	-0.12496	0.078367	0.180993	protein_coding
ENSG0000(	WHRN	595.0161	-0.18792	0.078376	0.180994	protein_coding
ENSG0000(	RASIP1	86.93449	0.226767	0.078382	0.180995	protein_coding
ENSG0000(	ENSG0000(	43.08297	-0.22585	0.078419	0.181068	processed_pseudogene
ENSG0000(	KCNQ1OT1	25806.72	0.08526	0.078461	0.181152	lncRNA
ENSG0000(	ENSG0000(	11.4211	0.352066	0.078472	0.181163	TEC
ENSG0000(	GS1-124K5	131.7329	0.142527	0.078479	0.181166	lncRNA
ENSG0000(	TRGV4	26.73855	-0.4461	0.078504	0.18121	TR_V_gene
ENSG0000(	ENSG0000(	30.01622	-0.29726	0.078536	0.181267	lncRNA
ENSG0000(	TDRD10	71.60076	-0.2133	0.07854	0.181267	protein_coding
ENSG0000(	ENSG0000(	54.97108	0.144202	0.078555	0.181289	processed_pseudogene
ENSG0000(	ENSG0000(	137.8325	0.187638	0.078601	0.181382	lncRNA
ENSG0000(	ENSG0000(	23.71272	0.199987	0.07864	0.181445	lncRNA
ENSG0000(	ENSG0000(	569.5904	0.086284	0.078639	0.181445	lncRNA
ENSG0000(	ZSCAN26	453.455	-0.11054	0.078646	0.181445	protein_coding
ENSG0000(	ENSG0000(	407.5181	-0.13455	0.078664	0.181474	lncRNA
ENSG0000(	ENSG0000(	150.8325	0.235292	0.078676	0.181487	lncRNA
ENSG0000(	PRR15L	2420.762	-0.37263	0.078683	0.181491	protein_coding
ENSG0000(	C19orf54	631.764	-0.16108	0.078729	0.181584	protein_coding
ENSG0000(	MROCKI	490.9365	-0.20919	0.078754	0.181628	lncRNA
ENSG0000(	ITGB2	1562.105	0.331614	0.078782	0.181679	protein_coding
ENSG0000(	P4HTM	1351.867	-0.07615	0.078789	0.181683	protein_coding
ENSG0000(	LINC02377	33.35531	-0.24359	0.07881	0.181717	lncRNA
ENSG0000(	CDKN1A	2292.176	-0.29223	0.078825	0.181738	protein_coding
ENSG0000(	ENSG0000(	423.7877	-0.11824	0.078832	0.181742	lncRNA
ENSG0000(	ATG10	587.422	-0.08917	0.078869	0.181813	protein_coding
ENSG0000(	LYVE1	159.8741	0.282656	0.078922	0.181923	protein_coding
ENSG0000(	NRBP1	1347.998	-0.07444	0.078975	0.182032	protein_coding
ENSG0000(	ENSG0000(	9.199833	0.335669	0.078984	0.182038	lncRNA
ENSG0000(	PIP4P2	109.9277	0.261043	0.079011	0.182087	protein_coding
ENSG0000(	KRT18P4	75.11503	-0.17357	0.07902	0.182096	processed_pseudogene
ENSG0000(	ENSG0000(	32.94628	0.191912	0.079064	0.182169	lncRNA
ENSG0000(	SYAP1	2659.016	-0.08568	0.079063	0.182169	protein_coding
ENSG0000(	ENSG0000(	220.19	-0.10874	0.079099	0.182211	lncRNA
ENSG0000(	ENSG0000(	252.055	0.108219	0.079096	0.182211	TEC

ENSG0000(	SLC15A1	10279.88	-0.46184	0.07909	0.182211	protein_coding
ENSG0000(	GVQW3	2345.198	0.054008	0.079105	0.182211	protein_coding
ENSG0000(	ENSG0000(	80.77749	0.140708	0.079131	0.182257	lncRNA
ENSG0000(	ASIC4	94.89764	0.124885	0.079152	0.182293	protein_coding
ENSG0000(	ENSG0000(	276.9055	0.095401	0.079161	0.182301	lncRNA
ENSG0000(	TMEM240	16.68529	-0.28265	0.079174	0.182317	protein_coding
ENSG0000(	HMCN1	727.735	-0.29495	0.0792	0.182363	protein_coding
ENSG0000(	LCT-AS1	18.37315	0.32339	0.079234	0.182429	lncRNA
ENSG0000(	MTRFR	4023.313	0.058305	0.079263	0.182482	protein_coding
ENSG0000(	ENSG0000(	459.002	-0.07377	0.079301	0.182549	lncRNA
ENSG0000(	MBTPS2	483.2702	0.083225	0.079304	0.182549	protein_coding
ENSG0000(	TMEM11	206.5297	-0.09219	0.079315	0.182562	protein_coding
ENSG0000(	ENSG0000(	168.4853	0.234356	0.079366	0.182666	TEC
ENSG0000(	ENSG0000(	75.56028	0.124586	0.079387	0.1827	lncRNA
ENSG0000(	ARHGEF39	652.4877	0.082101	0.07942	0.182764	protein_coding
ENSG0000(	LINC01951	62.74647	0.161867	0.079433	0.182779	lncRNA
ENSG0000(	ZADH2	2385.041	-0.09395	0.079442	0.182787	protein_coding
ENSG0000(	TPRG1	114.848	0.174637	0.079472	0.182841	protein_coding
ENSG0000(	ATP6V0D1-	1065.563	0.120933	0.079496	0.18288	lncRNA
ENSG0000(	MEX3A	63.93809	0.23566	0.0795	0.18288	protein_coding
ENSG0000(	ALOX12P1	20.10183	0.220231	0.079526	0.182894	unprocessed_pseudogene
ENSG0000(	CILP2	10.19501	-0.658	0.079524	0.182894	protein_coding
ENSG0000(	ENSG0000(	72.13488	-0.15823	0.079535	0.182894	lncRNA
ENSG0000(	GRM6	440.9103	0.071352	0.079518	0.182894	protein_coding
ENSG0000(	NCF1C	170.1628	0.403271	0.079535	0.182894	unprocessed_pseudogene
ENSG0000(	SORCS1	203.541	-0.2421	0.079552	0.182919	protein_coding
ENSG0000(	CFAP58	15.40959	0.457899	0.079558	0.18292	protein_coding
ENSG0000(	ENSG0000(	34.67333	0.463561	0.079583	0.182944	lncRNA
ENSG0000(	OR2J2	51.42447	-0.18454	0.07958	0.182944	protein_coding
ENSG0000(	SMYD3	513.53	0.132963	0.079586	0.182944	protein_coding
ENSG0000(	ENSG0000(	29.19674	0.217458	0.0796	0.182963	lncRNA
ENSG0000(	ENSG0000(	42.11293	0.193267	0.079623	0.183003	lncRNA
ENSG0000(	C1QBP	281.9162	-0.15509	0.079634	0.183014	protein_coding
ENSG0000(	MGMT	487.6737	0.129321	0.079649	0.183021	protein_coding
ENSG0000(	SH2B3	1657.069	0.205475	0.079643	0.183021	protein_coding
ENSG0000(	ENSG0000(	64.50255	-0.16347	0.079663	0.183036	lncRNA
ENSG0000(	TAS2R13	211.1496	0.195974	0.079667	0.183036	protein_coding
ENSG0000(	ROBO3	193.3784	0.247664	0.079674	0.183041	protein_coding
ENSG0000(	ENSG0000(	60.66667	0.266299	0.079696	0.18307	TEC
ENSG0000(	SNORA57	479.0579	0.227799	0.079699	0.18307	snoRNA
ENSG0000(	TMEM198	104.5563	-0.33556	0.079726	0.183116	protein_coding
ENSG0000(	ZNF717	830.9088	0.076793	0.079731	0.183116	protein_coding
ENSG0000(	ENSG0000(	201.5717	-0.12456	0.079738	0.183119	protein_coding
ENSG0000(	MIR548L	10.56489	0.32102	0.079751	0.183138	miRNA
ENSG0000(	ENSG0000(	54.81953	0.174806	0.079774	0.183177	lncRNA
ENSG0000(	TSBP1-AS1	10992.24	-0.07418	0.07978	0.183177	lncRNA
ENSG0000(	MIR497HG	205.9448	0.174578	0.079796	0.1832	lncRNA

ENSG0000(	DYNLT3	340.7907	-0.10686	0.07983	0.183211	protein_coding
ENSG0000(	ENSG0000(	51.40834	0.168506	0.079809	0.183211	lncRNA
ENSG0000(	ENSG0000(	44.85212	0.344846	0.079824	0.183211	lncRNA
ENSG0000(	ENSG0000(	68.16599	0.168193	0.079826	0.183211	lncRNA
ENSG0000(	KCNK13	22.97168	0.379245	0.079818	0.183211	protein_coding
ENSG0000(	C3orf14	46.21003	-0.27931	0.07984	0.18322	protein_coding
ENSG0000(	ENSG0000(	1804.72	0.057133	0.079853	0.183237	lncRNA
ENSG0000(	PYCARD-AS	31.91525	0.224332	0.079864	0.183249	lncRNA
ENSG0000(	ENSG0000(	12.97424	0.36895	0.079889	0.183294	lncRNA
ENSG0000(	ENSG0000(	83.36265	-0.11102	0.079899	0.183303	lncRNA
ENSG0000(	MTMR6	580.8596	0.1206	0.079916	0.183328	protein_coding
ENSG0000(	UBQLN1-AS	49.87656	0.180041	0.07993	0.183348	lncRNA
ENSG0000(	ELP4	2024.526	0.082292	0.079978	0.18343	protein_coding
ENSG0000(	ENSG0000(	137.5642	-0.10128	0.079973	0.18343	lncRNA
ENSG0000(	A2ML1	270.2769	0.088299	0.080081	0.183597	protein_coding
ENSG0000(	ENSG0000(	15.59276	0.3179	0.080072	0.183597	snoRNA
ENSG0000(	ENSG0000(	240.5475	-0.17583	0.080074	0.183597	lncRNA
ENSG0000(	ENSG0000(	146.0178	-0.13157	0.080073	0.183597	lncRNA
ENSG0000(	TEX9	1672.983	0.1087	0.080059	0.183597	protein_coding
ENSG0000(	ZMAT3	1569.163	0.121655	0.080085	0.183597	protein_coding
ENSG0000(	EIF5B	1317.231	0.091504	0.08011	0.183639	protein_coding
ENSG0000(	CD46	6224.529	-0.0818	0.08012	0.183649	protein_coding
ENSG0000(	ENSG0000(	208.9389	0.110111	0.080126	0.183649	lncRNA
ENSG0000(	ENSG0000(	128.8921	-0.13532	0.080193	0.18379	lncRNA
ENSG0000(	C2CD4D-AS	35.3671	0.240868	0.080212	0.18382	lncRNA
ENSG0000(	SENP5	2849.846	-0.05518	0.080224	0.183835	protein_coding
ENSG0000(	SNORD19	13.08088	0.36226	0.080237	0.18385	snoRNA
ENSG0000(	ENSG0000(	9.512986	0.367055	0.080257	0.183883	TEC
ENSG0000(	CYP46A1	1089.886	0.093489	0.080299	0.183966	protein_coding
ENSG0000(	ARHGDIA	1918.786	-0.14847	0.080307	0.183971	protein_coding
ENSG0000(	ASCC1	924.528	0.111678	0.080356	0.184069	protein_coding
ENSG0000(	ENSG0000(	44.94127	0.185836	0.080374	0.184099	lncRNA
ENSG0000(	SPATA2	466.133	-0.14784	0.080453	0.184265	protein_coding
ENSG0000(	ADAM20	192.8483	0.177878	0.08049	0.184305	protein_coding
ENSG0000(	CYP3A43	190.2567	-0.09526	0.080494	0.184305	protein_coding
ENSG0000(	MIR2052H(	51.97597	0.175614	0.080491	0.184305	lncRNA
ENSG0000(	TMEM42	455.5348	0.073249	0.080482	0.184305	protein_coding
ENSG0000(	FOXP4	1100.112	-0.12105	0.080539	0.184395	protein_coding
ENSG0000(	ENSG0000(	58.70992	0.139262	0.080569	0.184451	lncRNA
ENSG0000(	ENSG0000(	17.32453	0.559257	0.080617	0.184546	lncRNA
ENSG0000(	TTC23	540.8071	-0.09665	0.080623	0.184546	protein_coding
ENSG0000(	ENSG0000(	60.41336	0.374484	0.080634	0.184557	lncRNA
ENSG0000(	ENSG0000(	34.41368	-0.15207	0.080639	0.184557	lncRNA
ENSG0000(	GJA4	38.08306	0.318603	0.080661	0.184581	protein_coding
ENSG0000(	IFI44	604.5771	0.353205	0.08066	0.184581	protein_coding
ENSG0000(	ENSG0000(	137.2588	-0.1346	0.080669	0.184585	processed_pseudogene
ENSG0000(	NAMPTP1	43.81715	0.738242	0.080701	0.184644	processed_pseudogene

ENSG0000(	ENSG0000(	75.03076	-0.16571	0.080707	0.184646	transcribed_unprocessed_pseudo
ENSG0000(	ARFGAP2	1621.756	-0.06495	0.080731	0.184647	protein_coding
ENSG0000(	ENSG0000(	838.649	0.064176	0.080731	0.184647	lncRNA
ENSG0000(	LINC02535	287.1769	-0.50721	0.080723	0.184647	lncRNA
ENSG0000(	QTRT1	641.5368	-0.09971	0.08073	0.184647	protein_coding
ENSG0000(	ENSG0000(	86.42864	0.279192	0.080754	0.184686	TEC
ENSG0000(	LRRC75B	439.4123	-0.28503	0.080771	0.184712	protein_coding
ENSG0000(	TMEM45B	2814.501	-0.32824	0.080795	0.184754	protein_coding
ENSG0000(	ENSG0000(	37.94428	0.474268	0.080802	0.184757	lncRNA
ENSG0000(	ARMC6	290.9054	-0.11893	0.080826	0.184784	protein_coding
ENSG0000(	IPO8	1806.687	-0.0855	0.080825	0.184784	protein_coding
ENSG0000(	ADAMTSL5	292.662	-0.30868	0.080841	0.184805	protein_coding
ENSG0000(	SEC11A	4198.389	0.049669	0.080872	0.184862	protein_coding
ENSG0000(	ENSG0000(	97.06307	0.15534	0.080883	0.184873	lncRNA
ENSG0000(	DUSP8	503.6118	-0.29348	0.080958	0.185007	protein_coding
ENSG0000(	ENSG0000(	94.36626	0.139145	0.080957	0.185007	lncRNA
ENSG0000(	TTC27	437.8233	-0.17353	0.080959	0.185007	protein_coding
ENSG0000(	LOXHD1	38.7094	0.282666	0.080973	0.185025	protein_coding
ENSG0000(	ENSG0000(	315.5579	0.207078	0.081005	0.185085	lncRNA
ENSG0000(	ENSG0000(	684.4871	0.147756	0.081041	0.185154	TEC
ENSG0000(	LEPROT	2298.109	0.072168	0.081096	0.185267	protein_coding
ENSG0000(	PTK6	1342.915	-0.26706	0.081138	0.185349	protein_coding
ENSG0000(	RNU6ATAC	9.012384	0.425214	0.081158	0.185382	snRNA
ENSG0000(	SLAMF6P1	32.50389	0.419252	0.081192	0.185446	unprocessed_pseudogene
ENSG0000(	FRG1	419.1501	-0.13213	0.081208	0.185469	protein_coding
ENSG0000(	FAM110C	1039.122	-0.30962	0.08122	0.185482	protein_coding
ENSG0000(	ENSG0000(	18.88853	-0.49378	0.081233	0.185486	processed_pseudogene
ENSG0000(	ENSG0000(	19.48859	0.357821	0.081233	0.185486	lncRNA
ENSG0000(	KRT8P4	19.1125	-0.59874	0.081245	0.1855	processed_pseudogene
ENSG0000(	CLCA2	41.45179	0.467027	0.081276	0.185543	protein_coding
ENSG0000(	NBEAL1	9381.626	-0.1382	0.081277	0.185543	protein_coding
ENSG0000(	NBPF26	1120.426	0.311685	0.081288	0.185543	protein_coding
ENSG0000(	SENP8	433.0533	0.11232	0.081285	0.185543	protein_coding
ENSG0000(	SEC16B	882.5321	-0.33473	0.08131	0.185582	protein_coding
ENSG0000(	PURG	29.01328	0.222091	0.081325	0.185602	protein_coding
ENSG0000(	ZNF222-DT	11.20768	0.36159	0.081358	0.185662	lncRNA
ENSG0000(	GOLGA6B	17.65558	0.771253	0.081441	0.18584	protein_coding
ENSG0000(	LINC02587	29.9528	0.197895	0.081483	0.185921	lncRNA
ENSG0000(	ENSG0000(	778.6443	-0.10373	0.081513	0.185963	lncRNA
ENSG0000(	ZBTB16	1750.718	0.376976	0.081507	0.185963	protein_coding
ENSG0000(	EFCAB12	529.6152	0.084403	0.081529	0.185986	protein_coding
ENSG0000(	HMGN2P17	31.13519	-0.28346	0.081538	0.185993	processed_pseudogene
ENSG0000(	DEPDC7	460.5351	-0.39232	0.08155	0.186007	protein_coding
ENSG0000(	FAM86C2P	83.38001	0.175754	0.081585	0.186072	transcribed_unprocessed_pseudo
ENSG0000(	LINC01825	80.80431	0.202409	0.081631	0.18615	lncRNA
ENSG0000(	WDR5B	577.85	0.081573	0.081628	0.18615	protein_coding
ENSG0000(	HHLA1	269.7447	0.186207	0.081646	0.186173	protein_coding

ENSG0000(	ENSG0000(	17.34356	-0.27099	0.081676	0.186227	snoRNA
ENSG0000(	ENSG0000(	498.9855	-0.07296	0.081723	0.18632	lncRNA
ENSG0000(	MRPL38	322.5839	-0.11612	0.08182	0.186529	protein_coding
ENSG0000(	ENSG0000(	21.55028	0.226158	0.081831	0.186541	lncRNA
ENSG0000(	PCMT1	341.0729	-0.11621	0.081869	0.186613	protein_coding
ENSG0000(	BNIP3P28	265.9497	-0.08933	0.081877	0.186619	processed_pseudogene
ENSG0000(	Vault	34.66907	0.232317	0.08189	0.186634	misc_RNA
ENSG0000(	MIR4473	12.40515	0.336934	0.081912	0.186671	miRNA
ENSG0000(	SMIM22	197.944	-0.30365	0.081956	0.186757	protein_coding
ENSG0000(	BMS1P11	14.69119	0.764935	0.081968	0.18677	unprocessed_pseudogene
ENSG0000(	ENSG0000(	19.68867	-0.33053	0.082033	0.186905	lncRNA
ENSG0000(	NNT	2699.207	0.065043	0.08207	0.186975	protein_coding
ENSG0000(	ENSG0000(	45.2455	-0.17152	0.082085	0.186995	processed_pseudogene
ENSG0000(	ENSG0000(	733.1196	-0.15022	0.08209	0.186995	lncRNA
ENSG0000(	STAMBPL1	1204.26	0.148	0.0821	0.187004	protein_coding
ENSG0000(	LMNB2	601.3447	0.135293	0.082107	0.187008	protein_coding
ENSG0000(	SRPX	39.74966	0.341349	0.082156	0.187105	protein_coding
ENSG0000(	MFSD12	873.7561	0.136889	0.082189	0.187166	protein_coding
ENSG0000(	ENSG0000(	26.94902	0.187891	0.082203	0.187184	lncRNA
ENSG0000(	LINC01843	176.2982	-0.2547	0.082219	0.187207	lncRNA
ENSG0000(	OR4Q3	19.21255	0.280604	0.082275	0.187321	protein_coding
ENSG0000(	ENSG0000(	117.7182	0.099077	0.082327	0.187427	transcribed_unprocessed_pseudo
ENSG0000(	CLRN1-AS1	107.2149	-0.13659	0.082351	0.187453	lncRNA
ENSG0000(	RNF113B	33.46435	0.311957	0.082348	0.187453	protein_coding
ENSG0000(	DPYSL4	14.5107	0.611892	0.082377	0.187483	protein_coding
ENSG0000(	IGLC6	8.803085	0.534014	0.082381	0.187483	IG_C_pseudogene
ENSG0000(	IL17REL	239.8104	-0.40625	0.082375	0.187483	protein_coding
ENSG0000(	ENSG0000(	14.39269	-0.39074	0.082405	0.187522	lncRNA
ENSG0000(	KIF21B	1449.424	0.20641	0.082433	0.187574	protein_coding
ENSG0000(	ENSG0000(	245.1309	0.151846	0.08245	0.187597	lncRNA
ENSG0000(	GTF2IRD1P	325.4771	0.07022	0.082462	0.187612	transcribed_unprocessed_pseudo
ENSG0000(	UNC93A	23.76961	-0.47818	0.082539	0.187774	protein_coding
ENSG0000(	LINC02889	677.347	0.104299	0.082563	0.187815	lncRNA
ENSG0000(	ENSG0000(	148.5766	0.114567	0.08261	0.187907	lncRNA
ENSG0000(	ZNF148	2202.473	-0.07746	0.082628	0.187935	protein_coding
ENSG0000(	ENSG0000(	60.6662	0.147951	0.082661	0.18797	lncRNA
ENSG0000(	IL10RB-DT	141.7894	0.097513	0.082649	0.18797	lncRNA
ENSG0000(	TGFB1	764.0969	0.230135	0.082658	0.18797	protein_coding
ENSG0000(	ENSG0000(	171.0672	0.123216	0.082749	0.188142	lncRNA
ENSG0000(	LINC02290	12.09037	0.305281	0.082743	0.188142	lncRNA
ENSG0000(	GRK1	9.790395	0.499932	0.082769	0.18816	protein_coding
ENSG0000(	HGD	388.8567	-0.34041	0.082764	0.18816	protein_coding
ENSG0000(	ENSG0000(	84.61843	0.393582	0.082786	0.188185	unprocessed_pseudogene
ENSG0000(	CNN3-DT	302.2036	0.117864	0.082814	0.188236	lncRNA
ENSG0000(	ENSG0000(	88.00178	0.308663	0.082843	0.188287	lncRNA
ENSG0000(	STAG3	1374.948	0.132941	0.082848	0.188287	protein_coding
ENSG0000(	SLC6A11	140.2294	0.092018	0.08287	0.188323	protein_coding

ENSG0000(	BTBD10P2	55.06947	0.148648	0.082923	0.188429	processed_pseudogene
ENSG0000(	ENSG0000(	181.7081	0.101257	0.082962	0.188494	lncRNA
ENSG0000(	INPP5E	687.0682	-0.10153	0.082963	0.188494	protein_coding
ENSG0000(	OGT	16851.63	0.105597	0.083058	0.188678	protein_coding
ENSG0000(	SUSD6	3990.678	0.124433	0.083062	0.188678	protein_coding
ENSG0000(	ZNF189	1344.997	0.065778	0.083061	0.188678	protein_coding
ENSG0000(	EIF4A2P2	64.17687	0.134053	0.083086	0.188717	processed_pseudogene
ENSG0000(	PAG1	5275.838	-0.14995	0.0831	0.188737	protein_coding
ENSG0000(	JMJD6	843.7352	-0.11217	0.083137	0.188807	protein_coding
ENSG0000(	ENSG0000(	66.29436	-0.39306	0.083155	0.188833	lncRNA
ENSG0000(	FNDC11	15.03801	-0.49464	0.0832	0.188901	protein_coding
ENSG0000(	TNFRSF11A	1629.392	-0.21039	0.083191	0.188901	protein_coding
ENSG0000(	ZNF3	1771.397	-0.08676	0.083202	0.188901	protein_coding
ENSG0000(	GLRA2	9.968005	-0.54676	0.08324	0.188972	protein_coding
ENSG0000(	ENSG0000(	284.639	-0.16089	0.083312	0.189123	lncRNA
ENSG0000(	ENSG0000(	11.29653	-0.33106	0.083324	0.189137	lncRNA
ENSG0000(	LINC02812	87.16933	0.181409	0.083353	0.189161	lncRNA
ENSG0000(	MED17	3241.109	-0.05185	0.083348	0.189161	protein_coding
ENSG0000(	TMEM126A	63.89185	-0.1899	0.083346	0.189161	protein_coding
ENSG0000(	CD1D	717.5282	-0.16901	0.083394	0.189241	protein_coding
ENSG0000(	LINC01517	253.3272	0.086527	0.083417	0.18927	lncRNA
ENSG0000(	SLC22A17	142.458	0.214236	0.083419	0.18927	protein_coding
ENSG0000(	ENSG0000(	120.9673	-0.20174	0.08343	0.189281	lncRNA
ENSG0000(	HS2ST1	914.9865	-0.08417	0.08345	0.189303	protein_coding
ENSG0000(	TPM2	537.6055	0.218415	0.083452	0.189303	protein_coding
ENSG0000(	ENSG0000(	42.17427	0.199152	0.083475	0.189316	lncRNA
ENSG0000(	SOX7	178.8861	0.10116	0.083467	0.189316	protein_coding
ENSG0000(	USP38-DT	137.2253	0.262607	0.083474	0.189316	lncRNA
ENSG0000(	TPSB2	342.7593	-0.21417	0.083498	0.189353	protein_coding
ENSG0000(	AP3M2	527.0399	0.120092	0.083508	0.18936	protein_coding
ENSG0000(	ENSG0000(	11.37394	0.380673	0.083513	0.18936	lncRNA
ENSG0000(	WDR91	1386.151	0.104815	0.083541	0.189411	protein_coding
ENSG0000(	B3GALT5-A	440.0858	0.087417	0.083572	0.189426	lncRNA
ENSG0000(	ENSG0000(	45.92885	-0.2672	0.083572	0.189426	transcribed_unprocessed_pseudo
ENSG0000(	SCARNA7	3580.598	-0.14328	0.083568	0.189426	scaRNA
ENSG0000(	SUN1	3100.337	-0.1051	0.083561	0.189426	protein_coding
ENSG0000(	ENSG0000(	617.6686	0.070559	0.083584	0.189431	lncRNA
ENSG0000(	ENSG0000(	21.73811	0.243328	0.083586	0.189431	lncRNA
ENSG0000(	RN7SKP195	17.19444	-0.33097	0.083594	0.189434	misc_RNA
ENSG0000(	GALNT16-A	12.72966	0.329701	0.083607	0.189451	lncRNA
ENSG0000(	COX16	343.9988	-0.1434	0.08365	0.189522	protein_coding
ENSG0000(	RNU6-1176	39.08243	0.277316	0.083647	0.189522	snRNA
ENSG0000(	SLC22A18A	129.8243	-0.34535	0.083677	0.189567	protein_coding
ENSG0000(	BORCS7	676.1353	-0.07119	0.083775	0.189778	protein_coding
ENSG0000(	SHROOM3	5098.632	-0.31296	0.083794	0.189807	protein_coding
ENSG0000(	ENSG0000(	10.49928	-0.76507	0.083814	0.18983	lncRNA
ENSG0000(	STOX1	100.763	-0.28615	0.083817	0.18983	protein_coding

ENSG0000(	PIAS4	348.771	-0.11671	0.083883	0.189967	protein_coding
ENSG0000(	ENSG0000(	112.2528	0.132755	0.083909	0.190013	lncRNA
ENSG0000(	NCBP2AS2	101.9862	-0.12052	0.083981	0.190162	protein_coding
ENSG0000(	ENSG0000(	36.13304	0.254099	0.084	0.190191	lncRNA
ENSG0000(	ENSG0000(	20.22955	-0.50376	0.084023	0.19023	lncRNA
ENSG0000(	FZD1	398.0793	-0.16878	0.084065	0.190311	protein_coding
ENSG0000(	LRRC24	27.71165	-0.25253	0.084093	0.190361	protein_coding
ENSG0000(	ENSG0000(	15.40563	-0.48194	0.084118	0.190397	lncRNA
ENSG0000(	ENSG0000(	17.14614	0.276121	0.084121	0.190397	lncRNA
ENSG0000(	SLC22A31	15.34514	0.486439	0.084155	0.190459	protein_coding
ENSG0000(	ENSG0000(	76.05174	0.1514	0.084202	0.190528	lncRNA
ENSG0000(	HHIP	946.6233	-0.33227	0.084203	0.190528	protein_coding
ENSG0000(	PTCH1	4074.857	-0.19801	0.084193	0.190528	protein_coding
ENSG0000(	DAG1	1339.171	-0.12534	0.084224	0.190561	protein_coding
ENSG0000(	ENSG0000(	14.14374	-0.37048	0.08424	0.19057	lncRNA
ENSG0000(	ENSG0000(	289.8501	-0.09348	0.084235	0.19057	lncRNA
ENSG0000(	ADAL	469.1992	0.099066	0.084279	0.190624	protein_coding
ENSG0000(	ADAM1B	81.32278	0.135318	0.084282	0.190624	unitary_pseudogene
ENSG0000(	RNU2-59P	122.9477	-0.24666	0.084281	0.190624	snRNA
ENSG0000(	ENSG0000(	114.5995	-0.11839	0.084301	0.190652	lncRNA
ENSG0000(	DCAF12	794.1509	-0.10678	0.084365	0.190783	protein_coding
ENSG0000(	EXOSC10	1417.511	0.072221	0.084381	0.190807	protein_coding
ENSG0000(	PIF1	643.2332	0.090482	0.084421	0.190882	protein_coding
ENSG0000(	MT-ATP8	1167.46	-0.28295	0.084469	0.190978	protein_coding
ENSG0000(	ACTR3	5347.19	-0.11201	0.084522	0.191085	protein_coding
ENSG0000(	ENSG0000(	29.90498	0.252535	0.084537	0.191104	lncRNA
ENSG0000(	LINC00596	96.58342	-0.13628	0.084576	0.191178	lncRNA
ENSG0000(	CNOT11	757.7651	-0.10909	0.084613	0.191248	protein_coding
ENSG0000(	NEK2	339.4083	-0.08281	0.084621	0.191253	protein_coding
ENSG0000(	SLC10A6	17.18054	0.346413	0.084641	0.191284	protein_coding
ENSG0000(	LINC01535	201.8875	0.151845	0.084668	0.191319	lncRNA
ENSG0000(	ZCCHC17	232.1166	-0.0906	0.084665	0.191319	protein_coding
ENSG0000(	ENSG0000(	26.15408	0.245205	0.084686	0.191345	lncRNA
ENSG0000(	ENSG0000(	848.6293	-0.17954	0.084709	0.191383	lncRNA
ENSG0000(	ENSG0000(	93.18244	-0.15786	0.084785	0.19154	lncRNA
ENSG0000(	KLRC1	211.2981	0.134631	0.08479	0.19154	protein_coding
ENSG0000(	ENSG0000(	16.46915	-0.26338	0.084801	0.191549	lncRNA
ENSG0000(	DIP2C	820.9319	0.142494	0.084824	0.191587	protein_coding
ENSG0000(	ENSG0000(	2118.699	0.078373	0.084894	0.191726	lncRNA
ENSG0000(	NCAPH	506.4548	0.111286	0.084897	0.191726	protein_coding
ENSG0000(	B4GALNT1	75.44469	-0.33584	0.08492	0.191764	protein_coding
ENSG0000(	ENSG0000(	20.99907	0.28696	0.08494	0.191796	lncRNA
ENSG0000(	ITGAM	378.2359	0.372059	0.084947	0.191797	protein_coding
ENSG0000(	ENSG0000(	129.4127	0.147702	0.084962	0.191817	lncRNA
ENSG0000(	E2F3P2	340.4776	-0.1063	0.084978	0.191839	processed_pseudogene
ENSG0000(	CPA2	28.8015	0.526888	0.085015	0.191909	protein_coding
ENSG0000(	ENSG0000(	17.52071	-0.5175	0.085035	0.191942	lncRNA

ENSG0000(	CKMT1B	942.0525	-0.32391	0.085047	0.191954	protein_coding
ENSG0000(	POLG	1983.836	0.13816	0.085054	0.191955	protein_coding
ENSG0000(	SYNJ2BP	3185.044	-0.12138	0.085071	0.191982	protein_coding
ENSG0000(	SNRPCP9	22.80198	0.333597	0.085188	0.192231	processed_pseudogene
ENSG0000(	ANTKMT	85.7916	-0.20165	0.085201	0.192247	protein_coding
ENSG0000(	ENSG0000(	9.291141	-0.457	0.085248	0.192338	lncRNA
ENSG0000(	ENSG0000(	71.97673	0.204289	0.085262	0.192356	lncRNA
ENSG0000(	ACTR5	217.9331	-0.11394	0.085281	0.192372	protein_coding
ENSG0000(	LINC00479	78.64661	-0.56616	0.085278	0.192372	lncRNA
ENSG0000(	DHX16	1245.536	-0.13286	0.085305	0.192398	protein_coding
ENSG0000(	S1PR3	810.6717	0.245274	0.085302	0.192398	protein_coding
ENSG0000(	CSPG4	358.6394	0.245631	0.085327	0.192408	protein_coding
ENSG0000(	DGKE	1297.396	0.082951	0.085326	0.192408	protein_coding
ENSG0000(	MST1L	834.5529	-0.39603	0.085331	0.192408	transcribed_unprocessed_pseudo
ENSG0000(	ZPBP2	94.81262	0.188089	0.085334	0.192408	protein_coding
ENSG0000(	ENSG0000(	10.96404	0.319662	0.085436	0.192625	lncRNA
ENSG0000(	ZKSCAN4	471.4344	0.066338	0.085443	0.192627	protein_coding
ENSG0000(	ENSG0000(	92.48704	-0.12033	0.08545	0.192629	lncRNA
ENSG0000(	IQGAP2	2566.851	-0.09555	0.085466	0.192651	protein_coding
ENSG0000(	IDH3G	432.2525	-0.09048	0.085484	0.192664	protein_coding
ENSG0000(	SMYD4	2125.274	-0.0664	0.08549	0.192664	protein_coding
ENSG0000(	ZFP69	111.9966	0.209252	0.085483	0.192664	protein_coding
ENSG0000(	SYDE2	687.2082	-0.15622	0.085548	0.19278	protein_coding
ENSG0000(	ENSG0000(	10.77853	0.369433	0.085567	0.19281	TEC
ENSG0000(	ENSG0000(	58.62805	-0.19102	0.085581	0.192829	lncRNA
ENSG0000(	LYSMD3	934.481	-0.09416	0.085593	0.19284	protein_coding
ENSG0000(	ENSG0000(	61.08787	0.149457	0.085608	0.192847	lncRNA
ENSG0000(	WBP2	1313.023	-0.09394	0.085606	0.192847	protein_coding
ENSG0000(	FRMD3	983.7889	-0.35641	0.085684	0.192991	protein_coding
ENSG0000(	PHB	506.0159	-0.13251	0.085683	0.192991	protein_coding
ENSG0000(	SPTLC3	1676.676	-0.12976	0.0857	0.193013	protein_coding
ENSG0000(	UBD	782.3319	0.511816	0.085722	0.193049	protein_coding
ENSG0000(	ENSG0000(	104.6617	-0.14014	0.085732	0.193053	lncRNA
ENSG0000(	LINC01060	303.6264	0.092216	0.085736	0.193053	lncRNA
ENSG0000(	MTRNR2L6	18.21362	0.351691	0.085751	0.193074	protein_coding
ENSG0000(	CPOX	469.9929	-0.10361	0.08576	0.19308	protein_coding
ENSG0000(	NKTR	14249.61	0.053327	0.085767	0.193083	protein_coding
ENSG0000(	CLNS1A	1270.821	-0.09643	0.085795	0.193131	protein_coding
ENSG0000(	MED29	854.1346	-0.0732	0.085838	0.193215	protein_coding
ENSG0000(	ENSG0000(	241.3583	-0.31077	0.08585	0.193227	lncRNA
ENSG0000(	ENSG0000(	180.3591	0.102702	0.085889	0.193301	lncRNA
ENSG0000(	ANOS1	145.9561	0.287936	0.085947	0.193418	protein_coding
ENSG0000(	PNMA1	335.9997	0.152425	0.086018	0.193564	protein_coding
ENSG0000(	SHISA3	98.92459	-0.5116	0.086028	0.193572	protein_coding
ENSG0000(	ENSG0000(	320.511	0.079855	0.086041	0.193589	processed_pseudogene
ENSG0000(	HBG2	10.06097	0.377375	0.086072	0.193643	protein_coding
ENSG0000(	CD244	135.5624	-0.21576	0.086097	0.193685	protein_coding

ENSG0000(	ZSCAN20	575.439	0.07742	0.086107	0.193694	protein_coding
ENSG0000(	NIFK-AS1	625.2557	0.087224	0.086115	0.193699	lncRNA
ENSG0000(	NDUFS8	867.0551	-0.07486	0.086146	0.193755	protein_coding
ENSG0000(	SNAP47	688.9915	0.081469	0.086173	0.193802	protein_coding
ENSG0000(	RPL23AP7	128.853	-0.20662	0.086195	0.193836	transcribed_processed_pseudoge
ENSG0000(	ENSG0000(	265.1994	0.128089	0.086227	0.193895	transcribed_processed_pseudoge
ENSG0000(	ENSG0000(	117.2291	0.270665	0.086248	0.193928	lncRNA
ENSG0000(	ENSG0000(	65.67284	-0.16465	0.086256	0.193934	lncRNA
ENSG0000(	LINC01451	28.26257	-0.31541	0.086282	0.193978	lncRNA
ENSG0000(	GZMM	23.17602	-0.38402	0.086329	0.19407	protein_coding
ENSG0000(	TRPC4AP	1849.57	0.082916	0.08635	0.194103	protein_coding
ENSG0000(	MIR4440	30.67782	0.239214	0.08639	0.194178	miRNA
ENSG0000(	ENSG0000(	194.6344	0.087309	0.08641	0.194196	lncRNA
ENSG0000(	YPEL1	466.9341	-0.13427	0.086407	0.194196	protein_coding
ENSG0000(	ENSG0000(	165.3697	0.091198	0.086462	0.1943	TEC
ENSG0000(	ENSG0000(	9.656212	0.35127	0.086508	0.194389	processed_pseudogene
ENSG0000(	ENSG0000(	755.0097	0.1005	0.086546	0.19446	lncRNA
ENSG0000(	CCL24	605.4255	0.170067	0.086585	0.194491	protein_coding
ENSG0000(	ISOC2	350.4966	-0.18387	0.086584	0.194491	protein_coding
ENSG0000(	SOX13	1684.274	-0.17641	0.08658	0.194491	protein_coding
ENSG0000(	ZNF774	1020.213	0.081589	0.086569	0.194491	protein_coding
ENSG0000(	APOBEC3C	607.6754	0.198282	0.086601	0.194513	protein_coding
ENSG0000(	ENSG0000(	104.4325	0.207692	0.086611	0.194524	lncRNA
ENSG0000(	ZBTB40-IT1	41.55654	0.171387	0.086649	0.194595	lncRNA
ENSG0000(	LINC01231	69.17706	0.119373	0.086709	0.194715	lncRNA
ENSG0000(	ENSG0000(	2464.198	-0.14311	0.08672	0.194725	lncRNA
ENSG0000(	LINC02458	12.37414	0.389429	0.08676	0.194801	lncRNA
ENSG0000(	ENSG0000(	40.03724	0.196016	0.086787	0.19482	lncRNA
ENSG0000(	GPNMB	910.8127	0.244229	0.086786	0.19482	protein_coding
ENSG0000(	LDHA	6301.543	-0.24258	0.086779	0.19482	protein_coding
ENSG0000(	ENSG0000(	18.18004	0.360326	0.086794	0.194823	TEC
ENSG0000(	STAC2	177.0886	0.124658	0.086807	0.194839	protein_coding
ENSG0000(	NIPBL	6576.883	-0.07586	0.086868	0.194961	protein_coding
ENSG0000(	WDR5	562.6012	-0.10605	0.08689	0.194997	protein_coding
ENSG0000(	ANG	157.7495	-0.27003	0.086927	0.195066	protein_coding
ENSG0000(	ENSG0000(	28.91074	-0.28967	0.086945	0.195091	TEC
ENSG0000(	AADACL3	21.6631	0.239385	0.086953	0.195096	protein_coding
ENSG0000(	CDC25C	72.65431	-0.24844	0.08698	0.195114	protein_coding
ENSG0000(	DHRS4-AS1	2323.847	-0.06104	0.086986	0.195114	lncRNA
ENSG0000(	MOCOS	1066.809	-0.21558	0.086984	0.195114	protein_coding
ENSG0000(	TRAJ50	12.34947	-0.49265	0.086983	0.195114	TR_J_gene
ENSG0000(	CD96	1161.361	-0.20891	0.087019	0.195176	protein_coding
ENSG0000(	CSNK2A2	2034.479	0.05422	0.087038	0.195185	protein_coding
ENSG0000(	ENSG0000(	10.45167	0.380692	0.087053	0.195185	lncRNA
ENSG0000(	FGD2	3171.05	0.239471	0.087044	0.195185	protein_coding
ENSG0000(	KBTBD11-C	163.8442	-0.33272	0.087045	0.195185	protein_coding
ENSG0000(	NPM1	3595.598	-0.14376	0.087055	0.195185	protein_coding

ENSG0000(	ENSG0000(	1613.557	0.060807	0.087089	0.195248	transcribed_processed_pseudoge
ENSG0000(	ZBTB20-AS	100.4585	0.262564	0.087106	0.195273	lncRNA
ENSG0000(	ENSG0000(	33.23127	0.160913	0.087137	0.195318	lncRNA
ENSG0000(	GPC2	216.3025	0.13573	0.087139	0.195318	protein_coding
ENSG0000(	NR5A2	2949.262	-0.25077	0.087153	0.195336	protein_coding
ENSG0000(	RBMX	4567.109	-0.07139	0.087201	0.195431	protein_coding
ENSG0000(	ENSG0000(	29.212	0.223164	0.087222	0.195463	lncRNA
ENSG0000(	CAMK1	265.9357	-0.09868	0.087229	0.195466	protein_coding
ENSG0000(	ENSG0000(	14.81229	0.286128	0.087243	0.195483	lncRNA
ENSG0000(	C17orf80	620.2017	-0.07289	0.087275	0.195525	protein_coding
ENSG0000(	ENSG0000(	270.472	0.099168	0.08728	0.195525	lncRNA
ENSG0000(	LINC01123	829.9415	-0.33551	0.087274	0.195525	lncRNA
ENSG0000(	CCL20	330.4557	-0.50833	0.087316	0.19559	protein_coding
ENSG0000(	ENSG0000(	327.2023	0.101052	0.08733	0.195608	lncRNA
ENSG0000(	ADAMTS17	529.0922	-0.2145	0.087347	0.195632	protein_coding
ENSG0000(	HSF2	187.1199	-0.13289	0.087362	0.195652	protein_coding
ENSG0000(	TCEA1P2	115.1694	-0.22384	0.087372	0.19566	processed_pseudogene
ENSG0000(	ENSG0000(	17.08607	0.305669	0.0874	0.195709	lncRNA
ENSG0000(	ADAMTS15	92.85028	0.242918	0.087409	0.195716	protein_coding
ENSG0000(	MAP4K4	3353.477	0.233397	0.08747	0.195837	protein_coding
ENSG0000(	LINC00265	1342.313	0.056705	0.087478	0.195842	lncRNA
ENSG0000(	MIR605	12.92375	0.345527	0.087534	0.195953	miRNA
ENSG0000(	RABL6	2100.225	-0.10227	0.087573	0.196027	protein_coding
ENSG0000(	PPP2R5B	374.3424	0.134598	0.087585	0.196039	protein_coding
ENSG0000(	SLC45A3	429.0001	0.183832	0.0877	0.196282	protein_coding
ENSG0000(	ENSG0000(	27.4294	-0.46234	0.087717	0.196306	lncRNA
ENSG0000(	DPH5	687.7935	0.126355	0.087773	0.196413	protein_coding
ENSG0000(	TCTA	902.5671	-0.05854	0.087777	0.196413	protein_coding
ENSG0000(	ADAM3B	19.84257	0.260509	0.087863	0.196578	processed_pseudogene
ENSG0000(	CSTF3-DT	352.7941	-0.08709	0.08786	0.196578	lncRNA
ENSG0000(	ZNF731P	2471.992	0.100288	0.087908	0.196664	transcribed_unprocessed_pseudo
ENSG0000(	MINPP1	226.2477	-0.14687	0.087919	0.196669	protein_coding
ENSG0000(	TM2D1	1916.783	-0.0573	0.087922	0.196669	protein_coding
ENSG0000(	MRFAP1	1192.102	-0.09977	0.087955	0.196728	protein_coding
ENSG0000(	ENSG0000(	81.20724	0.190408	0.087987	0.196771	lncRNA
ENSG0000(	ENSG0000(	612.8195	0.089996	0.087983	0.196771	TEC
ENSG0000(	WBP1LP2	12.86105	0.327862	0.088022	0.196835	processed_pseudogene
ENSG0000(	RNH1	967.5325	0.087157	0.088039	0.19686	protein_coding
ENSG0000(	DTX1	692.8268	-0.38768	0.088064	0.196903	protein_coding
ENSG0000(	KCNT2	244.0943	0.231058	0.088094	0.196955	protein_coding
ENSG0000(	ENSG0000(	42.76466	0.256974	0.088121	0.197001	lncRNA
ENSG0000(	HYI-AS1	11.56649	-0.35744	0.088203	0.197172	lncRNA
ENSG0000(	PDCL3P4	1664.448	-0.06494	0.088253	0.197268	transcribed_processed_pseudoge
ENSG0000(	UBTD2	741.3971	-0.18531	0.088314	0.197391	protein_coding
ENSG0000(	CHTOP	2718.133	0.043735	0.088359	0.197477	protein_coding
ENSG0000(	GNAZ	51.07919	-0.26113	0.08837	0.197488	protein_coding
ENSG0000(	ENSG0000(	167.0793	0.091258	0.088382	0.197501	TEC

ENSG0000(	ENSG0000(	86.67754	-0.23613	0.08848	0.197706	lncRNA
ENSG0000(	ECHDC2	6452.281	-0.18434	0.08852	0.197755	protein_coding
ENSG0000(	SEC22C	1619.635	-0.09441	0.088521	0.197755	protein_coding
ENSG0000(	SUSD5	93.14379	-0.13163	0.088518	0.197755	protein_coding
ENSG0000(	ENSG0000(	14.81013	0.29187	0.088536	0.197774	lncRNA
ENSG0000(	PCDHA11	13.14034	-0.36762	0.088548	0.197787	protein_coding
ENSG0000(	ENSG0000(	21.11724	0.332882	0.088557	0.197794	lncRNA
ENSG0000(	LIX1L	1326.165	-0.11718	0.088574	0.197817	protein_coding
ENSG0000(	MLYCD	2758.991	0.078389	0.088594	0.197847	protein_coding
ENSG0000(	SNRPCP11	12.58356	0.275475	0.088633	0.197921	processed_pseudogene
ENSG0000(	ENSG0000(	130.3801	0.165908	0.088661	0.197969	lncRNA
ENSG0000(	ENSG0000(	88.712	0.122392	0.088714	0.198074	lncRNA
ENSG0000(	BEND3	238.4053	0.139541	0.088732	0.1981	protein_coding
ENSG0000(	COX8A	475.8145	-0.22741	0.088757	0.198143	protein_coding
ENSG0000(	RNF215	460.9085	0.136505	0.088764	0.198143	protein_coding
ENSG0000(	IZUMO1	260.0338	0.079663	0.088801	0.198198	protein_coding
ENSG0000(	TOM1L2	1745.93	-0.09248	0.0888	0.198198	protein_coding
ENSG0000(	DCTPP1	126.4607	-0.15	0.088915	0.198438	protein_coding
ENSG0000(	APOC1	77.11934	0.294811	0.088936	0.198459	protein_coding
ENSG0000(	ENSG0000(	14.79466	0.26015	0.088943	0.198459	lncRNA
ENSG0000(	SLC6A18	17.26739	-0.66113	0.088943	0.198459	protein_coding
ENSG0000(	KLKB1	285.4333	-0.40168	0.088958	0.198477	protein_coding
ENSG0000(	PRKACB	1324.098	-0.18052	0.088986	0.198528	protein_coding
ENSG0000(	DELE1	2004.588	0.072806	0.089038	0.198628	protein_coding
ENSG0000(	ENSG0000(	586.4086	-0.065	0.089059	0.198662	TEC
ENSG0000(	LINC02504	71.79835	0.189998	0.089142	0.198832	lncRNA
ENSG0000(	ENSG0000(	195.4996	-0.56841	0.089164	0.19884	lncRNA
ENSG0000(	LRRC29	113.6318	0.121815	0.089163	0.19884	transcribed_unitary_pseudogene
ENSG0000(	MYO1D	4534.713	-0.21301	0.089164	0.19884	protein_coding
ENSG0000(	PGAP1	2476.702	-0.19801	0.089173	0.198845	protein_coding
ENSG0000(	PKD1P6	1059.437	-0.10115	0.089199	0.19889	transcribed_unprocessed_pseudo
ENSG0000(	KLRK1	693.577	-0.19946	0.089207	0.198893	protein_coding
ENSG0000(	RNU4ATAC	37.40988	-0.21051	0.08926	0.198997	snRNA
ENSG0000(	ANKMY1	1562.283	-0.10036	0.089317	0.199109	protein_coding
ENSG0000(	LINC02141	261.929	0.114989	0.089362	0.199197	lncRNA
ENSG0000(	SNX6	994.0279	-0.11673	0.089369	0.199198	protein_coding
ENSG0000(	TYR	32.35398	0.182331	0.089418	0.199279	protein_coding
ENSG0000(	ZNF408	146.5799	-0.13662	0.089417	0.199279	protein_coding
ENSG0000(	AHCTF1	2475.527	-0.09263	0.089499	0.199444	protein_coding
ENSG0000(	ENSG0000(	99.78655	0.115034	0.089531	0.199488	lncRNA
ENSG0000(	PAX8	175.5741	-0.34638	0.089531	0.199488	protein_coding
ENSG0000(	HP1BP3	7785.001	-0.06565	0.08958	0.199584	protein_coding
ENSG0000(	NCOA1	3070.325	-0.08312	0.089602	0.199619	protein_coding
ENSG0000(	ENSG0000(	39.01993	0.238007	0.089668	0.199738	snoRNA
ENSG0000(	ENSG0000(	22.54295	-0.22202	0.089662	0.199738	lncRNA
ENSG0000(	C1orf105	105.3632	0.152932	0.08984	0.200096	protein_coding
ENSG0000(	CYTH1	4170.895	0.129077	0.089842	0.200096	protein_coding

ENSG0000(	ENSG0000(	402.7327	-0.07634	0.089904	0.200221	lncRNA
ENSG0000(	KRTCAP2	778.908	-0.11978	0.089954	0.200317	protein_coding
ENSG0000(	IL10RA	2892.64	0.266047	0.089973	0.20034	protein_coding
ENSG0000(	KASH5	59.95627	0.235183	0.089977	0.20034	protein_coding
ENSG0000(	ENSG0000(	133.1027	-0.09036	0.090003	0.200384	lncRNA
ENSG0000(	ST7L	805.0916	-0.07045	0.090021	0.20041	protein_coding
ENSG0000(	CEBPB-AS1	456.0596	0.097982	0.090078	0.200511	lncRNA
ENSG0000(	DCPS	520.1221	0.151709	0.090086	0.200511	protein_coding
ENSG0000(	PPP1R18	1174.315	0.275927	0.090083	0.200511	protein_coding
ENSG0000(	CTBP2P8	64.01409	0.204365	0.09012	0.200546	processed_pseudogene
ENSG0000(	SNAP91	61.56555	-0.56742	0.090118	0.200546	protein_coding
ENSG0000(	SYPL2	59.22077	0.305582	0.090113	0.200546	protein_coding
ENSG0000(	CTNNBIP1	256.3298	0.130113	0.090173	0.200648	protein_coding
ENSG0000(	ENSG0000(	22.94943	0.250392	0.090197	0.200689	lncRNA
ENSG0000(	ENSG0000(	118.0653	0.14432	0.090225	0.200736	lncRNA
ENSG0000(	ENSG0000(	13.51577	0.322764	0.090262	0.200796	lncRNA
ENSG0000(	POLR2G	232.6499	-0.13111	0.090265	0.200796	protein_coding
ENSG0000(	SLC45A4	732.0404	0.116287	0.090274	0.200802	protein_coding
ENSG0000(	NUTM2A-A	6008.959	-0.09243	0.090303	0.200853	lncRNA
ENSG0000(	MOB4P1	10.02008	-0.46962	0.090365	0.200978	processed_pseudogene
ENSG0000(	CRACD	3147.616	-0.28866	0.090411	0.201065	protein_coding
ENSG0000(	LCA10	209.7338	0.107946	0.090417	0.201065	lncRNA
ENSG0000(	SDHC	597.1889	-0.11171	0.090464	0.201154	protein_coding
ENSG0000(	ENSG0000(	27.15186	0.20433	0.090479	0.201161	lncRNA
ENSG0000(	TOR1AIP2	11999.55	-0.22142	0.090475	0.201161	protein_coding
ENSG0000(	TKT	1204.779	0.121155	0.090496	0.201184	protein_coding
ENSG0000(	ENSG0000(	199.6938	0.308887	0.090508	0.201195	lncRNA
ENSG0000(	LINC02076	54.5494	0.172122	0.090548	0.201256	lncRNA
ENSG0000(	POLR3K	543.8982	-0.08156	0.090546	0.201256	protein_coding
ENSG0000(	PPP2CA-DT	38.22848	0.175507	0.090585	0.201325	lncRNA
ENSG0000(	GHRL	46.06975	0.264462	0.090623	0.201393	protein_coding
ENSG0000(	LGALS3BP	2353.188	-0.20316	0.090661	0.20145	protein_coding
ENSG0000(	LINGO4	21.13258	-0.31691	0.090658	0.20145	protein_coding
ENSG0000(	UFSP2	256.8979	-0.14678	0.090683	0.201484	protein_coding
ENSG0000(	CPM	1579.692	0.093826	0.090706	0.201523	protein_coding
ENSG0000(	TMEM145	382.3598	-0.11514	0.090721	0.201542	protein_coding
ENSG0000(	LAGE3P1	51.72437	0.218535	0.090729	0.201545	processed_pseudogene
ENSG0000(	NET1	2096.986	-0.26499	0.090768	0.201603	protein_coding
ENSG0000(	RN7SL170F	10.75976	-0.32932	0.090767	0.201603	misc_RNA
ENSG0000(	PNISR	9931.649	0.070062	0.090783	0.201623	protein_coding
ENSG0000(	PLA2G2A	2363.017	0.41463	0.090803	0.201639	protein_coding
ENSG0000(	RN7SL558F	128.8166	0.117716	0.090803	0.201639	misc_RNA
ENSG0000(	MIR6832	9.126181	-0.39681	0.090821	0.201664	miRNA
ENSG0000(	LANCL1-AS	33.2245	0.185969	0.090852	0.201704	lncRNA
ENSG0000(	SLC22A11	99.1436	0.137807	0.090849	0.201704	protein_coding
ENSG0000(	ENSG0000(	14.86732	-0.34583	0.09089	0.201775	lncRNA
ENSG0000(	CBSL	101.7891	-0.88213	0.091055	0.202109	protein_coding

ENSG0000(	HID1	1143.786	0.194999	0.09106	0.202109	protein_coding
ENSG0000(	LINC02047	16.08904	0.260469	0.09105	0.202109	lncRNA
ENSG0000(	ADGRA3	1656.938	-0.14163	0.091085	0.202135	protein_coding
ENSG0000(	ENSG0000(	34.41499	0.236607	0.091081	0.202135	lncRNA
ENSG0000(	HNF4A	7673.574	-0.3034	0.091091	0.202136	protein_coding
ENSG0000(	MTCYBP44	31.07469	-0.6583	0.091114	0.202158	processed_pseudogene
ENSG0000(	TBKBP1	216.6501	0.198263	0.09111	0.202158	protein_coding
ENSG0000(	ENSG0000(	1100.491	-0.05373	0.091133	0.202186	lncRNA
ENSG0000(	NHLRC1	106.7648	0.110032	0.0912	0.20232	protein_coding
ENSG0000(	GABARAP	841.7969	-0.1773	0.09121	0.202328	protein_coding
ENSG0000(	ENSG0000(	194.7192	0.121164	0.091231	0.20236	lncRNA
ENSG0000(	OR4D9	1025.12	0.051312	0.091303	0.202505	protein_coding
ENSG0000(	ENSG0000(	178.3334	0.110805	0.091337	0.202538	lncRNA
ENSG0000(	HDAC1P1	46.14318	-0.30611	0.091325	0.202538	processed_pseudogene
ENSG0000(	VAMP8	638.5924	-0.22646	0.091331	0.202538	protein_coding
ENSG0000(	MYH14	6389.366	-0.31114	0.091358	0.20257	protein_coding
ENSG0000(	TLE6	15.49586	0.404224	0.09137	0.202582	protein_coding
ENSG0000(	LINC00604	40.39501	-0.17793	0.091393	0.202619	lncRNA
ENSG0000(	LINC00907	650.8583	0.076917	0.091414	0.202653	lncRNA
ENSG0000(	ENSG0000(	143.0317	0.135584	0.091423	0.202657	TEC
ENSG0000(	PPP4R1L	782.8243	0.09395	0.091479	0.202751	transcribed_unprocessed_pseudo
ENSG0000(	PTTG1IP	3122.128	-0.168	0.091484	0.202751	protein_coding
ENSG0000(	TEDC1	329.9821	-0.15229	0.091485	0.202751	protein_coding
ENSG0000(	LARGE-IT1	50.14308	0.24586	0.091528	0.202812	lncRNA
ENSG0000(	NMUR1	280.3902	-0.12803	0.091531	0.202812	protein_coding
ENSG0000(	SLCO2A1	970.9766	0.204808	0.091524	0.202812	protein_coding
ENSG0000(	WDR93	195.9855	0.097967	0.091562	0.202867	protein_coding
ENSG0000(	ABHD14A-/	266.3978	-0.35921	0.0916	0.202937	protein_coding
ENSG0000(	FAM167A	166.8728	0.372893	0.091631	0.202975	protein_coding
ENSG0000(	TOX2	107.3152	0.303212	0.091627	0.202975	protein_coding
ENSG0000(	SMARCE1P	49.04754	-0.1746	0.091639	0.202979	processed_pseudogene
ENSG0000(	DDB2	1050.052	0.074638	0.091685	0.203068	protein_coding
ENSG0000(	ENSG0000(	9.020232	0.301368	0.091699	0.203084	lncRNA
ENSG0000(	ENSG0000(	19.26525	0.268065	0.091743	0.203167	lncRNA
ENSG0000(	ENSG0000(	147.522	0.111789	0.091759	0.203188	lncRNA
ENSG0000(	FAM43A	175.745	0.275583	0.091782	0.203224	protein_coding
ENSG0000(	RNF14	1330.149	-0.08298	0.091874	0.203414	protein_coding
ENSG0000(	GOLGA7	608.0735	-0.09458	0.091887	0.203429	protein_coding
ENSG0000(	DQX1	934.8576	-0.32086	0.091908	0.203461	protein_coding
ENSG0000(	RTN4R	27.4086	-0.26245	0.091942	0.203522	protein_coding
ENSG0000(	KLHL6-AS1	201.2594	0.123889	0.09195	0.203525	lncRNA
ENSG0000(	ENSG0000(	83.4839	0.142839	0.091962	0.203538	lncRNA
ENSG0000(	OSBPL9	2220.35	-0.07726	0.092024	0.203646	protein_coding
ENSG0000(	TBC1D13	738.1396	-0.10976	0.09202	0.203646	protein_coding
ENSG0000(	WWP1	2161.53	-0.08259	0.092034	0.203654	protein_coding
ENSG0000(	MXRA5Y	13.3321	0.617352	0.092058	0.203693	unprocessed_pseudogene
ENSG0000(	MAPKAP1	1331.499	0.050087	0.09211	0.203792	protein_coding

ENSG0000(	TSPEAR-AS	181.8732	-0.10999	0.092224	0.204031	lncRNA
ENSG0000(	ENSG0000(	19.60786	0.278057	0.092258	0.204093	lncRNA
ENSG0000(	FIS1	485.6661	-0.09938	0.092283	0.204118	protein_coding
ENSG0000(	LINC01120	15.73333	0.426032	0.092277	0.204118	lncRNA
ENSG0000(	CABLES2	252.0995	0.14146	0.09234	0.20423	protein_coding
ENSG0000(	C2CD2	1339.834	0.149649	0.09235	0.204239	protein_coding
ENSG0000(	TEX29	16.35113	0.277442	0.092389	0.204308	protein_coding
ENSG0000(	WWOX	1642.82	0.067086	0.092394	0.204308	protein_coding
ENSG0000(	ENSG0000(	24.84041	0.267743	0.092427	0.204322	processed_pseudogene
ENSG0000(	KIF1A	124.4417	-0.41823	0.092409	0.204322	protein_coding
ENSG0000(	MAST2	3073.85	-0.23553	0.092425	0.204322	protein_coding
ENSG0000(	TBX18-AS1	16.19265	0.286516	0.092424	0.204322	lncRNA
ENSG0000(	ANAPC10P	29.84891	0.217341	0.092522	0.204517	processed_pseudogene
ENSG0000(	C6orf141	397.7111	-0.11647	0.092549	0.204563	protein_coding
ENSG0000(	ENSG0000(	71.85909	0.163379	0.092563	0.204579	lncRNA
ENSG0000(	RANP4	31.70099	0.189985	0.092592	0.20463	processed_pseudogene
ENSG0000(	PPP1R1A	10.5441	0.431808	0.092603	0.20464	protein_coding
ENSG0000(	CASP14	157.2968	0.101822	0.092622	0.204668	protein_coding
ENSG0000(	FHIT	708.3621	-0.105	0.092668	0.204754	protein_coding
ENSG0000(	PCNX4	4473.457	0.061388	0.092722	0.20486	protein_coding
ENSG0000(	ENSG0000(	352.7766	0.082809	0.092731	0.204865	lncRNA
ENSG0000(	ENSG0000(	207.7559	0.084861	0.092748	0.204889	lncRNA
ENSG0000(	SLC18A2	180.5128	0.261294	0.092755	0.204889	protein_coding
ENSG0000(	BAIAP2L1	1453.694	0.226282	0.092788	0.204948	protein_coding
ENSG0000(	CWC25	1149.874	-0.08755	0.092838	0.205034	protein_coding
ENSG0000(	SLC22A14	10.66652	0.483754	0.09284	0.205034	protein_coding
ENSG0000(	RNU6-322F	17.54689	0.385413	0.092885	0.205118	snRNA
ENSG0000(	COX6B2	176.1766	0.109514	0.092905	0.205148	protein_coding
ENSG0000(	ESAM-AS1	121.181	0.125787	0.092918	0.205149	lncRNA
ENSG0000(	PIK3CD-AS2	78.8627	0.11243	0.092915	0.205149	lncRNA
ENSG0000(	CFP	486.8231	0.189073	0.093023	0.205367	protein_coding
ENSG0000(	DNAJC30	347.4027	0.076023	0.093054	0.205421	protein_coding
ENSG0000(	SH3TC2-DT	39.01962	0.4066	0.093072	0.205445	lncRNA
ENSG0000(	RAB42	259.6188	0.112849	0.093108	0.205511	protein_coding
ENSG0000(	ENSG0000(	66.01377	0.14717	0.093115	0.205511	lncRNA
ENSG0000(	ENSG0000(	73.51451	0.13628	0.093135	0.205541	lncRNA
ENSG0000(	ENSG0000(	35.74382	0.182833	0.093207	0.205672	lncRNA
ENSG0000(	PKN1	740.7455	-0.13857	0.093207	0.205672	protein_coding
ENSG0000(	FOXP2	1076.704	-0.2077	0.09322	0.205687	protein_coding
ENSG0000(	TMEM192	3244.41	-0.04455	0.093242	0.205721	protein_coding
ENSG0000(	FCAMR	89.97945	-0.49339	0.093268	0.205763	protein_coding
ENSG0000(	LRRC49	547.8335	-0.09103	0.093279	0.205772	protein_coding
ENSG0000(	THAP2	213.3254	-0.10784	0.093289	0.205781	protein_coding
ENSG0000(	ENSG0000(	87.77292	-0.12839	0.093296	0.205782	lncRNA
ENSG0000(	HOXD9	15.59769	0.450776	0.093305	0.205786	protein_coding
ENSG0000(	ZNF143	483.721	-0.11732	0.093323	0.205813	protein_coding
ENSG0000(	PTMA	4784.478	-0.1256	0.093344	0.205844	protein_coding

ENSG0000(	MKRN1	2677.131	-0.09508	0.093353	0.205851	protein_coding
ENSG0000(	CITED4	13.77889	0.327414	0.093388	0.205898	protein_coding
ENSG0000(	XKR4	997.0967	-0.11472	0.093386	0.205898	protein_coding
ENSG0000(	TMEM220-	286.1236	-0.31715	0.093403	0.205916	lncRNA
ENSG0000(	NDUFV3	2241.605	-0.04525	0.093412	0.205923	protein_coding
ENSG0000(	FAM181B	114.3476	0.132807	0.09349	0.206079	protein_coding
ENSG0000(	ENSG0000(	9.979775	-0.35196	0.09351	0.206096	lncRNA
ENSG0000(	ENSG0000(	220.8215	0.082711	0.093509	0.206096	lncRNA
ENSG0000(	B3GNT2	483.3256	0.097102	0.09355	0.206168	protein_coding
ENSG0000(	TENM3	235.9047	0.360103	0.093602	0.206268	protein_coding
ENSG0000(	ACOXL-AS1	26.35958	0.283618	0.093612	0.206276	lncRNA
ENSG0000(	LONRF3	166.8958	-0.21133	0.093692	0.206424	protein_coding
ENSG0000(	RN7SL229F	12.34795	0.34444	0.093691	0.206424	misc_RNA
ENSG0000(	ENSG0000(	10.94973	0.350151	0.0937	0.206427	lncRNA
ENSG0000(	CFHR2	218.2442	-0.15081	0.093726	0.206455	protein_coding
ENSG0000(	NCOR1	11044.75	-0.07933	0.093721	0.206455	protein_coding
ENSG0000(	ENSG0000(	412.9534	0.114184	0.093742	0.206475	TEC
ENSG0000(	NRIP1	4662.946	-0.14582	0.093824	0.206641	protein_coding
ENSG0000(	MTMR10	2661.39	-0.08274	0.093849	0.206683	protein_coding
ENSG0000(	EBLN2	337.3954	0.101146	0.093871	0.206716	protein_coding
ENSG0000(	PTPDC1	412.1259	0.109347	0.093899	0.206764	protein_coding
ENSG0000(	RNU6-1003	23.33917	0.462011	0.093982	0.206931	snRNA
ENSG0000(	ENSG0000(	71.55928	0.129831	0.09399	0.206936	lncRNA
ENSG0000(	SIGIRR	780.6297	-0.1202	0.09401	0.206966	protein_coding
ENSG0000(	ULK4P1	14.12792	0.543052	0.094041	0.207011	transcribed_unprocessed_pseudo
ENSG0000(	VENTXP1	470.8258	0.092981	0.094044	0.207011	transcribed_processed_pseudoge
ENSG0000(	ENSG0000(	5376.136	-0.10218	0.094064	0.207033	lncRNA
ENSG0000(	RANBP3L	60.5207	-0.19791	0.094067	0.207033	protein_coding
ENSG0000(	MUTYH	897.4447	-0.05502	0.094121	0.207136	protein_coding
ENSG0000(	STK40	944.0041	0.129304	0.094164	0.207217	protein_coding
ENSG0000(	MET	1081.839	-0.20565	0.09418	0.207237	protein_coding
ENSG0000(	FRS3	175.8888	0.110788	0.09421	0.207286	protein_coding
ENSG0000(	NPSR1-AS1	55.59635	0.197406	0.094215	0.207286	lncRNA
ENSG0000(	LINC02209	19.46221	0.296872	0.094259	0.207368	lncRNA
ENSG0000(	ENSG0000(	1379.581	-0.0753	0.094311	0.207468	TEC
ENSG0000(	ENSG0000(	112.268	0.140242	0.094347	0.207532	lncRNA
ENSG0000(	ACTG1P14	149.5341	0.126897	0.094356	0.207539	processed_pseudogene
ENSG0000(	C1orf167	244.7702	0.102637	0.094375	0.207566	protein_coding
ENSG0000(	HPN-AS1	148.4195	0.118167	0.094412	0.20762	lncRNA
ENSG0000(	ZNF808	2266.513	-0.07589	0.094413	0.20762	protein_coding
ENSG0000(	ENSG0000(	330.8387	0.0838	0.094475	0.207742	lncRNA
ENSG0000(	RN7SKP38	17.88079	0.215159	0.094495	0.207773	misc_RNA
ENSG0000(	ENSG0000(	8.879194	0.435927	0.094521	0.207815	lncRNA
ENSG0000(	ATP1A2	296.1219	-0.18798	0.094578	0.207867	protein_coding
ENSG0000(	EMC2	1057.124	-0.07121	0.094559	0.207867	protein_coding
ENSG0000(	ENSG0000(	174.9084	0.079929	0.094568	0.207867	lncRNA
ENSG0000(	PMPCA	954.9464	-0.07971	0.094569	0.207867	protein_coding

ENSG0000(	RNU6ATAC	24.54773	-0.25748	0.094576	0.207867	snRNA
ENSG0000(	RPSAP58	755.3868	-0.18947	0.094586	0.207869	protein_coding
ENSG0000(	ENSG0000(	26.13649	0.28932	0.094597	0.207881	lncRNA
ENSG0000(	CCT7	652.549	-0.0958	0.094629	0.207935	protein_coding
ENSG0000(	CA5B	1584.183	0.106908	0.09472	0.208119	protein_coding
ENSG0000(	ENSG0000(	52.27003	0.207738	0.094726	0.208119	lncRNA
ENSG0000(	MUC3A	32076.69	-0.41294	0.094763	0.208187	protein_coding
ENSG0000(	ENSG0000(	69.96384	0.122582	0.094814	0.208269	lncRNA
ENSG0000(	KRT8P52	31.74831	-0.20352	0.094808	0.208269	processed_pseudogene
ENSG0000(	ENSG0000(	178.9034	0.123745	0.094821	0.20827	lncRNA
ENSG0000(	PPP3CA	1095.733	0.102226	0.094846	0.208296	protein_coding
ENSG0000(	RNF4	1987.454	-0.05435	0.094843	0.208296	protein_coding
ENSG0000(	ENSG0000(	9.808652	0.309544	0.094868	0.208312	lncRNA
ENSG0000(	ENSG0000(	134.2028	0.10621	0.094898	0.208312	lncRNA
ENSG0000(	ENSG0000(	402.4356	0.097569	0.094926	0.208312	lncRNA
ENSG0000(	KDM5C	3702.239	0.063674	0.094921	0.208312	protein_coding
ENSG0000(	KRT18P1	9.50199	0.303298	0.094895	0.208312	processed_pseudogene
ENSG0000(	KRT72	17.69576	0.495514	0.094913	0.208312	protein_coding
ENSG0000(	LCTL	311.3603	0.073016	0.094908	0.208312	protein_coding
ENSG0000(	MIA2	4935.441	-0.20656	0.094923	0.208312	protein_coding
ENSG0000(	MXI1	941.5926	-0.26571	0.094915	0.208312	protein_coding
ENSG0000(	RECQL	867.3857	0.106085	0.094876	0.208312	protein_coding
ENSG0000(	RN7SL331F	9.855871	0.303433	0.09489	0.208312	misc_RNA
ENSG0000(	ENSG0000(	78.35148	0.138157	0.094933	0.208313	lncRNA
ENSG0000(	ENSG0000(	127.3976	-0.18856	0.094956	0.208349	lncRNA
ENSG0000(	METTL27	65.29693	-0.29308	0.094962	0.208349	protein_coding
ENSG0000(	GCATP1	49.68393	-0.28388	0.09501	0.208424	processed_pseudogene
ENSG0000(	PPWD1	1246.526	0.076137	0.09501	0.208424	protein_coding
ENSG0000(	ENSG0000(	60.59904	-0.14871	0.09508	0.208544	lncRNA
ENSG0000(	HECA	2776.521	-0.08194	0.095077	0.208544	protein_coding
ENSG0000(	ZFAND5	3138.935	0.101606	0.095085	0.208544	protein_coding
ENSG0000(	RELB	574.4026	0.170471	0.095094	0.20855	protein_coding
ENSG0000(	ENSG0000(	16.98916	1.227792	0.095102	0.208554	lncRNA
ENSG0000(	PTGER4	858.8828	0.111527	0.09513	0.2086	protein_coding
ENSG0000(	IL17RC	519.6281	-0.20883	0.095148	0.208626	protein_coding
ENSG0000(	CAHM	19.0796	0.291028	0.095206	0.208672	lncRNA
ENSG0000(	CDAN1	581.404	-0.10005	0.09519	0.208672	protein_coding
ENSG0000(	DNAJB9	377.561	0.166502	0.095203	0.208672	protein_coding
ENSG0000(	LMAN2L	266.2989	-0.11293	0.095201	0.208672	protein_coding
ENSG0000(	RFC1	1356.382	-0.08653	0.095202	0.208672	protein_coding
ENSG0000(	TTLL6	341.1104	-0.20423	0.095209	0.208672	protein_coding
ENSG0000(	MT1L	11.75591	0.471417	0.095236	0.208716	transcribed_unprocessed_pseudo
ENSG0000(	FAM83D	124.1142	0.145052	0.0953	0.208842	protein_coding
ENSG0000(	CRTC3-AS1	75.7754	-0.13207	0.095354	0.208945	lncRNA
ENSG0000(	MTCO2P19	22.59346	0.201869	0.095379	0.208985	processed_pseudogene
ENSG0000(	F11R	3547.804	-0.18693	0.095386	0.208987	protein_coding
ENSG0000(	VPREB3	134.4373	-0.64133	0.095415	0.209036	protein_coding

ENSG0000(	NMNAT2	70.76029	0.238695	0.09543	0.209054	protein_coding
ENSG0000(	MIR590	31.52336	-0.19806	0.095493	0.209178	miRNA
ENSG0000(	ENSG0000(	97.22086	0.177741	0.095523	0.209229	lncRNA
ENSG0000(	FCRL3	1465.848	-0.29716	0.09554	0.209236	protein_coding
ENSG0000(	PBRM1	3117.988	0.05264	0.095536	0.209236	protein_coding
ENSG0000(	ENSG0000(	21.07307	0.220391	0.095628	0.209416	lncRNA
ENSG0000(	ENSG0000(	22.15187	0.193818	0.095671	0.209496	lncRNA
ENSG0000(	GNG11	135.6516	0.200208	0.095696	0.209505	protein_coding
ENSG0000(	HSBP1	964.7785	-0.09441	0.095686	0.209505	protein_coding
ENSG0000(	RAD23A	776.5189	-0.07078	0.095694	0.209505	protein_coding
ENSG0000(	TOLLIP	884.8376	-0.0976	0.09574	0.209588	protein_coding
ENSG0000(	MIR548V	18.93033	0.367989	0.095748	0.209591	miRNA
ENSG0000(	ITGA10	344.4344	0.06868	0.095793	0.209675	protein_coding
ENSG0000(	ADGRG7	1827.128	-0.31363	0.095827	0.209715	protein_coding
ENSG0000(	ENSG0000(	405.7408	0.153221	0.095824	0.209715	lncRNA
ENSG0000(	TASP1	353.9757	-0.1112	0.095832	0.209715	protein_coding
ENSG0000(	PTOV1	1164.358	-0.07927	0.095853	0.209747	protein_coding
ENSG0000(	ENSG0000(	32.3935	0.205776	0.095919	0.209795	lncRNA
ENSG0000(	FLT1	1108.426	0.10191	0.09593	0.209795	protein_coding
ENSG0000(	FZD3	676.404	0.097965	0.095915	0.209795	protein_coding
ENSG0000(	IGLV3-25	63.20665	0.448796	0.095889	0.209795	IG_V_gene
ENSG0000(	MAP1B	710.2258	0.235056	0.095905	0.209795	protein_coding
ENSG0000(	RALGAPA1	4192.282	0.060549	0.095907	0.209795	protein_coding
ENSG0000(	RN7SL647F	23.42851	0.239045	0.095908	0.209795	misc_RNA
ENSG0000(	TEKT4	85.73634	0.377549	0.095922	0.209795	protein_coding
ENSG0000(	ZNF799	803.3792	-0.09121	0.095935	0.209795	protein_coding
ENSG0000(	ENSG0000(	63.24249	0.165591	0.095948	0.209809	lncRNA
ENSG0000(	RAD51	108.706	-0.21235	0.095987	0.20988	protein_coding
ENSG0000(	ENSG0000(	16.35503	0.354319	0.096005	0.209905	lncRNA
ENSG0000(	ENSG0000(	207.82	0.170064	0.096043	0.209973	lncRNA
ENSG0000(	ENSG0000(	478.2973	0.097687	0.096064	0.210004	lncRNA
ENSG0000(	GNG10	292.3893	0.192371	0.096103	0.210062	protein_coding
ENSG0000(	RPL36AP43	46.82884	-0.25214	0.0961	0.210062	processed_pseudogene
ENSG0000(	NIPSNAP2	925.1558	-0.08563	0.096122	0.210089	protein_coding
ENSG0000(	ENSG0000(	162.4922	0.106176	0.096142	0.210116	lncRNA
ENSG0000(	ENSG0000(	32.20383	0.242413	0.096204	0.210238	lncRNA
ENSG0000(	RAB4B	1121.754	0.074345	0.096283	0.210381	protein_coding
ENSG0000(	ZNF688	338.9062	0.10899	0.09628	0.210381	protein_coding
ENSG0000(	ENSG0000(	12.0236	0.315181	0.096348	0.210509	lncRNA
ENSG0000(	ENSG0000(	136.8981	0.174086	0.096373	0.210549	lncRNA
ENSG0000(	AVPI1	151.4622	-0.28493	0.096402	0.210569	protein_coding
ENSG0000(	RNU1-91P	44.84464	0.196267	0.096402	0.210569	snRNA
ENSG0000(	SLC2A9	351.2662	0.201569	0.096401	0.210569	protein_coding
ENSG0000(	ARHGAP27	849.498	0.095847	0.096417	0.210583	lncRNA
ENSG0000(	ENSG0000(	39.111	-0.50238	0.096422	0.210583	lncRNA
ENSG0000(	GOLGA2P7	1571.485	0.183914	0.096435	0.210597	transcribed_unprocessed_pseudo
ENSG0000(	ENSG0000(	16.71813	-0.26463	0.096462	0.210642	lncRNA

ENSG0000(	NEXMIF	108.073	-0.18831	0.096482	0.21067	protein_coding
ENSG0000(	EDRF1-DT	49.5138	0.229455	0.096505	0.210707	lncRNA
ENSG0000(	PRDM11	603.5082	-0.12974	0.096547	0.210784	protein_coding
ENSG0000(	CPNE8	358.1749	0.149131	0.096576	0.210832	protein_coding
ENSG0000(	CCNE1	57.63279	0.235323	0.096594	0.210856	protein_coding
ENSG0000(	ACY3	323.4107	-0.37783	0.096642	0.210946	protein_coding
ENSG0000(	CHD1L	865.4432	-0.10003	0.096667	0.210987	protein_coding
ENSG0000(	KIAA2012-/	238.7721	0.098175	0.096737	0.211125	lncRNA
ENSG0000(	ACTBP4	57.20432	-0.17725	0.096791	0.211199	processed_pseudogene
ENSG0000(	ENSG0000(	43.038	0.198904	0.096785	0.211199	lncRNA
ENSG0000(	LINC02648	13.02737	-0.51263	0.096789	0.211199	lncRNA
ENSG0000(	SEC14L6	558.5836	0.070398	0.096807	0.211218	protein_coding
ENSG0000(	ENSG0000(	49.15559	0.277953	0.096841	0.211278	TEC
ENSG0000(	ABTB2	531.162	-0.29033	0.096873	0.211333	protein_coding
ENSG0000(	TADA1	265.3294	-0.10617	0.096924	0.21143	protein_coding
ENSG0000(	HDGFL2	610.3136	-0.07262	0.096958	0.211489	protein_coding
ENSG0000(	ENSG0000(	11.48195	-0.35125	0.096965	0.21149	lncRNA
ENSG0000(	CASK	3510.989	-0.06035	0.096996	0.211542	protein_coding
ENSG0000(	DDX11L9	9.960161	0.728979	0.097002	0.211542	transcribed_unprocessed_pseudo
ENSG0000(	FNIP1	2129.911	0.076635	0.097027	0.211581	protein_coding
ENSG0000(	NCR1	154.1614	0.118796	0.097041	0.211582	protein_coding
ENSG0000(	ZNF788P	1103.015	-0.08417	0.09704	0.211582	transcribed_unprocessed_pseudo
ENSG0000(	C1DP5	9.331977	0.416764	0.097058	0.211595	processed_pseudogene
ENSG0000(	ENSG0000(	454.0432	0.081998	0.09706	0.211595	lncRNA
ENSG0000(	ENSG0000(	10.28566	0.353792	0.097119	0.211708	lncRNA
ENSG0000(	PGLS-DT	422.8052	0.070556	0.097165	0.21178	lncRNA
ENSG0000(	PHLDB1	4405.739	0.107329	0.097159	0.21178	protein_coding
ENSG0000(	SEMA6A-A!	98.28386	-0.27382	0.097186	0.211812	lncRNA
ENSG0000(	ENSG0000(	131.1266	-0.1105	0.097195	0.211816	lncRNA
ENSG0000(	ENSG0000(	21.5555	0.238842	0.097214	0.211842	processed_pseudogene
ENSG0000(	ENSG0000(	193.8938	0.113533	0.097296	0.212006	lncRNA
ENSG0000(	SERPINA5	23.44199	-0.62029	0.097342	0.212093	protein_coding
ENSG0000(	MAPRE3	144.9751	0.148256	0.097358	0.212112	protein_coding
ENSG0000(	SART3	1393.819	0.068436	0.097382	0.212149	protein_coding
ENSG0000(	WDR97	348.9002	-0.10488	0.097412	0.212202	protein_coding
ENSG0000(	ENSG0000(	291.0279	0.187436	0.097438	0.212228	lncRNA
ENSG0000(	LINC02302	189.6066	0.096521	0.097435	0.212228	lncRNA
ENSG0000(	MSH6	1341.432	-0.10802	0.097454	0.212248	protein_coding
ENSG0000(	SSTR2	1359.502	0.061332	0.097467	0.212263	protein_coding
ENSG0000(	LRRC51	591.1988	-0.12896	0.097483	0.212282	protein_coding
ENSG0000(	NBR1	4474.87	-0.14494	0.097551	0.212402	protein_coding
ENSG0000(	PRPF3	2307.636	-0.05023	0.097549	0.212402	protein_coding
ENSG0000(	ENSG0000(	186.0038	-0.1282	0.097581	0.212425	lncRNA
ENSG0000(	LRRN3	131.7202	-0.33333	0.097586	0.212425	protein_coding
ENSG0000(	RNF141	1465.413	-0.11479	0.097574	0.212425	protein_coding
ENSG0000(	SNORC	250.8553	0.223001	0.097589	0.212425	protein_coding
ENSG0000(	DUXAP10	775.9039	0.269626	0.097637	0.212515	transcribed_processed_pseudoge

ENSG0000(	REXO2	396.1232	0.104285	0.09765	0.212528	protein_coding
ENSG0000(	ENSG0000(	699.0868	0.07256	0.097658	0.21253	lncRNA
ENSG0000(	GPR35	3061.44	-0.24967	0.097699	0.212606	protein_coding
ENSG0000(	ENSG0000(	94.56117	-0.14947	0.09773	0.212658	lncRNA
ENSG0000(	ENSG0000(	45.87333	-0.19533	0.097846	0.212895	lncRNA
ENSG0000(	PLAGL1	1167.909	0.144848	0.097852	0.212895	protein_coding
ENSG0000(	ENSG0000(	13.96991	0.350403	0.097888	0.212957	lncRNA
ENSG0000(	BMP2K	3680.398	-0.10693	0.097944	0.213049	protein_coding
ENSG0000(	HDAC2	1356.936	-0.08605	0.097948	0.213049	protein_coding
ENSG0000(	SALRNA2	18.96256	0.215976	0.09795	0.213049	lncRNA
ENSG0000(	LINC02179	56.61629	0.165342	0.097973	0.213084	lncRNA
ENSG0000(	OR10V3P	19.89259	0.242064	0.098004	0.213108	unprocessed_pseudogene
ENSG0000(	PSMB7	355.5066	-0.10105	0.098	0.213108	protein_coding
ENSG0000(	RUFY2	996.6901	-0.06645	0.097993	0.213108	protein_coding
ENSG0000(	CNPY4	298.7337	-0.08806	0.098021	0.213115	protein_coding
ENSG0000(	ENSG0000(	851.6087	0.080216	0.098019	0.213115	lncRNA
ENSG0000(	SKI	1938.998	-0.12602	0.098041	0.213143	protein_coding
ENSG0000(	ENSG0000(	21.49808	0.220467	0.098078	0.213169	lncRNA
ENSG0000(	ENSG0000(	51.05198	0.160304	0.098069	0.213169	lncRNA
ENSG0000(	ESCO2	717.6208	-0.14461	0.09808	0.213169	protein_coding
ENSG0000(	UBE2R2	1425.163	-0.10715	0.098073	0.213169	protein_coding
ENSG0000(	ENSG0000(	24.913	-0.51101	0.098115	0.213227	TEC
ENSG0000(	GLB1L	362.7064	-0.1013	0.098127	0.213227	protein_coding
ENSG0000(	MIR3159	31.0402	-0.19935	0.098124	0.213227	miRNA
ENSG0000(	ARMCX5	545.3208	0.079413	0.098204	0.213365	protein_coding
ENSG0000(	RN7SL293F	11.233	0.304328	0.0982	0.213365	misc_RNA
ENSG0000(	MT-TT	70.15453	0.601653	0.09823	0.213407	Mt_tRNA
ENSG0000(	LINC02014	19.249	0.271177	0.098316	0.213579	lncRNA
ENSG0000(	TAF13	1035.695	-0.07233	0.098384	0.213712	protein_coding
ENSG0000(	YEATS2	2034.125	0.099164	0.098493	0.213935	protein_coding
ENSG0000(	RRN3P3	1002.422	-0.06057	0.098511	0.213959	transcribed_unprocessed_pseudo
ENSG0000(	TRIM80P	11.91578	-0.34853	0.098558	0.214046	unitary_pseudogene
ENSG0000(	ENSG0000(	12.53231	0.322847	0.098577	0.214072	lncRNA
ENSG0000(	C8orf86	306.499	-0.06297	0.098602	0.214113	lncRNA
ENSG0000(	ENSG0000(	11.31062	0.390796	0.098635	0.214129	lncRNA
ENSG0000(	ENSG0000(	282.2911	-0.13914	0.098624	0.214129	lncRNA
ENSG0000(	SPATA17	425.0811	-0.09344	0.09863	0.214129	protein_coding
ENSG0000(	TTI2	245.4202	-0.10168	0.098637	0.214129	protein_coding
ENSG0000(	GALNT11	714.7639	0.079103	0.09873	0.214316	protein_coding
ENSG0000(	GGPS1	778.6339	-0.08591	0.098776	0.214398	protein_coding
ENSG0000(	YME1L1	2443.281	-0.05729	0.098781	0.214398	protein_coding
ENSG0000(	RYBP	1290.443	-0.07833	0.098799	0.214422	protein_coding
ENSG0000(	ENSG0000(	18.42561	0.264971	0.098848	0.214513	lncRNA
ENSG0000(	FAM47E-ST	156.0537	-0.26685	0.098958	0.214737	protein_coding
ENSG0000(	ENSG0000(	24.27385	0.218683	0.099102	0.215036	lncRNA
ENSG0000(	DNAJB6P8	7.821127	0.388526	0.099123	0.215042	transcribed_processed_pseudoge
ENSG0000(	DUSP8P5	115.2232	0.140981	0.099126	0.215042	processed_pseudogene

ENSG0000(	ENSG0000(	26.1596	-0.20871	0.099116	0.215042	lncRNA
ENSG0000(	ENSG0000(	9.345995	0.354604	0.099133	0.215042	lncRNA
ENSG0000(	N4BP3	145.3722	-0.26662	0.099153	0.215071	protein_coding
ENSG0000(	BMS1P8	76.8996	0.203605	0.099171	0.215096	transcribed_unprocessed_pseudo
ENSG0000(	ENSG0000(	151.7906	0.122432	0.099246	0.215244	transcribed_unprocessed_pseudo
ENSG0000(	ERP27	117.897	0.317904	0.099264	0.215268	protein_coding
ENSG0000(	FTSJ3	506.9295	-0.11576	0.099277	0.215282	protein_coding
ENSG0000(	NECTIN3	1332.461	-0.2061	0.0993	0.215317	protein_coding
ENSG0000(	ENSG0000(	44.70237	0.151382	0.099309	0.215321	lncRNA
ENSG0000(	HNF1A	830.4804	-0.22689	0.099316	0.215321	protein_coding
ENSG0000(	KIAA1143	1237.945	-0.0534	0.099323	0.215321	protein_coding
ENSG0000(	CYBA	1342.186	0.212523	0.099331	0.215325	protein_coding
ENSG0000(	ENSG0000(	19.66726	-0.37195	0.099343	0.215335	lncRNA
ENSG0000(	LINC02260	165.0389	0.163939	0.099356	0.21535	lncRNA
ENSG0000(	ENSG0000(	384.8678	-0.09462	0.099364	0.215351	lncRNA
ENSG0000(	ENSG0000(	56.05885	-0.21261	0.099468	0.215562	lncRNA
ENSG0000(	SP110	3355.073	0.263153	0.099485	0.215585	protein_coding
ENSG0000(	LINC02266	18.82554	0.267855	0.099585	0.215785	lncRNA
ENSG0000(	ETFBKMT	632.901	-0.10785	0.099599	0.215801	protein_coding
ENSG0000(	DRD4	33.26237	0.213798	0.099608	0.215806	protein_coding
ENSG0000(	PINK1	851.9431	-0.18289	0.099742	0.216081	protein_coding
ENSG0000(	CYB5R3	1999.296	-0.07863	0.099758	0.2161	protein_coding
ENSG0000(	RN7SL767F	181.467	-0.22579	0.099778	0.21613	misc_RNA
ENSG0000(	CERS6	4180.813	-0.18893	0.099842	0.216244	protein_coding
ENSG0000(	NR6A1	898.8624	-0.21062	0.099844	0.216244	protein_coding
ENSG0000(	ENSG0000(	211.1148	0.117932	0.099874	0.216292	TEC
ENSG0000(	C10orf53	522.5914	0.144482	0.099881	0.216293	protein_coding
ENSG0000(	ENSG0000(	182.073	-0.11057	0.099915	0.216351	lncRNA
ENSG0000(	LINC01612	74.08331	0.192791	0.100065	0.216661	lncRNA
ENSG0000(	ENSG0000(	42.02688	-0.19571	0.100072	0.216662	lncRNA
ENSG0000(	ENSG0000(	240.486	-0.09413	0.100121	0.216753	TEC
ENSG0000(	HCN4	10.4931	-0.38268	0.100195	0.216898	protein_coding
ENSG0000(	ENSG0000(	10.04535	0.361306	0.100205	0.216906	lncRNA
ENSG0000(	MAVS	8548.369	0.059386	0.100223	0.21693	protein_coding
ENSG0000(	ACOT8	586.5939	-0.09667	0.100254	0.216955	protein_coding
ENSG0000(	BUD13	326.9663	-0.07705	0.10026	0.216955	protein_coding
ENSG0000(	ENSG0000(	417.9644	0.070892	0.100262	0.216955	TEC
ENSG0000(	TNRC6B	11041.2	-0.08336	0.100244	0.216955	protein_coding
ENSG0000(	GRIN2A	376.0895	-0.38684	0.100286	0.216967	protein_coding
ENSG0000(	HLA-F-AS1	276.551	0.136202	0.100279	0.216967	lncRNA
ENSG0000(	MIR222HG	621.1262	0.153628	0.100288	0.216967	lncRNA
ENSG0000(	ENSG0000(	94.55099	0.30996	0.100299	0.216974	lncRNA
ENSG0000(	ADAMTSL3	1731.642	-0.10531	0.100319	0.217004	protein_coding
ENSG0000(	RRM1	583.1994	-0.08862	0.100337	0.217027	protein_coding
ENSG0000(	S100B	49.99246	0.222922	0.100364	0.217071	protein_coding
ENSG0000(	ODAD3	86.79298	0.144217	0.100433	0.217206	protein_coding
ENSG0000(	MIDEAS	4773.541	-0.0731	0.10045	0.217227	protein_coding

ENSG0000(	GLRX	2642.172	-0.23144	0.100463	0.21724	protein_coding
ENSG0000(	RNASE10	91.54674	0.183412	0.100492	0.217288	protein_coding
ENSG0000(	ENSG0000(	29.29213	0.190032	0.100524	0.217343	lncRNA
ENSG0000(	ENSG0000(	23.33884	0.322995	0.100567	0.217421	lncRNA
ENSG0000(	LINC01527	9.305527	0.438635	0.100699	0.21766	lncRNA
ENSG0000(	PRKAR1AP:	39.89721	0.2248	0.100689	0.21766	processed_pseudogene
ENSG0000(	RAB5IF	416.6695	0.157279	0.100698	0.21766	protein_coding
ENSG0000(	DMXL1	4369.323	-0.08503	0.100746	0.217747	protein_coding
ENSG0000(	SRGAP1	2296.169	0.103123	0.100776	0.217797	protein_coding
ENSG0000(	DCAF13	1192.053	0.080055	0.100795	0.217823	protein_coding
ENSG0000(	ENSG0000(	54.31384	0.212691	0.100806	0.217833	lncRNA
ENSG0000(	RTL6	620.8748	-0.08639	0.100872	0.21796	protein_coding
ENSG0000(	CD101	205.8213	-0.23117	0.100955	0.218117	protein_coding
ENSG0000(	ZNF652-AS	623.2779	-0.07747	0.100958	0.218117	lncRNA
ENSG0000(	ENSG0000(	83.1771	0.231928	0.100986	0.218161	lncRNA
ENSG0000(	CGAS	826.7303	0.118433	0.100993	0.218162	protein_coding
ENSG0000(	ENSG0000(	42.45208	0.182121	0.101022	0.218209	lncRNA
ENSG0000(	PDE4D	2065.74	0.105089	0.101039	0.218231	protein_coding
ENSG0000(	ADAT3	117.3837	0.173104	0.101056	0.218252	protein_coding
ENSG0000(	SCO1	2625.463	-0.09566	0.101082	0.218294	protein_coding
ENSG0000(	FAM240A	15.43064	0.2665	0.101137	0.218398	protein_coding
ENSG0000(	CKAP2	475.4426	-0.16007	0.101186	0.218445	protein_coding
ENSG0000(	DNAH7	1002.174	-0.18697	0.101189	0.218445	protein_coding
ENSG0000(	ENSG0000(	15.92046	0.324594	0.101176	0.218445	processed_pseudogene
ENSG0000(	KIAA1671	7279.419	-0.26062	0.101194	0.218445	protein_coding
ENSG0000(	RN7SL564F	96.34092	-0.32302	0.101174	0.218445	misc_RNA
ENSG0000(	BPHL	1030.007	-0.17874	0.101212	0.218455	protein_coding
ENSG0000(	MIR8086	85.5436	-0.13773	0.101212	0.218455	miRNA
ENSG0000(	ENSG0000(	55.49652	0.155934	0.101224	0.218456	lncRNA
ENSG0000(	GRID2IP	58.88435	-0.18697	0.101233	0.218456	protein_coding
ENSG0000(	LRRC20	79.47076	0.16851	0.101233	0.218456	protein_coding
ENSG0000(	ENSG0000(	27.02802	-0.3665	0.101361	0.2187	protein_coding
ENSG0000(	LINC02102	24.1685	0.311426	0.101364	0.2187	lncRNA
ENSG0000(	TGDS	252.8802	-0.08813	0.101367	0.2187	protein_coding
ENSG0000(	ADH7	15.45083	0.524701	0.101456	0.218861	protein_coding
ENSG0000(	BAZ2A	6173.808	-0.05804	0.101451	0.218861	protein_coding
ENSG0000(	ENSG0000(	29.74391	0.174959	0.101542	0.219017	lncRNA
ENSG0000(	SLC49A3	181.6833	0.142814	0.101537	0.219017	protein_coding
ENSG0000(	ENSG0000(	214.1283	0.093866	0.101593	0.219112	lncRNA
ENSG0000(	ENSG0000(	24.81959	0.322022	0.101608	0.219128	processed_pseudogene
ENSG0000(	ENSG0000(	257.855	0.16248	0.101621	0.219128	lncRNA
ENSG0000(	ENSG0000(	30.42292	0.228423	0.101621	0.219128	lncRNA
ENSG0000(	HMGN3-AS	82.53176	0.145322	0.101644	0.219162	lncRNA
ENSG0000(	FGF18	9.906136	-0.33087	0.101697	0.21926	protein_coding
ENSG0000(	ADPRS	162.7291	-0.11742	0.101762	0.219371	protein_coding
ENSG0000(	MTFP1	62.75421	0.165614	0.101759	0.219371	protein_coding
ENSG0000(	NUDT8	36.41861	-0.26248	0.101842	0.219529	protein_coding

ENSG0000(	NARF-AS1	169.4497	0.094235	0.101874	0.219582	lncRNA
ENSG0000(	EFHD1	94.96241	-0.12667	0.101907	0.21964	protein_coding
ENSG0000(	RNU6-1255	11.76297	0.29812	0.101929	0.219671	snRNA
ENSG0000(	ENSG0000(	23.05283	-0.20964	0.101939	0.219677	lncRNA
ENSG0000(	ENSG0000(	16.35842	0.274976	0.101961	0.21971	lncRNA
ENSG0000(	ENSG0000(	11.34437	0.331023	0.102001	0.219782	lncRNA
ENSG0000(	LINC02128	16.02308	0.295792	0.102051	0.219873	lncRNA
ENSG0000(	ENSG0000(	159.7479	0.53137	0.102063	0.219874	lncRNA
ENSG0000(	HSPA13	315.1363	0.19021	0.102065	0.219874	protein_coding
ENSG0000(	C22orf39	1260.863	0.057109	0.102132	0.219989	protein_coding
ENSG0000(	PSMD6-AS2	800.4934	0.098371	0.102131	0.219989	lncRNA
ENSG0000(	HOXC10	18.19874	0.377632	0.102178	0.220072	protein_coding
ENSG0000(	RSU1	686.36	0.126806	0.102194	0.220091	protein_coding
ENSG0000(	PCDH19	109.6251	-0.32859	0.102204	0.220097	protein_coding
ENSG0000(	ENSG0000(	139.745	0.102562	0.10222	0.220117	lncRNA
ENSG0000(	GAR1	125.3035	-0.10576	0.102272	0.220215	protein_coding
ENSG0000(	CCPG1	2168.279	-0.07048	0.102313	0.220289	protein_coding
ENSG0000(	DCTN2	2548.424	-0.08535	0.102338	0.220316	protein_coding
ENSG0000(	ENSG0000(	130.9858	-0.16719	0.10234	0.220316	lncRNA
ENSG0000(	ENSG0000(	10.30847	0.317811	0.10237	0.220355	lncRNA
ENSG0000(	PHBP2	52.39773	0.287087	0.102372	0.220355	processed_pseudogene
ENSG0000(	CCDC8	262.2689	0.091907	0.102393	0.220385	protein_coding
ENSG0000(	TRDMT1	1519.906	0.148313	0.102436	0.220463	protein_coding
ENSG0000(	ENSG0000(	52.60095	0.138087	0.102458	0.220494	lncRNA
ENSG0000(	ENSG0000(	16.45735	0.39619	0.102503	0.220532	transcribed_processed_pseudoge
ENSG0000(	MHENCR	254.1971	0.176081	0.102485	0.220532	lncRNA
ENSG0000(	PLSCR3	229.4143	0.18738	0.102503	0.220532	protein_coding
ENSG0000(	SLC66A1	466.2693	-0.12652	0.102499	0.220532	protein_coding
ENSG0000(	GLYATL2	10.03867	-0.35845	0.102516	0.220543	protein_coding
ENSG0000(	DDX56	1419.901	-0.09596	0.102531	0.220561	protein_coding
ENSG0000(	ENSG0000(	10.77625	0.323155	0.102554	0.220595	lncRNA
ENSG0000(	CEACAM8	163.7024	0.104353	0.102595	0.220654	protein_coding
ENSG0000(	LINC02547	357.8401	0.085091	0.102594	0.220654	lncRNA
ENSG0000(	SMTN	1794.604	0.173459	0.102602	0.220654	protein_coding
ENSG0000(	BEX4	167.7144	-0.14751	0.102705	0.22086	protein_coding
ENSG0000(	ENSG0000(	13.77477	-0.25436	0.102752	0.220917	lncRNA
ENSG0000(	MALLP2	28.68439	-0.34396	0.102748	0.220917	transcribed_unprocessed_pseudo
ENSG0000(	RN7SL806F	58.69872	0.135274	0.102744	0.220917	misc_RNA
ENSG0000(	ENSG0000(	20.63706	-0.40418	0.102777	0.220918	lncRNA
ENSG0000(	ENSG0000(	16.33012	0.339208	0.102773	0.220918	lncRNA
ENSG0000(	FAM225A	39.07886	1.43589	0.102771	0.220918	lncRNA
ENSG0000(	PMS1	728.1776	0.09746	0.102781	0.220918	protein_coding
ENSG0000(	C8orf34	504.4603	0.084804	0.102788	0.220918	protein_coding
ENSG0000(	HEPH	8109.172	-0.28911	0.102875	0.22109	protein_coding
ENSG0000(	EIF5AP3	32.49772	-0.15349	0.102919	0.221169	processed_pseudogene
ENSG0000(	RN7SL278F	56.15494	0.180951	0.102939	0.221198	misc_RNA
ENSG0000(	NOL9	2602.201	-0.06977	0.102967	0.221243	protein_coding

ENSG0000(	BTG1-DT	295.6562	0.26834	0.103014	0.221313	lncRNA
ENSG0000(	ENSG0000(	30.30728	0.294716	0.103009	0.221313	lncRNA
ENSG0000(	ENSG0000(	30.62248	-0.16744	0.103062	0.221401	lncRNA
ENSG0000(	TTBK2-AS1	64.47963	0.127072	0.103094	0.221455	lncRNA
ENSG0000(	ENSG0000(	10.73806	-0.59576	0.103122	0.2215	processed_pseudogene
ENSG0000(	EGFR	2126.734	-0.17384	0.103156	0.221543	protein_coding
ENSG0000(	OR6J1	268.0812	-0.07641	0.103156	0.221543	protein_coding
ENSG0000(	TRBV29OR!	41.80805	-0.39815	0.103188	0.221597	TR_V_pseudogene
ENSG0000(	CCDC142	1744.446	-0.07028	0.103249	0.2217	protein_coding
ENSG0000(	LINC01890	11.30023	0.389105	0.10325	0.2217	lncRNA
ENSG0000(	ENSG0000(	532.8232	0.055388	0.103268	0.221724	lncRNA
ENSG0000(	CTRL	25.23965	0.329044	0.103304	0.221785	protein_coding
ENSG0000(	FAM229B	161.0924	0.095564	0.103391	0.221958	protein_coding
ENSG0000(	C7orf25	220.5591	0.165744	0.103409	0.22198	protein_coding
ENSG0000(	MTCO1P19	16.30884	-0.3047	0.103418	0.221984	processed_pseudogene
ENSG0000(	PEX5L	194.9478	0.118048	0.103443	0.222024	protein_coding
ENSG0000(	RN7SL688F	31.08885	-0.21528	0.103453	0.222029	misc_RNA
ENSG0000(	DDX42	2912.642	-0.04172	0.103532	0.222169	protein_coding
ENSG0000(	ENSG0000(	1022.206	-0.07698	0.103526	0.222169	lncRNA
ENSG0000(	RN7SL724F	15.09121	-0.26491	0.103556	0.222204	misc_RNA
ENSG0000(	ENSG0000(	2830.563	0.075122	0.103601	0.222286	lncRNA
ENSG0000(	PIN1	963.7263	-0.07316	0.103683	0.222447	protein_coding
ENSG0000(	ENSG0000(	12.00775	-0.26259	0.103701	0.22247	lncRNA
ENSG0000(	BPTF	8274.924	-0.08318	0.103724	0.222504	protein_coding
ENSG0000(	ODR4	933.305	-0.08856	0.103743	0.22253	protein_coding
ENSG0000(	CCL3-AS1	102.9655	-0.17479	0.103755	0.222542	lncRNA
ENSG0000(	MUC13	7773.438	-0.36477	0.103776	0.22257	protein_coding
ENSG0000(	AMZ2P1	237.9556	0.139759	0.103815	0.22262	transcribed_unprocessed_pseudo
ENSG0000(	LINC01095	18.92921	0.279478	0.103807	0.22262	lncRNA
ENSG0000(	RACGAP1	343.4825	0.157586	0.103825	0.22262	protein_coding
ENSG0000(	TMEM203	255.4577	-0.10156	0.103827	0.22262	protein_coding
ENSG0000(	RTN4IP1	299.9292	-0.1667	0.103841	0.222636	protein_coding
ENSG0000(	ENSG0000(	151.5115	0.202453	0.103871	0.222683	TEC
ENSG0000(	CLIC4	1168.445	0.236247	0.103897	0.222724	protein_coding
ENSG0000(	ID2-AS1	446.7356	-0.4915	0.103906	0.222728	lncRNA
ENSG0000(	SNORA10	140.426	0.231749	0.103924	0.222752	snoRNA
ENSG0000(	FOXI2	16.46163	-0.3557	0.103934	0.222759	protein_coding
ENSG0000(	ENSG0000(	95.17179	-0.2105	0.10396	0.222798	lncRNA
ENSG0000(	ENSG0000(	267.9064	0.0798	0.103981	0.222828	lncRNA
ENSG0000(	LINC02388	501.3646	0.08562	0.103994	0.222842	lncRNA
ENSG0000(	CHAF1A	453.6863	0.154246	0.104131	0.223088	protein_coding
ENSG0000(	ENSG0000(	233.7454	-0.09882	0.104121	0.223088	lncRNA
ENSG0000(	PCNX3	1898.15	-0.10085	0.10413	0.223088	protein_coding
ENSG0000(	ATP6AP2	1181.215	-0.07115	0.10414	0.223094	protein_coding
ENSG0000(	TPPP3	66.32532	-0.34784	0.104179	0.223163	protein_coding
ENSG0000(	SAMD12-A:	41.97374	0.316361	0.10419	0.22317	lncRNA
ENSG0000(	GRM7	107.2645	0.230985	0.104237	0.223251	protein_coding

ENSG0000(	PLCH1	800.0517	0.262607	0.104242	0.223251	protein_coding
ENSG0000(	FOSL2	5024.689	0.210827	0.104266	0.223287	protein_coding
ENSG0000(	TUT1	332.9099	0.122382	0.10432	0.223388	protein_coding
ENSG0000(	KCNN4	311.9001	0.353567	0.104348	0.223432	protein_coding
ENSG0000(	LINC00366	107.9204	0.113579	0.104401	0.223515	lncRNA
ENSG0000(	LSM11	628.6732	-0.05554	0.104399	0.223515	protein_coding
ENSG0000(	ENSG0000(	25.8031	0.23376	0.104408	0.223515	TEC
ENSG0000(	DAP3	1172.034	-0.06797	0.10442	0.223527	protein_coding
ENSG0000(	PKD2L2-DT	3557.452	0.071407	0.104454	0.223584	lncRNA
ENSG0000(	SNX18P23	18.097	-0.4083	0.104532	0.223735	processed_pseudogene
ENSG0000(	MALRD1	8386.746	-0.2942	0.104621	0.2239	protein_coding
ENSG0000(	NISCH	3459.908	0.048168	0.104623	0.2239	protein_coding
ENSG0000(	ENSG0000(	1547.004	0.096132	0.104634	0.223908	lncRNA
ENSG0000(	SUGT1-DT	43.94216	0.160851	0.104646	0.223919	lncRNA
ENSG0000(	ENSG0000(	36.95988	0.264408	0.104692	0.224002	TEC
ENSG0000(	ENSG0000(	26.82574	-0.25414	0.104765	0.224143	lncRNA
ENSG0000(	CYP20A1	3959.328	-0.04708	0.104795	0.224168	protein_coding
ENSG0000(	ENSG0000(	34.0794	3.515388	0.104798	0.224168	processed_pseudogene
ENSG0000(	NDUFAF4P	12.92081	-0.30863	0.104786	0.224168	processed_pseudogene
ENSG0000(	AFAP1L2	393.1606	0.167391	0.104806	0.224169	protein_coding
ENSG0000(	TFPT	82.53469	-0.14127	0.104846	0.22424	protein_coding
ENSG0000(	BTBD11	263.579	0.207988	0.104904	0.224334	protein_coding
ENSG0000(	WRNIP1	531.2981	-0.06737	0.104904	0.224334	protein_coding
ENSG0000(	BRAF	4562.443	0.062284	0.104979	0.224463	protein_coding
ENSG0000(	ENSG0000(	20.99828	0.268559	0.104976	0.224463	lncRNA
ENSG0000(	LINC00997	223.1385	0.163212	0.105014	0.224522	lncRNA
ENSG0000(	ZC2HC1C	249.2562	0.090928	0.105042	0.224567	protein_coding
ENSG0000(	ARMC8	1829.339	-0.07154	0.10507	0.224613	protein_coding
ENSG0000(	YWHAQP5	360.9162	0.076336	0.105112	0.224686	processed_pseudogene
ENSG0000(	EFNA5	255.1932	0.259486	0.10513	0.224709	protein_coding
ENSG0000(	NOTCH2NL	112.8462	0.34226	0.105136	0.224709	protein_coding
ENSG0000(	SGCA	66.6026	-0.20277	0.105155	0.224734	protein_coding
ENSG0000(	ENSG0000(	394.3935	0.06817	0.105197	0.224808	lncRNA
ENSG0000(	ALOX5	957.9124	0.236849	0.105218	0.224821	protein_coding
ENSG0000(	PVRIG	77.48512	0.221831	0.105217	0.224821	protein_coding
ENSG0000(	RRN3P2	415.7405	0.185743	0.105242	0.224857	lncRNA
ENSG0000(	MX1	3702.547	0.222919	0.105252	0.224865	protein_coding
ENSG0000(	LINC02250	26.01781	0.202737	0.105262	0.224871	lncRNA
ENSG0000(	KCTD9	1392.178	-0.07536	0.105297	0.22493	protein_coding
ENSG0000(	LRRC70	72.22203	0.208779	0.105312	0.224946	protein_coding
ENSG0000(	COPG2IT1	490.7172	0.239984	0.105325	0.22496	lncRNA
ENSG0000(	SLC9A6	926.2971	-0.08746	0.10534	0.224975	protein_coding
ENSG0000(	PPP1R3B	1079.241	0.121642	0.105348	0.224978	protein_coding
ENSG0000(	BRMS1L	91.32681	0.183293	0.105376	0.225022	protein_coding
ENSG0000(	HIGD2A	382.5654	-0.12334	0.105422	0.225094	protein_coding
ENSG0000(	JAKMIP2	340.272	-0.09209	0.105424	0.225094	protein_coding
ENSG0000(	LINC01044	52.63416	-0.15386	0.105465	0.225167	lncRNA

ENSG0000(	URAD	132.6748	-0.31771	0.105502	0.225231	protein_coding
ENSG0000(	CACNB1	262.5307	0.187084	0.105516	0.225245	protein_coding
ENSG0000(	DTX3	454.9441	0.153302	0.105583	0.225373	protein_coding
ENSG0000(	ADGRG6	1018.588	0.222377	0.105592	0.225376	protein_coding
ENSG0000(	LINC02340	92.66984	-0.12738	0.105623	0.225426	lncRNA
ENSG0000(	TINCR	371.0281	-0.09647	0.105649	0.225468	protein_coding
ENSG0000(	CFAP221	522.9731	0.072719	0.105673	0.225486	protein_coding
ENSG0000(	ENSG0000(	792.6285	-0.0989	0.105675	0.225486	lncRNA
ENSG0000(	ENSG0000(	47.50552	0.149351	0.105679	0.225486	lncRNA
ENSG0000(	CDH8	335.4374	0.105816	0.105687	0.225488	protein_coding
ENSG0000(	ENSG0000(	51.12631	0.181464	0.105745	0.225597	lncRNA
ENSG0000(	ALG3P1	9.877045	0.333428	0.105753	0.225597	processed_pseudogene
ENSG0000(	AKIP1	513.2434	-0.07191	0.105774	0.225623	protein_coding
ENSG0000(	ENSG0000(	64.60132	-0.18055	0.105779	0.225623	lncRNA
ENSG0000(	PCIF1	483.1329	-0.10353	0.105827	0.225694	protein_coding
ENSG0000(	RNU6-681F	28.48657	-0.25424	0.105823	0.225694	snRNA
ENSG0000(	NAA40	1604.942	-0.10521	0.105874	0.22578	protein_coding
ENSG0000(	AGL	1009.284	-0.09133	0.10595	0.225911	protein_coding
ENSG0000(	NUDCD3	1936.977	-0.06887	0.105948	0.225911	protein_coding
ENSG0000(	ENSG0000(	70.39407	0.193175	0.106064	0.226138	lncRNA
ENSG0000(	FOXK2	1412.322	0.061922	0.106092	0.226183	protein_coding
ENSG0000(	ENSG0000(	11.8611	0.321337	0.106107	0.226195	lncRNA
ENSG0000(	ZNF337	1046.589	0.108988	0.106112	0.226195	protein_coding
ENSG0000(	BEAN1-AS1	24.73954	0.240713	0.106154	0.22627	lncRNA
ENSG0000(	SMC2	566.3686	-0.16974	0.106176	0.2263	protein_coding
ENSG0000(	ENSG0000(	101.5877	0.118177	0.10619	0.226316	lncRNA
ENSG0000(	SHOX	645.5525	-0.09192	0.106246	0.22642	protein_coding
ENSG0000(	ENSG0000(	11.77037	-0.30622	0.106261	0.226436	unprocessed_pseudogene
ENSG0000(	ENSG0000(	8.524796	0.353286	0.106288	0.226479	TEC
ENSG0000(	RNU6-1045	15.26462	0.36432	0.10633	0.226553	snRNA
ENSG0000(	CHCHD5	314.9266	-0.09444	0.10634	0.226559	protein_coding
ENSG0000(	ENSG0000(	264.9446	0.079981	0.106377	0.226575	lncRNA
ENSG0000(	ENSG0000(	280.4842	-0.08651	0.106355	0.226575	lncRNA
ENSG0000(	ENSG0000(	282.0347	0.080805	0.106388	0.226575	lncRNA
ENSG0000(	PDXK	5163.33	-0.15781	0.106391	0.226575	protein_coding
ENSG0000(	PTPRD	2571.914	-0.31665	0.106367	0.226575	protein_coding
ENSG0000(	SHOX2	13.67896	0.403215	0.106385	0.226575	protein_coding
ENSG0000(	FBXO31	1497.095	-0.13958	0.106402	0.226583	protein_coding
ENSG0000(	ATIC	540.8679	-0.11984	0.10642	0.226606	protein_coding
ENSG0000(	OR2L3	58.14186	0.232591	0.106439	0.226626	protein_coding
ENSG0000(	SLC25A27	468.7266	0.146599	0.106444	0.226626	protein_coding
ENSG0000(	TRAF3IP2	1743.075	-0.08274	0.106472	0.22667	protein_coding
ENSG0000(	ENSG0000(	116.1018	0.147302	0.106489	0.226676	lncRNA
ENSG0000(	GTF2H5	762.1459	-0.1	0.106482	0.226676	protein_coding
ENSG0000(	CFAP44	1286.543	0.110048	0.106533	0.226755	protein_coding
ENSG0000(	SAMM50	837.37	-0.08046	0.106555	0.226786	protein_coding
ENSG0000(	ENSG0000(	126.914	0.120239	0.106632	0.22692	lncRNA

ENSG0000(	HPAT5	96.65112	0.136305	0.106629	0.22692	lncRNA
ENSG0000(	ENSG0000(	159.3501	-0.10944	0.106645	0.226932	lncRNA
ENSG0000(	ENSG0000(	134.9852	-0.09393	0.106703	0.227041	lncRNA
ENSG0000(	ZSCAN4	27.44511	0.246008	0.106723	0.227068	protein_coding
ENSG0000(	ENSG0000(	99.79387	0.147292	0.106749	0.227107	lncRNA
ENSG0000(	INPP4A	3131.966	0.145652	0.106783	0.227165	protein_coding
ENSG0000(	CAVIN2	193.528	-0.18759	0.106851	0.227263	protein_coding
ENSG0000(	EMP3	137.57	0.249806	0.106851	0.227263	protein_coding
ENSG0000(	LINC02242	336.0102	0.081495	0.106837	0.227263	lncRNA
ENSG0000(	ENSG0000(	13.28474	0.253808	0.106889	0.227283	lncRNA
ENSG0000(	ENSG0000(	21.77474	0.336951	0.106877	0.227283	processed_pseudogene
ENSG0000(	ENSG0000(	210.4859	0.091782	0.106889	0.227283	TEC
ENSG0000(	MYOM1	234.3739	-0.14522	0.106886	0.227283	protein_coding
ENSG0000(	ENSG0000(	282.9588	0.078615	0.106966	0.22743	lncRNA
ENSG0000(	ENSG0000(	130.3931	-0.10766	0.107052	0.227582	lncRNA
ENSG0000(	MANCR	233.8046	-0.14325	0.107051	0.227582	lncRNA
ENSG0000(	UBE2E1	632.3207	0.098575	0.107072	0.227611	protein_coding
ENSG0000(	ENSG0000(	136.2207	0.232789	0.107093	0.227624	lncRNA
ENSG0000(	ENSG0000(	163.1586	0.087747	0.107093	0.227624	lncRNA
ENSG0000(	SH3YL1	2044.595	-0.10104	0.107154	0.227739	protein_coding
ENSG0000(	NBPF20	314.8802	-0.16254	0.107178	0.227775	protein_coding
ENSG0000(	USP19	1226.836	-0.0847	0.107199	0.227788	protein_coding
ENSG0000(	ZNNT1	505.3975	-0.12532	0.107194	0.227788	lncRNA
ENSG0000(	FBXO22	2847.048	0.080318	0.107212	0.227791	protein_coding
ENSG0000(	IQCA1	40.28386	0.270401	0.107215	0.227791	protein_coding
ENSG0000(	INIP	1159.883	-0.07005	0.107248	0.227846	protein_coding
ENSG0000(	CMSS1	1624.747	0.076148	0.107257	0.22785	protein_coding
ENSG0000(	ETNK1	4917.168	0.12849	0.107292	0.227908	protein_coding
ENSG0000(	PRSS12	871.8459	-0.26452	0.1073	0.22791	protein_coding
ENSG0000(	CRYZP1	88.0344	0.278324	0.107348	0.227981	processed_pseudogene
ENSG0000(	EREG	504.1776	-0.11618	0.107347	0.227981	protein_coding
ENSG0000(	ENSG0000(	35.85274	0.177581	0.107401	0.228078	lncRNA
ENSG0000(	APC	3589.355	-0.13455	0.107415	0.228094	protein_coding
ENSG0000(	CTNNA1P1	23.90321	-0.38143	0.107531	0.228325	processed_pseudogene
ENSG0000(	CNKSR2	1306.022	0.132498	0.107543	0.228329	protein_coding
ENSG0000(	ENSG0000(	10.16376	0.370449	0.107548	0.228329	lncRNA
ENSG0000(	DACT3	1059.018	0.051534	0.107581	0.228367	protein_coding
ENSG0000(	ENSG0000(	36.53766	-0.17422	0.107592	0.228367	processed_pseudogene
ENSG0000(	ENSG0000(	11.96372	-0.29232	0.107595	0.228367	TEC
ENSG0000(	ITSN1	1860.357	-0.12042	0.10758	0.228367	protein_coding
ENSG0000(	RC3H1-IT1	204.9926	-0.08104	0.107663	0.228496	lncRNA
ENSG0000(	TIGAR	1155.134	0.134411	0.107691	0.228541	protein_coding
ENSG0000(	ENSG0000(	53.68887	-0.14269	0.107704	0.228554	pseudogene
ENSG0000(	ENSG0000(	622.9718	-0.06642	0.107731	0.228594	lncRNA
ENSG0000(	ENSG0000(	29.09319	-0.16678	0.107783	0.22866	lncRNA
ENSG0000(	OR2M4	394.46	-0.07102	0.107783	0.22866	protein_coding
ENSG0000(	RNF157	1179.97	-0.10772	0.107784	0.22866	protein_coding

ENSG0000(	SOS1-IT1	59.34599	0.785773	0.107812	0.228704	lncRNA
ENSG0000(	DDAH1	1270.42	-0.2426	0.107826	0.228719	protein_coding
ENSG0000(	ENSG0000(	12.48733	-0.56796	0.107887	0.228834	lncRNA
ENSG0000(	RFLNB	585.0374	-0.20423	0.107937	0.228908	protein_coding
ENSG0000(	ZNF230	435.2275	0.068275	0.107935	0.228908	protein_coding
ENSG0000(	H1-5	250.0035	-0.29069	0.107954	0.228929	protein_coding
ENSG0000(	ENSG0000(	1250.66	0.050981	0.10799	0.228982	lncRNA
ENSG0000(	ORC1	173.7079	0.136323	0.107994	0.228982	protein_coding
ENSG0000(	C7	1402.103	-0.38461	0.108023	0.228997	protein_coding
ENSG0000(	ENSG0000(	69.90928	0.131301	0.108016	0.228997	lncRNA
ENSG0000(	ENSG0000(	784.6204	0.088261	0.108014	0.228997	lncRNA
ENSG0000(	LAMB4	172.1375	-0.182	0.108048	0.229035	protein_coding
ENSG0000(	IKBKE	720.9677	0.167766	0.108073	0.229074	protein_coding
ENSG0000(	MBD1	1969.173	-0.06221	0.108115	0.229144	protein_coding
ENSG0000(	NEK7	1132.185	-0.09006	0.108121	0.229144	protein_coding
ENSG0000(	OR2T10	30.20357	0.599231	0.108175	0.229241	protein_coding
ENSG0000(	SNORD116	13.0683	0.331433	0.108181	0.229241	snoRNA
ENSG0000(	JMJD1C	7839.471	-0.07453	0.108235	0.229294	protein_coding
ENSG0000(	LINC01503	1129.921	-0.07443	0.108233	0.229294	lncRNA
ENSG0000(	MAN1B1	1649.144	-0.06253	0.108232	0.229294	protein_coding
ENSG0000(	TMEM272	252.3604	0.352299	0.108233	0.229294	protein_coding
ENSG0000(	CTNS	494.5956	0.112354	0.108245	0.229299	protein_coding
ENSG0000(	ENSG0000(	13.67204	0.323096	0.108267	0.229311	TEC
ENSG0000(	GID8	861.377	-0.08706	0.108272	0.229311	protein_coding
ENSG0000(	LOXL1-AS1	527.897	-0.09402	0.108266	0.229311	lncRNA
ENSG0000(	ZNF555	832.2247	0.05942	0.108304	0.229363	protein_coding
ENSG0000(	IGF2BP3	434.9318	-0.17325	0.108357	0.229446	protein_coding
ENSG0000(	METTL23	265.2877	-0.0898	0.108358	0.229446	protein_coding
ENSG0000(	LINC00523	10.28398	0.408691	0.108379	0.229459	lncRNA
ENSG0000(	ZNF778	1024.022	0.129775	0.108377	0.229459	protein_coding
ENSG0000(	CACFD1	552.3111	-0.16741	0.108412	0.22948	protein_coding
ENSG0000(	ENSG0000(	58.99349	-0.1932	0.108425	0.22948	lncRNA
ENSG0000(	FAM151B-I	619.1133	0.074433	0.108425	0.22948	lncRNA
ENSG0000(	IMPA2	609.133	-0.1145	0.108424	0.22948	protein_coding
ENSG0000(	PPIAP89	9.674878	0.357967	0.108406	0.22948	processed_pseudogene
ENSG0000(	ENSG0000(	2819.257	0.305393	0.108442	0.229501	miRNA
ENSG0000(	ENSG0000(	98.17378	0.10707	0.10855	0.229698	lncRNA
ENSG0000(	PDE9A-AS1	49.25735	0.165817	0.108544	0.229698	lncRNA
ENSG0000(	FAM83F	3214.913	-0.29265	0.108574	0.229734	protein_coding
ENSG0000(	GSTA4	264.8246	-0.13186	0.108594	0.229753	protein_coding
ENSG0000(	WDR62	627.0587	0.06905	0.108598	0.229753	protein_coding
ENSG0000(	MIR663A	3289.526	0.316553	0.108615	0.229773	miRNA
ENSG0000(	TOLLIP-DT	15.54946	0.269168	0.108663	0.22986	lncRNA
ENSG0000(	ENSG0000(	16.22433	0.293173	0.108688	0.229898	transcribed_processed_pseudoge
ENSG0000(	RAB8A	1791.86	-0.10779	0.108741	0.229994	protein_coding
ENSG0000(	ENSG0000(	8.550559	-0.33634	0.108825	0.230156	lncRNA
ENSG0000(	TRAJ45	20.97266	-0.41522	0.108857	0.230208	TR_J_gene

ENSG0000(	CAVIN2-AS	79.73603	0.131142	0.108874	0.230214	lncRNA
ENSG0000(	FASTKD3	277.2829	-0.12602	0.108872	0.230214	protein_coding
ENSG0000(	FAM66E	43.32714	0.203672	0.10889	0.230233	lncRNA
ENSG0000(	EIF3E	6285.302	-0.08184	0.108932	0.230299	protein_coding
ENSG0000(	MANEAL	64.29957	0.199045	0.108936	0.230299	protein_coding
ENSG0000(	MSRA-DT	186.4462	0.105725	0.108995	0.230407	lncRNA
ENSG0000(	LINC00381	62.53374	0.150904	0.109055	0.23052	lncRNA
ENSG0000(	TRIAP1	108.5267	-0.13849	0.109075	0.230545	protein_coding
ENSG0000(	RBCK1	1733.253	0.085217	0.109139	0.230666	protein_coding
ENSG0000(	ENSG0000(	18.06339	0.251502	0.109155	0.230684	lncRNA
ENSG0000(	ENSG0000(	13.41758	-0.26897	0.109204	0.230761	unprocessed_pseudogene
ENSG0000(	MAPKAPK5	522.2311	-0.07146	0.109208	0.230761	lncRNA
ENSG0000(	PPRC1	1009.582	0.162257	0.109213	0.230761	protein_coding
ENSG0000(	SEC61A1	2824.755	0.111986	0.109256	0.230836	protein_coding
ENSG0000(	PPP1R14D	312.9495	-0.32766	0.109285	0.230882	protein_coding
ENSG0000(	RN7SL284F	66.70985	0.16579	0.10933	0.230961	misc_RNA
ENSG0000(	MAP3K14-/	425.5716	0.081034	0.109364	0.231018	lncRNA
ENSG0000(	RSF1	3191.393	-0.06508	0.109424	0.231129	protein_coding
ENSG0000(	LAIR1	2103.247	0.19955	0.109469	0.231209	protein_coding
ENSG0000(	MRPL12	136.9877	-0.167	0.109492	0.231209	protein_coding
ENSG0000(	RBFOX1	442.7306	0.084418	0.109487	0.231209	protein_coding
ENSG0000(	RN7SL97P	9.967177	-0.28552	0.109482	0.231209	misc_RNA
ENSG0000(	CCDC186	2763.638	-0.12117	0.109549	0.231301	protein_coding
ENSG0000(	POLK	1777.066	0.06803	0.10955	0.231301	protein_coding
ENSG0000(	CGN	4245.605	-0.32552	0.10957	0.231328	protein_coding
ENSG0000(	KAT8	1759.546	-0.05344	0.109609	0.231389	protein_coding
ENSG0000(	NF2	1451.055	-0.04979	0.109621	0.231389	protein_coding
ENSG0000(	ZNF578	1037.502	0.061958	0.109615	0.231389	protein_coding
ENSG0000(	APOF	72.12707	0.142866	0.109642	0.231406	protein_coding
ENSG0000(	ENSG0000(	142.9985	-0.09988	0.10965	0.231406	lncRNA
ENSG0000(	STARD7-AS	464.7588	0.080585	0.109651	0.231406	lncRNA
ENSG0000(	INTS12	323.2024	-0.09376	0.109669	0.231429	protein_coding
ENSG0000(	SYT5	344.5572	0.081207	0.109685	0.231448	protein_coding
ENSG0000(	ENSG0000(	310.4221	0.07056	0.109713	0.23149	lncRNA
ENSG0000(	ENSG0000(	41.5284	0.182685	0.10973	0.231512	lncRNA
ENSG0000(	ENSG0000(	15.23279	-0.53858	0.10976	0.231559	lncRNA
ENSG0000(	ENSG0000(	643.1895	-0.11836	0.109774	0.231569	lncRNA
ENSG0000(	FGB	93.6212	0.305138	0.10978	0.231569	protein_coding
ENSG0000(	ENSG0000(	9.729539	0.361932	0.109841	0.231667	processed_pseudogene
ENSG0000(	ZSCAN18	1305.517	-0.07987	0.109838	0.231667	protein_coding
ENSG0000(	VN1R1	70.63482	0.156889	0.109853	0.231677	protein_coding
ENSG0000(	ENSG0000(	1004.619	-0.05153	0.10988	0.23172	lncRNA
ENSG0000(	MIR4451	11.5141	0.522609	0.109901	0.231748	miRNA
ENSG0000(	ENSG0000(	9.220506	0.343393	0.109917	0.231767	snoRNA
ENSG0000(	PCDHA9	255.5187	-0.08076	0.109935	0.231788	protein_coding
ENSG0000(	LETR1	576.9369	0.117161	0.109951	0.231807	lncRNA
ENSG0000(	LINC00689	188.1956	-0.12216	0.109978	0.231833	lncRNA

ENSG0000(	TAS2R10	33.62286	0.240302	0.109974	0.231833	protein_coding
ENSG0000(	LINC01358	1063.687	0.077875	0.109999	0.231861	lncRNA
ENSG0000(	RCC2	1754.097	0.137195	0.110026	0.231903	protein_coding
ENSG0000(	ADGB	98.99439	0.1883	0.110071	0.231932	protein_coding
ENSG0000(	ENSG0000(	132.4863	0.131147	0.110075	0.231932	lncRNA
ENSG0000(	ENSG0000(	8.052052	-0.51756	0.110072	0.231932	lncRNA
ENSG0000(	ENSG0000(	60.38662	0.122171	0.110062	0.231932	lncRNA
ENSG0000(	SLITRK3	43.70731	-0.21756	0.110077	0.231932	protein_coding
ENSG0000(	FLJ43315	55.58692	0.253434	0.110086	0.231936	transcribed_unprocessed_pseudo
ENSG0000(	LYSMD1	229.5204	-0.1374	0.110095	0.231939	protein_coding
ENSG0000(	ENSG0000(	30.85728	0.190221	0.110117	0.23197	lncRNA
ENSG0000(	BRAFP1	13.48507	0.360168	0.110133	0.231983	unprocessed_pseudogene
ENSG0000(	FBXO28	1052.479	-0.07214	0.110138	0.231983	protein_coding
ENSG0000(	ENSG0000(	9.170847	0.390196	0.110231	0.232163	lncRNA
ENSG0000(	ENSG0000(	76.82814	0.111398	0.110249	0.232186	lncRNA
ENSG0000(	GATD3B	781.028	0.13455	0.110266	0.232206	protein_coding
ENSG0000(	GNPDA1	416.2767	-0.12603	0.110308	0.23228	protein_coding
ENSG0000(	ENSG0000(	62.70917	0.179291	0.110324	0.232298	lncRNA
ENSG0000(	MIR5687	25.68537	0.259035	0.110333	0.232302	miRNA
ENSG0000(	ENSG0000(	177.9241	-0.72973	0.110377	0.232378	protein_coding
ENSG0000(	ENSG0000(	46.05971	0.150881	0.110437	0.232488	processed_pseudogene
ENSG0000(	HDAC1P2	37.08804	0.216355	0.110461	0.232523	transcribed_processed_pseudoge
ENSG0000(	ENSG0000(	16.67178	0.32311	0.110469	0.232524	lncRNA
ENSG0000(	ENSG0000(	13.00829	0.29754	0.110511	0.232597	lncRNA
ENSG0000(	LINC00113	60.21182	0.158865	0.110583	0.232733	lncRNA
ENSG0000(	KIF16B	2083.917	-0.23356	0.110621	0.232798	protein_coding
ENSG0000(	ENSG0000(	142.0351	0.129781	0.11064	0.232808	lncRNA
ENSG0000(	MMP1	987.23	2.393831	0.110638	0.232808	protein_coding
ENSG0000(	H2BC11	112.7273	-0.22013	0.110666	0.232847	protein_coding
ENSG0000(	ENSG0000(	70.99486	0.133558	0.110693	0.232888	lncRNA
ENSG0000(	ENSG0000(	96.78425	0.122646	0.110702	0.232891	lncRNA
ENSG0000(	ENSG0000(	29.84121	0.26401	0.110743	0.232961	lncRNA
ENSG0000(	UBE2O	792.6621	0.084714	0.110757	0.232977	protein_coding
ENSG0000(	DNAJA1	1024.813	-0.11519	0.110775	0.232998	protein_coding
ENSG0000(	SMG7-AS1	136.5978	0.087803	0.110795	0.233025	lncRNA
ENSG0000(	LONRF1	500.715	0.128605	0.110812	0.233046	protein_coding
ENSG0000(	ENSG0000(	92.96746	-0.13716	0.110855	0.233119	lncRNA
ENSG0000(	ENSG0000(	200.1106	-0.0972	0.110862	0.233119	lncRNA
ENSG0000(	ATP5MJ	1029.415	-0.09383	0.11087	0.233119	protein_coding
ENSG0000(	PCDHA4	2899.553	-0.08156	0.1109	0.233151	protein_coding
ENSG0000(	UBE2Z	2685.94	0.076928	0.110899	0.233151	protein_coding
ENSG0000(	SQOR	556.7421	0.167336	0.110921	0.233181	protein_coding
ENSG0000(	ENSG0000(	197.2565	0.179431	0.110934	0.233186	lncRNA
ENSG0000(	TRAJ35	25.12787	-0.3596	0.110938	0.233186	TR_J_gene
ENSG0000(	RN7SL482F	43.75014	0.176915	0.110951	0.233196	misc_RNA
ENSG0000(	CLNK	475.6207	0.116248	0.110982	0.233232	protein_coding
ENSG0000(	ENSG0000(	52.32032	0.169454	0.110979	0.233232	lncRNA

ENSG0000(	ENSG0000(	114.8445	0.122081	0.111003	0.23326	lncRNA
ENSG0000(	KLHL29	644.962	0.216167	0.111081	0.233408	protein_coding
ENSG0000(	RPS29P12	67.90286	-0.38698	0.111099	0.233429	processed_pseudogene
ENSG0000(	MRPS30-D	385.4322	0.101909	0.111157	0.233537	lncRNA
ENSG0000(	HCN1	153.3447	-0.38324	0.11118	0.233569	protein_coding
ENSG0000(	SLC49A4	378.305	0.103312	0.111252	0.233706	protein_coding
ENSG0000(	DEGS1	670.9633	-0.11222	0.111303	0.23378	protein_coding
ENSG0000(	STEAP2-AS	10.02493	0.366877	0.111299	0.23378	lncRNA
ENSG0000(	TAF4B	435.047	0.281917	0.111323	0.233807	protein_coding
ENSG0000(	ENSG0000(	141.7122	0.174998	0.111345	0.233837	lncRNA
ENSG0000(	RASA2	1746.277	0.142327	0.111354	0.233841	protein_coding
ENSG0000(	RNU6-1079	19.48016	-0.22674	0.111399	0.233919	snRNA
ENSG0000(	ENSG0000(	19.90927	-0.29852	0.111454	0.234004	lncRNA
ENSG0000(	LHX1-DT	20.43332	0.210593	0.111449	0.234004	lncRNA
ENSG0000(	ENSG0000(	12.58915	0.285592	0.1116	0.234289	lncRNA
ENSG0000(	LINC00940	10.47808	0.338889	0.111605	0.234289	lncRNA
ENSG0000(	GATM	3625.41	-0.402	0.111673	0.234418	protein_coding
ENSG0000(	ENSG0000(	38.2331	-0.17682	0.111706	0.234472	lncRNA
ENSG0000(	C19orf53	199.5872	-0.10832	0.111726	0.234498	protein_coding
ENSG0000(	SLC47A2	95.03669	-0.25139	0.111749	0.23453	protein_coding
ENSG0000(	CYB561A3	3020.821	-0.0882	0.111771	0.234545	protein_coding
ENSG0000(	RBKS	426.0263	-0.22979	0.111768	0.234545	protein_coding
ENSG0000(	MIR7515H(	21.12671	-0.23781	0.111792	0.234573	lncRNA
ENSG0000(	RABGGTA	281.5995	-0.10307	0.111815	0.234605	protein_coding
ENSG0000(	ENSG0000(	68.10064	-0.17752	0.111855	0.234672	lncRNA
ENSG0000(	ENSG0000(	234.6703	0.091884	0.111862	0.234672	lncRNA
ENSG0000(	ARX	36.05542	0.703726	0.111919	0.234776	protein_coding
ENSG0000(	COX20	1361.773	-0.06127	0.111936	0.234791	protein_coding
ENSG0000(	LINC00314	40.68387	0.239222	0.11194	0.234791	lncRNA
ENSG0000(	ITPRIPL1	272.8124	0.117255	0.111968	0.234834	protein_coding
ENSG0000(	ENSG0000(	13.10117	-0.38698	0.112041	0.234968	lncRNA
ENSG0000(	ZNF681	484.1439	-0.13498	0.112047	0.234968	protein_coding
ENSG0000(	HASPIN	87.1662	0.188381	0.112076	0.235013	protein_coding
ENSG0000(	TMEM52B	208.6759	-0.15852	0.112094	0.235034	protein_coding
ENSG0000(	ENSG0000(	11.99259	0.338003	0.112133	0.235094	unprocessed_pseudogene
ENSG0000(	ENSG0000(	18.63512	-0.36216	0.112143	0.235094	lncRNA
ENSG0000(	ENSG0000(	1033.301	0.244602	0.112152	0.235094	transcribed_unprocessed_pseudo
ENSG0000(	ENSG0000(	1681.489	-0.10723	0.112151	0.235094	lncRNA
ENSG0000(	ENSG0000(	32.16491	0.166307	0.112205	0.23519	lncRNA
ENSG0000(	URB1	1234.804	0.14902	0.112224	0.235214	protein_coding
ENSG0000(	GDNF	229.634	0.15149	0.112249	0.235251	protein_coding
ENSG0000(	SEPTIN7P1	1553.796	-0.09924	0.112273	0.235286	transcribed_unprocessed_pseudo
ENSG0000(	AXL	862.5934	0.114045	0.112372	0.235476	protein_coding
ENSG0000(	KHDC4	3012.222	-0.05543	0.11238	0.235477	protein_coding
ENSG0000(	RN7SKP275	15.51275	-0.409	0.112492	0.235697	misc_RNA
ENSG0000(	INTS9-AS1	118.7111	-0.13618	0.112516	0.235732	lncRNA
ENSG0000(	ENSG0000(	125.53	-0.23728	0.112539	0.235763	processed_pseudogene

ENSG0000(	MSANTD1	182.5541	-0.08331	0.112546	0.235763	protein_coding
ENSG0000(	ENSG0000(	15.23906	0.225207	0.112635	0.235778	lncRNA
ENSG0000(	ENSG0000(	204.7104	0.133331	0.112566	0.235778	lncRNA
ENSG0000(	ENSG0000(	104.3277	0.157925	0.112581	0.235778	lncRNA
ENSG0000(	FAM230C	103.6591	0.263169	0.112623	0.235778	lncRNA
ENSG0000(	HNRNPA1P	14.55713	-0.42034	0.112611	0.235778	processed_pseudogene
ENSG0000(	LINC01560	89.09537	0.135739	0.112594	0.235778	lncRNA
ENSG0000(	NEMF	2752.474	-0.05873	0.112616	0.235778	protein_coding
ENSG0000(	SETMAR	271.0897	0.117826	0.112632	0.235778	protein_coding
ENSG0000(	SLC30A2	141.5925	0.159425	0.112635	0.235778	protein_coding
ENSG0000(	TRAV8-4	10.88914	-0.55964	0.112586	0.235778	TR_V_gene
ENSG0000(	UBL7	176.0894	-0.15132	0.112606	0.235778	protein_coding
ENSG0000(	CCSER1	1737.296	-0.11036	0.112647	0.235786	protein_coding
ENSG0000(	ELF3-AS1	1102.835	0.115377	0.112678	0.235819	lncRNA
ENSG0000(	USP37	1412.684	-0.07082	0.112675	0.235819	protein_coding
ENSG0000(	ENSG0000(	43.90556	0.304229	0.112716	0.235883	protein_coding
ENSG0000(	RXRG	17.26932	-0.4245	0.112724	0.235884	protein_coding
ENSG0000(	RN7SL861P	19.66904	0.25536	0.11279	0.236007	misc_RNA
ENSG0000(	TP53TG5	11.98579	0.385544	0.1128	0.236012	protein_coding
ENSG0000(	ENSG0000(	112.6836	0.301516	0.112812	0.236023	lncRNA
ENSG0000(	RASSF5	1549.636	0.282346	0.112828	0.236039	protein_coding
ENSG0000(	SLC12A6	4189.48	-0.10104	0.11287	0.236111	protein_coding
ENSG0000(	OXA1L	1950.272	0.044477	0.112892	0.236142	protein_coding
ENSG0000(	GLT8D1	521.5456	-0.06711	0.112948	0.236228	protein_coding
ENSG0000(	RGS7BP	80.9305	-0.27101	0.112947	0.236228	protein_coding
ENSG0000(	ENSG0000(	22.43562	0.202059	0.113025	0.236343	lncRNA
ENSG0000(	LRIG3-DT	24.65075	0.312271	0.113025	0.236343	lncRNA
ENSG0000(	MDM1	392.4577	0.131864	0.113019	0.236343	protein_coding
ENSG0000(	SLC7A4	21.55594	-0.37567	0.113103	0.23649	protein_coding
ENSG0000(	CZIB-DT	640.7319	0.065632	0.113114	0.236497	lncRNA
ENSG0000(	ZFP42	363.8349	0.059882	0.11313	0.236514	protein_coding
ENSG0000(	IGFL2-AS1	161.1105	0.100077	0.113139	0.236517	lncRNA
ENSG0000(	NR3C2	4576.346	-0.26621	0.113166	0.236559	protein_coding
ENSG0000(	HMG20B	857.6734	0.083989	0.113185	0.236583	protein_coding
ENSG0000(	SKAP2	1662.038	-0.06222	0.11325	0.236702	protein_coding
ENSG0000(	ENSG0000(	1341.302	0.075544	0.113282	0.236754	transcribed_unprocessed_pseudo
ENSG0000(	RNU4-12P	20.57756	0.264692	0.113329	0.236837	snRNA
ENSG0000(	RLN1	11.2225	0.487111	0.113389	0.236946	protein_coding
ENSG0000(	ENSG0000(	110.4884	0.303336	0.113407	0.236968	transcribed_processed_pseudoge
ENSG0000(	ENSG0000(	25.64273	0.229847	0.113439	0.237002	lncRNA
ENSG0000(	SUMO1	738.3814	-0.12195	0.113432	0.237002	protein_coding
ENSG0000(	ENSG0000(	339.7299	0.086503	0.113457	0.237009	lncRNA
ENSG0000(	ENSG0000(	84.7889	-0.13837	0.113456	0.237009	lncRNA
ENSG0000(	CALHM1	65.43743	0.13978	0.113493	0.237054	protein_coding
ENSG0000(	ENSG0000(	192.9064	-0.10244	0.113492	0.237054	lncRNA
ENSG0000(	ENSG0000(	163.2031	0.20963	0.113559	0.237129	protein_coding
ENSG0000(	HMBS	206.3736	0.146409	0.113543	0.237129	protein_coding

ENSG0000(	L3MBTL4	667.4804	0.112376	0.113555	0.237129	protein_coding
ENSG0000(	NPTX1	68.76143	-0.33606	0.113555	0.237129	protein_coding
ENSG0000(	GSTK1	3002.677	-0.07969	0.113629	0.237258	protein_coding
ENSG0000(	ENSG0000(	88.66429	-0.1217	0.113642	0.237269	lncRNA
ENSG0000(	CD63-AS1	68.05563	0.124952	0.113702	0.237295	lncRNA
ENSG0000(	ENSG0000(	10.24256	0.323213	0.113705	0.237295	lncRNA
ENSG0000(	ENSG0000(	17.56846	0.251886	0.113695	0.237295	lncRNA
ENSG0000(	GALNT16	636.9333	-0.11633	0.113666	0.237295	protein_coding
ENSG0000(	KCTD15	378.7241	0.166874	0.113679	0.237295	protein_coding
ENSG0000(	LINC02623	9.611349	-0.41752	0.113704	0.237295	lncRNA
ENSG0000(	PEG3	130.0928	-0.3108	0.113707	0.237295	protein_coding
ENSG0000(	ZNF300P1	170.0019	-0.29113	0.113774	0.237419	transcribed_unprocessed_pseudo
ENSG0000(	PABPC1P1:	53.01897	0.130536	0.113785	0.237428	processed_pseudogene
ENSG0000(	CHORDC1P	60.24505	0.122844	0.113823	0.23749	processed_pseudogene
ENSG0000(	SLC9A3R1-/	27.80523	-0.42423	0.113845	0.23752	lncRNA
ENSG0000(	ENSG0000(	29.24987	0.34976	0.113884	0.237587	lncRNA
ENSG0000(	NAT1	235.4981	-0.18954	0.113911	0.237628	protein_coding
ENSG0000(	IGKV1OR2-	10.49928	0.401672	0.113922	0.237634	IG_V_gene
ENSG0000(	LRP12	649.2032	0.104824	0.113939	0.237654	protein_coding
ENSG0000(	TRAF3IP1	566.7483	-0.06988	0.113949	0.23766	protein_coding
ENSG0000(	MYBPC1	19.40894	0.566359	0.114042	0.237838	protein_coding
ENSG0000(	APOE	456.3535	0.227088	0.114077	0.237895	protein_coding
ENSG0000(	ITGAV	2012.42	0.1156	0.114124	0.237976	protein_coding
ENSG0000(	ENSG0000(	36.44974	0.178505	0.114147	0.238006	lncRNA
ENSG0000(	RNF152	2297.7	0.29918	0.114153	0.238006	protein_coding
ENSG0000(	ENSG0000(	110.7169	-0.25694	0.114196	0.238079	processed_pseudogene
ENSG0000(	ENSG0000(	22.7206	0.239226	0.114314	0.23831	lncRNA
ENSG0000(	GOT2P7	42.16625	0.191259	0.114367	0.238404	processed_pseudogene
ENSG0000(	FAM76B	650.4598	-0.0846	0.114404	0.238466	protein_coding
ENSG0000(	MIR5190	22.66549	-0.22894	0.114414	0.238471	miRNA
ENSG0000(	RGS6	126.4406	0.193569	0.114431	0.238491	protein_coding
ENSG0000(	ENSG0000(	222.2422	0.101982	0.114498	0.238615	lncRNA
ENSG0000(	CBWD3	1816.352	0.105954	0.114517	0.238639	protein_coding
ENSG0000(	AARS1	2056.179	0.069526	0.11454	0.238669	protein_coding
ENSG0000(	PSMB6	353.7004	-0.08555	0.114568	0.238714	protein_coding
ENSG0000(	NOMO1	963.6104	-0.15766	0.114587	0.238737	protein_coding
ENSG0000(	GUSBP5	24.51763	0.372833	0.114639	0.23883	transcribed_processed_pseudoge
ENSG0000(	ENSG0000(	191.7234	-0.56457	0.114681	0.238868	unprocessed_pseudogene
ENSG0000(	EXTL3	1447.741	-0.0811	0.114688	0.238868	protein_coding
ENSG0000(	NRG4	181.9248	0.188937	0.114674	0.238868	protein_coding
ENSG0000(	ZNF676	384.1671	-0.07429	0.114677	0.238868	protein_coding
ENSG0000(	ATRNL1	139.3193	-0.17484	0.114706	0.238871	protein_coding
ENSG0000(	ENSG0000(	789.3403	0.077902	0.114697	0.238871	lncRNA
ENSG0000(	PDSS2	583.4765	-0.10565	0.114712	0.238871	protein_coding
ENSG0000(	PARK7P1	58.84568	0.178845	0.114741	0.238914	processed_pseudogene
ENSG0000(	ENSG0000(	438.6985	-0.09243	0.11478	0.238941	lncRNA
ENSG0000(	IRX2	17.59884	0.241728	0.114772	0.238941	protein_coding

ENSG0000(	POLR3DP1	26.18143	0.300275	0.114783	0.238941	processed_pseudogene
ENSG0000(	SEPTIN7	5018.974	-0.08085	0.114767	0.238941	protein_coding
ENSG0000(	ENSG0000(	41.04899	0.197807	0.114868	0.239084	unprocessed_pseudogene
ENSG0000(	HECW2-AS	39.79393	-0.28024	0.11489	0.239084	lncRNA
ENSG0000(	MTRNR2L8	67.86396	-0.85484	0.114889	0.239084	protein_coding
ENSG0000(	RTBDN	167.3936	-0.10304	0.11487	0.239084	protein_coding
ENSG0000(	TNFSF15	1288.43	-0.20875	0.114876	0.239084	protein_coding
ENSG0000(	TRIM44	3999.964	-0.06431	0.114905	0.239099	protein_coding
ENSG0000(	ENSG0000(	520.4058	0.076785	0.11494	0.239157	lncRNA
ENSG0000(	CABIN1	2762.946	0.100903	0.115041	0.23935	protein_coding
ENSG0000(	ENSG0000(	48.07433	-0.18846	0.115057	0.239352	lncRNA
ENSG0000(	FAM135A	3348.809	-0.12779	0.115051	0.239352	protein_coding
ENSG0000(	GOLGA2P8	18.17314	0.318854	0.115088	0.239394	transcribed_unprocessed_pseudo
ENSG0000(	RBM19	1679.037	0.117977	0.115092	0.239394	protein_coding
ENSG0000(	DNASE1L1	412.6785	-0.07093	0.115149	0.239497	protein_coding
ENSG0000(	CCDC82	3152.178	0.077943	0.11519	0.239566	protein_coding
ENSG0000(	ENSG0000(	9.329184	0.314671	0.115206	0.239583	transcribed_unprocessed_pseudo
ENSG0000(	ENSG0000(	9.86548	0.299318	0.115234	0.239603	lncRNA
ENSG0000(	HSCB	384.9154	-0.06874	0.115232	0.239603	protein_coding
ENSG0000(	PLSCR4	475.1936	0.154414	0.115238	0.239603	protein_coding
ENSG0000(	NKG7	68.14343	-0.25827	0.115278	0.23967	protein_coding
ENSG0000(	ECE1-AS1	42.81146	0.367627	0.11529	0.239678	lncRNA
ENSG0000(	ASMTL	274.3818	-0.12248	0.115308	0.239681	protein_coding
ENSG0000(	ENSG0000(	35.49939	0.194937	0.115314	0.239681	TEC
ENSG0000(	NECTIN2	860.2336	0.180462	0.115306	0.239681	protein_coding
ENSG0000(	LINC02484	57.19105	-0.24691	0.115386	0.239816	lncRNA
ENSG0000(	ADGRE4P	244.0505	0.163228	0.115415	0.239844	transcribed_unprocessed_pseudo
ENSG0000(	RIN1	266.1684	0.105182	0.115416	0.239844	protein_coding
ENSG0000(	SERP2	42.54065	-0.21111	0.11544	0.239879	protein_coding
ENSG0000(	RPSAP9	37.4972	-0.35714	0.115521	0.240031	processed_pseudogene
ENSG0000(	ENSG0000(	8.34313	0.324335	0.115589	0.240104	lncRNA
ENSG0000(	ENSG0000(	28.04905	0.20905	0.115566	0.240104	lncRNA
ENSG0000(	NEK3	866.9866	0.204521	0.115581	0.240104	protein_coding
ENSG0000(	RN7SL390F	26.49292	0.241243	0.115602	0.240104	misc_RNA
ENSG0000(	SLIT2	448.6887	-0.21818	0.115597	0.240104	protein_coding
ENSG0000(	THBS4	21.47312	0.334685	0.115584	0.240104	protein_coding
ENSG0000(	ENSG0000(	27.34453	-0.25286	0.115628	0.240128	lncRNA
ENSG0000(	HCN3	470.0574	-0.22297	0.115628	0.240128	protein_coding
ENSG0000(	ENSG0000(	170.734	-0.09522	0.115735	0.240333	lncRNA
ENSG0000(	ENSG0000(	193.9547	0.098819	0.115762	0.240373	lncRNA
ENSG0000(	RN7SL789F	30.71819	0.170013	0.115769	0.240373	misc_RNA
ENSG0000(	S1PR4	156.5762	0.368297	0.115777	0.240373	protein_coding
ENSG0000(	RNF216P1	1265.433	0.091657	0.115806	0.240418	lncRNA
ENSG0000(	TM9SF1	493.0901	0.069264	0.11582	0.240432	protein_coding
ENSG0000(	RNY3P11	10.88622	0.348254	0.115861	0.240501	misc_RNA
ENSG0000(	OSR1	99.12965	-0.26098	0.11587	0.240503	protein_coding
ENSG0000(	BLACE	17.71218	-0.20068	0.115984	0.240707	lncRNA

ENSG0000(	ENSG0000(	72.47411	0.145326	0.115981	0.240707	TEC
ENSG0000(	GLRA3	164.651	-0.23832	0.116003	0.240733	protein_coding
ENSG0000(	ENSG0000(	10.36756	0.327204	0.116061	0.240837	lncRNA
ENSG0000(	PDXDC2P	758.1031	-0.16792	0.116077	0.240854	transcribed_unprocessed_pseudo
ENSG0000(	ENSG0000(	69.21676	-0.21009	0.116127	0.240942	processed_pseudogene
ENSG0000(	OR51M1	299.9701	0.091598	0.116166	0.241007	protein_coding
ENSG0000(	YBX1P4	9.624995	-0.29406	0.116241	0.241145	processed_pseudogene
ENSG0000(	ENSG0000(	8.522124	0.373386	0.116275	0.241184	lncRNA
ENSG0000(	SNORD46	37.60974	0.227898	0.116268	0.241184	snoRNA
ENSG0000(	ENSG0000(	29.83164	-0.21418	0.116294	0.241208	lncRNA
ENSG0000(	OR2S1P	140.4494	0.118009	0.116319	0.241245	transcribed_unprocessed_pseudo
ENSG0000(	MAGI1-IT1	680.6469	-0.17238	0.116332	0.241256	lncRNA
ENSG0000(	PHKA2-AS1	34.68828	-0.24953	0.116355	0.241288	lncRNA
ENSG0000(	ENSG0000(	56.63359	0.174056	0.11638	0.241324	lncRNA
ENSG0000(	THRB-AS1	19.06988	0.287611	0.116401	0.241351	lncRNA
ENSG0000(	ENSG0000(	68.71797	-0.31516	0.116418	0.241358	unprocessed_pseudogene
ENSG0000(	ENSG0000(	11.28	-0.36777	0.116435	0.241358	processed_pseudogene
ENSG0000(	ENSG0000(	57.44442	0.143424	0.116424	0.241358	lncRNA
ENSG0000(	SCARF2	104.8789	0.154349	0.11643	0.241358	protein_coding
ENSG0000(	LINC01873	1687.534	0.098179	0.116495	0.241467	lncRNA
ENSG0000(	ENSG0000(	51.56345	0.428523	0.116519	0.2415	TEC
ENSG0000(	GAS8	329.1237	0.140026	0.116572	0.241595	protein_coding
ENSG0000(	CCNJ	331.7882	0.089617	0.116631	0.241688	protein_coding
ENSG0000(	SLC35D2	1967.548	-0.1853	0.116632	0.241688	protein_coding
ENSG0000(	CSRP3	32.64705	0.140447	0.116705	0.241821	protein_coding
ENSG0000(	ENSG0000(	416.7988	0.082141	0.116832	0.241999	lncRNA
ENSG0000(	MEI1	665.3854	0.127884	0.116836	0.241999	protein_coding
ENSG0000(	NRIP2	89.55161	0.2993	0.11682	0.241999	protein_coding
ENSG0000(	REM1	44.08286	0.215821	0.116833	0.241999	protein_coding
ENSG0000(	TJP1	3109.859	-0.16952	0.116815	0.241999	protein_coding
ENSG0000(	TRBV7-2	15.25994	-0.41538	0.116835	0.241999	TR_V_gene
ENSG0000(	CDK18	1121.114	-0.15669	0.116852	0.242011	protein_coding
ENSG0000(	SHFL	1348.633	0.082688	0.116858	0.242011	protein_coding
ENSG0000(	ENSG0000(	316.7855	-0.13216	0.116895	0.242074	lncRNA
ENSG0000(	RP2	434.3017	-0.09379	0.116949	0.242169	protein_coding
ENSG0000(	WWTR1-AS	100.2575	0.10709	0.116968	0.242193	lncRNA
ENSG0000(	POT1-AS1	1178.735	0.062927	0.117021	0.242285	lncRNA
ENSG0000(	ENSG0000(	93.17934	-0.36628	0.117048	0.242326	lncRNA
ENSG0000(	CYP2F1	20.37365	-0.2154	0.117228	0.242669	protein_coding
ENSG0000(	ENSG0000(	24.58682	0.224488	0.117237	0.242669	lncRNA
ENSG0000(	ENSG0000(	47.78887	0.337156	0.117231	0.242669	lncRNA
ENSG0000(	ENSG0000(	41.41247	0.163577	0.117264	0.242709	lncRNA
ENSG0000(	FXYD3	931.8849	-0.35647	0.117275	0.242717	protein_coding
ENSG0000(	FBXW2	3927.98	0.050881	0.117316	0.242784	protein_coding
ENSG0000(	ENOX2	307.5959	-0.12903	0.117353	0.242812	protein_coding
ENSG0000(	ENSG0000(	19.10126	0.284102	0.117359	0.242812	processed_pseudogene
ENSG0000(	IER5L	71.72834	0.17381	0.11736	0.242812	protein_coding

ENSG0000(	TIPIN	320.896	-0.09241	0.117346	0.242812	protein_coding
ENSG0000(	ENSG0000(	65.82027	0.13832	0.117372	0.242822	lncRNA
ENSG0000(	GZMA	80.31569	-0.29792	0.117401	0.242859	protein_coding
ENSG0000(	MTCO1P40	127.3695	-0.77698	0.117409	0.242859	processed_pseudogene
ENSG0000(	PCDHGA7	70.94013	-0.16998	0.117413	0.242859	protein_coding
ENSG0000(	BEND3P1	19.50905	0.260918	0.117455	0.242886	transcribed_processed_pseudoge
ENSG0000(	ENSG0000(	2033.464	-0.03964	0.117465	0.242886	lncRNA
ENSG0000(	ENSG0000(	11.94256	0.350139	0.117447	0.242886	lncRNA
ENSG0000(	RNU6-396F	11.2289	-0.2854	0.117461	0.242886	snRNA
ENSG0000(	SEPTIN4	645.6648	0.143838	0.117451	0.242886	protein_coding
ENSG0000(	TSEN34	250.6621	0.098046	0.117486	0.242913	protein_coding
ENSG0000(	ENSG0000(	53.43201	0.133186	0.117544	0.243017	lncRNA
ENSG0000(	LINC01836	398.5826	-0.07986	0.117564	0.243042	lncRNA
ENSG0000(	NAP1L5	31.2744	0.206228	0.117585	0.243069	protein_coding
ENSG0000(	PP2672	84.38713	-0.11664	0.117598	0.243081	lncRNA
ENSG0000(	LINC02042	263.5214	0.071223	0.117656	0.243186	lncRNA
ENSG0000(	CALCB	37.46924	0.179534	0.11772	0.243301	protein_coding
ENSG0000(	GON7	113.5896	-0.11892	0.117765	0.243378	protein_coding
ENSG0000(	SNAPC2	62.2906	-0.1753	0.117773	0.243378	protein_coding
ENSG0000(	LINC01106	1409.745	-0.27766	0.117793	0.243389	lncRNA
ENSG0000(	SIGLEC11	221.2666	0.099454	0.117786	0.243389	protein_coding
ENSG0000(	RXYLT1	836.7832	0.083226	0.117814	0.243415	protein_coding
ENSG0000(	ENSG0000(	330.3736	0.075198	0.117846	0.243465	lncRNA
ENSG0000(	LYPD5	549.0209	0.075262	0.117854	0.243466	protein_coding
ENSG0000(	RNU2-11P	12.03296	-0.31209	0.117861	0.243466	snRNA
ENSG0000(	ENSG0000(	52.68891	0.171778	0.117902	0.243505	lncRNA
ENSG0000(	ENSG0000(	8.470863	0.406481	0.117889	0.243505	lncRNA
ENSG0000(	TCAF1	1434.352	0.12256	0.117904	0.243505	protein_coding
ENSG0000(	CLEC18C	51.36874	-0.34063	0.117923	0.243524	protein_coding
ENSG0000(	UNG	278.8712	-0.12482	0.117928	0.243524	protein_coding
ENSG0000(	F11	110.438	-0.34613	0.117945	0.243544	protein_coding
ENSG0000(	CTH	149.0612	-0.15368	0.117983	0.243605	protein_coding
ENSG0000(	LINC01694	1050.437	0.125946	0.117997	0.243618	lncRNA
ENSG0000(	KLRC3	66.94741	-0.26127	0.118091	0.243795	protein_coding
ENSG0000(	PHF5CP	29.90699	0.259924	0.118107	0.243798	processed_pseudogene
ENSG0000(	ZNF767P	1544.331	0.069791	0.118103	0.243798	transcribed_unprocessed_pseudo
ENSG0000(	PRELID1P1	9.08162	-0.41071	0.118126	0.24381	transcribed_processed_pseudoge
ENSG0000(	TCF3	1377.812	-0.12341	0.118136	0.24381	protein_coding
ENSG0000(	ZNF577	1941.265	0.063155	0.118129	0.24381	protein_coding
ENSG0000(	ATP10A	1068.403	0.174163	0.118235	0.243982	protein_coding
ENSG0000(	ESYT1	1181.237	-0.20812	0.118235	0.243982	protein_coding
ENSG0000(	FBXO40	73.69157	0.113732	0.118278	0.244054	protein_coding
ENSG0000(	LINC01229	53.24055	0.231066	0.118317	0.244103	lncRNA
ENSG0000(	TOMM34	146.5461	-0.11938	0.118312	0.244103	protein_coding
ENSG0000(	TRIM63	15.48585	0.309504	0.118325	0.244104	protein_coding
ENSG0000(	ENSG0000(	45.10177	0.15545	0.118338	0.244114	lncRNA
ENSG0000(	IFIT5	684.9773	0.145405	0.11836	0.244143	protein_coding

ENSG0000(	H4C11	122.4871	-0.40571	0.118376	0.244162	protein_coding
ENSG0000(	CNBD2	227.0782	-0.10276	0.118405	0.244205	protein_coding
ENSG0000(	NSDHL	111.6369	-0.15959	0.118437	0.24425	protein_coding
ENSG0000(	SMPD1	388.8917	-0.17646	0.118447	0.24425	protein_coding
ENSG0000(	USP12P1	18.99932	0.205833	0.11845	0.24425	processed_pseudogene
ENSG0000(	ENSG0000(	36.5431	0.201464	0.118464	0.244262	lncRNA
ENSG0000(	GLYATL1	37.04726	0.228277	0.118575	0.244459	protein_coding
ENSG0000(	MIR6129	8.615104	0.365047	0.118575	0.244459	miRNA
ENSG0000(	ENSG0000(	301.2379	0.188867	0.118595	0.244474	lncRNA
ENSG0000(	LRRC37A11	90.47015	0.185764	0.118598	0.244474	transcribed_unprocessed_pseudo
ENSG0000(	RBM45	482.5945	-0.08329	0.118644	0.244554	protein_coding
ENSG0000(	B9D2	47.6725	-0.15662	0.11867	0.244559	protein_coding
ENSG0000(	ENSG0000(	1140.936	0.088498	0.11867	0.244559	lncRNA
ENSG0000(	JAK3	3325.715	0.325575	0.118668	0.244559	protein_coding
ENSG0000(	ABHD11	554.3704	-0.11671	0.118707	0.24462	protein_coding
ENSG0000(	ENSG0000(	43.71574	0.895557	0.118747	0.244686	lncRNA
ENSG0000(	GTF2E1	360.1863	-0.09601	0.11878	0.244737	protein_coding
ENSG0000(	ENSG0000(	38.81958	0.196797	0.118825	0.244797	lncRNA
ENSG0000(	SNORA44	111.1595	0.215049	0.118822	0.244797	snoRNA
ENSG0000(	SHLD1	197.1173	-0.11109	0.11884	0.244813	protein_coding
ENSG0000(	ENSG0000(	1163.023	0.059622	0.11889	0.244884	lncRNA
ENSG0000(	FAM66C	480.4371	0.112497	0.118886	0.244884	lncRNA
ENSG0000(	DMBT1L1	103.9102	-0.09304	0.119007	0.24511	transcribed_unitary_pseudogene
ENSG0000(	ANKRD17-[	41.2157	0.21745	0.119078	0.24524	lncRNA
ENSG0000(	MEGF6	725.8324	-0.19957	0.119102	0.245273	protein_coding
ENSG0000(	ENSG0000(	115.9218	-0.14814	0.119126	0.245307	lncRNA
ENSG0000(	ENSG0000(	150.2367	-0.11427	0.119151	0.245341	lncRNA
ENSG0000(	POU5F1P5	92.70359	-0.1291	0.119159	0.245342	processed_pseudogene
ENSG0000(	ENSG0000(	103.6653	0.172052	0.119187	0.245384	lncRNA
ENSG0000(	TRAJ12	37.29751	-0.36776	0.119244	0.245486	TR_J_gene
ENSG0000(	ENSG0000(	353.6593	-0.24224	0.119273	0.245511	lncRNA
ENSG0000(	FAM110D	216.6851	0.119075	0.119267	0.245511	protein_coding
ENSG0000(	MARS2	230.1205	0.11549	0.11928	0.245511	protein_coding
ENSG0000(	MIR3680-1	15.72112	0.327633	0.119311	0.24556	miRNA
ENSG0000(	LINC00235	162.4362	0.119063	0.119406	0.245738	lncRNA
ENSG0000(	RCN3	799.7308	-0.22661	0.119515	0.245946	protein_coding
ENSG0000(	ADAM3A	53.44364	0.175792	0.119539	0.24598	transcribed_unitary_pseudogene
ENSG0000(	ENSG0000(	45.87587	-0.18871	0.119562	0.246011	processed_pseudogene
ENSG0000(	ENSG0000(	397.896	0.086918	0.1196	0.246074	lncRNA
ENSG0000(	ENSG0000(	121.4181	0.104177	0.119629	0.246101	processed_pseudogene
ENSG0000(	RAB3C	194.0743	0.29197	0.119622	0.246101	protein_coding
ENSG0000(	HNRNPA2E	15786.95	0.053264	0.119728	0.246288	protein_coding
ENSG0000(	ENSG0000(	136.2449	-0.12718	0.119739	0.246295	lncRNA
ENSG0000(	ENSG0000(	249.346	0.105482	0.119757	0.246307	lncRNA
ENSG0000(	ENSG0000(	246.3232	0.085481	0.11976	0.246307	lncRNA
ENSG0000(	ENSG0000(	15.01007	0.269829	0.119786	0.24633	lncRNA
ENSG0000(	MC5R	16.47018	0.216534	0.119787	0.24633	protein_coding

ENSG0000(	PPM1K	3420.823	0.200244	0.11985	0.246443	protein_coding
ENSG0000(	IGLV1-36	11.74556	0.676207	0.119876	0.246479	IG_V_gene
ENSG0000(	SFR1	190.2169	-0.1004	0.119944	0.246603	protein_coding
ENSG0000(	LINC01908	15.16077	0.258789	0.119962	0.246608	lncRNA
ENSG0000(	MYL6	3445.639	-0.1351	0.119958	0.246608	protein_coding
ENSG0000(	RPSAP70	13.14029	0.317991	0.11999	0.246649	processed_pseudogene
ENSG0000(	PPIAP11	10.32912	0.331984	0.120021	0.246697	processed_pseudogene
ENSG0000(	LINC01716	32.44989	0.21484	0.120029	0.246698	lncRNA
ENSG0000(	H3C11	109.2136	-0.21897	0.120042	0.246709	protein_coding
ENSG0000(	LENG8	13161	0.066737	0.120058	0.246725	protein_coding
ENSG0000(	LPP	18540.24	0.084355	0.120067	0.246728	protein_coding
ENSG0000(	SCARNA8	36.37149	0.187002	0.120142	0.24685	scaRNA
ENSG0000(	ZNF793-AS	17.32126	-0.22723	0.120136	0.24685	lncRNA
ENSG0000(	MTERF2	535.8892	0.112423	0.120151	0.246852	protein_coding
ENSG0000(	ENSG0000(	12.95191	0.293266	0.120222	0.246982	lncRNA
ENSG0000(	MEIG1	33.91769	-0.26146	0.120258	0.247039	protein_coding
ENSG0000(	NIPAL4	18.76035	0.429973	0.120292	0.247094	protein_coding
ENSG0000(	CPNE3	1520.782	-0.11804	0.120332	0.247159	protein_coding
ENSG0000(	GPR18	165.1339	-0.35102	0.12035	0.247163	protein_coding
ENSG0000(	PCSK1	259.588	0.315922	0.120343	0.247163	protein_coding
ENSG0000(	CMAS	481.8419	-0.1882	0.120384	0.247218	protein_coding
ENSG0000(	STK10	2590.309	0.130197	0.120393	0.24722	protein_coding
ENSG0000(	ENSG0000(	51.05593	0.145874	0.120455	0.247331	lncRNA
ENSG0000(	ENSG0000(	16.79841	0.252739	0.120468	0.247342	lncRNA
ENSG0000(	ENSG0000(	11.65996	0.278967	0.120482	0.247354	lncRNA
ENSG0000(	ACD	125.2151	-0.15501	0.120517	0.247379	protein_coding
ENSG0000(	MRPL41	157.4202	-0.1325	0.120511	0.247379	protein_coding
ENSG0000(	PIP5KL1	113.7637	0.095474	0.120509	0.247379	protein_coding
ENSG0000(	BCKDK	337.3151	-0.11601	0.12053	0.247388	protein_coding
ENSG0000(	OSBPL1A	902.5681	0.070175	0.120545	0.247403	protein_coding
ENSG0000(	ENSG0000(	34.31812	0.189896	0.120566	0.247412	lncRNA
ENSG0000(	ENSG0000(	11.76404	0.307709	0.120571	0.247412	lncRNA
ENSG0000(	PGM1	601.3729	-0.1244	0.120573	0.247412	protein_coding
ENSG0000(	PSMA1	792.3596	0.092669	0.120593	0.247437	protein_coding
ENSG0000(	ENSG0000(	180.7395	0.113701	0.120676	0.247578	lncRNA
ENSG0000(	RNU6ATAC	41.86114	0.237644	0.120677	0.247578	snRNA
ENSG0000(	ENSG0000(	67.6684	0.284511	0.120708	0.247613	lncRNA
ENSG0000(	KBTBD2	1769.17	0.072858	0.12071	0.247613	protein_coding
ENSG0000(	ENSG0000(	203.6672	0.069718	0.120757	0.247694	lncRNA
ENSG0000(	ENSG0000(	560.8887	0.071515	0.120787	0.24771	lncRNA
ENSG0000(	ENSG0000(	45.25963	0.260429	0.120805	0.24771	TEC
ENSG0000(	KRBA2	2724.535	0.03956	0.120798	0.24771	protein_coding
ENSG0000(	LINC01266	275.2238	0.103059	0.120793	0.24771	lncRNA
ENSG0000(	RN7SKP228	71.80997	-0.26287	0.120786	0.24771	misc_RNA
ENSG0000(	CCNT1	1695.581	-0.07293	0.120892	0.247842	protein_coding
ENSG0000(	CDC14B	1168.478	-0.14757	0.120891	0.247842	protein_coding
ENSG0000(	LINC01567	207.0963	-0.09988	0.120892	0.247842	lncRNA

ENSG0000(	CALML4	1102.084	-0.25068	0.120976	0.247933	protein_coding
ENSG0000(	ENSG0000(	10.18908	0.334245	0.12097	0.247933	lncRNA
ENSG0000(	ENSG0000(	302.0639	0.257152	0.120968	0.247933	protein_coding
ENSG0000(	HNRNPCP6	12.35284	-0.33085	0.120945	0.247933	processed_pseudogene
ENSG0000(	YAF2	1926.429	0.058684	0.120964	0.247933	protein_coding
ENSG0000(	ENSG0000(	10.247	-0.52194	0.121007	0.247981	unprocessed_pseudogene
ENSG0000(	GJA3	8.875232	0.507358	0.121019	0.247988	protein_coding
ENSG0000(	LINC00539	128.943	0.153639	0.121085	0.248108	lncRNA
ENSG0000(	ENSG0000(	37.61543	-0.18569	0.121134	0.248192	lncRNA
ENSG0000(	ATP6V1G1I	10.76115	0.311476	0.121156	0.248206	processed_pseudogene
ENSG0000(	CYP3A5	21945.25	-0.34535	0.121156	0.248206	protein_coding
ENSG0000(	ENSG0000(	143.5374	0.110771	0.121214	0.248274	lncRNA
ENSG0000(	LINC00920	37.25572	0.229558	0.121203	0.248274	lncRNA
ENSG0000(	PHF5A	254.2387	-0.08163	0.121211	0.248274	protein_coding
ENSG0000(	GNB3	65.78482	0.259551	0.121254	0.248341	protein_coding
ENSG0000(	ENSG0000(	302.6071	-0.11228	0.121322	0.248449	lncRNA
ENSG0000(	STK35	1176.17	-0.06573	0.121315	0.248449	protein_coding
ENSG0000(	ENSG0000(	75.33379	0.235508	0.121341	0.248471	lncRNA
ENSG0000(	MIR3667H(	952.8808	0.256959	0.121363	0.248499	lncRNA
ENSG0000(	ENSG0000(	102.6722	-0.1561	0.121404	0.248567	lncRNA
ENSG0000(	ENSG0000(	275.7645	-0.07867	0.121414	0.248573	lncRNA
ENSG0000(	ANKRD45	322.3877	-0.09881	0.121423	0.248574	protein_coding
ENSG0000(	ASAP3	594.2294	0.089439	0.121431	0.248574	protein_coding
ENSG0000(	TMEM139	531.7142	-0.29377	0.121496	0.248691	protein_coding
ENSG0000(	LINC00837	33.47742	0.196788	0.121564	0.248815	lncRNA
ENSG0000(	LYPLA1	917.7867	-0.05827	0.121601	0.248857	protein_coding
ENSG0000(	NLRP3P1	12.12534	0.452018	0.121599	0.248857	unprocessed_pseudogene
ENSG0000(	ENSG0000(	23.32999	-0.20568	0.121627	0.248894	lncRNA
ENSG0000(	PDE4DIPP7	66.27981	0.188847	0.121651	0.248928	transcribed_unprocessed_pseudo
ENSG0000(	CNTN6	12.78597	0.308605	0.121709	0.249031	protein_coding
ENSG0000(	ENSG0000(	7.973405	-0.41156	0.121725	0.249046	TEC
ENSG0000(	PXN	3279.528	0.144448	0.121779	0.24914	protein_coding
ENSG0000(	TMEM63C	114.1418	-0.35208	0.12187	0.249311	protein_coding
ENSG0000(	AKT2	3971.025	-0.05215	0.121932	0.249373	protein_coding
ENSG0000(	EEF1A1P14	20.73622	0.303302	0.121912	0.249373	processed_pseudogene
ENSG0000(	ENSG0000(	80.46099	0.26107	0.121923	0.249373	protein_coding
ENSG0000(	GTSF1	91.6428	0.146892	0.121925	0.249373	protein_coding
ENSG0000(	ENSG0000(	9.878008	-0.26512	0.12207	0.24964	lncRNA
ENSG0000(	DNAAF1	576.5298	0.063865	0.12214	0.24975	protein_coding
ENSG0000(	ENSG0000(	14.31008	-0.54835	0.12214	0.24975	protein_coding
ENSG0000(	BTK	684.7387	0.337829	0.122157	0.249769	protein_coding
ENSG0000(	ENSG0000(	17.15038	0.192009	0.12217	0.24978	lncRNA
ENSG0000(	PIEZO2	1432.48	0.071236	0.122255	0.249937	protein_coding
ENSG0000(	ACAA2	1576.6	-0.18761	0.12228	0.249957	protein_coding
ENSG0000(	FIP1L1	955.6834	-0.05359	0.122281	0.249957	protein_coding
ENSG0000(	PNPLA8	664.4086	0.129706	0.122308	0.249996	protein_coding
ENSG0000(	SPAG9	4985.604	-0.09622	0.122317	0.249999	protein_coding

ENSG0000(	DGAT2-DT	15.7961	0.223742	0.122337	0.250007	lncRNA
ENSG0000(	OR2T12	1359.455	-0.08769	0.122332	0.250007	protein_coding
ENSG0000(	ENSG0000(	124.0303	0.324689	0.122374	0.250067	lncRNA
ENSG0000(	H3P3	8.686506	0.328083	0.122394	0.25009	processed_pseudogene
ENSG0000(	PLXNB2	5140.297	-0.16126	0.122403	0.250093	protein_coding
ENSG0000(	ENSG0000(	275.153	0.137217	0.122515	0.250305	lncRNA
ENSG0000(	ENSG0000(	131.3688	0.267637	0.122628	0.250519	protein_coding
ENSG0000(	ZFYVE28	1027.746	-0.19412	0.122641	0.250531	protein_coding
ENSG0000(	SLC9A1	1177.423	-0.13344	0.122677	0.250587	protein_coding
ENSG0000(	ABCC4	1196.005	0.109948	0.122689	0.250597	protein_coding
ENSG0000(	TRBC1	494.2872	-0.30391	0.12271	0.250622	TR_C_gene
ENSG0000(	ENSG0000(	9.913793	-0.56551	0.122743	0.250673	unprocessed_pseudogene
ENSG0000(	EMCN	473.3704	0.099056	0.122763	0.250698	protein_coding
ENSG0000(	HTR2A	106.9073	0.184723	0.122771	0.250698	protein_coding
ENSG0000(	OGFOD3	740.3478	-0.07389	0.122789	0.250719	protein_coding
ENSG0000(	APOLD1	509.3063	0.191473	0.122813	0.250749	protein_coding
ENSG0000(	ENSG0000(	11.3135	0.305444	0.12282	0.250749	lncRNA
ENSG0000(	CAPN14	48.31925	0.292237	0.122829	0.250751	protein_coding
ENSG0000(	ZNF233	217.9848	0.092052	0.122862	0.250802	protein_coding
ENSG0000(	LCMT1	323.8061	-0.12532	0.122909	0.250866	protein_coding
ENSG0000(	LINC01144	53.44426	0.162143	0.122907	0.250866	lncRNA
ENSG0000(	ENSG0000(	19.58721	-0.31292	0.123001	0.251021	processed_pseudogene
ENSG0000(	ENSG0000(	18.11968	0.308661	0.122994	0.251021	lncRNA
ENSG0000(	TRAJ2	19.63777	-0.36815	0.12304	0.251085	TR_J_gene
ENSG0000(	ENSG0000(	34.82066	-0.41153	0.123103	0.251181	protein_coding
ENSG0000(	KIF2C	143.8885	0.247679	0.123101	0.251181	protein_coding
ENSG0000(	ENSG0000(	106.1102	-0.18143	0.123119	0.251192	unprocessed_pseudogene
ENSG0000(	MAGOH2P	65.57669	-0.29598	0.123124	0.251192	transcribed_processed_pseudoge
ENSG0000(	KCTD1	292.4597	0.089954	0.1232	0.25133	protein_coding
ENSG0000(	ENSG0000(	27.87772	0.208067	0.123231	0.251377	lncRNA
ENSG0000(	DUX4L19	13.35857	0.604998	0.123287	0.251475	unprocessed_pseudogene
ENSG0000(	TRIM5	1015.729	0.098441	0.123311	0.251508	protein_coding
ENSG0000(	ENSG0000(	25.75427	0.190875	0.123362	0.251596	lncRNA
ENSG0000(	NETO1-DT	23.28628	0.474181	0.123432	0.25171	lncRNA
ENSG0000(	YPEL5	1265.371	-0.07589	0.123434	0.25171	protein_coding
ENSG0000(	GABRD	473.1884	0.071715	0.123462	0.251749	protein_coding
ENSG0000(	TAOK3	3083.637	-0.08754	0.123536	0.251885	protein_coding
ENSG0000(	ENSG0000(	109.7653	0.098165	0.123555	0.251907	lncRNA
ENSG0000(	TOP1P1	49.94443	-0.29362	0.123617	0.252018	processed_pseudogene
ENSG0000(	NDUFV2	1101.429	0.061402	0.123634	0.252035	protein_coding
ENSG0000(	ENSG0000(	26.81717	0.342314	0.123657	0.252066	processed_pseudogene
ENSG0000(	ENSG0000(	733.5251	-0.09656	0.123688	0.252096	lncRNA
ENSG0000(	ZNF132	235.1373	-0.11307	0.123684	0.252096	protein_coding
ENSG0000(	FBXO30	772.9629	0.059442	0.12376	0.252211	protein_coding
ENSG0000(	RN7SL19P	20.04362	0.235095	0.123755	0.252211	misc_RNA
ENSG0000(	KIAA1958	1471.05	-0.11851	0.123788	0.252252	protein_coding
ENSG0000(	RN7SL502F	33.06456	0.207538	0.123809	0.252277	misc_RNA

ENSG0000(	ENSG0000(	86.18499	-0.11525	0.123823	0.252289	lncRNA
ENSG0000(	UCK2	1195.853	-0.17627	0.12383	0.252289	protein_coding
ENSG0000(	ENSG0000(	27.86929	0.160597	0.123946	0.252508	lncRNA
ENSG0000(	ATG16L2	2797.082	0.121884	0.123981	0.252532	protein_coding
ENSG0000(	ENSG0000(	85.22594	0.155996	0.123983	0.252532	lncRNA
ENSG0000(	ENSG0000(	855.3879	0.074072	0.123995	0.252532	lncRNA
ENSG0000(	ENSG0000(	463.6811	0.060919	0.123984	0.252532	lncRNA
ENSG0000(	PRKAR2A	1893.464	-0.06246	0.123998	0.252532	protein_coding
ENSG0000(	ENSG0000(	76.41995	0.135715	0.124027	0.252575	lncRNA
ENSG0000(	LINC02894	217.5525	-0.12352	0.124053	0.252611	lncRNA
ENSG0000(	DEFB105A	290.1989	-0.08529	0.1241	0.252675	protein_coding
ENSG0000(	HNF1B	1003.248	-0.24987	0.124099	0.252675	protein_coding
ENSG0000(	AQP4-AS1	344.7718	0.080579	0.124119	0.252692	lncRNA
ENSG0000(	ENSG0000(	37.9207	-0.18366	0.124124	0.252692	lncRNA
ENSG0000(	MIR657	16.56133	-0.47577	0.124155	0.252739	miRNA
ENSG0000(	ANKRD36B	34.966	0.196858	0.124206	0.252826	transcribed_processed_pseudoge
ENSG0000(	GPR31	15.39533	0.35122	0.124219	0.252836	protein_coding
ENSG0000(	ZDHHC4	510.3728	0.058126	0.124273	0.252929	protein_coding
ENSG0000(	OR52V1P	14.4136	0.290035	0.12431	0.252989	unprocessed_pseudogene
ENSG0000(	ARF1P2	103.1037	-0.11567	0.12436	0.253074	processed_pseudogene
ENSG0000(	MYCT1	137.0242	0.16247	0.124375	0.253089	protein_coding
ENSG0000(	PTDSS1	860.968	-0.1508	0.124398	0.253118	protein_coding
ENSG0000(	NCAPG2	533.8224	0.180939	0.124415	0.253137	protein_coding
ENSG0000(	ANKRD34A	44.70548	-0.24622	0.12456	0.253415	protein_coding
ENSG0000(	ENSG0000(	107.5418	0.143487	0.124605	0.25349	lncRNA
ENSG0000(	ENSG0000(	18.56767	0.206094	0.124634	0.253534	lncRNA
ENSG0000(	ENSG0000(	73.44096	0.15753	0.124667	0.253577	lncRNA
ENSG0000(	KITLG	855.1399	-0.14018	0.124688	0.253577	protein_coding
ENSG0000(	SLC30A5	1182.247	-0.06071	0.124676	0.253577	protein_coding
ENSG0000(	TMEM60	106.2555	-0.11645	0.124683	0.253577	protein_coding
ENSG0000(	ENSG0000(	13.0834	-0.33415	0.124742	0.253639	lncRNA
ENSG0000(	ENSG0000(	66.82485	-0.30917	0.124733	0.253639	lncRNA
ENSG0000(	RNU6-233F	33.64922	0.155706	0.124742	0.253639	snRNA
ENSG0000(	ENSG0000(	404.1988	0.061707	0.124781	0.253702	lncRNA
ENSG0000(	ATP9B	6706.612	-0.08315	0.124821	0.253749	protein_coding
ENSG0000(	ENSG0000(	9.709063	0.319829	0.124818	0.253749	lncRNA
ENSG0000(	APH1A	1076.342	-0.08466	0.124979	0.254039	protein_coding
ENSG0000(	SERTAD1	230.7766	0.099612	0.124973	0.254039	protein_coding
ENSG0000(	MSTN	9.282045	0.32813	0.125013	0.25409	protein_coding
ENSG0000(	SNORA3B	123.4971	0.179631	0.125042	0.254133	snoRNA
ENSG0000(	ID4	269.0076	-0.18738	0.125113	0.254262	protein_coding
ENSG0000(	ZBTB20-AS	12.47426	0.352137	0.125195	0.25441	lncRNA
ENSG0000(	ZNF879	248.4307	-0.1047	0.125234	0.254475	protein_coding
ENSG0000(	AMIGO2	226.5476	0.207029	0.125291	0.254541	protein_coding
ENSG0000(	CBX8	93.09827	0.185293	0.12529	0.254541	protein_coding
ENSG0000(	ENSG0000(	103.0601	-0.14191	0.125285	0.254541	lncRNA
ENSG0000(	RCVRN	21.34479	0.262907	0.125325	0.254594	protein_coding

ENSG0000(	ENSG0000(	271.4864	-0.08909	0.125379	0.254681	lncRNA
ENSG0000(	ENSG0000(	19.84686	0.212478	0.125384	0.254681	lncRNA
ENSG0000(	RN7SL606F	40.56112	0.183145	0.125402	0.254701	misc_RNA
ENSG0000(	ENSG0000(	12.58557	0.378008	0.125438	0.254748	lncRNA
ENSG0000(	ENSG0000(	43.00682	0.171601	0.125441	0.254748	lncRNA
ENSG0000(	CDC42SE1	3880.617	0.153797	0.125462	0.254757	protein_coding
ENSG0000(	ENSG0000(	491.7985	0.058909	0.125459	0.254757	lncRNA
ENSG0000(	CCNT2	3045.385	-0.06654	0.125512	0.254841	protein_coding
ENSG0000(	EDNRB-AS1	251.1228	-0.11366	0.125523	0.254849	lncRNA
ENSG0000(	ENSG0000(	311.6273	0.119447	0.125545	0.254875	lncRNA
ENSG0000(	RN7SL494F	17.80776	0.244856	0.125554	0.254878	misc_RNA
ENSG0000(	RRAGA	341.6007	-0.08778	0.125569	0.254892	protein_coding
ENSG0000(	HRH1	534.3089	-0.23833	0.125579	0.254897	protein_coding
ENSG0000(	SAE1	634.5333	-0.08573	0.125622	0.254967	protein_coding
ENSG0000(	GRB14	34.23486	-0.34203	0.125668	0.255043	protein_coding
ENSG0000(	NTPCR	1795.581	-0.10022	0.125707	0.255106	protein_coding
ENSG0000(	NLRP10	323.2835	0.066561	0.125748	0.255173	protein_coding
ENSG0000(	ENSG0000(	12.07108	-0.28653	0.125769	0.2552	processed_pseudogene
ENSG0000(	MLC1	10.54867	0.530687	0.12579	0.255209	protein_coding
ENSG0000(	RUBCN	2692.36	0.1107	0.125789	0.255209	protein_coding
ENSG0000(	ADGRL3	1380.393	-0.20868	0.125853	0.255304	protein_coding
ENSG0000(	NPIPB14P	271.3426	-0.30662	0.12585	0.255304	transcribed_unprocessed_pseudo
ENSG0000(	ASPDH	11.10339	0.413574	0.125891	0.255348	protein_coding
ENSG0000(	DPP3-DT	406.0434	0.080763	0.125884	0.255348	lncRNA
ENSG0000(	ITPRID2-DT	175.2902	0.095918	0.125939	0.255428	lncRNA
ENSG0000(	ENSG0000(	26.51793	0.172393	0.125979	0.255494	lncRNA
ENSG0000(	IL20RB	85.43895	0.190503	0.126001	0.255522	protein_coding
ENSG0000(	ENSG0000(	52.36881	0.165353	0.126045	0.255529	processed_pseudogene
ENSG0000(	ENSG0000(	24.15105	-0.30972	0.126019	0.255529	processed_pseudogene
ENSG0000(	ENSG0000(	559.2539	0.102751	0.126043	0.255529	lncRNA
ENSG0000(	EPB41L1	508.9124	-0.22033	0.126039	0.255529	protein_coding
ENSG0000(	RNU1-89P	10.53628	-0.41721	0.126023	0.255529	snRNA
ENSG0000(	ENSG0000(	36.45659	-0.16552	0.126055	0.255533	lncRNA
ENSG0000(	ENSG0000(	161.8509	0.130256	0.126104	0.255616	lncRNA
ENSG0000(	SRPRB	459.9043	0.135651	0.126179	0.255751	protein_coding
ENSG0000(	ENSG0000(	180.3575	0.114607	0.126199	0.255776	lncRNA
ENSG0000(	ENSG0000(	623.2822	0.067216	0.12626	0.255866	lncRNA
ENSG0000(	GORASP2	921.5393	0.084536	0.126276	0.255866	protein_coding
ENSG0000(	LINC01638	139.7826	-0.10735	0.12627	0.255866	lncRNA
ENSG0000(	MORN1	639.8204	-0.05625	0.126275	0.255866	protein_coding
ENSG0000(	ENSG0000(	25.05213	0.353784	0.126329	0.255954	lncRNA
ENSG0000(	STXBP5L	50.85955	0.377588	0.126336	0.255954	protein_coding
ENSG0000(	LAMP2	1889.459	-0.07029	0.126344	0.255955	protein_coding
ENSG0000(	RN7SKP24(	25.83969	-0.23848	0.126417	0.256085	misc_RNA
ENSG0000(	LINC02891	59.19824	-0.1443	0.126516	0.256269	lncRNA
ENSG0000(	ENSG0000(	139.2888	0.119721	0.126526	0.256272	lncRNA
ENSG0000(	HTRA2	255.0027	-0.12371	0.126543	0.256292	protein_coding

ENSG0000(	LINC02030	19.90515	0.235958	0.126561	0.25631	lncRNA
ENSG0000(	NDUFS5P1	47.46728	-0.25417	0.126569	0.25631	processed_pseudogene
ENSG0000(	NARS1	1302.272	-0.0884	0.126622	0.256402	protein_coding
ENSG0000(	ENSG0000(	9.567889	0.318136	0.126637	0.256414	lncRNA
ENSG0000(	KRI1	712.1074	-0.11627	0.126644	0.256414	protein_coding
ENSG0000(	A1BG-AS1	291.8182	0.149153	0.126677	0.256456	lncRNA
ENSG0000(	ACKR2	364.5138	-0.07867	0.126713	0.256456	protein_coding
ENSG0000(	ENSG0000(	61.80736	-0.19024	0.126713	0.256456	lncRNA
ENSG0000(	ENSG0000(	18.68821	0.270567	0.126694	0.256456	lncRNA
ENSG0000(	ENSG0000(	16.77009	0.283249	0.126714	0.256456	TEC
ENSG0000(	SCN1A-AS1	763.6722	-0.05867	0.126695	0.256456	lncRNA
ENSG0000(	HYMAI	188.8312	0.13031	0.126796	0.256606	lncRNA
ENSG0000(	ENSG0000(	36.26789	0.236205	0.126807	0.256611	lncRNA
ENSG0000(	SLCO4A1-A	16.29577	0.36381	0.126838	0.256658	lncRNA
ENSG0000(	SLCO5A1-A	49.77544	0.202781	0.126854	0.256674	lncRNA
ENSG0000(	ENSG0000(	11.58905	0.280992	0.126874	0.256697	lncRNA
ENSG0000(	ENSG0000(	7.710613	-0.50768	0.126915	0.256747	lncRNA
ENSG0000(	IGHV3-35	20.19623	0.371549	0.126927	0.256747	IG_V_gene
ENSG0000(	OSGEP	1525.349	-0.06694	0.126912	0.256747	protein_coding
ENSG0000(	PTPRVP	235.7398	-0.15806	0.126931	0.256747	unitary_pseudogene
ENSG0000(	ENSG0000(	335.8178	0.085672	0.127059	0.256989	lncRNA
ENSG0000(	ENSG0000(	143.4727	0.092021	0.127098	0.257052	processed_pseudogene
ENSG0000(	EFTUD2	1499.834	-0.06178	0.127138	0.257116	protein_coding
ENSG0000(	C19orf71	71.17461	-0.15153	0.127187	0.257199	protein_coding
ENSG0000(	LUC7L2	2891.347	0.067856	0.127245	0.257298	protein_coding
ENSG0000(	ENSG0000(	42.47036	0.203089	0.127268	0.257328	lncRNA
ENSG0000(	ENSG0000(	50.67333	0.458784	0.127316	0.25741	lncRNA
ENSG0000(	ENSG0000(	142.7946	0.091382	0.127334	0.257421	lncRNA
ENSG0000(	PLA2G10	67.96092	0.256975	0.127346	0.257421	protein_coding
ENSG0000(	TMTC4	551.4685	-0.12144	0.127342	0.257421	protein_coding
ENSG0000(	CAPN13	2085.472	-0.38814	0.127442	0.257584	protein_coding
ENSG0000(	RNU6-82P	57.28386	-0.21945	0.127443	0.257584	snRNA
ENSG0000(	CCDC40	437.3599	0.083253	0.127466	0.257597	protein_coding
ENSG0000(	LBX2-AS1	304.4693	-0.23964	0.127463	0.257597	lncRNA
ENSG0000(	ENSG0000(	15.15276	0.562582	0.127491	0.257631	lncRNA
ENSG0000(	ENSG0000(	133.6094	0.130112	0.127514	0.257662	lncRNA
ENSG0000(	ENSG0000(	58.65938	-0.14477	0.127547	0.257662	lncRNA
ENSG0000(	ENSG0000(	65.44904	0.236696	0.127542	0.257662	lncRNA
ENSG0000(	MPV17L2	122.758	0.109227	0.127533	0.257662	protein_coding
ENSG0000(	TRIM24	1127.34	-0.089	0.127536	0.257662	protein_coding
ENSG0000(	CYB5D1	62.59007	0.235913	0.127577	0.25769	protein_coding
ENSG0000(	ENSG0000(	141.7066	0.19102	0.127575	0.25769	lncRNA
ENSG0000(	ZFAT	736.6242	0.153397	0.127586	0.257691	protein_coding
ENSG0000(	ELMOD1	346.1383	0.069061	0.127624	0.257751	protein_coding
ENSG0000(	HEXIM2	42.98066	0.15418	0.127639	0.257765	protein_coding
ENSG0000(	SLC47A1	447.1549	0.066449	0.127673	0.257818	protein_coding
ENSG0000(	ENSG0000(	16.55001	0.290132	0.127775	0.258006	lncRNA

ENSG0000(	ENSG0000(	9.980192	-0.48861	0.127812	0.258065	processed_pseudogene
ENSG0000(	GSPT2	132.5648	0.175888	0.127914	0.258239	protein_coding
ENSG0000(	MIR3619	36.87251	0.238766	0.127909	0.258239	miRNA
ENSG0000(	CLEC16A	2600.967	-0.06156	0.127961	0.258299	protein_coding
ENSG0000(	TMEM123	2168.007	-0.1517	0.127961	0.258299	protein_coding
ENSG0000(	ENSG0000(	14.74097	0.363716	0.127991	0.258344	lncRNA
ENSG0000(	RBMXL1	828.258	0.064061	0.128125	0.258597	protein_coding
ENSG0000(	IFITM1	576.5976	0.226345	0.128133	0.258598	protein_coding
ENSG0000(	CCDC187	24.79273	0.337074	0.128159	0.258633	protein_coding
ENSG0000(	ENSG0000(	30.17984	-0.81504	0.128206	0.25871	transcribed_processed_pseudoge
ENSG0000(	OR4G1P	11.40555	-0.4341	0.128224	0.25873	transcribed_unprocessed_pseudo
ENSG0000(	PTCHD3	59.61048	0.148785	0.12824	0.258737	polymorphic_pseudogene
ENSG0000(	ZNF541	17.18138	0.322224	0.128244	0.258737	protein_coding
ENSG0000(	ANO3	49.97554	0.136506	0.128296	0.25881	protein_coding
ENSG0000(	RN7SKP165	9.047131	0.366186	0.128292	0.25881	misc_RNA
ENSG0000(	FITM2	1104.727	-0.04986	0.128315	0.258828	protein_coding
ENSG0000(	MCM7	1143.621	-0.08784	0.128321	0.258828	protein_coding
ENSG0000(	CNIH4	1120.496	-0.12299	0.128344	0.258857	protein_coding
ENSG0000(	ENSG0000(	303.6531	0.0797	0.128373	0.2589	lncRNA
ENSG0000(	ENSG0000(	18.21139	0.20353	0.128394	0.258922	lncRNA
ENSG0000(	ENSG0000(	132.2942	0.197998	0.128401	0.258922	lncRNA
ENSG0000(	SDF2L1	100.3808	-0.18967	0.128435	0.258974	protein_coding
ENSG0000(	PSMA2	1320.027	-0.11536	0.12848	0.259032	protein_coding
ENSG0000(	SH2B2	103.9657	0.321225	0.128472	0.259032	protein_coding
ENSG0000(	NCAPD2	1218.183	-0.09708	0.128493	0.259041	protein_coding
ENSG0000(	LINC01546	637.429	0.070553	0.128529	0.259094	lncRNA
ENSG0000(	SLC4A2	830.3925	-0.11021	0.128535	0.259094	protein_coding
ENSG0000(	COL4A5	480.9679	0.118174	0.128634	0.259276	protein_coding
ENSG0000(	HEY1	57.96323	0.205907	0.128714	0.259404	protein_coding
ENSG0000(	TIAL1	3025.337	-0.08589	0.128713	0.259404	protein_coding
ENSG0000(	ENSG0000(	16.61972	0.266272	0.12876	0.25948	lncRNA
ENSG0000(	ENSG0000(	30.90791	-0.16493	0.128794	0.259532	processed_pseudogene
ENSG0000(	ENSG0000(	10.34787	0.277043	0.128873	0.259674	lncRNA
ENSG0000(	ENSG0000(	230.2314	0.123395	0.128888	0.259688	lncRNA
ENSG0000(	CEBPZOS	1301.043	-0.06617	0.128907	0.259709	protein_coding
ENSG0000(	LINC02117	59.00487	-0.14606	0.129026	0.259933	lncRNA
ENSG0000(	ENSG0000(	710.2117	0.082513	0.129053	0.259971	TEC
ENSG0000(	RGS10	253.245	-0.16953	0.129062	0.259972	protein_coding
ENSG0000(	ITLN2	404.4838	-0.53944	0.129071	0.259973	protein_coding
ENSG0000(	BASP1	423.2519	0.292035	0.129139	0.260094	protein_coding
ENSG0000(	ENSG0000(	62.71508	0.121521	0.129169	0.260138	TEC
ENSG0000(	FBXO4	232.4316	0.096386	0.129192	0.260168	protein_coding
ENSG0000(	PLP2	305.7654	0.167052	0.129201	0.26017	protein_coding
ENSG0000(	GNG2	969.6538	0.18438	0.129217	0.260184	protein_coding
ENSG0000(	CCDC102A	61.75325	-0.20502	0.129252	0.26021	protein_coding
ENSG0000(	MIEF1	1406.754	-0.0723	0.129254	0.26021	protein_coding
ENSG0000(	STXBP5-AS	884.962	0.163825	0.129249	0.26021	lncRNA

ENSG0000(	NFKBIA	1937.376	0.2232	0.129271	0.260226	protein_coding
ENSG0000(	ACKR3	226.912	-0.15563	0.129284	0.260237	protein_coding
ENSG0000(	SCARNA2	6930.954	0.194116	0.129311	0.260275	scaRNA
ENSG0000(	HSD11B1L	215.1665	0.120468	0.129336	0.260308	protein_coding
ENSG0000(	ENSG0000(	13.04128	0.261052	0.129359	0.260337	lncRNA
ENSG0000(	SNORA65	121.8083	0.171874	0.129374	0.26035	snoRNA
ENSG0000(	ENSG0000(	41.98951	0.173914	0.12941	0.260406	lncRNA
ENSG0000(	TSPYL5	235.2352	0.173489	0.129493	0.260557	protein_coding
ENSG0000(	TROAP-AS1	22.00731	0.192212	0.129545	0.260646	lncRNA
ENSG0000(	PMS2P6	286.1841	0.250151	0.129572	0.260684	unprocessed_pseudogene
ENSG0000(	LINC01285	1345.125	0.045624	0.129613	0.260732	lncRNA
ENSG0000(	RNU4-84P	10.88236	-0.29941	0.129609	0.260732	snRNA
ENSG0000(	KLRC4-KLRI	61.89139	-0.31239	0.129629	0.260748	protein_coding
ENSG0000(	C11orf52	91.16536	-0.3063	0.129688	0.26085	protein_coding
ENSG0000(	ENSG0000(	149.6089	0.103856	0.129752	0.260961	lncRNA
ENSG0000(	ENSG0000(	78.71922	0.159929	0.129782	0.261005	lncRNA
ENSG0000(	HIF1AP1	8.931087	0.266081	0.129795	0.261016	processed_pseudogene
ENSG0000(	CRTAM	311.141	-0.08696	0.129892	0.261159	protein_coding
ENSG0000(	ENSG0000(	387.9468	-0.06531	0.129887	0.261159	lncRNA
ENSG0000(	SLC17A6-D	144.7789	0.220223	0.129876	0.261159	lncRNA
ENSG0000(	ENSG0000(	151.2578	-0.0794	0.129914	0.261187	lncRNA
ENSG0000(	HSPA5	2760.815	-0.16229	0.129943	0.261229	protein_coding
ENSG0000(	TRBV18	13.93623	-0.40409	0.129976	0.261279	TR_V_gene
ENSG0000(	DPP7	522.1086	-0.16047	0.129985	0.26128	protein_coding
ENSG0000(	CIB2	135.675	-0.30112	0.130004	0.261285	protein_coding
ENSG0000(	IGLV3-27	9.333139	0.593576	0.129999	0.261285	IG_V_gene
ENSG0000(	NDUFA4	630.3587	-0.083	0.130027	0.261315	protein_coding
ENSG0000(	KDM6A	10413.32	-0.08624	0.130071	0.261386	protein_coding
ENSG0000(	ENSG0000(	46.35529	0.295382	0.130083	0.261394	lncRNA
ENSG0000(	ENSG0000(	81.81077	0.353896	0.130109	0.261429	lncRNA
ENSG0000(	LINC01004	1348.151	0.069895	0.130131	0.261458	lncRNA
ENSG0000(	UBE2R2-AS	105.617	0.197234	0.130152	0.261483	lncRNA
ENSG0000(	TMEM167E	550.3955	-0.0661	0.130167	0.261496	lncRNA
ENSG0000(	RNA5S1	534.3638	0.221946	0.130199	0.261543	rRNA
ENSG0000(	ENSG0000(	64.83813	0.153215	0.130251	0.261614	lncRNA
ENSG0000(	LINC02505	48.36327	0.16891	0.130246	0.261614	lncRNA
ENSG0000(	EPC1	2894.008	-0.05502	0.130288	0.261658	protein_coding
ENSG0000(	HCG18	7424.472	0.057227	0.130298	0.261658	lncRNA
ENSG0000(	NXPH3	27.60538	0.27434	0.13029	0.261658	protein_coding
ENSG0000(	PABPC4	3089.89	-0.06043	0.130347	0.261741	protein_coding
ENSG0000(	ENSG0000(	14.79467	0.243152	0.13036	0.26175	lncRNA
ENSG0000(	GTPBP3	535.8092	0.090351	0.130435	0.261866	protein_coding
ENSG0000(	METTL6	1044.34	0.054232	0.13044	0.261866	protein_coding
ENSG0000(	MRPL58	81.60578	-0.12734	0.130455	0.261866	protein_coding
ENSG0000(	TCEAL7	13.67882	0.284885	0.130444	0.261866	protein_coding
ENSG0000(	ZNF154	1460.339	0.164585	0.130459	0.261866	protein_coding
ENSG0000(	ENSG0000(	149.8778	-0.14635	0.130497	0.261926	lncRNA

ENSG0000(	ENSG0000(	975.2869	-0.06263	0.130531	0.261929	protein_coding
ENSG0000(	RPS3AP38	341.8678	0.086144	0.130532	0.261929	processed_pseudogene
ENSG0000(	TRAV13-1	18.01108	-0.37998	0.130528	0.261929	TR_V_gene
ENSG0000(	XKR9	1930.909	-0.14852	0.130512	0.261929	protein_coding
ENSG0000(	ENSG0000(	76.45841	0.140641	0.130549	0.261946	lncRNA
ENSG0000(	AP3S2	1995.84	-0.07191	0.130591	0.262013	protein_coding
ENSG0000(	ENSG0000(	41.11502	0.244522	0.13074	0.262295	unprocessed_pseudogene
ENSG0000(	ENSG0000(	64.09391	0.320034	0.130771	0.262341	lncRNA
ENSG0000(	ENSG0000(	532.5307	0.078804	0.130808	0.262398	TEC
ENSG0000(	FPGT	643.2155	-0.09415	0.130856	0.262478	protein_coding
ENSG0000(	ENSG0000(	23.17337	0.289054	0.130885	0.262519	lncRNA
ENSG0000(	H2AC16	72.77675	-0.29316	0.130951	0.262635	protein_coding
ENSG0000(	RN7SL423F	13.073	0.287121	0.13099	0.262696	misc_RNA
ENSG0000(	MPRIP	7884.718	-0.07477	0.131031	0.262763	protein_coding
ENSG0000(	AFDN	9453.959	-0.20671	0.131066	0.262798	protein_coding
ENSG0000(	SERPINI1	63.2583	0.191475	0.131061	0.262798	protein_coding
ENSG0000(	GLUD1P2	66.96009	-0.1499	0.131084	0.262819	transcribed_unprocessed_pseudo
ENSG0000(	ACTG1P17	356.6458	0.10846	0.131104	0.262843	transcribed_processed_pseudoge
ENSG0000(	TNN	21.15397	0.354108	0.131115	0.262848	protein_coding
ENSG0000(	UBE2S	162.2986	0.14737	0.131144	0.262888	protein_coding
ENSG0000(	MAK16	395.4931	-0.14949	0.131259	0.263102	protein_coding
ENSG0000(	STOX2	509.3292	0.169989	0.131282	0.263132	protein_coding
ENSG0000(	ENSG0000(	294.544	-0.06449	0.131296	0.263143	lncRNA
ENSG0000(	ENSG0000(	532.0783	-0.10485	0.131328	0.26319	transcribed_unprocessed_pseudo
ENSG0000(	ALG1L13P	369.7829	0.080155	0.131393	0.263277	transcribed_unprocessed_pseudo
ENSG0000(	LINC00571	97.47697	0.122787	0.131388	0.263277	lncRNA
ENSG0000(	SFPQ	10259.3	-0.0448	0.131396	0.263277	protein_coding
ENSG0000(	ENSG0000(	310.8375	0.313208	0.131432	0.263332	lncRNA
ENSG0000(	PGPEP1	3647.086	-0.1577	0.131525	0.263501	protein_coding
ENSG0000(	TPGS2	1909.363	0.065638	0.131534	0.263503	protein_coding
ENSG0000(	FXYD1	28.10162	-0.31212	0.131565	0.263548	protein_coding
ENSG0000(	ENSG0000(	161.0738	-0.11072	0.131619	0.26364	lncRNA
ENSG0000(	ENSG0000(	26.86282	0.256818	0.131677	0.263725	lncRNA
ENSG0000(	GDA	12670.94	-0.35168	0.131684	0.263725	protein_coding
ENSG0000(	UBE2I	1895.282	-0.07404	0.131687	0.263725	protein_coding
ENSG0000(	ENSG0000(	51.56459	-0.25941	0.131754	0.263844	processed_pseudogene
ENSG0000(	STK17A	1787.065	-0.20001	0.131763	0.263844	protein_coding
ENSG0000(	ENSG0000(	18.35798	0.29394	0.131877	0.264056	processed_pseudogene
ENSG0000(	DNAJB4	393.6743	-0.09186	0.132008	0.264301	protein_coding
ENSG0000(	AFAP1	1448.569	0.078482	0.132026	0.26432	protein_coding
ENSG0000(	AGR2	1977.95	0.311448	0.13217	0.264591	protein_coding
ENSG0000(	FXYD2	119.7919	0.232464	0.132191	0.264617	protein_coding
ENSG0000(	ENSG0000(	83.82032	-0.12879	0.132259	0.264737	protein_coding
ENSG0000(	AIDAP2	220.5366	0.218167	0.132314	0.264829	transcribed_processed_pseudoge
ENSG0000(	ENSG0000(	168.4032	0.22428	0.132334	0.264829	lncRNA
ENSG0000(	ENSG0000(	27.66454	0.235241	0.132339	0.264829	lncRNA
ENSG0000(	ROR1-AS1	2282.662	-0.05797	0.132335	0.264829	lncRNA

ENSG0000(	ENSG0000(	29.10933	0.16883	0.132385	0.264888	lncRNA
ENSG0000(	HIC1	798.2868	-0.14123	0.132382	0.264888	protein_coding
ENSG0000(	DDIT4L	14.68373	0.371039	0.132405	0.264902	protein_coding
ENSG0000(	RN7SKP37	16.97127	-0.27425	0.132409	0.264902	misc_RNA
ENSG0000(	ENSG0000(	219.0878	0.096155	0.13242	0.264906	lncRNA
ENSG0000(	ENSG0000(	161.0059	0.072582	0.132477	0.264988	lncRNA
ENSG0000(	RNU6-360F	10.01275	0.373688	0.132474	0.264988	snRNA
ENSG0000(	ASB7	523.6019	-0.07728	0.132583	0.265169	protein_coding
ENSG0000(	SERINC2	1460.892	-0.28448	0.132585	0.265169	protein_coding
ENSG0000(	MTHFD2P1	198.341	0.096548	0.132694	0.265372	transcribed_processed_pseudoge
ENSG0000(	ENSG0000(	13.61084	0.222994	0.132774	0.265502	lncRNA
ENSG0000(	H4C1	62.81603	-0.24293	0.132776	0.265502	protein_coding
ENSG0000(	ENSG0000(	162.729	0.240315	0.132837	0.265606	TEC
ENSG0000(	CDC27P1	19.36451	0.52568	0.132882	0.265648	processed_pseudogene
ENSG0000(	CENPBD1	289.5413	0.074154	0.132877	0.265648	transcribed_unitary_pseudogene
ENSG0000(	HCG20	190.8413	-0.08984	0.132883	0.265648	lncRNA
ENSG0000(	ENSG0000(	86.01473	0.164658	0.132924	0.265714	lncRNA
ENSG0000(	CYP51A1-A	102.5779	-0.11129	0.132953	0.265753	lncRNA
ENSG0000(	ENSG0000(	10.44627	0.329465	0.132966	0.265764	transcribed_processed_pseudoge
ENSG0000(	ENSG0000(	40.25931	-0.23307	0.132994	0.265802	lncRNA
ENSG0000(	LINC01058	21.52676	0.388616	0.133018	0.265833	lncRNA
ENSG0000(	ENSG0000(	83.64066	-0.10423	0.133045	0.265853	lncRNA
ENSG0000(	GSTO2	1510.384	0.108233	0.133037	0.265853	protein_coding
ENSG0000(	ENSG0000(	17.00142	0.329871	0.133081	0.265908	lncRNA
ENSG0000(	DISP1	1449.843	-0.12419	0.133111	0.265952	protein_coding
ENSG0000(	TMEM154	1996.486	0.2557	0.133121	0.265955	protein_coding
ENSG0000(	ENSG0000(	49.4607	0.197345	0.133181	0.266058	lncRNA
ENSG0000(	ENSG0000(	9137.988	-0.07484	0.133227	0.266133	lncRNA
ENSG0000(	EFHD2	1859.728	-0.15265	0.133237	0.266136	protein_coding
ENSG0000(	HNRNPC	6288.705	0.083932	0.133259	0.266163	protein_coding
ENSG0000(	DLK2	62.81786	-0.13394	0.133271	0.266171	protein_coding
ENSG0000(	RN7SKP205	9.767239	-0.29131	0.133303	0.266202	misc_RNA
ENSG0000(	SLC35E2A	580.5873	-0.08932	0.133304	0.266202	transcribed_unprocessed_pseudo
ENSG0000(	ENSG0000(	15.46694	0.335639	0.133319	0.266216	processed_pseudogene
ENSG0000(	RN7SL619F	255.0419	-0.08602	0.133347	0.266255	misc_RNA
ENSG0000(	SEC24AP1	127.3897	0.185735	0.133356	0.266255	processed_pseudogene
ENSG0000(	RPL21P10	57.9037	0.140348	0.133371	0.266269	processed_pseudogene
ENSG0000(	EPS8L1	183.5146	0.265851	0.133399	0.266296	protein_coding
ENSG0000(	PPP1R11P2	14.4043	0.250886	0.133402	0.266296	processed_pseudogene
ENSG0000(	SDHAF4	372.7587	-0.06709	0.133412	0.266301	protein_coding
ENSG0000(	ENSG0000(	698.0741	0.050276	0.133446	0.266352	TEC
ENSG0000(	ENSG0000(	13.99017	-0.27061	0.133456	0.266355	processed_pseudogene
ENSG0000(	ENSG0000(	27.00176	0.178733	0.133483	0.266391	lncRNA
ENSG0000(	ENSG0000(	19.34674	0.202828	0.133541	0.266489	lncRNA
ENSG0000(	TRIP13	214.592	0.097158	0.133602	0.266596	protein_coding
ENSG0000(	THUMPD3-	3684.346	0.051311	0.133618	0.26661	lncRNA
ENSG0000(	CCNC	1124.921	0.051307	0.133666	0.266616	protein_coding

ENSG0000(	ENSG0000(	97.2711	-0.34214	0.133648	0.266616	processed_pseudogene
ENSG0000(	ENSG0000(	57.15437	-0.12421	0.13367	0.266616	lncRNA
ENSG0000(	LOX	166.9384	0.24092	0.133639	0.266616	protein_coding
ENSG0000(	LRGUK	61.18625	0.267795	0.133643	0.266616	protein_coding
ENSG0000(	TRAV8-3	20.86197	-0.40616	0.133672	0.266616	TR_V_gene
ENSG0000(	ENSG0000(	26.64342	0.186722	0.13369	0.266635	lncRNA
ENSG0000(	ENSG0000(	311.5189	-0.07953	0.133736	0.266684	TEC
ENSG0000(	GVINP1	4493.683	0.238889	0.133727	0.266684	transcribed_unprocessed_pseudo
ENSG0000(	RUBCNL	2820.928	0.228082	0.13374	0.266684	protein_coding
ENSG0000(	MAGI3	2438.35	-0.23023	0.133758	0.266704	protein_coding
ENSG0000(	HOXB2	95.00853	0.166912	0.133786	0.266742	protein_coding
ENSG0000(	CCDC134	1382.537	0.066214	0.13391	0.266956	protein_coding
ENSG0000(	ENSG0000(	22.11525	0.191924	0.133909	0.266956	lncRNA
ENSG0000(	MSRB3	693.7469	0.094196	0.133941	0.267	protein_coding
ENSG0000(	ANKRD44-I	750.3171	0.220534	0.133951	0.267003	lncRNA
ENSG0000(	ECEL1P2	31.9213	0.276825	0.134006	0.267097	transcribed_unprocessed_pseudo
ENSG0000(	LINC02611	542.0106	-0.06448	0.13403	0.267128	lncRNA
ENSG0000(	ENSG0000(	8.204235	-0.30218	0.134079	0.267208	lncRNA
ENSG0000(	AGT	275.7696	-0.24038	0.134129	0.267291	protein_coding
ENSG0000(	ENSG0000(	28.47083	0.245627	0.134203	0.267337	lncRNA
ENSG0000(	ENSG0000(	16.26852	0.260396	0.134174	0.267337	lncRNA
ENSG0000(	EXOSC4	71.29003	-0.13964	0.134198	0.267337	protein_coding
ENSG0000(	HMGN3	276.537	-0.14599	0.13419	0.267337	protein_coding
ENSG0000(	SNORD17	2246.263	-0.22228	0.134195	0.267337	snoRNA
ENSG0000(	TMEM239	163.0901	-0.10502	0.134181	0.267337	protein_coding
ENSG0000(	GLOD5	18.91746	-0.37711	0.134212	0.267339	protein_coding
ENSG0000(	PRKACB-DT	21.28905	0.263386	0.134247	0.26739	lncRNA
ENSG0000(	ENSG0000(	106.4097	0.343893	0.134281	0.267442	lncRNA
ENSG0000(	ENSG0000(	122.182	0.15364	0.134316	0.267495	TEC
ENSG0000(	ENSG0000(	9.637261	0.387181	0.134355	0.267555	processed_pseudogene
ENSG0000(	ENSG0000(	200.5321	0.075026	0.134406	0.267589	lncRNA
ENSG0000(	KLF2P2	29.3734	0.206199	0.134386	0.267589	processed_pseudogene
ENSG0000(	NOX5	418.9538	-0.1253	0.134393	0.267589	protein_coding
ENSG0000(	THOC3-AS1	16.54239	0.207693	0.134406	0.267589	lncRNA
ENSG0000(	ENSG0000(	70.8025	0.116791	0.134422	0.267604	lncRNA
ENSG0000(	ENSG0000(	282.3587	0.069137	0.134456	0.267654	lncRNA
ENSG0000(	ENSG0000(	12.21041	0.319193	0.134489	0.267703	unprocessed_pseudogene
ENSG0000(	DHRS9	334.8681	0.355995	0.134523	0.267737	protein_coding
ENSG0000(	ENSG0000(	22.31381	0.231658	0.134523	0.267737	lncRNA
ENSG0000(	ENSG0000(	40.04945	0.223945	0.134535	0.267745	transcribed_unprocessed_pseudo
ENSG0000(	PICALM	4541.758	-0.08777	0.134556	0.267769	protein_coding
ENSG0000(	LINC00879	12.5056	0.259943	0.134593	0.267826	lncRNA
ENSG0000(	LINC01730	21.52002	0.230151	0.134639	0.267901	lncRNA
ENSG0000(	ENSG0000(	740.0665	0.051272	0.134682	0.267952	lncRNA
ENSG0000(	ENSG0000(	374.7952	0.08579	0.134677	0.267952	lncRNA
ENSG0000(	ENSG0000(	124.3511	0.097701	0.134716	0.268003	lncRNA
ENSG0000(	RNA5SP104	20.34869	-0.23727	0.134733	0.268019	rRNA_pseudogene

ENSG0000(	ENSG0000(	308.5542	-0.08275	0.134821	0.268177	lncRNA
ENSG0000(	ILDR2	161.3643	0.197634	0.134849	0.268217	protein_coding
ENSG0000(	ENSG0000(	212.3973	-0.08045	0.134878	0.268258	lncRNA
ENSG0000(	ENSG0000(	122.3504	0.170615	0.134957	0.268397	lncRNA
ENSG0000(	HCG27	780.3022	0.136742	0.134966	0.268398	lncRNA
ENSG0000(	ENSG0000(	116.5137	0.113797	0.134991	0.268432	lncRNA
ENSG0000(	ENSG0000(	32.84419	-0.19449	0.135006	0.268445	lncRNA
ENSG0000(	DNAH3	249.9584	0.091367	0.13505	0.268514	protein_coding
ENSG0000(	RAB3GAP2	4177.51	0.039034	0.135165	0.268727	protein_coding
ENSG0000(	RNASEH2B	4572.272	0.131901	0.135184	0.268748	protein_coding
ENSG0000(	ZNF454-DT	35.14679	-0.16209	0.135194	0.268751	lncRNA
ENSG0000(	HES1	276.1324	-0.22163	0.135222	0.268789	protein_coding
ENSG0000(	SNX29	2232.825	0.1622	0.135286	0.2689	protein_coding
ENSG0000(	ENSG0000(	28.17195	0.238422	0.135313	0.268936	lncRNA
ENSG0000(	B3GAT3	402.6918	-0.09743	0.135465	0.26922	protein_coding
ENSG0000(	ENSG0000(	2139.58	-0.09371	0.135476	0.269227	lncRNA
ENSG0000(	SUGCT	220.2549	-0.29897	0.13552	0.269297	protein_coding
ENSG0000(	LINC01128	2848.641	0.064629	0.135564	0.269361	lncRNA
ENSG0000(	RENBP	332.8784	-0.08033	0.13557	0.269361	protein_coding
ENSG0000(	ENSG0000(	30.64472	0.256183	0.13562	0.269444	lncRNA
ENSG0000(	BMP8A	480.0526	0.222299	0.135645	0.269459	protein_coding
ENSG0000(	PRDM16	158.9077	-0.12875	0.13564	0.269459	protein_coding
ENSG0000(	TBILA	653.38	-0.27304	0.135655	0.269463	lncRNA
ENSG0000(	TARS2	665.3012	-0.06537	0.135669	0.269474	protein_coding
ENSG0000(	ENSG0000(	15.20703	0.271432	0.135716	0.269545	lncRNA
ENSG0000(	SGO1	312.8972	-0.09761	0.13573	0.269545	protein_coding
ENSG0000(	WDR26	4691.664	0.041262	0.13573	0.269545	protein_coding
ENSG0000(	DYNLT5	23.08421	0.317713	0.135783	0.26961	protein_coding
ENSG0000(	ENSG0000(	199.7602	0.112914	0.135772	0.26961	lncRNA
ENSG0000(	RNF44	1792.679	-0.12759	0.135789	0.26961	protein_coding
ENSG0000(	FRG1FP	14.73788	0.358178	0.135849	0.269711	unprocessed_pseudogene
ENSG0000(	ENSG0000(	42.8409	0.329088	0.13589	0.269777	processed_pseudogene
ENSG0000(	RBM42	245.6479	-0.09067	0.135937	0.269853	protein_coding
ENSG0000(	IQCG	523.7389	0.071238	0.135997	0.269955	protein_coding
ENSG0000(	ENSG0000(	1227.746	0.087099	0.136051	0.270037	lncRNA
ENSG0000(	PGM5P2	466.6371	0.101949	0.136064	0.270037	transcribed_unprocessed_pseudo
ENSG0000(	RN7SL16P	60.2976	0.12628	0.136057	0.270037	misc_RNA
ENSG0000(	ENSG0000(	11.21293	0.303819	0.136108	0.270074	misc_RNA
ENSG0000(	PANCR	46.76365	0.268578	0.136099	0.270074	lncRNA
ENSG0000(	SYNE1	6885.597	0.153807	0.136103	0.270074	protein_coding
ENSG0000(	ETNPPL	68.65763	0.12226	0.13616	0.270147	protein_coding
ENSG0000(	FBLN5	378.8789	0.142399	0.136162	0.270147	protein_coding
ENSG0000(	KC6	31.36263	0.242676	0.136179	0.270162	lncRNA
ENSG0000(	RNF20	672.2602	-0.09103	0.136189	0.270166	protein_coding
ENSG0000(	LINC00858	17.69196	0.312328	0.136215	0.270201	lncRNA
ENSG0000(	RPL18A	2174.699	-0.14763	0.136296	0.270343	protein_coding
ENSG0000(	ENSG0000(	14.04271	-0.21094	0.136367	0.270468	processed_pseudogene

ENSG0000(	SLCO1A2	372.0957	0.109773	0.136376	0.270468	protein_coding
ENSG0000(	LINC01215	578.421	0.184155	0.13655	0.270763	lncRNA
ENSG0000(	LPCAT1	1102.461	0.210357	0.136547	0.270763	protein_coding
ENSG0000(	RNU6ATAC	11.73625	0.284445	0.136535	0.270763	snRNA
ENSG0000(	ZNF564	854.2261	-0.0972	0.136625	0.270895	protein_coding
ENSG0000(	MIR553	16.76018	0.281189	0.136654	0.270934	miRNA
ENSG0000(	ENSG0000(	34.45019	0.145893	0.136711	0.271031	lncRNA
ENSG0000(	ENSG0000(	18.87787	0.215243	0.136731	0.271035	lncRNA
ENSG0000(	ENSG0000(	25.3372	0.221713	0.136727	0.271035	lncRNA
ENSG0000(	FUT8-AS1	67.16241	0.194398	0.136792	0.271141	lncRNA
ENSG0000(	TTLL1	44.48482	-0.18116	0.136823	0.271184	protein_coding
ENSG0000(	MYO7B	7784.241	-0.28088	0.136833	0.271187	protein_coding
ENSG0000(	ARHGEF25	213.6424	0.174056	0.1369	0.271293	protein_coding
ENSG0000(	MT1E	592.6847	-0.36845	0.136904	0.271293	protein_coding
ENSG0000(	OTOAP1	41.53545	0.250489	0.136915	0.271299	transcribed_unprocessed_pseudo
ENSG0000(	ENSG0000(	14.75395	-0.24919	0.136924	0.2713	lncRNA
ENSG0000(	GOLGA8T	61.31728	-0.57014	0.136966	0.271364	protein_coding
ENSG0000(	ANLN	348.1861	0.222805	0.136997	0.271411	protein_coding
ENSG0000(	SAMD11	158.673	-0.21002	0.137009	0.271416	protein_coding
ENSG0000(	RAE1	536.8279	-0.06578	0.137031	0.271444	protein_coding
ENSG0000(	LINC01116	364.7573	0.079673	0.137051	0.271448	lncRNA
ENSG0000(	LINC02058	31.40695	0.160522	0.137044	0.271448	lncRNA
ENSG0000(	RPS12P5	34.62714	0.210235	0.137153	0.271633	processed_pseudogene
ENSG0000(	ENSG0000(	610.8913	0.073568	0.137192	0.271694	lncRNA
ENSG0000(	ERBB3	8604.858	-0.20332	0.137212	0.271716	protein_coding
ENSG0000(	ZNF850	1299.865	-0.07037	0.137259	0.271792	protein_coding
ENSG0000(	SGCD	384.2111	0.180906	0.13732	0.271897	protein_coding
ENSG0000(	ENSG0000(	1116.801	0.068441	0.137332	0.271902	lncRNA
ENSG0000(	USE1	711.1049	0.054633	0.137364	0.271949	protein_coding
ENSG0000(	RPS12P27	97.95812	-0.13167	0.137373	0.271949	processed_pseudogene
ENSG0000(	MGC4859	26.22431	0.201789	0.137503	0.27219	lncRNA
ENSG0000(	ENSG0000(	80.62856	-0.40563	0.137522	0.272196	lncRNA
ENSG0000(	PALM3	29.11133	-0.28128	0.137523	0.272196	protein_coding
ENSG0000(	LINC00551	52.79544	0.178878	0.137542	0.272216	lncRNA
ENSG0000(	ENSG0000(	10.38283	0.306538	0.137645	0.272403	transcribed_unprocessed_pseudo
ENSG0000(	HTRA4	401.5914	0.089598	0.137664	0.272423	protein_coding
ENSG0000(	ENSG0000(	21.12415	0.199652	0.137735	0.272545	lncRNA
ENSG0000(	SPIN2B	104.2135	0.149485	0.137756	0.27257	protein_coding
ENSG0000(	EIF2S3B	49.67833	-0.18871	0.137799	0.27261	protein_coding
ENSG0000(	ENSG0000(	13.10677	-0.32189	0.137799	0.27261	TEC
ENSG0000(	ENSG0000(	129.6494	0.121144	0.137802	0.27261	TEC
ENSG0000(	PRRG1	372.8223	0.133564	0.13782	0.272628	protein_coding
ENSG0000(	ENSG0000(	10.31039	0.275795	0.137857	0.272686	lncRNA
ENSG0000(	CDK6	3133.745	-0.11098	0.137908	0.272769	protein_coding
ENSG0000(	ENSG0000(	22.46977	0.18338	0.137967	0.272868	processed_pseudogene
ENSG0000(	CMTM3	300.9608	0.173002	0.137983	0.272883	protein_coding
ENSG0000(	CA13	2248.955	-0.2463	0.137995	0.272889	protein_coding

ENSG0000(	ZNF256	271.9059	0.122144	0.138021	0.272924	protein_coding
ENSG0000(	OIP5	26.23257	0.249398	0.138039	0.272942	protein_coding
ENSG0000(	FAM3B	1235.175	-0.27097	0.138081	0.273008	protein_coding
ENSG0000(	OR2AG1	139.4679	0.092493	0.138116	0.27306	protein_coding
ENSG0000(	ENSG0000(	25.34887	0.204739	0.138256	0.273269	lncRNA
ENSG0000(	ITGAD	80.54526	0.167882	0.138235	0.273269	protein_coding
ENSG0000(	MIR8075	91.9008	-0.16032	0.138256	0.273269	miRNA
ENSG0000(	SS18L1	894.0613	-0.08053	0.138251	0.273269	protein_coding
ENSG0000(	POT1	497.4472	0.165336	0.138269	0.273277	protein_coding
ENSG0000(	LINC01963	247.3771	0.116263	0.138291	0.273286	lncRNA
ENSG0000(	ZNF581	125.5411	-0.14634	0.138288	0.273286	protein_coding
ENSG0000(	ENSG0000(	14.98345	-0.22619	0.138344	0.273361	lncRNA
ENSG0000(	PRKCE	1194.014	0.111158	0.138354	0.273361	protein_coding
ENSG0000(	RAPSN	29.86371	0.248968	0.138355	0.273361	protein_coding
ENSG0000(	CCDC78	37.82529	-0.38801	0.138378	0.273389	protein_coding
ENSG0000(	ENSG0000(	1397.101	0.236504	0.138466	0.273545	lncRNA
ENSG0000(	AFTPH	2054.721	-0.0524	0.138482	0.273561	protein_coding
ENSG0000(	HBG1	47.8885	0.201453	0.138594	0.27375	protein_coding
ENSG0000(	SIGLEC10-A	11.2126	0.318062	0.138596	0.27375	lncRNA
ENSG0000(	MIR3942	7.759931	0.365193	0.138696	0.273915	miRNA
ENSG0000(	STAG1-DT	125.2668	0.087974	0.138691	0.273915	lncRNA
ENSG0000(	ENSG0000(	178.0095	0.105823	0.138729	0.273963	lncRNA
ENSG0000(	MTRNR2L4	307.0498	0.081937	0.138762	0.273993	protein_coding
ENSG0000(	ZNF852	621.9109	0.075779	0.13876	0.273993	protein_coding
ENSG0000(	LINC01234	753.8255	0.049864	0.138782	0.274016	lncRNA
ENSG0000(	ENSG0000(	25.25163	0.178644	0.138827	0.274071	lncRNA
ENSG0000(	ENSG0000(	18.29625	0.205535	0.138821	0.274071	lncRNA
ENSG0000(	ENSG0000(	141.5984	-0.08824	0.138874	0.274128	TEC
ENSG0000(	LINC02163	103.699	0.175496	0.138867	0.274128	lncRNA
ENSG0000(	NDUFAF6	564.3692	0.074036	0.139047	0.274452	protein_coding
ENSG0000(	MAD2L1BP	242.1653	-0.09531	0.139082	0.274487	protein_coding
ENSG0000(	ZBTB8B	1669.416	-0.05164	0.139077	0.274487	protein_coding
ENSG0000(	ENSG0000(	99.37468	0.11152	0.139146	0.274597	lncRNA
ENSG0000(	LINC02313	15.31882	-0.34877	0.139166	0.274619	lncRNA
ENSG0000(	ENSG0000(	60.78612	-0.17548	0.139203	0.274674	lncRNA
ENSG0000(	ENSG0000(	10.00983	0.288158	0.139284	0.274817	lncRNA
ENSG0000(	ENSG0000(	184.7844	0.08073	0.139322	0.274876	transcribed_unitary_pseudogene
ENSG0000(	ENSG0000(	101.4461	-0.11302	0.139349	0.274911	lncRNA
ENSG0000(	ENSG0000(	317.6749	-0.08036	0.139379	0.274936	lncRNA
ENSG0000(	ENSG0000(	262.9056	0.088347	0.139379	0.274936	lncRNA
ENSG0000(	ENSG0000(	74.58197	-0.13392	0.139398	0.274956	lncRNA
ENSG0000(	RN7SL712F	21.70737	0.20208	0.139408	0.274959	misc_RNA
ENSG0000(	ENSG0000(	113.2275	0.163202	0.139491	0.275105	lncRNA
ENSG0000(	ENSG0000(	24.69889	0.231841	0.139519	0.275144	lncRNA
ENSG0000(	ENSG0000(	9.494861	0.286675	0.139545	0.275178	TEC
ENSG0000(	ENSG0000(	126.148	0.091133	0.139646	0.275324	lncRNA
ENSG0000(	MMADHC-I	345.4216	-0.28195	0.139644	0.275324	lncRNA

ENSG0000(	THAP12P7	170.5356	-0.13072	0.139639	0.275324	processed_pseudogene
ENSG0000(	ENSG0000(	18.76867	-0.23543	0.139689	0.275391	lncRNA
ENSG0000(	ENSG0000(	97.4215	0.129537	0.139707	0.27541	lncRNA
ENSG0000(	EMX2OS	817.4974	0.052248	0.139748	0.275475	lncRNA
ENSG0000(	ENSG0000(	155.1628	-0.08832	0.139769	0.275489	lncRNA
ENSG0000(	ENSG0000(	1393.599	-0.18515	0.139777	0.275489	lncRNA
ENSG0000(	ENSG0000(	504.4184	-0.07207	0.139782	0.275489	lncRNA
ENSG0000(	B4GALT3	473.7457	0.103175	0.139836	0.275578	protein_coding
ENSG0000(	ENSG0000(	161.1941	-0.08528	0.139879	0.275645	protein_coding
ENSG0000(	PRCC	743.8316	0.057619	0.139903	0.275669	protein_coding
ENSG0000(	TNFAIP1	707.3149	0.10336	0.139908	0.275669	protein_coding
ENSG0000(	ENSG0000(	75.20121	-0.11259	0.139917	0.27567	lncRNA
ENSG0000(	ENSG0000(	64.57425	0.115437	0.139933	0.275683	lncRNA
ENSG0000(	RNU6-16P	90.07154	0.17659	0.139947	0.275694	snRNA
ENSG0000(	ENSG0000(	67.82796	0.129753	0.139976	0.275717	lncRNA
ENSG0000(	LINC02202	414.5832	-0.10926	0.139973	0.275717	lncRNA
ENSG0000(	SLC20A2	1523.873	-0.12072	0.140056	0.275857	protein_coding
ENSG0000(	EOMES	97.93901	-0.31798	0.140067	0.27586	protein_coding
ENSG0000(	USP10	1518.31	-0.05647	0.140097	0.275903	protein_coding
ENSG0000(	MRPL49P1	11.35495	0.280024	0.140173	0.276036	processed_pseudogene
ENSG0000(	CDC23	491.818	-0.09709	0.140244	0.276157	protein_coding
ENSG0000(	ENSG0000(	25.91154	0.205651	0.140294	0.276231	lncRNA
ENSG0000(	ENSG0000(	37.78719	0.23094	0.140316	0.276231	TEC
ENSG0000(	GPR19	30.59623	0.322588	0.140313	0.276231	protein_coding
ENSG0000(	TMEM117	562.9376	-0.22496	0.140305	0.276231	protein_coding
ENSG0000(	ENSG0000(	377.0859	0.095699	0.140374	0.276327	transcribed_unprocessed_pseudo
ENSG0000(	RPS3P2	134.2403	0.103241	0.140415	0.276391	processed_pseudogene
ENSG0000(	MT1G	586.048	-0.40333	0.140425	0.276394	protein_coding
ENSG0000(	RPS26P42	37.96331	0.14739	0.140486	0.276496	processed_pseudogene
ENSG0000(	PRKRIP1	853.6385	0.088292	0.140506	0.276518	protein_coding
ENSG0000(	ENSG0000(	80.53764	0.149434	0.140581	0.276632	lncRNA
ENSG0000(	WDR46	274.9632	-0.12596	0.140575	0.276632	protein_coding
ENSG0000(	LINC01659	120.8151	-0.19082	0.140624	0.276699	lncRNA
ENSG0000(	ENSG0000(	72.60955	0.151879	0.140647	0.276727	TEC
ENSG0000(	UBE2FP1	80.4053	-0.16392	0.140686	0.276786	processed_pseudogene
ENSG0000(	ENSG0000(	25.89921	0.220492	0.140704	0.276804	TEC
ENSG0000(	CRYBB2P1	2039.727	-0.08929	0.140739	0.276839	transcribed_unprocessed_pseudo
ENSG0000(	LRP10	4108.112	-0.14146	0.140732	0.276839	protein_coding
ENSG0000(	ECEL1P1	9.024745	0.516408	0.140908	0.277154	unprocessed_pseudogene
ENSG0000(	RNF144A	959.0123	0.156086	0.14095	0.27722	protein_coding
ENSG0000(	ENSG0000(	10.9463	0.335845	0.140984	0.277251	lncRNA
ENSG0000(	ENSG0000(	62.15104	0.136335	0.140978	0.277251	lncRNA
ENSG0000(	INAFM1	58.75866	0.118728	0.141128	0.2775	protein_coding
ENSG0000(	ZNF708	1364.028	-0.07676	0.14112	0.2775	protein_coding
ENSG0000(	TIMM23B	1260.252	0.049987	0.141187	0.277598	protein_coding
ENSG0000(	LRIG2-DT	629.1354	-0.07771	0.141205	0.277604	lncRNA
ENSG0000(	MTND4P12	193.6866	-0.5159	0.141207	0.277604	processed_pseudogene

ENSG0000(	ELOVL7	1090.15	-0.18982	0.141236	0.277634	protein_coding
ENSG0000(	TRPC7-AS1	44.11319	-0.32318	0.14124	0.277634	lncRNA
ENSG0000(	ENSG0000(	50.84797	-0.12796	0.141331	0.277761	lncRNA
ENSG0000(	LINC00824	200.5513	-0.07793	0.141316	0.277761	lncRNA
ENSG0000(	SLC6A13	41.85913	0.326039	0.141327	0.277761	protein_coding
ENSG0000(	TAOK1	6369.983	-0.07002	0.141396	0.277871	protein_coding
ENSG0000(	ETF1P2	57.39801	0.149882	0.141443	0.277946	processed_pseudogene
ENSG0000(	RNU6-48P	9.390952	0.457878	0.141517	0.278073	snRNA
ENSG0000(	ENSG0000(	154.9052	0.107873	0.141529	0.278081	lncRNA
ENSG0000(	ENSG0000(	119.8821	-0.5925	0.141643	0.278253	protein_coding
ENSG0000(	RNU6ATAC	41.18631	0.213936	0.14164	0.278253	snRNA
ENSG0000(	SOWAHCP5	129.0341	0.300981	0.14164	0.278253	processed_pseudogene
ENSG0000(	ENSG0000(	145.033	0.164011	0.141653	0.278253	lncRNA
ENSG0000(	DDR1	2744.333	-0.21568	0.141698	0.278307	protein_coding
ENSG0000(	LINC02767	89.29693	0.150927	0.141692	0.278307	lncRNA
ENSG0000(	LDHBP1	162.9819	-0.09519	0.141738	0.278318	processed_pseudogene
ENSG0000(	LINC01847	28.91381	0.206788	0.141736	0.278318	lncRNA
ENSG0000(	PAPLN	2507.811	0.257012	0.141719	0.278318	protein_coding
ENSG0000(	VSTM2A	54.39311	-0.19459	0.141722	0.278318	protein_coding
ENSG0000(	ENSG0000(	14.05649	-0.30302	0.141755	0.278328	lncRNA
ENSG0000(	KLHL26	302.0824	0.113022	0.141761	0.278328	protein_coding
ENSG0000(	TESMIN	208.6867	-0.13414	0.141776	0.278339	protein_coding
ENSG0000(	ENSG0000(	16.45413	0.789186	0.141878	0.278523	lncRNA
ENSG0000(	MUC16	213.3408	0.486398	0.141927	0.278601	protein_coding
ENSG0000(	FAM149A	592.3184	0.107618	0.142054	0.27882	protein_coding
ENSG0000(	LSM1	165.7891	-0.11159	0.142058	0.27882	protein_coding
ENSG0000(	ZNF597	237.2152	0.113367	0.142065	0.27882	protein_coding
ENSG0000(	SSX7	8.627188	-0.48294	0.142106	0.278883	protein_coding
ENSG0000(	SPON1-AS1	14.55289	-0.25231	0.14212	0.278894	lncRNA
ENSG0000(	ENSG0000(	19.5727	0.27828	0.142169	0.278973	processed_pseudogene
ENSG0000(	ENSG0000(	781.9446	-0.09414	0.14221	0.279036	lncRNA
ENSG0000(	LINC02261	318.9184	0.067342	0.142227	0.27905	lncRNA
ENSG0000(	ACADSB	2345.062	0.059265	0.142243	0.279066	protein_coding
ENSG0000(	ACSL3	1751.463	0.07853	0.142315	0.279188	protein_coding
ENSG0000(	MIR3176	19.0784	-0.27379	0.142341	0.279223	miRNA
ENSG0000(	ATXN2	4997.637	0.042282	0.142356	0.279234	protein_coding
ENSG0000(	ENSG0000(	72.93317	-0.12206	0.142392	0.279261	lncRNA
ENSG0000(	RNU1-134F	57.47112	0.132049	0.14238	0.279261	snRNA
ENSG0000(	SMG5	2083.387	-0.0867	0.142396	0.279261	protein_coding
ENSG0000(	RN7SKP217	28.98029	-0.29345	0.142405	0.279262	misc_RNA
ENSG0000(	KLHL17	199.8004	0.133871	0.142485	0.279383	protein_coding
ENSG0000(	PDCD6	2774.24	0.062599	0.142478	0.279383	protein_coding
ENSG0000(	ENSG0000(	15.53908	0.275998	0.142504	0.279404	lncRNA
ENSG0000(	NPIPB13	1245.605	0.13457	0.142537	0.279451	protein_coding
ENSG0000(	ENSG0000(	1013.48	0.06734	0.142577	0.279511	TEC
ENSG0000(	ENSG0000(	22.51215	-0.2906	0.142619	0.279577	lncRNA
ENSG0000(	NCOA7	1537.38	0.093777	0.142631	0.279584	protein_coding

ENSG0000(	ENSG0000(	171.3863	-0.06896	0.142667	0.279633	lncRNA
ENSG0000(	ENSG0000(	54.73153	0.207259	0.142675	0.279633	lncRNA
ENSG0000(	CD69	343.1883	0.294363	0.142728	0.27972	protein_coding
ENSG0000(	GSDMD	1406.872	0.084437	0.142745	0.279737	protein_coding
ENSG0000(	GPC5	18.94315	0.440559	0.14277	0.279768	protein_coding
ENSG0000(	ENSG0000(	26.09078	0.220421	0.142798	0.279789	processed_pseudogene
ENSG0000(	PPP2R2D	1767.398	-0.07775	0.142795	0.279789	protein_coding
ENSG0000(	BNIP3P37	97.51225	-0.10999	0.142854	0.279864	processed_pseudogene
ENSG0000(	C13orf46	121.5298	0.298605	0.142862	0.279864	protein_coding
ENSG0000(	Metazoa_S	22427.26	0.041128	0.142863	0.279864	misc_RNA
ENSG0000(	ADAM8	556.4637	0.246205	0.142874	0.279867	protein_coding
ENSG0000(	LINC01119	63.56188	0.160159	0.142901	0.279886	lncRNA
ENSG0000(	ST3GAL3-A	50.2653	0.163587	0.1429	0.279886	lncRNA
ENSG0000(	NEK5	790.2731	0.072651	0.142939	0.279942	protein_coding
ENSG0000(	BCL3	1281.551	-0.19071	0.142968	0.279983	protein_coding
ENSG0000(	COL20A1	39.00956	0.175012	0.143011	0.280034	protein_coding
ENSG0000(	REPS2	882.83	0.088537	0.143012	0.280034	protein_coding
ENSG0000(	RNU6-341F	40.29212	0.177022	0.14306	0.28011	snRNA
ENSG0000(	COA5	740.0708	-0.05433	0.143146	0.280261	protein_coding
ENSG0000(	AFF1	7121.167	0.094984	0.143169	0.280289	protein_coding
ENSG0000(	RPL24P8	10.97466	-0.39744	0.143197	0.280326	processed_pseudogene
ENSG0000(	FNIP2	1758.716	0.103703	0.143215	0.280343	protein_coding
ENSG0000(	GDPD4	17.40222	0.290503	0.143266	0.280408	protein_coding
ENSG0000(	HIVEP1	3772.831	0.075128	0.143262	0.280408	protein_coding
ENSG0000(	ZNF720	1609.437	0.074248	0.143295	0.280448	protein_coding
ENSG0000(	ASNSD1	402.1598	-0.10501	0.143388	0.280614	protein_coding
ENSG0000(	LINC01081	26.53586	0.193092	0.143438	0.280676	lncRNA
ENSG0000(	RNU6-122F	35.42836	-0.28553	0.143436	0.280676	snRNA
ENSG0000(	ENSG0000(	57.27385	0.149466	0.143472	0.280725	lncRNA
ENSG0000(	MIR193BH(	1455.225	0.072223	0.143548	0.280856	lncRNA
ENSG0000(	ENSG0000(	39.16599	0.495796	0.143571	0.280883	lncRNA
ENSG0000(	ENSG0000(	13.27028	-0.26315	0.143589	0.280901	lncRNA
ENSG0000(	CRACDL	251.593	-0.24439	0.143633	0.28097	protein_coding
ENSG0000(	LINC02148	8.885384	0.373896	0.143646	0.280977	lncRNA
ENSG0000(	FAM106C	33.0352	0.527599	0.143715	0.281095	lncRNA
ENSG0000(	COX10-AS1	2335.667	-0.05573	0.143732	0.281111	lncRNA
ENSG0000(	BDH1	2884.736	-0.11121	0.143744	0.281117	protein_coding
ENSG0000(	ENSG0000(	354.302	0.137888	0.143789	0.281189	lncRNA
ENSG0000(	ENAM	10.98973	0.523779	0.143883	0.281349	protein_coding
ENSG0000(	ENSG0000(	13.97215	0.464223	0.143889	0.281349	lncRNA
ENSG0000(	ENSG0000(	88.99918	0.111981	0.143977	0.281504	lncRNA
ENSG0000(	KANK2	1660.356	0.061997	0.144007	0.281544	protein_coding
ENSG0000(	ENSG0000(	105.5023	0.368249	0.144052	0.281616	protein_coding
ENSG0000(	ENSG0000(	32.70356	0.203811	0.144097	0.281685	lncRNA
ENSG0000(	ENSG0000(	18.91349	0.225859	0.144149	0.281769	lncRNA
ENSG0000(	LIN28B	15.82117	0.258854	0.144158	0.28177	protein_coding
ENSG0000(	PRR23A	198.6025	-0.07264	0.14417	0.281776	protein_coding

ENSG0000(	IQCH-AS1	1114.874	-0.07298	0.144179	0.281776	lncRNA
ENSG0000(	PLOD2	1576.346	-0.2477	0.144224	0.281821	protein_coding
ENSG0000(	TPM3P9	777.3854	0.078884	0.144229	0.281821	transcribed_processed_pseudoge
ENSG0000(	ZKSCAN8	2084.574	0.064362	0.14422	0.281821	protein_coding
ENSG0000(	ENSG0000(	161.8989	0.082266	0.144335	0.282011	transcribed_processed_pseudoge
ENSG0000(	SPDYA	207.0937	0.130919	0.144397	0.282115	protein_coding
ENSG0000(	ACTL6A	357.7466	-0.10905	0.144413	0.282128	protein_coding
ENSG0000(	FLRT3	207.3231	0.315936	0.144433	0.282143	protein_coding
ENSG0000(	RPS12P4	9.811139	0.360752	0.144438	0.282143	processed_pseudogene
ENSG0000(	FAM91A1	1753.585	0.054326	0.144484	0.282197	protein_coding
ENSG0000(	OR7E25P	714.2262	-0.05292	0.144484	0.282197	transcribed_unprocessed_pseudo
ENSG0000(	PIK3CG	1220.836	0.210559	0.14453	0.282268	protein_coding
ENSG0000(	ENSG0000(	9.403359	0.319462	0.144622	0.282431	lncRNA
ENSG0000(	ENSG0000(	12.0777	0.507908	0.144658	0.282484	lncRNA
ENSG0000(	TRBC2	637.3787	-0.28762	0.144733	0.282612	TR_C_gene
ENSG0000(	ENSG0000(	425.1098	-0.07347	0.144769	0.282665	lncRNA
ENSG0000(	NANOGP8	70.1396	0.141544	0.14481	0.282711	protein_coding
ENSG0000(	WDR41	906.5836	0.104246	0.144808	0.282711	protein_coding
ENSG0000(	LINC01456	9.546303	0.315271	0.144854	0.282779	lncRNA
ENSG0000(	DUXAP8	436.9212	0.229146	0.144911	0.28287	lncRNA
ENSG0000(	WDR75	950.2557	-0.09925	0.144918	0.28287	protein_coding
ENSG0000(	ENSG0000(	13128.85	-0.10226	0.145001	0.283013	lncRNA
ENSG0000(	ENSG0000(	251.0477	0.149129	0.145028	0.283049	lncRNA
ENSG0000(	COPS6	515.3199	-0.06229	0.14504	0.283055	protein_coding
ENSG0000(	ENSG0000(	142.294	0.230142	0.145112	0.283159	lncRNA
ENSG0000(	PIEZO1	3030.904	0.11066	0.145105	0.283159	protein_coding
ENSG0000(	ENSG0000(	87.61505	0.125032	0.14513	0.28316	lncRNA
ENSG0000(	SLCO2B1	5098.564	-0.09525	0.145128	0.28316	protein_coding
ENSG0000(	RWDD2B	281.1558	-0.10153	0.145161	0.283194	protein_coding
ENSG0000(	TRIM68	504.5117	0.07191	0.145166	0.283194	protein_coding
ENSG0000(	ENSG0000(	388.582	-0.09483	0.145194	0.283233	lncRNA
ENSG0000(	C20orf203	73.18426	0.379791	0.145206	0.283238	protein_coding
ENSG0000(	CDKL5	4099.393	0.062208	0.145233	0.283273	protein_coding
ENSG0000(	FBXO48	660.0675	-0.05954	0.14525	0.283289	protein_coding
ENSG0000(	MIR548AJ2	22.15075	0.236255	0.14526	0.283291	miRNA
ENSG0000(	MAGED4B	170.702	0.255218	0.145296	0.283326	protein_coding
ENSG0000(	RN7SL236F	31.57384	0.165837	0.145288	0.283326	misc_RNA
ENSG0000(	SLC7A13	19.43014	-0.1738	0.145307	0.283329	protein_coding
ENSG0000(	NECAP1	1941.133	-0.07025	0.145335	0.283367	protein_coding
ENSG0000(	ARSL	359.1517	-0.11392	0.145373	0.283406	protein_coding
ENSG0000(	SPDYE1	18.90684	0.370867	0.14537	0.283406	protein_coding
ENSG0000(	SLC16A13	157.2803	0.101634	0.145382	0.283407	protein_coding
ENSG0000(	CTAGE3P	63.0086	-0.29281	0.145417	0.283439	processed_pseudogene
ENSG0000(	PHAX	1219.185	-0.04452	0.145415	0.283439	protein_coding
ENSG0000(	ZNF236	1447.749	-0.10852	0.145433	0.283453	protein_coding
ENSG0000(	SNORA28	77.533	0.127075	0.145477	0.283522	snoRNA
ENSG0000(	CA3	302.172	-0.307	0.145603	0.283715	protein_coding

ENSG0000(	DTYMK	285.2364	-0.10086	0.145603	0.283715	protein_coding
ENSG0000(	ENSG0000(	50.87743	0.183165	0.145596	0.283715	lncRNA
ENSG0000(	TUBBP5	45.05837	0.380785	0.145633	0.283754	transcribed_unprocessed_pseudo
ENSG0000(	ENSG0000(	9.212306	0.28007	0.145684	0.283818	processed_pseudogene
ENSG0000(	ENSG0000(	65.12207	-0.17572	0.145682	0.283818	lncRNA
ENSG0000(	RN7SL118F	71.9475	-0.13492	0.14572	0.283871	misc_RNA
ENSG0000(	ENSG0000(	159.9826	-0.28308	0.145751	0.283897	lncRNA
ENSG0000(	HYDIN	336.376	0.129459	0.145745	0.283897	protein_coding
ENSG0000(	BET1	827.4717	-0.06784	0.145803	0.28398	protein_coding
ENSG0000(	ENSG0000(	16.43593	0.232813	0.145872	0.284098	lncRNA
ENSG0000(	HABP4	189.1919	0.157165	0.145893	0.28412	protein_coding
ENSG0000(	ZNRF2P2	43.87784	-0.19345	0.145979	0.28427	transcribed_processed_pseudoge
ENSG0000(	LINC01427	3905.488	-0.08318	0.146007	0.284308	lncRNA
ENSG0000(	H3C6	317.0818	-0.16439	0.146085	0.284424	protein_coding
ENSG0000(	OXTR	133.4281	0.130552	0.146076	0.284424	protein_coding
ENSG0000(	CCDC6	1966.176	-0.07845	0.146105	0.284445	protein_coding
ENSG0000(	ENSG0000(	99.02935	0.122185	0.14619	0.284593	lncRNA
ENSG0000(	ENSG0000(	38.05018	0.131295	0.146214	0.284623	lncRNA
ENSG0000(	PLA2G4B	385.4285	-0.15421	0.146224	0.284624	protein_coding
ENSG0000(	ENSG0000(	10.03499	-0.30934	0.146262	0.284658	lncRNA
ENSG0000(	PARD3	1061.584	-0.15419	0.146268	0.284658	protein_coding
ENSG0000(	ZNF219	460.2474	-0.18603	0.146262	0.284658	protein_coding
ENSG0000(	CORO1A	2355.917	-0.28516	0.146311	0.284707	protein_coding
ENSG0000(	GK-AS1	44.87176	0.133738	0.146312	0.284707	lncRNA
ENSG0000(	NWD1	528.6449	0.124224	0.146333	0.28473	protein_coding
ENSG0000(	CDX2	1690.32	-0.27625	0.146401	0.284846	protein_coding
ENSG0000(	WWC3	1410.053	0.091921	0.146432	0.284888	protein_coding
ENSG0000(	TRIP10	875.6679	-0.13154	0.146475	0.284954	protein_coding
ENSG0000(	DNAJC24	873.281	0.077383	0.146499	0.284984	protein_coding
ENSG0000(	ENSG0000(	13.24902	-0.25494	0.146567	0.285081	lncRNA
ENSG0000(	NT5DC4	73.77008	0.394552	0.146559	0.285081	protein_coding
ENSG0000(	ADCYAP1R1	139.16	0.28718	0.14659	0.28509	protein_coding
ENSG0000(	PRKCG	87.54552	-0.23149	0.146587	0.28509	protein_coding
ENSG0000(	ENSG0000(	18.09646	0.201831	0.146608	0.285094	lncRNA
ENSG0000(	RN7SKP283	31.13889	0.263276	0.14661	0.285094	misc_RNA
ENSG0000(	LINC02227	15.7679	0.429539	0.146667	0.285188	lncRNA
ENSG0000(	BCL9	527.3562	0.119026	0.146706	0.285246	protein_coding
ENSG0000(	CITED2	664.1269	-0.15968	0.146724	0.285263	protein_coding
ENSG0000(	ANKRD20A	9.025877	0.330992	0.146781	0.285357	unprocessed_pseudogene
ENSG0000(	SIRT7	1683.879	-0.10738	0.146792	0.285359	protein_coding
ENSG0000(	ORC6	10128.16	0.069487	0.146832	0.285421	protein_coding
ENSG0000(	TPM1	9951.187	0.131183	0.146848	0.285434	protein_coding
ENSG0000(	RHOXF1-AS	170.7919	0.105965	0.146971	0.285655	lncRNA
ENSG0000(	ENSG0000(	128.7923	0.121437	0.146989	0.285672	lncRNA
ENSG0000(	ENSG0000(	175.9533	0.207472	0.147042	0.285757	TEC
ENSG0000(	EFHB	78.28032	0.201068	0.147104	0.285857	protein_coding
ENSG0000(	MMS19	1141.669	0.071035	0.147111	0.285857	protein_coding

ENSG0000(	LINC01007	26.70515	0.32932	0.147153	0.28592	lncRNA
ENSG0000(	ERN2	1523.904	0.249096	0.147211	0.286016	protein_coding
ENSG0000(	ENSG0000(	43.82473	0.236812	0.147236	0.286047	lncRNA
ENSG0000(	RC3H1	4378.576	-0.08165	0.147307	0.286167	protein_coding
ENSG0000(	MLLT6	9594.73	-0.06708	0.147412	0.286354	protein_coding
ENSG0000(	CYTIP	1340.704	0.258307	0.147491	0.286488	protein_coding
ENSG0000(	ZNF93	425.0606	0.127407	0.147532	0.286551	protein_coding
ENSG0000(	ADGRV1	1256.58	0.079721	0.147635	0.286733	protein_coding
ENSG0000(	ENSG0000(	24.85358	-0.20231	0.147648	0.286741	snoRNA
ENSG0000(	KRT8P12	87.02009	-0.14146	0.147696	0.286816	transcribed_processed_pseudoge
ENSG0000(	BTG1P1	26.20475	-0.33545	0.14771	0.286827	processed_pseudogene
ENSG0000(	ENSG0000(	122.4657	0.108271	0.147736	0.286859	lncRNA
ENSG0000(	RASGRF2-A	152.3327	0.091509	0.147746	0.28686	lncRNA
ENSG0000(	ENSG0000(	208.944	0.096856	0.147781	0.28691	lncRNA
ENSG0000(	ENSG0000(	166.4273	0.108911	0.147815	0.286946	lncRNA
ENSG0000(	ENSG0000(	895.3645	-0.06595	0.147817	0.286946	TEC
ENSG0000(	RN7SL754F	34.05578	0.138143	0.147831	0.286954	misc_RNA
ENSG0000(	AKT3	2945.869	0.115788	0.147855	0.286965	protein_coding
ENSG0000(	ENSG0000(	120.7353	-0.08957	0.147847	0.286965	protein_coding
ENSG0000(	ENSG0000(	190.4973	-0.19042	0.147899	0.286965	protein_coding
ENSG0000(	PTGIS	534.7907	0.081393	0.147886	0.286965	protein_coding
ENSG0000(	TMEM161A	191.0651	0.135057	0.147897	0.286965	protein_coding
ENSG0000(	WRN	1103.723	0.106864	0.1479	0.286965	protein_coding
ENSG0000(	ZDHHC18	978.0014	0.118414	0.147898	0.286965	protein_coding
ENSG0000(	PIK3C2B	3645.182	-0.07733	0.147928	0.287001	protein_coding
ENSG0000(	ANKHD1-D	47.06792	0.151921	0.147939	0.287005	lncRNA
ENSG0000(	ENSG0000(	58.1512	-0.36527	0.147952	0.287012	lncRNA
ENSG0000(	ENSG0000(	135.4407	0.176634	0.14796	0.287012	lncRNA
ENSG0000(	DYNC2H1	1423.984	0.129398	0.148081	0.287193	protein_coding
ENSG0000(	EPN2-AS1	82.20274	-0.10943	0.148082	0.287193	lncRNA
ENSG0000(	GRM3-AS1	16.01274	0.340909	0.14809	0.287193	lncRNA
ENSG0000(	WDR44	654.243	0.079538	0.148065	0.287193	protein_coding
ENSG0000(	RN7SL396F	126.6747	0.19734	0.148161	0.28731	misc_RNA
ENSG0000(	RN7SL625F	83.22062	0.116668	0.148169	0.28731	misc_RNA
ENSG0000(	ADPRH	200.1213	0.243587	0.14819	0.287333	protein_coding
ENSG0000(	COLEC11	104.4912	0.119251	0.148215	0.287363	protein_coding
ENSG0000(	OR2D2	21.13986	0.305379	0.148261	0.287435	protein_coding
ENSG0000(	ENSG0000(	287.6229	0.078301	0.148282	0.287458	lncRNA
ENSG0000(	ENSG0000(	1008.332	-0.06372	0.148303	0.287471	lncRNA
ENSG0000(	ZNF529-AS	661.926	0.074084	0.148307	0.287471	lncRNA
ENSG0000(	FAM245B	132.2032	0.10155	0.148334	0.287486	lncRNA
ENSG0000(	ITK	2204.829	0.177952	0.148351	0.287486	protein_coding
ENSG0000(	MTND2P2	34.78695	0.174117	0.148336	0.287486	processed_pseudogene
ENSG0000(	SAP30L-AS	1233.369	-0.06464	0.148346	0.287486	lncRNA
ENSG0000(	CLDN9	11.42556	-0.2956	0.148371	0.287507	protein_coding
ENSG0000(	GDAP1	5689.075	0.062638	0.148384	0.287514	protein_coding
ENSG0000(	HEATR5A-D	264.3552	-0.1154	0.148452	0.28761	lncRNA

ENSG0000(	KRT8P39	55.03846	-0.17846	0.148446	0.28761	processed_pseudogene
ENSG0000(	ATF7IP	6708.176	0.114322	0.148469	0.287623	protein_coding
ENSG0000(	ENSG0000(	52.69823	0.146931	0.148476	0.287623	lncRNA
ENSG0000(	ENSG0000(	79.15907	0.16	0.148518	0.287685	lncRNA
ENSG0000(	COQ3	83.87894	-0.12268	0.14858	0.287787	protein_coding
ENSG0000(	SYT11	503.4859	0.128655	0.148646	0.287899	protein_coding
ENSG0000(	CXorf65	39.82776	0.562032	0.148705	0.287959	protein_coding
ENSG0000(	ENSG0000(	93.14697	0.141115	0.148688	0.287959	lncRNA
ENSG0000(	TRBV27	12.88233	-0.40392	0.148705	0.287959	TR_V_gene
ENSG0000(	FGGY	861.4381	-0.12377	0.148749	0.288026	protein_coding
ENSG0000(	ENSG0000(	118.6207	0.127507	0.148798	0.288105	lncRNA
ENSG0000(	IL33	373.5404	0.167761	0.148812	0.288113	protein_coding
ENSG0000(	RN7SL204F	103.7979	0.1011	0.148872	0.288211	misc_RNA
ENSG0000(	ENSG0000(	92.90735	0.117047	0.148933	0.288276	lncRNA
ENSG0000(	ENSG0000(	492.689	0.075875	0.14892	0.288276	lncRNA
ENSG0000(	ENSG0000(	156.7954	0.091086	0.148925	0.288276	lncRNA
ENSG0000(	LINC01809	10.75765	0.262805	0.148973	0.288337	lncRNA
ENSG0000(	SHISA5	1264.131	0.080794	0.148989	0.288351	protein_coding
ENSG0000(	ENSG0000(	24.67535	0.169064	0.149088	0.288524	lncRNA
ENSG0000(	SCYL2P1	246.6001	-0.10198	0.14912	0.288568	processed_pseudogene
ENSG0000(	CILP	91.6338	0.274648	0.149149	0.288607	protein_coding
ENSG0000(	TAF12	2480.899	0.072897	0.14922	0.288726	protein_coding
ENSG0000(	TRMT11	1045.939	-0.09126	0.149249	0.288764	protein_coding
ENSG0000(	RNU1-83P	99.64476	0.339413	0.14926	0.288768	snRNA
ENSG0000(	BARD1	850.3723	0.125565	0.149296	0.288802	protein_coding
ENSG0000(	ENSG0000(	45.66366	-0.29925	0.149294	0.288802	lncRNA
ENSG0000(	ENSG0000(	105.1788	0.086897	0.149409	0.288987	lncRNA
ENSG0000(	PCDH11Y	693.6108	-0.20807	0.14941	0.288987	protein_coding
ENSG0000(	ENSG0000(	645.3951	0.084822	0.14946	0.289066	protein_coding
ENSG0000(	ENSG0000(	99.35163	-0.10026	0.149477	0.289081	lncRNA
ENSG0000(	ALKBH3	212.9238	-0.1133	0.149604	0.289308	protein_coding
ENSG0000(	TRBJ1-1	8.509293	0.542631	0.149615	0.289313	TR_J_gene
ENSG0000(	ALG1L	64.07937	0.13641	0.149667	0.289395	transcribed_unprocessed_pseudo
ENSG0000(	RAB10	1704.492	-0.09924	0.149684	0.289411	protein_coding
ENSG0000(	MCM6	452.696	-0.1642	0.149753	0.289526	protein_coding
ENSG0000(	MYCL	262.3641	0.096684	0.149775	0.289551	protein_coding
ENSG0000(	HSD17B7	859.0178	0.08117	0.149819	0.289612	protein_coding
ENSG0000(	TGFA-IT1	14.60175	-0.28475	0.149825	0.289612	lncRNA
ENSG0000(	LINC02826	226.516	0.151547	0.149858	0.289639	lncRNA
ENSG0000(	TAS2R19	261.1012	0.121883	0.149853	0.289639	protein_coding
ENSG0000(	ACVR1B	1522.727	-0.18323	0.149886	0.289676	protein_coding
ENSG0000(	ENTR1	506.1414	-0.12337	0.149914	0.289712	protein_coding
ENSG0000(	LINC01778	25.17005	-0.18027	0.149944	0.289754	lncRNA
ENSG0000(	MIR3671	19.35127	-0.21986	0.149967	0.289763	miRNA
ENSG0000(	RN7SL452F	15.89727	0.224168	0.149959	0.289763	misc_RNA
ENSG0000(	ENSG0000(	20.54837	0.239569	0.14998	0.289769	lncRNA
ENSG0000(	C16orf72	9986.982	0.037752	0.150001	0.289792	protein_coding

ENSG0000(	ENSG0000(	66.4545	-0.29851	0.150033	0.289836	lncRNA
ENSG0000(	CCDC124	227.1739	-0.07306	0.150131	0.290008	protein_coding
ENSG0000(	ENSG0000(	780.5969	0.126926	0.150159	0.290044	lncRNA
ENSG0000(	CCDC117	906.9033	-0.07651	0.150177	0.29006	protein_coding
ENSG0000(	LINC01205	806.7012	-0.05835	0.150207	0.290101	lncRNA
ENSG0000(	BEAN1	130.4934	0.136485	0.150274	0.290212	protein_coding
ENSG0000(	HOATZ	204.6554	-0.10632	0.150291	0.290228	protein_coding
ENSG0000(	ZMAT2	312.9053	-0.08664	0.150329	0.290283	protein_coding
ENSG0000(	CAAP1	361.6807	-0.09077	0.150345	0.290284	protein_coding
ENSG0000(	HCFC1R1	100.0442	0.139297	0.150348	0.290284	protein_coding
ENSG0000(	OPRL1	66.54234	0.244619	0.150383	0.290334	protein_coding
ENSG0000(	A4GALT	312.3698	0.10356	0.150402	0.290335	protein_coding
ENSG0000(	GSTT4	38.94331	-0.15979	0.150401	0.290335	protein_coding
ENSG0000(	SNHG5	3395.195	-0.10811	0.150425	0.290363	lncRNA
ENSG0000(	ZNF561-AS	1690.291	0.043612	0.150436	0.290365	lncRNA
ENSG0000(	ENSG0000(	9.568816	0.300982	0.150466	0.290406	lncRNA
ENSG0000(	TRGV3	20.32231	-0.32721	0.150481	0.290417	TR_V_gene
ENSG0000(	GJB2	215.9242	-0.21458	0.150505	0.290445	protein_coding
ENSG0000(	DNAJC11	2498.007	0.036008	0.150564	0.290541	protein_coding
ENSG0000(	PDIA3P1	484.4384	-0.07214	0.150644	0.290677	transcribed_processed_pseudoge
ENSG0000(	HCG4	8.328618	0.370259	0.150664	0.290681	unprocessed_pseudogene
ENSG0000(	INTS14	550.5471	0.07317	0.150658	0.290681	protein_coding
ENSG0000(	ENSG0000(	37.71619	0.150815	0.150677	0.290687	lncRNA
ENSG0000(	SC5D	1458.074	-0.09104	0.150748	0.290808	protein_coding
ENSG0000(	ENSG0000(	21.50785	0.18569	0.150968	0.291214	lncRNA
ENSG0000(	ENSG0000(	124.5649	0.133008	0.15102	0.291297	lncRNA
ENSG0000(	ATP1A1	17347.95	-0.28003	0.151033	0.291304	protein_coding
ENSG0000(	ENSG0000(	24.73859	0.319244	0.151105	0.2914	processed_pseudogene
ENSG0000(	TMEM164	1973.673	-0.1235	0.151111	0.2914	protein_coding
ENSG0000(	VTCN1	11.99806	0.354707	0.151096	0.2914	protein_coding
ENSG0000(	APRT	234.6449	-0.12977	0.151199	0.291524	protein_coding
ENSG0000(	ENSG0000(	221.4965	0.089469	0.151203	0.291524	lncRNA
ENSG0000(	REXO5	151.2563	0.093429	0.151189	0.291524	protein_coding
ENSG0000(	ENSG0000(	14.45315	-0.25908	0.151238	0.291575	lncRNA
ENSG0000(	ZNF668	164.8332	0.144533	0.151249	0.291577	protein_coding
ENSG0000(	ENSG0000(	98.01654	0.215557	0.151314	0.291685	lncRNA
ENSG0000(	ARMT1	244.497	-0.10659	0.151338	0.291698	protein_coding
ENSG0000(	ENSG0000(	48.26821	-0.15039	0.151339	0.291698	lncRNA
ENSG0000(	DCTN3	387.0614	-0.09199	0.15135	0.2917	protein_coding
ENSG0000(	LINC01993	292.2792	0.094599	0.15139	0.291742	lncRNA
ENSG0000(	UPRT	367.2982	-0.07366	0.151387	0.291742	protein_coding
ENSG0000(	AAR2	700.5097	0.052293	0.151468	0.291785	protein_coding
ENSG0000(	CABP1-DT	104.9032	-0.13683	0.151462	0.291785	lncRNA
ENSG0000(	ENSG0000(	347.1966	-0.10001	0.151463	0.291785	lncRNA
ENSG0000(	ENSG0000(	208.1102	-0.09338	0.151438	0.291785	lncRNA
ENSG0000(	ENSG0000(	9.287607	0.941165	0.151449	0.291785	transcribed_unprocessed_pseudo
ENSG0000(	IGFALS	8.054008	0.325143	0.151439	0.291785	protein_coding

ENSG0000(	TSSK6	27.85921	0.187065	0.151498	0.291825	protein_coding
ENSG0000(	ZMYM5	874.3552	0.059299	0.151515	0.291841	protein_coding
ENSG0000(	TBC1D2	384.3516	0.150072	0.151559	0.291907	protein_coding
ENSG0000(	ENSG0000(	745.739	-0.05473	0.151653	0.292052	lncRNA
ENSG0000(	TSSC4	189.8309	0.132132	0.151649	0.292052	protein_coding
ENSG0000(	ADGRL2	1631.806	0.120078	0.151695	0.292116	protein_coding
ENSG0000(	ENSG0000(	35.72887	0.152193	0.151711	0.29212	lncRNA
ENSG0000(	NR2E3	36.29772	-0.32043	0.151716	0.29212	protein_coding
ENSG0000(	ELF5	109.3025	0.104659	0.151774	0.292214	protein_coding
ENSG0000(	HMBOX1-I	53.59241	0.106434	0.151854	0.292351	lncRNA
ENSG0000(	ENSG0000(	223.2395	-0.07152	0.151925	0.292468	lncRNA
ENSG0000(	OLA1P2	275.5203	0.074647	0.151951	0.292502	processed_pseudogene
ENSG0000(	ENSG0000(	46.48388	-0.23072	0.152006	0.29259	processed_pseudogene
ENSG0000(	ENSG0000(	9.882318	0.262198	0.152097	0.292747	lncRNA
ENSG0000(	CCDC191	448.8953	0.08482	0.152226	0.292838	protein_coding
ENSG0000(	CCDC192	15.05435	0.217521	0.15224	0.292838	protein_coding
ENSG0000(	DLEU7	563.6124	0.077382	0.152212	0.292838	protein_coding
ENSG0000(	ENSG0000(	176.1573	0.090129	0.152215	0.292838	lncRNA
ENSG0000(	ENSG0000(	82.2467	-0.32546	0.152217	0.292838	lncRNA
ENSG0000(	ENSG0000(	262.8432	0.090837	0.152181	0.292838	TEC
ENSG0000(	NPM1P37	126.983	-0.11232	0.152233	0.292838	processed_pseudogene
ENSG0000(	PPP2R5C	4416.361	0.090733	0.152165	0.292838	protein_coding
ENSG0000(	RPL36A	1787.138	-0.16456	0.152207	0.292838	protein_coding
ENSG0000(	SH3BP5-AS	2109.356	0.064087	0.152239	0.292838	lncRNA
ENSG0000(	TTLL12	431.8766	-0.12393	0.152247	0.292838	protein_coding
ENSG0000(	C1orf122	90.93777	0.168735	0.152285	0.292894	protein_coding
ENSG0000(	PXDC1	408.1796	-0.1862	0.15235	0.293	protein_coding
ENSG0000(	PHF2	911.4365	0.06182	0.152365	0.293012	protein_coding
ENSG0000(	SERPINA10	830.3659	0.139107	0.152385	0.293014	protein_coding
ENSG0000(	SOHLH2	34.77478	0.183845	0.152385	0.293014	protein_coding
ENSG0000(	MMP2-AS1	140.633	0.312063	0.152445	0.293111	lncRNA
ENSG0000(	CPEB2	1195.765	-0.1393	0.152462	0.293126	protein_coding
ENSG0000(	PDCD6IPP2	374.5868	0.077022	0.152481	0.293145	transcribed_unprocessed_pseudo
ENSG0000(	SDF4	1773.866	-0.12254	0.1525	0.293163	protein_coding
ENSG0000(	ENSG0000(	15.33926	0.209037	0.152661	0.293369	lncRNA
ENSG0000(	ENSG0000(	155.1239	0.097733	0.152662	0.293369	lncRNA
ENSG0000(	FAT2	29.977	0.321211	0.152653	0.293369	protein_coding
ENSG0000(	MIR4802	34.85519	-0.29247	0.152631	0.293369	miRNA
ENSG0000(	TTC13	1001.496	-0.10129	0.152624	0.293369	protein_coding
ENSG0000(	WIPF1	3968.302	0.205484	0.152654	0.293369	protein_coding
ENSG0000(	ENSG0000(	27.36974	0.195185	0.152738	0.293496	lncRNA
ENSG0000(	ENSG0000(	11.02949	-0.38376	0.152804	0.293605	transcribed_unprocessed_pseudo
ENSG0000(	NEDD9	4241.749	0.090155	0.152869	0.293712	protein_coding
ENSG0000(	ZBTB7C	244.8772	0.214604	0.152894	0.293743	protein_coding
ENSG0000(	ANKRD18B	70.11678	-0.18437	0.152955	0.293841	protein_coding
ENSG0000(	CCDC83	12.01036	-0.29964	0.152966	0.293845	protein_coding
ENSG0000(	RNU6-1305	9.800583	0.316266	0.153003	0.29388	snRNA

ENSG0000(	ZNF785	1626.287	-0.04782	0.152996	0.29388	protein_coding
ENSG0000(	ENSG0000(	20.04088	0.226954	0.153035	0.293924	lncRNA
ENSG0000(	ZNF815P	150.825	0.19803	0.153049	0.293932	transcribed_unprocessed_pseudo
ENSG0000(	C2orf50	732.2621	-0.04509	0.153106	0.294024	protein_coding
ENSG0000(	SARDH	199.1095	-0.16335	0.15312	0.294034	protein_coding
ENSG0000(	ENSG0000(	12.60992	0.253886	0.153139	0.294052	lncRNA
ENSG0000(	ZNF552	902.4965	-0.1227	0.153159	0.294072	protein_coding
ENSG0000(	ENSG0000(	258.1513	0.360096	0.153214	0.294161	lncRNA
ENSG0000(	RANBP1	412.0778	-0.11578	0.153236	0.294182	protein_coding
ENSG0000(	ZSCAN29	1496.952	-0.06507	0.153245	0.294182	protein_coding
ENSG0000(	ARVCF	213.6688	0.146167	0.153269	0.294211	protein_coding
ENSG0000(	TMX4	1213.968	-0.09075	0.153284	0.294223	protein_coding
ENSG0000(	LINC01394	13.82603	0.310268	0.153301	0.294237	lncRNA
ENSG0000(	EPB41L2	4488.03	0.064788	0.153314	0.294244	protein_coding
ENSG0000(	C21orf58	518.3971	0.107946	0.153343	0.294263	protein_coding
ENSG0000(	DUSP2	128.7657	0.351665	0.153338	0.294263	protein_coding
ENSG0000(	RPS23P6	42.53943	0.275625	0.153386	0.294328	processed_pseudogene
ENSG0000(	LINC01695	850.7353	0.076735	0.153423	0.294382	lncRNA
ENSG0000(	BEX2	34.6551	-0.23171	0.153455	0.294425	protein_coding
ENSG0000(	ENSG0000(	186.0545	0.080265	0.153503	0.294469	TEC
ENSG0000(	LINC02418	139.7143	-0.10286	0.153506	0.294469	lncRNA
ENSG0000(	ZNF606	1140.628	0.058821	0.153499	0.294469	protein_coding
ENSG0000(	IL31RA	424.1909	0.075602	0.153536	0.294502	protein_coding
ENSG0000(	PCDH20	57.66095	0.442489	0.153542	0.294502	protein_coding
ENSG0000(	MAP2K1	877.6242	0.094263	0.153587	0.294571	protein_coding
ENSG0000(	DRP2	47.42925	0.206547	0.153616	0.294608	protein_coding
ENSG0000(	DUT-AS1	577.9822	-0.06189	0.153697	0.294746	lncRNA
ENSG0000(	FABP7	57.36736	0.124975	0.153728	0.294776	protein_coding
ENSG0000(	ROCK1P1	741.0795	0.417111	0.153731	0.294776	transcribed_unprocessed_pseudo
ENSG0000(	ENSG0000(	136.8563	-0.08663	0.153837	0.294942	lncRNA
ENSG0000(	GXYLT1P4	17.72088	0.356453	0.153836	0.294942	transcribed_processed_pseudoge
ENSG0000(	ENSG0000(	137.247	0.212109	0.153901	0.295047	protein_coding
ENSG0000(	OR5B21	21.19806	0.336939	0.153933	0.295091	protein_coding
ENSG0000(	ENSG0000(	35.29389	0.343152	0.153991	0.295166	lncRNA
ENSG0000(	RNU2-30P	18.96805	-0.2157	0.153984	0.295166	snRNA
ENSG0000(	ENSG0000(	34.89024	0.390094	0.154019	0.295201	lncRNA
ENSG0000(	CBWD5	2703.043	-0.10232	0.154039	0.295222	protein_coding
ENSG0000(	TSPAN13	756.1944	-0.14219	0.15405	0.295225	protein_coding
ENSG0000(	ASPN	162.6936	0.175715	0.154091	0.295287	protein_coding
ENSG0000(	ENSG0000(	94.80795	-0.12397	0.154109	0.295303	lncRNA
ENSG0000(	ENSG0000(	71.03035	0.23706	0.154193	0.295429	lncRNA
ENSG0000(	ENSG0000(	27.01947	0.163901	0.154194	0.295429	lncRNA
ENSG0000(	SLU7	933.6815	-0.08045	0.154297	0.295609	protein_coding
ENSG0000(	ENHO	28.16573	-0.21403	0.154324	0.295642	protein_coding
ENSG0000(	MFGE8	1130.912	0.192307	0.154335	0.295645	protein_coding
ENSG0000(	ENSG0000(	154.3091	-0.13451	0.154369	0.295693	lncRNA
ENSG0000(	ENSG0000(	28.66151	0.232797	0.154402	0.295737	lncRNA

ENSG0000(	RNU1-104F	17.1954	-0.38625	0.154422	0.295758	snRNA
ENSG0000(	ENSG0000(	216.3984	-0.0979	0.154531	0.295949	lncRNA
ENSG0000(	MAP3K11	3095.182	-0.0805	0.154593	0.296049	protein_coding
ENSG0000(	DCLK2	245.4051	0.154647	0.154658	0.29613	protein_coding
ENSG0000(	FAM66B	281.1355	0.157084	0.154673	0.29613	lncRNA
ENSG0000(	PROCA1	184.7408	0.150632	0.154646	0.29613	protein_coding
ENSG0000(	RNU7-181F	17.01559	0.301198	0.154668	0.29613	snRNA
ENSG0000(	ATP8A2P3	19.04471	0.208359	0.154699	0.29614	unprocessed_pseudogene
ENSG0000(	LINC02586	11.25134	0.306495	0.154709	0.29614	lncRNA
ENSG0000(	NPM1P41	30.35473	-0.5135	0.154715	0.29614	processed_pseudogene
ENSG0000(	YTHDF3-DT	9.421958	0.254554	0.154715	0.29614	lncRNA
ENSG0000(	NUFIP2	6983.319	0.04912	0.154755	0.296198	protein_coding
ENSG0000(	ENSG0000(	25.25321	0.361161	0.154771	0.29621	unprocessed_pseudogene
ENSG0000(	SATB2-AS1	158.7748	-0.28206	0.154805	0.296257	lncRNA
ENSG0000(	RNU6-1157	25.20098	0.17949	0.154826	0.296279	snRNA
ENSG0000(	CDR2-DT	141.1004	0.073914	0.154849	0.296305	lncRNA
ENSG0000(	RBM7	1013.587	-0.08772	0.154902	0.29639	protein_coding
ENSG0000(	ENSG0000(	17.99752	-0.26565	0.154932	0.29641	lncRNA
ENSG0000(	UQCRHL	249.2183	0.067184	0.154928	0.29641	protein_coding
ENSG0000(	MIR4538	19.54223	0.436138	0.154945	0.296418	miRNA
ENSG0000(	ENSG0000(	24.11501	-0.21576	0.155154	0.2968	processed_pseudogene
ENSG0000(	RN7SL690F	272.3866	0.085123	0.155171	0.296813	misc_RNA
ENSG0000(	ENSG0000(	12.97677	0.258075	0.155199	0.296838	lncRNA
ENSG0000(	NPTXR	206.9081	-0.14118	0.155202	0.296838	protein_coding
ENSG0000(	ZDHHC22	48.25792	0.213343	0.155243	0.296897	protein_coding
ENSG0000(	ZMYM6	1861.111	-0.04712	0.155284	0.296957	protein_coding
ENSG0000(	C8orf44-SG	31.35364	-0.30299	0.155314	0.296997	protein_coding
ENSG0000(	C1QTNF7	65.01602	0.298117	0.155383	0.297096	protein_coding
ENSG0000(	FAM124B	107.076	0.108715	0.155385	0.297096	protein_coding
ENSG0000(	ENSG0000(	298.8736	0.088495	0.155451	0.297206	lncRNA
ENSG0000(	EFCAB6-DT	49.97163	0.156604	0.155464	0.297212	lncRNA
ENSG0000(	ARHGEF33	46.32119	0.234782	0.155507	0.297257	protein_coding
ENSG0000(	CELF2	3422.795	0.177577	0.155501	0.297257	protein_coding
ENSG0000(	ENSG0000(	144.551	-0.09641	0.155521	0.297257	lncRNA
ENSG0000(	ZFP69B	83.60336	0.194614	0.155525	0.297257	protein_coding
ENSG0000(	GTPBP4	907.5871	0.094535	0.155541	0.297269	protein_coding
ENSG0000(	SLC22A2	107.9111	0.160162	0.155575	0.297315	protein_coding
ENSG0000(	ENSG0000(	196.3212	0.101835	0.155612	0.297351	lncRNA
ENSG0000(	TXNL4B	429.8159	-0.07452	0.155603	0.297351	protein_coding
ENSG0000(	ENSG0000(	20.01535	-0.27284	0.155665	0.297416	processed_pseudogene
ENSG0000(	LINC00654	660.0267	-0.12863	0.155658	0.297416	lncRNA
ENSG0000(	SLIT3	608.8571	-0.17854	0.155736	0.297533	protein_coding
ENSG0000(	CYSLTR1	2827.799	-0.08171	0.155747	0.297537	protein_coding
ENSG0000(	ZDHHC16	614.2488	0.062847	0.15577	0.297562	protein_coding
ENSG0000(	TMPRSS12	186.2675	-0.09	0.155788	0.297579	protein_coding
ENSG0000(	LINC01415	12.57325	0.321247	0.155864	0.297706	lncRNA
ENSG0000(	ENSG0000(	33.90619	0.15281	0.155946	0.297845	lncRNA

ENSG0000(	ENSG0000(	66.89508	0.160074	0.155962	0.297857	lncRNA
ENSG0000(	NEK6	1894.419	-0.05896	0.156023	0.297954	protein_coding
ENSG0000(	UTP15	422.811	0.114995	0.156035	0.297959	protein_coding
ENSG0000(	NCOA4P4	10.49722	0.302149	0.156191	0.29824	processed_pseudogene
ENSG0000(	GIMAP1	800.0455	0.166358	0.156211	0.298248	protein_coding
ENSG0000(	RCOR3	2613.31	-0.07825	0.156215	0.298248	protein_coding
ENSG0000(	MTCO3P19	13.44738	0.2299	0.156229	0.298259	processed_pseudogene
ENSG0000(	CYP2U1-AS	369.4882	-0.15853	0.156256	0.298292	lncRNA
ENSG0000(	NACAD	53.30124	-0.26784	0.156288	0.298317	protein_coding
ENSG0000(	RN7SL819P	69.0444	-0.13695	0.156286	0.298317	misc_RNA
ENSG0000(	ENSG0000(	235.3852	0.067976	0.156341	0.298396	lncRNA
ENSG0000(	MIR581	23.40445	0.214039	0.156349	0.298396	miRNA
ENSG0000(	ENSG0000(	14.65436	0.385615	0.15637	0.2984	processed_pseudogene
ENSG0000(	PANK2-AS1	58.86175	0.13141	0.15637	0.2984	lncRNA
ENSG0000(	ENSG0000(	251.229	0.180469	0.156455	0.298526	lncRNA
ENSG0000(	IL17RD	693.5238	-0.09119	0.156448	0.298526	protein_coding
ENSG0000(	ENSG0000(	49.4481	0.140142	0.156465	0.298528	lncRNA
ENSG0000(	GPD2	1544.031	-0.13834	0.156484	0.298545	protein_coding
ENSG0000(	ENSG0000(	17.75121	0.211022	0.156529	0.298614	lncRNA
ENSG0000(	ENSG0000(	19.92187	0.741118	0.156629	0.298787	transcribed_unprocessed_pseudo
ENSG0000(	INHBE	80.02071	-0.10193	0.156649	0.298797	protein_coding
ENSG0000(	KCNC3	352.2964	0.246998	0.156653	0.298797	protein_coding
ENSG0000(	GSTZ1	709.1893	0.09392	0.156673	0.298816	protein_coding
ENSG0000(	ENSG0000(	9.954177	0.28228	0.156725	0.29888	lncRNA
ENSG0000(	ENSG0000(	27.73054	-0.27719	0.156726	0.29888	lncRNA
ENSG0000(	ENSG0000(	32.49831	0.158956	0.156739	0.298887	lncRNA
ENSG0000(	PDCL3	70.58567	0.138523	0.156754	0.298898	protein_coding
ENSG0000(	ENSG0000(	250.5826	0.075507	0.156815	0.298996	TEC
ENSG0000(	DESI2	1020.576	-0.07241	0.156837	0.299006	protein_coding
ENSG0000(	IFFO2	374.5753	0.143773	0.156839	0.299006	protein_coding
ENSG0000(	MFAP3	1449.411	0.053097	0.156856	0.299021	protein_coding
ENSG0000(	ENSG0000(	37.21664	0.174317	0.156876	0.299023	lncRNA
ENSG0000(	SERTM2	12.30493	0.443485	0.156869	0.299023	protein_coding
ENSG0000(	ENSG0000(	306.8368	0.06872	0.15696	0.299146	lncRNA
ENSG0000(	ZNF787	404.3204	-0.08388	0.15696	0.299146	protein_coding
ENSG0000(	RBM28	3319.557	0.073943	0.156982	0.299169	protein_coding
ENSG0000(	ENSG0000(	192.9612	0.084955	0.157033	0.29924	lncRNA
ENSG0000(	NDUFV2-AS	63.72711	0.164832	0.157038	0.29924	lncRNA
ENSG0000(	LINC00692	26.37733	0.146775	0.157173	0.299479	lncRNA
ENSG0000(	ENSG0000(	79.66725	0.127355	0.15721	0.29953	lncRNA
ENSG0000(	MED21	627.9401	0.126423	0.157219	0.29953	protein_coding
ENSG0000(	ENSG0000(	263.8505	0.088495	0.157261	0.299594	lncRNA
ENSG0000(	ENSG0000(	388.1422	0.080307	0.157299	0.299632	lncRNA
ENSG0000(	ENSG0000(	243.3801	0.062095	0.157301	0.299632	lncRNA
ENSG0000(	ENSG0000(	31.56797	0.201937	0.157405	0.299812	TEC
ENSG0000(	ENSG0000(	61.81369	0.187764	0.157416	0.299815	lncRNA
ENSG0000(	ENSG0000(	155.1818	0.110675	0.157426	0.299817	lncRNA

ENSG0000(	PREX1	1826.391	0.216496	0.157442	0.299828	protein_coding
ENSG0000(	C5orf51	860.5583	-0.08149	0.157527	0.299973	protein_coding
ENSG0000(	RN7SL301F	77.82651	0.149101	0.15754	0.29998	misc_RNA
ENSG0000(	GUSBP3	1826.445	-0.0954	0.157623	0.300118	transcribed_unprocessed_pseudo
ENSG0000(	ENSG0000(	565.2952	0.073328	0.157653	0.300157	lncRNA
ENSG0000(	ATP11A-AS	76.07062	0.104496	0.157671	0.300174	lncRNA
ENSG0000(	CDH10	21.92204	-0.19185	0.157681	0.300175	protein_coding
ENSG0000(	LRRC37A6F	26.48932	-0.27281	0.157747	0.300283	transcribed_processed_pseudoge
ENSG0000(	ENSG0000(	43.93967	-0.17532	0.157762	0.300292	processed_pseudogene
ENSG0000(	ENSG0000(	27.59973	-0.17804	0.157787	0.300322	lncRNA
ENSG0000(	C1orf21	2987.725	-0.19132	0.157817	0.300361	protein_coding
ENSG0000(	SEC14L5	222.9566	-0.11023	0.157828	0.300363	protein_coding
ENSG0000(	ENSG0000(	34.66417	-0.12394	0.157939	0.300539	lncRNA
ENSG0000(	ST20	191.7291	-0.0908	0.157935	0.300539	protein_coding
ENSG0000(	TBC1D30	529.0325	-0.08881	0.157963	0.300567	protein_coding
ENSG0000(	ENSG0000(	29.40289	-0.16071	0.158011	0.30064	processed_pseudogene
ENSG0000(	DPRXP3	71.4455	0.13731	0.158059	0.300707	processed_pseudogene
ENSG0000(	LINC00276	512.7057	0.073923	0.158066	0.300707	lncRNA
ENSG0000(	CCDC92B	257.13	-0.07375	0.158124	0.300793	protein_coding
ENSG0000(	DMKN	224.2522	0.112509	0.158129	0.300793	protein_coding
ENSG0000(	SNHG3	1924.57	0.159112	0.158165	0.300842	lncRNA
ENSG0000(	FAM131A	1002.865	-0.06123	0.158199	0.300888	protein_coding
ENSG0000(	SLC16A6	147.2941	0.17212	0.158316	0.301093	protein_coding
ENSG0000(	ZNF572	134.2633	0.115602	0.158379	0.301194	protein_coding
ENSG0000(	ETF1	1310.62	0.068147	0.15839	0.301198	protein_coding
ENSG0000(	ENSG0000(	13.43126	-0.20449	0.158408	0.301213	lncRNA
ENSG0000(	ENSG0000(	60.21271	-0.12118	0.158436	0.301249	lncRNA
ENSG0000(	RPL34-DT	272.3626	0.082954	0.158486	0.301326	lncRNA
ENSG0000(	ANKRD20A	115.9882	0.160453	0.158534	0.301384	transcribed_unprocessed_pseudo
ENSG0000(	SDR16C6P	15.22191	-0.23574	0.158536	0.301384	transcribed_unitary_pseudogene
ENSG0000(	MRPL57	669.0105	0.040125	0.158564	0.301419	protein_coding
ENSG0000(	SLC25A30	1015.246	-0.08954	0.158598	0.301448	protein_coding
ENSG0000(	ZNF660	612.9915	0.112452	0.158597	0.301448	protein_coding
ENSG0000(	ENSG0000(	256.6027	0.070878	0.158618	0.301467	lncRNA
ENSG0000(	ARMCX7P	27.90922	0.62808	0.15873	0.301662	transcribed_unprocessed_pseudo
ENSG0000(	PLEKHA6	2796.246	-0.19997	0.158773	0.301725	protein_coding
ENSG0000(	ENSG0000(	295.3934	-0.09874	0.158786	0.301732	TEC
ENSG0000(	ST3GAL5-A	33.18171	0.26785	0.158823	0.301783	lncRNA
ENSG0000(	CEP76	687.7843	-0.07631	0.158844	0.301806	protein_coding
ENSG0000(	ENSG0000(	24.7667	0.188402	0.158912	0.301917	lncRNA
ENSG0000(	ENSG0000(	18.88426	0.236744	0.158965	0.301986	protein_coding
ENSG0000(	SLF1	515.427	-0.08503	0.158967	0.301986	protein_coding
ENSG0000(	RPS6KB2-A	35.12336	-0.31109	0.158987	0.302004	lncRNA
ENSG0000(	AOX2P	88.8913	0.102916	0.159	0.302011	transcribed_unprocessed_pseudo
ENSG0000(	YBX1P8	32.44894	0.358379	0.159023	0.302036	processed_pseudogene
ENSG0000(	ENSG0000(	173.6262	-0.12526	0.159102	0.302168	lncRNA
ENSG0000(	ENSG0000(	127.0296	0.288219	0.15918	0.302298	transcribed_unprocessed_pseudo

ENSG0000(	ENSG0000(	16.40758	-0.35616	0.159267	0.302446	lncRNA
ENSG0000(	ENSG0000(	22.41789	0.196888	0.159285	0.302452	lncRNA
ENSG0000(	ITGB1	6244.085	0.091274	0.15929	0.302452	protein_coding
ENSG0000(	TIMM10B	1436.318	-0.05515	0.159408	0.302659	protein_coding
ENSG0000(	MUS81	831.7007	-0.06617	0.159466	0.30275	protein_coding
ENSG0000(	LINC01273	65.24565	-0.13381	0.159485	0.302767	lncRNA
ENSG0000(	C7orf61	22.3187	-0.24435	0.15952	0.302815	protein_coding
ENSG0000(	CDK11B	797.6719	-0.11056	0.159555	0.302864	protein_coding
ENSG0000(	ENSG0000(	346.3867	-0.07534	0.159577	0.302889	lncRNA
ENSG0000(	WDR43	716.8688	-0.09637	0.159622	0.302955	protein_coding
ENSG0000(	CALHM2	185.2199	0.150149	0.159728	0.303119	protein_coding
ENSG0000(	FOXD2-AS1	73.78151	0.163596	0.159727	0.303119	lncRNA
ENSG0000(	ENSG0000(	170.3478	-0.32345	0.159768	0.303176	lncRNA
ENSG0000(	NPAS3	197.8415	0.121064	0.159863	0.30334	protein_coding
ENSG0000(	ENSG0000(	387.423	0.095263	0.15989	0.303372	lncRNA
ENSG0000(	HOXA10	182.7777	-0.20319	0.159936	0.303426	protein_coding
ENSG0000(	IKZF1	4316.097	0.263046	0.159938	0.303426	protein_coding
ENSG0000(	ENSG0000(	9813.603	-0.07929	0.159962	0.303436	lncRNA
ENSG0000(	FAM214B	590.5255	0.137914	0.159959	0.303436	protein_coding
ENSG0000(	ENSG0000(	12.48314	0.233687	0.160005	0.303498	lncRNA
ENSG0000(	ENSG0000(	17.28715	-0.51527	0.160031	0.303526	lncRNA
ENSG0000(	MUC2	84257.82	0.320293	0.160039	0.303526	protein_coding
ENSG0000(	MANF	406.7575	0.135709	0.160109	0.303642	protein_coding
ENSG0000(	ENSG0000(	12.70325	0.304258	0.160154	0.303708	lncRNA
ENSG0000(	MAP3K9-D	26.61282	0.189471	0.160175	0.30373	lncRNA
ENSG0000(	AXIN1	885.747	-0.08053	0.160237	0.303755	protein_coding
ENSG0000(	CD3E	677.3242	-0.24021	0.160246	0.303755	protein_coding
ENSG0000(	ENSG0000(	60.86342	0.1141	0.160209	0.303755	lncRNA
ENSG0000(	LINC00488	65.79729	-0.15801	0.160242	0.303755	lncRNA
ENSG0000(	RPL36AP54	16.71142	0.212472	0.160238	0.303755	processed_pseudogene
ENSG0000(	SMPD4	2160.687	0.065617	0.160235	0.303755	protein_coding
ENSG0000(	DCP1B	421.35	-0.06354	0.160435	0.304051	protein_coding
ENSG0000(	IBTK	2303.061	0.063524	0.160441	0.304051	protein_coding
ENSG0000(	THRIL	158.1306	-0.09	0.160414	0.304051	lncRNA
ENSG0000(	YIPF2	269.2845	-0.13341	0.160432	0.304051	protein_coding
ENSG0000(	ENSG0000(	213.7942	0.124067	0.160452	0.304054	lncRNA
ENSG0000(	ZNF277	593.0118	-0.05348	0.160483	0.304094	protein_coding
ENSG0000(	RBPMS-AS:	90.93963	0.126837	0.160514	0.304135	lncRNA
ENSG0000(	BMP8B-AS:	96.01052	0.108174	0.160529	0.304139	lncRNA
ENSG0000(	ENSG0000(	15.78624	-0.3486	0.160535	0.304139	lncRNA
ENSG0000(	MRPL14	149.8255	-0.10818	0.160547	0.304143	protein_coding
ENSG0000(	LINC01539	22.1559	0.355361	0.16063	0.304268	lncRNA
ENSG0000(	PRXL2C	889.6741	-0.06993	0.160636	0.304268	protein_coding
ENSG0000(	ZP2	64.56202	0.147043	0.160642	0.304268	protein_coding
ENSG0000(	BCL2L10	16.34618	-0.31193	0.16066	0.304283	protein_coding
ENSG0000(	H3C12	67.14626	-0.2709	0.1607	0.304341	protein_coding
ENSG0000(	LINC00222	78.16135	0.147735	0.160773	0.30446	lncRNA

ENSG0000(	FERMT3	569.8194	0.179075	0.160834	0.304557	protein_coding
ENSG0000(	POU5F1P6	18.68553	0.410988	0.160909	0.304682	unprocessed_pseudogene
ENSG0000(	PDZD7	586.0233	-0.19344	0.16094	0.304722	protein_coding
ENSG0000(	DTNA	502.0525	0.127763	0.160965	0.304748	protein_coding
ENSG0000(	ENSG0000(	1660.46	-0.11784	0.160973	0.304748	lncRNA
ENSG0000(	RPL39P5	12.03949	0.280155	0.161037	0.304852	transcribed_processed_pseudoge
ENSG0000(	ENSG0000(	16.88756	-0.97034	0.161071	0.304878	protein_coding
ENSG0000(	FGD6	2852.128	-0.0942	0.161067	0.304878	protein_coding
ENSG0000(	ENSG0000(	45.3589	-0.39702	0.161149	0.305007	lncRNA
ENSG0000(	RPS4XP8	15.9797	-0.45588	0.161206	0.305096	processed_pseudogene
ENSG0000(	SOX9-AS1	1193.462	0.069683	0.161276	0.305212	lncRNA
ENSG0000(	SLC35A4	695.2655	-0.08678	0.161301	0.30524	protein_coding
ENSG0000(	KCNAB2	2162.773	0.223944	0.161313	0.305244	protein_coding
ENSG0000(	ENSG0000(	42.97297	0.195816	0.161341	0.305269	processed_pseudogene
ENSG0000(	PGAP4	308.7826	-0.19954	0.161345	0.305269	protein_coding
ENSG0000(	B3GNT7	1153.451	-0.2656	0.161373	0.305285	protein_coding
ENSG0000(	FBXO5	302.4688	-0.0941	0.161373	0.305285	protein_coding
ENSG0000(	TGFBRAP1	1000.704	-0.05658	0.161406	0.30533	protein_coding
ENSG0000(	ENSG0000(	25.18417	0.251553	0.161441	0.305377	lncRNA
ENSG0000(	LINC02018	1710.552	0.080848	0.161454	0.305383	lncRNA
ENSG0000(	RFX7	1828.665	0.085058	0.161468	0.305392	protein_coding
ENSG0000(	SOX2-OT	1297.711	0.039013	0.161478	0.305392	lncRNA
ENSG0000(	ENSG0000(	28.22852	0.183622	0.161507	0.305419	lncRNA
ENSG0000(	HAS2-AS1	83.10926	-0.12669	0.161512	0.305419	lncRNA
ENSG0000(	PPIAP70	33.10824	0.168344	0.161533	0.30544	processed_pseudogene
ENSG0000(	SCARA3	394.0196	-0.16579	0.161633	0.305611	protein_coding
ENSG0000(	ENSG0000(	68.24676	-0.12269	0.161676	0.305674	lncRNA
ENSG0000(	C17orf67	108.6986	-0.18349	0.161714	0.305699	protein_coding
ENSG0000(	ENSG0000(	10.13321	-0.36514	0.161756	0.305699	transcribed_unprocessed_pseudo
ENSG0000(	ENSG0000(	42.04472	-0.24062	0.161724	0.305699	lncRNA
ENSG0000(	HAO2	10.87058	0.379445	0.161728	0.305699	protein_coding
ENSG0000(	NUP98	3389.294	0.067649	0.16174	0.305699	protein_coding
ENSG0000(	TADA3	870.7175	-0.05399	0.161757	0.305699	protein_coding
ENSG0000(	ZC3H11A	3821.213	0.07114	0.161714	0.305699	protein_coding
ENSG0000(	ENSG0000(	13.1694	-0.23081	0.1618	0.305762	lncRNA
ENSG0000(	ENSG0000(	30.67485	0.19874	0.161878	0.305891	lncRNA
ENSG0000(	ENSG0000(	10.84767	-0.28386	0.161971	0.305988	lncRNA
ENSG0000(	ENSG0000(	97.09927	0.114234	0.161943	0.305988	lncRNA
ENSG0000(	LGI4	97.2727	0.218852	0.161978	0.305988	protein_coding
ENSG0000(	PPIL1	136.8555	0.167611	0.161967	0.305988	protein_coding
ENSG0000(	TMOD2	1388.722	0.100628	0.161951	0.305988	protein_coding
ENSG0000(	BAGE2	60.71908	0.166881	0.161993	0.305999	transcribed_unprocessed_pseudo
ENSG0000(	ENSG0000(	284.5473	0.095347	0.162004	0.306	lncRNA
ENSG0000(	ENSG0000(	54.24096	0.150005	0.162095	0.306155	lncRNA
ENSG0000(	RFC4	238.3785	-0.12656	0.162108	0.306162	protein_coding
ENSG0000(	ENSG0000(	73.27164	0.132392	0.16222	0.306354	lncRNA
ENSG0000(	ENSG0000(	34.57136	-0.26765	0.162271	0.306433	lncRNA

ENSG0000(	SNORD116	18.88686	0.580727	0.162286	0.306441	snoRNA
ENSG0000(	HSD17B4	5811.428	-0.06242	0.162324	0.306477	protein_coding
ENSG0000(	SLC44A1	3748.157	-0.12356	0.162315	0.306477	protein_coding
ENSG0000(	TCF7L1	240.1148	-0.0878	0.162374	0.306552	protein_coding
ENSG0000(	ADNP-AS1	46.30029	-0.11633	0.16241	0.306603	lncRNA
ENSG0000(	ENSG0000(	39.65732	-0.2082	0.162497	0.306729	lncRNA
ENSG0000(	ENSG0000(	73.72773	0.11351	0.162496	0.306729	lncRNA
ENSG0000(	ATRIP	265.9876	0.138036	0.162531	0.306738	protein_coding
ENSG0000(	SEC14L4	210.1007	0.073844	0.162529	0.306738	protein_coding
ENSG0000(	TOR1B	549.8866	-0.09771	0.162515	0.306738	protein_coding
ENSG0000(	ENSG0000(	13.34973	0.296926	0.162541	0.306739	processed_pseudogene
ENSG0000(	PWWP2A	1341.744	-0.05931	0.162652	0.30693	protein_coding
ENSG0000(	PCAT6	15.22894	0.302044	0.162748	0.307093	lncRNA
ENSG0000(	METTL14-D	307.8412	0.085598	0.162761	0.307099	lncRNA
ENSG0000(	ACTN2	205.523	0.105294	0.162874	0.307254	protein_coding
ENSG0000(	CEP295	1716.228	0.057046	0.162882	0.307254	protein_coding
ENSG0000(	ELOF1	434.734	-0.05455	0.16288	0.307254	protein_coding
ENSG0000(	RESF1	11160.49	-0.09486	0.162872	0.307254	protein_coding
ENSG0000(	TMEM238L	241.7421	-0.27795	0.162919	0.307307	protein_coding
ENSG0000(	RPL4	5781.28	-0.14033	0.16298	0.307403	protein_coding
ENSG0000(	BMP4	388.4566	-0.15309	0.163011	0.307443	protein_coding
ENSG0000(	DHX40	635.4262	-0.06897	0.163052	0.307465	protein_coding
ENSG0000(	RPS29P14	22.09749	-0.22819	0.163052	0.307465	processed_pseudogene
ENSG0000(	TUBGCP3	1182.646	-0.04792	0.163047	0.307465	protein_coding
ENSG0000(	ENSG0000(	36.90657	0.170486	0.16311	0.307556	lncRNA
ENSG0000(	ATAD5	762.0255	0.101217	0.163167	0.307627	protein_coding
ENSG0000(	ENSG0000(	24.22661	0.435342	0.163165	0.307627	TEC
ENSG0000(	MORC2-AS	126.1816	0.155903	0.163199	0.307649	lncRNA
ENSG0000(	RHNO1	397.9102	-0.06751	0.163197	0.307649	protein_coding
ENSG0000(	CYB5B	2871.354	-0.08299	0.163222	0.307675	protein_coding
ENSG0000(	SHISA8	16.91476	-0.44019	0.163239	0.307689	protein_coding
ENSG0000(	LINC00678	39.70841	-0.15892	0.163269	0.307719	lncRNA
ENSG0000(	YEATS2-AS:	44.96946	-0.15461	0.163275	0.307719	lncRNA
ENSG0000(	ENSG0000(	17.05008	0.193626	0.163294	0.307719	lncRNA
ENSG0000(	WDR86-AS	42.5311	0.205007	0.163288	0.307719	lncRNA
ENSG0000(	CMA1	8.760178	-0.50073	0.163344	0.307794	protein_coding
ENSG0000(	LRRC37B	1083.201	-0.07795	0.163379	0.307842	protein_coding
ENSG0000(	PCDHB10	14.89289	0.28289	0.163425	0.307892	protein_coding
ENSG0000(	ZNF860	217.9853	-0.31709	0.163418	0.307892	protein_coding
ENSG0000(	RNU1-148F	18.57164	0.334601	0.163437	0.307895	snRNA
ENSG0000(	HLA-DPB1	2843.122	0.192594	0.163471	0.307942	protein_coding
ENSG0000(	PPIL2	2635.399	0.077111	0.163482	0.307943	protein_coding
ENSG0000(	TRGV10	34.50655	-0.21175	0.163497	0.307954	TR_V_gene
ENSG0000(	CNGA3	32.69326	-0.4578	0.163542	0.308019	protein_coding
ENSG0000(	BRD4	4059.826	0.049205	0.1636	0.308092	protein_coding
ENSG0000(	FAR1	2372.566	0.068698	0.163597	0.308092	protein_coding
ENSG0000(	IGHV3-33-2	9.738359	0.396809	0.163649	0.308166	IG_V_pseudogene

ENSG0000(	ENSG0000(	43.15393	-0.30559	0.16369	0.308206	lncRNA
ENSG0000(	SERPINE2	702.9039	-0.15161	0.163683	0.308206	protein_coding
ENSG0000(	ERO1B	2224.773	0.078585	0.163703	0.308212	protein_coding
ENSG0000(	RN7SL623F	28.61813	0.152515	0.163729	0.308243	misc_RNA
ENSG0000(	LINC00687	163.6919	0.084156	0.163743	0.308251	lncRNA
ENSG0000(	TERF2IP	852.9968	-0.05658	0.163757	0.308259	protein_coding
ENSG0000(	NKPD1	86.57254	0.104849	0.163774	0.308272	protein_coding
ENSG0000(	COL6A4P2	83.22273	0.239906	0.163845	0.308387	transcribed_unitary_pseudogene
ENSG0000(	TFF3	1104.698	0.308203	0.163876	0.308427	protein_coding
ENSG0000(	INTS6L	1496.511	0.120661	0.163886	0.308428	protein_coding
ENSG0000(	INTS2	896.8696	-0.06612	0.163922	0.308476	protein_coding
ENSG0000(	ARHGAP6	212.108	0.13526	0.163956	0.308509	protein_coding
ENSG0000(	INTS9	325.9837	-0.09393	0.163968	0.308509	protein_coding
ENSG0000(	MTCYBP3	103.1057	0.117414	0.163963	0.308509	processed_pseudogene
ENSG0000(	DPH7	1109.251	0.08697	0.164013	0.308575	protein_coding
ENSG0000(	LIN52	826.3129	0.070898	0.164044	0.308616	protein_coding
ENSG0000(	RAB18	2296.953	-0.06995	0.164079	0.308656	protein_coding
ENSG0000(	TMEM232	137.9947	0.118745	0.164086	0.308656	protein_coding
ENSG0000(	ENSG0000(	17.27764	0.306845	0.164102	0.308659	lncRNA
ENSG0000(	MICOS10P2	8.742388	0.27306	0.164107	0.308659	processed_pseudogene
ENSG0000(	COPB1	1937.359	-0.07099	0.164123	0.308666	protein_coding
ENSG0000(	ENSG0000(	39.79406	0.181362	0.16413	0.308666	lncRNA
ENSG0000(	JADRR	427.8439	0.092133	0.164177	0.308736	lncRNA
ENSG0000(	ENSG0000(	257.7369	0.063806	0.164227	0.308774	processed_pseudogene
ENSG0000(	ENSG0000(	997.8119	0.262488	0.164229	0.308774	lncRNA
ENSG0000(	JAGN1	327.6244	-0.08114	0.164236	0.308774	protein_coding
ENSG0000(	SSR4	565.2141	0.181079	0.16423	0.308774	protein_coding
ENSG0000(	ARRDC1	911.767	-0.10065	0.164277	0.308831	protein_coding
ENSG0000(	ENSG0000(	1172.102	0.050102	0.164317	0.308888	lncRNA
ENSG0000(	ENSG0000(	806.2305	0.083821	0.164346	0.308924	lncRNA
ENSG0000(	GRAMD1B	2635.315	-0.19842	0.164455	0.309112	protein_coding
ENSG0000(	CELF6	140.3519	-0.15112	0.164526	0.309212	protein_coding
ENSG0000(	GRN	1464.984	-0.10512	0.164538	0.309212	protein_coding
ENSG0000(	TMEM255A	63.37986	-0.38898	0.164531	0.309212	protein_coding
ENSG0000(	RNU6-593F	17.03998	-0.25773	0.164574	0.309261	snRNA
ENSG0000(	APOD	52.89753	-0.18281	0.16465	0.309338	protein_coding
ENSG0000(	CBWD1	3776.445	0.065615	0.164648	0.309338	protein_coding
ENSG0000(	CFAP97D1	540.085	-0.05241	0.16463	0.309338	protein_coding
ENSG0000(	ENSG0000(	57.36268	0.124615	0.164664	0.309338	lncRNA
ENSG0000(	PNLDC1	15.45409	-0.33644	0.164659	0.309338	protein_coding
ENSG0000(	ENSG0000(	133.4169	0.084018	0.164698	0.30935	lncRNA
ENSG0000(	SLC25A53P	11.7328	0.313413	0.164697	0.30935	processed_pseudogene
ENSG0000(	STRCP1	21.3622	0.436152	0.164707	0.30935	transcribed_unprocessed_pseudo
ENSG0000(	TIPRL	701.6118	-0.05471	0.16471	0.30935	protein_coding
ENSG0000(	POP7	78.56557	-0.14653	0.164733	0.309374	protein_coding
ENSG0000(	CLEC12A-A	2669.134	0.122756	0.164759	0.309387	lncRNA
ENSG0000(	TFRC	4163.545	0.060813	0.164757	0.309387	protein_coding

ENSG0000(	ENSG0000(	118.7021	0.132851	0.164785	0.309399	lncRNA
ENSG0000(	TMEM120E	1465.159	-0.09931	0.164779	0.309399	protein_coding
ENSG0000(	ENSG0000(	58.59947	0.105339	0.1648	0.309409	lncRNA
ENSG0000(	LRRC52-AS	31.34588	-0.34133	0.16482	0.309427	lncRNA
ENSG0000(	PCCA-AS1	18.37397	0.234472	0.164835	0.309438	lncRNA
ENSG0000(	ENSG0000(	107.6341	0.122448	0.164875	0.309495	processed_pseudogene
ENSG0000(	LIMS3	102.4602	0.234186	0.164916	0.309553	protein_coding
ENSG0000(	PDXP-DT	21.10345	-0.29623	0.164928	0.309556	lncRNA
ENSG0000(	ENSG0000(	83.95616	-0.11246	0.16495	0.309579	lncRNA
ENSG0000(	ENSG0000(	40.04616	-0.22172	0.16502	0.309692	lncRNA
ENSG0000(	TTC4	251.5402	-0.10671	0.16506	0.309749	protein_coding
ENSG0000(	SATB2	1847.649	-0.21188	0.165086	0.309781	protein_coding
ENSG0000(	ENSG0000(	676.5495	0.074206	0.165125	0.309834	lncRNA
ENSG0000(	GLRX3	902.2012	-0.07275	0.16516	0.309863	protein_coding
ENSG0000(	IGLJ2	13.92089	0.448922	0.165152	0.309863	IG_J_gene
ENSG0000(	ZNF394	2643.383	0.046304	0.165185	0.309892	protein_coding
ENSG0000(	LCMT1-AS1	312.6117	0.095861	0.165241	0.309978	lncRNA
ENSG0000(	POGK	1778.3	-0.05585	0.165267	0.310001	protein_coding
ENSG0000(	SPINK1	516.8053	0.279574	0.165273	0.310001	protein_coding
ENSG0000(	ENSG0000(	17.12773	0.245359	0.165303	0.310025	lncRNA
ENSG0000(	POLR1A	2495.202	0.088498	0.165305	0.310025	protein_coding
ENSG0000(	ZNF846	613.6937	-0.07112	0.165344	0.310079	protein_coding
ENSG0000(	ENSG0000(	85.93986	0.098112	0.165386	0.31012	lncRNA
ENSG0000(	MANSC4	100.6591	0.111929	0.165383	0.31012	protein_coding
ENSG0000(	GPR75	174.9056	0.126889	0.165437	0.310198	protein_coding
ENSG0000(	PDE2A	251.1251	-0.14073	0.16549	0.310278	protein_coding
ENSG0000(	NDE1	1722.796	0.127381	0.165505	0.310289	protein_coding
ENSG0000(	CDYL	635.4995	0.057258	0.165546	0.310347	protein_coding
ENSG0000(	ENSG0000(	28.69524	-0.18423	0.16562	0.310467	lncRNA
ENSG0000(	RNU6-10P	12.91336	0.272295	0.165697	0.310592	snRNA
ENSG0000(	DHH	81.7978	0.136252	0.165744	0.310663	protein_coding
ENSG0000(	PPIAP30	27.87381	-0.20288	0.165791	0.310733	processed_pseudogene
ENSG0000(	DZIP3	1200.639	0.084766	0.165825	0.310777	protein_coding
ENSG0000(	TMEM52	12.0303	-0.35341	0.165913	0.310924	protein_coding
ENSG0000(	PTDSS2	423.0028	-0.09092	0.165958	0.31099	protein_coding
ENSG0000(	ENSG0000(	46.05444	-0.14716	0.166017	0.311064	lncRNA
ENSG0000(	STMND1	284.5585	0.060424	0.166016	0.311064	protein_coding
ENSG0000(	LINC01885	45.23325	0.144956	0.166134	0.311265	lncRNA
ENSG0000(	ELAPOR2	295.7515	-0.21733	0.166156	0.311266	protein_coding
ENSG0000(	ENSG0000(	38.79234	0.148457	0.16615	0.311266	lncRNA
ENSG0000(	GALNT13	106.6724	0.313721	0.166164	0.311266	protein_coding
ENSG0000(	ENSG0000(	96.26021	0.152411	0.166251	0.311357	lncRNA
ENSG0000(	NDE1P2	105.0946	0.09547	0.166234	0.311357	processed_pseudogene
ENSG0000(	PRSS8	1887.196	-0.25703	0.166253	0.311357	protein_coding
ENSG0000(	RPS3AP37	20.21792	0.23348	0.16623	0.311357	processed_pseudogene
ENSG0000(	SLCO4C1	134.3966	-0.1671	0.166358	0.311536	protein_coding
ENSG0000(	ANKDD1A	389.8869	0.15604	0.166369	0.311538	protein_coding

ENSG0000(	RPE	1332.169	-0.0572	0.166391	0.311561	protein_coding
ENSG0000(	DNAAF4	170.3745	0.08197	0.166406	0.311569	protein_coding
ENSG0000(	CLU	1349.089	-0.29081	0.166496	0.31172	protein_coding
ENSG0000(	ENSG0000(	952.7307	-0.07982	0.166526	0.311758	lncRNA
ENSG0000(	CARM1P1	21.21168	-0.18896	0.166544	0.311772	transcribed_unprocessed_pseudo
ENSG0000(	ENSG0000(	124.9711	0.118448	0.166608	0.311856	lncRNA
ENSG0000(	VEGFC	88.75306	0.168011	0.166608	0.311856	protein_coding
ENSG0000(	GLTPD2	10.79939	-0.41627	0.166649	0.311914	protein_coding
ENSG0000(	ENSG0000(	59.68103	-0.77678	0.16671	0.311972	protein_coding
ENSG0000(	SATB1	3173.259	0.094319	0.166709	0.311972	protein_coding
ENSG0000(	ZBBX	38.97305	0.199569	0.166702	0.311972	protein_coding
ENSG0000(	DDX50	463.905	-0.10216	0.166815	0.312083	protein_coding
ENSG0000(	ENSG0000(	42.33688	0.37284	0.166819	0.312083	lncRNA
ENSG0000(	FAM111A-I	264.4335	0.10692	0.166789	0.312083	lncRNA
ENSG0000(	MIR5094	16.21714	0.228622	0.166807	0.312083	miRNA
ENSG0000(	PIGU	225.256	-0.10994	0.166811	0.312083	protein_coding
ENSG0000(	SLC39A14	4244.374	-0.21064	0.166887	0.312193	protein_coding
ENSG0000(	DCAF4L2	11.14412	0.295718	0.166901	0.312199	protein_coding
ENSG0000(	LINC02533	169.3939	0.070603	0.166927	0.312212	lncRNA
ENSG0000(	LRRC47	704.7393	-0.08261	0.166927	0.312212	protein_coding
ENSG0000(	LAMA3	2612.963	-0.23738	0.166953	0.312232	protein_coding
ENSG0000(	LINC00894	1925.351	-0.07997	0.166958	0.312232	lncRNA
ENSG0000(	ENSG0000(	222.3491	-0.07912	0.167014	0.312251	lncRNA
ENSG0000(	ENSG0000(	548.3605	-0.05349	0.167016	0.312251	lncRNA
ENSG0000(	LINC01549	160.5063	-0.14572	0.166981	0.312251	lncRNA
ENSG0000(	MLIP-AS1	124.7166	-0.09968	0.16699	0.312251	lncRNA
ENSG0000(	OR2A5	672.9544	0.098088	0.167028	0.312251	protein_coding
ENSG0000(	SP5	17.49256	0.373308	0.167019	0.312251	protein_coding
ENSG0000(	ENSG0000(	19.39071	0.206473	0.167128	0.31242	lncRNA
ENSG0000(	CYRIB	2052.205	0.080043	0.167139	0.312422	protein_coding
ENSG0000(	FHDC1	855.8554	-0.1975	0.167155	0.312434	protein_coding
ENSG0000(	LINC01852	59.27463	-0.20297	0.167184	0.31247	lncRNA
ENSG0000(	UBAP2	1634.012	-0.05526	0.167215	0.312509	protein_coding
ENSG0000(	ENSG0000(	151.1032	0.146918	0.167294	0.31259	lncRNA
ENSG0000(	MB21D2	262.4239	-0.21065	0.167298	0.31259	protein_coding
ENSG0000(	RN7SL180F	69.91248	0.139937	0.167297	0.31259	misc_RNA
ENSG0000(	SLC23A2	1591.736	0.156265	0.167297	0.31259	protein_coding
ENSG0000(	ENSG0000(	14.64099	0.254789	0.167326	0.312604	lncRNA
ENSG0000(	PHACTR2	2651.425	-0.05805	0.167316	0.312604	protein_coding
ENSG0000(	LINC01297	916.5879	0.259983	0.167377	0.312682	lncRNA
ENSG0000(	CASC17	140.0564	-0.11413	0.167388	0.312685	lncRNA
ENSG0000(	ENSG0000(	698.2131	0.055271	0.167413	0.3127	protein_coding
ENSG0000(	MARCHF1C	56.85549	-0.11196	0.167417	0.3127	lncRNA
ENSG0000(	AGAP9	1403.428	0.243536	0.167435	0.312703	protein_coding
ENSG0000(	RNU1-61P	17.12363	0.311838	0.167438	0.312703	snRNA
ENSG0000(	EIF2AK3-DT	126.6176	0.091216	0.1675	0.312766	lncRNA
ENSG0000(	NIFK	331.9406	-0.10742	0.167497	0.312766	protein_coding

ENSG0000(	PPM1L-DT	66.91028	0.151503	0.167508	0.312766	lncRNA
ENSG0000(	TPRG1-AS2	25.00014	0.177255	0.167511	0.312766	lncRNA
ENSG0000(	SNAPC4	829.4666	-0.05705	0.167524	0.312771	protein_coding
ENSG0000(	MAEA	1972.632	-0.05131	0.167539	0.31278	protein_coding
ENSG0000(	ANKLE1	1138.224	0.160635	0.167584	0.312846	protein_coding
ENSG0000(	LRRC75A	2007.826	-0.25736	0.167601	0.312858	protein_coding
ENSG0000(	SUPT20H	1830.543	-0.03961	0.167652	0.312937	protein_coding
ENSG0000(	ACAP3	1515.286	-0.11111	0.167699	0.312986	protein_coding
ENSG0000(	MSANTD3-	17.52272	0.583353	0.167695	0.312986	protein_coding
ENSG0000(	ENSG0000(	62.08175	0.143228	0.167735	0.313017	processed_pseudogene
ENSG0000(	POC1B	2438.948	-0.04722	0.16773	0.313017	protein_coding
ENSG0000(	CAB39L	212.8126	0.131415	0.167768	0.313059	protein_coding
ENSG0000(	TSC22D4	883.6573	-0.0787	0.167812	0.313123	protein_coding
ENSG0000(	RAPH1	3575.974	-0.1441	0.167823	0.313126	protein_coding
ENSG0000(	BFAR	1125.468	-0.05815	0.167891	0.313216	protein_coding
ENSG0000(	CARD8	4139.883	0.136453	0.167882	0.313216	protein_coding
ENSG0000(	CABLES1	354.0102	-0.19477	0.167928	0.313265	protein_coding
ENSG0000(	CTSO	1475.998	0.065501	0.167944	0.313267	protein_coding
ENSG0000(	SHARPIN	368.9528	-0.08475	0.167948	0.313267	protein_coding
ENSG0000(	RPA3	126.8872	-0.13568	0.167976	0.313299	protein_coding
ENSG0000(	CASC20	33.10021	0.142244	0.168019	0.313326	lncRNA
ENSG0000(	ENSG0000(	14.19622	0.239102	0.168007	0.313326	lncRNA
ENSG0000(	ZC2HC1B	10.62726	0.352657	0.16802	0.313326	protein_coding
ENSG0000(	ENSG0000(	35.19757	0.149231	0.168051	0.313366	lncRNA
ENSG0000(	OR2T2	22.61368	0.56155	0.168069	0.313379	protein_coding
ENSG0000(	ENSG0000(	55.18963	0.186195	0.168107	0.313413	processed_pseudogene
ENSG0000(	RAB5B	3029.479	-0.04437	0.168117	0.313413	protein_coding
ENSG0000(	TBCB	463.1924	-0.07443	0.168104	0.313413	protein_coding
ENSG0000(	ZNF682	355.3357	0.130386	0.168135	0.313428	protein_coding
ENSG0000(	ENSG0000(	786.7434	0.166846	0.16819	0.313513	lncRNA
ENSG0000(	ENSG0000(	22.4057	0.180104	0.16823	0.313566	lncRNA
ENSG0000(	ENSG0000(	237.8345	0.075986	0.168238	0.313566	lncRNA
ENSG0000(	FKBPL	39.63289	0.133815	0.16829	0.313644	protein_coding
ENSG0000(	LTA4H	1831.91	0.071055	0.168303	0.31365	protein_coding
ENSG0000(	MAP2K5-D	98.18373	0.10161	0.168313	0.31365	lncRNA
ENSG0000(	ENSG0000(	17.22934	0.396107	0.168418	0.313827	lncRNA
ENSG0000(	ENSG0000(	172.6513	0.113513	0.168471	0.313902	lncRNA
ENSG0000(	PCBP2-OT1	69.08636	-0.10626	0.168479	0.313902	lncRNA
ENSG0000(	RN7SL116F	107.9906	-0.21461	0.168513	0.313948	misc_RNA
ENSG0000(	SLC16A1	771.2127	-0.11466	0.168581	0.314056	protein_coding
ENSG0000(	ENSG0000(	51.50134	0.135594	0.168611	0.314094	lncRNA
ENSG0000(	ENSG0000(	89.80219	-0.2167	0.168633	0.314095	protein_coding
ENSG0000(	MTR	4292.935	0.076247	0.168642	0.314095	protein_coding
ENSG0000(	RNU6-1266	12.09481	0.261995	0.168624	0.314095	snRNA
ENSG0000(	ZGLP1	50.81067	-0.20657	0.168658	0.314106	protein_coding
ENSG0000(	AOC2	87.679	0.102747	0.168719	0.314202	protein_coding
ENSG0000(	DENND10	956.9011	0.065898	0.168753	0.314247	protein_coding

ENSG0000(	RSL24D1	783.7113	-0.05213	0.16882	0.314352	protein_coding
ENSG0000(	FEZ1	569.7347	0.065627	0.168969	0.314595	protein_coding
ENSG0000(	KDF1	140.6299	-0.24433	0.16897	0.314595	protein_coding
ENSG0000(	SOCS4	1172.374	0.0922	0.168993	0.314619	protein_coding
ENSG0000(	ZNF16	588.5192	0.070031	0.169047	0.314701	protein_coding
ENSG0000(	ENSG0000(	420.7174	-0.05506	0.169084	0.314751	lncRNA
ENSG0000(	ABHD2	4765.751	0.092472	0.169145	0.314845	protein_coding
ENSG0000(	LINC02069	170.5036	-0.08062	0.169154	0.314845	lncRNA
ENSG0000(	ENSG0000(	14.62996	0.224113	0.169187	0.314887	lncRNA
ENSG0000(	LINC00987	114.6554	0.18715	0.169225	0.314939	lncRNA
ENSG0000(	SGSM1	438.3118	-0.12462	0.169255	0.314976	protein_coding
ENSG0000(	CASP2	2006.587	-0.05244	0.169309	0.315003	protein_coding
ENSG0000(	ENSG0000(	144.6469	0.154073	0.169288	0.315003	TEC
ENSG0000(	ENSG0000(	89.07976	-0.08851	0.169304	0.315003	TEC
ENSG0000(	SORL1	10276.4	-0.12594	0.169308	0.315003	protein_coding
ENSG0000(	FBXO33	479.5967	-0.07954	0.169339	0.315039	protein_coding
ENSG0000(	ITM2C	1870.401	0.176673	0.169521	0.315341	protein_coding
ENSG0000(	ZNF875	2319.789	-0.05503	0.169513	0.315341	protein_coding
ENSG0000(	WDFY4	4609.314	0.308179	0.169546	0.315368	protein_coding
ENSG0000(	RSC1A1	210.3324	-0.23911	0.169558	0.315373	protein_coding
ENSG0000(	ENSG0000(	52.03406	0.193559	0.169604	0.315439	lncRNA
ENSG0000(	ERC2	42.47664	0.175395	0.169728	0.315614	protein_coding
ENSG0000(	NUP93	958.0904	0.083014	0.169727	0.315614	protein_coding
ENSG0000(	PTCSC2	214.2096	0.098518	0.169724	0.315614	lncRNA
ENSG0000(	SNORA77	21.91677	0.228845	0.169786	0.315704	snoRNA
ENSG0000(	MAP2K3	959.2719	-0.11064	0.169809	0.315728	protein_coding
ENSG0000(	MECR	423.1502	0.078037	0.169824	0.315736	protein_coding
ENSG0000(	GLIPR1	984.8556	0.197744	0.169907	0.315872	protein_coding
ENSG0000(	ANAPC4	1264.198	-0.05738	0.169948	0.315931	protein_coding
ENSG0000(	RN7SL60P	21.01007	0.183775	0.170041	0.316085	misc_RNA
ENSG0000(	ENSG0000(	10.13357	-0.40624	0.170085	0.316129	lncRNA
ENSG0000(	OSBPL5	694.6525	0.125332	0.170085	0.316129	protein_coding
ENSG0000(	ODAPH	284.4049	-0.15071	0.170145	0.316222	protein_coding
ENSG0000(	IQCM	343.0972	-0.0954	0.17021	0.316324	protein_coding
ENSG0000(	TMC2	582.9187	0.095574	0.170243	0.316367	protein_coding
ENSG0000(	C3orf52	1543.894	-0.24257	0.170273	0.316403	protein_coding
ENSG0000(	ENSG0000(	17.16417	-0.27588	0.170327	0.316484	lncRNA
ENSG0000(	TIGD7	274.6903	0.112764	0.170394	0.316591	protein_coding
ENSG0000(	ENSG0000(	45.50994	0.172088	0.170405	0.316592	lncRNA
ENSG0000(	AGBL2	287.5103	-0.09888	0.170467	0.316671	protein_coding
ENSG0000(	ZZEF1	14059.4	-0.1642	0.17046	0.316671	protein_coding
ENSG0000(	ENSG0000(	639.5596	0.053136	0.170593	0.316886	lncRNA
ENSG0000(	TMEM119	648.2228	0.125979	0.170623	0.316913	protein_coding
ENSG0000(	ZNF514	2254.346	0.084946	0.170628	0.316913	protein_coding
ENSG0000(	KDM4D	12.9572	0.256456	0.170652	0.31694	protein_coding
ENSG0000(	FAXDC2	869.631	0.0851	0.17071	0.31701	protein_coding
ENSG0000(	ULK3	1756.1	-0.19579	0.170702	0.31701	protein_coding

ENSG0000(	U2AF1L5	1964.609	0.292964	0.170745	0.317055	protein_coding
ENSG0000(	ENSG0000(	8.600266	0.318755	0.17082	0.317176	lncRNA
ENSG0000(	MMRN1	303.7023	0.227109	0.170868	0.317247	protein_coding
ENSG0000(	LRIT3	30.47897	0.179327	0.170903	0.317293	protein_coding
ENSG0000(	BRF1	1136.472	0.05162	0.170919	0.317298	protein_coding
ENSG0000(	RBM18	488.4466	-0.0856	0.170936	0.317298	protein_coding
ENSG0000(	VIRMA-DT	199.997	0.106015	0.17093	0.317298	lncRNA
ENSG0000(	ENSG0000(	18.90205	0.195037	0.17097	0.317344	lncRNA
ENSG0000(	RNU1-1	403903.8	-0.09388	0.171027	0.31743	snRNA
ENSG0000(	SNORA58B	33.89221	0.167976	0.171037	0.317431	snoRNA
ENSG0000(	FZD2	137.662	0.1209	0.171088	0.317488	protein_coding
ENSG0000(	LINC00526	85.75484	-0.22402	0.17108	0.317488	lncRNA
ENSG0000(	ZNF114	191.6682	-0.12485	0.171102	0.317495	protein_coding
ENSG0000(	ADRM1	294.9492	-0.12253	0.171135	0.317538	protein_coding
ENSG0000(	ENSG0000(	133.7968	-0.08486	0.17117	0.317584	lncRNA
ENSG0000(	ENSG0000(	18.49577	-0.20844	0.171228	0.317636	lncRNA
ENSG0000(	SEMA5B	274.9912	0.091711	0.171222	0.317636	protein_coding
ENSG0000(	YTHDC1	2363.324	-0.05201	0.171229	0.317636	protein_coding
ENSG0000(	ENSG0000(	105.147	-0.11497	0.171365	0.317846	lncRNA
ENSG0000(	ENSG0000(	224.3038	0.070038	0.171372	0.317846	TEC
ENSG0000(	NRXN3	687.2194	0.165785	0.171355	0.317846	protein_coding
ENSG0000(	CFAP46	12.12869	0.480256	0.171428	0.31793	protein_coding
ENSG0000(	SPACA7	39.35385	0.135092	0.171438	0.31793	protein_coding
ENSG0000(	RN7SL706F	21.75437	-0.29736	0.171459	0.317951	misc_RNA
ENSG0000(	DOCK2	3778.529	0.233901	0.171492	0.317982	protein_coding
ENSG0000(	ENSG0000(	1248.564	0.049424	0.171496	0.317982	lncRNA
ENSG0000(	NFIX	926.7074	-0.15365	0.171516	0.318001	protein_coding
ENSG0000(	ENSG0000(	11.86856	0.230257	0.171551	0.318047	lncRNA
ENSG0000(	NXT1-AS1	8.550931	0.342837	0.17157	0.318062	lncRNA
ENSG0000(	MARCHF7	3308.71	-0.05449	0.171603	0.318105	protein_coding
ENSG0000(	RN7SKP15(	14.49608	-0.33946	0.171624	0.318126	misc_RNA
ENSG0000(	NRDE2	2969.319	-0.09118	0.171689	0.318227	protein_coding
ENSG0000(	FAM47E	2145.349	-0.12179	0.171745	0.318312	protein_coding
ENSG0000(	TOMM40L	298.2788	-0.11178	0.17177	0.31834	protein_coding
ENSG0000(	COL14A1	1268.158	0.249296	0.171855	0.318367	protein_coding
ENSG0000(	ELP1	2883.631	0.041395	0.171817	0.318367	protein_coding
ENSG0000(	ENSG0000(	132.2067	-0.16757	0.171842	0.318367	lncRNA
ENSG0000(	ENSG0000(	217.1627	0.115604	0.17185	0.318367	lncRNA
ENSG0000(	EYA1	85.25248	-0.13936	0.171808	0.318367	protein_coding
ENSG0000(	HJURP	426.711	0.108867	0.171834	0.318367	protein_coding
ENSG0000(	P2RY11	268.883	-0.14986	0.171795	0.318367	protein_coding
ENSG0000(	TRAC	581.0986	-0.24602	0.171875	0.318385	TR_C_gene
ENSG0000(	RNY1	1004.793	-0.16105	0.17192	0.31845	misc_RNA
ENSG0000(	ENSG0000(	30.78739	-0.37944	0.171964	0.318512	lncRNA
ENSG0000(	PGGHG	4679.498	-0.1236	0.171987	0.318536	protein_coding
ENSG0000(	ENSG0000(	45.68548	-0.14593	0.172082	0.318668	lncRNA
ENSG0000(	LINC01522	9.590331	0.452583	0.172089	0.318668	lncRNA

ENSG0000(	PRKAG2	2890.624	-0.0776	0.172075	0.318668	protein_coding
ENSG0000(	ENSG0000(	25.97017	0.263711	0.172151	0.318766	lncRNA
ENSG0000(	ENSG0000(	52.68047	-0.13813	0.172215	0.318865	lncRNA
ENSG0000(	FER	1725.459	0.05854	0.17224	0.318893	protein_coding
ENSG0000(	ENSG0000(	71.42662	-0.13813	0.172252	0.318896	lncRNA
ENSG0000(	CDKN1C	68.74689	0.160771	0.172296	0.31894	protein_coding
ENSG0000(	FAM199X	1268.665	-0.06834	0.172296	0.31894	protein_coding
ENSG0000(	ENSG0000(	20.20405	0.244142	0.172359	0.319038	lncRNA
ENSG0000(	DCAKD	1544.791	0.055096	0.172382	0.319043	protein_coding
ENSG0000(	TEX41	396.6109	0.10925	0.172379	0.319043	lncRNA
ENSG0000(	ENSG0000(	43.72163	-0.24552	0.172395	0.319044	lncRNA
ENSG0000(	PSMB3	251.6895	-0.11305	0.172403	0.319044	protein_coding
ENSG0000(	LSM5	506.9239	0.088416	0.172425	0.319066	protein_coding
ENSG0000(	TLE2	300.171	-0.09693	0.172435	0.319066	protein_coding
ENSG0000(	CDC16	1468.478	-0.06766	0.172458	0.31907	protein_coding
ENSG0000(	ZSWIM8	3281.977	-0.1134	0.172454	0.31907	protein_coding
ENSG0000(	LINC00463	44.6398	0.133246	0.172482	0.319081	lncRNA
ENSG0000(	PCDHAC2	61.10728	-0.44297	0.172484	0.319081	protein_coding
ENSG0000(	CHRDL2	58.34786	1.104016	0.172517	0.319123	protein_coding
ENSG0000(	AGAP1	2502.362	-0.18389	0.172553	0.319137	protein_coding
ENSG0000(	ENSG0000(	20.21806	0.276097	0.17254	0.319137	lncRNA
ENSG0000(	ENSG0000(	13.10678	0.544779	0.172554	0.319137	transcribed_unprocessed_pseudo
ENSG0000(	ENSG0000(	47.39561	0.120121	0.172576	0.319159	lncRNA
ENSG0000(	SLC29A2	243.7497	0.125553	0.172597	0.319179	protein_coding
ENSG0000(	ENSG0000(	42.62959	-0.22009	0.172623	0.319189	lncRNA
ENSG0000(	NR1I2	2703.088	-0.27625	0.172618	0.319189	protein_coding
ENSG0000(	LRBA	8110.143	0.044839	0.172782	0.319465	protein_coding
ENSG0000(	CCDC136	227.6752	0.1557	0.172884	0.319578	protein_coding
ENSG0000(	CECR3	70.55481	-0.27054	0.172869	0.319578	lncRNA
ENSG0000(	MED12	3624.277	-0.0495	0.17288	0.319578	protein_coding
ENSG0000(	PATE2	40.7151	0.188901	0.172858	0.319578	protein_coding
ENSG0000(	RN7SL248F	11.93324	0.282056	0.172965	0.319709	misc_RNA
ENSG0000(	OR7A17	541.5044	-0.05688	0.173027	0.319805	protein_coding
ENSG0000(	PPHLN1	3462.145	-0.05918	0.173071	0.319867	protein_coding
ENSG0000(	PJA1	156.5174	0.193088	0.173082	0.319868	protein_coding
ENSG0000(	KEL	116.2282	-0.33513	0.173103	0.319889	protein_coding
ENSG0000(	LRRC38	48.01208	0.176843	0.173154	0.319964	protein_coding
ENSG0000(	ENSG0000(	99.5858	0.112782	0.173297	0.320191	processed_pseudogene
ENSG0000(	NSL1	1962.225	-0.06295	0.173291	0.320191	protein_coding
ENSG0000(	ENSG0000(	19.08664	0.20402	0.173381	0.320327	lncRNA
ENSG0000(	NBPF9	10200.28	0.090116	0.173396	0.320336	protein_coding
ENSG0000(	TMEM127	2757.931	-0.10756	0.17342	0.320362	protein_coding
ENSG0000(	LIN7B	267.714	-0.08573	0.173494	0.320479	protein_coding
ENSG0000(	LIN54	811.4146	-0.09011	0.173532	0.320532	protein_coding
ENSG0000(	RAB11B-AS	84.43323	-0.13709	0.173606	0.320649	lncRNA
ENSG0000(	KYAT1	148.3507	-0.14825	0.173636	0.320686	protein_coding
ENSG0000(	ENSG0000(	20.06706	0.207691	0.173683	0.320754	lncRNA

ENSG0000(	WDR83	253.4432	0.077885	0.173727	0.320817	protein_coding
ENSG0000(	BLOC1S2	379.339	-0.09703	0.173785	0.320904	protein_coding
ENSG0000(	RN7SL608F	97.0738	-0.10058	0.173815	0.320941	misc_RNA
ENSG0000(	EBAG9	302.4503	-0.07148	0.173884	0.321049	protein_coding
ENSG0000(	SNORA68B	9.088361	0.2911	0.173942	0.321137	snoRNA
ENSG0000(	ZNF699	1059.374	0.061482	0.173996	0.321219	protein_coding
ENSG0000(	NFYB	452.0549	-0.06873	0.174033	0.321268	protein_coding
ENSG0000(	TMEM191A	13.40732	-0.27737	0.174075	0.321327	transcribed_unprocessed_pseudo
ENSG0000(	C5orf22	518.9904	-0.07086	0.174102	0.321359	protein_coding
ENSG0000(	ENSG0000(	119.292	0.119509	0.17413	0.321391	lncRNA
ENSG0000(	ENSG0000(	37.45382	0.148116	0.174237	0.321569	TEC
ENSG0000(	MVB12B	642.2254	-0.09709	0.174276	0.321623	protein_coding
ENSG0000(	ENSG0000(	109.4058	-0.10371	0.174373	0.321688	lncRNA
ENSG0000(	ENSG0000(	102.1064	0.172135	0.174337	0.321688	lncRNA
ENSG0000(	LINC01946	13.33631	-0.25461	0.174364	0.321688	lncRNA
ENSG0000(	NOP14	687.1139	-0.07602	0.174364	0.321688	protein_coding
ENSG0000(	PPARGC1B	2854.074	0.132707	0.174344	0.321688	protein_coding
ENSG0000(	RN7SL538F	39.81735	0.143897	0.174342	0.321688	misc_RNA
ENSG0000(	SNHG25	56.01298	-0.23704	0.174416	0.32175	lncRNA
ENSG0000(	ENSG0000(	9.099133	0.286755	0.174481	0.321821	lncRNA
ENSG0000(	GNA12	1015.791	0.111811	0.174486	0.321821	protein_coding
ENSG0000(	UBE2J2	1017.533	-0.04415	0.174467	0.321821	protein_coding
ENSG0000(	CACNA2D3	63.74935	-0.31867	0.174622	0.322038	protein_coding
ENSG0000(	S1PR2	787.9452	0.127466	0.174624	0.322038	protein_coding
ENSG0000(	ENSG0000(	191.1128	0.108594	0.174685	0.322132	lncRNA
ENSG0000(	ENSG0000(	57.51061	0.129449	0.174701	0.322143	lncRNA
ENSG0000(	MAPK6-DT	17.7089	0.211064	0.174762	0.322236	lncRNA
ENSG0000(	ENSG0000(	24.65493	0.161036	0.17484	0.322361	lncRNA
ENSG0000(	CFAP298-T	1016.167	-0.0749	0.174868	0.322393	protein_coding
ENSG0000(	ENSG0000(	199.209	-0.10289	0.174878	0.322393	lncRNA
ENSG0000(	EGF	55.43109	0.307089	0.174975	0.322497	protein_coding
ENSG0000(	ENSG0000(	10.19989	0.250329	0.174948	0.322497	lncRNA
ENSG0000(	MRPL36	70.26126	-0.10925	0.174966	0.322497	protein_coding
ENSG0000(	PDE4C	694.5648	-0.06186	0.17497	0.322497	protein_coding
ENSG0000(	PHAF1	879.6876	0.055156	0.174986	0.322499	protein_coding
ENSG0000(	SAXO1	119.5006	0.167779	0.175056	0.322609	protein_coding
ENSG0000(	H3C2	157.9535	-0.24822	0.175111	0.322691	protein_coding
ENSG0000(	CFAP43	36.77534	-0.23288	0.175171	0.322783	protein_coding
ENSG0000(	ENSG0000(	12.18678	-0.27785	0.175224	0.322862	TEC
ENSG0000(	ERCC8	3245.297	0.038799	0.17525	0.322891	protein_coding
ENSG0000(	ENSG0000(	357.0937	0.054375	0.175345	0.323046	TEC
ENSG0000(	COPB2	4640.394	0.059017	0.175435	0.323156	protein_coding
ENSG0000(	COPZ1	973.5113	-0.08501	0.175456	0.323156	protein_coding
ENSG0000(	ENSG0000(	37.26748	0.510108	0.175444	0.323156	processed_pseudogene
ENSG0000(	TCEAL1	80.92435	-0.12904	0.175449	0.323156	protein_coding
ENSG0000(	UBN2	4678.406	0.045625	0.175444	0.323156	protein_coding
ENSG0000(	DERL2	996.9413	0.05795	0.1755	0.323219	protein_coding

ENSG0000(	NEDD4L	8210.82	-0.21081	0.175532	0.323245	protein_coding
ENSG0000(	NUDT5	902.5674	0.05629	0.175535	0.323245	protein_coding
ENSG0000(	BANCR	21.48643	0.193994	0.175568	0.323286	lncRNA
ENSG0000(	IL10RB	1127.029	-0.10793	0.175578	0.323286	protein_coding
ENSG0000(	ZNF248	1144.882	0.057168	0.17559	0.323289	protein_coding
ENSG0000(	RGS22	37.61963	0.201373	0.175613	0.323313	protein_coding
ENSG0000(	SAR1A	2394.782	0.065486	0.175629	0.323324	protein_coding
ENSG0000(	ENSG0000(	32.65232	0.205768	0.175643	0.323331	lncRNA
ENSG0000(	SLC12A1	68.95912	0.115029	0.175657	0.323337	protein_coding
ENSG0000(	ATPSCKMT	547.4471	0.072073	0.175692	0.323383	protein_coding
ENSG0000(	PDE8A	2393.858	-0.0935	0.175744	0.323461	protein_coding
ENSG0000(	RN7SL635F	13.55709	-0.25609	0.175818	0.323578	misc_RNA
ENSG0000(	RNF34	1196.774	0.05217	0.175836	0.323591	protein_coding
ENSG0000(	ENSG0000(	14.64408	0.254105	0.175957	0.323796	lncRNA
ENSG0000(	ASPA	2068.636	-0.14561	0.176002	0.323859	protein_coding
ENSG0000(	OR1A1	1287.159	-0.06221	0.176041	0.323913	protein_coding
ENSG0000(	CNOT10	1320.291	-0.04214	0.176091	0.323985	protein_coding
ENSG0000(	NTM-AS1	84.79038	-0.09371	0.176111	0.324004	lncRNA
ENSG0000(	ENSG0000(	345.7141	-0.12112	0.176129	0.324007	lncRNA
ENSG0000(	PLAC8	2079.695	0.150435	0.176134	0.324007	protein_coding
ENSG0000(	ENSG0000(	382.7379	-0.05913	0.176145	0.324009	lncRNA
ENSG0000(	NCAM2	53.13189	-0.31509	0.176156	0.32401	protein_coding
ENSG0000(	GPRC5B	380.98	-0.16382	0.176191	0.324056	protein_coding
ENSG0000(	ENSG0000(	21.88333	-0.2352	0.176232	0.324112	lncRNA
ENSG0000(	PDCD1	82.96214	-0.32153	0.176265	0.324141	protein_coding
ENSG0000(	SLC44A3	472.1166	-0.21173	0.176268	0.324141	protein_coding
ENSG0000(	ENSG0000(	29.97416	-0.31221	0.176279	0.324142	lncRNA
ENSG0000(	ENPP2	1045.364	-0.17464	0.176313	0.324185	protein_coding
ENSG0000(	CCL22	686.2191	-0.17102	0.176382	0.324294	protein_coding
ENSG0000(	TNIK	3115.647	-0.14339	0.176394	0.324296	protein_coding
ENSG0000(	ENSG0000(	69.91907	0.11361	0.176453	0.324387	lncRNA
ENSG0000(	PODN	235.0336	0.14656	0.176468	0.324395	protein_coding
ENSG0000(	RPAP1	736.5403	0.07919	0.176506	0.324446	protein_coding
ENSG0000(	LINC01250	12.10737	0.275024	0.176523	0.324458	lncRNA
ENSG0000(	MIR3941	9.9835	-0.25349	0.176534	0.324459	miRNA
ENSG0000(	ENSG0000(	576.2878	-0.06056	0.176553	0.324476	lncRNA
ENSG0000(	ENSG0000(	14.38566	0.289855	0.176575	0.324498	lncRNA
ENSG0000(	PLBD1	366.5818	-0.11947	0.176621	0.324562	protein_coding
ENSG0000(	CDON	652.4544	0.066948	0.176664	0.324622	protein_coding
ENSG0000(	ENSG0000(	185.8819	0.07811	0.176674	0.324622	lncRNA
ENSG0000(	ACTR3C	1738.703	-0.10459	0.176695	0.324623	protein_coding
ENSG0000(	MIR4292	15.16671	-0.2781	0.176689	0.324623	miRNA
ENSG0000(	PAPSS2	2292.705	-0.24173	0.176717	0.324645	protein_coding
ENSG0000(	LINC01841	42.57103	0.179143	0.176786	0.324752	lncRNA
ENSG0000(	LAGE3	43.39493	-0.1677	0.176828	0.324809	protein_coding
ENSG0000(	ARHGAP19	12.21524	0.38051	0.176911	0.324927	protein_coding
ENSG0000(	ENSG0000(	254.2214	-0.07942	0.176912	0.324927	lncRNA

ENSG0000(	GRB2	2282.39	0.071388	0.17706	0.325179	protein_coding
ENSG0000(	RHOG	455.4457	0.097104	0.17712	0.325257	protein_coding
ENSG0000(	ZFP2	136.2467	-0.1087	0.177123	0.325257	protein_coding
ENSG0000(	SCOC-AS1	263.7035	-0.12261	0.177165	0.325315	lncRNA
ENSG0000(	GTF2IRD1	593.8563	0.148954	0.177185	0.325333	protein_coding
ENSG0000(	MIRLET7BH	1968.437	0.092634	0.177241	0.325416	lncRNA
ENSG0000(	ENSG0000(	217.048	0.080551	0.177293	0.325455	lncRNA
ENSG0000(	HSPE1P10	102.8555	-0.09866	0.17729	0.325455	processed_pseudogene
ENSG0000(	LIN7A	403.0539	-0.19173	0.177273	0.325455	protein_coding
ENSG0000(	ENSG0000(	11.6748	0.266418	0.177344	0.325511	lncRNA
ENSG0000(	SNORA52	204.1123	0.154142	0.17734	0.325511	snoRNA
ENSG0000(	DMRTC1B	39.33763	0.243969	0.177382	0.325563	protein_coding
ENSG0000(	FTLP6	171.1873	-0.08169	0.177481	0.325675	processed_pseudogene
ENSG0000(	RIDA	103.4932	-0.12692	0.177465	0.325675	protein_coding
ENSG0000(	SV2C	275.9266	-0.12455	0.177485	0.325675	protein_coding
ENSG0000(	TOR2A	214.5734	-0.1096	0.177483	0.325675	protein_coding
ENSG0000(	ENSG0000(	290.2944	-0.28665	0.17751	0.325702	protein_coding
ENSG0000(	ENSG0000(	24.17478	0.254948	0.1776	0.325829	lncRNA
ENSG0000(	TRMT2A	563.7901	-0.09428	0.177598	0.325829	protein_coding
ENSG0000(	GTF3C1	1878.404	-0.07519	0.177616	0.325837	protein_coding
ENSG0000(	TAF10	794.1144	-0.11636	0.177625	0.325837	protein_coding
ENSG0000(	ENSG0000(	147.4747	0.085688	0.177755	0.326052	lncRNA
ENSG0000(	FUNDC1	63.59253	-0.1275	0.177763	0.326052	protein_coding
ENSG0000(	IL21R	1004.462	0.283338	0.177784	0.326072	protein_coding
ENSG0000(	RPS29P19	11.912	-0.25889	0.177851	0.326175	processed_pseudogene
ENSG0000(	ENSG0000(	99.256	0.307635	0.177868	0.326187	protein_coding
ENSG0000(	DHX35	638.3375	0.055655	0.177882	0.326195	protein_coding
ENSG0000(	ENSG0000(	30.61951	0.194088	0.177896	0.326201	lncRNA
ENSG0000(	ENSG0000(	33.84649	0.190794	0.178	0.326354	lncRNA
ENSG0000(	SCYGR4	126.6354	0.084238	0.177991	0.326354	protein_coding
ENSG0000(	RN7SL67P	40.66935	0.202668	0.178024	0.326379	misc_RNA
ENSG0000(	ENSG0000(	441.7632	0.073516	0.178054	0.326401	lncRNA
ENSG0000(	PKD1P1	1195.076	-0.24657	0.178057	0.326401	unprocessed_pseudogene
ENSG0000(	PIGK	311.6943	-0.08218	0.178083	0.326431	protein_coding
ENSG0000(	ENSG0000(	462.4163	-0.08336	0.178115	0.326471	lncRNA
ENSG0000(	GRIK2	1067.377	-0.13753	0.178193	0.326594	protein_coding
ENSG0000(	CFAP69	2215.306	0.061763	0.178238	0.326657	protein_coding
ENSG0000(	GPR26	11.73195	0.303857	0.178305	0.326762	protein_coding
ENSG0000(	ENSG0000(	86.16202	-0.11687	0.178372	0.326865	lncRNA
ENSG0000(	ENSG0000(	234.732	0.122813	0.178413	0.326921	lncRNA
ENSG0000(	ENSG0000(	10.40163	0.265266	0.178427	0.326928	lncRNA
ENSG0000(	ATRX	6881.638	0.105795	0.178473	0.326993	protein_coding
ENSG0000(	CD200R1	131.5757	-0.19253	0.178547	0.327052	protein_coding
ENSG0000(	ENSG0000(	239.9884	0.064781	0.178546	0.327052	lncRNA
ENSG0000(	FMO2	100.1748	0.391205	0.178538	0.327052	protein_coding
ENSG0000(	PIGB	861.749	0.055984	0.178539	0.327052	protein_coding
ENSG0000(	TUSC3	181.8293	0.113702	0.178586	0.327105	protein_coding

ENSG0000(	PCDHA13	12.29126	-0.35156	0.178607	0.327125	protein_coding
ENSG0000(	ENSG0000(	34.76572	0.19637	0.178646	0.327177	processed_pseudogene
ENSG0000(	ENSG0000(	38.08141	-0.20209	0.178667	0.327196	lncRNA
ENSG0000(	TPTEP1	1369.375	0.07708	0.178696	0.32723	transcribed_unprocessed_pseudo
ENSG0000(	ENSG0000(	224.5856	0.077739	0.178753	0.327316	lncRNA
ENSG0000(	RSRC1	2208.823	0.047295	0.178775	0.327336	protein_coding
ENSG0000(	LEO1	628.7313	0.071822	0.178789	0.327343	protein_coding
ENSG0000(	MGAT1	2725.521	0.056285	0.178911	0.327549	protein_coding
ENSG0000(	CZIB	639.4679	-0.05991	0.178943	0.327587	protein_coding
ENSG0000(	ENSG0000(	419.2811	0.056828	0.178987	0.32763	lncRNA
ENSG0000(	RBMS2P1	9.438665	0.325401	0.178985	0.32763	processed_pseudogene
ENSG0000(	CDK4P1	85.31864	0.11749	0.179052	0.327692	processed_pseudogene
ENSG0000(	CEP135	1150.055	0.089557	0.179045	0.327692	protein_coding
ENSG0000(	PDC-AS1	659.1622	0.054282	0.179037	0.327692	lncRNA
ENSG0000(	MORF4L2-/	82.83999	0.115279	0.179064	0.327695	lncRNA
ENSG0000(	ENSG0000(	262.1794	-0.11303	0.17911	0.32776	lncRNA
ENSG0000(	FXYD7	10.04951	0.400857	0.179128	0.327773	protein_coding
ENSG0000(	BEND4	413.7838	-0.23983	0.179139	0.327774	protein_coding
ENSG0000(	ZNF665	1201.75	-0.06198	0.179161	0.327796	protein_coding
ENSG0000(	GOLM2	2224.478	0.044294	0.179196	0.327841	protein_coding
ENSG0000(	ENSG0000(	301.8023	0.070628	0.179298	0.328009	lncRNA
ENSG0000(	AAAS	546.0206	0.067863	0.17932	0.32803	protein_coding
ENSG0000(	C12orf4	875.2023	-0.05791	0.17934	0.328046	protein_coding
ENSG0000(	ENSG0000(	423.8932	-0.09388	0.179351	0.328048	lncRNA
ENSG0000(	FURIN	1419.299	0.121274	0.17937	0.328064	protein_coding
ENSG0000(	AMMECR1I	988.2479	0.058891	0.179424	0.328129	protein_coding
ENSG0000(	PDF	284.1605	-0.12068	0.179431	0.328129	protein_coding
ENSG0000(	RNF31	764.5335	-0.07087	0.179441	0.328129	protein_coding
ENSG0000(	ZNF17	508.3787	0.084591	0.179447	0.328129	protein_coding
ENSG0000(	LIMA1	5562.597	-0.15511	0.179525	0.328238	protein_coding
ENSG0000(	MMP20	10.71186	-0.33816	0.179528	0.328238	protein_coding
ENSG0000(	CGA	35.90371	-0.14417	0.179594	0.328341	protein_coding
ENSG0000(	ENSG0000(	89.79978	-0.1142	0.179618	0.328365	lncRNA
ENSG0000(	HNRNPA1P	83.87813	0.101469	0.179663	0.328427	processed_pseudogene
ENSG0000(	ENSG0000(	24.30577	-0.17506	0.179687	0.328453	lncRNA
ENSG0000(	ENSG0000(	32.63695	0.167171	0.1797	0.328458	lncRNA
ENSG0000(	AUNIP	180.044	0.087206	0.179765	0.328558	protein_coding
ENSG0000(	GUSBP14	517.4408	0.316517	0.179778	0.328563	unprocessed_pseudogene
ENSG0000(	SOX6	4341.234	-0.24317	0.179807	0.328596	protein_coding
ENSG0000(	ENSG0000(	20.65342	0.218864	0.179847	0.328624	lncRNA
ENSG0000(	HEXB	738.5033	-0.09571	0.179842	0.328624	protein_coding
ENSG0000(	TMEM219	894.8889	-0.13184	0.179854	0.328624	protein_coding
ENSG0000(	ENSG0000(	34.01311	0.203167	0.179899	0.328688	lncRNA
ENSG0000(	LAT2	1228.191	0.150005	0.17992	0.328707	protein_coding
ENSG0000(	LINC02583	9.056794	0.324538	0.179941	0.328727	lncRNA
ENSG0000(	ENSG0000(	24.12756	-0.30045	0.179961	0.328744	lncRNA
ENSG0000(	PELO	554.6761	0.077555	0.179983	0.328766	protein_coding

ENSG0000(	CYP2T1P	161.9634	-0.08235	0.180131	0.329016	transcribed_unitary_pseudogene
ENSG0000(	ENSG0000(	9.498895	-0.32099	0.180163	0.329017	processed_pseudogene
ENSG0000(	ENSG0000(	30.74168	-0.30852	0.180157	0.329017	lncRNA
ENSG0000(	LINC02575	130.1521	0.071299	0.180156	0.329017	lncRNA
ENSG0000(	DNAJC6	96.18575	0.165386	0.180196	0.329059	protein_coding
ENSG0000(	ANXA2R	156.5856	0.223826	0.180303	0.329235	protein_coding
ENSG0000(	PNO1	377.2437	-0.0764	0.180412	0.329396	protein_coding
ENSG0000(	RN7SL128F	135.3549	-0.07682	0.18041	0.329396	misc_RNA
ENSG0000(	SNORA84	50.94196	-0.19908	0.180423	0.329397	snoRNA
ENSG0000(	RN7SL113F	55.03473	0.133122	0.18046	0.329445	misc_RNA
ENSG0000(	BTD	2492.921	-0.08143	0.180542	0.329576	protein_coding
ENSG0000(	TRIM45	237.1207	0.103888	0.180577	0.329622	protein_coding
ENSG0000(	RBMY2FP	53.50502	0.155632	0.180672	0.329759	transcribed_unprocessed_pseudo
ENSG0000(	RN7SKP253	28.97484	0.177176	0.180674	0.329759	misc_RNA
ENSG0000(	ENSG0000(	102.7456	-0.0984	0.180705	0.329798	TEC
ENSG0000(	RAX2	161.0844	0.111768	0.180729	0.329823	protein_coding
ENSG0000(	ENSG0000(	93.35255	-0.15648	0.180742	0.329826	lncRNA
ENSG0000(	MAP3K1	2419.905	-0.13094	0.180761	0.329843	protein_coding
ENSG0000(	DUS4L-BCA	291.9281	0.09103	0.180783	0.329864	protein_coding
ENSG0000(	RAB11FIP2	873.026	0.088761	0.18092	0.330094	protein_coding
ENSG0000(	PRICKLE2-A	163.7681	-0.08176	0.180954	0.330137	lncRNA
ENSG0000(	ENSG0000(	124.9526	0.119138	0.180984	0.330172	lncRNA
ENSG0000(	MPHOSPH6	244.3144	-0.10516	0.181042	0.330259	protein_coding
ENSG0000(	MEOX2	33.37385	0.378087	0.181069	0.330289	protein_coding
ENSG0000(	FAM74A6	202.2722	0.106105	0.181092	0.330312	transcribed_processed_pseudoge
ENSG0000(	ENSG0000(	76.78054	0.126557	0.181141	0.330365	lncRNA
ENSG0000(	ENSG0000(	11.52035	0.263477	0.181142	0.330365	lncRNA
ENSG0000(	B3GALT1	166.0218	0.108602	0.181158	0.330376	protein_coding
ENSG0000(	ENSG0000(	46.37911	-0.12207	0.1812	0.330414	lncRNA
ENSG0000(	ENSG0000(	215.2166	-0.25876	0.1812	0.330414	lncRNA
ENSG0000(	ENSG0000(	23.73852	0.170906	0.181311	0.330597	lncRNA
ENSG0000(	ENSG0000(	282.0182	0.081588	0.181322	0.330598	lncRNA
ENSG0000(	ENSG0000(	25.46749	-0.17891	0.181346	0.330603	lncRNA
ENSG0000(	MSRB1	184.7206	0.180172	0.181344	0.330603	protein_coding
ENSG0000(	ENSG0000(	130.8678	0.268908	0.181497	0.33086	transcribed_unprocessed_pseudo
ENSG0000(	ENSG0000(	568.9535	0.150599	0.181608	0.331043	lncRNA
ENSG0000(	ENSG0000(	29.93758	-0.32556	0.18169	0.331173	unprocessed_pseudogene
ENSG0000(	EIF2D	1039.827	-0.05176	0.181751	0.331265	protein_coding
ENSG0000(	IMP4	556.7862	-0.08652	0.181788	0.331314	protein_coding
ENSG0000(	MON2	5786.188	0.048514	0.181844	0.331397	protein_coding
ENSG0000(	GYPC	502.8909	0.214238	0.181889	0.331459	protein_coding
ENSG0000(	ZSCAN12	1276.794	0.056197	0.181925	0.331505	protein_coding
ENSG0000(	LINC00877	544.302	0.113906	0.182062	0.331736	lncRNA
ENSG0000(	CAMK2D	2697.997	-0.07875	0.182073	0.331736	protein_coding
ENSG0000(	RTEL1	846.4717	0.068141	0.1821	0.331766	protein_coding
ENSG0000(	ENSG0000(	313.4644	0.064927	0.182144	0.331829	lncRNA
ENSG0000(	C4B	702.1549	0.306537	0.182211	0.33192	protein_coding

ENSG0000(	LINC02057	135.1923	0.119968	0.182216	0.33192	lncRNA
ENSG0000(	GLI1	239.4902	-0.16424	0.182238	0.331942	protein_coding
ENSG0000(	ACER1	91.60456	0.105327	0.182273	0.331986	protein_coding
ENSG0000(	SMG9	1341.568	0.078191	0.182347	0.332101	protein_coding
ENSG0000(	RDM1	98.42156	0.198409	0.182443	0.332257	protein_coding
ENSG0000(	METRNL	564.6391	-0.18567	0.182466	0.332261	protein_coding
ENSG0000(	ODF2	891.7638	0.083765	0.182456	0.332261	protein_coding
ENSG0000(	ENSG0000(	182.6731	-0.15031	0.182549	0.332393	lncRNA
ENSG0000(	GTF2IRD2	2459.37	-0.07554	0.182631	0.332515	protein_coding
ENSG0000(	LINC02897	201.8112	-0.09449	0.182637	0.332515	lncRNA
ENSG0000(	TRMO	404.2378	0.108143	0.182686	0.332584	protein_coding
ENSG0000(	ENSG0000(	316.9407	-0.06201	0.18274	0.332663	lncRNA
ENSG0000(	CSRP2	645.4066	0.16156	0.182758	0.332677	protein_coding
ENSG0000(	NIF3L1	262.1553	-0.07589	0.182776	0.332691	protein_coding
ENSG0000(	ENSG0000(	16.42414	0.235849	0.182818	0.332747	lncRNA
ENSG0000(	LINC01482	322.0386	0.076501	0.182863	0.332811	lncRNA
ENSG0000(	ACOX3	495.9119	0.088315	0.18294	0.332932	protein_coding
ENSG0000(	RFTN1P1	70.13091	-0.10976	0.183005	0.33303	transcribed_unprocessed_pseudo
ENSG0000(	LARP1P1	26.51225	-0.30481	0.183052	0.333077	processed_pseudogene
ENSG0000(	RAB39B	116.925	0.248906	0.183045	0.333077	protein_coding
ENSG0000(	SRP72	1156.227	-0.07643	0.183082	0.333112	protein_coding
ENSG0000(	CEP164P1	234.5652	0.082258	0.183282	0.333437	transcribed_processed_pseudoge
ENSG0000(	CUTALP	2053.676	0.169594	0.183279	0.333437	transcribed_unitary_pseudogene
ENSG0000(	ZNF763	511.1887	0.096	0.183316	0.33348	protein_coding
ENSG0000(	ENSG0000(	29.51745	-0.19905	0.183367	0.333554	lncRNA
ENSG0000(	ANO7L1	52.29826	0.22159	0.183426	0.333643	transcribed_unprocessed_pseudo
ENSG0000(	LINC01137	204.4458	0.126675	0.183448	0.333663	lncRNA
ENSG0000(	ENSG0000(	55.43657	0.115877	0.183524	0.333762	lncRNA
ENSG0000(	ENSG0000(	133.0224	0.187805	0.183517	0.333762	lncRNA
ENSG0000(	CDC27	1801.355	0.075051	0.183566	0.333811	protein_coding
ENSG0000(	GAD2	14.89362	0.406774	0.183571	0.333811	protein_coding
ENSG0000(	MRPL46	423.0502	-0.08947	0.183598	0.33384	protein_coding
ENSG0000(	RFX3	1231.928	0.125617	0.183623	0.333866	protein_coding
ENSG0000(	DTD1	356.9756	0.081873	0.183738	0.334056	protein_coding
ENSG0000(	FPGT-TNNI	132.4653	-0.14313	0.18378	0.334112	protein_coding
ENSG0000(	ENSG0000(	124.811	0.092384	0.183797	0.334124	lncRNA
ENSG0000(	ADCY7	2527.257	0.152242	0.18385	0.334202	protein_coding
ENSG0000(	CLDN7P1	353.7771	0.06676	0.183886	0.334247	processed_pseudogene
ENSG0000(	ENSG0000(	261.5556	0.159443	0.183937	0.334322	lncRNA
ENSG0000(	CIAO1	2184.183	-0.03399	0.184008	0.33443	protein_coding
ENSG0000(	ENSG0000(	47.01614	-0.13802	0.184023	0.334439	lncRNA
ENSG0000(	ENSG0000(	8.619925	0.276327	0.184207	0.33472	lncRNA
ENSG0000(	GOLGA4	8918.303	-0.12318	0.184196	0.33472	protein_coding
ENSG0000(	NFXL1	549.4701	0.080426	0.18421	0.33472	protein_coding
ENSG0000(	STK11IP	417.1123	-0.08178	0.184242	0.334759	protein_coding
ENSG0000(	CDT1	243.8735	-0.0882	0.184283	0.334815	protein_coding
ENSG0000(	TAF5L	638.8921	0.071311	0.184327	0.334875	protein_coding

ENSG0000(	PAN3-AS1	147.1886	0.100423	0.184364	0.334923	lncRNA
ENSG0000(	SNORD3E	16.22762	0.224558	0.184454	0.335068	snoRNA
ENSG0000(	TAPBP	7395.693	0.067901	0.184467	0.335071	protein_coding
ENSG0000(	ITLN1	530.8595	0.347643	0.184505	0.335121	protein_coding
ENSG0000(	LTBR	2483.456	-0.08542	0.18452	0.335129	protein_coding
ENSG0000(	ACTR3-AS1	171.0763	0.167447	0.184534	0.335135	lncRNA
ENSG0000(	ENSG0000(	48.68813	0.12801	0.184589	0.3352	lncRNA
ENSG0000(	ENSG0000(	921.7416	0.066915	0.184591	0.3352	lncRNA
ENSG0000(	KRT5	12.46558	0.546567	0.184627	0.335231	protein_coding
ENSG0000(	RPL32P1	77.13226	0.291381	0.184629	0.335231	processed_pseudogene
ENSG0000(	ENSG0000(	121.6682	0.075885	0.184681	0.335305	lncRNA
ENSG0000(	IRAG1-AS1	14.11189	0.355942	0.184779	0.335444	lncRNA
ENSG0000(	RIMS4	10.87933	-0.33367	0.184776	0.335444	protein_coding
ENSG0000(	ZNF687	1022.138	-0.05642	0.184811	0.335483	protein_coding
ENSG0000(	ENSG0000(	99.0866	0.284088	0.184845	0.335526	lncRNA
ENSG0000(	TNFRSF10D	460.34	0.09134	0.184888	0.335584	protein_coding
ENSG0000(	CAND1	3972.794	-0.03939	0.184899	0.335585	protein_coding
ENSG0000(	LLPH	1700.746	0.042252	0.184985	0.335723	protein_coding
ENSG0000(	ENSG0000(	53.50153	-0.14942	0.185025	0.335775	lncRNA
ENSG0000(	HSBP1P1	9.917349	0.245015	0.185049	0.3358	processed_pseudogene
ENSG0000(	EPCAM-DT	261.8511	-0.14556	0.185068	0.335807	lncRNA
ENSG0000(	LINC02204	34.38459	-0.17721	0.185074	0.335807	lncRNA
ENSG0000(	ENSG0000(	252.4023	-0.07557	0.185093	0.335822	lncRNA
ENSG0000(	KBTBD3	185.6819	-0.10537	0.185141	0.335889	protein_coding
ENSG0000(	ENSG0000(	32.05137	0.138131	0.185168	0.335918	lncRNA
ENSG0000(	FAM111B	620.9605	-0.11548	0.185238	0.335988	protein_coding
ENSG0000(	LVRN	41.73701	0.965813	0.185231	0.335988	protein_coding
ENSG0000(	PRPF19-DT	16.47436	0.229349	0.185237	0.335988	lncRNA
ENSG0000(	ARHGEF19	137.3839	0.137762	0.185251	0.335991	protein_coding
ENSG0000(	SMIM8	863.3498	0.075531	0.185305	0.33607	protein_coding
ENSG0000(	DIDO1	4700.768	-0.04015	0.185408	0.336218	protein_coding
ENSG0000(	MAP3K21	885.1986	-0.21102	0.185401	0.336218	protein_coding
ENSG0000(	PLAAT4	590.5227	0.148696	0.185436	0.336249	protein_coding
ENSG0000(	CTSLP3	80.50033	0.138791	0.185472	0.336296	transcribed_unprocessed_pseudo
ENSG0000(	AIFM2	449.5842	-0.08427	0.185493	0.336303	protein_coding
ENSG0000(	SATB1-AS1	552.4746	0.263181	0.185497	0.336303	lncRNA
ENSG0000(	RGPD8	1144.672	0.162089	0.185629	0.336522	protein_coding
ENSG0000(	RN7SL30P	29.36372	0.155461	0.185643	0.336529	misc_RNA
ENSG0000(	CASZ1	2794.381	-0.21152	0.185864	0.336893	protein_coding
ENSG0000(	CDCA2	189.0958	-0.18796	0.185865	0.336893	protein_coding
ENSG0000(	TRBJ1-3	15.31285	0.358527	0.185879	0.336899	TR_J_gene
ENSG0000(	BRI3	1183.663	-0.09551	0.186035	0.337143	protein_coding
ENSG0000(	ENSG0000(	15.08821	0.255339	0.186029	0.337143	unprocessed_pseudogene
ENSG0000(	AGA-DT	150.0552	0.100549	0.186093	0.337228	lncRNA
ENSG0000(	ZNF503-AS	31.92093	0.21591	0.186272	0.337533	lncRNA
ENSG0000(	TINAGL1	1286.835	-0.18783	0.186288	0.337542	protein_coding
ENSG0000(	CYP2C19	3129.259	-0.26761	0.18634	0.337604	protein_coding

ENSG0000(	ENSG0000(	19.51859	0.23568	0.186343	0.337604	processed_pseudogene
ENSG0000(	MAP3K15	288.3868	0.058859	0.186432	0.337745	protein_coding
ENSG0000(	ENSG0000(	169.2525	0.077794	0.186444	0.337748	lncRNA
ENSG0000(	NBPF6	83.18379	0.365714	0.186549	0.337918	protein_coding
ENSG0000(	ENSG0000(	74.94982	0.121998	0.186565	0.337928	lncRNA
ENSG0000(	EGFR-AS1	215.4095	-0.19628	0.18659	0.337954	lncRNA
ENSG0000(	ZBTB5	687.2399	-0.05628	0.186631	0.338009	protein_coding
ENSG0000(	ENSG0000(	10.67189	0.230517	0.186666	0.338053	lncRNA
ENSG0000(	HLA-Z	9.807036	-0.30752	0.186697	0.33809	unprocessed_pseudogene
ENSG0000(	ENSG0000(	172.6637	-0.1125	0.186716	0.338104	lncRNA
ENSG0000(	ENSG0000(	12.3588	0.239294	0.186732	0.338113	lncRNA
ENSG0000(	ENSG0000(	16.30482	0.18809	0.18683	0.338273	scaRNA
ENSG0000(	ATP6V1G2	35.0924	-0.2028	0.186855	0.338291	protein_coding
ENSG0000(	TCF4	5696.055	0.152511	0.186862	0.338291	protein_coding
ENSG0000(	EMX1	17.68143	-0.3523	0.186873	0.338292	protein_coding
ENSG0000(	PRKCZ-AS1	22.95164	0.201315	0.186961	0.338431	lncRNA
ENSG0000(	TYW3	605.4041	0.065472	0.186995	0.338473	protein_coding
ENSG0000(	HDAC7	2129.091	0.181168	0.187075	0.338552	protein_coding
ENSG0000(	ISCU	1258.895	-0.07742	0.187075	0.338552	protein_coding
ENSG0000(	METTL14	1303.427	-0.06792	0.18707	0.338552	protein_coding
ENSG0000(	NOS1	738.3685	0.044985	0.187081	0.338552	protein_coding
ENSG0000(	SYT2	341.7555	0.13544	0.187105	0.338574	protein_coding
ENSG0000(	ENSG0000(	129.899	-0.07718	0.187115	0.338574	lncRNA
ENSG0000(	RFTN2	508.1751	0.085926	0.187151	0.33862	protein_coding
ENSG0000(	TRIM49D1	381.5497	0.086955	0.187201	0.338671	protein_coding
ENSG0000(	VPS8	2207.217	0.078557	0.187197	0.338671	protein_coding
ENSG0000(	ENSG0000(	3596.948	-0.06364	0.187277	0.338789	lncRNA
ENSG0000(	TFAP2E-AS	27.00396	0.369138	0.187296	0.338803	lncRNA
ENSG0000(	PCDHGA10	226.5934	0.095237	0.187336	0.338858	protein_coding
ENSG0000(	ENSG0000(	18.80635	0.197377	0.187369	0.338898	lncRNA
ENSG0000(	AK3P3	55.2832	-0.1202	0.187423	0.338976	processed_pseudogene
ENSG0000(	CIR1	714.1391	0.064562	0.187463	0.339029	protein_coding
ENSG0000(	CARM1	757.5475	0.062168	0.187474	0.339029	protein_coding
ENSG0000(	COG6	687.4911	-0.08288	0.187523	0.339097	protein_coding
ENSG0000(	SMAP1	871.0189	-0.10594	0.187556	0.339139	protein_coding
ENSG0000(	PTPRN2-AS	38.90707	0.201267	0.187624	0.339238	lncRNA
ENSG0000(	PUS7L	3429.169	0.042214	0.187633	0.339238	protein_coding
ENSG0000(	EIF3M	1423.143	-0.08636	0.187652	0.339254	protein_coding
ENSG0000(	ENSG0000(	10.47478	0.338424	0.187683	0.33929	lncRNA
ENSG0000(	SLC37A1	2018.871	0.160763	0.18772	0.339337	protein_coding
ENSG0000(	MFSD14C	6655.697	0.045266	0.187739	0.339351	transcribed_unprocessed_pseudo
ENSG0000(	CPED1	675.3458	0.11115	0.187785	0.339375	protein_coding
ENSG0000(	ENSG0000(	9.223686	-0.48861	0.187805	0.339375	lncRNA
ENSG0000(	ENSG0000(	18.16005	0.360797	0.187765	0.339375	unprocessed_pseudogene
ENSG0000(	MRPS6	611.2543	-0.07905	0.187797	0.339375	protein_coding
ENSG0000(	RN7SL478F	21.44386	-0.2131	0.187776	0.339375	misc_RNA
ENSG0000(	ATP7A	3524.258	0.035412	0.187935	0.339589	protein_coding

ENSG0000(	ENSG0000(	36.62495	0.213769	0.18802	0.339704	processed_pseudogene
ENSG0000(	PIGX	728.9943	0.058116	0.188017	0.339704	protein_coding
ENSG0000(	ENSG0000(	603.5252	-0.064	0.188067	0.339738	lncRNA
ENSG0000(	GRIA2	16.85476	0.526976	0.188073	0.339738	protein_coding
ENSG0000(	IRS4	63.70628	0.101752	0.188071	0.339738	protein_coding
ENSG0000(	LINC02840	1000.152	-0.05641	0.188082	0.339738	lncRNA
ENSG0000(	SNORA63D	31.95137	0.166692	0.188146	0.339834	snoRNA
ENSG0000(	ZNF519	1815.798	0.073227	0.188188	0.339891	protein_coding
ENSG0000(	ENSG0000(	87.69658	0.089733	0.188251	0.339984	lncRNA
ENSG0000(	TMTC2	1098.687	0.164589	0.188352	0.340149	protein_coding
ENSG0000(	POLG-DT	8.564528	-0.23637	0.188438	0.340283	lncRNA
ENSG0000(	UBASH3A	350.7563	0.223859	0.188525	0.340421	protein_coding
ENSG0000(	LINC02256	282.5365	-0.09141	0.188545	0.34043	lncRNA
ENSG0000(	NBDY	148.1745	-0.10534	0.188552	0.34043	protein_coding
ENSG0000(	DISP3	20.96718	0.327503	0.1886	0.340493	protein_coding
ENSG0000(	HTN3	269.0513	-0.07922	0.188608	0.340493	protein_coding
ENSG0000(	ENSG0000(	124.8696	0.180048	0.188658	0.340564	lncRNA
ENSG0000(	SCARNA9	2738.78	-0.07834	0.188683	0.340589	lncRNA
ENSG0000(	SYCP2L	49.56646	0.280311	0.188699	0.340599	protein_coding
ENSG0000(	ENSG0000(	81.51525	0.12267	0.188773	0.340711	lncRNA
ENSG0000(	ENSG0000(	84.60568	-0.34839	0.188783	0.340711	protein_coding
ENSG0000(	ENSG0000(	52.07642	0.169952	0.188859	0.340829	lncRNA
ENSG0000(	ENSG0000(	10.61566	-0.26677	0.188908	0.340899	lncRNA
ENSG0000(	SLC2A1-AS:	472.8991	0.057044	0.188946	0.340928	lncRNA
ENSG0000(	THSD4	1587.965	-0.2604	0.188937	0.340928	protein_coding
ENSG0000(	ENSG0000(	2374.433	-0.08731	0.188958	0.34093	TEC
ENSG0000(	MRPS34	199.9219	-0.10927	0.18905	0.341077	protein_coding
ENSG0000(	ENSG0000(	218.4238	-0.08839	0.189071	0.341088	lncRNA
ENSG0000(	LINC01989	8.891614	0.388782	0.189078	0.341088	lncRNA
ENSG0000(	ENSG0000(	19.30126	0.175604	0.189106	0.341119	lncRNA
ENSG0000(	RYK	1430.301	-0.05427	0.189195	0.34126	protein_coding
ENSG0000(	ENSG0000(	66.39173	0.175654	0.189211	0.341269	lncRNA
ENSG0000(	ENSG0000(	34.42779	-0.14978	0.189289	0.34137	lncRNA
ENSG0000(	SNORA59B	54.01783	0.108832	0.189282	0.34137	snoRNA
ENSG0000(	ENSG0000(	140.6525	-0.08218	0.189304	0.341378	lncRNA
ENSG0000(	ENSG0000(	391.9572	0.083707	0.189323	0.341393	TEC
ENSG0000(	CCDC196	16.16356	0.193156	0.189353	0.341407	protein_coding
ENSG0000(	GSK3B	3962.715	0.046569	0.18935	0.341407	protein_coding
ENSG0000(	CLTB	598.4291	-0.16223	0.189379	0.341436	protein_coding
ENSG0000(	ENSG0000(	83.3383	0.111982	0.18939	0.341436	lncRNA
ENSG0000(	MFSD10	433.8503	-0.09026	0.189424	0.341477	protein_coding
ENSG0000(	BMS1P4-A(	20.1753	0.436933	0.189444	0.341494	lncRNA
ENSG0000(	KAZN	696.1951	-0.17855	0.189461	0.341504	protein_coding
ENSG0000(	CFL1P5	50.5358	-0.18328	0.189476	0.341508	processed_pseudogene
ENSG0000(	ENSG0000(	221.1059	-0.0735	0.189485	0.341508	lncRNA
ENSG0000(	GSN	3216.877	-0.07924	0.189535	0.341558	protein_coding
ENSG0000(	LINC02811	19.10634	-0.21227	0.189534	0.341558	lncRNA

ENSG0000(	ZNF791	3256.583	-0.05322	0.189545	0.341558	protein_coding
ENSG0000(	HIVEP2	2746.606	0.127929	0.189565	0.341562	protein_coding
ENSG0000(	TYRO3P	323.7667	-0.09094	0.189568	0.341562	processed_pseudogene
ENSG0000(	MED19	83.07166	-0.11376	0.189614	0.341605	protein_coding
ENSG0000(	SNRNP40	526.8632	-0.07551	0.18961	0.341605	protein_coding
ENSG0000(	ALG6	537.7386	-0.06533	0.189738	0.34173	protein_coding
ENSG0000(	ENSG0000(	11.1717	0.259464	0.189695	0.34173	processed_pseudogene
ENSG0000(	ENSG0000(	99.19727	-0.11066	0.189733	0.34173	lncRNA
ENSG0000(	EXT1	4161.785	-0.14473	0.189724	0.34173	protein_coding
ENSG0000(	PTGFRN	1247.446	-0.1678	0.189711	0.34173	protein_coding
ENSG0000(	MIR200CH(	85.93206	-0.21396	0.189765	0.341759	lncRNA
ENSG0000(	LINC01977	224.705	0.082655	0.189782	0.341769	lncRNA
ENSG0000(	ENSG0000(	142.7271	-0.10161	0.189793	0.341771	processed_pseudogene
ENSG0000(	ENSG0000(	8.1337	-0.53844	0.18988	0.341888	lncRNA
ENSG0000(	TRIM61	230.0367	0.103198	0.189877	0.341888	protein_coding
ENSG0000(	ENSG0000(	211.9706	0.107882	0.189932	0.341947	TEC
ENSG0000(	MMEL1	75.1565	-0.33854	0.189935	0.341947	protein_coding
ENSG0000(	PIK3C2A	3150.891	0.065507	0.189945	0.341947	protein_coding
ENSG0000(	RPL32P13	24.62852	-0.28041	0.189972	0.341975	processed_pseudogene
ENSG0000(	CRYM	27.98724	-0.31227	0.189986	0.34198	protein_coding
ENSG0000(	AMH	42.96393	-0.19409	0.190081	0.342118	protein_coding
ENSG0000(	FAM27C	1493.048	-0.12179	0.190083	0.342118	lncRNA
ENSG0000(	PCDHGB8P	224.7173	-0.07338	0.190154	0.342225	transcribed_unitary_pseudogene
ENSG0000(	ENSG0000(	9.172784	0.375267	0.190169	0.342232	lncRNA
ENSG0000(	DENND1B	2626.741	-0.13808	0.190187	0.342246	protein_coding
ENSG0000(	ANKRD16	467.5842	0.068201	0.190219	0.342284	protein_coding
ENSG0000(	GARRE1	2054.158	0.058299	0.190249	0.342317	protein_coding
ENSG0000(	DIP2C-AS1	474.3638	-0.23126	0.190264	0.342325	lncRNA
ENSG0000(	ENSG0000(	18.97873	-0.19712	0.19036	0.342478	lncRNA
ENSG0000(	ENSG0000(	25.32851	0.154123	0.190433	0.342591	lncRNA
ENSG0000(	ENSG0000(	19.96355	0.278487	0.190464	0.342626	lncRNA
ENSG0000(	ATP2B2	13.28651	0.403258	0.190508	0.342646	protein_coding
ENSG0000(	DIS3L2	1649.391	-0.05278	0.190528	0.342646	protein_coding
ENSG0000(	ENSG0000(	14.78721	0.233393	0.190573	0.342646	lncRNA
ENSG0000(	ENSG0000(	103.3693	0.140146	0.190546	0.342646	lncRNA
ENSG0000(	ENSG0000(	167.0699	0.085568	0.190564	0.342646	lncRNA
ENSG0000(	GTF2H4	164.3658	-0.11848	0.190507	0.342646	protein_coding
ENSG0000(	HNRNPA1L	68.66654	0.381838	0.190548	0.342646	protein_coding
ENSG0000(	MTX1	238.5325	0.094778	0.190566	0.342646	protein_coding
ENSG0000(	SUPT3H	284.3543	0.085888	0.19051	0.342646	protein_coding
ENSG0000(	ENSG0000(	35.01975	0.157403	0.190586	0.342651	lncRNA
ENSG0000(	FEN1	159.252	0.162224	0.190601	0.342657	protein_coding
ENSG0000(	HLTF	701.2925	-0.08886	0.19065	0.342727	protein_coding
ENSG0000(	RN7SL38P	49.9132	0.113808	0.190672	0.342747	misc_RNA
ENSG0000(	TMED7-TIC	88.79464	-0.16933	0.190747	0.342861	protein_coding
ENSG0000(	ATAD2B	3690.22	-0.04817	0.190781	0.342886	protein_coding
ENSG0000(	NBEAP1	43.63386	0.162661	0.190782	0.342886	transcribed_unprocessed_pseudo

ENSG0000(	DBH-AS1	113.4849	0.286958	0.190912	0.3431	lncRNA
ENSG0000(	ENSG0000(	441.5543	-0.086	0.190964	0.343173	TEC
ENSG0000(	SNORA33	112.8425	0.121407	0.190981	0.343185	snoRNA
ENSG0000(	ENSG0000(	28.04375	0.173428	0.191035	0.343243	lncRNA
ENSG0000(	LINC01970	501.2453	0.049658	0.191035	0.343243	lncRNA
ENSG0000(	DDX3Y	1086.451	0.392162	0.19106	0.34325	protein_coding
ENSG0000(	ENSG0000(	13.9669	0.6083	0.191061	0.34325	unprocessed_pseudogene
ENSG0000(	ENSG0000(	22.04279	0.215755	0.191095	0.343272	lncRNA
ENSG0000(	HGH1	187.6082	-0.10226	0.19109	0.343272	protein_coding
ENSG0000(	C16orf95-D	17.35634	-0.19192	0.191123	0.343289	lncRNA
ENSG0000(	RNU4-51P	27.85637	0.267241	0.191126	0.343289	snRNA
ENSG0000(	GDPD5	549.1737	0.145125	0.191168	0.343345	protein_coding
ENSG0000(	GRK3	1742.267	0.184873	0.191238	0.343451	protein_coding
ENSG0000(	ENSG0000(	38.44383	0.133266	0.19125	0.343452	lncRNA
ENSG0000(	ENSG0000(	238.1979	-0.07582	0.191266	0.343455	lncRNA
ENSG0000(	LINC02569	83.15419	-0.13453	0.191284	0.343455	lncRNA
ENSG0000(	TAF7	1036.87	-0.0484	0.191275	0.343455	protein_coding
ENSG0000(	ENSG0000(	272.4697	-0.18255	0.191329	0.343464	lncRNA
ENSG0000(	ENSG0000(	12.13086	0.302006	0.191314	0.343464	lncRNA
ENSG0000(	MOB3A	1678.994	-0.11173	0.191316	0.343464	protein_coding
ENSG0000(	PARM1	1280.113	0.153453	0.191333	0.343464	protein_coding
ENSG0000(	CAPN10	937.3485	-0.06131	0.191354	0.343482	protein_coding
ENSG0000(	ENSG0000(	606.1576	0.093635	0.191369	0.34349	lncRNA
ENSG0000(	EEF1A1P3	309.6051	-0.08175	0.191421	0.343541	processed_pseudogene
ENSG0000(	ENSG0000(	42.95204	-0.28754	0.191414	0.343541	lncRNA
ENSG0000(	LRRC2-AS1	10.64243	0.361258	0.19143	0.343541	lncRNA
ENSG0000(	TUBG1	468.4908	-0.07718	0.191445	0.343548	protein_coding
ENSG0000(	ENSG0000(	76.77669	0.130556	0.191518	0.343659	lncRNA
ENSG0000(	BRIP1	3054.026	0.044457	0.191536	0.343672	protein_coding
ENSG0000(	POLR3A	1489.438	0.035905	0.191584	0.343738	protein_coding
ENSG0000(	OLFM4	11071.46	0.298765	0.191623	0.343789	protein_coding
ENSG0000(	SNORA16A	76.47645	0.185881	0.191639	0.343797	snoRNA
ENSG0000(	ENSG0000(	29.58341	0.165938	0.191673	0.343839	lncRNA
ENSG0000(	ENSG0000(	30.56323	0.161048	0.191718	0.3439	lncRNA
ENSG0000(	ST6GALNA(	871.4852	0.145763	0.191729	0.3439	protein_coding
ENSG0000(	LINC01949	1136.325	0.062235	0.191811	0.344028	lncRNA
ENSG0000(	MKX-AS1	51.86467	-0.11733	0.191844	0.344068	lncRNA
ENSG0000(	BCL7C	353.3142	-0.07189	0.191875	0.344104	protein_coding
ENSG0000(	NBPF19	5728.315	0.157691	0.1919	0.344128	protein_coding
ENSG0000(	TMEM62	848.8301	0.050612	0.191922	0.344148	protein_coding
ENSG0000(	FCRLA	300.7181	-0.38473	0.191948	0.344176	protein_coding
ENSG0000(	LINC00612	48.83057	0.147115	0.19198	0.344214	lncRNA
ENSG0000(	RN7SKP3	17.96531	0.216255	0.192078	0.344354	misc_RNA
ENSG0000(	ZNF837	20.45997	0.17611	0.19208	0.344354	protein_coding
ENSG0000(	GGN	35.62397	-0.33136	0.192111	0.34437	protein_coding
ENSG0000(	KLF2P4	110.9977	-0.10652	0.192109	0.34437	processed_pseudogene
ENSG0000(	FOXA1	238.7938	-0.21182	0.192141	0.344405	protein_coding

ENSG0000(	ENSG0000(	37.81314	0.13204	0.192158	0.344414	lncRNA
ENSG0000(	ARHGAP42	179.7866	-0.0786	0.192178	0.344432	processed_pseudogene
ENSG0000(	ENSG0000(	49.40709	0.161207	0.192231	0.344507	lncRNA
ENSG0000(	ATG4A	279.922	-0.12478	0.192287	0.344568	protein_coding
ENSG0000(	ENSG0000(	213.2734	-0.09417	0.192277	0.344568	lncRNA
ENSG0000(	SH3RF2	2224.274	-0.20792	0.192319	0.344605	protein_coding
ENSG0000(	SNORA64	122.5937	0.180494	0.192362	0.344663	snoRNA
ENSG0000(	ENSG0000(	104.6547	-0.0911	0.192425	0.344756	lncRNA
ENSG0000(	TBC1D19	228.1239	0.113816	0.192439	0.344761	protein_coding
ENSG0000(	SIX3	35.05212	0.210307	0.192459	0.344778	protein_coding
ENSG0000(	RADX	177.2155	-0.1186	0.192673	0.345142	protein_coding
ENSG0000(	DCX	63.88762	0.117471	0.192728	0.345221	protein_coding
ENSG0000(	MTATP8P1	8.735339	0.291015	0.192753	0.345246	unprocessed_pseudogene
ENSG0000(	C1QTNF2	131.8709	-0.08599	0.192895	0.345461	protein_coding
ENSG0000(	PPFIA3	175.2934	-0.19151	0.192885	0.345461	protein_coding
ENSG0000(	ISL1	35.98905	0.187569	0.192933	0.34551	protein_coding
ENSG0000(	LINC00330	186.1112	-0.08154	0.192967	0.345551	lncRNA
ENSG0000(	DCST1-AS1	28.77834	-0.23073	0.193132	0.345827	lncRNA
ENSG0000(	ENSG0000(	132.5525	-0.12582	0.193166	0.345869	lncRNA
ENSG0000(	RHPN1	163.5071	0.216574	0.193246	0.345992	protein_coding
ENSG0000(	ENSG0000(	284.7738	-0.10146	0.193344	0.346148	lncRNA
ENSG0000(	ABCA9	900.5215	0.09274	0.193377	0.346167	protein_coding
ENSG0000(	ENSG0000(	328.9486	-0.40905	0.193373	0.346167	protein_coding
ENSG0000(	OR5H1	123.1433	0.105925	0.19347	0.346314	protein_coding
ENSG0000(	IL5RA	75.39478	0.191879	0.193509	0.346364	protein_coding
ENSG0000(	LINC00659	29.07189	0.270087	0.19359	0.346489	lncRNA
ENSG0000(	ENSG0000(	25.08471	-0.21614	0.193701	0.346658	lncRNA
ENSG0000(	KRT40	12.67425	-0.28597	0.193706	0.346658	protein_coding
ENSG0000(	C11orf71	288.4498	0.083761	0.193721	0.346664	protein_coding
ENSG0000(	ENSG0000(	8.266051	-0.27785	0.193738	0.346675	lncRNA
ENSG0000(	STK24	4097.223	-0.09826	0.193796	0.346759	protein_coding
ENSG0000(	POLH-AS1	194.1556	0.076021	0.19382	0.346782	lncRNA
ENSG0000(	ENSG0000(	376.2482	0.150521	0.193845	0.346807	TEC
ENSG0000(	GNAT1	161.6604	0.147439	0.193924	0.346929	protein_coding
ENSG0000(	ADRB1	161.8072	-0.18806	0.193975	0.346975	protein_coding
ENSG0000(	ANKRD20A	150.7796	-0.37927	0.193987	0.346975	transcribed_unprocessed_pseudo
ENSG0000(	ARL4C	973.2514	0.213661	0.194005	0.346975	protein_coding
ENSG0000(	C1orf174	785.3893	-0.06758	0.194021	0.346975	protein_coding
ENSG0000(	CDH4	985.3872	0.2102	0.194012	0.346975	protein_coding
ENSG0000(	ENSG0000(	16.05325	-0.27018	0.194021	0.346975	unprocessed_pseudogene
ENSG0000(	ENSG0000(	32.27332	-0.13958	0.194027	0.346975	lncRNA
ENSG0000(	NME9	104.4988	0.104053	0.19414	0.347144	protein_coding
ENSG0000(	ZNF90P3	94.34742	0.11597	0.194143	0.347144	processed_pseudogene
ENSG0000(	CENPBD1P	1152.728	-0.04603	0.19418	0.347171	transcribed_processed_pseudoge
ENSG0000(	RAPGEF1	3596.042	0.09529	0.194176	0.347171	protein_coding
ENSG0000(	KIAA2012	402.977	-0.0532	0.194194	0.347175	protein_coding
ENSG0000(	RNU4-50P	10.09125	0.274775	0.194225	0.347191	snRNA

ENSG0000(	U8	18.76561	0.224722	0.194215	0.347191	snoRNA
ENSG0000(	ENSG0000(	249.6184	-0.1742	0.194291	0.347271	lncRNA
ENSG0000(	SRMS	21.92649	0.382931	0.194285	0.347271	protein_coding
ENSG0000(	ENSG0000(	13.47684	-0.31002	0.194329	0.347318	lncRNA
ENSG0000(	ENSG0000(	28.70139	0.268148	0.194352	0.347339	lncRNA
ENSG0000(	ENSG0000(	98.64189	0.101238	0.194424	0.347417	lncRNA
ENSG0000(	ENSG0000(	21.46687	0.247581	0.194428	0.347417	lncRNA
ENSG0000(	UPK3BL1	935.435	0.160682	0.194424	0.347417	protein_coding
ENSG0000(	CREBL2	1058.916	-0.07483	0.194518	0.347558	protein_coding
ENSG0000(	ITGB6	667.7583	0.193157	0.194546	0.347588	protein_coding
ENSG0000(	SMUG1	856.6148	-0.05037	0.194572	0.347615	protein_coding
ENSG0000(	ZNF608	1325.475	-0.11388	0.194593	0.347633	protein_coding
ENSG0000(	CCNA2	205.2723	-0.14726	0.194607	0.347637	protein_coding
ENSG0000(	SNORA67	63.05371	0.168858	0.194746	0.347867	snoRNA
ENSG0000(	KCTD14	74.93755	0.195123	0.194779	0.347905	protein_coding
ENSG0000(	ENSG0000(	104.3606	-0.11464	0.194803	0.347928	lncRNA
ENSG0000(	VKORC1L1	1191.245	-0.04074	0.194824	0.347947	protein_coding
ENSG0000(	BANP	1048.496	0.056559	0.194845	0.347956	protein_coding
ENSG0000(	CNKSR1	498.6772	-0.14555	0.194851	0.347956	protein_coding
ENSG0000(	ADA	137.5216	0.213917	0.194866	0.347962	protein_coding
ENSG0000(	ENSG0000(	24.42179	0.196908	0.194991	0.348166	transcribed_processed_pseudoge
ENSG0000(	OR2H2	152.1829	0.130283	0.195091	0.348324	protein_coding
ENSG0000(	GOLGB1	8702.083	0.073919	0.195124	0.348363	protein_coding
ENSG0000(	ENSG0000(	487.345	0.047069	0.195136	0.348366	TEC
ENSG0000(	ENSG0000(	419.2667	0.055868	0.195168	0.348392	lncRNA
ENSG0000(	HOXA-AS2	114.3557	0.132905	0.195186	0.348392	lncRNA
ENSG0000(	PDS5B	1768.334	-0.05114	0.195195	0.348392	protein_coding
ENSG0000(	SLC31A1	2326.816	-0.09178	0.195195	0.348392	protein_coding
ENSG0000(	ENSG0000(	114.9311	0.0833	0.195235	0.348443	lncRNA
ENSG0000(	CRYBA2	12.42933	-0.32271	0.195252	0.348455	protein_coding
ENSG0000(	SMG8	237.9529	0.082213	0.195268	0.348463	protein_coding
ENSG0000(	ENSG0000(	130.2317	0.081752	0.195365	0.348617	lncRNA
ENSG0000(	MUC12	3438.682	0.340258	0.195383	0.348629	protein_coding
ENSG0000(	SPATA20	665.03	0.127776	0.195397	0.348635	protein_coding
ENSG0000(	SF3B5	165.0631	-0.13755	0.195422	0.348659	protein_coding
ENSG0000(	CFAP92	1561.939	0.074837	0.195466	0.348698	protein_coding
ENSG0000(	ENSG0000(	10.33765	-0.23255	0.195464	0.348698	processed_pseudogene
ENSG0000(	CUZD1	36.52904	0.207795	0.195625	0.348962	protein_coding
ENSG0000(	IL23A	19.04472	0.335136	0.195697	0.349051	protein_coding
ENSG0000(	SAMD4B	2845.226	-0.04206	0.195708	0.349051	protein_coding
ENSG0000(	TXN2	400.5699	-0.0885	0.195708	0.349051	protein_coding
ENSG0000(	CENPS	72.37198	0.14984	0.195729	0.349066	protein_coding
ENSG0000(	NANOS1	228.9837	-0.07232	0.195739	0.349066	protein_coding
ENSG0000(	ENSG0000(	9.001875	0.28092	0.195788	0.349135	unprocessed_pseudogene
ENSG0000(	CTBP1	3071.817	0.037869	0.19582	0.349171	protein_coding
ENSG0000(	ENSG0000(	8.742621	0.265071	0.195831	0.349172	lncRNA
ENSG0000(	SNORA46	181.4671	0.147478	0.195926	0.349304	snoRNA

ENSG0000(	TRAJ13	45.51367	-0.26905	0.195928	0.349304	TR_J_gene
ENSG0000(	RCC1L	423.3106	-0.06009	0.196036	0.349477	protein_coding
ENSG0000(	ADAM17	2326.519	0.070748	0.196047	0.349477	protein_coding
ENSG0000(	RN7SL424F	15.80455	0.213343	0.196134	0.349612	misc_RNA
ENSG0000(	CD82	1590.86	-0.07719	0.196197	0.349704	protein_coding
ENSG0000(	TBC1D29P	163.7636	-0.12621	0.196215	0.349717	transcribed_unprocessed_pseudo
ENSG0000(	LINC01361	369.1091	0.093502	0.196256	0.349771	lncRNA
ENSG0000(	SRRM1	3576.608	-0.03234	0.19632	0.349865	protein_coding
ENSG0000(	TEKT4P2	853.4053	0.246847	0.19635	0.349898	transcribed_unprocessed_pseudo
ENSG0000(	ENSG0000(	122.6563	0.084605	0.196461	0.350077	lncRNA
ENSG0000(	ENSG0000(	8.844656	0.31073	0.196498	0.350102	lncRNA
ENSG0000(	HENMT1	214.9329	0.092119	0.196497	0.350102	protein_coding
ENSG0000(	ACAP2	3379.196	-0.05633	0.196517	0.350116	protein_coding
ENSG0000(	HERPUD2	890.0859	0.074093	0.196587	0.350208	protein_coding
ENSG0000(	SVOPL	19.94329	0.410497	0.196591	0.350208	protein_coding
ENSG0000(	MFSD3	166.6944	-0.12329	0.196649	0.350293	protein_coding
ENSG0000(	ENSG0000(	63.49651	0.169004	0.196723	0.350404	lncRNA
ENSG0000(	ENSG0000(	1118.248	0.060202	0.196738	0.350411	lncRNA
ENSG0000(	MARK2	2851.409	-0.05567	0.196782	0.35047	protein_coding
ENSG0000(	OPCML	21.59511	0.523377	0.196954	0.350756	protein_coding
ENSG0000(	SHBG	31.66412	-0.39053	0.196971	0.350766	protein_coding
ENSG0000(	MSH2-OT1	29.93353	0.285822	0.197	0.350799	lncRNA
ENSG0000(	ENSG0000(	19.80753	-0.22279	0.197028	0.3508	lncRNA
ENSG0000(	ENSG0000(	527.3845	0.084689	0.197021	0.3508	protein_coding
ENSG0000(	STING1	1114.482	0.126982	0.197045	0.3508	protein_coding
ENSG0000(	TLCD3A	131.6632	0.161054	0.197038	0.3508	protein_coding
ENSG0000(	UCP3	100.6305	0.152628	0.197078	0.350832	protein_coding
ENSG0000(	ZNF180	483.5579	-0.07361	0.197086	0.350832	protein_coding
ENSG0000(	CACNB2	1846.136	0.097508	0.197111	0.350858	protein_coding
ENSG0000(	CDH2	88.14364	0.12435	0.197163	0.350861	protein_coding
ENSG0000(	IDH2-DT	65.72203	-0.10396	0.197156	0.350861	lncRNA
ENSG0000(	IRAK1BP1	514.1757	-0.10102	0.197155	0.350861	protein_coding
ENSG0000(	PTPRT	144.946	-0.30779	0.197126	0.350861	protein_coding
ENSG0000(	TVP23C-CD	21.88768	0.579675	0.197169	0.350861	protein_coding
ENSG0000(	ENSG0000(	3727.312	0.023336	0.197198	0.350882	lncRNA
ENSG0000(	HNRNPA1P	12.39932	0.233453	0.197203	0.350882	transcribed_unprocessed_pseudo
ENSG0000(	ENSG0000(	18.3736	0.313895	0.197233	0.350917	lncRNA
ENSG0000(	ENSG0000(	93.54844	-0.09937	0.197311	0.351035	lncRNA
ENSG0000(	POU2F2	1503.418	-0.26119	0.197346	0.351078	protein_coding
ENSG0000(	ENSG0000(	14.87211	0.192161	0.197376	0.35111	lncRNA
ENSG0000(	ENSG0000(	220.6819	-0.18114	0.197472	0.351223	transcribed_unprocessed_pseudo
ENSG0000(	KCNK1	404.9617	0.191576	0.197464	0.351223	protein_coding
ENSG0000(	LINC01098	29.88942	0.181841	0.197464	0.351223	lncRNA
ENSG0000(	CAP1	2723.005	-0.0953	0.197496	0.351232	protein_coding
ENSG0000(	ENSG0000(	14.61481	0.223526	0.1975	0.351232	lncRNA
ENSG0000(	TRIM73	1093.731	-0.07289	0.197511	0.351233	protein_coding
ENSG0000(	ENSG0000(	346.6294	0.059927	0.197524	0.351236	lncRNA

ENSG0000(	SUGT1P4-S	101.7945	-0.23322	0.197557	0.351275	lncRNA
ENSG0000(	ENSG0000(	624.1791	0.040956	0.197593	0.351318	lncRNA
ENSG0000(	ENSG0000(	68.97072	0.113474	0.197611	0.35133	lncRNA
ENSG0000(	PPP1R12B	6201.079	0.063763	0.197742	0.351543	protein_coding
ENSG0000(	MN1	134.22	0.200822	0.197804	0.351615	protein_coding
ENSG0000(	SLC2A14	89.18205	-0.1162	0.197816	0.351615	protein_coding
ENSG0000(	TTYH2	357.0117	0.16606	0.197813	0.351615	protein_coding
ENSG0000(	ZNF599	405.4924	-0.05939	0.197829	0.351619	protein_coding
ENSG0000(	ENSG0000(	113.9637	-0.14629	0.197845	0.351628	lncRNA
ENSG0000(	ENSG0000(	25.09045	0.192654	0.197936	0.35177	lncRNA
ENSG0000(	SECISBP2L	2595.255	-0.04668	0.197989	0.351844	protein_coding
ENSG0000(	SEC62	2022.781	-0.06447	0.198022	0.351882	protein_coding
ENSG0000(	AZI2	704.9293	0.092323	0.198125	0.352047	protein_coding
ENSG0000(	PCDHGB5	101.1275	-0.12004	0.198252	0.352252	protein_coding
ENSG0000(	LMO2	215.5505	0.173792	0.198319	0.35233	protein_coding
ENSG0000(	SNORD116	20.12241	0.170822	0.198308	0.35233	snoRNA
ENSG0000(	ACOT1	32.02477	-0.39047	0.198403	0.352369	protein_coding
ENSG0000(	ENSG0000(	294.7071	-0.06229	0.198407	0.352369	lncRNA
ENSG0000(	ENSG0000(	38.24418	0.127898	0.198394	0.352369	TEC
ENSG0000(	ENSG0000(	352.9683	0.055098	0.198395	0.352369	TEC
ENSG0000(	OGDHL	29.22518	-0.29481	0.198354	0.352369	protein_coding
ENSG0000(	RHBDL3	28.72958	0.251883	0.198407	0.352369	protein_coding
ENSG0000(	ENSG0000(	192.8585	-0.09007	0.198446	0.352418	lncRNA
ENSG0000(	ENSG0000(	35.88856	0.125364	0.198482	0.352462	lncRNA
ENSG0000(	ENSG0000(	15.08473	-0.17388	0.198534	0.352534	lncRNA
ENSG0000(	ZNF8	1999.167	0.033615	0.198635	0.352694	protein_coding
ENSG0000(	DDX50P1	373.3788	-0.07009	0.198703	0.352795	processed_pseudogene
ENSG0000(	ENSG0000(	15.30656	-0.49688	0.198751	0.352859	lncRNA
ENSG0000(	ITGB5	651.0169	-0.14769	0.19879	0.352889	protein_coding
ENSG0000(	PRCD	156.5046	0.135425	0.19878	0.352889	protein_coding
ENSG0000(	ATG2B	3629.603	-0.08597	0.198873	0.35301	protein_coding
ENSG0000(	ATP7B	2471.414	-0.12293	0.198881	0.35301	protein_coding
ENSG0000(	DIPK1B	76.74448	0.190839	0.198916	0.35305	protein_coding
ENSG0000(	VNN1	869.4731	0.309769	0.198925	0.35305	protein_coding
ENSG0000(	ENSG0000(	125.2931	0.186344	0.198994	0.353151	lncRNA
ENSG0000(	FMNL3	4030.525	0.185197	0.199039	0.353181	protein_coding
ENSG0000(	PLB1	4447.728	-0.38293	0.199044	0.353181	protein_coding
ENSG0000(	RNU6-25P	19.50136	0.761911	0.199042	0.353181	snRNA
ENSG0000(	CWF19L1	966.1823	0.07585	0.199067	0.353202	protein_coding
ENSG0000(	ARMCX1	109.1767	0.109055	0.199153	0.353335	protein_coding
ENSG0000(	ARFGEF2	4431.594	-0.0623	0.199165	0.353337	protein_coding
ENSG0000(	KIF2A	2923.37	-0.06215	0.199245	0.353457	protein_coding
ENSG0000(	ENSG0000(	652.7602	0.04416	0.199264	0.353472	lncRNA
ENSG0000(	KCNT1	232.4649	0.290198	0.199341	0.353588	protein_coding
ENSG0000(	FEZ2	1815.174	-0.07941	0.19943	0.353726	protein_coding
ENSG0000(	ENSG0000(	361.0356	-0.06044	0.199511	0.353849	lncRNA
ENSG0000(	BBIP1	1205.978	0.070537	0.199565	0.353925	protein_coding

ENSG0000(	ENSG0000(	68.01073	-0.10616	0.19961	0.353987	lncRNA
ENSG0000(	ZNF771	153.3281	0.070411	0.199684	0.354097	protein_coding
ENSG0000(	NAGA	757.456	-0.09257	0.199696	0.354098	protein_coding
ENSG0000(	TMOD1	107.5125	0.187974	0.199743	0.354161	protein_coding
ENSG0000(	ENSG0000(	5430.568	0.058097	0.199848	0.354304	lncRNA
ENSG0000(	ENSG0000(	768.0056	-0.07841	0.199868	0.354304	TEC
ENSG0000(	OR13I1P	62.18497	-0.10653	0.199851	0.354304	unprocessed_pseudogene
ENSG0000(	SNTB1	551.2671	0.095082	0.199858	0.354304	protein_coding
ENSG0000(	OR14L1P	30.43914	0.160296	0.199929	0.354389	protein_coding
ENSG0000(	ZNF365	15.57153	0.294001	0.199938	0.354389	protein_coding
ENSG0000(	USP13	1760.59	-0.15532	0.199969	0.354423	protein_coding
ENSG0000(	DNAJC5G	37.92378	0.146722	0.199984	0.354429	protein_coding
ENSG0000(	HOXA7	119.002	0.184322	0.200101	0.354617	protein_coding
ENSG0000(	NDUFV2P1	8.606207	-0.34679	0.200114	0.354621	processed_pseudogene
ENSG0000(	INAVA	4276.364	-0.19608	0.200132	0.354631	protein_coding
ENSG0000(	BCORL1	599.5696	-0.1252	0.200209	0.354748	protein_coding
ENSG0000(	FAM91A3P	31.28727	0.196201	0.200235	0.354775	transcribed_processed_pseudoge
ENSG0000(	ASXL3	227.9807	-0.1374	0.200307	0.354882	protein_coding
ENSG0000(	ENSG0000(	165.0581	0.090093	0.200325	0.354894	lncRNA
ENSG0000(	TTC3	9433.501	0.049894	0.200395	0.354999	protein_coding
ENSG0000(	EPS8	2848.401	-0.17811	0.200414	0.355012	protein_coding
ENSG0000(	NELFCD	1084.782	-0.05321	0.20048	0.355109	protein_coding
ENSG0000(	GIT2	4519.217	0.055543	0.200558	0.355227	protein_coding
ENSG0000(	ENSG0000(	8.927357	0.333405	0.200588	0.35526	lncRNA
ENSG0000(	TMPO-AS1	104.7956	0.128976	0.200683	0.355408	lncRNA
ENSG0000(	CCDC15-DT	104.1451	0.100005	0.200699	0.355416	lncRNA
ENSG0000(	ENSG0000(	657.841	0.118565	0.200755	0.355496	lncRNA
ENSG0000(	C6orf62	2878.735	-0.0763	0.200833	0.355612	protein_coding
ENSG0000(	ENSG0000(	225.68	-0.07639	0.200843	0.355612	lncRNA
ENSG0000(	BTRC	967.528	-0.05123	0.200893	0.355679	protein_coding
ENSG0000(	LINC02093	35.60498	-0.128	0.200903	0.355679	lncRNA
ENSG0000(	PPP5C	811.2487	-0.06732	0.201002	0.355833	protein_coding
ENSG0000(	MAP3K14	1233.544	-0.06738	0.20104	0.355881	protein_coding
ENSG0000(	ENSG0000(	31.09936	0.164688	0.201112	0.355987	lncRNA
ENSG0000(	ENSG0000(	42.26087	0.149813	0.201143	0.356024	lncRNA
ENSG0000(	CACNG5	550.2262	0.04657	0.201222	0.356127	protein_coding
ENSG0000(	MAP2K6	2758.743	0.096752	0.201224	0.356127	protein_coding
ENSG0000(	EIF1B-AS1	3847.232	0.05044	0.201254	0.356137	lncRNA
ENSG0000(	LINC00431	29.41073	0.22819	0.201264	0.356137	transcribed_unprocessed_pseudo
ENSG0000(	PTPN11	3302.078	-0.04894	0.201249	0.356137	protein_coding
ENSG0000(	FOXG1-AS1	24.34695	0.14918	0.201278	0.356141	lncRNA
ENSG0000(	MAP6	59.32318	0.185233	0.201404	0.356344	protein_coding
ENSG0000(	ME3	393.8531	-0.12207	0.201418	0.35635	protein_coding
ENSG0000(	ENSG0000(	136.8936	0.084502	0.201446	0.356379	lncRNA
ENSG0000(	USP54	2270.74	-0.13732	0.201458	0.356381	protein_coding
ENSG0000(	ATP2A3	4349.975	-0.13837	0.201493	0.356422	protein_coding
ENSG0000(	ENSG0000(	210.5789	-0.058	0.201518	0.356423	lncRNA

ENSG0000(	ENSG0000(	324.7765	0.081559	0.201539	0.356423	lncRNA
ENSG0000(	ENSG0000(	77.60164	0.120516	0.201524	0.356423	lncRNA
ENSG0000(	MAFK	1101.854	-0.10492	0.201536	0.356423	protein_coding
ENSG0000(	TM4SF20	2193.592	-0.30137	0.201571	0.356459	protein_coding
ENSG0000(	CPLX4	16.08767	0.214134	0.201606	0.356502	protein_coding
ENSG0000(	KIDINS220	6101.124	0.037643	0.201632	0.356527	protein_coding
ENSG0000(	FAM53C	1339.568	0.040296	0.201647	0.356535	protein_coding
ENSG0000(	ATE1-AS1	32.55843	-0.21645	0.201671	0.356544	transcribed_unitary_pseudogene
ENSG0000(	ENSG0000(	57.14844	0.125415	0.201698	0.356544	lncRNA
ENSG0000(	GBF1	8292.451	0.147641	0.201696	0.356544	protein_coding
ENSG0000(	ITGA2	1571.44	0.187723	0.201688	0.356544	protein_coding
ENSG0000(	CAMK2A	417.0836	-0.05764	0.201799	0.356703	protein_coding
ENSG0000(	RNU1-109F	19.36673	0.206602	0.201827	0.356732	snRNA
ENSG0000(	ENSG0000(	151.2979	0.073902	0.201862	0.356775	lncRNA
ENSG0000(	ENSG0000(	644.9926	0.154477	0.201879	0.356785	transcribed_unprocessed_pseudo
ENSG0000(	LINC02516	35.7959	0.228709	0.201897	0.356797	lncRNA
ENSG0000(	EIF3C	2989.655	-0.08471	0.201946	0.356802	protein_coding
ENSG0000(	ENSG0000(	375.6648	-0.1103	0.201916	0.356802	lncRNA
ENSG0000(	GUCY1B2	26.93219	0.397883	0.201938	0.356802	transcribed_unitary_pseudogene
ENSG0000(	SP3	4870.022	-0.05274	0.201928	0.356802	protein_coding
ENSG0000(	RNU6-883F	16.95428	0.272554	0.201958	0.356804	snRNA
ENSG0000(	ENSG0000(	79.62898	0.117986	0.202062	0.356967	lncRNA
ENSG0000(	BICD2	1011.307	0.149189	0.202091	0.356999	protein_coding
ENSG0000(	OR2AP1	106.2313	-0.12539	0.202146	0.357077	protein_coding
ENSG0000(	ENSG0000(	84.76228	0.098822	0.202178	0.357112	lncRNA
ENSG0000(	CD27	213.8688	-0.31013	0.202192	0.357117	protein_coding
ENSG0000(	ENSG0000(	186.5269	0.216413	0.20221	0.35713	TEC
ENSG0000(	EMC1	3159.104	-0.03295	0.2023	0.357229	protein_coding
ENSG0000(	ENSG0000(	16.21972	0.297293	0.202296	0.357229	lncRNA
ENSG0000(	ENSG0000(	32.57114	0.225103	0.202282	0.357229	lncRNA
ENSG0000(	RNU6-466F	27.57339	-0.20453	0.202333	0.357266	snRNA
ENSG0000(	CTU2	110.3279	-0.14392	0.202356	0.357287	protein_coding
ENSG0000(	ENSG0000(	30.11398	0.157551	0.202394	0.35733	lncRNA
ENSG0000(	KMT2E-AS1	106.8829	0.096135	0.202403	0.35733	lncRNA
ENSG0000(	ARL8B	1124.951	-0.0918	0.202531	0.357536	protein_coding
ENSG0000(	WBP11	838.6887	-0.05609	0.202549	0.357547	protein_coding
ENSG0000(	GCDH	277.5096	-0.10505	0.202591	0.357602	protein_coding
ENSG0000(	ASXL2	3343.562	-0.0559	0.202726	0.357819	protein_coding
ENSG0000(	RORB	118.8538	-0.11855	0.202751	0.357843	protein_coding
ENSG0000(	CCDC27	164.5388	-0.08602	0.202897	0.358082	protein_coding
ENSG0000(	CDC37	1049.012	0.064383	0.202933	0.358126	protein_coding
ENSG0000(	ZBTB10	705.2761	0.169689	0.20297	0.358171	protein_coding
ENSG0000(	PPP1R3G	167.5132	0.084938	0.203033	0.358262	protein_coding
ENSG0000(	MNX1	428.5415	-0.21935	0.203055	0.35828	protein_coding
ENSG0000(	DYNC2I1	1308.905	-0.12866	0.203188	0.358434	protein_coding
ENSG0000(	ENSG0000(	14.26137	-0.23765	0.203182	0.358434	lncRNA
ENSG0000(	ENSG0000(	17.2553	0.269976	0.203156	0.358434	lncRNA

ENSG0000(	ENSG0000(	355.6424	-0.06544	0.203167	0.358434	TEC
ENSG0000(	NCR3	25.01501	-0.32156	0.203209	0.358451	protein_coding
ENSG0000(	MICB	440.1993	0.069632	0.203227	0.358464	protein_coding
ENSG0000(	USP48	2607.556	0.064306	0.203266	0.358513	protein_coding
ENSG0000(	RN7SL856F	13.12655	-0.24372	0.203387	0.358705	misc_RNA
ENSG0000(	FBXW12	91.88861	0.086173	0.203423	0.358749	protein_coding
ENSG0000(	MIR6739	9.666141	-0.30474	0.203472	0.358815	miRNA
ENSG0000(	AURKA	232.3876	-0.10721	0.203526	0.358887	protein_coding
ENSG0000(	ENSG0000(	68.65064	-0.09182	0.203557	0.358887	lncRNA
ENSG0000(	RN7SL74P	16.63345	0.170768	0.203558	0.358887	misc_RNA
ENSG0000(	SKIV2L	1291.355	-0.09244	0.203538	0.358887	protein_coding
ENSG0000(	ENSG0000(	167.4091	0.074692	0.203578	0.358902	lncRNA
ENSG0000(	CYP1B1-AS	2139.963	0.093788	0.203726	0.359122	lncRNA
ENSG0000(	LYST-AS1	81.38457	0.099331	0.203723	0.359122	lncRNA
ENSG0000(	LRRC37A15	71.06214	0.205785	0.203743	0.359133	processed_pseudogene
ENSG0000(	MAPK6	2533.921	-0.12816	0.203838	0.35928	protein_coding
ENSG0000(	ENSG0000(	57.87897	-0.1309	0.203868	0.359312	lncRNA
ENSG0000(	ENSG0000(	8.683663	-0.45761	0.20388	0.359314	lncRNA
ENSG0000(	TTC5	829.7412	0.069393	0.203907	0.35934	protein_coding
ENSG0000(	SPACA6	389.5975	0.069586	0.20402	0.35952	protein_coding
ENSG0000(	ENSG0000(	57.39615	0.158558	0.204075	0.359597	unprocessed_pseudogene
ENSG0000(	LINC01346	22.24817	0.24958	0.204194	0.359766	lncRNA
ENSG0000(	NLN	1002.663	-0.07073	0.204192	0.359766	protein_coding
ENSG0000(	COPE	599.5409	-0.07951	0.204244	0.359796	protein_coding
ENSG0000(	ENSG0000(	12.51512	0.433187	0.204224	0.359796	protein_coding
ENSG0000(	ENSG0000(	233.3167	0.100248	0.204273	0.359796	lncRNA
ENSG0000(	FREM2	111.719	-0.11397	0.20428	0.359796	protein_coding
ENSG0000(	IFT81	809.6431	0.073711	0.204265	0.359796	protein_coding
ENSG0000(	LINC02235	117.3691	-0.09045	0.204274	0.359796	lncRNA
ENSG0000(	ENSG0000(	70.85563	0.122372	0.204313	0.359835	processed_pseudogene
ENSG0000(	GM2A	1599.433	0.188233	0.204387	0.359945	protein_coding
ENSG0000(	ENSG0000(	51.74343	0.203232	0.204466	0.360061	lncRNA
ENSG0000(	ENSG0000(	149.7391	0.151385	0.204476	0.360061	lncRNA
ENSG0000(	ENSG0000(	266.9632	-0.08375	0.2045	0.360065	lncRNA
ENSG0000(	KIF4CP	14.71413	0.308994	0.204501	0.360065	processed_pseudogene
ENSG0000(	ENSG0000(	27.22467	0.235237	0.204547	0.360127	lncRNA
ENSG0000(	THUMPD3F	58.78082	-0.27393	0.204592	0.360186	unprocessed_pseudogene
ENSG0000(	MGRN1	2601.896	-0.04761	0.204627	0.360207	protein_coding
ENSG0000(	PDE6D	269.4866	0.064693	0.204627	0.360207	protein_coding
ENSG0000(	ENSG0000(	346.6395	-0.07285	0.204654	0.360235	lncRNA
ENSG0000(	ENSG0000(	288.6923	-0.06122	0.204705	0.36027	lncRNA
ENSG0000(	SGCB	769.4587	-0.09704	0.204688	0.36027	protein_coding
ENSG0000(	VXN	12.23534	0.396811	0.204709	0.36027	protein_coding
ENSG0000(	POLE2	99.42647	0.134515	0.204743	0.360291	protein_coding
ENSG0000(	SHPRH	4315.505	-0.05189	0.204739	0.360291	protein_coding
ENSG0000(	RNU6-678F	9.81334	0.222898	0.204776	0.360327	snRNA
ENSG0000(	RDH8	14.66798	0.192202	0.204815	0.360377	protein_coding

ENSG0000(	ERCC1	948.3525	-0.05959	0.204941	0.360558	protein_coding
ENSG0000(	UTP25	941.4493	0.077087	0.204938	0.360558	protein_coding
ENSG0000(	TMEM97	220.197	-0.09509	0.205095	0.360809	protein_coding
ENSG0000(	ENSG0000(	42.19059	0.143786	0.205118	0.360828	lncRNA
ENSG0000(	ENSG0000(	9.003994	0.260215	0.205134	0.360837	lncRNA
ENSG0000(	LINC01530	30.66356	-0.24095	0.205187	0.360909	lncRNA
ENSG0000(	SERINC5	4151.743	0.066617	0.205243	0.360988	protein_coding
ENSG0000(	ENSG0000(	90.36365	0.100515	0.205255	0.360989	lncRNA
ENSG0000(	COQ6	1302.667	-0.0385	0.205314	0.361074	protein_coding
ENSG0000(	ENSG0000(	62.63372	-0.14799	0.205361	0.361136	lncRNA
ENSG0000(	ZBED2	19.58131	0.452935	0.205486	0.361335	protein_coding
ENSG0000(	GLI2	264.6856	0.127934	0.205557	0.361439	protein_coding
ENSG0000(	ENSG0000(	245.4367	-0.08265	0.205659	0.3616	lncRNA
ENSG0000(	PEAK1	7441.683	-0.06689	0.205686	0.361626	protein_coding
ENSG0000(	ENSG0000(	193.3538	-0.07209	0.20572	0.361665	lncRNA
ENSG0000(	MBOAT7	1534.599	-0.11535	0.205746	0.361671	protein_coding
ENSG0000(	ZNF257	325.8311	-0.14938	0.205744	0.361671	protein_coding
ENSG0000(	BNIP2	2314.783	-0.06849	0.205811	0.361765	protein_coding
ENSG0000(	ANKRD31	36.81648	0.215819	0.205915	0.361874	protein_coding
ENSG0000(	B3GNT3	842.0746	-0.21937	0.205933	0.361874	protein_coding
ENSG0000(	LINC01563	363.8424	0.071508	0.205942	0.361874	lncRNA
ENSG0000(	LINC02717	351.183	0.071828	0.205937	0.361874	lncRNA
ENSG0000(	RN7SL580F	23.58303	-0.20707	0.205928	0.361874	misc_RNA
ENSG0000(	SMIM1	9.983929	0.273993	0.205914	0.361874	protein_coding
ENSG0000(	ENSG0000(	17.62046	-0.40124	0.205963	0.361891	protein_coding
ENSG0000(	LINC01816	11.25642	0.256558	0.206002	0.361939	lncRNA
ENSG0000(	ENSG0000(	96.17513	-0.12281	0.206031	0.361951	transcribed_processed_pseudoge
ENSG0000(	NXT1	56.01271	0.144527	0.20603	0.361951	protein_coding
ENSG0000(	BLID	24.74657	0.288234	0.20606	0.361961	protein_coding
ENSG0000(	DOCK7	3874.682	0.092701	0.206051	0.361961	protein_coding
ENSG0000(	ENSG0000(	23.25495	0.199804	0.206089	0.361991	lncRNA
ENSG0000(	ENSG0000(	11.4169	-0.53901	0.206136	0.362053	lncRNA
ENSG0000(	ZNRF3-AS1	380.9293	0.063239	0.206163	0.362081	lncRNA
ENSG0000(	CA1	55.6391	-0.39067	0.206199	0.362085	protein_coding
ENSG0000(	ENSG0000(	20.15638	-0.25591	0.206187	0.362085	TEC
ENSG0000(	IFT43	284.4517	0.089122	0.2062	0.362085	protein_coding
ENSG0000(	ENSG0000(	245.8675	-0.10009	0.206256	0.362163	TEC
ENSG0000(	INSRR	11.91736	-0.37498	0.206345	0.362299	protein_coding
ENSG0000(	TMEM250	511.8369	-0.08145	0.206371	0.362326	protein_coding
ENSG0000(	TBC1D8-AS	48.8401	0.141037	0.206396	0.362349	lncRNA
ENSG0000(	BIN2	947.0975	0.171747	0.206461	0.362409	protein_coding
ENSG0000(	ENSG0000(	14.068	0.210343	0.206458	0.362409	lncRNA
ENSG0000(	ZNF35	187.4721	0.09369	0.206465	0.362409	protein_coding
ENSG0000(	RN7SL812F	30.79665	0.165593	0.206657	0.362726	misc_RNA
ENSG0000(	ENSG0000(	70.66299	0.090847	0.20669	0.362745	processed_pseudogene
ENSG0000(	ENSG0000(	81.61333	-0.15453	0.206692	0.362745	TEC
ENSG0000(	PATZ1	644.4946	-0.10296	0.206702	0.362745	protein_coding

ENSG0000(	ENSG0000(	65.62949	-0.09752	0.206758	0.362822	lncRNA
ENSG0000(	LINC02246	260.6955	0.076644	0.206796	0.362869	lncRNA
ENSG0000(	ANKRD55	37.14029	0.265104	0.206841	0.36287	protein_coding
ENSG0000(	GXYLT1P3	13.43995	0.588976	0.206843	0.36287	processed_pseudogene
ENSG0000(	RN7SL181F	24.46292	0.176294	0.206815	0.36287	misc_RNA
ENSG0000(	RN7SL492F	16.47923	0.206091	0.206832	0.36287	misc_RNA
ENSG0000(	TRAIP	79.6065	-0.16243	0.206897	0.362945	protein_coding
ENSG0000(	ARHGAP21	3939.663	-0.10167	0.206971	0.36303	protein_coding
ENSG0000(	ENSG0000(	108.2841	0.102034	0.20698	0.36303	processed_pseudogene
ENSG0000(	PMCHL1	95.36742	-0.15878	0.206961	0.36303	transcribed_unprocessed_pseudo
ENSG0000(	PDPK2P	495.8239	0.077617	0.207048	0.363129	transcribed_unprocessed_pseudo
ENSG0000(	ENSG0000(	360.6311	0.070125	0.20717	0.363264	lncRNA
ENSG0000(	ENSG0000(	999.4685	0.048962	0.207183	0.363264	TEC
ENSG0000(	LINC00304	18.01974	0.306952	0.207175	0.363264	lncRNA
ENSG0000(	NADK	1862.412	0.064302	0.207157	0.363264	protein_coding
ENSG0000(	TENT2	2326.914	0.05525	0.207145	0.363264	protein_coding
ENSG0000(	ELOBP4	20.57325	0.199905	0.207227	0.363322	processed_pseudogene
ENSG0000(	ENSG0000(	35.76873	0.193909	0.207269	0.363375	lncRNA
ENSG0000(	GAB1	2119.039	-0.06538	0.207392	0.363571	protein_coding
ENSG0000(	EIF2S2	631.534	-0.07567	0.20749	0.363702	protein_coding
ENSG0000(	FAM234B	398.4895	-0.08295	0.207485	0.363702	protein_coding
ENSG0000(	GPR63	495.079	0.097514	0.207542	0.363773	protein_coding
ENSG0000(	AP1G2-AS1	671.1215	0.065341	0.207597	0.363839	lncRNA
ENSG0000(	RASD2	116.9911	0.163513	0.207603	0.363839	protein_coding
ENSG0000(	RALGPS1	3281.908	-0.17623	0.207639	0.36387	protein_coding
ENSG0000(	RNU6-540F	11.35691	0.258968	0.207644	0.36387	snRNA
ENSG0000(	DARS1-AS1	1092.587	-0.06193	0.207675	0.363881	lncRNA
ENSG0000(	ENSG0000(	80.81188	0.149851	0.207685	0.363881	lncRNA
ENSG0000(	SF1	5320.075	-0.05835	0.207683	0.363881	protein_coding
ENSG0000(	PRAL	1786.208	0.104704	0.207702	0.363891	TEC
ENSG0000(	ENSG0000(	219.5765	-0.08745	0.207726	0.363913	lncRNA
ENSG0000(	ENSG0000(	265.101	-0.07742	0.207751	0.363936	lncRNA
ENSG0000(	TAB3	2712.463	0.036566	0.207765	0.36394	protein_coding
ENSG0000(	RN7SL163F	78.0467	0.086959	0.207871	0.364106	misc_RNA
ENSG0000(	ENSG0000(	9.170959	0.292149	0.207938	0.364145	processed_pseudogene
ENSG0000(	ENSG0000(	10.20527	0.233036	0.207939	0.364145	lncRNA
ENSG0000(	ENSG0000(	372.9671	0.049539	0.207912	0.364145	lncRNA
ENSG0000(	RNF111	1442.333	0.047591	0.20792	0.364145	protein_coding
ENSG0000(	ZNF517	336.1891	0.058163	0.207963	0.364166	protein_coding
ENSG0000(	ENSG0000(	44.34521	-0.14578	0.207981	0.364177	lncRNA
ENSG0000(	ZC3H7B	3226.026	0.036869	0.208031	0.364244	protein_coding
ENSG0000(	ENSG0000(	146.8875	0.096999	0.208105	0.364325	TEC
ENSG0000(	KCNB2	34.83485	0.232196	0.20811	0.364325	protein_coding
ENSG0000(	ODAD1	393.439	-0.08459	0.208111	0.364325	protein_coding
ENSG0000(	ATP6V0CP4	31.28764	0.205461	0.208177	0.36442	processed_pseudogene
ENSG0000(	CETN2	80.88629	0.127451	0.208244	0.364516	protein_coding
ENSG0000(	RPAIN	2713.694	-0.05372	0.208289	0.364555	protein_coding

ENSG0000(	SNORD101	19.34494	0.202434	0.208284	0.364555	snoRNA
ENSG0000(	LINC01608	12691.42	-0.06624	0.208414	0.364753	lncRNA
ENSG0000(	MTO1	3289.734	-0.04209	0.208475	0.364841	protein_coding
ENSG0000(	ENSG0000(	663.4372	0.066825	0.208496	0.364857	lncRNA
ENSG0000(	SLC17A7	136.1792	-0.10744	0.208537	0.364908	protein_coding
ENSG0000(	LINC02556	25.96562	-0.1462	0.208551	0.364911	lncRNA
ENSG0000(	SPEG	528.3062	0.151323	0.208612	0.364999	protein_coding
ENSG0000(	AAGAB	902.0345	0.064258	0.208783	0.365227	protein_coding
ENSG0000(	ENSG0000(	14.83422	0.289406	0.208789	0.365227	lncRNA
ENSG0000(	ENSG0000(	9.561121	0.446058	0.208767	0.365227	lncRNA
ENSG0000(	OR11A1	521.6794	-0.09835	0.208784	0.365227	protein_coding
ENSG0000(	FAM230J	48.69354	0.258368	0.20888	0.365366	lncRNA
ENSG0000(	ENSG0000(	356.7374	0.087156	0.208915	0.365403	lncRNA
ENSG0000(	TTC30B	146.5457	0.092131	0.208924	0.365403	protein_coding
ENSG0000(	KLC1	5710.576	-0.04331	0.208947	0.365423	protein_coding
ENSG0000(	ENSG0000(	153.3866	0.168609	0.208972	0.365446	protein_coding
ENSG0000(	NPC1	2270.319	0.068792	0.208998	0.365471	protein_coding
ENSG0000(	GRM5	17.63525	0.407433	0.209151	0.365719	protein_coding
ENSG0000(	RNU2-66P	10.1055	0.230224	0.209178	0.365745	snRNA
ENSG0000(	LINC02068	255.4472	0.115577	0.209258	0.365864	lncRNA
ENSG0000(	ENSG0000(	245.8712	0.107595	0.20933	0.36597	lncRNA
ENSG0000(	CELF1	5515.231	0.053164	0.209466	0.366189	protein_coding
ENSG0000(	FAM86B2	10.27845	0.525129	0.209518	0.366259	protein_coding
ENSG0000(	ADAMTS5	531.3826	0.177809	0.209565	0.366301	protein_coding
ENSG0000(	ENSG0000(	9.91269	0.333203	0.209556	0.366301	lncRNA
ENSG0000(	HDGFL3P1	119.5464	-0.10144	0.209637	0.366393	processed_pseudogene
ENSG0000(	HES2	720.0139	-0.06504	0.209641	0.366393	protein_coding
ENSG0000(	ABHD4	612.0671	-0.17729	0.209713	0.366498	protein_coding
ENSG0000(	LINC01609	2080.447	-0.09782	0.209779	0.366593	lncRNA
ENSG0000(	ENSG0000(	134.6244	0.086814	0.209808	0.366602	lncRNA
ENSG0000(	ITCH	3444.728	-0.06249	0.209807	0.366602	protein_coding
ENSG0000(	FAAP100	856.9702	0.074336	0.209929	0.366773	protein_coding
ENSG0000(	OR6C75	494.8597	0.058498	0.209926	0.366773	protein_coding
ENSG0000(	ENSG0000(	9.759039	0.318708	0.209947	0.366785	lncRNA
ENSG0000(	RNU1-73P	16.30854	0.216964	0.20998	0.366802	snRNA
ENSG0000(	TNFRSF10A	545.1243	-0.13959	0.20998	0.366802	protein_coding
ENSG0000(	ENSG0000(	19.50183	0.27424	0.210062	0.366884	TEC
ENSG0000(	HNRNPL	3227.885	0.059363	0.210043	0.366884	protein_coding
ENSG0000(	LINC01247	310.2018	-0.07039	0.210055	0.366884	lncRNA
ENSG0000(	TMEM45A	38049.3	-0.0636	0.210113	0.366952	protein_coding
ENSG0000(	ENSG0000(	92.06007	0.160545	0.210147	0.366978	lncRNA
ENSG0000(	ENSG0000(	55.29075	0.338337	0.210151	0.366978	protein_coding
ENSG0000(	RN7SL546P	38.3311	0.154925	0.210181	0.36701	misc_RNA
ENSG0000(	SLC12A5-A:	14.16795	0.209404	0.210331	0.367251	lncRNA
ENSG0000(	LILRA4	33.20604	0.393269	0.210412	0.367372	protein_coding
ENSG0000(	SLC44A3-A:	741.9518	0.132907	0.210448	0.367416	transcribed_processed_pseudoge
ENSG0000(	SNORD6	11.00024	0.237167	0.210494	0.367476	snoRNA

ENSG0000(	ENSG0000(	23.34135	0.196149	0.210526	0.367512	lncRNA
ENSG0000(	USP9YP3	59.84899	0.113073	0.21057	0.367567	unprocessed_pseudogene
ENSG0000(	LINC02666	21.81206	0.179933	0.210606	0.367594	lncRNA
ENSG0000(	POPDC2	106.7103	0.15591	0.210609	0.367594	protein_coding
ENSG0000(	ENSG0000(	37.63238	0.132677	0.210629	0.367608	lncRNA
ENSG0000(	FHAD1-AS1	85.15267	-0.10411	0.21064	0.367608	lncRNA
ENSG0000(	PSMD6-AS1	198.8957	-0.0825	0.210661	0.367623	lncRNA
ENSG0000(	METTL2B	2373.638	-0.04517	0.210687	0.367634	protein_coding
ENSG0000(	VPS26A	687.6028	-0.10187	0.21069	0.367634	protein_coding
ENSG0000(	UFD1	2041.342	0.043829	0.210702	0.367635	protein_coding
ENSG0000(	HES4	25.62855	0.185635	0.210794	0.367754	protein_coding
ENSG0000(	LINC01725	743.6657	-0.12668	0.210806	0.367754	lncRNA
ENSG0000(	TLCD2	2597.242	-0.12727	0.210806	0.367754	protein_coding
ENSG0000(	ENSG0000(	57.13652	-0.12533	0.210837	0.367788	transcribed_unprocessed_pseudo
ENSG0000(	SRP54	1548.443	0.063809	0.210888	0.367857	protein_coding
ENSG0000(	DNAJC9	548.9995	-0.07255	0.210912	0.367878	protein_coding
ENSG0000(	KIF6	797.548	-0.06154	0.210973	0.367965	protein_coding
ENSG0000(	C19orf47	456.4773	-0.05674	0.210997	0.367966	protein_coding
ENSG0000(	RNU1-70P	69.27288	-0.39314	0.210994	0.367966	snRNA
ENSG0000(	SLC15A3	1053.11	0.197718	0.211028	0.368	protein_coding
ENSG0000(	UNC13D	1131.639	0.161139	0.211086	0.368081	protein_coding
ENSG0000(	ENSG0000(	260.4463	0.082324	0.211197	0.368234	lncRNA
ENSG0000(	MIR194-1	20.33744	-0.37176	0.211189	0.368234	miRNA
ENSG0000(	ATP8B5P	39.15595	0.171131	0.211241	0.368273	transcribed_unprocessed_pseudo
ENSG0000(	MT1JP	48.26605	0.341876	0.211243	0.368273	transcribed_unprocessed_pseudo
ENSG0000(	ENSG0000(	25.0394	-0.35372	0.211306	0.368355	lncRNA
ENSG0000(	ZCCHC14-D	41.73716	-0.21018	0.211313	0.368355	lncRNA
ENSG0000(	MTIF3	512.8097	-0.06483	0.211367	0.368427	protein_coding
ENSG0000(	ENSG0000(	26.71388	0.175806	0.211398	0.368451	lncRNA
ENSG0000(	RGL2	1019.031	0.096035	0.211404	0.368451	protein_coding
ENSG0000(	LINC02217	223.3885	0.067625	0.211434	0.368484	lncRNA
ENSG0000(	ABCC9	4797.643	0.057078	0.211459	0.368506	protein_coding
ENSG0000(	CCDC24	337.8345	-0.07971	0.211483	0.368528	protein_coding
ENSG0000(	DAP-DT	14.94827	-0.2207	0.211529	0.368587	lncRNA
ENSG0000(	AP5M1	2514.638	-0.079	0.211627	0.368717	protein_coding
ENSG0000(	ENSG0000(	89.1567	0.168	0.211619	0.368717	lncRNA
ENSG0000(	TPM3P8	9.206095	0.419038	0.211643	0.368725	processed_pseudogene
ENSG0000(	MYD88	1283.2	0.089456	0.211714	0.368787	protein_coding
ENSG0000(	RN7SKP134	20.80632	0.238609	0.211706	0.368787	misc_RNA
ENSG0000(	RNU6-171F	69.60553	-0.19602	0.211691	0.368787	snRNA
ENSG0000(	ENSG0000(	40.23595	-0.36062	0.211728	0.368792	lncRNA
ENSG0000(	RXRA	2587.742	-0.13371	0.211796	0.368889	protein_coding
ENSG0000(	TNFRSF10A	19.34904	0.219845	0.211808	0.36889	lncRNA
ENSG0000(	VSIR	1690.589	-0.08984	0.211834	0.368915	protein_coding
ENSG0000(	ENSG0000(	49.9607	0.142842	0.211871	0.368925	lncRNA
ENSG0000(	FRG1DP	29.25591	-0.31267	0.211875	0.368925	unprocessed_pseudogene
ENSG0000(	SLC7A14	804.5286	-0.06227	0.211858	0.368925	protein_coding

ENSG0000(	MAP4K2	1046.291	-0.17135	0.211975	0.369079	protein_coding
ENSG0000(	SETBP1	1971.363	0.159281	0.21206	0.369206	protein_coding
ENSG0000(	LINC01230	33.76589	0.144977	0.212145	0.369335	lncRNA
ENSG0000(	ANKRD13A	2767.875	0.057006	0.212165	0.369349	protein_coding
ENSG0000(	RN7SL5P	59.10122	-0.17293	0.212185	0.369363	misc_RNA
ENSG0000(	ENSG0000(	235.7522	0.172316	0.212287	0.36948	lncRNA
ENSG0000(	ENSG0000(	548.0129	-0.07021	0.212282	0.36948	lncRNA
ENSG0000(	ZNF260	1025.421	-0.08342	0.212283	0.36948	protein_coding
ENSG0000(	ENSG0000(	200.5497	0.085278	0.212363	0.369591	lncRNA
ENSG0000(	ITPR3	5481.291	-0.09867	0.212403	0.36964	protein_coding
ENSG0000(	RN7SL784F	233.276	-0.08221	0.212455	0.36971	misc_RNA
ENSG0000(	LINC00293	14.15693	0.3117	0.212501	0.36977	lncRNA
ENSG0000(	DUS4L	254.645	0.102388	0.212515	0.369773	protein_coding
ENSG0000(	CTXN2-AS1	30.58311	0.147034	0.212536	0.36979	lncRNA
ENSG0000(	ENSG0000(	15.00619	0.271027	0.212601	0.369883	lncRNA
ENSG0000(	FCHO1	623.5392	-0.10911	0.212774	0.370163	protein_coding
ENSG0000(	RBFA	770.6335	-0.07511	0.212962	0.370471	protein_coding
ENSG0000(	MIR3174	19.83265	0.19161	0.213003	0.370492	miRNA
ENSG0000(	PTAR1	3551.383	0.036293	0.212989	0.370492	protein_coding
ENSG0000(	SRC	2337.575	-0.15364	0.213018	0.370492	protein_coding
ENSG0000(	TAS2R1	48.89163	0.207845	0.213022	0.370492	protein_coding
ENSG0000(	ENSG0000(	16.03653	-0.22974	0.213059	0.370536	lncRNA
ENSG0000(	BBS12	192.6249	-0.16741	0.213128	0.370616	protein_coding
ENSG0000(	PLEKHB1	137.7806	0.199601	0.213118	0.370616	protein_coding
ENSG0000(	SGSM3	1367.84	0.084623	0.21315	0.370634	protein_coding
ENSG0000(	CIC	2227.218	0.087553	0.21323	0.370744	protein_coding
ENSG0000(	ZNF19	763.6946	0.06999	0.213237	0.370744	protein_coding
ENSG0000(	ENSG0000(	11.7818	-0.25317	0.213267	0.370775	processed_pseudogene
ENSG0000(	LINC00886	284.1565	0.119427	0.213301	0.370815	lncRNA
ENSG0000(	TRADD	377.4771	0.064182	0.213329	0.370842	protein_coding
ENSG0000(	ENSG0000(	13.50396	0.224892	0.213386	0.370922	lncRNA
ENSG0000(	LINC02157	62.43782	-0.11832	0.213441	0.370997	lncRNA
ENSG0000(	ADAD2	46.37311	0.14722	0.213482	0.371047	protein_coding
ENSG0000(	ENSG0000(	543.3371	-0.06129	0.213585	0.371205	lncRNA
ENSG0000(	ENSG0000(	29.77304	0.150339	0.213668	0.371248	lncRNA
ENSG0000(	ENSG0000(	78.08807	-0.11143	0.213642	0.371248	TEC
ENSG0000(	FHIP1B	3109.495	-0.14971	0.213668	0.371248	protein_coding
ENSG0000(	LINC02635	75.52511	0.108832	0.213666	0.371248	lncRNA
ENSG0000(	MYLK	6077.403	0.063538	0.213665	0.371248	protein_coding
ENSG0000(	PTPN5	38.91044	0.154255	0.213696	0.371273	protein_coding
ENSG0000(	SLC45A1	18.88444	0.198692	0.213707	0.371273	protein_coding
ENSG0000(	MPZL1	2001.509	0.077593	0.213724	0.371283	protein_coding
ENSG0000(	MYL6B-AS1	19.36193	0.19746	0.213761	0.371327	lncRNA
ENSG0000(	ENSG0000(	81.55058	-0.09275	0.213862	0.37146	lncRNA
ENSG0000(	GAA	651.2814	0.107277	0.213878	0.37146	protein_coding
ENSG0000(	KLF5	5727.169	-0.22547	0.213889	0.37146	protein_coding
ENSG0000(	NEPNP	12.10373	0.26623	0.213889	0.37146	unitary_pseudogene

ENSG0000(	RNASEH2C	616.534	-0.07748	0.213897	0.37146	protein_coding
ENSG0000(	ENSG0000(	14.41094	0.229009	0.213991	0.371604	lncRNA
ENSG0000(	ENO1P3	62.8357	0.180906	0.214034	0.371637	processed_pseudogene
ENSG0000(	PIANP	23.67033	0.290994	0.214025	0.371637	protein_coding
ENSG0000(	RN7SL472F	38.57868	0.156573	0.214144	0.371807	misc_RNA
ENSG0000(	C1orf74	164.4044	0.094454	0.214157	0.371809	protein_coding
ENSG0000(	ENSG0000(	20.46091	0.529427	0.214213	0.371875	unprocessed_pseudogene
ENSG0000(	NANOGP1	201.854	0.079828	0.214218	0.371875	transcribed_unprocessed_pseudo
ENSG0000(	CHST9	155.0008	-0.09599	0.214249	0.371908	protein_coding
ENSG0000(	LINC02751	14.56556	-0.23495	0.214267	0.371918	lncRNA
ENSG0000(	SQLE	279.5509	0.128325	0.214298	0.371951	protein_coding
ENSG0000(	AFF2-IT1	12.97463	-0.44361	0.214339	0.371973	lncRNA
ENSG0000(	ENSG0000(	45.69977	0.126336	0.214345	0.371973	lncRNA
ENSG0000(	WASHC5-A	15.44552	0.192843	0.214333	0.371973	lncRNA
ENSG0000(	LINC01771	14.97145	0.247882	0.214365	0.371987	lncRNA
ENSG0000(	STK17B	5852.044	-0.12128	0.214417	0.372057	protein_coding
ENSG0000(	SLC25A17	407.3563	-0.06564	0.214492	0.372165	protein_coding
ENSG0000(	DEFA5	8781.773	-0.37325	0.214632	0.37238	protein_coding
ENSG0000(	RN7SKP16(	35.34769	-0.14971	0.214639	0.37238	misc_RNA
ENSG0000(	ENSG0000(	20.7305	0.174852	0.214691	0.37245	lncRNA
ENSG0000(	ENSG0000(	71.43827	0.149846	0.214748	0.37251	processed_pseudogene
ENSG0000(	ENSG0000(	34.01787	0.146958	0.21475	0.37251	protein_coding
ENSG0000(	ENSG0000(	54.88074	0.090249	0.214767	0.372519	lncRNA
ENSG0000(	PPP2R5E	1461.365	-0.03456	0.214879	0.372673	protein_coding
ENSG0000(	WASF2	4227.419	-0.04624	0.214879	0.372673	protein_coding
ENSG0000(	ENSG0000(	20.94814	0.251007	0.214922	0.372706	lncRNA
ENSG0000(	PPP3CC	460.2048	-0.12323	0.214915	0.372706	protein_coding
ENSG0000(	ENSG0000(	278.9611	0.066027	0.215187	0.373102	lncRNA
ENSG0000(	ENSG0000(	12.49499	0.313561	0.215182	0.373102	lncRNA
ENSG0000(	RN7SL23P	31.09261	0.135318	0.215207	0.373102	misc_RNA
ENSG0000(	SLC5A7	11.41287	-0.27649	0.215209	0.373102	protein_coding
ENSG0000(	WDR45B	1291.671	0.052774	0.215181	0.373102	protein_coding
ENSG0000(	DDX25	438.9069	0.053803	0.215268	0.373163	protein_coding
ENSG0000(	NTMT1	277.4361	0.055373	0.21526	0.373163	protein_coding
ENSG0000(	RSPH10B2	83.06395	0.397634	0.215283	0.373168	protein_coding
ENSG0000(	SNORD11	15.59474	0.2644	0.215431	0.373404	snoRNA
ENSG0000(	CHERP	740.8708	-0.04702	0.21553	0.373508	protein_coding
ENSG0000(	ENSG0000(	4449.982	0.056838	0.21555	0.373508	lncRNA
ENSG0000(	TMEM185/	510.4076	-0.0671	0.215541	0.373508	protein_coding
ENSG0000(	TTTY5	8.755094	-0.38228	0.215514	0.373508	lncRNA
ENSG0000(	ZNF18	370.0333	0.070272	0.215548	0.373508	protein_coding
ENSG0000(	ENSG0000(	75.45262	0.085149	0.215588	0.373542	lncRNA
ENSG0000(	ENSG0000(	65.42331	-0.15007	0.215605	0.373542	lncRNA
ENSG0000(	SRSF2	2559.607	-0.07021	0.215597	0.373542	protein_coding
ENSG0000(	ZCCHC2	1632.129	-0.08104	0.215629	0.373563	protein_coding
ENSG0000(	ENSG0000(	24.12239	0.185809	0.21565	0.373578	lncRNA
ENSG0000(	MERTK	647.1524	-0.13666	0.215683	0.373615	protein_coding

ENSG0000(	ZNF26	4940.024	0.056292	0.215712	0.373644	protein_coding
ENSG0000(	ANAPC1	2075.812	-0.06373	0.215756	0.3737	protein_coding
ENSG0000(	OR8D4	147.8039	-0.0857	0.215818	0.373788	protein_coding
ENSG0000(	ENSG0000(	600.7615	-0.04664	0.215889	0.373889	lncRNA
ENSG0000(	KREMEN2	12.68193	-0.32002	0.215918	0.373898	protein_coding
ENSG0000(	USO1	2057.411	0.073973	0.215909	0.373898	protein_coding
ENSG0000(	ENSG0000(	18.61693	0.215489	0.216011	0.374038	processed_pseudogene
ENSG0000(	ZNF90P1	71.09675	-0.12275	0.216109	0.374188	processed_pseudogene
ENSG0000(	CHTF8	472.0358	-0.13562	0.216181	0.37429	protein_coding
ENSG0000(	MAP3K6	372.904	-0.11744	0.216204	0.37429	protein_coding
ENSG0000(	ZRANB1	1256.218	-0.05629	0.216195	0.37429	protein_coding
ENSG0000(	ENSG0000(	230.1622	-0.09177	0.216233	0.37432	lncRNA
ENSG0000(	ATP1A4	360.8319	-0.06229	0.216254	0.374337	protein_coding
ENSG0000(	ZNF501	167.4293	0.118513	0.216289	0.374377	protein_coding
ENSG0000(	SYCE2	25.12733	0.178438	0.216322	0.374414	protein_coding
ENSG0000(	TNNT1	52.87292	-0.13874	0.216359	0.374457	protein_coding
ENSG0000(	PTGER3	166.0563	-0.20672	0.216387	0.374484	protein_coding
ENSG0000(	ENSG0000(	104.904	0.132354	0.21643	0.374538	lncRNA
ENSG0000(	ENSG0000(	247.6225	0.101466	0.216477	0.374578	lncRNA
ENSG0000(	TBCAP3	33.6534	0.150454	0.21647	0.374578	transcribed_processed_pseudoge
ENSG0000(	ENSG0000(	107.6966	0.20726	0.216527	0.374644	lncRNA
ENSG0000(	ENSG0000(	124.8795	-0.15174	0.216605	0.374758	lncRNA
ENSG0000(	GSDMC	133.6788	0.169199	0.216667	0.374845	protein_coding
ENSG0000(	ENSG0000(	11.30627	0.195857	0.216706	0.374893	lncRNA
ENSG0000(	ENSG0000(	40.77132	0.197446	0.216876	0.375166	lncRNA
ENSG0000(	LINC01309	53.03466	0.14699	0.216888	0.375166	lncRNA
ENSG0000(	TFDP1	803.163	0.076938	0.216905	0.375175	protein_coding
ENSG0000(	SSBP3	1316.174	-0.06733	0.21699	0.375301	protein_coding
ENSG0000(	TEKT2	38.08732	-0.15054	0.217024	0.375339	protein_coding
ENSG0000(	SALL1	173.2336	0.173188	0.217053	0.375369	protein_coding
ENSG0000(	LINC02898	458.994	-0.07131	0.21709	0.375413	lncRNA
ENSG0000(	ARHGAP22	566.4561	0.092298	0.217129	0.375426	protein_coding
ENSG0000(	COX18	1170.072	0.081533	0.217133	0.375426	protein_coding
ENSG0000(	RHD	286.5338	-0.06161	0.217112	0.375426	protein_coding
ENSG0000(	FAM182A	346.6108	0.07473	0.217147	0.375428	lncRNA
ENSG0000(	CDRT1	254.8995	0.170314	0.217263	0.375608	protein_coding
ENSG0000(	ENSG0000(	67.35503	-0.09544	0.217295	0.375643	TEC
ENSG0000(	ENSG0000(	11.91981	0.235206	0.217324	0.375655	lncRNA
ENSG0000(	MAGEA6-D	69.41645	0.108707	0.217326	0.375655	lncRNA
ENSG0000(	ENSG0000(	197.2583	0.080579	0.217357	0.375688	lncRNA
ENSG0000(	ENSG0000(	66.03884	0.109169	0.217394	0.375731	lncRNA
ENSG0000(	LINC02671	114.9301	0.122274	0.217408	0.375736	lncRNA
ENSG0000(	CDCA7	780.7978	-0.18697	0.217484	0.375826	protein_coding
ENSG0000(	EIF4EBP1	72.08528	0.150646	0.21748	0.375826	protein_coding
ENSG0000(	FKBP5	3462.036	0.199539	0.217552	0.375923	protein_coding
ENSG0000(	ENSG0000(	88.03255	-0.19789	0.217569	0.375931	protein_coding
ENSG0000(	ENSG0000(	297.4645	-0.05866	0.217608	0.375978	lncRNA

ENSG0000(	ENSG0000(	780.0521	0.135199	0.217667	0.376059	transcribed_unprocessed_pseudo
ENSG0000(	NR4A1	728.7508	0.139912	0.217707	0.376108	protein_coding
ENSG0000(	RN7SKP181	87.40151	0.124679	0.217776	0.376206	misc_RNA
ENSG0000(	ENSG0000(	63.03925	-0.4043	0.217799	0.376225	TEC
ENSG0000(	C17orf49	211.2237	0.175774	0.217841	0.376277	protein_coding
ENSG0000(	ANXA8	50.32869	-0.24937	0.217891	0.376322	protein_coding
ENSG0000(	LINC00598	652.0117	-0.08903	0.217886	0.376322	lncRNA
ENSG0000(	LINC02899	238.1145	0.135735	0.21797	0.376438	lncRNA
ENSG0000(	ENSG0000(	16.05681	0.316646	0.217985	0.376444	TEC
ENSG0000(	CGREF1	81.0304	0.166093	0.218156	0.376589	protein_coding
ENSG0000(	ENSG0000(	69.41287	0.143245	0.218085	0.376589	lncRNA
ENSG0000(	ENSG0000(	365.7892	0.09256	0.218165	0.376589	lncRNA
ENSG0000(	GMEB1	1412.3	-0.04278	0.218102	0.376589	protein_coding
ENSG0000(	ITGB4	2658.626	-0.18569	0.218158	0.376589	protein_coding
ENSG0000(	TARS1-DT	81.03065	0.17571	0.21816	0.376589	lncRNA
ENSG0000(	TMOD4	119.3329	-0.11266	0.218138	0.376589	protein_coding
ENSG0000(	TRIM59	868.0686	-0.06686	0.218126	0.376589	protein_coding
ENSG0000(	ENSG0000(	31.31484	-0.2431	0.218211	0.376648	lncRNA
ENSG0000(	ENSG0000(	20.70065	0.198987	0.218324	0.376814	lncRNA
ENSG0000(	ENSG0000(	117.1983	-0.2658	0.218331	0.376814	lncRNA
ENSG0000(	BRD3OS	458.9037	-0.09531	0.218478	0.377046	protein_coding
ENSG0000(	ASXL1	4176.622	0.069621	0.218505	0.377073	protein_coding
ENSG0000(	DCHS1	1455.051	0.143054	0.218595	0.377188	protein_coding
ENSG0000(	FGL2	2653.389	0.135887	0.218591	0.377188	protein_coding
ENSG0000(	ENSG0000(	44.20733	0.159514	0.218636	0.377237	lncRNA
ENSG0000(	ENSG0000(	101.8074	0.117806	0.218712	0.377339	lncRNA
ENSG0000(	ENSG0000(	9.288271	0.329116	0.218719	0.377339	processed_pseudogene
ENSG0000(	SGTB	701.0565	0.104897	0.218776	0.377416	protein_coding
ENSG0000(	LINC00937	2195.874	0.11161	0.218802	0.37744	lncRNA
ENSG0000(	FAHD2B	177.0816	-0.0876	0.218816	0.377444	protein_coding
ENSG0000(	ENSG0000(	18.20826	0.175293	0.218891	0.377512	lncRNA
ENSG0000(	MIR1281	16.43733	0.239783	0.218882	0.377512	miRNA
ENSG0000(	USP53	3573.929	0.114468	0.218877	0.377512	protein_coding
ENSG0000(	RAD17	725.2204	-0.07353	0.218914	0.37753	protein_coding
ENSG0000(	FN3K	593.7704	-0.09296	0.218933	0.377543	protein_coding
ENSG0000(	CES3	1372.501	0.209936	0.219015	0.377575	protein_coding
ENSG0000(	CLSTN1	2257.611	-0.08648	0.219115	0.377575	protein_coding
ENSG0000(	CYP27C1	427.9302	0.056673	0.219096	0.377575	protein_coding
ENSG0000(	EIF4E1B	46.58774	0.106335	0.21909	0.377575	protein_coding
ENSG0000(	ENSG0000(	421.9102	-0.13245	0.219032	0.377575	protein_coding
ENSG0000(	ENSG0000(	239.6814	-0.06447	0.219114	0.377575	lncRNA
ENSG0000(	ENSG0000(	662.1086	-0.06596	0.219067	0.377575	lncRNA
ENSG0000(	ENSG0000(	37.43527	0.126404	0.219004	0.377575	lncRNA
ENSG0000(	GCAT	117.383	0.104823	0.21899	0.377575	protein_coding
ENSG0000(	GLG1	5321.21	0.0361	0.219033	0.377575	protein_coding
ENSG0000(	PDE4DIPP2	2572.255	0.058856	0.2191	0.377575	transcribed_unprocessed_pseudo
ENSG0000(	TBX1	116.3411	0.118637	0.219058	0.377575	protein_coding

ENSG0000(	TRAJ39	34.26631	0.251796	0.21912	0.377575	TR_J_gene
ENSG0000(	UHRF1	533.0442	-0.0761	0.219118	0.377575	protein_coding
ENSG0000(	ENSG0000(	97.60412	0.100233	0.219177	0.377589	lncRNA
ENSG0000(	HOMEZ	860.5248	0.073504	0.219176	0.377589	protein_coding
ENSG0000(	RN7SL589F	49.37	0.133093	0.219188	0.377589	misc_RNA
ENSG0000(	SNORD71	23.67328	0.154881	0.219168	0.377589	snoRNA
ENSG0000(	YWHAH-AS	676.4418	0.041029	0.219165	0.377589	lncRNA
ENSG0000(	PELI3	171.4643	0.101232	0.219248	0.377663	protein_coding
ENSG0000(	SLC44A4	2361.488	-0.21577	0.219255	0.377663	protein_coding
ENSG0000(	LARS2	901.1892	0.072424	0.219298	0.377718	protein_coding
ENSG0000(	DIRAS1	27.93682	-0.22033	0.219342	0.377773	protein_coding
ENSG0000(	RN7SL45P	115.3108	0.082687	0.219389	0.377833	misc_RNA
ENSG0000(	ENSG0000(	64.67205	0.124151	0.219407	0.37784	lncRNA
ENSG0000(	ERGIC3	1750.228	-0.06345	0.219417	0.37784	protein_coding
ENSG0000(	PIGN	5426.113	-0.04823	0.219655	0.378229	protein_coding
ENSG0000(	ZNF451-AS	226.2899	-0.07795	0.21972	0.37832	lncRNA
ENSG0000(	GADD45GII	83.89347	-0.11143	0.219789	0.378419	protein_coding
ENSG0000(	RN7SL255F	23.7348	0.188869	0.219817	0.378445	misc_RNA
ENSG0000(	ENSG0000(	135.9736	-0.10809	0.219892	0.378554	lncRNA
ENSG0000(	ENSG0000(	140.2405	-0.08413	0.219964	0.378656	lncRNA
ENSG0000(	ENSG0000(	26.67168	-0.14437	0.220142	0.37886	lncRNA
ENSG0000(	MTMR14	1223.727	-0.05496	0.220119	0.37886	protein_coding
ENSG0000(	PNPLA7	1094.765	-0.15097	0.220136	0.37886	protein_coding
ENSG0000(	RAB35	717.9269	0.063849	0.220113	0.37886	protein_coding
ENSG0000(	TAMM41	983.8918	-0.05251	0.220096	0.37886	protein_coding
ENSG0000(	SNORA66	113.9072	0.13628	0.220194	0.37893	snoRNA
ENSG0000(	GOLGA8B	4908.047	0.139218	0.220291	0.379076	protein_coding
ENSG0000(	ENSG0000(	165.9192	0.190143	0.22036	0.379152	TEC
ENSG0000(	KCNH1	229.2026	0.069769	0.220349	0.379152	protein_coding
ENSG0000(	A2M-AS1	311.5737	-0.21591	0.220473	0.379326	lncRNA
ENSG0000(	ENSG0000(	167.0233	-0.0641	0.220532	0.379407	lncRNA
ENSG0000(	ENSG0000(	303.9642	-0.06654	0.2206	0.379503	lncRNA
ENSG0000(	RABL2A	1010.262	-0.06753	0.220614	0.379507	protein_coding
ENSG0000(	FAM215B	758.464	0.147156	0.220649	0.379545	lncRNA
ENSG0000(	CKS1BP1	17.90418	-0.26638	0.220681	0.379573	processed_pseudogene
ENSG0000(	ENSG0000(	106.3844	-0.08883	0.220689	0.379573	lncRNA
ENSG0000(	ENSG0000(	70.27982	0.186147	0.220792	0.37973	lncRNA
ENSG0000(	RN7SKP211	11.71983	-0.25932	0.220813	0.379746	misc_RNA
ENSG0000(	AASDHPPT	611.9043	-0.06065	0.220971	0.379997	protein_coding
ENSG0000(	ENSG0000(	14.71795	-0.2204	0.221009	0.380004	protein_coding
ENSG0000(	GK5	4711.97	-0.09377	0.221012	0.380004	protein_coding
ENSG0000(	PLD4	87.01061	-0.28171	0.221005	0.380004	protein_coding
ENSG0000(	MIR378E	20.91344	0.269411	0.221079	0.380078	miRNA
ENSG0000(	OBSCN	4521.547	-0.10526	0.221071	0.380078	protein_coding
ENSG0000(	ENSG0000(	18.31257	0.227451	0.221155	0.380157	lncRNA
ENSG0000(	ENSG0000(	185.825	0.141541	0.221147	0.380157	lncRNA
ENSG0000(	WDR81	1553.445	0.085082	0.221161	0.380157	protein_coding

ENSG0000(	DLGAP1-AS	196.8247	-0.06434	0.22118	0.380162	lncRNA
ENSG0000(	USP3	2507.815	0.031198	0.221196	0.380162	protein_coding
ENSG0000(	ZNF638	6903.058	0.041454	0.2212	0.380162	protein_coding
ENSG0000(	MAML2	2205.573	0.095222	0.221233	0.380198	protein_coding
ENSG0000(	KDM1A	3201.26	0.033361	0.221331	0.380335	protein_coding
ENSG0000(	PAK4	1425.635	-0.10224	0.221337	0.380335	protein_coding
ENSG0000(	MICOS10	1060.979	-0.06017	0.22136	0.380353	protein_coding
ENSG0000(	MIR570	119.9249	0.19866	0.221385	0.380355	miRNA
ENSG0000(	RN7SL720F	53.61808	-0.34941	0.22138	0.380355	misc_RNA
ENSG0000(	COMMD8	84.42257	-0.12955	0.22149	0.38037	protein_coding
ENSG0000(	ENSG0000(	317.8464	-0.21085	0.221482	0.38037	lncRNA
ENSG0000(	ENSG0000(	15.72375	0.776204	0.221468	0.38037	lncRNA
ENSG0000(	IL18	830.6953	0.086338	0.221445	0.38037	protein_coding
ENSG0000(	MED7	372.9837	0.0457	0.221483	0.38037	protein_coding
ENSG0000(	MIA3	3453.2	-0.04995	0.221461	0.38037	protein_coding
ENSG0000(	PPP1R14BF	29.85755	0.19166	0.221413	0.38037	processed_pseudogene
ENSG0000(	TRIM60P14	28.60999	0.184719	0.221419	0.38037	processed_pseudogene
ENSG0000(	LINC01554	54.53772	0.128308	0.221543	0.38044	lncRNA
ENSG0000(	IL21-AS1	17.07156	0.47322	0.22161	0.380535	lncRNA
ENSG0000(	IMPA1	1485.57	-0.1306	0.221664	0.380585	protein_coding
ENSG0000(	VSTM4	1011.478	0.059609	0.221662	0.380585	protein_coding
ENSG0000(	MALT1	2781.558	0.085588	0.221697	0.380622	protein_coding
ENSG0000(	OR5BA1P	612.4284	-0.04662	0.221731	0.380659	transcribed_unprocessed_pseudo
ENSG0000(	CDR2L	331.8677	-0.21068	0.221828	0.380805	protein_coding
ENSG0000(	BIRC5	564.5802	0.054222	0.221871	0.380851	protein_coding
ENSG0000(	CSTA	29.51093	0.337358	0.221879	0.380851	protein_coding
ENSG0000(	ABTB1	1056.531	0.074508	0.221984	0.38101	protein_coding
ENSG0000(	GAMT	197.2311	0.066756	0.222018	0.381048	protein_coding
ENSG0000(	ENSG0000(	45.29866	-0.1183	0.222137	0.381232	lncRNA
ENSG0000(	CELSR2	274.1633	-0.14159	0.222205	0.381327	protein_coding
ENSG0000(	ATP6V0B	483.9068	-0.10513	0.22228	0.381415	protein_coding
ENSG0000(	B3GNTL1P1	13.46257	0.214707	0.222274	0.381415	processed_pseudogene
ENSG0000(	CD52	333.2812	-0.27261	0.222451	0.381646	protein_coding
ENSG0000(	CD5L	11.80313	-0.38294	0.222429	0.381646	protein_coding
ENSG0000(	HAT1	909.9356	-0.0502	0.222475	0.381646	protein_coding
ENSG0000(	HCG9	60.7812	0.145156	0.222484	0.381646	lncRNA
ENSG0000(	RNU4-86P	56.88997	0.121583	0.22246	0.381646	snRNA
ENSG0000(	SDHAF3	64.94826	-0.14318	0.2225	0.381646	protein_coding
ENSG0000(	SLC25A47	10.50667	0.324452	0.222498	0.381646	protein_coding
ENSG0000(	LRP5	2775.226	-0.18328	0.222514	0.38165	protein_coding
ENSG0000(	LINC01499	105.375	-0.08521	0.22261	0.381793	lncRNA
ENSG0000(	ENSG0000(	41.00607	0.138068	0.2227	0.381928	lncRNA
ENSG0000(	ENSG0000(	70.27645	-0.38226	0.222826	0.382123	processed_pseudogene
ENSG0000(	MTCO1P12	1550.715	-0.66321	0.2229	0.382229	unprocessed_pseudogene
ENSG0000(	ENSG0000(	40.71865	-0.12762	0.222966	0.382321	lncRNA
ENSG0000(	CCDC158	277.6481	-0.068	0.222995	0.38235	protein_coding
ENSG0000(	CARS2	1713.373	-0.05279	0.223022	0.382362	protein_coding

ENSG0000(	ENSG0000(	822.9861	0.083275	0.223026	0.382362	lncRNA
ENSG0000(	ECT2	648.8183	-0.08312	0.223124	0.382508	protein_coding
ENSG0000(	C1orf112	392.7821	-0.10108	0.223166	0.38256	protein_coding
ENSG0000(	ZNF641	1513.886	0.086436	0.223179	0.382562	protein_coding
ENSG0000(	ENSG0000(	165.1431	0.067801	0.223206	0.382587	lncRNA
ENSG0000(	CD109	460.5204	0.10346	0.223276	0.382687	protein_coding
ENSG0000(	ENSG0000(	62.28941	0.147598	0.223357	0.382804	lncRNA
ENSG0000(	DNAJB12	951.9317	-0.05396	0.223455	0.382909	protein_coding
ENSG0000(	ENSG0000(	15.2136	0.242489	0.223445	0.382909	lncRNA
ENSG0000(	ZNF586	2086.581	0.061434	0.223454	0.382909	protein_coding
ENSG0000(	ZBTB1	2086.152	0.074107	0.223598	0.383133	protein_coding
ENSG0000(	CNOT4	1559.62	0.043118	0.223627	0.383163	protein_coding
ENSG0000(	AZU1	182.4725	-0.08844	0.223748	0.383308	protein_coding
ENSG0000(	DTHD1	245.5705	-0.24642	0.223743	0.383308	protein_coding
ENSG0000(	ZDHHC19	13.01148	0.316259	0.223735	0.383308	protein_coding
ENSG0000(	ENSG0000(	15.17673	0.232481	0.223838	0.38342	lncRNA
ENSG0000(	ENSG0000(	221.7469	0.062536	0.223832	0.38342	lncRNA
ENSG0000(	NUDT4	3779.069	-0.10575	0.223996	0.383669	protein_coding
ENSG0000(	AP2M1	1692.977	-0.08986	0.224049	0.383697	protein_coding
ENSG0000(	ENSG0000(	57.58404	0.128204	0.224045	0.383697	lncRNA
ENSG0000(	RN7SKP72	9.474087	-0.29909	0.224049	0.383697	misc_RNA
ENSG0000(	LINC01586	92.06658	0.133152	0.224089	0.383745	lncRNA
ENSG0000(	PSTK	275.762	-0.0672	0.224102	0.383747	protein_coding
ENSG0000(	HLA-W	23.52704	0.223578	0.224153	0.383813	unprocessed_pseudogene
ENSG0000(	ENSG0000(	29.59729	-0.15268	0.22418	0.383838	lncRNA
ENSG0000(	LINC02520	60.59001	-0.454	0.2242	0.383841	lncRNA
ENSG0000(	SPAG1	134.0504	0.143859	0.224206	0.383841	protein_coding
ENSG0000(	HACD1	297.2249	0.072541	0.224392	0.384118	protein_coding
ENSG0000(	POLE3	305.459	-0.09372	0.224391	0.384118	protein_coding
ENSG0000(	IFITM3P3	8.970198	-0.26537	0.224499	0.384281	processed_pseudogene
ENSG0000(	FKBP1A	2853.571	0.074955	0.224533	0.384317	protein_coding
ENSG0000(	CNDP1	255.8691	-0.06894	0.224573	0.384345	protein_coding
ENSG0000(	CYB5RL	2439.301	0.047683	0.224563	0.384345	protein_coding
ENSG0000(	ENSG0000(	9.852974	-0.23127	0.22461	0.384375	lncRNA
ENSG0000(	ENSG0000(	286.5449	0.091248	0.224651	0.384375	lncRNA
ENSG0000(	ENSG0000(	24.20498	0.21568	0.224652	0.384375	lncRNA
ENSG0000(	KLF4	1437.102	-0.25323	0.224648	0.384375	protein_coding
ENSG0000(	MIR3140	20.2548	0.238595	0.224642	0.384375	miRNA
ENSG0000(	ORAI1	438.8813	-0.07149	0.224747	0.384499	protein_coding
ENSG0000(	ZFYVE27	1639.475	-0.08869	0.224749	0.384499	protein_coding
ENSG0000(	ENSG0000(	535.838	0.097884	0.2248	0.384566	lncRNA
ENSG0000(	ENSG0000(	45.41341	-0.19155	0.224843	0.384598	TEC
ENSG0000(	RNU6-786F	10.89862	-0.22411	0.224839	0.384598	snRNA
ENSG0000(	ENSG0000(	139.5491	0.101596	0.224863	0.38461	lncRNA
ENSG0000(	ENSG0000(	735.5816	-0.3864	0.224947	0.384734	TEC
ENSG0000(	THAP10	20.61769	-0.20236	0.224989	0.384785	protein_coding
ENSG0000(	ZNF423	261.2002	0.140326	0.225099	0.384951	protein_coding

ENSG0000(	ENSG0000(	423.6027	-0.14249	0.225129	0.384983	lncRNA
ENSG0000(	ZNF497	96.80157	-0.10335	0.225215	0.385109	protein_coding
ENSG0000(	DNAJB14	3155.975	0.043437	0.225285	0.38519	protein_coding
ENSG0000(	RN7SL740F	91.59293	0.099188	0.225287	0.38519	misc_RNA
ENSG0000(	OR7A19P	88.23737	0.121697	0.225304	0.385199	unprocessed_pseudogene
ENSG0000(	PABPC1L	840.0515	0.129515	0.225325	0.385213	protein_coding
ENSG0000(	CHRNA5	289.8997	0.069755	0.225374	0.385255	protein_coding
ENSG0000(	RNU2-18P	13.63302	-0.19118	0.225362	0.385255	snRNA
ENSG0000(	ENSG0000(	123.477	-0.08008	0.225423	0.385276	lncRNA
ENSG0000(	OR2G6	1205.477	-0.06144	0.225406	0.385276	protein_coding
ENSG0000(	RNVU1-1	97.28884	0.270966	0.225414	0.385276	snRNA
ENSG0000(	ENSG0000(	57.83535	0.10988	0.225441	0.385285	lncRNA
ENSG0000(	CEP72	282.3916	0.129431	0.225497	0.38536	protein_coding
ENSG0000(	ENSG0000(	91.96296	-0.08723	0.225558	0.385422	lncRNA
ENSG0000(	RN7SL242F	15.01097	-0.1818	0.225547	0.385422	misc_RNA
ENSG0000(	IGFBP4	1570.9	0.17623	0.225593	0.385461	protein_coding
ENSG0000(	RBPMS2	61.24092	0.154008	0.225784	0.385768	protein_coding
ENSG0000(	RNF14P1	58.71809	0.118285	0.225821	0.385809	processed_pseudogene
ENSG0000(	ENSG0000(	741.5428	0.082453	0.225867	0.385867	lncRNA
ENSG0000(	CMTM7	502.4649	0.225197	0.225906	0.385892	protein_coding
ENSG0000(	ENSG0000(	13.28045	-0.20069	0.225895	0.385892	miRNA
ENSG0000(	ATF6	2704.426	0.049496	0.225943	0.385922	protein_coding
ENSG0000(	AVPR1B	37.26645	-0.29176	0.225954	0.385922	protein_coding
ENSG0000(	RNU4-38P	90.65111	-0.21462	0.22596	0.385922	snRNA
ENSG0000(	RASGRF1	254.146	-0.22933	0.226	0.38597	protein_coding
ENSG0000(	ZNF225	745.7605	0.046889	0.226022	0.385985	protein_coding
ENSG0000(	ENSG0000(	30.69866	0.120978	0.226041	0.385997	lncRNA
ENSG0000(	CXCR4	1343.948	0.360504	0.226073	0.38603	protein_coding
ENSG0000(	ENSG0000(	5166.939	-0.02521	0.226102	0.386059	lncRNA
ENSG0000(	ENSG0000(	235.3426	0.05929	0.22612	0.386069	lncRNA
ENSG0000(	ENSG0000(	163.2798	0.090283	0.226182	0.386155	transcribed_processed_pseudoge
ENSG0000(	IL13RA1	3213.972	-0.14564	0.226203	0.38617	protein_coding
ENSG0000(	DAPK3	426.112	-0.08543	0.226248	0.386225	protein_coding
ENSG0000(	PELP1	751.1621	-0.11567	0.226336	0.386355	protein_coding
ENSG0000(	ENSG0000(	515.7512	0.149905	0.226375	0.386357	lncRNA
ENSG0000(	ENSG0000(	115.0295	0.104531	0.226353	0.386357	lncRNA
ENSG0000(	ENSG0000(	94.86396	0.102199	0.226371	0.386357	lncRNA
ENSG0000(	NIPA2	1053.366	-0.0641	0.226458	0.386479	protein_coding
ENSG0000(	PIBF1	901.6449	-0.06681	0.22652	0.386564	protein_coding
ENSG0000(	HS6ST2	10.24072	0.383558	0.226555	0.386602	protein_coding
ENSG0000(	ENSG0000(	12.66863	0.212516	0.226595	0.38665	lncRNA
ENSG0000(	ENSG0000(	348.4525	-0.07723	0.226608	0.386651	protein_coding
ENSG0000(	TRMT6	172.3239	-0.0955	0.226672	0.386739	protein_coding
ENSG0000(	TNFRSF10E	2380.651	0.09597	0.226706	0.386777	protein_coding
ENSG0000(	ENSG0000(	12.21726	0.223201	0.226723	0.386784	processed_pseudogene
ENSG0000(	ENSG0000(	9.485844	0.279398	0.226755	0.386798	lncRNA
ENSG0000(	KTN1-AS1	684.1605	0.036774	0.226748	0.386798	lncRNA

ENSG0000(	MTMR12	1129.218	-0.05528	0.226805	0.386862	protein_coding
ENSG0000(	LINC02710	328.9838	0.051518	0.22687	0.386952	lncRNA
ENSG0000(	ENSG0000(	70.43003	0.111058	0.226902	0.386986	lncRNA
ENSG0000(	ENSG0000(	83.94642	0.111073	0.226929	0.387005	lncRNA
ENSG0000(	TBC1D23	973.0753	-0.08953	0.226938	0.387005	protein_coding
ENSG0000(	RN7SL40P	47.27699	0.114745	0.226992	0.387076	misc_RNA
ENSG0000(	ENSG0000(	26.92792	0.165963	0.227016	0.387095	misc_RNA
ENSG0000(	LINC02295	12.16287	0.489112	0.227046	0.387126	lncRNA
ENSG0000(	WDHD1	215.8833	0.160628	0.227103	0.387202	protein_coding
ENSG0000(	DGUOK-AS	54.47214	-0.14967	0.227219	0.387357	lncRNA
ENSG0000(	EEF1A1P13	70.47868	-0.16377	0.227256	0.387357	processed_pseudogene
ENSG0000(	ENSG0000(	743.0598	0.053238	0.227247	0.387357	lncRNA
ENSG0000(	OXLD1	259.2041	0.071162	0.227266	0.387357	protein_coding
ENSG0000(	RBP1	201.1274	-0.09515	0.227268	0.387357	protein_coding
ENSG0000(	TSPO	226.0844	0.137669	0.227261	0.387357	protein_coding
ENSG0000(	SACS	3103.87	0.071444	0.227321	0.387428	protein_coding
ENSG0000(	RNA5SP168	13.117	0.198326	0.22734	0.387438	rRNA_pseudogene
ENSG0000(	GPR89B	1477.253	-0.10377	0.227357	0.387447	protein_coding
ENSG0000(	BMS1P17	93.53704	0.183317	0.227451	0.387565	transcribed_unprocessed_pseudo
ENSG0000(	IFNAR2	1885.741	0.102642	0.227443	0.387565	protein_coding
ENSG0000(	KRT8P15	14.21805	0.229208	0.227578	0.387761	processed_pseudogene
ENSG0000(	CCNF	388.2657	0.109955	0.227613	0.387785	protein_coding
ENSG0000(	ENSG0000(	167.19	-0.07851	0.227617	0.387785	lncRNA
ENSG0000(	DYNLT2	239.9728	0.07575	0.227681	0.387835	protein_coding
ENSG0000(	ENSG0000(	29.50051	0.152853	0.227684	0.387835	lncRNA
ENSG0000(	SCARNA5	2562.543	-0.11379	0.227666	0.387835	scaRNA
ENSG0000(	ENSG0000(	36.13321	0.125634	0.227724	0.387851	lncRNA
ENSG0000(	GPR37L1	481.8933	0.070021	0.227722	0.387851	protein_coding
ENSG0000(	IGKV1-39	20.78751	1.042916	0.22773	0.387851	IG_V_gene
ENSG0000(	ENSG0000(	31.16444	0.20074	0.227769	0.387877	transcribed_unitary_pseudogene
ENSG0000(	FRAT2	326.1001	0.085057	0.227769	0.387877	protein_coding
ENSG0000(	COMP	193.0189	0.066814	0.227904	0.388064	protein_coding
ENSG0000(	ENSG0000(	760.7757	-0.05284	0.227899	0.388064	lncRNA
ENSG0000(	EIF4EBP3	63.93187	0.142869	0.227921	0.388072	protein_coding
ENSG0000(	KLRC2	108.9025	-0.22822	0.227987	0.388164	protein_coding
ENSG0000(	HOXD8	63.18862	0.157191	0.228053	0.388233	protein_coding
ENSG0000(	LINC00324	145.2215	-0.09847	0.228053	0.388233	lncRNA
ENSG0000(	PIKFYVE	3758.408	-0.06812	0.228137	0.388355	protein_coding
ENSG0000(	ANKEF1	633.36	-0.09773	0.228167	0.388386	protein_coding
ENSG0000(	ENSG0000(	687.4559	0.052598	0.228211	0.388439	lncRNA
ENSG0000(	ENSG0000(	151.9057	0.127763	0.228352	0.388655	lncRNA
ENSG0000(	ENSG0000(	1380.178	-0.03303	0.228362	0.388655	lncRNA
ENSG0000(	TSPOAP1	1041.562	-0.11917	0.228422	0.388736	protein_coding
ENSG0000(	KLK1	99.00584	-0.25211	0.228452	0.388765	protein_coding
ENSG0000(	GUCY1B1	260.0314	0.242733	0.228491	0.388811	protein_coding
ENSG0000(	SILC1	1274.708	0.070973	0.228549	0.38889	lncRNA
ENSG0000(	EEF1DP7	47.68329	-0.14423	0.228572	0.388907	transcribed_processed_pseudoge

ENSG0000(	CNTNAP3P	10.68142	0.304298	0.228635	0.388929	unprocessed_pseudogene
ENSG0000(	FAM30A	3202.37	0.318891	0.228603	0.388929	lncRNA
ENSG0000(	SIGLEC8	280.4554	-0.07103	0.228623	0.388929	protein_coding
ENSG0000(	TGS1	797.7115	-0.06124	0.228623	0.388929	protein_coding
ENSG0000(	OLFML1	185.0943	0.104833	0.228657	0.388947	protein_coding
ENSG0000(	C17orf75	1197.581	0.044312	0.228712	0.389019	protein_coding
ENSG0000(	ENSG0000(	79.97495	0.104955	0.228733	0.389034	lncRNA
ENSG0000(	ENSG0000(	14.94148	0.204337	0.228771	0.389056	lncRNA
ENSG0000(	ENSG0000(	9.92796	0.298319	0.228769	0.389056	lncRNA
ENSG0000(	LAG3	84.7433	-0.14465	0.228785	0.389059	protein_coding
ENSG0000(	ENSG0000(	49.44843	-0.41241	0.228843	0.389124	processed_pseudogene
ENSG0000(	GLA	898.6217	-0.04974	0.228848	0.389124	protein_coding
ENSG0000(	CYP4F35P	85.95705	0.171111	0.228886	0.389131	transcribed_unprocessed_pseudo
ENSG0000(	ENSG0000(	127.7296	-0.08448	0.228901	0.389131	lncRNA
ENSG0000(	LINC01220	203.0358	0.070812	0.228877	0.389131	lncRNA
ENSG0000(	SNORA20	168.9141	-0.15376	0.228894	0.389131	snoRNA
ENSG0000(	MRPS21	544.2122	-0.0445	0.228947	0.389187	protein_coding
ENSG0000(	ENSG0000(	207.5343	-0.07397	0.229036	0.389235	lncRNA
ENSG0000(	ENSG0000(	21.4014	0.196131	0.229021	0.389235	lncRNA
ENSG0000(	HLA-DPA1	3945.771	0.176587	0.229007	0.389235	protein_coding
ENSG0000(	RN7SKP50	61.50608	-0.17802	0.22902	0.389235	misc_RNA
ENSG0000(	SOX18	62.32407	0.217858	0.229035	0.389235	protein_coding
ENSG0000(	GORASP1	1446.398	0.060693	0.22908	0.389267	protein_coding
ENSG0000(	PRC1	368.8144	0.067105	0.229074	0.389267	protein_coding
ENSG0000(	PPIAP16	12.53488	0.226778	0.229113	0.389282	processed_pseudogene
ENSG0000(	PPIAP91	11.72439	-0.22364	0.229126	0.389282	processed_pseudogene
ENSG0000(	RN7SL198F	84.8274	0.088352	0.229119	0.389282	misc_RNA
ENSG0000(	AGBL5	311.9952	-0.05697	0.229171	0.389338	protein_coding
ENSG0000(	LINC00671	128.7026	-0.10662	0.229203	0.389371	lncRNA
ENSG0000(	FDPSP4	9.819879	-0.2689	0.229282	0.389483	processed_pseudogene
ENSG0000(	ENSG0000(	76.993	-0.09787	0.229295	0.389485	lncRNA
ENSG0000(	ENOPH1	284.3236	-0.09989	0.229356	0.389551	protein_coding
ENSG0000(	OR2T11	77.37594	0.251378	0.229359	0.389551	protein_coding
ENSG0000(	RAC1	2507.091	-0.10104	0.229417	0.389629	protein_coding
ENSG0000(	NOXRED1	148.0692	0.11553	0.22945	0.389664	protein_coding
ENSG0000(	ENSG0000(	119.3692	0.076047	0.229498	0.389726	lncRNA
ENSG0000(	ATP8B1	2752.965	-0.14056	0.229512	0.389728	protein_coding
ENSG0000(	RNU6-32P	16.05092	0.244007	0.229531	0.38974	snRNA
ENSG0000(	RNU4-72P	22.53221	0.168192	0.229556	0.389759	snRNA
ENSG0000(	OXA1L-DT	479.2148	0.048226	0.229595	0.389805	lncRNA
ENSG0000(	RN7SL653F	18.35191	0.201779	0.229659	0.389894	misc_RNA
ENSG0000(	ENSG0000(	27.42218	-0.33523	0.229718	0.389971	lncRNA
ENSG0000(	MROH3P	339.6637	-0.25237	0.229764	0.390029	transcribed_unitary_pseudogene
ENSG0000(	BUB1	352.2106	0.172093	0.229781	0.390037	protein_coding
ENSG0000(	GINS3	368.8042	-0.10204	0.22984	0.390096	protein_coding
ENSG0000(	PIWIL3	132.1703	-0.0718	0.229831	0.390096	protein_coding
ENSG0000(	POU2F1	2434.964	0.090288	0.22988	0.390143	protein_coding

ENSG0000(	SNORD8	43.82816	0.168267	0.229946	0.390232	snoRNA
ENSG0000(	PTPN18	3001.442	-0.07718	0.229959	0.390234	protein_coding
ENSG0000(	MYLIP	659.5003	-0.08885	0.230028	0.39033	protein_coding
ENSG0000(	ULBP1	33.40229	0.148336	0.230047	0.390341	protein_coding
ENSG0000(	MIR429	20.17427	-0.31948	0.230082	0.39038	miRNA
ENSG0000(	CARD14	600.1262	0.065204	0.230165	0.390481	protein_coding
ENSG0000(	NOS1AP	446.7277	0.15115	0.230166	0.390481	protein_coding
ENSG0000(	TTLL4	1043.507	0.060683	0.230189	0.390498	protein_coding
ENSG0000(	MEX3D	123.2354	-0.12581	0.230253	0.390585	protein_coding
ENSG0000(	PPM1K-DT	91.79682	0.116923	0.230267	0.390589	lncRNA
ENSG0000(	ENSG0000(	65.77376	0.256401	0.23032	0.390657	lncRNA
ENSG0000(	CCDC9B	1101.139	-0.12249	0.230412	0.390792	protein_coding
ENSG0000(	LINC01582	15.96413	0.233673	0.230424	0.390792	lncRNA
ENSG0000(	CACNA1C-/	51.93224	0.210822	0.230452	0.390797	lncRNA
ENSG0000(	TAF15	6105.507	0.047414	0.230451	0.390797	protein_coding
ENSG0000(	DOP1A	2880.688	-0.07573	0.23047	0.390806	protein_coding
ENSG0000(	ENSG0000(	234.4278	0.090469	0.230531	0.390848	TEC
ENSG0000(	LINC01360	77.14117	-0.1106	0.23051	0.390848	lncRNA
ENSG0000(	NEDD1	447.0886	-0.10338	0.230531	0.390848	protein_coding
ENSG0000(	GART	1284.895	0.043874	0.23055	0.390857	protein_coding
ENSG0000(	WFDC21P	23.26891	0.292	0.230572	0.390875	transcribed_unitary_pseudogene
ENSG0000(	ENSG0000(	35.11391	-0.38472	0.230684	0.391043	lncRNA
ENSG0000(	ENSG0000(	263.3406	0.066849	0.230745	0.391105	TEC
ENSG0000(	MRPS18A	223.2723	-0.07768	0.230738	0.391105	protein_coding
ENSG0000(	KIFC1	303.7763	0.125162	0.230786	0.391152	protein_coding
ENSG0000(	SLC5A9	2445.465	-0.3216	0.230812	0.391177	protein_coding
ENSG0000(	CHAMP1	815.8146	-0.06941	0.230866	0.391222	protein_coding
ENSG0000(	NUBP1	198.6998	-0.07863	0.230876	0.391222	protein_coding
ENSG0000(	RN7SL678F	12.42534	-0.22518	0.230863	0.391222	misc_RNA
ENSG0000(	POMK	2567.138	-0.04713	0.230891	0.391226	protein_coding
ENSG0000(	TRIM23	456.6495	-0.06312	0.230931	0.391273	protein_coding
ENSG0000(	CA14	228.1407	-0.06895	0.230962	0.391282	protein_coding
ENSG0000(	DDX20	425.8694	-0.06775	0.230957	0.391282	protein_coding
ENSG0000(	NACC1	658.1482	-0.0717	0.231018	0.391357	protein_coding
ENSG0000(	ENSG0000(	34.62503	0.181529	0.231047	0.391384	lncRNA
ENSG0000(	ENSG0000(	23.55063	-0.2943	0.231181	0.391591	TEC
ENSG0000(	PIAS1	3222.407	-0.0474	0.23121	0.391618	protein_coding
ENSG0000(	CLMN	3873.356	0.093444	0.231245	0.391656	protein_coding
ENSG0000(	ENSG0000(	267.1979	0.079855	0.231266	0.391672	TEC
ENSG0000(	RNU6-953F	13.1457	-0.49223	0.231404	0.391885	snRNA
ENSG0000(	CFAP157	142.9068	-0.08319	0.231439	0.391922	protein_coding
ENSG0000(	NAP1L1	4046.98	-0.13066	0.231465	0.391945	protein_coding
ENSG0000(	C20orf202	34.37192	0.241519	0.231484	0.391956	protein_coding
ENSG0000(	PLEKHG1	2960.255	0.11082	0.231513	0.391985	protein_coding
ENSG0000(	MIR4713H(	17.89567	-0.21042	0.231574	0.392055	lncRNA
ENSG0000(	RN7SL474F	217.6813	0.059707	0.231592	0.392055	misc_RNA
ENSG0000(	RN7SL667F	350.0248	0.066962	0.231587	0.392055	misc_RNA

ENSG0000(	KIT	790.7318	-0.1394	0.231611	0.392067	protein_coding
ENSG0000(	SNORD38C	10.41995	0.209523	0.231698	0.392193	snoRNA
ENSG0000(	RNF7	736.8315	-0.08846	0.231716	0.392202	protein_coding
ENSG0000(	ZGPAT	602.0241	-0.07989	0.231734	0.392211	protein_coding
ENSG0000(	LCK	623.7533	-0.15871	0.231787	0.39228	protein_coding
ENSG0000(	TAF1A	274.9624	0.098136	0.231806	0.392291	protein_coding
ENSG0000(	ENSG0000(	153.8767	0.105333	0.231885	0.392404	lncRNA
ENSG0000(	NOP16	160.4974	-0.08725	0.231959	0.392508	protein_coding
ENSG0000(	HSPD1P13	82.28317	-0.25845	0.232045	0.392633	processed_pseudogene
ENSG0000(	ENSG0000(	66.58066	-0.1127	0.232063	0.392642	processed_pseudogene
ENSG0000(	ADCY3	785.57	0.114593	0.23215	0.392768	protein_coding
ENSG0000(	KRT18P37	11.9851	-0.2545	0.232177	0.392791	processed_pseudogene
ENSG0000(	ARHGEF35	365.5347	-0.21156	0.232239	0.392876	protein_coding
ENSG0000(	ENSG0000(	12.19538	-1.60256	0.232268	0.392904	lncRNA
ENSG0000(	CD63	1103.528	-0.13792	0.232324	0.392918	protein_coding
ENSG0000(	HOXA9	410.9376	-0.13909	0.232321	0.392918	protein_coding
ENSG0000(	LINC01964	61.05382	0.138208	0.232292	0.392918	lncRNA
ENSG0000(	LPGAT1-AS	16.73615	0.220216	0.232335	0.392918	lncRNA
ENSG0000(	TSPYL2	757.0435	0.144149	0.232339	0.392918	protein_coding
ENSG0000(	ENSG0000(	14.52037	0.233381	0.232358	0.39293	lncRNA
ENSG0000(	ENSG0000(	16.489	0.196715	0.232418	0.39301	lncRNA
ENSG0000(	OXER1	250.5793	-0.20081	0.232436	0.393019	protein_coding
ENSG0000(	ADTRP	2003.918	-0.20067	0.232463	0.393023	protein_coding
ENSG0000(	POC1A	82.74575	-0.13029	0.232454	0.393023	protein_coding
ENSG0000(	ENSG0000(	26.08691	-0.1419	0.232585	0.393208	lncRNA
ENSG0000(	AZGP1	121.302	-0.18505	0.232611	0.39321	protein_coding
ENSG0000(	ENSG0000(	9.239502	-0.38832	0.232607	0.39321	processed_pseudogene
ENSG0000(	ADAMTSL2	178.8066	-0.10417	0.232647	0.393248	protein_coding
ENSG0000(	ENSG0000(	24.73608	-0.13945	0.232659	0.393248	processed_pseudogene
ENSG0000(	ENSG0000(	40.23906	-0.16077	0.232817	0.393494	protein_coding
ENSG0000(	ENSG0000(	614.8774	0.094438	0.232854	0.3935	lncRNA
ENSG0000(	GYPB	35.99494	0.14348	0.232865	0.3935	protein_coding
ENSG0000(	NPY4R	180.4706	-0.26478	0.23287	0.3935	protein_coding
ENSG0000(	RN7SKP56	30.8125	0.149139	0.232864	0.3935	misc_RNA
ENSG0000(	ENSG0000(	91.50263	0.091182	0.232888	0.393509	TEC
ENSG0000(	LINC02646	13.34133	0.25686	0.232954	0.393599	lncRNA
ENSG0000(	ENSG0000(	15.14223	0.354524	0.233018	0.393686	lncRNA
ENSG0000(	ENSG0000(	41.28974	0.138867	0.233086	0.393718	lncRNA
ENSG0000(	ENSG0000(	34.39785	0.271811	0.23305	0.393718	processed_pseudogene
ENSG0000(	LINC02617	22.87404	0.210576	0.233074	0.393718	lncRNA
ENSG0000(	RPL12P21	15.04433	0.225389	0.23307	0.393718	processed_pseudogene
ENSG0000(	RN7SL322F	28.38026	-0.16186	0.233109	0.393735	misc_RNA
ENSG0000(	LINC01148	82.39846	0.134977	0.233203	0.393866	lncRNA
ENSG0000(	TM4SF1	342.5793	-0.15859	0.233212	0.393866	protein_coding
ENSG0000(	ENSG0000(	10.66723	0.322066	0.233318	0.394024	lncRNA
ENSG0000(	ENSG0000(	5053.692	0.085456	0.233393	0.39413	lncRNA
ENSG0000(	ENSG0000(	297.1858	0.079796	0.233412	0.394141	lncRNA

ENSG0000(	PVR	1153.133	-0.12645	0.233434	0.394157	protein_coding
ENSG0000(	HSPA4L	1171.595	0.064044	0.233458	0.394176	protein_coding
ENSG0000(	ENSG0000(	280.7179	0.126149	0.233478	0.394189	lncRNA
ENSG0000(	CSAG3	13.16821	-0.3071	0.233529	0.394232	protein_coding
ENSG0000(	EGLN2	2313.681	0.062454	0.233529	0.394232	protein_coding
ENSG0000(	KCNMA1-A	320.3553	0.056809	0.233603	0.394337	lncRNA
ENSG0000(	ENSG0000(	52.47097	-0.11813	0.233637	0.394373	TEC
ENSG0000(	ENSG0000(	10.16168	-0.23115	0.233724	0.394498	misc_RNA
ENSG0000(	CTAGE9	13.96779	-1.43901	0.233784	0.394578	protein_coding
ENSG0000(	TBC1D7	646.0509	0.064807	0.233883	0.394724	protein_coding
ENSG0000(	ENSG0000(	134.3407	-0.17366	0.233901	0.394733	transcribed_processed_pseudoge
ENSG0000(	CRIPT	2586.333	0.04556	0.233925	0.394754	protein_coding
ENSG0000(	PRCP	2616.713	0.06401	0.23397	0.394807	protein_coding
ENSG0000(	ENSG0000(	132.3074	-0.08066	0.234042	0.394909	lncRNA
ENSG0000(	ZNF83	3143.97	0.086394	0.234076	0.394944	protein_coding
ENSG0000(	ZNF33A	4136.779	0.037774	0.234226	0.395176	protein_coding
ENSG0000(	ENSG0000(	226.7708	0.149568	0.234275	0.395195	lncRNA
ENSG0000(	HNRNPLL	1003.905	-0.05822	0.234267	0.395195	protein_coding
ENSG0000(	MEST	351.9434	-0.14229	0.234272	0.395195	protein_coding
ENSG0000(	DMXL1-DT	126.664	-0.08845	0.234293	0.395205	lncRNA
ENSG0000(	PYROXD2	317.5384	-0.07859	0.234368	0.395311	protein_coding
ENSG0000(	IGSF11	81.84946	-0.17391	0.234456	0.395437	protein_coding
ENSG0000(	HSPE1P13	242.7241	0.095361	0.23448	0.395457	processed_pseudogene
ENSG0000(	LRRC74B	647.8088	0.073572	0.234588	0.395618	protein_coding
ENSG0000(	POLM	1196.915	0.05925	0.234634	0.395655	protein_coding
ENSG0000(	SHANK1	47.01185	0.233974	0.234635	0.395655	protein_coding
ENSG0000(	ENSG0000(	247.9534	0.080553	0.234709	0.395758	TEC
ENSG0000(	ENSG0000(	157.602	0.103332	0.234783	0.395862	lncRNA
ENSG0000(	ENSG0000(	8.993654	0.267478	0.234816	0.395896	processed_pseudogene
ENSG0000(	AKAP7	409.6418	-0.07618	0.234849	0.395919	protein_coding
ENSG0000(	ENSG0000(	8.603268	0.280635	0.234855	0.395919	lncRNA
ENSG0000(	ENSG0000(	20.64289	0.294995	0.234908	0.395967	lncRNA
ENSG0000(	MIF4GD	281.8884	-0.06414	0.234898	0.395967	protein_coding
ENSG0000(	SELL	930.0834	0.404874	0.235035	0.396159	protein_coding
ENSG0000(	MKRN3	191.6411	-0.11161	0.235078	0.396211	protein_coding
ENSG0000(	BST1	470.3266	-0.14073	0.235139	0.396292	protein_coding
ENSG0000(	ENSG0000(	2748.183	-0.07512	0.235211	0.396329	lncRNA
ENSG0000(	F8	256.0991	0.106117	0.235208	0.396329	protein_coding
ENSG0000(	GATA2-AS1	47.95753	0.135046	0.235203	0.396329	lncRNA
ENSG0000(	RNU6-28P	9.875533	-0.269	0.235199	0.396329	snRNA
ENSG0000(	TRAJ25	24.59018	0.268022	0.235291	0.396442	TR_J_gene
ENSG0000(	RN7SL213F	10.55222	0.241157	0.235314	0.39646	misc_RNA
ENSG0000(	RBM15	2561.319	0.051331	0.235402	0.396578	protein_coding
ENSG0000(	SIM2	280.73	0.08834	0.235409	0.396578	protein_coding
ENSG0000(	MTRNR2L1	22.02235	0.568232	0.235487	0.396688	protein_coding
ENSG0000(	ENSG0000(	19.87285	0.181869	0.235503	0.396693	misc_RNA
ENSG0000(	ENSG0000(	33.07474	0.183817	0.235551	0.396754	TEC

ENSG0000(	L2HGDH	2436.769	-0.04633	0.235613	0.396836	protein_coding
ENSG0000(	TMEM106E	3350.704	-0.05558	0.235653	0.396882	protein_coding
ENSG0000(	MSL2	1968.636	-0.05694	0.235742	0.397012	protein_coding
ENSG0000(	THTPA	426.8271	0.068015	0.235889	0.397237	protein_coding
ENSG0000(	LINC01180	182.4373	0.07161	0.235975	0.397362	lncRNA
ENSG0000(	USP32P1	784.2199	0.276706	0.236017	0.397411	transcribed_unprocessed_pseudo
ENSG0000(	LRRC43	32.41418	0.119975	0.236166	0.397641	protein_coding
ENSG0000(	ENSG0000(	52.21845	0.118462	0.236218	0.397685	lncRNA
ENSG0000(	NUDT3	3590.341	0.046242	0.236208	0.397685	protein_coding
ENSG0000(	PAQR8	1930.591	-0.12979	0.236295	0.397793	protein_coding
ENSG0000(	CENPE	629.8338	0.179241	0.236421	0.397984	protein_coding
ENSG0000(	ENSG0000(	24.07564	0.154795	0.236474	0.398032	TEC
ENSG0000(	MRPS23	818.6721	-0.06249	0.236467	0.398032	protein_coding
ENSG0000(	HCFC1	2353.781	0.069009	0.236568	0.398168	protein_coding
ENSG0000(	EML5	300.2102	0.10544	0.236622	0.398234	protein_coding
ENSG0000(	ENSG0000(	30.33167	0.148085	0.236658	0.398234	lncRNA
ENSG0000(	LINC01376	111.1624	-0.10117	0.236638	0.398234	lncRNA
ENSG0000(	LRPPRC	6226.709	-0.04069	0.236647	0.398234	protein_coding
ENSG0000(	PLOD3	966.0008	-0.05883	0.236684	0.398256	protein_coding
ENSG0000(	LAMC2	935.4895	-0.16584	0.23672	0.398297	protein_coding
ENSG0000(	RASGRP3	2647.917	-0.07639	0.236767	0.398354	protein_coding
ENSG0000(	SYTL4	1166.701	0.111484	0.236792	0.398376	protein_coding
ENSG0000(	ST13P15	28.7742	0.248758	0.236841	0.398436	processed_pseudogene
ENSG0000(	BABAM2	567.7518	0.055131	0.236923	0.398532	protein_coding
ENSG0000(	RAB29	1245.753	-0.05856	0.236915	0.398532	protein_coding
ENSG0000(	IDI1	719.355	-0.09219	0.237069	0.398754	protein_coding
ENSG0000(	LINC01900	9.499488	-0.21899	0.23708	0.398754	lncRNA
ENSG0000(	ENSG0000(	782.8893	0.083588	0.237137	0.398828	lncRNA
ENSG0000(	RNU1-42P	17.25667	-0.21937	0.237197	0.398908	snRNA
ENSG0000(	CLPTM1L	1903.45	0.09005	0.237234	0.398948	protein_coding
ENSG0000(	ENSG0000(	155.8713	-2.39659	0.237308	0.398987	protein_coding
ENSG0000(	ENSG0000(	14.20012	0.21075	0.237286	0.398987	lncRNA
ENSG0000(	SMC1B	9.846018	-0.29539	0.237298	0.398987	protein_coding
ENSG0000(	UBA3	921.176	-0.08393	0.237278	0.398987	protein_coding
ENSG0000(	RGMA	745.5001	0.183296	0.237378	0.399084	protein_coding
ENSG0000(	ENSG0000(	51.84817	0.146727	0.237409	0.399094	processed_pseudogene
ENSG0000(	ENSG0000(	343.7947	-0.09483	0.237404	0.399094	TEC
ENSG0000(	GUK1	1211.983	0.071653	0.237486	0.399201	protein_coding
ENSG0000(	ENSG0000(	66.82275	0.084696	0.23755	0.399288	lncRNA
ENSG0000(	ENSG0000(	14.31823	-0.20943	0.237603	0.39935	processed_pseudogene
ENSG0000(	KCNS3	142.3486	0.225407	0.237625	0.39935	protein_coding
ENSG0000(	LRRC4C	41.03824	0.211574	0.237621	0.39935	protein_coding
ENSG0000(	NDUFAF7	716.9185	-0.05277	0.237663	0.399392	protein_coding
ENSG0000(	TRGV5	11.81623	-0.32546	0.237685	0.399409	TR_V_gene
ENSG0000(	ENSG0000(	15.84912	0.189145	0.237743	0.399485	lncRNA
ENSG0000(	GATAD2B	2876.608	0.102195	0.237852	0.399646	protein_coding
ENSG0000(	ENSG0000(	276.0917	-0.09054	0.238031	0.399861	lncRNA

ENSG0000(	ENSG0000(	10.94585	-0.89966	0.238017	0.399861	protein_coding
ENSG0000(	SLC25A30-/	8.619095	0.337453	0.238029	0.399861	lncRNA
ENSG0000(	SNX33	2120.979	-0.08578	0.23801	0.399861	protein_coding
ENSG0000(	COPS9	121.4917	-0.11106	0.238058	0.399866	protein_coding
ENSG0000(	ENSG0000(	10.09262	0.195906	0.238071	0.399866	lncRNA
ENSG0000(	ENSG0000(	12.92816	0.231163	0.238062	0.399866	lncRNA
ENSG0000(	TCF25	3282.762	0.042267	0.238227	0.400106	protein_coding
ENSG0000(	PTPRZ1	176.7175	-0.15277	0.238278	0.400169	protein_coding
ENSG0000(	ACTA2	1313.153	0.132064	0.238333	0.400229	protein_coding
ENSG0000(	CDC42BPA	4629.494	-0.1488	0.238351	0.400229	protein_coding
ENSG0000(	MIR3648-1	2396.366	0.26026	0.23834	0.400229	miRNA
ENSG0000(	GALC	1326.273	-0.03816	0.238395	0.400281	protein_coding
ENSG0000(	CDA	122.7182	-0.30166	0.238412	0.400288	protein_coding
ENSG0000(	TEX13C	15.69574	0.22532	0.23844	0.400314	protein_coding
ENSG0000(	LINC02240	77.20185	0.107678	0.238455	0.400318	lncRNA
ENSG0000(	ENSG0000(	102.5915	0.099208	0.238518	0.400403	lncRNA
ENSG0000(	ENSG0000(	75.63875	0.202938	0.238561	0.400432	TEC
ENSG0000(	FUT8	2237.441	0.044937	0.238552	0.400432	protein_coding
ENSG0000(	RND3	933.5172	0.163037	0.238586	0.400452	protein_coding
ENSG0000(	ENSG0000(	110.616	0.085367	0.238643	0.400527	lncRNA
ENSG0000(	SLC4A1AP	1066.381	-0.04453	0.238662	0.400537	protein_coding
ENSG0000(	ZNF319	257.118	0.116941	0.238714	0.400603	protein_coding
ENSG0000(	MAP3K2-D	576.2512	0.054434	0.238738	0.400622	lncRNA
ENSG0000(	RPL9P3	22.20396	0.172102	0.238751	0.400623	processed_pseudogene
ENSG0000(	ENSG0000(	29.88287	0.179505	0.238865	0.400771	lncRNA
ENSG0000(	PAX6	487.1871	0.120026	0.238865	0.400771	protein_coding
ENSG0000(	KRT17P5	33.3836	0.469429	0.238913	0.40083	transcribed_unprocessed_pseudo
ENSG0000(	PMS2	1192.72	-0.04917	0.238971	0.400907	protein_coding
ENSG0000(	LIPH	952.5377	-0.17412	0.239008	0.400948	protein_coding
ENSG0000(	IFITM3P6	13.80249	0.321321	0.239041	0.400981	transcribed_processed_pseudoge
ENSG0000(	ENSG0000(	932.7431	0.046527	0.239076	0.40101	lncRNA
ENSG0000(	PMS2P1	2644.622	-0.03816	0.239084	0.40101	transcribed_unprocessed_pseudo
ENSG0000(	ENSG0000(	50.07099	0.10396	0.239109	0.401032	processed_pseudogene
ENSG0000(	ABCB6	164.9719	-0.11954	0.239132	0.401049	protein_coding
ENSG0000(	MOBP	377.9588	0.081464	0.239174	0.401098	protein_coding
ENSG0000(	P2RY10	1521.995	-0.09397	0.239213	0.401142	protein_coding
ENSG0000(	TAF9B	152.6397	-0.09805	0.239265	0.401207	protein_coding
ENSG0000(	ENSG0000(	237.7641	0.067572	0.239294	0.401214	lncRNA
ENSG0000(	ZNF598	583.8109	0.085808	0.239292	0.401214	protein_coding
ENSG0000(	TTF1	452.5665	-0.07316	0.2394	0.401369	protein_coding
ENSG0000(	ENSG0000(	187.0702	-0.08187	0.239413	0.40137	transcribed_processed_pseudoge
ENSG0000(	ENSG0000(	167.4185	-0.12624	0.23945	0.401396	lncRNA
ENSG0000(	EPPK1	2788.707	-0.22194	0.239467	0.401396	protein_coding
ENSG0000(	RN7SKP11S	11.83767	0.291948	0.239454	0.401396	misc_RNA
ENSG0000(	VDR	2896.406	-0.17811	0.239504	0.401438	protein_coding
ENSG0000(	OR56A4	684.6903	-0.06873	0.239537	0.401471	protein_coding
ENSG0000(	ENSG0000(	61.28547	0.125475	0.239604	0.401562	lncRNA

ENSG0000(	ENSG0000(	792.8971	-0.10472	0.239631	0.401586	TEC
ENSG0000(	CPNE7	169.1372	-0.21687	0.239658	0.401596	protein_coding
ENSG0000(	ENSG0000(	85.35975	0.103519	0.239663	0.401596	processed_pseudogene
ENSG0000(	ONECUT1	18.41099	0.251731	0.239699	0.401636	protein_coding
ENSG0000(	DNAJC19P5	104.8255	-0.10568	0.239861	0.401864	processed_pseudogene
ENSG0000(	LINC02239	10.01734	0.241753	0.239849	0.401864	lncRNA
ENSG0000(	LIN9	150.8215	-0.11064	0.239925	0.40195	protein_coding
ENSG0000(	DTD2	563.6211	0.06565	0.239938	0.40195	protein_coding
ENSG0000(	LINC01780	9.926959	0.246526	0.239979	0.401977	lncRNA
ENSG0000(	RN7SL68P	23.7075	0.152692	0.239973	0.401977	misc_RNA
ENSG0000(	CCT6B	270.783	0.085859	0.240089	0.402139	protein_coding
ENSG0000(	TNPO2	1425.1	-0.06786	0.240155	0.402228	protein_coding
ENSG0000(	DARS2	737.4781	-0.03815	0.240218	0.402313	protein_coding
ENSG0000(	ENSG0000(	104.8337	0.083057	0.240242	0.402331	lncRNA
ENSG0000(	IRAK2	455.3431	0.106955	0.240267	0.402352	protein_coding
ENSG0000(	CFHR3	161.2013	0.087353	0.240395	0.402544	protein_coding
ENSG0000(	ENSG0000(	7.578922	0.968864	0.240486	0.402675	lncRNA
ENSG0000(	ENSG0000(	37.77314	-0.19995	0.240527	0.402723	snoRNA
ENSG0000(	PCDH11X	518.357	-0.19321	0.240582	0.402794	protein_coding
ENSG0000(	RN7SL748F	10.53984	0.20735	0.240627	0.402848	misc_RNA
ENSG0000(	ENSG0000(	481.5332	0.053387	0.240695	0.402919	lncRNA
ENSG0000(	NF1	7497.081	0.051386	0.240684	0.402919	protein_coding
ENSG0000(	ZNF600	1334.917	-0.08657	0.240747	0.402984	protein_coding
ENSG0000(	ENSG0000(	321.4194	-0.05635	0.24076	0.402985	lncRNA
ENSG0000(	ENSG0000(	271.4867	0.070564	0.240796	0.403024	lncRNA
ENSG0000(	SH3GLB1	2419.904	0.066645	0.240836	0.40307	protein_coding
ENSG0000(	ENSG0000(	75.81793	0.094534	0.240888	0.403135	lncRNA
ENSG0000(	GSKIP	952.5703	-0.17165	0.240965	0.403243	protein_coding
ENSG0000(	ENSG0000(	34.70611	-0.18384	0.240984	0.403253	lncRNA
ENSG0000(	ENSG0000(	72.63777	-0.09561	0.241053	0.403332	lncRNA
ENSG0000(	RN7SL760F	40.31955	0.11592	0.241057	0.403332	misc_RNA
ENSG0000(	ENSG0000(	497.6915	-0.05114	0.241078	0.403345	lncRNA
ENSG0000(	LINC00200	175.0711	0.073867	0.241093	0.403349	lncRNA
ENSG0000(	ENSG0000(	299.92	-0.19671	0.241219	0.403522	lncRNA
ENSG0000(	ULK4P2	45.4411	0.201372	0.241222	0.403522	transcribed_unprocessed_pseudo
ENSG0000(	ENSG0000(	13.80612	0.359862	0.241392	0.403699	processed_pseudogene
ENSG0000(	ENSG0000(	28.72935	0.160704	0.241382	0.403699	lncRNA
ENSG0000(	RO60	2100.206	-0.04266	0.241367	0.403699	protein_coding
ENSG0000(	SPATA2P1	69.87292	0.132884	0.241376	0.403699	processed_pseudogene
ENSG0000(	TMEM273	573.616	0.133642	0.241358	0.403699	protein_coding
ENSG0000(	VSIG2	47.54494	0.305565	0.24142	0.403726	protein_coding
ENSG0000(	CDC14C	263.1035	0.064142	0.241486	0.403804	protein_coding
ENSG0000(	MIR3908	15.80856	-0.19724	0.241493	0.403804	miRNA
ENSG0000(	RNU4-53P	14.24928	-0.20476	0.241547	0.403874	snRNA
ENSG0000(	ENSG0000(	467.47	-0.21804	0.241585	0.403914	processed_pseudogene
ENSG0000(	ZNF554	1499.286	-0.03673	0.241668	0.404033	protein_coding
ENSG0000(	OSBPL10	1583.292	-0.12748	0.241687	0.404042	protein_coding

ENSG0000(	ERI3	339.2101	-0.08391	0.241728	0.40409	protein_coding
ENSG0000(	ACKR4	119.7075	0.196561	0.241836	0.404249	protein_coding
ENSG0000(	ORC5	281.3214	-0.07076	0.241862	0.404271	protein_coding
ENSG0000(	TMEM71	256.46	0.195462	0.241916	0.40434	protein_coding
ENSG0000(	SYT15	552.9605	0.087647	0.241952	0.404379	protein_coding
ENSG0000(	ENSG0000(	9.475074	-0.2176	0.242026	0.404474	lncRNA
ENSG0000(	OSBPL11	710.4994	-0.06062	0.242035	0.404474	protein_coding
ENSG0000(	ENSG0000(	35.57456	0.305327	0.242051	0.404479	lncRNA
ENSG0000(	CREB3	235.2774	-0.09947	0.242124	0.404559	protein_coding
ENSG0000(	MYLK4	89.79977	0.116919	0.242115	0.404559	protein_coding
ENSG0000(	LIMD1-AS1	476.9667	-0.06104	0.242165	0.404588	lncRNA
ENSG0000(	PARG	1041.208	0.047327	0.242167	0.404588	protein_coding
ENSG0000(	ENSG0000(	15.93953	-0.31392	0.242213	0.404622	protein_coding
ENSG0000(	PKMYT1	126.3363	0.141789	0.242207	0.404622	protein_coding
ENSG0000(	PCAT7	357.1709	-0.08978	0.242255	0.40467	lncRNA
ENSG0000(	LINC02156	54.53214	-0.11961	0.24229	0.404706	lncRNA
ENSG0000(	DDX54	545.3088	-0.08561	0.242462	0.404909	protein_coding
ENSG0000(	ENSG0000(	501.6279	0.07887	0.242454	0.404909	lncRNA
ENSG0000(	GFM1	2389.363	-0.0432	0.242457	0.404909	protein_coding
ENSG0000(	HSPA8P18	24.22613	0.24257	0.242457	0.404909	processed_pseudogene
ENSG0000(	PLEKHA3P1	15.02027	0.243197	0.242598	0.405114	processed_pseudogene
ENSG0000(	ENSG0000(	437.8233	0.07482	0.242635	0.405139	lncRNA
ENSG0000(	ENSG0000(	71.42556	0.114993	0.242639	0.405139	lncRNA
ENSG0000(	OTUD7B	1954.482	-0.07999	0.242664	0.40516	protein_coding
ENSG0000(	UBAP1	707.4182	-0.05932	0.24271	0.405216	protein_coding
ENSG0000(	CLN8-AS1	535.3862	0.053021	0.242736	0.405234	lncRNA
ENSG0000(	ENSG0000(	595.4588	-0.05233	0.242747	0.405234	lncRNA
ENSG0000(	HYDIN2	253.7467	-0.19576	0.2428	0.4053	transcribed_unprocessed_pseudo
ENSG0000(	SRPK3	104.0272	0.108993	0.242883	0.405419	protein_coding
ENSG0000(	PIK3IP1	1697.43	0.164322	0.242925	0.405467	protein_coding
ENSG0000(	AGAP4	963.0992	-0.09171	0.242967	0.405495	protein_coding
ENSG0000(	PAPPA-AS1	97.63781	0.156812	0.24296	0.405495	lncRNA
ENSG0000(	ENSG0000(	100.3558	-0.12893	0.243062	0.405588	lncRNA
ENSG0000(	ENSG0000(	13.0672	-0.23667	0.243053	0.405588	lncRNA
ENSG0000(	ENSG0000(	154.9057	-0.08003	0.243051	0.405588	lncRNA
ENSG0000(	SNHG14	17184.23	0.066008	0.243086	0.405607	lncRNA
ENSG0000(	TIMM17A	973.6051	0.052623	0.243105	0.405617	protein_coding
ENSG0000(	HNRNPA0	1363.906	-0.05141	0.243124	0.405627	protein_coding
ENSG0000(	ENSG0000(	55.4274	0.15139	0.24316	0.405665	TEC
ENSG0000(	ORMDL3	1317.479	0.074579	0.2432	0.405711	protein_coding
ENSG0000(	HBS1L	1491.752	-0.0531	0.243222	0.405712	protein_coding
ENSG0000(	RN7SL361F	10.67468	0.211031	0.243226	0.405712	misc_RNA
ENSG0000(	ENSG0000(	39.8493	-0.15008	0.24328	0.405759	processed_pseudogene
ENSG0000(	RN7SL618F	16.31863	0.210342	0.243272	0.405759	misc_RNA
ENSG0000(	LINC02374	24.77988	-0.14506	0.243364	0.405878	lncRNA
ENSG0000(	ELOVL5	1554.171	0.168094	0.243392	0.405882	protein_coding
ENSG0000(	ENSG0000(	14.50311	-0.18866	0.243391	0.405882	lncRNA

ENSG0000(	ENSG0000(	76.77064	0.10142	0.243445	0.40592	lncRNA
ENSG0000(	ENSG0000(	145.7646	0.116983	0.243438	0.40592	lncRNA
ENSG0000(	FAM216A	240.4302	-0.08317	0.243476	0.40592	protein_coding
ENSG0000(	NCKAP5-IT:	68.6471	-0.27216	0.243474	0.40592	lncRNA
ENSG0000(	RPS3AP29	25.18142	-0.27777	0.24348	0.40592	processed_pseudogene
ENSG0000(	ENSG0000(	9.331802	0.239719	0.243511	0.40595	processed_pseudogene
ENSG0000(	GPR21	154.1404	0.067685	0.243568	0.406023	protein_coding
ENSG0000(	ENSG0000(	185.9237	0.063806	0.243598	0.406031	lncRNA
ENSG0000(	PPIAL4H	35.24286	0.303524	0.243595	0.406031	protein_coding
ENSG0000(	ENSG0000(	38.59276	-0.12209	0.243655	0.406091	processed_pseudogene
ENSG0000(	SEC14L3	68.17854	-0.085	0.24366	0.406091	protein_coding
ENSG0000(	SCARB1	2114.162	0.055292	0.243693	0.406125	protein_coding
ENSG0000(	FILIP1L	1102.309	0.118581	0.243739	0.40618	protein_coding
ENSG0000(	DCLRE1A	289.9775	-0.10082	0.243765	0.406203	protein_coding
ENSG0000(	ENSG0000(	14.83377	0.216924	0.243843	0.406289	lncRNA
ENSG0000(	FAM169B	100.3574	0.106273	0.243836	0.406289	transcribed_unitary_pseudogene
ENSG0000(	PABPC5	27.03391	0.215133	0.243865	0.406304	protein_coding
ENSG0000(	LINC00239	9.623268	0.24014	0.243887	0.406308	lncRNA
ENSG0000(	UCKL1-AS1	335.8892	0.059076	0.243893	0.406308	lncRNA
ENSG0000(	ENSG0000(	29.38392	0.141609	0.243985	0.40644	lncRNA
ENSG0000(	TRAJ36	43.68452	0.244963	0.244094	0.406599	TR_J_gene
ENSG0000(	ENSG0000(	21.56132	-0.23893	0.244258	0.406808	lncRNA
ENSG0000(	ENSG0000(	85.04207	0.110696	0.244243	0.406808	lncRNA
ENSG0000(	LONP1	687.4427	-0.05865	0.244245	0.406808	protein_coding
ENSG0000(	AKAP12	990.5426	0.105445	0.244318	0.406886	protein_coding
ENSG0000(	ABCD2	275.9493	0.188783	0.244396	0.406923	protein_coding
ENSG0000(	CCDC26	3244.934	0.044607	0.244384	0.406923	lncRNA
ENSG0000(	COBLL1	2286.041	0.079098	0.244393	0.406923	protein_coding
ENSG0000(	ENSG0000(	53.69658	0.149661	0.24441	0.406923	lncRNA
ENSG0000(	ENSG0000(	12.60871	0.203325	0.244424	0.406923	lncRNA
ENSG0000(	GTF2IP13	567.1156	0.119062	0.244388	0.406923	transcribed_unprocessed_pseudo
ENSG0000(	NT5C3AP2	140.0924	-0.18462	0.24443	0.406923	processed_pseudogene
ENSG0000(	SESN1	890.654	-0.10121	0.244466	0.40696	protein_coding
ENSG0000(	LINC02766	157.0482	-0.07735	0.244605	0.407164	lncRNA
ENSG0000(	VCAM1	817.3069	-0.20751	0.244614	0.407164	protein_coding
ENSG0000(	ENSG0000(	136.5719	-0.07353	0.244638	0.407183	lncRNA
ENSG0000(	ENSG0000(	3419.485	0.084652	0.244672	0.407214	transcribed_unprocessed_pseudo
ENSG0000(	NCKAP5	1083.422	-0.23998	0.244683	0.407214	protein_coding
ENSG0000(	ENSG0000(	16.80875	-0.1978	0.24479	0.407371	transcribed_processed_pseudoge
ENSG0000(	TVP23B	1311.057	0.059384	0.244876	0.407493	protein_coding
ENSG0000(	ENSG0000(	34.44503	-0.50879	0.2449	0.407511	lncRNA
ENSG0000(	ENSG0000(	44.5501	0.404119	0.244932	0.407543	snoRNA
ENSG0000(	PKP1	48.27286	-0.22269	0.245071	0.407754	protein_coding
ENSG0000(	RN7SL201F	13.16013	-0.22211	0.245164	0.407886	misc_RNA
ENSG0000(	SERTAD2	1155.893	-0.09015	0.245225	0.407966	protein_coding
ENSG0000(	CDC37L1	310.2291	-0.07255	0.245369	0.408184	protein_coding
ENSG0000(	TIMMDC1-	54.71716	0.109427	0.245475	0.408338	lncRNA

ENSG0000(	ZNF81	1431.388	0.063975	0.245533	0.408413	protein_coding
ENSG0000(	OR2AT4	1496.285	0.060478	0.245576	0.408443	protein_coding
ENSG0000(	SLC22A9	113.6653	0.081997	0.245565	0.408443	protein_coding
ENSG0000(	ENSG0000(	29.9658	0.27444	0.24563	0.40851	lncRNA
ENSG0000(	CIT	1498.906	0.076865	0.245714	0.408618	protein_coding
ENSG0000(	NRAV	630.7993	0.037446	0.245721	0.408618	lncRNA
ENSG0000(	TPBGL-AS1	35.21332	0.150662	0.245756	0.408655	lncRNA
ENSG0000(	MRPS17P1	560.3947	-0.05675	0.245773	0.408662	processed_pseudogene
ENSG0000(	ENSG0000(	23.91329	0.160499	0.245876	0.408811	lncRNA
ENSG0000(	SWI5	194.3486	-0.06921	0.245956	0.408923	protein_coding
ENSG0000(	UNGP1	97.00289	-0.07417	0.246008	0.408988	processed_pseudogene
ENSG0000(	TUB	185.916	0.13444	0.246024	0.408993	protein_coding
ENSG0000(	TIMMDC1	570.231	0.043531	0.246041	0.408999	protein_coding
ENSG0000(	B3GAT1-DT	1247.734	-0.26002	0.246103	0.409081	lncRNA
ENSG0000(	ENSG0000(	53.76761	-0.18279	0.246126	0.409098	processed_pseudogene
ENSG0000(	BECN1	1793.451	-0.04881	0.246147	0.40911	protein_coding
ENSG0000(	CA2	179.1878	0.504763	0.246219	0.409187	protein_coding
ENSG0000(	TSPAN1	1562.796	0.252699	0.246217	0.409187	protein_coding
ENSG0000(	ENSG0000(	112.8396	0.083097	0.246264	0.40924	lncRNA
ENSG0000(	ENSG0000(	13.29444	0.215819	0.246304	0.409286	lncRNA
ENSG0000(	ENSG0000(	14.58378	-0.36036	0.246329	0.409305	lncRNA
ENSG0000(	COQ9	789.9247	-0.09426	0.246441	0.409448	protein_coding
ENSG0000(	FLRT1	21.27838	0.252145	0.246436	0.409448	protein_coding
ENSG0000(	C16orf74	120.8537	0.135492	0.246524	0.409565	protein_coding
ENSG0000(	BSG-AS1	24.24991	0.177606	0.246635	0.409706	lncRNA
ENSG0000(	COQ8A	535.0229	0.105029	0.246624	0.409706	protein_coding
ENSG0000(	ENSG0000(	13.2185	0.225044	0.24666	0.409709	lncRNA
ENSG0000(	ENSG0000(	46.6334	-0.30503	0.246663	0.409709	unprocessed_pseudogene
ENSG0000(	ANXA2	5095.077	0.121178	0.246699	0.409726	protein_coding
ENSG0000(	STK31	220.1533	0.104381	0.246692	0.409726	protein_coding
ENSG0000(	CYB561D1	795.7076	-0.06353	0.24689	0.409911	protein_coding
ENSG0000(	DNAJC5B	169.7499	-0.11823	0.246883	0.409911	protein_coding
ENSG0000(	ENSG0000(	14.57373	0.252198	0.246901	0.409911	lncRNA
ENSG0000(	ENSG0000(	83.89747	0.090943	0.246888	0.409911	lncRNA
ENSG0000(	MIR31HG	99.15765	0.164738	0.246848	0.409911	lncRNA
ENSG0000(	PRRT1B	12.49628	-0.311	0.246895	0.409911	protein_coding
ENSG0000(	TENM2	54.68943	0.279987	0.246848	0.409911	protein_coding
ENSG0000(	RN7SKP30	69.54578	-0.10012	0.247011	0.410071	misc_RNA
ENSG0000(	ENSG0000(	113.3519	0.085287	0.247157	0.41024	protein_coding
ENSG0000(	RNU6-925F	10.07179	-0.22535	0.247129	0.41024	snRNA
ENSG0000(	RPS26P20	10.32502	0.222379	0.247154	0.41024	processed_pseudogene
ENSG0000(	RXRB	1116.219	-0.04439	0.247165	0.41024	protein_coding
ENSG0000(	ENSG0000(	111.9	-0.21435	0.247203	0.410281	lncRNA
ENSG0000(	ENSG0000(	65.63854	0.101171	0.247444	0.410401	lncRNA
ENSG0000(	ENSG0000(	16.31234	-0.17914	0.247433	0.410401	lncRNA
ENSG0000(	ENSG0000(	21.07357	0.187137	0.247417	0.410401	lncRNA
ENSG0000(	ENSG0000(	16.72402	0.189566	0.247438	0.410401	TEC

ENSG0000(	ERCC2	443.6952	0.073481	0.247434	0.410401	protein_coding
ENSG0000(	GSG1	451.21	0.074448	0.247377	0.410401	protein_coding
ENSG0000(	HOXA3	542.9763	-0.1488	0.247332	0.410401	protein_coding
ENSG0000(	HPS5	1472.982	0.062564	0.247367	0.410401	protein_coding
ENSG0000(	KNTC1	2410.025	0.084676	0.24739	0.410401	protein_coding
ENSG0000(	LINC00278	1674.658	-0.14399	0.247338	0.410401	lncRNA
ENSG0000(	MIPEPP3	79.53739	0.1141	0.247373	0.410401	transcribed_unprocessed_pseudo
ENSG0000(	MIR548C	67.71537	0.133057	0.247324	0.410401	miRNA
ENSG0000(	PLEKHS1	2201.633	-0.18314	0.247368	0.410401	protein_coding
ENSG0000(	PCBP4	380.5762	-0.08754	0.247515	0.410496	protein_coding
ENSG0000(	CACNA1I	101.0732	0.241475	0.247547	0.410528	protein_coding
ENSG0000(	CLN5	4506.169	0.040912	0.247573	0.410535	protein_coding
ENSG0000(	LBX1-AS1	37.8133	-0.15515	0.24759	0.410535	lncRNA
ENSG0000(	TPD52L2	1503.43	0.074656	0.247586	0.410535	protein_coding
ENSG0000(	RNU2-63P	12.33776	0.283194	0.247675	0.410653	snRNA
ENSG0000(	CIB1	718.0277	-0.1312	0.247695	0.410665	protein_coding
ENSG0000(	HAX1	378.2231	-0.07175	0.247722	0.410688	protein_coding
ENSG0000(	C1orf226	327.8097	0.163506	0.24775	0.410714	protein_coding
ENSG0000(	ENSG0000(	214.0413	-0.07279	0.247838	0.410838	lncRNA
ENSG0000(	FANCG	257.2868	0.106109	0.247874	0.410854	protein_coding
ENSG0000(	MAU2	2888.775	-0.04469	0.247862	0.410854	protein_coding
ENSG0000(	ARPC4	1325.396	0.052247	0.247914	0.4109	protein_coding
ENSG0000(	GPSM2	2455.978	-0.04485	0.247971	0.410971	protein_coding
ENSG0000(	ENSG0000(	184.5112	-0.08362	0.248015	0.411	lncRNA
ENSG0000(	ENSG0000(	15.17183	-0.22154	0.24801	0.411	transcribed_unprocessed_pseudo
ENSG0000(	ENSG0000(	198.8683	-0.15655	0.248027	0.411	lncRNA
ENSG0000(	ZNF266	2445.751	0.086904	0.248042	0.411002	protein_coding
ENSG0000(	ENSG0000(	264.1484	0.053664	0.248064	0.411017	lncRNA
ENSG0000(	SELENBP1	1552.475	-0.17416	0.248168	0.411147	protein_coding
ENSG0000(	SLC2A10	404.8186	0.137203	0.248158	0.411147	protein_coding
ENSG0000(	PHBP8	58.58209	-0.29973	0.248198	0.411174	processed_pseudogene
ENSG0000(	RNF114	2176.62	0.048716	0.248236	0.411216	protein_coding
ENSG0000(	SMG1-DT	43.78199	-0.12671	0.248253	0.411222	lncRNA
ENSG0000(	CTDP1	746.2259	0.059357	0.248274	0.411235	protein_coding
ENSG0000(	GALNT12	629.4111	-0.18861	0.248303	0.411247	protein_coding
ENSG0000(	HMGB1P5	88.40668	0.279114	0.24832	0.411247	transcribed_processed_pseudoge
ENSG0000(	LINC02080	1145.988	0.032847	0.248308	0.411247	lncRNA
ENSG0000(	HSD17B1	23.51468	-0.12417	0.248347	0.411271	protein_coding
ENSG0000(	NCAPD3	1587.947	0.062882	0.248384	0.411304	protein_coding
ENSG0000(	RN7SL440F	251.8869	-0.07658	0.248393	0.411304	misc_RNA
ENSG0000(	ZDHHC9	1029.931	-0.1636	0.248514	0.411483	protein_coding
ENSG0000(	HSPB11	539.708	-0.05647	0.248536	0.411498	protein_coding
ENSG0000(	ENSG0000(	14.38455	0.228652	0.248569	0.411531	lncRNA
ENSG0000(	RN7SL488F	108.5041	-0.09697	0.248626	0.411603	misc_RNA
ENSG0000(	BIN1	871.8276	-0.10483	0.248759	0.411802	protein_coding
ENSG0000(	MUC20	2151.803	-0.14733	0.248833	0.411898	protein_coding
ENSG0000(	RN7SL792F	70.6246	0.100463	0.248844	0.411898	misc_RNA

ENSG0000(	ALG14	1627.665	0.115147	0.248869	0.411918	protein_coding
ENSG0000(	INPPL1	2203.102	-0.06034	0.248896	0.411941	protein_coding
ENSG0000(	LINC01924	83.92639	-0.1156	0.249022	0.412129	lncRNA
ENSG0000(	OR56A1	323.7505	0.066332	0.249102	0.41222	protein_coding
ENSG0000(	RBM33	6234.657	-0.03909	0.249104	0.41222	protein_coding
ENSG0000(	ENSG0000(	10.7327	-0.24727	0.249165	0.4123	processed_pseudogene
ENSG0000(	ENSG0000(	95.41977	0.126169	0.249205	0.412301	TEC
ENSG0000(	ENSG0000(	161.2507	0.121492	0.249198	0.412301	lncRNA
ENSG0000(	H4C13	71.57411	-0.1702	0.24919	0.412301	protein_coding
ENSG0000(	ENSG0000(	115.9025	0.07483	0.249299	0.412435	lncRNA
ENSG0000(	ENSG0000(	1814.782	0.160364	0.249318	0.412445	lncRNA
ENSG0000(	ICOS	223.4343	0.262282	0.249445	0.412603	protein_coding
ENSG0000(	LINC02137	52.81554	-0.09678	0.249453	0.412603	lncRNA
ENSG0000(	RPUSD2	170.4139	0.077271	0.24945	0.412603	protein_coding
ENSG0000(	TSC1	3579.379	0.034891	0.249598	0.412822	protein_coding
ENSG0000(	WDR1	4016.903	0.052378	0.24965	0.412886	protein_coding
ENSG0000(	ENSG0000(	476.6525	0.079223	0.249677	0.412909	lncRNA
ENSG0000(	SYMPK	3553.367	0.042152	0.249716	0.412951	protein_coding
ENSG0000(	TOP3A	1523.282	-0.04424	0.249746	0.41298	protein_coding
ENSG0000(	ENSG0000(	12.01425	0.399312	0.249825	0.41305	unprocessed_pseudogene
ENSG0000(	ENSG0000(	109.1008	0.156088	0.249821	0.41305	lncRNA
ENSG0000(	ENSG0000(	216.5719	-0.22926	0.249828	0.41305	lncRNA
ENSG0000(	BNIP3P5	46.30067	0.131164	0.249967	0.413252	processed_pseudogene
ENSG0000(	ENSG0000(	35.43392	0.310451	0.24999	0.413252	IG_V_gene
ENSG0000(	HES6	101.0121	0.176065	0.249983	0.413252	protein_coding
ENSG0000(	CACNB3	710.6691	0.08023	0.250021	0.413283	protein_coding
ENSG0000(	ENSG0000(	202.2882	-0.0574	0.250069	0.413339	lncRNA
ENSG0000(	ENSG0000(	103.9575	0.125001	0.250087	0.413347	unprocessed_pseudogene
ENSG0000(	ENSG0000(	41.63544	-0.12295	0.250103	0.413353	processed_pseudogene
ENSG0000(	PLEKHA7	3743.415	-0.18221	0.250203	0.413497	protein_coding
ENSG0000(	FUT6	2598.853	-0.22784	0.250268	0.413581	protein_coding
ENSG0000(	KXD1	1117.515	0.043298	0.250303	0.413618	protein_coding
ENSG0000(	GNAI2	1926.914	0.117175	0.250338	0.413654	protein_coding
ENSG0000(	ZNF207	13118.46	-0.03118	0.250402	0.413738	protein_coding
ENSG0000(	B4GALT2	279.1919	0.108235	0.250598	0.413892	protein_coding
ENSG0000(	ENSG0000(	9.305312	-0.30943	0.250528	0.413892	transcribed_unprocessed_pseudo
ENSG0000(	ENSG0000(	47.78627	0.271403	0.25058	0.413892	lncRNA
ENSG0000(	MAP2K5	682.7114	-0.06324	0.2506	0.413892	protein_coding
ENSG0000(	MKRN2	1350.871	-0.03573	0.250569	0.413892	protein_coding
ENSG0000(	OR4K16P	79.77992	0.072018	0.250587	0.413892	transcribed_unprocessed_pseudo
ENSG0000(	SEC61G	304.5315	-0.11658	0.250557	0.413892	protein_coding
ENSG0000(	USP24	4439.734	-0.06514	0.250545	0.413892	protein_coding
ENSG0000(	ENSG0000(	159.6984	0.100792	0.250648	0.41395	protein_coding
ENSG0000(	KLHL7	990.3655	0.058927	0.250682	0.413984	protein_coding
ENSG0000(	ENSG0000(	40.37703	0.137042	0.250698	0.413986	lncRNA
ENSG0000(	GRWD1	470.0204	0.078649	0.250709	0.413986	protein_coding
ENSG0000(	CCDC173	14.93617	-0.33752	0.250772	0.414067	protein_coding

ENSG0000(	SETD1B	2196.754	0.04912	0.250796	0.414085	protein_coding
ENSG0000(	DNM2	10581.2	-0.05326	0.250959	0.414333	protein_coding
ENSG0000(	SRPK1	2085.92	0.064201	0.250974	0.414336	protein_coding
ENSG0000(	ARL3	127.5588	-0.09371	0.251	0.414336	protein_coding
ENSG0000(	OR2F1	199.6798	0.070047	0.250992	0.414336	protein_coding
ENSG0000(	ENSG0000(	23.03572	-0.19029	0.251084	0.414453	lncRNA
ENSG0000(	PACSIN1	42.76463	-0.33441	0.251133	0.414512	protein_coding
ENSG0000(	ENSG0000(	210.009	-0.07724	0.251196	0.414586	lncRNA
ENSG0000(	TMEM104	455.3963	0.093873	0.251205	0.414586	protein_coding
ENSG0000(	ENSG0000(	29.84155	-0.13538	0.251248	0.414637	lncRNA
ENSG0000(	MIR3975	29.69794	0.148369	0.251341	0.414767	miRNA
ENSG0000(	TYRO3	1460.202	-0.03304	0.251385	0.414819	protein_coding
ENSG0000(	ENSG0000(	11.54008	0.221609	0.251425	0.41482	lncRNA
ENSG0000(	NRSN1	56.56511	-0.13657	0.251416	0.41482	protein_coding
ENSG0000(	PLEKHG4	358.554	0.09239	0.251424	0.41482	protein_coding
ENSG0000(	RN7SKP154	63.08865	-0.24209	0.251444	0.41483	misc_RNA
ENSG0000(	MARK1	1079.168	0.044948	0.251547	0.414978	protein_coding
ENSG0000(	RN7SKP103	24.93397	-0.24541	0.251565	0.414986	misc_RNA
ENSG0000(	ZNF358	237.7841	-0.1166	0.251597	0.415016	protein_coding
ENSG0000(	CDNF	55.26344	0.145879	0.251665	0.415094	protein_coding
ENSG0000(	ENSG0000(	554.5357	0.226032	0.25167	0.415094	protein_coding
ENSG0000(	RPS6KA3	4446.268	-0.03998	0.251753	0.415209	protein_coding
ENSG0000(	CTBS	636.1212	0.057585	0.251852	0.41535	protein_coding
ENSG0000(	COILP1	9.838541	0.205725	0.251981	0.415526	processed_pseudogene
ENSG0000(	ENSG0000(	19.11704	-0.17543	0.252021	0.415526	lncRNA
ENSG0000(	ENSG0000(	294.2441	0.172159	0.251989	0.415526	TEC
ENSG0000(	RNF133	34.97835	0.157215	0.252024	0.415526	protein_coding
ENSG0000(	ZBTB32	52.05993	0.312908	0.252025	0.415526	protein_coding
ENSG0000(	PSMF1	1806.728	-0.0706	0.252042	0.415533	protein_coding
ENSG0000(	CTXND1	161.844	0.080219	0.252076	0.415567	protein_coding
ENSG0000(	ENSG0000(	11.36688	0.259222	0.252108	0.415598	lncRNA
ENSG0000(	FLNA	10853.13	0.139577	0.252226	0.415771	protein_coding
ENSG0000(	ATP11B	3756.478	-0.04142	0.252249	0.415788	protein_coding
ENSG0000(	PHKA1P1	34.80703	0.16606	0.252337	0.415911	processed_pseudogene
ENSG0000(	ZDHHC24	1317.839	-0.05555	0.252377	0.415955	protein_coding
ENSG0000(	ENSG0000(	26.21484	0.148018	0.252408	0.415962	lncRNA
ENSG0000(	MAPK13	2862.82	-0.10676	0.252395	0.415962	protein_coding
ENSG0000(	CENPN-AS1	393.0775	0.048402	0.252504	0.416094	lncRNA
ENSG0000(	ENSG0000(	90.83857	0.089369	0.252515	0.416094	lncRNA
ENSG0000(	STXBP4	1219.472	-0.06829	0.252599	0.416212	protein_coding
ENSG0000(	IRGQ	2122.99	-0.03732	0.252677	0.416318	protein_coding
ENSG0000(	ENSG0000(	202.4626	0.07472	0.25276	0.416353	lncRNA
ENSG0000(	ENSG0000(	28.4725	-0.18812	0.252764	0.416353	lncRNA
ENSG0000(	LINC02916	42.28777	0.115861	0.252759	0.416353	lncRNA
ENSG0000(	RNU6-116(	9.431833	-0.36515	0.25276	0.416353	snRNA
ENSG0000(	SELENOV	13.41496	0.184855	0.252749	0.416353	protein_coding
ENSG0000(	CAPG	291.7088	0.16753	0.252848	0.41647	protein_coding

ENSG0000(	AP1M1	4103.415	-0.07281	0.252872	0.416487	protein_coding
ENSG0000(	ENSG0000(	115.3488	-0.07691	0.252947	0.416589	lncRNA
ENSG0000(	ADM5	9.632433	-0.25287	0.253106	0.416828	protein_coding
ENSG0000(	TIMM8A	343.0975	0.056248	0.253229	0.41701	protein_coding
ENSG0000(	REV3L	4602.111	0.052135	0.253277	0.417067	protein_coding
ENSG0000(	SCIMP	1158.871	0.108146	0.253308	0.417079	protein_coding
ENSG0000(	TEN1	50.5835	-0.13519	0.25331	0.417079	protein_coding
ENSG0000(	ENSG0000(	13.55325	0.216309	0.253367	0.417128	lncRNA
ENSG0000(	PCTP	399.0083	0.07121	0.253362	0.417128	protein_coding
ENSG0000(	ENSG0000(	59.00966	0.08567	0.25347	0.417225	lncRNA
ENSG0000(	RN7SL434F	28.20838	0.128392	0.253492	0.417225	misc_RNA
ENSG0000(	TACC2	2758.703	-0.08732	0.253474	0.417225	protein_coding
ENSG0000(	TINF2	691.5488	-0.06756	0.253491	0.417225	protein_coding
ENSG0000(	TMPRSS6	28.20051	0.240472	0.253482	0.417225	protein_coding
ENSG0000(	ENSG0000(	21.28722	0.203781	0.253592	0.417357	lncRNA
ENSG0000(	RNF139-DT	521.278	0.054405	0.253599	0.417357	lncRNA
ENSG0000(	ENSG0000(	55.67111	-0.09458	0.253613	0.417359	lncRNA
ENSG0000(	ENSG0000(	18.42619	-0.19409	0.253648	0.417395	lncRNA
ENSG0000(	COSMOC	25.69997	-0.24157	0.253687	0.417436	lncRNA
ENSG0000(	PRMT3	603.2929	-0.05487	0.253734	0.417492	protein_coding
ENSG0000(	ENSG0000(	16.45925	0.172205	0.253791	0.417565	lncRNA
ENSG0000(	AADACL2-A	22.9931	0.170585	0.253814	0.417575	lncRNA
ENSG0000(	C5orf60	107.8225	-0.09615	0.253866	0.417575	lncRNA
ENSG0000(	ENSG0000(	74.72472	0.1378	0.253872	0.417575	lncRNA
ENSG0000(	FASLG	83.66841	-0.11705	0.253883	0.417575	protein_coding
ENSG0000(	LINC01254	144.0373	0.065094	0.253853	0.417575	lncRNA
ENSG0000(	NUP58	1500.842	0.077091	0.253824	0.417575	protein_coding
ENSG0000(	ZNF136	987.9181	0.075672	0.25389	0.417575	protein_coding
ENSG0000(	ENSG0000(	71.93651	0.096445	0.253938	0.417611	lncRNA
ENSG0000(	TTR	17.14104	0.184099	0.253935	0.417611	protein_coding
ENSG0000(	ENSG0000(	115.745	-0.08004	0.254084	0.417807	lncRNA
ENSG0000(	LINC01588	2314.973	0.080383	0.254079	0.417807	lncRNA
ENSG0000(	ENSG0000(	44.40713	0.114804	0.254102	0.417815	lncRNA
ENSG0000(	HEPACAM2	831.5219	-0.24782	0.254118	0.417819	protein_coding
ENSG0000(	RN7SL836F	26.47896	-0.1675	0.25414	0.417832	misc_RNA
ENSG0000(	CARMAL	98.32487	-0.08579	0.254154	0.417835	lncRNA
ENSG0000(	CTBP2P10	28.12439	0.202701	0.254181	0.417857	processed_pseudogene
ENSG0000(	KCMF1	1489.01	-0.07483	0.25421	0.417882	protein_coding
ENSG0000(	SNORD15B	419.0759	-0.12625	0.254249	0.417904	snoRNA
ENSG0000(	TRIM41	2777.09	0.033374	0.254237	0.417904	protein_coding
ENSG0000(	CLPB	2163.782	0.063353	0.254348	0.418044	protein_coding
ENSG0000(	ENSG0000(	17.70749	0.235075	0.254365	0.41805	lncRNA
ENSG0000(	RAB6B	46.29607	0.242795	0.254387	0.418064	protein_coding
ENSG0000(	ADARB2	186.1826	-0.25752	0.254407	0.418076	protein_coding
ENSG0000(	ENSG0000(	12.63381	0.307159	0.254461	0.418121	processed_pseudogene
ENSG0000(	POLR2J3	13001.9	0.079345	0.254455	0.418121	protein_coding
ENSG0000(	DYM	2046.613	0.042897	0.254503	0.418124	protein_coding

ENSG0000(	ENSG0000(	11.00115	0.212803	0.254499	0.418124	lncRNA
ENSG0000(	SLC25A14P	10.09894	0.311976	0.254489	0.418124	processed_pseudogene
ENSG0000(	ENSG0000(	530.6622	0.083504	0.254578	0.418226	lncRNA
ENSG0000(	XRCC4	127.2234	0.075203	0.2546	0.418241	protein_coding
ENSG0000(	CABYR	83.06695	0.103785	0.254615	0.418244	protein_coding
ENSG0000(	ENSG0000(	42.94789	-0.10729	0.254639	0.41826	lncRNA
ENSG0000(	ENSG0000(	23.37905	0.198829	0.254682	0.418287	lncRNA
ENSG0000(	ENSG0000(	19.36853	0.149961	0.254669	0.418287	lncRNA
ENSG0000(	ENSG0000(	27.42576	-0.17195	0.254787	0.418439	lncRNA
ENSG0000(	SENP1	693.772	-0.05992	0.254847	0.418515	protein_coding
ENSG0000(	ZNF343	567.3975	0.045624	0.254997	0.41874	protein_coding
ENSG0000(	GDF5	26.50857	0.146762	0.255111	0.418904	protein_coding
ENSG0000(	SEC22A	871.6808	-0.05272	0.255128	0.418911	protein_coding
ENSG0000(	ENSG0000(	66.45353	0.125255	0.255145	0.418917	lncRNA
ENSG0000(	ENSG0000(	794.5652	0.063578	0.255186	0.41896	TEC
ENSG0000(	RAB28	319.2044	0.090012	0.255198	0.41896	protein_coding
ENSG0000(	LINC02438	20.97204	-0.15851	0.255231	0.418992	lncRNA
ENSG0000(	GLMP	497.8031	-0.0727	0.255289	0.419012	protein_coding
ENSG0000(	POU4F1	11.53225	0.348185	0.255296	0.419012	protein_coding
ENSG0000(	ZNF404	71.90679	-0.1631	0.255287	0.419012	protein_coding
ENSG0000(	ZNF730	38.38362	-0.21284	0.255286	0.419012	protein_coding
ENSG0000(	ZNF556	1507.69	-0.04523	0.255367	0.419106	protein_coding
ENSG0000(	ECT2L	60.07943	0.12963	0.255481	0.419273	protein_coding
ENSG0000(	IGSF22	20.39197	0.272359	0.255525	0.419322	protein_coding
ENSG0000(	ENSG0000(	477.8732	-0.06982	0.255573	0.419379	protein_coding
ENSG0000(	ENSG0000(	12.72532	0.214937	0.255631	0.419453	transcribed_processed_pseudoge
ENSG0000(	ENSG0000(	225.1286	-0.09808	0.255695	0.419536	lncRNA
ENSG0000(	UBE4B	2192.931	-0.04484	0.255719	0.419553	protein_coding
ENSG0000(	LAMA1	304.6758	-0.1499	0.255752	0.419585	protein_coding
ENSG0000(	PRLHR	127.2572	0.065901	0.255815	0.419649	protein_coding
ENSG0000(	ZNF534	573.9264	0.046691	0.255817	0.419649	protein_coding
ENSG0000(	GIMAP4	699.228	0.174507	0.255849	0.419679	protein_coding
ENSG0000(	RN7SL200F	62.97579	-0.10016	0.255934	0.419775	misc_RNA
ENSG0000(	TBC1D5	2702.377	-0.05767	0.25593	0.419775	protein_coding
ENSG0000(	PRMT5-DT	29.08007	0.207227	0.25611	0.420042	lncRNA
ENSG0000(	APCDD1L-D	170.1406	0.066886	0.256158	0.420051	lncRNA
ENSG0000(	DOK7	21.03166	-0.33254	0.256169	0.420051	protein_coding
ENSG0000(	GOLPH3L	934.1201	-0.08444	0.256164	0.420051	protein_coding
ENSG0000(	TRMT1	429.6523	0.119636	0.256159	0.420051	protein_coding
ENSG0000(	UNK	1310.148	-0.06749	0.256269	0.420194	protein_coding
ENSG0000(	ENSG0000(	1548.909	0.035071	0.256305	0.42023	lncRNA
ENSG0000(	ZBED6	6863.307	0.042316	0.256318	0.42023	protein_coding
ENSG0000(	NGDN	401.1111	-0.05567	0.256396	0.420336	protein_coding
ENSG0000(	PEG10	91.65444	-0.2226	0.256415	0.420346	protein_coding
ENSG0000(	LINC00477	37.17811	0.106892	0.256458	0.420394	lncRNA
ENSG0000(	ENSG0000(	486.4786	-0.06233	0.256477	0.420403	lncRNA
ENSG0000(	C1orf158	351.184	0.04816	0.256497	0.420413	protein_coding

ENSG0000(	ALKBH6	577.9724	-0.04758	0.256523	0.420434	protein_coding
ENSG0000(	RPH3A	206.2875	-0.09065	0.256565	0.420481	protein_coding
ENSG0000(	DNAJA4-DT	21.19606	-0.17157	0.256647	0.420594	lncRNA
ENSG0000(	LINC02593	199.5337	0.160679	0.256681	0.420627	lncRNA
ENSG0000(	E2F8	220.5118	-0.15877	0.256716	0.420664	protein_coding
ENSG0000(	ZPLD1	71.23249	-0.106	0.256879	0.420908	protein_coding
ENSG0000(	BSPRY	227.1155	0.103151	0.256911	0.420938	protein_coding
ENSG0000(	RREB1	6932.01	0.054854	0.257011	0.42108	protein_coding
ENSG0000(	ENSG0000(	8.941373	0.302499	0.257058	0.421132	lncRNA
ENSG0000(	TRAP1	771.9842	-0.06206	0.257069	0.421132	protein_coding
ENSG0000(	PIFO	11.6893	0.322163	0.257185	0.4213	protein_coding
ENSG0000(	NMRAL2P	114.6822	0.208942	0.257201	0.421305	transcribed_unprocessed_pseudo
ENSG0000(	DPM3	60.60245	-0.10513	0.257235	0.421339	protein_coding
ENSG0000(	PCSK1N	46.9193	0.160254	0.257393	0.421576	protein_coding
ENSG0000(	UTP4	446.279	0.097901	0.257413	0.421587	protein_coding
ENSG0000(	ENSG0000(	390.3325	0.049635	0.257487	0.421642	lncRNA
ENSG0000(	ENSG0000(	19.4198	0.17971	0.25748	0.421642	lncRNA
ENSG0000(	KIZ-AS1	227.007	0.082323	0.257472	0.421642	lncRNA
ENSG0000(	ENSG0000(	14.80662	0.229545	0.257519	0.421672	lncRNA
ENSG0000(	ENSG0000(	69.89573	-0.12627	0.257557	0.421712	lncRNA
ENSG0000(	ENSG0000(	85.75016	-0.16855	0.257575	0.421719	processed_pseudogene
ENSG0000(	ENSG0000(	175.636	0.078929	0.257592	0.421726	lncRNA
ENSG0000(	ENSG0000(	55.91395	-0.09135	0.257614	0.421739	lncRNA
ENSG0000(	ENKUR	86.05113	0.247893	0.257685	0.421834	protein_coding
ENSG0000(	ENSG0000(	41.67546	0.102283	0.257708	0.421849	lncRNA
ENSG0000(	TPTE2P6	85.42573	-0.11551	0.25788	0.42211	transcribed_unprocessed_pseudo
ENSG0000(	ASB18	416.5687	-0.04534	0.257966	0.422167	protein_coding
ENSG0000(	ENSG0000(	10.80906	0.374336	0.257939	0.422167	lncRNA
ENSG0000(	MTND5P11	28.23248	-0.34876	0.257976	0.422167	processed_pseudogene
ENSG0000(	PDIA3	4842.647	-0.05886	0.257979	0.422167	protein_coding
ENSG0000(	SNORD21	17.0274	0.27282	0.257982	0.422167	snoRNA
ENSG0000(	FGF12	217.4546	-0.07529	0.258053	0.422243	protein_coding
ENSG0000(	KLHL30	137.8954	-0.08341	0.258055	0.422243	protein_coding
ENSG0000(	A2MP1	21.10622	0.352137	0.258078	0.422258	transcribed_unprocessed_pseudo
ENSG0000(	ENSG0000(	11.28964	-0.28217	0.258118	0.422293	protein_coding
ENSG0000(	NKD2	67.35008	-0.15752	0.258139	0.422293	protein_coding
ENSG0000(	SUPT16H	1610.217	-0.04399	0.258129	0.422293	protein_coding
ENSG0000(	RNU2-70P	17.0532	-0.23969	0.258198	0.422368	snRNA
ENSG0000(	TP63	104.3109	-0.10483	0.25828	0.42248	protein_coding
ENSG0000(	SNORD62A	46.712	-0.12211	0.258409	0.422668	snoRNA
ENSG0000(	MRPS16	1128.615	-0.04063	0.258454	0.42272	protein_coding
ENSG0000(	ENSG0000(	248.1916	0.062699	0.258495	0.422765	TEC
ENSG0000(	SENP3	476.3428	0.079733	0.258568	0.422863	protein_coding
ENSG0000(	ENSG0000(	20.89239	0.158574	0.258612	0.422912	processed_pseudogene
ENSG0000(	FGF11	195.7104	-0.09476	0.25876	0.423089	protein_coding
ENSG0000(	GRAMD4P7	17.48444	0.222192	0.258756	0.423089	processed_pseudogene
ENSG0000(	HELLS	1653.866	0.123846	0.258757	0.423089	protein_coding

ENSG0000(	MICAL2	4336.888	-0.12212	0.258807	0.423143	protein_coding
ENSG0000(	NPIPB2	251.7994	-0.11746	0.258836	0.423169	protein_coding
ENSG0000(	GJD4	24.69229	0.150547	0.258932	0.423304	protein_coding
ENSG0000(	ENSG0000(	11.09822	0.23328	0.258959	0.423327	lncRNA
ENSG0000(	ENSG0000(	125.8094	0.069596	0.258977	0.423334	lncRNA
ENSG0000(	ZKSCAN7-A	10.26102	-0.23791	0.258993	0.423337	lncRNA
ENSG0000(	NPIPA9	524.0495	-0.08093	0.259029	0.423375	protein_coding
ENSG0000(	ENSG0000(	3975.87	0.079446	0.259073	0.423414	TEC
ENSG0000(	ZBTB11	1611.937	-0.04498	0.25908	0.423414	protein_coding
ENSG0000(	ENSG0000(	44.34946	-0.14328	0.259108	0.423416	lncRNA
ENSG0000(	KIF23	412.3122	0.070218	0.259108	0.423416	protein_coding
ENSG0000(	DEF6	944.3184	0.177085	0.259153	0.423467	protein_coding
ENSG0000(	ENSG0000(	151.0247	0.070649	0.259215	0.423535	lncRNA
ENSG0000(	PTGER4P2-	175.7951	0.095518	0.259221	0.423535	lncRNA
ENSG0000(	CD3D	194.5983	-0.19876	0.25925	0.42356	protein_coding
ENSG0000(	C5orf63	599.9538	-0.06548	0.259367	0.423729	protein_coding
ENSG0000(	ZFP14	1798.967	-0.04609	0.259397	0.423756	protein_coding
ENSG0000(	OTUD1	334.3386	-0.08886	0.259458	0.423833	protein_coding
ENSG0000(	ENSG0000(	8.238197	-0.37883	0.259546	0.423956	lncRNA
ENSG0000(	ENSG0000(	116.8013	0.084486	0.259587	0.423979	lncRNA
ENSG0000(	GOLGA8O	298.3886	0.318688	0.259587	0.423979	protein_coding
ENSG0000(	MAMDC2-/	161.4048	0.089765	0.259676	0.424102	lncRNA
ENSG0000(	ENSG0000(	40.37122	-0.12892	0.259761	0.424219	TEC
ENSG0000(	CCNB1IP1	631.9455	-0.04114	0.25991	0.424376	protein_coding
ENSG0000(	CLEC17A	619.7916	-0.22632	0.259895	0.424376	protein_coding
ENSG0000(	ENSG0000(	262.7641	-0.0667	0.259886	0.424376	lncRNA
ENSG0000(	SRSF4	5018.991	0.028177	0.259911	0.424376	protein_coding
ENSG0000(	SMPD4BP	906.3359	0.052883	0.259963	0.424439	transcribed_processed_pseudoge
ENSG0000(	AARD	319.826	0.054205	0.260078	0.424564	protein_coding
ENSG0000(	ENSG0000(	59.29515	0.117271	0.260064	0.424564	lncRNA
ENSG0000(	SNX18P10	10.0531	-0.31562	0.26008	0.424564	processed_pseudogene
ENSG0000(	CTSV	458.4143	0.061133	0.260147	0.424651	protein_coding
ENSG0000(	ENSG0000(	19.785	0.181412	0.260208	0.424729	lncRNA
ENSG0000(	ENSG0000(	9.335995	0.223052	0.260254	0.424781	lncRNA
ENSG0000(	ZNF536	286.7974	0.131372	0.260348	0.424913	protein_coding
ENSG0000(	MEF2D	1827.366	-0.05779	0.260364	0.424917	protein_coding
ENSG0000(	ENSG0000(	20.81771	-0.17508	0.26043	0.424988	lncRNA
ENSG0000(	SOCS2-AS1	600.9984	0.0509	0.260435	0.424988	lncRNA
ENSG0000(	ENSG0000(	78.17788	-0.08699	0.260452	0.424994	lncRNA
ENSG0000(	SNORA58	21.48726	0.149786	0.260498	0.425048	snoRNA
ENSG0000(	ENSG0000(	81.79819	0.109464	0.260526	0.425065	lncRNA
ENSG0000(	PPIC	339.7564	-0.11946	0.260536	0.425065	protein_coding
ENSG0000(	KRAS	2121.294	-0.06014	0.260624	0.425166	protein_coding
ENSG0000(	MTCP1	77.10673	0.118222	0.260617	0.425166	protein_coding
ENSG0000(	NUB1	2137.178	0.059088	0.260666	0.425212	protein_coding
ENSG0000(	DHX29	772.6577	-0.0805	0.260851	0.425492	protein_coding
ENSG0000(	TMEM258	316.2681	-0.08374	0.260883	0.425521	protein_coding

ENSG0000(	LTB4R	562.8988	0.089649	0.260976	0.425651	protein_coding
ENSG0000(	ENSG0000(	2997.018	-0.06805	0.260998	0.425666	lncRNA
ENSG0000(	TTLL2	22.72325	0.177876	0.261126	0.425852	protein_coding
ENSG0000(	SCN4A	45.55664	-0.22308	0.261164	0.425875	protein_coding
ENSG0000(	UBE2D3-AS	40.688	-0.14448	0.261167	0.425875	lncRNA
ENSG0000(	RN7SL664P	14.82714	0.200264	0.261207	0.425917	misc_RNA
ENSG0000(	HOXC9	46.62708	0.144722	0.261296	0.426026	protein_coding
ENSG0000(	RANBP3	1489.709	-0.03776	0.2613	0.426026	protein_coding
ENSG0000(	DFFBP1	35.69256	-0.12915	0.261334	0.426039	processed_pseudogene
ENSG0000(	NT5DC3	1015.844	0.056702	0.261336	0.426039	protein_coding
ENSG0000(	ARHGEF6	1740.155	0.130481	0.261421	0.426156	protein_coding
ENSG0000(	ENSG0000(	86.30202	0.099178	0.261454	0.426189	lncRNA
ENSG0000(	ENSG0000(	33.07957	-0.16287	0.261581	0.426373	lncRNA
ENSG0000(	PAFAH1B2	1952.751	-0.03543	0.261636	0.426417	protein_coding
ENSG0000(	RNU6-377F	14.38061	-0.15121	0.261648	0.426417	snRNA
ENSG0000(	SORD	838.8705	0.082758	0.26163	0.426417	protein_coding
ENSG0000(	TNFSF12-TI	24.52069	1.28442	0.261685	0.426454	protein_coding
ENSG0000(	LINC01492	339.962	-0.07166	0.261754	0.426545	lncRNA
ENSG0000(	ENSG0000(	34.59653	0.156783	0.261771	0.42655	lncRNA
ENSG0000(	LAIR2	13.73234	0.3492	0.261818	0.426605	protein_coding
ENSG0000(	TRNAU1AP	723.854	0.050984	0.261902	0.42672	protein_coding
ENSG0000(	ASH1L-IT1	68.62241	0.100549	0.261961	0.426794	lncRNA
ENSG0000(	ENSG0000(	37.64528	-0.12139	0.262012	0.426855	transcribed_unprocessed_pseudo
ENSG0000(	CLN3	2755.684	-0.04049	0.262028	0.426859	protein_coding
ENSG0000(	HERC2P5	1192.567	0.083082	0.262105	0.426941	transcribed_unprocessed_pseudo
ENSG0000(	MAB21L2	221.4808	-0.11493	0.262103	0.426941	protein_coding
ENSG0000(	UVSSA	3785.214	0.077134	0.262156	0.427001	protein_coding
ENSG0000(	CCNB2	149.9955	-0.16237	0.262196	0.427044	protein_coding
ENSG0000(	B3GALT1-A	13.53453	0.201117	0.262234	0.427062	lncRNA
ENSG0000(	ZNF286A	776.2172	0.104023	0.262222	0.427062	protein_coding
ENSG0000(	ENSG0000(	78.93589	-0.18468	0.262281	0.427095	lncRNA
ENSG0000(	LNCARSR	310.0715	-0.08857	0.262281	0.427095	lncRNA
ENSG0000(	EFCAB7	200.6103	-0.12522	0.262323	0.427118	protein_coding
ENSG0000(	MICD	58.57141	-0.14634	0.262314	0.427118	unprocessed_pseudogene
ENSG0000(	ZC3HAV1	3959.155	0.075673	0.262359	0.427155	protein_coding
ENSG0000(	SCD	1296.593	0.174294	0.262374	0.427157	protein_coding
ENSG0000(	LINC02419	115.3783	-0.07525	0.262596	0.427486	lncRNA
ENSG0000(	RN7SL96P	207.315	-0.06843	0.262603	0.427486	misc_RNA
ENSG0000(	TPTE	20.92178	0.181795	0.262717	0.42765	protein_coding
ENSG0000(	VENTXP4	127.1971	0.067455	0.262746	0.427654	processed_pseudogene
ENSG0000(	WBP11P2	367.6284	-0.05497	0.262736	0.427654	processed_pseudogene
ENSG0000(	LRRC17	38.19115	0.16887	0.262766	0.427663	protein_coding
ENSG0000(	PIGQ	783.2555	-0.12285	0.262798	0.427694	protein_coding
ENSG0000(	LINC02629	17.84945	-0.17616	0.262824	0.427714	lncRNA
ENSG0000(	KLHL34	81.83752	-0.17881	0.262858	0.427746	protein_coding
ENSG0000(	PPP2R1B	2510.004	-0.12809	0.262894	0.427783	protein_coding
ENSG0000(	SMO	235.016	0.098357	0.262907	0.427783	protein_coding

ENSG0000(	CCDC62	232.8301	-0.08551	0.262943	0.427818	protein_coding
ENSG0000(	TLR6	1437.42	0.217761	0.263017	0.427918	protein_coding
ENSG0000(	EIF2AK2	4135.348	0.110661	0.263268	0.428303	protein_coding
ENSG0000(	ARIH2OS	195.9621	-0.06604	0.263322	0.428369	lncRNA
ENSG0000(	DPY19L2P3	189.4597	0.078046	0.263356	0.428381	transcribed_unprocessed_pseudo
ENSG0000(	KRT15	399.6508	0.098011	0.263353	0.428381	protein_coding
ENSG0000(	RAB40B	507.785	-0.10761	0.263407	0.42844	protein_coding
ENSG0000(	DCAF13P3	72.45116	-0.10312	0.263441	0.428475	processed_pseudogene
ENSG0000(	ENSG0000(	141.4184	0.074705	0.26352	0.42858	lncRNA
ENSG0000(	MAPRE2	1108.472	-0.06891	0.263674	0.428783	protein_coding
ENSG0000(	MT1X	449.8342	-0.27606	0.263665	0.428783	protein_coding
ENSG0000(	TRPT1	292.4131	-0.09674	0.263685	0.428783	protein_coding
ENSG0000(	LINC02351	18.85677	0.376946	0.263867	0.429056	lncRNA
ENSG0000(	ENSG0000(	13.82255	0.436655	0.263978	0.429115	lncRNA
ENSG0000(	HSPE1P28	44.51799	0.118386	0.263936	0.429115	processed_pseudogene
ENSG0000(	PLK2	446.6368	0.148461	0.263948	0.429115	protein_coding
ENSG0000(	SLC38A2	3928.485	0.078948	0.26396	0.429115	protein_coding
ENSG0000(	YES1	1752.41	0.072481	0.263985	0.429115	protein_coding
ENSG0000(	ZNF398	895.7729	-0.04358	0.263962	0.429115	protein_coding
ENSG0000(	PPIAP41	190.4764	-0.06891	0.264063	0.42922	processed_pseudogene
ENSG0000(	ENSG0000(	11.11917	0.214054	0.264112	0.429278	lncRNA
ENSG0000(	SDC4	872.4924	-0.13491	0.264187	0.429377	protein_coding
ENSG0000(	TNS2	1641.812	-0.08713	0.264271	0.429492	protein_coding
ENSG0000(	LINC02487	13.35722	0.226759	0.264334	0.429572	lncRNA
ENSG0000(	ACTN4	5962.502	-0.12233	0.264372	0.429612	protein_coding
ENSG0000(	ENPP7	247.7248	-0.31732	0.264435	0.429692	protein_coding
ENSG0000(	ENSG0000(	38.57626	0.109359	0.264501	0.429776	lncRNA
ENSG0000(	ENSG0000(	35.14658	0.121682	0.264568	0.429863	lncRNA
ENSG0000(	ENSG0000(	63.62669	0.094238	0.264642	0.429962	miRNA
ENSG0000(	CPE	139.914	-0.17321	0.264748	0.430091	protein_coding
ENSG0000(	ENSG0000(	96.53785	0.132828	0.264749	0.430091	lncRNA
ENSG0000(	TTC33	503.5518	0.055523	0.264884	0.430288	protein_coding
ENSG0000(	OR10K2	70.75818	0.089994	0.264903	0.430296	protein_coding
ENSG0000(	PROSER2-A	75.24592	-0.08152	0.264936	0.430329	lncRNA
ENSG0000(	C8orf88	22.41601	0.169295	0.264995	0.430402	protein_coding
ENSG0000(	CEL	60.11573	-0.21514	0.265039	0.430451	protein_coding
ENSG0000(	ENSG0000(	40.58476	0.169396	0.265179	0.430612	lncRNA
ENSG0000(	GTF2H2C	1656.527	0.089853	0.265154	0.430612	protein_coding
ENSG0000(	ZNF502	218.1158	0.089453	0.265175	0.430612	protein_coding
ENSG0000(	MAGEH1	101.3592	0.158817	0.265241	0.430691	protein_coding
ENSG0000(	ENSG0000(	740.1418	0.056759	0.265314	0.430763	lncRNA
ENSG0000(	ENSG0000(	24.11041	0.164841	0.265299	0.430763	lncRNA
ENSG0000(	UNC5B	571.6673	-0.1047	0.265327	0.430763	protein_coding
ENSG0000(	LINC02682	41.24951	0.182612	0.265359	0.430794	lncRNA
ENSG0000(	STAT5B	2345.699	0.065924	0.265379	0.430803	protein_coding
ENSG0000(	LILRB5	675.4727	-0.10843	0.265402	0.430819	protein_coding
ENSG0000(	ENSG0000(	20.74324	0.17862	0.265432	0.430844	lncRNA

ENSG0000(	PDPK1	3372.227	-0.0558	0.265479	0.4309	protein_coding
ENSG0000(	IGHMBP2	1301.229	-0.07408	0.265497	0.430906	protein_coding
ENSG0000(	ENSG0000(	10.38211	-0.19617	0.265517	0.430916	lncRNA
ENSG0000(	ENSG0000(	28.82442	0.327249	0.265699	0.431189	lncRNA
ENSG0000(	CCR7	982.2182	0.379823	0.265727	0.43119	protein_coding
ENSG0000(	NAT9	487.8907	0.057909	0.265714	0.43119	protein_coding
ENSG0000(	CYSTM1	576.2486	-0.13575	0.265882	0.431354	protein_coding
ENSG0000(	NDUFB9	2326.861	-0.05471	0.265873	0.431354	protein_coding
ENSG0000(	NTNG1	79.26988	-0.2369	0.26586	0.431354	protein_coding
ENSG0000(	WDR53	113.7174	0.100133	0.265845	0.431354	protein_coding
ENSG0000(	TWF2	303.2218	-0.08007	0.265933	0.431414	protein_coding
ENSG0000(	RNU4-78P	36.32505	-0.15382	0.265981	0.431469	snRNA
ENSG0000(	ATL1	2279.724	0.042477	0.266067	0.431542	protein_coding
ENSG0000(	BCL2L12	73.14304	0.162049	0.266046	0.431542	protein_coding
ENSG0000(	RNU6-90P	33.66181	0.155159	0.266056	0.431542	snRNA
ENSG0000(	ENSG0000(	24.66175	0.178427	0.266221	0.43177	lncRNA
ENSG0000(	ZNF669	488.5829	-0.05561	0.266276	0.431837	protein_coding
ENSG0000(	ENSG0000(	21.26162	0.161305	0.266335	0.431909	lncRNA
ENSG0000(	ABHD8	58.70928	0.190197	0.266403	0.431954	protein_coding
ENSG0000(	ENSG0000(	63.48041	0.097244	0.266389	0.431954	lncRNA
ENSG0000(	KRT2	11.89231	0.364147	0.266391	0.431954	protein_coding
ENSG0000(	LPAR3	107.3315	0.076225	0.266452	0.432011	protein_coding
ENSG0000(	ACSM6	47.5301	0.11924	0.266487	0.432045	protein_coding
ENSG0000(	CD177	97.50837	0.603882	0.266574	0.432142	protein_coding
ENSG0000(	USP32P3	2015.435	0.057106	0.266568	0.432142	transcribed_unprocessed_pseudo
ENSG0000(	ENSG0000(	57.78608	-0.1038	0.26662	0.432194	lncRNA
ENSG0000(	ADAMTS7P	21.68699	0.40074	0.266659	0.432203	transcribed_unprocessed_pseudo
ENSG0000(	ENSG0000(	3064.357	0.076959	0.266666	0.432203	transcribed_unprocessed_pseudo
ENSG0000(	ENSG0000(	86.79047	0.076841	0.266666	0.432203	lncRNA
ENSG0000(	BLOC1S5	489.5546	-0.05764	0.266717	0.432218	protein_coding
ENSG0000(	ENSG0000(	48.78604	0.108717	0.266712	0.432218	lncRNA
ENSG0000(	WDR70	930.5858	0.080212	0.266691	0.432218	protein_coding
ENSG0000(	FAM126B	3123.422	-0.06914	0.266734	0.432223	protein_coding
ENSG0000(	CDC123	311.2996	-0.09228	0.266841	0.432374	protein_coding
ENSG0000(	ABCC12	60.45037	0.164369	0.266863	0.432388	protein_coding
ENSG0000(	ENSG0000(	95.95154	-0.11127	0.266915	0.432405	lncRNA
ENSG0000(	ENSG0000(	254.0736	0.089601	0.266891	0.432405	lncRNA
ENSG0000(	RN7SL444P	94.34589	0.111466	0.266904	0.432405	misc_RNA
ENSG0000(	ENSG0000(	13.1677	-0.17907	0.266942	0.432419	lncRNA
ENSG0000(	OR8G1	96.18585	0.106567	0.266951	0.432419	protein_coding
ENSG0000(	KCTD9P4	55.68468	-0.15155	0.266998	0.43245	processed_pseudogene
ENSG0000(	MVD	413.2611	-0.07822	0.266997	0.43245	protein_coding
ENSG0000(	ENSG0000(	49.58523	0.109288	0.26704	0.432495	lncRNA
ENSG0000(	LPAL2	376.2457	0.10249	0.26708	0.432495	transcribed_unprocessed_pseudo
ENSG0000(	RNF157-AS	244.3018	-0.08175	0.26708	0.432495	lncRNA
ENSG0000(	SLC7A2-IT1	69.34211	0.103501	0.267064	0.432495	TEC
ENSG0000(	ENSG0000(	142.4778	-0.06555	0.267102	0.432508	lncRNA

ENSG0000(	PHF20L1	3522.799	0.038821	0.267117	0.43251	protein_coding
ENSG0000(	ASPM	1300.801	0.142333	0.267138	0.432522	protein_coding
ENSG0000(	LINC01287	197.2953	0.06871	0.267151	0.432522	lncRNA
ENSG0000(	ENSG0000(	35.66793	-0.17713	0.267192	0.432546	lncRNA
ENSG0000(	LINC00891	1722.078	0.066051	0.267194	0.432546	lncRNA
ENSG0000(	XRN2	974.7587	-0.0705	0.267233	0.432588	protein_coding
ENSG0000(	RN7SL728F	9.558623	0.233105	0.267257	0.432604	misc_RNA
ENSG0000(	ENSG0000(	95.39091	-0.07504	0.267322	0.432665	lncRNA
ENSG0000(	MIR941-1	67.61985	-0.17888	0.267322	0.432665	miRNA
ENSG0000(	ENSG0000(	11.48772	-0.19036	0.267384	0.432703	transcribed_processed_pseudoge
ENSG0000(	ENSG0000(	61.09505	0.139575	0.267387	0.432703	processed_pseudogene
ENSG0000(	KCNAB3	257.9874	-0.07154	0.267379	0.432703	protein_coding
ENSG0000(	SSBP1	1093.548	-0.03871	0.267418	0.432731	protein_coding
ENSG0000(	FAM169A	138.863	0.190723	0.267507	0.432833	protein_coding
ENSG0000(	ZDHHC5	2137.825	-0.06774	0.267508	0.432833	protein_coding
ENSG0000(	ENSG0000(	287.2298	0.046879	0.267591	0.432944	TEC
ENSG0000(	HELZ	5689.056	-0.04315	0.267679	0.433064	protein_coding
ENSG0000(	COLCA1	3232.712	-0.06063	0.267719	0.433106	lncRNA
ENSG0000(	KLHDC10	1554.071	-0.06148	0.267742	0.433122	protein_coding
ENSG0000(	CLCN3	2421.316	-0.06591	0.267766	0.433138	protein_coding
ENSG0000(	ENSG0000(	79.00533	0.086316	0.267898	0.433285	lncRNA
ENSG0000(	ENSG0000(	89.64176	0.080347	0.267871	0.433285	lncRNA
ENSG0000(	LINC02174	17.74896	0.145952	0.267896	0.433285	lncRNA
ENSG0000(	ENSG0000(	34.98134	0.13685	0.267941	0.433332	TEC
ENSG0000(	ENSG0000(	59.00504	0.109857	0.267976	0.433367	lncRNA
ENSG0000(	ENSG0000(	18.1651	0.213022	0.268021	0.433418	lncRNA
ENSG0000(	ENSG0000(	189.4449	0.062707	0.268215	0.433709	lncRNA
ENSG0000(	CCAT2	10.21714	0.265097	0.268251	0.43372	lncRNA
ENSG0000(	ENSG0000(	492.4044	-0.0524	0.268263	0.43372	lncRNA
ENSG0000(	LINC02021	827.9781	0.078143	0.268246	0.43372	lncRNA
ENSG0000(	GCM1	134.1576	0.078947	0.268283	0.43373	protein_coding
ENSG0000(	ENSG0000(	26.17078	0.267806	0.26833	0.433783	lncRNA
ENSG0000(	ENSG0000(	130.1898	-0.06094	0.268355	0.4338	lncRNA
ENSG0000(	ENSG0000(	385.2215	0.115906	0.268368	0.4338	lncRNA
ENSG0000(	RPL7AP52	77.98187	-0.25905	0.268392	0.433817	processed_pseudogene
ENSG0000(	ENSG0000(	62.57218	0.122606	0.268448	0.433885	TEC
ENSG0000(	EIF4BP6	19.33884	-0.24474	0.268472	0.433902	processed_pseudogene
ENSG0000(	BMS1P23	1921.103	0.064779	0.268582	0.434035	transcribed_unprocessed_pseudo
ENSG0000(	SOCS6	1472.164	-0.16667	0.268579	0.434035	protein_coding
ENSG0000(	SMIM43	14.61525	0.230243	0.268598	0.434038	protein_coding
ENSG0000(	ENSG0000(	116.812	-0.09421	0.268658	0.434114	lncRNA
ENSG0000(	ENSG0000(	27.01781	-0.31115	0.268701	0.434161	lncRNA
ENSG0000(	SSR2	2057.903	0.060166	0.268728	0.434182	protein_coding
ENSG0000(	ENSG0000(	20.11388	0.153431	0.268745	0.434186	lncRNA
ENSG0000(	LINC01252	241.765	0.134566	0.268831	0.434304	lncRNA
ENSG0000(	ENSG0000(	14.94326	0.192645	0.268861	0.43433	lncRNA
ENSG0000(	ENSG0000(	79.50597	-0.09635	0.268981	0.434479	lncRNA

ENSG0000(	LINC01258	31.6495	0.131085	0.268981	0.434479	lncRNA
ENSG0000(	ACACA	1843.886	0.071607	0.269012	0.434485	protein_coding
ENSG0000(	ENSG0000(	98.54084	0.093948	0.268999	0.434485	TEC
ENSG0000(	RFPL1S	510.8711	0.069172	0.269058	0.434536	lncRNA
ENSG0000(	CYP7A1	13.71915	0.191964	0.269077	0.434545	protein_coding
ENSG0000(	RN7SL1	278095.6	0.156982	0.269103	0.434564	misc_RNA
ENSG0000(	ENSG0000(	54.48305	0.09564	0.269162	0.434637	processed_pseudogene
ENSG0000(	ENSG0000(	12.46859	0.565655	0.269187	0.434655	protein_coding
ENSG0000(	OAF	1285.964	-0.11283	0.26925	0.434713	protein_coding
ENSG0000(	RASL10B	109.5506	0.09448	0.269261	0.434713	protein_coding
ENSG0000(	TMED2	2411.611	-0.07462	0.269264	0.434713	protein_coding
ENSG0000(	AGAP12P	190.2651	-0.35104	0.269344	0.434814	transcribed_unprocessed_pseudo
ENSG0000(	PAK2	2728.413	-0.05653	0.269368	0.434814	protein_coding
ENSG0000(	RCSD1	2559.563	0.149084	0.269367	0.434814	protein_coding
ENSG0000(	C11orf94	15.41937	-0.19701	0.26949	0.4349	protein_coding
ENSG0000(	ENSG0000(	41.57836	0.114549	0.269454	0.4349	lncRNA
ENSG0000(	FAM71D	19.55058	0.207144	0.269477	0.4349	protein_coding
ENSG0000(	TFG	4803.233	-0.10377	0.269469	0.4349	protein_coding
ENSG0000(	ZNF320	2383.455	-0.05582	0.26948	0.4349	protein_coding
ENSG0000(	ENSG0000(	80.1202	0.125767	0.269516	0.434913	lncRNA
ENSG0000(	INGX	197.6866	-0.07086	0.269525	0.434913	transcribed_processed_pseudoge
ENSG0000(	ARSD	1791.887	-0.05729	0.269754	0.435178	protein_coding
ENSG0000(	FBXW11P1	29.84136	0.183078	0.269746	0.435178	processed_pseudogene
ENSG0000(	POLR2J4	784.8834	-0.04319	0.269741	0.435178	lncRNA
ENSG0000(	RNU6-759F	48.54508	-0.11727	0.269759	0.435178	snRNA
ENSG0000(	TTC26	288.8288	-0.06872	0.269719	0.435178	protein_coding
ENSG0000(	ARID3B	936.9873	-0.04231	0.269777	0.435185	protein_coding
ENSG0000(	ENSG0000(	842.6923	0.045625	0.26989	0.435323	lncRNA
ENSG0000(	RNY1P12	11.03383	0.206571	0.26989	0.435323	misc_RNA
ENSG0000(	WDR31	983.4543	-0.0355	0.269924	0.435355	protein_coding
ENSG0000(	NPIPA3	34.82577	-0.20531	0.270019	0.435487	protein_coding
ENSG0000(	ENSG0000(	349.5831	0.063194	0.270053	0.435496	lncRNA
ENSG0000(	STAC	22.74773	0.324446	0.27004	0.435496	protein_coding
ENSG0000(	ENSG0000(	52.0046	-0.1047	0.270148	0.435626	lncRNA
ENSG0000(	ENSG0000(	19.05745	0.158418	0.270171	0.435628	lncRNA
ENSG0000(	ZNF503	157.3652	0.115339	0.270176	0.435628	protein_coding
ENSG0000(	ENSG0000(	23.74627	-0.25766	0.270311	0.435806	lncRNA
ENSG0000(	RPS27AP12	66.9264	-0.11167	0.270314	0.435806	processed_pseudogene
ENSG0000(	ENSG0000(	128.9719	-0.07216	0.270345	0.435834	lncRNA
ENSG0000(	CD226	1312.593	0.140129	0.270398	0.435897	protein_coding
ENSG0000(	HSPD1P6	25.07846	0.138214	0.270433	0.43593	transcribed_processed_pseudoge
ENSG0000(	CCDC174	673.1499	-0.04269	0.270511	0.436012	protein_coding
ENSG0000(	MYCBP	273.8051	-0.10337	0.270504	0.436012	protein_coding
ENSG0000(	ENSG0000(	65.29158	0.087371	0.270614	0.436155	lncRNA
ENSG0000(	CCNB3	23.74367	0.313115	0.270648	0.436165	protein_coding
ENSG0000(	ENSG0000(	137.0491	0.089349	0.270646	0.436165	TEC
ENSG0000(	MTMR9	1445.211	0.082458	0.270797	0.436384	protein_coding

ENSG0000(	RIPOR2	4301.737	0.261353	0.270827	0.43641	protein_coding
ENSG0000(	GGT3P	164.0515	-0.5065	0.270853	0.43643	transcribed_unprocessed_pseudo
ENSG0000(	CCT6P3	516.781	-0.08404	0.270996	0.436615	transcribed_unprocessed_pseudo
ENSG0000(	ENSG0000(	12.15391	-0.20401	0.270993	0.436615	lncRNA
ENSG0000(	ENSG0000(	63.87387	-0.13341	0.271046	0.436673	protein_coding
ENSG0000(	MMP25-AS	1099.243	-0.07045	0.271127	0.436782	lncRNA
ENSG0000(	ENSG0000(	1083.261	-0.04441	0.271241	0.43692	TEC
ENSG0000(	RN7SL797F	18.40306	0.17605	0.271238	0.43692	misc_RNA
ENSG0000(	ENSG0000(	10.13449	0.228391	0.271259	0.436926	lncRNA
ENSG0000(	ENSG0000(	85.82412	-0.08456	0.2713	0.43697	lncRNA
ENSG0000(	HAPLN4	1313.712	-0.36974	0.271327	0.436991	protein_coding
ENSG0000(	LINC01513	294.8566	0.056781	0.271456	0.437176	lncRNA
ENSG0000(	DIPK2A	457.3934	0.099705	0.271515	0.437249	protein_coding
ENSG0000(	ANKRD18C	21.15345	0.228433	0.271558	0.437275	unprocessed_pseudogene
ENSG0000(	TRBJ1-2	19.90946	0.30873	0.271544	0.437275	TR_J_gene
ENSG0000(	CNR1	443.8837	-0.34006	0.271582	0.437289	protein_coding
ENSG0000(	GMFB	1322.244	-0.05574	0.271595	0.437289	protein_coding
ENSG0000(	ENSG0000(	55.51636	0.237539	0.271672	0.437344	lncRNA
ENSG0000(	ENSG0000(	294.8548	-0.0827	0.27167	0.437344	lncRNA
ENSG0000(	OR8R1P	12.94009	0.270858	0.271684	0.437344	unprocessed_pseudogene
ENSG0000(	SLC4A5	528.8855	0.051998	0.27166	0.437344	protein_coding
ENSG0000(	AGPAT5	664.9037	-0.11098	0.271739	0.437409	protein_coding
ENSG0000(	HNRNPU	21911.09	-0.02948	0.27178	0.437452	protein_coding
ENSG0000(	HMGB1P1	46.64123	-0.09725	0.27186	0.437515	protein_coding
ENSG0000(	LINC02259	9.128408	0.291086	0.271857	0.437515	lncRNA
ENSG0000(	RPL22P1	190.2256	-0.20456	0.271845	0.437515	processed_pseudogene
ENSG0000(	MXRA7	1511.211	0.090901	0.271952	0.43761	protein_coding
ENSG0000(	PFDN4	81.72807	-0.10137	0.271937	0.43761	protein_coding
ENSG0000(	PLAC8L1	48.97926	0.118355	0.271961	0.43761	protein_coding
ENSG0000(	CXorf38	1487.362	0.038987	0.272177	0.437935	protein_coding
ENSG0000(	ENSG0000(	25.44924	0.124366	0.272195	0.437942	lncRNA
ENSG0000(	DVL3	1895.092	-0.05052	0.272221	0.437962	protein_coding
ENSG0000(	ENSG0000(	87.1179	0.108985	0.272423	0.438263	lncRNA
ENSG0000(	ENSG0000(	165.3655	0.102533	0.272447	0.43828	processed_pseudogene
ENSG0000(	ADAM32	4850.973	0.046851	0.272535	0.438379	protein_coding
ENSG0000(	MEAF6	1327.869	-0.05215	0.272536	0.438379	protein_coding
ENSG0000(	LINC01551	1239.443	0.091696	0.272613	0.43848	lncRNA
ENSG0000(	ENSG0000(	113.3365	-0.08421	0.272677	0.438516	lncRNA
ENSG0000(	HHIPL2	18.4824	0.274924	0.272665	0.438516	protein_coding
ENSG0000(	PUSL1	101.1317	-0.08743	0.272676	0.438516	protein_coding
ENSG0000(	ENSG0000(	58.85964	0.082618	0.272711	0.438549	lncRNA
ENSG0000(	PARD6A	14.65282	-0.22415	0.272777	0.438632	protein_coding
ENSG0000(	ENSG0000(	18.12307	-0.17853	0.272803	0.438651	processed_pseudogene
ENSG0000(	ENSG0000(	173.5474	0.10217	0.273003	0.438951	lncRNA
ENSG0000(	ENSG0000(	227.822	-0.18181	0.273056	0.438982	lncRNA
ENSG0000(	LINC01515	1375.421	0.090578	0.273038	0.438982	lncRNA
ENSG0000(	PAXBP1-AS	1340.228	0.043822	0.273065	0.438982	lncRNA

ENSG0000(	ENSG0000(	36.1452	0.133247	0.273103	0.439021	lncRNA
ENSG0000(	DEK	3010.169	-0.03973	0.273134	0.439027	protein_coding
ENSG0000(	PLCG1-AS1	620.0579	0.049505	0.273132	0.439027	lncRNA
ENSG0000(	CCDC183	61.40443	-0.23016	0.273248	0.439188	protein_coding
ENSG0000(	TSPAN32	266.8036	0.256468	0.273318	0.439278	protein_coding
ENSG0000(	CFAP74	690.7069	0.054995	0.273347	0.439302	protein_coding
ENSG0000(	ENSG0000(	106.8757	-0.0709	0.273378	0.43933	lncRNA
ENSG0000(	ENSG0000(	16.10855	0.262011	0.273537	0.439518	lncRNA
ENSG0000(	SMARCA2	6681.181	0.068595	0.273531	0.439518	protein_coding
ENSG0000(	TMEM68	703.9074	-0.04248	0.273522	0.439518	protein_coding
ENSG0000(	PRPF38B	2866.564	0.039673	0.273705	0.439764	protein_coding
ENSG0000(	NSUN2	1386.703	-0.066	0.273798	0.439893	protein_coding
ENSG0000(	ENSG0000(	37.45417	0.132212	0.273813	0.439893	lncRNA
ENSG0000(	SREK1IP1	911.8306	-0.0421	0.273843	0.439919	protein_coding
ENSG0000(	RN7SL543F	26.00057	0.13138	0.273883	0.439961	misc_RNA
ENSG0000(	ENSG0000(	245.9771	0.102788	0.273948	0.440021	TEC
ENSG0000(	TYW1B	282.2058	-0.08112	0.273935	0.440021	protein_coding
ENSG0000(	RPS25P3	17.35426	0.174941	0.273981	0.440051	processed_pseudogene
ENSG0000(	CHSY3	60.42798	0.172849	0.274025	0.4401	protein_coding
ENSG0000(	LUZP1	3982.775	-0.11864	0.274081	0.440168	protein_coding
ENSG0000(	ENSG0000(	234.765	-0.06819	0.274139	0.440238	lncRNA
ENSG0000(	LINC02297	13.9851	0.51687	0.274154	0.44024	lncRNA
ENSG0000(	GAPT	310.0689	-0.24561	0.274178	0.440255	protein_coding
ENSG0000(	ENSG0000(	18.65246	-0.21069	0.274229	0.440315	lncRNA
ENSG0000(	KLHL6	3540.362	0.181148	0.274277	0.440369	protein_coding
ENSG0000(	ENSG0000(	20.34429	-0.31805	0.274319	0.440414	lncRNA
ENSG0000(	ZNF282	556.2125	-0.08988	0.274509	0.440697	protein_coding
ENSG0000(	ENSG0000(	67.63059	-0.18285	0.274584	0.44075	lncRNA
ENSG0000(	SLC9A3-AS:	708.5592	0.157898	0.274574	0.44075	lncRNA
ENSG0000(	ZNF431	2930.03	-0.06111	0.274566	0.44075	protein_coding
ENSG0000(	SAMD8	2075.782	0.037425	0.274648	0.440831	protein_coding
ENSG0000(	MKLN1	6323.906	-0.04868	0.274753	0.440977	protein_coding
ENSG0000(	ENSG0000(	44.06862	0.121546	0.274804	0.441036	lncRNA
ENSG0000(	ENSG0000(	20.28209	-0.16164	0.274837	0.441061	lncRNA
ENSG0000(	RN7SL143F	12.35581	-0.21959	0.274847	0.441061	misc_RNA
ENSG0000(	NME1	241.7519	0.098577	0.274875	0.441068	protein_coding
ENSG0000(	RN7SL521F	15.04589	0.165807	0.27488	0.441068	misc_RNA
ENSG0000(	LNX1	467.0662	-0.19446	0.274899	0.441076	protein_coding
ENSG0000(	TIPARP	1063.999	0.101214	0.274975	0.441175	protein_coding
ENSG0000(	DPYSL3	1693.776	0.154876	0.275043	0.441194	protein_coding
ENSG0000(	ENSG0000(	10.2049	0.205084	0.275035	0.441194	processed_pseudogene
ENSG0000(	EWSR1	7837.321	0.021177	0.275034	0.441194	protein_coding
ENSG0000(	RAB33B	551.2068	-0.05481	0.275014	0.441194	protein_coding
ENSG0000(	ENSG0000(	57.64788	0.427136	0.275104	0.44127	protein_coding
ENSG0000(	ENSG0000(	6.75975	0.393873	0.275148	0.441295	lncRNA
ENSG0000(	NEIL2	196.1804	0.116729	0.275134	0.441295	protein_coding
ENSG0000(	APOBEC3G	1336.744	0.097157	0.275191	0.441297	protein_coding

ENSG0000(	CCDC65	16.08485	-0.29522	0.27519	0.441297	protein_coding
ENSG0000(	FAM98B	1146.424	0.050666	0.275175	0.441297	protein_coding
ENSG0000(	LINC02015	39.73212	-0.16519	0.275267	0.441388	lncRNA
ENSG0000(	TBC1D20	2002.252	-0.04461	0.275276	0.441388	protein_coding
ENSG0000(	ENSG0000(	337.1496	-0.12511	0.275297	0.441398	lncRNA
ENSG0000(	ENSG0000(	63.17196	0.104651	0.27531	0.441398	lncRNA
ENSG0000(	ENSG0000(	920.8628	0.083418	0.275386	0.441497	lncRNA
ENSG0000(	FMN2	71.28841	0.131523	0.275422	0.441532	protein_coding
ENSG0000(	PMS2P3	318.7055	-0.10248	0.275466	0.441581	transcribed_unprocessed_pseudo
ENSG0000(	RN7SKP36	66.7587	-0.17315	0.275497	0.441601	misc_RNA
ENSG0000(	SWAP70	2116.009	-0.10733	0.275507	0.441601	protein_coding
ENSG0000(	UNC45B	38.08007	0.129849	0.275559	0.441663	protein_coding
ENSG0000(	MEF2C	1923.631	0.224545	0.275574	0.441664	protein_coding
ENSG0000(	RBMY2EP	24.43691	-0.37472	0.275644	0.441754	transcribed_unprocessed_pseudo
ENSG0000(	ENSG0000(	31.62834	0.168989	0.275665	0.441765	lncRNA
ENSG0000(	ENSG0000(	208.7085	0.065774	0.275716	0.441824	lncRNA
ENSG0000(	SLC39A1	634.7137	-0.0653	0.275735	0.441826	protein_coding
ENSG0000(	TRIM62	291.5451	0.076298	0.275745	0.441826	protein_coding
ENSG0000(	LINC02427	429.3397	-0.04479	0.275793	0.441881	lncRNA
ENSG0000(	PIK3IP1-DT	22.51994	0.164021	0.275932	0.442081	lncRNA
ENSG0000(	PCDHGB9P	65.46321	0.114661	0.275989	0.442149	unprocessed_pseudogene
ENSG0000(	ENSG0000(	302.1219	0.064929	0.276088	0.442286	lncRNA
ENSG0000(	BRWD1-AS	19.99856	-0.18676	0.276129	0.442329	lncRNA
ENSG0000(	GPATCH2	1123.685	0.056638	0.27626	0.442515	protein_coding
ENSG0000(	R3HCC1L	1153.975	0.039129	0.276281	0.442527	protein_coding
ENSG0000(	RPL7L1P2	106.3105	-0.25341	0.276303	0.44254	processed_pseudogene
ENSG0000(	C12orf50	2313.534	0.056828	0.276322	0.442548	protein_coding
ENSG0000(	ENSG0000(	167.8034	0.068901	0.276521	0.442844	lncRNA
ENSG0000(	SYNDIG1	87.5962	-0.14256	0.276705	0.443117	protein_coding
ENSG0000(	IFI27L1	197.8385	0.091914	0.276769	0.443192	protein_coding
ENSG0000(	PKHD1L1	1018.828	-0.14938	0.276781	0.443192	protein_coding
ENSG0000(	MOCS3	500.6035	-0.0432	0.276803	0.443205	protein_coding
ENSG0000(	DISC1FP1	21.23259	0.170257	0.276849	0.443244	lncRNA
ENSG0000(	FOXP4-AS1	12.08117	0.291887	0.276855	0.443244	lncRNA
ENSG0000(	B2M	27372.78	0.105498	0.276909	0.443268	protein_coding
ENSG0000(	ENSG0000(	65.45058	-0.11022	0.27694	0.443268	lncRNA
ENSG0000(	ENSG0000(	1337.746	-0.077	0.276913	0.443268	lncRNA
ENSG0000(	PLPPR4	37.03587	0.197596	0.27693	0.443268	protein_coding
ENSG0000(	SFTPD-AS1	965.0219	0.033265	0.276891	0.443268	lncRNA
ENSG0000(	JMJD4	403.7732	-0.07538	0.276965	0.443285	protein_coding
ENSG0000(	ARHGEF26	678.963	-0.13606	0.276992	0.443286	protein_coding
ENSG0000(	SEMA4B	1668.451	0.091978	0.276994	0.443286	protein_coding
ENSG0000(	HERC1	7995.781	-0.05722	0.277032	0.443325	protein_coding
ENSG0000(	ENSG0000(	64.04548	0.099528	0.277123	0.443415	lncRNA
ENSG0000(	ENSG0000(	135.922	0.068153	0.277131	0.443415	lncRNA
ENSG0000(	ENSG0000(	325.8403	0.07271	0.277107	0.443415	lncRNA
ENSG0000(	ENSG0000(	216.6359	-0.06178	0.277164	0.443446	lncRNA

ENSG0000(	LINC01881	618.7122	0.061833	0.277188	0.443462	transcribed_unprocessed_pseudo
ENSG0000(	ENSG0000(	25.05791	0.229184	0.27726	0.443555	lncRNA
ENSG0000(	MALAT1	1197999	0.066546	0.277432	0.443806	lncRNA
ENSG0000(	ENSG0000(	9.429788	-0.35272	0.277468	0.443842	lncRNA
ENSG0000(	CHD6	2938.652	0.085877	0.277579	0.443996	protein_coding
ENSG0000(	CHRM4	114.8216	-0.232	0.277609	0.444023	protein_coding
ENSG0000(	ENSG0000(	61.14223	-0.19528	0.277655	0.444074	lncRNA
ENSG0000(	FAM149B1	774.2143	0.046934	0.277824	0.44432	protein_coding
ENSG0000(	TGM1	60.20111	-0.13347	0.277841	0.444326	protein_coding
ENSG0000(	ENSG0000(	12.57559	0.223328	0.27788	0.444365	TEC
ENSG0000(	ENSG0000(	221.009	-0.0489	0.277944	0.444444	transcribed_unprocessed_pseudo
ENSG0000(	RNU6-767F	21.08393	0.214484	0.277989	0.444495	snRNA
ENSG0000(	ENSG0000(	73.34223	-0.13069	0.278033	0.444543	processed_pseudogene
ENSG0000(	THOP1	557.2641	-0.07854	0.278049	0.444545	protein_coding
ENSG0000(	ENSG0000(	28.1355	0.145968	0.278108	0.44455	lncRNA
ENSG0000(	HDX	173.5104	0.124053	0.278086	0.44455	protein_coding
ENSG0000(	NOSTRIN	930.1364	-0.14212	0.278073	0.44455	protein_coding
ENSG0000(	SLC25A16	2739.552	-0.068	0.278101	0.44455	protein_coding
ENSG0000(	HLA-DQB2	216.7426	-0.1811	0.278204	0.444663	protein_coding
ENSG0000(	SNORA77B	34.9337	0.161683	0.278207	0.444663	snoRNA
ENSG0000(	ENSG0000(	95.14451	0.084295	0.278255	0.444716	lncRNA
ENSG0000(	CHORDC1	2503.529	-0.05536	0.278334	0.444821	protein_coding
ENSG0000(	ENSG0000(	66.69803	0.09083	0.278425	0.4449	lncRNA
ENSG0000(	LINC00378	39.20604	0.157368	0.278426	0.4449	lncRNA
ENSG0000(	SCHIP1	620.4853	0.048081	0.278411	0.4449	protein_coding
ENSG0000(	YPEL3	1185.013	-0.06255	0.278482	0.444967	protein_coding
ENSG0000(	LINC00032	111.7405	-0.10905	0.278501	0.444975	transcribed_unprocessed_pseudo
ENSG0000(	ENSG0000(	124.7405	0.081143	0.278548	0.445005	protein_coding
ENSG0000(	L3MBTL4-A	893.5764	0.088833	0.278541	0.445005	lncRNA
ENSG0000(	USPL1	1157.067	-0.05194	0.278594	0.445055	protein_coding
ENSG0000(	GPR158	49.19489	-0.23631	0.278744	0.445272	protein_coding
ENSG0000(	ENSG0000(	22.20823	-0.14799	0.27883	0.445387	snoRNA
ENSG0000(	ZNF229	150.2791	-0.12721	0.27889	0.445461	protein_coding
ENSG0000(	STARD8	442.7695	0.058578	0.279009	0.445628	protein_coding
ENSG0000(	DEPDC4	529.6772	-0.06023	0.279043	0.445637	protein_coding
ENSG0000(	USP41	16.84392	0.397131	0.279041	0.445637	protein_coding
ENSG0000(	ENSG0000(	187.463	0.074292	0.279066	0.445651	lncRNA
ENSG0000(	GSS	991.8953	-0.04253	0.2791	0.445683	protein_coding
ENSG0000(	EXOSC3P1	28.39686	0.15348	0.279127	0.445704	processed_pseudogene
ENSG0000(	UBR4	13018.03	-0.03374	0.279143	0.445706	protein_coding
ENSG0000(	ZBED5-AS1	239.0682	0.05989	0.279179	0.445741	lncRNA
ENSG0000(	ARR3	20.29445	0.314344	0.27923	0.4458	protein_coding
ENSG0000(	CDRT15P4	23.88169	0.201904	0.279289	0.445849	transcribed_unprocessed_pseudo
ENSG0000(	LZTR1	1610.783	0.046811	0.279284	0.445849	protein_coding
ENSG0000(	LINC01647	125.298	0.078549	0.279353	0.445928	lncRNA
ENSG0000(	BCO1	373.372	0.247336	0.279406	0.445974	protein_coding
ENSG0000(	SLC22A4	286.1246	-0.21127	0.279409	0.445974	protein_coding

ENSG0000(	PRKAG2-AS	25.69136	0.153564	0.279489	0.446079	lncRNA
ENSG0000(	FAM220A	191.2237	-0.09804	0.279528	0.446118	protein_coding
ENSG0000(	TIPARP-AS:	19.40648	0.169689	0.279545	0.446122	lncRNA
ENSG0000(	PRAMENP	40.90114	-0.29583	0.279655	0.446275	transcribed_unprocessed_pseudo
ENSG0000(	PTK2	3621.286	-0.0378	0.279716	0.44635	protein_coding
ENSG0000(	EDA	129.4861	0.075269	0.279755	0.44639	protein_coding
ENSG0000(	ENSG0000(	11.34736	0.215113	0.279798	0.446436	lncRNA
ENSG0000(	EPHA10	897.5477	-0.15978	0.279924	0.446614	protein_coding
ENSG0000(	ENSG0000(	14.26974	-0.79505	0.279946	0.446626	lncRNA
ENSG0000(	TP53	1241.714	-0.05408	0.279974	0.446649	protein_coding
ENSG0000(	CXXC5	685.9275	-0.05324	0.280074	0.446786	protein_coding
ENSG0000(	ENSG0000(	10.9463	0.209706	0.280107	0.446815	lncRNA
ENSG0000(	GREM2	412.905	-0.16761	0.280181	0.446889	protein_coding
ENSG0000(	RASSF7	523.1047	-0.1447	0.28018	0.446889	protein_coding
ENSG0000(	RAB23	229.1788	0.112236	0.280203	0.446901	protein_coding
ENSG0000(	ENSG0000(	140.322	0.067467	0.28023	0.446922	lncRNA
ENSG0000(	ZBTB45	206.0179	0.072284	0.280261	0.446949	protein_coding
ENSG0000(	DSTN	1286.526	-0.11043	0.280414	0.44717	protein_coding
ENSG0000(	COA1	2776.988	0.06538	0.280443	0.447188	protein_coding
ENSG0000(	GALNT7	1984.718	-0.11922	0.280454	0.447188	protein_coding
ENSG0000(	ENSG0000(	40.30142	0.129038	0.280497	0.447233	lncRNA
ENSG0000(	MRPS31P5	810.9857	0.067909	0.280539	0.447278	transcribed_unprocessed_pseudo
ENSG0000(	PEX2	1022.075	-0.05306	0.280567	0.4473	protein_coding
ENSG0000(	LINC01307	151.1535	0.077023	0.280594	0.44732	lncRNA
ENSG0000(	ENSG0000(	11.32593	0.214593	0.280635	0.447341	lncRNA
ENSG0000(	ENSG0000(	53.45925	0.116761	0.280634	0.447341	lncRNA
ENSG0000(	ZPLD2P	328.3984	0.064609	0.28085	0.44766	transcribed_unprocessed_pseudo
ENSG0000(	LINC02108	522.5408	0.041147	0.280901	0.447683	lncRNA
ENSG0000(	PRRT3	265.2758	0.059274	0.280898	0.447683	protein_coding
ENSG0000(	TPT1P10	84.84687	-0.13743	0.280907	0.447683	processed_pseudogene
ENSG0000(	ENSG0000(	101.705	-0.10072	0.280968	0.447758	lncRNA
ENSG0000(	NCBP2-AS1	19.081	0.154785	0.281045	0.447858	lncRNA
ENSG0000(	RNF2	318.9775	-0.08698	0.281092	0.44791	protein_coding
ENSG0000(	GMDS-DT	5699.719	0.055012	0.281117	0.447928	lncRNA
ENSG0000(	MAP1S	562.7605	-0.05396	0.281137	0.447936	protein_coding
ENSG0000(	RAB6C	8.31439	-0.37447	0.281172	0.44797	protein_coding
ENSG0000(	ENSG0000(	36.76232	-0.13783	0.281219	0.448022	lncRNA
ENSG0000(	ZNF195	2769.587	-0.04438	0.281317	0.448155	protein_coding
ENSG0000(	ENSG0000(	11.47823	0.215002	0.281406	0.448274	lncRNA
ENSG0000(	RAF1	6138.793	-0.03245	0.281453	0.448304	protein_coding
ENSG0000(	ZKSCAN7	347.9875	-0.05644	0.281448	0.448304	protein_coding
ENSG0000(	CCDC77	262.498	0.05724	0.281556	0.448423	protein_coding
ENSG0000(	COMT	1016.495	0.049775	0.281547	0.448423	protein_coding
ENSG0000(	APCDD1L	11.50607	0.410619	0.281646	0.448498	protein_coding
ENSG0000(	DYNC1I2	1260.052	-0.07036	0.281629	0.448498	protein_coding
ENSG0000(	MTPAP	1351.103	-0.03776	0.281644	0.448498	protein_coding
ENSG0000(	ENSG0000(	94.45442	-0.08833	0.281698	0.448559	lncRNA

ENSG0000(	ENSG0000(	41.43482	-0.11768	0.281722	0.448572	lncRNA
ENSG0000(	ENSG0000(	288.347	0.059013	0.281735	0.448572	lncRNA
ENSG0000(	ENSG0000(	2773.716	0.028308	0.281856	0.448743	lncRNA
ENSG0000(	ENSG0000(	535.3787	-0.07009	0.281909	0.448803	unprocessed_pseudogene
ENSG0000(	PRR4	91.386	0.124946	0.281945	0.448838	protein_coding
ENSG0000(	ALG10B	1448.435	0.054259	0.281994	0.448884	protein_coding
ENSG0000(	PHC2-AS1	9.024837	0.224552	0.282002	0.448884	lncRNA
ENSG0000(	ENSG0000(	8.993979	-0.23394	0.282079	0.448972	lncRNA
ENSG0000(	LINC01747	25.54168	0.18493	0.282086	0.448972	lncRNA
ENSG0000(	VPS33B-DT	396.8118	-0.06011	0.2821	0.448972	lncRNA
ENSG0000(	ENSG0000(	52.89712	0.144337	0.282173	0.449065	processed_pseudogene
ENSG0000(	RAP1GAP	931.3539	-0.19784	0.282265	0.449189	protein_coding
ENSG0000(	ENSG0000(	49.11658	0.162968	0.282411	0.449398	lncRNA
ENSG0000(	CR2	1557.523	-0.34631	0.282444	0.449409	protein_coding
ENSG0000(	ENSG0000(	66.52075	0.071259	0.282446	0.449409	lncRNA
ENSG0000(	ENSG0000(	54.67635	-0.13176	0.282507	0.449484	transcribed_processed_pseudoge
ENSG0000(	ENSG0000(	440.9501	-0.05918	0.282604	0.449615	TEC
ENSG0000(	PTRH1	331.1068	0.061113	0.282637	0.449644	protein_coding
ENSG0000(	FSD1	261.4825	0.076934	0.282736	0.449777	protein_coding
ENSG0000(	PCED1B	409.5829	0.226702	0.282763	0.449777	protein_coding
ENSG0000(	TRAJ23	51.1788	-0.22503	0.282756	0.449777	TR_J_gene
ENSG0000(	ENSG0000(	108.8456	-0.08544	0.28278	0.449778	lncRNA
ENSG0000(	TRIM36-IT1	71.21901	-0.23856	0.282792	0.449778	lncRNA
ENSG0000(	LINC02476	152.9698	-0.08817	0.282837	0.449826	lncRNA
ENSG0000(	H3P37	261.5423	0.051149	0.283144	0.450292	transcribed_unprocessed_pseudo
ENSG0000(	ENSG0000(	13.19794	0.178733	0.283217	0.450363	TEC
ENSG0000(	SAP25	143.6993	-0.13599	0.283204	0.450363	protein_coding
ENSG0000(	SNORD19C	12.86023	0.221725	0.283277	0.450435	snoRNA
ENSG0000(	ENSG0000(	688.2945	-0.13188	0.283349	0.450528	lncRNA
ENSG0000(	ENSG0000(	16.43597	-0.16012	0.283454	0.450671	lncRNA
ENSG0000(	LCP1	4217.688	0.205297	0.283485	0.450684	protein_coding
ENSG0000(	RTN3	1408.534	-0.07925	0.283491	0.450684	protein_coding
ENSG0000(	ENSG0000(	49.36685	-0.12264	0.283585	0.450789	lncRNA
ENSG0000(	PTPN22	843.0814	-0.08462	0.283577	0.450789	protein_coding
ENSG0000(	FRMPD1	267.9379	0.051421	0.283607	0.4508	protein_coding
ENSG0000(	ENSG0000(	81.36213	0.163357	0.283657	0.450839	transcribed_unprocessed_pseudo
ENSG0000(	HRC	20.69328	0.239273	0.28366	0.450839	protein_coding
ENSG0000(	CDK5RAP2	3505.026	0.064643	0.283705	0.450887	protein_coding
ENSG0000(	ENSG0000(	115.1416	-0.0701	0.283724	0.450887	lncRNA
ENSG0000(	GADD45A	224.4457	-0.14721	0.283733	0.450887	protein_coding
ENSG0000(	DST	28332.29	-0.10242	0.283824	0.450918	protein_coding
ENSG0000(	ENSG0000(	140.7405	-0.06673	0.283779	0.450918	transcribed_processed_pseudoge
ENSG0000(	ENSG0000(	13.24705	-0.18497	0.283824	0.450918	lncRNA
ENSG0000(	ENSG0000(	10.99781	0.23272	0.283786	0.450918	lncRNA
ENSG0000(	NAA16	956.2474	0.067464	0.283798	0.450918	protein_coding
ENSG0000(	LINC02440	10.58765	0.21643	0.283846	0.450931	lncRNA
ENSG0000(	CSAD	1402.044	-0.04209	0.283886	0.450971	protein_coding

ENSG0000(	LINC01138	814.6478	0.079498	0.283952	0.451054	lncRNA
ENSG0000(	ANP32E	647.3657	-0.09766	0.283985	0.45106	protein_coding
ENSG0000(	DPY19L2P2	1166.27	0.059045	0.283999	0.45106	transcribed_unprocessed_pseudo
ENSG0000(	MRTFB	3622.379	0.043235	0.283984	0.45106	protein_coding
ENSG0000(	SLC10A5P1	25.00954	-0.26481	0.284023	0.451076	unprocessed_pseudogene
ENSG0000(	GSE1	3103.578	0.042029	0.284197	0.451328	protein_coding
ENSG0000(	ENSG0000(	152.3956	0.07975	0.284367	0.451554	lncRNA
ENSG0000(	NDUFA12	2560.81	-0.04611	0.284361	0.451554	protein_coding
ENSG0000(	LCN12	113.7147	-0.08428	0.284391	0.451569	protein_coding
ENSG0000(	ENSG0000(	280.6284	-0.04964	0.284456	0.451649	lncRNA
ENSG0000(	CENPH	156.8451	-0.08508	0.284548	0.451772	protein_coding
ENSG0000(	HAUS6	923.8871	0.059576	0.284619	0.451862	protein_coding
ENSG0000(	ARHGEF10	825.794	0.096139	0.284646	0.451882	protein_coding
ENSG0000(	ENSG0000(	26.45169	0.132176	0.284695	0.451938	lncRNA
ENSG0000(	PIR	164.0201	-0.10262	0.284807	0.452093	protein_coding
ENSG0000(	ENSG0000(	8.523079	0.317899	0.284839	0.452121	lncRNA
ENSG0000(	APEX2	398.4905	-0.06293	0.284872	0.452149	protein_coding
ENSG0000(	ENSG0000(	97.96906	-0.107	0.284961	0.452259	lncRNA
ENSG0000(	ENSG0000(	11.95686	0.503703	0.284969	0.452259	protein_coding
ENSG0000(	RNU1-82P	12.93677	-0.23073	0.285006	0.452294	snRNA
ENSG0000(	ENSG0000(	88.78687	0.076942	0.28507	0.45235	TEC
ENSG0000(	ENSG0000(	22.59855	0.160622	0.285062	0.45235	lncRNA
ENSG0000(	ATP1B2	226.9142	0.059238	0.285139	0.452417	protein_coding
ENSG0000(	ATP8A2	777.112	0.04543	0.285141	0.452417	protein_coding
ENSG0000(	C1orf189	8.222513	0.244077	0.285285	0.452614	protein_coding
ENSG0000(	ENSG0000(	1670.029	0.054876	0.285293	0.452614	lncRNA
ENSG0000(	M1AP	77.75419	0.075213	0.285312	0.452621	protein_coding
ENSG0000(	ENSG0000(	82.28934	0.101089	0.285329	0.452624	processed_pseudogene
ENSG0000(	ENPP7P4	18.26012	0.242519	0.285411	0.452732	transcribed_unprocessed_pseudo
ENSG0000(	GPAA1P2	196.4719	-0.13185	0.285449	0.45277	transcribed_processed_pseudoge
ENSG0000(	CEP250	2464.7	-0.07869	0.285486	0.452783	protein_coding
ENSG0000(	FAM238A	18.63737	0.204899	0.285481	0.452783	transcribed_unprocessed_pseudo
ENSG0000(	NPRL3	503.8327	-0.07609	0.285517	0.452787	protein_coding
ENSG0000(	RN7SL753F	18.73801	0.157587	0.285518	0.452787	misc_RNA
ENSG0000(	RHOQP1	36.64318	-0.1064	0.285549	0.452807	processed_pseudogene
ENSG0000(	RN7SL363F	11.50156	-0.20863	0.285559	0.452807	misc_RNA
ENSG0000(	CCDC112	178.8632	-0.08556	0.285618	0.452878	protein_coding
ENSG0000(	SLC4A10	114.3822	0.135548	0.285639	0.452889	protein_coding
ENSG0000(	ZNF786	332.6777	0.070393	0.285692	0.45295	protein_coding
ENSG0000(	AMPD3	1177.932	0.085048	0.28575	0.453019	protein_coding
ENSG0000(	FOXN3-AS1	16.91787	0.17191	0.285884	0.453208	lncRNA
ENSG0000(	ANKRD29	537.0637	-0.16206	0.285899	0.453209	protein_coding
ENSG0000(	TAS2R64P	482.3743	0.109088	0.285918	0.453216	transcribed_unprocessed_pseudo
ENSG0000(	ENSG0000(	160.3439	-0.0991	0.286019	0.453312	lncRNA
ENSG0000(	GPR157	921.0658	0.065689	0.286022	0.453312	protein_coding
ENSG0000(	RN7SL8P	17.50667	-0.15003	0.28601	0.453312	misc_RNA
ENSG0000(	ENSG0000(	343.3012	-0.08583	0.286194	0.453563	lncRNA

ENSG0000(	MEF2C-AS1	315.9324	0.162309	0.286231	0.453579	lncRNA
ENSG0000(	MIR548AA1	11.75198	0.250871	0.286233	0.453579	miRNA
ENSG0000(	ENSG0000(	116.4523	-0.1344	0.286261	0.453601	lncRNA
ENSG0000(	ENSG0000(	194.2707	0.074274	0.286285	0.453615	lncRNA
ENSG0000(	ABCB9	970.8341	0.047176	0.286376	0.453646	protein_coding
ENSG0000(	CD48	885.9139	0.204343	0.286375	0.453646	protein_coding
ENSG0000(	ENSG0000(	39.67921	-0.11787	0.286339	0.453646	lncRNA
ENSG0000(	MBD4	1005.681	-0.08366	0.28635	0.453646	protein_coding
ENSG0000(	RASA4	2090.568	-0.126	0.286339	0.453646	protein_coding
ENSG0000(	PTPRC	8133.581	0.196166	0.286486	0.453797	protein_coding
ENSG0000(	ENSG0000(	50.13737	-0.11047	0.286517	0.453824	lncRNA
ENSG0000(	KCTD21-AS	571.5099	-0.04989	0.286573	0.453867	lncRNA
ENSG0000(	MMP17	112.3473	0.175717	0.286572	0.453867	protein_coding
ENSG0000(	CH17-340M	19.62544	0.149856	0.286618	0.453892	lncRNA
ENSG0000(	ENSG0000(	27.29749	-0.11754	0.286615	0.453892	lncRNA
ENSG0000(	ENSG0000(	290.9157	0.075142	0.286664	0.453942	lncRNA
ENSG0000(	ENSG0000(	835.6798	0.049598	0.28671	0.453992	TEC
ENSG0000(	ENSG0000(	10.57866	-0.21344	0.286854	0.454197	lncRNA
ENSG0000(	ENSG0000(	172.05	0.118155	0.287088	0.454546	lncRNA
ENSG0000(	AFF2	200.9823	-0.31829	0.287117	0.454568	protein_coding
ENSG0000(	PYCR3	233.7238	0.092884	0.28717	0.45463	protein_coding
ENSG0000(	COG2	1332.435	-0.04931	0.28719	0.454638	protein_coding
ENSG0000(	LINC01972	71.98553	-0.08781	0.287211	0.454648	lncRNA
ENSG0000(	ENSG0000(	14.35218	0.30639	0.287344	0.454722	protein_coding
ENSG0000(	ENSG0000(	68.02024	-0.11103	0.287313	0.454722	lncRNA
ENSG0000(	ENSG0000(	20.72081	0.167157	0.287301	0.454722	lncRNA
ENSG0000(	LINC00506	18373.88	-0.06189	0.287331	0.454722	lncRNA
ENSG0000(	PNMA2	306.8418	0.060882	0.287295	0.454722	protein_coding
ENSG0000(	TXN	330.0691	-0.14762	0.287323	0.454722	protein_coding
ENSG0000(	SHROOM1	1308.759	-0.15026	0.287399	0.454786	protein_coding
ENSG0000(	ACBD6	1151.812	0.055132	0.287414	0.454787	protein_coding
ENSG0000(	ENSG0000(	295.0612	0.094013	0.287484	0.454875	TEC
ENSG0000(	ENSG0000(	14.84403	0.247639	0.287543	0.454946	lncRNA
ENSG0000(	RN7SL337P	27.99602	-0.27177	0.287584	0.454987	misc_RNA
ENSG0000(	RN7SL32P	207.9895	-0.06591	0.287648	0.455058	misc_RNA
ENSG0000(	TMEM50B	1842.505	0.059367	0.287658	0.455058	protein_coding
ENSG0000(	ENSG0000(	11.50589	-0.23868	0.287732	0.455154	lncRNA
ENSG0000(	NSUN5	398.2063	-0.06783	0.287811	0.455255	protein_coding
ENSG0000(	KATNAL1	778.0693	-0.09033	0.287888	0.455354	protein_coding
ENSG0000(	ENSG0000(	119.6124	-0.09804	0.287989	0.455447	lncRNA
ENSG0000(	LCAT	254.1704	0.088869	0.287963	0.455447	protein_coding
ENSG0000(	S100A16	448.4945	-0.15008	0.28799	0.455447	protein_coding
ENSG0000(	ENSG0000(	17.37237	-0.15138	0.288008	0.455452	processed_pseudogene
ENSG0000(	RN7SL764P	12.46289	0.216406	0.288066	0.455522	misc_RNA
ENSG0000(	CCNH	2241.25	0.038196	0.288154	0.455631	protein_coding
ENSG0000(	ENSG0000(	299.5191	-0.04838	0.288164	0.455631	lncRNA
ENSG0000(	LINC00649	1812.11	0.059467	0.288256	0.455753	lncRNA

ENSG0000(	C5orf67	16.07494	-0.27961	0.288288	0.45578	lncRNA
ENSG0000(	IGLV5-52	31.71284	0.190946	0.288314	0.455799	IG_V_gene
ENSG0000(	SNORA40B	18.26404	0.206531	0.288357	0.455844	snoRNA
ENSG0000(	ENSG0000(	272.9528	-0.08674	0.288414	0.455883	lncRNA
ENSG0000(	ICA1L	767.4157	-0.05095	0.288422	0.455883	protein_coding
ENSG0000(	LINC00393	23.84258	-0.23011	0.288425	0.455883	lncRNA
ENSG0000(	RPS26	1258.638	-0.17622	0.288446	0.455893	protein_coding
ENSG0000(	NECTIN3-A	51.62506	-0.16428	0.288513	0.455977	lncRNA
ENSG0000(	WNT3	219.6472	0.143345	0.288534	0.455988	protein_coding
ENSG0000(	ZNF880	408.7128	-0.06276	0.28862	0.4561	protein_coding
ENSG0000(	ENSG0000(	48.01928	0.169235	0.288693	0.456192	TEC
ENSG0000(	LINC02000	14.40086	-0.30059	0.288752	0.456263	lncRNA
ENSG0000(	TIMP4	12.55265	0.253741	0.288795	0.456308	protein_coding
ENSG0000(	TAS2R63P	109.1899	0.10984	0.28881	0.456309	unprocessed_pseudogene
ENSG0000(	FAM78B	107.0505	0.102799	0.288887	0.456407	protein_coding
ENSG0000(	KDELR2	2522.324	-0.11266	0.28903	0.45661	protein_coding
ENSG0000(	ENSG0000(	133.7381	0.168488	0.289093	0.456688	lncRNA
ENSG0000(	RN7SL220F	137.3179	-0.10137	0.289128	0.456719	misc_RNA
ENSG0000(	PRPF40B	375.5757	0.084683	0.289211	0.456827	protein_coding
ENSG0000(	LINC01569	947.5269	0.044131	0.289254	0.456872	lncRNA
ENSG0000(	DLGAP1	124.5649	0.114133	0.289316	0.456925	protein_coding
ENSG0000(	E4F1	643.6001	-0.05968	0.289307	0.456925	protein_coding
ENSG0000(	ENSG0000(	24.67803	0.142873	0.289388	0.457015	lncRNA
ENSG0000(	LINC02550	11.4629	0.273506	0.28941	0.457027	lncRNA
ENSG0000(	LINC01704	19.11056	0.154881	0.289429	0.457034	lncRNA
ENSG0000(	ENSG0000(	34.71356	-0.3243	0.289543	0.457169	lncRNA
ENSG0000(	SLC25A13	585.7175	-0.08847	0.289542	0.457169	protein_coding
ENSG0000(	MC2R	362.6812	0.114061	0.289569	0.457187	protein_coding
ENSG0000(	MND1	15.75269	0.303561	0.289651	0.457293	protein_coding
ENSG0000(	TRAJ38	36.59172	-0.20689	0.289707	0.457359	TR_J_gene
ENSG0000(	LINC00869	906.9754	-0.07289	0.289743	0.457393	transcribed_unprocessed_pseudo
ENSG0000(	ENSG0000(	28.80663	-0.13115	0.289832	0.45751	lncRNA
ENSG0000(	ZNF75A	1582.197	0.04933	0.289853	0.45752	protein_coding
ENSG0000(	ENSG0000(	14.24841	-0.18726	0.289911	0.45759	lncRNA
ENSG0000(	ENSG0000(	97.72329	0.159621	0.289956	0.457591	lncRNA
ENSG0000(	OR5M2P	185.4513	0.07065	0.289935	0.457591	transcribed_unprocessed_pseudo
ENSG0000(	TRIM74	33.09347	-0.38355	0.289952	0.457591	protein_coding
ENSG0000(	GPX1	403.3742	0.112369	0.290088	0.457765	protein_coding
ENSG0000(	WNK1	7551.837	-0.04821	0.290095	0.457765	protein_coding
ENSG0000(	TRMT10A	167.7394	-0.11356	0.290181	0.457878	protein_coding
ENSG0000(	LINC00888	159.1433	0.061387	0.290227	0.457927	transcribed_unprocessed_pseudo
ENSG0000(	AGBL3	176.6899	-0.12874	0.290268	0.457969	protein_coding
ENSG0000(	ALKAL2	32.19245	-0.21878	0.290351	0.458064	protein_coding
ENSG0000(	ZNF12	1304.4	-0.06289	0.290357	0.458064	protein_coding
ENSG0000(	ENSG0000(	197.2165	-0.06424	0.290473	0.458108	lncRNA
ENSG0000(	ENSG0000(	37.23068	0.184613	0.290457	0.458108	lncRNA
ENSG0000(	ENSG0000(	19.70757	0.155337	0.290465	0.458108	lncRNA

ENSG0000(	INO80D	5016.883	-0.03619	0.290457	0.458108	protein_coding
ENSG0000(	KNSTRN	270.8648	0.074157	0.290426	0.458108	protein_coding
ENSG0000(	STK26	281.728	0.160305	0.290424	0.458108	protein_coding
ENSG0000(	ZNF518B	1567.139	-0.04544	0.290487	0.458108	protein_coding
ENSG0000(	FASTKD5	965.285	-0.03638	0.290563	0.458186	protein_coding
ENSG0000(	RBL2	4865.763	-0.07084	0.290565	0.458186	protein_coding
ENSG0000(	NLGN4Y-AS	21.48791	0.129187	0.290648	0.458293	lncRNA
ENSG0000(	BYSL	84.98269	0.127099	0.290694	0.458343	protein_coding
ENSG0000(	ADPRM	234.0739	-0.08072	0.290748	0.458405	protein_coding
ENSG0000(	CFAP410	573.888	0.100357	0.290811	0.458481	protein_coding
ENSG0000(	ENSG0000(	199.8718	0.06944	0.290876	0.458555	processed_pseudogene
ENSG0000(	TBC1D1	2119.396	0.053352	0.290887	0.458555	protein_coding
ENSG0000(	ENSG0000(	21.30718	0.159614	0.290946	0.458626	lncRNA
ENSG0000(	GNPNAT1	751.0941	0.066836	0.291016	0.458713	protein_coding
ENSG0000(	ABCA9-AS1	11.60762	-0.19447	0.291097	0.458818	lncRNA
ENSG0000(	XIAP-AS1	67.97028	0.095008	0.291129	0.458845	lncRNA
ENSG0000(	SYVN1	2013.903	-0.09989	0.291146	0.458848	protein_coding
ENSG0000(	ERCC3	1346.318	0.055165	0.291252	0.458994	protein_coding
ENSG0000(	WASH7P	87.1709	-0.27775	0.291311	0.459063	unprocessed_pseudogene
ENSG0000(	SNORA70B	22.63543	0.143361	0.291487	0.459317	snoRNA
ENSG0000(	CA8	150.595	0.149396	0.291644	0.459536	protein_coding
ENSG0000(	ENSG0000(	893.6785	0.066525	0.291684	0.459536	lncRNA
ENSG0000(	MELTF	1254.735	-0.12662	0.291677	0.459536	protein_coding
ENSG0000(	NDST4	29.9886	-0.15124	0.291681	0.459536	protein_coding
ENSG0000(	ENSG0000(	15.28129	-0.1743	0.291721	0.459556	lncRNA
ENSG0000(	TMEM200A	286.7709	-0.13259	0.291726	0.459556	protein_coding
ENSG0000(	CYREN	640.5568	0.124454	0.291766	0.45959	protein_coding
ENSG0000(	ENSG0000(	94.92309	-0.07294	0.291777	0.45959	lncRNA
ENSG0000(	HKDC1	2439.201	-0.20992	0.291803	0.459609	protein_coding
ENSG0000(	ENSG0000(	56.58833	0.093735	0.291855	0.459668	lncRNA
ENSG0000(	ENSG0000(	87.66521	0.083354	0.291897	0.459711	lncRNA
ENSG0000(	ENSG0000(	210.4225	0.049644	0.292041	0.459914	lncRNA
ENSG0000(	LINC01872	15.37064	-0.16536	0.292056	0.459914	lncRNA
ENSG0000(	ENSG0000(	40.45729	0.128339	0.292183	0.460092	unprocessed_pseudogene
ENSG0000(	ENSG0000(	69.68849	0.144947	0.292274	0.460211	lncRNA
ENSG0000(	ENSG0000(	52.84633	0.086522	0.292387	0.460344	lncRNA
ENSG0000(	ENSG0000(	434.7478	0.056634	0.29238	0.460344	lncRNA
ENSG0000(	HDAC11	260.0271	-0.12985	0.292436	0.460398	protein_coding
ENSG0000(	ENSG0000(	228.9293	0.091145	0.292492	0.46044	lncRNA
ENSG0000(	ZC3HAV1L	292.3246	-0.05044	0.29248	0.46044	protein_coding
ENSG0000(	ABHD14A	178.0459	-0.06221	0.292511	0.460447	protein_coding
ENSG0000(	ENSG0000(	99.98622	-0.08829	0.292538	0.460466	lncRNA
ENSG0000(	LINC02158	24.39609	-0.18206	0.292624	0.46058	lncRNA
ENSG0000(	NR2C1	942.9182	0.054818	0.292641	0.460582	protein_coding
ENSG0000(	SGO1-AS1	653.3706	0.053793	0.292657	0.460586	lncRNA
ENSG0000(	NATD1	392.6014	-0.06121	0.292722	0.46066	protein_coding
ENSG0000(	RNASEK	520.2778	-0.07975	0.292734	0.46066	protein_coding

ENSG0000(	ENSG0000(	69.48656	0.113165	0.292858	0.460832	protein_coding
ENSG0000(	ENSG0000(	26.50494	0.141014	0.293134	0.461224	TEC
ENSG0000(	NGFR	71.31385	-0.16372	0.293136	0.461224	protein_coding
ENSG0000(	NUMA1	9069.334	-0.04575	0.293212	0.461321	protein_coding
ENSG0000(	STEAP3	357.2625	-0.18146	0.293233	0.461331	protein_coding
ENSG0000(	ENSG0000(	77.25276	-0.08737	0.293329	0.461454	TEC
ENSG0000(	ENSG0000(	45.08047	-0.20558	0.293341	0.461454	unprocessed_pseudogene
ENSG0000(	ENSG0000(	247.852	-0.05943	0.293365	0.461454	lncRNA
ENSG0000(	LRRC23	66.2203	-0.12575	0.29337	0.461454	protein_coding
ENSG0000(	ENSG0000(	34.93873	0.122422	0.293409	0.461491	lncRNA
ENSG0000(	CASC16	15.36076	-0.26549	0.293429	0.461501	lncRNA
ENSG0000(	ENSG0000(	15.13886	0.328175	0.293468	0.461538	processed_pseudogene
ENSG0000(	RELA-DT	692.8497	-0.04274	0.293518	0.461594	lncRNA
ENSG0000(	C5	267.3833	-0.08957	0.293628	0.461744	protein_coding
ENSG0000(	COPG1	1826.884	0.062111	0.29372	0.461865	protein_coding
ENSG0000(	LINC02406	15.73372	-0.16855	0.293738	0.461871	lncRNA
ENSG0000(	RUNX3	1113.928	0.138316	0.29376	0.461883	protein_coding
ENSG0000(	TIGD4	23.06849	-0.15376	0.293828	0.461966	protein_coding
ENSG0000(	ENSG0000(	21.23314	-0.18996	0.293877	0.461999	lncRNA
ENSG0000(	NSD3	5752.297	0.034715	0.293892	0.461999	protein_coding
ENSG0000(	TRIM72	1377.862	0.035884	0.293892	0.461999	protein_coding
ENSG0000(	GJC2	24.85469	-0.16443	0.294022	0.462179	protein_coding
ENSG0000(	DOCK5	8467.164	-0.12845	0.294109	0.462293	protein_coding
ENSG0000(	CDCA8	153.9813	0.127343	0.294163	0.462355	protein_coding
ENSG0000(	LINC01755	16.5728	0.210479	0.294182	0.462362	lncRNA
ENSG0000(	C1orf56	48.06074	-0.19497	0.29423	0.462368	protein_coding
ENSG0000(	ENSG0000(	33.9806	0.138864	0.294214	0.462368	lncRNA
ENSG0000(	GNG7	458.9233	0.141992	0.294224	0.462368	protein_coding
ENSG0000(	ENSG0000(	52.77254	0.215424	0.294249	0.462374	lncRNA
ENSG0000(	ENSG0000(	49.56552	-0.11369	0.294299	0.46243	lncRNA
ENSG0000(	LOXL4	102.4629	0.110331	0.294405	0.462573	protein_coding
ENSG0000(	ENSG0000(	35.97816	-0.11753	0.294457	0.462633	snoRNA
ENSG0000(	UBR5-DT	255.6631	0.106656	0.294568	0.462784	lncRNA
ENSG0000(	ENSG0000(	8.855391	0.213875	0.294629	0.46281	lncRNA
ENSG0000(	FUT2	1951.843	-0.14006	0.294621	0.46281	protein_coding
ENSG0000(	LINC00570	71.23601	-0.1521	0.294602	0.46281	lncRNA
ENSG0000(	SNRNP25	92.65592	-0.12237	0.294679	0.462866	protein_coding
ENSG0000(	AVEN	305.8623	0.063133	0.294748	0.462892	protein_coding
ENSG0000(	ENSG0000(	31.1177	0.2687	0.294755	0.462892	lncRNA
ENSG0000(	GTF2H3	741.1357	-0.04357	0.29473	0.462892	protein_coding
ENSG0000(	SMIM11A	864.6181	0.132731	0.294738	0.462892	protein_coding
ENSG0000(	FEV	18.93698	0.24864	0.294849	0.463017	protein_coding
ENSG0000(	LINC02160	54.40426	-0.10094	0.294899	0.463072	lncRNA
ENSG0000(	ENSG0000(	45.06304	0.115209	0.294944	0.46312	lncRNA
ENSG0000(	UNC45A	798.2075	-0.03923	0.294965	0.46313	protein_coding
ENSG0000(	CD81-AS1	8.589261	0.245295	0.294992	0.463149	lncRNA
ENSG0000(	OR10J1	130.9677	-0.07894	0.29504	0.463202	protein_coding

ENSG0000(	APPBP2-DT	88.68084	-0.073	0.295135	0.463328	lncRNA
ENSG0000(	EFCC1	208.3012	0.130117	0.295162	0.463348	protein_coding
ENSG0000(	ENSG0000(	14.53014	-0.39287	0.295269	0.463469	protein_coding
ENSG0000(	VPS11	1019.203	-0.04771	0.295258	0.463469	protein_coding
ENSG0000(	RAB7A	2492.744	-0.05435	0.295285	0.463471	protein_coding
ENSG0000(	GOLT1B	489.5682	0.08784	0.295316	0.463497	protein_coding
ENSG0000(	AFF3	2395.77	-0.16519	0.295333	0.463499	protein_coding
ENSG0000(	ENSG0000(	10.24108	0.22958	0.295508	0.463751	lncRNA
ENSG0000(	ENSG0000(	218.9965	-0.06583	0.29555	0.463794	lncRNA
ENSG0000(	SNORA71C	17.51762	0.20163	0.29566	0.463943	snoRNA
ENSG0000(	RAB8B	1477.358	-0.0872	0.29568	0.463953	protein_coding
ENSG0000(	ENSG0000(	37.76095	0.1051	0.295715	0.463969	lncRNA
ENSG0000(	FBXO7	2589.67	0.038319	0.295735	0.463969	protein_coding
ENSG0000(	RPP38-DT	162.2578	0.066172	0.295732	0.463969	lncRNA
ENSG0000(	ENSG0000(	86.84802	0.112291	0.295764	0.463991	lncRNA
ENSG0000(	STX5	640.362	0.038481	0.295805	0.464033	protein_coding
ENSG0000(	MAP9	185.9398	0.197578	0.295923	0.464155	protein_coding
ENSG0000(	MASTL	804.7872	0.066497	0.295957	0.464155	protein_coding
ENSG0000(	SHKBP1	705.2947	0.072984	0.295936	0.464155	protein_coding
ENSG0000(	UGT8	2127.933	-0.11776	0.295956	0.464155	protein_coding
ENSG0000(	ZNF629	693.4941	0.079293	0.295928	0.464155	protein_coding
ENSG0000(	ENSG0000(	103.5079	0.085211	0.296049	0.464255	protein_coding
ENSG0000(	TTC14	3880.232	0.039685	0.29605	0.464255	protein_coding
ENSG0000(	TTBK2	1736.742	0.067503	0.296131	0.464341	protein_coding
ENSG0000(	ZBTB37	4082.437	0.064666	0.296134	0.464341	protein_coding
ENSG0000(	LINC02805	178.1507	-0.05995	0.296216	0.464427	lncRNA
ENSG0000(	MB	24.76962	-0.27988	0.296218	0.464427	protein_coding
ENSG0000(	STYX	919.3492	-0.07031	0.296273	0.46449	protein_coding
ENSG0000(	LINC02731	159.4913	0.074081	0.29634	0.464571	lncRNA
ENSG0000(	ENSG0000(	118.0102	-0.16411	0.296439	0.464703	protein_coding
ENSG0000(	ZNF43	1866.89	-0.07093	0.296479	0.464743	protein_coding
ENSG0000(	ENSG0000(	162.171	0.092004	0.296532	0.464802	lncRNA
ENSG0000(	LINC02052	37.79	0.109362	0.296551	0.46481	lncRNA
ENSG0000(	PIGO	863.3455	0.055799	0.29659	0.464848	protein_coding
ENSG0000(	RN7SL766F	13.49572	0.191289	0.296605	0.464848	misc_RNA
ENSG0000(	SLC39A3	449.695	0.041091	0.296665	0.464914	protein_coding
ENSG0000(	WDR55	1535.848	0.035974	0.296677	0.464914	protein_coding
ENSG0000(	S100A13	239.4049	-0.10499	0.296756	0.465015	protein_coding
ENSG0000(	ENSG0000(	31.25031	0.1736	0.296815	0.465044	lncRNA
ENSG0000(	ENSG0000(	20.24063	0.134988	0.296819	0.465044	lncRNA
ENSG0000(	ENSG0000(	31.91215	0.154252	0.296791	0.465044	lncRNA
ENSG0000(	ENSG0000(	14.44939	-0.39845	0.296878	0.465044	TEC
ENSG0000(	ERBIN-DT	20.70792	-0.1294	0.296868	0.465044	lncRNA
ENSG0000(	ERFL	35.88769	0.21689	0.296867	0.465044	protein_coding
ENSG0000(	NPAP1	523.3495	0.056085	0.296869	0.465044	protein_coding
ENSG0000(	ENSG0000(	50.02191	-0.09605	0.296961	0.465152	lncRNA
ENSG0000(	CTNNA1-AS	9.783962	0.187082	0.297022	0.465224	lncRNA

ENSG0000(	QSER1	2211.509	-0.04445	0.297066	0.465269	protein_coding
ENSG0000(	TRAJ24	36.609	0.240396	0.297179	0.465423	TR_J_gene
ENSG0000(	MIR558	16.79346	-0.16736	0.297226	0.465474	miRNA
ENSG0000(	RNU6-126F	21.49784	0.166405	0.297254	0.465494	snRNA
ENSG0000(	DDX11-AS1	78.02889	-0.13555	0.297284	0.465494	lncRNA
ENSG0000(	ENSG0000(	39.63444	0.150319	0.297279	0.465494	lncRNA
ENSG0000(	DDX52	1988.766	-0.03787	0.297321	0.465507	protein_coding
ENSG0000(	ENSG0000(	237.539	0.059438	0.297319	0.465507	lncRNA
ENSG0000(	CLLU1	202.7755	0.071259	0.29739	0.465591	lncRNA
ENSG0000(	ACAD9	2635.592	0.036129	0.29742	0.465592	protein_coding
ENSG0000(	RABIF	451.9713	-0.04313	0.29742	0.465592	protein_coding
ENSG0000(	ENSG0000(	21.99356	-0.14837	0.297537	0.465729	lncRNA
ENSG0000(	ENSG0000(	85.25368	-0.07704	0.297531	0.465729	lncRNA
ENSG0000(	ZNF862	1312.945	-0.07388	0.297554	0.465733	protein_coding
ENSG0000(	MBNL1	10440.55	-0.05807	0.297606	0.465792	protein_coding
ENSG0000(	C5AR2	1438.761	0.032771	0.297623	0.465795	protein_coding
ENSG0000(	ENSG0000(	37.71373	0.129025	0.297659	0.465827	lncRNA
ENSG0000(	NUBPL	1056.928	-0.04067	0.297691	0.465854	protein_coding
ENSG0000(	A2ML1-AS2	15.55811	0.165758	0.297815	0.466003	lncRNA
ENSG0000(	TRAJ9	20.98606	-0.26562	0.297816	0.466003	TR_J_gene
ENSG0000(	HHIP-AS1	14.34954	-0.19694	0.297903	0.466093	lncRNA
ENSG0000(	KLHL15	362.4191	-0.06535	0.297892	0.466093	protein_coding
ENSG0000(	PGAP6	931.8992	-0.10876	0.298	0.4662	protein_coding
ENSG0000(	SLC15A4	922.3663	0.084099	0.297988	0.4662	protein_coding
ENSG0000(	SEMA4C	1233.836	0.064159	0.298022	0.466211	protein_coding
ENSG0000(	SLC16A11	20.35569	-0.20975	0.29806	0.466247	protein_coding
ENSG0000(	OR7E115P	14.38006	0.240302	0.298105	0.466294	unprocessed_pseudogene
ENSG0000(	ENSG0000(	17.14912	-0.35637	0.298132	0.466297	TEC
ENSG0000(	GNAO1-DT	1217.443	-0.03683	0.298137	0.466297	lncRNA
ENSG0000(	DERPC	438.6655	0.156782	0.29822	0.466405	protein_coding
ENSG0000(	NRIR	222.7529	-0.10673	0.298272	0.466463	lncRNA
ENSG0000(	METTL15	761.0782	0.056981	0.298328	0.466522	protein_coding
ENSG0000(	NDUFAF2P	93.48961	0.097883	0.298354	0.466522	processed_pseudogene
ENSG0000(	TYMS	328.0141	0.1044	0.298346	0.466522	protein_coding
ENSG0000(	RRN3P1	1716.099	-0.06338	0.298379	0.466537	transcribed_unprocessed_pseudo
ENSG0000(	MIR5706	16.14087	-0.14544	0.298423	0.466584	miRNA
ENSG0000(	ENSG0000(	33.84661	0.156892	0.298443	0.466591	transcribed_unprocessed_pseudo
ENSG0000(	FBXL16	48.77398	0.149113	0.298465	0.466602	protein_coding
ENSG0000(	MRPL53	106.3776	-0.07395	0.298484	0.466609	protein_coding
ENSG0000(	SMIM10L2I	25.53791	0.134759	0.298625	0.466806	protein_coding
ENSG0000(	KRT73-AS1	119.9984	-0.11413	0.298654	0.466829	lncRNA
ENSG0000(	ENSG0000(	40.56341	0.275428	0.29869	0.466861	lncRNA
ENSG0000(	ADGRF2	9.584118	0.207502	0.298732	0.466904	transcribed_unitary_pseudogene
ENSG0000(	ERICH6B	15.78548	0.168083	0.298759	0.466923	protein_coding
ENSG0000(	ZNF689	239.6534	-0.06021	0.298793	0.466953	protein_coding
ENSG0000(	CYP4F24P	29.73782	-0.39148	0.298815	0.466964	unprocessed_pseudogene
ENSG0000(	C11orf65	53.05077	-0.12345	0.298865	0.46702	protein_coding

ENSG0000(	ENSG0000(	568.5951	0.052384	0.298898	0.467048	lncRNA
ENSG0000(	ENSG0000(	16.61854	-0.18051	0.298923	0.467063	lncRNA
ENSG0000(	MAP3K9	936.6957	-0.06206	0.29894	0.467068	protein_coding
ENSG0000(	FAM50B	58.08519	0.105032	0.299045	0.467207	protein_coding
ENSG0000(	KLHL7-DT	698.8475	0.052633	0.29911	0.467287	lncRNA
ENSG0000(	USP8	7396.5	-0.05013	0.299158	0.467338	protein_coding
ENSG0000(	DDX51	1015.434	0.049958	0.299194	0.46737	protein_coding
ENSG0000(	BIK	39.0305	-0.18803	0.299222	0.467391	protein_coding
ENSG0000(	ENSG0000(	13.87964	0.177723	0.299295	0.467482	lncRNA
ENSG0000(	ENSG0000(	834.798	0.046617	0.299315	0.46749	lncRNA
ENSG0000(	PSMD5	765.1107	-0.04078	0.299383	0.46757	protein_coding
ENSG0000(	SPTBN1	16433.6	-0.06724	0.299395	0.46757	protein_coding
ENSG0000(	DRAXIN	757.7243	0.046532	0.299433	0.467605	protein_coding
ENSG0000(	ENSG0000(	50.62699	0.085867	0.2995	0.467686	lncRNA
ENSG0000(	NDUFAF3	187.1335	-0.07387	0.299517	0.46769	protein_coding
ENSG0000(	SNHG15	364.8875	0.108702	0.299556	0.467728	lncRNA
ENSG0000(	UNKL	1221.961	-0.07608	0.299575	0.467735	protein_coding
ENSG0000(	ENSG0000(	15.32599	0.276478	0.299648	0.4678	lncRNA
ENSG0000(	ENSG0000(	57.63189	-0.15555	0.299662	0.4678	lncRNA
ENSG0000(	ENSG0000(	26.86146	0.147606	0.299654	0.4678	TEC
ENSG0000(	ENSG0000(	20.54769	0.155252	0.299737	0.467894	lncRNA
ENSG0000(	ZNF513	414.2298	0.067729	0.299758	0.467905	protein_coding
ENSG0000(	ZNF559-ZN	168.1484	-0.12193	0.299774	0.467906	protein_coding
ENSG0000(	ENSG0000(	18.41709	0.175709	0.299837	0.467981	lncRNA
ENSG0000(	ENSG0000(	108.9664	0.088644	0.299884	0.468008	lncRNA
ENSG0000(	RPL7L1P10	30.96433	0.125711	0.299883	0.468008	processed_pseudogene
ENSG0000(	ENSG0000(	451.5458	-0.11335	0.2999	0.46801	lncRNA
ENSG0000(	ENSG0000(	323.917	0.066889	0.300056	0.468184	lncRNA
ENSG0000(	EXOC1	1363.554	0.047104	0.300042	0.468184	protein_coding
ENSG0000(	SDHA	4776.204	-0.07077	0.300056	0.468184	protein_coding
ENSG0000(	F10-AS1	35.08059	0.108786	0.300086	0.468207	lncRNA
ENSG0000(	MCF2L	2539.106	0.117873	0.300102	0.468209	protein_coding
ENSG0000(	TMEM33	2494.712	-0.03968	0.300201	0.46834	protein_coding
ENSG0000(	OCRL	1379.498	-0.10828	0.300221	0.468348	protein_coding
ENSG0000(	TAB3-AS1	42.25335	-0.09806	0.30026	0.468386	lncRNA
ENSG0000(	MMP23B	53.8593	0.147699	0.300322	0.468459	protein_coding
ENSG0000(	ENSG0000(	354.1506	-0.0425	0.300423	0.46857	lncRNA
ENSG0000(	HERC2P8	26.67739	0.263468	0.300416	0.46857	transcribed_unprocessed_pseudo
ENSG0000(	ENSG0000(	10.31352	-0.30047	0.30052	0.468676	processed_pseudogene
ENSG0000(	USP27X	76.8392	-0.09488	0.300517	0.468676	protein_coding
ENSG0000(	ITGA6	5598.145	-0.11821	0.300566	0.468725	protein_coding
ENSG0000(	ENSG0000(	17.02036	0.159461	0.30068	0.468879	lncRNA
ENSG0000(	ACTG2	483.7735	0.130191	0.300761	0.468981	protein_coding
ENSG0000(	BCL9L	2587.834	-0.07048	0.300832	0.469033	protein_coding
ENSG0000(	EEF1A1P12	34.95524	-0.16167	0.300839	0.469033	processed_pseudogene
ENSG0000(	LINC00460	24.03453	0.168311	0.300837	0.469033	lncRNA
ENSG0000(	TNS1-AS1	96.11265	-0.08855	0.300859	0.469042	lncRNA

ENSG0000(	ENSG0000(	46.52162	0.134706	0.300881	0.469052	snoRNA
ENSG0000(	ENSG0000(	54.75815	0.263651	0.301045	0.469216	lncRNA
ENSG0000(	ENSG0000(	395.9872	0.058958	0.301042	0.469216	TEC
ENSG0000(	ENSG0000(	65.92774	0.188938	0.301022	0.469216	processed_pseudogene
ENSG0000(	ETNK2	48.6099	0.116471	0.301005	0.469216	protein_coding
ENSG0000(	SH3BP5	901.4677	0.079847	0.301077	0.469243	protein_coding
ENSG0000(	CARD19	359.1636	0.070111	0.301134	0.469307	protein_coding
ENSG0000(	ENSG0000(	17.91759	0.196767	0.301157	0.469309	processed_pseudogene
ENSG0000(	ENSG0000(	134.8413	-0.06607	0.301164	0.469309	lncRNA
ENSG0000(	ZNF324	566.6839	-0.07219	0.301243	0.469408	protein_coding
ENSG0000(	ENSG0000(	18.50779	0.13806	0.301309	0.469471	lncRNA
ENSG0000(	LARP7	1040.044	0.047315	0.301313	0.469471	protein_coding
ENSG0000(	ENSG0000(	10.64924	0.216785	0.3014	0.469584	lncRNA
ENSG0000(	IL9R	124.1592	-0.24064	0.301442	0.469625	protein_coding
ENSG0000(	ENSG0000(	16.77202	0.138169	0.30161	0.469841	lncRNA
ENSG0000(	ENSG0000(	766.4376	0.062289	0.301599	0.469841	protein_coding
ENSG0000(	ENSG0000(	73.34235	-0.08726	0.30163	0.469848	lncRNA
ENSG0000(	ENSG0000(	50.18479	-0.09489	0.301701	0.46992	processed_pseudogene
ENSG0000(	PHF20	1574.67	0.079054	0.301723	0.46992	protein_coding
ENSG0000(	SMIM31	1070.256	-0.10856	0.301713	0.46992	protein_coding
ENSG0000(	TALDO1	699.4145	0.069955	0.301735	0.46992	protein_coding
ENSG0000(	LINC00308	15.33161	-0.16507	0.301869	0.470105	lncRNA
ENSG0000(	CENPC	2512.276	-0.05214	0.301955	0.470215	protein_coding
ENSG0000(	COQ10A	367.2762	0.061808	0.301995	0.470239	protein_coding
ENSG0000(	XRCC2	1065.332	0.039159	0.302	0.470239	protein_coding
ENSG0000(	ENSG0000(	12.21048	0.187153	0.302191	0.470512	lncRNA
ENSG0000(	ILK	1035.427	-0.04803	0.302237	0.470549	protein_coding
ENSG0000(	SNORA5A	78.13799	-0.10255	0.302244	0.470549	snoRNA
ENSG0000(	SNX25	816.7598	-0.06335	0.302316	0.470638	protein_coding
ENSG0000(	CAB39	2179.313	-0.0563	0.302386	0.470724	protein_coding
ENSG0000(	EFEMP1	297.3753	0.158911	0.302455	0.470777	protein_coding
ENSG0000(	ITFG2-AS1	30.6496	-0.13764	0.302465	0.470777	lncRNA
ENSG0000(	SIT1	127.933	-0.2323	0.302451	0.470777	protein_coding
ENSG0000(	CCDC167	33.52061	0.160068	0.302485	0.470783	protein_coding
ENSG0000(	FYN	1993.495	0.147547	0.302499	0.470783	protein_coding
ENSG0000(	ENSG0000(	244.1246	0.067684	0.302575	0.470878	lncRNA
ENSG0000(	ENSG0000(	135.2143	0.065147	0.302686	0.471005	lncRNA
ENSG0000(	HLA-DRB1	4056.469	-0.10286	0.302675	0.471005	protein_coding
ENSG0000(	CCL23	11.57587	-0.42015	0.302719	0.471032	protein_coding
ENSG0000(	LINC00331	149.3959	-0.08537	0.302752	0.471061	lncRNA
ENSG0000(	ENSG0000(	43.54572	-0.09391	0.302785	0.471088	lncRNA
ENSG0000(	ENSG0000(	19.02981	-0.23647	0.302862	0.471186	lncRNA
ENSG0000(	ENSG0000(	11.16849	0.19953	0.302897	0.471196	lncRNA
ENSG0000(	FAM50A	318.6105	-0.08032	0.302899	0.471196	protein_coding
ENSG0000(	ENSG0000(	81.55794	-0.0887	0.302993	0.471278	lncRNA
ENSG0000(	ENSG0000(	24.04324	-0.22333	0.302993	0.471278	processed_pseudogene
ENSG0000(	MCM4	800.8104	-0.07556	0.302997	0.471278	protein_coding

ENSG0000(	AMT	534.6509	0.080184	0.303065	0.471362	protein_coding
ENSG0000(	SERF2	2556.357	-0.06476	0.303118	0.471421	protein_coding
ENSG0000(	ENSG0000(	8.915803	0.256032	0.303143	0.471436	lncRNA
ENSG0000(	ENSG0000(	18.75017	0.17298	0.30326	0.471594	miRNA
ENSG0000(	RNU6-1223	7.860734	-0.21332	0.303339	0.471686	snRNA
ENSG0000(	YIPF3	651.7572	-0.0542	0.303349	0.471686	protein_coding
ENSG0000(	TMEM40	183.6045	-0.05484	0.303395	0.471735	protein_coding
ENSG0000(	ENSG0000(	13.10044	-0.54119	0.303446	0.471768	lncRNA
ENSG0000(	MIR6780B	8.718982	0.25161	0.303433	0.471768	miRNA
ENSG0000(	ENSG0000(	19.95285	0.20408	0.303471	0.471783	lncRNA
ENSG0000(	ENSG0000(	48.02327	0.115319	0.303503	0.471809	lncRNA
ENSG0000(	KCTD13-DT	318.0887	-0.05187	0.303518	0.471809	lncRNA
ENSG0000(	ATG9B	38.41886	0.212888	0.303591	0.471822	protein_coding
ENSG0000(	CAPZA2	2141.016	-0.08171	0.303568	0.471822	protein_coding
ENSG0000(	DNAJC10	8111.996	0.06916	0.303577	0.471822	protein_coding
ENSG0000(	HAND2-AS1	1039.138	0.043596	0.303601	0.471822	lncRNA
ENSG0000(	LTN1	2363.916	-0.03747	0.303616	0.471822	protein_coding
ENSG0000(	OPN3	286.6924	-0.08027	0.303608	0.471822	protein_coding
ENSG0000(	KRT8P31	30.3345	-0.17912	0.303635	0.471828	processed_pseudogene
ENSG0000(	ENSG0000(	197.2569	-0.08311	0.303669	0.471845	lncRNA
ENSG0000(	RNU2-6P	43.15283	0.138381	0.303676	0.471845	snRNA
ENSG0000(	GATA3	138.16	0.163444	0.303714	0.471882	protein_coding
ENSG0000(	STPG3-AS1	212.9548	-0.20114	0.303732	0.471887	lncRNA
ENSG0000(	UXS1	483.9968	-0.1119	0.303764	0.471913	protein_coding
ENSG0000(	ENSG0000(	128.6578	0.073982	0.303828	0.471989	lncRNA
ENSG0000(	LINC02752	13.31306	0.229929	0.30386	0.472015	lncRNA
ENSG0000(	BAD	167.5486	-0.08019	0.303892	0.472041	protein_coding
ENSG0000(	ABCC6	1353.65	-0.19851	0.303932	0.47208	protein_coding
ENSG0000(	ENSG0000(	215.8936	-0.05903	0.30397	0.472116	lncRNA
ENSG0000(	RPL23AP87	25.1594	0.467752	0.304078	0.472261	transcribed_processed_pseudoge
ENSG0000(	ENSG0000(	507.1265	0.063555	0.30414	0.472334	lncRNA
ENSG0000(	CETN3	181.8917	-0.06963	0.304251	0.472453	protein_coding
ENSG0000(	ENSG0000(	425.5284	-0.04208	0.304277	0.472453	lncRNA
ENSG0000(	IGLON5	64.26895	0.118603	0.304277	0.472453	protein_coding
ENSG0000(	TMEM35A	43.84685	-0.12843	0.304268	0.472453	protein_coding
ENSG0000(	ENSG0000(	13.07783	0.170273	0.304402	0.472554	lncRNA
ENSG0000(	ENSG0000(	113.9387	-0.05832	0.304398	0.472554	lncRNA
ENSG0000(	MDH1	951.0228	-0.04721	0.304385	0.472554	protein_coding
ENSG0000(	ZNF777	148.9015	-0.06895	0.304368	0.472554	protein_coding
ENSG0000(	ENSG0000(	31.47873	0.174862	0.304418	0.472556	processed_pseudogene
ENSG0000(	ENSG0000(	42.60318	0.137349	0.304474	0.47262	lncRNA
ENSG0000(	IL27RA	439.8082	-0.16447	0.304598	0.472788	protein_coding
ENSG0000(	PRH2	64.47415	0.159629	0.304636	0.472824	protein_coding
ENSG0000(	ENSG0000(	18.8096	-0.16687	0.30468	0.47287	lncRNA
ENSG0000(	HSP90AB4P	94.55111	-0.08667	0.304858	0.473122	processed_pseudogene
ENSG0000(	ENSG0000(	354.9029	0.06852	0.304947	0.473238	lncRNA
ENSG0000(	ENSG0000(	63.7138	-0.08085	0.305072	0.473408	lncRNA

ENSG0000(	INSYN1	215.2559	-0.05014	0.305107	0.473439	protein_coding
ENSG0000(	PSMD13	906.4734	-0.04201	0.305132	0.473454	protein_coding
ENSG0000(	MIF-AS1	108.263	0.376405	0.305157	0.473469	lncRNA
ENSG0000(	C14orf178	67.36463	-0.10066	0.305323	0.473703	lncRNA
ENSG0000(	ENSG0000(	690.0198	-0.13477	0.305337	0.473703	lncRNA
ENSG0000(	ENGASE	2647.055	-0.08762	0.305441	0.47384	protein_coding
ENSG0000(	ENSG0000(	132.4362	-0.08687	0.305477	0.473873	miRNA
ENSG0000(	JMJD7-PLA	1436.489	0.061151	0.305515	0.473909	protein_coding
ENSG0000(	HLA-A	10888.38	0.097812	0.305636	0.474065	protein_coding
ENSG0000(	LRRC55	47.7661	0.201345	0.305646	0.474065	protein_coding
ENSG0000(	GPR146	59.38638	0.154291	0.305664	0.474069	protein_coding
ENSG0000(	ENSG0000(	10.48341	-0.23011	0.305715	0.474125	processed_pseudogene
ENSG0000(	IGF2BP1	132.8109	0.089979	0.305788	0.474214	protein_coding
ENSG0000(	ENSG0000(	52.55703	0.135752	0.305862	0.474274	lncRNA
ENSG0000(	LINC00674	1098.043	-0.03848	0.305868	0.474274	transcribed_unprocessed_pseudo
ENSG0000(	PRKCQ-AS1	734.6712	0.14145	0.305871	0.474274	lncRNA
ENSG0000(	CLDN5	73.31522	-0.1725	0.305919	0.474322	protein_coding
ENSG0000(	ENSG0000(	133.8718	0.097355	0.305932	0.474322	lncRNA
ENSG0000(	MZT2B	164.6402	-0.08183	0.306022	0.474438	protein_coding
ENSG0000(	ENSG0000(	11.12182	0.226101	0.306082	0.474469	lncRNA
ENSG0000(	ENSG0000(	12.84149	0.215563	0.306089	0.474469	TEC
ENSG0000(	RNF223	63.82848	0.095661	0.306102	0.474469	protein_coding
ENSG0000(	SMARCAL1	585.9231	0.054286	0.306072	0.474469	protein_coding
ENSG0000(	ENSG0000(	113.7998	0.089309	0.306156	0.474506	lncRNA
ENSG0000(	PTK2B	3566.687	-0.05838	0.306154	0.474506	protein_coding
ENSG0000(	BUD13P1	14.17737	-0.18806	0.306309	0.4747	processed_pseudogene
ENSG0000(	RN7SKP62	46.91945	0.192015	0.306312	0.4747	misc_RNA
ENSG0000(	ENSG0000(	39.0878	0.108455	0.306335	0.474712	transcribed_processed_pseudoge
ENSG0000(	DMTN	118.3449	-0.12396	0.306381	0.474761	protein_coding
ENSG0000(	ENSG0000(	24.73672	0.139473	0.306421	0.4748	lncRNA
ENSG0000(	CRTAP	1228.811	-0.07333	0.306524	0.474889	protein_coding
ENSG0000(	DHX38	1173.382	-0.038	0.306498	0.474889	protein_coding
ENSG0000(	MRPL9	261.1714	-0.07861	0.306519	0.474889	protein_coding
ENSG0000(	AMOTL1	881.9714	0.114689	0.306565	0.474906	protein_coding
ENSG0000(	ENSG0000(	265.6694	0.057293	0.30656	0.474906	lncRNA
ENSG0000(	MINDY1	721.4387	-0.07059	0.30661	0.474938	protein_coding
ENSG0000(	PPP1R15A	896.1643	-0.09812	0.306616	0.474938	protein_coding
ENSG0000(	LINC01115	157.6014	0.082522	0.306661	0.474985	lncRNA
ENSG0000(	PEX10	258.7857	0.0718	0.306692	0.47501	protein_coding
ENSG0000(	CLTC	6620.628	-0.0521	0.306753	0.475081	protein_coding
ENSG0000(	MICA	1332.352	-0.10813	0.306803	0.475134	protein_coding
ENSG0000(	ILDR1	170.2641	0.076922	0.306883	0.475216	protein_coding
ENSG0000(	RPL10P16	18.44187	0.319543	0.306886	0.475216	processed_pseudogene
ENSG0000(	DLGAP2	75.91343	0.225279	0.306932	0.475241	protein_coding
ENSG0000(	MYT1	118.1752	-0.09801	0.306918	0.475241	protein_coding
ENSG0000(	SLC25A14	171.9232	-0.05614	0.30695	0.475246	protein_coding
ENSG0000(	LINC02724	115.6946	0.063077	0.307123	0.475467	lncRNA

ENSG0000(	SLC6A17	22.63074	0.231898	0.307115	0.475467	protein_coding
ENSG0000(	TMEM64	589.7543	-0.06403	0.307242	0.475627	protein_coding
ENSG0000(	CHST14	94.43029	0.134148	0.307305	0.475701	protein_coding
ENSG0000(	GLIPR1L2	744.5493	0.05544	0.307406	0.475834	protein_coding
ENSG0000(	CLCN1	132.0023	-0.33589	0.307425	0.47584	protein_coding
ENSG0000(	BBS9	936.2586	0.058003	0.307466	0.475881	protein_coding
ENSG0000(	ARL17A	2799.398	0.156994	0.307515	0.475934	protein_coding
ENSG0000(	ENSG0000(	332.8643	0.052051	0.307599	0.47604	TEC
ENSG0000(	CBL	2920.176	-0.05883	0.30776	0.476124	protein_coding
ENSG0000(	CYP4F30P	48.19827	0.224131	0.307752	0.476124	transcribed_unprocessed_pseudo
ENSG0000(	ENSG0000(	38.54859	-0.12635	0.307691	0.476124	lncRNA
ENSG0000(	NKX3-1	45.18053	0.161903	0.307719	0.476124	protein_coding
ENSG0000(	NYNRIN	642.231	-0.10508	0.307696	0.476124	protein_coding
ENSG0000(	RN7SKP178	14.29651	-0.2142	0.307745	0.476124	misc_RNA
ENSG0000(	SGK1	4566.515	0.239677	0.307706	0.476124	protein_coding
ENSG0000(	CBLN1	11.92525	-0.27525	0.307804	0.47617	protein_coding
ENSG0000(	ENSG0000(	11.23256	-0.21627	0.307826	0.476179	lncRNA
ENSG0000(	UCHL3	90.8656	-0.09727	0.307855	0.476179	protein_coding
ENSG0000(	ZUP1	281.5186	0.065718	0.307849	0.476179	protein_coding
ENSG0000(	CASC9	461.1609	0.108529	0.307917	0.476204	lncRNA
ENSG0000(	ENSG0000(	22.45537	0.298797	0.307888	0.476204	lncRNA
ENSG0000(	GTF2B	261.7618	-0.0573	0.307914	0.476204	protein_coding
ENSG0000(	ENSG0000(	91.25217	0.129606	0.308053	0.476367	lncRNA
ENSG0000(	ENSG0000(	19.6397	0.183136	0.308046	0.476367	lncRNA
ENSG0000(	PDZD11	184.8189	-0.08205	0.308135	0.476448	protein_coding
ENSG0000(	TMEM132(	27.25397	0.199806	0.308124	0.476448	protein_coding
ENSG0000(	ENSG0000(	279.2486	-0.07522	0.308196	0.476519	lncRNA
ENSG0000(	ENSG0000(	120.9364	-0.07594	0.308326	0.476696	lncRNA
ENSG0000(	NRBF2P6	33.85821	0.164618	0.308342	0.476697	processed_pseudogene
ENSG0000(	NIM1K	158.6198	-0.07178	0.30838	0.476734	protein_coding
ENSG0000(	ENSG0000(	99.9422	0.071089	0.308431	0.47675	lncRNA
ENSG0000(	NAP1L3	21.452	0.166391	0.308426	0.47675	protein_coding
ENSG0000(	SPATA33	732.49	-0.10205	0.308436	0.47675	protein_coding
ENSG0000(	ENSG0000(	57.15589	0.11448	0.308581	0.476927	lncRNA
ENSG0000(	ENSG0000(	347.7366	0.047071	0.30861	0.476927	lncRNA
ENSG0000(	GNRH2	21.05364	0.167121	0.308582	0.476927	protein_coding
ENSG0000(	GTF2H2B	171.3387	-0.18648	0.308612	0.476927	transcribed_unprocessed_pseudo
ENSG0000(	ENSG0000(	59.97092	0.224634	0.308661	0.476956	TEC
ENSG0000(	ITGA7	15157.78	-0.07533	0.30866	0.476956	protein_coding
ENSG0000(	ZNF350-AS	290.9836	0.108643	0.308791	0.477135	lncRNA
ENSG0000(	ENSG0000(	91.32182	-0.1013	0.309003	0.477438	lncRNA
ENSG0000(	INAFM2	273.3174	0.093177	0.309042	0.477475	protein_coding
ENSG0000(	ENSG0000(	248.9994	-0.05763	0.309071	0.477491	lncRNA
ENSG0000(	NHP2	185.5586	-0.07711	0.309083	0.477491	protein_coding
ENSG0000(	GPN2	1315.752	-0.08467	0.309104	0.4775	protein_coding
ENSG0000(	MKRN2OS	390.7495	-0.04925	0.309134	0.477523	protein_coding
ENSG0000(	ENSG0000(	1912.678	0.06748	0.309174	0.477563	lncRNA

ENSG0000(	CEP85	527.0878	0.068529	0.309299	0.477721	protein_coding
ENSG0000(	ENSG0000(	141.1796	-0.0694	0.309311	0.477721	lncRNA
ENSG0000(	TRPM2	647.0155	0.132776	0.309323	0.477721	protein_coding
ENSG0000(	ITPR2	6154.353	0.064376	0.309354	0.477728	protein_coding
ENSG0000(	NAP1L4	2341.658	-0.03643	0.309357	0.477728	protein_coding
ENSG0000(	CCDC51	84.914	0.079574	0.309409	0.477785	protein_coding
ENSG0000(	LINC02527	15.07122	0.174549	0.309477	0.477866	lncRNA
ENSG0000(	COA6-AS1	26.48683	-0.1452	0.309514	0.47788	lncRNA
ENSG0000(	FDFT1	1464.704	-0.05068	0.309516	0.47788	protein_coding
ENSG0000(	ENSG0000(	70.62801	0.090202	0.309602	0.477989	processed_pseudogene
ENSG0000(	ZNF526	731.218	0.043023	0.309632	0.478013	protein_coding
ENSG0000(	CRYZP2	8.079093	0.229771	0.309717	0.478092	processed_pseudogene
ENSG0000(	ENSG0000(	218.7576	0.095673	0.309706	0.478092	lncRNA
ENSG0000(	MORN4	298.1361	-0.05168	0.309729	0.478092	protein_coding
ENSG0000(	LYSMD2	98.52236	-0.13443	0.309857	0.478266	protein_coding
ENSG0000(	NSFL1C	1195.647	0.050628	0.309929	0.478353	protein_coding
ENSG0000(	MCM3AP	3884.063	0.043849	0.309967	0.478388	protein_coding
ENSG0000(	ENSG0000(	169.3622	-0.0568	0.31	0.478414	lncRNA
ENSG0000(	GPHN	598.9688	-0.06383	0.310014	0.478414	protein_coding
ENSG0000(	LRCOL1	10.99857	-0.23211	0.31003	0.478415	protein_coding
ENSG0000(	EPS15P1	68.93334	0.093273	0.310064	0.478445	transcribed_processed_pseudoge
ENSG0000(	ENSG0000(	301.106	0.065868	0.310158	0.478566	lncRNA
ENSG0000(	Y_RNA	3862.505	0.065286	0.310174	0.478567	misc_RNA
ENSG0000(	HSPA12A	2054.989	0.062394	0.31022	0.478598	protein_coding
ENSG0000(	OR1AB1P	72.84023	-0.07939	0.310235	0.478598	unprocessed_pseudogene
ENSG0000(	PRNP	694.1315	-0.11194	0.310239	0.478598	protein_coding
ENSG0000(	TAX1BP3	641.1856	-0.17922	0.31028	0.478636	protein_coding
ENSG0000(	RN7SL846F	17.89715	0.178918	0.310358	0.478734	misc_RNA
ENSG0000(	ENSG0000(	143.6745	-0.09841	0.310433	0.478803	lncRNA
ENSG0000(	GUSBP9	832.7059	-0.19004	0.310423	0.478803	unprocessed_pseudogene
ENSG0000(	RN7SL18P	50.73929	0.108306	0.310523	0.478918	misc_RNA
ENSG0000(	AKR1C7P	18.81904	-0.20188	0.310546	0.47893	transcribed_unprocessed_pseudo
ENSG0000(	ENSG0000(	769.7075	-0.06092	0.310723	0.47918	lncRNA
ENSG0000(	ENSG0000(	8.237843	0.375219	0.310782	0.4792	lncRNA
ENSG0000(	KIAA1549L	31.56383	0.21536	0.310778	0.4792	protein_coding
ENSG0000(	RPUSD3	910.7167	-0.0478	0.310782	0.4792	protein_coding
ENSG0000(	KLHL28	948.3095	0.042229	0.310812	0.479224	protein_coding
ENSG0000(	TMEM229E	789.6554	0.070325	0.310856	0.479268	protein_coding
ENSG0000(	ENSG0000(	58.88407	0.105501	0.310939	0.479372	lncRNA
ENSG0000(	GBE1	389.6721	0.076456	0.310995	0.479434	protein_coding
ENSG0000(	TRIM40	315.4953	-0.25507	0.311067	0.479523	protein_coding
ENSG0000(	FAM53B	2493.316	-0.05519	0.311119	0.479579	protein_coding
ENSG0000(	ENSG0000(	48.25028	-0.11265	0.311153	0.479608	lncRNA
ENSG0000(	CCDC116	20.57005	0.204067	0.311196	0.47965	protein_coding
ENSG0000(	LINC00664	529.3394	-0.0362	0.311235	0.479665	lncRNA
ENSG0000(	OTULINL	1418.702	-0.06561	0.311223	0.479665	protein_coding
ENSG0000(	LINC00958	354.1077	-0.04135	0.311283	0.479715	lncRNA

ENSG0000(	VPS33B	335.8761	-0.04137	0.311305	0.479725	protein_coding
ENSG0000(	SOS2	3178.015	-0.07898	0.311322	0.479728	protein_coding
ENSG0000(	ENSG0000(	39.83736	0.248116	0.311398	0.479821	lncRNA
ENSG0000(	MOXD1	160.7045	0.155018	0.311448	0.479868	protein_coding
ENSG0000(	PSMG3-AS:	270.593	0.07178	0.311459	0.479868	lncRNA
ENSG0000(	ENSG0000(	99.40128	-0.09488	0.311532	0.479916	processed_pseudogene
ENSG0000(	ENSG0000(	81.41871	0.096881	0.311547	0.479916	lncRNA
ENSG0000(	MYO5C	1500.9	0.135326	0.311566	0.479916	protein_coding
ENSG0000(	RPL13AP3	9.932503	0.257513	0.311556	0.479916	transcribed_processed_pseudoge
ENSG0000(	TOP1	3082.936	0.048846	0.311547	0.479916	protein_coding
ENSG0000(	RNU6-876F	13.98786	0.281206	0.311608	0.479957	snRNA
ENSG0000(	BCL2L2-PAI	147.8651	0.156595	0.311705	0.48006	protein_coding
ENSG0000(	PTCD2	1085.526	-0.04613	0.311696	0.48006	protein_coding
ENSG0000(	G6PD	228.7853	0.07374	0.311825	0.48022	protein_coding
ENSG0000(	TRIML2	517.9043	0.048594	0.311878	0.48028	protein_coding
ENSG0000(	KCTD21	386.491	-0.05258	0.31196	0.480369	protein_coding
ENSG0000(	NKIRAS2	1048.58	0.037826	0.311967	0.480369	protein_coding
ENSG0000(	C14orf132	751.4409	0.067197	0.312003	0.480378	protein_coding
ENSG0000(	YIF1A	299.29	-0.0905	0.311994	0.480378	protein_coding
ENSG0000(	ADAMTS9-,	31.63072	-0.24476	0.312141	0.480423	lncRNA
ENSG0000(	BCL2L14	1318.588	-0.14755	0.312125	0.480423	protein_coding
ENSG0000(	EMBP1	797.4721	0.072952	0.312113	0.480423	transcribed_unprocessed_pseudo
ENSG0000(	ENSG0000(	10.85872	-0.33719	0.31209	0.480423	lncRNA
ENSG0000(	ENSG0000(	566.887	0.045801	0.312163	0.480423	lncRNA
ENSG0000(	ENSG0000(	20.4448	0.155681	0.312159	0.480423	lncRNA
ENSG0000(	OR2A3P	127.5806	0.084332	0.312129	0.480423	transcribed_unprocessed_pseudo
ENSG0000(	RGS3	1553.796	-0.0809	0.312069	0.480423	protein_coding
ENSG0000(	RNLS	99.98356	-0.14086	0.31217	0.480423	protein_coding
ENSG0000(	ENSG0000(	25.03236	-0.13039	0.312212	0.480442	lncRNA
ENSG0000(	ENSG0000(	12.65156	0.205239	0.312205	0.480442	lncRNA
ENSG0000(	ADGRF3	60.04996	0.208281	0.312402	0.48071	protein_coding
ENSG0000(	SIAH2-AS1	77.45859	-0.11949	0.312426	0.480723	lncRNA
ENSG0000(	ENSG0000(	162.2192	-0.17456	0.312477	0.480754	lncRNA
ENSG0000(	MAP3K7	1428.516	-0.03262	0.312462	0.480754	protein_coding
ENSG0000(	ZCWPW2	76.4765	0.089048	0.312496	0.480761	protein_coding
ENSG0000(	NANOGP11	10.53423	-0.29286	0.312526	0.480784	lncRNA
ENSG0000(	TMC7	719.5036	0.053822	0.312709	0.481041	protein_coding
ENSG0000(	RAMP2-AS:	611.3365	0.044941	0.312765	0.481104	lncRNA
ENSG0000(	HNRNPR	3953.799	-0.04689	0.312788	0.481117	protein_coding
ENSG0000(	IPO8P1	12.19923	-0.24849	0.312825	0.48115	processed_pseudogene
ENSG0000(	ANGPTL1	117.8645	-0.18344	0.312874	0.481166	protein_coding
ENSG0000(	ENSG0000(	133.4192	0.073934	0.312896	0.481166	lncRNA
ENSG0000(	LINC02211	43.51572	0.117436	0.312851	0.481166	lncRNA
ENSG0000(	MFSD2A	964.6164	-0.21163	0.312897	0.481166	protein_coding
ENSG0000(	SIVA1	690.4386	-0.05636	0.313008	0.481314	protein_coding
ENSG0000(	LINC01035	9.533298	0.219947	0.313025	0.481317	lncRNA
ENSG0000(	ENSG0000(	206.8969	-0.07558	0.313056	0.48134	lncRNA

ENSG0000(	MPL	249.3564	-0.04971	0.313102	0.481387	protein_coding
ENSG0000(	SMC6	2374.465	0.045108	0.313231	0.481538	protein_coding
ENSG0000(	TGFB1I1	384.4134	0.092659	0.313216	0.481538	protein_coding
ENSG0000(	SLC9C1	33.57777	0.253818	0.313358	0.481711	protein_coding
ENSG0000(	ENSG0000(	501.8127	0.065064	0.313411	0.481769	TEC
ENSG0000(	ENSG0000(	20.77489	0.163003	0.313467	0.481807	lncRNA
ENSG0000(	PAK3	359.8691	0.10336	0.313463	0.481807	protein_coding
ENSG0000(	NOVA1	444.2371	-0.08093	0.313547	0.481907	protein_coding
ENSG0000(	LINC00992	21.67751	-0.1828	0.31361	0.48198	lncRNA
ENSG0000(	TRBJ2-7	26.13129	0.313733	0.31365	0.482018	TR_J_gene
ENSG0000(	LRFN1	150.4607	0.090435	0.313671	0.482022	protein_coding
ENSG0000(	MACIR	440.58	-0.15421	0.313683	0.482022	protein_coding
ENSG0000(	ENSG0000(	61.8544	-0.09332	0.313818	0.482113	lncRNA
ENSG0000(	ENSG0000(	1498.143	0.042294	0.313782	0.482113	transcribed_unprocessed_pseudo
ENSG0000(	LARS1	5949.371	-0.04328	0.313812	0.482113	protein_coding
ENSG0000(	RRM1-AS1	21.91957	0.177262	0.313834	0.482113	lncRNA
ENSG0000(	SMG1P1	14948.22	-0.06372	0.313795	0.482113	transcribed_unprocessed_pseudo
ENSG0000(	SP8	31.10266	-0.36344	0.313826	0.482113	protein_coding
ENSG0000(	ENSG0000(	196.6987	-0.0508	0.313932	0.48224	lncRNA
ENSG0000(	ENSG0000(	244.0168	0.055901	0.313982	0.482294	TEC
ENSG0000(	DNAH9	161.1463	0.059162	0.314031	0.482338	protein_coding
ENSG0000(	ENSG0000(	72.21962	0.074663	0.314042	0.482338	lncRNA
ENSG0000(	ENSG0000(	24.72482	0.166976	0.314072	0.482338	lncRNA
ENSG0000(	TBC1D8B	6205.391	-0.0565	0.314064	0.482338	protein_coding
ENSG0000(	ENSG0000(	63.55427	0.163311	0.314119	0.482374	lncRNA
ENSG0000(	TNIP1	2854.341	0.074679	0.314127	0.482374	protein_coding
ENSG0000(	ENSG0000(	9.916241	0.18033	0.314165	0.482406	lncRNA
ENSG0000(	GNMT	17.23249	-0.22324	0.314178	0.482406	protein_coding
ENSG0000(	DLG1	5403.02	-0.05587	0.314246	0.482477	protein_coding
ENSG0000(	ELMO1	2584.153	0.11954	0.314255	0.482477	protein_coding
ENSG0000(	LINC02444	2253.796	-0.07358	0.314307	0.482534	lncRNA
ENSG0000(	ENSG0000(	12.00443	-0.20322	0.314324	0.482537	lncRNA
ENSG0000(	C3orf70	645.976	0.077631	0.314524	0.48282	protein_coding
ENSG0000(	GNL2	1207.854	0.049572	0.314552	0.482839	protein_coding
ENSG0000(	ZNF420	278.7549	-0.08051	0.314573	0.482848	protein_coding
ENSG0000(	ENSG0000(	8755.221	0.067303	0.314599	0.482865	lncRNA
ENSG0000(	TBCC	306.3128	-0.04926	0.314792	0.483137	protein_coding
ENSG0000(	PPIAP19	102.8103	-0.13526	0.314809	0.483139	processed_pseudogene
ENSG0000(	ENSG0000(	521.5022	0.044564	0.315	0.483408	lncRNA
ENSG0000(	STK25	1704.577	-0.0404	0.315026	0.483425	protein_coding
ENSG0000(	ENSG0000(	59.21315	-0.10291	0.315064	0.48346	protein_coding
ENSG0000(	ENSG0000(	14.41567	0.214409	0.315144	0.48356	transcribed_processed_pseudoge
ENSG0000(	ABI3BP	1507.776	0.095858	0.315269	0.483727	protein_coding
ENSG0000(	KIF18B	612.3208	0.079351	0.315373	0.483864	protein_coding
ENSG0000(	ENSG0000(	62.36966	0.137826	0.315402	0.483875	lncRNA
ENSG0000(	PCDH10	23.77328	0.186304	0.315411	0.483875	protein_coding
ENSG0000(	ENSG0000(	28.3981	0.166448	0.315444	0.483902	lncRNA

ENSG0000(	HEATR3	1348.9	-0.04374	0.315495	0.483957	protein_coding
ENSG0000(	ENSG0000(	12.10039	-0.44934	0.315614	0.484116	protein_coding
ENSG0000(	ZNF776	1430.517	-0.05503	0.315658	0.484159	protein_coding
ENSG0000(	RN7SL304F	12.05534	0.175626	0.315755	0.484284	misc_RNA
ENSG0000(	ENSG0000(	81.86911	-0.11768	0.315846	0.48431	lncRNA
ENSG0000(	FOXP1-AS1	223.2635	-0.0464	0.315848	0.48431	lncRNA
ENSG0000(	HLA-DRA	6385.996	-0.10916	0.315815	0.48431	protein_coding
ENSG0000(	OTX2P2	8.793824	0.338499	0.315828	0.48431	processed_pseudogene
ENSG0000(	TK2	1333.018	0.06665	0.315788	0.48431	protein_coding
ENSG0000(	ENSG0000(	9.071027	0.200572	0.315953	0.484359	processed_pseudogene
ENSG0000(	HCAR1	576.0672	-0.04226	0.315943	0.484359	protein_coding
ENSG0000(	LRCH1	1855.677	0.049089	0.315909	0.484359	protein_coding
ENSG0000(	RPH3AL	539.2872	0.1166	0.315942	0.484359	protein_coding
ENSG0000(	WNT2B	1421.292	0.097676	0.315957	0.484359	protein_coding
ENSG0000(	RASL10A	43.27136	-0.11418	0.316118	0.484583	protein_coding
ENSG0000(	BMP8B	390.9439	-0.17862	0.316162	0.484626	protein_coding
ENSG0000(	SCAF1	743.1178	-0.05814	0.316179	0.484629	protein_coding
ENSG0000(	EFNB3	28.4714	-0.16632	0.316222	0.484671	protein_coding
ENSG0000(	LINC00957	910.7254	0.044369	0.316282	0.484738	lncRNA
ENSG0000(	ZNF575	85.44195	-0.10738	0.316391	0.484883	protein_coding
ENSG0000(	ENSG0000(	292.897	-0.29425	0.316604	0.485185	lncRNA
ENSG0000(	ENSG0000(	118.2976	-0.06652	0.31667	0.485228	processed_pseudogene
ENSG0000(	FLYWCH1	1310.777	-0.06284	0.316678	0.485228	protein_coding
ENSG0000(	PRPH	15.12037	0.234768	0.316654	0.485228	protein_coding
ENSG0000(	ENSG0000(	131.2778	0.113073	0.316698	0.485236	lncRNA
ENSG0000(	ENSG0000(	26.46788	0.19018	0.316791	0.485354	lncRNA
ENSG0000(	ABLIM2	221.4259	0.15056	0.316925	0.485535	protein_coding
ENSG0000(	ENSG0000(	26.62978	0.131111	0.316977	0.485546	snoRNA
ENSG0000(	HSPB6	29.65004	0.251678	0.316967	0.485546	protein_coding
ENSG0000(	PON3	195.9369	-0.09319	0.316978	0.485546	protein_coding
ENSG0000(	ENSG0000(	53.32923	0.082574	0.317089	0.48568	lncRNA
ENSG0000(	ENSG0000(	671.6745	-0.03186	0.317096	0.48568	lncRNA
ENSG0000(	OXCT1	595.0781	0.080625	0.317132	0.485711	protein_coding
ENSG0000(	FAM151B	185.2115	-0.06883	0.317167	0.485741	protein_coding
ENSG0000(	ATP2C2-AS	204.7248	-0.13874	0.317269	0.48585	lncRNA
ENSG0000(	VN1R42P	14.32774	-0.20833	0.317266	0.48585	processed_pseudogene
ENSG0000(	FANCD2OS	94.69739	-0.10488	0.317302	0.485877	protein_coding
ENSG0000(	ENSG0000(	11.90943	0.217674	0.317325	0.485889	lncRNA
ENSG0000(	FAM86B1	93.31044	0.085484	0.317397	0.485975	protein_coding
ENSG0000(	RNU6-619F	22.40931	-0.1648	0.317438	0.486014	snRNA
ENSG0000(	ENSG0000(	26.10176	0.123314	0.317495	0.486078	lncRNA
ENSG0000(	ENSG0000(	27.63197	0.122815	0.317542	0.486102	lncRNA
ENSG0000(	SYNGAP1-A	63.85494	-0.10352	0.317534	0.486102	lncRNA
ENSG0000(	ATG10-IT1	166.1662	-0.06413	0.317587	0.486148	lncRNA
ENSG0000(	RET	88.68682	0.182622	0.317659	0.486234	protein_coding
ENSG0000(	NENF	144.7508	0.078961	0.31771	0.486289	protein_coding
ENSG0000(	ENSG0000(	120.886	-0.06243	0.318069	0.486815	lncRNA

ENSG0000(	MRPL32	382.5555	-0.06808	0.318094	0.486829	protein_coding
ENSG0000(	ENSG0000(	65.53339	-0.08234	0.318182	0.486941	lncRNA
ENSG0000(	ADK	619.0928	0.087473	0.318219	0.486973	protein_coding
ENSG0000(	ENSG0000(	18.61172	-0.26265	0.318257	0.487007	lncRNA
ENSG0000(	CCDC121	66.26446	0.105875	0.318292	0.487011	protein_coding
ENSG0000(	RN7SL503F	20.30215	0.127928	0.318306	0.487011	misc_RNA
ENSG0000(	STK4	4848.992	0.101954	0.318276	0.487011	protein_coding
ENSG0000(	USP33	3147.154	-0.03191	0.318333	0.487029	protein_coding
ENSG0000(	JAM2	714.7284	-0.06242	0.318408	0.48712	protein_coding
ENSG0000(	BTBD9	3560.949	-0.07768	0.318463	0.487182	protein_coding
ENSG0000(	DMTF1	5086.217	-0.05986	0.318492	0.487202	protein_coding
ENSG0000(	FAM224A	82.0245	0.716147	0.318541	0.487253	lncRNA
ENSG0000(	ENSG0000(	723.4787	0.052481	0.318575	0.487258	TEC
ENSG0000(	FAF2	1274.291	0.042794	0.31857	0.487258	protein_coding
ENSG0000(	ENSG0000(	98.23921	0.186028	0.318637	0.487269	lncRNA
ENSG0000(	GPR107	2211.548	0.06162	0.318644	0.487269	protein_coding
ENSG0000(	PRORY	85.69104	-0.12073	0.318639	0.487269	lncRNA
ENSG0000(	REG4	3854.138	0.215567	0.318622	0.487269	protein_coding
ENSG0000(	CUL5	1417.975	0.062679	0.318695	0.487315	protein_coding
ENSG0000(	SNORD116	14.34527	0.287241	0.318705	0.487315	snoRNA
ENSG0000(	NOS3	348.7415	0.09265	0.318734	0.487335	protein_coding
ENSG0000(	ENSG0000(	9.561519	-0.23204	0.318805	0.487406	lncRNA
ENSG0000(	LINC00392	35.89445	0.196891	0.318811	0.487406	lncRNA
ENSG0000(	LINC01121	217.4847	0.060984	0.31884	0.487417	lncRNA
ENSG0000(	LITAFD	166.5114	-0.0673	0.318849	0.487417	protein_coding
ENSG0000(	AGAP7P	89.58146	0.259646	0.318902	0.487473	unprocessed_pseudogene
ENSG0000(	DCDC2C	78.96827	0.079795	0.318952	0.487527	protein_coding
ENSG0000(	FAM13A	2797.319	-0.15568	0.318988	0.487559	protein_coding
ENSG0000(	DNAH17-A!	270.1858	0.060796	0.319053	0.487592	lncRNA
ENSG0000(	ENSG0000(	10.21367	0.195577	0.319057	0.487592	lncRNA
ENSG0000(	TUBB4A	22.45989	-0.17929	0.319035	0.487592	protein_coding
ENSG0000(	GABRA2	1142.67	-0.20634	0.319089	0.487604	protein_coding
ENSG0000(	SSTR3	62.27065	0.307943	0.319095	0.487604	protein_coding
ENSG0000(	RNU6-251F	34.09902	-0.20294	0.319139	0.487647	snRNA
ENSG0000(	S100PBP	2779.46	0.045584	0.319198	0.487714	protein_coding
ENSG0000(	ENSG0000(	37.19337	0.194413	0.319259	0.487783	lncRNA
ENSG0000(	ARHGAP42	1195.103	-0.09494	0.319335	0.48784	protein_coding
ENSG0000(	HYKK	1291.121	-0.05553	0.319343	0.48784	protein_coding
ENSG0000(	NUDT21	1807.524	-0.02839	0.31934	0.48784	protein_coding
ENSG0000(	FOXO6	11.28706	-0.2167	0.319434	0.487956	protein_coding
ENSG0000(	ANKDD1B	86.20798	0.142966	0.31947	0.487964	protein_coding
ENSG0000(	ZNF204P	214.654	0.111972	0.319458	0.487964	transcribed_unprocessed_pseudo
ENSG0000(	NR1H4	1624.34	-0.19834	0.319512	0.488003	protein_coding
ENSG0000(	H2AX	193.9632	-0.11524	0.319625	0.488129	protein_coding
ENSG0000(	PTPRU	117.282	0.233754	0.319618	0.488129	protein_coding
ENSG0000(	ENSG0000(	9.691611	0.21919	0.319673	0.488175	lncRNA
ENSG0000(	PCDHA3	14.89609	0.246686	0.319686	0.488175	protein_coding

ENSG0000(	OR7C1	971.5548	0.054443	0.319726	0.488212	protein_coding
ENSG0000(	RHBDD1	1277.411	0.055865	0.319742	0.488213	protein_coding
ENSG0000(	APIP	487.8062	-0.0813	0.319775	0.48824	protein_coding
ENSG0000(	LINC02636	19.50664	0.163559	0.31985	0.488331	lncRNA
ENSG0000(	ENSG0000(	163.3211	-0.06994	0.319975	0.488492	processed_pseudogene
ENSG0000(	RBM12	3582.338	-0.04793	0.319986	0.488492	protein_coding
ENSG0000(	SLC30A6-D	242.2854	-0.05266	0.320003	0.488494	lncRNA
ENSG0000(	MAP4K3-D	2118.043	-0.05657	0.320058	0.488507	lncRNA
ENSG0000(	SPCS2	843.0634	-0.08856	0.32004	0.488507	protein_coding
ENSG0000(	TMEM54	759.0677	-0.1709	0.320057	0.488507	protein_coding
ENSG0000(	EXOSC7	540.4948	0.057211	0.320083	0.488521	protein_coding
ENSG0000(	TTPA	68.27821	-0.0934	0.320162	0.488618	protein_coding
ENSG0000(	SFTPB	188.7908	0.076005	0.320196	0.488623	protein_coding
ENSG0000(	VRTN	75.80371	-0.08238	0.320185	0.488623	protein_coding
ENSG0000(	ENSG0000(	14.63427	-0.21598	0.320228	0.488648	transcribed_unprocessed_pseudo
ENSG0000(	ALDH16A1	903.1396	-0.05791	0.320345	0.488803	protein_coding
ENSG0000(	ENSG0000(	11.71216	-0.3609	0.320445	0.48887	processed_pseudogene
ENSG0000(	ENSG0000(	205.2219	-0.05646	0.320418	0.48887	lncRNA
ENSG0000(	ENSG0000(	66.95763	-0.09161	0.320435	0.48887	lncRNA
ENSG0000(	POC1B-GAI	108.242	0.366368	0.320452	0.48887	protein_coding
ENSG0000(	ENSG0000(	54.86213	0.091606	0.320489	0.488904	lncRNA
ENSG0000(	CCDC107	105.5605	-0.09178	0.320634	0.489034	protein_coding
ENSG0000(	DNAJC18	666.8393	-0.04989	0.320608	0.489034	protein_coding
ENSG0000(	ENSG0000(	3326.112	-0.06586	0.320592	0.489034	lncRNA
ENSG0000(	ENSG0000(	58.36616	-0.08143	0.320637	0.489034	processed_pseudogene
ENSG0000(	ENSG0000(	521.7936	-0.05675	0.320784	0.489236	lncRNA
ENSG0000(	NPIPA1	5968.077	0.04439	0.320912	0.489398	protein_coding
ENSG0000(	UBA5	2079.409	-0.04028	0.320922	0.489398	protein_coding
ENSG0000(	PTPRD-AS1	521.5675	-0.07257	0.321003	0.489451	lncRNA
ENSG0000(	TRIM8	1881.12	-0.04702	0.321003	0.489451	protein_coding
ENSG0000(	WASH3P	761.0721	-0.06805	0.321003	0.489451	transcribed_unprocessed_pseudo
ENSG0000(	EOLA1	559.2157	0.051977	0.321024	0.489459	protein_coding
ENSG0000(	ENSG0000(	230.4889	-0.04568	0.321043	0.489464	lncRNA
ENSG0000(	RNU6-750F	31.47841	0.167944	0.321079	0.489496	snRNA
ENSG0000(	PAQR3	1168.564	-0.10619	0.321164	0.489602	protein_coding
ENSG0000(	SDHAP3	2304.384	0.064933	0.321272	0.489742	transcribed_unprocessed_pseudo
ENSG0000(	C2-AS1	48.57395	0.078748	0.321454	0.489964	lncRNA
ENSG0000(	ELAC1	433.2339	-0.04214	0.321495	0.489964	protein_coding
ENSG0000(	ENSG0000(	684.4326	0.06154	0.321442	0.489964	lncRNA
ENSG0000(	ENSG0000(	9.998324	-0.24044	0.321495	0.489964	processed_pseudogene
ENSG0000(	PPP1R13B-	82.441	0.096234	0.321483	0.489964	lncRNA
ENSG0000(	KIF9-AS1	944.7011	-0.04202	0.321538	0.490006	lncRNA
ENSG0000(	ENSG0000(	100.0297	0.098406	0.321657	0.490164	lncRNA
ENSG0000(	RPS26P47	9.709341	0.364516	0.321704	0.490211	processed_pseudogene
ENSG0000(	PLPP3	1156.516	0.085002	0.321722	0.490216	protein_coding
ENSG0000(	ENSG0000(	26.29255	0.197986	0.321802	0.49029	processed_pseudogene
ENSG0000(	GOLGA5	801.8793	0.057123	0.321798	0.49029	protein_coding

ENSG0000(	ENSG0000(	317.7138	0.055126	0.321829	0.490307	lncRNA
ENSG0000(	C6orf163	476.8805	0.060355	0.32187	0.490323	protein_coding
ENSG0000(	ENSG0000(	35.70413	-0.39859	0.321856	0.490323	processed_pseudogene
ENSG0000(	C19orf44	193.8529	0.079011	0.321973	0.490456	protein_coding
ENSG0000(	CARF	1158.35	0.04256	0.322028	0.490515	protein_coding
ENSG0000(	ENSG0000(	9.825962	0.185559	0.322079	0.490545	processed_pseudogene
ENSG0000(	SLC35F6	1257.5	0.04037	0.322072	0.490545	protein_coding
ENSG0000(	GTF3C5	721.9375	-0.04318	0.32215	0.49063	protein_coding
ENSG0000(	MPV17L	1640.932	0.043927	0.322171	0.490638	protein_coding
ENSG0000(	ENSG0000(	9.670986	0.160874	0.322199	0.490657	lncRNA
ENSG0000(	ENSG0000(	14.35212	0.160046	0.322271	0.49072	processed_pseudogene
ENSG0000(	H3-3B	3360.327	0.063619	0.322266	0.49072	protein_coding
ENSG0000(	ENSG0000(	11.82008	0.21248	0.322324	0.490777	lncRNA
ENSG0000(	ENSG0000(	897.379	-0.04673	0.322433	0.490918	lncRNA
ENSG0000(	KIAA1755	896.6179	0.124184	0.322456	0.490929	protein_coding
ENSG0000(	RBM6	7035.242	0.040328	0.32256	0.491064	protein_coding
ENSG0000(	ENSG0000(	87.65585	0.094917	0.322708	0.491257	lncRNA
ENSG0000(	RARA-AS1	52.51445	-0.13389	0.322733	0.491257	lncRNA
ENSG0000(	SEL1L3	5435.226	-0.06384	0.322729	0.491257	protein_coding
ENSG0000(	EPB41L3	3269.463	-0.15707	0.322824	0.491324	protein_coding
ENSG0000(	HPS6	221.2391	-0.07025	0.3228	0.491324	protein_coding
ENSG0000(	SNRPB2	249.1751	-0.08961	0.322808	0.491324	protein_coding
ENSG0000(	ENSG0000(	179.1791	0.064165	0.322946	0.491462	lncRNA
ENSG0000(	LINC02594	125.665	-0.06424	0.32294	0.491462	lncRNA
ENSG0000(	ENSG0000(	11.18567	0.248747	0.322989	0.491504	lncRNA
ENSG0000(	EMD	221.4996	-0.06422	0.323135	0.491655	protein_coding
ENSG0000(	TRAV29DV!	10.48458	-0.2753	0.323109	0.491655	TR_V_gene
ENSG0000(	ZBED5	2079.406	-0.06207	0.32312	0.491655	protein_coding
ENSG0000(	BBS1	759.906	0.030945	0.323197	0.491661	protein_coding
ENSG0000(	EAF2	252.2494	0.168573	0.323204	0.491661	protein_coding
ENSG0000(	HNRNPA3	7480.202	0.034183	0.323219	0.491661	protein_coding
ENSG0000(	LINC01141	77.67345	-0.09366	0.323233	0.491661	lncRNA
ENSG0000(	RNVU1-27	81.74337	-0.09489	0.32319	0.491661	snRNA
ENSG0000(	SMIM4	203.5713	-0.08096	0.323163	0.491661	protein_coding
ENSG0000(	ENSG0000(	10.86662	0.195491	0.323265	0.491687	processed_pseudogene
ENSG0000(	TM9SF3	5357.062	-0.08089	0.323338	0.491773	protein_coding
ENSG0000(	ENSG0000(	65.81077	0.069292	0.323417	0.491864	lncRNA
ENSG0000(	RN7SL581F	24.20314	0.141221	0.323428	0.491864	misc_RNA
ENSG0000(	RN7SL376F	123.7718	-0.07143	0.323497	0.491944	misc_RNA
ENSG0000(	FAM117A	510.9035	-0.08277	0.323513	0.491945	protein_coding
ENSG0000(	FAM193B	2216.105	-0.04309	0.32355	0.491977	protein_coding
ENSG0000(	TBC1D3D	80.6409	0.312431	0.323628	0.492072	protein_coding
ENSG0000(	SFT2D3	578.3809	0.033273	0.323749	0.492232	protein_coding
ENSG0000(	HEATR4	101.8322	0.119629	0.323799	0.492285	protein_coding
ENSG0000(	EGFL7	397.5873	0.127567	0.323827	0.492304	protein_coding
ENSG0000(	ENSG0000(	2926.398	0.056008	0.323956	0.492476	transcribed_unprocessed_pseudo
ENSG0000(	CDC42P4	16.90323	0.151975	0.324026	0.492559	processed_pseudogene

ENSG0000(	ABI2	2780.732	0.057023	0.324076	0.492602	protein_coding
ENSG0000(	RHOA-IT1	111.1219	0.073836	0.324086	0.492602	lncRNA
ENSG0000(	RAG1	154.556	-0.20055	0.324333	0.492953	protein_coding
ENSG0000(	MAB21L1	9.018609	-0.22711	0.32435	0.492956	protein_coding
ENSG0000(	PMS2P7	42.08333	0.123515	0.324448	0.49308	unprocessed_pseudogene
ENSG0000(	FAM177A1	2316.759	-0.04794	0.324574	0.493249	protein_coding
ENSG0000(	ENSG0000(	93.42092	0.056698	0.324626	0.49328	lncRNA
ENSG0000(	TMC4	824.3052	-0.17573	0.324618	0.49328	protein_coding
ENSG0000(	MPRIP-AS1	33.57261	-0.10278	0.324645	0.493286	lncRNA
ENSG0000(	D2HGDH	1456.919	-0.05982	0.324701	0.49334	protein_coding
ENSG0000(	ENSG0000(	35.42348	0.106703	0.324713	0.49334	lncRNA
ENSG0000(	PRR12	1625.69	-0.07153	0.324739	0.493357	protein_coding
ENSG0000(	ENSG0000(	163.1329	0.067678	0.324813	0.493445	lncRNA
ENSG0000(	RN7SL239F	96.60049	0.091344	0.32495	0.493629	misc_RNA
ENSG0000(	MDC1	1307.047	0.097567	0.324995	0.493674	protein_coding
ENSG0000(	ENSG0000(	746.3218	0.034499	0.325039	0.493693	lncRNA
ENSG0000(	MZF1-AS1	415.0027	0.056493	0.325038	0.493693	lncRNA
ENSG0000(	FAM166B	17.28972	-0.17085	0.325058	0.493698	protein_coding
ENSG0000(	B4GALT5	1224.789	0.073774	0.325095	0.493708	protein_coding
ENSG0000(	CUX1	3341.72	0.032842	0.325081	0.493708	protein_coding
ENSG0000(	MCAT	95.62345	-0.09427	0.325134	0.493742	protein_coding
ENSG0000(	TRIM3	1518.679	-0.16466	0.325158	0.493754	protein_coding
ENSG0000(	TRAJ1	32.75546	0.226539	0.325184	0.49377	TR_J_gene
ENSG0000(	ENSG0000(	21.56757	0.187538	0.325233	0.493821	lncRNA
ENSG0000(	TCEA3	786.4766	-0.1641	0.325291	0.493885	protein_coding
ENSG0000(	ENSG0000(	295.642	-0.06951	0.325398	0.494001	lncRNA
ENSG0000(	IGKV2D-40	11.01731	0.508956	0.325398	0.494001	IG_V_gene
ENSG0000(	TBX2	652.9591	0.133458	0.325417	0.494005	protein_coding
ENSG0000(	SNAP25	1627.398	0.058785	0.32553	0.494153	protein_coding
ENSG0000(	ENSG0000(	179.5403	-0.13831	0.325574	0.49418	protein_coding
ENSG0000(	ENSG0000(	90.17099	-0.07885	0.325579	0.49418	TEC
ENSG0000(	ZNF561	1142.927	-0.03747	0.325605	0.494195	protein_coding
ENSG0000(	ENSG0000(	23.0366	-0.11658	0.325668	0.494243	lncRNA
ENSG0000(	ZNF839	869.0127	-0.04301	0.325658	0.494243	protein_coding
ENSG0000(	ENSG0000(	50.46735	0.167876	0.325722	0.494267	lncRNA
ENSG0000(	ENSG0000(	97.92214	0.21114	0.325746	0.494267	lncRNA
ENSG0000(	LY6G6E	181.9979	0.091499	0.325715	0.494267	protein_coding
ENSG0000(	NPFF	66.61496	-0.08019	0.325731	0.494267	protein_coding
ENSG0000(	H2AL1MP	16.83377	-0.22339	0.325765	0.494271	unprocessed_pseudogene
ENSG0000(	ENSG0000(	26.3805	0.139665	0.325808	0.494314	snoRNA
ENSG0000(	ASH1L-AS1	203.9173	0.064239	0.325919	0.494458	lncRNA
ENSG0000(	ENSG0000(	144.8751	-0.06777	0.326006	0.494544	lncRNA
ENSG0000(	METTL5	366.8883	-0.03877	0.326007	0.494544	protein_coding
ENSG0000(	RECK	275.5793	0.077675	0.326034	0.49456	protein_coding
ENSG0000(	RPL4P1	9.135214	0.294277	0.326136	0.494691	processed_pseudogene
ENSG0000(	ENSG0000(	134.9867	-0.05696	0.326232	0.494766	lncRNA
ENSG0000(	LINC01793	18.64661	-0.15133	0.32623	0.494766	lncRNA

ENSG0000(	MPLKIP	2043.957	-0.02827	0.326208	0.494766	protein_coding
ENSG0000(	ENSG0000(	596.4508	-0.09503	0.326311	0.494838	lncRNA
ENSG0000(	LINC01697	668.5687	0.049684	0.326298	0.494838	lncRNA
ENSG0000(	MED16	592.5582	0.07811	0.326364	0.494894	protein_coding
ENSG0000(	LINC01486	84.31151	-0.06461	0.326427	0.494966	lncRNA
ENSG0000(	ENSG0000(	192.6187	0.060268	0.326474	0.494967	lncRNA
ENSG0000(	LINC00896	92.59999	0.086859	0.326466	0.494967	lncRNA
ENSG0000(	SHCBP1	636.0031	0.070876	0.326457	0.494967	protein_coding
ENSG0000(	ENSG0000(	245.9413	-0.0723	0.326641	0.495195	lncRNA
ENSG0000(	ENSG0000(	307.6411	-0.08493	0.326741	0.495252	lncRNA
ENSG0000(	RNU6-672F	10.15655	0.25245	0.326704	0.495252	snRNA
ENSG0000(	RUVBL1	445.9321	-0.07349	0.32673	0.495252	protein_coding
ENSG0000(	ZNF543	589.3237	0.059216	0.326719	0.495252	protein_coding
ENSG0000(	DENND1A	2131.692	-0.07479	0.326832	0.495366	protein_coding
ENSG0000(	LINC00511	6403.624	-0.1998	0.32685	0.495369	lncRNA
ENSG0000(	LINC00426	946.9529	0.075154	0.326867	0.495371	lncRNA
ENSG0000(	ENSG0000(	283.544	-0.04834	0.326885	0.495374	lncRNA
ENSG0000(	ENSG0000(	188.9347	-0.09295	0.326918	0.495401	lncRNA
ENSG0000(	ENSG0000(	10.39249	-0.29906	0.327001	0.495498	protein_coding
ENSG0000(	ENSG0000(	9.275692	0.339702	0.327029	0.495498	lncRNA
ENSG0000(	IPP	727.7789	-0.06027	0.327014	0.495498	protein_coding
ENSG0000(	ENSG0000(	29.80377	-0.15303	0.327177	0.495696	lncRNA
ENSG0000(	MGME1	584.2821	0.057509	0.3272	0.495696	protein_coding
ENSG0000(	SIN3B	2074.49	0.043701	0.327207	0.495696	protein_coding
ENSG0000(	ENSG0000(	174.5285	-0.06534	0.327239	0.495697	lncRNA
ENSG0000(	PCDHGA12	113.3827	0.116954	0.327226	0.495697	protein_coding
ENSG0000(	NRAS	1330.1	-0.05193	0.327269	0.495718	protein_coding
ENSG0000(	KCNMB2-A	1188.53	-0.04659	0.327295	0.495734	lncRNA
ENSG0000(	CCL19	516.5447	-0.34479	0.327346	0.495763	protein_coding
ENSG0000(	CCND2	6864.381	0.086298	0.327342	0.495763	protein_coding
ENSG0000(	ENSG0000(	37.12961	0.10495	0.327381	0.495792	lncRNA
ENSG0000(	STYK1	441.0308	-0.18593	0.327535	0.496002	protein_coding
ENSG0000(	RASGRF2	778.0183	0.121623	0.327653	0.496133	protein_coding
ENSG0000(	TTC36	44.16767	-0.20552	0.32764	0.496133	protein_coding
ENSG0000(	ENSG0000(	187.9306	0.067257	0.3277	0.49618	TEC
ENSG0000(	CSTF1	826.6332	-0.04882	0.327869	0.496283	protein_coding
ENSG0000(	DNTTIP1	187.9829	-0.06512	0.327863	0.496283	protein_coding
ENSG0000(	ENSG0000(	12.43973	0.172632	0.327828	0.496283	processed_pseudogene
ENSG0000(	FCF1P1	47.44465	0.086674	0.327858	0.496283	processed_pseudogene
ENSG0000(	FLOT2	837.3993	0.114326	0.327795	0.496283	protein_coding
ENSG0000(	INPP5F	1133.236	0.093345	0.327881	0.496283	protein_coding
ENSG0000(	LINC01516	26.64744	-0.10724	0.327872	0.496283	lncRNA
ENSG0000(	WNK4	641.3407	-0.0849	0.327894	0.496283	protein_coding
ENSG0000(	ENSG0000(	8.966617	0.203006	0.327911	0.496285	lncRNA
ENSG0000(	ABCC10	1806.319	-0.04378	0.327933	0.496295	protein_coding
ENSG0000(	LINC01532	29.80373	0.122959	0.32796	0.496311	lncRNA
ENSG0000(	SLC1A4	785.2616	-0.07253	0.328058	0.496436	protein_coding

ENSG0000(	TAF1A-AS1	41.65668	-0.10179	0.32819	0.496612	lncRNA
ENSG0000(	CCDC144A	2773.024	0.093433	0.32824	0.496638	protein_coding
ENSG0000(	ENSG0000(	238.8359	-0.05875	0.328254	0.496638	lncRNA
ENSG0000(	ENSG0000(	10.51263	0.199731	0.328255	0.496638	snoRNA
ENSG0000(	ARIH1	5215.8	-0.04243	0.328355	0.496766	protein_coding
ENSG0000(	PLEKHA2	3742.913	-0.08733	0.32837	0.496766	protein_coding
ENSG0000(	TMEM30BI	92.36663	-0.13318	0.328441	0.496848	processed_pseudogene
ENSG0000(	ENSG0000(	21.69368	-0.13981	0.328636	0.497078	lncRNA
ENSG0000(	GRIK1-AS1	400.7804	0.046101	0.32864	0.497078	lncRNA
ENSG0000(	SPAG7	625.7696	0.038733	0.328631	0.497078	protein_coding
ENSG0000(	ENSG0000(	50.41109	0.127496	0.328694	0.497136	lncRNA
ENSG0000(	DCK	406.9027	-0.14346	0.328926	0.497462	protein_coding
ENSG0000(	ENSG0000(	179.8657	0.06955	0.32896	0.49749	lncRNA
ENSG0000(	GPN1	298.8912	-0.04518	0.329086	0.497609	protein_coding
ENSG0000(	INSL4	30.21415	-0.16756	0.329081	0.497609	protein_coding
ENSG0000(	THPO	15.72425	0.305823	0.329059	0.497609	protein_coding
ENSG0000(	CSTF2	195.6368	-0.09082	0.329136	0.497637	protein_coding
ENSG0000(	ENSG0000(	107.7973	0.132167	0.329133	0.497637	TEC
ENSG0000(	LRRC37A7F	186.1442	0.055033	0.329215	0.497732	transcribed_processed_pseudoge
ENSG0000(	ENSG0000(	17.68588	0.224814	0.329233	0.497736	lncRNA
ENSG0000(	ENSG0000(	79.27172	-0.12687	0.329399	0.497917	TEC
ENSG0000(	LIPJ	16.7924	-0.21401	0.3294	0.497917	protein_coding
ENSG0000(	POTEM	317.4281	0.099423	0.329396	0.497917	protein_coding
ENSG0000(	NAIPP1	131.6403	-0.10091	0.329428	0.497936	transcribed_unprocessed_pseudo
ENSG0000(	HAUS8	571.7923	-0.04759	0.329456	0.497954	protein_coding
ENSG0000(	FBXO11	4285.798	-0.03172	0.329617	0.498173	protein_coding
ENSG0000(	ENSG0000(	42.62692	0.12477	0.329654	0.498193	lncRNA
ENSG0000(	ENSG0000(	29.06489	0.125894	0.329662	0.498193	lncRNA
ENSG0000(	LINC02691	65.77028	0.089454	0.329712	0.498227	lncRNA
ENSG0000(	RNU1-67P	237.3015	0.186177	0.32973	0.498227	snRNA
ENSG0000(	SCLY	1622.822	0.033312	0.329732	0.498227	protein_coding
ENSG0000(	HMGN5	46.06628	0.132848	0.329763	0.49825	protein_coding
ENSG0000(	ZNF439	1773.241	-0.05876	0.329781	0.498253	protein_coding
ENSG0000(	DNAJB7	350.3218	-0.12707	0.329868	0.49836	protein_coding
ENSG0000(	DLGAP1-AS	667.6873	0.048399	0.329914	0.498406	lncRNA
ENSG0000(	ENSG0000(	159.0121	-0.06886	0.32997	0.498419	lncRNA
ENSG0000(	ENSG0000(	293.3775	0.06894	0.329946	0.498419	lncRNA
ENSG0000(	ESRRA	1414.828	-0.10256	0.329957	0.498419	protein_coding
ENSG0000(	CLIP3	305.0685	0.122874	0.330032	0.498466	protein_coding
ENSG0000(	RNU6-1053	32.54895	0.112045	0.33003	0.498466	snRNA
ENSG0000(	ORC3	603.5457	-0.04417	0.330074	0.498506	protein_coding
ENSG0000(	LAMA5	2491.235	-0.08878	0.330139	0.498561	protein_coding
ENSG0000(	LRATD1	2709.586	-0.13665	0.330143	0.498561	protein_coding
ENSG0000(	IP6K1	988.1217	-0.04604	0.330196	0.498617	protein_coding
ENSG0000(	KLB	358.844	-0.05241	0.330221	0.498631	protein_coding
ENSG0000(	RNU1-124F	22.09925	0.122981	0.33032	0.498757	snRNA
ENSG0000(	DUSP16	1674.533	-0.08532	0.330359	0.498792	protein_coding

ENSG0000(	CAV2	566.467	-0.05203	0.330433	0.498847	protein_coding
ENSG0000(	HESX1	34.47984	-0.10962	0.330443	0.498847	protein_coding
ENSG0000(	LINC01720	128.7797	0.094114	0.330426	0.498847	lncRNA
ENSG0000(	EIF1AY	334.0114	-0.09644	0.330564	0.499006	protein_coding
ENSG0000(	ENSG0000(	12.76933	0.213069	0.33066	0.499127	lncRNA
ENSG0000(	SULT1C2	810.7566	0.144722	0.330724	0.499199	protein_coding
ENSG0000(	ENSG0000(	20.80811	0.163503	0.33078	0.499236	processed_pseudogene
ENSG0000(	ENSG0000(	521.7631	0.106114	0.330768	0.499236	lncRNA
ENSG0000(	LINC01005	24.96063	-0.2245	0.330818	0.499269	lncRNA
ENSG0000(	DPT	86.44584	-0.30129	0.330974	0.499481	protein_coding
ENSG0000(	RNASEL	1107.468	0.048508	0.331042	0.49956	protein_coding
ENSG0000(	ENSG0000(	185.4852	-0.05079	0.331088	0.499605	lncRNA
ENSG0000(	TUBB	3095.641	-0.0917	0.331105	0.499607	protein_coding
ENSG0000(	LINC00589	17.65736	0.228712	0.331128	0.499618	lncRNA
ENSG0000(	ENSG0000(	808.3914	-0.03541	0.331218	0.499682	lncRNA
ENSG0000(	ENSG0000(	314.1225	0.044927	0.331218	0.499682	lncRNA
ENSG0000(	ENSG0000(	914.9099	0.032277	0.331198	0.499682	lncRNA
ENSG0000(	ENSG0000(	62.16334	-0.10261	0.331329	0.499826	processed_pseudogene
ENSG0000(	LINC02453	146.2971	-0.08507	0.331363	0.499853	lncRNA
ENSG0000(	PCDHGB4	91.84323	-0.14956	0.331438	0.499942	protein_coding
ENSG0000(	CORO1B	1260.989	-0.10767	0.331508	0.49996	protein_coding
ENSG0000(	ENSG0000(	47.92675	-0.13105	0.331513	0.49996	lncRNA
ENSG0000(	RNF130	934.9076	0.067871	0.331485	0.49996	protein_coding
ENSG0000(	RNF152P1	47.75032	0.094893	0.331474	0.49996	processed_pseudogene
ENSG0000(	ENSG0000(	143.6291	-0.1004	0.331572	0.500024	lncRNA
ENSG0000(	ENSG0000(	19.95749	-0.1443	0.33159	0.500027	processed_pseudogene
ENSG0000(	TMEM208	141.161	-0.08426	0.331649	0.500092	protein_coding
ENSG0000(	ENSG0000(	36.92879	0.133943	0.331683	0.500095	lncRNA
ENSG0000(	ENSG0000(	111.249	-0.11208	0.331677	0.500095	lncRNA
ENSG0000(	SSPN	303.9866	0.163226	0.331735	0.50015	protein_coding
ENSG0000(	PRR14	582.4412	-0.05733	0.331792	0.500212	protein_coding
ENSG0000(	ATP6V1D	822.3431	0.052731	0.331832	0.500224	protein_coding
ENSG0000(	KCNC1	74.61921	-0.07908	0.331818	0.500224	protein_coding
ENSG0000(	RNU6-1016	184.8189	0.092949	0.331865	0.50025	snRNA
ENSG0000(	ENSG0000(	66.027	0.267935	0.331933	0.500305	lncRNA
ENSG0000(	MGAT2	612.7024	0.056168	0.331929	0.500305	protein_coding
ENSG0000(	ENSG0000(	54.06143	0.140817	0.332005	0.50039	lncRNA
ENSG0000(	C19orf48	496.1701	-0.0608	0.332112	0.500479	transcribed_unitary_pseudogene
ENSG0000(	EDRF1	1483.332	-0.03206	0.332104	0.500479	protein_coding
ENSG0000(	ENSG0000(	21.35876	-0.14807	0.332112	0.500479	lncRNA
ENSG0000(	ENSG0000(	99.278	0.104388	0.332233	0.500638	lncRNA
ENSG0000(	NUDT13	148.4616	0.086576	0.332253	0.500644	protein_coding
ENSG0000(	RAD21-AS1	202.943	0.074834	0.332325	0.500728	lncRNA
ENSG0000(	UBQLN3	28.48448	0.194278	0.332476	0.500933	protein_coding
ENSG0000(	ENSG0000(	11.15962	0.168268	0.332512	0.500963	lncRNA
ENSG0000(	IGBP1-AS1	162.7599	-0.0608	0.332648	0.501143	lncRNA
ENSG0000(	WNT11	80.07002	0.239989	0.332836	0.501402	protein_coding

ENSG0000(	ENSG0000(	9.749739	-0.24893	0.332935	0.5015	lncRNA
ENSG0000(	FAM166A	19.20945	-0.27615	0.332948	0.5015	protein_coding
ENSG0000(	TTC23L	21.55306	0.257203	0.332923	0.5015	protein_coding
ENSG0000(	CEP57L1	2596.237	0.028219	0.333159	0.501673	protein_coding
ENSG0000(	FSD2	221.1012	0.082754	0.333143	0.501673	protein_coding
ENSG0000(	GNAO1	506.4758	0.087849	0.333143	0.501673	protein_coding
ENSG0000(	HOOK3	5136.269	0.042135	0.333156	0.501673	protein_coding
ENSG0000(	IFNAR1	2558.82	-0.04302	0.333112	0.501673	protein_coding
ENSG0000(	UBP1	2936.272	0.038055	0.333123	0.501673	protein_coding
ENSG0000(	PGLYRP4	16.14611	0.313929	0.333176	0.501674	protein_coding
ENSG0000(	ENSG0000(	28.16911	0.153866	0.333332	0.501814	lncRNA
ENSG0000(	FAM162B	9.036995	0.19715	0.333302	0.501814	protein_coding
ENSG0000(	MBD2	1706.963	-0.05738	0.333322	0.501814	protein_coding
ENSG0000(	PRKCB	2773.274	0.185024	0.333331	0.501814	protein_coding
ENSG0000(	ABCG1	1297.333	-0.10822	0.333452	0.501946	protein_coding
ENSG0000(	RN7SL156F	55.94998	0.086521	0.333446	0.501946	misc_RNA
ENSG0000(	BTBD7P1	197.5706	0.059551	0.333478	0.501961	processed_pseudogene
ENSG0000(	NUTM2D	111.4063	0.120331	0.333504	0.501977	protein_coding
ENSG0000(	DCBLD2	738.4907	-0.04564	0.333525	0.501984	protein_coding
ENSG0000(	RPH3AL-AS	21.38126	0.202387	0.333554	0.502004	lncRNA
ENSG0000(	SATL1	713.0225	-0.06455	0.333595	0.502041	protein_coding
ENSG0000(	TIGD3	24.48546	-0.21432	0.333693	0.502165	protein_coding
ENSG0000(	ENSG0000(	161.2815	-0.05264	0.333716	0.502176	lncRNA
ENSG0000(	ENSG0000(	24.40236	0.628789	0.333786	0.502257	lncRNA
ENSG0000(	ENSG0000(	347.5571	-0.06301	0.33381	0.502269	lncRNA
ENSG0000(	ENSG0000(	249.5527	-0.04282	0.333875	0.502343	lncRNA
ENSG0000(	ENSG0000(	314.877	-0.04389	0.333933	0.502406	protein_coding
ENSG0000(	PPARGC1A	1797.052	-0.19484	0.333954	0.502413	protein_coding
ENSG0000(	BRD2	5460.211	-0.03155	0.334015	0.502469	protein_coding
ENSG0000(	OSBPL2	2708.353	-0.04701	0.334022	0.502469	protein_coding
ENSG0000(	RPF2	303.3965	-0.06984	0.334054	0.502493	protein_coding
ENSG0000(	SPINDOC	183.0787	0.096588	0.334124	0.50255	protein_coding
ENSG0000(	YIPF7	70.3389	-0.10163	0.334114	0.50255	protein_coding
ENSG0000(	FAM162A	1327.827	0.052569	0.334149	0.502563	protein_coding
ENSG0000(	ENSG0000(	927.4483	-0.02958	0.334218	0.502643	TEC
ENSG0000(	ARNT	1927.189	-0.03707	0.334366	0.502842	protein_coding
ENSG0000(	SCOC	617.8349	-0.08065	0.334607	0.50318	protein_coding
ENSG0000(	RGL3	103.4779	0.123552	0.334708	0.503307	protein_coding
ENSG0000(	CAMTA2	1387.458	-0.0565	0.334764	0.503369	protein_coding
ENSG0000(	AP1M2P1	14.10125	-0.14936	0.3349	0.503549	processed_pseudogene
ENSG0000(	DNM1P51	629.5392	0.097501	0.334948	0.503583	transcribed_unprocessed_pseudo
ENSG0000(	ENSG0000(	186.9635	0.06009	0.334955	0.503583	lncRNA
ENSG0000(	ZNF451	3766.501	-0.03528	0.335023	0.503662	protein_coding
ENSG0000(	AP1S3	1428.896	0.035025	0.335086	0.503666	protein_coding
ENSG0000(	DOC2B	444.0448	0.118849	0.335095	0.503666	protein_coding
ENSG0000(	LRIG3	1142.189	0.112697	0.335106	0.503666	protein_coding
ENSG0000(	NIN	3413.027	0.127741	0.3351	0.503666	protein_coding

ENSG0000(	WAS	365.5904	0.146576	0.335055	0.503666	protein_coding
ENSG0000(	ENSG0000(	236.573	-0.1391	0.335143	0.503698	lncRNA
ENSG0000(	RNU6-137F	13.03071	-0.17297	0.335176	0.503724	snRNA
ENSG0000(	ENSG0000(	358.2648	-0.04975	0.335257	0.503801	lncRNA
ENSG0000(	ZNF253	724.0668	-0.08408	0.335259	0.503801	protein_coding
ENSG0000(	LINC02763	19.56163	-0.15148	0.335347	0.503908	lncRNA
ENSG0000(	EEF1DP2	27.03037	0.173327	0.335416	0.50394	processed_pseudogene
ENSG0000(	ENSG0000(	34.19731	-0.19522	0.335414	0.50394	lncRNA
ENSG0000(	MTRES1	423.6084	-0.03999	0.335393	0.50394	protein_coding
ENSG0000(	ENSG0000(	172.4307	0.054125	0.335504	0.504048	lncRNA
ENSG0000(	DDX39A	645.7772	-0.05386	0.33556	0.50406	protein_coding
ENSG0000(	ENSG0000(	198.4897	0.083782	0.335554	0.50406	lncRNA
ENSG0000(	OR7E94P	396.199	0.073473	0.335544	0.50406	transcribed_unprocessed_pseudo
ENSG0000(	C7orf26	442.8868	-0.05297	0.335585	0.504063	protein_coding
ENSG0000(	ENSG0000(	290.8602	-0.04921	0.335594	0.504063	lncRNA
ENSG0000(	RERGL	9.96571	-0.27979	0.335646	0.504116	protein_coding
ENSG0000(	ENSG0000(	161.0324	0.071119	0.335666	0.504123	lncRNA
ENSG0000(	LRRC8A	615.5078	-0.07716	0.335706	0.504159	protein_coding
ENSG0000(	POLR3H	673.9233	0.068873	0.335749	0.504185	protein_coding
ENSG0000(	ZNF70	1508.173	0.043018	0.335755	0.504185	protein_coding
ENSG0000(	GRAMD1A-	194.8705	0.056985	0.335827	0.504269	lncRNA
ENSG0000(	ENSG0000(	86.64945	0.083041	0.335946	0.504417	lncRNA
ENSG0000(	TTC32	244.4478	-0.04885	0.335958	0.504417	protein_coding
ENSG0000(	ENSG0000(	174.8327	-0.07019	0.336054	0.504538	lncRNA
ENSG0000(	ENSG0000(	40.94205	-0.16289	0.336141	0.50462	lncRNA
ENSG0000(	SLC44A2	1677.691	-0.09253	0.336127	0.50462	protein_coding
ENSG0000(	ENSG0000(	52.0946	-0.08552	0.336179	0.504629	lncRNA
ENSG0000(	ENSG0000(	21.66663	-0.12759	0.336167	0.504629	processed_pseudogene
ENSG0000(	ENSG0000(	38.5602	-0.11033	0.336199	0.504635	lncRNA
ENSG0000(	ENSG0000(	169.7059	0.060993	0.336277	0.504728	lncRNA
ENSG0000(	MIR2116	11.5285	-0.22294	0.336387	0.504863	miRNA
ENSG0000(	SIGLEC17P	47.93258	0.157088	0.336399	0.504863	transcribed_unprocessed_pseudo
ENSG0000(	ENSG0000(	900.0051	0.087623	0.336504	0.504997	protein_coding
ENSG0000(	RANBP2	6342.55	-0.04073	0.336593	0.505106	protein_coding
ENSG0000(	ENSG0000(	41.59374	-0.09554	0.336655	0.505175	lncRNA
ENSG0000(	MFSD8	1442.451	-0.0419	0.336678	0.505185	protein_coding
ENSG0000(	IL10	159.2389	0.063543	0.336701	0.505196	protein_coding
ENSG0000(	GTSE1	387.5	0.066423	0.336799	0.505273	protein_coding
ENSG0000(	HAP1	701.2443	0.044854	0.336817	0.505273	protein_coding
ENSG0000(	IGKV1D-8	11.41601	0.325642	0.336814	0.505273	IG_V_gene
ENSG0000(	RNF19B	1129.646	0.108913	0.336802	0.505273	protein_coding
ENSG0000(	TMOD3	4269.331	0.0413	0.336863	0.505318	protein_coding
ENSG0000(	ENSG0000(	9.805633	0.193845	0.336946	0.505408	lncRNA
ENSG0000(	ENSG0000(	80.35523	0.072056	0.336955	0.505408	lncRNA
ENSG0000(	NOL4	18.39619	-0.29759	0.337031	0.505499	protein_coding
ENSG0000(	LINC01858	195.3357	-0.05019	0.337052	0.505505	lncRNA
ENSG0000(	ENSG0000(	1147.422	-0.05415	0.337106	0.505563	lncRNA

ENSG0000(	ENSG0000(	34.34342	-0.27972	0.33714	0.50559	protein_coding
ENSG0000(	MIR3679	35.75598	-0.14816	0.337187	0.505626	miRNA
ENSG0000(	PGGT1B	1994.253	-0.04306	0.337228	0.505626	protein_coding
ENSG0000(	PTEN	6196.731	-0.0539	0.33721	0.505626	protein_coding
ENSG0000(	RSL24D1P8	17.1314	0.152968	0.337217	0.505626	processed_pseudogene
ENSG0000(	ENSG0000(	22.45446	0.152599	0.337248	0.505631	lncRNA
ENSG0000(	LINC00408	33.12228	0.113289	0.337275	0.505648	lncRNA
ENSG0000(	MOV10	2435.804	0.049751	0.33735	0.505736	protein_coding
ENSG0000(	MITD1	1253.528	-0.04069	0.337412	0.505805	protein_coding
ENSG0000(	SPSB2	149.3938	-0.07068	0.337434	0.505813	protein_coding
ENSG0000(	ENSG0000(	176.0482	0.065964	0.337497	0.505864	lncRNA
ENSG0000(	PIP5K1C	1122.594	0.093305	0.337499	0.505864	protein_coding
ENSG0000(	ZNF410	1096.04	-0.04168	0.337563	0.505935	protein_coding
ENSG0000(	ENSG0000(	390.7263	0.077434	0.337636	0.50602	TEC
ENSG0000(	ZCCHC7	1225.152	-0.06341	0.337659	0.506031	protein_coding
ENSG0000(	RNU6-611F	39.14639	-0.1088	0.337769	0.506171	snRNA
ENSG0000(	OR6Y1	569.2995	0.057763	0.337801	0.506195	protein_coding
ENSG0000(	CDC27P3	30.40597	0.343262	0.337911	0.506337	processed_pseudogene
ENSG0000(	TBC1D22B	638.7853	-0.08041	0.337955	0.506378	protein_coding
ENSG0000(	ENSG0000(	67.12029	0.074776	0.337982	0.506395	lncRNA
ENSG0000(	DPY19L3-D	58.98903	0.095448	0.338005	0.506405	lncRNA
ENSG0000(	RYR3-DT	67.52688	0.144528	0.33804	0.506432	lncRNA
ENSG0000(	KAZN-AS1	24.6935	0.134466	0.338062	0.506442	lncRNA
ENSG0000(	RN7SKP80	68.70955	-0.13535	0.338112	0.506492	misc_RNA
ENSG0000(	PCDH18	1094.054	0.122627	0.338207	0.506611	protein_coding
ENSG0000(	BARX2	369.1186	-0.12382	0.338291	0.506688	protein_coding
ENSG0000(	LINC02038	41.32514	-0.20598	0.338307	0.506688	lncRNA
ENSG0000(	TRIM52-AS	254.1569	0.050937	0.338276	0.506688	lncRNA
ENSG0000(	P3H2	156.0476	0.078567	0.338334	0.506699	protein_coding
ENSG0000(	SPSB1	567.8649	0.133899	0.338346	0.506699	protein_coding
ENSG0000(	GDF7	209.2442	0.237523	0.338557	0.506991	protein_coding
ENSG0000(	ENSG0000(	21.71782	-0.14793	0.338606	0.50704	lncRNA
ENSG0000(	ENSG0000(	24.05945	0.129696	0.338648	0.507054	lncRNA
ENSG0000(	H2AZ1-DT	253.4144	-0.05175	0.33864	0.507054	lncRNA
ENSG0000(	MYLK-AS1	503.7748	0.056791	0.338738	0.507166	lncRNA
ENSG0000(	ENSG0000(	189.3063	-0.04633	0.338788	0.507174	lncRNA
ENSG0000(	POLL	1015	0.028983	0.338792	0.507174	protein_coding
ENSG0000(	TAB1	561.9509	-0.0541	0.338777	0.507174	protein_coding
ENSG0000(	PLGRKT	186.1018	-0.06975	0.338825	0.507199	protein_coding
ENSG0000(	NUCKS1	3198.91	-0.07676	0.338927	0.507328	protein_coding
ENSG0000(	ENSG0000(	543.2847	0.049419	0.339008	0.507425	lncRNA
ENSG0000(	ENSG0000(	25.2732	0.119518	0.339075	0.507477	lncRNA
ENSG0000(	RN7SL342F	51.77062	0.094907	0.339074	0.507477	misc_RNA
ENSG0000(	MROH6	247.8071	0.142959	0.339297	0.507786	protein_coding
ENSG0000(	BAK1P1	16.35312	0.183573	0.339335	0.507794	processed_pseudogene
ENSG0000(	ENSG0000(	17.65565	0.138207	0.339321	0.507794	lncRNA
ENSG0000(	ENSG0000(	117.3226	-0.08988	0.33936	0.507807	lncRNA

ENSG0000(	BOP1	186.7413	0.098892	0.339545	0.507969	protein_coding
ENSG0000(	ENSG0000(	21.15667	0.192865	0.339536	0.507969	lncRNA
ENSG0000(	MIR200A	11.0026	-0.23746	0.339535	0.507969	miRNA
ENSG0000(	PDPR	3593.151	0.09102	0.339549	0.507969	protein_coding
ENSG0000(	PRO1804	245.538	-0.16635	0.339537	0.507969	TEC
ENSG0000(	ENSG0000(	190.2962	0.077896	0.3396	0.508005	lncRNA
ENSG0000(	PCYOX1L	707.3809	0.081186	0.339605	0.508005	protein_coding
ENSG0000(	CDKL2	36.77632	0.219347	0.339643	0.508037	protein_coding
ENSG0000(	B3GALNT1	438.8743	-0.06999	0.339761	0.508165	protein_coding
ENSG0000(	FHL2	1673.029	0.094741	0.339745	0.508165	protein_coding
ENSG0000(	CEP19	281.4712	-0.05389	0.339795	0.508187	protein_coding
ENSG0000(	FOXN2	1156.32	-0.05864	0.339807	0.508187	protein_coding
ENSG0000(	ENSG0000(	398.7339	0.03364	0.339977	0.508416	lncRNA
ENSG0000(	ZNF790	430.3974	-0.05607	0.340029	0.50847	protein_coding
ENSG0000(	C8orf17	97.81454	0.055976	0.340058	0.508489	TEC
ENSG0000(	ENSG0000(	466.2704	0.072934	0.340114	0.508549	lncRNA
ENSG0000(	CEP63	2603.831	0.029865	0.340174	0.508614	protein_coding
ENSG0000(	CNP	980.1419	0.058703	0.340273	0.508718	protein_coding
ENSG0000(	NFRKB	1217.128	-0.04034	0.340276	0.508718	protein_coding
ENSG0000(	PRMT7	1464.899	0.077496	0.340334	0.508781	protein_coding
ENSG0000(	IER3IP1	559.491	-0.04137	0.340365	0.508804	protein_coding
ENSG0000(	UICLM	26.08147	0.373307	0.340395	0.508824	lncRNA
ENSG0000(	ABHD13	953.0827	-0.03731	0.340512	0.508972	protein_coding
ENSG0000(	ENSG0000(	11.57043	0.189398	0.340526	0.508972	lncRNA
ENSG0000(	KLK14	45.2569	-0.15394	0.340548	0.50898	protein_coding
ENSG0000(	RAD51B	641.2293	-0.05828	0.340606	0.509043	protein_coding
ENSG0000(	LINC01827	17.88145	0.136679	0.340635	0.509062	lncRNA
ENSG0000(	POLD2	289.5725	-0.09911	0.340666	0.509084	protein_coding
ENSG0000(	ACOT13	425.7459	-0.08554	0.34069	0.509096	protein_coding
ENSG0000(	BPTFP1	123.0837	0.124136	0.340765	0.509184	unprocessed_pseudogene
ENSG0000(	ENSG0000(	162.4111	-0.06304	0.340794	0.509204	lncRNA
ENSG0000(	KRIT1	5728.62	0.025497	0.340835	0.509241	protein_coding
ENSG0000(	ENSG0000(	23.01111	0.14152	0.340853	0.509242	lncRNA
ENSG0000(	RBMX2	253.0642	0.058978	0.340901	0.50929	protein_coding
ENSG0000(	RNF150	542.9624	-0.20429	0.340924	0.509301	protein_coding
ENSG0000(	CFAP97	1282.075	0.040059	0.34095	0.509315	protein_coding
ENSG0000(	ZDHHC17	1859.469	0.042091	0.340975	0.509329	protein_coding
ENSG0000(	SNORA38B	57.36129	0.123386	0.341026	0.509381	snoRNA
ENSG0000(	ENSG0000(	589.5308	0.040179	0.341089	0.509427	lncRNA
ENSG0000(	MTARC1	884.3629	0.088561	0.341088	0.509427	protein_coding
ENSG0000(	ERI2	596.3512	0.055109	0.341139	0.509477	protein_coding
ENSG0000(	ENSG0000(	13.27385	-0.14882	0.341166	0.509493	lncRNA
ENSG0000(	ENSG0000(	3178.447	0.024213	0.341182	0.509493	lncRNA
ENSG0000(	RN7SL596F	133.5672	-0.06209	0.341243	0.509548	misc_RNA
ENSG0000(	TAOK2	2288.863	-0.04904	0.341251	0.509548	protein_coding
ENSG0000(	VWC2	93.38303	0.09105	0.34128	0.509567	protein_coding
ENSG0000(	IFI27	1444.178	0.160044	0.341353	0.509652	protein_coding

ENSG0000(	PXYLP1	1151.266	-0.07868	0.341386	0.509677	protein_coding
ENSG0000(	SNORA22	45.11326	-0.14607	0.341407	0.509684	snoRNA
ENSG0000(	ZNRF2	929.5015	-0.06873	0.341501	0.5098	protein_coding
ENSG0000(	CORO2A	2597.665	-0.11671	0.341668	0.510006	protein_coding
ENSG0000(	ZNF292	8756.779	-0.06166	0.341672	0.510006	protein_coding
ENSG0000(	MCM3AP-A	326.6514	0.059005	0.341694	0.510013	lncRNA
ENSG0000(	RN7SL123F	244.7962	-0.0512	0.341708	0.510013	misc_RNA
ENSG0000(	ALOX15	74.3489	0.122394	0.341726	0.510016	protein_coding
ENSG0000(	CAPZA1	3160.36	0.062047	0.341755	0.510034	protein_coding
ENSG0000(	DFFA	2558.04	-0.02169	0.341805	0.51007	protein_coding
ENSG0000(	ENSG0000(	11.02978	-0.13953	0.34186	0.51007	lncRNA
ENSG0000(	ENSG0000(	22.2855	0.161902	0.341848	0.51007	lncRNA
ENSG0000(	MFSD4A	288.5851	-0.07733	0.341832	0.51007	protein_coding
ENSG0000(	WASHC2C	2157.555	-0.04877	0.341826	0.51007	protein_coding
ENSG0000(	ENSG0000(	63.65161	0.085961	0.34189	0.510091	lncRNA
ENSG0000(	STPG1	726.4955	-0.07951	0.341917	0.510107	protein_coding
ENSG0000(	SELENOS	862.002	-0.07947	0.341972	0.510165	protein_coding
ENSG0000(	ENSG0000(	17.03702	-0.27454	0.342011	0.510199	lncRNA
ENSG0000(	ENSG0000(	185.086	0.066567	0.342053	0.510237	lncRNA
ENSG0000(	ENSG0000(	32.24668	-0.11307	0.342239	0.510487	lncRNA
ENSG0000(	THRAP3P3	26.64338	0.171904	0.342253	0.510487	processed_pseudogene
ENSG0000(	DOCK9	2624.209	0.051353	0.342335	0.510586	protein_coding
ENSG0000(	ENSG0000(	122.0778	-0.05614	0.342552	0.510885	lncRNA
ENSG0000(	VIM	3858.937	0.116535	0.34262	0.510963	protein_coding
ENSG0000(	ENSG0000(	16.67721	0.143199	0.342647	0.510979	lncRNA
ENSG0000(	DHX8	1684.636	0.055128	0.34277	0.511137	protein_coding
ENSG0000(	ENSG0000(	46.22055	0.099176	0.342813	0.511177	lncRNA
ENSG0000(	ENSG0000(	10.54625	0.22482	0.342893	0.511273	lncRNA
ENSG0000(	RBIS	520.2265	0.038417	0.342955	0.51134	protein_coding
ENSG0000(	LHPP	376.1105	-0.08528	0.343015	0.511406	protein_coding
ENSG0000(	KMO	441.2911	0.217411	0.343092	0.511496	protein_coding
ENSG0000(	GSTM3	726.6867	-0.05157	0.343132	0.511532	protein_coding
ENSG0000(	MIR4480	20.6734	0.157028	0.343157	0.511534	miRNA
ENSG0000(	TMEM67	1364.248	0.044389	0.343166	0.511534	protein_coding
ENSG0000(	ENSG0000(	47.94192	-0.20694	0.343207	0.511555	protein_coding
ENSG0000(	ENSG0000(	292.971	-0.04221	0.343228	0.511555	lncRNA
ENSG0000(	GFOD1	1681.978	-0.0773	0.343222	0.511555	protein_coding
ENSG0000(	ENSG0000(	18.82057	0.134661	0.343264	0.511583	protein_coding
ENSG0000(	KLHL9	1755.264	0.024204	0.343281	0.511584	protein_coding
ENSG0000(	PSD2	12.34415	0.325117	0.343442	0.5118	protein_coding
ENSG0000(	LMTK2	2575.63	-0.09613	0.343497	0.511858	protein_coding
ENSG0000(	CPLANE2	57.10422	0.136811	0.343551	0.511915	protein_coding
ENSG0000(	MAGEA10	181.0079	-0.04761	0.343597	0.511958	protein_coding
ENSG0000(	ENSG0000(	12.64329	0.180684	0.343744	0.512125	lncRNA
ENSG0000(	MEMO1	971.9035	-0.06512	0.343733	0.512125	protein_coding
ENSG0000(	ZNF239	120.5932	0.124387	0.343757	0.512125	protein_coding
ENSG0000(	ENSG0000(	25.34897	0.141964	0.343891	0.5123	lncRNA

ENSG0000(	DENND2C	806.8432	0.041857	0.343986	0.512409	protein_coding
ENSG0000(	PTRHD1	108.4485	-0.10136	0.344002	0.512409	protein_coding
ENSG0000(	ZGRF1	912.0733	-0.04587	0.344013	0.512409	protein_coding
ENSG0000(	C12orf54	8.440881	0.213925	0.344137	0.51257	protein_coding
ENSG0000(	ENSG0000(	21.18809	-0.18161	0.344188	0.512621	lncRNA
ENSG0000(	ENSG0000(	9.968822	0.239704	0.344436	0.512966	lncRNA
ENSG0000(	ENSG0000(	514.7147	-0.05943	0.344517	0.513024	lncRNA
ENSG0000(	KIF18A	201.133	0.09452	0.344521	0.513024	protein_coding
ENSG0000(	PXDNL	36.08473	-0.16252	0.344523	0.513024	protein_coding
ENSG0000(	LINC00092	221.423	0.058959	0.344607	0.513124	lncRNA
ENSG0000(	ENSG0000(	318.0839	0.070644	0.344629	0.513133	lncRNA
ENSG0000(	SUZ12P1	1429.445	-0.03788	0.344683	0.513188	transcribed_unprocessed_pseudo
ENSG0000(	ENSG0000(	39.82725	-0.1062	0.344717	0.513191	lncRNA
ENSG0000(	SLC39A7	1011.604	0.081323	0.344704	0.513191	protein_coding
ENSG0000(	ENSG0000(	196.3386	-0.09066	0.344756	0.513201	lncRNA
ENSG0000(	POLDIP3	1093.781	-0.03873	0.344755	0.513201	protein_coding
ENSG0000(	TMEM74	14.71327	0.205052	0.344849	0.513315	protein_coding
ENSG0000(	MUL1	226.4676	-0.0638	0.345006	0.513523	protein_coding
ENSG0000(	RBBP6	3204.255	-0.0395	0.345047	0.513561	protein_coding
ENSG0000(	MIR3135A	10.82931	-0.1761	0.34511	0.513631	miRNA
ENSG0000(	PGM5	633.4759	-0.10944	0.34523	0.513784	protein_coding
ENSG0000(	ENSG0000(	13.56688	-0.26649	0.34534	0.513924	lncRNA
ENSG0000(	QRICH2	217.3523	-0.06694	0.345384	0.513964	protein_coding
ENSG0000(	RN7SL614P	22.63373	0.136466	0.34541	0.51398	misc_RNA
ENSG0000(	PTGES3L-A/	12.68431	-0.34617	0.345465	0.514036	protein_coding
ENSG0000(	SMIM5	1738.009	0.201073	0.345521	0.514096	protein_coding
ENSG0000(	LINC02392	80.38305	0.091374	0.345567	0.51414	lncRNA
ENSG0000(	ENSG0000(	60.32658	0.083146	0.345684	0.514291	lncRNA
ENSG0000(	LINC00974	9.81192	0.227364	0.34573	0.514335	lncRNA
ENSG0000(	ZNRF3-IT1	57.11789	0.15247	0.345775	0.514377	lncRNA
ENSG0000(	APOH	13.66751	-0.20758	0.345856	0.514472	protein_coding
ENSG0000(	HMGB3P22	10.29766	0.219069	0.345876	0.514478	transcribed_processed_pseudoge
ENSG0000(	MSTO2P	47.27512	0.172554	0.345937	0.514521	unprocessed_pseudogene
ENSG0000(	SEC23B	759.8145	-0.05372	0.345923	0.514521	protein_coding
ENSG0000(	TARM1	60.7439	0.10127	0.346022	0.514623	protein_coding
ENSG0000(	DES	464.1273	-0.22586	0.34617	0.514798	protein_coding
ENSG0000(	ENSG0000(	35.49206	0.12352	0.346173	0.514798	lncRNA
ENSG0000(	ENSG0000(	536.0147	-0.0733	0.34622	0.514828	protein_coding
ENSG0000(	H2BU1	437.7915	0.068788	0.346225	0.514828	protein_coding
ENSG0000(	MRPL24	184.634	0.062882	0.34626	0.514855	protein_coding
ENSG0000(	ENSG0000(	37.73373	-0.13878	0.346491	0.515174	lncRNA
ENSG0000(	ZNF702P	463.0997	-0.05185	0.34658	0.515282	transcribed_processed_pseudoge
ENSG0000(	CENPW	31.22128	-0.14877	0.346608	0.515299	protein_coding
ENSG0000(	TAPT1-AS1	409.7965	0.055267	0.346695	0.515405	lncRNA
ENSG0000(	RPS10-NUI	322.8749	-0.13396	0.346756	0.515471	protein_coding
ENSG0000(	ENSG0000(	23.97831	0.148407	0.346897	0.515608	lncRNA
ENSG0000(	IFNGR1	1823.226	-0.06988	0.346882	0.515608	protein_coding

ENSG0000(	ZNF264	3165.973	0.045412	0.346884	0.515608	protein_coding
ENSG0000(	RGPD4	52.36788	0.296692	0.346952	0.515666	protein_coding
ENSG0000(	ENSG0000(	387.8545	0.124689	0.347187	0.51599	protein_coding
ENSG0000(	ENSG0000(	80.58693	-0.29125	0.347278	0.516101	processed_pseudogene
ENSG0000(	ANTXRL	14.20917	0.297735	0.347363	0.516204	protein_coding
ENSG0000(	ENSG0000(	22.87896	0.135226	0.347462	0.516326	lncRNA
ENSG0000(	KIF1B	4748.062	-0.07812	0.347492	0.516326	protein_coding
ENSG0000(	LINC01036	208.6854	0.07923	0.347495	0.516326	lncRNA
ENSG0000(	ENSG0000(	11.22121	0.195344	0.347523	0.516344	lncRNA
ENSG0000(	ENSG0000(	114.3548	-1.00057	0.347615	0.516454	protein_coding
ENSG0000(	LMX1B	241.6591	0.077627	0.34763	0.516454	protein_coding
ENSG0000(	UGT2B29P	23.00233	0.322003	0.347651	0.516461	unprocessed_pseudogene
ENSG0000(	GCG	481.8686	-0.19681	0.347743	0.516546	protein_coding
ENSG0000(	NOXO1	62.59078	-0.10784	0.347758	0.516546	protein_coding
ENSG0000(	RN7SL344F	84.67195	0.102878	0.347744	0.516546	misc_RNA
ENSG0000(	ENSG0000(	19.57466	0.122287	0.347778	0.516552	lncRNA
ENSG0000(	DBN1	254.2483	0.118089	0.3479	0.516681	protein_coding
ENSG0000(	HLA-K	230.7154	-0.18692	0.347909	0.516681	unprocessed_pseudogene
ENSG0000(	RFC2	477.7402	-0.04168	0.347914	0.516681	protein_coding
ENSG0000(	ENSG0000(	20.75921	-0.16258	0.347947	0.516705	protein_coding
ENSG0000(	ENSG0000(	30.55664	-0.10747	0.347985	0.516737	lncRNA
ENSG0000(	ENSG0000(	13.46518	0.179223	0.348148	0.516955	lncRNA
ENSG0000(	HDHD3	1680.39	-0.13081	0.348178	0.516976	protein_coding
ENSG0000(	SSH2	4403.909	-0.04758	0.34821	0.516999	protein_coding
ENSG0000(	RN7SKP173	30.7867	-0.11859	0.348283	0.517058	misc_RNA
ENSG0000(	RNU2-64P	16.21161	-0.24052	0.348274	0.517058	snRNA
ENSG0000(	MIR7854	12.42442	0.194133	0.348395	0.5172	miRNA
ENSG0000(	EDIL3-DT	1463.285	0.030835	0.348432	0.51723	lncRNA
ENSG0000(	ZC3H7A	1911.278	-0.02711	0.34857	0.517411	protein_coding
ENSG0000(	SYT1	75.78311	-0.16432	0.348657	0.517515	protein_coding
ENSG0000(	ENSG0000(	44.78268	-0.11957	0.348738	0.517611	processed_pseudogene
ENSG0000(	SLC25A40	901.6019	0.060217	0.348774	0.517641	protein_coding
ENSG0000(	ALG9	1153.373	0.03251	0.348816	0.517655	protein_coding
ENSG0000(	OTX2-AS1	323.1114	0.078027	0.348805	0.517655	lncRNA
ENSG0000(	ENSG0000(	12.58974	0.160023	0.348864	0.517676	lncRNA
ENSG0000(	STAG3L2	1834.277	0.040134	0.348856	0.517676	transcribed_unprocessed_pseudo
ENSG0000(	ATP13A4	277.0181	-0.15053	0.348904	0.517677	protein_coding
ENSG0000(	ENSG0000(	18.95887	0.157533	0.348914	0.517677	lncRNA
ENSG0000(	ESM1	13.19028	-0.26797	0.348882	0.517677	protein_coding
ENSG0000(	ADM2	343.2514	-0.06568	0.348937	0.517688	protein_coding
ENSG0000(	ENSG0000(	94.17772	0.079106	0.348957	0.517693	lncRNA
ENSG0000(	IKBKG	818.6043	0.047879	0.349063	0.517825	protein_coding
ENSG0000(	C12orf60	307.1298	0.04724	0.349141	0.517863	protein_coding
ENSG0000(	ENSG0000(	634.1868	0.043062	0.349145	0.517863	TEC
ENSG0000(	GPSM3	539.8784	0.160532	0.349133	0.517863	protein_coding
ENSG0000(	RN7SL356F	58.90384	0.085652	0.349165	0.517863	misc_RNA
ENSG0000(	TDRD12	163.9075	0.06429	0.34917	0.517863	protein_coding

ENSG0000(	ENSG0000(	54.05399	0.095317	0.349276	0.517971	lncRNA
ENSG0000(	MFSD5	165.9337	-0.09034	0.349268	0.517971	protein_coding
ENSG0000(	CFAP299	111.046	-0.07515	0.3493	0.517983	protein_coding
ENSG0000(	FUT11	772.8895	0.044257	0.349641	0.518464	protein_coding
ENSG0000(	ENSG0000(	154.5048	-0.07656	0.349775	0.518637	lncRNA
ENSG0000(	TMIE	14.19076	0.206639	0.349792	0.518637	protein_coding
ENSG0000(	ZNF490	1275.963	-0.06502	0.349808	0.518637	protein_coding
ENSG0000(	ENSG0000(	29.6622	-0.11739	0.349838	0.518657	lncRNA
ENSG0000(	ENSG0000(	25.40742	-0.10871	0.350035	0.518925	lncRNA
ENSG0000(	HEATR1	1533.342	-0.08271	0.350155	0.519078	protein_coding
ENSG0000(	MYH9	17012.25	0.031839	0.35023	0.519092	protein_coding
ENSG0000(	NEURL1B	1960.957	-0.15832	0.350203	0.519092	protein_coding
ENSG0000(	PRKAB1	1947.737	0.05414	0.35022	0.519092	protein_coding
ENSG0000(	PRKG1	1379.306	-0.07857	0.350228	0.519092	protein_coding
ENSG0000(	ENSG0000(	19.92946	0.123519	0.350261	0.519114	lncRNA
ENSG0000(	SYNCRIP	4294.478	-0.03275	0.350284	0.519124	protein_coding
ENSG0000(	RPS6KA4	438.0922	-0.07166	0.350346	0.519169	protein_coding
ENSG0000(	SHOC1	17.40377	0.684804	0.350348	0.519169	protein_coding
ENSG0000(	OR8J3	44.90153	-0.1366	0.350385	0.5192	protein_coding
ENSG0000(	ENSG0000(	575.6297	-0.0498	0.350485	0.519274	lncRNA
ENSG0000(	ENSG0000(	10.30846	-0.22158	0.35046	0.519274	lncRNA
ENSG0000(	GLCE	1084.146	-0.09595	0.350484	0.519274	protein_coding
ENSG0000(	HLA-DOA	1408.019	0.157737	0.350505	0.51928	protein_coding
ENSG0000(	SRPRA	1610.098	0.044675	0.350533	0.519298	protein_coding
ENSG0000(	ENSG0000(	29.56874	-0.11763	0.350589	0.519355	lncRNA
ENSG0000(	ENSG0000(	24.81017	0.12413	0.350605	0.519355	lncRNA
ENSG0000(	ENSG0000(	8.828221	-0.27576	0.350643	0.519387	processed_pseudogene
ENSG0000(	PTPN21	926.2738	-0.1193	0.350786	0.519574	protein_coding
ENSG0000(	DENND6A	1454.333	-0.05518	0.350876	0.519669	protein_coding
ENSG0000(	FOXL2NB	776.3445	-0.05061	0.350883	0.519669	protein_coding
ENSG0000(	ENSG0000(	699.591	-0.11526	0.350927	0.519709	lncRNA
ENSG0000(	GAS5-AS1	47.8912	0.098475	0.350974	0.519755	lncRNA
ENSG0000(	PLA2G6	822.4177	-0.0541	0.350995	0.519761	protein_coding
ENSG0000(	ABCC6P2	139.6576	-0.18158	0.351211	0.520009	transcribed_unprocessed_pseudo
ENSG0000(	MCCD1P2	8.63724	0.243104	0.351193	0.520009	unprocessed_pseudogene
ENSG0000(	PSMD10	240.8951	0.057151	0.351197	0.520009	protein_coding
ENSG0000(	CELF3	83.21376	-0.13282	0.351263	0.520061	protein_coding
ENSG0000(	LINC00964	344.3016	0.048992	0.35128	0.520061	lncRNA
ENSG0000(	ENSG0000(	443.3993	0.078637	0.351307	0.520073	lncRNA
ENSG0000(	ISLR2	145.5841	0.087429	0.351321	0.520073	protein_coding
ENSG0000(	ENSG0000(	29.86582	0.192637	0.35134	0.520077	protein_coding
ENSG0000(	ABHD12B	198.7512	0.054724	0.351434	0.520169	protein_coding
ENSG0000(	OPA3	2227.353	0.032476	0.351436	0.520169	protein_coding
ENSG0000(	ENSG0000(	607.472	-0.03953	0.351506	0.520228	lncRNA
ENSG0000(	ENSG0000(	60.31034	0.181792	0.351508	0.520228	TEC
ENSG0000(	C3orf38	573.0321	-0.05395	0.35169	0.520448	protein_coding
ENSG0000(	ENSG0000(	319.7397	-0.04991	0.351687	0.520448	lncRNA

ENSG0000(	ZNF583	545.9894	0.063242	0.351718	0.520465	protein_coding
ENSG0000(	TXNRD2	872.4533	-0.03461	0.351789	0.520545	protein_coding
ENSG0000(	GRAMD4P3	685.575	-0.13454	0.351829	0.520572	processed_pseudogene
ENSG0000(	MED28-DT	233.0535	-0.05207	0.351839	0.520572	lncRNA
ENSG0000(	CLCN3P1	446.4014	0.06429	0.351959	0.520723	transcribed_unprocessed_pseudo
ENSG0000(	ENSG0000(	26.47523	0.131686	0.351997	0.520756	lncRNA
ENSG0000(	CDV3	2873.464	0.070702	0.352043	0.520772	protein_coding
ENSG0000(	ENSG0000(	15.16597	-0.21549	0.352058	0.520772	lncRNA
ENSG0000(	HAGHL	120.141	-0.13016	0.352036	0.520772	protein_coding
ENSG0000(	TATDN2	1947.322	-0.05167	0.352101	0.520788	protein_coding
ENSG0000(	TCAM1P	210.3347	0.062716	0.352102	0.520788	transcribed_unitary_pseudogene
ENSG0000(	MIR6891	12.07921	0.239476	0.352182	0.520882	miRNA
ENSG0000(	ENSG0000(	8.858454	0.219256	0.352225	0.520898	processed_pseudogene
ENSG0000(	PRSS27	505.1406	0.052421	0.352221	0.520898	protein_coding
ENSG0000(	ENSG0000(	14.64315	0.408147	0.352302	0.520963	lncRNA
ENSG0000(	TMEM38A	300.4889	-0.04515	0.352291	0.520963	protein_coding
ENSG0000(	EP300	6507.105	-0.07881	0.352577	0.521345	protein_coding
ENSG0000(	GGTLC2	174.7613	0.09198	0.352635	0.521406	protein_coding
ENSG0000(	PCK1	7648.723	-0.31723	0.352655	0.521412	protein_coding
ENSG0000(	PGM5P4-A	110.2613	0.073198	0.352759	0.521541	lncRNA
ENSG0000(	CARD11	918.199	-0.1945	0.352781	0.521549	protein_coding
ENSG0000(	UNC13B	2679.665	0.119812	0.352814	0.521573	protein_coding
ENSG0000(	FAAP20	1044.739	0.047017	0.35291	0.52169	protein_coding
ENSG0000(	RFWD3	1131.491	-0.04317	0.352959	0.521738	protein_coding
ENSG0000(	IRF2BP1	210.7039	-0.07251	0.352983	0.521748	protein_coding
ENSG0000(	ENSG0000(	57.41733	0.081082	0.353007	0.521761	lncRNA
ENSG0000(	COIL	409.4514	0.045685	0.353156	0.521931	protein_coding
ENSG0000(	TTTY9B	11.56549	0.162496	0.353142	0.521931	lncRNA
ENSG0000(	LRRFIP2	3436.629	-0.07638	0.353235	0.522024	protein_coding
ENSG0000(	ENSG0000(	510.3121	0.0388	0.353296	0.522072	lncRNA
ENSG0000(	ENSG0000(	15.72118	0.175506	0.353301	0.522072	lncRNA
ENSG0000(	AGAP14P	23.99914	0.411785	0.353354	0.522126	unprocessed_pseudogene
ENSG0000(	DDX59	668.7033	0.058151	0.353461	0.52226	protein_coding
ENSG0000(	PTER	506.1781	-0.06022	0.353617	0.522465	protein_coding
ENSG0000(	ENSG0000(	15.34238	0.160781	0.353645	0.522483	lncRNA
ENSG0000(	ENSG0000(	138.0701	-0.06765	0.353712	0.52255	processed_pseudogene
ENSG0000(	ENSG0000(	167.8396	0.062227	0.353724	0.52255	lncRNA
ENSG0000(	ENSG0000(	56.4093	0.163378	0.353935	0.522806	processed_pseudogene
ENSG0000(	MIR1296	18.22414	-0.15818	0.353947	0.522806	miRNA
ENSG0000(	RN7SL577F	50.11178	0.098583	0.353914	0.522806	misc_RNA
ENSG0000(	RN7SKP187	73.11808	-0.21243	0.35401	0.522875	misc_RNA
ENSG0000(	ESYT3	305.2223	-0.0618	0.354054	0.52289	protein_coding
ENSG0000(	P2RX4	2244.31	-0.05914	0.354037	0.52289	protein_coding
ENSG0000(	ENSG0000(	9.443965	0.229881	0.354089	0.522918	lncRNA
ENSG0000(	CLDN16	165.4783	-0.07537	0.354122	0.522921	protein_coding
ENSG0000(	ENSG0000(	8.897037	0.228042	0.354141	0.522921	processed_pseudogene
ENSG0000(	ENSG0000(	292.2336	-0.04722	0.354129	0.522921	lncRNA

ENSG0000(	CCNI2	141.5733	0.124221	0.354184	0.52296	protein_coding
ENSG0000(	ENSG0000(	15.0863	0.180772	0.354251	0.522986	lncRNA
ENSG0000(	FNDC5	19.52299	0.309938	0.35424	0.522986	protein_coding
ENSG0000(	RELCH	3281.901	-0.03309	0.35425	0.522986	protein_coding
ENSG0000(	ENSG0000(	10.57091	0.211354	0.354315	0.523056	lncRNA
ENSG0000(	AGBL4	198.6082	-0.06284	0.354451	0.523231	protein_coding
ENSG0000(	PSEN1	3298.003	-0.05999	0.354498	0.523277	protein_coding
ENSG0000(	ENSG0000(	143.6332	0.075057	0.354545	0.523297	lncRNA
ENSG0000(	SUDS3P1	9.245039	-0.24487	0.354529	0.523297	processed_pseudogene
ENSG0000(	HSD17B1-A	355.1662	0.050297	0.354661	0.523443	lncRNA
ENSG0000(	SMARCC2	3930.448	-0.02463	0.354714	0.523498	protein_coding
ENSG0000(	ENSG0000(	242.0885	0.233356	0.35474	0.523511	lncRNA
ENSG0000(	FAM66D	259.6428	0.117578	0.354882	0.523672	lncRNA
ENSG0000(	LMF1	658.6052	-0.06998	0.354868	0.523672	protein_coding
ENSG0000(	ENSG0000(	12.09232	0.194272	0.354904	0.523679	TEC
ENSG0000(	ENSG0000(	16.2068	-0.16835	0.354992	0.523785	transcribed_processed_pseudoge
ENSG0000(	ENSG0000(	77.49083	0.118379	0.355183	0.524042	lncRNA
ENSG0000(	SPN	2375.986	-0.05185	0.355251	0.524117	protein_coding
ENSG0000(	FNBP4	5006.706	0.030537	0.355286	0.524145	protein_coding
ENSG0000(	ENSG0000(	16.39893	0.156131	0.355317	0.524166	lncRNA
ENSG0000(	RRP9	77.58888	-0.10113	0.355336	0.524169	protein_coding
ENSG0000(	ENSG0000(	9.230999	-0.18241	0.355424	0.52425	lncRNA
ENSG0000(	ERVMER34	82.10579	-0.08505	0.355417	0.52425	protein_coding
ENSG0000(	CERS5	1377.182	-0.05024	0.35553	0.524382	protein_coding
ENSG0000(	ENSG0000(	132.5117	0.118582	0.35559	0.524422	transcribed_unprocessed_pseudo
ENSG0000(	SLC16A12	218.5971	0.051701	0.355579	0.524422	protein_coding
ENSG0000(	C2orf80	41.26995	0.083603	0.355662	0.524436	protein_coding
ENSG0000(	GUCA1B	174.697	0.063059	0.355666	0.524436	protein_coding
ENSG0000(	LINC01687	228.3507	0.10208	0.355667	0.524436	lncRNA
ENSG0000(	PACRG	26.10825	0.190465	0.355658	0.524436	protein_coding
ENSG0000(	NR2E1	14.80041	-0.21683	0.355768	0.524487	protein_coding
ENSG0000(	RCC1	396.8335	-0.07346	0.35573	0.524487	protein_coding
ENSG0000(	RN7SL864F	8.838316	-0.21146	0.35575	0.524487	misc_RNA
ENSG0000(	SESN2	494.2764	-0.13899	0.355761	0.524487	protein_coding
ENSG0000(	CRYM-AS1	306.5063	-0.0554	0.355952	0.524611	transcribed_unitary_pseudogene
ENSG0000(	DCAF10	1924.946	0.035437	0.355869	0.524611	protein_coding
ENSG0000(	ENSG0000(	60.50917	0.123536	0.355932	0.524611	lncRNA
ENSG0000(	ENSG0000(	66.26932	0.072697	0.355922	0.524611	lncRNA
ENSG0000(	GTF2IP14	19.46871	0.164719	0.355945	0.524611	unprocessed_pseudogene
ENSG0000(	KDELR1	1281.311	-0.059	0.355902	0.524611	protein_coding
ENSG0000(	WNK2	3848.152	-0.15821	0.356005	0.524665	protein_coding
ENSG0000(	ENSG0000(	36.15015	0.200274	0.356036	0.524686	lncRNA
ENSG0000(	HOTAIRM1	308.5873	0.083819	0.356055	0.52469	lncRNA
ENSG0000(	RPS6KL1	476.8826	-0.04375	0.356103	0.524737	protein_coding
ENSG0000(	ENSG0000(	148.8073	-0.05915	0.356127	0.524747	lncRNA
ENSG0000(	C1QTNF5	106.7201	0.119898	0.356181	0.524777	protein_coding
ENSG0000(	ECEL1	14.22841	-0.34309	0.356178	0.524777	protein_coding

ENSG0000(	POFUT2	1082.808	0.06977	0.356248	0.524852	protein_coding
ENSG0000(	ENSG0000(	48.97405	0.146563	0.35627	0.52486	lncRNA
ENSG0000(	RAB27B	583.0424	0.146431	0.356323	0.524888	protein_coding
ENSG0000(	TACC3	1733.17	0.07098	0.356317	0.524888	protein_coding
ENSG0000(	ENSG0000(	62.37496	0.081554	0.356358	0.524916	processed_pseudogene
ENSG0000(	LINC02348	241.3418	0.042872	0.356429	0.524936	lncRNA
ENSG0000(	NOP56	973.1336	-0.07457	0.356428	0.524936	protein_coding
ENSG0000(	SULT1C4	278.022	0.043081	0.356438	0.524936	protein_coding
ENSG0000(	TRAJ17	45.26274	-0.19921	0.356439	0.524936	TR_J_gene
ENSG0000(	ENSG0000(	29.98005	-0.16005	0.356459	0.524941	lncRNA
ENSG0000(	ENSG0000(	10.5956	0.223703	0.356495	0.52497	misc_RNA
ENSG0000(	SEC24C	2400.152	0.035718	0.356522	0.524985	protein_coding
ENSG0000(	NMB	12.63588	0.190282	0.356592	0.525064	protein_coding
ENSG0000(	ENSG0000(	188.0698	-0.06847	0.356688	0.525157	lncRNA
ENSG0000(	ENSG0000(	12.80569	0.180763	0.356683	0.525157	lncRNA
ENSG0000(	C11orf21	281.0204	0.207208	0.356721	0.525179	protein_coding
ENSG0000(	MRNIP	2231.209	-0.05791	0.35679	0.525258	protein_coding
ENSG0000(	ENSG0000(	55.38061	0.107983	0.356821	0.525278	protein_coding
ENSG0000(	ENSG0000(	14.77047	-0.25518	0.356872	0.525329	lncRNA
ENSG0000(	SBSPON	215.0895	-0.06339	0.356918	0.525372	protein_coding
ENSG0000(	ENSG0000(	14.51183	0.179537	0.35697	0.525422	lncRNA
ENSG0000(	NOS2P3	123.303	0.050142	0.356985	0.525422	transcribed_unprocessed_pseudo
ENSG0000(	ST8SIA1	1310.607	0.10342	0.357075	0.525529	protein_coding
ENSG0000(	TBC1D22A	1860.523	0.044563	0.3571	0.525541	protein_coding
ENSG0000(	SART1	661.8665	-0.06766	0.357131	0.525563	protein_coding
ENSG0000(	MCM3	670.8714	-0.07965	0.357223	0.525657	protein_coding
ENSG0000(	RRP36	633.1728	0.042671	0.357228	0.525657	protein_coding
ENSG0000(	ENSG0000(	43.32437	0.0824	0.357265	0.525687	lncRNA
ENSG0000(	CMC2	1591.859	0.049423	0.357416	0.525877	protein_coding
ENSG0000(	LONP2	5073.116	-0.04628	0.357436	0.525877	protein_coding
ENSG0000(	MBNL1-AS:	204.6556	0.085874	0.357445	0.525877	lncRNA
ENSG0000(	ENSG0000(	103.0278	-0.1108	0.357549	0.526006	lncRNA
ENSG0000(	NTRK3	659.3156	0.063193	0.357623	0.52609	protein_coding
ENSG0000(	ARHGEF5	2090.048	-0.16894	0.357756	0.526236	protein_coding
ENSG0000(	CASP7	1349.711	-0.10909	0.357748	0.526236	protein_coding
ENSG0000(	ENSG0000(	1157.61	0.085939	0.357824	0.526287	protein_coding
ENSG0000(	SEMA6D	3990.602	-0.16007	0.357812	0.526287	protein_coding
ENSG0000(	ENSG0000(	36.33268	0.115755	0.35789	0.52636	lncRNA
ENSG0000(	CRISPLD1	27.80192	0.229245	0.357935	0.526401	protein_coding
ENSG0000(	CRADD	431.3686	-0.07526	0.357995	0.526464	protein_coding
ENSG0000(	RALGAPA1I	373.891	-0.08373	0.358011	0.526464	processed_pseudogene
ENSG0000(	EFCAB2	1035.873	-0.0786	0.358087	0.526477	protein_coding
ENSG0000(	FAM226B	91.43096	0.164415	0.358045	0.526477	lncRNA
ENSG0000(	SLC25A45	1655.318	0.04091	0.358084	0.526477	protein_coding
ENSG0000(	SSH3	548.7027	-0.12077	0.358085	0.526477	protein_coding
ENSG0000(	TACSTD2	19.45235	0.228644	0.35813	0.526516	protein_coding
ENSG0000(	ARMC10	1067.32	-0.03898	0.358258	0.52656	protein_coding

ENSG0000(	PPA1	1476.407	0.063649	0.358194	0.52656	protein_coding
ENSG0000(	RNA5SP473	31.7458	0.103985	0.358243	0.52656	rRNA_pseudogene
ENSG0000(	SH3RF1	1545.008	-0.14458	0.358241	0.52656	protein_coding
ENSG0000(	TEDC2-AS1	468.8037	0.0466	0.358197	0.52656	lncRNA
ENSG0000(	TLK2	2910.794	0.030845	0.35826	0.52656	protein_coding
ENSG0000(	ABRA	15.25944	0.139258	0.358447	0.526785	protein_coding
ENSG0000(	ZMYND10	12.20381	0.218104	0.358448	0.526785	protein_coding
ENSG0000(	ZMYND8	2791.073	0.060789	0.358463	0.526785	protein_coding
ENSG0000(	ENSG0000(	1893.133	0.062631	0.358584	0.526938	lncRNA
ENSG0000(	YWHAG	1817.266	-0.05763	0.358687	0.527065	protein_coding
ENSG0000(	GAPDHP72	60.8546	-0.07555	0.358782	0.52718	transcribed_processed_pseudoge
ENSG0000(	NKRF	408.3566	0.068312	0.358819	0.527209	protein_coding
ENSG0000(	RFPL3S	736.3603	-0.0401	0.358839	0.527215	lncRNA
ENSG0000(	LRRIQ4	45.58875	0.104379	0.358929	0.527322	protein_coding
ENSG0000(	ARHGEF28	943.28	0.103143	0.359044	0.527451	protein_coding
ENSG0000(	ENSG0000(	10.93752	-0.18604	0.35905	0.527451	lncRNA
ENSG0000(	ENSG0000(	197.6518	-0.28225	0.35907	0.527456	lncRNA
ENSG0000(	ERI3-IT1	92.22998	0.085211	0.359292	0.527756	lncRNA
ENSG0000(	ZNF112	210.5202	-0.08073	0.359372	0.527849	protein_coding
ENSG0000(	RNU6-1152	17.93967	0.190839	0.359432	0.527912	snRNA
ENSG0000(	ENSG0000(	172.1488	0.071448	0.359514	0.528008	lncRNA
ENSG0000(	ENSG0000(	67.5623	0.173432	0.359577	0.528078	lncRNA
ENSG0000(	PCDHB16	127.3383	0.063061	0.359607	0.528096	protein_coding
ENSG0000(	MIR548AQ	16.98045	-0.16475	0.35972	0.528238	miRNA
ENSG0000(	ENSG0000(	94.82492	0.088709	0.359799	0.528329	lncRNA
ENSG0000(	USP40	1582.999	0.058421	0.359828	0.528347	protein_coding
ENSG0000(	PLXNA1	1517.113	0.087147	0.359855	0.528362	protein_coding
ENSG0000(	EIF3CL	549.8682	-0.15313	0.359956	0.528439	protein_coding
ENSG0000(	ENSG0000(	147.8555	0.055498	0.359958	0.528439	lncRNA
ENSG0000(	RETREG1	1640.501	-0.08829	0.359955	0.528439	protein_coding
ENSG0000(	SPACA9	266.6235	-0.05299	0.359986	0.528456	protein_coding
ENSG0000(	GOLGA8DP	15.70236	0.237232	0.360041	0.528487	transcribed_unprocessed_pseudo
ENSG0000(	PACS2	1864.742	-0.04872	0.360037	0.528487	protein_coding
ENSG0000(	EMG1	919.3719	-0.05853	0.360112	0.528542	protein_coding
ENSG0000(	ENSG0000(	686.8212	0.075071	0.360136	0.528542	lncRNA
ENSG0000(	ITCH-AS1	31.32538	0.111227	0.360146	0.528542	lncRNA
ENSG0000(	MAPK7	469.6707	0.075621	0.360122	0.528542	protein_coding
ENSG0000(	ENSG0000(	69.74821	0.087233	0.360201	0.528598	lncRNA
ENSG0000(	CDIN1	502.4112	0.046981	0.360269	0.528625	protein_coding
ENSG0000(	CEP170B	2223.511	-0.1291	0.360256	0.528625	protein_coding
ENSG0000(	ENSG0000(	491.9079	0.060937	0.360249	0.528625	lncRNA
ENSG0000(	ENSG0000(	3968.82	0.031921	0.360302	0.528648	lncRNA
ENSG0000(	EIF4E2	2828.562	-0.03417	0.360367	0.52872	protein_coding
ENSG0000(	RASSF2	2062.225	0.175346	0.360474	0.528852	protein_coding
ENSG0000(	OVCA2	24.26128	0.322544	0.360562	0.528956	protein_coding
ENSG0000(	NLGN1	54.45795	0.178226	0.360594	0.528978	protein_coding
ENSG0000(	ENSG0000(	102.0776	0.080171	0.360841	0.529316	lncRNA

ENSG0000(	ENSG0000(	40.82033	0.176192	0.360881	0.529351	lncRNA
ENSG0000(	RNF208	26.00004	-0.13568	0.361003	0.529504	protein_coding
ENSG0000(	ENSG0000(	20.44256	0.164207	0.361023	0.52951	processed_pseudogene
ENSG0000(	ANKRD23	141.3894	-0.07065	0.361061	0.529541	protein_coding
ENSG0000(	ENSG0000(	305.2935	0.039895	0.361181	0.529692	lncRNA
ENSG0000(	ENSG0000(	96.29602	-0.08328	0.361243	0.529734	TEC
ENSG0000(	RN7SL524F	878.6566	0.042899	0.361227	0.529734	misc_RNA
ENSG0000(	RN7SL733F	15.27665	0.17752	0.361288	0.529774	misc_RNA
ENSG0000(	C4A-AS1	34.67945	0.103455	0.361326	0.529781	lncRNA
ENSG0000(	HSD3B2	135.9622	0.264985	0.361343	0.529781	protein_coding
ENSG0000(	PRND	202.624	-0.05284	0.361321	0.529781	protein_coding
ENSG0000(	RPS20P33	33.77866	0.102307	0.361399	0.529839	processed_pseudogene
ENSG0000(	MIER1	1819.474	0.034815	0.361527	0.530002	protein_coding
ENSG0000(	LTB4R2	313.9857	-0.06025	0.361551	0.530012	protein_coding
ENSG0000(	NEMP1	1006.121	0.083625	0.36162	0.530089	protein_coding
ENSG0000(	POC1B-AS1	1130.576	0.074402	0.361681	0.530154	lncRNA
ENSG0000(	APTR	1155.241	0.056396	0.361781	0.530238	lncRNA
ENSG0000(	CLCA1	14694.92	-0.2153	0.361789	0.530238	protein_coding
ENSG0000(	IRAK4	1989.755	0.04102	0.361757	0.530238	protein_coding
ENSG0000(	CFAP20DC	199.6977	-0.08541	0.361918	0.530397	protein_coding
ENSG0000(	DYRK4	365.5376	0.070977	0.361948	0.530397	protein_coding
ENSG0000(	TOGARAM1	2084.79	-0.05483	0.361941	0.530397	protein_coding
ENSG0000(	UPK3BL2	668.5087	0.099841	0.362047	0.530518	protein_coding
ENSG0000(	ENSG0000(	217.7457	0.047492	0.362099	0.530525	TEC
ENSG0000(	ENSG0000(	108.6983	0.05822	0.362092	0.530525	TEC
ENSG0000(	PLD5	119.2275	0.077579	0.362103	0.530525	protein_coding
ENSG0000(	UACA	2766.77	-0.12222	0.362155	0.530576	protein_coding
ENSG0000(	OR4E1	31.76649	-0.1264	0.362232	0.530665	protein_coding
ENSG0000(	ENSG0000(	28.79306	-0.16833	0.362267	0.530692	transcribed_unprocessed_pseudo
ENSG0000(	LINC00629	305.9515	0.081562	0.362307	0.530725	lncRNA
ENSG0000(	HMGN1P15	10.27759	0.188138	0.362355	0.530767	processed_pseudogene
ENSG0000(	ITIH4	135.0337	0.140537	0.362369	0.530767	protein_coding
ENSG0000(	ENSG0000(	29.69184	0.118029	0.36249	0.53092	lncRNA
ENSG0000(	ENSG0000(	30.17882	0.131948	0.362728	0.531243	lncRNA
ENSG0000(	SMC4	2308.772	-0.04948	0.362746	0.531245	protein_coding
ENSG0000(	C21orf62-A	1888.999	0.033582	0.36278	0.53127	lncRNA
ENSG0000(	ARPC5	3173.096	-0.05714	0.362933	0.531388	protein_coding
ENSG0000(	ENSG0000(	60.69227	0.072775	0.362895	0.531388	lncRNA
ENSG0000(	RNVU1-4	31.74104	-0.14346	0.362925	0.531388	snRNA
ENSG0000(	RPL18P10	10.44407	-0.19012	0.362962	0.531388	processed_pseudogene
ENSG0000(	SLC25A25	639.4304	-0.08261	0.36295	0.531388	protein_coding
ENSG0000(	UBE2W	1528.386	0.047598	0.362897	0.531388	protein_coding
ENSG0000(	ENSG0000(	14.01813	0.204409	0.362987	0.5314	lncRNA
ENSG0000(	ENSG0000(	2784.984	-0.0531	0.363224	0.531723	lncRNA
ENSG0000(	ENSG0000(	55.37868	0.090316	0.363301	0.531781	lncRNA
ENSG0000(	GOLGA8Q	76.47739	-0.20622	0.363331	0.531781	protein_coding
ENSG0000(	PDE8B	124.2676	0.148121	0.363309	0.531781	protein_coding

ENSG0000(	SNX18P12	44.67654	-0.1532	0.36332	0.531781	processed_pseudogene
ENSG0000(	SDHDP6	8.877045	0.341709	0.363471	0.53196	processed_pseudogene
ENSG0000(	ARHGAP11	14.07413	0.303497	0.363608	0.532137	protein_coding
ENSG0000(	ENSG0000(	47.34087	0.117752	0.363701	0.532227	processed_pseudogene
ENSG0000(	PHF1	804.6241	-0.05165	0.363703	0.532227	protein_coding
ENSG0000(	RSPH6A	9.047108	-0.19938	0.363768	0.532297	protein_coding
ENSG0000(	ZCCHC9	913.5765	-0.06221	0.363798	0.532315	protein_coding
ENSG0000(	TAF1L	121.1581	0.054181	0.363851	0.532368	protein_coding
ENSG0000(	LRRC9	32.07167	-0.36909	0.363879	0.532384	protein_coding
ENSG0000(	THAP5	1191.776	-0.03224	0.363918	0.532417	protein_coding
ENSG0000(	SYP	557.5277	0.056299	0.363942	0.532427	protein_coding
ENSG0000(	ENSG0000(	44.6719	0.092946	0.364164	0.53261	lncRNA
ENSG0000(	ENSG0000(	34.10007	0.162225	0.364163	0.53261	lncRNA
ENSG0000(	MRPS31P4	34.36971	0.179584	0.364175	0.53261	transcribed_unprocessed_pseudo
ENSG0000(	MUC20P1	229.0305	-0.2351	0.364185	0.53261	unprocessed_pseudogene
ENSG0000(	PPFIA2-AS1	24.84264	0.135209	0.364131	0.53261	lncRNA
ENSG0000(	RNU2-5P	9.731242	-0.1481	0.364176	0.53261	snRNA
ENSG0000(	XCL1	13.5489	-0.19864	0.36412	0.53261	protein_coding
ENSG0000(	CDC6	281.9867	0.104016	0.364471	0.532986	protein_coding
ENSG0000(	ZNF783	921.5946	0.055722	0.364476	0.532986	protein_coding
ENSG0000(	FMC1	224.8047	-0.04796	0.364625	0.533178	protein_coding
ENSG0000(	ZNF426-DT	46.60035	0.087832	0.364703	0.533269	lncRNA
ENSG0000(	HIGD1AP14	40.94512	0.201448	0.364763	0.533331	processed_pseudogene
ENSG0000(	ZNF711	189.6666	0.067097	0.364784	0.533337	protein_coding
ENSG0000(	ZNF155	256.5254	0.078798	0.364807	0.533347	protein_coding
ENSG0000(	ENSG0000(	236.5557	0.06213	0.364885	0.533419	lncRNA
ENSG0000(	RN7SL865P	14.24134	0.122897	0.364891	0.533419	misc_RNA
ENSG0000(	ATOH8	234.038	0.10442	0.364913	0.533421	protein_coding
ENSG0000(	ENSG0000(	378.8	0.043871	0.364926	0.533421	lncRNA
ENSG0000(	ABCA13	367.2303	-0.07274	0.365011	0.533496	protein_coding
ENSG0000(	TEX26-AS1	219.1418	0.045266	0.365003	0.533496	lncRNA
ENSG0000(	DLG5	2201.213	-0.09037	0.365036	0.533507	protein_coding
ENSG0000(	TRANK1	7255.734	0.080403	0.365063	0.533522	protein_coding
ENSG0000(	PLEKHA4	214.6787	0.086507	0.365203	0.533702	protein_coding
ENSG0000(	ENSG0000(	13.61302	-0.1524	0.365258	0.533757	lncRNA
ENSG0000(	CERS3-AS1	13.90139	0.260939	0.365402	0.533919	lncRNA
ENSG0000(	ENSG0000(	20.48385	0.155707	0.365388	0.533919	lncRNA
ENSG0000(	MTCO2P2	16.24058	-0.4123	0.365466	0.533987	processed_pseudogene
ENSG0000(	MAMSTR	43.69516	0.110971	0.365495	0.534005	protein_coding
ENSG0000(	ENSG0000(	56.20999	0.079352	0.365569	0.534062	lncRNA
ENSG0000(	ENSG0000(	23.74601	-0.12999	0.365585	0.534062	lncRNA
ENSG0000(	H2AJ	368.3483	-0.07231	0.365566	0.534062	protein_coding
ENSG0000(	ENSG0000(	9.468819	-0.25148	0.365657	0.534143	lncRNA
ENSG0000(	EML4	5456.619	-0.04063	0.365718	0.534178	protein_coding
ENSG0000(	ENSG0000(	19.1324	0.147764	0.365732	0.534178	lncRNA
ENSG0000(	ENSG0000(	8.599505	0.207792	0.36571	0.534178	lncRNA
ENSG0000(	ENSG0000(	20.87879	0.096985	0.365763	0.534179	lncRNA

ENSG0000(	ENSG0000(	148.0274	0.058081	0.365788	0.534179	lncRNA
ENSG0000(	ERVE-1	276.9683	0.076093	0.365809	0.534179	lncRNA
ENSG0000(	FCHSD2	2005.526	0.137008	0.365835	0.534179	protein_coding
ENSG0000(	ITGAE	852.2843	-0.04795	0.36582	0.534179	protein_coding
ENSG0000(	MIR4434	9.565179	-0.20123	0.365795	0.534179	miRNA
ENSG0000(	ENSG0000(	205.4885	-0.05439	0.365964	0.534319	lncRNA
ENSG0000(	SCARNA12	784.1728	0.067178	0.365947	0.534319	snoRNA
ENSG0000(	MIR573	39.08522	-0.11614	0.36606	0.534433	miRNA
ENSG0000(	HNRNPH1	16783.87	-0.03672	0.366079	0.534437	protein_coding
ENSG0000(	SLX9	92.05424	-0.08249	0.36611	0.534457	protein_coding
ENSG0000(	MED27	512.7065	0.05344	0.36613	0.534462	protein_coding
ENSG0000(	HMGB1P41	42.90405	-0.14108	0.366257	0.534623	processed_pseudogene
ENSG0000(	SLC6A1	663.7757	0.041411	0.366305	0.534647	protein_coding
ENSG0000(	THUMPD3	657.1722	-0.05127	0.366308	0.534647	protein_coding
ENSG0000(	ENSG0000(	78.81869	0.132382	0.366422	0.534764	lncRNA
ENSG0000(	LINC02181	70.15455	0.069714	0.366421	0.534764	lncRNA
ENSG0000(	FAM136A	728.6319	0.038826	0.366486	0.534833	protein_coding
ENSG0000(	REELD1	54.20972	0.153415	0.366554	0.534908	protein_coding
ENSG0000(	RDM1P1	248.845	0.130155	0.366664	0.535043	transcribed_unprocessed_pseudo
ENSG0000(	TUFT1	675.6136	-0.12295	0.366762	0.535161	protein_coding
ENSG0000(	DENR	1202.605	-0.03193	0.366801	0.535194	protein_coding
ENSG0000(	ENSG0000(	316.1693	0.110414	0.36689	0.53525	transcribed_unprocessed_pseudo
ENSG0000(	RABGEF1	595.0624	0.055647	0.366883	0.53525	protein_coding
ENSG0000(	ZSCAN22	458.1456	-0.04821	0.366883	0.53525	protein_coding
ENSG0000(	ENSG0000(	9.083436	0.180574	0.36693	0.535254	transcribed_unprocessed_pseudo
ENSG0000(	ENSG0000(	65.62865	0.122863	0.366962	0.535254	protein_coding
ENSG0000(	LINC02915	39.82279	0.097531	0.366916	0.535254	lncRNA
ENSG0000(	RNY1P16	84.80973	-0.14647	0.366948	0.535254	misc_RNA
ENSG0000(	LOH12CR2	238.5762	-0.06477	0.367048	0.535356	lncRNA
ENSG0000(	QDPR	195.4393	-0.07336	0.367074	0.535368	protein_coding
ENSG0000(	ENSG0000(	13.25178	0.162726	0.367196	0.535522	lncRNA
ENSG0000(	ENSG0000(	255.9235	-0.04938	0.367243	0.535566	lncRNA
ENSG0000(	SP140L	2080.789	0.062854	0.367408	0.535782	protein_coding
ENSG0000(	AGO2	4386.415	-0.04589	0.367515	0.535843	protein_coding
ENSG0000(	KCNA7	497.7785	0.034992	0.367468	0.535843	protein_coding
ENSG0000(	SLC7A6	2944.466	0.096141	0.367519	0.535843	protein_coding
ENSG0000(	WDR27	2582.243	0.038499	0.367494	0.535843	protein_coding
ENSG0000(	LPAR2	366.7675	0.087487	0.367568	0.53589	protein_coding
ENSG0000(	IHO1	399.4574	-0.06803	0.367798	0.536201	protein_coding
ENSG0000(	ISCA2	600.0537	-0.03801	0.367828	0.536212	protein_coding
ENSG0000(	RNU2-7P	15.21212	-0.14419	0.36784	0.536212	snRNA
ENSG0000(	ENSG0000(	109.5925	-0.07786	0.3679	0.536275	lncRNA
ENSG0000(	ENSG0000(	22.14547	0.128634	0.367961	0.536296	TEC
ENSG0000(	PIK3R6	262.8842	-0.09004	0.367974	0.536296	protein_coding
ENSG0000(	THSD4-AS1	78.4822	0.098611	0.367944	0.536296	lncRNA
ENSG0000(	TM2D3	1316.488	0.039368	0.367982	0.536296	protein_coding
ENSG0000(	ENSG0000(	155.8544	0.075778	0.368033	0.536321	lncRNA

ENSG0000(	NUP43	1706.935	-0.04466	0.368023	0.536321	protein_coding
ENSG0000(	ENSG0000(	35.64985	0.135818	0.368108	0.536406	lncRNA
ENSG0000(	ENSG0000(	80.1725	0.089033	0.368203	0.536518	lncRNA
ENSG0000(	ENSG0000(	59.8518	-0.08887	0.368357	0.536665	lncRNA
ENSG0000(	ENSG0000(	168.6058	0.055808	0.368372	0.536665	lncRNA
ENSG0000(	MTURN	718.548	0.069148	0.368335	0.536665	protein_coding
ENSG0000(	U6	37.26549	0.157457	0.368371	0.536665	snRNA
ENSG0000(	CD180	998.4859	-0.21141	0.368424	0.536717	protein_coding
ENSG0000(	IGHV5-78	8.167898	-0.3315	0.368534	0.536851	IG_V_pseudogene
ENSG0000(	ENSG0000(	38.58771	0.107134	0.368591	0.53691	unprocessed_pseudogene
ENSG0000(	CDCA5	124.7222	0.141547	0.368749	0.537065	protein_coding
ENSG0000(	ENSG0000(	16.37613	0.243286	0.368751	0.537065	lncRNA
ENSG0000(	ENSG0000(	271.4358	0.0433	0.368733	0.537065	lncRNA
ENSG0000(	ENSG0000(	19.21897	0.513245	0.368765	0.537065	protein_coding
ENSG0000(	ENSG0000(	159.341	0.151458	0.368805	0.537092	lncRNA
ENSG0000(	LINC01903	64.38201	-0.10861	0.368818	0.537092	lncRNA
ENSG0000(	ENSG0000(	99.0532	-0.18618	0.368877	0.537152	transcribed_processed_pseudoge
ENSG0000(	RNASE4	324.5779	-0.12643	0.368895	0.537155	protein_coding
ENSG0000(	ATP6V1C2	22.70484	0.200098	0.368926	0.537174	protein_coding
ENSG0000(	G2E3-AS1	127.8245	-0.04974	0.368985	0.537236	lncRNA
ENSG0000(	ENSG0000(	72.89445	0.112914	0.369039	0.537289	processed_pseudogene
ENSG0000(	C17orf100	121.0171	-0.0767	0.369297	0.537641	protein_coding
ENSG0000(	GOLGA8M	97.75957	0.152072	0.369361	0.537697	protein_coding
ENSG0000(	SLC35E1	2522.218	0.028265	0.36937	0.537697	protein_coding
ENSG0000(	ENSG0000(	162.8348	0.269278	0.369401	0.537715	lncRNA
ENSG0000(	ENSG0000(	129.2575	0.060155	0.369434	0.537715	protein_coding
ENSG0000(	GPR39	183.3325	-0.12799	0.369426	0.537715	protein_coding
ENSG0000(	ERVK9-11	834.137	0.046433	0.369556	0.537868	lncRNA
ENSG0000(	SLC25A46	1614.902	-0.03473	0.369583	0.537882	protein_coding
ENSG0000(	TUBE1	848.5078	0.036268	0.369623	0.537916	protein_coding
ENSG0000(	ENSG0000(	21.26545	0.123097	0.369743	0.53806	lncRNA
ENSG0000(	TUBGCP2	2714.355	0.023051	0.369756	0.53806	protein_coding
ENSG0000(	MANSC1	1462.985	-0.03056	0.369796	0.538093	protein_coding
ENSG0000(	PSD4	5973.073	-0.07473	0.369818	0.5381	protein_coding
ENSG0000(	TMSB4Y	30.0299	0.185506	0.370098	0.538458	protein_coding
ENSG0000(	WT1-AS	102.0681	-0.08796	0.370088	0.538458	lncRNA
ENSG0000(	ENSG0000(	100.614	0.065763	0.370144	0.538466	lncRNA
ENSG0000(	LRRCC1	378.3653	-0.07449	0.370135	0.538466	protein_coding
ENSG0000(	PSMD14	526.4853	-0.06333	0.370155	0.538466	protein_coding
ENSG0000(	ENSG0000(	10.17233	-0.38517	0.370215	0.538529	lncRNA
ENSG0000(	SNORA54	584.1585	0.104825	0.370267	0.538579	snoRNA
ENSG0000(	SLC35F4	42.20565	0.120862	0.370468	0.538847	protein_coding
ENSG0000(	ENSG0000(	83.31814	0.087078	0.3705	0.538853	TEC
ENSG0000(	PTCD1	630.4093	0.045257	0.370506	0.538853	protein_coding
ENSG0000(	ENSG0000(	74.84459	0.119259	0.370567	0.538891	lncRNA
ENSG0000(	SH2D4A	430.168	-0.12304	0.370565	0.538891	protein_coding
ENSG0000(	PLEKHG5	587.8625	-0.09182	0.3706	0.538907	protein_coding

ENSG0000(	SHC1	2050.881	0.059092	0.370612	0.538907	protein_coding
ENSG0000(	ZCCHC18	94.62674	0.15949	0.370634	0.538914	protein_coding
ENSG0000(	NDUFA2	295.6216	-0.05476	0.370775	0.539094	protein_coding
ENSG0000(	RHPN2	789.1408	-0.10426	0.370797	0.539102	protein_coding
ENSG0000(	FNBP1P1	303.2645	0.066067	0.370853	0.539157	processed_pseudogene
ENSG0000(	PODXL2	73.78775	0.108131	0.370878	0.539169	protein_coding
ENSG0000(	WAC-AS1	1020.721	-0.04256	0.370926	0.539215	lncRNA
ENSG0000(	ENSG0000(	50.99028	0.144328	0.370961	0.539223	lncRNA
ENSG0000(	ENSG0000(	22.18235	0.141344	0.370966	0.539223	lncRNA
ENSG0000(	ENSG0000(	103.4139	0.110097	0.37099	0.539228	lncRNA
ENSG0000(	ENSG0000(	331.2313	-0.23124	0.371011	0.539228	lncRNA
ENSG0000(	RGS8	11.70013	0.390649	0.371038	0.539228	protein_coding
ENSG0000(	TMEM269	178.2542	-0.06234	0.371038	0.539228	protein_coding
ENSG0000(	ENSG0000(	9.726217	0.219475	0.371065	0.539242	lncRNA
ENSG0000(	ENSG0000(	69.35213	-0.08382	0.371217	0.539393	lncRNA
ENSG0000(	FRG1GP	35.22227	-0.21011	0.371191	0.539393	unprocessed_pseudogene
ENSG0000(	HNRNPA1P	20.29705	-0.14212	0.371237	0.539393	processed_pseudogene
ENSG0000(	LPIN2	3067.351	0.102627	0.371235	0.539393	protein_coding
ENSG0000(	FRMPD3	145.0542	0.069079	0.371267	0.539411	protein_coding
ENSG0000(	ENSG0000(	808.1255	-0.11263	0.371294	0.539426	lncRNA
ENSG0000(	ADO	423.8479	-0.05026	0.371375	0.539519	protein_coding
ENSG0000(	ENSG0000(	240.7843	-0.17373	0.37142	0.539549	lncRNA
ENSG0000(	ZNF594	873.2102	0.073809	0.37143	0.539549	protein_coding
ENSG0000(	ENSG0000(	40.53519	-0.13249	0.371461	0.539569	lncRNA
ENSG0000(	FAM174A	61.6165	0.110435	0.371503	0.539605	protein_coding
ENSG0000(	CFAP65	52.26671	0.263538	0.371569	0.539673	protein_coding
ENSG0000(	TRMT44	630.7269	0.049191	0.371584	0.539673	protein_coding
ENSG0000(	CDH9	29.5663	0.105187	0.37166	0.539758	protein_coding
ENSG0000(	SMIM13	308.4475	-0.04638	0.371698	0.539764	protein_coding
ENSG0000(	SUV39H1	99.47129	0.086868	0.371695	0.539764	protein_coding
ENSG0000(	ENSG0000(	17.56928	0.137486	0.371716	0.539765	lncRNA
ENSG0000(	MCTS2P	22.47462	-0.46738	0.371734	0.539766	protein_coding
ENSG0000(	ENSG0000(	27.60265	-0.53892	0.371939	0.540039	protein_coding
ENSG0000(	PPP3CB-AS	1013.415	-0.03513	0.371956	0.54004	lncRNA
ENSG0000(	C21orf91	713.4096	0.077685	0.371991	0.540064	protein_coding
ENSG0000(	CCNE2	108.3476	0.099516	0.372132	0.540195	protein_coding
ENSG0000(	CSMD1	162.7075	0.169194	0.372109	0.540195	protein_coding
ENSG0000(	SDCBP2-AS	432.3325	0.091343	0.372124	0.540195	lncRNA
ENSG0000(	NXF3	89.75782	0.183242	0.372518	0.54073	protein_coding
ENSG0000(	EPHB2	1326.755	0.121114	0.37257	0.540773	protein_coding
ENSG0000(	TPSAB1	200.9047	-0.17925	0.372582	0.540773	protein_coding
ENSG0000(	DYNC1LI2-I	213.1645	0.04094	0.372648	0.540844	lncRNA
ENSG0000(	SRXN1	242.1191	-0.11002	0.372724	0.54093	protein_coding
ENSG0000(	TMEM249	17.82045	-0.16047	0.372765	0.540965	protein_coding
ENSG0000(	ENSG0000(	384.3758	0.036375	0.372851	0.54104	lncRNA
ENSG0000(	LRRC59	1127.403	0.078203	0.372835	0.54104	protein_coding
ENSG0000(	PRKCI	1021.265	-0.05563	0.372898	0.541082	protein_coding

ENSG0000(	FAM13B-A!	11.40725	0.193719	0.372945	0.541126	lncRNA
ENSG0000(	CAMK4	1566.03	-0.15657	0.372985	0.541159	protein_coding
ENSG0000(	FAM131B	34.58453	0.154906	0.37302	0.541185	protein_coding
ENSG0000(	ENSG0000(	216.3611	-0.06106	0.373038	0.541186	TEC
ENSG0000(	ENSG0000(	131.2955	-0.0673	0.373081	0.541223	lncRNA
ENSG0000(	ENSG0000(	38.19499	-0.12062	0.373275	0.541481	lncRNA
ENSG0000(	ENSG0000(	17.26548	-0.16881	0.373351	0.541541	lncRNA
ENSG0000(	VRK1	17517.74	0.04955	0.373337	0.541541	protein_coding
ENSG0000(	CYB561	1006.65	0.085424	0.373379	0.541556	protein_coding
ENSG0000(	ENSG0000(	10.42669	0.192117	0.37345	0.541598	lncRNA
ENSG0000(	GATD3A	390.0253	-0.09428	0.373425	0.541598	protein_coding
ENSG0000(	WDR3	1002.084	-0.05362	0.373459	0.541598	protein_coding
ENSG0000(	LINC01840	229.9144	-0.04778	0.373546	0.541674	lncRNA
ENSG0000(	MT1M	93.55112	-0.2955	0.373531	0.541674	protein_coding
ENSG0000(	BCRP2	166.8045	0.074869	0.373625	0.541763	transcribed_unprocessed_pseudo
ENSG0000(	ENSG0000(	123.4269	-0.15737	0.373705	0.541854	processed_pseudogene
ENSG0000(	ANKRD20A	435.3159	0.112032	0.373842	0.541979	protein_coding
ENSG0000(	BLOC1S4	137.1763	-0.05153	0.373843	0.541979	protein_coding
ENSG0000(	TTC9C	528.5142	-0.04732	0.373815	0.541979	protein_coding
ENSG0000(	GPRACR	164.2484	0.059199	0.37392	0.542066	lncRNA
ENSG0000(	SETD1A	686.8964	-0.06517	0.373993	0.542147	protein_coding
ENSG0000(	ITPKC	591.8189	-0.09535	0.374021	0.542163	protein_coding
ENSG0000(	RPGRIP1	132.768	0.07848	0.374088	0.542235	protein_coding
ENSG0000(	ENSG0000(	57.22857	0.13053	0.374113	0.542246	protein_coding
ENSG0000(	ENSG0000(	23.48752	-0.21076	0.374217	0.542372	processed_pseudogene
ENSG0000(	ENSG0000(	246.9078	-0.03668	0.374357	0.54255	lncRNA
ENSG0000(	ANAPC7	1257.707	0.050594	0.374383	0.54255	protein_coding
ENSG0000(	EFCAB6-AS	1596.107	-0.03749	0.374392	0.54255	lncRNA
ENSG0000(	PENK-AS1	2139.002	-0.05467	0.374449	0.542609	lncRNA
ENSG0000(	ENSG0000(	16.44599	0.143452	0.374552	0.542623	lncRNA
ENSG0000(	ENSG0000(	62.15362	-0.09005	0.374483	0.542623	lncRNA
ENSG0000(	GPC1	188.8089	-0.1022	0.374614	0.542623	protein_coding
ENSG0000(	LINC02421	32.91386	0.195319	0.37453	0.542623	lncRNA
ENSG0000(	MED15	3423.847	-0.03421	0.37455	0.542623	protein_coding
ENSG0000(	NAT8L	24.44913	-0.18936	0.374518	0.542623	protein_coding
ENSG0000(	SEMA4D	3766.577	0.109284	0.374592	0.542623	protein_coding
ENSG0000(	SLC9A9	707.2007	0.122276	0.374568	0.542623	protein_coding
ENSG0000(	TTN	11210.57	0.1805	0.374602	0.542623	protein_coding
ENSG0000(	ENSG0000(	56.42142	-0.22458	0.374644	0.542642	protein_coding
ENSG0000(	SAMMSON	519.9068	-0.03572	0.374711	0.542714	lncRNA
ENSG0000(	AGAP6	2500.761	0.053752	0.374872	0.542857	protein_coding
ENSG0000(	BHLHE40-A	656.9578	0.031873	0.374866	0.542857	lncRNA
ENSG0000(	ENSG0000(	41.88317	0.407779	0.374879	0.542857	unprocessed_pseudogene
ENSG0000(	TRAJ40	47.00733	-0.18001	0.374866	0.542857	TR_J_gene
ENSG0000(	ENSG0000(	20.64714	-0.14368	0.374974	0.542907	lncRNA
ENSG0000(	ENSG0000(	43.87169	0.076289	0.374983	0.542907	lncRNA
ENSG0000(	RNVU1-28	330.2963	-0.23963	0.374937	0.542907	snRNA

ENSG0000(	RPL12P38	58.44936	0.087546	0.374972	0.542907	transcribed_processed_pseudoge
ENSG0000(	ENSG0000(	183.3721	0.088622	0.375089	0.543037	processed_pseudogene
ENSG0000(	ENSG0000(	129.7212	-0.0485	0.375122	0.543059	lncRNA
ENSG0000(	ENSG0000(	83.99271	-0.06846	0.375174	0.543068	lncRNA
ENSG0000(	KMT5C	542.4253	0.054029	0.375154	0.543068	protein_coding
ENSG0000(	RAP1GDS1	893.6033	0.04304	0.37518	0.543068	protein_coding
ENSG0000(	TRBJ1-6	27.46595	-0.25676	0.375321	0.543223	TR_J_gene
ENSG0000(	UBAP1L	1328.715	-0.04843	0.375316	0.543223	protein_coding
ENSG0000(	RHOT1	1201.239	-0.05467	0.375478	0.543425	protein_coding
ENSG0000(	RCN1P2	16.52452	0.218777	0.375613	0.543594	processed_pseudogene
ENSG0000(	AGO3	6095.094	0.033987	0.375667	0.543649	protein_coding
ENSG0000(	ENO2	453.0149	0.089559	0.375694	0.543662	protein_coding
ENSG0000(	ENSG0000(	341.1071	0.179016	0.375797	0.543786	lncRNA
ENSG0000(	ENSG0000(	123.8053	-0.12128	0.375868	0.543864	TEC
ENSG0000(	TSNAX	1138.581	-0.03791	0.375957	0.543968	protein_coding
ENSG0000(	ENSG0000(	93.28841	0.262755	0.375975	0.543969	protein_coding
ENSG0000(	YRDCP1	14.83369	-0.2528	0.376152	0.5442	processed_pseudogene
ENSG0000(	SOGA1	2419.997	0.055104	0.376258	0.544329	protein_coding
ENSG0000(	HDHD5	486.4096	-0.06145	0.376313	0.544383	protein_coding
ENSG0000(	ENSG0000(	48.05824	0.169897	0.376363	0.54443	lncRNA
ENSG0000(	C19orf73	10.4123	-0.18192	0.376431	0.544472	protein_coding
ENSG0000(	FMNL1-DT	852.3106	0.072678	0.376444	0.544472	lncRNA
ENSG0000(	SUV39H2-D	61.36517	0.07298	0.376461	0.544472	lncRNA
ENSG0000(	ZNF436	637.5262	-0.08316	0.376417	0.544472	protein_coding
ENSG0000(	FKBP14-AS	12.69202	0.206407	0.376596	0.544642	lncRNA
ENSG0000(	TRAM1	1615.526	-0.07205	0.376637	0.544676	protein_coding
ENSG0000(	YLPM1	4987.881	0.048213	0.376654	0.544676	protein_coding
ENSG0000(	CLEC1B	16.67127	0.18055	0.376833	0.544885	protein_coding
ENSG0000(	OR8B8	83.42484	0.063776	0.376827	0.544885	protein_coding
ENSG0000(	ENSG0000(	49.58256	0.085286	0.376939	0.545012	protein_coding
ENSG0000(	LINC02338	229.6124	-0.04812	0.376955	0.545012	lncRNA
ENSG0000(	ENSG0000(	14.62887	0.130546	0.377003	0.545032	lncRNA
ENSG0000(	HBA1	36.55177	0.374813	0.377	0.545032	protein_coding
ENSG0000(	DSCAML1	89.44279	0.202386	0.377196	0.545284	protein_coding
ENSG0000(	ABCA5	8148.026	-0.12959	0.377267	0.545313	protein_coding
ENSG0000(	OR9G1	12.50987	0.148396	0.377245	0.545313	protein_coding
ENSG0000(	P2RX5-TAX	1337.659	-0.0735	0.377261	0.545313	protein_coding
ENSG0000(	KCNRG	86.06961	0.077648	0.377408	0.545492	protein_coding
ENSG0000(	ENSG0000(	66.98002	-0.07985	0.377448	0.545524	processed_pseudogene
ENSG0000(	C10orf143	283.0816	-0.05955	0.377568	0.545659	protein_coding
ENSG0000(	RN7SL336P	42.5639	0.086136	0.377576	0.545659	misc_RNA
ENSG0000(	CD79A	448.0887	-0.28203	0.377599	0.545667	protein_coding
ENSG0000(	NCOR2	4253.253	0.039054	0.377751	0.545862	protein_coding
ENSG0000(	ATP6V0C	478.919	-0.06221	0.377773	0.545868	protein_coding
ENSG0000(	ENSG0000(	17.09892	0.145532	0.377852	0.545918	scaRNA
ENSG0000(	ENSG0000(	242.5155	0.05134	0.377929	0.545918	TEC
ENSG0000(	ENSG0000(	79.16869	-0.07085	0.37783	0.545918	TEC

ENSG0000(	ENSG0000(	274.861	0.046393	0.377905	0.545918	lncRNA
ENSG0000(	GINS1	433.1129	0.058554	0.377878	0.545918	protein_coding
ENSG0000(	MMS22L	2428.025	-0.03793	0.377924	0.545918	protein_coding
ENSG0000(	RPL26L1	57.74749	-0.0882	0.377869	0.545918	protein_coding
ENSG0000(	APOBEC3B	33.31145	0.193844	0.377964	0.545944	protein_coding
ENSG0000(	ENSG0000(	1838.705	0.036779	0.377999	0.545953	lncRNA
ENSG0000(	ENSG0000(	47.14982	0.090111	0.378004	0.545953	lncRNA
ENSG0000(	ENSG0000(	190.0006	-0.05367	0.378233	0.546249	lncRNA
ENSG0000(	LINC01922	147.0028	-0.04819	0.378244	0.546249	lncRNA
ENSG0000(	DHX32	633.6677	0.086869	0.378268	0.546249	protein_coding
ENSG0000(	SMNDC1	652.8157	-0.04125	0.378279	0.546249	protein_coding
ENSG0000(	RNU12	668.0302	0.099118	0.378308	0.546266	snRNA
ENSG0000(	POU5F1B	258.9217	-0.03739	0.378432	0.54642	protein_coding
ENSG0000(	ENSG0000(	14.93708	0.187505	0.378504	0.546498	lncRNA
ENSG0000(	SUN3	30.51229	-0.13852	0.37855	0.54654	protein_coding
ENSG0000(	NPIPB8	42.84617	0.244087	0.378571	0.546545	protein_coding
ENSG0000(	ZNF613	259.7487	-0.05815	0.378616	0.546585	protein_coding
ENSG0000(	FCHSD1	1196.739	-0.05805	0.378648	0.546606	protein_coding
ENSG0000(	ECI2-DT	44.62364	-0.13771	0.378675	0.54662	lncRNA
ENSG0000(	RN7SKP115	29.39927	-0.13085	0.378758	0.546715	misc_RNA
ENSG0000(	CELF2-DT	688.877	0.044832	0.378843	0.546789	lncRNA
ENSG0000(	ENSG0000(	18.29353	0.134386	0.378844	0.546789	lncRNA
ENSG0000(	ENSG0000(	299.9602	0.052618	0.378918	0.546871	processed_pseudogene
ENSG0000(	PITRM1	2391.453	0.025793	0.379005	0.546971	protein_coding
ENSG0000(	MFSD6L	60.80933	0.091925	0.379051	0.547013	protein_coding
ENSG0000(	SERTAD4	97.30266	-0.1426	0.37911	0.547073	protein_coding
ENSG0000(	PDE12	2043.362	-0.02536	0.379148	0.547096	protein_coding
ENSG0000(	RNA5SP217	9.89841	0.187401	0.379164	0.547096	rRNA_pseudogene
ENSG0000(	SPAG5	663.0655	-0.09348	0.379178	0.547096	protein_coding
ENSG0000(	ENSG0000(	178.1701	-0.07152	0.379235	0.547128	lncRNA
ENSG0000(	RNF123	1131.232	-0.05201	0.379232	0.547128	protein_coding
ENSG0000(	TNFRSF10A	422.917	-0.05091	0.379349	0.547268	lncRNA
ENSG0000(	ENSG0000(	157.7931	0.056385	0.379372	0.547275	lncRNA
ENSG0000(	TDRKH	157.3195	0.084619	0.379429	0.547332	protein_coding
ENSG0000(	DDOST	932.8215	-0.0524	0.379471	0.547368	protein_coding
ENSG0000(	ENSG0000(	232.8653	-0.04373	0.379523	0.547419	lncRNA
ENSG0000(	PPM1J	30.06927	0.171545	0.37957	0.547461	protein_coding
ENSG0000(	ENSG0000(	13.40154	0.172027	0.379596	0.547473	lncRNA
ENSG0000(	SFT2D1	718.9343	-0.0457	0.379668	0.547552	protein_coding
ENSG0000(	DPYD-IT1	91.4059	0.075577	0.379692	0.547561	lncRNA
ENSG0000(	ENSG0000(	559.7981	0.043147	0.37973	0.547569	lncRNA
ENSG0000(	SUSD1	471.7229	-0.06781	0.379732	0.547569	protein_coding
ENSG0000(	PLN	122.4498	0.101751	0.379804	0.547623	protein_coding
ENSG0000(	RAB40AL	13.00095	0.146309	0.379797	0.547623	protein_coding
ENSG0000(	HERC3	2003.751	-0.04122	0.379839	0.547648	protein_coding
ENSG0000(	ARSI	31.2115	0.20264	0.379899	0.54771	protein_coding
ENSG0000(	TRPC7	11.64945	0.252422	0.379951	0.54776	protein_coding

ENSG0000(	ENSG0000(	47.07423	0.085266	0.380028	0.54781	lncRNA
ENSG0000(	ENSG0000(	45.71763	0.1053	0.380056	0.54781	lncRNA
ENSG0000(	FIZ1	250.3655	0.060303	0.38004	0.54781	protein_coding
ENSG0000(	PLCL2	1576.849	-0.0497	0.380056	0.54781	protein_coding
ENSG0000(	LINC00365	35.03501	-0.17646	0.380117	0.547873	lncRNA
ENSG0000(	ADNP2	1065.44	0.103465	0.380312	0.548105	protein_coding
ENSG0000(	NLRP8	96.66825	-0.0592	0.380303	0.548105	protein_coding
ENSG0000(	ADCK1	289.2535	-0.05138	0.380404	0.548212	protein_coding
ENSG0000(	CTNNA2	131.3669	0.069255	0.380471	0.548283	protein_coding
ENSG0000(	ENSG0000(	14.59906	0.128978	0.380557	0.548374	lncRNA
ENSG0000(	ENSG0000(	263.6178	0.036406	0.380569	0.548374	lncRNA
ENSG0000(	ATP6AP1	1309.707	0.056562	0.380691	0.548526	protein_coding
ENSG0000(	ENSG0000(	819.2324	0.041129	0.380729	0.548554	lncRNA
ENSG0000(	GOT2	966.8445	-0.03432	0.380844	0.548671	protein_coding
ENSG0000(	RPL39P36	15.22641	0.131662	0.380837	0.548671	processed_pseudogene
ENSG0000(	NCMAP-DT	53.8058	0.098129	0.380943	0.548787	lncRNA
ENSG0000(	SFXN2	1368.12	0.058401	0.38096	0.548787	protein_coding
ENSG0000(	SMIM32	60.68379	-0.21445	0.381068	0.548918	protein_coding
ENSG0000(	SNORD116	23.94071	0.180596	0.381118	0.548965	snoRNA
ENSG0000(	ENSG0000(	9.36415	0.262421	0.381217	0.549082	lncRNA
ENSG0000(	YARS2	416.7471	-0.03989	0.381678	0.549721	protein_coding
ENSG0000(	ENSG0000(	49.32	0.092223	0.381839	0.549927	lncRNA
ENSG0000(	FOXO1	2201.748	0.08352	0.381894	0.549982	protein_coding
ENSG0000(	ENSG0000(	15.15326	0.450742	0.381924	0.55	unprocessed_pseudogene
ENSG0000(	ENSG0000(	150.568	-0.05072	0.381947	0.550008	transcribed_processed_pseudoge
ENSG0000(	TP53BP2	970.6697	0.054295	0.381985	0.550038	protein_coding
ENSG0000(	ZNF623	1698.479	0.025228	0.382116	0.550201	protein_coding
ENSG0000(	EMSY-DT	180.528	-0.05139	0.382152	0.550228	lncRNA
ENSG0000(	LINC01496	387.2239	-0.04642	0.38218	0.550243	lncRNA
ENSG0000(	ENSG0000(	513.6937	0.046609	0.382215	0.550268	lncRNA
ENSG0000(	KLF17	68.9886	-0.06962	0.382244	0.550284	protein_coding
ENSG0000(	RNU6-789F	18.26118	0.158711	0.382294	0.550331	snRNA
ENSG0000(	STS	1270.381	-0.0483	0.382312	0.550332	protein_coding
ENSG0000(	CCNL1	7555.777	-0.06889	0.382395	0.550376	protein_coding
ENSG0000(	MAN2A1	4080.669	-0.04271	0.382392	0.550376	protein_coding
ENSG0000(	TBC1D26	52.70414	0.20286	0.382385	0.550376	protein_coding
ENSG0000(	ENSG0000(	375.1771	0.037513	0.382462	0.550447	lncRNA
ENSG0000(	ENSG0000(	261.1082	0.043067	0.38256	0.550563	lncRNA
ENSG0000(	ENSG0000(	115.2279	0.091966	0.38261	0.550586	lncRNA
ENSG0000(	IMP3	446.7304	-0.04099	0.38261	0.550586	protein_coding
ENSG0000(	OR6C6	48.42132	0.090397	0.382711	0.550681	protein_coding
ENSG0000(	RN7SL617F	23.35753	0.115744	0.382697	0.550681	misc_RNA
ENSG0000(	ENSG0000(	188.2946	-0.06253	0.382841	0.5508	lncRNA
ENSG0000(	RN7SKP23	260.3883	0.03256	0.382847	0.5508	TEC
ENSG0000(	SMIM14	3154.355	0.030387	0.38283	0.5508	protein_coding
ENSG0000(	DPM2	350.9536	0.046471	0.382973	0.550956	protein_coding
ENSG0000(	RNU6-1284	16.26343	0.142596	0.383044	0.551033	snRNA

ENSG0000(	NDST3	97.76512	-0.0845	0.383107	0.551099	protein_coding
ENSG0000(	STMN3	319.4709	0.150582	0.383199	0.551206	protein_coding
ENSG0000(	SLC30A9	1715.288	-0.04075	0.38325	0.551254	protein_coding
ENSG0000(	COL19A1	731.2166	-0.11986	0.383449	0.551516	protein_coding
ENSG0000(	TMX1	990.4884	-0.0433	0.383483	0.551539	protein_coding
ENSG0000(	CFAP70	441.6416	0.057086	0.383532	0.551584	protein_coding
ENSG0000(	ENSG0000(	48.27233	-0.09172	0.383573	0.551618	lncRNA
ENSG0000(	ENSG0000(	110.1754	-0.06687	0.383614	0.551652	lncRNA
ENSG0000(	MTCYBP32	47.67345	0.097895	0.383696	0.551744	processed_pseudogene
ENSG0000(	SEMA7A	412.5867	-0.19901	0.38373	0.551769	protein_coding
ENSG0000(	USP6NL	1560.426	0.063075	0.38389	0.551973	protein_coding
ENSG0000(	TMEM53	403.4431	-0.07276	0.383921	0.551993	protein_coding
ENSG0000(	SLC6A16	293.2606	-0.08966	0.383957	0.552019	protein_coding
ENSG0000(	ENSG0000(	61.39373	-0.06716	0.384154	0.552277	lncRNA
ENSG0000(	MGA	5415.331	0.038639	0.384234	0.552367	protein_coding
ENSG0000(	ENSG0000(	25.42308	-0.11547	0.384343	0.552498	lncRNA
ENSG0000(	FRG2	32.41528	0.131591	0.384385	0.552508	protein_coding
ENSG0000(	HCP5B	109.5554	-0.14271	0.384375	0.552508	lncRNA
ENSG0000(	SPIC	14.70141	-0.31399	0.384417	0.552529	protein_coding
ENSG0000(	POP1	198.4328	0.119251	0.384535	0.552673	protein_coding
ENSG0000(	COQ8B	2174.722	0.024753	0.3847	0.55276	protein_coding
ENSG0000(	CTTNBP2NI	2462.859	0.081584	0.384696	0.55276	protein_coding
ENSG0000(	CYP2C9	1582.098	-0.15412	0.384658	0.55276	protein_coding
ENSG0000(	ENSG0000(	114.3411	-0.06443	0.384677	0.55276	lncRNA
ENSG0000(	SEMA6A-A!	135.9769	0.107795	0.384665	0.55276	lncRNA
ENSG0000(	TAF6L	196.8811	-0.05985	0.384648	0.55276	protein_coding
ENSG0000(	TUBA3FP	328.7848	0.048631	0.384825	0.552913	lncRNA
ENSG0000(	CD53	2170.97	0.180675	0.384875	0.552931	protein_coding
ENSG0000(	ENSG0000(	39.37748	0.08902	0.384878	0.552931	processed_pseudogene
ENSG0000(	ENSG0000(	88.4589	0.147896	0.384942	0.552931	protein_coding
ENSG0000(	ENSG0000(	392.2093	0.128054	0.38492	0.552931	protein_coding
ENSG0000(	POLG2	813.7866	0.07059	0.384896	0.552931	protein_coding
ENSG0000(	RNU6-255F	8.779477	-0.27432	0.384932	0.552931	snRNA
ENSG0000(	ENSG0000(	24.25937	-0.11386	0.3851	0.553132	processed_pseudogene
ENSG0000(	PLXNA3	2484.481	-0.09771	0.385164	0.553199	protein_coding
ENSG0000(	ENSG0000(	36.51837	0.13821	0.385232	0.553246	lncRNA
ENSG0000(	ENSG0000(	23.6651	0.174309	0.385218	0.553246	lncRNA
ENSG0000(	TSR1	938.1408	-0.05258	0.385451	0.553536	protein_coding
ENSG0000(	XIAPP3	54.84923	0.095795	0.385489	0.553566	processed_pseudogene
ENSG0000(	TROAP	271.3159	0.082674	0.38553	0.553598	protein_coding
ENSG0000(	SNORA41	15.27362	0.166659	0.385599	0.553673	snoRNA
ENSG0000(	ANP32A	2080.364	0.034238	0.385647	0.553703	protein_coding
ENSG0000(	ENSG0000(	13.94598	0.184117	0.385656	0.553703	lncRNA
ENSG0000(	RASSF9	265.3195	-0.07427	0.385695	0.553709	protein_coding
ENSG0000(	SLC39A9	2104.196	-0.04441	0.385682	0.553709	protein_coding
ENSG0000(	ENSG0000(	491.3127	-0.06469	0.385752	0.553753	lncRNA
ENSG0000(	ENSG0000(	38.99898	0.081317	0.385796	0.553753	lncRNA

ENSG0000(	RNU6-479F	10.63546	-0.18268	0.385783	0.553753	snRNA
ENSG0000(	TBC1D10B	1412.213	-0.02529	0.385794	0.553753	protein_coding
ENSG0000(	SCUBE1-AS	203.2521	-0.04808	0.386035	0.554071	lncRNA
ENSG0000(	RN7SL153F	25.14997	0.127602	0.3861	0.55414	misc_RNA
ENSG0000(	SMG1P6	356.8139	-0.10696	0.386267	0.554353	unprocessed_pseudogene
ENSG0000(	KLF9	1086.011	0.098787	0.386383	0.554467	protein_coding
ENSG0000(	RNU6-1111	18.00458	0.140228	0.386399	0.554467	snRNA
ENSG0000(	RNU6-942F	73.9076	0.097718	0.386368	0.554467	snRNA
ENSG0000(	ARHGAP30	1881.375	0.144644	0.386453	0.55452	protein_coding
ENSG0000(	ENSG0000(	11.12615	0.202722	0.386534	0.554599	lncRNA
ENSG0000(	LINCR-0001	291.4986	-0.10544	0.386543	0.554599	lncRNA
ENSG0000(	TBC1D3B	290.7194	-0.09219	0.386614	0.554674	protein_coding
ENSG0000(	ENSG0000(	27.2601	0.131197	0.386714	0.554793	lncRNA
ENSG0000(	ENSG0000(	8.596337	-0.21582	0.386768	0.554794	lncRNA
ENSG0000(	TRABD	692.1137	0.064927	0.386742	0.554794	protein_coding
ENSG0000(	TRAPPC2B	192.6215	-0.06001	0.386759	0.554794	protein_coding
ENSG0000(	CNN3	995.7683	-0.07023	0.386914	0.554856	protein_coding
ENSG0000(	ENSG0000(	8334.223	-0.05796	0.386916	0.554856	lncRNA
ENSG0000(	ENSG0000(	26.03731	-0.09775	0.386862	0.554856	lncRNA
ENSG0000(	ENSG0000(	329.4389	0.043616	0.386904	0.554856	lncRNA
ENSG0000(	ESF1	387.263	-0.05867	0.386831	0.554856	protein_coding
ENSG0000(	RN7SL814F	20.41331	0.130455	0.386897	0.554856	misc_RNA
ENSG0000(	ENSG0000(	11.63413	-0.13922	0.38704	0.555009	lncRNA
ENSG0000(	KCTD5P1	35.33869	-0.10662	0.387096	0.555063	processed_pseudogene
ENSG0000(	ENSG0000(	933.8058	0.07744	0.387153	0.555069	lncRNA
ENSG0000(	PCDHGA8	541.2758	-0.04308	0.387148	0.555069	protein_coding
ENSG0000(	PPP4C	511.928	-0.05349	0.387127	0.555069	protein_coding
ENSG0000(	ITSN2	3679.733	-0.04348	0.387217	0.555136	protein_coding
ENSG0000(	ENSG0000(	11.15694	0.159765	0.387264	0.555152	lncRNA
ENSG0000(	SCIRT	119.8878	0.067365	0.38725	0.555152	lncRNA
ENSG0000(	KLHDC1	229.8912	-0.05298	0.387358	0.555262	protein_coding
ENSG0000(	FAM72C	31.87739	0.483763	0.387572	0.555543	protein_coding
ENSG0000(	ENSG0000(	29.54512	-0.14651	0.387606	0.555566	lncRNA
ENSG0000(	ENSG0000(	67.79986	-0.07579	0.387625	0.555569	lncRNA
ENSG0000(	RN7SL504F	20.21628	0.130372	0.387677	0.555617	misc_RNA
ENSG0000(	NKAPD1	1104.727	0.033475	0.387881	0.555885	protein_coding
ENSG0000(	NUP37	293.8085	0.041405	0.387973	0.555992	protein_coding
ENSG0000(	DHCR24	988.1691	-0.10879	0.388057	0.556086	protein_coding
ENSG0000(	ADI1	2932.611	-0.07306	0.38816	0.556209	protein_coding
ENSG0000(	ARHGEF9-I	25.92644	0.125368	0.388201	0.556211	lncRNA
ENSG0000(	BRWD3	3036.256	-0.04576	0.388214	0.556211	protein_coding
ENSG0000(	CCDC171	685.8383	-0.07265	0.388189	0.556211	protein_coding
ENSG0000(	ENSG0000(	10.10759	0.191894	0.388258	0.556248	lncRNA
ENSG0000(	ENSG0000(	10.11619	0.188415	0.388286	0.556263	lncRNA
ENSG0000(	ENSG0000(	63.30306	-0.07899	0.388355	0.556337	lncRNA
ENSG0000(	C10orf67	87.47663	0.064935	0.388394	0.556362	protein_coding
ENSG0000(	RNPS1	2344.078	-0.02178	0.388408	0.556362	protein_coding

ENSG0000(	ENSG0000(	284.1843	-0.08343	0.388492	0.556432	lncRNA
ENSG0000(	EQTN	22.92476	-0.16188	0.388477	0.556432	protein_coding
ENSG0000(	ENSG0000(	26.65573	-0.10309	0.388537	0.55647	unprocessed_pseudogene
ENSG0000(	ENSG0000(	70.13186	-0.09518	0.388572	0.556496	lncRNA
ENSG0000(	ENSG0000(	17.30799	0.130682	0.388596	0.556505	lncRNA
ENSG0000(	PKD1P4	945.9913	-0.09101	0.388618	0.556511	transcribed_unprocessed_pseudo
ENSG0000(	AGAP3	1216.646	0.046291	0.388643	0.556522	protein_coding
ENSG0000(	ENSG0000(	552.4627	0.071212	0.388728	0.556618	transcribed_unprocessed_pseudo
ENSG0000(	ENSG0000(	81.13292	-0.06177	0.388792	0.556621	TEC
ENSG0000(	FAM242A	171.04	0.119656	0.388787	0.556621	lncRNA
ENSG0000(	PDIK1L	410.5061	-0.04835	0.388801	0.556621	protein_coding
ENSG0000(	TPT1P5	14.16414	0.167517	0.388795	0.556621	processed_pseudogene
ENSG0000(	ZNF805	1512.373	-0.03617	0.38882	0.556623	protein_coding
ENSG0000(	ENSG0000(	20.81795	0.14041	0.388908	0.556725	lncRNA
ENSG0000(	MAGI1	1947.567	-0.1088	0.388941	0.556745	protein_coding
ENSG0000(	ENSG0000(	21.37125	-0.14227	0.388995	0.556788	processed_pseudogene
ENSG0000(	TSGA13	155.776	0.053931	0.389006	0.556788	protein_coding
ENSG0000(	ENSG0000(	46.66487	-0.07566	0.389173	0.556908	processed_pseudogene
ENSG0000(	ENSG0000(	13.50223	-0.48551	0.389178	0.556908	lncRNA
ENSG0000(	SETP9	14.81496	0.232146	0.389123	0.556908	transcribed_processed_pseudoge
ENSG0000(	TCOF1	1359.755	-0.05035	0.389128	0.556908	protein_coding
ENSG0000(	TSPAN6	854.4215	0.055714	0.389159	0.556908	protein_coding
ENSG0000(	ENSG0000(	12.01226	-0.15662	0.389234	0.556962	lncRNA
ENSG0000(	ENSG0000(	274.7567	-0.12093	0.389391	0.557137	lncRNA
ENSG0000(	IGFLR1	318.2436	0.079122	0.389382	0.557137	protein_coding
ENSG0000(	WBP1L	1773.78	-0.03493	0.389479	0.557237	protein_coding
ENSG0000(	PLCD1	248.4057	-0.0944	0.389579	0.557323	protein_coding
ENSG0000(	TMEM109	415.7323	-0.10228	0.389592	0.557323	protein_coding
ENSG0000(	ZER1	1279.944	-0.09593	0.389561	0.557323	protein_coding
ENSG0000(	RN7SL835F	98.69642	0.06188	0.389709	0.557441	misc_RNA
ENSG0000(	ZC3H11B	18.6565	0.152889	0.389694	0.557441	protein_coding
ENSG0000(	ENSG0000(	74.5484	0.071544	0.389865	0.557638	lncRNA
ENSG0000(	RNU6-414F	17.69143	-0.1529	0.389947	0.55773	snRNA
ENSG0000(	ZNF503-AS	129.2717	0.137712	0.389972	0.55774	lncRNA
ENSG0000(	AMER1	627.6647	0.036824	0.390001	0.557741	protein_coding
ENSG0000(	ENSG0000(	14.90602	0.190189	0.390022	0.557741	lncRNA
ENSG0000(	PHF23	530.3058	-0.06113	0.390026	0.557741	protein_coding
ENSG0000(	RNU6-94P	9.418715	0.220508	0.390093	0.557812	snRNA
ENSG0000(	C11orf58	3095.455	0.028667	0.390186	0.557919	protein_coding
ENSG0000(	ENSG0000(	488.5511	-0.04242	0.390228	0.557954	lncRNA
ENSG0000(	EHMT2-AS:	510.5226	-0.17551	0.390264	0.557955	lncRNA
ENSG0000(	PRKAA2	167.9159	0.090376	0.390247	0.557955	protein_coding
ENSG0000(	IDUA	386.7453	-0.06575	0.390373	0.558086	protein_coding
ENSG0000(	ENSG0000(	213.5875	0.049681	0.390583	0.558361	lncRNA
ENSG0000(	ENSG0000(	301.0661	0.034238	0.39077	0.558603	lncRNA
ENSG0000(	CCDC34	97.68612	0.069543	0.390859	0.558704	protein_coding
ENSG0000(	LINC01545	25.80104	-0.09002	0.390912	0.558713	lncRNA

ENSG0000(	RAD54B	108.1902	0.123904	0.390896	0.558713	protein_coding
ENSG0000(	TGM5	16.35968	0.159807	0.390919	0.558713	protein_coding
ENSG0000(	LINC01911	19.66772	0.13835	0.39102	0.558833	lncRNA
ENSG0000(	MBOAT1	1579.809	-0.07549	0.391116	0.558945	protein_coding
ENSG0000(	ELAVL1	1664.679	-0.027	0.391156	0.558956	protein_coding
ENSG0000(	SLC25A52	27.91645	-0.12669	0.391159	0.558956	protein_coding
ENSG0000(	MAPKAPK5	4140.464	-0.04509	0.391212	0.559006	protein_coding
ENSG0000(	ENSG0000(	113.1185	-0.0719	0.39124	0.559021	lncRNA
ENSG0000(	ENSG0000(	137.9079	0.061872	0.391374	0.559164	lncRNA
ENSG0000(	LINC01340	46.73	0.191684	0.391376	0.559164	lncRNA
ENSG0000(	WNK3	189.7562	0.085469	0.391404	0.55918	protein_coding
ENSG0000(	ENSG0000(	113.2377	0.123965	0.391524	0.559236	lncRNA
ENSG0000(	FOXI3	61.2339	-0.07859	0.391539	0.559236	protein_coding
ENSG0000(	KIAA0232	2434.902	0.075074	0.391555	0.559236	protein_coding
ENSG0000(	KIAA0753	778.0086	0.04992	0.391499	0.559236	protein_coding
ENSG0000(	NEURL1	842.9062	-0.10432	0.391568	0.559236	protein_coding
ENSG0000(	RNPC3	2450.083	-0.03921	0.391465	0.559236	protein_coding
ENSG0000(	ZXDC	2158.942	-0.04013	0.391565	0.559236	protein_coding
ENSG0000(	TBC1D26-A	18.74214	-0.11072	0.391662	0.559345	lncRNA
ENSG0000(	ATXN7L2	185.9251	0.079359	0.391774	0.559465	protein_coding
ENSG0000(	RNU6-936F	15.92497	0.133924	0.391782	0.559465	snRNA
ENSG0000(	ENSG0000(	8.957231	-0.22448	0.391824	0.5595	lncRNA
ENSG0000(	ENSG0000(	29.86179	0.117744	0.391869	0.559539	lncRNA
ENSG0000(	DAP	1561.774	-0.06548	0.391917	0.559581	protein_coding
ENSG0000(	SNORA75	39.36127	0.141897	0.391954	0.559609	snoRNA
ENSG0000(	BRD7	1588.553	-0.02648	0.391973	0.559611	protein_coding
ENSG0000(	ENSG0000(	175.6523	-0.06511	0.392033	0.559672	lncRNA
ENSG0000(	ENSG0000(	54.38845	0.128492	0.392074	0.559701	lncRNA
ENSG0000(	ENSG0000(	38.85445	-0.11363	0.392089	0.559701	lncRNA
ENSG0000(	SLC25A47P	18.72269	-0.22926	0.392124	0.559725	processed_pseudogene
ENSG0000(	IQCE	1246.494	-0.0693	0.39215	0.559737	protein_coding
ENSG0000(	MCF2L-AS1	9.774325	-0.18611	0.392204	0.559742	lncRNA
ENSG0000(	NPY1R	47.57387	-0.19105	0.392201	0.559742	protein_coding
ENSG0000(	POLR3GL	194.5655	0.055414	0.392206	0.559742	protein_coding
ENSG0000(	QRICH1	1923.974	-0.03197	0.392243	0.559768	protein_coding
ENSG0000(	CHML	1745.897	-0.04475	0.392274	0.559787	protein_coding
ENSG0000(	LINC00927	85.13165	0.057011	0.392323	0.559831	lncRNA
ENSG0000(	ENSG0000(	26.20371	0.101282	0.392474	0.559997	lncRNA
ENSG0000(	SNORA70E	17.95672	0.21836	0.392472	0.559997	snoRNA
ENSG0000(	RPL35AP36	17.91844	0.119421	0.392529	0.56005	processed_pseudogene
ENSG0000(	ENSG0000(	241.9533	0.048329	0.392586	0.560105	lncRNA
ENSG0000(	MVK	323.3835	-0.06224	0.392669	0.560199	protein_coding
ENSG0000(	LINC01782	45.29069	-0.07655	0.392703	0.560222	lncRNA
ENSG0000(	GIMAP5	1739.65	0.124827	0.392794	0.560327	protein_coding
ENSG0000(	ENSG0000(	244.3717	0.054456	0.392818	0.560335	lncRNA
ENSG0000(	ASB1	1043.037	0.061823	0.392863	0.560349	protein_coding
ENSG0000(	ENSG0000(	12.55914	0.210074	0.39286	0.560349	TEC

ENSG0000(	CCDC197	12.63235	0.164698	0.392927	0.560397	protein_coding
ENSG0000(	HIC2	576.8525	-0.04313	0.392972	0.560397	protein_coding
ENSG0000(	MED28	5400.817	-0.0386	0.392983	0.560397	protein_coding
ENSG0000(	RNY4P7	28.42029	0.129346	0.392959	0.560397	misc_RNA
ENSG0000(	TNFRSF6B	64.48808	2.185871	0.392986	0.560397	protein_coding
ENSG0000(	ENSG0000(	36.92166	0.094012	0.393181	0.56065	lncRNA
ENSG0000(	BCL10	955.1279	0.073662	0.393233	0.56069	protein_coding
ENSG0000(	ENSG0000(	43.05079	0.105295	0.393254	0.56069	lncRNA
ENSG0000(	ZNF362	469.2347	0.10465	0.393262	0.56069	protein_coding
ENSG0000(	GRIK1	8.982109	0.204416	0.393328	0.56072	protein_coding
ENSG0000(	MIR7150	10.36756	0.224066	0.393309	0.56072	miRNA
ENSG0000(	OLFM2	44.8007	0.148859	0.393337	0.56072	protein_coding
ENSG0000(	SEC62-AS1	146.57	-0.07312	0.393424	0.560819	lncRNA
ENSG0000(	SNX18P3	629.9174	0.098853	0.393481	0.560875	transcribed_unprocessed_pseudo
ENSG0000(	SEPTIN14P:	20.83679	-0.1805	0.393563	0.560966	transcribed_unprocessed_pseudo
ENSG0000(	ENSG0000(	28.81641	0.109309	0.393638	0.561027	lncRNA
ENSG0000(	FGF17	12.87976	0.191611	0.393642	0.561027	protein_coding
ENSG0000(	MTDH	2821.555	-0.05066	0.393667	0.561038	protein_coding
ENSG0000(	DISC1-IT1	427.6978	-0.05362	0.393767	0.56113	lncRNA
ENSG0000(	STX3	2099.372	-0.10067	0.393762	0.56113	protein_coding
ENSG0000(	ENSG0000(	58.32685	0.080206	0.393822	0.561183	lncRNA
ENSG0000(	DIAPH3	384.3649	0.058121	0.393856	0.561206	protein_coding
ENSG0000(	EPHA1-AS1	644.1809	0.092709	0.39391	0.561257	lncRNA
ENSG0000(	SOCS3-DT	68.63281	0.09005	0.393964	0.561309	lncRNA
ENSG0000(	ENSG0000(	42.51831	0.225381	0.394067	0.561423	protein_coding
ENSG0000(	SNORD14E	19.42806	-0.12804	0.394079	0.561423	snoRNA
ENSG0000(	ARGFXP1	25.30103	-0.10055	0.394267	0.561665	processed_pseudogene
ENSG0000(	ENSG0000(	14.64903	-0.14937	0.394315	0.561685	lncRNA
ENSG0000(	RPS3AP34	74.27056	0.165574	0.394317	0.561685	processed_pseudogene
ENSG0000(	ACTL10	21.70171	0.134878	0.39434	0.561693	protein_coding
ENSG0000(	ENSG0000(	8.410489	-0.16882	0.394372	0.561712	snoRNA
ENSG0000(	TNFRSF12A	184.0792	-0.16262	0.394393	0.561717	protein_coding
ENSG0000(	ENSG0000(	28.35812	-0.22735	0.394537	0.561871	transcribed_unprocessed_pseudo
ENSG0000(	FAM86FP	10.65993	0.181831	0.394525	0.561871	transcribed_unprocessed_pseudo
ENSG0000(	NAPA	921.7555	-0.05427	0.394644	0.561998	protein_coding
ENSG0000(	RN7SL602F	584.0173	0.054206	0.394733	0.562099	misc_RNA
ENSG0000(	PRDM14	93.5471	0.069262	0.394754	0.562104	protein_coding
ENSG0000(	LINC01362	18.99228	0.174597	0.39479	0.56213	lncRNA
ENSG0000(	FAM153A	178.5601	0.265803	0.394836	0.562149	protein_coding
ENSG0000(	LYAR	92.859	0.104955	0.394839	0.562149	protein_coding
ENSG0000(	ENSG0000(	28.41433	-0.12654	0.394913	0.562229	lncRNA
ENSG0000(	ASIC1	89.00925	-0.13445	0.39495	0.562231	protein_coding
ENSG0000(	NEPRO	1049.303	0.041477	0.394948	0.562231	protein_coding
ENSG0000(	ENSG0000(	38.96913	0.089945	0.394988	0.562259	unprocessed_pseudogene
ENSG0000(	TAF7L	18.62421	-0.14009	0.39511	0.562409	protein_coding
ENSG0000(	TNNI1	123.7237	0.072277	0.39516	0.562453	protein_coding
ENSG0000(	PYCR2	916.8437	-0.03918	0.395331	0.562672	protein_coding

ENSG0000(	ENSG0000(	15.98937	0.165901	0.395411	0.562749	lncRNA
ENSG0000(	NLGN4Y	272.9979	-0.10095	0.395457	0.562749	protein_coding
ENSG0000(	PGAM1P11	52.73456	-0.08447	0.395446	0.562749	processed_pseudogene
ENSG0000(	SPRING1	1234.927	-0.05326	0.395453	0.562749	protein_coding
ENSG0000(	PSAP	6397.854	0.082793	0.395603	0.562932	protein_coding
ENSG0000(	LINC01952	39.16237	0.07986	0.395624	0.562935	lncRNA
ENSG0000(	ENSG0000(	855.858	0.031854	0.395703	0.563024	lncRNA
ENSG0000(	ENSG0000(	103.0705	-0.05934	0.395783	0.563073	lncRNA
ENSG0000(	MOSPD3	57.07031	-0.07683	0.395782	0.563073	protein_coding
ENSG0000(	NRIP3	357.0745	-0.04721	0.395792	0.563073	protein_coding
ENSG0000(	BDP1	3646.304	0.046283	0.395822	0.56309	protein_coding
ENSG0000(	SNORA59A	13.38152	0.209259	0.395843	0.563094	snoRNA
ENSG0000(	ANKUB1	160.5192	0.053807	0.395937	0.563204	protein_coding
ENSG0000(	GPATCH4	290.5668	-0.06311	0.395957	0.563207	protein_coding
ENSG0000(	GRAPLDR	372.4523	0.237369	0.396063	0.563331	lncRNA
ENSG0000(	RPF1	249.3402	-0.06195	0.39614	0.563416	protein_coding
ENSG0000(	MSH5-SAP(	192.7953	-0.10864	0.39617	0.563433	protein_coding
ENSG0000(	C20orf204	147.2016	-0.09102	0.396237	0.563486	protein_coding
ENSG0000(	TRAPPC9	2354.456	-0.03592	0.396243	0.563486	protein_coding
ENSG0000(	VPS37B	1547.753	0.047238	0.396301	0.563543	protein_coding
ENSG0000(	TNFSF9	28.38995	-0.10649	0.396429	0.5637	protein_coding
ENSG0000(	ZNF551	824.3558	0.060689	0.396448	0.563701	protein_coding
ENSG0000(	ENSG0000(	354.1283	0.099364	0.396469	0.563705	transcribed_unprocessed_pseudo
ENSG0000(	PSMD4	701.7249	-0.03553	0.396488	0.563707	protein_coding
ENSG0000(	FAM107A	179.3631	-0.12398	0.396528	0.563717	protein_coding
ENSG0000(	PSMA5	905.8529	-0.05222	0.396531	0.563717	protein_coding
ENSG0000(	ANKRD18A	454.9734	-0.10029	0.396642	0.56385	protein_coding
ENSG0000(	ENSG0000(	29.7323	-0.14911	0.396677	0.563875	lncRNA
ENSG0000(	ENSG0000(	29.70033	0.105688	0.396795	0.563991	transcribed_unitary_pseudogene
ENSG0000(	IQCC	204.1698	0.045351	0.396792	0.563991	protein_coding
ENSG0000(	SIDT1	1764.263	0.046215	0.396838	0.564027	protein_coding
ENSG0000(	ENSG0000(	15.21382	0.173962	0.396949	0.564159	lncRNA
ENSG0000(	RASA4CP	529.3593	-0.08523	0.396996	0.564201	transcribed_unprocessed_pseudo
ENSG0000(	E2F5-DT	151.3641	0.047117	0.397063	0.564245	lncRNA
ENSG0000(	NCOA6	3552.09	-0.04861	0.397057	0.564245	protein_coding
ENSG0000(	USP32P2	578.0125	0.105767	0.397154	0.564349	transcribed_unprocessed_pseudo
ENSG0000(	ENSG0000(	114.1012	0.073407	0.397241	0.564447	lncRNA
ENSG0000(	TMEM47	208.1188	-0.10811	0.397322	0.564536	protein_coding
ENSG0000(	CRY1	376.608	0.0997	0.397401	0.564623	protein_coding
ENSG0000(	ADAMTS10	598.007	0.118092	0.3975	0.564738	protein_coding
ENSG0000(	ENSG0000(	18.23795	-0.13666	0.397647	0.564921	lncRNA
ENSG0000(	AP4B1-AS1	113.3905	0.065878	0.397694	0.564922	lncRNA
ENSG0000(	ENSG0000(	10.697	0.175147	0.397693	0.564922	lncRNA
ENSG0000(	RGPD4-AS1	549.559	-0.0867	0.397701	0.564922	lncRNA
ENSG0000(	POGZ	4697.568	-0.02069	0.39773	0.564938	protein_coding
ENSG0000(	AIF1L	83.09046	0.137331	0.398043	0.565306	protein_coding
ENSG0000(	CAPN9	528.8822	-0.19717	0.398025	0.565306	protein_coding

ENSG0000(	RNU6-1093	38.10727	-0.18974	0.398035	0.565306	snRNA
ENSG0000(	ENSG0000(	110.1453	-0.06857	0.398063	0.565309	lncRNA
ENSG0000(	LINC02474	32.88126	-0.1393	0.398085	0.565315	lncRNA
ENSG0000(	ENSG0000(	9.1939	-0.22914	0.398143	0.565371	lncRNA
ENSG0000(	TAC4	586.5948	0.07681	0.398361	0.565655	protein_coding
ENSG0000(	SPRED1	1862.457	-0.0747	0.398432	0.565731	protein_coding
ENSG0000(	PPT1	794.3232	0.102811	0.398564	0.565893	protein_coding
ENSG0000(	MIR619	36.56758	-0.10121	0.398619	0.56592	miRNA
ENSG0000(	ZNF316	1333.9	0.057442	0.398612	0.56592	protein_coding
ENSG0000(	RRP7A	1959.848	0.072845	0.398699	0.566008	protein_coding
ENSG0000(	ENSG0000(	10.62532	-0.23375	0.398793	0.56609	processed_pseudogene
ENSG0000(	ENSG0000(	420.8684	-0.07466	0.398776	0.56609	lncRNA
ENSG0000(	ENSG0000(	21.52274	-0.26854	0.398815	0.566095	lncRNA
ENSG0000(	RPL41P2	14.53104	0.223791	0.398902	0.566194	processed_pseudogene
ENSG0000(	ENSG0000(	8.508047	-0.16908	0.398962	0.566228	snoRNA
ENSG0000(	ENSG0000(	42.0502	0.175596	0.398958	0.566228	lncRNA
ENSG0000(	RN7SL445F	9.444802	-0.16265	0.399087	0.56638	misc_RNA
ENSG0000(	ENSG0000(	32.77212	-0.08761	0.39911	0.566387	lncRNA
ENSG0000(	ENSG0000(	492.3063	0.03633	0.399132	0.566393	lncRNA
ENSG0000(	ENSG0000(	275.583	0.039499	0.399228	0.566402	lncRNA
ENSG0000(	ENSG0000(	11.6944	0.179284	0.399214	0.566402	lncRNA
ENSG0000(	IGF2BP2	1628.423	-0.14544	0.399196	0.566402	protein_coding
ENSG0000(	KCTD17	131.8388	0.104186	0.399181	0.566402	protein_coding
ENSG0000(	LINC01504	654.5318	-0.12415	0.399172	0.566402	lncRNA
ENSG0000(	DUX4L35	11.94409	0.210097	0.399286	0.56641	unprocessed_pseudogene
ENSG0000(	EIF2B2	512.0649	0.040606	0.399254	0.56641	protein_coding
ENSG0000(	KRT18P15	80.23391	0.100041	0.399288	0.56641	processed_pseudogene
ENSG0000(	GPR135	297.3513	0.069369	0.399362	0.566464	protein_coding
ENSG0000(	RN7SKP296	23.68412	0.158173	0.399361	0.566464	misc_RNA
ENSG0000(	MACROH2/	9804.661	0.035165	0.399437	0.56652	protein_coding
ENSG0000(	SH2D1A	220.5841	0.168721	0.399423	0.56652	protein_coding
ENSG0000(	ENSG0000(	34.59098	0.476795	0.399545	0.566648	protein_coding
ENSG0000(	ENSG0000(	12.00912	0.143905	0.399583	0.56665	processed_pseudogene
ENSG0000(	SNORA78	11.84159	0.207918	0.399575	0.56665	snoRNA
ENSG0000(	ENSG0000(	27.74594	-0.09928	0.3997	0.566791	lncRNA
ENSG0000(	SLC35E2B	6630.323	0.0332	0.39978	0.566879	protein_coding
ENSG0000(	MTHFD1	1837.703	0.035662	0.399813	0.5669	protein_coding
ENSG0000(	CENPN	925.4944	0.029069	0.399858	0.566938	protein_coding
ENSG0000(	ENSG0000(	84.02813	0.220397	0.399926	0.567009	lncRNA
ENSG0000(	ENSG0000(	87.76196	0.051381	0.39997	0.567021	lncRNA
ENSG0000(	PROM1	2314.169	-0.1695	0.399963	0.567021	protein_coding
ENSG0000(	ERVK-28	10.37354	-0.28718	0.399996	0.567032	lncRNA
ENSG0000(	ENSG0000(	443.5936	0.046142	0.400059	0.567095	lncRNA
ENSG0000(	CTSG	28.61196	-0.24358	0.400082	0.567102	protein_coding
ENSG0000(	ENSG0000(	25.7259	-0.43356	0.400278	0.567329	protein_coding
ENSG0000(	ENSG0000(	15.9867	0.134455	0.400296	0.567329	lncRNA
ENSG0000(	TPX2	348.2104	0.117163	0.400293	0.567329	protein_coding

ENSG0000(	FAM138D	47.18204	0.08999	0.400378	0.567395	lncRNA
ENSG0000(	TLCD5	433.8018	0.047698	0.400366	0.567395	protein_coding
ENSG0000(	LINC02765	22.43309	-0.09723	0.40041	0.567408	lncRNA
ENSG0000(	TBL2	820.5326	-0.03385	0.400423	0.567408	protein_coding
ENSG0000(	ENSG0000(	13.16912	0.168092	0.400494	0.567456	lncRNA
ENSG0000(	ENSG0000(	32.86761	0.097157	0.40049	0.567456	lncRNA
ENSG0000(	COL4A6	71.42327	-0.17752	0.400654	0.56762	protein_coding
ENSG0000(	ENSG0000(	156.2348	0.058538	0.400654	0.56762	lncRNA
ENSG0000(	WDR36	1075.871	-0.06884	0.400663	0.56762	protein_coding
ENSG0000(	ENSG0000(	21.10143	0.305624	0.400687	0.567628	lncRNA
ENSG0000(	ENSG0000(	52.98051	0.091137	0.400862	0.56785	lncRNA
ENSG0000(	ENSG0000(	31.76865	-0.09969	0.400927	0.567916	lncRNA
ENSG0000(	COL4A4	756.9893	0.076315	0.40114	0.567988	protein_coding
ENSG0000(	DTX4	1971.758	-0.08696	0.40105	0.567988	protein_coding
ENSG0000(	GNAS	8679.957	-0.04224	0.401074	0.567988	protein_coding
ENSG0000(	MPPED2	141.4898	0.085945	0.401092	0.567988	protein_coding
ENSG0000(	PJVK	233.4742	0.052299	0.4011	0.567988	protein_coding
ENSG0000(	PTGR2	1262.634	-0.05835	0.401102	0.567988	protein_coding
ENSG0000(	QSOX2	789.0398	0.088815	0.401126	0.567988	protein_coding
ENSG0000(	STX1A	117.8433	0.092216	0.401124	0.567988	protein_coding
ENSG0000(	ZNF615	471.066	-0.04352	0.40104	0.567988	protein_coding
ENSG0000(	ANKS6	632.072	-0.06219	0.401287	0.568171	protein_coding
ENSG0000(	MAP4K3	1029.679	-0.06681	0.401342	0.568223	protein_coding
ENSG0000(	RN7SL149F	33.53589	0.094521	0.401374	0.568243	misc_RNA
ENSG0000(	KTI12	114.4936	0.06279	0.401421	0.56826	protein_coding
ENSG0000(	SIAE	1164.764	-0.06988	0.401421	0.56826	protein_coding
ENSG0000(	ZNF687-AS	11.84886	-0.16418	0.401469	0.568301	lncRNA
ENSG0000(	AIMP1	1846.959	0.030128	0.40164	0.568411	protein_coding
ENSG0000(	BET1-AS1	15.13945	-0.12674	0.401703	0.568411	lncRNA
ENSG0000(	C2orf76	69.025	0.066506	0.40162	0.568411	protein_coding
ENSG0000(	DCTN1-AS1	90.86757	0.054851	0.401616	0.568411	lncRNA
ENSG0000(	EML1	677.6692	0.046394	0.401709	0.568411	protein_coding
ENSG0000(	ENSG0000(	77.76883	-0.06706	0.401565	0.568411	lncRNA
ENSG0000(	ENSG0000(	16.61712	0.162702	0.401681	0.568411	lncRNA
ENSG0000(	KIN	1302.661	-0.03843	0.401674	0.568411	protein_coding
ENSG0000(	RPRD1A	1270.865	0.030397	0.401689	0.568411	protein_coding
ENSG0000(	CCDC175	40.36866	-0.21754	0.401798	0.56842	protein_coding
ENSG0000(	CLGN	10.49464	-0.25256	0.401799	0.56842	protein_coding
ENSG0000(	SMAD4	5232.366	-0.02669	0.401805	0.56842	protein_coding
ENSG0000(	STARD4	2094.842	0.086887	0.401736	0.56842	protein_coding
ENSG0000(	XIAP	3888.313	0.059538	0.401775	0.56842	protein_coding
ENSG0000(	RNU4ATAC	123.9151	0.105744	0.401889	0.568488	snRNA
ENSG0000(	SLC25A36	2824.738	0.043574	0.401878	0.568488	protein_coding
ENSG0000(	GINS2	189.6049	-0.08875	0.401945	0.568542	protein_coding
ENSG0000(	AP2B1	2535.194	0.032903	0.402035	0.568642	protein_coding
ENSG0000(	CIAO3	1128.948	-0.03317	0.402065	0.568657	protein_coding
ENSG0000(	ENSG0000(	13.33851	0.177275	0.402081	0.568657	lncRNA

ENSG0000(	SGO2	623.3904	-0.04156	0.402117	0.568682	protein_coding
ENSG0000(	SNRPCP19	12.65489	0.173516	0.402183	0.56875	processed_pseudogene
ENSG0000(	ENSG0000(	52.56624	0.116836	0.402283	0.568866	lncRNA
ENSG0000(	LINC02441	16.64028	-0.26338	0.402577	0.569255	lncRNA
ENSG0000(	ENSG0000(	71.16921	-0.07261	0.402641	0.56932	lncRNA
ENSG0000(	RELL2	139.4238	0.09882	0.402679	0.569349	protein_coding
ENSG0000(	SLC6A7	320.5554	-0.14	0.402709	0.569366	protein_coding
ENSG0000(	AGAP1-IT1	307.4979	0.118737	0.40287	0.569567	lncRNA
ENSG0000(	NOMO2	585.4757	-0.09662	0.402946	0.569649	protein_coding
ENSG0000(	LMLN2	15.22389	0.126572	0.403003	0.569705	protein_coding
ENSG0000(	ENSG0000(	33.85049	-0.10281	0.403027	0.569713	lncRNA
ENSG0000(	EED	768.437	-0.04682	0.403124	0.569825	protein_coding
ENSG0000(	ENSG0000(	21.33437	-0.1547	0.403166	0.569849	lncRNA
ENSG0000(	INE2	78.75378	-0.06594	0.403195	0.569849	lncRNA
ENSG0000(	MIR4280H(	2706.901	0.035953	0.403179	0.569849	lncRNA
ENSG0000(	ENSG0000(	14.66463	-0.21168	0.403409	0.570118	lncRNA
ENSG0000(	MYBBP1A	953.7096	0.068437	0.403428	0.570118	protein_coding
ENSG0000(	SLC25A51P	455.5866	-0.04542	0.403458	0.570118	transcribed_unprocessed_pseudo
ENSG0000(	STON2	1780.482	-0.06787	0.403449	0.570118	protein_coding
ENSG0000(	ENSG0000(	140.977	-0.05971	0.40354	0.570207	lncRNA
ENSG0000(	CENPU	180.8979	0.103913	0.403644	0.57033	protein_coding
ENSG0000(	ENSG0000(	60.67312	-0.07277	0.403682	0.570346	lncRNA
ENSG0000(	FGGY-DT	212.6567	-0.04499	0.403692	0.570346	lncRNA
ENSG0000(	GSDMB	5143.166	0.087792	0.403752	0.57038	protein_coding
ENSG0000(	OR7E104P	145.3168	0.102435	0.40375	0.57038	transcribed_unprocessed_pseudo
ENSG0000(	RPL11P1	41.01506	0.151232	0.403798	0.570419	processed_pseudogene
ENSG0000(	CASTOR1	59.55135	0.120453	0.404	0.57068	protein_coding
ENSG0000(	COMMD5	246.5378	-0.03981	0.404038	0.570708	protein_coding
ENSG0000(	ENSG0000(	252.7072	-0.05026	0.40406	0.570712	lncRNA
ENSG0000(	FGF14-IT1	39.72555	0.19947	0.404171	0.570818	lncRNA
ENSG0000(	MDFIC	1060.35	0.097219	0.404165	0.570818	protein_coding
ENSG0000(	ENSG0000(	370.1062	0.034396	0.40419	0.57082	lncRNA
ENSG0000(	CDC20	105.2161	0.126917	0.404212	0.570826	protein_coding
ENSG0000(	ENSG0000(	287.0052	0.068646	0.404285	0.570903	lncRNA
ENSG0000(	REXO1L1P	294.3526	0.19687	0.404341	0.570956	transcribed_processed_pseudoge
ENSG0000(	ARMH1	146.9538	-0.12283	0.404502	0.571158	protein_coding
ENSG0000(	MRPL34	321.1977	-0.05065	0.404573	0.571232	protein_coding
ENSG0000(	PARP12	2550.724	0.065606	0.404654	0.571321	protein_coding
ENSG0000(	MED11	410.7986	-0.03854	0.404684	0.571331	protein_coding
ENSG0000(	RUNDC3A	223.1828	0.064121	0.404697	0.571331	protein_coding
ENSG0000(	OBSCN-AS1	78.66618	0.118002	0.40474	0.571366	lncRNA
ENSG0000(	ENSG0000(	28.37002	0.125389	0.40486	0.571458	TEC
ENSG0000(	IMPDH1P8	22.292	-0.12558	0.404842	0.571458	processed_pseudogene
ENSG0000(	NDRG4	144.4208	0.09503	0.404854	0.571458	protein_coding
ENSG0000(	ENSG0000(	17.70137	0.140595	0.404914	0.571509	lncRNA
ENSG0000(	ENSG0000(	164.5818	0.05746	0.40501	0.571548	lncRNA
ENSG0000(	GLMN	214.5999	0.089921	0.405014	0.571548	protein_coding

ENSG0000(	HMGB1P1§	13.57954	-0.16114	0.405	0.571548	processed_pseudogene
ENSG0000(	THOC6	138.421	-0.06933	0.405002	0.571548	protein_coding
ENSG0000(	ENSG0000(	38.22157	-0.11962	0.40504	0.571559	lncRNA
ENSG0000(	SUGCT-AS1	187.0971	0.055617	0.405171	0.571717	lncRNA
ENSG0000(	ATF4P3	32.43732	-0.13039	0.405206	0.571729	processed_pseudogene
ENSG0000(	ENSG0000(	46.55315	0.101975	0.405219	0.571729	lncRNA
ENSG0000(	OR4E2	43.10258	-0.0933	0.405234	0.571729	protein_coding
ENSG0000(	HLA-H	776.7617	-0.11541	0.405455	0.572016	unprocessed_pseudogene
ENSG0000(	SDF2	542.8437	0.042888	0.405493	0.572045	protein_coding
ENSG0000(	ENSG0000(	188.5105	0.049644	0.405558	0.57211	lncRNA
ENSG0000(	A1BG	716.0167	0.034159	0.405607	0.572138	protein_coding
ENSG0000(	PATJ-DT	8.501075	0.171259	0.405614	0.572138	lncRNA
ENSG0000(	CA5BP1	219.5219	-0.04943	0.405654	0.572169	transcribed_unprocessed_pseudo
ENSG0000(	ENSG0000(	340.0296	-0.07285	0.405729	0.572249	lncRNA
ENSG0000(	NCBP3	4324.736	-0.03898	0.405749	0.572252	protein_coding
ENSG0000(	BSND	196.4512	-0.0393	0.405849	0.572367	protein_coding
ENSG0000(	RNU6-310F	11.68536	-0.16616	0.405901	0.572415	snRNA
ENSG0000(	ENSG0000(	180.7542	-0.04965	0.406036	0.57258	lncRNA
ENSG0000(	LINC01992	225.2952	-0.0376	0.40621	0.572799	lncRNA
ENSG0000(	COLCA2	156.051	-0.11939	0.406278	0.572869	protein_coding
ENSG0000(	ENSG0000(	14.35681	0.17107	0.40635	0.572919	lncRNA
ENSG0000(	LAMTOR5P	29.66174	-0.13369	0.406342	0.572919	processed_pseudogene
ENSG0000(	ENSG0000(	46.63655	-0.09807	0.406377	0.572932	lncRNA
ENSG0000(	CAPN5	2913.646	-0.14186	0.406396	0.572934	protein_coding
ENSG0000(	TAF1C	1446.296	-0.04513	0.40652	0.573057	protein_coding
ENSG0000(	TSTD3	1486.773	0.030336	0.406511	0.573057	protein_coding
ENSG0000(	TRAPPC13	605.7669	-0.04778	0.406542	0.573062	protein_coding
ENSG0000(	CEACAM21	120.2277	0.147613	0.406601	0.57312	protein_coding
ENSG0000(	ENSG0000(	55.72782	-0.09292	0.406919	0.57353	lncRNA
ENSG0000(	PBX1	5560.269	-0.08254	0.406929	0.57353	protein_coding
ENSG0000(	ENSG0000(	18.9716	0.12981	0.406972	0.573539	transcribed_processed_pseudoge
ENSG0000(	ENSG0000(	57.31472	0.070742	0.406967	0.573539	lncRNA
ENSG0000(	ENSG0000(	94.06906	-0.05305	0.407036	0.573605	lncRNA
ENSG0000(	PIGAP1	92.55517	0.09885	0.407057	0.573609	processed_pseudogene
ENSG0000(	AMMECR1	654.5703	0.044213	0.407178	0.573753	protein_coding
ENSG0000(	ENSG0000(	18.40498	0.100416	0.407209	0.573771	lncRNA
ENSG0000(	DHX37	609.666	0.045228	0.407238	0.573787	protein_coding
ENSG0000(	FAM9C	424.5461	0.053135	0.407278	0.573818	protein_coding
ENSG0000(	BVES	468.9764	0.033301	0.407307	0.573833	protein_coding
ENSG0000(	ENSG0000(	131.2333	0.047777	0.407372	0.573898	lncRNA
ENSG0000(	ENSG0000(	513.2194	-0.05452	0.40745	0.573905	lncRNA
ENSG0000(	ENSG0000(	13.7372	0.151533	0.407417	0.573905	lncRNA
ENSG0000(	GGT6	1175.933	-0.11741	0.407432	0.573905	protein_coding
ENSG0000(	PLA2G2C	132.7378	0.102339	0.40744	0.573905	protein_coding
ENSG0000(	ADD3-AS1	571.115	-0.05555	0.407506	0.573933	lncRNA
ENSG0000(	ENSG0000(	613.2201	0.041799	0.407497	0.573933	lncRNA
ENSG0000(	ASB9	42.52852	-0.0947	0.407536	0.57395	protein_coding

ENSG0000(	ENSG0000(	190.886	0.074126	0.407587	0.573996	lncRNA
ENSG0000(	SAG	1135.892	-0.0217	0.407638	0.574042	protein_coding
ENSG0000(	SLC7A8	680.6016	-0.11194	0.407715	0.574125	protein_coding
ENSG0000(	FAM177B	435.8634	0.114492	0.407794	0.574211	protein_coding
ENSG0000(	MCMBP	1002.265	-0.0385	0.407826	0.57423	protein_coding
ENSG0000(	ENSG0000(	20.36961	0.131939	0.407896	0.574268	lncRNA
ENSG0000(	ENSG0000(	9.209205	-0.17574	0.407896	0.574268	processed_pseudogene
ENSG0000(	ENSG0000(	176.3184	-0.07708	0.407908	0.574268	protein_coding
ENSG0000(	MYRIP	209.7134	0.101182	0.408017	0.574388	protein_coding
ENSG0000(	SHISA6	9.807722	-0.32267	0.40803	0.574388	protein_coding
ENSG0000(	ENSG0000(	67.89876	0.092181	0.408052	0.574393	TEC
ENSG0000(	ENSG0000(	8.172667	-0.20196	0.408143	0.574497	lncRNA
ENSG0000(	ENSG0000(	282.8574	0.060813	0.408203	0.574504	lncRNA
ENSG0000(	STOM	2472.193	0.096077	0.408199	0.574504	protein_coding
ENSG0000(	ZNF747	678.4146	-0.02825	0.408178	0.574504	protein_coding
ENSG0000(	ENSG0000(	10.95374	0.14042	0.408274	0.574552	lncRNA
ENSG0000(	PBK	46.23627	-0.15676	0.408267	0.574552	protein_coding
ENSG0000(	GALNT9	113.7699	-0.05462	0.408315	0.574556	protein_coding
ENSG0000(	ICAM2	390.0856	-0.08616	0.408313	0.574556	protein_coding
ENSG0000(	PICART1	28.90477	-0.11589	0.408331	0.574556	lncRNA
ENSG0000(	NSFP1	354.377	-0.53109	0.408362	0.574573	unprocessed_pseudogene
ENSG0000(	KIAA1210	71.15562	-0.14745	0.408448	0.574669	protein_coding
ENSG0000(	H2AZ2P1	159.5787	-0.05301	0.408505	0.574724	transcribed_processed_pseudoge
ENSG0000(	IGSF9B	728.9327	-0.13769	0.408604	0.574811	protein_coding
ENSG0000(	RN7SL775F	58.96848	0.084512	0.4086	0.574811	misc_RNA
ENSG0000(	SNX1	4233.8	-0.07286	0.40872	0.574924	protein_coding
ENSG0000(	TRAPPC6A	58.22975	-0.09653	0.408716	0.574924	protein_coding
ENSG0000(	ENSG0000(	245.0524	-0.04603	0.408888	0.575134	lncRNA
ENSG0000(	CIART	274.4077	-0.05746	0.409036	0.575316	protein_coding
ENSG0000(	ZNF732	14.86938	-0.24354	0.409054	0.575316	protein_coding
ENSG0000(	NLRP11	85.01342	-0.09365	0.40928	0.575608	protein_coding
ENSG0000(	ENSG0000(	160.5583	0.062446	0.409335	0.575609	lncRNA
ENSG0000(	ENSG0000(	140.5316	0.06461	0.409304	0.575609	lncRNA
ENSG0000(	NAALAD2	114.0195	0.086912	0.409332	0.575609	protein_coding
ENSG0000(	MIR17HG	410.2905	0.085014	0.409429	0.575715	lncRNA
ENSG0000(	CABP7	36.93468	0.228171	0.409471	0.575749	protein_coding
ENSG0000(	ENSG0000(	64.66913	-0.13202	0.409535	0.575813	lncRNA
ENSG0000(	ENSG0000(	9.47233	-0.40296	0.409638	0.575932	processed_pseudogene
ENSG0000(	TAF1B	354.8544	0.059828	0.40968	0.575965	protein_coding
ENSG0000(	LINC00449	98.29094	-0.0564	0.409782	0.576083	lncRNA
ENSG0000(	PLD6	199.8389	-0.06746	0.409801	0.576083	protein_coding
ENSG0000(	ADGRF5P1	91.30985	-0.09327	0.409859	0.576089	transcribed_processed_pseudoge
ENSG0000(	ENSG0000(	90.09241	0.066759	0.409837	0.576089	lncRNA
ENSG0000(	NUP42	802.0958	-0.03724	0.409848	0.576089	protein_coding
ENSG0000(	ENSG0000(	181.8884	-0.05089	0.409905	0.576124	lncRNA
ENSG0000(	GCNA	414.0294	-0.05077	0.409957	0.576124	protein_coding
ENSG0000(	RECQL4	188.7214	0.120653	0.409952	0.576124	protein_coding

ENSG0000(	ZFP1	175.2798	-0.08967	0.409937	0.576124	protein_coding
ENSG0000(	ENSG0000(	188.1366	-0.07486	0.410023	0.576189	lncRNA
ENSG0000(	LRPAP1	2213.705	-0.03717	0.41004	0.576189	protein_coding
ENSG0000(	ENSG0000(	191.3781	-0.06013	0.410089	0.576206	lncRNA
ENSG0000(	SCAI	1971.807	-0.04737	0.410077	0.576206	protein_coding
ENSG0000(	FBN3	18.50665	0.335924	0.410183	0.576295	protein_coding
ENSG0000(	SNX9	3218.713	-0.0453	0.410189	0.576295	protein_coding
ENSG0000(	TUBA1B	1950.92	-0.09098	0.410228	0.576325	protein_coding
ENSG0000(	ENSG0000(	56.2712	0.09495	0.410383	0.576517	lncRNA
ENSG0000(	PAK1	1830.828	0.047495	0.410413	0.576533	protein_coding
ENSG0000(	ENSG0000(	13.64128	0.149186	0.41049	0.576563	lncRNA
ENSG0000(	ENSG0000(	545.9673	-0.0491	0.41048	0.576563	lncRNA
ENSG0000(	ENSG0000(	513.1507	-0.03856	0.410458	0.576563	lncRNA
ENSG0000(	ENSG0000(	60.23043	0.106962	0.410764	0.576923	lncRNA
ENSG0000(	ENSG0000(	1181.912	0.043533	0.410814	0.576967	lncRNA
ENSG0000(	ENSG0000(	256.8929	-0.08356	0.410895	0.577042	lncRNA
ENSG0000(	ENSG0000(	171.4982	0.050833	0.410904	0.577042	lncRNA
ENSG0000(	CFLAR-AS1	432.6475	-0.03753	0.410971	0.57711	lncRNA
ENSG0000(	TMEM214	1121.494	0.042206	0.411019	0.577152	protein_coding
ENSG0000(	ENSG0000(	257.0408	-0.06362	0.411055	0.577169	lncRNA
ENSG0000(	ENSG0000(	42.03317	0.088901	0.411067	0.577169	lncRNA
ENSG0000(	OGFR	1461.643	0.045951	0.411131	0.577232	protein_coding
ENSG0000(	OASL	554.1198	0.062538	0.411181	0.577251	protein_coding
ENSG0000(	TUBGCP6	3071.5	-0.04368	0.41117	0.577251	protein_coding
ENSG0000(	ENSG0000(	37.15905	-0.07767	0.41126	0.577311	lncRNA
ENSG0000(	TRAJ41	18.47333	0.197925	0.411252	0.577311	TR_J_gene
ENSG0000(	CACNA2D4	273.5855	0.089567	0.411286	0.577322	protein_coding
ENSG0000(	MRPL20-D	358.9302	0.04309	0.41144	0.577511	lncRNA
ENSG0000(	KLHL18	749.8187	0.053451	0.411466	0.577522	protein_coding
ENSG0000(	ANKRD13B	65.92099	0.11286	0.411499	0.577543	protein_coding
ENSG0000(	SLC4A8	1577.168	-0.07517	0.411535	0.577568	protein_coding
ENSG0000(	TCHH	14.48653	-0.17088	0.411578	0.577602	protein_coding
ENSG0000(	LINC01583	24.29462	0.135123	0.411679	0.577692	lncRNA
ENSG0000(	XDH	2732.998	-0.14188	0.411677	0.577692	protein_coding
ENSG0000(	HSPB8	141.9427	-0.13309	0.411707	0.577695	protein_coding
ENSG0000(	LRIG1	1366.194	0.071817	0.411753	0.577695	protein_coding
ENSG0000(	PNMA8C	302.786	-0.03838	0.411754	0.577695	protein_coding
ENSG0000(	RNASET2	6226.618	0.085496	0.411752	0.577695	protein_coding
ENSG0000(	ENSG0000(	35.78098	0.092935	0.411861	0.577819	lncRNA
ENSG0000(	ENSG0000(	204.0486	0.045437	0.411881	0.577822	lncRNA
ENSG0000(	CD1A	17.51185	0.274975	0.411916	0.577846	protein_coding
ENSG0000(	CLEC2D	3876.844	0.148785	0.411969	0.577893	protein_coding
ENSG0000(	ENSG0000(	197.4649	-0.04987	0.412056	0.577988	lncRNA
ENSG0000(	ZNF22	322.5682	0.080451	0.412073	0.577988	protein_coding
ENSG0000(	GCSH	318.7156	0.044631	0.412161	0.578086	protein_coding
ENSG0000(	LINC01683	125.7736	-0.0647	0.412461	0.578479	lncRNA
ENSG0000(	PIGT	3461.852	-0.01808	0.412478	0.578479	protein_coding

ENSG0000(	LINC02595	294.8181	0.084604	0.41258	0.578597	lncRNA
ENSG0000(	SMAD2	12991.28	-0.02267	0.412646	0.578663	protein_coding
ENSG0000(	CASC6	162.1631	-0.06565	0.412801	0.578854	lncRNA
ENSG0000(	RANGAP1	1148.185	0.038032	0.412865	0.578867	protein_coding
ENSG0000(	SH3D19	4143.664	-0.12364	0.412837	0.578867	protein_coding
ENSG0000(	SNORA6	124.2181	0.100523	0.41286	0.578867	snoRNA
ENSG0000(	ZSWIM1	591.3264	-0.02503	0.412922	0.578921	protein_coding
ENSG0000(	FKBP6	210.3487	0.034462	0.413003	0.578984	protein_coding
ENSG0000(	RNY3	1668.323	-0.11201	0.412998	0.578984	misc_RNA
ENSG0000(	GALR1	610.98	0.045297	0.413053	0.579015	protein_coding
ENSG0000(	LINC00852	303.9743	0.071085	0.413062	0.579015	lncRNA
ENSG0000(	PEX11G	42.47553	-0.13137	0.413101	0.579044	protein_coding
ENSG0000(	CDH26	126.0965	-0.07917	0.413219	0.579157	protein_coding
ENSG0000(	MST1	726.7051	-0.13697	0.41321	0.579157	protein_coding
ENSG0000(	GPR153	630.8129	0.125412	0.413249	0.579174	protein_coding
ENSG0000(	AGBL1	243.6862	0.07379	0.413281	0.57918	protein_coding
ENSG0000(	TAF11	2204.673	-0.02256	0.41329	0.57918	protein_coding
ENSG0000(	ENSG0000(	18.4194	0.158299	0.413355	0.579245	lncRNA
ENSG0000(	ENSG0000(	360.7628	-0.07521	0.413454	0.579358	lncRNA
ENSG0000(	ENSG0000(	16.14371	-0.25373	0.413518	0.579422	lncRNA
ENSG0000(	TTC36-AS1	18.56668	0.144459	0.413543	0.579431	lncRNA
ENSG0000(	ARNT2	561.4687	0.039873	0.413629	0.579526	protein_coding
ENSG0000(	ENSG0000(	33.28477	0.153443	0.413658	0.579541	lncRNA
ENSG0000(	ZNF396	590.7064	-0.03358	0.413712	0.57959	protein_coding
ENSG0000(	ENSG0000(	13.8636	0.130482	0.413841	0.579717	lncRNA
ENSG0000(	NDNF	64.77971	-0.12225	0.413824	0.579717	protein_coding
ENSG0000(	ZCCHC8	1827.905	-0.02904	0.413858	0.579717	protein_coding
ENSG0000(	ANAPC2	1171.662	0.03057	0.413997	0.579861	protein_coding
ENSG0000(	ENSG0000(	11.85343	0.147671	0.413989	0.579861	lncRNA
ENSG0000(	ENSG0000(	202.1831	-0.04709	0.414052	0.579886	lncRNA
ENSG0000(	ZNF782	1002.483	-0.03644	0.414051	0.579886	protein_coding
ENSG0000(	HYLS1	237.0049	-0.0376	0.414074	0.579891	protein_coding
ENSG0000(	MRPS10	714.6995	-0.02865	0.414195	0.580035	protein_coding
ENSG0000(	ANKAR	634.7669	0.043659	0.414397	0.580221	protein_coding
ENSG0000(	ENSG0000(	169.0183	0.061029	0.414399	0.580221	lncRNA
ENSG0000(	PTGER2	203.9979	0.065339	0.414401	0.580221	protein_coding
ENSG0000(	SGMS1	1089.031	-0.04423	0.414373	0.580221	protein_coding
ENSG0000(	MAP7D3	542.6252	0.074925	0.414444	0.580255	protein_coding
ENSG0000(	ENSG0000(	31.34205	0.098687	0.414555	0.580384	lncRNA
ENSG0000(	ENSG0000(	10.40193	-0.25595	0.414641	0.580478	processed_pseudogene
ENSG0000(	MYSM1	4319.829	0.025062	0.414807	0.580685	protein_coding
ENSG0000(	LINC01488	12.5487	0.171265	0.414847	0.580715	lncRNA
ENSG0000(	ENSG0000(	40.43486	0.088725	0.414896	0.580759	lncRNA
ENSG0000(	IFITM4P	17.49689	0.153976	0.414978	0.580848	processed_pseudogene
ENSG0000(	ENSG0000(	78.53406	0.129398	0.415026	0.580889	protein_coding
ENSG0000(	ENSG0000(	185.3029	-0.05799	0.415126	0.580935	lncRNA
ENSG0000(	MTCO3P21	53.26409	-0.09278	0.415133	0.580935	unprocessed_pseudogene

ENSG0000(	SPINK5	33.39816	0.196173	0.41513	0.580935	protein_coding
ENSG0000(	WNT9A	115.5769	-0.08042	0.415109	0.580935	protein_coding
ENSG0000(	SLC22A20P	259.5515	0.05845	0.415164	0.580953	transcribed_unitary_pseudogene
ENSG0000(	ENSG0000(	77.58633	-0.07146	0.415251	0.581049	TEC
ENSG0000(	DIS3	2362.867	0.031626	0.415413	0.58125	protein_coding
ENSG0000(	ENSG0000(	188.4973	0.066494	0.415434	0.581254	unprocessed_pseudogene
ENSG0000(	ENSG0000(	187.1323	0.05213	0.415522	0.581351	lncRNA
ENSG0000(	SLC66A3	256.1929	0.067358	0.415558	0.581376	protein_coding
ENSG0000(	FAR2P1	298.2372	0.043232	0.415627	0.581446	transcribed_unprocessed_pseudo
ENSG0000(	ENSG0000(	9.79437	0.22277	0.415647	0.581448	lncRNA
ENSG0000(	LINC01431	57.69477	0.089316	0.415673	0.581458	lncRNA
ENSG0000(	CCDC138	234.3032	-0.06649	0.415722	0.581465	protein_coding
ENSG0000(	ENSG0000(	35.78794	-0.1142	0.415704	0.581465	lncRNA
ENSG0000(	PHC1	1140.763	0.080494	0.415733	0.581465	protein_coding
ENSG0000(	JPT1	654.5898	-0.08797	0.415759	0.581475	protein_coding
ENSG0000(	ENSG0000(	89.86774	0.115788	0.415789	0.581492	lncRNA
ENSG0000(	ENSG0000(	1217.368	0.047755	0.416026	0.581778	lncRNA
ENSG0000(	ENSG0000(	14.18887	0.156635	0.416049	0.581778	TEC
ENSG0000(	ENSG0000(	13.71862	-0.13524	0.416043	0.581778	lncRNA
ENSG0000(	ENSG0000(	38.96916	0.094987	0.416088	0.581807	lncRNA
ENSG0000(	SCAMP4	1439.195	0.030997	0.416115	0.581819	protein_coding
ENSG0000(	BCOR	1564.759	-0.03531	0.416194	0.581828	protein_coding
ENSG0000(	SNORA24	112.6387	0.088804	0.416181	0.581828	snoRNA
ENSG0000(	TSPAN5	528.4123	0.078589	0.416176	0.581828	protein_coding
ENSG0000(	ZEB1-AS1	215.9664	0.074048	0.416196	0.581828	lncRNA
ENSG0000(	ENSG0000(	10.85937	0.144529	0.416236	0.581859	lncRNA
ENSG0000(	CAD	1364.441	0.034352	0.416313	0.581941	protein_coding
ENSG0000(	AJUBA	180.6339	0.068168	0.416372	0.581998	protein_coding
ENSG0000(	ANXA7	701.8993	-0.04981	0.416609	0.582264	protein_coding
ENSG0000(	BLOC1S6	17415.69	-0.04301	0.416606	0.582264	protein_coding
ENSG0000(	COLGALT2	49.75055	-0.13601	0.416635	0.582264	protein_coding
ENSG0000(	ENSG0000(	10.26886	0.171061	0.416637	0.582264	lncRNA
ENSG0000(	ENSG0000(	300.6831	0.044891	0.416672	0.582287	lncRNA
ENSG0000(	RN7SKP13(	16.42294	-0.16738	0.416709	0.582287	misc_RNA
ENSG0000(	RNU6-53P	9.900338	-0.21354	0.416704	0.582287	snRNA
ENSG0000(	ZC3H8	1134.552	-0.05414	0.416742	0.582307	protein_coding
ENSG0000(	RAB30	1373.348	0.107997	0.416914	0.582522	protein_coding
ENSG0000(	ENSG0000(	41.26758	0.120426	0.416998	0.582588	lncRNA
ENSG0000(	ENSG0000(	272.4611	-0.05931	0.416988	0.582588	lncRNA
ENSG0000(	ENSG0000(	221.1567	0.043748	0.417053	0.582638	TEC
ENSG0000(	RN7SL825F	18.56428	0.107701	0.417071	0.582638	misc_RNA
ENSG0000(	RPS15AP3(	219.453	0.122179	0.417139	0.582681	processed_pseudogene
ENSG0000(	ZNF142	1587.465	0.038664	0.417122	0.582681	protein_coding
ENSG0000(	ENSG0000(	1228.495	0.049387	0.417173	0.582687	lncRNA
ENSG0000(	TTC39C	1219.975	0.070086	0.41718	0.582687	protein_coding
ENSG0000(	ENSG0000(	104.228	-0.07433	0.417245	0.582753	lncRNA
ENSG0000(	CD22	3167.679	-0.23278	0.417281	0.582776	protein_coding

ENSG0000(	NKAPP1	529.0834	0.037031	0.41749	0.583042	transcribed_processed_pseudoge
ENSG0000(	TIMM50	1556.864	0.032182	0.417533	0.583077	protein_coding
ENSG0000(	RN7SL689F	25.92554	0.10731	0.417578	0.583114	misc_RNA
ENSG0000(	ENSG0000(	132.0192	-0.05963	0.417758	0.583339	lncRNA
ENSG0000(	SPAG8	53.72949	0.1325	0.417842	0.583431	protein_coding
ENSG0000(	ENSG0000(	17.91717	0.185377	0.417883	0.583461	TEC
ENSG0000(	ENSG0000(	44.2713	-0.07245	0.418007	0.583609	lncRNA
ENSG0000(	HMG20A	1298.96	0.02743	0.418092	0.583703	protein_coding
ENSG0000(	DPH2	253.2085	0.08197	0.418133	0.583734	protein_coding
ENSG0000(	ENSG0000(	19.56624	0.13374	0.418213	0.583819	lncRNA
ENSG0000(	FAM83H	818.8135	-0.10208	0.418242	0.583834	protein_coding
ENSG0000(	CHGB	293.8176	-0.06219	0.418299	0.583888	protein_coding
ENSG0000(	ZNF135	377.9772	0.064314	0.418344	0.583925	protein_coding
ENSG0000(	ENSG0000(	23.53092	0.094341	0.418407	0.583987	lncRNA
ENSG0000(	LINC01224	726.6023	-0.05925	0.418506	0.584099	lncRNA
ENSG0000(	ENSG0000(	38.62949	-0.07901	0.418528	0.584104	lncRNA
ENSG0000(	ENSG0000(	108.5415	0.074528	0.418613	0.584197	lncRNA
ENSG0000(	OR52A1	220.249	0.054566	0.418665	0.584243	protein_coding
ENSG0000(	ENSG0000(	113.9679	-0.06599	0.418755	0.584344	processed_pseudogene
ENSG0000(	DNALI1	146.5955	0.065303	0.418904	0.584496	protein_coding
ENSG0000(	ENSG0000(	537.1625	0.035085	0.418893	0.584496	transcribed_unprocessed_pseudo
ENSG0000(	ENSG0000(	932.5226	-0.05602	0.41892	0.584496	lncRNA
ENSG0000(	EXTL1	25.45972	-0.20708	0.418978	0.58455	protein_coding
ENSG0000(	NAPSB	438.67	-0.18943	0.419148	0.584762	transcribed_unprocessed_pseudo
ENSG0000(	ENSG0000(	57.59345	-0.08895	0.419167	0.584763	lncRNA
ENSG0000(	RN7SL468F	24.12292	-0.11528	0.419206	0.58479	misc_RNA
ENSG0000(	RAD9B	237.0747	0.050678	0.419273	0.584858	protein_coding
ENSG0000(	ZNF471	1825.047	0.049735	0.419316	0.584893	protein_coding
ENSG0000(	MMP21	24.49027	0.106011	0.419377	0.584951	protein_coding
ENSG0000(	GOSR1	2909.392	0.019618	0.419425	0.584967	protein_coding
ENSG0000(	SH3GLB2	2360	-0.05208	0.419414	0.584967	protein_coding
ENSG0000(	KRTAP3-1	21.26964	0.118095	0.41947	0.585004	protein_coding
ENSG0000(	DCXR	130.2088	-0.09516	0.41963	0.585147	protein_coding
ENSG0000(	ENSG0000(	136.0129	-0.06211	0.41962	0.585147	lncRNA
ENSG0000(	KIAA1109	15935.52	-0.03945	0.419614	0.585147	protein_coding
ENSG0000(	RPLP0	4268.639	-0.08644	0.419647	0.585147	protein_coding
ENSG0000(	ENSG0000(	75.69191	0.062726	0.419742	0.585254	lncRNA
ENSG0000(	TOP1MT	148.3443	-0.10007	0.419817	0.585332	protein_coding
ENSG0000(	ENSG0000(	641.2008	-0.02876	0.41986	0.585366	lncRNA
ENSG0000(	GDF11	530.051	0.073027	0.41992	0.585424	protein_coding
ENSG0000(	ENSG0000(	55.92028	-0.0912	0.420102	0.585644	lncRNA
ENSG0000(	KIF3C	146.6323	0.100139	0.420115	0.585644	protein_coding
ENSG0000(	RN7SL166F	72.83974	-0.07714	0.420155	0.585674	misc_RNA
ENSG0000(	ENSG0000(	252.2224	-0.10145	0.420215	0.585732	lncRNA
ENSG0000(	ENSG0000(	72.61197	-0.06303	0.420307	0.585762	lncRNA
ENSG0000(	ENSG0000(	12.07574	0.127203	0.420311	0.585762	lncRNA
ENSG0000(	MIR98	8.721643	0.162637	0.420283	0.585762	miRNA

ENSG0000(	RAET1K	34.95824	-0.09326	0.420282	0.585762	transcribed_unprocessed_pseudo
ENSG0000(	NUDT18	233.9151	-0.0659	0.420383	0.585836	protein_coding
ENSG0000(	LINC02046	57.1951	0.084672	0.420416	0.585856	lncRNA
ENSG0000(	ENSG0000(	24.00685	-0.20006	0.420453	0.585883	processed_pseudogene
ENSG0000(	RHOBTB1	456.1042	0.083349	0.420544	0.585983	protein_coding
ENSG0000(	RNU6-212F	29.14037	0.152261	0.420734	0.586221	snRNA
ENSG0000(	ZNF584	414.6368	-0.05476	0.42104	0.586622	protein_coding
ENSG0000(	CHST1	210.7715	0.052346	0.421113	0.586697	protein_coding
ENSG0000(	TRAV4	14.9583	-0.22121	0.421201	0.586794	TR_V_gene
ENSG0000(	ENSG0000(	87.31483	-0.06086	0.421256	0.586809	lncRNA
ENSG0000(	ENSG0000(	102.8334	-0.05432	0.421305	0.586809	lncRNA
ENSG0000(	ENSG0000(	29.97655	0.108569	0.421303	0.586809	lncRNA
ENSG0000(	HNRNPD-D	24.53148	-0.10967	0.421285	0.586809	lncRNA
ENSG0000(	RRAD	27.48701	-0.13232	0.421277	0.586809	protein_coding
ENSG0000(	WWP2	2358.026	-0.03463	0.421392	0.586905	protein_coding
ENSG0000(	SAAL1	174.9673	0.072355	0.421414	0.58691	protein_coding
ENSG0000(	IZUMO2	361.394	0.037102	0.42148	0.586975	protein_coding
ENSG0000(	DNAAF8	12.58384	-0.20066	0.421587	0.587099	protein_coding
ENSG0000(	PRSS1	105.7237	-0.13248	0.421618	0.587116	protein_coding
ENSG0000(	ZNF814	3152.645	0.044311	0.421742	0.587262	protein_coding
ENSG0000(	TRIM16	636.2706	0.058201	0.421893	0.587446	protein_coding
ENSG0000(	ARHGAP11	94.686	-0.0682	0.421947	0.587497	lncRNA
ENSG0000(	PLEKHG4B	395.1877	-0.05261	0.421979	0.587514	protein_coding
ENSG0000(	DGAT2	411.7361	-0.13637	0.422074	0.587622	protein_coding
ENSG0000(	HDAC9	1087.106	0.150595	0.422178	0.587714	protein_coding
ENSG0000(	TMEM132E	39.15314	0.085004	0.422174	0.587714	lncRNA
ENSG0000(	ENSG0000(	14.20591	0.128453	0.422227	0.587756	lncRNA
ENSG0000(	ENSG0000(	8.794528	0.233992	0.42232	0.587857	processed_pseudogene
ENSG0000(	RN7SL338F	17.29933	0.127494	0.422337	0.587857	misc_RNA
ENSG0000(	MED4-AS1	10.52066	0.153551	0.422428	0.587912	lncRNA
ENSG0000(	PNKP	1006.24	-0.05253	0.422432	0.587912	protein_coding
ENSG0000(	RALGAPB	4633.789	-0.03641	0.422404	0.587912	protein_coding
ENSG0000(	RALA	852.1133	-0.05827	0.422586	0.5881	protein_coding
ENSG0000(	ENSG0000(	815.6119	0.042807	0.422639	0.588148	lncRNA
ENSG0000(	ENSG0000(	36.56055	0.127061	0.422677	0.588156	lncRNA
ENSG0000(	LGR6	64.12198	-0.13607	0.422696	0.588156	protein_coding
ENSG0000(	LMAN1L	10.35881	0.242806	0.422706	0.588156	protein_coding
ENSG0000(	TRAPPC3L	497.5161	0.038935	0.42272	0.588156	protein_coding
ENSG0000(	GPRC5C	567.6176	-0.11472	0.422757	0.588182	protein_coding
ENSG0000(	DEPDC1P1	20.67374	-0.13937	0.4229	0.588355	processed_pseudogene
ENSG0000(	IFT88	727.6545	-0.0449	0.422945	0.588385	protein_coding
ENSG0000(	LINC01905	93.23132	0.06852	0.422959	0.588385	lncRNA
ENSG0000(	HIPK1	3160.795	0.031675	0.423028	0.588455	protein_coding
ENSG0000(	SLC8A3	59.28159	0.129666	0.423085	0.588509	protein_coding
ENSG0000(	ENSG0000(	430.0837	-0.15738	0.423138	0.588557	processed_pseudogene
ENSG0000(	MAFG	1001.24	-0.05864	0.423303	0.58876	protein_coding
ENSG0000(	SPPL2A	4314.659	-0.0487	0.423328	0.588769	protein_coding

ENSG0000(	AADACL2	32.01951	-0.16318	0.423586	0.58909	protein_coding
ENSG0000(	DLL4	376.1808	-0.0822	0.423596	0.58909	protein_coding
ENSG0000(	GRID1	185.8735	-0.07234	0.423691	0.589196	protein_coding
ENSG0000(	PDE3A	1630.584	-0.11905	0.423769	0.589252	protein_coding
ENSG0000(	UBXN7	2940.892	-0.03414	0.423766	0.589252	protein_coding
ENSG0000(	ENSG0000(	55.70784	-0.08969	0.4238	0.589259	lncRNA
ENSG0000(	USP27X-DT	13.02257	0.142446	0.423812	0.589259	lncRNA
ENSG0000(	TSC22D1	4012.381	-0.06788	0.423912	0.589373	protein_coding
ENSG0000(	SNORA37	204.6989	0.075887	0.424	0.589469	snoRNA
ENSG0000(	PLEKHA5	3198.62	0.056281	0.424044	0.589504	protein_coding
ENSG0000(	CDKN1B	1805.281	0.030125	0.424131	0.589599	protein_coding
ENSG0000(	RN7SKP186	182.9437	-0.11087	0.424188	0.589635	misc_RNA
ENSG0000(	TMEM183A	1042.97	-0.03643	0.424194	0.589635	protein_coding
ENSG0000(	ENSG0000(	16.6071	-0.11871	0.424219	0.589643	lncRNA
ENSG0000(	ENSG0000(	25.05966	-0.16431	0.424253	0.589665	lncRNA
ENSG0000(	RAD1	984.5865	0.03625	0.424323	0.589736	protein_coding
ENSG0000(	MIR6769A	10.77257	-0.17828	0.424408	0.589828	miRNA
ENSG0000(	ENSG0000(	513.1311	-0.05762	0.424532	0.589974	lncRNA
ENSG0000(	ENSG0000(	33.00731	0.115421	0.424621	0.590055	lncRNA
ENSG0000(	MIR624	26.01498	0.127667	0.424665	0.590055	miRNA
ENSG0000(	PSMC5	800.4221	-0.04508	0.424648	0.590055	protein_coding
ENSG0000(	TBCK	2042.814	-0.03254	0.424663	0.590055	protein_coding
ENSG0000(	ATP6V1B1-	18.49196	0.144573	0.424704	0.590083	lncRNA
ENSG0000(	CBX5	5865.767	0.057651	0.425017	0.590492	protein_coding
ENSG0000(	PSMC2	919.5552	-0.03077	0.425137	0.590633	protein_coding
ENSG0000(	SEPSECS	1736.075	-0.07359	0.425157	0.590635	protein_coding
ENSG0000(	ZNF549	1060.99	0.040519	0.425271	0.590767	protein_coding
ENSG0000(	APOC1P1	62.40493	-0.08442	0.425393	0.590884	transcribed_unprocessed_pseudo
ENSG0000(	ENSG0000(	32.88281	-0.09753	0.42539	0.590884	lncRNA
ENSG0000(	ENSG0000(	8.673699	0.176135	0.425467	0.590961	lncRNA
ENSG0000(	UBE2D4	570.1639	0.053333	0.425496	0.590975	protein_coding
ENSG0000(	ENSG0000(	4697.335	0.017434	0.425567	0.591048	lncRNA
ENSG0000(	ENSG0000(	125.6842	0.066835	0.425636	0.591117	lncRNA
ENSG0000(	ANKFN1	183.4639	-0.18839	0.425718	0.59118	protein_coding
ENSG0000(	SEPSECS-AS	3279.267	0.042762	0.425701	0.59118	lncRNA
ENSG0000(	TRAJ6	37.5425	0.168606	0.425762	0.591213	TR_J_gene
ENSG0000(	ENSG0000(	14.47102	-0.1431	0.425859	0.591323	lncRNA
ENSG0000(	NRM	94.40835	0.102797	0.425894	0.591345	protein_coding
ENSG0000(	ENSG0000(	2526.467	0.040886	0.425922	0.591358	TEC
ENSG0000(	ENSG0000(	2276.65	-0.13312	0.425952	0.591361	lncRNA
ENSG0000(	TADA2A	931.4979	0.035055	0.425962	0.591361	protein_coding
ENSG0000(	GBP7	10.42852	0.26513	0.426067	0.591482	protein_coding
ENSG0000(	TMEM63B	727.4449	-0.07968	0.426129	0.591541	protein_coding
ENSG0000(	ENSG0000(	117.2503	0.174561	0.426171	0.591561	lncRNA
ENSG0000(	ENSG0000(	52.52404	0.078999	0.426218	0.591561	lncRNA
ENSG0000(	RN7SL523P	17.37753	0.140777	0.426216	0.591561	misc_RNA
ENSG0000(	SLC22A13	117.9569	0.064433	0.42619	0.591561	protein_coding

ENSG0000(	CD2BP2-DT	52.55465	-0.10485	0.426388	0.59177	lncRNA
ENSG0000(	DCAF5	2531.618	0.018123	0.426427	0.591799	protein_coding
ENSG0000(	AKNAD1	35.10056	-0.09119	0.426473	0.591813	protein_coding
ENSG0000(	ENSG0000(	49.40643	0.102074	0.426475	0.591813	lncRNA
ENSG0000(	DANT2	1335.561	0.098932	0.426507	0.591831	lncRNA
ENSG0000(	STX18	910.8168	-0.03909	0.426609	0.591946	protein_coding
ENSG0000(	CEP290	2804.928	-0.03582	0.426685	0.592026	protein_coding
ENSG0000(	ENSG0000(	62.3839	-0.07428	0.426718	0.592046	lncRNA
ENSG0000(	FBRSL1	1598.25	-0.04102	0.426796	0.592127	protein_coding
ENSG0000(	COL6A5	204.7477	0.155412	0.426825	0.592142	protein_coding
ENSG0000(	LINC02506	31.11833	0.09778	0.426898	0.592218	lncRNA
ENSG0000(	C9orf64	541.551	-0.04448	0.426978	0.592303	protein_coding
ENSG0000(	B3GALT9	635.3427	-0.02706	0.42702	0.592308	protein_coding
ENSG0000(	GPAT2	69.80192	0.187554	0.427019	0.592308	protein_coding
ENSG0000(	PSIP1	1260.814	-0.05991	0.427099	0.592392	protein_coding
ENSG0000(	ENSG0000(	9.726817	0.164108	0.427134	0.592414	lncRNA
ENSG0000(	PSMB2	1105.37	0.047821	0.427169	0.592437	protein_coding
ENSG0000(	CTNNA2-AS	179.5426	-0.0834	0.427273	0.592451	lncRNA
ENSG0000(	ENSG0000(	23.12724	-0.13962	0.42721	0.592451	lncRNA
ENSG0000(	IRAG2	1156.643	-0.17581	0.427254	0.592451	protein_coding
ENSG0000(	OR10AH1P	303.1063	0.035878	0.427255	0.592451	transcribed_unprocessed_pseudo
ENSG0000(	PGAM1	1253.501	-0.06045	0.427244	0.592451	protein_coding
ENSG0000(	AP1AR-DT	36.57988	0.099175	0.427402	0.592567	lncRNA
ENSG0000(	ENSG0000(	12.353	0.15613	0.427407	0.592567	lncRNA
ENSG0000(	ENSG0000(	36.71159	-0.10533	0.427413	0.592567	lncRNA
ENSG0000(	AVIL	608.2584	0.05736	0.427496	0.592575	protein_coding
ENSG0000(	CAMK2B	85.36175	0.115393	0.42748	0.592575	protein_coding
ENSG0000(	ENSG0000(	404.6924	0.037932	0.427513	0.592575	lncRNA
ENSG0000(	FAM189B	620.7555	0.029207	0.427478	0.592575	protein_coding
ENSG0000(	LCA5L	118.4501	-0.06276	0.427482	0.592575	protein_coding
ENSG0000(	ENSG0000(	145.8614	-0.12463	0.427576	0.592608	TEC
ENSG0000(	HYOU1	1693	0.064031	0.427571	0.592608	protein_coding
ENSG0000(	PPP1R3E	1569.064	0.050712	0.427593	0.592608	protein_coding
ENSG0000(	RNU4ATAC	24.46985	-0.09673	0.42766	0.592674	snRNA
ENSG0000(	ENSG0000(	166.8675	0.048226	0.427839	0.592871	lncRNA
ENSG0000(	ZNF823	338.8937	-0.08168	0.427837	0.592871	protein_coding
ENSG0000(	TRMU	1123.239	-0.03361	0.427862	0.592875	protein_coding
ENSG0000(	FZD6	305.9434	0.067482	0.427937	0.592917	protein_coding
ENSG0000(	LINC02466	66.43554	0.084016	0.427938	0.592917	lncRNA
ENSG0000(	SYNRG	4645.686	0.048651	0.427948	0.592917	protein_coding
ENSG0000(	ENSG0000(	64.16508	-0.40777	0.428026	0.592998	lncRNA
ENSG0000(	SNORA50A	97.34006	-0.06244	0.428045	0.593	snoRNA
ENSG0000(	RNU6-482F	14.1504	-0.14741	0.428093	0.593039	snRNA
ENSG0000(	ENSG0000(	102.4282	-0.05304	0.428114	0.593042	lncRNA
ENSG0000(	ENSG0000(	22.8112	-0.12513	0.428169	0.593093	unprocessed_pseudogene
ENSG0000(	LIN28B-AS1	596.9242	-0.03215	0.42821	0.593123	lncRNA
ENSG0000(	ZNF335	1342.659	0.033868	0.428235	0.593132	protein_coding

ENSG0000(	SRSF10	4976.377	-0.03252	0.428312	0.593213	protein_coding
ENSG0000(	ENSG0000(	12.60861	0.166915	0.428403	0.593242	lncRNA
ENSG0000(	GMIP	1108.946	0.083183	0.428409	0.593242	protein_coding
ENSG0000(	NPAP1P6	12.97952	0.283033	0.428379	0.593242	processed_pseudogene
ENSG0000(	RASSF1	828.3437	0.071859	0.428373	0.593242	protein_coding
ENSG0000(	OSGEPL1	141.0913	-0.07834	0.428463	0.593291	protein_coding
ENSG0000(	YIPF4	5515.741	0.027068	0.428615	0.593475	protein_coding
ENSG0000(	LINC00462	13.98196	0.11525	0.428709	0.59358	lncRNA
ENSG0000(	CCDC141	1376.466	0.148425	0.428839	0.593733	protein_coding
ENSG0000(	ENSG0000(	12.06877	-0.27544	0.428893	0.593782	transcribed_processed_pseudoge
ENSG0000(	NPAT	2291.28	-0.05816	0.428946	0.593829	protein_coding
ENSG0000(	ENSG0000(	15.3612	0.193979	0.428986	0.593859	processed_pseudogene
ENSG0000(	PPP2R3B	548.2199	0.066706	0.429088	0.593974	protein_coding
ENSG0000(	CKAP5	1634.19	-0.05014	0.429281	0.594163	protein_coding
ENSG0000(	ENSG0000(	23.76091	0.108802	0.429275	0.594163	lncRNA
ENSG0000(	IL21R-AS1	126.8992	0.053724	0.429251	0.594163	lncRNA
ENSG0000(	ENSG0000(	58.09894	-0.08554	0.429331	0.594205	lncRNA
ENSG0000(	ENSG0000(	411.6687	0.051736	0.429409	0.594287	lncRNA
ENSG0000(	ENSG0000(	308.5977	-0.10105	0.429438	0.594302	lncRNA
ENSG0000(	KDM5C-IT1	140.8226	0.053055	0.429563	0.594449	lncRNA
ENSG0000(	CARMIL3	89.56007	-0.12313	0.429715	0.594633	protein_coding
ENSG0000(	AP3D1	2433.409	0.038446	0.429812	0.59474	protein_coding
ENSG0000(	ENSG0000(	352.5739	-0.12638	0.429857	0.594765	lncRNA
ENSG0000(	LINC02023	103.3128	0.082495	0.429905	0.594765	lncRNA
ENSG0000(	NDUFS6	146.7868	-0.07849	0.429897	0.594765	protein_coding
ENSG0000(	RN7SKP213	24.89866	-0.10571	0.429873	0.594765	misc_RNA
ENSG0000(	ENSG0000(	241.0367	0.067497	0.429952	0.594804	TEC
ENSG0000(	ENSG0000(	135.0738	-0.1679	0.429977	0.594813	lncRNA
ENSG0000(	TSPAN18	258.1591	0.118148	0.430031	0.594862	protein_coding
ENSG0000(	LAPTM5	2896.498	0.143761	0.430131	0.594947	protein_coding
ENSG0000(	MARCHF1	1227.023	0.162844	0.43012	0.594947	protein_coding
ENSG0000(	ENSG0000(	30.3194	0.115724	0.430233	0.595036	lncRNA
ENSG0000(	FBXL15	115.043	-0.06849	0.430216	0.595036	protein_coding
ENSG0000(	ENSG0000(	17.52365	0.112797	0.430373	0.595203	TEC
ENSG0000(	RPL7L1P3	140.8721	-0.1132	0.430402	0.595218	processed_pseudogene
ENSG0000(	ENSG0000(	283.9685	0.092213	0.430473	0.595289	transcribed_unprocessed_pseudo
ENSG0000(	LINC01089	1052.885	-0.04245	0.43065	0.595482	lncRNA
ENSG0000(	SLC2A12	160.9192	-0.2234	0.430634	0.595482	protein_coding
ENSG0000(	OTUD6B	416.8657	0.044613	0.4307	0.595498	protein_coding
ENSG0000(	SLC26A1	132.7774	-0.16567	0.430693	0.595498	protein_coding
ENSG0000(	ENSA	2521.905	0.020691	0.430871	0.595709	protein_coding
ENSG0000(	RNY3P12	12.38149	0.16763	0.430896	0.595718	misc_RNA
ENSG0000(	ENSG0000(	26.77818	-0.09139	0.430955	0.595773	lncRNA
ENSG0000(	ENSG0000(	53.55943	0.089571	0.431029	0.595849	lncRNA
ENSG0000(	FBXL19	601.1881	0.050133	0.431081	0.595869	protein_coding
ENSG0000(	YRDC	118.5989	0.048295	0.431069	0.595869	protein_coding
ENSG0000(	ENSG0000(	22.46158	0.115363	0.431207	0.595995	lncRNA

ENSG0000(	MIR3183	15.54087	0.152182	0.43121	0.595995	miRNA
ENSG0000(	ENSG0000(	10.12921	-0.15596	0.431275	0.596007	lncRNA
ENSG0000(	PNPT1P1	71.66316	0.101599	0.431272	0.596007	processed_pseudogene
ENSG0000(	TMEM139-	154.7723	0.046402	0.431257	0.596007	lncRNA
ENSG0000(	ENSG0000(	105.9302	-0.17239	0.431413	0.596171	lncRNA
ENSG0000(	ENSG0000(	147.782	0.061936	0.431517	0.596288	transcribed_unprocessed_pseudo
ENSG0000(	ENSG0000(	69.59916	0.085898	0.431576	0.596343	lncRNA
ENSG0000(	SIAH1	840.7339	0.03733	0.431673	0.596452	protein_coding
ENSG0000(	ENSG0000(	15.85231	0.119357	0.431732	0.596507	lncRNA
ENSG0000(	LINC01020	93.09054	0.060293	0.431761	0.596521	lncRNA
ENSG0000(	ENSG0000(	81.28094	0.06634	0.431835	0.596571	lncRNA
ENSG0000(	SYNE2	13942.41	-0.03287	0.431827	0.596571	protein_coding
ENSG0000(	BTBD6	529.0849	-0.0446	0.431884	0.596601	protein_coding
ENSG0000(	RPL23AP66	61.63354	-0.08738	0.431895	0.596601	processed_pseudogene
ENSG0000(	LMOD1	473.0403	0.113901	0.43197	0.596652	protein_coding
ENSG0000(	MAEL	80.63759	0.070656	0.431953	0.596652	protein_coding
ENSG0000(	KCNQ4	54.9464	-0.10484	0.432016	0.59669	protein_coding
ENSG0000(	LINC01055	59.06431	-0.06171	0.432084	0.596758	lncRNA
ENSG0000(	ENSG0000(	1272.769	-0.10718	0.432105	0.596761	lncRNA
ENSG0000(	ENSG0000(	22.39615	-0.11041	0.432132	0.596772	lncRNA
ENSG0000(	ENSG0000(	839.2221	0.329047	0.432194	0.59678	lncRNA
ENSG0000(	MTCO2P22	12.58201	-0.52311	0.432195	0.59678	processed_pseudogene
ENSG0000(	USP34	11361.87	-0.02563	0.432162	0.59678	protein_coding
ENSG0000(	CARHSP1	1068.557	0.038106	0.432315	0.596912	protein_coding
ENSG0000(	ENSG0000(	32.27948	-0.10914	0.432333	0.596912	lncRNA
ENSG0000(	ZNF429	1619.065	-0.02915	0.432347	0.596912	protein_coding
ENSG0000(	DNTTIP2	2437.756	0.037138	0.432424	0.596992	protein_coding
ENSG0000(	LINC00698	32.48452	0.087207	0.432643	0.597242	lncRNA
ENSG0000(	LINC02249	218.654	0.055919	0.432662	0.597242	lncRNA
ENSG0000(	RSRP1	7805.599	-0.03031	0.432639	0.597242	protein_coding
ENSG0000(	ENSG0000(	45.02199	0.152995	0.432684	0.597244	lncRNA
ENSG0000(	ENSG0000(	205.0701	-0.04026	0.43272	0.597244	lncRNA
ENSG0000(	TAGLN	1672.36	0.084654	0.432714	0.597244	protein_coding
ENSG0000(	HRCT1	50.95943	-0.21504	0.432822	0.597358	protein_coding
ENSG0000(	ZSWIM9	193.7226	0.087491	0.43294	0.597495	protein_coding
ENSG0000(	ENSG0000(	114.7512	0.045818	0.433069	0.597646	lncRNA
ENSG0000(	C6orf52	8.946428	0.159433	0.4331	0.597663	protein_coding
ENSG0000(	ADCY10	96.26951	-0.17457	0.433253	0.597796	protein_coding
ENSG0000(	ENSG0000(	41.96145	-0.26436	0.433246	0.597796	protein_coding
ENSG0000(	MIR604	34.11144	0.120279	0.433242	0.597796	miRNA
ENSG0000(	ENSG0000(	16.32116	-0.11055	0.433357	0.597887	lncRNA
ENSG0000(	GRM8-AS1	25.70196	0.10584	0.433354	0.597887	lncRNA
ENSG0000(	CEMIP2	4676.442	-0.04932	0.433413	0.597904	protein_coding
ENSG0000(	ENSG0000(	16.43016	-0.09956	0.433426	0.597904	processed_pseudogene
ENSG0000(	EZH2	1207.764	0.058399	0.433425	0.597904	protein_coding
ENSG0000(	EXD3	735.8859	-0.05954	0.433559	0.598009	protein_coding
ENSG0000(	PGAM4	34.81278	0.129097	0.433552	0.598009	protein_coding

ENSG0000(	PLAC9	13.69294	-0.16195	0.433559	0.598009	protein_coding
ENSG0000(	USP2-AS1	448.7144	0.034588	0.433597	0.598035	lncRNA
ENSG0000(	LSP1	1432.585	-0.1032	0.433743	0.598209	protein_coding
ENSG0000(	ZNF441	661.8759	-0.05353	0.433762	0.598209	protein_coding
ENSG0000(	PGK1	2452.235	-0.05828	0.433792	0.598225	protein_coding
ENSG0000(	ENSG0000(	2206.9	0.075736	0.433816	0.598233	lncRNA
ENSG0000(	C3orf35	2188.2	0.044338	0.433886	0.598302	lncRNA
ENSG0000(	GHRLOS	597.1466	0.072333	0.433931	0.598338	lncRNA
ENSG0000(	SNTG2	65.50733	-0.11887	0.433981	0.598381	protein_coding
ENSG0000(	ENSG0000(	26.57319	-0.1153	0.434131	0.598561	lncRNA
ENSG0000(	PAQR4	153.7496	0.088216	0.434347	0.598808	protein_coding
ENSG0000(	RN7SL132P	53.01218	0.072998	0.434336	0.598808	misc_RNA
ENSG0000(	ITGB1BP2	71.64095	-0.08113	0.434431	0.598897	protein_coding
ENSG0000(	AP1S2	449.3178	0.087391	0.434599	0.599102	protein_coding
ENSG0000(	MMRN2	333.5674	0.075839	0.43464	0.599133	protein_coding
ENSG0000(	ARHGAP19	659.8098	-0.05215	0.434666	0.599142	protein_coding
ENSG0000(	ENSG0000(	13.60823	-0.21345	0.434761	0.599221	TEC
ENSG0000(	NPAS1	8.783221	-0.22199	0.434762	0.599221	protein_coding
ENSG0000(	NEFH	22.87233	-0.13909	0.434861	0.599332	protein_coding
ENSG0000(	ENSG0000(	261.5052	0.038877	0.434889	0.599344	lncRNA
ENSG0000(	MTMR2	954.884	0.044842	0.434939	0.599387	protein_coding
ENSG0000(	INO80E	2959.559	-0.04219	0.435023	0.599477	protein_coding
ENSG0000(	ENSG0000(	134.1564	-0.05666	0.435148	0.599622	TEC
ENSG0000(	ENSG0000(	1380.698	-0.06261	0.435274	0.599718	lncRNA
ENSG0000(	ENSG0000(	21.17934	0.115916	0.435245	0.599718	lncRNA
ENSG0000(	ENSG0000(	99.78397	-0.08234	0.435271	0.599718	lncRNA
ENSG0000(	DAB2IP	1799.405	-0.09231	0.435355	0.599777	protein_coding
ENSG0000(	VTI1A	2116.763	-0.02443	0.435347	0.599777	protein_coding
ENSG0000(	ENSG0000(	12.52272	-0.13752	0.43548	0.599844	lncRNA
ENSG0000(	LINC00326	11.96261	0.160727	0.435445	0.599844	lncRNA
ENSG0000(	NPHP3	3023.198	0.033244	0.435473	0.599844	protein_coding
ENSG0000(	USP17L15	16.92906	0.403983	0.435427	0.599844	protein_coding
ENSG0000(	DGKG	196.0505	-0.10794	0.435653	0.600057	protein_coding
ENSG0000(	LINC01054	18.50498	-0.12318	0.435696	0.600089	lncRNA
ENSG0000(	RRP1	477.3094	-0.04231	0.435763	0.600156	protein_coding
ENSG0000(	ENSG0000(	16.21197	0.121438	0.435803	0.600184	lncRNA
ENSG0000(	KMT5A	908.1053	0.046256	0.435878	0.60026	protein_coding
ENSG0000(	BTBD1	1133.695	-0.05873	0.435942	0.600311	protein_coding
ENSG0000(	ENSG0000(	195.7258	0.043721	0.43603	0.600311	lncRNA
ENSG0000(	GGT7	405.0222	0.049618	0.435995	0.600311	protein_coding
ENSG0000(	HERC2P3	1209.704	0.061607	0.436048	0.600311	transcribed_unprocessed_pseudo
ENSG0000(	RPL32P2	14.39252	-0.13793	0.435986	0.600311	processed_pseudogene
ENSG0000(	RUNDC1	1771.215	-0.0285	0.435979	0.600311	protein_coding
ENSG0000(	TRIM46	188.6525	0.055155	0.436024	0.600311	protein_coding
ENSG0000(	NDUFV1	1230.194	-0.04478	0.436106	0.600339	protein_coding
ENSG0000(	RPL37A-DT	254.1306	-0.0528	0.436094	0.600339	lncRNA
ENSG0000(	ENSG0000(	20.36439	0.159097	0.436192	0.600431	lncRNA

ENSG0000(	ATP6V1G2-	12.64966	0.460953	0.436304	0.600553	protein_coding
ENSG0000(	ENSG0000(	8.571812	-0.48083	0.436338	0.600553	protein_coding
ENSG0000(	ENSG0000(	45.30759	0.177983	0.436326	0.600553	lncRNA
ENSG0000(	RNU1-19P	89.78066	0.087827	0.436361	0.600559	snRNA
ENSG0000(	PCDHB14	91.95137	0.066161	0.436644	0.600921	protein_coding
ENSG0000(	GALNTL5	25.87145	0.117676	0.436809	0.601097	protein_coding
ENSG0000(	PSPH	448.7363	-0.04506	0.436806	0.601097	protein_coding
ENSG0000(	H2BE1	9.802255	-0.16836	0.436866	0.601149	protein_coding
ENSG0000(	CYTH2	2365.097	-0.05236	0.436921	0.601198	protein_coding
ENSG0000(	FNDC10	23.30772	-0.13123	0.437059	0.601322	protein_coding
ENSG0000(	MIR4422H(	12.51864	0.273282	0.437034	0.601322	lncRNA
ENSG0000(	UBE2E3	465.6584	-0.05023	0.437068	0.601322	protein_coding
ENSG0000(	RN7SL190F	152.7205	-0.05067	0.437132	0.601383	misc_RNA
ENSG0000(	PRANCR	1508.277	0.031611	0.437294	0.601581	lncRNA
ENSG0000(	MIR548AA2	57.80087	-0.075	0.437444	0.601761	miRNA
ENSG0000(	HELQ	543.4456	0.048615	0.437558	0.60189	protein_coding
ENSG0000(	TMEM18	852.6018	0.068543	0.437636	0.601972	protein_coding
ENSG0000(	RN7SL57P	86.49839	0.059966	0.43773	0.602075	misc_RNA
ENSG0000(	RNF180	261.7712	0.046563	0.437801	0.602146	protein_coding
ENSG0000(	MEF2A	2200.584	-0.03489	0.437893	0.602246	protein_coding
ENSG0000(	ENSG0000(	28.14145	-0.11067	0.438054	0.602442	processed_pseudogene
ENSG0000(	RN7SKP149	9.036844	-0.19061	0.438111	0.602468	misc_RNA
ENSG0000(	TAS2R43	149.9287	-0.11501	0.438112	0.602468	protein_coding
ENSG0000(	OR3A2	104.5486	0.070662	0.438262	0.602648	protein_coding
ENSG0000(	ENSG0000(	14.33908	0.152479	0.438357	0.602726	lncRNA
ENSG0000(	SLC36A2	319.7365	-0.03876	0.43835	0.602726	protein_coding
ENSG0000(	FMR1-IT1	163.3055	-0.06322	0.438476	0.602864	lncRNA
ENSG0000(	TUT4	3995.585	0.073628	0.438499	0.602869	protein_coding
ENSG0000(	MTND4P3C	174.2201	-0.04585	0.43869	0.60308	unprocessed_pseudogene
ENSG0000(	RPL23P2	12.59642	0.139413	0.438684	0.60308	processed_pseudogene
ENSG0000(	ENSG0000(	743.4388	0.032449	0.438845	0.603266	lncRNA
ENSG0000(	TEX21P	546.4781	-0.04316	0.438867	0.60327	transcribed_unitary_pseudogene
ENSG0000(	PLCD3	585.1593	0.083881	0.439023	0.603459	protein_coding
ENSG0000(	ENSG0000(	37.67989	0.117357	0.439046	0.603464	TEC
ENSG0000(	ENSG0000(	12.19199	0.212207	0.439113	0.603529	protein_coding
ENSG0000(	ENSG0000(	26.79238	0.201528	0.439215	0.603642	lncRNA
ENSG0000(	NDUFB2-AS	82.25765	0.059724	0.439264	0.603668	lncRNA
ENSG0000(	TRIM2	4678.395	-0.08698	0.439272	0.603668	protein_coding
ENSG0000(	SPOP	1729.646	-0.02426	0.439384	0.603796	protein_coding
ENSG0000(	FAM161A	646.0584	0.059728	0.439427	0.603828	protein_coding
ENSG0000(	CCR4	737.7084	0.051123	0.439527	0.60387	protein_coding
ENSG0000(	CTPS2	1005.024	-0.06024	0.439533	0.60387	protein_coding
ENSG0000(	LINC02576	273.5963	0.062608	0.439512	0.60387	lncRNA
ENSG0000(	SNORD67	75.21197	-0.09222	0.439519	0.60387	snoRNA
ENSG0000(	PAPOLG	1238.519	0.038961	0.439671	0.604032	protein_coding
ENSG0000(	GDI1	1654.414	0.037247	0.439751	0.604115	protein_coding
ENSG0000(	ENSG0000(	61.66221	-0.26428	0.439773	0.604119	protein_coding

ENSG0000(	TASOR	3319.762	-0.03467	0.439813	0.604148	protein_coding
ENSG0000(	FBXO30-DT	2082.66	0.043598	0.439907	0.604224	lncRNA
ENSG0000(	LINC01489	24.90559	0.122784	0.439892	0.604224	lncRNA
ENSG0000(	FER1L6	1133.152	-0.17894	0.440185	0.604539	protein_coding
ENSG0000(	HOPX	454.0755	-0.03995	0.440177	0.604539	protein_coding
ENSG0000(	LMNTD2-A	62.22012	0.125177	0.440208	0.604539	lncRNA
ENSG0000(	RPS6P25	37.00686	-0.10585	0.440213	0.604539	processed_pseudogene
ENSG0000(	ENSG0000(	19.06964	0.203579	0.440236	0.604545	lncRNA
ENSG0000(	IVD	1869.798	0.048299	0.440306	0.604611	protein_coding
ENSG0000(	MIR326	13.90982	-0.16525	0.440323	0.604611	miRNA
ENSG0000(	ENSG0000(	51.17266	0.076303	0.440513	0.604768	lncRNA
ENSG0000(	ENSG0000(	8.486743	0.220703	0.440513	0.604768	lncRNA
ENSG0000(	RN7SL817F	33.67874	0.084285	0.440493	0.604768	misc_RNA
ENSG0000(	RNU6-1025	16.82323	-0.13867	0.440508	0.604768	snRNA
ENSG0000(	ENSG0000(	13.28613	0.16393	0.440642	0.604917	lncRNA
ENSG0000(	ENSG0000(	565.5619	0.047641	0.440664	0.604922	lncRNA
ENSG0000(	ENSG0000(	74.59871	-0.10415	0.440705	0.604925	lncRNA
ENSG0000(	LINC01419	13.61405	0.186575	0.440687	0.604925	lncRNA
ENSG0000(	LHX4	36.84581	0.127135	0.440949	0.605228	protein_coding
ENSG0000(	MIR562	11.84671	0.13306	0.440988	0.605228	miRNA
ENSG0000(	NCF1B	252.0171	0.167537	0.440975	0.605228	transcribed_unprocessed_pseudo
ENSG0000(	RN7SL78P	11.18169	0.193152	0.441003	0.605228	misc_RNA
ENSG0000(	IGIP	694.6167	0.034187	0.441084	0.605314	protein_coding
ENSG0000(	KLF13	3633.618	-0.04361	0.441135	0.60533	protein_coding
ENSG0000(	NOG	23.56384	0.27735	0.441125	0.60533	protein_coding
ENSG0000(	H3P13	155.3297	0.041606	0.441246	0.605457	processed_pseudogene
ENSG0000(	ENSG0000(	329.7155	0.046511	0.441333	0.605524	lncRNA
ENSG0000(	PGAM1P5	106.7353	0.060624	0.441329	0.605524	transcribed_processed_pseudoge
ENSG0000(	CREB3L4	221.3936	0.044633	0.441404	0.605568	protein_coding
ENSG0000(	IGLJ1	17.07587	0.288619	0.441396	0.605568	IG_J_gene
ENSG0000(	CBR4	1347.192	0.037613	0.44146	0.605608	protein_coding
ENSG0000(	ENSG0000(	76.82615	-0.10148	0.441471	0.605608	transcribed_unprocessed_pseudo
ENSG0000(	TRAV8-6	14.9533	-0.19472	0.441504	0.605626	TR_V_gene
ENSG0000(	ENSG0000(	1058.194	-0.03463	0.441562	0.605653	lncRNA
ENSG0000(	ENSG0000(	36.44771	-0.09954	0.441568	0.605653	TEC
ENSG0000(	ENSG0000(	22.27066	0.080604	0.441591	0.605653	lncRNA
ENSG0000(	PRMT6	254.913	-0.06663	0.4416	0.605653	protein_coding
ENSG0000(	BRCA1	1223.933	-0.02752	0.441717	0.605737	protein_coding
ENSG0000(	ENSG0000(	284.5441	-0.11292	0.441716	0.605737	lncRNA
ENSG0000(	MBD5	3773.071	0.043619	0.441731	0.605737	protein_coding
ENSG0000(	PCSK5	18429.5	0.125025	0.441739	0.605737	protein_coding
ENSG0000(	ENSG0000(	98.11771	0.060197	0.441782	0.60577	lncRNA
ENSG0000(	ATP6V1F	179.2084	-0.06185	0.44189	0.605865	protein_coding
ENSG0000(	ENSG0000(	359.1474	-0.06228	0.441878	0.605865	transcribed_processed_pseudoge
ENSG0000(	UTP11	700.6082	-0.04542	0.442127	0.606164	protein_coding
ENSG0000(	BCL2L15	4273.936	-0.11243	0.44217	0.606196	protein_coding
ENSG0000(	ENSG0000(	232.4497	0.055528	0.442192	0.606201	lncRNA

ENSG0000(	ENSG0000(	25.32848	0.121486	0.442229	0.606226	lncRNA
ENSG0000(	ENSG0000(	53.19926	0.090834	0.442375	0.606397	lncRNA
ENSG0000(	PPP1R1B	593.0593	0.140084	0.442393	0.606397	protein_coding
ENSG0000(	CRIP2	251.7722	-0.09452	0.442498	0.606415	protein_coding
ENSG0000(	L1CAM	120.452	-0.11633	0.442513	0.606415	protein_coding
ENSG0000(	NIPSNAP1	590.0705	-0.03177	0.442508	0.606415	protein_coding
ENSG0000(	REEP1	280.8471	-0.05794	0.442509	0.606415	protein_coding
ENSG0000(	RNU11-6P	10.0675	0.189965	0.442521	0.606415	snRNA
ENSG0000(	SPECC1L	2337.126	-0.03225	0.442427	0.606415	protein_coding
ENSG0000(	CLIP2	1702.463	0.036409	0.44262	0.606423	protein_coding
ENSG0000(	ENSG0000(	123.3927	-0.06415	0.442688	0.606423	lncRNA
ENSG0000(	ENSG0000(	47.07354	0.492069	0.442662	0.606423	lncRNA
ENSG0000(	ENSG0000(	2156.977	0.043431	0.442701	0.606423	TEC
ENSG0000(	EVI2B	993.8819	0.144697	0.442689	0.606423	protein_coding
ENSG0000(	L3MBTL3	404.0104	0.137452	0.442647	0.606423	protein_coding
ENSG0000(	LINC01208	118.6289	0.08794	0.442628	0.606423	lncRNA
ENSG0000(	LSM3	409.751	-0.03006	0.442667	0.606423	protein_coding
ENSG0000(	SCG3	125.1456	0.071765	0.442647	0.606423	protein_coding
ENSG0000(	CYCSP34	98.1124	0.084634	0.44273	0.606437	processed_pseudogene
ENSG0000(	CTSH	4017.692	0.092007	0.44282	0.606533	protein_coding
ENSG0000(	RN7SL591F	28.71344	-0.10171	0.443067	0.606845	misc_RNA
ENSG0000(	SCRN1	704.1654	0.114103	0.443107	0.606874	protein_coding
ENSG0000(	AZGP1P1	22.05604	-0.14391	0.443201	0.60691	transcribed_unprocessed_pseudo
ENSG0000(	ENSG0000(	62.58293	-0.07154	0.443183	0.60691	lncRNA
ENSG0000(	ENSG0000(	39.97189	0.081961	0.443268	0.60691	lncRNA
ENSG0000(	HPCAL1	1950.613	-0.06607	0.443237	0.60691	protein_coding
ENSG0000(	PCDHB15	60.28624	0.113499	0.443204	0.60691	protein_coding
ENSG0000(	PCDHB2	80.53035	-0.06395	0.443264	0.60691	protein_coding
ENSG0000(	SOWAHCP2	183.5059	0.166636	0.443231	0.60691	processed_pseudogene
ENSG0000(	LINC00882	1812.814	-0.04572	0.443432	0.607108	lncRNA
ENSG0000(	C4orf48	29.65244	-0.14081	0.443495	0.607116	protein_coding
ENSG0000(	C8orf34-AS	482.9338	0.066891	0.443459	0.607116	lncRNA
ENSG0000(	FXN	989.1262	0.028872	0.443496	0.607116	protein_coding
ENSG0000(	ENSG0000(	172.2379	-0.04165	0.443535	0.607144	transcribed_processed_pseudoge
ENSG0000(	LRRC37A9F	223.2919	0.037189	0.443626	0.607241	processed_pseudogene
ENSG0000(	ENSG0000(	176.3843	0.062441	0.443667	0.607271	lncRNA
ENSG0000(	IQSEC3	102.7555	0.168846	0.443767	0.607381	protein_coding
ENSG0000(	MLLT11	123.4791	-0.05351	0.443812	0.607417	protein_coding
ENSG0000(	RN7SL778F	39.35259	0.079627	0.443861	0.607457	misc_RNA
ENSG0000(	RN7SL526F	104.0993	-0.05835	0.443942	0.607542	misc_RNA
ENSG0000(	ENSG0000(	13.59963	0.450042	0.443982	0.60757	lncRNA
ENSG0000(	CTPS1	2014.026	0.073759	0.444017	0.607592	protein_coding
ENSG0000(	TMEM175	599.7976	-0.05686	0.444045	0.607604	protein_coding
ENSG0000(	ARHGAP24	937.883	0.160458	0.444162	0.607737	protein_coding
ENSG0000(	RFC5	269.8677	-0.05262	0.444188	0.607746	protein_coding
ENSG0000(	ENSG0000(	359.9786	-0.06041	0.444255	0.607792	lncRNA
ENSG0000(	NEK11	161.5358	0.067886	0.44426	0.607792	protein_coding

ENSG0000(	LINC01692	22.82242	0.114641	0.444325	0.607855	lncRNA
ENSG0000(	LINC02267	152.5693	0.082097	0.444414	0.607951	lncRNA
ENSG0000(	RAB1A	1983.602	0.06498	0.444577	0.608147	protein_coding
ENSG0000(	ENSG0000(	24.61268	-0.09643	0.444647	0.608217	lncRNA
ENSG0000(	ENSG0000(	45.14869	0.08182	0.444758	0.608342	lncRNA
ENSG0000(	ENSG0000(	219.4632	-0.06575	0.445143	0.608789	TEC
ENSG0000(	JADE2	2735.211	-0.04636	0.445142	0.608789	protein_coding
ENSG0000(	LINC01209	246.2453	-0.0627	0.44512	0.608789	lncRNA
ENSG0000(	ARHGAP9	1399.697	0.131092	0.445374	0.608946	protein_coding
ENSG0000(	CCDC180	756.9037	0.068754	0.44535	0.608946	protein_coding
ENSG0000(	ENSG0000(	70.19062	-0.07935	0.445325	0.608946	lncRNA
ENSG0000(	ENSG0000(	355.0916	0.070957	0.445365	0.608946	lncRNA
ENSG0000(	HARS1	2969.989	0.026587	0.445285	0.608946	protein_coding
ENSG0000(	MTFR2P1	48.4905	0.10782	0.445365	0.608946	processed_pseudogene
ENSG0000(	ENSG0000(	296.3822	0.060261	0.445408	0.608966	lncRNA
ENSG0000(	ENSG0000(	29.60466	-0.08309	0.445453	0.608975	lncRNA
ENSG0000(	ENSG0000(	41.49223	0.08419	0.445449	0.608975	TEC
ENSG0000(	ENSG0000(	262.3311	0.048954	0.44552	0.60904	lncRNA
ENSG0000(	ZRANB2-AS	12.12411	0.108204	0.445544	0.609047	lncRNA
ENSG0000(	PPP2R5A	1114.099	-0.0485	0.445609	0.60911	protein_coding
ENSG0000(	CNNM2	1456.532	-0.05484	0.445693	0.609198	protein_coding
ENSG0000(	AK5	180.8753	0.093926	0.445827	0.609328	protein_coding
ENSG0000(	ENSG0000(	55.67012	0.247668	0.445809	0.609328	lncRNA
ENSG0000(	RBBP8	472.4129	-0.06777	0.44589	0.609388	protein_coding
ENSG0000(	ENSG0000(	177.1733	0.071853	0.445913	0.609393	lncRNA
ENSG0000(	ENSG0000(	13.06883	0.126419	0.446081	0.609596	lncRNA
ENSG0000(	ARL14EP	556.2525	-0.08735	0.446296	0.60981	protein_coding
ENSG0000(	ENSG0000(	29.32668	0.094981	0.446287	0.60981	lncRNA
ENSG0000(	TP53RK	428.5624	0.047389	0.446292	0.60981	protein_coding
ENSG0000(	IRF7	531.9853	0.096127	0.446559	0.610144	protein_coding
ENSG0000(	JRK	1810.261	0.041075	0.446611	0.610188	protein_coding
ENSG0000(	ENSG0000(	52.09575	-0.12163	0.446798	0.61039	lncRNA
ENSG0000(	ENSG0000(	21.47389	-0.10795	0.446796	0.61039	lncRNA
ENSG0000(	CHMP7	1706.576	-0.10417	0.446877	0.610445	protein_coding
ENSG0000(	ENSG0000(	30.62954	0.090645	0.446867	0.610445	processed_pseudogene
ENSG0000(	FAM74A4	8.838615	0.254318	0.446932	0.610467	transcribed_processed_pseudoge
ENSG0000(	RGPD5	7016.289	-0.03742	0.446913	0.610467	protein_coding
ENSG0000(	NDST2	911.013	0.054021	0.447206	0.610816	protein_coding
ENSG0000(	SCX	37.68637	-0.11123	0.447377	0.611022	protein_coding
ENSG0000(	ACSS2	3206.48	-0.10731	0.447498	0.6111	protein_coding
ENSG0000(	EEF1A1P10	52.32948	-0.09336	0.447499	0.6111	processed_pseudogene
ENSG0000(	LINC02606	275.135	-0.03761	0.447479	0.6111	lncRNA
ENSG0000(	SORT1	3211.484	-0.08547	0.447512	0.6111	protein_coding
ENSG0000(	ANKRD12	6788.526	-0.03196	0.447647	0.611153	protein_coding
ENSG0000(	GRHPR	1210.758	-0.03106	0.447638	0.611153	protein_coding
ENSG0000(	GTF2IP11	64.03428	0.054735	0.447617	0.611153	unprocessed_pseudogene
ENSG0000(	SLC1A2	1164.762	-0.04292	0.447602	0.611153	protein_coding

ENSG0000(	TRBV5-1	20.07578	-0.20864	0.447575	0.611153	TR_V_gene
ENSG0000(	AMD1	2824.549	-0.03069	0.44774	0.611211	protein_coding
ENSG0000(	ENSG0000(	10.68006	0.148255	0.447748	0.611211	lncRNA
ENSG0000(	ENSG0000(	43.93385	-0.11087	0.447738	0.611211	lncRNA
ENSG0000(	ENSG0000(	1174.711	0.059742	0.447959	0.611472	lncRNA
ENSG0000(	DCDC1	215.7166	0.041665	0.448042	0.611507	protein_coding
ENSG0000(	ENSG0000(	56.20001	-0.09373	0.448039	0.611507	lncRNA
ENSG0000(	KCNV1	649.0897	0.048055	0.448011	0.611507	protein_coding
ENSG0000(	ZKSCAN1	7719.858	0.028862	0.448134	0.611605	protein_coding
ENSG0000(	SNHG11	237.6761	0.05863	0.448315	0.611825	lncRNA
ENSG0000(	ENSG0000(	11.61986	-0.15388	0.448343	0.611837	lncRNA
ENSG0000(	OR1M1	293.7732	0.037853	0.448532	0.612069	protein_coding
ENSG0000(	ARG2	165.1314	-0.22686	0.448577	0.612077	protein_coding
ENSG0000(	ENSG0000(	15.70425	0.194924	0.448575	0.612077	TEC
ENSG0000(	DPP6	984.1947	0.111873	0.448659	0.612163	protein_coding
ENSG0000(	DDA1	613.9924	-0.04908	0.448729	0.612232	protein_coding
ENSG0000(	MRLN	227.1313	-0.04716	0.44884	0.612357	protein_coding
ENSG0000(	WDR33	4112.91	-0.02628	0.449001	0.61255	protein_coding
ENSG0000(	USP39	560.2634	-0.02919	0.449047	0.612586	protein_coding
ENSG0000(	ATP2A1-AS	8.918441	0.183491	0.449122	0.612662	lncRNA
ENSG0000(	FBXO21	986.3187	-0.03626	0.449145	0.612667	protein_coding
ENSG0000(	MRPL33	189.1647	0.060041	0.449222	0.612692	protein_coding
ENSG0000(	SFMBT1	753.2167	-0.05544	0.449193	0.612692	protein_coding
ENSG0000(	ZNF646	1354.27	-0.05354	0.449206	0.612692	protein_coding
ENSG0000(	EEF2KMT	86.59885	0.083729	0.449412	0.612923	protein_coding
ENSG0000(	RN7SL636F	10.19973	-0.16082	0.44943	0.612923	misc_RNA
ENSG0000(	SLC41A2	1980.475	-0.12894	0.449469	0.61295	protein_coding
ENSG0000(	HMGB3	284.3261	0.076946	0.449524	0.612997	protein_coding
ENSG0000(	SLC25A5-A:	64.45553	0.092513	0.449563	0.613015	lncRNA
ENSG0000(	TREML1	26.44176	0.240437	0.449576	0.613015	protein_coding
ENSG0000(	ZDHHC15	512.1876	0.03944	0.449786	0.613275	protein_coding
ENSG0000(	C4orf19	1490.175	-0.06367	0.449831	0.613302	protein_coding
ENSG0000(	GATAD1	2950.292	0.027672	0.449845	0.613302	protein_coding
ENSG0000(	ENSG0000(	168.3842	-0.05314	0.449868	0.613308	lncRNA
ENSG0000(	DISP2	1538.37	0.086607	0.45	0.613461	protein_coding
ENSG0000(	NLRP2	462.8065	0.049542	0.450029	0.613474	protein_coding
ENSG0000(	GLS2	23.7035	-0.17756	0.450062	0.613476	protein_coding
ENSG0000(	TPSD1	29.39034	-0.27882	0.45007	0.613476	protein_coding
ENSG0000(	PFKP	2217.748	-0.08552	0.450167	0.613582	protein_coding
ENSG0000(	ENSG0000(	296.1073	-0.04053	0.450239	0.613628	lncRNA
ENSG0000(	GRM7-AS2	8.403642	0.15345	0.450226	0.613628	lncRNA
ENSG0000(	ENSG0000(	63.13445	-0.06922	0.450494	0.613922	processed_pseudogene
ENSG0000(	PBX4	117.2175	0.104741	0.450483	0.613922	protein_coding
ENSG0000(	CRIM1	1816.889	0.068317	0.450666	0.614131	protein_coding
ENSG0000(	FAM98A	1826.04	0.026134	0.450697	0.614146	protein_coding
ENSG0000(	ITGB7	586.8656	-0.10236	0.450869	0.614353	protein_coding
ENSG0000(	LINC01185	162.9904	0.052483	0.450947	0.614433	lncRNA

ENSG0000(	PIGF	569.4284	0.034493	0.450967	0.614433	protein_coding
ENSG0000(	RPS12P26	119.5726	-0.0827	0.45109	0.614575	transcribed_processed_pseudoge
ENSG0000(	ENSG0000(	10.28072	-0.16465	0.451135	0.614609	processed_pseudogene
ENSG0000(	ENSG0000(	16.50024	0.192976	0.451447	0.614945	lncRNA
ENSG0000(	GBP6	434.9972	-0.03039	0.451479	0.614945	protein_coding
ENSG0000(	LINC01267	100.5326	0.094302	0.451434	0.614945	lncRNA
ENSG0000(	NIT1	1491.662	-0.06387	0.451469	0.614945	protein_coding
ENSG0000(	UROS	1094.079	-0.02519	0.45146	0.614945	protein_coding
ENSG0000(	ENSG0000(	15.90313	-0.12141	0.45161	0.615098	TEC
ENSG0000(	SNORA49	224.4625	-0.07941	0.451773	0.615293	snoRNA
ENSG0000(	CSPG4P12	1594.353	0.055338	0.451854	0.615376	transcribed_unprocessed_pseudo
ENSG0000(	RN7SKP91	9.253019	0.18684	0.452023	0.615579	misc_RNA
ENSG0000(	EIF3EP2	121.6748	0.046895	0.452097	0.615624	processed_pseudogene
ENSG0000(	MAP7D1	744.4845	0.05482	0.452114	0.615624	protein_coding
ENSG0000(	PCGF6	348.7804	-0.04622	0.452109	0.615624	protein_coding
ENSG0000(	ENSG0000(	216.6622	0.04157	0.452163	0.615664	lncRNA
ENSG0000(	ENSG0000(	358.0825	0.033437	0.452219	0.615687	lncRNA
ENSG0000(	NGF-AS1	14.07025	-0.13533	0.452207	0.615687	lncRNA
ENSG0000(	MAP2	130.2671	0.112469	0.452316	0.615766	protein_coding
ENSG0000(	MIR3127	9.01834	-0.17835	0.452316	0.615766	miRNA
ENSG0000(	LRRC1	1147.651	-0.10051	0.452381	0.615778	protein_coding
ENSG0000(	PNPT1	1819.752	0.045309	0.452362	0.615778	protein_coding
ENSG0000(	PTH2R	28.32854	0.160612	0.452384	0.615778	protein_coding
ENSG0000(	EDEM2	429.7881	-0.04814	0.45257	0.615921	protein_coding
ENSG0000(	ENSG0000(	28.5093	0.09801	0.452567	0.615921	lncRNA
ENSG0000(	ENSG0000(	224.5644	-0.03687	0.452635	0.615921	lncRNA
ENSG0000(	ENSG0000(	10.7741	-0.66156	0.452645	0.615921	protein_coding
ENSG0000(	ENSG0000(	69.72057	0.088364	0.452614	0.615921	lncRNA
ENSG0000(	GAPDHP33	10.84788	0.169838	0.452622	0.615921	processed_pseudogene
ENSG0000(	LIMCH1	787.6778	0.076684	0.452543	0.615921	protein_coding
ENSG0000(	SNX18P8	71.72886	0.163702	0.452645	0.615921	unprocessed_pseudogene
ENSG0000(	CAPS	423.5155	0.043936	0.452673	0.615925	protein_coding
ENSG0000(	ENSG0000(	171.4724	0.046837	0.452735	0.615925	lncRNA
ENSG0000(	NIPA2P4	20.79835	-0.16546	0.452746	0.615925	processed_pseudogene
ENSG0000(	PAWR	3515.454	0.047127	0.452714	0.615925	protein_coding
ENSG0000(	ZNF579	72.1818	0.089988	0.45271	0.615925	protein_coding
ENSG0000(	ENSG0000(	23.62257	-0.1299	0.452786	0.615953	lncRNA
ENSG0000(	UBE2V1	2254.716	0.02133	0.452812	0.615962	protein_coding
ENSG0000(	ENSG0000(	11.86711	0.162286	0.452985	0.616098	processed_pseudogene
ENSG0000(	ENSG0000(	57.50028	-0.06502	0.452948	0.616098	lncRNA
ENSG0000(	ZFP28	238.4613	0.083344	0.452977	0.616098	protein_coding
ENSG0000(	ZNF202	460.4539	0.060411	0.45299	0.616098	protein_coding
ENSG0000(	CCDC39	478.4559	0.038808	0.453019	0.6161	protein_coding
ENSG0000(	IFNE	49.71216	-0.13282	0.453031	0.6161	protein_coding
ENSG0000(	ENSG0000(	96.54971	0.055609	0.45315	0.616193	lncRNA
ENSG0000(	RPL21P7	26.37193	-0.25633	0.453158	0.616193	processed_pseudogene
ENSG0000(	ZNF205	111.2656	0.092802	0.453126	0.616193	protein_coding

ENSG0000(	ENSG0000(	879.0111	0.044998	0.453215	0.616244	TEC
ENSG0000(	COX7A2P1	20.00889	0.108491	0.453288	0.616316	processed_pseudogene
ENSG0000(	ENSG0000(	23.20008	0.109465	0.453415	0.61644	lncRNA
ENSG0000(	ENSG0000(	11.07608	-0.14852	0.453476	0.61644	processed_pseudogene
ENSG0000(	FGF14	125.6215	0.170934	0.453425	0.61644	protein_coding
ENSG0000(	LINC00485	116.6016	-0.05974	0.453445	0.61644	lncRNA
ENSG0000(	SMAD6	890.5604	0.070602	0.453467	0.61644	protein_coding
ENSG0000(	CARNMT1-	15.22344	-0.12561	0.453509	0.616455	lncRNA
ENSG0000(	PNN	4158.334	0.047321	0.453526	0.616455	protein_coding
ENSG0000(	ZBTB8A	1758.01	-0.02593	0.453575	0.616494	protein_coding
ENSG0000(	IKZF3	6504.49	-0.12022	0.453624	0.616534	protein_coding
ENSG0000(	ENSG0000(	61.01629	-0.08406	0.453692	0.616599	transcribed_unprocessed_pseudo
ENSG0000(	ENSG0000(	21.47117	-0.13525	0.453786	0.616701	lncRNA
ENSG0000(	ENSG0000(	170.166	0.121943	0.453889	0.616815	lncRNA
ENSG0000(	MTND1P1(	97.64106	-0.05176	0.453926	0.616838	processed_pseudogene
ENSG0000(	ENSG0000(	16.19319	0.151926	0.453952	0.616847	lncRNA
ENSG0000(	PLEKHF1	145.6833	-0.08882	0.454007	0.616895	protein_coding
ENSG0000(	ENSG0000(	161.0621	-0.04542	0.454091	0.616982	lncRNA
ENSG0000(	HSBP1L1	406.401	-0.06125	0.454203	0.617081	protein_coding
ENSG0000(	RPUSD4	814.8706	0.024625	0.454187	0.617081	protein_coding
ENSG0000(	FBLN7	321.481	0.092717	0.45464	0.617648	protein_coding
ENSG0000(	ENSG0000(	22.06293	-0.09894	0.454829	0.617879	lncRNA
ENSG0000(	LINC00461	560.6392	0.062263	0.454895	0.617941	lncRNA
ENSG0000(	ARHGAP4	1810.364	0.111258	0.455126	0.618198	protein_coding
ENSG0000(	FAR2P3	119.6251	-0.07499	0.455143	0.618198	transcribed_unprocessed_pseudo
ENSG0000(	ZFAND2A	184.3065	-0.07293	0.455116	0.618198	protein_coding
ENSG0000(	IGHE	20.74168	0.172598	0.455205	0.618253	IG_C_gene
ENSG0000(	RNF220	1250.504	-0.02074	0.455223	0.618253	protein_coding
ENSG0000(	GOLGA6L2	36.07496	0.078173	0.455244	0.618255	protein_coding
ENSG0000(	ENSG0000(	26.72684	-0.09494	0.455279	0.618277	lncRNA
ENSG0000(	GDPGP1	1069.972	0.025939	0.45535	0.618334	protein_coding
ENSG0000(	HNRNPA1P	47.47786	0.083814	0.45536	0.618334	processed_pseudogene
ENSG0000(	ABCC6P1	558.3462	-0.1503	0.455505	0.618482	transcribed_unprocessed_pseudo
ENSG0000(	RN7SL862F	9.064885	-0.16301	0.455509	0.618482	misc_RNA
ENSG0000(	ENSG0000(	176.1279	0.05538	0.455601	0.61858	lncRNA
ENSG0000(	FAM133DP	11.77887	0.267054	0.455802	0.618823	processed_pseudogene
ENSG0000(	RNU6-1237	14.50485	-0.11273	0.455819	0.618823	snRNA
ENSG0000(	ENSG0000(	136.9024	-0.04912	0.45584	0.618825	lncRNA
ENSG0000(	ZNF497-AS	43.63595	-0.102	0.455978	0.618986	lncRNA
ENSG0000(	ADCK2	206.3657	-0.07029	0.45604	0.619043	protein_coding
ENSG0000(	ENSG0000(	46.04941	0.13563	0.456252	0.619253	lncRNA
ENSG0000(	ST3GAL3	2398.088	-0.03186	0.456253	0.619253	protein_coding
ENSG0000(	TCEAL3	63.00554	0.076311	0.456243	0.619253	protein_coding
ENSG0000(	KIAA1586	278.2078	-0.06441	0.456397	0.619395	protein_coding
ENSG0000(	RN7SL76P	29.63273	-0.06787	0.456397	0.619395	misc_RNA
ENSG0000(	ENSG0000(	23.23331	0.117564	0.456424	0.619404	lncRNA
ENSG0000(	KCNC2	63.03732	-0.10215	0.456491	0.619442	protein_coding

ENSG0000(	SPNS1	870.8369	0.026975	0.45648	0.619442	protein_coding
ENSG0000(	DRC7	13.8151	0.184774	0.456512	0.619444	protein_coding
ENSG0000(	IL6ST	8362.02	-0.06682	0.456763	0.619758	protein_coding
ENSG0000(	ENSG0000(	10.63846	0.148404	0.456931	0.619959	lncRNA
ENSG0000(	ENSG0000(	10.47119	-0.18627	0.456959	0.61997	lncRNA
ENSG0000(	ENSG0000(	36.47754	-0.07976	0.456997	0.619995	lncRNA
ENSG0000(	SMIM35	95.21575	0.091949	0.457067	0.620063	protein_coding
ENSG0000(	FAM86MP	9.58281	0.162135	0.457116	0.620103	unprocessed_pseudogene
ENSG0000(	CCNY-AS1	556.5724	-0.02907	0.457232	0.620153	lncRNA
ENSG0000(	DCAF16	2539.855	-0.02806	0.457178	0.620153	protein_coding
ENSG0000(	LINC01337	8.940625	-0.15226	0.457226	0.620153	lncRNA
ENSG0000(	TRAJ11	34.0925	-0.17579	0.457197	0.620153	TR_J_gene
ENSG0000(	RPS26P18	25.56191	0.117092	0.457257	0.620161	processed_pseudogene
ENSG0000(	ANKRD13D	1105.497	0.068382	0.457346	0.620254	protein_coding
ENSG0000(	IPO11	630.2586	0.052063	0.45745	0.620369	protein_coding
ENSG0000(	PDK4	2184.662	0.208453	0.457474	0.620376	protein_coding
ENSG0000(	BAG5	1118.648	-0.027	0.457553	0.620456	protein_coding
ENSG0000(	LINC01502	132.7228	0.057288	0.457712	0.620592	lncRNA
ENSG0000(	OR4F13P	67.53599	-0.07342	0.457703	0.620592	transcribed_unprocessed_pseudo
ENSG0000(	SNORD88A	8.764883	0.157166	0.457696	0.620592	snoRNA
ENSG0000(	EXOC6	703.6628	0.048793	0.457737	0.620598	protein_coding
ENSG0000(	ATP8B4	781.2907	0.044599	0.457837	0.620622	protein_coding
ENSG0000(	HLA-T	28.80347	-0.18181	0.45783	0.620622	unprocessed_pseudogene
ENSG0000(	IGHV3-36	13.61347	0.212974	0.457853	0.620622	IG_V_pseudogene
ENSG0000(	RMI2	185.2982	0.100249	0.457802	0.620622	protein_coding
ENSG0000(	SEPHS1	549.5217	-0.04465	0.457839	0.620622	protein_coding
ENSG0000(	PREP	1444.853	-0.0522	0.45789	0.620645	protein_coding
ENSG0000(	ADRB2	170.1981	0.070124	0.457934	0.620679	protein_coding
ENSG0000(	ENSG0000(	113.5412	-0.04869	0.457983	0.620689	lncRNA
ENSG0000(	ENSG0000(	20.73658	0.103232	0.458001	0.620689	lncRNA
ENSG0000(	LMNB1	664.7129	0.094706	0.457999	0.620689	protein_coding
ENSG0000(	ENSG0000(	1848.809	0.070473	0.458052	0.620732	lncRNA
ENSG0000(	HPCA	10.25565	0.217299	0.458129	0.620756	protein_coding
ENSG0000(	LINC02525	14.2291	0.147917	0.458099	0.620756	lncRNA
ENSG0000(	RNY1P9	25.20179	0.105738	0.45811	0.620756	misc_RNA
ENSG0000(	ENSG0000(	22.3124	-0.10126	0.458157	0.620767	processed_pseudogene
ENSG0000(	ANKFY1	3238.836	0.030952	0.458236	0.620848	protein_coding
ENSG0000(	BCAR4	9.981586	0.193275	0.458308	0.620918	lncRNA
ENSG0000(	AGAP5	914.822	0.059318	0.458374	0.620981	protein_coding
ENSG0000(	CNN2	1890.875	0.071922	0.458426	0.621025	protein_coding
ENSG0000(	ENSG0000(	327.9377	0.038014	0.458448	0.621027	lncRNA
ENSG0000(	RBMX2P3	12.92647	0.138408	0.458467	0.621027	processed_pseudogene
ENSG0000(	ENSG0000(	277.304	0.075646	0.45856	0.6211	lncRNA
ENSG0000(	SWSAP1	395.1141	0.025832	0.458553	0.6211	protein_coding
ENSG0000(	CERS4	614.5618	-0.10421	0.458628	0.621112	protein_coding
ENSG0000(	CSPG5	16.04636	0.170457	0.458619	0.621112	protein_coding
ENSG0000(	ENSG0000(	994.9327	-0.04596	0.458615	0.621112	TEC

ENSG0000(	ENSG0000(	994.2085	-0.03502	0.458686	0.621163	lncRNA
ENSG0000(	HRH4	192.0611	0.049125	0.458794	0.621283	protein_coding
ENSG0000(	ENSG0000(	614.9042	-0.03655	0.458925	0.621434	lncRNA
ENSG0000(	ENSG0000(	26.14741	0.096387	0.458952	0.621443	lncRNA
ENSG0000(	F12	21.36345	0.173479	0.459001	0.621456	protein_coding
ENSG0000(	STX10	380.067	-0.04373	0.458996	0.621456	protein_coding
ENSG0000(	MUC6	765.6226	1.75803	0.459022	0.621458	protein_coding
ENSG0000(	ENSG0000(	8.680256	0.145579	0.459095	0.62153	lncRNA
ENSG0000(	FASN	1658.519	0.140212	0.459117	0.621533	protein_coding
ENSG0000(	ABI3	526.7236	-0.06222	0.459273	0.621718	protein_coding
ENSG0000(	ENSG0000(	523.6117	0.031099	0.459321	0.621756	lncRNA
ENSG0000(	ENSG0000(	109.2212	0.053877	0.459479	0.621916	lncRNA
ENSG0000(	SNORA2B	32.1316	0.09334	0.459473	0.621916	snoRNA
ENSG0000(	SRP68	1051.103	0.023189	0.459575	0.62202	protein_coding
ENSG0000(	WASHC3	395.2655	0.041174	0.459612	0.622043	protein_coding
ENSG0000(	ENSG0000(	56.14372	-0.0849	0.459639	0.622052	transcribed_processed_pseudoge
ENSG0000(	GGTA1	112.7687	-0.0825	0.45976	0.62219	protein_coding
ENSG0000(	IPO4	238.9667	0.081456	0.459793	0.622207	protein_coding
ENSG0000(	ADH1C	614.7104	-0.16756	0.459973	0.622424	protein_coding
ENSG0000(	RN7SL443F	19.26301	0.173074	0.460013	0.622451	misc_RNA
ENSG0000(	ACAT2	169.2415	-0.07097	0.460067	0.622471	protein_coding
ENSG0000(	ACSL6	558.1206	0.049462	0.460059	0.622471	protein_coding
ENSG0000(	ISG15	91.07539	0.169116	0.460098	0.622487	protein_coding
ENSG0000(	MSI1	189.0007	0.087498	0.460127	0.622499	protein_coding
ENSG0000(	RN7SL674F	372.696	-0.11788	0.460154	0.62251	misc_RNA
ENSG0000(	ZNF221	314.8892	-0.039	0.460194	0.622537	protein_coding
ENSG0000(	ENSG0000(	94.3351	-0.06649	0.460253	0.622589	lncRNA
ENSG0000(	TAS2R6P	60.59787	-0.07505	0.460548	0.622962	processed_pseudogene
ENSG0000(	ENSG0000(	16.18111	-0.14546	0.460663	0.62309	lncRNA
ENSG0000(	SAMD3	257.4973	-0.06993	0.460754	0.623186	protein_coding
ENSG0000(	RNVU1-3	10.46734	0.15902	0.460775	0.623189	snRNA
ENSG0000(	LIX1-AS1	63.98619	0.180509	0.460882	0.623307	lncRNA
ENSG0000(	ENSG0000(	121.0746	-0.07485	0.460929	0.623344	lncRNA
ENSG0000(	MANBA	1163.164	0.074128	0.460988	0.623397	protein_coding
ENSG0000(	CHRM1	19.13898	0.202342	0.461029	0.623399	protein_coding
ENSG0000(	ENSG0000(	88.04185	0.055181	0.461022	0.623399	lncRNA
ENSG0000(	ENSG0000(	18.61364	-0.18363	0.461077	0.623436	lncRNA
ENSG0000(	ENSG0000(	44.66918	0.093362	0.461118	0.623439	processed_pseudogene
ENSG0000(	LEPROTL1	1180.326	-0.05524	0.461115	0.623439	protein_coding
ENSG0000(	ENSG0000(	58.13548	0.075164	0.461145	0.623448	lncRNA
ENSG0000(	DHX15	4103.514	-0.0203	0.461219	0.623495	protein_coding
ENSG0000(	SLX1B	89.52327	-0.33681	0.461218	0.623495	protein_coding
ENSG0000(	ENSG0000(	5466.569	-0.03423	0.461387	0.623684	lncRNA
ENSG0000(	LINC02599	39.07691	-0.14652	0.461398	0.623684	lncRNA
ENSG0000(	LINC01441	192.0594	-0.05458	0.461476	0.623761	lncRNA
ENSG0000(	BRINP3	581.2438	-0.14246	0.461641	0.623872	protein_coding
ENSG0000(	CHTF18	300.8291	0.092535	0.461643	0.623872	protein_coding

ENSG0000(	CPSF4	492.4331	0.037541	0.461664	0.623872	protein_coding
ENSG0000(	ENSG0000(	28.01444	0.117842	0.461589	0.623872	lncRNA
ENSG0000(	MAST4-AS1	11.11809	0.157482	0.461616	0.623872	lncRNA
ENSG0000(	TRAV14DV4	11.65816	-0.21364	0.461676	0.623872	TR_V_gene
ENSG0000(	RN7SL277P	32.05625	0.114895	0.461703	0.623882	misc_RNA
ENSG0000(	RMND5B	1227.863	0.025902	0.461742	0.623907	protein_coding
ENSG0000(	MAPK8IP2	169.7577	0.046916	0.461774	0.623924	protein_coding
ENSG0000(	CAPRIN2	1620.627	-0.03429	0.461897	0.624061	protein_coding
ENSG0000(	ENSG0000(	27.32768	-0.11368	0.461915	0.624061	lncRNA
ENSG0000(	BRWD1-AS	44.17291	-0.07135	0.46202	0.624176	lncRNA
ENSG0000(	MIR4312	18.38384	0.111189	0.4621	0.624257	miRNA
ENSG0000(	BLOC1S3	374.9129	-0.03708	0.462168	0.624299	protein_coding
ENSG0000(	ENSG0000(	29.19002	-0.08193	0.462171	0.624299	lncRNA
ENSG0000(	HSPA8P14	14.7953	0.115293	0.462208	0.624323	processed_pseudogene
ENSG0000(	SPNS2	498.2323	0.091044	0.462232	0.624329	protein_coding
ENSG0000(	XKR7	12.21439	0.146497	0.462254	0.624331	protein_coding
ENSG0000(	HMGB2	429.688	-0.08668	0.462383	0.624452	protein_coding
ENSG0000(	LINC01801	45.94621	0.141332	0.462364	0.624452	lncRNA
ENSG0000(	ENSG0000(	10.79303	0.319512	0.462406	0.624456	unprocessed_pseudogene
ENSG0000(	ALKBH4	332.9608	-0.03511	0.462466	0.624457	protein_coding
ENSG0000(	ENSG0000(	272.5055	-0.04913	0.462454	0.624457	lncRNA
ENSG0000(	GSTCD-AS1	11.7794	-0.1729	0.462434	0.624457	lncRNA
ENSG0000(	RN7SKP127	13.73641	-0.26831	0.462498	0.624474	misc_RNA
ENSG0000(	WNT7A	59.10164	-0.11701	0.462669	0.624678	protein_coding
ENSG0000(	ENSG0000(	44.02509	0.06873	0.462838	0.624879	lncRNA
ENSG0000(	ENSG0000(	367.1916	-0.03327	0.462903	0.62494	lncRNA
ENSG0000(	ALDH4A1	88.43109	0.096938	0.463059	0.625124	protein_coding
ENSG0000(	EIPR1	401.2904	-0.05481	0.463139	0.625158	protein_coding
ENSG0000(	ENSG0000(	15.18353	0.128516	0.463144	0.625158	lncRNA
ENSG0000(	USP44	67.59765	-0.18829	0.463144	0.625158	protein_coding
ENSG0000(	ENSG0000(	54.49627	-0.09578	0.463205	0.625166	lncRNA
ENSG0000(	LINC01304	244.4939	-0.05417	0.463209	0.625166	lncRNA
ENSG0000(	LINC02544	11.39214	-0.11832	0.463185	0.625166	lncRNA
ENSG0000(	ENSG0000(	9.173626	-0.16811	0.463255	0.625201	lncRNA
ENSG0000(	ENSG0000(	11.8988	-0.14708	0.463386	0.625325	TEC
ENSG0000(	PIP5K1B	1814.155	-0.09797	0.463369	0.625325	protein_coding
ENSG0000(	SNORD3B-1	112.8434	0.185818	0.463499	0.62545	snoRNA
ENSG0000(	CUEDC2	101.2335	0.075626	0.463616	0.625474	protein_coding
ENSG0000(	ENSG0000(	11.70886	0.166536	0.463602	0.625474	lncRNA
ENSG0000(	KRBA1	189.9508	-0.09609	0.463571	0.625474	protein_coding
ENSG0000(	WASH8P	426.2673	0.137496	0.46359	0.625474	unprocessed_pseudogene
ENSG0000(	ZNF174	155.0812	-0.04283	0.46359	0.625474	protein_coding
ENSG0000(	MTRNR2L1	13.52987	0.155628	0.463667	0.625514	protein_coding
ENSG0000(	TRIM54	11.8421	-0.17146	0.463685	0.625514	protein_coding
ENSG0000(	LYRM2	1301.778	-0.02346	0.463706	0.625515	protein_coding
ENSG0000(	ENSG0000(	14.78261	0.110586	0.463777	0.625522	lncRNA
ENSG0000(	KDM3A	3061.242	-0.05814	0.463788	0.625522	protein_coding

ENSG0000(	LINC02186	13.45883	0.131407	0.463738	0.625522	lncRNA
ENSG0000(	VSX1	25.3956	0.090931	0.463791	0.625522	protein_coding
ENSG0000(	SKAP1	360.0129	-0.11805	0.463835	0.625555	protein_coding
ENSG0000(	EFCAB14	9228.023	-0.03527	0.463862	0.625561	protein_coding
ENSG0000(	ZFP90	1129.928	0.076778	0.463879	0.625561	protein_coding
ENSG0000(	TASOR2	3963.426	-0.04137	0.463982	0.625673	protein_coding
ENSG0000(	RN7SL851F	10.76544	-0.12892	0.464014	0.625689	misc_RNA
ENSG0000(	ENSG0000(	19.3453	0.104612	0.464104	0.625784	lncRNA
ENSG0000(	ENSG0000(	166.6962	-0.07474	0.464169	0.625844	lncRNA
ENSG0000(	DEFA6	3077.16	-0.21617	0.464201	0.625861	protein_coding
ENSG0000(	BRWD1-IT1	72.62261	-0.05668	0.464282	0.625916	lncRNA
ENSG0000(	LINC00871	15.12409	0.122225	0.464273	0.625916	lncRNA
ENSG0000(	LDLRAD4	10170.06	-0.03923	0.464303	0.625918	protein_coding
ENSG0000(	ZNHIT2	33.44389	0.093928	0.464323	0.625918	protein_coding
ENSG0000(	DYNLT2B	54.88199	-0.10017	0.464434	0.625978	protein_coding
ENSG0000(	ENSG0000(	41.72578	0.082111	0.464402	0.625978	lncRNA
ENSG0000(	FAM8A1	1537.393	0.065624	0.464435	0.625978	protein_coding
ENSG0000(	RABGGTB	2094.031	0.036241	0.464447	0.625978	protein_coding
ENSG0000(	LINC02619	31.81718	0.085647	0.464484	0.626001	lncRNA
ENSG0000(	ENSG0000(	54.7584	-0.14393	0.464592	0.62612	lncRNA
ENSG0000(	KDM2B-DT	18.06146	0.172189	0.464648	0.626168	lncRNA
ENSG0000(	OXCT2	56.65793	-0.15581	0.464806	0.626355	protein_coding
ENSG0000(	P2RX1	671.9563	0.070506	0.464873	0.626418	protein_coding
ENSG0000(	RBM26	3450.747	0.029184	0.464982	0.626539	protein_coding
ENSG0000(	ARHGAP33	326.4071	-0.08492	0.465068	0.626628	protein_coding
ENSG0000(	LINC00662	3343.799	0.030183	0.465139	0.626697	lncRNA
ENSG0000(	ENSG0000(	23.6153	0.121027	0.465221	0.62678	processed_pseudogene
ENSG0000(	ENSG0000(	57.25494	0.121157	0.46535	0.626786	lncRNA
ENSG0000(	ENSG0000(	48.92533	-0.06533	0.465384	0.626786	lncRNA
ENSG0000(	ENSG0000(	8.118248	-0.15694	0.465381	0.626786	processed_pseudogene
ENSG0000(	ENSG0000(	148.0611	0.046463	0.465269	0.626786	TEC
ENSG0000(	GUSBP2	1415.405	-0.04091	0.465338	0.626786	transcribed_unprocessed_pseudo
ENSG0000(	MPHOSPH9	1897.253	0.04758	0.465328	0.626786	protein_coding
ENSG0000(	RN7SKP35	12.13983	-0.19348	0.465301	0.626786	misc_RNA
ENSG0000(	TMEM184(	110.4298	-0.0483	0.465311	0.626786	lncRNA
ENSG0000(	LENG9	147.6296	0.084941	0.465439	0.626807	protein_coding
ENSG0000(	TAF9	1543.155	0.017746	0.46544	0.626807	protein_coding
ENSG0000(	SPTBN4	62.39315	0.107376	0.465477	0.62683	protein_coding
ENSG0000(	ARGFX	1205.373	-0.02402	0.465641	0.627024	protein_coding
ENSG0000(	ULK1	1126.712	-0.04649	0.465697	0.627073	protein_coding
ENSG0000(	ENSG0000(	163.9894	-0.05256	0.465823	0.627215	lncRNA
ENSG0000(	ENSG0000(	25.85409	0.097743	0.465869	0.627224	lncRNA
ENSG0000(	LY6G5B	985.187	-0.0353	0.465851	0.627224	protein_coding
ENSG0000(	ENSG0000(	9.932553	0.141283	0.465981	0.627321	lncRNA
ENSG0000(	SNHG27	131.5021	0.084763	0.465977	0.627321	lncRNA
ENSG0000(	HLTF-AS1	35.35978	0.107327	0.466458	0.627937	lncRNA
ENSG0000(	ENSG0000(	578.5938	0.031394	0.466589	0.62802	lncRNA

ENSG0000(	ENSG0000(	297.5803	0.054542	0.466549	0.62802	lncRNA
ENSG0000(	NONO	4119.729	0.032229	0.466568	0.62802	protein_coding
ENSG0000(	TMEM221	48.55319	0.0734	0.466599	0.62802	protein_coding
ENSG0000(	ENSG0000(	122.1803	0.275347	0.466812	0.628265	protein_coding
ENSG0000(	PI4K2A	485.9044	0.043568	0.466821	0.628265	protein_coding
ENSG0000(	ENSG0000(	261.4705	-0.03865	0.466935	0.628391	lncRNA
ENSG0000(	CHMP1B2F	236.7473	-0.1072	0.467063	0.628509	transcribed_unitary_pseudogene
ENSG0000(	RAB33B-AS	2548.385	0.030359	0.467049	0.628509	lncRNA
ENSG0000(	GYG2P1	221.2858	-0.08702	0.467205	0.628674	transcribed_unprocessed_pseudo
ENSG0000(	VDAC3	879.4728	-0.03546	0.467268	0.628731	protein_coding
ENSG0000(	ZSCAN31	154.2467	-0.08779	0.467459	0.628961	protein_coding
ENSG0000(	NCBP2	1108.27	-0.03219	0.467572	0.629086	protein_coding
ENSG0000(	ENSG0000(	78.04144	-0.0805	0.467616	0.62912	processed_pseudogene
ENSG0000(	CFAP418	256.8247	-0.04795	0.467681	0.62918	protein_coding
ENSG0000(	ARHGEF37	592.9536	-0.08748	0.467904	0.629346	protein_coding
ENSG0000(	CA10	15.50162	-0.27551	0.467828	0.629346	protein_coding
ENSG0000(	ENSG0000(	31.11734	-0.09679	0.467898	0.629346	lncRNA
ENSG0000(	LINC02321	101.1994	0.076319	0.467888	0.629346	lncRNA
ENSG0000(	TIMM8B	153.4764	-0.04951	0.467852	0.629346	protein_coding
ENSG0000(	BFSP2-AS1	52.93183	0.113249	0.467999	0.629411	lncRNA
ENSG0000(	ENSG0000(	347.1651	0.103058	0.468013	0.629411	lncRNA
ENSG0000(	LINC00967	118.009	-0.12203	0.46801	0.629411	lncRNA
ENSG0000(	EXOC3L2	397.3122	0.042346	0.468059	0.629446	protein_coding
ENSG0000(	NUDT2	130.2116	-0.05172	0.468195	0.629602	protein_coding
ENSG0000(	DBF4	347.5946	-0.05882	0.468278	0.629687	protein_coding
ENSG0000(	ENSG0000(	113.8111	-0.03634	0.468328	0.629728	lncRNA
ENSG0000(	DRICH1	48.52016	-0.10953	0.468389	0.629783	protein_coding
ENSG0000(	CHEK1	392.4862	-0.04212	0.468464	0.62983	protein_coding
ENSG0000(	ENSG0000(	18.71847	-0.11138	0.468464	0.62983	lncRNA
ENSG0000(	PCDHGB1	37.96934	-0.1106	0.468504	0.629856	protein_coding
ENSG0000(	PGM2	398.3047	0.056302	0.46855	0.629892	protein_coding
ENSG0000(	RNU4-24P	45.32018	0.089512	0.468631	0.629973	snRNA
ENSG0000(	ZNF486	561.3822	0.05446	0.46866	0.629986	protein_coding
ENSG0000(	ENSG0000(	9.688246	-0.15764	0.468714	0.630031	lncRNA
ENSG0000(	ENSG0000(	12.86416	0.144509	0.468738	0.630037	lncRNA
ENSG0000(	ATP5MC1P	10.35521	0.195677	0.468937	0.630271	processed_pseudogene
ENSG0000(	SNORA79B	208.9413	-0.06441	0.468952	0.630271	snoRNA
ENSG0000(	PPIAL4E	11.87345	-0.51793	0.469092	0.630431	protein_coding
ENSG0000(	ENSG0000(	321.5689	0.04607	0.469171	0.630511	lncRNA
ENSG0000(	ENSG0000(	845.0908	0.023482	0.469196	0.630515	lncRNA
ENSG0000(	TTLL1-AS1	234.3307	0.033663	0.469214	0.630515	lncRNA
ENSG0000(	MIR661	10.58451	0.177731	0.469515	0.630893	miRNA
ENSG0000(	FAM118B	448.693	0.054728	0.469762	0.631193	protein_coding
ENSG0000(	RN7SL397F	33.50652	0.067423	0.469779	0.631193	misc_RNA
ENSG0000(	TMEM191(	224.8273	-0.04628	0.469866	0.631284	protein_coding
ENSG0000(	MLLT3	1082.764	-0.05269	0.469899	0.631302	protein_coding
ENSG0000(	TRNT1	948.366	0.034018	0.470033	0.631454	protein_coding

ENSG0000(	NAF1	336.3737	0.04885	0.470082	0.631494	protein_coding
ENSG0000(	SEH1L	1619.264	-0.03039	0.470192	0.631613	protein_coding
ENSG0000(	ENSG0000(	11.77792	0.111254	0.470249	0.631664	lncRNA
ENSG0000(	CTCF-DT	93.76867	-0.12835	0.470392	0.631805	lncRNA
ENSG0000(	LINC02269	90.12124	0.064379	0.470395	0.631805	lncRNA
ENSG0000(	SIGLEC15	219.5086	-0.05693	0.470477	0.631889	protein_coding
ENSG0000(	ACSM2B	391.968	0.062931	0.470557	0.63197	protein_coding
ENSG0000(	MTND5P15	20.11485	0.101027	0.470585	0.63198	processed_pseudogene
ENSG0000(	ENSG0000(	86.29514	0.190842	0.47061	0.631987	lncRNA
ENSG0000(	DR1	3280.599	-0.02443	0.470658	0.632008	protein_coding
ENSG0000(	GNB1L	209.6979	-0.08919	0.470666	0.632008	protein_coding
ENSG0000(	CHURC1	2171.476	-0.03043	0.470805	0.632058	protein_coding
ENSG0000(	ENSG0000(	46.36494	0.117654	0.470963	0.632058	processed_pseudogene
ENSG0000(	ENSG0000(	3099.563	0.04166	0.470908	0.632058	lncRNA
ENSG0000(	ENSG0000(	528.7051	0.028258	0.470979	0.632058	lncRNA
ENSG0000(	FAM153B	282.2595	0.079825	0.470918	0.632058	protein_coding
ENSG0000(	FLOT1	959.2971	-0.04698	0.470892	0.632058	protein_coding
ENSG0000(	GK	2993.588	0.138817	0.470895	0.632058	protein_coding
ENSG0000(	MIR548AZ	12.42004	0.151756	0.470963	0.632058	miRNA
ENSG0000(	OR8B9P	608.097	0.032443	0.470739	0.632058	transcribed_unprocessed_pseudo
ENSG0000(	PHKG1	298.8878	0.046057	0.470813	0.632058	protein_coding
ENSG0000(	SPC25	558.4531	-0.04436	0.470953	0.632058	protein_coding
ENSG0000(	SPRYD3	509.6957	-0.03739	0.470984	0.632058	protein_coding
ENSG0000(	THAP1	142.1792	-0.04862	0.470848	0.632058	protein_coding
ENSG0000(	ZNF391	132.0395	0.123279	0.470801	0.632058	protein_coding
ENSG0000(	COMMD9	621.7425	0.04347	0.471078	0.632157	protein_coding
ENSG0000(	RN7SKP193	19.81164	0.104125	0.471126	0.632195	misc_RNA
ENSG0000(	ALKBH2	89.38317	0.077179	0.47139	0.632522	protein_coding
ENSG0000(	PNPLA5	66.97392	0.062393	0.471576	0.632744	protein_coding
ENSG0000(	RNVU1-31	1588.611	0.062398	0.47173	0.632925	snRNA
ENSG0000(	FAM241A	29940.83	-0.03841	0.471826	0.632964	protein_coding
ENSG0000(	PDCL3P2	26.65133	-0.11463	0.47186	0.632964	processed_pseudogene
ENSG0000(	PLS3-AS1	148.5193	-0.04422	0.471813	0.632964	lncRNA
ENSG0000(	RNGTT	822.4047	0.036132	0.471783	0.632964	protein_coding
ENSG0000(	UBXN2B	961.0075	0.038114	0.471859	0.632964	protein_coding
ENSG0000(	PCDHGA6	649.5185	-0.03046	0.471885	0.63297	protein_coding
ENSG0000(	IQGAP1	5880.996	0.031225	0.47192	0.632991	protein_coding
ENSG0000(	PDCD10	527.9295	-0.0429	0.471948	0.633001	protein_coding
ENSG0000(	ESRG	448.4292	0.061591	0.47201	0.633055	lncRNA
ENSG0000(	S100A1	11.88354	0.195433	0.472029	0.633055	protein_coding
ENSG0000(	FTCD	15.94892	-0.18855	0.472108	0.633133	protein_coding
ENSG0000(	PSMA3	652.7998	0.0289	0.472127	0.633133	protein_coding
ENSG0000(	SIAH3	118.7791	-0.05008	0.472161	0.633151	protein_coding
ENSG0000(	PRDM2	3723.805	-0.03599	0.472201	0.633178	protein_coding
ENSG0000(	CDH15	121.2442	0.051737	0.472236	0.633198	protein_coding
ENSG0000(	RELA	1030.054	0.037144	0.472369	0.63335	protein_coding
ENSG0000(	BRD3	910.0748	-0.04361	0.472397	0.63336	protein_coding

ENSG0000(	COA7	1048.151	0.018855	0.472764	0.633685	protein_coding
ENSG0000(	ENSG0000(	66.00524	0.059685	0.472779	0.633685	lncRNA
ENSG0000(	ENSG0000(	25.0482	-0.16797	0.472685	0.633685	lncRNA
ENSG0000(	ENSG0000(	29.2065	0.099624	0.472766	0.633685	lncRNA
ENSG0000(	ENSG0000(	169.6134	0.137266	0.472677	0.633685	lncRNA
ENSG0000(	ENSG0000(	61.97538	-0.06978	0.472755	0.633685	lncRNA
ENSG0000(	SON	17246.93	0.018532	0.472753	0.633685	protein_coding
ENSG0000(	ELOVL2-AS	222.6174	-0.07094	0.472975	0.633893	lncRNA
ENSG0000(	ENSG0000(	23.92601	-0.1675	0.472971	0.633893	lncRNA
ENSG0000(	GEMIN7	214.6172	0.039175	0.473044	0.633958	protein_coding
ENSG0000(	C12orf56	16.05829	-0.17115	0.473283	0.634198	protein_coding
ENSG0000(	C3orf20	33.06601	0.137218	0.473253	0.634198	protein_coding
ENSG0000(	RNU6-610F	27.18418	-0.14045	0.47328	0.634198	snRNA
ENSG0000(	ENSG0000(	14.28779	0.154615	0.473563	0.634546	lncRNA
ENSG0000(	RASAL3	993.7005	-0.12759	0.473649	0.634634	protein_coding
ENSG0000(	MKI67	3111.504	0.092837	0.473719	0.634701	protein_coding
ENSG0000(	LINC02251	59.57926	-0.15292	0.473763	0.634733	lncRNA
ENSG0000(	LPO	209.7477	-0.05403	0.473853	0.634826	protein_coding
ENSG0000(	CSNK1G2	1124.688	0.044363	0.473926	0.634897	protein_coding
ENSG0000(	ENSG0000(	70.3959	0.073637	0.474076	0.635071	lncRNA
ENSG0000(	ELAVL4	38.92261	0.101288	0.474141	0.635132	protein_coding
ENSG0000(	LINC00114	108.3319	-0.05592	0.474198	0.63518	lncRNA
ENSG0000(	ENSG0000(	26.50452	0.100829	0.474246	0.635217	lncRNA
ENSG0000(	ENSG0000(	155.7482	-0.05334	0.474313	0.635281	lncRNA
ENSG0000(	GAB2	1223.715	0.05285	0.474481	0.635425	protein_coding
ENSG0000(	RN7SL430F	50.58901	-0.07007	0.474472	0.635425	misc_RNA
ENSG0000(	TMEM37	1420.003	-0.16609	0.474469	0.635425	protein_coding
ENSG0000(	NSF	1251.413	-0.06165	0.474563	0.635507	protein_coding
ENSG0000(	THSD7B	21.97261	-0.21187	0.474642	0.635586	protein_coding
ENSG0000(	CRB2	109.3396	-0.17469	0.474681	0.635611	protein_coding
ENSG0000(	SDCCAG8	1336.7	-0.02494	0.474757	0.635686	protein_coding
ENSG0000(	TYW5	2551.596	0.032591	0.474897	0.635846	protein_coding
ENSG0000(	WDR90	1250.124	0.031816	0.475043	0.636015	protein_coding
ENSG0000(	GAPDHP47	28.32786	-0.13559	0.475138	0.636061	processed_pseudogene
ENSG0000(	PIGP	461.8855	-0.04515	0.475117	0.636061	protein_coding
ENSG0000(	RNU6-1099	17.77085	0.121682	0.475122	0.636061	snRNA
ENSG0000(	NT5M	30.29169	0.108785	0.475197	0.636113	protein_coding
ENSG0000(	ENSG0000(	112.8731	0.056068	0.475288	0.636155	lncRNA
ENSG0000(	METTL24	359.4204	0.051429	0.475289	0.636155	protein_coding
ENSG0000(	MYO5A	5508.544	0.062807	0.475286	0.636155	protein_coding
ENSG0000(	TMPRSS4	2854.063	-0.12058	0.475381	0.636252	protein_coding
ENSG0000(	EIF5AL1	108.6589	0.106806	0.475507	0.636393	protein_coding
ENSG0000(	SLFNL1-AS1	828.8026	0.063454	0.475569	0.636449	lncRNA
ENSG0000(	ENSG0000(	4564.024	0.034473	0.475614	0.636456	lncRNA
ENSG0000(	FUCA2	916.9313	-0.03899	0.475615	0.636456	protein_coding
ENSG0000(	TTLL10	121.3174	-0.06184	0.475645	0.636469	protein_coding
ENSG0000(	TIA1	3528.101	-0.02863	0.475667	0.636472	protein_coding

ENSG0000(	ENSG0000(	55.97082	0.063582	0.475837	0.636673	lncRNA
ENSG0000(	MAP1LC3B	996.2105	-0.04447	0.475915	0.636749	protein_coding
ENSG0000(	H1-0	1310.769	-0.10385	0.475951	0.636771	protein_coding
ENSG0000(	PPT2	357.764	0.064223	0.475974	0.636775	protein_coding
ENSG0000(	RASD1	125.188	0.143484	0.476065	0.636869	protein_coding
ENSG0000(	RCOR2	20.55813	0.138415	0.476219	0.637048	protein_coding
ENSG0000(	TIMP3	1071.239	0.107873	0.476331	0.63717	protein_coding
ENSG0000(	SETD2	7365.356	-0.01602	0.476362	0.637185	protein_coding
ENSG0000(	PAQR5-DT	256.5251	-0.04049	0.476413	0.637226	lncRNA
ENSG0000(	ENSG0000(	28.52886	-0.08809	0.476436	0.63723	lncRNA
ENSG0000(	ENSG0000(	13.8179	0.140677	0.47653	0.637329	lncRNA
ENSG0000(	AJUBA-DT	12.50281	0.154431	0.476606	0.637403	lncRNA
ENSG0000(	NUP210	3186.2	-0.09559	0.476648	0.637432	protein_coding
ENSG0000(	APBB1IP	784.7178	0.127012	0.47667	0.637435	protein_coding
ENSG0000(	ENSG0000(	146.7894	0.041536	0.476739	0.637499	TEC
ENSG0000(	LINC02808	23.38304	-0.11601	0.476761	0.637502	lncRNA
ENSG0000(	RGS7	883.6315	0.023817	0.476792	0.637517	protein_coding
ENSG0000(	HSPA1A	551.4832	-0.10917	0.476943	0.637664	protein_coding
ENSG0000(	ISYNA1	142.3703	0.093086	0.476923	0.637664	protein_coding
ENSG0000(	EEF1A1P19	36.51035	-0.09807	0.477054	0.637759	processed_pseudogene
ENSG0000(	MTND5P5	93.54952	0.25487	0.477036	0.637759	processed_pseudogene
ENSG0000(	RNASEH1-/	544.2496	-0.03395	0.477111	0.637781	lncRNA
ENSG0000(	ZNF280C	331.2018	-0.05037	0.477106	0.637781	protein_coding
ENSG0000(	ENSG0000(	72.4512	-0.07048	0.477173	0.637809	TEC
ENSG0000(	FBXO36-IT1	18.16829	0.116889	0.477158	0.637809	lncRNA
ENSG0000(	WDR47	322.0137	-0.05082	0.477231	0.637833	protein_coding
ENSG0000(	ZNF274	1795.686	0.033158	0.477229	0.637833	protein_coding
ENSG0000(	FCRL2	2088.611	-0.14823	0.477254	0.637836	protein_coding
ENSG0000(	TBC1D25	333.0929	0.047568	0.477333	0.637916	protein_coding
ENSG0000(	SNX17	844.2139	-0.02777	0.477408	0.637989	protein_coding
ENSG0000(	ENSG0000(	19.36232	-0.10392	0.477489	0.63807	unprocessed_pseudogene
ENSG0000(	ENSG0000(	35.20957	-0.22335	0.477588	0.638175	processed_pseudogene
ENSG0000(	CRHR2	16.69816	0.191419	0.47763	0.638203	protein_coding
ENSG0000(	ENSG0000(	420.473	-0.09514	0.477651	0.638206	lncRNA
ENSG0000(	SKIL	4040.214	-0.05368	0.477677	0.638213	protein_coding
ENSG0000(	SERPINB11	18.60494	0.123604	0.47772	0.638242	protein_coding
ENSG0000(	ENSG0000(	20.77859	-0.09106	0.477934	0.638502	lncRNA
ENSG0000(	RN7SKP95	122.6305	-0.05842	0.477982	0.638539	misc_RNA
ENSG0000(	RIOX1	246.0615	0.049462	0.478025	0.638569	protein_coding
ENSG0000(	GPR55	122.3734	-0.08351	0.478097	0.638639	protein_coding
ENSG0000(	ENSG0000(	251.1075	-0.04034	0.47815	0.638639	lncRNA
ENSG0000(	MIDN	2245.9	-0.06821	0.478158	0.638639	protein_coding
ENSG0000(	VPS13D	9454.676	0.025597	0.478139	0.638639	protein_coding
ENSG0000(	CEP83	726.4902	0.049227	0.478265	0.638714	protein_coding
ENSG0000(	ENSG0000(	13.45173	0.107446	0.478275	0.638714	lncRNA
ENSG0000(	NCAM1	627.8471	-0.07263	0.478246	0.638714	protein_coding
ENSG0000(	LINC01047	35.09527	-0.08472	0.478307	0.63873	lncRNA

ENSG0000(	CAMTA1-D	10.47445	-0.12586	0.478344	0.638745	lncRNA
ENSG0000(	ENSG0000(	8.52456	0.147463	0.478413	0.638745	processed_pseudogene
ENSG0000(	ENSG0000(	8.715021	-0.16858	0.478394	0.638745	lncRNA
ENSG0000(	GALE	575.9371	-0.0842	0.47842	0.638745	protein_coding
ENSG0000(	KPTN	72.83896	-0.06785	0.478367	0.638745	protein_coding
ENSG0000(	GALNT5	1804.653	0.11495	0.478459	0.63877	protein_coding
ENSG0000(	ESCO1	1506.255	-0.03238	0.478533	0.638841	protein_coding
ENSG0000(	ANKRD36B	2262.339	0.045749	0.478661	0.638908	protein_coding
ENSG0000(	CSTL1	97.71562	0.059335	0.478724	0.638908	protein_coding
ENSG0000(	ENSG0000(	339.7583	-0.04033	0.478658	0.638908	protein_coding
ENSG0000(	ENSG0000(	1033.278	-0.04957	0.478696	0.638908	lncRNA
ENSG0000(	ENSG0000(	11.10685	0.133675	0.478658	0.638908	lncRNA
ENSG0000(	MUSK	565.4254	-0.11942	0.47872	0.638908	protein_coding
ENSG0000(	TMEM121	13.44029	0.212378	0.478679	0.638908	protein_coding
ENSG0000(	ENSG0000(	20.27093	-0.11526	0.478766	0.638936	lncRNA
ENSG0000(	ENSG0000(	249.9748	-0.03761	0.478822	0.638983	lncRNA
ENSG0000(	ENSG0000(	43.14381	0.168974	0.47891	0.639073	lncRNA
ENSG0000(	ENSG0000(	21.63362	0.103399	0.478976	0.639105	lncRNA
ENSG0000(	NAA10	413.4916	-0.03919	0.479014	0.639105	protein_coding
ENSG0000(	RAB38	41.87425	0.08192	0.478961	0.639105	protein_coding
ENSG0000(	ZNF662	1271.829	0.036258	0.479012	0.639105	protein_coding
ENSG0000(	CCDC183-A	205.4553	-0.05097	0.479058	0.63912	lncRNA
ENSG0000(	ENSG0000(	66.96269	0.05146	0.479095	0.63912	lncRNA
ENSG0000(	GIPC3	114.8427	0.08647	0.479096	0.63912	protein_coding
ENSG0000(	UMAD1	542.3216	-0.05179	0.479107	0.63912	protein_coding
ENSG0000(	BIRC6-AS2	28.88284	-0.09027	0.479175	0.639184	transcribed_unprocessed_pseudo
ENSG0000(	ZFY	241.676	-0.20984	0.479218	0.639215	protein_coding
ENSG0000(	ESPNL	57.83535	0.166447	0.479245	0.639216	protein_coding
ENSG0000(	SMAD1	990.9542	0.044323	0.47926	0.639216	protein_coding
ENSG0000(	ENSG0000(	180.3479	-0.29629	0.479325	0.639227	transcribed_processed_pseudoge
ENSG0000(	ENSG0000(	176.1205	0.042679	0.479319	0.639227	TEC
ENSG0000(	LRRK1	5800.489	0.048361	0.479329	0.639227	protein_coding
ENSG0000(	AKAP8L	2032.606	-0.02495	0.479459	0.639353	protein_coding
ENSG0000(	ENSG0000(	138.8409	-0.06041	0.479464	0.639353	lncRNA
ENSG0000(	ENSG0000(	163.5942	0.046049	0.47953	0.639414	lncRNA
ENSG0000(	ENSG0000(	90.35904	0.048529	0.479624	0.639466	lncRNA
ENSG0000(	SCARNA21	851.0488	-0.09413	0.479628	0.639466	scaRNA
ENSG0000(	TRAPPC12	2606.369	-0.02225	0.47963	0.639466	protein_coding
ENSG0000(	ENSG0000(	19.47199	-0.10808	0.479669	0.639491	lncRNA
ENSG0000(	CAPN11	14.97901	0.174677	0.479772	0.639602	protein_coding
ENSG0000(	AXDND1	46.1701	0.174679	0.47983	0.639652	protein_coding
ENSG0000(	EYS	961.7711	-0.03179	0.479891	0.639705	protein_coding
ENSG0000(	TRAV8-2	13.83056	0.172312	0.479955	0.639764	TR_V_gene
ENSG0000(	TRAJ20	17.29782	0.16993	0.479984	0.639776	TR_J_gene
ENSG0000(	LINC00924	155.1392	0.064876	0.480018	0.639781	lncRNA
ENSG0000(	SCYL2	1653.235	-0.04725	0.480039	0.639781	protein_coding
ENSG0000(	WDPCP	2957.596	0.037276	0.480049	0.639781	protein_coding

ENSG0000(	FAM78A	946.3014	0.105225	0.480118	0.639846	protein_coding
ENSG0000(	RN7SL677F	46.37272	0.079624	0.480179	0.6399	misc_RNA
ENSG0000(	ENSG0000(	119.867	0.056356	0.480213	0.639918	lncRNA
ENSG0000(	MVP	4936.804	-0.06144	0.480283	0.639984	protein_coding
ENSG0000(	CHCHD6	69.8824	-0.07931	0.480351	0.640022	protein_coding
ENSG0000(	ENSG0000(	10.72796	-0.15669	0.480333	0.640022	lncRNA
ENSG0000(	PDCL	410.8549	-0.04001	0.480375	0.640026	protein_coding
ENSG0000(	LINC01405	392.7589	0.036056	0.480423	0.640063	lncRNA
ENSG0000(	LINC01392	9.409583	0.143382	0.480493	0.640111	lncRNA
ENSG0000(	LINC02257	21.51929	0.097905	0.48052	0.640111	lncRNA
ENSG0000(	SCARNA6	688.3295	-0.06995	0.480506	0.640111	scaRNA
ENSG0000(	ENSG0000(	167.8721	-0.07875	0.48056	0.640136	processed_pseudogene
ENSG0000(	LINC02610	50.17988	0.162962	0.480579	0.640136	lncRNA
ENSG0000(	ZNF214	126.078	-0.09307	0.480651	0.640204	protein_coding
ENSG0000(	ABCA17P	44.50077	0.108102	0.480751	0.640274	transcribed_unitary_pseudogene
ENSG0000(	ARL9	103.2389	-0.06445	0.480738	0.640274	protein_coding
ENSG0000(	CNTRL	4571.202	0.093636	0.480785	0.640274	protein_coding
ENSG0000(	PIMREG	83.42395	0.07698	0.480765	0.640274	protein_coding
ENSG0000(	PI4K2B	701.8139	-0.0614	0.480823	0.640298	protein_coding
ENSG0000(	SUPT5H	2578.787	-0.02879	0.480972	0.640469	protein_coding
ENSG0000(	PSKH1	855.9144	-0.05054	0.481059	0.640558	protein_coding
ENSG0000(	AP1B1P1	25.16187	-0.10275	0.481115	0.640605	transcribed_unprocessed_pseudo
ENSG0000(	INCENP	449.0019	-0.06067	0.481198	0.640688	protein_coding
ENSG0000(	WDR87	25.73573	0.243937	0.481269	0.640756	protein_coding
ENSG0000(	ENSG0000(	123.454	-0.04097	0.481361	0.640852	lncRNA
ENSG0000(	DNAJC15	1062.523	-0.04393	0.481384	0.640856	protein_coding
ENSG0000(	ENSG0000(	578.8081	0.062013	0.481415	0.640865	lncRNA
ENSG0000(	TRIM7	169.3984	0.048752	0.481432	0.640865	protein_coding
ENSG0000(	ENSG0000(	85.04133	0.051644	0.481534	0.640971	lncRNA
ENSG0000(	NHEG1	54.59322	0.05343	0.481552	0.640971	lncRNA
ENSG0000(	ASTE1	441.6682	-0.07746	0.481619	0.640995	protein_coding
ENSG0000(	ENSG0000(	29.26647	-0.10723	0.481666	0.640995	transcribed_processed_pseudoge
ENSG0000(	ENSG0000(	146.9083	-0.04677	0.481668	0.640995	lncRNA
ENSG0000(	ENSG0000(	9.652722	-0.13618	0.481648	0.640995	lncRNA
ENSG0000(	ENSG0000(	2407.027	0.035943	0.481672	0.640995	lncRNA
ENSG0000(	LINC00424	43.52323	0.067994	0.481816	0.641159	lncRNA
ENSG0000(	TXNDC9	223.4507	-0.0457	0.481884	0.641223	protein_coding
ENSG0000(	ZNF510	946.397	0.0417	0.481909	0.641229	protein_coding
ENSG0000(	TXNIP	10045.98	-0.06391	0.481975	0.64129	protein_coding
ENSG0000(	LINC02696	46.73657	0.11533	0.48202	0.641296	lncRNA
ENSG0000(	RPS9	2095.25	-0.0576	0.482011	0.641296	protein_coding
ENSG0000(	ENSG0000(	81.17689	0.050595	0.482054	0.641305	lncRNA
ENSG0000(	MYH15	32.75449	0.145285	0.482068	0.641305	protein_coding
ENSG0000(	SEC13	1536.817	-0.06102	0.482117	0.641344	protein_coding
ENSG0000(	ADARB1	1322.113	0.094679	0.482242	0.64143	protein_coding
ENSG0000(	FUT4	1571.979	-0.03886	0.482243	0.64143	protein_coding
ENSG0000(	RN7SL847F	76.21017	-0.05123	0.48223	0.64143	misc_RNA

ENSG0000(	LINC01748	678.5541	0.048375	0.482491	0.641733	lncRNA
ENSG0000(	COX5AP2	16.83501	0.139998	0.482574	0.641788	processed_pseudogene
ENSG0000(	SPDEF	212.2233	0.15002	0.482555	0.641788	protein_coding
ENSG0000(	ENSG0000(	33.27744	-0.11105	0.482626	0.641808	processed_pseudogene
ENSG0000(	ENSG0000(	209.9951	0.073585	0.48265	0.641808	lncRNA
ENSG0000(	RN7SL2	255465.2	-0.09219	0.482649	0.641808	misc_RNA
ENSG0000(	SLC2A4	212.0834	0.064902	0.482672	0.641811	protein_coding
ENSG0000(	ENSG0000(	192.7716	-0.08487	0.482715	0.64184	lncRNA
ENSG0000(	HSD3BP2	13.51261	0.227659	0.482792	0.641851	unprocessed_pseudogene
ENSG0000(	MRPS7	449.8071	-0.02964	0.482773	0.641851	protein_coding
ENSG0000(	OGFOD1	717.9405	0.04749	0.482804	0.641851	protein_coding
ENSG0000(	ZFP82	1060.651	0.044295	0.482778	0.641851	protein_coding
ENSG0000(	ABHD17A	1775.164	-0.03668	0.482874	0.641863	protein_coding
ENSG0000(	ENSG0000(	133.8927	-0.06614	0.482859	0.641863	lncRNA
ENSG0000(	USP17L2	25.89171	0.22904	0.48286	0.641863	protein_coding
ENSG0000(	ENSG0000(	12.28616	0.138674	0.482957	0.641946	lncRNA
ENSG0000(	LINC00581	74.12123	-0.0668	0.483112	0.642125	lncRNA
ENSG0000(	ENSG0000(	26.10463	0.078321	0.483174	0.64218	lncRNA
ENSG0000(	PROSER3	1091.341	0.027853	0.483222	0.642216	protein_coding
ENSG0000(	ERP44	3120.539	0.022286	0.483258	0.642237	protein_coding
ENSG0000(	LINC00402	411.7226	-0.15423	0.483304	0.642272	lncRNA
ENSG0000(	ENSG0000(	88.95715	-0.06862	0.483357	0.642287	lncRNA
ENSG0000(	ENSG0000(	136.0601	-0.05902	0.483339	0.642287	lncRNA
ENSG0000(	ENSG0000(	50.02485	-0.09382	0.483398	0.642315	lncRNA
ENSG0000(	RN7SL660F	40.5649	0.081342	0.483527	0.64246	misc_RNA
ENSG0000(	SNORD94	555.768	0.085199	0.483718	0.642686	snoRNA
ENSG0000(	LINC01807	73.70022	0.078267	0.483742	0.642691	lncRNA
ENSG0000(	LINC02507	40.82704	-0.07425	0.483775	0.642708	lncRNA
ENSG0000(	UFL1	918.8185	-0.04525	0.48383	0.642754	protein_coding
ENSG0000(	ENSG0000(	152.2236	0.04126	0.483904	0.642825	lncRNA
ENSG0000(	ARPC1B	1734.139	0.038001	0.484152	0.643127	protein_coding
ENSG0000(	AP4B1	1255.394	0.034857	0.48425	0.643141	protein_coding
ENSG0000(	ENSG0000(	383.6298	-0.04121	0.484233	0.643141	TEC
ENSG0000(	ENSG0000(	40.61556	-0.07245	0.484221	0.643141	lncRNA
ENSG0000(	OR2AK2	10.73496	0.218526	0.484228	0.643141	protein_coding
ENSG0000(	UBASH3B	575.3716	0.072713	0.484264	0.643141	protein_coding
ENSG0000(	C12orf57	217.5845	-0.0642	0.484463	0.643378	protein_coding
ENSG0000(	ENSG0000(	81.18133	0.087071	0.484551	0.643386	lncRNA
ENSG0000(	MIR3681H(	25.95386	-0.21544	0.48453	0.643386	lncRNA
ENSG0000(	PLK1	380.4196	0.049915	0.484531	0.643386	protein_coding
ENSG0000(	SLX4	872.2898	0.035993	0.484536	0.643386	protein_coding
ENSG0000(	MIR646HG	1081.598	0.044785	0.484585	0.643405	lncRNA
ENSG0000(	ZNF479	162.5626	-0.05023	0.484627	0.643433	protein_coding
ENSG0000(	MIX23P1	13.53616	-0.16825	0.484662	0.643453	processed_pseudogene
ENSG0000(	NAA20	299.0515	0.033689	0.484737	0.643524	protein_coding
ENSG0000(	ENSG0000(	245.6496	-0.08524	0.484792	0.643571	lncRNA
ENSG0000(	RN7SKP85	10.76484	-0.16073	0.484823	0.643585	misc_RNA

ENSG0000(	DNAJB2	827.0823	-0.03617	0.484946	0.64372	protein_coding
ENSG0000(	ENSG0000(	13.10736	0.103528	0.485027	0.643748	lncRNA
ENSG0000(	LYG1	44.56557	-0.07449	0.485028	0.643748	protein_coding
ENSG0000(	PPP1R2	1290.883	-0.04511	0.485003	0.643748	protein_coding
ENSG0000(	KCTD2	543.7794	0.027224	0.485089	0.643774	protein_coding
ENSG0000(	RN7SL321F	33.10936	-0.08932	0.485083	0.643774	misc_RNA
ENSG0000(	ENSG0000(	319.8152	0.040598	0.485115	0.643783	lncRNA
ENSG0000(	ZNF324B	241.8628	0.040457	0.485138	0.643786	protein_coding
ENSG0000(	ENSG0000(	32.96598	-0.0954	0.485192	0.64383	lncRNA
ENSG0000(	ELOA-AS1	373.2003	-0.03802	0.48525	0.643853	lncRNA
ENSG0000(	FOXF1	434.9884	0.065888	0.485235	0.643853	protein_coding
ENSG0000(	ENSG0000(	308.2351	-0.04402	0.485349	0.643927	processed_pseudogene
ENSG0000(	ENSG0000(	24.66493	0.146023	0.485347	0.643927	lncRNA
ENSG0000(	TRAJ3	40.28356	0.157276	0.485367	0.643927	TR_J_gene
ENSG0000(	TAS2R30	856.9201	0.053817	0.485404	0.643949	protein_coding
ENSG0000(	ZFP36L1	5452.456	-0.03876	0.485437	0.643965	protein_coding
ENSG0000(	LINC01895	33.86014	0.071237	0.485469	0.643981	lncRNA
ENSG0000(	MAPRE3-AS	352.0682	0.03815	0.485512	0.64401	lncRNA
ENSG0000(	RNU6-858F	8.04421	0.169097	0.485557	0.644042	snRNA
ENSG0000(	ZNF593	31.82607	0.112992	0.485577	0.644042	protein_coding
ENSG0000(	FHOD1	508.4659	0.04669	0.48568	0.644122	protein_coding
ENSG0000(	KCNJ12	44.76695	0.096038	0.485739	0.644122	protein_coding
ENSG0000(	KCTD5	570.7473	0.040177	0.485709	0.644122	protein_coding
ENSG0000(	MPP2	51.74884	-0.11749	0.485726	0.644122	protein_coding
ENSG0000(	RSU1P3	187.6951	0.091564	0.485696	0.644122	processed_pseudogene
ENSG0000(	ENSG0000(	759.5577	0.047691	0.485825	0.644155	lncRNA
ENSG0000(	HS1BP3	1106.519	0.069794	0.485793	0.644155	protein_coding
ENSG0000(	SAMD5	1394.899	-0.06383	0.485811	0.644155	protein_coding
ENSG0000(	ENSG0000(	29.54887	-0.07749	0.485858	0.644171	lncRNA
ENSG0000(	ENSG0000(	13.70285	0.121963	0.486142	0.644521	lncRNA
ENSG0000(	RBMXP2	9.289897	0.268473	0.486205	0.644571	processed_pseudogene
ENSG0000(	XPC-AS1	54.01204	0.08961	0.486221	0.644571	lncRNA
ENSG0000(	LTBP1	619.878	-0.07522	0.486302	0.644652	protein_coding
ENSG0000(	ZBTB34	846.7374	0.022109	0.486369	0.644713	protein_coding
ENSG0000(	ENSG0000(	60.84357	0.075407	0.486396	0.644722	lncRNA
ENSG0000(	RN7SKP137	13.69946	-0.15782	0.486448	0.644764	misc_RNA
ENSG0000(	ENSG0000(	9.863578	0.129436	0.486496	0.644784	lncRNA
ENSG0000(	FAM21EP	136.6439	0.050618	0.486504	0.644784	transcribed_unprocessed_pseudo
ENSG0000(	ENSG0000(	12.56356	0.148545	0.486617	0.644906	lncRNA
ENSG0000(	ENSG0000(	111.1118	0.05129	0.48665	0.644923	lncRNA
ENSG0000(	LINC01637	51.60486	0.113949	0.486674	0.644928	lncRNA
ENSG0000(	NCBP2L	15.65984	0.12674	0.486703	0.644938	protein_coding
ENSG0000(	ENSG0000(	20.93858	-0.11321	0.486885	0.645153	lncRNA
ENSG0000(	JARID2	1322.512	0.035253	0.486934	0.645191	protein_coding
ENSG0000(	ENSG0000(	50.80298	-0.06927	0.486986	0.645232	lncRNA
ENSG0000(	BRD1	1631.741	-0.034	0.487083	0.645333	protein_coding
ENSG0000(	ITGA4	3809.812	0.112691	0.487114	0.645348	protein_coding

ENSG0000(	RPS3AP26	39.41672	-0.16297	0.487227	0.645444	processed_pseudogene
ENSG0000(	SLA2	358.2036	-0.06456	0.487225	0.645444	protein_coding
ENSG0000(	MAP3K7CL	400.5567	0.045631	0.487275	0.645452	protein_coding
ENSG0000(	MIA-RAB4E	8.340452	0.293559	0.487265	0.645452	protein_coding
ENSG0000(	ENSG0000(	277.1127	0.0477	0.487415	0.645601	lncRNA
ENSG0000(	ENSG0000(	228.8719	-0.03159	0.487428	0.645601	lncRNA
ENSG0000(	CNPY2	1318.944	0.024497	0.487564	0.645729	protein_coding
ENSG0000(	IGFL2	86.46736	0.049492	0.487566	0.645729	protein_coding
ENSG0000(	KRT8P34	31.43063	-0.186	0.487614	0.645766	processed_pseudogene
ENSG0000(	LINC02818	9.888955	-0.19025	0.487637	0.645769	lncRNA
ENSG0000(	DKFZP434A	36.3394	0.099136	0.487663	0.645776	lncRNA
ENSG0000(	ZNF667-AS	828.6405	0.04062	0.487701	0.645799	lncRNA
ENSG0000(	ENSG0000(	1183.295	0.034874	0.487864	0.645933	lncRNA
ENSG0000(	LINC01529	8.402105	0.185382	0.487828	0.645933	transcribed_unitary_pseudogene
ENSG0000(	RNY1P11	27.8154	0.163702	0.487858	0.645933	misc_RNA
ENSG0000(	KDSR-DT	8.806052	0.114772	0.4879	0.645955	lncRNA
ENSG0000(	ENSG0000(	26.83078	-0.10764	0.488219	0.646349	misc_RNA
ENSG0000(	SCARNA18	33.75935	-0.08399	0.488284	0.646367	snoRNA
ENSG0000(	SULT1E1	577.5873	-0.11043	0.488272	0.646367	protein_coding
ENSG0000(	ZNF563	405.223	-0.04292	0.488294	0.646367	protein_coding
ENSG0000(	CMKLR2	97.39169	-0.04892	0.48832	0.646374	protein_coding
ENSG0000(	NSMCE1-DT	502.581	0.031152	0.488416	0.646474	lncRNA
ENSG0000(	JKAMP	915.3186	0.031153	0.488491	0.646546	protein_coding
ENSG0000(	ENSG0000(	19.2986	0.118516	0.488515	0.646551	processed_pseudogene
ENSG0000(	CCP110	1582.061	0.048199	0.488705	0.646776	protein_coding
ENSG0000(	TRMT9B	1578.156	-0.04501	0.488752	0.646811	protein_coding
ENSG0000(	ALDH3B1	1132.904	-0.06858	0.488811	0.646862	protein_coding
ENSG0000(	SLC9A7	1810.387	0.070899	0.488847	0.646881	protein_coding
ENSG0000(	ZNF433-AS	1371.074	-0.02666	0.488879	0.646897	lncRNA
ENSG0000(	PON2	1524.743	-0.0686	0.488939	0.646949	protein_coding
ENSG0000(	ENSG0000(	41.4203	0.110842	0.489069	0.647094	lncRNA
ENSG0000(	ENSG0000(	11.95203	0.130914	0.489184	0.647219	lncRNA
ENSG0000(	KIFC3	2087.577	-0.10108	0.489347	0.647408	protein_coding
ENSG0000(	MKNK1	2591.568	0.022309	0.489408	0.647461	protein_coding
ENSG0000(	LINC01936	301.9381	-0.02869	0.489449	0.647489	lncRNA
ENSG0000(	ANGPT4	113.7964	-0.05335	0.489549	0.647593	protein_coding
ENSG0000(	SBNO1-AS1	34.77621	-0.09493	0.489589	0.647619	lncRNA
ENSG0000(	ENSG0000(	97.56303	-0.08088	0.489796	0.647865	lncRNA
ENSG0000(	CAMK1D	1940.502	0.04621	0.489822	0.647873	protein_coding
ENSG0000(	MORF4L2	1637.712	0.047004	0.489871	0.64791	protein_coding
ENSG0000(	ZNF761	1160.699	0.028469	0.48996	0.648001	protein_coding
ENSG0000(	ARSF	21.85421	-0.09163	0.490125	0.648179	protein_coding
ENSG0000(	ENSG0000(	430.1372	-0.03173	0.490136	0.648179	lncRNA
ENSG0000(	PCDHGC3	243.0477	0.083486	0.490272	0.648332	protein_coding
ENSG0000(	AMZ2	695.1347	0.039591	0.490373	0.648411	protein_coding
ENSG0000(	GREP1	348.5999	0.038343	0.490356	0.648411	protein_coding
ENSG0000(	KRBOX4	720.4484	0.028901	0.490415	0.648423	protein_coding

ENSG0000(	MYPOP	185.5894	0.046747	0.490443	0.648423	protein_coding
ENSG0000(	STX12	972.1459	0.035398	0.490444	0.648423	protein_coding
ENSG0000(	ENSG0000(	297.4212	0.091131	0.490524	0.648502	TEC
ENSG0000(	DPEP2	432.1741	0.112264	0.490566	0.648504	protein_coding
ENSG0000(	RBM3	2445.597	-0.04034	0.490564	0.648504	protein_coding
ENSG0000(	ENSG0000(	45.39088	-0.06758	0.490685	0.648579	misc_RNA
ENSG0000(	MTCO3P17	11.69657	0.154842	0.490671	0.648579	unprocessed_pseudogene
ENSG0000(	PRDM5	559.7874	0.035654	0.490684	0.648579	protein_coding
ENSG0000(	LERFS	184.0135	0.115899	0.490748	0.648634	lncRNA
ENSG0000(	LINC02802	96.24032	-0.08487	0.490818	0.64868	lncRNA
ENSG0000(	RN7SL850P	97.67582	-0.05813	0.490824	0.64868	misc_RNA
ENSG0000(	DHTKD1	1522.234	0.053304	0.490902	0.648757	protein_coding
ENSG0000(	ZNF546	967.0282	-0.03775	0.490973	0.648824	protein_coding
ENSG0000(	LINC00870	168.4635	0.075699	0.491233	0.649016	lncRNA
ENSG0000(	LINC01194	22.43408	0.119403	0.491189	0.649016	lncRNA
ENSG0000(	LMAN2	785.4895	-0.04915	0.491144	0.649016	protein_coding
ENSG0000(	RNASE6	147.3671	0.114097	0.49121	0.649016	protein_coding
ENSG0000(	TTPAL	1188.356	0.028239	0.491228	0.649016	protein_coding
ENSG0000(	ZNF674	874.2935	-0.02898	0.491243	0.649016	protein_coding
ENSG0000(	RN7SKP264	8.285385	0.14915	0.491366	0.649131	misc_RNA
ENSG0000(	USP20	901.7942	0.055485	0.491371	0.649131	protein_coding
ENSG0000(	ENSG0000(	146.2446	-0.04448	0.491437	0.649164	lncRNA
ENSG0000(	GSG1L	28.38978	-0.11238	0.491421	0.649164	protein_coding
ENSG0000(	ARHGEF7	3259.828	-0.02745	0.491462	0.64917	protein_coding
ENSG0000(	NARS2	326.0661	-0.06204	0.491525	0.649226	protein_coding
ENSG0000(	ENSG0000(	19.87932	-0.10136	0.491559	0.649244	lncRNA
ENSG0000(	FABP3	9.385233	0.175774	0.491635	0.649289	protein_coding
ENSG0000(	NR2F2	891.4423	0.055164	0.491624	0.649289	protein_coding
ENSG0000(	CDC40	1032.945	-0.04177	0.491657	0.649292	protein_coding
ENSG0000(	ENSG0000(	82.65133	-0.11281	0.491741	0.649375	lncRNA
ENSG0000(	ENSG0000(	63.55602	0.08376	0.491841	0.649454	lncRNA
ENSG0000(	MIX23P3	63.55303	-0.09868	0.491842	0.649454	processed_pseudogene
ENSG0000(	ENSG0000(	719.3273	0.037244	0.491888	0.649488	transcribed_unprocessed_pseudo
ENSG0000(	ENSG0000(	201.0802	-0.84008	0.491981	0.649583	protein_coding
ENSG0000(	DNAH1	3345.226	-0.05061	0.492057	0.64963	protein_coding
ENSG0000(	UFL1-AS1	169.903	0.051701	0.492058	0.64963	lncRNA
ENSG0000(	ENSG0000(	16.075	0.094388	0.492176	0.649759	lncRNA
ENSG0000(	LRRC37A2	1585.706	0.080592	0.492201	0.649765	protein_coding
ENSG0000(	CALB1	98.62438	0.090716	0.49225	0.649802	protein_coding
ENSG0000(	ATP11C	1150.189	0.040655	0.492302	0.649838	protein_coding
ENSG0000(	ENSG0000(	8.480151	-0.15063	0.492319	0.649838	lncRNA
ENSG0000(	AS3MT	503.0611	-0.02866	0.492478	0.650021	protein_coding
ENSG0000(	NXPE1	1220.891	0.156745	0.49265	0.650216	protein_coding
ENSG0000(	PITX1	103.9392	0.104716	0.492667	0.650216	protein_coding
ENSG0000(	ZNF428	131.0395	-0.05724	0.492712	0.650249	protein_coding
ENSG0000(	BANF1	216.9586	0.059198	0.492823	0.650286	protein_coding
ENSG0000(	CTNNA1	4288.194	-0.05605	0.492809	0.650286	protein_coding

ENSG0000(	ENSG0000(	117.1068	-0.05419	0.492805	0.650286	lncRNA
ENSG0000(	MTCYBP18	30.11588	-0.34401	0.492781	0.650286	processed_pseudogene
ENSG0000(	PSTPIP1	623.2825	0.089957	0.492859	0.650306	protein_coding
ENSG0000(	LINC01006	850.8977	0.035536	0.492944	0.650389	lncRNA
ENSG0000(	LINC02024	20.57122	-0.15469	0.49297	0.650389	lncRNA
ENSG0000(	MUC21	81.5182	-0.04954	0.492983	0.650389	protein_coding
ENSG0000(	AMY2B	1298.517	0.050219	0.493131	0.650501	protein_coding
ENSG0000(	ENSG0000(	48.72813	-0.06452	0.493099	0.650501	lncRNA
ENSG0000(	PEAK3	114.0865	0.066204	0.493116	0.650501	protein_coding
ENSG0000(	PARP11-AS	29.3634	0.143354	0.493181	0.650541	lncRNA
ENSG0000(	FSIP2-AS2	8.58868	0.214161	0.493205	0.650545	lncRNA
ENSG0000(	ENSG0000(	9.063013	0.150159	0.493384	0.650704	processed_pseudogene
ENSG0000(	ENSG0000(	10.83798	0.198941	0.493381	0.650704	lncRNA
ENSG0000(	MAPT	335.2134	-0.07332	0.493388	0.650704	protein_coding
ENSG0000(	ENSG0000(	128.3319	0.060651	0.493479	0.650784	lncRNA
ENSG0000(	LINC02405	327.8356	-0.06577	0.493489	0.650784	lncRNA
ENSG0000(	PRKCH	1780.425	0.052829	0.493561	0.650851	protein_coding
ENSG0000(	SCAT1	473.3523	-0.03959	0.49359	0.650862	lncRNA
ENSG0000(	IZUMO4	39.69218	-0.11501	0.493622	0.650877	protein_coding
ENSG0000(	MYCN	27.98855	0.114495	0.493667	0.650909	protein_coding
ENSG0000(	LINC00173	328.1329	0.045916	0.493789	0.651043	lncRNA
ENSG0000(	ENSG0000(	209.8353	-0.06025	0.493829	0.651057	transcribed_unprocessed_pseudo
ENSG0000(	TMEM74B	578.7068	-0.13156	0.493841	0.651057	protein_coding
ENSG0000(	FAM222B	1384.25	-0.04327	0.493886	0.651059	protein_coding
ENSG0000(	LINC02832	616.8058	0.0339	0.493885	0.651059	lncRNA
ENSG0000(	SOCS5P4	138.7262	-0.08576	0.493905	0.651059	processed_pseudogene
ENSG0000(	ENSG0000(	128.3367	-0.04226	0.493956	0.651072	lncRNA
ENSG0000(	RN7SL362F	30.11033	-0.08533	0.493955	0.651072	misc_RNA
ENSG0000(	ENSG0000(	116.3349	0.054568	0.494016	0.651124	lncRNA
ENSG0000(	NUDT17	49.97082	0.090184	0.49413	0.651246	protein_coding
ENSG0000(	RNA5SP434	17.52234	-0.13957	0.494283	0.651422	rRNA_pseudogene
ENSG0000(	ENSG0000(	16.58027	0.100013	0.494309	0.651428	lncRNA
ENSG0000(	CNTNAP1	353.0855	-0.04766	0.494348	0.651452	protein_coding
ENSG0000(	LINC02199	308.4838	0.040543	0.494369	0.651453	lncRNA
ENSG0000(	ENSG0000(	254.4267	-0.03522	0.494556	0.651601	lncRNA
ENSG0000(	LRRC66	928.5586	-0.11988	0.494564	0.651601	protein_coding
ENSG0000(	MIR3131	9.603093	-0.2142	0.494556	0.651601	miRNA
ENSG0000(	PARVG	2252.986	0.118459	0.494535	0.651601	protein_coding
ENSG0000(	RNU6-834F	14.96944	-0.11572	0.494592	0.651611	snRNA
ENSG0000(	IRGM	95.34702	-0.06283	0.494721	0.651754	protein_coding
ENSG0000(	LINC01910	176.7044	-0.0372	0.494828	0.651813	lncRNA
ENSG0000(	MXD1	5053.164	0.121917	0.4948	0.651813	protein_coding
ENSG0000(	NUDT9P1	21.51722	0.116092	0.494812	0.651813	processed_pseudogene
ENSG0000(	FLI1	1455.909	0.086891	0.494936	0.651927	protein_coding
ENSG0000(	ENSG0000(	265.0583	-0.0275	0.495026	0.652018	lncRNA
ENSG0000(	GRK6	1087.318	-0.077	0.495184	0.652199	protein_coding
ENSG0000(	NICN1	503.7203	0.066164	0.495208	0.652204	protein_coding

ENSG0000(	DENND1C	814.7403	-0.12052	0.495254	0.65221	protein_coding
ENSG0000(	WNT10A	62.69635	0.097324	0.49525	0.65221	protein_coding
ENSG0000(	INTS10	1640.324	0.025518	0.495305	0.652251	protein_coding
ENSG0000(	RN7SL665F	27.49867	0.108429	0.49556	0.652558	misc_RNA
ENSG0000(	RYR2	365.5874	0.119526	0.495668	0.652674	protein_coding
ENSG0000(	ENSG0000(	25.88728	0.157456	0.495795	0.652813	TEC
ENSG0000(	TRAPPC6B	1156.21	-0.02258	0.495853	0.652862	protein_coding
ENSG0000(	R3HDM2-D	46.39738	0.066444	0.495934	0.652942	lncRNA
ENSG0000(	ENSG0000(	31.75602	-0.10506	0.496008	0.652992	lncRNA
ENSG0000(	RAET1E	324.0926	0.045424	0.496014	0.652992	protein_coding
ENSG0000(	ENSG0000(	22.67119	0.10492	0.496216	0.65323	lncRNA
ENSG0000(	ENSG0000(	109.8788	-0.04399	0.496236	0.65323	TEC
ENSG0000(	GALNT8	425.1241	0.119416	0.496287	0.65327	protein_coding
ENSG0000(	XPO4	1493.72	0.057001	0.496384	0.65337	protein_coding
ENSG0000(	ENSG0000(	49.19131	-0.06522	0.496417	0.653387	lncRNA
ENSG0000(	ENSG0000(	310.6545	0.047766	0.496717	0.653754	lncRNA
ENSG0000(	CXXC4-AS1	574.0093	0.040598	0.496747	0.653766	lncRNA
ENSG0000(	ENSG0000(	32.25619	0.129949	0.496837	0.653831	misc_RNA
ENSG0000(	ENSG0000(	374.8447	-0.03192	0.496838	0.653831	TEC
ENSG0000(	ENSG0000(	54.09675	-0.06579	0.496927	0.653922	lncRNA
ENSG0000(	EHD1	1421.613	-0.04439	0.496973	0.653954	protein_coding
ENSG0000(	LINC00658	35.15131	-0.07139	0.496996	0.653958	lncRNA
ENSG0000(	ENSG0000(	194.3376	0.049619	0.497195	0.654114	processed_pseudogene
ENSG0000(	ENSG0000(	145.0049	0.065241	0.497188	0.654114	transcribed_unprocessed_pseudo
ENSG0000(	PPM1L	555.0319	0.047096	0.497143	0.654114	protein_coding
ENSG0000(	RUNX2-AS1	162.5356	-0.03956	0.497198	0.654114	lncRNA
ENSG0000(	CLASP1	2706.674	0.029555	0.497281	0.654196	protein_coding
ENSG0000(	PUM1	2744.463	-0.0199	0.497332	0.654235	protein_coding
ENSG0000(	CRAMP1	1385.497	0.040468	0.497397	0.654294	protein_coding
ENSG0000(	ENSG0000(	12.89385	0.135906	0.49745	0.654308	lncRNA
ENSG0000(	RN7SL714F	26.28066	0.076459	0.49745	0.654308	misc_RNA
ENSG0000(	PKN3	123.8615	-0.09581	0.497486	0.654329	protein_coding
ENSG0000(	ENSG0000(	10.18658	-0.17887	0.497547	0.654382	lncRNA
ENSG0000(	LINC00670	1060.496	0.055492	0.497569	0.654384	lncRNA
ENSG0000(	ABCC3	3933.572	0.056705	0.497884	0.65447	protein_coding
ENSG0000(	AUTS2	3262.372	0.02742	0.497831	0.65447	protein_coding
ENSG0000(	C15orf48	573.7086	-0.12369	0.497774	0.65447	protein_coding
ENSG0000(	ENSG0000(	21.24425	-0.08843	0.49779	0.65447	lncRNA
ENSG0000(	ENSG0000(	111.9818	0.061121	0.49781	0.65447	lncRNA
ENSG0000(	ENSG0000(	190.6603	0.078578	0.497867	0.65447	lncRNA
ENSG0000(	ENSG0000(	9.142877	0.144097	0.497721	0.65447	lncRNA
ENSG0000(	HSD17B12	1357.464	0.039212	0.497793	0.65447	protein_coding
ENSG0000(	HTR3E-AS1	38.67291	0.081308	0.497855	0.65447	lncRNA
ENSG0000(	RNA5SP527	10.39276	-0.12135	0.497782	0.65447	rRNA
ENSG0000(	SEC24A	2306.67	-0.07491	0.497847	0.65447	protein_coding
ENSG0000(	SLC5A5	251.5027	-0.04761	0.497844	0.65447	protein_coding
ENSG0000(	ADSS1	313.8055	0.044801	0.497956	0.654538	protein_coding

ENSG0000(	ITPRID2	5294.319	-0.07568	0.498018	0.654577	protein_coding
ENSG0000(	TRAV13-2	8.238881	-0.20524	0.498028	0.654577	TR_V_gene
ENSG0000(	GLB1	703.807	0.034708	0.498167	0.654709	protein_coding
ENSG0000(	OR6M2P	56.26927	0.062082	0.49817	0.654709	transcribed_unprocessed_pseudo
ENSG0000(	ZBTB48	785.6899	-0.04949	0.498202	0.654724	protein_coding
ENSG0000(	CPPED1	1377.159	-0.05498	0.498297	0.654822	protein_coding
ENSG0000(	DCLK1	383.9289	-0.05922	0.498397	0.654871	protein_coding
ENSG0000(	ENSG0000(	33.41535	0.131342	0.498373	0.654871	lncRNA
ENSG0000(	LINC01979	18.83983	0.126784	0.498384	0.654871	lncRNA
ENSG0000(	RN7SL294F	12.85826	-0.11114	0.498496	0.654974	misc_RNA
ENSG0000(	PTTG2	26.21635	0.142625	0.498546	0.655012	protein_coding
ENSG0000(	LINC02027	331.4492	-0.04293	0.498615	0.655076	lncRNA
ENSG0000(	LINC01289	105.7097	0.04551	0.498686	0.655141	lncRNA
ENSG0000(	TNFSF18	60.59742	-0.08366	0.498783	0.655241	protein_coding
ENSG0000(	ENSG0000(	26.3105	-0.13542	0.498934	0.655413	TEC
ENSG0000(	RASGEF1C	21.8035	-0.19344	0.499239	0.655786	protein_coding
ENSG0000(	CR1	2838.427	0.183838	0.499309	0.655836	protein_coding
ENSG0000(	ENSG0000(	173.637	-0.0372	0.499319	0.655836	lncRNA
ENSG0000(	ENSG0000(	9.81971	-0.14331	0.499342	0.65584	snoRNA
ENSG0000(	ENSG0000(	27.74829	0.118779	0.499523	0.65605	lncRNA
ENSG0000(	ENSG0000(	102.227	-0.08014	0.499563	0.656075	transcribed_unprocessed_pseudo
ENSG0000(	MIR3145	28.23549	-0.16683	0.499645	0.656156	miRNA
ENSG0000(	APLF	235.493	0.057393	0.499695	0.656193	protein_coding
ENSG0000(	LRFN3	292.6634	-0.05339	0.499875	0.656402	protein_coding
ENSG0000(	ENSG0000(	492.757	-0.02827	0.499925	0.65644	TEC
ENSG0000(	C20orf27	117.7078	0.072802	0.500046	0.656572	protein_coding
ENSG0000(	SLC4A4	1238.095	-0.119	0.500067	0.656573	protein_coding
ENSG0000(	ENSG0000(	82.15011	0.046856	0.500125	0.656593	TEC
ENSG0000(	P4HA2	996.4823	-0.08078	0.500113	0.656593	protein_coding
ENSG0000(	XPO1	10039.55	0.030283	0.500312	0.656811	protein_coding
ENSG0000(	POGLUT2P	187.3889	0.107877	0.500357	0.656844	processed_pseudogene
ENSG0000(	ENSG0000(	8.413452	0.144733	0.500547	0.657061	lncRNA
ENSG0000(	HSDL1	460.8761	-0.04192	0.500585	0.657061	protein_coding
ENSG0000(	PTENP1	28.97958	-0.11748	0.500583	0.657061	transcribed_processed_pseudoge
ENSG0000(	FGD5P1	185.9769	0.038173	0.50062	0.65708	transcribed_unprocessed_pseudo
ENSG0000(	ENSG0000(	353.6623	0.02778	0.500652	0.657095	TEC
ENSG0000(	ENSG0000(	23.9455	-0.09665	0.500774	0.657227	lncRNA
ENSG0000(	MINCR	95.4698	-0.06914	0.500823	0.657237	lncRNA
ENSG0000(	SCARNA10	7085.218	-0.05789	0.500813	0.657237	snoRNA
ENSG0000(	MRPS27	910.6723	0.072422	0.50093	0.657349	protein_coding
ENSG0000(	CLP1	92.89323	0.059144	0.501036	0.657433	protein_coding
ENSG0000(	RN7SL328F	94.94555	-0.05721	0.501033	0.657433	misc_RNA
ENSG0000(	DBET	95.90207	0.206465	0.5011	0.65749	lncRNA
ENSG0000(	ENSG0000(	107.0901	-0.04916	0.501138	0.657513	lncRNA
ENSG0000(	RPS3AP13	15.33117	-0.10294	0.501264	0.657632	processed_pseudogene
ENSG0000(	SIX4	230.8316	0.052192	0.501271	0.657632	protein_coding
ENSG0000(	DENND4A	3057.92	0.067108	0.501356	0.657694	protein_coding

ENSG0000(	ENSG0000(	151.1928	-0.0476	0.50136	0.657694	TEC
ENSG0000(	ANKRD20A	286.9483	-0.13009	0.501479	0.657824	protein_coding
ENSG0000(	RN7SL507F	45.12689	0.073886	0.501514	0.657842	misc_RNA
ENSG0000(	LINC02325	243.9515	0.10122	0.501624	0.657959	lncRNA
ENSG0000(	ENSG0000(	59.57283	0.113504	0.501769	0.658121	lncRNA
ENSG0000(	ENSG0000(	10.22153	-0.32981	0.501888	0.65825	lncRNA
ENSG0000(	ENSG0000(	9.487658	0.138312	0.501967	0.658327	processed_pseudogene
ENSG0000(	ENSG0000(	14.34044	0.190934	0.502055	0.65836	TEC
ENSG0000(	MAK	75.77186	0.052904	0.502055	0.65836	protein_coding
ENSG0000(	ZNF582	73.20938	0.088195	0.502018	0.65836	protein_coding
ENSG0000(	PRDM6	120.3909	0.160613	0.502139	0.658443	protein_coding
ENSG0000(	ENSG0000(	18.31091	-0.15575	0.502211	0.658482	processed_pseudogene
ENSG0000(	PDE7A	4414.374	0.117346	0.502197	0.658482	protein_coding
ENSG0000(	SPICE1	1308.536	0.029772	0.502244	0.658498	protein_coding
ENSG0000(	ENSG0000(	47.56765	0.079089	0.502345	0.658603	TEC
ENSG0000(	ATF2	1306.574	0.026559	0.502495	0.658725	protein_coding
ENSG0000(	ENSG0000(	31.46746	-0.13478	0.502471	0.658725	lncRNA
ENSG0000(	FCRL1	1921.832	0.214873	0.502501	0.658725	protein_coding
ENSG0000(	ENSG0000(	66.91774	0.095613	0.502586	0.658809	lncRNA
ENSG0000(	ENSG0000(	56.7464	0.053819	0.502661	0.65888	lncRNA
ENSG0000(	C1orf54	74.25968	0.085035	0.502725	0.658908	protein_coding
ENSG0000(	KCNK10	326.8622	0.084663	0.502707	0.658908	protein_coding
ENSG0000(	XRRA1	1059.06	-0.09069	0.502762	0.658929	protein_coding
ENSG0000(	ADRA2B	23.30941	0.127311	0.502884	0.659062	protein_coding
ENSG0000(	ENSG0000(	109.4651	0.043791	0.503015	0.659207	lncRNA
[truncated: 626,641 more chars]
