# Supplementary material for: Linking gene expression to clinical outcomes in pediatric Crohn’s disease using machine learning
Source: Sci Rep. 2024 Feb 1;14:2667. doi: 10.1038/s41598-024-52678-0 (PMC10834600; doi:10.1038/s41598-024-52678-0)
Supplement: Supplementary file 3 — Supplementary Table 3. [file 41598_2024_52678_MOESM3_ESM.pdf]

ensembl	gene	baseMean	log2FoldCh	pvalue	padj	biotype
ENSG0000(	MMP1	1477.89	-5.19384	1.55E-09	2.14E-05	protein_coding
ENSG0000(	UCN2	141.6038	-5.23193	1.23E-09	2.14E-05	protein_coding
ENSG0000(	CXCL5	940.5693	-4.60218	1.52E-08	0.00014	protein_coding
ENSG0000(	ZNF527	889.8036	0.349682	5.74E-08	0.000395	protein_coding
ENSG0000(	CXCL8	545.5149	-3.51853	2.47E-07	0.001361	protein_coding
ENSG0000(	CHI3L1	1260.523	-3.05874	4.29E-07	0.001493	protein_coding
ENSG0000(	CLEC4D	48.68216	-3.11818	3.98E-07	0.001493	protein_coding
ENSG0000(	MFSD4A	425.1746	0.964643	4.34E-07	0.001493	protein_coding
ENSG0000(	CEMIP	971.8043	-2.70477	6.76E-07	0.001859	protein_coding
ENSG0000(	FOXP3	80.70535	-1.38398	6.40E-07	0.001859	protein_coding
ENSG0000(	S100A9	190.5847	-3.55262	9.22E-07	0.002306	protein_coding
ENSG0000(	AQP9	332.6059	-3.53495	1.34E-06	0.003069	protein_coding
ENSG0000(	HCAR3	356.0569	-3.73449	1.63E-06	0.003194	protein_coding
ENSG0000(	S100A8	164.5596	-3.84872	1.54E-06	0.003194	protein_coding
ENSG0000(	ENSG0000(	61.68473	-4.1136	1.95E-06	0.003569	lncRNA
ENSG0000(	CXCL6	140.3422	-3.30966	2.14E-06	0.003673	protein_coding
ENSG0000(	MIR4307H(	137.08	0.68939	2.64E-06	0.004228	lncRNA
ENSG0000(	PLAU	780.7818	-2.0484	2.77E-06	0.004228	protein_coding
ENSG0000(	CARD16	295.7179	-0.87836	3.11E-06	0.004356	protein_coding
ENSG0000(	HCAR2	263.0826	-3.35794	3.50E-06	0.004356	protein_coding
ENSG0000(	PSMB9	1084.327	-0.66679	3.22E-06	0.004356	protein_coding
ENSG0000(	SLC22A15	255.6354	-1.00246	3.64E-06	0.004356	protein_coding
ENSG0000(	WNT5A	1229.648	-1.59707	3.39E-06	0.004356	protein_coding
ENSG0000(	GBP2	2203.676	-1.00339	4.29E-06	0.00492	protein_coding
ENSG0000(	INHBA	943.9139	-2.99693	4.63E-06	0.00509	protein_coding
ENSG0000(	STOM	2449.033	-0.96812	4.91E-06	0.005198	protein_coding
ENSG0000(	IFI30	1012.542	-1.13912	5.23E-06	0.005334	protein_coding
ENSG0000(	SLC7A5	488.3092	-1.13004	5.51E-06	0.00541	protein_coding
ENSG0000(	ALPL	182.8713	-2.22822	6.26E-06	0.005941	protein_coding
ENSG0000(	IL1B	1161.73	-2.84425	6.54E-06	0.005996	protein_coding
ENSG0000(	PRPF3	1942.882	0.196787	6.85E-06	0.00608	protein_coding
ENSG0000(	ACKR4	45.17205	-1.59352	8.66E-06	0.00732	protein_coding
ENSG0000(	TIMP1	360.1655	-1.86275	8.78E-06	0.00732	protein_coding
ENSG0000(	FCGR3A	535.218	-1.78789	1.23E-05	0.009695	protein_coding
ENSG0000(	MIR4435-2	4751.222	-1.10596	1.21E-05	0.009695	lncRNA
ENSG0000(	MTCO3P23	25.64282	-3.31644	1.31E-05	0.010008	processed_pseudogene
ENSG0000(	CXCL9	1222.245	-2.40842	1.44E-05	0.010739	protein_coding
ENSG0000(	IL4I1	180.4504	-1.20368	1.56E-05	0.011294	protein_coding
ENSG0000(	ENSG0000(	331.6653	-0.78518	1.69E-05	0.011907	lncRNA
ENSG0000(	PTGES3P1	82.74427	-1.07577	1.80E-05	0.012355	processed_pseudogene
ENSG0000(	APOL2	1955.485	-0.76289	1.96E-05	0.012566	protein_coding
ENSG0000(	LDHD	211.5404	1.34764	1.91E-05	0.012566	protein_coding
ENSG0000(	NOS2	2645.448	-2.18178	1.92E-05	0.012566	protein_coding
ENSG0000(	GLCCI1	1460.565	-0.6195	2.01E-05	0.012576	protein_coding
ENSG0000(	ENSG0000(	49.17065	-1.39364	2.13E-05	0.012993	lncRNA
ENSG0000(	APOL1	1611	-0.95774	2.41E-05	0.014437	protein_coding

ENSG0000(	ENSG0000(	22.44373	-1.69439	2.54E-05	0.014585	lncRNA
ENSG0000(	MTCO1P12	1057.394	2.929617	2.53E-05	0.014585	unprocessed_pseudogene
ENSG0000(	CSF3R	1794.158	-1.89911	2.87E-05	0.01464	protein_coding
ENSG0000(	ENSG0000(	76.99436	-1.58363	2.86E-05	0.01464	lncRNA
ENSG0000(	G0S2	185.2751	-2.56027	2.80E-05	0.01464	protein_coding
ENSG0000(	MMP2	1774.06	-1.65656	2.77E-05	0.01464	protein_coding
ENSG0000(	PRDM1	2713.273	-0.8617	2.81E-05	0.01464	protein_coding
ENSG0000(	WARS1	3723.437	-1.09065	2.78E-05	0.01464	protein_coding
ENSG0000(	MTCYBP23	45.82411	-3.26122	3.09E-05	0.015444	processed_pseudogene
ENSG0000(	CEP128	986.2695	-0.84485	3.34E-05	0.015845	protein_coding
ENSG0000(	LILRB4	427.0581	-1.19897	3.31E-05	0.015845	protein_coding
ENSG0000(	OR8D4	186.7109	0.660703	3.23E-05	0.015845	protein_coding
ENSG0000(	CFB	903.1202	-1.28147	3.54E-05	0.016512	protein_coding
ENSG0000(	EFEMP2	318.8738	-0.70589	3.83E-05	0.016984	protein_coding
ENSG0000(	LINC02362	2061.192	-0.66421	3.81E-05	0.016984	lncRNA
ENSG0000(	TREM1	234.6843	-2.90025	3.78E-05	0.016984	protein_coding
ENSG0000(	IL18RAP	190.8295	-1.19451	3.95E-05	0.017242	protein_coding
ENSG0000(	MMP12	261.5272	-2.06024	4.09E-05	0.017572	protein_coding
ENSG0000(	ENSG0000(	98.38212	2.174982	4.24E-05	0.017593	lncRNA
ENSG0000(	IGFBP7	656.7417	-0.88447	4.27E-05	0.017593	protein_coding
ENSG0000(	TNIP3	646.7035	-1.95395	4.28E-05	0.017593	protein_coding
ENSG0000(	COL4A1	3396.954	-1.22944	4.48E-05	0.018116	protein_coding
ENSG0000(	COL7A1	3643.895	-2.15023	4.65E-05	0.018264	protein_coding
ENSG0000(	CPXM1	95.51819	-1.67901	4.58E-05	0.018264	protein_coding
ENSG0000(	OR7E11P	73.75288	0.860105	4.72E-05	0.018282	lncRNA
ENSG0000(	PDPN	382.2197	-1.21054	4.88E-05	0.018658	protein_coding
ENSG0000(	DNAJB9	397.4053	-0.60934	5.14E-05	0.018861	protein_coding
ENSG0000(	IL1RN	385.1968	-2.56343	5.22E-05	0.018861	protein_coding
ENSG0000(	LOXL1	106.1534	-1.24891	5.12E-05	0.018861	protein_coding
ENSG0000(	MAPK12	271.6361	-0.57577	5.28E-05	0.018861	protein_coding
ENSG0000(	RHBDD1	978.6838	-0.34889	5.17E-05	0.018861	protein_coding
ENSG0000(	CEL	41.02052	0.739796	6.62E-05	0.020377	protein_coding
ENSG0000(	CXCL1	671.6755	-1.77535	5.92E-05	0.020377	protein_coding
ENSG0000(	DMBT1	11428.1	-2.02828	6.02E-05	0.020377	protein_coding
ENSG0000(	DUOXA2	431.2114	-2.80288	6.74E-05	0.020377	protein_coding
ENSG0000(	FGR	371.5782	-1.33811	6.71E-05	0.020377	protein_coding
ENSG0000(	FHL3	130.8624	-1.02575	6.07E-05	0.020377	protein_coding
ENSG0000(	GPR84	53.51324	-2.45972	6.65E-05	0.020377	protein_coding
ENSG0000(	HLA-L	1323.212	-0.25661	6.54E-05	0.020377	transcribed_unprocessed_pseudo
ENSG0000(	IL6	91.60872	-2.74622	6.25E-05	0.020377	protein_coding
ENSG0000(	PSME2	964.0034	-0.52264	6.25E-05	0.020377	protein_coding
ENSG0000(	SELENOO	342.3648	0.329943	6.32E-05	0.020377	protein_coding
ENSG0000(	TGFBI	1750.221	-0.8928	6.58E-05	0.020377	protein_coding
ENSG0000(	TIFA	372.0652	-0.87426	6.35E-05	0.020377	protein_coding
ENSG0000(	TMEM158	120.04	-1.84619	6.48E-05	0.020377	protein_coding
ENSG0000(	HLA-DQA1	2139.204	-0.73921	6.96E-05	0.020714	protein_coding
ENSG0000(	ZNF585B	1910.411	0.454379	7.00E-05	0.020714	protein_coding

ENSG0000(	SOCS1	172.6493	-1.12854	7.22E-05	0.020971	protein_coding
ENSG0000(	UBD	390.8252	-1.6217	7.24E-05	0.020971	protein_coding
ENSG0000(	SGIP1	1097.1	-1.30543	7.34E-05	0.021048	protein_coding
ENSG0000(	TRPV4	44.34123	-1.06119	7.47E-05	0.021173	protein_coding
ENSG0000(	CHIAP2	76.00755	-0.50868	7.68E-05	0.021568	transcribed_unprocessed_pseudo
ENSG0000(	CREB5	1026.05	-2.08616	7.78E-05	0.021632	protein_coding
ENSG0000(	ABCC1	2496.63	-0.33932	8.17E-05	0.022488	protein_coding
ENSG0000(	GAS1	144.2554	-2.72375	8.51E-05	0.02317	protein_coding
ENSG0000(	STC1	163.2447	-2.01453	8.82E-05	0.023556	protein_coding
ENSG0000(	TFAP2C	52.39307	-1.90896	8.81E-05	0.023556	protein_coding
ENSG0000(	CYTOR	2268.39	-1.03013	9.25E-05	0.024469	lncRNA
ENSG0000(	CCL4	109.4613	-1.39729	0.000104	0.025333	protein_coding
ENSG0000(	CCN4	648.8474	-1.85293	0.000105	0.025333	protein_coding
ENSG0000(	CD274	499.0241	-1.45984	9.77E-05	0.025333	protein_coding
ENSG0000(	CENPA	54.43132	-0.68211	0.000107	0.025333	protein_coding
ENSG0000(	CXCL11	84.54923	-2.19968	0.000107	0.025333	protein_coding
ENSG0000(	ENSG0000(	198.7461	-1.19339	0.000105	0.025333	lncRNA
ENSG0000(	IRF1	6852.134	-0.64757	0.000106	0.025333	protein_coding
ENSG0000(	PIM2	2555.17	-0.93233	0.000103	0.025333	protein_coding
ENSG0000(	PLEKHS1	689.732	-1.08373	0.000103	0.025333	protein_coding
ENSG0000(	PTGS2	400.3005	-2.04478	0.000106	0.025333	protein_coding
ENSG0000(	RNF24	1125.698	-0.82765	0.000105	0.025333	protein_coding
ENSG0000(	RTEL1-TNFI	803.5973	-0.53307	0.000108	0.025333	protein_coding
ENSG0000(	SNORD3B-2	102.4429	-0.99101	0.000104	0.025333	snoRNA
ENSG0000(	EVA1B	41.00131	-0.90782	0.000111	0.025822	protein_coding
ENSG0000(	OSM	148.8206	-2.55477	0.000112	0.025961	protein_coding
ENSG0000(	C4orf3	675.0138	-0.42729	0.000115	0.02633	protein_coding
ENSG0000(	LRRC7	1450.224	0.412807	0.000116	0.026372	protein_coding
ENSG0000(	BATF	62.41082	-1.26478	0.000121	0.026882	protein_coding
ENSG0000(	HLA-DRB1	2135.3	-0.75701	0.000121	0.026882	protein_coding
ENSG0000(	PLEK	1720.996	-1.71262	0.000121	0.026882	protein_coding
ENSG0000(	ANGPTL2	391.1149	-1.49203	0.000126	0.027578	protein_coding
ENSG0000(	ENSG0000(	100.6716	0.922356	0.000126	0.027578	lncRNA
ENSG0000(	CTSB	6844.181	-0.63629	0.000131	0.027887	protein_coding
ENSG0000(	ENSG0000(	108.4832	-1.04733	0.00013	0.027887	lncRNA
ENSG0000(	ENSG0000(	51.45989	-2.36508	0.000134	0.027887	lncRNA
ENSG0000(	GFPT2	147.0897	-1.92131	0.000135	0.027887	protein_coding
ENSG0000(	MEDAG	26.0095	-2.96303	0.000136	0.027887	protein_coding
ENSG0000(	RIPOR3	852.1239	-0.87352	0.000134	0.027887	protein_coding
ENSG0000(	TMTC1	916.0569	-0.54977	0.000135	0.027887	protein_coding
ENSG0000(	WNK4	798.9053	0.57263	0.000131	0.027887	protein_coding
ENSG0000(	PRSS22	95.03269	-1.93178	0.000139	0.02805	protein_coding
ENSG0000(	STC2	94.4772	-2.19624	0.000138	0.02805	protein_coding
ENSG0000(	AGT	176.9127	-1.03836	0.000149	0.02853	protein_coding
ENSG0000(	BCL6	1465.578	-1.37515	0.000144	0.02853	protein_coding
ENSG0000(	COL4A2	4031.048	-0.89525	0.00015	0.02853	protein_coding
ENSG0000(	DYSF	898.5982	-1.4278	0.000146	0.02853	protein_coding

ENSG0000(	FCGR1B	261.8219	-1.92875	0.000142	0.02853	protein_coding
ENSG0000(	KLHL36	2386.779	0.191689	0.000147	0.02853	protein_coding
ENSG0000(	SGCB	582.2567	-0.42651	0.00015	0.02853	protein_coding
ENSG0000(	SNX10	254.6103	-1.15816	0.00015	0.02853	protein_coding
ENSG0000(	TAP1	2412.133	-0.68885	0.000148	0.02853	protein_coding
ENSG0000(	ENSG0000(	390.9787	-0.81335	0.000155	0.028884	lncRNA
ENSG0000(	TLR8	180.7284	-1.5082	0.000155	0.028884	protein_coding
ENSG0000(	ZNF888	926.4772	0.436785	0.000155	0.028884	protein_coding
ENSG0000(	FSTL1	2452.29	-0.98039	0.000157	0.028953	protein_coding
ENSG0000(	MMP25	464.9674	-1.49375	0.000159	0.029193	protein_coding
ENSG0000(	GBP1	2784.335	-1.30488	0.000163	0.029623	protein_coding
ENSG0000(	IDO1	701.284	-1.41471	0.000168	0.030433	protein_coding
ENSG0000(	ENSG0000(	36.35731	1.106116	0.00017	0.030629	lncRNA
ENSG0000(	C2	932.7865	-0.49601	0.000172	0.030687	protein_coding
ENSG0000(	MTCO1P11	338.372	-0.62834	0.000173	0.030745	unprocessed_pseudogene
ENSG0000(	ENSG0000(	1180.643	1.053522	0.000175	0.030819	lncRNA
ENSG0000(	GBP5	3461.195	-1.45316	0.000176	0.030819	protein_coding
ENSG0000(	ENSG0000(	27.46419	-1.12033	0.000178	0.030984	lncRNA
ENSG0000(	VCAN	2828.091	-1.12539	0.00018	0.031184	protein_coding
ENSG0000(	LILRB2	491.2724	-1.11925	0.000182	0.031257	protein_coding
ENSG0000(	ATP2B4	2687.605	-0.55287	0.000183	0.031292	protein_coding
ENSG0000(	TNFAIP2	3614.643	-0.79458	0.000187	0.03175	protein_coding
ENSG0000(	NME8	47.59527	-1.22264	0.00019	0.032098	protein_coding
ENSG0000(	ELL2	1719.152	-0.74399	0.000192	0.032191	protein_coding
ENSG0000(	ASAP1	1636.562	-0.67743	0.000195	0.032589	protein_coding
ENSG0000(	KANK4	201.9425	0.512662	0.000199	0.032951	protein_coding
ENSG0000(	RAB34	166.4338	-0.6468	0.0002	0.032951	protein_coding
ENSG0000(	FCER1G	279.2882	-0.75969	0.000202	0.032971	protein_coding
ENSG0000(	GRINA	767.7691	-0.63329	0.000205	0.032971	protein_coding
ENSG0000(	LINC02421	56.27195	1.461524	0.000206	0.032971	lncRNA
ENSG0000(	MIR31HG	26.66952	-2.03543	0.000204	0.032971	lncRNA
ENSG0000(	SEC14L1	2303.147	-0.56978	0.000206	0.032971	protein_coding
ENSG0000(	BCL2A1	103.442	-1.65652	0.000208	0.033012	protein_coding
ENSG0000(	GLRX	1281.716	-0.51502	0.000209	0.033012	protein_coding
ENSG0000(	TYROBP	124.1647	-1.0582	0.00021	0.033012	protein_coding
ENSG0000(	CASP1	1319.599	-0.57479	0.000212	0.033122	protein_coding
ENSG0000(	GBP4	2775.407	-0.78626	0.000217	0.033528	protein_coding
ENSG0000(	MACROD1	126.444	0.69959	0.000217	0.033528	protein_coding
ENSG0000(	ENKUR	85.91818	-1.7816	0.000223	0.034251	protein_coding
ENSG0000(	IGKV1-27	235.0755	-1.31712	0.000224	0.034251	IG_V_gene
ENSG0000(	MTATP6P1	109.7467	-0.72346	0.000235	0.035762	unprocessed_pseudogene
ENSG0000(	CXCL10	294.991	-1.93318	0.000239	0.035867	protein_coding
ENSG0000(	TYMP	1595.846	-0.82265	0.000238	0.035867	protein_coding
ENSG0000(	RAB31	1185.935	-1.03067	0.00024	0.035945	protein_coding
ENSG0000(	ENSG0000(	42.13044	-1.01242	0.000247	0.036377	lncRNA
ENSG0000(	STAT3	6670.441	-0.39358	0.000247	0.036377	protein_coding
ENSG0000(	TCF7L1-IT1	25.5314	-0.75098	0.000246	0.036377	lncRNA

ENSG0000(	HTRA3	143.7839	-1.13604	0.000251	0.03674	protein_coding
ENSG0000(	ARPC1B	1321.934	-0.28867	0.000255	0.036757	protein_coding
ENSG0000(	ENSG0000(	37.56437	-0.89549	0.000254	0.036757	lncRNA
ENSG0000(	TGM2	2142.358	-0.96069	0.000253	0.036757	protein_coding
ENSG0000(	ICAM1	1250.987	-1.1249	0.000258	0.036949	protein_coding
ENSG0000(	ENSG0000(	87.6976	-1.0268	0.000263	0.037404	lncRNA
ENSG0000(	RHOQ	1282.457	-0.76482	0.000264	0.037404	protein_coding
ENSG0000(	GJA1	356.9126	-0.7784	0.000265	0.037426	protein_coding
ENSG0000(	GZMB	26.16575	-1.5275	0.00027	0.037852	protein_coding
ENSG0000(	C1S	2510.281	-0.57033	0.000274	0.037864	protein_coding
ENSG0000(	MUC16	140.4572	-1.77377	0.000273	0.037864	protein_coding
ENSG0000(	PRKAG2	2245.314	0.23491	0.000272	0.037864	protein_coding
ENSG0000(	EMILIN1	690.0499	-0.58284	0.000284	0.038207	protein_coding
ENSG0000(	ENSG0000(	20.22192	-2.29742	0.000283	0.038207	lncRNA
ENSG0000(	ENSG0000(	824.067	0.929211	0.000279	0.038207	lncRNA
ENSG0000(	FAP	195.361	-2.05012	0.000284	0.038207	protein_coding
ENSG0000(	LAMC3	520.0911	-0.53259	0.000281	0.038207	protein_coding
ENSG0000(	STK25	1497.609	0.226596	0.000285	0.038207	protein_coding
ENSG0000(	SAA2	383.8846	-1.40384	0.000288	0.038356	protein_coding
ENSG0000(	TNFRSF18	53.4623	-0.88932	0.000289	0.038356	protein_coding
ENSG0000(	ALDH1L2	836.7796	-0.74249	0.000291	0.038443	protein_coding
ENSG0000(	RUNX1	3658.164	-0.50475	0.000292	0.038447	protein_coding
ENSG0000(	SDC2	268.3497	-0.65801	0.000295	0.038593	protein_coding
ENSG0000(	CRYM-AS1	432.3538	0.453301	0.000297	0.038606	transcribed_unitary_pseudogene
ENSG0000(	ZNF263	753.7675	0.240735	0.000297	0.038606	protein_coding
ENSG0000(	KARS1	787.5053	-0.30956	0.000302	0.038866	protein_coding
ENSG0000(	TMEM132/	520.4563	-1.30537	0.000301	0.038866	protein_coding
ENSG0000(	ENPP7P11	86.10217	0.95724	0.000324	0.040463	processed_pseudogene
ENSG0000(	IL1A	73.78367	-2.43763	0.000321	0.040463	protein_coding
ENSG0000(	MIR503HG	84.75409	-1.14377	0.000316	0.040463	lncRNA
ENSG0000(	NAMPT	15642.57	-1.36172	0.000322	0.040463	protein_coding
ENSG0000(	RAB40B	601.3518	0.526046	0.00032	0.040463	protein_coding
ENSG0000(	VIP	34.12069	-2.10358	0.000321	0.040463	protein_coding
ENSG0000(	ENSG0000(	51.98602	-1.14916	0.000326	0.040588	lncRNA
ENSG0000(	ACSL4	1827.441	-1.16003	0.000331	0.040869	protein_coding
ENSG0000(	IFITM2	311.2097	-0.88281	0.000331	0.040869	protein_coding
ENSG0000(	FAM225A	41.04784	-2.20413	0.000333	0.040915	lncRNA
ENSG0000(	ENSG0000(	21.42601	0.865774	0.00034	0.040982	lncRNA
ENSG0000(	ENSG0000(	288.9741	0.452047	0.000341	0.040982	lncRNA
ENSG0000(	FPR2	120.6235	-0.96363	0.000346	0.040982	protein_coding
ENSG0000(	HAPLN3	448.5369	-1.15124	0.00034	0.040982	protein_coding
ENSG0000(	HRH2	232.0931	-1.16135	0.000342	0.040982	protein_coding
ENSG0000(	MXRA5	1820.284	-0.92173	0.000345	0.040982	protein_coding
ENSG0000(	SPCS3	2771.995	-0.32459	0.000337	0.040982	protein_coding
ENSG0000(	TAT-AS1	126.9254	0.368937	0.000345	0.040982	lncRNA
ENSG0000(	CAPN13	380.8411	1.091172	0.000354	0.041661	protein_coding
ENSG0000(	LINC01709	48.01262	-2.22263	0.000354	0.041661	lncRNA

ENSG0000(	GRPEL2	441.1361	-0.38648	0.000357	0.04176	protein_coding
ENSG0000(	CD40	552.1221	-0.71421	0.000363	0.042267	protein_coding
ENSG0000(	CFLAR	22206.92	-0.33107	0.000367	0.042633	protein_coding
ENSG0000(	PCDHB2	85.47439	0.730149	0.000371	0.042893	protein_coding
ENSG0000(	ENSG0000(	1197.848	0.882119	0.000381	0.043474	TEC
ENSG0000(	SRGN	552.0832	-1.00774	0.000379	0.043474	protein_coding
ENSG0000(	ZNF727	364.0609	0.458371	0.00038	0.043474	protein_coding
ENSG0000(	FAM72D	49.41159	-1.70487	0.00039	0.04439	protein_coding
ENSG0000(	NID2	511.9104	-0.67933	0.000393	0.044546	protein_coding
ENSG0000(	ACSL1	1597.242	-1.50458	0.000404	0.045002	protein_coding
ENSG0000(	CIITA	10495.99	-0.66985	0.000401	0.045002	protein_coding
ENSG0000(	ENSG0000(	326.6637	-0.74146	0.000412	0.045002	processed_pseudogene
ENSG0000(	FER1L4	1380.516	-0.57234	0.000411	0.045002	transcribed_unitary_pseudogene
ENSG0000(	GPC2	191.4306	0.367013	0.000411	0.045002	protein_coding
ENSG0000(	IL2RA	524.1101	-0.92815	0.000409	0.045002	protein_coding
ENSG0000(	LMO4	960.9457	-0.43827	0.000408	0.045002	protein_coding
ENSG0000(	RGS4	44.57317	-1.1482	0.000405	0.045002	protein_coding
ENSG0000(	SP6	129.9876	-0.99014	0.000405	0.045002	protein_coding
ENSG0000(	CLDN8	26.56363	2.756798	0.000422	0.045519	protein_coding
ENSG0000(	MEIS3	169.7428	-0.57522	0.000421	0.045519	protein_coding
ENSG0000(	NRSN2-AS1	1651.993	0.27599	0.00042	0.045519	lncRNA
ENSG0000(	EDNRA	216.5174	-1.18014	0.000429	0.046063	protein_coding
ENSG0000(	CD74	10752.4	-0.6883	0.000439	0.046188	protein_coding
ENSG0000(	GBP1P1	165.2146	-1.21783	0.00044	0.046188	transcribed_unprocessed_pseudo
ENSG0000(	IGHGP	404.9385	-2.10985	0.00044	0.046188	IG_C_pseudogene
ENSG0000(	KIF26B	257.9296	-1.57306	0.000434	0.046188	protein_coding
ENSG0000(	MNDA	128.7852	-1.6284	0.000437	0.046188	protein_coding
ENSG0000(	SLAMF7	1842.875	-0.67065	0.000437	0.046188	protein_coding
ENSG0000(	ADAMTS14	795.8547	-0.60837	0.000446	0.046325	protein_coding
ENSG0000(	CXCR6	172.7365	-0.7184	0.000446	0.046325	protein_coding
ENSG0000(	SPARC	2952.624	-1.07882	0.000444	0.046325	protein_coding
ENSG0000(	AMZ2	600.5126	-0.32022	0.000452	0.04675	protein_coding
ENSG0000(	DCC	167.5172	-1.07045	0.000454	0.04682	protein_coding
ENSG0000(	KIRREL1	966.5615	-0.6851	0.000459	0.046964	protein_coding
ENSG0000(	MYEOV	221.1367	-1.10637	0.000459	0.046964	protein_coding
ENSG0000(	ARHGAP10	627.7447	-0.44601	0.000464	0.04731	protein_coding
ENSG0000(	CD80	341.2526	-1.06064	0.000467	0.04731	protein_coding
ENSG0000(	TACSTD2	29.05283	-1.50288	0.000468	0.04731	protein_coding
ENSG0000(	CCDC14	6773.38	0.342942	0.000476	0.047573	protein_coding
ENSG0000(	ENSG0000(	246.6353	-1.36553	0.000473	0.047573	lncRNA
ENSG0000(	TOGARAM:	714.3002	-0.21515	0.000474	0.047573	protein_coding
ENSG0000(	DDR2	1762.333	-0.68683	0.000477	0.047578	protein_coding
ENSG0000(	LAG3	54.56288	-0.93801	0.000483	0.047912	protein_coding
ENSG0000(	VWF	2166.404	-0.87161	0.000484	0.047912	protein_coding
ENSG0000(	FGF7	633.2764	-1.31782	0.000487	0.048021	protein_coding
ENSG0000(	TNFRSF6B	132.4268	-4.04113	0.000495	0.048653	protein_coding
ENSG0000(	MTND1P11	136.8423	-0.71656	0.0005	0.048979	unprocessed_pseudogene

ENSG0000(	A2M	3751.745	-0.48224	0.000507	0.049259	protein_coding
ENSG0000(	C5orf15	454.1209	-0.42206	0.000506	0.049259	protein_coding
ENSG0000(	FAM157A	779.9033	-1.51044	0.000514	0.049791	transcribed_unprocessed_pseudo
ENSG0000(	TWIST1	24.40407	-1.87933	0.000519	0.050116	protein_coding
ENSG0000(	IRAK3	2053.086	-0.84308	0.000522	0.050259	protein_coding
ENSG0000(	ADAM12	398.649	-1.43841	0.000546	0.050553	protein_coding
ENSG0000(	CDK10	2213.924	0.256519	0.000545	0.050553	protein_coding
ENSG0000(	HK3	306.2257	-1.30281	0.000532	0.050553	protein_coding
ENSG0000(	HLA-DRA	3176.921	-0.76756	0.000549	0.050553	protein_coding
ENSG0000(	IGKJ3	71.37415	-1.11691	0.000539	0.050553	IG_J_gene
ENSG0000(	LYPD1	43.14013	-1.40064	0.000537	0.050553	protein_coding
ENSG0000(	MTCO3P11	153.2359	-0.63381	0.00053	0.050553	unprocessed_pseudogene
ENSG0000(	NALCN	384.9373	0.244001	0.000541	0.050553	protein_coding
ENSG0000(	NCF2	333.4052	-1.2123	0.00053	0.050553	protein_coding
ENSG0000(	NEURL1	637.5517	0.530004	0.000541	0.050553	protein_coding
ENSG0000(	SPCS2	856.6476	-0.40655	0.000549	0.050553	protein_coding
ENSG0000(	SPRED3	232.4	-0.65367	0.000539	0.050553	protein_coding
ENSG0000(	TRIM69	1648.154	-0.41236	0.000543	0.050553	protein_coding
ENSG0000(	C1QTNF5	87.04533	-0.71065	0.000573	0.052061	protein_coding
ENSG0000(	LILRB1	1075.622	-0.67659	0.000574	0.052061	protein_coding
ENSG0000(	PLEKHO2	653.6159	-0.66032	0.000575	0.052061	protein_coding
ENSG0000(	RPS16P5	76.76654	-0.85991	0.00057	0.052061	transcribed_processed_pseudoge
ENSG0000(	SLC6A14	511.303	-1.84725	0.000569	0.052061	protein_coding
ENSG0000(	POLRMT	491.0396	0.392237	0.000578	0.052094	protein_coding
ENSG0000(	TAPBP	6052.56	-0.3173	0.000581	0.05221	protein_coding
ENSG0000(	ENSG0000(	849.3245	0.810273	0.000583	0.052257	TEC
ENSG0000(	CALU	2240.715	-0.46126	0.000586	0.052354	protein_coding
ENSG0000(	NAIPP3	380.5558	-0.53461	0.000592	0.05274	transcribed_unprocessed_pseudo
ENSG0000(	SH3PXD2B	1127.777	-0.94301	0.000605	0.053696	protein_coding
ENSG0000(	ENSG0000(	22.43813	-3.05106	0.000608	0.053748	transcribed_unitary_pseudogene
ENSG0000(	ENSG0000(	30.10965	0.929169	0.000613	0.053903	lncRNA
ENSG0000(	LCP2	2094.597	-0.78914	0.000611	0.053903	protein_coding
ENSG0000(	C1R	1373.83	-0.71821	0.000623	0.054255	protein_coding
ENSG0000(	IGLV1-44	289.1504	-1.41714	0.000622	0.054255	IG_V_gene
ENSG0000(	NEU3	1237.104	-0.36269	0.000623	0.054255	protein_coding
ENSG0000(	ENSG0000(	378.5516	1.146773	0.00063	0.054641	lncRNA
ENSG0000(	ADGRG3	164.9417	-1.82588	0.000637	0.054805	protein_coding
ENSG0000(	CYREN	540.6421	0.486834	0.000637	0.054805	protein_coding
ENSG0000(	ENSG0000(	263.2078	1.293459	0.000634	0.054805	lncRNA
ENSG0000(	SELP	132.9064	-1.39449	0.000646	0.055353	protein_coding
ENSG0000(	EBI3	40.46305	-1.07143	0.000655	0.055632	protein_coding
ENSG0000(	ENSG0000(	53.94601	-1.06599	0.000655	0.055632	processed_pseudogene
ENSG0000(	SERPINE1	391.3406	-1.16636	0.000655	0.055632	protein_coding
ENSG0000(	CDKL2	62.98967	0.965304	0.000668	0.055686	protein_coding
ENSG0000(	COG2	1324.182	0.310663	0.000668	0.055686	protein_coding
ENSG0000(	LAX1	764.152	-0.72261	0.000665	0.055686	protein_coding
ENSG0000(	LILRA6	174.6669	-1.2954	0.000668	0.055686	protein_coding

ENSG0000(	RGS5	2110.386	-0.43713	0.000666	0.055686	protein_coding
ENSG0000(	UBE2L6	715.2849	-0.76063	0.000668	0.055686	protein_coding
ENSG0000(	CEBPB	381.3917	-0.8412	0.000684	0.055723	protein_coding
ENSG0000(	DIO2	144.9089	-1.22005	0.000675	0.055723	protein_coding
ENSG0000(	NRDC	2643.174	-0.20175	0.000687	0.055723	protein_coding
ENSG0000(	PGLYRP3	669.516	0.88163	0.000683	0.055723	protein_coding
ENSG0000(	PLAAT4	318.1081	-0.82937	0.000684	0.055723	protein_coding
ENSG0000(	POU2AF1	2829.523	-0.69035	0.000678	0.055723	protein_coding
ENSG0000(	SLED1	92.29162	-1.77642	0.000684	0.055723	transcribed_processed_pseudoge
ENSG0000(	TBCEL-TEC	38.51289	-0.72294	0.000675	0.055723	protein_coding
ENSG0000(	TLR4	2175.11	-0.76879	0.000686	0.055723	protein_coding
ENSG0000(	LRRK2	1770.079	-0.92644	0.000692	0.055793	protein_coding
ENSG0000(	TNFRSF8	39.00393	-1.26318	0.00069	0.055793	protein_coding
ENSG0000(	GAL3ST2	176.9352	1.093767	0.000701	0.056378	protein_coding
ENSG0000(	SEC24A	1584.989	-0.37365	0.000707	0.056698	protein_coding
ENSG0000(	NCF1B	178.0878	-0.9159	0.000711	0.056715	transcribed_unprocessed_pseudo
ENSG0000(	SIRPA	758.6407	-0.86835	0.000711	0.056715	protein_coding
ENSG0000(	CD93	1402.918	-0.76297	0.00073	0.057018	protein_coding
ENSG0000(	CDC16	1240.166	0.202616	0.000728	0.057018	protein_coding
ENSG0000(	ENSG0000(	288.4627	0.378292	0.000723	0.057018	lncRNA
ENSG0000(	FAM89A	79.96057	-0.74469	0.000729	0.057018	protein_coding
ENSG0000(	MBD2	1555.591	-0.27881	0.000718	0.057018	protein_coding
ENSG0000(	SFMBT2	1868.831	-0.4279	0.000719	0.057018	protein_coding
ENSG0000(	WDR31	878.9672	0.196358	0.000727	0.057018	protein_coding
ENSG0000(	ZNF321P	814.6076	0.328024	0.00074	0.057684	transcribed_processed_pseudoge
ENSG0000(	LTK	177.7537	0.893744	0.000744	0.057807	protein_coding
ENSG0000(	SELPLG	305.3104	-0.80827	0.000749	0.058042	protein_coding
ENSG0000(	SLC6A6	1578.19	-0.7237	0.000752	0.058106	protein_coding
ENSG0000(	C3AR1	176.5517	-0.87985	0.000754	0.058126	protein_coding
ENSG0000(	XBP1	5220.655	-0.71651	0.000756	0.058126	protein_coding
ENSG0000(	HLA-DPA1	2413.987	-0.67554	0.00076	0.058278	protein_coding
ENSG0000(	NBN	1108.326	-0.439	0.000771	0.058746	protein_coding
ENSG0000(	NOS2P1	288.8583	-0.99906	0.00077	0.058746	transcribed_unprocessed_pseudo
ENSG0000(	TCIRG1	2070.966	-0.37	0.000775	0.058871	protein_coding
ENSG0000(	HNRNPK	4043.717	-0.15261	0.000777	0.05888	protein_coding
ENSG0000(	ENSG0000(	223.9568	-0.57656	0.000787	0.05929	lncRNA
ENSG0000(	FBXO30	742.5584	-0.1963	0.000786	0.05929	protein_coding
ENSG0000(	PLCH2	252.1467	0.670932	0.000791	0.059427	protein_coding
ENSG0000(	GPR153	471.4714	0.668672	0.000795	0.059578	protein_coding
ENSG0000(	ARAF	855.5409	0.255923	0.000804	0.060014	protein_coding
ENSG0000(	CD38	1698.21	-0.62893	0.000811	0.060014	protein_coding
ENSG0000(	PTPRE	2059.814	-0.44946	0.000806	0.060014	protein_coding
ENSG0000(	TNC	2616.525	-1.87543	0.000811	0.060014	protein_coding
ENSG0000(	YBX1P8	20.64078	-1.0821	0.000808	0.060014	processed_pseudogene
ENSG0000(	BST2	252.9716	-0.81624	0.000833	0.06147	protein_coding
ENSG0000(	IL1R1	2234.871	-0.67656	0.000839	0.061533	protein_coding
ENSG0000(	PRAG1	741.1323	0.691965	0.000838	0.061533	protein_coding

ENSG0000(	ADM	483.7507	-1.12752	0.000846	0.061634	protein_coding
ENSG0000(	FCGR2A	1213.538	-1.30041	0.000849	0.061634	protein_coding
ENSG0000(	NABP1	7534.041	-0.64205	0.000848	0.061634	protein_coding
ENSG0000(	SMIM13	252.938	-0.33544	0.000844	0.061634	protein_coding
ENSG0000(	ENSG0000(	773.9713	0.395518	0.000853	0.061728	lncRNA
ENSG0000(	P4HA1	522.5543	-0.54468	0.00086	0.062122	protein_coding
ENSG0000(	ENSG0000(	165.7683	0.32566	0.000869	0.062587	lncRNA
ENSG0000(	IGKV2-28	710.4315	-1.34697	0.000872	0.062641	IG_V_gene
ENSG0000(	LAMC1	1786.645	-0.53638	0.000883	0.063285	protein_coding
ENSG0000(	MMP9	330.2451	-1.40469	0.0009	0.064301	protein_coding
ENSG0000(	SOCS3	1213.366	-1.41328	0.000902	0.064301	protein_coding
ENSG0000(	FNDC3B	6328.533	-0.43478	0.00091	0.064682	protein_coding
ENSG0000(	S100A11	260.4314	-0.96719	0.000912	0.064691	protein_coding
ENSG0000(	EFEMP1	172.69	-0.91815	0.000929	0.065678	protein_coding
ENSG0000(	ENSG0000(	75.54723	-0.96559	0.000934	0.065713	TEC
ENSG0000(	GLIS2	122.8577	-0.84013	0.000932	0.065713	protein_coding
ENSG0000(	ENSG0000(	1123.271	-0.46142	0.000939	0.065901	lncRNA
ENSG0000(	CXCL3	481.7693	-1.25148	0.000948	0.066026	protein_coding
ENSG0000(	CYRIB	1701.451	-0.25395	0.000955	0.066026	protein_coding
ENSG0000(	PRR16	207.8757	-0.66064	0.00095	0.066026	protein_coding
ENSG0000(	PTPRN2	324.3822	0.741397	0.000952	0.066026	protein_coding
ENSG0000(	RPL36AP33	41.02771	-0.87345	0.000955	0.066026	processed_pseudogene
ENSG0000(	TMEM140	552.8631	-0.56912	0.000949	0.066026	protein_coding
ENSG0000(	DESI1	1366.004	-0.25733	0.00096	0.066059	protein_coding
ENSG0000(	IGKV2D-29	88.58132	-1.35204	0.000959	0.066059	IG_V_gene
ENSG0000(	SNX18	713.061	-0.43926	0.000965	0.066206	protein_coding
ENSG0000(	FBN1	1866.409	-0.74484	0.000977	0.066832	protein_coding
ENSG0000(	COL3A1	9976.124	-1.05384	0.000987	0.067414	protein_coding
ENSG0000(	NRP1	1809.674	-0.44277	0.000993	0.067462	protein_coding
ENSG0000(	TBK1	2620.992	-0.18883	0.000991	0.067462	protein_coding
ENSG0000(	BRSK1	139.79	-0.63491	0.001005	0.067728	protein_coding
ENSG0000(	DUOX2	9704.057	-2.14453	0.001007	0.067728	protein_coding
ENSG0000(	LINC00279	57.28766	1.026802	0.001005	0.067728	lncRNA
ENSG0000(	RFX6	25.47988	1.283843	0.001005	0.067728	protein_coding
ENSG0000(	ENSG0000(	531.9925	0.196246	0.001021	0.068113	lncRNA
ENSG0000(	ENSG0000(	112.1741	-1.46002	0.001016	0.068113	lncRNA
ENSG0000(	PHC2	1054.261	-0.4532	0.001023	0.068113	protein_coding
ENSG0000(	PTK7	827.1412	-0.46639	0.001025	0.068113	protein_coding
ENSG0000(	STXBP5-AS	654.4259	0.417472	0.00102	0.068113	lncRNA
ENSG0000(	ADAMTS4	1455.162	-0.87083	0.001033	0.068492	protein_coding
ENSG0000(	ENSG0000(	32.51636	-0.87152	0.001053	0.069665	processed_pseudogene
ENSG0000(	GSTO2	1558.479	0.345565	0.001061	0.069889	protein_coding
ENSG0000(	LIMK1	1071.907	-0.31899	0.001063	0.069889	protein_coding
ENSG0000(	RPP38-DT	179.0763	0.328528	0.001064	0.069889	lncRNA
ENSG0000(	JAKMIP1	29.23567	-1.02884	0.001076	0.070511	protein_coding
ENSG0000(	IGHG1	9187.07	-1.84983	0.00109	0.071253	IG_C_gene
ENSG0000(	LAP3	948.3666	-0.71104	0.001107	0.071782	protein_coding

ENSG0000(	LOX	150.6844	-0.88045	0.001107	0.071782	protein_coding
ENSG0000(	LYZ	3847.424	-0.97044	0.001103	0.071782	protein_coding
ENSG0000(	VASN	97.71207	-0.92867	0.001109	0.071782	protein_coding
ENSG0000(	BICC1	540.1963	-0.81658	0.00114	0.072968	protein_coding
ENSG0000(	CACNA2D2	99.34328	0.915216	0.001137	0.072968	protein_coding
ENSG0000(	ENSG0000(	49.00183	-2.62225	0.001133	0.072968	processed_pseudogene
ENSG0000(	MITF	450.898	-0.38276	0.001139	0.072968	protein_coding
ENSG0000(	ZNF497-AS	51.09438	0.680719	0.00114	0.072968	lncRNA
ENSG0000(	HCK	503.2876	-0.73179	0.00115	0.073417	protein_coding
ENSG0000(	RAB23	239.6353	-0.45733	0.001163	0.073737	protein_coding
ENSG0000(	THRB	444.1879	1.140981	0.001161	0.073737	protein_coding
ENSG0000(	TRAFD1	890.0086	-0.46092	0.001163	0.073737	protein_coding
ENSG0000(	ADGRE3	58.29923	-1.32492	0.001173	0.073755	protein_coding
ENSG0000(	CHST14	72.03159	-0.51551	0.001168	0.073755	protein_coding
ENSG0000(	ENSG0000(	1146.318	-0.71024	0.001174	0.073755	lncRNA
ENSG0000(	SEL1L	3730.75	-0.38181	0.001174	0.073755	protein_coding
ENSG0000(	CXCL2	203.0692	-1.27915	0.001181	0.074023	protein_coding
ENSG0000(	CPNE7	95.50559	-0.89098	0.001189	0.074074	protein_coding
ENSG0000(	ENSG0000(	55.53558	-1.23395	0.00119	0.074074	lncRNA
ENSG0000(	ENSG0000(	145.2543	-1.8128	0.001188	0.074074	lncRNA
ENSG0000(	CCL2	308.0571	-0.88188	0.0012	0.074162	protein_coding
ENSG0000(	IER5L	66.34539	-0.66078	0.001208	0.074162	protein_coding
ENSG0000(	MMP14	1353.544	-0.70346	0.001207	0.074162	protein_coding
ENSG0000(	MRAS	330.5963	-0.7471	0.001205	0.074162	protein_coding
ENSG0000(	TMEM106/	1248.028	-0.29068	0.001194	0.074162	protein_coding
ENSG0000(	VEGFB	266.3481	-0.43569	0.001208	0.074162	protein_coding
ENSG0000(	APOL3	1450.395	-0.63034	0.001215	0.07442	protein_coding
ENSG0000(	DNM1P34	49.30153	-0.80953	0.001224	0.074472	transcribed_unprocessed_pseudo
ENSG0000(	ENSG0000(	134.8306	-0.62835	0.001225	0.074472	lncRNA
ENSG0000(	FBXW7	1947.171	-0.34897	0.001229	0.074472	protein_coding
ENSG0000(	IGKV3D-20	78.20122	-1.38971	0.001229	0.074472	IG_V_gene
ENSG0000(	IL11	357.6162	-1.60633	0.001221	0.074472	protein_coding
ENSG0000(	PNOC	69.12116	-0.8668	0.001232	0.074472	protein_coding
ENSG0000(	HGF	263.1239	-0.86508	0.001238	0.074666	protein_coding
ENSG0000(	SRPX2	797.4218	-0.83722	0.00124	0.074666	protein_coding
ENSG0000(	MAP1A	251.4394	-0.73213	0.00125	0.075069	protein_coding
ENSG0000(	BNIP3	226.4082	-0.59266	0.001254	0.075181	protein_coding
ENSG0000(	ADAMTS9	970.7552	-0.67131	0.001272	0.075271	protein_coding
ENSG0000(	ADGRE2	1546.083	-0.63407	0.001269	0.075271	protein_coding
ENSG0000(	ANKDD1B	361.597	0.706636	0.00126	0.075271	protein_coding
ENSG0000(	BATF2	499.8992	-0.84259	0.001275	0.075271	protein_coding
ENSG0000(	BTN3A3	2479.076	-0.39944	0.001279	0.075271	protein_coding
ENSG0000(	CCDC134	1291.465	-0.19833	0.001276	0.075271	protein_coding
ENSG0000(	CKAP2L	379.0148	-0.37863	0.00128	0.075271	protein_coding
ENSG0000(	MDK	355.1569	-0.87744	0.001266	0.075271	protein_coding
ENSG0000(	PKM	2962.415	-0.42804	0.001278	0.075271	protein_coding
ENSG0000(	DCST2	66.47777	0.769454	0.001307	0.075859	protein_coding

ENSG0000(	IGHV1-18	511.1366	-1.32448	0.001296	0.075859	IG_V_gene
ENSG0000(	LILRB3	577.6766	-1.1618	0.001305	0.075859	protein_coding
ENSG0000(	PAF1	244.6931	-0.32063	0.001301	0.075859	protein_coding
ENSG0000(	RBPMS	2359.95	-0.58403	0.001304	0.075859	protein_coding
ENSG0000(	ZFHX2	167.5604	-0.63633	0.001302	0.075859	protein_coding
ENSG0000(	DUSP4	366.4921	-0.98764	0.001311	0.075911	protein_coding
ENSG0000(	ELK3	777.4805	-0.5392	0.001313	0.075914	protein_coding
ENSG0000(	LUM	686.1132	-1.26376	0.001333	0.0767	protein_coding
ENSG0000(	RN7SL597F	45.26752	-0.44343	0.00133	0.0767	misc_RNA
ENSG0000(	ENSG0000(	597.2673	-0.19843	0.00134	0.076921	lncRNA
ENSG0000(	GPR176	161.4469	-1.12315	0.001342	0.076921	protein_coding
ENSG0000(	COL6A3	10588.83	-0.90139	0.001348	0.076947	protein_coding
ENSG0000(	GPRIN1	37.63482	-0.75364	0.001347	0.076947	protein_coding
ENSG0000(	ENSG0000(	21.43941	-0.84018	0.001356	0.077228	lncRNA
ENSG0000(	HOXA11-AS	122.252	1.52882	0.001359	0.07726	lncRNA
ENSG0000(	APOL6	6271.413	-0.39063	0.001372	0.077785	protein_coding
ENSG0000(	LPL	109.0372	-1.16685	0.001374	0.077785	protein_coding
ENSG0000(	LIMS3	88.29422	-0.87845	0.001378	0.077828	protein_coding
ENSG0000(	SERPINA3	96.6935	-1.79125	0.00138	0.077828	protein_coding
ENSG0000(	ST6GALNA(	1174.132	0.533155	0.001395	0.078463	protein_coding
ENSG0000(	TICAM2	196.5687	-0.9036	0.001398	0.078483	protein_coding
ENSG0000(	SUN1	3281.797	0.309422	0.001402	0.078565	protein_coding
ENSG0000(	LINC01303	77.56211	-1.18968	0.001415	0.078987	lncRNA
ENSG0000(	OLFM4	7612.067	-1.39762	0.001415	0.078987	protein_coding
ENSG0000(	CUL5	1271.778	0.317952	0.001432	0.079407	protein_coding
ENSG0000(	HSD17B13	358.2646	0.319489	0.001434	0.079407	protein_coding
ENSG0000(	LASTR	652.457	0.788477	0.001431	0.079407	lncRNA
ENSG0000(	LINC02327	260.3631	0.250383	0.001427	0.079407	lncRNA
ENSG0000(	CCR2	255.8546	-0.78527	0.001441	0.079467	protein_coding
ENSG0000(	ENSG0000(	418.6054	-0.66574	0.001441	0.079467	lncRNA
ENSG0000(	PDCD1LG2	102.6067	-0.9474	0.001451	0.079679	protein_coding
ENSG0000(	POLL	947.2733	0.199665	0.001449	0.079679	protein_coding
ENSG0000(	FERMT2	804.7749	-0.4908	0.001465	0.080315	protein_coding
ENSG0000(	PVR	1171.884	0.381511	0.001472	0.080505	protein_coding
ENSG0000(	CDHR3	715.571	0.383313	0.001485	0.080891	protein_coding
ENSG0000(	ENSG0000(	198.3721	0.274849	0.001487	0.080891	lncRNA
ENSG0000(	SHANK1	46.7109	-1.04454	0.001491	0.080891	protein_coding
ENSG0000(	UBTD1	23.40246	-1.30349	0.001489	0.080891	protein_coding
ENSG0000(	ADGRA2	744.7408	-0.58603	0.001511	0.08128	protein_coding
ENSG0000(	CDH11	1411.87	-0.60852	0.001511	0.08128	protein_coding
ENSG0000(	ENSG0000(	90.34513	0.776064	0.001509	0.08128	lncRNA
ENSG0000(	GPR4	140.5747	-0.71398	0.001516	0.08128	protein_coding
ENSG0000(	LINC00472	1322.255	-0.37211	0.001513	0.08128	lncRNA
ENSG0000(	PALM2AKA	2394.09	-0.53516	0.001515	0.08128	protein_coding
ENSG0000(	CASP8	1834.938	-0.29995	0.001528	0.081617	protein_coding
ENSG0000(	COL1A1	6614.344	-1.21882	0.001528	0.081617	protein_coding
ENSG0000(	MAPKAPK2	1445.398	-0.54569	0.001537	0.081933	protein_coding

ENSG0000(	FPR1	187.2142	-1.53583	0.00154	0.081976	protein_coding
ENSG0000(	CYRIA	569.5961	-0.65275	0.001548	0.082231	protein_coding
ENSG0000(	DNAH10	60.98162	0.887951	0.001557	0.082414	protein_coding
ENSG0000(	PEA15	835.0444	-0.59616	0.001564	0.082414	protein_coding
ENSG0000(	PTPRO	1048.605	0.52564	0.001559	0.082414	protein_coding
ENSG0000(	SPECC1L	2448.877	0.402145	0.001563	0.082414	protein_coding
ENSG0000(	COPB1	1472.545	-0.22918	0.001576	0.082742	protein_coding
ENSG0000(	MIR223HG	1078.778	-1.04721	0.001576	0.082742	lncRNA
ENSG0000(	AEBP1	1185.171	-0.81273	0.001589	0.082777	protein_coding
ENSG0000(	CXCR2	251.9435	-1.25334	0.001597	0.082777	protein_coding
ENSG0000(	DAPP1	938.9848	-0.70758	0.001584	0.082777	protein_coding
ENSG0000(	ENSG0000(	110.1247	-0.45575	0.001598	0.082777	lncRNA
ENSG0000(	ENSG0000(	19.48655	-1.81204	0.001593	0.082777	lncRNA
ENSG0000(	MTCO1P40	100.0774	2.123284	0.001594	0.082777	processed_pseudogene
ENSG0000(	PPP1R3D	264.1365	-0.27171	0.001589	0.082777	protein_coding
ENSG0000(	ICAM5	41.34784	-0.95906	0.001609	0.083198	protein_coding
ENSG0000(	MIR34AHG	674.0521	-0.79771	0.001624	0.083809	lncRNA
ENSG0000(	GGT5	975.195	-0.45705	0.001629	0.083943	protein_coding
ENSG0000(	B3GALT5	2975.296	0.99801	0.001653	0.084552	protein_coding
ENSG0000(	CCR1	220.9482	-0.88764	0.001646	0.084552	protein_coding
ENSG0000(	CHI3L2	150.7394	-0.94819	0.001653	0.084552	protein_coding
ENSG0000(	MSC	78.12696	-0.96063	0.00165	0.084552	protein_coding
ENSG0000(	TMEM80	773.4389	0.299953	0.001667	0.085097	protein_coding
ENSG0000(	FAM153B	233.1051	0.620306	0.001684	0.085505	protein_coding
ENSG0000(	MTND4P14	166.6851	-0.58944	0.001681	0.085505	unprocessed_pseudogene
ENSG0000(	THY1	583.3784	-0.97846	0.001682	0.085505	protein_coding
ENSG0000(	ATOX1	683.9339	-0.35963	0.001706	0.085794	protein_coding
ENSG0000(	ENSG0000(	131.4718	-0.82374	0.001705	0.085794	processed_pseudogene
ENSG0000(	P3H1	1478.192	-0.24087	0.001698	0.085794	protein_coding
ENSG0000(	RIMKLA	526.6471	0.621654	0.001703	0.085794	protein_coding
ENSG0000(	TPST2	929.6364	-0.36699	0.001704	0.085794	protein_coding
ENSG0000(	NFE2	40.48912	-2.3325	0.001713	0.086	protein_coding
ENSG0000(	ZFP64	561.6012	0.222242	0.001719	0.08613	protein_coding
ENSG0000(	NPL	558.8327	-0.57107	0.001728	0.086296	protein_coding
ENSG0000(	ZNF701	1203.655	0.301301	0.001727	0.086296	protein_coding
ENSG0000(	ENSG0000(	31.4997	-1.79018	0.001742	0.086662	lncRNA
ENSG0000(	ENSG0000(	299.6247	0.310386	0.00174	0.086662	lncRNA
ENSG0000(	CLEC4A	75.06193	-0.84893	0.001746	0.086726	protein_coding
ENSG0000(	MMP25-AS	815.2691	-0.24657	0.001752	0.086873	lncRNA
ENSG0000(	ENSG0000(	20.21845	-1.0816	0.001758	0.086915	lncRNA
ENSG0000(	IGHV4-34	326.1964	-1.40423	0.00176	0.086915	IG_V_gene
ENSG0000(	BICD1	914.7841	-0.58453	0.001771	0.087024	protein_coding
ENSG0000(	LINC01140	211.1324	-0.40195	0.001766	0.087024	lncRNA
ENSG0000(	VWC2	89.18215	0.53461	0.001771	0.087024	protein_coding
ENSG0000(	PCOLCE	258.7008	-0.82038	0.001776	0.087058	protein_coding
ENSG0000(	SDK1	775.4704	-0.83775	0.001778	0.087058	protein_coding
ENSG0000(	WASF1	70.62477	-0.8051	0.001787	0.087305	protein_coding

ENSG0000(	ENSG0000(	78.63569	-2.27682	0.001816	0.08828	protein_coding
ENSG0000(	EXOC3	1518.989	0.285675	0.001811	0.08828	protein_coding
ENSG0000(	TDO2	271.832	-1.20112	0.001816	0.08828	protein_coding
ENSG0000(	CSF2RB	1531.611	-0.84188	0.00183	0.088794	protein_coding
ENSG0000(	FLT3LG	567.3118	-0.24913	0.001847	0.089277	protein_coding
ENSG0000(	MYLK3	2047.758	0.356535	0.00185	0.089277	protein_coding
ENSG0000(	TRNP1	36.32564	-1.2617	0.001845	0.089277	protein_coding
ENSG0000(	H19	81.20465	-1.62493	0.001854	0.089344	lncRNA
ENSG0000(	ACER2	222.7269	0.633131	0.001894	0.090439	protein_coding
ENSG0000(	BGN	545.0922	-1.08051	0.001906	0.090439	protein_coding
ENSG0000(	CHRDL2	48.3571	-1.76944	0.001973	0.090439	protein_coding
ENSG0000(	CTSC	7992.021	-0.39593	0.001918	0.090439	protein_coding
ENSG0000(	DBN1	212.3418	-0.71235	0.001955	0.090439	protein_coding
ENSG0000(	ENSG0000(	101.5569	-0.64486	0.001981	0.090439	lncRNA
ENSG0000(	ESR1	178.3256	-0.83551	0.001937	0.090439	protein_coding
ENSG0000(	FAM20A	490.8297	-1.05002	0.001983	0.090439	protein_coding
ENSG0000(	GTF2IRD2	2210.989	0.305113	0.001964	0.090439	protein_coding
ENSG0000(	IGHG4	1074.846	-1.78931	0.0019	0.090439	IG_C_gene
ENSG0000(	LINC02605	2151.058	-0.52577	0.001975	0.090439	lncRNA
ENSG0000(	LPGAT1	2445.623	-0.35714	0.001889	0.090439	protein_coding
ENSG0000(	MRPS35-D	153.3299	-0.33795	0.001898	0.090439	lncRNA
ENSG0000(	MSANTD3	220.6833	-0.57506	0.001946	0.090439	protein_coding
ENSG0000(	MSH3	1621.697	0.183577	0.001914	0.090439	protein_coding
ENSG0000(	NLRC4	109.9525	-0.8877	0.001977	0.090439	protein_coding
ENSG0000(	PA2G4P4	94.14698	0.543742	0.001952	0.090439	processed_pseudogene
ENSG0000(	PHLDA1	1328.158	-0.78455	0.001929	0.090439	protein_coding
ENSG0000(	PLA1A	40.58008	-1.38918	0.001985	0.090439	protein_coding
ENSG0000(	RAP1GAP	547.8914	0.688358	0.001963	0.090439	protein_coding
ENSG0000(	RDH11	768.0874	-0.32556	0.001904	0.090439	protein_coding
ENSG0000(	RN7SL16P	64.26236	0.361939	0.001939	0.090439	misc_RNA
ENSG0000(	RNF213	31025.85	-0.52993	0.001921	0.090439	protein_coding
ENSG0000(	SAA1	60.1126	-2.18212	0.001956	0.090439	protein_coding
ENSG0000(	SERPINA1	1451.373	-0.93403	0.001977	0.090439	protein_coding
ENSG0000(	SETD7	1742.378	-0.28193	0.001929	0.090439	protein_coding
ENSG0000(	SIGLEC9	119.5575	-0.77204	0.001908	0.090439	protein_coding
ENSG0000(	SLAMF8	488.9217	-0.72697	0.001889	0.090439	protein_coding
ENSG0000(	SLC31A2	301.3952	-0.62848	0.001975	0.090439	protein_coding
ENSG0000(	SPI1	362.7869	-0.82917	0.001899	0.090439	protein_coding
ENSG0000(	Telomerase	226.2362	0.527907	0.001975	0.090439	misc_RNA
ENSG0000(	TRIM45	199.7765	0.314723	0.001981	0.090439	protein_coding
ENSG0000(	TXNDC5	6470.785	-0.64655	0.001917	0.090439	protein_coding
ENSG0000(	CCDC71L	750.0503	-0.71437	0.001997	0.090454	protein_coding
ENSG0000(	DHRS4	220.4219	0.425938	0.002	0.090454	protein_coding
ENSG0000(	FSCN1	279.8122	-0.72003	0.001991	0.090454	protein_coding
ENSG0000(	KSR1	1961.644	-0.3473	0.002002	0.090454	protein_coding
ENSG0000(	PRRT1B	26.094	1.324672	0.001997	0.090454	protein_coding
ENSG0000(	ANO2	142.2708	-0.87527	0.002013	0.090811	protein_coding

ENSG0000(	CLIC2	204.0905	-0.47583	0.002018	0.090868	protein_coding
ENSG0000(	SYNGR3	31.53306	-1.12118	0.002022	0.090883	protein_coding
ENSG0000(	CTNNA1P1	29.15	0.882633	0.002083	0.091317	processed_pseudogene
ENSG0000(	ENSG0000(	20.35302	-1.44964	0.002082	0.091317	transcribed_unprocessed_pseudo
ENSG0000(	ENSG0000(	24.52367	0.89962	0.002064	0.091317	TEC
ENSG0000(	ENSG0000(	41.54449	0.441089	0.002092	0.091317	pseudogene
ENSG0000(	FAM157B	149.9346	-1.7379	0.002086	0.091317	transcribed_unprocessed_pseudo
ENSG0000(	FGF2	284.6394	-0.73713	0.002045	0.091317	protein_coding
ENSG0000(	GAL3ST1	54.19551	0.695669	0.002042	0.091317	protein_coding
ENSG0000(	IGHV3-11	305.0062	-0.92185	0.002035	0.091317	IG_V_gene
ENSG0000(	ING5	1780.746	0.280936	0.002066	0.091317	protein_coding
ENSG0000(	LINC01871	70.28032	-0.70391	0.002084	0.091317	lncRNA
ENSG0000(	LINC02197	743.1309	0.650008	0.002084	0.091317	lncRNA
ENSG0000(	LMCD1	731.6171	-0.95257	0.002078	0.091317	protein_coding
ENSG0000(	MDGA1	367.3411	-0.8222	0.002092	0.091317	protein_coding
ENSG0000(	MYO1F	1349.637	-0.48372	0.002058	0.091317	protein_coding
ENSG0000(	NEU4	291.748	1.072783	0.00207	0.091317	protein_coding
ENSG0000(	PHAF1	776.4111	0.199207	0.002094	0.091317	protein_coding
ENSG0000(	PIM1	756.0042	-0.463	0.002082	0.091317	protein_coding
ENSG0000(	PROK2	51.76904	-2.26547	0.002094	0.091317	protein_coding
ENSG0000(	SARM1	1152.81	0.32161	0.002053	0.091317	protein_coding
ENSG0000(	CAPS2	618.7922	-0.20012	0.0021	0.09141	protein_coding
ENSG0000(	GNLY	215.8103	-0.74782	0.002103	0.09141	protein_coding
ENSG0000(	ADGRF5	619.9995	-0.68267	0.002111	0.091565	protein_coding
ENSG0000(	KIFC3	862.0883	-0.47265	0.002115	0.091565	protein_coding
ENSG0000(	MCTP1	694.3348	-0.69511	0.002118	0.091565	protein_coding
ENSG0000(	TTYH3	1630.18	-0.43803	0.00212	0.091565	protein_coding
ENSG0000(	MIR100HG	3263.411	0.705734	0.002126	0.091675	lncRNA
ENSG0000(	STX2	428.5674	-0.38292	0.002136	0.09198	protein_coding
ENSG0000(	CAV1	467.8296	-0.74714	0.002144	0.092178	protein_coding
ENSG0000(	ENSG0000(	50.16062	-0.68739	0.002152	0.092216	protein_coding
ENSG0000(	NRCAM	206.5745	-1.54039	0.002155	0.092216	protein_coding
ENSG0000(	TRAM2	2519.612	-0.39357	0.002153	0.092216	protein_coding
ENSG0000(	ADGRL4	191.4862	-0.61868	0.002172	0.092272	protein_coding
ENSG0000(	DDHD1	4018.079	-0.24214	0.002162	0.092272	protein_coding
ENSG0000(	ENSG0000(	134.0852	-0.49366	0.002196	0.092272	TEC
ENSG0000(	IGHV3-23	1815.472	-0.9629	0.002194	0.092272	IG_V_gene
ENSG0000(	IGKC	23414.37	-0.97639	0.002201	0.092272	IG_C_gene
ENSG0000(	IL15RA	427.0964	-0.44455	0.002163	0.092272	protein_coding
ENSG0000(	KCNA2	397.6128	-0.5373	0.002189	0.092272	protein_coding
ENSG0000(	LTA4H	1305.517	-0.38633	0.002186	0.092272	protein_coding
ENSG0000(	MCFD2	3841.257	-0.14986	0.002198	0.092272	protein_coding
ENSG0000(	MRPS25	2625.094	0.326902	0.002184	0.092272	protein_coding
ENSG0000(	PALMD	220.1988	-0.65563	0.002198	0.092272	protein_coding
ENSG0000(	PDK1	5574.438	-0.52394	0.00221	0.092272	protein_coding
ENSG0000(	RUNX2	972.4923	-0.52754	0.002208	0.092272	protein_coding
ENSG0000(	SLC2A5	650.5214	-0.36251	0.002185	0.092272	protein_coding

ENSG0000(	STAG3L1	441.1561	0.263887	0.002208	0.092272	transcribed_unprocessed_pseudo
ENSG0000(	TIMP2	2047.311	-0.70943	0.00217	0.092272	protein_coding
ENSG0000(	DLEU2	2897.495	-0.22609	0.002224	0.092414	lncRNA
ENSG0000(	ENSG0000(	450.0242	-1.2659	0.002227	0.092414	lncRNA
ENSG0000(	PHTF1	723.7859	-0.32837	0.002218	0.092414	protein_coding
ENSG0000(	RBM17	1435.187	-0.14909	0.002221	0.092414	protein_coding
ENSG0000(	TANK	1317.24	-0.30836	0.002233	0.092524	protein_coding
ENSG0000(	ENSG0000(	19.59279	-0.9677	0.002264	0.093679	lncRNA
ENSG0000(	ITGA8	768.8018	-0.5236	0.002278	0.094087	protein_coding
ENSG0000(	NCOA7	1494.401	-0.40482	0.002295	0.094573	protein_coding
ENSG0000(	TPP1	2311.364	-0.3652	0.002296	0.094573	protein_coding
ENSG0000(	ENSG0000(	61.90592	-0.83558	0.002306	0.094698	lncRNA
ENSG0000(	RGS16	124.5844	-1.13041	0.002306	0.094698	protein_coding
ENSG0000(	CFAP97	1185.769	-0.20021	0.002328	0.095166	protein_coding
ENSG0000(	ENSG0000(	236.1913	-0.43979	0.00233	0.095166	lncRNA
ENSG0000(	NKG7	40.69264	-0.96308	0.002331	0.095166	protein_coding
ENSG0000(	PRRX1	600.1433	-1.65605	0.002329	0.095166	protein_coding
ENSG0000(	GPR68	110.0258	-0.87381	0.002335	0.095177	protein_coding
ENSG0000(	B3GNT9	147.8267	-0.47583	0.002348	0.095421	protein_coding
ENSG0000(	HPRT1	130.2051	-0.44052	0.002348	0.095421	protein_coding
ENSG0000(	CLDN1	127.1754	-1.4549	0.002363	0.095892	protein_coding
ENSG0000(	HOXC4	39.24899	-0.95374	0.002376	0.096258	protein_coding
ENSG0000(	ADAMTS2	729.7361	-0.84747	0.002385	0.096423	protein_coding
ENSG0000(	LINC01572	523.3826	0.299108	0.002387	0.096423	lncRNA
ENSG0000(	PXDC1	167.1438	-0.55349	0.00239	0.096423	protein_coding
ENSG0000(	ELOVL5	1042.966	-0.56809	0.002406	0.096585	protein_coding
ENSG0000(	OSMR	1132.163	-0.63815	0.002402	0.096585	protein_coding
ENSG0000(	SERPING1	1543.286	-0.50463	0.002405	0.096585	protein_coding
ENSG0000(	VNN2	189.6989	-0.85713	0.002408	0.096585	protein_coding
ENSG0000(	GNS	1814.979	-0.42591	0.002417	0.096746	protein_coding
ENSG0000(	ZNF283	1224.534	0.191315	0.002419	0.096746	protein_coding
ENSG0000(	B3GNT7	532.7	0.883881	0.002447	0.097704	protein_coding
ENSG0000(	WWTR1	661.9374	-0.45645	0.002457	0.097969	protein_coding
ENSG0000(	ALOX5AP	92.03889	-0.90806	0.002461	0.097977	protein_coding
ENSG0000(	COL24A1	216.3508	-0.98265	0.002494	0.098671	protein_coding
ENSG0000(	IGHG2	4617.385	-1.66693	0.0025	0.098671	IG_C_gene
ENSG0000(	MGAM	473.1548	-1.22714	0.0025	0.098671	protein_coding
ENSG0000(	OR52N5	34.88336	-1.34072	0.002482	0.098671	protein_coding
ENSG0000(	PI15	660.6201	-2.15293	0.002502	0.098671	protein_coding
ENSG0000(	PON3	181.3231	0.390272	0.0025	0.098671	protein_coding
ENSG0000(	SDCBP	1334.264	-0.45135	0.002503	0.098671	protein_coding
ENSG0000(	ANKRD40	1113.983	0.200377	0.002507	0.098677	protein_coding
ENSG0000(	CALD1	3006.116	-0.63381	0.002518	0.098677	protein_coding
ENSG0000(	ENSG0000(	249.9839	-0.51485	0.002512	0.098677	lncRNA
ENSG0000(	PSME1	558.6512	-0.42712	0.002514	0.098677	protein_coding
ENSG0000(	FCGR2B	1301.596	-0.83909	0.002527	0.098896	protein_coding
ENSG0000(	ENSG0000(	1319.024	0.16531	0.002564	0.099913	lncRNA

ENSG0000(	IGKV3-15	735.554	-1.08747	0.002558	0.099913	IG_V_gene
ENSG0000(	NQO1-DT	36.19535	-0.5741	0.002562	0.099913	lncRNA
ENSG0000(	FBLN5	285.8287	-0.48529	0.002575	0.100054	protein_coding
ENSG0000(	SPSB3	491.9597	0.299637	0.002574	0.100054	protein_coding
ENSG0000(	D2HGDH	1425.263	0.310184	0.002587	0.100107	protein_coding
ENSG0000(	PEAR1	308.7639	-0.47712	0.002583	0.100107	protein_coding
ENSG0000(	RRN3P3	1191.501	0.254505	0.002586	0.100107	transcribed_unprocessed_pseudo
ENSG0000(	ENSG0000(	33.26409	-0.80301	0.00261	0.100865	lncRNA
ENSG0000(	COG1	1087.791	0.242349	0.002616	0.100961	protein_coding
ENSG0000(	ENSG0000(	624.5819	0.345219	0.002622	0.101025	lncRNA
ENSG0000(	SETBP1-DT	94.33898	-0.3871	0.002638	0.101516	lncRNA
ENSG0000(	MSX2	23.94035	-1.53378	0.002647	0.101554	protein_coding
ENSG0000(	PAK2	2199.7	-0.25675	0.002646	0.101554	protein_coding
ENSG0000(	MTCO2P11	127.9494	-0.58591	0.002659	0.101901	unprocessed_pseudogene
ENSG0000(	ENSG0000(	532.1081	-0.35657	0.002668	0.101938	lncRNA
ENSG0000(	PCSK5	18065.26	0.749064	0.002667	0.101938	protein_coding
ENSG0000(	CPTP	348.3596	0.529202	0.002684	0.102259	protein_coding
ENSG0000(	ENTPD1	5707.977	-0.42828	0.002682	0.102259	protein_coding
ENSG0000(	LINC02384	612.4226	-0.50874	0.002695	0.102538	lncRNA
ENSG0000(	IGKV3-20	2065.851	-0.99835	0.002707	0.102629	IG_V_gene
ENSG0000(	MIAT	3682.614	-0.47154	0.002708	0.102629	lncRNA
ENSG0000(	TMEM185E	1008.049	-0.38324	0.002708	0.102629	protein_coding
ENSG0000(	GASK1B-AS	305.4744	-0.52822	0.002715	0.102759	lncRNA
ENSG0000(	ANKRD18A	464.8054	0.668996	0.002723	0.102861	protein_coding
ENSG0000(	RORA	2130.878	-0.32306	0.002726	0.102861	protein_coding
ENSG0000(	CDK14	591.2803	-0.63539	0.002756	0.102897	protein_coding
ENSG0000(	CHST2	306.5965	-0.74238	0.002731	0.102897	protein_coding
ENSG0000(	ENSG0000(	96.2763	-0.633	0.002736	0.102897	lncRNA
ENSG0000(	ENSG0000(	59.61858	-0.59012	0.002754	0.102897	lncRNA
ENSG0000(	EVC2	192.7116	-0.51002	0.002749	0.102897	protein_coding
ENSG0000(	PGK1	2136.689	-0.35442	0.00275	0.102897	protein_coding
ENSG0000(	PRKAR2B	384.1965	-0.56789	0.002744	0.102897	protein_coding
ENSG0000(	SLC16A2	173.712	-0.49527	0.002741	0.102897	protein_coding
ENSG0000(	PSMA6	1587.357	-0.25284	0.00277	0.10328	protein_coding
ENSG0000(	BRWD1	7645.445	0.170186	0.002785	0.103692	protein_coding
ENSG0000(	PACS2	1768.742	0.216029	0.002794	0.103746	protein_coding
ENSG0000(	VSIG2	545.863	0.797402	0.002798	0.103746	protein_coding
ENSG0000(	ZNF69	797.1409	0.375017	0.002796	0.103746	protein_coding
ENSG0000(	ENSG0000(	310.7685	0.32098	0.002814	0.10418	lncRNA
ENSG0000(	DCBLD1	1098.021	-0.47257	0.002821	0.104313	protein_coding
ENSG0000(	SESTD1	1241.458	-0.51737	0.002838	0.104671	protein_coding
ENSG0000(	ST3GAL2	2076.669	-0.45327	0.002837	0.104671	protein_coding
ENSG0000(	CTSH	2605.467	-0.53883	0.002853	0.105075	protein_coding
ENSG0000(	LARP6	207.9483	-0.55196	0.002859	0.105148	protein_coding
ENSG0000(	REG3A	443.4115	-1.82601	0.002865	0.105221	protein_coding
ENSG0000(	ANTXR1	2286.952	-0.67909	0.002897	0.105254	protein_coding
ENSG0000(	CMTM7	358.542	-0.50721	0.002904	0.105254	protein_coding

ENSG0000(	DPYD	1551.336	-0.43527	0.002885	0.105254	protein_coding
ENSG0000(	ITPRID2-DT	170.1074	0.367642	0.002872	0.105254	lncRNA
ENSG0000(	MAB21L4	112.1764	0.615174	0.002892	0.105254	protein_coding
ENSG0000(	MTND4P12	251.7524	1.52222	0.002903	0.105254	processed_pseudogene
ENSG0000(	SAT1	7020.84	-0.53065	0.002895	0.105254	protein_coding
ENSG0000(	SNAPC2	60.9197	-0.73263	0.002874	0.105254	protein_coding
ENSG0000(	SULF1	1201.711	-0.67858	0.002896	0.105254	protein_coding
ENSG0000(	TMEM129	555.8559	0.346184	0.002879	0.105254	protein_coding
ENSG0000(	IGHV1-46	234.0758	-1.09377	0.002917	0.105594	IG_V_gene
ENSG0000(	APOL4	663.6821	-0.50852	0.00295	0.106634	protein_coding
ENSG0000(	LMOD3	468.0568	0.277686	0.002953	0.106634	protein_coding
ENSG0000(	ENSG0000(	2049.235	0.616989	0.00297	0.107032	lncRNA
ENSG0000(	ZFAT	662.6495	-0.36846	0.002972	0.107032	protein_coding
ENSG0000(	CEBPD	375.3756	-0.54594	0.002977	0.107047	protein_coding
ENSG0000(	ANKRD22	656.1071	-0.65362	0.003013	0.107854	protein_coding
ENSG0000(	ANXA13	470.9211	0.313317	0.00301	0.107854	protein_coding
ENSG0000(	ENSG0000(	676.7009	0.181244	0.003015	0.107854	lncRNA
ENSG0000(	LILRA1	70.58874	-1.10341	0.003007	0.107854	protein_coding
ENSG0000(	P2RY8	566.6974	-0.6867	0.00302	0.10789	protein_coding
ENSG0000(	PSMA3	549.3922	-0.20247	0.003027	0.108016	protein_coding
ENSG0000(	HSPA6	275.9986	-1.28659	0.003031	0.108034	protein_coding
ENSG0000(	ADAMTS6	237.5817	-0.73216	0.00304	0.108189	protein_coding
ENSG0000(	ENSG0000(	32.23729	0.567658	0.003054	0.108542	lncRNA
ENSG0000(	ENSG0000(	822.1143	0.810968	0.003069	0.108668	lncRNA
ENSG0000(	ENSG0000(	122.8696	-0.77095	0.003068	0.108668	lncRNA
ENSG0000(	MTND5P14	126.5305	-0.61293	0.003066	0.108668	unprocessed_pseudogene
ENSG0000(	ERAP2	2564.459	-0.40917	0.003081	0.108964	protein_coding
ENSG0000(	CCDC85B	45.15847	-0.57996	0.00309	0.108991	protein_coding
ENSG0000(	CHST15	1822.223	-0.63686	0.003094	0.108991	protein_coding
ENSG0000(	HLA-V	1819.656	0.672782	0.003091	0.108991	transcribed_unprocessed_pseudo
ENSG0000(	DENND5B	1751.413	-0.33513	0.003099	0.108999	protein_coding
ENSG0000(	ENSG0000(	27.06247	-0.95324	0.00311	0.108999	processed_pseudogene
ENSG0000(	ENSG0000(	28.46332	-1.53507	0.003122	0.108999	lncRNA
ENSG0000(	GJD3	114.3711	-0.727	0.003112	0.108999	protein_coding
ENSG0000(	IGF2BP3	373.2292	-0.50812	0.003111	0.108999	protein_coding
ENSG0000(	RGMB	2039.144	0.62279	0.003119	0.108999	protein_coding
ENSG0000(	RPEP4	63.23797	-0.51824	0.003119	0.108999	processed_pseudogene
ENSG0000(	BACH1-IT1	192.1303	-0.5521	0.003132	0.109067	lncRNA
ENSG0000(	PCMTD1	2719.832	-0.24879	0.003129	0.109067	protein_coding
ENSG0000(	ENSG0000(	472.464	0.16861	0.003185	0.11079	lncRNA
ENSG0000(	IGF2	748.1226	-0.84059	0.003195	0.110829	protein_coding
ENSG0000(	MINPP1	183.5472	0.446794	0.003193	0.110829	protein_coding
ENSG0000(	C4orf50	100.4284	-0.55703	0.00321	0.110954	protein_coding
ENSG0000(	ENSG0000(	22.63483	1.273675	0.003213	0.110954	processed_pseudogene
ENSG0000(	ENSG0000(	248.9187	-0.35708	0.003214	0.110954	lncRNA
ENSG0000(	GK	1410.207	-0.98785	0.003212	0.110954	protein_coding
ENSG0000(	CYP7B1	688.204	-0.8459	0.003231	0.111256	protein_coding

ENSG0000(	DEFA5	433.6034	-1.93788	0.003228	0.111256	protein_coding
ENSG0000(	GALNT2	1263.616	-0.32291	0.003249	0.111321	protein_coding
ENSG0000(	NCF1	568.5955	-0.56673	0.003242	0.111321	protein_coding
ENSG0000(	NID1	1339.118	-0.56926	0.003246	0.111321	protein_coding
ENSG0000(	PDGFRB	1889.794	-0.86716	0.003243	0.111321	protein_coding
ENSG0000(	ENSG0000(	26.56938	-1.14319	0.003259	0.111512	unprocessed_pseudogene
ENSG0000(	PMS2P4	1838.501	0.14626	0.003264	0.111566	transcribed_unprocessed_pseudo
ENSG0000(	TAGAP	607.4962	-1.10136	0.003268	0.111566	protein_coding
ENSG0000(	C1QB	475.8066	-0.72582	0.00328	0.111816	protein_coding
ENSG0000(	CDC42EP2	195.0756	-0.51853	0.003309	0.11219	protein_coding
ENSG0000(	CEMIP2	4713.148	0.281325	0.003315	0.11219	protein_coding
ENSG0000(	CLIC6	364.6564	-0.51729	0.003299	0.11219	protein_coding
ENSG0000(	GUSBP4	146.4963	0.467677	0.003306	0.11219	transcribed_unprocessed_pseudo
ENSG0000(	RANBP9	650.0733	-0.23693	0.003312	0.11219	protein_coding
ENSG0000(	SNX18P3	619.8729	0.522419	0.003308	0.11219	transcribed_unprocessed_pseudo
ENSG0000(	GPR180	553.3458	-0.3701	0.003323	0.112303	protein_coding
ENSG0000(	NPM1P40	82.40871	0.523969	0.003327	0.112303	processed_pseudogene
ENSG0000(	ENO1	2554.783	-0.31235	0.003342	0.112667	protein_coding
ENSG0000(	CST7	34.51609	-1.05598	0.003356	0.113009	protein_coding
ENSG0000(	ADGRV1	1341.991	0.299142	0.003374	0.113225	protein_coding
ENSG0000(	ENSG0000(	90.53887	0.529641	0.003375	0.113225	lncRNA
ENSG0000(	JAK2	2073.927	-0.46466	0.00337	0.113225	protein_coding
ENSG0000(	ZDHHC23	1763.486	0.578483	0.003386	0.113456	protein_coding
ENSG0000(	ZEB2	7405.336	-0.44051	0.003392	0.113515	protein_coding
ENSG0000(	TP53INP1	3753.937	-0.45987	0.003396	0.113516	protein_coding
ENSG0000(	DYRK3	493.7619	-0.57518	0.003406	0.11357	protein_coding
ENSG0000(	RANGRF	80.97336	-0.43654	0.003406	0.11357	protein_coding
ENSG0000(	MROH6	285.2127	-0.63141	0.00342	0.113857	protein_coding
ENSG0000(	ZNF774	1283.729	0.311476	0.003423	0.113857	protein_coding
ENSG0000(	SLC4A8	1103.738	-0.46372	0.003442	0.114216	protein_coding
ENSG0000(	ZYX	1279.696	-0.44662	0.003439	0.114216	protein_coding
ENSG0000(	ENSG0000(	30.57472	-0.74728	0.003446	0.114239	lncRNA
ENSG0000(	PDZK1IP1	290.6239	-1.21899	0.00346	0.114541	protein_coding
ENSG0000(	ZNF700	1733.021	0.22718	0.003477	0.114982	protein_coding
ENSG0000(	CD68	522.2024	-0.59855	0.003494	0.115395	protein_coding
ENSG0000(	SAMSN1	467.179	-0.8521	0.003507	0.11569	protein_coding
ENSG0000(	CHMP6	290.1572	0.339136	0.003517	0.115754	protein_coding
ENSG0000(	LINC01270	121.0066	-0.74297	0.003517	0.115754	lncRNA
ENSG0000(	MS4A14	193.3721	-0.74009	0.003532	0.116101	protein_coding
ENSG0000(	IGKV4-1	993.9572	-0.91071	0.003542	0.116278	IG_V_gene
ENSG0000(	SEC61A1	2714.005	-0.30267	0.003553	0.116372	protein_coding
ENSG0000(	SLC44A5	198.2123	-0.96621	0.003549	0.116372	protein_coding
ENSG0000(	BHLHE40	1183.663	-0.5124	0.003558	0.116397	protein_coding
ENSG0000(	BIRC3	6878.088	-0.55867	0.003589	0.116976	protein_coding
ENSG0000(	PDE4B	1119.471	-1.04685	0.003587	0.116976	protein_coding
ENSG0000(	SF3B2	1837.708	-0.20246	0.003585	0.116976	protein_coding
ENSG0000(	IGSF6	754.2821	-0.46325	0.003594	0.117025	protein_coding

ENSG0000(	PCDH17	315.2635	-0.98857	0.003608	0.117183	protein_coding
ENSG0000(	TAS1R3	68.27597	-0.89502	0.003604	0.117183	protein_coding
ENSG0000(	BACH1-IT2	208.3681	-0.5726	0.003697	0.117252	lncRNA
ENSG0000(	CCDC162P	596.8417	0.596166	0.003656	0.117252	transcribed_unitary_pseudogene
ENSG0000(	CDCA4	192.4295	-0.31055	0.003637	0.117252	protein_coding
ENSG0000(	CLMP	983.8414	-0.44198	0.003684	0.117252	protein_coding
ENSG0000(	CYSLTR2	308.3279	-0.44807	0.003679	0.117252	protein_coding
ENSG0000(	ENSG0000(	94.80883	-0.66451	0.00363	0.117252	lncRNA
ENSG0000(	ENSG0000(	47.94231	0.980265	0.00367	0.117252	unprocessed_pseudogene
ENSG0000(	ENSG0000(	95.42483	-0.50055	0.003689	0.117252	lncRNA
ENSG0000(	HTRA1	181.2314	-0.92238	0.003681	0.117252	protein_coding
ENSG0000(	IGHV3-66	138.2631	-0.93112	0.00366	0.117252	IG_V_gene
ENSG0000(	IGKV1-16	217.716	-1.0608	0.003615	0.117252	IG_V_gene
ENSG0000(	KANSL3	3232.503	0.13634	0.003699	0.117252	protein_coding
ENSG0000(	KDR	437.6059	-0.6449	0.003656	0.117252	protein_coding
ENSG0000(	LGI1	417.6912	0.441025	0.003675	0.117252	protein_coding
ENSG0000(	LPCAT1	832.3918	-0.55819	0.003659	0.117252	protein_coding
ENSG0000(	PDIA3	3913.836	-0.24414	0.003683	0.117252	protein_coding
ENSG0000(	PRDM15	1127.33	-0.32671	0.003634	0.117252	protein_coding
ENSG0000(	PTPN1	1584.595	-0.37478	0.003628	0.117252	protein_coding
ENSG0000(	RASEF	2054.874	0.433174	0.003691	0.117252	protein_coding
ENSG0000(	RLIM	2598.015	-0.20182	0.003627	0.117252	protein_coding
ENSG0000(	TTLL6	307.2222	0.607649	0.003699	0.117252	protein_coding
ENSG0000(	GRIN3A	175.7841	-0.87446	0.00372	0.117633	protein_coding
ENSG0000(	LDB2	452.7473	-0.45392	0.00372	0.117633	protein_coding
ENSG0000(	ITGAX	2911.693	-0.72449	0.00374	0.118009	protein_coding
ENSG0000(	JAML	1989.508	-0.37785	0.003745	0.118009	protein_coding
ENSG0000(	MCC	918.3995	-0.53787	0.003738	0.118009	protein_coding
ENSG0000(	CEACAM4	44.89549	-1.15582	0.003764	0.11836	protein_coding
ENSG0000(	MMP28	209.239	0.761933	0.003764	0.11836	protein_coding
ENSG0000(	SLC35B1	613.1023	-0.24378	0.003771	0.118426	protein_coding
ENSG0000(	PDLIM7	573.4984	-0.68918	0.003804	0.119328	protein_coding
ENSG0000(	BHLHA15	225.2883	-0.74862	0.003809	0.11935	protein_coding
ENSG0000(	ENSG0000(	48.30701	0.547644	0.003864	0.12094	transcribed_processed_pseudoge
ENSG0000(	CDK18	793.8403	0.355757	0.003871	0.12096	protein_coding
ENSG0000(	IGDCC4	554.1009	-0.70632	0.003873	0.12096	protein_coding
ENSG0000(	IGKV3OR2-	26.67123	-1.49175	0.003885	0.121053	IG_V_gene
ENSG0000(	KCNJ10	248.2299	-0.74863	0.003884	0.121053	protein_coding
ENSG0000(	ATP11B	3432.663	-0.18143	0.003899	0.121083	protein_coding
ENSG0000(	COL18A1	1684.538	-0.60496	0.003896	0.121083	protein_coding
ENSG0000(	LY96	29.66428	-1.04312	0.003891	0.121083	protein_coding
ENSG0000(	DUSP10	196.6031	-0.5611	0.00392	0.121437	protein_coding
ENSG0000(	FILIP1L	901.2728	-0.49288	0.003928	0.121437	protein_coding
ENSG0000(	PCDH18	1107.838	-0.56462	0.003922	0.121437	protein_coding
ENSG0000(	TRABD2A	3325.818	0.558588	0.003925	0.121437	protein_coding
ENSG0000(	ENSG0000(	165.5198	-0.75613	0.003941	0.121699	lncRNA
ENSG0000(	DSE	1515.962	-0.4039	0.003951	0.121864	protein_coding

ENSG0000(	SHTN1	1271.033	0.349974	0.004	0.123225	protein_coding
ENSG0000(	COL1A2	6143.197	-1.04175	0.004022	0.123281	protein_coding
ENSG0000(	ENSG0000(	44.48331	-0.65611	0.004033	0.123281	lncRNA
ENSG0000(	ENSG0000(	114.4472	-0.66497	0.00402	0.123281	lncRNA
ENSG0000(	ENSG0000(	134.5575	0.70102	0.004036	0.123281	TEC
ENSG0000(	NRP2	1107.53	-0.51555	0.004042	0.123281	protein_coding
ENSG0000(	PIPSL	205.6504	0.814273	0.004032	0.123281	transcribed_processed_pseudoge
ENSG0000(	SLC2A10	482.3704	0.401129	0.004028	0.123281	protein_coding
ENSG0000(	SPHK1	161.4872	-1.0024	0.004039	0.123281	protein_coding
ENSG0000(	TACR1	54.12049	-1.62995	0.004016	0.123281	protein_coding
ENSG0000(	DCXR	129.635	0.510608	0.004058	0.123643	protein_coding
ENSG0000(	SPART	555.5039	-0.37902	0.004065	0.123713	protein_coding
ENSG0000(	ADAMTS5	377.2729	-0.6836	0.004071	0.123765	protein_coding
ENSG0000(	GALNT18	117.5105	-0.54955	0.004082	0.123964	protein_coding
ENSG0000(	STAT1	8541.16	-0.75216	0.004087	0.123972	protein_coding
ENSG0000(	LATS2	458.7142	-0.48103	0.004092	0.123977	protein_coding
ENSG0000(	IRF1-AS1	4570.758	-0.32635	0.004113	0.124498	lncRNA
ENSG0000(	PTP4A3	285.9378	-0.78342	0.004124	0.124677	protein_coding
ENSG0000(	IGLC2	7019.747	-0.93774	0.00414	0.125026	IG_C_gene
ENSG0000(	SPAG4	97.55739	-0.70025	0.004153	0.12527	protein_coding
ENSG0000(	TP53I11	1237.368	0.248872	0.004174	0.125772	protein_coding
ENSG0000(	ME1	195.013	-0.63494	0.00418	0.125826	protein_coding
ENSG0000(	LINGO1	24.26105	-1.31615	0.004186	0.125858	protein_coding
ENSG0000(	COL5A2	1683.451	-0.67491	0.004203	0.126245	protein_coding
ENSG0000(	CFI	582.9622	-0.49401	0.004244	0.126445	protein_coding
ENSG0000(	IGKV3-11	1058.642	-1.16898	0.004247	0.126445	IG_V_gene
ENSG0000(	LINC01002	1597.085	-0.65066	0.004235	0.126445	lncRNA
ENSG0000(	QKI	2197.961	-0.37152	0.004224	0.126445	protein_coding
ENSG0000(	RANP3	19.53308	-0.7682	0.00422	0.126445	processed_pseudogene
ENSG0000(	RAP2A	807.8017	-0.27998	0.00424	0.126445	protein_coding
ENSG0000(	TUBB	2513.777	-0.37718	0.004234	0.126445	protein_coding
ENSG0000(	ZNF281	781.7071	-0.4558	0.004222	0.126445	protein_coding
ENSG0000(	GIMAP8	778.8795	-0.42333	0.004261	0.12659	protein_coding
ENSG0000(	PPM1E	22.12451	-1.12977	0.004258	0.12659	protein_coding
ENSG0000(	ENSG0000(	62.92089	-0.78365	0.004268	0.126657	lncRNA
ENSG0000(	ASAH2B	586.9037	0.32313	0.004286	0.126787	protein_coding
ENSG0000(	ATP2C2	2072.172	0.7537	0.004284	0.126787	protein_coding
ENSG0000(	IGKJ5	88.29295	-0.83683	0.004284	0.126787	IG_J_gene
ENSG0000(	ENSG0000(	27.09821	0.568892	0.004303	0.127165	lncRNA
ENSG0000(	MACROD2	554.2913	0.199489	0.00431	0.127239	protein_coding
ENSG0000(	PPIB	1718.963	-0.39214	0.004317	0.127298	protein_coding
ENSG0000(	TNRC18	4091.267	0.150163	0.004331	0.127584	protein_coding
ENSG0000(	ENSG0000(	39.42584	-0.83914	0.004349	0.127947	lncRNA
ENSG0000(	MXD4	1413.942	0.165496	0.004353	0.127947	protein_coding
ENSG0000(	ABCB1	590.3908	0.768145	0.004367	0.12821	protein_coding
ENSG0000(	DOP1B	3013.161	0.325038	0.004371	0.12821	protein_coding
ENSG0000(	ZNF451	3232.466	-0.17022	0.004396	0.128795	protein_coding

ENSG0000(	PLEKHM2	782.864	-0.34622	0.004411	0.129098	protein_coding
ENSG0000(	SLC43A3	406.2068	-0.54316	0.004428	0.129376	protein_coding
ENSG0000(	TWIST2	60.35765	-1.16828	0.00443	0.129376	protein_coding
ENSG0000(	PICALM	3342.5	-0.29686	0.004442	0.12947	protein_coding
ENSG0000(	ZNF469	325.3549	-1.09914	0.00444	0.12947	protein_coding
ENSG0000(	BATF3	108.9429	-0.85663	0.004455	0.129691	protein_coding
ENSG0000(	SIRPB1	458.4752	-0.70845	0.00446	0.129702	protein_coding
ENSG0000(	RASGRP4	250.386	-0.40692	0.004488	0.130369	protein_coding
ENSG0000(	WDFY1	2478.89	-0.23878	0.004492	0.130369	protein_coding
ENSG0000(	VAMP7	354.0896	-0.31183	0.004507	0.130668	protein_coding
ENSG0000(	ARGLU1	5468.305	0.13809	0.004523	0.130975	protein_coding
ENSG0000(	PLXND1	2033.908	-0.54855	0.004528	0.130984	protein_coding
ENSG0000(	LINC01465	22.0842	-0.91782	0.004535	0.131058	lncRNA
ENSG0000(	ENSG0000(	430.615	-0.30227	0.004547	0.131255	lncRNA
ENSG0000(	CA11	33.08665	-0.87442	0.004557	0.13139	protein_coding
ENSG0000(	LINC02328	206.8567	-0.23739	0.004571	0.13139	lncRNA
ENSG0000(	LYN	1657.559	-0.54303	0.00457	0.13139	protein_coding
ENSG0000(	MKRN3	196.2269	-0.36379	0.004575	0.13139	protein_coding
ENSG0000(	ST3GAL1	1953.4	-0.53181	0.004565	0.13139	protein_coding
ENSG0000(	ABLIM3	365.077	-0.6305	0.004581	0.131426	protein_coding
ENSG0000(	IL21R	723.0845	-0.71543	0.004602	0.131879	protein_coding
ENSG0000(	PPP1R18	767.8675	-0.65243	0.004613	0.132073	protein_coding
ENSG0000(	HCLS1	1834.151	-0.48815	0.004634	0.132532	protein_coding
ENSG0000(	ENSG0000(	84.53392	-0.58686	0.004639	0.132545	lncRNA
ENSG0000(	CREB3L2	4973.675	-0.44764	0.004645	0.132579	protein_coding
ENSG0000(	PAN3	2344.069	0.181052	0.004662	0.132763	protein_coding
ENSG0000(	TRAJ31	22.98203	-0.86555	0.004659	0.132763	TR_J_gene
ENSG0000(	EPHB1	108.5658	-0.85236	0.004679	0.132974	protein_coding
ENSG0000(	MUC5AC	227.535	-2.39299	0.004678	0.132974	protein_coding
ENSG0000(	SEMA4D	2743.569	-0.39395	0.004688	0.132974	protein_coding
ENSG0000(	SLC12A3	57.85083	-0.82725	0.004684	0.132974	protein_coding
ENSG0000(	FNDC3A	3240.846	-0.21015	0.004699	0.133008	protein_coding
ENSG0000(	MDM2	3341.266	-0.39271	0.004698	0.133008	protein_coding
ENSG0000(	ANKRD65	74.3365	-0.60808	0.004712	0.133135	protein_coding
ENSG0000(	ARL5A	1547.867	-0.2294	0.004713	0.133135	protein_coding
ENSG0000(	XDH	2523.104	-0.85223	0.004721	0.13322	protein_coding
ENSG0000(	CMKLR1	721.8322	-0.5615	0.004735	0.133479	protein_coding
ENSG0000(	ANXA5	800.495	-0.55013	0.004749	0.133594	protein_coding
ENSG0000(	COL10A1	19.67291	-1.91241	0.004747	0.133594	protein_coding
ENSG0000(	LCP1	2654.246	-0.66892	0.004774	0.133941	protein_coding
ENSG0000(	MAFB	435.6148	-0.5776	0.004774	0.133941	protein_coding
ENSG0000(	SPAG1	430.9665	0.477478	0.004776	0.133941	protein_coding
ENSG0000(	PLCB1-IT1	36.0907	-0.81677	0.004789	0.134164	lncRNA
ENSG0000(	IGHV3-21	611.3763	-1.03896	0.004812	0.134404	IG_V_gene
ENSG0000(	IGHV3-53	175.9141	-0.98974	0.004809	0.134404	IG_V_gene
ENSG0000(	TRIB2	1173.758	-0.75325	0.004812	0.134404	protein_coding
ENSG0000(	GTDC1	1338.492	-0.43626	0.004824	0.134472	protein_coding

ENSG0000(	KATNIP	1557.204	0.251189	0.004822	0.134472	protein_coding
ENSG0000(	LINC02430	27.53518	1.083753	0.00484	0.134762	lncRNA
ENSG0000(	ENSG0000(	121.7761	0.354793	0.004869	0.135454	lncRNA
ENSG0000(	B2M	20597.1	-0.48361	0.004887	0.135818	protein_coding
ENSG0000(	ENSG0000(	83.65337	0.869305	0.004933	0.136528	lncRNA
ENSG0000(	RFLNB	454.3936	-0.44709	0.00493	0.136528	protein_coding
ENSG0000(	RNASEK	397.7559	-0.37714	0.004925	0.136528	protein_coding
ENSG0000(	SLC35A5	819.2719	-0.21467	0.004925	0.136528	protein_coding
ENSG0000(	ENSG0000(	52.15196	-1.40912	0.00495	0.136761	lncRNA
ENSG0000(	SLC12A1	66.82241	0.482354	0.004951	0.136761	protein_coding
ENSG0000(	ANKFN1	74.37831	1.006639	0.005025	0.13722	protein_coding
ENSG0000(	ARNTL2	821.015	-0.61704	0.005023	0.13722	protein_coding
ENSG0000(	CCL3	115.3759	-1.2149	0.00501	0.13722	protein_coding
ENSG0000(	COLGALT1	1755.933	-0.25828	0.004985	0.13722	protein_coding
ENSG0000(	ENSG0000(	60.06269	-0.60451	0.005026	0.13722	lncRNA
ENSG0000(	ENSG0000(	252.1431	-0.37388	0.004978	0.13722	lncRNA
ENSG0000(	ENSG0000(	1076.922	0.172076	0.005027	0.13722	lncRNA
ENSG0000(	IGLV6-57	111.6895	-1.12166	0.004981	0.13722	IG_V_gene
ENSG0000(	NSUN5P2	265.9348	0.328427	0.004992	0.13722	transcribed_unprocessed_pseudo
ENSG0000(	POM121L9	147.9857	-0.75416	0.005028	0.13722	transcribed_unprocessed_pseudo
ENSG0000(	SSH3	601.3045	0.461191	0.005026	0.13722	protein_coding
ENSG0000(	STEAP4	1398.792	-0.93412	0.005024	0.13722	protein_coding
ENSG0000(	SUCNR1	51.91739	-0.91296	0.005042	0.137479	protein_coding
ENSG0000(	CA7	206.3132	1.404192	0.005052	0.137604	protein_coding
ENSG0000(	ASPHD2	164.1514	-0.55872	0.00507	0.137845	protein_coding
ENSG0000(	RAI14	1001.888	-0.64578	0.005068	0.137845	protein_coding
ENSG0000(	CLDN2	101.1741	-1.81867	0.005083	0.137901	protein_coding
ENSG0000(	FFAR2	391.7081	-0.73035	0.00508	0.137901	protein_coding
ENSG0000(	FOXN2	797.2351	-0.37587	0.0051	0.138234	protein_coding
ENSG0000(	ENSG0000(	299.1974	-0.45813	0.005117	0.138575	lncRNA
ENSG0000(	ENSG0000(	596.407	0.674437	0.005128	0.138731	lncRNA
ENSG0000(	P2RY13	299.5423	-0.79714	0.005139	0.138884	protein_coding
ENSG0000(	EVI2B	722.9698	-0.53029	0.005169	0.13957	protein_coding
ENSG0000(	ATP6V1G1	715.666	-0.21366	0.005193	0.139834	protein_coding
ENSG0000(	CLEC6A	21.32684	-1.82799	0.005194	0.139834	protein_coding
ENSG0000(	PML	2843.936	-0.44732	0.005187	0.139834	protein_coding
ENSG0000(	LYPD9P	20.34211	0.774263	0.0052	0.139842	transcribed_unitary_pseudogene
ENSG0000(	ENSG0000(	117.6801	-0.6595	0.005227	0.140236	TEC
ENSG0000(	FCRL5	4638.086	-0.64308	0.005224	0.140236	protein_coding
ENSG0000(	TBX21	56.22542	-0.73868	0.00523	0.140236	protein_coding
ENSG0000(	ENSG0000(	26.60683	0.644149	0.005238	0.140331	lncRNA
ENSG0000(	B3GNT4	50.04515	0.505112	0.005292	0.141125	protein_coding
ENSG0000(	ENSG0000(	106.0096	0.399528	0.005289	0.141125	lncRNA
ENSG0000(	HCG9	57.80296	0.563805	0.005283	0.141125	lncRNA
ENSG0000(	PELATON	267.7576	-0.74581	0.005294	0.141125	lncRNA
ENSG0000(	VNN3	440.6664	-0.46277	0.005292	0.141125	protein_coding
ENSG0000(	DOCK4	1511.865	-0.51202	0.00531	0.141153	protein_coding

ENSG0000(	IL1RAP	1132.215	-0.26734	0.005307	0.141153	protein_coding
ENSG0000(	TLR1	655.3146	-0.69538	0.0053	0.141153	protein_coding
ENSG0000(	SMARCE1	2316.08	-0.16028	0.005322	0.141319	protein_coding
ENSG0000(	CALR	2567.658	-0.31246	0.005396	0.14137	protein_coding
ENSG0000(	CCL18	190.6475	-1.8664	0.005389	0.14137	protein_coding
ENSG0000(	DENND2B	2575.454	-0.33314	0.005346	0.14137	protein_coding
ENSG0000(	ENSG0000(	71.14155	-0.54121	0.005351	0.14137	lncRNA
ENSG0000(	ENSG0000(	122.1701	0.318489	0.00539	0.14137	lncRNA
ENSG0000(	ENSG0000(	509.5511	0.211288	0.005381	0.14137	lncRNA
ENSG0000(	ENSG0000(	34.76714	-0.71266	0.005368	0.14137	lncRNA
ENSG0000(	ETV1	131.5166	-0.62262	0.005391	0.14137	protein_coding
ENSG0000(	LCA5	103.2703	-0.46791	0.005394	0.14137	protein_coding
ENSG0000(	MRPL48	594.0447	0.211621	0.005374	0.14137	protein_coding
ENSG0000(	PFKFB3	1744.844	-0.72581	0.005395	0.14137	protein_coding
ENSG0000(	PLEKHA4	188.5853	-0.43529	0.005416	0.14137	protein_coding
ENSG0000(	RASSF4	2500.292	-0.4471	0.005399	0.14137	protein_coding
ENSG0000(	RNF144B	558.4142	-0.76953	0.005358	0.14137	protein_coding
ENSG0000(	THBD	205.1318	-0.83029	0.005412	0.14137	protein_coding
ENSG0000(	UTRN	10341.99	-0.24462	0.005405	0.14137	protein_coding
ENSG0000(	ZBTB10	1249.527	0.287498	0.005409	0.14137	protein_coding
ENSG0000(	ZDHHC11	182.5878	0.556564	0.00539	0.14137	protein_coding
ENSG0000(	CA6	233.0669	0.226481	0.005435	0.141461	protein_coding
ENSG0000(	ENSG0000(	44.78173	-0.54646	0.005434	0.141461	lncRNA
ENSG0000(	HLA-DMB	2116.325	-0.51578	0.005428	0.141461	protein_coding
ENSG0000(	SOD2	45345.69	-0.5985	0.005445	0.14159	protein_coding
ENSG0000(	ATG4D	460.1011	0.458073	0.00546	0.141699	protein_coding
ENSG0000(	NECTIN2	892.1609	-0.36607	0.005458	0.141699	protein_coding
ENSG0000(	SHMT2	349.157	-0.37907	0.005468	0.141699	protein_coding
ENSG0000(	TPH1	1146.321	0.457138	0.00547	0.141699	protein_coding
ENSG0000(	ENSG0000(	1399.361	-0.47949	0.005492	0.141906	TEC
ENSG0000(	GPX7	87.44716	-0.68231	0.005484	0.141906	protein_coding
ENSG0000(	PXDN	1154.233	-0.51315	0.005493	0.141906	protein_coding
ENSG0000(	CSMD2	323.7748	-0.66844	0.00551	0.142079	protein_coding
ENSG0000(	NUPR1	1916.523	0.261224	0.005506	0.142079	protein_coding
ENSG0000(	ENSG0000(	37.7106	1.145024	0.005524	0.142161	processed_pseudogene
ENSG0000(	RGS18	85.97983	-0.71762	0.005522	0.142161	protein_coding
ENSG0000(	MDFIC	870.0639	-0.39515	0.005538	0.142399	protein_coding
ENSG0000(	CASP4	1604.067	-0.53104	0.005554	0.142561	protein_coding
ENSG0000(	CAVIN1	658.7927	-0.61332	0.00556	0.142561	protein_coding
ENSG0000(	ENSG0000(	80.50113	-0.42235	0.005559	0.142561	lncRNA
ENSG0000(	ENSG0000(	174.6536	0.256603	0.005577	0.142853	TEC
ENSG0000(	ALDH7A1	2269.705	0.153201	0.005601	0.142896	protein_coding
ENSG0000(	CYP1B1	88.50931	-1.28258	0.005594	0.142896	protein_coding
ENSG0000(	DIS3L2	1544.483	0.121379	0.005607	0.142896	protein_coding
ENSG0000(	ENSG0000(	606.1758	0.362585	0.0056	0.142896	lncRNA
ENSG0000(	ENSG0000(	255.6716	0.567022	0.005587	0.142896	TEC
ENSG0000(	SYT7	380.7147	0.596845	0.005609	0.142896	protein_coding

ENSG0000(	HDAC7	1900.386	-0.44441	0.005626	0.14304	protein_coding
ENSG0000(	RN7SL440F	224.7168	0.313928	0.005623	0.14304	misc_RNA
ENSG0000(	DGCR8	1509.439	0.231749	0.005647	0.143451	protein_coding
ENSG0000(	HSP90B1	6402.277	-0.36542	0.005659	0.143501	protein_coding
ENSG0000(	IFFO1	555.9658	-0.31394	0.005659	0.143501	protein_coding
ENSG0000(	ENSG0000(	33.51667	-0.88654	0.005672	0.143566	lncRNA
ENSG0000(	FMO2	72.19443	-1.24986	0.005668	0.143566	protein_coding
ENSG0000(	ROR2	303.1351	-0.77201	0.005687	0.143817	protein_coding
ENSG0000(	SLC2A6	266.1732	-0.59922	0.005701	0.144031	protein_coding
ENSG0000(	ODF3B	288.414	-0.61267	0.00571	0.144124	protein_coding
ENSG0000(	TICAM2-AS	61.32996	-0.88933	0.005718	0.14418	lncRNA
ENSG0000(	CTU2	101.1472	0.458263	0.005744	0.14431	protein_coding
ENSG0000(	ENSG0000(	203.9555	0.259195	0.00574	0.14431	lncRNA
ENSG0000(	ENSG0000(	51.71625	0.544893	0.005749	0.14431	lncRNA
ENSG0000(	LDHA	3740.153	-0.35845	0.00574	0.14431	protein_coding
ENSG0000(	TRIM40	502.4766	-1.13453	0.005745	0.14431	protein_coding
ENSG0000(	ENSG0000(	447.6226	0.314312	0.005755	0.144343	lncRNA
ENSG0000(	ZNF525	678.6237	0.288308	0.005835	0.146206	protein_coding
ENSG0000(	UBE2E2	174.948	-0.5966	0.005859	0.146683	protein_coding
ENSG0000(	CXCR3	44.81507	-0.77492	0.005871	0.146797	protein_coding
ENSG0000(	HOMER1	174.3823	-0.50217	0.005875	0.146797	protein_coding
ENSG0000(	ITGA5	1310.524	-0.86868	0.00591	0.147535	protein_coding
ENSG0000(	PECAM1	2375.299	-0.58506	0.005921	0.147677	protein_coding
ENSG0000(	GSDMC	122.143	-0.70089	0.00593	0.147768	protein_coding
ENSG0000(	SAMD4A	1478.87	-0.51265	0.005952	0.148197	protein_coding
ENSG0000(	DTNB	313.5652	-0.34228	0.005969	0.148492	protein_coding
ENSG0000(	DENND3-A:	379.5451	-0.33312	0.005995	0.148712	lncRNA
ENSG0000(	ENSG0000(	62.05342	-0.92888	0.005985	0.148712	TEC
ENSG0000(	ZNF787	432.6271	0.222992	0.005992	0.148712	protein_coding
ENSG0000(	AXIN1	954.8949	0.305393	0.006031	0.149091	protein_coding
ENSG0000(	CHRNA1	33.92217	0.650806	0.006016	0.149091	protein_coding
ENSG0000(	DERL1	1838.568	-0.20011	0.006064	0.149091	protein_coding
ENSG0000(	ENSG0000(	136.8888	-0.98705	0.00604	0.149091	lncRNA
ENSG0000(	ENSG0000(	478.0312	-0.50998	0.006024	0.149091	lncRNA
ENSG0000(	ENSG0000(	70.03562	-0.5197	0.006069	0.149091	lncRNA
ENSG0000(	FKBP11	1353.511	-0.3184	0.006042	0.149091	protein_coding
ENSG0000(	PSMB3	213.5297	-0.36386	0.006063	0.149091	protein_coding
ENSG0000(	RN7SL558F	131.2898	0.299074	0.006069	0.149091	misc_RNA
ENSG0000(	TSPAN9	390.1292	-0.5987	0.006053	0.149091	protein_coding
ENSG0000(	ZNF765	1468.858	0.182314	0.006052	0.149091	protein_coding
ENSG0000(	CBR3-AS1	2489.644	0.144538	0.00609	0.149336	lncRNA
ENSG0000(	SOCS3-DT	63.28043	-0.5197	0.006086	0.149336	lncRNA
ENSG0000(	PYY	79.6285	1.313708	0.006102	0.149496	protein_coding
ENSG0000(	CTHRC1	44.14771	-1.30552	0.006117	0.149684	protein_coding
ENSG0000(	RPTOR	1515.971	0.161485	0.006121	0.149684	protein_coding
ENSG0000(	ENSG0000(	986.9582	-0.43074	0.006142	0.150069	lncRNA
ENSG0000(	TTC6	215.1087	0.331896	0.006167	0.150551	protein_coding

ENSG0000(	CGNL1	368.8263	0.373545	0.006199	0.150891	protein_coding
ENSG0000(	GMPPA	848.9963	-0.25315	0.006218	0.150891	protein_coding
ENSG0000(	GNPTAB	2279.785	0.198636	0.006203	0.150891	protein_coding
ENSG0000(	GRID1	188.4395	-0.39071	0.006216	0.150891	protein_coding
ENSG0000(	HLA-DMA	931.104	-0.48257	0.006219	0.150891	protein_coding
ENSG0000(	SLC29A3	151.5017	-0.33774	0.006205	0.150891	protein_coding
ENSG0000(	ZNF479	136.1032	0.315269	0.006207	0.150891	protein_coding
ENSG0000(	YIPF5	569.062	-0.27287	0.006237	0.151187	protein_coding
ENSG0000(	PPP5C	789.1062	0.196744	0.00625	0.151374	protein_coding
ENSG0000(	ENSG0000(	265.6896	0.37762	0.006263	0.151434	lncRNA
ENSG0000(	LINC02334	734.6955	0.215183	0.006264	0.151434	lncRNA
ENSG0000(	ATP11C	979.0635	-0.27858	0.006273	0.15153	protein_coding
ENSG0000(	IGKV1-12	309.1525	-1.09419	0.006289	0.151783	IG_V_gene
ENSG0000(	IGHV4-39	727.7659	-1.07014	0.00633	0.152642	IG_V_gene
ENSG0000(	IGKJ1	110.8791	-0.93657	0.006338	0.152698	IG_J_gene
ENSG0000(	ENSG0000(	24.96655	1.168945	0.006355	0.152831	lncRNA
ENSG0000(	FAM157C	1281.954	-0.66334	0.006363	0.152831	lncRNA
ENSG0000(	PPP1R9A	1379.718	0.584627	0.006363	0.152831	protein_coding
ENSG0000(	SMIM3	164.8559	-0.50881	0.006366	0.152831	protein_coding
ENSG0000(	GPR132	443.1376	-0.62934	0.00639	0.153205	protein_coding
ENSG0000(	TP73	36.99116	-0.93934	0.006393	0.153205	protein_coding
ENSG0000(	RAP1GAP2	1206.153	0.494371	0.006404	0.153349	protein_coding
ENSG0000(	ENSG0000(	36.00149	0.594605	0.006415	0.153475	lncRNA
ENSG0000(	CDKN2A	62.57449	-0.53948	0.00644	0.153776	protein_coding
ENSG0000(	ENSG0000(	25.28977	-0.72992	0.006443	0.153776	TEC
ENSG0000(	MAPT	302.6896	0.628036	0.006445	0.153776	protein_coding
ENSG0000(	COL12A1	2781.958	-1.18978	0.006461	0.154042	protein_coding
ENSG0000(	IPO13	415.695	-0.30157	0.006482	0.154405	protein_coding
ENSG0000(	ENSG0000(	32.63018	1.005334	0.006503	0.154642	lncRNA
ENSG0000(	TOP1	2986.147	-0.22443	0.006499	0.154642	protein_coding
ENSG0000(	IGHG3	3310.377	-1.68601	0.006511	0.154692	IG_C_gene
ENSG0000(	SOBP	216.4454	-0.50986	0.006534	0.155101	protein_coding
ENSG0000(	PPM1M	290.1568	-0.47774	0.006557	0.155369	protein_coding
ENSG0000(	SCO2	259.2082	-0.63737	0.006551	0.155369	protein_coding
ENSG0000(	LCNL1	132.0452	-0.88211	0.006571	0.155583	protein_coding
ENSG0000(	FMNL3	2883.166	-0.54054	0.006588	0.155856	protein_coding
ENSG0000(	MGAT1	2213.649	-0.21399	0.0066	0.155864	protein_coding
ENSG0000(	PLEKHG2	2029.932	-0.45656	0.006595	0.155864	protein_coding
ENSG0000(	TOR1B	614.9281	0.321579	0.006622	0.156255	protein_coding
ENSG0000(	CLCA2	41.42731	1.05468	0.006649	0.156607	protein_coding
ENSG0000(	MR1	1366.79	-0.24863	0.006648	0.156607	protein_coding
ENSG0000(	IGLC7	75.31715	-1.56989	0.006659	0.156708	IG_C_gene
ENSG0000(	MADCAM1	62.0823	-0.78148	0.006664	0.156708	protein_coding
ENSG0000(	ENSG0000(	129.3272	-0.42792	0.006675	0.156814	lncRNA
ENSG0000(	LOXL3	181.4636	-0.37076	0.00669	0.157045	protein_coding
ENSG0000(	CPB2-AS1	841.2911	0.2752	0.006716	0.157129	lncRNA
ENSG0000(	DMAP1	1314.677	0.171789	0.00671	0.157129	protein_coding

ENSG0000(	GJA5	54.47823	-0.96821	0.006703	0.157129	protein_coding
ENSG0000(	WDR27	2676.372	0.171219	0.006712	0.157129	protein_coding
ENSG0000(	DGAT2	269.6652	-0.78538	0.006768	0.157924	protein_coding
ENSG0000(	ENSG0000(	106.2112	0.362416	0.006767	0.157924	lncRNA
ENSG0000(	SIGLEC12	51.29971	-0.85116	0.006761	0.157924	protein_coding
ENSG0000(	FCGR1A	138.7199	-1.38788	0.006791	0.158327	protein_coding
ENSG0000(	IPO5P1	891.4948	0.476716	0.006816	0.15879	transcribed_processed_pseudoge
ENSG0000(	RIT1	856.0033	-0.23463	0.006837	0.159129	protein_coding
ENSG0000(	ENSG0000(	88.95779	0.954669	0.006845	0.159161	unprocessed_pseudogene
ENSG0000(	NALF1-IT1	32.84923	-1.2818	0.00685	0.159161	lncRNA
ENSG0000(	MIB1	1899.031	-0.17552	0.006866	0.159418	protein_coding
ENSG0000(	ENSG0000(	88.02044	-0.42079	0.006878	0.159556	lncRNA
ENSG0000(	ENSG0000(	319.0159	0.377646	0.006894	0.159782	TEC
ENSG0000(	ENSG0000(	248.8678	0.536602	0.006901	0.159797	lncRNA
ENSG0000(	SEC22C	1065.639	-0.23126	0.006906	0.159797	protein_coding
ENSG0000(	ENSG0000(	101.4154	0.361273	0.006917	0.159903	lncRNA
ENSG0000(	TNFRSF1B	1245.741	-0.46345	0.006922	0.159903	protein_coding
ENSG0000(	CHST11	833.5051	-0.59273	0.006937	0.159967	protein_coding
ENSG0000(	RNVU1-4	22.50016	-0.75273	0.006936	0.159967	snRNA
ENSG0000(	ENSG0000(	127.8113	0.70678	0.006953	0.160206	lncRNA
ENSG0000(	ACO1	1047.363	-0.26969	0.007004	0.160677	protein_coding
ENSG0000(	ENSG0000(	316.1696	-0.31303	0.007005	0.160677	lncRNA
ENSG0000(	FAM95C	238.7914	0.65558	0.006988	0.160677	protein_coding
ENSG0000(	HLA-E	8731.832	-0.31049	0.007012	0.160677	protein_coding
ENSG0000(	NIBAN1	1023.884	-0.38097	0.007021	0.160677	protein_coding
ENSG0000(	SH3BGRL	642.635	-0.3574	0.006992	0.160677	protein_coding
ENSG0000(	SP140L	1658.515	-0.24901	0.007026	0.160677	protein_coding
ENSG0000(	TTL	2557.362	-0.26201	0.007023	0.160677	protein_coding
ENSG0000(	UBASH3B	442.9762	-0.40349	0.006997	0.160677	protein_coding
ENSG0000(	CBLN3	193.5483	-0.67392	0.007042	0.160779	protein_coding
ENSG0000(	CYB5R3	1666.262	-0.21675	0.00704	0.160779	protein_coding
ENSG0000(	TXLNB	109.7344	-0.75075	0.007051	0.160852	protein_coding
ENSG0000(	NRN1	60.54982	-0.64491	0.007058	0.16087	protein_coding
ENSG0000(	AADACL3	25.27473	-0.6605	0.007079	0.161215	protein_coding
ENSG0000(	ZNF770	993.3544	-0.16304	0.007086	0.161256	protein_coding
ENSG0000(	HMGB1P41	47.35057	0.566059	0.007106	0.161577	processed_pseudogene
ENSG0000(	B3GALT5-A	413.5356	0.377696	0.00712	0.161755	lncRNA
ENSG0000(	RN7SL381F	167.0305	0.302126	0.00713	0.161849	misc_RNA
ENSG0000(	GNPDA2	232.8772	-0.28257	0.00715	0.162159	protein_coding
ENSG0000(	GNA15	69.18811	-0.86148	0.00721	0.1634	protein_coding
ENSG0000(	SUPV3L1	585.6083	0.227645	0.007225	0.16359	protein_coding
ENSG0000(	DNASE2	323.9039	-0.26484	0.00724	0.163673	protein_coding
ENSG0000(	NBPF10	1383.559	0.394026	0.007238	0.163673	protein_coding
ENSG0000(	CES1P1	25.97652	-0.76089	0.007296	0.164104	transcribed_unprocessed_pseudo
ENSG0000(	ENSG0000(	139.0386	-0.95701	0.007295	0.164104	lncRNA
ENSG0000(	H2AC21	43.8557	-0.71888	0.007284	0.164104	protein_coding
ENSG0000(	RN7SL559F	101.6824	-0.34348	0.007313	0.164104	misc_RNA

ENSG0000(	RNF135	418.2076	0.34301	0.007298	0.164104	protein_coding
ENSG0000(	RPS3AP32	20.59289	-0.9617	0.007276	0.164104	processed_pseudogene
ENSG0000(	SSR1	3376.989	-0.253	0.007309	0.164104	protein_coding
ENSG0000(	UBE2Z	2247.306	-0.19774	0.007307	0.164104	protein_coding
ENSG0000(	ZFHX4	164.1003	-0.38471	0.007297	0.164104	protein_coding
ENSG0000(	ENSG0000(	21.00806	-1.28881	0.007366	0.165169	lncRNA
ENSG0000(	ENSG0000(	51.44255	-0.73471	0.007378	0.165298	lncRNA
ENSG0000(	GRK5	761.324	-0.35075	0.007392	0.165472	protein_coding
ENSG0000(	CNTLN	725.4253	-0.34484	0.007402	0.165536	protein_coding
ENSG0000(	TPST1	257.2433	-0.62341	0.007407	0.165536	protein_coding
ENSG0000(	ENSG0000(	43.89002	-0.56998	0.007417	0.165626	lncRNA
ENSG0000(	STXBP4	962.6037	-0.27672	0.007423	0.165634	protein_coding
ENSG0000(	CPEB4	2667.678	-0.46913	0.007436	0.165647	protein_coding
ENSG0000(	DGKD	2896.979	0.302363	0.007432	0.165647	protein_coding
ENSG0000(	FGL2	1526.429	-0.45736	0.00748	0.166498	protein_coding
ENSG0000(	ENSG0000(	742.6007	0.192926	0.007506	0.16668	lncRNA
ENSG0000(	ENSG0000(	1497.179	0.173827	0.007504	0.16668	lncRNA
ENSG0000(	TXNDC11	1408.187	-0.33334	0.0075	0.16668	protein_coding
ENSG0000(	ANKRD62	194.5933	0.336256	0.007526	0.166706	protein_coding
ENSG0000(	COL2A1	32.58574	1.314284	0.007516	0.166706	protein_coding
ENSG0000(	DHRS13	182.3021	-0.43909	0.007533	0.166706	protein_coding
ENSG0000(	KIRREL1-IT1	38.94298	-0.8578	0.007527	0.166706	lncRNA
ENSG0000(	MME	390.5492	-0.96707	0.007538	0.166706	protein_coding
ENSG0000(	ENSG0000(	383.1657	-0.30007	0.007567	0.166949	lncRNA
ENSG0000(	EXOC3L2	383.4047	-0.24953	0.007561	0.166949	protein_coding
ENSG0000(	PKD1	3972.05	0.17401	0.007565	0.166949	protein_coding
ENSG0000(	ZNF213	347.2845	0.285346	0.00759	0.167326	protein_coding
ENSG0000(	GNAI2	1393.619	-0.4495	0.007598	0.167361	protein_coding
ENSG0000(	ENSG0000(	295.5719	0.306477	0.007611	0.167519	lncRNA
ENSG0000(	GSEC	243.0558	-0.37107	0.007618	0.167546	lncRNA
ENSG0000(	CYBB	2280.514	-0.56482	0.007636	0.167737	protein_coding
ENSG0000(	SEMA4A	525.9711	-0.55277	0.007639	0.167737	protein_coding
ENSG0000(	ZFP36L1	3776.566	-0.34799	0.007663	0.168129	protein_coding
ENSG0000(	NSMF	615.5148	0.340463	0.007695	0.168548	protein_coding
ENSG0000(	TBC1D12	1004.273	0.262004	0.007694	0.168548	protein_coding
ENSG0000(	FER1L6	1002.958	0.652865	0.007735	0.169287	protein_coding
ENSG0000(	EDNRB	481.8352	-0.34238	0.00775	0.1695	protein_coding
ENSG0000(	APLNR	235.6835	-0.77219	0.007761	0.169586	protein_coding
ENSG0000(	ATXN1	4497.416	-0.20762	0.007804	0.170399	protein_coding
ENSG0000(	ADAMTS12	267.7094	-0.85498	0.007846	0.170719	protein_coding
ENSG0000(	ENSG0000(	156.538	1.419286	0.007852	0.170719	lncRNA
ENSG0000(	ENSG0000(	24.21829	-0.94601	0.007853	0.170719	TEC
ENSG0000(	IL18R1	515.1345	-0.49511	0.007852	0.170719	protein_coding
ENSG0000(	RNU4-62P	36.30039	-0.71588	0.007856	0.170719	snRNA
ENSG0000(	SHISA5	1021.956	-0.33348	0.007845	0.170719	protein_coding
ENSG0000(	ENSG0000(	221.9137	-0.40303	0.007867	0.170824	TEC
ENSG0000(	IGHV3-48	584.7723	-1.02507	0.007877	0.170907	IG_V_gene

ENSG0000(	ADD3-AS1	424.4215	0.245	0.007925	0.171735	lncRNA
ENSG0000(	ZDHHC7	1840.437	0.258269	0.007928	0.171735	protein_coding
ENSG0000(	MTCO1P21	40.01318	-3.54714	0.007974	0.172467	unprocessed_pseudogene
ENSG0000(	PPA1	1185.776	-0.25391	0.007971	0.172467	protein_coding
ENSG0000(	BTN2A2	1184.602	-0.39745	0.008008	0.172661	protein_coding
ENSG0000(	CLEC4E	367.0252	-0.5012	0.007998	0.172661	protein_coding
ENSG0000(	KIF25	102.6109	-0.56948	0.00799	0.172661	protein_coding
ENSG0000(	LINC02816	19.49358	-0.66302	0.008002	0.172661	lncRNA
ENSG0000(	ITPRIP	706.2578	-0.69179	0.00804	0.172938	protein_coding
ENSG0000(	LINC02547	312.9718	0.179906	0.008033	0.172938	lncRNA
ENSG0000(	ZNF471	1793.284	0.27861	0.008037	0.172938	protein_coding
ENSG0000(	TSC1	3308.379	0.114743	0.00805	0.173018	protein_coding
ENSG0000(	AHR	2213.307	-0.34433	0.008148	0.174779	protein_coding
ENSG0000(	BACH1	2768.851	-0.34424	0.008152	0.174779	protein_coding
ENSG0000(	HERPUD1	3715.914	-0.29023	0.008147	0.174779	protein_coding
ENSG0000(	RDX	696.1187	-0.44205	0.008169	0.174779	protein_coding
ENSG0000(	RNF8	439.8317	-0.2358	0.00816	0.174779	protein_coding
ENSG0000(	ZBED8	132.5615	-0.33985	0.00817	0.174779	protein_coding
ENSG0000(	DPP4	803.9287	-0.43316	0.008194	0.174881	protein_coding
ENSG0000(	KCNJ6	949.6471	0.300711	0.008195	0.174881	protein_coding
ENSG0000(	MSN	2627.854	-0.39296	0.00819	0.174881	protein_coding
ENSG0000(	SLC7A11	962.8652	-0.50123	0.0082	0.174881	protein_coding
ENSG0000(	ENSG0000(	138.0833	-0.61417	0.008207	0.174894	lncRNA
ENSG0000(	FSIP1	101.7612	1.212836	0.008216	0.174957	protein_coding
ENSG0000(	TFEC	1055.559	-0.84414	0.008223	0.174974	protein_coding
ENSG0000(	ENSG0000(	89.92698	-1.04929	0.008236	0.175098	lncRNA
ENSG0000(	CIZ1	1224.571	-0.19236	0.00825	0.175263	protein_coding
ENSG0000(	ILK	974.7083	-0.18697	0.008272	0.1756	protein_coding
ENSG0000(	SCML2P2	45.87722	0.488646	0.008287	0.175779	processed_pseudogene
ENSG0000(	NBPF11	1145.196	0.273826	0.008294	0.175806	protein_coding
ENSG0000(	NXN	596.2741	-0.43946	0.008317	0.176154	protein_coding
ENSG0000(	ACTN1	3847.692	-0.38889	0.00834	0.176454	protein_coding
ENSG0000(	ENG	718.4994	-0.56974	0.008344	0.176454	protein_coding
ENSG0000(	BEST4	278.7927	1.137587	0.008354	0.176515	protein_coding
ENSG0000(	HLA-C	8538.547	-0.41849	0.00839	0.176877	protein_coding
ENSG0000(	RANBP10	932.8173	0.134245	0.008389	0.176877	protein_coding
ENSG0000(	SPATS1	443.9915	0.306894	0.008389	0.176877	protein_coding
ENSG0000(	LAMP3	547.3144	-0.70053	0.008407	0.177097	protein_coding
ENSG0000(	ENSG0000(	1125.874	0.159963	0.008456	0.177997	lncRNA
ENSG0000(	ENSG0000(	21.10668	-0.97737	0.008464	0.177997	lncRNA
ENSG0000(	ID2-AS1	284.2657	0.993127	0.008475	0.177997	lncRNA
ENSG0000(	RN7SKP56	23.54074	-0.77671	0.008475	0.177997	misc_RNA
ENSG0000(	LINC02159	21.14616	-0.87153	0.008496	0.178296	lncRNA
ENSG0000(	PTGDR	680.2191	0.567992	0.008517	0.178468	protein_coding
ENSG0000(	PTPRM	1649.778	-0.30201	0.008512	0.178468	protein_coding
ENSG0000(	LRRC32	371.979	-0.50397	0.008524	0.178482	protein_coding
ENSG0000(	RN7SL510F	394.1212	0.241315	0.00855	0.178885	misc_RNA

ENSG0000(	GIHCG	152.5742	-0.30177	0.008563	0.179024	lncRNA
ENSG0000(	METTL24	278.2819	-0.24084	0.008588	0.179399	protein_coding
ENSG0000(	NR6A1	733.0766	0.418026	0.008612	0.179647	protein_coding
ENSG0000(	PDIA6	1235.39	-0.3299	0.008613	0.179647	protein_coding
ENSG0000(	HLX	265.9522	-0.53586	0.008626	0.179796	protein_coding
ENSG0000(	ENSG0000(	169.7675	0.321352	0.008649	0.179996	lncRNA
ENSG0000(	LINC02580	220.1288	0.513198	0.008649	0.179996	lncRNA
ENSG0000(	F3	2722.595	-0.57134	0.00866	0.18005	protein_coding
ENSG0000(	PCSK7	11572.88	0.168145	0.008665	0.18005	protein_coding
ENSG0000(	ANAPC1P1	106.973	-0.29713	0.008691	0.180083	transcribed_unprocessed_pseudo
ENSG0000(	ENSG0000(	263.8362	-0.45928	0.008678	0.180083	lncRNA
ENSG0000(	ENSG0000(	858.6088	0.852753	0.008686	0.180083	lncRNA
ENSG0000(	MTRNR2L5	107.2986	0.338019	0.008693	0.180083	protein_coding
ENSG0000(	SNX20	493.3303	-0.54488	0.008707	0.180249	protein_coding
ENSG0000(	VAV3	1724.445	0.350265	0.008736	0.180714	protein_coding
ENSG0000(	ENSG0000(	76.51338	0.480192	0.008754	0.180948	lncRNA
ENSG0000(	IGHV5-51	395.4065	-0.88172	0.008791	0.181568	IG_V_gene
ENSG0000(	EOGT	620.2309	-0.36641	0.008811	0.181696	protein_coding
ENSG0000(	SH2D3A	1796.991	0.213699	0.008817	0.181696	protein_coding
ENSG0000(	SLCO3A1	970.6142	-0.41312	0.008816	0.181696	protein_coding
ENSG0000(	ZNF496	1718.041	0.153473	0.008845	0.182139	protein_coding
ENSG0000(	RASGRP1	522.9607	-0.59466	0.008859	0.182288	protein_coding
ENSG0000(	DSCAML1	83.46506	0.868641	0.008873	0.182442	protein_coding
ENSG0000(	AJAP1	178.4259	-0.83507	0.008897	0.182572	protein_coding
ENSG0000(	FZD8	119.7273	0.493983	0.008892	0.182572	protein_coding
ENSG0000(	MOBP	440.9842	0.417716	0.008899	0.182572	protein_coding
ENSG0000(	PLIN4	62.67206	-1.10364	0.008944	0.183364	protein_coding
ENSG0000(	ENSG0000(	36.09739	0.506048	0.008961	0.183573	lncRNA
ENSG0000(	ABI3	359.3046	-0.36472	0.009108	0.183706	protein_coding
ENSG0000(	ANKDD1A	351.1046	-0.33012	0.009053	0.183706	protein_coding
ENSG0000(	ENSG0000(	351.3804	0.175635	0.009064	0.183706	lncRNA
ENSG0000(	ENSG0000(	287.3574	0.401461	0.008999	0.183706	lncRNA
ENSG0000(	ENSG0000(	65.19714	0.382599	0.008985	0.183706	lncRNA
ENSG0000(	ENSG0000(	63.86402	0.380261	0.009047	0.183706	lncRNA
ENSG0000(	GK5	3810.103	0.190346	0.009083	0.183706	protein_coding
ENSG0000(	GRK5-IT1	172.2409	-0.36025	0.009065	0.183706	lncRNA
ENSG0000(	GRM7	131.8973	0.53029	0.009072	0.183706	protein_coding
ENSG0000(	LINC01679	29.44779	-0.96862	0.009016	0.183706	lncRNA
ENSG0000(	LRRK2-DT	51.91708	-0.62486	0.009025	0.183706	lncRNA
ENSG0000(	MIER2	342.9432	-0.27342	0.009103	0.183706	protein_coding
ENSG0000(	NLGN2	615.8309	-0.54082	0.009044	0.183706	protein_coding
ENSG0000(	SASH1	1163.942	-0.26657	0.009098	0.183706	protein_coding
ENSG0000(	SCARA3	264.7597	-0.52485	0.009038	0.183706	protein_coding
ENSG0000(	SGTA	1114.287	0.158301	0.009016	0.183706	protein_coding
ENSG0000(	SLITRK3	35.23229	-0.49727	0.009009	0.183706	protein_coding
ENSG0000(	SNORA50A	82.7971	0.331195	0.009048	0.183706	snoRNA
ENSG0000(	SSR3	2473.868	-0.25319	0.009096	0.183706	protein_coding

ENSG0000(	SYTL2	9237.682	0.610609	0.009062	0.183706	protein_coding
ENSG0000(	TULP4	3069.846	0.164666	0.009083	0.183706	protein_coding
ENSG0000(	COL15A1	865.5437	-0.57337	0.009169	0.18481	protein_coding
ENSG0000(	FAM189B	537.6952	-0.19984	0.009182	0.184935	protein_coding
ENSG0000(	CEP295NL	171.5947	-0.47928	0.009213	0.185019	protein_coding
ENSG0000(	GMCL1	496.4681	0.257639	0.009213	0.185019	protein_coding
ENSG0000(	PSMC2	747.7628	-0.22327	0.009201	0.185019	protein_coding
ENSG0000(	RHOJ	385.2287	-0.45644	0.009204	0.185019	protein_coding
ENSG0000(	CMTM3	222.9565	-0.46423	0.009283	0.185067	protein_coding
ENSG0000(	ENSG0000(	261.0155	-0.28428	0.009256	0.185067	lncRNA
ENSG0000(	ENSG0000(	218.4471	-0.47734	0.00928	0.185067	lncRNA
ENSG0000(	ENSG0000(	200.3431	0.247097	0.009283	0.185067	lncRNA
ENSG0000(	H2BC11	86.72679	-0.661	0.009228	0.185067	protein_coding
ENSG0000(	NFKB1	1300.499	-0.24889	0.009277	0.185067	protein_coding
ENSG0000(	PTCSC1	41.6264	-0.62427	0.009235	0.185067	lncRNA
ENSG0000(	SH2D2A	126.5483	-0.4748	0.009262	0.185067	protein_coding
ENSG0000(	SNX19	4990.288	-0.23174	0.009262	0.185067	protein_coding
ENSG0000(	UNC5CL	276.0995	-0.62675	0.009263	0.185067	protein_coding
ENSG0000(	FKBP15	2543.406	-0.15392	0.009291	0.185086	protein_coding
ENSG0000(	BOC	557.466	-0.47622	0.009306	0.185261	protein_coding
ENSG0000(	SIGLEC7	37.65911	-0.81281	0.009318	0.185355	protein_coding
ENSG0000(	CTDNEP1	796.4655	-0.23051	0.00934	0.185666	protein_coding
ENSG0000(	ENSG0000(	26.41452	-0.52789	0.009362	0.185951	lncRNA
ENSG0000(	SEC24D	1867.702	-0.39794	0.009368	0.185951	protein_coding
ENSG0000(	ENSG0000(	53.63614	-0.51599	0.009377	0.186006	lncRNA
ENSG0000(	NMI	429.9671	-0.38137	0.009384	0.186013	protein_coding
ENSG0000(	C20orf204	101.7834	0.396482	0.009408	0.186343	protein_coding
ENSG0000(	LTBP3	1934.969	-0.27848	0.009415	0.186354	protein_coding
ENSG0000(	ENSG0000(	263.5559	-0.23427	0.009429	0.186491	lncRNA
ENSG0000(	SMOX	159.0663	-0.65212	0.009448	0.186725	protein_coding
ENSG0000(	P2RX7	782.3145	-0.34019	0.009477	0.187042	protein_coding
ENSG0000(	PCNX1	4636.478	-0.34661	0.009471	0.187042	protein_coding
ENSG0000(	ANO7	1908.491	0.686632	0.009544	0.187962	protein_coding
ENSG0000(	FOXQ1	97.32275	-0.83263	0.009548	0.187962	protein_coding
ENSG0000(	KCNN3	612.4371	-0.53112	0.009551	0.187962	protein_coding
ENSG0000(	MTHFD2	2494.191	-0.23825	0.00954	0.187962	protein_coding
ENSG0000(	KLF7	1260.523	-0.53581	0.009564	0.188087	protein_coding
ENSG0000(	CCDC88A	4720.546	-0.24717	0.009581	0.18828	protein_coding
ENSG0000(	CPZ	55.77118	-1.27915	0.0096	0.188515	protein_coding
ENSG0000(	CHRM4	26.45948	-1.21587	0.009612	0.188571	protein_coding
ENSG0000(	CREG1	865.018	-0.34564	0.009616	0.188571	protein_coding
ENSG0000(	STX11	381.5442	-0.45004	0.00963	0.188705	protein_coding
ENSG0000(	CD59	4526.782	-0.43351	0.009665	0.189049	protein_coding
ENSG0000(	DCN	1601.372	-0.5835	0.009671	0.189049	protein_coding
ENSG0000(	ENSG0000(	173.0172	0.241574	0.009695	0.189049	lncRNA
ENSG0000(	EXD3	837.5018	0.341618	0.009671	0.189049	protein_coding
ENSG0000(	IFI16	1680.661	-0.57108	0.009701	0.189049	protein_coding

ENSG0000(	LINC-PINT	3951.448	-0.32524	0.009712	0.189049	lncRNA
ENSG0000(	LINC01659	90.76143	0.400273	0.009707	0.189049	lncRNA
ENSG0000(	PSG2	163.5903	0.534423	0.009716	0.189049	protein_coding
ENSG0000(	SNORA58B	37.62917	-0.45794	0.009716	0.189049	snoRNA
ENSG0000(	YWHAEP7	56.07791	-0.61561	0.009677	0.189049	transcribed_unprocessed_pseudo
ENSG0000(	CBLL1P1	150.1518	0.264053	0.009769	0.18911	processed_pseudogene
ENSG0000(	CIP2A	266.9484	0.486929	0.009775	0.18911	protein_coding
ENSG0000(	ENSG0000(	26.20521	-0.66257	0.009781	0.18911	lncRNA
ENSG0000(	ENSG0000(	213.6293	-0.4535	0.009765	0.18911	lncRNA
ENSG0000(	IGHV3-7	1259.264	-0.7792	0.009767	0.18911	IG_V_gene
ENSG0000(	LINC01005	36.08606	0.965875	0.009753	0.18911	lncRNA
ENSG0000(	MYO3B-AS	545.3424	-0.12975	0.009781	0.18911	lncRNA
ENSG0000(	RNF214	523.9268	-0.17885	0.009748	0.18911	protein_coding
ENSG0000(	SYNJ2	2226.851	0.145202	0.00974	0.18911	protein_coding
ENSG0000(	ERBB4	78.29465	-1.60192	0.009806	0.189388	protein_coding
ENSG0000(	GTPBP6	352.3739	0.274864	0.009809	0.189388	protein_coding
ENSG0000(	AMPH	85.59666	-0.87258	0.009879	0.189419	protein_coding
ENSG0000(	ANKRD34A	29.01209	-0.87149	0.009865	0.189419	protein_coding
ENSG0000(	COL27A1	2078.152	-0.47698	0.009882	0.189419	protein_coding
ENSG0000(	ENSG0000(	46.85436	-0.86695	0.009874	0.189419	lncRNA
ENSG0000(	ENSG0000(	2532.861	-0.27732	0.009869	0.189419	lncRNA
ENSG0000(	ENSG0000(	250.8358	0.208304	0.009903	0.189419	lncRNA
ENSG0000(	IFFO2	421.0687	-0.38136	0.009856	0.189419	protein_coding
ENSG0000(	IGHV3-73	187.82	-1.01598	0.009855	0.189419	IG_V_gene
ENSG0000(	MBL1P	49.89704	-0.66171	0.009907	0.189419	transcribed_unitary_pseudogene
ENSG0000(	NINJ2-AS1	300.9445	-0.33677	0.00989	0.189419	lncRNA
ENSG0000(	RAB30	1211.574	-0.39804	0.009871	0.189419	protein_coding
ENSG0000(	RNPC3	2081.151	0.201351	0.009903	0.189419	protein_coding
ENSG0000(	TMEM165	2188.914	-0.33851	0.009863	0.189419	protein_coding
ENSG0000(	TNFSF14	620.7696	-0.54578	0.009875	0.189419	protein_coding
ENSG0000(	ENSG0000(	160.5968	0.270585	0.009926	0.189645	transcribed_processed_pseudoge
ENSG0000(	ENSG0000(	94.48855	-0.46212	0.009941	0.189796	lncRNA
ENSG0000(	MIR181A1I	488.5289	-0.43215	0.009983	0.190472	lncRNA
ENSG0000(	REXO2	438.5124	-0.24318	0.009991	0.190481	protein_coding
ENSG0000(	HNRNPH2	604.7907	-0.20923	0.010008	0.190685	protein_coding
ENSG0000(	CTSS	3542.505	-0.20725	0.010016	0.190692	protein_coding
ENSG0000(	AIDA	996.4649	-0.24659	0.010052	0.190728	protein_coding
ENSG0000(	PRDX4	110.9484	-0.55142	0.010036	0.190728	protein_coding
ENSG0000(	PTAFR	703.5451	-0.46284	0.010052	0.190728	protein_coding
ENSG0000(	RN7SL141F	118.5056	-0.44793	0.010044	0.190728	misc_RNA
ENSG0000(	SLC28A3	162.1593	-1.11789	0.010048	0.190728	protein_coding
ENSG0000(	HSPG2	7127.632	-0.33956	0.010065	0.190846	protein_coding
ENSG0000(	FRG1DP	23.2935	-1.06622	0.010086	0.191099	unprocessed_pseudogene
ENSG0000(	IGKV1-33	534.6025	-1.10698	0.010125	0.191534	IG_V_gene
ENSG0000(	LINC01050	336.2881	-0.28619	0.010129	0.191534	lncRNA
ENSG0000(	RFLNA	247.45	0.641889	0.010128	0.191534	protein_coding
ENSG0000(	PDE5A	1885.566	0.384117	0.010141	0.191621	protein_coding

ENSG0000(	RGL1	740.9472	-0.34693	0.010161	0.191857	protein_coding
ENSG0000(	BTG2	2718.62	-0.49419	0.010172	0.191948	protein_coding
ENSG0000(	RFX5	1338.927	-0.30998	0.010193	0.192214	protein_coding
ENSG0000(	PCAT18	219.3425	0.257182	0.010203	0.192267	lncRNA
ENSG0000(	CHSY3	46.33474	-0.8412	0.010215	0.192356	protein_coding
ENSG0000(	FSTL3	185.55	-0.56336	0.010226	0.192437	protein_coding
ENSG0000(	ERG	328.031	-0.47202	0.010243	0.192615	protein_coding
ENSG0000(	ENSG0000(	47.63957	-0.90989	0.010288	0.193342	lncRNA
ENSG0000(	PRR14	467.5839	-0.25332	0.010301	0.193456	protein_coding
ENSG0000(	SERPINB5	123.3822	-0.95304	0.010327	0.193799	protein_coding
ENSG0000(	ENSG0000(	170.0614	-0.31692	0.010338	0.193872	lncRNA
ENSG0000(	LDLRAD3	262.817	-0.5577	0.010355	0.193989	protein_coding
ENSG0000(	PSMA2	928.6881	-0.25103	0.010358	0.193989	protein_coding
ENSG0000(	ENSG0000(	99.25411	-0.5022	0.010385	0.194226	lncRNA
ENSG0000(	ENSG0000(	78.98105	-0.48081	0.010383	0.194226	lncRNA
ENSG0000(	CNNM2	1820.362	0.383714	0.010418	0.194314	protein_coding
ENSG0000(	IFNG-AS1	484.9697	-0.61026	0.010417	0.194314	lncRNA
ENSG0000(	M6PR	1243.921	-0.2372	0.010408	0.194314	protein_coding
ENSG0000(	ZEB1	1554.662	-0.44556	0.01041	0.194314	protein_coding
ENSG0000(	ENSG0000(	22.24941	-0.74418	0.01043	0.194416	lncRNA
ENSG0000(	ENSG0000(	326.1631	0.177814	0.010449	0.194633	processed_pseudogene
ENSG0000(	PPIL2	2464.938	0.237893	0.01046	0.194701	protein_coding
ENSG0000(	ANKRD36B	4886.543	-0.43111	0.010494	0.194833	transcribed_unprocessed_pseudo
ENSG0000(	ARFGAP3	724.8163	-0.33901	0.010495	0.194833	protein_coding
ENSG0000(	ENSG0000(	38.10057	-0.58149	0.010495	0.194833	lncRNA
ENSG0000(	ZNF697	389.4384	-0.4434	0.01049	0.194833	protein_coding
ENSG0000(	ENSG0000(	464.4278	0.234654	0.010519	0.195032	protein_coding
ENSG0000(	ENSG0000(	196.0067	0.266852	0.010527	0.195032	lncRNA
ENSG0000(	RN7SL204F	87.73259	0.283797	0.010525	0.195032	misc_RNA
ENSG0000(	IGKV2-29	58.13426	-2.18525	0.010542	0.195169	IG_V_pseudogene
ENSG0000(	GPR141	966.7169	-0.26233	0.010577	0.195695	protein_coding
ENSG0000(	FAAH	509.7831	0.538827	0.010601	0.196006	protein_coding
ENSG0000(	ANAPC4	1096.054	0.196437	0.010643	0.196394	protein_coding
ENSG0000(	DERL3	1298.927	-0.57548	0.010637	0.196394	protein_coding
ENSG0000(	RCN1	873.081	-0.3215	0.010637	0.196394	protein_coding
ENSG0000(	ADGRL2	1312.59	-0.34409	0.010666	0.196679	protein_coding
ENSG0000(	C3orf18	150.5321	-0.32383	0.01068	0.196811	protein_coding
ENSG0000(	PPP1R26-A	639.5862	0.358845	0.010692	0.196893	lncRNA
ENSG0000(	HS3ST3B1	548.5869	-0.50445	0.010703	0.196957	protein_coding
ENSG0000(	CHRNE	32.00343	-0.64957	0.010721	0.197167	protein_coding
ENSG0000(	TRIM47	229.7712	-0.50558	0.010738	0.197341	protein_coding
ENSG0000(	AMOTL1	749.083	-0.44614	0.010763	0.197456	protein_coding
ENSG0000(	ARRB2	1015.114	-0.46412	0.010783	0.197456	protein_coding
ENSG0000(	CARD17	20.4101	-1.10025	0.01079	0.197456	transcribed_unprocessed_pseudo
ENSG0000(	LINC00654	518.9878	0.288992	0.010799	0.197456	lncRNA
ENSG0000(	LINC01607	164.4421	0.334518	0.010787	0.197456	lncRNA
ENSG0000(	OSBPL8	1342.824	-0.41054	0.010779	0.197456	protein_coding

ENSG0000(	PCDHGB3	114.6347	0.435083	0.010769	0.197456	protein_coding
ENSG0000(	SERPINB9	1483.741	-0.51822	0.010801	0.197456	protein_coding
ENSG0000(	GEMIN7-AS	142.6024	0.25941	0.010821	0.197679	lncRNA
ENSG0000(	ENSG0000(	19.48975	0.796763	0.010858	0.198217	lncRNA
ENSG0000(	ENTPD8	375.31	0.728026	0.01087	0.198306	protein_coding
ENSG0000(	DENND3	4231.953	-0.39308	0.010919	0.198815	protein_coding
ENSG0000(	PIK3R3	1230.184	-0.25034	0.010913	0.198815	protein_coding
ENSG0000(	U3	206.4954	-0.34988	0.010906	0.198815	snoRNA
ENSG0000(	MMP13	47.41345	-1.78169	0.010955	0.199328	protein_coding
ENSG0000(	SNAI1	30.64276	-1.0599	0.010967	0.199429	protein_coding
ENSG0000(	CRMP1	47.90566	-0.72127	0.010982	0.199555	protein_coding
ENSG0000(	PTPA	962.2395	-0.16317	0.011024	0.200193	protein_coding
ENSG0000(	CD14	219.7815	-0.61784	0.011031	0.200197	protein_coding
ENSG0000(	CDC42EP4	983.8795	0.272555	0.011039	0.200203	protein_coding
ENSG0000(	ENSG0000(	194.5558	0.465485	0.011062	0.200225	lncRNA
ENSG0000(	ENSG0000(	496.5162	0.188653	0.011051	0.200225	lncRNA
ENSG0000(	NR4A3	214.4211	-1.09857	0.011061	0.200225	protein_coding
ENSG0000(	NLGN4X	179.3663	-0.4591	0.01108	0.200421	protein_coding
ENSG0000(	LRRN2	308.1871	0.891487	0.011107	0.200646	protein_coding
ENSG0000(	TRAF3	2379.131	-0.35647	0.011104	0.200646	protein_coding
ENSG0000(	PSAP	5653.263	-0.36123	0.011118	0.200707	protein_coding
ENSG0000(	ENSG0000(	28.61191	-0.77035	0.011157	0.200884	lncRNA
ENSG0000(	ENSG0000(	70.12062	0.427674	0.01115	0.200884	lncRNA
ENSG0000(	MPV17	953.6266	-0.20263	0.011147	0.200884	protein_coding
ENSG0000(	TPM2	441.7997	-0.56768	0.011139	0.200884	protein_coding
ENSG0000(	SPATA2	457.5394	0.29987	0.011177	0.201107	protein_coding
ENSG0000(	CDH5	432.4813	-0.5647	0.01119	0.20118	protein_coding
ENSG0000(	HLA-DPB1	1841.585	-0.41042	0.011198	0.20118	protein_coding
ENSG0000(	TLR2	1095.12	-0.68477	0.011203	0.20118	protein_coding
ENSG0000(	CASP1P2	28.04766	-0.98131	0.011236	0.20165	unprocessed_pseudogene
ENSG0000(	BAIAP2L2	1496.939	0.483373	0.011271	0.201876	protein_coding
ENSG0000(	ENSG0000(	451.3625	0.145205	0.011266	0.201876	TEC
ENSG0000(	FLOT1	865.346	-0.25672	0.011261	0.201876	protein_coding
ENSG0000(	TUSC3	162.8983	-0.48449	0.011299	0.202249	protein_coding
ENSG0000(	ENSG0000(	649.1053	0.198443	0.01132	0.202368	lncRNA
ENSG0000(	PNRC1	2045.138	-0.32187	0.011313	0.202368	protein_coding
ENSG0000(	ENSG0000(	52.61785	-0.71741	0.011356	0.20282	lncRNA
ENSG0000(	GPR65	373.17	-0.48129	0.011367	0.20282	protein_coding
ENSG0000(	PLVAP	772.5199	-0.48869	0.011368	0.20282	protein_coding
ENSG0000(	SUCLA2	629.0065	0.235723	0.011375	0.20282	protein_coding
ENSG0000(	TUBB6	308.8534	-0.53033	0.011383	0.202832	protein_coding
ENSG0000(	ELL	718.7204	-0.32991	0.011396	0.202892	protein_coding
ENSG0000(	ENSG0000(	479.1788	0.153552	0.011416	0.202892	lncRNA
ENSG0000(	SEC22B	3218.351	-0.1735	0.011414	0.202892	protein_coding
ENSG0000(	VPS37B	2146.718	0.243171	0.011408	0.202892	protein_coding
ENSG0000(	PAWR	4213.184	0.283179	0.01143	0.203006	protein_coding
ENSG0000(	NDUFV1	1318.262	0.265649	0.011466	0.203526	protein_coding

ENSG0000(	TMEM39A	1033.125	-0.28632	0.011488	0.203774	protein_coding
ENSG0000(	PCDHGB8P	224.6232	0.213995	0.011498	0.203825	transcribed_unitary_pseudogene
ENSG0000(	TOR3A	849.2738	-0.19473	0.01151	0.203911	protein_coding
ENSG0000(	LENG9	153.3421	0.38069	0.011532	0.204156	protein_coding
ENSG0000(	RNU1-46P	31.13846	-0.60202	0.011559	0.204502	snRNA
ENSG0000(	DLG4	841.3395	-0.32085	0.011574	0.204636	protein_coding
ENSG0000(	ENSG0000(	273.9489	-0.31253	0.011584	0.204693	lncRNA
ENSG0000(	IFITM1	522.9842	-0.60851	0.011601	0.204865	protein_coding
ENSG0000(	BRCA2	1135.166	-0.20593	0.01164	0.205043	protein_coding
ENSG0000(	ENSG0000(	302.1138	-0.54094	0.011642	0.205043	lncRNA
ENSG0000(	PDGFRA	3452.441	-0.30944	0.011649	0.205043	protein_coding
ENSG0000(	SEPTIN2	4497.206	-0.18235	0.011644	0.205043	protein_coding
ENSG0000(	ZNF736P9Y	28.52222	0.473054	0.011648	0.205043	transcribed_processed_pseudoge
ENSG0000(	CDHR1	1983.376	0.926181	0.011696	0.205056	protein_coding
ENSG0000(	CEP170B	3061.031	0.498019	0.011698	0.205056	protein_coding
ENSG0000(	ENSG0000(	62.1525	-0.46371	0.011669	0.205056	lncRNA
ENSG0000(	ERH	322.7493	-0.3709	0.011691	0.205056	protein_coding
ENSG0000(	OTOP2	120.8761	1.421717	0.011699	0.205056	protein_coding
ENSG0000(	TCF12	2598.702	0.139287	0.011702	0.205056	protein_coding
ENSG0000(	TNFRSF10C	140.1154	-1.18714	0.011689	0.205056	protein_coding
ENSG0000(	ENSG0000(	885.076	-0.33264	0.011715	0.205164	lncRNA
ENSG0000(	ARHGAP5-/	31.64355	0.586162	0.011778	0.205694	lncRNA
ENSG0000(	GPATCH3	161.2273	0.346857	0.011785	0.205694	protein_coding
ENSG0000(	LAMA5	2138.369	-0.30173	0.01179	0.205694	protein_coding
ENSG0000(	RPL10L	49.64935	0.957728	0.011788	0.205694	protein_coding
ENSG0000(	SEC61B	272.0151	-0.34508	0.01178	0.205694	protein_coding
ENSG0000(	WFDC2	46.63199	1.027521	0.011763	0.205694	protein_coding
ENSG0000(	APCDD1	429.3799	-0.40755	0.011819	0.205721	protein_coding
ENSG0000(	ENSG0000(	63.40616	-1.09895	0.011812	0.205721	processed_pseudogene
ENSG0000(	RNF19B	1027.285	-0.39051	0.011822	0.205721	protein_coding
ENSG0000(	SERPINH1	1229.519	-0.51473	0.011822	0.205721	protein_coding
ENSG0000(	BTN3A1	3549.427	-0.30183	0.011856	0.206039	protein_coding
ENSG0000(	CSGALNAC	366.2516	-0.6386	0.011863	0.206039	protein_coding
ENSG0000(	UNC13B	3509.977	0.435551	0.011849	0.206039	protein_coding
ENSG0000(	CD300LF	142.2378	-0.53493	0.011908	0.206129	protein_coding
ENSG0000(	DRAM1	904.8263	-0.36762	0.011928	0.206129	protein_coding
ENSG0000(	ENSG0000(	285.1591	0.261833	0.01193	0.206129	lncRNA
ENSG0000(	MAGI2	1654.496	-0.39188	0.011899	0.206129	protein_coding
ENSG0000(	NEMP2	539.9886	-0.41469	0.011916	0.206129	protein_coding
ENSG0000(	SAMMSON	455.4016	0.164538	0.01191	0.206129	lncRNA
ENSG0000(	THEMIS2	1244.344	-0.54942	0.011932	0.206129	protein_coding
ENSG0000(	TTC3P1	140.8654	0.387747	0.011929	0.206129	processed_pseudogene
ENSG0000(	ZBP1	961.9195	-0.53979	0.011935	0.206129	protein_coding
ENSG0000(	ENSG0000(	491.0492	0.300882	0.011954	0.206324	transcribed_unprocessed_pseudo
ENSG0000(	CPVL	197.6283	-0.48697	0.011987	0.206492	protein_coding
ENSG0000(	PDZD7	275.9768	-0.26223	0.012001	0.206492	protein_coding
ENSG0000(	RAD52	702.6363	0.349256	0.012001	0.206492	protein_coding

ENSG0000(	TMEM255E	298.8505	-0.58786	0.011977	0.206492	protein_coding
ENSG0000(	ZNF66	2006.523	0.227322	0.011979	0.206492	protein_coding
ENSG0000(	ENSG0000(	31.57223	-0.5674	0.012017	0.206629	lncRNA
ENSG0000(	C1orf226	439.8028	0.438263	0.012029	0.206709	protein_coding
ENSG0000(	KYNU	5818.684	-0.39943	0.012038	0.206739	protein_coding
ENSG0000(	TUBGCP3	972.5043	-0.14358	0.012051	0.206829	protein_coding
ENSG0000(	PSTPIP2	645.1205	-0.6326	0.012061	0.206864	protein_coding
ENSG0000(	CIBAR1	234.1647	-0.40434	0.012082	0.207049	protein_coding
ENSG0000(	ENSG0000(	21.89906	-0.84667	0.012087	0.207049	lncRNA
ENSG0000(	SLC22A23	3094.981	0.466275	0.012094	0.207049	protein_coding
ENSG0000(	MMD	328.538	-0.3531	0.012111	0.207086	protein_coding
ENSG0000(	VPS51	947.8584	0.233597	0.012109	0.207086	protein_coding
ENSG0000(	ENSG0000(	42.27692	-0.51007	0.012121	0.207132	protein_coding
ENSG0000(	MRGPRF	99.66767	-0.77252	0.012139	0.207308	protein_coding
ENSG0000(	CEP44	766.1488	0.186624	0.012197	0.207331	protein_coding
ENSG0000(	ETV4	74.65851	-0.57163	0.01219	0.207331	protein_coding
ENSG0000(	MCAM	814.9689	-0.54358	0.01219	0.207331	protein_coding
ENSG0000(	MTRNR2L1	41.35684	-1.21767	0.012171	0.207331	protein_coding
ENSG0000(	PCDHB13	248.0905	0.192119	0.012201	0.207331	protein_coding
ENSG0000(	PTGES	313.796	-0.61138	0.012196	0.207331	protein_coding
ENSG0000(	TMEM72	107.759	1.15828	0.012151	0.207331	protein_coding
ENSG0000(	TRAF4	1110.578	0.30606	0.012163	0.207331	protein_coding
ENSG0000(	C9orf131	125.161	-0.30014	0.012225	0.207436	protein_coding
ENSG0000(	CMTM4	2644.147	0.454372	0.012229	0.207436	protein_coding
ENSG0000(	OGFR	1095.864	-0.27181	0.01223	0.207436	protein_coding
ENSG0000(	NCF1C	120.7827	-0.77007	0.01225	0.207653	unprocessed_pseudogene
ENSG0000(	TRPV2	308.1526	-0.46836	0.012272	0.207899	protein_coding
ENSG0000(	DNM3OS	328.036	-0.53796	0.012306	0.208221	lncRNA
ENSG0000(	MFGE8	1302.237	-0.419	0.012306	0.208221	protein_coding
ENSG0000(	SLC35B3	701.0556	0.214541	0.012332	0.208528	protein_coding
ENSG0000(	SMIM29	62.53775	-0.501	0.012358	0.208841	protein_coding
ENSG0000(	SGSM3-AS1	100.7233	0.324316	0.012367	0.208863	lncRNA
ENSG0000(	ALPK2	462.3788	-0.94661	0.012382	0.208986	protein_coding
ENSG0000(	IGLV1-40	341.3424	-1.06167	0.012391	0.209013	IG_V_gene
ENSG0000(	IRS1	921.4173	-0.4898	0.012401	0.209052	protein_coding
ENSG0000(	ENSG0000(	497.1306	-0.39257	0.012428	0.209133	TEC
ENSG0000(	P3H3	233.7396	-0.3361	0.012415	0.209133	protein_coding
ENSG0000(	RSPO1	58.37519	-0.5361	0.012425	0.209133	protein_coding
ENSG0000(	CTDSPL	1050.573	0.369366	0.012466	0.20951	protein_coding
ENSG0000(	SLAMF1	418.2745	-0.59157	0.012461	0.20951	protein_coding
ENSG0000(	COL6A1	3832.514	-0.48247	0.012485	0.209703	protein_coding
ENSG0000(	LRRC26	116.6413	0.786767	0.012522	0.210198	protein_coding
ENSG0000(	ADAM20P1	125.3285	-0.38669	0.012564	0.210768	transcribed_unprocessed_pseudo
ENSG0000(	ENSG0000(	189.2992	-0.37432	0.0126	0.211045	lncRNA
ENSG0000(	LRP8	1196.733	-0.37219	0.012604	0.211045	protein_coding
ENSG0000(	NECTIN1	622.8152	0.431098	0.012603	0.211045	protein_coding
ENSG0000(	ENSG0000(	46.04701	-1.19774	0.012647	0.211644	lncRNA

ENSG0000(	ELMO3	330.0709	0.492755	0.012691	0.21185	protein_coding
ENSG0000(	ETV6	3052.649	-0.19878	0.012689	0.21185	protein_coding
ENSG0000(	GADD45B	195.8167	-0.53949	0.012698	0.21185	protein_coding
ENSG0000(	HAVCR2	509.3864	-0.29908	0.012692	0.21185	protein_coding
ENSG0000(	JCAD	381.1804	-0.56678	0.012685	0.21185	protein_coding
ENSG0000(	IL1RL1	575.5022	-0.67151	0.012718	0.212058	protein_coding
ENSG0000(	ATP6V1B2	1741.569	-0.29151	0.012831	0.212267	protein_coding
ENSG0000(	BMERB1	174.0762	-0.48129	0.012796	0.212267	protein_coding
ENSG0000(	EMILIN3	29.19012	-0.73903	0.01281	0.212267	protein_coding
ENSG0000(	ENSG0000(	113.6363	-0.3208	0.012825	0.212267	lncRNA
ENSG0000(	GM2A	1268.535	-0.41378	0.012826	0.212267	protein_coding
ENSG0000(	GUCY1A1	754.6612	-0.80953	0.012821	0.212267	protein_coding
ENSG0000(	MFSD4B	6114.621	0.170874	0.012826	0.212267	protein_coding
ENSG0000(	NVL	961.9446	0.188008	0.012826	0.212267	protein_coding
ENSG0000(	RFTN1	1120.974	-0.47074	0.012816	0.212267	protein_coding
ENSG0000(	RNU6-1283	30.96365	-0.54287	0.01281	0.212267	snRNA
ENSG0000(	SIGLEC10	859.5305	-0.22736	0.012782	0.212267	protein_coding
ENSG0000(	SPON2	805.2891	-0.39518	0.012802	0.212267	protein_coding
ENSG0000(	ZNF793	1841.347	0.144745	0.012762	0.212267	protein_coding
ENSG0000(	ENSG0000(	172.7455	-0.2167	0.012859	0.212483	lncRNA
ENSG0000(	LRRC63	31.86649	-0.56315	0.012859	0.212483	protein_coding
ENSG0000(	PRICKLE2	1993.648	-0.27434	0.012868	0.212495	protein_coding
ENSG0000(	SND1-IT1	744.1096	-0.27609	0.012883	0.212619	lncRNA
ENSG0000(	ENSG0000(	20.62774	-0.8708	0.012899	0.212621	misc_RNA
ENSG0000(	ITGB2	1009.166	-0.53951	0.012895	0.212621	protein_coding
ENSG0000(	ENSG0000(	360.0126	-0.71837	0.012914	0.212743	TEC
ENSG0000(	AIF1	114.0962	-0.59535	0.012982	0.213237	protein_coding
ENSG0000(	CCDC117	802.0058	-0.23237	0.012988	0.213237	protein_coding
ENSG0000(	CD248	88.91666	-0.85379	0.01299	0.213237	protein_coding
ENSG0000(	SLC16A3	1673.314	-0.41024	0.012979	0.213237	protein_coding
ENSG0000(	ST6GAL1	4029.81	-0.47958	0.01298	0.213237	protein_coding
ENSG0000(	VOPP1	2588.535	-0.30424	0.012956	0.213237	protein_coding
ENSG0000(	CCDC171	618.4192	0.313666	0.013034	0.213448	protein_coding
ENSG0000(	CLEC14A	102.7194	-0.52103	0.013033	0.213448	protein_coding
ENSG0000(	DYNLT1	976.5091	-0.274	0.013015	0.213448	protein_coding
ENSG0000(	ENSG0000(	955.3815	0.203504	0.013019	0.213448	TEC
ENSG0000(	ENSG0000(	22.52835	0.879032	0.013053	0.213543	lncRNA
ENSG0000(	KLHL6	3273.818	-0.35412	0.013055	0.213543	protein_coding
ENSG0000(	DTX2P1-UF	4032.395	0.268703	0.013104	0.213575	lncRNA
ENSG0000(	ENSG0000(	309.4801	0.256391	0.013112	0.213575	lncRNA
ENSG0000(	FBLN7	244.8756	-0.30184	0.013074	0.213575	protein_coding
ENSG0000(	FOXP2	801.7468	0.402831	0.013074	0.213575	protein_coding
ENSG0000(	GIT2	3138.191	-0.19424	0.013102	0.213575	protein_coding
ENSG0000(	SLC8A1	1434.73	-0.49219	0.013103	0.213575	protein_coding
ENSG0000(	ZSCAN9	468.8839	0.192869	0.013095	0.213575	protein_coding
ENSG0000(	ADGRE5	1657.617	-0.28106	0.013177	0.213747	protein_coding
ENSG0000(	ASIC2	26.88663	1.234871	0.013146	0.213747	protein_coding

ENSG0000(	COPA	3367.406	-0.19921	0.013142	0.213747	protein_coding
ENSG0000(	ENSG0000(	96.35281	0.322465	0.013176	0.213747	lncRNA
ENSG0000(	SLC35A1	511.8329	0.237693	0.013162	0.213747	protein_coding
ENSG0000(	SLC39A1	518.1703	-0.26474	0.013166	0.213747	protein_coding
ENSG0000(	ZNF181	599.6016	0.203983	0.01313	0.213747	protein_coding
ENSG0000(	LAMB1	7142.877	-0.29036	0.013199	0.213874	protein_coding
ENSG0000(	MCEMP1	20.77034	-2.13114	0.0132	0.213874	protein_coding
ENSG0000(	ENSG0000(	333.579	0.209876	0.013252	0.214588	transcribed_unprocessed_pseudo
ENSG0000(	CLIC4	1245.124	-0.45827	0.013283	0.214847	protein_coding
ENSG0000(	ENSG0000(	20.51737	-0.81166	0.013283	0.214847	lncRNA
ENSG0000(	CD84	2278.713	-0.45157	0.013297	0.214869	protein_coding
ENSG0000(	ST3GAL6	394.4063	-0.37151	0.0133	0.214869	protein_coding
ENSG0000(	ARID5A	548.9336	-0.55861	0.013341	0.215267	protein_coding
ENSG0000(	ENSG0000(	148.1108	0.300559	0.013342	0.215267	TEC
ENSG0000(	GATAD2B	2598.923	-0.38029	0.013349	0.215267	protein_coding
ENSG0000(	PLEKHO1	1411.987	-0.45682	0.013377	0.215596	protein_coding
ENSG0000(	DUSP6	1296.998	-0.38262	0.013398	0.215818	protein_coding
ENSG0000(	GPR137B	360.5719	-0.31346	0.013422	0.215822	protein_coding
ENSG0000(	IGHV3-15	847.763	-0.82608	0.01342	0.215822	IG_V_gene
ENSG0000(	ZSCAN23	124.6548	0.526462	0.013418	0.215822	protein_coding
ENSG0000(	ABR	7296.886	0.368774	0.013459	0.215954	protein_coding
ENSG0000(	ADO	355.6631	-0.24003	0.013462	0.215954	protein_coding
ENSG0000(	LINC01320	475.6065	0.232248	0.013442	0.215954	lncRNA
ENSG0000(	WWC2	508.1609	-0.42085	0.013455	0.215954	protein_coding
ENSG0000(	DAP	1164.572	-0.24642	0.013512	0.216385	protein_coding
ENSG0000(	IGLC3	3318.362	-0.9802	0.013508	0.216385	IG_C_gene
ENSG0000(	RNF145	1445.863	-0.30778	0.013512	0.216385	protein_coding
ENSG0000(	CCDC69	1292.122	-0.28973	0.013528	0.216515	protein_coding
ENSG0000(	EMILIN2	645.1318	-0.39384	0.013551	0.216654	protein_coding
ENSG0000(	ENSG0000(	30.98371	-1.01822	0.013553	0.216654	lncRNA
ENSG0000(	ENSG0000(	206.0887	-0.32408	0.013595	0.217153	TEC
ENSG0000(	IQCM	486.549	0.36288	0.013608	0.217153	protein_coding
ENSG0000(	ZSWIM4	227.5911	-0.5435	0.013605	0.217153	protein_coding
ENSG0000(	PEX26	7700.911	0.350803	0.013627	0.217342	protein_coding
ENSG0000(	BRF1	1007.709	0.136117	0.013719	0.217684	protein_coding
ENSG0000(	ENSG0000(	180.8799	-0.31041	0.013709	0.217684	lncRNA
ENSG0000(	ENSG0000(	21.61433	-0.72502	0.013691	0.217684	lncRNA
ENSG0000(	ENSG0000(	158.3737	0.512033	0.013686	0.217684	lncRNA
ENSG0000(	IGHV3-49	245.8385	-0.88859	0.013705	0.217684	IG_V_gene
ENSG0000(	KPNB1	3879.807	-0.17275	0.013682	0.217684	protein_coding
ENSG0000(	PPP3CC	327.7246	-0.42239	0.013728	0.217684	protein_coding
ENSG0000(	SLC15A2	1117.607	-0.37903	0.013725	0.217684	protein_coding
ENSG0000(	SPATS2	2163.053	-0.17802	0.013669	0.217684	protein_coding
ENSG0000(	UCHL5	797.1203	-0.1938	0.013688	0.217684	protein_coding
ENSG0000(	ENSG0000(	28.35766	-0.56302	0.013749	0.217806	lncRNA
ENSG0000(	HSPE1P18	95.94095	-0.44426	0.013751	0.217806	processed_pseudogene
ENSG0000(	FAM171B	139.7491	-0.47567	0.013808	0.218234	protein_coding

ENSG0000(	OTOAP1	51.19495	-0.60703	0.01381	0.218234	transcribed_unprocessed_pseudo
ENSG0000(	RAB1AP1	343.2796	-0.82332	0.0138	0.218234	processed_pseudogene
ENSG0000(	SFXN3	633.726	-0.37824	0.013808	0.218234	protein_coding
ENSG0000(	DGKG	156.0666	-0.50223	0.013834	0.218428	protein_coding
ENSG0000(	POU2F2	937.6511	-0.60857	0.01384	0.218428	protein_coding
ENSG0000(	ST3GAL5	2259.91	-0.38354	0.013846	0.218428	protein_coding
ENSG0000(	IGKV1D-17	23.857	-1.97078	0.013871	0.218688	IG_V_gene
ENSG0000(	PTPRG	2448.393	-0.21415	0.013889	0.218852	protein_coding
ENSG0000(	CPNE5	590.2883	-0.51313	0.013937	0.219484	protein_coding
ENSG0000(	DKK3	480.4331	-0.51303	0.013964	0.219572	protein_coding
ENSG0000(	ENSG0000(	251.251	0.262318	0.013982	0.219572	lncRNA
ENSG0000(	ENSG0000(	158.9664	0.220349	0.013983	0.219572	lncRNA
ENSG0000(	H3-3A	3572.008	-0.29281	0.01398	0.219572	protein_coding
ENSG0000(	TGFBR1	1158.858	-0.36189	0.013981	0.219572	protein_coding
ENSG0000(	HSD17B14	28.57029	-0.57444	0.01407	0.220194	protein_coding
ENSG0000(	PFKFB4	695.3911	-1.237	0.014063	0.220194	protein_coding
ENSG0000(	RALGDS	2316.065	-0.27917	0.014069	0.220194	protein_coding
ENSG0000(	TUBA1A	243.3179	-0.57035	0.014033	0.220194	protein_coding
ENSG0000(	UAP1L1	575.6856	-0.23959	0.014048	0.220194	protein_coding
ENSG0000(	ZNF675	1109.672	0.142581	0.014039	0.220194	protein_coding
ENSG0000(	ENSG0000(	491.5197	0.249893	0.014084	0.22028	TEC
ENSG0000(	GASK1B	805.5285	-0.51361	0.014139	0.220566	protein_coding
ENSG0000(	ITPKA	118.1163	0.790458	0.014142	0.220566	protein_coding
ENSG0000(	MROH7	1926.785	0.554097	0.014137	0.220566	protein_coding
ENSG0000(	ZNF707	376.7897	-0.24658	0.014142	0.220566	protein_coding
ENSG0000(	ZNF782	860.6666	0.215549	0.014134	0.220566	protein_coding
ENSG0000(	PXMP2	207.7403	0.506762	0.014203	0.221383	protein_coding
ENSG0000(	LITAF	3710.726	-0.48188	0.014237	0.221393	protein_coding
ENSG0000(	NRM	83.92429	-0.45368	0.014225	0.221393	protein_coding
ENSG0000(	OGFRP1	305.4119	0.237575	0.01426	0.221393	lncRNA
ENSG0000(	PPP1R9B	936.3039	-0.34029	0.014246	0.221393	protein_coding
ENSG0000(	RTN1	103.1181	-0.51904	0.014252	0.221393	protein_coding
ENSG0000(	STRA6	270.6816	-0.61893	0.01425	0.221393	protein_coding
ENSG0000(	TOX4P1	32.86308	-0.56612	0.014252	0.221393	processed_pseudogene
ENSG0000(	GRB7	288.2295	0.510282	0.01432	0.222201	protein_coding
ENSG0000(	ALOX5	626.9224	-0.60414	0.01436	0.222698	protein_coding
ENSG0000(	IGHV3-74	674.5731	-0.76603	0.01438	0.222891	IG_V_gene
ENSG0000(	FYN	1353.288	-0.5147	0.0144	0.223076	protein_coding
ENSG0000(	PPT1	672.1719	-0.4149	0.014412	0.223135	protein_coding
ENSG0000(	ABHD2	4137.798	-0.25717	0.014437	0.223395	protein_coding
ENSG0000(	PTGIR	446.5269	-0.3555	0.014455	0.223423	protein_coding
ENSG0000(	XPR1	1068.278	-0.22668	0.014454	0.223423	protein_coding
ENSG0000(	ENSG0000(	135.7328	-0.3434	0.014515	0.223724	lncRNA
ENSG0000(	ENSG0000(	339.5877	-0.22966	0.014501	0.223724	lncRNA
ENSG0000(	HGSNAT	1785.526	0.13544	0.014504	0.223724	protein_coding
ENSG0000(	RTL5	79.31241	-0.36054	0.014508	0.223724	protein_coding
ENSG0000(	ZNF329	719.5539	0.172386	0.014514	0.223724	protein_coding

ENSG0000(	DDX12P	149.9651	0.344469	0.014583	0.224145	unprocessed_pseudogene
ENSG0000(	ENSG0000(	164.8017	0.70906	0.014577	0.224145	lncRNA
ENSG0000(	ENSG0000(	224.6028	0.21206	0.014575	0.224145	lncRNA
ENSG0000(	ENSG0000(	344.8345	-0.22297	0.014553	0.224145	lncRNA
ENSG0000(	MESP2	43.18296	-0.48577	0.014572	0.224145	protein_coding
ENSG0000(	ENSG0000(	33.10788	-0.68291	0.014597	0.224227	lncRNA
ENSG0000(	ENSG0000(	42.68869	0.693688	0.014619	0.224438	TEC
ENSG0000(	CASP4LP	579.7865	-0.49212	0.014655	0.224459	transcribed_unprocessed_pseudo
ENSG0000(	ENSG0000(	57.08986	-0.65158	0.014651	0.224459	lncRNA
ENSG0000(	FAM83A	99.70818	-0.89375	0.014643	0.224459	protein_coding
ENSG0000(	MAP3K3	852.8974	-0.27195	0.014661	0.224459	protein_coding
ENSG0000(	MMP17	86.5794	-0.51006	0.014629	0.224459	protein_coding
ENSG0000(	DAW1	164.3732	0.493294	0.014689	0.22477	protein_coding
ENSG0000(	BLCAP	2436.861	0.174968	0.014738	0.225137	protein_coding
ENSG0000(	HIF1A	2705.535	-0.4325	0.014726	0.225137	protein_coding
ENSG0000(	LMAN1	2012.073	-0.35549	0.014735	0.225137	protein_coding
ENSG0000(	RHBDL2	474.8467	0.300054	0.014761	0.22537	protein_coding
ENSG0000(	CHAC1	70.07193	-0.80713	0.014775	0.225376	protein_coding
ENSG0000(	ENSG0000(	2096.755	-0.30823	0.014803	0.225376	TEC
ENSG0000(	ENSG0000(	406.2514	0.185405	0.014781	0.225376	lncRNA
ENSG0000(	FBXL5	1646.333	-0.34704	0.0148	0.225376	protein_coding
ENSG0000(	TBX19	213.3239	-0.32306	0.014801	0.225376	protein_coding
ENSG0000(	BCAT1	449.2705	-0.38843	0.014822	0.225414	protein_coding
ENSG0000(	NDST1	2211.901	-0.23833	0.014817	0.225414	protein_coding
ENSG0000(	MRTFB	3325.93	0.138947	0.014848	0.225686	protein_coding
ENSG0000(	ORC2	1365.13	0.143079	0.014888	0.22618	protein_coding
ENSG0000(	CRACR2A	2262.64	0.391679	0.014954	0.226232	protein_coding
ENSG0000(	IGLV4-60	37.24478	-1.39682	0.014938	0.226232	IG_V_gene
ENSG0000(	MFHAS1	1205.968	0.248209	0.014929	0.226232	protein_coding
ENSG0000(	PDE4DIP	5119.752	-0.27599	0.014947	0.226232	protein_coding
ENSG0000(	PLXNA2	3584.767	0.503104	0.014902	0.226232	protein_coding
ENSG0000(	PRKD1	248.227	-0.34247	0.014958	0.226232	protein_coding
ENSG0000(	RTEL1	805.8074	-0.21278	0.014936	0.226232	protein_coding
ENSG0000(	ZMAT5	343.3033	-0.19487	0.014929	0.226232	protein_coding
ENSG0000(	SLC16A9	460.8918	1.018642	0.014996	0.22669	protein_coding
ENSG0000(	LNP1	55.15439	-0.52919	0.015024	0.22699	protein_coding
ENSG0000(	ENSG0000(	55.70652	-0.37972	0.015035	0.227025	lncRNA
ENSG0000(	DNAJB11	1341.22	-0.26443	0.015094	0.22779	protein_coding
ENSG0000(	PHLDB1	5429.915	0.140623	0.015124	0.228114	protein_coding
ENSG0000(	ENSG0000(	603.0721	0.267887	0.015134	0.228148	lncRNA
ENSG0000(	IGKV1-5	1103.561	-0.77088	0.015144	0.22817	IG_V_gene
ENSG0000(	ENSG0000(	296.9277	-0.31605	0.015196	0.228828	lncRNA
ENSG0000(	NEIL2	176.452	-0.32002	0.015205	0.228833	protein_coding
ENSG0000(	MTCO1P1	23.21766	0.689928	0.015222	0.228843	processed_pseudogene
ENSG0000(	PSMB8	663.6223	-0.30906	0.015222	0.228843	protein_coding
ENSG0000(	QPCT	40.10925	-0.71371	0.015258	0.229258	protein_coding
ENSG0000(	SLC17A6-D	125.8329	0.603074	0.015268	0.229292	lncRNA

ENSG0000(	ATF5	328.2603	-0.3052	0.015314	0.229293	protein_coding
ENSG0000(	ENSG0000(	36.98231	-0.67454	0.015278	0.229293	lncRNA
ENSG0000(	IGFBP4	1508.081	-0.55612	0.0153	0.229293	protein_coding
ENSG0000(	IGHEP2	73.94316	-0.52402	0.015311	0.229293	IG_C_pseudogene
ENSG0000(	MAP3K8	572.8206	-0.46238	0.015318	0.229293	protein_coding
ENSG0000(	STK11	1244.875	0.200704	0.015302	0.229293	protein_coding
ENSG0000(	ICAM3	233.8639	-0.44974	0.01533	0.229346	protein_coding
ENSG0000(	POLR2H	1010.166	0.234951	0.015373	0.229853	protein_coding
ENSG0000(	LY75	2036.149	-0.26156	0.015397	0.230087	protein_coding
ENSG0000(	ITGAV	2101.306	-0.3213	0.015424	0.230373	protein_coding
ENSG0000(	ENSG0000(	24.15195	-0.54822	0.015447	0.230427	lncRNA
ENSG0000(	ENSG0000(	213.8391	0.24668	0.01544	0.230427	lncRNA
ENSG0000(	HTR2A	102.5208	0.50597	0.015453	0.230427	protein_coding
ENSG0000(	ECI1	323.8763	0.310519	0.015467	0.230437	protein_coding
ENSG0000(	RAB18	2019.53	-0.24966	0.01547	0.230437	protein_coding
ENSG0000(	CHIT1	151.738	-0.71407	0.015485	0.23052	protein_coding
ENSG0000(	HSPA5	2403.447	-0.32408	0.015493	0.23052	protein_coding
ENSG0000(	OLFM1	121.617	-0.79104	0.015518	0.230768	protein_coding
ENSG0000(	PWWP2B	441.6802	0.464752	0.01558	0.231577	protein_coding
ENSG0000(	EMID1	349.7576	-0.38834	0.015632	0.232089	protein_coding
ENSG0000(	NUDT5	789.6479	-0.20309	0.015625	0.232089	protein_coding
ENSG0000(	CABLES2	267.8592	0.2835	0.015661	0.232391	protein_coding
ENSG0000(	DNM1	1262.081	-0.56759	0.015737	0.2334	protein_coding
ENSG0000(	TNFRSF25	1159.557	-0.22644	0.015779	0.233894	protein_coding
ENSG0000(	CYP26B1	177.6131	-1.0417	0.015792	0.233967	protein_coding
ENSG0000(	THRB-IT1	330.5833	0.476895	0.015814	0.234167	lncRNA
ENSG0000(	CBX6	1517.316	-0.35012	0.015836	0.234177	protein_coding
ENSG0000(	CDV3	3238.212	-0.21091	0.015849	0.234177	protein_coding
ENSG0000(	CPA6	78.20387	0.663331	0.015847	0.234177	protein_coding
ENSG0000(	ENSG0000(	201.1306	0.312631	0.015845	0.234177	lncRNA
ENSG0000(	LAT2	932.4363	-0.36439	0.015865	0.234288	protein_coding
ENSG0000(	PLA2G2A	759.0276	-1.30634	0.015885	0.23445	protein_coding
ENSG0000(	BPGM	151.6379	-0.37937	0.015901	0.234522	protein_coding
ENSG0000(	MASP1	424.5262	-0.83062	0.015906	0.234522	protein_coding
ENSG0000(	CCDC136	162.2421	-0.43231	0.016017	0.234917	protein_coding
ENSG0000(	EEPD1	348.1596	0.379038	0.016027	0.234917	protein_coding
ENSG0000(	ENSG0000(	47.69873	0.401285	0.015942	0.234917	lncRNA
ENSG0000(	ENSG0000(	34.95456	0.785943	0.015982	0.234917	processed_pseudogene
ENSG0000(	GRIN2D	37.76954	-0.69984	0.015971	0.234917	protein_coding
ENSG0000(	HOXA3	594.2623	0.395691	0.016037	0.234917	protein_coding
ENSG0000(	IGKV1D-16	40.43581	-1.1353	0.016034	0.234917	IG_V_gene
ENSG0000(	MTA1	1117.068	0.153679	0.015979	0.234917	protein_coding
ENSG0000(	PARP8	1836.83	-0.41371	0.015975	0.234917	protein_coding
ENSG0000(	PLCD3	2321.889	0.736221	0.016031	0.234917	protein_coding
ENSG0000(	PSMA5	726.9062	-0.23159	0.015996	0.234917	protein_coding
ENSG0000(	PSMC1	734.5094	-0.27865	0.016037	0.234917	protein_coding
ENSG0000(	RAD17P1	132.7381	0.611365	0.016044	0.234917	processed_pseudogene

ENSG0000(	KIAA0040	941.6643	-0.36161	0.016067	0.23512	protein_coding
ENSG0000(	SCARNA22	66.57097	0.511673	0.016088	0.235309	scaRNA
ENSG0000(	MCOLN2	728.3397	0.593977	0.016112	0.235401	protein_coding
ENSG0000(	SUGP2	5301.957	0.17097	0.016105	0.235401	protein_coding
ENSG0000(	ATP11A	1723.076	-0.2839	0.01613	0.23542	protein_coding
ENSG0000(	TOMM5	519.4841	-0.2544	0.016129	0.23542	protein_coding
ENSG0000(	C3orf80	22.43584	-0.72657	0.016177	0.235906	protein_coding
ENSG0000(	FPR3	453.4401	-0.41031	0.016189	0.235906	protein_coding
ENSG0000(	MDN1	4436.117	0.223401	0.016185	0.235906	protein_coding
ENSG0000(	ENSG0000(	193.4366	0.285146	0.016201	0.235957	lncRNA
ENSG0000(	ENSG0000(	745.8468	-0.25041	0.016277	0.236816	lncRNA
ENSG0000(	ENSG0000(	769.6352	0.128511	0.016275	0.236816	lncRNA
ENSG0000(	CPNE2	868.6694	0.310762	0.016299	0.237006	protein_coding
ENSG0000(	GRB2	1894.882	-0.20307	0.01631	0.23704	protein_coding
ENSG0000(	ENSG0000(	21.85634	-0.85553	0.016353	0.237421	transcribed_unprocessed_pseudo
ENSG0000(	OR5AU1	409.3316	0.216719	0.016352	0.237421	protein_coding
ENSG0000(	NUDT19P5	20.66098	-0.82159	0.016406	0.237812	processed_pseudogene
ENSG0000(	SH2D6	131.2573	1.060164	0.016401	0.237812	protein_coding
ENSG0000(	SUCLG2P4	69.82087	0.318503	0.016399	0.237812	processed_pseudogene
ENSG0000(	GPR85	3595.644	0.491136	0.016448	0.238287	protein_coding
ENSG0000(	SEC14L3	57.18913	0.332568	0.016482	0.238415	protein_coding
ENSG0000(	SELENOM	400.2851	-0.40974	0.016478	0.238415	protein_coding
ENSG0000(	ZNF26	4487.814	0.13714	0.016471	0.238415	protein_coding
ENSG0000(	UBA2	1032.711	-0.2999	0.016494	0.238454	protein_coding
ENSG0000(	PCA3	610.0411	0.202883	0.016525	0.238783	lncRNA
ENSG0000(	PABPC5	26.23207	-0.69531	0.016552	0.239037	protein_coding
ENSG0000(	PODN	205.4187	-0.43909	0.016579	0.239305	protein_coding
ENSG0000(	ENSG0000(	114.5842	0.266047	0.016622	0.239808	lncRNA
ENSG0000(	AOX1	128.1734	-1.32778	0.016678	0.239858	protein_coding
ENSG0000(	ENSG0000(	107.6329	0.293476	0.016668	0.239858	lncRNA
ENSG0000(	OAZ2	1899.558	-0.30997	0.016675	0.239858	protein_coding
ENSG0000(	PANX1	295.773	-0.4485	0.016675	0.239858	protein_coding
ENSG0000(	SNAI2	330.5983	-0.50594	0.016655	0.239858	protein_coding
ENSG0000(	STX18	787.7595	-0.18469	0.016635	0.239858	protein_coding
ENSG0000(	SBF2-AS1	1552.617	0.14095	0.016722	0.240359	lncRNA
ENSG0000(	ENSG0000(	150.5212	0.63632	0.016806	0.241452	processed_pseudogene
ENSG0000(	BTK	472.5366	-0.59662	0.016865	0.241576	protein_coding
ENSG0000(	ENSG0000(	160.6066	0.258466	0.016868	0.241576	lncRNA
ENSG0000(	EYA3	1805.538	-0.1394	0.016866	0.241576	protein_coding
ENSG0000(	RETNLB	252.3619	0.917335	0.016855	0.241576	protein_coding
ENSG0000(	SSR4	594.7133	-0.51342	0.016849	0.241576	protein_coding
ENSG0000(	TMED7	1130.655	-0.26907	0.016841	0.241576	protein_coding
ENSG0000(	ENSG0000(	148.6925	0.207197	0.016877	0.241579	lncRNA
ENSG0000(	CHST9	133.7553	0.317908	0.016891	0.241586	protein_coding
ENSG0000(	ZNF320	2243.765	0.192013	0.016895	0.241586	protein_coding
ENSG0000(	PLEKHA8P1	197.9758	-0.22672	0.016916	0.241698	transcribed_processed_pseudoge
ENSG0000(	TNS3	5088.681	-0.24029	0.01692	0.241698	protein_coding

ENSG0000(	ENSG0000(	380.535	-0.3513	0.016932	0.241744	lncRNA
ENSG0000(	C6orf223	118.9709	-0.79404	0.01697	0.24194	lncRNA
ENSG0000(	MLXIP	11284.1	0.27595	0.016987	0.24194	protein_coding
ENSG0000(	RPL21P44	87.62363	-0.44226	0.01699	0.24194	processed_pseudogene
ENSG0000(	SAMD12-A:	68.4108	0.581607	0.016964	0.24194	lncRNA
ENSG0000(	SLC23A2	1457.316	-0.28604	0.016984	0.24194	protein_coding
ENSG0000(	CEACAM19	522.5166	0.221442	0.017027	0.241943	protein_coding
ENSG0000(	IGKV2-30	348.0621	-0.82551	0.017027	0.241943	IG_V_gene
ENSG0000(	LINC02023	502.4494	0.65659	0.017005	0.241943	lncRNA
ENSG0000(	MIR5194	36.38923	-0.56311	0.017015	0.241943	miRNA
ENSG0000(	MTFMT	744.6414	0.157606	0.017034	0.241943	protein_coding
ENSG0000(	ENSG0000(	30.83618	0.883179	0.017159	0.242894	lncRNA
ENSG0000(	GMPR2	575.2416	-0.24047	0.017163	0.242894	protein_coding
ENSG0000(	INSM1	22.94041	0.835982	0.017117	0.242894	protein_coding
ENSG0000(	NOD2	386.6934	-0.66385	0.017131	0.242894	protein_coding
ENSG0000(	TMED8	1419.132	-0.18754	0.017144	0.242894	protein_coding
ENSG0000(	TMEM200E	108.1625	-0.48782	0.017127	0.242894	protein_coding
ENSG0000(	TRPS1	1378.105	-0.52341	0.017161	0.242894	protein_coding
ENSG0000(	LAMB2	1286.546	-0.41106	0.017204	0.242971	protein_coding
ENSG0000(	MOB3C	1093.436	-0.22799	0.017212	0.242971	protein_coding
ENSG0000(	SBNO2	2199.558	-0.34909	0.01718	0.242971	protein_coding
ENSG0000(	ZNF267	1018.183	-0.34984	0.017211	0.242971	protein_coding
ENSG0000(	ZNF875	2116.238	0.140307	0.017208	0.242971	protein_coding
ENSG0000(	PTPN7	820.5445	-0.35686	0.017232	0.243126	protein_coding
ENSG0000(	PCBD2	1757.322	0.256899	0.017273	0.243568	protein_coding
ENSG0000(	PACC1	142.7542	-0.56849	0.017306	0.243913	protein_coding
ENSG0000(	CX3CL1	522.9373	-0.26554	0.017353	0.244222	protein_coding
ENSG0000(	ENSG0000(	38.17658	-0.54007	0.017354	0.244222	lncRNA
ENSG0000(	ZNF630	414.5137	0.221133	0.017354	0.244222	protein_coding
ENSG0000(	MECP2	3544.998	-0.28951	0.01737	0.244323	protein_coding
ENSG0000(	PTPN12	1975.513	-0.21837	0.01739	0.244468	protein_coding
ENSG0000(	CHKA	1457.219	0.471818	0.017463	0.244498	protein_coding
ENSG0000(	DIPK2B	596.6874	-0.44175	0.017455	0.244498	protein_coding
ENSG0000(	ENSG0000(	41.01466	-0.81987	0.017445	0.244498	processed_pseudogene
ENSG0000(	ENSG0000(	143.5235	0.202294	0.017464	0.244498	TEC
ENSG0000(	ENSG0000(	116.6471	0.522084	0.017458	0.244498	lncRNA
ENSG0000(	FAS	4240.382	-0.24022	0.017446	0.244498	protein_coding
ENSG0000(	IGLV4-69	84.82529	-0.83508	0.017476	0.244498	IG_V_gene
ENSG0000(	LINC02119	78.83209	0.404064	0.017435	0.244498	lncRNA
ENSG0000(	MYO7A	452.0413	-0.32014	0.017418	0.244498	protein_coding
ENSG0000(	RPS3P2	117.3664	0.215431	0.017481	0.244498	processed_pseudogene
ENSG0000(	ENSG0000(	58.86146	-0.35983	0.017491	0.244521	TEC
ENSG0000(	MYO5C	2821.131	0.459487	0.017513	0.244571	protein_coding
ENSG0000(	SINHCAFP3	462.6694	0.363554	0.017506	0.244571	processed_pseudogene
ENSG0000(	FYB1	2588.841	-0.52456	0.017536	0.244741	protein_coding
ENSG0000(	NSFL1C	1002.717	-0.19249	0.017542	0.244741	protein_coding
ENSG0000(	LINC02199	259.5969	-0.24607	0.017618	0.245552	lncRNA

ENSG0000(	RFX8	28.09707	-1.42377	0.01761	0.245552	protein_coding
ENSG0000(	ATE1-AS1	33.27598	0.656241	0.017646	0.24568	transcribed_unitary_pseudogene
ENSG0000(	HRG	123.7642	0.459912	0.017637	0.24568	protein_coding
ENSG0000(	CNTN4	389.0748	0.605792	0.017674	0.245861	protein_coding
ENSG0000(	ENSG0000(	1147.332	0.508265	0.017676	0.245861	lncRNA
ENSG0000(	AK1	602.1432	0.457245	0.01779	0.246134	protein_coding
ENSG0000(	ATP7B	1822.149	0.279517	0.017786	0.246134	protein_coding
ENSG0000(	CPD	3008.116	-0.18222	0.017794	0.246134	protein_coding
ENSG0000(	ENSG0000(	149.2787	0.256245	0.017781	0.246134	lncRNA
ENSG0000(	ENSG0000(	20.77976	-0.64855	0.017792	0.246134	TEC
ENSG0000(	ENSG0000(	577.022	-0.21074	0.017788	0.246134	protein_coding
ENSG0000(	ENSG0000(	27.71055	-0.59226	0.017787	0.246134	lncRNA
ENSG0000(	GAK	4799.323	0.170603	0.01778	0.246134	protein_coding
ENSG0000(	SIRPB2	469.2087	-0.55831	0.017727	0.246134	protein_coding
ENSG0000(	SULF2	1464.963	-0.29173	0.017735	0.246134	protein_coding
ENSG0000(	ZNRF3	403.6821	0.310651	0.017784	0.246134	protein_coding
ENSG0000(	COL5A3	612.0552	-0.52558	0.017883	0.246165	protein_coding
ENSG0000(	ENSG0000(	294.8938	0.30205	0.017836	0.246165	TEC
ENSG0000(	ENSG0000(	191.6487	0.514077	0.017851	0.246165	lncRNA
ENSG0000(	GALNT8	295.4375	0.703831	0.01784	0.246165	protein_coding
ENSG0000(	IRF2	771.2513	-0.25336	0.017879	0.246165	protein_coding
ENSG0000(	LINC00877	490.0325	-0.22401	0.017886	0.246165	lncRNA
ENSG0000(	PTGDS	498.4383	-0.63682	0.017867	0.246165	protein_coding
ENSG0000(	RAB1A	1822.411	-0.22308	0.017869	0.246165	protein_coding
ENSG0000(	RBM15	2125.087	0.154387	0.017816	0.246165	protein_coding
ENSG0000(	ZNF440	3122.976	0.214227	0.017815	0.246165	protein_coding
ENSG0000(	IGHV1-2	426.8302	-1.13003	0.017906	0.24631	IG_V_gene
ENSG0000(	TRMT112	180.1573	-0.40186	0.017915	0.24631	protein_coding
ENSG0000(	DOK3	614.8898	-0.50351	0.017926	0.246312	protein_coding
ENSG0000(	ENSG0000(	23.01782	-0.63819	0.017995	0.246312	lncRNA
ENSG0000(	HNF1A-AS1	2908.986	0.480594	0.017982	0.246312	lncRNA
ENSG0000(	MAP3K20	1536.923	-0.28114	0.017962	0.246312	protein_coding
ENSG0000(	METTL25	312.9127	0.283525	0.017988	0.246312	protein_coding
ENSG0000(	MLPH	2140.865	0.400389	0.017936	0.246312	protein_coding
ENSG0000(	NUB1	1524.223	-0.21933	0.017967	0.246312	protein_coding
ENSG0000(	SDCBP2-AS	438.2039	-0.27772	0.017964	0.246312	lncRNA
ENSG0000(	SFXN4	269.3872	0.410738	0.017979	0.246312	protein_coding
ENSG0000(	GOLT1B	457.6102	-0.3564	0.018016	0.246475	protein_coding
ENSG0000(	RTN4IP1	311.9893	0.463956	0.018047	0.246775	protein_coding
ENSG0000(	RNU7-119F	19.78304	-0.82568	0.018058	0.246808	snRNA
ENSG0000(	ANP32E	447.688	-0.36928	0.018079	0.246911	protein_coding
ENSG0000(	SHPK	991.7888	0.266172	0.018084	0.246911	protein_coding
ENSG0000(	TLN2	2903.839	0.419158	0.018097	0.24697	protein_coding
ENSG0000(	MAGI2-AS3	1231.723	-0.29724	0.018116	0.247101	lncRNA
ENSG0000(	DLC1	1104.97	-0.28841	0.018138	0.247286	protein_coding
ENSG0000(	DEPP1	383.7474	-0.59336	0.018188	0.247406	protein_coding
ENSG0000(	FAM20C	572.8271	-0.64997	0.018162	0.247406	protein_coding

ENSG0000(	RN7SL65P	24.63352	-0.56678	0.018184	0.247406	misc_RNA
ENSG0000(	TMED10	3375.669	-0.23448	0.018192	0.247406	protein_coding
ENSG0000(	TMEM30A	1978.289	-0.28858	0.018192	0.247406	protein_coding
ENSG0000(	AFG1L	361.9566	0.22923	0.018206	0.247466	protein_coding
ENSG0000(	AMMECR1I	982.9774	0.146736	0.018246	0.247633	protein_coding
ENSG0000(	BLACAT1	77.34745	-1.04036	0.018239	0.247633	protein_coding
ENSG0000(	BSND	173.0882	-0.20846	0.018244	0.247633	protein_coding
ENSG0000(	IGKV6D-21	21.0479	-1.48952	0.018254	0.247633	IG_V_gene
ENSG0000(	ENSG0000(	158.8141	0.374868	0.018294	0.2479	processed_pseudogene
ENSG0000(	ROBO1	1401.949	-0.43474	0.018287	0.2479	protein_coding
ENSG0000(	ZNF503-AS	23.50302	-0.65614	0.018301	0.2479	lncRNA
ENSG0000(	DHX15	4314.077	0.176408	0.018358	0.2481	protein_coding
ENSG0000(	IGHV4-59	771.6875	-0.95692	0.018338	0.2481	IG_V_gene
ENSG0000(	LCN2	1187.23	-1.19511	0.01836	0.2481	protein_coding
ENSG0000(	SH3RF3	418.5416	-0.41612	0.018333	0.2481	protein_coding
ENSG0000(	TRAM1	1262.578	-0.37346	0.01836	0.2481	protein_coding
ENSG0000(	ENSG0000(	76.48062	-0.63655	0.01843	0.248124	lncRNA
ENSG0000(	PBX2	1446.28	-0.24998	0.018392	0.248124	protein_coding
ENSG0000(	PIK3AP1	1010.7	-0.54752	0.018432	0.248124	protein_coding
ENSG0000(	PRNP	507.7812	-0.44299	0.018434	0.248124	protein_coding
ENSG0000(	SPIB	1026.037	0.399062	0.018391	0.248124	protein_coding
ENSG0000(	STARD10	734.3996	0.400246	0.018373	0.248124	protein_coding
ENSG0000(	TYSND1	830.4182	0.201183	0.018429	0.248124	protein_coding
ENSG0000(	ZNF324	512.8528	0.202195	0.018405	0.248124	protein_coding
ENSG0000(	ENSG0000(	489.4739	0.247007	0.018451	0.248228	processed_pseudogene
ENSG0000(	ENSG0000(	165.1909	-0.36478	0.018461	0.248239	lncRNA
ENSG0000(	AXIN2	1081.552	0.465834	0.018503	0.248276	protein_coding
ENSG0000(	ELMO2	770.7615	-0.3261	0.018485	0.248276	protein_coding
ENSG0000(	HSF4	194.093	0.439159	0.018527	0.248276	protein_coding
ENSG0000(	INPP4A	3053.457	-0.1839	0.01851	0.248276	protein_coding
ENSG0000(	PHF21A	4573.214	-0.28637	0.018527	0.248276	protein_coding
ENSG0000(	RNASEH1	1111.601	-0.1625	0.018496	0.248276	protein_coding
ENSG0000(	SPICE1	1335.487	0.194869	0.018525	0.248276	protein_coding
ENSG0000(	ENSG0000(	83.49239	-0.60281	0.018559	0.248277	lncRNA
ENSG0000(	KCNJ15	2283.634	-0.64502	0.018549	0.248277	protein_coding
ENSG0000(	LINC02268	929.886	0.28989	0.018546	0.248277	lncRNA
ENSG0000(	NCBP1	1746.104	0.172312	0.018563	0.248277	protein_coding
ENSG0000(	B4GALNT1	57.50196	-0.66025	0.018622	0.248464	protein_coding
ENSG0000(	DOCK2	2708.229	-0.4255	0.018605	0.248464	protein_coding
ENSG0000(	ENSG0000(	146.4514	0.272003	0.018606	0.248464	lncRNA
ENSG0000(	KLHL17	187.0687	-0.32181	0.018613	0.248464	protein_coding
ENSG0000(	SYDE1	186.8485	-0.46768	0.018597	0.248464	protein_coding
ENSG0000(	H2AC20	129.444	-0.42173	0.018645	0.248645	protein_coding
ENSG0000(	ENSG0000(	46.02832	1.23201	0.018695	0.249194	lncRNA
ENSG0000(	CANT1	2514.335	0.350186	0.018723	0.249444	protein_coding
ENSG0000(	IGHV3-72	215.5563	-0.84115	0.01879	0.250099	IG_V_gene
ENSG0000(	MRPL19	787.9514	-0.24155	0.018784	0.250099	protein_coding

ENSG0000(	AFF2	95.05989	-0.95675	0.018896	0.250902	protein_coding
ENSG0000(	COX6CP17	36.35976	-0.52036	0.018898	0.250902	processed_pseudogene
ENSG0000(	LGALS1	210.134	-0.85818	0.018923	0.250902	protein_coding
ENSG0000(	MPND	199.5843	0.424133	0.018867	0.250902	protein_coding
ENSG0000(	NTN4	441.424	0.443671	0.01891	0.250902	protein_coding
ENSG0000(	SMAGP	928.882	0.290833	0.01887	0.250902	protein_coding
ENSG0000(	TCAF1P1	47.07359	0.543616	0.018921	0.250902	unprocessed_pseudogene
ENSG0000(	ZNF502	221.7539	0.304922	0.018923	0.250902	protein_coding
ENSG0000(	DEPDC1P2	57.56422	0.411964	0.018947	0.251047	processed_pseudogene
ENSG0000(	FLRT3	210.7849	-0.89107	0.018953	0.251047	protein_coding
ENSG0000(	TMEM26	122.9624	-0.45743	0.018962	0.251049	protein_coding
ENSG0000(	GALNT12	881.5421	0.54175	0.018986	0.251123	protein_coding
ENSG0000(	SETD4	1020.27	0.211816	0.018977	0.251123	protein_coding
ENSG0000(	RIMBP2	159.9098	0.524215	0.018998	0.251166	protein_coding
ENSG0000(	ABCD1	287.643	-0.26975	0.019096	0.251972	protein_coding
ENSG0000(	ENSG0000(	23.08544	-0.74981	0.019085	0.251972	misc_RNA
ENSG0000(	POGLUT3	755.0996	-0.19179	0.019074	0.251972	protein_coding
ENSG0000(	TMEM109	287.7954	-0.39342	0.019087	0.251972	protein_coding
ENSG0000(	ENSG0000(	26.09033	-0.65324	0.019126	0.25208	lncRNA
ENSG0000(	GALNT15	593.6241	-0.22639	0.019123	0.25208	protein_coding
ENSG0000(	IL17RB	297.3645	0.574821	0.019136	0.25208	protein_coding
ENSG0000(	LAMTOR5-/	762.2122	0.159118	0.019141	0.25208	lncRNA
ENSG0000(	ENSG0000(	367.7096	-0.34967	0.019167	0.252252	TEC
ENSG0000(	VWA1	338.4272	-0.41917	0.019172	0.252252	protein_coding
ENSG0000(	ARL14	513.3453	0.62212	0.01919	0.252282	protein_coding
ENSG0000(	MIRLET7IH	1087.953	-0.40141	0.019193	0.252282	lncRNA
ENSG0000(	FUNDC1	58.12623	-0.35939	0.01922	0.252526	protein_coding
ENSG0000(	APBA2	493.877	-0.40281	0.019234	0.25259	protein_coding
ENSG0000(	ENSG0000(	34.36296	-0.8284	0.019255	0.252618	lncRNA
ENSG0000(	SND1	2539.458	-0.2608	0.019252	0.252618	protein_coding
ENSG0000(	TARM1	49.07871	0.552676	0.019271	0.252709	protein_coding
ENSG0000(	C1QA	488.4944	-0.58265	0.019287	0.252741	protein_coding
ENSG0000(	ENSG0000(	63.05117	0.324647	0.019309	0.252741	lncRNA
ENSG0000(	MEG3	5058.022	-0.51077	0.019319	0.252741	lncRNA
ENSG0000(	ST6GALNA(	1501.565	0.563053	0.019308	0.252741	protein_coding
ENSG0000(	TDRKH-AS1	160.5652	0.269559	0.019319	0.252741	lncRNA
ENSG0000(	NPTX2	60.4568	-1.24984	0.019338	0.252871	protein_coding
ENSG0000(	DOC2B	499.5451	-0.69439	0.019489	0.254141	protein_coding
ENSG0000(	ENSG0000(	44.56007	1.090015	0.019456	0.254141	protein_coding
ENSG0000(	GRIPAP1	1512.723	-0.13603	0.019466	0.254141	protein_coding
ENSG0000(	LINC01678	21.19463	-0.7895	0.019491	0.254141	lncRNA
ENSG0000(	RALGPS2-A	32.69396	-0.6158	0.019468	0.254141	lncRNA
ENSG0000(	RNF149	2275.772	-0.36658	0.019486	0.254141	protein_coding
ENSG0000(	TCEAL9	147.7265	-0.35177	0.019513	0.25431	protein_coding
ENSG0000(	C4orf48	31.24282	0.801563	0.01955	0.254675	protein_coding
ENSG0000(	P2RY14	249.6021	-0.43609	0.019614	0.255258	protein_coding
ENSG0000(	TBCEL	965.673	-0.24446	0.019612	0.255258	protein_coding

ENSG0000(	EIF2S3	2761.667	-0.14029	0.019624	0.255266	protein_coding
ENSG0000(	SP140	692.8257	-0.54486	0.019669	0.255733	protein_coding
ENSG0000(	BNIP3L	871.6255	-0.29566	0.019705	0.255954	protein_coding
ENSG0000(	LINC02058	26.38608	0.42903	0.019702	0.255954	lncRNA
ENSG0000(	GNAS	7666.241	-0.1857	0.01973	0.256159	protein_coding
ENSG0000(	ZNF521	524.2918	-0.60682	0.019786	0.256776	protein_coding
ENSG0000(	SCRIB	1034.983	0.297737	0.019818	0.257064	protein_coding
ENSG0000(	ADGRL1-AS	296.8719	-0.25609	0.019831	0.257108	lncRNA
ENSG0000(	ENSG0000(	54.16226	-0.4683	0.019843	0.25714	processed_pseudogene
ENSG0000(	ARFGEF3	1873.437	0.386424	0.01989	0.257591	protein_coding
ENSG0000(	RPL26L1	53.69537	-0.47612	0.019896	0.257591	protein_coding
ENSG0000(	LIMS1	1949.632	-0.23515	0.019925	0.257839	protein_coding
ENSG0000(	SEZ6L2	85.1601	-0.52568	0.019945	0.257984	protein_coding
ENSG0000(	INTS1	1873.836	0.194524	0.019955	0.257995	protein_coding
ENSG0000(	MCOLN3	340.1549	0.22464	0.019966	0.258008	protein_coding
ENSG0000(	SUSD6	3266.042	-0.31133	0.019993	0.258233	protein_coding
ENSG0000(	TNS1	3875.825	-0.41081	0.020023	0.258508	protein_coding
ENSG0000(	EBNA1BP2	231.2153	-0.24723	0.020071	0.258885	protein_coding
ENSG0000(	ZNF197-AS	41.42025	0.532549	0.020067	0.258885	lncRNA
ENSG0000(	BACH1-IT3	61.64764	-0.61255	0.020115	0.25921	lncRNA
ENSG0000(	PPP4R3A	2373.34	0.142424	0.020115	0.25921	protein_coding
ENSG0000(	MAP3K6	353.2884	-0.26854	0.020126	0.259226	protein_coding
ENSG0000(	AGAP2-AS1	21.62637	-0.83614	0.020185	0.259376	lncRNA
ENSG0000(	COBLL1	1954.511	-0.21685	0.020162	0.259376	protein_coding
ENSG0000(	FKBP10	660.1697	-0.29587	0.020178	0.259376	protein_coding
ENSG0000(	LINC01180	161.4823	0.235326	0.020158	0.259376	lncRNA
ENSG0000(	ZNF844	1747.241	0.17679	0.020172	0.259376	protein_coding
ENSG0000(	PORCN	118.5297	-0.44833	0.02022	0.25958	protein_coding
ENSG0000(	ZNRD2	56.82539	-0.42846	0.020218	0.25958	protein_coding
ENSG0000(	ACP5	319.7814	-0.40308	0.020236	0.259672	protein_coding
ENSG0000(	BTBD7P1	194.2546	0.242776	0.020318	0.259919	processed_pseudogene
ENSG0000(	CLK3	2845.934	0.170742	0.020336	0.259919	protein_coding
ENSG0000(	CYTH4	1426.966	-0.47468	0.020301	0.259919	protein_coding
ENSG0000(	ENSG0000(	86.87882	-0.66056	0.02034	0.259919	lncRNA
ENSG0000(	PSMB4	432.5923	-0.25167	0.020306	0.259919	protein_coding
ENSG0000(	SEC11C	619.9884	-0.29133	0.020329	0.259919	protein_coding
ENSG0000(	SHC1	1836.646	-0.2379	0.020327	0.259919	protein_coding
ENSG0000(	SMARCA5	1477.037	-0.23393	0.02031	0.259919	protein_coding
ENSG0000(	ZNF132	194.859	0.329724	0.020305	0.259919	protein_coding
ENSG0000(	ENSG0000(	195.2528	0.329949	0.020396	0.26031	protein_coding
ENSG0000(	IGHV3-35	25.50932	-0.87345	0.020394	0.26031	IG_V_gene
ENSG0000(	PTPN23-DT	176.0742	-0.29824	0.020399	0.26031	lncRNA
ENSG0000(	ENSG0000(	850.3691	-0.14724	0.020416	0.260406	lncRNA
ENSG0000(	FMNL2	1463.417	-0.26658	0.020471	0.260856	protein_coding
ENSG0000(	MTND5P2	84.74376	-0.37527	0.020462	0.260856	processed_pseudogene
ENSG0000(	PDGFA	3819.305	0.409787	0.020545	0.261579	protein_coding
ENSG0000(	TBKBP1	190.5327	-0.42597	0.020546	0.261579	protein_coding

ENSG0000(	ENSG0000(	222.9276	0.187649	0.020567	0.261716	TEC
ENSG0000(	PSTPIP1	391.7952	-0.40145	0.020589	0.26188	protein_coding
ENSG0000(	CDC42SE1	3447.177	-0.23114	0.02066	0.261883	protein_coding
ENSG0000(	FGF7P3	37.85505	0.610018	0.02062	0.261883	transcribed_unprocessed_pseudo
ENSG0000(	HEPACAM2	592.2992	0.777582	0.020665	0.261883	protein_coding
ENSG0000(	IGHV3-64D	141.1974	-1.49736	0.020658	0.261883	IG_V_gene
ENSG0000(	MEI1	665.7346	-0.27698	0.02064	0.261883	protein_coding
ENSG0000(	MEOX1	94.75283	-0.86626	0.020643	0.261883	protein_coding
ENSG0000(	OTX2-AS1	289.5607	0.307348	0.020637	0.261883	lncRNA
ENSG0000(	TCHP	777.5229	-0.22762	0.020654	0.261883	protein_coding
ENSG0000(	RPS26P21	63.78259	-0.4012	0.020688	0.262042	processed_pseudogene
ENSG0000(	ENSG0000(	379.5993	-0.22909	0.020717	0.262295	lncRNA
ENSG0000(	ENSG0000(	233.9785	-0.19781	0.020729	0.26233	lncRNA
ENSG0000(	EEF1AKMT1	409.5503	-0.25744	0.020764	0.262611	protein_coding
ENSG0000(	ENSG0000(	146.6827	-0.29227	0.02078	0.262611	lncRNA
ENSG0000(	PER2	1276.657	0.35301	0.020792	0.262611	protein_coding
ENSG0000(	PTGFR	167.0674	-0.8976	0.020799	0.262611	protein_coding
ENSG0000(	TENM4	433.5371	-0.89659	0.020797	0.262611	protein_coding
ENSG0000(	ENSG0000(	23.0193	-0.63423	0.020827	0.262839	lncRNA
ENSG0000(	FGD2	2263.214	-0.36483	0.020859	0.263129	protein_coding
ENSG0000(	ARL15	422.9672	0.33733	0.020875	0.26318	protein_coding
ENSG0000(	ENSG0000(	687.9434	-0.19141	0.02089	0.26318	protein_coding
ENSG0000(	MBOAT1	1943.581	0.401584	0.020902	0.26318	protein_coding
ENSG0000(	RN7SL587P	57.78704	0.313164	0.020896	0.26318	misc_RNA
ENSG0000(	ABCA5	5143.256	0.322677	0.020964	0.263412	protein_coding
ENSG0000(	BASP1	355.7265	-0.55497	0.020933	0.263412	protein_coding
ENSG0000(	CCL4L2	41.89632	-1.03088	0.020968	0.263412	protein_coding
ENSG0000(	ENSG0000(	81.46601	0.300857	0.020964	0.263412	lncRNA
ENSG0000(	STK32B	42.44834	-0.67373	0.020954	0.263412	protein_coding
ENSG0000(	ETV7	172.3518	-0.55808	0.021003	0.263572	protein_coding
ENSG0000(	GANAB	2362.144	-0.16928	0.021014	0.263572	protein_coding
ENSG0000(	IGHV3-62	21.98138	-0.85744	0.021019	0.263572	IG_V_pseudogene
ENSG0000(	OR6D1P	744.9615	0.215297	0.021009	0.263572	transcribed_unitary_pseudogene
ENSG0000(	C1RL-AS1	1850.044	-0.24795	0.021058	0.263762	lncRNA
ENSG0000(	RPSAP48	255.4269	0.504041	0.021048	0.263762	processed_pseudogene
ENSG0000(	XBP1P1	71.70914	-0.35106	0.021072	0.263762	processed_pseudogene
ENSG0000(	ZNF296	41.88494	0.440247	0.021073	0.263762	protein_coding
ENSG0000(	AMT	471.8757	0.344498	0.021088	0.263836	protein_coding
ENSG0000(	BLVRA	93.82241	-0.54512	0.021161	0.264101	protein_coding
ENSG0000(	CALHM6	60.69115	-0.84164	0.021167	0.264101	protein_coding
ENSG0000(	DPP10-AS1	45.75239	0.675681	0.021164	0.264101	lncRNA
ENSG0000(	ENTPD7	1152.875	-0.33345	0.021176	0.264101	protein_coding
ENSG0000(	IGKV1-17	256.4196	-0.84573	0.021133	0.264101	IG_V_gene
ENSG0000(	LNX1-AS2	229.1783	-0.19408	0.021153	0.264101	lncRNA
ENSG0000(	TSHZ2	866.2652	-0.4473	0.021169	0.264101	protein_coding
ENSG0000(	ADAMTSL4	390.0295	-0.41928	0.021216	0.264335	protein_coding
ENSG0000(	LINC02037	145.1511	-0.28595	0.021222	0.264335	lncRNA

ENSG0000(	TCF4	5652.396	-0.313	0.021224	0.264335	protein_coding
ENSG0000(	AHNAK	36123.91	0.301051	0.021247	0.264394	protein_coding
ENSG0000(	LINC02887	501.1776	-0.50415	0.021248	0.264394	lncRNA
ENSG0000(	ENSG0000(	45.42085	-0.50308	0.021275	0.264609	lncRNA
ENSG0000(	CCDC85C	1425.113	0.365965	0.021285	0.264621	protein_coding
ENSG0000(	ENSG0000(	43.8492	-0.52411	0.021305	0.264625	snoRNA
ENSG0000(	MICB	376.1999	-0.20179	0.021298	0.264625	protein_coding
ENSG0000(	ADAM9	2512.618	-0.33417	0.021319	0.264678	protein_coding
ENSG0000(	MAGT1	1718.058	-0.15372	0.021362	0.26509	protein_coding
ENSG0000(	ITGA2	1642.362	-0.62615	0.021397	0.265375	protein_coding
ENSG0000(	MAN2C1	2862.8	0.174996	0.021404	0.265375	protein_coding
ENSG0000(	PGD	573.1857	-0.36001	0.021418	0.265424	protein_coding
ENSG0000(	ENSG0000(	4431.687	-0.16978	0.021449	0.265568	TEC
ENSG0000(	TMEM121E	20.50975	-0.82208	0.021439	0.265568	protein_coding
ENSG0000(	DENND1A	1759.941	-0.19646	0.02147	0.26571	protein_coding
ENSG0000(	JAK3	2340.986	-0.57175	0.021502	0.265986	protein_coding
ENSG0000(	TXNL1	859.3204	-0.15187	0.02152	0.266093	protein_coding
ENSG0000(	ABCA7	1080.274	-0.28428	0.021583	0.26639	protein_coding
ENSG0000(	DHRS12	155.0096	0.296474	0.021573	0.26639	protein_coding
ENSG0000(	METTL2A	1905.438	0.171924	0.021583	0.26639	protein_coding
ENSG0000(	PDE4DIPP7	58.52451	-0.50296	0.021567	0.26639	transcribed_unprocessed_pseudo
ENSG0000(	DPYSL2	1488.844	-0.2711	0.021621	0.266645	protein_coding
ENSG0000(	GYPC	348.6842	-0.43493	0.021623	0.266645	protein_coding
ENSG0000(	CNN2	1657.86	-0.24686	0.021696	0.267431	protein_coding
ENSG0000(	ENSG0000(	67.50266	-0.7402	0.021723	0.267636	lncRNA
ENSG0000(	COA8	726.2366	0.186762	0.021749	0.26766	protein_coding
ENSG0000(	SLFN11	1300.794	-0.45393	0.021754	0.26766	protein_coding
ENSG0000(	ZGPAT	529.7055	0.262043	0.021746	0.26766	protein_coding
ENSG0000(	ENSG0000(	27.94293	0.690212	0.021768	0.26771	lncRNA
ENSG0000(	ENSG0000(	98.57212	0.344786	0.021806	0.268069	lncRNA
ENSG0000(	LILRA2	416.4391	-0.25747	0.021838	0.268335	protein_coding
ENSG0000(	TIMM23B-/	745.9653	-0.19737	0.021865	0.268549	protein_coding
ENSG0000(	LINC01004	2098.6	0.315224	0.021876	0.268565	lncRNA
ENSG0000(	ENDOD1	962.0887	0.371289	0.021893	0.268659	protein_coding
ENSG0000(	ENSG0000(	43.63296	-0.82517	0.021908	0.268717	lncRNA
ENSG0000(	ARHGAP23	462.0311	-0.5308	0.02192	0.268746	protein_coding
ENSG0000(	RC3H2	2770.498	-0.1557	0.021942	0.268781	protein_coding
ENSG0000(	WDSUB1	176.8578	0.295153	0.02194	0.268781	protein_coding
ENSG0000(	COL23A1	139.4233	-0.60331	0.021978	0.269093	protein_coding
ENSG0000(	CRTAP	989.0171	-0.28647	0.022009	0.269126	protein_coding
ENSG0000(	FEN1	168.388	-0.37749	0.021996	0.269126	protein_coding
ENSG0000(	MAP1S	515.8099	-0.18585	0.022012	0.269126	protein_coding
ENSG0000(	USP30	482.4028	0.290205	0.02202	0.269126	protein_coding
ENSG0000(	ADAM19	2511.392	-0.45434	0.022065	0.269319	protein_coding
ENSG0000(	ENSG0000(	60.45035	0.353432	0.02205	0.269319	lncRNA
ENSG0000(	NFKBIZ	3512.374	-0.37427	0.022064	0.269319	protein_coding
ENSG0000(	ENSG0000(	127.6428	0.236774	0.02209	0.269384	lncRNA

ENSG0000(	H2BC18	162.0336	-0.42276	0.022084	0.269384	protein_coding
ENSG0000(	TMEM258	297.7718	-0.26566	0.022115	0.269577	protein_coding
ENSG0000(	ENSG0000(	287.8676	-0.21556	0.022158	0.269744	lncRNA
ENSG0000(	ENSG0000(	26.50013	0.692047	0.022145	0.269744	lncRNA
ENSG0000(	GRAMD1A	1327.235	-0.42748	0.022151	0.269744	protein_coding
ENSG0000(	ZNRF1	691.1249	0.205013	0.022168	0.269747	protein_coding
ENSG0000(	CNRIP1	217.6579	-0.39965	0.02221	0.269871	protein_coding
ENSG0000(	NCK1-DT	538.1008	-0.15956	0.022216	0.269871	lncRNA
ENSG0000(	TNFSF13B	326.4719	-0.42943	0.022197	0.269871	protein_coding
ENSG0000(	TRAPPC11	1170.476	-0.21123	0.022218	0.269871	protein_coding
ENSG0000(	CACFD1	615.999	0.354673	0.022289	0.269899	protein_coding
ENSG0000(	FBRSL1	1358.536	0.173581	0.022284	0.269899	protein_coding
ENSG0000(	OMP	47.05892	0.812728	0.022251	0.269899	protein_coding
ENSG0000(	SDF2	555.9162	0.180622	0.022286	0.269899	protein_coding
ENSG0000(	SELENBP1	5220.319	0.800612	0.022248	0.269899	protein_coding
ENSG0000(	TINAG	167.0437	0.704844	0.022262	0.269899	protein_coding
ENSG0000(	TSHZ3	396.531	-0.53389	0.02224	0.269899	protein_coding
ENSG0000(	ENSG0000(	23.91246	-1.00266	0.022328	0.270224	lncRNA
ENSG0000(	FZD4	1031.509	-0.27425	0.022335	0.270224	protein_coding
ENSG0000(	IGLV2-23	651.2344	-0.82265	0.022364	0.270252	IG_V_gene
ENSG0000(	LINC00930	98.38932	0.435217	0.022377	0.270252	lncRNA
ENSG0000(	RIC1	2345.334	-0.1367	0.022362	0.270252	protein_coding
ENSG0000(	SIRT1	543.4881	0.267624	0.022375	0.270252	protein_coding
ENSG0000(	AKT1S1	845.4916	-0.32187	0.022425	0.270374	protein_coding
ENSG0000(	CHSY1	1033.528	-0.43734	0.022461	0.270374	protein_coding
ENSG0000(	ENSG0000(	3100.283	0.454353	0.022437	0.270374	processed_pseudogene
ENSG0000(	ENSG0000(	36.61249	-0.51235	0.022403	0.270374	TEC
ENSG0000(	RBMS1	1910.661	-0.38277	0.022462	0.270374	protein_coding
ENSG0000(	SRP72	1078.026	-0.2377	0.022466	0.270374	protein_coding
ENSG0000(	UMPS	1023.754	0.239905	0.022465	0.270374	protein_coding
ENSG0000(	WWC3	1184.36	-0.32343	0.022419	0.270374	protein_coding
ENSG0000(	PRDM8	121.0002	-0.68658	0.022479	0.270422	protein_coding
ENSG0000(	LCLAT1	2147.7	0.125564	0.02251	0.270673	protein_coding
ENSG0000(	DARS1-AS1	948.7797	-0.15036	0.022536	0.270806	lncRNA
ENSG0000(	PACS1	1692.391	-0.25527	0.022541	0.270806	protein_coding
ENSG0000(	UNC13D	930.6803	-0.3604	0.022555	0.270861	protein_coding
ENSG0000(	GAS7	1333.293	-0.34133	0.022574	0.270962	protein_coding
ENSG0000(	CD53	1356.535	-0.53556	0.022645	0.271343	protein_coding
ENSG0000(	ENSG0000(	411.216	-0.28772	0.022643	0.271343	TEC
ENSG0000(	KLHL31	61.37224	0.498321	0.02262	0.271343	protein_coding
ENSG0000(	SNORA38B	51.03835	0.413821	0.022629	0.271343	snoRNA
ENSG0000(	CLCN3P1	331.7938	-0.22769	0.02267	0.271407	transcribed_unprocessed_pseudo
ENSG0000(	EAF2	263.2079	-0.36599	0.022668	0.271407	protein_coding
ENSG0000(	ITPR1	4249.003	-0.3804	0.022682	0.271433	protein_coding
ENSG0000(	FAM83G	1269.563	0.384678	0.022692	0.271441	protein_coding
ENSG0000(	AMPD2	774.8764	-0.3229	0.022735	0.271492	protein_coding
ENSG0000(	H2BW2	24.93914	-0.61541	0.022736	0.271492	protein_coding

ENSG0000(	HCG11	851.9498	-0.15646	0.022708	0.271492	lncRNA
ENSG0000(	OSTC	469.6166	-0.22185	0.022732	0.271492	protein_coding
ENSG0000(	GTF2A1	868.8281	-0.20707	0.022748	0.271513	protein_coding
ENSG0000(	TRAPPC14	655.0783	0.299331	0.022795	0.271842	protein_coding
ENSG0000(	ZFP3	851.6462	0.237659	0.022786	0.271842	protein_coding
ENSG0000(	RNPS1	2184.777	0.10606	0.022869	0.27261	protein_coding
ENSG0000(	DNAAF5	618.2582	0.296971	0.02288	0.272624	protein_coding
ENSG0000(	ERMN	171.8016	-0.43906	0.022912	0.272869	protein_coding
ENSG0000(	LOXL2	604.0483	-0.35344	0.022921	0.272869	protein_coding
ENSG0000(	COTL1	1610.008	-0.29211	0.023089	0.274637	protein_coding
ENSG0000(	DYNC2I2	250.4148	0.314171	0.02308	0.274637	protein_coding
ENSG0000(	ARHGEF3	559.9463	-0.42306	0.023165	0.274697	protein_coding
ENSG0000(	BCL6B	161.3805	-0.60828	0.023126	0.274697	protein_coding
ENSG0000(	ENSG0000(	149.924	-0.4866	0.023174	0.274697	lncRNA
ENSG0000(	ENSG0000(	202.0158	-0.26998	0.023116	0.274697	lncRNA
ENSG0000(	GMFG	306.9461	-0.34245	0.023138	0.274697	protein_coding
ENSG0000(	H2AC14	68.8472	-0.57876	0.023172	0.274697	protein_coding
ENSG0000(	IGLV7-46	110.5403	-1.03712	0.023151	0.274697	IG_V_gene
ENSG0000(	RSPO3	120.2797	-1.04469	0.023119	0.274697	protein_coding
ENSG0000(	CTSK	1665.514	-0.31258	0.023244	0.275046	protein_coding
ENSG0000(	LINC02046	54.67172	0.441557	0.023237	0.275046	lncRNA
ENSG0000(	MYOSLID	174.3672	-0.27446	0.023243	0.275046	lncRNA
ENSG0000(	PLXNC1	1409.811	-0.54429	0.023215	0.275046	protein_coding
ENSG0000(	FAM161B	325.4293	0.204526	0.023259	0.275114	protein_coding
ENSG0000(	MEFV	689.5103	-0.38169	0.023285	0.275298	protein_coding
ENSG0000(	EBF3	61.66479	-0.97434	0.023333	0.275641	protein_coding
ENSG0000(	ENSG0000(	132.6359	-0.30355	0.023334	0.275641	TEC
ENSG0000(	MIOS-DT	92.94077	-0.48074	0.023354	0.275761	lncRNA
ENSG0000(	ENSG0000(	34.06263	-0.66611	0.023375	0.275785	lncRNA
ENSG0000(	ENSG0000(	71.23724	-0.87276	0.023396	0.275785	lncRNA
ENSG0000(	IGHV4-28	119.776	-0.84797	0.023391	0.275785	IG_V_gene
ENSG0000(	ZNF385D	353.8786	-0.6974	0.023392	0.275785	protein_coding
ENSG0000(	ENSG0000(	71.67561	0.6808	0.023466	0.276489	lncRNA
ENSG0000(	SMIM14	3051.584	0.145025	0.023518	0.276977	protein_coding
ENSG0000(	TPTE2	34.58331	-0.59004	0.023558	0.277311	protein_coding
ENSG0000(	ZNF91	6337.352	0.299265	0.023566	0.277311	protein_coding
ENSG0000(	CD300E	711.676	-0.85061	0.023584	0.277399	protein_coding
ENSG0000(	MTMR6	554.7247	-0.26059	0.02361	0.277467	protein_coding
ENSG0000(	NHSL2	935.3688	-0.47775	0.02361	0.277467	protein_coding
ENSG0000(	IGKV2-24	182.8356	-0.93937	0.023655	0.277879	IG_V_gene
ENSG0000(	ENSG0000(	2534.563	0.259317	0.023669	0.277929	lncRNA
ENSG0000(	CACNA1C-I	52.29763	-0.93615	0.023764	0.278329	lncRNA
ENSG0000(	ENSG0000(	221.8393	-0.33994	0.023739	0.278329	lncRNA
ENSG0000(	HSPA12B	81.02957	-0.56292	0.023722	0.278329	protein_coding
ENSG0000(	OVCH1	33.13258	-1.05965	0.02375	0.278329	protein_coding
ENSG0000(	RFC3	209.8144	-0.28282	0.023756	0.278329	protein_coding
ENSG0000(	ZNF622	215.3134	0.277915	0.023753	0.278329	protein_coding

ENSG0000(	ANGPT1	196.8627	-0.52175	0.02382	0.278785	protein_coding
ENSG0000(	CD33	290.1185	-0.32505	0.023839	0.278785	protein_coding
ENSG0000(	E2F3	671.83	-0.17191	0.023844	0.278785	protein_coding
ENSG0000(	PDCD5	312.844	-0.26002	0.023854	0.278785	protein_coding
ENSG0000(	RPS3P7	54.97978	-0.39136	0.023824	0.278785	processed_pseudogene
ENSG0000(	ITM2B	2592.477	-0.34879	0.023902	0.279229	protein_coding
ENSG0000(	ENSG0000(	21.27648	0.551094	0.023934	0.279484	lncRNA
ENSG0000(	TES	1362.015	0.125125	0.023955	0.279616	protein_coding
ENSG0000(	ARMCX6	181.4404	-0.30717	0.024003	0.27992	protein_coding
ENSG0000(	CYB5R4	2975.845	-0.13691	0.023993	0.27992	protein_coding
ENSG0000(	RGS10	165.7062	-0.31709	0.024012	0.27992	protein_coding
ENSG0000(	CAPN9	473.5705	0.770591	0.024048	0.279982	protein_coding
ENSG0000(	FGFR3	843.9281	0.596937	0.024036	0.279982	protein_coding
ENSG0000(	KHDRBS1	1480.074	-0.16095	0.024046	0.279982	protein_coding
ENSG0000(	FHAD1-AS1	74.82189	0.293293	0.024069	0.280115	lncRNA
ENSG0000(	CPNE4	25.22619	-1.36701	0.024097	0.280205	protein_coding
ENSG0000(	POU2F3	248.6598	0.261122	0.024089	0.280205	protein_coding
ENSG0000(	ENSG0000(	51.7727	-0.53645	0.024132	0.280339	protein_coding
ENSG0000(	ENSG0000(	39.59978	0.793214	0.024139	0.280339	lncRNA
ENSG0000(	HLA-DQB1	1804.556	-0.53975	0.024135	0.280339	protein_coding
ENSG0000(	FAM114A2	487.7611	-0.24603	0.024177	0.280653	protein_coding
ENSG0000(	MT2A	404.5526	-0.62336	0.024197	0.280768	protein_coding
ENSG0000(	ASS1	2155.116	-0.56781	0.024228	0.280823	protein_coding
ENSG0000(	CD48	549.0436	-0.52275	0.02422	0.280823	protein_coding
ENSG0000(	STRIT1	79.71475	0.347213	0.024232	0.280823	protein_coding
ENSG0000(	GPR12	88.61346	0.309948	0.024262	0.28105	protein_coding
ENSG0000(	ENSG0000(	2488.559	0.189464	0.024318	0.281348	lncRNA
ENSG0000(	EPOP	52.62248	-0.61204	0.024312	0.281348	protein_coding
ENSG0000(	LINC02641	104.4328	-0.36965	0.024301	0.281348	lncRNA
ENSG0000(	ENSG0000(	26.08812	-0.87764	0.024337	0.281449	lncRNA
ENSG0000(	DAAM1	1311.168	0.154906	0.024351	0.281493	protein_coding
ENSG0000(	RPS14P4	42.15465	-0.87394	0.024384	0.281748	processed_pseudogene
ENSG0000(	BHLHB9P1	36.17952	-0.69066	0.024403	0.281855	processed_pseudogene
ENSG0000(	ENSG0000(	225.0893	0.324298	0.024433	0.281968	lncRNA
ENSG0000(	UBE2J1	2384.019	-0.27699	0.024428	0.281968	protein_coding
ENSG0000(	PSMA7	413.2064	-0.20877	0.024464	0.282202	protein_coding
ENSG0000(	CEP95	1584.791	0.135401	0.024514	0.282269	protein_coding
ENSG0000(	CLPB	2055.601	-0.17939	0.024549	0.282269	protein_coding
ENSG0000(	LRRC3	213.439	0.277916	0.024502	0.282269	protein_coding
ENSG0000(	MEA1	209.0125	-0.27093	0.024521	0.282269	protein_coding
ENSG0000(	PLEKHN1	66.58626	-0.71233	0.0245	0.282269	protein_coding
ENSG0000(	SLITRK5	298.3779	0.238364	0.024499	0.282269	protein_coding
ENSG0000(	TMEM119	511.3439	-0.31168	0.024552	0.282269	protein_coding
ENSG0000(	ZNF618	732.889	-0.32896	0.024543	0.282269	protein_coding
ENSG0000(	DYNC1I2	1107.244	0.224636	0.024616	0.282281	protein_coding
ENSG0000(	ENSG0000(	825.3355	0.185104	0.024604	0.282281	lncRNA
ENSG0000(	ENSG0000(	201.061	0.59718	0.024594	0.282281	lncRNA

ENSG0000(	NFIL3	319.2943	-0.4332	0.024606	0.282281	protein_coding
ENSG0000(	PTPRU	118.7865	-0.74696	0.024625	0.282281	protein_coding
ENSG0000(	RARRES2	379.4697	-0.56283	0.024581	0.282281	protein_coding
ENSG0000(	SRPX	31.7656	-0.73514	0.024578	0.282281	protein_coding
ENSG0000(	ASH1L-IT1	59.06697	-0.35308	0.024705	0.282516	lncRNA
ENSG0000(	CBFA2T2	3167.111	0.175814	0.024694	0.282516	protein_coding
ENSG0000(	CEACAM6	6073.026	-0.72579	0.024668	0.282516	protein_coding
ENSG0000(	ENSG0000(	79.92286	0.285491	0.024657	0.282516	lncRNA
ENSG0000(	H2BC10	67.24095	-0.54501	0.024707	0.282516	protein_coding
ENSG0000(	PDIA4	1335.262	-0.37332	0.024696	0.282516	protein_coding
ENSG0000(	DUSP22	1353.444	-0.26381	0.024748	0.282865	protein_coding
ENSG0000(	CDRT1	208.1219	-0.43946	0.024772	0.283031	protein_coding
ENSG0000(	CAV2	480.7648	-0.25563	0.024807	0.283196	protein_coding
ENSG0000(	CHST5	541.4055	0.489981	0.024805	0.283196	protein_coding
ENSG0000(	PHLDA3	273.8952	-0.26363	0.024835	0.283396	protein_coding
ENSG0000(	NIPA2	1037.997	0.17234	0.024851	0.283455	protein_coding
ENSG0000(	DEPTOR	210.3542	0.542495	0.02488	0.283473	protein_coding
ENSG0000(	SPINK8	39.13616	-0.43611	0.024871	0.283473	protein_coding
ENSG0000(	ZNF446	187.4107	0.271788	0.024883	0.283473	protein_coding
ENSG0000(	LINC01505	145.1635	0.272744	0.024907	0.283485	lncRNA
ENSG0000(	SAXO2	102.8098	0.659716	0.024915	0.283485	protein_coding
ENSG0000(	SLC13A3	103.1002	-0.32341	0.024913	0.283485	protein_coding
ENSG0000(	GRB14	22.45436	0.709889	0.02493	0.283538	protein_coding
ENSG0000(	RUSC1	552.9333	0.286897	0.024981	0.283996	protein_coding
ENSG0000(	CSGALNAC	921.4568	-0.42022	0.025008	0.284186	protein_coding
ENSG0000(	ENSG0000(	862.6812	0.332824	0.025027	0.284286	lncRNA
ENSG0000(	ZNF415P1	45.87894	-0.80072	0.025059	0.284536	processed_pseudogene
ENSG0000(	ENSG0000(	55.7708	0.343893	0.025097	0.284558	lncRNA
ENSG0000(	ENSG0000(	173.6473	-0.26096	0.025141	0.284558	lncRNA
ENSG0000(	ENSG0000(	123.0746	0.233412	0.025096	0.284558	lncRNA
ENSG0000(	FXYD5	288.3266	-0.43991	0.025131	0.284558	protein_coding
ENSG0000(	LINC01358	1001.679	-0.17335	0.025087	0.284558	lncRNA
ENSG0000(	MAML3	956.7075	0.313527	0.025144	0.284558	protein_coding
ENSG0000(	PJA2	2842.589	-0.12803	0.025111	0.284558	protein_coding
ENSG0000(	SSUH2	140.4618	0.393225	0.025128	0.284558	protein_coding
ENSG0000(	HEG1	1440.124	-0.41639	0.025157	0.284592	protein_coding
ENSG0000(	HLA-A	8378.283	-0.35781	0.025224	0.284763	protein_coding
ENSG0000(	IP6K2	2287.859	0.260273	0.025217	0.284763	protein_coding
ENSG0000(	LINC01864	109.2779	-0.34869	0.025211	0.284763	lncRNA
ENSG0000(	LINC02595	156.7852	-0.53757	0.02522	0.284763	lncRNA
ENSG0000(	TM6SF1	57.19682	-0.61819	0.025197	0.284763	protein_coding
ENSG0000(	VDAC1P13	28.73994	0.839981	0.025235	0.284768	processed_pseudogene
ENSG0000(	PATL1	1191.163	-0.19988	0.025261	0.284945	protein_coding
ENSG0000(	ADGRF1	1441.174	-0.46836	0.02532	0.285294	protein_coding
ENSG0000(	ATG14	1209.204	0.14559	0.025354	0.285294	protein_coding
ENSG0000(	ENSG0000(	58.41612	0.412425	0.025333	0.285294	processed_pseudogene
ENSG0000(	ENSG0000(	252.2851	-0.28081	0.025331	0.285294	lncRNA

ENSG0000(	ENSG0000(	502.0784	-0.16936	0.025346	0.285294	lncRNA
ENSG0000(	XPOT	1435.087	-0.21345	0.025354	0.285294	protein_coding
ENSG0000(	ENSG0000(	82.48762	0.477816	0.025378	0.285332	TEC
ENSG0000(	ZBTB47	295.6865	-0.35427	0.025378	0.285332	protein_coding
ENSG0000(	ENSG0000(	21.67737	-0.55412	0.025466	0.286199	lncRNA
ENSG0000(	RHOT2	1298.64	0.243888	0.025494	0.286344	protein_coding
ENSG0000(	TRERF1	896.7198	-0.25669	0.0255	0.286344	protein_coding
ENSG0000(	PI4KAP2	1892.587	0.205432	0.025516	0.286412	transcribed_unitary_pseudogene
ENSG0000(	ENSG0000(	250.7805	0.189948	0.025541	0.286464	lncRNA
ENSG0000(	FAM185A	201.9208	0.226247	0.025537	0.286464	protein_coding
ENSG0000(	ENSG0000(	1200.382	-0.15578	0.025582	0.286749	TEC
ENSG0000(	XRRA1	928.0909	-0.43455	0.025588	0.286749	protein_coding
ENSG0000(	PRDM6	133.2374	-0.74141	0.0256	0.286765	protein_coding
ENSG0000(	DENND5A	2770.81	-0.37222	0.025619	0.286868	protein_coding
ENSG0000(	CCL3L3	47.87721	-1.10939	0.025645	0.286908	protein_coding
ENSG0000(	CMAHP	1392.105	-0.43543	0.025636	0.286908	transcribed_unitary_pseudogene
ENSG0000(	MOXD1	102.0908	-0.60113	0.025675	0.286908	protein_coding
ENSG0000(	PPP3CB-AS	895.3608	0.143552	0.025671	0.286908	lncRNA
ENSG0000(	TMEM61	19.91188	0.741491	0.025654	0.286908	protein_coding
ENSG0000(	IGFBP5	4543.121	-0.84783	0.025686	0.286913	protein_coding
ENSG0000(	COL8A1	952.9075	-0.37013	0.025721	0.287033	protein_coding
ENSG0000(	ENSG0000(	83.85311	0.309824	0.025716	0.287033	lncRNA
ENSG0000(	ENSG0000(	44.39681	-0.47332	0.025728	0.287033	lncRNA
ENSG0000(	ENSG0000(	293.8374	-0.19972	0.025746	0.28712	lncRNA
ENSG0000(	CACNA2D1	275.9677	-0.49211	0.025757	0.287128	protein_coding
ENSG0000(	ATP10D	1087.256	-0.24061	0.025816	0.28767	protein_coding
ENSG0000(	RNF166	542.5102	-0.39737	0.02586	0.287973	protein_coding
ENSG0000(	TMEM116	1160.138	0.179428	0.025864	0.287973	protein_coding
ENSG0000(	ENSG0000(	34.59936	-0.77813	0.025897	0.288101	protein_coding
ENSG0000(	MYO9B	4817.046	-0.16656	0.025889	0.288101	protein_coding
ENSG0000(	ECM1	150.2686	-0.43487	0.025929	0.288222	protein_coding
ENSG0000(	ENSG0000(	125.964	0.228928	0.025921	0.288222	lncRNA
ENSG0000(	CD27-AS1	764.6058	-0.18343	0.025989	0.288322	lncRNA
ENSG0000(	ENPEP	140.6038	-0.79999	0.026074	0.288322	protein_coding
ENSG0000(	F2R	648.8618	-0.40464	0.026019	0.288322	protein_coding
ENSG0000(	KSR2	341.5752	0.405456	0.025981	0.288322	protein_coding
ENSG0000(	MTHFD1L	348.4792	-0.38774	0.026061	0.288322	protein_coding
ENSG0000(	NR1I3	111.9163	0.248069	0.025957	0.288322	protein_coding
ENSG0000(	OVOL1	415.7073	0.52971	0.026008	0.288322	protein_coding
ENSG0000(	PET117	128.1558	0.392072	0.02606	0.288322	protein_coding
ENSG0000(	PTPRC	5105.108	-0.51636	0.026008	0.288322	protein_coding
ENSG0000(	PYGB	2878.392	0.441246	0.025983	0.288322	protein_coding
ENSG0000(	SPATC1L	35.29109	-0.90634	0.026036	0.288322	protein_coding
ENSG0000(	TMEM8B	1396.252	0.285253	0.026074	0.288322	protein_coding
ENSG0000(	ZNF300P1	116.447	0.626987	0.026065	0.288322	transcribed_unprocessed_pseudo
ENSG0000(	CTDSPL2P1	171.4499	-0.28561	0.026086	0.288335	processed_pseudogene
ENSG0000(	CANX	6507.87	-0.29034	0.026117	0.288354	protein_coding

ENSG0000(	CYB561D1	612.3273	0.14261	0.026177	0.288354	protein_coding
ENSG0000(	ENSG0000(	118.819	-0.66884	0.026155	0.288354	lncRNA
ENSG0000(	ENSG0000(	312.6194	0.236888	0.026152	0.288354	lncRNA
ENSG0000(	NFKBIA	1674.163	-0.37513	0.02615	0.288354	protein_coding
ENSG0000(	PSMD6-AS2	710.9307	-0.20187	0.026187	0.288354	lncRNA
ENSG0000(	RIPK2	203.2452	-0.47621	0.026115	0.288354	protein_coding
ENSG0000(	SLC38A2	3929.191	-0.27805	0.026176	0.288354	protein_coding
ENSG0000(	SLC9B2	252.0459	-0.45474	0.026125	0.288354	protein_coding
ENSG0000(	VANGL2	268.4047	0.378066	0.026192	0.288354	protein_coding
ENSG0000(	HS1BP3	1113.957	0.393552	0.026232	0.288679	protein_coding
ENSG0000(	ENSG0000(	250.2784	0.183327	0.026251	0.288739	TEC
ENSG0000(	NEO1	2700.131	0.41388	0.026259	0.288739	protein_coding
ENSG0000(	ENSG0000(	746.7927	-0.16509	0.026284	0.288906	lncRNA
ENSG0000(	C11orf54	1376.137	0.190893	0.026323	0.289104	protein_coding
ENSG0000(	STEAP1B	111.2012	-0.87066	0.026322	0.289104	protein_coding
ENSG0000(	ENSG0000(	970.4266	0.233566	0.026368	0.289369	processed_pseudogene
ENSG0000(	GCDH	266.6436	0.269697	0.026358	0.289369	protein_coding
ENSG0000(	EMX2	567.6744	0.468713	0.026503	0.290031	protein_coding
ENSG0000(	HSPE1P13	216.8625	-0.2199	0.026502	0.290031	processed_pseudogene
ENSG0000(	PILRA	115.6422	-0.51738	0.026466	0.290031	protein_coding
ENSG0000(	SLC1A3	1141.698	-0.8519	0.026507	0.290031	protein_coding
ENSG0000(	SURF6	844.3012	0.190356	0.026506	0.290031	protein_coding
ENSG0000(	TMED2	2144.186	-0.23418	0.026444	0.290031	protein_coding
ENSG0000(	UNC13A	168.2878	-0.54036	0.026521	0.290031	protein_coding
ENSG0000(	ZNF106	4007.579	-0.1137	0.026524	0.290031	protein_coding
ENSG0000(	ZSWIM5	466.3802	0.339735	0.026504	0.290031	protein_coding
ENSG0000(	EHBP1L1	6229.489	-0.25285	0.026564	0.290353	protein_coding
ENSG0000(	PSMF1	1515.211	-0.18578	0.026603	0.290666	protein_coding
ENSG0000(	LAPTM4A	631.8211	-0.31921	0.026628	0.290713	protein_coding
ENSG0000(	PRAF2	77.33557	-0.52398	0.026623	0.290713	protein_coding
ENSG0000(	BAG1	1733.511	0.281305	0.026668	0.290983	protein_coding
ENSG0000(	FSCN2	35.10419	0.515276	0.026674	0.290983	protein_coding
ENSG0000(	ENTPD3	54.00573	-0.62712	0.026687	0.291005	protein_coding
ENSG0000(	MLKL	1543.472	-0.3348	0.026722	0.291272	protein_coding
ENSG0000(	KNSTRN	266.686	-0.20364	0.026733	0.291277	protein_coding
ENSG0000(	NLRC5	7656.555	-0.29811	0.026757	0.291423	protein_coding
ENSG0000(	RAC2	740.6762	-0.48561	0.026774	0.291494	protein_coding
ENSG0000(	ARHGEF6	1266.496	-0.31017	0.026793	0.291585	protein_coding
ENSG0000(	ENSG0000(	383.2026	0.402563	0.026845	0.291732	protein_coding
ENSG0000(	TERT	200.0474	0.410577	0.026844	0.291732	protein_coding
ENSG0000(	TMEM35A	43.47979	0.477967	0.026835	0.291732	protein_coding
ENSG0000(	ULBP3	463.2314	0.199105	0.026849	0.291732	protein_coding
ENSG0000(	ENSG0000(	261.5456	0.181182	0.026865	0.291788	unprocessed_pseudogene
ENSG0000(	SELENOF	670.4889	-0.21382	0.026886	0.291909	protein_coding
ENSG0000(	ZNF33B	2021.635	0.229159	0.026911	0.29206	protein_coding
ENSG0000(	GPX8	289.4497	-0.42295	0.026931	0.29216	protein_coding
ENSG0000(	LAPTM5	1918.849	-0.48479	0.026949	0.29224	protein_coding

ENSG0000(	IGHM	7818.414	-0.73881	0.026964	0.292289	IG_C_gene
ENSG0000(	PNPLA7	938.5198	0.361556	0.02698	0.29235	protein_coding
ENSG0000(	PRXL2B	499.9001	0.277091	0.02702	0.292666	protein_coding
ENSG0000(	ENSG0000(	97.72181	0.620291	0.027035	0.292668	lncRNA
ENSG0000(	SLC2A3	1257.379	-0.66303	0.027041	0.292668	protein_coding
ENSG0000(	LUNAR1	61.27355	0.366301	0.027067	0.292832	lncRNA
ENSG0000(	EHBP1	1146.211	-0.16892	0.027106	0.292942	protein_coding
ENSG0000(	GREM1	5830.831	-0.83988	0.027097	0.292942	protein_coding
ENSG0000(	MS4A6E	40.423	-0.62266	0.027126	0.292942	protein_coding
ENSG0000(	TMEM204	146.9376	-0.40601	0.027112	0.292942	protein_coding
ENSG0000(	TTC34	599.1999	-0.19572	0.027131	0.292942	protein_coding
ENSG0000(	ENSG0000(	70.83714	-0.32335	0.027174	0.293048	lncRNA
ENSG0000(	IL10RA	2396.883	-0.38008	0.027169	0.293048	protein_coding
ENSG0000(	ITGA1	3539.3	-0.21446	0.027176	0.293048	protein_coding
ENSG0000(	ZNF816	318.2213	0.228893	0.027183	0.293048	protein_coding
ENSG0000(	MAPKAPK5	527.6028	0.171634	0.027227	0.293411	lncRNA
ENSG0000(	TIFAB	329.7854	-0.50676	0.027245	0.293482	protein_coding
ENSG0000(	ZNF790-AS	1001.285	0.131715	0.027255	0.293482	lncRNA
ENSG0000(	ARIH2OS	164.0312	-0.17018	0.027361	0.294158	lncRNA
ENSG0000(	ENSG0000(	64.56931	-0.40482	0.027358	0.294158	lncRNA
ENSG0000(	HOXA13	242.5011	1.711762	0.027339	0.294158	protein_coding
ENSG0000(	NCKAP1L	2306.702	-0.29888	0.027345	0.294158	protein_coding
ENSG0000(	CYP2R1	633.2022	0.236042	0.027472	0.294189	protein_coding
ENSG0000(	DET1	1021.489	-0.35801	0.027416	0.294189	protein_coding
ENSG0000(	EFCAB5	259.7184	0.249824	0.027502	0.294189	protein_coding
ENSG0000(	EMC3	836.2261	0.122324	0.027508	0.294189	protein_coding
ENSG0000(	ENSG0000(	423.7982	-0.28463	0.027464	0.294189	transcribed_unprocessed_pseudo
ENSG0000(	ENSG0000(	248.3336	-0.17077	0.027513	0.294189	lncRNA
ENSG0000(	ENSG0000(	38.9832	-0.90306	0.027511	0.294189	IG_V_gene
ENSG0000(	FARSB	1189.251	0.188562	0.027422	0.294189	protein_coding
ENSG0000(	HAGLR	619.1968	0.441806	0.027488	0.294189	lncRNA
ENSG0000(	MEX3C	1116.259	-0.18003	0.027401	0.294189	protein_coding
ENSG0000(	RGS2	400.8226	-0.61231	0.027453	0.294189	protein_coding
ENSG0000(	SEPTIN4-AS	207.3288	-0.27874	0.027467	0.294189	lncRNA
ENSG0000(	UBXN10	149.4057	0.449934	0.027437	0.294189	protein_coding
ENSG0000(	ZNF195	2637.557	0.165907	0.027508	0.294189	protein_coding
ENSG0000(	C2CD4B	41.51945	-0.82825	0.027536	0.294202	protein_coding
ENSG0000(	SV2A	165.4561	-0.34053	0.027534	0.294202	protein_coding
ENSG0000(	ARL14EP	450.1763	-0.30582	0.027557	0.294308	protein_coding
ENSG0000(	SETD5	10943.79	-0.11866	0.027597	0.294628	protein_coding
ENSG0000(	ACYP1	300.252	0.228662	0.02772	0.295213	protein_coding
ENSG0000(	ENSG0000(	217.704	-0.37678	0.027712	0.295213	lncRNA
ENSG0000(	ENSG0000(	44.04544	0.392457	0.027668	0.295213	lncRNA
ENSG0000(	GMNN	250.8828	0.390425	0.027703	0.295213	protein_coding
ENSG0000(	IGKV3-7	22.18777	-1.11961	0.027701	0.295213	IG_V_gene
ENSG0000(	POMP	226.8642	-0.28891	0.027727	0.295213	protein_coding
ENSG0000(	STX1B	207.5194	-0.3738	0.027687	0.295213	protein_coding

ENSG0000(	ACTB	31247.49	-0.30514	0.027761	0.295313	protein_coding
ENSG0000(	ENSG0000(	126.3994	0.267985	0.027771	0.295313	lncRNA
ENSG0000(	ENSG0000(	631.8051	-0.16133	0.027797	0.295313	lncRNA
ENSG0000(	ERC2-IT1	97.56602	0.20807	0.027796	0.295313	lncRNA
ENSG0000(	TBRG4	1101.331	0.220812	0.027769	0.295313	protein_coding
ENSG0000(	TCEAL8	154.2506	-0.34014	0.027801	0.295313	protein_coding
ENSG0000(	MAP1B	711.7448	-0.47922	0.027814	0.29534	protein_coding
ENSG0000(	ENSG0000(	74.74401	-0.43922	0.02787	0.29548	lncRNA
ENSG0000(	HIPK2	4512.435	-0.16815	0.027866	0.29548	protein_coding
ENSG0000(	NINJ1	343.7053	-0.42812	0.027849	0.29548	protein_coding
ENSG0000(	PI4K2A	338.4769	-0.23437	0.027865	0.29548	protein_coding
ENSG0000(	FGFR1	1828.266	-0.24154	0.027899	0.295552	protein_coding
ENSG0000(	RGCC	87.81698	-0.52673	0.027888	0.295552	protein_coding
ENSG0000(	PRKY	1612.358	0.293913	0.027931	0.295777	transcribed_unitary_pseudogene
ENSG0000(	PCDH12	117.0584	-0.66508	0.027946	0.295826	protein_coding
ENSG0000(	IGHV2-5	289.3141	-0.7875	0.027982	0.295976	IG_V_gene
ENSG0000(	SLC36A2	283.8157	0.176075	0.027976	0.295976	protein_coding
ENSG0000(	COL5A1	3166.557	-0.45376	0.02802	0.296011	protein_coding
ENSG0000(	ENSG0000(	101.2032	-0.58487	0.028025	0.296011	lncRNA
ENSG0000(	EOMES	55.76646	-0.64393	0.028028	0.296011	protein_coding
ENSG0000(	VCAM1	489.5086	-0.48372	0.027999	0.296011	protein_coding
ENSG0000(	ENSG0000(	110.3062	-0.41554	0.028078	0.29642	lncRNA
ENSG0000(	HM13	3057.533	-0.2178	0.028091	0.296444	protein_coding
ENSG0000(	GSAP	1398.596	-0.34711	0.028109	0.296525	protein_coding
ENSG0000(	SPACA6	347.2344	-0.25079	0.028121	0.296536	protein_coding
ENSG0000(	ENSG0000(	463.0709	-0.19509	0.028139	0.296565	lncRNA
ENSG0000(	H4C6	47.31549	-0.62293	0.028148	0.296565	protein_coding
ENSG0000(	PDLIM4	82.46176	-0.77984	0.028156	0.296565	protein_coding
ENSG0000(	CLCN4	137.5738	-0.58753	0.028218	0.297105	protein_coding
ENSG0000(	C4BPB	934.2021	-0.46699	0.028329	0.298043	protein_coding
ENSG0000(	ENSG0000(	36.55707	-0.5116	0.028325	0.298043	lncRNA
ENSG0000(	LINC00668	591.9419	0.786274	0.028366	0.298318	lncRNA
ENSG0000(	ZBTB42	183.6283	0.240467	0.028403	0.298591	protein_coding
ENSG0000(	ARAP1	2405.185	-0.20911	0.028462	0.299014	protein_coding
ENSG0000(	ENSG0000(	43.20746	-0.44016	0.028465	0.299014	processed_pseudogene
ENSG0000(	TRPM6	1812.52	0.767628	0.028513	0.299412	protein_coding
ENSG0000(	ABI1	1304.058	-0.13873	0.028554	0.299497	protein_coding
ENSG0000(	CELF3	49.42187	0.522791	0.028554	0.299497	protein_coding
ENSG0000(	ENSG0000(	230.1978	0.251837	0.028533	0.299497	lncRNA
ENSG0000(	PURG	24.76718	-0.58855	0.028603	0.299893	protein_coding
ENSG0000(	ENSG0000(	4324.398	0.123393	0.02862	0.299955	lncRNA
ENSG0000(	UNC5C-AS1	29.47537	-0.4802	0.028646	0.30012	lncRNA
ENSG0000(	RHBDF2	1428.335	-0.3096	0.028658	0.300124	protein_coding
ENSG0000(	SAA4	26.31689	-1.79035	0.028683	0.30016	protein_coding
ENSG0000(	ZC3H12B	389.281	0.217256	0.028682	0.30016	protein_coding
ENSG0000(	ENSG0000(	120.8362	0.352993	0.02879	0.30059	transcribed_processed_pseudoge
ENSG0000(	FBXL2	465.2518	-0.20226	0.028749	0.30059	protein_coding

ENSG0000(	NIPSNAP1	475.484	0.175617	0.028747	0.30059	protein_coding
ENSG0000(	SMIM19	261.3066	0.314645	0.028817	0.30059	protein_coding
ENSG0000(	SPTBN4	46.50279	0.690479	0.028805	0.30059	protein_coding
ENSG0000(	STAT5A	874.58	-0.29326	0.028822	0.30059	protein_coding
ENSG0000(	TTC39A	956.4961	0.418718	0.028807	0.30059	protein_coding
ENSG0000(	WNK2	3714.191	0.530594	0.028809	0.30059	protein_coding
ENSG0000(	ZFYVE21	1120.386	0.181165	0.028786	0.30059	protein_coding
ENSG0000(	ENSG0000(	156.3173	0.224864	0.028846	0.300727	lncRNA
ENSG0000(	ARRDC4	1408.304	0.429028	0.028867	0.300828	protein_coding
ENSG0000(	SLC5A1	739.3706	-0.54006	0.028878	0.300828	protein_coding
ENSG0000(	MACF1	28602.77	-0.16442	0.028894	0.300884	protein_coding
ENSG0000(	CCT8	711.8497	-0.17286	0.028915	0.300986	protein_coding
ENSG0000(	PEG3	279.6747	-0.83202	0.02893	0.301027	protein_coding
ENSG0000(	PLEKHJ1	1019.363	0.279901	0.028998	0.301505	protein_coding
ENSG0000(	USP32P2	575.0807	-0.4247	0.028991	0.301505	transcribed_unprocessed_pseudo
ENSG0000(	ANKRD28	2147.844	-0.25058	0.02912	0.301648	protein_coding
ENSG0000(	BCAS1	3030.192	0.487637	0.029032	0.301648	protein_coding
ENSG0000(	E2F3-IT1	102.8984	-0.31721	0.02906	0.301648	lncRNA
ENSG0000(	LARGE1	1874.926	0.28691	0.029098	0.301648	protein_coding
ENSG0000(	LBR	1503.273	0.203736	0.029034	0.301648	protein_coding
ENSG0000(	LINC00940	21.92756	0.753021	0.029088	0.301648	lncRNA
ENSG0000(	POSTN	1674.161	-0.59773	0.02906	0.301648	protein_coding
ENSG0000(	RAMP2	42.77231	-0.54205	0.02908	0.301648	protein_coding
ENSG0000(	RCBTB2	430.2955	-0.29795	0.029108	0.301648	protein_coding
ENSG0000(	RUNX1T1	836.8621	-0.26304	0.029132	0.301648	protein_coding
ENSG0000(	ZNF467	96.84721	-0.44817	0.029125	0.301648	protein_coding
ENSG0000(	ENSG0000(	4583.399	-0.32792	0.029176	0.301706	lncRNA
ENSG0000(	LINC01146	237.9034	-0.33921	0.029181	0.301706	lncRNA
ENSG0000(	PLPP7	147.38	-0.37864	0.029168	0.301706	protein_coding
ENSG0000(	SPPL2B	2571.108	0.20151	0.029159	0.301706	protein_coding
ENSG0000(	ENSG0000(	37.04081	-0.60376	0.029203	0.301709	processed_pseudogene
ENSG0000(	NNMT	502.4307	-0.65002	0.029204	0.301709	protein_coding
ENSG0000(	NCR3LG1	1162.148	0.38082	0.029295	0.302536	protein_coding
ENSG0000(	KRBA2	2502.233	0.106526	0.029317	0.302648	protein_coding
ENSG0000(	EPOR	93.81002	0.346671	0.029332	0.302692	protein_coding
ENSG0000(	ENSG0000(	128.1731	-0.21878	0.029352	0.302782	lncRNA
ENSG0000(	GRHL3	24.27245	-1.08372	0.029367	0.302825	protein_coding
ENSG0000(	ENSG0000(	251.6319	0.543205	0.029405	0.302997	lncRNA
ENSG0000(	PGAP3	534.9155	0.37275	0.029405	0.302997	protein_coding
ENSG0000(	TSPAN11	710.0021	-0.52093	0.029418	0.30301	protein_coding
ENSG0000(	ERN2	2841.007	0.477851	0.029457	0.30322	protein_coding
ENSG0000(	MAF	1403.895	-0.26696	0.029468	0.30322	protein_coding
ENSG0000(	MYO1B	2076.638	-0.21978	0.029471	0.30322	protein_coding
ENSG0000(	AFTPH-DT	27.65239	-0.55933	0.029486	0.303255	lncRNA
ENSG0000(	ENSG0000(	79.95978	-0.43256	0.029518	0.303362	lncRNA
ENSG0000(	VAMP1	839.8159	-0.34267	0.029508	0.303362	protein_coding
ENSG0000(	RAB3B	1055.372	0.240477	0.029557	0.303647	protein_coding

ENSG0000(	ZBTB33	614.5198	-0.15038	0.029584	0.303811	protein_coding
ENSG0000(	PLIN1	80.42745	0.501968	0.029601	0.303869	protein_coding
ENSG0000(	IGHV1-24	79.77372	-1.40168	0.029632	0.303972	IG_V_gene
ENSG0000(	ZNF808	2110.012	0.220069	0.029633	0.303972	protein_coding
ENSG0000(	CCDC32	1698.777	-0.20056	0.029665	0.304016	protein_coding
ENSG0000(	CLEC5A	64.31588	-0.99406	0.029693	0.304016	protein_coding
ENSG0000(	EEF1A2	135.3759	0.297738	0.029743	0.304016	protein_coding
ENSG0000(	EXPH5	2792.336	0.55031	0.029819	0.304016	protein_coding
ENSG0000(	GSG1	444.1367	-0.18103	0.029727	0.304016	protein_coding
ENSG0000(	H3-3B	3303.303	-0.23591	0.029787	0.304016	protein_coding
ENSG0000(	HDAC10	490.9924	0.210312	0.029818	0.304016	protein_coding
ENSG0000(	KCNK10	469.5536	0.482217	0.029825	0.304016	protein_coding
ENSG0000(	LINC02682	49.40227	-0.58595	0.029764	0.304016	lncRNA
ENSG0000(	MEF2B	98.77621	-0.47035	0.029703	0.304016	protein_coding
ENSG0000(	MYO1G	771.8937	-0.42595	0.029649	0.304016	protein_coding
ENSG0000(	NLRP14	77.40604	-0.53106	0.029774	0.304016	protein_coding
ENSG0000(	NUAK1	323.59	-0.35936	0.029748	0.304016	protein_coding
ENSG0000(	NUMA1	7661.271	0.107496	0.029805	0.304016	protein_coding
ENSG0000(	SPDEF	186.4116	0.61333	0.029776	0.304016	protein_coding
ENSG0000(	UBR4	10700.13	-0.12004	0.029706	0.304016	protein_coding
ENSG0000(	ZNF141	3241.923	0.148871	0.029791	0.304016	protein_coding
ENSG0000(	SYT14	460.2098	0.266644	0.029844	0.304095	protein_coding
ENSG0000(	JCHAIN	13854.35	-0.66163	0.029935	0.304917	protein_coding
ENSG0000(	BCO2	407.5034	0.211826	0.029971	0.305169	protein_coding
ENSG0000(	ASAP1-IT2	356.2156	-0.22215	0.030029	0.305312	lncRNA
ENSG0000(	LRR1	219.8284	0.324578	0.030027	0.305312	protein_coding
ENSG0000(	PPIL4	493.1096	-0.14122	0.03003	0.305312	protein_coding
ENSG0000(	RTEL1P1	233.4492	-0.28015	0.030003	0.305312	transcribed_processed_pseudoge
ENSG0000(	ZDHHC11B	378.5343	0.498536	0.030081	0.305717	protein_coding
ENSG0000(	EFR3A	1373.734	-0.17094	0.030155	0.306359	protein_coding
ENSG0000(	ENSG0000(	35.92227	-0.67346	0.030189	0.306536	lncRNA
ENSG0000(	NDUFA6-D	446.6532	0.246927	0.030195	0.306536	lncRNA
ENSG0000(	ENSG0000(	133.3539	0.227613	0.030264	0.30672	lncRNA
ENSG0000(	ENSG0000(	26.7725	-0.52511	0.030255	0.30672	lncRNA
ENSG0000(	HTRA4	340.8491	-0.26067	0.030268	0.30672	protein_coding
ENSG0000(	NCDN	293.9397	-0.26044	0.030245	0.30672	protein_coding
ENSG0000(	TDRD7	788.7546	0.267239	0.030225	0.30672	protein_coding
ENSG0000(	NFE2L3	493.5522	-0.26042	0.030308	0.307013	protein_coding
ENSG0000(	RIN3	979.9697	-0.25596	0.030339	0.307213	protein_coding
ENSG0000(	MSI2	4558.113	-0.18703	0.030366	0.307328	protein_coding
ENSG0000(	ZNF189	1114.633	-0.1829	0.030373	0.307328	protein_coding
ENSG0000(	LAMP2	1581.228	-0.18818	0.030414	0.307519	protein_coding
ENSG0000(	SYT5	359.5205	0.230313	0.030403	0.307519	protein_coding
ENSG0000(	ENSG0000(	58.10311	0.572615	0.030461	0.307878	lncRNA
ENSG0000(	METTL9	1047.58	-0.20881	0.03048	0.307959	protein_coding
ENSG0000(	ECE1-AS1	32.34639	-0.61573	0.030516	0.308048	lncRNA
ENSG0000(	MAPKAPK5	4147.531	0.203489	0.030523	0.308048	protein_coding

ENSG0000(	RAB8B	817.8123	-0.43011	0.030506	0.308048	protein_coding
ENSG0000(	NBEAL1	8854.968	0.237753	0.03054	0.30811	protein_coding
ENSG0000(	CCDC22	158.0807	0.290019	0.030606	0.308328	protein_coding
ENSG0000(	NFATC4	570.6698	-0.484	0.030577	0.308328	protein_coding
ENSG0000(	OR51M1	260.1902	0.242295	0.030589	0.308328	protein_coding
ENSG0000(	TIMP3	937.3721	-0.38324	0.030603	0.308328	protein_coding
ENSG0000(	CADM1	464.172	-0.45729	0.03066	0.308752	protein_coding
ENSG0000(	DLGAP5	174.6829	-0.46857	0.030706	0.308994	protein_coding
ENSG0000(	HECTD4	7161.225	0.111535	0.030696	0.308994	protein_coding
ENSG0000(	LINC02696	41.83194	-0.70426	0.030734	0.309157	lncRNA
ENSG0000(	ERVFRD-3	52.79832	-0.41674	0.030754	0.309246	transcribed_processed_pseudoge
ENSG0000(	TM4SF1	421.8193	-0.69418	0.030772	0.309321	protein_coding
ENSG0000(	BACE2	1580.255	-0.44602	0.030835	0.309838	protein_coding
ENSG0000(	ENSG0000(	589.4621	0.098368	0.030944	0.310823	lncRNA
ENSG0000(	LINC02551	307.2538	0.176133	0.030999	0.311203	lncRNA
ENSG0000(	VN1R83P	70.28839	-0.34032	0.031005	0.311203	unprocessed_pseudogene
ENSG0000(	RN7SL145P	140.7024	0.215824	0.031056	0.311378	misc_RNA
ENSG0000(	UGGT1	3829.324	-0.15327	0.031054	0.311378	protein_coding
ENSG0000(	ZNF969P	482.617	0.220595	0.031039	0.311378	processed_pseudogene
ENSG0000(	ENSG0000(	49.61297	-0.57502	0.031112	0.311825	lncRNA
ENSG0000(	ENSG0000(	95.55911	0.478688	0.031169	0.312227	processed_pseudogene
ENSG0000(	TMEM25	195.7513	-0.31065	0.031183	0.312227	protein_coding
ENSG0000(	TSHZ1	748.3006	0.276844	0.031186	0.312227	protein_coding
ENSG0000(	ENSG0000(	153.4456	-0.24678	0.031219	0.312392	lncRNA
ENSG0000(	GDPD5	393.346	-0.3844	0.031226	0.312392	protein_coding
ENSG0000(	ENSG0000(	319.981	-0.18451	0.031292	0.312613	lncRNA
ENSG0000(	ENSG0000(	33.53196	-0.65161	0.031304	0.312613	lncRNA
ENSG0000(	ENSG0000(	45.32398	0.319831	0.031278	0.312613	lncRNA
ENSG0000(	LINC02236	23.51467	0.45038	0.031292	0.312613	lncRNA
ENSG0000(	NRSN2	99.41057	-0.43782	0.031304	0.312613	protein_coding
ENSG0000(	NUDT3	3082.588	-0.11308	0.031324	0.312692	protein_coding
ENSG0000(	H3-2	509.8588	-0.30754	0.03135	0.312726	protein_coding
ENSG0000(	MINAR2	402.0664	0.221068	0.031347	0.312726	protein_coding
ENSG0000(	TVP23CP1	36.10866	-0.71466	0.031419	0.313302	processed_pseudogene
ENSG0000(	PDXDC2P-N	3321.596	0.258009	0.031434	0.313339	lncRNA
ENSG0000(	AEN	597.6132	-0.3201	0.031484	0.31337	protein_coding
ENSG0000(	CASZ1	1750.705	0.343323	0.031506	0.31337	protein_coding
ENSG0000(	LAMA4	3134.292	-0.24026	0.031499	0.31337	protein_coding
ENSG0000(	NGDN	377.3874	0.196116	0.031502	0.31337	protein_coding
ENSG0000(	SLC10A7	1058.471	-0.23082	0.031484	0.31337	protein_coding
ENSG0000(	TMC4	893.1463	0.484075	0.031496	0.31337	protein_coding
ENSG0000(	ENSG0000(	435.8303	-0.24304	0.031584	0.314032	lncRNA
ENSG0000(	IGKV1D-39	1166.808	-0.89714	0.031609	0.314171	IG_V_gene
ENSG0000(	PELO	452.0183	-0.2617	0.031622	0.314188	protein_coding
ENSG0000(	ATP6V1A	1173.584	-0.21884	0.031648	0.314221	protein_coding
ENSG0000(	MYBPC3	31.61374	-0.69326	0.031645	0.314221	protein_coding
ENSG0000(	ENSG0000(	39.07402	0.376448	0.031674	0.31425	lncRNA

ENSG0000(	IGHJ4	817.6478	-0.66343	0.031667	0.31425	IG_J_gene
ENSG0000(	ENSG0000(	305.5963	-0.42638	0.031709	0.314483	lncRNA
ENSG0000(	GYPE	73.73165	-0.36284	0.031757	0.314847	protein_coding
ENSG0000(	DDX55	1070.983	0.121698	0.031771	0.314868	protein_coding
ENSG0000(	MRPL27	444.0238	0.261267	0.031782	0.314868	protein_coding
ENSG0000(	GUSB	873.9902	0.137905	0.031808	0.315008	protein_coding
ENSG0000(	XRN1	5189.729	-0.19733	0.031827	0.315087	protein_coding
ENSG0000(	LMO2	178.1587	-0.49469	0.031879	0.315483	protein_coding
ENSG0000(	AGA	121.7748	-0.35251	0.031941	0.315986	protein_coding
ENSG0000(	FAM189A1	163.5781	0.770017	0.032018	0.316634	protein_coding
ENSG0000(	FBXL6	341.2111	0.298312	0.032052	0.316743	protein_coding
ENSG0000(	SLC41A1	467.8513	-0.43226	0.032047	0.316743	protein_coding
ENSG0000(	ENSG0000(	63.00655	-0.35668	0.032072	0.31683	lncRNA
ENSG0000(	NLRP6	62.08259	-0.79391	0.032095	0.316943	protein_coding
ENSG0000(	DGKQ	971.082	0.31166	0.032119	0.317068	protein_coding
ENSG0000(	ENSG0000(	32.2701	0.610686	0.032143	0.317178	processed_pseudogene
ENSG0000(	SLC19A2	329.9914	0.302239	0.032154	0.317178	protein_coding
ENSG0000(	ENSG0000(	58.46077	-0.30947	0.032191	0.317437	lncRNA
ENSG0000(	CLEC18C	69.20881	0.675899	0.032282	0.31799	protein_coding
ENSG0000(	ENSG0000(	38.23554	0.441886	0.032264	0.31799	lncRNA
ENSG0000(	ENSG0000(	76.25664	-0.36801	0.032294	0.31799	lncRNA
ENSG0000(	MAMDC2-/	207.6937	0.362959	0.032285	0.31799	lncRNA
ENSG0000(	LRRC55	38.73815	-0.58658	0.032361	0.318422	protein_coding
ENSG0000(	OLFML2B	124.7929	-0.87528	0.032358	0.318422	protein_coding
ENSG0000(	ENSG0000(	71.42309	0.270624	0.032387	0.318564	lncRNA
ENSG0000(	METTL16	1389.187	0.123412	0.032431	0.318888	protein_coding
ENSG0000(	SLCO5A1	515.5021	-0.39572	0.032455	0.319004	protein_coding
ENSG0000(	RN7SL740F	91.07641	-0.30867	0.032509	0.31942	misc_RNA
ENSG0000(	ENSG0000(	138.0257	-0.54367	0.032588	0.31992	TEC
ENSG0000(	PEAK1	7154.621	0.16594	0.032603	0.31992	protein_coding
ENSG0000(	TRMT1	405.8241	0.289503	0.032606	0.31992	protein_coding
ENSG0000(	UTP25	921.7545	0.140161	0.032588	0.31992	protein_coding
ENSG0000(	OR10J1	114.8835	0.294792	0.032642	0.320159	protein_coding
ENSG0000(	SLC11A2	2025.586	-0.20277	0.032748	0.321084	protein_coding
ENSG0000(	RTRAF	957.5337	-0.24701	0.032773	0.321098	protein_coding
ENSG0000(	ZNF497	85.56227	0.331423	0.032762	0.321098	protein_coding
ENSG0000(	APOBEC3G	1085.952	-0.21732	0.032822	0.321279	protein_coding
ENSG0000(	FOXC1	27.67124	-0.72837	0.032826	0.321279	protein_coding
ENSG0000(	LINC01081	27.6291	-0.58396	0.032823	0.321279	lncRNA
ENSG0000(	HEATR3	1236.391	-0.13548	0.032845	0.321347	protein_coding
ENSG0000(	DLST	1887.184	0.108622	0.032897	0.321746	protein_coding
ENSG0000(	ADCY4	884.435	-0.33532	0.032917	0.321821	protein_coding
ENSG0000(	ADRB2	150.5857	-0.32071	0.03301	0.321934	protein_coding
ENSG0000(	ENSG0000(	150.3108	-0.3459	0.032975	0.321934	TEC
ENSG0000(	NBPF8	972.9589	-0.32971	0.03297	0.321934	transcribed_unprocessed_pseudo
ENSG0000(	P2RX4	2281.994	0.23686	0.032956	0.321934	protein_coding
ENSG0000(	SLC16A1-A:	2708.11	-0.19252	0.033	0.321934	lncRNA

ENSG0000(	ST7-OT4	291.4347	-0.22813	0.032998	0.321934	lncRNA
ENSG0000(	WASL-DT	189.3859	0.296373	0.032954	0.321934	lncRNA
ENSG0000(	ILVBL	558.8034	0.290762	0.03305	0.322203	protein_coding
ENSG0000(	LINC01876	328.6607	0.467593	0.033061	0.322203	lncRNA
ENSG0000(	LINC02488	38.55194	-0.35454	0.033133	0.322788	lncRNA
ENSG0000(	ENSG0000(	72.87589	-0.38458	0.033149	0.32283	lncRNA
ENSG0000(	HTATSF1P2	27.33485	-0.72536	0.033172	0.32283	processed_pseudogene
ENSG0000(	IGKV1-9	372.0923	-0.83279	0.033163	0.32283	IG_V_gene
ENSG0000(	PIWIL1	97.41794	-0.3125	0.033185	0.322838	protein_coding
ENSG0000(	U2	7839.89	-1.37558	0.033227	0.323131	snRNA
ENSG0000(	ITGAE	631.3654	-0.16502	0.033272	0.323459	protein_coding
ENSG0000(	EGR2	142.2471	-0.66808	0.033303	0.323532	protein_coding
ENSG0000(	TWSG1	382.2139	-0.2713	0.033293	0.323532	protein_coding
ENSG0000(	AHSA2P	4638.331	0.117442	0.033337	0.323596	transcribed_unitary_pseudogene
ENSG0000(	C3	2465.835	-0.5263	0.033401	0.323596	protein_coding
ENSG0000(	CDKN2B-AS	1840.642	0.35359	0.033366	0.323596	lncRNA
ENSG0000(	DNAJC11	2793.473	0.189772	0.033331	0.323596	protein_coding
ENSG0000(	ECHDC2	4572.504	0.234235	0.033439	0.323596	protein_coding
ENSG0000(	FBXW4	918.2316	0.158543	0.033438	0.323596	protein_coding
ENSG0000(	GLRA2	20.94703	0.88345	0.03338	0.323596	protein_coding
ENSG0000(	HIP1R	1590.529	0.229808	0.033427	0.323596	protein_coding
ENSG0000(	MEGF9	744.3386	-0.26754	0.03343	0.323596	protein_coding
ENSG0000(	RBBP8	445.7216	-0.27376	0.033406	0.323596	protein_coding
ENSG0000(	SRF	676.994	-0.21157	0.033397	0.323596	protein_coding
ENSG0000(	RNU7-40P	25.24823	-0.6473	0.033454	0.323621	snRNA
ENSG0000(	ENO2	376.6862	-0.27455	0.033483	0.323754	protein_coding
ENSG0000(	MAMLD1	131.551	-0.43847	0.033503	0.323754	protein_coding
ENSG0000(	UACA	2342.333	-0.23311	0.033499	0.323754	protein_coding
ENSG0000(	DARS1	650.3576	-0.20111	0.033568	0.323812	protein_coding
ENSG0000(	DIP2A	3840.876	0.103242	0.033525	0.323812	protein_coding
ENSG0000(	ENSG0000(	90.60792	-0.34993	0.033537	0.323812	processed_pseudogene
ENSG0000(	GCA	406.203	-0.5644	0.033548	0.323812	protein_coding
ENSG0000(	ZSWIM8	2152.593	-0.18773	0.033557	0.323812	protein_coding
ENSG0000(	ADAMTSL4	760.1745	-0.377	0.033652	0.324051	lncRNA
ENSG0000(	CCDC88B	2650.062	-0.29015	0.033672	0.324051	protein_coding
ENSG0000(	CD44	6236.685	-0.45864	0.03369	0.324051	protein_coding
ENSG0000(	ENSG0000(	798.1291	0.169424	0.033734	0.324051	lncRNA
ENSG0000(	ENSG0000(	206.4365	-0.30591	0.033729	0.324051	lncRNA
ENSG0000(	GFAP	317.2219	0.225904	0.033616	0.324051	protein_coding
ENSG0000(	MAEL	74.3291	0.328005	0.033665	0.324051	protein_coding
ENSG0000(	NEIL3	111.7101	-0.29647	0.033698	0.324051	protein_coding
ENSG0000(	NQO2	150.5634	-0.37023	0.033681	0.324051	protein_coding
ENSG0000(	PPL	157.0885	0.582242	0.033618	0.324051	protein_coding
ENSG0000(	SALRNA3	192.3386	-0.2081	0.033717	0.324051	lncRNA
ENSG0000(	STRADA	1693.113	-0.15581	0.033709	0.324051	protein_coding
ENSG0000(	SEMA7A	228.6432	-0.60806	0.033752	0.324113	protein_coding
ENSG0000(	IGHV5-10-1	116.6399	-1.66774	0.033766	0.324137	IG_V_gene

ENSG0000(	ENSG0000(	42.25979	-0.38365	0.033794	0.324163	lncRNA
ENSG0000(	ENSG0000(	26.38856	-0.87451	0.033787	0.324163	lncRNA
ENSG0000(	RNF111	1179.704	-0.19813	0.033804	0.324163	protein_coding
ENSG0000(	PLEKHA6	3379.695	0.435198	0.033839	0.324378	protein_coding
ENSG0000(	CEBPZOS	1119.682	0.148905	0.033906	0.324822	protein_coding
ENSG0000(	LINC01198	418.3007	0.188276	0.03392	0.324822	lncRNA
ENSG0000(	SPATC1	22.98428	-0.83473	0.033914	0.324822	protein_coding
ENSG0000(	ATP6V1F	135.332	-0.36314	0.033988	0.324924	protein_coding
ENSG0000(	CHST3	324.5143	-0.3661	0.034	0.324924	protein_coding
ENSG0000(	ISX	657.5502	0.698207	0.033944	0.324924	protein_coding
ENSG0000(	OSCP1	41.06351	-0.51243	0.034	0.324924	protein_coding
ENSG0000(	SLC27A1	1730.17	-0.13962	0.033995	0.324924	protein_coding
ENSG0000(	ZFP41	668.0873	0.188518	0.034002	0.324924	protein_coding
ENSG0000(	PHF24	45.60076	-1.01284	0.03404	0.325062	protein_coding
ENSG0000(	WASHC3	320.9435	-0.2286	0.034033	0.325062	protein_coding
ENSG0000(	NOVA1-DT	78.90318	0.709945	0.034063	0.325117	lncRNA
ENSG0000(	RPSAP36	239.6638	0.224595	0.034069	0.325117	transcribed_processed_pseudoge
ENSG0000(	RNU6ATAC	169.9921	-0.3179	0.034099	0.32529	snRNA
ENSG0000(	ENSG0000(	99.11188	0.312208	0.03413	0.325476	lncRNA
ENSG0000(	MSRB1	161.3064	-0.40948	0.034157	0.325618	protein_coding
ENSG0000(	ENSG0000(	221.2854	0.214575	0.034172	0.325622	lncRNA
ENSG0000(	ENSG0000(	27717.26	0.647976	0.034182	0.325622	lncRNA
ENSG0000(	TXNRD1	1229.236	-0.35312	0.034193	0.325622	protein_coding
ENSG0000(	CTLA4	225.0705	-0.68336	0.034212	0.325685	protein_coding
ENSG0000(	EAF1	979.8694	-0.13981	0.034227	0.32572	protein_coding
ENSG0000(	ZNF37A	3024.878	-0.15523	0.034244	0.325772	protein_coding
ENSG0000(	TBILA	649.4724	0.406935	0.034295	0.326136	lncRNA
ENSG0000(	ADAMTS7F	89.26662	-0.38694	0.034332	0.326328	transcribed_unprocessed_pseudo
ENSG0000(	IGLV2-14	1274.614	-0.71579	0.034349	0.326328	IG_V_gene
ENSG0000(	P2RY4	53.78285	0.439686	0.03435	0.326328	protein_coding
ENSG0000(	CASD1	1145.863	0.257591	0.034371	0.326412	protein_coding
ENSG0000(	PARP14	11767.42	-0.46812	0.03443	0.326861	protein_coding
ENSG0000(	ALPK3	200.5512	-0.49271	0.034542	0.327073	protein_coding
ENSG0000(	CCAR2	1015.742	0.214522	0.034502	0.327073	protein_coding
ENSG0000(	DIP2C-AS1	450.7794	0.484176	0.034473	0.327073	lncRNA
ENSG0000(	ENSG0000(	607.5667	0.554387	0.034548	0.327073	lncRNA
ENSG0000(	KCNK5	936.8838	0.49634	0.034517	0.327073	protein_coding
ENSG0000(	MTRNR2L7	33.90893	-0.49162	0.034541	0.327073	protein_coding
ENSG0000(	PPM1B	2063.069	0.126122	0.034527	0.327073	protein_coding
ENSG0000(	S1PR3	751.1616	-0.4945	0.03448	0.327073	protein_coding
ENSG0000(	ABCA2	1312.375	0.357879	0.034566	0.327074	protein_coding
ENSG0000(	CLUHP3	1754.079	0.140952	0.034573	0.327074	transcribed_unprocessed_pseudo
ENSG0000(	ENSG0000(	137.1407	0.502712	0.034583	0.327074	lncRNA
ENSG0000(	GS1-24F4.2	450.7564	0.260596	0.034609	0.327095	lncRNA
ENSG0000(	TAF1B	313.8724	-0.23285	0.034601	0.327095	protein_coding
ENSG0000(	ENSG0000(	20.92656	0.443219	0.03463	0.327177	lncRNA
ENSG0000(	PSKH1	760.8921	0.278084	0.034648	0.327231	protein_coding

ENSG0000(	AVPR1A	130.2644	-0.49134	0.034688	0.327304	protein_coding
ENSG0000(	ENSG0000(	83.67568	-0.22666	0.034691	0.327304	lncRNA
ENSG0000(	HSPA8P4	19.64436	-0.69494	0.034678	0.327304	processed_pseudogene
ENSG0000(	SLC38A7	722.3012	-0.18505	0.034723	0.327495	protein_coding
ENSG0000(	EMX2OS	763.343	0.144463	0.034736	0.327508	lncRNA
ENSG0000(	JAZF1	307.0892	-0.38188	0.034776	0.327736	protein_coding
ENSG0000(	LINC01152	89.54243	0.220863	0.034784	0.327736	lncRNA
ENSG0000(	LPAR5	416.3559	0.255704	0.034829	0.328047	protein_coding
ENSG0000(	METRN	116.5409	0.535501	0.034851	0.328139	protein_coding
ENSG0000(	ENSG0000(	23.20187	0.488786	0.034882	0.328314	lncRNA
ENSG0000(	ENSG0000(	40.40981	0.387413	0.034925	0.328607	lncRNA
ENSG0000(	ENSG0000(	53.88347	-0.46337	0.034947	0.3287	lncRNA
ENSG0000(	SORBS2	1186.252	0.276898	0.034958	0.3287	protein_coding
ENSG0000(	LAMC2	1260.698	-0.52865	0.034984	0.328829	protein_coding
ENSG0000(	YARS1	1911.252	-0.13352	0.035014	0.328994	protein_coding
ENSG0000(	GOLGA2	2814.434	-0.12656	0.035036	0.329091	protein_coding
ENSG0000(	MEF2C-AS1	285.6866	-0.37305	0.035066	0.329264	lncRNA
ENSG0000(	SP2-AS1	153.4111	0.182871	0.03512	0.329544	lncRNA
ENSG0000(	TAC3	34.84195	0.549223	0.035118	0.329544	protein_coding
ENSG0000(	H2BC14	56.22714	-0.5163	0.035161	0.32982	protein_coding
ENSG0000(	ENSG0000(	41.45576	-0.77773	0.035192	0.329995	lncRNA
ENSG0000(	RN7SL751F	57.95037	-0.5221	0.03528	0.330705	misc_RNA
ENSG0000(	RNF183	58.65629	-0.86709	0.035307	0.330851	protein_coding
ENSG0000(	ENSG0000(	184.9606	-0.40916	0.035338	0.331026	lncRNA
ENSG0000(	CFH	1329.25	-0.39596	0.035372	0.331232	protein_coding
ENSG0000(	ATAD2B	3084.813	0.121976	0.035441	0.331494	protein_coding
ENSG0000(	ENSG0000(	4831.192	-0.30336	0.035423	0.331494	lncRNA
ENSG0000(	ENSG0000(	644.1135	0.135784	0.035448	0.331494	TEC
ENSG0000(	ENSG0000(	39.23742	0.599284	0.035427	0.331494	lncRNA
ENSG0000(	IFT122	1887.036	0.158077	0.035474	0.331545	protein_coding
ENSG0000(	SLC30A4	1419.147	0.127723	0.035478	0.331545	protein_coding
ENSG0000(	LINC01102	191.1632	0.228599	0.035491	0.331557	lncRNA
ENSG0000(	ENSG0000(	21.56206	0.611572	0.035573	0.331867	lncRNA
ENSG0000(	RFX7	1404.424	-0.19382	0.035546	0.331867	protein_coding
ENSG0000(	SLX1B-SULT	893.1042	0.184918	0.035572	0.331867	lncRNA
ENSG0000(	VIPR1	2968.643	0.606005	0.035555	0.331867	protein_coding
ENSG0000(	ENSG0000(	2266.086	-0.26116	0.035608	0.33191	transcribed_unprocessed_pseudo
ENSG0000(	ISLR	95.52587	-0.66188	0.035626	0.33191	protein_coding
ENSG0000(	MAPK13	2482.7	0.213377	0.035683	0.33191	protein_coding
ENSG0000(	PADI2	3867.394	0.74008	0.035672	0.33191	protein_coding
ENSG0000(	PGAP1	1148.093	0.280401	0.035686	0.33191	protein_coding
ENSG0000(	SERPINB1	2424.699	-0.24385	0.03562	0.33191	protein_coding
ENSG0000(	SNORA38	87.36865	0.363934	0.035676	0.33191	snoRNA
ENSG0000(	ST3GAL1-D	32.86032	-0.5524	0.035661	0.33191	lncRNA
ENSG0000(	STEAP2	2161.8	-0.3456	0.03567	0.33191	protein_coding
ENSG0000(	CCDC89	110.8424	0.556024	0.036007	0.331986	protein_coding
ENSG0000(	CD302	657.357	-0.24936	0.035873	0.331986	protein_coding

ENSG0000(	DPY30	270.8929	0.347541	0.035813	0.331986	protein_coding
ENSG0000(	DUSP7	310.8142	-0.2993	0.035809	0.331986	protein_coding
ENSG0000(	ENSG0000(	103.5113	-0.3509	0.035939	0.331986	lncRNA
ENSG0000(	ENSG0000(	28.63247	-0.47185	0.036027	0.331986	lncRNA
ENSG0000(	ENSG0000(	29.53817	-0.64254	0.035981	0.331986	lncRNA
ENSG0000(	ENSG0000(	87.5118	-0.48348	0.035831	0.331986	lncRNA
ENSG0000(	ENSG0000(	621.4173	-0.22984	0.036084	0.331986	TEC
ENSG0000(	ENSG0000(	478.9932	0.211928	0.035723	0.331986	lncRNA
ENSG0000(	ENSG0000(	144.0843	0.174193	0.035867	0.331986	lncRNA
ENSG0000(	ENSG0000(	39.37172	-0.42775	0.03601	0.331986	lncRNA
ENSG0000(	FAM186A	186.1818	0.13778	0.036043	0.331986	protein_coding
ENSG0000(	FRMD4A	1402.351	-0.30123	0.035907	0.331986	protein_coding
ENSG0000(	KBTBD12	366.373	0.377963	0.035812	0.331986	protein_coding
ENSG0000(	KIF23-AS1	148.0946	-0.38581	0.035984	0.331986	lncRNA
ENSG0000(	KLF11	320.9671	0.328868	0.03611	0.331986	protein_coding
ENSG0000(	LINC02013	675.7063	-0.18123	0.036092	0.331986	lncRNA
ENSG0000(	MIB2	902.6743	0.259347	0.036015	0.331986	protein_coding
ENSG0000(	MTND6P5	62.29669	-0.53213	0.035951	0.331986	unprocessed_pseudogene
ENSG0000(	NT5DC2	229.2995	-0.41497	0.0361	0.331986	protein_coding
ENSG0000(	OLFML3	133.8748	-0.48874	0.036091	0.331986	protein_coding
ENSG0000(	OXR1	1427.312	0.195693	0.03612	0.331986	protein_coding
ENSG0000(	PAPLN	1909.805	-0.43805	0.036041	0.331986	protein_coding
ENSG0000(	PLS3	371.7721	-0.31893	0.035763	0.331986	protein_coding
ENSG0000(	PPIF	961.9009	-0.39159	0.035978	0.331986	protein_coding
ENSG0000(	PRC1-AS1	193.5687	0.298507	0.035835	0.331986	lncRNA
ENSG0000(	PRF1	112.0761	-0.54278	0.036114	0.331986	protein_coding
ENSG0000(	RHPN2	1076.895	0.388932	0.035862	0.331986	protein_coding
ENSG0000(	RPP21	128.9585	0.328583	0.035999	0.331986	protein_coding
ENSG0000(	TAS2R10	40.94305	0.564537	0.035865	0.331986	protein_coding
ENSG0000(	TCAF2	1268.222	-0.20345	0.035932	0.331986	protein_coding
ENSG0000(	TMC3-AS1	1299.45	-0.34156	0.036032	0.331986	lncRNA
ENSG0000(	TMEM167/	1849.068	-0.1447	0.035736	0.331986	protein_coding
ENSG0000(	WIPF1	2980.138	-0.35512	0.036102	0.331986	protein_coding
ENSG0000(	ZMYND15	84.02685	-0.50476	0.036128	0.331986	protein_coding
ENSG0000(	TEX9	1320.071	-0.18782	0.036146	0.332037	protein_coding
ENSG0000(	DPP8	2144.377	-0.11279	0.036214	0.332111	protein_coding
ENSG0000(	LAMA2	1221.595	-0.24854	0.036189	0.332111	protein_coding
ENSG0000(	SAP25	81.75192	-0.58661	0.036214	0.332111	protein_coding
ENSG0000(	SLA	1743.861	-0.61226	0.036211	0.332111	protein_coding
ENSG0000(	SLC10A2	116.2826	-0.38733	0.036193	0.332111	protein_coding
ENSG0000(	PLEKHA2	2376.232	-0.25199	0.036296	0.332748	protein_coding
ENSG0000(	ENSG0000(	96.68597	-0.2257	0.036381	0.333138	lncRNA
ENSG0000(	RN7SL619F	209.839	0.160709	0.036372	0.333138	misc_RNA
ENSG0000(	RYR3	587.9678	0.591543	0.036387	0.333138	protein_coding
ENSG0000(	SPP2	115.3766	0.390397	0.036356	0.333138	protein_coding
ENSG0000(	LBH	620.7461	-0.43778	0.036417	0.333191	protein_coding
ENSG0000(	RNF152P1	39.92017	0.433835	0.036405	0.333191	processed_pseudogene

ENSG0000(	CRISPLD1	24.02351	-1.12323	0.036478	0.333508	protein_coding
ENSG0000(	DIDO1	3681.001	0.088108	0.036472	0.333508	protein_coding
ENSG0000(	EPM2AIP1	2274.224	-0.16598	0.036517	0.333508	protein_coding
ENSG0000(	MTPN	1637.387	-0.2549	0.036524	0.333508	protein_coding
ENSG0000(	RN7SKP11(	25.22919	-0.94215	0.036511	0.333508	misc_RNA
ENSG0000(	TECTA	73.38609	-0.58138	0.036496	0.333508	protein_coding
ENSG0000(	AFG3L2	2091.226	0.249699	0.036598	0.334068	protein_coding
ENSG0000(	TCIM	778.7044	-0.48449	0.03662	0.334155	protein_coding
ENSG0000(	TNFSF10	2229.836	-0.33995	0.036638	0.334214	protein_coding
ENSG0000(	ENSG0000(	123.6722	0.282952	0.036652	0.334232	lncRNA
ENSG0000(	FHIP1B	3141.294	0.317948	0.03681	0.335116	protein_coding
ENSG0000(	KIF2A	2120.726	-0.187	0.036805	0.335116	protein_coding
ENSG0000(	PKDCC	718.6212	0.408693	0.036777	0.335116	protein_coding
ENSG0000(	SEMA4G	1577.094	0.487309	0.036797	0.335116	protein_coding
ENSG0000(	SHLD2	968.086	0.167363	0.036804	0.335116	protein_coding
ENSG0000(	CD81	1445.868	-0.30684	0.036902	0.335626	protein_coding
ENSG0000(	DUXAP8	381.4924	-0.50115	0.036917	0.335626	lncRNA
ENSG0000(	NEK3	963.1226	0.427378	0.036924	0.335626	protein_coding
ENSG0000(	STARD13	1223.773	-0.20464	0.036927	0.335626	protein_coding
ENSG0000(	TBXAS1	529.7468	-0.31851	0.036909	0.335626	protein_coding
ENSG0000(	FBXW5	1031.731	0.234366	0.036941	0.335636	protein_coding
ENSG0000(	RRS1-DT	260.6601	0.24412	0.03702	0.336138	lncRNA
ENSG0000(	SURF4	1955.13	-0.21187	0.037015	0.336138	protein_coding
ENSG0000(	CCR5	262.5874	-0.39219	0.03712	0.33621	protein_coding
ENSG0000(	ENSG0000(	226.4674	-0.30198	0.037099	0.33621	TEC
ENSG0000(	ENSG0000(	40.08878	-0.5464	0.037114	0.33621	lncRNA
ENSG0000(	ENSG0000(	33.27036	0.407653	0.037111	0.33621	lncRNA
ENSG0000(	FOXP4	775.9871	0.241497	0.037116	0.33621	protein_coding
ENSG0000(	GLB1L2	643.9794	0.46711	0.037044	0.33621	protein_coding
ENSG0000(	IGF1	641.9772	-0.2844	0.037126	0.33621	protein_coding
ENSG0000(	TMEM213	560.3213	0.122395	0.037068	0.33621	protein_coding
ENSG0000(	ARHGAP44	260.2955	0.253255	0.03728	0.33636	lncRNA
ENSG0000(	CBL	2357.023	-0.17434	0.037273	0.33636	protein_coding
ENSG0000(	CLU	788.0192	-0.57596	0.037208	0.33636	protein_coding
ENSG0000(	DDIT4	711.9266	-0.53027	0.03727	0.33636	protein_coding
ENSG0000(	ENSG0000(	61.24196	-0.31933	0.037289	0.33636	lncRNA
ENSG0000(	GATA6	596.9752	0.407921	0.037217	0.33636	protein_coding
ENSG0000(	H3C8	82.84787	-0.48649	0.037226	0.33636	protein_coding
ENSG0000(	HR	1060.953	0.492263	0.037191	0.33636	protein_coding
ENSG0000(	MIR4500H(	535.1372	0.268282	0.037166	0.33636	lncRNA
ENSG0000(	SMCR8	2118.727	0.110212	0.037269	0.33636	protein_coding
ENSG0000(	WAS	253.9473	-0.39222	0.037205	0.33636	protein_coding
ENSG0000(	XIRP1	23.72574	-1.99157	0.037279	0.33636	protein_coding
ENSG0000(	PRR13P2	29.57127	-0.49005	0.037317	0.336503	processed_pseudogene
ENSG0000(	FJX1	21.6942	-1.02247	0.03744	0.337503	protein_coding
ENSG0000(	GEM	441.1901	-0.53681	0.037469	0.337591	protein_coding
ENSG0000(	UCK2	886.2503	0.247983	0.037475	0.337591	protein_coding

ENSG0000(	HYI	488.0237	0.278395	0.037513	0.337701	protein_coding
ENSG0000(	NCF4	325.1492	-0.38793	0.037528	0.337701	protein_coding
ENSG0000(	STUM	325.6565	0.649891	0.037531	0.337701	protein_coding
ENSG0000(	TBC1D15	1372.392	-0.16474	0.037536	0.337701	protein_coding
ENSG0000(	TTYH2	278.4397	-0.40526	0.037572	0.337911	protein_coding
ENSG0000(	ARX	41.25494	1.465681	0.037633	0.338354	protein_coding
ENSG0000(	ENSG0000(	117.5194	0.226877	0.037693	0.338668	processed_pseudogene
ENSG0000(	ENSG0000(	234.0079	0.407436	0.037685	0.338668	lncRNA
ENSG0000(	IPP	602.4308	0.235364	0.037714	0.338749	protein_coding
ENSG0000(	PURB	2288.194	-0.09345	0.037761	0.339056	protein_coding
ENSG0000(	TRIM13	1468.761	0.154515	0.037806	0.33935	protein_coding
ENSG0000(	ANAPC10	285.0738	-0.1867	0.037822	0.339387	protein_coding
ENSG0000(	COL6A2	3706.067	-0.41675	0.037916	0.34002	protein_coding
ENSG0000(	ENSG0000(	288.9695	-0.34844	0.037942	0.34002	protein_coding
ENSG0000(	PRDM4	1010.514	0.190208	0.037935	0.34002	protein_coding
ENSG0000(	SCUBE2	308.9508	0.318359	0.037926	0.34002	protein_coding
ENSG0000(	RPS3AP19	108.1882	0.510458	0.037983	0.34018	processed_pseudogene
ENSG0000(	ZNF20	318.194	0.186456	0.037985	0.34018	protein_coding
ENSG0000(	ENSG0000(	25.92746	-0.54353	0.03802	0.340258	lncRNA
ENSG0000(	FAH	412.9569	0.169197	0.038013	0.340258	protein_coding
ENSG0000(	TFPI	3579.374	-0.18385	0.03803	0.340258	protein_coding
ENSG0000(	MFSD3	254.1806	0.442361	0.038075	0.340439	protein_coding
ENSG0000(	TBXA2R	271.1254	-0.19191	0.038069	0.340439	protein_coding
ENSG0000(	AKTIP	652.5909	0.15303	0.038097	0.340443	protein_coding
ENSG0000(	S1PR1	386.8432	-0.45716	0.038101	0.340443	protein_coding
ENSG0000(	DNAJC24	829.8179	-0.16216	0.038157	0.340836	protein_coding
ENSG0000(	NWD2	36.74241	0.88511	0.03817	0.340842	protein_coding
ENSG0000(	SLC35F6	1196.336	-0.16918	0.038196	0.340966	protein_coding
ENSG0000(	GCNA	355.1762	-0.1886	0.038219	0.341034	protein_coding
ENSG0000(	INPP5K	662.7907	0.153917	0.038241	0.341034	protein_coding
ENSG0000(	TMEM192	2860.83	0.083848	0.038236	0.341034	protein_coding
ENSG0000(	EHD3	232.5721	-0.40338	0.038267	0.341059	protein_coding
ENSG0000(	TMEM163	142.2121	-0.91158	0.038269	0.341059	protein_coding
ENSG0000(	CXCR2P1	299.1097	-0.63481	0.038301	0.341236	transcribed_unprocessed_pseudo
ENSG0000(	PRKN	628.3478	0.221541	0.038316	0.341262	protein_coding
ENSG0000(	ENSG0000(	202.4747	0.20109	0.038368	0.341327	lncRNA
ENSG0000(	GNPDA1	301.0985	-0.27723	0.038373	0.341327	protein_coding
ENSG0000(	NUDT14	204.775	0.281132	0.038344	0.341327	protein_coding
ENSG0000(	TAS2R50	80.44201	0.399616	0.038356	0.341327	protein_coding
ENSG0000(	SARS2	193.4058	0.302339	0.038405	0.341502	protein_coding
ENSG0000(	ENSG0000(	102.195	-0.31098	0.038421	0.341529	lncRNA
ENSG0000(	BPHL	617.1496	0.175631	0.038518	0.342284	protein_coding
ENSG0000(	ADAMTS1(	484.1391	-0.40951	0.038577	0.342312	protein_coding
ENSG0000(	BORCS8	1983.718	0.200515	0.038579	0.342312	protein_coding
ENSG0000(	ENSG0000(	39.65073	0.420781	0.038584	0.342312	transcribed_unprocessed_pseudo
ENSG0000(	PSMB2	990.8249	-0.21599	0.03857	0.342312	protein_coding
ENSG0000(	YEATS2	1845.18	-0.19996	0.038553	0.342312	protein_coding

ENSG0000(	INTS6L	1711.517	0.205044	0.038664	0.342918	protein_coding
ENSG0000(	C3orf86	43.86128	-0.72321	0.038704	0.343156	transcribed_unitary_pseudogene
ENSG0000(	FARP1	4278.013	-0.13396	0.038751	0.343467	protein_coding
ENSG0000(	ENSG0000(	410.5622	-0.3262	0.038819	0.343625	lncRNA
ENSG0000(	GSTM3	605.2582	0.129416	0.038796	0.343625	protein_coding
ENSG0000(	SSC5D	263.7256	-0.35833	0.038811	0.343625	protein_coding
ENSG0000(	TNFAIP3	2639.806	-0.45168	0.038812	0.343625	protein_coding
ENSG0000(	ARCN1	2100.282	-0.21056	0.038878	0.343824	protein_coding
ENSG0000(	ENSG0000(	112.6381	0.256305	0.038861	0.343824	lncRNA
ENSG0000(	SNCA	72.78252	-0.43415	0.038891	0.343824	protein_coding
ENSG0000(	YJEFN3	303.27	0.286784	0.038882	0.343824	protein_coding
ENSG0000(	CD86	236.8737	-0.50157	0.038991	0.34419	protein_coding
ENSG0000(	ENSG0000(	3100.206	-0.25898	0.039008	0.34419	transcribed_unprocessed_pseudo
ENSG0000(	ENSG0000(	1468.526	0.198477	0.039007	0.34419	lncRNA
ENSG0000(	MB21D2	94.27492	-0.48136	0.038976	0.34419	protein_coding
ENSG0000(	NPIPA7	224.8649	0.323533	0.038978	0.34419	protein_coding
ENSG0000(	RPS3AP48	29.8676	0.779683	0.038994	0.34419	processed_pseudogene
ENSG0000(	SERPINF1	573.9561	-0.27245	0.039039	0.344352	protein_coding
ENSG0000(	ASCC3	2888.075	-0.14563	0.03906	0.344425	protein_coding
ENSG0000(	XYLB	766.7088	0.22816	0.039083	0.344519	protein_coding
ENSG0000(	NT5C	282.7583	0.290875	0.039098	0.344542	protein_coding
ENSG0000(	ENSG0000(	355.4525	0.221315	0.039115	0.344551	lncRNA
ENSG0000(	TMEM176/	684.7123	-0.28997	0.039124	0.344551	protein_coding
ENSG0000(	ENSG0000(	333.6877	0.140186	0.039164	0.344683	TEC
ENSG0000(	ZCCHC4	532.4464	0.147488	0.039152	0.344683	protein_coding
ENSG0000(	ENSG0000(	482.6906	-0.39073	0.039189	0.344794	protein_coding
ENSG0000(	DPM3	53.96527	-0.35151	0.03926	0.3452	protein_coding
ENSG0000(	SNORD33	267.875	-0.37141	0.039258	0.3452	snoRNA
ENSG0000(	FAM174B	440.9863	0.173041	0.039317	0.345586	protein_coding
ENSG0000(	PLEKHF1	93.82433	-0.39871	0.039335	0.345633	protein_coding
ENSG0000(	ANG	102.4741	0.591025	0.03939	0.3457	protein_coding
ENSG0000(	CHDH	1157.2	0.349087	0.039377	0.3457	protein_coding
ENSG0000(	FOXF1	457.6475	-0.32302	0.039405	0.3457	protein_coding
ENSG0000(	PLEKHM1P	4631.867	0.110177	0.039405	0.3457	transcribed_unprocessed_pseudo
ENSG0000(	ZNF366	502.1295	-0.3637	0.039371	0.3457	protein_coding
ENSG0000(	ENSG0000(	540.2908	-0.21221	0.039486	0.34586	lncRNA
ENSG0000(	ENSG0000(	255.2951	0.418795	0.039481	0.34586	TEC
ENSG0000(	GET4	865.3168	0.257374	0.039446	0.34586	protein_coding
ENSG0000(	LINC02694	743.6643	-0.19146	0.039472	0.34586	lncRNA
ENSG0000(	SNX17	710.7439	-0.13592	0.039462	0.34586	protein_coding
ENSG0000(	TMEM209	738.4198	0.190655	0.039552	0.346323	protein_coding
ENSG0000(	PCDHB1	737.9409	0.142373	0.039581	0.346365	protein_coding
ENSG0000(	ZNF264	2854.607	-0.14341	0.039582	0.346365	protein_coding
ENSG0000(	C1orf159	516.4122	0.209893	0.039624	0.346542	protein_coding
ENSG0000(	DHRSX	654.4837	0.159511	0.03964	0.346542	protein_coding
ENSG0000(	ZNF556	1311.326	0.134651	0.039628	0.346542	protein_coding
ENSG0000(	DTX2P1	208.1292	0.520703	0.039682	0.346805	transcribed_unprocessed_pseudo

ENSG0000(	ENSG0000(	88.36546	-0.48675	0.039695	0.346806	lncRNA
ENSG0000(	TNFAIP1	647.5527	-0.22202	0.039747	0.347041	protein_coding
ENSG0000(	VIL1	2468.151	0.527903	0.03974	0.347041	protein_coding
ENSG0000(	HLA-B	15493.48	-0.31719	0.039776	0.347131	protein_coding
ENSG0000(	NAAA	1177.879	0.323861	0.039783	0.347131	protein_coding
ENSG0000(	ANKRD20A	436.8536	0.385812	0.039868	0.347574	protein_coding
ENSG0000(	AP3B2	366.8446	-0.24388	0.039882	0.347574	protein_coding
ENSG0000(	ENSG0000(	30.44818	-0.81984	0.039884	0.347574	lncRNA
ENSG0000(	ENSG0000(	80.31019	0.242835	0.03986	0.347574	lncRNA
ENSG0000(	ENSG0000(	81.63138	0.260319	0.039908	0.347676	lncRNA
ENSG0000(	C19orf18	37.87477	0.430139	0.03994	0.347786	protein_coding
ENSG0000(	FAM3C	531.7061	-0.29641	0.039946	0.347786	protein_coding
ENSG0000(	CD7	176.3013	-0.45394	0.039979	0.347958	protein_coding
ENSG0000(	CLUH	1682.553	0.358709	0.04	0.347958	protein_coding
ENSG0000(	ERMAP	841.797	0.262183	0.040004	0.347958	protein_coding
ENSG0000(	PPP3R1	904.8297	-0.13194	0.040039	0.348156	protein_coding
ENSG0000(	C2orf72	773.3029	0.486683	0.040062	0.348243	protein_coding
ENSG0000(	SIPA1L1	2989.232	-0.17163	0.0401	0.348463	protein_coding
ENSG0000(	LGALS8	2258.891	-0.15207	0.040115	0.348484	protein_coding
ENSG0000(	PLCD1	426.803	0.52481	0.040133	0.3485	protein_coding
ENSG0000(	TCEA1	1214.938	-0.1724	0.040142	0.3485	protein_coding
ENSG0000(	NKIRAS1	193.0302	-0.23234	0.040176	0.348678	protein_coding
ENSG0000(	SRGAP2C	664.44	-0.2878	0.040241	0.349132	protein_coding
ENSG0000(	ENSG0000(	533.9902	0.105385	0.040259	0.349186	lncRNA
ENSG0000(	LINC02894	195.7776	0.30406	0.040291	0.349352	lncRNA
ENSG0000(	DAB2	2153.955	-0.26912	0.040356	0.349627	protein_coding
ENSG0000(	ENSG0000(	209.9738	-0.1903	0.040386	0.349627	lncRNA
ENSG0000(	MAP7D1	631.231	-0.23448	0.040385	0.349627	protein_coding
ENSG0000(	PILRB	3275.723	0.185191	0.040347	0.349627	protein_coding
ENSG0000(	WBP1	684.0282	0.135261	0.040386	0.349627	protein_coding
ENSG0000(	CDK13	7458.237	0.097663	0.04048	0.350216	protein_coding
ENSG0000(	SSTR1	76.28457	0.537901	0.040479	0.350216	protein_coding
ENSG0000(	FGD1	118.9393	-0.36075	0.040544	0.350598	protein_coding
ENSG0000(	GLT8D2	132.8072	-0.36788	0.04055	0.350598	protein_coding
ENSG0000(	ZNF443	333.4867	0.279536	0.040564	0.350611	protein_coding
ENSG0000(	KLRA1P	131.7793	0.296453	0.04061	0.350899	transcribed_unprocessed_pseudo
ENSG0000(	GTPBP1	3309.175	-0.20591	0.040653	0.350972	protein_coding
ENSG0000(	PDF	274.1935	0.276589	0.040645	0.350972	protein_coding
ENSG0000(	SLC22A1	87.98043	-0.42183	0.040657	0.350972	protein_coding
ENSG0000(	CSF1	897.487	-0.24995	0.040728	0.351196	protein_coding
ENSG0000(	ENSG0000(	61.79662	0.339259	0.040705	0.351196	lncRNA
ENSG0000(	NCOA3	3548.99	-0.1753	0.040724	0.351196	protein_coding
ENSG0000(	PARP9	2539.673	-0.43227	0.04074	0.351196	protein_coding
ENSG0000(	ZNF703	881.931	0.404146	0.040747	0.351196	protein_coding
ENSG0000(	BTNL8	905.9836	0.528402	0.040791	0.351469	protein_coding
ENSG0000(	SEPSECS-A:	2667.367	0.167856	0.04081	0.351519	lncRNA
ENSG0000(	LINC02408	161.4122	0.378722	0.04087	0.351926	lncRNA

ENSG0000(	ENSG0000(	35.04208	0.458634	0.040885	0.351946	lncRNA
ENSG0000(	ANGEL1	1145.241	-0.19265	0.040951	0.352037	protein_coding
ENSG0000(	ENSG0000(	81.34698	-0.40119	0.040954	0.352037	TEC
ENSG0000(	ENSG0000(	31.49175	0.472019	0.04097	0.352037	TEC
ENSG0000(	ENSG0000(	684.1472	-0.1527	0.041005	0.352037	lncRNA
ENSG0000(	ENSG0000(	33.5275	0.46547	0.04101	0.352037	lncRNA
ENSG0000(	ENSG0000(	212.4659	0.222074	0.040979	0.352037	lncRNA
ENSG0000(	FAM221A	1685.171	0.122329	0.040998	0.352037	protein_coding
ENSG0000(	SMG1P3	12908.92	0.201232	0.041005	0.352037	transcribed_unprocessed_pseudo
ENSG0000(	WNK1	5699.858	-0.16583	0.040929	0.352037	protein_coding
ENSG0000(	LINC00648	1487.523	0.261474	0.041058	0.352339	lncRNA
ENSG0000(	PIK3R5	767.6056	-0.41844	0.041089	0.352489	protein_coding
ENSG0000(	ANO5	534.0002	0.274667	0.041358	0.352961	protein_coding
ENSG0000(	CTIF	551.7041	-0.27415	0.041275	0.352961	protein_coding
ENSG0000(	EGFR-AS1	104.46	-0.418	0.04117	0.352961	lncRNA
ENSG0000(	ENSG0000(	37.18245	-0.39665	0.041328	0.352961	lncRNA
ENSG0000(	ENSG0000(	228.3068	-0.96813	0.041294	0.352961	processed_pseudogene
ENSG0000(	ENSG0000(	3114.402	-0.14687	0.041224	0.352961	lncRNA
ENSG0000(	ENSG0000(	559.5406	-0.22818	0.041361	0.352961	lncRNA
ENSG0000(	ENSG0000(	4277.78	-0.22542	0.041299	0.352961	TEC
ENSG0000(	ENSG0000(	527.0854	-0.1821	0.04134	0.352961	TEC
ENSG0000(	HHIPL1	315.3128	-0.3025	0.041179	0.352961	protein_coding
ENSG0000(	IGLV3-21	266.9085	-0.93925	0.041277	0.352961	IG_V_gene
ENSG0000(	LRTM1	248.5689	0.428225	0.0413	0.352961	protein_coding
ENSG0000(	OACYLP	32.42139	-0.92094	0.041303	0.352961	transcribed_unitary_pseudogene
ENSG0000(	PRRC2B	5497.921	-0.14795	0.041257	0.352961	protein_coding
ENSG0000(	RRM2	1208.301	-0.24355	0.041375	0.352961	protein_coding
ENSG0000(	RSBN1	919.7698	0.195775	0.041192	0.352961	protein_coding
ENSG0000(	SP1	5671.121	-0.21047	0.041362	0.352961	protein_coding
ENSG0000(	ZNF599	338.4029	0.138036	0.041309	0.352961	protein_coding
ENSG0000(	ENSG0000(	84.70877	-0.28912	0.041493	0.353858	lncRNA
ENSG0000(	TEX12	22.496	0.584445	0.041507	0.353873	protein_coding
ENSG0000(	PRO1804	142.4956	0.448322	0.041554	0.354162	TEC
ENSG0000(	B4GALT1-A	265.8191	0.210451	0.041576	0.354173	lncRNA
ENSG0000(	NTNG2	130.3682	-0.53132	0.041581	0.354173	protein_coding
ENSG0000(	JPH1	218.4935	0.411968	0.041595	0.35418	protein_coding
ENSG0000(	IGHJ3	105.0982	-0.68224	0.041656	0.35442	IG_J_gene
ENSG0000(	LINC00707	206.7914	-0.2072	0.041668	0.35442	lncRNA
ENSG0000(	RPS20P33	25.20529	0.499675	0.04168	0.35442	processed_pseudogene
ENSG0000(	SLC11A1	779.6474	-0.47679	0.041676	0.35442	protein_coding
ENSG0000(	ZNF280B	214.5263	0.247571	0.041687	0.35442	protein_coding
ENSG0000(	PGM5P2	463.5521	0.206373	0.041749	0.35483	transcribed_unprocessed_pseudo
ENSG0000(	ENSG0000(	93.56232	-0.40152	0.041765	0.354858	lncRNA
ENSG0000(	PPP2R2B	318.6608	-0.32494	0.041782	0.354892	protein_coding
ENSG0000(	PRKAG2-AS	31.17465	0.479619	0.041808	0.3549	lncRNA
ENSG0000(	SRSF6	5844.593	0.104502	0.041799	0.3549	protein_coding
ENSG0000(	KCTD11	237.8341	-0.29477	0.04183	0.354976	protein_coding

ENSG0000(	CSTF2T	570.4255	-0.20183	0.041947	0.35557	protein_coding
ENSG0000(	ENSG0000(	284.3164	-0.21778	0.041957	0.35557	lncRNA
ENSG0000(	IGLV9-49	22.20282	-1.21647	0.041963	0.35557	IG_V_gene
ENSG0000(	MRPS5	2543.758	0.102751	0.041974	0.35557	protein_coding
ENSG0000(	RS1	41.76994	-0.56585	0.04192	0.35557	protein_coding
ENSG0000(	SDCBP2	1056.115	0.517097	0.041978	0.35557	protein_coding
ENSG0000(	ENSG0000(	56.28294	0.586081	0.042012	0.355728	lncRNA
ENSG0000(	MTCL1	464.9598	-0.44209	0.04204	0.355728	protein_coding
ENSG0000(	PHF19	428.8499	-0.28432	0.042048	0.355728	protein_coding
ENSG0000(	PSMB6	312.5384	-0.20822	0.042026	0.355728	protein_coding
ENSG0000(	FXR1	1616.764	-0.16542	0.042115	0.356026	protein_coding
ENSG0000(	SELENOS	809.5614	-0.14596	0.042106	0.356026	protein_coding
ENSG0000(	SNORA60	47.48277	0.321233	0.042122	0.356026	snoRNA
ENSG0000(	GGA1	2588.09	0.097845	0.042155	0.356084	protein_coding
ENSG0000(	LYST	5000.833	-0.25031	0.042155	0.356084	protein_coding
ENSG0000(	ENSG0000(	811.0237	-0.1966	0.042189	0.356261	TEC
ENSG0000(	ENSG0000(	26.49344	0.422145	0.042237	0.356455	lncRNA
ENSG0000(	ENTPD4	3606.18	-0.16896	0.042238	0.356455	protein_coding
ENSG0000(	LAMP5	26.55274	-0.93324	0.042276	0.35667	protein_coding
ENSG0000(	GPX2	1055.792	-0.44158	0.042333	0.356724	protein_coding
ENSG0000(	HSD17B12	1452.208	-0.18561	0.042347	0.356724	protein_coding
ENSG0000(	IL20RB	98.73447	-0.36515	0.042309	0.356724	protein_coding
ENSG0000(	NUDT13	122.531	0.305428	0.042297	0.356724	protein_coding
ENSG0000(	ST6GALNA(	93.82895	-0.89102	0.042336	0.356724	protein_coding
ENSG0000(	ENSG0000(	109.2036	0.361965	0.042372	0.356821	protein_coding
ENSG0000(	ENSG0000(	262.3608	0.218517	0.042391	0.35687	lncRNA
ENSG0000(	ARGFX	1074.508	0.126487	0.042432	0.356938	protein_coding
ENSG0000(	ISYNA1	120.6156	-0.36363	0.042438	0.356938	protein_coding
ENSG0000(	MYRIP	248.4489	0.418457	0.042417	0.356938	protein_coding
ENSG0000(	NPIPB11	644.4529	0.318338	0.042464	0.356988	protein_coding
ENSG0000(	RN7SL819F	53.38268	0.342181	0.042469	0.356988	misc_RNA
ENSG0000(	CPA4	367.2183	0.272314	0.042562	0.35718	protein_coding
ENSG0000(	DGKI	106.5361	-0.74812	0.042511	0.35718	protein_coding
ENSG0000(	ENSG0000(	63.80121	0.501182	0.042558	0.35718	lncRNA
ENSG0000(	ENSG0000(	434.0295	-0.22464	0.042559	0.35718	lncRNA
ENSG0000(	GUCY1A2	220.5634	-0.56779	0.042596	0.35718	protein_coding
ENSG0000(	HDC	312.2094	-0.64022	0.042579	0.35718	protein_coding
ENSG0000(	IGKJ4	153.5227	-0.59527	0.042591	0.35718	IG_J_gene
ENSG0000(	TMEM98	975.304	0.222116	0.042527	0.35718	protein_coding
ENSG0000(	ENSG0000(	207.801	0.38947	0.042634	0.357279	lncRNA
ENSG0000(	ENSG0000(	41.3837	-0.37284	0.042622	0.357279	lncRNA
ENSG0000(	CTAGE6	22.72242	-1.42299	0.042679	0.357548	protein_coding
ENSG0000(	ENSG0000(	66.71996	-0.4016	0.042694	0.35756	lncRNA
ENSG0000(	B3GAT2	42.02927	0.290061	0.042716	0.357642	protein_coding
ENSG0000(	ACTR3	4397.125	-0.17294	0.042752	0.357829	protein_coding
ENSG0000(	CCDC167	31.68303	-0.45011	0.042771	0.357831	protein_coding
ENSG0000(	FARS2	331.709	0.219523	0.042791	0.357831	protein_coding

ENSG0000(	OSMR-DT	214.2699	-0.22102	0.042804	0.357831	lncRNA
ENSG0000(	STON1	538.701	-0.36838	0.042785	0.357831	protein_coding
ENSG0000(	DRAXIN	673.4123	-0.10757	0.042824	0.357891	protein_coding
ENSG0000(	PPP1R16B	1280.266	-0.43127	0.04284	0.357916	protein_coding
ENSG0000(	AIM2	161.1005	-0.459	0.042855	0.357928	protein_coding
ENSG0000(	ANKRD26P	24.13043	-1.15208	0.042905	0.357978	unprocessed_pseudogene
ENSG0000(	CACNA1E	185.1047	-0.9464	0.042926	0.357978	protein_coding
ENSG0000(	ENSG0000(	508.6665	-0.13996	0.042906	0.357978	TEC
ENSG0000(	OR2D2	26.30581	-0.70673	0.042924	0.357978	protein_coding
ENSG0000(	SYT17	421.2392	0.269042	0.042901	0.357978	protein_coding
ENSG0000(	KIF21B	1343.896	-0.22109	0.04294	0.35799	protein_coding
ENSG0000(	DAZAP1	3229.816	0.110318	0.042989	0.358089	protein_coding
ENSG0000(	F2RL2	220.4186	-0.37793	0.043	0.358089	protein_coding
ENSG0000(	RPN2	1145.392	-0.26372	0.043004	0.358089	protein_coding
ENSG0000(	WDR45	1022.818	-0.33841	0.042986	0.358089	protein_coding
ENSG0000(	AVPR1B	25.44719	0.701336	0.043021	0.358118	protein_coding
ENSG0000(	IRF8	1320.177	-0.29426	0.043041	0.35818	protein_coding
ENSG0000(	ABRAXAS1	985.4229	0.106791	0.04308	0.358382	protein_coding
ENSG0000(	NUS1	1140.415	-0.17233	0.043091	0.358382	protein_coding
ENSG0000(	DUSP5	829.1302	-0.46048	0.04312	0.358511	protein_coding
ENSG0000(	CDC25B	1069.847	-0.36401	0.043193	0.358647	protein_coding
ENSG0000(	ENSG0000(	30.75139	-0.42889	0.043198	0.358647	lncRNA
ENSG0000(	ENSG0000(	218.2745	0.345474	0.04323	0.358647	lncRNA
ENSG0000(	ENSG0000(	31.95789	-1.00018	0.043233	0.358647	lncRNA
ENSG0000(	FAM136A	724.0158	0.156465	0.04328	0.358647	protein_coding
ENSG0000(	FASTKD1	1456.247	0.256436	0.043197	0.358647	protein_coding
ENSG0000(	NBEAP2	24.47372	-0.72092	0.043241	0.358647	transcribed_unprocessed_pseudo
ENSG0000(	RNU6-1003	21.85899	-0.7908	0.043263	0.358647	snRNA
ENSG0000(	SNHG18	328.5392	0.365591	0.043271	0.358647	lncRNA
ENSG0000(	SPRY4	1418.656	-0.31832	0.043263	0.358647	protein_coding
ENSG0000(	ZNF804A	38.16902	-0.48065	0.043186	0.358647	protein_coding
ENSG0000(	PAIP1	419.7426	-0.25571	0.043327	0.358932	protein_coding
ENSG0000(	C5orf51	780.1151	-0.17885	0.043372	0.359138	protein_coding
ENSG0000(	DOC2A	332.1237	0.299366	0.043378	0.359138	protein_coding
ENSG0000(	TBCE	1562.255	0.107222	0.043419	0.359367	protein_coding
ENSG0000(	MAD2L1P1	21.28005	0.606617	0.043444	0.359465	processed_pseudogene
ENSG0000(	CRLS1	846.0349	0.191683	0.043465	0.359522	protein_coding
ENSG0000(	ENSG0000(	53.07505	0.331828	0.04349	0.359522	processed_pseudogene
ENSG0000(	MIF	144.5266	-0.37135	0.043485	0.359522	protein_coding
ENSG0000(	AP3D1	2400.379	-0.18989	0.043526	0.359606	protein_coding
ENSG0000(	ENSG0000(	47.38172	-0.47277	0.043618	0.359606	lncRNA
ENSG0000(	ENSG0000(	24.49619	0.606647	0.043588	0.359606	lncRNA
ENSG0000(	IGFLR1	250.1866	-0.23152	0.043567	0.359606	protein_coding
ENSG0000(	ITGB3	796.8327	-0.35574	0.043634	0.359606	protein_coding
ENSG0000(	LFNG	683.3827	0.290582	0.043657	0.359606	protein_coding
ENSG0000(	MTRNR2L6	20.65883	-0.67523	0.043596	0.359606	protein_coding
ENSG0000(	NOTCH3	1011.044	-0.23465	0.043569	0.359606	protein_coding

ENSG0000(	PPP4R1	1374.842	-0.21595	0.043651	0.359606	protein_coding
ENSG0000(	SIGLEC15	188.1079	-0.20905	0.043622	0.359606	protein_coding
ENSG0000(	SLCO4C1	139.7037	0.349996	0.043563	0.359606	protein_coding
ENSG0000(	ZNF90	573.3176	0.194849	0.043519	0.359606	protein_coding
ENSG0000(	GPNMB	974.5189	-0.38922	0.043682	0.359703	protein_coding
ENSG0000(	ACYP2	459.8446	-0.14833	0.043736	0.359848	protein_coding
ENSG0000(	ANKRD13D	922.2711	-0.20002	0.043817	0.359848	protein_coding
ENSG0000(	C3orf52	1601.252	0.387087	0.043746	0.359848	protein_coding
ENSG0000(	CD58	449.9267	-0.20007	0.043815	0.359848	protein_coding
ENSG0000(	DNAJC8	611.4594	-0.11604	0.043736	0.359848	protein_coding
ENSG0000(	ENSG0000(	153.7222	0.2459	0.043805	0.359848	lncRNA
ENSG0000(	HOXA-AS2	90.06505	0.351893	0.043812	0.359848	lncRNA
ENSG0000(	NDUFC2	418.0072	-0.34131	0.043803	0.359848	protein_coding
ENSG0000(	USP7	3115.351	0.140275	0.043757	0.359848	protein_coding
ENSG0000(	CAP2P1	127.7115	0.365627	0.043913	0.360138	transcribed_processed_pseudoge
ENSG0000(	CILK1	837.7763	0.187617	0.043936	0.360138	protein_coding
ENSG0000(	CLEC12A	142.367	-0.53383	0.043937	0.360138	protein_coding
ENSG0000(	LERFS	189.0815	0.543711	0.043944	0.360138	lncRNA
ENSG0000(	MAGOH2P	36.70429	0.625598	0.043957	0.360138	transcribed_processed_pseudoge
ENSG0000(	OPRK1	96.91807	0.291116	0.04395	0.360138	protein_coding
ENSG0000(	PCDHGA7	66.74083	0.437996	0.043915	0.360138	protein_coding
ENSG0000(	UGCG	1632.613	-0.26441	0.043893	0.360138	protein_coding
ENSG0000(	FMNL1	1152.154	-0.33654	0.043977	0.360191	protein_coding
ENSG0000(	CCDC51	84.30158	-0.24758	0.044038	0.360268	protein_coding
ENSG0000(	ENSG0000(	547.6939	0.187113	0.044036	0.360268	lncRNA
ENSG0000(	LHFPL6	373.5413	-0.34611	0.044014	0.360268	protein_coding
ENSG0000(	PRKAR1A	3189.526	-0.20371	0.044029	0.360268	protein_coding
ENSG0000(	EP400P1	1805.201	-0.21277	0.044056	0.360302	transcribed_unprocessed_pseudo
ENSG0000(	DUOXA1	42.46979	-0.76752	0.044138	0.360546	protein_coding
ENSG0000(	EFNB3	29.56717	-0.65896	0.044135	0.360546	protein_coding
ENSG0000(	HILPDA	314.1609	-0.26164	0.044131	0.360546	protein_coding
ENSG0000(	LONRF3	210.2644	0.343017	0.044116	0.360546	protein_coding
ENSG0000(	ELK1	366.8962	-0.24526	0.044192	0.360715	protein_coding
ENSG0000(	GID8	765.8114	0.197109	0.044201	0.360715	protein_coding
ENSG0000(	LINC01541	127.5445	0.301638	0.044192	0.360715	lncRNA
ENSG0000(	SPACA7	33.74168	0.348057	0.044211	0.360715	protein_coding
ENSG0000(	TRPM4	5355.963	0.443532	0.044271	0.361097	protein_coding
ENSG0000(	ZNF526	644.7946	0.146832	0.044288	0.36113	protein_coding
ENSG0000(	ENSG0000(	54.31185	-0.32521	0.044346	0.361172	lncRNA
ENSG0000(	KCNS2	42.54445	0.63916	0.044331	0.361172	protein_coding
ENSG0000(	MRPL37P1	111.973	-0.32536	0.044343	0.361172	processed_pseudogene
ENSG0000(	TBC1D32	1072.742	0.19783	0.044345	0.361172	protein_coding
ENSG0000(	CCDC153	72.63443	-0.2892	0.044364	0.361217	protein_coding
ENSG0000(	UGT1A8	206.4353	0.773983	0.044406	0.36145	protein_coding
ENSG0000(	ADCY5	386.8067	0.381476	0.044434	0.361467	protein_coding
ENSG0000(	ARHGAP25	823.1452	-0.33698	0.044424	0.361467	protein_coding
ENSG0000(	ENSG0000(	188.266	0.231778	0.044454	0.36148	lncRNA

ENSG0000(	LINC00907	540.1218	0.170887	0.044462	0.36148	lncRNA
ENSG0000(	ETV5	232.7443	-0.34513	0.044507	0.361734	protein_coding
ENSG0000(	ENSG0000(	237.1347	0.197031	0.044562	0.362067	lncRNA
ENSG0000(	ST6GALNA(	272.7641	-0.39507	0.044574	0.362067	protein_coding
ENSG0000(	TMEM234	577.277	0.175844	0.044596	0.362139	protein_coding
ENSG0000(	CLSTN3	1116.315	-0.35623	0.044623	0.362198	protein_coding
ENSG0000(	GAPDHP47	20.4995	-0.61216	0.04463	0.362198	processed_pseudogene
ENSG0000(	TTC38	1051.477	0.208526	0.044765	0.363188	protein_coding
ENSG0000(	TNFRSF21	1857.48	0.322102	0.044797	0.363342	protein_coding
ENSG0000(	ENSG0000(	99.81678	-0.30833	0.044863	0.363663	TEC
ENSG0000(	UBA6	2223.219	-0.14091	0.044856	0.363663	protein_coding
ENSG0000(	KCNJ3	286.1341	0.245838	0.044882	0.363679	protein_coding
ENSG0000(	ZNF571-AS	323.016	0.181419	0.044891	0.363679	lncRNA
ENSG0000(	AKR1B1	764.6923	-0.21052	0.045017	0.363744	protein_coding
ENSG0000(	ARID3A	1181.301	-0.23455	0.045004	0.363744	protein_coding
ENSG0000(	ENSG0000(	542.6031	0.143692	0.045007	0.363744	lncRNA
ENSG0000(	ENSG0000(	52.05071	-0.3457	0.045005	0.363744	lncRNA
ENSG0000(	GAL3ST4	168.1788	0.337779	0.044919	0.363744	protein_coding
ENSG0000(	MAP3K21	851.89	0.406676	0.045019	0.363744	protein_coding
ENSG0000(	OSCAR	66.55412	-0.55452	0.044979	0.363744	protein_coding
ENSG0000(	RWDD3	345.862	-0.17125	0.044952	0.363744	protein_coding
ENSG0000(	TMPRSS4	5741.108	0.419961	0.04499	0.363744	protein_coding
ENSG0000(	ENSG0000(	42.2398	-0.38882	0.04506	0.363915	lncRNA
ENSG0000(	LCK	373.124	-0.2982	0.045066	0.363915	protein_coding
ENSG0000(	DPYSL3	1141.103	-0.50155	0.045105	0.364051	protein_coding
ENSG0000(	LINC00641	1861.381	-0.19961	0.045123	0.364051	lncRNA
ENSG0000(	RTKN	510.3088	0.277559	0.045117	0.364051	protein_coding
ENSG0000(	ENSG0000(	183.1553	0.204388	0.045235	0.364833	transcribed_processed_pseudoge
ENSG0000(	IZUMO2	328.2832	0.14274	0.045254	0.364833	protein_coding
ENSG0000(	OTULIN	2637.431	-0.13879	0.045282	0.364833	protein_coding
ENSG0000(	P4HB	5358.185	-0.24166	0.045265	0.364833	protein_coding
ENSG0000(	SLC12A8	542.8036	0.318799	0.045286	0.364833	protein_coding
ENSG0000(	CLEC7A	897.4987	-0.33847	0.045429	0.365135	protein_coding
ENSG0000(	ENSG0000(	178.1619	-0.19687	0.045378	0.365135	lncRNA
ENSG0000(	ENSG0000(	114.6134	0.248793	0.045371	0.365135	TEC
ENSG0000(	ENSG0000(	1611.343	-0.1392	0.04543	0.365135	lncRNA
ENSG0000(	NBAT1	27.48937	-0.55146	0.045406	0.365135	lncRNA
ENSG0000(	NR1H3	1065.37	-0.33084	0.045386	0.365135	protein_coding
ENSG0000(	PHOSPHO1	20.80145	-0.98575	0.045423	0.365135	protein_coding
ENSG0000(	TGFBR2	3346.659	-0.2439	0.045367	0.365135	protein_coding
ENSG0000(	ZSWIM8-A!	44.07112	-0.77862	0.04551	0.365671	lncRNA
ENSG0000(	ARHGDIB	963.4515	-0.42827	0.045538	0.365684	protein_coding
ENSG0000(	ENSG0000(	425.2475	0.136599	0.045529	0.365684	lncRNA
ENSG0000(	ENSG0000(	80.62621	0.34431	0.045568	0.365723	lncRNA
ENSG0000(	MUC2	37659.5	0.64527	0.045569	0.365723	protein_coding
ENSG0000(	KLHL5	2023.376	-0.35572	0.045586	0.365753	protein_coding
ENSG0000(	LINC02371	348.7214	-0.15053	0.045618	0.365898	lncRNA

ENSG0000(	APH1B	365.0423	-0.34293	0.045688	0.366349	protein_coding
ENSG0000(	CD34	842.9087	-0.31521	0.04574	0.366349	protein_coding
ENSG0000(	ENSG0000(	85.0009	-0.54387	0.045713	0.366349	lncRNA
ENSG0000(	KMT5AP3	42.65666	0.676759	0.04574	0.366349	processed_pseudogene
ENSG0000(	STX6	1071.919	-0.18694	0.045725	0.366349	protein_coding
ENSG0000(	ACVR2A	655.3484	0.308512	0.045825	0.366465	protein_coding
ENSG0000(	APBB1IP	496.3094	-0.43883	0.045838	0.366465	protein_coding
ENSG0000(	CFP	407.6729	-0.2778	0.045857	0.366465	protein_coding
ENSG0000(	DHRS3	664.5521	0.246464	0.045843	0.366465	protein_coding
ENSG0000(	ENSG0000(	31.35738	0.358001	0.045847	0.366465	lncRNA
ENSG0000(	ENSG0000(	2038.16	-0.16677	0.045802	0.366465	lncRNA
ENSG0000(	OR52N1	152.85	-0.56482	0.045861	0.366465	protein_coding
ENSG0000(	PTPN21	1074.236	0.335752	0.045857	0.366465	protein_coding
ENSG0000(	MICALL1	1031.705	-0.12262	0.04588	0.366508	protein_coding
ENSG0000(	CCDC175	26.66002	0.943891	0.046024	0.367121	protein_coding
ENSG0000(	CCP110	1306.741	-0.22165	0.045971	0.367121	protein_coding
ENSG0000(	MAT1A	33.40807	-1.20949	0.046015	0.367121	protein_coding
ENSG0000(	PRX	170.6032	-0.37034	0.046005	0.367121	protein_coding
ENSG0000(	RILPL2	129.2354	-0.47645	0.046012	0.367121	protein_coding
ENSG0000(	ENSG0000(	347.792	0.192211	0.04604	0.367149	lncRNA
ENSG0000(	C15orf62	20.9018	-0.53719	0.046116	0.367218	protein_coding
ENSG0000(	DIPK1A	263.1739	-0.30565	0.046091	0.367218	protein_coding
ENSG0000(	ONECUT3	321.3963	-0.1718	0.046063	0.367218	protein_coding
ENSG0000(	SP3	4226.314	-0.15622	0.046076	0.367218	protein_coding
ENSG0000(	TMPRSS3	112.5444	-0.44799	0.046109	0.367218	protein_coding
ENSG0000(	NCSTN	903.1534	-0.12827	0.0462	0.367784	protein_coding
ENSG0000(	VSIR	1108.406	-0.2911	0.046246	0.36804	protein_coding
ENSG0000(	PATJ	5449.144	0.293212	0.046302	0.36838	protein_coding
ENSG0000(	BID	654.688	-0.17459	0.046428	0.368729	protein_coding
ENSG0000(	CACUL1	2700.495	-0.14989	0.046368	0.368729	protein_coding
ENSG0000(	CHGA	506.0871	0.677152	0.046407	0.368729	protein_coding
ENSG0000(	CYTIP	1002.876	-0.45548	0.046457	0.368729	protein_coding
ENSG0000(	ENSG0000(	286.133	-0.19738	0.046374	0.368729	lncRNA
ENSG0000(	ENSG0000(	401.6527	-0.15757	0.046455	0.368729	protein_coding
ENSG0000(	LINC01091	469.7565	0.176994	0.046452	0.368729	lncRNA
ENSG0000(	SVOP	491.7317	0.164263	0.046402	0.368729	protein_coding
ENSG0000(	TRIO	7743.565	0.12714	0.046466	0.368729	protein_coding
ENSG0000(	DEPDC4	478.8747	0.184237	0.046521	0.369054	protein_coding
ENSG0000(	ELAPOR1	1746.29	0.410874	0.046544	0.369107	protein_coding
ENSG0000(	ENSG0000(	49.87179	0.253835	0.046595	0.369107	lncRNA
ENSG0000(	LINC02899	192.6104	0.409334	0.046569	0.369107	lncRNA
ENSG0000(	PDHA1	1453.505	0.263834	0.04657	0.369107	protein_coding
ENSG0000(	RN7SL3	4529.514	0.596429	0.046594	0.369107	misc_RNA
ENSG0000(	ANK1	146.3191	-0.51532	0.046827	0.369463	protein_coding
ENSG0000(	CATSPERG	624.6582	0.253536	0.046813	0.369463	protein_coding
ENSG0000(	DALRD3	159.7091	0.271527	0.046804	0.369463	protein_coding
ENSG0000(	ENSG0000(	145.4105	0.177625	0.046689	0.369463	lncRNA

ENSG0000(	ENSG0000(	141.7324	0.390905	0.04675	0.369463	lncRNA
ENSG0000(	HYPK	1943.819	0.114369	0.046701	0.369463	protein_coding
ENSG0000(	JPT2	924.6654	0.183346	0.046819	0.369463	protein_coding
ENSG0000(	LENG8	10978.02	0.119496	0.046733	0.369463	protein_coding
ENSG0000(	LINC01342	137.0755	0.530396	0.046725	0.369463	lncRNA
ENSG0000(	MBD4	779.3317	-0.20492	0.046719	0.369463	protein_coding
ENSG0000(	PHLPP1	863.8164	0.193452	0.046659	0.369463	protein_coding
ENSG0000(	PTGR2	1100.585	0.178934	0.046816	0.369463	protein_coding
ENSG0000(	SNORD116	22.47488	-0.68838	0.046775	0.369463	snoRNA
ENSG0000(	VKORC1	129.6163	-0.31541	0.046806	0.369463	protein_coding
ENSG0000(	PIGN	4875.538	0.116282	0.046848	0.369522	protein_coding
ENSG0000(	SECTM1	631.8752	-0.53071	0.046863	0.36953	protein_coding
ENSG0000(	ENSG0000(	46.08817	0.379447	0.046907	0.369727	TEC
ENSG0000(	LRRC75A	1277.156	0.515543	0.046915	0.369727	protein_coding
ENSG0000(	C11orf96	180.2289	-0.54717	0.046984	0.369783	protein_coding
ENSG0000(	CCN2	786.5663	-0.64079	0.046975	0.369783	protein_coding
ENSG0000(	ENSG0000(	30.57567	0.425639	0.046989	0.369783	processed_pseudogene
ENSG0000(	MIR3945H(	152.2022	-0.53314	0.046967	0.369783	lncRNA
ENSG0000(	STON2	1829.922	0.260514	0.046941	0.369783	protein_coding
ENSG0000(	MARVELD1	450.9718	0.316526	0.047055	0.370188	protein_coding
ENSG0000(	SLCO1C1	62.54898	0.592469	0.047067	0.370188	protein_coding
ENSG0000(	TMEM44	972.8491	0.248177	0.047109	0.370408	protein_coding
ENSG0000(	SERPINE2	716.5242	-0.6064	0.047127	0.370446	protein_coding
ENSG0000(	RASGEF1B	1809.862	-0.26592	0.047154	0.370551	protein_coding
ENSG0000(	LINC01370	326.0444	-0.21645	0.047168	0.370557	lncRNA
ENSG0000(	NXPE3	1584.4	-0.22735	0.047198	0.370686	protein_coding
ENSG0000(	ARPC2	4777.19	-0.16664	0.047223	0.370777	protein_coding
ENSG0000(	EDEM1	3144.899	-0.22899	0.047268	0.370812	protein_coding
ENSG0000(	PSME3	969.5498	-0.15551	0.047259	0.370812	protein_coding
ENSG0000(	TM2D3	1188.492	-0.11952	0.047254	0.370812	protein_coding
ENSG0000(	CARNS1	90.73838	-0.47796	0.047325	0.370945	protein_coding
ENSG0000(	LIMD2	685.1872	-0.46956	0.047315	0.370945	protein_coding
ENSG0000(	TESK2	295.1243	0.307408	0.047317	0.370945	protein_coding
ENSG0000(	ENSG0000(	362.5254	-0.27562	0.047355	0.370953	lncRNA
ENSG0000(	ENSG0000(	688.0628	-0.2377	0.047358	0.370953	transcribed_unprocessed_pseudo
ENSG0000(	TMTC2	969.0742	0.189403	0.047367	0.370953	protein_coding
ENSG0000(	SLC25A51P	327.8893	-0.13992	0.047424	0.371254	transcribed_unprocessed_pseudo
ENSG0000(	TMEM86A	187.0812	-0.36598	0.047432	0.371254	protein_coding
ENSG0000(	ENSG0000(	28.71699	0.60159	0.047457	0.371264	lncRNA
ENSG0000(	LINC01716	32.18029	0.40535	0.047474	0.371264	lncRNA
ENSG0000(	LUC7L3	4716.671	0.156109	0.047461	0.371264	protein_coding
ENSG0000(	ADAM8	399.1522	-0.56589	0.047548	0.371289	protein_coding
ENSG0000(	ENSG0000(	43.49299	-0.49146	0.04754	0.371289	lncRNA
ENSG0000(	ESRRA	1616.725	0.345052	0.047552	0.371289	protein_coding
ENSG0000(	GOLGA8B	3628.634	-0.33082	0.047514	0.371289	protein_coding
ENSG0000(	OXTR	125.4949	-0.3075	0.047558	0.371289	protein_coding
ENSG0000(	SUMF1	750.1139	0.156319	0.047554	0.371289	protein_coding

ENSG0000(	CERCAM	470.3619	-0.23452	0.047606	0.371454	protein_coding
ENSG0000(	DEGS1	421.8548	-0.27578	0.0476	0.371454	protein_coding
ENSG0000(	BARD1	792.5518	-0.29743	0.04769	0.371467	protein_coding
ENSG0000(	ENSG0000(	392.2553	0.273011	0.047703	0.371467	lncRNA
ENSG0000(	EVC	661.4072	-0.33086	0.047667	0.371467	protein_coding
ENSG0000(	MAMDC4	645.8973	0.372523	0.047694	0.371467	protein_coding
ENSG0000(	RNU7-45P	80.67146	-0.39714	0.047657	0.371467	snRNA
ENSG0000(	SIL1	466.8717	-0.19263	0.047667	0.371467	protein_coding
ENSG0000(	SPIN1	1888.557	0.174076	0.047676	0.371467	protein_coding
ENSG0000(	ENSG0000(	145.4105	-0.22391	0.047725	0.371499	TEC
ENSG0000(	ID3	540.692	0.351707	0.047744	0.371499	protein_coding
ENSG0000(	SAFB	1671.276	0.137246	0.047747	0.371499	protein_coding
ENSG0000(	ENSG0000(	111.7384	0.259349	0.047773	0.371592	lncRNA
ENSG0000(	DDO	43.5406	0.489702	0.047796	0.371637	protein_coding
ENSG0000(	ENSG0000(	51.79713	-0.58762	0.047805	0.371637	TEC
ENSG0000(	MAP2K1	802.2655	-0.20497	0.047826	0.371652	protein_coding
ENSG0000(	RN7SL718F	1199.585	0.222694	0.047847	0.371652	misc_RNA
ENSG0000(	RNU6-43P	22.29687	-0.48548	0.047848	0.371652	snRNA
ENSG0000(	LINC01291	70.50738	-0.82671	0.047942	0.37228	lncRNA
ENSG0000(	ENSG0000(	155.1856	-0.48689	0.047986	0.372512	lncRNA
ENSG0000(	RELT	769.6556	-0.46238	0.048002	0.372535	protein_coding
ENSG0000(	CHAD	124.195	0.740663	0.048048	0.372786	protein_coding
ENSG0000(	ARHGEF17	1308.458	-0.29807	0.048101	0.373091	protein_coding
ENSG0000(	ENSG0000(	220.5287	0.419726	0.048169	0.373147	lncRNA
ENSG0000(	ENSG0000(	61.58594	-0.32291	0.04819	0.373147	lncRNA
ENSG0000(	IGLC1	3348.225	-0.69149	0.048181	0.373147	IG_C_gene
ENSG0000(	KIAA1755	737.1242	-0.40847	0.048138	0.373147	protein_coding
ENSG0000(	LINC00470	469.5206	0.195041	0.048173	0.373147	lncRNA
ENSG0000(	SRM	207.0985	-0.24146	0.048169	0.373147	protein_coding
ENSG0000(	IGHJ3P	473.9207	-0.45008	0.048216	0.373249	IG_J_pseudogene
ENSG0000(	ANKRD44-I	556.0315	-0.32356	0.048272	0.373472	lncRNA
ENSG0000(	WWTR1-AS	93.15982	0.227761	0.048267	0.373472	lncRNA
ENSG0000(	ENSG0000(	72.49579	-0.35968	0.048293	0.373525	TEC
ENSG0000(	TMEM168	2485.63	0.20059	0.048306	0.373526	protein_coding
ENSG0000(	CRIP2	193.5173	-0.41832	0.048363	0.373847	protein_coding
ENSG0000(	NAA35	1267.463	0.135702	0.048389	0.373847	protein_coding
ENSG0000(	PMS2P1	2497.312	0.087849	0.048378	0.373847	transcribed_unprocessed_pseudo
ENSG0000(	ENSG0000(	27.39398	0.627612	0.048417	0.373852	lncRNA
ENSG0000(	FURIN	1273.201	-0.29154	0.048413	0.373852	protein_coding
ENSG0000(	ENSG0000(	604.3143	0.400157	0.048453	0.374026	lncRNA
ENSG0000(	DUS1L	902.3391	0.223729	0.04866	0.374334	protein_coding
ENSG0000(	ENSG0000(	20.43861	-0.50957	0.048645	0.374334	lncRNA
ENSG0000(	ENSG0000(	177.4009	-0.29231	0.048668	0.374334	lncRNA
ENSG0000(	ENSG0000(	124.2092	-0.34817	0.04863	0.374334	lncRNA
ENSG0000(	GUCY2C	1125.57	0.439966	0.048578	0.374334	protein_coding
ENSG0000(	IGLV5-45	64.7227	-0.72393	0.048623	0.374334	IG_V_gene
ENSG0000(	PDXDC2P	589.5129	0.263355	0.048669	0.374334	transcribed_unprocessed_pseudo

ENSG0000(	POPDC2	88.0857	-0.38952	0.048594	0.374334	protein_coding
ENSG0000(	PSMC3	264.5236	-0.24526	0.048563	0.374334	protein_coding
ENSG0000(	RN7SL391P	77.74153	0.325791	0.048646	0.374334	misc_RNA
ENSG0000(	SGSM1	377.6371	0.397317	0.048666	0.374334	protein_coding
ENSG0000(	SNX14	5761.294	0.066744	0.048563	0.374334	protein_coding
ENSG0000(	TSPAN9-IT1	20.43411	-0.78582	0.048631	0.374334	lncRNA
ENSG0000(	GUSBP3	1567.866	0.21431	0.048708	0.374523	transcribed_unprocessed_pseudo
ENSG0000(	DNPEP	1357.22	0.189242	0.048748	0.374711	protein_coding
ENSG0000(	FBXL13	64.26285	-0.61033	0.048765	0.374711	protein_coding
ENSG0000(	FKBP4	684.9385	0.230379	0.048773	0.374711	protein_coding
ENSG0000(	ENSG0000(	28.46712	-0.60715	0.048804	0.374842	lncRNA
ENSG0000(	LINC00667	2118.282	0.170449	0.048827	0.374919	lncRNA
ENSG0000(	ARL8A	178.9014	-0.2877	0.048888	0.375177	protein_coding
ENSG0000(	IFT57	351.1362	0.309186	0.048884	0.375177	protein_coding
ENSG0000(	TFE3	949.4486	-0.21412	0.048962	0.375536	protein_coding
ENSG0000(	ZNF529	973.965	0.142503	0.048958	0.375536	protein_coding
ENSG0000(	ERN1	4559.809	-0.25404	0.048977	0.375544	protein_coding
ENSG0000(	ENSG0000(	415.5803	0.213845	0.048999	0.375611	transcribed_unprocessed_pseudo
ENSG0000(	CHD9	6738.115	0.136048	0.049022	0.375679	protein_coding
ENSG0000(	ENSG0000(	19.78509	0.56201	0.049054	0.375824	lncRNA
ENSG0000(	C4BPA	73.03278	-0.79168	0.049074	0.375866	protein_coding
ENSG0000(	ENSG0000(	37.54918	0.357213	0.0491	0.375963	lncRNA
ENSG0000(	MYO19	2723.813	0.178124	0.049137	0.376143	protein_coding
ENSG0000(	BNC2	1105.467	-0.41738	0.049188	0.37632	protein_coding
ENSG0000(	ENSG0000(	323.7432	-0.24344	0.049184	0.37632	lncRNA
ENSG0000(	CEP57L1	2490.284	-0.09991	0.04923	0.376331	protein_coding
ENSG0000(	ESR2	1073.739	-0.2377	0.04922	0.376331	protein_coding
ENSG0000(	ZFTA	415.8837	-0.28986	0.049228	0.376331	protein_coding
ENSG0000(	LINC01474	79.36804	-0.33937	0.04925	0.376379	lncRNA
ENSG0000(	ANXA6	1116.932	-0.35207	0.049275	0.376385	protein_coding
ENSG0000(	CNTNAP1	317.2934	-0.23511	0.049278	0.376385	protein_coding
ENSG0000(	ASAH2	336.9605	0.465934	0.049315	0.376508	protein_coding
ENSG0000(	ENSG0000(	222.8915	-0.20718	0.049327	0.376508	lncRNA
ENSG0000(	IKZF1	2939.529	-0.41817	0.049337	0.376508	protein_coding
ENSG0000(	POU6F2	96.6419	0.352466	0.049349	0.376508	protein_coding
ENSG0000(	ZCCHC17	206.5874	-0.18012	0.049397	0.376767	protein_coding
ENSG0000(	FES	291.1735	-0.30685	0.049421	0.376852	protein_coding
ENSG0000(	FRG1GP	24.06603	-0.7273	0.049455	0.377003	unprocessed_pseudogene
ENSG0000(	APOF	64.09664	0.259223	0.049527	0.377449	protein_coding
ENSG0000(	ENSG0000(	506.9176	-0.22161	0.049598	0.377883	lncRNA
ENSG0000(	OR4F13P	53.9671	-0.32118	0.049634	0.378053	transcribed_unprocessed_pseudo
ENSG0000(	ARID5B	2458.269	-0.28869	0.049713	0.37822	protein_coding
ENSG0000(	ENSG0000(	28.35459	0.614398	0.049722	0.37822	lncRNA
ENSG0000(	ENSG0000(	24.94823	0.418472	0.049725	0.37822	lncRNA
ENSG0000(	LINC00824	171.1726	0.202747	0.049674	0.37822	lncRNA
ENSG0000(	PRKCB	1755.515	-0.40116	0.049685	0.37822	protein_coding
ENSG0000(	MSL3	1397.476	-0.21856	0.049772	0.37837	protein_coding

ENSG0000(	SYNGAP1	977.5405	-0.48889	0.049765	0.37837	protein_coding
ENSG0000(	ADAMTSL4	134.3952	-0.37588	0.049913	0.378396	lncRNA
ENSG0000(	EHD1	1113.93	-0.23469	0.049853	0.378396	protein_coding
ENSG0000(	ENSG0000(	23.5724	-0.39976	0.049898	0.378396	lncRNA
ENSG0000(	IQCB1	756.1507	-0.14824	0.049801	0.378396	protein_coding
ENSG0000(	ROBO4	319.7042	-0.38423	0.049907	0.378396	protein_coding
ENSG0000(	RORC	648.1472	0.261235	0.049815	0.378396	protein_coding
ENSG0000(	ST8SIA4	1189.469	-0.33829	0.049837	0.378396	protein_coding
ENSG0000(	SUGT1P4-S	81.73362	0.60361	0.049877	0.378396	lncRNA
ENSG0000(	TTC39A-AS	50.68237	0.580328	0.049885	0.378396	lncRNA
ENSG0000(	ZNF775	67.3472	0.446136	0.049836	0.378396	protein_coding
ENSG0000(	ENSG0000(	47.30525	0.421818	0.049935	0.37846	lncRNA
ENSG0000(	BEX3	106.2953	-0.35197	0.049958	0.37853	protein_coding
ENSG0000(	ENSG0000(	34.12363	-0.4305	0.049976	0.378565	lncRNA
ENSG0000(	CACNA1C	1312.706	-0.44387	0.049992	0.378579	protein_coding
ENSG0000(	ENSG0000(	256.8886	0.20583	0.050083	0.378693	lncRNA
ENSG0000(	GEMIN6	1079.572	0.11642	0.050074	0.378693	protein_coding
ENSG0000(	IGHV3-33	661.4973	-0.83935	0.050029	0.378693	IG_V_gene
ENSG0000(	ITPA	299.827	0.181606	0.050044	0.378693	protein_coding
ENSG0000(	MIR429	30.18439	0.658892	0.05009	0.378693	miRNA
ENSG0000(	NTPCR	1664.023	0.220304	0.050074	0.378693	protein_coding
ENSG0000(	ATG4B	1323.183	0.167039	0.050156	0.379094	protein_coding
ENSG0000(	PARP2	304.4555	-0.17368	0.050186	0.379215	protein_coding
ENSG0000(	BISPR	555.292	-0.28353	0.050234	0.379471	lncRNA
ENSG0000(	RN7SL329F	77.49543	-0.26569	0.050272	0.379655	misc_RNA
ENSG0000(	ENSG0000(	26.37707	-0.56588	0.050294	0.379675	lncRNA
ENSG0000(	ROBO3	183.5782	-0.38127	0.050302	0.379675	protein_coding
ENSG0000(	ACAD10	1460.025	0.138664	0.050476	0.379793	protein_coding
ENSG0000(	ALS2CL	2254.101	0.285966	0.050519	0.379793	protein_coding
ENSG0000(	ARNTL	792.0286	-0.28807	0.050364	0.379793	protein_coding
ENSG0000(	DEK	2376.745	-0.13306	0.050468	0.379793	protein_coding
ENSG0000(	ENSG0000(	1392.663	0.178246	0.050397	0.379793	protein_coding
ENSG0000(	ENSG0000(	42.89325	-0.79322	0.050519	0.379793	lncRNA
ENSG0000(	ENSG0000(	114.2495	0.507056	0.050525	0.379793	processed_pseudogene
ENSG0000(	ENSG0000(	154.2532	0.254293	0.050445	0.379793	lncRNA
ENSG0000(	ENSG0000(	79.69026	-0.45552	0.050407	0.379793	lncRNA
ENSG0000(	ENSG0000(	57.45344	-0.39605	0.050402	0.379793	lncRNA
ENSG0000(	FOXA2	113.7323	0.588002	0.050491	0.379793	protein_coding
ENSG0000(	IRAG1	1024.12	-0.29926	0.05049	0.379793	protein_coding
ENSG0000(	MGAT4B	2781.713	0.350589	0.050481	0.379793	protein_coding
ENSG0000(	TPM4	8825.15	-0.26026	0.050495	0.379793	protein_coding
ENSG0000(	USP13	1465.418	-0.45848	0.05045	0.379793	protein_coding
ENSG0000(	AKAP9	11731.41	0.139056	0.050543	0.379802	protein_coding
ENSG0000(	INSR	3964.715	0.270703	0.050554	0.379802	protein_coding
ENSG0000(	KCND3	776.9816	-0.41219	0.050629	0.380266	protein_coding
ENSG0000(	LINC02613	281.9132	0.197097	0.050648	0.380302	lncRNA
ENSG0000(	ZFX	2058.686	-0.1929	0.050665	0.380325	protein_coding

ENSG0000(	ENSG0000(	98.89008	-0.46675	0.050713	0.380468	lncRNA
ENSG0000(	KLHDC2	966.7462	0.2129	0.050718	0.380468	protein_coding
ENSG0000(	MNX1	371.8122	0.569221	0.050725	0.380468	protein_coding
ENSG0000(	COL14A1	1243.355	-0.48648	0.050744	0.380504	protein_coding
ENSG0000(	ENSG0000(	21.6642	0.400094	0.050776	0.380538	lncRNA
ENSG0000(	FLI1	1124.648	-0.28723	0.050772	0.380538	protein_coding
ENSG0000(	AGER	158.2076	-0.38461	0.050816	0.380631	protein_coding
ENSG0000(	ENSG0000(	599.9173	-0.18689	0.050804	0.380631	protein_coding
ENSG0000(	FOXO4	344.2474	0.292308	0.050834	0.380657	protein_coding
ENSG0000(	FAM3B	458.6143	-0.69804	0.051069	0.382315	protein_coding
ENSG0000(	ENSG0000(	326.4268	0.200398	0.051213	0.383235	lncRNA
ENSG0000(	ENSG0000(	323.0047	0.275697	0.05122	0.383235	lncRNA
ENSG0000(	OPHN1	6679.998	0.160636	0.05126	0.38339	protein_coding
ENSG0000(	ZNF507	1974.763	0.120488	0.051268	0.38339	protein_coding
ENSG0000(	DNAH11	316.9192	-0.165	0.051325	0.383454	protein_coding
ENSG0000(	S100A4	107.8532	-0.60923	0.051333	0.383454	protein_coding
ENSG0000(	SLC25A39	931.2541	0.16925	0.051327	0.383454	protein_coding
ENSG0000(	VAMP4	576.5991	-0.1635	0.051304	0.383454	protein_coding
ENSG0000(	AJM1	328.8327	0.377421	0.051397	0.383644	protein_coding
ENSG0000(	ATP6V1C1	1111.801	-0.19446	0.051427	0.383644	protein_coding
ENSG0000(	GRIP2	152.7709	-0.50437	0.051431	0.383644	protein_coding
ENSG0000(	IFIT2	543.8504	-0.89426	0.051442	0.383644	protein_coding
ENSG0000(	PARD6B	809.8212	0.236833	0.05144	0.383644	protein_coding
ENSG0000(	UFL1	692.8462	-0.23336	0.051404	0.383644	protein_coding
ENSG0000(	PITPNB	1141.567	-0.14304	0.051469	0.383743	protein_coding
ENSG0000(	TMEM63C	224.6539	0.760575	0.051501	0.383776	protein_coding
ENSG0000(	WDR70	779.0681	0.222074	0.0515	0.383776	protein_coding
ENSG0000(	CD55	2354.798	-0.5861	0.051543	0.383985	protein_coding
ENSG0000(	ADCK5	220.902	0.351313	0.051561	0.384015	protein_coding
ENSG0000(	CD276	555.3039	-0.3714	0.0516	0.384204	protein_coding
ENSG0000(	ENSG0000(	109.7999	-0.64828	0.051618	0.384231	transcribed_processed_pseudoge
ENSG0000(	NARF	1408.819	-0.12657	0.051676	0.384554	protein_coding
ENSG0000(	OR8A1	545.1983	-0.13995	0.051713	0.384728	protein_coding
ENSG0000(	TRPC1	160.4254	-0.36348	0.051762	0.384993	protein_coding
ENSG0000(	MS4A4E	1367.252	0.142143	0.051783	0.385038	protein_coding
ENSG0000(	ABHD15	554.283	0.14817	0.051827	0.385159	protein_coding
ENSG0000(	SORBS3	657.8265	0.313696	0.051818	0.385159	protein_coding
ENSG0000(	CDK17	1217.181	-0.23544	0.051903	0.385618	protein_coding
ENSG0000(	ENSG0000(	1362.417	-0.48905	0.051919	0.385639	lncRNA
ENSG0000(	IRF4	2342.963	-0.45161	0.051943	0.385713	protein_coding
ENSG0000(	TMEM187	51.18502	0.495682	0.051973	0.385826	protein_coding
ENSG0000(	FIBIN	63.72938	-0.80933	0.051996	0.385893	protein_coding
ENSG0000(	LINC02346	355.0735	-0.2114	0.052125	0.386643	lncRNA
ENSG0000(	TM7SF2	172.8594	0.374524	0.052116	0.386643	protein_coding
ENSG0000(	HNRNPA2B	14135.92	-0.10769	0.052144	0.386679	protein_coding
ENSG0000(	CLIP4	1944.382	-0.25764	0.052236	0.386841	protein_coding
ENSG0000(	IER3	714.9353	-0.4236	0.052211	0.386841	protein_coding

ENSG0000(	LINC02843	150.4404	0.249803	0.052208	0.386841	lncRNA
ENSG0000(	TMED2-DT	2386.433	0.078144	0.052199	0.386841	lncRNA
ENSG0000(	ZG16	2387.653	0.735845	0.052229	0.386841	protein_coding
ENSG0000(	EMSLR	953.2581	0.117026	0.052313	0.386975	lncRNA
ENSG0000(	ENSG0000(	118.8075	0.230899	0.05232	0.386975	lncRNA
ENSG0000(	NEDD4L	7696.149	0.365752	0.052279	0.386975	protein_coding
ENSG0000(	PRORY	66.62588	-0.39168	0.052314	0.386975	lncRNA
ENSG0000(	TUG1	9046.333	0.070198	0.052324	0.386975	protein_coding
ENSG0000(	ADGRA3	2536.962	0.321676	0.052474	0.387319	protein_coding
ENSG0000(	ARMCX3	888.5024	-0.23146	0.052589	0.387319	protein_coding
ENSG0000(	CSTA	21.77312	-0.75353	0.052698	0.387319	protein_coding
ENSG0000(	ENSG0000(	108.6301	0.486557	0.052723	0.387319	lncRNA
ENSG0000(	ENSG0000(	158.1556	0.215098	0.052494	0.387319	transcribed_processed_pseudoge
ENSG0000(	ENSG0000(	37.27693	0.364705	0.052481	0.387319	lncRNA
ENSG0000(	ENSG0000(	23.78514	0.550663	0.052568	0.387319	lncRNA
ENSG0000(	ENSG0000(	341.6751	-0.13357	0.052576	0.387319	lncRNA
ENSG0000(	ENSG0000(	20.53367	-0.53233	0.052638	0.387319	unprocessed_pseudogene
ENSG0000(	ENSG0000(	22.17568	-0.54597	0.052532	0.387319	lncRNA
ENSG0000(	FAM110B	124.1789	-0.49016	0.052538	0.387319	protein_coding
ENSG0000(	FAT4	2451.632	-0.2175	0.052659	0.387319	protein_coding
ENSG0000(	FCGR2C	610.5865	-0.62863	0.052486	0.387319	polymorphic_pseudogene
ENSG0000(	IGLV1-47	200.501	-0.71536	0.052488	0.387319	IG_V_gene
ENSG0000(	IQSEC1	2078.579	-0.35485	0.052526	0.387319	protein_coding
ENSG0000(	MAGED2	334.937	-0.34697	0.052643	0.387319	protein_coding
ENSG0000(	OPRL1	48.77828	-0.43522	0.052695	0.387319	protein_coding
ENSG0000(	PLA2G10	132.521	0.468584	0.052404	0.387319	protein_coding
ENSG0000(	RAB11FIP5	486.3496	-0.23184	0.052614	0.387319	protein_coding
ENSG0000(	REM1	28.08194	-0.60559	0.052458	0.387319	protein_coding
ENSG0000(	SLC9A8	1346.785	0.191153	0.052669	0.387319	protein_coding
ENSG0000(	TEK	210.3296	-0.37694	0.052396	0.387319	protein_coding
ENSG0000(	THUMPD1	1731.63	0.132429	0.05266	0.387319	protein_coding
ENSG0000(	TRIT1	710.7888	0.227145	0.052579	0.387319	protein_coding
ENSG0000(	ZNF248	1089.061	0.139054	0.05272	0.387319	protein_coding
ENSG0000(	GIMAP4	418.227	-0.42162	0.052806	0.38752	protein_coding
ENSG0000(	OTUD5	754.4844	-0.16026	0.052781	0.38752	protein_coding
ENSG0000(	SH2B2	84.53124	-0.5204	0.0528	0.38752	protein_coding
ENSG0000(	UBR1	1988.957	-0.18041	0.052789	0.38752	protein_coding
ENSG0000(	FBXO10	313.9378	-0.3325	0.052868	0.387866	protein_coding
ENSG0000(	RABL6	2174.076	0.22828	0.052885	0.38789	protein_coding
ENSG0000(	MRC2	705.7645	-0.42709	0.052948	0.38825	protein_coding
ENSG0000(	ENSG0000(	81.49328	-0.31055	0.052986	0.388268	lncRNA
ENSG0000(	PDE8A	2885.639	0.284777	0.052979	0.388268	protein_coding
ENSG0000(	PGGHG	1917.678	-0.36556	0.052993	0.388268	protein_coding
ENSG0000(	C12orf60	287.7277	0.157199	0.053254	0.388647	protein_coding
ENSG0000(	CHN1	396.1476	-0.34556	0.053208	0.388647	protein_coding
ENSG0000(	CLCN7	1365.873	-0.17734	0.053074	0.388647	protein_coding
ENSG0000(	ENSG0000(	22.41187	0.571084	0.053344	0.388647	lncRNA

ENSG0000(	ENSG0000(	155.5503	0.370103	0.053265	0.388647	lncRNA
ENSG0000(	ENSG0000(	166.6034	-0.23609	0.053315	0.388647	lncRNA
ENSG0000(	ENSG0000(	43.11739	0.456349	0.053181	0.388647	lncRNA
ENSG0000(	EOLA2	249.8249	0.212709	0.053319	0.388647	protein_coding
ENSG0000(	FAM117B	1195.113	0.200458	0.05333	0.388647	protein_coding
ENSG0000(	GATA6-AS1	93.32357	0.594975	0.053268	0.388647	lncRNA
ENSG0000(	H6PD	3105.914	-0.1563	0.053248	0.388647	protein_coding
ENSG0000(	KCNN2	55.45889	0.303669	0.05337	0.388647	protein_coding
ENSG0000(	KDM5B	4684.023	0.103821	0.053321	0.388647	protein_coding
ENSG0000(	MESP1	20.39048	0.835007	0.053121	0.388647	protein_coding
ENSG0000(	NMRK1	640.5809	-0.22043	0.053107	0.388647	protein_coding
ENSG0000(	NOX1	406.4385	0.723617	0.053238	0.388647	protein_coding
ENSG0000(	POLR2J4	666.9753	0.150059	0.053328	0.388647	lncRNA
ENSG0000(	PPP1R26	464.4457	0.292563	0.053362	0.388647	protein_coding
ENSG0000(	RP2	362.2506	-0.2121	0.053294	0.388647	protein_coding
ENSG0000(	RPL21P99	20.65921	-0.69323	0.053349	0.388647	processed_pseudogene
ENSG0000(	SLC38A5	135.751	-0.42447	0.053269	0.388647	protein_coding
ENSG0000(	STARD4	1281.577	-0.26208	0.05328	0.388647	protein_coding
ENSG0000(	TMEM33	2225.098	-0.09535	0.053096	0.388647	protein_coding
ENSG0000(	LRRC23	61.08337	-0.35238	0.053413	0.38865	protein_coding
ENSG0000(	MSC-AS1	760.6667	-0.32757	0.053405	0.38865	lncRNA
ENSG0000(	POGLUT2	49.06592	-0.54643	0.053411	0.38865	protein_coding
ENSG0000(	DNAJA1	882.1792	-0.20656	0.053468	0.388891	protein_coding
ENSG0000(	ENSG0000(	277.6603	0.214225	0.053488	0.388891	lncRNA
ENSG0000(	PMP22	1174.203	-0.28387	0.053477	0.388891	protein_coding
ENSG0000(	NRAV	689.3343	0.091132	0.053514	0.388979	lncRNA
ENSG0000(	ATP6V1C2	29.86529	0.788334	0.053572	0.389194	protein_coding
ENSG0000(	SLC25A25-/	4343.91	0.25891	0.05357	0.389194	lncRNA
ENSG0000(	GZF1	342.6835	-0.24517	0.053591	0.389227	protein_coding
ENSG0000(	PRDM14	73.84106	-0.32664	0.053645	0.389519	protein_coding
ENSG0000(	CAVIN3	44.84977	-0.60951	0.053684	0.389597	protein_coding
ENSG0000(	IL7R	3020.85	-0.3971	0.053683	0.389597	protein_coding
ENSG0000(	ARHGAP9	903.1631	-0.4446	0.053721	0.389638	protein_coding
ENSG0000(	FOXA3	258.0359	0.434763	0.053713	0.389638	protein_coding
ENSG0000(	GPC1	283.7656	0.382697	0.053732	0.389638	protein_coding
ENSG0000(	ENSG0000(	170.6965	0.215121	0.053766	0.389775	lncRNA
ENSG0000(	CDH8	274.221	0.220477	0.053815	0.389865	protein_coding
ENSG0000(	ENSG0000(	63.94184	-0.38675	0.05384	0.389865	lncRNA
ENSG0000(	PKHD1L1	760.3641	-0.46552	0.053794	0.389865	protein_coding
ENSG0000(	SLC4A11	47.28467	-0.87023	0.053863	0.389865	protein_coding
ENSG0000(	TAB3	2562.604	0.10361	0.053844	0.389865	protein_coding
ENSG0000(	TMEM107	482.61	-0.13358	0.053852	0.389865	protein_coding
ENSG0000(	ZCCHC24	470.1218	-0.34094	0.053891	0.389964	protein_coding
ENSG0000(	FAM124A	286.0638	-0.3299	0.053914	0.390006	protein_coding
ENSG0000(	OXA1L	1813.693	-0.11812	0.053939	0.390006	protein_coding
ENSG0000(	TAF9	1509.034	0.14724	0.053934	0.390006	protein_coding
ENSG0000(	TNFRSF4	55.53792	-0.61348	0.053959	0.390045	protein_coding

ENSG0000(	ENSG0000(	287.4743	-0.32537	0.053997	0.390055	lncRNA
ENSG0000(	ENSG0000(	333.1726	0.275988	0.054003	0.390055	lncRNA
ENSG0000(	ZFYVE9	1258.818	0.187574	0.053974	0.390055	protein_coding
ENSG0000(	ENSG0000(	68.82363	0.242425	0.054127	0.390746	TEC
ENSG0000(	PATL2	487.3853	-0.3176	0.054122	0.390746	protein_coding
ENSG0000(	ENSG0000(	90.15002	-0.42983	0.054192	0.390913	lncRNA
ENSG0000(	ENSG0000(	355.0989	-0.26175	0.054194	0.390913	lncRNA
ENSG0000(	ENSG0000(	922.3007	0.201595	0.054235	0.390913	lncRNA
ENSG0000(	MIR663AH(	50134.41	0.474726	0.054224	0.390913	lncRNA
ENSG0000(	TUBA1B	1803.241	-0.26179	0.054235	0.390913	protein_coding
ENSG0000(	ZNFX1	3212.968	-0.20753	0.054172	0.390913	protein_coding
ENSG0000(	IGLV1-51	393.5471	-0.77651	0.054264	0.391019	IG_V_gene
ENSG0000(	RPS6KA6	964.6049	0.317734	0.054318	0.391224	protein_coding
ENSG0000(	TNF	107.4137	-0.57555	0.054321	0.391224	protein_coding
ENSG0000(	RABEPK	514.9322	0.135816	0.054338	0.391243	protein_coding
ENSG0000(	ATP2C1	1768.238	-0.19271	0.054356	0.391244	protein_coding
ENSG0000(	ENSG0000(	48.13087	0.638862	0.054369	0.391244	lncRNA
ENSG0000(	HMG20B	829.404	0.158312	0.054408	0.391244	protein_coding
ENSG0000(	INTS11	1488.345	0.129883	0.054424	0.391244	protein_coding
ENSG0000(	PRDX1	727.2872	-0.30354	0.054389	0.391244	protein_coding
ENSG0000(	RAB5A	1062.468	-0.17608	0.05443	0.391244	protein_coding
ENSG0000(	SCN9A	1225.03	0.272802	0.054437	0.391244	protein_coding
ENSG0000(	ENSG0000(	98.4012	-0.24077	0.054469	0.391303	lncRNA
ENSG0000(	RNFT1-DT	30.51595	-0.64775	0.054474	0.391303	lncRNA
ENSG0000(	EDN1	308.9481	0.478537	0.054607	0.392154	protein_coding
ENSG0000(	ACAP1	1282.242	-0.27615	0.054855	0.392254	protein_coding
ENSG0000(	B3GALT4	244.2268	0.189543	0.054658	0.392254	protein_coding
ENSG0000(	CD72	251.248	-0.54608	0.054988	0.392254	protein_coding
ENSG0000(	CNST	1820.316	0.249174	0.054811	0.392254	protein_coding
ENSG0000(	CYBC1	3281.81	-0.24412	0.054904	0.392254	protein_coding
ENSG0000(	DNAJC3	2139.511	-0.19404	0.054881	0.392254	protein_coding
ENSG0000(	DUSP14	42.46151	-0.74928	0.054954	0.392254	protein_coding
ENSG0000(	ENSG0000(	169.5239	0.215693	0.054907	0.392254	lncRNA
ENSG0000(	ENSG0000(	1644.756	-0.10871	0.05495	0.392254	lncRNA
ENSG0000(	ENSG0000(	78.40504	0.525815	0.054991	0.392254	processed_pseudogene
ENSG0000(	ENSG0000(	143.0195	0.174388	0.054689	0.392254	lncRNA
ENSG0000(	ENSG0000(	19.59951	-0.5346	0.054967	0.392254	lncRNA
ENSG0000(	ESRP2	1527.102	0.460532	0.054876	0.392254	protein_coding
ENSG0000(	G2E3-AS1	116.9536	0.205302	0.054704	0.392254	lncRNA
ENSG0000(	GIPC1	1181.448	0.331224	0.054731	0.392254	protein_coding
ENSG0000(	KIF12	171.1381	0.533901	0.054822	0.392254	protein_coding
ENSG0000(	LINC01436	28.85604	-0.56296	0.054944	0.392254	lncRNA
ENSG0000(	LINC01675	43.9548	-0.32219	0.054717	0.392254	lncRNA
ENSG0000(	PANTR1	242.1789	0.148708	0.054976	0.392254	lncRNA
ENSG0000(	RAB42	249.6862	-0.17174	0.054718	0.392254	protein_coding
ENSG0000(	RSL24D1	709.3365	-0.15155	0.05496	0.392254	protein_coding
ENSG0000(	SLC25A6	1869.841	0.204124	0.054908	0.392254	protein_coding

ENSG0000(	TCF7L1	223.7118	-0.18225	0.054816	0.392254	protein_coding
ENSG0000(	TMEM41A	1311.695	0.116352	0.054861	0.392254	protein_coding
ENSG0000(	WDR86	60.12232	-0.60241	0.054771	0.392254	protein_coding
ENSG0000(	ZNF517	294.6345	0.148751	0.054829	0.392254	protein_coding
ENSG0000(	PITPNC1	907.4009	-0.31537	0.055026	0.392398	protein_coding
ENSG0000(	DIRC3-AS1	24.36011	0.668089	0.055054	0.392499	lncRNA
ENSG0000(	DOCK8	4705.585	-0.29716	0.055098	0.392508	protein_coding
ENSG0000(	HHAT	281.3527	0.24535	0.055081	0.392508	protein_coding
ENSG0000(	RN7SL785F	72.08253	0.259664	0.055098	0.392508	misc_RNA
ENSG0000(	RAB11B-AS	68.65698	0.378751	0.055143	0.392724	lncRNA
ENSG0000(	CRLF2	38.75471	-0.50711	0.055179	0.392831	protein_coding
ENSG0000(	NSUN7	418.6088	0.336926	0.055187	0.392831	protein_coding
ENSG0000(	ZBTB7C	718.5175	0.445647	0.055209	0.392888	protein_coding
ENSG0000(	DPY19L3-D	44.71437	-0.35314	0.055245	0.392999	lncRNA
ENSG0000(	ENSG0000(	54.81806	-0.46867	0.055259	0.392999	lncRNA
ENSG0000(	RN7SL353F	141.3231	0.229295	0.055282	0.392999	misc_RNA
ENSG0000(	UCHL1	22.65523	-0.86067	0.055274	0.392999	protein_coding
ENSG0000(	IGLV2-8	670.2586	-0.82592	0.055313	0.393124	IG_V_gene
ENSG0000(	HIPK3	2769.523	-0.16293	0.055353	0.3933	protein_coding
ENSG0000(	OR7A19P	85.22879	-0.29428	0.055383	0.393413	unprocessed_pseudogene
ENSG0000(	LINC00460	22.69122	-0.51663	0.055413	0.393425	lncRNA
ENSG0000(	RNF13	763.1318	-0.17401	0.055407	0.393425	protein_coding
ENSG0000(	RN7SL517F	163.1205	-0.2422	0.055438	0.393501	misc_RNA
ENSG0000(	MTFR2	56.47966	-0.39916	0.055453	0.393504	protein_coding
ENSG0000(	TDRKH	154.4795	-0.33815	0.055469	0.393519	protein_coding
ENSG0000(	STK19	354.3271	-0.16083	0.055488	0.393551	protein_coding
ENSG0000(	ENSG0000(	229.1315	-0.42953	0.055531	0.393556	lncRNA
ENSG0000(	KLHL21	1026.191	-0.16121	0.055511	0.393556	protein_coding
ENSG0000(	SLC35C1	712.9069	0.244095	0.055526	0.393556	protein_coding
ENSG0000(	ANKRD20A	504.2092	0.275508	0.05556	0.393655	transcribed_unprocessed_pseudo
ENSG0000(	ARHGEF38	842.2308	0.264803	0.055619	0.393714	protein_coding
ENSG0000(	CCDC170	228.2659	-0.23589	0.055602	0.393714	protein_coding
ENSG0000(	ENSG0000(	40.5993	-0.39587	0.055663	0.393714	lncRNA
ENSG0000(	ENSG0000(	24.0125	-0.50906	0.055656	0.393714	lncRNA
ENSG0000(	ENSG0000(	444.4921	0.409393	0.055675	0.393714	lncRNA
ENSG0000(	ENSG0000(	150.7869	0.228282	0.055672	0.393714	lncRNA
ENSG0000(	IGHV1-69D	307.5295	-0.93411	0.055683	0.393714	IG_V_gene
ENSG0000(	TMEM263	816.2259	-0.23465	0.055629	0.393714	protein_coding
ENSG0000(	BTBD6	517.77	0.216322	0.055743	0.393718	protein_coding
ENSG0000(	ENSG0000(	144.923	0.4707	0.055709	0.393718	lncRNA
ENSG0000(	FAM201A	88.95804	0.334073	0.055783	0.393718	lncRNA
ENSG0000(	GIMAP5	1201.197	-0.37492	0.055754	0.393718	protein_coding
ENSG0000(	LYSMD4	463.277	0.29675	0.055772	0.393718	protein_coding
ENSG0000(	MPEG1	2460.66	-0.29788	0.055775	0.393718	protein_coding
ENSG0000(	SERP1	1749.399	-0.15957	0.055741	0.393718	protein_coding
ENSG0000(	CLASRP	772.2712	-0.12563	0.055919	0.394038	protein_coding
ENSG0000(	ENSG0000(	427.6059	0.188841	0.055897	0.394038	processed_pseudogene

ENSG0000(	ENSG0000(	92.40158	0.260236	0.055914	0.394038	lncRNA
ENSG0000(	ITGB2-AS1	309.4597	-0.47632	0.055887	0.394038	lncRNA
ENSG0000(	MFSD6	1506.647	0.178398	0.055912	0.394038	protein_coding
ENSG0000(	NPIPB14P	194.4198	0.509013	0.055929	0.394038	transcribed_unprocessed_pseudo
ENSG0000(	TSFM	274.197	-0.17878	0.055849	0.394038	protein_coding
ENSG0000(	RN7SKP78	97.74154	-0.39489	0.055947	0.394061	misc_RNA
ENSG0000(	ADAMTS7	273.0097	-0.40265	0.055963	0.394076	protein_coding
ENSG0000(	ENSG0000(	169.8644	0.261069	0.056041	0.394327	lncRNA
ENSG0000(	LINC00456	244.0833	-0.20342	0.056035	0.394327	lncRNA
ENSG0000(	PPP2R5C	3984.412	-0.14292	0.056042	0.394327	protein_coding
ENSG0000(	CXorf38	1232.369	-0.11012	0.056062	0.394366	protein_coding
ENSG0000(	MED12	3219.075	0.086698	0.056079	0.394385	protein_coding
ENSG0000(	RN7SL660F	37.51021	-0.37029	0.056107	0.394484	misc_RNA
ENSG0000(	SLX4IP	1000.469	0.150453	0.05613	0.394542	protein_coding
ENSG0000(	FAM219A	467.4991	-0.23425	0.056145	0.394553	protein_coding
ENSG0000(	ENSG0000(	161.4739	-0.21235	0.056241	0.395038	lncRNA
ENSG0000(	MIR155HG	138.4749	-0.6096	0.056243	0.395038	lncRNA
ENSG0000(	ENSG0000(	26.21174	-0.51582	0.056303	0.395253	lncRNA
ENSG0000(	GDI2	2186.063	-0.15439	0.056293	0.395253	protein_coding
ENSG0000(	RGL3	162.615	0.302833	0.056326	0.395319	protein_coding
ENSG0000(	GNG11	111.0145	-0.4549	0.056341	0.395323	protein_coding
ENSG0000(	ENSG0000(	21.8033	-0.49966	0.056385	0.395527	lncRNA
ENSG0000(	ABCA10	571.4134	0.377454	0.056475	0.395648	protein_coding
ENSG0000(	ATP13A3	4531.737	-0.24725	0.056442	0.395648	protein_coding
ENSG0000(	DAZL	23.60865	-1.31029	0.056457	0.395648	protein_coding
ENSG0000(	ENSG0000(	274.6376	0.171878	0.056602	0.395648	lncRNA
ENSG0000(	ENSG0000(	75.16459	-0.71803	0.056534	0.395648	lncRNA
ENSG0000(	FBXL12	509.5266	0.155871	0.056497	0.395648	protein_coding
ENSG0000(	GABARAPL	808.6287	-0.25561	0.056478	0.395648	protein_coding
ENSG0000(	IGLV3-1	326.6658	-0.82129	0.056534	0.395648	IG_V_gene
ENSG0000(	KAT2B	1175.626	-0.21842	0.056491	0.395648	protein_coding
ENSG0000(	LAIR1	1454.353	-0.29008	0.056563	0.395648	protein_coding
ENSG0000(	LINC00261	553.9866	0.442418	0.056553	0.395648	lncRNA
ENSG0000(	NRARP	1154.735	0.432475	0.056582	0.395648	protein_coding
ENSG0000(	SNORA70D	32.39945	0.74507	0.056515	0.395648	snoRNA
ENSG0000(	SNX3	533.0409	-0.28738	0.056603	0.395648	protein_coding
ENSG0000(	EGLN3	1300.971	-0.51592	0.056678	0.39585	protein_coding
ENSG0000(	ENSG0000(	1990.782	0.194904	0.056702	0.39585	transcribed_unprocessed_pseudo
ENSG0000(	ENSG0000(	648.5483	0.21152	0.056704	0.39585	transcribed_unprocessed_pseudo
ENSG0000(	PSMD5	677.1886	0.169652	0.056671	0.39585	protein_coding
ENSG0000(	TYRO3P	273.5916	0.193457	0.056679	0.39585	processed_pseudogene
ENSG0000(	ENSG0000(	37.75455	0.400673	0.056728	0.395917	lncRNA
ENSG0000(	GPN1	290.1583	0.25809	0.05675	0.395972	protein_coding
ENSG0000(	CFAP20DC	216.9229	0.269722	0.056793	0.396077	protein_coding
ENSG0000(	MAMDC2	127.3196	0.582666	0.056794	0.396077	protein_coding
ENSG0000(	FAM83F	3071.09	0.368834	0.056817	0.396133	protein_coding
ENSG0000(	CLEC11A	50.53988	-0.36672	0.056851	0.396219	protein_coding

ENSG0000(	ENSG0000(	35.96975	-0.35323	0.056872	0.396219	lncRNA
ENSG0000(	ENSG0000(	136.2886	-0.17368	0.056863	0.396219	lncRNA
ENSG0000(	LINC01080	32.66034	0.665632	0.056905	0.396346	lncRNA
ENSG0000(	BCKDHA	185.2538	0.243363	0.057007	0.396758	protein_coding
ENSG0000(	ERO1A	1773.975	-0.30757	0.056999	0.396758	protein_coding
ENSG0000(	LINC02608	60.9037	0.517512	0.056982	0.396758	lncRNA
ENSG0000(	ENSG0000(	77.94536	-0.35206	0.05709	0.397232	lncRNA
ENSG0000(	NOL8	1349.981	-0.14023	0.057158	0.397605	protein_coding
ENSG0000(	DEFB105A	248.6907	0.205932	0.057258	0.397752	protein_coding
ENSG0000(	ENSG0000(	22.72123	-1.03959	0.057245	0.397752	processed_pseudogene
ENSG0000(	HUNK	686.2916	0.233623	0.057201	0.397752	protein_coding
ENSG0000(	LRRIQ4	56.97984	-0.33013	0.057236	0.397752	protein_coding
ENSG0000(	OR4K2	167.3849	0.303539	0.057243	0.397752	protein_coding
ENSG0000(	PIM3	1124.172	-0.23184	0.057272	0.397752	protein_coding
ENSG0000(	ZHX2	783.0397	-0.29025	0.05728	0.397752	protein_coding
ENSG0000(	EEF1A1P3	228.4966	0.238988	0.057334	0.397873	processed_pseudogene
ENSG0000(	ENSG0000(	426.8299	-0.24838	0.057384	0.397873	lncRNA
ENSG0000(	FLJ36000	54.90822	0.484175	0.057384	0.397873	lncRNA
ENSG0000(	IGHV1-3	303.7109	-0.9674	0.057363	0.397873	IG_V_gene
ENSG0000(	MARCHF4	46.66929	-0.39882	0.057381	0.397873	protein_coding
ENSG0000(	SYT8	42.1314	0.572832	0.057336	0.397873	protein_coding
ENSG0000(	ENSG0000(	170.7773	0.226204	0.057445	0.398012	lncRNA
ENSG0000(	ENSG0000(	346.0033	-0.17876	0.057462	0.398012	TEC
ENSG0000(	NXPE2	566.702	0.558709	0.057452	0.398012	protein_coding
ENSG0000(	SLC7A1	1403.545	-0.30486	0.057435	0.398012	protein_coding
ENSG0000(	KLC2	351.1892	-0.27654	0.057523	0.398332	protein_coding
ENSG0000(	LINC02163	112.5433	0.330309	0.057685	0.39935	lncRNA
ENSG0000(	FDX2	40.73151	-0.44331	0.057748	0.399485	protein_coding
ENSG0000(	MALT1	2402.894	-0.19446	0.05774	0.399485	protein_coding
ENSG0000(	SH3BP2	5081.45	-0.19304	0.057731	0.399485	protein_coding
ENSG0000(	CREM	321.403	-0.21944	0.057778	0.399494	protein_coding
ENSG0000(	ZNF234	852.1473	0.115487	0.05777	0.399494	protein_coding
ENSG0000(	CNNM4	2697.854	0.457727	0.057854	0.399716	protein_coding
ENSG0000(	ENSG0000(	164.1594	-0.29166	0.057853	0.399716	lncRNA
ENSG0000(	ENSG0000(	153.123	0.252856	0.057842	0.399716	lncRNA
ENSG0000(	LINC00839	393.754	-0.19494	0.057871	0.399739	lncRNA
ENSG0000(	LINC01209	197.1903	0.271489	0.057911	0.399914	lncRNA
ENSG0000(	ENSG0000(	393.7009	-0.09086	0.057964	0.400179	lncRNA
ENSG0000(	DHX32	803.0175	0.282739	0.058012	0.400407	protein_coding
ENSG0000(	GEMIN4	504.3851	0.196456	0.058056	0.400509	protein_coding
ENSG0000(	IFNAR2	1463.197	-0.22811	0.05805	0.400509	protein_coding
ENSG0000(	C1QC	531.4411	-0.44812	0.058086	0.400618	protein_coding
ENSG0000(	FANCD2	1315.382	-0.28586	0.05813	0.400718	protein_coding
ENSG0000(	GXYLT2	467.5311	0.25652	0.058128	0.400718	protein_coding
ENSG0000(	FLT1	1113.163	-0.23938	0.058293	0.400937	protein_coding
ENSG0000(	GRIA3	139.6359	0.508785	0.058288	0.400937	protein_coding
ENSG0000(	IHH	504.9748	0.539041	0.058205	0.400937	protein_coding

ENSG0000(	LINC00842	70.6726	0.346184	0.058197	0.400937	lncRNA
ENSG0000(	LZTS1	160.3392	-0.39885	0.058238	0.400937	protein_coding
ENSG0000(	MVP	4906.979	0.20971	0.058307	0.400937	protein_coding
ENSG0000(	PAPSS1	344.5578	-0.32579	0.058193	0.400937	protein_coding
ENSG0000(	SAV1	566.3935	-0.25984	0.058265	0.400937	protein_coding
ENSG0000(	SNAP25-AS	658.7391	0.190681	0.058283	0.400937	lncRNA
ENSG0000(	STRADB	627.2488	0.320994	0.058296	0.400937	protein_coding
ENSG0000(	AHCYL2	5140.843	0.46168	0.058373	0.401291	protein_coding
ENSG0000(	C5orf34	74.11522	-0.41389	0.058442	0.401545	protein_coding
ENSG0000(	ENSG0000(	26.62015	-0.43507	0.058461	0.401545	transcribed_unitary_pseudogene
ENSG0000(	ENSG0000(	525.1411	0.108004	0.058469	0.401545	TEC
ENSG0000(	XPO6	2614.861	-0.23366	0.058431	0.401545	protein_coding
ENSG0000(	IGLV3-10	63.12225	-0.99826	0.058512	0.401742	IG_V_gene
ENSG0000(	WBP11	716.9726	-0.16931	0.058542	0.401845	protein_coding
ENSG0000(	RNF115	3347.044	-0.11779	0.058579	0.402004	protein_coding
ENSG0000(	PCDH19	214.017	0.512076	0.05861	0.402113	protein_coding
ENSG0000(	ENSG0000(	30.80202	0.593925	0.058639	0.402116	lncRNA
ENSG0000(	LINC01006	1206.649	0.232676	0.058639	0.402116	lncRNA
ENSG0000(	CAPZB	5747.093	-0.12244	0.058692	0.402178	protein_coding
ENSG0000(	GCLM	854.1445	-0.17143	0.05868	0.402178	protein_coding
ENSG0000(	RN7SKP30	54.45589	-0.31247	0.058667	0.402178	misc_RNA
ENSG0000(	TBC1D14	2163.568	0.160661	0.058765	0.402576	protein_coding
ENSG0000(	ENSG0000(	312.1816	-0.17679	0.05879	0.402649	lncRNA
ENSG0000(	HPS5	1008.648	-0.11711	0.05885	0.402958	protein_coding
ENSG0000(	LINC02035	515.7765	-0.17494	0.058909	0.403258	lncRNA
ENSG0000(	CACNB2	1510.835	0.202241	0.058926	0.403274	protein_coding
ENSG0000(	ENSG0000(	67.25789	0.472974	0.058992	0.40334	lncRNA
ENSG0000(	LRATD1	3057.884	0.359903	0.059009	0.40334	protein_coding
ENSG0000(	PDZD4	84.72094	0.335475	0.058997	0.40334	protein_coding
ENSG0000(	RALB	1036.494	-0.28037	0.058976	0.40334	protein_coding
ENSG0000(	ZNF721	1561.581	0.154591	0.058991	0.40334	protein_coding
ENSG0000(	DCX	56.26374	0.321201	0.059081	0.403451	protein_coding
ENSG0000(	ENSG0000(	64.93854	-0.24304	0.059107	0.403451	lncRNA
ENSG0000(	LINC00466	77.28996	0.307721	0.059101	0.403451	lncRNA
ENSG0000(	PPM1L	904.2358	0.318014	0.05908	0.403451	protein_coding
ENSG0000(	PPP4R4	22.80109	-0.83135	0.059113	0.403451	protein_coding
ENSG0000(	TKT	1167.23	-0.2169	0.0591	0.403451	protein_coding
ENSG0000(	ENSG0000(	50.84247	0.405422	0.059191	0.403577	lncRNA
ENSG0000(	ENSG0000(	29.47218	-0.54718	0.059199	0.403577	lncRNA
ENSG0000(	FMO1	41.9491	-0.99563	0.059159	0.403577	protein_coding
ENSG0000(	MRPS6	569.3849	0.170665	0.059205	0.403577	protein_coding
ENSG0000(	PISD	903.6162	0.146874	0.059191	0.403577	protein_coding
ENSG0000(	LINC01637	110.4588	0.53676	0.059343	0.404418	lncRNA
ENSG0000(	ENSG0000(	38.99081	-0.91147	0.059408	0.404602	lncRNA
ENSG0000(	ENSG0000(	2183.82	-0.25842	0.059442	0.404602	lncRNA
ENSG0000(	GPER1	50.55076	0.519601	0.059443	0.404602	protein_coding
ENSG0000(	LINC02501	80.86664	0.26789	0.059434	0.404602	lncRNA

ENSG0000(	MEF2C	1946.538	-0.35525	0.059404	0.404602	protein_coding
ENSG0000(	BTNL3	1185.944	0.575074	0.059519	0.404826	protein_coding
ENSG0000(	ENSG0000(	134.1345	-0.20076	0.059511	0.404826	lncRNA
ENSG0000(	ENSG0000(	224.0684	0.202127	0.05952	0.404826	lncRNA
ENSG0000(	CNN3	760.5619	-0.18175	0.059571	0.404844	protein_coding
ENSG0000(	ENSG0000(	3381.055	0.067817	0.059555	0.404844	lncRNA
ENSG0000(	HECA	2144.701	-0.14334	0.059582	0.404844	protein_coding
ENSG0000(	LTN1	2192.921	0.133787	0.059574	0.404844	protein_coding
ENSG0000(	ERCC6	1712.568	0.163542	0.059607	0.404915	protein_coding
ENSG0000(	SHKBP1	606.5384	-0.19263	0.059625	0.404941	protein_coding
ENSG0000(	GOLGA3	7043.542	0.125304	0.059641	0.404944	protein_coding
ENSG0000(	MRFAP1	934.1809	-0.21655	0.059657	0.404958	protein_coding
ENSG0000(	PREX1	1438.408	-0.38259	0.059705	0.405082	protein_coding
ENSG0000(	STAT2	4510.834	-0.28334	0.059703	0.405082	protein_coding
ENSG0000(	STRIP2	537.7597	-0.41975	0.05973	0.405151	protein_coding
ENSG0000(	ENSG0000(	267.8944	-0.23913	0.059746	0.405158	lncRNA
ENSG0000(	THAP7	198.5311	0.218066	0.05979	0.405356	protein_coding
ENSG0000(	GAGE1	24.50818	0.529181	0.059823	0.405379	protein_coding
ENSG0000(	TIGIT	290.0406	-0.54034	0.059812	0.405379	protein_coding
ENSG0000(	ENSG0000(	132.326	-0.20636	0.059877	0.405469	TEC
ENSG0000(	ERCC5	2712.617	0.121851	0.05991	0.405469	protein_coding
ENSG0000(	POP4	271.0204	-0.18255	0.059898	0.405469	protein_coding
ENSG0000(	REXO4	294.8733	0.230175	0.059874	0.405469	protein_coding
ENSG0000(	RUFY4	86.53158	-0.59235	0.059883	0.405469	protein_coding
ENSG0000(	ENSG0000(	121.6785	0.28244	0.060011	0.405939	lncRNA
ENSG0000(	HIPK1	2956.571	-0.14152	0.060018	0.405939	protein_coding
ENSG0000(	SNHG30	329.7227	0.139515	0.060023	0.405939	lncRNA
ENSG0000(	ENSG0000(	28.28596	-0.47707	0.060108	0.406275	lncRNA
ENSG0000(	ENSG0000(	56.46694	0.267611	0.060251	0.406275	lncRNA
ENSG0000(	ENSG0000(	108.3199	-0.26109	0.06018	0.406275	TEC
ENSG0000(	ENSG0000(	58.91113	-0.3848	0.060255	0.406275	TEC
ENSG0000(	ENSG0000(	146.2888	-0.21419	0.060137	0.406275	lncRNA
ENSG0000(	FAM238C	218.8397	-0.24214	0.060207	0.406275	transcribed_unprocessed_pseudo
ENSG0000(	GRIN2A	344.9257	0.656545	0.060261	0.406275	protein_coding
ENSG0000(	ISOC1	224.1853	-0.22387	0.060253	0.406275	protein_coding
ENSG0000(	LINC01414	165.8957	-0.19085	0.060263	0.406275	lncRNA
ENSG0000(	PLCG2	2221.141	-0.36126	0.060133	0.406275	protein_coding
ENSG0000(	SCN1B	593.4674	-0.30842	0.06019	0.406275	protein_coding
ENSG0000(	TRIM9	198.0878	0.572087	0.060265	0.406275	protein_coding
ENSG0000(	XKR4	857.474	0.159917	0.060188	0.406275	protein_coding
ENSG0000(	HCP5	5834.329	-0.21286	0.06029	0.406324	lncRNA
ENSG0000(	TNFSF15	742.2132	-0.26433	0.060302	0.406324	protein_coding
ENSG0000(	HLA-DOA	981.6701	-0.36464	0.060394	0.406759	protein_coding
ENSG0000(	MRM3	137.3689	0.231217	0.060396	0.406759	protein_coding
ENSG0000(	ENSG0000(	20.71634	0.500865	0.060493	0.407212	lncRNA
ENSG0000(	GAS5-AS1	42.71417	-0.29581	0.060489	0.407212	lncRNA
ENSG0000(	NR2F1-AS1	2853.013	-0.11146	0.060523	0.407317	lncRNA

ENSG0000(	ENSG0000(	265.3991	0.240566	0.060563	0.407484	lncRNA
ENSG0000(	MAVS	11336.57	0.205213	0.060596	0.407609	protein_coding
ENSG0000(	IDH3B	424.6711	0.231714	0.060662	0.407951	protein_coding
ENSG0000(	AKIRIN2	670.1711	-0.18859	0.060718	0.408043	protein_coding
ENSG0000(	ENSG0000(	26.39068	-0.72306	0.060691	0.408043	lncRNA
ENSG0000(	SNRPD1	1169.732	0.105393	0.060729	0.408043	protein_coding
ENSG0000(	TMEM231	729.9386	0.160456	0.060735	0.408043	protein_coding
ENSG0000(	DUSP8	481.9508	0.356602	0.060775	0.408213	protein_coding
ENSG0000(	ENSG0000(	40.44763	-0.43057	0.060809	0.408343	lncRNA
ENSG0000(	RAB26	118.1019	0.526179	0.060857	0.408522	protein_coding
ENSG0000(	SNORD3D	19239.24	0.467048	0.060865	0.408522	snoRNA
ENSG0000(	DPP10	220.8768	0.393281	0.060931	0.408604	protein_coding
ENSG0000(	FERMT3	417.5048	-0.30206	0.060952	0.408604	protein_coding
ENSG0000(	MTND5P15	33.75343	-0.48871	0.060943	0.408604	processed_pseudogene
ENSG0000(	POLR2K	191.8058	-0.19235	0.060948	0.408604	protein_coding
ENSG0000(	TMEM63A	4966.959	0.188922	0.060913	0.408604	protein_coding
ENSG0000(	DACT2	31.71483	0.635105	0.060972	0.408631	protein_coding
ENSG0000(	SRGAP1	3204.618	0.233504	0.060986	0.408631	protein_coding
ENSG0000(	APOBEC3C	455.9964	-0.27684	0.061086	0.408904	protein_coding
ENSG0000(	IL7	202.2851	-0.28926	0.061075	0.408904	protein_coding
ENSG0000(	ITGAM	330.6118	-0.63202	0.061076	0.408904	protein_coding
ENSG0000(	TRO	175.9922	-0.43669	0.061076	0.408904	protein_coding
ENSG0000(	C1orf105	87.02585	-0.20234	0.061103	0.408922	protein_coding
ENSG0000(	MARCHF3	637.2203	0.208324	0.061154	0.409128	protein_coding
ENSG0000(	ZNF566-AS	82.36848	0.225145	0.061164	0.409128	lncRNA
ENSG0000(	CAPN14	45.15524	-0.62178	0.061229	0.409451	protein_coding
ENSG0000(	GPR78	126.4964	-0.34007	0.061242	0.409451	protein_coding
ENSG0000(	ENSG0000(	444.5588	-0.14465	0.061274	0.409566	lncRNA
ENSG0000(	CCDC142	1383.159	0.124115	0.061388	0.409667	protein_coding
ENSG0000(	ENSG0000(	73.00915	0.283436	0.061345	0.409667	lncRNA
ENSG0000(	ENSG0000(	203.0165	-0.22836	0.061438	0.409667	TEC
ENSG0000(	FAM76A	717.5738	0.229933	0.061456	0.409667	protein_coding
ENSG0000(	HIC2	581.2076	0.144926	0.061434	0.409667	protein_coding
ENSG0000(	IL12A-AS1	347.5833	-0.16019	0.061376	0.409667	lncRNA
ENSG0000(	MCF2L	2712.278	0.271573	0.061447	0.409667	protein_coding
ENSG0000(	MCL1	12374.46	-0.23754	0.06139	0.409667	protein_coding
ENSG0000(	ORAI1	338.9752	-0.16223	0.061473	0.409667	protein_coding
ENSG0000(	POP1	212.5995	-0.38006	0.061483	0.409667	protein_coding
ENSG0000(	RPL17P10	39.43587	-0.48902	0.061478	0.409667	processed_pseudogene
ENSG0000(	VIM	3151.525	-0.39522	0.061368	0.409667	protein_coding
ENSG0000(	WASF3	120.4029	-0.45985	0.061349	0.409667	protein_coding
ENSG0000(	ENSG0000(	333.6339	0.178112	0.061528	0.4098	lncRNA
ENSG0000(	ENSG0000(	609.8347	0.137175	0.061533	0.4098	lncRNA
ENSG0000(	MAPK1IP1L	2908.396	-0.1183	0.061635	0.410386	protein_coding
ENSG0000(	LINC01484	696.6393	-0.32851	0.061755	0.41098	lncRNA
ENSG0000(	SCML1	707.0489	0.240883	0.061751	0.41098	protein_coding
ENSG0000(	OR14L1P	30.24803	-0.47066	0.061839	0.411444	protein_coding

ENSG0000(	TP53I3	823.8347	0.307556	0.061929	0.411945	protein_coding
ENSG0000(	ADRB1	100.1704	0.434988	0.061978	0.412135	protein_coding
ENSG0000(	PCDHGA12	111.2751	0.335022	0.061988	0.412135	protein_coding
ENSG0000(	IGHV3-30	936.1447	-0.59617	0.06204	0.412343	IG_V_gene
ENSG0000(	SHROOM2	402.5295	0.339383	0.062049	0.412343	protein_coding
ENSG0000(	GPRC5C	650.6498	0.381755	0.062129	0.412772	protein_coding
ENSG0000(	ENSG0000(	59.82656	0.550525	0.062149	0.412804	lncRNA
ENSG0000(	ENSG0000(	46.11315	-0.29546	0.062209	0.413008	lncRNA
ENSG0000(	TMPRSS5	59.09834	-0.53578	0.062198	0.413008	protein_coding
ENSG0000(	ENSG0000(	82.41142	-0.36992	0.062244	0.413135	lncRNA
ENSG0000(	BUB1	342.7393	-0.28649	0.062306	0.413272	protein_coding
ENSG0000(	POGLUT2P	160.5749	0.374034	0.062304	0.413272	processed_pseudogene
ENSG0000(	SNX30	2364.608	0.286201	0.062309	0.413272	protein_coding
ENSG0000(	ENSG0000(	115.8222	-0.23003	0.062333	0.413328	lncRNA
ENSG0000(	ENSG0000(	102.9763	-0.32412	0.062351	0.413348	lncRNA
ENSG0000(	ARHGAP29	1526.754	-0.17389	0.062505	0.413549	protein_coding
ENSG0000(	CYP4F3	571.9223	-0.32987	0.062446	0.413549	protein_coding
ENSG0000(	DEPDC5	2848.275	0.108294	0.062485	0.413549	protein_coding
ENSG0000(	H2AX	184.3121	-0.25591	0.062504	0.413549	protein_coding
ENSG0000(	LINC01297	1240.246	0.457249	0.062516	0.413549	lncRNA
ENSG0000(	RAB39B	88.64811	-0.38525	0.062471	0.413549	protein_coding
ENSG0000(	SDF4	1431.647	0.175519	0.062504	0.413549	protein_coding
ENSG0000(	SPNS2	540.8265	-0.44154	0.062439	0.413549	protein_coding
ENSG0000(	USP6NL	1339.564	-0.12084	0.062465	0.413549	protein_coding
ENSG0000(	ARFRP1	1714.339	0.12311	0.062558	0.41363	protein_coding
ENSG0000(	GOT1	517.3376	0.348653	0.062559	0.41363	protein_coding
ENSG0000(	CNOT10	1172.05	0.114579	0.062599	0.413796	protein_coding
ENSG0000(	RN7SL683P	104.9144	-0.26103	0.062617	0.413819	misc_RNA
ENSG0000(	ENSG0000(	159.0217	0.168014	0.062764	0.414249	lncRNA
ENSG0000(	ENSG0000(	36.57534	-0.66597	0.062747	0.414249	lncRNA
ENSG0000(	EPB41L1	1085.032	0.428436	0.062707	0.414249	protein_coding
ENSG0000(	HNRNPA1P	89.90724	0.68072	0.062768	0.414249	processed_pseudogene
ENSG0000(	SUSD1	522.8194	0.235396	0.062745	0.414249	protein_coding
ENSG0000(	TPK1	888.9041	-0.31516	0.062773	0.414249	protein_coding
ENSG0000(	KLF7-IT1	123.3094	-0.44708	0.062802	0.41434	lncRNA
ENSG0000(	PAQR5	1416.243	0.457858	0.062844	0.414519	protein_coding
ENSG0000(	ADCYAP1	32.75219	-0.92447	0.063174	0.414581	protein_coding
ENSG0000(	AGAP3	944.5176	-0.18712	0.06308	0.414581	protein_coding
ENSG0000(	BRD1	1371.16	0.130916	0.063177	0.414581	protein_coding
ENSG0000(	BZW2	581.2352	0.22114	0.062954	0.414581	protein_coding
ENSG0000(	CRTC2	630.6505	-0.17564	0.062876	0.414581	protein_coding
ENSG0000(	DARS2	700.2168	0.117652	0.063152	0.414581	protein_coding
ENSG0000(	ENSG0000(	2486.359	-0.18484	0.0632	0.414581	lncRNA
ENSG0000(	ENSG0000(	87.86869	-0.36206	0.0631	0.414581	lncRNA
ENSG0000(	ENSG0000(	37.16993	0.399718	0.062956	0.414581	lncRNA
ENSG0000(	ENSG0000(	155.7718	-0.21638	0.063051	0.414581	TEC
ENSG0000(	ENSG0000(	77.09976	0.263136	0.06309	0.414581	lncRNA

ENSG0000(	FHIP1A	1921.526	0.429184	0.063144	0.414581	protein_coding
ENSG0000(	IKBIP	331.2143	-0.27173	0.063131	0.414581	protein_coding
ENSG0000(	ISL2	25.31399	-0.5382	0.063167	0.414581	protein_coding
ENSG0000(	LGALS17A	30.30115	-0.68687	0.062964	0.414581	transcribed_unprocessed_pseudo
ENSG0000(	PMPCA	824.7076	0.122416	0.062894	0.414581	protein_coding
ENSG0000(	PRPF40B	348.1706	-0.19811	0.063196	0.414581	protein_coding
ENSG0000(	RNU1-100F	27.00998	-0.55236	0.063163	0.414581	snRNA
ENSG0000(	SLC28A2	918.3296	0.189134	0.063078	0.414581	protein_coding
ENSG0000(	TEF	373.9326	0.418293	0.063126	0.414581	protein_coding
ENSG0000(	TMEM176E	843.946	-0.24756	0.063188	0.414581	protein_coding
ENSG0000(	TNFAIP8	1551.353	-0.24842	0.063164	0.414581	protein_coding
ENSG0000(	UBXN4	3375.319	-0.1239	0.063191	0.414581	protein_coding
ENSG0000(	NPY4R	184.4446	0.812183	0.063277	0.414991	protein_coding
ENSG0000(	TRMT2B	1504.866	0.122383	0.063326	0.415212	protein_coding
ENSG0000(	ACP2	519.1462	-0.20554	0.063556	0.415252	protein_coding
ENSG0000(	CHD6	2751.274	0.15126	0.063499	0.415252	protein_coding
ENSG0000(	CNP	858.4151	-0.16205	0.063418	0.415252	protein_coding
ENSG0000(	GK-IT1	141.446	-0.51084	0.06352	0.415252	lncRNA
ENSG0000(	KCTD14	64.9193	-0.505	0.063528	0.415252	protein_coding
ENSG0000(	NAP1L1	3168.681	-0.23972	0.063412	0.415252	protein_coding
ENSG0000(	PABPC1L	734.6611	-0.20949	0.063432	0.415252	protein_coding
ENSG0000(	PODXL	900.8856	-0.30759	0.063449	0.415252	protein_coding
ENSG0000(	RBP1	171.7974	-0.31023	0.063362	0.415252	protein_coding
ENSG0000(	RRAGA	290.532	-0.1746	0.063479	0.415252	protein_coding
ENSG0000(	TMEM178A	75.79642	0.379778	0.063559	0.415252	protein_coding
ENSG0000(	TNFRSF12A	108.4349	-0.61341	0.063488	0.415252	protein_coding
ENSG0000(	TRAJ40	27.32779	-0.61333	0.063466	0.415252	TR_J_gene
ENSG0000(	UXS1	377.0169	-0.26568	0.063457	0.415252	protein_coding
ENSG0000(	ZNF273	919.5439	0.130755	0.063415	0.415252	protein_coding
ENSG0000(	RASSF1	691.6324	-0.17124	0.063588	0.415348	protein_coding
ENSG0000(	EFNA3	40.75167	0.578343	0.063618	0.415392	protein_coding
ENSG0000(	ENSG0000(	192.0719	0.183394	0.063625	0.415392	lncRNA
ENSG0000(	COPZ2	60.37298	-0.50243	0.063696	0.415652	protein_coding
ENSG0000(	IL6ST	7073.731	-0.2531	0.063689	0.415652	protein_coding
ENSG0000(	NXNL2	428.4016	-0.20443	0.063711	0.415652	protein_coding
ENSG0000(	ENSG0000(	121.4666	0.17878	0.063744	0.415676	lncRNA
ENSG0000(	ENSG0000(	35.39562	0.420995	0.06373	0.415676	lncRNA
ENSG0000(	ENSG0000(	314.9736	0.129091	0.063789	0.41587	lncRNA
ENSG0000(	BRWD1-AS	36.62025	0.392663	0.063812	0.415903	lncRNA
ENSG0000(	CASP3	845.1973	-0.17163	0.06384	0.415903	protein_coding
ENSG0000(	ENSG0000(	459.4778	0.158372	0.06384	0.415903	lncRNA
ENSG0000(	POM121L7	149.7966	-0.36705	0.063855	0.415903	processed_pseudogene
ENSG0000(	AFAP1L1	434.2229	-0.34407	0.063946	0.416105	protein_coding
ENSG0000(	BEST1	939.3857	-0.28054	0.063939	0.416105	protein_coding
ENSG0000(	ENSG0000(	86.09245	0.373759	0.063935	0.416105	lncRNA
ENSG0000(	SLC1A4	831.3192	-0.21652	0.06391	0.416105	protein_coding
ENSG0000(	NMT2	1278.391	0.190954	0.063989	0.416287	protein_coding

ENSG0000(	PDZD3	899.0301	0.578358	0.064073	0.41673	protein_coding
ENSG0000(	ENSG0000(	252.1594	0.174444	0.064121	0.416901	lncRNA
ENSG0000(	PIWIL4	469.9696	0.300122	0.064129	0.416901	protein_coding
ENSG0000(	ARPC5	2707.039	-0.16859	0.064225	0.417166	protein_coding
ENSG0000(	FCGBP	59306.86	0.455989	0.064259	0.417166	protein_coding
ENSG0000(	NPIPB10P	69.48059	0.377819	0.064237	0.417166	unprocessed_pseudogene
ENSG0000(	PCDHGC3	252.6332	-0.30704	0.064261	0.417166	protein_coding
ENSG0000(	ZNF41	853.983	0.167625	0.064232	0.417166	protein_coding
ENSG0000(	ZNF57	260.4901	0.385291	0.064203	0.417166	protein_coding
ENSG0000(	BTBD10	762.7906	0.170072	0.064295	0.417187	protein_coding
ENSG0000(	ENSG0000(	453.0689	0.14051	0.064292	0.417187	lncRNA
ENSG0000(	AADAT	114.9451	0.257576	0.064461	0.417683	protein_coding
ENSG0000(	DNAJC9	481.3769	-0.20699	0.064457	0.417683	protein_coding
ENSG0000(	EIF2S3B	42.79504	0.396387	0.064415	0.417683	protein_coding
ENSG0000(	ENSG0000(	73.76454	-0.29676	0.064389	0.417683	lncRNA
ENSG0000(	ENSG0000(	298.9707	-0.16002	0.064485	0.417683	lncRNA
ENSG0000(	ENSG0000(	203.0202	0.187589	0.064508	0.417683	lncRNA
ENSG0000(	GCSAM	154.6964	-0.47471	0.064474	0.417683	protein_coding
ENSG0000(	SIDT1	1584.89	0.209176	0.064502	0.417683	protein_coding
ENSG0000(	STK32A	115.8783	-0.56855	0.064465	0.417683	protein_coding
ENSG0000(	RPS3AP14	32.94396	0.876229	0.064523	0.417684	processed_pseudogene
ENSG0000(	ARHGAP31	1568.678	-0.26588	0.064639	0.417781	protein_coding
ENSG0000(	CORO6	76.30794	-0.41118	0.064642	0.417781	protein_coding
ENSG0000(	ENSG0000(	112.3998	-0.46546	0.064591	0.417781	lncRNA
ENSG0000(	ENSG0000(	23.78799	-0.52888	0.064659	0.417781	lncRNA
ENSG0000(	GNE	1671.034	0.246859	0.064659	0.417781	protein_coding
ENSG0000(	LRRC8C	901.7848	-0.32057	0.06464	0.417781	protein_coding
ENSG0000(	NR2F6	718.4708	0.342177	0.064592	0.417781	protein_coding
ENSG0000(	SPINK1	372.4048	-0.53114	0.064656	0.417781	protein_coding
ENSG0000(	BEAN1	138.1156	-0.35118	0.064718	0.417824	protein_coding
ENSG0000(	DNAJB5	141.9895	-0.3368	0.064697	0.417824	protein_coding
ENSG0000(	ENSG0000(	401.3967	0.10826	0.064712	0.417824	lncRNA
ENSG0000(	FAM126A	2198.822	-0.28644	0.064727	0.417824	protein_coding
ENSG0000(	IL12RB2	356.519	-0.27047	0.064747	0.417857	protein_coding
ENSG0000(	ENSG0000(	106.201	0.245908	0.064769	0.417901	lncRNA
ENSG0000(	ENSG0000(	1000.425	0.167945	0.064806	0.417943	TEC
ENSG0000(	SLCO4A1	210.237	-0.32657	0.064794	0.417943	protein_coding
ENSG0000(	HMSD	22.47404	-0.68217	0.064883	0.418338	protein_coding
ENSG0000(	ADAR	8832.96	-0.18115	0.064977	0.41861	protein_coding
ENSG0000(	AZI2	609.1006	-0.24898	0.064967	0.41861	protein_coding
ENSG0000(	ENSG0000(	56.01785	-0.33594	0.064999	0.41861	lncRNA
ENSG0000(	ENSG0000(	50.67361	-0.41014	0.065002	0.41861	TEC
ENSG0000(	KIAA2026	3349.924	0.146314	0.065012	0.41861	protein_coding
ENSG0000(	RAB6D	51.20042	0.566604	0.065016	0.41861	protein_coding
ENSG0000(	RPIA	242.7415	0.180028	0.065032	0.418617	protein_coding
ENSG0000(	CDKN2C	73.87591	-0.3906	0.065088	0.418698	protein_coding
ENSG0000(	CYP4F2	73.58569	0.596337	0.065115	0.418698	protein_coding

ENSG0000(	ENSG0000(	52.69385	0.423199	0.065136	0.418698	lncRNA
ENSG0000(	IFIT1	335.6059	0.571409	0.065136	0.418698	protein_coding
ENSG0000(	LINC00620	57.57675	0.30267	0.06511	0.418698	lncRNA
ENSG0000(	PVT1	2735.111	-0.26412	0.065086	0.418698	lncRNA
ENSG0000(	C9orf72	1024.541	-0.28059	0.065176	0.418856	protein_coding
ENSG0000(	ENSG0000(	1378.523	0.118847	0.065193	0.418866	TEC
ENSG0000(	ENSG0000(	1492.642	0.113513	0.065255	0.418996	lncRNA
ENSG0000(	ENSG0000(	70.46727	0.296533	0.065282	0.418996	lncRNA
ENSG0000(	NDUFAF1	634.3482	0.122296	0.065289	0.418996	protein_coding
ENSG0000(	TRMT9B	1695.837	0.205139	0.065279	0.418996	protein_coding
ENSG0000(	ZNF100	898.5159	0.160734	0.065249	0.418996	protein_coding
ENSG0000(	PLXNB1	2418.499	0.256629	0.065305	0.418997	protein_coding
ENSG0000(	APOBEC3A	344.6988	-0.69197	0.065345	0.419021	protein_coding
ENSG0000(	B3GAT3	327.59	-0.18136	0.065384	0.419021	protein_coding
ENSG0000(	CBLC	483.9718	0.468744	0.065338	0.419021	protein_coding
ENSG0000(	KLRG1	441.1992	-0.13988	0.065373	0.419021	protein_coding
ENSG0000(	TCP10L3	192.9094	-0.28305	0.065357	0.419021	transcribed_unprocessed_pseudo
ENSG0000(	GPSM2	2337.914	0.142335	0.065411	0.419043	protein_coding
ENSG0000(	LINC02857	71.2645	-0.3217	0.065418	0.419043	lncRNA
ENSG0000(	ENSG0000(	216.4469	0.167836	0.065447	0.419128	lncRNA
ENSG0000(	IGHJ5	482.9851	-0.49199	0.065462	0.419128	IG_J_gene
ENSG0000(	CDKN2D	47.70428	-0.38754	0.06554	0.419369	protein_coding
ENSG0000(	ENSG0000(	290.2227	-0.17406	0.065564	0.419369	lncRNA
ENSG0000(	ENSG0000(	40.51946	-0.8008	0.065605	0.419369	TEC
ENSG0000(	LINC01123	452.2588	0.509329	0.065593	0.419369	lncRNA
ENSG0000(	LINC01962	279.7089	0.149642	0.065622	0.419369	lncRNA
ENSG0000(	LINC02395	428.3799	0.157674	0.065608	0.419369	lncRNA
ENSG0000(	MAFK	949.2036	0.176891	0.065614	0.419369	protein_coding
ENSG0000(	SLC25A22	277.6571	-0.22822	0.0656	0.419369	protein_coding
ENSG0000(	SNIP1	1037.695	-0.10127	0.065655	0.419486	protein_coding
ENSG0000(	LINC02511	148.2278	0.19591	0.065694	0.419634	lncRNA
ENSG0000(	ENSG0000(	2902.551	0.19062	0.065737	0.419716	transcribed_unprocessed_pseudo
ENSG0000(	IFT52	173.8047	0.307123	0.065723	0.419716	protein_coding
ENSG0000(	LINC02019	67.85035	0.257477	0.065772	0.41984	lncRNA
ENSG0000(	ENSG0000(	311.8853	-0.2775	0.065833	0.419939	TEC
ENSG0000(	RN7SL828F	28.09738	-0.34444	0.065822	0.419939	misc_RNA
ENSG0000(	UFM1	1553.347	-0.15875	0.065804	0.419939	protein_coding
ENSG0000(	DLG1	5410.548	0.162978	0.065994	0.420665	protein_coding
ENSG0000(	ELP1	2650.941	0.071183	0.065974	0.420665	protein_coding
ENSG0000(	ENSG0000(	28.23861	0.41786	0.066002	0.420665	lncRNA
ENSG0000(	OBI1-AS1	19.48022	-0.85377	0.066008	0.420665	lncRNA
ENSG0000(	CSRP2	554.6199	-0.36314	0.066116	0.421043	protein_coding
ENSG0000(	ENSG0000(	27.15687	0.451961	0.066094	0.421043	TEC
ENSG0000(	ENSG0000(	428.3182	0.12341	0.066128	0.421043	TEC
ENSG0000(	SLC25A4	244.9506	0.283438	0.066127	0.421043	protein_coding
ENSG0000(	ENSG0000(	258.0919	-0.23478	0.066178	0.421111	lncRNA
ENSG0000(	ENSG0000(	841.0439	0.22654	0.066185	0.421111	lncRNA

ENSG0000(	TMEM14C	305.8918	0.268913	0.066184	0.421111	protein_coding
ENSG0000(	ARAP3	369.2315	-0.2741	0.066301	0.421749	protein_coding
ENSG0000(	CPB1	43.56131	0.544813	0.066378	0.422145	protein_coding
ENSG0000(	ENSG0000(	43.10778	0.482771	0.066442	0.422203	lncRNA
ENSG0000(	ENSG0000(	71.58668	-0.23232	0.06643	0.422203	TEC
ENSG0000(	SYNGAP1-A	55.99678	-0.27023	0.066449	0.422203	lncRNA
ENSG0000(	XPO5	991.7122	-0.17588	0.066417	0.422203	protein_coding
ENSG0000(	ENSG0000(	88.14837	0.222006	0.066524	0.422489	processed_pseudogene
ENSG0000(	GJC2	20.14324	-0.61543	0.066513	0.422489	protein_coding
ENSG0000(	CCL15	167.898	0.402524	0.066595	0.422842	protein_coding
ENSG0000(	ANKS6	584.6184	0.184485	0.066665	0.422957	protein_coding
ENSG0000(	PLPPR2	191.9498	-0.26082	0.066654	0.422957	protein_coding
ENSG0000(	STPG4	94.90856	0.352532	0.066675	0.422957	protein_coding
ENSG0000(	ZNF140	505.091	0.155452	0.066639	0.422957	protein_coding
ENSG0000(	MRPL50	345.7263	-0.17787	0.066729	0.42303	protein_coding
ENSG0000(	PCDHB19P	41.20226	0.31266	0.066732	0.42303	transcribed_unprocessed_pseudo
ENSG0000(	SERINC5	5561.697	0.228708	0.066748	0.42303	protein_coding
ENSG0000(	TEX35	258.1809	-0.20766	0.066738	0.42303	protein_coding
ENSG0000(	ALMS1	4471.059	0.10774	0.066796	0.423047	protein_coding
ENSG0000(	DNAH1	2623.964	0.223518	0.066903	0.423047	protein_coding
ENSG0000(	ENSG0000(	242.0437	0.266845	0.066835	0.423047	lncRNA
ENSG0000(	ENSG0000(	72.00971	0.549918	0.066831	0.423047	lncRNA
ENSG0000(	FAM83E	3536.614	0.350123	0.066935	0.423047	protein_coding
ENSG0000(	ITSN1	1848.722	0.153721	0.066861	0.423047	protein_coding
ENSG0000(	SDC3	1641.71	-0.27597	0.066906	0.423047	protein_coding
ENSG0000(	SFMBT1	727.8252	0.2403	0.066789	0.423047	protein_coding
ENSG0000(	SMAD7	627.8704	0.274573	0.066934	0.423047	protein_coding
ENSG0000(	TMEM141	465.2302	0.373389	0.066885	0.423047	protein_coding
ENSG0000(	TSPYL5	194.6645	-0.27434	0.066828	0.423047	protein_coding
ENSG0000(	ULK3	1178.637	0.283953	0.0669	0.423047	protein_coding
ENSG0000(	ABTB2	311.6735	0.390213	0.067027	0.423232	protein_coding
ENSG0000(	FFAR4	515.563	0.242451	0.06705	0.423232	protein_coding
ENSG0000(	MC1R	673.9367	0.237032	0.067032	0.423232	protein_coding
ENSG0000(	MMP24OS	888.5008	0.166208	0.067092	0.423232	protein_coding
ENSG0000(	MT-TM	178.4305	-0.4853	0.067015	0.423232	Mt_tRNA
ENSG0000(	NUDCD1	817.3117	-0.18269	0.067103	0.423232	protein_coding
ENSG0000(	RASGRF1	162.6234	-0.40312	0.067096	0.423232	protein_coding
ENSG0000(	RN7SL317P	135.5334	0.22117	0.06709	0.423232	misc_RNA
ENSG0000(	RNF175	25.19639	-0.6932	0.067061	0.423232	protein_coding
ENSG0000(	AGFG2	1609.052	0.318636	0.067208	0.423337	protein_coding
ENSG0000(	FADS1	785.2533	-0.33354	0.067144	0.423337	protein_coding
ENSG0000(	ICA1	1070.644	0.233201	0.067211	0.423337	protein_coding
ENSG0000(	KCNJ8	36.35892	-0.76677	0.067196	0.423337	protein_coding
ENSG0000(	ORAI2	3465.711	-0.13812	0.067212	0.423337	protein_coding
ENSG0000(	SLITRK4	227.9344	0.317839	0.067151	0.423337	protein_coding
ENSG0000(	LINC00663	409.8801	0.198418	0.067259	0.423349	lncRNA
ENSG0000(	SNHG11	224.7454	0.309473	0.067257	0.423349	lncRNA

ENSG0000(	STAG3L3	1882.123	0.110033	0.067276	0.423349	transcribed_unprocessed_pseudo
ENSG0000(	STRN3	1096.593	-0.10687	0.067262	0.423349	protein_coding
ENSG0000(	RN7SKP50	46.09217	-0.41532	0.067292	0.423353	misc_RNA
ENSG0000(	ENSG0000(	34.72088	0.389409	0.067358	0.423674	lncRNA
ENSG0000(	RNU6-681F	30.0815	-0.44472	0.067456	0.424194	snRNA
ENSG0000(	MYO1C	3164.455	0.186501	0.06749	0.424211	protein_coding
ENSG0000(	PIGQ	724.9862	-0.25661	0.067484	0.424211	protein_coding
ENSG0000(	CYB5A	1377.665	0.174986	0.067587	0.424214	protein_coding
ENSG0000(	DCK	275.0836	-0.30477	0.067561	0.424214	protein_coding
ENSG0000(	DDR1	2977.537	0.304254	0.067557	0.424214	protein_coding
ENSG0000(	ENSG0000(	66.51192	0.442079	0.067631	0.424214	lncRNA
ENSG0000(	ENSG0000(	1886.49	-0.11735	0.067626	0.424214	lncRNA
ENSG0000(	KDM5D	2121.884	-0.23493	0.06763	0.424214	protein_coding
ENSG0000(	RN7SL589F	49.91865	0.271456	0.067583	0.424214	misc_RNA
ENSG0000(	RPL7L1P2	48.79483	0.558867	0.067602	0.424214	processed_pseudogene
ENSG0000(	TMEM167E	481.828	0.134737	0.067579	0.424214	lncRNA
ENSG0000(	ZMIZ1-AS1	114.0005	-0.27398	0.067644	0.424214	lncRNA
ENSG0000(	ENSG0000(	80.66873	-0.28381	0.067662	0.424229	lncRNA
ENSG0000(	CLRN3	377.7566	0.404976	0.067752	0.424384	protein_coding
ENSG0000(	DDX3Y	982.1969	0.665614	0.067733	0.424384	protein_coding
ENSG0000(	ENSG0000(	347.3407	-0.17328	0.067795	0.424384	lncRNA
ENSG0000(	IGLJ3	23.66632	-0.68211	0.067773	0.424384	IG_J_gene
ENSG0000(	MLLT3	1225.288	0.210347	0.067767	0.424384	protein_coding
ENSG0000(	TRG-AS1	1258.334	-0.27522	0.06774	0.424384	lncRNA
ENSG0000(	XKRX	99.35966	0.333961	0.06779	0.424384	protein_coding
ENSG0000(	ARL17A	2644.462	-0.34772	0.06785	0.424636	protein_coding
ENSG0000(	ENSG0000(	126.4738	-0.21385	0.068022	0.425611	lncRNA
ENSG0000(	CHIC2	194.4883	-0.2378	0.068089	0.425937	protein_coding
ENSG0000(	AKAP8	1238.937	0.102534	0.068193	0.426036	protein_coding
ENSG0000(	ATXN7	4934.81	-0.10987	0.068223	0.426036	protein_coding
ENSG0000(	B3GALT9	559.5507	0.128061	0.068242	0.426036	protein_coding
ENSG0000(	CCRL2	208.2699	-0.2875	0.068185	0.426036	protein_coding
ENSG0000(	DTX4	2505.644	0.315793	0.06826	0.426036	protein_coding
ENSG0000(	ENSG0000(	148.9245	-0.21121	0.068199	0.426036	lncRNA
ENSG0000(	ITFG1	1019.754	-0.13968	0.068157	0.426036	protein_coding
ENSG0000(	MDFI	45.10095	-0.85689	0.068247	0.426036	protein_coding
ENSG0000(	POMGNT1	935.3816	0.190475	0.068249	0.426036	protein_coding
ENSG0000(	RELB	499.1199	-0.29277	0.068236	0.426036	protein_coding
ENSG0000(	ENSG0000(	220.329	0.154473	0.068286	0.426074	TEC
ENSG0000(	POTEB3	98.29108	0.366848	0.068297	0.426074	protein_coding
ENSG0000(	PLSCR1	2211.735	-0.26355	0.06833	0.426184	protein_coding
ENSG0000(	ENSG0000(	707.7422	0.122166	0.068362	0.426195	lncRNA
ENSG0000(	SIGLEC16	548.9592	-0.1091	0.068363	0.426195	polymorphic_pseudogene
ENSG0000(	CHEK1	368.8064	-0.20487	0.068402	0.426244	protein_coding
ENSG0000(	SLC25A45	1315.363	-0.14828	0.068393	0.426244	protein_coding
ENSG0000(	ADAMTSL1	758.9428	0.393108	0.068671	0.426323	protein_coding
ENSG0000(	ASAH1	2648.813	-0.14675	0.068569	0.426323	protein_coding

ENSG0000(	BLZF2P	87.69005	-0.39406	0.068697	0.426323	unprocessed_pseudogene
ENSG0000(	CDC7	259.1512	-0.31975	0.068656	0.426323	protein_coding
ENSG0000(	CHERP	648.8257	0.155235	0.068698	0.426323	protein_coding
ENSG0000(	ENSG0000(	30.62095	0.397468	0.068485	0.426323	lncRNA
ENSG0000(	ENSG0000(	35.66467	-0.381	0.06864	0.426323	lncRNA
ENSG0000(	ENSG0000(	300.0021	0.190353	0.068849	0.426323	lncRNA
ENSG0000(	ENSG0000(	295.2	0.257215	0.068764	0.426323	lncRNA
ENSG0000(	ENSG0000(	64.78647	0.319861	0.06883	0.426323	lncRNA
ENSG0000(	ENSG0000(	141.2982	0.344697	0.068887	0.426323	TEC
ENSG0000(	GCNT2	1076.738	0.262507	0.068837	0.426323	protein_coding
ENSG0000(	IGIP	554.5153	0.122789	0.068856	0.426323	protein_coding
ENSG0000(	IGLV3-25	158.0602	-0.86714	0.068461	0.426323	IG_V_gene
ENSG0000(	IRAK2	303.5501	-0.35797	0.068882	0.426323	protein_coding
ENSG0000(	KBTBD11-A	182.0584	0.238915	0.068758	0.426323	lncRNA
ENSG0000(	KCNE1	427.4862	-0.34821	0.068679	0.426323	protein_coding
ENSG0000(	KCNIP4	59.20112	-0.6493	0.068864	0.426323	protein_coding
ENSG0000(	LEPROT	2169.142	-0.14479	0.068853	0.426323	protein_coding
ENSG0000(	LRP6	2234.854	0.255986	0.068621	0.426323	protein_coding
ENSG0000(	LSM11	579.2386	0.133047	0.068654	0.426323	protein_coding
ENSG0000(	LY6E-DT	168.2374	0.220654	0.068614	0.426323	lncRNA
ENSG0000(	OST4	328.2595	-0.25543	0.068893	0.426323	protein_coding
ENSG0000(	PHRF1	1452.864	0.147939	0.068846	0.426323	protein_coding
ENSG0000(	PRR22	85.73533	0.332253	0.068481	0.426323	protein_coding
ENSG0000(	RAVER2	2545.051	0.406151	0.068895	0.426323	protein_coding
ENSG0000(	RIPK2-DT	13850.66	-0.13701	0.068875	0.426323	lncRNA
ENSG0000(	SNX13	3866.697	0.200362	0.068778	0.426323	protein_coding
ENSG0000(	STK24-AS1	54.17677	0.269241	0.068449	0.426323	lncRNA
ENSG0000(	TPRG1	125.0639	-0.33154	0.068851	0.426323	protein_coding
ENSG0000(	ZNF438	298.8566	-0.24944	0.068858	0.426323	protein_coding
ENSG0000(	TNIP1	2426.159	-0.1905	0.068913	0.426338	protein_coding
ENSG0000(	MRPS18B	301.5713	-0.18175	0.068972	0.426609	protein_coding
ENSG0000(	RASSF8-AS:	716.559	-0.17408	0.069	0.426684	lncRNA
ENSG0000(	ENSG0000(	148.955	0.170835	0.069096	0.426992	lncRNA
ENSG0000(	ODC1-DT	653.868	-0.14563	0.069095	0.426992	lncRNA
ENSG0000(	RASSF5	1099.9	-0.39203	0.069068	0.426992	protein_coding
ENSG0000(	MTRES1	367.5428	0.168421	0.069121	0.427049	protein_coding
ENSG0000(	GUSBP9	610.9826	0.445611	0.069143	0.427093	unprocessed_pseudogene
ENSG0000(	CDCA7	606.4224	0.3059	0.069227	0.427173	protein_coding
ENSG0000(	ENSG0000(	37.30388	-0.42486	0.069197	0.427173	lncRNA
ENSG0000(	HEBP2P1	110.9094	0.347416	0.069234	0.427173	processed_pseudogene
ENSG0000(	IL1RAPL1	44.0578	0.463538	0.069255	0.427173	protein_coding
ENSG0000(	NAGPA	491.5783	-0.14165	0.069265	0.427173	protein_coding
ENSG0000(	STX1A	76.65695	-0.39684	0.069208	0.427173	protein_coding
ENSG0000(	TMEM147	146.7653	-0.31567	0.06924	0.427173	protein_coding
ENSG0000(	ANKH	3080.439	0.304201	0.069321	0.427281	protein_coding
ENSG0000(	CENPV	527.6126	0.230528	0.069309	0.427281	protein_coding
ENSG0000(	ZNF799	704.0759	0.178497	0.069329	0.427281	protein_coding

ENSG0000(	SYTL4	1905.749	0.263408	0.069457	0.427975	protein_coding
ENSG0000(	LINC00880	50.06823	-0.29089	0.0695	0.428142	lncRNA
ENSG0000(	ENSG0000(	31.33416	-0.30481	0.069522	0.428181	lncRNA
ENSG0000(	ENSG0000(	350.6238	0.367945	0.069546	0.428237	lncRNA
ENSG0000(	HSPA13	333.2623	-0.28909	0.069568	0.428273	protein_coding
ENSG0000(	FASTKD5	818.9845	0.09209	0.069729	0.428605	protein_coding
ENSG0000(	HLA-F	2581.192	-0.27421	0.069743	0.428605	protein_coding
ENSG0000(	MYBL2	204.7139	-0.36889	0.06978	0.428605	protein_coding
ENSG0000(	PIF1	611.1578	-0.13578	0.069751	0.428605	protein_coding
ENSG0000(	PWP1	356.268	-0.15353	0.069739	0.428605	protein_coding
ENSG0000(	RN7SKP16	76.72834	-0.38231	0.069793	0.428605	misc_RNA
ENSG0000(	SLC4A3	39.97894	-0.66819	0.069717	0.428605	protein_coding
ENSG0000(	STK38	2771.845	0.219588	0.06976	0.428605	protein_coding
ENSG0000(	TAP2	2284.928	-0.29708	0.06979	0.428605	protein_coding
ENSG0000(	TIE1	434.9871	-0.25747	0.069644	0.428605	protein_coding
ENSG0000(	ZBTB4	1413.064	-0.17699	0.069657	0.428605	protein_coding
ENSG0000(	CR1	1601.46	-0.65287	0.069847	0.428669	protein_coding
ENSG0000(	LINC01482	287.2743	-0.16927	0.069831	0.428669	lncRNA
ENSG0000(	TRIM21	388.9165	-0.2837	0.06985	0.428669	protein_coding
ENSG0000(	SORCS2	127.4339	-0.62006	0.069963	0.429266	protein_coding
ENSG0000(	ENSG0000(	74.86939	0.2856	0.06999	0.429336	lncRNA
ENSG0000(	IL2RB	473.5239	-0.27015	0.070014	0.429388	protein_coding
ENSG0000(	ABHD11	658.7642	0.273692	0.070656	0.429689	protein_coding
ENSG0000(	ASLP1	106.1476	0.246865	0.070619	0.429689	unprocessed_pseudogene
ENSG0000(	ATP8B2	1358.218	-0.22972	0.070657	0.429689	protein_coding
ENSG0000(	CALB1	75.51276	0.417754	0.070642	0.429689	protein_coding
ENSG0000(	CBX2	65.67459	-0.56947	0.070432	0.429689	protein_coding
ENSG0000(	CLEC10A	146.1281	-0.37138	0.070299	0.429689	protein_coding
ENSG0000(	COQ7	553.9332	0.181319	0.070476	0.429689	protein_coding
ENSG0000(	COX19	1141.811	0.130835	0.070485	0.429689	protein_coding
ENSG0000(	CYP4X1	30.98272	-0.63789	0.070546	0.429689	protein_coding
ENSG0000(	DES	203.2479	0.728644	0.070646	0.429689	protein_coding
ENSG0000(	DHX30	1167.622	0.124913	0.070273	0.429689	protein_coding
ENSG0000(	DIO3OS	308.2078	0.432401	0.070591	0.429689	lncRNA
ENSG0000(	DOP1A	2185.102	0.179462	0.070188	0.429689	protein_coding
ENSG0000(	ENPP7P4	25.38491	0.730331	0.070634	0.429689	transcribed_unprocessed_pseudo
ENSG0000(	ENSG0000(	21.00042	-0.4757	0.070425	0.429689	lncRNA
ENSG0000(	ENSG0000(	848.5538	-0.2187	0.070196	0.429689	lncRNA
ENSG0000(	ENSG0000(	21.74536	0.864036	0.070365	0.429689	lncRNA
ENSG0000(	FAM171A1	772.7178	0.244772	0.070313	0.429689	protein_coding
ENSG0000(	FAM230C	121.775	0.440797	0.070261	0.429689	lncRNA
ENSG0000(	GABRR2	473.9129	-0.23701	0.070442	0.429689	protein_coding
ENSG0000(	GRAMD2B	1077.744	0.268542	0.07062	0.429689	protein_coding
ENSG0000(	KDELR3	159.117	-0.44558	0.070514	0.429689	protein_coding
ENSG0000(	KLK1	153.0003	0.627947	0.070119	0.429689	protein_coding
ENSG0000(	MGAT2	541.3173	-0.18903	0.07051	0.429689	protein_coding
ENSG0000(	MMUT	407.1396	0.194633	0.070184	0.429689	protein_coding

ENSG0000(	MRPL34	304.2165	0.17739	0.07055	0.429689	protein_coding
ENSG0000(	MTMR11	3783.111	0.389164	0.07025	0.429689	protein_coding
ENSG0000(	OR2B11	339.5008	0.177925	0.070464	0.429689	protein_coding
ENSG0000(	PIGU	233.2274	0.263426	0.07031	0.429689	protein_coding
ENSG0000(	PRDX6	1200.601	0.357075	0.070152	0.429689	protein_coding
ENSG0000(	RAB11FIP1	3982.383	0.203093	0.070533	0.429689	protein_coding
ENSG0000(	RN7SL690F	230.4141	0.161188	0.07062	0.429689	misc_RNA
ENSG0000(	RPL17P50	42.2138	-0.39232	0.070254	0.429689	processed_pseudogene
ENSG0000(	RPS6KA2	808.2234	-0.262	0.070452	0.429689	protein_coding
ENSG0000(	SAMD14	134.4617	-0.49668	0.070387	0.429689	protein_coding
ENSG0000(	STK3	1349.471	-0.1786	0.070355	0.429689	protein_coding
ENSG0000(	TRPM5	72.40176	0.549497	0.070209	0.429689	protein_coding
ENSG0000(	YPEL4	55.0489	-0.51932	0.07019	0.429689	protein_coding
ENSG0000(	ADCK1	279.7207	0.204436	0.070676	0.429712	protein_coding
ENSG0000(	SRMS	49.59974	-0.72589	0.070703	0.429782	protein_coding
ENSG0000(	SORL1-AS1	278.0225	0.239557	0.070742	0.429924	lncRNA
ENSG0000(	GSE1	3649.83	0.143389	0.070805	0.430211	protein_coding
ENSG0000(	BLNK	1832.519	-0.16658	0.070827	0.430249	protein_coding
ENSG0000(	HTRA2	194.7535	-0.22133	0.070919	0.430532	protein_coding
ENSG0000(	LINC00345	361.1277	-0.16233	0.070921	0.430532	lncRNA
ENSG0000(	TRDMT1	1178.203	-0.1897	0.070921	0.430532	protein_coding
ENSG0000(	ENSG0000(	739.4975	-0.38128	0.070996	0.430618	lncRNA
ENSG0000(	ENSG0000(	53.85857	0.346052	0.070997	0.430618	lncRNA
ENSG0000(	ENSG0000(	344.328	0.143535	0.071014	0.430618	lncRNA
ENSG0000(	IGKV3D-15	61.44707	-0.99773	0.071029	0.430618	IG_V_gene
ENSG0000(	LINC00534	1026.338	0.10216	0.07096	0.430618	lncRNA
ENSG0000(	SSR2	1817.923	-0.18432	0.071014	0.430618	protein_coding
ENSG0000(	PLA2G2D	451.9175	-0.43147	0.071151	0.431173	protein_coding
ENSG0000(	ZNF554	1329.786	0.115653	0.071152	0.431173	protein_coding
ENSG0000(	RGS11	298.5945	0.233555	0.071248	0.431649	protein_coding
ENSG0000(	USP48	2382.97	-0.16706	0.071262	0.431649	protein_coding
ENSG0000(	BAIAP3	152.3099	0.425308	0.071797	0.431668	protein_coding
ENSG0000(	BEND3	330.7942	0.252398	0.071787	0.431668	protein_coding
ENSG0000(	BOLA1	63.0982	-0.36355	0.071572	0.431668	protein_coding
ENSG0000(	CDHR5	4129.311	0.528074	0.071396	0.431668	protein_coding
ENSG0000(	CDK11A	710.2714	0.201385	0.071664	0.431668	protein_coding
ENSG0000(	CORO7	1298.066	-0.12282	0.07143	0.431668	protein_coding
ENSG0000(	CYP2U1-AS	309.8824	0.228753	0.071651	0.431668	lncRNA
ENSG0000(	DDT	595.6958	0.225709	0.071819	0.431668	protein_coding
ENSG0000(	ECHDC3	50.73217	-0.54878	0.071428	0.431668	protein_coding
ENSG0000(	ENSG0000(	92.75413	-0.68658	0.071589	0.431668	unprocessed_pseudogene
ENSG0000(	ENSG0000(	54.00224	0.283614	0.071539	0.431668	lncRNA
ENSG0000(	ENSG0000(	664.1211	-0.3872	0.07145	0.431668	TEC
ENSG0000(	ENSG0000(	125.5799	0.193186	0.071854	0.431668	lncRNA
ENSG0000(	ENSG0000(	26.11347	0.621813	0.071763	0.431668	lncRNA
ENSG0000(	ENSG0000(	64.36074	0.302374	0.071436	0.431668	lncRNA
ENSG0000(	ENSG0000(	794.2286	0.132613	0.071728	0.431668	lncRNA

ENSG0000(	ENSG0000(	79.44177	-0.65928	0.071552	0.431668	transcribed_unprocessed_pseudo
ENSG0000(	ENSG0000(	50.97751	-0.46247	0.071416	0.431668	lncRNA
ENSG0000(	EVI2A	383.4412	-0.33885	0.071505	0.431668	protein_coding
ENSG0000(	KLHL2	373.0463	-0.24511	0.071702	0.431668	protein_coding
ENSG0000(	LINC01750	54.90414	-0.5901	0.071562	0.431668	lncRNA
ENSG0000(	LINC02617	23.81055	-0.48316	0.071865	0.431668	lncRNA
ENSG0000(	LSS	1485.287	-0.23989	0.07169	0.431668	protein_coding
ENSG0000(	MIR325HG	254.5168	-0.18306	0.07174	0.431668	lncRNA
ENSG0000(	MIR5192	19.86498	0.470121	0.071875	0.431668	miRNA
ENSG0000(	MIR581	23.68507	0.39684	0.071669	0.431668	miRNA
ENSG0000(	N6AMT1	612.0795	0.192473	0.07181	0.431668	protein_coding
ENSG0000(	NHEG1	49.20583	0.321933	0.071863	0.431668	lncRNA
ENSG0000(	PCBP3	296.7311	-0.4042	0.071493	0.431668	protein_coding
ENSG0000(	PCOLCE-AS	38.84273	-0.35022	0.071366	0.431668	lncRNA
ENSG0000(	PLEKHH1	3872.76	0.309309	0.071877	0.431668	protein_coding
ENSG0000(	RASGRF2	575.4163	-0.41869	0.071361	0.431668	protein_coding
ENSG0000(	SH3TC2	580.6198	-0.21766	0.071543	0.431668	protein_coding
ENSG0000(	STARD3NL	732.1334	0.239558	0.071316	0.431668	protein_coding
ENSG0000(	TNFRSF11A	1342.906	0.39013	0.071484	0.431668	protein_coding
ENSG0000(	UBE2W	1418.88	-0.15615	0.071697	0.431668	protein_coding
ENSG0000(	URGCP	1895.686	0.197159	0.071603	0.431668	protein_coding
ENSG0000(	ZFYVE27	1437.966	0.175829	0.071662	0.431668	protein_coding
ENSG0000(	ZNF426	2386.058	0.108964	0.071427	0.431668	protein_coding
ENSG0000(	COBL	1448.159	0.417	0.071899	0.43171	protein_coding
ENSG0000(	ALKBH5	821.8469	-0.17871	0.071933	0.431818	protein_coding
ENSG0000(	AASDHPPT	482.3191	-0.1468	0.072038	0.432165	protein_coding
ENSG0000(	ENSG0000(	674.2442	0.113145	0.07202	0.432165	protein_coding
ENSG0000(	IGHV3OR16	32.20131	-0.74118	0.072031	0.432165	IG_V_gene
ENSG0000(	LNCTAM34	25.70614	-0.73379	0.072149	0.432635	lncRNA
ENSG0000(	LRG1	530.8495	-0.20656	0.072157	0.432635	protein_coding
ENSG0000(	PPP1R9A-A	151.9922	0.213065	0.072163	0.432635	lncRNA
ENSG0000(	MRTFA	1155.114	-0.18942	0.072184	0.432643	protein_coding
ENSG0000(	TPO	149.4812	0.420901	0.072196	0.432643	protein_coding
ENSG0000(	TECPR2	916.733	-0.27602	0.072232	0.432766	protein_coding
ENSG0000(	CD300A	286.7337	-0.34528	0.072336	0.433008	protein_coding
ENSG0000(	ENSG0000(	26.6924	0.371117	0.072291	0.433008	lncRNA
ENSG0000(	VAC14	1366.61	-0.19467	0.07233	0.433008	protein_coding
ENSG0000(	VWA5B2	39.96232	0.596416	0.072318	0.433008	protein_coding
ENSG0000(	SLC22A18A	227.3714	0.493112	0.072372	0.433129	protein_coding
ENSG0000(	HK2	6348.314	-0.36989	0.07243	0.433386	protein_coding
ENSG0000(	ENSG0000(	183.7403	0.170469	0.072552	0.43383	lncRNA
ENSG0000(	SIPA1	739.7919	-0.26503	0.072541	0.43383	protein_coding
ENSG0000(	STK4	3969.565	-0.1907	0.072548	0.43383	protein_coding
ENSG0000(	DYNLL2-DT	182.2242	0.162497	0.072574	0.433859	lncRNA
ENSG0000(	GUCA2A	416.5165	0.869436	0.072589	0.433859	protein_coding
ENSG0000(	ENSG0000(	121.8535	0.24257	0.072627	0.433922	lncRNA
ENSG0000(	TXLNG	524.1738	0.140286	0.072631	0.433922	protein_coding

ENSG0000(	CHP2	1850.468	0.456516	0.072647	0.433924	protein_coding
ENSG0000(	ACADS	664.7919	0.402856	0.072697	0.433994	protein_coding
ENSG0000(	CLEC16A	2259.221	-0.13139	0.072729	0.433994	protein_coding
ENSG0000(	DNM1P46	167.182	-0.22543	0.072753	0.433994	transcribed_unprocessed_pseudo
ENSG0000(	ENSG0000(	23.74009	-0.5033	0.072739	0.433994	lncRNA
ENSG0000(	ENSG0000(	30.1728	0.309064	0.072746	0.433994	lncRNA
ENSG0000(	WNT5B	276.5967	-0.24556	0.072719	0.433994	protein_coding
ENSG0000(	ENSG0000(	88.75639	0.219801	0.072818	0.434287	lncRNA
ENSG0000(	SIRT5	1683.463	0.125036	0.072855	0.434392	protein_coding
ENSG0000(	ZBED4	1165.565	0.104316	0.072867	0.434392	protein_coding
ENSG0000(	ADGRE1	129.9194	-0.51448	0.072902	0.434504	protein_coding
ENSG0000(	LAMB4	120.2784	-0.17784	0.072962	0.434766	protein_coding
ENSG0000(	SET	2358.038	-0.15642	0.073053	0.435218	protein_coding
ENSG0000(	ENSG0000(	717.1265	0.137649	0.073148	0.435691	lncRNA
ENSG0000(	GDNF-AS1	419.2292	0.128018	0.073231	0.435995	lncRNA
ENSG0000(	RHOV	20.06451	0.571015	0.073227	0.435995	protein_coding
ENSG0000(	KDM4A-AS	381.7325	-0.21236	0.073269	0.436126	lncRNA
ENSG0000(	DRC3	188.4817	0.164258	0.073316	0.4362	protein_coding
ENSG0000(	ENSG0000(	42.19494	0.394641	0.073316	0.4362	lncRNA
ENSG0000(	HOXA6	284.6232	0.326462	0.073345	0.4362	protein_coding
ENSG0000(	IGLV3-19	265.4047	-0.80356	0.073334	0.4362	IG_V_gene
ENSG0000(	ENSG0000(	118.8269	0.20776	0.073454	0.436756	lncRNA
ENSG0000(	ENSG0000(	515.3694	0.172591	0.073525	0.436894	lncRNA
ENSG0000(	ENSG0000(	43.44407	-0.33892	0.073522	0.436894	lncRNA
ENSG0000(	ENSG0000(	38.12333	-0.5069	0.073495	0.436894	lncRNA
ENSG0000(	MCM10	206.1414	-0.37669	0.073556	0.436986	protein_coding
ENSG0000(	LINC00967	141.2294	0.433342	0.073614	0.437232	lncRNA
ENSG0000(	DIRAS3	27.89837	-0.58057	0.073729	0.437315	protein_coding
ENSG0000(	IGKV1-6	145.5076	-0.63848	0.073663	0.437315	IG_V_gene
ENSG0000(	MIGA2	1225.037	0.250638	0.0737	0.437315	protein_coding
ENSG0000(	PLTP	190.0919	-0.36376	0.073676	0.437315	protein_coding
ENSG0000(	SNORD13	794.1021	-0.26891	0.073739	0.437315	snoRNA
ENSG0000(	SNRPGP14	26.17279	-0.45684	0.073662	0.437315	processed_pseudogene
ENSG0000(	SUN2	2048.918	0.168531	0.073736	0.437315	protein_coding
ENSG0000(	CD300C	30.43824	-0.54893	0.073796	0.437404	protein_coding
ENSG0000(	ENSG0000(	96.23915	-0.4534	0.073785	0.437404	TEC
ENSG0000(	IKZF2	1102.611	-0.23235	0.073802	0.437404	protein_coding
ENSG0000(	GPR160P2	116.5786	0.220347	0.073878	0.437479	processed_pseudogene
ENSG0000(	MT-TH	136.3838	-0.7817	0.073834	0.437479	Mt_tRNA
ENSG0000(	RN7SKP93	26.12763	0.546563	0.073872	0.437479	misc_RNA
ENSG0000(	ZP2	65.52452	-0.26355	0.073868	0.437479	protein_coding
ENSG0000(	TLR9	56.61355	-1.09404	0.073904	0.437542	protein_coding
ENSG0000(	ENSG0000(	25.27006	0.371317	0.073984	0.437837	lncRNA
ENSG0000(	STIM2	1259.291	-0.14379	0.073986	0.437837	protein_coding
ENSG0000(	ZNF285	236.6464	0.328926	0.074044	0.438084	protein_coding
ENSG0000(	ENSG0000(	76.02426	-0.28714	0.07408	0.438112	lncRNA
ENSG0000(	MINAR1	99.66812	-0.38224	0.074071	0.438112	protein_coding

ENSG0000(	DYNLT5	27.0415	-0.66608	0.074108	0.438153	protein_coding
ENSG0000(	ENSG0000(	24.56766	-0.64241	0.074119	0.438153	lncRNA
ENSG0000(	ENSG0000(	23.093	-0.7434	0.074164	0.438216	lncRNA
ENSG0000(	NAF1	337.081	0.158359	0.074172	0.438216	protein_coding
ENSG0000(	STAG3L5P	1521.608	0.134496	0.074178	0.438216	transcribed_unprocessed_pseudo
ENSG0000(	REP15	274.1368	0.46502	0.074271	0.438673	protein_coding
ENSG0000(	PRND	182.9849	0.158619	0.074385	0.439255	protein_coding
ENSG0000(	ENSG0000(	175.0578	0.205122	0.074422	0.439284	lncRNA
ENSG0000(	MRPL20-AS	274.7605	-0.2108	0.074409	0.439284	lncRNA
ENSG0000(	ENSG0000(	336.3101	0.164071	0.07447	0.439286	lncRNA
ENSG0000(	FAM91A1	1542.813	-0.11771	0.074455	0.439286	protein_coding
ENSG0000(	MUC12	33623.21	0.491494	0.074459	0.439286	protein_coding
ENSG0000(	PARD3B	1259.926	0.280191	0.074495	0.439337	protein_coding
ENSG0000(	ARSD	1667.154	0.155514	0.074612	0.439346	protein_coding
ENSG0000(	CARD6	384.0161	-0.20953	0.07453	0.439346	protein_coding
ENSG0000(	CTSL	629.1108	-0.31907	0.074563	0.439346	protein_coding
ENSG0000(	ENSG0000(	29.10022	0.652113	0.074631	0.439346	lncRNA
ENSG0000(	ENSG0000(	108.3873	0.256239	0.074626	0.439346	protein_coding
ENSG0000(	HPS4	1940.724	0.086999	0.074627	0.439346	protein_coding
ENSG0000(	MKLN1	5134.43	-0.12371	0.07464	0.439346	protein_coding
ENSG0000(	RHOQP1	37.08379	0.328326	0.074582	0.439346	processed_pseudogene
ENSG0000(	STAP2	937.0196	0.268499	0.074525	0.439346	protein_coding
ENSG0000(	LINC01133	1615.661	0.362132	0.074688	0.439439	lncRNA
ENSG0000(	MYCBP	262.6717	-0.29181	0.074674	0.439439	protein_coding
ENSG0000(	DPPA4	3448.557	0.442807	0.074727	0.439575	protein_coding
ENSG0000(	AKAP11	3673.808	0.090495	0.074794	0.439607	protein_coding
ENSG0000(	ATG9A	700.7292	-0.18601	0.074838	0.439607	protein_coding
ENSG0000(	CA12	7349.586	0.400248	0.074835	0.439607	protein_coding
ENSG0000(	CD244	79.57107	-0.32228	0.074856	0.439607	protein_coding
ENSG0000(	ENSG0000(	218.3741	0.176201	0.074924	0.439607	lncRNA
ENSG0000(	LINC01886	140.9393	0.257572	0.074908	0.439607	lncRNA
ENSG0000(	LINC02193	771.9455	-0.25659	0.074814	0.439607	lncRNA
ENSG0000(	PPCDC	521.7723	-0.19365	0.074776	0.439607	protein_coding
ENSG0000(	PPIAP30	19.67623	0.558628	0.074846	0.439607	processed_pseudogene
ENSG0000(	RRP1B	624.5698	-0.15483	0.074924	0.439607	protein_coding
ENSG0000(	SPAG8	36.40931	-0.51497	0.074888	0.439607	protein_coding
ENSG0000(	TMEM220-	151.4498	0.359936	0.074864	0.439607	lncRNA
ENSG0000(	SNHG7	620.677	0.169882	0.075032	0.440143	lncRNA
ENSG0000(	CPQ	533.8999	-0.18847	0.07508	0.440253	protein_coding
ENSG0000(	SLCO1A2	352.6254	0.187998	0.075082	0.440253	protein_coding
ENSG0000(	CCT8P1	100.211	0.502591	0.075165	0.440552	processed_pseudogene
ENSG0000(	SPINT1	1681.799	0.394339	0.07516	0.440552	protein_coding
ENSG0000(	CDADC1	184.0506	0.221944	0.075222	0.440667	protein_coding
ENSG0000(	CNR1	205.2915	0.555706	0.075233	0.440667	protein_coding
ENSG0000(	ENSG0000(	389.1292	0.261935	0.075224	0.440667	processed_pseudogene
ENSG0000(	LINC00328	42.67824	0.505898	0.075272	0.440801	processed_pseudogene
ENSG0000(	KCNA3	440.6445	-0.34715	0.075367	0.441261	protein_coding

ENSG0000(	MEGF10	65.0014	-0.77443	0.075385	0.441272	protein_coding
ENSG0000(	ENPP7P8	128.2242	0.489434	0.075553	0.441317	transcribed_unprocessed_pseudo
ENSG0000(	ENSG0000(	166.6627	0.580266	0.075423	0.441317	lncRNA
ENSG0000(	FOXD2-AS1	333.5661	0.417297	0.075506	0.441317	lncRNA
ENSG0000(	HSF1	785.4993	0.130157	0.07551	0.441317	protein_coding
ENSG0000(	MAP4K4	2739.205	-0.28509	0.075444	0.441317	protein_coding
ENSG0000(	MARVELD2	1248.795	0.23075	0.075474	0.441317	protein_coding
ENSG0000(	MYH15	41.00873	-0.53213	0.075465	0.441317	protein_coding
ENSG0000(	PACERR	34.76	-0.502	0.075543	0.441317	lncRNA
ENSG0000(	SNORA52	217.9284	0.354117	0.075476	0.441317	snoRNA
ENSG0000(	ZNF571	618.7503	-0.151	0.075541	0.441317	protein_coding
ENSG0000(	ENSG0000(	19.93658	1.011867	0.075569	0.44132	lncRNA
ENSG0000(	CNGB3	40.56877	0.608197	0.075624	0.441549	protein_coding
ENSG0000(	C2orf68	1557.172	0.093423	0.075704	0.441553	protein_coding
ENSG0000(	ENSG0000(	37.09789	0.276034	0.075673	0.441553	lncRNA
ENSG0000(	KC6	36.98051	0.474202	0.075712	0.441553	lncRNA
ENSG0000(	NCOA6	3369.599	0.134858	0.075721	0.441553	protein_coding
ENSG0000(	PFDN5	968.9419	-0.18488	0.075691	0.441553	protein_coding
ENSG0000(	PSTK	249.6107	0.19152	0.075713	0.441553	protein_coding
ENSG0000(	ZBED2	22.26167	-0.99215	0.075774	0.441766	protein_coding
ENSG0000(	IFNGR1	1193.487	-0.21385	0.075795	0.441772	protein_coding
ENSG0000(	JUNB	1356.388	-0.32183	0.075807	0.441772	protein_coding
ENSG0000(	CCNI	3009.301	-0.1476	0.076013	0.441991	protein_coding
ENSG0000(	ENSG0000(	44.08646	0.421565	0.075975	0.441991	processed_pseudogene
ENSG0000(	ENSG0000(	277.6811	0.148528	0.075978	0.441991	processed_pseudogene
ENSG0000(	ENSG0000(	162.5334	-0.22202	0.075982	0.441991	lncRNA
ENSG0000(	ENSG0000(	37.28132	0.311443	0.076021	0.441991	lncRNA
ENSG0000(	FGFR1OP2	1301.933	-0.14988	0.076021	0.441991	protein_coding
ENSG0000(	FZR1	1039.764	0.180188	0.075912	0.441991	protein_coding
ENSG0000(	NDUFA10	3649.205	0.13649	0.075896	0.441991	protein_coding
ENSG0000(	PLEKHM1	2305.701	0.111525	0.076008	0.441991	protein_coding
ENSG0000(	RHOA	3136.659	-0.19531	0.075908	0.441991	protein_coding
ENSG0000(	TFCP2	957.2869	0.124331	0.076021	0.441991	protein_coding
ENSG0000(	KDM2A	4544.495	-0.13502	0.07604	0.442003	protein_coding
ENSG0000(	MIPOL1	4376.448	0.146065	0.076063	0.442047	protein_coding
ENSG0000(	ZNF317	763.8731	0.137764	0.076086	0.442087	protein_coding
ENSG0000(	ACO2	2923.032	0.231984	0.076277	0.442294	protein_coding
ENSG0000(	ANKRD20A	118.7275	-0.24862	0.076239	0.442294	transcribed_unprocessed_pseudo
ENSG0000(	ENSG0000(	40.05943	0.405793	0.076184	0.442294	lncRNA
ENSG0000(	ENSG0000(	624.7914	0.216189	0.076215	0.442294	protein_coding
ENSG0000(	GALNT10	2301.275	-0.14567	0.076273	0.442294	protein_coding
ENSG0000(	IGLV2-11	515.184	-0.64614	0.076184	0.442294	IG_V_gene
ENSG0000(	MIR497HG	206.1745	-0.20527	0.076283	0.442294	lncRNA
ENSG0000(	MPST	2734.526	0.251548	0.076256	0.442294	protein_coding
ENSG0000(	TADA2A	779.9203	-0.16229	0.076165	0.442294	protein_coding
ENSG0000(	VRK2	395.8064	-0.20551	0.076245	0.442294	protein_coding
ENSG0000(	LINC01533	67.23043	0.384536	0.076304	0.442325	lncRNA

ENSG0000(	MTSS1	1113.124	0.209632	0.076339	0.442437	protein_coding
ENSG0000(	ENSG0000(	346.4678	-0.14264	0.07647	0.442915	lncRNA
ENSG0000(	TCAF2P1	57.43319	-0.37692	0.076464	0.442915	transcribed_unprocessed_pseudo
ENSG0000(	TTC28-AS1	1358.495	0.102668	0.076448	0.442915	lncRNA
ENSG0000(	PCDH1	1985.994	0.30863	0.076487	0.442917	protein_coding
ENSG0000(	AACSP1	85.60699	-0.26514	0.076548	0.442947	transcribed_unprocessed_pseudo
ENSG0000(	AKAP1	2820.438	0.349717	0.076576	0.442947	protein_coding
ENSG0000(	ENSG0000(	1396.614	-0.18787	0.076556	0.442947	lncRNA
ENSG0000(	ENSG0000(	73.63743	0.243798	0.076617	0.442947	TEC
ENSG0000(	FAM3D	970.9592	0.412938	0.076603	0.442947	protein_coding
ENSG0000(	LYPD6B	46.16548	-0.6979	0.076629	0.442947	protein_coding
ENSG0000(	MIXL1	107.7048	-0.22805	0.076529	0.442947	protein_coding
ENSG0000(	PLPP6	356.6089	0.187625	0.076598	0.442947	protein_coding
ENSG0000(	TMEM214	1146.234	-0.17302	0.076637	0.442947	protein_coding
ENSG0000(	ANP32A	1642.545	-0.14681	0.076721	0.443064	protein_coding
ENSG0000(	ENSG0000(	78.44068	-0.3083	0.076697	0.443064	lncRNA
ENSG0000(	GATA2	182.0248	-0.44916	0.076676	0.443064	protein_coding
ENSG0000(	IQANK1	549.4324	0.178196	0.076746	0.443064	protein_coding
ENSG0000(	NFYB	387.2931	-0.14905	0.076758	0.443064	protein_coding
ENSG0000(	TOR1A	626.0758	0.12599	0.076764	0.443064	protein_coding
ENSG0000(	USP8	5962.943	0.107529	0.07677	0.443064	protein_coding
ENSG0000(	ENSG0000(	39.69701	-0.43172	0.076831	0.443326	lncRNA
ENSG0000(	FABP7P1	24.5702	-0.48052	0.076852	0.443355	processed_pseudogene
ENSG0000(	FAM189A2	149.1122	0.339212	0.076907	0.44358	protein_coding
ENSG0000(	MCMDC2	116.5389	0.216959	0.076942	0.443689	protein_coding
ENSG0000(	SMAP2	1488.748	-0.41066	0.076973	0.443705	protein_coding
ENSG0000(	SYCP2	192.5965	-0.33833	0.076977	0.443705	protein_coding
ENSG0000(	NEUROD1	441.6979	0.20809	0.077011	0.443806	protein_coding
ENSG0000(	MIR4538	20.03767	-0.76361	0.077064	0.444016	miRNA
ENSG0000(	SPDL1	230.8054	-0.25544	0.077122	0.44426	protein_coding
ENSG0000(	CINP	643.9314	0.149491	0.077156	0.444268	protein_coding
ENSG0000(	RTL8C	270.7047	-0.25888	0.077149	0.444268	protein_coding
ENSG0000(	DHDDS	1845.559	0.198007	0.077195	0.444403	protein_coding
ENSG0000(	TRAJ18	24.98009	-0.55565	0.077255	0.444655	TR_J_gene
ENSG0000(	DNAJC28	103.1536	0.201678	0.077416	0.444928	protein_coding
ENSG0000(	ENSG0000(	154.884	0.150096	0.077432	0.444928	TEC
ENSG0000(	KBTBD6	1878.737	0.249333	0.077404	0.444928	protein_coding
ENSG0000(	LINC01843	181.4442	0.347851	0.077365	0.444928	lncRNA
ENSG0000(	PSMB10	255.5509	-0.25611	0.077426	0.444928	protein_coding
ENSG0000(	RCBTB1	490.7947	-0.22628	0.077336	0.444928	protein_coding
ENSG0000(	SCAP	1976.541	0.197764	0.077382	0.444928	protein_coding
ENSG0000(	VASH1	965.1799	-0.292	0.077354	0.444928	protein_coding
ENSG0000(	FTH1P3	22.19662	-0.51403	0.077487	0.444967	processed_pseudogene
ENSG0000(	KLHL7-DT	655.5393	-0.15239	0.077497	0.444967	lncRNA
ENSG0000(	LONP1	654.7957	-0.10508	0.077481	0.444967	protein_coding
ENSG0000(	SPINK4	234.0873	-0.91777	0.077504	0.444967	protein_coding
ENSG0000(	GUCY1B1	168.1312	-0.50274	0.077605	0.445277	protein_coding

ENSG0000(	PDZD2	475.346	-0.24119	0.077606	0.445277	protein_coding
ENSG0000(	RCL1	530.6716	-0.1622	0.077592	0.445277	protein_coding
ENSG0000(	RNPEPL1	1123.088	0.22508	0.077623	0.44528	protein_coding
ENSG0000(	DEDD	597.9281	-0.16264	0.077668	0.445342	protein_coding
ENSG0000(	ENSG0000(	42.58831	0.319618	0.077682	0.445342	processed_pseudogene
ENSG0000(	SMG1P4	976.8451	0.590729	0.077664	0.445342	transcribed_unprocessed_pseudo
ENSG0000(	LAMTOR4	395.716	0.157488	0.077724	0.445487	protein_coding
ENSG0000(	RASSF8	550.1032	-0.42445	0.077745	0.445513	protein_coding
ENSG0000(	NUCB1	1116.013	-0.21317	0.077767	0.445549	protein_coding
ENSG0000(	CYYR1	187.2531	-0.37059	0.077831	0.445824	protein_coding
ENSG0000(	ENSG0000(	188.9149	0.176003	0.077857	0.44588	lncRNA
ENSG0000(	SAMD13	190.1004	0.380775	0.077894	0.445995	protein_coding
ENSG0000(	GRK3	1274.529	-0.29687	0.077921	0.446058	protein_coding
ENSG0000(	ENSG0000(	396.0678	-0.1904	0.077967	0.446205	lncRNA
ENSG0000(	SALL1	493.2046	-0.41029	0.077979	0.446205	protein_coding
ENSG0000(	ENSG0000(	48.21155	0.295944	0.078069	0.446352	TEC
ENSG0000(	HERC6	623.3307	0.439826	0.078069	0.446352	protein_coding
ENSG0000(	NFAM1	563.7421	-0.35187	0.07806	0.446352	protein_coding
ENSG0000(	RALGAPA1	5035.35	0.158328	0.078045	0.446352	protein_coding
ENSG0000(	ENSG0000(	37.96487	0.315565	0.078106	0.446376	TEC
ENSG0000(	TTC7A	1003.91	-0.24159	0.078104	0.446376	protein_coding
ENSG0000(	DDAH2	364.5664	0.347349	0.078153	0.446474	protein_coding
ENSG0000(	ENSG0000(	31.69087	0.424958	0.078156	0.446474	lncRNA
ENSG0000(	ENSG0000(	57.87315	0.278688	0.078219	0.446744	lncRNA
ENSG0000(	H2AC12	74.09071	-0.52313	0.07835	0.447303	protein_coding
ENSG0000(	POM121	2011.556	0.094155	0.078334	0.447303	protein_coding
ENSG0000(	RCAN1	1501.183	-0.34351	0.078384	0.447314	protein_coding
ENSG0000(	SLC30A7	2100.299	-0.14956	0.078377	0.447314	protein_coding
ENSG0000(	AKAP12	733.2479	-0.28353	0.078434	0.447507	protein_coding
ENSG0000(	ITGAL	2660.869	-0.25808	0.078476	0.447654	protein_coding
ENSG0000(	ENSG0000(	171.6263	0.204632	0.07853	0.447681	lncRNA
ENSG0000(	ENSG0000(	85.61733	-0.27491	0.078535	0.447681	TEC
ENSG0000(	LINC00324	97.31643	-0.23551	0.078562	0.447681	lncRNA
ENSG0000(	RRP9	75.98717	0.243979	0.078547	0.447681	protein_coding
ENSG0000(	SLC36A1	2023.744	0.305609	0.078511	0.447681	protein_coding
ENSG0000(	ERCC6L2	4283.288	0.088105	0.078659	0.448139	protein_coding
ENSG0000(	SASH3	586.0049	-0.38747	0.078678	0.448157	protein_coding
ENSG0000(	ENSG0000(	481.4428	0.150245	0.078722	0.448315	TEC
ENSG0000(	HINT2P1	19.94839	0.815882	0.078745	0.448351	processed_pseudogene
ENSG0000(	LINC02587	32.5182	-0.41668	0.078762	0.448355	lncRNA
ENSG0000(	NAMPTP1	43.44283	-1.31417	0.078816	0.44857	processed_pseudogene
ENSG0000(	ADCY6	4031.438	0.308741	0.078865	0.448573	protein_coding
ENSG0000(	ENSG0000(	314.9624	-0.14875	0.078842	0.448573	TEC
ENSG0000(	ISL1-DT	39.78214	0.61569	0.07886	0.448573	lncRNA
ENSG0000(	CDC42BPG	1368.943	0.364967	0.07891	0.448623	protein_coding
ENSG0000(	ENSG0000(	169.0648	0.215865	0.078923	0.448623	lncRNA
ENSG0000(	FANK1	110.4142	0.35009	0.078907	0.448623	protein_coding

ENSG0000(	ENSG0000(	115.4568	0.227171	0.078956	0.448716	lncRNA
ENSG0000(	VSIG10	2044.331	0.270218	0.078998	0.448864	protein_coding
ENSG0000(	ERI3	339.0125	0.156559	0.079106	0.449107	protein_coding
ENSG0000(	LINC02606	255.9258	0.164465	0.079084	0.449107	lncRNA
ENSG0000(	OR4K17	1142.307	-0.15075	0.079095	0.449107	protein_coding
ENSG0000(	ZBED9-AS1	32.44184	0.498447	0.079104	0.449107	lncRNA
ENSG0000(	ENSG0000(	88.01075	-0.29	0.079135	0.449176	TEC
ENSG0000(	THSD7A	241.8979	-0.54504	0.079152	0.449184	protein_coding
ENSG0000(	USH2A	44.62654	1.050224	0.079174	0.449216	protein_coding
ENSG0000(	DCAKD	1579.286	0.149934	0.079194	0.449237	protein_coding
ENSG0000(	ENSG0000(	169.4882	-0.19596	0.079219	0.449284	lncRNA
ENSG0000(	GREP1	296.1107	-0.19752	0.079257	0.449331	protein_coding
ENSG0000(	MED20	388.2928	0.161655	0.07926	0.449331	protein_coding
ENSG0000(	ENSG0000(	137.5277	-0.2378	0.079297	0.449448	lncRNA
ENSG0000(	KLHL8	895.8304	0.120582	0.079316	0.449466	protein_coding
ENSG0000(	ENSG0000(	466.1274	-0.14642	0.079391	0.449622	lncRNA
ENSG0000(	ENSG0000(	128.6613	-0.2126	0.079409	0.449622	lncRNA
ENSG0000(	SLC25A26	2642.094	0.126548	0.079361	0.449622	protein_coding
ENSG0000(	USP30-AS1	42.37729	-0.4503	0.079395	0.449622	lncRNA
ENSG0000(	ENSG0000(	25.75005	0.334585	0.079433	0.44966	lncRNA
ENSG0000(	ENSG0000(	153.6992	0.200565	0.079481	0.449749	lncRNA
ENSG0000(	FBXO31	1208.047	0.213926	0.079473	0.449749	protein_coding
ENSG0000(	FAM220A	177.8468	-0.26415	0.079508	0.449808	protein_coding
ENSG0000(	RUNX3	779.6965	-0.32256	0.079538	0.449885	protein_coding
ENSG0000(	PJA1	120.6958	-0.34166	0.079573	0.449903	protein_coding
ENSG0000(	YWHAG	1855.339	-0.18566	0.079566	0.449903	protein_coding
ENSG0000(	ATG3	562.091	-0.21621	0.079677	0.450117	protein_coding
ENSG0000(	CROCCP3	865.8892	-0.12386	0.079665	0.450117	transcribed_unprocessed_pseudo
ENSG0000(	ENSG0000(	509.4249	0.138203	0.079658	0.450117	lncRNA
ENSG0000(	RSPH3	1347.897	0.141412	0.079642	0.450117	protein_coding
ENSG0000(	KLF7P1	20.51592	-0.55376	0.079717	0.450157	processed_pseudogene
ENSG0000(	RGPD4-AS1	540.8587	0.26031	0.079708	0.450157	lncRNA
ENSG0000(	CSDE1	6094.426	-0.10759	0.079745	0.450227	protein_coding
ENSG0000(	CCDC159	347.6993	0.130984	0.079772	0.450286	protein_coding
ENSG0000(	CD2	253.1036	-0.3653	0.079968	0.450652	protein_coding
ENSG0000(	COPG2	276.497	0.182107	0.079965	0.450652	protein_coding
ENSG0000(	ENPP2	751.1983	-0.35459	0.079979	0.450652	protein_coding
ENSG0000(	ENSG0000(	91.52161	0.496666	0.079892	0.450652	lncRNA
ENSG0000(	GAPDH	5358.395	-0.26223	0.080001	0.450652	protein_coding
ENSG0000(	NDUFB2	955.4028	0.138215	0.079989	0.450652	protein_coding
ENSG0000(	PTPRVP	151.6452	-0.25667	0.079966	0.450652	unitary_pseudogene
ENSG0000(	RNF44	1516.611	0.143265	0.079911	0.450652	protein_coding
ENSG0000(	RRN3P2	325.2509	-0.22749	0.07995	0.450652	lncRNA
ENSG0000(	ZNF236	1279.673	0.199181	0.079867	0.450652	protein_coding
ENSG0000(	NOMO3	2310.101	-0.11921	0.080049	0.450768	protein_coding
ENSG0000(	SPOCD1	224.8283	-0.31922	0.080054	0.450768	protein_coding
ENSG0000(	CCN1	729.0281	-0.79011	0.080097	0.450914	protein_coding

ENSG0000(	ENSG0000(	23.1376	0.377975	0.08014	0.450976	lncRNA
ENSG0000(	ENSG0000(	51.71141	0.27849	0.08013	0.450976	processed_pseudogene
ENSG0000(	ESRP1	2561.17	0.40044	0.08017	0.451053	protein_coding
ENSG0000(	ENSG0000(	25.8669	-0.517	0.080225	0.451268	lncRNA
ENSG0000(	ENSG0000(	233.4322	0.183131	0.080277	0.451466	lncRNA
ENSG0000(	GDI1	1432.67	-0.13981	0.08033	0.451672	protein_coding
ENSG0000(	ENSG0000(	142.3239	0.201978	0.080383	0.451877	lncRNA
ENSG0000(	SRPRA	1360.133	-0.16835	0.080414	0.451962	protein_coding
ENSG0000(	PIGM	922.7183	0.117805	0.080433	0.451976	protein_coding
ENSG0000(	PREX2	811.8438	-0.2619	0.080479	0.452141	protein_coding
ENSG0000(	C2orf80	34.00997	-0.40086	0.08058	0.452468	protein_coding
ENSG0000(	LDHB	540.1727	-0.29965	0.08059	0.452468	protein_coding
ENSG0000(	RN7SL543F	26.79283	0.345496	0.080603	0.452468	misc_RNA
ENSG0000(	SF3B4	789.1618	0.318848	0.080564	0.452468	protein_coding
ENSG0000(	UBE2L5	106.1478	-0.26931	0.080631	0.452533	protein_coding
ENSG0000(	DPF1	181.0153	-0.17615	0.08068	0.452625	protein_coding
ENSG0000(	ENSG0000(	1677.642	0.429415	0.080671	0.452625	lncRNA
ENSG0000(	LMTK3	102.197	-0.40829	0.080729	0.452625	protein_coding
ENSG0000(	MRRF	1891.1	0.162418	0.080726	0.452625	protein_coding
ENSG0000(	PPP1R14BF	24.41791	0.514602	0.080718	0.452625	processed_pseudogene
ENSG0000(	CARD8	3231.139	-0.23502	0.080763	0.452721	protein_coding
ENSG0000(	LAMP1	1837.828	-0.19765	0.080792	0.452789	protein_coding
ENSG0000(	KAT6B	4782.09	0.083462	0.080814	0.452821	protein_coding
ENSG0000(	BAG2	388.3564	-0.24352	0.080895	0.452967	protein_coding
ENSG0000(	HORMAD1	74.74899	-0.45167	0.080899	0.452967	protein_coding
ENSG0000(	INTS3	3379.68	-0.09164	0.080882	0.452967	protein_coding
ENSG0000(	TNS2	1316.049	-0.16637	0.080906	0.452967	protein_coding
ENSG0000(	MOCS2-DT	898.6498	-0.16588	0.081001	0.453407	lncRNA
ENSG0000(	ENSG0000(	193.612	0.178742	0.081057	0.453631	lncRNA
ENSG0000(	EBF4	171.9193	-0.31989	0.08108	0.453667	protein_coding
ENSG0000(	ZNF607	379.5808	0.284982	0.081152	0.453978	protein_coding
ENSG0000(	ANXA3	523.9871	-0.28224	0.081205	0.454007	protein_coding
ENSG0000(	ENSG0000(	88.9226	-0.33737	0.081208	0.454007	lncRNA
ENSG0000(	SGK2	2560.037	0.604802	0.081184	0.454007	protein_coding
ENSG0000(	UTP6	1353.43	0.121178	0.081224	0.454007	protein_coding
ENSG0000(	PDGFB	228.8062	-0.37517	0.081319	0.454447	protein_coding
ENSG0000(	MANF	459.2783	-0.24852	0.081414	0.454703	protein_coding
ENSG0000(	PLEKHB2	2483.107	0.152989	0.081406	0.454703	protein_coding
ENSG0000(	ZFP92	81.46193	-0.4014	0.081396	0.454703	protein_coding
ENSG0000(	TBC1D23	741.818	-0.16497	0.081462	0.454877	protein_coding
ENSG0000(	ADARB1	1010.347	-0.33317	0.081495	0.454969	protein_coding
ENSG0000(	SPIRE2	2549.976	0.283551	0.081515	0.454987	protein_coding
ENSG0000(	CKB	1234.05	0.805137	0.081592	0.455326	protein_coding
ENSG0000(	PTPN13	780.8206	-0.3102	0.08162	0.455393	protein_coding
ENSG0000(	ENSG0000(	34.06959	0.403671	0.081649	0.455461	lncRNA
ENSG0000(	ENSG0000(	35.38606	0.355399	0.081701	0.455656	lncRNA
ENSG0000(	ADAMTS8	22.18426	0.568828	0.081862	0.455771	protein_coding

ENSG0000(	APOE	454.7716	-0.28606	0.081858	0.455771	protein_coding
ENSG0000(	CHRFAM7A	102.1837	-0.36206	0.081887	0.455771	protein_coding
ENSG0000(	CNNM3	1021.078	0.154667	0.081827	0.455771	protein_coding
ENSG0000(	ENSG0000(	266.4611	0.153572	0.081877	0.455771	lncRNA
ENSG0000(	ENSG0000(	268.1707	-0.22189	0.081822	0.455771	lncRNA
ENSG0000(	MZB1	814.6511	-0.38466	0.08178	0.455771	protein_coding
ENSG0000(	NLRP3	308.4305	-0.46808	0.081744	0.455771	protein_coding
ENSG0000(	TIPRL	604.7038	0.143088	0.081795	0.455771	protein_coding
ENSG0000(	ZNF107	2465.438	-0.2242	0.081798	0.455771	protein_coding
ENSG0000(	ZBTB43	1748.773	0.107425	0.081929	0.455914	protein_coding
ENSG0000(	RN7SKP151	41.36134	-0.36422	0.081984	0.456128	misc_RNA
ENSG0000(	CREBZF	3201.883	0.108896	0.082051	0.456409	protein_coding
ENSG0000(	LINC01489	19.97438	0.500791	0.082138	0.4568	lncRNA
ENSG0000(	FAM30A	2740.13	-0.34409	0.082184	0.45696	lncRNA
ENSG0000(	ENSG0000(	40.28888	1.158872	0.082221	0.457075	processed_pseudogene
ENSG0000(	CENPNP1	262.2164	0.162761	0.082301	0.457153	processed_pseudogene
ENSG0000(	FOXP1-IT1	783.9715	0.181681	0.082283	0.457153	lncRNA
ENSG0000(	GMIP	855.8397	-0.22502	0.082258	0.457153	protein_coding
ENSG0000(	ZNF600	1095.946	0.187805	0.082291	0.457153	protein_coding
ENSG0000(	CRELD2	713.7291	-0.20498	0.082403	0.457496	protein_coding
ENSG0000(	ENSG0000(	65.77585	0.355689	0.082413	0.457496	lncRNA
ENSG0000(	GEMIN8	607.9159	0.137014	0.082411	0.457496	protein_coding
ENSG0000(	PCDH11X	380.2336	0.299528	0.082463	0.457538	protein_coding
ENSG0000(	RABGAP1L-	1026.734	0.102487	0.082452	0.457538	lncRNA
ENSG0000(	SUCLG1	928.9358	0.210721	0.082475	0.457538	protein_coding
ENSG0000(	TMF1	2083.733	-0.19391	0.082487	0.457538	protein_coding
ENSG0000(	ENSG0000(	366.6967	-0.2144	0.082504	0.457541	TEC
ENSG0000(	ZNF524	137.9251	0.221931	0.08253	0.457591	protein_coding
ENSG0000(	MTM1	2240.385	0.17698	0.082616	0.457974	protein_coding
ENSG0000(	LINC01012	583.853	0.092227	0.08267	0.458184	lncRNA
ENSG0000(	SELENOW	2408.728	0.194058	0.082703	0.458273	protein_coding
ENSG0000(	ENPP5	111.855	0.521538	0.082774	0.458576	protein_coding
ENSG0000(	ARHGAP22	553.171	-0.1745	0.082804	0.45865	protein_coding
ENSG0000(	LINC00958	311.7705	0.133452	0.082831	0.458666	lncRNA
ENSG0000(	UBASH3A	204.9454	-0.36512	0.082841	0.458666	protein_coding
ENSG0000(	C1orf54	58.90221	-0.38029	0.082915	0.458683	protein_coding
ENSG0000(	FAM83B	1894.963	0.32679	0.08296	0.458683	protein_coding
ENSG0000(	GUCA2B	87.27517	0.974434	0.082896	0.458683	protein_coding
ENSG0000(	KCNA6	75.95768	0.431584	0.082955	0.458683	protein_coding
ENSG0000(	LINC01683	122.1571	-0.20351	0.082933	0.458683	lncRNA
ENSG0000(	LINC02250	26.17825	0.365065	0.08295	0.458683	lncRNA
ENSG0000(	MED17	2793.935	0.061316	0.082887	0.458683	protein_coding
ENSG0000(	GABRE	3061.368	-0.23574	0.083017	0.458905	protein_coding
ENSG0000(	CFTR	3848.114	0.347553	0.083053	0.459009	protein_coding
ENSG0000(	SRPRB	476.4426	-0.24164	0.083091	0.459127	protein_coding
ENSG0000(	MICE	94.26915	-0.35198	0.083179	0.459524	transcribed_unprocessed_pseudo
ENSG0000(	ENSG0000(	81.73974	0.368745	0.083243	0.459576	lncRNA

ENSG0000(	LNC-LBCS	103.6877	-0.37488	0.083247	0.459576	lncRNA
ENSG0000(	SH3RF1	1786.127	0.339629	0.083239	0.459576	protein_coding
ENSG0000(	YPEL3-DT	22.16474	-0.49968	0.083256	0.459576	lncRNA
ENSG0000(	ENSG0000(	88.35826	-0.27327	0.083304	0.459661	lncRNA
ENSG0000(	SFR1	144.6604	-0.24841	0.0833	0.459661	protein_coding
ENSG0000(	ENSG0000(	35.19482	0.459247	0.083352	0.459781	lncRNA
ENSG0000(	USP15	4169.67	-0.18807	0.08336	0.459781	protein_coding
ENSG0000(	ENSG0000(	35.57896	-0.37992	0.083428	0.459817	lncRNA
ENSG0000(	ENSG0000(	103.7486	-0.30005	0.08341	0.459817	lncRNA
ENSG0000(	MYO10	4848.753	0.212469	0.083386	0.459817	protein_coding
ENSG0000(	RNU6-522F	45.90486	-0.37496	0.083433	0.459817	snRNA
ENSG0000(	ENSG0000(	109.9128	-0.32076	0.083518	0.459827	lncRNA
ENSG0000(	IQSEC3	62.26474	-0.75038	0.083515	0.459827	protein_coding
ENSG0000(	RNASE10	79.24018	0.290402	0.083484	0.459827	protein_coding
ENSG0000(	SUSD2	33.91272	-0.48782	0.083518	0.459827	protein_coding
ENSG0000(	TNS4	502.6343	-0.4648	0.083465	0.459827	protein_coding
ENSG0000(	DAAM2-AS	159.7238	-0.61457	0.083585	0.4601	lncRNA
ENSG0000(	ALKBH6	519.9307	-0.1373	0.083657	0.46013	protein_coding
ENSG0000(	AMOTL2	400.7718	-0.31043	0.083635	0.46013	protein_coding
ENSG0000(	DSP	6488.197	0.33578	0.083642	0.46013	protein_coding
ENSG0000(	PIK3CD	1159.859	-0.43165	0.083622	0.46013	protein_coding
ENSG0000(	ATAT1	412.6629	0.148231	0.083752	0.460355	protein_coding
ENSG0000(	CES3	2576.158	0.38361	0.083752	0.460355	protein_coding
ENSG0000(	DDX39B-AS	90.01676	0.275032	0.083733	0.460355	lncRNA
ENSG0000(	ENSG0000(	104.9447	-0.3004	0.083765	0.460355	lncRNA
ENSG0000(	ACSF3	3028.635	0.110914	0.083804	0.460388	protein_coding
ENSG0000(	ZNF569	282.684	0.126608	0.083791	0.460388	protein_coding
ENSG0000(	SPSB2	130.8321	0.248179	0.083843	0.460508	protein_coding
ENSG0000(	SLC25A17	333.688	-0.16517	0.083872	0.460576	protein_coding
ENSG0000(	KRTAP3-1	22.88842	-0.41896	0.083898	0.460625	protein_coding
ENSG0000(	SEPTIN4	651.5583	-0.28866	0.083968	0.460918	protein_coding
ENSG0000(	ENSG0000(	290.1905	-0.22582	0.084062	0.461345	lncRNA
ENSG0000(	BRMS1	216.3004	0.190914	0.084093	0.461424	protein_coding
ENSG0000(	RN7SL134F	313.6747	0.1883	0.084153	0.461473	misc_RNA
ENSG0000(	RN7SL455F	217.3443	0.143275	0.084153	0.461473	misc_RNA
ENSG0000(	SLC12A6	2446.667	-0.21985	0.084152	0.461473	protein_coding
ENSG0000(	ENSG0000(	223.3621	-0.17486	0.084214	0.461718	lncRNA
ENSG0000(	CD82	756.3913	-0.37717	0.08436	0.461804	protein_coding
ENSG0000(	ENSG0000(	20.51188	0.609901	0.084274	0.461804	TEC
ENSG0000(	ENSG0000(	21.58883	0.651907	0.084317	0.461804	lncRNA
ENSG0000(	IKZF3	4125.203	-0.30249	0.08428	0.461804	protein_coding
ENSG0000(	NRG1	2759.85	-0.41596	0.084333	0.461804	protein_coding
ENSG0000(	SLC39A12-/	334.9598	-0.15112	0.084306	0.461804	lncRNA
ENSG0000(	TLE4	1154.657	-0.15531	0.084309	0.461804	protein_coding
ENSG0000(	ZNRF2P2	34.07046	0.398196	0.084364	0.461804	transcribed_processed_pseudoge
ENSG0000(	TSNARE1	369.3337	0.211746	0.084387	0.461837	protein_coding
ENSG0000(	ING1	644.0523	-0.10615	0.084443	0.46205	protein_coding

ENSG0000(	PAFAH2	831.9203	0.125088	0.084487	0.462202	protein_coding
ENSG0000(	DNAJC27-A	506.997	0.209111	0.084562	0.462365	lncRNA
ENSG0000(	GCM1	138.2994	0.199013	0.084557	0.462365	protein_coding
ENSG0000(	SLC37A4	710.7418	0.253509	0.084567	0.462365	protein_coding
ENSG0000(	DTX2	1719.826	0.17028	0.084596	0.46243	protein_coding
ENSG0000(	RN7SL368F	113.532	-0.54806	0.084624	0.462489	misc_RNA
ENSG0000(	SIX5	171.7717	-0.25345	0.084653	0.462557	protein_coding
ENSG0000(	ENSG0000(	86.85262	0.19496	0.084704	0.462743	lncRNA
ENSG0000(	ATF6	2278.567	-0.10755	0.084755	0.462932	protein_coding
ENSG0000(	EPS8L2	2421.49	0.399104	0.084785	0.463003	protein_coding
ENSG0000(	ENSG0000(	21.12469	-0.48531	0.084872	0.463321	lncRNA
ENSG0000(	ENSG0000(	223.3194	-0.22225	0.084911	0.463321	lncRNA
ENSG0000(	LINC02780	73.28618	-0.29893	0.084901	0.463321	lncRNA
ENSG0000(	NTM-AS1	75.3269	0.222206	0.084881	0.463321	lncRNA
ENSG0000(	ENSG0000(	103.7988	0.260524	0.08494	0.463386	transcribed_unprocessed_pseudo
ENSG0000(	ERP44	2776.78	-0.08997	0.084964	0.463429	protein_coding
ENSG0000(	PPIEL	869.953	0.11878	0.085007	0.463572	transcribed_unprocessed_pseudo
ENSG0000(	MMP7	34.27913	-0.87892	0.085032	0.463616	protein_coding
ENSG0000(	ZFP36	3279.979	-0.31866	0.085049	0.463618	protein_coding
ENSG0000(	ENSG0000(	2044.066	-0.10611	0.085114	0.463877	TEC
ENSG0000(	ACOXL	258.7137	-0.26507	0.08521	0.463985	protein_coding
ENSG0000(	ACSM2A	400.2735	-0.1961	0.085319	0.463985	protein_coding
ENSG0000(	ACTR2	3832.591	-0.15419	0.085281	0.463985	protein_coding
ENSG0000(	ENSG0000(	83.27421	0.374587	0.085215	0.463985	lncRNA
ENSG0000(	ENSG0000(	89.47069	-0.23424	0.085317	0.463985	TEC
ENSG0000(	GALNT14	38.29338	-0.73966	0.085155	0.463985	protein_coding
ENSG0000(	GOSR2	3318.479	-0.08472	0.085278	0.463985	protein_coding
ENSG0000(	H2BC13	46.87227	-0.47506	0.085213	0.463985	protein_coding
ENSG0000(	PRPF19	435.6725	-0.17577	0.08533	0.463985	protein_coding
ENSG0000(	TARBP1	2664.515	0.165725	0.085244	0.463985	protein_coding
ENSG0000(	TMCC2	61.20171	-0.42185	0.085243	0.463985	protein_coding
ENSG0000(	U8	20.74579	-0.4982	0.085336	0.463985	snoRNA
ENSG0000(	PSMA4	712.4099	-0.22454	0.085375	0.464104	protein_coding
ENSG0000(	ENSG0000(	22.38056	-0.43376	0.08547	0.464202	lncRNA
ENSG0000(	ENSG0000(	85.2106	0.292177	0.085435	0.464202	lncRNA
ENSG0000(	ENSG0000(	20.15964	-0.62031	0.085438	0.464202	lncRNA
ENSG0000(	ENSG0000(	260.0577	0.140789	0.085498	0.464202	TEC
ENSG0000(	ENSG0000(	62.54194	-0.3069	0.085496	0.464202	lncRNA
ENSG0000(	MTMR8	252.2251	0.322089	0.085484	0.464202	protein_coding
ENSG0000(	PLAUR	1195.847	-0.3932	0.085511	0.464202	protein_coding
ENSG0000(	BNIP5	329.7074	0.505212	0.085666	0.46424	protein_coding
ENSG0000(	BTN2A3P	581.5263	-0.13969	0.085667	0.46424	transcribed_unprocessed_pseudo
ENSG0000(	ELOVL7	473.245	-0.22727	0.085648	0.46424	protein_coding
ENSG0000(	ENSG0000(	334.7639	0.174084	0.08567	0.46424	lncRNA
ENSG0000(	FSIP2-AS1	74.11862	0.271011	0.085569	0.46424	lncRNA
ENSG0000(	HMGN5	53.72362	0.423759	0.085628	0.46424	protein_coding
ENSG0000(	MTND1P23	91.13668	1.921579	0.085536	0.46424	unprocessed_pseudogene

ENSG0000(	PTPRK	3334.499	0.190666	0.085571	0.46424	protein_coding
ENSG0000(	SLC22A17	142.7969	-0.31319	0.085638	0.46424	protein_coding
ENSG0000(	ENSG0000(	37.8016	0.426866	0.085701	0.464249	lncRNA
ENSG0000(	HIVEP3	1067.005	-0.20519	0.085722	0.464249	protein_coding
ENSG0000(	TPRKBP2	43.36312	-0.34935	0.08571	0.464249	processed_pseudogene
ENSG0000(	ENSG0000(	56.19228	0.271087	0.085745	0.464283	lncRNA
ENSG0000(	SEPTIN12	21.39677	0.467602	0.085781	0.464386	protein_coding
ENSG0000(	DAB2IP	2390.454	0.278722	0.08585	0.464638	protein_coding
ENSG0000(	ENSG0000(	73.53916	0.222209	0.085861	0.464638	TEC
ENSG0000(	LINC01288	31.33675	0.349145	0.085937	0.464953	lncRNA
ENSG0000(	SEC61G-DT	151.1008	0.185111	0.085994	0.465175	lncRNA
ENSG0000(	POLN	509.9702	-0.21844	0.086012	0.465179	protein_coding
ENSG0000(	LIPA	688.1927	-0.31445	0.086035	0.465209	protein_coding
ENSG0000(	FAF1	1069.004	0.198613	0.086117	0.465563	protein_coding
ENSG0000(	NOL9	2319.403	0.108681	0.086162	0.465713	protein_coding
ENSG0000(	GUSBP14	410.4449	0.659563	0.086184	0.465744	unprocessed_pseudogene
ENSG0000(	EGR3	324.7109	-0.50478	0.08622	0.465845	protein_coding
ENSG0000(	CCNJL	1018.913	0.259443	0.086324	0.465991	protein_coding
ENSG0000(	ENSG0000(	254.7077	0.166012	0.086299	0.465991	lncRNA
ENSG0000(	ENSG0000(	51.85167	-0.49405	0.086366	0.465991	lncRNA
ENSG0000(	GGPS1	631.675	-0.14905	0.086345	0.465991	protein_coding
ENSG0000(	GRM3-AS1	21.51877	0.680783	0.086282	0.465991	lncRNA
ENSG0000(	HNF1B	994.2356	0.402859	0.086292	0.465991	protein_coding
ENSG0000(	MANEA	737.3598	-0.23451	0.086349	0.465991	protein_coding
ENSG0000(	HOXA5	509.4365	0.355544	0.086399	0.466082	protein_coding
ENSG0000(	ENSG0000(	226.5428	0.186996	0.086431	0.466163	lncRNA
ENSG0000(	GBA2	2777.637	0.215645	0.086525	0.466508	protein_coding
ENSG0000(	RINT1	535.5331	0.166181	0.086529	0.466508	protein_coding
ENSG0000(	C1QTNF1-A	140.5045	0.258695	0.086664	0.466779	lncRNA
ENSG0000(	ENAH	1764.223	-0.26685	0.086608	0.466779	protein_coding
ENSG0000(	ENSG0000(	130.4853	-0.29471	0.086653	0.466779	lncRNA
ENSG0000(	LINC01506	136.2867	0.211532	0.086657	0.466779	lncRNA
ENSG0000(	MT-TC	122.523	-0.74662	0.086659	0.466779	Mt_tRNA
ENSG0000(	FAM225B	128.6084	-0.29191	0.086684	0.466795	lncRNA
ENSG0000(	C5orf34-AS	53.41259	0.317708	0.086795	0.467116	lncRNA
ENSG0000(	ENSG0000(	53.03822	-0.26053	0.086794	0.467116	lncRNA
ENSG0000(	NSL1	1406.048	-0.17795	0.086784	0.467116	protein_coding
ENSG0000(	ANK3	3789.94	0.303208	0.086962	0.467927	protein_coding
ENSG0000(	NAE1	734.0656	0.099502	0.086983	0.467948	protein_coding
ENSG0000(	CXCL16	488.0323	-0.26938	0.087032	0.468117	protein_coding
ENSG0000(	LINC02158	23.70756	0.494822	0.087088	0.468245	lncRNA
ENSG0000(	REC8	375.4293	-0.27801	0.087107	0.468245	protein_coding
ENSG0000(	UBE2D3	5489.212	-0.10854	0.087101	0.468245	protein_coding
ENSG0000(	CPLX1	31.68981	-0.44737	0.087203	0.468321	protein_coding
ENSG0000(	ENSG0000(	87.2669	-0.28089	0.087164	0.468321	lncRNA
ENSG0000(	LTBP2	728.5458	-0.52592	0.087161	0.468321	protein_coding
ENSG0000(	PHF13	265.7552	-0.28076	0.087204	0.468321	protein_coding

ENSG0000(	SAMD9L	4726.45	-0.43425	0.087206	0.468321	protein_coding
ENSG0000(	C17orf113	329.0463	0.155139	0.087313	0.46843	protein_coding
ENSG0000(	DDX19A-D	137.1639	0.199508	0.087343	0.46843	lncRNA
ENSG0000(	ENSG0000(	337.471	0.356739	0.087383	0.46843	transcribed_processed_pseudoge
ENSG0000(	ENSG0000(	57.76153	-0.2775	0.087251	0.46843	lncRNA
ENSG0000(	ENSG0000(	41.27493	0.28428	0.087397	0.46843	lncRNA
ENSG0000(	ENSG0000(	7417.145	0.19956	0.087349	0.46843	lncRNA
ENSG0000(	ENSG0000(	176.5644	0.509439	0.087388	0.46843	lncRNA
ENSG0000(	IL12RB1	635.3608	-0.17985	0.08728	0.46843	protein_coding
ENSG0000(	LINC02542	80.3016	0.30082	0.087365	0.46843	lncRNA
ENSG0000(	SPTBN5	272.9327	-0.60536	0.087301	0.46843	protein_coding
ENSG0000(	NECAB1	51.79333	0.433033	0.087517	0.468983	protein_coding
ENSG0000(	AKAP5	878.3457	0.191636	0.087537	0.469002	protein_coding
ENSG0000(	RFESD	54.71926	-0.45571	0.087615	0.469326	protein_coding
ENSG0000(	USP54	2770.323	0.256598	0.087655	0.469452	protein_coding
ENSG0000(	CES1	61.94475	-0.48157	0.087685	0.46952	protein_coding
ENSG0000(	ENTPD5	4233.223	0.374458	0.087758	0.469728	protein_coding
ENSG0000(	STAG3L5P-	472.6335	0.407902	0.087755	0.469728	lncRNA
ENSG0000(	GGNBP1	99.6504	-0.25896	0.087776	0.46973	transcribed_unitary_pseudogene
ENSG0000(	ENSG0000(	1725.917	0.176924	0.087793	0.46973	lncRNA
ENSG0000(	AGK	1521.511	-0.10956	0.087863	0.469739	protein_coding
ENSG0000(	EFCAB15P	19.85631	0.537767	0.087852	0.469739	transcribed_unitary_pseudogene
ENSG0000(	INSIG2	647.5626	-0.19788	0.087828	0.469739	protein_coding
ENSG0000(	ZNF230-DT	48.00452	0.237699	0.087832	0.469739	lncRNA
ENSG0000(	ABLIM2	252.0961	0.27192	0.087944	0.469746	protein_coding
ENSG0000(	ENSG0000(	203.0077	0.220369	0.087949	0.469746	lncRNA
ENSG0000(	FTLP14	52.42051	0.402726	0.087911	0.469746	processed_pseudogene
ENSG0000(	H2BC7	83.40092	-0.31717	0.087884	0.469746	protein_coding
ENSG0000(	RNU1-1	332725.3	0.173827	0.087941	0.469746	snRNA
ENSG0000(	FAT3	226.0528	0.305556	0.087988	0.469844	protein_coding
ENSG0000(	LYSMD2	52.95772	-0.339	0.088019	0.469844	protein_coding
ENSG0000(	SLC25A35	212.2611	-0.2051	0.088008	0.469844	protein_coding
ENSG0000(	CCNC	1058.702	-0.12792	0.088137	0.469944	protein_coding
ENSG0000(	ENSG0000(	96.03383	0.501872	0.088067	0.469944	transcribed_processed_pseudoge
ENSG0000(	FAM21EP	123.824	0.206445	0.088162	0.469944	transcribed_unprocessed_pseudo
ENSG0000(	HMBOX1-I	54.81697	-0.22293	0.088174	0.469944	lncRNA
ENSG0000(	LCN15	665.8246	-0.18652	0.088107	0.469944	protein_coding
ENSG0000(	LCT	22.1511	-0.65375	0.088097	0.469944	protein_coding
ENSG0000(	RAPGEFL1	3336.953	0.426036	0.088164	0.469944	protein_coding
ENSG0000(	TTC19	1908.244	0.155989	0.088103	0.469944	protein_coding
ENSG0000(	SYT11	409.7409	-0.24	0.088212	0.470052	protein_coding
ENSG0000(	ENSG0000(	191.6828	0.244372	0.088305	0.470186	lncRNA
ENSG0000(	SDAD1	532.6295	-0.2156	0.088281	0.470186	protein_coding
ENSG0000(	SLC38A6	621.3365	-0.19255	0.088277	0.470186	protein_coding
ENSG0000(	SSTR2	1274.577	0.129376	0.088299	0.470186	protein_coding
ENSG0000(	PSMD7	473.4245	-0.16226	0.088336	0.47026	protein_coding
ENSG0000(	CSF1R	927.1707	-0.21636	0.088365	0.470324	protein_coding

ENSG0000(	C1orf198	390.3732	-0.37988	0.088418	0.470514	protein_coding
ENSG0000(	DCAF6	1731.838	0.117339	0.088506	0.470597	protein_coding
ENSG0000(	ENSG0000(	118.6181	0.208163	0.088497	0.470597	lncRNA
ENSG0000(	NAV1	3147.91	-0.12215	0.088468	0.470597	protein_coding
ENSG0000(	RAD23B	1565.297	-0.13109	0.088519	0.470597	protein_coding
ENSG0000(	RING1	372.4823	-0.1295	0.088516	0.470597	protein_coding
ENSG0000(	ENSG0000(	23.08156	0.501382	0.08858	0.470657	lncRNA
ENSG0000(	ENSG0000(	34.67523	-0.52557	0.088603	0.470657	TEC
ENSG0000(	IRF9	2007.468	-0.20956	0.088641	0.470657	protein_coding
ENSG0000(	KIAA1586	212.3752	-0.26206	0.088581	0.470657	protein_coding
ENSG0000(	KIF1C	3410.822	0.197203	0.088653	0.470657	protein_coding
ENSG0000(	LHX6	70.34441	-0.54167	0.088762	0.470657	protein_coding
ENSG0000(	MAD2L1BP	249.357	0.205731	0.088785	0.470657	protein_coding
ENSG0000(	RNU6-1016	194.7237	-0.25972	0.088684	0.470657	snRNA
ENSG0000(	SYVN1	1679.404	-0.2026	0.088787	0.470657	protein_coding
ENSG0000(	TEX261	746.1554	-0.21529	0.088775	0.470657	protein_coding
ENSG0000(	TMEM185/	427.6293	-0.16136	0.088771	0.470657	protein_coding
ENSG0000(	TOX3	873.2904	0.339547	0.088785	0.470657	protein_coding
ENSG0000(	TRIM3	1204.839	0.321879	0.088597	0.470657	protein_coding
ENSG0000(	TRMT61A	250.3127	0.190645	0.088646	0.470657	protein_coding
ENSG0000(	UEVLD	1234.154	0.095995	0.088744	0.470657	protein_coding
ENSG0000(	CASP10	2912.212	-0.19734	0.08884	0.470845	protein_coding
ENSG0000(	CNIH1	708.0609	-0.20279	0.08887	0.470914	protein_coding
ENSG0000(	ENSG0000(	41.8277	-0.35625	0.088904	0.470951	lncRNA
ENSG0000(	ENSG0000(	62.31458	-0.24269	0.088929	0.470951	lncRNA
ENSG0000(	ENSG0000(	108.6769	-0.30173	0.088938	0.470951	processed_pseudogene
ENSG0000(	KDM3B	3237.377	0.102693	0.088947	0.470951	protein_coding
ENSG0000(	NNT	2552.016	0.104821	0.08898	0.470951	protein_coding
ENSG0000(	SLC16A13	107.3155	-0.22731	0.088978	0.470951	protein_coding
ENSG0000(	ADIPOR1	829.588	-0.1694	0.089056	0.470963	protein_coding
ENSG0000(	ENSG0000(	70.07078	0.212674	0.089106	0.470963	lncRNA
ENSG0000(	ENSG0000(	456.6564	0.121234	0.089085	0.470963	TEC
ENSG0000(	HERC2P4	234.9537	-0.28095	0.089027	0.470963	transcribed_unprocessed_pseudo
ENSG0000(	HMGN2P28	29.53815	0.441837	0.089119	0.470963	processed_pseudogene
ENSG0000(	LINC02688	62.23668	0.434259	0.089002	0.470963	lncRNA
ENSG0000(	TSGA13	142.171	0.203115	0.089034	0.470963	protein_coding
ENSG0000(	ZNF227	513.1991	-0.12817	0.08907	0.470963	protein_coding
ENSG0000(	ENSG0000(	232.2057	0.265424	0.089147	0.471021	lncRNA
ENSG0000(	MOB3B	1791.448	0.152186	0.08922	0.47132	protein_coding
ENSG0000(	ENSG0000(	28.11234	-0.3624	0.089245	0.471358	processed_pseudogene
ENSG0000(	SPG7	15288.94	0.15933	0.089298	0.471546	protein_coding
ENSG0000(	EIF3I	2326.076	-0.07659	0.089369	0.471572	protein_coding
ENSG0000(	ENSG0000(	24.14303	-0.54917	0.089362	0.471572	lncRNA
ENSG0000(	ENSG0000(	1694.208	-0.10623	0.089371	0.471572	lncRNA
ENSG0000(	ITCH-AS1	24.33295	-0.41732	0.089363	0.471572	lncRNA
ENSG0000(	BMS1P22	24.10018	-0.98959	0.089403	0.471651	transcribed_unprocessed_pseudo
ENSG0000(	AGAP1	2522.179	0.264811	0.089797	0.471666	protein_coding

ENSG0000(	ATL2	1206.27	0.119593	0.089484	0.471666	protein_coding
ENSG0000(	C6orf120	523.1663	-0.11245	0.089505	0.471666	protein_coding
ENSG0000(	CISD3	459.8747	0.257201	0.089637	0.471666	protein_coding
ENSG0000(	CXorf65	26.29666	-0.7223	0.089807	0.471666	protein_coding
ENSG0000(	DPY19L2P2	1092.972	0.12908	0.089605	0.471666	transcribed_unprocessed_pseudo
ENSG0000(	DYNC1LI2	6306.082	0.173824	0.089636	0.471666	protein_coding
ENSG0000(	ENSG0000(	21.41564	-0.57235	0.089641	0.471666	lncRNA
ENSG0000(	ENSG0000(	131.3344	0.235247	0.089756	0.471666	lncRNA
ENSG0000(	ENSG0000(	485.6469	0.12011	0.089818	0.471666	lncRNA
ENSG0000(	ENSG0000(	159.612	-0.19722	0.089748	0.471666	lncRNA
ENSG0000(	ENSG0000(	50.52869	0.232951	0.089474	0.471666	lncRNA
ENSG0000(	IGKV3D-11	24.42992	-0.97619	0.089698	0.471666	IG_V_gene
ENSG0000(	IQCC	192.8796	0.171096	0.08981	0.471666	protein_coding
ENSG0000(	KIF21A	817.9819	0.221106	0.089662	0.471666	protein_coding
ENSG0000(	LIF	411.696	-0.26999	0.089425	0.471666	protein_coding
ENSG0000(	LPIN1	2857.559	-0.20466	0.08955	0.471666	protein_coding
ENSG0000(	MED13L	8431.212	-0.1008	0.089477	0.471666	protein_coding
ENSG0000(	MFAP3	1395.458	-0.09487	0.089571	0.471666	protein_coding
ENSG0000(	NECTIN4	215.938	-0.43781	0.089689	0.471666	protein_coding
ENSG0000(	RBFOX2	2714.361	-0.15752	0.089771	0.471666	protein_coding
ENSG0000(	RUBCN	2173.458	-0.14579	0.089793	0.471666	protein_coding
ENSG0000(	SETMAR	284.844	0.179293	0.089762	0.471666	protein_coding
ENSG0000(	SLC12A4	951.68	-0.24246	0.089781	0.471666	protein_coding
ENSG0000(	ENSG0000(	118.1887	0.211126	0.089839	0.471691	lncRNA
ENSG0000(	SAMHD1	3579.087	-0.19164	0.089875	0.471788	protein_coding
ENSG0000(	CMTM6	1324.144	-0.20746	0.090051	0.471962	protein_coding
ENSG0000(	DDIAS	153.8497	-0.24547	0.090034	0.471962	protein_coding
ENSG0000(	ECM2	70.21432	-0.61225	0.090007	0.471962	protein_coding
ENSG0000(	ENSG0000(	393.9219	-0.11638	0.089994	0.471962	TEC
ENSG0000(	ENSG0000(	20.96508	0.360254	0.090065	0.471962	lncRNA
ENSG0000(	EXOC3L1	39.80145	-0.47945	0.090111	0.471962	protein_coding
ENSG0000(	HMGB3P9	166.336	-0.18172	0.090114	0.471962	processed_pseudogene
ENSG0000(	IRAG2	742.0175	-0.3967	0.090008	0.471962	protein_coding
ENSG0000(	IREB2	2706.03	0.082029	0.090071	0.471962	protein_coding
ENSG0000(	RHOA-IT1	108.8117	0.180337	0.089955	0.471962	lncRNA
ENSG0000(	RXFP1	39.95249	0.503352	0.09008	0.471962	protein_coding
ENSG0000(	SMIM30	154.3007	-0.2213	0.090089	0.471962	protein_coding
ENSG0000(	CRYZL2P	2533.194	0.202713	0.090154	0.471992	transcribed_unitary_pseudogene
ENSG0000(	ENSG0000(	11615.44	0.2051	0.090144	0.471992	lncRNA
ENSG0000(	CCDC43	296.0464	-0.18341	0.090174	0.472007	protein_coding
ENSG0000(	ENSG0000(	387.4257	0.135063	0.090198	0.472043	lncRNA
ENSG0000(	METAP1	920.8055	0.161803	0.090257	0.472259	protein_coding
ENSG0000(	EVPL	627.0756	0.223364	0.09028	0.472291	protein_coding
ENSG0000(	A4GALT	270.0878	-0.19183	0.090336	0.472315	protein_coding
ENSG0000(	CNOT9	1221.949	-0.13831	0.090331	0.472315	protein_coding
ENSG0000(	PPIA	4821.643	-0.18989	0.090322	0.472315	protein_coding
ENSG0000(	ACBD7	890.1107	-0.08768	0.090379	0.472359	protein_coding

ENSG0000(	PATE2	34.19469	-0.39288	0.090365	0.472359	protein_coding
ENSG0000(	CSK	1089.788	-0.16657	0.090412	0.472361	protein_coding
ENSG0000(	LINC01106	887.7448	0.344804	0.090448	0.472361	lncRNA
ENSG0000(	LRPPRC	6367.956	0.127559	0.090445	0.472361	protein_coding
ENSG0000(	PMEPA1	933.7092	-0.33776	0.090422	0.472361	protein_coding
ENSG0000(	LY86	40.77879	-0.53321	0.090526	0.472681	protein_coding
ENSG0000(	ACACA	1919.908	0.165424	0.09057	0.472821	protein_coding
ENSG0000(	LINC02009	60.14268	-0.36561	0.090608	0.472929	lncRNA
ENSG0000(	ENSG0000(	34.12606	0.340156	0.090651	0.472974	lncRNA
ENSG0000(	RMDN3	551.0217	-0.14865	0.090634	0.472974	protein_coding
ENSG0000(	OTULIN-DT	131.0332	-0.27376	0.090689	0.473084	lncRNA
ENSG0000(	ENSG0000(	75.27988	0.259252	0.090718	0.473144	lncRNA
ENSG0000(	AIF1L	82.16809	-0.43768	0.09079	0.473341	protein_coding
ENSG0000(	UGT1A10	581.4115	0.624405	0.090776	0.473341	protein_coding
ENSG0000(	ZC2HC1C	259.8734	0.170007	0.090809	0.473349	protein_coding
ENSG0000(	FAM41C	89.71067	-0.51697	0.090839	0.473416	lncRNA
ENSG0000(	LITAFD	153.0705	-0.16488	0.090921	0.473416	protein_coding
ENSG0000(	PSMD14	527.4743	-0.19942	0.090924	0.473416	protein_coding
ENSG0000(	SMPDL3B	147.0933	0.334316	0.0909	0.473416	protein_coding
ENSG0000(	SNORA63D	29.30922	-0.40001	0.090925	0.473416	snoRNA
ENSG0000(	UBLCP1	320.7563	-0.18117	0.090923	0.473416	protein_coding
ENSG0000(	SFSWAP	2689.484	0.119352	0.090962	0.473518	protein_coding
ENSG0000(	WDR81	1287.386	-0.2028	0.091083	0.474058	protein_coding
ENSG0000(	CARD11	453.0295	-0.44441	0.091116	0.474125	protein_coding
ENSG0000(	RNF144A	1136.28	-0.23613	0.09113	0.474125	protein_coding
ENSG0000(	HMGB1P21	45.97922	0.357216	0.09117	0.474245	processed_pseudogene
ENSG0000(	N4BP2L2-IT	1519.519	-0.14032	0.091233	0.47448	lncRNA
ENSG0000(	CCDC102A	43.67491	-0.46169	0.09133	0.474628	protein_coding
ENSG0000(	HOTTIP	233.3535	1.542742	0.091292	0.474628	lncRNA
ENSG0000(	LIPT2	70.30668	0.270538	0.091321	0.474628	protein_coding
ENSG0000(	PTPRF	8019.802	0.387442	0.091298	0.474628	protein_coding
ENSG0000(	PAX6	536.8626	0.38271	0.09137	0.474744	protein_coding
ENSG0000(	ENSG0000(	182.1242	0.197969	0.09144	0.47484	lncRNA
ENSG0000(	ENSG0000(	553.0656	0.16537	0.091406	0.47484	lncRNA
ENSG0000(	GAB1	2549.95	0.152777	0.091457	0.47484	protein_coding
ENSG0000(	SLC39A5	787.6995	0.453398	0.091457	0.47484	protein_coding
ENSG0000(	MROH1	2878.929	0.173637	0.09149	0.474918	protein_coding
ENSG0000(	XRCC4	119.0155	-0.27337	0.091598	0.475393	protein_coding
ENSG0000(	ENSG0000(	21.77658	-0.56842	0.091671	0.4755	lncRNA
ENSG0000(	ZCCHC18	61.75889	-0.40675	0.091656	0.4755	protein_coding
ENSG0000(	ZRANB2-AS	283.3769	0.274754	0.091669	0.4755	lncRNA
ENSG0000(	DHX16	885.9162	0.132949	0.091736	0.47566	protein_coding
ENSG0000(	ENSG0000(	20.48703	0.502515	0.091731	0.47566	lncRNA
ENSG0000(	DHX9	1622.233	-0.15155	0.091853	0.475911	protein_coding
ENSG0000(	DNAJB2	732.2654	-0.13139	0.091814	0.475911	protein_coding
ENSG0000(	ENSG0000(	733.5549	-0.15923	0.091866	0.475911	lncRNA
ENSG0000(	ENSG0000(	22.10804	0.411785	0.09182	0.475911	lncRNA

ENSG0000(	HFM1	41.56766	-0.68159	0.09189	0.475911	protein_coding
ENSG0000(	NUP88	1168.84	0.121652	0.09194	0.475911	protein_coding
ENSG0000(	RNH1	997.5937	0.138801	0.091916	0.475911	protein_coding
ENSG0000(	YES1	1644.934	-0.12291	0.091941	0.475911	protein_coding
ENSG0000(	ZNF250	701.8629	0.156045	0.091898	0.475911	protein_coding
ENSG0000(	TUBGCP4	1771.875	0.146795	0.091961	0.475926	protein_coding
ENSG0000(	PSMB1	734.0092	-0.14253	0.092001	0.476044	protein_coding
ENSG0000(	CHCHD2	589.0406	-0.22792	0.092055	0.476057	protein_coding
ENSG0000(	ENSG0000(	201.0673	0.169041	0.092071	0.476057	lncRNA
ENSG0000(	PHACTR1	1263.656	-0.18414	0.092069	0.476057	protein_coding
ENSG0000(	SATB2	6941.278	0.447661	0.092073	0.476057	protein_coding
ENSG0000(	IGLL5	1292.973	-0.47043	0.092126	0.476242	protein_coding
ENSG0000(	ENSG0000(	200.2171	-0.22007	0.092158	0.476253	lncRNA
ENSG0000(	ZMAT3	1311.478	-0.18396	0.092162	0.476253	protein_coding
ENSG0000(	ANXA1	679.208	-0.56948	0.092219	0.476258	protein_coding
ENSG0000(	ENSG0000(	23.23553	0.462368	0.092214	0.476258	lncRNA
ENSG0000(	MYO1D	6293.906	0.307928	0.092228	0.476258	protein_coding
ENSG0000(	ORMDL3	1040.409	-0.22026	0.092233	0.476258	protein_coding
ENSG0000(	ZNF611	1254.834	0.123283	0.09225	0.476258	protein_coding
ENSG0000(	ACOT11	4160.881	0.260383	0.092367	0.476349	protein_coding
ENSG0000(	ENSG0000(	262.0822	0.20864	0.092365	0.476349	lncRNA
ENSG0000(	ENSG0000(	545.3948	0.137781	0.092367	0.476349	lncRNA
ENSG0000(	PREPL	1534.91	0.118939	0.092371	0.476349	protein_coding
ENSG0000(	RFPL1S	470.9737	0.149349	0.092351	0.476349	lncRNA
ENSG0000(	TMED9	886.385	-0.14024	0.09234	0.476349	protein_coding
ENSG0000(	BTN2A1	1144.729	-0.12865	0.09251	0.476804	protein_coding
ENSG0000(	ENSG0000(	28.89564	0.405862	0.092556	0.476804	lncRNA
ENSG0000(	NELFB	425.785	0.199541	0.092578	0.476804	protein_coding
ENSG0000(	PCBP1	1129.406	-0.14564	0.092578	0.476804	protein_coding
ENSG0000(	PLEKHG3	2994.273	0.229986	0.092484	0.476804	protein_coding
ENSG0000(	SLC29A2	378.0231	0.332443	0.092581	0.476804	protein_coding
ENSG0000(	SRGAP2	2322.363	-0.21052	0.092548	0.476804	protein_coding
ENSG0000(	ENSG0000(	92.98344	0.368613	0.092732	0.477312	transcribed_unprocessed_pseudo
ENSG0000(	ENSG0000(	421.7534	0.145921	0.092712	0.477312	lncRNA
ENSG0000(	ENSG0000(	65.54869	0.216373	0.092716	0.477312	lncRNA
ENSG0000(	LINC00894	1761.422	0.146137	0.092766	0.477399	lncRNA
ENSG0000(	PELI3	153.4154	-0.20304	0.09285	0.47774	protein_coding
ENSG0000(	ZKSCAN8P1	68.19437	0.327689	0.092869	0.477749	protein_coding
ENSG0000(	KCNK2	19.49239	-0.57036	0.092907	0.47778	protein_coding
ENSG0000(	RAPSN	32.74408	-0.50609	0.092909	0.47778	protein_coding
ENSG0000(	ENSG0000(	107.9222	0.259993	0.092935	0.477822	lncRNA
ENSG0000(	ENSG0000(	57.2979	-0.27978	0.092965	0.477886	lncRNA
ENSG0000(	EPPK1	2898.908	0.347223	0.092983	0.477892	protein_coding
ENSG0000(	CBY1	310.1225	0.149984	0.093022	0.477975	protein_coding
ENSG0000(	FGF13-AS1	46.17502	-0.27363	0.093034	0.477975	lncRNA
ENSG0000(	LINC01623	280.5876	-0.18737	0.093052	0.477977	lncRNA
ENSG0000(	IGHV3-13	88.96953	-0.67777	0.093092	0.478095	IG_V_gene

ENSG0000(	LINC02656	261.9796	-0.35662	0.093125	0.478128	lncRNA
ENSG0000(	RN7SL66P	77.93312	0.221405	0.093133	0.478128	misc_RNA
ENSG0000(	ATXN7L2	148.8538	-0.2331	0.093157	0.478139	protein_coding
ENSG0000(	ENSG0000(	152.515	-0.17235	0.093201	0.478139	lncRNA
ENSG0000(	LRRC10	48.32744	0.225039	0.093222	0.478139	protein_coding
ENSG0000(	PLA2G15	399.4673	0.156164	0.093223	0.478139	protein_coding
ENSG0000(	TREML2	253.7714	-0.3565	0.09319	0.478139	protein_coding
ENSG0000(	RTN4R	22.47917	-0.58507	0.093253	0.478205	protein_coding
ENSG0000(	PCDHA9	234.803	0.133976	0.093293	0.478324	protein_coding
ENSG0000(	ENSG0000(	404.1037	0.233424	0.093404	0.478804	lncRNA
ENSG0000(	ENSG0000(	60.3789	0.325099	0.093458	0.478989	lncRNA
ENSG0000(	ACBD3	927.6763	-0.14757	0.093501	0.47912	protein_coding
ENSG0000(	GPRIN2	2290.406	0.267367	0.093627	0.479668	protein_coding
ENSG0000(	RND1	496.9603	-0.32572	0.093643	0.479668	protein_coding
ENSG0000(	CLMN	6914.05	0.258828	0.09373	0.479974	protein_coding
ENSG0000(	CYLD	3077.181	-0.22225	0.093741	0.479974	protein_coding
ENSG0000(	MIR3936H(	1965.307	0.252305	0.093755	0.479974	lncRNA
ENSG0000(	PRORSD1P	278.0693	0.162759	0.093812	0.480177	transcribed_unitary_pseudogene
ENSG0000(	GFOD1	1539.195	0.205199	0.093832	0.48019	protein_coding
ENSG0000(	ABHD16A	520.4221	-0.15936	0.093934	0.480361	protein_coding
ENSG0000(	ENSG0000(	30.52367	-0.39284	0.093906	0.480361	lncRNA
ENSG0000(	ENSG0000(	118.4218	0.207996	0.093935	0.480361	lncRNA
ENSG0000(	EPG5	2630.423	-0.13741	0.093932	0.480361	protein_coding
ENSG0000(	DEPDC1P1	52.05138	0.504897	0.094082	0.480962	processed_pseudogene
ENSG0000(	NEK4	1018.34	-0.15459	0.094088	0.480962	protein_coding
ENSG0000(	SORD	941.4152	-0.28768	0.094126	0.481069	protein_coding
ENSG0000(	ARMC7	294.4942	0.225471	0.094203	0.481104	protein_coding
ENSG0000(	ENSG0000(	19.99529	-0.58604	0.094159	0.481104	transcribed_processed_pseudoge
ENSG0000(	ENSG0000(	75.12969	-0.20025	0.094196	0.481104	lncRNA
ENSG0000(	OR7E104P	93.16463	0.340625	0.094169	0.481104	transcribed_unprocessed_pseudo
ENSG0000(	ENSG0000(	135.1194	0.194486	0.094243	0.481221	lncRNA
ENSG0000(	CAPN10	839.6862	0.097352	0.094295	0.481308	protein_coding
ENSG0000(	OTUD7B	2070.713	0.160466	0.094287	0.481308	protein_coding
ENSG0000(	ACVR1	905.3256	-0.1819	0.094503	0.481564	protein_coding
ENSG0000(	CNOT6L	2291.648	-0.16695	0.094451	0.481564	protein_coding
ENSG0000(	EMC1	2871.471	0.059745	0.094508	0.481564	protein_coding
ENSG0000(	ENSG0000(	36.04056	0.33359	0.094573	0.481564	lncRNA
ENSG0000(	ENSG0000(	53.66136	0.260701	0.094424	0.481564	lncRNA
ENSG0000(	FBXO7	1970.824	-0.09684	0.09445	0.481564	protein_coding
ENSG0000(	FCGRT	2437.818	0.328402	0.0945	0.481564	protein_coding
ENSG0000(	GNG10	226.0028	-0.4795	0.094386	0.481564	protein_coding
ENSG0000(	HOMER3	186.2262	-0.24057	0.094547	0.481564	protein_coding
ENSG0000(	PCLAF	678.8059	0.153996	0.094444	0.481564	protein_coding
ENSG0000(	RNY4	157.6239	-0.33369	0.094571	0.481564	misc_RNA
ENSG0000(	SHARPIN	341.8921	0.119675	0.094368	0.481564	protein_coding
ENSG0000(	VAMP5	92.11185	-0.33027	0.094555	0.481564	protein_coding
ENSG0000(	ENSG0000(	46.53498	-0.36756	0.094649	0.481774	lncRNA

ENSG0000(	NPC2	433.8945	-0.25537	0.094641	0.481774	protein_coding
ENSG0000(	NUTM2A	49.12604	0.93528	0.094702	0.481951	protein_coding
ENSG0000(	VIPR1-AS1	200.9187	0.191795	0.094729	0.482001	lncRNA
ENSG0000(	ENSG0000(	234.8938	0.134437	0.094777	0.482158	lncRNA
ENSG0000(	ENSG0000(	111.4955	-0.26083	0.094896	0.482671	lncRNA
ENSG0000(	DHX35	657.5149	0.135281	0.094945	0.482784	protein_coding
ENSG0000(	ENSG0000(	26.25437	0.366286	0.094988	0.482784	lncRNA
ENSG0000(	ENSG0000(	199.2721	-0.26791	0.094977	0.482784	lncRNA
ENSG0000(	TLCD5	332.9325	-0.17092	0.094974	0.482784	protein_coding
ENSG0000(	ENSG0000(	91.40875	0.193877	0.09505	0.483008	lncRNA
ENSG0000(	LSM1	153.4091	-0.21962	0.09514	0.483286	protein_coding
ENSG0000(	RN7SL76P	24.52086	0.366214	0.095137	0.483286	misc_RNA
ENSG0000(	TENM3	130.4162	-0.75995	0.095161	0.483309	protein_coding
ENSG0000(	PSAT1	97.3879	-0.59948	0.095184	0.483314	protein_coding
ENSG0000(	RPL23AP49	22.79573	0.429899	0.095198	0.483314	transcribed_processed_pseudoge
ENSG0000(	ASXL3-DT	39.47477	0.345484	0.095483	0.48343	lncRNA
ENSG0000(	BFSP2-AS1	44.90712	-0.36099	0.095503	0.48343	lncRNA
ENSG0000(	C2CD5	2354.108	0.194885	0.095478	0.48343	protein_coding
ENSG0000(	EFCAB6-DT	47.13805	0.275396	0.095519	0.48343	lncRNA
ENSG0000(	ENSG0000(	25.40793	0.443208	0.095405	0.48343	lncRNA
ENSG0000(	ENSG0000(	43.68737	-0.38101	0.095371	0.48343	lncRNA
ENSG0000(	ENSG0000(	215.3824	-0.4094	0.095243	0.48343	lncRNA
ENSG0000(	LINC01232	2600.885	-0.10241	0.095336	0.48343	lncRNA
ENSG0000(	LINC01606	97.30262	0.264802	0.095474	0.48343	lncRNA
ENSG0000(	MFSD12	689.475	-0.18377	0.09545	0.48343	protein_coding
ENSG0000(	NHSL1	3101.418	0.35199	0.095335	0.48343	protein_coding
ENSG0000(	RARRES1	168.8649	-0.45209	0.095503	0.48343	protein_coding
ENSG0000(	RPL9	2111.721	0.263031	0.095337	0.48343	protein_coding
ENSG0000(	SH3GLB2	2903.165	0.217243	0.095299	0.48343	protein_coding
ENSG0000(	SH3PXD2A	5656.909	0.166479	0.09546	0.48343	protein_coding
ENSG0000(	SLC30A8	158.0678	0.224594	0.0953	0.48343	protein_coding
ENSG0000(	SRA1	474.4444	-0.15285	0.095355	0.48343	protein_coding
ENSG0000(	RAB43	1028.65	-0.12561	0.095545	0.483473	protein_coding
ENSG0000(	KCTD9	1423.382	0.153005	0.095609	0.483707	protein_coding
ENSG0000(	NBPF26	956.4126	-0.46786	0.095629	0.483717	protein_coding
ENSG0000(	NPHP3-ACA	21.78015	-1.40439	0.095668	0.483737	protein_coding
ENSG0000(	SNORD89	310.6132	-0.22655	0.095662	0.483737	snoRNA
ENSG0000(	OFD1	3224.524	0.119279	0.095686	0.483739	protein_coding
ENSG0000(	FBXO6	208.0132	-0.20349	0.095705	0.483749	protein_coding
ENSG0000(	KIAA0825	1423.485	0.132554	0.095749	0.483817	protein_coding
ENSG0000(	NUDT15	411.5302	0.190009	0.095754	0.483817	protein_coding
ENSG0000(	TTC21A	424.2263	0.170146	0.0958	0.483958	protein_coding
ENSG0000(	DPF3	646.8706	0.327049	0.095894	0.484273	protein_coding
ENSG0000(	SLC25A46	1459.211	-0.09657	0.095897	0.484273	protein_coding
ENSG0000(	ENSG0000(	121.9197	0.20663	0.095931	0.484356	unprocessed_pseudogene
ENSG0000(	CKLF-CMTN	47.86863	-0.46044	0.096025	0.484473	protein_coding
ENSG0000(	ENSG0000(	185.165	0.141174	0.095991	0.484473	lncRNA

ENSG0000(	H3P13	152.8605	0.180213	0.096008	0.484473	processed_pseudogene
ENSG0000(	ZNF219	377.2045	0.293333	0.095986	0.484473	protein_coding
ENSG0000(	ENSG0000(	129.9064	-0.25221	0.096055	0.484478	lncRNA
ENSG0000(	OSR2	55.92212	-0.5982	0.096061	0.484478	protein_coding
ENSG0000(	C11orf98	63.97462	-0.51375	0.096173	0.4846	protein_coding
ENSG0000(	CCL11	109.6121	-0.59503	0.096118	0.4846	protein_coding
ENSG0000(	ENSG0000(	70.09251	0.257698	0.096169	0.4846	lncRNA
ENSG0000(	ENSG0000(	636.2889	-0.17169	0.09616	0.4846	lncRNA
ENSG0000(	SLC25A37	5697.864	-0.23531	0.096164	0.4846	protein_coding
ENSG0000(	AK9	658.3308	0.135748	0.096254	0.484859	protein_coding
ENSG0000(	ILF3	5223.376	0.11557	0.096267	0.484859	protein_coding
ENSG0000(	ZNF687	855.9839	0.113697	0.096277	0.484859	protein_coding
ENSG0000(	BCL2L1	1032.736	-0.19419	0.09634	0.484906	protein_coding
ENSG0000(	MRPL33	180.9599	-0.21564	0.096305	0.484906	protein_coding
ENSG0000(	ZBTB44-DT	44.81967	0.365152	0.096338	0.484906	lncRNA
ENSG0000(	PGS1	1725.509	-0.17707	0.096391	0.485078	protein_coding
ENSG0000(	C21orf91	597.4682	-0.20351	0.096495	0.485343	protein_coding
ENSG0000(	EPSTI1	1308.61	-0.42948	0.096528	0.485343	protein_coding
ENSG0000(	FAM234A	1045.089	0.210762	0.096471	0.485343	protein_coding
ENSG0000(	PARPBP	306.3885	-0.17085	0.096532	0.485343	protein_coding
ENSG0000(	ZNF460-AS	290.9533	-0.12818	0.096515	0.485343	lncRNA
ENSG0000(	ENSG0000(	263.6829	0.410204	0.096555	0.485354	processed_pseudogene
ENSG0000(	FASTK	873.1853	0.155321	0.09657	0.485354	protein_coding
ENSG0000(	ENSG0000(	241.9819	0.207662	0.096598	0.485405	TEC
ENSG0000(	DENND5B-/	19.66929	-0.56398	0.096722	0.485659	lncRNA
ENSG0000(	DNMT1	2602.603	-0.14811	0.096759	0.485659	protein_coding
ENSG0000(	ENSG0000(	22.97426	0.472705	0.096772	0.485659	lncRNA
ENSG0000(	LINC01705	40.3623	-0.49349	0.096675	0.485659	lncRNA
ENSG0000(	PIK3R1	2618.725	0.159308	0.096763	0.485659	protein_coding
ENSG0000(	SLC39A13	362.0218	-0.2431	0.096737	0.485659	protein_coding
ENSG0000(	TSPAN19	263.3768	0.270981	0.096723	0.485659	protein_coding
ENSG0000(	AXL	772.1251	-0.2024	0.097629	0.485835	protein_coding
ENSG0000(	C17orf78	21.52077	-1.65352	0.097604	0.485835	protein_coding
ENSG0000(	CMTM1	63.48984	-0.3386	0.09742	0.485835	protein_coding
ENSG0000(	COX6B1	504.7805	0.261431	0.097121	0.485835	protein_coding
ENSG0000(	CSTL1	83.73472	0.261397	0.097074	0.485835	protein_coding
ENSG0000(	ELP3	562.8422	-0.11206	0.097263	0.485835	protein_coding
ENSG0000(	ENSG0000(	58.54576	0.427544	0.097499	0.485835	processed_pseudogene
ENSG0000(	ENSG0000(	1423.459	-0.11574	0.097346	0.485835	lncRNA
ENSG0000(	ENSG0000(	1720.713	0.148346	0.097534	0.485835	lncRNA
ENSG0000(	ENSG0000(	24.68139	-0.39264	0.097491	0.485835	lncRNA
ENSG0000(	ENSG0000(	263.9232	0.174584	0.097543	0.485835	lncRNA
ENSG0000(	ENSG0000(	664.155	0.218192	0.09762	0.485835	protein_coding
ENSG0000(	ENSG0000(	390.1282	0.236193	0.097149	0.485835	lncRNA
ENSG0000(	ENSG0000(	218.9186	0.18729	0.097707	0.485835	TEC
ENSG0000(	ENSG0000(	164.6038	0.262482	0.097076	0.485835	lncRNA
ENSG0000(	ENSG0000(	388.8063	-0.12611	0.09732	0.485835	lncRNA

ENSG0000(	ENSG0000(	261.3075	0.129299	0.097389	0.485835	lncRNA
ENSG0000(	ENSG0000(	34.92237	-0.31738	0.097131	0.485835	lncRNA
ENSG0000(	ENSG0000(	210.7946	0.134301	0.097161	0.485835	lncRNA
ENSG0000(	FAM106A	1418.935	-0.18137	0.097671	0.485835	lncRNA
ENSG0000(	FICD	265.1099	-0.25967	0.097547	0.485835	protein_coding
ENSG0000(	FRG1	310.9409	-0.20454	0.097619	0.485835	protein_coding
ENSG0000(	FUT11	648.6751	-0.12512	0.096832	0.485835	protein_coding
ENSG0000(	GPATCH2	1020.696	-0.12218	0.097662	0.485835	protein_coding
ENSG0000(	GPR52	343.9292	-0.17144	0.097519	0.485835	protein_coding
ENSG0000(	INSL6	91.56853	0.355442	0.097221	0.485835	protein_coding
ENSG0000(	KIF9	99.05784	0.352088	0.097515	0.485835	protein_coding
ENSG0000(	LINC00635	295.0303	0.151399	0.097638	0.485835	lncRNA
ENSG0000(	LINC01137	158.5745	-0.2934	0.096887	0.485835	lncRNA
ENSG0000(	LINC02898	398.2357	-0.138	0.097141	0.485835	lncRNA
ENSG0000(	MAPT-AS1	338.1518	0.15138	0.097578	0.485835	lncRNA
ENSG0000(	MGAT3	261.3859	-0.32939	0.097523	0.485835	protein_coding
ENSG0000(	MIR9-3HG	205.7257	-0.3294	0.096964	0.485835	lncRNA
ENSG0000(	MPP1	399.4569	-0.255	0.096986	0.485835	protein_coding
ENSG0000(	MYB	1400.118	0.321742	0.097595	0.485835	protein_coding
ENSG0000(	NDRG2	1462.305	0.190519	0.097403	0.485835	protein_coding
ENSG0000(	NPR1	90.67533	-0.57699	0.097601	0.485835	protein_coding
ENSG0000(	OBSCN-AS1	67.15694	0.436781	0.097243	0.485835	lncRNA
ENSG0000(	OR52K3P	154.5352	-0.2654	0.097089	0.485835	transcribed_unprocessed_pseudo
ENSG0000(	PHGDH	377.9583	-0.17525	0.097021	0.485835	protein_coding
ENSG0000(	PODXL2	99.87132	0.374778	0.097648	0.485835	protein_coding
ENSG0000(	PTGES2	1126.996	0.119687	0.097668	0.485835	protein_coding
ENSG0000(	RN7SL625F	75.02998	-0.23005	0.097168	0.485835	misc_RNA
ENSG0000(	RPL21P123	230.9973	-0.27994	0.097208	0.485835	transcribed_processed_pseudoge
ENSG0000(	RTP4	129.1643	-0.33531	0.097693	0.485835	protein_coding
ENSG0000(	SCEL	26.23456	0.742906	0.0976	0.485835	protein_coding
ENSG0000(	TEDC1	325.5339	0.266127	0.097678	0.485835	protein_coding
ENSG0000(	TMEM134	770.8228	0.107077	0.096845	0.485835	protein_coding
ENSG0000(	TSPAN2	127.8305	-0.40976	0.097612	0.485835	protein_coding
ENSG0000(	UGT1A13P	232.1563	0.630017	0.097056	0.485835	unprocessed_pseudogene
ENSG0000(	XIAPP2	237.9464	0.188371	0.09702	0.485835	processed_pseudogene
ENSG0000(	LINC02347	81.14447	0.24609	0.097742	0.485921	lncRNA
ENSG0000(	ENSG0000(	411.9028	0.54631	0.09778	0.485935	protein_coding
ENSG0000(	ENSG0000(	68.43351	-0.41326	0.09778	0.485935	lncRNA
ENSG0000(	CDH7	197.3262	0.154174	0.097832	0.486016	protein_coding
ENSG0000(	ENSG0000(	92.68043	-0.2597	0.097829	0.486016	TEC
ENSG0000(	ENSG0000(	180.6565	0.155847	0.097898	0.486073	lncRNA
ENSG0000(	GNGT2	31.48335	-0.44847	0.097884	0.486073	protein_coding
ENSG0000(	GUK1	1130.395	-0.13323	0.097886	0.486073	protein_coding
ENSG0000(	LINC02293	81.77373	0.86246	0.097914	0.486073	lncRNA
ENSG0000(	ENSG0000(	20.01226	-1.10739	0.098042	0.486358	lncRNA
ENSG0000(	ENSG0000(	701.6452	0.088528	0.098017	0.486358	TEC
ENSG0000(	MICU2	583.0008	0.133344	0.098041	0.486358	protein_coding

ENSG0000(	NRBP2	1204.618	0.191848	0.098042	0.486358	protein_coding
ENSG0000(	NARS2	306.2189	0.270096	0.098094	0.486471	protein_coding
ENSG0000(	PDE6C	46.48499	-0.30978	0.098101	0.486471	protein_coding
ENSG0000(	RAI1	1307.475	-0.18203	0.098135	0.486516	protein_coding
ENSG0000(	S100A6	1551.498	-0.36265	0.098145	0.486516	protein_coding
ENSG0000(	MIR4280H(	2560.518	0.099217	0.098165	0.486526	lncRNA
ENSG0000(	ENSG0000(	24.04188	0.48282	0.098226	0.486699	processed_pseudogene
ENSG0000(	RASSF2	1392.433	-0.38357	0.098235	0.486699	protein_coding
ENSG0000(	RNU6-946F	25.73369	-0.37826	0.098267	0.486771	snRNA
ENSG0000(	CCDC201	21.50303	-0.40478	0.098364	0.487083	protein_coding
ENSG0000(	TRAF1	982.9741	-0.421	0.098366	0.487083	protein_coding
ENSG0000(	CDK15	87.31296	-0.42537	0.098575	0.487095	protein_coding
ENSG0000(	EIF2AK1	1349.81	0.173467	0.098404	0.487095	protein_coding
ENSG0000(	ENSG0000(	45.79195	-0.78308	0.098598	0.487095	transcribed_unprocessed_pseudo
ENSG0000(	ENSG0000(	31.45108	-0.49666	0.098558	0.487095	lncRNA
ENSG0000(	ENSG0000(	112.0065	0.16382	0.098458	0.487095	lncRNA
ENSG0000(	FAM72B	79.23171	-0.49152	0.098496	0.487095	protein_coding
ENSG0000(	ITGB1	6327.064	-0.12045	0.09851	0.487095	protein_coding
ENSG0000(	LINC01814	2878.412	0.19556	0.098485	0.487095	lncRNA
ENSG0000(	LINC02472	28.91796	-0.32771	0.098595	0.487095	lncRNA
ENSG0000(	NXPE1	6984.127	0.500401	0.09843	0.487095	protein_coding
ENSG0000(	PPP2R2D	1393.597	0.112517	0.098566	0.487095	protein_coding
ENSG0000(	PSMD2	824.2258	-0.17362	0.098497	0.487095	protein_coding
ENSG0000(	SLC19A3	1060.338	0.231725	0.098585	0.487095	protein_coding
ENSG0000(	ENSG0000(	25.64976	0.39538	0.098705	0.487537	snoRNA
ENSG0000(	CBX4	311.9175	-0.24987	0.098767	0.487752	protein_coding
ENSG0000(	PNPLA8	823.7411	-0.17253	0.098815	0.487903	protein_coding
ENSG0000(	ENSG0000(	178.6887	0.185857	0.098892	0.488197	lncRNA
ENSG0000(	ADGRG7	345.2109	-0.49502	0.099016	0.488506	protein_coding
ENSG0000(	PLXNA1	1387.79	-0.26402	0.099043	0.488506	protein_coding
ENSG0000(	RAB15	642.5211	0.26855	0.098991	0.488506	protein_coding
ENSG0000(	TCEA3	1306.169	0.428222	0.099043	0.488506	protein_coding
ENSG0000(	ZDHHC1	107.29	0.237295	0.099042	0.488506	protein_coding
ENSG0000(	DENND1C	451.9841	-0.33486	0.099155	0.488966	protein_coding
ENSG0000(	MSL1	2785.33	-0.11222	0.099178	0.488995	protein_coding
ENSG0000(	RECK	221.2295	-0.22779	0.099251	0.489115	protein_coding
ENSG0000(	SLC18A2	142.6336	-0.42271	0.099233	0.489115	protein_coding
ENSG0000(	ZNF503	129.0552	-0.32637	0.099256	0.489115	protein_coding
ENSG0000(	CDH9	27.22775	0.44002	0.099384	0.489133	protein_coding
ENSG0000(	DMAC2	417.9086	0.157783	0.099356	0.489133	protein_coding
ENSG0000(	GRAPLDR	180.6577	-0.48609	0.099371	0.489133	lncRNA
ENSG0000(	LINC02614	2306.046	0.173937	0.0993	0.489133	lncRNA
ENSG0000(	MXD1	5174.53	-0.32538	0.099371	0.489133	protein_coding
ENSG0000(	PAN3-AS1	129.7587	-0.20073	0.099355	0.489133	lncRNA
ENSG0000(	TGFB3	241.1548	-0.63399	0.099296	0.489133	protein_coding
ENSG0000(	ELL3	255.5413	0.28573	0.099471	0.489264	protein_coding
ENSG0000(	ENSG0000(	31.22486	-0.41947	0.099489	0.489264	TEC

ENSG0000(	RSL1D1	1326.836	-0.14895	0.09946	0.489264	protein_coding
ENSG0000(	RUVBL1	374.5984	-0.19917	0.099499	0.489264	protein_coding
ENSG0000(	ZNF284	371.0144	0.108047	0.099479	0.489264	protein_coding
ENSG0000(	ENSG0000(	69.52087	-0.25064	0.099609	0.489353	lncRNA
ENSG0000(	FAAP24	661.4763	0.096619	0.099599	0.489353	protein_coding
ENSG0000(	HNF4A	7451.637	0.354695	0.099542	0.489353	protein_coding
ENSG0000(	RALGPS2	2661.198	-0.18801	0.099624	0.489353	protein_coding
ENSG0000(	SLFN12	311.7139	-0.32303	0.099595	0.489353	protein_coding
ENSG0000(	SUFU	489.7646	-0.16596	0.099589	0.489353	protein_coding
ENSG0000(	HERC2P2	5070.5	-0.12882	0.0997	0.489637	transcribed_unprocessed_pseudo
ENSG0000(	B3GALT1	565.8558	0.398988	0.09984	0.489755	protein_coding
ENSG0000(	CDH4	1184.278	0.358763	0.099863	0.489755	protein_coding
ENSG0000(	ENSG0000(	50.41825	-0.25373	0.099801	0.489755	lncRNA
ENSG0000(	ENSG0000(	570.7591	0.111149	0.099789	0.489755	lncRNA
ENSG0000(	ENSG0000(	98.21804	0.241182	0.099876	0.489755	pseudogene
ENSG0000(	GABPB1-IT:	1362.765	-0.15794	0.099858	0.489755	lncRNA
ENSG0000(	PRCD	153.1689	-0.28617	0.099793	0.489755	protein_coding
ENSG0000(	SEC31A	4044.866	-0.12933	0.099884	0.489755	protein_coding
ENSG0000(	TOM1L1	679.5605	0.242968	0.09985	0.489755	protein_coding
ENSG0000(	BEST2	223.8724	0.495436	0.10001	0.489934	protein_coding
ENSG0000(	ENSG0000(	115.7152	0.22007	0.099942	0.489934	lncRNA
ENSG0000(	LINC00458	129.5378	0.185567	0.100002	0.489934	lncRNA
ENSG0000(	NR3C2	5626.6	0.346298	0.099994	0.489934	protein_coding
ENSG0000(	OARD1	742.0686	-0.1314	0.099961	0.489934	protein_coding
ENSG0000(	CAPZA1	3057.663	-0.13003	0.100179	0.490075	protein_coding
ENSG0000(	IGHV6-1	126.3741	-0.76472	0.100089	0.490075	IG_V_gene
ENSG0000(	MRPS10	635.2327	-0.11411	0.100156	0.490075	protein_coding
ENSG0000(	MRS2	1032.069	0.127489	0.100178	0.490075	protein_coding
ENSG0000(	MT-TF	101.863	-0.56388	0.100119	0.490075	Mt_tRNA
ENSG0000(	PPP2R2A	1412.169	-0.11024	0.100181	0.490075	protein_coding
ENSG0000(	TMEM200/	276.4168	0.472349	0.100062	0.490075	protein_coding
ENSG0000(	ZBTB8A	1667.054	0.122538	0.100168	0.490075	protein_coding
ENSG0000(	EPN3	246.308	0.458414	0.100199	0.490077	protein_coding
ENSG0000(	THYN1	164.42	-0.18812	0.100236	0.490168	protein_coding
ENSG0000(	CAMK2N1	1411.253	0.413336	0.100304	0.490417	protein_coding
ENSG0000(	CCL22	497.394	-0.30884	0.10036	0.490481	protein_coding
ENSG0000(	ENSG0000(	68.14325	0.253745	0.100361	0.490481	lncRNA
ENSG0000(	LINC01781	20.07122	0.490118	0.100371	0.490481	lncRNA
ENSG0000(	FAM110A	165.4747	-0.19874	0.100477	0.490738	protein_coding
ENSG0000(	LINC02826	158.7717	0.239674	0.100456	0.490738	lncRNA
ENSG0000(	MALSU1	217.4715	-0.15978	0.100468	0.490738	protein_coding
ENSG0000(	TRIM38	3917.096	-0.12905	0.10054	0.490959	protein_coding
ENSG0000(	SYTL1	847.6049	-0.22762	0.100586	0.491095	protein_coding
ENSG0000(	APOO	128.7774	-0.28494	0.100697	0.491264	protein_coding
ENSG0000(	EIF4E	1056.019	-0.1	0.100691	0.491264	protein_coding
ENSG0000(	GPR35	2506.432	0.298632	0.100669	0.491264	protein_coding
ENSG0000(	TTTY2B	70.83928	0.455127	0.100653	0.491264	lncRNA

ENSG0000(	ZNF845	603.3642	0.127395	0.10071	0.491264	protein_coding
ENSG0000(	ENSG0000(	1034.902	0.220267	0.100749	0.491367	lncRNA
ENSG0000(	CCT7	597.4781	-0.1393	0.100802	0.491502	protein_coding
ENSG0000(	ENSG0000(	208.4284	0.185711	0.100866	0.491502	TEC
ENSG0000(	JAM3	356.66	-0.28215	0.100841	0.491502	protein_coding
ENSG0000(	SEC63	1652.403	-0.16262	0.100884	0.491502	protein_coding
ENSG0000(	TNNT1	46.38906	0.395938	0.10086	0.491502	protein_coding
ENSG0000(	ZNF695	85.53665	0.371931	0.100822	0.491502	protein_coding
ENSG0000(	VPS28	729.8131	0.150121	0.100913	0.491559	protein_coding
ENSG0000(	MFSD6L	64.11156	0.298299	0.100937	0.491589	protein_coding
ENSG0000(	CHMP4C	303.5046	0.192658	0.101009	0.491853	protein_coding
ENSG0000(	ENSG0000(	388.065	0.192602	0.101194	0.492144	transcribed_processed_pseudoge
ENSG0000(	ENSG0000(	764.1973	0.154276	0.101163	0.492144	lncRNA
ENSG0000(	ENSG0000(	136.9037	0.219082	0.101183	0.492144	lncRNA
ENSG0000(	ENSG0000(	169.7962	0.223732	0.101133	0.492144	lncRNA
ENSG0000(	MARCHF11	64.5508	0.243311	0.101105	0.492144	lncRNA
ENSG0000(	NOP10	194.5679	-0.24118	0.101184	0.492144	protein_coding
ENSG0000(	TMC1	819.531	-0.13918	0.101139	0.492144	protein_coding
ENSG0000(	NANOGP4	72.51945	0.379389	0.101215	0.492158	transcribed_processed_pseudoge
ENSG0000(	AIFM2	527.9612	0.208368	0.101476	0.492379	protein_coding
ENSG0000(	CLEC1A	117.7107	-0.26415	0.101371	0.492379	protein_coding
ENSG0000(	CXCL13	504.6487	-1.08893	0.10145	0.492379	protein_coding
ENSG0000(	ENSG0000(	33.97828	0.799914	0.101529	0.492379	lncRNA
ENSG0000(	IRF2BP2	2671.67	0.14551	0.101499	0.492379	protein_coding
ENSG0000(	LINC01515	1581.498	0.216066	0.101399	0.492379	lncRNA
ENSG0000(	MROH7-TT	297.4126	0.331568	0.101452	0.492379	protein_coding
ENSG0000(	NGEF	673.4701	0.294234	0.101387	0.492379	protein_coding
ENSG0000(	NRK	62.09836	0.589708	0.101481	0.492379	protein_coding
ENSG0000(	PPP1R8	370.5804	-0.13797	0.101445	0.492379	protein_coding
ENSG0000(	SLC20A2	1905.935	0.235531	0.101286	0.492379	protein_coding
ENSG0000(	SLIT3-AS2	444.4556	0.246354	0.101512	0.492379	lncRNA
ENSG0000(	TCF3P1	74.75347	-0.41304	0.101319	0.492379	processed_pseudogene
ENSG0000(	WASH7P	79.63096	0.707433	0.101375	0.492379	unprocessed_pseudogene
ENSG0000(	ZNF575	231.0285	0.49855	0.101497	0.492379	protein_coding
ENSG0000(	ENSG0000(	46.68159	-0.2952	0.101611	0.492397	lncRNA
ENSG0000(	LINC00870	176.4536	0.270909	0.101572	0.492397	lncRNA
ENSG0000(	MTG2	1348.307	-0.07938	0.101622	0.492397	protein_coding
ENSG0000(	SLC29A1	241.4748	-0.28231	0.101592	0.492397	protein_coding
ENSG0000(	ZNF704	2134.366	0.412022	0.101552	0.492397	protein_coding
ENSG0000(	LDLRAP1	1430.204	0.223885	0.101671	0.492546	protein_coding
ENSG0000(	LY6E	279.0766	-0.45044	0.101689	0.492546	protein_coding
ENSG0000(	DENND2A	804.6233	-0.12513	0.101719	0.492604	protein_coding
ENSG0000(	CFAP65	31.74606	0.620666	0.101804	0.49261	protein_coding
ENSG0000(	ENSG0000(	191.0439	0.215314	0.101881	0.49261	lncRNA
ENSG0000(	ENSG0000(	486.4513	0.194735	0.101758	0.49261	TEC
ENSG0000(	ENSG0000(	64.6617	0.459627	0.101793	0.49261	lncRNA
ENSG0000(	ENSG0000(	113.9361	0.204207	0.101914	0.49261	lncRNA

ENSG0000(	FAM184B	560.685	0.284868	0.101831	0.49261	protein_coding
ENSG0000(	LPAR6	755.5463	-0.20916	0.101917	0.49261	protein_coding
ENSG0000(	NOLC1	758.3947	-0.15514	0.101893	0.49261	protein_coding
ENSG0000(	PDE1B	645.7958	-0.15849	0.101767	0.49261	protein_coding
ENSG0000(	RNU4-13P	20.92396	-0.42933	0.101893	0.49261	snRNA
ENSG0000(	RPA4	100.9722	0.186675	0.101834	0.49261	protein_coding
ENSG0000(	ENSG0000(	22.7688	-0.38358	0.101995	0.492852	lncRNA
ENSG0000(	TIMM44	447.7864	0.191758	0.102003	0.492852	protein_coding
ENSG0000(	BLOC1S2	276.7336	-0.21251	0.102052	0.493005	protein_coding
ENSG0000(	KIR3DX1	748.1424	-0.08448	0.102078	0.493041	transcribed_unprocessed_pseudo
ENSG0000(	AFDN	6394.315	0.164543	0.10212	0.493157	protein_coding
ENSG0000(	DAGLA	438.0689	0.327375	0.102143	0.493181	protein_coding
ENSG0000(	DOCK11	1050.059	-0.33812	0.102238	0.493424	protein_coding
ENSG0000(	ENSG0000(	20.49647	0.352132	0.102247	0.493424	lncRNA
ENSG0000(	FLRT2	2564.63	-0.42404	0.10223	0.493424	protein_coding
ENSG0000(	TRIB3	56.93329	-0.48325	0.102294	0.493481	protein_coding
ENSG0000(	ZNF492	208.6162	0.196189	0.102287	0.493481	protein_coding
ENSG0000(	CNPPD1	784.6828	0.210155	0.102335	0.493589	protein_coding
ENSG0000(	NNT-AS1	2173.771	0.069484	0.102362	0.493632	lncRNA
ENSG0000(	ENKD1	127.7083	0.221892	0.102395	0.493709	protein_coding
ENSG0000(	JADRR	361.7868	0.160989	0.102415	0.493716	lncRNA
ENSG0000(	PDP1	628.3058	-0.29048	0.102463	0.493862	protein_coding
ENSG0000(	RB1	987.0204	-0.21204	0.102491	0.493908	protein_coding
ENSG0000(	CARMN	13847.95	-0.29613	0.102548	0.493931	lncRNA
ENSG0000(	DHRS1	553.2281	0.15664	0.102619	0.493931	protein_coding
ENSG0000(	ENSG0000(	1627.177	0.112265	0.102568	0.493931	lncRNA
ENSG0000(	EPS15	2637.18	-0.11556	0.102674	0.493931	protein_coding
ENSG0000(	HCG25	1060.46	0.115801	0.102569	0.493931	lncRNA
ENSG0000(	NUTM2E	84.53174	0.354385	0.102647	0.493931	protein_coding
ENSG0000(	PRSS1	107.7873	0.400458	0.102613	0.493931	protein_coding
ENSG0000(	RAB6B	134.9463	0.588865	0.10264	0.493931	protein_coding
ENSG0000(	SMPD4BP	808.4545	0.106121	0.102613	0.493931	transcribed_processed_pseudoge
ENSG0000(	TFCP2L1	3757.06	0.408762	0.102693	0.493931	protein_coding
ENSG0000(	TMEM64	516.2007	-0.18863	0.102688	0.493931	protein_coding
ENSG0000(	ZNF215	508.1257	-0.24614	0.10273	0.494023	protein_coding
ENSG0000(	H3C6	247.7536	0.21433	0.102758	0.494072	protein_coding
ENSG0000(	BST1	250.8412	-0.18174	0.102874	0.494192	protein_coding
ENSG0000(	ENSG0000(	123.5699	0.301842	0.102838	0.494192	transcribed_processed_pseudoge
ENSG0000(	ENSG0000(	69.30263	0.22917	0.102922	0.494192	lncRNA
ENSG0000(	ENSG0000(	31.97369	0.427134	0.102915	0.494192	lncRNA
ENSG0000(	MPDZ	688.0842	-0.32895	0.102802	0.494192	protein_coding
ENSG0000(	PHETA1	293.6013	0.244522	0.102927	0.494192	protein_coding
ENSG0000(	RECQL	674.412	-0.17874	0.102873	0.494192	protein_coding
ENSG0000(	ZNF709	252.9317	-0.34418	0.10287	0.494192	protein_coding
ENSG0000(	DGCR5	652.6258	0.1485	0.102992	0.494421	lncRNA
ENSG0000(	MESD	1532.779	-0.0957	0.103011	0.494422	protein_coding
ENSG0000(	COMT	864.3867	-0.13574	0.103032	0.494438	protein_coding

ENSG0000(	ENSG0000(	51.81849	0.428278	0.103087	0.49461	transcribed_processed_pseudoge
ENSG0000(	MOV10L1	128.9204	-0.36217	0.103104	0.49461	protein_coding
ENSG0000(	ENSG0000(	483.6486	-0.17759	0.103168	0.494834	lncRNA
ENSG0000(	ABHD18	1369.894	0.108413	0.103245	0.494848	protein_coding
ENSG0000(	ALDH3B1	677.722	-0.23389	0.103216	0.494848	protein_coding
ENSG0000(	ENSG0000(	141.9873	0.273813	0.103279	0.494848	lncRNA
ENSG0000(	ENSG0000(	677.684	0.220799	0.103196	0.494848	lncRNA
ENSG0000(	ENSG0000(	116.177	-0.2086	0.103247	0.494848	lncRNA
ENSG0000(	LINC01873	1566.81	0.189543	0.103269	0.494848	lncRNA
ENSG0000(	ANKS4B	1484.299	0.25396	0.103358	0.495054	protein_coding
ENSG0000(	ENSG0000(	272.5383	0.202318	0.103358	0.495054	lncRNA
ENSG0000(	NKX3-1	31.74436	-0.46613	0.103425	0.495117	protein_coding
ENSG0000(	PELI1	1624.344	-0.16027	0.103424	0.495117	protein_coding
ENSG0000(	PLCL1	290.2034	-0.27018	0.103395	0.495117	protein_coding
ENSG0000(	SYN3	1305.149	-0.16748	0.103467	0.495146	protein_coding
ENSG0000(	TPRG1-AS2	19.98499	-0.39954	0.103461	0.495146	lncRNA
ENSG0000(	TSPYL4	380.2735	-0.15949	0.103492	0.495178	protein_coding
ENSG0000(	LINC01782	35.56015	0.277518	0.103563	0.49543	lncRNA
ENSG0000(	ENSG0000(	2019.238	0.191551	0.103621	0.495538	TEC
ENSG0000(	RNU6-920F	31.92959	-0.38019	0.103612	0.495538	snRNA
ENSG0000(	PATL1-DT	272.0545	-0.17224	0.103642	0.495552	lncRNA
ENSG0000(	CARHSP1	978.8665	-0.17294	0.103703	0.49569	protein_coding
ENSG0000(	FOXRED1	1288.317	-0.12726	0.103707	0.49569	protein_coding
ENSG0000(	ENSG0000(	216.4414	0.146687	0.103741	0.495766	lncRNA
ENSG0000(	LINC02893	387.4414	0.268118	0.103803	0.495975	lncRNA
ENSG0000(	CASS4	232.2384	-0.25998	0.103843	0.496082	protein_coding
ENSG0000(	GAB2	1241.054	-0.20177	0.10387	0.496123	protein_coding
ENSG0000(	FAM177A1	2328.166	0.139641	0.103895	0.496145	protein_coding
ENSG0000(	ZIM3	150.1137	0.163979	0.103911	0.496145	protein_coding
ENSG0000(	HSPA8P3	151.2833	0.326216	0.103929	0.496147	processed_pseudogene
ENSG0000(	DUX4L26	27.22087	0.768797	0.104155	0.496578	processed_pseudogene
ENSG0000(	ENSG0000(	2356.134	0.13159	0.104062	0.496578	lncRNA
ENSG0000(	ENSG0000(	173.4674	0.129045	0.104182	0.496578	lncRNA
ENSG0000(	FBXO5	269.4598	-0.17378	0.104125	0.496578	protein_coding
ENSG0000(	HKDC1	1075.397	-0.4989	0.10404	0.496578	protein_coding
ENSG0000(	HPSE2	180.2476	0.479153	0.104176	0.496578	protein_coding
ENSG0000(	KIF22	1547.743	0.133443	0.104122	0.496578	protein_coding
ENSG0000(	MIR196A1	85.23165	0.426892	0.104098	0.496578	miRNA
ENSG0000(	PLPP3	1132.718	-0.20933	0.104129	0.496578	protein_coding
ENSG0000(	ENSG0000(	34.89261	0.308852	0.104241	0.496683	lncRNA
ENSG0000(	GTF2IP1	1389.677	-0.20709	0.104258	0.496683	transcribed_unprocessed_pseudo
ENSG0000(	MRPS22	1163.801	0.114922	0.104246	0.496683	protein_coding
ENSG0000(	SERPINB9P	917.2756	-0.17456	0.104283	0.496718	lncRNA
ENSG0000(	NT5DC1	564.2588	0.207596	0.104359	0.496991	protein_coding
ENSG0000(	BLOC1S1	335.8802	0.218649	0.104464	0.497062	protein_coding
ENSG0000(	ENSG0000(	838.3623	0.237075	0.104451	0.497062	lncRNA
ENSG0000(	LRCH1	1362.509	-0.14258	0.104435	0.497062	protein_coding

ENSG0000(	NUTF2	444.5527	-0.15489	0.104424	0.497062	protein_coding
ENSG0000(	OR10G4	303.8493	-0.18318	0.10445	0.497062	protein_coding
ENSG0000(	DUXAP10	614.4231	-0.38458	0.104489	0.497097	transcribed_processed_pseudoge
ENSG0000(	SLA2	249.2898	-0.18953	0.104512	0.497119	protein_coding
ENSG0000(	C2orf27A	257.9042	-0.42447	0.104632	0.497445	lncRNA
ENSG0000(	DNAH14	575.6331	0.250692	0.104779	0.497445	protein_coding
ENSG0000(	ENSG0000(	633.4299	0.160076	0.104778	0.497445	lncRNA
ENSG0000(	ENSG0000(	75.29801	-0.2355	0.104795	0.497445	lncRNA
ENSG0000(	ENSG0000(	97.21178	0.192482	0.104778	0.497445	lncRNA
ENSG0000(	MARK2P4	47.1375	0.396432	0.104619	0.497445	processed_pseudogene
ENSG0000(	MAST4	1892.634	-0.17214	0.104798	0.497445	protein_coding
ENSG0000(	MPHOSPH9	1573.295	-0.12879	0.104787	0.497445	protein_coding
ENSG0000(	PLCG1	2251.129	-0.1389	0.104643	0.497445	protein_coding
ENSG0000(	RNU6-118F	34.7543	-0.36123	0.104658	0.497445	snRNA
ENSG0000(	SELENOH	393.8568	-0.13439	0.104681	0.497445	protein_coding
ENSG0000(	YWHAZP10	23.59395	0.414006	0.10473	0.497445	processed_pseudogene
ENSG0000(	CTDSPL2	1543.354	0.089204	0.105026	0.497587	protein_coding
ENSG0000(	DUT	320.6747	-0.16603	0.104846	0.497587	protein_coding
ENSG0000(	ENSG0000(	21.77313	0.411365	0.104978	0.497587	lncRNA
ENSG0000(	ENSG0000(	28.65024	0.40374	0.104957	0.497587	lncRNA
ENSG0000(	ENSG0000(	46.98035	-0.28235	0.104991	0.497587	lncRNA
ENSG0000(	ENSG0000(	614.2801	0.112204	0.105011	0.497587	transcribed_processed_pseudoge
ENSG0000(	HLA-DOB	229.5722	-0.47878	0.104934	0.497587	protein_coding
ENSG0000(	PTDSS1	685.0299	-0.18659	0.104978	0.497587	protein_coding
ENSG0000(	QARS1	1505.991	0.138043	0.104975	0.497587	protein_coding
ENSG0000(	RPL5P13	62.69482	0.27407	0.104912	0.497587	processed_pseudogene
ENSG0000(	RRN3P1	1587.678	0.089845	0.104903	0.497587	transcribed_unprocessed_pseudo
ENSG0000(	CDX1	956.275	0.376144	0.105061	0.497587	protein_coding
ENSG0000(	GKAP1	103.497	0.30427	0.105129	0.497587	protein_coding
ENSG0000(	H2AC4	28.76548	-0.42836	0.10516	0.497587	protein_coding
ENSG0000(	KCTD21	344.1106	-0.1324	0.105129	0.497587	protein_coding
ENSG0000(	MBNL2	1762.7	-0.12034	0.105135	0.497587	protein_coding
ENSG0000(	MCUB	174.4533	-0.36674	0.105171	0.497587	protein_coding
ENSG0000(	TMSB10	3002.978	-0.22906	0.105118	0.497587	protein_coding
ENSG0000(	TOM1	764.0075	-0.22357	0.105164	0.497587	protein_coding
ENSG0000(	CLCN2	1336.214	0.335656	0.105225	0.497756	protein_coding
ENSG0000(	AIFM3	949.1905	0.509593	0.105317	0.49791	protein_coding
ENSG0000(	ARHGAP11	449.9406	-0.23445	0.105471	0.49791	protein_coding
ENSG0000(	CYP4F11	265.5988	0.380222	0.105547	0.49791	protein_coding
ENSG0000(	ENSG0000(	25.90176	0.571689	0.105519	0.49791	lncRNA
ENSG0000(	ENSG0000(	319.4461	0.130305	0.105504	0.49791	lncRNA
ENSG0000(	EPS8	3124.217	0.241606	0.105356	0.49791	protein_coding
ENSG0000(	FYTTD1	1620.656	-0.10681	0.105542	0.49791	protein_coding
ENSG0000(	KCP	425.908	-0.14805	0.105455	0.49791	protein_coding
ENSG0000(	LINC02284	127.2349	-0.26953	0.105475	0.49791	lncRNA
ENSG0000(	LPAL2	358.0111	-0.23625	0.105406	0.49791	transcribed_unprocessed_pseudo
ENSG0000(	MT-ND6	1654.644	-0.5505	0.105404	0.49791	protein_coding

ENSG0000(	NMNAT2	60.76007	-0.41635	0.105539	0.49791	protein_coding
ENSG0000(	POU5F1	650.3209	0.237459	0.105389	0.49791	protein_coding
ENSG0000(	PRRC2C	10309.7	-0.11485	0.10546	0.49791	protein_coding
ENSG0000(	RN7SL328F	80.90701	-0.24766	0.105531	0.49791	misc_RNA
ENSG0000(	ZBTB2	277.8813	-0.14436	0.105478	0.49791	protein_coding
ENSG0000(	DCP2	2551.106	-0.16337	0.105583	0.497921	protein_coding
ENSG0000(	FGF7P6	69.65259	-0.41875	0.105586	0.497921	transcribed_unprocessed_pseudo
ENSG0000(	ZNF3	1408.721	0.114876	0.105605	0.497926	protein_coding
ENSG0000(	ENSG0000(	239.2792	-0.1382	0.105624	0.49793	lncRNA
ENSG0000(	U2AF1L4	489.6839	0.127313	0.105735	0.498282	protein_coding
ENSG0000(	ZMYND12	24.91581	0.515147	0.105724	0.498282	protein_coding
ENSG0000(	CA1	1065.535	0.823452	0.105759	0.498311	protein_coding
ENSG0000(	ENSG0000(	33.60332	0.744478	0.105795	0.498397	lncRNA
ENSG0000(	PCSK4	58.77908	0.475075	0.105844	0.498542	protein_coding
ENSG0000(	PMFBP1	212.2307	0.379638	0.105878	0.498615	protein_coding
ENSG0000(	ENSG0000(	125.3468	-0.35798	0.105956	0.498787	TEC
ENSG0000(	FEZ2	1708.836	0.143467	0.105942	0.498787	protein_coding
ENSG0000(	MINK1	3002.209	0.183025	0.105969	0.498787	protein_coding
ENSG0000(	ENSG0000(	108.4443	0.245114	0.106048	0.499074	lncRNA
ENSG0000(	EIF3K	958.7075	0.124895	0.106106	0.499177	protein_coding
ENSG0000(	HES2	726.301	0.156208	0.106095	0.499177	protein_coding
ENSG0000(	C1orf131	576.4917	0.117973	0.106179	0.499311	protein_coding
ENSG0000(	ENSG0000(	99.48147	-0.20798	0.106189	0.499311	protein_coding
ENSG0000(	LINC02465	83.76013	0.292724	0.106161	0.499311	lncRNA
ENSG0000(	ADAMTS1	937.3714	-0.35133	0.106326	0.499719	protein_coding
ENSG0000(	ADHFE1	202.9962	0.230666	0.106379	0.499719	protein_coding
ENSG0000(	CSNK1E	2443.154	-0.12067	0.106349	0.499719	protein_coding
ENSG0000(	DTNBP1	324.6479	-0.13441	0.106439	0.499719	protein_coding
ENSG0000(	FAM168A	1249.312	-0.18152	0.106418	0.499719	protein_coding
ENSG0000(	GNAI1	1414.776	0.168549	0.106394	0.499719	protein_coding
ENSG0000(	IFT88	693.3654	0.155568	0.106361	0.499719	protein_coding
ENSG0000(	RGSL1	82.87461	0.430533	0.10633	0.499719	protein_coding
ENSG0000(	ZNF681	479.7243	0.168837	0.106429	0.499719	protein_coding
ENSG0000(	ENSG0000(	61.55243	-0.29194	0.10654	0.500049	processed_pseudogene
ENSG0000(	FRAS1	253.2088	0.389576	0.106564	0.500049	protein_coding
ENSG0000(	MTCO1P25	175.6469	0.19925	0.106564	0.500049	unprocessed_pseudogene
ENSG0000(	ENSG0000(	27.94414	0.529314	0.106642	0.500332	lncRNA
ENSG0000(	HBP1	1519.619	-0.12449	0.106694	0.500489	protein_coding
ENSG0000(	MMADHC	455.0053	-0.16293	0.10673	0.500519	protein_coding
ENSG0000(	WHAMMP3	1177.249	-0.10253	0.106755	0.500519	transcribed_unprocessed_pseudo
ENSG0000(	ZNF8-DT	192.5487	-0.29238	0.106738	0.500519	lncRNA
ENSG0000(	LEPROTL1	974.6152	-0.10952	0.106792	0.500606	protein_coding
ENSG0000(	P2RY1	1136.587	0.359993	0.106871	0.500893	protein_coding
ENSG0000(	SEPTIN7	4025.12	-0.09958	0.106911	0.500994	protein_coding
ENSG0000(	ADGRG1	970.2483	0.188149	0.106966	0.501114	protein_coding
ENSG0000(	AGTRAP	191.7941	-0.27322	0.106991	0.501114	protein_coding
ENSG0000(	ENSG0000(	792.7958	0.151676	0.106987	0.501114	processed_pseudogene

ENSG0000(	ELMO1	1868.547	-0.25228	0.107098	0.501527	protein_coding
ENSG0000(	ENSG0000(	71.18806	-0.27922	0.107212	0.501778	processed_pseudogene
ENSG0000(	ENSG0000(	101.4981	-0.26322	0.107217	0.501778	lncRNA
ENSG0000(	ENSG0000(	50.88473	-0.39683	0.107227	0.501778	lncRNA
ENSG0000(	LINC02125	26.41651	-0.40279	0.107207	0.501778	lncRNA
ENSG0000(	POMK	2524.874	0.168443	0.10726	0.501778	protein_coding
ENSG0000(	PPFIA4	116.3061	-0.46138	0.107279	0.501778	protein_coding
ENSG0000(	YPEL2	2347.149	-0.24068	0.107272	0.501778	protein_coding
ENSG0000(	ENSG0000(	261.5315	0.196573	0.107382	0.50188	lncRNA
ENSG0000(	ENSG0000(	363.5259	0.125754	0.107357	0.50188	TEC
ENSG0000(	HINFP	2420.336	0.077416	0.107342	0.50188	protein_coding
ENSG0000(	PMF1	399.565	0.102307	0.107344	0.50188	protein_coding
ENSG0000(	VENTXP4	117.4491	-0.18353	0.107392	0.50188	processed_pseudogene
ENSG0000(	CHD7	4452.313	0.142332	0.107592	0.502302	protein_coding
ENSG0000(	GUSBP13	263.9236	0.464738	0.107569	0.502302	unprocessed_pseudogene
ENSG0000(	LINC01725	478.0681	0.260572	0.10758	0.502302	lncRNA
ENSG0000(	NAT1	295.1624	0.258098	0.107566	0.502302	protein_coding
ENSG0000(	NUGGC	716.679	-0.15877	0.107543	0.502302	protein_coding
ENSG0000(	ZZEF1	10094.5	0.223511	0.107583	0.502302	protein_coding
ENSG0000(	GTF2IRD1	658.7882	-0.22063	0.107612	0.502311	protein_coding
ENSG0000(	PPP2R3A	1264.282	0.191392	0.107631	0.502317	protein_coding
ENSG0000(	ENSG0000(	1455.484	0.204293	0.107671	0.50233	lncRNA
ENSG0000(	KLHL29	572.2645	-0.25298	0.10767	0.50233	protein_coding
ENSG0000(	C2orf88	1276.099	0.312362	0.107819	0.502338	protein_coding
ENSG0000(	ENSG0000(	37.30352	0.316119	0.107805	0.502338	lncRNA
ENSG0000(	NAIP	3982.367	-0.24486	0.107855	0.502338	protein_coding
ENSG0000(	OR2A3P	125.5954	-0.18017	0.107793	0.502338	transcribed_unprocessed_pseudo
ENSG0000(	PIP4K2A	1176.468	-0.23771	0.107704	0.502338	protein_coding
ENSG0000(	PTGES3L	401.4292	0.16434	0.107841	0.502338	protein_coding
ENSG0000(	SEMA4B	1846.69	-0.21742	0.107751	0.502338	protein_coding
ENSG0000(	SLC25A1	439.0822	0.256802	0.107741	0.502338	protein_coding
ENSG0000(	SLC6A7	487.2721	0.406811	0.107713	0.502338	protein_coding
ENSG0000(	ZNF204P	295.6085	0.281357	0.107783	0.502338	transcribed_unprocessed_pseudo
ENSG0000(	ELF3	5389.887	0.347279	0.107893	0.502388	protein_coding
ENSG0000(	ENSG0000(	216.1851	0.126163	0.107921	0.502388	processed_pseudogene
ENSG0000(	SLX1B	55.36241	1.552349	0.107905	0.502388	protein_coding
ENSG0000(	ENSG0000(	246.7212	0.46938	0.107996	0.502422	processed_pseudogene
ENSG0000(	FABP5P3	30.71145	0.388378	0.108019	0.502422	transcribed_processed_pseudoge
ENSG0000(	GDF6	55.70649	0.500045	0.107996	0.502422	protein_coding
ENSG0000(	HOXB-AS1	48.84266	-0.38962	0.108017	0.502422	lncRNA
ENSG0000(	NSD1	6228.916	-0.08923	0.107987	0.502422	protein_coding
ENSG0000(	ACSM2B	372.6968	-0.2336	0.108106	0.502487	protein_coding
ENSG0000(	ASRGL1	524.6991	-0.24077	0.10808	0.502487	protein_coding
ENSG0000(	KLLN	307.7583	0.298411	0.108105	0.502487	protein_coding
ENSG0000(	PPP4R2	1347.926	0.120921	0.108083	0.502487	protein_coding
ENSG0000(	LINC01730	22.66326	-0.52422	0.108139	0.502556	lncRNA
ENSG0000(	ENSG0000(	1680.846	0.084539	0.10821	0.502715	protein_coding

ENSG0000(	TCEAL3	58.04718	-0.33408	0.108208	0.502715	protein_coding
ENSG0000(	FBXL17	727.9063	0.180154	0.108292	0.503011	protein_coding
ENSG0000(	ATP6V0E1	574.5675	-0.22188	0.10842	0.503119	protein_coding
ENSG0000(	ENSG0000(	3208.671	0.173722	0.108455	0.503119	transcribed_unprocessed_pseudo
ENSG0000(	ENSG0000(	199.3055	0.469663	0.108418	0.503119	protein_coding
ENSG0000(	ENSG0000(	43.46648	-0.34265	0.108453	0.503119	lncRNA
ENSG0000(	ENSG0000(	25.79044	0.384839	0.108351	0.503119	lncRNA
ENSG0000(	FAM168B	1869.381	0.121592	0.1085	0.503119	protein_coding
ENSG0000(	FNDC4	22.53179	-0.65988	0.108398	0.503119	protein_coding
ENSG0000(	FREM1	470.3711	-0.45875	0.108517	0.503119	protein_coding
ENSG0000(	FSBP	608.9542	-0.16818	0.108509	0.503119	protein_coding
ENSG0000(	LEMD1-DT	331.5538	0.138152	0.108475	0.503119	lncRNA
ENSG0000(	RHOT1P1	49.3091	0.530797	0.108372	0.503119	processed_pseudogene
ENSG0000(	CCT6P3	472.0982	-0.15865	0.10857	0.503266	transcribed_unprocessed_pseudo
ENSG0000(	CLEC12A-A	2487.354	0.202489	0.108624	0.503266	lncRNA
ENSG0000(	ENSG0000(	38.86024	-0.41323	0.108593	0.503266	lncRNA
ENSG0000(	ENSG0000(	129.9141	0.169278	0.108625	0.503266	lncRNA
ENSG0000(	FRMD1	1795.344	0.559857	0.108658	0.503266	protein_coding
ENSG0000(	SNORD118	944.3904	-0.32813	0.108658	0.503266	snoRNA
ENSG0000(	ENSG0000(	1013.52	-0.42915	0.108702	0.503383	unprocessed_pseudogene
ENSG0000(	ALDH3A1	49.95634	0.577702	0.108869	0.50353	protein_coding
ENSG0000(	C13orf42	195.3888	0.17656	0.108936	0.50353	protein_coding
ENSG0000(	C16orf86	26.01024	-0.43756	0.108953	0.50353	protein_coding
ENSG0000(	C5orf24	1143.218	-0.08702	0.109142	0.50353	protein_coding
ENSG0000(	CASK	2833.134	0.096965	0.108863	0.50353	protein_coding
ENSG0000(	CMAS	466.458	0.24928	0.109183	0.50353	protein_coding
ENSG0000(	CRISPLD2	1834.823	-0.28978	0.108808	0.50353	protein_coding
ENSG0000(	ENSG0000(	79.24961	0.347862	0.109012	0.50353	lncRNA
ENSG0000(	ENSG0000(	103.9045	-0.50999	0.108879	0.50353	lncRNA
ENSG0000(	ENSG0000(	4633.107	0.338964	0.10912	0.50353	lncRNA
ENSG0000(	ENSG0000(	39.82844	0.367214	0.109055	0.50353	lncRNA
ENSG0000(	ENSG0000(	130.7491	0.1586	0.109097	0.50353	lncRNA
ENSG0000(	ILRUN	1502.037	-0.14112	0.109147	0.50353	protein_coding
ENSG0000(	KCNK17	20.10075	-0.48154	0.108934	0.50353	protein_coding
ENSG0000(	LEPR	280.9282	-0.24651	0.108884	0.50353	protein_coding
ENSG0000(	LST1	524.1833	-0.22394	0.108964	0.50353	protein_coding
ENSG0000(	MZF1	1386.119	0.099635	0.109181	0.50353	protein_coding
ENSG0000(	NRG4	186.0916	0.31488	0.10906	0.50353	protein_coding
ENSG0000(	PABPC1	9067.449	-0.14114	0.109209	0.50353	protein_coding
ENSG0000(	PCCA-DT	348.0285	0.140078	0.10914	0.50353	lncRNA
ENSG0000(	PCYT1B	215.6271	0.214737	0.108761	0.50353	protein_coding
ENSG0000(	PFDN1	610.4416	0.252854	0.109192	0.50353	protein_coding
ENSG0000(	PRPF39	791.3863	-0.12317	0.10909	0.50353	protein_coding
ENSG0000(	SEC13	1033.462	-0.13968	0.108832	0.50353	protein_coding
ENSG0000(	SLC52A3	600.5198	0.317766	0.109172	0.50353	protein_coding
ENSG0000(	ZNF439	1694.749	0.147226	0.108858	0.50353	protein_coding
ENSG0000(	GPRC5B	275.9384	-0.39429	0.109256	0.50366	protein_coding

ENSG0000(	HNRNPUL1	2625.161	-0.08663	0.109335	0.503939	protein_coding
ENSG0000(	MTF1	1412.218	-0.16328	0.109389	0.504021	protein_coding
ENSG0000(	TMEM171	361.5287	0.551773	0.109388	0.504021	protein_coding
ENSG0000(	ENSG0000(	237.6482	-0.13978	0.109506	0.504307	TEC
ENSG0000(	ENSG0000(	21.8242	-0.32418	0.109493	0.504307	lncRNA
ENSG0000(	STAT4	346.1193	-0.26873	0.109492	0.504307	protein_coding
ENSG0000(	CLCN5	988.2275	0.1461	0.10957	0.504433	protein_coding
ENSG0000(	DBNL	4610.764	-0.13751	0.109556	0.504433	protein_coding
ENSG0000(	BOLA2-SM(	985.1103	0.190914	0.10968	0.504519	protein_coding
ENSG0000(	CAMSAP3	686.7766	0.363785	0.109665	0.504519	protein_coding
ENSG0000(	ENSG0000(	125.0583	0.236049	0.109632	0.504519	lncRNA
ENSG0000(	ENSG0000(	134.356	0.278513	0.109693	0.504519	lncRNA
ENSG0000(	ENSG0000(	21.5696	0.37962	0.109727	0.504519	lncRNA
ENSG0000(	KCNMB3	443.0272	-0.13975	0.109699	0.504519	protein_coding
ENSG0000(	SOCS5	987.004	-0.08394	0.109736	0.504519	protein_coding
ENSG0000(	TBC1D2B	3012.186	-0.16676	0.109675	0.504519	protein_coding
ENSG0000(	ENSG0000(	204.2349	-0.1223	0.109795	0.504625	lncRNA
ENSG0000(	YAP1	1593.081	-0.13994	0.109786	0.504625	protein_coding
ENSG0000(	CAMK2G	2044.039	0.162388	0.109845	0.504682	protein_coding
ENSG0000(	ENSG0000(	532.7185	0.142489	0.109881	0.504682	lncRNA
ENSG0000(	FOSL1	78.24077	-0.34033	0.109842	0.504682	protein_coding
ENSG0000(	RASSF3	1416.958	-0.15775	0.109866	0.504682	protein_coding
ENSG0000(	ENSG0000(	10276.07	0.135242	0.109913	0.504743	TEC
ENSG0000(	SLCO1B3	26.23961	-0.93791	0.11	0.505061	protein_coding
ENSG0000(	LINC00892	26.24753	-0.59033	0.110097	0.505366	lncRNA
ENSG0000(	NCKAP5L	637.9028	-0.2032	0.110122	0.505366	protein_coding
ENSG0000(	NR1D2	900.8209	0.239547	0.110115	0.505366	protein_coding
ENSG0000(	ENSG0000(	32.1615	0.396368	0.110409	0.505966	processed_pseudogene
ENSG0000(	ENSG0000(	72.22102	0.28049	0.11035	0.505966	lncRNA
ENSG0000(	FBXO48	568.9055	0.125636	0.110329	0.505966	protein_coding
ENSG0000(	GPR160P1	92.26502	0.234158	0.110377	0.505966	processed_pseudogene
ENSG0000(	LINC02376	158.0909	0.364887	0.110418	0.505966	lncRNA
ENSG0000(	NUDT16L2I	332.5705	0.218697	0.110343	0.505966	transcribed_unitary_pseudogene
ENSG0000(	PPP6R3	3400.489	0.068985	0.110313	0.505966	protein_coding
ENSG0000(	RBFOX3	341.6124	0.168892	0.11028	0.505966	protein_coding
ENSG0000(	RN7SL163F	70.76753	0.238155	0.110402	0.505966	misc_RNA
ENSG0000(	ALG11	3735.474	0.069249	0.110453	0.506014	protein_coding
ENSG0000(	ENSG0000(	66.89477	0.399054	0.110557	0.506014	transcribed_processed_pseudoge
ENSG0000(	ENSG0000(	704.9994	-0.10839	0.110493	0.506014	lncRNA
ENSG0000(	MIR4300H(	137.2074	0.170857	0.110522	0.506014	lncRNA
ENSG0000(	MTMR10	1993.574	0.082664	0.11049	0.506014	protein_coding
ENSG0000(	OR4E1	27.8506	0.401156	0.110534	0.506014	protein_coding
ENSG0000(	RELL2	98.44977	-0.25092	0.110543	0.506014	protein_coding
ENSG0000(	ENSG0000(	387.3122	0.235512	0.110594	0.506099	lncRNA
ENSG0000(	DIP2A-IT1	59.38861	0.245575	0.110758	0.506394	lncRNA
ENSG0000(	ENSG0000(	26.42372	0.489347	0.110769	0.506394	lncRNA
ENSG0000(	ENSG0000(	210.6168	-0.17514	0.110753	0.506394	lncRNA

ENSG0000(	ENSG0000(	152.7014	-0.17997	0.110746	0.506394	lncRNA
ENSG0000(	PRRT3	232.4089	-0.15559	0.110725	0.506394	protein_coding
ENSG0000(	UCP2	731.5093	-0.3163	0.110765	0.506394	protein_coding
ENSG0000(	BCL2L14	914.1643	0.271379	0.110942	0.506429	protein_coding
ENSG0000(	CSRNP3	301.4138	0.31418	0.110924	0.506429	protein_coding
ENSG0000(	ENSG0000(	156.2173	0.168122	0.110925	0.506429	TEC
ENSG0000(	ENSG0000(	100.7391	0.209493	0.110842	0.506429	lncRNA
ENSG0000(	FADS2	565.3896	-0.31445	0.110864	0.506429	protein_coding
ENSG0000(	HOXC6	51.59186	-0.67616	0.110889	0.506429	protein_coding
ENSG0000(	OR2V2	305.0819	0.131187	0.110803	0.506429	protein_coding
ENSG0000(	RAD21	3004.032	-0.09809	0.110921	0.506429	protein_coding
ENSG0000(	SNORA71A	107.621	0.420755	0.110934	0.506429	snoRNA
ENSG0000(	ENSG0000(	24.13063	-0.76025	0.111006	0.506512	lncRNA
ENSG0000(	ENSG0000(	47.45376	0.294231	0.111031	0.506512	lncRNA
ENSG0000(	FA2H	416.985	0.364263	0.111108	0.506512	protein_coding
ENSG0000(	HOXD4	126.6497	0.337344	0.111053	0.506512	protein_coding
ENSG0000(	LINC01948	313.7123	-0.16107	0.111069	0.506512	lncRNA
ENSG0000(	PSG5	72.6784	0.325925	0.111103	0.506512	protein_coding
ENSG0000(	SRCAP	4227.735	0.098072	0.111083	0.506512	protein_coding
ENSG0000(	UGT1A6	871.5942	0.58095	0.11099	0.506512	protein_coding
ENSG0000(	CAMTA2	1175.301	-0.13792	0.111159	0.506628	protein_coding
ENSG0000(	DLEU7	548.675	-0.1245	0.1112	0.506628	protein_coding
ENSG0000(	H3C2	116.4362	-0.36477	0.111207	0.506628	protein_coding
ENSG0000(	ZNF540	321.784	0.199937	0.111187	0.506628	protein_coding
ENSG0000(	BAG6	1983.389	0.136525	0.111307	0.506936	protein_coding
ENSG0000(	CASP6	396.5238	0.205536	0.111332	0.506936	protein_coding
ENSG0000(	ENSG0000(	167.5271	0.171218	0.111328	0.506936	lncRNA
ENSG0000(	NRBF2	275.1091	-0.15131	0.111367	0.506936	protein_coding
ENSG0000(	TMEM125	209.5081	0.38116	0.111365	0.506936	protein_coding
ENSG0000(	ENSG0000(	172.9923	-0.2096	0.111441	0.507106	lncRNA
ENSG0000(	ENSG0000(	74.75909	-0.23672	0.111423	0.507106	lncRNA
ENSG0000(	EGFL7	315.516	-0.36676	0.111563	0.507158	protein_coding
ENSG0000(	ENSG0000(	160.9583	0.163063	0.111506	0.507158	lncRNA
ENSG0000(	POLR2I	50.13386	-0.27181	0.111546	0.507158	protein_coding
ENSG0000(	RAPGEF3	789.87	-0.27645	0.111511	0.507158	protein_coding
ENSG0000(	TRIM62	246.8363	0.178996	0.111473	0.507158	protein_coding
ENSG0000(	WBP2NL	438.2365	0.152372	0.111555	0.507158	protein_coding
ENSG0000(	ENSG0000(	44.1951	0.411131	0.111594	0.507204	TEC
ENSG0000(	LRRCC1	388.5331	0.238205	0.11161	0.507204	protein_coding
ENSG0000(	SIAE	1425.485	0.230336	0.111642	0.50725	protein_coding
ENSG0000(	USP31	1087.834	-0.20149	0.111657	0.50725	protein_coding
ENSG0000(	KHDRBS2	37.89712	0.504253	0.111683	0.507285	protein_coding
ENSG0000(	EXTL3	1188.587	-0.13202	0.111705	0.507301	protein_coding
ENSG0000(	GOT2P6	49.57615	0.345437	0.111744	0.507393	processed_pseudogene
ENSG0000(	KIAA1522	3531.17	0.320426	0.111771	0.507435	protein_coding
ENSG0000(	ENSG0000(	361.0274	0.168827	0.111831	0.507575	transcribed_unprocessed_pseudo
ENSG0000(	TMX4	816.2354	-0.1809	0.111839	0.507575	protein_coding

ENSG0000(	ENSG0000(	55.0571	-0.25323	0.111928	0.507733	lncRNA
ENSG0000(	OR8B8	75.58614	0.218441	0.111929	0.507733	protein_coding
ENSG0000(	STT3A	1479.676	-0.14306	0.111926	0.507733	protein_coding
ENSG0000(	ENSG0000(	24.8757	-0.37703	0.111958	0.507779	lncRNA
ENSG0000(	ENSG0000(	84.15471	0.189946	0.112028	0.508011	lncRNA
ENSG0000(	ST7	314.2207	-0.19355	0.112113	0.508313	protein_coding
ENSG0000(	MAGI1-IT1	685.6093	0.257638	0.112143	0.508365	lncRNA
ENSG0000(	ENSG0000(	126.9196	0.1554	0.112167	0.508393	lncRNA
ENSG0000(	COG8	1248.467	0.09254	0.112319	0.508599	protein_coding
ENSG0000(	ENSG0000(	710.5839	0.229625	0.112379	0.508599	protein_coding
ENSG0000(	ENSG0000(	1370.315	0.264379	0.112252	0.508599	lncRNA
ENSG0000(	ESYT2	2338.316	0.089582	0.112273	0.508599	protein_coding
ENSG0000(	HCG27	579.4648	-0.18339	0.112342	0.508599	lncRNA
ENSG0000(	MGLL	11350.47	0.383744	0.112332	0.508599	protein_coding
ENSG0000(	MYO1A	2205.957	0.403224	0.112355	0.508599	protein_coding
ENSG0000(	PON2	1375.363	0.184236	0.112374	0.508599	protein_coding
ENSG0000(	ZNF326	2782.681	0.173886	0.112286	0.508599	protein_coding
ENSG0000(	DOK4	1039.88	0.281891	0.112503	0.508639	protein_coding
ENSG0000(	ENSG0000(	50.37866	0.357679	0.112424	0.508639	lncRNA
ENSG0000(	ENSG0000(	50.78689	0.276881	0.112494	0.508639	lncRNA
ENSG0000(	ITPR2	4450.634	-0.17985	0.112511	0.508639	protein_coding
ENSG0000(	LINC02177	212.0781	0.259722	0.112517	0.508639	lncRNA
ENSG0000(	PROCR	314.6849	-0.1577	0.112437	0.508639	protein_coding
ENSG0000(	SGCE	869.0049	-0.11428	0.112483	0.508639	protein_coding
ENSG0000(	ENSG0000(	1400.49	0.169501	0.112636	0.509006	lncRNA
ENSG0000(	KLHL25	503.2808	0.194893	0.112635	0.509006	protein_coding
ENSG0000(	FGF23	387.9955	0.31348	0.112674	0.509098	protein_coding
ENSG0000(	ENSG0000(	36.75466	0.279083	0.112725	0.509242	lncRNA
ENSG0000(	MYZAP	221.3262	0.476649	0.112769	0.509359	protein_coding
ENSG0000(	ZNF768	468.4312	0.272135	0.112837	0.509582	protein_coding
ENSG0000(	ENSG0000(	35.13744	0.616444	0.112898	0.509693	transcribed_unitary_pseudogene
ENSG0000(	TENT5C	3719.651	-0.33411	0.112899	0.509693	protein_coding
ENSG0000(	ENSG0000(	29.84922	0.280219	0.113015	0.510134	lncRNA
ENSG0000(	IYD	1123.562	0.223322	0.113101	0.510438	protein_coding
ENSG0000(	DLGAP3	21.12092	0.544545	0.113185	0.510733	protein_coding
ENSG0000(	KYAT1	98.95144	-0.28808	0.113268	0.510856	protein_coding
ENSG0000(	LEO1	565.0682	0.142274	0.113253	0.510856	protein_coding
ENSG0000(	TUB	158.9714	-0.28289	0.113259	0.510856	protein_coding
ENSG0000(	ENSG0000(	199.3864	0.185364	0.11334	0.511098	protein_coding
ENSG0000(	CLOCK	1637.826	-0.16175	0.113425	0.51123	protein_coding
ENSG0000(	LINC02008	118.1103	-0.17736	0.113411	0.51123	lncRNA
ENSG0000(	PRSS27	488.3294	-0.18078	0.113412	0.51123	protein_coding
ENSG0000(	GRIN2C	35.91248	-0.29885	0.113543	0.511676	protein_coding
ENSG0000(	ENSG0000(	669.8921	0.141986	0.113581	0.511765	lncRNA
ENSG0000(	ENSG0000(	133.627	0.638993	0.113695	0.512193	lncRNA
ENSG0000(	MEG9	58.13128	-0.44346	0.113798	0.512326	lncRNA
ENSG0000(	SLC39A6	289.8821	-0.28193	0.113783	0.512326	protein_coding

ENSG0000(	SREBF2	3148.972	0.157087	0.113798	0.512326	protein_coding
ENSG0000(	TP53RK	379.3664	-0.13596	0.113788	0.512326	protein_coding
ENSG0000(	IGHV4-31	178.7049	-0.68709	0.11384	0.512346	IG_V_gene
ENSG0000(	SMYD3-IT1	112.6177	-0.21679	0.113836	0.512346	lncRNA
ENSG0000(	WTIP	3033.904	-0.1266	0.11386	0.51235	protein_coding
ENSG0000(	RCN3	598.5756	0.355509	0.11388	0.512357	protein_coding
ENSG0000(	ZC3H12A	912.8143	-0.32476	0.113899	0.512358	protein_coding
ENSG0000(	KIAA1210	54.24009	0.249065	0.113934	0.512434	protein_coding
ENSG0000(	NFATC1	520.4193	-0.38878	0.11398	0.512555	protein_coding
ENSG0000(	ATOH1	314.323	0.477566	0.114064	0.512596	protein_coding
ENSG0000(	LINC01570	50.4477	0.234211	0.114094	0.512596	lncRNA
ENSG0000(	RN7SL541F	60.54663	0.44357	0.114071	0.512596	misc_RNA
ENSG0000(	SLC49A3	183.1917	-0.24569	0.114059	0.512596	protein_coding
ENSG0000(	WDR41	842.7472	-0.11845	0.114101	0.512596	protein_coding
ENSG0000(	ZPLD1	69.78627	-0.24729	0.114095	0.512596	protein_coding
ENSG0000(	ENSG0000(	22.08578	-0.423	0.114135	0.512668	lncRNA
ENSG0000(	ENSG0000(	22.35143	0.40647	0.114171	0.512746	lncRNA
ENSG0000(	IGLV8-61	145.2722	-0.8061	0.114204	0.512807	IG_V_gene
ENSG0000(	SKAP1	216.7897	-0.29618	0.114256	0.512958	protein_coding
ENSG0000(	LLGL1	380.8442	-0.16104	0.114282	0.512994	protein_coding
ENSG0000(	ENSG0000(	116.5378	-0.20468	0.114317	0.513065	lncRNA
ENSG0000(	ENSG0000(	49.39349	0.341078	0.114405	0.513375	transcribed_unprocessed_pseudo
ENSG0000(	BMP3	461.5491	0.533937	0.114518	0.513466	protein_coding
ENSG0000(	ENSG0000(	204.4158	0.19384	0.11446	0.513466	lncRNA
ENSG0000(	GLUD1P3	215.9556	-0.22559	0.114537	0.513466	transcribed_unprocessed_pseudo
ENSG0000(	GPX1	354.3816	-0.2446	0.114492	0.513466	protein_coding
ENSG0000(	KCNMB4	143.4407	0.229471	0.114525	0.513466	protein_coding
ENSG0000(	RN7SL154F	43.17611	0.288139	0.114506	0.513466	misc_RNA
ENSG0000(	SMAD5	1670.994	0.146214	0.114575	0.513551	protein_coding
ENSG0000(	ARRDC1	988.3755	0.188195	0.114643	0.513581	protein_coding
ENSG0000(	ENSG0000(	115.7914	0.210437	0.114607	0.513581	lncRNA
ENSG0000(	LINC01001	1422.686	-0.29261	0.114666	0.513581	lncRNA
ENSG0000(	MANSC4	98.70419	0.209828	0.114675	0.513581	protein_coding
ENSG0000(	PHBP19	283.5265	0.17274	0.114652	0.513581	transcribed_processed_pseudoge
ENSG0000(	SLC5A10	32.37854	0.375115	0.11476	0.513796	protein_coding
ENSG0000(	TPM1	10472.51	0.191436	0.114754	0.513796	protein_coding
ENSG0000(	ENSG0000(	176.5441	0.154483	0.114812	0.513863	lncRNA
ENSG0000(	ENSG0000(	22.68569	-0.42923	0.114799	0.513863	lncRNA
ENSG0000(	LINC01055	61.69151	0.250762	0.11489	0.514128	lncRNA
ENSG0000(	EP300	5814.339	-0.20676	0.114967	0.514387	protein_coding
ENSG0000(	CTSF	228.0848	-0.17476	0.115018	0.514449	protein_coding
ENSG0000(	PPT2-EGFL8	170.1639	0.141218	0.115004	0.514449	protein_coding
ENSG0000(	CACNA1G	113.8295	-0.19242	0.115096	0.514632	protein_coding
ENSG0000(	GRTP1	624.2062	0.274953	0.115082	0.514632	protein_coding
ENSG0000(	G3BP2	2815.442	0.115897	0.115207	0.514961	protein_coding
ENSG0000(	HAL	88.90095	-0.4222	0.115201	0.514961	protein_coding
ENSG0000(	CCND1	1053.82	-0.24065	0.115249	0.515065	protein_coding

ENSG0000(	CARD14	519.5853	-0.1454	0.115415	0.515252	protein_coding
ENSG0000(	CASC9	459.303	0.235641	0.115331	0.515252	lncRNA
ENSG0000(	ENOSF1	4654.637	0.141812	0.115401	0.515252	protein_coding
ENSG0000(	ENSG0000(	26.0292	0.664346	0.115357	0.515252	lncRNA
ENSG0000(	IRS3P	172.2778	0.275886	0.115392	0.515252	processed_pseudogene
ENSG0000(	MAP2K4P1	41.85217	0.278194	0.115433	0.515252	transcribed_processed_pseudoge
ENSG0000(	PSMC4	499.1745	-0.12156	0.115441	0.515252	protein_coding
ENSG0000(	SHC1P2	169.0833	0.176711	0.115329	0.515252	processed_pseudogene
ENSG0000(	ENSG0000(	37.57618	0.30938	0.11551	0.515273	lncRNA
ENSG0000(	ENSG0000(	42.42092	-0.27416	0.115493	0.515273	lncRNA
ENSG0000(	ENSG0000(	75.61438	-0.2055	0.115521	0.515273	lncRNA
ENSG0000(	USH1C	1728.945	0.334084	0.115509	0.515273	protein_coding
ENSG0000(	H3C12	46.77629	-0.39691	0.115551	0.515278	protein_coding
ENSG0000(	MYOCD	437.9439	-0.37916	0.115559	0.515278	protein_coding
ENSG0000(	CCDC38	94.87543	0.233968	0.11565	0.515398	protein_coding
ENSG0000(	CEP164	1112.364	-0.12579	0.115647	0.515398	protein_coding
ENSG0000(	EMP2	4289.797	0.125707	0.115674	0.515398	protein_coding
ENSG0000(	ENSG0000(	182.3661	-0.1903	0.115628	0.515398	lncRNA
ENSG0000(	MT-TA	139.3882	-0.72128	0.11568	0.515398	Mt_tRNA
ENSG0000(	HERC2P8	24.23838	0.800256	0.115711	0.515455	transcribed_unprocessed_pseudo
ENSG0000(	ENSG0000(	57.29752	0.34432	0.115766	0.515563	TEC
ENSG0000(	OXA1L-DT	438.9826	0.11263	0.115773	0.515563	lncRNA
ENSG0000(	BORCS8-M	35.78685	-0.50131	0.115905	0.515734	protein_coding
ENSG0000(	ENSG0000(	34.19102	0.63771	0.115867	0.515734	processed_pseudogene
ENSG0000(	ENSG0000(	274.7147	0.155146	0.115883	0.515734	transcribed_processed_pseudoge
ENSG0000(	ENSG0000(	745.7618	0.256299	0.115899	0.515734	lncRNA
ENSG0000(	RPA2	171.5683	-0.21591	0.115839	0.515734	protein_coding
ENSG0000(	KLHL3	691.3675	-0.20714	0.115948	0.51584	protein_coding
ENSG0000(	CCDC102B	512.8047	-0.24101	0.116032	0.516098	protein_coding
ENSG0000(	CD83	476.1635	-0.51516	0.116062	0.516098	protein_coding
ENSG0000(	ENSG0000(	213.7928	0.14984	0.116048	0.516098	lncRNA
ENSG0000(	HSPA1L	34.89559	-0.34838	0.116141	0.516197	protein_coding
ENSG0000(	ITGA7	13676.43	-0.15503	0.116135	0.516197	protein_coding
ENSG0000(	SOX5	437.306	-0.34831	0.116107	0.516197	protein_coding
ENSG0000(	ENSG0000(	180.8266	-0.17267	0.116203	0.51624	lncRNA
ENSG0000(	GLO1	399.1667	-0.2355	0.116185	0.51624	protein_coding
ENSG0000(	IGHA1	20237.14	-0.53491	0.116207	0.51624	IG_C_gene
ENSG0000(	ENSG0000(	27.61106	0.343093	0.116246	0.51633	lncRNA
ENSG0000(	NOXA1	239.7083	0.347372	0.116289	0.516439	protein_coding
ENSG0000(	CNEP1R1	563.5782	-0.14636	0.116374	0.516573	protein_coding
ENSG0000(	GTSE1-DT	47.77185	-0.33225	0.11638	0.516573	lncRNA
ENSG0000(	LINC01856	272.1133	0.205888	0.116394	0.516573	lncRNA
ENSG0000(	ZCCHC2	1187.334	0.209086	0.116393	0.516573	protein_coding
ENSG0000(	ENSG0000(	35.26603	0.311578	0.116442	0.516621	lncRNA
ENSG0000(	ENSG0000(	35.64318	0.336602	0.116441	0.516621	lncRNA
ENSG0000(	ENSG0000(	22.6764	0.500556	0.116505	0.516816	lncRNA
ENSG0000(	ENSG0000(	367.0167	0.154965	0.116617	0.517146	lncRNA

ENSG0000(	LINC01496	334.5436	0.153791	0.116605	0.517146	lncRNA
ENSG0000(	CWH43	209.3016	0.638493	0.116667	0.517285	protein_coding
ENSG0000(	PWRN2	32.77072	-1.00548	0.116734	0.517497	lncRNA
ENSG0000(	AVPI1	197.661	0.399141	0.116824	0.517811	protein_coding
ENSG0000(	ITIH4	118.553	-0.28576	0.116894	0.517875	protein_coding
ENSG0000(	LRIG3	1311.158	0.274045	0.116883	0.517875	protein_coding
ENSG0000(	MPP4	143.819	-0.219	0.116859	0.517875	protein_coding
ENSG0000(	EID1	573.7335	-0.21448	0.116969	0.518121	protein_coding
ENSG0000(	UGT1A7	1204.065	0.500557	0.116997	0.518164	protein_coding
ENSG0000(	AGAP7P	58.88121	-0.95999	0.117335	0.518644	unprocessed_pseudogene
ENSG0000(	C1GALT1C1	213.736	-0.20685	0.1173	0.518644	protein_coding
ENSG0000(	CHRDL1	169.1574	0.381433	0.11724	0.518644	protein_coding
ENSG0000(	CHST10	66.55382	-0.45801	0.117342	0.518644	protein_coding
ENSG0000(	DENND4A	2307.024	-0.20752	0.117351	0.518644	protein_coding
ENSG0000(	ENSG0000(	88.06567	0.20492	0.117161	0.518644	lncRNA
ENSG0000(	ENSG0000(	271.4917	0.343711	0.11726	0.518644	lncRNA
ENSG0000(	ESAM	224.1458	-0.34205	0.117294	0.518644	protein_coding
ENSG0000(	GCNT1	1399.366	0.235272	0.117319	0.518644	protein_coding
ENSG0000(	NOTCH2NL	117.3547	-0.53761	0.11728	0.518644	protein_coding
ENSG0000(	NUP37	277.0543	-0.15595	0.117187	0.518644	protein_coding
ENSG0000(	NXPE4	1686.909	0.588079	0.11728	0.518644	protein_coding
ENSG0000(	PUF60	566.6405	0.152011	0.117323	0.518644	protein_coding
ENSG0000(	HNF1A	434.3265	0.21623	0.117374	0.518662	protein_coding
ENSG0000(	STX5	577.0333	-0.10951	0.117395	0.51867	protein_coding
ENSG0000(	BDH1	3410.883	0.24926	0.117439	0.518697	protein_coding
ENSG0000(	ENSG0000(	74.78088	0.223784	0.117421	0.518697	lncRNA
ENSG0000(	TRIM27	2225.277	-0.13228	0.117457	0.518697	protein_coding
ENSG0000(	AP5M1	2395.204	0.163299	0.117489	0.518753	protein_coding
ENSG0000(	DNAAF4	154.9536	0.163125	0.117567	0.518993	protein_coding
ENSG0000(	ENSG0000(	24.44235	-0.33743	0.117607	0.518993	lncRNA
ENSG0000(	PHLPP2	4127.059	0.453587	0.117595	0.518993	protein_coding
ENSG0000(	TCEANC2	1665.585	0.088782	0.117619	0.518993	protein_coding
ENSG0000(	ENSG0000(	262.4328	0.138568	0.117703	0.519137	lncRNA
ENSG0000(	LINC01619	143.0274	-0.29866	0.117683	0.519137	lncRNA
ENSG0000(	MMRN1	131.7547	-0.65736	0.117708	0.519137	protein_coding
ENSG0000(	ENSG0000(	929.9603	0.411131	0.117784	0.51939	transcribed_unprocessed_pseudo
ENSG0000(	H4C3	142.1209	-0.32971	0.117847	0.519548	protein_coding
ENSG0000(	ZNF354B	586.8875	0.100635	0.117858	0.519548	protein_coding
ENSG0000(	H2AC17	84.12052	-0.33242	0.117885	0.519585	protein_coding
ENSG0000(	CASP5	546.0967	-0.51332	0.117932	0.519707	protein_coding
ENSG0000(	ABCB4	201.0844	-0.57048	0.11803	0.520009	protein_coding
ENSG0000(	C4B	522.1433	-0.57336	0.118054	0.520009	protein_coding
ENSG0000(	ENSG0000(	25.86222	0.302371	0.118057	0.520009	lncRNA
ENSG0000(	ENSG0000(	229.9082	0.257563	0.118127	0.520103	lncRNA
ENSG0000(	LINC02627	63.54324	0.263371	0.118135	0.520103	lncRNA
ENSG0000(	NBPF14	4284.304	0.190068	0.118134	0.520103	protein_coding
ENSG0000(	CARF	953.2744	0.128509	0.118175	0.520115	protein_coding

ENSG0000(	RIPK4	213.3439	0.391208	0.118171	0.520115	protein_coding
ENSG0000(	ENSG0000(	606.573	0.118273	0.118228	0.520195	processed_pseudogene
ENSG0000(	ENSG0000(	119.197	0.194322	0.118233	0.520195	TEC
ENSG0000(	UPF3B	446.6352	-0.11423	0.11825	0.520195	protein_coding
ENSG0000(	ENSG0000(	137.5028	-0.27748	0.11838	0.520599	lncRNA
ENSG0000(	HDAC1P1	138.1726	0.514302	0.118373	0.520599	processed_pseudogene
ENSG0000(	CCDC137	231.8297	-0.20556	0.118539	0.520759	protein_coding
ENSG0000(	FGF10	122.1989	-0.31527	0.118471	0.520759	protein_coding
ENSG0000(	LILRA5	241.5947	-0.36713	0.118496	0.520759	protein_coding
ENSG0000(	MICAL2	7006.952	0.25474	0.118505	0.520759	protein_coding
ENSG0000(	TMEM170/	1752.205	0.160582	0.118541	0.520759	protein_coding
ENSG0000(	TMLHE	717.5474	0.110889	0.118549	0.520759	protein_coding
ENSG0000(	ZNF836	569.6919	0.132654	0.118438	0.520759	protein_coding
ENSG0000(	MARK3	7731.973	-0.07352	0.118615	0.520967	protein_coding
ENSG0000(	FAM215A	61.33034	0.246542	0.118694	0.521064	lncRNA
ENSG0000(	MELTF	678.5921	-0.21012	0.118672	0.521064	protein_coding
ENSG0000(	RAMP3	175.9916	-0.40338	0.118691	0.521064	protein_coding
ENSG0000(	AUNIP	170.3283	0.12795	0.118789	0.521178	protein_coding
ENSG0000(	DMRT2	46.62265	0.565929	0.11879	0.521178	protein_coding
ENSG0000(	ENSG0000(	98.89331	0.156745	0.118796	0.521178	lncRNA
ENSG0000(	FKBP4P1	36.61808	0.46708	0.118796	0.521178	processed_pseudogene
ENSG0000(	ENSG0000(	72.86707	0.264342	0.118832	0.521256	unprocessed_pseudogene
ENSG0000(	WDR93	178.8908	0.200967	0.118904	0.521487	protein_coding
ENSG0000(	ENSG0000(	179.7805	-0.30077	0.118958	0.52164	lncRNA
ENSG0000(	ENSG0000(	136.8322	0.220387	0.119017	0.521759	lncRNA
ENSG0000(	ERP27	79.20985	-0.4025	0.119023	0.521759	protein_coding
ENSG0000(	GOLGA4-A!	475.0556	-0.11131	0.119075	0.52178	lncRNA
ENSG0000(	RN7SL288F	60.99941	-0.35613	0.11908	0.52178	misc_RNA
ENSG0000(	RTN2	97.37555	-0.23426	0.119085	0.52178	protein_coding
ENSG0000(	COQ8A	514.7587	0.189164	0.119257	0.521902	protein_coding
ENSG0000(	CTTNBP2	251.556	0.297921	0.119386	0.521902	protein_coding
ENSG0000(	EFCAB6	257.5374	-0.20016	0.119329	0.521902	protein_coding
ENSG0000(	ENSG0000(	90.66927	0.24688	0.119342	0.521902	lncRNA
ENSG0000(	ENSG0000(	56.1889	-0.65413	0.119403	0.521902	TEC
ENSG0000(	FAM177B	175.9246	0.397343	0.11938	0.521902	protein_coding
ENSG0000(	HTATSF1	333.2745	0.211484	0.11926	0.521902	protein_coding
ENSG0000(	INMT-MINI	20.51727	1.043976	0.119218	0.521902	protein_coding
ENSG0000(	KCTD12	1400.089	-0.24213	0.119246	0.521902	protein_coding
ENSG0000(	LINC02241	508.1134	-0.13416	0.119429	0.521902	lncRNA
ENSG0000(	MIR3174	21.52655	0.409488	0.11928	0.521902	miRNA
ENSG0000(	MYH10	1813.467	-0.15458	0.119359	0.521902	protein_coding
ENSG0000(	NOS3	337.3354	-0.265	0.119417	0.521902	protein_coding
ENSG0000(	NUDT18	152.6852	0.171315	0.119435	0.521902	protein_coding
ENSG0000(	PHKA2	1375.478	0.164724	0.119346	0.521902	protein_coding
ENSG0000(	SMCO4	226.1392	-0.19535	0.119296	0.521902	protein_coding
ENSG0000(	SNTN	206.7379	0.174001	0.119165	0.521902	protein_coding
ENSG0000(	ENSG0000(	120.878	0.13772	0.119458	0.521918	lncRNA

ENSG0000(	ARPP19	2458.118	-0.13046	0.119505	0.522043	protein_coding
ENSG0000(	ENSG0000(	27.96009	-0.32253	0.11954	0.522113	TEC
ENSG0000(	FRG1JP	29.38875	-0.46923	0.11967	0.522489	unprocessed_pseudogene
ENSG0000(	LINC02127	19.83499	-0.41414	0.119683	0.522489	lncRNA
ENSG0000(	NR2F2-AS1	578.5628	-0.23592	0.11967	0.522489	lncRNA
ENSG0000(	ENSG0000(	50.30554	-0.30733	0.119723	0.522578	lncRNA
ENSG0000(	GALNT5	1676.547	0.360674	0.119811	0.522882	protein_coding
ENSG0000(	BCKDK	291.8619	-0.20309	0.120026	0.523734	protein_coding
ENSG0000(	ANKRD20A	741.9044	0.272509	0.120421	0.523827	transcribed_unprocessed_pseudo
ENSG0000(	BCDIN3D	581.5546	0.156461	0.120248	0.523827	protein_coding
ENSG0000(	C1orf35	139.7472	0.202907	0.120129	0.523827	protein_coding
ENSG0000(	CAAP1	400.1419	0.182895	0.120413	0.523827	protein_coding
ENSG0000(	CDRT15P6	39.85604	0.409511	0.120266	0.523827	transcribed_unprocessed_pseudo
ENSG0000(	COPS6	441.0857	-0.10964	0.120103	0.523827	protein_coding
ENSG0000(	DDX28	88.42485	-0.21232	0.120212	0.523827	protein_coding
ENSG0000(	ENDOV	654.5437	0.128944	0.12021	0.523827	protein_coding
ENSG0000(	ENSG0000(	99.95957	0.20629	0.120327	0.523827	unprocessed_pseudogene
ENSG0000(	ENSG0000(	19.87577	-0.45915	0.120168	0.523827	lncRNA
ENSG0000(	HTN3	294.2314	0.165493	0.120428	0.523827	protein_coding
ENSG0000(	KLF4	3286.509	0.370884	0.12039	0.523827	protein_coding
ENSG0000(	MUC1	3116.046	-0.40076	0.120421	0.523827	protein_coding
ENSG0000(	PKP4	1592.845	-0.14988	0.120286	0.523827	protein_coding
ENSG0000(	PPP1R16A	1298.136	0.213675	0.120086	0.523827	protein_coding
ENSG0000(	PSMD9	1833.504	0.056147	0.120379	0.523827	protein_coding
ENSG0000(	RPL23AP66	63.91544	-0.29057	0.12037	0.523827	processed_pseudogene
ENSG0000(	SWI5	137.3736	-0.2023	0.120142	0.523827	protein_coding
ENSG0000(	UBAC2-AS1	202.672	0.145118	0.120297	0.523827	lncRNA
ENSG0000(	ZNF846	589.8981	0.166206	0.120259	0.523827	protein_coding
ENSG0000(	GYG2	127.285	0.359004	0.120616	0.52415	protein_coding
ENSG0000(	MAPK15	20.23897	-0.65245	0.120593	0.52415	protein_coding
ENSG0000(	MLH3	2055.039	0.122117	0.120594	0.52415	protein_coding
ENSG0000(	MTTP	40.31951	-1.15198	0.120561	0.52415	protein_coding
ENSG0000(	RNF157	1043.117	0.243729	0.120526	0.52415	protein_coding
ENSG0000(	SEMA6A-AS	129.1567	0.498582	0.120598	0.52415	lncRNA
ENSG0000(	HYLS1	208.9607	0.137669	0.120659	0.524254	protein_coding
ENSG0000(	ELP4	1932.185	-0.0857	0.120689	0.524302	protein_coding
ENSG0000(	ENSG0000(	20.32743	0.424264	0.120733	0.524369	lncRNA
ENSG0000(	PLEKHG6	1006.962	0.467203	0.120743	0.524369	protein_coding
ENSG0000(	ARSB	755.8401	-0.16724	0.120769	0.524398	protein_coding
ENSG0000(	ENSG0000(	1140.486	0.235767	0.120884	0.524483	TEC
ENSG0000(	IKBKB	3719.834	0.085997	0.120877	0.524483	protein_coding
ENSG0000(	PLD2	451.1119	-0.1746	0.120836	0.524483	protein_coding
ENSG0000(	TAPT1-AS1	330.5769	0.192875	0.120865	0.524483	lncRNA
ENSG0000(	TRIM36	738.1354	0.364727	0.120809	0.524483	protein_coding
ENSG0000(	APOD	40.10041	0.427056	0.12095	0.524545	protein_coding
ENSG0000(	EHD2	609.552	-0.27353	0.120927	0.524545	protein_coding
ENSG0000(	EPAS1	4193.557	-0.21784	0.120974	0.524545	protein_coding

ENSG0000(	RN7SL665F	31.8608	-0.47791	0.120963	0.524545	misc_RNA
ENSG0000(	LRFN5	126.0826	-0.66393	0.121041	0.524672	protein_coding
ENSG0000(	NT5DC4	66.78641	-0.68603	0.121038	0.524672	protein_coding
ENSG0000(	IL15	1026.2	-0.18711	0.121096	0.524824	protein_coding
ENSG0000(	CHPF2	1897.873	-0.10311	0.121136	0.524917	protein_coding
ENSG0000(	HACD3	2027.852	-0.09892	0.121165	0.524961	protein_coding
ENSG0000(	ZNF252P	1277.541	0.128969	0.121188	0.524977	transcribed_unprocessed_pseudo
ENSG0000(	ENSG0000(	45.57866	-0.48091	0.121261	0.525208	lncRNA
ENSG0000(	CRACR2B	592.9211	0.289552	0.121299	0.525291	protein_coding
ENSG0000(	H1-10	430.4344	-0.18132	0.121359	0.52547	protein_coding
ENSG0000(	ENSG0000(	21.21589	-0.5361	0.121422	0.525557	lncRNA
ENSG0000(	MAP3K14-/	368.0199	0.145583	0.121443	0.525557	lncRNA
ENSG0000(	MSH5-SAP(	183.9712	0.300815	0.121466	0.525557	protein_coding
ENSG0000(	PDXDC1	5240.394	0.167541	0.121475	0.525557	protein_coding
ENSG0000(	PLK3	400.4318	-0.22545	0.121411	0.525557	protein_coding
ENSG0000(	FIBCD1	43.28956	0.789758	0.121564	0.525693	protein_coding
ENSG0000(	KLF17	67.36384	-0.20987	0.121529	0.525693	protein_coding
ENSG0000(	TRPV3	857.5322	0.299371	0.121561	0.525693	protein_coding
ENSG0000(	ENSG0000(	531.8857	0.151485	0.121646	0.525729	lncRNA
ENSG0000(	ENSG0000(	203.2053	0.136659	0.121698	0.525729	TEC
ENSG0000(	FRYL	15194.18	0.221029	0.121706	0.525729	protein_coding
ENSG0000(	GNAI3	2178.923	-0.07477	0.121681	0.525729	protein_coding
ENSG0000(	PER3	925.6246	0.332672	0.121594	0.525729	protein_coding
ENSG0000(	RAPGEF4	197.7648	-0.32591	0.121668	0.525729	protein_coding
ENSG0000(	TAB1	440.6924	0.169843	0.121692	0.525729	protein_coding
ENSG0000(	ENSG0000(	312.6856	0.141645	0.121817	0.525809	TEC
ENSG0000(	ENSG0000(	1035.509	0.072869	0.121858	0.525809	lncRNA
ENSG0000(	NCOA1	2217.33	-0.13781	0.12183	0.525809	protein_coding
ENSG0000(	PDCL3P5	24.50752	-0.47487	0.121796	0.525809	processed_pseudogene
ENSG0000(	PIWIL3	115.483	0.181	0.121847	0.525809	protein_coding
ENSG0000(	SCAMP5	302.2785	-0.35233	0.121803	0.525809	protein_coding
ENSG0000(	UQCRFS1-D	159.2991	-0.1482	0.121796	0.525809	lncRNA
ENSG0000(	DMTN	224.4505	0.298092	0.121879	0.525815	protein_coding
ENSG0000(	MYO9A	3622.164	0.170272	0.121913	0.525883	protein_coding
ENSG0000(	KCNN1	146.1212	-0.18181	0.121949	0.525955	protein_coding
ENSG0000(	TMTC4	470.6709	0.202604	0.122026	0.526205	protein_coding
ENSG0000(	ENSG0000(	2597.898	-0.12339	0.122068	0.526221	TEC
ENSG0000(	VEGFC	48.70154	-0.64099	0.122065	0.526221	protein_coding
ENSG0000(	ENSG0000(	61.70994	-0.40012	0.122124	0.526317	lncRNA
ENSG0000(	SYNE1	5328.009	-0.21061	0.122129	0.526317	protein_coding
ENSG0000(	ENSG0000(	48.20064	0.401294	0.122178	0.526398	lncRNA
ENSG0000(	SLC45A3	409.1346	0.231947	0.122186	0.526398	protein_coding
ENSG0000(	KLF3	5167.735	0.210274	0.122239	0.526544	protein_coding
ENSG0000(	ENSG0000(	660.5471	0.118862	0.122265	0.526574	lncRNA
ENSG0000(	LINC01304	216.7782	0.133478	0.122316	0.526711	lncRNA
ENSG0000(	ENSG0000(	285.5147	0.198413	0.122374	0.526876	lncRNA
ENSG0000(	ENSG0000(	31.94239	-0.31853	0.122415	0.526905	lncRNA

ENSG0000(	ZNF268	1761.226	0.14838	0.122419	0.526905	protein_coding
ENSG0000(	ARHGAP8	1166.441	0.282252	0.122536	0.526925	protein_coding
ENSG0000(	C8orf82	281.1627	0.230333	0.122578	0.526925	protein_coding
ENSG0000(	ENSG0000(	69.56045	-0.21842	0.122691	0.526925	lncRNA
ENSG0000(	ENSG0000(	41.26702	-0.38027	0.122582	0.526925	lncRNA
ENSG0000(	ENSG0000(	45.24335	0.373765	0.122445	0.526925	lncRNA
ENSG0000(	GAA	549.8072	0.231344	0.122636	0.526925	protein_coding
ENSG0000(	KLRC4-KLRI	36.71923	-0.56383	0.122542	0.526925	protein_coding
ENSG0000(	NUP50	2620.469	-0.09971	0.122684	0.526925	protein_coding
ENSG0000(	PLSCR3	189.2036	-0.28833	0.122602	0.526925	protein_coding
ENSG0000(	RBFA	774.1672	0.113448	0.122589	0.526925	protein_coding
ENSG0000(	SH2D1B	44.13897	-0.41276	0.122636	0.526925	protein_coding
ENSG0000(	SHANK2-AS	195.3206	-0.16428	0.122659	0.526925	lncRNA
ENSG0000(	SLC25A11	427.5757	0.163838	0.122518	0.526925	protein_coding
ENSG0000(	ZFAND3	1248.633	-0.12513	0.12247	0.526925	protein_coding
ENSG0000(	LINC00899	277.3481	0.179161	0.122782	0.527152	lncRNA
ENSG0000(	MIX23	601.987	0.28366	0.122764	0.527152	protein_coding
ENSG0000(	ENSG0000(	85.277	-0.22924	0.122819	0.527227	lncRNA
ENSG0000(	NCS1	130.0752	-0.61916	0.122838	0.527228	protein_coding
ENSG0000(	ATAD3C	522.754	0.253151	0.122899	0.527407	protein_coding
ENSG0000(	ENSG0000(	153.8833	-0.16365	0.122961	0.527499	lncRNA
ENSG0000(	LINC02281	95.26728	0.232884	0.122978	0.527499	lncRNA
ENSG0000(	WAKMAR2	267.1549	-0.19034	0.122972	0.527499	lncRNA
ENSG0000(	CHCHD3	815.369	0.126051	0.123039	0.527512	protein_coding
ENSG0000(	FUT8-AS1	54.30193	-0.36866	0.123002	0.527512	lncRNA
ENSG0000(	TMEM218	829.177	-0.13165	0.123034	0.527512	protein_coding
ENSG0000(	ABCC3	8252.916	0.288641	0.123105	0.527553	protein_coding
ENSG0000(	ANO7L1	64.21407	0.382322	0.123106	0.527553	transcribed_unprocessed_pseudo
ENSG0000(	APOB	441.224	-1.04375	0.123304	0.527553	protein_coding
ENSG0000(	ATG7	1674.314	-0.12311	0.123387	0.527553	protein_coding
ENSG0000(	CHST1	211.9185	-0.18311	0.123293	0.527553	protein_coding
ENSG0000(	ENSG0000(	49.66005	0.340863	0.123205	0.527553	lncRNA
ENSG0000(	ENSG0000(	22.27256	-0.61325	0.123144	0.527553	lncRNA
ENSG0000(	ENSG0000(	23.50648	-0.46194	0.123154	0.527553	lncRNA
ENSG0000(	ENSG0000(	194.0517	-0.13423	0.123102	0.527553	lncRNA
ENSG0000(	INTS6	5114.53	-0.10169	0.123352	0.527553	protein_coding
ENSG0000(	NME1	274.8648	-0.19611	0.123394	0.527553	protein_coding
ENSG0000(	NPM3	33.5039	-0.38311	0.123343	0.527553	protein_coding
ENSG0000(	PCDHGC4	60.26907	0.29817	0.123296	0.527553	protein_coding
ENSG0000(	RBBP4P1	115.3671	0.343473	0.123283	0.527553	processed_pseudogene
ENSG0000(	RPP25L	86.03955	0.319094	0.123381	0.527553	protein_coding
ENSG0000(	SLC40A1	3414.228	-0.34052	0.123372	0.527553	protein_coding
ENSG0000(	TLCD1	82.32515	0.239875	0.123356	0.527553	protein_coding
ENSG0000(	UBE2FP1	70.91344	0.3172	0.123311	0.527553	processed_pseudogene
ENSG0000(	ENSG0000(	134.0114	-0.16846	0.123446	0.527697	lncRNA
ENSG0000(	ALG14	1504.148	0.254913	0.123532	0.527898	protein_coding
ENSG0000(	PLCE1	9312.432	0.326964	0.123527	0.527898	protein_coding

ENSG0000(	C17orf49	180.9463	-0.30145	0.123566	0.527962	protein_coding
ENSG0000(	COQ10B	665.3737	0.108559	0.123698	0.528278	protein_coding
ENSG0000(	ENSG0000(	49.16353	0.341246	0.123672	0.528278	lncRNA
ENSG0000(	TET3	3212.225	0.126206	0.12369	0.528278	protein_coding
ENSG0000(	TLR6	993.1335	-0.31012	0.123789	0.528586	protein_coding
ENSG0000(	GSTT2B	25.26957	-0.80055	0.123854	0.528605	protein_coding
ENSG0000(	PP2672	71.58104	0.243111	0.12387	0.528605	lncRNA
ENSG0000(	SLFN13	1457.328	-0.241	0.123862	0.528605	protein_coding
ENSG0000(	ZNF436-AS	162.5723	-0.18106	0.123851	0.528605	lncRNA
ENSG0000(	MRPL15	230.585	0.214886	0.123941	0.528824	protein_coding
ENSG0000(	CLSTN1	2509.992	0.200047	0.123962	0.528834	protein_coding
ENSG0000(	ENSG0000(	121.9444	-0.30884	0.124043	0.529094	lncRNA
ENSG0000(	HMBOX1	1984.899	-0.08572	0.124123	0.529272	protein_coding
ENSG0000(	MLX	1306.687	0.157504	0.124118	0.529272	protein_coding
ENSG0000(	KRT18P4	55.45668	0.351525	0.124241	0.529696	processed_pseudogene
ENSG0000(	ABCD2	189.3608	-0.35307	0.124273	0.529749	protein_coding
ENSG0000(	AAGAB	960.992	0.128454	0.124304	0.529799	protein_coding
ENSG0000(	SSBP4	299.9272	0.245955	0.124428	0.530163	protein_coding
ENSG0000(	ZFP62	1339.471	0.135421	0.124419	0.530163	protein_coding
ENSG0000(	ADM2	250.0203	-0.19755	0.12446	0.530216	protein_coding
ENSG0000(	ENSG0000(	422.1354	0.224727	0.124537	0.530402	lncRNA
ENSG0000(	SLC35E2A	447.5415	0.144514	0.124542	0.530402	transcribed_unprocessed_pseudo
ENSG0000(	EMP3	92.69818	-0.36601	0.124572	0.530429	protein_coding
ENSG0000(	IL18BP	760.1218	-0.15978	0.124589	0.530429	protein_coding
ENSG0000(	SRSF5	4370.765	0.117469	0.124606	0.530429	protein_coding
ENSG0000(	ASL	2046.899	0.171593	0.124692	0.530437	protein_coding
ENSG0000(	ENSG0000(	658.4522	0.139829	0.124752	0.530437	lncRNA
ENSG0000(	ENSG0000(	1045.314	-0.1275	0.12472	0.530437	lncRNA
ENSG0000(	GIN1	311.2864	0.160598	0.124678	0.530437	protein_coding
ENSG0000(	LIFR-AS1	722.4981	-0.12053	0.124745	0.530437	lncRNA
ENSG0000(	NFIA	15819.63	0.156591	0.124776	0.530437	protein_coding
ENSG0000(	SEC24C	2102.355	-0.1089	0.124781	0.530437	protein_coding
ENSG0000(	SLC5A8	68.63369	-0.4909	0.124732	0.530437	protein_coding
ENSG0000(	WDHD1	233.0712	-0.23976	0.124731	0.530437	protein_coding
ENSG0000(	ENSG0000(	231.4227	0.189575	0.124836	0.530587	lncRNA
ENSG0000(	ZNF662	1326.476	0.147603	0.124956	0.531015	protein_coding
ENSG0000(	C16orf72	9313.127	-0.0825	0.125171	0.531209	protein_coding
ENSG0000(	CCDC80	1420.86	-0.34642	0.125085	0.531209	protein_coding
ENSG0000(	ENSG0000(	173.8144	-0.24306	0.125187	0.531209	lncRNA
ENSG0000(	ERFE	71.94577	-0.41022	0.125195	0.531209	protein_coding
ENSG0000(	FKBP14	1534.384	-0.11514	0.125165	0.531209	protein_coding
ENSG0000(	GPR179	22.58038	0.704463	0.125128	0.531209	protein_coding
ENSG0000(	LINC02288	215.8553	0.218339	0.125076	0.531209	lncRNA
ENSG0000(	NPHP4	554.8881	0.158892	0.125123	0.531209	protein_coding
ENSG0000(	RENO1	348.5406	0.147036	0.125082	0.531209	lncRNA
ENSG0000(	RPS12P27	57.17771	-0.27236	0.125056	0.531209	processed_pseudogene
ENSG0000(	LINC02506	28.15579	0.384777	0.125265	0.531425	lncRNA

ENSG0000(	TCEAL4	197.1197	-0.23789	0.125292	0.531457	protein_coding
ENSG0000(	GJB1	646.4698	0.24754	0.125315	0.531472	protein_coding
ENSG0000(	LIPH	1821.043	0.345033	0.125344	0.531515	protein_coding
ENSG0000(	FZD4-DT	23.61768	0.488808	0.125416	0.531737	lncRNA
ENSG0000(	PSMB8-AS1	524.9425	-0.15783	0.125453	0.531809	lncRNA
ENSG0000(	UPK3BL1	764.4443	0.298601	0.125472	0.531809	protein_coding
ENSG0000(	MRAP2	190.9471	-0.25806	0.125533	0.531986	protein_coding
ENSG0000(	C8orf34-AS	463.9362	-0.21537	0.125613	0.532073	lncRNA
ENSG0000(	ENSG0000(	76.44942	0.187579	0.12563	0.532073	lncRNA
ENSG0000(	ETHE1	1246.706	0.311215	0.125622	0.532073	protein_coding
ENSG0000(	HPS3	836.7334	-0.16747	0.125611	0.532073	protein_coding
ENSG0000(	ENSG0000(	42.23524	-0.24553	0.125758	0.532531	lncRNA
ENSG0000(	SNAPC4	709.9444	0.114932	0.125814	0.532687	protein_coding
ENSG0000(	BCL9L	1659.449	-0.1893	0.125906	0.532994	protein_coding
ENSG0000(	MIR663B	1384.457	0.396585	0.125925	0.532994	miRNA
ENSG0000(	ENSG0000(	688.3699	-0.19692	0.126047	0.533344	transcribed_processed_pseudoge
ENSG0000(	ENSG0000(	42.41538	-0.31161	0.12603	0.533344	lncRNA
ENSG0000(	PTRH2	690.2581	0.107296	0.126094	0.533458	protein_coding
ENSG0000(	SERINC3	2114.985	-0.10229	0.126113	0.533458	protein_coding
ENSG0000(	EIF1B	181.4165	-0.18895	0.126136	0.533474	protein_coding
ENSG0000(	ENSG0000(	768.5523	0.088915	0.126181	0.533585	lncRNA
ENSG0000(	ARHGEF40	1706.956	0.210551	0.126302	0.533605	protein_coding
ENSG0000(	BAG4	609.695	-0.11218	0.126289	0.533605	protein_coding
ENSG0000(	ENSG0000(	27.74776	-0.52853	0.126282	0.533605	lncRNA
ENSG0000(	ENSG0000(	392.0833	-0.13792	0.126277	0.533605	TEC
ENSG0000(	PECR	337.198	0.168637	0.126267	0.533605	protein_coding
ENSG0000(	SNORA22	36.88625	0.359252	0.126249	0.533605	snoRNA
ENSG0000(	ENSG0000(	340.3302	-0.10679	0.126397	0.533894	lncRNA
ENSG0000(	ENSG0000(	105.5767	-0.31104	0.12641	0.533894	lncRNA
ENSG0000(	NUP210	2109.15	-0.19527	0.12644	0.533941	protein_coding
ENSG0000(	LINC00698	29.83756	-0.4355	0.126482	0.533958	lncRNA
ENSG0000(	NXT2	82.39939	-0.36375	0.126483	0.533958	protein_coding
ENSG0000(	BICRAL	1471.564	0.095581	0.126546	0.534061	protein_coding
ENSG0000(	ENSG0000(	388.0966	0.189499	0.126545	0.534061	lncRNA
ENSG0000(	CDYL2	524.0438	-0.27149	0.126611	0.534251	protein_coding
ENSG0000(	EFCAB11	811.3514	0.113045	0.126649	0.534331	protein_coding
ENSG0000(	ENSG0000(	85.7821	0.220096	0.126734	0.534363	TEC
ENSG0000(	ENSG0000(	31.94145	-0.30709	0.126728	0.534363	lncRNA
ENSG0000(	RPL37P18	73.69233	0.356788	0.126718	0.534363	processed_pseudogene
ENSG0000(	YARS2	364.6676	0.114277	0.12671	0.534363	protein_coding
ENSG0000(	BCL7A	922.4254	0.19778	0.126755	0.534368	protein_coding
ENSG0000(	HNRNPA1P	3284.454	0.103974	0.126784	0.534385	processed_pseudogene
ENSG0000(	LSP1P5	28.23429	-0.5428	0.126806	0.534385	lncRNA
ENSG0000(	MANBAL	313.6517	-0.15985	0.126817	0.534385	protein_coding
ENSG0000(	CDK20	267.9154	0.32241	0.126908	0.534638	protein_coding
ENSG0000(	UBE2H	2070.905	-0.12469	0.126936	0.534638	protein_coding
ENSG0000(	ZNF839	689.8066	0.110544	0.12693	0.534638	protein_coding

ENSG0000(	MYO5A	4525.364	-0.15033	0.127043	0.535009	protein_coding
ENSG0000(	PTGER4	1011.163	0.19909	0.127093	0.535139	protein_coding
ENSG0000(	ENSG0000(	124.9516	-0.16753	0.127154	0.535245	lncRNA
ENSG0000(	ENSG0000(	62.74381	0.240182	0.127158	0.535245	lncRNA
ENSG0000(	GRIN2B	1195.803	-0.84093	0.127189	0.535294	protein_coding
ENSG0000(	ENSG0000(	594.7376	-0.11425	0.127242	0.535407	lncRNA
ENSG0000(	LINC01933	97.00126	-0.24642	0.127255	0.535407	lncRNA
ENSG0000(	WDR55	1650.549	0.097892	0.127302	0.535441	protein_coding
ENSG0000(	ZNF302	2953.654	0.263465	0.127296	0.535441	protein_coding
ENSG0000(	ENSG0000(	33.27279	0.325761	0.127325	0.535456	lncRNA
ENSG0000(	ASAP3	714.791	0.151288	0.127364	0.535541	protein_coding
ENSG0000(	DIAPH2	2455.212	0.131733	0.127428	0.535728	protein_coding
ENSG0000(	TMC7	742.4111	0.178772	0.127457	0.535768	protein_coding
ENSG0000(	SLC4A4	4436.345	0.501011	0.127504	0.535801	protein_coding
ENSG0000(	ZBTB7A	2879.433	0.172785	0.127499	0.535801	protein_coding
ENSG0000(	CD164	6482.578	0.153524	0.127591	0.535832	protein_coding
ENSG0000(	ENSG0000(	20.32708	0.513883	0.127609	0.535832	lncRNA
ENSG0000(	OAF	1217.852	0.197829	0.127581	0.535832	protein_coding
ENSG0000(	RASAL3	543.134	-0.34212	0.127603	0.535832	protein_coding
ENSG0000(	RNF122	160.2346	-0.23762	0.127592	0.535832	protein_coding
ENSG0000(	E4F1	606.1685	0.153172	0.127666	0.535872	protein_coding
ENSG0000(	IL1R2	252.3055	-0.48245	0.127656	0.535872	protein_coding
ENSG0000(	ZNF613	248.3463	0.166572	0.127677	0.535872	protein_coding
ENSG0000(	ARRDC2	951.9593	-0.19372	0.127746	0.535983	protein_coding
ENSG0000(	ENSG0000(	168.5348	0.144965	0.127762	0.535983	lncRNA
ENSG0000(	GUSBP2	1200.243	0.15415	0.127732	0.535983	transcribed_unprocessed_pseudo
ENSG0000(	NFKBIE	239.3768	-0.31935	0.127782	0.535989	protein_coding
ENSG0000(	CEP126	716.0909	0.146538	0.127835	0.536128	protein_coding
ENSG0000(	FLJ31356	192.0509	0.161922	0.127948	0.536518	lncRNA
ENSG0000(	B4GALNT3	2171.807	0.321613	0.128111	0.536528	protein_coding
ENSG0000(	CNDP2	2736.364	-0.11816	0.128016	0.536528	protein_coding
ENSG0000(	ENSG0000(	422.6242	-0.17227	0.128079	0.536528	lncRNA
ENSG0000(	ENSG0000(	1445.871	0.240741	0.128043	0.536528	protein_coding
ENSG0000(	ENSG0000(	173.3279	0.124236	0.128085	0.536528	lncRNA
ENSG0000(	ESAM-AS1	155.7991	0.232749	0.128017	0.536528	lncRNA
ENSG0000(	GSTZ1	717.393	0.159211	0.128125	0.536528	protein_coding
ENSG0000(	LSR	732.6679	0.284421	0.128099	0.536528	protein_coding
ENSG0000(	PAUPAR	183.6958	0.15483	0.128093	0.536528	lncRNA
ENSG0000(	BCAN-AS1	302.8529	0.209631	0.128169	0.536603	lncRNA
ENSG0000(	GSTM4	585.3893	0.269159	0.128224	0.536603	protein_coding
ENSG0000(	NEMP1	848.4927	-0.14815	0.128224	0.536603	protein_coding
ENSG0000(	STX12	969.6527	-0.15023	0.128218	0.536603	protein_coding
ENSG0000(	ZPBP2	91.01758	-0.24077	0.128241	0.536603	protein_coding
ENSG0000(	CIRBP	4694.897	0.10577	0.128284	0.536701	protein_coding
ENSG0000(	ENSG0000(	120.3121	0.178047	0.128342	0.536864	lncRNA
ENSG0000(	CPPED1	1035.448	-0.17525	0.128375	0.536894	protein_coding
ENSG0000(	PTK6	1725.294	0.284377	0.128388	0.536894	protein_coding

ENSG0000(	YPEL3	798.9576	-0.16761	0.128459	0.537105	protein_coding
ENSG0000(	ARHGEF15	220.0342	-0.20472	0.128482	0.537122	protein_coding
ENSG0000(	ATAD1	1299.06	0.133107	0.128542	0.537208	protein_coding
ENSG0000(	MYBL1	208.1476	-0.33829	0.128537	0.537208	protein_coding
ENSG0000(	FIS1	361.5728	0.112634	0.128676	0.537548	protein_coding
ENSG0000(	TFPI2	52.06837	-0.6696	0.128682	0.537548	protein_coding
ENSG0000(	ZNF491	273.1279	0.200066	0.128668	0.537548	protein_coding
ENSG0000(	ANKRD42	445.3348	-0.1284	0.128977	0.537881	protein_coding
ENSG0000(	CASC2	596.6318	0.129738	0.128939	0.537881	lncRNA
ENSG0000(	CPA3	156.2639	-0.45856	0.128829	0.537881	protein_coding
ENSG0000(	DEGS2	228.3563	0.379601	0.128964	0.537881	protein_coding
ENSG0000(	DISC1	1095.257	-0.16347	0.128971	0.537881	protein_coding
ENSG0000(	ENSG0000(	89.09777	-0.24993	0.128942	0.537881	lncRNA
ENSG0000(	ENSG0000(	89.35292	0.188482	0.128989	0.537881	lncRNA
ENSG0000(	MEF2A	1896.862	-0.11337	0.128851	0.537881	protein_coding
ENSG0000(	MYLIP	646.1229	0.201047	0.128953	0.537881	protein_coding
ENSG0000(	SIGLEC5	48.37615	-0.73899	0.128949	0.537881	protein_coding
ENSG0000(	TMEM184E	1197.464	-0.20851	0.128877	0.537881	protein_coding
ENSG0000(	UGT8	1642.44	0.23547	0.128996	0.537881	protein_coding
ENSG0000(	ALCAM	556.9098	-0.2322	0.129035	0.537962	protein_coding
ENSG0000(	EDRF1	1299.906	0.101722	0.129078	0.537978	protein_coding
ENSG0000(	ENSG0000(	103.8636	-0.305	0.129063	0.537978	lncRNA
ENSG0000(	GGTLC2	148.8652	0.200692	0.129102	0.537995	protein_coding
ENSG0000(	CORO1C	1985.487	-0.13497	0.129717	0.538116	protein_coding
ENSG0000(	CYP2J2	292.9481	0.396415	0.129513	0.538116	protein_coding
ENSG0000(	DOCK7	4017.34	0.197	0.129391	0.538116	protein_coding
ENSG0000(	ENSG0000(	314360	0.359059	0.12938	0.538116	processed_pseudogene
ENSG0000(	ENSG0000(	328.6998	0.139818	0.129641	0.538116	lncRNA
ENSG0000(	ENSG0000(	115.0652	-0.32388	0.129696	0.538116	lncRNA
ENSG0000(	ENSG0000(	741.31	0.092514	0.129587	0.538116	TEC
ENSG0000(	ENSG0000(	46.86167	0.469705	0.129406	0.538116	TEC
ENSG0000(	ENSG0000(	72.83798	-0.33196	0.129706	0.538116	lncRNA
ENSG0000(	FAM227A	919.1099	0.096541	0.129587	0.538116	protein_coding
ENSG0000(	FMO5	864.5488	0.257732	0.129267	0.538116	protein_coding
ENSG0000(	HES1	483.7067	0.273782	0.129502	0.538116	protein_coding
ENSG0000(	IL17RA	2031.276	-0.18773	0.129661	0.538116	protein_coding
ENSG0000(	INHBB	42.97461	-0.48844	0.129657	0.538116	protein_coding
ENSG0000(	LINC02301	1230.29	0.131131	0.129183	0.538116	lncRNA
ENSG0000(	MGC16275	62.25162	-0.23027	0.129375	0.538116	lncRNA
ENSG0000(	MPDU1	533.0758	0.12261	0.129697	0.538116	protein_coding
ENSG0000(	RASA1	1185.834	0.074754	0.129305	0.538116	protein_coding
ENSG0000(	RFX3	1091.626	-0.18595	0.129688	0.538116	protein_coding
ENSG0000(	RILP	193.1195	0.282314	0.129448	0.538116	protein_coding
ENSG0000(	SAMD12	4605.157	0.11452	0.129619	0.538116	protein_coding
ENSG0000(	SDF2L1	93.03542	-0.26073	0.129189	0.538116	protein_coding
ENSG0000(	SEMA3E	326.6518	0.377966	0.129563	0.538116	protein_coding
ENSG0000(	SETX	8223.813	-0.12258	0.129601	0.538116	protein_coding

ENSG0000(	SLC9A6	736.7188	0.131931	0.129159	0.538116	protein_coding
ENSG0000(	TEX26-AS1	193.9633	0.165252	0.129704	0.538116	lncRNA
ENSG0000(	TRMT11	694.8749	-0.11609	0.129565	0.538116	protein_coding
ENSG0000(	WNT2B	2023.336	-0.22382	0.129582	0.538116	protein_coding
ENSG0000(	ZNF532	2314.187	-0.1209	0.129214	0.538116	protein_coding
ENSG0000(	ZNF7	1779.434	0.076014	0.129553	0.538116	protein_coding
ENSG0000(	ANGPT2	468.9653	-0.15591	0.129783	0.53827	protein_coding
ENSG0000(	ENSG0000(	52.12899	-0.38485	0.129952	0.53827	lncRNA
ENSG0000(	ENSG0000(	120.036	0.215749	0.12997	0.53827	lncRNA
ENSG0000(	ENSG0000(	76.3879	-0.27301	0.129968	0.53827	TEC
ENSG0000(	ENSG0000(	26.07279	-0.40104	0.129901	0.53827	lncRNA
ENSG0000(	HMGN1P1(	23.04104	-0.35713	0.129962	0.53827	processed_pseudogene
ENSG0000(	ITGA3	2343.193	0.191044	0.129963	0.53827	protein_coding
ENSG0000(	ITPRIPL1	237.243	-0.1628	0.129896	0.53827	protein_coding
ENSG0000(	REPIN1	1918.99	0.11659	0.129833	0.53827	protein_coding
ENSG0000(	RPL4P4	20.98216	-0.66906	0.129935	0.53827	processed_pseudogene
ENSG0000(	TSPAN7	577.9462	0.399077	0.129886	0.53827	protein_coding
ENSG0000(	RSPO2	228.3288	0.202652	0.130066	0.538513	protein_coding
ENSG0000(	SCARNA13	1531.553	0.164301	0.130068	0.538513	scaRNA
ENSG0000(	ACTR1B	684.5852	0.11195	0.130113	0.53855	protein_coding
ENSG0000(	ENSG0000(	65.4048	-0.78991	0.130155	0.53855	lncRNA
ENSG0000(	NPAT	1759.725	-0.13798	0.130139	0.53855	protein_coding
ENSG0000(	SLC6A20	660.0639	-0.28211	0.130129	0.53855	protein_coding
ENSG0000(	APIP	505.7374	-0.18934	0.130347	0.53864	protein_coding
ENSG0000(	DUXAP9	337.3569	-0.26767	0.130508	0.53864	transcribed_processed_pseudoge
ENSG0000(	EIF2S2	579.9491	-0.17263	0.130509	0.53864	protein_coding
ENSG0000(	ENSG0000(	67.52424	0.359367	0.130456	0.53864	lncRNA
ENSG0000(	ENSG0000(	458.8527	0.345101	0.130323	0.53864	protein_coding
ENSG0000(	ENSG0000(	1641.437	-0.12972	0.130363	0.53864	TEC
ENSG0000(	FTL	6252.483	-0.25084	0.130482	0.53864	protein_coding
ENSG0000(	GPHN	627.7847	0.18059	0.13046	0.53864	protein_coding
ENSG0000(	GPSM3	319.6999	-0.34673	0.130321	0.53864	protein_coding
ENSG0000(	GRHL2	1294.02	0.222521	0.130397	0.53864	protein_coding
ENSG0000(	MRLN	220.2669	0.172482	0.130467	0.53864	protein_coding
ENSG0000(	MSRA	371.0137	0.138308	0.130549	0.53864	protein_coding
ENSG0000(	MTMR12	927.0315	-0.11035	0.130516	0.53864	protein_coding
ENSG0000(	PAK4	1437.317	0.156989	0.130338	0.53864	protein_coding
ENSG0000(	PCSK6	2324.526	0.281993	0.130198	0.53864	protein_coding
ENSG0000(	SMIM22	241.8434	0.340577	0.130469	0.53864	protein_coding
ENSG0000(	TAB2	2593.276	-0.10547	0.130235	0.53864	protein_coding
ENSG0000(	TTC16	237.2435	-0.14217	0.130544	0.53864	protein_coding
ENSG0000(	ZNF552	973.7137	0.204713	0.130507	0.53864	protein_coding
ENSG0000(	ENSG0000(	67.23371	-0.28746	0.130601	0.538774	lncRNA
ENSG0000(	SELL	499.8758	-0.68588	0.130677	0.539006	protein_coding
ENSG0000(	CLEC12B	29.36571	-0.68476	0.130795	0.539332	protein_coding
ENSG0000(	PCDH15	62.47812	-0.4505	0.130789	0.539332	protein_coding
ENSG0000(	CGAS	740.2395	-0.15518	0.130926	0.539558	protein_coding

ENSG0000(	GGACT	160.5515	-0.29107	0.130896	0.539558	protein_coding
ENSG0000(	ILDR1	265.9928	0.25123	0.130928	0.539558	protein_coding
ENSG0000(	MAGED4B	108.7596	-0.43465	0.130911	0.539558	protein_coding
ENSG0000(	TTC27	387.3406	0.243513	0.130999	0.53977	protein_coding
ENSG0000(	C16orf87	542.7229	-0.12061	0.131098	0.540096	protein_coding
ENSG0000(	HOXB5	747.3079	0.319104	0.131128	0.540142	protein_coding
ENSG0000(	ENSG0000(	196.8836	0.188359	0.131213	0.540348	protein_coding
ENSG0000(	LINC00566	30.63414	-0.30786	0.131238	0.540348	lncRNA
ENSG0000(	NEXN-AS1	125.5056	0.322711	0.131226	0.540348	lncRNA
ENSG0000(	ENSG0000(	1222.863	0.092895	0.131334	0.540663	lncRNA
ENSG0000(	C5orf60	99.34232	-0.24093	0.131424	0.54069	lncRNA
ENSG0000(	FGF13	2658.468	-0.09639	0.131435	0.54069	protein_coding
ENSG0000(	MKNK2	2320.522	0.211394	0.131449	0.54069	protein_coding
ENSG0000(	RBPJ	2836.317	-0.12477	0.131458	0.54069	protein_coding
ENSG0000(	SETD1A	563.1005	0.203957	0.13137	0.54069	protein_coding
ENSG0000(	TRIM73	867.301	0.129588	0.131421	0.54069	protein_coding
ENSG0000(	TRPC4	96.05104	-0.46521	0.131564	0.541044	protein_coding
ENSG0000(	ATP13A4	868.1555	0.4352	0.131814	0.541088	protein_coding
ENSG0000(	ELAPOR2	318.4215	0.35183	0.131759	0.541088	protein_coding
ENSG0000(	ENSG0000(	80.03373	0.231406	0.131645	0.541088	lncRNA
ENSG0000(	ENSG0000(	42.85011	-0.2817	0.13178	0.541088	lncRNA
ENSG0000(	ENSG0000(	75.62447	-0.26952	0.131628	0.541088	lncRNA
ENSG0000(	ENSG0000(	642.2554	0.082439	0.131811	0.541088	lncRNA
ENSG0000(	ENSG0000(	27.89293	-0.47544	0.131643	0.541088	lncRNA
ENSG0000(	EXD2	484.2914	0.164415	0.131673	0.541088	protein_coding
ENSG0000(	FMR1-AS1	24.34212	-0.42347	0.1318	0.541088	lncRNA
ENSG0000(	MOB2	625.3399	0.170056	0.131796	0.541088	protein_coding
ENSG0000(	MT-TY	147.8173	-0.76637	0.131681	0.541088	Mt_tRNA
ENSG0000(	SREBF1	1284.835	0.197801	0.13183	0.541088	protein_coding
ENSG0000(	TAS2R20	285.9119	0.19676	0.131695	0.541088	protein_coding
ENSG0000(	KIZ	543.2148	-0.20445	0.131928	0.541326	protein_coding
ENSG0000(	MANBA	885.0262	-0.17613	0.131914	0.541326	protein_coding
ENSG0000(	MAPKAPK3	396.292	-0.18568	0.131952	0.541344	protein_coding
ENSG0000(	ENSG0000(	38.44927	0.338151	0.132003	0.541356	lncRNA
ENSG0000(	RN7SL308F	60.58801	0.306203	0.131988	0.541356	misc_RNA
ENSG0000(	ZNF23	693.9413	0.158605	0.132014	0.541356	protein_coding
ENSG0000(	COL16A1	1159.806	-0.26992	0.132183	0.541501	protein_coding
ENSG0000(	DANT2	1878.575	0.225601	0.132131	0.541501	lncRNA
ENSG0000(	DUSP2	99.51279	-0.47091	0.132171	0.541501	protein_coding
ENSG0000(	ENSG0000(	268.034	-0.20991	0.132187	0.541501	lncRNA
ENSG0000(	PLXDC2	2109.802	0.155254	0.132085	0.541501	protein_coding
ENSG0000(	TTC30B	138.5606	-0.19631	0.132108	0.541501	protein_coding
ENSG0000(	UPP1	1604.32	-0.26311	0.132144	0.541501	protein_coding
ENSG0000(	ADSS1	287.8169	0.143358	0.132258	0.541712	protein_coding
ENSG0000(	ENSG0000(	235.8416	0.128787	0.132306	0.541828	lncRNA
ENSG0000(	VCL	3815.55	-0.17617	0.132329	0.541842	protein_coding
ENSG0000(	ENSG0000(	341.9411	0.194838	0.132444	0.542038	TEC

ENSG0000(	PIP4K2C	560.0956	0.203643	0.132417	0.542038	protein_coding
ENSG0000(	PTENP1-AS	127.6911	-0.15775	0.132466	0.542038	lncRNA
ENSG0000(	SUGCT-AS1	160.7283	0.1542	0.132475	0.542038	lncRNA
ENSG0000(	ZNF736	1352.592	0.161339	0.132423	0.542038	protein_coding
ENSG0000(	ENSG0000(	155.2811	0.176178	0.132571	0.54209	lncRNA
ENSG0000(	GVINP1	3035.366	-0.29218	0.132569	0.54209	transcribed_unprocessed_pseudo
ENSG0000(	LINC01733	184.9402	0.185173	0.132565	0.54209	lncRNA
ENSG0000(	PCDH9	3078.279	0.359505	0.132606	0.54209	protein_coding
ENSG0000(	TEAD2	329.7842	-0.21706	0.132517	0.54209	protein_coding
ENSG0000(	ZNF764	337.6429	0.166767	0.13259	0.54209	protein_coding
ENSG0000(	ENSG0000(	88.92621	0.261608	0.132638	0.542138	lncRNA
ENSG0000(	ENSG0000(	384.5336	-0.11676	0.132738	0.542468	lncRNA
ENSG0000(	ACSM1	25.48817	0.410988	0.132888	0.542796	protein_coding
ENSG0000(	ENSG0000(	234.6525	2.011834	0.132843	0.542796	lncRNA
ENSG0000(	ENSG0000(	164.2655	-0.19691	0.132909	0.542796	lncRNA
ENSG0000(	HCG24	64.29048	-0.23676	0.13292	0.542796	lncRNA
ENSG0000(	LINC02303	61.68679	-0.24067	0.132874	0.542796	lncRNA
ENSG0000(	QTRT1	661.8156	0.145309	0.132955	0.542796	protein_coding
ENSG0000(	SERGEF	345.1484	-0.12448	0.132957	0.542796	protein_coding
ENSG0000(	TMEM150E	195.0417	-0.22106	0.132983	0.542824	protein_coding
ENSG0000(	ENSG0000(	29.37873	0.535041	0.133075	0.543037	lncRNA
ENSG0000(	LINC00266-	37.41377	-0.29225	0.133082	0.543037	lncRNA
ENSG0000(	RN7SL336F	30.48663	-0.29563	0.133095	0.543037	misc_RNA
ENSG0000(	ENSG0000(	1430.381	0.192607	0.133159	0.543219	lncRNA
ENSG0000(	OR56A4	622.2492	-0.10275	0.133239	0.543464	protein_coding
ENSG0000(	ENSG0000(	48.01643	-0.31461	0.133299	0.5435	lncRNA
ENSG0000(	FN3KRP	396.2703	0.161855	0.133302	0.5435	protein_coding
ENSG0000(	PACSIN1	23.00951	-0.73195	0.133345	0.5435	protein_coding
ENSG0000(	RN7SL750F	42.1042	0.314955	0.133346	0.5435	misc_RNA
ENSG0000(	THAP5	1003.924	-0.11544	0.133332	0.5435	protein_coding
ENSG0000(	ENSG0000(	184.872	0.150673	0.133508	0.543996	lncRNA
ENSG0000(	KIAA0930	1162.043	-0.23098	0.133499	0.543996	protein_coding
ENSG0000(	EIF5AL1	107.2015	-0.269	0.133559	0.544125	protein_coding
ENSG0000(	AUTS2	2614.758	0.111729	0.133918	0.544243	protein_coding
ENSG0000(	CNIH3-AS2	1055.532	-0.12458	0.133772	0.544243	lncRNA
ENSG0000(	CX3CR1	41.86775	-0.45006	0.133685	0.544243	protein_coding
ENSG0000(	CYP24A1	58.76666	-0.96762	0.133816	0.544243	protein_coding
ENSG0000(	ENSG0000(	174.1582	0.217301	0.133894	0.544243	lncRNA
ENSG0000(	ENSG0000(	1388.529	0.115285	0.133944	0.544243	lncRNA
ENSG0000(	ENSG0000(	620.6373	0.283376	0.133962	0.544243	transcribed_processed_pseudoge
ENSG0000(	ENSG0000(	27.5894	0.377465	0.13384	0.544243	lncRNA
ENSG0000(	ENSG0000(	29.76856	-0.41047	0.133735	0.544243	processed_pseudogene
ENSG0000(	ENSG0000(	88.1781	0.205454	0.133751	0.544243	lncRNA
ENSG0000(	ENSG0000(	27.65004	0.590495	0.133727	0.544243	lncRNA
ENSG0000(	FAAP100	743.6226	0.128437	0.133986	0.544243	protein_coding
ENSG0000(	HIRA	723.7425	-0.13747	0.133779	0.544243	protein_coding
ENSG0000(	LINC01160	255.7917	-0.12332	0.134003	0.544243	lncRNA

ENSG0000(	RAB3C	115.3561	-0.52565	0.133967	0.544243	protein_coding
ENSG0000(	RRN3	637.7506	0.134466	0.133774	0.544243	protein_coding
ENSG0000(	RSPRY1	1551.598	0.108908	0.133979	0.544243	protein_coding
ENSG0000(	SAYSD1	345.1077	0.107594	0.133865	0.544243	protein_coding
ENSG0000(	STAG3L4	543.3551	0.162262	0.133709	0.544243	transcribed_unprocessed_pseudo
ENSG0000(	WFDC8	89.04309	-0.18238	0.133654	0.544243	protein_coding
ENSG0000(	ZNF578	983.0575	0.074011	0.133897	0.544243	protein_coding
ENSG0000(	BSG	2850.937	0.3542	0.134041	0.544271	protein_coding
ENSG0000(	MLLT11	108.62	-0.25507	0.13405	0.544271	protein_coding
ENSG0000(	DZIP1	614.438	-0.24064	0.134169	0.544675	protein_coding
ENSG0000(	MKKS	678.9024	0.181109	0.134211	0.544703	protein_coding
ENSG0000(	RBM48	809.1546	0.098061	0.134216	0.544703	protein_coding
ENSG0000(	SIN3A	1958.417	0.106596	0.134333	0.545098	protein_coding
ENSG0000(	NONOP2	78.49912	0.35948	0.134437	0.545441	processed_pseudogene
ENSG0000(	ALDH5A1	864.5441	0.135564	0.134504	0.545524	protein_coding
ENSG0000(	CA5BP1	201.8341	0.147832	0.134547	0.545524	transcribed_unprocessed_pseudo
ENSG0000(	HTT-AS	88.56219	0.176744	0.134565	0.545524	lncRNA
ENSG0000(	PARP1	1963.249	-0.15876	0.134577	0.545524	protein_coding
ENSG0000(	TIGD2	274.8336	-0.15641	0.134513	0.545524	protein_coding
ENSG0000(	TMCO4	590.4463	0.156951	0.134543	0.545524	protein_coding
ENSG0000(	LINC00384	21.89538	-0.50032	0.134612	0.545587	lncRNA
ENSG0000(	ENSG0000(	20.43462	-1.18198	0.13468	0.545784	lncRNA
ENSG0000(	LGR4-AS1	29.1035	0.347279	0.134711	0.545827	lncRNA
ENSG0000(	GRM3	38.12965	0.346724	0.134733	0.545838	protein_coding
ENSG0000(	ZNF714	1506.396	0.105241	0.134812	0.546077	protein_coding
ENSG0000(	COX18	1058.337	-0.13202	0.134896	0.546095	protein_coding
ENSG0000(	DOLPP1	272.7401	0.26794	0.134896	0.546095	protein_coding
ENSG0000(	PCAT1	782.9975	0.10407	0.134866	0.546095	lncRNA
ENSG0000(	RWDD1	651.2503	-0.12644	0.134893	0.546095	protein_coding
ENSG0000(	ENSG0000(	55.22845	-0.32491	0.134922	0.546118	lncRNA
ENSG0000(	GUCY1B2	43.7062	0.874824	0.13495	0.546153	transcribed_unitary_pseudogene
ENSG0000(	ZDHHC13	1271.539	0.177889	0.134975	0.546174	protein_coding
ENSG0000(	GRAMD1B	655.1364	-0.29609	0.135106	0.546623	protein_coding
ENSG0000(	ENSG0000(	394.4822	0.114101	0.135138	0.546674	lncRNA
ENSG0000(	ATG13	1376.346	-0.08847	0.135223	0.546694	protein_coding
ENSG0000(	CEP97	1033.539	-0.18299	0.135198	0.546694	protein_coding
ENSG0000(	ENSG0000(	671.7645	0.14622	0.135183	0.546694	lncRNA
ENSG0000(	RAB4A	457.5417	0.208883	0.135218	0.546694	protein_coding
ENSG0000(	CES2	2843.013	0.273163	0.135245	0.546702	protein_coding
ENSG0000(	ACY1	576.66	0.163554	0.135289	0.546722	protein_coding
ENSG0000(	GTF3C6	205.8225	-0.21312	0.135289	0.546722	protein_coding
ENSG0000(	INF2	3995.78	0.192281	0.135316	0.546751	protein_coding
ENSG0000(	ZNF287	268.7567	-0.2971	0.13537	0.546885	protein_coding
ENSG0000(	ENSG0000(	34.19056	-0.29072	0.13541	0.546967	lncRNA
ENSG0000(	PLD5	113.6777	0.261372	0.135535	0.547392	protein_coding
ENSG0000(	ENSG0000(	96.77231	-0.17549	0.135582	0.547481	lncRNA
ENSG0000(	ENSG0000(	279.7095	0.125581	0.135597	0.547481	lncRNA

ENSG0000(	ENSG0000(	404.0452	0.153265	0.135627	0.547521	lncRNA
ENSG0000(	ADAMTSL3	1395.389	0.189281	0.135771	0.547524	protein_coding
ENSG0000(	ADSS2	715.3899	-0.09724	0.135721	0.547524	protein_coding
ENSG0000(	AKNAD1	31.67104	0.359463	0.135832	0.547524	protein_coding
ENSG0000(	BTG3-AS1	29.72944	0.403916	0.135838	0.547524	lncRNA
ENSG0000(	FAM9B	172.7077	0.148347	0.135778	0.547524	protein_coding
ENSG0000(	HEXD	582.8473	0.113879	0.135745	0.547524	protein_coding
ENSG0000(	HIGD1AP14	25.8546	-0.54033	0.135709	0.547524	processed_pseudogene
ENSG0000(	RASSF7	600.2141	0.258812	0.135866	0.547524	protein_coding
ENSG0000(	SCYL1	520.7739	-0.13426	0.135859	0.547524	protein_coding
ENSG0000(	STMN1	729.8071	-0.16979	0.135746	0.547524	protein_coding
ENSG0000(	TEX41	357.4305	-0.19363	0.13583	0.547524	lncRNA
ENSG0000(	THAP12	1278.877	0.115803	0.135858	0.547524	protein_coding
ENSG0000(	MAGEH1	90.78897	-0.34382	0.136023	0.548077	protein_coding
ENSG0000(	HEATR4	88.12431	0.309657	0.136122	0.548237	protein_coding
ENSG0000(	POLR2KP1	22.78427	-0.31464	0.136125	0.548237	processed_pseudogene
ENSG0000(	SH3D19	3481.395	0.235042	0.136129	0.548237	protein_coding
ENSG0000(	TMED5	1660.181	-0.11997	0.136142	0.548237	protein_coding
ENSG0000(	PLSCR2	190.5004	-0.271	0.136165	0.548246	protein_coding
ENSG0000(	CEP104	1842.436	0.095648	0.13621	0.548267	protein_coding
ENSG0000(	ZMYM5	730.1806	-0.14539	0.136195	0.548267	protein_coding
ENSG0000(	BAZ1A	2103.685	-0.13011	0.136281	0.548312	protein_coding
ENSG0000(	DEF6	657.8672	-0.25707	0.136271	0.548312	protein_coding
ENSG0000(	MRPL20	595.1054	0.124108	0.136243	0.548312	protein_coding
ENSG0000(	NIM1K	148.6826	0.155316	0.136304	0.548327	protein_coding
ENSG0000(	ENSG0000(	118.8655	0.156645	0.136378	0.548482	lncRNA
ENSG0000(	ENSG0000(	4961.268	0.127874	0.136502	0.548482	lncRNA
ENSG0000(	RASIP1	74.75286	-0.3635	0.136493	0.548482	protein_coding
ENSG0000(	SGO1-AS1	579.6454	0.10379	0.13648	0.548482	lncRNA
ENSG0000(	SLC25A32	581.7168	-0.14012	0.136478	0.548482	protein_coding
ENSG0000(	SLC44A1	5364.603	0.208867	0.136405	0.548482	protein_coding
ENSG0000(	SNORA73	50.33834	0.232074	0.136493	0.548482	snoRNA
ENSG0000(	SNX1	3170.031	0.215917	0.136386	0.548482	protein_coding
ENSG0000(	ENSG0000(	25.75646	-0.38956	0.136523	0.548484	lncRNA
ENSG0000(	ENSG0000(	37.9552	0.508666	0.136614	0.548771	lncRNA
ENSG0000(	ENSG0000(	40.0812	0.324455	0.136726	0.54914	transcribed_processed_pseudoge
ENSG0000(	TAS2R46	106.5787	0.215405	0.136758	0.549187	protein_coding
ENSG0000(	ECSIT	907.0255	0.115278	0.136779	0.54919	protein_coding
ENSG0000(	UGDH-AS1	8162.583	0.126371	0.136821	0.549281	lncRNA
ENSG0000(	ENSG0000(	20.07705	0.413804	0.136869	0.549392	lncRNA
ENSG0000(	TAS2R5	90.72175	0.234634	0.136896	0.549422	protein_coding
ENSG0000(	RENBP	268.9766	-0.14806	0.136956	0.549551	protein_coding
ENSG0000(	SLC35A3	7412.417	0.195292	0.136968	0.549551	protein_coding
ENSG0000(	COQ9	847.0861	0.227507	0.137068	0.549787	protein_coding
ENSG0000(	CPOX	333.5427	-0.16576	0.137086	0.549787	protein_coding
ENSG0000(	EIF4EBP3	67.66119	0.296001	0.137132	0.549787	protein_coding
ENSG0000(	ENSG0000(	385.0599	0.131275	0.137167	0.549787	lncRNA

ENSG0000(	ITGA11	854.5179	-0.27407	0.137167	0.549787	protein_coding
ENSG0000(	TBC1D13	528.627	0.116302	0.137127	0.549787	protein_coding
ENSG0000(	ZNF823	405.5591	0.253428	0.137154	0.549787	protein_coding
ENSG0000(	MAP3K5	1246.76	-0.15408	0.137188	0.549792	protein_coding
ENSG0000(	MAPK8	1210.669	-0.11074	0.137294	0.550055	protein_coding
ENSG0000(	NDUFB4	292.5583	-0.19779	0.137286	0.550055	protein_coding
ENSG0000(	BARX2	326.0782	0.28539	0.137335	0.550142	protein_coding
ENSG0000(	CEP85L	1094.826	-0.27123	0.137394	0.550294	protein_coding
ENSG0000(	UBA6-DT	1352.116	-0.1146	0.137444	0.550416	lncRNA
ENSG0000(	SYNPR	25.98369	-0.42378	0.137474	0.550456	protein_coding
ENSG0000(	SNORA74A	309.4253	0.316835	0.137604	0.550898	snoRNA
ENSG0000(	ENSG0000(	427.3907	-0.14644	0.137625	0.550902	TEC
ENSG0000(	AATF	939.0358	0.092821	0.137725	0.550915	protein_coding
ENSG0000(	AKT3	2381.72	-0.15929	0.137782	0.550915	protein_coding
ENSG0000(	ENSG0000(	26.68332	-0.4767	0.137829	0.550915	lncRNA
ENSG0000(	ENSG0000(	81.8207	0.228814	0.137734	0.550915	lncRNA
ENSG0000(	ENSG0000(	137.3555	-0.18853	0.137661	0.550915	lncRNA
ENSG0000(	ENSG0000(	149.5513	-0.15302	0.137805	0.550915	lncRNA
ENSG0000(	OSBPL3	1513.178	-0.18301	0.137819	0.550915	protein_coding
ENSG0000(	PTGES2-AS	627.404	-0.12211	0.137811	0.550915	lncRNA
ENSG0000(	RNY3	1541.076	-0.25871	0.137762	0.550915	misc_RNA
ENSG0000(	TMEM126E	356.4284	0.115452	0.137822	0.550915	protein_coding
ENSG0000(	RIMBP3	38.24217	-0.68467	0.137858	0.550953	protein_coding
ENSG0000(	CROT	507.7727	0.235804	0.138078	0.551499	protein_coding
ENSG0000(	MT-TE	158.332	-0.68989	0.138095	0.551499	Mt_tRNA
ENSG0000(	PTPRS	1121.99	-0.20453	0.138069	0.551499	protein_coding
ENSG0000(	TMEM108	163.7716	-0.39972	0.138084	0.551499	protein_coding
ENSG0000(	ZDHHC8P1	204.5873	0.164696	0.138055	0.551499	transcribed_unprocessed_pseudo
ENSG0000(	ZBTB22	387.2695	0.152858	0.138149	0.551632	protein_coding
ENSG0000(	NR5A2	1643.154	0.246063	0.138176	0.55166	protein_coding
ENSG0000(	C1orf162	282.0416	-0.27855	0.138258	0.55169	protein_coding
ENSG0000(	NFKB2	868.3811	-0.24607	0.138263	0.55169	protein_coding
ENSG0000(	SEPTIN6	1978.343	-0.28465	0.138235	0.55169	protein_coding
ENSG0000(	SMIM8	954.5033	0.126498	0.138209	0.55169	protein_coding
ENSG0000(	DNAH5	254.724	-0.18574	0.138473	0.551822	protein_coding
ENSG0000(	ENSG0000(	245.8277	0.157785	0.138348	0.551822	transcribed_unprocessed_pseudo
ENSG0000(	ENSG0000(	139.2619	-0.25467	0.138447	0.551822	lncRNA
ENSG0000(	ENSG0000(	82.95166	0.293035	0.138468	0.551822	transcribed_unprocessed_pseudo
ENSG0000(	ENSG0000(	175.0591	0.139828	0.138497	0.551822	lncRNA
ENSG0000(	FCN1	730.3591	-0.21997	0.13834	0.551822	protein_coding
ENSG0000(	KYAT3	305.8309	-0.14752	0.138363	0.551822	protein_coding
ENSG0000(	SERPINI1	38.51843	-0.41002	0.138432	0.551822	protein_coding
ENSG0000(	UXT-AS1	708.8144	0.080791	0.138484	0.551822	lncRNA
ENSG0000(	ZNF541	22.17152	0.610897	0.13846	0.551822	protein_coding
ENSG0000(	TMUB1	243.8868	0.147203	0.13855	0.551952	protein_coding
ENSG0000(	ENSG0000(	38.85976	0.379079	0.138599	0.552067	lncRNA
ENSG0000(	DNAAF9	653.8068	-0.15395	0.138765	0.552621	protein_coding

ENSG0000(	ENSG0000(	267.4703	0.142843	0.138778	0.552621	lncRNA
ENSG0000(	AGTPBP1	680.0825	-0.20677	0.138836	0.55272	protein_coding
ENSG0000(	LINC01524	171.1359	0.179914	0.13885	0.55272	lncRNA
ENSG0000(	PRRG2	97.49528	0.322103	0.138863	0.55272	protein_coding
ENSG0000(	GBP3	1590.523	-0.24484	0.138917	0.552774	protein_coding
ENSG0000(	ZNF865	228.4437	0.149008	0.138901	0.552774	protein_coding
ENSG0000(	ENSG0000(	206.8801	0.369463	0.138944	0.552801	lncRNA
ENSG0000(	ENSG0000(	58.6294	0.315344	0.138978	0.552858	transcribed_processed_pseudoge
ENSG0000(	ENSG0000(	83.20744	0.204798	0.139019	0.552942	lncRNA
ENSG0000(	SULT1C4	230.8002	0.149508	0.139077	0.553091	protein_coding
ENSG0000(	CTNND1	18944.54	0.202284	0.139135	0.553241	protein_coding
ENSG0000(	CFHR1	70.98607	0.32902	0.139165	0.55328	protein_coding
ENSG0000(	STIL	887.5215	0.149231	0.139211	0.553306	protein_coding
ENSG0000(	ZHX3	1430.911	-0.20253	0.139203	0.553306	protein_coding
ENSG0000(	ENSG0000(	20.55059	0.442552	0.139339	0.553687	lncRNA
ENSG0000(	LINC01638	117.5582	-0.22834	0.139347	0.553687	lncRNA
ENSG0000(	ENSG0000(	97.97349	0.188839	0.139456	0.554038	lncRNA
ENSG0000(	ENSG0000(	25.8339	-0.48738	0.139545	0.554313	lncRNA
ENSG0000(	HAUS7	174.5185	-0.23917	0.139566	0.554316	protein_coding
ENSG0000(	CCDC88C	2809.612	-0.1211	0.139631	0.55443	protein_coding
ENSG0000(	ENSG0000(	275.8291	0.11052	0.139655	0.55443	lncRNA
ENSG0000(	MAD2L2	72.32981	-0.32154	0.139637	0.55443	protein_coding
ENSG0000(	CCDC187	31.59153	0.453578	0.139696	0.55446	protein_coding
ENSG0000(	MIPEPP3	86.09975	-0.23821	0.13972	0.55446	transcribed_unprocessed_pseudo
ENSG0000(	RPS3AP12	52.57849	0.29719	0.139724	0.55446	processed_pseudogene
ENSG0000(	ENSG0000(	64.21811	-0.22568	0.13987	0.554881	lncRNA
ENSG0000(	LINC02316	28.47241	0.33194	0.139864	0.554881	lncRNA
ENSG0000(	CD2BP2-DT	43.88554	0.284308	0.13992	0.554922	lncRNA
ENSG0000(	STAC	23.60935	-0.72844	0.139905	0.554922	protein_coding
ENSG0000(	CREB3L1	1002.855	0.208759	0.139946	0.554944	protein_coding
ENSG0000(	ENSG0000(	491.0885	0.10332	0.139985	0.555018	lncRNA
ENSG0000(	C1QTNF12	50.84032	0.423806	0.140062	0.555034	protein_coding
ENSG0000(	ENSG0000(	94.66595	-0.19138	0.14009	0.555034	lncRNA
ENSG0000(	JUN	1566.769	0.285958	0.140035	0.555034	protein_coding
ENSG0000(	LPAR1	797.1637	-0.15398	0.140088	0.555034	protein_coding
ENSG0000(	SMIM32	40.1844	0.425669	0.14001	0.555034	protein_coding
ENSG0000(	BTBD10P2	48.3726	-0.33606	0.140221	0.55517	processed_pseudogene
ENSG0000(	ENSG0000(	567.5783	0.135918	0.140225	0.55517	lncRNA
ENSG0000(	ENSG0000(	24.81435	0.687697	0.1402	0.55517	lncRNA
ENSG0000(	ENSG0000(	91.9635	0.190786	0.140159	0.55517	lncRNA
ENSG0000(	RDH13	2018.804	0.189561	0.1402	0.55517	protein_coding
ENSG0000(	IQCA1	32.68835	-0.54613	0.140307	0.555334	protein_coding
ENSG0000(	PTBP1	3084.762	0.086476	0.140307	0.555334	protein_coding
ENSG0000(	ACSBG1	376.5747	0.182949	0.140457	0.555344	protein_coding
ENSG0000(	ACTG1P9	261.5046	0.119328	0.140504	0.555344	processed_pseudogene
ENSG0000(	AK3	2148.841	0.19372	0.140579	0.555344	protein_coding
ENSG0000(	ENPP6	211.9859	0.197604	0.140428	0.555344	protein_coding

ENSG0000(	ENSG0000(	61.81516	0.242867	0.140592	0.555344	lncRNA
ENSG0000(	ENSG0000(	202.223	0.114655	0.140579	0.555344	lncRNA
ENSG0000(	ENSG0000(	21.16617	-0.33463	0.140516	0.555344	lncRNA
ENSG0000(	ENSG0000(	172.7481	0.167758	0.140517	0.555344	TEC
ENSG0000(	FUT6	1751.357	0.295307	0.140484	0.555344	protein_coding
ENSG0000(	HDLBP	6910.398	-0.12256	0.140395	0.555344	protein_coding
ENSG0000(	NFAT5	8529.487	-0.10463	0.140518	0.555344	protein_coding
ENSG0000(	RNF207	1182.625	0.173514	0.140537	0.555344	protein_coding
ENSG0000(	TUBE1	783.9408	0.103929	0.14037	0.555344	protein_coding
ENSG0000(	ZNF436	583.0949	-0.25914	0.140509	0.555344	protein_coding
ENSG0000(	MDM1	351.8843	-0.19416	0.140644	0.555389	protein_coding
ENSG0000(	SLC34A2	165.4269	0.202192	0.140629	0.555389	protein_coding
ENSG0000(	SLC9A7	1337.861	-0.17915	0.140677	0.55544	protein_coding
ENSG0000(	ENSG0000(	33.97567	0.276604	0.140762	0.555697	processed_pseudogene
ENSG0000(	HMGN2P17	27.26967	-0.41694	0.140796	0.555751	processed_pseudogene
ENSG0000(	CACNA2D4	178.6612	-0.23236	0.140916	0.555985	protein_coding
ENSG0000(	ENSG0000(	56.73548	-0.26319	0.140901	0.555985	lncRNA
ENSG0000(	HARBI1	236.4233	-0.14685	0.140879	0.555985	protein_coding
ENSG0000(	PIGG	2009.813	0.08177	0.141012	0.556214	protein_coding
ENSG0000(	RNF43	2186.789	0.276287	0.141027	0.556214	protein_coding
ENSG0000(	SHISA4	26.01269	-0.43948	0.141035	0.556214	protein_coding
ENSG0000(	MRPL52	605.3604	-0.11508	0.141161	0.55663	protein_coding
ENSG0000(	RABGAP1L	5069.592	-0.09473	0.141206	0.556729	protein_coding
ENSG0000(	ENSG0000(	26.2816	-0.38342	0.14132	0.55694	lncRNA
ENSG0000(	NOA1	268.7559	0.186428	0.141284	0.55694	protein_coding
ENSG0000(	OPLAH	327.5538	0.318888	0.141304	0.55694	protein_coding
ENSG0000(	ABLIM1	5397.409	0.120447	0.141382	0.557103	protein_coding
ENSG0000(	KANK1	4748.177	0.184491	0.141432	0.557142	protein_coding
ENSG0000(	PTS	448.202	-0.14531	0.141419	0.557142	protein_coding
ENSG0000(	ENSG0000(	100.3304	0.174193	0.141585	0.557585	processed_pseudogene
ENSG0000(	OIP5-AS1	5636.7	0.057938	0.141567	0.557585	lncRNA
ENSG0000(	ATP8B1	4945.114	0.270553	0.141713	0.557693	protein_coding
ENSG0000(	ENSG0000(	47.15177	-0.75545	0.141685	0.557693	lncRNA
ENSG0000(	ENSG0000(	29.34471	-0.64478	0.14165	0.557693	lncRNA
ENSG0000(	SCD	1559.876	-0.38858	0.141732	0.557693	protein_coding
ENSG0000(	TIGD5	255.9607	0.143374	0.141734	0.557693	protein_coding
ENSG0000(	TMEM242	358.4727	0.159542	0.141686	0.557693	protein_coding
ENSG0000(	ARHGAP26	7040.2	-0.12615	0.141766	0.557739	protein_coding
ENSG0000(	APOBEC3B	75.4034	0.442013	0.141914	0.557852	protein_coding
ENSG0000(	COMMD8	64.77847	-0.26181	0.142079	0.557852	protein_coding
ENSG0000(	DCP1A	1364.787	-0.08392	0.141941	0.557852	protein_coding
ENSG0000(	ENSG0000(	21.52237	-0.47509	0.141939	0.557852	lncRNA
ENSG0000(	ENSG0000(	165.8622	-0.13968	0.141917	0.557852	lncRNA
ENSG0000(	LINC01275	60.32099	-0.2488	0.141828	0.557852	lncRNA
ENSG0000(	LINC02624	99.1748	0.199225	0.142078	0.557852	lncRNA
ENSG0000(	LMF1	537.2175	0.158991	0.142066	0.557852	protein_coding
ENSG0000(	LSG1	1034.589	0.139309	0.141991	0.557852	protein_coding

ENSG0000(	SEL1L3	4359.149	-0.14112	0.141934	0.557852	protein_coding
ENSG0000(	TBL1Y	19.64945	-0.64685	0.141992	0.557852	protein_coding
ENSG0000(	TMEM179E	242.205	-0.1576	0.141976	0.557852	protein_coding
ENSG0000(	TPT1P8	145.9072	0.21057	0.142075	0.557852	transcribed_processed_pseudoge
ENSG0000(	TRAF3IP2	2101.903	0.152251	0.142063	0.557852	protein_coding
ENSG0000(	ENSG0000(	483.4961	0.114861	0.14214	0.558012	lncRNA
ENSG0000(	FLNC	218.2993	-0.42871	0.142205	0.55819	protein_coding
ENSG0000(	ENSG0000(	39.38808	0.242992	0.142248	0.558208	lncRNA
ENSG0000(	GABRP	884.5562	-0.14255	0.142251	0.558208	protein_coding
ENSG0000(	P4HTM	1092.002	0.120678	0.142271	0.558208	protein_coding
ENSG0000(	WARS2	327.0521	0.175709	0.142344	0.558415	protein_coding
ENSG0000(	MFSD14B	875.7409	-0.12086	0.142425	0.558554	protein_coding
ENSG0000(	POGK	1665.048	0.077226	0.142412	0.558554	protein_coding
ENSG0000(	TAL1	162.6716	-0.33317	0.14244	0.558554	protein_coding
ENSG0000(	ADA	104.9003	-0.33604	0.142476	0.558614	protein_coding
ENSG0000(	ENSG0000(	80.48958	-0.22855	0.142568	0.558895	lncRNA
ENSG0000(	ENSG0000(	31.19198	-0.39175	0.142664	0.559147	lncRNA
ENSG0000(	LARP4B	5041.714	0.073309	0.142673	0.559147	protein_coding
ENSG0000(	MINDY1	660.9065	0.15149	0.14277	0.559448	protein_coding
ENSG0000(	ENSG0000(	185.7796	0.171569	0.142794	0.559463	lncRNA
ENSG0000(	ENSG0000(	413.3952	0.214286	0.142878	0.559712	lncRNA
ENSG0000(	ENSG0000(	159.781	-0.23758	0.14302	0.559879	lncRNA
ENSG0000(	FPGS	461.1842	-0.13808	0.143011	0.559879	protein_coding
ENSG0000(	OR4P4	106.8996	0.217491	0.143022	0.559879	protein_coding
ENSG0000(	PPFIA2	119.039	-0.55903	0.142988	0.559879	protein_coding
ENSG0000(	RNU4-51P	21.96851	-0.49864	0.142974	0.559879	snRNA
ENSG0000(	ENSG0000(	236.3166	0.126755	0.143106	0.559912	lncRNA
ENSG0000(	ENSG0000(	138.7869	0.183333	0.143186	0.559912	protein_coding
ENSG0000(	NECAB3	401.3337	0.208466	0.143206	0.559912	protein_coding
ENSG0000(	NHLRC3	1672.524	0.131989	0.143056	0.559912	protein_coding
ENSG0000(	NPIPB15	7267.828	0.117067	0.143189	0.559912	protein_coding
ENSG0000(	SCARNA12	887.8648	0.168846	0.143177	0.559912	snoRNA
ENSG0000(	SIPA1L2	3221.885	0.233985	0.143193	0.559912	protein_coding
ENSG0000(	TAF4	820.648	0.163067	0.143214	0.559912	protein_coding
ENSG0000(	TEP1	5839.643	0.132724	0.143105	0.559912	protein_coding
ENSG0000(	ENSG0000(	172.7299	-0.17145	0.14327	0.559971	lncRNA
ENSG0000(	ENSG0000(	143.1908	0.249964	0.143263	0.559971	processed_pseudogene
ENSG0000(	CLIP2	2245.009	0.163873	0.143436	0.560187	protein_coding
ENSG0000(	DHRS11	2142.643	0.373229	0.143395	0.560187	protein_coding
ENSG0000(	ENSG0000(	439.7682	0.109744	0.143382	0.560187	lncRNA
ENSG0000(	ENSG0000(	25.42663	-0.40907	0.143417	0.560187	misc_RNA
ENSG0000(	ENSG0000(	170.1	0.148464	0.143447	0.560187	lncRNA
ENSG0000(	ENSG0000(	95.03373	-0.20192	0.143347	0.560187	lncRNA
ENSG0000(	ENSG0000(	338.3898	-0.14875	0.143595	0.560686	lncRNA
ENSG0000(	CELF2	2634.543	-0.18001	0.143756	0.560695	protein_coding
ENSG0000(	DUSP18	578.2808	0.133602	0.143684	0.560695	protein_coding
ENSG0000(	ENSG0000(	80.75476	0.195185	0.143705	0.560695	lncRNA

ENSG0000(	EPHX2	1340.952	0.183065	0.143669	0.560695	protein_coding
ENSG0000(	ERMP1	2625.167	0.122048	0.143761	0.560695	protein_coding
ENSG0000(	EXD1	204.7775	0.144949	0.14365	0.560695	protein_coding
ENSG0000(	GLT8D1	424.9738	-0.1288	0.14362	0.560695	protein_coding
ENSG0000(	LINC00955	65.6137	0.851934	0.143751	0.560695	lncRNA
ENSG0000(	ENSG0000(	29.75818	0.426149	0.143847	0.560736	lncRNA
ENSG0000(	ENSG0000(	197.6096	0.132144	0.143827	0.560736	TEC
ENSG0000(	NLGN1	43.17259	-0.45264	0.143853	0.560736	protein_coding
ENSG0000(	PDP2	1241.119	0.124923	0.143825	0.560736	protein_coding
ENSG0000(	ADAMTS9-,	26.17015	-0.48089	0.143989	0.561007	lncRNA
ENSG0000(	API5P2	29.52574	-0.52346	0.144041	0.561007	processed_pseudogene
ENSG0000(	DPP6	1031.484	0.274041	0.144085	0.561007	protein_coding
ENSG0000(	ENSG0000(	92.19257	0.22206	0.144085	0.561007	lncRNA
ENSG0000(	ENSG0000(	199.4612	0.139226	0.14406	0.561007	lncRNA
ENSG0000(	GPC6	379.1161	-0.30424	0.144005	0.561007	protein_coding
ENSG0000(	MAP4K3-D	1751.469	0.101983	0.143962	0.561007	lncRNA
ENSG0000(	PLEKHA1	2386.038	0.079324	0.144065	0.561007	protein_coding
ENSG0000(	GALNT6	730.2243	-0.24189	0.144128	0.561015	protein_coding
ENSG0000(	KPNA3	641.6788	-0.1148	0.14412	0.561015	protein_coding
ENSG0000(	KLHL32	57.56968	0.394817	0.14419	0.561176	protein_coding
ENSG0000(	PSMD11	1306.793	-0.10323	0.144351	0.561566	protein_coding
ENSG0000(	SLC25A33	488.1458	0.155923	0.144342	0.561566	protein_coding
ENSG0000(	TLDC2	510.1668	0.396124	0.144321	0.561566	protein_coding
ENSG0000(	FAM83H	972.3997	0.229349	0.144395	0.561578	protein_coding
ENSG0000(	TUNAR	144.2346	-0.15669	0.144395	0.561578	protein_coding
ENSG0000(	MTARC1	1650.648	0.2816	0.144423	0.561606	protein_coding
ENSG0000(	ENSG0000(	90.36487	0.188803	0.144476	0.561636	lncRNA
ENSG0000(	ENSG0000(	19.75599	0.34972	0.144512	0.561636	lncRNA
ENSG0000(	ENSG0000(	188.3825	-0.43692	0.144499	0.561636	TEC
ENSG0000(	RPSAP58	1717.946	0.291132	0.144503	0.561636	protein_coding
ENSG0000(	ENSG0000(	74.70606	-0.21953	0.144596	0.561646	lncRNA
ENSG0000(	ENSG0000(	34.28191	-0.3251	0.144601	0.561646	lncRNA
ENSG0000(	MID1IP1	541.5411	-0.15807	0.144543	0.561646	protein_coding
ENSG0000(	NOXO1	93.42415	0.303481	0.144617	0.561646	protein_coding
ENSG0000(	TRAJ10	25.84961	-0.48382	0.144558	0.561646	TR_J_gene
ENSG0000(	ENSG0000(	45.71576	-0.21621	0.144659	0.561731	lncRNA
ENSG0000(	FBXL7	295.6568	-0.20708	0.144702	0.561816	protein_coding
ENSG0000(	TAS2R4	725.2962	0.126977	0.144768	0.561996	protein_coding
ENSG0000(	ENSG0000(	33.81222	-0.30471	0.144801	0.562045	TEC
ENSG0000(	LINC01235	150.2433	-0.23824	0.144836	0.562101	lncRNA
ENSG0000(	ENSG0000(	38.48112	-0.28346	0.14486	0.562113	processed_pseudogene
ENSG0000(	LINC02866	22.20828	-0.27058	0.145072	0.562856	lncRNA
ENSG0000(	ACKR3	145.9166	-0.33141	0.145121	0.562966	protein_coding
ENSG0000(	ENSG0000(	193.1582	0.197039	0.145172	0.563088	lncRNA
ENSG0000(	MIR200B	28.0868	0.53602	0.145224	0.563093	miRNA
ENSG0000(	PEG10	86.68916	-0.48743	0.145235	0.563093	protein_coding
ENSG0000(	PPARG	4262.059	0.346476	0.145231	0.563093	protein_coding

ENSG0000(	CLDN18	92.29866	-0.37217	0.145286	0.563212	protein_coding
ENSG0000(	IGHV3-43	173.7645	-0.56062	0.145321	0.563269	IG_V_gene
ENSG0000(	CACNA1D	1631.801	0.221564	0.145384	0.563434	protein_coding
ENSG0000(	ENSG0000(	86.16476	-0.37446	0.14541	0.563453	lncRNA
ENSG0000(	LINC02343	37.6925	0.398631	0.145449	0.563527	lncRNA
ENSG0000(	BRWD1-IT1	61.77655	0.228211	0.145544	0.563541	lncRNA
ENSG0000(	ENSG0000(	184.141	0.152919	0.145555	0.563541	lncRNA
ENSG0000(	ENSG0000(	51.45372	0.252275	0.145505	0.563541	lncRNA
ENSG0000(	GNB1	3209.24	-0.09952	0.145511	0.563541	protein_coding
ENSG0000(	RPS29P12	21.93428	0.55829	0.145533	0.563541	processed_pseudogene
ENSG0000(	SNRK	2000.287	-0.13799	0.145656	0.563852	protein_coding
ENSG0000(	RN7SL711F	45.07106	-0.23033	0.145764	0.564188	misc_RNA
ENSG0000(	MYCL	254.3609	0.194764	0.14585	0.564365	protein_coding
ENSG0000(	PARK7P1	53.15155	-0.2578	0.145832	0.564365	processed_pseudogene
ENSG0000(	ENSG0000(	39.76084	0.341691	0.145905	0.564419	protein_coding
ENSG0000(	POMT1	1725.208	0.139579	0.1459	0.564419	protein_coding
ENSG0000(	FPGT	582.9675	0.177267	0.145954	0.56452	protein_coding
ENSG0000(	UQCR11	367.6183	0.224696	0.145973	0.56452	protein_coding
ENSG0000(	ENSG0000(	19.67286	-0.33721	0.146061	0.564746	lncRNA
ENSG0000(	SIGLEC14	883.4396	-0.18362	0.146072	0.564746	protein_coding
ENSG0000(	CYP2W1	35.97752	0.374494	0.146146	0.564796	protein_coding
ENSG0000(	OVOL2	265.8978	0.394409	0.146134	0.564796	protein_coding
ENSG0000(	VMP1	16557.43	-0.18853	0.146128	0.564796	protein_coding
ENSG0000(	C16orf74	127.6553	-0.22884	0.146181	0.564813	protein_coding
ENSG0000(	DPEP1	62.60882	-0.62175	0.146195	0.564813	protein_coding
ENSG0000(	LGR4	2078.928	0.235647	0.146212	0.564813	protein_coding
ENSG0000(	ENSG0000(	377.1549	0.143194	0.146272	0.564886	lncRNA
ENSG0000(	OLFML1	133.0878	-0.25803	0.146266	0.564886	protein_coding
ENSG0000(	ALG13	2115.073	-0.2234	0.146656	0.564978	protein_coding
ENSG0000(	ATXN2L	2824.641	-0.11131	0.146549	0.564978	protein_coding
ENSG0000(	CASC15	2623.349	-0.10959	0.146563	0.564978	lncRNA
ENSG0000(	DUSP23	76.55144	0.25271	0.14669	0.564978	protein_coding
ENSG0000(	ENSG0000(	366.1539	0.12869	0.146545	0.564978	TEC
ENSG0000(	GLUL	6826.87	-0.24804	0.146486	0.564978	protein_coding
ENSG0000(	LINC01796	86.11874	-0.18619	0.146653	0.564978	lncRNA
ENSG0000(	LINC01968	139.156	0.161489	0.146599	0.564978	lncRNA
ENSG0000(	MAGI3	2601.567	0.268941	0.146414	0.564978	protein_coding
ENSG0000(	MTCO3P39	53.86411	0.478211	0.146635	0.564978	processed_pseudogene
ENSG0000(	PCDHA10	458.5982	0.123633	0.146389	0.564978	protein_coding
ENSG0000(	PP7080	5381.148	0.599617	0.146409	0.564978	lncRNA
ENSG0000(	RASA4B	1233.365	-0.25422	0.146707	0.564978	protein_coding
ENSG0000(	RNU6-433F	24.2727	-0.38498	0.146668	0.564978	snRNA
ENSG0000(	SEC61G	284.6707	-0.18546	0.146605	0.564978	protein_coding
ENSG0000(	SLC43A2	1292.676	-0.216	0.146548	0.564978	protein_coding
ENSG0000(	TAS2R15P	198.848	0.213127	0.14641	0.564978	unprocessed_pseudogene
ENSG0000(	TGFBR3	585.1661	-0.23313	0.146575	0.564978	protein_coding
ENSG0000(	TMEM106E	2914.496	-0.12657	0.146436	0.564978	protein_coding

ENSG0000(	ZFP14	1357.493	0.100308	0.146463	0.564978	protein_coding
ENSG0000(	IGKJ2	24.39601	-0.64017	0.146771	0.565003	IG_J_gene
ENSG0000(	NEDD9	3177.948	-0.2107	0.146767	0.565003	protein_coding
ENSG0000(	UTP15	433.417	-0.17771	0.146775	0.565003	protein_coding
ENSG0000(	ENSG0000(	400.7653	0.082443	0.146816	0.565004	lncRNA
ENSG0000(	ZFP36L2	4981.388	0.159095	0.146805	0.565004	protein_coding
ENSG0000(	ENSG0000(	87.0582	-0.45343	0.146933	0.565375	lncRNA
ENSG0000(	CERS5	1186.027	-0.11369	0.147064	0.565564	protein_coding
ENSG0000(	ENSG0000(	178.2012	-0.43188	0.147026	0.565564	protein_coding
ENSG0000(	FITM2	1007.168	0.10203	0.147073	0.565564	protein_coding
ENSG0000(	LARP1P1	38.08534	0.536632	0.147085	0.565564	processed_pseudogene
ENSG0000(	SNX21	384.0067	0.142669	0.147054	0.565564	protein_coding
ENSG0000(	PRDM16-D	29.66617	0.558087	0.147139	0.565692	lncRNA
ENSG0000(	HOMER2	444.9089	-0.16852	0.14724	0.565842	protein_coding
ENSG0000(	KLHL20	500.8247	0.129994	0.147201	0.565842	protein_coding
ENSG0000(	MARF1	3271.922	0.117611	0.147237	0.565842	protein_coding
ENSG0000(	C11orf80	668.8524	-0.16379	0.147267	0.565869	protein_coding
ENSG0000(	PLCB1	547.3031	-0.33111	0.147289	0.565874	protein_coding
ENSG0000(	ARHGAP32	4492.321	0.229614	0.147422	0.565909	protein_coding
ENSG0000(	ENSG0000(	234.6447	0.137924	0.147379	0.565909	lncRNA
ENSG0000(	KRT18P5	63.73734	-0.33901	0.147409	0.565909	processed_pseudogene
ENSG0000(	NAA15	1648.808	0.102407	0.147345	0.565909	protein_coding
ENSG0000(	SORBS1	1269.672	0.264586	0.147343	0.565909	protein_coding
ENSG0000(	ZNF850	1117.043	0.116781	0.147418	0.565909	protein_coding
ENSG0000(	CYB5B	2768.685	0.135168	0.14745	0.565939	protein_coding
ENSG0000(	ARL16	592.3437	0.123301	0.147555	0.566176	protein_coding
ENSG0000(	ARL6IP1	1694.341	-0.14162	0.147606	0.566176	protein_coding
ENSG0000(	FAM66C	418.658	-0.1215	0.147541	0.566176	lncRNA
ENSG0000(	RIC8B	887.1961	0.1561	0.147615	0.566176	protein_coding
ENSG0000(	ZNF876P	172.5007	-0.14984	0.147595	0.566176	transcribed_unprocessed_pseudo
ENSG0000(	RTN4	2309.503	-0.12835	0.14769	0.566387	protein_coding
ENSG0000(	SLC24A2	153.2759	0.164151	0.147817	0.566795	protein_coding
ENSG0000(	EID3	121.6069	-0.27473	0.147965	0.567282	protein_coding
ENSG0000(	KLHL30-AS:	163.4296	-0.18737	0.147989	0.567295	lncRNA
ENSG0000(	ENSG0000(	48.6547	0.226549	0.148016	0.567318	lncRNA
ENSG0000(	HEATR6-DT	231.3773	-0.13314	0.148043	0.567342	lncRNA
ENSG0000(	H4C9	41.04111	-0.50354	0.14812	0.567531	protein_coding
ENSG0000(	SRGAP3	1108.152	-0.08802	0.148133	0.567531	protein_coding
ENSG0000(	LINC01127	86.00402	-0.30486	0.148162	0.567562	lncRNA
ENSG0000(	MARCHF2	335.3263	0.138452	0.148198	0.567621	protein_coding
ENSG0000(	CTBP2P8	68.53687	0.322402	0.148223	0.567639	processed_pseudogene
ENSG0000(	RPL7P14	64.55489	0.328761	0.148274	0.567752	processed_pseudogene
ENSG0000(	GLYCTK	1647.02	0.216071	0.148427	0.567867	protein_coding
ENSG0000(	LINC01559	1394.613	0.570727	0.148447	0.567867	lncRNA
ENSG0000(	LINC02594	109.5422	0.153809	0.148445	0.567867	lncRNA
ENSG0000(	NEAT1	401928	-0.18282	0.148469	0.567867	lncRNA
ENSG0000(	RPAP3-DT	269.9046	-0.22537	0.148345	0.567867	lncRNA

ENSG0000(	SHD	41.83573	0.57416	0.148451	0.567867	protein_coding
ENSG0000(	ZBTB32	36.42628	-0.53584	0.148387	0.567867	protein_coding
ENSG0000(	ZNF654	771.9862	-0.13589	0.148394	0.567867	protein_coding
ENSG0000(	KLC3	298.4255	-0.15343	0.148503	0.567919	protein_coding
ENSG0000(	ENSG0000(	4607.309	0.053598	0.148557	0.567969	lncRNA
ENSG0000(	HEXD-IT1	876.6517	0.078047	0.148583	0.567969	lncRNA
ENSG0000(	LINC02915	29.4169	-0.36945	0.148576	0.567969	lncRNA
ENSG0000(	POLR1F	223.6005	-0.18937	0.148599	0.567969	protein_coding
ENSG0000(	CAP1	2765.283	-0.16681	0.148674	0.56818	protein_coding
ENSG0000(	MTARC2	276.6347	0.253813	0.148722	0.568285	protein_coding
ENSG0000(	PLXDC1	977.9899	-0.22335	0.148762	0.568356	protein_coding
ENSG0000(	COPB2	4199.65	-0.07527	0.148807	0.568371	protein_coding
ENSG0000(	ENSG0000(	59.03213	-0.27511	0.148804	0.568371	lncRNA
ENSG0000(	FBXO34	2344.364	0.21233	0.148835	0.568398	protein_coding
ENSG0000(	RPS6P20	139.6127	0.24201	0.148889	0.568528	unprocessed_pseudogene
ENSG0000(	ENSG0000(	149.9141	-0.13628	0.14898	0.568716	TEC
ENSG0000(	IGHV2-70D	45.62818	-0.84217	0.148979	0.568716	IG_V_gene
ENSG0000(	CR2	625.2577	-0.62632	0.149024	0.568806	protein_coding
ENSG0000(	ARHGAP26	101.6806	-0.24345	0.149123	0.568874	lncRNA
ENSG0000(	ENSG0000(	495.095	0.106905	0.149125	0.568874	lncRNA
ENSG0000(	ENSG0000(	66.03596	0.309406	0.149068	0.568874	lncRNA
ENSG0000(	SGSH	1050.363	0.138623	0.149123	0.568874	protein_coding
ENSG0000(	SH3YL1	1879.895	0.154973	0.149183	0.569016	protein_coding
ENSG0000(	FADS3	780.7289	-0.2097	0.149234	0.569134	protein_coding
ENSG0000(	USP35	332.9556	-0.21209	0.149256	0.569138	protein_coding
ENSG0000(	EIPR1	378.5034	-0.13851	0.14928	0.569149	protein_coding
ENSG0000(	ENSG0000(	69.88731	0.200972	0.149332	0.569271	lncRNA
ENSG0000(	ADAP1	1112.532	0.277422	0.149358	0.569291	protein_coding
ENSG0000(	RMC1	461.2166	-0.12117	0.149379	0.569291	protein_coding
ENSG0000(	PSME4	2822.727	-0.12735	0.149463	0.569532	protein_coding
ENSG0000(	GPD1L	1520.684	0.280763	0.149606	0.569921	protein_coding
ENSG0000(	NDE1	1448.199	-0.14634	0.149606	0.569921	protein_coding
ENSG0000(	ARHGEF5	2407.966	0.358726	0.149678	0.569961	protein_coding
ENSG0000(	LINC02441	62.82775	0.571984	0.149686	0.569961	lncRNA
ENSG0000(	MARS1	1451.27	-0.16143	0.1497	0.569961	protein_coding
ENSG0000(	REG1A	2166.228	-3.52642	0.149697	0.569961	protein_coding
ENSG0000(	IGKV1-8	33.88528	-0.81156	0.149748	0.570066	IG_V_gene
ENSG0000(	ZFAND4	604.373	0.138636	0.149778	0.570101	protein_coding
ENSG0000(	AIFM1	1850.566	0.074622	0.149826	0.570206	protein_coding
ENSG0000(	ACVR1C	520.222	0.382053	0.149925	0.570235	protein_coding
ENSG0000(	LINC01943	59.75968	-0.6474	0.149879	0.570235	lncRNA
ENSG0000(	RAB27A	1500.323	-0.1684	0.149933	0.570235	protein_coding
ENSG0000(	RNF31	680.8238	-0.15595	0.149937	0.570235	protein_coding
ENSG0000(	SHCBP1	615.4641	-0.14517	0.149925	0.570235	protein_coding
ENSG0000(	ALPK1	2895.823	-0.12962	0.150046	0.570419	protein_coding
ENSG0000(	MRFAP1L1	661.8985	-0.10372	0.150048	0.570419	protein_coding
ENSG0000(	NASP	2506.354	-0.09145	0.150034	0.570419	protein_coding

ENSG0000(	ARHGAP44	1962.128	0.368225	0.150083	0.570473	protein_coding
ENSG0000(	ERBB3	6618.401	0.217449	0.150161	0.570611	protein_coding
ENSG0000(	PPP1R12C	1788.327	0.116397	0.150149	0.570611	protein_coding
ENSG0000(	SKIL	3388.941	-0.16106	0.150187	0.570632	protein_coding
ENSG0000(	GLS	3501.527	-0.13686	0.150364	0.570997	protein_coding
ENSG0000(	MIR573	42.08416	0.288852	0.150366	0.570997	miRNA
ENSG0000(	MOV10	2042.447	-0.08886	0.150335	0.570997	protein_coding
ENSG0000(	TSHR	801.4173	-0.13973	0.150321	0.570997	protein_coding
ENSG0000(	NIPAL2	2049.096	0.209677	0.150439	0.571195	protein_coding
ENSG0000(	FMR1	2395.68	0.135179	0.15048	0.571225	protein_coding
ENSG0000(	MARVELD3	1780.36	0.261009	0.150488	0.571225	protein_coding
ENSG0000(	TEPP	112.8199	-0.25286	0.150566	0.571292	protein_coding
ENSG0000(	ZNF254	1150.168	0.113111	0.150568	0.571292	protein_coding
ENSG0000(	ZNF610	181.4466	0.158331	0.150565	0.571292	protein_coding
ENSG0000(	EGFLAM	152.2094	-0.23786	0.150595	0.571315	protein_coding
ENSG0000(	ANKRD30B	14371.97	0.310984	0.150753	0.571678	protein_coding
ENSG0000(	ENSG0000(	206.8664	-0.26732	0.150737	0.571678	lncRNA
ENSG0000(	MAGIX	282.9509	0.173002	0.150718	0.571678	protein_coding
ENSG0000(	MZF1-AS1	390.928	0.138599	0.150819	0.571847	lncRNA
ENSG0000(	ENSG0000(	265.9658	-0.2905	0.150852	0.571893	lncRNA
ENSG0000(	MED28	4366.781	0.10037	0.15088	0.571921	protein_coding
ENSG0000(	CRNKL1	504.1365	0.152737	0.150965	0.572087	protein_coding
ENSG0000(	ENSG0000(	214.1047	-0.16213	0.150949	0.572087	TEC
ENSG0000(	ENSG0000(	77.48895	-0.28538	0.151012	0.572186	lncRNA
ENSG0000(	GRM4	110.6918	-0.21341	0.151056	0.572242	protein_coding
ENSG0000(	SRRM4	215.485	0.239796	0.151068	0.572242	protein_coding
ENSG0000(	ARF4-AS1	49.4007	0.269663	0.151108	0.572314	lncRNA
ENSG0000(	CD207	25.45059	0.576018	0.151178	0.572422	protein_coding
ENSG0000(	TRMT44	619.1785	0.121814	0.151163	0.572422	protein_coding
ENSG0000(	NPIPA5	72.21385	-0.84784	0.15123	0.57254	protein_coding
ENSG0000(	ENSG0000(	241.25	0.112577	0.151306	0.572748	lncRNA
ENSG0000(	DROSHA	1301.96	0.114441	0.151328	0.572753	protein_coding
ENSG0000(	ENSG0000(	658.073	-0.1499	0.151405	0.572965	lncRNA
ENSG0000(	ATP9A	2892.815	0.257341	0.151651	0.573301	protein_coding
ENSG0000(	CCDC191	386.7177	0.147184	0.151623	0.573301	protein_coding
ENSG0000(	ENSG0000(	27.9508	0.436506	0.151583	0.573301	lncRNA
ENSG0000(	ENSG0000(	71.78022	0.233755	0.15166	0.573301	lncRNA
ENSG0000(	ENSG0000(	135.9042	-0.31432	0.151688	0.573301	lncRNA
ENSG0000(	ENSG0000(	134.1412	-0.30808	0.151702	0.573301	lncRNA
ENSG0000(	ENSG0000(	138.0617	-0.20533	0.151685	0.573301	lncRNA
ENSG0000(	H2AZ1	408.3199	-0.22754	0.151675	0.573301	protein_coding
ENSG0000(	OTUD6A	59.85795	-0.23398	0.151533	0.573301	protein_coding
ENSG0000(	TKFC	595.6962	0.150428	0.151633	0.573301	protein_coding
ENSG0000(	ENSG0000(	562.1699	0.133231	0.151851	0.573391	lncRNA
ENSG0000(	ENSG0000(	38.34137	-0.34531	0.151835	0.573391	lncRNA
ENSG0000(	ENSG0000(	293.0882	-0.10093	0.151768	0.573391	lncRNA
ENSG0000(	GTF2IP23	25.41144	0.398384	0.151841	0.573391	unprocessed_pseudogene

ENSG0000(	LRIF1	381.4206	-0.14789	0.151839	0.573391	protein_coding
ENSG0000(	PLOD1	745.2706	-0.10932	0.151819	0.573391	protein_coding
ENSG0000(	ENSG0000(	648.4139	-0.13803	0.151944	0.573552	transcribed_unprocessed_pseudo
ENSG0000(	ENSG0000(	637.2586	0.132191	0.151947	0.573552	TEC
ENSG0000(	GPI	4600.968	-0.09698	0.151956	0.573552	protein_coding
ENSG0000(	CEP120	1328.32	-0.0958	0.152106	0.573724	protein_coding
ENSG0000(	HID1-AS1	51.64056	0.234331	0.152049	0.573724	lncRNA
ENSG0000(	IGHV2-70	66.80721	-0.88932	0.152049	0.573724	IG_V_gene
ENSG0000(	KLHL7	896.2458	0.129115	0.152105	0.573724	protein_coding
ENSG0000(	TET1	504.2504	-0.1589	0.152119	0.573724	protein_coding
ENSG0000(	WWC1	805.8057	-0.24026	0.152127	0.573724	protein_coding
ENSG0000(	ENSG0000(	1156.616	0.105361	0.152288	0.57381	lncRNA
ENSG0000(	ENSG0000(	223.082	0.159296	0.152296	0.57381	TEC
ENSG0000(	FAM47E-ST	109.1403	0.325305	0.15228	0.57381	protein_coding
ENSG0000(	HMGB1P39	106.3226	0.172593	0.152268	0.57381	processed_pseudogene
ENSG0000(	HOXA10	292.3116	0.242082	0.152207	0.57381	protein_coding
ENSG0000(	MED30	64.40715	-0.24659	0.152278	0.57381	protein_coding
ENSG0000(	SUPT3H	240.5213	-0.1369	0.152172	0.57381	protein_coding
ENSG0000(	DDX20	365.6989	0.151291	0.152364	0.573884	protein_coding
ENSG0000(	ENSG0000(	165.7567	0.148205	0.152406	0.573884	lncRNA
ENSG0000(	ENSG0000(	45.91937	0.295253	0.152412	0.573884	TEC
ENSG0000(	ENSG0000(	171.4628	-0.15765	0.15242	0.573884	lncRNA
ENSG0000(	FUBP3	1142.375	-0.10398	0.152399	0.573884	protein_coding
ENSG0000(	IGHV2-26	52.72964	-0.65482	0.152486	0.574011	IG_V_gene
ENSG0000(	TBPL2	22.55709	0.436164	0.152516	0.574011	protein_coding
ENSG0000(	UIMC1	981.2459	0.117517	0.152516	0.574011	protein_coding
ENSG0000(	ENSG0000(	20.02896	0.39659	0.152538	0.574016	lncRNA
ENSG0000(	DIS3L	1052.162	0.089198	0.15256	0.574019	protein_coding
ENSG0000(	CCDC183	146.6319	0.561402	0.152624	0.574025	protein_coding
ENSG0000(	ENSG0000(	622.5011	0.097721	0.152616	0.574025	lncRNA
ENSG0000(	HSPB7	25.99779	-0.61275	0.152602	0.574025	protein_coding
ENSG0000(	GARS1	2804.996	-0.11957	0.152716	0.574063	protein_coding
ENSG0000(	PGLS-DT	391.9273	0.093755	0.152696	0.574063	lncRNA
ENSG0000(	RN7SKP104	42.91754	0.477595	0.152718	0.574063	misc_RNA
ENSG0000(	RN7SL535P	84.67454	-0.22946	0.152657	0.574063	misc_RNA
ENSG0000(	ARHGAP27	4742.443	0.155571	0.152791	0.574181	protein_coding
ENSG0000(	UBQLN1-AS	46.28994	-0.26827	0.152777	0.574181	lncRNA
ENSG0000(	CDC42SE2	3207.797	-0.18179	0.152874	0.574403	protein_coding
ENSG0000(	ENSG0000(	94.73748	0.17897	0.152925	0.574403	TEC
ENSG0000(	ENSG0000(	1046.842	0.136332	0.152933	0.574403	lncRNA
ENSG0000(	TENT2	1982.422	-0.09495	0.152906	0.574403	protein_coding
ENSG0000(	ENSG0000(	255.0217	0.146434	0.152969	0.574459	TEC
ENSG0000(	ENSG0000(	35.41552	0.38276	0.153024	0.574509	lncRNA
ENSG0000(	FAM230A	98.14102	0.209555	0.15302	0.574509	lncRNA
ENSG0000(	ENSG0000(	94.09679	0.251534	0.153171	0.574539	lncRNA
ENSG0000(	LRFN4	224.2182	0.226429	0.153188	0.574539	protein_coding
ENSG0000(	RPL22L1	163.9314	-0.20943	0.153127	0.574539	protein_coding

ENSG0000(	SLC8B1	1290.309	0.110501	0.153173	0.574539	protein_coding
ENSG0000(	SMPD3	1349.438	0.195912	0.15322	0.574539	protein_coding
ENSG0000(	TMEM19	1182.71	-0.09748	0.15309	0.574539	protein_coding
ENSG0000(	TMIGD1	319.7031	0.688536	0.153126	0.574539	protein_coding
ENSG0000(	UCKL1	563.1923	0.172064	0.153209	0.574539	protein_coding
ENSG0000(	VPS35L	1641.92	0.072653	0.153126	0.574539	protein_coding
ENSG0000(	ANKRD20A	261.2335	0.50884	0.153266	0.574553	protein_coding
ENSG0000(	FLNA	8246.615	-0.26523	0.15326	0.574553	protein_coding
ENSG0000(	ENSG0000(	142.6636	0.260691	0.153336	0.574737	lncRNA
ENSG0000(	ENSG0000(	185.957	0.116265	0.153408	0.574916	lncRNA
ENSG0000(	ENSG0000(	1182.929	0.066795	0.153425	0.574916	lncRNA
ENSG0000(	ITGB1BP2	64.82341	-0.25285	0.153568	0.575296	protein_coding
ENSG0000(	THRAP3	2027.237	-0.11251	0.153562	0.575296	protein_coding
ENSG0000(	ACSF2	1026.621	0.309578	0.153661	0.575403	protein_coding
ENSG0000(	ALOX15	77.09785	-0.58313	0.153651	0.575403	protein_coding
ENSG0000(	MAILR	683.802	-0.19002	0.15363	0.575403	lncRNA
ENSG0000(	MTCYBP3	108.4637	-0.22312	0.153681	0.575403	processed_pseudogene
ENSG0000(	ENSG0000(	21.40915	-0.37439	0.153747	0.575495	lncRNA
ENSG0000(	ENSG0000(	62.31511	-0.36937	0.153741	0.575495	lncRNA
ENSG0000(	ENSG0000(	60.43412	0.276763	0.153797	0.575606	lncRNA
ENSG0000(	ENSG0000(	136.3276	-0.188	0.153845	0.575706	lncRNA
ENSG0000(	GC	25.49452	-1.59174	0.153885	0.575761	protein_coding
ENSG0000(	SEPTIN5	44.91122	-0.45514	0.153902	0.575761	protein_coding
ENSG0000(	COPDA1	20.01439	-0.70277	0.154053	0.57603	lncRNA
ENSG0000(	ENSG0000(	324.358	0.17858	0.154099	0.57603	lncRNA
ENSG0000(	ENSG0000(	318.8491	0.103397	0.154018	0.57603	TEC
ENSG0000(	NAALADL1	148.4397	0.50018	0.15409	0.57603	protein_coding
ENSG0000(	PLA2G7	730.764	-0.29355	0.15406	0.57603	protein_coding
ENSG0000(	ZFAND2B	399.8911	0.165473	0.154051	0.57603	protein_coding
ENSG0000(	CHODL	67.98106	0.211629	0.154166	0.576058	protein_coding
ENSG0000(	ENSG0000(	336.8336	0.276482	0.154193	0.576058	lncRNA
ENSG0000(	ENSG0000(	41.54461	-0.39245	0.154211	0.576058	lncRNA
ENSG0000(	RN7SKP97	44.64609	-0.25558	0.154204	0.576058	misc_RNA
ENSG0000(	TPTE2P1	377.1773	0.130067	0.154189	0.576058	transcribed_unprocessed_pseudo
ENSG0000(	ARHGAP15	1205.761	-0.25348	0.154687	0.57676	protein_coding
ENSG0000(	BIVM	1561.127	0.0696	0.154618	0.57676	protein_coding
ENSG0000(	DTX1	258.4905	-0.50456	0.154434	0.57676	protein_coding
ENSG0000(	ENSG0000(	98.1349	0.22408	0.154796	0.57676	lncRNA
ENSG0000(	ENSG0000(	33.49237	-0.28918	0.154482	0.57676	lncRNA
ENSG0000(	ENSG0000(	31.57578	0.296148	0.154623	0.57676	TEC
ENSG0000(	ENSG0000(	31.23013	-0.43558	0.154537	0.57676	lncRNA
ENSG0000(	ERO1B	1819.042	-0.134	0.154797	0.57676	protein_coding
ENSG0000(	FCHSD2	1459.254	-0.24175	0.154575	0.57676	protein_coding
ENSG0000(	FOLH1	143.9123	-0.60796	0.154577	0.57676	protein_coding
ENSG0000(	LINC02800	74.42787	-0.36627	0.154743	0.57676	lncRNA
ENSG0000(	MAS1	367.5776	0.147346	0.154638	0.57676	protein_coding
ENSG0000(	MIR3140	20.9532	-0.43461	0.154494	0.57676	miRNA

ENSG0000(	MIR8086	79.36491	0.185121	0.15467	0.57676	miRNA
ENSG0000(	NANOG	670.1418	0.117081	0.154726	0.57676	protein_coding
ENSG0000(	PFKL	3374.194	0.224052	0.154661	0.57676	protein_coding
ENSG0000(	SOX18	52.30067	-0.44788	0.154705	0.57676	protein_coding
ENSG0000(	SULT2B1	96.84381	-0.22954	0.154611	0.57676	protein_coding
ENSG0000(	SWAP70	1445.694	-0.19634	0.154798	0.57676	protein_coding
ENSG0000(	ZNF311	103.2299	0.305874	0.15487	0.576952	protein_coding
ENSG0000(	DPH6-DT	21.80087	0.636285	0.155013	0.577404	lncRNA
ENSG0000(	AOC3	280.5927	-0.28771	0.155368	0.577492	protein_coding
ENSG0000(	CLASP1	2213.052	-0.1029	0.1554	0.577492	protein_coding
ENSG0000(	ENSG0000(	825.0131	0.095771	0.155348	0.577492	lncRNA
ENSG0000(	ENSG0000(	483.8712	-0.17059	0.155334	0.577492	lncRNA
ENSG0000(	ENSG0000(	171.7045	0.16343	0.155281	0.577492	lncRNA
ENSG0000(	ENSG0000(	49.64734	-0.25144	0.155344	0.577492	lncRNA
ENSG0000(	GAPDHP72	49.83685	-0.21153	0.155214	0.577492	transcribed_processed_pseudoge
ENSG0000(	LCAT	231.5582	-0.20158	0.155118	0.577492	protein_coding
ENSG0000(	LINC00449	87.65608	0.210597	0.155141	0.577492	lncRNA
ENSG0000(	LINC01346	30.93527	0.323114	0.155456	0.577492	lncRNA
ENSG0000(	PHF5GP	67.91267	0.30075	0.155411	0.577492	processed_pseudogene
ENSG0000(	PXK	911.3905	-0.18981	0.155192	0.577492	protein_coding
ENSG0000(	RBM45	405.9646	0.169097	0.155242	0.577492	protein_coding
ENSG0000(	RNF103	2995.764	0.190972	0.155201	0.577492	protein_coding
ENSG0000(	SF3A3	641.6713	-0.15251	0.155441	0.577492	protein_coding
ENSG0000(	SMC4	1994.173	-0.14002	0.155139	0.577492	protein_coding
ENSG0000(	SPAG5	546.1236	-0.17742	0.155226	0.577492	protein_coding
ENSG0000(	TERF2	998.5308	0.102359	0.155272	0.577492	protein_coding
ENSG0000(	WIPF3	196.7847	0.175828	0.155452	0.577492	protein_coding
ENSG0000(	ZNF350-AS	277.0124	0.184605	0.155147	0.577492	lncRNA
ENSG0000(	C17orf97	74.86467	0.4565	0.15555	0.577584	protein_coding
ENSG0000(	FAM98A	1592.578	-0.08605	0.15559	0.577584	protein_coding
ENSG0000(	LINC02801	116.2703	0.149983	0.155607	0.577584	lncRNA
ENSG0000(	ME3	729.3974	0.306084	0.155533	0.577584	protein_coding
ENSG0000(	PRKAR2A-A	826.9983	0.07324	0.155601	0.577584	lncRNA
ENSG0000(	RPS15AP10	121.3782	0.191869	0.155522	0.577584	processed_pseudogene
ENSG0000(	BANP	972.6591	-0.11038	0.155696	0.577634	protein_coding
ENSG0000(	CCDC112	265.3349	0.21828	0.155672	0.577634	protein_coding
ENSG0000(	ENSG0000(	311.268	0.184703	0.155928	0.577634	processed_pseudogene
ENSG0000(	ENSG0000(	137.3674	-0.20603	0.155729	0.577634	lncRNA
ENSG0000(	ENSG0000(	53.96953	0.301	0.155878	0.577634	lncRNA
ENSG0000(	IDH3G	399.485	0.127259	0.15581	0.577634	protein_coding
ENSG0000(	ITGA9-AS1	1397.405	0.096694	0.155783	0.577634	lncRNA
ENSG0000(	KIDINS220	5615.953	0.067845	0.155891	0.577634	protein_coding
ENSG0000(	LIMA1	4792.615	0.166638	0.155866	0.577634	protein_coding
ENSG0000(	PCGF5	1601.994	-0.13546	0.155886	0.577634	protein_coding
ENSG0000(	RN7SL555F	36.0342	0.381436	0.155663	0.577634	misc_RNA
ENSG0000(	SLC26A4	180.0576	-0.39944	0.155933	0.577634	protein_coding
ENSG0000(	TMEM250	503.113	0.163895	0.15577	0.577634	protein_coding

ENSG0000(	ZCRB1	748.6544	-0.08192	0.155731	0.577634	protein_coding
ENSG0000(	ZNF490	986.5341	-0.14989	0.155935	0.577634	protein_coding
ENSG0000(	ENSG0000(	235.8983	-0.23824	0.156008	0.577672	TEC
ENSG0000(	FGF5	348.2795	0.108557	0.155998	0.577672	protein_coding
ENSG0000(	TJP1	3278.449	0.20388	0.155969	0.577672	protein_coding
ENSG0000(	ENSG0000(	67.00953	0.221626	0.15603	0.577674	lncRNA
ENSG0000(	ANKRD37	85.49612	-0.32524	0.156194	0.577968	protein_coding
ENSG0000(	CD37	988.4609	-0.43871	0.156214	0.577968	protein_coding
ENSG0000(	EVA1A	53.10157	-0.61307	0.156198	0.577968	protein_coding
ENSG0000(	MIR4733H(	57.19583	-0.23242	0.156206	0.577968	lncRNA
ENSG0000(	WT1-AS	97.88186	-0.23347	0.156156	0.577968	lncRNA
ENSG0000(	ENSG0000(	342.9326	0.112331	0.156245	0.578002	lncRNA
ENSG0000(	ADIRF-AS1	375.5726	0.154122	0.156355	0.578025	lncRNA
ENSG0000(	ALAS1	687.778	-0.21109	0.156302	0.578025	protein_coding
ENSG0000(	CCNY	958.4622	-0.12468	0.156351	0.578025	protein_coding
ENSG0000(	ENSG0000(	69.53359	0.217381	0.156461	0.578025	lncRNA
ENSG0000(	ENSG0000(	21.03786	0.568659	0.156366	0.578025	unprocessed_pseudogene
ENSG0000(	ENSG0000(	127.2368	-0.35274	0.156483	0.578025	lncRNA
ENSG0000(	EVL	4197.483	-0.26203	0.156503	0.578025	protein_coding
ENSG0000(	NAP1L5	27.3697	-0.40736	0.15635	0.578025	protein_coding
ENSG0000(	RAB30-DT	2555.305	-0.09749	0.15642	0.578025	lncRNA
ENSG0000(	UTP11	632.9114	0.157533	0.156464	0.578025	protein_coding
ENSG0000(	YES1P1	98.22773	-0.3547	0.156401	0.578025	processed_pseudogene
ENSG0000(	ZNF75D	1177.957	0.086945	0.156499	0.578025	protein_coding
ENSG0000(	IL17RD	580.1829	0.246725	0.156593	0.578217	protein_coding
ENSG0000(	LINC01186	29.53757	-0.445	0.156597	0.578217	lncRNA
ENSG0000(	CHTOP	2642.421	0.079702	0.156668	0.578222	protein_coding
ENSG0000(	CTU1	70.64596	-0.19142	0.156657	0.578222	protein_coding
ENSG0000(	ENSG0000(	34.94258	0.263263	0.156676	0.578222	lncRNA
ENSG0000(	ENSG0000(	605.8415	-0.10518	0.156759	0.578222	lncRNA
ENSG0000(	ENSG0000(	183.799	0.171714	0.156741	0.578222	lncRNA
ENSG0000(	KIFC2	1131.829	0.251993	0.156804	0.578222	protein_coding
ENSG0000(	KRT80	46.49073	-0.3463	0.15674	0.578222	protein_coding
ENSG0000(	NOMO2	505.3396	-0.22826	0.15673	0.578222	protein_coding
ENSG0000(	SCIMP	886.2557	-0.15834	0.156808	0.578222	protein_coding
ENSG0000(	ST18	183.3763	0.174055	0.156774	0.578222	protein_coding
ENSG0000(	CCNYL1	932.6607	0.213354	0.156869	0.578297	protein_coding
ENSG0000(	SEPTIN14	461.8168	-0.18081	0.156871	0.578297	protein_coding
ENSG0000(	GLTP	1297.965	0.156442	0.156924	0.578349	protein_coding
ENSG0000(	STX3	2447.471	0.212478	0.156927	0.578349	protein_coding
ENSG0000(	CRYZ	186.458	-0.24052	0.157083	0.578844	protein_coding
ENSG0000(	KIF26A	101.4096	0.380065	0.157104	0.578844	protein_coding
ENSG0000(	LINC02535	93.41688	0.559936	0.157161	0.578901	lncRNA
ENSG0000(	TSSK3	49.67541	-0.2694	0.157161	0.578901	protein_coding
ENSG0000(	ARF4	1039.174	-0.18167	0.157268	0.578909	protein_coding
ENSG0000(	ENSG0000(	395.5186	0.212896	0.157235	0.578909	lncRNA
ENSG0000(	ENSG0000(	288.4368	-0.17293	0.157259	0.578909	lncRNA

ENSG0000(	SLC44A3	548.9716	0.324178	0.157251	0.578909	protein_coding
ENSG0000(	TUBGCP6	2513.061	0.098582	0.157187	0.578909	protein_coding
ENSG0000(	DSG3	125.0355	-0.34568	0.157388	0.579003	protein_coding
ENSG0000(	ENSG0000(	87.26968	0.193825	0.157404	0.579003	lncRNA
ENSG0000(	ENSG0000(	46.22302	-0.32106	0.157413	0.579003	lncRNA
ENSG0000(	MAB21L3	13032.68	0.118883	0.15742	0.579003	protein_coding
ENSG0000(	PTPRJ	3195.139	-0.15006	0.157337	0.579003	protein_coding
ENSG0000(	ROCK1P1	1032.253	0.499018	0.157325	0.579003	transcribed_unprocessed_pseudo
ENSG0000(	LINC01892	182.2815	0.155213	0.157476	0.579131	lncRNA
ENSG0000(	CNOT3	985.6156	-0.14906	0.15752	0.579153	protein_coding
ENSG0000(	IGHMBP2	1111.225	0.141682	0.157524	0.579153	protein_coding
ENSG0000(	ENSG0000(	134.8817	0.158522	0.15766	0.579313	lncRNA
ENSG0000(	IDO2	441.8342	-0.14793	0.157702	0.579313	protein_coding
ENSG0000(	RN7SKP204	43.56334	0.344682	0.157685	0.579313	misc_RNA
ENSG0000(	SFT2D3	495.6237	0.084202	0.157604	0.579313	protein_coding
ENSG0000(	ST6GAL2	320.7748	0.148745	0.157658	0.579313	protein_coding
ENSG0000(	WNT9A	92.68222	0.274129	0.157664	0.579313	protein_coding
ENSG0000(	ZNF592	1889.002	0.105353	0.157715	0.579313	protein_coding
ENSG0000(	ALG9	1028.98	-0.09909	0.157909	0.579399	protein_coding
ENSG0000(	CD300LG	87.48413	-0.28315	0.157808	0.579399	protein_coding
ENSG0000(	CFL2	401.5508	-0.19127	0.157775	0.579399	protein_coding
ENSG0000(	ENSG0000(	38.60119	-0.27432	0.157884	0.579399	lncRNA
ENSG0000(	GNA11	3256.831	0.27239	0.157841	0.579399	protein_coding
ENSG0000(	LINC00706	148.3732	0.200961	0.157873	0.579399	lncRNA
ENSG0000(	OVCH1-AS1	449.887	-0.19676	0.157852	0.579399	lncRNA
ENSG0000(	PROKR2	241.4313	-0.11747	0.157928	0.579399	protein_coding
ENSG0000(	RBMXL1	813.6235	-0.09526	0.157917	0.579399	protein_coding
ENSG0000(	IGSF5	252.7945	-0.17638	0.15798	0.579513	protein_coding
ENSG0000(	LDHAL6A	46.73092	0.236467	0.158047	0.579682	protein_coding
ENSG0000(	ENSG0000(	291.0354	0.117932	0.158081	0.579728	lncRNA
ENSG0000(	ARHGEF10I	2697.275	0.213046	0.158181	0.579941	protein_coding
ENSG0000(	CD247	303.2591	-0.33623	0.15818	0.579941	protein_coding
ENSG0000(	ENSG0000(	47.81488	-0.34307	0.158257	0.580065	lncRNA
ENSG0000(	TAF1A-AS1	35.02632	0.268699	0.158251	0.580065	lncRNA
ENSG0000(	HAT1	789.0982	-0.11827	0.158324	0.580235	protein_coding
ENSG0000(	DMAC2L	1035.663	0.095744	0.158427	0.580241	protein_coding
ENSG0000(	ENSG0000(	406.9153	-0.17107	0.158375	0.580241	TEC
ENSG0000(	NMUR1	192.4767	0.14579	0.158423	0.580241	protein_coding
ENSG0000(	PMM1	483.7572	0.240901	0.158356	0.580241	protein_coding
ENSG0000(	ZNF783	851.3445	0.087631	0.158432	0.580241	protein_coding
ENSG0000(	ASPN	131.9305	-0.2492	0.158586	0.580343	protein_coding
ENSG0000(	ENSG0000(	106.2539	0.200982	0.158551	0.580343	lncRNA
ENSG0000(	ENSG0000(	746.1404	-0.10424	0.158511	0.580343	lncRNA
ENSG0000(	GTF2IRD2B	1675.146	0.104076	0.158556	0.580343	protein_coding
ENSG0000(	LSP1	1026.059	-0.16978	0.158573	0.580343	protein_coding
ENSG0000(	TAGLN2	1276.402	-0.17949	0.158582	0.580343	protein_coding
ENSG0000(	ACTR6	274.5795	0.121806	0.158632	0.580365	protein_coding

ENSG0000(	ALPP	39.61593	-0.32068	0.158634	0.580365	protein_coding
ENSG0000(	BIN2	695.3271	-0.23477	0.158747	0.580622	protein_coding
ENSG0000(	MED27	455.169	-0.12868	0.158737	0.580622	protein_coding
ENSG0000(	HMGB1P49	20.6111	0.488887	0.158794	0.580702	processed_pseudogene
ENSG0000(	WHAMMP2	1081.058	-0.10159	0.158811	0.580702	transcribed_unprocessed_pseudo
ENSG0000(	ASB6	698.113	0.080482	0.158869	0.580756	protein_coding
ENSG0000(	CAPG	289.4369	-0.24229	0.158907	0.580756	protein_coding
ENSG0000(	EFL1	729.1924	0.202589	0.158965	0.580756	protein_coding
ENSG0000(	ENSG0000(	71.196	-0.28083	0.158994	0.580756	lncRNA
ENSG0000(	HPN	162.4513	-0.16727	0.158858	0.580756	protein_coding
ENSG0000(	IMPA2	754.2639	0.198944	0.158961	0.580756	protein_coding
ENSG0000(	SHROOM1	1637.202	0.277951	0.158934	0.580756	protein_coding
ENSG0000(	SIGIRR	715.194	0.147198	0.158987	0.580756	protein_coding
ENSG0000(	VWA8	1345.587	0.136193	0.159027	0.580796	protein_coding
ENSG0000(	LINC01247	258.7508	0.115315	0.159075	0.580896	lncRNA
ENSG0000(	TRAJ37	24.84555	-0.37739	0.159116	0.58097	TR_J_gene
ENSG0000(	HYKK	964.2034	0.079275	0.159247	0.581215	protein_coding
ENSG0000(	PARM1	3864.886	0.248618	0.159236	0.581215	protein_coding
ENSG0000(	SLC35D1	2691.751	0.216996	0.159227	0.581215	protein_coding
ENSG0000(	IGHA2	10938.33	-0.49687	0.159274	0.581238	IG_C_gene
ENSG0000(	ENSG0000(	108.1329	0.209355	0.159307	0.581281	lncRNA
ENSG0000(	ENSG0000(	100.7348	-0.18581	0.159425	0.581479	lncRNA
ENSG0000(	PKD2	708.6432	-0.21049	0.15942	0.581479	protein_coding
ENSG0000(	PRPS2	332.5226	-0.21615	0.159405	0.581479	protein_coding
ENSG0000(	CA4	1225.622	0.534937	0.159458	0.581523	protein_coding
ENSG0000(	SLBP	243.5988	-0.15848	0.159508	0.581628	protein_coding
ENSG0000(	ENSG0000(	600.2406	0.154536	0.159555	0.581692	lncRNA
ENSG0000(	MAP7D2	52.80395	0.465828	0.159568	0.581692	protein_coding
ENSG0000(	DOK2	146.0936	-0.22726	0.159615	0.581788	protein_coding
ENSG0000(	ENSG0000(	168.9534	0.179017	0.159638	0.581793	lncRNA
ENSG0000(	BACE1	676.9596	-0.12949	0.159693	0.581915	protein_coding
ENSG0000(	B4GALT1	3290.807	-0.2506	0.159726	0.58196	protein_coding
ENSG0000(	BCCIP	392.8928	-0.12264	0.159855	0.582271	protein_coding
ENSG0000(	CAPN5	3530.204	0.272034	0.159836	0.582271	protein_coding
ENSG0000(	DNAJC12	53.34657	0.284649	0.159915	0.582271	protein_coding
ENSG0000(	FAM180A	26.57381	-0.44185	0.159917	0.582271	protein_coding
ENSG0000(	PHPT1	425.9751	0.211344	0.159882	0.582271	protein_coding
ENSG0000(	COA7	1123.841	0.100211	0.160021	0.582298	protein_coding
ENSG0000(	ENSG0000(	38.37777	0.45971	0.16003	0.582298	lncRNA
ENSG0000(	HMGB3P4	179.2399	-0.1414	0.159974	0.582298	processed_pseudogene
ENSG0000(	SAR1B	4423.03	-0.05855	0.160014	0.582298	protein_coding
ENSG0000(	SYP	438.0472	0.124516	0.159953	0.582298	protein_coding
ENSG0000(	BICD2	896.2347	-0.14111	0.160119	0.582467	protein_coding
ENSG0000(	WDR6	2674.62	0.12831	0.160114	0.582467	protein_coding
ENSG0000(	AAR2	670.4238	0.09666	0.160327	0.582606	protein_coding
ENSG0000(	ARHGAP42	1368.661	0.219888	0.160319	0.582606	protein_coding
ENSG0000(	DLGAP1	105.6147	0.286865	0.160281	0.582606	protein_coding

ENSG0000(	ENSG0000(	94.62209	0.238659	0.160207	0.582606	lncRNA
ENSG0000(	ENSG0000(	52.31805	0.21867	0.160309	0.582606	lncRNA
ENSG0000(	SOS1	5673.03	-0.08301	0.160195	0.582606	protein_coding
ENSG0000(	SPDYA	273.2782	0.169712	0.160265	0.582606	protein_coding
ENSG0000(	STK40	978.773	-0.1542	0.160236	0.582606	protein_coding
ENSG0000(	FBXO41	507.6952	-0.25933	0.160381	0.582726	protein_coding
ENSG0000(	ENSG0000(	289.6415	0.119657	0.160469	0.582779	lncRNA
ENSG0000(	MCRIP2	1104.314	0.263128	0.16048	0.582779	protein_coding
ENSG0000(	MT-TK	85.14843	-0.59409	0.160422	0.582779	Mt_tRNA
ENSG0000(	Y_RNA	3626.905	-0.14968	0.160459	0.582779	misc_RNA
ENSG0000(	CCR3	73.4286	-0.3238	0.160537	0.582908	protein_coding
ENSG0000(	PRKCZ	974.1012	0.173259	0.160564	0.582929	protein_coding
ENSG0000(	ARPIN	3034.83	0.147079	0.160605	0.583002	protein_coding
ENSG0000(	ENSG0000(	86.5256	-0.20979	0.160699	0.583187	lncRNA
ENSG0000(	MFF-DT	1175.855	0.080986	0.16072	0.583187	lncRNA
ENSG0000(	MUL1	220.9232	0.184927	0.160691	0.583187	protein_coding
ENSG0000(	ENSG0000(	213.7779	0.153516	0.160777	0.58324	lncRNA
ENSG0000(	TMEM63B	774.9044	0.188161	0.160761	0.58324	protein_coding
ENSG0000(	GJA6P	31.15823	-0.29167	0.160821	0.583246	processed_pseudogene
ENSG0000(	LGI2	99.3302	-0.50587	0.160817	0.583246	protein_coding
ENSG0000(	XRCC5	2399.982	-0.08017	0.16087	0.583346	protein_coding
ENSG0000(	ENSG0000(	111.9951	-0.24097	0.1609	0.583381	lncRNA
ENSG0000(	EFCAB8	51.94068	-0.44906	0.160994	0.583638	protein_coding
ENSG0000(	ENC1	3153.887	-0.16807	0.161156	0.583638	protein_coding
ENSG0000(	ENSG0000(	258.3793	-0.13655	0.16113	0.583638	TEC
ENSG0000(	GOLGA6L4	241.8702	-0.24871	0.161076	0.583638	protein_coding
ENSG0000(	RGS17	399.9307	-0.10738	0.161162	0.583638	protein_coding
ENSG0000(	UGT1A2P	94.73787	0.61449	0.161161	0.583638	unprocessed_pseudogene
ENSG0000(	VWCE	105.8286	-0.17158	0.161128	0.583638	protein_coding
ENSG0000(	ZNF25	397.6077	-0.15621	0.161131	0.583638	protein_coding
ENSG0000(	ZNF428	120.5877	-0.17964	0.161108	0.583638	protein_coding
ENSG0000(	RN7SL847F	60.7819	0.224046	0.161233	0.583742	misc_RNA
ENSG0000(	TBC1D2	466.0731	0.177776	0.161226	0.583742	protein_coding
ENSG0000(	CD180	567.8038	-0.39734	0.161285	0.583773	protein_coding
ENSG0000(	CUTALP	1917.682	0.247801	0.16128	0.583773	transcribed_unitary_pseudogene
ENSG0000(	GADD45G	51.41358	-0.31258	0.16132	0.583826	protein_coding
ENSG0000(	API5	1261.348	-0.10234	0.161351	0.583859	protein_coding
ENSG0000(	AARSD1	606.0229	0.126342	0.161484	0.584062	protein_coding
ENSG0000(	CYS1	40.84809	0.417733	0.161492	0.584062	protein_coding
ENSG0000(	DRAM2	774.5185	-0.16664	0.161475	0.584062	protein_coding
ENSG0000(	VPS54	1473.903	-0.04928	0.16146	0.584062	protein_coding
ENSG0000(	ALDH4A1	87.44715	-0.2505	0.162002	0.584111	protein_coding
ENSG0000(	BEND7	664.9759	0.169385	0.16162	0.584111	protein_coding
ENSG0000(	CUX2	22.5013	-0.79417	0.161991	0.584111	protein_coding
ENSG0000(	ENGASE	2527.753	0.149579	0.161981	0.584111	protein_coding
ENSG0000(	ENSG0000(	211.8789	-0.16265	0.161967	0.584111	lncRNA
ENSG0000(	ENSG0000(	793.0592	0.140601	0.161599	0.584111	lncRNA

ENSG0000(	ENSG0000(	25.71968	-0.31122	0.161865	0.584111	lncRNA
ENSG0000(	ENSG0000(	231.3229	-0.15887	0.162046	0.584111	lncRNA
ENSG0000(	ENSG0000(	81.26403	0.217216	0.161654	0.584111	lncRNA
ENSG0000(	ENSG0000(	78.28723	-0.26449	0.161979	0.584111	TEC
ENSG0000(	ENSG0000(	20.63423	-0.36245	0.1621	0.584111	TEC
ENSG0000(	ENSG0000(	66.06937	-0.239	0.16186	0.584111	TEC
ENSG0000(	ENSG0000(	57.07298	0.211528	0.161797	0.584111	lncRNA
ENSG0000(	ENSG0000(	112.5917	0.202718	0.162081	0.584111	lncRNA
ENSG0000(	ENSG0000(	223.4106	0.157329	0.161556	0.584111	lncRNA
ENSG0000(	FOXK2	1414.243	0.117124	0.161846	0.584111	protein_coding
ENSG0000(	LDB1	1713.431	0.093907	0.161819	0.584111	protein_coding
ENSG0000(	MEIS1	1085.272	-0.29331	0.162041	0.584111	protein_coding
ENSG0000(	METTL7B	183.9175	0.443692	0.161869	0.584111	protein_coding
ENSG0000(	MMAA	503.9863	-0.15558	0.161556	0.584111	protein_coding
ENSG0000(	RN7SL116F	75.25762	-0.33648	0.161859	0.584111	misc_RNA
ENSG0000(	SCHIP1	543.0454	-0.12735	0.161916	0.584111	protein_coding
ENSG0000(	SEMA3D	232.3154	0.143386	0.162012	0.584111	protein_coding
ENSG0000(	SMC2	499.764	-0.20565	0.161913	0.584111	protein_coding
ENSG0000(	SOGA1	2136.239	-0.13656	0.162094	0.584111	protein_coding
ENSG0000(	TINAGL1	1493.955	0.203449	0.162024	0.584111	protein_coding
ENSG0000(	USP34	9324.632	-0.07973	0.161956	0.584111	protein_coding
ENSG0000(	WDR43	666.7418	0.138548	0.161821	0.584111	protein_coding
ENSG0000(	ABCG4	25.88991	0.366628	0.162123	0.584119	protein_coding
ENSG0000(	ATP5MC1	470.3233	0.223368	0.162177	0.584235	protein_coding
ENSG0000(	CD27	118.5005	-0.36351	0.162205	0.584263	protein_coding
ENSG0000(	AMFR	978.3748	-0.12003	0.162419	0.584506	protein_coding
ENSG0000(	ENSG0000(	500.5193	0.085249	0.162385	0.584506	lncRNA
ENSG0000(	ENSG0000(	76.41591	0.157647	0.162427	0.584506	lncRNA
ENSG0000(	ENSG0000(	239.2178	0.107408	0.162462	0.584506	lncRNA
ENSG0000(	GASK1A	8362.602	0.159144	0.162401	0.584506	protein_coding
ENSG0000(	IMPDH1	366.8704	-0.22236	0.162464	0.584506	protein_coding
ENSG0000(	PGLS	395.2578	0.117202	0.162416	0.584506	protein_coding
ENSG0000(	PSMD1	3191.376	-0.11154	0.162428	0.584506	protein_coding
ENSG0000(	ZSCAN2	404.7249	0.169146	0.162362	0.584506	protein_coding
ENSG0000(	RGS13	160.1001	-0.30193	0.162487	0.584513	protein_coding
ENSG0000(	DOCK1	4442.153	0.231907	0.162718	0.584585	protein_coding
ENSG0000(	ENSG0000(	139.9421	0.145992	0.162529	0.584585	lncRNA
ENSG0000(	ENSG0000(	54.01222	0.527995	0.162733	0.584585	lncRNA
ENSG0000(	ENSG0000(	23.24143	0.340649	0.162636	0.584585	lncRNA
ENSG0000(	KLK4	106.2424	0.380878	0.162732	0.584585	protein_coding
ENSG0000(	LINC00937	2273.374	0.206064	0.162826	0.584585	lncRNA
ENSG0000(	LPCAT2	682.1264	-0.22274	0.162808	0.584585	protein_coding
ENSG0000(	PKD1L2	839.9171	-0.08155	0.162653	0.584585	polymorphic_pseudogene
ENSG0000(	RN7SKP23	240.3109	0.1016	0.162648	0.584585	TEC
ENSG0000(	RPS12P3	27.42238	0.34024	0.162679	0.584585	processed_pseudogene
ENSG0000(	RTCA-AS1	7072.091	0.097285	0.162753	0.584585	lncRNA
ENSG0000(	SIPA1L3	3739.876	0.142588	0.162635	0.584585	protein_coding

ENSG0000(	SMPD2	208.6849	0.170261	0.162782	0.584585	protein_coding
ENSG0000(	SNORD110	24.14699	-0.35166	0.1626	0.584585	snoRNA
ENSG0000(	STK24	4768.857	-0.18388	0.162821	0.584585	protein_coding
ENSG0000(	B3GNT5	1846.166	0.232679	0.162874	0.584585	protein_coding
ENSG0000(	CRTC3	1747.406	-0.14597	0.162881	0.584585	protein_coding
ENSG0000(	CTXN2-AS1	29.12873	-0.35853	0.162961	0.584585	lncRNA
ENSG0000(	ENSG0000(	96.02747	0.14781	0.162917	0.584585	lncRNA
ENSG0000(	ENSG0000(	338.502	0.1173	0.162953	0.584585	TEC
ENSG0000(	FAM181A-/	360.7384	0.094472	0.162948	0.584585	lncRNA
ENSG0000(	NADSYN1	4549.869	0.131588	0.162975	0.584585	protein_coding
ENSG0000(	A1CF	1821.366	0.36455	0.163082	0.584642	protein_coding
ENSG0000(	ENSG0000(	38.62384	0.294912	0.163117	0.584642	lncRNA
ENSG0000(	EPB41L2	4944.92	-0.1481	0.163062	0.584642	protein_coding
ENSG0000(	ERLEC1	2411.082	-0.09963	0.163118	0.584642	protein_coding
ENSG0000(	HACD4	687.4414	-0.14347	0.163076	0.584642	protein_coding
ENSG0000(	RGPD2	601.6228	-0.83463	0.163071	0.584642	protein_coding
ENSG0000(	ENSG0000(	23.84985	-0.38382	0.163163	0.584726	processed_pseudogene
ENSG0000(	ENSG0000(	423.7056	0.181964	0.163228	0.584808	lncRNA
ENSG0000(	LINC00501	253.5425	0.141325	0.163219	0.584808	lncRNA
ENSG0000(	CALCOCO1	2010.053	-0.11233	0.163271	0.584883	protein_coding
ENSG0000(	RBM26-AS:	544.1777	-0.10232	0.163309	0.584943	lncRNA
ENSG0000(	USP27X	63.19961	-0.23068	0.16333	0.584943	protein_coding
ENSG0000(	ENSG0000(	260.9894	0.130312	0.163369	0.585005	lncRNA
ENSG0000(	CACTIN	662.8823	0.099561	0.163436	0.585171	protein_coding
ENSG0000(	CDKN1C	46.75658	-0.31365	0.163488	0.585196	protein_coding
ENSG0000(	ENSG0000(	22.18348	0.361443	0.163563	0.585196	lncRNA
ENSG0000(	ENSG0000(	111.8412	0.284648	0.163568	0.585196	unprocessed_pseudogene
ENSG0000(	MZT1	74.41329	-0.25017	0.163571	0.585196	protein_coding
ENSG0000(	NUDT12	229.6872	0.297409	0.16355	0.585196	protein_coding
ENSG0000(	TRAPPC12	2098.924	0.065936	0.163482	0.585196	protein_coding
ENSG0000(	ENSG0000(	71.40592	-0.30946	0.163678	0.585429	lncRNA
ENSG0000(	SLC22A18	1045.344	0.301345	0.163676	0.585429	protein_coding
ENSG0000(	FOXD2	260.5448	0.351926	0.163772	0.585689	protein_coding
ENSG0000(	ENSG0000(	67.76549	0.176242	0.163863	0.585758	lncRNA
ENSG0000(	ENSG0000(	151.7886	0.162849	0.16392	0.585758	lncRNA
ENSG0000(	ENSG0000(	4221.12	0.045863	0.16383	0.585758	lncRNA
ENSG0000(	GSTO1	141.3784	-0.23113	0.163882	0.585758	protein_coding
ENSG0000(	SMIM36	202.4058	-0.15076	0.163861	0.585758	protein_coding
ENSG0000(	ZNF791	2831.886	0.118488	0.163916	0.585758	protein_coding
ENSG0000(	OLFML2A	587.7793	-0.1579	0.164011	0.586008	protein_coding
ENSG0000(	ARMCX2	224.2566	-0.29724	0.164073	0.586044	protein_coding
ENSG0000(	P4HA2	674.4849	0.143216	0.164054	0.586044	protein_coding
ENSG0000(	PSMC3IP	77.56861	-0.2418	0.164085	0.586044	protein_coding
ENSG0000(	NME3	132.7773	0.204421	0.164204	0.586241	protein_coding
ENSG0000(	SNCAIP	294.6888	-0.27328	0.164166	0.586241	protein_coding
ENSG0000(	ZKSCAN1	7298.935	0.098882	0.164194	0.586241	protein_coding
ENSG0000(	ARHGEF2	3559.336	0.111883	0.164285	0.586454	protein_coding

ENSG0000(	SPRY2	742.4542	0.213579	0.164326	0.586526	protein_coding
ENSG0000(	ENSG0000(	695.7684	0.099125	0.164375	0.586548	lncRNA
ENSG0000(	FLII	1527.375	0.123049	0.164368	0.586548	protein_coding
ENSG0000(	AGO2	4339.444	-0.11822	0.164513	0.586897	protein_coding
ENSG0000(	BTN3A2	4245.486	-0.16561	0.164533	0.586897	protein_coding
ENSG0000(	SHANK3	2014.827	-0.24943	0.164551	0.586897	protein_coding
ENSG0000(	SRP54-AS1	314.015	-0.12289	0.164558	0.586897	transcribed_unitary_pseudogene
ENSG0000(	MYL6B	219.5177	-0.13481	0.164581	0.586902	protein_coding
ENSG0000(	ENSG0000(	29.06566	0.299387	0.164647	0.587012	lncRNA
ENSG0000(	SOD3	110.4443	-0.27664	0.164654	0.587012	protein_coding
ENSG0000(	ARL5B	1358.316	-0.1385	0.164689	0.587059	protein_coding
ENSG0000(	ZNF710	990.6049	0.146883	0.164711	0.587063	protein_coding
ENSG0000(	ERRFI1	2559.569	-0.34729	0.164753	0.587137	protein_coding
ENSG0000(	DVL1	613.1542	0.181682	0.164801	0.587198	protein_coding
ENSG0000(	EIF4E2	2748.442	0.100237	0.164864	0.587198	protein_coding
ENSG0000(	ENSG0000(	50.0778	0.344561	0.164877	0.587198	lncRNA
ENSG0000(	ENSG0000(	140.4927	-0.21554	0.164836	0.587198	lncRNA
ENSG0000(	ZDBF2	722.0585	-0.18376	0.164828	0.587198	protein_coding
ENSG0000(	ENSG0000(	54.14923	0.229928	0.164908	0.587232	lncRNA
ENSG0000(	ZBED6CL	413.3412	-0.184	0.165	0.587483	protein_coding
ENSG0000(	CDR2L	81.06516	-0.38502	0.165058	0.587536	protein_coding
ENSG0000(	NBPF12	1665.447	0.144335	0.165056	0.587536	protein_coding
ENSG0000(	ENSG0000(	2681.554	-0.08538	0.165123	0.587608	lncRNA
ENSG0000(	PLCG1-AS1	542.1573	0.091259	0.165144	0.587608	lncRNA
ENSG0000(	ZBTB20	8074.053	-0.11379	0.165159	0.587608	protein_coding
ENSG0000(	ZNF880	335.6447	0.112694	0.165163	0.587608	protein_coding
ENSG0000(	GLUD1	4143.826	0.091317	0.165189	0.587622	protein_coding
ENSG0000(	DNMBP	2739.525	0.20907	0.165377	0.587932	protein_coding
ENSG0000(	ENSG0000(	656.1727	0.08115	0.165337	0.587932	lncRNA
ENSG0000(	LINC01909	176.6848	0.144783	0.165404	0.587932	lncRNA
ENSG0000(	RN7SL728F	31.75892	0.418811	0.16534	0.587932	misc_RNA
ENSG0000(	TEX11	47.65526	0.33685	0.165359	0.587932	protein_coding
ENSG0000(	TUFT1	680.2854	0.223568	0.165391	0.587932	protein_coding
ENSG0000(	C2CD2	1182.611	-0.17497	0.165456	0.587958	protein_coding
ENSG0000(	ENSG0000(	625.852	0.222033	0.165497	0.587958	lncRNA
ENSG0000(	ENSG0000(	37.44989	-0.36294	0.165496	0.587958	transcribed_processed_pseudoge
ENSG0000(	LONP2	4109.732	0.085818	0.165452	0.587958	protein_coding
ENSG0000(	BIRC2	2487.056	-0.12766	0.165631	0.587979	protein_coding
ENSG0000(	CACNG2	22.24507	0.518126	0.165638	0.587979	protein_coding
ENSG0000(	CARD8-AS1	207.0793	-0.17045	0.165529	0.587979	lncRNA
ENSG0000(	ENSG0000(	20.64496	0.467379	0.165633	0.587979	lncRNA
ENSG0000(	ENSG0000(	23.44973	-0.32716	0.16563	0.587979	lncRNA
ENSG0000(	NUBPL	916.6656	0.089652	0.165652	0.587979	protein_coding
ENSG0000(	ZNF80	59.61278	0.353313	0.165622	0.587979	protein_coding
ENSG0000(	ACBD4	417.7183	0.145122	0.16593	0.58813	protein_coding
ENSG0000(	ENSG0000(	58.32635	0.258178	0.1659	0.58813	lncRNA
ENSG0000(	ENSG0000(	22.79676	-0.39046	0.165725	0.58813	lncRNA

ENSG0000(	LINC00390	119.4823	-0.21899	0.165878	0.58813	lncRNA
ENSG0000(	LMNTD2	86.62843	0.220248	0.165753	0.58813	protein_coding
ENSG0000(	RAMACL	172.1434	-0.29879	0.16591	0.58813	protein_coding
ENSG0000(	RGS19	193.2256	-0.28763	0.165761	0.58813	protein_coding
ENSG0000(	STXBP2	1117.56	0.147432	0.165793	0.58813	protein_coding
ENSG0000(	SYDE2	554.1474	0.172755	0.165901	0.58813	protein_coding
ENSG0000(	TSPYL2	575.1204	-0.19316	0.165887	0.58813	protein_coding
ENSG0000(	VPS13C	11413.85	-0.10271	0.16592	0.58813	protein_coding
ENSG0000(	ENSG0000(	63.17704	0.261744	0.165989	0.588264	lncRNA
ENSG0000(	ENSG0000(	26.81134	0.413033	0.166054	0.588417	lncRNA
ENSG0000(	NIT2	1180.664	-0.08525	0.166142	0.588654	protein_coding
ENSG0000(	ALDOB	558.1268	-0.68676	0.166271	0.588746	protein_coding
ENSG0000(	AMOT	385.9061	-0.30138	0.166234	0.588746	protein_coding
ENSG0000(	ENSG0000(	25.68556	0.539123	0.166296	0.588746	unprocessed_pseudogene
ENSG0000(	GEMIN7	187.8467	-0.20337	0.166199	0.588746	protein_coding
ENSG0000(	HSPE1	299.6335	-0.17528	0.166292	0.588746	protein_coding
ENSG0000(	PTPN11	3014.143	-0.08667	0.166265	0.588746	protein_coding
ENSG0000(	SCAMP2	1933.493	0.178339	0.166319	0.588751	protein_coding
ENSG0000(	RNY1	805.4919	-0.21695	0.16641	0.588921	misc_RNA
ENSG0000(	TIMM13	317.8583	0.186836	0.166393	0.588921	protein_coding
ENSG0000(	FOXA1	520.1259	0.305018	0.166444	0.588964	protein_coding
ENSG0000(	SERINC1	1795.355	-0.10987	0.166491	0.589055	protein_coding
ENSG0000(	ADAMTS16	698.8763	0.126317	0.166537	0.589066	lncRNA
ENSG0000(	SRP14	783.669	-0.14235	0.166518	0.589066	protein_coding
ENSG0000(	C7orf25	206.7907	-0.17813	0.166717	0.589309	protein_coding
ENSG0000(	CUL1	1023.702	-0.10296	0.166699	0.589309	protein_coding
ENSG0000(	HK1	1360.572	-0.12855	0.166711	0.589309	protein_coding
ENSG0000(	PABPC4L	27.48695	-0.37614	0.166648	0.589309	protein_coding
ENSG0000(	SH3BP5	741.6546	-0.1737	0.166734	0.589309	protein_coding
ENSG0000(	SLC25A21-/	35.86438	0.329481	0.166676	0.589309	lncRNA
ENSG0000(	LINC02503	3778.666	0.171932	0.166786	0.589418	lncRNA
ENSG0000(	KRBOX4	632.0044	-0.12136	0.166824	0.589477	protein_coding
ENSG0000(	ENSG0000(	536.9729	0.108887	0.166846	0.589479	lncRNA
ENSG0000(	CORO1A	1251.843	-0.30402	0.166871	0.58949	protein_coding
ENSG0000(	BSCL2	755.5261	-0.09846	0.167143	0.589698	protein_coding
ENSG0000(	CARMIL2	343.485	-0.34742	0.167091	0.589698	protein_coding
ENSG0000(	ENSG0000(	81.62381	-0.23749	0.16709	0.589698	lncRNA
ENSG0000(	ENSG0000(	120.0412	0.397212	0.167132	0.589698	lncRNA
ENSG0000(	ENSG0000(	52.49023	-0.26783	0.167144	0.589698	lncRNA
ENSG0000(	ENSG0000(	151.9107	-0.15497	0.167007	0.589698	lncRNA
ENSG0000(	LINC02073	470.1912	-0.08958	0.167159	0.589698	lncRNA
ENSG0000(	RER1	2384.871	0.076003	0.167047	0.589698	protein_coding
ENSG0000(	THAP2	155.4292	-0.17537	0.166967	0.589698	protein_coding
ENSG0000(	TNFSF18	59.15257	-0.23132	0.167165	0.589698	protein_coding
ENSG0000(	TSC22D4	696.3947	-0.13386	0.167037	0.589698	protein_coding
ENSG0000(	ENSG0000(	120.9304	-0.2369	0.167221	0.589742	lncRNA
ENSG0000(	ENSG0000(	401.8951	0.113393	0.167212	0.589742	lncRNA

ENSG0000(	RN7SL615F	65.89382	-0.21324	0.167305	0.589964	misc_RNA
ENSG0000(	ENSG0000(	20.64454	-0.51931	0.16737	0.590103	lncRNA
ENSG0000(	GALNT7	1845.678	0.208693	0.167387	0.590103	protein_coding
ENSG0000(	ANKRD44	3362.738	-0.24793	0.167672	0.5904	protein_coding
ENSG0000(	ARHGAP30	1188.132	-0.28578	0.167772	0.5904	protein_coding
ENSG0000(	B9D1	240.2171	-0.1867	0.167605	0.5904	protein_coding
ENSG0000(	C4orf19	1474.923	0.161958	0.167689	0.5904	protein_coding
ENSG0000(	DPH6	815.8663	0.110926	0.167546	0.5904	protein_coding
ENSG0000(	ENSG0000(	77.94589	-0.14576	0.167733	0.5904	lncRNA
ENSG0000(	GPR173	221.0777	-0.17289	0.167553	0.5904	protein_coding
ENSG0000(	KATNBL1P2	55.70243	-0.32604	0.167696	0.5904	processed_pseudogene
ENSG0000(	MTFR2P1	44.40495	-0.26585	0.167522	0.5904	processed_pseudogene
ENSG0000(	NANP	198.2214	-0.129	0.167663	0.5904	protein_coding
ENSG0000(	NCAPD2	899.4083	0.162564	0.16758	0.5904	protein_coding
ENSG0000(	RN7SL424F	22.08279	0.353469	0.167765	0.5904	misc_RNA
ENSG0000(	ZBTB48	646.9258	0.138964	0.167679	0.5904	protein_coding
ENSG0000(	ZNF615	404.4992	-0.15542	0.167734	0.5904	protein_coding
ENSG0000(	ENSG0000(	61.58454	0.27267	0.167806	0.590444	lncRNA
ENSG0000(	OR6U2P	261.7883	0.11928	0.167891	0.590666	transcribed_unprocessed_pseudo
ENSG0000(	MT-TQ	166.5607	-0.74603	0.167921	0.590698	Mt_tRNA
ENSG0000(	ENSG0000(	490.8964	0.091085	0.168003	0.590805	lncRNA
ENSG0000(	RPS6P25	33.07374	0.321984	0.16798	0.590805	processed_pseudogene
ENSG0000(	USP9YP4	27.71258	0.271181	0.168016	0.590805	unprocessed_pseudogene
ENSG0000(	ENSG0000(	296.7432	0.101749	0.16806	0.59081	lncRNA
ENSG0000(	TCF19	346.589	-0.17521	0.168044	0.59081	protein_coding
ENSG0000(	UBE3B	1984.394	0.0847	0.168101	0.590878	protein_coding
ENSG0000(	ARF5	385.0735	0.153023	0.168132	0.59091	protein_coding
ENSG0000(	MIEN1	222.3062	0.236446	0.168176	0.590916	protein_coding
ENSG0000(	TMEM273	458.2731	-0.21207	0.168166	0.590916	protein_coding
ENSG0000(	ENSG0000(	138.1568	0.488062	0.168202	0.59093	unprocessed_pseudogene
ENSG0000(	KCNT1	183.1807	-0.39325	0.168238	0.590981	protein_coding
ENSG0000(	ARVCF	229.3034	0.328235	0.168303	0.591054	protein_coding
ENSG0000(	ENSG0000(	753.0981	-0.18726	0.168323	0.591054	lncRNA
ENSG0000(	SLC3A1	792.8357	0.236489	0.168321	0.591054	protein_coding
ENSG0000(	ACR	28.76248	0.499425	0.16842	0.591242	protein_coding
ENSG0000(	AP4S1	1467.547	0.063821	0.168399	0.591242	protein_coding
ENSG0000(	ALG6	443.6937	0.128336	0.168507	0.591322	protein_coding
ENSG0000(	ENSG0000(	174.2201	0.144464	0.168485	0.591322	lncRNA
ENSG0000(	TMSB15B-A	155.2571	-0.2225	0.168506	0.591322	lncRNA
ENSG0000(	UGT1A4	264.6282	0.53348	0.168564	0.591447	protein_coding
ENSG0000(	OR8B9P	540.46	0.113997	0.168618	0.591562	transcribed_unprocessed_pseudo
ENSG0000(	ACOT9	1520.023	-0.1024	0.168797	0.591672	protein_coding
ENSG0000(	CERNA2	421.0892	-0.19149	0.168686	0.591672	lncRNA
ENSG0000(	COL28A1	626.8891	-0.16277	0.168791	0.591672	protein_coding
ENSG0000(	ENSG0000(	36.48491	0.321762	0.168791	0.591672	lncRNA
ENSG0000(	MRPL53	95.24042	-0.24857	0.168776	0.591672	protein_coding
ENSG0000(	RNLS	83.79118	0.268976	0.168728	0.591672	protein_coding

ENSG0000(	SNX31	20.44879	0.358261	0.1688	0.591672	protein_coding
ENSG0000(	ANKRD16	446.2448	-0.11682	0.169641	0.592016	protein_coding
ENSG0000(	AOAH-IT1	147.4437	-0.24816	0.169579	0.592016	lncRNA
ENSG0000(	APPBP2-DT	73.41088	0.192238	0.169236	0.592016	lncRNA
ENSG0000(	ARHGEF37	612.8187	0.273531	0.169017	0.592016	protein_coding
ENSG0000(	ATAD3B	978.8923	0.13639	0.169061	0.592016	protein_coding
ENSG0000(	CMIP	3450.884	-0.10031	0.169602	0.592016	protein_coding
ENSG0000(	DCAF1	875.3054	0.085277	0.169121	0.592016	protein_coding
ENSG0000(	DOLK	85.27861	-0.22259	0.169563	0.592016	protein_coding
ENSG0000(	ENSG0000(	43.88949	0.275802	0.169146	0.592016	lncRNA
ENSG0000(	ENSG0000(	254.9073	-0.20559	0.169056	0.592016	lncRNA
ENSG0000(	ENSG0000(	194.1617	0.229592	0.168951	0.592016	lncRNA
ENSG0000(	ENSG0000(	69.05983	0.389642	0.169165	0.592016	lncRNA
ENSG0000(	FBRS	1776.337	-0.10781	0.169381	0.592016	protein_coding
ENSG0000(	FRG1HP	1288.411	-0.12412	0.16918	0.592016	transcribed_unprocessed_pseudo
ENSG0000(	GPATCH11	461.0503	-0.13619	0.169071	0.592016	protein_coding
ENSG0000(	KALRN	2227.812	0.125582	0.169479	0.592016	protein_coding
ENSG0000(	LINC01503	1005.816	-0.12288	0.169351	0.592016	lncRNA
ENSG0000(	LINC02291	54.80058	-0.25453	0.169646	0.592016	lncRNA
ENSG0000(	MANCR	192.5998	0.178332	0.169469	0.592016	lncRNA
ENSG0000(	METTL21C	193.8537	0.211142	0.169652	0.592016	protein_coding
ENSG0000(	MGAT5	2800.671	0.159829	0.169425	0.592016	protein_coding
ENSG0000(	MIR194-2H	4114.286	0.285849	0.169502	0.592016	lncRNA
ENSG0000(	MIR4441	129.3033	-0.21292	0.169364	0.592016	miRNA
ENSG0000(	MYLK4	92.92815	0.282993	0.169075	0.592016	protein_coding
ENSG0000(	NBPF19	4781.727	0.211533	0.16927	0.592016	protein_coding
ENSG0000(	OR2A25	57.63653	0.242487	0.169184	0.592016	protein_coding
ENSG0000(	PID1	600.4266	-0.20192	0.169439	0.592016	protein_coding
ENSG0000(	PRR15	828.189	0.425177	0.16944	0.592016	protein_coding
ENSG0000(	PYHIN1	427.0036	-0.18753	0.169533	0.592016	protein_coding
ENSG0000(	SIRT7	1754.577	0.151104	0.169079	0.592016	protein_coding
ENSG0000(	SPINT1-AS1	117.8636	0.299506	0.169002	0.592016	lncRNA
ENSG0000(	TIPARP	1041.025	-0.17881	0.169349	0.592016	protein_coding
ENSG0000(	TSPAN31	948.948	-0.07911	0.169516	0.592016	protein_coding
ENSG0000(	VWA5B1	229.8174	0.16345	0.169337	0.592016	protein_coding
ENSG0000(	XRN2	803.6829	-0.12261	0.169457	0.592016	protein_coding
ENSG0000(	ENSG0000(	88.53945	-0.21913	0.169684	0.592053	lncRNA
ENSG0000(	ZMYM4	1647.913	-0.14986	0.16971	0.592068	protein_coding
ENSG0000(	LGALS9B	183.742	0.394656	0.169755	0.592151	protein_coding
ENSG0000(	CD9	1711.492	0.270709	0.169863	0.59223	protein_coding
ENSG0000(	ENSG0000(	35.0846	-0.24687	0.169971	0.59223	lncRNA
ENSG0000(	ENSG0000(	64.28397	0.205172	0.169928	0.59223	lncRNA
ENSG0000(	ENSG0000(	54.1958	0.249782	0.169867	0.59223	lncRNA
ENSG0000(	LINC01327	31.78765	-0.24977	0.169967	0.59223	lncRNA
ENSG0000(	MYSM1	4201.143	0.093221	0.169861	0.59223	protein_coding
ENSG0000(	NARF-AS2	126.5911	-0.23475	0.169923	0.59223	lncRNA
ENSG0000(	SLC12A2	5341.153	0.234398	0.169853	0.59223	protein_coding

ENSG0000(	ZNF44	1528.06	0.155507	0.169945	0.59223	protein_coding
ENSG0000(	LRRTM4	342.2974	0.13606	0.170027	0.592349	protein_coding
ENSG0000(	KANSL1L	1024.966	-0.10759	0.170079	0.592381	protein_coding
ENSG0000(	ZNF814	3152.26	0.105007	0.170069	0.592381	protein_coding
ENSG0000(	LRP2	103.7804	-0.41324	0.170178	0.592651	protein_coding
ENSG0000(	CEP350	4397.356	-0.12936	0.170535	0.592984	protein_coding
ENSG0000(	DAPK1	1365.465	-0.18279	0.1703	0.592984	protein_coding
ENSG0000(	ENSG0000(	28.76893	0.38781	0.170834	0.592984	lncRNA
ENSG0000(	ENSG0000(	2224.584	0.133786	0.170705	0.592984	lncRNA
ENSG0000(	ENSG0000(	727.1889	0.199944	0.170752	0.592984	lncRNA
ENSG0000(	ENSG0000(	21.38777	-0.46343	0.170753	0.592984	TEC
ENSG0000(	ENSG0000(	147.5718	-0.16148	0.170506	0.592984	lncRNA
ENSG0000(	ENSG0000(	100.6793	-0.16828	0.170412	0.592984	lncRNA
ENSG0000(	ENSG0000(	970.0004	0.132933	0.170452	0.592984	lncRNA
ENSG0000(	FAT2	25.54788	-0.48851	0.170433	0.592984	protein_coding
ENSG0000(	FBXO22	2447.659	-0.12294	0.170461	0.592984	protein_coding
ENSG0000(	HHEX	143.8496	-0.35446	0.170686	0.592984	protein_coding
ENSG0000(	ID2	476.6645	0.238505	0.170646	0.592984	protein_coding
ENSG0000(	INHBA-AS1	153.9795	0.190653	0.170329	0.592984	lncRNA
ENSG0000(	JADE3	258.9264	0.179724	0.17055	0.592984	protein_coding
ENSG0000(	LGALS9C	652.1019	0.426141	0.170382	0.592984	protein_coding
ENSG0000(	LGALSL	625.6354	0.283187	0.170701	0.592984	protein_coding
ENSG0000(	MIR1267	31.90463	-0.34307	0.170812	0.592984	miRNA
ENSG0000(	MIR2052H(	47.34351	-0.28702	0.170729	0.592984	lncRNA
ENSG0000(	OSGIN2	294.5829	-0.21138	0.170701	0.592984	protein_coding
ENSG0000(	RPL26P30	180.2131	-0.1751	0.170807	0.592984	transcribed_processed_pseudoge
ENSG0000(	RRAS	139.2666	-0.24801	0.170762	0.592984	protein_coding
ENSG0000(	TPPP	111.4537	0.264332	0.170833	0.592984	protein_coding
ENSG0000(	TPSB2	263.476	-0.28315	0.170461	0.592984	protein_coding
ENSG0000(	TVP23A	167.1845	-0.25793	0.170733	0.592984	protein_coding
ENSG0000(	ZDHHC20	1620.744	-0.11948	0.170799	0.592984	protein_coding
ENSG0000(	LYPLAL1	239.8254	-0.17563	0.170897	0.593128	protein_coding
ENSG0000(	RBX1	429.8557	-0.13982	0.170951	0.593164	protein_coding
ENSG0000(	SEC1P	105.3185	-0.20655	0.17095	0.593164	transcribed_unitary_pseudogene
ENSG0000(	ENSG0000(	199.8971	-0.14521	0.170989	0.593221	lncRNA
ENSG0000(	CCR5AS	244.9657	-0.13752	0.171077	0.593294	lncRNA
ENSG0000(	ENSG0000(	108.0441	0.187315	0.171075	0.593294	lncRNA
ENSG0000(	ENSG0000(	24.64619	0.425986	0.17112	0.593294	lncRNA
ENSG0000(	GIMAP6	451.388	-0.21704	0.17111	0.593294	protein_coding
ENSG0000(	TPRG1L	644.1698	0.208182	0.171139	0.593294	protein_coding
ENSG0000(	ZNF432	823.3986	0.144292	0.171078	0.593294	protein_coding
ENSG0000(	ENSG0000(	75.27863	0.201907	0.171274	0.593335	processed_pseudogene
ENSG0000(	ENSG0000(	117.6249	-0.1644	0.171281	0.593335	lncRNA
ENSG0000(	ENSG0000(	181.6454	0.153411	0.171221	0.593335	lncRNA
ENSG0000(	ENSG0000(	109.9724	-0.20584	0.171256	0.593335	lncRNA
ENSG0000(	RN7SL457F	22.97311	-0.30059	0.171193	0.593335	misc_RNA
ENSG0000(	TENT4A	991.0391	-0.12319	0.171218	0.593335	protein_coding

ENSG0000(	MLANA	278.7309	0.169944	0.171331	0.593437	protein_coding
ENSG0000(	HNRNPA1P	89.10397	-0.19344	0.171365	0.593479	processed_pseudogene
ENSG0000(	ENSG0000(	416.7595	0.153821	0.171436	0.59365	lncRNA
ENSG0000(	ENSG0000(	457.6136	0.136058	0.17155	0.593794	lncRNA
ENSG0000(	ENSG0000(	43.19169	-0.29979	0.171503	0.593794	TEC
ENSG0000(	FAM3A	801.1478	0.133193	0.171541	0.593794	protein_coding
ENSG0000(	FOXF2	154.6474	-0.23318	0.171633	0.593794	protein_coding
ENSG0000(	GTF2IP11	49.57366	0.230555	0.171648	0.593794	unprocessed_pseudogene
ENSG0000(	IGHJ6	222.7873	-0.36535	0.171672	0.593794	IG_J_gene
ENSG0000(	TM4SF20	235.028	-0.57959	0.171666	0.593794	protein_coding
ENSG0000(	ZNF449	360.1962	-0.14343	0.171603	0.593794	protein_coding
ENSG0000(	ZSCAN12	1200.821	0.079244	0.17167	0.593794	protein_coding
ENSG0000(	SRP19	1898.568	-0.0899	0.171836	0.594288	protein_coding
ENSG0000(	ENSG0000(	699.3371	0.158062	0.171863	0.594305	lncRNA
ENSG0000(	ENSG0000(	422.2557	0.13017	0.171964	0.59458	protein_coding
ENSG0000(	ENSG0000(	3705.242	0.154422	0.171994	0.594608	TEC
ENSG0000(	ENSG0000(	46.30029	-0.22123	0.172159	0.595105	lncRNA
ENSG0000(	ENSG0000(	41.80035	-0.41933	0.17219	0.595138	lncRNA
ENSG0000(	ENSG0000(	40.8412	-0.25948	0.172237	0.595224	lncRNA
ENSG0000(	ALMS1P1	400.2504	0.146754	0.172335	0.595241	transcribed_unprocessed_pseudo
ENSG0000(	ENSG0000(	222.4108	0.180172	0.172359	0.595241	TEC
ENSG0000(	LINC01016	984.6669	-0.12186	0.172458	0.595241	lncRNA
ENSG0000(	LINC02298	313.6455	0.191291	0.172396	0.595241	lncRNA
ENSG0000(	MIR3679	25.21946	0.404871	0.172316	0.595241	miRNA
ENSG0000(	MORC4	5424.935	0.112728	0.172367	0.595241	protein_coding
ENSG0000(	NAGK	2997.812	-0.11293	0.172448	0.595241	protein_coding
ENSG0000(	PITPNM3	980.9463	0.235321	0.172365	0.595241	protein_coding
ENSG0000(	SEMA3B	897.3517	0.277708	0.172434	0.595241	protein_coding
ENSG0000(	SYT10	59.42573	0.457234	0.172448	0.595241	protein_coding
ENSG0000(	EXOC7	3543.839	0.093227	0.172545	0.595329	protein_coding
ENSG0000(	TMEM243	394.4199	-0.19795	0.172548	0.595329	protein_coding
ENSG0000(	WDFY4	2938.827	-0.33414	0.172524	0.595329	protein_coding
ENSG0000(	ENSG0000(	28.30986	0.251342	0.172644	0.595517	lncRNA
ENSG0000(	GTF3A	462.7179	-0.17253	0.172665	0.595517	protein_coding
ENSG0000(	H2AC11	114.9187	-0.25799	0.172668	0.595517	protein_coding
ENSG0000(	DHFR2	391.0638	-0.14029	0.172722	0.595627	protein_coding
ENSG0000(	PRKD3	1679.675	-0.16331	0.172812	0.595866	protein_coding
ENSG0000(	CYP4F26P	140.8516	0.179378	0.172929	0.59606	lncRNA
ENSG0000(	DACT1	244.2083	-0.19976	0.173075	0.59606	protein_coding
ENSG0000(	ENSG0000(	85.98449	-0.21182	0.172917	0.59606	lncRNA
ENSG0000(	ENSG0000(	334.8821	0.158153	0.173133	0.59606	lncRNA
ENSG0000(	ENSG0000(	197.2626	0.130939	0.173043	0.59606	lncRNA
ENSG0000(	EPHB3	489.8216	0.33575	0.173017	0.59606	protein_coding
ENSG0000(	LINC01356	827.0553	-0.1104	0.173063	0.59606	lncRNA
ENSG0000(	NBDY	128.0141	-0.21106	0.173066	0.59606	protein_coding
ENSG0000(	RUNDC3A-/	789.1963	0.074845	0.173054	0.59606	lncRNA
ENSG0000(	SMIM7	1564.195	0.068408	0.172969	0.59606	protein_coding

ENSG0000(	SNORA74B	243.2011	0.294884	0.173098	0.59606	snoRNA
ENSG0000(	SRC	1785.021	0.136357	0.173114	0.59606	protein_coding
ENSG0000(	ZNF275	1023.417	-0.13832	0.17315	0.59606	protein_coding
ENSG0000(	TMEM154	1326.75	-0.27072	0.173366	0.596728	protein_coding
ENSG0000(	CD8B	119.5659	-0.36265	0.173435	0.596782	protein_coding
ENSG0000(	CTSO	1151.459	-0.09217	0.173426	0.596782	protein_coding
ENSG0000(	ENSG0000(	22.56983	0.396781	0.173468	0.596782	snoRNA
ENSG0000(	SNORD101	21.64837	-0.42711	0.173462	0.596782	snoRNA
ENSG0000(	ITPK1	1293.748	0.150884	0.173578	0.597084	protein_coding
ENSG0000(	RRP8	1185.32	0.099232	0.17364	0.597221	protein_coding
ENSG0000(	TBCCD1	606.1183	-0.14063	0.173665	0.597232	protein_coding
ENSG0000(	ENSG0000(	31.08055	-0.4256	0.173734	0.597321	lncRNA
ENSG0000(	KLF2-DT	27.05467	-0.33414	0.173714	0.597321	lncRNA
ENSG0000(	ENSG0000(	773.6733	0.139806	0.17379	0.59744	lncRNA
ENSG0000(	DTX3	366.2957	-0.16189	0.173847	0.597462	protein_coding
ENSG0000(	ENSG0000(	125.2189	-0.17613	0.173878	0.597462	lncRNA
ENSG0000(	RPS19BP1	505.8012	0.101992	0.173852	0.597462	protein_coding
ENSG0000(	TTBK2	1386.299	-0.14615	0.173883	0.597462	protein_coding
ENSG0000(	CA15P1	22.01409	-0.39198	0.174012	0.597532	unitary_pseudogene
ENSG0000(	ENSG0000(	45.42629	-0.30559	0.173962	0.597532	lncRNA
ENSG0000(	FAM24B	76.91593	0.276785	0.173985	0.597532	protein_coding
ENSG0000(	HSBP1L1	643.2693	0.244261	0.173992	0.597532	protein_coding
ENSG0000(	N4BP2L1	1059.542	-0.15945	0.173938	0.597532	protein_coding
ENSG0000(	DSCAM-AS	38.53064	-0.29536	0.174224	0.597569	lncRNA
ENSG0000(	ENSG0000(	148.1667	0.220912	0.174161	0.597569	lncRNA
ENSG0000(	ENSG0000(	45.52667	0.250668	0.174186	0.597569	lncRNA
ENSG0000(	ENSG0000(	258.9782	0.122902	0.174192	0.597569	lncRNA
ENSG0000(	FOXRED2	796.9796	-0.09155	0.174229	0.597569	protein_coding
ENSG0000(	INSL3	34.81395	-0.32264	0.174131	0.597569	protein_coding
ENSG0000(	LINC02483	21.58433	-0.38696	0.174108	0.597569	lncRNA
ENSG0000(	MAT2B	879.6724	-0.158	0.174172	0.597569	protein_coding
ENSG0000(	NEK1	1385.527	-0.08691	0.174207	0.597569	protein_coding
ENSG0000(	ZBED1	1158.793	0.143395	0.17424	0.597569	protein_coding
ENSG0000(	CMSS1	1533.075	-0.0739	0.174396	0.597658	protein_coding
ENSG0000(	ENSG0000(	33.87885	0.391775	0.174357	0.597658	lncRNA
ENSG0000(	LMBR1L	1224.562	-0.09143	0.174382	0.597658	protein_coding
ENSG0000(	NUMB	3708.269	-0.07806	0.174363	0.597658	protein_coding
ENSG0000(	PGAP2	431.0409	0.155568	0.174379	0.597658	protein_coding
ENSG0000(	RASA3	887.2316	-0.19688	0.17429	0.597658	protein_coding
ENSG0000(	HDAC8	3733.523	-0.10088	0.174457	0.597789	protein_coding
ENSG0000(	BBS10	247.9627	-0.16678	0.174669	0.597997	protein_coding
ENSG0000(	EHMT2	1127.712	-0.08186	0.174668	0.597997	protein_coding
ENSG0000(	ENSG0000(	41.75941	-0.41528	0.174587	0.597997	lncRNA
ENSG0000(	ENSG0000(	719.187	0.290145	0.174631	0.597997	lncRNA
ENSG0000(	GLRA3	121.5284	0.2565	0.174647	0.597997	protein_coding
ENSG0000(	LINC02482	241.2126	0.147387	0.174572	0.597997	lncRNA
ENSG0000(	PRAMEF9	41.99149	0.245818	0.174611	0.597997	protein_coding

ENSG0000(	ELOVL1	599.6878	0.156517	0.174778	0.598002	protein_coding
ENSG0000(	GOLGA8M	66.65566	-0.4728	0.174747	0.598002	protein_coding
ENSG0000(	KIAA0087	69.76673	0.183115	0.17478	0.598002	lncRNA
ENSG0000(	MXD3	239.9519	-0.20816	0.17474	0.598002	protein_coding
ENSG0000(	VPS52	1001.57	0.093566	0.174764	0.598002	protein_coding
ENSG0000(	BCYRN1	429.2199	0.156499	0.175058	0.598208	scRNA
ENSG0000(	ELOF1	397.8873	0.13303	0.174987	0.598208	protein_coding
ENSG0000(	ENSG0000(	30.76452	-0.40681	0.17502	0.598208	lncRNA
ENSG0000(	ENSG0000(	80.68307	0.275891	0.174966	0.598208	lncRNA
ENSG0000(	ENSG0000(	155.9175	0.161955	0.175079	0.598208	TEC
ENSG0000(	FNBP1L	1659.036	0.178479	0.175011	0.598208	protein_coding
ENSG0000(	NAA60	2004.47	0.086649	0.174961	0.598208	protein_coding
ENSG0000(	PCCA	2029.025	0.120384	0.175021	0.598208	protein_coding
ENSG0000(	THBS4-AS1	299.9904	0.178998	0.175047	0.598208	lncRNA
ENSG0000(	USP29	33.36484	0.284921	0.175036	0.598208	protein_coding
ENSG0000(	ZNF584	384.9482	0.148896	0.174913	0.598208	protein_coding
ENSG0000(	RN7SKP13:	34.72878	0.390536	0.175118	0.598267	misc_RNA
ENSG0000(	ATP23	578.4277	0.145373	0.175203	0.598483	protein_coding
ENSG0000(	NPNT	1415.933	0.261186	0.175262	0.59861	protein_coding
ENSG0000(	ENSG0000(	234.2062	-0.14532	0.175374	0.59867	lncRNA
ENSG0000(	ENSG0000(	35.01103	-0.7671	0.175343	0.59867	lncRNA
ENSG0000(	FRA10AC1	1063.583	0.137746	0.175413	0.59867	protein_coding
ENSG0000(	PKNOX1	1080.817	0.09154	0.175432	0.59867	protein_coding
ENSG0000(	PRICKLE3	244.0773	0.167449	0.175406	0.59867	protein_coding
ENSG0000(	RN7SL600F	268.4532	-0.26075	0.175424	0.59867	misc_RNA
ENSG0000(	SDR42E2	446.5722	-0.12528	0.17543	0.59867	protein_coding
ENSG0000(	LAT	706.3818	-0.15709	0.175494	0.598732	protein_coding
ENSG0000(	SNORA80B	66.40949	0.254928	0.175481	0.598732	snoRNA
ENSG0000(	STK17B	3473.288	-0.21668	0.175621	0.599093	protein_coding
ENSG0000(	CAVIN2	116.242	-0.27216	0.175698	0.599182	protein_coding
ENSG0000(	ENSG0000(	411.3004	-0.132	0.175734	0.599182	lncRNA
ENSG0000(	LINC02422	196.5429	-0.44431	0.175731	0.599182	lncRNA
ENSG0000(	SNORA17B	180.7146	0.257888	0.17573	0.599182	snoRNA
ENSG0000(	AGPAT1	467.7094	-0.13438	0.175849	0.599194	protein_coding
ENSG0000(	ENSG0000(	41.82303	-0.33008	0.175857	0.599194	lncRNA
ENSG0000(	ENSG0000(	121.9343	-0.15098	0.17581	0.599194	processed_pseudogene
ENSG0000(	LINC02454	40.77863	-0.3175	0.175888	0.599194	lncRNA
ENSG0000(	NEXN	311.698	-0.19325	0.175824	0.599194	protein_coding
ENSG0000(	RBM38-AS:	53.49497	-0.23866	0.17589	0.599194	lncRNA
ENSG0000(	RGPD1	234.9545	-0.74972	0.175775	0.599194	protein_coding
ENSG0000(	DOCK5	6538.303	0.148213	0.17595	0.599277	protein_coding
ENSG0000(	TUT4	2925.782	-0.15047	0.175958	0.599277	protein_coding
ENSG0000(	ENSG0000(	557.3746	0.093593	0.176024	0.599353	lncRNA
ENSG0000(	LINC00639	576.8697	-0.12554	0.176005	0.599353	lncRNA
ENSG0000(	GPC4	382.5608	-0.22942	0.176075	0.599452	protein_coding
ENSG0000(	SNED1	1341.139	-0.21024	0.176102	0.59947	protein_coding
ENSG0000(	PPARGC1A	2220.483	0.414219	0.176196	0.599646	protein_coding

ENSG0000(	PPP1R14B	323.5459	-0.17129	0.176197	0.599646	protein_coding
ENSG0000(	ENSG0000(	238.943	0.124576	0.17631	0.59988	TEC
ENSG0000(	MSANTD1	156.6171	0.153283	0.176302	0.59988	protein_coding
ENSG0000(	ENSG0000(	271.3374	0.164913	0.176392	0.600088	lncRNA
ENSG0000(	ABO	3388.416	0.178187	0.176575	0.600356	protein_coding
ENSG0000(	CTBP1	2872.88	0.065344	0.176616	0.600356	protein_coding
ENSG0000(	ENSG0000(	24.10783	-0.43351	0.176568	0.600356	lncRNA
ENSG0000(	ENSG0000(	20.35278	0.345353	0.176643	0.600356	lncRNA
ENSG0000(	FAR2P1	324.7475	0.132508	0.176592	0.600356	transcribed_unprocessed_pseudo
ENSG0000(	SSRP1	811.7083	-0.14101	0.176496	0.600356	protein_coding
ENSG0000(	TAF8	2555.772	-0.05967	0.176549	0.600356	protein_coding
ENSG0000(	TGIF2	598.6577	-0.20108	0.176646	0.600356	protein_coding
ENSG0000(	POLR1C	562.9389	0.09847	0.176668	0.600357	protein_coding
ENSG0000(	FAM182A	298.5148	0.132572	0.176772	0.600495	lncRNA
ENSG0000(	LANCL3	456.6961	0.320434	0.176765	0.600495	protein_coding
ENSG0000(	PITPNA-AS:	20.86231	-0.37133	0.176774	0.600495	lncRNA
ENSG0000(	NRXN2	213.2071	-0.33479	0.176816	0.600511	protein_coding
ENSG0000(	TSTD1	239.1821	0.225303	0.176823	0.600511	protein_coding
ENSG0000(	EIF1	2651.551	-0.10332	0.176885	0.600641	protein_coding
ENSG0000(	LINC02848	584.5159	0.081557	0.176915	0.600641	lncRNA
ENSG0000(	SV2B	207.0923	0.24046	0.176926	0.600641	protein_coding
ENSG0000(	EFCAB2	871.2672	0.197369	0.176949	0.600644	protein_coding
ENSG0000(	ARL10	1667.747	-0.15015	0.177005	0.600685	protein_coding
ENSG0000(	ZC3H8	970.5681	0.111323	0.176984	0.600685	protein_coding
ENSG0000(	BYSL	95.45952	-0.18664	0.177063	0.600734	protein_coding
ENSG0000(	ENSG0000(	27.46861	0.450059	0.17705	0.600734	lncRNA
ENSG0000(	CERS6	2642.396	0.177641	0.177175	0.600745	protein_coding
ENSG0000(	ENSG0000(	47.73775	-0.28418	0.177156	0.600745	processed_pseudogene
ENSG0000(	ENSG0000(	955.2236	-0.08684	0.177131	0.600745	TEC
ENSG0000(	FDCSP	106.2366	-0.81066	0.177116	0.600745	protein_coding
ENSG0000(	RIPOR2	2414.008	-0.41639	0.177143	0.600745	protein_coding
ENSG0000(	ADCY2	954.5046	-0.11523	0.177267	0.600833	protein_coding
ENSG0000(	ENSG0000(	384.0951	-0.13354	0.177235	0.600833	lncRNA
ENSG0000(	OR10H1	1075.861	0.108895	0.177263	0.600833	protein_coding
ENSG0000(	SYNE3	2355.526	-0.15404	0.177294	0.600852	protein_coding
ENSG0000(	ENSG0000(	178.0862	-0.2114	0.177361	0.601003	TEC
ENSG0000(	ENSG0000(	689.4576	0.14922	0.1774	0.60104	lncRNA
ENSG0000(	FRMD6	457.6756	-0.26495	0.177437	0.60104	protein_coding
ENSG0000(	RN7SKP253	33.13971	0.329245	0.177421	0.60104	misc_RNA
ENSG0000(	TTN-AS1	2188.665	-0.1019	0.177478	0.601105	lncRNA
ENSG0000(	DEF8	965.1191	-0.18437	0.177575	0.601287	protein_coding
ENSG0000(	RN7SL749F	99.99896	-0.2585	0.177575	0.601287	misc_RNA
ENSG0000(	ENSG0000(	225.4103	0.246972	0.177685	0.601477	protein_coding
ENSG0000(	FLJ40194	309.9734	-0.20813	0.177697	0.601477	lncRNA
ENSG0000(	MICD	47.46255	0.255848	0.177686	0.601477	unprocessed_pseudogene
ENSG0000(	C21orf62-A	1642.481	0.056163	0.177801	0.601541	lncRNA
ENSG0000(	CEP76	535.8113	0.090617	0.177749	0.601541	protein_coding

ENSG0000(	DNM2	12753.42	0.165486	0.177825	0.601541	protein_coding
ENSG0000(	RPAP2	9175.001	0.094938	0.177825	0.601541	protein_coding
ENSG0000(	ZNF843	285.2319	0.103346	0.177807	0.601541	protein_coding
ENSG0000(	ADPGK	2700.997	-0.10799	0.178153	0.601591	protein_coding
ENSG0000(	CIBAR1P2	134.487	0.184994	0.178113	0.601591	transcribed_processed_pseudoge
ENSG0000(	EML3	816.1482	-0.14807	0.17824	0.601591	protein_coding
ENSG0000(	ENSG0000(	130.5354	-0.2614	0.178001	0.601591	lncRNA
ENSG0000(	ENSG0000(	71.36729	0.261454	0.178108	0.601591	lncRNA
ENSG0000(	ENSG0000(	30.2975	0.489038	0.178137	0.601591	processed_pseudogene
ENSG0000(	ENSG0000(	87.08975	-0.19953	0.177862	0.601591	lncRNA
ENSG0000(	ENSG0000(	191.0091	-0.13636	0.178321	0.601591	TEC
ENSG0000(	ENSG0000(	216.7127	0.124293	0.178202	0.601591	lncRNA
ENSG0000(	ENSG0000(	33.89926	-0.26575	0.178045	0.601591	lncRNA
ENSG0000(	FAM21FP	39.66723	-0.42143	0.17823	0.601591	transcribed_unprocessed_pseudo
ENSG0000(	KCNK13	21.24894	-0.46294	0.178088	0.601591	protein_coding
ENSG0000(	LRRC14	783.7378	0.125754	0.178071	0.601591	protein_coding
ENSG0000(	LSM10	131.8041	-0.20905	0.17804	0.601591	protein_coding
ENSG0000(	MGP	292.3773	-0.30475	0.178298	0.601591	protein_coding
ENSG0000(	MIIP	202.6801	-0.18006	0.178304	0.601591	protein_coding
ENSG0000(	SIDT2	1628.9	0.12053	0.178081	0.601591	protein_coding
ENSG0000(	SPINK5	93.12484	0.424149	0.178251	0.601591	protein_coding
ENSG0000(	STOX1	72.69657	0.339002	0.177939	0.601591	protein_coding
ENSG0000(	TIMM50	1485.69	0.094923	0.178286	0.601591	protein_coding
ENSG0000(	WASH6P	2378.824	0.119894	0.178263	0.601591	protein_coding
ENSG0000(	XCR1	143.5635	-0.34026	0.177975	0.601591	protein_coding
ENSG0000(	ANKRD36B	37.42011	-0.27067	0.178372	0.601666	transcribed_processed_pseudoge
ENSG0000(	ENSG0000(	272.4268	-0.13505	0.178389	0.601666	TEC
ENSG0000(	ZNF513	431.434	0.146863	0.178409	0.601666	protein_coding
ENSG0000(	ENSG0000(	98.84635	0.197289	0.178498	0.601817	lncRNA
ENSG0000(	KDM4A	1549.495	0.154397	0.178494	0.601817	protein_coding
ENSG0000(	AOX2P	90.98874	0.151544	0.178524	0.601826	transcribed_unprocessed_pseudo
ENSG0000(	ENSG0000(	38.36108	-0.37264	0.178581	0.601826	TEC
ENSG0000(	GOLGA6L9	1073.437	0.153204	0.178558	0.601826	protein_coding
ENSG0000(	YTHDC2	1794.633	0.109671	0.178588	0.601826	protein_coding
ENSG0000(	CELF4	109.4937	0.333639	0.178614	0.601839	protein_coding
ENSG0000(	ENSG0000(	617.8841	0.103021	0.17871	0.601924	lncRNA
ENSG0000(	IDUA	330.2091	0.192744	0.178745	0.601924	protein_coding
ENSG0000(	PIDD1	484.0239	0.108876	0.178663	0.601924	protein_coding
ENSG0000(	QSER1	2248.999	-0.0967	0.17874	0.601924	protein_coding
ENSG0000(	SNORD14C	84.60243	-0.25585	0.178748	0.601924	snoRNA
ENSG0000(	CEBPA-DT	142.3222	0.303868	0.178797	0.602015	lncRNA
ENSG0000(	LGALS14	51.62593	0.226683	0.178881	0.602224	protein_coding
ENSG0000(	ACD	106.2408	-0.27262	0.178903	0.602226	protein_coding
ENSG0000(	SGK3	1381.791	0.073423	0.178931	0.602245	protein_coding
ENSG0000(	KIAA0895	150.4638	-0.17428	0.178954	0.602249	protein_coding
ENSG0000(	ENSG0000(	43.28788	0.252178	0.178976	0.60225	lncRNA
ENSG0000(	ATP5F1D	451.6952	0.187826	0.179048	0.602344	protein_coding

ENSG0000(	ENSG0000(	31.9086	0.256699	0.179038	0.602344	TEC
ENSG0000(	GPR174	1367.255	0.292369	0.179124	0.602526	protein_coding
ENSG0000(	GNB4	1259.534	-0.15578	0.179193	0.602685	protein_coding
ENSG0000(	LINC01341	232.7688	0.132402	0.179263	0.602846	lncRNA
ENSG0000(	EML2	930.5092	-0.09214	0.179288	0.602858	protein_coding
ENSG0000(	C2CD4A	213.114	-0.23597	0.179419	0.602944	protein_coding
ENSG0000(	ENSG0000(	493.7714	0.097054	0.179471	0.602944	lncRNA
ENSG0000(	ENSG0000(	197.8974	-0.40974	0.179472	0.602944	lncRNA
ENSG0000(	NEXMIF	99.11589	0.219779	0.179489	0.602944	protein_coding
ENSG0000(	PIGW	294.0294	0.132219	0.179465	0.602944	protein_coding
ENSG0000(	POLD1	288.8758	0.191614	0.179447	0.602944	protein_coding
ENSG0000(	RBM5	7097.071	0.052131	0.179439	0.602944	protein_coding
ENSG0000(	SYCP3	264.1943	0.150883	0.179462	0.602944	protein_coding
ENSG0000(	ENSG0000(	108.194	0.163212	0.179523	0.602953	lncRNA
ENSG0000(	HLF	167.3601	-0.33181	0.179648	0.602953	protein_coding
ENSG0000(	MAP1LC3A	173.2296	0.212478	0.179622	0.602953	protein_coding
ENSG0000(	MAP2K3	817.5184	-0.16397	0.179673	0.602953	protein_coding
ENSG0000(	RNF113A	81.56122	-0.22553	0.179623	0.602953	protein_coding
ENSG0000(	SYNJ1	943.0366	-0.11759	0.179546	0.602953	protein_coding
ENSG0000(	TCAF1	1263.51	0.149649	0.179655	0.602953	protein_coding
ENSG0000(	TMEM232	129.6426	0.245148	0.179689	0.602953	protein_coding
ENSG0000(	WDR62	587.2694	-0.09439	0.179573	0.602953	protein_coding
ENSG0000(	AP1G2-AS1	600.7876	-0.10943	0.179774	0.603091	lncRNA
ENSG0000(	HSPA8	4532.11	-0.1573	0.179756	0.603091	protein_coding
ENSG0000(	ENSG0000(	44.95118	-0.27403	0.179844	0.603105	lncRNA
ENSG0000(	TMEM62	772.2602	-0.09205	0.17982	0.603105	protein_coding
ENSG0000(	ZNF563	376.5062	0.109163	0.179825	0.603105	protein_coding
ENSG0000(	BPIFB1	107.1841	-0.33787	0.179899	0.603142	protein_coding
ENSG0000(	RAP1B	5359.683	-0.09763	0.179897	0.603142	protein_coding
ENSG0000(	ENSG0000(	25.41501	0.524636	0.179989	0.603371	lncRNA
ENSG0000(	ENSG0000(	58.17326	0.21375	0.180088	0.603419	lncRNA
ENSG0000(	KRBA1	140.3957	-0.26992	0.180091	0.603419	protein_coding
ENSG0000(	RASA2-IT1	107.4592	-0.16171	0.180031	0.603419	lncRNA
ENSG0000(	SMG5	1855.805	0.111167	0.180085	0.603419	protein_coding
ENSG0000(	ASCC1	860.1584	-0.15051	0.180184	0.603655	protein_coding
ENSG0000(	H2BC15	402.0775	-0.2494	0.18034	0.603937	protein_coding
ENSG0000(	NDUFS7	1118.015	0.105323	0.180356	0.603937	protein_coding
ENSG0000(	RN7SL149F	27.83272	0.36489	0.180294	0.603937	misc_RNA
ENSG0000(	SLC22A5	4382.568	0.325866	0.180353	0.603937	protein_coding
ENSG0000(	RN7SL473F	179.6246	-0.23235	0.18042	0.60408	misc_RNA
ENSG0000(	CDH6	396.2853	-0.24008	0.180532	0.604381	protein_coding
ENSG0000(	ENSG0000(	40.88053	0.346273	0.180558	0.604395	lncRNA
ENSG0000(	BAG3	250.837	-0.19682	0.180631	0.604419	protein_coding
ENSG0000(	PSMA1	774.2693	-0.10448	0.180602	0.604419	protein_coding
ENSG0000(	SLC35E2B	5282.654	0.079781	0.180621	0.604419	protein_coding
ENSG0000(	SALL2	89.24369	-0.30217	0.180698	0.604568	protein_coding
ENSG0000(	CCNB1IP1	558.1185	0.106794	0.180804	0.604665	protein_coding

ENSG0000(	SLC36A3	96.9204	0.163327	0.180784	0.604665	protein_coding
ENSG0000(	SLC38A11	199.2345	0.258515	0.180815	0.604665	protein_coding
ENSG0000(	ZNF271P	991.4644	-0.07245	0.180764	0.604665	transcribed_unitary_pseudogene
ENSG0000(	ARMC6	299.6429	0.155024	0.180849	0.604681	protein_coding
ENSG0000(	B3GNT6	894.2575	0.292308	0.180886	0.604681	protein_coding
ENSG0000(	PPM1N	912.6474	0.307919	0.180883	0.604681	protein_coding
ENSG0000(	ENSG0000(	110.2456	0.20059	0.181011	0.604806	lncRNA
ENSG0000(	GPR39	413.8044	0.351596	0.180947	0.604806	protein_coding
ENSG0000(	LRRC37A16	35.51319	0.52956	0.180994	0.604806	transcribed_unprocessed_pseudo
ENSG0000(	SLC16A5	542.6956	0.259542	0.181008	0.604806	protein_coding
ENSG0000(	DAGLB	633.36	0.109329	0.181165	0.604856	protein_coding
ENSG0000(	ENSG0000(	216.8185	0.11984	0.181137	0.604856	transcribed_processed_pseudoge
ENSG0000(	ENSG0000(	190.6518	0.168048	0.181258	0.604856	lncRNA
ENSG0000(	ENSG0000(	72.01932	0.227131	0.181178	0.604856	lncRNA
ENSG0000(	ENSG0000(	283.7235	0.127729	0.181363	0.604856	processed_pseudogene
ENSG0000(	ENSG0000(	20.18199	0.365615	0.181103	0.604856	lncRNA
ENSG0000(	ENSG0000(	92.92527	-0.19393	0.181098	0.604856	lncRNA
ENSG0000(	ENSG0000(	51.89561	0.2623	0.1814	0.604856	lncRNA
ENSG0000(	INPP5D	2024.024	-0.24506	0.181333	0.604856	protein_coding
ENSG0000(	KIF16B	2372.709	0.258286	0.181353	0.604856	protein_coding
ENSG0000(	LYSMD3	874.6855	-0.12332	0.181357	0.604856	protein_coding
ENSG0000(	Metazoa_S	19947.54	0.058559	0.181224	0.604856	misc_RNA
ENSG0000(	RASA4CP	328.8781	-0.17721	0.181388	0.604856	transcribed_unprocessed_pseudo
ENSG0000(	RGS7BP	52.19026	-0.32882	0.181255	0.604856	protein_coding
ENSG0000(	SPOCK1	58.61362	-0.5104	0.181156	0.604856	protein_coding
ENSG0000(	TOMM20	1407.338	-0.10423	0.181383	0.604856	protein_coding
ENSG0000(	TREX2	24.2758	0.393335	0.18118	0.604856	protein_coding
ENSG0000(	BMP2K-DT	69.9983	0.170566	0.181429	0.60486	lncRNA
ENSG0000(	KLHDC10	1434.441	0.110697	0.181445	0.60486	protein_coding
ENSG0000(	FBXO2	71.17926	-0.24414	0.181468	0.604864	protein_coding
ENSG0000(	ENSG0000(	145.2878	0.154156	0.181586	0.60501	lncRNA
ENSG0000(	ICAM2	308.3687	-0.16781	0.181547	0.60501	protein_coding
ENSG0000(	MYO15B	9647.3	0.197976	0.1816	0.60501	protein_coding
ENSG0000(	RN7SL525F	150.2437	0.156511	0.181578	0.60501	misc_RNA
ENSG0000(	ENSG0000(	508.7748	-0.10874	0.181655	0.605121	lncRNA
ENSG0000(	C1orf21	2371.087	0.207439	0.18175	0.605216	protein_coding
ENSG0000(	HIP1	1202.972	-0.21815	0.181717	0.605216	protein_coding
ENSG0000(	STING1	1194.256	-0.14838	0.181746	0.605216	protein_coding
ENSG0000(	ARMT1	191.5965	-0.15022	0.181844	0.605243	protein_coding
ENSG0000(	ENSG0000(	264.942	0.101342	0.181834	0.605243	lncRNA
ENSG0000(	GCOM1	481.8802	0.373064	0.181868	0.605243	protein_coding
ENSG0000(	LINC02540	180.1076	0.158507	0.181854	0.605243	lncRNA
ENSG0000(	RNU6-100F	24.2314	-0.29698	0.181841	0.605243	snRNA
ENSG0000(	ENSG0000(	265.9524	0.139692	0.182012	0.605649	lncRNA
ENSG0000(	ENSG0000(	76.04574	-0.15989	0.182073	0.605758	lncRNA
ENSG0000(	FAM102A	2372.301	0.170117	0.182088	0.605758	protein_coding
ENSG0000(	ENSG0000(	70.10098	-0.2133	0.182125	0.605805	lncRNA

ENSG0000(	FHL2	2286.079	-0.18286	0.182173	0.605867	protein_coding
ENSG0000(	GCG	73.19618	0.557652	0.182216	0.605867	protein_coding
ENSG0000(	PDCL3P4	1393.516	0.064689	0.182231	0.605867	transcribed_processed_pseudoge
ENSG0000(	PRKCH	1383.431	-0.14675	0.182197	0.605867	protein_coding
ENSG0000(	PKP3	691.9221	0.287899	0.182276	0.605942	protein_coding
ENSG0000(	ENSG0000(	1869.933	0.104726	0.182327	0.606039	lncRNA
ENSG0000(	ATG16L2	2542.366	-0.13209	0.182434	0.606322	protein_coding
ENSG0000(	CYB5D1	53.78361	-0.40938	0.182511	0.606364	protein_coding
ENSG0000(	EDA	183.701	0.271343	0.182601	0.606364	protein_coding
ENSG0000(	ENSG0000(	100.9302	-0.2154	0.182536	0.606364	lncRNA
ENSG0000(	ENSG0000(	25.51093	-0.35183	0.182551	0.606364	processed_pseudogene
ENSG0000(	LINC00887	207.2112	0.181544	0.182578	0.606364	lncRNA
ENSG0000(	PTPN22	612.6151	-0.15486	0.182486	0.606364	protein_coding
ENSG0000(	STXBP5L	30.52284	0.564916	0.182582	0.606364	protein_coding
ENSG0000(	SLC44A4	3198.86	0.256809	0.182671	0.606521	protein_coding
ENSG0000(	PIP4P2	93.49263	-0.41191	0.182789	0.606768	protein_coding
ENSG0000(	RN7SL714F	26.96778	0.261657	0.182775	0.606768	misc_RNA
ENSG0000(	ENSG0000(	99.96036	0.275091	0.182863	0.606887	lncRNA
ENSG0000(	MIR3976H(	125.4936	0.227907	0.182869	0.606887	lncRNA
ENSG0000(	CATSPER2	1514.312	0.170002	0.183007	0.607132	protein_coding
ENSG0000(	CDC42EP5	232.4767	0.309256	0.182984	0.607132	protein_coding
ENSG0000(	TUBBP10	22.65889	0.912263	0.183009	0.607132	processed_pseudogene
ENSG0000(	ENSG0000(	48.82772	-0.23912	0.183044	0.607175	TEC
ENSG0000(	EPCAM-DT	217.9708	0.191219	0.183069	0.607185	lncRNA
ENSG0000(	TMEM82	41.68167	0.429959	0.183092	0.607186	protein_coding
ENSG0000(	FER	1611.338	-0.10075	0.183131	0.607242	protein_coding
ENSG0000(	ENSG0000(	26.72358	0.364824	0.183195	0.607308	lncRNA
ENSG0000(	REG4	676.1251	-0.68557	0.183184	0.607308	protein_coding
ENSG0000(	DTX3L	3018.34	-0.15637	0.183356	0.607331	protein_coding
ENSG0000(	ENSG0000(	56.41581	-0.26729	0.183257	0.607331	lncRNA
ENSG0000(	ENSG0000(	26.16399	0.282695	0.183339	0.607331	lncRNA
ENSG0000(	GARNL3	353.3412	0.170865	0.183348	0.607331	protein_coding
ENSG0000(	LRRC56	211.94	0.218428	0.183324	0.607331	protein_coding
ENSG0000(	SLC20A1	9222.584	0.417894	0.183273	0.607331	protein_coding
ENSG0000(	WBP11P2	328.9946	0.108671	0.18326	0.607331	processed_pseudogene
ENSG0000(	NOL4L	3098.325	0.130403	0.183418	0.607389	protein_coding
ENSG0000(	TEX30	60.79966	-0.26696	0.1834	0.607389	protein_coding
ENSG0000(	SNHG5	2854.775	-0.12161	0.183461	0.60746	lncRNA
ENSG0000(	SEC23A	2523.946	0.100787	0.183487	0.607471	protein_coding
ENSG0000(	ENSG0000(	61.32825	-0.32508	0.183526	0.607529	lncRNA
ENSG0000(	USP2	420.2544	0.521175	0.183554	0.607549	protein_coding
ENSG0000(	LINC00987	88.15094	-0.24479	0.183626	0.607713	lncRNA
ENSG0000(	LINC00485	109.5897	0.188737	0.183655	0.607735	lncRNA
ENSG0000(	ENSG0000(	203.1018	-0.15912	0.183752	0.607765	lncRNA
ENSG0000(	ENSG0000(	37.75926	-0.3676	0.183745	0.607765	lncRNA
ENSG0000(	INPP5J	460.0401	0.459094	0.183737	0.607765	protein_coding
ENSG0000(	ZADH2	1856.632	0.115985	0.183703	0.607765	protein_coding

ENSG0000(	ADAM20	155.8449	-0.1975	0.183789	0.607813	protein_coding
ENSG0000(	CCDC113	285.6016	0.142336	0.183818	0.607836	protein_coding
ENSG0000(	ENSG0000(	55.12211	-0.29235	0.183841	0.607839	processed_pseudogene
ENSG0000(	ASB13	408.7223	0.225817	0.18392	0.607952	protein_coding
ENSG0000(	TGFB1	543.0279	-0.24001	0.183963	0.607952	protein_coding
ENSG0000(	TRAF7	843.6611	0.144867	0.183963	0.607952	protein_coding
ENSG0000(	XIAP	3998.267	-0.16931	0.183954	0.607952	protein_coding
ENSG0000(	CENPW	25.70612	-0.41272	0.184104	0.608215	protein_coding
ENSG0000(	ENSG0000(	248.5621	0.157531	0.184078	0.608215	lncRNA
ENSG0000(	FAM110C	769.9748	0.280703	0.184109	0.608215	protein_coding
ENSG0000(	CDKN2B	1412.375	0.46887	0.184214	0.608422	protein_coding
ENSG0000(	SCN4B	108.1701	0.316577	0.184216	0.608422	protein_coding
ENSG0000(	ENSG0000(	148.6499	0.116225	0.184278	0.608552	TEC
ENSG0000(	ZNF239	125.7493	-0.22872	0.184335	0.60867	protein_coding
ENSG0000(	AMPD3	1100.601	-0.17407	0.184451	0.608979	protein_coding
ENSG0000(	BMPR2	2899.679	-0.15289	0.18462	0.609049	protein_coding
ENSG0000(	ENSG0000(	35.5109	-0.45811	0.184672	0.609049	processed_pseudogene
ENSG0000(	ENSG0000(	112.8494	0.158983	0.184652	0.609049	lncRNA
ENSG0000(	ENSG0000(	59.84853	0.234384	0.184582	0.609049	lncRNA
ENSG0000(	GZMK	23.95948	-0.59628	0.184625	0.609049	protein_coding
ENSG0000(	NCAPH2	469.9088	-0.15682	0.184625	0.609049	protein_coding
ENSG0000(	NRIP2	75.31398	-0.34224	0.184633	0.609049	protein_coding
ENSG0000(	PLSCR4	530.3815	0.168881	0.184504	0.609049	protein_coding
ENSG0000(	SPR	154.9793	0.285697	0.18457	0.609049	protein_coding
ENSG0000(	ATF2	1156.555	-0.09277	0.184719	0.609133	protein_coding
ENSG0000(	LGI4	247.7355	0.414668	0.184763	0.609205	protein_coding
ENSG0000(	IGLV7-43	90.35573	-0.50477	0.184788	0.609215	IG_V_gene
ENSG0000(	PLG	113.2246	-0.27986	0.184813	0.609223	protein_coding
ENSG0000(	AP1S2	370.6664	-0.17877	0.186108	0.609791	protein_coding
ENSG0000(	BAIAP2	307.1485	0.184606	0.185422	0.609791	protein_coding
ENSG0000(	BCL3	1025.35	-0.1788	0.186168	0.609791	protein_coding
ENSG0000(	CEACAMP1	21.46771	-0.32942	0.185027	0.609791	transcribed_processed_pseudoge
ENSG0000(	CNOT7	1370.483	0.120568	0.185676	0.609791	protein_coding
ENSG0000(	EIF2B1	636.1699	-0.07898	0.185249	0.609791	protein_coding
ENSG0000(	ENSG0000(	144.1282	0.149309	0.185804	0.609791	processed_pseudogene
ENSG0000(	ENSG0000(	102.3701	0.192344	0.185856	0.609791	processed_pseudogene
ENSG0000(	ENSG0000(	337.7193	0.395688	0.18596	0.609791	lncRNA
ENSG0000(	ENSG0000(	24.43959	-0.50632	0.185786	0.609791	lncRNA
ENSG0000(	ENSG0000(	77.55446	-0.20553	0.18569	0.609791	lncRNA
ENSG0000(	ENSG0000(	35.93574	0.576109	0.186114	0.609791	lncRNA
ENSG0000(	ENSG0000(	140.1308	0.17361	0.185184	0.609791	lncRNA
ENSG0000(	ENSG0000(	282.2909	0.202392	0.18519	0.609791	lncRNA
ENSG0000(	ENSG0000(	86.76133	0.44877	0.185848	0.609791	transcribed_processed_pseudoge
ENSG0000(	ENSG0000(	96.96528	-0.23136	0.186041	0.609791	lncRNA
ENSG0000(	ENSG0000(	189.935	0.361362	0.185259	0.609791	protein_coding
ENSG0000(	ENSG0000(	50.53293	-0.19399	0.185605	0.609791	lncRNA
ENSG0000(	ENSG0000(	111.7792	0.166503	0.185386	0.609791	lncRNA

ENSG0000(	ENSG0000(	2933.018	-0.08163	0.18554	0.609791	lncRNA
ENSG0000(	ENSG0000(	114.7931	0.190886	0.186107	0.609791	lncRNA
ENSG0000(	ENSG0000(	51.92985	0.353704	0.185789	0.609791	lncRNA
ENSG0000(	EPS8L1	485.0859	-0.33893	0.185658	0.609791	protein_coding
ENSG0000(	F11-AS1	196.2245	0.197939	0.186182	0.609791	lncRNA
ENSG0000(	FTSJ3	441.4455	-0.12456	0.185541	0.609791	protein_coding
ENSG0000(	GPR1-AS	84.89003	-0.24263	0.185809	0.609791	lncRNA
ENSG0000(	HDDC2	404.9155	-0.12714	0.185911	0.609791	protein_coding
ENSG0000(	IGBP1-AS1	137.8593	0.140839	0.185198	0.609791	lncRNA
ENSG0000(	KLF3-AS1	1230.232	0.097805	0.185079	0.609791	lncRNA
ENSG0000(	KRT15	385.8467	0.242074	0.185492	0.609791	protein_coding
ENSG0000(	LECT2	170.2709	-0.16604	0.186152	0.609791	protein_coding
ENSG0000(	MATK	146.1975	-0.14932	0.185227	0.609791	protein_coding
ENSG0000(	MCM9	981.7669	0.122213	0.18575	0.609791	protein_coding
ENSG0000(	OR13I1P	45.44267	-0.24672	0.185117	0.609791	unprocessed_pseudogene
ENSG0000(	PKP2	3486.143	0.274241	0.186151	0.609791	protein_coding
ENSG0000(	PLEC	14251.41	0.188218	0.185478	0.609791	protein_coding
ENSG0000(	PPP4R3B-D	23.7125	0.296916	0.185918	0.609791	lncRNA
ENSG0000(	RABIF	413.941	0.08983	0.186014	0.609791	protein_coding
ENSG0000(	RDH5	388.9583	0.209652	0.185461	0.609791	protein_coding
ENSG0000(	RNF14	1153.667	0.084506	0.185719	0.609791	protein_coding
ENSG0000(	SERPINB6	6281.509	-0.17516	0.185263	0.609791	protein_coding
ENSG0000(	SLC37A3	1366.968	0.065385	0.185941	0.609791	protein_coding
ENSG0000(	SNHG29	2020.681	0.150738	0.185468	0.609791	lncRNA
ENSG0000(	SOX17	30.23632	-0.44059	0.185654	0.609791	protein_coding
ENSG0000(	SRPK2	1050.754	-0.11758	0.185492	0.609791	protein_coding
ENSG0000(	STX18-IT1	53.42854	-0.18403	0.186154	0.609791	lncRNA
ENSG0000(	SUDS3	1751.238	0.08312	0.186096	0.609791	protein_coding
ENSG0000(	TACC3	1538.377	-0.09819	0.185331	0.609791	protein_coding
ENSG0000(	TCL1A	133.6206	-0.62822	0.185642	0.609791	protein_coding
ENSG0000(	TMBIM7P	27.26133	0.395878	0.185342	0.609791	transcribed_unitary_pseudogene
ENSG0000(	TMEM102	101.4631	0.229681	0.185876	0.609791	protein_coding
ENSG0000(	TPM4P1	27.00801	-0.3982	0.185952	0.609791	processed_pseudogene
ENSG0000(	TRIM52	1880.116	0.090904	0.185648	0.609791	protein_coding
ENSG0000(	ZNF639	1176.302	-0.1147	0.186021	0.609791	protein_coding
ENSG0000(	IFI44L	2139.531	0.65161	0.186224	0.609856	protein_coding
ENSG0000(	C1QTNF3-A	36.39666	0.705913	0.186361	0.60994	protein_coding
ENSG0000(	CHST6	1034.225	0.091646	0.186344	0.60994	protein_coding
ENSG0000(	ENSG0000(	77.33697	0.275212	0.18635	0.60994	lncRNA
ENSG0000(	RBIS	447.4516	-0.12686	0.186323	0.60994	protein_coding
ENSG0000(	RIPOR1	690.8911	-0.24125	0.18631	0.60994	protein_coding
ENSG0000(	RN7SL362F	29.51764	0.308637	0.186399	0.609993	misc_RNA
ENSG0000(	ENSG0000(	21.24326	0.285539	0.186648	0.610166	lncRNA
ENSG0000(	ENSG0000(	374.9177	0.114784	0.186674	0.610166	lncRNA
ENSG0000(	ENSG0000(	41.0787	-0.29263	0.186478	0.610166	lncRNA
ENSG0000(	ENSG0000(	22.76155	-0.33419	0.186575	0.610166	lncRNA
ENSG0000(	HNRNPA3	7058.133	-0.06082	0.186659	0.610166	protein_coding

ENSG0000(	MESTIT1	101.6544	0.148646	0.186575	0.610166	lncRNA
ENSG0000(	RPL39L	31.74428	-0.34605	0.186652	0.610166	protein_coding
ENSG0000(	SNORD55	32.44436	0.406678	0.186585	0.610166	snoRNA
ENSG0000(	UNGP1	91.09589	-0.16579	0.186551	0.610166	processed_pseudogene
ENSG0000(	VAV1	652.7549	-0.18296	0.186647	0.610166	protein_coding
ENSG0000(	PIP4P1	623.2125	0.133095	0.186735	0.610295	protein_coding
ENSG0000(	SMARCA4	3413.8	0.065194	0.186768	0.610331	protein_coding
ENSG0000(	ALDH1A2	94.47114	-0.25729	0.186991	0.610414	protein_coding
ENSG0000(	ENSG0000(	943.6903	-0.08358	0.186958	0.610414	lncRNA
ENSG0000(	ENSG0000(	19.83694	-0.51967	0.186876	0.610414	lncRNA
ENSG0000(	FAM162A	1835.231	0.167392	0.186907	0.610414	protein_coding
ENSG0000(	HEYL	181.1789	-0.24129	0.186994	0.610414	protein_coding
ENSG0000(	LINC00643	229.4335	-0.23656	0.18687	0.610414	transcribed_unitary_pseudogene
ENSG0000(	MAT2A	7099.613	0.127025	0.186967	0.610414	protein_coding
ENSG0000(	SCRN3	735.3307	0.122167	0.186988	0.610414	protein_coding
ENSG0000(	STOML2	257.748	-0.19162	0.186965	0.610414	protein_coding
ENSG0000(	YME1L1	2187.221	-0.07362	0.187043	0.610503	protein_coding
ENSG0000(	CFAP44	1466.759	0.147713	0.187085	0.610519	protein_coding
ENSG0000(	ENSG0000(	111.6038	1.051403	0.187096	0.610519	transcribed_processed_pseudoge
ENSG0000(	PDK3	3558.099	-0.0922	0.187115	0.610519	protein_coding
ENSG0000(	ACAD11	1033.366	-0.14347	0.187156	0.610547	protein_coding
ENSG0000(	EXOC3L4	599.1523	-0.20382	0.187168	0.610547	protein_coding
ENSG0000(	ENSG0000(	21.22521	-0.79761	0.187268	0.610754	processed_pseudogene
ENSG0000(	ENSG0000(	243.9149	0.164361	0.187275	0.610754	lncRNA
ENSG0000(	CDK16	952.3597	0.113736	0.187324	0.610839	protein_coding
ENSG0000(	NMNAT3	13865.36	0.116217	0.187362	0.61089	protein_coding
ENSG0000(	GNB5	1098.627	-0.14366	0.187415	0.610992	protein_coding
ENSG0000(	WDR7	1751.133	-0.10837	0.187439	0.610997	protein_coding
ENSG0000(	NETO1-DT	26.75244	0.571222	0.187527	0.611138	lncRNA
ENSG0000(	RBM43	591.2353	-0.1242	0.187508	0.611138	protein_coding
ENSG0000(	ENSG0000(	1723.395	0.135798	0.187644	0.611308	lncRNA
ENSG0000(	PHBP8	35.66206	0.416254	0.187645	0.611308	processed_pseudogene
ENSG0000(	RN7SL399F	67.68088	0.262213	0.187616	0.611308	misc_RNA
ENSG0000(	CCDC106	62.19092	0.277914	0.187712	0.611452	protein_coding
ENSG0000(	EBF2	72.47253	-0.25459	0.187751	0.611508	protein_coding
ENSG0000(	LPXN	629.4682	-0.17092	0.187828	0.611685	protein_coding
ENSG0000(	SCNM1	673.3104	-0.07195	0.187906	0.611869	protein_coding
ENSG0000(	ENSG0000(	200.8259	0.139799	0.188012	0.611971	lncRNA
ENSG0000(	PANK2	1993.666	-0.10719	0.188027	0.611971	protein_coding
ENSG0000(	SOCS2-AS1	518.1529	0.092903	0.188026	0.611971	lncRNA
ENSG0000(	UBE2R2-AS	75.14669	-0.27856	0.188013	0.611971	lncRNA
ENSG0000(	ANKRD18B	65.05142	0.351439	0.188072	0.612047	protein_coding
ENSG0000(	CCL24	480.6701	-0.27483	0.188162	0.61221	protein_coding
ENSG0000(	EBF1	714.9469	-0.29096	0.188256	0.61221	protein_coding
ENSG0000(	ENSG0000(	23.02191	-0.57121	0.188181	0.61221	lncRNA
ENSG0000(	ENSG0000(	817.4575	-0.14084	0.188254	0.61221	transcribed_unprocessed_pseudo
ENSG0000(	LRWD1	350.2903	-0.16299	0.188248	0.61221	protein_coding

ENSG0000(	TSPOAP1-A	218.2711	-0.32432	0.188198	0.61221	lncRNA
ENSG0000(	BLOC1S6	15255.92	0.12976	0.188291	0.612252	protein_coding
ENSG0000(	ENSG0000(	1183.986	0.084441	0.188331	0.61231	processed_pseudogene
ENSG0000(	ADORA1	47.84183	-0.43675	0.188393	0.612357	protein_coding
ENSG0000(	CCDC144CI	435.8959	-0.12653	0.188435	0.612357	transcribed_processed_pseudoge
ENSG0000(	MRPS9	181.15	0.170946	0.188382	0.612357	protein_coding
ENSG0000(	ZNF182	434.9039	0.150061	0.188429	0.612357	protein_coding
ENSG0000(	COL9A2	279.541	0.249563	0.188474	0.612412	protein_coding
ENSG0000(	ENSG0000(	55.65039	0.258789	0.188499	0.612412	lncRNA
ENSG0000(	RAMAC	116.4923	-0.27826	0.188518	0.612412	protein_coding
ENSG0000(	H4C15	408.3779	0.328334	0.188558	0.612453	protein_coding
ENSG0000(	LPIN3	1685.192	0.236513	0.188576	0.612453	protein_coding
ENSG0000(	C1orf167	223.9077	-0.13379	0.188717	0.6126	protein_coding
ENSG0000(	ENSG0000(	61.26135	0.249481	0.188697	0.6126	TEC
ENSG0000(	ENSG0000(	29.59701	-0.32852	0.188718	0.6126	lncRNA
ENSG0000(	LINC02302	183.3391	-0.14176	0.188648	0.6126	lncRNA
ENSG0000(	LINC02701	49.49103	-0.34703	0.188732	0.6126	lncRNA
ENSG0000(	CEP70	638.4809	0.204039	0.188801	0.612722	protein_coding
ENSG0000(	PARVB	1196.422	-0.15096	0.188814	0.612722	protein_coding
ENSG0000(	LINC01676	22.44262	0.330766	0.188846	0.612753	lncRNA
ENSG0000(	PEX1	1081.74	0.10297	0.188915	0.612905	protein_coding
ENSG0000(	BRAT1	604.0351	0.11857	0.188975	0.612973	protein_coding
ENSG0000(	EIF2S2P2	20.9889	0.439218	0.188981	0.612973	processed_pseudogene
ENSG0000(	PCDHGA3	31.719	0.41592	0.189014	0.613008	protein_coding
ENSG0000(	ABL1	2474.317	0.121898	0.189037	0.61301	protein_coding
ENSG0000(	ENSG0000(	165.9336	0.205974	0.189093	0.613049	lncRNA
ENSG0000(	JAK1	4065.05	-0.12295	0.18908	0.613049	protein_coding
ENSG0000(	ENSG0000(	75.4975	0.211313	0.189213	0.613292	lncRNA
ENSG0000(	ENSG0000(	66.31489	0.220366	0.189195	0.613292	protein_coding
ENSG0000(	C22orf46	453.8687	-0.11514	0.189356	0.613322	transcribed_unitary_pseudogene
ENSG0000(	CRACDL	190.3127	0.267983	0.189324	0.613322	protein_coding
ENSG0000(	CTSZ	4373.222	-0.13299	0.189303	0.613322	protein_coding
ENSG0000(	OPRD1	669.722	0.07995	0.189337	0.613322	protein_coding
ENSG0000(	TAX1BP3	692.6567	0.287201	0.189315	0.613322	protein_coding
ENSG0000(	TGFBRAP1	897.37	0.07272	0.189355	0.613322	protein_coding
ENSG0000(	ALAD	1033.354	0.14846	0.189392	0.613366	protein_coding
ENSG0000(	APBA1	432.752	0.137238	0.189685	0.613545	protein_coding
ENSG0000(	CDKN1B	1541.108	-0.11303	0.189567	0.613545	protein_coding
ENSG0000(	CELA2A	87.43855	0.152399	0.189631	0.613545	protein_coding
ENSG0000(	CHRNA7	647.5016	0.13843	0.189946	0.613545	protein_coding
ENSG0000(	EFNA5	550.8713	0.210812	0.189743	0.613545	protein_coding
ENSG0000(	ENSG0000(	183.7126	-0.16013	0.189932	0.613545	lncRNA
ENSG0000(	ENSG0000(	154.4336	-0.17007	0.189696	0.613545	lncRNA
ENSG0000(	ENSG0000(	94.46505	0.211621	0.189652	0.613545	lncRNA
ENSG0000(	FHAD1	304.296	-0.22229	0.189909	0.613545	protein_coding
ENSG0000(	FLJ31104	347.7868	-0.11603	0.189661	0.613545	lncRNA
ENSG0000(	KCNC3	266.169	-0.22115	0.189841	0.613545	protein_coding

ENSG0000(	PDCD4	4540.488	0.236302	0.189716	0.613545	protein_coding
ENSG0000(	RN7SL820F	29.5656	0.335505	0.18992	0.613545	misc_RNA
ENSG0000(	SH2D1A	147.5304	-0.33869	0.189824	0.613545	protein_coding
ENSG0000(	SLC24A1	548.8283	-0.15671	0.18979	0.613545	protein_coding
ENSG0000(	SMIM10L1	442.4695	-0.11675	0.189939	0.613545	protein_coding
ENSG0000(	SYNC	273.173	0.169972	0.18996	0.613545	protein_coding
ENSG0000(	TJP2	3349.778	0.176142	0.189632	0.613545	protein_coding
ENSG0000(	TMEM101	206.7195	0.149401	0.189825	0.613545	protein_coding
ENSG0000(	TMEM87A	1526.677	0.115371	0.189762	0.613545	protein_coding
ENSG0000(	TNFAIP8L1	995.6448	-0.0798	0.189883	0.613545	protein_coding
ENSG0000(	UQCRC1	780.8047	0.217086	0.189708	0.613545	protein_coding
ENSG0000(	ZNF131	1344.627	0.071143	0.189776	0.613545	protein_coding
ENSG0000(	BEND5	38.50298	0.35334	0.190103	0.613781	protein_coding
ENSG0000(	ENSG0000(	403.083	-0.16934	0.190127	0.613781	lncRNA
ENSG0000(	ENSG0000(	587.9995	0.188569	0.190089	0.613781	transcribed_unprocessed_pseudo
ENSG0000(	LINC01828	157.9806	0.155797	0.190164	0.613781	lncRNA
ENSG0000(	LINC02455	20.52321	0.318536	0.190071	0.613781	lncRNA
ENSG0000(	USP24	3524.814	0.091847	0.190167	0.613781	protein_coding
ENSG0000(	DHX33	642.0253	0.082961	0.190213	0.613787	protein_coding
ENSG0000(	GCC1	503.1122	0.117676	0.190203	0.613787	protein_coding
ENSG0000(	GSDMD	1190.912	-0.14809	0.190258	0.61381	protein_coding
ENSG0000(	ZC3H10	614.7123	-0.1346	0.190265	0.61381	protein_coding
ENSG0000(	ENSG0000(	142.633	0.161363	0.190317	0.613863	TEC
ENSG0000(	ENSG0000(	215.6481	0.258614	0.190326	0.613863	lncRNA
ENSG0000(	SUMO2	1522.081	-0.177	0.190364	0.613915	protein_coding
ENSG0000(	CD3E	336.5406	-0.34072	0.190437	0.614006	protein_coding
ENSG0000(	MBOAT2	1664.499	0.195901	0.190436	0.614006	protein_coding
ENSG0000(	CHCHD5	252.666	0.14367	0.190493	0.614094	protein_coding
ENSG0000(	MT-TW	65.96859	-0.7326	0.190532	0.614094	Mt_tRNA
ENSG0000(	ST8SIA1	1121.835	-0.15008	0.190524	0.614094	protein_coding
ENSG0000(	CDYLP1	62.05551	-0.23417	0.190803	0.614284	unprocessed_pseudogene
ENSG0000(	CTRL	21.96611	-0.42269	0.190636	0.614284	protein_coding
ENSG0000(	ENSG0000(	899.4606	-0.08867	0.190777	0.614284	lncRNA
ENSG0000(	ENSG0000(	75.42463	0.244015	0.19068	0.614284	processed_pseudogene
ENSG0000(	ENSG0000(	235.9676	-0.1131	0.19066	0.614284	protein_coding
ENSG0000(	PAPPA	707.263	-0.35306	0.190756	0.614284	protein_coding
ENSG0000(	PGAM1	1088.552	-0.16198	0.190788	0.614284	protein_coding
ENSG0000(	SLC25A21	181.185	0.133695	0.190752	0.614284	protein_coding
ENSG0000(	SPPL3	1620.657	0.076168	0.190814	0.614284	protein_coding
ENSG0000(	UHRF1BP1l	1032.597	-0.10773	0.190716	0.614284	protein_coding
ENSG0000(	TBCK	1707.259	0.094378	0.190871	0.614397	protein_coding
ENSG0000(	EDIL3-DT	1399.064	0.073985	0.190949	0.614434	lncRNA
ENSG0000(	ENSG0000(	42.60084	0.39034	0.190951	0.614434	lncRNA
ENSG0000(	GFER	230.119	0.209338	0.190972	0.614434	protein_coding
ENSG0000(	GLDN	111.7694	0.436831	0.190931	0.614434	protein_coding
ENSG0000(	MTCO2P2	21.40666	0.790513	0.191027	0.614538	processed_pseudogene
ENSG0000(	RN7SL278F	52.83078	-0.26295	0.191058	0.614565	misc_RNA

ENSG0000(	SNORA80D	23.60063	0.384056	0.191091	0.6146	snoRNA
ENSG0000(	PCDHGA10	304.7203	0.15123	0.191148	0.614713	protein_coding
ENSG0000(	SNX22	526.6592	-0.21728	0.191182	0.614748	protein_coding
ENSG0000(	ADAT3	116.7668	0.19006	0.191211	0.614772	protein_coding
ENSG0000(	ALOX12P2	228.0829	-0.21709	0.191379	0.614773	transcribed_unprocessed_pseudo
ENSG0000(	DYNLL2	1806.88	-0.09954	0.191318	0.614773	protein_coding
ENSG0000(	ENSG0000(	131.2155	0.263727	0.191315	0.614773	unprocessed_pseudogene
ENSG0000(	ENSG0000(	34.83853	-0.28335	0.191326	0.614773	lncRNA
ENSG0000(	LINC00989	164.0884	-0.18516	0.191339	0.614773	lncRNA
ENSG0000(	LRP1B	66.4699	-0.66758	0.19127	0.614773	protein_coding
ENSG0000(	RN7SL774F	223.7465	0.156177	0.191351	0.614773	misc_RNA
ENSG0000(	RNF223	97.3626	0.200953	0.19139	0.614773	protein_coding
ENSG0000(	ENSG0000(	151.134	0.165532	0.19142	0.614787	lncRNA
ENSG0000(	GPBP1L1	3822.624	0.098612	0.191444	0.614787	protein_coding
ENSG0000(	PPP2R5E	1458.489	0.06981	0.191462	0.614787	protein_coding
ENSG0000(	DOCK9	2126.708	-0.12079	0.19164	0.615287	protein_coding
ENSG0000(	IGKV5-2	20.75056	-0.5065	0.191664	0.615291	IG_V_gene
ENSG0000(	GLIS1	38.13092	-0.35432	0.191701	0.615341	protein_coding
ENSG0000(	ENSG0000(	174.4612	0.138856	0.191818	0.615429	lncRNA
ENSG0000(	ENSG0000(	30.61267	-0.33216	0.191782	0.615429	processed_pseudogene
ENSG0000(	HRAS	229.0167	0.284234	0.191765	0.615429	protein_coding
ENSG0000(	RNF167	545.1622	-0.14107	0.191807	0.615429	protein_coding
ENSG0000(	BTG1-DT	232.144	-0.30915	0.191913	0.615622	lncRNA
ENSG0000(	ENSG0000(	32.22347	-0.2341	0.191923	0.615622	lncRNA
ENSG0000(	E2F6	349.9904	-0.15965	0.19201	0.615685	protein_coding
ENSG0000(	MRPS17	590.2871	0.07698	0.191966	0.615685	protein_coding
ENSG0000(	TRAJ39	24.1651	-0.43051	0.192005	0.615685	TR_J_gene
ENSG0000(	ZSCAN22	411.9263	0.093655	0.192043	0.615719	protein_coding
ENSG0000(	ENSG0000(	184.919	-0.14199	0.19211	0.615837	lncRNA
ENSG0000(	ENSG0000(	75.73737	0.184669	0.19213	0.615837	lncRNA
ENSG0000(	EXOC5	1267.966	-0.09651	0.192183	0.615837	protein_coding
ENSG0000(	GGCT	259.2786	-0.17404	0.192151	0.615837	protein_coding
ENSG0000(	LINC01923	412.0546	0.145112	0.192192	0.615837	lncRNA
ENSG0000(	ATP13A1	1142.392	-0.11291	0.192226	0.615874	protein_coding
ENSG0000(	ENSG0000(	236.1378	-0.21353	0.192312	0.616009	lncRNA
ENSG0000(	TNFAIP8L2	74.55701	-0.28374	0.192312	0.616009	protein_coding
ENSG0000(	MPIG6B	149.8808	0.17173	0.192343	0.616035	protein_coding
ENSG0000(	DYNLT3	308.0748	-0.14602	0.19241	0.616035	protein_coding
ENSG0000(	ENSG0000(	64.03583	-0.25052	0.192406	0.616035	protein_coding
ENSG0000(	GLRA1	64.30723	-0.21148	0.192376	0.616035	protein_coding
ENSG0000(	ENSG0000(	287.8832	0.135197	0.192468	0.616147	lncRNA
ENSG0000(	ENSG0000(	121.6842	-0.4061	0.192504	0.616191	lncRNA
ENSG0000(	NEK2	283.4563	0.116693	0.192588	0.616391	protein_coding
ENSG0000(	ENSG0000(	412.6098	0.101649	0.192691	0.616647	lncRNA
ENSG0000(	BZW1	2362.653	-0.12575	0.192718	0.616663	protein_coding
ENSG0000(	NPC1L1	32.96329	-0.28058	0.192751	0.616696	protein_coding
ENSG0000(	SNORA51	20.51152	-0.33083	0.192778	0.616711	snoRNA

ENSG0000(	BAZ2B	7377.899	-0.07444	0.192848	0.616724	protein_coding
ENSG0000(	ENSG0000(	154.8284	-0.12492	0.192849	0.616724	lncRNA
ENSG0000(	MIR4539	65.6815	-0.45736	0.192815	0.616724	miRNA
ENSG0000(	AKR1E2	100.0681	0.283857	0.19294	0.616748	protein_coding
ENSG0000(	CDIPT	415.4969	0.135952	0.192948	0.616748	protein_coding
ENSG0000(	ENSG0000(	19.53923	0.428069	0.192884	0.616748	lncRNA
ENSG0000(	GOT2P7	57.13878	-0.25571	0.192969	0.616748	processed_pseudogene
ENSG0000(	RNU6-476F	20.39972	-0.37746	0.192953	0.616748	snRNA
ENSG0000(	ZC3HC1	189.5573	0.144281	0.192996	0.616762	protein_coding
ENSG0000(	ENSG0000(	87.94657	-0.15888	0.193089	0.616916	lncRNA
ENSG0000(	RNF41	1540.86	-0.08683	0.193082	0.616916	protein_coding
ENSG0000(	CENPBD1	271.9834	0.108045	0.193589	0.617294	transcribed_unitary_pseudogene
ENSG0000(	EFNA1	453.1147	0.280131	0.193384	0.617294	protein_coding
ENSG0000(	ENSG0000(	69.81938	-0.31971	0.193509	0.617294	lncRNA
ENSG0000(	ENSG0000(	642.5064	-0.14752	0.193364	0.617294	lncRNA
ENSG0000(	ENSG0000(	223.8472	0.21513	0.193514	0.617294	lncRNA
ENSG0000(	ENSG0000(	303.6248	-0.12048	0.193366	0.617294	lncRNA
ENSG0000(	ENSG0000(	48.56906	-0.19248	0.193647	0.617294	lncRNA
ENSG0000(	ENSG0000(	414.5669	0.105911	0.193483	0.617294	lncRNA
ENSG0000(	EPS8L3	2840.63	0.274643	0.193529	0.617294	protein_coding
ENSG0000(	FCAMR	42.15661	-0.56432	0.193619	0.617294	protein_coding
ENSG0000(	HOXA9	640.2426	0.245087	0.19357	0.617294	protein_coding
ENSG0000(	KANSL1L-A	583.5374	-0.08455	0.193285	0.617294	lncRNA
ENSG0000(	LINC02074	78.43818	-0.17414	0.193326	0.617294	lncRNA
ENSG0000(	NDRG4	150.147	-0.23786	0.193352	0.617294	protein_coding
ENSG0000(	PRKACA	972.9703	-0.11365	0.193641	0.617294	protein_coding
ENSG0000(	SNORD104	43.08269	-0.27804	0.193599	0.617294	snoRNA
ENSG0000(	STAG2	5806.135	0.164653	0.193455	0.617294	protein_coding
ENSG0000(	TRIM7-AS1	223.1772	0.168941	0.193515	0.617294	lncRNA
ENSG0000(	ZNF534	534.5175	0.103989	0.193545	0.617294	protein_coding
ENSG0000(	ZNF587	5606.97	0.105815	0.193656	0.617294	protein_coding
ENSG0000(	ENSG0000(	56.50674	-0.1771	0.193806	0.617413	lncRNA
ENSG0000(	FHL5	73.18171	-0.38715	0.193764	0.617413	protein_coding
ENSG0000(	LINC01703	102.0962	0.15208	0.193802	0.617413	lncRNA
ENSG0000(	MORF4L1	2026.799	-0.08741	0.19372	0.617413	protein_coding
ENSG0000(	NSUN5P1	4051.486	0.097366	0.193782	0.617413	transcribed_unprocessed_pseudo
ENSG0000(	POLR2F	441.6988	0.103557	0.193929	0.617735	protein_coding
ENSG0000(	CAMK2A	398.4014	0.12684	0.194062	0.617943	protein_coding
ENSG0000(	IQCH-AS1	1014.352	0.133746	0.194021	0.617943	lncRNA
ENSG0000(	SLK	2434.721	0.144415	0.194056	0.617943	protein_coding
ENSG0000(	ENSG0000(	108.5525	0.153804	0.194088	0.617957	lncRNA
ENSG0000(	ENSG0000(	27.74769	-0.31172	0.194234	0.618348	lncRNA
ENSG0000(	ERBB2	3724.109	0.241407	0.194327	0.618465	protein_coding
ENSG0000(	FAM81A	114.9249	0.370316	0.194326	0.618465	protein_coding
ENSG0000(	KIF5B	3053.081	-0.10861	0.194363	0.618465	protein_coding
ENSG0000(	LINC01783	97.63302	-0.25427	0.194343	0.618465	lncRNA
ENSG0000(	RITA1	168.1696	0.160509	0.194383	0.618465	protein_coding

ENSG0000(	PRKACB	1890.193	0.158513	0.19443	0.618471	protein_coding
ENSG0000(	UBE2E1	655.4904	-0.1199	0.194416	0.618471	protein_coding
ENSG0000(	ACSS2	3728.481	0.259062	0.194623	0.618513	protein_coding
ENSG0000(	ARHGAP11	69.73849	0.193086	0.194608	0.618513	lncRNA
ENSG0000(	CCDC71	208.418	0.151587	0.194592	0.618513	protein_coding
ENSG0000(	CNTD1	20.67455	0.451943	0.194601	0.618513	protein_coding
ENSG0000(	ENSG0000(	65.44243	-0.28055	0.194585	0.618513	lncRNA
ENSG0000(	ENSG0000(	33.81013	0.320735	0.194554	0.618513	lncRNA
ENSG0000(	HSPE1P28	46.17918	0.226619	0.194502	0.618513	processed_pseudogene
ENSG0000(	NAA40	1207.447	0.084574	0.194602	0.618513	protein_coding
ENSG0000(	AP2M1	1448.984	-0.12957	0.194828	0.618671	protein_coding
ENSG0000(	ENSG0000(	48.76551	0.289699	0.194803	0.618671	lncRNA
ENSG0000(	NCK1	823.7036	-0.09146	0.19483	0.618671	protein_coding
ENSG0000(	NKILA	45.08813	0.395669	0.1948	0.618671	lncRNA
ENSG0000(	PPIL1	135.7777	-0.22162	0.19476	0.618671	protein_coding
ENSG0000(	SNN	259.6834	-0.29833	0.194743	0.618671	protein_coding
ENSG0000(	TMC5	3825.837	-0.29757	0.194789	0.618671	protein_coding
ENSG0000(	OSER1	464.9317	0.109257	0.194889	0.618788	protein_coding
ENSG0000(	DCAF4L1	108.3518	-0.18097	0.194959	0.618867	protein_coding
ENSG0000(	ENSG0000(	31.16168	0.298173	0.194953	0.618867	transcribed_unprocessed_pseudo
ENSG0000(	ADAMTS16	41.13699	-0.36001	0.195078	0.618952	protein_coding
ENSG0000(	ENSG0000(	180.9859	-0.1598	0.195089	0.618952	lncRNA
ENSG0000(	MIR27B	19.71748	0.502772	0.195026	0.618952	miRNA
ENSG0000(	MYL9	487.3539	-0.29985	0.195048	0.618952	protein_coding
ENSG0000(	XIAP-AS1	59.94099	-0.22785	0.195098	0.618952	lncRNA
ENSG0000(	CENPQ	42.99975	-0.33491	0.195239	0.619252	protein_coding
ENSG0000(	KHDC4	2881.397	0.0857	0.195301	0.619252	protein_coding
ENSG0000(	OCRL	1535.59	0.168947	0.195275	0.619252	protein_coding
ENSG0000(	PTPRG-AS1	279.3589	0.130875	0.195306	0.619252	lncRNA
ENSG0000(	TEX2	1161.698	0.11937	0.195265	0.619252	protein_coding
ENSG0000(	RPS6KB2-A	20.27888	-0.47684	0.19538	0.619417	lncRNA
ENSG0000(	ADNP-AS1	43.87372	0.215118	0.195628	0.619631	lncRNA
ENSG0000(	FMO4	185.3439	0.277796	0.195588	0.619631	protein_coding
ENSG0000(	GREB1	686.0404	-0.18947	0.195616	0.619631	protein_coding
ENSG0000(	OTOG	58.9599	-0.2249	0.195474	0.619631	protein_coding
ENSG0000(	QRFPR	126.2586	0.163759	0.195552	0.619631	protein_coding
ENSG0000(	RPL26P6	49.25276	0.193796	0.195555	0.619631	processed_pseudogene
ENSG0000(	SLAMF6	454.1351	-0.25602	0.195589	0.619631	protein_coding
ENSG0000(	TBC1D29P	128.9044	0.201776	0.19562	0.619631	transcribed_unprocessed_pseudo
ENSG0000(	OR2L2	581.4302	0.144681	0.195664	0.619674	protein_coding
ENSG0000(	CELF5	3988.115	0.320078	0.195769	0.619934	protein_coding
ENSG0000(	MX1	3196.895	0.315231	0.195804	0.619975	protein_coding
ENSG0000(	PHLDB2	1052.339	-0.24565	0.195927	0.620261	protein_coding
ENSG0000(	PIN4	318.9351	0.102788	0.19594	0.620261	protein_coding
ENSG0000(	CD99L2	547.4077	-0.10944	0.196171	0.62028	protein_coding
ENSG0000(	ENSG0000(	222.1945	0.369865	0.196045	0.62028	lncRNA
ENSG0000(	ENSG0000(	1951.038	0.114859	0.196167	0.62028	lncRNA

ENSG0000(	ENSG0000(	975.6927	0.057884	0.196081	0.62028	lncRNA
ENSG0000(	INE1	516.8865	-0.09784	0.196101	0.62028	lncRNA
ENSG0000(	LINC02599	59.81249	-0.34432	0.196055	0.62028	lncRNA
ENSG0000(	LLGL2	4733.248	0.271978	0.196065	0.62028	protein_coding
ENSG0000(	SLC26A6	1517.797	0.191986	0.196029	0.62028	protein_coding
ENSG0000(	TREX1	69.69978	-0.35057	0.196127	0.62028	protein_coding
ENSG0000(	ZNF286B	313.111	0.128831	0.196145	0.62028	transcribed_unprocessed_pseudo
ENSG0000(	LINC02200	149.4897	0.180762	0.196308	0.620642	lncRNA
ENSG0000(	PYCR1	198.7583	-0.29276	0.196379	0.620795	protein_coding
ENSG0000(	IMPA1	1436.991	0.17513	0.196433	0.620895	protein_coding
ENSG0000(	ENSG0000(	40.73057	-0.67429	0.19647	0.62094	lncRNA
ENSG0000(	CAPN15	2845.39	0.139775	0.196857	0.621576	protein_coding
ENSG0000(	ENSG0000(	198.9882	0.133097	0.196855	0.621576	processed_pseudogene
ENSG0000(	GCH1	548.0916	-0.22798	0.196838	0.621576	protein_coding
ENSG0000(	HUWE1	7987.869	-0.06306	0.196875	0.621576	protein_coding
ENSG0000(	LDAH	551.3467	-0.12874	0.196744	0.621576	protein_coding
ENSG0000(	LINC01949	988.4355	0.122654	0.196853	0.621576	lncRNA
ENSG0000(	MIR3681H(	26.46036	-0.57285	0.196871	0.621576	lncRNA
ENSG0000(	TAF11	1872.289	0.078471	0.196846	0.621576	protein_coding
ENSG0000(	TSPAN4	280.6796	-0.17111	0.196796	0.621576	protein_coding
ENSG0000(	LRATD2	767.3644	0.17233	0.196906	0.621603	protein_coding
ENSG0000(	FOXM1	812.2637	-0.0984	0.196952	0.621678	protein_coding
ENSG0000(	ATP9B	5978.843	0.098672	0.197296	0.62208	protein_coding
ENSG0000(	C14orf132	688.0343	-0.14649	0.197304	0.62208	protein_coding
ENSG0000(	CIC	1909.117	0.13447	0.19735	0.62208	protein_coding
ENSG0000(	ENSG0000(	330.9158	0.121511	0.197185	0.62208	lncRNA
ENSG0000(	ENSG0000(	144.6247	0.176667	0.197317	0.62208	transcribed_unprocessed_pseudo
ENSG0000(	EZR	3818.413	0.18984	0.197324	0.62208	protein_coding
ENSG0000(	GGH	326.5441	0.221214	0.197253	0.62208	protein_coding
ENSG0000(	NT5DC3	1003.887	0.119042	0.197199	0.62208	protein_coding
ENSG0000(	PPP1R13L	790.0444	-0.11761	0.197351	0.62208	protein_coding
ENSG0000(	UBE3C	2888.678	0.078417	0.197163	0.62208	protein_coding
ENSG0000(	WASL	1817.343	0.200805	0.197104	0.62208	protein_coding
ENSG0000(	ZNF771	151.2895	0.119705	0.197135	0.62208	protein_coding
ENSG0000(	ACOT8	531.2405	0.173669	0.197653	0.622109	protein_coding
ENSG0000(	APOC1	79.46739	-0.33833	0.197663	0.622109	protein_coding
ENSG0000(	CCDC144B	3263.281	-0.1725	0.19779	0.622109	transcribed_unprocessed_pseudo
ENSG0000(	CTPS1	1379.846	-0.13261	0.197638	0.622109	protein_coding
ENSG0000(	ENSG0000(	88.10359	0.164989	0.197486	0.622109	lncRNA
ENSG0000(	ENSG0000(	174.3884	-0.13873	0.197453	0.622109	TEC
ENSG0000(	ENSG0000(	32.26904	0.251137	0.197695	0.622109	lncRNA
ENSG0000(	ERF	510.9331	0.158324	0.197428	0.622109	protein_coding
ENSG0000(	ERI1	551.0182	-0.179	0.197424	0.622109	protein_coding
ENSG0000(	GPATCH1	533.0715	0.108507	0.197752	0.622109	protein_coding
ENSG0000(	GPR89B	820.1701	-0.09527	0.197788	0.622109	protein_coding
ENSG0000(	IDH3A	1694.343	0.131307	0.197455	0.622109	protein_coding
ENSG0000(	MRPL22	285.2289	-0.11025	0.197775	0.622109	protein_coding

ENSG0000(	PLPP5	1424.686	-0.13785	0.197768	0.622109	protein_coding
ENSG0000(	RGS14	250.3323	-0.21778	0.197595	0.622109	protein_coding
ENSG0000(	RNU6-1176	33.62612	-0.30184	0.197618	0.622109	snRNA
ENSG0000(	SCARF1	746.6012	-0.21028	0.197715	0.622109	protein_coding
ENSG0000(	SLC26A2	27433.03	0.505287	0.197653	0.622109	protein_coding
ENSG0000(	ST8SIA5	268.0461	0.161128	0.197761	0.622109	protein_coding
ENSG0000(	MOSPD1	99.88009	-0.18937	0.197817	0.622123	protein_coding
ENSG0000(	TEAD4	82.56918	-0.25977	0.197872	0.622225	protein_coding
ENSG0000(	IRF2-DT	634.0833	-0.10583	0.197906	0.622261	lncRNA
ENSG0000(	ENSG0000(	27.33038	-0.50923	0.197935	0.622281	lncRNA
ENSG0000(	DDX10P1	66.51801	-0.26211	0.198012	0.622381	processed_pseudogene
ENSG0000(	ENSG0000(	187.3378	0.162458	0.198011	0.622381	lncRNA
ENSG0000(	CELF6	126.7324	0.194535	0.198153	0.622753	protein_coding
ENSG0000(	AGAP2	352.4778	-0.28068	0.198243	0.622753	protein_coding
ENSG0000(	ENSG0000(	45.43253	0.201672	0.198216	0.622753	TEC
ENSG0000(	ENSG0000(	186.9949	0.247176	0.198235	0.622753	TEC
ENSG0000(	SLC15A3	820.6533	-0.26998	0.198208	0.622753	protein_coding
ENSG0000(	H3C11	82.56323	-0.28558	0.198319	0.622849	protein_coding
ENSG0000(	LINC02516	50.00111	-0.48067	0.198307	0.622849	lncRNA
ENSG0000(	CD151	1364.418	0.133372	0.198372	0.622872	protein_coding
ENSG0000(	ENSG0000(	327.7789	0.12052	0.19835	0.622872	lncRNA
ENSG0000(	ZBTB46	389.2813	-0.1781	0.19858	0.623455	protein_coding
ENSG0000(	CLEC9A	29.74993	-0.40325	0.198651	0.623607	protein_coding
ENSG0000(	ENSG0000(	489.913	-0.11296	0.198674	0.623608	TEC
ENSG0000(	ASXL3	212.2437	0.194427	0.198858	0.623624	protein_coding
ENSG0000(	ENSG0000(	55.06214	0.246608	0.198727	0.623624	lncRNA
ENSG0000(	ENSG0000(	77.63072	0.2184	0.198847	0.623624	lncRNA
ENSG0000(	ENSG0000(	117.1774	0.17389	0.198897	0.623624	lncRNA
ENSG0000(	ENSG0000(	144.2953	-0.25731	0.198861	0.623624	TEC
ENSG0000(	HNRNPM	1493.257	-0.08217	0.198906	0.623624	protein_coding
ENSG0000(	LRP5L	672.7835	0.148017	0.198808	0.623624	transcribed_unprocessed_pseudo
ENSG0000(	MICAL3	6291.973	0.157625	0.198885	0.623624	protein_coding
ENSG0000(	RNF165	315.6939	0.29511	0.198727	0.623624	protein_coding
ENSG0000(	TNFRSF9	867.2599	-0.15526	0.198774	0.623624	protein_coding
ENSG0000(	PPP1R2	1003.479	-0.09718	0.199007	0.62387	protein_coding
ENSG0000(	ENSG0000(	940.4596	0.07713	0.199049	0.623898	TEC
ENSG0000(	LGALS4	6721.021	0.364619	0.199061	0.623898	protein_coding
ENSG0000(	DDX49	199.7668	-0.19501	0.199095	0.623931	protein_coding
ENSG0000(	ENSG0000(	126.1909	0.149006	0.199263	0.624119	lncRNA
ENSG0000(	GATB	474.8832	0.140521	0.199268	0.624119	protein_coding
ENSG0000(	GIMAP7	176.1553	-0.29313	0.199287	0.624119	protein_coding
ENSG0000(	MYCBP2-AS	36.36114	0.290643	0.199263	0.624119	lncRNA
ENSG0000(	SP2-DT	797.1294	-0.12778	0.199291	0.624119	lncRNA
ENSG0000(	TST	862.4545	0.351418	0.199285	0.624119	protein_coding
ENSG0000(	ENSG0000(	145.9507	-0.14381	0.199333	0.624179	lncRNA
ENSG0000(	EHMT1	4283.042	-0.12229	0.199445	0.624223	protein_coding
ENSG0000(	ENSG0000(	102.5365	-0.19947	0.199549	0.624223	lncRNA

ENSG0000(	IRF6	1424.814	0.274862	0.199535	0.624223	protein_coding
ENSG0000(	LINC02504	65.99888	0.254817	0.199403	0.624223	lncRNA
ENSG0000(	PPP1R1B	1208.807	0.322584	0.199422	0.624223	protein_coding
ENSG0000(	RBM7	890.4732	-0.1155	0.199544	0.624223	protein_coding
ENSG0000(	SLC66A1	340.2193	0.104264	0.199494	0.624223	protein_coding
ENSG0000(	TFG	3628.133	-0.09165	0.199482	0.624223	protein_coding
ENSG0000(	ZNF48	203.3875	-0.14984	0.199551	0.624223	protein_coding
ENSG0000(	UGT1A5	216.7936	0.516638	0.199715	0.624666	protein_coding
ENSG0000(	ADD3	2668.202	0.116201	0.199871	0.624824	protein_coding
ENSG0000(	CALCRL	407.9213	-0.15935	0.199972	0.624824	protein_coding
ENSG0000(	CNIH4	1033.018	-0.15848	0.199996	0.624824	protein_coding
ENSG0000(	ENSG0000(	55.81557	-0.20728	0.199915	0.624824	lncRNA
ENSG0000(	ENSG0000(	24.27466	0.347148	0.199937	0.624824	lncRNA
ENSG0000(	ENSG0000(	295.8934	0.137951	0.199839	0.624824	lncRNA
ENSG0000(	ENSG0000(	189.9407	-0.13297	0.199983	0.624824	lncRNA
ENSG0000(	ENSG0000(	304.3474	0.205227	0.199916	0.624824	lncRNA
ENSG0000(	LINC01089	907.5218	0.099994	0.199999	0.624824	lncRNA
ENSG0000(	MT-TL2	80.64994	-0.66045	0.200015	0.624824	Mt_tRNA
ENSG0000(	UQCRC2	2204.463	0.109684	0.199919	0.624824	protein_coding
ENSG0000(	ARHGAP42	149.1416	-0.17148	0.200219	0.624872	processed_pseudogene
ENSG0000(	ENSG0000(	60.11236	0.294369	0.200091	0.624872	lncRNA
ENSG0000(	ENSG0000(	103.8452	0.205631	0.200258	0.624872	lncRNA
ENSG0000(	ENSG0000(	4415.452	-0.11582	0.200238	0.624872	lncRNA
ENSG0000(	IFT140	465.5259	0.127784	0.200218	0.624872	protein_coding
ENSG0000(	SGTB	587.1324	-0.14647	0.200186	0.624872	protein_coding
ENSG0000(	TCERG1L	25.98205	0.448311	0.200205	0.624872	protein_coding
ENSG0000(	TRIP13	186.3299	0.170433	0.200239	0.624872	protein_coding
ENSG0000(	TUBG2	189.4029	-0.18518	0.200089	0.624872	protein_coding
ENSG0000(	YIF1B	383.2851	-0.11622	0.200229	0.624872	protein_coding
ENSG0000(	ANPEP	7425.612	-0.47102	0.200313	0.624974	protein_coding
ENSG0000(	PLA2R1	1897.791	0.244805	0.200339	0.624984	protein_coding
ENSG0000(	RIBC1	28.7762	0.409938	0.200368	0.625002	protein_coding
ENSG0000(	SLC9A3	15413.62	0.545334	0.200394	0.625012	protein_coding
ENSG0000(	ENSG0000(	141.7171	-0.15074	0.200472	0.625043	lncRNA
ENSG0000(	ENSG0000(	1043.7	-0.09389	0.200517	0.625043	lncRNA
ENSG0000(	PKD1P6-NF	69.04323	0.41587	0.200508	0.625043	lncRNA
ENSG0000(	PRANCR	1394.567	0.078149	0.200453	0.625043	lncRNA
ENSG0000(	TBCA	271.4979	-0.14751	0.200439	0.625043	protein_coding
ENSG0000(	ENSG0000(	112.1566	-0.15264	0.200559	0.625051	lncRNA
ENSG0000(	HNRNPA1P	21.81804	0.594308	0.200565	0.625051	processed_pseudogene
ENSG0000(	DYNC1LI2-I	197.526	0.158007	0.200904	0.625112	lncRNA
ENSG0000(	ENSG0000(	22.354	0.276787	0.200915	0.625112	lncRNA
ENSG0000(	ENSG0000(	111.9535	-0.21779	0.200687	0.625112	lncRNA
ENSG0000(	ENSG0000(	40.94383	0.199052	0.2008	0.625112	lncRNA
ENSG0000(	ENSG0000(	66.26273	-0.18088	0.200912	0.625112	processed_pseudogene
ENSG0000(	ENSG0000(	22.41921	0.343488	0.200629	0.625112	lncRNA
ENSG0000(	ENSG0000(	313.2947	0.107314	0.200866	0.625112	lncRNA

ENSG0000(	ENSG0000(	436.0698	0.249404	0.200866	0.625112	lncRNA
ENSG0000(	ENSG0000(	281.4174	0.123774	0.200697	0.625112	lncRNA
ENSG0000(	FAM107B	2704.898	-0.12789	0.200711	0.625112	protein_coding
ENSG0000(	HSP90AB4I	77.37991	0.227046	0.200908	0.625112	processed_pseudogene
ENSG0000(	MAPK9	1119.333	0.081447	0.200713	0.625112	protein_coding
ENSG0000(	NCBP2AS2	110.9415	0.172788	0.200926	0.625112	protein_coding
ENSG0000(	TRIM10	152.347	0.324279	0.200748	0.625112	protein_coding
ENSG0000(	UBTF	1524.896	0.093479	0.200841	0.625112	protein_coding
ENSG0000(	PGAM1P6	37.24419	0.253012	0.200966	0.625168	processed_pseudogene
ENSG0000(	ENSG0000(	381.0259	0.102894	0.201037	0.625246	lncRNA
ENSG0000(	TRIM68	578.188	-0.13204	0.201034	0.625246	protein_coding
ENSG0000(	SNAP25	1377.949	-0.17057	0.201067	0.62527	protein_coding
ENSG0000(	PAGR1	1642.99	0.115608	0.201118	0.625356	protein_coding
ENSG0000(	ACACB	3225.672	0.122081	0.201286	0.625658	protein_coding
ENSG0000(	KCNG3	261.4058	0.334484	0.201266	0.625658	protein_coding
ENSG0000(	MIA2	2675.516	0.125626	0.201288	0.625658	protein_coding
ENSG0000(	NUP43	1458.08	0.081235	0.201306	0.625658	protein_coding
ENSG0000(	ENSG0000(	27.9392	-0.508	0.201508	0.626216	lncRNA
ENSG0000(	MAST4-IT1	44.29419	0.263127	0.201559	0.626232	lncRNA
ENSG0000(	OR5AS1	2809.886	0.126004	0.20155	0.626232	protein_coding
ENSG0000(	ENSG0000(	24.78858	0.388958	0.201641	0.626321	lncRNA
ENSG0000(	SLC39A8	1549.005	0.205325	0.201638	0.626321	protein_coding
ENSG0000(	THUMPD3-	3624.614	0.069713	0.201679	0.626321	lncRNA
ENSG0000(	WDR74	393.0265	-0.14538	0.201671	0.626321	protein_coding
ENSG0000(	ANO10	1112.954	0.181308	0.201793	0.626464	protein_coding
ENSG0000(	CHRD	326.9228	-0.17379	0.201749	0.626464	protein_coding
ENSG0000(	ENSG0000(	66.77981	0.174662	0.201783	0.626464	lncRNA
ENSG0000(	LINC00265	1173.171	0.077288	0.201841	0.626504	lncRNA
ENSG0000(	SENP5	2750.878	0.077872	0.201851	0.626504	protein_coding
ENSG0000(	TRAJ26	34.54093	-0.39584	0.201883	0.626532	TR_J_gene
ENSG0000(	ENSG0000(	61.06444	0.217724	0.201985	0.62659	lncRNA
ENSG0000(	ENSG0000(	46.36708	-0.27569	0.201978	0.62659	TEC
ENSG0000(	PEX10	264.0292	0.184281	0.201993	0.62659	protein_coding
ENSG0000(	SEC62	1686.884	-0.10908	0.201943	0.62659	protein_coding
ENSG0000(	DGKZ	1461.038	-0.13597	0.202115	0.626827	protein_coding
ENSG0000(	NFKBID	403.1164	-0.16044	0.20211	0.626827	protein_coding
ENSG0000(	SLC17A7	152.9801	0.172628	0.202149	0.626861	protein_coding
ENSG0000(	ENSG0000(	131.4941	0.221086	0.202244	0.627061	lncRNA
ENSG0000(	ENSG0000(	336.1557	0.097113	0.202259	0.627061	lncRNA
ENSG0000(	CSNK1D	3767.865	0.095634	0.202365	0.627119	protein_coding
ENSG0000(	ENSG0000(	358.6755	0.687943	0.202407	0.627119	lncRNA
ENSG0000(	ETS1	4231.589	-0.18572	0.20231	0.627119	protein_coding
ENSG0000(	GLI4	525.3554	0.114041	0.202384	0.627119	protein_coding
ENSG0000(	RN7SL74P	26.57986	-0.30125	0.202414	0.627119	misc_RNA
ENSG0000(	SLC10A5	251.3285	0.362923	0.202333	0.627119	protein_coding
ENSG0000(	RN7SL815F	22.14084	0.315278	0.202462	0.627149	misc_RNA
ENSG0000(	SLC33A1	2060.647	-0.10449	0.202469	0.627149	protein_coding

ENSG0000(	ENSG0000(	171.0966	0.186643	0.202652	0.627431	lncRNA
ENSG0000(	GABBR2	75.704	-0.46388	0.202632	0.627431	protein_coding
ENSG0000(	KLHL6-AS1	196.7741	-0.15448	0.202603	0.627431	lncRNA
ENSG0000(	UBE2V1	1893.694	-0.07229	0.20264	0.627431	protein_coding
ENSG0000(	ARHGEF16	1082.958	0.277473	0.20274	0.627443	protein_coding
ENSG0000(	ARHGEF2-A	139.2654	-0.19035	0.202722	0.627443	lncRNA
ENSG0000(	ENSG0000(	224.4279	0.168347	0.202747	0.627443	lncRNA
ENSG0000(	MTND1P10	86.84666	0.166631	0.202687	0.627443	processed_pseudogene
ENSG0000(	ENSG0000(	33.30296	0.434431	0.20282	0.627598	lncRNA
ENSG0000(	ZNF8	1821.015	0.051192	0.202891	0.627748	protein_coding
ENSG0000(	ENSG0000(	165.1788	0.148548	0.203057	0.62775	lncRNA
ENSG0000(	ENSG0000(	325.8214	-0.11823	0.203097	0.62775	lncRNA
ENSG0000(	ENSG0000(	116.5505	0.25241	0.203096	0.62775	lncRNA
ENSG0000(	IVD	1787.321	0.102569	0.203038	0.62775	protein_coding
ENSG0000(	LAPTM4B	560.1054	-0.16238	0.202948	0.62775	protein_coding
ENSG0000(	LINC01996	48.66728	0.20761	0.202944	0.62775	lncRNA
ENSG0000(	MEG8	2200.857	-0.15418	0.203089	0.62775	lncRNA
ENSG0000(	STUB1	250.5692	0.21012	0.203027	0.62775	protein_coding
ENSG0000(	TBC1D10C	688.2938	-0.29558	0.203019	0.62775	protein_coding
ENSG0000(	NAT9	489.0497	0.109114	0.203122	0.627758	protein_coding
ENSG0000(	ENSG0000(	30.10643	0.394612	0.203209	0.627898	lncRNA
ENSG0000(	STK39	1187.409	0.199165	0.203213	0.627898	protein_coding
ENSG0000(	ACADSB	2254.069	-0.07107	0.203254	0.627914	protein_coding
ENSG0000(	RN7SKP237	21.30679	0.380817	0.203264	0.627914	misc_RNA
ENSG0000(	A2M-AS1	200.8671	-0.34041	0.203388	0.628026	lncRNA
ENSG0000(	ENSG0000(	59.59195	-0.22901	0.203477	0.628026	lncRNA
ENSG0000(	ENSG0000(	31.7106	-0.27425	0.203514	0.628026	lncRNA
ENSG0000(	ENSG0000(	60.94684	-0.46398	0.203418	0.628026	protein_coding
ENSG0000(	ENSG0000(	50.86526	-0.34768	0.203529	0.628026	lncRNA
ENSG0000(	MAST2	3600.128	0.283023	0.203473	0.628026	protein_coding
ENSG0000(	MEPCE	542.0071	0.124618	0.203433	0.628026	protein_coding
ENSG0000(	MEX3B	45.88987	-0.35202	0.203495	0.628026	protein_coding
ENSG0000(	TTC24	213.906	-0.27337	0.203403	0.628026	protein_coding
ENSG0000(	ZNF417	1916.688	0.124297	0.203406	0.628026	protein_coding
ENSG0000(	IGKV2D-40	20.34277	-1.2915	0.203565	0.628069	IG_V_gene
ENSG0000(	ENSG0000(	70.9526	0.183036	0.203629	0.628194	lncRNA
ENSG0000(	MFAP2	63.11608	-0.51977	0.20368	0.628282	protein_coding
ENSG0000(	ENSG0000(	32.6302	-0.26778	0.203769	0.628361	lncRNA
ENSG0000(	ENSG0000(	75.9872	0.251935	0.203774	0.628361	lncRNA
ENSG0000(	RASL12	115.7902	-0.2885	0.203771	0.628361	protein_coding
ENSG0000(	MIR663A	3283.032	0.314738	0.203801	0.628372	miRNA
ENSG0000(	ENSG0000(	27.65988	0.280832	0.203843	0.628432	processed_pseudogene
ENSG0000(	CYP4F12	2132.742	0.368347	0.204022	0.628558	protein_coding
ENSG0000(	GLI2	249.1643	-0.27991	0.204056	0.628558	protein_coding
ENSG0000(	GTPBP3	478.56	0.139763	0.20404	0.628558	protein_coding
ENSG0000(	MCPH1-DT	567.2036	0.166826	0.203969	0.628558	lncRNA
ENSG0000(	OR4E2	36.39356	0.239349	0.204067	0.628558	protein_coding

ENSG0000(	RBM24	28.60542	0.394923	0.204014	0.628558	protein_coding
ENSG0000(	SMPD4	2042.193	0.079972	0.203984	0.628558	protein_coding
ENSG0000(	ZNF747	578.2298	0.073123	0.203941	0.628558	protein_coding
ENSG0000(	ENSG0000(	26.46136	-0.44678	0.204147	0.628625	lncRNA
ENSG0000(	ENSG0000(	624.4023	-0.08116	0.204157	0.628625	TEC
ENSG0000(	SEMA6B	333.7612	-0.12091	0.204126	0.628625	protein_coding
ENSG0000(	MOB1A	3213.038	-0.0823	0.204185	0.628641	protein_coding
ENSG0000(	GPR119	219.3643	0.118326	0.204256	0.628791	protein_coding
ENSG0000(	ENSG0000(	43.89014	0.245828	0.204313	0.628824	lncRNA
ENSG0000(	SVIL-AS1	1306.128	0.079688	0.204301	0.628824	transcribed_unprocessed_pseudo
ENSG0000(	ENSG0000(	399.1333	-0.34567	0.204463	0.629216	processed_pseudogene
ENSG0000(	SGMS1	641.1366	-0.16379	0.204535	0.629365	protein_coding
ENSG0000(	B3GNTL1	304.3985	-0.18657	0.204566	0.629391	protein_coding
ENSG0000(	IGHV3-69-1	72.98512	-0.61168	0.204671	0.629646	IG_V_pseudogene
ENSG0000(	RBBP4	4025.692	-0.0796	0.204731	0.629759	protein_coding
ENSG0000(	FAAH2	195.1288	0.20394	0.204814	0.629873	protein_coding
ENSG0000(	LINC01191	93.35048	-0.28193	0.204797	0.629873	lncRNA
ENSG0000(	LINC02844	48.1292	0.230859	0.204921	0.630133	lncRNA
ENSG0000(	CASP2	1770.792	-0.07289	0.205019	0.63015	protein_coding
ENSG0000(	FCHO1	469.72	-0.1201	0.204991	0.63015	protein_coding
ENSG0000(	INSYN2B	22.41208	-0.54975	0.204976	0.63015	protein_coding
ENSG0000(	OGFRL1	1290.161	-0.20693	0.205009	0.63015	protein_coding
ENSG0000(	MTMR3	2870.549	0.119041	0.205155	0.6305	protein_coding
ENSG0000(	ENSG0000(	129.8291	0.396097	0.205274	0.630548	processed_pseudogene
ENSG0000(	ENSG0000(	100.093	0.286854	0.205218	0.630548	lncRNA
ENSG0000(	ENSG0000(	201.3415	0.131233	0.205332	0.630548	lncRNA
ENSG0000(	ENSG0000(	48.27931	-0.24745	0.205288	0.630548	lncRNA
ENSG0000(	GLIPR1	739.6416	-0.26967	0.20532	0.630548	protein_coding
ENSG0000(	MN1	124.4616	-0.34191	0.205303	0.630548	protein_coding
ENSG0000(	ZNF222	76.70467	-0.21858	0.205289	0.630548	protein_coding
ENSG0000(	ENSG0000(	5843.476	0.15646	0.205436	0.630571	lncRNA
ENSG0000(	ENSG0000(	469.2655	-0.08474	0.205591	0.630571	lncRNA
ENSG0000(	FANCA	826.1502	-0.15465	0.205489	0.630571	protein_coding
ENSG0000(	HDHD3	1466.036	0.233112	0.20555	0.630571	protein_coding
ENSG0000(	HOXD8	72.57672	-0.27191	0.205573	0.630571	protein_coding
ENSG0000(	KIF5A	390.1127	-0.26526	0.205554	0.630571	protein_coding
ENSG0000(	RPS3AP26	68.74329	0.472687	0.205565	0.630571	processed_pseudogene
ENSG0000(	SNHG26	768.4847	-0.22301	0.205564	0.630571	lncRNA
ENSG0000(	STYK1	587.8737	0.260288	0.205591	0.630571	protein_coding
ENSG0000(	TGFB1I1	329.561	-0.20339	0.205446	0.630571	protein_coding
ENSG0000(	UGT1A9	396.4557	0.439925	0.205402	0.630571	protein_coding
ENSG0000(	TOP3A	1450.459	0.083457	0.205684	0.630717	protein_coding
ENSG0000(	ZNF512B	370.3562	-0.1905	0.205684	0.630717	protein_coding
ENSG0000(	CPEB2	764.4609	0.164415	0.205826	0.630843	protein_coding
ENSG0000(	HCAR1	493.772	0.082826	0.205801	0.630843	protein_coding
ENSG0000(	LINC00565	409.1694	-0.09752	0.205778	0.630843	lncRNA
ENSG0000(	LINC01224	935.9066	0.169106	0.20584	0.630843	lncRNA

ENSG0000(	RPUSD2	173.1096	0.189823	0.205767	0.630843	protein_coding
ENSG0000(	UFD1	1868.233	-0.07827	0.205925	0.631034	protein_coding
ENSG0000(	ENSG0000(	19.9092	0.339433	0.206033	0.6312	lncRNA
ENSG0000(	LINC01500	33.42628	-0.34113	0.206049	0.6312	lncRNA
ENSG0000(	UBA7	1779.369	-0.12482	0.206021	0.6312	protein_coding
ENSG0000(	HACD1	266.5489	0.110258	0.206252	0.631711	protein_coding
ENSG0000(	OSTF1	344.8365	-0.16163	0.206261	0.631711	protein_coding
ENSG0000(	ENSG0000(	55.18176	0.278804	0.206451	0.632222	transcribed_processed_pseudoge
ENSG0000(	RAB40C	696.796	0.233976	0.206487	0.632261	protein_coding
ENSG0000(	ENSG0000(	43.5258	0.235801	0.20656	0.632414	lncRNA
ENSG0000(	RAD51AP1	116.7253	-0.28423	0.206636	0.632578	protein_coding
ENSG0000(	KMO	282.3581	-0.3157	0.206702	0.632708	protein_coding
ENSG0000(	ENSG0000(	26.82657	-0.30826	0.20673	0.632723	lncRNA
ENSG0000(	CDH23	286.8077	-0.23869	0.206823	0.632727	protein_coding
ENSG0000(	ENSG0000(	94.04215	-0.16769	0.206771	0.632727	lncRNA
ENSG0000(	HSPB11	417.1338	-0.11159	0.206799	0.632727	protein_coding
ENSG0000(	OR2J2	44.71309	0.245753	0.206801	0.632727	protein_coding
ENSG0000(	PLAA	562.17	0.121212	0.206873	0.632811	protein_coding
ENSG0000(	CALHM2	146.8403	-0.28613	0.206962	0.632902	protein_coding
ENSG0000(	ENSG0000(	230.9637	0.141682	0.206949	0.632902	lncRNA
ENSG0000(	SNAPC1	446.7527	0.114519	0.206972	0.632902	protein_coding
ENSG0000(	INTS4	939.7956	0.083811	0.207026	0.632926	protein_coding
ENSG0000(	JPH4	21.17835	-0.37739	0.207014	0.632926	protein_coding
ENSG0000(	ENSG0000(	149.4606	0.154863	0.207075	0.632935	lncRNA
ENSG0000(	ENSG0000(	220.1559	0.115654	0.207072	0.632935	lncRNA
ENSG0000(	RETSAT	1981.878	0.271994	0.20722	0.633308	protein_coding
ENSG0000(	ENSG0000(	51.64606	0.248959	0.207291	0.633346	lncRNA
ENSG0000(	ENSG0000(	25.90728	-0.25043	0.207326	0.633346	lncRNA
ENSG0000(	ISCU	960.3886	-0.13051	0.207309	0.633346	protein_coding
ENSG0000(	KIAA1109	14205.83	0.085975	0.207317	0.633346	protein_coding
ENSG0000(	LINC02649	425.7908	-0.18101	0.207347	0.633346	lncRNA
ENSG0000(	AIDAP2	157.6515	0.172584	0.207446	0.633446	transcribed_processed_pseudoge
ENSG0000(	CCND2	5666.749	-0.13323	0.207446	0.633446	protein_coding
ENSG0000(	CHURC1	1749.24	-0.08565	0.207595	0.633446	protein_coding
ENSG0000(	EEF2K	2263.264	0.112364	0.207451	0.633446	protein_coding
ENSG0000(	ENSG0000(	1069.46	-0.08857	0.207591	0.633446	lncRNA
ENSG0000(	LINC01670	122.5979	0.338523	0.207611	0.633446	lncRNA
ENSG0000(	PARK7	545.4477	-0.09591	0.207544	0.633446	protein_coding
ENSG0000(	SOS1-IT1	60.58929	-1.00014	0.207566	0.633446	lncRNA
ENSG0000(	TACO1	262.1979	0.164703	0.207594	0.633446	protein_coding
ENSG0000(	TRHDE	387.1572	0.361401	0.207567	0.633446	protein_coding
ENSG0000(	RNF222	220.7171	-0.11088	0.207637	0.633456	protein_coding
ENSG0000(	ENSG0000(	256.6737	-0.11633	0.207795	0.63384	TEC
ENSG0000(	ZNF135	339.9425	0.13788	0.207809	0.63384	protein_coding
ENSG0000(	CHCHD6	70.07308	0.215432	0.207892	0.633843	protein_coding
ENSG0000(	LINC01473	506.7082	0.147512	0.207899	0.633843	lncRNA
ENSG0000(	MSH6	1149.225	-0.08763	0.207911	0.633843	protein_coding

ENSG0000(	NDUFAF3	144.2389	-0.19496	0.207934	0.633843	protein_coding
ENSG0000(	SVEP1	677.9194	-0.26426	0.207924	0.633843	protein_coding
ENSG0000(	VDR	2150.094	0.210095	0.207948	0.633843	protein_coding
ENSG0000(	FAM229A	365.6482	0.154761	0.208054	0.634096	protein_coding
ENSG0000(	ARL4C	762.4038	-0.23805	0.208158	0.634115	protein_coding
ENSG0000(	CRB3	234.7878	0.302827	0.208199	0.634115	protein_coding
ENSG0000(	FAT1	11663.47	0.153864	0.208188	0.634115	protein_coding
ENSG0000(	FXYD3	3077.895	0.38842	0.208119	0.634115	protein_coding
ENSG0000(	HIBADH	316.5201	-0.15755	0.208142	0.634115	protein_coding
ENSG0000(	PMAIP1	78.05058	-0.2968	0.208109	0.634115	protein_coding
ENSG0000(	SLU7	684.2598	-0.12377	0.20823	0.63414	protein_coding
ENSG0000(	ENSG0000(	196.682	0.141297	0.208271	0.634182	lncRNA
ENSG0000(	ENSG0000(	44.50367	-0.28104	0.20829	0.634182	TEC
ENSG0000(	ENSG0000(	562.1445	0.123722	0.208362	0.634262	lncRNA
ENSG0000(	MT-TS2	85.31457	-0.49409	0.208342	0.634262	Mt_tRNA
ENSG0000(	ENSG0000(	775.9359	0.107617	0.208453	0.634469	protein_coding
ENSG0000(	ENSG0000(	69.98222	-0.23242	0.208502	0.634548	lncRNA
ENSG0000(	CASC19	839.1648	-0.18825	0.208741	0.634874	lncRNA
ENSG0000(	ENSG0000(	49.06828	-0.21165	0.208677	0.634874	lncRNA
ENSG0000(	FOXD4L5	123.0964	-0.18484	0.208724	0.634874	protein_coding
ENSG0000(	HLA-DRB5	648.2974	-0.4314	0.20871	0.634874	protein_coding
ENSG0000(	MMADHC-I	209.3478	0.282273	0.208748	0.634874	lncRNA
ENSG0000(	RSPH10B	282.2643	0.103928	0.208739	0.634874	protein_coding
ENSG0000(	LINC00336	136.254	-0.19266	0.208845	0.634886	lncRNA
ENSG0000(	LURAP1	164.9306	-0.14141	0.208802	0.634886	protein_coding
ENSG0000(	MAP4K2	697.6333	-0.17952	0.208874	0.634886	protein_coding
ENSG0000(	NAA16	756.1106	-0.14269	0.20889	0.634886	protein_coding
ENSG0000(	PRDM7	233.6595	-0.13302	0.208786	0.634886	protein_coding
ENSG0000(	SMARCD1	938.6	-0.1144	0.208873	0.634886	protein_coding
ENSG0000(	ENSG0000(	19.6135	0.826166	0.208941	0.634899	processed_pseudogene
ENSG0000(	GNG2	785.3241	-0.19695	0.20894	0.634899	protein_coding
ENSG0000(	ATP8B3	203.3473	-0.15121	0.209067	0.635073	protein_coding
ENSG0000(	ENSG0000(	44.70437	-0.27211	0.209053	0.635073	lncRNA
ENSG0000(	ENSG0000(	234.1587	0.231313	0.20906	0.635073	TEC
ENSG0000(	ENSG0000(	55.26084	-0.23328	0.209093	0.635081	processed_pseudogene
ENSG0000(	DUSP16	1615.198	0.157608	0.209194	0.635119	protein_coding
ENSG0000(	PLA2G12A	1278.003	0.145063	0.209198	0.635119	protein_coding
ENSG0000(	PUM2	4124.076	0.08043	0.209191	0.635119	protein_coding
ENSG0000(	RN7SL801F	81.28683	0.187053	0.209181	0.635119	misc_RNA
ENSG0000(	CCDC28A	132.866	-0.2096	0.20927	0.635269	protein_coding
ENSG0000(	COMMD6	173.0177	0.175373	0.209312	0.635327	protein_coding
ENSG0000(	MLIP-AS1	123.2128	-0.14056	0.209407	0.635545	lncRNA
ENSG0000(	ENSG0000(	96.11624	-0.17852	0.209563	0.635837	lncRNA
ENSG0000(	OSBPL10-A	54.87874	0.247339	0.209573	0.635837	lncRNA
ENSG0000(	RETREG1	1519.962	0.15525	0.209569	0.635837	protein_coding
ENSG0000(	CLPTM1	643.0093	-0.1193	0.209616	0.635864	protein_coding
ENSG0000(	ENSG0000(	25.52254	-0.31115	0.209697	0.635864	lncRNA

ENSG0000(	ENSG0000(	25.13673	-0.33869	0.209685	0.635864	lncRNA
ENSG0000(	ENSG0000(	62.61509	0.185772	0.20967	0.635864	lncRNA
ENSG0000(	GRK7	157.8052	0.101221	0.209656	0.635864	protein_coding
ENSG0000(	NMT1	1203.091	-0.11759	0.209803	0.636044	protein_coding
ENSG0000(	OSBPL2	2805.312	0.098072	0.20978	0.636044	protein_coding
ENSG0000(	SIKE1	1202.551	-0.09801	0.209835	0.636072	protein_coding
ENSG0000(	MARCHF1	755.6869	-0.29018	0.209862	0.636082	protein_coding
ENSG0000(	EIF5A2	182.7326	-0.17736	0.209997	0.636294	protein_coding
ENSG0000(	ENSG0000(	109.6828	0.180196	0.209971	0.636294	lncRNA
ENSG0000(	LINC01546	594.6149	-0.09707	0.210024	0.636294	lncRNA
ENSG0000(	PARVG	1401.62	-0.24221	0.210016	0.636294	protein_coding
ENSG0000(	BRINP3	259.922	0.360203	0.210145	0.636316	protein_coding
ENSG0000(	ENSG0000(	30.2791	0.36455	0.210111	0.636316	lncRNA
ENSG0000(	ID4	232.5398	-0.20217	0.21017	0.636316	protein_coding
ENSG0000(	IKBKE	599.5497	-0.13902	0.210157	0.636316	protein_coding
ENSG0000(	LRRC51	519.3278	0.137682	0.210086	0.636316	protein_coding
ENSG0000(	MORN2	27.85512	-0.30573	0.210129	0.636316	protein_coding
ENSG0000(	ENSG0000(	83.0138	-0.2209	0.210201	0.636338	transcribed_unprocessed_pseudo
ENSG0000(	C1orf158	327.213	0.104492	0.21031	0.63636	protein_coding
ENSG0000(	ENSG0000(	371.1296	-0.09535	0.210324	0.63636	lncRNA
ENSG0000(	OR2AG2	185.3003	-0.16483	0.2103	0.63636	protein_coding
ENSG0000(	RBM18	407.5698	0.110639	0.210235	0.63636	protein_coding
ENSG0000(	RGMB-AS1	51.87906	0.425255	0.210266	0.63636	lncRNA
ENSG0000(	CGN	4391.605	0.309062	0.210365	0.636408	protein_coding
ENSG0000(	ZNF385C	314.4503	-0.11191	0.210386	0.636408	protein_coding
ENSG0000(	KCNA7	451.3148	0.07602	0.210417	0.636434	protein_coding
ENSG0000(	GSK3B	3512.632	-0.06058	0.210455	0.636453	protein_coding
ENSG0000(	KATNAL1	609.8212	-0.13807	0.21047	0.636453	protein_coding
ENSG0000(	IL3RA	120.6304	-0.3283	0.210515	0.636471	protein_coding
ENSG0000(	SFTA1P	161.6578	-0.20794	0.210522	0.636471	lncRNA
ENSG0000(	ZNF121	3325.704	0.085081	0.210593	0.636614	protein_coding
ENSG0000(	KIF4A	201.3966	0.283176	0.210617	0.636619	protein_coding
ENSG0000(	POMGNT2	128.2124	0.163599	0.210678	0.636733	protein_coding
ENSG0000(	UBFD1	1105.392	-0.0998	0.210797	0.637022	protein_coding
ENSG0000(	STK11IP	409.9603	0.142223	0.210859	0.637139	protein_coding
ENSG0000(	SATB2-AS1	969.3244	0.395931	0.210909	0.637219	lncRNA
ENSG0000(	LINC00672	844.6092	0.123369	0.210964	0.637318	protein_coding
ENSG0000(	PLD6	162.1922	0.125737	0.211012	0.637372	protein_coding
ENSG0000(	TUBA1C	905.9171	0.184971	0.211029	0.637372	protein_coding
ENSG0000(	ENSG0000(	94.42146	0.485528	0.211124	0.637449	protein_coding
ENSG0000(	ENSG0000(	158.897	-0.13779	0.211103	0.637449	lncRNA
ENSG0000(	ENSG0000(	156.3899	0.123667	0.211082	0.637449	lncRNA
ENSG0000(	ATP1B3	1232.813	0.230475	0.211493	0.637465	protein_coding
ENSG0000(	CCNF	382.7463	-0.14437	0.211236	0.637465	protein_coding
ENSG0000(	CD47	11796.89	0.222206	0.211556	0.637465	protein_coding
ENSG0000(	CNPY3	334.542	-0.17675	0.211359	0.637465	protein_coding
ENSG0000(	DLK2	47.79685	0.171921	0.211829	0.637465	protein_coding

ENSG0000(	E2F8	249.6929	0.256341	0.211357	0.637465	protein_coding
ENSG0000(	ENSG0000(	285.2108	0.263333	0.211896	0.637465	processed_pseudogene
ENSG0000(	ENSG0000(	38.77409	0.188977	0.211223	0.637465	lncRNA
ENSG0000(	ENSG0000(	795.4755	0.122455	0.211967	0.637465	lncRNA
ENSG0000(	ENSG0000(	26.1082	-0.73554	0.211374	0.637465	protein_coding
ENSG0000(	ENSG0000(	34.96925	-0.33676	0.211582	0.637465	lncRNA
ENSG0000(	ENSG0000(	201.3185	0.117806	0.211588	0.637465	lncRNA
ENSG0000(	ENSG0000(	152.7973	0.134482	0.21167	0.637465	lncRNA
ENSG0000(	ENSG0000(	51.54333	-0.28282	0.211846	0.637465	TEC
ENSG0000(	ENSG0000(	65.41383	-0.33188	0.211876	0.637465	TEC
ENSG0000(	ENSG0000(	144.1337	0.13491	0.21155	0.637465	lncRNA
ENSG0000(	ENSG0000(	24.20194	-0.32631	0.211297	0.637465	lncRNA
ENSG0000(	ENSG0000(	87.20773	-0.20105	0.21135	0.637465	lncRNA
ENSG0000(	ENSG0000(	19.74539	0.436796	0.211605	0.637465	lncRNA
ENSG0000(	EXOSC7	501.1727	0.091154	0.211527	0.637465	protein_coding
ENSG0000(	GHRL	34.47997	0.399039	0.211252	0.637465	protein_coding
ENSG0000(	KRI1	549.0717	-0.12909	0.211896	0.637465	protein_coding
ENSG0000(	LINC02593	228.2304	-0.25926	0.211982	0.637465	lncRNA
ENSG0000(	MMP15	935.0304	0.28643	0.211586	0.637465	protein_coding
ENSG0000(	MTND5P11	31.51012	0.471194	0.211728	0.637465	processed_pseudogene
ENSG0000(	NANOGP7	25.0234	-0.45343	0.211898	0.637465	transcribed_processed_pseudoge
ENSG0000(	NDUFB9	2338.213	0.108721	0.211981	0.637465	protein_coding
ENSG0000(	NSUN3	1375.918	0.131109	0.211413	0.637465	protein_coding
ENSG0000(	PCDHGB4	94.54404	0.257604	0.211272	0.637465	protein_coding
ENSG0000(	RN7SL477F	73.47689	0.1661	0.211968	0.637465	misc_RNA
ENSG0000(	RNF4	1690.229	0.067171	0.211691	0.637465	protein_coding
ENSG0000(	SHROOM3-	24.16137	0.424733	0.211986	0.637465	lncRNA
ENSG0000(	TFIP11	487.5818	-0.11136	0.211877	0.637465	protein_coding
ENSG0000(	TVP23C	2612.362	-0.103	0.211827	0.637465	protein_coding
ENSG0000(	ZC3HAV1	3306.787	-0.1228	0.211678	0.637465	protein_coding
ENSG0000(	ZNF84	1610.904	0.093149	0.211442	0.637465	protein_coding
ENSG0000(	ZSCAN20	564.4302	0.104948	0.211583	0.637465	protein_coding
ENSG0000(	DNAJB6	3048.94	-0.10961	0.212115	0.637645	protein_coding
ENSG0000(	RNU6-638F	70.22307	-0.31459	0.212079	0.637645	snRNA
ENSG0000(	TRIM8	1275.099	-0.1467	0.212116	0.637645	protein_coding
ENSG0000(	BICDL2	2118.057	0.283152	0.212202	0.637651	protein_coding
ENSG0000(	COLCA1	3369.014	0.109078	0.212249	0.637651	lncRNA
ENSG0000(	DDI2	3154.401	-0.08613	0.212242	0.637651	protein_coding
ENSG0000(	DDX39B	9104.654	0.060283	0.212174	0.637651	protein_coding
ENSG0000(	ENSG0000(	795.1082	0.133404	0.212227	0.637651	lncRNA
ENSG0000(	ENSG0000(	321.2643	-0.12802	0.212257	0.637651	lncRNA
ENSG0000(	ENSG0000(	50.88356	-0.2803	0.212424	0.638016	lncRNA
ENSG0000(	RNF126	388.7524	0.139007	0.212425	0.638016	protein_coding
ENSG0000(	ENSG0000(	25.77591	-0.25998	0.212455	0.638039	lncRNA
ENSG0000(	PSMB7	298.0603	-0.13603	0.212499	0.638099	protein_coding
ENSG0000(	GUSBP11	2423.928	0.114775	0.212576	0.638261	lncRNA
ENSG0000(	ENSG0000(	46.36568	0.17392	0.212641	0.638317	lncRNA

ENSG0000(	TOM1L2	1879.121	0.13725	0.212628	0.638317	protein_coding
ENSG0000(	ENSG0000(	75.80266	0.207566	0.212727	0.638435	transcribed_processed_pseudoge
ENSG0000(	ENSG0000(	89.42992	-0.31153	0.212716	0.638435	protein_coding
ENSG0000(	ENSG0000(	39.98229	-0.26217	0.212753	0.638446	lncRNA
ENSG0000(	CENPS	60.35862	0.195821	0.212828	0.638601	protein_coding
ENSG0000(	ENSG0000(	77.10555	-0.31779	0.212909	0.638636	TEC
ENSG0000(	ENSG0000(	97.39183	-0.15216	0.212866	0.638636	lncRNA
ENSG0000(	LRRC25	213.0391	-0.17276	0.21291	0.638636	protein_coding
ENSG0000(	RNU6ATAC	27.77018	-0.29927	0.212948	0.638682	snRNA
ENSG0000(	ZBTB3	625.9493	0.114096	0.212974	0.638691	protein_coding
ENSG0000(	FAM204A	1235.508	0.129192	0.21314	0.639118	protein_coding
ENSG0000(	ENSG0000(	22.16635	-0.25067	0.213236	0.639196	lncRNA
ENSG0000(	ENSG0000(	162.3402	0.125327	0.213225	0.639196	TEC
ENSG0000(	ZNF28	717.8077	0.14637	0.213211	0.639196	protein_coding
ENSG0000(	ENSG0000(	21.14007	0.270852	0.213274	0.639241	lncRNA
ENSG0000(	ZNF614	272.4714	0.149638	0.213327	0.639331	protein_coding
ENSG0000(	EIF5B	1264.667	-0.12044	0.213546	0.639568	protein_coding
ENSG0000(	ENSG0000(	263.9133	0.141075	0.213519	0.639568	processed_pseudogene
ENSG0000(	ENSG0000(	519.974	0.161121	0.213523	0.639568	protein_coding
ENSG0000(	ENSG0000(	58.10619	-0.28113	0.213543	0.639568	lncRNA
ENSG0000(	LIG3	1831.157	0.092654	0.213526	0.639568	protein_coding
ENSG0000(	PITRM1-AS	59.38385	0.36466	0.213442	0.639568	lncRNA
ENSG0000(	ENSG0000(	131.1703	-0.18926	0.213625	0.639667	lncRNA
ENSG0000(	MRPL11	124.6889	0.201803	0.213602	0.639667	protein_coding
ENSG0000(	LINC02750	28.7452	-0.29064	0.213651	0.639674	lncRNA
ENSG0000(	PRPF6	647.8365	0.116218	0.213707	0.639772	protein_coding
ENSG0000(	ENSG0000(	1257.801	-0.08753	0.213762	0.639867	transcribed_processed_pseudoge
ENSG0000(	HELB	809.1488	-0.21269	0.213945	0.640345	protein_coding
ENSG0000(	ENSG0000(	46.10385	0.194762	0.214128	0.640694	lncRNA
ENSG0000(	PYGM	273.318	0.135412	0.214131	0.640694	protein_coding
ENSG0000(	ST13	1083.203	-0.14206	0.214115	0.640694	protein_coding
ENSG0000(	ENSG0000(	36.57201	-0.30459	0.214193	0.640809	lncRNA
ENSG0000(	CHUK	557.9912	-0.09046	0.214227	0.640842	protein_coding
ENSG0000(	LEFTY1	219.8187	0.364787	0.214259	0.640866	protein_coding
ENSG0000(	ENSG0000(	178.1577	0.118247	0.214287	0.64088	lncRNA
ENSG0000(	CAP2	51.84493	-0.32336	0.214352	0.640964	protein_coding
ENSG0000(	CIAO1	1985.345	0.057454	0.214501	0.640964	protein_coding
ENSG0000(	ENSG0000(	93.32709	0.132852	0.214381	0.640964	lncRNA
ENSG0000(	ENSG0000(	22.26251	0.311156	0.214501	0.640964	lncRNA
ENSG0000(	ENSG0000(	55.9889	-0.19648	0.214485	0.640964	lncRNA
ENSG0000(	HLA-DPB2	107.9431	-0.17899	0.214471	0.640964	transcribed_unprocessed_pseudo
ENSG0000(	HYMAI	308.7047	0.173156	0.214409	0.640964	lncRNA
ENSG0000(	RBMY2FP	50.9728	0.210297	0.214434	0.640964	transcribed_unprocessed_pseudo
ENSG0000(	CXCL12	1270.728	-0.29098	0.214709	0.641028	protein_coding
ENSG0000(	DOCK10	2815.792	-0.19544	0.214641	0.641028	protein_coding
ENSG0000(	ENSG0000(	128.6984	-0.13141	0.214645	0.641028	lncRNA
ENSG0000(	ENSG0000(	402.5577	0.131055	0.214697	0.641028	lncRNA

ENSG0000(	ENSG0000(	2183.6	0.088823	0.214698	0.641028	TEC
ENSG0000(	MOGAT3	959.2082	0.231171	0.214657	0.641028	protein_coding
ENSG0000(	RAB13	538.5196	-0.18691	0.214651	0.641028	protein_coding
ENSG0000(	RBMS2	2662.211	-0.15495	0.214682	0.641028	protein_coding
ENSG0000(	ENSG0000(	70.57554	0.196266	0.214787	0.641192	lncRNA
ENSG0000(	SPDYE10P	313.9941	0.128521	0.214852	0.641315	protein_coding
ENSG0000(	TIMM22	175.6171	0.167753	0.214932	0.641485	protein_coding
ENSG0000(	ENSG0000(	54.72491	-0.23903	0.215031	0.641622	lncRNA
ENSG0000(	GVQW3	2194.845	0.068046	0.215006	0.641622	protein_coding
ENSG0000(	LINC01625	137.1326	0.142874	0.215054	0.641622	lncRNA
ENSG0000(	PLEKHG4	377.8073	0.11796	0.215071	0.641622	protein_coding
ENSG0000(	ENSG0000(	163.7483	0.174665	0.215102	0.641645	lncRNA
ENSG0000(	RGPD8	960.6624	-0.25443	0.215204	0.641739	protein_coding
ENSG0000(	RHBDL3	88.42327	0.372403	0.215179	0.641739	protein_coding
ENSG0000(	SPDYE18	45.30592	0.268115	0.215193	0.641739	protein_coding
ENSG0000(	COX6B2	486.7573	0.279195	0.215294	0.641811	protein_coding
ENSG0000(	LINC00189	51.44449	-0.27135	0.215303	0.641811	lncRNA
ENSG0000(	RN7SL25P	75.25866	0.186514	0.215321	0.641811	misc_RNA
ENSG0000(	SMIM38	63.79937	-0.44173	0.215302	0.641811	protein_coding
ENSG0000(	ASTE1	311.5759	0.172562	0.215645	0.641948	protein_coding
ENSG0000(	BCRP2	149.1956	0.184334	0.21573	0.641948	transcribed_unprocessed_pseudo
ENSG0000(	ENSG0000(	235.0525	-0.15867	0.21574	0.641948	lncRNA
ENSG0000(	ENSG0000(	25.78737	-0.29294	0.215648	0.641948	lncRNA
ENSG0000(	ENSG0000(	139.0593	0.143564	0.215731	0.641948	lncRNA
ENSG0000(	ENSG0000(	19.77217	0.368314	0.215606	0.641948	lncRNA
ENSG0000(	FBXW4P1	99.54144	0.176696	0.215604	0.641948	processed_pseudogene
ENSG0000(	FTSJ1	277.8426	-0.17844	0.21564	0.641948	protein_coding
ENSG0000(	INTS5	230.3316	0.138323	0.215403	0.641948	protein_coding
ENSG0000(	LAMTOR5P	24.85494	0.350795	0.215728	0.641948	processed_pseudogene
ENSG0000(	LINC02478	19.48573	-0.43105	0.215506	0.641948	lncRNA
ENSG0000(	LRRC49	475.4936	0.134684	0.215483	0.641948	protein_coding
ENSG0000(	MACC1	1540.52	0.193691	0.215456	0.641948	protein_coding
ENSG0000(	PIK3CG	998.1552	-0.22725	0.215583	0.641948	protein_coding
ENSG0000(	SCARNA3	70.9675	0.164243	0.215557	0.641948	scaRNA
ENSG0000(	SERPINB8	318.9733	-0.19266	0.215621	0.641948	protein_coding
ENSG0000(	CPE	102.2725	0.314574	0.2158	0.642016	protein_coding
ENSG0000(	TRIM2	5724.248	0.199374	0.21581	0.642016	protein_coding
ENSG0000(	EDN3	448.1671	0.409964	0.21593	0.642096	protein_coding
ENSG0000(	ENSG0000(	167.6809	-0.13418	0.215926	0.642096	lncRNA
ENSG0000(	ENSG0000(	294.794	0.099133	0.21592	0.642096	lncRNA
ENSG0000(	SLC5A3	4277.283	0.099219	0.215876	0.642096	protein_coding
ENSG0000(	LOXL1-AS1	449.5375	-0.10087	0.216128	0.642546	lncRNA
ENSG0000(	SLC25A3	3610.77	0.117557	0.216119	0.642546	protein_coding
ENSG0000(	IGSF9	1205.796	0.456177	0.216182	0.642566	protein_coding
ENSG0000(	ZMIZ2	2873.87	-0.0755	0.216173	0.642566	protein_coding
ENSG0000(	ADAMTS13	181.1437	0.25317	0.216482	0.642625	protein_coding
ENSG0000(	ENSG0000(	28.33707	0.27134	0.216386	0.642625	lncRNA

ENSG0000(	ENSG0000(	132.7781	-0.18617	0.216495	0.642625	processed_pseudogene
ENSG0000(	ENSG0000(	133.3112	0.14718	0.216507	0.642625	lncRNA
ENSG0000(	ENSG0000(	81.42183	0.190943	0.216529	0.642625	lncRNA
ENSG0000(	GTF2I-AS1	187.4421	-0.1281	0.216527	0.642625	lncRNA
ENSG0000(	KIF3A	563.8007	-0.1381	0.21643	0.642625	protein_coding
ENSG0000(	LINC00299	965.1789	-0.12798	0.216432	0.642625	lncRNA
ENSG0000(	OGFOD1	655.5227	-0.11159	0.216323	0.642625	protein_coding
ENSG0000(	PTRH1	311.5693	-0.11416	0.216399	0.642625	protein_coding
ENSG0000(	RIOX2	807.5413	-0.14264	0.216381	0.642625	protein_coding
ENSG0000(	RN7SL835F	78.48399	-0.16943	0.216452	0.642625	misc_RNA
ENSG0000(	RNU4ATAC	129.5132	-0.22106	0.216404	0.642625	snRNA
ENSG0000(	ZNF470-DT	73.22387	0.22251	0.216522	0.642625	lncRNA
ENSG0000(	LDAF1	393.4603	0.131379	0.216624	0.64284	protein_coding
ENSG0000(	ATL3	1992.624	-0.08822	0.21675	0.642874	protein_coding
ENSG0000(	CD79B	180.6274	-0.48525	0.216799	0.642874	protein_coding
ENSG0000(	ENSG0000(	44.62441	-0.26178	0.216788	0.642874	TEC
ENSG0000(	HMGN3-AS	85.90007	0.17833	0.216735	0.642874	lncRNA
ENSG0000(	LINC01704	33.59228	-0.34825	0.216757	0.642874	lncRNA
ENSG0000(	LNCARSR	271.3035	0.154002	0.216661	0.642874	lncRNA
ENSG0000(	TUBB3	776.8654	-0.11643	0.216799	0.642874	protein_coding
ENSG0000(	LRSAM1	1295.344	-0.08105	0.216951	0.643253	protein_coding
ENSG0000(	CATSPER2F	279.0164	0.123817	0.217067	0.643389	transcribed_unprocessed_pseudo
ENSG0000(	ENSG0000(	24.53906	-0.29828	0.217038	0.643389	lncRNA
ENSG0000(	KCTD9P1	47.82754	-0.28966	0.21705	0.643389	transcribed_processed_pseudoge
ENSG0000(	ENSG0000(	23.70539	0.369137	0.217096	0.643407	lncRNA
ENSG0000(	C9orf152	513.2759	0.336322	0.217221	0.643568	protein_coding
ENSG0000(	CADPS2	677.4814	-0.14925	0.217206	0.643568	protein_coding
ENSG0000(	ENSG0000(	98.07	-0.17887	0.217184	0.643568	lncRNA
ENSG0000(	ZNF516	1344.529	-0.14701	0.217256	0.643602	protein_coding
ENSG0000(	ENSG0000(	26.51664	0.335872	0.217334	0.643695	lncRNA
ENSG0000(	ENSG0000(	276.3127	-0.10677	0.217327	0.643695	lncRNA
ENSG0000(	ENSG0000(	100.1179	0.125542	0.217438	0.643838	lncRNA
ENSG0000(	ENSG0000(	372.1282	0.145266	0.217499	0.643838	lncRNA
ENSG0000(	ENSG0000(	81.31738	0.175967	0.217472	0.643838	lncRNA
ENSG0000(	P2RX1	610.5089	-0.15664	0.217488	0.643838	protein_coding
ENSG0000(	PRAP1	166.8187	0.41916	0.21749	0.643838	protein_coding
ENSG0000(	BVES-AS1	99.27024	-0.20988	0.217644	0.643917	lncRNA
ENSG0000(	ENSG0000(	578.2247	0.226843	0.217603	0.643917	TEC
ENSG0000(	IRGM	81.66231	-0.18599	0.217564	0.643917	protein_coding
ENSG0000(	LRRC2	685.3528	0.074625	0.217666	0.643917	protein_coding
ENSG0000(	SLC35B2	314.5604	-0.15053	0.217584	0.643917	protein_coding
ENSG0000(	UBE2Q2	427.8275	-0.18749	0.217619	0.643917	protein_coding
ENSG0000(	KLB	317.3758	0.14443	0.217706	0.64392	protein_coding
ENSG0000(	SEMA3C	981.2704	0.176042	0.217714	0.64392	protein_coding
ENSG0000(	ENSG0000(	60.63573	0.360533	0.217743	0.643936	lncRNA
ENSG0000(	INMT	384.3579	0.141002	0.21777	0.643948	protein_coding
ENSG0000(	C6orf136	316.4463	0.231879	0.217812	0.643985	protein_coding

ENSG0000(	ENSG0000(	83.63685	0.168077	0.217829	0.643985	lncRNA
ENSG0000(	ENDOG	35.73939	0.318607	0.217922	0.644047	protein_coding
ENSG0000(	ETF1	1405.786	-0.09301	0.217944	0.644047	protein_coding
ENSG0000(	RPL10P9	47.84527	0.811866	0.217943	0.644047	processed_pseudogene
ENSG0000(	TENM3-AS:	198.8314	0.114338	0.217889	0.644047	lncRNA
ENSG0000(	TEPSIN	735.0114	0.105384	0.217968	0.64405	protein_coding
ENSG0000(	DACT3-AS1	21.72734	-0.39194	0.218018	0.644058	lncRNA
ENSG0000(	ZNRF3-IT1	67.8876	0.325757	0.218003	0.644058	lncRNA
ENSG0000(	C8orf44	538.5398	0.099097	0.218165	0.644183	lncRNA
ENSG0000(	ENSG0000(	95.86533	0.158413	0.218177	0.644183	lncRNA
ENSG0000(	FAM234B	279.1026	-0.19865	0.218099	0.644183	protein_coding
ENSG0000(	LINC01409	1399.363	-0.09493	0.218132	0.644183	lncRNA
ENSG0000(	PTGS1	610.0127	-0.26008	0.218123	0.644183	protein_coding
ENSG0000(	H4C13	56.62413	-0.27059	0.218206	0.6442	protein_coding
ENSG0000(	LINC00424	35.83692	-0.24818	0.218279	0.644276	lncRNA
ENSG0000(	RAB19	650.1265	0.092224	0.218275	0.644276	protein_coding
ENSG0000(	RPE	1155.915	-0.08824	0.218357	0.644436	protein_coding
ENSG0000(	ENSG0000(	478.3079	0.104859	0.218736	0.644488	processed_pseudogene
ENSG0000(	ENSG0000(	593.2961	0.082696	0.218636	0.644488	lncRNA
ENSG0000(	ENSG0000(	271.5904	0.117474	0.218545	0.644488	lncRNA
ENSG0000(	ENSG0000(	77.1561	0.197722	0.218484	0.644488	lncRNA
ENSG0000(	ENSG0000(	40.13258	-0.25587	0.21863	0.644488	lncRNA
ENSG0000(	ENSG0000(	35.00044	-0.30307	0.218709	0.644488	lncRNA
ENSG0000(	FRG1-DT	2182.929	-0.14342	0.21843	0.644488	lncRNA
ENSG0000(	GCAWKR	152.6366	0.157309	0.218739	0.644488	lncRNA
ENSG0000(	HNRNPA1P	49.35645	-0.22756	0.218398	0.644488	processed_pseudogene
ENSG0000(	IDE	2227.781	-0.09156	0.218674	0.644488	protein_coding
ENSG0000(	ITPRID2	6717.589	0.197691	0.218667	0.644488	protein_coding
ENSG0000(	KLHDC9	31.49223	0.407721	0.218664	0.644488	protein_coding
ENSG0000(	LINC01134	415.5212	0.156514	0.218749	0.644488	lncRNA
ENSG0000(	NAP1L6P	150.7115	0.145149	0.21865	0.644488	transcribed_processed_pseudoge
ENSG0000(	PAQR5-DT	219.7147	-0.12836	0.21851	0.644488	lncRNA
ENSG0000(	ZBTB6	304.9144	-0.09547	0.21862	0.644488	protein_coding
ENSG0000(	BMP8A	424.6797	-0.19375	0.218866	0.644658	protein_coding
ENSG0000(	ENSG0000(	430.3364	0.116274	0.218837	0.644658	TEC
ENSG0000(	MDH1	901.0054	0.101039	0.218917	0.644658	protein_coding
ENSG0000(	NSA2	458.3584	0.196161	0.218881	0.644658	protein_coding
ENSG0000(	SHH	275.4257	-0.30222	0.218924	0.644658	protein_coding
ENSG0000(	ZNF43	1647.921	0.086743	0.218962	0.6447	protein_coding
ENSG0000(	ENSG0000(	32.48704	-0.27448	0.219028	0.644825	processed_pseudogene
ENSG0000(	NFXL1	525.8551	-0.13948	0.219072	0.644843	protein_coding
ENSG0000(	RAB11B	606.8181	0.110765	0.219081	0.644843	protein_coding
ENSG0000(	ALOX15B	73.43068	-0.45807	0.219176	0.644854	protein_coding
ENSG0000(	BBC3	186.7872	-0.2201	0.219215	0.644854	protein_coding
ENSG0000(	CSNK1A1	3268.237	-0.0684	0.219305	0.644854	protein_coding
ENSG0000(	ENSG0000(	62.97139	-0.18536	0.219126	0.644854	lncRNA
ENSG0000(	FDXR	105.8385	-0.2381	0.219221	0.644854	protein_coding

ENSG0000(	HSP90AB1	2337.448	-0.16862	0.219231	0.644854	protein_coding
ENSG0000(	KCNQ3	107.2122	-0.2388	0.219319	0.644854	protein_coding
ENSG0000(	MAP3K7CL	319.3292	0.124438	0.219313	0.644854	protein_coding
ENSG0000(	MATN2	985.8535	0.212001	0.219262	0.644854	protein_coding
ENSG0000(	NBPF3	1286.356	-0.12495	0.219199	0.644854	protein_coding
ENSG0000(	PPARA	4791.764	0.140166	0.219355	0.644892	protein_coding
ENSG0000(	ANO1	282.2862	0.215115	0.219401	0.644957	protein_coding
ENSG0000(	NOL6	662.0285	-0.11931	0.219424	0.644957	protein_coding
ENSG0000(	CD44-AS1	136.6131	0.198272	0.219582	0.645008	lncRNA
ENSG0000(	ENSG0000(	55.52224	0.173336	0.219494	0.645008	lncRNA
ENSG0000(	EPB41	4651.239	0.064721	0.219488	0.645008	protein_coding
ENSG0000(	LINC02253	345.2412	-0.13	0.219558	0.645008	lncRNA
ENSG0000(	PCDHA5	54.82049	-0.22908	0.219533	0.645008	protein_coding
ENSG0000(	RAB38	38.18727	-0.25741	0.219581	0.645008	protein_coding
ENSG0000(	ADAMTS19	31.00233	0.610769	0.219961	0.645018	protein_coding
ENSG0000(	ENSG0000(	21.46432	0.359352	0.21996	0.645018	lncRNA
ENSG0000(	ENSG0000(	29.3653	-0.39488	0.219656	0.645018	lncRNA
ENSG0000(	ENSG0000(	53.50285	0.263348	0.219898	0.645018	lncRNA
ENSG0000(	ENSG0000(	129.9787	-0.12062	0.219803	0.645018	lncRNA
ENSG0000(	FBXW11P1	23.09997	0.465687	0.219872	0.645018	processed_pseudogene
ENSG0000(	KNDC1	275.4943	-0.18908	0.219831	0.645018	protein_coding
ENSG0000(	LINC00922	32.17242	0.277949	0.219653	0.645018	lncRNA
ENSG0000(	LINC01475	29.69747	0.334664	0.219761	0.645018	lncRNA
ENSG0000(	OR1A1	1159.815	0.104478	0.219822	0.645018	protein_coding
ENSG0000(	PAOX	96.25612	-0.23433	0.219807	0.645018	protein_coding
ENSG0000(	RHBG	219.8699	-0.13227	0.219714	0.645018	protein_coding
ENSG0000(	RNU1-134P	49.68352	-0.23003	0.219687	0.645018	snRNA
ENSG0000(	SNX18P8	71.71222	0.663265	0.219947	0.645018	unprocessed_pseudogene
ENSG0000(	YOD1	710.6314	-0.12617	0.219855	0.645018	protein_coding
ENSG0000(	ZNF628	106.1266	0.307317	0.219921	0.645018	protein_coding
ENSG0000(	SMARCA1	270.1693	-0.24494	0.22001	0.645095	protein_coding
ENSG0000(	HSD17B11	1090.865	0.186794	0.220065	0.645116	protein_coding
ENSG0000(	OR9M1P	58.02333	0.222652	0.220046	0.645116	transcribed_unprocessed_pseudo
ENSG0000(	ENSG0000(	20.0566	0.354552	0.220093	0.645132	lncRNA
ENSG0000(	NOS1AP	576.1246	0.208116	0.220312	0.645485	protein_coding
ENSG0000(	PRXL2A	1311.635	0.186012	0.220328	0.645485	protein_coding
ENSG0000(	R3HDM4	677.7579	-0.20969	0.220241	0.645485	protein_coding
ENSG0000(	SHISAL2A	378.3957	-0.2442	0.220302	0.645485	protein_coding
ENSG0000(	UBXN2B	830.4058	-0.12015	0.220331	0.645485	protein_coding
ENSG0000(	TSEN2	2913.097	0.154423	0.220388	0.645584	protein_coding
ENSG0000(	TMEM201	309.1527	0.142506	0.220457	0.645715	protein_coding
ENSG0000(	CCDC163	224.9678	-0.12615	0.220482	0.645721	protein_coding
ENSG0000(	SULT1A2	377.7042	0.351388	0.220513	0.645744	protein_coding
ENSG0000(	ENSG0000(	19.74971	0.344212	0.220553	0.645792	lncRNA
ENSG0000(	BCLAF3	1742.531	0.120906	0.220625	0.645933	protein_coding
ENSG0000(	CCNB3P1	179.2537	-0.12611	0.220889	0.646001	processed_pseudogene
ENSG0000(	ENSG0000(	75.39262	0.329192	0.220818	0.646001	processed_pseudogene

ENSG0000(	ENSG0000(	62.24871	-0.28607	0.220866	0.646001	lncRNA
ENSG0000(	ENSG0000(	29.32288	-0.34243	0.221024	0.646001	lncRNA
ENSG0000(	ENSG0000(	62.99308	0.173469	0.220815	0.646001	lncRNA
ENSG0000(	ENSG0000(	29.79727	-0.33786	0.220924	0.646001	lncRNA
ENSG0000(	ENSG0000(	34.73245	-0.31945	0.220962	0.646001	lncRNA
ENSG0000(	FHIP2A	738.3353	-0.17696	0.220874	0.646001	protein_coding
ENSG0000(	IFIT3	1054.559	-0.38537	0.221007	0.646001	protein_coding
ENSG0000(	INAFM1	58.40401	0.181235	0.220714	0.646001	protein_coding
ENSG0000(	LINC02071	206.9694	0.101278	0.220935	0.646001	lncRNA
ENSG0000(	RN7SL356F	44.17726	-0.21806	0.220933	0.646001	misc_RNA
ENSG0000(	SNORA49	184.661	0.173892	0.220718	0.646001	snoRNA
ENSG0000(	SOD1	587.9188	-0.17038	0.220842	0.646001	protein_coding
ENSG0000(	TMSB4X	9322.43	-0.1417	0.221008	0.646001	protein_coding
ENSG0000(	VPS13B-DT	128.5912	0.111276	0.22075	0.646001	lncRNA
ENSG0000(	ENSG0000(	40.50287	0.264928	0.22108	0.646029	processed_pseudogene
ENSG0000(	ENSG0000(	177.2518	0.130516	0.22106	0.646029	lncRNA
ENSG0000(	MIR3142H(	450.005	-0.27493	0.221174	0.646233	lncRNA
ENSG0000(	APOOL	863.371	0.076505	0.221256	0.64636	protein_coding
ENSG0000(	PCED1B	281.2757	-0.3269	0.221264	0.64636	protein_coding
ENSG0000(	ZNF609	3638.58	-0.07025	0.221338	0.646507	protein_coding
ENSG0000(	ANKRD11	6280.971	-0.06193	0.221385	0.646577	protein_coding
ENSG0000(	ENSG0000(	40.70866	-0.26628	0.221462	0.646661	lncRNA
ENSG0000(	ENSG0000(	162.4393	0.124251	0.221485	0.646661	TEC
ENSG0000(	FAM111A-I	240.225	0.168966	0.221483	0.646661	lncRNA
ENSG0000(	GABRA1	56.70954	0.325964	0.221516	0.646685	protein_coding
ENSG0000(	SLC17A4	4204.08	0.454482	0.221559	0.64674	protein_coding
ENSG0000(	ADH7	25.63652	-0.53912	0.221692	0.646923	protein_coding
ENSG0000(	ENSG0000(	102.6891	-0.191	0.22168	0.646923	TEC
ENSG0000(	HNRNPDL	3033.869	-0.11101	0.221669	0.646923	protein_coding
ENSG0000(	ENSG0000(	34.47486	-0.28452	0.221734	0.646978	lncRNA
ENSG0000(	SLC15A4	816.8669	-0.11328	0.221813	0.64714	protein_coding
ENSG0000(	ENSG0000(	39.59961	0.255599	0.221838	0.647142	lncRNA
ENSG0000(	ENSG0000(	140.4867	-0.1426	0.221878	0.647192	processed_pseudogene
ENSG0000(	NBPF9	9495.814	0.10233	0.221946	0.647253	protein_coding
ENSG0000(	NPY6R	230.0597	0.2962	0.221937	0.647253	transcribed_unitary_pseudogene
ENSG0000(	ENSG0000(	40.55212	0.455526	0.222017	0.647282	lncRNA
ENSG0000(	ENSG0000(	64.64665	0.2005	0.222003	0.647282	lncRNA
ENSG0000(	TSPAN5	420.8334	-0.21241	0.222027	0.647282	protein_coding
ENSG0000(	FCHO2	855.494	-0.12248	0.222069	0.647337	protein_coding
ENSG0000(	ENSG0000(	490.4269	0.092044	0.222103	0.647366	lncRNA
ENSG0000(	SLC16A4	289.8693	-0.17297	0.222146	0.647399	protein_coding
ENSG0000(	TAOK1	5669.558	-0.08322	0.222191	0.647399	protein_coding
ENSG0000(	TNR	177.9567	0.215922	0.222169	0.647399	protein_coding
ENSG0000(	TPM1-AS	250.8663	0.337728	0.222229	0.647399	lncRNA
ENSG0000(	WASHC2A	1920.617	0.165964	0.222232	0.647399	protein_coding
ENSG0000(	DIABLO	907.6439	0.062769	0.222297	0.647463	protein_coding
ENSG0000(	TACC2	3352.906	0.161065	0.222301	0.647463	protein_coding

ENSG0000(	CYC1	594.8524	0.201424	0.22247	0.647477	protein_coding
ENSG0000(	DCTN4	1715.819	0.081344	0.222513	0.647477	protein_coding
ENSG0000(	ENSG0000(	250.7631	0.119475	0.222425	0.647477	lncRNA
ENSG0000(	ENSG0000(	62.39156	0.342021	0.222486	0.647477	lncRNA
ENSG0000(	FBXO38-DT	14258.82	0.115768	0.222564	0.647477	lncRNA
ENSG0000(	H4C8	294.8873	-0.24668	0.222389	0.647477	protein_coding
ENSG0000(	LINC00391	29.47728	0.262257	0.222559	0.647477	lncRNA
ENSG0000(	MFSD2B	62.99684	-0.20091	0.222437	0.647477	protein_coding
ENSG0000(	MTPAP	1248.446	0.082646	0.222338	0.647477	protein_coding
ENSG0000(	PDXK	4387.393	0.115872	0.222534	0.647477	protein_coding
ENSG0000(	WASH8P	355.8384	0.374108	0.222399	0.647477	unprocessed_pseudogene
ENSG0000(	ENSG0000(	76.74578	-0.17148	0.222652	0.647663	TEC
ENSG0000(	MIR8485	36.95431	-0.31579	0.2227	0.647697	miRNA
ENSG0000(	PHC1	857.4634	-0.1953	0.22271	0.647697	protein_coding
ENSG0000(	BTBD2	720.6476	0.1247	0.222839	0.647933	protein_coding
ENSG0000(	ENSG0000(	905.3599	0.127719	0.222815	0.647933	lncRNA
ENSG0000(	THUMPD3	616.9879	0.123259	0.222867	0.647945	protein_coding
ENSG0000(	DDA1	598.9475	0.160022	0.222936	0.64801	protein_coding
ENSG0000(	ITPRIPL2	2189.289	-0.12126	0.222934	0.64801	protein_coding
ENSG0000(	SREK1	4868.184	0.083898	0.222964	0.648022	protein_coding
ENSG0000(	PGM5P3-A	126.9285	0.166401	0.223024	0.648129	lncRNA
ENSG0000(	CEBPA	574.915	0.225204	0.223157	0.648177	protein_coding
ENSG0000(	ENSG0000(	75.20886	-0.20015	0.223271	0.648177	lncRNA
ENSG0000(	ENSG0000(	238.0583	0.122991	0.223265	0.648177	lncRNA
ENSG0000(	ITGAD	70.49978	0.283132	0.223266	0.648177	protein_coding
ENSG0000(	KAZALD1	178.8394	0.267371	0.223276	0.648177	protein_coding
ENSG0000(	MRPL45P2	650.5582	0.083936	0.223072	0.648177	transcribed_unprocessed_pseudo
ENSG0000(	SHANK2	980.1857	0.224549	0.223173	0.648177	protein_coding
ENSG0000(	TACC1	4995.749	0.093811	0.223158	0.648177	protein_coding
ENSG0000(	ZNF562	4758.727	0.082811	0.223184	0.648177	protein_coding
ENSG0000(	ZNF805	1286.055	0.073972	0.22324	0.648177	protein_coding
ENSG0000(	CD177	2133.474	0.586576	0.223348	0.648192	protein_coding
ENSG0000(	DLGAP1-AS	93.42627	-0.16864	0.223423	0.648192	lncRNA
ENSG0000(	ENSG0000(	41.48268	-0.30337	0.223412	0.648192	processed_pseudogene
ENSG0000(	NME6	1531.861	0.074777	0.223409	0.648192	protein_coding
ENSG0000(	PDCD6IPP1	183.5974	-0.21256	0.22341	0.648192	transcribed_unprocessed_pseudo
ENSG0000(	SPIDR	1712.797	-0.08615	0.223375	0.648192	protein_coding
ENSG0000(	RNF113B	35.22635	-0.38426	0.223464	0.648243	protein_coding
ENSG0000(	ENSG0000(	67.34937	0.938456	0.223554	0.6483	protein_coding
ENSG0000(	FAM86C2P	121.2696	0.181025	0.223528	0.6483	transcribed_unprocessed_pseudo
ENSG0000(	SLC2A14	72.26119	0.213158	0.223551	0.6483	protein_coding
ENSG0000(	ACTN2	197.1729	0.212429	0.2239	0.648331	protein_coding
ENSG0000(	AP3M1	1360.481	-0.08198	0.223772	0.648331	protein_coding
ENSG0000(	CACNB1	433.712	-0.20772	0.223677	0.648331	protein_coding
ENSG0000(	CACNB4	1099.554	-0.1493	0.223995	0.648331	protein_coding
ENSG0000(	CASTOR1	60.90591	-0.31112	0.223815	0.648331	protein_coding
ENSG0000(	CEP89	1401.422	-0.10963	0.223811	0.648331	protein_coding

ENSG0000(	ENSG0000(	29.56625	-0.31223	0.223971	0.648331	lncRNA
ENSG0000(	ENSG0000(	791.7331	0.072852	0.223751	0.648331	lncRNA
ENSG0000(	ENSG0000(	36.7739	-0.25146	0.224154	0.648331	lncRNA
ENSG0000(	ENSG0000(	24.15866	0.499067	0.224178	0.648331	processed_pseudogene
ENSG0000(	ENSG0000(	66.10049	-0.23354	0.223988	0.648331	lncRNA
ENSG0000(	ENSG0000(	2935.743	0.056659	0.22409	0.648331	lncRNA
ENSG0000(	ENSG0000(	133.6562	0.168711	0.223998	0.648331	TEC
ENSG0000(	ENSG0000(	452.5477	-0.13512	0.223877	0.648331	lncRNA
ENSG0000(	NDST3	85.97792	0.19942	0.22366	0.648331	protein_coding
ENSG0000(	PDSS2	517.1415	0.129476	0.224167	0.648331	protein_coding
ENSG0000(	PLAT	1454.468	-0.17439	0.22403	0.648331	protein_coding
ENSG0000(	RAB20	235.5249	-0.17918	0.224071	0.648331	protein_coding
ENSG0000(	REEP4	255.9678	0.158031	0.224141	0.648331	protein_coding
ENSG0000(	RN7SL602F	513.028	0.156308	0.224037	0.648331	misc_RNA
ENSG0000(	SLC16A14	191.7291	-0.22215	0.224027	0.648331	protein_coding
ENSG0000(	TMEM143	206.2488	-0.15176	0.223751	0.648331	protein_coding
ENSG0000(	TMX1	869.522	-0.09138	0.223875	0.648331	protein_coding
ENSG0000(	UBE2C	108.9723	-0.22873	0.223923	0.648331	protein_coding
ENSG0000(	ZMYM6	1693.65	0.069778	0.223948	0.648331	protein_coding
ENSG0000(	ZNF593	32.01524	-0.26269	0.223816	0.648331	protein_coding
ENSG0000(	TNNT3	20.25667	-0.5278	0.224264	0.648514	protein_coding
ENSG0000(	ARPC3	884.4667	-0.13379	0.224354	0.648542	protein_coding
ENSG0000(	CCNL2	5193.849	0.060564	0.224374	0.648542	protein_coding
ENSG0000(	ENSG0000(	24.15317	-0.37139	0.224325	0.648542	protein_coding
ENSG0000(	ENSG0000(	266.5876	0.14554	0.224344	0.648542	lncRNA
ENSG0000(	FAM167A	122.0499	-0.42589	0.224392	0.648542	protein_coding
ENSG0000(	CTD-3080P	68.60575	0.251086	0.22443	0.648583	lncRNA
ENSG0000(	CERT1	2194.025	0.096068	0.224473	0.64864	protein_coding
ENSG0000(	LINC00616	25.91707	0.292483	0.224558	0.648817	lncRNA
ENSG0000(	SKAP2	1274.189	-0.07628	0.224599	0.648867	protein_coding
ENSG0000(	CYP21A1P	66.86917	-0.24439	0.224661	0.648912	transcribed_unprocessed_pseudo
ENSG0000(	ENSG0000(	817.6829	0.118036	0.22465	0.648912	lncRNA
ENSG0000(	COQ3	74.38038	0.218905	0.224744	0.648975	protein_coding
ENSG0000(	ENSG0000(	358.7546	-0.16487	0.224746	0.648975	lncRNA
ENSG0000(	NUMBL	579.2158	-0.12247	0.224754	0.648975	protein_coding
ENSG0000(	BOLA3-AS1	150.9006	-0.13922	0.224943	0.648991	lncRNA
ENSG0000(	ENSG0000(	336.3151	0.096645	0.225285	0.648991	processed_pseudogene
ENSG0000(	ENSG0000(	32.91257	0.279022	0.225294	0.648991	lncRNA
ENSG0000(	ENSG0000(	48.03507	0.187547	0.225236	0.648991	lncRNA
ENSG0000(	ENSG0000(	86.68127	-0.14682	0.225303	0.648991	transcribed_unprocessed_pseudo
ENSG0000(	ENSG0000(	89.64258	-0.16812	0.225326	0.648991	lncRNA
ENSG0000(	ENSG0000(	64.02341	0.350868	0.225198	0.648991	lncRNA
ENSG0000(	EPHA6	526.6399	0.126883	0.224881	0.648991	protein_coding
ENSG0000(	EPN1	5600.854	0.127951	0.224914	0.648991	protein_coding
ENSG0000(	FSIP2	11226.1	0.096378	0.224958	0.648991	protein_coding
ENSG0000(	HMGN1P8	188.1557	-0.14203	0.225307	0.648991	processed_pseudogene
ENSG0000(	HNRNPC	5487.892	-0.1063	0.224856	0.648991	protein_coding

ENSG0000(	IST1	3037.741	0.078	0.225292	0.648991	protein_coding
ENSG0000(	LIN37	213.2475	0.14185	0.224868	0.648991	protein_coding
ENSG0000(	NOL4	29.70414	0.497573	0.224977	0.648991	protein_coding
ENSG0000(	PDK2	478.7981	0.194975	0.225262	0.648991	protein_coding
ENSG0000(	PRR15L	1741.414	0.273082	0.225122	0.648991	protein_coding
ENSG0000(	RN7SKP189	20.97811	0.357447	0.225078	0.648991	misc_RNA
ENSG0000(	RN7SL48P	311.0417	0.100561	0.225111	0.648991	misc_RNA
ENSG0000(	RPS10P7	749.0526	0.075353	0.224929	0.648991	transcribed_processed_pseudoge
ENSG0000(	SNORA11	82.53998	-0.21164	0.225012	0.648991	snoRNA
ENSG0000(	TEX10	791.8114	-0.11556	0.225306	0.648991	protein_coding
ENSG0000(	ZNF408	147.215	-0.17059	0.225112	0.648991	protein_coding
ENSG0000(	ZNF580	1017.887	-0.12446	0.225279	0.648991	protein_coding
ENSG0000(	HSD17B8	84.47128	0.205848	0.225379	0.649008	protein_coding
ENSG0000(	SYK	1883.772	-0.14899	0.225379	0.649008	protein_coding
ENSG0000(	FASN	1787.896	0.371787	0.22541	0.649031	protein_coding
ENSG0000(	ENSG0000(	210.3036	0.196245	0.225453	0.649085	protein_coding
ENSG0000(	APOC4	29.25826	-0.33448	0.225526	0.649105	protein_coding
ENSG0000(	ENSG0000(	52.1134	0.189622	0.225531	0.649105	lncRNA
ENSG0000(	ENSG0000(	81.32487	-0.20095	0.225554	0.649105	TEC
ENSG0000(	OR2G6	1143.873	0.071555	0.225494	0.649105	protein_coding
ENSG0000(	PPP1R12B	6089.742	0.106171	0.225597	0.649161	protein_coding
ENSG0000(	ENSG0000(	151.4448	-0.11837	0.225691	0.649218	lncRNA
ENSG0000(	IL1RL2	45.04593	-0.38607	0.22565	0.649218	protein_coding
ENSG0000(	SERPINB10	195.7674	0.133682	0.225676	0.649218	protein_coding
ENSG0000(	SUV39H2-D	51.50348	-0.2353	0.225711	0.649218	lncRNA
ENSG0000(	IL27RA	298.3246	-0.19774	0.225792	0.649381	protein_coding
ENSG0000(	ZBTB7B	2515.323	0.23181	0.22583	0.649425	protein_coding
ENSG0000(	ENSG0000(	170.6811	0.194972	0.225921	0.64955	lncRNA
ENSG0000(	RSU1P2	100.6631	-0.16613	0.225909	0.64955	transcribed_unprocessed_pseudo
ENSG0000(	EIF4EBP2	4144.262	0.175734	0.225988	0.649673	protein_coding
ENSG0000(	DNAJC17	730.4061	0.106218	0.226117	0.649914	protein_coding
ENSG0000(	ENSG0000(	506.3534	0.130691	0.226119	0.649914	lncRNA
ENSG0000(	FAM218A	44.25036	0.269503	0.226255	0.64992	lncRNA
ENSG0000(	FOXO1	1962.391	-0.17139	0.226216	0.64992	protein_coding
ENSG0000(	GDPD3	260.765	0.27581	0.226197	0.64992	protein_coding
ENSG0000(	GPAA1P2	80.52588	-0.16235	0.226245	0.64992	transcribed_processed_pseudoge
ENSG0000(	LINC01702	71.11796	0.22873	0.226263	0.64992	lncRNA
ENSG0000(	TIMELESS	538.6202	-0.13685	0.226216	0.64992	protein_coding
ENSG0000(	MSTO1	504.0578	-0.14131	0.226297	0.649921	protein_coding
ENSG0000(	RN7SKP118	98.71441	0.317338	0.22631	0.649921	misc_RNA
ENSG0000(	C15orf48	845.5021	0.243775	0.226439	0.649925	protein_coding
ENSG0000(	EFCC1	198.8205	-0.21722	0.226439	0.649925	protein_coding
ENSG0000(	ENSG0000(	94.46279	0.173193	0.226421	0.649925	lncRNA
ENSG0000(	ENSG0000(	113.8246	0.173507	0.226434	0.649925	lncRNA
ENSG0000(	GALNS	1464.785	0.087647	0.226477	0.649925	protein_coding
ENSG0000(	IL20RA	230.635	-0.33773	0.226352	0.649925	protein_coding
ENSG0000(	PTCD1	576.3525	0.104433	0.226454	0.649925	protein_coding

ENSG0000(	DNM1P41	191.343	-0.14618	0.226602	0.65015	TEC
ENSG0000(	MPZ	83.82081	-0.21101	0.226599	0.65015	protein_coding
ENSG0000(	BOK-AS1	45.98262	0.22103	0.227188	0.650957	lncRNA
ENSG0000(	ENSG0000(	129.5672	0.16957	0.227133	0.650957	lncRNA
ENSG0000(	ENSG0000(	28.82197	-0.28483	0.22715	0.650957	lncRNA
ENSG0000(	ENSG0000(	71.41391	-0.30682	0.227055	0.650957	protein_coding
ENSG0000(	ENSG0000(	20.61115	0.502702	0.227041	0.650957	lncRNA
ENSG0000(	FAM226B	61.52006	0.213711	0.227024	0.650957	lncRNA
ENSG0000(	GIMAP1	589.9952	-0.18623	0.227215	0.650957	protein_coding
ENSG0000(	INPP5F	969.7865	-0.15995	0.227031	0.650957	protein_coding
ENSG0000(	PHYKPL	6702.364	0.111924	0.22706	0.650957	protein_coding
ENSG0000(	SERPINA10	832.8038	-0.16896	0.227183	0.650957	protein_coding
ENSG0000(	SF1	4487.22	0.083871	0.227177	0.650957	protein_coding
ENSG0000(	SNORA46	165.3147	0.216456	0.226918	0.650957	snoRNA
ENSG0000(	TMEM182	1413.028	0.137796	0.226932	0.650957	protein_coding
ENSG0000(	ZNF8-ERVK	183.931	-0.13614	0.227203	0.650957	lncRNA
ENSG0000(	FZD5	4848.827	0.280656	0.22727	0.651046	protein_coding
ENSG0000(	LPCAT4	1431.155	0.164546	0.227313	0.651103	protein_coding
ENSG0000(	RN7SL396F	135.4376	-0.26418	0.227345	0.651126	misc_RNA
ENSG0000(	ARG2	46.00906	-0.44254	0.227434	0.651314	protein_coding
ENSG0000(	ARHGAP5	4210.881	0.11835	0.227628	0.651684	protein_coding
ENSG0000(	MSH5	1337.755	0.07628	0.227611	0.651684	protein_coding
ENSG0000(	ZNF624	323.447	-0.1493	0.227635	0.651684	protein_coding
ENSG0000(	LINC00607	206.699	0.136614	0.227701	0.651806	lncRNA
ENSG0000(	THAP12P9	25.57276	0.340429	0.22777	0.651936	processed_pseudogene
ENSG0000(	SLC45A4	945.6498	-0.16243	0.22783	0.65204	protein_coding
ENSG0000(	ENSG0000(	283.7078	-0.14529	0.227901	0.652108	lncRNA
ENSG0000(	PLA2G4D	194.6008	0.171721	0.227884	0.652108	protein_coding
ENSG0000(	ENSG0000(	78.32276	-0.18602	0.228067	0.652176	lncRNA
ENSG0000(	ENSG0000(	227.2109	0.113092	0.228025	0.652176	lncRNA
ENSG0000(	ENSG0000(	82.0097	-0.23143	0.228056	0.652176	lncRNA
ENSG0000(	H2BC17	47.31628	-0.35078	0.227955	0.652176	protein_coding
ENSG0000(	SCIN	2004.267	0.29764	0.22805	0.652176	protein_coding
ENSG0000(	SNORA77B	35.60859	0.334393	0.227975	0.652176	snoRNA
ENSG0000(	ENSG0000(	173.2282	-0.10779	0.228154	0.652355	lncRNA
ENSG0000(	MSANTD2	694.5442	0.097098	0.228217	0.652419	protein_coding
ENSG0000(	TDRD5	65.57919	0.361002	0.228223	0.652419	protein_coding
ENSG0000(	ATP6AP1L	468.3185	-0.10652	0.228376	0.652425	transcribed_unitary_pseudogene
ENSG0000(	CTNNB1	5944.993	-0.0966	0.228371	0.652425	protein_coding
ENSG0000(	ENSG0000(	44.19611	0.207735	0.22836	0.652425	transcribed_processed_pseudoge
ENSG0000(	FBLN1	1638.633	-0.14126	0.22845	0.652425	protein_coding
ENSG0000(	GSG1L	29.41082	-0.29083	0.228264	0.652425	protein_coding
ENSG0000(	GSTT4	35.19439	0.263788	0.228454	0.652425	protein_coding
ENSG0000(	HYAL3	26.41799	0.403595	0.228396	0.652425	protein_coding
ENSG0000(	SOCS4	1170.871	-0.16875	0.228376	0.652425	protein_coding
ENSG0000(	SOCS6	1337.244	0.168109	0.228463	0.652425	protein_coding
ENSG0000(	WSB1	7639.968	-0.10757	0.228335	0.652425	protein_coding

ENSG0000(	ATP2A3	3818.874	0.114749	0.22857	0.652625	protein_coding
ENSG0000(	LINC01825	69.16345	0.189603	0.228604	0.652625	lncRNA
ENSG0000(	LYRM9	213.8687	0.173582	0.228595	0.652625	protein_coding
ENSG0000(	ENSG0000(	124.7901	0.139957	0.228669	0.652677	lncRNA
ENSG0000(	ZXDC	1934.664	0.078929	0.228651	0.652677	protein_coding
ENSG0000(	ZC3H7B	2644.265	-0.06693	0.228694	0.652678	protein_coding
ENSG0000(	ACIN1	5810.835	-0.04629	0.228841	0.652983	protein_coding
ENSG0000(	ENSG0000(	20.11692	-0.37495	0.228848	0.652983	transcribed_processed_pseudoge
ENSG0000(	COG7	793.0237	0.09398	0.229015	0.653256	protein_coding
ENSG0000(	DHODH	754.7527	0.091509	0.229	0.653256	protein_coding
ENSG0000(	ENSG0000(	558.1164	-0.09562	0.228981	0.653256	lncRNA
ENSG0000(	WDR37	1231.782	-0.07788	0.229098	0.653427	protein_coding
ENSG0000(	TFR2	247.6215	-0.1194	0.229143	0.653487	protein_coding
ENSG0000(	ARL3	94.21664	-0.24215	0.229172	0.653502	protein_coding
ENSG0000(	ENSG0000(	56.1035	-0.22572	0.229324	0.653799	lncRNA
ENSG0000(	ENSG0000(	46.10431	-0.30429	0.229318	0.653799	lncRNA
ENSG0000(	ENSG0000(	85.83388	-0.17221	0.229433	0.653975	lncRNA
ENSG0000(	MPZL2	785.3738	0.162403	0.22942	0.653975	protein_coding
ENSG0000(	POLR3A	1279.096	0.04895	0.229549	0.654237	protein_coding
ENSG0000(	AMDHD2	560.9183	0.102584	0.229653	0.654416	protein_coding
ENSG0000(	ENSG0000(	263.583	0.234721	0.229659	0.654416	lncRNA
ENSG0000(	DNAJB12	867.9711	0.07381	0.229735	0.654429	protein_coding
ENSG0000(	IBTK	2203.83	-0.08678	0.229724	0.654429	protein_coding
ENSG0000(	TMEM238	60.07526	0.265697	0.229734	0.654429	protein_coding
ENSG0000(	ENSG0000(	779.3834	0.102483	0.229808	0.654461	TEC
ENSG0000(	IGHV1-69-2	29.06296	-1.20644	0.229833	0.654461	IG_V_gene
ENSG0000(	MMRN2	295.273	-0.18643	0.229866	0.654461	protein_coding
ENSG0000(	NEDD1	395.3685	-0.14131	0.229856	0.654461	protein_coding
ENSG0000(	SLC39A4	402.9836	0.154724	0.229858	0.654461	protein_coding
ENSG0000(	G6PC2	22.12866	0.420905	0.229931	0.65458	protein_coding
ENSG0000(	CNTN2	365.2456	0.092248	0.229972	0.65463	protein_coding
ENSG0000(	RN7SKP127	31.31766	0.61347	0.230029	0.654723	misc_RNA
ENSG0000(	IER3IP1	452.5798	-0.11248	0.230076	0.654791	protein_coding
ENSG0000(	ZNF608	1375.204	0.138253	0.230164	0.654972	protein_coding
ENSG0000(	SEPTIN14P1	22.62519	0.459595	0.23023	0.655091	transcribed_unprocessed_pseudo
ENSG0000(	ANKRD23	106.7152	0.180157	0.230457	0.65517	protein_coding
ENSG0000(	ARMC2	131.2003	0.2306	0.230617	0.65517	protein_coding
ENSG0000(	CCSER1	1183.755	0.149171	0.230714	0.65517	protein_coding
ENSG0000(	CDK19	1225.637	0.119295	0.230451	0.65517	protein_coding
ENSG0000(	DAP3	1051.493	-0.07853	0.230585	0.65517	protein_coding
ENSG0000(	ENSG0000(	160.2572	-0.13372	0.230323	0.65517	lncRNA
ENSG0000(	ENSG0000(	218.5538	0.125688	0.23069	0.65517	lncRNA
ENSG0000(	ENSG0000(	26.53968	-0.35055	0.230842	0.65517	lncRNA
ENSG0000(	ENSG0000(	128.9097	0.144577	0.230496	0.65517	lncRNA
ENSG0000(	ENSG0000(	273.2802	0.098287	0.230323	0.65517	lncRNA
ENSG0000(	ENSG0000(	271.7141	0.319821	0.230861	0.65517	TEC
ENSG0000(	ENSG0000(	31.4595	-0.35624	0.230643	0.65517	processed_pseudogene

ENSG0000(	ENSG0000(	347.5339	0.099717	0.230927	0.65517	lncRNA
ENSG0000(	ENSG0000(	2423.831	0.126211	0.230948	0.65517	lncRNA
ENSG0000(	ENSG0000(	62.17104	0.324276	0.230446	0.65517	lncRNA
ENSG0000(	FAM43A	358.5335	-0.31184	0.230775	0.65517	protein_coding
ENSG0000(	GALNTL5	22.19084	0.318628	0.230703	0.65517	protein_coding
ENSG0000(	HMGXB3	1477.494	0.110523	0.230686	0.65517	protein_coding
ENSG0000(	JAKMIP2	282.3033	-0.12525	0.230472	0.65517	protein_coding
ENSG0000(	LINC01044	42.73761	0.209369	0.23033	0.65517	lncRNA
ENSG0000(	LINC01426	314.6612	-0.14053	0.23089	0.65517	lncRNA
ENSG0000(	PKD1P5	698.4861	0.340568	0.230919	0.65517	transcribed_unprocessed_pseudo
ENSG0000(	RNF10	2587.163	0.079799	0.230565	0.65517	protein_coding
ENSG0000(	RNF32	77.42808	0.291301	0.230811	0.65517	protein_coding
ENSG0000(	ROM1	19.84011	-0.3516	0.23066	0.65517	protein_coding
ENSG0000(	SRSF3	4351.473	0.042079	0.230591	0.65517	protein_coding
ENSG0000(	TMEM47	172.5765	-0.22146	0.230912	0.65517	protein_coding
ENSG0000(	TTLL12	651.4522	0.182031	0.230359	0.65517	protein_coding
ENSG0000(	VWDE	77.80494	0.458269	0.23086	0.65517	protein_coding
ENSG0000(	RPS4X	3100.408	-0.14437	0.231031	0.655338	protein_coding
ENSG0000(	RPL32P32	22.97868	-0.43097	0.231093	0.655446	processed_pseudogene
ENSG0000(	ENSG0000(	560.4612	0.092645	0.231119	0.655451	lncRNA
ENSG0000(	INE2	73.189	0.183618	0.231155	0.655488	lncRNA
ENSG0000(	ENSG0000(	182.7283	-0.3917	0.231294	0.655678	unprocessed_pseudogene
ENSG0000(	ENSG0000(	82.79297	-0.39117	0.231259	0.655678	lncRNA
ENSG0000(	ENSG0000(	88.04586	0.243129	0.231292	0.655678	lncRNA
ENSG0000(	HEXA	3162.672	-0.07761	0.23132	0.655685	protein_coding
ENSG0000(	CRADD	321.9572	-0.12965	0.231384	0.655789	protein_coding
ENSG0000(	EAPP	249.2886	0.130613	0.231405	0.655789	protein_coding
ENSG0000(	LINC01347	260.7212	0.164436	0.231477	0.655928	transcribed_unprocessed_pseudo
ENSG0000(	C10orf143	249.5903	0.108597	0.231579	0.655929	protein_coding
ENSG0000(	ENSG0000(	70.26222	0.241559	0.231589	0.655929	unprocessed_pseudogene
ENSG0000(	ENSG0000(	49.94363	0.182667	0.231597	0.655929	lncRNA
ENSG0000(	LINC02005	29.02697	-0.30806	0.231559	0.655929	lncRNA
ENSG0000(	PDE1C	967.265	-0.16362	0.231515	0.655929	protein_coding
ENSG0000(	CASQ1	38.27879	-0.3357	0.231634	0.655944	protein_coding
ENSG0000(	ENSG0000(	807.8138	-0.175	0.23165	0.655944	protein_coding
ENSG0000(	DUSP12	130.2903	-0.1714	0.231966	0.65596	protein_coding
ENSG0000(	ENSG0000(	542.0476	0.074325	0.231936	0.65596	lncRNA
ENSG0000(	ENSG0000(	732.7723	-0.08933	0.231992	0.65596	lncRNA
ENSG0000(	ENSG0000(	303.027	0.100655	0.231813	0.65596	lncRNA
ENSG0000(	FTX	19355.92	-0.0502	0.23184	0.65596	lncRNA
ENSG0000(	IGHV4-61	380.0589	-0.46655	0.23186	0.65596	IG_V_gene
ENSG0000(	IGLV2-18	90.34648	-0.48358	0.231932	0.65596	IG_V_gene
ENSG0000(	LINC01562	848.3511	0.059894	0.23177	0.65596	lncRNA
ENSG0000(	LINC02895	5668.118	0.149388	0.231953	0.65596	lncRNA
ENSG0000(	PCDHGB9P	71.37918	0.240694	0.231774	0.65596	unprocessed_pseudogene
ENSG0000(	RMDN2-AS	49.65945	-0.23906	0.231803	0.65596	lncRNA
ENSG0000(	SPTY2D1	724.3726	-0.09138	0.231709	0.65596	protein_coding

ENSG0000(	ST20-AS1	826.0135	-0.08928	0.231803	0.65596	lncRNA
ENSG0000(	TRAPPC10	2478.552	0.096307	0.232013	0.65596	protein_coding
ENSG0000(	USP4	2454.06	-0.06938	0.232002	0.65596	protein_coding
ENSG0000(	ACVR1B	1412.714	0.184087	0.232229	0.656502	protein_coding
ENSG0000(	ENSG0000(	42.79746	0.242048	0.232309	0.65666	unprocessed_pseudogene
ENSG0000(	CYB561D2	203.0456	0.141523	0.232422	0.656913	protein_coding
ENSG0000(	APLP2	3587.484	0.096814	0.232447	0.656917	protein_coding
ENSG0000(	STBD1	228.7826	0.471092	0.232471	0.656917	protein_coding
ENSG0000(	ANKFY1	2987.224	-0.0965	0.232636	0.656918	protein_coding
ENSG0000(	CCDC34	97.8643	-0.18312	0.232527	0.656918	protein_coding
ENSG0000(	CHRM1	22.38795	0.619135	0.232588	0.656918	protein_coding
ENSG0000(	RN7SL110F	25.58153	-0.29088	0.232638	0.656918	misc_RNA
ENSG0000(	SAP18	687.5876	-0.11024	0.23257	0.656918	protein_coding
ENSG0000(	SLC50A1	764.1862	-0.08943	0.232612	0.656918	protein_coding
ENSG0000(	WDFY3-AS2	393.7789	-0.08093	0.23254	0.656918	lncRNA
ENSG0000(	CYB5AP2	184.1521	-0.11482	0.232711	0.656984	processed_pseudogene
ENSG0000(	ENSG0000(	63.40902	0.347229	0.232733	0.656984	lncRNA
ENSG0000(	MFSD5	160.3784	0.230294	0.232708	0.656984	protein_coding
ENSG0000(	EIF3C	2834.636	0.127242	0.232842	0.657089	protein_coding
ENSG0000(	ENSG0000(	28.72851	0.441916	0.232812	0.657089	lncRNA
ENSG0000(	MVD	421.5243	0.184227	0.232841	0.657089	protein_coding
ENSG0000(	RXRA	2306.38	0.151332	0.232906	0.657202	protein_coding
ENSG0000(	GABRB3	288.6047	0.356227	0.232937	0.657221	protein_coding
ENSG0000(	CSGALNACT	72.44351	-0.13615	0.233199	0.657421	lncRNA
ENSG0000(	E2F7	157.351	-0.24626	0.233195	0.657421	protein_coding
ENSG0000(	EFCAB1	38.44243	0.282914	0.233032	0.657421	protein_coding
ENSG0000(	ENSG0000(	35.84889	-0.32381	0.233172	0.657421	lncRNA
ENSG0000(	KCTD17	173.5279	-0.17219	0.233136	0.657421	protein_coding
ENSG0000(	MTERF3	182.2715	-0.18121	0.233153	0.657421	protein_coding
ENSG0000(	OR51E2	165.1824	0.152543	0.233178	0.657421	protein_coding
ENSG0000(	RAX2	166.5229	-0.16448	0.233166	0.657421	protein_coding
ENSG0000(	CRB2	142.9946	0.226845	0.233368	0.657475	protein_coding
ENSG0000(	DGAT1	898.766	0.181619	0.233334	0.657475	protein_coding
ENSG0000(	ENSG0000(	49.73482	0.297922	0.233385	0.657475	lncRNA
ENSG0000(	ENSG0000(	277.5036	0.135843	0.233292	0.657475	lncRNA
ENSG0000(	NPDC1	1215.887	0.202441	0.233284	0.657475	protein_coding
ENSG0000(	PAM	1015	-0.16654	0.233248	0.657475	protein_coding
ENSG0000(	RASGRP3	2296.691	-0.10862	0.23334	0.657475	protein_coding
ENSG0000(	ZNF711	150.1899	0.194881	0.233428	0.657528	protein_coding
ENSG0000(	CCL19	378.2278	-0.57359	0.233488	0.657629	protein_coding
ENSG0000(	ENSG0000(	57.70948	0.165652	0.233623	0.657659	TEC
ENSG0000(	FGB	92.96194	-0.45616	0.233583	0.657659	protein_coding
ENSG0000(	LINC01393	44.93152	0.19275	0.233639	0.657659	lncRNA
ENSG0000(	OIT3	27.01478	-0.30385	0.233642	0.657659	protein_coding
ENSG0000(	PALS1	2625.053	0.130564	0.23353	0.657659	protein_coding
ENSG0000(	RN7SL127F	70.44562	0.205331	0.233627	0.657659	misc_RNA
ENSG0000(	SGSM3	1267.29	0.1309	0.233666	0.65766	protein_coding

ENSG0000(	SIRPG	154.425	-0.34167	0.23369	0.65766	protein_coding
ENSG0000(	WDR44	524.5842	-0.10885	0.233716	0.657665	protein_coding
ENSG0000(	ALOX15P1	450.5054	-0.09991	0.233853	0.657848	transcribed_unprocessed_pseudo
ENSG0000(	ASAP2	2034.121	0.171413	0.233805	0.657848	protein_coding
ENSG0000(	PRODH	173.1053	0.255245	0.233834	0.657848	protein_coding
ENSG0000(	FAM53C	1251.897	-0.07548	0.233926	0.657987	protein_coding
ENSG0000(	EIF3B	1663.103	0.089647	0.23402	0.658047	protein_coding
ENSG0000(	JMJD1C	5513.648	-0.09021	0.233992	0.658047	protein_coding
ENSG0000(	MIR1-1HG	42.6859	0.402405	0.234027	0.658047	lncRNA
ENSG0000(	OAZ3	207.981	-0.13467	0.234067	0.658047	protein_coding
ENSG0000(	TMEM115	270.4951	0.150858	0.234053	0.658047	protein_coding
ENSG0000(	CYP2C18	638.3408	0.390745	0.234093	0.658053	protein_coding
ENSG0000(	ENSG0000(	281.4513	0.100647	0.234219	0.658194	TEC
ENSG0000(	IL21R-AS1	108.4131	-0.13818	0.234187	0.658194	lncRNA
ENSG0000(	KIAA1671	7443.896	0.243689	0.234225	0.658194	protein_coding
ENSG0000(	RHPN2P1	69.35604	0.164294	0.234239	0.658194	unprocessed_pseudogene
ENSG0000(	CEP41	735.2952	0.080621	0.234487	0.658223	protein_coding
ENSG0000(	CFAP410	496.6362	0.17858	0.234335	0.658223	protein_coding
ENSG0000(	ENSG0000(	55.83632	0.205719	0.234361	0.658223	lncRNA
ENSG0000(	EPHA4	415.7128	0.175246	0.234466	0.658223	protein_coding
ENSG0000(	LINC00482	201.9916	0.260211	0.234294	0.658223	lncRNA
ENSG0000(	MMACHC	677.0608	0.101387	0.234488	0.658223	protein_coding
ENSG0000(	SDE2	688.1786	-0.08745	0.234408	0.658223	protein_coding
ENSG0000(	SHB	571.6853	-0.16544	0.234336	0.658223	protein_coding
ENSG0000(	SNORA73A	3377.702	0.214533	0.234438	0.658223	snoRNA
ENSG0000(	UBE4A	2462.903	0.057073	0.234447	0.658223	protein_coding
ENSG0000(	CNKSR3	3710.073	0.186397	0.234768	0.658248	protein_coding
ENSG0000(	ENO3	178.9242	-0.14017	0.234782	0.658248	protein_coding
ENSG0000(	ENSG0000(	81.49327	0.143268	0.234764	0.658248	lncRNA
ENSG0000(	ENSG0000(	103.1224	-0.47235	0.23474	0.658248	lncRNA
ENSG0000(	ENSG0000(	149.1524	0.192492	0.234795	0.658248	lncRNA
ENSG0000(	ENSG0000(	192.1373	0.159816	0.234832	0.658248	lncRNA
ENSG0000(	FABP5P9	80.49174	-0.23495	0.23459	0.658248	transcribed_processed_pseudoge
ENSG0000(	INTS9-AS1	99.83555	0.209463	0.234747	0.658248	lncRNA
ENSG0000(	MTND1P32	41.44386	0.233927	0.234543	0.658248	processed_pseudogene
ENSG0000(	NETO2	989.7431	0.212148	0.234722	0.658248	protein_coding
ENSG0000(	PFKFB2	3820.257	0.184576	0.234827	0.658248	protein_coding
ENSG0000(	RAB28	317.0719	0.18497	0.234755	0.658248	protein_coding
ENSG0000(	SNORD14A	29.59174	-0.29355	0.234732	0.658248	snoRNA
ENSG0000(	TRIM58	434.8487	0.164576	0.234582	0.658248	protein_coding
ENSG0000(	ENSG0000(	124.1243	-0.29151	0.234894	0.658336	lncRNA
ENSG0000(	HEPH	6105.472	0.236816	0.234935	0.658336	protein_coding
ENSG0000(	POLR3GL	142.7306	-0.17803	0.234915	0.658336	protein_coding
ENSG0000(	ANKHD1-D	49.98808	-0.19936	0.235048	0.658449	lncRNA
ENSG0000(	ENSG0000(	85.02649	-0.20778	0.235025	0.658449	lncRNA
ENSG0000(	ENSG0000(	31.83614	0.265153	0.235009	0.658449	protein_coding
ENSG0000(	ENSG0000(	162.8376	0.184559	0.235073	0.658454	lncRNA

ENSG0000(	ELOVL2-AS	209.3383	-0.15932	0.235097	0.658455	lncRNA
ENSG0000(	CEP55	157.0705	-0.19159	0.235251	0.658482	protein_coding
ENSG0000(	KLHL30	120.7754	-0.14885	0.235172	0.658482	protein_coding
ENSG0000(	LINC01149	66.65454	-0.20084	0.235245	0.658482	lncRNA
ENSG0000(	PLAC8L1	41.21476	-0.23407	0.235207	0.658482	protein_coding
ENSG0000(	STMN4	45.66206	-0.24156	0.235146	0.658482	protein_coding
ENSG0000(	TMEM14B	837.9562	-0.07074	0.23522	0.658482	protein_coding
ENSG0000(	TRIP6	297.1132	-0.13174	0.235345	0.658679	protein_coding
ENSG0000(	TMEM259	2075.883	0.098261	0.235399	0.658763	protein_coding
ENSG0000(	CALHM5	153.4959	-0.21739	0.235494	0.658962	protein_coding
ENSG0000(	LINC01761	1106.97	-0.06067	0.235534	0.659006	lncRNA
ENSG0000(	RNU1-67P	273.7926	-0.36612	0.235565	0.659027	snRNA
ENSG0000(	ENSG0000(	265.497	0.114881	0.235746	0.659362	lncRNA
ENSG0000(	HOXA7	245.6558	0.256859	0.235781	0.659362	protein_coding
ENSG0000(	TMEM175	479.3072	0.124912	0.235728	0.659362	protein_coding
ENSG0000(	ZBED9	183.3808	0.198821	0.235761	0.659362	protein_coding
ENSG0000(	ENSG0000(	35.40879	0.251956	0.235828	0.659426	lncRNA
ENSG0000(	TMTC3	539.2157	-0.1161	0.235906	0.659578	protein_coding
ENSG0000(	APPL2	2538.471	0.195717	0.235984	0.659624	protein_coding
ENSG0000(	ARHGEF26	335.8583	0.154388	0.236199	0.659624	protein_coding
ENSG0000(	CEP135	925.825	-0.11724	0.236016	0.659624	protein_coding
ENSG0000(	DPH2	267.4157	-0.14809	0.236068	0.659624	protein_coding
ENSG0000(	ENSG0000(	84.96027	0.192616	0.236123	0.659624	lncRNA
ENSG0000(	ENSG0000(	116.4506	0.264437	0.235989	0.659624	lncRNA
ENSG0000(	ENSG0000(	29.66434	0.305794	0.23621	0.659624	lncRNA
ENSG0000(	ENSG0000(	41.50693	-0.21287	0.236149	0.659624	lncRNA
ENSG0000(	ENSG0000(	24.86296	0.319447	0.236116	0.659624	TEC
ENSG0000(	ENSG0000(	440.8019	-0.09448	0.236134	0.659624	lncRNA
ENSG0000(	MECOM	3465.978	0.195533	0.236192	0.659624	protein_coding
ENSG0000(	RFC5	263.52	-0.12826	0.236186	0.659624	protein_coding
ENSG0000(	BRWD3	2490.525	-0.12754	0.236528	0.659651	protein_coding
ENSG0000(	DIRAS1	31.1247	-0.43393	0.236524	0.659651	protein_coding
ENSG0000(	ENSG0000(	34.75777	0.258169	0.236489	0.659651	lncRNA
ENSG0000(	ENSG0000(	27.46945	-0.2649	0.236546	0.659651	lncRNA
ENSG0000(	ENSG0000(	483.255	0.130616	0.236556	0.659651	lncRNA
ENSG0000(	ENSG0000(	38.43272	0.451394	0.236412	0.659651	lncRNA
ENSG0000(	ENSG0000(	24.78262	0.315362	0.236439	0.659651	lncRNA
ENSG0000(	ENSG0000(	794.9949	0.093094	0.236414	0.659651	TEC
ENSG0000(	ENSG0000(	39.82745	-0.23474	0.236248	0.659651	lncRNA
ENSG0000(	ENSG0000(	164.4912	0.14162	0.236306	0.659651	lncRNA
ENSG0000(	PRDM11	502.7947	0.118424	0.236474	0.659651	protein_coding
ENSG0000(	SLC2A9	290.5835	-0.13982	0.236457	0.659651	protein_coding
ENSG0000(	TMEM223	115.4148	0.138913	0.236356	0.659651	protein_coding
ENSG0000(	ZMYM1	605.0678	0.13631	0.236378	0.659651	protein_coding
ENSG0000(	C20orf203	61.59437	0.428077	0.236612	0.659674	protein_coding
ENSG0000(	LY6K	98.31777	-0.14762	0.23659	0.659674	protein_coding
ENSG0000(	ATP8B4	744.6275	-0.14571	0.236712	0.659694	protein_coding

ENSG0000(	ENSG0000(	39.18861	0.274663	0.236651	0.659694	lncRNA
ENSG0000(	RAB7A	2334.811	-0.10605	0.236702	0.659694	protein_coding
ENSG0000(	SMIM24	44.12598	-0.4524	0.23673	0.659694	protein_coding
ENSG0000(	SPINDOC	142.2831	-0.197	0.236739	0.659694	protein_coding
ENSG0000(	LIMK2	3568.785	-0.16404	0.236776	0.659732	protein_coding
ENSG0000(	ENSG0000(	1036.533	0.296043	0.237009	0.659989	lncRNA
ENSG0000(	ENSG0000(	33.69846	-0.26568	0.236971	0.659989	lncRNA
ENSG0000(	ENSG0000(	725.9869	0.075492	0.237013	0.659989	lncRNA
ENSG0000(	FAF2	1204.636	-0.08254	0.236993	0.659989	protein_coding
ENSG0000(	GOLGA5	653.7944	-0.11616	0.236922	0.659989	protein_coding
ENSG0000(	KPNA4P1	94.44688	0.222197	0.236982	0.659989	processed_pseudogene
ENSG0000(	CBLL1-AS1	35.50129	0.334543	0.237201	0.660127	lncRNA
ENSG0000(	ENSG0000(	77.49429	-0.16537	0.23718	0.660127	lncRNA
ENSG0000(	ETNPPL	57.02788	0.188927	0.237192	0.660127	protein_coding
ENSG0000(	OR11G2	186.5709	0.128423	0.237191	0.660127	protein_coding
ENSG0000(	PIH1D1	474.6047	0.083773	0.237165	0.660127	protein_coding
ENSG0000(	SLC41A3-A:	62.53236	0.196367	0.237206	0.660127	lncRNA
ENSG0000(	ENSG0000(	157.0848	-0.27107	0.237322	0.660382	transcribed_unprocessed_pseudo
ENSG0000(	RIN2	2159.051	0.12311	0.237447	0.660663	protein_coding
ENSG0000(	ENSG0000(	21.80102	-0.53023	0.237526	0.660816	lncRNA
ENSG0000(	ENSG0000(	5226.735	0.112903	0.237643	0.660941	lncRNA
ENSG0000(	MAN1B1	1341.834	0.08818	0.237638	0.660941	protein_coding
ENSG0000(	SAC3D1	38.63768	0.29176	0.237641	0.660941	protein_coding
ENSG0000(	AGPAT2	791.4972	0.303845	0.237691	0.660999	protein_coding
ENSG0000(	CETN4P	134.5113	0.294939	0.237712	0.660999	transcribed_unitary_pseudogene
ENSG0000(	DCHS1	1211.853	-0.18847	0.237784	0.661	protein_coding
ENSG0000(	EDAR	80.21147	0.264828	0.237748	0.661	protein_coding
ENSG0000(	RPL32P1	53.7975	-0.36158	0.237774	0.661	processed_pseudogene
ENSG0000(	LINC01206	1506.683	0.063257	0.237812	0.661012	lncRNA
ENSG0000(	ENSG0000(	722.259	0.082701	0.237856	0.661067	lncRNA
ENSG0000(	ENSG0000(	281.8885	0.14233	0.237943	0.661175	lncRNA
ENSG0000(	RAB3GAP2	3670.306	-0.04227	0.237938	0.661175	protein_coding
ENSG0000(	CHD5	21.68864	-0.34212	0.238108	0.661361	protein_coding
ENSG0000(	ENSG0000(	60.26142	0.333946	0.238084	0.661361	lncRNA
ENSG0000(	ENSG0000(	123.2855	0.128672	0.238144	0.661361	processed_pseudogene
ENSG0000(	ENSG0000(	301.0689	0.092275	0.238154	0.661361	lncRNA
ENSG0000(	ESYT1	916.8942	-0.17006	0.238049	0.661361	protein_coding
ENSG0000(	LINC01721	396.7547	-0.10175	0.238145	0.661361	lncRNA
ENSG0000(	ALDH16A1	759.561	0.094128	0.238251	0.661428	protein_coding
ENSG0000(	CTBS	539.1148	-0.13442	0.238215	0.661428	protein_coding
ENSG0000(	STAM2	1251.027	0.061044	0.238235	0.661428	protein_coding
ENSG0000(	ENSG0000(	126.3279	0.209779	0.238299	0.661429	lncRNA
ENSG0000(	PWWP3A	1730.789	0.086039	0.238291	0.661429	protein_coding
ENSG0000(	ADGRL1	601.7567	0.193272	0.238511	0.661452	protein_coding
ENSG0000(	ANKRD7	26.06962	0.379313	0.238668	0.661452	protein_coding
ENSG0000(	DDX42	2429.599	-0.06396	0.23863	0.661452	protein_coding
ENSG0000(	ELAVL2	71.39824	0.220007	0.238539	0.661452	protein_coding

ENSG0000(	ENSG0000(	3294.26	-0.07471	0.238458	0.661452	lncRNA
ENSG0000(	ENSG0000(	182.0502	0.107353	0.238661	0.661452	lncRNA
ENSG0000(	ENSG0000(	350.3841	0.094758	0.238431	0.661452	lncRNA
ENSG0000(	ENSG0000(	85.84497	-0.14647	0.238661	0.661452	lncRNA
ENSG0000(	EPHB2	1606.601	0.205616	0.238647	0.661452	protein_coding
ENSG0000(	GLUD1P2	61.33108	0.210897	0.238562	0.661452	transcribed_unprocessed_pseudo
ENSG0000(	GSDME	140.129	-0.37286	0.238527	0.661452	protein_coding
ENSG0000(	LINC00381	52.31281	-0.20415	0.238543	0.661452	lncRNA
ENSG0000(	LINC01259	43.2121	0.257404	0.238538	0.661452	lncRNA
ENSG0000(	SBDS	471.0707	0.160277	0.238545	0.661452	protein_coding
ENSG0000(	URI1	1021.989	0.08871	0.238366	0.661452	protein_coding
ENSG0000(	NIPAL1	1182.636	0.238729	0.238742	0.66159	protein_coding
ENSG0000(	BMPER	186.5672	0.17521	0.238846	0.661745	protein_coding
ENSG0000(	ENSG0000(	162.9103	0.163173	0.238846	0.661745	lncRNA
ENSG0000(	SCNN1A	3360.705	0.2193	0.238874	0.661757	protein_coding
ENSG0000(	DNAJC1	373.0626	-0.14863	0.238946	0.661888	protein_coding
ENSG0000(	LNX2	1015.048	0.15756	0.239037	0.661941	protein_coding
ENSG0000(	MIR548XH(	100.0213	0.160412	0.239015	0.661941	lncRNA
ENSG0000(	PLK2	436.8741	-0.23392	0.239035	0.661941	protein_coding
ENSG0000(	SCOC	632.0938	-0.13841	0.2391	0.662048	protein_coding
ENSG0000(	ABL2	3270.326	-0.12462	0.23922	0.662055	protein_coding
ENSG0000(	CBFB	909.2918	-0.08306	0.239232	0.662055	protein_coding
ENSG0000(	ENSG0000(	27.83831	0.28497	0.239319	0.662055	lncRNA
ENSG0000(	ENSG0000(	194.2045	0.128024	0.239296	0.662055	protein_coding
ENSG0000(	ENSG0000(	231.8264	-0.13224	0.23917	0.662055	lncRNA
ENSG0000(	ENSG0000(	20.9102	0.4139	0.239257	0.662055	transcribed_unprocessed_pseudo
ENSG0000(	NRDE2	2456.507	0.110204	0.239243	0.662055	protein_coding
ENSG0000(	OGDH	1870.748	0.14942	0.239285	0.662055	protein_coding
ENSG0000(	TMEM88	37.48707	-0.33547	0.239132	0.662055	protein_coding
ENSG0000(	TUBAL3	176.9379	0.397359	0.239398	0.662208	protein_coding
ENSG0000(	SPSB1	429.171	-0.13077	0.239492	0.662401	protein_coding
ENSG0000(	C11orf65	41.98593	0.271462	0.239551	0.662481	protein_coding
ENSG0000(	ODF2L	4437.852	-0.08055	0.239569	0.662481	protein_coding
ENSG0000(	ENSG0000(	20.51478	-0.48811	0.239603	0.662508	processed_pseudogene
ENSG0000(	LINC02318	159.4409	-0.14483	0.239667	0.662553	lncRNA
ENSG0000(	RN7SL623F	31.63943	0.26495	0.239659	0.662553	misc_RNA
ENSG0000(	ADAMTS18	58.17697	-0.52123	0.239738	0.662641	protein_coding
ENSG0000(	RN7SKP16(	25.37127	-0.31166	0.239747	0.662641	misc_RNA
ENSG0000(	F12	21.10774	-0.50303	0.239802	0.662725	protein_coding
ENSG0000(	C17orf75	1044.042	0.080396	0.240149	0.662958	protein_coding
ENSG0000(	EIF2B2	457.872	-0.1022	0.240077	0.662958	protein_coding
ENSG0000(	ENSG0000(	267.2242	0.106087	0.240059	0.662958	lncRNA
ENSG0000(	GABRR3	68.83937	-0.19153	0.240231	0.662958	protein_coding
ENSG0000(	LMTK2	2669.053	0.175438	0.240151	0.662958	protein_coding
ENSG0000(	LPAR2	333.8856	-0.21716	0.240054	0.662958	protein_coding
ENSG0000(	LYL1	256.9075	-0.24124	0.24022	0.662958	protein_coding
ENSG0000(	MTFR1L	1275.696	0.141023	0.239964	0.662958	protein_coding

ENSG0000(	PHF11	1052.135	-0.1235	0.239949	0.662958	protein_coding
ENSG0000(	PRSS36	40.70108	0.343508	0.240261	0.662958	protein_coding
ENSG0000(	RN7SL622P	169.3033	0.143534	0.240139	0.662958	misc_RNA
ENSG0000(	RNF19A	2703.426	-0.09235	0.24027	0.662958	protein_coding
ENSG0000(	SELENON	1018.131	-0.13104	0.240173	0.662958	protein_coding
ENSG0000(	SP110	2229.362	-0.26306	0.239974	0.662958	protein_coding
ENSG0000(	TIMM8B	152.5932	-0.1575	0.240272	0.662958	protein_coding
ENSG0000(	ZNF396	527.3709	0.080436	0.240242	0.662958	protein_coding
ENSG0000(	UNC119	684.5459	-0.11875	0.240365	0.663148	protein_coding
ENSG0000(	CMBL	1397.695	0.258029	0.240433	0.66325	protein_coding
ENSG0000(	CTDSP1	1835.716	0.093257	0.240473	0.66325	protein_coding
ENSG0000(	DLEC1	250.7114	0.193133	0.240587	0.66325	protein_coding
ENSG0000(	ENSG0000(	108.0694	0.276725	0.240594	0.66325	lncRNA
ENSG0000(	ENSG0000(	136.0985	0.155633	0.24054	0.66325	lncRNA
ENSG0000(	FBXO40	74.72761	0.157205	0.240535	0.66325	protein_coding
ENSG0000(	MTMR14	1068.851	-0.10534	0.240535	0.66325	protein_coding
ENSG0000(	NUAK2	829.4729	0.126206	0.240552	0.66325	protein_coding
ENSG0000(	ENSG0000(	28.47143	-0.29436	0.240721	0.66328	lncRNA
ENSG0000(	NDUFA3	436.0035	0.115455	0.240726	0.66328	protein_coding
ENSG0000(	PAPSS2	2591.458	0.239626	0.240713	0.66328	protein_coding
ENSG0000(	USP49	1007.955	-0.08028	0.240724	0.66328	protein_coding
ENSG0000(	ZNF544	1666.857	0.093034	0.24072	0.66328	protein_coding
ENSG0000(	MIR941-1	46.42952	0.346283	0.240773	0.663343	miRNA
ENSG0000(	CPB2	449.0943	-0.13706	0.240946	0.663651	protein_coding
ENSG0000(	NBR1	3358.208	0.095173	0.240941	0.663651	protein_coding
ENSG0000(	ZNF441	518.2672	-0.11543	0.240957	0.663651	protein_coding
ENSG0000(	LINC01568	566.3537	0.102816	0.24107	0.663829	lncRNA
ENSG0000(	ZNF649	486.5909	0.11657	0.241058	0.663829	protein_coding
ENSG0000(	PPDPF	879.481	0.240419	0.24114	0.663888	protein_coding
ENSG0000(	ZNHIT3	894.7122	0.07436	0.241119	0.663888	protein_coding
ENSG0000(	ENSG0000(	210.5345	0.126845	0.241287	0.663894	lncRNA
ENSG0000(	ENSG0000(	110.6833	0.124768	0.241271	0.663894	lncRNA
ENSG0000(	RNASEH2C	473.6709	-0.09351	0.241278	0.663894	protein_coding
ENSG0000(	TMEM132E	293.2506	0.149536	0.241199	0.663894	protein_coding
ENSG0000(	TMEM230	854.4485	-0.09873	0.241279	0.663894	protein_coding
ENSG0000(	WDFY3	3987.253	-0.12925	0.241175	0.663894	protein_coding
ENSG0000(	OR10H5	917.3669	0.072648	0.241323	0.663928	protein_coding
ENSG0000(	ANKMY2	448.4868	0.149032	0.241486	0.664053	protein_coding
ENSG0000(	AP1M2	335.6988	0.257464	0.241493	0.664053	protein_coding
ENSG0000(	ENSG0000(	46.68037	0.260694	0.241535	0.664053	transcribed_processed_pseudoge
ENSG0000(	ENSG0000(	23.36574	0.28122	0.241575	0.664053	lncRNA
ENSG0000(	ENSG0000(	121.4683	0.175772	0.241545	0.664053	lncRNA
ENSG0000(	ENSG0000(	53.99835	-0.18399	0.241599	0.664053	lncRNA
ENSG0000(	FRG1CP	1124.666	-0.13248	0.241711	0.664053	transcribed_unprocessed_pseudo
ENSG0000(	INGX	172.1313	0.137698	0.241755	0.664053	transcribed_processed_pseudoge
ENSG0000(	PEPD	285.2498	-0.20992	0.241643	0.664053	protein_coding
ENSG0000(	RN7SL275P	534.5768	-0.08984	0.241713	0.664053	misc_RNA

ENSG0000(	SEPTIN7P1:	976.4653	-0.09442	0.241733	0.664053	transcribed_unprocessed_pseudo
ENSG0000(	SNHG16	1701.79	-0.10195	0.24173	0.664053	lncRNA
ENSG0000(	TBC1D9B	3212.34	0.07364	0.241676	0.664053	protein_coding
ENSG0000(	TPSG1	170.743	0.314384	0.241576	0.664053	protein_coding
ENSG0000(	USP38-DT	101.3982	-0.22769	0.241734	0.664053	lncRNA
ENSG0000(	ZNF383	1762.389	0.088045	0.241732	0.664053	protein_coding
ENSG0000(	ENSG0000(	25.05191	0.326003	0.241893	0.664299	lncRNA
ENSG0000(	TRIM33	2266.305	-0.06093	0.241889	0.664299	protein_coding
ENSG0000(	OGDHL	40.67746	-0.46701	0.241922	0.664313	protein_coding
ENSG0000(	LINC02447	283.186	0.106523	0.241977	0.664399	lncRNA
ENSG0000(	C19orf25	838.5405	0.08874	0.242078	0.664609	protein_coding
ENSG0000(	SLC12A5	35.16721	-0.36074	0.242159	0.664766	protein_coding
ENSG0000(	CCDC154	45.737	-0.35781	0.242208	0.664835	protein_coding
ENSG0000(	CFDP1	674.8735	0.086441	0.242248	0.664877	protein_coding
ENSG0000(	APC	2450.106	0.080985	0.242374	0.664926	protein_coding
ENSG0000(	ATXN1-AS1	102.1965	-0.18561	0.242382	0.664926	lncRNA
ENSG0000(	CHM	879.4503	-0.09297	0.242336	0.664926	protein_coding
ENSG0000(	ENSG0000(	167.2719	0.10473	0.24231	0.664926	processed_pseudogene
ENSG0000(	ENSG0000(	791.3959	-0.19021	0.242435	0.664926	lncRNA
ENSG0000(	ENSG0000(	3294.655	0.086198	0.242426	0.664926	lncRNA
ENSG0000(	ENSG0000(	122.0352	0.145349	0.242393	0.664926	lncRNA
ENSG0000(	TOE1	192.4867	0.128213	0.242473	0.66494	protein_coding
ENSG0000(	WASIR2	56.73189	-0.67189	0.242488	0.66494	lncRNA
ENSG0000(	MAGED1	1458.085	-0.12567	0.242597	0.665172	protein_coding
ENSG0000(	ENSG0000(	121.7284	-0.15188	0.242692	0.665366	lncRNA
ENSG0000(	ENSG0000(	45.29803	-0.16722	0.242843	0.665673	lncRNA
ENSG0000(	HSPA7	251.7507	-0.33073	0.242852	0.665673	unprocessed_pseudogene
ENSG0000(	RDM1P1	245.0858	-0.21582	0.242899	0.665733	transcribed_unprocessed_pseudo
ENSG0000(	FUT8	1722.186	-0.11705	0.242944	0.665792	protein_coding
ENSG0000(	RNU6-140F	22.62565	0.5922	0.242968	0.665792	snRNA
ENSG0000(	MIR3648-1	2613.926	0.350588	0.243009	0.665795	miRNA
ENSG0000(	PRRT1	83.82806	0.294217	0.243018	0.665795	protein_coding
ENSG0000(	SNHG31	619.9474	0.132721	0.243059	0.665841	lncRNA
ENSG0000(	ARHGEF10	813.4081	-0.15446	0.243336	0.666268	protein_coding
ENSG0000(	BEND3P1	23.89066	0.295289	0.24329	0.666268	transcribed_processed_pseudoge
ENSG0000(	ENSG0000(	165.9901	0.490901	0.24333	0.666268	lncRNA
ENSG0000(	ENSG0000(	74.84241	0.190702	0.243329	0.666268	lncRNA
ENSG0000(	TRIB1	1684.809	-0.17489	0.243273	0.666268	protein_coding
ENSG0000(	ADPRH	148.7051	-0.24267	0.243509	0.666417	protein_coding
ENSG0000(	CRY2	1113.005	0.099504	0.243487	0.666417	protein_coding
ENSG0000(	ENSG0000(	54.65261	0.187433	0.243426	0.666417	lncRNA
ENSG0000(	SCN4A	42.32589	0.543688	0.243512	0.666417	protein_coding
ENSG0000(	SPP1	273.4855	-0.22077	0.243481	0.666417	protein_coding
ENSG0000(	ENSG0000(	703.6628	0.24572	0.243572	0.666472	protein_coding
ENSG0000(	WDR26	4132.199	-0.06712	0.24358	0.666472	protein_coding
ENSG0000(	KCNJ11	205.1157	0.137687	0.243621	0.666516	protein_coding
ENSG0000(	DCTN1-AS1	88.70231	-0.11481	0.24368	0.666526	lncRNA

ENSG0000(	ENSG0000(	44.91922	0.204067	0.243687	0.666526	lncRNA
ENSG0000(	LRRC18	45.98466	0.1957	0.243697	0.666526	protein_coding
ENSG0000(	ENSG0000(	114.6756	-0.1387	0.24376	0.666633	lncRNA
ENSG0000(	ENSG0000(	39.43257	0.218652	0.244033	0.666702	lncRNA
ENSG0000(	ENSG0000(	1071.75	-0.08402	0.243933	0.666702	lncRNA
ENSG0000(	ENSG0000(	249.2979	0.111778	0.243994	0.666702	lncRNA
ENSG0000(	ENSG0000(	83.70441	0.154589	0.244065	0.666702	lncRNA
ENSG0000(	GDNF	251.3944	-0.21101	0.244011	0.666702	protein_coding
ENSG0000(	KANK3	86.82168	-0.22787	0.243919	0.666702	protein_coding
ENSG0000(	MROH3P	344.1943	0.352415	0.244076	0.666702	transcribed_unitary_pseudogene
ENSG0000(	MRPL2	290.27	0.112291	0.243959	0.666702	protein_coding
ENSG0000(	NIFK	300.3251	0.118568	0.243907	0.666702	protein_coding
ENSG0000(	PYROXD1	693.0137	0.12739	0.243966	0.666702	protein_coding
ENSG0000(	RSRC2	3057.931	0.061037	0.244045	0.666702	protein_coding
ENSG0000(	YY1AP1	1767.162	-0.05382	0.243836	0.666702	protein_coding
ENSG0000(	CDC37L1-D	22.21163	0.29953	0.244242	0.666889	lncRNA
ENSG0000(	NCL	2778.897	-0.09693	0.244195	0.666889	protein_coding
ENSG0000(	RPL35P6	37.41224	0.283967	0.244197	0.666889	processed_pseudogene
ENSG0000(	TAMM41	888.6167	0.089986	0.244224	0.666889	protein_coding
ENSG0000(	ENSG0000(	19.54301	0.3589	0.244337	0.667042	lncRNA
ENSG0000(	WDR24	160.8161	-0.12294	0.244346	0.667042	protein_coding
ENSG0000(	MYO6	4192.867	0.140258	0.24444	0.667233	protein_coding
ENSG0000(	PNPLA5	56.95794	-0.17253	0.244508	0.667353	protein_coding
ENSG0000(	ENSG0000(	49.48674	-0.30465	0.244599	0.667534	lncRNA
ENSG0000(	TPTE2P2	439.5729	0.096044	0.244677	0.667679	transcribed_unprocessed_pseudo
ENSG0000(	RINL	471.5097	-0.11138	0.244792	0.667928	protein_coding
ENSG0000(	PGAM1P5	94.59313	0.158166	0.244875	0.668022	transcribed_processed_pseudoge
ENSG0000(	TOP2A	937.3857	-0.21328	0.244858	0.668022	protein_coding
ENSG0000(	ENSG0000(	33.40616	0.305012	0.2449	0.668024	lncRNA
ENSG0000(	MON2	5499.16	0.077811	0.24495	0.668028	protein_coding
ENSG0000(	RFC1	995.1127	-0.10133	0.244945	0.668028	protein_coding
ENSG0000(	SCARNA2	6993.464	0.289287	0.245012	0.66813	scaRNA
ENSG0000(	ARHGEF25	181.2044	-0.2133	0.245144	0.668326	protein_coding
ENSG0000(	ENSG0000(	97.20443	0.218236	0.24527	0.668326	lncRNA
ENSG0000(	LINC02102	27.62435	0.400047	0.245322	0.668326	lncRNA
ENSG0000(	METTL22	1191.404	-0.07395	0.245321	0.668326	protein_coding
ENSG0000(	MRPL42	2965.084	-0.10824	0.245326	0.668326	protein_coding
ENSG0000(	PLN	98.1601	-0.26135	0.245283	0.668326	protein_coding
ENSG0000(	PRC1	355.6237	-0.11882	0.245169	0.668326	protein_coding
ENSG0000(	RBM26	2963.205	0.072899	0.245251	0.668326	protein_coding
ENSG0000(	SNX2	1190.737	-0.12924	0.245152	0.668326	protein_coding
ENSG0000(	UGT1A3	198.7309	0.435911	0.245198	0.668326	protein_coding
ENSG0000(	ENSG0000(	39.88396	0.37204	0.245367	0.668369	TEC
ENSG0000(	SS18L2	224.0372	-0.13705	0.245456	0.668547	protein_coding
ENSG0000(	TM4SF19	95.57461	-0.15659	0.24559	0.668845	protein_coding
ENSG0000(	LINC01451	40.80834	0.349216	0.245709	0.669101	lncRNA
ENSG0000(	CNIH3	181.2673	0.09778	0.245768	0.669172	protein_coding

ENSG0000(	ENSG0000(	21.72425	0.297342	0.245832	0.669172	lncRNA
ENSG0000(	RN7SKP80	64.05227	0.214506	0.245801	0.669172	misc_RNA
ENSG0000(	RNF152	2404.877	0.266618	0.245813	0.669172	protein_coding
ENSG0000(	UST	410.3323	-0.16318	0.245933	0.669383	protein_coding
ENSG0000(	BRK1	381.8326	-0.15907	0.246013	0.669418	protein_coding
ENSG0000(	MARCHF7	2715.788	-0.08219	0.246012	0.669418	protein_coding
ENSG0000(	MCPH1	3383.497	-0.05232	0.246019	0.669418	protein_coding
ENSG0000(	ITGA6-AS1	41.24342	-0.30425	0.246122	0.669581	lncRNA
ENSG0000(	OR7E25P	616.1271	0.075671	0.246128	0.669581	transcribed_unprocessed_pseudo
ENSG0000(	ENSG0000(	1155.347	-0.22859	0.24616	0.669585	lncRNA
ENSG0000(	ENSG0000(	135.8052	0.203769	0.246178	0.669585	lncRNA
ENSG0000(	FMN1	4590.415	0.194052	0.246206	0.669594	protein_coding
ENSG0000(	ENSG0000(	134.2689	0.13893	0.246305	0.669665	lncRNA
ENSG0000(	ENSG0000(	422.9342	0.096201	0.246267	0.669665	lncRNA
ENSG0000(	LRP5	1887.631	0.209401	0.246296	0.669665	protein_coding
ENSG0000(	TANGO6	841.069	-0.09728	0.246349	0.669718	protein_coding
ENSG0000(	ATG4A	257.7795	-0.16406	0.246445	0.669771	protein_coding
ENSG0000(	C9orf85	705.1961	0.066478	0.246408	0.669771	protein_coding
ENSG0000(	MIR4480	26.97917	0.285392	0.246439	0.669771	miRNA
ENSG0000(	TCTN2	582.1284	0.083722	0.246466	0.669771	protein_coding
ENSG0000(	GLRA4	212.6301	0.276431	0.246505	0.669813	transcribed_unitary_pseudogene
ENSG0000(	C1orf216	271.6342	-0.11653	0.246645	0.669927	protein_coding
ENSG0000(	COPG2IT1	705.5798	0.250148	0.246639	0.669927	lncRNA
ENSG0000(	PPIL6	285.8462	0.111425	0.246608	0.669927	protein_coding
ENSG0000(	SNORA63	1403.517	0.193833	0.246622	0.669927	snoRNA
ENSG0000(	LIMCH1	491.0549	-0.17456	0.246681	0.669959	protein_coding
ENSG0000(	GPR137	294.5548	0.111095	0.246809	0.670226	protein_coding
ENSG0000(	MYH7B	347.3432	0.098302	0.246828	0.670226	protein_coding
ENSG0000(	AZIN1	1622.908	-0.10931	0.24704	0.670284	protein_coding
ENSG0000(	C2CD2L	1256.683	0.122233	0.247003	0.670284	protein_coding
ENSG0000(	DENND6A-	174.5587	0.104091	0.247192	0.670284	lncRNA
ENSG0000(	EIF6	450.4941	0.165558	0.246972	0.670284	protein_coding
ENSG0000(	ENSG0000(	45.06901	0.229543	0.247171	0.670284	processed_pseudogene
ENSG0000(	ENSG0000(	25.13891	0.293153	0.247046	0.670284	lncRNA
ENSG0000(	ENSG0000(	27.71332	0.35671	0.247118	0.670284	lncRNA
ENSG0000(	KCNK1	582.5017	0.197514	0.247097	0.670284	protein_coding
ENSG0000(	MFAP3L	136.5737	-0.24976	0.247136	0.670284	protein_coding
ENSG0000(	MMP24	20.34598	0.394832	0.247104	0.670284	protein_coding
ENSG0000(	RN7SL37P	38.90694	0.197335	0.247195	0.670284	misc_RNA
ENSG0000(	RN7SL836F	22.43148	0.312424	0.24703	0.670284	misc_RNA
ENSG0000(	TEN1	39.53955	-0.261	0.247215	0.670284	protein_coding
ENSG0000(	TTF1	375.5846	-0.14151	0.246981	0.670284	protein_coding
ENSG0000(	UGT2B17	2332.337	0.520665	0.247155	0.670284	protein_coding
ENSG0000(	PMS2	1056.186	0.083394	0.247264	0.670352	protein_coding
ENSG0000(	RN7SKP239	30.53224	0.232196	0.247341	0.670494	misc_RNA
ENSG0000(	NOTCH4	1139.256	-0.12335	0.247396	0.67053	protein_coding
ENSG0000(	PRKDC	5058.35	-0.11829	0.247403	0.67053	protein_coding

ENSG0000(	NDUFAF2P	78.8853	-0.17022	0.247433	0.670545	processed_pseudogene
ENSG0000(	CATSPER3	29.17283	0.38771	0.247552	0.670609	protein_coding
ENSG0000(	CRYBG3	4083.522	-0.08388	0.247652	0.670609	protein_coding
ENSG0000(	DHH	85.51878	-0.20446	0.247563	0.670609	protein_coding
ENSG0000(	ENSG0000(	45.3364	0.216005	0.247603	0.670609	lncRNA
ENSG0000(	ENSG0000(	235.8547	0.140133	0.247567	0.670609	lncRNA
ENSG0000(	ENSG0000(	238.3266	0.080412	0.247485	0.670609	lncRNA
ENSG0000(	POLE3	269.1174	-0.12423	0.247597	0.670609	protein_coding
ENSG0000(	SYCP1	215.184	-0.12235	0.247637	0.670609	protein_coding
ENSG0000(	ENSG0000(	177.0974	0.135542	0.247753	0.670704	TEC
ENSG0000(	PCDH20	203.3711	0.517696	0.247748	0.670704	protein_coding
ENSG0000(	WDR48	978.4831	0.110998	0.24776	0.670704	protein_coding
ENSG0000(	ENSG0000(	20.60361	-0.36349	0.247874	0.670948	processed_pseudogene
ENSG0000(	ENSG0000(	177.4145	-0.17479	0.247995	0.67121	lncRNA
ENSG0000(	ATP8A2	739.1611	0.085388	0.248047	0.671219	protein_coding
ENSG0000(	ZNF395	1343.574	0.110469	0.248043	0.671219	protein_coding
ENSG0000(	ENSG0000(	49.29008	-0.27198	0.248161	0.671393	lncRNA
ENSG0000(	F2RL1	1232.481	0.271681	0.248154	0.671393	protein_coding
ENSG0000(	CBWD2	822.9567	-0.10489	0.248297	0.671496	protein_coding
ENSG0000(	ICAM4	124.9028	-0.14403	0.248259	0.671496	protein_coding
ENSG0000(	NCKAP5	323.0403	0.247877	0.248294	0.671496	protein_coding
ENSG0000(	RN7SKP24(	24.25103	0.330063	0.248246	0.671496	misc_RNA
ENSG0000(	CDC14B	808.9365	0.122971	0.24843	0.671637	protein_coding
ENSG0000(	DDHD1-DT	19.78493	-0.36358	0.248405	0.671637	lncRNA
ENSG0000(	HSP90B2P	70.55888	0.209159	0.248446	0.671637	processed_pseudogene
ENSG0000(	SNRPGP9	20.63155	-0.45863	0.248404	0.671637	processed_pseudogene
ENSG0000(	MTDH	2303.257	-0.14618	0.248531	0.671733	protein_coding
ENSG0000(	ZMIZ1	3735.723	-0.11202	0.248509	0.671733	protein_coding
ENSG0000(	LINC01671	145.7422	-0.16113	0.248636	0.671886	lncRNA
ENSG0000(	SUGT1P2	81.3045	0.147547	0.248619	0.671886	processed_pseudogene
ENSG0000(	DELE1	2102.449	0.06856	0.248783	0.672218	protein_coding
ENSG0000(	GPR183	200.965	-0.31344	0.248808	0.67222	protein_coding
ENSG0000(	ATF4P3	28.06286	0.387392	0.248906	0.67222	processed_pseudogene
ENSG0000(	ENSG0000(	87.65745	0.175804	0.248845	0.67222	lncRNA
ENSG0000(	FABP6-AS1	459.0822	-0.15544	0.248863	0.67222	lncRNA
ENSG0000(	LPCAT3	970.8953	0.15067	0.248903	0.67222	protein_coding
ENSG0000(	HOXA2	133.3167	0.251111	0.248982	0.672359	protein_coding
ENSG0000(	ADCK2	194.1613	0.167644	0.249036	0.672439	protein_coding
ENSG0000(	ZKSCAN8	1884.47	-0.07077	0.24913	0.672627	protein_coding
ENSG0000(	TXLNA	1773.612	-0.08553	0.249223	0.67281	protein_coding
ENSG0000(	SLC26A3	12942.05	0.463938	0.249259	0.672842	protein_coding
ENSG0000(	GRPEL1	271.7741	0.108366	0.24931	0.672848	protein_coding
ENSG0000(	KIF27	472.4735	0.115326	0.249299	0.672848	protein_coding
ENSG0000(	FGD3	873.8129	-0.25267	0.249406	0.673043	protein_coding
ENSG0000(	MBD5	3130.194	-0.10357	0.249437	0.673059	protein_coding
ENSG0000(	ENSG0000(	46.65887	-0.2129	0.249536	0.673243	lncRNA
ENSG0000(	MMGT1	555.8034	-0.10667	0.249554	0.673243	protein_coding

ENSG0000(	B3GNT2	494.9955	0.143791	0.249611	0.67328	protein_coding
ENSG0000(	EML4	3986.604	-0.08642	0.249881	0.67328	protein_coding
ENSG0000(	ENSG0000(	159.191	0.236638	0.249706	0.67328	lncRNA
ENSG0000(	EPHA1	451.9536	0.095824	0.24974	0.67328	protein_coding
ENSG0000(	KCTD5P1	33.48763	0.286546	0.249885	0.67328	processed_pseudogene
ENSG0000(	LINC00598	574.7466	0.116953	0.249822	0.67328	lncRNA
ENSG0000(	LRRC8D	683.101	0.117029	0.249744	0.67328	protein_coding
ENSG0000(	MRPS17P1	521.1274	0.112721	0.249817	0.67328	processed_pseudogene
ENSG0000(	NUP35	338.6996	0.119607	0.249811	0.67328	protein_coding
ENSG0000(	P2RX5	533.1281	-0.3982	0.249859	0.67328	protein_coding
ENSG0000(	RPS15AP6	101.0723	-0.17694	0.249701	0.67328	processed_pseudogene
ENSG0000(	ULBP1	26.86304	-0.21806	0.24986	0.67328	protein_coding
ENSG0000(	USP39	512.3424	0.095474	0.249886	0.67328	protein_coding
ENSG0000(	ENSG0000(	33.50916	-0.29926	0.249945	0.673298	protein_coding
ENSG0000(	FAM50B	39.88429	-0.27066	0.249922	0.673298	protein_coding
ENSG0000(	PRR5-ARH(	129.0988	0.223467	0.249966	0.673298	protein_coding
ENSG0000(	MRPL32	332.0038	-0.11694	0.249991	0.673298	protein_coding
ENSG0000(	ENSG0000(	7341.964	0.414794	0.250131	0.673611	lncRNA
ENSG0000(	ENSG0000(	24.83252	0.2753	0.250312	0.673977	transcribed_processed_pseudoge
ENSG0000(	ENSG0000(	203.7872	0.150342	0.250341	0.673977	lncRNA
ENSG0000(	YDJC	140.4024	0.139152	0.250337	0.673977	protein_coding
ENSG0000(	ZMYM3	710.336	-0.12272	0.25037	0.673989	protein_coding
ENSG0000(	ENSG0000(	355.2047	0.109084	0.250608	0.673997	transcribed_processed_pseudoge
ENSG0000(	FGFR2	3388.013	0.29299	0.250574	0.673997	protein_coding
ENSG0000(	GGA3	1372.102	0.079832	0.250682	0.673997	protein_coding
ENSG0000(	INPP4B	1912.371	-0.13989	0.250572	0.673997	protein_coding
ENSG0000(	LINC00412	58.78726	-0.24341	0.250661	0.673997	lncRNA
ENSG0000(	MLLT6	6895.341	-0.08629	0.250508	0.673997	protein_coding
ENSG0000(	OR6A2	90.62336	-0.30614	0.250457	0.673997	protein_coding
ENSG0000(	PPP1R7	327.7448	-0.12361	0.250597	0.673997	protein_coding
ENSG0000(	RBL2	3510.207	0.114389	0.250691	0.673997	protein_coding
ENSG0000(	SEZ6L	27.51205	0.481492	0.250616	0.673997	protein_coding
ENSG0000(	SLC18B1	273.6541	0.171824	0.250515	0.673997	protein_coding
ENSG0000(	SNORD15B	321.2823	0.191038	0.250667	0.673997	snoRNA
ENSG0000(	STRN	2574.835	0.103502	0.250497	0.673997	protein_coding
ENSG0000(	ARNT	1733.974	0.085943	0.250741	0.674047	protein_coding
ENSG0000(	ENSG0000(	223.2065	0.139176	0.250759	0.674047	lncRNA
ENSG0000(	MASP2	60.60467	0.218471	0.250811	0.674121	protein_coding
ENSG0000(	DNAH6	365.4962	0.119541	0.251002	0.674371	protein_coding
ENSG0000(	ENSG0000(	327.4837	0.107398	0.25093	0.674371	lncRNA
ENSG0000(	ZCWPW2	70.7929	0.213738	0.251	0.674371	protein_coding
ENSG0000(	ZRANB3	624.0339	-0.1446	0.250997	0.674371	protein_coding
ENSG0000(	LINC02225	256.2065	-0.12618	0.251157	0.674722	lncRNA
ENSG0000(	RNU1-43P	27.54918	0.377986	0.251204	0.674783	snRNA
ENSG0000(	ALG10	213.5363	-0.13585	0.251397	0.674977	protein_coding
ENSG0000(	ATP6V1H	475.4334	-0.07975	0.251414	0.674977	protein_coding
ENSG0000(	C8orf33	577.3759	0.092309	0.251423	0.674977	protein_coding

ENSG0000(	ENSG0000(	23.52791	-0.29125	0.251337	0.674977	lncRNA
ENSG0000(	ENSG0000(	42.49534	0.203778	0.251407	0.674977	lncRNA
ENSG0000(	ENSG0000(	174.3284	-0.38011	0.251369	0.674977	lncRNA
ENSG0000(	GPR171	103.6008	-0.34698	0.251541	0.675049	protein_coding
ENSG0000(	HAP1	648.7092	-0.09354	0.251549	0.675049	protein_coding
ENSG0000(	PHC3	5847.193	-0.06722	0.251544	0.675049	protein_coding
ENSG0000(	SLC7A7	582.6423	-0.17954	0.251494	0.675049	protein_coding
ENSG0000(	BCAR3	593.102	0.121646	0.251646	0.67509	protein_coding
ENSG0000(	ENSG0000(	91.86959	-0.16951	0.251622	0.67509	lncRNA
ENSG0000(	GPR161	423.231	-0.27929	0.251662	0.67509	protein_coding
ENSG0000(	RPS26	1010.981	0.23531	0.251636	0.67509	protein_coding
ENSG0000(	ENSG0000(	126.4563	-0.16141	0.251695	0.675114	lncRNA
ENSG0000(	RAB32	110.8389	0.170558	0.25191	0.675623	protein_coding
ENSG0000(	ENSG0000(	20.95144	-0.52846	0.252047	0.675732	lncRNA
ENSG0000(	ENSG0000(	61.53061	-0.17785	0.252049	0.675732	lncRNA
ENSG0000(	NFIA-AS2	672.5362	0.089249	0.252031	0.675732	lncRNA
ENSG0000(	NGFR	53.69522	-0.28705	0.252019	0.675732	protein_coding
ENSG0000(	CDK2AP1	1184.245	-0.1379	0.252082	0.675756	protein_coding
ENSG0000(	ENSG0000(	534.7956	0.103666	0.252107	0.675758	lncRNA
ENSG0000(	OR5M2P	174.6582	0.108188	0.252147	0.675799	transcribed_unprocessed_pseudo
ENSG0000(	FILIP1	298.7888	-0.14344	0.252206	0.675826	protein_coding
ENSG0000(	RPS3AP38	346.2252	-0.1453	0.252194	0.675826	processed_pseudogene
ENSG0000(	CNKSR1	520.0372	0.189762	0.252241	0.675854	protein_coding
ENSG0000(	ENSG0000(	326.8926	0.077104	0.252367	0.676124	lncRNA
ENSG0000(	RBM27	908.0695	-0.15148	0.252402	0.676153	protein_coding
ENSG0000(	C10orf95-A	1990.416	0.043557	0.252499	0.676218	lncRNA
ENSG0000(	NDUFA13	385.4267	0.132152	0.252471	0.676218	protein_coding
ENSG0000(	RPL11P3	331.9305	-0.11654	0.2525	0.676218	processed_pseudogene
ENSG0000(	AKIRIN1	1012.155	-0.09017	0.253463	0.676496	protein_coding
ENSG0000(	ALDH1A3-A	479.6951	0.117687	0.253076	0.676496	lncRNA
ENSG0000(	AMY2B	1120.569	0.14213	0.252728	0.676496	protein_coding
ENSG0000(	B3GNT8	224.5232	0.33944	0.252848	0.676496	protein_coding
ENSG0000(	C2orf76	53.00963	-0.17193	0.252777	0.676496	protein_coding
ENSG0000(	CADPS	505.224	0.198451	0.253589	0.676496	protein_coding
ENSG0000(	EFNA4	109.8577	0.215079	0.253667	0.676496	protein_coding
ENSG0000(	ELMOD2	613.4608	-0.0817	0.253232	0.676496	protein_coding
ENSG0000(	ENSG0000(	30.28251	0.232207	0.253654	0.676496	lncRNA
ENSG0000(	ENSG0000(	137.0486	0.191911	0.253757	0.676496	processed_pseudogene
ENSG0000(	ENSG0000(	42.99397	-0.20581	0.253027	0.676496	lncRNA
ENSG0000(	ENSG0000(	58.37928	-0.30344	0.252847	0.676496	lncRNA
ENSG0000(	ENSG0000(	1141.78	0.101227	0.252915	0.676496	lncRNA
ENSG0000(	ENSG0000(	27.67443	-0.27073	0.253872	0.676496	lncRNA
ENSG0000(	ENSG0000(	36.35438	0.308829	0.253253	0.676496	lncRNA
ENSG0000(	ENSG0000(	25.01202	-0.24403	0.253028	0.676496	lncRNA
ENSG0000(	ENSG0000(	118.6103	-0.36201	0.252666	0.676496	lncRNA
ENSG0000(	ENSG0000(	28.08337	-0.27466	0.253388	0.676496	lncRNA
ENSG0000(	ENSG0000(	779.105	0.076427	0.253817	0.676496	lncRNA

ENSG0000(	FAHD2B	150.6666	0.162429	0.253491	0.676496	protein_coding
ENSG0000(	GALNT11	643.3214	-0.10024	0.25347	0.676496	protein_coding
ENSG0000(	GLE1	1865.133	0.037396	0.252812	0.676496	protein_coding
ENSG0000(	GLIS3	1173.698	-0.31687	0.253244	0.676496	protein_coding
ENSG0000(	GNAT1	164.3863	-0.22404	0.253586	0.676496	protein_coding
ENSG0000(	HHLA2	2501.347	0.344444	0.253883	0.676496	protein_coding
ENSG0000(	LINC02394	100.6766	-0.17038	0.25337	0.676496	lncRNA
ENSG0000(	MAP3K2-D	485.1824	-0.08498	0.253081	0.676496	lncRNA
ENSG0000(	MAP7	947.7862	0.197132	0.253128	0.676496	protein_coding
ENSG0000(	MATN1	30.73085	-0.3236	0.252839	0.676496	protein_coding
ENSG0000(	MPI	1086.94	0.122271	0.25377	0.676496	protein_coding
ENSG0000(	MT-TD	115.8514	-0.57058	0.253877	0.676496	Mt_tRNA
ENSG0000(	MT-TG	56.12972	-0.69658	0.252794	0.676496	Mt_tRNA
ENSG0000(	NAA10	353.7003	-0.10085	0.253093	0.676496	protein_coding
ENSG0000(	NANS	1478.661	0.158153	0.253202	0.676496	protein_coding
ENSG0000(	NAPEPLD	1914.784	0.158618	0.252933	0.676496	protein_coding
ENSG0000(	PIP5K1C	1080.766	-0.18299	0.253032	0.676496	protein_coding
ENSG0000(	PRPF38B	2496.945	-0.08218	0.253824	0.676496	protein_coding
ENSG0000(	PTPN18	3495.304	0.138621	0.253481	0.676496	protein_coding
ENSG0000(	RBM25	5883.893	0.052473	0.253753	0.676496	protein_coding
ENSG0000(	RN7SL789F	27.81734	-0.31138	0.253572	0.676496	misc_RNA
ENSG0000(	RRM1	491.1895	-0.10419	0.253144	0.676496	protein_coding
ENSG0000(	SCARF2	85.04804	-0.22401	0.25344	0.676496	protein_coding
ENSG0000(	SCARNA4	165.0876	0.136581	0.252808	0.676496	scaRNA
ENSG0000(	SFTPA2	22.98189	-0.53835	0.253428	0.676496	protein_coding
ENSG0000(	SLC24A4	729.7335	-0.12735	0.25336	0.676496	protein_coding
ENSG0000(	SLC31A1	1736.088	-0.10183	0.253334	0.676496	protein_coding
ENSG0000(	SNHG17	2109.962	0.077386	0.252901	0.676496	lncRNA
ENSG0000(	STRN4	1057.249	-0.08048	0.253278	0.676496	protein_coding
ENSG0000(	SYN2	22.64129	-0.42713	0.2529	0.676496	protein_coding
ENSG0000(	SYTL5	359.1464	0.300993	0.253859	0.676496	protein_coding
ENSG0000(	UQCRFS1	750.4936	0.172195	0.253675	0.676496	protein_coding
ENSG0000(	ZNF300	279.3021	0.132417	0.253827	0.676496	protein_coding
ENSG0000(	ENSG0000(	50.41699	-0.20714	0.25391	0.676503	lncRNA
ENSG0000(	ABCD3	1873.981	0.160522	0.254089	0.676587	protein_coding
ENSG0000(	C1orf52	405.0738	0.109905	0.254326	0.676587	protein_coding
ENSG0000(	CKMT1B	1309.644	0.277589	0.25421	0.676587	protein_coding
ENSG0000(	ENSG0000(	152.4656	-0.13652	0.254034	0.676587	lncRNA
ENSG0000(	ENSG0000(	79.81638	-0.21159	0.254206	0.676587	lncRNA
ENSG0000(	ENSG0000(	221.2083	-0.18008	0.254266	0.676587	lncRNA
ENSG0000(	ERG28	215.0479	0.128747	0.254255	0.676587	protein_coding
ENSG0000(	H3C13	35.3357	-0.38661	0.254354	0.676587	protein_coding
ENSG0000(	HBA1	27.66778	-1.10568	0.254123	0.676587	protein_coding
ENSG0000(	LIN7A	145.1162	-0.36875	0.254067	0.676587	protein_coding
ENSG0000(	LINC00904	179.5772	0.086062	0.254155	0.676587	lncRNA
ENSG0000(	LINC01340	43.84454	0.419004	0.254247	0.676587	lncRNA
ENSG0000(	OR7D4	384.1116	0.086733	0.254093	0.676587	protein_coding

ENSG0000(	PAPLN-AS1	396.7718	0.0818	0.254315	0.676587	lncRNA
ENSG0000(	TALDO1	640.3746	-0.11821	0.254175	0.676587	protein_coding
ENSG0000(	TPGS2	1779.933	-0.07382	0.25436	0.676587	protein_coding
ENSG0000(	UVSSA	3277.633	0.118964	0.254205	0.676587	protein_coding
ENSG0000(	RELCH	3271.581	0.103254	0.254396	0.676619	protein_coding
ENSG0000(	ENSG0000(	138.6347	-0.12586	0.254592	0.676878	lncRNA
ENSG0000(	GLI3	515.1502	-0.32424	0.254577	0.676878	protein_coding
ENSG0000(	SYTL3	188.7033	-0.22286	0.254534	0.676878	protein_coding
ENSG0000(	TRAPPC3	631.3651	-0.07969	0.254553	0.676878	protein_coding
ENSG0000(	ADORA2A	104.8029	-0.21308	0.254692	0.677014	protein_coding
ENSG0000(	CSAD	1195.168	0.076929	0.254937	0.677014	protein_coding
ENSG0000(	ENSG0000(	43.3708	0.178789	0.254829	0.677014	lncRNA
ENSG0000(	ENSG0000(	215.1458	-0.11283	0.25503	0.677014	lncRNA
ENSG0000(	ENSG0000(	145.4577	-0.14753	0.255013	0.677014	TEC
ENSG0000(	ENSG0000(	32.29039	-0.40489	0.255037	0.677014	lncRNA
ENSG0000(	MAP3K12	1049.227	-0.1184	0.254734	0.677014	protein_coding
ENSG0000(	NACAD	57.61104	-0.45037	0.255028	0.677014	protein_coding
ENSG0000(	PCDH10-DT	147.6672	-0.14558	0.254886	0.677014	lncRNA
ENSG0000(	PGPEP1	2753.426	0.12999	0.254748	0.677014	protein_coding
ENSG0000(	RGS17P1	88.68833	0.18213	0.254835	0.677014	processed_pseudogene
ENSG0000(	SLC41A2	1539.055	0.177672	0.254899	0.677014	protein_coding
ENSG0000(	SNRK-AS1	188.9076	0.24676	0.254848	0.677014	lncRNA
ENSG0000(	TIGD7	266.6462	-0.15888	0.254837	0.677014	protein_coding
ENSG0000(	ZEB2-AS1	63.39234	-0.60311	0.255015	0.677014	lncRNA
ENSG0000(	ZNF423	327.8919	-0.21963	0.25473	0.677014	protein_coding
ENSG0000(	SLIT3	493.0574	-0.18424	0.25507	0.677038	protein_coding
ENSG0000(	DHFR	846.9505	-0.10854	0.255121	0.677077	protein_coding
ENSG0000(	SLC22A20P	286.6577	-0.15205	0.255134	0.677077	transcribed_unitary_pseudogene
ENSG0000(	HDAC9	1010.719	-0.15684	0.255261	0.67725	protein_coding
ENSG0000(	NAALADL2	1426.769	0.183432	0.255273	0.67725	protein_coding
ENSG0000(	WDTC1	1136.85	0.074063	0.255257	0.67725	protein_coding
ENSG0000(	KRT18P15	58.95114	0.253294	0.255299	0.677254	processed_pseudogene
ENSG0000(	RNVU1-1	121.4457	-0.49669	0.255379	0.677398	snRNA
ENSG0000(	TSEN15	1600.764	-0.08186	0.255444	0.677507	protein_coding
ENSG0000(	CYP2D6	94.83367	-0.36423	0.255721	0.677547	protein_coding
ENSG0000(	ENSG0000(	80.24831	-0.16707	0.255679	0.677547	lncRNA
ENSG0000(	ENSG0000(	36.39854	0.187483	0.255541	0.677547	processed_pseudogene
ENSG0000(	ENSG0000(	21.70993	0.323794	0.25553	0.677547	lncRNA
ENSG0000(	ENSG0000(	48.76838	-0.20899	0.25549	0.677547	lncRNA
ENSG0000(	ENSG0000(	537.1761	0.087926	0.255776	0.677547	lncRNA
ENSG0000(	FNDC1	153.6728	-0.53976	0.255774	0.677547	protein_coding
ENSG0000(	GTF2A2	522.6311	0.122887	0.255626	0.677547	protein_coding
ENSG0000(	LINC00539	108.3032	-0.22159	0.255802	0.677547	lncRNA
ENSG0000(	MIR571	60.16642	0.213102	0.255599	0.677547	miRNA
ENSG0000(	OR2T11	49.27404	-0.36082	0.255731	0.677547	protein_coding
ENSG0000(	PURPL	377.2323	0.111172	0.255678	0.677547	lncRNA
ENSG0000(	RALGPS1	1672.402	0.164888	0.255804	0.677547	protein_coding

ENSG0000(	TBX2-AS1	43.45871	-0.27816	0.255778	0.677547	lncRNA
ENSG0000(	ENSG0000(	158.4022	-0.17469	0.255909	0.677728	lncRNA
ENSG0000(	RC3H1	3654.588	0.097249	0.255946	0.677728	protein_coding
ENSG0000(	WRAP73	520.6965	0.118415	0.255939	0.677728	protein_coding
ENSG0000(	ENSG0000(	52.59065	0.211407	0.256023	0.677801	lncRNA
ENSG0000(	NDUFV2	1011.529	-0.07146	0.255999	0.677801	protein_coding
ENSG0000(	ENSG0000(	100.8631	0.249434	0.256061	0.677835	lncRNA
ENSG0000(	CD1D	355.8611	-0.16944	0.256358	0.678361	protein_coding
ENSG0000(	ENSG0000(	133.4669	0.148917	0.256308	0.678361	lncRNA
ENSG0000(	METTL13	510.6061	0.109997	0.256324	0.678361	protein_coding
ENSG0000(	XKR9	1098.745	-0.14328	0.256344	0.678361	protein_coding
ENSG0000(	ATXN7L1	878.7936	0.096271	0.256498	0.678371	protein_coding
ENSG0000(	BRPF3	1779.923	0.170321	0.256513	0.678371	protein_coding
ENSG0000(	CC2D1A	2382.642	0.117737	0.256589	0.678371	protein_coding
ENSG0000(	CRCP	988.601	0.097845	0.256475	0.678371	protein_coding
ENSG0000(	ENSG0000(	66.38364	0.156898	0.256633	0.678371	lncRNA
ENSG0000(	ENSG0000(	80.99316	0.138051	0.25642	0.678371	lncRNA
ENSG0000(	ENSG0000(	181.5469	0.13563	0.256601	0.678371	lncRNA
ENSG0000(	FMNL1-DT	673.2808	-0.11696	0.2566	0.678371	lncRNA
ENSG0000(	PITX1	221.7624	0.212027	0.256587	0.678371	protein_coding
ENSG0000(	PRAM1	269.4588	-0.13047	0.256575	0.678371	protein_coding
ENSG0000(	SLC26A4-A:	2954.638	0.116793	0.25663	0.678371	lncRNA
ENSG0000(	LINC02849	39.84136	0.204361	0.256682	0.678437	lncRNA
ENSG0000(	PTP4A2	3711.965	-0.08674	0.256713	0.678452	protein_coding
ENSG0000(	ENSG0000(	112.1714	0.137423	0.256772	0.678544	lncRNA
ENSG0000(	ENSG0000(	161.7699	0.144496	0.256895	0.678802	lncRNA
ENSG0000(	MFNG	417.4869	-0.14703	0.256966	0.678926	protein_coding
ENSG0000(	RN7SL147F	44.79765	-0.18551	0.257018	0.678997	misc_RNA
ENSG0000(	BAHD1	771.7126	0.13511	0.257151	0.679047	protein_coding
ENSG0000(	ENSG0000(	52.03079	-0.26063	0.25722	0.679047	lncRNA
ENSG0000(	ENSG0000(	1163.876	0.057719	0.257234	0.679047	TEC
ENSG0000(	H1-5	178.48	-0.29755	0.257121	0.679047	protein_coding
ENSG0000(	LINC01762	118.3923	0.133392	0.257204	0.679047	lncRNA
ENSG0000(	NWD1	591.0711	-0.12676	0.25721	0.679047	protein_coding
ENSG0000(	PIH1D2	57.63022	0.197084	0.25712	0.679047	protein_coding
ENSG0000(	PPAT	329.8773	-0.10645	0.257074	0.679047	protein_coding
ENSG0000(	P4HA3	113.0534	-0.24826	0.257294	0.67914	protein_coding
ENSG0000(	ENSG0000(	21.83851	0.261309	0.257372	0.679281	lncRNA
ENSG0000(	CHAF1A	490.7479	-0.13206	0.257535	0.679449	protein_coding
ENSG0000(	ENSG0000(	26.06132	0.2476	0.257518	0.679449	lncRNA
ENSG0000(	MRPL41	165.7481	0.189611	0.257469	0.679449	protein_coding
ENSG0000(	POLR3D	871.6169	-0.08489	0.257491	0.679449	protein_coding
ENSG0000(	ENSG0000(	23.79759	0.296878	0.257643	0.679669	lncRNA
ENSG0000(	DOCK6	1610.606	0.1191	0.257806	0.679825	protein_coding
ENSG0000(	ENSG0000(	486.6428	0.439048	0.257825	0.679825	lncRNA
ENSG0000(	LINC01258	24.24105	-0.32638	0.257753	0.679825	lncRNA
ENSG0000(	LMAN2	707.0228	-0.13469	0.257744	0.679825	protein_coding

ENSG0000(	RYR3-DT	74.09336	0.254921	0.257823	0.679825	lncRNA
ENSG0000(	ZNF518B	1332.332	-0.104	0.257888	0.679925	protein_coding
ENSG0000(	ZNF589	1026.158	-0.1574	0.257937	0.67999	protein_coding
ENSG0000(	ENTPD2	116.9189	0.338991	0.258029	0.680167	protein_coding
ENSG0000(	GADL1	768.2047	0.086275	0.258104	0.680298	protein_coding
ENSG0000(	STX8	515.1484	0.06796	0.258138	0.680323	protein_coding
ENSG0000(	ACP3	258.5191	-0.23979	0.258197	0.680347	protein_coding
ENSG0000(	ENSG0000(	537.3344	0.135487	0.258193	0.680347	lncRNA
ENSG0000(	MCCC2	3384.719	0.052594	0.258452	0.680889	protein_coding
ENSG0000(	TAS2R43	124.4147	0.25749	0.258445	0.680889	protein_coding
ENSG0000(	ENSG0000(	50.72586	0.215933	0.25854	0.680896	lncRNA
ENSG0000(	FCGR1CP	19.77745	-0.75865	0.258486	0.680896	unprocessed_pseudogene
ENSG0000(	TMED4	1804.312	-0.06025	0.258553	0.680896	protein_coding
ENSG0000(	UPK1B	82.46864	0.172003	0.258547	0.680896	protein_coding
ENSG0000(	EPPIN	473.8651	-0.09789	0.258585	0.680913	protein_coding
ENSG0000(	GTF2H3	693.3934	0.075169	0.258668	0.681001	protein_coding
ENSG0000(	NEMF	2319.241	0.075807	0.258663	0.681001	protein_coding
ENSG0000(	SEMA6A	4504.37	0.242727	0.258788	0.681254	protein_coding
ENSG0000(	MT-TP	124.6713	-0.58754	0.258834	0.681308	Mt_tRNA
ENSG0000(	SHFL	1068.074	-0.10765	0.258965	0.681589	protein_coding
ENSG0000(	ENSG0000(	25.64097	-0.30136	0.25902	0.681668	lncRNA
ENSG0000(	FN1	2923.318	-0.4168	0.259145	0.681932	protein_coding
ENSG0000(	ABCA8	1181.491	0.300946	0.259234	0.682017	protein_coding
ENSG0000(	ENSG0000(	21.79727	-0.27193	0.259376	0.682017	lncRNA
ENSG0000(	ENSG0000(	1205.879	0.103468	0.259323	0.682017	lncRNA
ENSG0000(	ENSG0000(	552.4862	0.086091	0.259316	0.682017	lncRNA
ENSG0000(	GSTCD	643.7742	-0.10714	0.259334	0.682017	protein_coding
ENSG0000(	PLEKHG1	2515.263	-0.11152	0.259363	0.682017	protein_coding
ENSG0000(	SNORD10	683.0242	0.207897	0.259368	0.682017	snoRNA
ENSG0000(	TMX2	827.5737	0.098047	0.25937	0.682017	protein_coding
ENSG0000(	CA8	173.0251	0.301505	0.259411	0.682045	protein_coding
ENSG0000(	ENSG0000(	143.3429	0.416125	0.259611	0.682206	unprocessed_pseudogene
ENSG0000(	ENSG0000(	44.64127	0.219606	0.25959	0.682206	lncRNA
ENSG0000(	ENSG0000(	160.9598	0.180591	0.259597	0.682206	protein_coding
ENSG0000(	ENSG0000(	42.9993	0.230834	0.259595	0.682206	lncRNA
ENSG0000(	LRRC31	654.2025	0.353101	0.259621	0.682206	protein_coding
ENSG0000(	SALRNA1	62.01167	-0.2562	0.259553	0.682206	lncRNA
ENSG0000(	AIMP2	211.5218	0.142789	0.259916	0.682309	protein_coding
ENSG0000(	C17orf58	67.2994	-0.18557	0.259928	0.682309	protein_coding
ENSG0000(	DUXAP7	60.61741	-0.14258	0.259836	0.682309	processed_pseudogene
ENSG0000(	ENSG0000(	53.39228	0.190031	0.259834	0.682309	lncRNA
ENSG0000(	ENSG0000(	220.2589	0.116862	0.259742	0.682309	lncRNA
ENSG0000(	ENSG0000(	73.74304	-0.17174	0.259752	0.682309	lncRNA
ENSG0000(	ENSG0000(	24.43891	0.259061	0.259983	0.682309	lncRNA
ENSG0000(	ENSG0000(	200.3748	-0.12712	0.259956	0.682309	TEC
ENSG0000(	ENSG0000(	86.8435	0.200474	0.259858	0.682309	TEC
ENSG0000(	HTR5A-AS1	121.1906	0.137717	0.259905	0.682309	lncRNA

ENSG0000(	LINC01266	289.8231	0.113884	0.259972	0.682309	lncRNA
ENSG0000(	LINC02574	23.29282	0.567534	0.259917	0.682309	lncRNA
ENSG0000(	MRPL37	307.6616	0.12212	0.259937	0.682309	protein_coding
ENSG0000(	EIF4E3	1924.669	-0.15316	0.260129	0.682496	protein_coding
ENSG0000(	ENSG0000(	139.5208	0.124393	0.260164	0.682496	lncRNA
ENSG0000(	LMNB2	634.8746	-0.15912	0.260178	0.682496	protein_coding
ENSG0000(	SRI	2819.619	0.323842	0.260132	0.682496	protein_coding
ENSG0000(	STK38L	1292.414	0.092075	0.260155	0.682496	protein_coding
ENSG0000(	PRTFDC1	122.5128	0.221971	0.260218	0.682537	protein_coding
ENSG0000(	ENSG0000(	44.04709	-0.29296	0.260249	0.682551	transcribed_processed_pseudoge
ENSG0000(	LINC00294	1047.17	0.126409	0.260371	0.682807	lncRNA
ENSG0000(	ANO9	3078.304	0.207339	0.2604	0.682817	protein_coding
ENSG0000(	ENSG0000(	173.2982	0.140483	0.260439	0.682855	lncRNA
ENSG0000(	DBR1	318.2012	-0.12606	0.260596	0.682904	protein_coding
ENSG0000(	ENSG0000(	252.3984	0.080215	0.260568	0.682904	lncRNA
ENSG0000(	LINC02271	325.8581	0.091565	0.260607	0.682904	lncRNA
ENSG0000(	RPS27AP12	54.56484	0.264926	0.260567	0.682904	processed_pseudogene
ENSG0000(	VDAC2	1136.443	0.169949	0.260499	0.682904	protein_coding
ENSG0000(	ZNF22	269.2949	-0.16631	0.260577	0.682904	protein_coding
ENSG0000(	ASCL2	55.79246	0.326461	0.260913	0.682994	protein_coding
ENSG0000(	CDRT4	807.6761	-0.11603	0.260938	0.682994	protein_coding
ENSG0000(	ENSG0000(	113.9753	0.177538	0.260885	0.682994	lncRNA
ENSG0000(	ENSG0000(	43.6783	0.243322	0.260752	0.682994	lncRNA
ENSG0000(	GNRH1	262.7726	0.11803	0.261067	0.682994	protein_coding
ENSG0000(	GPR139	20.1615	-0.41911	0.261062	0.682994	protein_coding
ENSG0000(	GPR160	1109.331	0.239678	0.260972	0.682994	protein_coding
ENSG0000(	HEPACAM	26.20053	-0.35725	0.260764	0.682994	protein_coding
ENSG0000(	HPS6	204.6584	0.11066	0.260689	0.682994	protein_coding
ENSG0000(	ITGA4	2593.725	-0.20448	0.260803	0.682994	protein_coding
ENSG0000(	LINC01973	29.84496	-0.29167	0.260793	0.682994	lncRNA
ENSG0000(	LINC02033	192.6739	-0.21822	0.260927	0.682994	lncRNA
ENSG0000(	LMNA	2187.514	0.208513	0.26102	0.682994	protein_coding
ENSG0000(	PURA	2890.225	0.107134	0.260926	0.682994	protein_coding
ENSG0000(	RBM4	2764.071	-0.06203	0.260699	0.682994	protein_coding
ENSG0000(	SLF1	385.2638	-0.142	0.261001	0.682994	protein_coding
ENSG0000(	THG1L	381.4418	0.093535	0.260983	0.682994	protein_coding
ENSG0000(	WDR59	1642.439	0.083154	0.261088	0.682994	protein_coding
ENSG0000(	ASB9	35.73227	-0.27489	0.261268	0.683092	protein_coding
ENSG0000(	ENSG0000(	189.1929	0.114155	0.261377	0.683092	protein_coding
ENSG0000(	ENSG0000(	145.1159	-0.13433	0.261423	0.683092	lncRNA
ENSG0000(	ENSG0000(	21.20209	-0.31366	0.261417	0.683092	lncRNA
ENSG0000(	FENDRR	2780.603	-0.15504	0.261177	0.683092	lncRNA
ENSG0000(	IKBKG	771.3431	-0.0916	0.261329	0.683092	protein_coding
ENSG0000(	ILDR2	253.8961	-0.26072	0.261353	0.683092	protein_coding
ENSG0000(	LINC00622	68.81445	-0.22775	0.261192	0.683092	lncRNA
ENSG0000(	PLK4	215.3822	-0.23833	0.26135	0.683092	protein_coding
ENSG0000(	PRSS3	457.6257	0.277485	0.261389	0.683092	protein_coding

ENSG0000(	S100A14	682.0203	0.265995	0.2612	0.683092	protein_coding
ENSG0000(	SLC25A30	857.1962	0.10183	0.261349	0.683092	protein_coding
ENSG0000(	TRIM37	546.6482	-0.13146	0.261471	0.683152	protein_coding
ENSG0000(	FOCAD	1353.629	-0.13763	0.261551	0.683232	protein_coding
ENSG0000(	PPP1CB	3457.889	0.124832	0.261544	0.683232	protein_coding
ENSG0000(	KLHL22	653.7716	-0.09706	0.26163	0.683244	protein_coding
ENSG0000(	SNRNP200	3904.489	-0.05806	0.261606	0.683244	protein_coding
ENSG0000(	UHRF1BP1	1590.336	-0.07237	0.261621	0.683244	protein_coding
ENSG0000(	CFAP299	97.8887	0.154443	0.261706	0.683314	protein_coding
ENSG0000(	DNAJC22	964.8244	0.177305	0.261732	0.683314	protein_coding
ENSG0000(	KCNH1	215.9741	0.125221	0.261722	0.683314	protein_coding
ENSG0000(	ROCR	74.55087	0.237086	0.261786	0.68339	lncRNA
ENSG0000(	PRH2	65.493	-0.22212	0.261926	0.683691	protein_coding
ENSG0000(	CCDC158	241.7722	0.133418	0.261967	0.683733	protein_coding
ENSG0000(	OR4D9	940.0433	0.065374	0.262041	0.683863	protein_coding
ENSG0000(	C4orf33	379.0466	-0.11525	0.262251	0.68389	protein_coding
ENSG0000(	DNAJC25-G	143.8581	-0.41033	0.262214	0.68389	protein_coding
ENSG0000(	ENSG0000(	78.54331	0.148551	0.262106	0.68389	lncRNA
ENSG0000(	ENSG0000(	85.75911	0.1599	0.262167	0.68389	transcribed_processed_pseudoge
ENSG0000(	ENSG0000(	149.2749	0.197875	0.262234	0.68389	lncRNA
ENSG0000(	TMEM131	4205.844	0.104864	0.26223	0.68389	protein_coding
ENSG0000(	TNFRSF19	125.9986	0.169763	0.262131	0.68389	protein_coding
ENSG0000(	UPF1	1949.513	0.076977	0.262182	0.68389	protein_coding
ENSG0000(	DTD1	333.9078	0.079501	0.262296	0.683943	protein_coding
ENSG0000(	ENSG0000(	128.0901	0.118235	0.262414	0.684121	lncRNA
ENSG0000(	LINC02393	43.41334	0.340079	0.262397	0.684121	lncRNA
ENSG0000(	ENSG0000(	70.19814	0.221542	0.262448	0.684145	lncRNA
ENSG0000(	SLC25A23	1179.921	0.241794	0.262504	0.684198	protein_coding
ENSG0000(	ZNF112	222.5127	0.151635	0.262518	0.684198	protein_coding
ENSG0000(	GBA	475.3239	-0.1284	0.262586	0.684245	protein_coding
ENSG0000(	NPSR1-AS1	63.32152	-0.27239	0.262571	0.684245	lncRNA
ENSG0000(	ENSG0000(	46.01639	-0.21225	0.262662	0.684248	lncRNA
ENSG0000(	ITCH-IT1	53.70096	-0.22088	0.262654	0.684248	lncRNA
ENSG0000(	TPGS1	619.2921	0.07085	0.26263	0.684248	protein_coding
ENSG0000(	ADCY7	2054.564	-0.11584	0.262778	0.684357	protein_coding
ENSG0000(	C4A	903.0071	-0.28165	0.262769	0.684357	protein_coding
ENSG0000(	LINC00550	96.57005	-0.27075	0.262755	0.684357	lncRNA
ENSG0000(	CLVS1	122.2256	0.12301	0.262927	0.684438	protein_coding
ENSG0000(	ENSG0000(	92.51175	-0.17906	0.262934	0.684438	lncRNA
ENSG0000(	ENSG0000(	92.29183	-0.1921	0.262909	0.684438	TEC
ENSG0000(	LINC02054	291.1049	0.09659	0.262878	0.684438	lncRNA
ENSG0000(	PPM1H	345.8205	-0.21462	0.262859	0.684438	protein_coding
ENSG0000(	LETM1	2919.902	0.178666	0.26296	0.684442	protein_coding
ENSG0000(	RN7SL200F	54.89728	-0.23486	0.262999	0.684479	misc_RNA
ENSG0000(	RMI1	313.1556	0.105487	0.263035	0.684508	protein_coding
ENSG0000(	MEIG1	28.06333	0.305201	0.263078	0.684554	protein_coding
ENSG0000(	ENSG0000(	1796.045	0.124354	0.263183	0.684763	lncRNA

ENSG0000(	CLHC1	620.7221	0.130234	0.263262	0.6848	protein_coding
ENSG0000(	MYDGF	217.1322	-0.1855	0.263269	0.6848	protein_coding
ENSG0000(	RPP38	230.3237	0.10898	0.263272	0.6848	protein_coding
ENSG0000(	ENSG0000(	333.1482	-0.17511	0.263301	0.68481	transcribed_unprocessed_pseudo
ENSG0000(	CYP19A1	26.49962	-0.82036	0.263357	0.684893	protein_coding
ENSG0000(	ENSG0000(	43.52409	0.212924	0.263414	0.684977	lncRNA
ENSG0000(	NFKBIB	621.2962	-0.07518	0.26347	0.685056	protein_coding
ENSG0000(	ENSG0000(	953.7041	0.148594	0.263576	0.685112	lncRNA
ENSG0000(	ENSG0000(	57.25562	0.204405	0.263559	0.685112	processed_pseudogene
ENSG0000(	ENSG0000(	24.10605	0.261819	0.263591	0.685112	lncRNA
ENSG0000(	PBXIP1	1090.108	-0.15046	0.263562	0.685112	protein_coding
ENSG0000(	TSKU	1294.49	-0.1179	0.26364	0.685174	protein_coding
ENSG0000(	ENSG0000(	44.433	0.188634	0.26375	0.685293	lncRNA
ENSG0000(	IARS2P1	146.5759	0.147219	0.263744	0.685293	processed_pseudogene
ENSG0000(	MAU2	2406.522	0.045989	0.263777	0.685293	protein_coding
ENSG0000(	MEN1	412.566	0.086431	0.263785	0.685293	protein_coding
ENSG0000(	BRD3OS	374.0137	-0.13639	0.263985	0.685545	protein_coding
ENSG0000(	ENSG0000(	21.78004	-0.35505	0.263928	0.685545	lncRNA
ENSG0000(	MAK16	361.0315	-0.14786	0.264006	0.685545	protein_coding
ENSG0000(	SPRYD7	407.2149	0.15479	0.263971	0.685545	protein_coding
ENSG0000(	ZNF689	198.8048	-0.13562	0.263942	0.685545	protein_coding
ENSG0000(	CTBP2	2628.025	0.113877	0.264081	0.68566	protein_coding
ENSG0000(	ENSG0000(	801.0571	0.094862	0.264173	0.68566	TEC
ENSG0000(	MYORG	2829.323	0.127528	0.264104	0.68566	protein_coding
ENSG0000(	NBPF1	6189.755	0.150006	0.26419	0.68566	protein_coding
ENSG0000(	PRMT1	771.9288	-0.09727	0.2642	0.68566	protein_coding
ENSG0000(	SMIM2-AS1	726.4994	0.286773	0.26418	0.68566	lncRNA
ENSG0000(	ENSG0000(	452.055	-0.08071	0.264253	0.685732	lncRNA
ENSG0000(	STX19	181.3088	0.272341	0.264315	0.685827	protein_coding
ENSG0000(	TIMM17A	998.5707	0.093572	0.264405	0.685996	protein_coding
ENSG0000(	TMCC1-DT	1995.095	-0.0829	0.26456	0.686334	lncRNA
ENSG0000(	EID2B	230.4973	-0.20247	0.264706	0.686583	protein_coding
ENSG0000(	RBM10	716.6417	0.108218	0.264682	0.686583	protein_coding
ENSG0000(	RN7SKP86	37.96692	0.336247	0.264743	0.686614	misc_RNA
ENSG0000(	LINC00551	55.69892	-0.21748	0.264902	0.686963	lncRNA
ENSG0000(	ENSG0000(	63.68847	0.224585	0.265003	0.687159	lncRNA
ENSG0000(	LONRF2	325.4172	0.12346	0.265057	0.687236	protein_coding
ENSG0000(	ENSG0000(	4134.371	0.088161	0.265186	0.687375	lncRNA
ENSG0000(	NCAPG	240.3825	-0.23504	0.265143	0.687375	protein_coding
ENSG0000(	OR7D2	483.1678	0.072756	0.265168	0.687375	protein_coding
ENSG0000(	SERTAD1	262.6884	0.122217	0.265222	0.687405	protein_coding
ENSG0000(	DYRK2	4568.776	0.161415	0.265486	0.687416	protein_coding
ENSG0000(	ENSG0000(	53.99132	0.16189	0.265576	0.687416	lncRNA
ENSG0000(	ENSG0000(	63.95963	0.174628	0.265626	0.687416	lncRNA
ENSG0000(	ENSG0000(	411.413	0.093514	0.265336	0.687416	lncRNA
ENSG0000(	ENSG0000(	346.1081	-0.15565	0.265458	0.687416	transcribed_unitary_pseudogene
ENSG0000(	ENSG0000(	40.47557	0.266193	0.265462	0.687416	lncRNA

ENSG0000(	FOXL2NB	743.0426	-0.1025	0.265604	0.687416	protein_coding
ENSG0000(	GRIP1	176.1929	0.246437	0.265555	0.687416	protein_coding
ENSG0000(	PCBP2	6941.112	0.047014	0.265411	0.687416	protein_coding
ENSG0000(	POU5F1B	222.6982	-0.11402	0.265287	0.687416	protein_coding
ENSG0000(	RMI2	172.9017	-0.16025	0.265368	0.687416	protein_coding
ENSG0000(	SGSM2	1658.714	0.098543	0.265627	0.687416	protein_coding
ENSG0000(	SURF1	204.9481	0.12453	0.265286	0.687416	protein_coding
ENSG0000(	THEMIS	343.7704	-0.21991	0.265566	0.687416	protein_coding
ENSG0000(	TIAM2	650.3751	-0.08218	0.265478	0.687416	protein_coding
ENSG0000(	XPO4	1554.399	0.122588	0.265614	0.687416	protein_coding
ENSG0000(	BHLHE22	40.53696	-0.45089	0.265791	0.687522	protein_coding
ENSG0000(	C8orf44-SG	21.52867	0.495544	0.265867	0.687522	protein_coding
ENSG0000(	CTCFL	594.7763	0.056411	0.265862	0.687522	protein_coding
ENSG0000(	ENSG0000(	27.24615	-0.2521	0.265854	0.687522	TEC
ENSG0000(	ENSG0000(	106.7278	0.137931	0.26582	0.687522	lncRNA
ENSG0000(	MARK4	518.7254	-0.08001	0.265693	0.687522	protein_coding
ENSG0000(	TRBC2	280.3154	-0.29671	0.265857	0.687522	TR_C_gene
ENSG0000(	UBE2F	682.4668	-0.08005	0.265832	0.687522	protein_coding
ENSG0000(	DIP2B	2374.556	0.075701	0.265912	0.687549	protein_coding
ENSG0000(	PANCR	47.43006	-0.31103	0.265953	0.687549	lncRNA
ENSG0000(	TMEM123	1955.516	-0.12527	0.265938	0.687549	protein_coding
ENSG0000(	C9orf78	992.6184	0.046006	0.266032	0.687625	protein_coding
ENSG0000(	TRPV6	61.88044	0.474697	0.266022	0.687625	protein_coding
ENSG0000(	B3GNT3	1250.637	0.238222	0.266245	0.687845	protein_coding
ENSG0000(	COA1	2449.543	-0.0867	0.266197	0.687845	protein_coding
ENSG0000(	ENSG0000(	66.40032	-0.45482	0.266163	0.687845	lncRNA
ENSG0000(	ENSG0000(	454.7852	0.10414	0.266236	0.687845	lncRNA
ENSG0000(	ENSG0000(	44.82376	0.255381	0.266234	0.687845	lncRNA
ENSG0000(	PCCB	615.5011	0.116966	0.266268	0.687845	protein_coding
ENSG0000(	ENSG0000(	80.41033	-0.20042	0.266299	0.687862	lncRNA
ENSG0000(	OR10A2	209.3976	0.130808	0.266399	0.688057	protein_coding
ENSG0000(	DDC	266.3447	0.312253	0.266438	0.688092	protein_coding
ENSG0000(	ACTR1A	1110.524	0.086544	0.267053	0.688199	protein_coding
ENSG0000(	CD99	1237.483	0.090366	0.26668	0.688199	protein_coding
ENSG0000(	CDK5	44.5532	-0.25007	0.267015	0.688199	protein_coding
ENSG0000(	CHID1	479.0922	0.106928	0.26706	0.688199	protein_coding
ENSG0000(	DGKE	1167.309	0.073737	0.267049	0.688199	protein_coding
ENSG0000(	ENSG0000(	175.6909	0.145769	0.266896	0.688199	lncRNA
ENSG0000(	ENSG0000(	152.9025	0.343428	0.26718	0.688199	transcribed_processed_pseudoge
ENSG0000(	ENSG0000(	59.07998	0.301601	0.266776	0.688199	protein_coding
ENSG0000(	ENSG0000(	207.5856	-0.19121	0.266601	0.688199	lncRNA
ENSG0000(	ENSG0000(	54.76716	0.221583	0.267069	0.688199	lncRNA
ENSG0000(	ENSG0000(	235.1902	0.119551	0.266756	0.688199	lncRNA
ENSG0000(	ENSG0000(	56.46733	0.22266	0.266619	0.688199	lncRNA
ENSG0000(	ENSG0000(	510.8002	0.090278	0.267174	0.688199	TEC
ENSG0000(	ENSG0000(	2491.802	0.046994	0.266755	0.688199	lncRNA
ENSG0000(	ENSG0000(	109.9227	-0.14338	0.26673	0.688199	lncRNA

ENSG0000(	ENSG0000(	127.7595	-0.20181	0.266908	0.688199	lncRNA
ENSG0000(	FBXO11	3706.016	-0.05575	0.267145	0.688199	protein_coding
ENSG0000(	FBXO43	176.8905	0.148657	0.266989	0.688199	protein_coding
ENSG0000(	GULOP	43.18937	0.386436	0.267161	0.688199	unitary_pseudogene
ENSG0000(	LINC00710	311.7043	0.136948	0.267093	0.688199	lncRNA
ENSG0000(	LINC01961	45.11466	0.221173	0.26684	0.688199	lncRNA
ENSG0000(	LRRFIP2	3396.4	0.129586	0.266651	0.688199	protein_coding
ENSG0000(	MAGI1	2000.382	0.213301	0.266734	0.688199	protein_coding
ENSG0000(	MPZL3	691.1192	0.102055	0.266844	0.688199	protein_coding
ENSG0000(	PRRT3-AS1	34.82198	0.201971	0.267127	0.688199	lncRNA
ENSG0000(	RNF217	944.8412	-0.09407	0.266662	0.688199	protein_coding
ENSG0000(	RNVU1-28	270.8716	-0.42105	0.267157	0.688199	snRNA
ENSG0000(	SULT2A1	180.7728	0.119994	0.266815	0.688199	protein_coding
ENSG0000(	HYAL2	305.8106	-0.18364	0.26724	0.68829	protein_coding
ENSG0000(	CIAO2A	450.1044	-0.13375	0.26751	0.688432	protein_coding
ENSG0000(	CYP2S1	294.1172	0.265642	0.267494	0.688432	protein_coding
ENSG0000(	ENSG0000(	115.0301	0.149441	0.267411	0.688432	lncRNA
ENSG0000(	LINC01797	20.77394	0.408242	0.26752	0.688432	lncRNA
ENSG0000(	NRXN1	780.5864	0.366334	0.267494	0.688432	protein_coding
ENSG0000(	P2RY6	2031.602	-0.12374	0.267421	0.688432	protein_coding
ENSG0000(	PNPT1	1541.871	0.12122	0.267429	0.688432	protein_coding
ENSG0000(	RAD50	2223.936	-0.09943	0.26749	0.688432	protein_coding
ENSG0000(	RNF146	768.1173	-0.09633	0.267411	0.688432	protein_coding
ENSG0000(	RGPD3	42.47797	-0.38883	0.267655	0.688691	protein_coding
ENSG0000(	SLC17A5	569.9242	0.160144	0.267671	0.688691	protein_coding
ENSG0000(	ENSG0000(	34.39376	0.401432	0.267765	0.68871	lncRNA
ENSG0000(	IL19	287.1655	-0.1295	0.267706	0.68871	protein_coding
ENSG0000(	RN7SL297F	32.54385	-0.27255	0.267813	0.68871	misc_RNA
ENSG0000(	RN7SL47P	45.67975	0.230827	0.267777	0.68871	misc_RNA
ENSG0000(	SCD5	1102.825	-0.09949	0.267829	0.68871	protein_coding
ENSG0000(	USP16	1717.343	0.052791	0.26779	0.68871	protein_coding
ENSG0000(	ACSL6	384.8585	0.109115	0.2681	0.688788	protein_coding
ENSG0000(	ANKRD49P	233.9147	-0.1102	0.268328	0.688788	processed_pseudogene
ENSG0000(	ATP5MJ	1000.142	0.119614	0.268189	0.688788	protein_coding
ENSG0000(	DNAJC5B	131.5857	-0.17918	0.268361	0.688788	protein_coding
ENSG0000(	ENSG0000(	51.27541	-0.16835	0.268368	0.688788	lncRNA
ENSG0000(	ENSG0000(	108.9756	-0.14459	0.268245	0.688788	lncRNA
ENSG0000(	ENSG0000(	50.20432	0.205275	0.267971	0.688788	lncRNA
ENSG0000(	ENSG0000(	234.3393	0.114659	0.268265	0.688788	TEC
ENSG0000(	ENSG0000(	461.2731	-0.12811	0.268061	0.688788	lncRNA
ENSG0000(	ENSG0000(	495.4131	0.079454	0.268122	0.688788	lncRNA
ENSG0000(	ENSG0000(	36.27109	-0.27138	0.26811	0.688788	lncRNA
ENSG0000(	INCA1	36.86123	0.21692	0.268251	0.688788	protein_coding
ENSG0000(	LINC00513	441.2668	0.095234	0.268049	0.688788	lncRNA
ENSG0000(	LINC02323	32.38694	-0.43378	0.268147	0.688788	lncRNA
ENSG0000(	LINC02877	137.2054	0.150387	0.268361	0.688788	lncRNA
ENSG0000(	LYRM7	1930.751	0.081901	0.268386	0.688788	protein_coding

ENSG0000(	MPHOSPH(	307.8102	-0.12324	0.268393	0.688788	protein_coding
ENSG0000(	ORC1	183.4908	-0.1534	0.26841	0.688788	protein_coding
ENSG0000(	SLC22A16	35.65007	-0.26989	0.268288	0.688788	protein_coding
ENSG0000(	SNORD116	30.04211	0.291633	0.268085	0.688788	snoRNA
ENSG0000(	TBC1D1	1845.937	-0.0722	0.268032	0.688788	protein_coding
ENSG0000(	UBE2Q1	598.9561	-0.17087	0.268137	0.688788	protein_coding
ENSG0000(	LINC00910	1864.399	-0.07852	0.268552	0.689063	lncRNA
ENSG0000(	PCDHGB2	90.50522	0.15799	0.268567	0.689063	protein_coding
ENSG0000(	YJU2B	684.8968	0.072121	0.268613	0.689117	protein_coding
ENSG0000(	APRT	210.1993	0.16353	0.268669	0.68914	protein_coding
ENSG0000(	PRKCD	982.4671	-0.14336	0.268693	0.68914	protein_coding
ENSG0000(	TMEM87B	1674.063	0.090343	0.268697	0.68914	protein_coding
ENSG0000(	ENSG0000(	121.7511	0.130718	0.268744	0.689195	lncRNA
ENSG0000(	ENSG0000(	668.7186	0.271755	0.26886	0.689429	lncRNA
ENSG0000(	C3orf20	32.74598	-0.28847	0.269092	0.689489	protein_coding
ENSG0000(	DNAJC10	6043.499	-0.11635	0.269081	0.689489	protein_coding
ENSG0000(	ENSG0000(	22.90165	-0.31122	0.268979	0.689489	processed_pseudogene
ENSG0000(	ENSG0000(	106.0889	0.152199	0.269124	0.689489	lncRNA
ENSG0000(	LAD1	1083.72	0.250789	0.269132	0.689489	protein_coding
ENSG0000(	NDUFS6	138.7009	0.200169	0.269134	0.689489	protein_coding
ENSG0000(	TMEM236	1992.307	0.215011	0.269052	0.689489	protein_coding
ENSG0000(	TNFSF13	516.1413	-0.18226	0.269013	0.689489	protein_coding
ENSG0000(	USB1	830.0237	-0.12367	0.269107	0.689489	protein_coding
ENSG0000(	ZNF503-AS	169.7319	0.319105	0.269	0.689489	lncRNA
ENSG0000(	GDF11	415.3117	-0.12605	0.269232	0.689611	protein_coding
ENSG0000(	NBEAL2	3786.635	-0.14136	0.269209	0.689611	protein_coding
ENSG0000(	ENSG0000(	23.98078	-0.31366	0.269341	0.689705	lncRNA
ENSG0000(	ENSG0000(	26.83625	-0.27646	0.269344	0.689705	lncRNA
ENSG0000(	WDR91	1314.494	-0.06939	0.269318	0.689705	protein_coding
ENSG0000(	ELF2P1	32.02266	-0.28	0.26939	0.689737	processed_pseudogene
ENSG0000(	ENSG0000(	138.913	0.126496	0.269469	0.689737	lncRNA
ENSG0000(	ENSG0000(	207.6568	-0.1228	0.269416	0.689737	lncRNA
ENSG0000(	KLRD1	3867.3	-0.06666	0.269532	0.689737	protein_coding
ENSG0000(	LINC02214	65.1667	0.154214	0.269452	0.689737	lncRNA
ENSG0000(	LINC02273	75.75681	-0.23339	0.269518	0.689737	lncRNA
ENSG0000(	PARD3	1247.117	0.146501	0.269504	0.689737	protein_coding
ENSG0000(	SCN3A	281.1341	0.216287	0.269561	0.689747	protein_coding
ENSG0000(	LACC1	705.862	-0.15108	0.269622	0.689839	protein_coding
ENSG0000(	C3orf35	1713.879	-0.04725	0.269747	0.690095	lncRNA
ENSG0000(	COX16	297.0582	0.166891	0.269819	0.690215	protein_coding
ENSG0000(	FARP2	4705.23	0.139254	0.269913	0.690391	protein_coding
ENSG0000(	ATP6V0D1-	874.9485	-0.0694	0.270376	0.690549	lncRNA
ENSG0000(	DCHS2	275.0869	0.212434	0.270277	0.690549	protein_coding
ENSG0000(	DUSP26	30.79133	-0.36149	0.27035	0.690549	protein_coding
ENSG0000(	ENSG0000(	462.3443	0.086196	0.270022	0.690549	lncRNA
ENSG0000(	ENSG0000(	91.63708	0.155613	0.270145	0.690549	lncRNA
ENSG0000(	ENSG0000(	141.8062	0.109168	0.270207	0.690549	lncRNA

ENSG0000(	ENSG0000(	45.07373	0.199547	0.270174	0.690549	pseudogene
ENSG0000(	ENSG0000(	86.72042	0.3566	0.270316	0.690549	lncRNA
ENSG0000(	ENSG0000(	412.8306	0.081103	0.270197	0.690549	lncRNA
ENSG0000(	ENSG0000(	881.5065	0.057937	0.270161	0.690549	lncRNA
ENSG0000(	HSD11B2	4652.442	0.377908	0.270376	0.690549	protein_coding
ENSG0000(	LEMD2	1261.925	-0.0553	0.270201	0.690549	protein_coding
ENSG0000(	NSMCE3	274.8043	0.087231	0.270363	0.690549	protein_coding
ENSG0000(	SNORA9	61.58737	0.247997	0.270254	0.690549	snoRNA
ENSG0000(	TOB1	874.7788	0.179354	0.270265	0.690549	protein_coding
ENSG0000(	ZMYND11	1003.6	-0.1037	0.270093	0.690549	protein_coding
ENSG0000(	DMBT1L1	99.48286	0.156585	0.270457	0.690691	transcribed_unitary_pseudogene
ENSG0000(	LACTB	460.9775	-0.12208	0.270544	0.690786	protein_coding
ENSG0000(	WEE2-AS1	198.9604	-0.16484	0.270541	0.690786	lncRNA
ENSG0000(	AMACR	579.014	0.275067	0.271015	0.690877	protein_coding
ENSG0000(	CLEC20A	22.31886	-0.22324	0.270657	0.690877	protein_coding
ENSG0000(	DNAH7	944.2375	0.158537	0.270935	0.690877	protein_coding
ENSG0000(	ENSG0000(	67.65506	0.199298	0.271093	0.690877	lncRNA
ENSG0000(	ENSG0000(	717.3726	-0.16118	0.270663	0.690877	processed_pseudogene
ENSG0000(	ENSG0000(	231.5686	-0.17147	0.270785	0.690877	lncRNA
ENSG0000(	ENSG0000(	53.65781	0.20367	0.271158	0.690877	transcribed_unprocessed_pseudo
ENSG0000(	ENSG0000(	118.1479	-0.19387	0.270912	0.690877	lncRNA
ENSG0000(	FAM104B	179.7956	0.204714	0.271049	0.690877	protein_coding
ENSG0000(	GJA4	37.81181	-0.34828	0.27114	0.690877	protein_coding
ENSG0000(	GSN	3436.558	0.092369	0.271109	0.690877	protein_coding
ENSG0000(	ITSN2	2897.055	-0.09053	0.2711	0.690877	protein_coding
ENSG0000(	KCNC2	48.51365	0.309766	0.270984	0.690877	protein_coding
ENSG0000(	KLHL15	289.0497	-0.12114	0.27085	0.690877	protein_coding
ENSG0000(	LINC00454	37.39485	-0.32324	0.270675	0.690877	lncRNA
ENSG0000(	LINC00921	315.5727	-0.10747	0.270803	0.690877	lncRNA
ENSG0000(	MED11	354.6424	-0.0979	0.270828	0.690877	protein_coding
ENSG0000(	PINK1	704.3336	0.127467	0.270807	0.690877	protein_coding
ENSG0000(	SNORA16A	75.36053	0.20075	0.271154	0.690877	snoRNA
ENSG0000(	SPOCK2	984.567	-0.28296	0.271097	0.690877	protein_coding
ENSG0000(	ZBED6	5432.945	-0.07597	0.270933	0.690877	protein_coding
ENSG0000(	ZBTB25	2591.342	-0.09233	0.270846	0.690877	protein_coding
ENSG0000(	ZHX1	1047.824	-0.08975	0.270793	0.690877	protein_coding
ENSG0000(	DNAL1	1110.702	0.104151	0.271228	0.690992	protein_coding
ENSG0000(	AMN	1216.257	0.326411	0.271291	0.691058	protein_coding
ENSG0000(	ENSG0000(	859.1366	0.09745	0.271304	0.691058	lncRNA
ENSG0000(	ENSG0000(	20.08769	0.317049	0.271395	0.691069	processed_pseudogene
ENSG0000(	ENSG0000(	52.30988	0.136256	0.271468	0.691069	lncRNA
ENSG0000(	ENSG0000(	452.4343	-0.0836	0.271515	0.691069	lncRNA
ENSG0000(	LINC02145	157.9616	0.174823	0.271525	0.691069	lncRNA
ENSG0000(	MAP4K1	555.1237	-0.12459	0.271533	0.691069	protein_coding
ENSG0000(	SCNN1B	587.2757	0.428318	0.271424	0.691069	protein_coding
ENSG0000(	SLC25A53	486.9568	-0.08765	0.271357	0.691069	protein_coding
ENSG0000(	WIZ	788.1881	0.112963	0.27156	0.691069	protein_coding

ENSG0000(	ZNF19	826.5175	0.146288	0.271555	0.691069	protein_coding
ENSG0000(	ZNF702P	449.0889	0.113771	0.271453	0.691069	transcribed_processed_pseudoge
ENSG0000(	CDS2	2623.569	-0.05933	0.271597	0.691077	protein_coding
ENSG0000(	COX20	1161.499	0.068286	0.271631	0.691077	protein_coding
ENSG0000(	ERBIN	6493.294	0.125513	0.271638	0.691077	protein_coding
ENSG0000(	CCDC6	1702.662	-0.08792	0.271774	0.691149	protein_coding
ENSG0000(	ENSG0000(	79.54627	0.180167	0.271801	0.691149	lncRNA
ENSG0000(	ENSG0000(	85.29836	0.159373	0.271817	0.691149	lncRNA
ENSG0000(	ENSG0000(	122.6856	-0.1661	0.2717	0.691149	TEC
ENSG0000(	KCNMA1	2795.511	0.16034	0.271804	0.691149	protein_coding
ENSG0000(	MTCO1P24	27.99328	-0.6321	0.271763	0.691149	processed_pseudogene
ENSG0000(	ERVK3-1	2124.229	-0.06831	0.271994	0.691216	protein_coding
ENSG0000(	HSPA12A	1981.649	0.081001	0.271955	0.691216	protein_coding
ENSG0000(	PKN2-AS1	292.4138	0.087019	0.2719	0.691216	lncRNA
ENSG0000(	RNF138	934.1609	0.152197	0.271919	0.691216	protein_coding
ENSG0000(	SUCLG2-AS	1708.998	0.070625	0.271967	0.691216	lncRNA
ENSG0000(	ZKSCAN5	651.8151	-0.06135	0.271994	0.691216	protein_coding
ENSG0000(	ENSG0000(	267.1117	-0.11304	0.272031	0.691247	lncRNA
ENSG0000(	GJB6	27.91023	-0.30557	0.272074	0.69129	protein_coding
ENSG0000(	CEP170	1558.522	-0.13077	0.272109	0.691315	protein_coding
ENSG0000(	ENSG0000(	47.40118	-0.30018	0.27217	0.691393	lncRNA
ENSG0000(	ZNF134	629.9617	-0.11405	0.272189	0.691393	protein_coding
ENSG0000(	DSC2	4337.717	0.238148	0.272218	0.691402	protein_coding
ENSG0000(	ZNF891	2602.786	0.060907	0.272294	0.69153	protein_coding
ENSG0000(	ENSG0000(	114.2616	-0.12008	0.272357	0.691562	lncRNA
ENSG0000(	SLC25A42	552.2054	-0.10006	0.272353	0.691562	protein_coding
ENSG0000(	MIR762HG	91.21209	-0.14794	0.272416	0.691648	lncRNA
ENSG0000(	ENSG0000(	32.40617	0.274223	0.272494	0.691783	lncRNA
ENSG0000(	ENSG0000(	123.4589	-0.15453	0.272568	0.691908	lncRNA
ENSG0000(	LINC02664	288.2821	0.123093	0.272631	0.692004	lncRNA
ENSG0000(	CDC34	254.7387	0.158609	0.272758	0.692096	protein_coding
ENSG0000(	LINC02709	74.59142	0.180652	0.272768	0.692096	lncRNA
ENSG0000(	MKRN2OS	327.3981	0.088942	0.272698	0.692096	protein_coding
ENSG0000(	PGBD4	1372.578	0.056362	0.272768	0.692096	protein_coding
ENSG0000(	LINC02018	1968.356	0.118284	0.272826	0.69215	lncRNA
ENSG0000(	PEX11A	550.5349	0.125361	0.27284	0.69215	protein_coding
ENSG0000(	CR1L	32.83733	-0.432	0.272971	0.69242	protein_coding
ENSG0000(	ARHGAP24	590.0409	-0.20273	0.273086	0.692583	protein_coding
ENSG0000(	ENSG0000(	41.79821	-0.25956	0.27307	0.692583	lncRNA
ENSG0000(	PREB	704.3035	-0.14306	0.273185	0.69277	protein_coding
ENSG0000(	NDST2	736.1191	-0.10917	0.273247	0.692863	protein_coding
ENSG0000(	TENM1	136.689	0.309514	0.273363	0.693093	protein_coding
ENSG0000(	GARRE1	2005.934	0.079233	0.273536	0.693149	protein_coding
ENSG0000(	PPP1R12A	2827.785	-0.08267	0.273496	0.693149	protein_coding
ENSG0000(	REG1B	419.8126	-2.89803	0.273505	0.693149	protein_coding
ENSG0000(	SECISBP2	4024.401	0.061798	0.273533	0.693149	protein_coding
ENSG0000(	SH2D3C	313.0424	-0.24645	0.273521	0.693149	protein_coding

ENSG0000(	USF3	3223.941	0.072274	0.273437	0.693149	protein_coding
ENSG0000(	SEPTIN1	444.6669	-0.19823	0.273608	0.693267	protein_coding
ENSG0000(	FAM118A	3260.042	0.267927	0.273707	0.693335	protein_coding
ENSG0000(	PDE10A	894.9766	-0.20173	0.27371	0.693335	protein_coding
ENSG0000(	PROM2	1080.921	0.172774	0.273668	0.693335	protein_coding
ENSG0000(	TRAPPC3L	489.5064	-0.07042	0.273811	0.693527	protein_coding
ENSG0000(	CLECL1	79.48545	-0.31053	0.273837	0.693528	transcribed_unitary_pseudogene
ENSG0000(	TMOD2	1076.332	-0.13282	0.273888	0.693594	protein_coding
ENSG0000(	GJC1	1114.586	-0.11787	0.273954	0.693697	protein_coding
ENSG0000(	PSG8-AS1	29.50663	-0.64263	0.273996	0.693739	lncRNA
ENSG0000(	NEU1	818.4006	0.124712	0.274087	0.693905	protein_coding
ENSG0000(	KEL	62.46537	-0.31228	0.27414	0.693964	protein_coding
ENSG0000(	LINC01011	229.5146	0.121715	0.274185	0.693964	lncRNA
ENSG0000(	WWOX	1522.898	-0.06923	0.274182	0.693964	protein_coding
ENSG0000(	CDC20B	321.5269	0.084182	0.274228	0.693993	protein_coding
ENSG0000(	HSPA2	418.5912	0.148575	0.274247	0.693993	protein_coding
ENSG0000(	MIR4453H(	497.8435	-0.10715	0.274295	0.694049	lncRNA
ENSG0000(	ABCB11	426.007	0.172702	0.274349	0.694122	protein_coding
ENSG0000(	DHPS	454.2908	0.084586	0.274525	0.694143	protein_coding
ENSG0000(	FAM221B	147.5306	-0.12279	0.274527	0.694143	protein_coding
ENSG0000(	FSTL4	59.93581	0.258011	0.274518	0.694143	protein_coding
ENSG0000(	RNF125	1347.466	0.097272	0.274458	0.694143	protein_coding
ENSG0000(	TSC22D1-A	279.7043	0.125976	0.274491	0.694143	lncRNA
ENSG0000(	TSEN54	596.1251	0.08825	0.274462	0.694143	protein_coding
ENSG0000(	ZNF558	1117.42	0.11699	0.274534	0.694143	protein_coding
ENSG0000(	ENSG0000(	99.11857	0.156463	0.274578	0.694191	lncRNA
ENSG0000(	TIMM29	247.8308	0.105122	0.274638	0.694279	protein_coding
ENSG0000(	TARS1-DT	90.0146	-0.27758	0.274717	0.694414	lncRNA
ENSG0000(	ANKS1B	192.3551	0.13077	0.27475	0.694421	protein_coding
ENSG0000(	ENSG0000(	548.9422	0.098613	0.27477	0.694421	lncRNA
ENSG0000(	LINC01441	174.5889	0.148086	0.274857	0.694558	lncRNA
ENSG0000(	SCYL2P1	198.8259	0.132183	0.274874	0.694558	processed_pseudogene
ENSG0000(	ATP5F1AP1	101.5554	-0.18242	0.274944	0.694617	processed_pseudogene
ENSG0000(	VPS37A	937.3553	-0.0864	0.274948	0.694617	protein_coding
ENSG0000(	C12orf76	384.8218	-0.11329	0.275152	0.694801	protein_coding
ENSG0000(	CNTRL	3253.108	-0.14771	0.275107	0.694801	protein_coding
ENSG0000(	ENOPH1	245.1297	-0.12123	0.27505	0.694801	protein_coding
ENSG0000(	ENSG0000(	487.3722	-0.0841	0.275138	0.694801	lncRNA
ENSG0000(	GOLGB1	8083.083	0.078779	0.275173	0.694801	protein_coding
ENSG0000(	LINC02803	93.90428	0.160648	0.27509	0.694801	lncRNA
ENSG0000(	KLRF1	32.14244	-0.3455	0.275297	0.694923	protein_coding
ENSG0000(	LRRC52-AS	22.02707	-0.45671	0.275289	0.694923	lncRNA
ENSG0000(	RIOK2	504.1113	-0.11346	0.275286	0.694923	protein_coding
ENSG0000(	SLC3A2	1059.712	-0.1029	0.275336	0.694958	protein_coding
ENSG0000(	RAPH1	3069.215	-0.11231	0.275424	0.695116	protein_coding
ENSG0000(	RN7SL265F	27.74064	0.294666	0.275471	0.695171	misc_RNA
ENSG0000(	LINC02160	53.08833	0.186605	0.275552	0.695248	lncRNA

ENSG0000(	MOCOS	535.2241	-0.10386	0.275528	0.695248	protein_coding
ENSG0000(	ENSG0000(	86.31856	0.162181	0.27559	0.695281	lncRNA
ENSG0000(	LINC02505	49.11993	0.234823	0.275692	0.695474	lncRNA
ENSG0000(	CARS1	2180.314	-0.07701	0.275744	0.695478	protein_coding
ENSG0000(	PHGR1	3565.348	0.346504	0.275744	0.695478	protein_coding
ENSG0000(	CYP3A7	83.36647	-0.33261	0.275821	0.695537	protein_coding
ENSG0000(	ENSG0000(	160.5439	0.206463	0.275799	0.695537	lncRNA
ENSG0000(	ENSG0000(	58.45457	0.163419	0.27588	0.695537	lncRNA
ENSG0000(	FAM13C	283.8379	-0.19933	0.275894	0.695537	protein_coding
ENSG0000(	RIMKLB	784.4995	-0.15457	0.275868	0.695537	protein_coding
ENSG0000(	RBM15-AS:	145.1512	-0.19214	0.275922	0.695544	lncRNA
ENSG0000(	ENSG0000(	25.7547	0.27297	0.276009	0.695699	lncRNA
ENSG0000(	HSPA8P18	22.23747	0.379391	0.2761	0.695802	processed_pseudogene
ENSG0000(	TANC2	1918.958	-0.14988	0.276088	0.695802	protein_coding
ENSG0000(	BLOC1S6P1	394.5786	0.103583	0.276229	0.695872	processed_pseudogene
ENSG0000(	FADS6	319.0033	-0.1257	0.276165	0.695872	protein_coding
ENSG0000(	PTPRD	1657	0.238871	0.276225	0.695872	protein_coding
ENSG0000(	ZNF565	130.9473	0.168782	0.276205	0.695872	protein_coding
ENSG0000(	CKLF	194.3372	-0.19508	0.276364	0.695958	protein_coding
ENSG0000(	ENSG0000(	54.05244	0.215086	0.276335	0.695958	lncRNA
ENSG0000(	ENSG0000(	143.8308	-0.129	0.276352	0.695958	lncRNA
ENSG0000(	LINC02515	333.2738	0.089106	0.276327	0.695958	lncRNA
ENSG0000(	CCPG1	1916.878	-0.07621	0.27643	0.696015	protein_coding
ENSG0000(	IGHV3-20	168.3142	-0.4643	0.276438	0.696015	IG_V_gene
ENSG0000(	BRME1	128.6958	0.264944	0.276481	0.696054	protein_coding
ENSG0000(	ENSG0000(	186.4644	0.119655	0.276504	0.696054	lncRNA
ENSG0000(	ZNF572	128.2509	0.130542	0.276613	0.696265	protein_coding
ENSG0000(	ENSG0000(	626.1474	0.242775	0.276679	0.696314	lncRNA
ENSG0000(	FAR1-IT1	75.71375	-0.19931	0.276759	0.696314	lncRNA
ENSG0000(	FBXL16	39.86361	0.33294	0.276744	0.696314	protein_coding
ENSG0000(	LINC01635	52.49956	-0.22043	0.276706	0.696314	lncRNA
ENSG0000(	RHOG	369.3397	-0.17511	0.276733	0.696314	protein_coding
ENSG0000(	ENSG0000(	492.5138	0.07808	0.276878	0.696422	lncRNA
ENSG0000(	ENSG0000(	79.38379	0.161747	0.276854	0.696422	TEC
ENSG0000(	TAFA2	248.3181	-0.18195	0.27684	0.696422	protein_coding
ENSG0000(	LINC02109	113.0755	0.142938	0.276952	0.696543	lncRNA
ENSG0000(	STPG1	739.9903	0.12806	0.276976	0.696543	protein_coding
ENSG0000(	HCFC1	2013.399	-0.07328	0.277076	0.696609	protein_coding
ENSG0000(	MTCO2P12	47.53323	-0.36312	0.277064	0.696609	unprocessed_pseudogene
ENSG0000(	SLC35F5	5355.07	-0.07676	0.277079	0.696609	protein_coding
ENSG0000(	ENSG0000(	36.73791	0.236083	0.277129	0.696663	lncRNA
ENSG0000(	SEC22B4P	49.53558	0.249544	0.277151	0.696663	transcribed_unprocessed_pseudo
ENSG0000(	ENSG0000(	129.2938	0.148112	0.277233	0.696678	lncRNA
ENSG0000(	FAM193B	1751.027	0.089748	0.277258	0.696678	protein_coding
ENSG0000(	PDE3A	1841.992	0.248653	0.277185	0.696678	protein_coding
ENSG0000(	TRIM60P14	35.23783	0.360812	0.277224	0.696678	processed_pseudogene
ENSG0000(	MLIP	102.1094	0.237987	0.27734	0.69682	protein_coding

ENSG0000(	ENSG0000(	78.61971	-0.15359	0.277395	0.696895	lncRNA
ENSG0000(	DCUN1D2	714.3568	0.103104	0.277502	0.697042	protein_coding
ENSG0000(	GTF2B	202.4392	-0.10998	0.277525	0.697042	protein_coding
ENSG0000(	NR3C1	1849.057	-0.17291	0.27753	0.697042	protein_coding
ENSG0000(	ENSG0000(	216.242	0.182373	0.277568	0.697074	lncRNA
ENSG0000(	ENSG0000(	127.616	0.140018	0.277673	0.697085	lncRNA
ENSG0000(	HMCN2	3081.576	-0.33353	0.277658	0.697085	protein_coding
ENSG0000(	POLR2B	1423.972	-0.08067	0.277656	0.697085	protein_coding
ENSG0000(	SNF8P1	165.767	-0.10934	0.277609	0.697085	processed_pseudogene
ENSG0000(	NEK6	1528.349	-0.09959	0.277778	0.697284	protein_coding
ENSG0000(	LINC00271	1047.072	-0.07762	0.277846	0.697391	lncRNA
ENSG0000(	SNHG32	383.3608	-0.1255	0.277871	0.697391	lncRNA
ENSG0000(	POTEC	87.06872	0.252662	0.277924	0.69746	protein_coding
ENSG0000(	RNF139-DT	506.8338	-0.09975	0.278004	0.697597	lncRNA
ENSG0000(	ADA2	1570.231	-0.10786	0.278302	0.697606	protein_coding
ENSG0000(	BTNL9	578.5346	0.120014	0.278325	0.697606	protein_coding
ENSG0000(	C1RL	1327.543	-0.11401	0.27841	0.697606	protein_coding
ENSG0000(	CATSPERD	26.52974	0.224114	0.278439	0.697606	protein_coding
ENSG0000(	ENSG0000(	44.79502	-0.2607	0.278437	0.697606	lncRNA
ENSG0000(	ENSG0000(	26.02753	0.480578	0.278168	0.697606	processed_pseudogene
ENSG0000(	ENSG0000(	808.9439	0.061097	0.278146	0.697606	TEC
ENSG0000(	ENSG0000(	178.0375	-0.09193	0.278292	0.697606	lncRNA
ENSG0000(	FBXO3	1502.686	-0.06386	0.278338	0.697606	protein_coding
ENSG0000(	LINC01819	95.36634	-0.26373	0.278065	0.697606	lncRNA
ENSG0000(	MT-ATP6	11595.09	-0.21547	0.278235	0.697606	protein_coding
ENSG0000(	MTHFD2P1	181.1065	0.128459	0.278436	0.697606	transcribed_processed_pseudoge
ENSG0000(	OR2A20P	278.0392	0.291499	0.278252	0.697606	transcribed_unprocessed_pseudo
ENSG0000(	OR52B5P	114.4419	0.143757	0.278291	0.697606	transcribed_unprocessed_pseudo
ENSG0000(	PPP2CA-DT	31.03158	-0.25746	0.278235	0.697606	lncRNA
ENSG0000(	TMCO1	1126.201	0.109427	0.278274	0.697606	protein_coding
ENSG0000(	VLDLR-AS1	429.5742	0.180787	0.278408	0.697606	lncRNA
ENSG0000(	RPL17	1992.896	-0.12277	0.278473	0.697629	protein_coding
ENSG0000(	CFAP70	404.1034	0.101818	0.278634	0.697639	protein_coding
ENSG0000(	MED15P9	279.3138	-0.10372	0.278599	0.697639	transcribed_unprocessed_pseudo
ENSG0000(	MRO	203.1878	0.130756	0.278613	0.697639	protein_coding
ENSG0000(	PCDHGA8	513.3021	0.075462	0.278568	0.697639	protein_coding
ENSG0000(	PLBD1-AS1	225.7282	0.165496	0.278655	0.697639	lncRNA
ENSG0000(	SEPTIN14P	59.52394	-0.26752	0.278596	0.697639	transcribed_unprocessed_pseudo
ENSG0000(	TRPM2	596.0025	-0.19122	0.278623	0.697639	protein_coding
ENSG0000(	ADAM15	919.1656	-0.14486	0.278715	0.697725	protein_coding
ENSG0000(	KRT8P12	83.83569	0.155227	0.278772	0.697805	transcribed_processed_pseudoge
ENSG0000(	FBXL18	2087.367	0.074333	0.278877	0.697845	protein_coding
ENSG0000(	MIR4458H(	156.6442	0.226451	0.278882	0.697845	lncRNA
ENSG0000(	RNF215	439.7434	-0.1517	0.278889	0.697845	protein_coding
ENSG0000(	STAG3L2	1727.295	0.070316	0.278889	0.697845	transcribed_unprocessed_pseudo
ENSG0000(	CHTF18	330.1837	0.190738	0.279017	0.697909	protein_coding
ENSG0000(	DDX58	2338.125	0.17086	0.278969	0.697909	protein_coding

ENSG0000(	E2F3P2	285.876	0.130509	0.279013	0.697909	processed_pseudogene
ENSG0000(	RMDN2	728.854	0.201322	0.278995	0.697909	protein_coding
ENSG0000(	SPINT2	1921.608	0.211334	0.279063	0.697961	protein_coding
ENSG0000(	BACE2-IT1	65.18684	-0.23409	0.279193	0.697998	lncRNA
ENSG0000(	ENSG0000(	50.97931	0.286753	0.279148	0.697998	protein_coding
ENSG0000(	ENSG0000(	28.18232	0.231879	0.279156	0.697998	lncRNA
ENSG0000(	ITGB6	1008.16	-0.24371	0.279168	0.697998	protein_coding
ENSG0000(	ZNF354C	267.4899	-0.12656	0.279204	0.697998	protein_coding
ENSG0000(	DST	27430.45	0.140504	0.279409	0.698232	protein_coding
ENSG0000(	ENSG0000(	32.50932	-0.31785	0.279465	0.698232	lncRNA
ENSG0000(	LINC00996	408.8848	-0.12443	0.279526	0.698232	lncRNA
ENSG0000(	MARCHF6	5915.477	0.074261	0.279521	0.698232	protein_coding
ENSG0000(	PRICKLE2-A	157.7099	0.125298	0.279337	0.698232	lncRNA
ENSG0000(	PRKG2	186.3554	0.436871	0.279378	0.698232	protein_coding
ENSG0000(	SPON1	538.9016	0.230927	0.279459	0.698232	protein_coding
ENSG0000(	STK32C	83.36282	-0.1674	0.279492	0.698232	protein_coding
ENSG0000(	TMPO-AS1	85.01343	-0.20657	0.279386	0.698232	lncRNA
ENSG0000(	CDH18	163.0032	0.125886	0.279578	0.698251	protein_coding
ENSG0000(	LPAR4	52.63346	0.211962	0.279609	0.698251	protein_coding
ENSG0000(	PDE1A	190.2127	0.153118	0.27961	0.698251	protein_coding
ENSG0000(	RNPEP	817.5152	0.135973	0.279661	0.698315	protein_coding
ENSG0000(	BEX2	23.71814	-0.32572	0.279708	0.698324	protein_coding
ENSG0000(	FBXL15	100.3886	0.171713	0.279716	0.698324	protein_coding
ENSG0000(	DGCR2	1536.547	0.092528	0.279795	0.698428	protein_coding
ENSG0000(	LYRM2	1194.601	0.064181	0.279808	0.698428	protein_coding
ENSG0000(	DGLUCY	3169.854	0.084885	0.279871	0.698523	protein_coding
ENSG0000(	ANKRD33B	350.3446	-0.26217	0.27996	0.698592	protein_coding
ENSG0000(	C1orf210	341.1187	0.198539	0.28003	0.698592	protein_coding
ENSG0000(	GOLGA2P5	2945.124	0.140615	0.280077	0.698592	transcribed_unprocessed_pseudo
ENSG0000(	H1-3	213.4376	-0.19876	0.280069	0.698592	protein_coding
ENSG0000(	KIAA1549	583.0598	0.089198	0.279929	0.698592	protein_coding
ENSG0000(	MTERF2	471.0247	0.103104	0.280062	0.698592	protein_coding
ENSG0000(	PHYH	268.1781	0.194613	0.279978	0.698592	protein_coding
ENSG0000(	AGA-DT	145.7498	0.132518	0.280114	0.698623	lncRNA
ENSG0000(	LINC02242	300.8374	0.100569	0.280156	0.698664	lncRNA
ENSG0000(	BMT2	221.1627	-0.14008	0.280274	0.698785	protein_coding
ENSG0000(	DPEP2	258.9305	-0.2106	0.280373	0.698785	protein_coding
ENSG0000(	ENSG0000(	19.54286	-0.49536	0.28047	0.698785	lncRNA
ENSG0000(	ENSG0000(	62.48613	-0.14965	0.28039	0.698785	lncRNA
ENSG0000(	ENSG0000(	474.4808	0.102238	0.280337	0.698785	lncRNA
ENSG0000(	KCTD7	843.4893	-0.07956	0.280484	0.698785	protein_coding
ENSG0000(	LMNB1	632.7987	-0.15731	0.280472	0.698785	protein_coding
ENSG0000(	MIER3	4262.799	0.339817	0.280365	0.698785	protein_coding
ENSG0000(	OTUD3	1247.224	-0.1538	0.280424	0.698785	protein_coding
ENSG0000(	SPRED2	1354.57	0.154902	0.280294	0.698785	protein_coding
ENSG0000(	ZSCAN26	360.4538	-0.12088	0.280274	0.698785	protein_coding
ENSG0000(	ENSG0000(	202.0464	-0.11199	0.280547	0.698815	lncRNA

ENSG0000(	KCNMB1	696.8502	-0.10631	0.280537	0.698815	protein_coding
ENSG0000(	ENSG0000(	56.3129	-0.33462	0.280595	0.69887	TEC
ENSG0000(	ENSG0000(	902.3073	0.078051	0.280713	0.699102	lncRNA
ENSG0000(	OR6C75	466.6204	0.096537	0.280769	0.699177	protein_coding
ENSG0000(	ADPRHL1	256.4707	0.119125	0.281062	0.699335	protein_coding
ENSG0000(	C6orf163	551.7027	0.106291	0.281017	0.699335	protein_coding
ENSG0000(	ENSG0000(	86.24828	0.317154	0.280986	0.699335	lncRNA
ENSG0000(	ENSG0000(	93.42	-0.13653	0.281048	0.699335	lncRNA
ENSG0000(	ENSG0000(	87.96662	0.125358	0.281162	0.699335	lncRNA
ENSG0000(	ENSG0000(	80.89152	0.286971	0.281006	0.699335	lncRNA
ENSG0000(	ENSG0000(	70.5575	0.159933	0.281047	0.699335	lncRNA
ENSG0000(	GRM8-AS1	21.14854	-0.3507	0.281115	0.699335	lncRNA
ENSG0000(	LINC02275	831.1269	-0.0731	0.281161	0.699335	lncRNA
ENSG0000(	ORC5	287.8828	0.108851	0.28116	0.699335	protein_coding
ENSG0000(	PDCD7	674.2451	-0.10292	0.281001	0.699335	protein_coding
ENSG0000(	SOAT1	1146.517	-0.09075	0.281028	0.699335	protein_coding
ENSG0000(	TDP2	1988.728	0.201612	0.281029	0.699335	protein_coding
ENSG0000(	RN7SKP102	59.36313	0.252198	0.281258	0.69951	misc_RNA
ENSG0000(	ENSG0000(	244.9146	0.204205	0.281355	0.699561	lncRNA
ENSG0000(	ENSG0000(	160.2977	0.135148	0.281352	0.699561	lncRNA
ENSG0000(	QSOX1	2573.946	-0.20712	0.281339	0.699561	protein_coding
ENSG0000(	ENSG0000(	664.7229	-0.17629	0.281474	0.699577	transcribed_unprocessed_pseudo
ENSG0000(	ENSG0000(	41.88653	-0.23986	0.281489	0.699577	lncRNA
ENSG0000(	LAYN	99.91235	-0.21132	0.281483	0.699577	protein_coding
ENSG0000(	LINC02693	660.2948	-0.13205	0.28143	0.699577	lncRNA
ENSG0000(	NPTN	1089.698	-0.09488	0.281419	0.699577	protein_coding
ENSG0000(	ENSG0000(	38.77975	-0.32054	0.281521	0.699595	lncRNA
ENSG0000(	ENSG0000(	120.1121	-0.11381	0.281707	0.69993	TEC
ENSG0000(	ERI2	588.6866	0.109678	0.2817	0.69993	protein_coding
ENSG0000(	TM9SF1	466.2238	0.093904	0.281801	0.7001	protein_coding
ENSG0000(	ENSG0000(	23.88701	0.355678	0.281903	0.700187	lncRNA
ENSG0000(	NAT2	56.76756	0.288906	0.281912	0.700187	protein_coding
ENSG0000(	PPM1A	2816.083	0.128983	0.281891	0.700187	protein_coding
ENSG0000(	ENSG0000(	128.0906	-0.12426	0.282026	0.700312	lncRNA
ENSG0000(	ENSG0000(	214.1493	-0.13022	0.282014	0.700312	lncRNA
ENSG0000(	SRRD	491.0187	0.094596	0.282039	0.700312	protein_coding
ENSG0000(	RNU1-16P	20.69457	0.324811	0.282127	0.700467	snRNA
ENSG0000(	CTXND1	160.4902	-0.11402	0.282195	0.700537	protein_coding
ENSG0000(	GPR21	175.3411	0.105902	0.282206	0.700537	protein_coding
ENSG0000(	C5orf22	477.9338	-0.0737	0.282249	0.700576	protein_coding
ENSG0000(	C8orf88	20.5369	-0.35131	0.282273	0.700576	protein_coding
ENSG0000(	KBTBD11	1362.472	0.234274	0.282376	0.70077	protein_coding
ENSG0000(	SLC13A5	499.5936	0.072706	0.282441	0.700868	protein_coding
ENSG0000(	ENSG0000(	84.24547	0.161016	0.282491	0.700929	processed_pseudogene
ENSG0000(	DECR2	580.4285	0.124364	0.282672	0.701187	protein_coding
ENSG0000(	ENSG0000(	579.3186	0.072417	0.282664	0.701187	lncRNA
ENSG0000(	ENSG0000(	46.66261	-0.24204	0.282665	0.701187	lncRNA

ENSG0000(	IGFL2-AS1	133.7726	-0.14152	0.28274	0.70123	lncRNA
ENSG0000(	NUP210L	24.52613	-0.23733	0.282738	0.70123	protein_coding
ENSG0000(	ENSG0000(	148.0511	0.171589	0.282817	0.701358	lncRNA
ENSG0000(	ENPP7	24.45928	-0.57639	0.282857	0.701393	protein_coding
ENSG0000(	WDR19	839.0235	-0.1023	0.282934	0.70152	protein_coding
ENSG0000(	ENSG0000(	25.92427	-0.25206	0.283015	0.701595	lncRNA
ENSG0000(	HYDIN2	185.8052	0.32429	0.283004	0.701595	transcribed_unprocessed_pseudo
ENSG0000(	ENSG0000(	120.305	0.136463	0.283119	0.701727	lncRNA
ENSG0000(	TNKS1BP1	2800.464	-0.1392	0.283099	0.701727	protein_coding
ENSG0000(	ETV3	1171.469	-0.09742	0.283151	0.701742	protein_coding
ENSG0000(	ATG4C	186.1179	-0.17659	0.283338	0.701881	protein_coding
ENSG0000(	C3orf36	91.6329	-0.29854	0.283271	0.701881	lncRNA
ENSG0000(	CDH1	5336.41	0.201218	0.283558	0.701881	protein_coding
ENSG0000(	DAB1	50.36682	-0.52563	0.283545	0.701881	protein_coding
ENSG0000(	EIF3J	853.5988	-0.11382	0.283811	0.701881	protein_coding
ENSG0000(	ENSG0000(	43.57762	-0.45677	0.283854	0.701881	processed_pseudogene
ENSG0000(	ENSG0000(	410.3906	-0.09961	0.283657	0.701881	lncRNA
ENSG0000(	ENSG0000(	91.47269	-0.16884	0.28341	0.701881	lncRNA
ENSG0000(	ENSG0000(	45.334	-0.20661	0.283608	0.701881	transcribed_unprocessed_pseudo
ENSG0000(	ENSG0000(	310.8311	-0.0971	0.28329	0.701881	lncRNA
ENSG0000(	ENSG0000(	55.90972	-0.16829	0.283702	0.701881	lncRNA
ENSG0000(	ENSG0000(	175.6012	0.095666	0.283938	0.701881	lncRNA
ENSG0000(	ENSG0000(	41.94758	-0.25128	0.283992	0.701881	lncRNA
ENSG0000(	FAM86B3P	380.248	0.108656	0.283337	0.701881	transcribed_unprocessed_pseudo
ENSG0000(	FAN1	3435.513	0.056498	0.28375	0.701881	protein_coding
ENSG0000(	GOLGA6L7	168.6388	-0.14256	0.283546	0.701881	protein_coding
ENSG0000(	HTR3E-AS1	30.76464	0.260713	0.283683	0.701881	lncRNA
ENSG0000(	MAZ	1281.786	0.093379	0.283435	0.701881	protein_coding
ENSG0000(	MRPL12	169.5914	0.200331	0.284034	0.701881	protein_coding
ENSG0000(	NCAPH	508.1906	-0.0915	0.283362	0.701881	protein_coding
ENSG0000(	PPP1R36	80.40713	0.277375	0.283879	0.701881	protein_coding
ENSG0000(	PROX1-AS1	729.947	0.1351	0.283621	0.701881	lncRNA
ENSG0000(	REELD1	44.96432	0.259658	0.283457	0.701881	protein_coding
ENSG0000(	RN7SKP173	28.73565	-0.24646	0.283865	0.701881	misc_RNA
ENSG0000(	SIAH2	376.0987	-0.10159	0.284013	0.701881	protein_coding
ENSG0000(	SMARCD3	198.5515	-0.18424	0.283322	0.701881	protein_coding
ENSG0000(	SNORD99	32.54776	-0.22431	0.284048	0.701881	snoRNA
ENSG0000(	SRCIN1	195.0242	0.187149	0.283897	0.701881	protein_coding
ENSG0000(	UHRF1	478.5476	-0.12617	0.283886	0.701881	protein_coding
ENSG0000(	UICLM	23.77201	-0.63006	0.283904	0.701881	lncRNA
ENSG0000(	USP19	1078.172	0.099502	0.283631	0.701881	protein_coding
ENSG0000(	USP40	1300.069	-0.09659	0.28376	0.701881	protein_coding
ENSG0000(	VAT1	336.0606	-0.24534	0.284005	0.701881	protein_coding
ENSG0000(	CENPF	1717.599	-0.10736	0.284139	0.702041	protein_coding
ENSG0000(	ANAPC1P2	337.4881	0.12998	0.284398	0.702275	lncRNA
ENSG0000(	ENSG0000(	70.29374	-0.15559	0.284483	0.702275	lncRNA
ENSG0000(	ENSG0000(	211.2059	0.190435	0.284489	0.702275	lncRNA

ENSG0000(	ENSG0000(	148.2026	-0.11926	0.284352	0.702275	TEC
ENSG0000(	ENSG0000(	51.54483	-0.19476	0.284417	0.702275	lncRNA
ENSG0000(	FUOM	85.22998	0.207274	0.284463	0.702275	protein_coding
ENSG0000(	HERC3	1561.469	0.098995	0.284414	0.702275	protein_coding
ENSG0000(	KAT5	528.3482	0.10575	0.284397	0.702275	protein_coding
ENSG0000(	PEAK3	105.1937	-0.18064	0.284279	0.702275	protein_coding
ENSG0000(	PRKAR2A	1843.831	0.093577	0.284407	0.702275	protein_coding
ENSG0000(	ENSG0000(	1093.915	-0.06547	0.284599	0.702485	lncRNA
ENSG0000(	ENSG0000(	2404.292	0.11157	0.284663	0.702549	lncRNA
ENSG0000(	LINC02747	158.4751	0.359893	0.284702	0.702549	lncRNA
ENSG0000(	ZNF425	175.6262	-0.1378	0.284691	0.702549	protein_coding
ENSG0000(	USP18	106.7056	0.359192	0.284796	0.702717	protein_coding
ENSG0000(	CYP27A1	228.274	-0.20916	0.28494	0.702948	protein_coding
ENSG0000(	TAS2R1	63.58862	-0.28073	0.284935	0.702948	protein_coding
ENSG0000(	CACNA1A	3321.782	0.048232	0.285013	0.703065	protein_coding
ENSG0000(	LINC00869	661.341	-0.11618	0.285062	0.703121	transcribed_unprocessed_pseudo
ENSG0000(	LINC02038	128.4281	0.30274	0.285163	0.703308	lncRNA
ENSG0000(	CSNK1G2P:	28.11729	-0.31286	0.285404	0.703777	processed_pseudogene
ENSG0000(	MMAB	637.6567	0.074629	0.285392	0.703777	protein_coding
ENSG0000(	PTGES3	2259.883	-0.08611	0.285446	0.703817	protein_coding
ENSG0000(	ANKRD6	473.4042	-0.09587	0.285557	0.703964	protein_coding
ENSG0000(	ENSG0000(	22.6817	-0.30722	0.285548	0.703964	lncRNA
ENSG0000(	LTA	23.39587	-0.479	0.285603	0.704015	protein_coding
ENSG0000(	SLC9A2	1923.599	0.361897	0.285678	0.704136	protein_coding
ENSG0000(	PDGFD	273.291	-0.24863	0.285736	0.704217	protein_coding
ENSG0000(	NT5M	31.29575	0.265876	0.285805	0.70426	protein_coding
ENSG0000(	RARG	267.5163	-0.16624	0.285783	0.70426	protein_coding
ENSG0000(	SZRD1	1482.841	-0.07897	0.285875	0.704371	protein_coding
ENSG0000(	ACAD9	2485.731	0.066344	0.28594	0.704402	protein_coding
ENSG0000(	PENK-AS1	1907.862	0.114647	0.285924	0.704402	lncRNA
ENSG0000(	RN7SL674F	320.9353	-0.27522	0.285965	0.704402	misc_RNA
ENSG0000(	MAPK3	1230.314	0.118771	0.286019	0.704473	protein_coding
ENSG0000(	LPP-AS2	160.7794	-0.10418	0.286105	0.704622	lncRNA
ENSG0000(	ENSG0000(	227.4786	-0.11733	0.286234	0.704875	lncRNA
ENSG0000(	ALG1L9P	890.4501	0.077238	0.286492	0.704952	lncRNA
ENSG0000(	ANAPC2	1133.885	0.075821	0.286617	0.704952	protein_coding
ENSG0000(	CPM	1998.55	0.091361	0.28643	0.704952	protein_coding
ENSG0000(	DENND11	3378.156	0.079622	0.286358	0.704952	protein_coding
ENSG0000(	ENSG0000(	60.57512	0.186451	0.286331	0.704952	lncRNA
ENSG0000(	ENSG0000(	96.68389	0.141844	0.286624	0.704952	lncRNA
ENSG0000(	ENSG0000(	98.63346	0.151079	0.286497	0.704952	lncRNA
ENSG0000(	IFI35	351.0079	-0.12998	0.286575	0.704952	protein_coding
ENSG0000(	LINC01267	90.87036	-0.21155	0.286621	0.704952	lncRNA
ENSG0000(	MSL2	1554.424	0.081753	0.286574	0.704952	protein_coding
ENSG0000(	NMRAL1	451.255	0.081163	0.286525	0.704952	protein_coding
ENSG0000(	RN7SL693F	214.1169	0.129949	0.286509	0.704952	misc_RNA
ENSG0000(	SHOX	607.1915	0.099666	0.286402	0.704952	protein_coding

ENSG0000(	SYT2	344.4075	0.203631	0.286522	0.704952	protein_coding
ENSG0000(	ANKRD45	297.0392	0.105962	0.287073	0.70498	protein_coding
ENSG0000(	CEP170P1	72.5523	-0.18662	0.287155	0.70498	transcribed_unprocessed_pseudo
ENSG0000(	ENSG0000(	22.20507	-0.26615	0.286982	0.70498	lncRNA
ENSG0000(	ENSG0000(	27.46396	0.317618	0.287233	0.70498	lncRNA
ENSG0000(	ENSG0000(	41.26203	0.244033	0.287237	0.70498	lncRNA
ENSG0000(	ENSG0000(	77.95118	-0.18382	0.287151	0.70498	lncRNA
ENSG0000(	ENSG0000(	53.3983	0.163209	0.286919	0.70498	lncRNA
ENSG0000(	ENSG0000(	161.2759	-0.12799	0.286871	0.70498	TEC
ENSG0000(	ENSG0000(	194.5038	0.138614	0.287135	0.70498	lncRNA
ENSG0000(	EPB41L4B	1751.491	0.325586	0.286896	0.70498	protein_coding
ENSG0000(	GPR158	42.17359	-0.35588	0.28725	0.70498	protein_coding
ENSG0000(	HM13-IT1	97.34439	-0.12748	0.287234	0.70498	lncRNA
ENSG0000(	MIR1-1HG-	130.9693	-0.15001	0.287135	0.70498	lncRNA
ENSG0000(	OLMALINC	19779.59	0.0755	0.286906	0.70498	lncRNA
ENSG0000(	PGF	514.178	-0.16238	0.287129	0.70498	protein_coding
ENSG0000(	PPP1R35	95.83553	0.140844	0.287167	0.70498	protein_coding
ENSG0000(	PYGO1	345.5355	-0.12264	0.286838	0.70498	protein_coding
ENSG0000(	RCC1	406.211	0.150286	0.286717	0.70498	protein_coding
ENSG0000(	RIOK3	2705.691	0.169798	0.286663	0.70498	protein_coding
ENSG0000(	RN7SL503F	20.61805	0.281876	0.286916	0.70498	misc_RNA
ENSG0000(	SLC25A16	2274.118	-0.06547	0.287012	0.70498	protein_coding
ENSG0000(	SNX18P12	29.30111	0.350585	0.287077	0.70498	processed_pseudogene
ENSG0000(	TMEM78	198.0343	0.108666	0.28711	0.70498	lncRNA
ENSG0000(	WEE1	1144.541	0.120084	0.286917	0.70498	protein_coding
ENSG0000(	ENSG0000(	34.5447	0.251519	0.287327	0.705106	lncRNA
ENSG0000(	ENSG0000(	24.26834	-0.21982	0.287406	0.705236	lncRNA
ENSG0000(	LINC02246	239.4426	0.131522	0.287432	0.705238	lncRNA
ENSG0000(	URAD	154.4765	0.340301	0.287501	0.705344	protein_coding
ENSG0000(	CHRNB1	757.2706	0.1372	0.287663	0.705566	protein_coding
ENSG0000(	ENSG0000(	44.36669	0.200626	0.287655	0.705566	processed_pseudogene
ENSG0000(	ENSG0000(	751.822	-0.07299	0.28782	0.705566	lncRNA
ENSG0000(	ENSG0000(	228.4221	-0.09248	0.287784	0.705566	protein_coding
ENSG0000(	GATD3B	766.0143	0.127865	0.287763	0.705566	protein_coding
ENSG0000(	GREM2	303.4219	0.228753	0.287792	0.705566	protein_coding
ENSG0000(	LINC00682	55.3708	-0.16685	0.287848	0.705566	lncRNA
ENSG0000(	SLC25A19	474.8836	-0.10935	0.287833	0.705566	protein_coding
ENSG0000(	SLC2A4RG	280.1632	0.171677	0.287685	0.705566	protein_coding
ENSG0000(	SNORA2C	201.68	0.193368	0.287845	0.705566	snoRNA
ENSG0000(	PELP1	654.4074	0.115085	0.288005	0.705889	protein_coding
ENSG0000(	ENSG0000(	24.63093	0.238403	0.288109	0.705953	processed_pseudogene
ENSG0000(	ENSG0000(	304.5855	0.096101	0.288061	0.705953	TEC
ENSG0000(	ZNF616	476.5097	0.120906	0.288103	0.705953	protein_coding
ENSG0000(	ENSG0000(	225.3574	-0.19905	0.288146	0.705982	TEC
ENSG0000(	SIK1	277.8887	-0.43988	0.288193	0.706034	protein_coding
ENSG0000(	BRPF3-AS1	190.6629	-0.10028	0.288307	0.706092	lncRNA
ENSG0000(	ENSG0000(	155.9562	0.226445	0.288343	0.706092	lncRNA

ENSG0000(	H2AL1MP	27.55097	0.346079	0.288281	0.706092	unprocessed_pseudogene
ENSG0000(	LINC02266	20.43354	-0.2556	0.288282	0.706092	lncRNA
ENSG0000(	TMEM88B	284.284	-0.09286	0.288345	0.706092	protein_coding
ENSG0000(	AQP7P1	30.90063	-0.44467	0.288567	0.706112	unprocessed_pseudogene
ENSG0000(	ARSF	22.61568	0.254514	0.288836	0.706112	protein_coding
ENSG0000(	C15orf39	1195.944	-0.12613	0.288933	0.706112	protein_coding
ENSG0000(	CLCF1	121.749	-0.26665	0.288788	0.706112	protein_coding
ENSG0000(	CTD-2194D	42.4827	0.209434	0.28908	0.706112	lncRNA
ENSG0000(	EIF3H	1104.579	-0.10635	0.288869	0.706112	protein_coding
ENSG0000(	ENSG0000(	255.363	-0.12685	0.288934	0.706112	transcribed_unprocessed_pseudo
ENSG0000(	ENSG0000(	25.72937	0.268399	0.288996	0.706112	lncRNA
ENSG0000(	ENSG0000(	58.20317	-0.16883	0.288738	0.706112	lncRNA
ENSG0000(	ENSG0000(	43.04859	-0.2064	0.289092	0.706112	lncRNA
ENSG0000(	ENSG0000(	38.15272	-0.20642	0.288618	0.706112	lncRNA
ENSG0000(	ENSG0000(	22.39958	0.275331	0.289079	0.706112	lncRNA
ENSG0000(	ENSG0000(	110.1363	-0.13903	0.288473	0.706112	lncRNA
ENSG0000(	ENSG0000(	22.25023	0.282649	0.288726	0.706112	lncRNA
ENSG0000(	ENSG0000(	131.8875	-0.23105	0.288839	0.706112	lncRNA
ENSG0000(	ENSG0000(	30.60598	-0.26299	0.289038	0.706112	lncRNA
ENSG0000(	FAHD2A	556.6371	0.100918	0.289151	0.706112	protein_coding
ENSG0000(	FBXO46	750.166	0.130569	0.289194	0.706112	protein_coding
ENSG0000(	ITFG2-AS1	23.58291	-0.24631	0.289108	0.706112	lncRNA
ENSG0000(	LINC00900	379.6474	0.124219	0.288439	0.706112	lncRNA
ENSG0000(	LINC01885	41.14306	-0.27985	0.289025	0.706112	lncRNA
ENSG0000(	MS4A12	1604.41	0.470325	0.288802	0.706112	protein_coding
ENSG0000(	PALS2	356.1056	-0.19673	0.28886	0.706112	protein_coding
ENSG0000(	PLCL2	1203.528	0.120923	0.2892	0.706112	protein_coding
ENSG0000(	PPP1R3G	143.4409	0.097917	0.288475	0.706112	protein_coding
ENSG0000(	PWAR5	40.1301	-2.12449	0.289022	0.706112	TEC
ENSG0000(	RAP2C-AS1	326.3098	-0.07806	0.289052	0.706112	lncRNA
ENSG0000(	RN7SL507F	46.45997	-0.19368	0.289164	0.706112	misc_RNA
ENSG0000(	RNF187	721.9453	-0.08202	0.288677	0.706112	protein_coding
ENSG0000(	SNORA2A	43.50355	0.187654	0.288449	0.706112	snoRNA
ENSG0000(	SSTR5-AS1	64.48702	-0.2557	0.289041	0.706112	lncRNA
ENSG0000(	UGT1A1	695.7615	0.166436	0.288935	0.706112	protein_coding
ENSG0000(	ZNF763	454.1026	0.126387	0.289095	0.706112	protein_coding
ENSG0000(	BCLAF1	4665.771	-0.07941	0.2894	0.706188	protein_coding
ENSG0000(	BNIP3P41	46.34021	-0.52118	0.289409	0.706188	transcribed_processed_pseudoge
ENSG0000(	ENSG0000(	133.2124	-0.27045	0.289328	0.706188	lncRNA
ENSG0000(	ENSG0000(	123.0602	0.148118	0.289368	0.706188	lncRNA
ENSG0000(	GPR19	27.27447	-0.38779	0.28934	0.706188	protein_coding
ENSG0000(	MTX1	251.5426	-0.10919	0.289471	0.706188	protein_coding
ENSG0000(	PHIP	4488.993	-0.05631	0.289488	0.706188	protein_coding
ENSG0000(	SLX1A	497.6072	-0.13748	0.289292	0.706188	protein_coding
ENSG0000(	UGT2A3	360.3895	0.427169	0.28946	0.706188	protein_coding
ENSG0000(	ZNF620	467.9263	0.101147	0.289467	0.706188	protein_coding
ENSG0000(	FNDC10	25.40397	0.310294	0.289514	0.70619	protein_coding

ENSG0000(	ENSG0000(	68.04844	-0.21957	0.289544	0.7062	lncRNA
ENSG0000(	TBX18	76.91024	-0.62917	0.289587	0.706242	protein_coding
ENSG0000(	ATRX	5621.501	-0.1269	0.289624	0.70627	protein_coding
ENSG0000(	COPS5	1799.192	0.064459	0.289692	0.706374	protein_coding
ENSG0000(	C11orf71	269.3212	-0.09601	0.289787	0.706426	protein_coding
ENSG0000(	ENSG0000(	915.4673	0.088569	0.289791	0.706426	lncRNA
ENSG0000(	ENSG0000(	275.9838	0.490771	0.289756	0.706426	protein_coding
ENSG0000(	ENSG0000(	38.22975	0.296499	0.289843	0.706427	processed_pseudogene
ENSG0000(	ENSG0000(	59.49697	-0.19749	0.289842	0.706427	lncRNA
ENSG0000(	FH	825.6347	0.104041	0.289913	0.70649	protein_coding
ENSG0000(	VLDLR	613.8643	0.192831	0.28992	0.70649	protein_coding
ENSG0000(	CD22	1434.43	-0.39082	0.290322	0.706511	protein_coding
ENSG0000(	CHP1	4107.978	0.201267	0.290205	0.706511	protein_coding
ENSG0000(	CLCC1	4960.672	-0.04282	0.290071	0.706511	protein_coding
ENSG0000(	ENSG0000(	24.19681	0.245176	0.2903	0.706511	transcribed_unitary_pseudogene
ENSG0000(	ENSG0000(	21.65555	0.477829	0.290117	0.706511	lncRNA
ENSG0000(	ENSG0000(	134.6941	0.14492	0.290402	0.706511	lncRNA
ENSG0000(	ENSG0000(	300.9901	0.123881	0.290244	0.706511	protein_coding
ENSG0000(	ENSG0000(	1298.055	0.108369	0.290113	0.706511	lncRNA
ENSG0000(	EPHA10	1784.4	0.251277	0.290144	0.706511	protein_coding
ENSG0000(	KRTAP5-AS	202.9624	0.227961	0.290077	0.706511	lncRNA
ENSG0000(	LINC01136	269.3723	0.115633	0.290306	0.706511	lncRNA
ENSG0000(	LINC02418	120.9119	0.117707	0.290133	0.706511	lncRNA
ENSG0000(	LRRC19	1314.042	0.249616	0.290063	0.706511	protein_coding
ENSG0000(	NDUFV1-D	237.8236	0.151735	0.290246	0.706511	lncRNA
ENSG0000(	NSMCE1-D	484.8077	-0.09335	0.290412	0.706511	lncRNA
ENSG0000(	OSBPL7	1228.084	0.149294	0.290364	0.706511	protein_coding
ENSG0000(	PARP3	353.8246	-0.13157	0.290441	0.706511	protein_coding
ENSG0000(	PRKCH-AS1	479.994	0.092352	0.290453	0.706511	lncRNA
ENSG0000(	SLC9A9	559.2005	-0.1784	0.289988	0.706511	protein_coding
ENSG0000(	TSPAN33	388.667	-0.19442	0.290468	0.706511	protein_coding
ENSG0000(	TXNDC15	1695.139	-0.12272	0.290188	0.706511	protein_coding
ENSG0000(	CPT1B	502.9488	0.144881	0.290591	0.706623	protein_coding
ENSG0000(	LINC01643	20.56956	-0.31168	0.290579	0.706623	lncRNA
ENSG0000(	PPFIA1	2319.003	0.064266	0.290542	0.706623	protein_coding
ENSG0000(	CIPC	1347.418	0.197087	0.290733	0.706876	protein_coding
ENSG0000(	ENSG0000(	387.2766	-0.07635	0.290798	0.706876	lncRNA
ENSG0000(	LINC01010	104.492	-0.13676	0.290785	0.706876	lncRNA
ENSG0000(	SMYD3	482.4746	-0.11221	0.290779	0.706876	protein_coding
ENSG0000(	ENSG0000(	26.94487	-0.27106	0.290904	0.707009	lncRNA
ENSG0000(	HELLS	1493.536	0.135817	0.290882	0.707009	protein_coding
ENSG0000(	IGHV4-4	243.5684	-0.52384	0.29094	0.707034	IG_V_gene
ENSG0000(	ENSG0000(	40.88112	0.206238	0.290977	0.707063	lncRNA
ENSG0000(	GABPB2	3335.81	0.050936	0.291013	0.707087	protein_coding
ENSG0000(	CYP3A43	169.9165	0.13549	0.291087	0.707143	protein_coding
ENSG0000(	PNPO	795.6358	0.070521	0.29107	0.707143	protein_coding
ENSG0000(	ENSG0000(	1303.949	-0.05686	0.291125	0.707145	lncRNA

ENSG0000(	PLD3	1321.077	-0.05284	0.291181	0.707145	protein_coding
ENSG0000(	RN7SL531F	165.3312	-0.09772	0.291152	0.707145	misc_RNA
ENSG0000(	ZDHHC9	604.0831	0.132517	0.291191	0.707145	protein_coding
ENSG0000(	ITPRID1	269.7754	-0.38467	0.29127	0.707274	protein_coding
ENSG0000(	ENSG0000(	37.00761	-0.19781	0.291308	0.707303	lncRNA
ENSG0000(	GAPLINC	410.4843	-0.08261	0.291353	0.707351	lncRNA
ENSG0000(	ENSG0000(	139.6392	-0.18241	0.291406	0.707352	lncRNA
ENSG0000(	GPR176-DT	169.4624	0.115925	0.29143	0.707352	lncRNA
ENSG0000(	NPIPB3	10529.51	0.082152	0.291393	0.707352	protein_coding
ENSG0000(	HAS2-AS1	69.671	0.185384	0.291578	0.707648	lncRNA
ENSG0000(	THRA	856.9106	0.125078	0.291646	0.707751	protein_coding
ENSG0000(	DCPS	492.1057	-0.12845	0.291782	0.707923	protein_coding
ENSG0000(	MAX	1613.504	-0.1004	0.291807	0.707923	protein_coding
ENSG0000(	RN7SL488F	95.18425	0.149118	0.291822	0.707923	misc_RNA
ENSG0000(	SPATA31A7	55.41708	0.163896	0.291846	0.707923	protein_coding
ENSG0000(	ZFYVE19	427.6863	0.148372	0.291792	0.707923	protein_coding
ENSG0000(	LINC00629	285.8614	-0.13265	0.291909	0.707951	lncRNA
ENSG0000(	OR2A9P	577.1851	0.182881	0.291901	0.707951	transcribed_unprocessed_pseudo
ENSG0000(	SERPINB12	182.5727	-0.10885	0.291977	0.708054	protein_coding
ENSG0000(	ENSG0000(	251.2813	-0.17629	0.292042	0.708149	lncRNA
ENSG0000(	ANKRD20A	126.2524	0.231062	0.292321	0.708372	transcribed_unprocessed_pseudo
ENSG0000(	DVL2	452.2744	-0.09414	0.292642	0.708372	protein_coding
ENSG0000(	ELN	598.1916	0.124385	0.292365	0.708372	protein_coding
ENSG0000(	ENSG0000(	20.73776	0.351626	0.29225	0.708372	lncRNA
ENSG0000(	ENSG0000(	28.66584	0.239796	0.292517	0.708372	lncRNA
ENSG0000(	ENSG0000(	29.67031	-0.24468	0.292563	0.708372	lncRNA
ENSG0000(	ENSG0000(	740.0918	-0.11611	0.292452	0.708372	lncRNA
ENSG0000(	ENSG0000(	276.3827	0.080829	0.292504	0.708372	lncRNA
ENSG0000(	GOLGA8A	6206.104	-0.14581	0.292564	0.708372	protein_coding
ENSG0000(	LACTB2	105.5752	-0.19231	0.292649	0.708372	protein_coding
ENSG0000(	LINC01088	1597.232	0.071402	0.29233	0.708372	lncRNA
ENSG0000(	LINC01537	867.1575	-0.07359	0.292333	0.708372	lncRNA
ENSG0000(	LNX1	356.3136	0.210195	0.292597	0.708372	protein_coding
ENSG0000(	LYSMD1	176.7132	-0.11097	0.292348	0.708372	protein_coding
ENSG0000(	METTL5	305.4773	-0.08464	0.292524	0.708372	protein_coding
ENSG0000(	NADK2	489.0167	-0.13857	0.292329	0.708372	protein_coding
ENSG0000(	NOTCH1	2980.293	0.116778	0.292513	0.708372	protein_coding
ENSG0000(	ODAD3	80.68272	-0.1671	0.292603	0.708372	protein_coding
ENSG0000(	PRCC	724.8083	-0.07674	0.292441	0.708372	protein_coding
ENSG0000(	UBAP1L	1053.051	-0.1029	0.292575	0.708372	protein_coding
ENSG0000(	ENSG0000(	45.24381	0.196144	0.292686	0.7084	lncRNA
ENSG0000(	CHL1	821.0586	-0.19528	0.292762	0.708459	protein_coding
ENSG0000(	ENSG0000(	168.2225	0.245868	0.292748	0.708459	lncRNA
ENSG0000(	MGST1	1389.427	0.187288	0.292852	0.708615	protein_coding
ENSG0000(	ENSG0000(	188.0362	0.087595	0.29291	0.70866	transcribed_unprocessed_pseudo
ENSG0000(	GIPR	752.0641	0.130274	0.292922	0.70866	protein_coding
ENSG0000(	B4GALT3	451.1234	-0.13104	0.293039	0.708809	protein_coding

ENSG0000(	MAPRE1	1008.327	-0.08679	0.293072	0.708809	protein_coding
ENSG0000(	OTUD7A	615.0191	0.165013	0.293087	0.708809	protein_coding
ENSG0000(	RPS29	2288.166	-0.13898	0.293074	0.708809	protein_coding
ENSG0000(	ENSG0000(	62.64206	0.199817	0.293191	0.708874	lncRNA
ENSG0000(	LHFPL3-AS:	19.84001	-0.32957	0.293179	0.708874	lncRNA
ENSG0000(	SNRNP27	210.1923	-0.1362	0.293179	0.708874	protein_coding
ENSG0000(	CTNND2	73.57712	-0.35031	0.293294	0.709016	protein_coding
ENSG0000(	RBM11	22.24673	0.339479	0.293302	0.709016	protein_coding
ENSG0000(	ENSG0000(	63.8339	0.196554	0.293377	0.709137	lncRNA
ENSG0000(	FMOD	368.1545	-0.25563	0.29346	0.709213	protein_coding
ENSG0000(	POLB	414.9713	0.104434	0.293449	0.709213	protein_coding
ENSG0000(	STX16-NPE	227.6499	-0.17586	0.293487	0.709216	protein_coding
ENSG0000(	NIFK-AS1	543.31	-0.0782	0.293538	0.709275	lncRNA
ENSG0000(	ABCC2	442.6094	0.149459	0.293956	0.709332	protein_coding
ENSG0000(	ASGR1	258.3753	0.139352	0.293868	0.709332	protein_coding
ENSG0000(	CLDND2	21.19686	0.338229	0.294047	0.709332	protein_coding
ENSG0000(	DMWD	1140.498	0.060964	0.293907	0.709332	protein_coding
ENSG0000(	ENSG0000(	22.8901	-0.25819	0.294197	0.709332	lncRNA
ENSG0000(	ENSG0000(	164.732	-0.10516	0.294106	0.709332	lncRNA
ENSG0000(	ENSG0000(	74.84843	0.154856	0.294199	0.709332	lncRNA
ENSG0000(	ENSG0000(	44.93159	-0.21448	0.293721	0.709332	lncRNA
ENSG0000(	ENSG0000(	134.9739	-0.12391	0.294206	0.709332	lncRNA
ENSG0000(	ENSG0000(	80.77469	-0.13137	0.293664	0.709332	lncRNA
ENSG0000(	FBXW12	86.41147	-0.1933	0.293724	0.709332	protein_coding
ENSG0000(	HTT	4314.201	0.061556	0.294055	0.709332	protein_coding
ENSG0000(	LINC02139	199.013	-0.09779	0.29418	0.709332	lncRNA
ENSG0000(	LSM6	287.7554	-0.10548	0.293895	0.709332	protein_coding
ENSG0000(	OTUB2	59.52324	-0.26299	0.293854	0.709332	protein_coding
ENSG0000(	PKHD1	224.6497	0.255371	0.293882	0.709332	protein_coding
ENSG0000(	POTEI	88.90022	0.186898	0.293706	0.709332	protein_coding
ENSG0000(	PTCH1	3942.377	0.170242	0.294123	0.709332	protein_coding
ENSG0000(	RAC3	26.32111	0.254447	0.294199	0.709332	protein_coding
ENSG0000(	RASSF6	1684.044	0.125294	0.29389	0.709332	protein_coding
ENSG0000(	TERF2IP	654.9862	-0.09485	0.294146	0.709332	protein_coding
ENSG0000(	VN1R80P	19.73811	-0.3019	0.293928	0.709332	unprocessed_pseudogene
ENSG0000(	WSCD2	51.19273	-0.34621	0.29404	0.709332	protein_coding
ENSG0000(	ZNF211	626.0942	-0.09429	0.294171	0.709332	protein_coding
ENSG0000(	ZNF672	640.1858	-0.06413	0.293853	0.709332	protein_coding
ENSG0000(	ACOX2	248.89	0.222923	0.294307	0.709428	protein_coding
ENSG0000(	ENSG0000(	133.1026	0.15302	0.294323	0.709428	lncRNA
ENSG0000(	SRP14P3	28.43088	-0.20902	0.294293	0.709428	processed_pseudogene
ENSG0000(	AKT2	3373.603	0.053717	0.294369	0.709478	protein_coding
ENSG0000(	IL12B	33.83515	-0.2261	0.294439	0.709522	protein_coding
ENSG0000(	KLF12	1466.437	-0.16378	0.294433	0.709522	protein_coding
ENSG0000(	ABCE1	780.8929	-0.12023	0.29467	0.709642	protein_coding
ENSG0000(	CHORDC1P	55.60085	-0.13349	0.294648	0.709642	processed_pseudogene
ENSG0000(	ENSG0000(	590.0232	-0.07974	0.294607	0.709642	lncRNA

ENSG0000(	LINC02603	40.96986	0.285464	0.294658	0.709642	lncRNA
ENSG0000(	ZBTB12	35.42681	-0.19885	0.294573	0.709642	protein_coding
ENSG0000(	ZCCHC3	356.153	-0.09595	0.294609	0.709642	protein_coding
ENSG0000(	ZNF486	493.7529	0.086506	0.294592	0.709642	protein_coding
ENSG0000(	ALK	33.18277	0.42802	0.294721	0.709674	protein_coding
ENSG0000(	ELAVL4	38.49614	-0.33284	0.294763	0.709674	protein_coding
ENSG0000(	ENSG0000(	70.40483	-0.16499	0.294816	0.709674	TEC
ENSG0000(	ENSG0000(	201.5296	0.132581	0.294773	0.709674	lncRNA
ENSG0000(	NATD1	303.3241	-0.11054	0.294797	0.709674	protein_coding
ENSG0000(	TDRD6	79.18881	0.27165	0.294838	0.709674	protein_coding
ENSG0000(	COX10	808.2852	0.130874	0.294886	0.709729	protein_coding
ENSG0000(	EGFEM1P	102.2454	0.202916	0.295181	0.710258	transcribed_unitary_pseudogene
ENSG0000(	ENSG0000(	250.8171	0.133276	0.295184	0.710258	lncRNA
ENSG0000(	PLPP2	1316.418	0.151066	0.29514	0.710258	protein_coding
ENSG0000(	CSDC2	41.70949	-0.36748	0.295234	0.710268	protein_coding
ENSG0000(	DDX18	1763.168	-0.07435	0.295239	0.710268	protein_coding
ENSG0000(	RNF139	494.2967	0.134089	0.295289	0.710324	protein_coding
ENSG0000(	GGA2	3395.229	-0.12336	0.295367	0.710451	protein_coding
ENSG0000(	GUCD1	1458.881	0.129379	0.295397	0.710462	protein_coding
ENSG0000(	ENSG0000(	703.5428	0.05731	0.295425	0.710466	TEC
ENSG0000(	FGGY	702.7025	0.13621	0.295453	0.71047	protein_coding
ENSG0000(	FDPSP3	99.37005	-0.23494	0.295498	0.710517	transcribed_processed_pseudoge
ENSG0000(	ANO8	385.3674	-0.08158	0.295675	0.710542	protein_coding
ENSG0000(	FAM102B	1523.954	0.088221	0.295559	0.710542	protein_coding
ENSG0000(	LINC01281	29.78807	-0.4455	0.29567	0.710542	lncRNA
ENSG0000(	MFN2	2352.866	0.096783	0.295715	0.710542	protein_coding
ENSG0000(	NT5C3AP2	75.56338	-0.2539	0.29571	0.710542	processed_pseudogene
ENSG0000(	RGP1	2348.062	0.092712	0.295648	0.710542	protein_coding
ENSG0000(	SLC24A3	196.7034	-0.17395	0.295625	0.710542	protein_coding
ENSG0000(	ZNF789	1480.055	0.07751	0.295646	0.710542	protein_coding
ENSG0000(	PCAT5	111.853	0.135998	0.295759	0.710586	lncRNA
ENSG0000(	LIPE	125.3726	0.181597	0.295818	0.710665	protein_coding
ENSG0000(	COP1	1290.714	0.07892	0.295885	0.710698	protein_coding
ENSG0000(	ENSG0000(	579.9817	0.538765	0.295878	0.710698	TEC
ENSG0000(	GRAPL	294.8512	-0.28756	0.295909	0.710698	protein_coding
ENSG0000(	GPATCH8	2345.098	-0.07137	0.29602	0.710903	protein_coding
ENSG0000(	PRIMPOL	224.0373	0.112411	0.296068	0.710956	protein_coding
ENSG0000(	CEP78	1364.837	-0.0985	0.296168	0.711134	protein_coding
ENSG0000(	ENSG0000(	1277.54	-0.07727	0.296207	0.711166	TEC
ENSG0000(	BCL2	3687.09	0.124643	0.296238	0.711179	protein_coding
ENSG0000(	SLC35G2	188.4333	-0.14838	0.296274	0.711203	protein_coding
ENSG0000(	KRT8P46	69.50342	0.207668	0.296322	0.711256	processed_pseudogene
ENSG0000(	ENSG0000(	180.5272	0.245788	0.296384	0.711285	lncRNA
ENSG0000(	LDHC	25.77801	0.264406	0.296412	0.711285	protein_coding
ENSG0000(	RPL35P3	25.12579	0.311453	0.296397	0.711285	processed_pseudogene
ENSG0000(	ENSG0000(	208.99	-0.12185	0.296481	0.711388	lncRNA
ENSG0000(	ENSG0000(	25.06641	0.329903	0.296532	0.711449	transcribed_unprocessed_pseudo

ENSG0000(	BIRC6-AS2	24.03021	0.24042	0.296722	0.711467	transcribed_unprocessed_pseudo
ENSG0000(	C2CD4D-AS	28.83173	-0.28113	0.296741	0.711467	lncRNA
ENSG0000(	CCNI2	199.5533	0.227071	0.297288	0.711467	protein_coding
ENSG0000(	CPEB2-DT	80.19906	0.193249	0.296826	0.711467	lncRNA
ENSG0000(	CTSW	123.4922	-0.15103	0.297402	0.711467	protein_coding
ENSG0000(	EFNB2	2156.328	0.184049	0.297082	0.711467	protein_coding
ENSG0000(	ENSG0000(	134.0605	0.152975	0.297036	0.711467	processed_pseudogene
ENSG0000(	ENSG0000(	247.177	0.132489	0.297431	0.711467	lncRNA
ENSG0000(	ENSG0000(	62.46834	-0.13738	0.296847	0.711467	processed_pseudogene
ENSG0000(	ENSG0000(	52.49652	-0.1789	0.297231	0.711467	lncRNA
ENSG0000(	ENSG0000(	287.9546	-0.08617	0.296848	0.711467	TEC
ENSG0000(	ENSG0000(	114.457	-0.1709	0.297276	0.711467	lncRNA
ENSG0000(	ENSG0000(	20.45263	-0.32346	0.297254	0.711467	lncRNA
ENSG0000(	ENSG0000(	136.3095	-0.22105	0.297261	0.711467	lncRNA
ENSG0000(	FGF10-AS1	20.39047	-0.45753	0.297403	0.711467	lncRNA
ENSG0000(	FRY-AS1	91.5103	-0.14981	0.297441	0.711467	lncRNA
ENSG0000(	IDI1	672.2156	-0.12247	0.297331	0.711467	protein_coding
ENSG0000(	IZUMO1	229.2282	-0.07997	0.297355	0.711467	protein_coding
ENSG0000(	LEKR1	122.4314	0.212396	0.297087	0.711467	protein_coding
ENSG0000(	LINC01852	45.73064	-0.23271	0.296977	0.711467	lncRNA
ENSG0000(	MBNL3	1558.425	0.131591	0.297024	0.711467	protein_coding
ENSG0000(	MEAK7	1382.844	0.095606	0.29678	0.711467	protein_coding
ENSG0000(	METTL27	69.55684	0.289324	0.297026	0.711467	protein_coding
ENSG0000(	MIR3150BH	103.5205	0.371116	0.297297	0.711467	lncRNA
ENSG0000(	NDUFB10	200.0277	0.202322	0.296939	0.711467	protein_coding
ENSG0000(	NDUFB8	534.395	0.100588	0.296954	0.711467	protein_coding
ENSG0000(	PTOV1	986.3949	-0.07419	0.297366	0.711467	protein_coding
ENSG0000(	RAPGEF5	2760.73	0.154038	0.296905	0.711467	protein_coding
ENSG0000(	SNX6	1036.392	0.082924	0.296566	0.711467	protein_coding
ENSG0000(	TMEM216	106.8503	0.151726	0.297444	0.711467	protein_coding
ENSG0000(	TOB2	1026.113	0.103445	0.296818	0.711467	protein_coding
ENSG0000(	TRAJ19	23.68307	-0.34335	0.296927	0.711467	TR_J_gene
ENSG0000(	ZIK1P1	265.8765	0.099663	0.296912	0.711467	processed_pseudogene
ENSG0000(	ZNF454	150.2626	0.127234	0.297079	0.711467	protein_coding
ENSG0000(	ZXDA	328.1266	0.113152	0.296899	0.711467	protein_coding
ENSG0000(	C17orf107	68.70345	-0.27051	0.297686	0.711803	protein_coding
ENSG0000(	ENSG0000(	172.7634	0.102158	0.297614	0.711803	lncRNA
ENSG0000(	KIF19	238.7786	-0.13638	0.297649	0.711803	protein_coding
ENSG0000(	NPY1R	174.6602	0.35746	0.297688	0.711803	protein_coding
ENSG0000(	LRRC29	76.86789	-0.17424	0.297722	0.71182	transcribed_unitary_pseudogene
ENSG0000(	ENSG0000(	25.88535	0.300943	0.2978	0.711946	lncRNA
ENSG0000(	CD163	1173.533	-0.12933	0.298044	0.712076	protein_coding
ENSG0000(	ENSG0000(	314.8144	-0.10043	0.297959	0.712076	lncRNA
ENSG0000(	ENSG0000(	44.61955	-0.27025	0.298113	0.712076	TEC
ENSG0000(	L3MBTL4-A	847.8542	-0.10997	0.298034	0.712076	lncRNA
ENSG0000(	LINC02392	82.59022	-0.14627	0.297938	0.712076	lncRNA
ENSG0000(	PSMC5	659.8882	-0.11752	0.297939	0.712076	protein_coding

ENSG0000(	SERF1B	444.5743	0.170701	0.298084	0.712076	protein_coding
ENSG0000(	TCEA2	124.907	-0.21081	0.298046	0.712076	protein_coding
ENSG0000(	TPRN	1576.572	0.267932	0.29808	0.712076	protein_coding
ENSG0000(	TRAPPC6B	1096.537	-0.07184	0.2981	0.712076	protein_coding
ENSG0000(	SPHK2	824.9595	0.124577	0.298142	0.712082	protein_coding
ENSG0000(	ENSG0000(	156.7763	0.110143	0.298191	0.712082	lncRNA
ENSG0000(	ISL1	27.70693	0.320332	0.298193	0.712082	protein_coding
ENSG0000(	LDHAL6CP	143.2796	0.128885	0.298303	0.71222	transcribed_processed_pseudoge
ENSG0000(	WASHC4	2411.343	-0.10549	0.298294	0.71222	protein_coding
ENSG0000(	ENSG0000(	51.54729	0.201742	0.298468	0.712244	lncRNA
ENSG0000(	KPNA6	2259.987	-0.05972	0.298339	0.712244	protein_coding
ENSG0000(	LYG1	36.11587	-0.24651	0.29845	0.712244	protein_coding
ENSG0000(	NIPBL-DT	1165.307	0.072196	0.298425	0.712244	lncRNA
ENSG0000(	PTPRH	2163.914	0.24507	0.298432	0.712244	protein_coding
ENSG0000(	ZRANB2	2556.602	0.093068	0.298416	0.712244	protein_coding
ENSG0000(	VCPIP1	2347.583	-0.05866	0.298498	0.712253	protein_coding
ENSG0000(	CC2D2A	990.775	-0.07074	0.298596	0.712309	protein_coding
ENSG0000(	CTNNA2	124.81	0.152154	0.298563	0.712309	protein_coding
ENSG0000(	ENSG0000(	31.46326	-0.2897	0.298599	0.712309	lncRNA
ENSG0000(	TXNIP	10477.27	0.138631	0.298625	0.712309	protein_coding
ENSG0000(	COL4A4	714.7897	-0.15932	0.298834	0.712372	protein_coding
ENSG0000(	ENSG0000(	160.5956	-0.13067	0.298736	0.712372	lncRNA
ENSG0000(	ENSG0000(	185.8595	0.083668	0.298765	0.712372	lncRNA
ENSG0000(	MIR600HG	1248.295	0.112165	0.29875	0.712372	lncRNA
ENSG0000(	PIGK	258.2437	-0.1066	0.298824	0.712372	protein_coding
ENSG0000(	SOWAHCP5	114.7629	0.350139	0.298858	0.712372	processed_pseudogene
ENSG0000(	SPECC1L-AI	372.0537	-0.1553	0.298746	0.712372	protein_coding
ENSG0000(	TRAJ38	22.41925	-0.37934	0.298855	0.712372	TR_J_gene
ENSG0000(	ANKRD50	1672.383	-0.10756	0.298898	0.712394	protein_coding
ENSG0000(	IL17RE	882.4469	0.179185	0.29892	0.712394	protein_coding
ENSG0000(	ENSG0000(	748.5284	0.068295	0.29898	0.712413	lncRNA
ENSG0000(	KIAA1614-/	66.7945	-0.15044	0.298961	0.712413	lncRNA
ENSG0000(	APTX	1758.702	0.049965	0.299205	0.712611	protein_coding
ENSG0000(	COL17A1	2264.92	0.247796	0.29919	0.712611	protein_coding
ENSG0000(	ENSG0000(	166.8653	0.125959	0.299219	0.712611	lncRNA
ENSG0000(	ENSG0000(	134.1454	-0.12876	0.299233	0.712611	lncRNA
ENSG0000(	ENSG0000(	272.6036	-0.11184	0.29914	0.712611	lncRNA
ENSG0000(	GCC2-AS1	19.78101	-0.27407	0.299244	0.712611	lncRNA
ENSG0000(	RSAD1	682.359	0.115863	0.299184	0.712611	protein_coding
ENSG0000(	ALG1	1603.453	0.040713	0.299445	0.712659	protein_coding
ENSG0000(	ECSCR	38.92903	-0.41496	0.299397	0.712659	protein_coding
ENSG0000(	ENSG0000(	544.3999	0.147191	0.299432	0.712659	lncRNA
ENSG0000(	ENSG0000(	45.85279	-0.42206	0.29935	0.712659	unprocessed_pseudogene
ENSG0000(	H4C4	113.443	-0.20699	0.299413	0.712659	protein_coding
ENSG0000(	NIBAN3	1728.875	-0.1398	0.299317	0.712659	protein_coding
ENSG0000(	SH3RF2	2949.576	0.221959	0.299441	0.712659	protein_coding
ENSG0000(	EDNRB-AS1	210.0561	0.143669	0.299531	0.712677	lncRNA

ENSG0000(	ENSG0000(	958.1409	-0.07321	0.29948	0.712677	lncRNA
ENSG0000(	WDR13	1650.236	0.093534	0.299512	0.712677	protein_coding
ENSG0000(	ENSG0000(	233.7866	0.120763	0.299568	0.712688	lncRNA
ENSG0000(	LINC02666	19.753	0.315972	0.299588	0.712688	lncRNA
ENSG0000(	SNUPN	484.3216	0.073537	0.299613	0.712688	protein_coding
ENSG0000(	ENSG0000(	445.8114	-0.09437	0.29966	0.712699	lncRNA
ENSG0000(	ENSG0000(	1083.671	-0.06607	0.299669	0.712699	lncRNA
ENSG0000(	ENSG0000(	88.15433	-0.14511	0.299808	0.712888	lncRNA
ENSG0000(	TAF1	2924.007	0.060958	0.299805	0.712888	protein_coding
ENSG0000(	ZNF577	1744.699	0.063387	0.299826	0.712888	protein_coding
ENSG0000(	ENSG0000(	180.1719	-0.10571	0.299853	0.712889	lncRNA
ENSG0000(	ENSG0000(	100.5937	-0.14415	0.299942	0.713039	lncRNA
ENSG0000(	DLGAP4	1504.111	-0.09122	0.300023	0.713047	protein_coding
ENSG0000(	ENSG0000(	27.644	-0.21426	0.299979	0.713047	lncRNA
ENSG0000(	HADHA	2196.677	0.11565	0.300018	0.713047	protein_coding
ENSG0000(	CFAP20	113.6921	-0.17082	0.300275	0.71332	protein_coding
ENSG0000(	DTHD1	158.7915	-0.36385	0.300316	0.71332	protein_coding
ENSG0000(	ENSG0000(	20.16413	0.361922	0.300319	0.71332	lncRNA
ENSG0000(	MED15	2766.06	-0.07911	0.300254	0.71332	protein_coding
ENSG0000(	MTBP	419.4959	0.084318	0.300191	0.71332	protein_coding
ENSG0000(	RBM22	668.4112	-0.06542	0.300193	0.71332	protein_coding
ENSG0000(	SMIM15	690.5086	-0.11322	0.300319	0.71332	protein_coding
ENSG0000(	DEDD2	494.222	0.124925	0.300497	0.713393	protein_coding
ENSG0000(	ENSG0000(	109.4752	0.140769	0.300399	0.713393	lncRNA
ENSG0000(	ENSG0000(	47.3688	0.182408	0.300505	0.713393	lncRNA
ENSG0000(	GGT7	368.6669	0.122323	0.30041	0.713393	protein_coding
ENSG0000(	IGLON5	53.78663	-0.19801	0.30046	0.713393	protein_coding
ENSG0000(	MPV17L	1417.478	0.073135	0.300466	0.713393	protein_coding
ENSG0000(	ANO6	889.1492	-0.1621	0.300679	0.713515	protein_coding
ENSG0000(	BSN	120.6323	0.246784	0.300686	0.713515	protein_coding
ENSG0000(	CLN3	2934.918	0.089426	0.300627	0.713515	protein_coding
ENSG0000(	ENSG0000(	61.40655	0.141747	0.300733	0.713515	lncRNA
ENSG0000(	ENSG0000(	74.64198	0.147363	0.300608	0.713515	lncRNA
ENSG0000(	ENSG0000(	22.00345	-0.25848	0.300738	0.713515	lncRNA
ENSG0000(	SRD5A1	1999.197	-0.073	0.300695	0.713515	protein_coding
ENSG0000(	BTBD3	1684.238	0.195713	0.300833	0.713555	protein_coding
ENSG0000(	CDK1	227.3337	-0.16797	0.300832	0.713555	protein_coding
ENSG0000(	DYNC2H1	1159.439	-0.20074	0.30079	0.713555	protein_coding
ENSG0000(	LINC02338	239.4158	0.101322	0.300945	0.713702	lncRNA
ENSG0000(	SCRN1	532.684	-0.17466	0.300947	0.713702	protein_coding
ENSG0000(	RPS3AP30	31.6509	0.295613	0.300978	0.713714	processed_pseudogene
ENSG0000(	ENSG0000(	139.4463	0.275965	0.30107	0.713866	lncRNA
ENSG0000(	RN7SL334F	23.63449	-0.3014	0.301094	0.713866	misc_RNA
ENSG0000(	GTF2H5	701.9003	0.121787	0.301155	0.713948	protein_coding
ENSG0000(	ELAVL3	94.51459	-0.23343	0.301335	0.714251	protein_coding
ENSG0000(	ENSG0000(	149.3603	0.140819	0.301316	0.714251	TEC
ENSG0000(	MBNL1-AS:	214.3724	0.154039	0.301363	0.714258	lncRNA

ENSG0000(	ENSG0000(	132.392	0.194716	0.301587	0.714537	lncRNA
ENSG0000(	ENSG0000(	649.0779	-0.06002	0.301575	0.714537	lncRNA
ENSG0000(	FBXL19-AS:	602.0285	0.08271	0.301603	0.714537	lncRNA
ENSG0000(	OXSM	150.5873	0.162753	0.301611	0.714537	protein_coding
ENSG0000(	REXO1	884.0939	0.124915	0.301537	0.714537	protein_coding
ENSG0000(	PPA2	943.8997	0.080599	0.301665	0.714604	protein_coding
ENSG0000(	LINC01215	439.5798	-0.14339	0.301723	0.71468	lncRNA
ENSG0000(	C18orf21	103.5103	-0.15798	0.302009	0.714948	protein_coding
ENSG0000(	DIRC3	287.211	-0.18573	0.302067	0.714948	lncRNA
ENSG0000(	DISC1-IT1	381.391	0.122725	0.30207	0.714948	lncRNA
ENSG0000(	ENSG0000(	614.3845	-0.07121	0.302058	0.714948	lncRNA
ENSG0000(	EPB41L5	1063.818	0.195933	0.301961	0.714948	protein_coding
ENSG0000(	LINC02890	61.77885	0.182912	0.302016	0.714948	lncRNA
ENSG0000(	NUDT16L1	197.0953	0.12698	0.301989	0.714948	protein_coding
ENSG0000(	PEBP1P2	20.78526	0.353418	0.301913	0.714948	processed_pseudogene
ENSG0000(	TYW1	533.5153	0.129698	0.30187	0.714948	protein_coding
ENSG0000(	ENSG0000(	153.4246	-0.11113	0.302252	0.715071	lncRNA
ENSG0000(	LINC00571	98.76049	-0.13714	0.302212	0.715071	lncRNA
ENSG0000(	NPM1P3	22.25194	0.477464	0.302152	0.715071	processed_pseudogene
ENSG0000(	PRPF4	479.6825	0.085602	0.302229	0.715071	protein_coding
ENSG0000(	SH3BGRL2	2698.539	0.230148	0.30223	0.715071	protein_coding
ENSG0000(	ENSG0000(	105.0639	0.120725	0.302391	0.715215	lncRNA
ENSG0000(	MSX1	52.66276	0.237479	0.302386	0.715215	protein_coding
ENSG0000(	UNC5D	62.44692	0.330929	0.30239	0.715215	protein_coding
ENSG0000(	MAP3K10	435.8155	-0.1075	0.302474	0.715226	protein_coding
ENSG0000(	RYR2	373.338	-0.40436	0.302456	0.715226	protein_coding
ENSG0000(	ZBTB21	679.7785	0.129463	0.302444	0.715226	protein_coding
ENSG0000(	ZNF767P	1354.689	-0.07721	0.3025	0.715226	transcribed_unprocessed_pseudo
ENSG0000(	ABHD14A	141.8177	-0.12973	0.302617	0.715246	protein_coding
ENSG0000(	AQP3	153.6197	-0.23341	0.302575	0.715246	protein_coding
ENSG0000(	DENND4B	1623.84	-0.09824	0.302657	0.715246	protein_coding
ENSG0000(	ENSG0000(	50.05703	0.212723	0.302537	0.715246	TEC
ENSG0000(	LRRD1	228.8863	0.094513	0.302661	0.715246	protein_coding
ENSG0000(	NDUFB7	127.4693	0.208113	0.302713	0.715246	protein_coding
ENSG0000(	RNASEL	1035.302	0.100564	0.302681	0.715246	protein_coding
ENSG0000(	SH3GL1P1	337.4029	-0.14575	0.302735	0.715246	transcribed_processed_pseudoge
ENSG0000(	YIPF1	296.3938	-0.0907	0.302742	0.715246	protein_coding
ENSG0000(	ASB8	705.2839	-0.07962	0.302912	0.715341	protein_coding
ENSG0000(	CLEC2B	374.3422	-0.20624	0.302899	0.715341	protein_coding
ENSG0000(	CLIP3	219.0995	-0.15811	0.302848	0.715341	protein_coding
ENSG0000(	FITM1	42.8975	0.216685	0.302909	0.715341	protein_coding
ENSG0000(	KPNA2	930.4086	-0.08751	0.302899	0.715341	protein_coding
ENSG0000(	ZNF358	176.3036	0.16573	0.302979	0.715438	protein_coding
ENSG0000(	ENSG0000(	225.3482	0.073084	0.303119	0.715553	lncRNA
ENSG0000(	H4C14	240.4736	-0.27406	0.303088	0.715553	protein_coding
ENSG0000(	SATL1	644.2674	0.115902	0.303056	0.715553	protein_coding
ENSG0000(	ZAP70	576.219	-0.2505	0.303132	0.715553	protein_coding

ENSG0000(	GCFC2	419.4947	-0.12139	0.303226	0.715712	protein_coding
ENSG0000(	MIR4537	159.8558	-0.28429	0.303329	0.715833	miRNA
ENSG0000(	MRPS14	468.0827	-0.07509	0.303316	0.715833	protein_coding
ENSG0000(	IMPDH1P8	23.08838	0.263889	0.303387	0.71591	processed_pseudogene
ENSG0000(	HNRNPR	3192.712	-0.09592	0.30344	0.715914	protein_coding
ENSG0000(	LUCAT1	2078.698	0.203765	0.303441	0.715914	lncRNA
ENSG0000(	ENSG0000(	812.322	0.081996	0.303578	0.715953	TEC
ENSG0000(	H2AZ1-DT	209.5555	0.083785	0.303588	0.715953	lncRNA
ENSG0000(	LINC00624	41.49769	0.21289	0.303493	0.715953	lncRNA
ENSG0000(	SLC25A34	485.1993	0.165306	0.30353	0.715953	protein_coding
ENSG0000(	ZNF286A	706.7862	-0.11775	0.303584	0.715953	protein_coding
ENSG0000(	ANK2-AS1	61.5301	0.138247	0.303719	0.71617	lncRNA
ENSG0000(	BCL7C	307.1983	0.09419	0.303765	0.71617	protein_coding
ENSG0000(	CTPS2	842.0488	0.089873	0.303807	0.71617	protein_coding
ENSG0000(	ENSG0000(	102.6175	-0.14909	0.303833	0.71617	lncRNA
ENSG0000(	ENSG0000(	79.15186	0.16049	0.303836	0.71617	lncRNA
ENSG0000(	RRP7BP	857.1286	0.121579	0.303747	0.71617	transcribed_unprocessed_pseudo
ENSG0000(	CERKL	112.715	0.150166	0.303879	0.716188	protein_coding
ENSG0000(	TASOR2	3760.21	0.103192	0.303896	0.716188	protein_coding
ENSG0000(	ATAD3A	219.9014	-0.19073	0.304027	0.716189	protein_coding
ENSG0000(	ENSG0000(	36.88247	-0.25941	0.304032	0.716189	lncRNA
ENSG0000(	ENSG0000(	37.96065	-0.40357	0.303976	0.716189	TEC
ENSG0000(	ENSG0000(	97.47638	0.123548	0.303953	0.716189	lncRNA
ENSG0000(	LINC01460	149.7027	-0.0924	0.304039	0.716189	lncRNA
ENSG0000(	TOR1AIP1	909.8755	-0.08651	0.304052	0.716189	protein_coding
ENSG0000(	ENSG0000(	55.52898	-0.21359	0.304188	0.716392	lncRNA
ENSG0000(	NUP214	2707.8	0.058872	0.30419	0.716392	protein_coding
ENSG0000(	ENSG0000(	371.6381	-0.09688	0.304333	0.716665	TEC
ENSG0000(	BACH2	718.1926	-0.40824	0.304409	0.716723	protein_coding
ENSG0000(	HYAL4	82.59747	0.174326	0.304389	0.716723	protein_coding
ENSG0000(	CD226	985.0459	-0.16242	0.304603	0.716794	protein_coding
ENSG0000(	CFAP157	135.9543	-0.12001	0.30458	0.716794	protein_coding
ENSG0000(	EHF	8611.364	0.19298	0.304576	0.716794	protein_coding
ENSG0000(	MIR8075	81.98001	0.127453	0.304501	0.716794	miRNA
ENSG0000(	NUDCD2	536.5713	-0.09699	0.304533	0.716794	protein_coding
ENSG0000(	PCDHB12	20.28716	-0.25741	0.304635	0.716794	protein_coding
ENSG0000(	TRABD	641.0704	0.097384	0.304635	0.716794	protein_coding
ENSG0000(	ZSCAN10	21.12709	0.296813	0.304648	0.716794	protein_coding
ENSG0000(	ATP6AP1	1241.836	-0.10283	0.304813	0.716845	protein_coding
ENSG0000(	ENSG0000(	71.92196	0.133258	0.304764	0.716845	lncRNA
ENSG0000(	FBXO42	1271.581	-0.05345	0.304826	0.716845	protein_coding
ENSG0000(	PAQR8	2375.923	0.214134	0.304747	0.716845	protein_coding
ENSG0000(	RGL4	61.61495	-0.39719	0.304801	0.716845	protein_coding
ENSG0000(	ZNF724	446.1814	0.105041	0.30471	0.716845	protein_coding
ENSG0000(	SERINC2	2102.82	0.219296	0.304875	0.716898	protein_coding
ENSG0000(	ENTPD6	2247.745	0.137392	0.305096	0.717204	protein_coding
ENSG0000(	LINC00886	238.2846	0.123462	0.305123	0.717204	lncRNA

ENSG0000(	PLEKHG7	211.0004	0.208628	0.305053	0.717204	protein_coding
ENSG0000(	RAB29	965.8817	-0.09107	0.305116	0.717204	protein_coding
ENSG0000(	ZNF217	3797.877	0.140663	0.305135	0.717204	protein_coding
ENSG0000(	ACAA1	1467.764	0.132668	0.30558	0.717344	protein_coding
ENSG0000(	ENSG0000(	47.14054	0.20609	0.305605	0.717344	lncRNA
ENSG0000(	ENSG0000(	277.0124	0.097293	0.305466	0.717344	lncRNA
ENSG0000(	ENSG0000(	30.12482	-0.21626	0.305679	0.717344	lncRNA
ENSG0000(	ENSG0000(	108.2859	-0.15415	0.305633	0.717344	lncRNA
ENSG0000(	ENSG0000(	32.91281	-0.24738	0.30569	0.717344	lncRNA
ENSG0000(	ENSG0000(	27.71921	-0.1984	0.305321	0.717344	lncRNA
ENSG0000(	GGT6	1516.85	0.212107	0.305616	0.717344	protein_coding
ENSG0000(	KANK2	1502.797	-0.08368	0.305501	0.717344	protein_coding
ENSG0000(	KAZN	663.7905	0.136125	0.30526	0.717344	protein_coding
ENSG0000(	MYCBP2-A!	52.25892	-0.16897	0.305347	0.717344	lncRNA
ENSG0000(	NUCKS1	2841.04	-0.10011	0.305655	0.717344	protein_coding
ENSG0000(	SETDB1	1583.339	-0.05096	0.305689	0.717344	protein_coding
ENSG0000(	SNORA26	109.4472	-0.17245	0.305566	0.717344	snoRNA
ENSG0000(	SNORA8	304.1141	-0.20581	0.305411	0.717344	snoRNA
ENSG0000(	TEDC2	29.57159	-0.36472	0.305626	0.717344	protein_coding
ENSG0000(	TPM3P9	721.4837	-0.06803	0.305584	0.717344	transcribed_processed_pseudoge
ENSG0000(	ZNF776	1230.673	0.109709	0.305317	0.717344	protein_coding
ENSG0000(	ZNNT1	389.521	-0.11776	0.305463	0.717344	lncRNA
ENSG0000(	ENSG0000(	121.7381	0.140071	0.305733	0.717384	lncRNA
ENSG0000(	ENSG0000(	21.50512	-0.33766	0.305779	0.717431	lncRNA
ENSG0000(	MYL5	454.4291	0.17636	0.305813	0.71745	protein_coding
ENSG0000(	RABL2B	1403.878	-0.07102	0.305855	0.717487	protein_coding
ENSG0000(	PHKB	3405.235	-0.05148	0.305886	0.717498	protein_coding
ENSG0000(	C11orf86	86.78695	0.416617	0.305949	0.7175	protein_coding
ENSG0000(	DONSON	339.2376	0.150641	0.306101	0.7175	protein_coding
ENSG0000(	ENSG0000(	37.88767	-0.26184	0.306185	0.7175	lncRNA
ENSG0000(	ENSG0000(	113.5053	-0.20062	0.306132	0.7175	lncRNA
ENSG0000(	ENSG0000(	29.63682	0.228333	0.306202	0.7175	TEC
ENSG0000(	ENSG0000(	101.9225	-0.12113	0.306041	0.7175	lncRNA
ENSG0000(	NUDT21	1489.602	0.071721	0.306226	0.7175	protein_coding
ENSG0000(	OR5H14	257.5861	-0.0969	0.306169	0.7175	protein_coding
ENSG0000(	PRELID3A	83.74434	-0.16718	0.306177	0.7175	protein_coding
ENSG0000(	PTPN6	1024.709	-0.1496	0.306189	0.7175	protein_coding
ENSG0000(	TNFAIP8L3	275.4678	0.098596	0.306131	0.7175	protein_coding
ENSG0000(	UGP2	2751.819	0.157118	0.306134	0.7175	protein_coding
ENSG0000(	USP25	1848.907	0.083292	0.306006	0.7175	protein_coding
ENSG0000(	ENSG0000(	83.68611	-0.17039	0.306273	0.717551	lncRNA
ENSG0000(	SNX29	2010.828	-0.1046	0.306316	0.717589	protein_coding
ENSG0000(	EIF2S2P1	118.203	0.215017	0.306484	0.717637	processed_pseudogene
ENSG0000(	ENSG0000(	246.6869	-0.10773	0.306519	0.717637	lncRNA
ENSG0000(	ENSG0000(	235.5003	0.128523	0.306433	0.717637	lncRNA
ENSG0000(	HMGB1P8	138.2478	0.114828	0.306384	0.717637	processed_pseudogene
ENSG0000(	OR55B1P	306.9776	-0.13832	0.30646	0.717637	transcribed_unitary_pseudogene

ENSG0000(	PSMG3-AS:	269.2241	0.139757	0.306514	0.717637	lncRNA
ENSG0000(	TMEM268	737.9396	-0.12252	0.306425	0.717637	protein_coding
ENSG0000(	ENSG0000(	32.86863	0.274679	0.306579	0.717716	lncRNA
ENSG0000(	ADSL	2460.048	0.076234	0.306731	0.717798	protein_coding
ENSG0000(	ANKRD40C	1078.384	0.161293	0.306697	0.717798	protein_coding
ENSG0000(	HOXA1	46.83257	-0.22845	0.306702	0.717798	protein_coding
ENSG0000(	LINC02766	127.9501	-0.10249	0.306744	0.717798	lncRNA
ENSG0000(	SLC4A10	190.5452	0.247857	0.306691	0.717798	protein_coding
ENSG0000(	CPT1A	4233.934	0.18728	0.306816	0.717832	protein_coding
ENSG0000(	ENSG0000(	45.44961	0.202744	0.306846	0.717832	lncRNA
ENSG0000(	FGD6	2522.644	-0.12722	0.306789	0.717832	protein_coding
ENSG0000(	FKBP5	3794.068	-0.29719	0.306915	0.717832	protein_coding
ENSG0000(	HOOK2	1328.433	0.121981	0.306869	0.717832	protein_coding
ENSG0000(	LINC02428	212.7571	0.104386	0.306892	0.717832	lncRNA
ENSG0000(	ARL4AP2	20.26503	-0.37576	0.30707	0.718082	processed_pseudogene
ENSG0000(	CBLL1	605.3688	-0.08022	0.307122	0.718082	protein_coding
ENSG0000(	ENSG0000(	225.6621	0.124506	0.307205	0.718082	transcribed_unprocessed_pseudo
ENSG0000(	ENSG0000(	748.3763	0.070743	0.307192	0.718082	lncRNA
ENSG0000(	ENSG0000(	22.5412	-0.39929	0.307077	0.718082	lncRNA
ENSG0000(	IL11RA	467.1936	0.108662	0.307189	0.718082	protein_coding
ENSG0000(	PCK1	2955.481	0.489313	0.307189	0.718082	protein_coding
ENSG0000(	AHCTF1P1	19.80872	0.526266	0.307242	0.718108	processed_pseudogene
ENSG0000(	C10orf53	579.6002	0.171367	0.30734	0.71815	protein_coding
ENSG0000(	GIPC3	81.93574	-0.22392	0.307365	0.71815	protein_coding
ENSG0000(	STK35	1003.58	0.068745	0.307289	0.71815	protein_coding
ENSG0000(	ZNF232-AS	112.5364	-0.15937	0.307343	0.71815	lncRNA
ENSG0000(	PCDHB14	91.71958	0.198908	0.307418	0.718152	protein_coding
ENSG0000(	USH1G	23.1581	0.341567	0.307398	0.718152	protein_coding
ENSG0000(	TOPORS	541.9658	-0.10151	0.307447	0.71816	protein_coding
ENSG0000(	A1BG-AS1	255.2496	-0.10665	0.3075	0.718222	lncRNA
ENSG0000(	LINC01902	879.4544	0.081145	0.307537	0.718248	lncRNA
ENSG0000(	ENSG0000(	1516.648	-0.06123	0.307762	0.71859	TEC
ENSG0000(	ILF3-DT	753.0829	0.058128	0.307758	0.71859	lncRNA
ENSG0000(	ZNF214	132.4903	0.197537	0.307729	0.71859	protein_coding
ENSG0000(	C10orf55	20.66378	-0.29116	0.307958	0.718623	lncRNA
ENSG0000(	CUL7	786.2469	0.100134	0.30793	0.718623	protein_coding
ENSG0000(	DMPK	1252.482	0.071837	0.307989	0.718623	protein_coding
ENSG0000(	EDC3	773.1473	0.095672	0.30791	0.718623	protein_coding
ENSG0000(	ENSG0000(	150.7631	-0.09366	0.307988	0.718623	lncRNA
ENSG0000(	ENSG0000(	44.70608	0.201984	0.308011	0.718623	lncRNA
ENSG0000(	GPM6B	95.64598	-0.30651	0.307803	0.718623	protein_coding
ENSG0000(	PPFIBP2	1623.027	0.09509	0.307965	0.718623	protein_coding
ENSG0000(	SV2C	245.277	0.109282	0.307966	0.718623	protein_coding
ENSG0000(	UHMK1	3038.745	-0.07391	0.3081	0.71877	protein_coding
ENSG0000(	ENSG0000(	497.9626	-0.06816	0.308203	0.718792	lncRNA
ENSG0000(	POTED	82.05084	0.177797	0.308193	0.718792	protein_coding
ENSG0000(	SAMD8	1652.856	-0.07921	0.308214	0.718792	protein_coding

ENSG0000(	ZMAT2	296.0362	-0.12025	0.308204	0.718792	protein_coding
ENSG0000(	CNTN3	614.8547	0.334772	0.308338	0.718958	protein_coding
ENSG0000(	SPRING1	1623.737	0.119445	0.308323	0.718958	protein_coding
ENSG0000(	ENSG0000(	86.60501	0.121136	0.308364	0.718959	lncRNA
ENSG0000(	ENSG0000(	2535.265	-0.08362	0.308434	0.719007	lncRNA
ENSG0000(	LINC00310	299.1236	-0.11404	0.308437	0.719007	lncRNA
ENSG0000(	FN3K	535.4681	0.108642	0.308528	0.719121	protein_coding
ENSG0000(	RPS4XP7	32.40361	0.468382	0.308538	0.719121	processed_pseudogene
ENSG0000(	CAGE1	86.03097	0.146919	0.308571	0.719136	protein_coding
ENSG0000(	MIS18A	85.4611	-0.1797	0.308618	0.719186	protein_coding
ENSG0000(	AP1M1	3324.411	-0.08473	0.308689	0.719255	protein_coding
ENSG0000(	PRRG1	443.575	-0.08845	0.3087	0.719255	protein_coding
ENSG0000(	CEP250	1980.783	0.110742	0.308828	0.719396	protein_coding
ENSG0000(	ENSG0000(	32.17314	0.200904	0.308839	0.719396	lncRNA
ENSG0000(	NEDD4	6884.548	0.141989	0.308796	0.719396	protein_coding
ENSG0000(	SPTLC1	1861.053	-0.06418	0.308865	0.719396	protein_coding
ENSG0000(	RPS3AP37	19.82636	-0.31678	0.308917	0.719456	processed_pseudogene
ENSG0000(	NDE1P2	98.81839	0.144937	0.308965	0.719507	processed_pseudogene
ENSG0000(	ENSG0000(	202.6395	0.107646	0.309047	0.719514	lncRNA
ENSG0000(	MISP3	231.3851	0.237072	0.30902	0.719514	protein_coding
ENSG0000(	TMEM238L	466.5186	0.275631	0.309031	0.719514	protein_coding
ENSG0000(	GPATCH4	316.3282	0.134226	0.30911	0.7196	protein_coding
ENSG0000(	FGFR4	546.0786	0.210417	0.309217	0.719605	protein_coding
ENSG0000(	MRPS30-DT	339.5707	-0.09526	0.309166	0.719605	lncRNA
ENSG0000(	PRKRIP1	727.0471	-0.07974	0.30921	0.719605	protein_coding
ENSG0000(	YBEY	191.8601	0.157821	0.309201	0.719605	protein_coding
ENSG0000(	CLEC4F	63.80782	0.357309	0.309259	0.719642	protein_coding
ENSG0000(	MAP1LC3C	35.07343	-0.30523	0.309286	0.719646	protein_coding
ENSG0000(	PGAP6	995.4988	0.175979	0.309358	0.71975	protein_coding
ENSG0000(	ENSG0000(	89.1329	0.12052	0.309415	0.719823	lncRNA
ENSG0000(	TARDBP	3071.745	0.060873	0.309455	0.719856	protein_coding
ENSG0000(	LINC02620	23.89546	0.227586	0.309528	0.719963	lncRNA
ENSG0000(	KRT18P61	20.674	0.285549	0.309567	0.719995	processed_pseudogene
ENSG0000(	ENSG0000(	551.167	-0.09185	0.309644	0.720051	lncRNA
ENSG0000(	PFKP	1563.004	-0.13486	0.309626	0.720051	protein_coding
ENSG0000(	DNAH10OS	279.4321	-0.08904	0.30971	0.720143	lncRNA
ENSG0000(	ENSG0000(	157.6175	0.122576	0.309822	0.720224	lncRNA
ENSG0000(	PLA2G6	678.3565	0.083168	0.30981	0.720224	protein_coding
ENSG0000(	ZNF442	587.2161	0.102375	0.309823	0.720224	protein_coding
ENSG0000(	ENSG0000(	324.2516	-0.11872	0.309912	0.72037	lncRNA
ENSG0000(	MEP1B	122.7396	0.42669	0.30998	0.720467	protein_coding
ENSG0000(	ABCG2	614.0614	0.487322	0.310097	0.720568	protein_coding
ENSG0000(	CMC4	31.5223	-0.46158	0.310226	0.720568	protein_coding
ENSG0000(	CRYZL2P-SE	670.5772	0.166123	0.310181	0.720568	lncRNA
ENSG0000(	DNAJB6P7	174.0573	0.114798	0.310068	0.720568	processed_pseudogene
ENSG0000(	ENSG0000(	30.11353	-0.27846	0.310195	0.720568	lncRNA
ENSG0000(	ENSG0000(	46.75637	-0.23098	0.310229	0.720568	lncRNA

ENSG0000(	ENSG0000(	189.612	0.096231	0.310233	0.720568	lncRNA
ENSG0000(	ZDHHC14	989.4385	0.07718	0.31019	0.720568	protein_coding
ENSG0000(	CA5A	20.7088	0.23827	0.310411	0.720897	protein_coding
ENSG0000(	KDM4C	2982.135	0.068285	0.310427	0.720897	protein_coding
ENSG0000(	ENSG0000(	36.061	0.230237	0.310507	0.721022	lncRNA
ENSG0000(	ENSG0000(	22.23186	0.244586	0.31061	0.721139	lncRNA
ENSG0000(	MAPKAP1	1217.768	-0.05569	0.310607	0.721139	protein_coding
ENSG0000(	ENSG0000(	97.17469	0.116156	0.310699	0.721284	processed_pseudogene
ENSG0000(	VILL	1598.87	0.185738	0.310822	0.721509	protein_coding
ENSG0000(	ENSG0000(	29.98014	0.27193	0.311055	0.721745	processed_pseudogene
ENSG0000(	ENSG0000(	118.465	0.165788	0.311033	0.721745	TEC
ENSG0000(	ENSG0000(	21.28297	0.274388	0.310996	0.721745	lncRNA
ENSG0000(	ENSG0000(	274.9874	0.119492	0.311023	0.721745	lncRNA
ENSG0000(	FBLN2	203.2371	-0.43924	0.311044	0.721745	protein_coding
ENSG0000(	CSTB	779.4378	-0.19671	0.311103	0.721764	protein_coding
ENSG0000(	IGHV3-64	92.33197	-1.5733	0.311141	0.721764	IG_V_gene
ENSG0000(	MAP3K11	2472.825	0.071544	0.311116	0.721764	protein_coding
ENSG0000(	CA14	211.9634	0.106548	0.311261	0.721904	protein_coding
ENSG0000(	DESI2	907.892	-0.08105	0.311253	0.721904	protein_coding
ENSG0000(	RNASEH2B	3758.626	-0.098	0.31128	0.721904	protein_coding
ENSG0000(	ENSG0000(	56.28067	-0.26417	0.311391	0.72204	TEC
ENSG0000(	ENSG0000(	44.11232	0.196988	0.311384	0.72204	lncRNA
ENSG0000(	ENSG0000(	64.84661	0.170684	0.311418	0.72204	lncRNA
ENSG0000(	ENSG0000(	116.9301	0.121846	0.311553	0.722143	lncRNA
ENSG0000(	HLCS	882.6775	0.109282	0.31156	0.722143	protein_coding
ENSG0000(	MIR200CH(	85.37119	0.280833	0.311567	0.722143	lncRNA
ENSG0000(	RPS29P14	23.02312	-0.31188	0.311563	0.722143	processed_pseudogene
ENSG0000(	ENSG0000(	670.3602	0.116905	0.31164	0.72217	lncRNA
ENSG0000(	ENSG0000(	41.6442	0.26991	0.311841	0.72217	lncRNA
ENSG0000(	GNRH2	22.75534	0.304867	0.311825	0.72217	protein_coding
ENSG0000(	LINC00934	71.23676	0.143641	0.311795	0.72217	lncRNA
ENSG0000(	REST	1886.868	0.057928	0.311637	0.72217	protein_coding
ENSG0000(	SLC7A6OS	949.3428	-0.08164	0.311776	0.72217	protein_coding
ENSG0000(	TXNL4B	319.8748	0.091423	0.311822	0.72217	protein_coding
ENSG0000(	ULK2	626.4317	0.144163	0.311803	0.72217	protein_coding
ENSG0000(	VPS35	1927.54	0.066501	0.311773	0.72217	protein_coding
ENSG0000(	ZDHHC20-I	67.87108	-0.27351	0.311764	0.72217	lncRNA
ENSG0000(	RPP14	1755.736	0.093062	0.311903	0.722253	protein_coding
ENSG0000(	ENSG0000(	70.04806	0.178166	0.311983	0.722273	lncRNA
ENSG0000(	RIN1	276.5084	-0.12923	0.311991	0.722273	protein_coding
ENSG0000(	TCEA1P2	60.01641	0.292444	0.311968	0.722273	processed_pseudogene
ENSG0000(	ZNF154	1183.923	-0.13201	0.312018	0.722275	protein_coding
ENSG0000(	OR5A1	843.4132	-0.08344	0.312078	0.722293	protein_coding
ENSG0000(	TRIP12	5892.641	-0.05937	0.312073	0.722293	protein_coding
ENSG0000(	ENSG0000(	245.2488	-0.10835	0.312123	0.722335	lncRNA
ENSG0000(	GOLGA2P7	1767.387	0.168707	0.312165	0.722373	transcribed_unprocessed_pseudo
ENSG0000(	ENSG0000(	20.70803	0.334984	0.312221	0.722442	lncRNA

ENSG0000(	PDCD6-DT	29.69235	-0.23046	0.312278	0.722511	lncRNA
ENSG0000(	ARRB1	2346.018	0.089754	0.31237	0.722543	protein_coding
ENSG0000(	HAR1B	117.831	0.123134	0.312368	0.722543	lncRNA
ENSG0000(	ZC3H12C	2032.808	0.135908	0.312365	0.722543	protein_coding
ENSG0000(	CDC25C	59.76965	-0.19571	0.312398	0.722547	protein_coding
ENSG0000(	ENSG0000(	21.09549	-0.28073	0.312468	0.722649	lncRNA
ENSG0000(	ENSG0000(	303.1252	0.081171	0.312512	0.722688	lncRNA
ENSG0000(	ANXA4	3275.541	-0.13124	0.312924	0.722716	protein_coding
ENSG0000(	ATP5F1E	944.0023	-0.09689	0.312942	0.722716	protein_coding
ENSG0000(	C10orf67	83.33788	0.14287	0.313026	0.722716	protein_coding
ENSG0000(	CDK8	400.7679	0.095296	0.312754	0.722716	protein_coding
ENSG0000(	CYBA	1155.676	-0.12585	0.312928	0.722716	protein_coding
ENSG0000(	DRAP1	126.0387	-0.17007	0.31275	0.722716	protein_coding
ENSG0000(	ENSG0000(	50.17364	0.178781	0.312735	0.722716	lncRNA
ENSG0000(	ENSG0000(	40.64506	0.225739	0.31296	0.722716	TEC
ENSG0000(	ENSG0000(	86.7497	0.1318	0.312922	0.722716	lncRNA
ENSG0000(	EVA1C	391.9797	0.138016	0.313049	0.722716	protein_coding
ENSG0000(	FZD1	341.6713	-0.14427	0.312702	0.722716	protein_coding
ENSG0000(	LINC00992	37.14721	0.305781	0.312967	0.722716	lncRNA
ENSG0000(	LINC01811	101.1653	-0.24544	0.312714	0.722716	lncRNA
ENSG0000(	LINC02910	34.50098	-0.28805	0.313041	0.722716	lncRNA
ENSG0000(	PXMP4	1396.934	0.039526	0.313043	0.722716	protein_coding
ENSG0000(	RCSD1	1941.568	-0.15992	0.312812	0.722716	protein_coding
ENSG0000(	RN7SL192P	44.57114	-0.19448	0.313014	0.722716	misc_RNA
ENSG0000(	RNVU1-2	1752.863	0.167219	0.312965	0.722716	snRNA
ENSG0000(	SPRY1	654.0397	-0.12202	0.312715	0.722716	protein_coding
ENSG0000(	XPO1	8793.634	0.052638	0.312775	0.722716	protein_coding
ENSG0000(	CHMP4BP1	90.00508	0.237451	0.313188	0.722762	processed_pseudogene
ENSG0000(	CLNK	366.9986	-0.10881	0.313111	0.722762	protein_coding
ENSG0000(	ENSG0000(	105.2449	-0.15334	0.31314	0.722762	TEC
ENSG0000(	HAUS3	1053.259	0.057536	0.313177	0.722762	protein_coding
ENSG0000(	POLR2G	170.7338	-0.15061	0.313212	0.722762	protein_coding
ENSG0000(	REL	4352.274	-0.09034	0.313253	0.722762	protein_coding
ENSG0000(	S100P	101.4611	-0.5243	0.31323	0.722762	protein_coding
ENSG0000(	BEAN1-AS1	23.47502	-0.27955	0.313454	0.72284	lncRNA
ENSG0000(	ENSG0000(	171.3261	-0.10336	0.313379	0.72284	transcribed_unprocessed_pseudo
ENSG0000(	ENSG0000(	123.1948	0.120718	0.313353	0.72284	lncRNA
ENSG0000(	ENSG0000(	30.00869	-0.2822	0.313346	0.72284	TEC
ENSG0000(	ENSG0000(	142.6842	-0.10787	0.313405	0.72284	lncRNA
ENSG0000(	HRAT92	150.039	-0.13904	0.313473	0.72284	lncRNA
ENSG0000(	MT-ND4	29128.74	-0.22758	0.31345	0.72284	protein_coding
ENSG0000(	RN7SKP103	53.88368	0.260526	0.313497	0.72284	misc_RNA
ENSG0000(	ENSG0000(	224.2663	0.108883	0.313568	0.72291	lncRNA
ENSG0000(	LINC-ROR	183.5462	0.139467	0.313602	0.72291	lncRNA
ENSG0000(	TRBV29-1	36.55738	0.253755	0.313606	0.72291	TR_V_gene
ENSG0000(	RNF130	941.9819	-0.1253	0.313637	0.722921	protein_coding
ENSG0000(	RIMS2	463.7473	0.115311	0.313795	0.723225	protein_coding

ENSG0000(	HEMK1	2790.703	0.050971	0.313897	0.723303	protein_coding
ENSG0000(	LINC01145	843.1602	-0.18748	0.313908	0.723303	transcribed_unprocessed_pseudo
ENSG0000(	LINC01910	153.8915	0.080918	0.313888	0.723303	lncRNA
ENSG0000(	ENSG0000(	167.1365	0.118942	0.313994	0.723401	transcribed_processed_pseudoge
ENSG0000(	PDZRN3	608.8956	-0.14875	0.314003	0.723401	protein_coding
ENSG0000(	ENSG0000(	186.7168	0.108514	0.314132	0.723545	lncRNA
ENSG0000(	ENSG0000(	99.36502	-0.17574	0.314143	0.723545	lncRNA
ENSG0000(	RSC1A1	133.1458	-0.27674	0.314145	0.723545	protein_coding
ENSG0000(	ENSG0000(	25.35465	0.273112	0.314329	0.723848	lncRNA
ENSG0000(	ENSG0000(	101.988	0.147359	0.314321	0.723848	lncRNA
ENSG0000(	EMD	192.5964	-0.12577	0.314421	0.723879	protein_coding
ENSG0000(	ENSG0000(	464.7469	0.080028	0.31442	0.723879	protein_coding
ENSG0000(	LINC01847	30.56527	-0.25864	0.314383	0.723879	lncRNA
ENSG0000(	ENSG0000(	382.4445	-0.11693	0.314458	0.723904	lncRNA
ENSG0000(	ENSG0000(	71.43358	0.18765	0.314602	0.724175	TEC
ENSG0000(	KDM7A-DT	818.4624	0.078495	0.314661	0.72425	lncRNA
ENSG0000(	ENSG0000(	631.9158	0.06686	0.314718	0.724259	protein_coding
ENSG0000(	ENSG0000(	148.061	0.109086	0.314693	0.724259	lncRNA
ENSG0000(	HECW2	828.8133	-0.16817	0.314764	0.724304	protein_coding
ENSG0000(	SERTAD2	1034.565	-0.11777	0.314839	0.724418	protein_coding
ENSG0000(	MIR548V	19.69787	0.27787	0.314879	0.724448	miRNA
ENSG0000(	ENSG0000(	22.5152	0.269455	0.314984	0.724629	lncRNA
ENSG0000(	OR4F17	1875.241	0.130693	0.315032	0.72463	protein_coding
ENSG0000(	WDR20	1180.801	0.074464	0.315037	0.72463	protein_coding
ENSG0000(	PLBD2	600.2597	-0.13711	0.315085	0.724679	protein_coding
ENSG0000(	CSPG4P10	253.692	0.197709	0.31514	0.724746	transcribed_unprocessed_pseudo
ENSG0000(	KBTBD11-C	107.5958	0.327688	0.315223	0.724876	protein_coding
ENSG0000(	CORO2B	50.15037	-0.24468	0.315319	0.724928	protein_coding
ENSG0000(	ELF1	1515.646	0.079995	0.315377	0.724928	protein_coding
ENSG0000(	ENSG0000(	102.4182	0.123606	0.315299	0.724928	lncRNA
ENSG0000(	PKIA-AS1	121.6721	-0.14516	0.315375	0.724928	lncRNA
ENSG0000(	TMEM248	1861.034	0.076367	0.315371	0.724928	protein_coding
ENSG0000(	UGDH	1893.639	0.219627	0.315408	0.724938	protein_coding
ENSG0000(	CEACAM22	382.0346	0.113481	0.315558	0.724945	transcribed_unprocessed_pseudo
ENSG0000(	ENSG0000(	123.8726	0.124179	0.315537	0.724945	lncRNA
ENSG0000(	FLRT1	23.19153	0.285132	0.315439	0.724945	protein_coding
ENSG0000(	FLYWCH1	1083.516	-0.10209	0.315558	0.724945	protein_coding
ENSG0000(	OLA1P3	1032.634	-0.04853	0.315596	0.724945	processed_pseudogene
ENSG0000(	SLC9A3R1	927.8921	0.193682	0.315582	0.724945	protein_coding
ENSG0000(	TRIM17	28.77262	0.296186	0.315534	0.724945	protein_coding
ENSG0000(	CD200R1	84.69719	-0.24327	0.315964	0.725357	protein_coding
ENSG0000(	ENSG0000(	823.6898	0.052012	0.315839	0.725357	lncRNA
ENSG0000(	ENSG0000(	142.7286	0.123663	0.315998	0.725357	lncRNA
ENSG0000(	FAM118B	658.5356	0.131782	0.316012	0.725357	protein_coding
ENSG0000(	KIAA1958	1177.96	0.102084	0.31594	0.725357	protein_coding
ENSG0000(	MRPS36	124.9288	0.242293	0.315842	0.725357	protein_coding
ENSG0000(	PRDX2	377.3847	0.140358	0.315887	0.725357	protein_coding

ENSG0000(	RIMS3	554.4405	-0.2029	0.315964	0.725357	protein_coding
ENSG0000(	TRA2B	6046.777	-0.06452	0.315883	0.725357	protein_coding
ENSG0000(	LINC00167	75.25437	0.126333	0.316082	0.725415	lncRNA
ENSG0000(	SSH1	3802.848	-0.10948	0.31609	0.725415	protein_coding
ENSG0000(	INTS7	687.947	0.126859	0.316131	0.725447	protein_coding
ENSG0000(	RNF5	255.4339	0.182207	0.316219	0.725589	protein_coding
ENSG0000(	ENSG0000(	56.68436	0.156442	0.316329	0.725721	lncRNA
ENSG0000(	WNT10A	67.38217	-0.2311	0.316307	0.725721	protein_coding
ENSG0000(	ENSG0000(	207.3867	0.097394	0.316378	0.725758	lncRNA
ENSG0000(	ENSG0000(	29.74591	-0.22933	0.316398	0.725758	lncRNA
ENSG0000(	CHCHD4	114.2278	0.168698	0.316466	0.725803	protein_coding
ENSG0000(	LINC00320	651.0545	-0.06172	0.316471	0.725803	lncRNA
ENSG0000(	ENSG0000(	34.78858	0.20477	0.316695	0.72589	lncRNA
ENSG0000(	ENSG0000(	36.19903	-0.27522	0.316573	0.72589	lncRNA
ENSG0000(	ENSG0000(	20.23592	0.270602	0.316719	0.72589	lncRNA
ENSG0000(	KLC4	1055.465	0.099953	0.316633	0.72589	protein_coding
ENSG0000(	LARP1B	1355.794	0.09142	0.316624	0.72589	protein_coding
ENSG0000(	PTPRN2-AS	26.22252	-0.27365	0.316668	0.72589	lncRNA
ENSG0000(	SCPEP1	513.5022	-0.11128	0.316553	0.72589	protein_coding
ENSG0000(	SP4	742.7844	-0.12574	0.316654	0.72589	protein_coding
ENSG0000(	ENSG0000(	447.6987	0.108414	0.316772	0.725951	TEC
ENSG0000(	NTAN1P2	59.59349	0.153991	0.316808	0.725973	processed_pseudogene
ENSG0000(	HMGCR	1923.49	0.119952	0.31684	0.725986	protein_coding
ENSG0000(	TTC13	961.7195	0.101426	0.316956	0.72619	protein_coding
ENSG0000(	TNRC6B	8482.863	0.068994	0.317017	0.726269	protein_coding
ENSG0000(	ABCA3	155.3398	0.167664	0.317127	0.726272	protein_coding
ENSG0000(	APBB3	568.3777	-0.09686	0.317229	0.726272	protein_coding
ENSG0000(	BBS2	1779.735	0.064582	0.317215	0.726272	protein_coding
ENSG0000(	ENSG0000(	32.29273	0.177148	0.317102	0.726272	lncRNA
ENSG0000(	KLKP1	24.44097	0.237475	0.317159	0.726272	transcribed_unprocessed_pseudo
ENSG0000(	NRROS	137.8281	-0.24	0.317198	0.726272	protein_coding
ENSG0000(	SCAMP4	1359.317	0.06973	0.317161	0.726272	protein_coding
ENSG0000(	ZFYVE26	2762.131	-0.05745	0.317141	0.726272	protein_coding
ENSG0000(	ABHD8	51.2965	-0.2337	0.317557	0.726572	protein_coding
ENSG0000(	CD320	294.628	0.126345	0.317561	0.726572	protein_coding
ENSG0000(	CDK5RAP3	3176.776	0.083808	0.317468	0.726572	protein_coding
ENSG0000(	COL4A6	174.9641	0.302207	0.31764	0.726572	protein_coding
ENSG0000(	CRNDE	282.3349	0.089082	0.317603	0.726572	lncRNA
ENSG0000(	CRYM	41.69554	0.331802	0.317556	0.726572	protein_coding
ENSG0000(	ENSG0000(	23.01889	0.254569	0.317514	0.726572	lncRNA
ENSG0000(	SACS	2596.676	-0.09292	0.317433	0.726572	protein_coding
ENSG0000(	SKA3	105.1707	-0.20639	0.3177	0.726572	protein_coding
ENSG0000(	SMG6-IT1	84.73624	-0.12285	0.317664	0.726572	TEC
ENSG0000(	SNORA80E	155.7633	-0.14063	0.317644	0.726572	snoRNA
ENSG0000(	SYBU	371.7468	0.258958	0.317703	0.726572	protein_coding
ENSG0000(	TLL1	165.9702	-0.27332	0.317539	0.726572	protein_coding
ENSG0000(	CA3	83.49161	-0.26577	0.317761	0.726643	protein_coding

ENSG0000(	BOLA3	422.1652	-0.13999	0.317847	0.726718	protein_coding
ENSG0000(	TYR	28.94891	-0.21613	0.317834	0.726718	protein_coding
ENSG0000(	REXO1L1P	361.7703	0.274484	0.317926	0.726839	transcribed_processed_pseudoge
ENSG0000(	EVI5	1730.576	0.076167	0.317955	0.726844	protein_coding
ENSG0000(	ENSG0000(	35.59468	0.183326	0.318027	0.72695	lncRNA
ENSG0000(	ATG2B	2574.355	-0.08067	0.318083	0.726957	protein_coding
ENSG0000(	SCCPDH	269.0693	0.110549	0.318059	0.726957	protein_coding
ENSG0000(	ADAMTS17	395.6611	0.188049	0.31825	0.727036	protein_coding
ENSG0000(	ENSG0000(	37.09715	-0.21987	0.318245	0.727036	lncRNA
ENSG0000(	ENSG0000(	162.409	-0.10594	0.318172	0.727036	lncRNA
ENSG0000(	FAM135A	3247.883	-0.11563	0.318239	0.727036	protein_coding
ENSG0000(	SNX25	748.9053	0.080133	0.318213	0.727036	protein_coding
ENSG0000(	ENSG0000(	413.6501	0.07823	0.318407	0.727273	lncRNA
ENSG0000(	NAPB	932.0624	-0.06812	0.318381	0.727273	protein_coding
ENSG0000(	WSCD1	666.7586	0.33189	0.318501	0.727427	protein_coding
ENSG0000(	ANKAR	612.4414	0.093327	0.318774	0.727459	protein_coding
ENSG0000(	ENTPD1-AS	3129.267	0.036292	0.318743	0.727459	lncRNA
ENSG0000(	FPGT-TNNI	136.6239	0.167436	0.318692	0.727459	protein_coding
ENSG0000(	GABRB2	338.673	0.117267	0.318752	0.727459	protein_coding
ENSG0000(	GOLGA5P1	91.58289	-0.27024	0.318703	0.727459	processed_pseudogene
ENSG0000(	IDH2	936.6261	-0.1037	0.318771	0.727459	protein_coding
ENSG0000(	KCNAB2	1672.049	-0.15689	0.31858	0.727459	protein_coding
ENSG0000(	PLAG1	271.8477	-0.17817	0.318703	0.727459	protein_coding
ENSG0000(	TRIM16L	408.3289	0.123509	0.318732	0.727459	transcribed_unprocessed_pseudo
ENSG0000(	TSPAN15	823	0.146905	0.318779	0.727459	protein_coding
ENSG0000(	ITGB5	658.4101	0.132362	0.318816	0.727484	protein_coding
ENSG0000(	IFIT5	501.2431	-0.19826	0.319012	0.727753	protein_coding
ENSG0000(	SCN1A	232.3578	0.126644	0.319013	0.727753	protein_coding
ENSG0000(	THSD4	1626.81	0.218886	0.318995	0.727753	protein_coding
ENSG0000(	AQP8	2664.393	0.704445	0.319098	0.727764	protein_coding
ENSG0000(	ENSG0000(	92.17391	0.204319	0.319062	0.727764	lncRNA
ENSG0000(	ENSG0000(	511.0248	0.13902	0.319085	0.727764	lncRNA
ENSG0000(	ENSG0000(	601.8555	0.056755	0.319164	0.727836	lncRNA
ENSG0000(	RN7SL351F	73.19521	-0.17382	0.319182	0.727836	misc_RNA
ENSG0000(	CCDC7	622.0237	0.108396	0.319323	0.727853	protein_coding
ENSG0000(	ENSG0000(	48.16483	-0.43019	0.319244	0.727853	lncRNA
ENSG0000(	ENSG0000(	266.096	-0.09025	0.319291	0.727853	lncRNA
ENSG0000(	ERFL	28.27529	0.249571	0.3193	0.727853	protein_coding
ENSG0000(	MCM2	206.5745	-0.20992	0.31935	0.727853	protein_coding
ENSG0000(	PGM5	462.2667	0.167839	0.319262	0.727853	protein_coding
ENSG0000(	SLC6A10P	128.2086	-0.27864	0.319375	0.727853	transcribed_unprocessed_pseudo
ENSG0000(	PANK3	6692.253	0.136613	0.319519	0.728023	protein_coding
ENSG0000(	PFN1	2002.82	-0.11743	0.319515	0.728023	protein_coding
ENSG0000(	SGPL1	2185.39	-0.07004	0.319529	0.728023	protein_coding
ENSG0000(	TRIM52-AS	216.7627	0.11107	0.319557	0.728027	lncRNA
ENSG0000(	ENSG0000(	30.71077	0.276521	0.319748	0.728342	lncRNA
ENSG0000(	ENSG0000(	110.2197	0.144412	0.319729	0.728342	lncRNA

ENSG0000(	ENSG0000(	201.7112	-0.13601	0.31984	0.72837	lncRNA
ENSG0000(	FUZ	113.4129	-0.1263	0.319834	0.72837	protein_coding
ENSG0000(	UTP4	438.2285	-0.1538	0.319804	0.72837	protein_coding
ENSG0000(	ENSG0000(	525.0864	0.064522	0.320028	0.728436	transcribed_processed_pseudoge
ENSG0000(	ENSG0000(	70.17057	-0.17705	0.320006	0.728436	TEC
ENSG0000(	GNPAT	565.3306	0.082582	0.319965	0.728436	protein_coding
ENSG0000(	GSPT2	170.1337	-0.12091	0.319993	0.728436	protein_coding
ENSG0000(	LINC02767	85.93348	0.167741	0.319997	0.728436	lncRNA
ENSG0000(	MFSD9	737.5439	0.129347	0.319918	0.728436	protein_coding
ENSG0000(	TMEM220	392.9842	0.162237	0.320092	0.728521	protein_coding
ENSG0000(	PCYT2	415.6723	0.173819	0.32015	0.728549	protein_coding
ENSG0000(	POLG	1865.521	0.128287	0.320157	0.728549	protein_coding
ENSG0000(	ERCC1	832.86	0.070629	0.320185	0.728553	protein_coding
ENSG0000(	CREB3L4	187.1672	0.111326	0.320282	0.728562	protein_coding
ENSG0000(	ENSG0000(	20.1157	0.261313	0.32022	0.728562	lncRNA
ENSG0000(	GPR137C	441.8548	0.097445	0.320256	0.728562	protein_coding
ENSG0000(	LINC01776	468.8551	-0.08099	0.320295	0.728562	lncRNA
ENSG0000(	PSMG1	183.8342	-0.11852	0.320435	0.728813	protein_coding
ENSG0000(	TRPM7	4422.449	0.059438	0.320458	0.728813	protein_coding
ENSG0000(	CBX3	1464.607	-0.07218	0.320536	0.728835	protein_coding
ENSG0000(	CDS1	679.3535	0.22586	0.320579	0.728835	protein_coding
ENSG0000(	DECR1	1724.526	-0.06153	0.320689	0.728835	protein_coding
ENSG0000(	ENSG0000(	27.17062	0.228245	0.320634	0.728835	lncRNA
ENSG0000(	IGFBP2	663.7346	-0.20621	0.320564	0.728835	protein_coding
ENSG0000(	NSD2	5553.751	0.094687	0.320585	0.728835	protein_coding
ENSG0000(	PHF1	588.4407	-0.10096	0.320668	0.728835	protein_coding
ENSG0000(	ST3GAL4	681.9413	-0.22929	0.320639	0.728835	protein_coding
ENSG0000(	TBC1D9	1185.005	-0.13089	0.320706	0.728835	protein_coding
ENSG0000(	ENSG0000(	37.13009	0.185691	0.320779	0.72888	lncRNA
ENSG0000(	ZNF17	501.6299	0.090352	0.320779	0.72888	protein_coding
ENSG0000(	KLHDC4	1499.406	0.124211	0.320811	0.728892	protein_coding
ENSG0000(	ENSG0000(	19.5989	0.253452	0.320896	0.72901	lncRNA
ENSG0000(	TRIM25	4497.266	-0.1236	0.320916	0.72901	protein_coding
ENSG0000(	RSPH1	97.70085	-0.20304	0.321082	0.729278	protein_coding
ENSG0000(	TRIM4	1237.612	-0.06045	0.321087	0.729278	protein_coding
ENSG0000(	ALG1L2	295.2512	0.154066	0.321125	0.729305	protein_coding
ENSG0000(	SEPTIN11	3397.987	-0.07329	0.32116	0.729325	protein_coding
ENSG0000(	ENSG0000(	32.89968	-0.2706	0.321266	0.729444	lncRNA
ENSG0000(	ICOS	151.2688	-0.29361	0.321254	0.729444	protein_coding
ENSG0000(	ENSG0000(	254.8914	0.095749	0.321298	0.729456	lncRNA
ENSG0000(	ENSG0000(	30.78249	-0.28321	0.321393	0.72959	snoRNA
ENSG0000(	MIR4802	34.61229	0.257812	0.32141	0.72959	miRNA
ENSG0000(	SLC25A27	468.3086	0.12873	0.321447	0.729614	protein_coding
ENSG0000(	ATL1	2119.064	0.050214	0.321674	0.729911	protein_coding
ENSG0000(	ENSG0000(	49.49568	-0.1845	0.321677	0.729911	lncRNA
ENSG0000(	ENSG0000(	71.90126	-0.18379	0.321711	0.729911	TEC
ENSG0000(	FAM215B	735.4367	-0.15531	0.321707	0.729911	lncRNA

ENSG0000(	PGP	333.9867	0.100214	0.321662	0.729911	protein_coding
ENSG0000(	CREBL2	834.433	-0.10422	0.321814	0.730025	protein_coding
ENSG0000(	ZFP28	234.0984	0.172182	0.321796	0.730025	protein_coding
ENSG0000(	ENSG0000(	63.85433	0.248876	0.321861	0.730071	transcribed_unprocessed_pseudo
ENSG0000(	ZNF397	3521.899	0.053674	0.321946	0.730205	protein_coding
ENSG0000(	AURKAIP1	193.1738	0.132194	0.322118	0.730255	protein_coding
ENSG0000(	CREB1	2167.456	-0.09428	0.32209	0.730255	protein_coding
ENSG0000(	CYP2F1	20.36659	-0.31051	0.322287	0.730255	protein_coding
ENSG0000(	ENSG0000(	421.3847	0.119628	0.322135	0.730255	transcribed_unprocessed_pseudo
ENSG0000(	GYG2P1	236.7141	0.158426	0.322166	0.730255	transcribed_unprocessed_pseudo
ENSG0000(	LRRC8C-DT	477.8132	-0.15689	0.322184	0.730255	lncRNA
ENSG0000(	SAP30-DT	850.9166	0.066433	0.322235	0.730255	lncRNA
ENSG0000(	SNORD71	19.64468	-0.26667	0.322205	0.730255	snoRNA
ENSG0000(	SPTA1	61.45654	-0.36941	0.322274	0.730255	protein_coding
ENSG0000(	SYNJ2-IT1	58.38098	-0.14014	0.322204	0.730255	lncRNA
ENSG0000(	TSC22D1	4090.554	0.117767	0.322226	0.730255	protein_coding
ENSG0000(	ZBTB5	622.7576	0.064601	0.322131	0.730255	protein_coding
ENSG0000(	FCRL1	888.1317	-0.41949	0.32232	0.73027	protein_coding
ENSG0000(	ENSG0000(	20.24242	0.360888	0.322458	0.730369	processed_pseudogene
ENSG0000(	ENSG0000(	107.9639	-0.12675	0.322585	0.730369	lncRNA
ENSG0000(	ENSG0000(	412.9645	0.075855	0.322483	0.730369	TEC
ENSG0000(	EXOSC10	1258.441	0.083734	0.322655	0.730369	protein_coding
ENSG0000(	HPS1	1415.569	0.071525	0.322529	0.730369	protein_coding
ENSG0000(	KMT5A	859.5143	0.078871	0.322577	0.730369	protein_coding
ENSG0000(	LINC01588	1851.053	-0.0803	0.322619	0.730369	lncRNA
ENSG0000(	LINC01778	23.31279	0.217843	0.322646	0.730369	lncRNA
ENSG0000(	RAPGEF1	3073.778	-0.10076	0.32258	0.730369	protein_coding
ENSG0000(	SLC35E4	275.5807	0.118519	0.322656	0.730369	protein_coding
ENSG0000(	ZNF56P	120.1338	-0.11676	0.32265	0.730369	transcribed_unprocessed_pseudo
ENSG0000(	SCLY	1269.1	0.050788	0.322695	0.730397	protein_coding
ENSG0000(	USP5	431.1573	0.138387	0.322725	0.730406	protein_coding
ENSG0000(	CAMK2B	92.59469	0.232769	0.322753	0.730408	protein_coding
ENSG0000(	OCIAD1	1301.569	-0.05004	0.322805	0.730468	protein_coding
ENSG0000(	PFN2	330.7795	-0.1533	0.32287	0.730552	protein_coding
ENSG0000(	ARHGAP35	3671.691	0.119812	0.323128	0.730735	protein_coding
ENSG0000(	KCND3-IT1	23.85007	-0.31484	0.323132	0.730735	lncRNA
ENSG0000(	LRRC37A6F	28.48362	0.338742	0.323136	0.730735	transcribed_processed_pseudoge
ENSG0000(	PLIN5	361.0739	0.086818	0.323133	0.730735	protein_coding
ENSG0000(	PRDX6-AS1	745.6102	0.109911	0.323103	0.730735	lncRNA
ENSG0000(	RPS6KA4	383.8104	0.114677	0.322987	0.730735	protein_coding
ENSG0000(	SLC5A9	68.41177	-0.41223	0.323047	0.730735	protein_coding
ENSG0000(	BTBD11	181.7282	-0.15421	0.323175	0.730762	protein_coding
ENSG0000(	ENSG0000(	474.3471	0.077263	0.323334	0.731012	lncRNA
ENSG0000(	TMEM150(	239.9894	0.168012	0.323338	0.731012	protein_coding
ENSG0000(	ENSG0000(	59.03218	-0.16927	0.323403	0.731098	lncRNA
ENSG0000(	RPL13AP12	47.993	-0.18998	0.323513	0.731287	processed_pseudogene
ENSG0000(	ENSG0000(	307.1403	0.075636	0.323592	0.731346	lncRNA

ENSG0000(	MARK1	1120.355	0.073095	0.323578	0.731346	protein_coding
ENSG0000(	C11orf21	204.076	-0.21642	0.323726	0.731422	protein_coding
ENSG0000(	ENSG0000(	28.0357	-0.25367	0.323724	0.731422	processed_pseudogene
ENSG0000(	NOL11	549.7459	0.130098	0.323704	0.731422	protein_coding
ENSG0000(	TRIQK	543.0405	0.104558	0.323732	0.731422	protein_coding
ENSG0000(	LAMTOR1	616.5508	0.071601	0.323761	0.731425	protein_coding
ENSG0000(	VPS45	881.7254	-0.0698	0.323787	0.731425	protein_coding
ENSG0000(	ENSG0000(	87.74164	0.16813	0.323958	0.73145	lncRNA
ENSG0000(	ENSG0000(	106.7112	-0.38429	0.323922	0.73145	protein_coding
ENSG0000(	FOXO3B	221.4999	-0.22076	0.32392	0.73145	protein_coding
ENSG0000(	OSR1	126.0674	0.257675	0.323887	0.73145	protein_coding
ENSG0000(	RBBP8NL	87.38943	0.317628	0.32387	0.73145	protein_coding
ENSG0000(	ZNF420	229.7689	-0.14124	0.323955	0.73145	protein_coding
ENSG0000(	KCND1	117.9566	-0.19172	0.324002	0.731491	protein_coding
ENSG0000(	EPHX4	46.67947	-0.17105	0.324097	0.731525	protein_coding
ENSG0000(	IL18	1451.402	-0.15623	0.324088	0.731525	protein_coding
ENSG0000(	NRADDP	25.02076	-0.31389	0.324048	0.731525	transcribed_unitary_pseudogene
ENSG0000(	ENSG0000(	25.53533	0.380824	0.324137	0.731556	lncRNA
ENSG0000(	CCDC150	697.5051	0.090294	0.324195	0.731625	protein_coding
ENSG0000(	ENSG0000(	145.5725	-0.10947	0.324221	0.731626	lncRNA
ENSG0000(	HYDIN	285.7138	0.144573	0.32427	0.731657	protein_coding
ENSG0000(	WLS	875.5169	0.129429	0.324288	0.731657	protein_coding
ENSG0000(	GSDMB	4540.15	-0.13774	0.32436	0.731673	protein_coding
ENSG0000(	LINC01936	269.4669	0.091848	0.324376	0.731673	lncRNA
ENSG0000(	RBM17P4	26.85262	0.237854	0.324364	0.731673	processed_pseudogene
ENSG0000(	ENSG0000(	24.13239	-0.24275	0.324417	0.731707	lncRNA
ENSG0000(	PCDHB18P	33.12977	0.352108	0.324455	0.731732	transcribed_unprocessed_pseudo
ENSG0000(	ADH5	1296.882	0.105458	0.324745	0.732099	protein_coding
ENSG0000(	CENPP	330.0885	-0.18062	0.324672	0.732099	protein_coding
ENSG0000(	DNAJC15	980.934	0.117494	0.324804	0.732099	protein_coding
ENSG0000(	SDHC	553.4796	-0.10379	0.324673	0.732099	protein_coding
ENSG0000(	SLC9C1	40.19649	-0.38949	0.324801	0.732099	protein_coding
ENSG0000(	TLE2	353.8213	0.148019	0.324723	0.732099	protein_coding
ENSG0000(	WDR97	280.6715	0.113441	0.324794	0.732099	protein_coding
ENSG0000(	WTAP	2193.175	-0.07801	0.324843	0.732128	protein_coding
ENSG0000(	ENSG0000(	128.2184	-0.09998	0.325049	0.732264	lncRNA
ENSG0000(	IFTAP	2502.629	0.062083	0.325063	0.732264	protein_coding
ENSG0000(	MOSMO	500.0566	-0.09302	0.324997	0.732264	protein_coding
ENSG0000(	NAPSA	62.65796	0.306818	0.324959	0.732264	protein_coding
ENSG0000(	SAXO1	106.5426	0.210857	0.324951	0.732264	protein_coding
ENSG0000(	TUBB4B	1124.299	0.16159	0.32503	0.732264	protein_coding
ENSG0000(	ENSG0000(	263.8858	-0.12598	0.325096	0.732278	transcribed_processed_pseudoge
ENSG0000(	HMGB2	356.7383	-0.12145	0.325178	0.732283	protein_coding
ENSG0000(	HS6ST2	21.21024	-0.45176	0.325172	0.732283	protein_coding
ENSG0000(	KRT8P39	41.42247	0.202095	0.32516	0.732283	processed_pseudogene
ENSG0000(	TICAM1	345.5519	0.179453	0.32527	0.73243	protein_coding
ENSG0000(	ENSG0000(	112.6229	-0.247	0.325463	0.732505	TEC

ENSG0000(	ING4	305.4592	0.111004	0.325438	0.732505	protein_coding
ENSG0000(	KIF13A	1884.778	0.097775	0.325452	0.732505	protein_coding
ENSG0000(	NLRP12	314.2233	-0.10274	0.325396	0.732505	protein_coding
ENSG0000(	RHBDD3	391.2714	-0.07514	0.325334	0.732505	protein_coding
ENSG0000(	TMEM170E	302.1042	-0.12022	0.325393	0.732505	protein_coding
ENSG0000(	ENSG0000(	208.5725	-0.09876	0.325515	0.732561	lncRNA
ENSG0000(	BAX	513.1864	-0.09015	0.325793	0.732688	protein_coding
ENSG0000(	EMCN	445.5101	-0.1173	0.325695	0.732688	protein_coding
ENSG0000(	ENSG0000(	25.24921	-0.3111	0.325703	0.732688	processed_pseudogene
ENSG0000(	ENSG0000(	104.4691	-0.16988	0.325798	0.732688	lncRNA
ENSG0000(	ENSG0000(	381.6384	-0.11795	0.325648	0.732688	lncRNA
ENSG0000(	HSD17B2	2062.24	0.268853	0.325811	0.732688	protein_coding
ENSG0000(	PPP1R13B	1862.11	0.115305	0.325802	0.732688	protein_coding
ENSG0000(	SCN2B	78.58032	0.128541	0.325755	0.732688	protein_coding
ENSG0000(	UBAC2	1095.388	-0.06068	0.325694	0.732688	protein_coding
ENSG0000(	ARHGEF9-I	29.67427	0.213818	0.326156	0.732869	lncRNA
ENSG0000(	ENSG0000(	23.91472	0.272928	0.326063	0.732869	lncRNA
ENSG0000(	ENSG0000(	136.2538	0.199062	0.325977	0.732869	lncRNA
ENSG0000(	ENSG0000(	424.3375	0.071312	0.326102	0.732869	TEC
ENSG0000(	ENSG0000(	2258.361	-0.06208	0.326069	0.732869	lncRNA
ENSG0000(	MRPL4	367.4735	0.079638	0.326158	0.732869	protein_coding
ENSG0000(	SIRT2	974.0594	-0.05721	0.32609	0.732869	protein_coding
ENSG0000(	SMYD5	239.6977	0.162542	0.325992	0.732869	protein_coding
ENSG0000(	TFB1M	379.7422	0.096965	0.326069	0.732869	protein_coding
ENSG0000(	TMEM184A	2064.507	0.20443	0.326116	0.732869	protein_coding
ENSG0000(	MRC1	588.4805	-0.19282	0.3262	0.732904	protein_coding
ENSG0000(	BEND4	182.1667	-0.25861	0.326302	0.732999	protein_coding
ENSG0000(	ENSG0000(	33.97797	0.227827	0.326282	0.732999	lncRNA
ENSG0000(	HERC2P10	69.5343	-0.29225	0.326322	0.732999	unprocessed_pseudogene
ENSG0000(	JDP2-AS1	62.79035	-0.19248	0.326356	0.733017	lncRNA
ENSG0000(	ENSG0000(	43.11179	-0.21179	0.32643	0.733061	pseudogene
ENSG0000(	LINC01967	473.5989	0.117854	0.326415	0.733061	lncRNA
ENSG0000(	ENSG0000(	143.1996	0.132968	0.326517	0.733199	processed_pseudogene
ENSG0000(	AMER3	114.363	0.160458	0.326578	0.733276	protein_coding
ENSG0000(	ENSG0000(	36.17673	0.292073	0.326707	0.733386	lncRNA
ENSG0000(	ENSG0000(	320.8925	0.139462	0.326708	0.733386	lncRNA
ENSG0000(	PFN1P2	106.0085	-0.17727	0.326669	0.733386	transcribed_processed_pseudoge
ENSG0000(	ARHGAP42	127.7369	0.131217	0.327079	0.734041	processed_pseudogene
ENSG0000(	KDM5A	4269.952	0.050062	0.327073	0.734041	protein_coding
ENSG0000(	LINC00923	2370.458	0.073153	0.327061	0.734041	lncRNA
ENSG0000(	ABCF1	1294.36	0.066053	0.327365	0.734136	protein_coding
ENSG0000(	ARL6IP5	904.6506	-0.09753	0.327871	0.734136	protein_coding
ENSG0000(	C1orf174	665.6731	0.079631	0.327573	0.734136	protein_coding
ENSG0000(	C9orf64	402.4324	0.060602	0.327671	0.734136	protein_coding
ENSG0000(	CHAC2	27.93799	0.256547	0.327774	0.734136	protein_coding
ENSG0000(	ENSG0000(	219.5447	-0.10536	0.327315	0.734136	lncRNA
ENSG0000(	ENSG0000(	21.97524	-0.24613	0.32791	0.734136	lncRNA

ENSG0000(	ENSG0000(	80.57028	0.273036	0.327423	0.734136	lncRNA
ENSG0000(	ENSG0000(	51.2534	0.220855	0.327864	0.734136	lncRNA
ENSG0000(	ENSG0000(	68.0183	0.155684	0.327697	0.734136	transcribed_processed_pseudoge
ENSG0000(	ENSG0000(	159.6394	0.099557	0.327323	0.734136	TEC
ENSG0000(	ENSG0000(	57.96574	0.18912	0.327909	0.734136	lncRNA
ENSG0000(	ENSG0000(	52.77799	0.259819	0.327801	0.734136	lncRNA
ENSG0000(	ENSG0000(	87.58117	0.139957	0.327976	0.734136	lncRNA
ENSG0000(	ENSG0000(	54.07918	-0.23546	0.327622	0.734136	lncRNA
ENSG0000(	ERICH1	2483.657	-0.11129	0.327708	0.734136	protein_coding
ENSG0000(	ESCO1	1335.954	0.114386	0.327254	0.734136	protein_coding
ENSG0000(	HCN1	128.9447	-0.32556	0.327814	0.734136	protein_coding
ENSG0000(	MAPK11	115.9933	-0.25897	0.327763	0.734136	protein_coding
ENSG0000(	NDOR1	520.2092	0.101516	0.3277	0.734136	protein_coding
ENSG0000(	P2RY2	978.1159	0.161486	0.32792	0.734136	protein_coding
ENSG0000(	QPRT	792.4602	0.073944	0.327273	0.734136	protein_coding
ENSG0000(	RELA	979.2842	-0.07755	0.327965	0.734136	protein_coding
ENSG0000(	RPL23AP53	118.9778	-0.12917	0.32765	0.734136	transcribed_processed_pseudoge
ENSG0000(	SEPTIN7P1⁄	1127.02	-0.10697	0.327733	0.734136	transcribed_unprocessed_pseudo
ENSG0000(	TFF3	898.2927	0.277841	0.327568	0.734136	protein_coding
ENSG0000(	TMEM126/	48.06687	0.229758	0.327363	0.734136	protein_coding
ENSG0000(	TRAPPC9	2260.556	0.089606	0.327226	0.734136	protein_coding
ENSG0000(	TTLL3	3920.053	0.057078	0.327549	0.734136	protein_coding
ENSG0000(	VWA5A	1126.13	0.112326	0.3276	0.734136	protein_coding
ENSG0000(	ZNF557	1327.25	0.053	0.327952	0.734136	protein_coding
ENSG0000(	ZSCAN16	135.0784	0.120309	0.327834	0.734136	protein_coding
ENSG0000(	BSDC1	2386.208	0.074026	0.328044	0.734169	protein_coding
ENSG0000(	PPP2R1A	1999.704	0.115994	0.328021	0.734169	protein_coding
ENSG0000(	AK5	135.862	-0.1701	0.328079	0.734187	protein_coding
ENSG0000(	CLDN4	2491.491	0.231959	0.32829	0.734521	protein_coding
ENSG0000(	ETNK1	14062.28	0.255044	0.328327	0.734521	protein_coding
ENSG0000(	OXCT1	413.7936	-0.12101	0.328327	0.734521	protein_coding
ENSG0000(	SLC22A4	72.99098	-0.31585	0.328334	0.734521	protein_coding
ENSG0000(	OR2A5	621.1704	-0.10967	0.32841	0.734571	protein_coding
ENSG0000(	SNORA73B	5071.794	0.145152	0.328405	0.734571	snoRNA
ENSG0000(	TMCC1	1091.45	-0.06359	0.32847	0.734644	protein_coding
ENSG0000(	VARS2	511.7623	0.11613	0.328562	0.734791	protein_coding
ENSG0000(	ENSG0000(	124.3223	0.170304	0.328596	0.734808	lncRNA
ENSG0000(	ENSG0000(	74.07534	-0.17593	0.328631	0.734817	lncRNA
ENSG0000(	ENSG0000(	396.1675	-0.08045	0.328665	0.734817	lncRNA
ENSG0000(	IATPR	137.7861	-0.1203	0.32868	0.734817	lncRNA
ENSG0000(	ELFN1	54.84693	-0.28776	0.328723	0.73483	protein_coding
ENSG0000(	WHSC1L2P	197.1375	0.099888	0.32874	0.73483	processed_pseudogene
ENSG0000(	HDAC1P2	52.56748	-0.21785	0.328772	0.734842	transcribed_processed_pseudoge
ENSG0000(	USP32P3	1989.45	-0.08229	0.328814	0.734876	transcribed_unprocessed_pseudo
ENSG0000(	ENSG0000(	44.51712	0.176816	0.328862	0.734879	lncRNA
ENSG0000(	ENSG0000(	20.71148	0.32115	0.32891	0.734879	lncRNA
ENSG0000(	MROH2A	71.8036	-0.6522	0.328922	0.734879	protein_coding

ENSG0000(	SHLD1	141.1687	0.115227	0.328884	0.734879	protein_coding
ENSG0000(	CLDN23	549.8152	0.316849	0.328949	0.734879	protein_coding
ENSG0000(	GART	1067.786	-0.06604	0.328985	0.734899	protein_coding
ENSG0000(	LINC02348	226.2535	-0.08365	0.329129	0.735124	lncRNA
ENSG0000(	NELFE	254.5436	0.121113	0.329139	0.735124	protein_coding
ENSG0000(	IL4R	3681.882	-0.10696	0.329252	0.735318	protein_coding
ENSG0000(	CDH15	114.4423	0.139554	0.329441	0.735465	protein_coding
ENSG0000(	ENSG0000(	350.7627	0.089127	0.329475	0.735465	lncRNA
ENSG0000(	MTNR1A	40.69407	0.369067	0.329478	0.735465	protein_coding
ENSG0000(	NDUFV2-A!	47.93907	-0.20791	0.329376	0.735465	lncRNA
ENSG0000(	RBAK	1199.829	-0.088	0.329417	0.735465	protein_coding
ENSG0000(	RPL11P1	37.93041	-0.18397	0.329361	0.735465	processed_pseudogene
ENSG0000(	PPIAP67	39.43548	0.232927	0.329509	0.735473	processed_pseudogene
ENSG0000(	ZNF737	1837.165	0.101119	0.329535	0.735473	protein_coding
ENSG0000(	ENSG0000(	159.1396	0.146248	0.329605	0.735569	lncRNA
ENSG0000(	ENSG0000(	63.31441	0.208954	0.329667	0.735647	lncRNA
ENSG0000(	HIGD2A	419.0038	0.153654	0.329729	0.735667	protein_coding
ENSG0000(	SSPOP	225.2031	0.171337	0.329715	0.735667	transcribed_unitary_pseudogene
ENSG0000(	CCDC18	682.9366	-0.12683	0.329893	0.735974	protein_coding
ENSG0000(	ADAMTS15	180.5461	-0.23042	0.329986	0.736119	protein_coding
ENSG0000(	AGO1	2140.291	-0.06331	0.33017	0.736345	protein_coding
ENSG0000(	ENSG0000(	8221.241	-0.04962	0.330136	0.736345	lncRNA
ENSG0000(	U2AF1L5	2008.396	0.303312	0.330194	0.736345	protein_coding
ENSG0000(	VCP	3689.33	-0.07611	0.330177	0.736345	protein_coding
ENSG0000(	CKAP4	863.9421	-0.13315	0.330334	0.736538	protein_coding
ENSG0000(	PARP12	1924.728	-0.14012	0.330311	0.736538	protein_coding
ENSG0000(	ATM	13713.18	-0.11241	0.330715	0.736596	protein_coding
ENSG0000(	CDCA4P4	39.98016	0.224897	0.330633	0.736596	unprocessed_pseudogene
ENSG0000(	DRG1	305.4555	0.10243	0.330488	0.736596	protein_coding
ENSG0000(	ENSG0000(	147.2814	0.112707	0.330527	0.736596	lncRNA
ENSG0000(	ENSG0000(	764.0852	0.062358	0.330608	0.736596	lncRNA
ENSG0000(	ENSG0000(	41.00759	-0.22384	0.330508	0.736596	lncRNA
ENSG0000(	ENSG0000(	200.0172	0.121856	0.330687	0.736596	TEC
ENSG0000(	ENSG0000(	361.7026	-0.16736	0.330733	0.736596	TEC
ENSG0000(	ERLIN2	1050.948	0.077	0.330595	0.736596	protein_coding
ENSG0000(	GOLGA6L2	37.75963	0.182109	0.330788	0.736596	protein_coding
ENSG0000(	HAND2	42.96591	-0.34671	0.330779	0.736596	protein_coding
ENSG0000(	MRPL10	365.477	-0.07466	0.330565	0.736596	protein_coding
ENSG0000(	MRPL51	164.2143	-0.19046	0.330769	0.736596	protein_coding
ENSG0000(	SCAT8	105.8481	0.10238	0.330766	0.736596	lncRNA
ENSG0000(	TGFB2	65.30209	-0.30956	0.33048	0.736596	protein_coding
ENSG0000(	TRMT2A	505.435	-0.09405	0.330767	0.736596	protein_coding
ENSG0000(	ATXN2	4903.716	-0.05794	0.330887	0.73662	protein_coding
ENSG0000(	ENSG0000(	90.54278	0.202492	0.330906	0.73662	processed_pseudogene
ENSG0000(	HSPH1	1176.852	-0.11896	0.330831	0.73662	protein_coding
ENSG0000(	LRRC66	432.5662	-0.22177	0.330878	0.73662	protein_coding
ENSG0000(	INPP5B	2108.233	-0.06291	0.330964	0.736689	protein_coding

ENSG0000(	PRR5L	4212.928	0.23178	0.331065	0.736854	protein_coding
ENSG0000(	ENSG0000(	65.04993	0.201805	0.331103	0.73688	lncRNA
ENSG0000(	ATR	4724.118	-0.05583	0.331582	0.736882	protein_coding
ENSG0000(	CCL28	2105.276	0.149817	0.331338	0.736882	protein_coding
ENSG0000(	CDH17	5845.238	0.219141	0.331447	0.736882	protein_coding
ENSG0000(	DIAPH1	2278.47	-0.08023	0.331266	0.736882	protein_coding
ENSG0000(	ENSG0000(	24.34108	0.215262	0.331384	0.736882	lncRNA
ENSG0000(	ENSG0000(	192.3799	-0.1134	0.331132	0.736882	TEC
ENSG0000(	ENSG0000(	23.64999	-0.32991	0.331465	0.736882	TEC
ENSG0000(	ENSG0000(	59.86009	0.154214	0.331684	0.736882	lncRNA
ENSG0000(	ENSG0000(	66.51099	0.165633	0.331556	0.736882	lncRNA
ENSG0000(	EXOSC4	70.33964	-0.15007	0.331455	0.736882	protein_coding
ENSG0000(	IQCE	1156.461	0.100734	0.331595	0.736882	protein_coding
ENSG0000(	LSM12	763.5862	-0.0693	0.331688	0.736882	protein_coding
ENSG0000(	MIR648	30.89486	0.289679	0.331449	0.736882	miRNA
ENSG0000(	POU2F1	2236.217	-0.09551	0.331595	0.736882	protein_coding
ENSG0000(	RDH14	174.9941	-0.09518	0.331312	0.736882	protein_coding
ENSG0000(	RERE	4138.094	0.060987	0.331693	0.736882	protein_coding
ENSG0000(	RPUSD1	207.8878	0.093218	0.331668	0.736882	protein_coding
ENSG0000(	SLC25A28	638.5982	-0.10728	0.331399	0.736882	protein_coding
ENSG0000(	SPTLC3	1248.103	0.119448	0.331248	0.736882	protein_coding
ENSG0000(	STS	1189.807	-0.10387	0.331533	0.736882	protein_coding
ENSG0000(	WIPF2	2759.823	0.111211	0.331365	0.736882	protein_coding
ENSG0000(	ZC3H12D	1469.566	-0.13699	0.33129	0.736882	protein_coding
ENSG0000(	AGGF1	811.4524	0.075444	0.331813	0.737058	protein_coding
ENSG0000(	ENSG0000(	53.88495	-0.2257	0.332237	0.737058	processed_pseudogene
ENSG0000(	ENSG0000(	28.57814	-0.35054	0.332282	0.737058	lncRNA
ENSG0000(	ENSG0000(	39.10833	0.266994	0.332263	0.737058	lncRNA
ENSG0000(	ENSG0000(	29.96107	-0.25354	0.332236	0.737058	lncRNA
ENSG0000(	ENSG0000(	114.2845	0.108001	0.332236	0.737058	lncRNA
ENSG0000(	ENSG0000(	284.8317	-0.08284	0.332121	0.737058	lncRNA
ENSG0000(	GGT8P	19.72367	0.462417	0.332138	0.737058	unprocessed_pseudogene
ENSG0000(	IGHJ2	33.09127	-0.40558	0.332179	0.737058	IG_J_gene
ENSG0000(	LYNX1	75.15706	-0.26149	0.331957	0.737058	protein_coding
ENSG0000(	MORC2	1398.895	0.071023	0.332148	0.737058	protein_coding
ENSG0000(	NANOGNB	24.43219	0.23249	0.3319	0.737058	protein_coding
ENSG0000(	PGBD5	287.4592	0.167867	0.332001	0.737058	protein_coding
ENSG0000(	RFX3-AS1	357.9761	0.077329	0.332084	0.737058	lncRNA
ENSG0000(	TRIM7	198.748	0.160163	0.332159	0.737058	protein_coding
ENSG0000(	TTC9	163.5632	-0.30487	0.332238	0.737058	protein_coding
ENSG0000(	USP9YP3	53.49247	0.153698	0.332095	0.737058	unprocessed_pseudogene
ENSG0000(	ZC3H4	1239.052	0.081141	0.332027	0.737058	protein_coding
ENSG0000(	ZNF784	102.2478	0.147593	0.332124	0.737058	protein_coding
ENSG0000(	DCLRE1B	125.9374	-0.17196	0.332348	0.737086	protein_coding
ENSG0000(	HES6	85.91575	0.260469	0.332335	0.737086	protein_coding
ENSG0000(	CAPN2	4949.079	0.111675	0.332523	0.7371	protein_coding
ENSG0000(	ENSG0000(	48.25737	-0.16421	0.332491	0.7371	lncRNA

ENSG0000(	ENSG0000(	667.3936	-0.06536	0.332415	0.7371	lncRNA
ENSG0000(	ENSG0000(	90.77591	0.131934	0.332541	0.7371	lncRNA
ENSG0000(	KIAA1614	382.9175	-0.12757	0.332454	0.7371	protein_coding
ENSG0000(	TMEM30B	1121.353	0.157901	0.332397	0.7371	protein_coding
ENSG0000(	TMUB2	1164.316	0.063034	0.332493	0.7371	protein_coding
ENSG0000(	CYP2G1P	74.78825	0.137121	0.332729	0.73714	transcribed_unprocessed_pseudo
ENSG0000(	ENSG0000(	62.7296	0.154276	0.332678	0.73714	processed_pseudogene
ENSG0000(	KLF3P1	62.80817	0.169991	0.332655	0.73714	processed_pseudogene
ENSG0000(	SYNE2	15013.1	0.070474	0.332774	0.73714	protein_coding
ENSG0000(	TMEM181	2456.088	0.065398	0.332694	0.73714	protein_coding
ENSG0000(	TMEM9B	467.329	0.128738	0.332764	0.73714	protein_coding
ENSG0000(	WDR5B	415.0405	-0.10298	0.332649	0.73714	protein_coding
ENSG0000(	XPNPEP1	1444.571	-0.09545	0.332665	0.73714	protein_coding
ENSG0000(	APBB1	199.5382	-0.15512	0.33282	0.737182	protein_coding
ENSG0000(	FGD5	811.3615	-0.11824	0.33294	0.737211	protein_coding
ENSG0000(	LINC02669	175.6319	0.104461	0.332928	0.737211	lncRNA
ENSG0000(	TUBD1	528.2698	0.091624	0.332923	0.737211	protein_coding
ENSG0000(	UBAC1	431.6508	0.127317	0.33291	0.737211	protein_coding
ENSG0000(	ADARB2	130.6642	-0.26314	0.333464	0.737312	protein_coding
ENSG0000(	APBA3	663.9562	-0.08495	0.333311	0.737312	protein_coding
ENSG0000(	CLIC5	5221.992	-0.21511	0.333642	0.737312	protein_coding
ENSG0000(	DDX10	1694.819	-0.08232	0.333622	0.737312	protein_coding
ENSG0000(	EHMT2-AS1	511.077	0.26315	0.333594	0.737312	lncRNA
ENSG0000(	ELK4	4221.614	-0.07857	0.33334	0.737312	protein_coding
ENSG0000(	ENSG0000(	218.5495	-0.14423	0.333321	0.737312	lncRNA
ENSG0000(	ENSG0000(	473.8737	-0.10876	0.333502	0.737312	lncRNA
ENSG0000(	ENSG0000(	133.5011	0.111722	0.333339	0.737312	lncRNA
ENSG0000(	ENSG0000(	73.58728	-0.17344	0.33357	0.737312	lncRNA
ENSG0000(	ENSG0000(	207.9814	0.130244	0.33361	0.737312	lncRNA
ENSG0000(	ENSG0000(	23.55559	-0.26807	0.333064	0.737312	lncRNA
ENSG0000(	ENSG0000(	171.889	0.100375	0.333598	0.737312	lncRNA
ENSG0000(	ENSG0000(	29.00226	-0.55525	0.333414	0.737312	protein_coding
ENSG0000(	HECTD3	1041.123	0.130637	0.333586	0.737312	protein_coding
ENSG0000(	IL33	286.9535	-0.20415	0.333416	0.737312	protein_coding
ENSG0000(	KCTD16	20.91345	0.34225	0.33359	0.737312	protein_coding
ENSG0000(	KRT73-AS1	98.56806	0.149996	0.333089	0.737312	lncRNA
ENSG0000(	PRKCG	66.78734	0.209545	0.333527	0.737312	protein_coding
ENSG0000(	PRMT5	409.8239	-0.16434	0.333656	0.737312	protein_coding
ENSG0000(	RBM3	2396.033	-0.06798	0.333368	0.737312	protein_coding
ENSG0000(	RGPD6	205.3226	0.274971	0.333127	0.737312	protein_coding
ENSG0000(	SH3RF3-AS	29.01587	-0.21752	0.333402	0.737312	lncRNA
ENSG0000(	SLC26A7	42.16407	0.330283	0.33334	0.737312	protein_coding
ENSG0000(	ZNF143	418.1366	-0.11329	0.333322	0.737312	protein_coding
ENSG0000(	MANEA-DT	79.92577	-0.1768	0.333687	0.737323	lncRNA
ENSG0000(	LIPG	2480.677	-0.15422	0.33374	0.737381	protein_coding
ENSG0000(	DHRSX-IT1	102.194	0.132053	0.333909	0.737459	lncRNA
ENSG0000(	ELN-AS1	27.28138	0.273523	0.333888	0.737459	lncRNA

ENSG0000(	ENSG0000(	967.0719	0.076193	0.333885	0.737459	lncRNA
ENSG0000(	OR51E1	33.47652	-0.30727	0.333862	0.737459	protein_coding
ENSG0000(	RN7SL850F	88.39157	0.170204	0.33391	0.737459	misc_RNA
ENSG0000(	ENSG0000(	39.43235	0.201957	0.334011	0.737507	unprocessed_pseudogene
ENSG0000(	ENSG0000(	62.40279	-0.20181	0.333984	0.737507	lncRNA
ENSG0000(	SLC25A10	254.0975	0.20958	0.334012	0.737507	protein_coding
ENSG0000(	ANKRD52	2316.766	-0.05803	0.334105	0.737653	protein_coding
ENSG0000(	PRIM2	610.8031	0.079628	0.334132	0.737653	protein_coding
ENSG0000(	ADAM23	83.8549	-0.23558	0.334563	0.73776	protein_coding
ENSG0000(	ARHGAP17	3219.906	-0.07674	0.334739	0.73776	protein_coding
ENSG0000(	ARHGEF35-	2889.603	-0.09768	0.334339	0.73776	lncRNA
ENSG0000(	ARL5C	69.37186	0.160654	0.334532	0.73776	protein_coding
ENSG0000(	CACNB3	1013.647	0.141384	0.334238	0.73776	protein_coding
ENSG0000(	DDX11	1693.082	-0.1031	0.334944	0.73776	protein_coding
ENSG0000(	DPP7	377.8184	-0.12649	0.334721	0.73776	protein_coding
ENSG0000(	ENSG0000(	502.1185	0.097747	0.334272	0.73776	lncRNA
ENSG0000(	ENSG0000(	81.95364	-0.15601	0.334705	0.73776	lncRNA
ENSG0000(	ENSG0000(	35.46369	-0.27704	0.335002	0.73776	lncRNA
ENSG0000(	ENSG0000(	75.2841	0.117355	0.334634	0.73776	lncRNA
ENSG0000(	ENSG0000(	57.30712	0.166318	0.334448	0.73776	lncRNA
ENSG0000(	ENSG0000(	224.0162	0.163329	0.334364	0.73776	transcribed_unprocessed_pseudo
ENSG0000(	ENSG0000(	141.3968	-0.11096	0.334624	0.73776	lncRNA
ENSG0000(	ENSG0000(	69.32949	0.148613	0.334894	0.73776	TEC
ENSG0000(	ENSG0000(	679.0927	-0.07056	0.334771	0.73776	TEC
ENSG0000(	IGHV4-55	74.82263	-0.39032	0.334462	0.73776	IG_V_pseudogene
ENSG0000(	LINC00314	32.31984	-0.31903	0.335038	0.73776	lncRNA
ENSG0000(	LINC02228	94.97002	-0.12823	0.334546	0.73776	lncRNA
ENSG0000(	MIX23P3	176.1514	0.268793	0.334718	0.73776	processed_pseudogene
ENSG0000(	NKIRAS2	960.74	-0.06898	0.334448	0.73776	protein_coding
ENSG0000(	NRIP3-DT	207.6027	0.096872	0.334971	0.73776	lncRNA
ENSG0000(	P3H4	670.1129	-0.07953	0.334672	0.73776	protein_coding
ENSG0000(	PCLO	1874.519	0.212429	0.334959	0.73776	protein_coding
ENSG0000(	PHF20L1	2945.518	-0.0653	0.334839	0.73776	protein_coding
ENSG0000(	PRRC2A	3251.916	-0.0604	0.334345	0.73776	protein_coding
ENSG0000(	SLC25A44	1849.448	0.114025	0.33481	0.73776	protein_coding
ENSG0000(	SUMO3	589.8379	0.08334	0.334872	0.73776	protein_coding
ENSG0000(	TARS1	882.6585	-0.10041	0.334785	0.73776	protein_coding
ENSG0000(	TBL1X	1256.18	-0.0602	0.335023	0.73776	protein_coding
ENSG0000(	TRIM71	53.41429	0.253707	0.334764	0.73776	protein_coding
ENSG0000(	TRMT10A	114.5733	-0.19646	0.334762	0.73776	protein_coding
ENSG0000(	RPS2P36	30.18713	-0.34193	0.335088	0.73781	processed_pseudogene
ENSG0000(	ENSG0000(	335.6117	-0.07592	0.335128	0.73784	lncRNA
ENSG0000(	AP2A1	1221.993	-0.07238	0.335165	0.737862	protein_coding
ENSG0000(	MAP6D1	30.37799	0.269449	0.335196	0.737871	protein_coding
ENSG0000(	ENSG0000(	198.2031	0.189026	0.33523	0.737887	TEC
ENSG0000(	OCIAD2	521.6453	0.207693	0.335264	0.737902	protein_coding
ENSG0000(	ABI3BP	1481.983	-0.15436	0.335352	0.737979	protein_coding

ENSG0000(	SNORD14D	23.65529	-0.46036	0.335347	0.737979	snoRNA
ENSG0000(	ZNF30	102.2695	0.165614	0.335414	0.738056	protein_coding
ENSG0000(	LINC00926	819.5014	-0.23904	0.335456	0.73809	lncRNA
ENSG0000(	LRRC57	1725.562	0.060443	0.335499	0.738124	protein_coding
ENSG0000(	RND2	138.0881	-0.12074	0.335544	0.738164	protein_coding
ENSG0000(	OPA1	2008.16	0.077848	0.33562	0.738164	protein_coding
ENSG0000(	PRDM2	3061.25	-0.08341	0.335584	0.738164	protein_coding
ENSG0000(	TREH	40.87157	0.33457	0.335624	0.738164	protein_coding
ENSG0000(	ABHD1	424.9666	0.077288	0.335754	0.738331	protein_coding
ENSG0000(	MIEF2	359.8257	0.103097	0.33574	0.738331	protein_coding
ENSG0000(	ZNF561-AS	1612.827	0.054583	0.335834	0.738448	lncRNA
ENSG0000(	CECR2	221.4529	-0.15003	0.335874	0.738474	protein_coding
ENSG0000(	CLDN7	3612.35	0.221903	0.33591	0.738474	protein_coding
ENSG0000(	ENSG0000(	46.59338	-0.18062	0.335926	0.738474	lncRNA
ENSG0000(	CCDC17	394.5768	0.140764	0.336012	0.738587	protein_coding
ENSG0000(	ENSG0000(	21.15147	0.267055	0.336138	0.738587	lncRNA
ENSG0000(	ENSG0000(	45.32535	-0.20594	0.336125	0.738587	TEC
ENSG0000(	RN7SL190P	129.9108	0.111972	0.336133	0.738587	misc_RNA
ENSG0000(	RPS25	1310.061	-0.13704	0.33611	0.738587	protein_coding
ENSG0000(	UBR7	406.1618	-0.12152	0.336117	0.738587	protein_coding
ENSG0000(	ENSG0000(	113.7057	-0.15825	0.336292	0.738782	lncRNA
ENSG0000(	LINC01993	281.2714	-0.11058	0.336308	0.738782	lncRNA
ENSG0000(	PALLD	2285.548	-0.11086	0.336287	0.738782	protein_coding
ENSG0000(	TRAJ3	21.73392	-0.37058	0.336353	0.738823	TR_J_gene
ENSG0000(	ENSG0000(	98.95621	0.11755	0.336385	0.738832	lncRNA
ENSG0000(	RAB8A	1462.664	0.06849	0.336481	0.738985	protein_coding
ENSG0000(	C12orf40	125.0081	0.121554	0.336554	0.739087	protein_coding
ENSG0000(	VPS33B	288.5761	0.086967	0.336586	0.739098	protein_coding
ENSG0000(	CYFIP2	1341.134	-0.19182	0.336624	0.739123	protein_coding
ENSG0000(	ENSG0000(	21.43156	-0.34547	0.336656	0.739134	lncRNA
ENSG0000(	COMMD2	660.1924	-0.06688	0.336778	0.739282	protein_coding
ENSG0000(	CSPG4	373.4877	-0.16608	0.336761	0.739282	protein_coding
ENSG0000(	ADAM22	317.3084	0.195292	0.337085	0.739296	protein_coding
ENSG0000(	ATP5PBP1	37.55741	-0.21847	0.337382	0.739296	processed_pseudogene
ENSG0000(	CHMP3	1705.836	0.08324	0.336926	0.739296	protein_coding
ENSG0000(	CPNE8	624.6449	-0.1403	0.337572	0.739296	protein_coding
ENSG0000(	CRACD	1226.944	0.179087	0.337531	0.739296	protein_coding
ENSG0000(	DDX52	1641.55	-0.04312	0.337442	0.739296	protein_coding
ENSG0000(	ENSG0000(	91.20867	-0.15737	0.336991	0.739296	processed_pseudogene
ENSG0000(	ENSG0000(	42.66987	0.249804	0.337574	0.739296	lncRNA
ENSG0000(	ENSG0000(	78.0776	0.107924	0.33745	0.739296	lncRNA
ENSG0000(	ENSG0000(	174.6309	0.11618	0.337392	0.739296	lncRNA
ENSG0000(	ENSG0000(	233.9826	-0.09017	0.33764	0.739296	lncRNA
ENSG0000(	ENSG0000(	460.8329	0.092114	0.337112	0.739296	lncRNA
ENSG0000(	ENSG0000(	84.32612	-0.16026	0.337084	0.739296	lncRNA
ENSG0000(	ENSG0000(	829.7693	0.114859	0.337532	0.739296	lncRNA
ENSG0000(	ENSG0000(	222.4908	0.295353	0.33773	0.739296	lncRNA

ENSG0000(	FECH	1158.252	-0.0644	0.337163	0.739296	protein_coding
ENSG0000(	GRK6	749.7661	-0.13058	0.337751	0.739296	protein_coding
ENSG0000(	IARS1	4270.201	-0.0729	0.337569	0.739296	protein_coding
ENSG0000(	IGF2BP2	1410.072	0.194758	0.337383	0.739296	protein_coding
ENSG0000(	LHFPL5	1257.179	-0.07792	0.337116	0.739296	protein_coding
ENSG0000(	LINC01121	209.0523	0.107964	0.337569	0.739296	lncRNA
ENSG0000(	LINC01732	22.18347	0.184964	0.337681	0.739296	lncRNA
ENSG0000(	LINC02227	22.26279	-0.39938	0.337336	0.739296	lncRNA
ENSG0000(	MAP3K4	1307.295	-0.10512	0.337671	0.739296	protein_coding
ENSG0000(	NUP153	1792.48	0.059368	0.337699	0.739296	protein_coding
ENSG0000(	NXT1	49.43733	-0.22554	0.337172	0.739296	protein_coding
ENSG0000(	PXN	3145.626	-0.10419	0.337688	0.739296	protein_coding
ENSG0000(	RAD18	568.8105	0.118779	0.337367	0.739296	protein_coding
ENSG0000(	RBBP7	1598.101	-0.06398	0.337608	0.739296	protein_coding
ENSG0000(	SH3KBP1	1250.032	-0.09507	0.337198	0.739296	protein_coding
ENSG0000(	SRD5A3	600.3926	-0.17941	0.33687	0.739296	protein_coding
ENSG0000(	STK31	189.3203	0.14269	0.337577	0.739296	protein_coding
ENSG0000(	STMN3	191.5409	-0.22737	0.337461	0.739296	protein_coding
ENSG0000(	TNFSF12	132.4377	-0.14705	0.33761	0.739296	protein_coding
ENSG0000(	UQCRH	737.4227	0.122236	0.33707	0.739296	protein_coding
ENSG0000(	VRTN	68.15363	-0.14778	0.336996	0.739296	protein_coding
ENSG0000(	ENOX1-AS1	73.79006	0.12564	0.337865	0.739487	lncRNA
ENSG0000(	MTX2	385.3212	0.116578	0.337947	0.739607	protein_coding
ENSG0000(	ENSG0000(	50.23182	0.227569	0.338004	0.739673	lncRNA
ENSG0000(	ENSG0000(	188.1721	0.128592	0.338128	0.739826	lncRNA
ENSG0000(	TMPRSS7	44.37355	0.177967	0.338119	0.739826	protein_coding
ENSG0000(	NOL12	597.2402	0.110413	0.338181	0.739882	protein_coding
ENSG0000(	POLD4	1321.064	0.106178	0.338207	0.739882	protein_coding
ENSG0000(	CLDN3	769.4022	0.241208	0.338462	0.740321	protein_coding
ENSG0000(	SLC35E3	5754.997	-0.06062	0.338445	0.740321	protein_coding
ENSG0000(	BGLAP	24.39849	0.296242	0.338572	0.740417	protein_coding
ENSG0000(	MIR3619	38.05604	0.203813	0.33856	0.740417	miRNA
ENSG0000(	SBF2	5477.373	-0.05264	0.338586	0.740417	protein_coding
ENSG0000(	ENSG0000(	60.16109	-0.24646	0.338798	0.740468	lncRNA
ENSG0000(	ENSG0000(	93.65067	0.160251	0.338731	0.740468	lncRNA
ENSG0000(	EPC1	2785.912	0.060842	0.338707	0.740468	protein_coding
ENSG0000(	GPCPD1	3732.118	-0.08984	0.338732	0.740468	protein_coding
ENSG0000(	KDM3AP1	21.10206	0.309388	0.338691	0.740468	processed_pseudogene
ENSG0000(	LRRN4CL	165.3997	-0.09123	0.338784	0.740468	protein_coding
ENSG0000(	SETD2	5956.84	0.040579	0.338782	0.740468	protein_coding
ENSG0000(	ZNF22-AS1	63.7812	-0.16428	0.338917	0.740669	lncRNA
ENSG0000(	ETAA1	540.6993	-0.09386	0.33898	0.740747	protein_coding
ENSG0000(	FCHSD1	865.9521	-0.09302	0.339011	0.740756	protein_coding
ENSG0000(	MRTO4	287.9977	0.10483	0.33908	0.740787	protein_coding
ENSG0000(	RN7SKP213	21.87236	0.254797	0.339105	0.740787	misc_RNA
ENSG0000(	VPS33A	1040.343	0.052355	0.339056	0.740787	protein_coding
ENSG0000(	ADAM17	2125.797	-0.07129	0.339196	0.7408	protein_coding

ENSG0000(	ENSG0000(	474.1019	-0.06414	0.339219	0.7408	lncRNA
ENSG0000(	OCLN	3034.849	0.107196	0.339193	0.7408	protein_coding
ENSG0000(	PROM1	3441.211	0.291007	0.339175	0.7408	protein_coding
ENSG0000(	FCF1P1	31.8749	-0.21501	0.339284	0.740883	processed_pseudogene
ENSG0000(	ZNF232	147.7923	0.169479	0.339338	0.740941	protein_coding
ENSG0000(	ENSG0000(	394.0689	-0.09032	0.339447	0.741014	lncRNA
ENSG0000(	ENSG0000(	22.75532	0.207753	0.33956	0.741014	lncRNA
ENSG0000(	ENSG0000(	665.7136	0.49611	0.339541	0.741014	protein_coding
ENSG0000(	ETFDH	3818.531	0.078911	0.339554	0.741014	protein_coding
ENSG0000(	KDM2B	1377.359	-0.12724	0.339462	0.741014	protein_coding
ENSG0000(	MAD2L1-D'	232.6007	-0.08206	0.339555	0.741014	lncRNA
ENSG0000(	PMEL	30.20745	-0.28304	0.339552	0.741014	protein_coding
ENSG0000(	ENSG0000(	220.144	0.080254	0.339749	0.741084	lncRNA
ENSG0000(	ENSG0000(	141.3556	0.21172	0.339716	0.741084	lncRNA
ENSG0000(	ENSG0000(	58.52216	0.173576	0.339763	0.741084	lncRNA
ENSG0000(	FYB2	88.87622	-0.1342	0.339689	0.741084	protein_coding
ENSG0000(	SAP30	59.30357	-0.23676	0.33971	0.741084	protein_coding
ENSG0000(	SNORA62	231.0068	-0.13391	0.33978	0.741084	snoRNA
ENSG0000(	SRSF4	4969.56	-0.04041	0.339769	0.741084	protein_coding
ENSG0000(	LINC01602	91.11567	0.130404	0.339838	0.741151	lncRNA
ENSG0000(	SIM2	262.4092	-0.10959	0.339884	0.741192	protein_coding
ENSG0000(	RPS19P3	78.41822	-0.14683	0.339945	0.741268	processed_pseudogene
ENSG0000(	ENSG0000(	1035.64	0.055854	0.34013	0.741339	lncRNA
ENSG0000(	LINC00857	132.2193	0.1156	0.34014	0.741339	lncRNA
ENSG0000(	MPHOSPH8	1931.465	0.0713	0.340132	0.741339	protein_coding
ENSG0000(	SERAC1	838.5334	0.091035	0.340047	0.741339	protein_coding
ENSG0000(	TSPAN3	5753.11	0.148855	0.340093	0.741339	protein_coding
ENSG0000(	UBA52	1231.139	-0.12621	0.340074	0.741339	protein_coding
ENSG0000(	ENSG0000(	22.55464	-0.29737	0.340228	0.741354	lncRNA
ENSG0000(	KIAA0100	4328.339	0.048717	0.340212	0.741354	protein_coding
ENSG0000(	SCARA5	584.0192	-0.18068	0.340188	0.741354	protein_coding
ENSG0000(	H2BC3	29.28151	-0.30123	0.340295	0.741442	protein_coding
ENSG0000(	STAM	388.5683	-0.10858	0.340335	0.741472	protein_coding
ENSG0000(	CHRNA5	239.3231	0.100869	0.340485	0.741739	protein_coding
ENSG0000(	FALEC	33.781	0.17671	0.340531	0.741781	lncRNA
ENSG0000(	RNU6-942F	84.49069	-0.17543	0.340618	0.741853	snRNA
ENSG0000(	ZNF773	1069.667	0.080335	0.340613	0.741853	protein_coding
ENSG0000(	ABCB6	157.7691	-0.20074	0.340722	0.741865	protein_coding
ENSG0000(	ENSG0000(	241.702	0.094181	0.340759	0.741865	lncRNA
ENSG0000(	PLIN3	782.6239	-0.13428	0.340655	0.741865	protein_coding
ENSG0000(	PNPLA2	1826.364	0.16871	0.340701	0.741865	protein_coding
ENSG0000(	TBX10	28.84036	0.646173	0.340736	0.741865	protein_coding
ENSG0000(	NBPF6	21.17375	0.59047	0.340792	0.741879	protein_coding
ENSG0000(	NRIP3	339.8746	0.091888	0.340865	0.74198	protein_coding
ENSG0000(	ENSG0000(	130.4252	-0.10672	0.340943	0.742016	lncRNA
ENSG0000(	ENSG0000(	51.7136	0.385222	0.340963	0.742016	TEC
ENSG0000(	SART3	1245.323	-0.06126	0.340943	0.742016	protein_coding

ENSG0000(	ENSG0000(	27.77697	-0.19858	0.341002	0.74203	lncRNA
ENSG0000(	SLC2A3P2	85.87735	0.156191	0.341023	0.74203	processed_pseudogene
ENSG0000(	ENSG0000(	481.6417	0.105918	0.341151	0.742249	lncRNA
ENSG0000(	EXOSC6	3986.623	0.042429	0.341192	0.742279	protein_coding
ENSG0000(	KCTD3	973.4051	0.083651	0.341251	0.74235	protein_coding
ENSG0000(	ENSG0000(	2330.025	-0.08921	0.341358	0.742524	lncRNA
ENSG0000(	SNRPN	395.7419	-0.11946	0.341401	0.742559	protein_coding
ENSG0000(	RNU6-652F	55.00701	0.234071	0.341455	0.742618	snRNA
ENSG0000(	LINC01882	22.2505	-0.29168	0.341528	0.742717	lncRNA
ENSG0000(	ADCY3	713.686	-0.14296	0.341707	0.742914	protein_coding
ENSG0000(	DNAI4	757.6911	0.171054	0.341747	0.742914	protein_coding
ENSG0000(	ENSG0000(	143.1825	0.227561	0.341754	0.742914	processed_pseudogene
ENSG0000(	SGCD	315.3795	-0.20724	0.341684	0.742914	protein_coding
ENSG0000(	ZNF319	203.5148	-0.1675	0.341656	0.742914	protein_coding
ENSG0000(	AOAH	1116.926	-0.12913	0.34191	0.74296	protein_coding
ENSG0000(	ENSG0000(	36.73304	-0.21359	0.341864	0.74296	lncRNA
ENSG0000(	ENSG0000(	177.838	-0.13887	0.341902	0.74296	TEC
ENSG0000(	PPWD1	1149.11	-0.06431	0.341819	0.74296	protein_coding
ENSG0000(	TP53INP2	2593.49	0.250289	0.341832	0.74296	protein_coding
ENSG0000(	NLRC3	1082.918	-0.15054	0.34197	0.743033	protein_coding
ENSG0000(	FAM120B	2089.407	0.05274	0.342053	0.743068	protein_coding
ENSG0000(	TPT1	12498.49	-0.1111	0.342067	0.743068	protein_coding
ENSG0000(	TTC4	237.1253	0.108926	0.342015	0.743068	protein_coding
ENSG0000(	ENSG0000(	514.034	-0.0861	0.342194	0.743099	unprocessed_pseudogene
ENSG0000(	TIMM8A	338.4186	0.093794	0.342161	0.743099	protein_coding
ENSG0000(	TMEM42	405.6265	-0.07795	0.342216	0.743099	protein_coding
ENSG0000(	TNKS	2488.091	-0.05383	0.342167	0.743099	protein_coding
ENSG0000(	WAC	3236.294	-0.05474	0.342216	0.743099	protein_coding
ENSG0000(	ZNF148	1846.339	-0.06291	0.342312	0.743248	protein_coding
ENSG0000(	ENSG0000(	255.4028	-0.08478	0.342394	0.74332	lncRNA
ENSG0000(	MLXIPL	446.8811	0.100132	0.3424	0.74332	protein_coding
ENSG0000(	FSCN3	31.26676	-0.30286	0.342554	0.743538	protein_coding
ENSG0000(	PCBP2-OT1	65.23845	0.16167	0.342552	0.743538	lncRNA
ENSG0000(	CATIP	254.8644	-0.08258	0.342976	0.743721	protein_coding
ENSG0000(	CTF1	38.07621	0.191967	0.342952	0.743721	protein_coding
ENSG0000(	EIF2B4	631.2156	0.080595	0.342926	0.743721	protein_coding
ENSG0000(	ENSG0000(	273.8597	0.1027	0.342718	0.743721	lncRNA
ENSG0000(	ENSG0000(	818.2074	-0.10049	0.342881	0.743721	protein_coding
ENSG0000(	FERMT1	1683.596	0.188679	0.343026	0.743721	protein_coding
ENSG0000(	IGLV3-9	49.41317	-0.53531	0.342939	0.743721	IG_V_gene
ENSG0000(	NUDCP1	50.66421	-0.18126	0.342939	0.743721	processed_pseudogene
ENSG0000(	OMD	87.48395	0.12178	0.342681	0.743721	protein_coding
ENSG0000(	OR7E122P	801.9448	0.073055	0.342978	0.743721	transcribed_unprocessed_pseudo
ENSG0000(	TOP2B	2071.438	-0.08035	0.342724	0.743721	protein_coding
ENSG0000(	WDR73	2463.134	0.072444	0.343035	0.743721	protein_coding
ENSG0000(	ZNF404	57.20133	0.236063	0.342747	0.743721	protein_coding
ENSG0000(	ZSCAN16-A	565.2608	-0.09656	0.34299	0.743721	lncRNA

ENSG0000(	ZSWIM7	445.9447	0.081318	0.343044	0.743721	protein_coding
ENSG0000(	ENSG0000(	261.4781	0.106006	0.34308	0.743741	lncRNA
ENSG0000(	ENSG0000(	20.24667	-0.24894	0.343209	0.743962	lncRNA
ENSG0000(	ENSG0000(	57.36122	0.176808	0.343384	0.744042	lncRNA
ENSG0000(	FNBP1	3289.593	-0.10627	0.343301	0.744042	protein_coding
ENSG0000(	HEY2	27.79227	-0.29632	0.343435	0.744042	protein_coding
ENSG0000(	MGC4859	25.71002	-0.26656	0.343328	0.744042	lncRNA
ENSG0000(	PPARGC1B	3772.918	0.160728	0.343404	0.744042	protein_coding
ENSG0000(	RN7SL233F	39.67308	-0.21457	0.343414	0.744042	misc_RNA
ENSG0000(	SYPL1	609.0768	-0.0876	0.343341	0.744042	protein_coding
ENSG0000(	ENSG0000(	149.9066	-0.11058	0.343544	0.744102	processed_pseudogene
ENSG0000(	ENSG0000(	217.0756	0.143795	0.343515	0.744102	transcribed_unprocessed_pseudo
ENSG0000(	MAP4	4304.313	-0.06509	0.343533	0.744102	protein_coding
ENSG0000(	PAEP	58.81978	0.162981	0.343601	0.744167	protein_coding
ENSG0000(	MRPS23	772.7057	-0.07444	0.34364	0.744192	protein_coding
ENSG0000(	CBX1	367.8563	-0.08563	0.343764	0.744234	protein_coding
ENSG0000(	CYP21A2	26.27706	-0.30323	0.343774	0.744234	protein_coding
ENSG0000(	ENSG0000(	329.3264	0.090554	0.343788	0.744234	lncRNA
ENSG0000(	ENSG0000(	30.95927	0.215315	0.343708	0.744234	protein_coding
ENSG0000(	NPM1	3117.777	-0.10462	0.343794	0.744234	protein_coding
ENSG0000(	ALG12	596.5492	0.119392	0.34385	0.744237	protein_coding
ENSG0000(	CDC14A	834.2944	0.172353	0.343847	0.744237	protein_coding
ENSG0000(	CEPT1	1618.354	-0.07707	0.344039	0.744237	protein_coding
ENSG0000(	ENSG0000(	65.07392	0.155406	0.344031	0.744237	lncRNA
ENSG0000(	INO80	1396.041	-0.08532	0.344017	0.744237	protein_coding
ENSG0000(	PCDHB3	29.32822	0.237136	0.343987	0.744237	protein_coding
ENSG0000(	RN7SKP25	155.3191	0.161214	0.344039	0.744237	misc_RNA
ENSG0000(	RTN3	1648.178	-0.10732	0.343988	0.744237	protein_coding
ENSG0000(	SH3BP1	875.3801	0.139348	0.344002	0.744237	protein_coding
ENSG0000(	ENSG0000(	59.17911	-0.17585	0.34414	0.74428	lncRNA
ENSG0000(	GPR83	213.6773	0.09535	0.344102	0.74428	protein_coding
ENSG0000(	H4C1	44.69436	-0.29192	0.344117	0.74428	protein_coding
ENSG0000(	ZNF137P	251.2639	0.166829	0.344199	0.744348	transcribed_unprocessed_pseudo
ENSG0000(	SULT1A1	2235.463	0.160724	0.344257	0.744415	protein_coding
ENSG0000(	NAPSB	188.268	-0.28621	0.344301	0.744442	transcribed_unprocessed_pseudo
ENSG0000(	SLC16A6	109.024	-0.2203	0.344323	0.744442	protein_coding
ENSG0000(	CERS2	951.9089	-0.09522	0.34472	0.744502	protein_coding
ENSG0000(	CLEC4M	117.8521	0.112558	0.344753	0.744502	protein_coding
ENSG0000(	EGFL8	502.4838	0.135852	0.344732	0.744502	protein_coding
ENSG0000(	ENSG0000(	137.3477	-0.17162	0.344919	0.744502	transcribed_unprocessed_pseudo
ENSG0000(	ENSG0000(	112.6371	0.109212	0.344484	0.744502	lncRNA
ENSG0000(	ENSG0000(	158.5167	0.164992	0.344884	0.744502	lncRNA
ENSG0000(	ENSG0000(	27.54928	0.210498	0.344792	0.744502	lncRNA
ENSG0000(	ENSG0000(	23.54521	0.266016	0.344899	0.744502	lncRNA
ENSG0000(	ENSG0000(	65.90048	-0.19268	0.34468	0.744502	transcribed_unprocessed_pseudo
ENSG0000(	ENSG0000(	114.8931	0.10235	0.344738	0.744502	lncRNA
ENSG0000(	ENSG0000(	148.0896	-0.11516	0.34438	0.744502	lncRNA

ENSG0000(	EPS15-AS1	76.95952	0.263473	0.344701	0.744502	lncRNA
ENSG0000(	LINC00476	1264.694	0.056101	0.344792	0.744502	lncRNA
ENSG0000(	LINC01905	86.89267	-0.11938	0.344613	0.744502	lncRNA
ENSG0000(	LINC02206	27.18412	-0.23002	0.344838	0.744502	lncRNA
ENSG0000(	MDP1	23.0171	-0.30687	0.344631	0.744502	protein_coding
ENSG0000(	MIS12	628.9357	0.126328	0.344575	0.744502	protein_coding
ENSG0000(	RN7SL778F	34.45237	0.219651	0.344841	0.744502	misc_RNA
ENSG0000(	RTCA	796.8261	0.07334	0.344724	0.744502	protein_coding
ENSG0000(	SIGLEC8	255.5826	0.095706	0.344627	0.744502	protein_coding
ENSG0000(	VEZT	2034.666	-0.05752	0.34447	0.744502	protein_coding
ENSG0000(	GOLGA2P1	125.5092	0.265298	0.345065	0.744642	transcribed_unprocessed_pseudo
ENSG0000(	H4-16	617.8609	0.11646	0.345046	0.744642	protein_coding
ENSG0000(	PPP4R3B	2491.22	0.066883	0.345014	0.744642	protein_coding
ENSG0000(	ENSG0000(	76.91253	0.204068	0.345106	0.744672	lncRNA
ENSG0000(	CDRT15P4	20.00478	-0.32649	0.345164	0.744738	transcribed_unprocessed_pseudo
ENSG0000(	ADH6	170.8818	0.271244	0.345328	0.744748	protein_coding
ENSG0000(	ANKRD27	883.5155	0.101207	0.345281	0.744748	protein_coding
ENSG0000(	C14orf119	508.3491	-0.10696	0.345349	0.744748	protein_coding
ENSG0000(	ENSG0000(	67.97916	0.163188	0.34529	0.744748	lncRNA
ENSG0000(	FAM120C	376.8041	0.149045	0.345358	0.744748	protein_coding
ENSG0000(	KMT2E-AS1	120.0834	0.094904	0.345285	0.744748	lncRNA
ENSG0000(	LETR1	546.1801	-0.09894	0.345347	0.744748	lncRNA
ENSG0000(	ATP1A3	246.4102	-0.1007	0.345394	0.744766	protein_coding
ENSG0000(	ENSG0000(	69.97237	0.157642	0.345519	0.744862	lncRNA
ENSG0000(	ENSG0000(	77.70018	0.177184	0.345489	0.744862	lncRNA
ENSG0000(	SIX4	194.1987	-0.14586	0.345511	0.744862	protein_coding
ENSG0000(	MAFTRR	104.8548	0.159248	0.345636	0.744997	lncRNA
ENSG0000(	MT-TS1	144.1761	-0.41534	0.345628	0.744997	Mt_tRNA
ENSG0000(	DHX37	576.1129	-0.07676	0.345712	0.745102	protein_coding
ENSG0000(	ASXL1	3095.099	-0.08455	0.345797	0.745227	protein_coding
ENSG0000(	ACTG1P14	139.0002	0.130272	0.345928	0.745275	processed_pseudogene
ENSG0000(	CACNA1B	55.68958	0.240653	0.345873	0.745275	protein_coding
ENSG0000(	SLFN12L	4969.321	-0.10334	0.345924	0.745275	protein_coding
ENSG0000(	ZNF583	504.6646	-0.12696	0.345923	0.745275	protein_coding
ENSG0000(	ENSG0000(	117.4974	-0.17473	0.346018	0.745354	lncRNA
ENSG0000(	MYCN	21.82821	0.305726	0.346018	0.745354	protein_coding
ENSG0000(	CNTROB	650.0133	0.068351	0.346214	0.745658	protein_coding
ENSG0000(	NPTN-IT1	640.6458	-0.0745	0.346203	0.745658	lncRNA
ENSG0000(	DDRGK1	278.621	-0.09481	0.346317	0.745705	protein_coding
ENSG0000(	LINC01318	54.38776	0.195532	0.346266	0.745705	lncRNA
ENSG0000(	WDR11	2449.278	0.054674	0.346308	0.745705	protein_coding
ENSG0000(	ATP1B4	140.0096	0.09921	0.346364	0.745749	protein_coding
ENSG0000(	LINC01036	195.2275	0.119417	0.346458	0.74583	lncRNA
ENSG0000(	SOCS5P4	80.71511	0.154322	0.346483	0.74583	processed_pseudogene
ENSG0000(	TRAF3IP3	1258.319	-0.21248	0.346469	0.74583	protein_coding
ENSG0000(	POF1B	1972.592	0.207059	0.346524	0.74586	protein_coding
ENSG0000(	ENSG0000(	102.1805	0.128548	0.346643	0.746049	lncRNA

ENSG0000(	H2BC9	115.3747	-0.15532	0.346667	0.746049	protein_coding
ENSG0000(	ARHGEF26-	1193.528	-0.05503	0.346716	0.74607	lncRNA
ENSG0000(	ENSG0000(	483.1058	-0.08846	0.346731	0.74607	TEC
ENSG0000(	ENSG0000(	38.51471	-0.19256	0.346794	0.746149	processed_pseudogene
ENSG0000(	ENSG0000(	33.89316	-0.2352	0.346839	0.746187	lncRNA
ENSG0000(	ANGPTL4	117.1561	-0.20853	0.34706	0.746213	protein_coding
ENSG0000(	EEFSEC	165.3636	0.13087	0.346881	0.746213	protein_coding
ENSG0000(	EMG1	777.9209	-0.07771	0.347001	0.746213	protein_coding
ENSG0000(	ENSG0000(	33.37886	0.201681	0.346955	0.746213	lncRNA
ENSG0000(	ENSG0000(	199.9528	-0.09675	0.347068	0.746213	lncRNA
ENSG0000(	SLC12A9	1877.987	-0.08048	0.347038	0.746213	protein_coding
ENSG0000(	SPATA24	31.67431	0.220148	0.346985	0.746213	protein_coding
ENSG0000(	TRMU	995.5731	0.063884	0.347015	0.746213	protein_coding
ENSG0000(	ENSG0000(	403.4064	0.078004	0.347143	0.746315	lncRNA
ENSG0000(	LINC01354	33.60834	0.27487	0.347177	0.74633	lncRNA
ENSG0000(	CCDC138	216.7056	-0.1132	0.347478	0.746401	protein_coding
ENSG0000(	CPLANE2	45.04784	0.207445	0.347526	0.746401	protein_coding
ENSG0000(	ENSG0000(	315.907	0.149454	0.347402	0.746401	protein_coding
ENSG0000(	ENSG0000(	19.68896	-0.28605	0.347255	0.746401	TEC
ENSG0000(	KCTD13-DT	266.4841	0.080654	0.347482	0.746401	lncRNA
ENSG0000(	LINC02179	43.32078	0.174319	0.347536	0.746401	lncRNA
ENSG0000(	OR2H2	151.7689	-0.11348	0.347427	0.746401	protein_coding
ENSG0000(	POGLUT1	1020.946	-0.09755	0.347419	0.746401	protein_coding
ENSG0000(	PTGES3L-A.	23.67819	0.426895	0.347311	0.746401	protein_coding
ENSG0000(	PYCR3	243.1316	0.116797	0.347362	0.746401	protein_coding
ENSG0000(	RLF	1033.34	-0.07448	0.347452	0.746401	protein_coding
ENSG0000(	RN7SL834F	198.0712	-0.16947	0.347381	0.746401	misc_RNA
ENSG0000(	OR6M2P	62.11406	0.119421	0.347656	0.746485	transcribed_unprocessed_pseudo
ENSG0000(	PIK3C2G	87.528	-0.19014	0.347649	0.746485	protein_coding
ENSG0000(	SNORA31	95.11842	0.139182	0.347683	0.746485	snoRNA
ENSG0000(	TRAK1	5009.294	0.099584	0.347617	0.746485	protein_coding
ENSG0000(	CHKB-CPT1	177.3768	0.212813	0.347739	0.746493	protein_coding
ENSG0000(	ENSG0000(	129.4181	0.139759	0.347769	0.746493	lncRNA
ENSG0000(	ENSG0000(	150.6651	0.12441	0.347823	0.746493	lncRNA
ENSG0000(	RNU1-148F	21.29853	-0.32012	0.347802	0.746493	snRNA
ENSG0000(	RPL7P49	47.42004	0.197437	0.347814	0.746493	processed_pseudogene
ENSG0000(	EMC10	2245.782	0.07092	0.347936	0.746494	protein_coding
ENSG0000(	ENSG0000(	121.8391	0.134306	0.347954	0.746494	lncRNA
ENSG0000(	MIDEAS	5310.106	0.113888	0.347906	0.746494	protein_coding
ENSG0000(	MRPL35	769.4253	0.119324	0.347959	0.746494	protein_coding
ENSG0000(	SRXN1	242.6706	-0.16603	0.347956	0.746494	protein_coding
ENSG0000(	ENSG0000(	2374.654	0.047398	0.348071	0.746572	lncRNA
ENSG0000(	MKRN1	2921.443	0.116616	0.348076	0.746572	protein_coding
ENSG0000(	PVRIG	60.38602	0.19258	0.348039	0.746572	protein_coding
ENSG0000(	CLDN16	149.5969	0.125487	0.348169	0.746674	protein_coding
ENSG0000(	ENSG0000(	527.1152	0.081132	0.348205	0.746674	lncRNA
ENSG0000(	PLA2G4B	351.1218	-0.19156	0.348197	0.746674	protein_coding

ENSG0000(	INVS	1234.747	-0.05887	0.348392	0.747017	protein_coding
ENSG0000(	LINC01179	135.3227	0.147133	0.348444	0.747068	lncRNA
ENSG0000(	AP3S2	2463.756	0.116291	0.348792	0.747295	protein_coding
ENSG0000(	ENSG0000(	21.93135	-0.22096	0.348984	0.747295	lncRNA
ENSG0000(	ENSG0000(	101.0475	0.225634	0.348754	0.747295	TEC
ENSG0000(	ENSG0000(	166.6308	-0.09738	0.348746	0.747295	lncRNA
ENSG0000(	ENSG0000(	62.05385	-0.20226	0.348626	0.747295	lncRNA
ENSG0000(	ENSG0000(	31.03652	0.379221	0.348981	0.747295	lncRNA
ENSG0000(	FKBP8	606.2649	-0.107	0.348771	0.747295	protein_coding
ENSG0000(	LINC02014	24.32805	0.218028	0.348693	0.747295	lncRNA
ENSG0000(	MAL	29.79416	0.341188	0.348894	0.747295	protein_coding
ENSG0000(	OLR1	194.7469	-0.11984	0.348738	0.747295	protein_coding
ENSG0000(	PHF3	8607.638	0.082299	0.348823	0.747295	protein_coding
ENSG0000(	PPP4C	475.4595	-0.09276	0.34897	0.747295	protein_coding
ENSG0000(	RAB2B	999.9193	-0.06891	0.34878	0.747295	protein_coding
ENSG0000(	RN7SL840F	20.93264	0.243012	0.348872	0.747295	misc_RNA
ENSG0000(	SLC4A1AP	943.9277	-0.05737	0.348924	0.747295	protein_coding
ENSG0000(	SPTSSB	19.71812	0.412521	0.348836	0.747295	protein_coding
ENSG0000(	LINC02388	508.7441	0.076384	0.349074	0.747429	lncRNA
ENSG0000(	MYO5B	3271.265	0.185993	0.349101	0.747429	protein_coding
ENSG0000(	ENSG0000(	67.52259	-0.17706	0.349143	0.74746	lncRNA
ENSG0000(	ADGRG6	878.69	-0.17489	0.350073	0.747479	protein_coding
ENSG0000(	ADTRP	2177.852	0.180214	0.350184	0.747479	protein_coding
ENSG0000(	BDNF	35.91394	-0.31338	0.349453	0.747479	protein_coding
ENSG0000(	CDK9	826.9436	0.072071	0.350127	0.747479	protein_coding
ENSG0000(	CYFIP1	2057.079	0.09573	0.349271	0.747479	protein_coding
ENSG0000(	DNAJA4	1187.473	0.13344	0.349207	0.747479	protein_coding
ENSG0000(	ENSG0000(	118.5322	0.112376	0.349512	0.747479	lncRNA
ENSG0000(	ENSG0000(	296.1976	-0.09996	0.349956	0.747479	lncRNA
ENSG0000(	ENSG0000(	83.75348	-0.10602	0.349481	0.747479	lncRNA
ENSG0000(	ENSG0000(	547.4329	0.067199	0.3492	0.747479	lncRNA
ENSG0000(	ENSG0000(	554.3037	-0.07738	0.349834	0.747479	lncRNA
ENSG0000(	ENSG0000(	27.5848	-0.19662	0.349353	0.747479	lncRNA
ENSG0000(	ENSG0000(	968.9596	0.192157	0.35011	0.747479	lncRNA
ENSG0000(	ENSG0000(	118.9167	-0.1297	0.349806	0.747479	lncRNA
ENSG0000(	ENSG0000(	254.3459	0.080707	0.349238	0.747479	lncRNA
ENSG0000(	ENSG0000(	79.41941	0.132754	0.349969	0.747479	lncRNA
ENSG0000(	ENSG0000(	363.4245	0.064705	0.350176	0.747479	lncRNA
ENSG0000(	ENSG0000(	33.63869	-0.24376	0.349345	0.747479	lncRNA
ENSG0000(	ENSG0000(	46.9495	-0.15779	0.349656	0.747479	lncRNA
ENSG0000(	FAM66D	218.8194	-0.20114	0.349909	0.747479	lncRNA
ENSG0000(	KIAA1328	2819.344	0.088167	0.349656	0.747479	protein_coding
ENSG0000(	KIFAP3	624.6134	0.102678	0.349957	0.747479	protein_coding
ENSG0000(	LINC00311	499.942	-0.08237	0.349812	0.747479	lncRNA
ENSG0000(	LINC01229	51.64445	-0.3114	0.349443	0.747479	lncRNA
ENSG0000(	LY9	801.1114	-0.1648	0.34965	0.747479	protein_coding
ENSG0000(	MT-ND5	18090.99	-0.18464	0.350086	0.747479	protein_coding

ENSG0000(	MT-TT	97.0484	-0.49122	0.349641	0.747479	Mt_tRNA
ENSG0000(	OSBPL10	1495.24	-0.08256	0.349723	0.747479	protein_coding
ENSG0000(	PCBP1-AS1	5568.038	-0.04296	0.349366	0.747479	lncRNA
ENSG0000(	PPP1R8P1	42.75784	-0.22851	0.349903	0.747479	processed_pseudogene
ENSG0000(	PSMC6	1456.843	-0.04308	0.349469	0.747479	protein_coding
ENSG0000(	SMG1P5	3220.412	0.252106	0.350004	0.747479	transcribed_unprocessed_pseudo
ENSG0000(	TNK1	265.1677	0.170299	0.349594	0.747479	protein_coding
ENSG0000(	TRAT1	77.37364	-0.30271	0.350169	0.747479	protein_coding
ENSG0000(	VAV2	808.9686	0.133668	0.349721	0.747479	protein_coding
ENSG0000(	ZNF200	996.5681	-0.0672	0.350147	0.747479	protein_coding
ENSG0000(	ZNF831	737.6657	-0.30413	0.350105	0.747479	protein_coding
ENSG0000(	ZRANB1	1063.152	-0.0879	0.34938	0.747479	protein_coding
ENSG0000(	ENSG0000(	101.5364	0.122992	0.350245	0.747493	lncRNA
ENSG0000(	NOP58	719.686	-0.10005	0.350223	0.747493	protein_coding
ENSG0000(	CCL3-AS1	79.3698	-0.18195	0.350329	0.747543	lncRNA
ENSG0000(	ENSG0000(	38.79608	0.210692	0.35035	0.747543	unprocessed_pseudogene
ENSG0000(	ENSG0000(	255.8092	0.083071	0.35031	0.747543	lncRNA
ENSG0000(	ENSG0000(	1268.759	0.058738	0.350399	0.747557	lncRNA
ENSG0000(	LENG1	74.4288	0.14914	0.35041	0.747557	protein_coding
ENSG0000(	AFF1	6983.528	0.071108	0.350505	0.7477	protein_coding
ENSG0000(	NUDT19	557.8447	0.073295	0.350555	0.747748	protein_coding
ENSG0000(	IRF5	340.8154	-0.12108	0.350589	0.747764	protein_coding
ENSG0000(	TRPM3	264.3718	-0.07783	0.350701	0.747927	protein_coding
ENSG0000(	UNC5C	497.3539	-0.16042	0.35072	0.747927	protein_coding
ENSG0000(	ANKZF1	1360.297	-0.05909	0.350803	0.748046	protein_coding
ENSG0000(	ENSG0000(	25.59322	0.300044	0.350872	0.748128	transcribed_unprocessed_pseudo
ENSG0000(	INSYN1	191.027	0.081666	0.35095	0.748128	protein_coding
ENSG0000(	SCAI	1643.783	0.082902	0.350923	0.748128	protein_coding
ENSG0000(	SEC14L4	193.0298	-0.09052	0.35091	0.748128	protein_coding
ENSG0000(	ABCA11P	230.4841	0.122768	0.351052	0.748248	transcribed_processed_pseudoge
ENSG0000(	ENSG0000(	270.6362	-0.1542	0.351088	0.748248	TEC
ENSG0000(	LINC01795	98.85974	0.149107	0.351088	0.748248	lncRNA
ENSG0000(	FCRL3	820.6407	-0.18699	0.351129	0.748277	protein_coding
ENSG0000(	CLEC17A	341.4497	-0.20298	0.351336	0.748434	protein_coding
ENSG0000(	ENSG0000(	187.8948	0.266638	0.351332	0.748434	lncRNA
ENSG0000(	GASAL1	106.8054	0.129076	0.351382	0.748434	lncRNA
ENSG0000(	LINC00205	736.1343	-0.145	0.35131	0.748434	lncRNA
ENSG0000(	ORMDL2	544.47	0.078376	0.351393	0.748434	protein_coding
ENSG0000(	PLCXD2	705.7565	0.106439	0.351356	0.748434	protein_coding
ENSG0000(	RPGR	897.6051	0.087687	0.351339	0.748434	protein_coding
ENSG0000(	KMT2C	17864.93	0.041951	0.351456	0.748511	protein_coding
ENSG0000(	EDA2R	69.15386	-0.25804	0.351576	0.748533	protein_coding
ENSG0000(	EIF3M	1218.333	-0.08737	0.351524	0.748533	protein_coding
ENSG0000(	MRPS31	321.7201	-0.08501	0.351522	0.748533	protein_coding
ENSG0000(	PPP1R3B	937.2831	-0.10636	0.351568	0.748533	protein_coding
ENSG0000(	PDE3B	2015.242	0.087544	0.351617	0.748563	protein_coding
ENSG0000(	NDUFV3	2036.713	0.057636	0.351682	0.748626	protein_coding

ENSG0000(	PIAS1	2473.688	-0.0662	0.351701	0.748626	protein_coding
ENSG0000(	ENSG0000(	157.0099	-0.14066	0.351746	0.748663	lncRNA
ENSG0000(	PKD2L2	204.6279	-0.12151	0.351855	0.748838	protein_coding
ENSG0000(	ENSG0000(	134.8893	0.211688	0.351977	0.749041	TEC
ENSG0000(	RNU6-1093	38.97524	0.296382	0.352075	0.749175	snRNA
ENSG0000(	ZNF415	224.4659	0.120249	0.352095	0.749175	protein_coding
ENSG0000(	CAMLG	1140.883	-0.07081	0.352123	0.749177	protein_coding
ENSG0000(	PIK3CA	1703.213	-0.07683	0.352152	0.749182	protein_coding
ENSG0000(	HIVEP2	1879.671	-0.16121	0.352235	0.749265	protein_coding
ENSG0000(	NTRK1	147.4254	-0.14001	0.352246	0.749265	protein_coding
ENSG0000(	ARSD-AS1	35.01173	0.340581	0.352547	0.749408	lncRNA
ENSG0000(	BROX	1662.362	0.106379	0.352722	0.749408	protein_coding
ENSG0000(	C10orf99	941.7011	0.280825	0.352568	0.749408	protein_coding
ENSG0000(	CD300LD	62.66253	0.135537	0.352561	0.749408	protein_coding
ENSG0000(	CDCA2	182.2652	0.199607	0.352686	0.749408	protein_coding
ENSG0000(	ENSG0000(	158.2613	-0.14606	0.35268	0.749408	lncRNA
ENSG0000(	ENSG0000(	133.3009	-0.09761	0.352723	0.749408	lncRNA
ENSG0000(	ENSG0000(	114.1261	0.114193	0.35262	0.749408	TEC
ENSG0000(	KCNJ16	98.51888	0.13649	0.352677	0.749408	protein_coding
ENSG0000(	MED14	2122.645	0.077903	0.352652	0.749408	protein_coding
ENSG0000(	MORC2-AS	138.9937	0.154529	0.352773	0.749408	lncRNA
ENSG0000(	MSRB2	208.9171	-0.12561	0.352647	0.749408	protein_coding
ENSG0000(	NAPG	1435.119	-0.06035	0.352753	0.749408	protein_coding
ENSG0000(	OCA2	23.32503	-0.40613	0.352777	0.749408	protein_coding
ENSG0000(	STARD4-AS	126.8423	0.126871	0.352518	0.749408	lncRNA
ENSG0000(	VIRMA-DT	165.1002	-0.14617	0.35276	0.749408	lncRNA
ENSG0000(	ZNF785	1421.34	0.044403	0.352736	0.749408	protein_coding
ENSG0000(	ENSG0000(	182.2083	0.125425	0.352877	0.749564	lncRNA
ENSG0000(	CAB39L	205.8993	0.141764	0.353024	0.749806	protein_coding
ENSG0000(	LINC01579	1379.078	-0.05866	0.353045	0.749806	lncRNA
ENSG0000(	RCC2	1805.988	-0.07578	0.353095	0.749854	protein_coding
ENSG0000(	ENSG0000(	183.0015	0.091607	0.353208	0.750035	lncRNA
ENSG0000(	ENSG0000(	73.51222	0.134017	0.353402	0.750157	lncRNA
ENSG0000(	ENSG0000(	72.31153	-0.19752	0.353399	0.750157	processed_pseudogene
ENSG0000(	ENSG0000(	127.8508	0.089575	0.35333	0.750157	lncRNA
ENSG0000(	HEATR6	467.6619	-0.09014	0.353374	0.750157	protein_coding
ENSG0000(	LINC00933	146.1602	0.103342	0.353311	0.750157	transcribed_unprocessed_pseudo
ENSG0000(	DOC2GP	30.60889	0.223701	0.353431	0.750162	transcribed_unitary_pseudogene
ENSG0000(	CDH8-AS1	97.30817	0.126567	0.353492	0.750193	lncRNA
ENSG0000(	EML5	316.2297	0.115137	0.353546	0.750193	protein_coding
ENSG0000(	PPP4R1L	722.6997	-0.08891	0.353508	0.750193	transcribed_unprocessed_pseudo
ENSG0000(	TRAPPC1	180.5993	-0.10966	0.353555	0.750193	protein_coding
ENSG0000(	CDC23	449.9396	-0.09277	0.353676	0.750345	protein_coding
ENSG0000(	DHRS9	2492.999	0.240353	0.353899	0.750345	protein_coding
ENSG0000(	ENSG0000(	543.5374	0.063618	0.353853	0.750345	lncRNA
ENSG0000(	ENSG0000(	134.5307	-0.20483	0.353898	0.750345	protein_coding
ENSG0000(	ENSG0000(	168.9446	-0.08629	0.35387	0.750345	lncRNA

ENSG0000(	GNPTG	920.8403	-0.06335	0.353801	0.750345	protein_coding
ENSG0000(	MCM3AP-/	319.5685	0.093781	0.353831	0.750345	lncRNA
ENSG0000(	MRPS27	879.4771	-0.13264	0.353893	0.750345	protein_coding
ENSG0000(	THEM4	1378.759	0.08083	0.353858	0.750345	protein_coding
ENSG0000(	ZER1	895.2477	0.119955	0.353811	0.750345	protein_coding
ENSG0000(	HPX	130.968	-0.26752	0.353983	0.750425	protein_coding
ENSG0000(	URB1	1328.798	-0.10686	0.353992	0.750425	protein_coding
ENSG0000(	IRX2	20.80707	-0.26763	0.354043	0.750477	protein_coding
ENSG0000(	TMEM161E	10368.97	0.074343	0.354116	0.750573	lncRNA
ENSG0000(	ENSG0000(	232.8608	-0.0723	0.354214	0.750723	lncRNA
ENSG0000(	RRP7A	1883.184	0.127588	0.354261	0.750765	protein_coding
ENSG0000(	ENSG0000(	83.42837	-0.1436	0.354435	0.750844	lncRNA
ENSG0000(	JAG1	2870.74	0.105991	0.354345	0.750844	protein_coding
ENSG0000(	LMNTD1	141.4663	0.125321	0.354416	0.750844	protein_coding
ENSG0000(	RAP1A	1194.007	-0.07587	0.354416	0.750844	protein_coding
ENSG0000(	UBE2G1	996.2722	-0.09281	0.354419	0.750844	protein_coding
ENSG0000(	ENSG0000(	56.66953	0.529637	0.354483	0.750847	lncRNA
ENSG0000(	TM4SF19-/	170.6694	0.152588	0.354491	0.750847	lncRNA
ENSG0000(	PIKFYVE	2746.495	-0.08948	0.354586	0.750991	protein_coding
ENSG0000(	CDK5RAP2	3106.072	-0.06531	0.354619	0.751004	protein_coding
ENSG0000(	NTAN1	713.7694	0.092801	0.354651	0.751013	protein_coding
ENSG0000(	ENSG0000(	65.04355	-0.15408	0.35468	0.751017	lncRNA
ENSG0000(	ENSG0000(	580.6423	-0.07857	0.354718	0.751038	TEC
ENSG0000(	C6orf141	438.9841	0.126977	0.354827	0.75118	protein_coding
ENSG0000(	ENSG0000(	172.0581	0.10108	0.354894	0.75118	lncRNA
ENSG0000(	H2AC16	47.70653	-0.26955	0.354886	0.75118	protein_coding
ENSG0000(	MUC13	6360.677	-0.21824	0.354845	0.75118	protein_coding
ENSG0000(	DYNLT2	222.9681	0.104281	0.354949	0.751181	protein_coding
ENSG0000(	TMEM45B	2762.585	0.196747	0.354931	0.751181	protein_coding
ENSG0000(	ASPH	4142.716	-0.101	0.354986	0.751201	protein_coding
ENSG0000(	ENSG0000(	35.79675	-0.18355	0.355038	0.751255	lncRNA
ENSG0000(	ARRDC5	205.1359	-0.21551	0.355143	0.751301	protein_coding
ENSG0000(	ENSG0000(	29.82774	0.214281	0.355188	0.751301	lncRNA
ENSG0000(	ENSG0000(	243.698	-0.11501	0.355271	0.751301	TEC
ENSG0000(	ENSG0000(	122.3335	0.110749	0.355168	0.751301	lncRNA
ENSG0000(	LINC01237	279.9446	0.117549	0.355205	0.751301	lncRNA
ENSG0000(	R3HCC1L	1132.25	0.061911	0.355203	0.751301	protein_coding
ENSG0000(	RANBP3	1196.837	-0.04895	0.355279	0.751301	protein_coding
ENSG0000(	ZNF132-DT	919.5576	0.058402	0.355278	0.751301	lncRNA
ENSG0000(	AGBL3	125.0158	-0.18028	0.355352	0.751393	protein_coding
ENSG0000(	ALPI	72.19533	0.482443	0.355661	0.751393	protein_coding
ENSG0000(	ATP13A2	609.6155	-0.10769	0.355637	0.751393	protein_coding
ENSG0000(	CHGB	224.7121	0.114406	0.355648	0.751393	protein_coding
ENSG0000(	DNAJC2	873.589	-0.0687	0.35554	0.751393	protein_coding
ENSG0000(	ENSG0000(	22.79656	-0.27827	0.355736	0.751393	lncRNA
ENSG0000(	ENSG0000(	27.55532	0.23657	0.355437	0.751393	TEC
ENSG0000(	ENSG0000(	164.6264	0.110412	0.355698	0.751393	TEC

ENSG0000(	ENSG0000(	199.4546	0.112376	0.355547	0.751393	lncRNA
ENSG0000(	FMC1	194.6311	-0.0926	0.355759	0.751393	protein_coding
ENSG0000(	LINC02754	2270.463	0.120157	0.35556	0.751393	lncRNA
ENSG0000(	MINCR	90.99991	-0.18467	0.355409	0.751393	lncRNA
ENSG0000(	PIBF1	795.7802	0.102867	0.355622	0.751393	protein_coding
ENSG0000(	PTCD2	940.6955	0.067476	0.355737	0.751393	protein_coding
ENSG0000(	RBMY2TP	101.5149	-0.30387	0.35543	0.751393	unprocessed_pseudogene
ENSG0000(	VANGL1	737.5078	0.180216	0.355627	0.751393	protein_coding
ENSG0000(	BCS1L	452.0648	0.086915	0.356419	0.751482	protein_coding
ENSG0000(	CLN8-AS1	494.6365	-0.06649	0.356358	0.751482	lncRNA
ENSG0000(	ENSG0000(	167.2994	-0.12156	0.356059	0.751482	lncRNA
ENSG0000(	ENSG0000(	350.0001	0.095252	0.356062	0.751482	lncRNA
ENSG0000(	ENSG0000(	51.7235	-0.19406	0.35643	0.751482	lncRNA
ENSG0000(	ENSG0000(	34.31985	-0.26348	0.356256	0.751482	lncRNA
ENSG0000(	ENSG0000(	109.9252	0.118106	0.356113	0.751482	lncRNA
ENSG0000(	ENSG0000(	136.2955	0.202346	0.35632	0.751482	lncRNA
ENSG0000(	ENSG0000(	197.6839	0.112027	0.356003	0.751482	lncRNA
ENSG0000(	ENSG0000(	45.28189	-0.18036	0.356314	0.751482	lncRNA
ENSG0000(	ENSG0000(	235.6974	0.243816	0.356053	0.751482	lncRNA
ENSG0000(	FUT4	1761.07	0.102746	0.356153	0.751482	protein_coding
ENSG0000(	GDPGP1	1018.892	0.059738	0.35631	0.751482	protein_coding
ENSG0000(	GPATCH2L	4946.661	-0.07138	0.356128	0.751482	protein_coding
ENSG0000(	H2AJ	440.6576	0.135053	0.356484	0.751482	protein_coding
ENSG0000(	IGKV1D-8	20.53193	-0.51625	0.356481	0.751482	IG_V_gene
ENSG0000(	LINC02042	248.6	0.089448	0.356402	0.751482	lncRNA
ENSG0000(	LINC02261	291.3683	0.072665	0.356063	0.751482	lncRNA
ENSG0000(	PTPN2	2713.53	-0.08033	0.355894	0.751482	protein_coding
ENSG0000(	S100A13	173.745	-0.18021	0.356031	0.751482	protein_coding
ENSG0000(	STAP1	110.3863	-0.36658	0.35592	0.751482	protein_coding
ENSG0000(	TBC1D3I	137.7875	0.224239	0.356444	0.751482	protein_coding
ENSG0000(	TCL6	590.9125	-0.11337	0.356266	0.751482	lncRNA
ENSG0000(	TPI1	1753.756	-0.1116	0.356386	0.751482	protein_coding
ENSG0000(	UGT2B15	133.6525	0.305482	0.356428	0.751482	protein_coding
ENSG0000(	FAM86HP	417.2701	0.106695	0.356541	0.751545	transcribed_unprocessed_pseudo
ENSG0000(	SOX9	617.2526	0.198008	0.356575	0.751558	protein_coding
ENSG0000(	ENSG0000(	139.2899	0.129536	0.35669	0.751686	processed_pseudogene
ENSG0000(	SLC7A2-IT1	62.75097	-0.13026	0.356684	0.751686	TEC
ENSG0000(	ACY3	62.63818	0.24191	0.356731	0.751714	protein_coding
ENSG0000(	CRKL	1589.587	0.053646	0.356818	0.751782	protein_coding
ENSG0000(	NCMAP-DT	42.08193	0.221351	0.356811	0.751782	lncRNA
ENSG0000(	ENSG0000(	755.3785	-0.11337	0.356961	0.75194	lncRNA
ENSG0000(	LINC00691	263.5486	-0.08632	0.357054	0.75194	lncRNA
ENSG0000(	LIX1L	1031.379	-0.09167	0.357057	0.75194	protein_coding
ENSG0000(	NCAM2	77.21772	0.339646	0.356992	0.75194	protein_coding
ENSG0000(	OR9K1P	86.58135	0.18062	0.357009	0.75194	transcribed_unitary_pseudogene
ENSG0000(	ZNF551	797.8155	0.091678	0.356993	0.75194	protein_coding
ENSG0000(	ENSG0000(	30.09293	0.183971	0.357104	0.751981	TEC

ENSG0000(	KAT2A	805.5723	0.104357	0.357131	0.751981	protein_coding
ENSG0000(	ENSG0000(	156.7538	-0.15772	0.357175	0.752017	transcribed_unprocessed_pseudo
ENSG0000(	ENSG0000(	23.01982	0.270103	0.357264	0.752138	transcribed_unprocessed_pseudo
ENSG0000(	LRRC17	39.10369	-0.20899	0.35729	0.752138	protein_coding
ENSG0000(	ZFAND2A-[	977.7036	-0.06297	0.357315	0.752138	lncRNA
ENSG0000(	PXN-AS1	385.4263	0.081129	0.357346	0.752146	lncRNA
ENSG0000(	NCAPD3	1416.802	-0.06849	0.357392	0.752185	protein_coding
ENSG0000(	RNU6-1053	30.62023	-0.19523	0.357472	0.752297	snRNA
ENSG0000(	ENSG0000(	28.76527	-0.26465	0.357838	0.752553	processed_pseudogene
ENSG0000(	ENSG0000(	470.9009	0.070617	0.357704	0.752553	lncRNA
ENSG0000(	ENSG0000(	105.4963	-0.11419	0.357809	0.752553	lncRNA
ENSG0000(	ENSG0000(	339.4464	0.111188	0.357867	0.752553	protein_coding
ENSG0000(	PCAT14	824.8453	0.08119	0.357816	0.752553	lncRNA
ENSG0000(	PTMA	4885.038	-0.09107	0.357669	0.752553	protein_coding
ENSG0000(	SNAI3	35.54639	-0.23162	0.357689	0.752553	protein_coding
ENSG0000(	SNX29P1	173.8176	0.167622	0.357782	0.752553	unprocessed_pseudogene
ENSG0000(	TBCD	2586.106	-0.06622	0.357855	0.752553	protein_coding
ENSG0000(	YEATS2-AS1	39.36473	0.192779	0.357834	0.752553	lncRNA
ENSG0000(	CA13	1068.754	0.154477	0.35792	0.75257	protein_coding
ENSG0000(	ENSG0000(	283.4773	-0.10293	0.35793	0.75257	lncRNA
ENSG0000(	ENSG0000(	141.1019	0.121916	0.358014	0.752671	lncRNA
ENSG0000(	TJP3	2019.157	0.211625	0.358033	0.752671	protein_coding
ENSG0000(	LINC01090	53.92672	-0.15506	0.358073	0.752681	lncRNA
ENSG0000(	RAD54L2	2159.911	-0.08415	0.358092	0.752681	protein_coding
ENSG0000(	CKMT1A	1320.762	0.214863	0.358199	0.75279	protein_coding
ENSG0000(	ENSG0000(	826.4726	0.166892	0.358182	0.75279	lncRNA
ENSG0000(	ATP6V1D	828.6615	0.072772	0.358278	0.75284	protein_coding
ENSG0000(	AURKA	224.9943	0.13024	0.358266	0.75284	protein_coding
ENSG0000(	ENSG0000(	2615.314	0.099011	0.358346	0.752908	lncRNA
ENSG0000(	ENSG0000(	84.59124	0.148727	0.358365	0.752908	lncRNA
ENSG0000(	ATP5MG	1470.149	0.083671	0.358503	0.753083	protein_coding
ENSG0000(	TRIM23	353.8314	-0.11686	0.358483	0.753083	protein_coding
ENSG0000(	C19orf12	738.9095	-0.0941	0.358581	0.753191	protein_coding
ENSG0000(	ENSG0000(	50.0182	-0.14785	0.35866	0.753298	lncRNA
ENSG0000(	CD96	692.7882	-0.16915	0.358726	0.753301	protein_coding
ENSG0000(	ENSG0000(	1727.514	-0.0667	0.358743	0.753301	lncRNA
ENSG0000(	ZNF550	510.2443	-0.09907	0.358714	0.753301	protein_coding
ENSG0000(	ENSG0000(	36.91595	-0.19685	0.358898	0.753383	lncRNA
ENSG0000(	IMPA1P1	23.10586	0.379975	0.358919	0.753383	transcribed_unprocessed_pseudo
ENSG0000(	NBAS	2121.304	-0.08901	0.358852	0.753383	protein_coding
ENSG0000(	TRIM6	118.9685	-0.12176	0.358917	0.753383	protein_coding
ENSG0000(	ZNF746	1527.046	-0.05122	0.358873	0.753383	protein_coding
ENSG0000(	ENSG0000(	42.19324	0.158996	0.358974	0.75344	lncRNA
ENSG0000(	CLEC18A	267.8316	0.1279	0.359099	0.753473	protein_coding
ENSG0000(	ENSG0000(	157.9327	-0.13487	0.359088	0.753473	lncRNA
ENSG0000(	RNF212	202.3722	0.125731	0.35904	0.753473	protein_coding
ENSG0000(	TTLL2	20.65853	0.280046	0.359051	0.753473	protein_coding

ENSG0000(	ENSG0000(	133.7517	0.100308	0.359198	0.753559	lncRNA
ENSG0000(	ENSG0000(	49.33954	0.138993	0.35925	0.753559	miRNA
ENSG0000(	HAUS8	530.8372	0.085076	0.359244	0.753559	protein_coding
ENSG0000(	POPDC3	24.564	-0.44763	0.359214	0.753559	protein_coding
ENSG0000(	CYCSP34	98.53328	0.16486	0.359277	0.75356	processed_pseudogene
ENSG0000(	PDXP	235.8134	0.185987	0.359333	0.753618	protein_coding
ENSG0000(	ENSG0000(	123.3338	0.12022	0.359457	0.753701	lncRNA
ENSG0000(	ENSG0000(	200.702	0.157537	0.359459	0.753701	lncRNA
ENSG0000(	LHX4	39.87082	0.327578	0.359595	0.753701	protein_coding
ENSG0000(	LINC00269	199.5615	0.100948	0.359568	0.753701	lncRNA
ENSG0000(	LINC01359	1002.584	0.083341	0.359517	0.753701	lncRNA
ENSG0000(	OSBPL9	1855.303	0.060425	0.359592	0.753701	protein_coding
ENSG0000(	SLC7A6	2528.532	-0.0747	0.359619	0.753701	protein_coding
ENSG0000(	TLCD4	1530.068	0.276058	0.359516	0.753701	protein_coding
ENSG0000(	ZNF318	1971.086	-0.12107	0.359503	0.753701	protein_coding
ENSG0000(	PLEKHH3	558.9819	-0.09832	0.359672	0.753733	protein_coding
ENSG0000(	WNT7B	168.4604	-0.1002	0.359689	0.753733	protein_coding
ENSG0000(	ENSG0000(	41.87887	0.182406	0.359747	0.753756	lncRNA
ENSG0000(	RNU6-444F	54.2008	-0.21378	0.359755	0.753756	snRNA
ENSG0000(	ENSG0000(	95.74199	0.101615	0.359874	0.753923	lncRNA
ENSG0000(	TNFSF9	24.79585	-0.1994	0.359889	0.753923	protein_coding
ENSG0000(	CHD4	3915.892	-0.06181	0.359965	0.753967	protein_coding
ENSG0000(	ENSG0000(	67.98577	-0.19049	0.359964	0.753967	lncRNA
ENSG0000(	LINC02117	50.50152	0.130018	0.360034	0.754054	lncRNA
ENSG0000(	MAPK10	3059.1	-0.06455	0.360082	0.754098	protein_coding
ENSG0000(	ENSG0000(	609.3983	0.069883	0.360197	0.754281	lncRNA
ENSG0000(	ENSG0000(	74.82081	-0.21251	0.360272	0.754286	lncRNA
ENSG0000(	HDAC6	1937.012	0.05039	0.360258	0.754286	protein_coding
ENSG0000(	HMGB1P1	47.53879	0.162166	0.360343	0.754286	protein_coding
ENSG0000(	MORN3	483.8845	-0.07358	0.360313	0.754286	protein_coding
ENSG0000(	PTTG1IP	2486.414	0.109977	0.360391	0.754286	protein_coding
ENSG0000(	SAP130	673.7187	0.077328	0.360369	0.754286	protein_coding
ENSG0000(	TMEM169	108.4829	0.118595	0.36029	0.754286	protein_coding
ENSG0000(	SMAD3	3402.855	-0.10103	0.360441	0.754332	protein_coding
ENSG0000(	DOCK3	37.276	0.252106	0.360478	0.754353	protein_coding
ENSG0000(	CAMK1	239.0316	-0.10064	0.360539	0.754422	protein_coding
ENSG0000(	CACNA1I	81.14504	-0.31625	0.360611	0.754515	protein_coding
ENSG0000(	ENSG0000(	31.8449	0.167535	0.360734	0.754647	lncRNA
ENSG0000(	ENSG0000(	97.09566	0.129856	0.360756	0.754647	lncRNA
ENSG0000(	FAM86JP	67.13254	0.184407	0.360706	0.754647	transcribed_unprocessed_pseudo
ENSG0000(	ENSG0000(	683.946	0.113306	0.360856	0.754769	lncRNA
ENSG0000(	MTCP1	77.39385	-0.12856	0.360869	0.754769	protein_coding
ENSG0000(	MARCKSL1	839.0921	-0.10856	0.360918	0.754814	protein_coding
ENSG0000(	MED29	688.7094	-0.07235	0.361011	0.754859	protein_coding
ENSG0000(	MYO7B	4805.493	0.169427	0.361022	0.754859	protein_coding
ENSG0000(	ZMYND8	2798.171	-0.1123	0.360976	0.754859	protein_coding
ENSG0000(	CDC6	395.6027	-0.13668	0.361091	0.754946	protein_coding

ENSG0000(	BCL2L2-PAI	122.3156	0.258929	0.361134	0.754979	protein_coding
ENSG0000(	ENSG0000(	24.3989	0.367486	0.361302	0.755199	lncRNA
ENSG0000(	ENTPD3-AS	556.9992	0.075026	0.361311	0.755199	lncRNA
ENSG0000(	UBE2R2	1062.368	-0.08049	0.361322	0.755199	protein_coding
ENSG0000(	PEX5L	191.634	0.129624	0.361441	0.75539	protein_coding
ENSG0000(	BAALC	52.15817	0.277955	0.36156	0.755479	protein_coding
ENSG0000(	ENSG0000(	1214.659	0.071527	0.361565	0.755479	lncRNA
ENSG0000(	KLHL42	1609.608	-0.09453	0.361525	0.755479	protein_coding
ENSG0000(	SNORA20	124.6515	-0.14712	0.361604	0.755485	snoRNA
ENSG0000(	TELO2	465.1692	0.096009	0.361623	0.755485	protein_coding
ENSG0000(	ENSG0000(	95.32489	-0.15434	0.36167	0.755526	lncRNA
ENSG0000(	PRKAR1B-A	21.6146	-0.28346	0.361761	0.755658	lncRNA
ENSG0000(	DGCR6	116.4398	0.174467	0.361855	0.75574	protein_coding
ENSG0000(	ENSG0000(	197.5855	0.095089	0.361852	0.75574	lncRNA
ENSG0000(	RNF168	1259.094	0.052474	0.361958	0.755896	protein_coding
ENSG0000(	OR7E47P	30.62598	0.252938	0.362052	0.756037	transcribed_unprocessed_pseudo
ENSG0000(	COPS3	283.1354	-0.1308	0.362213	0.756055	protein_coding
ENSG0000(	ENSG0000(	23.28776	0.279872	0.362317	0.756055	lncRNA
ENSG0000(	ENSG0000(	166.4517	0.139127	0.362141	0.756055	lncRNA
ENSG0000(	ENSG0000(	28.3429	0.365122	0.362119	0.756055	lncRNA
ENSG0000(	ENSG0000(	25.38965	-0.23992	0.362295	0.756055	lncRNA
ENSG0000(	FABP7	50.85324	-0.16541	0.362368	0.756055	protein_coding
ENSG0000(	FAM199X	1222.647	-0.07198	0.362384	0.756055	protein_coding
ENSG0000(	GRWD1	461.6479	0.081635	0.36217	0.756055	protein_coding
ENSG0000(	MATR3	5459.746	-0.05783	0.362191	0.756055	protein_coding
ENSG0000(	PUDP	324.105	0.097268	0.362391	0.756055	protein_coding
ENSG0000(	SDAD1-AS1	233.7391	0.112257	0.362345	0.756055	lncRNA
ENSG0000(	USP42	624.4479	0.06571	0.362248	0.756055	protein_coding
ENSG0000(	ENSG0000(	28.1342	-0.27067	0.362457	0.756103	lncRNA
ENSG0000(	NTNG1	46.04185	-0.38669	0.362493	0.756103	protein_coding
ENSG0000(	TMEM239	151.2568	0.097949	0.362496	0.756103	protein_coding
ENSG0000(	DCTN6	298.3163	-0.0808	0.36265	0.756365	protein_coding
ENSG0000(	CEP131	168.0513	0.160196	0.362714	0.756442	protein_coding
ENSG0000(	SLC30A3	285.9593	-0.09274	0.362885	0.756741	protein_coding
ENSG0000(	BAALC-AS1	396.5731	-0.07842	0.362918	0.756754	lncRNA
ENSG0000(	CLN5	4205.873	-0.04775	0.362975	0.756754	protein_coding
ENSG0000(	ENSG0000(	41.4062	0.180589	0.362989	0.756754	lncRNA
ENSG0000(	TXNRD2	717.7629	-0.06149	0.363001	0.756754	protein_coding
ENSG0000(	ENSG0000(	27.58602	0.189563	0.363048	0.756795	lncRNA
ENSG0000(	ENSG0000(	116.424	0.100016	0.36315	0.75695	lncRNA
ENSG0000(	RBM14-RBI	598.7222	0.094433	0.363319	0.757245	protein_coding
ENSG0000(	ENSG0000(	166.4927	0.073279	0.363352	0.757255	transcribed_processed_pseudoge
ENSG0000(	ARID4B	2345.9	-0.07331	0.363606	0.757335	protein_coding
ENSG0000(	DEFA6	123.2365	-1.53806	0.363481	0.757335	protein_coding
ENSG0000(	ENSG0000(	48.49958	0.144747	0.363797	0.757335	lncRNA
ENSG0000(	ENSG0000(	226.5266	0.088077	0.363783	0.757335	lncRNA
ENSG0000(	ENSG0000(	151.369	0.09875	0.363495	0.757335	lncRNA

ENSG0000(	ENSG0000(	22.12048	0.21304	0.363521	0.757335	lncRNA
ENSG0000(	FHDC1	889.8932	0.162607	0.363668	0.757335	protein_coding
ENSG0000(	GBF1	7790.644	-0.16025	0.363605	0.757335	protein_coding
ENSG0000(	IFI44	473.0565	0.340172	0.363909	0.757335	protein_coding
ENSG0000(	KIN	1135.754	0.067728	0.363724	0.757335	protein_coding
ENSG0000(	LARP7	1052.185	0.078491	0.363552	0.757335	protein_coding
ENSG0000(	MCU	1331.67	0.113307	0.363888	0.757335	protein_coding
ENSG0000(	MRPL39	97.89399	0.133095	0.363913	0.757335	protein_coding
ENSG0000(	PPP1R14D	300.107	0.217682	0.363767	0.757335	protein_coding
ENSG0000(	PRMT2	1838.777	-0.07996	0.363675	0.757335	protein_coding
ENSG0000(	SFN	755.711	0.207167	0.363833	0.757335	protein_coding
ENSG0000(	STAC3	96.31499	0.145104	0.36347	0.757335	protein_coding
ENSG0000(	WDR4	400.0198	0.113118	0.363805	0.757335	protein_coding
ENSG0000(	WDR54	238.4382	-0.09198	0.363616	0.757335	protein_coding
ENSG0000(	C1orf116	815.1378	0.199754	0.364004	0.757353	protein_coding
ENSG0000(	LINC01249	81.92328	0.17741	0.363964	0.757353	lncRNA
ENSG0000(	PDE7B	472.5518	0.108685	0.363986	0.757353	protein_coding
ENSG0000(	DAB1-AS1	65.74136	0.126661	0.364042	0.757353	lncRNA
ENSG0000(	ENSG0000(	35.82103	0.162036	0.364059	0.757353	lncRNA
ENSG0000(	AATK	414.1463	-0.16668	0.364333	0.7575	protein_coding
ENSG0000(	FAM200B	548.5814	0.065216	0.364332	0.7575	protein_coding
ENSG0000(	LARGE2	52.363	-0.26545	0.364187	0.7575	protein_coding
ENSG0000(	PAQR6	99.36391	0.143993	0.364312	0.7575	protein_coding
ENSG0000(	PLOD2	1770.854	-0.17198	0.364197	0.7575	protein_coding
ENSG0000(	PPP1R1C	48.26642	-0.15348	0.36435	0.7575	protein_coding
ENSG0000(	RELN	722.251	0.067271	0.364276	0.7575	protein_coding
ENSG0000(	ZNF813	684.1315	0.065442	0.364273	0.7575	protein_coding
ENSG0000(	ENSG0000(	58.87048	0.25698	0.364431	0.757554	processed_pseudogene
ENSG0000(	ENSG0000(	36.1134	-0.23312	0.364416	0.757554	lncRNA
ENSG0000(	CCR7	538.1253	-0.40589	0.36489	0.757692	protein_coding
ENSG0000(	CYP11A1	42.82367	-0.22282	0.364598	0.757692	protein_coding
ENSG0000(	ENSG0000(	27.80372	-0.21213	0.364596	0.757692	processed_pseudogene
ENSG0000(	ENSG0000(	57.77702	0.220153	0.364949	0.757692	lncRNA
ENSG0000(	ENSG0000(	68.33874	0.278237	0.364688	0.757692	lncRNA
ENSG0000(	ENSG0000(	33.76226	-0.18698	0.3647	0.757692	lncRNA
ENSG0000(	ITGB1-DT	139.6669	0.107261	0.364673	0.757692	lncRNA
ENSG0000(	LINC01567	179.022	-0.08833	0.364558	0.757692	lncRNA
ENSG0000(	LINC02517	165.799	0.132929	0.364774	0.757692	lncRNA
ENSG0000(	NECTIN3	1076.24	0.133942	0.364895	0.757692	protein_coding
ENSG0000(	NOX4	311.8662	-0.134	0.364965	0.757692	protein_coding
ENSG0000(	ODAPH	207.9289	0.150649	0.364764	0.757692	protein_coding
ENSG0000(	PTPN9	1345.786	-0.10391	0.364813	0.757692	protein_coding
ENSG0000(	STARD5	1118.721	0.102257	0.364902	0.757692	protein_coding
ENSG0000(	TAF1D	2849.804	-0.06778	0.364612	0.757692	protein_coding
ENSG0000(	UBALD1	356.3124	-0.10585	0.364818	0.757692	protein_coding
ENSG0000(	ZNF547	138.8176	-0.11209	0.364933	0.757692	protein_coding
ENSG0000(	GSS	1045.543	0.07616	0.365003	0.757713	protein_coding

ENSG0000(	TMEM145	336.3361	-0.13333	0.365094	0.757845	protein_coding
ENSG0000(	EIF5A	1394.023	-0.10436	0.365142	0.757865	protein_coding
ENSG0000(	ENSG0000(	219.377	-0.10957	0.365187	0.757865	TEC
ENSG0000(	GFOD2	2214.155	0.057158	0.365161	0.757865	protein_coding
ENSG0000(	ENSG0000(	829.6455	-0.06815	0.365243	0.757923	TEC
ENSG0000(	GRID2IP	68.42681	0.174642	0.365298	0.757923	protein_coding
ENSG0000(	LINC01269	35.25481	0.171703	0.365278	0.757923	lncRNA
ENSG0000(	PCAT7	339.2689	0.124322	0.365325	0.757923	lncRNA
ENSG0000(	ENSG0000(	275.3513	-0.07736	0.365371	0.757962	lncRNA
ENSG0000(	DTL	242.3048	-0.18867	0.365416	0.757972	protein_coding
ENSG0000(	STMND1	265.5508	0.07849	0.365431	0.757972	protein_coding
ENSG0000(	ENSG0000(	48.78829	0.186401	0.365476	0.758001	lncRNA
ENSG0000(	MYT1	89.76996	0.114274	0.3655	0.758001	protein_coding
ENSG0000(	MAN1A1	1627.096	-0.11276	0.365548	0.758043	protein_coding
ENSG0000(	MT-TR	74.41644	-0.5761	0.365658	0.758215	Mt_tRNA
ENSG0000(	ENSG0000(	26.25875	-0.2506	0.365718	0.758282	lncRNA
ENSG0000(	MAP2K5	708.5775	-0.0913	0.365754	0.758299	protein_coding
ENSG0000(	TSGA10	435.9654	0.096839	0.365786	0.758307	protein_coding
ENSG0000(	ABCC9	4440.893	-0.05005	0.365906	0.758311	protein_coding
ENSG0000(	ACP7	338.634	-0.0885	0.366764	0.758311	protein_coding
ENSG0000(	ARHGAP19	514.5104	-0.09752	0.366939	0.758311	protein_coding
ENSG0000(	ATXN7L3B	1634.233	0.061543	0.366371	0.758311	protein_coding
ENSG0000(	B4GALNT2	6989.261	0.189268	0.365887	0.758311	protein_coding
ENSG0000(	DEAF1	729.1713	0.068412	0.367	0.758311	protein_coding
ENSG0000(	ENSG0000(	973.302	0.056057	0.366697	0.758311	protein_coding
ENSG0000(	ENSG0000(	232.5976	0.109999	0.366822	0.758311	lncRNA
ENSG0000(	ENSG0000(	88.39867	-0.30833	0.366674	0.758311	lncRNA
ENSG0000(	ENSG0000(	41.8396	-0.14912	0.366879	0.758311	lncRNA
ENSG0000(	ENSG0000(	167.6769	0.152675	0.366854	0.758311	lncRNA
ENSG0000(	ENSG0000(	106.0499	0.200111	0.367081	0.758311	lncRNA
ENSG0000(	ENSG0000(	41.65876	0.155149	0.366706	0.758311	transcribed_processed_pseudoge
ENSG0000(	ENSG0000(	456.073	0.079402	0.36674	0.758311	TEC
ENSG0000(	ENSG0000(	178.4223	0.145519	0.366693	0.758311	lncRNA
ENSG0000(	ENSG0000(	85.66758	-0.20374	0.366558	0.758311	lncRNA
ENSG0000(	ENSG0000(	32.01792	0.409405	0.366929	0.758311	lncRNA
ENSG0000(	ENSG0000(	1131.149	-0.12288	0.366063	0.758311	lncRNA
ENSG0000(	FAM124B	101.824	-0.13799	0.36673	0.758311	protein_coding
ENSG0000(	FAM86C1P	469.7098	-0.07111	0.366067	0.758311	transcribed_unprocessed_pseudo
ENSG0000(	GMFB	1267.736	-0.07552	0.365865	0.758311	protein_coding
ENSG0000(	HMGN3	228.6115	-0.12487	0.366417	0.758311	protein_coding
ENSG0000(	KATNA1	171.656	0.10282	0.366767	0.758311	protein_coding
ENSG0000(	LARS1	5410.562	0.04717	0.366592	0.758311	protein_coding
ENSG0000(	LINC00484	136.3581	-0.13628	0.367063	0.758311	lncRNA
ENSG0000(	LINC02055	663.585	0.069644	0.366714	0.758311	lncRNA
ENSG0000(	MED12L	139.8094	-0.1578	0.365821	0.758311	protein_coding
ENSG0000(	MEIS3P1	66.84171	0.163866	0.366229	0.758311	processed_pseudogene
ENSG0000(	NME2	644.5056	-0.10827	0.365979	0.758311	protein_coding

ENSG0000(	NUDT1	113.1543	0.132104	0.366972	0.758311	protein_coding
ENSG0000(	PCED1B-AS	714.3742	-0.11309	0.366405	0.758311	lncRNA
ENSG0000(	PDZK1	77.96912	0.218091	0.366659	0.758311	protein_coding
ENSG0000(	PNMA2	255.9505	-0.11474	0.366637	0.758311	protein_coding
ENSG0000(	POLM	987.4877	0.05694	0.366127	0.758311	protein_coding
ENSG0000(	RN7SL346F	33.61015	-0.3462	0.366251	0.758311	misc_RNA
ENSG0000(	RN7SL38P	46.43229	-0.17796	0.366645	0.758311	misc_RNA
ENSG0000(	RSF1-IT1	59.91856	0.109453	0.365934	0.758311	lncRNA
ENSG0000(	RSU1	767.151	-0.09757	0.366751	0.758311	protein_coding
ENSG0000(	RWDD2A	216.3388	-0.1284	0.366065	0.758311	protein_coding
ENSG0000(	SHISAL1	308.9071	-0.19044	0.366729	0.758311	protein_coding
ENSG0000(	SLC38A4	232.1962	0.47693	0.366778	0.758311	protein_coding
ENSG0000(	SOX10	61.29716	0.238322	0.367067	0.758311	protein_coding
ENSG0000(	TASL	202.6845	-0.13209	0.366421	0.758311	protein_coding
ENSG0000(	TMEM70	140.5924	-0.16049	0.366733	0.758311	protein_coding
ENSG0000(	TRIM44	3098.795	0.060045	0.366934	0.758311	protein_coding
ENSG0000(	TTC8	420.2444	-0.09953	0.366538	0.758311	protein_coding
ENSG0000(	TUSC8	121.1918	0.190286	0.367088	0.758311	lncRNA
ENSG0000(	ZBTB40-IT1	34.36342	-0.17448	0.367111	0.758311	lncRNA
ENSG0000(	CYGB	251.3149	-0.18667	0.367194	0.758426	protein_coding
ENSG0000(	PANK1	280.6551	0.206501	0.367244	0.758474	protein_coding
ENSG0000(	ENSG0000(	123.3634	0.110162	0.36732	0.75849	lncRNA
ENSG0000(	ENSG0000(	215.5456	0.090766	0.367335	0.75849	lncRNA
ENSG0000(	KCTD9P4	43.26856	0.174968	0.367343	0.75849	processed_pseudogene
ENSG0000(	LINC01620	23.66574	-0.28438	0.367363	0.75849	lncRNA
ENSG0000(	ENSG0000(	49.39428	0.326905	0.367437	0.758529	lncRNA
ENSG0000(	NMNAT1	1346.286	0.058926	0.367432	0.758529	protein_coding
ENSG0000(	USP1	612.3412	0.076652	0.367473	0.758547	protein_coding
ENSG0000(	ENSG0000(	21.51075	0.252516	0.367511	0.758568	lncRNA
ENSG0000(	ENSG0000(	53.65576	-0.27356	0.367588	0.758613	lncRNA
ENSG0000(	VGLL4	1450.188	-0.0545	0.367582	0.758613	protein_coding
ENSG0000(	PPT2	388.8826	0.115325	0.367708	0.758805	protein_coding
ENSG0000(	NAA80	82.68827	0.159709	0.367823	0.758944	protein_coding
ENSG0000(	ZNF667	858.9223	0.058039	0.367831	0.758944	protein_coding
ENSG0000(	ARFGEF1	4833.618	0.067113	0.36797	0.759118	protein_coding
ENSG0000(	RP9P	176.3306	0.093883	0.367946	0.759118	transcribed_unprocessed_pseudo
ENSG0000(	CRIM1	1953.444	0.100273	0.368177	0.759286	protein_coding
ENSG0000(	EIF4G2	10546.15	0.064039	0.368154	0.759286	protein_coding
ENSG0000(	GTF2E2	395.9675	-0.09234	0.3682	0.759286	protein_coding
ENSG0000(	MSMO1	548.301	0.147926	0.368107	0.759286	protein_coding
ENSG0000(	RN7SL792F	67.14976	0.114029	0.368186	0.759286	misc_RNA
ENSG0000(	RNU6-98P	24.81751	-0.28057	0.368217	0.759286	snRNA
ENSG0000(	ACTE1P	193.249	0.09368	0.368934	0.75952	transcribed_unitary_pseudogene
ENSG0000(	CERS4	399.367	-0.16407	0.368994	0.75952	protein_coding
ENSG0000(	ENSG0000(	39.62221	-0.21665	0.368761	0.75952	lncRNA
ENSG0000(	ENSG0000(	24.83899	-0.23751	0.368567	0.75952	lncRNA
ENSG0000(	ENSG0000(	101.2209	-0.12821	0.368685	0.75952	lncRNA

ENSG0000(	ENSG0000(	70.24203	-0.1675	0.368629	0.75952	processed_pseudogene
ENSG0000(	ENSG0000(	30.50574	-0.21529	0.368861	0.75952	TEC
ENSG0000(	ENSG0000(	69.93863	-0.26547	0.368399	0.75952	lncRNA
ENSG0000(	FBXO30-DT	1914.163	0.097776	0.368604	0.75952	lncRNA
ENSG0000(	FDFT1	1440.38	0.114435	0.368978	0.75952	protein_coding
ENSG0000(	GDAP1	5227.607	-0.04997	0.368893	0.75952	protein_coding
ENSG0000(	HNRNPA1P	48.52324	0.20914	0.368815	0.75952	processed_pseudogene
ENSG0000(	L2HGDH	2384.335	0.057779	0.36894	0.75952	protein_coding
ENSG0000(	LINC00504	8671.753	0.081686	0.36892	0.75952	lncRNA
ENSG0000(	MEP1A	1122.268	0.297901	0.368636	0.75952	protein_coding
ENSG0000(	MLF1	110.1308	0.120786	0.368515	0.75952	protein_coding
ENSG0000(	RN7SL842P	44.3505	-0.16227	0.368419	0.75952	misc_RNA
ENSG0000(	SLAIN1	66.11446	-0.23703	0.368951	0.75952	protein_coding
ENSG0000(	TBRG1	1907.665	0.046336	0.3688	0.75952	protein_coding
ENSG0000(	TMC2	554.3213	-0.1004	0.368581	0.75952	protein_coding
ENSG0000(	TMEM81	47.22093	0.179043	0.368943	0.75952	protein_coding
ENSG0000(	TPR	4093.588	0.066278	0.36893	0.75952	protein_coding
ENSG0000(	ZDHHC12	214.1196	0.170714	0.368779	0.75952	protein_coding
ENSG0000(	ZNF718	692.5233	0.07411	0.368616	0.75952	protein_coding
ENSG0000(	MIR222HG	730.8887	-0.16917	0.369028	0.759534	lncRNA
ENSG0000(	LINC01234	658.3068	0.050063	0.369321	0.760024	lncRNA
ENSG0000(	SMIM31	786.9123	0.097034	0.369296	0.760024	protein_coding
ENSG0000(	BBS5	406.1902	-0.09258	0.369588	0.760088	protein_coding
ENSG0000(	ENSG0000(	221.6103	0.087529	0.369534	0.760088	TEC
ENSG0000(	ENSG0000(	120.0116	-0.10627	0.369665	0.760088	TEC
ENSG0000(	ENSG0000(	28.49939	-0.17365	0.369548	0.760088	lncRNA
ENSG0000(	ENSG0000(	25.61549	-0.25684	0.369427	0.760088	lncRNA
ENSG0000(	FLCN	2401.197	-0.08836	0.369543	0.760088	protein_coding
ENSG0000(	G3BP1P1	26.84522	-0.23376	0.369684	0.760088	processed_pseudogene
ENSG0000(	GOPC	1384.597	0.060582	0.369629	0.760088	protein_coding
ENSG0000(	LOXL4	69.41404	-0.16787	0.369387	0.760088	protein_coding
ENSG0000(	NGLY1	1608.248	-0.06893	0.369517	0.760088	protein_coding
ENSG0000(	SOX8	71.11354	0.313824	0.369487	0.760088	protein_coding
ENSG0000(	TRPA1	780.2064	-0.16374	0.369662	0.760088	protein_coding
ENSG0000(	BORA	243.868	-0.11037	0.369763	0.760089	protein_coding
ENSG0000(	ENSG0000(	44.35371	0.222342	0.369801	0.760089	unprocessed_pseudogene
ENSG0000(	ENSG0000(	190.6438	-0.1041	0.369812	0.760089	lncRNA
ENSG0000(	LINC00235	163.3627	0.100895	0.369787	0.760089	lncRNA
ENSG0000(	PTPDC1	543.7637	0.077359	0.369822	0.760089	protein_coding
ENSG0000(	TFDP1	830.751	0.092499	0.369911	0.760215	protein_coding
ENSG0000(	ENSG0000(	202.1496	0.117329	0.369967	0.760215	lncRNA
ENSG0000(	SCYL2	1731.301	0.081503	0.369941	0.760215	protein_coding
ENSG0000(	ENSG0000(	24.18458	0.320354	0.369994	0.760215	lncRNA
ENSG0000(	ENSG0000(	35.66615	-0.18743	0.370224	0.760291	lncRNA
ENSG0000(	ENSG0000(	126.6243	-0.15007	0.370171	0.760291	lncRNA
ENSG0000(	ENSG0000(	229.8598	0.082516	0.370205	0.760291	lncRNA
ENSG0000(	ENSG0000(	328.6095	0.070946	0.370201	0.760291	TEC

ENSG0000(	HID1	927.7525	0.105819	0.370125	0.760291	protein_coding
ENSG0000(	PA2G4	714.2536	-0.08731	0.370221	0.760291	protein_coding
ENSG0000(	RFK	719.0354	-0.15643	0.370221	0.760291	protein_coding
ENSG0000(	BHMT2	409.2057	-0.1314	0.3704	0.760368	protein_coding
ENSG0000(	CADM3-AS	179.4033	-0.21999	0.370384	0.760368	lncRNA
ENSG0000(	ENSG0000(	24.51158	0.654809	0.370336	0.760368	transcribed_unprocessed_pseudo
ENSG0000(	LINC00888	123.8427	-0.12841	0.370377	0.760368	transcribed_unprocessed_pseudo
ENSG0000(	SLC25A43	486.926	0.085364	0.370311	0.760368	protein_coding
ENSG0000(	TNFRSF11B	116.4207	-0.19601	0.37044	0.760394	protein_coding
ENSG0000(	ADGRG2	464.5935	0.096693	0.370528	0.760493	protein_coding
ENSG0000(	GRAP2	617.3714	-0.17084	0.370676	0.760493	protein_coding
ENSG0000(	RAB5IF	483.9774	-0.12779	0.37064	0.760493	protein_coding
ENSG0000(	SLC8A1-AS:	299.0619	0.106334	0.370603	0.760493	lncRNA
ENSG0000(	SRGAP2B	804.7251	-0.11127	0.370623	0.760493	protein_coding
ENSG0000(	SULT1A3	662.4162	0.112041	0.370642	0.760493	protein_coding
ENSG0000(	UBA3	687.7748	-0.09263	0.370682	0.760493	protein_coding
ENSG0000(	BBX	1794.933	-0.09316	0.370818	0.760587	protein_coding
ENSG0000(	CRIP3	59.52305	-0.34326	0.370984	0.760587	protein_coding
ENSG0000(	ENSG0000(	43.03201	-0.24607	0.370937	0.760587	lncRNA
ENSG0000(	ENSG0000(	24.27082	-0.25838	0.370949	0.760587	lncRNA
ENSG0000(	LINC00540	99.44451	-0.30097	0.371005	0.760587	lncRNA
ENSG0000(	LIX1L-AS1	187.4207	0.104905	0.370966	0.760587	lncRNA
ENSG0000(	LYPLA1	813.2529	-0.07575	0.370823	0.760587	protein_coding
ENSG0000(	OSGIN1	291.8449	-0.13699	0.370906	0.760587	protein_coding
ENSG0000(	PAXBP1-AS	1141.327	-0.05114	0.370929	0.760587	lncRNA
ENSG0000(	SUGT1	2975.58	-0.05007	0.370896	0.760587	protein_coding
ENSG0000(	GABARAPL:	513.129	-0.09515	0.371078	0.760681	protein_coding
ENSG0000(	AFDN-DT	535.293	0.096399	0.371131	0.760732	lncRNA
ENSG0000(	APEX1	589.0567	-0.08159	0.371343	0.760742	protein_coding
ENSG0000(	C3orf14	52.82239	-0.2198	0.37134	0.760742	protein_coding
ENSG0000(	DNHD1	1704.906	-0.07264	0.37138	0.760742	protein_coding
ENSG0000(	ENSG0000(	23.96031	0.364773	0.371281	0.760742	processed_pseudogene
ENSG0000(	ENSG0000(	64.06934	-0.14384	0.371442	0.760742	lncRNA
ENSG0000(	ENSG0000(	163.081	0.176573	0.371229	0.760742	TEC
ENSG0000(	GPR82	857.5646	0.087449	0.371324	0.760742	protein_coding
ENSG0000(	LDHAL6FP	33.9498	0.235724	0.371447	0.760742	processed_pseudogene
ENSG0000(	MORF4L2	1555.6	-0.07996	0.371467	0.760742	protein_coding
ENSG0000(	MPC2	631.1618	0.11198	0.371214	0.760742	protein_coding
ENSG0000(	ZNF629	678.9306	0.093499	0.3714	0.760742	protein_coding
ENSG0000(	ZNF806	195.346	0.093752	0.371271	0.760742	transcribed_unprocessed_pseudo
ENSG0000(	ABHD12	773.8646	-0.07336	0.371507	0.76076	protein_coding
ENSG0000(	DAND5	266.109	0.080091	0.371587	0.76076	protein_coding
ENSG0000(	ENSG0000(	172.6917	0.119541	0.371563	0.76076	lncRNA
ENSG0000(	WFDC11	112.1674	-0.1269	0.371552	0.76076	protein_coding
ENSG0000(	TPT1-AS1	3679.444	0.069369	0.371645	0.760823	lncRNA
ENSG0000(	HMG20A	1248.322	-0.06	0.371703	0.760828	protein_coding
ENSG0000(	TFF1	140.3154	-0.42717	0.371691	0.760828	protein_coding

ENSG0000(	ENSG0000(	272.983	0.07479	0.371771	0.760911	lncRNA
ENSG0000(	ENSG0000(	363.7642	0.060369	0.371986	0.761294	lncRNA
ENSG0000(	SNORD62A	37.81291	-0.17071	0.372068	0.761405	snoRNA
ENSG0000(	LDLRAD2	92.64275	-0.24893	0.372211	0.761641	protein_coding
ENSG0000(	ENSG0000(	53.78353	0.150959	0.372268	0.761701	lncRNA
ENSG0000(	TMEM106(	947.6015	0.124938	0.372327	0.761766	protein_coding
ENSG0000(	APAF1	1660.262	0.088425	0.37281	0.761846	protein_coding
ENSG0000(	CDCP1	2596.119	-0.13708	0.372802	0.761846	protein_coding
ENSG0000(	DICER1-AS1	990.8196	-0.06913	0.372764	0.761846	lncRNA
ENSG0000(	DMRTC1B	33.97244	0.226924	0.372464	0.761846	protein_coding
ENSG0000(	DNAJC6	138.147	-0.22761	0.372686	0.761846	protein_coding
ENSG0000(	EEF1B2	1133.924	-0.11808	0.372415	0.761846	protein_coding
ENSG0000(	ENSG0000(	30.90456	-0.1985	0.372684	0.761846	lncRNA
ENSG0000(	ENSG0000(	38.33885	-0.17409	0.372677	0.761846	lncRNA
ENSG0000(	ENSG0000(	28.64839	-0.21401	0.372494	0.761846	lncRNA
ENSG0000(	ENSG0000(	269.374	-0.06549	0.372597	0.761846	TEC
ENSG0000(	ENSG0000(	266.4752	0.090539	0.372564	0.761846	lncRNA
ENSG0000(	FRMD8	799.9442	-0.1032	0.372728	0.761846	protein_coding
ENSG0000(	IGFL4	2464.293	0.11961	0.372764	0.761846	protein_coding
ENSG0000(	LRP4	2142.84	-0.23934	0.372475	0.761846	protein_coding
ENSG0000(	RNU4-40P	31.27687	0.231096	0.372551	0.761846	snRNA
ENSG0000(	TFDP2	2599.583	0.084586	0.372566	0.761846	protein_coding
ENSG0000(	ARHGAP28	1290.915	-0.10209	0.372855	0.761882	protein_coding
ENSG0000(	CCM2L	55.78269	-0.21811	0.373105	0.762279	protein_coding
ENSG0000(	ENSG0000(	155.2511	-0.0989	0.373115	0.762279	unprocessed_pseudogene
ENSG0000(	KIF1A	146.9146	0.365575	0.373132	0.762279	protein_coding
ENSG0000(	CHRNA2	76.09038	0.189875	0.373225	0.762406	protein_coding
ENSG0000(	CROCC2	27.11336	0.343563	0.37325	0.762406	protein_coding
ENSG0000(	S1PR5	41.46104	-0.19332	0.37335	0.762553	protein_coding
ENSG0000(	ABHD12B	241.4506	0.123143	0.373474	0.762582	protein_coding
ENSG0000(	ENSG0000(	109.4985	-0.21428	0.373453	0.762582	lncRNA
ENSG0000(	ENSG0000(	187.3231	-0.09456	0.373403	0.762582	lncRNA
ENSG0000(	TCF20	3437.86	0.066822	0.373455	0.762582	protein_coding
ENSG0000(	SNORA47	87.16563	0.191176	0.373519	0.762616	snoRNA
ENSG0000(	ENSG0000(	49.16143	-0.14281	0.373669	0.762866	lncRNA
ENSG0000(	ENSG0000(	26.41747	0.209908	0.373801	0.76308	lncRNA
ENSG0000(	ADAT1	2207.973	-0.04883	0.373858	0.763082	protein_coding
ENSG0000(	ERI3-IT1	105.8337	0.108893	0.373857	0.763082	lncRNA
ENSG0000(	CD209	444.8666	-0.16411	0.373976	0.763116	protein_coding
ENSG0000(	ELOBP4	30.60882	0.230149	0.374019	0.763116	processed_pseudogene
ENSG0000(	ENSG0000(	29.39535	0.282455	0.373944	0.763116	processed_pseudogene
ENSG0000(	OR2A7	1135.338	0.246203	0.374038	0.763116	protein_coding
ENSG0000(	SLFN14	393.3478	0.075361	0.373935	0.763116	protein_coding
ENSG0000(	TRGC1	112.2173	-0.18059	0.374054	0.763116	TR_C_gene
ENSG0000(	TRIM26	1312.674	-0.06961	0.374069	0.763116	protein_coding
ENSG0000(	MT-TI	98.32123	-0.41218	0.374131	0.763129	Mt_tRNA
ENSG0000(	UBQLNL	29.1306	-0.26304	0.374116	0.763129	protein_coding

ENSG0000(	KCTD21-AS	494.8175	0.067691	0.374283	0.763382	lncRNA
ENSG0000(	SLC25A40	848.9613	0.081334	0.374314	0.76339	protein_coding
ENSG0000(	ENSG0000(	94.30152	0.095326	0.374378	0.763406	transcribed_unprocessed_pseudo
ENSG0000(	MVB12B	837.2492	0.126775	0.374362	0.763406	protein_coding
ENSG0000(	BSN-DT	352.3759	-0.07092	0.374566	0.763413	lncRNA
ENSG0000(	CABP1	225.6401	0.183198	0.374527	0.763413	protein_coding
ENSG0000(	CTSV	440.5353	0.089885	0.374754	0.763413	protein_coding
ENSG0000(	EIF4G1	4010.569	-0.0656	0.374978	0.763413	protein_coding
ENSG0000(	ELF5	92.68627	0.108072	0.374944	0.763413	protein_coding
ENSG0000(	EML1	581.5019	-0.08966	0.374738	0.763413	protein_coding
ENSG0000(	ENSG0000(	78.19947	0.253631	0.374722	0.763413	lncRNA
ENSG0000(	ENSG0000(	21.12614	0.255319	0.374438	0.763413	lncRNA
ENSG0000(	ENSG0000(	74.72346	0.217712	0.374786	0.763413	lncRNA
ENSG0000(	ENSG0000(	46.2457	-0.18482	0.374692	0.763413	lncRNA
ENSG0000(	ENSG0000(	445.0176	0.063109	0.375013	0.763413	lncRNA
ENSG0000(	ENSG0000(	165.4251	0.099539	0.374975	0.763413	lncRNA
ENSG0000(	ERVW-1	1741.463	-0.06784	0.374603	0.763413	protein_coding
ENSG0000(	FAM185BP	99.97371	-0.14811	0.374565	0.763413	transcribed_unprocessed_pseudo
ENSG0000(	KLHDC7B	61.0444	-0.27442	0.374697	0.763413	protein_coding
ENSG0000(	RAD9A	952.8162	-0.05664	0.375019	0.763413	protein_coding
ENSG0000(	RPS23	1748.261	-0.14214	0.374558	0.763413	protein_coding
ENSG0000(	RPSAP21	21.34615	0.261549	0.37484	0.763413	processed_pseudogene
ENSG0000(	SHISA9	6971.236	-0.09129	0.374889	0.763413	protein_coding
ENSG0000(	SPTBN1	16546.01	0.072279	0.374772	0.763413	protein_coding
ENSG0000(	TMIGD2	122.7843	-0.11955	0.374836	0.763413	protein_coding
ENSG0000(	TSPEAR-AS	157.2761	0.106785	0.374578	0.763413	lncRNA
ENSG0000(	WFDC3	72.30384	0.174404	0.374876	0.763413	protein_coding
ENSG0000(	DYNC1H1	14218.74	-0.0333	0.375079	0.763478	protein_coding
ENSG0000(	CELSR3	228.6814	-0.20689	0.375291	0.763667	protein_coding
ENSG0000(	ENSG0000(	22.27851	-0.20651	0.375366	0.763667	lncRNA
ENSG0000(	RND3	1289.072	-0.14183	0.3753	0.763667	protein_coding
ENSG0000(	THSD1	93.82883	-0.15786	0.375344	0.763667	protein_coding
ENSG0000(	TRAJ29	21.98334	-0.31668	0.375321	0.763667	TR_J_gene
ENSG0000(	USE1	654.8657	0.055692	0.375338	0.763667	protein_coding
ENSG0000(	ZFAND1	546.005	0.093595	0.375227	0.763667	protein_coding
ENSG0000(	KHDC1	320.6398	-0.066	0.375486	0.763855	protein_coding
ENSG0000(	ENSG0000(	46.82975	0.253407	0.375564	0.763958	lncRNA
ENSG0000(	ENSG0000(	408.1027	0.088969	0.3757	0.764117	lncRNA
ENSG0000(	ENSG0000(	106.8471	0.109508	0.37568	0.764117	lncRNA
ENSG0000(	NLRP1	2106.333	-0.14033	0.375726	0.764117	protein_coding
ENSG0000(	ZNF582-DT	208.2605	-0.09828	0.375882	0.764378	lncRNA
ENSG0000(	KLF2P2	29.24771	0.233415	0.375935	0.764429	processed_pseudogene
ENSG0000(	ENSG0000(	24.09077	0.242969	0.375993	0.76449	lncRNA
ENSG0000(	NR2F1	213.7373	-0.18885	0.376176	0.764805	protein_coding
ENSG0000(	APOC1P1	58.6997	-0.19995	0.376561	0.764865	transcribed_unprocessed_pseudo
ENSG0000(	C1orf74	282.2442	0.225897	0.376698	0.764865	protein_coding
ENSG0000(	CD2AP	2771.535	0.121997	0.377011	0.764865	protein_coding

ENSG0000(	EEF2	9039.686	0.094287	0.376484	0.764865	protein_coding
ENSG0000(	ENSG0000(	121.867	-0.09812	0.376745	0.764865	lncRNA
ENSG0000(	ENSG0000(	506.5975	0.0846	0.376478	0.764865	lncRNA
ENSG0000(	ENSG0000(	257.1797	-0.18515	0.376605	0.764865	processed_pseudogene
ENSG0000(	ENSG0000(	29.79515	-0.23002	0.376694	0.764865	lncRNA
ENSG0000(	ENSG0000(	36.24514	0.253103	0.376928	0.764865	lncRNA
ENSG0000(	ENSG0000(	168.8806	0.101006	0.376563	0.764865	lncRNA
ENSG0000(	FAM78A	619.4786	-0.15892	0.376396	0.764865	protein_coding
ENSG0000(	HHLA3	85.31548	-0.10739	0.376307	0.764865	lncRNA
ENSG0000(	HNRNPA0	1164.282	-0.07137	0.376243	0.764865	protein_coding
ENSG0000(	JAM2	606.0722	0.073928	0.376987	0.764865	protein_coding
ENSG0000(	KHDRBS3	188.2633	0.146991	0.376911	0.764865	protein_coding
ENSG0000(	KIAA0232	2953.626	0.123126	0.376471	0.764865	protein_coding
ENSG0000(	KLHL23	381.1927	0.129076	0.37689	0.764865	protein_coding
ENSG0000(	MAP9	219.0033	0.221677	0.376929	0.764865	protein_coding
ENSG0000(	MIER1	2137.682	0.077757	0.376856	0.764865	protein_coding
ENSG0000(	MRPS30	1035.461	0.065708	0.376595	0.764865	protein_coding
ENSG0000(	NFIC	1908.412	-0.07372	0.376572	0.764865	protein_coding
ENSG0000(	OSBP	1401.325	0.064977	0.376754	0.764865	protein_coding
ENSG0000(	PNPLA6	892.7321	-0.08855	0.376833	0.764865	protein_coding
ENSG0000(	RN7SL284F	61.56949	-0.18384	0.376493	0.764865	misc_RNA
ENSG0000(	SIT1	59.87696	-0.28098	0.376838	0.764865	protein_coding
ENSG0000(	SNRPG	234.0162	-0.09125	0.376956	0.764865	protein_coding
ENSG0000(	TGFBR3L	117.2141	0.107938	0.376415	0.764865	protein_coding
ENSG0000(	TTC21B	4510.866	0.049799	0.376976	0.764865	protein_coding
ENSG0000(	UBE2M	183.3704	0.123994	0.376925	0.764865	protein_coding
ENSG0000(	GABRG2	2028.091	0.068057	0.37714	0.764981	protein_coding
ENSG0000(	LINC01138	603.8494	-0.11421	0.377137	0.764981	lncRNA
ENSG0000(	SAR1A	2136.024	-0.07611	0.377152	0.764981	protein_coding
ENSG0000(	SENP8	412.1238	0.082416	0.377199	0.76502	protein_coding
ENSG0000(	ATN1	2037.167	-0.08505	0.377262	0.765035	protein_coding
ENSG0000(	ENSG0000(	58.61715	1.256134	0.37725	0.765035	lncRNA
ENSG0000(	ATP5PF	244.3732	-0.1517	0.377419	0.765242	protein_coding
ENSG0000(	LINC01435	165.5647	-0.10714	0.377412	0.765242	lncRNA
ENSG0000(	LINC02809	237.0515	-0.08745	0.377468	0.765284	lncRNA
ENSG0000(	CP	439.7744	-0.14451	0.37753	0.765296	protein_coding
ENSG0000(	ENSG0000(	119.4363	-0.10499	0.377519	0.765296	lncRNA
ENSG0000(	ENSG0000(	62.0117	-0.12634	0.377572	0.765325	lncRNA
ENSG0000(	GPS2	354.7632	-0.10852	0.377617	0.765361	protein_coding
ENSG0000(	ENSG0000(	253.2382	0.158299	0.377703	0.765479	lncRNA
ENSG0000(	RPL24	1110.92	-0.11725	0.377765	0.765547	protein_coding
ENSG0000(	ENSG0000(	58.10665	0.150736	0.377801	0.765563	lncRNA
ENSG0000(	ENSG0000(	1616.807	0.090325	0.377935	0.76568	lncRNA
ENSG0000(	LPO	184.0631	0.113664	0.377926	0.76568	protein_coding
ENSG0000(	RNVU1-33	29.47197	-0.17587	0.37797	0.76568	snRNA
ENSG0000(	ROS1	265.1587	0.143114	0.377956	0.76568	protein_coding
ENSG0000(	POLA2	700.31	-0.06377	0.378027	0.765739	protein_coding

ENSG0000(	RNF185	684.1537	0.092133	0.378097	0.765825	protein_coding
ENSG0000(	C7	631.0357	0.346142	0.378227	0.765844	protein_coding
ENSG0000(	CALM3	1967.369	-0.10962	0.378222	0.765844	protein_coding
ENSG0000(	MEST	421.3388	0.171226	0.378165	0.765844	protein_coding
ENSG0000(	TMEM53	416.4467	0.118534	0.378246	0.765844	protein_coding
ENSG0000(	ZNF124	1353.441	0.074383	0.378135	0.765844	protein_coding
ENSG0000(	KIAA1549L	48.08513	-0.53476	0.378303	0.765904	protein_coding
ENSG0000(	APOBEC3F	809.0323	-0.11344	0.378656	0.766224	protein_coding
ENSG0000(	ENSG0000(	755.4974	-0.10666	0.378496	0.766224	lncRNA
ENSG0000(	ENSG0000(	54.01252	-0.15	0.378654	0.766224	lncRNA
ENSG0000(	KLF16	332.4123	0.104532	0.378553	0.766224	protein_coding
ENSG0000(	NOP14-AS1	501.01	0.073713	0.37852	0.766224	lncRNA
ENSG0000(	RPL34	1389.849	-0.11452	0.378629	0.766224	protein_coding
ENSG0000(	TFEB	741.8804	-0.09277	0.378604	0.766224	protein_coding
ENSG0000(	ENSG0000(	91.96871	0.131437	0.378723	0.766247	lncRNA
ENSG0000(	LUZP2	145.4872	0.157602	0.378707	0.766247	protein_coding
ENSG0000(	TM9SF2	1797.599	-0.07181	0.378775	0.766296	protein_coding
ENSG0000(	IFI27	2117.493	-0.19772	0.378841	0.766372	protein_coding
ENSG0000(	RN7SKP193	20.12575	0.19025	0.378932	0.7665	misc_RNA
ENSG0000(	ENSG0000(	181.5436	0.082518	0.378971	0.766522	lncRNA
ENSG0000(	CCDC124	215.1977	-0.11335	0.379131	0.766525	protein_coding
ENSG0000(	ENSG0000(	26.07675	-0.16913	0.379195	0.766525	transcribed_unitary_pseudogene
ENSG0000(	ENSG0000(	104.3727	0.143441	0.379025	0.766525	lncRNA
ENSG0000(	ENSG0000(	81.05379	0.150747	0.37914	0.766525	lncRNA
ENSG0000(	ENSG0000(	285.3749	-0.09604	0.37919	0.766525	transcribed_processed_pseudoge
ENSG0000(	ENSG0000(	475.9855	-0.07329	0.379059	0.766525	lncRNA
ENSG0000(	PDE6A	1182.473	0.064829	0.379188	0.766525	protein_coding
ENSG0000(	PRXL2C	766.7598	0.071042	0.379086	0.766525	protein_coding
ENSG0000(	ENSG0000(	20.55411	-0.26006	0.379238	0.766556	lncRNA
ENSG0000(	SYNDIG1	48.04944	-0.25544	0.379289	0.766604	protein_coding
ENSG0000(	ENSG0000(	36.76867	0.178335	0.379325	0.76662	lncRNA
ENSG0000(	BDP1	3409.622	0.070025	0.379391	0.766657	protein_coding
ENSG0000(	ZNF705E	26.34346	0.233117	0.379399	0.766657	protein_coding
ENSG0000(	ARPC5L	438.7209	-0.10113	0.379717	0.766791	protein_coding
ENSG0000(	C1orf141	46.1562	0.192924	0.379593	0.766791	protein_coding
ENSG0000(	CEACAM5	23072.17	-0.20453	0.379703	0.766791	protein_coding
ENSG0000(	ENSG0000(	36.93865	0.15205	0.379515	0.766791	lncRNA
ENSG0000(	JPX	6605.214	-0.05515	0.379632	0.766791	lncRNA
ENSG0000(	LINC02746	115.9656	0.190109	0.379638	0.766791	lncRNA
ENSG0000(	PPP1R21	759.9681	-0.07121	0.379605	0.766791	protein_coding
ENSG0000(	RAB25	447.3306	0.184806	0.379588	0.766791	protein_coding
ENSG0000(	TTC33	444.1723	0.070509	0.379672	0.766791	protein_coding
ENSG0000(	FKBP1B	75.44346	-0.18428	0.379763	0.766828	protein_coding
ENSG0000(	ENSG0000(	30.17499	0.192943	0.37985	0.766949	lncRNA
ENSG0000(	SBNO1-AS1	27.12769	0.253421	0.379907	0.76698	lncRNA
ENSG0000(	UBC	4210.275	-0.11992	0.379922	0.76698	protein_coding
ENSG0000(	ENSG0000(	134.5537	0.101746	0.379972	0.767026	TEC

ENSG0000(	ARMCX7P	32.01407	0.567243	0.380135	0.76722	transcribed_unprocessed_pseudo
ENSG0000(	HERC1	6381.083	0.054631	0.38018	0.76722	protein_coding
ENSG0000(	SPCS1	954.2579	-0.10581	0.380104	0.76722	protein_coding
ENSG0000(	TWSG1-DT	2991.647	0.037557	0.380172	0.76722	lncRNA
ENSG0000(	FRMD6-AS:	271.1932	-0.07167	0.380324	0.767337	lncRNA
ENSG0000(	GPRASP2	137.0466	-0.13834	0.380349	0.767337	protein_coding
ENSG0000(	SLC5A2	21.63176	-0.38002	0.380306	0.767337	protein_coding
ENSG0000(	ZNF101	1953.007	-0.07349	0.380285	0.767337	protein_coding
ENSG0000(	ENSG0000(	351.0237	0.110758	0.380392	0.767343	TEC
ENSG0000(	RRAGD	162.2586	-0.18609	0.380408	0.767343	protein_coding
ENSG0000(	CCNL1	5550.24	-0.06359	0.380591	0.767591	protein_coding
ENSG0000(	DCDC1	235.5784	-0.09552	0.380626	0.767591	protein_coding
ENSG0000(	ENSG0000(	44.43931	0.169105	0.380688	0.767591	lncRNA
ENSG0000(	ENSG0000(	42.67726	-0.1628	0.3807	0.767591	protein_coding
ENSG0000(	GTF2F2	438.5537	-0.08355	0.380866	0.767591	protein_coding
ENSG0000(	GTF2H2	1578.171	-0.1199	0.380856	0.767591	protein_coding
ENSG0000(	KBTBD4	174.1064	-0.10819	0.38085	0.767591	protein_coding
ENSG0000(	NCMAP	469.1738	0.064642	0.380849	0.767591	protein_coding
ENSG0000(	OR6Y1	557.4658	-0.06198	0.380665	0.767591	protein_coding
ENSG0000(	PACSIN2	1986.625	0.096064	0.380858	0.767591	protein_coding
ENSG0000(	SCNN1D	102.2935	-0.17623	0.38065	0.767591	protein_coding
ENSG0000(	TMEM44-A	156.06	0.116301	0.380759	0.767591	lncRNA
ENSG0000(	CDK13-DT	98.75754	0.101793	0.381221	0.767621	lncRNA
ENSG0000(	ENSG0000(	30.06123	-0.24247	0.381215	0.767621	lncRNA
ENSG0000(	ENSG0000(	57.42926	-0.16267	0.381094	0.767621	TEC
ENSG0000(	ENSG0000(	105.6881	0.105336	0.381006	0.767621	lncRNA
ENSG0000(	HSPD1P11	302.9396	0.122187	0.380998	0.767621	processed_pseudogene
ENSG0000(	KDF1	138.5181	0.214866	0.381156	0.767621	protein_coding
ENSG0000(	MPV17L2	131.1779	-0.13386	0.381244	0.767621	protein_coding
ENSG0000(	MRPL40	119.8622	-0.10646	0.381138	0.767621	protein_coding
ENSG0000(	NIT1	1168.766	0.081036	0.381233	0.767621	protein_coding
ENSG0000(	PI4KAP1	697.2925	0.211812	0.381015	0.767621	transcribed_unprocessed_pseudo
ENSG0000(	PPEF1	58.66618	0.193697	0.381019	0.767621	protein_coding
ENSG0000(	RHOU	820.3447	0.222281	0.381166	0.767621	protein_coding
ENSG0000(	ZNF641	1288.927	-0.09324	0.381197	0.767621	protein_coding
ENSG0000(	PSD	72.61929	-0.17583	0.38133	0.767739	protein_coding
ENSG0000(	CBFA2T3	450.1857	-0.16587	0.381414	0.767751	protein_coding
ENSG0000(	ENSG0000(	36.97193	-0.30577	0.381491	0.767751	transcribed_unprocessed_pseudo
ENSG0000(	FADD	172.9446	0.128585	0.381503	0.767751	protein_coding
ENSG0000(	FAM153CP	2734.89	0.053703	0.381456	0.767751	lncRNA
ENSG0000(	OAZ1	1894.711	-0.09949	0.381475	0.767751	protein_coding
ENSG0000(	PGAP4	489.7414	0.177021	0.381502	0.767751	protein_coding
ENSG0000(	ENSG0000(	66.99896	0.176827	0.381537	0.767763	TEC
ENSG0000(	PCDHGB5	120.442	0.142952	0.381588	0.767809	protein_coding
ENSG0000(	DIMT1	1307.003	0.04706	0.381622	0.767821	protein_coding
ENSG0000(	RN7SL580F	23.55076	0.230312	0.381652	0.767826	misc_RNA
ENSG0000(	ACSM6	51.19151	-0.13776	0.38197	0.767829	protein_coding

ENSG0000(	AS3MT	422.5697	0.062274	0.382005	0.767829	protein_coding
ENSG0000(	ATP2B1-AS	121.3658	-0.12595	0.381931	0.767829	lncRNA
ENSG0000(	BAZ1A-AS1	49.15676	-0.13177	0.381873	0.767829	lncRNA
ENSG0000(	DNAH3	220.8749	0.080976	0.381824	0.767829	protein_coding
ENSG0000(	ELOB	775.6521	-0.14026	0.381912	0.767829	protein_coding
ENSG0000(	ENSG0000(	462.4383	-0.09029	0.3817	0.767829	lncRNA
ENSG0000(	ENSG0000(	47.6284	0.152667	0.381957	0.767829	lncRNA
ENSG0000(	IDS	2408.521	-0.08106	0.382016	0.767829	protein_coding
ENSG0000(	PRSS12	1812.962	0.18712	0.382044	0.767829	protein_coding
ENSG0000(	PSMG3	308.0279	-0.06658	0.381732	0.767829	protein_coding
ENSG0000(	SMPDL3A	479.6774	0.14505	0.381908	0.767829	protein_coding
ENSG0000(	TNPO1P1	138.8912	-0.1155	0.381872	0.767829	processed_pseudogene
ENSG0000(	ZNF414	470.8114	0.15953	0.382022	0.767829	protein_coding
ENSG0000(	GRB10	1062.276	-0.12287	0.382083	0.767847	protein_coding
ENSG0000(	LINC02020	72.35653	-0.14177	0.382118	0.767847	lncRNA
ENSG0000(	USP37	1153.831	0.080721	0.382137	0.767847	protein_coding
ENSG0000(	UHRF1BP1I	35.6172	0.194894	0.382344	0.768206	lncRNA
ENSG0000(	BRCC3	731.3883	0.070028	0.382482	0.768246	protein_coding
ENSG0000(	DACH2	26.5153	-0.21245	0.382522	0.768246	protein_coding
ENSG0000(	ENSG0000(	117.1873	0.208463	0.382484	0.768246	lncRNA
ENSG0000(	GPBAR1	37.44805	-0.15395	0.382531	0.768246	protein_coding
ENSG0000(	HP1BP3	6929.905	0.060056	0.382479	0.768246	protein_coding
ENSG0000(	PAXBP1	1982.143	0.07281	0.382432	0.768246	protein_coding
ENSG0000(	ENSG0000(	69.01876	0.140767	0.382565	0.768258	lncRNA
ENSG0000(	PIGR	90049.21	0.206916	0.382694	0.76846	protein_coding
ENSG0000(	B3GAT1-DT	1595.819	0.277587	0.382805	0.768481	lncRNA
ENSG0000(	CCDC120	517.2673	0.083692	0.382733	0.768481	protein_coding
ENSG0000(	CDON	525.848	0.097908	0.382844	0.768481	protein_coding
ENSG0000(	ENSG0000(	56.29094	0.146184	0.382835	0.768481	lncRNA
ENSG0000(	KNTC1	2468.869	-0.0768	0.382811	0.768481	protein_coding
ENSG0000(	AP5B1	1442.497	-0.08505	0.382973	0.768488	protein_coding
ENSG0000(	BCORL1	418.549	0.137197	0.383114	0.768488	protein_coding
ENSG0000(	C6orf62	2242.677	-0.07179	0.382904	0.768488	protein_coding
ENSG0000(	ENSG0000(	237.8975	-0.09416	0.382876	0.768488	lncRNA
ENSG0000(	ERVE-1	261.6964	0.118726	0.383191	0.768488	lncRNA
ENSG0000(	LINC00115	157.9441	0.110407	0.38321	0.768488	lncRNA
ENSG0000(	LINC02188	140.0477	-0.09488	0.383171	0.768488	lncRNA
ENSG0000(	LINC02234	37.23485	-0.19295	0.38296	0.768488	lncRNA
ENSG0000(	LRRC43	31.94169	0.155715	0.383201	0.768488	protein_coding
ENSG0000(	OSGEP	1296.719	0.060954	0.383043	0.768488	protein_coding
ENSG0000(	PHLDB3	521.7296	0.144145	0.383142	0.768488	protein_coding
ENSG0000(	POU5F2	1506.459	-0.04913	0.383089	0.768488	protein_coding
ENSG0000(	RN7SL679F	25.13015	0.167574	0.383195	0.768488	misc_RNA
ENSG0000(	WDCP	228.494	0.137701	0.383304	0.768619	protein_coding
ENSG0000(	XACT	120986.6	0.096105	0.383377	0.768709	lncRNA
ENSG0000(	LINC02345	32.58652	-0.23956	0.38343	0.76876	lncRNA
ENSG0000(	SNORA14A	20.51391	-0.20576	0.383573	0.768991	snoRNA

ENSG0000(	RP9	703.657	0.047652	0.383633	0.769049	protein_coding
ENSG0000(	USP3	2228.784	0.047197	0.383658	0.769049	protein_coding
ENSG0000(	CENPN-AS1	362.1508	-0.06492	0.38375	0.769066	lncRNA
ENSG0000(	CPSF1	1778.33	0.145402	0.383739	0.769066	protein_coding
ENSG0000(	VCPKMT	385.673	0.075647	0.383699	0.769066	protein_coding
ENSG0000(	ENSG0000(	167.5072	-0.11903	0.383786	0.769082	lncRNA
ENSG0000(	GABBR1	1499.694	-0.11841	0.383831	0.769116	protein_coding
ENSG0000(	AQP6	103.4061	0.088698	0.383954	0.769306	protein_coding
ENSG0000(	ENSG0000(	56.93576	0.174661	0.384067	0.769477	lncRNA
ENSG0000(	CCDC107	79.08652	-0.13042	0.384183	0.769641	protein_coding
ENSG0000(	LINC02904	318.274	-0.0785	0.384233	0.769641	lncRNA
ENSG0000(	PHF8	1543.827	0.060328	0.384209	0.769641	protein_coding
ENSG0000(	ENSG0000(	45.23864	-0.16824	0.384281	0.769654	lncRNA
ENSG0000(	ENSG0000(	107.7263	-0.17457	0.384296	0.769654	lncRNA
ENSG0000(	ANKRD17	5431.053	0.044052	0.384551	0.769852	protein_coding
ENSG0000(	ARFIP2	1061.372	0.067709	0.384605	0.769852	protein_coding
ENSG0000(	COMTD1	121.0609	0.161415	0.384495	0.769852	protein_coding
ENSG0000(	EGFL6	19.65738	-1.53195	0.384537	0.769852	protein_coding
ENSG0000(	ENSG0000(	85.05648	-0.17327	0.384646	0.769852	lncRNA
ENSG0000(	FZD6	340.0089	0.085571	0.384634	0.769852	protein_coding
ENSG0000(	LGSN	262.6797	0.087783	0.384639	0.769852	protein_coding
ENSG0000(	SKAP1-AS1	264.0023	0.084526	0.384489	0.769852	lncRNA
ENSG0000(	ZSCAN18	1097.665	0.068802	0.384558	0.769852	protein_coding
ENSG0000(	MAGEE1	37.81137	-0.22196	0.384704	0.769856	protein_coding
ENSG0000(	TEX14	57.11442	-0.17503	0.384679	0.769856	protein_coding
ENSG0000(	ENSG0000(	65.09062	-0.10556	0.38474	0.769872	lncRNA
ENSG0000(	ENSG0000(	374.3455	-0.06011	0.38491	0.769931	lncRNA
ENSG0000(	FSD1L	465.7373	-0.08284	0.384884	0.769931	protein_coding
ENSG0000(	KLRC2	74.47157	0.20429	0.384867	0.769931	protein_coding
ENSG0000(	METTL21AI	108.6408	0.10999	0.384815	0.769931	processed_pseudogene
ENSG0000(	SLC17A8	38.6868	0.367786	0.384856	0.769931	protein_coding
ENSG0000(	ENSG0000(	25.1975	0.218649	0.385046	0.770091	protein_coding
ENSG0000(	FGFRL1	336.3412	0.13402	0.385033	0.770091	protein_coding
ENSG0000(	UPB1	363.2058	0.080331	0.385202	0.770348	protein_coding
ENSG0000(	ADORA2B	530.0033	0.177857	0.385457	0.770633	protein_coding
ENSG0000(	CCDC125	4193.464	-0.03091	0.385472	0.770633	protein_coding
ENSG0000(	ENSG0000(	88.25726	0.128747	0.385429	0.770633	lncRNA
ENSG0000(	PRPF8	4650.033	-0.05119	0.385485	0.770633	protein_coding
ENSG0000(	SNX29P2	250.4723	-0.18756	0.385395	0.770633	transcribed_unprocessed_pseudo
ENSG0000(	ENSG0000(	130.5649	0.08803	0.385539	0.770685	lncRNA
ENSG0000(	DPRXP4	92.28885	0.127189	0.385616	0.770784	processed_pseudogene
ENSG0000(	MIR4432H(	248.6145	-0.18162	0.385719	0.770934	lncRNA
ENSG0000(	LINC02798	123.2241	0.109493	0.385784	0.770951	lncRNA
ENSG0000(	MAL2	1874.696	0.222672	0.385794	0.770951	protein_coding
ENSG0000(	OLAH	109.7687	-0.10963	0.385812	0.770951	protein_coding
ENSG0000(	ENSG0000(	38.94884	-0.17599	0.385889	0.77105	lncRNA
ENSG0000(	ENSG0000(	47.46686	0.132235	0.385919	0.771054	lncRNA

ENSG0000(	ELFN2	25.58897	-0.44641	0.386243	0.771108	lncRNA
ENSG0000(	ENSG0000(	49.67047	-0.14514	0.386033	0.771108	lncRNA
ENSG0000(	ENSG0000(	119.6024	0.124813	0.386117	0.771108	lncRNA
ENSG0000(	ENSG0000(	80.02693	-0.14084	0.386147	0.771108	lncRNA
ENSG0000(	ENSG0000(	21.61864	-0.33053	0.386091	0.771108	lncRNA
ENSG0000(	FAM120A	5602.673	0.067069	0.385981	0.771108	protein_coding
ENSG0000(	LINC01858	205.2645	0.093344	0.386193	0.771108	lncRNA
ENSG0000(	SLC27A4	467.3903	0.191584	0.386255	0.771108	protein_coding
ENSG0000(	SPANXA2-(	1271.588	0.044328	0.386043	0.771108	lncRNA
ENSG0000(	TRIM56	7169.997	-0.05457	0.386247	0.771108	protein_coding
ENSG0000(	ZNF678	723.0481	0.09942	0.386128	0.771108	protein_coding
ENSG0000(	FTLP10	23.6015	0.22345	0.386346	0.771123	transcribed_processed_pseudoge
ENSG0000(	ICMT	989.7298	-0.0854	0.386342	0.771123	protein_coding
ENSG0000(	TRIOBP	1986.019	0.077227	0.386325	0.771123	protein_coding
ENSG0000(	GAS1RR	100.2283	-0.14656	0.386411	0.771141	lncRNA
ENSG0000(	SEC14L1P1	114.0957	0.141519	0.386386	0.771141	processed_pseudogene
ENSG0000(	DCAF4	442.5837	0.097195	0.386489	0.771185	protein_coding
ENSG0000(	TMEM51-A	870.7554	0.078699	0.386468	0.771185	lncRNA
ENSG0000(	TIMMDC1-	47.04262	-0.12636	0.386551	0.771252	lncRNA
ENSG0000(	CFAP69	2123.475	-0.06896	0.386774	0.771295	protein_coding
ENSG0000(	COL6A4P2	52.09278	0.308761	0.386803	0.771295	transcribed_unitary_pseudogene
ENSG0000(	COQ8B	1866.688	-0.05033	0.386825	0.771295	protein_coding
ENSG0000(	ENSG0000(	225.0897	0.09719	0.386616	0.771295	lncRNA
ENSG0000(	ENSG0000(	39.41757	0.19941	0.386749	0.771295	lncRNA
ENSG0000(	EPRS1	1327.759	-0.09399	0.38677	0.771295	protein_coding
ENSG0000(	FBL	418.3596	0.092156	0.386746	0.771295	protein_coding
ENSG0000(	GCN1	2464.131	-0.08721	0.386752	0.771295	protein_coding
ENSG0000(	HCN3	393.3758	0.191214	0.386771	0.771295	protein_coding
ENSG0000(	HHIP	926.7528	0.225054	0.386917	0.771422	protein_coding
ENSG0000(	ENSG0000(	190.7027	0.100742	0.387018	0.771439	processed_pseudogene
ENSG0000(	ENSG0000(	130.8378	0.082935	0.38696	0.771439	lncRNA
ENSG0000(	GPN2	1220.401	0.129916	0.387074	0.771439	protein_coding
ENSG0000(	MAP1LC3B	1072.982	-0.10121	0.387088	0.771439	protein_coding
ENSG0000(	MCEE	223.1752	-0.11724	0.386995	0.771439	protein_coding
ENSG0000(	PMPCB	1008.636	0.063526	0.387103	0.771439	protein_coding
ENSG0000(	ZNF33BP1	107.1473	0.149732	0.387121	0.771439	processed_pseudogene
ENSG0000(	ASMTL-AS1	399.8924	0.106241	0.387265	0.771463	lncRNA
ENSG0000(	ENSG0000(	127.2616	-0.12305	0.387358	0.771463	lncRNA
ENSG0000(	ENSG0000(	62.36581	0.140808	0.387302	0.771463	lncRNA
ENSG0000(	PEX6	360.5214	0.108205	0.387283	0.771463	protein_coding
ENSG0000(	SHOC2	2855.678	-0.05408	0.387351	0.771463	protein_coding
ENSG0000(	SLPI	54.85687	-0.38224	0.387304	0.771463	protein_coding
ENSG0000(	SNORA5C	81.6044	0.140465	0.387289	0.771463	snoRNA
ENSG0000(	UBQLN2	643.4723	-0.07372	0.387295	0.771463	protein_coding
ENSG0000(	LINC02680	511.7802	-0.06762	0.387471	0.771578	lncRNA
ENSG0000(	PGM2L1	1142.492	-0.07168	0.387458	0.771578	protein_coding
ENSG0000(	AZU1	166.5645	0.101731	0.387651	0.771657	protein_coding

ENSG0000(	DBIL5P2	72.27226	-0.14204	0.387794	0.771657	transcribed_processed_pseudoge
ENSG0000(	ENSG0000(	204.9908	-0.07086	0.387661	0.771657	TEC
ENSG0000(	ENSG0000(	474.1095	0.142259	0.3878	0.771657	transcribed_unprocessed_pseudo
ENSG0000(	ENSG0000(	81.20503	0.307797	0.387624	0.771657	lncRNA
ENSG0000(	ENSG0000(	691.6353	0.100963	0.387813	0.771657	lncRNA
ENSG0000(	RFWD3	1008.155	-0.07542	0.387807	0.771657	protein_coding
ENSG0000(	RN7SL220F	132.8883	-0.12148	0.387631	0.771657	misc_RNA
ENSG0000(	SNX9	2991.915	-0.07798	0.387546	0.771657	protein_coding
ENSG0000(	TLCD4-RWI	128.0149	0.186575	0.387711	0.771657	protein_coding
ENSG0000(	TLL2	264.5283	0.075008	0.38782	0.771657	protein_coding
ENSG0000(	RN7SKP70	65.79445	0.115714	0.38787	0.771701	misc_RNA
ENSG0000(	AZGP1	65.58202	-0.21157	0.387949	0.771735	protein_coding
ENSG0000(	RBL1	776.2955	-0.09485	0.387924	0.771735	protein_coding
ENSG0000(	SNORD17	1382.734	0.178959	0.38798	0.771735	snoRNA
ENSG0000(	SPIRE1	341.8061	-0.12774	0.387999	0.771735	protein_coding
ENSG0000(	BCAT2	325.2659	0.114875	0.388131	0.771792	protein_coding
ENSG0000(	ENSG0000(	105.1081	-0.12488	0.388114	0.771792	lncRNA
ENSG0000(	RIPK1	2758.547	0.070354	0.388135	0.771792	protein_coding
ENSG0000(	TAC4	588.1228	-0.14955	0.38814	0.771792	protein_coding
ENSG0000(	ANKRD9	1729.375	0.17887	0.388221	0.771897	protein_coding
ENSG0000(	ATP6V0C	502.6552	-0.11346	0.388524	0.771901	protein_coding
ENSG0000(	BVES	435.7305	-0.07354	0.388451	0.771901	protein_coding
ENSG0000(	C1orf122	94.8364	-0.16911	0.388285	0.771901	protein_coding
ENSG0000(	CAPN1	2000.561	0.096718	0.38886	0.771901	protein_coding
ENSG0000(	DHX40	567.7662	0.071632	0.388562	0.771901	protein_coding
ENSG0000(	ECPAS	3573.013	0.065134	0.388596	0.771901	protein_coding
ENSG0000(	ENSG0000(	285.0846	0.054347	0.388924	0.771901	lncRNA
ENSG0000(	ENSG0000(	71.05167	-0.14548	0.388619	0.771901	processed_pseudogene
ENSG0000(	ENSG0000(	102.1534	0.125483	0.388843	0.771901	lncRNA
ENSG0000(	ENSG0000(	156.0835	0.096156	0.388921	0.771901	TEC
ENSG0000(	ENSG0000(	278.012	0.085144	0.388259	0.771901	transcribed_unprocessed_pseudo
ENSG0000(	ENSG0000(	20.69417	0.27698	0.388671	0.771901	lncRNA
ENSG0000(	ENSG0000(	281.9849	0.173804	0.388589	0.771901	lncRNA
ENSG0000(	HS3ST3A1	99.61937	-0.30186	0.388394	0.771901	protein_coding
ENSG0000(	IGKV1-39	41.44264	-2.47427	0.38886	0.771901	IG_V_gene
ENSG0000(	LGR6	74.39604	0.22155	0.388886	0.771901	protein_coding
ENSG0000(	LINC01787	100.4468	-0.14577	0.388329	0.771901	lncRNA
ENSG0000(	MIR7-3HG	125.6959	0.133233	0.388582	0.771901	lncRNA
ENSG0000(	NDUFA9	3777.902	0.067983	0.388411	0.771901	protein_coding
ENSG0000(	NEK5	755.5042	0.070639	0.388389	0.771901	protein_coding
ENSG0000(	POLA1	1490.476	0.053992	0.388757	0.771901	protein_coding
ENSG0000(	RN7SL524F	739.2635	0.076244	0.388828	0.771901	misc_RNA
ENSG0000(	STAT5B	1841.586	-0.08904	0.388815	0.771901	protein_coding
ENSG0000(	TMEM45A	34548.9	-0.04639	0.388687	0.771901	protein_coding
ENSG0000(	ZBED3	297.2945	0.183981	0.388592	0.771901	protein_coding
ENSG0000(	NRIP1	3961.597	0.104329	0.388955	0.771905	protein_coding
ENSG0000(	CTSE	362.1789	-0.2478	0.389044	0.771916	protein_coding

ENSG0000(	HULC	1786.18	0.041585	0.389032	0.771916	lncRNA
ENSG0000(	PPP1R10	2327.03	0.058544	0.389032	0.771916	protein_coding
ENSG0000(	DCLK2	285.9093	0.154781	0.389453	0.772145	protein_coding
ENSG0000(	ENSG0000(	29.88207	0.315293	0.38945	0.772145	lncRNA
ENSG0000(	GID4	698.6457	-0.05444	0.389299	0.772145	protein_coding
ENSG0000(	GOLGA7B	68.96893	-0.21329	0.389459	0.772145	protein_coding
ENSG0000(	HSP90AA1	3633.888	-0.08803	0.38932	0.772145	protein_coding
ENSG0000(	INTS13	293.4328	0.144907	0.389465	0.772145	protein_coding
ENSG0000(	LINC02607	77.05319	0.134553	0.389466	0.772145	lncRNA
ENSG0000(	MMP3	1128.017	-3.16318	0.389321	0.772145	protein_coding
ENSG0000(	NEURL1B	1812.218	0.193003	0.389443	0.772145	protein_coding
ENSG0000(	RN7SKP95	113.423	0.110271	0.389377	0.772145	misc_RNA
ENSG0000(	RN7SL254F	24.79261	-0.24709	0.389479	0.772145	misc_RNA
ENSG0000(	SYF2	500.6333	-0.07349	0.389496	0.772145	protein_coding
ENSG0000(	ADAMDEC1	2170.634	-0.18392	0.389687	0.772365	protein_coding
ENSG0000(	HAUS4	297.7868	-0.0992	0.389692	0.772365	protein_coding
ENSG0000(	HAUS6	745.0033	-0.07334	0.389643	0.772365	protein_coding
ENSG0000(	AMIGO1	147.1048	0.156137	0.389815	0.772403	protein_coding
ENSG0000(	CDKL1	2372.169	0.200702	0.390048	0.772403	protein_coding
ENSG0000(	CFHR3	142.519	-0.08675	0.389833	0.772403	protein_coding
ENSG0000(	ENSG0000(	147.7606	0.087957	0.390037	0.772403	lncRNA
ENSG0000(	ENSG0000(	129.491	0.15407	0.390039	0.772403	TEC
ENSG0000(	ENSG0000(	105.1848	0.089825	0.3899	0.772403	lncRNA
ENSG0000(	ENSG0000(	173.9974	0.105031	0.389966	0.772403	lncRNA
ENSG0000(	FAM186B	28.52947	-0.24993	0.390027	0.772403	protein_coding
ENSG0000(	LRRC69	2885.445	0.168622	0.38998	0.772403	protein_coding
ENSG0000(	MERTK	617.0554	0.140996	0.389813	0.772403	protein_coding
ENSG0000(	MTRNR2L1	39.15654	-0.32732	0.389923	0.772403	protein_coding
ENSG0000(	ZDHHC18	1033.154	-0.11944	0.389782	0.772403	protein_coding
ENSG0000(	SRFBP1	432.3426	-0.08325	0.390081	0.772412	protein_coding
ENSG0000(	RNF208	24.57373	-0.21767	0.390167	0.772528	protein_coding
ENSG0000(	ENSG0000(	134.442	0.10637	0.390431	0.77259	lncRNA
ENSG0000(	ENSG0000(	296.4197	0.150612	0.390359	0.77259	lncRNA
ENSG0000(	INPP5E	547.8998	0.071931	0.390507	0.77259	protein_coding
ENSG0000(	KCNRG	86.05619	-0.14855	0.390364	0.77259	protein_coding
ENSG0000(	LINC01940	54.97128	-0.15522	0.390494	0.77259	lncRNA
ENSG0000(	MST1P2	60.96308	-0.25769	0.390257	0.77259	unprocessed_pseudogene
ENSG0000(	NAA11	34.44027	-0.22342	0.390468	0.77259	protein_coding
ENSG0000(	NYNRIN	701.1459	0.151662	0.390474	0.77259	protein_coding
ENSG0000(	PLK5	466.199	-0.0726	0.390364	0.77259	protein_coding
ENSG0000(	RORB	99.73752	0.132571	0.390242	0.77259	protein_coding
ENSG0000(	RPS10-NUD	288.027	0.249423	0.390451	0.77259	protein_coding
ENSG0000(	BABAM1	516.875	0.054216	0.39067	0.7728	protein_coding
ENSG0000(	LINC01424	75.88876	-0.12949	0.390652	0.7728	lncRNA
ENSG0000(	BANCR	24.98054	-0.25122	0.390767	0.772825	lncRNA
ENSG0000(	ENSG0000(	136.198	0.117077	0.390763	0.772825	lncRNA
ENSG0000(	ZC3H14	2942.793	0.061814	0.39073	0.772825	protein_coding

ENSG0000(	DNPH1	119.2914	-0.15364	0.390825	0.772885	protein_coding
ENSG0000(	ENSG0000(	688.1477	0.078412	0.39093	0.772981	lncRNA
ENSG0000(	RAB6A	1811.102	-0.06249	0.390975	0.772981	protein_coding
ENSG0000(	RGL2	828.4979	-0.10341	0.390916	0.772981	protein_coding
ENSG0000(	RNU2-6P	39.3145	-0.20797	0.390986	0.772981	snRNA
ENSG0000(	ENSG0000(	112.6284	-0.13771	0.39107	0.773037	lncRNA
ENSG0000(	ENSG0000(	42.9411	-0.18843	0.391051	0.773037	lncRNA
ENSG0000(	ALG9-IT1	37.15317	-0.20858	0.391719	0.773041	lncRNA
ENSG0000(	BSG-AS1	39.17641	0.240809	0.391582	0.773041	lncRNA
ENSG0000(	ECHS1	473.1713	0.167645	0.391555	0.773041	protein_coding
ENSG0000(	ENSG0000(	22.79656	-0.21641	0.391668	0.773041	lncRNA
ENSG0000(	ENSG0000(	346.5517	-0.05755	0.391566	0.773041	lncRNA
ENSG0000(	ENSG0000(	367.215	-0.24575	0.391532	0.773041	lncRNA
ENSG0000(	ENSG0000(	44.80331	-0.23216	0.391509	0.773041	lncRNA
ENSG0000(	ENSG0000(	2370.984	-0.17227	0.391282	0.773041	miRNA
ENSG0000(	ENSG0000(	651.2107	0.050722	0.391168	0.773041	lncRNA
ENSG0000(	FCSK	912.8367	0.081414	0.391482	0.773041	protein_coding
ENSG0000(	HPCAL1	1389.767	0.094143	0.391149	0.773041	protein_coding
ENSG0000(	KCTD15	407.7494	-0.11414	0.391713	0.773041	protein_coding
ENSG0000(	LINC00174	1843.169	-0.05801	0.391326	0.773041	lncRNA
ENSG0000(	LINC01411	134.9949	-0.14441	0.391379	0.773041	lncRNA
ENSG0000(	LINC02671	115.7197	0.119954	0.391418	0.773041	lncRNA
ENSG0000(	MXRA8	305.9228	-0.15252	0.391381	0.773041	protein_coding
ENSG0000(	PCM1	4768.739	0.056385	0.391385	0.773041	protein_coding
ENSG0000(	PDCL3	61.44798	-0.14872	0.391415	0.773041	protein_coding
ENSG0000(	PSMA3-AS1	4764.832	0.046533	0.391685	0.773041	lncRNA
ENSG0000(	RNF227	660.546	0.057374	0.39136	0.773041	protein_coding
ENSG0000(	RPEP3	39.97803	-0.16556	0.391314	0.773041	processed_pseudogene
ENSG0000(	STARD7-AS	464.6192	0.074365	0.391499	0.773041	lncRNA
ENSG0000(	TOMM70	981.5529	0.070396	0.391621	0.773041	protein_coding
ENSG0000(	ENSG0000(	34.93815	0.17945	0.391748	0.773042	lncRNA
ENSG0000(	RMND5A	1951.577	0.130755	0.391797	0.773084	protein_coding
ENSG0000(	ISG20	2232.057	-0.17682	0.391849	0.773131	protein_coding
ENSG0000(	ENSG0000(	41.72575	-0.27188	0.391905	0.773187	lncRNA
ENSG0000(	EIF3J-DT	562.8016	0.081868	0.392005	0.773273	lncRNA
ENSG0000(	ENSG0000(	131.6985	0.119483	0.391983	0.773273	lncRNA
ENSG0000(	APPBP2	1434.843	-0.04722	0.392284	0.773302	protein_coding
ENSG0000(	CTNNBL1	338.6784	-0.09176	0.392265	0.773302	protein_coding
ENSG0000(	ENSG0000(	470.2717	0.078847	0.392135	0.773302	lncRNA
ENSG0000(	ENSG0000(	360.8656	-0.07752	0.392151	0.773302	lncRNA
ENSG0000(	ENSG0000(	275.1821	-0.07161	0.39218	0.773302	TEC
ENSG0000(	ENSG0000(	288.0474	0.064636	0.392182	0.773302	protein_coding
ENSG0000(	ENSG0000(	26.00936	0.245492	0.392211	0.773302	lncRNA
ENSG0000(	GALNTL6	38.57815	-0.3192	0.392301	0.773302	protein_coding
ENSG0000(	NADK	1948.916	0.063705	0.392289	0.773302	protein_coding
ENSG0000(	PIK3C2A	2809.851	-0.07058	0.392239	0.773302	protein_coding
ENSG0000(	CXCR1	75.36365	-2.01585	0.392407	0.7734	protein_coding

ENSG0000(	HSD17B7P2	148.3098	-0.11813	0.392382	0.7734	transcribed_unprocessed_pseudo
ENSG0000(	MMP23B	41.74659	-0.20764	0.392468	0.773466	protein_coding
ENSG0000(	ADGRB3	122.5932	-0.17936	0.392524	0.77349	protein_coding
ENSG0000(	AP2A2	2312.872	0.060833	0.39257	0.77349	protein_coding
ENSG0000(	ASTN2	4808.639	-0.052	0.392568	0.77349	protein_coding
ENSG0000(	FNBP1P1	256.2678	0.08576	0.392593	0.77349	processed_pseudogene
ENSG0000(	ENSG0000(	1284.024	-0.06195	0.392654	0.7735	lncRNA
ENSG0000(	ZNF343	534.5132	0.053024	0.39265	0.7735	protein_coding
ENSG0000(	CAPN12	227.3629	-0.15369	0.392711	0.773556	protein_coding
ENSG0000(	ANKRD42-I	856.7148	0.064579	0.392884	0.773613	lncRNA
ENSG0000(	COPS4	237.6476	-0.09953	0.392842	0.773613	protein_coding
ENSG0000(	ENSG0000(	48.18405	0.153528	0.392901	0.773613	lncRNA
ENSG0000(	ENSG0000(	536.0835	0.062183	0.392876	0.773613	lncRNA
ENSG0000(	LINC02558	850.7072	-0.06202	0.392811	0.773613	lncRNA
ENSG0000(	PDE9A-AS1	55.51083	0.16662	0.392909	0.773613	lncRNA
ENSG0000(	ARHGAP4	1372.998	-0.12021	0.393036	0.773654	protein_coding
ENSG0000(	ENSG0000(	233.3082	0.073266	0.39307	0.773654	lncRNA
ENSG0000(	ENSG0000(	96.56496	0.136011	0.393066	0.773654	lncRNA
ENSG0000(	ENSG0000(	35.24513	-0.33332	0.393029	0.773654	lncRNA
ENSG0000(	RBM42	211.8241	-0.09524	0.393	0.773654	protein_coding
ENSG0000(	ANGPTL6	31.38431	-0.26753	0.393204	0.773727	protein_coding
ENSG0000(	ENSG0000(	360.1106	0.070359	0.393208	0.773727	lncRNA
ENSG0000(	FLNB	26585.38	0.154649	0.393143	0.773727	protein_coding
ENSG0000(	RAB3A	27.84081	-0.22751	0.393219	0.773727	protein_coding
ENSG0000(	ENSG0000(	212.4263	0.094668	0.393265	0.773738	lncRNA
ENSG0000(	PCDHAC1	28.25342	-0.28636	0.393281	0.773738	protein_coding
ENSG0000(	ENSG0000(	326.2858	-0.06624	0.393331	0.773781	lncRNA
ENSG0000(	ENSG0000(	55.51063	0.194176	0.393434	0.773908	processed_pseudogene
ENSG0000(	ZNF114	147.6667	0.11602	0.393452	0.773908	protein_coding
ENSG0000(	ENSG0000(	84.48169	-0.22891	0.393502	0.773916	transcribed_unprocessed_pseudo
ENSG0000(	OR6K1P	71.43586	0.095866	0.393512	0.773916	unprocessed_pseudogene
ENSG0000(	DAAM2	1078.104	-0.08511	0.393654	0.774139	protein_coding
ENSG0000(	ENSG0000(	24.23608	0.853921	0.393731	0.774235	unprocessed_pseudogene
ENSG0000(	PKP1	28.41081	-0.35513	0.393802	0.774319	protein_coding
ENSG0000(	HVCN1	431.8368	-0.17198	0.393898	0.774453	protein_coding
ENSG0000(	ENSG0000(	108.6094	-0.27415	0.393965	0.774459	lncRNA
ENSG0000(	NUDCD3	1642.349	0.067849	0.393969	0.774459	protein_coding
ENSG0000(	TMEM177	262.5542	0.119081	0.393985	0.774459	protein_coding
ENSG0000(	AKAP13	14296.94	-0.06763	0.394054	0.774472	protein_coding
ENSG0000(	EDIL3	725.9708	0.183058	0.394189	0.774472	protein_coding
ENSG0000(	ENSG0000(	329.1311	-0.19743	0.39409	0.774472	lncRNA
ENSG0000(	LINC02016	108.619	-0.15055	0.394185	0.774472	lncRNA
ENSG0000(	MTSS2	512.7529	-0.12437	0.394181	0.774472	protein_coding
ENSG0000(	MYL10	72.12583	-0.12822	0.394163	0.774472	protein_coding
ENSG0000(	SPATA32	33.85731	0.23143	0.39406	0.774472	protein_coding
ENSG0000(	AMIGO2	149.9013	-0.19769	0.394316	0.774496	protein_coding
ENSG0000(	IBA57	1647.486	0.050435	0.394338	0.774496	protein_coding

ENSG0000(	PDCD6IPP2	335.2673	-0.08137	0.394342	0.774496	transcribed_unprocessed_pseudo
ENSG0000(	RAD54L	218.1769	-0.1073	0.394263	0.774496	protein_coding
ENSG0000(	RPEL1	20.85023	0.236918	0.394236	0.774496	protein_coding
ENSG0000(	FUT3	2841.33	0.156309	0.394435	0.774567	protein_coding
ENSG0000(	WDR17	371.4121	0.107205	0.394419	0.774567	protein_coding
ENSG0000(	BLMH	696.8831	-0.06495	0.394469	0.77458	protein_coding
ENSG0000(	ENSG0000(	202.37	-0.0872	0.394557	0.774698	lncRNA
ENSG0000(	FAM216B	209.7643	0.140195	0.394622	0.774768	protein_coding
ENSG0000(	ENSG0000(	91.27529	-0.2685	0.394799	0.774903	lncRNA
ENSG0000(	KCNG1	378.7518	-0.09463	0.394745	0.774903	protein_coding
ENSG0000(	UGT2A1	24.47569	0.235884	0.394803	0.774903	protein_coding
ENSG0000(	VBP1	178.4245	-0.12783	0.394754	0.774903	protein_coding
ENSG0000(	CHAF1B	970.5213	0.046739	0.394865	0.77497	protein_coding
ENSG0000(	ENSG0000(	308.5941	0.098001	0.394983	0.774974	lncRNA
ENSG0000(	KAT14	389.904	0.114358	0.394996	0.774974	protein_coding
ENSG0000(	MCM3	638.3915	-0.08986	0.394923	0.774974	protein_coding
ENSG0000(	THAP3	144.368	0.113612	0.394985	0.774974	protein_coding
ENSG0000(	TRAK2	1621.981	0.091598	0.395008	0.774974	protein_coding
ENSG0000(	DCDC2C	79.06042	-0.11844	0.395124	0.775146	protein_coding
ENSG0000(	FCF1P5	26.63607	0.248277	0.395291	0.775346	processed_pseudogene
ENSG0000(	GSPT1	2231.432	0.08007	0.395278	0.775346	protein_coding
ENSG0000(	RNU6-653F	22.55203	-0.23569	0.39531	0.775346	snRNA
ENSG0000(	ENSG0000(	1171.099	0.125441	0.395408	0.775427	lncRNA
ENSG0000(	SIRPB3P	22.24151	-0.34215	0.39538	0.775427	unprocessed_pseudogene
ENSG0000(	C22orf39	1113.82	0.042512	0.395615	0.775573	protein_coding
ENSG0000(	ENSG0000(	40.76853	-0.20648	0.395612	0.775573	processed_pseudogene
ENSG0000(	ENSG0000(	32.04551	0.234772	0.395727	0.775573	lncRNA
ENSG0000(	ENSG0000(	545.5462	0.065616	0.395736	0.775573	lncRNA
ENSG0000(	ENSG0000(	260.8274	0.06813	0.395661	0.775573	lncRNA
ENSG0000(	LIN9	121.3224	0.17576	0.395557	0.775573	protein_coding
ENSG0000(	LINC01829	26.02334	0.230834	0.39565	0.775573	lncRNA
ENSG0000(	NDUFS5	201.8719	-0.12697	0.395525	0.775573	protein_coding
ENSG0000(	RN7SL743F	53.13656	0.159041	0.395702	0.775573	misc_RNA
ENSG0000(	C12orf73	125.8426	-0.12078	0.395949	0.775665	protein_coding
ENSG0000(	C6orf89	1740.12	-0.04823	0.396072	0.775665	protein_coding
ENSG0000(	EMC7	187.3253	-0.10763	0.396026	0.775665	protein_coding
ENSG0000(	ENSG0000(	3280.509	0.039704	0.396093	0.775665	transcribed_unprocessed_pseudo
ENSG0000(	ENSG0000(	37.62529	-0.24849	0.395961	0.775665	lncRNA
ENSG0000(	ENSG0000(	58.38565	0.122239	0.39592	0.775665	lncRNA
ENSG0000(	ENSG0000(	1208.393	0.087804	0.395966	0.775665	lncRNA
ENSG0000(	FST	104.063	0.150305	0.396088	0.775665	protein_coding
ENSG0000(	LINC00906	301.767	0.070531	0.396	0.775665	lncRNA
ENSG0000(	MOB3A	1290.15	-0.09118	0.396017	0.775665	protein_coding
ENSG0000(	RHOBTB2	399.1	0.178307	0.39602	0.775665	protein_coding
ENSG0000(	ENSG0000(	21.54104	0.234198	0.396157	0.775735	lncRNA
ENSG0000(	WDR87	24.55184	-0.48114	0.396246	0.775854	protein_coding
ENSG0000(	CHST7	44.41375	-0.21872	0.396308	0.775919	protein_coding

ENSG0000(	ARID1A	5220.403	-0.03592	0.396512	0.775934	protein_coding
ENSG0000(	BMS1P14	137.0967	-0.21818	0.396559	0.775934	lncRNA
ENSG0000(	CBWD3	1551.49	0.084465	0.396412	0.775934	protein_coding
ENSG0000(	ENSG0000(	195.0961	0.105579	0.396597	0.775934	lncRNA
ENSG0000(	ENSG0000(	85.63483	-0.11086	0.396555	0.775934	lncRNA
ENSG0000(	ENSG0000(	255.6878	0.083669	0.396501	0.775934	lncRNA
ENSG0000(	GSK3A	902.6538	0.055567	0.396577	0.775934	protein_coding
ENSG0000(	NDUFS5P1	43.82576	0.207176	0.396415	0.775934	processed_pseudogene
ENSG0000(	RNU6-1284	19.69695	-0.24406	0.39646	0.775934	snRNA
ENSG0000(	STRIP1	975.7363	0.053678	0.396548	0.775934	protein_coding
ENSG0000(	ZNF514	2049.315	0.074166	0.396705	0.77609	protein_coding
ENSG0000(	CDA	64.74201	-0.32413	0.396771	0.776094	protein_coding
ENSG0000(	DUOX1	392.5218	0.081247	0.396783	0.776094	protein_coding
ENSG0000(	ENSG0000(	100.5527	-0.13222	0.39682	0.776094	lncRNA
ENSG0000(	RN7SL760F	37.47396	-0.1592	0.396799	0.776094	misc_RNA
ENSG0000(	ERCC2	408.7436	-0.08228	0.397022	0.776381	protein_coding
ENSG0000(	MCF2L2	1274.755	-0.05736	0.397051	0.776381	protein_coding
ENSG0000(	RAB35	702.694	0.066323	0.39705	0.776381	protein_coding
ENSG0000(	ENSG0000(	159.8763	0.226854	0.397097	0.776416	lncRNA
ENSG0000(	CENPBD1P	1041.691	-0.04854	0.397128	0.77642	transcribed_processed_pseudoge
ENSG0000(	ENSG0000(	91.3542	-0.26048	0.39721	0.776524	lncRNA
ENSG0000(	ADAM3B	28.08401	-0.25027	0.397377	0.776627	processed_pseudogene
ENSG0000(	CLIP1	4091.534	-0.04725	0.397319	0.776627	protein_coding
ENSG0000(	PLCXD3	93.5461	0.213227	0.397369	0.776627	protein_coding
ENSG0000(	THNSL2	628.9247	-0.20842	0.397403	0.776627	protein_coding
ENSG0000(	TSC22D2	2372.809	0.102797	0.397373	0.776627	protein_coding
ENSG0000(	ENSG0000(	33.36356	-0.21028	0.397433	0.776631	lncRNA
ENSG0000(	UNC45B	32.69784	0.140846	0.397464	0.776637	protein_coding
ENSG0000(	CREB3	181.6591	-0.09581	0.397654	0.77667	protein_coding
ENSG0000(	ENSG0000(	114.3019	-0.09751	0.39762	0.77667	lncRNA
ENSG0000(	ENSG0000(	21.84966	0.23261	0.397736	0.77667	lncRNA
ENSG0000(	ENSG0000(	30.61664	0.214154	0.397616	0.77667	processed_pseudogene
ENSG0000(	ENSG0000(	167.0278	-0.08991	0.397662	0.77667	lncRNA
ENSG0000(	LINC01502	113.187	0.106006	0.397696	0.77667	lncRNA
ENSG0000(	LINC02080	1146.017	0.045706	0.39765	0.77667	lncRNA
ENSG0000(	RABL2A	978.8732	0.072994	0.397708	0.77667	protein_coding
ENSG0000(	WDR86-AS	49.95957	-0.21322	0.397597	0.77667	lncRNA
ENSG0000(	DDX51	900.9523	0.057288	0.397853	0.776823	protein_coding
ENSG0000(	NPAS3	173.6747	-0.13235	0.397931	0.776823	protein_coding
ENSG0000(	NUP62	1559.232	-0.06747	0.397927	0.776823	protein_coding
ENSG0000(	SH3TC1	847.5123	-0.18953	0.397937	0.776823	protein_coding
ENSG0000(	SNORA30	19.7283	0.301414	0.397955	0.776823	snoRNA
ENSG0000(	ACTG2	533.7503	0.20987	0.398172	0.776872	protein_coding
ENSG0000(	ALKBH4	298.6917	-0.06938	0.398342	0.776872	protein_coding
ENSG0000(	ENSG0000(	171.7403	-0.08359	0.39846	0.776872	lncRNA
ENSG0000(	ENSG0000(	48.45289	-0.1577	0.398228	0.776872	lncRNA
ENSG0000(	ENSG0000(	61.52131	0.126243	0.398428	0.776872	lncRNA

ENSG0000(	ENSG0000(	36.83407	-0.22135	0.398407	0.776872	lncRNA
ENSG0000(	ENSG0000(	715.5058	-0.06301	0.398124	0.776872	lncRNA
ENSG0000(	ENSG0000(	552.4069	0.062954	0.398419	0.776872	lncRNA
ENSG0000(	ENSG0000(	39.37196	-0.20445	0.398375	0.776872	lncRNA
ENSG0000(	FRS2	860.6774	0.081692	0.398443	0.776872	protein_coding
ENSG0000(	GATA3	89.27478	-0.19132	0.398124	0.776872	protein_coding
ENSG0000(	HOPX	354.6495	-0.08385	0.398307	0.776872	protein_coding
ENSG0000(	MT-TV	191.4962	-0.23846	0.398357	0.776872	Mt_tRNA
ENSG0000(	PRORP	1517.292	0.071311	0.398191	0.776872	protein_coding
ENSG0000(	SLIRP	366.0965	-0.07984	0.398246	0.776872	protein_coding
ENSG0000(	ZFYVE16	3563.288	-0.06675	0.3984	0.776872	protein_coding
ENSG0000(	ZNF207	11038.24	-0.0487	0.398159	0.776872	protein_coding
ENSG0000(	ENSG0000(	76.78247	0.182759	0.398602	0.777014	processed_pseudogene
ENSG0000(	ENSG0000(	175.749	0.096644	0.398615	0.777014	lncRNA
ENSG0000(	GPAM	443.4044	0.087216	0.398698	0.777014	protein_coding
ENSG0000(	LEF1-AS1	182.093	0.184809	0.398718	0.777014	lncRNA
ENSG0000(	MCRIP1	283.5584	-0.11223	0.398731	0.777014	protein_coding
ENSG0000(	METTL25B	416.505	0.081246	0.398672	0.777014	protein_coding
ENSG0000(	RN7SL799F	42.62957	0.159677	0.398634	0.777014	misc_RNA
ENSG0000(	ANTXRLP1	78.3039	-0.27946	0.398799	0.777034	unprocessed_pseudogene
ENSG0000(	C2orf83	154.5928	0.106508	0.398871	0.777034	transcribed_unprocessed_pseudo
ENSG0000(	LINC00674	894.7761	0.056754	0.398848	0.777034	transcribed_unprocessed_pseudo
ENSG0000(	PCSK1	106.6864	-0.37506	0.398882	0.777034	protein_coding
ENSG0000(	WASHC1	325.4677	0.168981	0.398813	0.777034	protein_coding
ENSG0000(	B3GALNT1	360.7829	-0.08414	0.399033	0.777154	protein_coding
ENSG0000(	PZP	33.61364	-0.23857	0.399027	0.777154	protein_coding
ENSG0000(	RN7SKP184	42.52356	-0.20958	0.399057	0.777154	misc_RNA
ENSG0000(	ZNF692	662.3661	-0.07286	0.399002	0.777154	protein_coding
ENSG0000(	ARID1B	10107.55	-0.05192	0.399246	0.777405	protein_coding
ENSG0000(	ENSG0000(	60.09911	0.229363	0.399297	0.777405	lncRNA
ENSG0000(	LCMT1	310.6383	0.096672	0.399299	0.777405	protein_coding
ENSG0000(	OR6C6	51.59654	0.108535	0.399294	0.777405	protein_coding
ENSG0000(	ENSG0000(	25.70165	-0.20188	0.399399	0.777512	lncRNA
ENSG0000(	MUC12-AS	58.60849	-0.16575	0.399439	0.777512	lncRNA
ENSG0000(	RN7SL681F	32.27595	0.22808	0.399413	0.777512	misc_RNA
ENSG0000(	ANKRD26	2300.81	0.04338	0.399475	0.777528	protein_coding
ENSG0000(	RPL7AP52	20.55703	0.351634	0.399578	0.777563	processed_pseudogene
ENSG0000(	SFPQ	9303.431	0.043909	0.399578	0.777563	protein_coding
ENSG0000(	STXBP1	721.2495	-0.10797	0.399575	0.777563	protein_coding
ENSG0000(	LIMD1-AS1	424.0238	-0.06914	0.399619	0.777588	lncRNA
ENSG0000(	KLK11	184.4221	-0.10415	0.399708	0.777706	protein_coding
ENSG0000(	ATAD2	766.616	-0.09619	0.399737	0.777707	protein_coding
ENSG0000(	GGT2	20.67588	0.432392	0.399872	0.777846	protein_coding
ENSG0000(	LINC02833	64.91345	-0.17496	0.399867	0.777846	lncRNA
ENSG0000(	SNORD32A	76.47227	-0.22792	0.399893	0.777846	snoRNA
ENSG0000(	NAXD	748.1636	0.066378	0.399977	0.777954	protein_coding
ENSG0000(	ENSG0000(	98.033	0.129401	0.400021	0.777985	lncRNA

ENSG0000(	SERBP1	2814.679	-0.07423	0.400083	0.77805	protein_coding
ENSG0000(	DZANK1	392.9817	0.059259	0.400117	0.778062	protein_coding
ENSG0000(	HADH	2324.166	0.12689	0.400157	0.778085	protein_coding
ENSG0000(	DCUN1D3	1132.748	0.171319	0.400194	0.778101	protein_coding
ENSG0000(	ASPSCR1	1131.373	0.049924	0.400271	0.778197	protein_coding
ENSG0000(	CACHD1	258.9009	-0.1402	0.40042	0.778221	protein_coding
ENSG0000(	CD52	148.7427	-0.26721	0.400389	0.778221	protein_coding
ENSG0000(	ENSG0000(	398.4773	-0.07367	0.400457	0.778221	lncRNA
ENSG0000(	ENSG0000(	20.22852	-0.1824	0.400454	0.778221	lncRNA
ENSG0000(	ENSG0000(	42.03386	-0.15117	0.400481	0.778221	lncRNA
ENSG0000(	SNORA71D	116.8556	0.169378	0.400456	0.778221	snoRNA
ENSG0000(	TBC1D8B	5368.261	0.076034	0.400373	0.778221	protein_coding
ENSG0000(	ZNF79	174.5218	0.107593	0.400551	0.778301	protein_coding
ENSG0000(	ENSG0000(	112.1726	-0.19499	0.400584	0.778303	lncRNA
ENSG0000(	ENSG0000(	737.7645	-0.07751	0.400637	0.778303	lncRNA
ENSG0000(	RN7SL732F	22.38659	-0.1858	0.400609	0.778303	misc_RNA
ENSG0000(	ENSG0000(	38.0541	-0.16817	0.400723	0.778398	lncRNA
ENSG0000(	MAML2	2389.937	0.074737	0.400743	0.778398	protein_coding
ENSG0000(	BCAR1	1583.224	0.093249	0.400912	0.77843	protein_coding
ENSG0000(	CIBAR2	54.9179	-0.22299	0.400853	0.77843	protein_coding
ENSG0000(	ENSG0000(	162.9887	0.088688	0.400909	0.77843	TEC
ENSG0000(	HSPE1P3	28.76181	-0.24094	0.400957	0.77843	processed_pseudogene
ENSG0000(	PDPK1	2783.59	0.045118	0.400825	0.77843	protein_coding
ENSG0000(	PPIG	2094.309	0.039613	0.400975	0.77843	protein_coding
ENSG0000(	TRIM22	3303.881	-0.15591	0.400985	0.77843	protein_coding
ENSG0000(	UBXN11	495.3	-0.08857	0.400812	0.77843	protein_coding
ENSG0000(	HLCS-IT1	53.72533	0.122748	0.401052	0.778504	lncRNA
ENSG0000(	DCUN1D4	1114.751	0.073526	0.40111	0.778507	protein_coding
ENSG0000(	GRK4	322.2514	-0.11434	0.401089	0.778507	protein_coding
ENSG0000(	ENSG0000(	48.43788	-0.19709	0.401158	0.778517	lncRNA
ENSG0000(	ENSG0000(	74.0304	0.126848	0.401227	0.778517	lncRNA
ENSG0000(	LTB4R	424.9802	-0.1225	0.401228	0.778517	protein_coding
ENSG0000(	PWP2	118.1535	0.27841	0.40118	0.778517	protein_coding
ENSG0000(	ENSG0000(	740.6335	0.054128	0.401271	0.778545	TEC
ENSG0000(	ENSG0000(	39.66435	-0.11475	0.401439	0.778794	lncRNA
ENSG0000(	TDRD9	69.69977	-0.33347	0.401456	0.778794	protein_coding
ENSG0000(	TOR2A	204.7952	0.127335	0.401485	0.778795	protein_coding
ENSG0000(	CACNG8	763.4809	0.059406	0.401583	0.778822	protein_coding
ENSG0000(	CLDN11	129.5149	-0.13462	0.401535	0.778822	protein_coding
ENSG0000(	ENSG0000(	141.0749	-0.12445	0.401584	0.778822	processed_pseudogene
ENSG0000(	CSNK1G2-A	23.37912	-0.24072	0.401643	0.77884	lncRNA
ENSG0000(	SMPD1	472.7763	0.186192	0.40165	0.77884	protein_coding
ENSG0000(	PTEN	5607.692	-0.07223	0.401743	0.778966	protein_coding
ENSG0000(	ENSG0000(	93.48252	0.114783	0.401787	0.778997	lncRNA
ENSG0000(	CLEC19A	317.2103	0.077206	0.402137	0.779457	protein_coding
ENSG0000(	LINC02132	38.99165	-0.16084	0.402099	0.779457	lncRNA
ENSG0000(	WDFY2	1746.393	-0.09156	0.402066	0.779457	protein_coding

ENSG0000(	ZNF233	199.778	-0.09442	0.402138	0.779457	protein_coding
ENSG0000(	BNIP3P37	77.98758	-0.13158	0.402174	0.779465	processed_pseudogene
ENSG0000(	CFAP300	108.4046	0.147001	0.402287	0.779465	protein_coding
ENSG0000(	DISP2	3835.457	0.30328	0.402262	0.779465	protein_coding
ENSG0000(	ENSG0000(	47.34055	0.380208	0.402267	0.779465	processed_pseudogene
ENSG0000(	LINC00276	481.7502	-0.06692	0.402312	0.779465	lncRNA
ENSG0000(	TECPR1	1338.407	0.077327	0.402204	0.779465	protein_coding
ENSG0000(	PDE4DIPP2	1739.46	-0.09275	0.402373	0.779473	transcribed_unprocessed_pseudo
ENSG0000(	USP47	2323.295	-0.05247	0.402364	0.779473	protein_coding
ENSG0000(	ENSG0000(	330.1828	-0.11985	0.402551	0.779734	lncRNA
ENSG0000(	PELI2	930.7906	-0.14191	0.402564	0.779734	protein_coding
ENSG0000(	ENSG0000(	104.0392	0.116678	0.402601	0.779751	lncRNA
ENSG0000(	ARL1	963.6785	-0.06743	0.402655	0.779801	protein_coding
ENSG0000(	ENSG0000(	44.75631	0.187767	0.402701	0.779835	lncRNA
ENSG0000(	CPSF2	2426.651	0.083798	0.40284	0.779845	protein_coding
ENSG0000(	ENSG0000(	34.76403	-0.21506	0.402848	0.779845	lncRNA
ENSG0000(	ENSG0000(	91.36209	0.181576	0.402835	0.779845	lncRNA
ENSG0000(	GPM6A	49.53805	0.21889	0.402773	0.779845	protein_coding
ENSG0000(	YIF1A	234.5988	0.105032	0.402749	0.779845	protein_coding
ENSG0000(	CRBN	1239.459	-0.04853	0.402889	0.779868	protein_coding
ENSG0000(	TDP1	504.7828	0.095828	0.402923	0.77988	protein_coding
ENSG0000(	ENSG0000(	65.82869	-0.25432	0.402983	0.779942	unprocessed_pseudogene
ENSG0000(	ENSG0000(	71.39544	0.102324	0.403118	0.780093	lncRNA
ENSG0000(	TTC39B	3658.536	-0.09681	0.403094	0.780093	protein_coding
ENSG0000(	ENSG0000(	109.9264	0.111624	0.403267	0.780164	lncRNA
ENSG0000(	ENSG0000(	57.4623	-0.10948	0.403188	0.780164	lncRNA
ENSG0000(	IGSF8	268.9804	0.110367	0.403296	0.780164	protein_coding
ENSG0000(	LINC01821	1904.189	0.113576	0.403286	0.780164	lncRNA
ENSG0000(	WNT7A	57.50692	0.220005	0.40325	0.780164	protein_coding
ENSG0000(	ENSG0000(	63.113	-0.16412	0.403396	0.780182	lncRNA
ENSG0000(	KCNQ5	238.4183	-0.23363	0.403358	0.780182	protein_coding
ENSG0000(	MBD1	1763.876	0.052628	0.403369	0.780182	protein_coding
ENSG0000(	NFS1	673.2526	0.065688	0.403419	0.780182	protein_coding
ENSG0000(	APOLD1	516.2755	-0.14496	0.40345	0.780187	protein_coding
ENSG0000(	LINC02685	34.88281	0.20677	0.403492	0.780213	lncRNA
ENSG0000(	C11orf52	47.13319	0.252966	0.403591	0.780295	protein_coding
ENSG0000(	COX5B	560.8752	0.121418	0.403589	0.780295	protein_coding
ENSG0000(	CHN2	995.8172	0.090648	0.403831	0.780325	protein_coding
ENSG0000(	CLN6	1447.273	-0.06137	0.403673	0.780325	protein_coding
ENSG0000(	CPEB3	594.9264	0.095735	0.403825	0.780325	protein_coding
ENSG0000(	EIF1AX	760.5341	-0.08115	0.403833	0.780325	protein_coding
ENSG0000(	ENSG0000(	295.8044	-0.06813	0.40367	0.780325	TEC
ENSG0000(	ENSG0000(	70.28372	-0.13735	0.403707	0.780325	lncRNA
ENSG0000(	KCNIP4-IT1	196.7806	0.100538	0.403757	0.780325	lncRNA
ENSG0000(	SNHG1	1198.167	-0.0855	0.403793	0.780325	lncRNA
ENSG0000(	ENSG0000(	26.94093	0.217237	0.403903	0.780405	lncRNA
ENSG0000(	KRR1	1284.599	0.063945	0.404076	0.780683	protein_coding

ENSG0000(	GINS3	310.0038	-0.12399	0.404115	0.780702	protein_coding
ENSG0000(	LSM14A	1793.586	-0.06081	0.404227	0.780702	protein_coding
ENSG0000(	RRM1-AS1	20.35881	-0.21728	0.404225	0.780702	lncRNA
ENSG0000(	SLC30A6-D	194.0854	0.074165	0.404143	0.780702	lncRNA
ENSG0000(	SLC43A1	169.0858	-0.14748	0.404177	0.780702	protein_coding
ENSG0000(	ENSG0000(	326.4557	-0.07992	0.404291	0.780716	TEC
ENSG0000(	ENSG0000(	109.0869	-0.13032	0.404274	0.780716	lncRNA
ENSG0000(	GATD1	2440.367	0.053726	0.404385	0.780837	protein_coding
ENSG0000(	SPATS2L	2852.39	0.109918	0.404411	0.780837	protein_coding
ENSG0000(	CLPP	539.0334	0.063403	0.404791	0.780883	protein_coding
ENSG0000(	ENSG0000(	270.3821	0.081008	0.404941	0.780883	lncRNA
ENSG0000(	ENSG0000(	40.80587	0.403387	0.404912	0.780883	lncRNA
ENSG0000(	FAAP20	922.1873	0.07754	0.404954	0.780883	protein_coding
ENSG0000(	FAM110D	212.1122	-0.12175	0.404666	0.780883	protein_coding
ENSG0000(	INO80E	2526.214	0.060656	0.404902	0.780883	protein_coding
ENSG0000(	KCNQ4	68.8831	0.174696	0.40458	0.780883	protein_coding
ENSG0000(	LINC01410	325.7354	0.135948	0.404548	0.780883	lncRNA
ENSG0000(	MFSD14A	402.0396	-0.16333	0.4048	0.780883	protein_coding
ENSG0000(	NDUFB5	977.4291	0.094174	0.404925	0.780883	protein_coding
ENSG0000(	NUDT7	108.9227	0.113332	0.404974	0.780883	protein_coding
ENSG0000(	ROCK2	3430.23	0.088802	0.404616	0.780883	protein_coding
ENSG0000(	SCAND1	81.86763	0.118289	0.404725	0.780883	protein_coding
ENSG0000(	SCGB3A2	440.7443	-0.10163	0.404559	0.780883	protein_coding
ENSG0000(	SFRP2	61.3513	-2.21845	0.404783	0.780883	protein_coding
ENSG0000(	STYX	723.2519	-0.09023	0.404472	0.780883	protein_coding
ENSG0000(	TLK2	2840.359	-0.04423	0.404762	0.780883	protein_coding
ENSG0000(	VAC14-AS1	304.9485	-0.07672	0.404604	0.780883	lncRNA
ENSG0000(	ZNF668	154.4084	0.126481	0.404884	0.780883	protein_coding
ENSG0000(	DNAJC7	3344.637	-0.0412	0.405055	0.780984	protein_coding
ENSG0000(	UAP1	1480.592	-0.13618	0.405171	0.781155	protein_coding
ENSG0000(	INAVA	3385.632	0.150451	0.405268	0.781286	protein_coding
ENSG0000(	LINC02207	418.2429	-0.07691	0.40533	0.781351	lncRNA
ENSG0000(	USP53	7835.36	0.16544	0.405527	0.781676	protein_coding
ENSG0000(	ENSG0000(	500.2934	0.063938	0.405629	0.781817	TEC
ENSG0000(	ENSG0000(	207.3116	-0.10449	0.405692	0.781884	lncRNA
ENSG0000(	PARD6G	270.6746	-0.07432	0.405738	0.781918	protein_coding
ENSG0000(	AK4P3	76.80523	-0.19041	0.405796	0.781975	protein_coding
ENSG0000(	LINC01748	714.2516	0.093257	0.405831	0.781988	lncRNA
ENSG0000(	NDRG1	6930.467	-0.15942	0.40596	0.782097	protein_coding
ENSG0000(	RANP8	24.16561	0.223883	0.405973	0.782097	processed_pseudogene
ENSG0000(	TRMT10B	519.9829	0.060246	0.405955	0.782097	protein_coding
ENSG0000(	ENSG0000(	88.55901	0.223686	0.406088	0.782099	transcribed_unprocessed_pseudo
ENSG0000(	ENSG0000(	59.89872	-0.13225	0.406016	0.782099	lncRNA
ENSG0000(	GABARAP	628.6397	-0.1259	0.40604	0.782099	protein_coding
ENSG0000(	LINC02030	22.66126	-0.22185	0.406069	0.782099	lncRNA
ENSG0000(	ENTR1	427.9947	0.108811	0.406124	0.782113	protein_coding
ENSG0000(	CDKAL1	913.5779	-0.05608	0.406193	0.782138	protein_coding

ENSG0000(	ENSG0000(	644.2616	-0.11103	0.406192	0.782138	TEC
ENSG0000(	ENSG0000(	2138.691	-0.12799	0.406244	0.782142	lncRNA
ENSG0000(	LINC02056	384.9713	-0.11121	0.406276	0.782142	lncRNA
ENSG0000(	SUPT6H	3074.428	-0.04115	0.40628	0.782142	protein_coding
ENSG0000(	LTB	176.5612	-0.24615	0.406349	0.782164	protein_coding
ENSG0000(	PIK3R4	789.4831	-0.11028	0.406321	0.782164	protein_coding
ENSG0000(	AFTPH	1783.663	0.038547	0.407114	0.782218	protein_coding
ENSG0000(	AGGF1P2	24.62213	0.285408	0.406545	0.782218	processed_pseudogene
ENSG0000(	ATF7IP	5266.378	-0.09613	0.406694	0.782218	protein_coding
ENSG0000(	CCNJ	332.3381	0.069385	0.406808	0.782218	protein_coding
ENSG0000(	CD4	694.9864	-0.13912	0.40706	0.782218	protein_coding
ENSG0000(	CNN3-DT	264.1762	0.088366	0.407018	0.782218	lncRNA
ENSG0000(	ELP6	461.8321	-0.05721	0.40662	0.782218	protein_coding
ENSG0000(	ENSG0000(	78.80365	0.147682	0.407133	0.782218	lncRNA
ENSG0000(	ENSG0000(	55.03178	0.152592	0.40702	0.782218	lncRNA
ENSG0000(	ENSG0000(	213.7645	0.121123	0.406934	0.782218	lncRNA
ENSG0000(	ENSG0000(	31.04462	0.23028	0.407202	0.782218	lncRNA
ENSG0000(	ENSG0000(	28.3051	-0.19584	0.407055	0.782218	lncRNA
ENSG0000(	ENSG0000(	74.52804	0.123282	0.406676	0.782218	lncRNA
ENSG0000(	ENSG0000(	60.5154	0.170851	0.406742	0.782218	lncRNA
ENSG0000(	ENSG0000(	198.3934	0.098003	0.407188	0.782218	lncRNA
ENSG0000(	ENSG0000(	133.3229	-0.11488	0.406544	0.782218	lncRNA
ENSG0000(	ENSG0000(	19.72295	-0.24786	0.407049	0.782218	lncRNA
ENSG0000(	ENSG0000(	649.5882	-0.05585	0.406648	0.782218	lncRNA
ENSG0000(	LINC02549	45.89429	-0.19231	0.406499	0.782218	lncRNA
ENSG0000(	MEIOC	315.6438	0.084338	0.407093	0.782218	protein_coding
ENSG0000(	MIR548Z	46.81116	0.148311	0.407164	0.782218	miRNA
ENSG0000(	PHLDA2	51.00681	-0.25629	0.407	0.782218	protein_coding
ENSG0000(	RNF170	1094.715	-0.05282	0.407062	0.782218	protein_coding
ENSG0000(	RPS26P42	29.27572	-0.17699	0.406603	0.782218	processed_pseudogene
ENSG0000(	STARD8	388.5313	-0.09964	0.406455	0.782218	protein_coding
ENSG0000(	STPG3-AS1	97.69802	0.19933	0.407092	0.782218	lncRNA
ENSG0000(	TBC1D22A-	165.6546	0.08117	0.406418	0.782218	lncRNA
ENSG0000(	USF2	1181.45	-0.0676	0.406657	0.782218	protein_coding
ENSG0000(	ZYG11B	1877.976	0.061506	0.406957	0.782218	protein_coding
ENSG0000(	UBE2L3	702.9641	-0.08429	0.407335	0.78242	protein_coding
ENSG0000(	ZNF385A	297.3451	-0.09953	0.407383	0.782457	protein_coding
ENSG0000(	SYMPK	3184.228	-0.06442	0.407525	0.782675	protein_coding
ENSG0000(	ABCA1	3610.286	-0.12712	0.407741	0.782728	protein_coding
ENSG0000(	BOK	153.8762	0.166871	0.407582	0.782728	protein_coding
ENSG0000(	ENSG0000(	65.58267	-0.17339	0.407709	0.782728	lncRNA
ENSG0000(	ENSG0000(	21.51515	0.203989	0.407752	0.782728	lncRNA
ENSG0000(	ENSG0000(	30.37201	-0.20822	0.407728	0.782728	transcribed_unprocessed_pseudo
ENSG0000(	MT-ND3	2125.085	-0.15716	0.407632	0.782728	protein_coding
ENSG0000(	NINL	283.3826	-0.12792	0.407705	0.782728	protein_coding
ENSG0000(	ACLY	1231.71	0.069589	0.408105	0.782908	protein_coding
ENSG0000(	CD40LG	119.3493	-0.16156	0.407978	0.782908	protein_coding

ENSG0000(	ENSG0000(	111.8488	-0.08911	0.408092	0.782908	lncRNA
ENSG0000(	ENSG0000(	54.76352	-0.14035	0.407995	0.782908	TEC
ENSG0000(	ENSG0000(	87.29447	0.147395	0.408076	0.782908	TEC
ENSG0000(	ENSG0000(	19.77904	-0.29106	0.407923	0.782908	lncRNA
ENSG0000(	ENSG0000(	888.1085	-0.06419	0.40813	0.782908	lncRNA
ENSG0000(	LINC01992	213.0762	0.069421	0.408044	0.782908	lncRNA
ENSG0000(	SETD1B	1880.224	0.053051	0.40798	0.782908	protein_coding
ENSG0000(	SPRY4-AS1	224.319	-0.11497	0.40799	0.782908	lncRNA
ENSG0000(	USP50	60.1001	0.161293	0.408187	0.782962	protein_coding
ENSG0000(	RNU6-1045	23.89689	-0.28907	0.408228	0.782988	snRNA
ENSG0000(	SLC9A3-AS:	1325.315	0.200515	0.408261	0.782997	lncRNA
ENSG0000(	RRP1	447.2593	0.065843	0.408428	0.783208	protein_coding
ENSG0000(	SUGT1P4-S	253.0387	0.098729	0.408413	0.783208	lncRNA
ENSG0000(	ENSG0000(	57.61452	-0.14391	0.408487	0.783266	lncRNA
ENSG0000(	KHSRP	1390.016	-0.07911	0.408532	0.783298	protein_coding
ENSG0000(	MS4A7	1263.244	-0.13609	0.408571	0.783317	protein_coding
ENSG0000(	LINC01231	67.36557	0.101211	0.408617	0.783351	lncRNA
ENSG0000(	CCDC26	2957.236	0.062014	0.408732	0.783391	lncRNA
ENSG0000(	CNGA1	112.4567	0.246489	0.40878	0.783391	protein_coding
ENSG0000(	FUCA1	1113.603	-0.16956	0.408703	0.783391	protein_coding
ENSG0000(	NPIPB8	34.88835	-0.34519	0.408758	0.783391	protein_coding
ENSG0000(	ZNF731P	2394.69	-0.04943	0.408729	0.783391	transcribed_unprocessed_pseudo
ENSG0000(	ENSG0000(	66.69892	-0.15175	0.408976	0.783421	lncRNA
ENSG0000(	ENSG0000(	20.15402	0.301755	0.408886	0.783421	lncRNA
ENSG0000(	ENSG0000(	96.57181	-0.14524	0.409017	0.783421	lncRNA
ENSG0000(	ESF1	391.4951	0.084883	0.409046	0.783421	protein_coding
ENSG0000(	FAIM	253.2507	0.090579	0.408957	0.783421	protein_coding
ENSG0000(	ITPR3	5663.681	0.08912	0.40895	0.783421	protein_coding
ENSG0000(	LINC02523	70.6499	0.114083	0.409052	0.783421	lncRNA
ENSG0000(	LRRFIP1P1	257.2853	-0.10366	0.408946	0.783421	processed_pseudogene
ENSG0000(	TPSAB1	147.5107	-0.27564	0.408861	0.783421	protein_coding
ENSG0000(	BTG3	172.5497	0.107991	0.409115	0.783487	protein_coding
ENSG0000(	ELF2	1967.509	-0.04839	0.409163	0.783514	protein_coding
ENSG0000(	ENSG0000(	370.2731	0.095162	0.409186	0.783514	lncRNA
ENSG0000(	MPLKIP	1836.664	0.042513	0.409235	0.783553	protein_coding
ENSG0000(	ENSG0000(	109.4955	-0.13303	0.409277	0.78358	lncRNA
ENSG0000(	HBB	85.11974	-0.40233	0.409339	0.783589	protein_coding
ENSG0000(	LINC02732	248.2437	0.072141	0.409336	0.783589	lncRNA
ENSG0000(	VPS9D1-AS	87.25267	-0.22228	0.409404	0.783659	lncRNA
ENSG0000(	MAP3K9-D	27.86259	-0.2387	0.409522	0.783775	lncRNA
ENSG0000(	SMNDC1	543.1546	0.074231	0.409506	0.783775	protein_coding
ENSG0000(	DPP4-DT	133.7574	-0.13288	0.409642	0.783782	lncRNA
ENSG0000(	ENSG0000(	436.5586	-0.07503	0.409664	0.783782	lncRNA
ENSG0000(	LINC00671	106.6118	-0.11398	0.409668	0.783782	lncRNA
ENSG0000(	LINC00963	2330.539	-0.06291	0.409638	0.783782	lncRNA
ENSG0000(	TTC41P	95.16611	-0.16998	0.409595	0.783782	transcribed_unitary_pseudogene
ENSG0000(	CASR	1240.396	-0.06662	0.410321	0.783788	protein_coding

ENSG0000(	CCDC97	648.0177	-0.06468	0.410197	0.783788	protein_coding
ENSG0000(	ENSG0000(	90.35908	0.128808	0.410394	0.783788	lncRNA
ENSG0000(	ENSG0000(	381.1659	0.081587	0.410278	0.783788	lncRNA
ENSG0000(	ENSG0000(	28.10513	-0.22068	0.410384	0.783788	lncRNA
ENSG0000(	ENSG0000(	54.25614	-0.16959	0.410351	0.783788	lncRNA
ENSG0000(	ENSG0000(	103.8603	0.087886	0.410507	0.783788	TEC
ENSG0000(	ENSG0000(	54.5638	0.13848	0.410137	0.783788	lncRNA
ENSG0000(	ENSG0000(	198.8096	0.085704	0.410557	0.783788	lncRNA
ENSG0000(	ENSG0000(	298.6791	0.090578	0.410049	0.783788	lncRNA
ENSG0000(	EXOC8	886.9984	-0.04739	0.41014	0.783788	protein_coding
ENSG0000(	GBGT1	464.6748	-0.12034	0.410577	0.783788	protein_coding
ENSG0000(	IL21	23.57813	-0.45333	0.410497	0.783788	protein_coding
ENSG0000(	LANCL1-AS	33.58732	-0.16975	0.410483	0.783788	lncRNA
ENSG0000(	LINC00440	52.92568	-0.16855	0.410374	0.783788	lncRNA
ENSG0000(	LINC00581	64.85592	0.122138	0.410175	0.783788	lncRNA
ENSG0000(	LINC01277	33.72591	-0.22304	0.410554	0.783788	lncRNA
ENSG0000(	LYVE1	68.8334	-0.2605	0.410587	0.783788	protein_coding
ENSG0000(	MLLT10	1557.901	0.066142	0.410069	0.783788	protein_coding
ENSG0000(	NIPAL3	1902.271	0.062525	0.409778	0.783788	protein_coding
ENSG0000(	OR2L3	48.23318	0.170612	0.410544	0.783788	protein_coding
ENSG0000(	OR7E94P	367.3725	-0.10443	0.410518	0.783788	transcribed_unprocessed_pseudo
ENSG0000(	PCYT1A	2129.024	-0.05782	0.409736	0.783788	protein_coding
ENSG0000(	PHF12	2750.176	-0.05101	0.410393	0.783788	protein_coding
ENSG0000(	POLE2	101.3489	-0.15453	0.410465	0.783788	protein_coding
ENSG0000(	RN7SL202P	54.96845	-0.15445	0.410439	0.783788	misc_RNA
ENSG0000(	RNU4ATAC	40.50283	-0.20121	0.410611	0.783788	snRNA
ENSG0000(	SCARNA6	554.1804	0.101768	0.410199	0.783788	scaRNA
ENSG0000(	SGMS1-AS1	792.4289	0.079392	0.410578	0.783788	lncRNA
ENSG0000(	TBL1XR1	6079.409	-0.09025	0.410285	0.783788	protein_coding
ENSG0000(	TXLNGY	4149.577	-0.17265	0.410334	0.783788	transcribed_unprocessed_pseudo
ENSG0000(	VPS29	1213.013	0.062207	0.410611	0.783788	protein_coding
ENSG0000(	ZNF602P	97.93567	0.099992	0.41036	0.783788	transcribed_unprocessed_pseudo
ENSG0000(	ENSG0000(	2238.27	-0.06948	0.41065	0.783809	lncRNA
ENSG0000(	ENSG0000(	449.9453	-0.05523	0.410967	0.784158	lncRNA
ENSG0000(	GAR1	119.3673	0.10973	0.411033	0.784158	protein_coding
ENSG0000(	MAN2B1	1270.443	-0.08626	0.410907	0.784158	protein_coding
ENSG0000(	MIR590	27.46294	0.237907	0.410946	0.784158	miRNA
ENSG0000(	SEC14L5	215.1626	-0.09489	0.410966	0.784158	protein_coding
ENSG0000(	SEPSECS	1198.565	0.081296	0.41103	0.784158	protein_coding
ENSG0000(	TCERG1	2510.628	0.055311	0.410989	0.784158	protein_coding
ENSG0000(	CDK4	557.0497	-0.08411	0.411232	0.784161	protein_coding
ENSG0000(	ENSG0000(	81.55106	-0.34417	0.411196	0.784161	protein_coding
ENSG0000(	ENSG0000(	109.3709	-0.17283	0.411234	0.784161	TEC
ENSG0000(	ITGB3BP	1863.968	-0.2569	0.411174	0.784161	protein_coding
ENSG0000(	KLF2	476.8176	-0.20145	0.411075	0.784161	protein_coding
ENSG0000(	MACROH2/	1577.218	-0.05321	0.411234	0.784161	protein_coding
ENSG0000(	SYT13	444.8003	0.174781	0.411135	0.784161	protein_coding

ENSG0000(	TTI1	925.7506	0.079394	0.411287	0.784207	protein_coding
ENSG0000(	ENSG0000(	65.008	-0.21853	0.411348	0.784215	protein_coding
ENSG0000(	SLC15A1	510.2976	0.178734	0.411343	0.784215	protein_coding
ENSG0000(	CASTOR2	673.1273	-0.06193	0.411475	0.784239	protein_coding
ENSG0000(	ILF2	447.6993	-0.10332	0.411462	0.784239	protein_coding
ENSG0000(	PDK4	1412.38	-0.28626	0.41144	0.784239	protein_coding
ENSG0000(	VDAC1	1808.318	-0.10433	0.411454	0.784239	protein_coding
ENSG0000(	ACBD6	1101.107	0.062815	0.411506	0.784244	protein_coding
ENSG0000(	DPY19L2P3	153.7339	-0.11728	0.411556	0.784285	transcribed_unprocessed_pseudo
ENSG0000(	BBS9	811.6685	-0.08192	0.411606	0.784326	protein_coding
ENSG0000(	PPP3CA	1036.822	-0.0841	0.41165	0.784356	protein_coding
ENSG0000(	ENSG0000(	309.7599	-0.06315	0.411859	0.784427	lncRNA
ENSG0000(	ENSG0000(	25.57456	-0.16239	0.411731	0.784427	lncRNA
ENSG0000(	ENSG0000(	197.1634	0.080174	0.41175	0.784427	lncRNA
ENSG0000(	FTCDNL1	344.582	0.108171	0.41183	0.784427	protein_coding
ENSG0000(	MRPL1	131.3339	0.12496	0.411853	0.784427	protein_coding
ENSG0000(	RPL23AP35	56.04159	0.158676	0.411844	0.784427	processed_pseudogene
ENSG0000(	ENSG0000(	107.3514	0.124031	0.412076	0.784502	lncRNA
ENSG0000(	ENSG0000(	613.2937	0.054324	0.411969	0.784502	lncRNA
ENSG0000(	LINC02280	831.671	0.058461	0.41204	0.784502	lncRNA
ENSG0000(	LINC02325	174.7114	-0.15361	0.412097	0.784502	lncRNA
ENSG0000(	MFAP1	187.0394	-0.07775	0.412084	0.784502	protein_coding
ENSG0000(	NTM	100.6191	-0.2046	0.412043	0.784502	protein_coding
ENSG0000(	RASAL1	302.6374	0.141462	0.412025	0.784502	protein_coding
ENSG0000(	NDUFS3	628.5113	0.120785	0.412135	0.784519	protein_coding
ENSG0000(	ENSG0000(	508.3979	-0.12444	0.412319	0.784566	lncRNA
ENSG0000(	ENSG0000(	19.49524	-0.25364	0.412202	0.784566	lncRNA
ENSG0000(	ENSG0000(	228.3736	0.162445	0.412325	0.784566	TEC
ENSG0000(	HSD11B1L	224.3991	-0.0962	0.412246	0.784566	protein_coding
ENSG0000(	LSP1P4	769.3107	-0.13999	0.412331	0.784566	transcribed_unprocessed_pseudo
ENSG0000(	TMEM39B	142.278	0.085277	0.412249	0.784566	protein_coding
ENSG0000(	CARNMT1	522.228	-0.07715	0.412639	0.784879	protein_coding
ENSG0000(	CCDC144N	311.3529	0.089933	0.412666	0.784879	transcribed_unprocessed_pseudo
ENSG0000(	CCNT2-AS1	281.6595	0.084523	0.412633	0.784879	lncRNA
ENSG0000(	ENSG0000(	60.74206	-0.145	0.412558	0.784879	lncRNA
ENSG0000(	SHROOM3	5841.342	0.204918	0.412585	0.784879	protein_coding
ENSG0000(	UBTD2	493.0008	-0.08077	0.412584	0.784879	protein_coding
ENSG0000(	HES4	28.69813	0.237835	0.412726	0.784938	protein_coding
ENSG0000(	AGO4	1468.206	-0.10293	0.41324	0.784974	protein_coding
ENSG0000(	ASXL2	2987.351	0.08544	0.412995	0.784974	protein_coding
ENSG0000(	DPH3	371.6038	-0.08991	0.413225	0.784974	protein_coding
ENSG0000(	DYNC2I1	882.639	0.075391	0.413146	0.784974	protein_coding
ENSG0000(	ENSG0000(	220.6852	0.081173	0.412874	0.784974	lncRNA
ENSG0000(	ENSG0000(	571.5838	-0.08018	0.413043	0.784974	TEC
ENSG0000(	ENSG0000(	284.2137	0.066555	0.413259	0.784974	TEC
ENSG0000(	ENSG0000(	225.9567	0.075631	0.413041	0.784974	lncRNA
ENSG0000(	ENSG0000(	23.62972	-0.24923	0.412887	0.784974	lncRNA

ENSG0000(	GPLD1	556.1472	0.061703	0.41314	0.784974	protein_coding
ENSG0000(	JKAMP	763.9594	-0.06365	0.413318	0.784974	protein_coding
ENSG0000(	LINC01609	1903.106	0.100064	0.413309	0.784974	lncRNA
ENSG0000(	LINC02175	764.4856	0.07285	0.413344	0.784974	lncRNA
ENSG0000(	MED22	1039.994	-0.06407	0.4128	0.784974	protein_coding
ENSG0000(	PTPN14	2203.618	-0.0728	0.412969	0.784974	protein_coding
ENSG0000(	RBM15B	1134.383	0.05764	0.413071	0.784974	protein_coding
ENSG0000(	SURF2	29.99111	-0.19017	0.413255	0.784974	protein_coding
ENSG0000(	SVIL2P	74.18079	-0.16548	0.41315	0.784974	transcribed_unprocessed_pseudo
ENSG0000(	VAMP3	1057.973	-0.07339	0.413081	0.784974	protein_coding
ENSG0000(	ZNF670-ZN	660.4369	-0.05702	0.413034	0.784974	protein_coding
ENSG0000(	ZSWIM6	1790.175	0.079289	0.41326	0.784974	protein_coding
ENSG0000(	KRT19	1697.122	0.200835	0.413373	0.784975	protein_coding
ENSG0000(	ENSG0000(	111.3621	-0.10557	0.413446	0.784987	lncRNA
ENSG0000(	HOXB6	1518.671	0.173294	0.413465	0.784987	protein_coding
ENSG0000(	PDCL	363.4978	-0.08522	0.413461	0.784987	protein_coding
ENSG0000(	CD3D	91.27405	-0.25151	0.41356	0.785067	protein_coding
ENSG0000(	ENSG0000(	134.6496	-0.08812	0.413593	0.785067	protein_coding
ENSG0000(	LINC02026	36.68937	0.221881	0.413575	0.785067	lncRNA
ENSG0000(	ENSG0000(	2192.559	-0.09249	0.41364	0.785097	transcribed_unprocessed_pseudo
ENSG0000(	ENSG0000(	23.26484	-0.27282	0.413751	0.785097	lncRNA
ENSG0000(	MAD2L1	315.2309	-0.12044	0.413671	0.785097	protein_coding
ENSG0000(	PDE4A	372.4059	-0.09423	0.413697	0.785097	protein_coding
ENSG0000(	TLN1	4368.332	-0.08803	0.41374	0.785097	protein_coding
ENSG0000(	ENSG0000(	39.50634	-0.20749	0.413795	0.785127	lncRNA
ENSG0000(	IL12A	20.86975	-0.22734	0.413844	0.785133	protein_coding
ENSG0000(	OGFOD3	642.7654	0.076923	0.413856	0.785133	protein_coding
ENSG0000(	OR10Z1	88.86336	-0.10835	0.413915	0.785192	protein_coding
ENSG0000(	HIGD1A	2593.462	0.183906	0.41395	0.785204	protein_coding
ENSG0000(	ENSG0000(	268.9752	-0.08136	0.414061	0.78536	TEC
ENSG0000(	ENSG0000(	239.0054	-0.09049	0.414237	0.785361	protein_coding
ENSG0000(	ENSG0000(	29.74554	-0.21876	0.414203	0.785361	lncRNA
ENSG0000(	FAM174A-I	35.94729	-0.19006	0.414245	0.785361	lncRNA
ENSG0000(	SMAD9	515.1896	0.117406	0.414247	0.785361	protein_coding
ENSG0000(	ST14	3622.652	0.172832	0.414161	0.785361	protein_coding
ENSG0000(	TCTA	786.8811	0.052909	0.414262	0.785361	protein_coding
ENSG0000(	VWA3A	21.51437	0.3538	0.414245	0.785361	protein_coding
ENSG0000(	ENSG0000(	278.2687	-0.07801	0.414304	0.785387	TEC
ENSG0000(	ENSG0000(	51.69462	-0.13408	0.414418	0.785442	processed_pseudogene
ENSG0000(	RNA5S1	632.4192	-0.20223	0.414416	0.785442	rRNA
ENSG0000(	SMG8	214.9128	-0.10473	0.414384	0.785442	protein_coding
ENSG0000(	MFSD2A	389.9735	-0.15734	0.414518	0.785469	protein_coding
ENSG0000(	PARGP1	988.5955	-0.06512	0.414517	0.785469	transcribed_unprocessed_pseudo
ENSG0000(	SPNS1	787.2095	-0.04981	0.414479	0.785469	protein_coding
ENSG0000(	ADGB	161.6109	0.191801	0.414719	0.785597	protein_coding
ENSG0000(	CCR12P	311.1096	0.097441	0.414794	0.785597	transcribed_unprocessed_pseudo
ENSG0000(	ENSG0000(	47.45988	-0.19344	0.414765	0.785597	lncRNA

ENSG0000(	ENSG0000(	70.92141	-0.12056	0.414855	0.785597	TEC
ENSG0000(	NDUFB11	192.9857	-0.13955	0.414692	0.785597	protein_coding
ENSG0000(	NES	376.8186	-0.11675	0.414694	0.785597	protein_coding
ENSG0000(	PRKAR2B-A	209.6604	0.191128	0.414846	0.785597	lncRNA
ENSG0000(	SNORA16B	22.55143	0.230277	0.41477	0.785597	snoRNA
ENSG0000(	TMEM225E	36.18413	-0.20759	0.414619	0.785597	protein_coding
ENSG0000(	VPS26BP1	141.3361	-0.10432	0.414871	0.785597	processed_pseudogene
ENSG0000(	ZNF790	339.685	-0.09522	0.414913	0.785622	protein_coding
ENSG0000(	LINC01185	146.5363	-0.11036	0.415012	0.785743	lncRNA
ENSG0000(	NAB1	1433.164	-0.05952	0.415034	0.785743	protein_coding
ENSG0000(	ATG5	727.9954	0.056817	0.415265	0.785789	protein_coding
ENSG0000(	ENSG0000(	25.49345	-0.21944	0.415287	0.785789	lncRNA
ENSG0000(	ENSG0000(	25.91768	0.209097	0.41519	0.785789	lncRNA
ENSG0000(	ENSG0000(	74.36981	-0.12849	0.415285	0.785789	lncRNA
ENSG0000(	ENSG0000(	41.74532	-0.18425	0.415158	0.785789	TEC
ENSG0000(	PEX16	281.7691	0.102317	0.415178	0.785789	protein_coding
ENSG0000(	RBPMS-AS1	78.75693	-0.11211	0.415225	0.785789	lncRNA
ENSG0000(	TSPAN5-DT	201.9909	-0.09466	0.415127	0.785789	lncRNA
ENSG0000(	ALDH9A1	494.7819	-0.07875	0.415379	0.7858	protein_coding
ENSG0000(	AMD1P4	154.8515	0.093646	0.415323	0.7858	processed_pseudogene
ENSG0000(	ENSG0000(	31.34888	-0.21153	0.415378	0.7858	lncRNA
ENSG0000(	VAT1L	124.9578	-0.21349	0.415418	0.785821	protein_coding
ENSG0000(	ENSG0000(	355.6489	-0.11068	0.415482	0.785835	lncRNA
ENSG0000(	MKNK1	2483.918	-0.04774	0.415456	0.785835	protein_coding
ENSG0000(	BMS1P4	858.7557	-0.05443	0.415579	0.785882	lncRNA
ENSG0000(	CRPPA	464.091	0.079303	0.415621	0.785882	protein_coding
ENSG0000(	GPRC5D-AS	209.6723	-0.1343	0.415547	0.785882	lncRNA
ENSG0000(	LTBP1	760.6105	-0.14227	0.41561	0.785882	protein_coding
ENSG0000(	EIF4EBP1	70.93326	-0.15332	0.415665	0.78591	protein_coding
ENSG0000(	NAGS	50.52306	-0.30663	0.415866	0.786236	protein_coding
ENSG0000(	CNOT2	2682.585	0.050814	0.4159	0.786245	protein_coding
ENSG0000(	EFCAB13	589.4811	0.104362	0.415965	0.786315	protein_coding
ENSG0000(	DCTD	552.1955	-0.08705	0.416229	0.78649	protein_coding
ENSG0000(	ENSG0000(	226.1514	0.135223	0.416096	0.78649	lncRNA
ENSG0000(	ENSG0000(	20.56181	0.214584	0.416223	0.78649	lncRNA
ENSG0000(	HIPK1-AS1	359.2643	0.064568	0.416206	0.78649	lncRNA
ENSG0000(	MAGEF1	204.0507	-0.11174	0.416182	0.78649	protein_coding
ENSG0000(	NUFIP2	6526.444	-0.03748	0.41617	0.78649	protein_coding
ENSG0000(	UBAP2	1667.862	0.047693	0.416277	0.786527	protein_coding
ENSG0000(	CREBBP	4967.112	-0.1128	0.416335	0.786568	protein_coding
ENSG0000(	ENSG0000(	93.09141	-0.10045	0.416358	0.786568	lncRNA
ENSG0000(	LINC02854	72.86944	0.259687	0.416385	0.786568	lncRNA
ENSG0000(	DNTTIP2	2239.208	-0.05453	0.416484	0.786587	protein_coding
ENSG0000(	ENSG0000(	28.3386	0.221601	0.416509	0.786587	lncRNA
ENSG0000(	NCBP3	4266.659	0.059298	0.416464	0.786587	protein_coding
ENSG0000(	UBE2N	1120.97	0.064102	0.416493	0.786587	protein_coding
ENSG0000(	FEM1B	1913.078	0.065683	0.416589	0.786608	protein_coding

ENSG0000(	LINC01449	106.7611	-0.09851	0.416606	0.786608	lncRNA
ENSG0000(	ZNF33A	3801.543	0.057854	0.416567	0.786608	protein_coding
ENSG0000(	NME7	455.5397	-0.08168	0.416796	0.786912	protein_coding
ENSG0000(	ENSG0000(	329.0888	-0.07416	0.416855	0.78697	lncRNA
ENSG0000(	RWDD2B	228.9151	-0.10589	0.416898	0.786996	protein_coding
ENSG0000(	PHBP2	59.07384	0.28742	0.416975	0.787089	processed_pseudogene
ENSG0000(	ERGIC2	1126.131	-0.08081	0.417008	0.787097	protein_coding
ENSG0000(	RNU2-1	19912.46	0.465153	0.417178	0.787363	snRNA
ENSG0000(	ENSG0000(	128.739	0.114091	0.417449	0.787488	processed_pseudogene
ENSG0000(	ENSG0000(	21.19445	0.224276	0.417439	0.787488	lncRNA
ENSG0000(	FCRL6	51.11409	-0.20793	0.417434	0.787488	protein_coding
ENSG0000(	KCNK12	166.5025	-0.17924	0.417473	0.787488	protein_coding
ENSG0000(	LRMDA	479.5238	-0.0937	0.41747	0.787488	protein_coding
ENSG0000(	ORC4	8989.524	0.04222	0.417316	0.787488	protein_coding
ENSG0000(	PARK7P2	42.20266	0.140688	0.417379	0.787488	processed_pseudogene
ENSG0000(	SERHL	170.8161	0.083297	0.417343	0.787488	transcribed_unitary_pseudogene
ENSG0000(	DYDC2	125.3688	-0.09095	0.417585	0.787543	protein_coding
ENSG0000(	ENSG0000(	21.12103	-0.21461	0.417674	0.787543	lncRNA
ENSG0000(	FAR1	2812.527	0.069954	0.417577	0.787543	protein_coding
ENSG0000(	KDM1A	2847.59	0.034624	0.41767	0.787543	protein_coding
ENSG0000(	MIR205HG	90.88652	-0.11118	0.41763	0.787543	lncRNA
ENSG0000(	NOTO	94.30109	0.11364	0.41763	0.787543	protein_coding
ENSG0000(	GATA5	33.62705	-0.62227	0.417703	0.787545	protein_coding
ENSG0000(	AGR3	165.5233	0.210166	0.417944	0.787945	protein_coding
ENSG0000(	EFHC1	2071.154	-0.09502	0.417997	0.78799	protein_coding
ENSG0000(	CPSF4	415.5272	-0.07049	0.418296	0.788247	protein_coding
ENSG0000(	HPN-AS1	145.0392	0.112819	0.418279	0.788247	lncRNA
ENSG0000(	KIRREL3	105.4278	0.095679	0.418174	0.788247	protein_coding
ENSG0000(	RDH16	758.5559	0.056831	0.418301	0.788247	protein_coding
ENSG0000(	RPN1	1431.598	-0.07023	0.418305	0.788247	protein_coding
ENSG0000(	XXYLT1	246.7885	-0.10613	0.418203	0.788247	protein_coding
ENSG0000(	LINC02267	129.1548	0.141523	0.418353	0.788283	lncRNA
ENSG0000(	ASPHD1	22.09877	-0.37474	0.418403	0.788324	protein_coding
ENSG0000(	DEXI	799.6235	-0.0661	0.41871	0.788363	protein_coding
ENSG0000(	ENSG0000(	322.4034	0.079401	0.418491	0.788363	protein_coding
ENSG0000(	ENSG0000(	49.06594	0.175803	0.418603	0.788363	lncRNA
ENSG0000(	ENSG0000(	28.79545	0.403805	0.418694	0.788363	protein_coding
ENSG0000(	ICE1	2110.328	0.058886	0.418672	0.788363	protein_coding
ENSG0000(	OTUD6B-AS	746.8251	-0.07089	0.418692	0.788363	lncRNA
ENSG0000(	PAPOLA-DT	323.3716	0.176709	0.418667	0.788363	lncRNA
ENSG0000(	PET100	257.0152	-0.11855	0.418478	0.788363	protein_coding
ENSG0000(	SNORA3B	126.803	-0.10956	0.418659	0.788363	snoRNA
ENSG0000(	ZNF696	589.2847	0.087991	0.418698	0.788363	protein_coding
ENSG0000(	BUB3	1665.083	-0.07046	0.419038	0.788575	protein_coding
ENSG0000(	CEP164P1	194.9504	-0.06956	0.419138	0.788575	transcribed_processed_pseudoge
ENSG0000(	DDX5	17365.58	-0.07838	0.419111	0.788575	protein_coding
ENSG0000(	ENSG0000(	47.94259	0.132496	0.418986	0.788575	lncRNA

ENSG0000(	ENSG0000(	43.30138	0.160253	0.419253	0.788575	lncRNA
ENSG0000(	FKBP1A	2933.152	-0.08363	0.419	0.788575	protein_coding
ENSG0000(	LINC00528	107.5797	-0.15969	0.419235	0.788575	lncRNA
ENSG0000(	LINC01020	82.78857	0.110946	0.418908	0.788575	lncRNA
ENSG0000(	MTCYBP32	52.12252	0.170376	0.419041	0.788575	processed_pseudogene
ENSG0000(	MUS81	712.0312	-0.05956	0.419059	0.788575	protein_coding
ENSG0000(	PALB2	455.772	-0.08454	0.419121	0.788575	protein_coding
ENSG0000(	PDC-AS1	611.2186	-0.04911	0.419211	0.788575	lncRNA
ENSG0000(	RBBP9	398.9829	-0.06564	0.419152	0.788575	protein_coding
ENSG0000(	SLC25A38	520.0036	0.086891	0.419213	0.788575	protein_coding
ENSG0000(	UQCC3	217.0518	0.067004	0.419178	0.788575	protein_coding
ENSG0000(	BDKRB1	81.03083	-0.12639	0.41936	0.788723	protein_coding
ENSG0000(	ENSG0000(	25.11651	0.224165	0.419394	0.788732	lncRNA
ENSG0000(	RN7SKP245	23.855	-0.29849	0.419425	0.788736	misc_RNA
ENSG0000(	ENSG0000(	91.62464	-0.15703	0.419501	0.788826	lncRNA
ENSG0000(	CA2	4408.692	0.280988	0.419603	0.788866	protein_coding
ENSG0000(	DYNLT2B	44.11184	-0.19518	0.419566	0.788866	protein_coding
ENSG0000(	GADD45GII	79.67445	-0.15232	0.419609	0.788866	protein_coding
ENSG0000(	CLEC3A	127.129	0.079627	0.419647	0.788884	protein_coding
ENSG0000(	ENSG0000(	345.7116	0.136994	0.419714	0.788956	lncRNA
ENSG0000(	INTS12	256.8975	-0.0749	0.419746	0.788964	protein_coding
ENSG0000(	H3C10	127.4131	-0.18677	0.419784	0.78898	protein_coding
ENSG0000(	C12orf50	2190.233	0.062984	0.419893	0.789077	protein_coding
ENSG0000(	FANCM	527.1126	0.090907	0.419886	0.789077	protein_coding
ENSG0000(	ENSG0000(	86.06839	0.116261	0.419984	0.789141	lncRNA
ENSG0000(	HDAC4	1515.443	-0.06502	0.420041	0.789141	protein_coding
ENSG0000(	MSH2	862.3588	-0.10263	0.419985	0.789141	protein_coding
ENSG0000(	TUBG1	426.3408	-0.09226	0.420037	0.789141	protein_coding
ENSG0000(	CHPT1	1519.954	0.126355	0.42009	0.789177	protein_coding
ENSG0000(	GTF2IP12	2797.889	-0.07961	0.420185	0.789302	transcribed_unprocessed_pseudo
ENSG0000(	CDKL5	4034.895	-0.05408	0.420347	0.789424	protein_coding
ENSG0000(	ENSG0000(	95.26842	0.104993	0.42035	0.789424	lncRNA
ENSG0000(	ENSG0000(	65.1723	-0.12742	0.420515	0.789424	lncRNA
ENSG0000(	ENSG0000(	50.57958	0.145527	0.420401	0.789424	TEC
ENSG0000(	LINC02436	107.0713	0.110292	0.420322	0.789424	lncRNA
ENSG0000(	MSRB3	595.9469	-0.13194	0.42038	0.789424	protein_coding
ENSG0000(	OR52A1	208.4126	0.105901	0.420488	0.789424	protein_coding
ENSG0000(	RNF133	36.69473	-0.2318	0.420493	0.789424	protein_coding
ENSG0000(	SLC17A9	793.6694	-0.19822	0.420505	0.789424	protein_coding
ENSG0000(	SYT1	59.08541	0.270317	0.420536	0.789424	protein_coding
ENSG0000(	ENSG0000(	647.1417	0.094002	0.420583	0.789458	lncRNA
ENSG0000(	ADCY9	1271	0.091927	0.421275	0.789463	protein_coding
ENSG0000(	ENSG0000(	910.0421	0.056295	0.420957	0.789463	TEC
ENSG0000(	ENSG0000(	135.6838	0.149935	0.420836	0.789463	TEC
ENSG0000(	ENSG0000(	151.5404	-0.10023	0.421054	0.789463	lncRNA
ENSG0000(	ENSG0000(	78.1279	0.23609	0.420912	0.789463	lncRNA
ENSG0000(	ENSG0000(	46.52401	0.12796	0.421271	0.789463	lncRNA

ENSG0000(	ENSG0000(	463.1227	0.126511	0.42082	0.789463	lncRNA
ENSG0000(	FER1L5	206.325	0.069779	0.421272	0.789463	protein_coding
ENSG0000(	FRMD3	428.0854	0.171868	0.420818	0.789463	protein_coding
ENSG0000(	GAS2L1	234.9626	-0.12749	0.421271	0.789463	protein_coding
ENSG0000(	GFM1	2173.953	-0.04391	0.420907	0.789463	protein_coding
ENSG0000(	GIGYF1	2201.825	0.063368	0.421108	0.789463	protein_coding
ENSG0000(	KLHL41	26.54018	-0.28255	0.421096	0.789463	protein_coding
ENSG0000(	LINC00221	209.7638	0.115666	0.42102	0.789463	lncRNA
ENSG0000(	LINC02610	39.9524	0.274668	0.42068	0.789463	lncRNA
ENSG0000(	OR7E38P	183.1171	0.103266	0.420812	0.789463	unprocessed_pseudogene
ENSG0000(	ORC6	10657.26	-0.06586	0.421229	0.789463	protein_coding
ENSG0000(	PHF5CP	26.88599	-0.18821	0.420824	0.789463	processed_pseudogene
ENSG0000(	PPP1R32	90.41369	-0.1641	0.420622	0.789463	protein_coding
ENSG0000(	RNF25	123.5246	0.134082	0.420974	0.789463	protein_coding
ENSG0000(	ST13P15	20.45682	-0.28565	0.420965	0.789463	processed_pseudogene
ENSG0000(	SUGCT	191.0773	0.208357	0.420735	0.789463	protein_coding
ENSG0000(	VSTM2B-D	211.9005	-0.10168	0.421045	0.789463	lncRNA
ENSG0000(	ZNF253	552.6768	-0.09862	0.421202	0.789463	protein_coding
ENSG0000(	ENSG0000(	32.26404	-0.22776	0.421354	0.789559	lncRNA
ENSG0000(	D21S2088E	70.08711	0.143467	0.421437	0.789579	lncRNA
ENSG0000(	DACH1	412.2674	0.120509	0.421442	0.789579	protein_coding
ENSG0000(	ENSG0000(	111.8379	0.22145	0.421537	0.789579	lncRNA
ENSG0000(	ENSG0000(	56.42657	0.265217	0.421477	0.789579	lncRNA
ENSG0000(	PIGB	756.0358	-0.04596	0.421512	0.789579	protein_coding
ENSG0000(	SPDYE16	58.87061	0.192764	0.421531	0.789579	protein_coding
ENSG0000(	FGF11	173.8065	-0.08872	0.421687	0.789752	protein_coding
ENSG0000(	GTPBP10	994.8702	-0.0781	0.421763	0.789752	protein_coding
ENSG0000(	RN7SL105P	47.89258	-0.21182	0.421773	0.789752	misc_RNA
ENSG0000(	RPL18A	1885.44	-0.09492	0.421769	0.789752	protein_coding
ENSG0000(	SHROOM4	1383.95	0.08733	0.42169	0.789752	protein_coding
ENSG0000(	LINC01965	217.8267	0.119379	0.421834	0.789759	lncRNA
ENSG0000(	PLGRKT	173.6993	-0.11062	0.42181	0.789759	protein_coding
ENSG0000(	LINC01252	222.1381	0.124236	0.421942	0.789907	lncRNA
ENSG0000(	MIR133A1H	22.72416	-0.21953	0.421979	0.789922	lncRNA
ENSG0000(	ENSG0000(	76.78244	-0.27199	0.422075	0.789941	lncRNA
ENSG0000(	MTMR7	20.22713	0.417999	0.422056	0.789941	protein_coding
ENSG0000(	P2RY12	20.36954	-0.352	0.422048	0.789941	protein_coding
ENSG0000(	MAPK14	2736.877	-0.07544	0.422109	0.78995	protein_coding
ENSG0000(	CCL13	56.97424	0.348294	0.422305	0.790059	protein_coding
ENSG0000(	E2F4	872.9518	0.063511	0.422287	0.790059	protein_coding
ENSG0000(	ENSG0000(	455.8316	0.056501	0.422413	0.790059	lncRNA
ENSG0000(	ENSG0000(	52.61701	0.128927	0.422357	0.790059	lncRNA
ENSG0000(	ENSG0000(	235.5246	-0.08012	0.422384	0.790059	TEC
ENSG0000(	ENSG0000(	97.1116	0.2513	0.422301	0.790059	lncRNA
ENSG0000(	ENSG0000(	168.9955	0.353253	0.422425	0.790059	lncRNA
ENSG0000(	ODC1	661.4227	0.124318	0.422326	0.790059	protein_coding
ENSG0000(	QRICH1	1642.645	0.046136	0.422322	0.790059	protein_coding

ENSG0000(	ENSG0000(	91.0618	0.101014	0.422555	0.790193	lncRNA
ENSG0000(	SNORD67	62.12529	-0.14911	0.422549	0.790193	snoRNA
ENSG0000(	ENSG0000(	156.0624	-0.08505	0.42272	0.790234	lncRNA
ENSG0000(	LINC00942	67.24217	0.178974	0.422718	0.790234	lncRNA
ENSG0000(	LRP1	10064.71	-0.08959	0.422687	0.790234	protein_coding
ENSG0000(	NDUFAF4	280.1621	-0.09762	0.422661	0.790234	protein_coding
ENSG0000(	TRGC2	100.4986	-0.18043	0.42271	0.790234	TR_C_gene
ENSG0000(	ENSG0000(	58.32917	0.190029	0.422894	0.790437	unprocessed_pseudogene
ENSG0000(	HINT2	144.9836	0.133479	0.422865	0.790437	protein_coding
ENSG0000(	PRKAR1B	226.1626	0.144796	0.422915	0.790437	protein_coding
ENSG0000(	ODAD1	333.3899	-0.07417	0.422965	0.790476	protein_coding
ENSG0000(	AKR1B15	487.388	-0.06271	0.422994	0.790477	protein_coding
ENSG0000(	DCTN1	1524.983	-0.05564	0.423049	0.790526	protein_coding
ENSG0000(	FARSA	234.0948	0.096893	0.423092	0.790554	protein_coding
ENSG0000(	BAK1	832.6726	-0.10573	0.423157	0.79058	protein_coding
ENSG0000(	JMJD8	313.3287	0.116342	0.423164	0.79058	protein_coding
ENSG0000(	NSMCE4A	577.234	0.07989	0.423229	0.790647	protein_coding
ENSG0000(	FLACC1	152.4593	0.1137	0.423272	0.790676	protein_coding
ENSG0000(	ENSG0000(	227.7003	0.081917	0.423461	0.790682	lncRNA
ENSG0000(	HDGFL2	548.5049	0.054999	0.423314	0.790682	protein_coding
ENSG0000(	LINC01922	130.8334	0.078146	0.423347	0.790682	lncRNA
ENSG0000(	MINDY4	323.8554	-0.08394	0.423438	0.790682	protein_coding
ENSG0000(	PHF23	419.9426	-0.09756	0.423505	0.790682	protein_coding
ENSG0000(	RN7SL138F	45.01429	-0.17045	0.423499	0.790682	misc_RNA
ENSG0000(	STEAP1	145.7271	-0.15321	0.423505	0.790682	protein_coding
ENSG0000(	WDR83	231.0719	-0.08436	0.423432	0.790682	protein_coding
ENSG0000(	ENSG0000(	65.95437	0.12048	0.423694	0.790864	lncRNA
ENSG0000(	ENSG0000(	232.0725	0.068238	0.423657	0.790864	lncRNA
ENSG0000(	ETF1P2	58.80091	0.133574	0.423718	0.790864	processed_pseudogene
ENSG0000(	SNRPB	775.3091	-0.07186	0.423667	0.790864	protein_coding
ENSG0000(	ANKRD20A	32.42626	0.408683	0.423849	0.791013	protein_coding
ENSG0000(	ENSG0000(	65.22726	0.136277	0.423862	0.791013	lncRNA
ENSG0000(	SMARCAD1	876.2906	0.081097	0.423884	0.791013	protein_coding
ENSG0000(	ZNF295-AS	220.6526	-0.09735	0.423984	0.791146	lncRNA
ENSG0000(	ACAP2-IT1	102.6041	-0.18287	0.424132	0.791191	lncRNA
ENSG0000(	ENSG0000(	67.80013	0.140003	0.424152	0.791191	lncRNA
ENSG0000(	LINC00649	1613.191	0.068839	0.424046	0.791191	lncRNA
ENSG0000(	OPA3	1975.884	0.039798	0.42412	0.791191	protein_coding
ENSG0000(	ZNF394	2297.742	0.041253	0.424134	0.791191	protein_coding
ENSG0000(	ENSG0000(	179.6828	0.114822	0.424285	0.791385	TEC
ENSG0000(	ARHGEF38-	63.01914	0.190412	0.424322	0.7914	lncRNA
ENSG0000(	HMGN1P1	82.9818	0.110226	0.424394	0.791402	processed_pseudogene
ENSG0000(	RNF216-IT1	45.48208	-0.21373	0.424409	0.791402	lncRNA
ENSG0000(	WDR12	1169.334	0.067164	0.424357	0.791402	protein_coding
ENSG0000(	ABITRAM	241.8266	0.095067	0.424483	0.791487	protein_coding
ENSG0000(	PHB	645.3699	0.107033	0.424548	0.791505	protein_coding
ENSG0000(	WIPI1	1116.034	0.068934	0.424551	0.791505	protein_coding

ENSG0000(	MBD6	1933.472	0.063874	0.424634	0.791607	protein_coding
ENSG0000(	ENSG0000(	112.5768	-0.12266	0.424739	0.791652	lncRNA
ENSG0000(	ENSG0000(	155.6892	0.080178	0.424745	0.791652	processed_pseudogene
ENSG0000(	PM20D2	705.6555	0.074555	0.424732	0.791652	protein_coding
ENSG0000(	ENSG0000(	34.98262	-0.14644	0.424852	0.791745	lncRNA
ENSG0000(	ENSG0000(	161.7434	0.210553	0.424828	0.791745	TEC
ENSG0000(	ENSG0000(	68.9838	-0.19197	0.424892	0.791766	lncRNA
ENSG0000(	ANGPTL1	46.91391	0.248898	0.424994	0.791771	protein_coding
ENSG0000(	CCDC40	375.538	-0.08458	0.424992	0.791771	protein_coding
ENSG0000(	ENSG0000(	97.52617	-0.11607	0.42501	0.791771	lncRNA
ENSG0000(	SEMA3A	367.6178	-0.19062	0.424996	0.791771	protein_coding
ENSG0000(	RTL6	564.4593	0.06184	0.425039	0.791771	protein_coding
ENSG0000(	LINC00159	192.3196	0.091977	0.425069	0.791774	lncRNA
ENSG0000(	ENSG0000(	134.228	-0.10567	0.425133	0.79184	TEC
ENSG0000(	C14orf93	301.6296	0.099057	0.425429	0.792029	protein_coding
ENSG0000(	DNLZ	40.28446	0.20672	0.425403	0.792029	protein_coding
ENSG0000(	ENSG0000(	39.75137	-0.17044	0.425445	0.792029	lncRNA
ENSG0000(	ENSG0000(	188.6963	-0.14048	0.425305	0.792029	TEC
ENSG0000(	ENSG0000(	305.142	-0.06929	0.425363	0.792029	lncRNA
ENSG0000(	ENSG0000(	53.62911	-0.13538	0.425435	0.792029	lncRNA
ENSG0000(	TSPAN1	4315.994	0.176465	0.425323	0.792029	protein_coding
ENSG0000(	ZNF559	964.6661	0.085665	0.425465	0.792029	protein_coding
ENSG0000(	ENSG0000(	57.60756	-0.15698	0.425573	0.792177	lncRNA
ENSG0000(	AQR	2084.699	-0.05267	0.425723	0.792199	protein_coding
ENSG0000(	ENSG0000(	176.9369	0.078045	0.425729	0.792199	lncRNA
ENSG0000(	ENSG0000(	41.77618	-0.15716	0.425659	0.792199	lncRNA
ENSG0000(	KANSL1	9256.113	-0.08108	0.425661	0.792199	protein_coding
ENSG0000(	POT1-AS1	1078.651	0.050183	0.425694	0.792199	lncRNA
ENSG0000(	RIC3	481.6804	0.102607	0.425767	0.792217	protein_coding
ENSG0000(	HSD17B10	342.037	0.08552	0.425831	0.792227	protein_coding
ENSG0000(	TRMT12	298.2601	-0.09669	0.425815	0.792227	protein_coding
ENSG0000(	FAM193A	1724.483	0.048977	0.425896	0.792241	protein_coding
ENSG0000(	KCNH8	140.6589	0.169011	0.42588	0.792241	protein_coding
ENSG0000(	TAGAP-AS1	1483.188	-0.03957	0.425934	0.792258	lncRNA
ENSG0000(	AMMECR1-	68.09754	0.127591	0.426281	0.792541	lncRNA
ENSG0000(	ATG16L1	940.9623	-0.05598	0.426255	0.792541	protein_coding
ENSG0000(	ENSG0000(	60.46341	-0.15997	0.426472	0.792541	processed_pseudogene
ENSG0000(	ENSG0000(	59.53954	-0.10766	0.426349	0.792541	lncRNA
ENSG0000(	ENSG0000(	48.19851	0.14554	0.426117	0.792541	lncRNA
ENSG0000(	ENSG0000(	41.08878	-0.1957	0.426349	0.792541	lncRNA
ENSG0000(	ENSG0000(	55.15426	0.187442	0.426426	0.792541	lncRNA
ENSG0000(	FAM169A	133.4562	0.191012	0.426489	0.792541	protein_coding
ENSG0000(	FBXL14	242.0633	0.142698	0.426214	0.792541	protein_coding
ENSG0000(	FBXO21	775.2051	-0.07078	0.426416	0.792541	protein_coding
ENSG0000(	GRIK1-AS1	355.1048	0.063473	0.426306	0.792541	lncRNA
ENSG0000(	RNF26	240.7593	-0.09678	0.426393	0.792541	protein_coding
ENSG0000(	SNX8	457.9245	-0.11522	0.426477	0.792541	protein_coding

ENSG0000(	WDR82	2312.021	-0.03968	0.426449	0.792541	protein_coding
ENSG0000(	AHCYL1	1624.949	-0.06254	0.426546	0.792579	protein_coding
ENSG0000(	EPN2	1445.702	-0.05854	0.426718	0.792579	protein_coding
ENSG0000(	GABPB1-AS	6495.468	0.090708	0.426608	0.792579	lncRNA
ENSG0000(	KIAA1217	2872.46	0.106911	0.426748	0.792579	protein_coding
ENSG0000(	KIAA2012	357.9679	0.058023	0.426769	0.792579	protein_coding
ENSG0000(	LINC02575	116.8929	0.086777	0.426747	0.792579	lncRNA
ENSG0000(	LINC02832	581.9214	0.066671	0.426664	0.792579	lncRNA
ENSG0000(	MYH14	6414.339	0.179882	0.426691	0.792579	protein_coding
ENSG0000(	RNU6-1005	26.89066	-0.21553	0.426639	0.792579	snRNA
ENSG0000(	RCVRN	23.53443	-0.22491	0.426811	0.792603	protein_coding
ENSG0000(	MOB1B	1212.407	-0.0717	0.426912	0.792738	protein_coding
ENSG0000(	ENSG0000(	583.3448	-0.07342	0.42702	0.792812	lncRNA
ENSG0000(	ENSG0000(	41.43002	0.160423	0.427125	0.792812	lncRNA
ENSG0000(	ENSG0000(	33.77938	-0.19043	0.427069	0.792812	TEC
ENSG0000(	ENSG0000(	261.2158	-0.09015	0.42712	0.792812	lncRNA
ENSG0000(	NINJ2	22.57265	0.24091	0.427009	0.792812	protein_coding
ENSG0000(	RRS1	133.8861	-0.13105	0.427099	0.792812	protein_coding
ENSG0000(	ENSG0000(	101.8033	0.218822	0.427174	0.79285	lncRNA
ENSG0000(	C7orf26	365.5117	0.062325	0.427406	0.793083	protein_coding
ENSG0000(	CNTFR	84.73296	0.440354	0.427493	0.793083	protein_coding
ENSG0000(	ENSG0000(	233.2179	-0.07708	0.427405	0.793083	lncRNA
ENSG0000(	ENSG0000(	198.4614	-0.11952	0.427501	0.793083	lncRNA
ENSG0000(	PAPPA-AS1	71.35505	0.122643	0.427491	0.793083	lncRNA
ENSG0000(	TEKT4	79.61854	-0.24866	0.427474	0.793083	protein_coding
ENSG0000(	TTBK1	109.6883	-0.14822	0.427366	0.793083	protein_coding
ENSG0000(	ENSG0000(	160.372	0.088829	0.427542	0.793104	lncRNA
ENSG0000(	B4GAT1	75.37281	0.167799	0.427804	0.793166	protein_coding
ENSG0000(	CTSA	3182.406	0.146254	0.427805	0.793166	protein_coding
ENSG0000(	LRRC58	1528.557	-0.05823	0.427669	0.793166	protein_coding
ENSG0000(	MNT	1688.91	0.062779	0.427707	0.793166	protein_coding
ENSG0000(	NR1D1	127.166	0.195076	0.427729	0.793166	protein_coding
ENSG0000(	PRR3	958.369	0.054169	0.427727	0.793166	protein_coding
ENSG0000(	RN7SL806F	50.54147	0.137758	0.427797	0.793166	misc_RNA
ENSG0000(	SOX2-OT	1299.486	0.036363	0.427682	0.793166	lncRNA
ENSG0000(	ENSG0000(	54.70151	0.202279	0.427844	0.793183	lncRNA
ENSG0000(	ACSS3	342.8355	-0.1108	0.428071	0.793283	protein_coding
ENSG0000(	ENSG0000(	297.0952	-0.05778	0.428046	0.793283	lncRNA
ENSG0000(	ENSG0000(	97.45249	0.091626	0.428025	0.793283	lncRNA
ENSG0000(	ENSG0000(	812.0401	0.054866	0.42801	0.793283	TEC
ENSG0000(	NUP62CL	66.84272	0.199436	0.428	0.793283	protein_coding
ENSG0000(	YWHAE	1923.149	-0.06477	0.428009	0.793283	protein_coding
ENSG0000(	AKR7L	928.3665	0.090803	0.428236	0.793326	polymorphic_pseudogene
ENSG0000(	CPSF6	3024.453	0.054077	0.42813	0.793326	protein_coding
ENSG0000(	CRYAB	66.52341	-0.2014	0.428165	0.793326	protein_coding
ENSG0000(	ENSG0000(	45.08083	0.171711	0.428219	0.793326	processed_pseudogene
ENSG0000(	TMEM50B	1367.258	-0.05125	0.428238	0.793326	protein_coding

ENSG0000(	LINC02821	57.36557	0.181664	0.428313	0.793412	lncRNA
ENSG0000(	ENSG0000(	32.53864	0.175127	0.428409	0.793429	lncRNA
ENSG0000(	TCP11L1	1033.654	0.133137	0.428358	0.793429	protein_coding
ENSG0000(	THOC1-DT	209.8573	0.068382	0.428397	0.793429	lncRNA
ENSG0000(	ENSG0000(	30.34161	-0.15221	0.428463	0.793475	processed_pseudogene
ENSG0000(	CCDC85A	20.65797	-0.33689	0.428669	0.793574	protein_coding
ENSG0000(	ENSG0000(	1310.402	-0.06056	0.428623	0.793574	lncRNA
ENSG0000(	ENSG0000(	109.831	-0.10875	0.42871	0.793574	lncRNA
ENSG0000(	ENSG0000(	318.0971	-0.05848	0.428647	0.793574	lncRNA
ENSG0000(	ENSG0000(	493.3836	-0.07041	0.428569	0.793574	lncRNA
ENSG0000(	PPIL3	596.2841	-0.04006	0.428741	0.793574	protein_coding
ENSG0000(	SCARNA5	1861.016	0.085727	0.428591	0.793574	scaRNA
ENSG0000(	SLC47A2	70.95398	-0.22616	0.428747	0.793574	protein_coding
ENSG0000(	INTS4P1	93.72974	-0.1185	0.428819	0.793655	transcribed_unprocessed_pseudo
ENSG0000(	CENPT	601.8518	0.085496	0.42897	0.793688	protein_coding
ENSG0000(	ENSG0000(	168.9718	0.084163	0.429033	0.793688	lncRNA
ENSG0000(	LIMS4	208.3132	0.208623	0.429068	0.793688	protein_coding
ENSG0000(	LINC01550	488.6793	0.11088	0.428959	0.793688	lncRNA
ENSG0000(	NSMAF	930.8952	0.065748	0.428978	0.793688	protein_coding
ENSG0000(	SLC25A51	564.6241	-0.04778	0.428886	0.793688	protein_coding
ENSG0000(	THAP6	1240.099	0.038508	0.429056	0.793688	protein_coding
ENSG0000(	TRAC	262.5554	-0.22832	0.428992	0.793688	TR_C_gene
ENSG0000(	RN7SL657F	30.40856	-0.43844	0.42922	0.793915	misc_RNA
ENSG0000(	RN7SL449F	23.5177	-0.22448	0.429268	0.793952	misc_RNA
ENSG0000(	CCDC152	132.3285	-0.15797	0.429376	0.793991	protein_coding
ENSG0000(	GNAZ	32.0213	-0.30459	0.429342	0.793991	protein_coding
ENSG0000(	MALL	3552.194	0.190391	0.429371	0.793991	protein_coding
ENSG0000(	HSDL2-AS1	440.5062	-0.05643	0.429479	0.794127	lncRNA
ENSG0000(	ENSG0000(	168.5956	-0.11409	0.429741	0.794355	lncRNA
ENSG0000(	ENSG0000(	113.8383	-0.12901	0.429797	0.794355	TEC
ENSG0000(	ENSG0000(	91.42302	-0.13865	0.42979	0.794355	lncRNA
ENSG0000(	ERGIC1	5099.358	0.057521	0.42975	0.794355	protein_coding
ENSG0000(	MKS1	1116.358	0.045922	0.429804	0.794355	protein_coding
ENSG0000(	MYOCOS	215.9863	0.083209	0.429795	0.794355	protein_coding
ENSG0000(	PCDH11Y	648.8557	0.113086	0.429694	0.794355	protein_coding
ENSG0000(	ENSG0000(	71.58659	0.14196	0.429882	0.794357	lncRNA
ENSG0000(	NAP1L4P1	94.82449	0.138905	0.429852	0.794357	processed_pseudogene
ENSG0000(	PDPK2P	416.8484	0.06902	0.429892	0.794357	transcribed_unprocessed_pseudo
ENSG0000(	H2AC13	96.15148	-0.17757	0.430073	0.794585	protein_coding
ENSG0000(	RN7SL236F	25.24599	0.149089	0.430048	0.794585	misc_RNA
ENSG0000(	GAS8	412.2456	-0.10922	0.430169	0.794709	protein_coding
ENSG0000(	ENSG0000(	211.2797	0.077292	0.430209	0.79473	lncRNA
ENSG0000(	R3HDM2-D	44.97439	0.157999	0.430304	0.794825	lncRNA
ENSG0000(	ZNF777	141.1639	0.119543	0.430318	0.794825	protein_coding
ENSG0000(	FBF1	291.0533	-0.10021	0.430369	0.794866	protein_coding
ENSG0000(	CTRB1	28.01204	-0.21859	0.430503	0.794911	protein_coding
ENSG0000(	DLX6-AS1	404.6745	0.091879	0.430631	0.794911	lncRNA

ENSG0000(	ENSG0000(	393.8297	-0.06766	0.43059	0.794911	lncRNA
ENSG0000(	ENSG0000(	33.71628	0.199926	0.430624	0.794911	lncRNA
ENSG0000(	ENSG0000(	78.4611	-0.10623	0.430704	0.794911	lncRNA
ENSG0000(	ENSG0000(	113.2236	0.084079	0.430687	0.794911	lncRNA
ENSG0000(	ENSG0000(	131.1847	0.125359	0.430712	0.794911	protein_coding
ENSG0000(	ENSG0000(	119.3709	-0.1042	0.4307	0.794911	lncRNA
ENSG0000(	ENSG0000(	52.74063	-0.14367	0.430643	0.794911	lncRNA
ENSG0000(	PITX1-AS1	117.5508	-0.15886	0.430503	0.794911	lncRNA
ENSG0000(	TSIX	9203.062	-0.05908	0.430628	0.794911	lncRNA
ENSG0000(	ARL2BP	346.0908	-0.08355	0.430809	0.795016	protein_coding
ENSG0000(	SNORA28	80.74454	0.125493	0.430826	0.795016	snoRNA
ENSG0000(	PCDHB16	134.296	-0.09144	0.430862	0.795028	protein_coding
ENSG0000(	HNRNPH1F	179.9465	-0.12846	0.430934	0.795108	processed_pseudogene
ENSG0000(	ESS2	206.2045	-0.09389	0.430966	0.795114	protein_coding
ENSG0000(	FANCB	106.7581	-0.19868	0.431063	0.795187	protein_coding
ENSG0000(	FKBP9P1	171.5079	0.086687	0.431045	0.795187	transcribed_unprocessed_pseudo
ENSG0000(	AGBL5-IT1	22.88365	-0.15368	0.431537	0.795189	lncRNA
ENSG0000(	ANKRD17-[	33.56705	0.165967	0.43148	0.795189	lncRNA
ENSG0000(	ANLN	425.5385	-0.13981	0.431378	0.795189	protein_coding
ENSG0000(	CNBD2	178.4822	0.088338	0.431459	0.795189	protein_coding
ENSG0000(	ENSG0000(	118.5994	-0.12862	0.431427	0.795189	lncRNA
ENSG0000(	ENSG0000(	69.66702	-0.1126	0.431468	0.795189	lncRNA
ENSG0000(	ENSG0000(	30.94528	0.18484	0.431147	0.795189	processed_pseudogene
ENSG0000(	ENSG0000(	151.7241	0.107056	0.43133	0.795189	lncRNA
ENSG0000(	ENSG0000(	55.34438	0.130458	0.431548	0.795189	TEC
ENSG0000(	ENSG0000(	556.1154	-0.07883	0.431435	0.795189	lncRNA
ENSG0000(	GPX4	358.5639	-0.11798	0.431269	0.795189	protein_coding
ENSG0000(	KLF5	6465.293	0.22692	0.431388	0.795189	protein_coding
ENSG0000(	LINC00862	117.4022	-0.14747	0.431206	0.795189	lncRNA
ENSG0000(	MYBBP1A	946.3574	0.075779	0.431582	0.795189	protein_coding
ENSG0000(	PSEN2	2751.214	0.064939	0.431446	0.795189	protein_coding
ENSG0000(	SLC22A31	20.0882	0.325932	0.431219	0.795189	protein_coding
ENSG0000(	SLC22A9	108.8307	0.10523	0.431585	0.795189	protein_coding
ENSG0000(	XAB2	403.847	0.070893	0.431134	0.795189	protein_coding
ENSG0000(	SIK3	1927.586	-0.04984	0.431684	0.795319	protein_coding
ENSG0000(	BMP7	260.8634	-0.1499	0.431769	0.795422	protein_coding
ENSG0000(	ENSG0000(	842.2979	0.047434	0.43182	0.795463	protein_coding
ENSG0000(	SMAD4	4246.171	0.037699	0.431904	0.795564	protein_coding
ENSG0000(	SNHG3	1943.638	-0.09154	0.432083	0.79584	lncRNA
ENSG0000(	ABCB7	871.2605	-0.06104	0.432175	0.79586	protein_coding
ENSG0000(	G6PC3	594.3967	-0.05415	0.432158	0.79586	protein_coding
ENSG0000(	NFATC2IP	2920.151	0.036619	0.43218	0.79586	protein_coding
ENSG0000(	ANKRD20A	250.6506	-0.37103	0.432378	0.796117	protein_coding
ENSG0000(	SNORA84	51.64273	0.149744	0.432367	0.796117	snoRNA
ENSG0000(	ENSG0000(	89.49792	-0.13481	0.432476	0.796148	lncRNA
ENSG0000(	SLC25A20	321.3216	0.149499	0.432444	0.796148	protein_coding
ENSG0000(	SUV39H1	86.53385	-0.13294	0.432482	0.796148	protein_coding

ENSG0000(	GDF7	196.4787	-0.22453	0.432576	0.796163	protein_coding
ENSG0000(	PDHB	997.2084	0.066199	0.432576	0.796163	protein_coding
ENSG0000(	RNU5D-1	19.53673	-0.27858	0.432571	0.796163	snRNA
ENSG0000(	SLC7A2	258.6753	-0.18723	0.432636	0.796167	protein_coding
ENSG0000(	VHL	2266.747	-0.0414	0.432621	0.796167	protein_coding
ENSG0000(	ENSG0000(	113.749	-0.07385	0.432669	0.796173	lncRNA
ENSG0000(	HECTD2	589.2177	0.062118	0.43272	0.796213	protein_coding
ENSG0000(	ENSG0000(	30.75711	0.218763	0.432903	0.796232	lncRNA
ENSG0000(	ENSG0000(	160.4235	0.074724	0.432857	0.796232	TEC
ENSG0000(	LDOC1	149.0652	0.102973	0.432892	0.796232	protein_coding
ENSG0000(	MB	57.51072	0.330738	0.432788	0.796232	protein_coding
ENSG0000(	PRR29	67.72458	-0.1486	0.432875	0.796232	protein_coding
ENSG0000(	SUMO4	137.9668	-0.0927	0.432817	0.796232	protein_coding
ENSG0000(	ZBTB41	632.7979	-0.07701	0.432993	0.79629	protein_coding
ENSG0000(	ZNF676	316.6322	0.055365	0.432986	0.79629	protein_coding
ENSG0000(	COLEC12	1155.001	0.076783	0.433283	0.796661	protein_coding
ENSG0000(	ENSG0000(	30.94794	0.381586	0.433307	0.796661	lncRNA
ENSG0000(	ENSG0000(	313.6923	-0.05669	0.433282	0.796661	lncRNA
ENSG0000(	ENSG0000(	29.46022	0.304743	0.43331	0.796661	lncRNA
ENSG0000(	MUCL1	110.1291	0.166734	0.433369	0.796716	protein_coding
ENSG0000(	ENSG0000(	667.5846	-0.06677	0.433532	0.796909	lncRNA
ENSG0000(	ENSG0000(	294.2821	-0.09966	0.433529	0.796909	lncRNA
ENSG0000(	PROCA1	145.3514	-0.14829	0.433567	0.796919	protein_coding
ENSG0000(	ALDH3A2	3391.657	0.081682	0.433776	0.796932	protein_coding
ENSG0000(	ENSG0000(	240.5894	0.077243	0.433734	0.796932	lncRNA
ENSG0000(	ENSG0000(	46.16182	-0.1344	0.433612	0.796932	unprocessed_pseudogene
ENSG0000(	ENSG0000(	28.46577	-0.19366	0.433693	0.796932	lncRNA
ENSG0000(	FLJ20021	25.31081	0.231104	0.433644	0.796932	lncRNA
ENSG0000(	GMDS-DT	5109.301	0.059962	0.433748	0.796932	lncRNA
ENSG0000(	SNX12	515.2651	-0.10024	0.433704	0.796932	protein_coding
ENSG0000(	C1orf109	294.4242	-0.07278	0.434176	0.797064	protein_coding
ENSG0000(	ENSG0000(	84.71836	0.216609	0.433947	0.797064	TEC
ENSG0000(	ENSG0000(	38.44393	0.212303	0.434268	0.797064	lncRNA
ENSG0000(	ENSG0000(	87.95599	-0.13397	0.434171	0.797064	lncRNA
ENSG0000(	EPHB4	593.7572	0.091065	0.43418	0.797064	protein_coding
ENSG0000(	HEXA-AS1	111.3751	0.112357	0.434054	0.797064	lncRNA
ENSG0000(	NTAQ1	446.6884	-0.07937	0.433946	0.797064	protein_coding
ENSG0000(	OR10A6	271.289	-0.10675	0.434107	0.797064	protein_coding
ENSG0000(	RPL23AP34	21.69108	-0.25883	0.43422	0.797064	processed_pseudogene
ENSG0000(	SEMA5A	3021.791	0.183086	0.434043	0.797064	protein_coding
ENSG0000(	SQSTM1	3081.254	0.103312	0.433973	0.797064	protein_coding
ENSG0000(	STEAP3	602.8302	0.165313	0.434282	0.797064	protein_coding
ENSG0000(	TMEM72-A	301.3591	0.0623	0.434283	0.797064	lncRNA
ENSG0000(	TRABD2B	89.04473	-0.20332	0.434081	0.797064	protein_coding
ENSG0000(	UTP14A	265.5405	0.090386	0.434263	0.797064	protein_coding
ENSG0000(	ENSG0000(	166.3288	0.090669	0.434422	0.797265	lncRNA
ENSG0000(	IAPP	202.2569	0.112976	0.43454	0.797421	protein_coding

ENSG0000(	LINC00605	38.66925	0.146729	0.434564	0.797421	lncRNA
ENSG0000(	ENSG0000(	23.48157	-0.19744	0.434636	0.797448	lncRNA
ENSG0000(	ENSG0000(	117.9616	-0.11142	0.434666	0.797448	lncRNA
ENSG0000(	ENSG0000(	43.12434	-0.19742	0.434658	0.797448	lncRNA
ENSG0000(	ENSG0000(	48.34685	0.151319	0.43472	0.797462	lncRNA
ENSG0000(	RN7SL342F	43.54243	-0.1512	0.434731	0.797462	misc_RNA
ENSG0000(	ARSG	908.4406	-0.05706	0.434778	0.797493	protein_coding
ENSG0000(	CCM2	1029.148	-0.07212	0.434877	0.797583	protein_coding
ENSG0000(	SNF8	817.0397	0.039871	0.434885	0.797583	protein_coding
ENSG0000(	SLC39A14	2678.157	0.108336	0.434924	0.797602	protein_coding
ENSG0000(	ENSG0000(	57.23347	-0.16068	0.435037	0.797738	lncRNA
ENSG0000(	ENSG0000(	194.6869	0.075689	0.435056	0.797738	lncRNA
ENSG0000(	ENSG0000(	21.69798	0.192555	0.435191	0.797856	lncRNA
ENSG0000(	ENSG0000(	25.89877	0.194942	0.435326	0.797856	lncRNA
ENSG0000(	ENSG0000(	1733.073	0.077915	0.435362	0.797856	TEC
ENSG0000(	GUSBP5	27.49697	0.239628	0.435393	0.797856	transcribed_processed_pseudoge
ENSG0000(	IL22RA2	28.08973	0.440431	0.43541	0.797856	protein_coding
ENSG0000(	LINC01094	455.4548	-0.05523	0.435274	0.797856	lncRNA
ENSG0000(	PPM1J	35.06439	-0.24461	0.435377	0.797856	protein_coding
ENSG0000(	PSEN1	3104.102	0.069084	0.435401	0.797856	protein_coding
ENSG0000(	SMO	195.1931	-0.13117	0.435366	0.797856	protein_coding
ENSG0000(	TMEM18	832.4231	0.094299	0.435352	0.797856	protein_coding
ENSG0000(	AMBRA1	1912.59	0.071011	0.435442	0.797862	protein_coding
ENSG0000(	ING2	111.6842	0.125734	0.435516	0.797933	protein_coding
ENSG0000(	SUMF2	1326.419	-0.04718	0.43554	0.797933	protein_coding
ENSG0000(	ENSG0000(	55.04563	0.163103	0.435595	0.797943	lncRNA
ENSG0000(	ENSG0000(	148.3232	0.078295	0.435603	0.797943	lncRNA
ENSG0000(	C2CD6	23.84358	0.454696	0.435716	0.798044	protein_coding
ENSG0000(	ENSG0000(	136.1581	0.113461	0.435707	0.798044	lncRNA
ENSG0000(	IQGAP2	2791.532	0.094391	0.435755	0.798063	protein_coding
ENSG0000(	ENSG0000(	128.9453	-0.09904	0.435808	0.798068	lncRNA
ENSG0000(	TBP	378.0794	0.061312	0.435816	0.798068	protein_coding
ENSG0000(	TIMM17B	341.9505	0.050297	0.435862	0.798099	protein_coding
ENSG0000(	PSMD4	573.9472	-0.0644	0.435952	0.798202	protein_coding
ENSG0000(	SLC49A4	374.3533	-0.10078	0.436005	0.798202	protein_coding
ENSG0000(	WDR61	589.7418	-0.05283	0.435984	0.798202	protein_coding
ENSG0000(	HECW1	149.2185	-0.10951	0.436088	0.798246	protein_coding
ENSG0000(	OR56A1	333.2839	0.081107	0.436072	0.798246	protein_coding
ENSG0000(	TADA2B	830.5624	0.038544	0.436195	0.79839	protein_coding
ENSG0000(	POFUT1	1241.755	0.071583	0.436261	0.798449	protein_coding
ENSG0000(	Vault	48.09786	-0.19045	0.436285	0.798449	misc_RNA
ENSG0000(	MRPS21	495.3988	-0.04567	0.436332	0.798482	protein_coding
ENSG0000(	CD300LD-A	156.1398	0.077258	0.436366	0.798491	lncRNA
ENSG0000(	ST8SIA6	314.8955	-0.111	0.436457	0.798604	protein_coding
ENSG0000(	ENSG0000(	48.47418	-0.13883	0.436535	0.798694	lncRNA
ENSG0000(	EIF2A	1186.417	-0.06685	0.436676	0.798897	protein_coding
ENSG0000(	ENSG0000(	480.4213	0.110522	0.436769	0.798964	lncRNA

ENSG0000(	ZNF644	2520.261	0.059006	0.43677	0.798964	protein_coding
ENSG0000(	EIF3G	634.9724	-0.0647	0.437212	0.799003	protein_coding
ENSG0000(	ENSG0000(	227.3283	-0.18589	0.436922	0.799003	lncRNA
ENSG0000(	ENSG0000(	1295.291	-0.36969	0.437227	0.799003	transcribed_processed_pseudoge
ENSG0000(	ENSG0000(	44.80428	0.182857	0.436931	0.799003	TEC
ENSG0000(	LGR5	268.451	0.271588	0.437088	0.799003	protein_coding
ENSG0000(	LRRC39	22.17881	-0.21547	0.437196	0.799003	protein_coding
ENSG0000(	NTF3	35.8523	-0.24349	0.437043	0.799003	protein_coding
ENSG0000(	OR7G2	217.3555	0.069074	0.437179	0.799003	protein_coding
ENSG0000(	RABGGTB	2155.323	-0.05102	0.437073	0.799003	protein_coding
ENSG0000(	SLC1A6	450.0176	0.055459	0.437052	0.799003	protein_coding
ENSG0000(	TMEM161E	1687.138	0.060562	0.437132	0.799003	protein_coding
ENSG0000(	TWF1	969.7204	-0.08856	0.437086	0.799003	protein_coding
ENSG0000(	UCKL1-AS1	313.0965	0.076912	0.437132	0.799003	lncRNA
ENSG0000(	UNC5B	1024.337	0.123233	0.437191	0.799003	protein_coding
ENSG0000(	ZNRD1ASP	2084.428	0.064642	0.437128	0.799003	transcribed_unitary_pseudogene
ENSG0000(	LINC01176	781.8148	0.09653	0.437301	0.799085	lncRNA
ENSG0000(	CCDC66	1374.228	-0.06079	0.437789	0.799183	protein_coding
ENSG0000(	CHKB-DT	337.8326	0.062659	0.437772	0.799183	lncRNA
ENSG0000(	CRB1	55.99246	-0.18868	0.437787	0.799183	protein_coding
ENSG0000(	DSEL	751.5356	-0.09595	0.437784	0.799183	protein_coding
ENSG0000(	EEF1AKMT:	840.7035	0.067801	0.437471	0.799183	protein_coding
ENSG0000(	ENSG0000(	108.6673	-0.20281	0.437655	0.799183	lncRNA
ENSG0000(	ENSG0000(	282.0759	0.077091	0.437726	0.799183	lncRNA
ENSG0000(	ENSG0000(	91.32883	0.115596	0.437776	0.799183	lncRNA
ENSG0000(	EPM2A	971.718	0.057686	0.43779	0.799183	protein_coding
ENSG0000(	ERV3-1	1375.204	-0.07307	0.43767	0.799183	protein_coding
ENSG0000(	MTND2P2	36.18855	-0.19156	0.437552	0.799183	processed_pseudogene
ENSG0000(	PRPS1	226.0127	-0.12517	0.437564	0.799183	protein_coding
ENSG0000(	SERTAD4	168.5124	0.202801	0.43778	0.799183	protein_coding
ENSG0000(	TNNI1	110.2748	0.10647	0.437436	0.799183	protein_coding
ENSG0000(	ZNF708	1058.754	0.056921	0.437614	0.799183	protein_coding
ENSG0000(	KRT12	173.5603	0.312043	0.437925	0.799357	protein_coding
ENSG0000(	LINC01653	148.5664	0.087933	0.437944	0.799357	lncRNA
ENSG0000(	ENSG0000(	50.60069	-0.15735	0.438018	0.799439	lncRNA
ENSG0000(	ASIC4	93.72947	-0.09607	0.43871	0.799505	protein_coding
ENSG0000(	CCR6	479.0401	-0.20963	0.438717	0.799505	protein_coding
ENSG0000(	CETN2	73.87251	-0.19175	0.438751	0.799505	protein_coding
ENSG0000(	CHRNB2	24.58978	-0.39446	0.438347	0.799505	protein_coding
ENSG0000(	CTAGE3P	33.72967	0.282541	0.438679	0.799505	processed_pseudogene
ENSG0000(	ENSG0000(	44.93128	0.161235	0.438439	0.799505	lncRNA
ENSG0000(	ENSG0000(	20.44242	0.203228	0.438153	0.799505	lncRNA
ENSG0000(	ENSG0000(	49.11869	0.120263	0.438527	0.799505	lncRNA
ENSG0000(	ENSG0000(	48.70061	-0.13086	0.438293	0.799505	lncRNA
ENSG0000(	ENSG0000(	100.2474	-0.10256	0.438437	0.799505	lncRNA
ENSG0000(	ENSG0000(	48.0817	0.102267	0.438705	0.799505	lncRNA
ENSG0000(	ENSG0000(	21.84864	-0.18801	0.43837	0.799505	lncRNA

ENSG0000(	ENSG0000(	204.0497	0.147105	0.438313	0.799505	TEC
ENSG0000(	ENSG0000(	400.417	0.126993	0.438674	0.799505	lncRNA
ENSG0000(	ENSG0000(	28.617	0.240515	0.438725	0.799505	lncRNA
ENSG0000(	GNL3	690.8205	0.087272	0.438378	0.799505	protein_coding
ENSG0000(	GRN	1538.075	-0.10499	0.43815	0.799505	protein_coding
ENSG0000(	HLTF	588.3635	0.086524	0.438232	0.799505	protein_coding
ENSG0000(	HOXA4	137.9718	0.138682	0.438439	0.799505	protein_coding
ENSG0000(	LINC02093	30.65905	-0.15161	0.438528	0.799505	lncRNA
ENSG0000(	LSAMP	610.0444	-0.15426	0.438579	0.799505	protein_coding
ENSG0000(	PPP6R1	1489.284	-0.05355	0.438699	0.799505	protein_coding
ENSG0000(	PTGDR2	76.65733	0.23019	0.438669	0.799505	protein_coding
ENSG0000(	SCUBE1	689.073	-0.13121	0.438157	0.799505	protein_coding
ENSG0000(	ENSG0000(	386.1081	-0.10779	0.438887	0.799557	transcribed_unprocessed_pseudo
ENSG0000(	GORAB	1552.037	0.047262	0.438891	0.799557	protein_coding
ENSG0000(	SKOR1	30.85717	0.21952	0.438835	0.799557	protein_coding
ENSG0000(	SMIM17	228.1427	0.09173	0.438896	0.799557	protein_coding
ENSG0000(	AFAP1-AS1	86.67613	-0.14111	0.438926	0.799558	lncRNA
ENSG0000(	ENSG0000(	91.61403	-0.108	0.439018	0.799627	lncRNA
ENSG0000(	IRF3	2003.977	0.046734	0.439022	0.799627	protein_coding
ENSG0000(	ZNF860	133.8021	-0.16442	0.439078	0.799677	protein_coding
ENSG0000(	ENSG0000(	34.18025	0.145079	0.439157	0.799767	lncRNA
ENSG0000(	EFNB1	772.5364	0.133962	0.439303	0.79998	protein_coding
ENSG0000(	TRDN-AS1	1088.006	0.061882	0.439344	0.800002	lncRNA
ENSG0000(	ENSG0000(	52.32228	0.116898	0.439398	0.800048	lncRNA
ENSG0000(	ADGRD2	391.2872	-0.06099	0.439486	0.800082	protein_coding
ENSG0000(	ENSG0000(	407.1087	0.11614	0.439533	0.800082	lncRNA
ENSG0000(	ENSG0000(	25.80494	-0.1787	0.439472	0.800082	lncRNA
ENSG0000(	UBE2QL1	33.3144	-0.2127	0.439507	0.800082	protein_coding
ENSG0000(	LDLRAD4	7615.932	-0.059	0.439594	0.80014	protein_coding
ENSG0000(	LINC02548	742.8535	0.062329	0.439665	0.800173	lncRNA
ENSG0000(	PRKAA2	238.9224	0.149018	0.43967	0.800173	protein_coding
ENSG0000(	GPALPP1	2414.673	0.053207	0.439795	0.800293	protein_coding
ENSG0000(	NR2E3	42.13895	0.262029	0.439795	0.800293	protein_coding
ENSG0000(	ENSG0000(	108.9962	0.143079	0.439849	0.800339	lncRNA
ENSG0000(	ENSG0000(	76.98537	0.102629	0.43992	0.800416	lncRNA
ENSG0000(	CHMP1B2F	218.6244	-0.13262	0.439965	0.800445	transcribed_unitary_pseudogene
ENSG0000(	IGFBP3	678.787	-0.17803	0.440018	0.800488	protein_coding
ENSG0000(	CD2BP2	693.685	0.049141	0.440182	0.800644	protein_coding
ENSG0000(	ENSG0000(	36.94711	-0.20246	0.440176	0.800644	lncRNA
ENSG0000(	GANC	1630.714	0.071518	0.440191	0.800644	protein_coding
ENSG0000(	EIF2D	922.2641	0.068859	0.440283	0.800704	protein_coding
ENSG0000(	TMEM35B	97.42237	-0.12903	0.440282	0.800704	protein_coding
ENSG0000(	MLH1	1290.112	-0.04603	0.440324	0.800727	protein_coding
ENSG0000(	PALD1	245.1802	-0.121	0.440383	0.800781	protein_coding
ENSG0000(	HACL1	253.5586	-0.09156	0.440419	0.800793	protein_coding
ENSG0000(	TRPC4AP	1569.805	-0.04881	0.440504	0.800895	protein_coding
ENSG0000(	LINC00836	587.7551	-0.05737	0.440576	0.800921	lncRNA

ENSG0000(	ZNF581	92.21931	0.141891	0.440548	0.800921	protein_coding
ENSG0000(	EMSY-DT	158.6806	0.085278	0.440613	0.800934	lncRNA
ENSG0000(	C5orf63	657.0022	0.11635	0.440666	0.800969	protein_coding
ENSG0000(	DHCR24	1332.796	0.151933	0.44069	0.800969	protein_coding
ENSG0000(	ENSG0000(	143.2452	0.090417	0.440824	0.801139	lncRNA
ENSG0000(	KAT7	3532.867	0.044777	0.440842	0.801139	protein_coding
ENSG0000(	TMEM256	82.27954	-0.13853	0.44089	0.801174	protein_coding
ENSG0000(	BLM	1201.023	0.069205	0.441	0.801212	protein_coding
ENSG0000(	ENSG0000(	217.925	0.096022	0.440988	0.801212	lncRNA
ENSG0000(	ENSG0000(	586.1948	-0.06065	0.441028	0.801212	lncRNA
ENSG0000(	IGKV6-21	47.40756	-0.28302	0.440945	0.801212	IG_V_gene
ENSG0000(	DIP2C	643.8283	-0.07175	0.441145	0.801266	protein_coding
ENSG0000(	LY6G6E	234.4932	0.141962	0.441107	0.801266	protein_coding
ENSG0000(	PEF1	466.4627	0.078742	0.441127	0.801266	protein_coding
ENSG0000(	ENSG0000(	314.1267	0.057833	0.4412	0.801313	lncRNA
ENSG0000(	DDX39A	599.0955	-0.05855	0.441408	0.801533	protein_coding
ENSG0000(	ENSG0000(	110.4638	-0.0991	0.441409	0.801533	protein_coding
ENSG0000(	MIR202HG	147.1145	-0.13026	0.441387	0.801533	lncRNA
ENSG0000(	ENSG0000(	22.21668	-0.21046	0.441731	0.802027	lncRNA
ENSG0000(	ENSG0000(	69.64108	-0.08362	0.441768	0.802027	lncRNA
ENSG0000(	S1PR2	784.8225	-0.11563	0.441751	0.802027	protein_coding
ENSG0000(	HEATR5A	2020.874	0.069112	0.441904	0.802221	protein_coding
ENSG0000(	MT-TL1	268.5569	-0.221	0.441934	0.802224	Mt_tRNA
ENSG0000(	VMAC	526.8666	0.060418	0.44202	0.802326	protein_coding
ENSG0000(	ENSG0000(	55.16398	-0.15283	0.442095	0.80241	lncRNA
ENSG0000(	ENSG0000(	42.34052	-0.16525	0.44237	0.802696	lncRNA
ENSG0000(	MRPL18	200.3305	-0.1005	0.442307	0.802696	protein_coding
ENSG0000(	RN7SL414F	23.48429	0.174849	0.442384	0.802696	misc_RNA
ENSG0000(	RN7SL434F	23.4774	-0.17814	0.442399	0.802696	misc_RNA
ENSG0000(	SLC9A7P1	52.66814	-0.12484	0.442323	0.802696	transcribed_processed_pseudoge
ENSG0000(	ENSG0000(	85.06132	0.12589	0.442469	0.802766	lncRNA
ENSG0000(	KLHDC8B	137.6998	-0.16893	0.442496	0.802766	protein_coding
ENSG0000(	ENSG0000(	171.6167	0.072502	0.442618	0.802775	transcribed_unitary_pseudogene
ENSG0000(	LINC02405	337.9124	-0.10751	0.44257	0.802775	lncRNA
ENSG0000(	TPCN2	896.2383	-0.06833	0.442613	0.802775	protein_coding
ENSG0000(	ZNF518A	4344.721	0.08196	0.442597	0.802775	protein_coding
ENSG0000(	RN7SL564F	85.23753	0.231541	0.442692	0.802857	misc_RNA
ENSG0000(	SHQ1	854.1768	0.102903	0.442822	0.803008	protein_coding
ENSG0000(	UQCC2	394.2997	0.121316	0.442834	0.803008	protein_coding
ENSG0000(	ZNF430	1280.626	-0.05386	0.442919	0.803109	protein_coding
ENSG0000(	IFNLR1	669.4379	0.140889	0.442993	0.803192	protein_coding
ENSG0000(	GXYLT1	856.9007	0.043833	0.44306	0.80326	protein_coding
ENSG0000(	GGNBP2	2416.189	0.054657	0.443188	0.803335	protein_coding
ENSG0000(	SNORD8	37.5454	-0.14371	0.443168	0.803335	snoRNA
ENSG0000(	ZNF382	1196.606	0.081892	0.443189	0.803335	protein_coding
ENSG0000(	ENSG0000(	353.6128	-0.16544	0.443303	0.803382	lncRNA
ENSG0000(	LINC00494	216.0997	0.187353	0.443295	0.803382	lncRNA

ENSG0000(	OXR1-AS1	34.93631	-0.14476	0.44326	0.803382	lncRNA
ENSG0000(	ENSG0000(	273.4103	0.066551	0.443374	0.803458	lncRNA
ENSG0000(	AP1AR-DT	27.7616	-0.17452	0.44359	0.803759	lncRNA
ENSG0000(	ENSG0000(	111.4448	-0.10245	0.443599	0.803759	processed_pseudogene
ENSG0000(	EXOG	868.6591	-0.07186	0.443644	0.80379	protein_coding
ENSG0000(	EIF4H	3081.384	-0.06328	0.443855	0.803907	protein_coding
ENSG0000(	FBLIM1	3415.678	0.129498	0.443833	0.803907	protein_coding
ENSG0000(	FCER2	103.2807	-0.37281	0.443817	0.803907	protein_coding
ENSG0000(	GPRACR	155.5923	0.086875	0.443807	0.803907	lncRNA
ENSG0000(	UBA5	1813.46	-0.05883	0.443855	0.803907	protein_coding
ENSG0000(	LINC01788	23.01608	0.201324	0.443895	0.803926	lncRNA
ENSG0000(	ENSG0000(	381.1701	-0.05626	0.444034	0.804091	lncRNA
ENSG0000(	ENSG0000(	26.78598	-0.17042	0.444044	0.804091	lncRNA
ENSG0000(	COA6	113.8748	0.129347	0.444127	0.804095	protein_coding
ENSG0000(	PSORS1C1	367.159	0.284741	0.444083	0.804095	protein_coding
ENSG0000(	RERE-AS1	25.43236	0.238685	0.444134	0.804095	lncRNA
ENSG0000(	ENSG0000(	1796.355	-0.03302	0.444191	0.804144	lncRNA
ENSG0000(	ARL8B	1009.032	-0.10189	0.444506	0.804223	protein_coding
ENSG0000(	CENPX	214.9854	0.125138	0.4451	0.804223	protein_coding
ENSG0000(	CEP83	863.6264	0.072128	0.444414	0.804223	protein_coding
ENSG0000(	CPLX2	26.36435	0.313436	0.444881	0.804223	protein_coding
ENSG0000(	CRIPT	2212.925	-0.04478	0.44517	0.804223	protein_coding
ENSG0000(	DBF4	347.0045	-0.12898	0.445025	0.804223	protein_coding
ENSG0000(	DDX19A	1424.377	0.037743	0.444749	0.804223	protein_coding
ENSG0000(	ENSG0000(	34.81279	0.198889	0.444803	0.804223	processed_pseudogene
ENSG0000(	ENSG0000(	200.6699	0.131832	0.444914	0.804223	lncRNA
ENSG0000(	ENSG0000(	754.8492	0.124291	0.444955	0.804223	lncRNA
ENSG0000(	ENSG0000(	92.00281	0.103944	0.445052	0.804223	lncRNA
ENSG0000(	ENSG0000(	45.77535	-0.18534	0.444896	0.804223	TEC
ENSG0000(	ENSG0000(	95.71646	-0.14359	0.444889	0.804223	TEC
ENSG0000(	ENSG0000(	334.261	0.079868	0.444939	0.804223	TEC
ENSG0000(	ENSG0000(	92.70358	0.115414	0.444875	0.804223	lncRNA
ENSG0000(	ENSG0000(	344.6961	0.068125	0.444563	0.804223	lncRNA
ENSG0000(	ENSG0000(	34.5628	0.243286	0.444278	0.804223	lncRNA
ENSG0000(	GET1	508.0994	-0.08861	0.444721	0.804223	protein_coding
ENSG0000(	GTF2IP4	803.7908	-0.23797	0.444635	0.804223	transcribed_processed_pseudoge
ENSG0000(	HOMER3-A	39.45326	-0.13127	0.444501	0.804223	lncRNA
ENSG0000(	IL24	81.90752	-2.06257	0.445061	0.804223	protein_coding
ENSG0000(	LMOD1	389.3807	-0.16041	0.445087	0.804223	protein_coding
ENSG0000(	MPC1	455.2489	0.110284	0.444588	0.804223	protein_coding
ENSG0000(	NAB2	159.936	-0.16529	0.444966	0.804223	protein_coding
ENSG0000(	P2RX5-TAX	1008.467	-0.08026	0.444694	0.804223	protein_coding
ENSG0000(	PDCD6	2564.651	0.045226	0.445144	0.804223	protein_coding
ENSG0000(	PLK1	363.8322	0.086937	0.445004	0.804223	protein_coding
ENSG0000(	SLC38A3	29.82435	-0.2762	0.44512	0.804223	protein_coding
ENSG0000(	SNAP29	1010.774	-0.04966	0.444769	0.804223	protein_coding
ENSG0000(	SYPL2	46.5221	-0.20979	0.444461	0.804223	protein_coding

ENSG0000(	TLK2P2	105.8266	0.113666	0.444691	0.804223	processed_pseudogene
ENSG0000(	TTC5	735.527	0.064094	0.444519	0.804223	protein_coding
ENSG0000(	HRCT1	131.3284	0.308029	0.445243	0.804302	protein_coding
ENSG0000(	SRSF1	4879.923	0.040952	0.445304	0.804361	protein_coding
ENSG0000(	LCORL	1938.876	-0.04688	0.44536	0.804408	protein_coding
ENSG0000(	ENSG0000(	24.43572	0.205887	0.44558	0.804753	unprocessed_pseudogene
ENSG0000(	MIX23P1	29.25376	0.33218	0.445715	0.804935	processed_pseudogene
ENSG0000(	ZSWIM1	550.9402	-0.06587	0.445739	0.804935	protein_coding
ENSG0000(	PIGL	2922.272	0.052719	0.44578	0.804956	protein_coding
ENSG0000(	ENSG0000(	160.4087	0.094413	0.445852	0.805024	lncRNA
ENSG0000(	LRP12	746.5748	0.083738	0.445876	0.805024	protein_coding
ENSG0000(	ENSG0000(	116.9408	0.132523	0.445952	0.805088	lncRNA
ENSG0000(	ENSG0000(	280.2983	-0.07152	0.446	0.805088	lncRNA
ENSG0000(	RN7SKP154	29.63505	0.227588	0.446	0.805088	misc_RNA
ENSG0000(	CD1C	88.32451	0.276769	0.446156	0.805197	protein_coding
ENSG0000(	ENSG0000(	31.08068	-0.13645	0.446206	0.805197	lncRNA
ENSG0000(	KCNH5	43.52793	0.149783	0.446131	0.805197	protein_coding
ENSG0000(	KCNK6	2879.287	0.044675	0.446206	0.805197	protein_coding
ENSG0000(	TTN	6883.752	-0.23064	0.446207	0.805197	protein_coding
ENSG0000(	ENSG0000(	105.1851	-0.20158	0.446342	0.805262	lncRNA
ENSG0000(	ENSG0000(	261.472	0.077673	0.446375	0.805262	lncRNA
ENSG0000(	FANCI	1502.7	-0.0785	0.446303	0.805262	protein_coding
ENSG0000(	IGFBP6	23.30667	0.267289	0.446389	0.805262	protein_coding
ENSG0000(	STAR	265.902	0.060426	0.446302	0.805262	protein_coding
ENSG0000(	ENSG0000(	52.50341	0.182784	0.446538	0.80532	lncRNA
ENSG0000(	ENSG0000(	40.57288	0.136544	0.446509	0.80532	lncRNA
ENSG0000(	NIN	2857.276	-0.08964	0.446501	0.80532	protein_coding
ENSG0000(	NPB	31.79613	-0.23725	0.446528	0.80532	protein_coding
ENSG0000(	ENSG0000(	154.6488	0.191895	0.446719	0.805506	protein_coding
ENSG0000(	RPL30P13	41.1877	-0.1737	0.446708	0.805506	processed_pseudogene
ENSG0000(	WDPCP	2574.323	0.059329	0.446729	0.805506	protein_coding
ENSG0000(	COX14	189.2317	-0.08517	0.447076	0.805612	protein_coding
ENSG0000(	DPH1	1199.48	-0.056	0.44689	0.805612	protein_coding
ENSG0000(	ENSG0000(	26.3757	-0.16332	0.44705	0.805612	processed_pseudogene
ENSG0000(	ENSG0000(	80.02913	0.133839	0.44711	0.805612	processed_pseudogene
ENSG0000(	ENSG0000(	1675.162	-0.05022	0.44711	0.805612	lncRNA
ENSG0000(	HCP5B	63.13545	-0.16464	0.447095	0.805612	lncRNA
ENSG0000(	NFATC2	1046.34	-0.1073	0.447058	0.805612	protein_coding
ENSG0000(	SCARNA16	309.6123	0.108491	0.446896	0.805612	scaRNA
ENSG0000(	SCLT1	1039.778	-0.05549	0.446821	0.805612	protein_coding
ENSG0000(	TNPO2	1368.825	-0.07441	0.447026	0.805612	protein_coding
ENSG0000(	ZNF138	357.0399	0.095379	0.447034	0.805612	protein_coding
ENSG0000(	RN7SL258P	59.32103	-0.11221	0.447191	0.805644	misc_RNA
ENSG0000(	SAMD11	162.5232	-0.19438	0.447216	0.805644	protein_coding
ENSG0000(	TTC12	411.2553	0.082282	0.447199	0.805644	protein_coding
ENSG0000(	ENSG0000(	4945.052	-0.03909	0.447325	0.805735	lncRNA
ENSG0000(	LINC00867	113.2086	0.152404	0.44732	0.805735	lncRNA

ENSG0000(	ABHD17C	1934.317	0.126807	0.447474	0.805779	protein_coding
ENSG0000(	ENSG0000(	88.48983	-0.10971	0.447496	0.805779	lncRNA
ENSG0000(	ENSG0000(	57.76742	0.115793	0.447459	0.805779	lncRNA
ENSG0000(	LINC00837	32.51548	0.212102	0.447457	0.805779	lncRNA
ENSG0000(	SLC22A3	186.3941	0.155557	0.44749	0.805779	protein_coding
ENSG0000(	NEB	657.9548	-0.0916	0.447539	0.805804	protein_coding
ENSG0000(	ENSG0000(	34.52543	0.148368	0.447588	0.805839	lncRNA
ENSG0000(	ACAT1	839.291	-0.06069	0.447814	0.806145	protein_coding
ENSG0000(	ENSG0000(	36.59121	-0.15696	0.447845	0.806145	lncRNA
ENSG0000(	TMEM132(	28.10775	-0.21716	0.447824	0.806145	protein_coding
ENSG0000(	ENSG0000(	65.92113	-0.14211	0.448115	0.806284	lncRNA
ENSG0000(	PKDREJ	65.29949	0.260688	0.448125	0.806284	protein_coding
ENSG0000(	POLR2E	636.2526	0.070745	0.448098	0.806284	protein_coding
ENSG0000(	PPP6R2	1717.484	0.041284	0.448061	0.806284	protein_coding
ENSG0000(	RPL23AP82	915.9275	-0.06495	0.448138	0.806284	transcribed_unprocessed_pseudo
ENSG0000(	SEC16A	4233.394	0.04985	0.448157	0.806284	protein_coding
ENSG0000(	SINHCAF	1225.184	0.044001	0.447979	0.806284	protein_coding
ENSG0000(	TMEM54	1391.321	0.162342	0.448147	0.806284	protein_coding
ENSG0000(	ENSG0000(	204.7785	-0.09283	0.448358	0.806385	lncRNA
ENSG0000(	ENSG0000(	54.40547	-0.15371	0.448266	0.806385	processed_pseudogene
ENSG0000(	ENSG0000(	544.4623	0.044522	0.44836	0.806385	TEC
ENSG0000(	FTLP6	137.7004	0.084235	0.448283	0.806385	processed_pseudogene
ENSG0000(	TMEM229E	448.8061	-0.11943	0.448305	0.806385	protein_coding
ENSG0000(	CIR1	581.8678	-0.04855	0.448448	0.806404	protein_coding
ENSG0000(	ENSG0000(	33.42609	-0.31059	0.448527	0.806404	transcribed_unprocessed_pseudo
ENSG0000(	MYCBPAP	613.0293	0.076996	0.448546	0.806404	protein_coding
ENSG0000(	TCTE1	38.17605	-0.18146	0.448546	0.806404	protein_coding
ENSG0000(	TMSB4XP4	29.59077	-0.18312	0.448531	0.806404	processed_pseudogene
ENSG0000(	ZNF407-AS	722.2223	-0.06276	0.448532	0.806404	lncRNA
ENSG0000(	ENSG0000(	8735.801	0.086582	0.448699	0.806521	lncRNA
ENSG0000(	HPYR1	31.62267	-0.19481	0.448659	0.806521	lncRNA
ENSG0000(	ZBTB8B	1427.647	0.050359	0.448687	0.806521	protein_coding
ENSG0000(	ENSG0000(	325.6355	-0.10132	0.448737	0.806536	TEC
ENSG0000(	PABIR2	920.3963	0.077256	0.448795	0.806589	protein_coding
ENSG0000(	DNAH2	292.7289	-0.07119	0.448842	0.806619	protein_coding
ENSG0000(	HOXB4	344.5721	-0.10295	0.448895	0.806663	protein_coding
ENSG0000(	ENSG0000(	104.1164	0.087366	0.449019	0.806664	lncRNA
ENSG0000(	LTBR	2194.19	0.056112	0.448929	0.806664	protein_coding
ENSG0000(	NFE2L2	2020.654	-0.0714	0.448998	0.806664	protein_coding
ENSG0000(	RNF20	565.8388	0.069512	0.449043	0.806664	protein_coding
ENSG0000(	ZNF598	555.9064	0.092287	0.449033	0.806664	protein_coding
ENSG0000(	APC2	33.53971	-0.17072	0.449279	0.806708	protein_coding
ENSG0000(	ENSG0000(	28.25045	-0.14954	0.449197	0.806708	lncRNA
ENSG0000(	ENSG0000(	266.2585	-0.07538	0.44936	0.806708	lncRNA
ENSG0000(	LINC01833	23.79799	0.198094	0.449114	0.806708	lncRNA
ENSG0000(	PLCH1	701.2216	0.142404	0.449238	0.806708	protein_coding
ENSG0000(	POLH	2011.635	0.041535	0.44928	0.806708	protein_coding

ENSG0000(	RNU6-233F	26.17151	-0.14609	0.449136	0.806708	snRNA
ENSG0000(	SCYGR4	114.6582	-0.06756	0.449203	0.806708	protein_coding
ENSG0000(	SLC27A5	2132.242	0.053781	0.449339	0.806708	protein_coding
ENSG0000(	STPG2	45.04873	0.17101	0.449306	0.806708	protein_coding
ENSG0000(	PLA2G4E-A	111.6973	0.071442	0.44941	0.806745	lncRNA
ENSG0000(	EEF1A1P5	78.09638	-0.44847	0.449478	0.806813	processed_pseudogene
ENSG0000(	INKA2	350.3909	-0.1072	0.449538	0.80687	protein_coding
ENSG0000(	ACAT2	218.4146	-0.14759	0.449711	0.806973	protein_coding
ENSG0000(	CFAP36	339.8942	-0.07908	0.449918	0.806973	protein_coding
ENSG0000(	ENSG0000(	41.11625	-0.11982	0.449835	0.806973	lncRNA
ENSG0000(	ENSG0000(	128.0641	0.123042	0.44991	0.806973	lncRNA
ENSG0000(	ENSG0000(	28.00798	-0.22853	0.449747	0.806973	lncRNA
ENSG0000(	GPT	737.7157	0.216211	0.449632	0.806973	protein_coding
ENSG0000(	HMGCLL1	30.44165	0.199537	0.449867	0.806973	protein_coding
ENSG0000(	NTN1	209.2489	-0.25346	0.449846	0.806973	protein_coding
ENSG0000(	SFT2D1	662.0633	-0.05605	0.449847	0.806973	protein_coding
ENSG0000(	TRPC3	152.0026	0.172198	0.449808	0.806973	protein_coding
ENSG0000(	UBE2D3-AS	37.343	0.204225	0.449899	0.806973	lncRNA
ENSG0000(	ENSG0000(	20.95918	-0.31111	0.450142	0.807033	processed_pseudogene
ENSG0000(	ENSG0000(	58.8858	0.13363	0.450051	0.807033	lncRNA
ENSG0000(	ENSG0000(	66.77059	0.117309	0.450069	0.807033	lncRNA
ENSG0000(	IGDCC3	54.47549	0.139673	0.450157	0.807033	protein_coding
ENSG0000(	PPP1R42	24.82135	-0.19577	0.450138	0.807033	protein_coding
ENSG0000(	TMEM150A	111.7036	0.125573	0.4501	0.807033	protein_coding
ENSG0000(	ZNF493	3340.815	0.049999	0.450146	0.807033	protein_coding
ENSG0000(	BAIAP2-DT	734.0083	0.058249	0.450261	0.807167	lncRNA
ENSG0000(	NPIPB5	14107.61	-0.05716	0.450329	0.807236	protein_coding
ENSG0000(	CYP4A11	61.78495	0.205748	0.450364	0.807245	protein_coding
ENSG0000(	EEF1DP7	34.00852	-0.18348	0.450498	0.807296	transcribed_processed_pseudoge
ENSG0000(	ENSG0000(	677.8565	0.054338	0.450475	0.807296	lncRNA
ENSG0000(	ENSG0000(	25.43869	0.169728	0.450508	0.807296	lncRNA
ENSG0000(	NKAP	1355.945	0.070048	0.45051	0.807296	protein_coding
ENSG0000(	SCARB1	2173.201	0.059007	0.450539	0.807296	protein_coding
ENSG0000(	DUS2	245.1043	0.089733	0.450601	0.807303	protein_coding
ENSG0000(	PPIAP72	30.14391	-0.19825	0.450581	0.807303	processed_pseudogene
ENSG0000(	H4C11	80.88709	-0.41913	0.45065	0.807338	protein_coding
ENSG0000(	PAXIP1-DT	321.5976	0.074592	0.450716	0.807402	lncRNA
ENSG0000(	ENSG0000(	190.0081	0.080057	0.45075	0.807411	lncRNA
ENSG0000(	DPYD-IT1	76.01301	0.157223	0.450937	0.807611	lncRNA
ENSG0000(	GAU1	102.4235	0.138727	0.45095	0.807611	lncRNA
ENSG0000(	ZNF710-AS	323.4998	-0.07702	0.45092	0.807611	lncRNA
ENSG0000(	TRIP4	476.0581	0.079496	0.451027	0.807697	protein_coding
ENSG0000(	ENSG0000(	59.7282	-0.13834	0.451197	0.807835	lncRNA
ENSG0000(	ENSG0000(	73.28488	-0.21367	0.451188	0.807835	lncRNA
ENSG0000(	FGFBP3	62.31113	0.142506	0.451337	0.807835	protein_coding
ENSG0000(	GNL2	1315.525	0.050682	0.451321	0.807835	protein_coding
ENSG0000(	HCFC1R1	76.36839	-0.11729	0.451186	0.807835	protein_coding

ENSG0000(	LAMTOR5	525.8471	-0.08198	0.451279	0.807835	protein_coding
ENSG0000(	SMG9	1255.815	-0.0639	0.451339	0.807835	protein_coding
ENSG0000(	SNORD83A	27.43514	-0.19656	0.451244	0.807835	snoRNA
ENSG0000(	RN7SL630F	28.84638	-0.21472	0.451377	0.80785	misc_RNA
ENSG0000(	ENSG0000(	271.6769	0.196036	0.45154	0.808089	lncRNA
ENSG0000(	ABCG8	712.8029	-0.04891	0.451616	0.808174	protein_coding
ENSG0000(	ALYREF	159.5524	0.107353	0.452594	0.808266	protein_coding
ENSG0000(	CCNT2	2865.66	0.061314	0.452525	0.808266	protein_coding
ENSG0000(	CSRNP1	487.28	-0.14336	0.452094	0.808266	protein_coding
ENSG0000(	ENSG0000(	42.0836	0.185982	0.452242	0.808266	lncRNA
ENSG0000(	ENSG0000(	325.8722	0.088728	0.452667	0.808266	lncRNA
ENSG0000(	ENSG0000(	28.71025	0.213562	0.452569	0.808266	lncRNA
ENSG0000(	ENSG0000(	78.13995	-0.16675	0.452652	0.808266	lncRNA
ENSG0000(	ENSG0000(	34.71093	-0.15605	0.452268	0.808266	misc_RNA
ENSG0000(	ENSG0000(	105.9795	-0.13379	0.45227	0.808266	pseudogene
ENSG0000(	FAM210B	627.6279	-0.06181	0.452194	0.808266	protein_coding
ENSG0000(	FAM245B	113.3861	0.098027	0.452497	0.808266	lncRNA
ENSG0000(	FLJ37453	202.2154	0.075576	0.452024	0.808266	lncRNA
ENSG0000(	FZD7	164.2318	-0.14322	0.452656	0.808266	protein_coding
ENSG0000(	HAVCR1	135.6111	0.19701	0.452374	0.808266	protein_coding
ENSG0000(	HOXA-AS3	113.3009	0.218671	0.452332	0.808266	lncRNA
ENSG0000(	IMPDH1P5	50.91511	0.210789	0.452309	0.808266	processed_pseudogene
ENSG0000(	JOSD1	2006.557	0.112942	0.452402	0.808266	protein_coding
ENSG0000(	KBTBD8	241.162	-0.15091	0.452092	0.808266	protein_coding
ENSG0000(	KRTCAP2	676.7998	-0.09161	0.452503	0.808266	protein_coding
ENSG0000(	LINC00378	34.93636	-0.18404	0.452463	0.808266	lncRNA
ENSG0000(	LINC00703	28.47946	-0.14188	0.452145	0.808266	lncRNA
ENSG0000(	LINC02311	365.2436	0.091282	0.452504	0.808266	lncRNA
ENSG0000(	LINC02530	32.65854	0.17265	0.452548	0.808266	lncRNA
ENSG0000(	MGST2	371.6988	0.112045	0.452583	0.808266	protein_coding
ENSG0000(	MT-ATP8	1144.843	-0.15589	0.451855	0.808266	protein_coding
ENSG0000(	NBEA	2030.244	0.087745	0.452069	0.808266	protein_coding
ENSG0000(	OGA	7932.579	0.052552	0.452041	0.808266	protein_coding
ENSG0000(	PIAS2	2561.936	0.063856	0.451843	0.808266	protein_coding
ENSG0000(	RALY-AS1	1218.243	0.03713	0.452365	0.808266	lncRNA
ENSG0000(	RARB	498.3045	-0.12067	0.452587	0.808266	protein_coding
ENSG0000(	TFRC	4713.415	0.072716	0.452619	0.808266	protein_coding
ENSG0000(	TMEM65	1733.032	0.067796	0.452358	0.808266	protein_coding
ENSG0000(	TMEM71	167.7326	-0.223	0.451793	0.808266	protein_coding
ENSG0000(	TOMM20P.	46.71583	-0.24366	0.452432	0.808266	processed_pseudogene
ENSG0000(	SNX18P13	22.41153	0.347379	0.452811	0.808471	processed_pseudogene
ENSG0000(	WDR11-AS	308.9734	0.131824	0.452888	0.808557	lncRNA
ENSG0000(	LINC01977	216.0611	0.073152	0.452961	0.808634	lncRNA
ENSG0000(	ENSG0000(	29.24022	-0.24324	0.453004	0.808639	lncRNA
ENSG0000(	ENSG0000(	286.3452	-0.10204	0.453052	0.808639	lncRNA
ENSG0000(	OTUD6B	385.1065	0.064633	0.453035	0.808639	protein_coding
ENSG0000(	ALDH1A1	489.8248	-0.16176	0.453214	0.808823	protein_coding

ENSG0000(	OR6C4	148.7148	0.077859	0.453204	0.808823	protein_coding
ENSG0000(	SKP2	1690.879	-0.05077	0.453264	0.808831	protein_coding
ENSG0000(	SLC35E1P1	41.71009	0.193664	0.453277	0.808831	processed_pseudogene
ENSG0000(	FAM104A	674.69	-0.07436	0.453309	0.808835	protein_coding
ENSG0000(	ENSG0000(	87.63778	-0.09938	0.453496	0.809017	lncRNA
ENSG0000(	FMO3	115.073	-0.13212	0.453499	0.809017	protein_coding
ENSG0000(	OXSR1	1343.463	0.059737	0.453472	0.809017	protein_coding
ENSG0000(	FAM172A	592.7899	-0.08184	0.453587	0.809122	protein_coding
ENSG0000(	ASB4	293.6538	-0.07752	0.453633	0.809152	protein_coding
ENSG0000(	EFCAB7	194.9805	-0.12444	0.453678	0.80918	protein_coding
ENSG0000(	CD63	1115.597	-0.11846	0.453715	0.809193	protein_coding
ENSG0000(	ENSG0000(	212.7523	0.110043	0.453758	0.809206	lncRNA
ENSG0000(	ENSG0000(	31.81504	-0.20734	0.453781	0.809206	lncRNA
ENSG0000(	ENSG0000(	47.1114	-0.17709	0.453851	0.809209	lncRNA
ENSG0000(	ENSG0000(	266.0613	0.080395	0.453835	0.809209	transcribed_processed_pseudoge
ENSG0000(	TRAJ13	24.13317	-0.22965	0.453871	0.809209	TR_J_gene
ENSG0000(	ENTPD4-DT	43.15888	0.138602	0.453975	0.809238	lncRNA
ENSG0000(	LINC01257	100.7122	0.094921	0.453939	0.809238	lncRNA
ENSG0000(	PSG8	190.9185	-0.18195	0.453957	0.809238	protein_coding
ENSG0000(	ATOH8	295.7821	-0.14117	0.454351	0.809435	protein_coding
ENSG0000(	ENSG0000(	505.2935	-0.06568	0.454332	0.809435	lncRNA
ENSG0000(	ENSG0000(	57.32898	0.18031	0.454198	0.809435	lncRNA
ENSG0000(	ENSG0000(	122.3792	-0.0933	0.454231	0.809435	lncRNA
ENSG0000(	EPDR1	150.5151	0.170915	0.454319	0.809435	protein_coding
ENSG0000(	GFRA2	112.4401	0.200218	0.45429	0.809435	protein_coding
ENSG0000(	MEF2D	1922.616	-0.08035	0.454236	0.809435	protein_coding
ENSG0000(	PAM16	459.2678	0.070425	0.454274	0.809435	protein_coding
ENSG0000(	SSX2IP	344.3972	0.105506	0.454163	0.809435	protein_coding
ENSG0000(	OR2I1P	104.5374	-0.11966	0.454445	0.809551	protein_coding
ENSG0000(	LINC02739	87.71589	-0.1346	0.454482	0.809563	lncRNA
ENSG0000(	MISP	3253.838	0.182016	0.454544	0.809574	protein_coding
ENSG0000(	PLEKHB1	101.2479	-0.15278	0.454547	0.809574	protein_coding
ENSG0000(	GNB2	942.8536	0.082375	0.454648	0.809701	protein_coding
ENSG0000(	ENSG0000(	408.9593	0.054975	0.45468	0.809706	lncRNA
ENSG0000(	CENPO	496.4081	0.068953	0.454979	0.809872	protein_coding
ENSG0000(	LGALS3	3858.477	0.156428	0.454946	0.809872	protein_coding
ENSG0000(	MAP3K1	1432.321	-0.09332	0.454863	0.809872	protein_coding
ENSG0000(	OVCH2	31.51032	-0.18004	0.454946	0.809872	protein_coding
ENSG0000(	SEC11A	3931.905	0.03637	0.454876	0.809872	protein_coding
ENSG0000(	TSSK4	45.33623	-0.14157	0.454979	0.809872	protein_coding
ENSG0000(	ZNF487	367.178	-0.10509	0.45492	0.809872	protein_coding
ENSG0000(	GZMA	40.10598	-0.29208	0.455206	0.810174	protein_coding
ENSG0000(	PPIAP71	26.57007	0.197674	0.455208	0.810174	processed_pseudogene
ENSG0000(	APP-DT	278.4437	0.068084	0.455284	0.81019	lncRNA
ENSG0000(	ATXN3	2135.003	0.03395	0.455432	0.81019	protein_coding
ENSG0000(	EIF1B-AS1	3285.311	-0.03966	0.455435	0.81019	lncRNA
ENSG0000(	ENSG0000(	55.29951	0.435674	0.455372	0.81019	protein_coding

ENSG0000(	ENSG0000(	162.8625	-0.08439	0.455376	0.81019	lncRNA
ENSG0000(	IQCG	454.9743	-0.0582	0.45551	0.81019	protein_coding
ENSG0000(	JRK	1634	-0.06513	0.455539	0.81019	protein_coding
ENSG0000(	LRIG1	1310.773	0.103062	0.455359	0.81019	protein_coding
ENSG0000(	MEMO1	874.1024	0.071874	0.455505	0.81019	protein_coding
ENSG0000(	POLD3	821.1184	-0.08211	0.455541	0.81019	protein_coding
ENSG0000(	S1PR4	94.30868	-0.23719	0.455269	0.81019	protein_coding
ENSG0000(	ENSG0000(	127.0026	0.079101	0.455626	0.810237	lncRNA
ENSG0000(	MEGF6	416.8579	-0.15	0.455607	0.810237	protein_coding
ENSG0000(	COL20A1	38.84456	0.178592	0.455772	0.810393	protein_coding
ENSG0000(	S100B	47.64374	-0.15742	0.455744	0.810393	protein_coding
ENSG0000(	ENSG0000(	465.1086	-0.07388	0.455826	0.810437	lncRNA
ENSG0000(	ATG12	1762.112	0.043353	0.455893	0.81045	protein_coding
ENSG0000(	EDRF1-AS1	34.77854	-0.1308	0.455868	0.81045	lncRNA
ENSG0000(	ENSG0000(	33.56005	-0.1733	0.45597	0.810535	transcribed_processed_pseudoge
ENSG0000(	RNA5-8SN2	880.5298	-0.22913	0.456008	0.81055	rRNA
ENSG0000(	ASPA	1148.453	0.04104	0.456425	0.810626	protein_coding
ENSG0000(	CCZ1B	803.7697	0.06861	0.456415	0.810626	protein_coding
ENSG0000(	CMC1	624.2722	0.071388	0.456295	0.810626	protein_coding
ENSG0000(	ENSG0000(	110.9273	-0.18427	0.456476	0.810626	protein_coding
ENSG0000(	ENSG0000(	104.1853	-0.13376	0.456262	0.810626	lncRNA
ENSG0000(	ENSG0000(	630.7015	-0.05391	0.456338	0.810626	lncRNA
ENSG0000(	IFRD2	293.2094	0.097969	0.456483	0.810626	protein_coding
ENSG0000(	PCF11	4650.669	-0.05233	0.45625	0.810626	protein_coding
ENSG0000(	PDCD4-AS1	147.7384	-0.0753	0.456365	0.810626	lncRNA
ENSG0000(	PHETA2	216.776	-0.10747	0.456443	0.810626	protein_coding
ENSG0000(	SBNO1	2793.831	0.047126	0.456391	0.810626	protein_coding
ENSG0000(	SMAD6	980.6788	-0.09943	0.456238	0.810626	protein_coding
ENSG0000(	TMEM233	88.67708	-0.10041	0.456344	0.810626	protein_coding
ENSG0000(	VPS11	885.2295	0.054949	0.456493	0.810626	protein_coding
ENSG0000(	WDR36	1017.109	-0.10323	0.456415	0.810626	protein_coding
ENSG0000(	ENSG0000(	239.8449	-0.07494	0.456669	0.810886	lncRNA
ENSG0000(	FBXO15	2246.048	-0.07504	0.456698	0.810886	protein_coding
ENSG0000(	GALNT3	665.2358	0.115463	0.456782	0.810931	protein_coding
ENSG0000(	LINC02883	35.61623	-0.17666	0.456778	0.810931	lncRNA
ENSG0000(	DLL4	291.7901	-0.13032	0.456837	0.810955	protein_coding
ENSG0000(	LDHBP1	139.0446	0.082131	0.456855	0.810955	processed_pseudogene
ENSG0000(	RN7SL652F	25.92249	-0.22509	0.456923	0.811024	misc_RNA
ENSG0000(	LINC01684	1051.641	-0.08065	0.456996	0.811058	lncRNA
ENSG0000(	PRDX2P3	50.4181	0.136518	0.457001	0.811058	processed_pseudogene
ENSG0000(	DCAF16	2227.335	0.047837	0.457149	0.811218	protein_coding
ENSG0000(	THAP4	414.5961	0.097942	0.457151	0.811218	protein_coding
ENSG0000(	ZMPSTE24	667.087	-0.14854	0.457204	0.811261	protein_coding
ENSG0000(	ENSG0000(	32.66472	0.196097	0.457241	0.811274	lncRNA
ENSG0000(	EXOSC2	2255.415	0.045255	0.457309	0.811343	protein_coding
ENSG0000(	POMZP3	179.1996	0.0864	0.457352	0.811366	protein_coding
ENSG0000(	AOC1	1216.172	0.156325	0.457595	0.811692	protein_coding

ENSG0000(	CCNYL4	20.1106	-0.22155	0.457576	0.811692	transcribed_unprocessed_pseudo
ENSG0000(	ENSG0000(	75.92959	-0.12053	0.457728	0.811741	lncRNA
ENSG0000(	ENSG0000(	174.0095	-0.07892	0.45767	0.811741	lncRNA
ENSG0000(	ENSG0000(	45.90781	-0.16637	0.45778	0.811741	lncRNA
ENSG0000(	KLHL14	77.546	-0.24525	0.45776	0.811741	protein_coding
ENSG0000(	NACA	2690.955	-0.06729	0.457829	0.811741	protein_coding
ENSG0000(	SNORA3A	205.437	0.104358	0.457803	0.811741	snoRNA
ENSG0000(	ZNF835	251.1202	0.069408	0.457828	0.811741	protein_coding
ENSG0000(	CHORDC1P	247.0816	-0.12542	0.457937	0.811781	transcribed_processed_pseudoge
ENSG0000(	FAM153A	137.6824	-0.31772	0.457994	0.811781	protein_coding
ENSG0000(	LINC01445	2081.394	0.064638	0.457962	0.811781	lncRNA
ENSG0000(	OIP5	28.89372	-0.22533	0.457999	0.811781	protein_coding
ENSG0000(	TP53I13	401.2129	0.09602	0.457947	0.811781	protein_coding
ENSG0000(	ENSG0000(	222.9549	0.065145	0.45806	0.811785	TEC
ENSG0000(	HELLPAR	65881.58	-0.0314	0.458057	0.811785	lncRNA
ENSG0000(	ENSG0000(	56.7598	0.208199	0.458137	0.811807	lncRNA
ENSG0000(	ENSG0000(	61.86859	0.147995	0.458178	0.811807	lncRNA
ENSG0000(	RN7SL621F	21.02769	0.206483	0.45822	0.811807	misc_RNA
ENSG0000(	RNU5E-6P	93.68684	-0.25663	0.458214	0.811807	snRNA
ENSG0000(	SUPT7L	1901.535	-0.03446	0.458135	0.811807	protein_coding
ENSG0000(	ENSG0000(	103.0823	-0.09361	0.458376	0.812031	lncRNA
ENSG0000(	CWC27	6464.569	-0.02625	0.458454	0.812117	protein_coding
ENSG0000(	ENSG0000(	1106.969	-0.04884	0.458484	0.812118	lncRNA
ENSG0000(	ENSG0000(	76.74683	0.173214	0.458518	0.812126	lncRNA
ENSG0000(	BTBD9	3317.453	0.116273	0.458676	0.812129	protein_coding
ENSG0000(	C1QTNF7	75.67241	0.222322	0.458567	0.812129	protein_coding
ENSG0000(	ENSG0000(	78.62628	-0.15667	0.458684	0.812129	pseudogene
ENSG0000(	FAM219B	1908.53	0.061141	0.458621	0.812129	protein_coding
ENSG0000(	LRRC37A2	1548.801	0.108848	0.458727	0.812129	protein_coding
ENSG0000(	SCIRT	111.0551	-0.11472	0.45864	0.812129	lncRNA
ENSG0000(	TRBC1	209.6623	-0.18558	0.458713	0.812129	TR_C_gene
ENSG0000(	ENSG0000(	48.80196	0.185927	0.458792	0.812193	lncRNA
ENSG0000(	ZNF660	569.5256	-0.08374	0.458838	0.812221	protein_coding
ENSG0000(	MACIR	738.0026	0.16082	0.458948	0.812328	protein_coding
ENSG0000(	SHLD3	96.77268	-0.11211	0.458957	0.812328	protein_coding
ENSG0000(	CENPC	1929.866	0.056839	0.459035	0.81235	protein_coding
ENSG0000(	ENSG0000(	72.39357	0.153751	0.459079	0.81235	lncRNA
ENSG0000(	JUP	2711.843	0.139918	0.459088	0.81235	protein_coding
ENSG0000(	OTUD1	335.107	0.105997	0.459004	0.81235	protein_coding
ENSG0000(	USP32	1484.232	-0.08154	0.459165	0.812435	protein_coding
ENSG0000(	RNU6-122(	21.52682	-0.2333	0.459215	0.812471	snRNA
ENSG0000(	ENSG0000(	45.27248	-0.12938	0.459268	0.812512	lncRNA
ENSG0000(	ENSG0000(	94.27868	0.114815	0.459352	0.812608	lncRNA
ENSG0000(	ENSG0000(	19.82675	-0.23937	0.459438	0.812637	lncRNA
ENSG0000(	FLJ42393	795.6585	-0.08243	0.45943	0.812637	TEC
ENSG0000(	NACC1	692.1499	-0.0784	0.459456	0.812637	protein_coding
ENSG0000(	LINC00632	1767.278	-0.12732	0.4595	0.812662	lncRNA

ENSG0000(	SLC1A1	386.3356	0.242572	0.459549	0.812696	protein_coding
ENSG0000(	RNF217-AS	510.4236	-0.049	0.459602	0.812738	lncRNA
ENSG0000(	ARRDC1-AS	621.9029	-0.04611	0.459776	0.81291	lncRNA
ENSG0000(	ENSG0000(	777.2893	-0.06771	0.45977	0.81291	lncRNA
ENSG0000(	ENSG0000(	121.0945	0.074221	0.459818	0.81291	lncRNA
ENSG0000(	MIGA1	1462.822	-0.03646	0.459815	0.81291	protein_coding
ENSG0000(	CLCN3	2429.029	0.069786	0.460212	0.812917	protein_coding
ENSG0000(	DNTTIP1	186.7399	-0.11192	0.460438	0.812917	protein_coding
ENSG0000(	DTNB-AS1	34.7418	-0.27019	0.460341	0.812917	lncRNA
ENSG0000(	ELAC2	787.6217	-0.06233	0.45996	0.812917	protein_coding
ENSG0000(	ENSG0000(	243.7677	0.129657	0.460281	0.812917	lncRNA
ENSG0000(	ENSG0000(	1220.114	0.038371	0.460383	0.812917	lncRNA
ENSG0000(	ENSG0000(	576.5042	0.050145	0.459967	0.812917	lncRNA
ENSG0000(	ENSG0000(	58.10169	0.126099	0.460417	0.812917	TEC
ENSG0000(	ENSG0000(	55.09599	0.132471	0.460402	0.812917	TEC
ENSG0000(	ENSG0000(	8914.658	0.070509	0.460051	0.812917	lncRNA
ENSG0000(	ERAP1	2444.856	-0.06357	0.46039	0.812917	protein_coding
ENSG0000(	GSKIP	1097.231	-0.12303	0.460429	0.812917	protein_coding
ENSG0000(	MIOS	1006.74	-0.05536	0.46039	0.812917	protein_coding
ENSG0000(	MTF2	1106.734	-0.05515	0.460041	0.812917	protein_coding
ENSG0000(	PKN2	2612.643	0.069662	0.460097	0.812917	protein_coding
ENSG0000(	PPP2CB	1210.154	-0.08934	0.460313	0.812917	protein_coding
ENSG0000(	RUFY2	976.9769	0.04747	0.460058	0.812917	protein_coding
ENSG0000(	TCAM1P	197.2613	0.094533	0.460442	0.812917	transcribed_unitary_pseudogene
ENSG0000(	TOB1-AS1	107.3179	-0.1241	0.460007	0.812917	lncRNA
ENSG0000(	UBR5-DT	236.1336	0.098329	0.459987	0.812917	lncRNA
ENSG0000(	ZBBX	38.1489	-0.19443	0.460437	0.812917	protein_coding
ENSG0000(	C21orf62	646.5625	0.067499	0.460542	0.812988	protein_coding
ENSG0000(	SLC26A10	49.90448	-0.19925	0.460523	0.812988	transcribed_unitary_pseudogene
ENSG0000(	ENSG0000(	255.515	-0.08023	0.460589	0.81302	lncRNA
ENSG0000(	ENSG0000(	245.5245	-0.09998	0.46074	0.813047	lncRNA
ENSG0000(	ENSG0000(	265.3099	-0.10286	0.46079	0.813047	TEC
ENSG0000(	ENSG0000(	490.9515	-0.06036	0.460775	0.813047	TEC
ENSG0000(	FAR2P3	124.7286	-0.13652	0.460774	0.813047	transcribed_unprocessed_pseudo
ENSG0000(	LINC01695	841.5331	0.056904	0.460773	0.813047	lncRNA
ENSG0000(	NAT14	49.01401	-0.16726	0.460811	0.813047	protein_coding
ENSG0000(	ZBTB45	191.4737	-0.08612	0.460802	0.813047	protein_coding
ENSG0000(	CCNE2	165.1469	-0.11867	0.460852	0.813058	protein_coding
ENSG0000(	ENSG0000(	140.8014	-0.13325	0.46092	0.813058	processed_pseudogene
ENSG0000(	ENSG0000(	28.16681	-0.15455	0.460926	0.813058	TEC
ENSG0000(	TUT1	287.5851	0.092622	0.460936	0.813058	protein_coding
ENSG0000(	ALG1L13P	333.5553	-0.07836	0.461158	0.813061	transcribed_unprocessed_pseudo
ENSG0000(	BMP8B-AS:	94.20408	0.09413	0.461098	0.813061	lncRNA
ENSG0000(	CEP19	237.7213	-0.08271	0.46112	0.813061	protein_coding
ENSG0000(	COCH	31.07178	-0.27334	0.461139	0.813061	protein_coding
ENSG0000(	ENSG0000(	32.17262	0.185718	0.46106	0.813061	lncRNA
ENSG0000(	ENSG0000(	252.3815	-0.10406	0.461047	0.813061	lncRNA

ENSG0000(	PRRG3	12044.97	0.245886	0.461173	0.813061	protein_coding
ENSG0000(	ZFY	265.9384	0.15369	0.461174	0.813061	protein_coding
ENSG0000(	APELA	60.49161	0.152294	0.461362	0.813133	protein_coding
ENSG0000(	ENSG0000(	129.138	-0.09384	0.46136	0.813133	lncRNA
ENSG0000(	JAZF1-AS1	84.59975	0.095469	0.461339	0.813133	lncRNA
ENSG0000(	MTCO2P19	23.40812	0.216828	0.461318	0.813133	processed_pseudogene
ENSG0000(	PUM3	585.86	-0.08243	0.461345	0.813133	protein_coding
ENSG0000(	ENSG0000(	23.08584	0.210606	0.461483	0.813241	lncRNA
ENSG0000(	UTS2R	817.4119	-0.11111	0.461468	0.813241	protein_coding
ENSG0000(	RADX	130.4082	0.106939	0.461559	0.813322	protein_coding
ENSG0000(	ENSG0000(	77.08316	0.126926	0.46161	0.813349	lncRNA
ENSG0000(	LINC01485	339.015	0.090618	0.461639	0.813349	lncRNA
ENSG0000(	MTCH2	849.6124	0.08889	0.461662	0.813349	protein_coding
ENSG0000(	LINC01978	190.9936	-0.0714	0.461739	0.813379	lncRNA
ENSG0000(	PPM1L-DT	59.02557	-0.13472	0.461727	0.813379	lncRNA
ENSG0000(	HDAC11	225.2674	0.116193	0.461872	0.813561	protein_coding
ENSG0000(	JUND	1301.37	0.124295	0.461948	0.813613	protein_coding
ENSG0000(	RBMX	3826.181	0.038824	0.46196	0.813613	protein_coding
ENSG0000(	ANKRD49	549.2317	-0.05712	0.46218	0.813679	protein_coding
ENSG0000(	ATP5MC3	1151.407	0.119609	0.462255	0.813679	protein_coding
ENSG0000(	CCNA2	201.416	-0.11225	0.462306	0.813679	protein_coding
ENSG0000(	ENSG0000(	39.96627	0.147795	0.462196	0.813679	processed_pseudogene
ENSG0000(	LIMS2	475.0805	-0.11749	0.462238	0.813679	protein_coding
ENSG0000(	PPIP5K2	6144.686	0.067889	0.462323	0.813679	protein_coding
ENSG0000(	PRKG1	1365.733	-0.08305	0.462088	0.813679	protein_coding
ENSG0000(	SLC13A4	145.0227	-0.10044	0.462316	0.813679	protein_coding
ENSG0000(	SPRYD3	412.3598	-0.06854	0.462296	0.813679	protein_coding
ENSG0000(	USP22	2813.529	0.051636	0.462141	0.813679	protein_coding
ENSG0000(	ZNF202	518.5735	0.078913	0.462194	0.813679	protein_coding
ENSG0000(	ENSG0000(	185.5607	0.303959	0.462382	0.813731	lncRNA
ENSG0000(	CBX5	5186.031	-0.05162	0.462859	0.813819	protein_coding
ENSG0000(	CPNE1	983.4195	-0.06155	0.462888	0.813819	protein_coding
ENSG0000(	EMB	867.6037	-0.18367	0.462587	0.813819	protein_coding
ENSG0000(	ENSG0000(	225.1702	0.092452	0.462512	0.813819	lncRNA
ENSG0000(	HARS1	3032.101	0.032574	0.46283	0.813819	protein_coding
ENSG0000(	IL16	1720.71	-0.13965	0.462851	0.813819	protein_coding
ENSG0000(	LINC01122	142.3268	0.112595	0.462905	0.813819	lncRNA
ENSG0000(	LINC01309	47.1579	0.16278	0.462746	0.813819	lncRNA
ENSG0000(	LINC02457	167.251	-0.08334	0.462642	0.813819	lncRNA
ENSG0000(	MST1L	490.2992	-0.29462	0.462622	0.813819	transcribed_unprocessed_pseudo
ENSG0000(	OCLNP1	29.2001	-0.46027	0.462757	0.813819	unprocessed_pseudogene
ENSG0000(	PSMG4	1273.306	0.040805	0.462511	0.813819	protein_coding
ENSG0000(	ROBO2	270.529	0.128363	0.462629	0.813819	protein_coding
ENSG0000(	SPATA5L1	185.1663	0.105317	0.462799	0.813819	protein_coding
ENSG0000(	TBC1D31	656.7423	-0.05669	0.462686	0.813819	protein_coding
ENSG0000(	ZSCAN31	177.5758	0.190284	0.462865	0.813819	protein_coding
ENSG0000(	ARHGEF34I	1899.19	0.221325	0.463175	0.813981	unprocessed_pseudogene

ENSG0000(	FOXI3	50.96258	0.150379	0.463086	0.813981	protein_coding
ENSG0000(	IGFBPL1	169.001	0.087312	0.463058	0.813981	protein_coding
ENSG0000(	LRFN1	157.3858	-0.15257	0.463175	0.813981	protein_coding
ENSG0000(	SMAD1	1004.198	0.064819	0.463175	0.813981	protein_coding
ENSG0000(	SSU72	2224.314	0.039551	0.463103	0.813981	protein_coding
ENSG0000(	ENSG0000(	57.19185	0.133769	0.463286	0.81406	lncRNA
ENSG0000(	ITIH1	34.48085	0.173846	0.463264	0.81406	protein_coding
ENSG0000(	LMLN	643.3771	0.069931	0.463368	0.81406	protein_coding
ENSG0000(	TPRA1	379.709	-0.06901	0.463344	0.81406	protein_coding
ENSG0000(	UPK1A-AS1	69.52944	-0.1179	0.463364	0.81406	lncRNA
ENSG0000(	ENSG0000(	62.24673	0.137002	0.463416	0.814092	lncRNA
ENSG0000(	ARL4A	1147.406	0.120314	0.463583	0.814281	protein_coding
ENSG0000(	SPATA7	227.8033	0.131162	0.463567	0.814281	protein_coding
ENSG0000(	CREG2	287.9953	-0.06679	0.463665	0.814373	protein_coding
ENSG0000(	ENSG0000(	94.26789	0.11132	0.463728	0.81438	lncRNA
ENSG0000(	HNF4G	1379.573	0.158659	0.463757	0.81438	protein_coding
ENSG0000(	RN7SK	343777.8	-0.06263	0.463706	0.81438	snRNA
ENSG0000(	COPB2-DT	544.8162	-0.07502	0.463843	0.814478	lncRNA
ENSG0000(	HEMGN	80.76722	0.146001	0.463946	0.814556	protein_coding
ENSG0000(	SMC1A	1850.913	0.074197	0.463941	0.814556	protein_coding
ENSG0000(	CCDC121	68.70325	-0.15439	0.463984	0.814567	protein_coding
ENSG0000(	LINC01378	120.345	0.15102	0.464042	0.814567	lncRNA
ENSG0000(	SLC1A7	49.16629	0.36636	0.464031	0.814567	protein_coding
ENSG0000(	DRICH1	52.05575	-0.1892	0.464074	0.814569	protein_coding
ENSG0000(	TAS2R31	202.9532	0.088062	0.464114	0.814569	protein_coding
ENSG0000(	VPS37C	575.6262	0.042975	0.464131	0.814569	protein_coding
ENSG0000(	ELOA-AS1	301.4368	-0.06698	0.464251	0.814688	lncRNA
ENSG0000(	VEGFA	5552.149	-0.1054	0.46428	0.814688	protein_coding
ENSG0000(	ZC2HC1A	234.6169	-0.10987	0.464288	0.814688	protein_coding
ENSG0000(	ENSG0000(	104.675	0.560613	0.464361	0.814764	protein_coding
ENSG0000(	ENSG0000(	91.74736	-0.2151	0.464505	0.814808	TEC
ENSG0000(	GABRD	450.8993	-0.05994	0.464464	0.814808	protein_coding
ENSG0000(	MAP3K14	864.6078	0.057543	0.464488	0.814808	protein_coding
ENSG0000(	RPP40	30.98808	-0.19452	0.464464	0.814808	protein_coding
ENSG0000(	AKR1C1	273.2232	0.128247	0.464739	0.814907	protein_coding
ENSG0000(	DVL3	1669.07	-0.05678	0.464724	0.814907	protein_coding
ENSG0000(	ENSG0000(	41.96414	0.123225	0.464688	0.814907	lncRNA
ENSG0000(	ENSG0000(	32.39302	-0.17604	0.464628	0.814907	lncRNA
ENSG0000(	LRCH3	3878.484	0.042644	0.464691	0.814907	protein_coding
ENSG0000(	YIPF7	81.16981	0.161037	0.4647	0.814907	protein_coding
ENSG0000(	AADACL2	22.05257	-0.22634	0.465103	0.81497	protein_coding
ENSG0000(	CHD2	8944.448	-0.06546	0.465275	0.81497	protein_coding
ENSG0000(	EEF1A1P12	32.06249	-0.24008	0.465308	0.81497	processed_pseudogene
ENSG0000(	ELOCP33	166.0879	0.087814	0.46499	0.81497	processed_pseudogene
ENSG0000(	ENSG0000(	164.7237	0.092971	0.465244	0.81497	lncRNA
ENSG0000(	ENSG0000(	104.1361	0.273341	0.465059	0.81497	protein_coding
ENSG0000(	ENSG0000(	29.47249	-0.17591	0.465022	0.81497	lncRNA

ENSG0000(	EPHB6	143.9403	-0.23649	0.464857	0.81497	protein_coding
ENSG0000(	GNG12-AS1	36.05	-0.22597	0.465166	0.81497	lncRNA
ENSG0000(	MED4	544.4566	-0.04672	0.465308	0.81497	protein_coding
ENSG0000(	OPN4	21.08253	0.331644	0.46525	0.81497	protein_coding
ENSG0000(	PRSS23	1522.357	-0.12282	0.46527	0.81497	protein_coding
ENSG0000(	SLC39A7	963.6336	-0.08009	0.465278	0.81497	protein_coding
ENSG0000(	SNAPC5	288.0028	0.066722	0.465154	0.81497	protein_coding
ENSG0000(	TMEM131L	920.5158	-0.16679	0.465073	0.81497	protein_coding
ENSG0000(	TNK2	1926.457	-0.08961	0.46504	0.81497	protein_coding
ENSG0000(	TTPA	115.9888	0.146439	0.464961	0.81497	protein_coding
ENSG0000(	VEPH1	42.8082	0.225066	0.46523	0.81497	protein_coding
ENSG0000(	BAP1	908.6162	0.053868	0.465354	0.814975	protein_coding
ENSG0000(	ZXDB	290.4747	-0.06349	0.46537	0.814975	protein_coding
ENSG0000(	ENSG0000(	2995.858	0.095345	0.46555	0.815083	lncRNA
ENSG0000(	ENSG0000(	419.857	0.059979	0.465533	0.815083	lncRNA
ENSG0000(	RPL36AP15	38.28952	-0.15068	0.465528	0.815083	processed_pseudogene
ENSG0000(	SLC16A1	1938.489	0.180864	0.46551	0.815083	protein_coding
ENSG0000(	ADAM5	32.4363	0.161817	0.465659	0.815116	transcribed_unitary_pseudogene
ENSG0000(	ENSG0000(	652.8298	0.058824	0.465747	0.815116	lncRNA
ENSG0000(	ENSG0000(	24.35186	0.182413	0.465631	0.815116	lncRNA
ENSG0000(	ENSG0000(	126.0606	-0.07948	0.465721	0.815116	lncRNA
ENSG0000(	ENSG0000(	107.2201	0.124848	0.465713	0.815116	lncRNA
ENSG0000(	RPL28	1708.844	-0.09497	0.465674	0.815116	protein_coding
ENSG0000(	CATIP-AS2	19.59131	-0.21006	0.466207	0.815351	lncRNA
ENSG0000(	ENSG0000(	75.48499	0.094294	0.466236	0.815351	lncRNA
ENSG0000(	ENSG0000(	211.5999	-0.09162	0.466197	0.815351	lncRNA
ENSG0000(	ENSG0000(	252.6469	-0.14112	0.466128	0.815351	TEC
ENSG0000(	ENSG0000(	893.3655	-0.07227	0.46608	0.815351	TEC
ENSG0000(	HSBP1	811.0005	-0.07141	0.466216	0.815351	protein_coding
ENSG0000(	HSH2D	1377.342	-0.08318	0.465941	0.815351	protein_coding
ENSG0000(	LINC00511	7404.483	0.169465	0.466149	0.815351	lncRNA
ENSG0000(	LINC02823	31.73314	0.144223	0.465993	0.815351	lncRNA
ENSG0000(	PLA2G4F	859.7823	0.190185	0.466226	0.815351	protein_coding
ENSG0000(	RPS26P28	48.3438	0.176184	0.466078	0.815351	processed_pseudogene
ENSG0000(	VAX1	23.83985	0.154836	0.466023	0.815351	protein_coding
ENSG0000(	ADPRM	150.4438	0.099276	0.466316	0.815437	protein_coding
ENSG0000(	EFR3B	344.9192	0.054662	0.466434	0.815482	protein_coding
ENSG0000(	ENSG0000(	1518.018	0.050223	0.466386	0.815482	TEC
ENSG0000(	PXYLP1	891.2106	0.080218	0.466462	0.815482	protein_coding
ENSG0000(	SAMD15	209.678	0.06254	0.46649	0.815482	protein_coding
ENSG0000(	SHC4	317.5391	-0.06824	0.466426	0.815482	protein_coding
ENSG0000(	ZNF266	2032.8	0.079709	0.466624	0.815665	protein_coding
ENSG0000(	ABCC11	105.6241	0.111901	0.466687	0.815724	protein_coding
ENSG0000(	SCUBE1-AS	173.7021	0.078391	0.466725	0.815738	lncRNA
ENSG0000(	RHEB	518.6445	-0.07057	0.466771	0.815767	protein_coding
ENSG0000(	ENSG0000(	308.2936	0.063324	0.467037	0.815921	lncRNA
ENSG0000(	FHIP1A-DT	43.30308	0.233772	0.467	0.815921	lncRNA

ENSG0000(	LINC00665	2225.377	0.039993	0.467018	0.815921	lncRNA
ENSG0000(	MGME1	457.901	0.06713	0.46697	0.815921	protein_coding
ENSG0000(	NSMCE1	249.4039	-0.09148	0.466916	0.815921	protein_coding
ENSG0000(	SPTB	231.6002	-0.07495	0.466993	0.815921	protein_coding
ENSG0000(	ENSG0000(	49.99392	-0.15876	0.467104	0.815934	lncRNA
ENSG0000(	TEAD1	2096.15	-0.0897	0.467096	0.815934	protein_coding
ENSG0000(	AFF4	5394.432	-0.04409	0.46728	0.815987	protein_coding
ENSG0000(	ENSG0000(	372.2436	0.0647	0.4672	0.815987	lncRNA
ENSG0000(	ENSG0000(	245.3274	0.065437	0.467255	0.815987	lncRNA
ENSG0000(	RHPN1	159.1595	0.171578	0.467298	0.815987	protein_coding
ENSG0000(	SGO1	265.5545	0.083377	0.467312	0.815987	protein_coding
ENSG0000(	ZNF185	151.8473	-0.16949	0.467286	0.815987	protein_coding
ENSG0000(	ZNF827	2587.931	-0.07882	0.467417	0.816118	protein_coding
ENSG0000(	SLC25A24	1301.733	0.102046	0.467474	0.816165	protein_coding
ENSG0000(	SLX1A-SUL	373.3315	0.245659	0.467509	0.816175	lncRNA
ENSG0000(	BRD3	720.217	0.064536	0.467737	0.816435	protein_coding
ENSG0000(	ENSG0000(	80.2998	0.232948	0.467747	0.816435	lncRNA
ENSG0000(	TDRD1	102.7617	0.09841	0.467718	0.816435	protein_coding
ENSG0000(	ACVR2B	1123.544	0.051596	0.46798	0.816455	protein_coding
ENSG0000(	ARTN	28.46157	-0.25429	0.467972	0.816455	protein_coding
ENSG0000(	ENSG0000(	467.1629	0.078812	0.467862	0.816455	lncRNA
ENSG0000(	ENSG0000(	31.89475	-0.16338	0.468021	0.816455	lncRNA
ENSG0000(	ENSG0000(	81.49435	0.097771	0.468106	0.816455	lncRNA
ENSG0000(	ENSG0000(	20.69319	0.256509	0.46806	0.816455	lncRNA
ENSG0000(	FBXL4	1489.972	0.045169	0.467958	0.816455	protein_coding
ENSG0000(	LINC02057	147.1087	0.078345	0.467907	0.816455	lncRNA
ENSG0000(	PAQR9	572.1435	0.256156	0.467808	0.816455	protein_coding
ENSG0000(	SLC2A8	270.8179	0.097789	0.468017	0.816455	protein_coding
ENSG0000(	TIMM10B	1192.893	-0.04978	0.468114	0.816455	protein_coding
ENSG0000(	ZNF671	303.6047	-0.12198	0.468077	0.816455	protein_coding
ENSG0000(	FOXG1-AS1	24.11399	0.238436	0.468241	0.816625	lncRNA
ENSG0000(	IRF2BPL	479.6916	-0.07727	0.468277	0.816635	protein_coding
ENSG0000(	PAQR4	181.3086	-0.12045	0.468312	0.816645	protein_coding
ENSG0000(	ENSG0000(	101.3816	-0.10154	0.468352	0.816663	lncRNA
ENSG0000(	C15orf61	203.8119	-0.10477	0.468653	0.817033	protein_coding
ENSG0000(	KAZN-AS1	26.97845	-0.1923	0.46864	0.817033	lncRNA
ENSG0000(	TEX45	122.3879	-0.10163	0.468644	0.817033	protein_coding
ENSG0000(	ATP1A4	316.207	-0.073	0.469188	0.817033	protein_coding
ENSG0000(	CRHBP	20.67413	-0.28455	0.468889	0.817033	protein_coding
ENSG0000(	ENSG0000(	103.7425	-0.10464	0.468892	0.817033	lncRNA
ENSG0000(	ENSG0000(	2109.246	0.146429	0.468894	0.817033	lncRNA
ENSG0000(	ENSG0000(	31.28324	-0.16155	0.46889	0.817033	lncRNA
ENSG0000(	ENSG0000(	21.49061	0.186325	0.469166	0.817033	lncRNA
ENSG0000(	MRPS16	1104.717	-0.05264	0.468815	0.817033	protein_coding
ENSG0000(	PAICS	1372.09	0.062594	0.468951	0.817033	protein_coding
ENSG0000(	PAPOLG	1054.224	0.07449	0.46896	0.817033	protein_coding
ENSG0000(	PIP5K1B	2590.61	0.128083	0.469004	0.817033	protein_coding

ENSG0000(	RBM12B	2315.498	-0.07549	0.469075	0.817033	protein_coding
ENSG0000(	RPL36AP43	66.37845	0.170389	0.468827	0.817033	processed_pseudogene
ENSG0000(	SERF1A	135.7829	-0.33506	0.469142	0.817033	protein_coding
ENSG0000(	TARS2	568.7052	0.0553	0.468874	0.817033	protein_coding
ENSG0000(	TBX4	53.78316	-0.12121	0.468892	0.817033	protein_coding
ENSG0000(	ZNF252P-A	243.5112	0.080434	0.469083	0.817033	lncRNA
ENSG0000(	ZNF528	1327.848	0.06667	0.469061	0.817033	protein_coding
ENSG0000(	ZNF883	115.1706	0.114196	0.469143	0.817033	transcribed_unprocessed_pseudo
ENSG0000(	ANKMY1	1366.388	0.06835	0.469369	0.817248	protein_coding
ENSG0000(	LINC02344	123.8786	-0.13241	0.469371	0.817248	lncRNA
ENSG0000(	CXADR	1446.845	0.158271	0.469407	0.817259	protein_coding
ENSG0000(	ZNF14	309.4988	0.078491	0.469597	0.817539	protein_coding
ENSG0000(	ADD1	5701.481	0.040095	0.469698	0.81761	protein_coding
ENSG0000(	ANKRD10-I	483.5324	-0.07492	0.469683	0.81761	lncRNA
ENSG0000(	ATXN1L	1808.96	-0.0512	0.469967	0.817628	protein_coding
ENSG0000(	ENSG0000(	108.7018	0.094956	0.470131	0.817628	processed_pseudogene
ENSG0000(	ENSG0000(	75.96984	0.086979	0.469972	0.817628	processed_pseudogene
ENSG0000(	ENSG0000(	45.88789	0.363999	0.469806	0.817628	protein_coding
ENSG0000(	HMGB1	3208.733	-0.05911	0.470138	0.817628	protein_coding
ENSG0000(	LINC00868	211.4906	-0.1071	0.470001	0.817628	lncRNA
ENSG0000(	MED25	1043.211	-0.04777	0.469867	0.817628	protein_coding
ENSG0000(	MLEC	6692.505	0.096134	0.47007	0.817628	protein_coding
ENSG0000(	MMP10	42.0219	-1.51845	0.469986	0.817628	protein_coding
ENSG0000(	NETO1	22.0599	-0.4876	0.469933	0.817628	protein_coding
ENSG0000(	PACSIN3	43.30829	-0.21696	0.470138	0.817628	protein_coding
ENSG0000(	REV3L-IT1	162.0592	0.103612	0.470132	0.817628	lncRNA
ENSG0000(	SH2B3	1409.381	-0.10649	0.469881	0.817628	protein_coding
ENSG0000(	THUMPD3I	50.25976	0.211007	0.470062	0.817628	unprocessed_pseudogene
ENSG0000(	ZNF627	506.5384	0.057946	0.470154	0.817628	protein_coding
ENSG0000(	PAQR9-AS1	385.5614	0.056332	0.470188	0.817636	lncRNA
ENSG0000(	RPL14	2365.599	-0.09035	0.470218	0.817636	protein_coding
ENSG0000(	AMZ2P1	194.1161	0.094909	0.470561	0.817854	transcribed_unprocessed_pseudo
ENSG0000(	CRYZP1	69.65906	0.170385	0.470532	0.817854	processed_pseudogene
ENSG0000(	GPN3	97.58425	-0.10668	0.470538	0.817854	protein_coding
ENSG0000(	KLF2P4	100.6235	-0.12605	0.470581	0.817854	processed_pseudogene
ENSG0000(	RN7SKP283	37.88919	0.164771	0.470578	0.817854	misc_RNA
ENSG0000(	RPL7P46	32.48797	0.164643	0.470423	0.817854	processed_pseudogene
ENSG0000(	SAMD4B	2525.443	0.043876	0.470436	0.817854	protein_coding
ENSG0000(	TP53TG1	483.892	-0.06761	0.470501	0.817854	lncRNA
ENSG0000(	EIF2AK3-DT	121.7088	0.089258	0.470613	0.817857	lncRNA
ENSG0000(	ENSG0000(	125.5831	-0.13176	0.47065	0.81787	lncRNA
ENSG0000(	PIMREG	88.45416	0.095923	0.4707	0.817905	protein_coding
ENSG0000(	ENSG0000(	68.8271	0.150893	0.470833	0.817982	lncRNA
ENSG0000(	IGKV1D-13	124.1808	-1.13119	0.470808	0.817982	IG_V_gene
ENSG0000(	RAI2	95.7903	-0.16878	0.470824	0.817982	protein_coding
ENSG0000(	PGM5-AS1	36.63743	0.137776	0.470866	0.817988	lncRNA
ENSG0000(	ARHGAP6	258.4435	-0.11262	0.470912	0.818016	protein_coding

ENSG0000(	AGRN	1321.422	-0.08411	0.471034	0.818134	protein_coding
ENSG0000(	ENSG0000(	1342.202	0.050973	0.47107	0.818134	protein_coding
ENSG0000(	ENSG0000(	20.76337	0.212238	0.471053	0.818134	lncRNA
ENSG0000(	OR52K1	399.5779	-0.06031	0.471099	0.818134	protein_coding
ENSG0000(	NTHL1	88.69652	0.103651	0.471174	0.818213	protein_coding
ENSG0000(	ABCC12	60.90453	0.134655	0.471276	0.818234	protein_coding
ENSG0000(	DPRXP2	41.96571	0.144312	0.471249	0.818234	transcribed_processed_pseudoge
ENSG0000(	KDELR2	2202.114	-0.08108	0.471268	0.818234	protein_coding
ENSG0000(	ZNF174	135.6356	-0.07779	0.471386	0.818375	protein_coding
ENSG0000(	UNC79	173.8331	0.076491	0.471438	0.818413	protein_coding
ENSG0000(	LINC00278	1685.896	-0.11795	0.471558	0.818569	lncRNA
ENSG0000(	CEP72	371.1009	0.139237	0.471662	0.81858	protein_coding
ENSG0000(	CYP4B1	54.22131	0.129336	0.471802	0.81858	protein_coding
ENSG0000(	ENSG0000(	50.87968	0.191496	0.471688	0.81858	TEC
ENSG0000(	ENSG0000(	245.5777	-0.06268	0.471661	0.81858	TEC
ENSG0000(	ENSG0000(	344.6761	0.097392	0.471676	0.81858	TEC
ENSG0000(	ENSG0000(	84.86398	0.147755	0.471739	0.81858	TEC
ENSG0000(	ENSG0000(	160.5949	0.191964	0.471797	0.81858	protein_coding
ENSG0000(	SEMA5B	256.8977	-0.09179	0.471768	0.81858	protein_coding
ENSG0000(	BBS7-DT	1002.069	-0.07178	0.47254	0.81863	lncRNA
ENSG0000(	CARD9	146.9838	-0.11877	0.472558	0.81863	protein_coding
ENSG0000(	CXCR5	178.32	-0.32641	0.472545	0.81863	protein_coding
ENSG0000(	ENSG0000(	21.11861	-0.2064	0.472246	0.81863	lncRNA
ENSG0000(	ENSG0000(	302.9645	0.050043	0.472328	0.81863	lncRNA
ENSG0000(	ENSG0000(	253.212	-0.08975	0.472269	0.81863	lncRNA
ENSG0000(	ENSG0000(	106.2117	0.163858	0.471883	0.81863	lncRNA
ENSG0000(	ENSG0000(	25.26377	-0.61064	0.472352	0.81863	protein_coding
ENSG0000(	ENSG0000(	94.81405	0.103613	0.472551	0.81863	lncRNA
ENSG0000(	ENSG0000(	129.0362	0.093092	0.472575	0.81863	lncRNA
ENSG0000(	ENSG0000(	124.5566	-0.09476	0.472169	0.81863	lncRNA
ENSG0000(	FAM66B	277.5334	-0.1008	0.472511	0.81863	lncRNA
ENSG0000(	FYCO1	1748.04	0.053397	0.47201	0.81863	protein_coding
ENSG0000(	GRSF1	1813.001	0.046162	0.472496	0.81863	protein_coding
ENSG0000(	HYOU1	1721.759	-0.08211	0.472392	0.81863	protein_coding
ENSG0000(	KIAA2013	787.9452	0.080794	0.47191	0.81863	protein_coding
ENSG0000(	KIT	732.8341	-0.1489	0.472557	0.81863	protein_coding
ENSG0000(	LINC02112	59.00287	-0.13325	0.472065	0.81863	lncRNA
ENSG0000(	PRTG	175.5727	0.167165	0.472342	0.81863	protein_coding
ENSG0000(	SAG	1003.544	0.036426	0.471945	0.81863	protein_coding
ENSG0000(	SPIN2B	97.32076	0.127795	0.472571	0.81863	protein_coding
ENSG0000(	SRARP	221.8204	0.055843	0.472058	0.81863	protein_coding
ENSG0000(	STIM1	1378.804	0.056064	0.472084	0.81863	protein_coding
ENSG0000(	TMEM269	172.7474	0.100544	0.472367	0.81863	protein_coding
ENSG0000(	ZBTB9	123.1302	-0.12427	0.47249	0.81863	protein_coding
ENSG0000(	TNPO3	1270.769	0.04269	0.472644	0.818698	protein_coding
ENSG0000(	ENSG0000(	60.97042	-0.10968	0.472684	0.818716	lncRNA
ENSG0000(	SMYD4	1644.207	-0.04863	0.472821	0.818901	protein_coding

ENSG0000(	ATF3	767.0405	-0.27178	0.472974	0.818954	protein_coding
ENSG0000(	EIF4G3	5558.663	0.078666	0.473018	0.818954	protein_coding
ENSG0000(	ENSG0000(	33.79833	0.145436	0.473171	0.818954	lncRNA
ENSG0000(	ENSG0000(	22.70925	-0.18887	0.472976	0.818954	TEC
ENSG0000(	LIN54	758.2145	-0.09537	0.47316	0.818954	protein_coding
ENSG0000(	LINC00964	389.2043	-0.06606	0.473179	0.818954	lncRNA
ENSG0000(	METTL21EI	127.9659	0.086451	0.473088	0.818954	transcribed_unprocessed_pseudo
ENSG0000(	PIGS	864.934	0.049732	0.472945	0.818954	protein_coding
ENSG0000(	PLAGL1	1814.694	0.086482	0.473053	0.818954	protein_coding
ENSG0000(	ZNF407	2630.093	-0.05025	0.472887	0.818954	protein_coding
ENSG0000(	ZNF596	289.8744	-0.07872	0.473178	0.818954	protein_coding
ENSG0000(	NFATC3	2035.948	-0.06134	0.47326	0.819043	protein_coding
ENSG0000(	FAM182B	65.28431	-0.14506	0.473332	0.8191	lncRNA
ENSG0000(	ZNF101P2	48.21236	-0.12471	0.473352	0.8191	processed_pseudogene
ENSG0000(	ENSG0000(	57.22934	-0.15414	0.473419	0.819105	lncRNA
ENSG0000(	FAM204BP	61.06256	0.172554	0.473474	0.819105	processed_pseudogene
ENSG0000(	LINC02728	152.6458	0.091246	0.473467	0.819105	lncRNA
ENSG0000(	ZNF488	24.6896	-0.33021	0.473445	0.819105	protein_coding
ENSG0000(	DERPC	386.9006	0.186823	0.473604	0.819278	protein_coding
ENSG0000(	ENSG0000(	256.2207	0.058018	0.473772	0.819295	lncRNA
ENSG0000(	ITGB4	2629.487	0.110986	0.473793	0.819295	protein_coding
ENSG0000(	MTRF1	395.2948	-0.10033	0.473682	0.819295	protein_coding
ENSG0000(	RMDN1	1000.462	-0.04761	0.473735	0.819295	protein_coding
ENSG0000(	RNU6-126I	22.84909	0.246374	0.473756	0.819295	snRNA
ENSG0000(	WDR90	1309.47	0.068434	0.473776	0.819295	protein_coding
ENSG0000(	ENSG0000(	930.1797	-0.05623	0.473912	0.81945	lncRNA
ENSG0000(	ENSG0000(	123.6815	-0.12106	0.473952	0.819467	lncRNA
ENSG0000(	CTBP1-DT	841.4307	0.061607	0.474148	0.819675	lncRNA
ENSG0000(	MCOLN1	486.2943	-0.05261	0.474152	0.819675	protein_coding
ENSG0000(	PTOV1-AS1	151.607	0.076528	0.474161	0.819675	lncRNA
ENSG0000(	ENSG0000(	198.0515	-0.09645	0.474349	0.81971	lncRNA
ENSG0000(	ENSG0000(	61.37053	0.118578	0.47436	0.81971	lncRNA
ENSG0000(	PMVK	92.08544	0.128037	0.474349	0.81971	protein_coding
ENSG0000(	RASAL2-AS	3171.072	-0.05372	0.474266	0.81971	lncRNA
ENSG0000(	SNORA6	130.5672	0.100033	0.47431	0.81971	snoRNA
ENSG0000(	ZNF74	390.3455	0.040523	0.474251	0.81971	protein_coding
ENSG0000(	CRTAM	287.05	0.068702	0.474466	0.81979	protein_coding
ENSG0000(	LINC01673	24.35868	-0.18817	0.474444	0.81979	lncRNA
ENSG0000(	CIDEB	132.3784	-0.12439	0.474639	0.819791	protein_coding
ENSG0000(	ENSG0000(	118.1299	-0.20004	0.474615	0.819791	protein_coding
ENSG0000(	ENSG0000(	22.49499	-0.38451	0.474528	0.819791	lncRNA
ENSG0000(	ENSG0000(	351.3699	-0.06733	0.474666	0.819791	lncRNA
ENSG0000(	ENSG0000(	96.93642	0.096767	0.474704	0.819791	lncRNA
ENSG0000(	GDA	3499.415	0.15918	0.474703	0.819791	protein_coding
ENSG0000(	HTD2	253.961	0.141588	0.474685	0.819791	protein_coding
ENSG0000(	WFDC1	1084.809	-0.07068	0.474705	0.819791	protein_coding
ENSG0000(	ISG20L2	1312.375	-0.05202	0.474752	0.81982	protein_coding

ENSG0000(	DUX4	45.47903	-0.21959	0.474843	0.81982	protein_coding
ENSG0000(	ENSG0000(	121.9188	0.093728	0.47483	0.81982	lncRNA
ENSG0000(	ENSG0000(	149.3777	0.079281	0.47487	0.81982	TEC
ENSG0000(	FAM131A	931.6507	0.052532	0.47496	0.81982	protein_coding
ENSG0000(	KIAA0753	862.316	0.064759	0.474948	0.81982	protein_coding
ENSG0000(	LINC01517	236.7133	-0.06463	0.474923	0.81982	lncRNA
ENSG0000(	SUNO1	75.54162	0.114454	0.47496	0.81982	lncRNA
ENSG0000(	NAV3	914.8089	0.087658	0.475032	0.819893	protein_coding
ENSG0000(	ENSG0000(	7612.645	-0.07115	0.475067	0.819901	lncRNA
ENSG0000(	EBP	208.5775	0.121043	0.475121	0.819943	protein_coding
ENSG0000(	C6orf132	1251.218	-0.08804	0.475407	0.81998	protein_coding
ENSG0000(	COQ10A	293.6729	-0.05944	0.475393	0.81998	protein_coding
ENSG0000(	ENSG0000(	131.636	0.110033	0.475325	0.81998	lncRNA
ENSG0000(	ENSG0000(	222.8811	-0.07921	0.475469	0.81998	TEC
ENSG0000(	ENSG0000(	36.58829	-0.20354	0.47547	0.81998	TEC
ENSG0000(	ENSG0000(	77.7729	0.085622	0.475402	0.81998	lncRNA
ENSG0000(	ENSG0000(	23.5793	0.162301	0.475188	0.81998	lncRNA
ENSG0000(	FAM95B1	975.9669	0.242332	0.475462	0.81998	transcribed_unprocessed_pseudo
ENSG0000(	HS6ST1	736.9647	-0.08694	0.475377	0.81998	protein_coding
ENSG0000(	ITK	1677.009	-0.12068	0.475216	0.81998	protein_coding
ENSG0000(	NDUFA1	206.2819	-0.15492	0.475427	0.81998	protein_coding
ENSG0000(	C12orf43	639.7539	0.041525	0.475611	0.819984	protein_coding
ENSG0000(	ENSG0000(	235.9097	-0.14205	0.475587	0.819984	lncRNA
ENSG0000(	ENSG0000(	121.659	0.079862	0.475622	0.819984	lncRNA
ENSG0000(	GMPR	92.8061	-0.11742	0.475612	0.819984	protein_coding
ENSG0000(	KCTD2	485.5259	-0.05781	0.475552	0.819984	protein_coding
ENSG0000(	ENSG0000(	170.0352	0.077545	0.475682	0.819985	lncRNA
ENSG0000(	RN7SL752F	215.4834	-0.15398	0.47566	0.819985	misc_RNA
ENSG0000(	ENSG0000(	417.4376	0.044021	0.475881	0.820034	lncRNA
ENSG0000(	HOOK1	1299.975	0.131206	0.475834	0.820034	protein_coding
ENSG0000(	KNG1	73.98579	0.143689	0.47587	0.820034	protein_coding
ENSG0000(	SNRPD2	227.8178	-0.07516	0.475743	0.820034	protein_coding
ENSG0000(	SPN	1927.383	-0.04388	0.475889	0.820034	protein_coding
ENSG0000(	ZBTB39	468.8254	-0.06151	0.47584	0.820034	protein_coding
ENSG0000(	LINC02782	28.57287	0.170438	0.475979	0.820086	lncRNA
ENSG0000(	ZNF398	802.0426	0.050016	0.475952	0.820086	protein_coding
ENSG0000(	SMC6	2443.256	-0.04724	0.476093	0.820232	protein_coding
ENSG0000(	CCDC61	142.2966	0.146375	0.476139	0.820258	protein_coding
ENSG0000(	ENSG0000(	111.4209	-0.09456	0.476257	0.820411	processed_pseudogene
ENSG0000(	ENSG0000(	26.43069	0.190004	0.476292	0.82042	transcribed_unitary_pseudogene
ENSG0000(	TMEM212	1713.512	0.061601	0.476351	0.82047	protein_coding
ENSG0000(	OLA1P2	243.6534	0.081882	0.476399	0.820502	processed_pseudogene
ENSG0000(	ENSG0000(	67.15539	-0.10578	0.476509	0.820588	lncRNA
ENSG0000(	PARTICL	45.12995	-0.14962	0.476484	0.820588	lncRNA
ENSG0000(	ENSG0000(	21.63755	0.413473	0.476796	0.820775	unprocessed_pseudogene
ENSG0000(	ENSG0000(	53.90149	0.130774	0.476752	0.820775	processed_pseudogene
ENSG0000(	ENSG0000(	60.86162	0.148175	0.476748	0.820775	lncRNA

ENSG0000(	ENSG0000(	63.84933	0.131294	0.47668	0.820775	lncRNA
ENSG0000(	ENSG0000(	27.55768	-0.21518	0.476794	0.820775	lncRNA
ENSG0000(	WASIR1	31.15068	0.704332	0.476772	0.820775	lncRNA
ENSG0000(	FBXW8	664.3955	0.072014	0.476902	0.820804	protein_coding
ENSG0000(	RUSC2	398.4285	-0.1032	0.476862	0.820804	protein_coding
ENSG0000(	ZMPSTE24-	121.214	0.08657	0.476903	0.820804	lncRNA
ENSG0000(	AGBL4	214.8781	-0.10109	0.477022	0.820958	protein_coding
ENSG0000(	ANKRD46	429.204	0.051453	0.477248	0.821043	protein_coding
ENSG0000(	CTNNBIP1	282.0384	-0.08976	0.477326	0.821043	protein_coding
ENSG0000(	ENSG0000(	73.67749	-0.11708	0.477549	0.821043	lncRNA
ENSG0000(	ENSG0000(	355.7621	0.053402	0.47741	0.821043	lncRNA
ENSG0000(	ENSG0000(	35.10538	-0.15523	0.477339	0.821043	lncRNA
ENSG0000(	ENSG0000(	111.2767	-0.12928	0.477284	0.821043	TEC
ENSG0000(	ENSG0000(	200.661	-0.08423	0.477247	0.821043	TEC
ENSG0000(	H2AZ2P1	161.5088	0.098946	0.477537	0.821043	transcribed_processed_pseudoge
ENSG0000(	HMX3	110.7114	0.087099	0.477442	0.821043	protein_coding
ENSG0000(	IKZF4	330.7986	-0.10047	0.477532	0.821043	protein_coding
ENSG0000(	MARCHF5	565.1787	0.067146	0.477305	0.821043	protein_coding
ENSG0000(	NOSTRIN	1007.6	0.120421	0.477451	0.821043	protein_coding
ENSG0000(	PPM1K-DT	80.21227	-0.12225	0.47732	0.821043	lncRNA
ENSG0000(	SF3B1	9969.629	-0.04237	0.477398	0.821043	protein_coding
ENSG0000(	UBL7-DT	3008.466	0.046388	0.477509	0.821043	lncRNA
ENSG0000(	WDR53	101.4607	-0.11652	0.477506	0.821043	protein_coding
ENSG0000(	ANKLE1	1577.092	0.170224	0.477741	0.821096	protein_coding
ENSG0000(	ENOX1	263.9687	0.13834	0.477767	0.821096	protein_coding
ENSG0000(	ENSG0000(	24.76083	-0.25248	0.477849	0.821096	lncRNA
ENSG0000(	MSH4	34.27854	0.219006	0.477836	0.821096	protein_coding
ENSG0000(	MYOF	2410.062	-0.0993	0.47773	0.821096	protein_coding
ENSG0000(	PEX3	150.8122	-0.1459	0.47781	0.821096	protein_coding
ENSG0000(	SMN2	386.1805	-0.21662	0.477774	0.821096	protein_coding
ENSG0000(	TBCC	299.498	0.072165	0.477661	0.821096	protein_coding
ENSG0000(	ZNF280D	2004.478	0.054815	0.477627	0.821096	protein_coding
ENSG0000(	CIDECP1	416.8325	0.044036	0.47808	0.821335	transcribed_unprocessed_pseudo
ENSG0000(	ENSG0000(	164.6764	0.072915	0.478137	0.821335	lncRNA
ENSG0000(	NOD1	824.2111	-0.08213	0.478137	0.821335	protein_coding
ENSG0000(	RN7SKP94	21.45811	0.215624	0.478081	0.821335	misc_RNA
ENSG0000(	TYW1B	252.2058	0.097887	0.478134	0.821335	protein_coding
ENSG0000(	CFLAR-AS1	375.9209	0.086337	0.4783	0.821461	lncRNA
ENSG0000(	ENSG0000(	101.5319	0.121875	0.478299	0.821461	protein_coding
ENSG0000(	WDR5-DT	41.30808	-0.15566	0.47827	0.821461	lncRNA
ENSG0000(	CD79A	226.2224	-0.25834	0.478456	0.821472	protein_coding
ENSG0000(	ENSG0000(	116.7434	0.129736	0.478429	0.821472	lncRNA
ENSG0000(	ENSG0000(	886.441	0.053126	0.478387	0.821472	lncRNA
ENSG0000(	TENT4B	584.2853	0.055258	0.478437	0.821472	protein_coding
ENSG0000(	THAP10	20.14352	-0.21332	0.478345	0.821472	protein_coding
ENSG0000(	ENSG0000(	42.51438	-0.27926	0.478543	0.821519	lncRNA
ENSG0000(	KPNA4	3603.376	0.042033	0.478522	0.821519	protein_coding

ENSG0000(	ENSG0000(	403.5123	0.052612	0.478579	0.821531	lncRNA
ENSG0000(	LINC02568	140.5753	0.146113	0.478722	0.821673	lncRNA
ENSG0000(	PPP1R14A	26.36155	0.219296	0.478693	0.821673	protein_coding
ENSG0000(	ATXN7L3	1157.773	-0.0744	0.479505	0.821715	protein_coding
ENSG0000(	BMS1P4-A(	21.7319	-0.4575	0.479069	0.821715	lncRNA
ENSG0000(	BSN-AS1	91.45609	0.089066	0.478988	0.821715	lncRNA
ENSG0000(	CD8A	156.1584	-0.20629	0.479553	0.821715	protein_coding
ENSG0000(	COMP	173.9212	0.074868	0.479201	0.821715	protein_coding
ENSG0000(	CYB5R2	239.3569	-0.13277	0.479187	0.821715	protein_coding
ENSG0000(	ENSG0000(	24.40921	-0.19057	0.479537	0.821715	lncRNA
ENSG0000(	ENSG0000(	113.4577	0.085531	0.479194	0.821715	lncRNA
ENSG0000(	ENSG0000(	87.77968	0.138049	0.479405	0.821715	lncRNA
ENSG0000(	ENSG0000(	62.27563	0.107248	0.479322	0.821715	lncRNA
ENSG0000(	ENSG0000(	893.7458	0.057338	0.479195	0.821715	protein_coding
ENSG0000(	ENSG0000(	281.7537	0.079455	0.478984	0.821715	protein_coding
ENSG0000(	ENSG0000(	68.62411	0.112356	0.479229	0.821715	lncRNA
ENSG0000(	ENSG0000(	20.04122	0.176902	0.479457	0.821715	lncRNA
ENSG0000(	ERC2	42.6182	-0.22957	0.47933	0.821715	protein_coding
ENSG0000(	GOLGA6L5I	272.4602	-0.09601	0.479358	0.821715	transcribed_unprocessed_pseudo
ENSG0000(	HGH1	186.5604	0.103446	0.47882	0.821715	protein_coding
ENSG0000(	KIZ-AS1	220.7319	0.073604	0.479552	0.821715	lncRNA
ENSG0000(	LINC00893	372.5588	0.098651	0.479264	0.821715	lncRNA
ENSG0000(	LINC02740	27.69074	0.153313	0.479033	0.821715	lncRNA
ENSG0000(	MPZL1	2092.6	-0.05083	0.478889	0.821715	protein_coding
ENSG0000(	NR2C2AP	77.73331	0.13844	0.479402	0.821715	protein_coding
ENSG0000(	PTK2B	2773.735	-0.06487	0.47936	0.821715	protein_coding
ENSG0000(	RN7SL556F	20.9678	-0.21855	0.479405	0.821715	misc_RNA
ENSG0000(	SDK2	409.5362	0.138305	0.479329	0.821715	protein_coding
ENSG0000(	TBX1	81.34871	-0.12456	0.478827	0.821715	protein_coding
ENSG0000(	UTP18	245.0572	-0.07886	0.479109	0.821715	protein_coding
ENSG0000(	ENSG0000(	1486.979	-0.05294	0.479662	0.821852	transcribed_processed_pseudoge
ENSG0000(	ENSG0000(	42.77363	0.141065	0.479817	0.822034	lncRNA
ENSG0000(	SPEG	387.9	-0.1889	0.479829	0.822034	protein_coding
ENSG0000(	FDPS	1088.552	-0.07614	0.479887	0.822083	protein_coding
ENSG0000(	AP4B1-AS1	101.8058	0.10514	0.480223	0.822122	lncRNA
ENSG0000(	CYLD-AS1	193.8547	-0.12909	0.480128	0.822122	lncRNA
ENSG0000(	ENSG0000(	285.0123	-0.07754	0.480012	0.822122	lncRNA
ENSG0000(	ENSG0000(	29.13141	-0.16759	0.480168	0.822122	lncRNA
ENSG0000(	ENSG0000(	264.0648	0.090885	0.480269	0.822122	lncRNA
ENSG0000(	EPHA1-AS1	470.7259	-0.08495	0.480074	0.822122	lncRNA
ENSG0000(	KDM3A	2153.412	0.060708	0.48009	0.822122	protein_coding
ENSG0000(	LRRC37A7F	168.6484	0.0667	0.480053	0.822122	transcribed_processed_pseudoge
ENSG0000(	PPP2R5B	367.8804	-0.10279	0.480045	0.822122	protein_coding
ENSG0000(	RN7SL91P	26.05551	0.155193	0.480133	0.822122	misc_RNA
ENSG0000(	RORB-AS1	48.24806	0.128035	0.480265	0.822122	lncRNA
ENSG0000(	TFPI2-DT	26.51192	0.151571	0.480254	0.822122	lncRNA
ENSG0000(	C19orf71	50.72638	0.158161	0.480302	0.822129	protein_coding

ENSG0000(	ATP5F1A	4887.517	0.067855	0.480469	0.822239	protein_coding
ENSG0000(	ENSG0000(	206.0318	0.078022	0.4804	0.822239	lncRNA
ENSG0000(	LGALS9	2149.428	0.092029	0.480486	0.822239	protein_coding
ENSG0000(	SCAMP3	278.0328	-0.08769	0.480446	0.822239	protein_coding
ENSG0000(	ENSG0000(	63.35191	-0.11022	0.480543	0.82227	lncRNA
ENSG0000(	TESK1	444.0795	0.065579	0.480594	0.82227	protein_coding
ENSG0000(	WWP1-AS1	279.3757	0.067484	0.480566	0.82227	lncRNA
ENSG0000(	ENSG0000(	743.9988	-0.06391	0.480771	0.822295	protein_coding
ENSG0000(	KCNB1	2155.578	-0.05643	0.480848	0.822295	protein_coding
ENSG0000(	MCAT	105.2829	0.141304	0.480706	0.822295	protein_coding
ENSG0000(	NPIPB6	81.17182	0.527188	0.480647	0.822295	protein_coding
ENSG0000(	OR5AN1	201.2751	-0.08384	0.480778	0.822295	protein_coding
ENSG0000(	RBM6	5987.695	0.041559	0.480813	0.822295	protein_coding
ENSG0000(	RCCD1-AS1	163.045	0.07187	0.480696	0.822295	lncRNA
ENSG0000(	SH2D4A	475.5497	0.127286	0.480838	0.822295	protein_coding
ENSG0000(	IL10	142.4231	-0.10801	0.480916	0.82236	protein_coding
ENSG0000(	FOXL1	186.0174	0.161132	0.481028	0.822399	protein_coding
ENSG0000(	MEX3D	128.0701	0.095831	0.481023	0.822399	protein_coding
ENSG0000(	PAPPA2	118.4999	0.31173	0.480976	0.822399	protein_coding
ENSG0000(	ENSG0000(	77.49935	-0.12388	0.481065	0.822411	protein_coding
ENSG0000(	ARMH1	97.28708	0.138519	0.481169	0.822532	protein_coding
ENSG0000(	DNMT3A	2985.168	-0.05082	0.48122	0.822532	protein_coding
ENSG0000(	SCAF4	1119.005	0.036787	0.481226	0.822532	protein_coding
ENSG0000(	ENSG0000(	97.94273	0.099613	0.481438	0.822845	lncRNA
ENSG0000(	ENSG0000(	26.20571	0.180237	0.481533	0.822848	lncRNA
ENSG0000(	ENSG0000(	54.96487	0.152679	0.481488	0.822848	lncRNA
ENSG0000(	NPTX1	48.69209	0.176125	0.481544	0.822848	protein_coding
ENSG0000(	OR2A1-AS1	305.5461	0.096386	0.48156	0.822848	lncRNA
ENSG0000(	FBXW11	1221.043	-0.06507	0.481611	0.822884	protein_coding
ENSG0000(	B4GALT7	410.9553	-0.06623	0.481829	0.822962	protein_coding
ENSG0000(	ENSG0000(	26.91	0.171668	0.481837	0.822962	lncRNA
ENSG0000(	ENSG0000(	307.5134	0.07069	0.481773	0.822962	lncRNA
ENSG0000(	ENSG0000(	223.9386	-0.07366	0.481955	0.822962	lncRNA
ENSG0000(	H3-3A-DT	74.54106	0.103691	0.481889	0.822962	lncRNA
ENSG0000(	IL13RA1	2349.587	-0.07552	0.481955	0.822962	protein_coding
ENSG0000(	IRAK1	1091.878	-0.06886	0.481705	0.822962	protein_coding
ENSG0000(	LINC01722	595.6867	0.092271	0.481819	0.822962	lncRNA
ENSG0000(	LRRC41	1933.183	-0.03234	0.48184	0.822962	protein_coding
ENSG0000(	TSSC4	179.2533	-0.08323	0.481944	0.822962	protein_coding
ENSG0000(	ARL13B	396.3735	-0.07122	0.482084	0.823005	protein_coding
ENSG0000(	ATPSCKMT	482.3625	0.066837	0.482101	0.823005	protein_coding
ENSG0000(	EIF2AK3	4122.554	-0.03827	0.482095	0.823005	protein_coding
ENSG0000(	PIK3R6	174.857	-0.09705	0.482083	0.823005	protein_coding
ENSG0000(	GRIA1	125.273	-0.09046	0.482161	0.823057	protein_coding
ENSG0000(	TMEM50A	770.3085	-0.07717	0.482198	0.82307	protein_coding
ENSG0000(	PCDHGB6	292.9719	0.061805	0.482281	0.823122	protein_coding
ENSG0000(	RN7SL390F	24.79713	-0.1881	0.482289	0.823122	misc_RNA

ENSG0000(	ENSG0000(	55.34775	0.114002	0.48236	0.823192	lncRNA
ENSG0000(	OXER1	112.6385	0.13814	0.482465	0.823321	protein_coding
ENSG0000(	ENSG0000(	41.66469	-0.14513	0.482511	0.823324	lncRNA
ENSG0000(	HSPA14	202.2212	0.08253	0.482527	0.823324	protein_coding
ENSG0000(	ENSG0000(	326.2385	0.05702	0.482741	0.823502	lncRNA
ENSG0000(	ENSG0000(	320.4415	0.053817	0.482775	0.823502	lncRNA
ENSG0000(	ENSG0000(	112.8529	-0.0762	0.482667	0.823502	lncRNA
ENSG0000(	IGLV2-5	23.52637	-0.30786	0.482744	0.823502	IG_V_pseudogene
ENSG0000(	LINC02532	275.4159	0.118266	0.482781	0.823502	lncRNA
ENSG0000(	CNPY4	247.2212	-0.0677	0.482855	0.823578	protein_coding
ENSG0000(	ARV1	234.7358	0.086607	0.484015	0.823578	protein_coding
ENSG0000(	ASF1B	116.0378	-0.14169	0.483151	0.823578	protein_coding
ENSG0000(	BRCA1	1122.615	-0.05081	0.483085	0.823578	protein_coding
ENSG0000(	C10orf105	27.42219	-0.20009	0.483276	0.823578	protein_coding
ENSG0000(	CCDC82	2920.718	-0.0615	0.483028	0.823578	protein_coding
ENSG0000(	CCDC92B	264.7667	-0.08217	0.483153	0.823578	protein_coding
ENSG0000(	CDHR4	329.3863	0.064081	0.483194	0.823578	protein_coding
ENSG0000(	DPYSL5	189.7232	0.063041	0.483076	0.823578	protein_coding
ENSG0000(	DUSP19	314.6824	0.075963	0.483917	0.823578	protein_coding
ENSG0000(	ENO1P3	68.20816	0.11208	0.483948	0.823578	processed_pseudogene
ENSG0000(	ENSG0000(	22.33101	0.255742	0.483943	0.823578	transcribed_unprocessed_pseudo
ENSG0000(	ENSG0000(	119.1019	-0.10352	0.483272	0.823578	transcribed_unprocessed_pseudo
ENSG0000(	ENSG0000(	28.99237	0.268512	0.484085	0.823578	lncRNA
ENSG0000(	ENSG0000(	21.49749	0.20414	0.483947	0.823578	lncRNA
ENSG0000(	ENSG0000(	675.0975	-0.0799	0.483332	0.823578	lncRNA
ENSG0000(	ENSG0000(	499.1115	-0.0923	0.48384	0.823578	lncRNA
ENSG0000(	ENSG0000(	32.80503	-0.21916	0.483335	0.823578	lncRNA
ENSG0000(	ENSG0000(	213.7196	-0.07681	0.484036	0.823578	TEC
ENSG0000(	ENSG0000(	591.0711	0.063943	0.483052	0.823578	TEC
ENSG0000(	ENSG0000(	46.84699	-0.15682	0.483377	0.823578	TEC
ENSG0000(	ENSG0000(	287.7284	-0.05936	0.484014	0.823578	lncRNA
ENSG0000(	ENSG0000(	114.0559	0.082858	0.483491	0.823578	lncRNA
ENSG0000(	FRY	1057.93	-0.15449	0.483583	0.823578	protein_coding
ENSG0000(	GABPB1	341.6992	-0.07294	0.483285	0.823578	protein_coding
ENSG0000(	HTR1D	92.96916	0.109692	0.483612	0.823578	protein_coding
ENSG0000(	KLHL13	84.39566	0.178438	0.483808	0.823578	protein_coding
ENSG0000(	KRIT1	4989.948	-0.02941	0.483318	0.823578	protein_coding
ENSG0000(	LINC00486	367.6893	-0.05488	0.483788	0.823578	lncRNA
ENSG0000(	LINC02619	27.02714	-0.16305	0.483605	0.823578	lncRNA
ENSG0000(	LINC02889	614.1774	-0.06952	0.483181	0.823578	lncRNA
ENSG0000(	MTUS1	5360.982	0.091191	0.484113	0.823578	protein_coding
ENSG0000(	OR3A2	94.20785	0.108502	0.483473	0.823578	protein_coding
ENSG0000(	OR8H1	123.536	0.106564	0.483693	0.823578	protein_coding
ENSG0000(	POMT2	1014.46	-0.06072	0.483808	0.823578	protein_coding
ENSG0000(	RN7SKP55	242.699	0.165789	0.483745	0.823578	misc_RNA
ENSG0000(	RN7SL96P	176.4851	0.072004	0.48366	0.823578	misc_RNA
ENSG0000(	RNF141	943.7269	-0.05843	0.483242	0.823578	protein_coding

ENSG0000(	RPUSD4	759.5853	0.037752	0.483468	0.823578	protein_coding
ENSG0000(	RXYLT1	817.1868	0.039432	0.484074	0.823578	protein_coding
ENSG0000(	SH2D7	39.89115	0.284675	0.48406	0.823578	protein_coding
ENSG0000(	SLC25A48	244.9824	0.143953	0.483085	0.823578	protein_coding
ENSG0000(	VPS41	1534.926	-0.05912	0.484053	0.823578	protein_coding
ENSG0000(	GOLGA8J	110.4842	0.099913	0.484257	0.82375	protein_coding
ENSG0000(	TCTN3	1494.892	0.049531	0.484273	0.82375	protein_coding
ENSG0000(	FAM151B	183.6627	0.106528	0.484402	0.823918	protein_coding
ENSG0000(	HCCS	199.5972	-0.12766	0.484452	0.823951	protein_coding
ENSG0000(	GRHPR	1028.942	-0.0545	0.484617	0.824131	protein_coding
ENSG0000(	PDE7A	2844.533	-0.11664	0.4846	0.824131	protein_coding
ENSG0000(	ENSG0000(	95.40413	0.12801	0.484647	0.824131	lncRNA
ENSG0000(	UROD	225.5859	0.076523	0.484718	0.8242	protein_coding
ENSG0000(	ADAM29	154.3336	0.10639	0.485043	0.82422	protein_coding
ENSG0000(	ADIRF	131.3694	0.239939	0.485038	0.82422	protein_coding
ENSG0000(	ENSG0000(	34.83889	-0.18707	0.484873	0.82422	lncRNA
ENSG0000(	ENSG0000(	114.4876	0.1749	0.485083	0.82422	processed_pseudogene
ENSG0000(	ENSG0000(	105.2314	0.115129	0.484831	0.82422	lncRNA
ENSG0000(	ENSG0000(	72.78902	-0.1108	0.484847	0.82422	lncRNA
ENSG0000(	ENSG0000(	80.5628	-0.09574	0.48492	0.82422	lncRNA
ENSG0000(	PDLIM1	1626.071	0.095156	0.485018	0.82422	protein_coding
ENSG0000(	RNU6-123F	29.28703	0.19074	0.484794	0.82422	snRNA
ENSG0000(	SDAD1P1	158.5003	0.076404	0.485089	0.82422	transcribed_processed_pseudoge
ENSG0000(	SNORD83B	30.92745	-0.21091	0.484952	0.82422	snoRNA
ENSG0000(	TBC1D22A-	26.56335	-0.15641	0.484963	0.82422	lncRNA
ENSG0000(	ENSG0000(	291.4758	-0.06079	0.485208	0.824372	TEC
ENSG0000(	TNFRSF10A	379.9117	0.054451	0.485247	0.824387	lncRNA
ENSG0000(	PTPN23	1032.1	0.074851	0.485329	0.824425	protein_coding
ENSG0000(	RET	75.34657	0.169889	0.4853	0.824425	protein_coding
ENSG0000(	RANP4	30.37022	-0.17538	0.48541	0.82451	processed_pseudogene
ENSG0000(	ENSG0000(	161.1805	1.760995	0.485504	0.824524	protein_coding
ENSG0000(	ENSG0000(	26.17873	-0.17751	0.485512	0.824524	lncRNA
ENSG0000(	PSMD3	996.9217	-0.07031	0.485535	0.824524	protein_coding
ENSG0000(	TPTE2P6	71.73084	0.108068	0.485538	0.824524	transcribed_unprocessed_pseudo
ENSG0000(	CABLES1	268.0443	0.123836	0.485713	0.824669	protein_coding
ENSG0000(	GAPVD1	2683.139	-0.04824	0.48571	0.824669	protein_coding
ENSG0000(	SLC6A12	46.01224	-0.22356	0.485705	0.824669	protein_coding
ENSG0000(	CDIN1	463.4641	-0.06424	0.485928	0.82478	protein_coding
ENSG0000(	ENSG0000(	82.59452	-0.15067	0.485887	0.82478	lncRNA
ENSG0000(	LINC02044	24.94723	0.173682	0.485831	0.82478	lncRNA
ENSG0000(	PDIA5	511.5641	-0.08171	0.485894	0.82478	protein_coding
ENSG0000(	SLC10A3	160.8022	0.090953	0.485918	0.82478	protein_coding
ENSG0000(	AP4B1	1173.212	0.055195	0.486112	0.824965	protein_coding
ENSG0000(	LINC02642	297.4763	0.051512	0.486159	0.824965	lncRNA
ENSG0000(	MIR5690	55.60709	-0.3446	0.486189	0.824965	miRNA
ENSG0000(	SERF2	2520.31	-0.07209	0.486217	0.824965	protein_coding
ENSG0000(	TBC1D10B	1285.411	0.040433	0.486204	0.824965	protein_coding

ENSG0000(	TECRL	125.4495	0.088216	0.486159	0.824965	protein_coding
ENSG0000(	TBC1D19	209.3147	-0.08524	0.486375	0.825183	protein_coding
ENSG0000(	L3MBTL1	1566.042	0.039251	0.486463	0.825183	protein_coding
ENSG0000(	RUNDC1	1781.787	0.047561	0.486443	0.825183	protein_coding
ENSG0000(	STOX2	688.9217	0.153152	0.486466	0.825183	protein_coding
ENSG0000(	MAOA	3526.156	0.201302	0.486572	0.825312	protein_coding
ENSG0000(	ENSG0000(	381.0579	-0.06963	0.486764	0.825384	lncRNA
ENSG0000(	ENSG0000(	171.0719	0.075744	0.486672	0.825384	lncRNA
ENSG0000(	ENSG0000(	143.5454	-0.13412	0.486748	0.825384	lncRNA
ENSG0000(	ENSG0000(	465.7246	-0.10068	0.486718	0.825384	lncRNA
ENSG0000(	RN7SL388F	32.01582	0.172969	0.48668	0.825384	misc_RNA
ENSG0000(	ENSG0000(	157.7545	0.072721	0.486823	0.825432	lncRNA
ENSG0000(	ENSG0000(	48.79166	0.152491	0.486891	0.825498	lncRNA
ENSG0000(	EIF4E1B	49.52845	-0.11827	0.48714	0.82559	protein_coding
ENSG0000(	ENSG0000(	98.97961	0.147139	0.486988	0.82559	processed_pseudogene
ENSG0000(	ENSG0000(	23.67873	-0.21605	0.487306	0.82559	protein_coding
ENSG0000(	ENSG0000(	59.18667	-0.12217	0.487225	0.82559	TEC
ENSG0000(	ENSG0000(	150.0435	-0.08593	0.487063	0.82559	lncRNA
ENSG0000(	GSR	1204.427	0.095266	0.487303	0.82559	protein_coding
ENSG0000(	HARS2	817.0176	0.049568	0.487084	0.82559	protein_coding
ENSG0000(	NCAPGP1	31.80108	0.172875	0.487273	0.82559	processed_pseudogene
ENSG0000(	NFYC-AS1	251.3839	-0.07505	0.487116	0.82559	lncRNA
ENSG0000(	PHF10	1280.286	-0.0743	0.487217	0.82559	protein_coding
ENSG0000(	VSIG10L2	31.19529	-0.1932	0.487232	0.82559	protein_coding
ENSG0000(	ZWILCH	278.6355	0.095209	0.487084	0.82559	protein_coding
ENSG0000(	FCRL2	1158.818	-0.1585	0.487398	0.825695	protein_coding
ENSG0000(	ENSG0000(	256.5695	0.089022	0.487487	0.825795	lncRNA
ENSG0000(	ENSG0000(	28.34764	-0.25421	0.48757	0.825885	lncRNA
ENSG0000(	LINC01624	28.81933	0.349238	0.487612	0.825906	lncRNA
ENSG0000(	LINC00355	331.916	0.057125	0.487722	0.82604	lncRNA
ENSG0000(	CLRN1	41.39866	0.136731	0.487878	0.826069	protein_coding
ENSG0000(	COL13A1	289.3092	-0.15175	0.487919	0.826069	protein_coding
ENSG0000(	ENSG0000(	72.98592	-0.11451	0.487799	0.826069	lncRNA
ENSG0000(	ENSG0000(	46.83614	0.11904	0.487927	0.826069	lncRNA
ENSG0000(	ENSG0000(	44.71106	-0.2355	0.487844	0.826069	protein_coding
ENSG0000(	ENSG0000(	28.31322	-0.14517	0.487981	0.826069	lncRNA
ENSG0000(	PTGER2	259.113	-0.10821	0.488009	0.826069	protein_coding
ENSG0000(	SSPN	328.9117	-0.15113	0.488001	0.826069	protein_coding
ENSG0000(	TMEM161/	189.0616	0.102819	0.487847	0.826069	protein_coding
ENSG0000(	ALG1L	74.47484	0.098777	0.488276	0.826277	transcribed_unprocessed_pseudo
ENSG0000(	ENSG0000(	52.51148	0.273337	0.48817	0.826277	lncRNA
ENSG0000(	ENSG0000(	251.2449	-0.06224	0.488282	0.826277	lncRNA
ENSG0000(	ENSG0000(	193.2507	0.170556	0.488271	0.826277	lncRNA
ENSG0000(	ZNF728	29.27117	0.197755	0.488279	0.826277	protein_coding
ENSG0000(	ARHGEF4	326.6437	-0.08932	0.48855	0.826419	protein_coding
ENSG0000(	CYLC2	33.39398	0.255998	0.488498	0.826419	protein_coding
ENSG0000(	CYTH3	385.6963	0.092815	0.488554	0.826419	protein_coding

ENSG0000(	EIF1AY	289.9622	0.088158	0.488481	0.826419	protein_coding
ENSG0000(	ENSG0000(	259.539	-0.05858	0.488462	0.826419	lncRNA
ENSG0000(	HMGA2	1263.564	0.128543	0.488521	0.826419	protein_coding
ENSG0000(	LINC01605	49.98762	0.230645	0.488576	0.826419	lncRNA
ENSG0000(	ADAMTS7P	32.69781	0.340839	0.488923	0.826701	transcribed_unprocessed_pseudo
ENSG0000(	DFFBP1	28.79829	-0.19465	0.48885	0.826701	processed_pseudogene
ENSG0000(	ENSG0000(	168.5825	0.148177	0.48888	0.826701	lncRNA
ENSG0000(	ENSG0000(	79.11778	0.104275	0.489062	0.826701	lncRNA
ENSG0000(	ENSG0000(	20.28625	0.209078	0.488987	0.826701	lncRNA
ENSG0000(	ENSG0000(	51.46215	-0.13014	0.488799	0.826701	lncRNA
ENSG0000(	GCHFR	36.51317	-0.2114	0.488986	0.826701	protein_coding
ENSG0000(	KMT2B	2297.048	0.070697	0.489048	0.826701	protein_coding
ENSG0000(	MYLK	5626.635	0.067664	0.488864	0.826701	protein_coding
ENSG0000(	RPS11	2737.079	-0.08504	0.489073	0.826701	protein_coding
ENSG0000(	VPS13D	9381.837	0.054455	0.489057	0.826701	protein_coding
ENSG0000(	ENSG0000(	159.9959	0.061532	0.489149	0.826752	lncRNA
ENSG0000(	RPL23A	1731.881	-0.06934	0.489164	0.826752	protein_coding
ENSG0000(	LINC02012	148.5967	0.113544	0.489296	0.826877	lncRNA
ENSG0000(	TEX22	76.78751	0.155461	0.489298	0.826877	protein_coding
ENSG0000(	CUL3	1989.382	0.046151	0.489408	0.826911	protein_coding
ENSG0000(	KRT8	8082.53	0.149086	0.489405	0.826911	protein_coding
ENSG0000(	RPS4XP5	187.0018	0.082845	0.489388	0.826911	transcribed_processed_pseudoge
ENSG0000(	ENSG0000(	74.15046	0.09728	0.489447	0.826927	lncRNA
ENSG0000(	CALHM1	62.27273	-0.09167	0.489703	0.826933	protein_coding
ENSG0000(	DKC1	490.5018	-0.08023	0.489522	0.826933	protein_coding
ENSG0000(	ENSG0000(	46.74136	0.164941	0.489749	0.826933	lncRNA
ENSG0000(	MALLP2	23.81665	0.244488	0.489747	0.826933	transcribed_unprocessed_pseudo
ENSG0000(	MAN1C1	907.8405	0.075833	0.489704	0.826933	protein_coding
ENSG0000(	NRL	131.5501	-0.12257	0.489634	0.826933	protein_coding
ENSG0000(	PAX5	1046.057	-0.19164	0.489693	0.826933	protein_coding
ENSG0000(	SCFD1	5378.454	-0.04054	0.489588	0.826933	protein_coding
ENSG0000(	TULP3	387.7944	-0.07518	0.489752	0.826933	protein_coding
ENSG0000(	ZNF720	1435.292	-0.0478	0.489504	0.826933	protein_coding
ENSG0000(	ABCA13	360.3553	-0.08802	0.490362	0.826948	protein_coding
ENSG0000(	CLNS1A	1132.705	0.068478	0.490594	0.826948	protein_coding
ENSG0000(	CUEDC1	487.9451	-0.05891	0.490073	0.826948	protein_coding
ENSG0000(	ENSG0000(	161.5818	0.071718	0.489942	0.826948	lncRNA
ENSG0000(	ENSG0000(	33.5349	0.242263	0.490579	0.826948	lncRNA
ENSG0000(	ENSG0000(	77.13724	0.132174	0.490464	0.826948	lncRNA
ENSG0000(	ENSG0000(	23.96621	-0.9734	0.490656	0.826948	protein_coding
ENSG0000(	ENSG0000(	255.4484	0.082823	0.490295	0.826948	lncRNA
ENSG0000(	ENSG0000(	104.3381	-0.08028	0.489846	0.826948	lncRNA
ENSG0000(	ENSG0000(	21.83272	-0.24233	0.490509	0.826948	lncRNA
ENSG0000(	ENSG0000(	210.602	0.097036	0.490479	0.826948	TEC
ENSG0000(	ENSG0000(	63.26235	-0.09538	0.490222	0.826948	lncRNA
ENSG0000(	ENSG0000(	196.164	-0.07348	0.490659	0.826948	lncRNA
ENSG0000(	GPR15	404.2544	0.229383	0.490626	0.826948	protein_coding

ENSG0000(	GPR18	76.55298	-0.2126	0.49031	0.826948	protein_coding
ENSG0000(	HSFX2	27.37593	-0.75613	0.490457	0.826948	protein_coding
ENSG0000(	LINC02076	50.11858	0.111533	0.490159	0.826948	lncRNA
ENSG0000(	MCPH1-AS	1290.454	0.045955	0.490357	0.826948	lncRNA
ENSG0000(	MILR1	344.9397	-0.07467	0.489994	0.826948	protein_coding
ENSG0000(	NBEAP1	42.34268	-0.16436	0.490051	0.826948	transcribed_unprocessed_pseudo
ENSG0000(	PTAR1	3508.565	0.046121	0.489824	0.826948	protein_coding
ENSG0000(	RAB24	398.5914	-0.09833	0.489941	0.826948	protein_coding
ENSG0000(	RASL11A	135.1167	-0.16296	0.490633	0.826948	protein_coding
ENSG0000(	RBMS3	1483.374	0.080087	0.490662	0.826948	protein_coding
ENSG0000(	RN7SL371F	341.6859	0.069668	0.490153	0.826948	misc_RNA
ENSG0000(	RPAIN	2486.89	0.038377	0.490224	0.826948	protein_coding
ENSG0000(	SEMA6A-AS	106.3886	-0.11095	0.490079	0.826948	lncRNA
ENSG0000(	TCF24	26.70719	0.193418	0.489959	0.826948	protein_coding
ENSG0000(	TNIP2	454.9995	-0.0646	0.490571	0.826948	protein_coding
ENSG0000(	TRIM66	1770.252	-0.09139	0.490434	0.826948	protein_coding
ENSG0000(	PRRC1	1241.961	-0.06262	0.490715	0.826987	protein_coding
ENSG0000(	MICOS13	313.4829	0.057297	0.490747	0.82699	protein_coding
ENSG0000(	COMMD7	261.691	0.079424	0.490815	0.827054	protein_coding
ENSG0000(	C18orf54	245.659	-0.12368	0.490952	0.827169	protein_coding
ENSG0000(	CDX2	2090.012	0.17687	0.491028	0.827169	protein_coding
ENSG0000(	DGUOK-AS	62.73015	0.165675	0.490971	0.827169	lncRNA
ENSG0000(	ENSG0000(	24.49868	-0.17099	0.49106	0.827169	lncRNA
ENSG0000(	HSP90AB7F	122.1683	0.086002	0.491071	0.827169	processed_pseudogene
ENSG0000(	HSPB8	104.7496	-0.18046	0.491094	0.827169	protein_coding
ENSG0000(	INSIG1	652.5799	-0.13209	0.491015	0.827169	protein_coding
ENSG0000(	POC1B	2087.954	-0.04031	0.491139	0.827194	protein_coding
ENSG0000(	LINC02025	55.74635	0.124614	0.491232	0.8273	lncRNA
ENSG0000(	ENSG0000(	57.48702	-0.13604	0.49132	0.827398	lncRNA
ENSG0000(	ENSG0000(	446.9351	0.04986	0.491397	0.827399	lncRNA
ENSG0000(	ENSG0000(	27.19851	-0.17259	0.491411	0.827399	lncRNA
ENSG0000(	GATM	429.6379	-0.11642	0.491386	0.827399	protein_coding
ENSG0000(	RAB17	589.9456	0.176528	0.491482	0.827468	protein_coding
ENSG0000(	LINC01226	1893.259	-0.05943	0.49156	0.827549	lncRNA
ENSG0000(	NHLRC4	21.26735	0.202974	0.491592	0.827551	protein_coding
ENSG0000(	ENSG0000(	49.11925	-0.13168	0.491634	0.827572	lncRNA
ENSG0000(	AARS1	1965.274	-0.07211	0.49185	0.827652	protein_coding
ENSG0000(	CCT4	534.1228	-0.08728	0.491758	0.827652	protein_coding
ENSG0000(	ENSG0000(	91.48132	-0.10017	0.491901	0.827652	lncRNA
ENSG0000(	ENSG0000(	21.78013	-0.18566	0.491918	0.827652	lncRNA
ENSG0000(	ENSG0000(	121.8428	-0.11017	0.491922	0.827652	lncRNA
ENSG0000(	HNRNPAB	1360.493	0.088736	0.491805	0.827652	protein_coding
ENSG0000(	WDR72	192.5266	0.078099	0.49192	0.827652	protein_coding
ENSG0000(	ZBTB37	3355.429	0.051364	0.491792	0.827652	protein_coding
ENSG0000(	ENSG0000(	57.2785	-0.08579	0.49205	0.827665	lncRNA
ENSG0000(	KCNC4	2014.569	0.0899	0.492016	0.827665	protein_coding
ENSG0000(	TPCN1	3572.722	0.074759	0.492038	0.827665	protein_coding

ENSG0000(	YTHDF1	702.5855	0.042621	0.492021	0.827665	protein_coding
ENSG0000(	TNRC6C	1283.733	0.080143	0.492092	0.827684	protein_coding
ENSG0000(	GRM1	81.07885	0.174881	0.492158	0.827694	protein_coding
ENSG0000(	RRAGB	242.67	-0.07326	0.49215	0.827694	protein_coding
ENSG0000(	ENSG0000(	147.7969	0.088672	0.492239	0.82773	protein_coding
ENSG0000(	PIGF	525.0562	0.065317	0.492217	0.82773	protein_coding
ENSG0000(	DYM	1700.748	-0.04939	0.492305	0.82779	protein_coding
ENSG0000(	C2orf74	105.2503	0.117996	0.492433	0.827803	protein_coding
ENSG0000(	MLYCD	2541.531	0.052562	0.492432	0.827803	protein_coding
ENSG0000(	PIGZ	1489.06	0.214327	0.492416	0.827803	protein_coding
ENSG0000(	PLRG1	505.6574	-0.05168	0.492346	0.827803	protein_coding
ENSG0000(	ENSG0000(	64.04006	-0.12624	0.492488	0.827845	lncRNA
ENSG0000(	CYP2C19	946.7245	0.147841	0.492544	0.827853	protein_coding
ENSG0000(	ENSG0000(	125.8299	0.072598	0.492553	0.827853	lncRNA
ENSG0000(	ENSG0000(	204.3877	-0.08286	0.492627	0.827876	lncRNA
ENSG0000(	ENSG0000(	152.4784	-0.09438	0.492625	0.827876	lncRNA
ENSG0000(	ENSG0000(	1004.533	-0.06367	0.492667	0.827893	TEC
ENSG0000(	ENSG0000(	365.0066	-0.06956	0.492798	0.828061	TEC
ENSG0000(	OBI1	252.0304	0.074774	0.492827	0.828061	protein_coding
ENSG0000(	NOSIP	526.0956	0.068369	0.492903	0.828088	protein_coding
ENSG0000(	PCK2	759.6686	0.110666	0.492904	0.828088	protein_coding
ENSG0000(	ZNF155	205.381	0.102858	0.492984	0.828131	protein_coding
ENSG0000(	ZNF606	987.946	0.040286	0.49299	0.828131	protein_coding
ENSG0000(	RN7SL166F	62.97646	-0.13883	0.493092	0.828252	misc_RNA
ENSG0000(	ZFPM2-AS1	87.84597	0.112243	0.493126	0.828259	lncRNA
ENSG0000(	CNOT1	6765.924	0.038418	0.493181	0.828301	protein_coding
ENSG0000(	DELEC1	656.9555	-0.05855	0.49353	0.828369	lncRNA
ENSG0000(	ENSG0000(	92.96524	0.0971	0.493386	0.828369	lncRNA
ENSG0000(	ENSG0000(	87.09673	0.108158	0.493429	0.828369	TEC
ENSG0000(	ENSG0000(	532.7078	-0.06708	0.493613	0.828369	protein_coding
ENSG0000(	FAM245A	97.79564	0.082911	0.493585	0.828369	lncRNA
ENSG0000(	KLHL33	887.7731	-0.0609	0.493512	0.828369	protein_coding
ENSG0000(	LINC00882	1644.998	-0.05178	0.493492	0.828369	lncRNA
ENSG0000(	LINC02101	29.67507	-0.24711	0.493366	0.828369	lncRNA
ENSG0000(	PUS7L	3239.989	-0.03978	0.493302	0.828369	protein_coding
ENSG0000(	TMOD3	4162.099	-0.0482	0.493556	0.828369	protein_coding
ENSG0000(	TNFRSF10E	1668.464	-0.06243	0.493461	0.828369	protein_coding
ENSG0000(	UBE3D	489.6583	0.061157	0.493598	0.828369	protein_coding
ENSG0000(	WWTR1-IT:	123.6205	-0.07116	0.493253	0.828369	lncRNA
ENSG0000(	IRF2BP1	157.3917	0.063501	0.493653	0.828386	protein_coding
ENSG0000(	CYP4F62P	23.08038	-0.54047	0.493684	0.828387	transcribed_unprocessed_pseudo
ENSG0000(	COMMD3	386.6025	0.074948	0.493827	0.828577	protein_coding
ENSG0000(	HMGB1P31	26.75444	-0.15169	0.493861	0.828583	processed_pseudogene
ENSG0000(	ACSL3	1885.85	-0.06717	0.494784	0.828593	protein_coding
ENSG0000(	ADAM32	4841.242	-0.04148	0.494144	0.828593	protein_coding
ENSG0000(	AMDHD1	65.92747	0.212786	0.494338	0.828593	protein_coding
ENSG0000(	APOBEC3B-	20.79397	0.214653	0.494423	0.828593	lncRNA

ENSG0000(	ATRIP	270.3129	0.067208	0.49429	0.828593	protein_coding
ENSG0000(	BTC	132.6476	0.134903	0.493972	0.828593	protein_coding
ENSG0000(	CCL8	117.2608	-0.22441	0.494194	0.828593	protein_coding
ENSG0000(	CDK5R1	163.6012	-0.19083	0.494573	0.828593	protein_coding
ENSG0000(	CSF3	136.9471	-2.1471	0.494072	0.828593	protein_coding
ENSG0000(	CYP2T1P	131.2824	-0.08045	0.494346	0.828593	transcribed_unitary_pseudogene
ENSG0000(	EDEM2	320.1399	-0.06627	0.494161	0.828593	protein_coding
ENSG0000(	ENSG0000(	205.5235	0.103351	0.494441	0.828593	lncRNA
ENSG0000(	ENSG0000(	45.51209	0.126168	0.494476	0.828593	lncRNA
ENSG0000(	ENSG0000(	636.8501	-0.0865	0.494686	0.828593	lncRNA
ENSG0000(	ENSG0000(	122.7464	-0.1447	0.494111	0.828593	lncRNA
ENSG0000(	ENSG0000(	109.5628	-0.37358	0.494434	0.828593	lncRNA
ENSG0000(	ENSG0000(	837.3207	-0.0632	0.494269	0.828593	TEC
ENSG0000(	ENSG0000(	222.8774	0.067126	0.494487	0.828593	lncRNA
ENSG0000(	ENSG0000(	43.36829	-0.13806	0.494428	0.828593	lncRNA
ENSG0000(	ENSG0000(	122.3471	0.087851	0.494489	0.828593	lncRNA
ENSG0000(	LINC01972	64.14788	-0.10662	0.49446	0.828593	lncRNA
ENSG0000(	LTF	122.6189	-0.30278	0.4948	0.828593	protein_coding
ENSG0000(	PDCD11	1016.81	-0.05143	0.494524	0.828593	protein_coding
ENSG0000(	POP7	70.8052	-0.10352	0.494672	0.828593	protein_coding
ENSG0000(	RARA-AS1	42.96882	-0.17611	0.494238	0.828593	lncRNA
ENSG0000(	RPLP2	1388.877	0.092772	0.494788	0.828593	protein_coding
ENSG0000(	RRM2B	469.1136	-0.09061	0.494408	0.828593	protein_coding
ENSG0000(	SAMM50	748.3915	0.06978	0.494655	0.828593	protein_coding
ENSG0000(	SEMA3F-AS	53.10205	0.116167	0.4948	0.828593	lncRNA
ENSG0000(	STK10	2046.281	-0.07055	0.494777	0.828593	protein_coding
ENSG0000(	SYCP2L	43.8803	-0.2608	0.494565	0.828593	protein_coding
ENSG0000(	CAMSAP1	676.1611	0.09246	0.495095	0.828918	protein_coding
ENSG0000(	DDX11L2	35.30662	0.312537	0.495144	0.828918	unprocessed_pseudogene
ENSG0000(	ENSG0000(	86.20254	0.19927	0.495361	0.828918	processed_pseudogene
ENSG0000(	ENSG0000(	116.9407	-0.07388	0.495399	0.828918	lncRNA
ENSG0000(	GTF3C2-AS	204.6639	0.056267	0.495417	0.828918	lncRNA
ENSG0000(	LINC00628	59.76807	0.157201	0.495334	0.828918	lncRNA
ENSG0000(	NAV2-AS5	31.82965	-0.16827	0.495103	0.828918	lncRNA
ENSG0000(	NR2F2	874.1928	-0.10795	0.495266	0.828918	protein_coding
ENSG0000(	PKD1L3	58.39042	-0.20897	0.495388	0.828918	protein_coding
ENSG0000(	PPP1CA	819.2354	0.07083	0.495316	0.828918	protein_coding
ENSG0000(	REXO5	139.8249	0.096332	0.495224	0.828918	protein_coding
ENSG0000(	SNORA57	440.2049	0.108237	0.495375	0.828918	snoRNA
ENSG0000(	TMPRSS2	6243.84	0.154914	0.495366	0.828918	protein_coding
ENSG0000(	TONSL	791.3049	0.051391	0.495096	0.828918	protein_coding
ENSG0000(	TPD52L1	875.0245	-0.05049	0.495474	0.828964	protein_coding
ENSG0000(	LINC02069	147.4309	-0.0782	0.495519	0.828988	lncRNA
ENSG0000(	AUP1	1135.162	0.053258	0.495645	0.829106	protein_coding
ENSG0000(	CYP27B1	76.82332	-0.25788	0.49577	0.829106	protein_coding
ENSG0000(	ENSG0000(	894.1475	0.052362	0.495714	0.829106	lncRNA
ENSG0000(	ENSG0000(	28.84417	-0.16254	0.49574	0.829106	TEC

ENSG0000(	HSPA8P15	53.37772	0.120534	0.495655	0.829106	processed_pseudogene
ENSG0000(	ICA1L	684.6262	0.068985	0.495751	0.829106	protein_coding
ENSG0000(	ENSG0000(	19.80881	-0.16618	0.495845	0.82913	lncRNA
ENSG0000(	LINC01879	28.72421	0.150053	0.495818	0.82913	lncRNA
ENSG0000(	ENSG0000(	120.918	0.098892	0.496077	0.829144	lncRNA
ENSG0000(	ENSG0000(	311.0731	0.086567	0.496118	0.829144	lncRNA
ENSG0000(	HCST	20.51512	-0.24142	0.495946	0.829144	protein_coding
ENSG0000(	IGBP1	359.2753	0.071872	0.49609	0.829144	protein_coding
ENSG0000(	RN7SL608F	74.11802	-0.0989	0.496036	0.829144	misc_RNA
ENSG0000(	SLC1A2	1074.048	0.064209	0.496124	0.829144	protein_coding
ENSG0000(	SPTAN1	7019.602	-0.06043	0.496008	0.829144	protein_coding
ENSG0000(	UNC50	373.4177	0.069372	0.496083	0.829144	protein_coding
ENSG0000(	VTA1	657.109	0.080322	0.49597	0.829144	protein_coding
ENSG0000(	CCT6P1	208.4893	0.088042	0.4963	0.829387	transcribed_unprocessed_pseudo
ENSG0000(	ENSG0000(	19.89171	0.179869	0.496412	0.829439	lncRNA
ENSG0000(	ENSG0000(	83.96483	-0.11411	0.496486	0.829439	TEC
ENSG0000(	HSD17B7	758.4106	-0.07264	0.496405	0.829439	protein_coding
ENSG0000(	LINC02021	852.7868	0.078854	0.496542	0.829439	lncRNA
ENSG0000(	RAB6C-AS1	49.68594	-0.11994	0.496437	0.829439	transcribed_unprocessed_pseudo
ENSG0000(	SLFN5	3294.32	-0.13864	0.496523	0.829439	protein_coding
ENSG0000(	SNORD116	47.25653	-0.17015	0.496452	0.829439	snoRNA
ENSG0000(	ENSG0000(	297.0167	-0.09467	0.496604	0.829442	lncRNA
ENSG0000(	MYO16	103.3654	-0.15692	0.496604	0.829442	protein_coding
ENSG0000(	ABHD14A-/	101.8041	0.144541	0.496742	0.829445	protein_coding
ENSG0000(	CATSPERB	616.1496	-0.13126	0.496682	0.829445	protein_coding
ENSG0000(	FLT4	263.0809	-0.15386	0.496757	0.829445	protein_coding
ENSG0000(	RAB4B	1078.286	0.058594	0.496734	0.829445	protein_coding
ENSG0000(	TM6SF2	79.55291	0.279052	0.496649	0.829445	protein_coding
ENSG0000(	ENSG0000(	78.46167	0.101432	0.496804	0.829474	TEC
ENSG0000(	ABCC6P1	326.6611	-0.17968	0.497261	0.829584	transcribed_unprocessed_pseudo
ENSG0000(	ALG8	1918.56	0.034388	0.497282	0.829584	protein_coding
ENSG0000(	CLIC1	1131.922	-0.10438	0.497261	0.829584	protein_coding
ENSG0000(	ENSG0000(	33.63151	0.155302	0.497241	0.829584	lncRNA
ENSG0000(	ENSG0000(	87.06426	0.091234	0.497085	0.829584	processed_pseudogene
ENSG0000(	ENSG0000(	44.93811	-0.13179	0.497273	0.829584	lncRNA
ENSG0000(	ENSG0000(	39.8285	-0.92103	0.497306	0.829584	processed_pseudogene
ENSG0000(	KIF9-AS1	813.6226	-0.03234	0.497247	0.829584	lncRNA
ENSG0000(	PANK2-AS1	44.47458	-0.13524	0.497036	0.829584	lncRNA
ENSG0000(	POLR3G	115.625	-0.09885	0.497313	0.829584	protein_coding
ENSG0000(	PRKXP1	384.5762	-0.1609	0.497084	0.829584	processed_pseudogene
ENSG0000(	RPL7AP22	65.07985	0.114764	0.497322	0.829584	processed_pseudogene
ENSG0000(	SLC44A2	1639.7	0.060604	0.497188	0.829584	protein_coding
ENSG0000(	TMEM198	57.73759	-0.14301	0.496953	0.829584	protein_coding
ENSG0000(	ZFP30	675.1175	0.063177	0.496919	0.829584	protein_coding
ENSG0000(	ENSG0000(	411.3859	0.070424	0.497399	0.829648	lncRNA
ENSG0000(	IL32	860.7264	-0.13768	0.497421	0.829648	protein_coding
ENSG0000(	ENSG0000(	191.335	0.069472	0.497452	0.829649	lncRNA

ENSG0000(	CAPN6	35.69553	-0.23611	0.49754	0.829711	protein_coding
ENSG0000(	ENSG0000(	190.9938	0.062125	0.497564	0.829711	lncRNA
ENSG0000(	SCO1	2264.065	-0.06597	0.497579	0.829711	protein_coding
ENSG0000(	ENSG0000(	59.05023	0.095968	0.497753	0.82984	lncRNA
ENSG0000(	LINC01532	22.17888	0.189287	0.497755	0.82984	lncRNA
ENSG0000(	SAMD1	440.6271	0.067071	0.497778	0.82984	protein_coding
ENSG0000(	USP28	739.3416	-0.07422	0.497691	0.82984	protein_coding
ENSG0000(	CCDC194	59.86763	-0.14136	0.497956	0.829909	protein_coding
ENSG0000(	CDNF	46.61359	0.13164	0.497924	0.829909	protein_coding
ENSG0000(	ENSG0000(	54.58102	-0.10417	0.49788	0.829909	lncRNA
ENSG0000(	PRLR	852.0055	0.166135	0.497969	0.829909	protein_coding
ENSG0000(	RPL36AL	449.4982	-0.09255	0.497912	0.829909	protein_coding
ENSG0000(	MAP6	51.34848	-0.19571	0.498041	0.829927	protein_coding
ENSG0000(	MOAP1	417.868	0.041475	0.49804	0.829927	protein_coding
ENSG0000(	MTND4P9	28.85023	0.18779	0.498072	0.829929	unprocessed_pseudogene
ENSG0000(	ENSG0000(	90.01366	0.087778	0.498356	0.829933	lncRNA
ENSG0000(	ENSG0000(	120.8495	-0.09206	0.498352	0.829933	processed_pseudogene
ENSG0000(	ENSG0000(	111.2069	-0.0878	0.498223	0.829933	lncRNA
ENSG0000(	ENSG0000(	49.01771	-0.13172	0.498313	0.829933	lncRNA
ENSG0000(	EXOC1	1357.156	0.05464	0.49829	0.829933	protein_coding
ENSG0000(	FUCA2	842.7169	0.04656	0.498391	0.829933	protein_coding
ENSG0000(	KCTD13	1131.085	0.057336	0.498304	0.829933	protein_coding
ENSG0000(	LINC00944	137.5154	0.092165	0.498119	0.829933	lncRNA
ENSG0000(	LINC01119	69.14228	0.179845	0.498406	0.829933	lncRNA
ENSG0000(	PPIAP2	28.37744	0.141133	0.498349	0.829933	processed_pseudogene
ENSG0000(	TSNAX	1013.517	-0.04734	0.498295	0.829933	protein_coding
ENSG0000(	ENSG0000(	418.104	-0.08611	0.498492	0.830002	lncRNA
ENSG0000(	POLH-AS1	184.1858	0.07952	0.498508	0.830002	lncRNA
ENSG0000(	TNFRSF14	2746.97	0.048099	0.498575	0.830063	protein_coding
ENSG0000(	ENSG0000(	657.8276	0.066475	0.4987	0.830198	lncRNA
ENSG0000(	LRRC37A5F	119.2843	0.061919	0.498716	0.830198	transcribed_unprocessed_pseudo
ENSG0000(	MYEF2	862.7355	0.062538	0.49875	0.830204	protein_coding
ENSG0000(	ENSG0000(	93.80561	0.080763	0.498795	0.830228	lncRNA
ENSG0000(	RNFT1	527.5129	0.068328	0.498923	0.830392	protein_coding
ENSG0000(	DBNDD1	68.83803	-0.16641	0.499119	0.830395	protein_coding
ENSG0000(	ENSG0000(	63.45354	-0.10287	0.499137	0.830395	lncRNA
ENSG0000(	ENSG0000(	663.0302	-0.04405	0.499077	0.830395	TEC
ENSG0000(	ENSG0000(	36.48487	0.121938	0.498975	0.830395	lncRNA
ENSG0000(	PHACTR3	272.6544	-0.06876	0.499055	0.830395	protein_coding
ENSG0000(	SLC16A7	1223.625	-0.0857	0.499085	0.830395	protein_coding
ENSG0000(	TM4SF18	107.2382	-0.13324	0.499082	0.830395	protein_coding
ENSG0000(	MYOM2	39.8462	0.222024	0.499283	0.830589	protein_coding
ENSG0000(	ENSG0000(	436.7268	0.089869	0.499419	0.830675	TEC
ENSG0000(	HOXD1	58.67319	0.212871	0.49941	0.830675	protein_coding
ENSG0000(	METTL2B	2365.028	0.043026	0.499455	0.830675	protein_coding
ENSG0000(	MYOT	42.51329	0.20898	0.499449	0.830675	protein_coding
ENSG0000(	ENSG0000(	50.4916	-0.10401	0.499502	0.830701	lncRNA

ENSG0000(	APOBEC1	51.44549	-0.23102	0.499779	0.830873	protein_coding
ENSG0000(	CHD1	3582.09	0.050453	0.499786	0.830873	protein_coding
ENSG0000(	ENSG0000(	22.57084	-0.13921	0.499719	0.830873	lncRNA
ENSG0000(	ENSG0000(	29.87125	0.213678	0.499733	0.830873	lncRNA
ENSG0000(	LRRC8E	159.6433	-0.06035	0.499722	0.830873	protein_coding
ENSG0000(	VSX1	26.14331	0.144581	0.499785	0.830873	protein_coding
ENSG0000(	CYP2B6	474.491	0.241902	0.49984	0.830912	protein_coding
ENSG0000(	RSBN1L	1567.161	-0.03983	0.499873	0.830916	protein_coding
ENSG0000(	SPATA18	256.6886	0.132657	0.499914	0.830934	protein_coding
ENSG0000(	ENSG0000(	86.4732	0.090071	0.499949	0.830943	lncRNA
ENSG0000(	BASP1-AS1	926.6978	0.039839	0.500043	0.830944	lncRNA
ENSG0000(	ENSG0000(	20.87001	-0.16663	0.500032	0.830944	lncRNA
ENSG0000(	ENSG0000(	203.8037	-0.05414	0.500077	0.830944	lncRNA
ENSG0000(	PSMD12	1044.586	-0.0684	0.500101	0.830944	protein_coding
ENSG0000(	STARD3	782.8314	0.072476	0.499991	0.830944	protein_coding
ENSG0000(	ENSG0000(	70.62502	0.119892	0.50014	0.83096	lncRNA
ENSG0000(	ADIPOR2	1919.015	0.066018	0.500196	0.831002	protein_coding
ENSG0000(	ENSG0000(	164.7874	0.07764	0.500239	0.831008	lncRNA
ENSG0000(	ULK4	636.9878	-0.05934	0.50026	0.831008	protein_coding
ENSG0000(	RPS28	850.6564	0.111509	0.500312	0.83102	protein_coding
ENSG0000(	VPS26A	654.7853	-0.10185	0.500328	0.83102	protein_coding
ENSG0000(	ARID3B	832.5277	0.042252	0.500374	0.831048	protein_coding
ENSG0000(	HS3ST1	353.4849	-0.15298	0.500406	0.83105	protein_coding
ENSG0000(	HOXB-AS3	24.96323	-0.19658	0.500502	0.831159	lncRNA
ENSG0000(	ENSG0000(	275.6567	-0.05434	0.500557	0.831199	TEC
ENSG0000(	ENSG0000(	278.1965	0.064927	0.500658	0.831273	lncRNA
ENSG0000(	MIR193BH(	1275.898	-0.06326	0.500661	0.831273	lncRNA
ENSG0000(	FHOD1	509.978	-0.10061	0.500693	0.831276	protein_coding
ENSG0000(	ITPKC	529.7219	-0.10005	0.500792	0.83136	protein_coding
ENSG0000(	OR3A3	98.61016	-0.0876	0.500804	0.83136	protein_coding
ENSG0000(	SORD2P	82.06713	-0.33858	0.500868	0.831416	transcribed_unprocessed_pseudo
ENSG0000(	RNU4ATAC	28.64191	-0.14814	0.500951	0.831503	snRNA
ENSG0000(	ARHGAP18	989.8896	-0.07912	0.50107	0.831543	protein_coding
ENSG0000(	C12orf29	286.6038	0.069133	0.501178	0.831543	protein_coding
ENSG0000(	ENSG0000(	61.80099	0.110303	0.501147	0.831543	lncRNA
ENSG0000(	NFIX	887.5858	0.106465	0.501181	0.831543	protein_coding
ENSG0000(	PARP10	1005.808	-0.08845	0.501021	0.831543	protein_coding
ENSG0000(	RPL9P7	25.53477	0.342719	0.501076	0.831543	processed_pseudogene
ENSG0000(	SI	773.4444	0.223371	0.501191	0.831543	protein_coding
ENSG0000(	TOP3B	723.2668	-0.04744	0.501217	0.831543	protein_coding
ENSG0000(	ANAPC13	249.0348	0.086547	0.501404	0.831555	protein_coding
ENSG0000(	EIF3F	2458.996	0.063587	0.50135	0.831555	protein_coding
ENSG0000(	ENSG0000(	97.24559	0.097474	0.501436	0.831555	lncRNA
ENSG0000(	IL10RB	927.9177	0.063964	0.501352	0.831555	protein_coding
ENSG0000(	MIR3975	27.35125	-0.19814	0.501413	0.831555	miRNA
ENSG0000(	RPAP3	544.2802	-0.06553	0.501419	0.831555	protein_coding
ENSG0000(	WBP2	984.2693	-0.04932	0.501432	0.831555	protein_coding

ENSG0000(	COPS7A	476.2447	-0.05138	0.501619	0.831615	protein_coding
ENSG0000(	ENSG0000(	78.92038	-0.10616	0.50162	0.831615	lncRNA
ENSG0000(	FOXP1	9101.246	0.055753	0.501617	0.831615	protein_coding
ENSG0000(	MYO3B	252.9604	0.054909	0.501623	0.831615	protein_coding
ENSG0000(	ZNF483	1126.177	0.043988	0.501618	0.831615	protein_coding
ENSG0000(	ENSG0000(	27.85892	0.152394	0.501762	0.831669	lncRNA
ENSG0000(	ENSG0000(	37.54234	-0.15208	0.501751	0.831669	lncRNA
ENSG0000(	GSTP1	536.3941	-0.10137	0.501807	0.831669	protein_coding
ENSG0000(	LINC00032	91.95102	0.147701	0.501699	0.831669	transcribed_unprocessed_pseudo
ENSG0000(	LVRN	43.91738	-0.96732	0.501793	0.831669	protein_coding
ENSG0000(	ZFP2	115.6797	0.097062	0.501849	0.831689	protein_coding
ENSG0000(	FCGR3B	283.541	-1.45197	0.501909	0.831738	protein_coding
ENSG0000(	HSFX1	23.22701	0.695132	0.502057	0.831933	protein_coding
ENSG0000(	RNF169	2062.756	-0.0412	0.502133	0.83201	protein_coding
ENSG0000(	FAM230G	208.8521	0.360354	0.502233	0.832125	lncRNA
ENSG0000(	AURKB	194.5038	-0.12953	0.502381	0.832166	protein_coding
ENSG0000(	CYBRD1	1436.28	-0.07441	0.502292	0.832166	protein_coding
ENSG0000(	ENSG0000(	22.87691	0.647791	0.502403	0.832166	lncRNA
ENSG0000(	ENSG0000(	1231.55	-0.03185	0.502322	0.832166	TEC
ENSG0000(	RN7SL736F	49.50591	-0.17774	0.502409	0.832166	misc_RNA
ENSG0000(	GALK1	78.91889	-0.11291	0.5025	0.832266	protein_coding
ENSG0000(	ARL4D	19.84077	0.209203	0.502594	0.83234	protein_coding
ENSG0000(	ENSG0000(	220.2208	-0.06415	0.50265	0.83234	lncRNA
ENSG0000(	GOLPH3L	1032.763	0.084312	0.502682	0.83234	protein_coding
ENSG0000(	METTL18	80.66089	-0.12359	0.502631	0.83234	protein_coding
ENSG0000(	PRDM10	636.3853	0.087786	0.502696	0.83234	protein_coding
ENSG0000(	TOX2	92.12193	-0.16492	0.502757	0.832392	protein_coding
ENSG0000(	KLK2	114.9575	-0.09197	0.50289	0.832561	protein_coding
ENSG0000(	IPCEF1	607.8117	-0.16639	0.502958	0.832623	protein_coding
ENSG0000(	ENSG0000(	30.86206	-0.17809	0.503025	0.832685	lncRNA
ENSG0000(	SON	13903.26	-0.03323	0.50306	0.832692	protein_coding
ENSG0000(	TRIM61	229.5007	0.064504	0.503341	0.833108	protein_coding
ENSG0000(	TMCO5B	321.135	0.300519	0.503509	0.833335	transcribed_unitary_pseudogene
ENSG0000(	ENSG0000(	121.656	-0.0853	0.503547	0.833349	lncRNA
ENSG0000(	AARD	288.1081	0.060372	0.50373	0.833351	protein_coding
ENSG0000(	ADCY10	59.01997	0.221911	0.503711	0.833351	protein_coding
ENSG0000(	NCOA4	2519.497	-0.07275	0.503724	0.833351	protein_coding
ENSG0000(	RASD1	79.65944	0.214061	0.503616	0.833351	protein_coding
ENSG0000(	RPF1	218.2093	-0.08284	0.503614	0.833351	protein_coding
ENSG0000(	SEMA3G	105.7327	0.159639	0.503721	0.833351	protein_coding
ENSG0000(	RN7SL754F	30.99552	-0.14289	0.503768	0.833363	misc_RNA
ENSG0000(	CDKN1A	1746.593	-0.11973	0.503824	0.833406	protein_coding
ENSG0000(	CHPF	329.3241	-0.12923	0.503955	0.833495	protein_coding
ENSG0000(	H2BC8	130.9766	-0.11456	0.503929	0.833495	protein_coding
ENSG0000(	ZNF646	1115.758	-0.08807	0.503969	0.833495	protein_coding
ENSG0000(	COPRS	82.71111	-0.11002	0.504092	0.833548	protein_coding
ENSG0000(	ENSG0000(	230.6458	-0.06068	0.504079	0.833548	lncRNA

ENSG0000(	HAUS5	400.9003	-0.07031	0.504085	0.833548	protein_coding
ENSG0000(	ENSG0000(	367.2005	-0.15383	0.504133	0.833566	lncRNA
ENSG0000(	ENSG0000(	137.5736	0.119434	0.504366	0.833701	processed_pseudogene
ENSG0000(	PTPRB	2478.904	-0.11007	0.504312	0.833701	protein_coding
ENSG0000(	SSBP1	959.5218	0.041822	0.504324	0.833701	protein_coding
ENSG0000(	TMC6	2879.803	-0.07772	0.504246	0.833701	protein_coding
ENSG0000(	TMEM260	1082.791	-0.04764	0.504348	0.833701	protein_coding
ENSG0000(	GLB1	671.8983	-0.0599	0.504439	0.833772	protein_coding
ENSG0000(	DXO	152.1168	-0.081	0.504513	0.833844	protein_coding
ENSG0000(	ENSG0000(	118.5813	-0.09678	0.50456	0.833872	lncRNA
ENSG0000(	ARHGAP12	1818.446	0.049456	0.50472	0.833886	protein_coding
ENSG0000(	ENSG0000(	23.50544	0.182616	0.504665	0.833886	lncRNA
ENSG0000(	ENSG0000(	310.0796	0.07823	0.5047	0.833886	lncRNA
ENSG0000(	PREP	1635.975	0.064826	0.504608	0.833886	protein_coding
ENSG0000(	SKI	1445.298	0.088345	0.504675	0.833886	protein_coding
ENSG0000(	AATBC	93.3737	-0.24205	0.50477	0.833899	lncRNA
ENSG0000(	C19orf38	49.80138	-0.15456	0.504819	0.833899	protein_coding
ENSG0000(	ENSG0000(	25.24945	-0.26959	0.504818	0.833899	processed_pseudogene
ENSG0000(	UBN2	4231.816	-0.02386	0.504906	0.833992	protein_coding
ENSG0000(	UBR3	2078.92	-0.05823	0.504938	0.833995	protein_coding
ENSG0000(	CAPN11	23.03402	-0.25252	0.505203	0.834034	protein_coding
ENSG0000(	CDH2	64.8942	-0.12114	0.50505	0.834034	protein_coding
ENSG0000(	GSTK1	2686.282	0.055673	0.505185	0.834034	protein_coding
ENSG0000(	KPNA2P3	231.816	0.145921	0.505206	0.834034	transcribed_unprocessed_pseudo
ENSG0000(	LIAS	2086.365	0.03809	0.505054	0.834034	protein_coding
ENSG0000(	LINC02217	198.7438	0.08445	0.505271	0.834034	lncRNA
ENSG0000(	LINC02453	121.2731	0.103735	0.505248	0.834034	lncRNA
ENSG0000(	LOH12CR2	218.4116	0.08921	0.505295	0.834034	lncRNA
ENSG0000(	MRI1	876.0869	0.057263	0.505188	0.834034	protein_coding
ENSG0000(	PPP1R37	820.9404	0.05486	0.505262	0.834034	protein_coding
ENSG0000(	RPL7L1P10	27.96521	-0.1885	0.505028	0.834034	processed_pseudogene
ENSG0000(	GRAMD4P3	637.9149	-0.1117	0.505436	0.834067	processed_pseudogene
ENSG0000(	IAH1	1000.658	0.047853	0.50543	0.834067	protein_coding
ENSG0000(	SPTY2D1OS	78.81693	0.106159	0.505428	0.834067	protein_coding
ENSG0000(	TMEM74B	142.5571	-0.11936	0.505351	0.834067	protein_coding
ENSG0000(	CCNE1	83.87643	0.108369	0.505571	0.834181	protein_coding
ENSG0000(	ENSG0000(	464.429	-0.04596	0.505649	0.834181	lncRNA
ENSG0000(	ENSG0000(	66.14354	-0.12032	0.505657	0.834181	lncRNA
ENSG0000(	FAXC	268.377	0.069399	0.505639	0.834181	protein_coding
ENSG0000(	NOL7	224.1786	0.084994	0.505593	0.834181	protein_coding
ENSG0000(	PRMT5-DT	25.86636	-0.23819	0.505734	0.834207	lncRNA
ENSG0000(	RN7SL502P	31.32249	-0.18743	0.505764	0.834207	misc_RNA
ENSG0000(	SALRNA2	20.08039	-0.16097	0.505752	0.834207	lncRNA
ENSG0000(	ALG2	567.7731	-0.06671	0.505962	0.834277	protein_coding
ENSG0000(	ANOS1	252.7783	-0.17387	0.506143	0.834277	protein_coding
ENSG0000(	C10orf88B	42.54416	-0.14322	0.5061	0.834277	transcribed_processed_pseudoge
ENSG0000(	CPN2	116.7331	-0.109	0.505976	0.834277	protein_coding

ENSG0000(	ENSG0000(	157.9225	0.183631	0.506005	0.834277	processed_pseudogene
ENSG0000(	ENSG0000(	20.4272	0.180334	0.50598	0.834277	transcribed_processed_pseudoge
ENSG0000(	ENSG0000(	20.1992	0.196554	0.506144	0.834277	lncRNA
ENSG0000(	ENSG0000(	489.9268	-0.05436	0.50596	0.834277	TEC
ENSG0000(	FMN2	72.38785	0.150863	0.506157	0.834277	protein_coding
ENSG0000(	MCM3AP	3350.262	0.042987	0.50615	0.834277	protein_coding
ENSG0000(	SSTR3	37.28114	-0.22246	0.50617	0.834277	protein_coding
ENSG0000(	TRAPPC5	1172.723	0.060709	0.505839	0.834277	protein_coding
ENSG0000(	ENSG0000(	617.9043	-0.09845	0.50621	0.834294	lncRNA
ENSG0000(	CLP1	80.76105	-0.11152	0.506311	0.83441	protein_coding
ENSG0000(	RN7SL606F	38.9631	-0.11744	0.506519	0.834652	misc_RNA
ENSG0000(	TBC1D22B	454.479	0.051902	0.506489	0.834652	protein_coding
ENSG0000(	SUB1	1996.21	-0.05198	0.506605	0.834744	protein_coding
ENSG0000(	PMS2CL	640.4923	0.05537	0.506644	0.834758	transcribed_unprocessed_pseudo
ENSG0000(	ENSG0000(	104.3057	-0.19869	0.506698	0.834796	lncRNA
ENSG0000(	SLC30A10	3134.03	0.299021	0.506826	0.834957	protein_coding
ENSG0000(	ENSG0000(	65.87992	0.081288	0.506897	0.835024	TEC
ENSG0000(	DBF4B	642.2257	-0.04756	0.507086	0.835089	protein_coding
ENSG0000(	ENSG0000(	221.5238	-0.05485	0.507179	0.835089	lncRNA
ENSG0000(	ENSG0000(	512.0539	-0.0614	0.507127	0.835089	lncRNA
ENSG0000(	ENSG0000(	52.94083	-0.112	0.507008	0.835089	lncRNA
ENSG0000(	MAPK1	2337.805	-0.05321	0.50712	0.835089	protein_coding
ENSG0000(	MRPL30	911.0214	-0.04813	0.507177	0.835089	protein_coding
ENSG0000(	SEPHS1	490.3287	0.075344	0.507133	0.835089	protein_coding
ENSG0000(	TRMO	381.2706	0.082027	0.507123	0.835089	protein_coding
ENSG0000(	ENSG0000(	137.717	0.080942	0.507211	0.835092	lncRNA
ENSG0000(	ATP6V1G1I	26.86991	0.19612	0.507434	0.835107	processed_pseudogene
ENSG0000(	ENSG0000(	242.3563	-0.08446	0.507463	0.835107	lncRNA
ENSG0000(	ENSG0000(	242.9961	0.05804	0.507388	0.835107	TEC
ENSG0000(	ENSG0000(	279.099	0.058974	0.507292	0.835107	lncRNA
ENSG0000(	H3C7	78.53362	-0.19549	0.507431	0.835107	protein_coding
ENSG0000(	KIF18B	631.3208	-0.07667	0.507342	0.835107	protein_coding
ENSG0000(	RHOT1	863.0767	-0.06027	0.507387	0.835107	protein_coding
ENSG0000(	RTBDN	158.0818	0.073539	0.507336	0.835107	protein_coding
ENSG0000(	ENSG0000(	99.21679	-0.09803	0.507517	0.835146	lncRNA
ENSG0000(	ENSG0000(	107.5148	0.079269	0.507865	0.835319	lncRNA
ENSG0000(	ENSG0000(	24.24863	0.166369	0.507871	0.835319	lncRNA
ENSG0000(	FNTA	3149.716	-0.03407	0.507766	0.835319	protein_coding
ENSG0000(	GK-AS1	40.86813	-0.1226	0.50786	0.835319	lncRNA
ENSG0000(	HMGB3	409.4086	0.100931	0.507886	0.835319	protein_coding
ENSG0000(	NUP58	1433.263	-0.06829	0.507779	0.835319	protein_coding
ENSG0000(	POLK	1448.885	-0.05962	0.507895	0.835319	protein_coding
ENSG0000(	RPL29P24	32.83723	0.130318	0.507671	0.835319	processed_pseudogene
ENSG0000(	TNS1-AS1	82.39886	0.147083	0.507871	0.835319	lncRNA
ENSG0000(	KRT26	52.50149	0.165741	0.507941	0.83534	protein_coding
ENSG0000(	MIR3667H(	781.1835	-0.14805	0.507969	0.83534	lncRNA
ENSG0000(	ANK2	3422.996	0.053952	0.508033	0.835396	protein_coding

ENSG0000(	ENSG0000(	94.18016	0.080699	0.508287	0.835399	lncRNA
ENSG0000(	ENSG0000(	141.974	0.073502	0.508118	0.835399	lncRNA
ENSG0000(	EYA1	79.01737	-0.1224	0.508162	0.835399	protein_coding
ENSG0000(	FBXO25	1075.102	0.09345	0.508308	0.835399	protein_coding
ENSG0000(	PMCHL1	80.22438	-0.12711	0.508247	0.835399	transcribed_unprocessed_pseudo
ENSG0000(	SH3GL3	55.90612	0.121761	0.508213	0.835399	protein_coding
ENSG0000(	SPAG17	25.81744	-0.43078	0.508149	0.835399	protein_coding
ENSG0000(	URB2	408.4906	-0.08732	0.508264	0.835399	protein_coding
ENSG0000(	USP45	747.0063	0.062806	0.50812	0.835399	protein_coding
ENSG0000(	ARHGEF12	7271.176	-0.03229	0.508507	0.835529	protein_coding
ENSG0000(	ENSG0000(	27.65586	-0.33593	0.508494	0.835529	processed_pseudogene
ENSG0000(	ENSG0000(	120.5308	0.075356	0.508483	0.835529	TEC
ENSG0000(	HOXB7	164.8291	-0.09275	0.508509	0.835529	protein_coding
ENSG0000(	CDC27	1608.2	-0.09262	0.508554	0.835554	protein_coding
ENSG0000(	TBC1D8-AS	43.62318	-0.13572	0.508617	0.835606	lncRNA
ENSG0000(	OSBPL11	851.1092	0.091832	0.508714	0.835716	protein_coding
ENSG0000(	CYP1B1-AS	2317.568	-0.06806	0.508821	0.835727	lncRNA
ENSG0000(	LINC02381	136.9247	-0.08609	0.508857	0.835727	lncRNA
ENSG0000(	LINC02681	127.5084	0.084862	0.508872	0.835727	lncRNA
ENSG0000(	SLC2A12	38.51938	0.216254	0.508845	0.835727	protein_coding
ENSG0000(	TTLL1	46.04586	0.107456	0.508827	0.835727	protein_coding
ENSG0000(	GDF5	25.82336	0.192104	0.508909	0.835737	protein_coding
ENSG0000(	ST20	160.3389	-0.08918	0.508993	0.835825	protein_coding
ENSG0000(	NFE2L3P1	45.21118	0.190638	0.50903	0.835836	processed_pseudogene
ENSG0000(	MYT1L	1948.83	-0.02193	0.509164	0.836006	protein_coding
ENSG0000(	ENSG0000(	126.5824	0.070274	0.509206	0.836025	TEC
ENSG0000(	SOX13	1265.212	-0.06205	0.509255	0.836055	protein_coding
ENSG0000(	ENSG0000(	142.0487	-0.09195	0.509347	0.836138	protein_coding
ENSG0000(	SMG7-AS1	132.9969	-0.0718	0.509396	0.836138	lncRNA
ENSG0000(	TGIF2P1	34.00808	-0.13719	0.509396	0.836138	processed_pseudogene
ENSG0000(	ADAMTSL2	147.7777	0.079771	0.509517	0.836186	protein_coding
ENSG0000(	ENSG0000(	211.831	0.087304	0.509473	0.836186	lncRNA
ENSG0000(	GTF3C5	600.5675	-0.04891	0.509492	0.836186	protein_coding
ENSG0000(	ZSWIM3	144.9914	0.100532	0.50955	0.836191	protein_coding
ENSG0000(	ENSG0000(	48.07179	0.154708	0.50971	0.836205	lncRNA
ENSG0000(	KIF15	221.3013	-0.13546	0.509667	0.836205	protein_coding
ENSG0000(	LINC00592	49.14206	-0.1657	0.509605	0.836205	lncRNA
ENSG0000(	RNF112	130.688	0.166684	0.509694	0.836205	protein_coding
ENSG0000(	SREBF2-AS:	49.84972	0.112415	0.509674	0.836205	lncRNA
ENSG0000(	ENSG0000(	33.28069	-0.14507	0.509889	0.836448	lncRNA
ENSG0000(	PCDHGA2	35.10427	0.154973	0.510116	0.836573	protein_coding
ENSG0000(	PHKG2	975.8117	0.052723	0.510072	0.836573	protein_coding
ENSG0000(	RN7SL605F	345.0986	-0.06575	0.510117	0.836573	misc_RNA
ENSG0000(	TCN2	179.4506	-0.14632	0.510041	0.836573	protein_coding
ENSG0000(	TXNDC9	209.5337	-0.08379	0.510061	0.836573	protein_coding
ENSG0000(	ENSG0000(	42.44419	-0.14619	0.510149	0.836575	lncRNA
ENSG0000(	NOC2LP1	24.40018	0.236617	0.510251	0.836643	transcribed_processed_pseudoge

ENSG0000(	SDHCP3	42.09599	-0.10185	0.510235	0.836643	processed_pseudogene
ENSG0000(	PNMA1	342.6667	-0.07049	0.510286	0.836651	protein_coding
ENSG0000(	ENSG0000(	91.27159	0.080188	0.510339	0.836688	lncRNA
ENSG0000(	ARIH1	4184.219	-0.05098	0.511008	0.836781	protein_coding
ENSG0000(	CEP63	2441.776	0.029324	0.510677	0.836781	protein_coding
ENSG0000(	DCAF12	743.0681	-0.04661	0.51073	0.836781	protein_coding
ENSG0000(	ENSG0000(	61.57093	-0.12393	0.510758	0.836781	lncRNA
ENSG0000(	ENSG0000(	70.45236	-0.14217	0.511126	0.836781	lncRNA
ENSG0000(	ENSG0000(	61.23338	0.14027	0.510845	0.836781	lncRNA
ENSG0000(	ENSG0000(	38.21934	-0.09036	0.510938	0.836781	lncRNA
ENSG0000(	ENSG0000(	33.97211	-0.16477	0.510574	0.836781	lncRNA
ENSG0000(	HBA2	83.7504	-0.34022	0.510843	0.836781	protein_coding
ENSG0000(	HOXA10-AS	62.62952	0.106204	0.510927	0.836781	lncRNA
ENSG0000(	KIF11	452.4306	-0.08781	0.510667	0.836781	protein_coding
ENSG0000(	LANCL1	681.2182	0.069583	0.510482	0.836781	protein_coding
ENSG0000(	LCOR	7222.795	0.070866	0.510719	0.836781	protein_coding
ENSG0000(	LINC02031	50.37609	-0.11223	0.511042	0.836781	lncRNA
ENSG0000(	LINC02257	19.75312	-0.21123	0.510545	0.836781	lncRNA
ENSG0000(	LINC02632	256.7274	-0.07233	0.510987	0.836781	lncRNA
ENSG0000(	LINC02896	84.80467	-0.07938	0.510813	0.836781	lncRNA
ENSG0000(	LTBP4	2130.057	0.072936	0.510892	0.836781	protein_coding
ENSG0000(	SOS2	2811.226	0.067441	0.511126	0.836781	protein_coding
ENSG0000(	STARD9	3168.558	0.039158	0.511113	0.836781	protein_coding
ENSG0000(	TMEM263-	22.30774	-0.24577	0.511003	0.836781	lncRNA
ENSG0000(	TMEM30A-	309.049	-0.0814	0.510621	0.836781	lncRNA
ENSG0000(	TSPO	326.0596	0.099834	0.510486	0.836781	protein_coding
ENSG0000(	ZNF717	721.8737	0.061118	0.510718	0.836781	protein_coding
ENSG0000(	CCDC68	1647.398	0.128223	0.511413	0.836937	protein_coding
ENSG0000(	ENSG0000(	37.51113	-0.16136	0.511405	0.836937	lncRNA
ENSG0000(	ENSG0000(	37.44737	-0.20014	0.511456	0.836937	lncRNA
ENSG0000(	ENSG0000(	161.2762	-0.07527	0.51135	0.836937	lncRNA
ENSG0000(	ENSG0000(	43.06549	-0.10781	0.51146	0.836937	lncRNA
ENSG0000(	ENSG0000(	169.3103	0.08747	0.511465	0.836937	TEC
ENSG0000(	HUS1	1184.815	0.071989	0.511373	0.836937	protein_coding
ENSG0000(	PEX12	71.61671	-0.1166	0.511348	0.836937	protein_coding
ENSG0000(	ENSG0000(	176.7975	0.069615	0.511502	0.836948	lncRNA
ENSG0000(	DNAAF10	634.1353	0.058275	0.511908	0.837015	protein_coding
ENSG0000(	ENSG0000(	107.9033	0.10475	0.511716	0.837015	processed_pseudogene
ENSG0000(	ENSG0000(	85.14448	-0.08937	0.511717	0.837015	lncRNA
ENSG0000(	ENSG0000(	96.579	-0.13013	0.511803	0.837015	lncRNA
ENSG0000(	ENSG0000(	85.4986	-0.14166	0.511646	0.837015	lncRNA
ENSG0000(	JTB	531.4213	-0.06937	0.511739	0.837015	protein_coding
ENSG0000(	LINC01290	23.56769	0.183013	0.51188	0.837015	lncRNA
ENSG0000(	LSM2	122.0363	-0.08855	0.511609	0.837015	protein_coding
ENSG0000(	PCDHGB1	42.29736	0.176326	0.51183	0.837015	protein_coding
ENSG0000(	RN7SL377P	123.1052	-0.07257	0.511835	0.837015	misc_RNA
ENSG0000(	RNU6-16P	93.67417	-0.12594	0.511856	0.837015	snRNA

ENSG0000(	SLC51A	560.9221	0.217762	0.511686	0.837015	protein_coding
ENSG0000(	PRKCI	1069.77	0.06146	0.511963	0.837055	protein_coding
ENSG0000(	TOMM40L	254.4716	-0.09021	0.51204	0.837132	protein_coding
ENSG0000(	LINC01667	59.66762	0.156459	0.512083	0.837151	transcribed_processed_pseudoge
ENSG0000(	ENSG0000(	24.57109	0.201838	0.512119	0.837156	lncRNA
ENSG0000(	NIPSNAP2	883.4499	0.055451	0.512146	0.837156	protein_coding
ENSG0000(	CENPK	534.5587	-0.06284	0.512178	0.837159	protein_coding
ENSG0000(	ENSG0000(	110.7178	-0.0958	0.512367	0.837363	lncRNA
ENSG0000(	ENSG0000(	201.1312	0.058568	0.512395	0.837363	lncRNA
ENSG0000(	TEKT2	40.22534	0.158101	0.512353	0.837363	protein_coding
ENSG0000(	TLR3	711.4227	-0.07873	0.512477	0.837448	protein_coding
ENSG0000(	SLC25A52	21.11414	-0.21419	0.512568	0.837547	protein_coding
ENSG0000(	DNASE1L1	365.9305	-0.0556	0.512653	0.837605	protein_coding
ENSG0000(	ENSG0000(	195.7827	0.085371	0.512695	0.837605	TEC
ENSG0000(	KNOP1	1237.305	-0.05316	0.512667	0.837605	protein_coding
ENSG0000(	ENSG0000(	127.5314	-0.08212	0.512789	0.837708	lncRNA
ENSG0000(	MED9	1647.719	0.051688	0.512825	0.837717	protein_coding
ENSG0000(	ENSG0000(	1594.938	-0.04702	0.512983	0.83778	TEC
ENSG0000(	LINC02785	91.84811	-0.16128	0.512985	0.83778	lncRNA
ENSG0000(	NT5C2	4722.094	0.084544	0.512977	0.83778	protein_coding
ENSG0000(	SPATA13	3699.865	0.062364	0.512927	0.83778	protein_coding
ENSG0000(	ANKRD20A	59.27982	0.243071	0.513123	0.83783	transcribed_unprocessed_pseudo
ENSG0000(	ENSG0000(	44.64806	0.491974	0.513174	0.83783	protein_coding
ENSG0000(	FBXO45	567.5289	-0.05772	0.513192	0.83783	protein_coding
ENSG0000(	PEMT	50.00404	-0.16497	0.513159	0.83783	protein_coding
ENSG0000(	PRELID1	603.2226	0.095092	0.513198	0.83783	protein_coding
ENSG0000(	XPNPEP2	73.77028	-0.32257	0.513154	0.83783	protein_coding
ENSG0000(	ARFGAP1	1666.705	-0.05128	0.51348	0.837845	protein_coding
ENSG0000(	CCT3	917.4208	-0.04214	0.513521	0.837845	protein_coding
ENSG0000(	CLTB	803.0618	0.116016	0.51332	0.837845	protein_coding
ENSG0000(	CRK	2170.818	0.039568	0.513395	0.837845	protein_coding
ENSG0000(	ENSG0000(	134.7182	0.086208	0.51345	0.837845	lncRNA
ENSG0000(	ENSG0000(	144.7116	0.084653	0.513488	0.837845	lncRNA
ENSG0000(	ENSG0000(	778.9683	-0.07583	0.513406	0.837845	lncRNA
ENSG0000(	EWSR1	6942.62	-0.03126	0.513494	0.837845	protein_coding
ENSG0000(	GINS4	633.8154	0.056983	0.513247	0.837845	protein_coding
ENSG0000(	HOXB9	1726.617	0.144021	0.513474	0.837845	protein_coding
ENSG0000(	LAGE3	36.90336	-0.1335	0.513542	0.837845	protein_coding
ENSG0000(	DLL1	515.3939	0.065307	0.513595	0.837882	protein_coding
ENSG0000(	GPA33	3272.544	0.168798	0.513789	0.837968	protein_coding
ENSG0000(	H4C2	118.5558	-0.13687	0.513708	0.837968	protein_coding
ENSG0000(	PMS2P3	246.7142	-0.1026	0.51379	0.837968	transcribed_unprocessed_pseudo
ENSG0000(	PRPH2	103.977	-0.12688	0.513801	0.837968	protein_coding
ENSG0000(	ZSCAN32	312.6041	0.053441	0.513712	0.837968	protein_coding
ENSG0000(	TRUB2	1604.038	0.033646	0.513843	0.837987	protein_coding
ENSG0000(	ENSG0000(	368872.1	0.292679	0.513971	0.838146	lncRNA
ENSG0000(	SNORA59B	58.51574	0.097166	0.514009	0.838159	snoRNA

ENSG0000(	ENSG0000(	157.1906	0.167902	0.514132	0.838161	lncRNA
ENSG0000(	IFT22	1477.822	0.034272	0.514097	0.838161	protein_coding
ENSG0000(	PEX11G	28.47126	0.172611	0.514075	0.838161	protein_coding
ENSG0000(	RN7SL650F	19.74702	0.381968	0.514121	0.838161	misc_RNA
ENSG0000(	ENSG0000(	129.5368	0.46743	0.514435	0.838555	lncRNA
ENSG0000(	MRNIP	1767.631	0.043964	0.514405	0.838555	protein_coding
ENSG0000(	ENSG0000(	491.7119	0.061156	0.514514	0.838585	lncRNA
ENSG0000(	RN7SL376F	98.40354	0.094343	0.514512	0.838585	misc_RNA
ENSG0000(	ENSG0000(	473.3779	0.062547	0.514599	0.838624	TEC
ENSG0000(	TIAM1	568.5244	-0.08852	0.514588	0.838624	protein_coding
ENSG0000(	GLMN	193.946	-0.09918	0.514669	0.838689	protein_coding
ENSG0000(	ACTR3C	1732.81	0.082726	0.514747	0.838715	protein_coding
ENSG0000(	IFNGR2	911.1177	-0.07373	0.514733	0.838715	protein_coding
ENSG0000(	NOP14	609.0585	-0.05655	0.514821	0.838737	protein_coding
ENSG0000(	TYK2	2467.141	-0.03662	0.514819	0.838737	protein_coding
ENSG0000(	ENSG0000(	869.0375	-0.03838	0.515007	0.838755	lncRNA
ENSG0000(	ENSG0000(	39.57181	0.115095	0.515066	0.838755	lncRNA
ENSG0000(	ENSG0000(	131.2139	0.080084	0.515063	0.838755	lncRNA
ENSG0000(	GLB1L	313.572	-0.06304	0.515076	0.838755	protein_coding
ENSG0000(	MS4A5	20.55007	0.209207	0.515073	0.838755	protein_coding
ENSG0000(	RN7SKP79	184.1823	0.105053	0.515044	0.838755	misc_RNA
ENSG0000(	SKP1	3004.845	-0.04738	0.515069	0.838755	protein_coding
ENSG0000(	SPIN4	162.3066	0.094779	0.515033	0.838755	protein_coding
ENSG0000(	ENSG0000(	136.2945	-0.07666	0.515212	0.838815	lncRNA
ENSG0000(	ENSG0000(	94.94962	-0.07691	0.515217	0.838815	lncRNA
ENSG0000(	ENSG0000(	62.13289	0.123109	0.515265	0.838815	lncRNA
ENSG0000(	GRIK3	125.194	-0.16041	0.515259	0.838815	protein_coding
ENSG0000(	OR6K3	100.5892	0.099495	0.515244	0.838815	protein_coding
ENSG0000(	ENSG0000(	109.2911	0.082005	0.515356	0.838891	miRNA
ENSG0000(	ENSG0000(	25.89102	-0.16546	0.515373	0.838891	processed_pseudogene
ENSG0000(	APLP1	153.4677	0.081442	0.515602	0.838999	protein_coding
ENSG0000(	COPS7B	1226.467	-0.0515	0.515509	0.838999	protein_coding
ENSG0000(	DDX31	346.5865	0.075678	0.515622	0.838999	protein_coding
ENSG0000(	ENSG0000(	90.4762	-0.09309	0.515497	0.838999	lncRNA
ENSG0000(	SPARCL1	794.6796	-0.13463	0.515592	0.838999	protein_coding
ENSG0000(	TMCC3	1706.678	0.133493	0.515571	0.838999	protein_coding
ENSG0000(	LMBR1	1407.521	-0.04443	0.515751	0.83916	protein_coding
ENSG0000(	PPHLN1	2841.507	0.042808	0.51584	0.839246	protein_coding
ENSG0000(	STIMATE	630.6156	-0.10336	0.515865	0.839246	protein_coding
ENSG0000(	ADAM33	548.1312	-0.12631	0.516044	0.839486	protein_coding
ENSG0000(	RHD	242.3099	0.057707	0.516086	0.839506	protein_coding
ENSG0000(	MYL6P4	22.75034	0.189507	0.516165	0.839585	processed_pseudogene
ENSG0000(	MRPL36	68.03328	-0.13127	0.516197	0.839587	protein_coding
ENSG0000(	CLEC18B	38.85785	0.324865	0.516532	0.839737	protein_coding
ENSG0000(	ENSG0000(	239.2647	-0.17036	0.516416	0.839737	lncRNA
ENSG0000(	ENSG0000(	331.0131	0.077291	0.516497	0.839737	lncRNA
ENSG0000(	NPRL3	422.8218	-0.06382	0.516533	0.839737	protein_coding

ENSG0000(	RD3	136.2182	0.072122	0.516341	0.839737	protein_coding
ENSG0000(	RNU6-750F	39.02393	0.111292	0.516442	0.839737	snRNA
ENSG0000(	SIAH3	105.3558	-0.08242	0.516406	0.839737	protein_coding
ENSG0000(	TTLL13P	25.53875	-0.27927	0.516403	0.839737	protein_coding
ENSG0000(	LRP10	3627.914	-0.08688	0.516567	0.839742	protein_coding
ENSG0000(	AIG1	711.7003	-0.0489	0.516607	0.839757	protein_coding
ENSG0000(	LINC02336	238.2905	-0.0581	0.516656	0.839787	lncRNA
ENSG0000(	SUPT20HL2	2916.921	0.197009	0.5167	0.83981	protein_coding
ENSG0000(	BBOF1	132.2801	0.121641	0.516808	0.83987	protein_coding
ENSG0000(	ENSG0000(	41.3602	-0.15551	0.516852	0.83987	lncRNA
ENSG0000(	ENSG0000(	201.9473	0.116647	0.51686	0.83987	lncRNA
ENSG0000(	SUOX	1230.038	-0.05201	0.516789	0.83987	protein_coding
ENSG0000(	CAPRIN1	3742.553	-0.03362	0.516989	0.839904	protein_coding
ENSG0000(	CYP46A1	1016.704	-0.05421	0.516976	0.839904	protein_coding
ENSG0000(	ENSG0000(	446.9136	-0.05652	0.517033	0.839904	lncRNA
ENSG0000(	FBN2	353.0895	-0.14418	0.516955	0.839904	protein_coding
ENSG0000(	MRPS26	144.7875	0.098402	0.517024	0.839904	protein_coding
ENSG0000(	SH3GLB1	2607.689	-0.05035	0.517075	0.839923	protein_coding
ENSG0000(	TCF23	609.5755	0.039593	0.51712	0.839946	protein_coding
ENSG0000(	MIR3659H(	30.63371	0.169077	0.517231	0.840063	lncRNA
ENSG0000(	SLC44A3-A:	1001.22	0.084831	0.517253	0.840063	transcribed_processed_pseudoge
ENSG0000(	ENSG0000(	138.9831	0.122331	0.517339	0.840087	lncRNA
ENSG0000(	LINC01144	48.89697	-0.11425	0.517418	0.840087	lncRNA
ENSG0000(	PLP1	89.41738	0.215298	0.51734	0.840087	protein_coding
ENSG0000(	SNORC	383.6549	0.114497	0.517421	0.840087	protein_coding
ENSG0000(	TP53BP1	2431.385	0.054006	0.517391	0.840087	protein_coding
ENSG0000(	EIF3FP3	33.48841	0.242322	0.517927	0.840215	processed_pseudogene
ENSG0000(	ENSG0000(	62.8238	-0.11962	0.51769	0.840215	lncRNA
ENSG0000(	ENSG0000(	255.8383	0.075455	0.517901	0.840215	lncRNA
ENSG0000(	ENSG0000(	149.3152	0.101981	0.517648	0.840215	lncRNA
ENSG0000(	ENSG0000(	398.2399	-0.05545	0.517873	0.840215	lncRNA
ENSG0000(	ENSG0000(	160.8865	0.084479	0.517739	0.840215	lncRNA
ENSG0000(	IFNAR1	2137.45	-0.0511	0.517901	0.840215	protein_coding
ENSG0000(	LINC00519	264.0369	-0.06121	0.517737	0.840215	lncRNA
ENSG0000(	PIAS4	309.2733	-0.07751	0.517665	0.840215	protein_coding
ENSG0000(	PTPRT	156.2409	-0.21979	0.517792	0.840215	protein_coding
ENSG0000(	RAB11A	2041.901	0.064885	0.517831	0.840215	protein_coding
ENSG0000(	ROR1	169.8317	0.124372	0.517674	0.840215	protein_coding
ENSG0000(	UBIAD1	482.4868	-0.05798	0.51779	0.840215	protein_coding
ENSG0000(	ZNF346-IT1	202.5524	0.056937	0.517631	0.840215	lncRNA
ENSG0000(	ENSG0000(	1136.092	0.040501	0.518078	0.84022	lncRNA
ENSG0000(	ENSG0000(	554.6476	-0.04024	0.518175	0.84022	lncRNA
ENSG0000(	ERICH6-AS:	899.5586	-0.05164	0.517981	0.84022	lncRNA
ENSG0000(	KIAA1143	1070.444	0.036514	0.518137	0.84022	protein_coding
ENSG0000(	LINC01443	286.8706	-0.07171	0.518154	0.84022	lncRNA
ENSG0000(	MRPL45	178.1208	0.07075	0.518005	0.84022	protein_coding
ENSG0000(	RN7SL784F	204	0.064177	0.518069	0.84022	misc_RNA

ENSG0000(	TINF2	609.0307	-0.06438	0.51813	0.84022	protein_coding
ENSG0000(	CDC123	273.7909	0.081868	0.519107	0.840349	protein_coding
ENSG0000(	CMTR1	1269.382	-0.07103	0.518737	0.840349	protein_coding
ENSG0000(	CPNE3	1330.397	0.083167	0.51896	0.840349	protein_coding
ENSG0000(	CSPG4P12	1449.219	-0.0583	0.51841	0.840349	transcribed_unprocessed_pseudo
ENSG0000(	DNM1L	1598.018	-0.04876	0.518461	0.840349	protein_coding
ENSG0000(	DPAGT1	459.1408	-0.088	0.518893	0.840349	protein_coding
ENSG0000(	EME1	68.69448	0.160394	0.518937	0.840349	protein_coding
ENSG0000(	ENSG0000(	330.1504	0.055259	0.51858	0.840349	lncRNA
ENSG0000(	ENSG0000(	35.14389	0.143498	0.518983	0.840349	lncRNA
ENSG0000(	ENSG0000(	116.0016	-0.16003	0.518649	0.840349	lncRNA
ENSG0000(	ENSG0000(	260.093	0.063713	0.518364	0.840349	lncRNA
ENSG0000(	ENSG0000(	165.0845	-0.06889	0.518942	0.840349	lncRNA
ENSG0000(	ENSG0000(	169.15	0.06356	0.518912	0.840349	lncRNA
ENSG0000(	ENSG0000(	80.95603	-0.09712	0.518652	0.840349	TEC
ENSG0000(	ENSG0000(	3153.357	0.061376	0.519139	0.840349	lncRNA
ENSG0000(	ENSG0000(	30.37193	0.157404	0.519081	0.840349	lncRNA
ENSG0000(	FAM174A	59.66402	-0.15524	0.518328	0.840349	protein_coding
ENSG0000(	LINC02363	254.9873	-0.05753	0.518971	0.840349	lncRNA
ENSG0000(	MCM5	1065.959	-0.07177	0.519105	0.840349	protein_coding
ENSG0000(	NR1I2	1900.247	0.150098	0.519123	0.840349	protein_coding
ENSG0000(	PCNX3	1623.501	0.078708	0.519129	0.840349	protein_coding
ENSG0000(	PPM1F	1221.552	-0.05371	0.519028	0.840349	protein_coding
ENSG0000(	RBCK1	1524.257	-0.05428	0.519021	0.840349	protein_coding
ENSG0000(	RBSN	1485.128	-0.04659	0.518527	0.840349	protein_coding
ENSG0000(	RHPN1-AS1	64.13769	-0.1349	0.518498	0.840349	lncRNA
ENSG0000(	RN7SL482F	42.50963	0.121735	0.51911	0.840349	misc_RNA
ENSG0000(	TARS3	578.1999	-0.06963	0.518405	0.840349	protein_coding
ENSG0000(	TRPC5	77.88002	0.091132	0.51914	0.840349	protein_coding
ENSG0000(	TRPM1	102.9401	0.080909	0.51909	0.840349	protein_coding
ENSG0000(	ZNF34	319.9059	-0.05493	0.519181	0.840366	protein_coding
ENSG0000(	CALML3-AS	225.7058	0.065057	0.519265	0.840403	lncRNA
ENSG0000(	TRPC7-AS1	47.44681	0.197185	0.519256	0.840403	lncRNA
ENSG0000(	ENSG0000(	28.22999	-0.16005	0.519409	0.840538	lncRNA
ENSG0000(	GYG1	945.1774	-0.06542	0.5194	0.840538	protein_coding
ENSG0000(	ENSG0000(	43.43451	-0.1239	0.519455	0.840563	lncRNA
ENSG0000(	LINC01344	229.9445	0.13953	0.519555	0.840631	lncRNA
ENSG0000(	ORAI3	257.1955	-0.09369	0.519558	0.840631	protein_coding
ENSG0000(	SCN11A	319.9196	0.096465	0.519718	0.84084	protein_coding
ENSG0000(	ENSG0000(	339.9117	-0.08203	0.519755	0.84085	lncRNA
ENSG0000(	CDT1	210.1302	-0.07836	0.520075	0.840877	protein_coding
ENSG0000(	COX8A	499.7593	0.126184	0.519812	0.840877	protein_coding
ENSG0000(	ENSG0000(	21.70013	-0.16847	0.520037	0.840877	lncRNA
ENSG0000(	HMMR	140.9521	0.138709	0.520136	0.840877	protein_coding
ENSG0000(	MC2R	357.6665	0.136587	0.520138	0.840877	protein_coding
ENSG0000(	OR5A2	626.7905	-0.04081	0.519908	0.840877	protein_coding
ENSG0000(	PARD6G-AS	107.3109	0.127823	0.520127	0.840877	lncRNA

ENSG0000(	PNMA8C	265.0313	0.054938	0.519951	0.840877	protein_coding
ENSG0000(	PRAL	1728.679	-0.08303	0.519964	0.840877	TEC
ENSG0000(	SCML4	1580.006	-0.10282	0.520009	0.840877	protein_coding
ENSG0000(	SLC27A2	247.7848	0.147888	0.519958	0.840877	protein_coding
ENSG0000(	TTC14	2950.224	-0.04289	0.519916	0.840877	protein_coding
ENSG0000(	NPR2	550.2133	0.066238	0.520173	0.840884	protein_coding
ENSG0000(	ENSG0000(	20.79591	0.21086	0.520214	0.8409	lncRNA
ENSG0000(	GLT1D1	70.57283	-1.22331	0.520248	0.840907	protein_coding
ENSG0000(	ADAM11	22.50162	-0.22767	0.520322	0.840976	protein_coding
ENSG0000(	SNHG22	2275.872	-0.06425	0.520406	0.841062	lncRNA
ENSG0000(	SSR4P1	27.94258	-0.18419	0.520484	0.841139	transcribed_processed_pseudoge
ENSG0000(	MANEAL	87.39917	0.130048	0.520656	0.841322	protein_coding
ENSG0000(	NDUFA7	251.5576	0.056631	0.520658	0.841322	protein_coding
ENSG0000(	ENSG0000(	367.5244	0.070133	0.520697	0.841335	lncRNA
ENSG0000(	CBX3P2	37.52279	-0.13826	0.52079	0.841337	transcribed_processed_pseudoge
ENSG0000(	ENSG0000(	116.4833	0.108296	0.52078	0.841337	transcribed_processed_pseudoge
ENSG0000(	EPHA3	310.6475	0.095387	0.520781	0.841337	protein_coding
ENSG0000(	ENSG0000(	31.0638	0.192115	0.520936	0.841445	lncRNA
ENSG0000(	HFE	639.9963	-0.0551	0.520948	0.841445	protein_coding
ENSG0000(	VN1R2	54.60086	-0.11229	0.520904	0.841445	protein_coding
ENSG0000(	RBM28	3154.318	-0.05514	0.521008	0.841493	protein_coding
ENSG0000(	HPAT5	98.74486	-0.07865	0.52114	0.841656	lncRNA
ENSG0000(	MTERF1	950.3966	-0.04351	0.521198	0.8417	protein_coding
ENSG0000(	ENSG0000(	159.2959	-0.10248	0.521294	0.841736	lncRNA
ENSG0000(	ENSG0000(	59.45058	0.224702	0.521312	0.841736	transcribed_unprocessed_pseudo
ENSG0000(	GTF2IP7	481.4089	0.060927	0.52129	0.841736	transcribed_unprocessed_pseudo
ENSG0000(	ENSG0000(	124.1747	-0.07656	0.521374	0.841737	lncRNA
ENSG0000(	FAM241A	26601.29	-0.03437	0.521362	0.841737	protein_coding
ENSG0000(	NEDD8	931.5608	-0.04079	0.521448	0.841808	protein_coding
ENSG0000(	LIME1	195.3994	0.115034	0.521567	0.841851	protein_coding
ENSG0000(	UBE2D4	549.0574	-0.07083	0.521521	0.841851	protein_coding
ENSG0000(	URM1	475.4803	0.071003	0.521538	0.841851	protein_coding
ENSG0000(	DYDC1	67.34133	-0.11564	0.52165	0.841887	protein_coding
ENSG0000(	PDZRN4	21.46015	-0.30639	0.521641	0.841887	protein_coding
ENSG0000(	CYP4F30P	43.03841	0.200395	0.521715	0.841893	transcribed_unprocessed_pseudo
ENSG0000(	PCSK6-AS1	54.12107	-0.13957	0.52169	0.841893	lncRNA
ENSG0000(	ENSG0000(	27.83794	0.222517	0.521751	0.841902	lncRNA
ENSG0000(	BAGE2	59.9251	0.150617	0.521883	0.841916	transcribed_unprocessed_pseudo
ENSG0000(	ENSG0000(	57.27303	0.128027	0.521822	0.841916	lncRNA
ENSG0000(	LINC01033	47.9501	0.166641	0.521793	0.841916	lncRNA
ENSG0000(	P2RY11	223.8752	0.096857	0.52186	0.841916	protein_coding
ENSG0000(	BRAF	4312.416	-0.03377	0.521961	0.841967	protein_coding
ENSG0000(	NSDHL	99.11106	-0.09358	0.521976	0.841967	protein_coding
ENSG0000(	ENSG0000(	31.32094	-0.13107	0.522033	0.842011	lncRNA
ENSG0000(	L3HYPDH	530.363	0.05941	0.522066	0.842014	protein_coding
ENSG0000(	DBNDD2	473.6915	0.100525	0.522138	0.842056	protein_coding
ENSG0000(	ENSG0000(	27.28067	0.152348	0.522214	0.842056	TEC

ENSG0000(	GNG7	415.1141	-0.084	0.522198	0.842056	protein_coding
ENSG0000(	SNORA79B	216.3763	0.099046	0.522198	0.842056	snoRNA
ENSG0000(	ENSG0000(	173.5261	0.089227	0.52247	0.84232	lncRNA
ENSG0000(	LINC01726	44.65865	-0.12845	0.522427	0.84232	lncRNA
ENSG0000(	RPL13P12	26.64586	0.669637	0.522459	0.84232	processed_pseudogene
ENSG0000(	ACVR2B-AS	223.6878	-0.04825	0.522574	0.842439	lncRNA
ENSG0000(	C3orf85	333.0975	0.172283	0.522868	0.842464	protein_coding
ENSG0000(	CTDP1	807.5293	0.06513	0.522763	0.842464	protein_coding
ENSG0000(	DDX11-AS1	92.86612	-0.10179	0.522887	0.842464	lncRNA
ENSG0000(	ENSG0000(	119.5003	-0.07632	0.522834	0.842464	lncRNA
ENSG0000(	ENSG0000(	499.1549	-0.11388	0.522728	0.842464	TEC
ENSG0000(	GSDMA	265.195	0.056758	0.52287	0.842464	protein_coding
ENSG0000(	LINC02196	82.27312	0.110487	0.522734	0.842464	lncRNA
ENSG0000(	LINC02245	479.5676	-0.06524	0.522896	0.842464	lncRNA
ENSG0000(	PCDHA4	2635.936	-0.0371	0.522678	0.842464	protein_coding
ENSG0000(	PKD1P2	290.6797	0.410246	0.522654	0.842464	unprocessed_pseudogene
ENSG0000(	ENSG0000(	43.41288	-0.11775	0.52294	0.842485	lncRNA
ENSG0000(	ENSG0000(	95.85179	-0.12931	0.523153	0.842749	lncRNA
ENSG0000(	HIRIP3	96.2437	-0.10312	0.523165	0.842749	protein_coding
ENSG0000(	MMP26	27.32136	0.172143	0.523279	0.842884	protein_coding
ENSG0000(	TERC	61.51872	-0.22499	0.523343	0.842938	lncRNA
ENSG0000(	TTC17	3394.828	0.043708	0.523502	0.843094	protein_coding
ENSG0000(	ZNF536	293.5283	0.111674	0.523492	0.843094	protein_coding
ENSG0000(	CDK2	553.095	-0.09595	0.52362	0.843137	protein_coding
ENSG0000(	ENSG0000(	116.0092	0.104141	0.523609	0.843137	lncRNA
ENSG0000(	TG	150.4824	-0.19682	0.523593	0.843137	protein_coding
ENSG0000(	ENSG0000(	44.66426	-0.11991	0.523759	0.843213	protein_coding
ENSG0000(	ENSG0000(	30.37252	-0.12779	0.523756	0.843213	lncRNA
ENSG0000(	LILRB5	580.2238	-0.11228	0.523753	0.843213	protein_coding
ENSG0000(	CARMIL3	64.88937	-0.18273	0.523919	0.843272	protein_coding
ENSG0000(	ENSG0000(	59.94986	-0.12375	0.523952	0.843272	lncRNA
ENSG0000(	ENSG0000(	167.4973	0.057277	0.523835	0.843272	lncRNA
ENSG0000(	FOXF2-DT	41.61489	0.134321	0.523871	0.843272	lncRNA
ENSG0000(	METTL14	1040.851	-0.04349	0.523982	0.843272	protein_coding
ENSG0000(	RN7SL178F	30.65763	0.118459	0.523979	0.843272	misc_RNA
ENSG0000(	SLC5A4	20.05242	-0.27476	0.52401	0.843272	protein_coding
ENSG0000(	LRP11	455.873	-0.07828	0.524146	0.84344	protein_coding
ENSG0000(	IL9RP3	45.87232	-0.1647	0.524209	0.843443	unprocessed_pseudogene
ENSG0000(	TCF3	1036.999	-0.0822	0.524192	0.843443	protein_coding
ENSG0000(	ENSG0000(	40.66702	-0.17897	0.524278	0.843484	lncRNA
ENSG0000(	MFAP5	179.7616	0.108539	0.524295	0.843484	protein_coding
ENSG0000(	ENSG0000(	36.9413	-0.1635	0.524422	0.843499	lncRNA
ENSG0000(	KATNAL2	782.8984	0.041622	0.524427	0.843499	protein_coding
ENSG0000(	RTL10	1111.775	-0.04753	0.524394	0.843499	protein_coding
ENSG0000(	SDC4	1060.787	-0.10198	0.524397	0.843499	protein_coding
ENSG0000(	ENSG0000(	344.4749	-0.04121	0.524619	0.843579	lncRNA
ENSG0000(	ENSG0000(	252.1946	-0.07426	0.524614	0.843579	TEC

ENSG0000(	GLRX2	504.0436	-0.05217	0.524596	0.843579	protein_coding
ENSG0000(	STK36	779.9701	-0.04792	0.52463	0.843579	protein_coding
ENSG0000(	UXT	148.6269	0.081546	0.524624	0.843579	protein_coding
ENSG0000(	RNU4-2	23346.16	0.07355	0.524666	0.843586	snRNA
ENSG0000(	C21orf58	532.6131	0.065763	0.524841	0.843693	protein_coding
ENSG0000(	COQ7-DT	74.78464	0.193101	0.52489	0.843693	lncRNA
ENSG0000(	IL31RA	378.1228	0.062808	0.524916	0.843693	protein_coding
ENSG0000(	SPEF2	695.8531	0.064674	0.524858	0.843693	protein_coding
ENSG0000(	TNKS2	1876.782	-0.03772	0.524849	0.843693	protein_coding
ENSG0000(	ZKSCAN3	1132.558	0.026118	0.524905	0.843693	protein_coding
ENSG0000(	SNORA1	51.41936	0.114683	0.52496	0.843714	snoRNA
ENSG0000(	PCDHB11	124.3256	0.067393	0.524994	0.843719	protein_coding
ENSG0000(	ADGRG4	63.63371	-0.09702	0.525415	0.84372	protein_coding
ENSG0000(	ASB18	381.8459	-0.04701	0.525228	0.84372	protein_coding
ENSG0000(	CLCNKB	107.8486	-0.07259	0.525406	0.84372	protein_coding
ENSG0000(	DDX19B	548.0375	0.055555	0.525285	0.84372	protein_coding
ENSG0000(	DNMT3B	90.08744	-0.10623	0.525423	0.84372	protein_coding
ENSG0000(	DTNA	391.2381	0.082645	0.525199	0.84372	protein_coding
ENSG0000(	ENSG0000(	152.26	0.084478	0.52507	0.84372	lncRNA
ENSG0000(	ENSG0000(	25.03394	0.149801	0.525282	0.84372	snoRNA
ENSG0000(	ENSG0000(	119.495	0.07691	0.525404	0.84372	lncRNA
ENSG0000(	ENSG0000(	26.47882	0.126964	0.525249	0.84372	lncRNA
ENSG0000(	ENSG0000(	529.7878	-0.06671	0.525367	0.84372	lncRNA
ENSG0000(	LAMB3	918.7863	0.144992	0.525105	0.84372	protein_coding
ENSG0000(	PHAX	1118.855	0.040452	0.525181	0.84372	protein_coding
ENSG0000(	RGS6	163.3685	-0.11434	0.525205	0.84372	protein_coding
ENSG0000(	EEF1AKMT	43.89607	0.138294	0.525587	0.843736	protein_coding
ENSG0000(	ENSG0000(	119.5823	0.10061	0.525548	0.843736	lncRNA
ENSG0000(	FOXO3	3556.022	0.06268	0.525538	0.843736	protein_coding
ENSG0000(	SLC6A15	332.3002	0.050378	0.525579	0.843736	protein_coding
ENSG0000(	VPS13A	8800.372	0.086673	0.525582	0.843736	protein_coding
ENSG0000(	ENSG0000(	76.02579	-0.11927	0.525647	0.843737	lncRNA
ENSG0000(	ENSG0000(	37.57132	0.130844	0.525649	0.843737	lncRNA
ENSG0000(	GUSBP1	2081.726	0.063053	0.525708	0.843782	transcribed_unprocessed_pseudo
ENSG0000(	TGOLN2	7602.892	-0.04016	0.525758	0.843814	protein_coding
ENSG0000(	ENSG0000(	41.1513	0.146197	0.525806	0.843842	lncRNA
ENSG0000(	ADAM28	3980.237	-0.08694	0.52591	0.843911	protein_coding
ENSG0000(	DSC3	178.2571	0.076919	0.525911	0.843911	protein_coding
ENSG0000(	ENSG0000(	37.26013	0.104391	0.52603	0.844053	lncRNA
ENSG0000(	ACSM3	548.4418	0.068423	0.526197	0.844273	protein_coding
ENSG0000(	KCNK3	410.2748	-0.05206	0.526266	0.844333	protein_coding
ENSG0000(	ZNF256	249.9371	0.082512	0.526352	0.844422	protein_coding
ENSG0000(	RN7SL364F	53.5652	0.112525	0.526457	0.844491	misc_RNA
ENSG0000(	SH3BP5-AS	1772.786	-0.04662	0.526442	0.844491	lncRNA
ENSG0000(	ENSG0000(	51.29644	0.113686	0.526493	0.844501	lncRNA
ENSG0000(	RASGRP2	753.9013	-0.19688	0.526591	0.844609	protein_coding
ENSG0000(	A1BG	645.4931	-0.04437	0.52676	0.844627	protein_coding

ENSG0000(	CDIP1	308.9274	-0.07068	0.526745	0.844627	protein_coding
ENSG0000(	COPS2	883.0083	-0.05316	0.526747	0.844627	protein_coding
ENSG0000(	DR1	2646.777	-0.02814	0.526788	0.844627	protein_coding
ENSG0000(	ENSG0000(	127.3159	0.065534	0.526851	0.844627	lncRNA
ENSG0000(	RNF181	137.9808	0.075688	0.526943	0.844627	protein_coding
ENSG0000(	RNU6-60P	38.67986	-0.14408	0.526836	0.844627	snRNA
ENSG0000(	RNU6-759F	41.75866	-0.12561	0.526941	0.844627	snRNA
ENSG0000(	RPL35	862.9377	-0.07911	0.526637	0.844627	protein_coding
ENSG0000(	SENP2	1370.165	0.03903	0.526925	0.844627	protein_coding
ENSG0000(	TSPAN8	3139.561	-0.12036	0.526737	0.844627	protein_coding
ENSG0000(	ULK4P2	46.03219	-0.14665	0.526971	0.844627	transcribed_unprocessed_pseudo
ENSG0000(	PCGF3	2221.505	0.065022	0.527013	0.844645	protein_coding
ENSG0000(	ENSG0000(	32.70223	-0.17367	0.527164	0.844838	processed_pseudogene
ENSG0000(	ENSG0000(	103.4442	-0.08589	0.527319	0.845038	lncRNA
ENSG0000(	STX7	3813.688	-0.06419	0.527405	0.845127	protein_coding
ENSG0000(	NPRL2	239.8398	0.079493	0.527449	0.845147	protein_coding
ENSG0000(	GCSH	347.5299	-0.05245	0.527538	0.845192	protein_coding
ENSG0000(	PARS2	95.25772	0.104737	0.527525	0.845192	protein_coding
ENSG0000(	YWHAZ	8416.756	0.054004	0.527587	0.84522	protein_coding
ENSG0000(	SMG1	27641.4	0.033926	0.527687	0.845332	protein_coding
ENSG0000(	ZUP1	221.0468	-0.08667	0.52774	0.845367	protein_coding
ENSG0000(	RFC4	209.4415	-0.06628	0.527944	0.845627	protein_coding
ENSG0000(	TAF9B	118.8753	-0.11393	0.527994	0.845627	protein_coding
ENSG0000(	WNT10B	67.26514	-0.16817	0.527973	0.845627	protein_coding
ENSG0000(	CCNB1	168.7005	-0.16117	0.528159	0.845671	protein_coding
ENSG0000(	ENSG0000(	1934.555	-0.05075	0.528176	0.845671	lncRNA
ENSG0000(	FBXL20	3260.551	-0.07129	0.528155	0.845671	protein_coding
ENSG0000(	HSDL2	591.1353	-0.09027	0.528168	0.845671	protein_coding
ENSG0000(	PDLIM2	1142.902	0.085041	0.528151	0.845671	protein_coding
ENSG0000(	ENSG0000(	391.3067	0.055426	0.528366	0.845926	processed_pseudogene
ENSG0000(	CNNM1	93.5973	0.126884	0.528616	0.846031	protein_coding
ENSG0000(	ENSG0000(	211.9599	0.069008	0.52857	0.846031	lncRNA
ENSG0000(	ENSG0000(	564.7386	-0.0539	0.528513	0.846031	lncRNA
ENSG0000(	LYPD8	1164.846	0.27636	0.528497	0.846031	protein_coding
ENSG0000(	NOP9	1143.836	0.04697	0.528615	0.846031	protein_coding
ENSG0000(	PDE4D	2497.563	0.064512	0.528613	0.846031	protein_coding
ENSG0000(	CSRNP2	1144.625	-0.04974	0.528682	0.846088	protein_coding
ENSG0000(	ENSG0000(	379.5346	0.055779	0.528833	0.846183	lncRNA
ENSG0000(	RN7SL18P	42.48793	-0.12644	0.528818	0.846183	misc_RNA
ENSG0000(	SESN1	882.3412	0.084078	0.528811	0.846183	protein_coding
ENSG0000(	IFI27L1	205.1616	-0.07638	0.5289	0.84624	protein_coding
ENSG0000(	SIRT6	460.6601	0.120035	0.528969	0.846252	protein_coding
ENSG0000(	SLC7A8	360.1855	-0.10377	0.528945	0.846252	protein_coding
ENSG0000(	TWF1P2	24.2598	-0.18926	0.529009	0.846267	processed_pseudogene
ENSG0000(	CCDC81	172.6124	0.06001	0.529134	0.84632	protein_coding
ENSG0000(	HEY1	82.75941	-0.13685	0.529118	0.84632	protein_coding
ENSG0000(	PCDH7	904.5945	-0.11459	0.529097	0.84632	protein_coding

ENSG0000(	ENSG0000(	111.0768	0.112198	0.52926	0.846323	lncRNA
ENSG0000(	ENSG0000(	227.8757	-0.05555	0.529215	0.846323	lncRNA
ENSG0000(	ENSG0000(	7222.983	-0.06153	0.529179	0.846323	lncRNA
ENSG0000(	TBX2	851.7134	-0.10099	0.529247	0.846323	protein_coding
ENSG0000(	ATP5PO	636.9486	0.100911	0.529322	0.846324	protein_coding
ENSG0000(	LINC02884	186.2265	0.140967	0.529316	0.846324	lncRNA
ENSG0000(	ENSG0000(	114.3904	0.075521	0.529398	0.846398	lncRNA
ENSG0000(	ENSG0000(	24.63672	0.150835	0.529496	0.846405	lncRNA
ENSG0000(	LINC00368	51.99403	-0.10152	0.529487	0.846405	lncRNA
ENSG0000(	SNORA64	127.4066	0.127534	0.529476	0.846405	snoRNA
ENSG0000(	LINC02822	2275.651	0.086652	0.529574	0.846482	lncRNA
ENSG0000(	ANKRD54	586.5875	0.043308	0.529627	0.846517	protein_coding
ENSG0000(	KANSL1-AS	283.181	0.067434	0.529757	0.846577	lncRNA
ENSG0000(	LINC02432	403.6404	-0.05328	0.529731	0.846577	lncRNA
ENSG0000(	NDUFAF8	148.1217	-0.07946	0.529701	0.846577	protein_coding
ENSG0000(	ENSG0000(	206.8272	-0.08987	0.529911	0.846774	lncRNA
ENSG0000(	NUP160	1464.135	0.046399	0.529981	0.846837	protein_coding
ENSG0000(	BCL9	560.4902	0.069965	0.530046	0.846842	protein_coding
ENSG0000(	HPF1	69.04168	-0.11997	0.53004	0.846842	protein_coding
ENSG0000(	C11orf1	716.2704	0.053095	0.530313	0.846943	protein_coding
ENSG0000(	CSPG5	20.6376	0.236675	0.530339	0.846943	protein_coding
ENSG0000(	ENSG0000(	136.1726	-0.08107	0.530231	0.846943	lncRNA
ENSG0000(	PPM1K	2532.41	-0.10202	0.530215	0.846943	protein_coding
ENSG0000(	RN7SL128F	114.085	0.097465	0.530332	0.846943	misc_RNA
ENSG0000(	SNORD68	56.37433	-0.17918	0.530355	0.846943	snoRNA
ENSG0000(	TUBB2A	145.6403	0.138343	0.530222	0.846943	protein_coding
ENSG0000(	UVRAG	1386.6	-0.05215	0.530243	0.846943	protein_coding
ENSG0000(	ELOVL6	1259.16	-0.13222	0.530473	0.846956	protein_coding
ENSG0000(	GREB1L	38.53965	-0.26607	0.530483	0.846956	protein_coding
ENSG0000(	TMEM164	1822.383	0.089677	0.530473	0.846956	protein_coding
ENSG0000(	ZFP69	105.8672	0.095861	0.530487	0.846956	protein_coding
ENSG0000(	CSPP1	5011.456	0.037939	0.530579	0.846961	protein_coding
ENSG0000(	DACT3	939.5529	-0.03413	0.530582	0.846961	protein_coding
ENSG0000(	SHLD2P1	49.69267	0.175367	0.53057	0.846961	transcribed_unprocessed_pseudo
ENSG0000(	DHX58	330.5503	-0.14074	0.530702	0.847005	protein_coding
ENSG0000(	DUSP5-DT	133.5559	0.073577	0.530686	0.847005	lncRNA
ENSG0000(	MRPL58	77.76956	-0.0988	0.530673	0.847005	protein_coding
ENSG0000(	BRF2	178.1355	-0.07757	0.530856	0.847105	protein_coding
ENSG0000(	ENSG0000(	87.30475	-0.11046	0.530854	0.847105	lncRNA
ENSG0000(	FBXO24	208.6466	-0.05921	0.530857	0.847105	protein_coding
ENSG0000(	ENSG0000(	247.1032	-0.0511	0.53095	0.847106	lncRNA
ENSG0000(	ENSG0000(	2693.957	-0.05422	0.530949	0.847106	lncRNA
ENSG0000(	RAMP2-AS:	570.6306	-0.04621	0.530914	0.847106	lncRNA
ENSG0000(	CYSTM1	673.3191	-0.10726	0.531079	0.847151	protein_coding
ENSG0000(	LINC02675	102.9442	-0.08056	0.531063	0.847151	lncRNA
ENSG0000(	RN7SL344F	51.37541	-0.10387	0.531101	0.847151	misc_RNA
ENSG0000(	UNC13C	24.91318	-0.35266	0.531042	0.847151	protein_coding

ENSG0000(	FRAT2	314.8506	0.07152	0.531297	0.847299	protein_coding
ENSG0000(	TAF1A	227.028	-0.07242	0.531305	0.847299	protein_coding
ENSG0000(	USP21P1	198.331	0.06762	0.531317	0.847299	processed_pseudogene
ENSG0000(	VPS26C	1569.716	0.034236	0.531283	0.847299	protein_coding
ENSG0000(	SLC23A3	489.6187	0.1453	0.531376	0.847343	protein_coding
ENSG0000(	ENSG0000(	36.50648	-0.11832	0.531471	0.847446	lncRNA
ENSG0000(	FLVCR1	1203.692	0.058275	0.531559	0.847537	protein_coding
ENSG0000(	PDZD9	224.0639	-0.06607	0.531598	0.84755	protein_coding
ENSG0000(	LINC01565	26.47251	0.159612	0.531756	0.847586	lncRNA
ENSG0000(	NDUFA4	579.667	0.064973	0.531688	0.847586	protein_coding
ENSG0000(	RAPGEF6	2424.453	-0.09565	0.531706	0.847586	protein_coding
ENSG0000(	TLE1	855.9134	0.081366	0.531755	0.847586	protein_coding
ENSG0000(	TMEM184(	875.9413	0.056292	0.531774	0.847586	protein_coding
ENSG0000(	INTS6-AS1	796.5671	0.050341	0.531834	0.847632	lncRNA
ENSG0000(	BTLA	455.9117	-0.20922	0.531873	0.847645	protein_coding
ENSG0000(	ENSG0000(	94.73679	0.076798	0.531973	0.847656	transcribed_unitary_pseudogene
ENSG0000(	ENSG0000(	41.21943	0.163236	0.531945	0.847656	lncRNA
ENSG0000(	PI4KA	3283.465	-0.04073	0.531955	0.847656	protein_coding
ENSG0000(	ABRAXAS2	328.3955	-0.05747	0.532022	0.847656	protein_coding
ENSG0000(	CLDN19	56.19405	-0.09907	0.532042	0.847656	protein_coding
ENSG0000(	ENSG0000(	46.73474	0.185083	0.532065	0.847656	lncRNA
ENSG0000(	CAD	1213.036	-0.04342	0.532183	0.847745	protein_coding
ENSG0000(	L3MBTL3	348.0703	-0.12325	0.532164	0.847745	protein_coding
ENSG0000(	C5AR2	1354.881	-0.04753	0.532373	0.847803	protein_coding
ENSG0000(	CRYBG1	4666.208	0.071845	0.532297	0.847803	protein_coding
ENSG0000(	RNASEK-C1	28.01823	0.773307	0.53228	0.847803	protein_coding
ENSG0000(	SEC23B	693.7777	-0.06956	0.532354	0.847803	protein_coding
ENSG0000(	ZMAT1	1000.438	0.082821	0.532335	0.847803	protein_coding
ENSG0000(	ENSG0000(	41.31427	0.1407	0.532412	0.847816	lncRNA
ENSG0000(	ARHGEF11	2577.377	0.064293	0.532478	0.847872	protein_coding
ENSG0000(	CD1E	80.76162	0.992939	0.532547	0.84789	protein_coding
ENSG0000(	PABPC4-AS	142.881	-0.07868	0.532551	0.84789	lncRNA
ENSG0000(	ENSG0000(	55.91387	0.121799	0.532676	0.848041	lncRNA
ENSG0000(	ABHD14B	420.4542	0.080877	0.534942	0.848083	protein_coding
ENSG0000(	ABHD6	364.0746	0.058303	0.533941	0.848083	protein_coding
ENSG0000(	ACOX1	4907.255	0.148718	0.534581	0.848083	protein_coding
ENSG0000(	ALDH18A1	1743.479	0.135747	0.533698	0.848083	protein_coding
ENSG0000(	ARIH2	2520.829	0.028676	0.535143	0.848083	protein_coding
ENSG0000(	ATP6V0A2	3108.849	0.025215	0.53296	0.848083	protein_coding
ENSG0000(	BCL7B	467.3439	-0.07205	0.534434	0.848083	protein_coding
ENSG0000(	CFHR2	173.2475	-0.0943	0.533034	0.848083	protein_coding
ENSG0000(	COL9A3	75.21345	0.117197	0.533449	0.848083	protein_coding
ENSG0000(	DDHD2	1698.868	0.0416	0.534457	0.848083	protein_coding
ENSG0000(	EFL1P1	72.73185	-0.11913	0.535054	0.848083	transcribed_unprocessed_pseudo
ENSG0000(	EGF	152.1889	0.242753	0.534014	0.848083	protein_coding
ENSG0000(	ENPP4	554.0304	0.075077	0.53435	0.848083	protein_coding
ENSG0000(	ENSG0000(	35.23011	0.11044	0.532812	0.848083	lncRNA

ENSG0000(	ENSG0000(	23.97699	-0.16663	0.534903	0.848083	processed_pseudogene
ENSG0000(	ENSG0000(	60.15583	0.104054	0.534562	0.848083	transcribed_processed_pseudoge
ENSG0000(	ENSG0000(	37.72152	0.120193	0.533485	0.848083	lncRNA
ENSG0000(	ENSG0000(	626.5038	0.171308	0.534638	0.848083	lncRNA
ENSG0000(	ENSG0000(	113.0935	0.087398	0.53465	0.848083	lncRNA
ENSG0000(	ENSG0000(	68.62705	0.101849	0.535101	0.848083	lncRNA
ENSG0000(	ENSG0000(	38.81934	-0.11886	0.534663	0.848083	lncRNA
ENSG0000(	ENSG0000(	27.50926	0.13875	0.535241	0.848083	processed_pseudogene
ENSG0000(	ENSG0000(	1512.024	-0.03813	0.534724	0.848083	lncRNA
ENSG0000(	ENSG0000(	38.15438	0.134526	0.533761	0.848083	lncRNA
ENSG0000(	ENSG0000(	91.31602	0.093671	0.535344	0.848083	lncRNA
ENSG0000(	ENSG0000(	29.89527	0.139397	0.533793	0.848083	lncRNA
ENSG0000(	ENSG0000(	71.29771	0.143064	0.534462	0.848083	lncRNA
ENSG0000(	ENSG0000(	96.13431	0.075207	0.533024	0.848083	TEC
ENSG0000(	ENSG0000(	4227.6	-0.03574	0.534855	0.848083	lncRNA
ENSG0000(	ENSG0000(	381.9045	-0.05016	0.535338	0.848083	lncRNA
ENSG0000(	ENSG0000(	63.51811	0.114383	0.534238	0.848083	lncRNA
ENSG0000(	ENSG0000(	26.92006	-0.14595	0.534334	0.848083	lncRNA
ENSG0000(	ENSG0000(	123.7038	0.069799	0.53413	0.848083	lncRNA
ENSG0000(	ENSG0000(	52.1514	0.119274	0.533152	0.848083	lncRNA
ENSG0000(	EZH2	1238.922	-0.05462	0.535289	0.848083	protein_coding
ENSG0000(	FAM230B	63.72628	0.258105	0.532859	0.848083	lncRNA
ENSG0000(	FOXO6-AS1	26.21992	-0.14055	0.534608	0.848083	lncRNA
ENSG0000(	FZD10-AS1	370.6964	-0.11289	0.533341	0.848083	lncRNA
ENSG0000(	GAS2L3	1150.049	0.085896	0.533541	0.848083	protein_coding
ENSG0000(	GJB2	399.7063	-0.11348	0.534124	0.848083	protein_coding
ENSG0000(	GNL1	710.7435	-0.06301	0.53455	0.848083	protein_coding
ENSG0000(	GRIN1	89.20364	0.216697	0.534852	0.848083	protein_coding
ENSG0000(	GTF2I	4938.11	0.040278	0.534078	0.848083	protein_coding
ENSG0000(	HECTD1	7416.883	0.02781	0.535215	0.848083	protein_coding
ENSG0000(	HOXB3	2211.491	0.098175	0.533833	0.848083	protein_coding
ENSG0000(	HOXD11	136.2062	-0.14926	0.53486	0.848083	protein_coding
ENSG0000(	HSD3B2	266.4136	0.38546	0.535385	0.848083	protein_coding
ENSG0000(	INO80D	4392.196	0.038081	0.534514	0.848083	protein_coding
ENSG0000(	LIN28A	350.5796	0.03925	0.534307	0.848083	protein_coding
ENSG0000(	LINC00638	94.70807	0.098903	0.535081	0.848083	lncRNA
ENSG0000(	LINC00971	88.47854	0.10791	0.533682	0.848083	lncRNA
ENSG0000(	LINC01376	104.8789	0.123799	0.533447	0.848083	lncRNA
ENSG0000(	LINC01586	91.46717	-0.13212	0.53465	0.848083	lncRNA
ENSG0000(	LINC02429	52.87305	-0.09307	0.534303	0.848083	lncRNA
ENSG0000(	LINC02723	138.4265	-0.07227	0.53335	0.848083	lncRNA
ENSG0000(	LSM14B	559.2121	0.049413	0.534285	0.848083	protein_coding
ENSG0000(	MORF4L2-A	79.57431	0.077576	0.535296	0.848083	lncRNA
ENSG0000(	MPPE1	1533.268	0.052806	0.533547	0.848083	protein_coding
ENSG0000(	NISCH	3016.603	-0.03004	0.534021	0.848083	protein_coding
ENSG0000(	NKAPD1	1015.308	0.044232	0.534586	0.848083	protein_coding
ENSG0000(	NSUN2	1181.921	-0.05205	0.535367	0.848083	protein_coding

ENSG0000(	OR14J1	3388.621	-0.04397	0.533992	0.848083	protein_coding
ENSG0000(	OSGEPL1	110.4165	-0.09045	0.533054	0.848083	protein_coding
ENSG0000(	P2RY10	1223.364	-0.06137	0.533076	0.848083	protein_coding
ENSG0000(	PCSEAT	170.8455	0.21113	0.533446	0.848083	lncRNA
ENSG0000(	PTPN5	36.85965	-0.16112	0.534212	0.848083	protein_coding
ENSG0000(	RNU6-130F	70.08054	-0.1255	0.534347	0.848083	snRNA
ENSG0000(	RRAGAP1-/	77.60291	0.097342	0.53311	0.848083	lncRNA
ENSG0000(	RRH	108.5406	-0.09592	0.534973	0.848083	protein_coding
ENSG0000(	SCAND2P	1980.023	0.042764	0.534162	0.848083	transcribed_unprocessed_pseudo
ENSG0000(	SLC25A15	612.5134	0.043901	0.535246	0.848083	protein_coding
ENSG0000(	SLC66A2	740.1479	0.080957	0.533734	0.848083	protein_coding
ENSG0000(	STEAP3-AS	21.10191	0.155971	0.53474	0.848083	lncRNA
ENSG0000(	SVBP	62.65301	0.125943	0.534143	0.848083	protein_coding
ENSG0000(	TFAP4	1185.409	0.102028	0.53371	0.848083	protein_coding
ENSG0000(	TMX3	1704.783	-0.03785	0.533498	0.848083	protein_coding
ENSG0000(	TROAP	296.2362	-0.08088	0.53332	0.848083	protein_coding
ENSG0000(	UBR5	5140.764	-0.03644	0.535039	0.848083	protein_coding
ENSG0000(	VMA21	911.997	-0.05155	0.535157	0.848083	protein_coding
ENSG0000(	WASF3-AS1	277.6324	0.054505	0.532799	0.848083	lncRNA
ENSG0000(	WTAPP1	93.49825	0.160002	0.534184	0.848083	transcribed_processed_pseudoge
ENSG0000(	XPNPEP3	2425.584	0.034388	0.53323	0.848083	protein_coding
ENSG0000(	XYLT1	1478.695	-0.08741	0.534673	0.848083	protein_coding
ENSG0000(	ZNF586	2029.281	0.048106	0.533185	0.848083	protein_coding
ENSG0000(	ZNF625	150.5314	0.068582	0.534026	0.848083	protein_coding
ENSG0000(	ZNF716	361.3406	0.072469	0.534526	0.848083	protein_coding
ENSG0000(	ZNHIT1	1267.922	0.044333	0.535231	0.848083	protein_coding
ENSG0000(	ABHD4	365.5935	-0.12109	0.535435	0.848114	protein_coding
ENSG0000(	LINC02431	46.49419	0.118488	0.535509	0.848133	lncRNA
ENSG0000(	SYT16	231.3235	0.087864	0.535493	0.848133	protein_coding
ENSG0000(	ENSG0000(	100.4353	0.075008	0.535628	0.848224	lncRNA
ENSG0000(	NDUFA4L2	44.74886	-0.24641	0.535624	0.848224	protein_coding
ENSG0000(	ENSG0000(	135.0667	-0.07029	0.535706	0.848234	lncRNA
ENSG0000(	FRZB	118.1658	0.134063	0.535757	0.848234	protein_coding
ENSG0000(	KCNAB1	24.93692	-0.25695	0.535683	0.848234	protein_coding
ENSG0000(	PTP4A1	3939.397	0.127816	0.535737	0.848234	protein_coding
ENSG0000(	ESPNL	25.21144	-0.30829	0.535842	0.84825	protein_coding
ENSG0000(	RIDA	91.50791	-0.11679	0.53586	0.84825	protein_coding
ENSG0000(	SBK1	732.0106	0.156909	0.535823	0.84825	protein_coding
ENSG0000(	PDCD1	54.159	-0.25099	0.535954	0.84835	protein_coding
ENSG0000(	ENSG0000(	189.6622	0.288418	0.536138	0.848592	lncRNA
ENSG0000(	ENSG0000(	35.79552	0.116832	0.536307	0.848619	TEC
ENSG0000(	ENSG0000(	36.94345	-0.14617	0.536201	0.848619	lncRNA
ENSG0000(	MCF2	24.09265	-0.24379	0.536325	0.848619	protein_coding
ENSG0000(	MEX3A	74.8143	0.152299	0.536321	0.848619	protein_coding
ENSG0000(	PKNOX2	921.0245	0.138626	0.53634	0.848619	protein_coding
ENSG0000(	SETD3	845.852	0.057121	0.536283	0.848619	protein_coding
ENSG0000(	POLE	2640.488	0.047964	0.536438	0.848725	protein_coding

ENSG0000(	CWC25	933.7078	-0.04385	0.536571	0.848887	protein_coding
ENSG0000(	GCNT3	8384.667	0.148566	0.536661	0.848932	protein_coding
ENSG0000(	GRAMD4	1645.877	0.104212	0.536635	0.848932	protein_coding
ENSG0000(	YJU2	129.2027	0.067794	0.536749	0.849022	protein_coding
ENSG0000(	PITHD1	399.6626	-0.0517	0.536782	0.849025	protein_coding
ENSG0000(	ENSG0000(	360.6634	0.086501	0.536878	0.849031	lncRNA
ENSG0000(	LINC01099	81.27578	0.086986	0.536851	0.849031	lncRNA
ENSG0000(	POU6F1	479.9341	-0.05876	0.536863	0.849031	protein_coding
ENSG0000(	ANKRD36	5919.335	0.05769	0.536966	0.84911	protein_coding
ENSG0000(	CKAP2	365.3458	-0.08294	0.53699	0.84911	protein_coding
ENSG0000(	CABP1-DT	81.50591	0.098414	0.537054	0.849162	lncRNA
ENSG0000(	ENSG0000(	130.7307	0.107656	0.5371	0.849187	lncRNA
ENSG0000(	LINC02157	50.25271	-0.12799	0.537173	0.849254	lncRNA
ENSG0000(	ENSG0000(	29.24891	0.131318	0.537324	0.849346	lncRNA
ENSG0000(	FUT1	458.1764	-0.06515	0.537271	0.849346	protein_coding
ENSG0000(	SNRNP25	94.16821	0.116171	0.537319	0.849346	protein_coding
ENSG0000(	ENSG0000(	42.18334	-0.12474	0.53757	0.849397	lncRNA
ENSG0000(	ENSG0000(	138.9782	-0.07238	0.537573	0.849397	TEC
ENSG0000(	ENSG0000(	253.9112	-0.07461	0.537511	0.849397	TEC
ENSG0000(	ENSG0000(	53.94254	0.088232	0.53752	0.849397	lncRNA
ENSG0000(	MPC1-DT	374.1249	0.040692	0.537542	0.849397	lncRNA
ENSG0000(	PPIE	1162.603	-0.06079	0.537438	0.849397	protein_coding
ENSG0000(	PRDX5	499.3892	0.117235	0.537464	0.849397	protein_coding
ENSG0000(	ENSG0000(	51.89475	0.285663	0.537627	0.849433	TEC
ENSG0000(	CMKLR2	82.5492	-0.08951	0.537749	0.84945	protein_coding
ENSG0000(	ENSG0000(	29.43982	-0.14846	0.537791	0.84945	snoRNA
ENSG0000(	ENSG0000(	81.11507	0.073651	0.537739	0.84945	TEC
ENSG0000(	ENSG0000(	783.6264	0.067424	0.537789	0.84945	TEC
ENSG0000(	ENSG0000(	177.5335	0.05766	0.537743	0.84945	lncRNA
ENSG0000(	LINC00392	30.59865	0.221587	0.53795	0.849651	lncRNA
ENSG0000(	ENSG0000(	53.01413	0.100048	0.538033	0.849689	lncRNA
ENSG0000(	NOL10	515.4617	0.063385	0.538097	0.849689	protein_coding
ENSG0000(	SNX15	655.0872	0.059177	0.538079	0.849689	protein_coding
ENSG0000(	THOP1	488.8589	0.059669	0.538055	0.849689	protein_coding
ENSG0000(	CDYL	755.4857	0.054758	0.538153	0.849723	protein_coding
ENSG0000(	NANOGP5	75.92642	0.120021	0.538195	0.849723	transcribed_processed_pseudoge
ENSG0000(	NEGR1	2789.619	-0.05539	0.538211	0.849723	protein_coding
ENSG0000(	BTBD1	944.8868	-0.0551	0.538333	0.849769	protein_coding
ENSG0000(	CUTC	164.225	-0.0815	0.538325	0.849769	protein_coding
ENSG0000(	HERPUD2	797.8543	0.065478	0.538275	0.849769	protein_coding
ENSG0000(	CCDC91	945.7226	0.051173	0.538371	0.849779	protein_coding
ENSG0000(	AKR1C3	429.6693	-0.15567	0.538438	0.849814	protein_coding
ENSG0000(	RN7SL23P	24.57123	-0.18494	0.538455	0.849814	misc_RNA
ENSG0000(	ENSG0000(	21.20939	-0.12969	0.538684	0.849884	lncRNA
ENSG0000(	IPO11	626.3948	0.060106	0.538584	0.849884	protein_coding
ENSG0000(	LINC00700	77.38654	-0.09399	0.538587	0.849884	lncRNA
ENSG0000(	LINC01913	107.6922	-0.08162	0.538678	0.849884	lncRNA

ENSG0000(	LRRC3B	35.46525	0.315045	0.538662	0.849884	protein_coding
ENSG0000(	NECAP2	1113.842	-0.05954	0.538624	0.849884	protein_coding
ENSG0000(	FANCD2OS	77.28886	0.112807	0.538756	0.849948	protein_coding
ENSG0000(	AACS	2608.337	0.035615	0.539144	0.850001	protein_coding
ENSG0000(	C7orf50	428.4744	0.060725	0.53915	0.850001	protein_coding
ENSG0000(	ENSG0000(	25.97725	-0.14828	0.539001	0.850001	processed_pseudogene
ENSG0000(	ENSG0000(	66.44857	0.15385	0.538896	0.850001	lncRNA
ENSG0000(	ENSG0000(	321.9524	-0.05748	0.539118	0.850001	TEC
ENSG0000(	ENSG0000(	203.7004	-0.05893	0.539119	0.850001	lncRNA
ENSG0000(	ENSG0000(	234.9899	0.053787	0.538903	0.850001	lncRNA
ENSG0000(	FTH1	13966.45	-0.12449	0.539191	0.850001	protein_coding
ENSG0000(	LARP4P	53.09254	0.098097	0.539096	0.850001	transcribed_processed_pseudoge
ENSG0000(	LINC01427	3558.048	-0.03887	0.539109	0.850001	lncRNA
ENSG0000(	RABGAP1	2285.141	0.038748	0.538836	0.850001	protein_coding
ENSG0000(	TBC1D16	1316.561	-0.09363	0.539165	0.850001	protein_coding
ENSG0000(	TOX4	1808.089	-0.04427	0.539116	0.850001	protein_coding
ENSG0000(	SEC14L2	138.4543	-0.1894	0.539234	0.85002	protein_coding
ENSG0000(	LINC02717	319.4355	-0.04168	0.539334	0.850081	lncRNA
ENSG0000(	SNRPC	184.9145	-0.07995	0.539321	0.850081	protein_coding
ENSG0000(	ATP1A1-AS	856.9351	0.066232	0.539981	0.850123	lncRNA
ENSG0000(	BBS7	245.4695	0.123251	0.539931	0.850123	protein_coding
ENSG0000(	COLCA2	196.9989	0.169255	0.540133	0.850123	protein_coding
ENSG0000(	COX7A2L	840.1441	0.055595	0.53997	0.850123	protein_coding
ENSG0000(	EBAG9	265.4926	0.05722	0.539488	0.850123	protein_coding
ENSG0000(	ENSG0000(	318.8629	-0.05309	0.539982	0.850123	lncRNA
ENSG0000(	ENSG0000(	100.5691	-0.08338	0.540047	0.850123	lncRNA
ENSG0000(	ENSG0000(	149.2637	-0.08085	0.539748	0.850123	lncRNA
ENSG0000(	ENSG0000(	443.5232	0.084288	0.539744	0.850123	lncRNA
ENSG0000(	ENSG0000(	25.42008	0.190501	0.539665	0.850123	lncRNA
ENSG0000(	ENSG0000(	44.85236	0.113537	0.540078	0.850123	lncRNA
ENSG0000(	ENSG0000(	97.21286	0.08358	0.539532	0.850123	lncRNA
ENSG0000(	ENSG0000(	163.5449	0.06619	0.539417	0.850123	lncRNA
ENSG0000(	ENSG0000(	3456.239	-0.03849	0.539721	0.850123	lncRNA
ENSG0000(	ENSG0000(	47.61519	0.128771	0.539553	0.850123	lncRNA
ENSG0000(	ENSG0000(	33.62723	-0.15449	0.540133	0.850123	lncRNA
ENSG0000(	FNIP1	1801.697	-0.04276	0.539926	0.850123	protein_coding
ENSG0000(	GGCX	2294.127	-0.03194	0.539926	0.850123	protein_coding
ENSG0000(	LINC01630	325.5949	-0.0649	0.539816	0.850123	lncRNA
ENSG0000(	MED18	434.0505	0.045512	0.539712	0.850123	protein_coding
ENSG0000(	MED6	636.6171	-0.04585	0.539491	0.850123	protein_coding
ENSG0000(	PSG9	600.1903	0.135342	0.54007	0.850123	protein_coding
ENSG0000(	RNU1-11P	370.336	0.13507	0.539648	0.850123	snRNA
ENSG0000(	RPL32P3	1700.271	0.041195	0.539464	0.850123	transcribed_unprocessed_pseudo
ENSG0000(	SPTSSA	617.1511	0.061249	0.539945	0.850123	protein_coding
ENSG0000(	ENSG0000(	954.3397	-0.06429	0.540461	0.850434	lncRNA
ENSG0000(	LACTB2-AS	756.1493	0.051042	0.540465	0.850434	lncRNA
ENSG0000(	LINC00943	126.1026	0.10115	0.540485	0.850434	lncRNA

ENSG0000(	TUBAP11	27.21658	-0.1286	0.540413	0.850434	processed_pseudogene
ENSG0000(	WDR35	375.3477	-0.10757	0.54046	0.850434	protein_coding
ENSG0000(	HLA-K	114.7506	-0.21224	0.540554	0.850445	unprocessed_pseudogene
ENSG0000(	THEM6	174.8513	0.116895	0.540542	0.850445	protein_coding
ENSG0000(	TWNK	172.6153	0.10857	0.540607	0.85048	protein_coding
ENSG0000(	CACNG5	509.3115	0.042764	0.540908	0.850491	protein_coding
ENSG0000(	CASKIN2	420.3254	0.116313	0.541048	0.850491	protein_coding
ENSG0000(	EGFLAM-A!	34.12196	0.155332	0.540733	0.850491	lncRNA
ENSG0000(	ENSG0000(	59.81532	0.103476	0.540787	0.850491	lncRNA
ENSG0000(	ENSG0000(	497.793	0.042216	0.540799	0.850491	lncRNA
ENSG0000(	ENSG0000(	302.1183	-0.05351	0.540701	0.850491	lncRNA
ENSG0000(	FASTKD2	1337.763	0.043803	0.540937	0.850491	protein_coding
ENSG0000(	FEM1C	1511.538	0.073987	0.541006	0.850491	protein_coding
ENSG0000(	LINC01837	53.74324	0.147457	0.540833	0.850491	lncRNA
ENSG0000(	LRRC1	1440.732	0.117589	0.54092	0.850491	protein_coding
ENSG0000(	PRKAB1	2517.47	0.077543	0.540968	0.850491	protein_coding
ENSG0000(	PROSER2-A	66.42222	0.082104	0.54104	0.850491	lncRNA
ENSG0000(	RN7SL624F	58.76431	0.102094	0.540943	0.850491	misc_RNA
ENSG0000(	VENTXP1	429.7864	0.047091	0.54078	0.850491	transcribed_processed_pseudoge
ENSG0000(	GLP2R	240.2716	0.198738	0.541105	0.850534	protein_coding
ENSG0000(	ITM2C	4982.106	0.127195	0.541141	0.850542	protein_coding
ENSG0000(	ENSG0000(	434.1662	0.136694	0.54124	0.85055	processed_pseudogene
ENSG0000(	ENSG0000(	1743.693	-0.084	0.541219	0.85055	lncRNA
ENSG0000(	ENSG0000(	22.33227	-1.07058	0.541199	0.85055	lncRNA
ENSG0000(	ATF7	2185.143	-0.04642	0.541477	0.850736	protein_coding
ENSG0000(	ENSG0000(	342.2529	0.056903	0.541481	0.850736	lncRNA
ENSG0000(	ENSG0000(	22.60623	-0.15244	0.54143	0.850736	processed_pseudogene
ENSG0000(	OR1C1	44.99782	0.139764	0.54148	0.850736	protein_coding
ENSG0000(	MOGAT2	1005.569	0.16508	0.541516	0.850741	protein_coding
ENSG0000(	ENSG0000(	238.2301	0.082019	0.541675	0.850798	lncRNA
ENSG0000(	KIF14	391.8429	-0.08866	0.541655	0.850798	protein_coding
ENSG0000(	LINC02466	60.44405	0.089802	0.541711	0.850798	lncRNA
ENSG0000(	RN7SL461F	61.10441	-0.10347	0.541738	0.850798	misc_RNA
ENSG0000(	VASH1-AS1	446.0293	-0.0471	0.541718	0.850798	lncRNA
ENSG0000(	ZNF334	474.1468	0.134188	0.541614	0.850798	protein_coding
ENSG0000(	ABCA6	1703.536	-0.06814	0.541842	0.850913	protein_coding
ENSG0000(	MAST3	1703.714	0.050146	0.541906	0.850966	protein_coding
ENSG0000(	ENSG0000(	94.46715	0.068918	0.542005	0.851024	lncRNA
ENSG0000(	LRRC20	78.2145	-0.10495	0.541989	0.851024	protein_coding
ENSG0000(	ABCC4	1181.975	-0.07498	0.542387	0.851027	protein_coding
ENSG0000(	AKR7A3	234.1401	0.146469	0.54266	0.851027	protein_coding
ENSG0000(	ATP6V0D2	202.2423	0.088585	0.543043	0.851027	protein_coding
ENSG0000(	C12orf4	846.289	0.051486	0.5429	0.851027	protein_coding
ENSG0000(	C3orf33	176.5485	0.094022	0.5429	0.851027	protein_coding
ENSG0000(	CNPY2	1185.462	-0.04155	0.543378	0.851027	protein_coding
ENSG0000(	DDX50	377.9111	-0.07583	0.542295	0.851027	protein_coding
ENSG0000(	DLG3	1568.649	0.103845	0.543285	0.851027	protein_coding

ENSG0000(	DMTF1	4543.354	-0.06218	0.542143	0.851027	protein_coding
ENSG0000(	DMXL1	3881.927	0.063304	0.542555	0.851027	protein_coding
ENSG0000(	ENSG0000(	27.48295	0.143473	0.542531	0.851027	lncRNA
ENSG0000(	ENSG0000(	100.239	0.07236	0.542995	0.851027	lncRNA
ENSG0000(	ENSG0000(	224.1415	0.058827	0.543035	0.851027	lncRNA
ENSG0000(	ENSG0000(	25.80273	0.169884	0.542322	0.851027	lncRNA
ENSG0000(	ENSG0000(	309.3747	0.062322	0.542803	0.851027	lncRNA
ENSG0000(	ENSG0000(	360.8846	0.050144	0.543231	0.851027	lncRNA
ENSG0000(	ENSG0000(	345.6656	-0.05003	0.543093	0.851027	lncRNA
ENSG0000(	ENSG0000(	77.38223	-0.08712	0.543093	0.851027	lncRNA
ENSG0000(	ENSG0000(	66.22347	0.23871	0.542436	0.851027	transcribed_unitary_pseudogene
ENSG0000(	ENSG0000(	137.9528	-0.10328	0.54292	0.851027	lncRNA
ENSG0000(	ENSG0000(	19.91634	0.169542	0.543399	0.851027	lncRNA
ENSG0000(	JMJD7	80.17034	0.129504	0.542507	0.851027	protein_coding
ENSG0000(	KCNMA1-A	284.4071	0.045449	0.54287	0.851027	lncRNA
ENSG0000(	LINC01618	107.8335	-0.09289	0.543192	0.851027	lncRNA
ENSG0000(	LINC01952	35.59758	-0.12904	0.542921	0.851027	lncRNA
ENSG0000(	LRRC59	1452.385	-0.07045	0.543281	0.851027	protein_coding
ENSG0000(	MSANTD4	424.9984	-0.06761	0.54336	0.851027	protein_coding
ENSG0000(	NOL3	463.7863	-0.06041	0.543128	0.851027	protein_coding
ENSG0000(	PBX3	518.0677	-0.09971	0.543388	0.851027	protein_coding
ENSG0000(	PMS2P6	300.3405	0.172561	0.542385	0.851027	unprocessed_pseudogene
ENSG0000(	PRR11	1555.013	0.046236	0.543161	0.851027	protein_coding
ENSG0000(	RFNG	270.4275	-0.07429	0.542673	0.851027	protein_coding
ENSG0000(	RPL21P65	40.4363	-0.13808	0.543019	0.851027	processed_pseudogene
ENSG0000(	SILC1	1179.719	-0.0528	0.54278	0.851027	lncRNA
ENSG0000(	SMS	394.8562	-0.08004	0.542524	0.851027	protein_coding
ENSG0000(	SNPH	155.5696	0.165263	0.542712	0.851027	protein_coding
ENSG0000(	SNRPA1-DT	78.91342	0.077087	0.543388	0.851027	lncRNA
ENSG0000(	SOX11	108.1155	0.115439	0.5427	0.851027	protein_coding
ENSG0000(	SQOR	1120.087	-0.08945	0.542574	0.851027	protein_coding
ENSG0000(	TMEM255/	49.4958	0.208639	0.543222	0.851027	protein_coding
ENSG0000(	TPTE	23.90431	-0.18534	0.542328	0.851027	protein_coding
ENSG0000(	WDR25	224.4476	0.069535	0.543024	0.851027	protein_coding
ENSG0000(	WNT3	147.7368	0.182202	0.542939	0.851027	protein_coding
ENSG0000(	YWHAH-AS	610.8872	-0.03666	0.543114	0.851027	lncRNA
ENSG0000(	ZNF780A	2144.766	-0.03237	0.542857	0.851027	protein_coding
ENSG0000(	LINC02324	1039.251	-0.03739	0.543451	0.85106	lncRNA
ENSG0000(	SOHLH2	36.0209	0.104976	0.54349	0.851073	protein_coding
ENSG0000(	OSER1-DT	968.2831	-0.03171	0.54367	0.851306	lncRNA
ENSG0000(	ENSG0000(	154.898	0.068215	0.543724	0.851341	lncRNA
ENSG0000(	INTS9	252.9661	0.072347	0.543822	0.851446	protein_coding
ENSG0000(	NTMT1	242.2923	-0.06432	0.543852	0.851446	protein_coding
ENSG0000(	ABCF2	675.4164	-0.07204	0.544106	0.85148	protein_coding
ENSG0000(	ENSG0000(	64.66175	0.110892	0.544103	0.85148	lncRNA
ENSG0000(	ENSG0000(	1710.238	0.029926	0.543995	0.85148	lncRNA
ENSG0000(	FBXL8	351.1793	0.062067	0.544084	0.85148	protein_coding

ENSG0000(	SCX	45.10405	0.176429	0.544121	0.85148	protein_coding
ENSG0000(	SLC35B4	798.2185	-0.06648	0.544112	0.85148	protein_coding
ENSG0000(	SOWAHB	413.1379	0.141896	0.543952	0.85148	protein_coding
ENSG0000(	TRMT5	340.0972	0.086207	0.543993	0.85148	protein_coding
ENSG0000(	ENSG0000(	23.42236	0.245752	0.544339	0.851747	processed_pseudogene
ENSG0000(	MYL3	213.1637	-0.05205	0.544354	0.851747	protein_coding
ENSG0000(	ABHD17B	442.3779	0.071929	0.544468	0.851837	protein_coding
ENSG0000(	ENSG0000(	26.25121	0.150604	0.544474	0.851837	TEC
ENSG0000(	ENSG0000(	114.0336	0.071733	0.544553	0.851865	lncRNA
ENSG0000(	ENSG0000(	79.14516	-0.08579	0.544544	0.851865	lncRNA
ENSG0000(	DBET	112.5852	0.255291	0.544622	0.851875	lncRNA
ENSG0000(	MIEF1	1233.448	0.052949	0.544613	0.851875	protein_coding
ENSG0000(	ENSG0000(	111.8831	0.061329	0.544692	0.851937	TEC
ENSG0000(	ENSG0000(	96.27143	0.099219	0.544976	0.8523	lncRNA
ENSG0000(	NPIPB7	782.3757	0.05841	0.545	0.8523	protein_coding
ENSG0000(	PRUNE1	340.5011	-0.06991	0.545017	0.8523	protein_coding
ENSG0000(	ENSG0000(	108.6691	0.127525	0.545127	0.852335	lncRNA
ENSG0000(	HSPA1B	1086.24	0.134381	0.545158	0.852335	protein_coding
ENSG0000(	PHF20	1407.517	-0.04852	0.54513	0.852335	protein_coding
ENSG0000(	ZDHHC5	2016.011	0.055057	0.545164	0.852335	protein_coding
ENSG0000(	ENSG0000(	93.06173	-0.09045	0.545255	0.852429	lncRNA
ENSG0000(	ENSG0000(	129.6939	-0.1013	0.545286	0.85243	lncRNA
ENSG0000(	F2RL3	560.5121	-0.06135	0.545345	0.852474	protein_coding
ENSG0000(	ENSG0000(	111.0843	0.0757	0.545481	0.852638	lncRNA
ENSG0000(	ENSG0000(	207.9975	-0.08799	0.545556	0.852667	lncRNA
ENSG0000(	ZFP90	954.3919	-0.10072	0.545562	0.852667	protein_coding
ENSG0000(	RAN	1160.938	-0.05411	0.545613	0.852698	protein_coding
ENSG0000(	AADACP1	110.6346	-0.20603	0.545975	0.852706	transcribed_unprocessed_pseudo
ENSG0000(	BTD	1801.877	0.037084	0.546122	0.852706	protein_coding
ENSG0000(	C17orf80	473.5043	0.057345	0.546003	0.852706	protein_coding
ENSG0000(	ENSG0000(	36.12552	0.114835	0.545857	0.852706	lncRNA
ENSG0000(	ENSG0000(	70.24972	0.123224	0.546108	0.852706	TEC
ENSG0000(	ENSG0000(	191.1151	-0.10544	0.546167	0.852706	lncRNA
ENSG0000(	ENSG0000(	22.71028	-0.15923	0.54644	0.852706	lncRNA
ENSG0000(	ENSG0000(	31.87707	0.141863	0.546435	0.852706	lncRNA
ENSG0000(	ENSG0000(	97.37611	-0.08382	0.546055	0.852706	lncRNA
ENSG0000(	ENSG0000(	553.1749	0.055184	0.546306	0.852706	lncRNA
ENSG0000(	ENSG0000(	35.42273	-0.16934	0.54586	0.852706	TEC
ENSG0000(	ENSG0000(	65.88364	0.086074	0.545798	0.852706	TEC
ENSG0000(	ENSG0000(	88.90792	0.076975	0.546436	0.852706	lncRNA
ENSG0000(	ENSG0000(	46.74044	0.165022	0.545672	0.852706	lncRNA
ENSG0000(	ENSG0000(	45.71184	-0.11583	0.546342	0.852706	lncRNA
ENSG0000(	ENSG0000(	86.14549	0.083055	0.546357	0.852706	lncRNA
ENSG0000(	ENSG0000(	311.4158	0.049097	0.546364	0.852706	lncRNA
ENSG0000(	FAM72C	42.99621	0.364132	0.546516	0.852706	protein_coding
ENSG0000(	IVNS1ABP	4627.279	-0.08527	0.545989	0.852706	protein_coding
ENSG0000(	JMJD6	675.1065	-0.07049	0.546198	0.852706	protein_coding

ENSG0000(	KLF6	8167.348	0.107468	0.545757	0.852706	protein_coding
ENSG0000(	PMS1	679.7951	-0.05696	0.545681	0.852706	protein_coding
ENSG0000(	POC1A	76.23422	-0.14939	0.546176	0.852706	protein_coding
ENSG0000(	PTGR1	1434.571	-0.0652	0.546302	0.852706	protein_coding
ENSG0000(	SCARNA11	33.24321	0.129015	0.546322	0.852706	scaRNA
ENSG0000(	SCGN	20.22839	0.219895	0.546486	0.852706	protein_coding
ENSG0000(	STRAP	463.8146	-0.07512	0.545785	0.852706	protein_coding
ENSG0000(	TSTD3	1364.706	0.040364	0.546264	0.852706	protein_coding
ENSG0000(	WDR46	253.0495	-0.08696	0.546482	0.852706	protein_coding
ENSG0000(	BACE1-AS	646.557	0.048902	0.546581	0.852758	lncRNA
ENSG0000(	LINC00298	37.07479	-0.15029	0.546776	0.853014	lncRNA
ENSG0000(	ENSG0000(	568.6041	-0.04367	0.546835	0.853057	lncRNA
ENSG0000(	GRM8	159.248	0.216145	0.546897	0.853106	protein_coding
ENSG0000(	ATP6AP2	1005.62	-0.04611	0.546983	0.853143	protein_coding
ENSG0000(	SNORD97	728.6349	0.162015	0.546973	0.853143	snoRNA
ENSG0000(	PNPLA3	263.7539	-0.08987	0.54703	0.853168	protein_coding
ENSG0000(	ENSG0000(	80.9156	0.067813	0.54723	0.853287	lncRNA
ENSG0000(	ENSG0000(	38.35169	-0.10598	0.547185	0.853287	lncRNA
ENSG0000(	ENSG0000(	858.5716	0.056444	0.547215	0.853287	lncRNA
ENSG0000(	SPINK9	107.4124	0.123281	0.547214	0.853287	protein_coding
ENSG0000(	LINC02687	98.64956	0.083082	0.547265	0.853293	lncRNA
ENSG0000(	ACOT2	104.2898	0.102411	0.547371	0.853365	protein_coding
ENSG0000(	ENSG0000(	488.0252	-0.04455	0.547373	0.853365	lncRNA
ENSG0000(	PGRMC1	723.6629	-0.10093	0.547412	0.853377	protein_coding
ENSG0000(	ENSG0000(	1374.753	0.050033	0.547507	0.853477	lncRNA
ENSG0000(	ANAPC10P	27.43046	0.129002	0.547605	0.85352	processed_pseudogene
ENSG0000(	ENSG0000(	97.84752	0.085994	0.547625	0.85352	lncRNA
ENSG0000(	NUTM2A-A	6066.107	0.047022	0.547628	0.85352	lncRNA
ENSG0000(	ZNF638	5945.429	-0.0383	0.547671	0.853538	protein_coding
ENSG0000(	ABCC8	568.1942	-0.03531	0.548282	0.853572	protein_coding
ENSG0000(	ANKRD29	311.6081	-0.12955	0.54832	0.853572	protein_coding
ENSG0000(	APTR	1110.026	0.043325	0.5483	0.853572	lncRNA
ENSG0000(	CCDC183-A	178.7009	0.07543	0.547775	0.853572	lncRNA
ENSG0000(	CRYBG2	434.1064	0.200731	0.548203	0.853572	protein_coding
ENSG0000(	EFCAB14	8390.499	-0.03373	0.547873	0.853572	protein_coding
ENSG0000(	ENSG0000(	32.01002	0.12215	0.548354	0.853572	snoRNA
ENSG0000(	ENSG0000(	27.75583	0.150643	0.548102	0.853572	lncRNA
ENSG0000(	ENSG0000(	626.3177	0.040497	0.548011	0.853572	lncRNA
ENSG0000(	ENSG0000(	114.9811	0.097334	0.548406	0.853572	lncRNA
ENSG0000(	ENSG0000(	89.833	-0.10154	0.547905	0.853572	lncRNA
ENSG0000(	ENSG0000(	370.1097	0.10195	0.548159	0.853572	protein_coding
ENSG0000(	ENSG0000(	74.83858	0.107126	0.547925	0.853572	lncRNA
ENSG0000(	FGF14-IT1	23.24138	0.245412	0.548093	0.853572	lncRNA
ENSG0000(	LINC00861	857.9481	-0.18164	0.548143	0.853572	lncRNA
ENSG0000(	LINC02289	72.98379	-0.1657	0.547954	0.853572	lncRNA
ENSG0000(	P2RX6	50.53804	0.119128	0.548141	0.853572	protein_coding
ENSG0000(	RNU1-83P	91.24552	-0.15526	0.54802	0.853572	snRNA

ENSG0000(	RNU6-122F	36.8422	-0.15262	0.547894	0.853572	snRNA
ENSG0000(	RPS18	2904.05	0.082722	0.548319	0.853572	protein_coding
ENSG0000(	SIX3	39.83561	0.126949	0.547874	0.853572	protein_coding
ENSG0000(	SOX9-AS1	1155.259	0.045417	0.548384	0.853572	lncRNA
ENSG0000(	TAOK3	2513.652	0.040668	0.548314	0.853572	protein_coding
ENSG0000(	ENSG0000(	96.21929	0.074501	0.548457	0.853599	lncRNA
ENSG0000(	TMEM212-	137.5982	0.07209	0.548485	0.853599	lncRNA
ENSG0000(	C2CD3	4499.171	0.023819	0.548705	0.853642	protein_coding
ENSG0000(	CDC42BPA	5101.962	0.08374	0.548556	0.853642	protein_coding
ENSG0000(	CEACAM3	440.5494	-0.11745	0.548629	0.853642	protein_coding
ENSG0000(	ENSG0000(	483.5578	-0.04031	0.54896	0.853642	lncRNA
ENSG0000(	ENSG0000(	104.0234	0.074781	0.549006	0.853642	lncRNA
ENSG0000(	ENSG0000(	71.04566	-0.10312	0.548715	0.853642	lncRNA
ENSG0000(	ENSG0000(	195.5273	-0.08348	0.548992	0.853642	lncRNA
ENSG0000(	ENSG0000(	480.6359	0.043309	0.54904	0.853642	TEC
ENSG0000(	ENSG0000(	329.784	-0.06467	0.548809	0.853642	protein_coding
ENSG0000(	INPP5A	458.2075	0.079299	0.548964	0.853642	protein_coding
ENSG0000(	KBTBD7	240.8646	-0.05591	0.549038	0.853642	protein_coding
ENSG0000(	LRRC47	571.5805	0.058791	0.549001	0.853642	protein_coding
ENSG0000(	NAT16	100.7169	-0.09952	0.548915	0.853642	protein_coding
ENSG0000(	NDUFB1	478.8081	0.061708	0.548721	0.853642	protein_coding
ENSG0000(	PRELP	420.2127	0.074346	0.548855	0.853642	protein_coding
ENSG0000(	RAD54B	116.9513	-0.11094	0.549003	0.853642	protein_coding
ENSG0000(	RALGAPA1I	292.1847	0.101079	0.54862	0.853642	processed_pseudogene
ENSG0000(	ENSG0000(	784.1002	0.04011	0.549155	0.853675	lncRNA
ENSG0000(	KDM7A	4479.335	0.05099	0.549154	0.853675	protein_coding
ENSG0000(	ZDHHC22	62.13927	-0.14123	0.549145	0.853675	protein_coding
ENSG0000(	RPL3P4	63.8411	1.37722	0.54925	0.853775	processed_pseudogene
ENSG0000(	ENSG0000(	20.1574	0.216514	0.549365	0.853833	lncRNA
ENSG0000(	FABP3	30.01015	-0.28616	0.54933	0.853833	protein_coding
ENSG0000(	RNF2	286.1966	0.06365	0.54938	0.853833	protein_coding
ENSG0000(	ENSG0000(	68.73153	0.120291	0.549414	0.853837	lncRNA
ENSG0000(	ENSG0000(	184.5436	-0.0688	0.549637	0.854136	lncRNA
ENSG0000(	ENSG0000(	92.48301	0.076359	0.549961	0.85435	lncRNA
ENSG0000(	ENSG0000(	131.628	0.063304	0.550024	0.85435	lncRNA
ENSG0000(	ENSG0000(	122.2598	0.186706	0.549945	0.85435	protein_coding
ENSG0000(	OXLD1	216.191	-0.0517	0.549886	0.85435	protein_coding
ENSG0000(	RAD21L1	31.80115	-0.1769	0.549908	0.85435	protein_coding
ENSG0000(	TEX264	606.0348	0.062651	0.550023	0.85435	protein_coding
ENSG0000(	UTP20	910.4762	0.078398	0.549994	0.85435	protein_coding
ENSG0000(	ZSCAN4	22.31885	0.168929	0.549904	0.85435	protein_coding
ENSG0000(	OSBPL1A	947.9842	0.05445	0.550219	0.854488	protein_coding
ENSG0000(	PKMYT1	138.7635	-0.11973	0.55024	0.854488	protein_coding
ENSG0000(	RN7SL577F	41.54523	-0.11953	0.550236	0.854488	misc_RNA
ENSG0000(	SMIM26	121.8314	-0.08495	0.550231	0.854488	protein_coding
ENSG0000(	SYS1-DBND	23.2028	-0.22037	0.550268	0.854488	protein_coding
ENSG0000(	MGAT4C	676.3561	0.076906	0.550428	0.854582	protein_coding

ENSG0000(	NLK	965.0473	-0.05487	0.550452	0.854582	protein_coding
ENSG0000(	RGR	295.0492	-0.06095	0.550444	0.854582	protein_coding
ENSG0000(	RPF2	303.2197	-0.06336	0.550376	0.854582	protein_coding
ENSG0000(	ADCY1	293.965	-0.0919	0.550604	0.854666	protein_coding
ENSG0000(	CASP9	407.8151	0.073026	0.550629	0.854666	protein_coding
ENSG0000(	ENSG0000(	89.65878	0.091319	0.550603	0.854666	lncRNA
ENSG0000(	LINC01772	557.6992	0.045709	0.550631	0.854666	lncRNA
ENSG0000(	CUL4B	11266.56	-0.02617	0.550698	0.854669	protein_coding
ENSG0000(	ITGB1BP1	532.0335	-0.06319	0.550703	0.854669	protein_coding
ENSG0000(	TAPBPL	814.6967	-0.06189	0.550726	0.854669	protein_coding
ENSG0000(	ACADM	2130.931	0.045441	0.551206	0.854705	protein_coding
ENSG0000(	AGR2	1951.8	0.192187	0.550976	0.854705	protein_coding
ENSG0000(	ATIC	456.6439	-0.0699	0.55127	0.854705	protein_coding
ENSG0000(	BRIP1	2819.986	0.044473	0.551159	0.854705	protein_coding
ENSG0000(	CLDN10	141.4343	0.071629	0.551032	0.854705	protein_coding
ENSG0000(	CLDND1	1080.499	-0.05499	0.551308	0.854705	protein_coding
ENSG0000(	DLAT	886.3642	0.059301	0.550919	0.854705	protein_coding
ENSG0000(	ENSG0000(	351.1254	0.049086	0.550802	0.854705	lncRNA
ENSG0000(	ENSG0000(	124.9358	-0.10191	0.551094	0.854705	lncRNA
ENSG0000(	ENSG0000(	25.0907	-0.40203	0.550932	0.854705	lncRNA
ENSG0000(	ENSG0000(	65.19667	-0.1182	0.550994	0.854705	lncRNA
ENSG0000(	ENSG0000(	67.96085	0.141747	0.551141	0.854705	lncRNA
ENSG0000(	GFM2	435.6517	-0.05058	0.551264	0.854705	protein_coding
ENSG0000(	IGFN1	85.96148	0.23789	0.551303	0.854705	protein_coding
ENSG0000(	NPFFR2	50.06798	-0.1127	0.551098	0.854705	protein_coding
ENSG0000(	PSMD6	1003.742	-0.03444	0.551052	0.854705	protein_coding
ENSG0000(	RNU6-407F	19.52402	0.169528	0.55103	0.854705	snRNA
ENSG0000(	ZEB1-AS1	187.0999	-0.0899	0.55101	0.854705	lncRNA
ENSG0000(	ENSG0000(	257.2911	0.060005	0.551374	0.854759	lncRNA
ENSG0000(	ATP5MC2	1016.542	0.069979	0.551448	0.854825	protein_coding
ENSG0000(	C19orf48	475.9071	-0.05985	0.551597	0.854865	transcribed_unitary_pseudogene
ENSG0000(	CFAP298	467.8138	-0.04961	0.55154	0.854865	protein_coding
ENSG0000(	IZUMO4	34.81827	0.204176	0.551629	0.854865	protein_coding
ENSG0000(	PTPRQ	29.17596	-0.428	0.551612	0.854865	protein_coding
ENSG0000(	TRAIP	81.92691	0.126149	0.551595	0.854865	protein_coding
ENSG0000(	ENSG0000(	74.91894	-0.11591	0.551777	0.855046	unprocessed_pseudogene
ENSG0000(	MYH11	3972.376	0.092743	0.55183	0.85508	protein_coding
ENSG0000(	AHDC1	837.1165	-0.0629	0.552066	0.855107	protein_coding
ENSG0000(	CFAP251	1352.111	-0.02394	0.552085	0.855107	protein_coding
ENSG0000(	ENSG0000(	141.6829	0.093033	0.552082	0.855107	lncRNA
ENSG0000(	FRRS1L	1563.322	-0.02699	0.55194	0.855107	protein_coding
ENSG0000(	KIF25-AS1	55.33528	0.105649	0.551971	0.855107	lncRNA
ENSG0000(	NORAD	6378.088	-0.04461	0.552011	0.855107	lncRNA
ENSG0000(	PLAAT3	237.8019	-0.08276	0.552096	0.855107	protein_coding
ENSG0000(	SUSD3	97.03234	-0.12645	0.552003	0.855107	protein_coding
ENSG0000(	ZFP1	134.8321	0.117881	0.552232	0.85527	protein_coding
ENSG0000(	VSTM2A	165.0258	0.182023	0.552298	0.855324	protein_coding

ENSG0000(	ALKBH7	95.36204	0.137648	0.552463	0.855386	protein_coding
ENSG0000(	MAP2K6	5016.997	0.107972	0.552435	0.855386	protein_coding
ENSG0000(	STX5-DT	56.29167	-0.10918	0.552441	0.855386	lncRNA
ENSG0000(	TEX15	27.11166	0.165936	0.552408	0.855386	protein_coding
ENSG0000(	ENSG0000(	256.2961	-0.0874	0.552564	0.855437	lncRNA
ENSG0000(	ENSG0000(	582.0614	0.057628	0.552589	0.855437	lncRNA
ENSG0000(	TPM3	7420.742	-0.04229	0.552538	0.855437	protein_coding
ENSG0000(	BECN1	1819.165	0.037389	0.552703	0.855539	protein_coding
ENSG0000(	CLSTN2	384.6604	-0.1296	0.552778	0.855539	protein_coding
ENSG0000(	EIF2B5	1057.84	0.046456	0.552802	0.855539	protein_coding
ENSG0000(	ENSG0000(	43.3322	0.123556	0.552775	0.855539	lncRNA
ENSG0000(	MELK	212.177	-0.08127	0.552841	0.855539	protein_coding
ENSG0000(	UCP3	92.06977	-0.1062	0.552823	0.855539	protein_coding
ENSG0000(	AOPEP	3029.785	-0.05018	0.552898	0.855565	protein_coding
ENSG0000(	ENSG0000(	34.41011	0.151306	0.552951	0.855565	TEC
ENSG0000(	HMGN2P4(	463.9748	0.087204	0.552942	0.855565	transcribed_processed_pseudoge
ENSG0000(	ENSG0000(	23.01233	-0.15659	0.552983	0.855566	lncRNA
ENSG0000(	CPED1	631.5287	-0.07036	0.553075	0.855611	protein_coding
ENSG0000(	ENSG0000(	217.1886	0.060681	0.55305	0.855611	lncRNA
ENSG0000(	ENSG0000(	34.56321	0.122942	0.553176	0.855706	lncRNA
ENSG0000(	MLF2	562.9063	-0.04961	0.553229	0.855706	protein_coding
ENSG0000(	TSR1	901.8813	-0.05329	0.553222	0.855706	protein_coding
ENSG0000(	ENSG0000(	27.68241	-0.1423	0.55326	0.855706	lncRNA
ENSG0000(	TRIM8-DT	22.69036	-0.13451	0.553313	0.85574	lncRNA
ENSG0000(	ENSG0000(	23.46258	-0.13444	0.553512	0.856	lncRNA
ENSG0000(	ENSG0000(	63.75303	-0.19656	0.553552	0.856013	lncRNA
ENSG0000(	ZNF788P	967.422	-0.04032	0.553585	0.856016	transcribed_unprocessed_pseudo
ENSG0000(	ENSG0000(	355.8226	0.042803	0.553643	0.856057	lncRNA
ENSG0000(	RUBCNL	2835.012	0.075657	0.553679	0.856065	protein_coding
ENSG0000(	PTCH2	107.787	0.107679	0.553727	0.856091	protein_coding
ENSG0000(	DGKH	4499.206	0.050558	0.553819	0.856134	protein_coding
ENSG0000(	ENSG0000(	19.47895	0.181905	0.553848	0.856134	lncRNA
ENSG0000(	MIR548AA:	54.29225	0.107761	0.553791	0.856134	miRNA
ENSG0000(	ENSG0000(	90.71108	-0.07572	0.553998	0.856317	lncRNA
ENSG0000(	ENSG0000(	245.2915	-0.07038	0.554276	0.856507	lncRNA
ENSG0000(	ENSG0000(	26.36888	0.174796	0.554203	0.856507	lncRNA
ENSG0000(	ENSG0000(	53.60832	0.105475	0.554244	0.856507	TEC
ENSG0000(	ENSG0000(	20.68792	0.193425	0.554266	0.856507	lncRNA
ENSG0000(	GTF3C2	1316.91	0.037122	0.554173	0.856507	protein_coding
ENSG0000(	POTEH	29.32668	-0.59092	0.554332	0.856545	protein_coding
ENSG0000(	DHRS7	484.842	-0.0393	0.554451	0.856607	protein_coding
ENSG0000(	LARP1	4904.635	-0.04863	0.554416	0.856607	protein_coding
ENSG0000(	MTR	3603.379	0.035198	0.554465	0.856607	protein_coding
ENSG0000(	DDX21	2131.598	-0.05033	0.554789	0.856626	protein_coding
ENSG0000(	ENSG0000(	68.5758	-0.09513	0.554753	0.856626	lncRNA
ENSG0000(	ENSG0000(	269.6113	-0.04675	0.554618	0.856626	processed_pseudogene
ENSG0000(	ENSG0000(	44.90391	-0.12957	0.55482	0.856626	lncRNA

ENSG0000(	ENSG0000(	1155.982	0.033521	0.55481	0.856626	TEC
ENSG0000(	ENSG0000(	177.7602	-0.07376	0.554604	0.856626	protein_coding
ENSG0000(	ENSG0000(	107.9614	1.120441	0.55479	0.856626	lncRNA
ENSG0000(	HPGDS	88.39663	-0.16123	0.554673	0.856626	protein_coding
ENSG0000(	LCN12	99.9513	0.082514	0.554704	0.856626	protein_coding
ENSG0000(	NPY4R2	93.02894	0.288533	0.554565	0.856626	protein_coding
ENSG0000(	RN7SL644F	157.5284	0.072559	0.554635	0.856626	misc_RNA
ENSG0000(	LINC02126	49.39607	-0.09709	0.554885	0.85663	lncRNA
ENSG0000(	RASA2	1588.507	-0.05289	0.554883	0.85663	protein_coding
ENSG0000(	TSPAN6	1128.541	0.078407	0.554927	0.856646	protein_coding
ENSG0000(	CSNK2A2	1907.333	-0.03647	0.555076	0.856719	protein_coding
ENSG0000(	ENSG0000(	112.0145	-0.0758	0.555098	0.856719	lncRNA
ENSG0000(	ENSG0000(	1226.801	-0.04856	0.55504	0.856719	lncRNA
ENSG0000(	GDPD1	843.09	0.049529	0.55505	0.856719	protein_coding
ENSG0000(	ENSG0000(	64.08256	-0.1086	0.555155	0.856757	lncRNA
ENSG0000(	AHCY	568.464	-0.07308	0.555654	0.856768	protein_coding
ENSG0000(	ENSG0000(	71.22667	-0.10535	0.555687	0.856768	lncRNA
ENSG0000(	ENSG0000(	76.68908	0.105793	0.555417	0.856768	lncRNA
ENSG0000(	ENSG0000(	93.35633	-0.09021	0.555483	0.856768	lncRNA
ENSG0000(	ENSG0000(	282.0173	-0.09108	0.555397	0.856768	lncRNA
ENSG0000(	FUBP1	3117.509	-0.05817	0.555629	0.856768	protein_coding
ENSG0000(	HNRNPA1P	52.53788	-0.17481	0.555614	0.856768	protein_coding
ENSG0000(	IGLV3-27	29.16579	-0.346	0.555318	0.856768	IG_V_gene
ENSG0000(	MT1JP	36.6512	0.277521	0.555523	0.856768	transcribed_unprocessed_pseudo
ENSG0000(	NARF-AS1	154.993	0.057954	0.555422	0.856768	lncRNA
ENSG0000(	OLFM2	30.66014	-0.18957	0.555304	0.856768	protein_coding
ENSG0000(	POU2F2-AS	20.92026	-0.25863	0.55542	0.856768	lncRNA
ENSG0000(	RBM20	273.9913	0.121345	0.555691	0.856768	protein_coding
ENSG0000(	RN7SL655F	69.31778	-0.08492	0.555342	0.856768	misc_RNA
ENSG0000(	RNF128	185.9066	0.144035	0.555641	0.856768	protein_coding
ENSG0000(	SUMO1	571.2277	-0.05741	0.555532	0.856768	protein_coding
ENSG0000(	UCK1	264.3176	0.083472	0.555673	0.856768	protein_coding
ENSG0000(	ENSG0000(	222.9119	-0.07279	0.555729	0.856779	lncRNA
ENSG0000(	CHKB	1249.017	0.049877	0.555876	0.856958	protein_coding
ENSG0000(	ASB16-AS1	761.9693	0.055459	0.5563	0.857002	lncRNA
ENSG0000(	ENSG0000(	43.89424	0.11595	0.556466	0.857002	lncRNA
ENSG0000(	ENSG0000(	26.86159	-0.18489	0.55644	0.857002	snoRNA
ENSG0000(	ENSG0000(	133.6531	0.072358	0.555975	0.857002	lncRNA
ENSG0000(	ENSG0000(	737.9261	-0.04309	0.556373	0.857002	TEC
ENSG0000(	ENSG0000(	23.54283	-0.14984	0.556438	0.857002	lncRNA
ENSG0000(	ENSG0000(	30.72481	0.210168	0.556019	0.857002	lncRNA
ENSG0000(	ERMARD	707.936	-0.04082	0.556251	0.857002	protein_coding
ENSG0000(	FAM178B	91.86006	-0.08454	0.556352	0.857002	protein_coding
ENSG0000(	IGHD	171.698	-0.28858	0.556181	0.857002	IG_C_gene
ENSG0000(	LINC01970	462.1785	0.036548	0.556031	0.857002	lncRNA
ENSG0000(	LINC02052	33.97511	0.105003	0.556287	0.857002	lncRNA
ENSG0000(	LINC02811	21.70495	-0.16996	0.556284	0.857002	lncRNA

ENSG0000(	MAFG	907.5593	-0.06622	0.55615	0.857002	protein_coding
ENSG0000(	MOK	1203.04	0.034386	0.55628	0.857002	protein_coding
ENSG0000(	POLR2L	138.602	-0.09499	0.556463	0.857002	protein_coding
ENSG0000(	SMC5-DT	212.5673	0.08553	0.55627	0.857002	lncRNA
ENSG0000(	ZBTB14	430.9228	-0.06516	0.556296	0.857002	protein_coding
ENSG0000(	CNTF	161.8486	0.068508	0.556641	0.857176	protein_coding
ENSG0000(	ENSG0000(	73.40779	-0.18295	0.556628	0.857176	lncRNA
ENSG0000(	ADIPOQ-AS	242.4628	-0.04697	0.556783	0.857285	lncRNA
ENSG0000(	BMPR1B	48.10658	-0.18394	0.556881	0.857285	protein_coding
ENSG0000(	ENSG0000(	62.95112	0.076949	0.55705	0.857285	processed_pseudogene
ENSG0000(	ENSG0000(	293.2915	0.089317	0.556883	0.857285	lncRNA
ENSG0000(	ENSG0000(	122.2405	0.087538	0.556922	0.857285	transcribed_unprocessed_pseudo
ENSG0000(	ENSG0000(	247.8004	0.127049	0.556783	0.857285	lncRNA
ENSG0000(	ENSG0000(	33.29741	-0.10468	0.55698	0.857285	lncRNA
ENSG0000(	KTI12	103.2463	-0.12362	0.556965	0.857285	protein_coding
ENSG0000(	LINC01697	614.1485	-0.05284	0.557055	0.857285	lncRNA
ENSG0000(	PSPC1	1383.285	0.040163	0.557032	0.857285	protein_coding
ENSG0000(	RCHY1	758.759	0.03753	0.557003	0.857285	protein_coding
ENSG0000(	LINC01807	70.74154	0.108206	0.557095	0.857299	lncRNA
ENSG0000(	ENSG0000(	203.2738	0.062344	0.557179	0.857381	lncRNA
ENSG0000(	ABCA4	33.43198	-0.77057	0.557378	0.857397	protein_coding
ENSG0000(	BCL2L12	72.4582	-0.15966	0.557439	0.857397	protein_coding
ENSG0000(	ENSG0000(	75.59582	-0.10003	0.557255	0.857397	TEC
ENSG0000(	ENSG0000(	25.46209	0.138535	0.557368	0.857397	lncRNA
ENSG0000(	FAM216A	198.8495	-0.05549	0.557438	0.857397	protein_coding
ENSG0000(	SLC47A1	448.0789	-0.05278	0.557314	0.857397	protein_coding
ENSG0000(	U2AF1	2265.93	-0.23451	0.557329	0.857397	protein_coding
ENSG0000(	UPK3BL2	557.9713	0.093501	0.557352	0.857397	protein_coding
ENSG0000(	CCZ1	1448.779	0.047486	0.557716	0.857439	protein_coding
ENSG0000(	DMGDH	94.40552	-0.08401	0.557705	0.857439	protein_coding
ENSG0000(	ENSG0000(	134.2059	0.095042	0.557513	0.857439	lncRNA
ENSG0000(	ENSG0000(	298.9279	0.15519	0.557584	0.857439	lncRNA
ENSG0000(	ENSG0000(	894.757	-0.04197	0.557627	0.857439	TEC
ENSG0000(	IPO7	1757.921	-0.05305	0.557705	0.857439	protein_coding
ENSG0000(	PRSS40A	238.685	-0.05788	0.557637	0.857439	transcribed_unprocessed_pseudo
ENSG0000(	TEDDM1	54.49328	0.091369	0.557574	0.857439	protein_coding
ENSG0000(	ENSG0000(	133.6914	0.083652	0.557914	0.857469	lncRNA
ENSG0000(	ENSG0000(	31.5107	0.15361	0.557883	0.857469	lncRNA
ENSG0000(	KRT18	1840.667	0.116771	0.557922	0.857469	protein_coding
ENSG0000(	LINC02254	2854.27	-0.03361	0.557918	0.857469	lncRNA
ENSG0000(	OTOGL	102.032	0.187267	0.557871	0.857469	protein_coding
ENSG0000(	UGT2B4	47.18409	0.092236	0.557882	0.857469	protein_coding
ENSG0000(	AQP4-AS1	340.0874	0.056775	0.558009	0.857484	lncRNA
ENSG0000(	CFL1P1	712.8298	0.053506	0.558102	0.857484	transcribed_unprocessed_pseudo
ENSG0000(	CHCHD10	530.5675	0.128929	0.55818	0.857484	protein_coding
ENSG0000(	EIF4A3	793.6226	0.037672	0.557984	0.857484	protein_coding
ENSG0000(	ENSG0000(	116.0954	-0.06828	0.558181	0.857484	lncRNA

ENSG0000(	ENSG0000(	56.51145	0.089235	0.55814	0.857484	lncRNA
ENSG0000(	RNASET2	5138.091	0.052761	0.558173	0.857484	protein_coding
ENSG0000(	SH3PXD2A-	45.19579	0.131776	0.558152	0.857484	lncRNA
ENSG0000(	METTL14-D	290.6991	0.058771	0.558226	0.857504	lncRNA
ENSG0000(	PRKCQ	180.8239	-0.13462	0.55827	0.857525	protein_coding
ENSG0000(	ENSG0000(	28.02031	-0.17199	0.558345	0.857591	lncRNA
ENSG0000(	ENSG0000(	25.9133	-0.15809	0.558416	0.857609	TEC
ENSG0000(	ENSG0000(	634.6004	-0.06669	0.558423	0.857609	lncRNA
ENSG0000(	FBXO38	1661.114	-0.03451	0.55845	0.857609	protein_coding
ENSG0000(	TRIM11	1708.235	0.040173	0.558493	0.857627	protein_coding
ENSG0000(	CECR3	89.63204	0.144005	0.558629	0.857737	lncRNA
ENSG0000(	DYNAP	283.1945	-0.04665	0.558665	0.857737	protein_coding
ENSG0000(	ENSG0000(	277.0251	0.04927	0.558723	0.857737	lncRNA
ENSG0000(	OR4K16P	73.70643	-0.07784	0.558641	0.857737	transcribed_unprocessed_pseudo
ENSG0000(	SNX7	290.6367	0.126294	0.558739	0.857737	protein_coding
ENSG0000(	TSPAN13	507.1476	0.081814	0.558751	0.857737	protein_coding
ENSG0000(	CEP162	552.7455	0.062912	0.559303	0.857771	protein_coding
ENSG0000(	CTTN-DT	120.2906	-0.10191	0.559257	0.857771	lncRNA
ENSG0000(	ENSG0000(	32.51056	0.148417	0.559266	0.857771	lncRNA
ENSG0000(	ENSG0000(	30.5777	0.15171	0.558889	0.857771	lncRNA
ENSG0000(	ENSG0000(	87.27107	-0.08253	0.559202	0.857771	lncRNA
ENSG0000(	ENSG0000(	45.42327	-0.14802	0.55906	0.857771	lncRNA
ENSG0000(	ENSG0000(	68.82274	-0.13167	0.558985	0.857771	lncRNA
ENSG0000(	ENSG0000(	190.1726	-0.11182	0.559022	0.857771	protein_coding
ENSG0000(	ENSG0000(	69.74962	0.165753	0.559247	0.857771	TEC
ENSG0000(	ENSG0000(	22.51815	-0.15558	0.559269	0.857771	lncRNA
ENSG0000(	ENSG0000(	19.72956	-0.15269	0.558903	0.857771	lncRNA
ENSG0000(	GALNT9	112.9061	-0.07786	0.558865	0.857771	protein_coding
ENSG0000(	LINC00840	89.49107	-0.08988	0.559241	0.857771	lncRNA
ENSG0000(	RFXAP	165.8785	0.076349	0.558985	0.857771	protein_coding
ENSG0000(	SCG3	121.5002	-0.09116	0.559301	0.857771	protein_coding
ENSG0000(	SORT1	2730.659	0.065944	0.558989	0.857771	protein_coding
ENSG0000(	ST7L	773.3176	0.042159	0.559247	0.857771	protein_coding
ENSG0000(	B3GALT6	183.2493	-0.07277	0.55959	0.857923	protein_coding
ENSG0000(	DNAJC13	2734.36	0.04084	0.559435	0.857923	protein_coding
ENSG0000(	FGFBP1	42.49022	0.205894	0.559514	0.857923	protein_coding
ENSG0000(	LINC02731	137.9282	-0.09516	0.559471	0.857923	lncRNA
ENSG0000(	PTCHD3	51.12835	0.093159	0.559536	0.857923	polymorphic_pseudogene
ENSG0000(	TRIM34	304.9165	0.061789	0.559589	0.857923	protein_coding
ENSG0000(	ENSG0000(	323.0574	0.046962	0.559653	0.857973	lncRNA
ENSG0000(	DNAH8	137.6178	-0.25829	0.559806	0.858016	protein_coding
ENSG0000(	HMBS	246.9612	0.073795	0.559756	0.858016	protein_coding
ENSG0000(	ISCA2	503.9575	0.041581	0.559805	0.858016	protein_coding
ENSG0000(	LINC00664	490.4906	0.047509	0.559755	0.858016	lncRNA
ENSG0000(	EMBP1	574.3853	0.061975	0.560109	0.858183	transcribed_unprocessed_pseudo
ENSG0000(	ENSG0000(	143.3545	0.072236	0.560133	0.858183	lncRNA
ENSG0000(	ENSG0000(	433.4558	-0.04392	0.560025	0.858183	lncRNA

ENSG0000(	ENSG0000(	339.9264	0.086284	0.560024	0.858183	lncRNA
ENSG0000(	MBOAT7	1140.237	0.061475	0.560122	0.858183	protein_coding
ENSG0000(	TAF5	461.7052	-0.04336	0.560111	0.858183	protein_coding
ENSG0000(	UBL3	790.669	0.071361	0.560033	0.858183	protein_coding
ENSG0000(	C19orf44	167.5994	0.075924	0.560274	0.858351	protein_coding
ENSG0000(	ACOX3	471.2789	-0.06402	0.560323	0.858376	protein_coding
ENSG0000(	ENSG0000(	214.7781	0.072779	0.560373	0.858376	lncRNA
ENSG0000(	ENSG0000(	320.3453	0.04269	0.560384	0.858376	lncRNA
ENSG0000(	ENSG0000(	187.5427	0.069486	0.560596	0.858541	lncRNA
ENSG0000(	ENSG0000(	168.2075	-0.06166	0.560564	0.858541	lncRNA
ENSG0000(	ENSG0000(	137.2212	0.070629	0.560599	0.858541	lncRNA
ENSG0000(	TRMT13	540.9646	0.059136	0.560617	0.858541	protein_coding
ENSG0000(	PTPN4	1420.235	0.059948	0.560665	0.858566	protein_coding
ENSG0000(	BMP8B	324.876	0.078814	0.560776	0.858628	protein_coding
ENSG0000(	ENSG0000(	149.9357	0.058751	0.560799	0.858628	TEC
ENSG0000(	WFIKKN1	91.21402	0.215693	0.56079	0.858628	protein_coding
ENSG0000(	AKR1A1	1113.937	-0.04058	0.560989	0.858645	protein_coding
ENSG0000(	ENSG0000(	429.4403	-0.05911	0.561059	0.858645	lncRNA
ENSG0000(	ENSG0000(	164.0294	-0.05517	0.561122	0.858645	lncRNA
ENSG0000(	ENSG0000(	25.17726	0.135606	0.560873	0.858645	lncRNA
ENSG0000(	ENSG0000(	264.2871	0.072605	0.560988	0.858645	lncRNA
ENSG0000(	FAM27E3	270.8083	-0.0867	0.560968	0.858645	lncRNA
ENSG0000(	FAM71F2	1447.729	-0.0445	0.561099	0.858645	protein_coding
ENSG0000(	GOLT1A	50.10662	0.199424	0.560903	0.858645	protein_coding
ENSG0000(	RN7SKP203	291.5267	-0.12817	0.561115	0.858645	misc_RNA
ENSG0000(	SRRM2	48484.95	0.039612	0.56111	0.858645	protein_coding
ENSG0000(	ENSG0000(	77.58688	-0.07985	0.56116	0.858646	lncRNA
ENSG0000(	H2AC18	133.1222	-2.15011	0.561185	0.858646	protein_coding
ENSG0000(	ARHGAP11	641.5502	-0.09459	0.561264	0.858677	protein_coding
ENSG0000(	ENSG0000(	34.61507	-0.26396	0.561267	0.858677	lncRNA
ENSG0000(	CPLANE1	3900.68	-0.0275	0.561356	0.858765	protein_coding
ENSG0000(	PDLIM3	923.1503	0.126969	0.561514	0.858958	protein_coding
ENSG0000(	CALCOCO2	3274.59	0.031762	0.56197	0.858987	protein_coding
ENSG0000(	DOK1	158.8471	-0.07153	0.562344	0.858987	protein_coding
ENSG0000(	DZIP1L	256.095	-0.10687	0.561712	0.858987	protein_coding
ENSG0000(	ELF4	2130.422	0.06794	0.562312	0.858987	protein_coding
ENSG0000(	ENSG0000(	256.0366	0.094323	0.562076	0.858987	unprocessed_pseudogene
ENSG0000(	ENSG0000(	92.09981	-0.07305	0.561915	0.858987	lncRNA
ENSG0000(	ENSG0000(	20.54316	-0.15148	0.562081	0.858987	lncRNA
ENSG0000(	ENSG0000(	20.98944	0.181215	0.562344	0.858987	lncRNA
ENSG0000(	ENSG0000(	973.6052	0.037478	0.561815	0.858987	protein_coding
ENSG0000(	ENSG0000(	38.9161	0.112475	0.562233	0.858987	lncRNA
ENSG0000(	GATA2-AS1	40.30101	0.153284	0.562091	0.858987	lncRNA
ENSG0000(	KDM4B	2219.649	0.038949	0.562019	0.858987	protein_coding
ENSG0000(	LINC00891	1672.764	-0.04931	0.561776	0.858987	lncRNA
ENSG0000(	LINC02269	79.1339	-0.07876	0.56184	0.858987	lncRNA
ENSG0000(	NACC2	879.7973	0.064066	0.561897	0.858987	protein_coding

ENSG0000(	NPHP3	2805.265	0.042386	0.562312	0.858987	protein_coding
ENSG0000(	PI4K2B	493.3621	0.067655	0.561626	0.858987	protein_coding
ENSG0000(	POLR2J	91.94318	0.083042	0.562078	0.858987	protein_coding
ENSG0000(	PRDM16	131.5936	-0.08682	0.562236	0.858987	protein_coding
ENSG0000(	RPL7AP60	50.54159	0.119619	0.562333	0.858987	processed_pseudogene
ENSG0000(	SPAST	1212.5	0.036838	0.562175	0.858987	protein_coding
ENSG0000(	STN1	702.4712	-0.05219	0.562202	0.858987	protein_coding
ENSG0000(	THOC6	110.0484	0.081527	0.562327	0.858987	protein_coding
ENSG0000(	TMEM138	1937.407	-0.03049	0.561813	0.858987	protein_coding
ENSG0000(	VRK3	1549.995	0.037718	0.561806	0.858987	protein_coding
ENSG0000(	ZNF32	130.9942	-0.08065	0.561578	0.858987	protein_coding
ENSG0000(	MRPL24	174.3682	0.075091	0.562438	0.859083	protein_coding
ENSG0000(	BIK	70.96451	0.142613	0.56247	0.859083	protein_coding
ENSG0000(	CENPJ	806.4471	-0.06938	0.562628	0.859278	protein_coding
ENSG0000(	CPEB1-AS1	701.7725	0.045977	0.562693	0.859301	lncRNA
ENSG0000(	ENSG0000(	265.1293	0.063766	0.562737	0.859301	lncRNA
ENSG0000(	RPL7L1P3	78.92886	0.095619	0.562727	0.859301	processed_pseudogene
ENSG0000(	CCAR1	2209.317	0.045729	0.563047	0.859508	protein_coding
ENSG0000(	ENSG0000(	279.7279	0.0436	0.563111	0.859508	lncRNA
ENSG0000(	ENSG0000(	25.58796	0.16662	0.56296	0.859508	lncRNA
ENSG0000(	MIR3139	19.74255	-0.18586	0.563046	0.859508	miRNA
ENSG0000(	MS4A4A	655.8746	0.050106	0.563122	0.859508	protein_coding
ENSG0000(	MYD88	1223.83	-0.06699	0.563088	0.859508	protein_coding
ENSG0000(	SLC25A41	44.48448	0.112798	0.562927	0.859508	protein_coding
ENSG0000(	ZNF18	335.4513	0.082246	0.563123	0.859508	protein_coding
ENSG0000(	ENSG0000(	70.57064	0.117367	0.563182	0.859551	transcribed_processed_pseudoge
ENSG0000(	CCT5	854.5114	0.046461	0.563292	0.859561	protein_coding
ENSG0000(	ENSG0000(	236.0879	-0.0508	0.563314	0.859561	lncRNA
ENSG0000(	ENSG0000(	157.9377	-0.07888	0.563312	0.859561	lncRNA
ENSG0000(	LINC02531	36.62365	0.120415	0.563301	0.859561	lncRNA
ENSG0000(	CSTF3	487.2734	-0.05514	0.563464	0.859689	protein_coding
ENSG0000(	ENSG0000(	688.6407	-0.03751	0.563585	0.859689	lncRNA
ENSG0000(	IMMT	1068.29	0.039629	0.563583	0.859689	protein_coding
ENSG0000(	LINC02340	79.45276	-0.08238	0.563558	0.859689	lncRNA
ENSG0000(	MYOM3	531.5957	-0.09291	0.563488	0.859689	protein_coding
ENSG0000(	ZNF772	552.451	-0.05883	0.563569	0.859689	protein_coding
ENSG0000(	ATG9B	34.42501	-0.20442	0.563649	0.859739	protein_coding
ENSG0000(	BPNT2	1685.323	-0.04155	0.564122	0.859967	protein_coding
ENSG0000(	EMC2	860.0971	-0.03792	0.564081	0.859967	protein_coding
ENSG0000(	ENSG0000(	20.37669	-0.23343	0.564205	0.859967	lncRNA
ENSG0000(	ENSG0000(	428.8781	-0.12279	0.564034	0.859967	lncRNA
ENSG0000(	ENSG0000(	479.0403	-0.05477	0.564007	0.859967	lncRNA
ENSG0000(	ENSG0000(	405.4226	-0.06691	0.563979	0.859967	lncRNA
ENSG0000(	ENSG0000(	393.4539	0.050104	0.56405	0.859967	lncRNA
ENSG0000(	ENSG0000(	65.51331	-0.10874	0.563928	0.859967	lncRNA
ENSG0000(	ERVFRD-1	31.87426	0.149559	0.564077	0.859967	protein_coding
ENSG0000(	LINC00642	138.1191	0.069711	0.564194	0.859967	lncRNA

ENSG0000(	RPS20P22	237.7237	-0.06334	0.56397	0.859967	transcribed_processed_pseudoge
ENSG0000(	SLC4A1	305.8562	0.056349	0.56402	0.859967	protein_coding
ENSG0000(	SNHG21	22.36356	0.165987	0.564198	0.859967	lncRNA
ENSG0000(	ENSG0000(	1411.791	-0.03119	0.564376	0.86018	transcribed_unprocessed_pseudo
ENSG0000(	RFPL3S	650.6074	-0.04539	0.564414	0.860191	lncRNA
ENSG0000(	C11orf24	593.6626	0.059599	0.564544	0.860293	protein_coding
ENSG0000(	DPY19L4	979.2442	0.041481	0.564587	0.860293	protein_coding
ENSG0000(	ENSG0000(	137.5961	-0.08269	0.56455	0.860293	lncRNA
ENSG0000(	HABP4	156.4288	-0.08789	0.564606	0.860293	protein_coding
ENSG0000(	ASB11	160.7431	0.054921	0.564859	0.860487	protein_coding
ENSG0000(	CSNK1G3	1130.118	-0.06883	0.564903	0.860487	protein_coding
ENSG0000(	ENSG0000(	27.76797	-0.14797	0.564861	0.860487	lncRNA
ENSG0000(	ENSG0000(	46.5978	-0.1468	0.564874	0.860487	lncRNA
ENSG0000(	LDC1P	31.22704	-0.19582	0.564817	0.860487	transcribed_unitary_pseudogene
ENSG0000(	LINC00852	275.7031	-0.07085	0.564922	0.860487	lncRNA
ENSG0000(	ENSG0000(	41.04116	-0.12655	0.565124	0.860748	lncRNA
ENSG0000(	CICP18	1010.463	0.053511	0.565299	0.860968	transcribed_processed_pseudoge
ENSG0000(	COG5	2127.25	-0.0451	0.565436	0.86099	protein_coding
ENSG0000(	ENSG0000(	734.864	0.039795	0.56552	0.86099	lncRNA
ENSG0000(	ENSG0000(	356.1564	-0.0467	0.565532	0.86099	lncRNA
ENSG0000(	LINC02397	773.7453	-0.21706	0.565458	0.86099	lncRNA
ENSG0000(	MUC19	57.30767	0.094306	0.565366	0.86099	polymorphic_pseudogene
ENSG0000(	NTN5	81.51777	0.069839	0.565533	0.86099	protein_coding
ENSG0000(	PDE11A	637.2248	-0.07525	0.565478	0.86099	protein_coding
ENSG0000(	ANAPC15	742.2867	0.040574	0.565783	0.861055	protein_coding
ENSG0000(	ANKRD26P	42.12058	0.127487	0.56579	0.861055	processed_pseudogene
ENSG0000(	ENSG0000(	21.04894	0.189946	0.566235	0.861055	transcribed_processed_pseudoge
ENSG0000(	ENSG0000(	43.40613	0.098124	0.566242	0.861055	processed_pseudogene
ENSG0000(	ENSG0000(	87.18174	-0.08444	0.565692	0.861055	lncRNA
ENSG0000(	ENSG0000(	97.4189	0.18421	0.566194	0.861055	lncRNA
ENSG0000(	ENSG0000(	33.06264	-0.17162	0.565912	0.861055	lncRNA
ENSG0000(	ENSG0000(	28.25403	-0.14898	0.56596	0.861055	lncRNA
ENSG0000(	ENSG0000(	40.39594	-0.15195	0.565791	0.861055	TEC
ENSG0000(	ENSG0000(	169.0696	-0.06624	0.566023	0.861055	lncRNA
ENSG0000(	ENSG0000(	148.7436	-0.06183	0.56589	0.861055	lncRNA
ENSG0000(	ERVV-2	71.64206	0.102507	0.566081	0.861055	protein_coding
ENSG0000(	EXT1	3479.201	-0.05137	0.565723	0.861055	protein_coding
ENSG0000(	GS1-124K5	133.0337	0.060067	0.566146	0.861055	lncRNA
ENSG0000(	IBA57-DT	40.37294	-0.11262	0.566327	0.861055	lncRNA
ENSG0000(	KLK3	113.2408	0.200324	0.56606	0.861055	protein_coding
ENSG0000(	MIOX	94.22492	-0.06482	0.566139	0.861055	protein_coding
ENSG0000(	MTHFD2L	673.1297	-0.05453	0.56631	0.861055	protein_coding
ENSG0000(	MXRA7	1179.835	-0.07505	0.566241	0.861055	protein_coding
ENSG0000(	PPOX	221.721	0.061203	0.566259	0.861055	protein_coding
ENSG0000(	RN7SL230F	46.35251	0.122242	0.56571	0.861055	misc_RNA
ENSG0000(	SDS	276.8559	-0.06167	0.566266	0.861055	protein_coding
ENSG0000(	SFT2D2	8607.331	-0.03711	0.565888	0.861055	protein_coding

ENSG0000(	SWT1	350.9559	0.070867	0.566137	0.861055	protein_coding
ENSG0000(	ENSG0000(	142.2185	0.070576	0.56638	0.861088	processed_pseudogene
ENSG0000(	AGFG1	2497.553	-0.03839	0.566701	0.861148	protein_coding
ENSG0000(	ENSG0000(	513.1157	0.037422	0.56669	0.861148	lncRNA
ENSG0000(	ENSG0000(	35.26077	-0.1244	0.566565	0.861148	processed_pseudogene
ENSG0000(	GRK2	2045.526	0.046908	0.56669	0.861148	protein_coding
ENSG0000(	MAP3K7	1229.616	0.043212	0.566619	0.861148	protein_coding
ENSG0000(	MRPS11	729.2602	-0.05191	0.566538	0.861148	protein_coding
ENSG0000(	NPIPB12	2836.22	0.086524	0.566521	0.861148	protein_coding
ENSG0000(	THOC7	199.7599	-0.07201	0.566695	0.861148	protein_coding
ENSG0000(	ZNF367	144.1177	-0.12588	0.566536	0.861148	protein_coding
ENSG0000(	ZNRF2P1	106.7205	-0.10867	0.566761	0.861192	transcribed_processed_pseudoge
ENSG0000(	FAM91A3P	29.26489	-0.1795	0.566839	0.861214	transcribed_processed_pseudoge
ENSG0000(	RN7SL172F	51.02841	-0.11145	0.566822	0.861214	misc_RNA
ENSG0000(	ARHGEF39	655.6781	-0.05817	0.566908	0.861225	protein_coding
ENSG0000(	ENSG0000(	73.24839	-0.10662	0.566888	0.861225	lncRNA
ENSG0000(	ABCF3	705.3238	0.059659	0.566987	0.861249	protein_coding
ENSG0000(	LRIG2	3555.843	0.052443	0.566964	0.861249	protein_coding
ENSG0000(	PFDN6	88.8493	-0.0773	0.567032	0.861271	protein_coding
ENSG0000(	ENSG0000(	130.0812	-0.07965	0.567115	0.861348	lncRNA
ENSG0000(	CLEC4C	49.9591	-0.11801	0.56729	0.861471	protein_coding
ENSG0000(	ENSG0000(	109.7187	0.073424	0.567274	0.861471	lncRNA
ENSG0000(	PIEZO1	3040.771	-0.06341	0.567275	0.861471	protein_coding
ENSG0000(	MIR570	114.0921	0.122298	0.567417	0.861617	miRNA
ENSG0000(	ENSG0000(	34.3616	-0.12393	0.567491	0.861634	lncRNA
ENSG0000(	ENSG0000(	151.9385	0.088334	0.567479	0.861634	protein_coding
ENSG0000(	ENSG0000(	36.70161	0.122813	0.567603	0.861684	TEC
ENSG0000(	NPIPB13	1194.67	0.074582	0.567588	0.861684	protein_coding
ENSG0000(	SNORD66	19.56332	-0.17441	0.567618	0.861684	snoRNA
ENSG0000(	SLFNL1	100.6621	-0.13555	0.567695	0.861754	protein_coding
ENSG0000(	ANXA11	6076.443	0.06447	0.568596	0.861775	protein_coding
ENSG0000(	AZIN2	1309.272	-0.11621	0.568313	0.861775	protein_coding
ENSG0000(	B4GALT4	1842.035	0.063877	0.568905	0.861775	protein_coding
ENSG0000(	BCAS4	145.5722	-0.11617	0.569511	0.861775	protein_coding
ENSG0000(	CCDC15	187.7074	-0.07417	0.568452	0.861775	protein_coding
ENSG0000(	CCN5	349.7404	0.089348	0.568285	0.861775	protein_coding
ENSG0000(	CYYR1-AS1	667.28	0.032961	0.569558	0.861775	lncRNA
ENSG0000(	DNAAF1	542.9646	-0.04886	0.569576	0.861775	protein_coding
ENSG0000(	ENSG0000(	953.7571	-0.07989	0.56958	0.861775	lncRNA
ENSG0000(	ENSG0000(	1896.187	0.059028	0.569349	0.861775	lncRNA
ENSG0000(	ENSG0000(	162.209	-0.07206	0.569709	0.861775	lncRNA
ENSG0000(	ENSG0000(	24.21453	0.125075	0.568384	0.861775	lncRNA
ENSG0000(	ENSG0000(	26.99346	0.119084	0.569687	0.861775	lncRNA
ENSG0000(	ENSG0000(	94.64226	0.088368	0.568674	0.861775	lncRNA
ENSG0000(	ENSG0000(	32.11736	0.131753	0.56892	0.861775	lncRNA
ENSG0000(	ENSG0000(	32.02551	0.117912	0.569776	0.861775	lncRNA
ENSG0000(	ENSG0000(	991.243	0.188147	0.569104	0.861775	miRNA

ENSG0000(	ENSG0000(	84.66805	-0.08989	0.568283	0.861775	TEC
ENSG0000(	ENSG0000(	58.96673	0.098379	0.568193	0.861775	TEC
ENSG0000(	ENSG0000(	97.27434	0.176019	0.567871	0.861775	protein_coding
ENSG0000(	ENSG0000(	78.47897	0.09002	0.567743	0.861775	lncRNA
ENSG0000(	ENSG0000(	59.73864	0.080801	0.569418	0.861775	lncRNA
ENSG0000(	ENSG0000(	106.4815	0.096261	0.568959	0.861775	lncRNA
ENSG0000(	EXOC6B	1638.477	0.084842	0.568649	0.861775	protein_coding
ENSG0000(	GHRLOS	541.8406	-0.06977	0.568896	0.861775	lncRNA
ENSG0000(	H2AZ2	2681.608	-0.04497	0.568744	0.861775	protein_coding
ENSG0000(	HELZ	4492.189	-0.03766	0.56926	0.861775	protein_coding
ENSG0000(	HJURP	434.4528	-0.08337	0.568852	0.861775	protein_coding
ENSG0000(	HNRNPA1P	41.32905	0.120015	0.568295	0.861775	processed_pseudogene
ENSG0000(	ITFG2	1401.196	0.047595	0.568687	0.861775	protein_coding
ENSG0000(	JADE2	1738.531	-0.05941	0.568163	0.861775	protein_coding
ENSG0000(	KIAA0408	497.5036	0.061945	0.569415	0.861775	protein_coding
ENSG0000(	LARS2	1059.271	-0.04553	0.569601	0.861775	protein_coding
ENSG0000(	LINC01307	147.1242	-0.05679	0.569647	0.861775	lncRNA
ENSG0000(	LINC01422	1243.952	-0.04279	0.56884	0.861775	lncRNA
ENSG0000(	LINC01504	551.9972	-0.10178	0.569064	0.861775	lncRNA
ENSG0000(	LOXHD1	31.61437	-0.20307	0.568287	0.861775	protein_coding
ENSG0000(	MAPK8IP1	120.066	0.218434	0.569588	0.861775	protein_coding
ENSG0000(	METRNL	605.1157	0.10411	0.568045	0.861775	protein_coding
ENSG0000(	NDUFA12	2420.374	-0.03899	0.569397	0.861775	protein_coding
ENSG0000(	NDUFA5	1613.747	0.059059	0.568648	0.861775	protein_coding
ENSG0000(	NT5C3B	383.2035	0.056839	0.568591	0.861775	protein_coding
ENSG0000(	NUP155	1343.689	0.057097	0.569323	0.861775	protein_coding
ENSG0000(	PDE6D	238.7309	-0.05109	0.569168	0.861775	protein_coding
ENSG0000(	PDZD11	170.5252	-0.07934	0.568699	0.861775	protein_coding
ENSG0000(	PPIP5K1	2385.962	0.070808	0.568529	0.861775	protein_coding
ENSG0000(	PPP1R12A-	194.7488	0.055872	0.568647	0.861775	lncRNA
ENSG0000(	PSG7	134.244	0.154233	0.5682	0.861775	protein_coding
ENSG0000(	RN7SL300F	35.57782	-0.11618	0.569549	0.861775	misc_RNA
ENSG0000(	RN7SL387F	161.7353	0.063867	0.568888	0.861775	misc_RNA
ENSG0000(	RNFT2	782.0429	0.036946	0.569002	0.861775	protein_coding
ENSG0000(	RPS14	2434.054	0.078463	0.569515	0.861775	protein_coding
ENSG0000(	RUSF1	912.127	-0.05404	0.568688	0.861775	protein_coding
ENSG0000(	SEM1	733.7615	-0.03347	0.569596	0.861775	protein_coding
ENSG0000(	SIAH2-AS1	85.83521	-0.14455	0.568073	0.861775	lncRNA
ENSG0000(	SLC45A2	46.77829	0.248071	0.569745	0.861775	protein_coding
ENSG0000(	STAU2-AS1	106.6734	-0.07443	0.568958	0.861775	lncRNA
ENSG0000(	TGS1	683.0957	0.06068	0.567955	0.861775	protein_coding
ENSG0000(	TMEM79	192.2714	0.056247	0.568974	0.861775	protein_coding
ENSG0000(	TNRC6A	4779.069	-0.03081	0.569301	0.861775	protein_coding
ENSG0000(	TSR2	469.6798	-0.0381	0.567821	0.861775	protein_coding
ENSG0000(	ULK1	838.4284	0.054144	0.569174	0.861775	protein_coding
ENSG0000(	VDAC3	863.0646	0.052819	0.569395	0.861775	protein_coding
ENSG0000(	VIRMA	1748.768	0.031822	0.568994	0.861775	protein_coding

ENSG0000(	ZNF160	2386.983	0.029324	0.568676	0.861775	protein_coding
ENSG0000(	ZNF76	734.8274	-0.07357	0.569746	0.861775	protein_coding
ENSG0000(	ENSG0000(	205.2131	0.084331	0.569831	0.86181	lncRNA
ENSG0000(	C5orf64	1460.572	-0.03172	0.570356	0.861816	lncRNA
ENSG0000(	ENSG0000(	90.40002	-0.07157	0.570243	0.861816	lncRNA
ENSG0000(	ENSG0000(	231.5006	-0.07175	0.570399	0.861816	lncRNA
ENSG0000(	ENSG0000(	32.29486	-0.1364	0.570056	0.861816	transcribed_processed_pseudoge
ENSG0000(	ENSG0000(	4912.333	-0.04069	0.5702	0.861816	lncRNA
ENSG0000(	ENSG0000(	58.6319	-0.10535	0.569904	0.861816	lncRNA
ENSG0000(	ENSG0000(	76.10277	0.152285	0.570126	0.861816	TEC
ENSG0000(	ENSG0000(	34.70171	-0.1049	0.570322	0.861816	lncRNA
ENSG0000(	ENSG0000(	113.5409	0.066316	0.569899	0.861816	lncRNA
ENSG0000(	FAM47E	1588.067	-0.04055	0.570245	0.861816	protein_coding
ENSG0000(	GOLPH3	1169.823	-0.05596	0.570254	0.861816	protein_coding
ENSG0000(	METTL1	114.6252	0.10747	0.57016	0.861816	protein_coding
ENSG0000(	MFN1	2111.473	-0.05122	0.570055	0.861816	protein_coding
ENSG0000(	RPSA	2556.272	0.061239	0.569979	0.861816	protein_coding
ENSG0000(	TTLL11	315.3012	-0.07255	0.570016	0.861816	protein_coding
ENSG0000(	YPEL5	1060.015	-0.06726	0.570325	0.861816	protein_coding
ENSG0000(	ZIK1	204.2167	-0.09811	0.570389	0.861816	protein_coding
ENSG0000(	ZNF77	122.8286	0.089808	0.57017	0.861816	protein_coding
ENSG0000(	EIF4A2P2	56.90308	0.116625	0.570481	0.86186	processed_pseudogene
ENSG0000(	ENSG0000(	33.17657	-0.19711	0.57049	0.86186	lncRNA
ENSG0000(	ENSG0000(	61.18745	0.089038	0.570583	0.861916	lncRNA
ENSG0000(	MFSD1	1005.383	0.050307	0.57059	0.861916	protein_coding
ENSG0000(	C1D	111.9411	-0.07112	0.570625	0.861921	protein_coding
ENSG0000(	ENSG0000(	83.00628	0.087486	0.57081	0.862154	lncRNA
ENSG0000(	APCDD1L-[	150.9903	-0.06673	0.571004	0.862211	lncRNA
ENSG0000(	ENSG0000(	488.5806	0.037857	0.570947	0.862211	lncRNA
ENSG0000(	ETFRF1	175.2202	-0.06918	0.570934	0.862211	protein_coding
ENSG0000(	RNU1-91P	51.39109	-0.12202	0.570965	0.862211	snRNA
ENSG0000(	SDHAP1	1245.153	0.084204	0.570996	0.862211	transcribed_unprocessed_pseudo
ENSG0000(	AKAP3	36.37236	-0.13287	0.571514	0.862402	protein_coding
ENSG0000(	AKAP8L	1762.842	-0.03836	0.571914	0.862402	protein_coding
ENSG0000(	ARHGEF9	2063.998	0.094631	0.571507	0.862402	protein_coding
ENSG0000(	BCAR3-AS1	318.4348	0.150995	0.571754	0.862402	lncRNA
ENSG0000(	CFAP52	43.47395	0.087587	0.571903	0.862402	protein_coding
ENSG0000(	DCAF15	333.0864	0.061201	0.571631	0.862402	protein_coding
ENSG0000(	ENSG0000(	36.6192	-0.13159	0.571777	0.862402	lncRNA
ENSG0000(	ENSG0000(	58.67581	-0.10403	0.57124	0.862402	TEC
ENSG0000(	ENSG0000(	139.1038	0.055909	0.571579	0.862402	TEC
ENSG0000(	ENSG0000(	35.95862	-0.15563	0.571384	0.862402	transcribed_unprocessed_pseudo
ENSG0000(	ENSG0000(	28.61568	-0.11896	0.571853	0.862402	lncRNA
ENSG0000(	ENSG0000(	113.5298	0.0602	0.571588	0.862402	lncRNA
ENSG0000(	ENSG0000(	273.1535	-0.09015	0.571544	0.862402	transcribed_unprocessed_pseudo
ENSG0000(	IL5RA	75.29223	-0.18022	0.57138	0.862402	protein_coding
ENSG0000(	JMY	1046.238	-0.06621	0.571932	0.862402	protein_coding

ENSG0000(	LINC01239	238.1173	-0.04729	0.571411	0.862402	lncRNA
ENSG0000(	LNCOC1	303.0579	0.057481	0.571455	0.862402	lncRNA
ENSG0000(	MMEL1	34.43079	-0.21337	0.571746	0.862402	protein_coding
ENSG0000(	MRPS18C	714.6178	0.040912	0.57156	0.862402	protein_coding
ENSG0000(	MTA3	1576.38	0.038416	0.57125	0.862402	protein_coding
ENSG0000(	PSENEN	246.392	0.070002	0.571946	0.862402	protein_coding
ENSG0000(	PUM1	2532.54	-0.03033	0.571254	0.862402	protein_coding
ENSG0000(	RPL7P41	62.06095	0.089127	0.571874	0.862402	processed_pseudogene
ENSG0000(	SHMT1	751.5784	0.085932	0.571684	0.862402	protein_coding
ENSG0000(	USP41	24.61048	0.307288	0.571798	0.862402	protein_coding
ENSG0000(	ZNF658B	110.4078	0.107204	0.571459	0.862402	transcribed_unprocessed_pseudo
ENSG0000(	NPLOC4	4322.814	-0.04666	0.571999	0.862434	protein_coding
ENSG0000(	ENSG0000(	440.0284	0.032724	0.572216	0.862714	lncRNA
ENSG0000(	ENSG0000(	630.1265	0.067548	0.572312	0.862763	lncRNA
ENSG0000(	LINC02232	19.63792	-0.22435	0.572287	0.862763	lncRNA
ENSG0000(	PHKA1	213.6168	0.08315	0.572371	0.862763	protein_coding
ENSG0000(	PNCK	30.02407	0.189328	0.572374	0.862763	protein_coding
ENSG0000(	CRAMP1	1137.224	0.051124	0.572438	0.862795	protein_coding
ENSG0000(	ENSG0000(	76.46172	0.11295	0.572489	0.862795	lncRNA
ENSG0000(	RAB7B	104.9569	0.142747	0.572473	0.862795	protein_coding
ENSG0000(	ENSG0000(	272.3188	0.045047	0.572521	0.862796	lncRNA
ENSG0000(	ARHGAP20	284.1216	0.100495	0.572785	0.863099	protein_coding
ENSG0000(	ZSCAN21	155.9904	0.09575	0.572771	0.863099	protein_coding
ENSG0000(	BRI3BP	2048.758	0.0528	0.572817	0.8631	protein_coding
ENSG0000(	IFIH1	657.8741	-0.12792	0.572882	0.863151	protein_coding
ENSG0000(	AKR7A2	301.5571	0.0808	0.572955	0.863161	protein_coding
ENSG0000(	ENSG0000(	189.6062	0.056212	0.572988	0.863161	lncRNA
ENSG0000(	LRRIQ1	97.98092	0.084981	0.573011	0.863161	protein_coding
ENSG0000(	USP17L2	30.80797	0.188243	0.573015	0.863161	protein_coding
ENSG0000(	ENSG0000(	42.88815	-0.13005	0.573171	0.863169	lncRNA
ENSG0000(	NOMO1	800.5403	-0.07511	0.573176	0.863169	protein_coding
ENSG0000(	TIMM23	295.0147	-0.0557	0.573125	0.863169	protein_coding
ENSG0000(	TMEM183/	1049.337	0.04601	0.57306	0.863169	protein_coding
ENSG0000(	ZCCHC14	1585.841	0.061425	0.573099	0.863169	protein_coding
ENSG0000(	ENSG0000(	71.30771	0.124848	0.573428	0.863294	lncRNA
ENSG0000(	ENSG0000(	50.62295	0.094619	0.573433	0.863294	lncRNA
ENSG0000(	EXTL2	272.0086	-0.07799	0.573473	0.863294	protein_coding
ENSG0000(	EYA4	70.05909	-0.13502	0.573479	0.863294	protein_coding
ENSG0000(	FAM72A	111.6254	-0.13337	0.573372	0.863294	protein_coding
ENSG0000(	KIF13B	5122.706	0.080902	0.573455	0.863294	protein_coding
ENSG0000(	NR4A2	326.7201	-0.15959	0.57341	0.863294	protein_coding
ENSG0000(	TXNL4A	1096.951	0.039629	0.573529	0.863322	protein_coding
ENSG0000(	ZNF669	435.1358	-0.04415	0.573595	0.863373	protein_coding
ENSG0000(	LRRC8D-DT	23.62187	-0.16031	0.573651	0.863411	lncRNA
ENSG0000(	CCDC189	71.45672	0.106105	0.574427	0.863458	protein_coding
ENSG0000(	CEP192	3037.271	0.053578	0.57457	0.863458	protein_coding
ENSG0000(	DDX41	464.2389	0.074287	0.574105	0.863458	protein_coding

ENSG0000(	ENSG0000(	70.57101	-0.08273	0.574169	0.863458	lncRNA
ENSG0000(	ENSG0000(	116.2426	0.110029	0.574746	0.863458	lncRNA
ENSG0000(	ENSG0000(	70.74665	0.210095	0.574022	0.863458	lncRNA
ENSG0000(	ENSG0000(	23.18297	0.176046	0.574643	0.863458	lncRNA
ENSG0000(	ENSG0000(	567.2854	0.032185	0.574349	0.863458	lncRNA
ENSG0000(	ENSG0000(	76.52916	-0.07072	0.573809	0.863458	lncRNA
ENSG0000(	ENSG0000(	136.1658	0.144547	0.574468	0.863458	lncRNA
ENSG0000(	ENSG0000(	167.768	0.07147	0.574313	0.863458	lncRNA
ENSG0000(	ENSG0000(	95.75236	0.340227	0.574604	0.863458	protein_coding
ENSG0000(	ENSG0000(	52.34736	0.10946	0.574782	0.863458	lncRNA
ENSG0000(	ENSG0000(	1924.557	0.028572	0.573802	0.863458	lncRNA
ENSG0000(	ENSG0000(	60.82079	-0.13799	0.574051	0.863458	lncRNA
ENSG0000(	GON7	115.6577	0.065125	0.574587	0.863458	protein_coding
ENSG0000(	GPR146	62.18776	0.111742	0.574287	0.863458	protein_coding
ENSG0000(	KLRC3	42.6785	-0.20045	0.574076	0.863458	protein_coding
ENSG0000(	KXD1	962.0501	-0.04025	0.574792	0.863458	protein_coding
ENSG0000(	LINC00173	269.4742	0.061463	0.574812	0.863458	lncRNA
ENSG0000(	LINC02840	930.2626	0.035172	0.57425	0.863458	lncRNA
ENSG0000(	MED16	572.4478	0.06845	0.574144	0.863458	protein_coding
ENSG0000(	MTIF2	669.8931	0.056375	0.574487	0.863458	protein_coding
ENSG0000(	MTMR9LP	409.5472	-0.08624	0.574148	0.863458	transcribed_unprocessed_pseudo
ENSG0000(	MTND2P28	799.2703	0.14896	0.574161	0.863458	unprocessed_pseudogene
ENSG0000(	NIF3L1	232.5576	0.052794	0.57388	0.863458	protein_coding
ENSG0000(	NIPSNAP3A	200.9898	0.090263	0.574086	0.863458	protein_coding
ENSG0000(	OR2M4	331.9001	0.041737	0.574788	0.863458	protein_coding
ENSG0000(	PBRM1	2822.953	0.031094	0.574542	0.863458	protein_coding
ENSG0000(	PHACTR2	2645.713	-0.03729	0.574471	0.863458	protein_coding
ENSG0000(	PPAN	444.154	0.056307	0.574379	0.863458	protein_coding
ENSG0000(	RPS16	1650.033	0.084469	0.574197	0.863458	protein_coding
ENSG0000(	SEC16B	257.4435	-0.11191	0.574329	0.863458	protein_coding
ENSG0000(	SPTLC2	3128.139	0.034233	0.574289	0.863458	protein_coding
ENSG0000(	WASHC2C	2327.202	0.082682	0.574166	0.863458	protein_coding
ENSG0000(	ZNF337	934.5488	0.047977	0.574734	0.863458	protein_coding
ENSG0000(	ONECUT2	72.81542	0.27197	0.574903	0.863515	protein_coding
ENSG0000(	TADA1	223.9369	0.05579	0.574913	0.863515	protein_coding
ENSG0000(	HMGA1P4	123.9795	-0.08669	0.574975	0.863561	lncRNA
ENSG0000(	EIF5AP3	26.15384	-0.12293	0.575261	0.863566	processed_pseudogene
ENSG0000(	ENSG0000(	81.66199	0.071478	0.575185	0.863566	lncRNA
ENSG0000(	ENSG0000(	178.0475	-0.08252	0.575119	0.863566	lncRNA
ENSG0000(	ENSG0000(	268.9404	0.051108	0.575162	0.863566	lncRNA
ENSG0000(	FLJ46284	193.3384	0.080214	0.575082	0.863566	lncRNA
ENSG0000(	HOMEZ	847.7656	-0.06186	0.575228	0.863566	protein_coding
ENSG0000(	LINC01786	32.406	0.107444	0.575126	0.863566	lncRNA
ENSG0000(	PLEKHA8	986.7851	0.049223	0.575235	0.863566	protein_coding
ENSG0000(	ZCCHC7	924.6003	-0.0521	0.575203	0.863566	protein_coding
ENSG0000(	ENSG0000(	48.30102	-0.11393	0.575365	0.863675	lncRNA
ENSG0000(	TRIM39	441.743	-0.05742	0.575416	0.863704	protein_coding

ENSG0000(	AKNA	4754.239	-0.07589	0.575545	0.863725	protein_coding
ENSG0000(	CCL5	835.457	-0.05369	0.575643	0.863725	protein_coding
ENSG0000(	CDC42	6474.001	-0.03847	0.575625	0.863725	protein_coding
ENSG0000(	GNG5	1192.239	-0.06724	0.575598	0.863725	protein_coding
ENSG0000(	LBHD1	594.5084	-0.03581	0.575527	0.863725	protein_coding
ENSG0000(	MTUS2	24.57546	0.763825	0.575568	0.863725	protein_coding
ENSG0000(	RN7SL706F	26.21765	-0.2411	0.575712	0.863725	misc_RNA
ENSG0000(	TOP1MT	146.0824	0.09627	0.575674	0.863725	protein_coding
ENSG0000(	ZNF223	277.9938	0.083342	0.575693	0.863725	protein_coding
ENSG0000(	CDC27P1	30.09751	0.281822	0.575766	0.863759	processed_pseudogene
ENSG0000(	DDN-AS1	39.08343	-0.15745	0.575825	0.863801	lncRNA
ENSG0000(	CACYBP	394.0736	-0.07617	0.576054	0.863814	protein_coding
ENSG0000(	ENSG0000(	97.22557	0.091624	0.575968	0.863814	lncRNA
ENSG0000(	MLLT1	778.9146	-0.07481	0.575936	0.863814	protein_coding
ENSG0000(	NPR3	80.86	0.113601	0.576028	0.863814	protein_coding
ENSG0000(	SCRG1	760.2164	0.050573	0.575956	0.863814	protein_coding
ENSG0000(	TRAJ7	19.88042	-0.19761	0.576052	0.863814	TR_J_gene
ENSG0000(	ZNF75A	1511.834	0.043289	0.575912	0.863814	protein_coding
ENSG0000(	BCL2L2	1704.747	0.048227	0.576091	0.863823	protein_coding
ENSG0000(	ENSG0000(	63.93346	-0.10188	0.576191	0.863926	lncRNA
ENSG0000(	AP2S1	195.5665	-0.08227	0.576957	0.864128	protein_coding
ENSG0000(	CEP112	812.06	-0.04306	0.576668	0.864128	protein_coding
ENSG0000(	CNPY2-AS1	186.1024	-0.08053	0.576613	0.864128	lncRNA
ENSG0000(	COMMD1	179.1021	0.069407	0.577023	0.864128	protein_coding
ENSG0000(	ENSG0000(	21.44975	0.158235	0.577444	0.864128	lncRNA
ENSG0000(	ENSG0000(	29.77431	0.184192	0.576576	0.864128	processed_pseudogene
ENSG0000(	ENSG0000(	141.2727	0.068437	0.577316	0.864128	lncRNA
ENSG0000(	ENSG0000(	162.7679	0.067428	0.576824	0.864128	lncRNA
ENSG0000(	ENSG0000(	95.22163	0.072276	0.577297	0.864128	lncRNA
ENSG0000(	ENSG0000(	47.69695	-0.09777	0.577181	0.864128	lncRNA
ENSG0000(	ENSG0000(	121.2559	-0.16165	0.576653	0.864128	transcribed_unprocessed_pseudo
ENSG0000(	ENSG0000(	20.12758	0.155032	0.577399	0.864128	lncRNA
ENSG0000(	ENSG0000(	29.0167	-0.14885	0.577461	0.864128	lncRNA
ENSG0000(	FLYWCH2	97.53527	-0.09472	0.576956	0.864128	protein_coding
ENSG0000(	GPS1	517.2926	0.06652	0.577446	0.864128	protein_coding
ENSG0000(	LINC00467	1257.412	0.037539	0.57681	0.864128	lncRNA
ENSG0000(	LINC01555	31.3554	-0.12077	0.577301	0.864128	lncRNA
ENSG0000(	MS4A6A	1708.327	-0.07833	0.577164	0.864128	protein_coding
ENSG0000(	NDUFS8	813.5158	-0.05595	0.577021	0.864128	protein_coding
ENSG0000(	NRF1	561.5859	-0.0526	0.576386	0.864128	protein_coding
ENSG0000(	PANK4	385.2468	0.051227	0.57692	0.864128	protein_coding
ENSG0000(	PBX4	105.3282	-0.09277	0.577016	0.864128	protein_coding
ENSG0000(	PLCD4	161.2137	0.079913	0.576867	0.864128	protein_coding
ENSG0000(	PLIN2	598.7491	-0.09077	0.577149	0.864128	protein_coding
ENSG0000(	PSMD10	214.0266	-0.05754	0.577003	0.864128	protein_coding
ENSG0000(	RFX1	436.0519	-0.07168	0.57747	0.864128	protein_coding
ENSG0000(	RN7SL45P	100.8759	-0.07285	0.57682	0.864128	misc_RNA

ENSG0000(	RNA5SP47:	27.32585	-0.09159	0.577373	0.864128	rRNA_pseudogene
ENSG0000(	RPL41	3932.124	-0.07424	0.577027	0.864128	protein_coding
ENSG0000(	SESN3	1991.061	0.116084	0.577488	0.864128	protein_coding
ENSG0000(	SH3D21	1012.643	-0.06678	0.577207	0.864128	protein_coding
ENSG0000(	USP32P1	865.8255	0.216084	0.576669	0.864128	transcribed_unprocessed_pseudo
ENSG0000(	VAPA	3031.791	0.051362	0.576882	0.864128	protein_coding
ENSG0000(	VEZF1	1198.708	-0.04194	0.577129	0.864128	protein_coding
ENSG0000(	YBX1	2530.243	-0.07654	0.576669	0.864128	protein_coding
ENSG0000(	ZFPM1	234.6567	-0.05946	0.577475	0.864128	protein_coding
ENSG0000(	ZSCAN5A	697.6432	-0.03843	0.577445	0.864128	protein_coding
ENSG0000(	ENSG0000(	186.5991	-0.06198	0.577573	0.864208	lncRNA
ENSG0000(	CC2D1B	1426.145	-0.07575	0.577874	0.864235	protein_coding
ENSG0000(	CNOT8	1089.658	0.036517	0.577874	0.864235	protein_coding
ENSG0000(	ENSG0000(	26.36043	-0.14482	0.577768	0.864235	lncRNA
ENSG0000(	ENSG0000(	41.8782	-0.08755	0.577694	0.864235	lncRNA
ENSG0000(	ENSG0000(	52.00383	0.081495	0.577727	0.864235	lncRNA
ENSG0000(	ENSG0000(	81.48203	0.087314	0.577848	0.864235	lncRNA
ENSG0000(	IFT27	861.9381	0.056762	0.577799	0.864235	protein_coding
ENSG0000(	SAPCD2	215.2211	0.120669	0.57776	0.864235	protein_coding
ENSG0000(	ZNF184	213.475	0.055959	0.577672	0.864235	protein_coding
ENSG0000(	BANK1	830.0862	-0.23112	0.578406	0.86432	protein_coding
ENSG0000(	CBX5P1	23.45195	-0.16473	0.57861	0.86432	processed_pseudogene
ENSG0000(	CDH24	154.9594	-0.08866	0.578622	0.86432	protein_coding
ENSG0000(	COL11A2	84.60042	0.143655	0.578369	0.86432	protein_coding
ENSG0000(	ENSG0000(	81.07739	0.107657	0.577997	0.86432	TEC
ENSG0000(	ENSG0000(	28.33088	0.215126	0.578068	0.86432	lncRNA
ENSG0000(	ENSG0000(	84.84173	0.075373	0.578369	0.86432	lncRNA
ENSG0000(	ENSG0000(	105.4778	-0.07065	0.578007	0.86432	lncRNA
ENSG0000(	FAM107A	195.7833	0.130531	0.57854	0.86432	protein_coding
ENSG0000(	FBH1	2320.951	-0.04052	0.578116	0.86432	protein_coding
ENSG0000(	GRIK2	708.7156	0.049337	0.578532	0.86432	protein_coding
ENSG0000(	KIAA0319L	2621.9	0.041182	0.578403	0.86432	protein_coding
ENSG0000(	LEAP2	118.1107	0.071251	0.578299	0.86432	protein_coding
ENSG0000(	LINC00343	1691.166	0.066941	0.57818	0.86432	lncRNA
ENSG0000(	LINC01823	97.11617	-0.05981	0.578339	0.86432	lncRNA
ENSG0000(	LINC02133	110.1694	-0.07942	0.5784	0.86432	lncRNA
ENSG0000(	MST1	259.0972	-0.08717	0.578092	0.86432	protein_coding
ENSG0000(	MYL12B	1409.75	-0.09256	0.578229	0.86432	protein_coding
ENSG0000(	NDFIP2	814.7293	0.097676	0.578374	0.86432	protein_coding
ENSG0000(	RPS27A	1550.002	-0.06827	0.578556	0.86432	protein_coding
ENSG0000(	TXK	497.4402	-0.08355	0.578559	0.86432	protein_coding
ENSG0000(	UCA1	256.2486	-0.06474	0.578484	0.86432	lncRNA
ENSG0000(	RNF157-AS	213.6523	0.052352	0.578664	0.864335	lncRNA
ENSG0000(	EML6	877.3062	-0.10297	0.578812	0.864416	protein_coding
ENSG0000(	ENSG0000(	124.4975	-0.05609	0.578798	0.864416	lncRNA
ENSG0000(	ENSG0000(	775.8718	-0.03408	0.578765	0.864416	lncRNA
ENSG0000(	APOBR	1819.776	0.072826	0.578896	0.864488	protein_coding

ENSG0000(	LINC02861	325.9367	-0.04612	0.578923	0.864488	lncRNA
ENSG0000(	ENSG0000(	35.24546	-0.2827	0.57905	0.864535	lncRNA
ENSG0000(	ENSG0000(	390.1594	-0.05801	0.57908	0.864535	lncRNA
ENSG0000(	GCATP1	38.11788	0.166594	0.57902	0.864535	processed_pseudogene
ENSG0000(	LRRIQ3	40.16825	0.174549	0.579022	0.864535	protein_coding
ENSG0000(	KCNS1	26.99319	0.163599	0.579154	0.864598	protein_coding
ENSG0000(	ATP11A-AS	64.22278	0.089285	0.579299	0.864627	lncRNA
ENSG0000(	MMP16	86.90144	0.20467	0.579247	0.864627	protein_coding
ENSG0000(	SNHG28	120.9789	0.117798	0.579283	0.864627	lncRNA
ENSG0000(	STARD13-A	125.5875	0.081908	0.579225	0.864627	lncRNA
ENSG0000(	ENSG0000(	42.91173	-1.1199	0.579433	0.864702	processed_pseudogene
ENSG0000(	ENSG0000(	257.6595	0.042442	0.579381	0.864702	lncRNA
ENSG0000(	ENSG0000(	92.65413	0.115653	0.57954	0.864702	lncRNA
ENSG0000(	ENSG0000(	1188.2	0.099155	0.579565	0.864702	lncRNA
ENSG0000(	ENSG0000(	328.7006	0.091478	0.579468	0.864702	lncRNA
ENSG0000(	ST3GAL6-A	399.8003	-0.058	0.579569	0.864702	lncRNA
ENSG0000(	TSPAN17	537.0364	0.052083	0.579509	0.864702	protein_coding
ENSG0000(	ZNF587B	3108.887	0.059839	0.579609	0.864715	protein_coding
ENSG0000(	ENSG0000(	231.7574	-0.05783	0.579723	0.864729	TEC
ENSG0000(	MCRS1	391.513	0.065068	0.57967	0.864729	protein_coding
ENSG0000(	PRSS55	32.3998	0.126739	0.579744	0.864729	protein_coding
ENSG0000(	SLC35C2	1272.062	-0.03397	0.579709	0.864729	protein_coding
ENSG0000(	DSCC1	47.97937	0.143672	0.579889	0.864881	protein_coding
ENSG0000(	RN7SL132F	45.76885	0.093359	0.579909	0.864881	misc_RNA
ENSG0000(	CBSL	50.08426	-0.39223	0.580565	0.864891	protein_coding
ENSG0000(	ENSG0000(	29.08471	-0.14627	0.580195	0.864891	lncRNA
ENSG0000(	ENSG0000(	348.3597	-0.05308	0.580352	0.864891	unprocessed_pseudogene
ENSG0000(	ENSG0000(	42.47646	0.153666	0.580265	0.864891	lncRNA
ENSG0000(	ENSG0000(	38.30275	0.150468	0.580063	0.864891	lncRNA
ENSG0000(	ENSG0000(	342.6404	0.065554	0.580343	0.864891	lncRNA
ENSG0000(	ENSG0000(	25.72504	-0.12899	0.580454	0.864891	lncRNA
ENSG0000(	ENSG0000(	48.66432	0.130913	0.58064	0.864891	lncRNA
ENSG0000(	ENSG0000(	193.6595	0.057789	0.580421	0.864891	lncRNA
ENSG0000(	ENSG0000(	170.4742	0.981726	0.580702	0.864891	lncRNA
ENSG0000(	ENSG0000(	93.02393	0.064658	0.580573	0.864891	lncRNA
ENSG0000(	EXOC2	801.2299	-0.04631	0.58018	0.864891	protein_coding
ENSG0000(	FAM86DP	446.061	0.045782	0.58008	0.864891	transcribed_unprocessed_pseudo
ENSG0000(	GALNT17	66.459	-0.11836	0.580138	0.864891	protein_coding
ENSG0000(	HMOX1	392.7818	-0.13389	0.579954	0.864891	protein_coding
ENSG0000(	KLHL26	278.4083	-0.07671	0.580678	0.864891	protein_coding
ENSG0000(	LINC01534	642.9466	-0.03862	0.580697	0.864891	lncRNA
ENSG0000(	LYAR	98.5176	-0.08465	0.580625	0.864891	protein_coding
ENSG0000(	MAST1	289.2452	0.053984	0.580585	0.864891	protein_coding
ENSG0000(	NSG1	215.8171	0.09327	0.580204	0.864891	protein_coding
ENSG0000(	STMP1	1023.922	0.030418	0.580597	0.864891	protein_coding
ENSG0000(	TCF7	1377.929	-0.1697	0.580647	0.864891	protein_coding
ENSG0000(	TYW3	563.0978	-0.0467	0.580308	0.864891	protein_coding

ENSG0000(	VPS39	2154.769	0.035432	0.580032	0.864891	protein_coding
ENSG0000(	ZFAND5	3568.607	-0.05494	0.580214	0.864891	protein_coding
ENSG0000(	TP73-AS1	1349.721	0.04123	0.580739	0.864899	transcribed_unitary_pseudogene
ENSG0000(	ENSG0000(	97.56591	0.072825	0.580788	0.864926	lncRNA
ENSG0000(	RGS9	416.3298	0.050617	0.580837	0.864952	protein_coding
ENSG0000(	ENSG0000(	35.93625	-0.13793	0.580904	0.865005	lncRNA
ENSG0000(	AFAP1L2	495.2696	0.081	0.580999	0.865006	protein_coding
ENSG0000(	ALKBH3	186.7037	0.079882	0.580957	0.865006	protein_coding
ENSG0000(	C1orf115	744.4623	0.138344	0.580983	0.865006	protein_coding
ENSG0000(	CCR4	637.9979	0.046532	0.581162	0.865202	protein_coding
ENSG0000(	CDK2AP2	348.6077	-0.07331	0.581219	0.86524	protein_coding
ENSG0000(	DNAJC9-AS	234.1091	0.049886	0.581253	0.865243	lncRNA
ENSG0000(	RPL7A	2842.481	-0.07776	0.581322	0.865299	protein_coding
ENSG0000(	TRIM32	393.0996	-0.05788	0.581422	0.865343	protein_coding
ENSG0000(	TRIM46	160.9609	-0.05682	0.581445	0.865343	protein_coding
ENSG0000(	USP44	38.1081	0.210732	0.581445	0.865343	protein_coding
ENSG0000(	ENSG0000(	29.09172	0.187716	0.581555	0.865459	lncRNA
ENSG0000(	ENSG0000(	30.39655	0.135263	0.581586	0.865459	lncRNA
ENSG0000(	ENSG0000(	29.00714	-0.10343	0.581654	0.865512	lncRNA
ENSG0000(	TNXB	1195.552	0.076259	0.581688	0.865516	protein_coding
ENSG0000(	C1QTNF1	260.3295	-0.12009	0.581748	0.865521	protein_coding
ENSG0000(	N4BP3	108.4759	-0.10088	0.581754	0.865521	protein_coding
ENSG0000(	ENSG0000(	202.3114	-0.072	0.58188	0.865525	lncRNA
ENSG0000(	ENSG0000(	101.1068	0.087474	0.581993	0.865525	processed_pseudogene
ENSG0000(	FKBP7	366.145	-0.04789	0.581955	0.865525	protein_coding
ENSG0000(	PRECSIT	871.5475	-0.03915	0.581954	0.865525	lncRNA
ENSG0000(	RPS6KA1	1945.136	0.066874	0.581833	0.865525	protein_coding
ENSG0000(	SPNS3	24.58643	-0.21625	0.581982	0.865525	protein_coding
ENSG0000(	SYNM	343.3426	-0.07704	0.582008	0.865525	protein_coding
ENSG0000(	VTRNA1-2	29.88422	0.174114	0.5818	0.865525	misc_RNA
ENSG0000(	RNF216P1	1112.439	-0.0386	0.582143	0.865678	lncRNA
ENSG0000(	DAXX	408.201	0.069172	0.582261	0.865761	protein_coding
ENSG0000(	SLC4A9	48.92891	-0.28094	0.582243	0.865761	protein_coding
ENSG0000(	DPM2	388.8311	-0.06174	0.582397	0.865916	protein_coding
ENSG0000(	THOC1	986.6608	-0.04541	0.582576	0.866135	protein_coding
ENSG0000(	ENSG0000(	28.44405	-0.13993	0.582724	0.86618	lncRNA
ENSG0000(	ENSG0000(	627.3095	-0.03334	0.582743	0.86618	lncRNA
ENSG0000(	LINS1	2034.819	-0.06651	0.582655	0.86618	protein_coding
ENSG0000(	LRRTM2	76.31081	0.129616	0.582778	0.86618	protein_coding
ENSG0000(	RAB39A	89.32649	-0.09856	0.582685	0.86618	protein_coding
ENSG0000(	UBE2D1	258.8831	-0.07007	0.582795	0.86618	protein_coding
ENSG0000(	FNIP2	2972.316	0.086669	0.582981	0.866409	protein_coding
ENSG0000(	CASC3	1557.834	0.02685	0.583084	0.866425	protein_coding
ENSG0000(	CSNK1G1	1797.95	-0.05534	0.583081	0.866425	protein_coding
ENSG0000(	MTA2	892.3024	-0.04416	0.583086	0.866425	protein_coding
ENSG0000(	LINC02733	79.57912	0.090318	0.583263	0.866642	lncRNA
ENSG0000(	ARMC9	2238.954	-0.06456	0.583346	0.866655	protein_coding

ENSG0000(	FAU	525.3457	-0.07639	0.583339	0.866655	protein_coding
ENSG0000(	MRPS7	447.1425	0.04442	0.583366	0.866655	protein_coding
ENSG0000(	CDKN2AIPI	245.2703	0.049914	0.583553	0.866816	protein_coding
ENSG0000(	ENSG0000(	352.3892	0.05218	0.583518	0.866816	lncRNA
ENSG0000(	ENSG0000(	22.09735	0.136229	0.583671	0.866816	lncRNA
ENSG0000(	ENSG0000(	66.62265	-0.0828	0.583827	0.866816	lncRNA
ENSG0000(	GET3	126.4673	-0.08531	0.583646	0.866816	protein_coding
ENSG0000(	MAGEB10	140.2291	-0.0678	0.583884	0.866816	protein_coding
ENSG0000(	OR4D1	123.2589	0.055856	0.583867	0.866816	protein_coding
ENSG0000(	PKN3	128.2911	0.14693	0.583851	0.866816	protein_coding
ENSG0000(	PLD5P1	464.3733	-0.04958	0.583876	0.866816	protein_coding
ENSG0000(	PLEKHM3	1764.442	0.033641	0.583642	0.866816	protein_coding
ENSG0000(	RFTN1P1	58.98282	0.079062	0.583829	0.866816	transcribed_unprocessed_pseudo
ENSG0000(	RPS3AP34	79.73976	0.174708	0.583883	0.866816	processed_pseudogene
ENSG0000(	ZNF236-DT	66.37191	0.068731	0.583698	0.866816	lncRNA
ENSG0000(	OR5BA1P	551.2387	0.036141	0.583917	0.866817	transcribed_unprocessed_pseudo
ENSG0000(	MT-ND4L	4638.839	-0.12016	0.583965	0.866842	protein_coding
ENSG0000(	KPNB1-DT	76.05955	-0.09601	0.58406	0.86689	lncRNA
ENSG0000(	WFDC12	35.18792	-0.09716	0.584053	0.86689	protein_coding
ENSG0000(	PCBP2P1	42.07882	0.126778	0.584112	0.86692	processed_pseudogene
ENSG0000(	ABCB10	477.4701	0.067811	0.584377	0.866957	protein_coding
ENSG0000(	C2orf81	60.52636	0.114965	0.584207	0.866957	protein_coding
ENSG0000(	CD6	685.1536	-0.08698	0.584389	0.866957	protein_coding
ENSG0000(	DNAAF6	106.2723	0.101742	0.584266	0.866957	protein_coding
ENSG0000(	ENSG0000(	53.06319	-0.09718	0.584335	0.866957	lncRNA
ENSG0000(	HERC2P7	751.6208	0.070426	0.584287	0.866957	unprocessed_pseudogene
ENSG0000(	KLF9	1173.937	-0.0803	0.584347	0.866957	protein_coding
ENSG0000(	LINC00574	185.0427	0.042656	0.58424	0.866957	lncRNA
ENSG0000(	ENSG0000(	125.3474	0.126024	0.584438	0.866983	lncRNA
ENSG0000(	ZDHHC3	3576.989	0.065115	0.584475	0.866991	protein_coding
ENSG0000(	ENSG0000(	54.36524	-0.10099	0.584554	0.867027	lncRNA
ENSG0000(	ENSG0000(	125.3921	-0.06077	0.584563	0.867027	lncRNA
ENSG0000(	ARMCX1	108.0143	-0.08048	0.584683	0.867078	protein_coding
ENSG0000(	ENSG0000(	164.2251	-0.07593	0.584633	0.867078	lncRNA
ENSG0000(	SIGLEC17P	38.09349	0.172013	0.584691	0.867078	transcribed_unprocessed_pseudo
ENSG0000(	ACTG1P17	332.0227	-0.05265	0.584773	0.867106	transcribed_processed_pseudoge
ENSG0000(	ENSG0000(	190.0786	-0.06059	0.584747	0.867106	lncRNA
ENSG0000(	PRRG4	980.4795	0.050831	0.584819	0.867127	protein_coding
ENSG0000(	DUS4L	204.6764	0.094298	0.58493	0.867244	protein_coding
ENSG0000(	ENSG0000(	55.51489	-0.13908	0.585124	0.867252	transcribed_processed_pseudoge
ENSG0000(	KDM5C-IT1	123.2915	0.059434	0.585101	0.867252	lncRNA
ENSG0000(	PLCXD1	1722.908	0.070691	0.585114	0.867252	protein_coding
ENSG0000(	TBC1D22A	1502.152	-0.03868	0.584979	0.867252	protein_coding
ENSG0000(	TPD52L2	1320.144	-0.0519	0.585049	0.867252	protein_coding
ENSG0000(	TUT7	2565.978	-0.05054	0.585101	0.867252	protein_coding
ENSG0000(	ENSG0000(	603.6808	-0.06378	0.585209	0.867331	lncRNA
ENSG0000(	CFD	149.7301	-0.08355	0.585268	0.867373	protein_coding

ENSG0000(	MAP4K3	823.57	0.056071	0.585628	0.867808	protein_coding
ENSG0000(	RN7SKP217	26.91283	0.149924	0.585657	0.867808	misc_RNA
ENSG0000(	UBN1	1763.511	0.048152	0.58561	0.867808	protein_coding
ENSG0000(	COMMD9	515.3661	-0.06295	0.58577	0.867859	protein_coding
ENSG0000(	ENSG0000(	51.46299	-0.11664	0.585741	0.867859	processed_pseudogene
ENSG0000(	ENSG0000(	111.5575	-0.11672	0.585944	0.867859	lncRNA
ENSG0000(	ENSG0000(	451.6004	0.037708	0.585829	0.867859	TEC
ENSG0000(	HEXIM2	39.54214	-0.09122	0.585892	0.867859	protein_coding
ENSG0000(	PCAT6	21.19536	-0.23819	0.585924	0.867859	lncRNA
ENSG0000(	RN7SL181F	24.53494	-0.11656	0.585822	0.867859	misc_RNA
ENSG0000(	TRIP10	885.5253	-0.06413	0.585858	0.867859	protein_coding
ENSG0000(	CD19	279.6175	-0.23027	0.586009	0.86791	protein_coding
ENSG0000(	AHSA1	530.2161	0.04687	0.586139	0.867968	protein_coding
ENSG0000(	ENSG0000(	62.22714	-0.08064	0.586138	0.867968	lncRNA
ENSG0000(	ZFP37	143.7117	-0.07344	0.586143	0.867968	protein_coding
ENSG0000(	ENSG0000(	64.52546	0.0923	0.586207	0.867969	lncRNA
ENSG0000(	GGT1	335.8032	-0.10289	0.586177	0.867969	protein_coding
ENSG0000(	COX7C	768.8974	-0.08392	0.58625	0.867971	protein_coding
ENSG0000(	ENSG0000(	126.9058	-0.07625	0.586271	0.867971	lncRNA
ENSG0000(	RNU6-1327	38.89408	-0.15111	0.586404	0.86812	snRNA
ENSG0000(	BICDL1	518.5435	-0.06919	0.586441	0.868129	protein_coding
ENSG0000(	PHLDA1-DT	24.56493	0.151305	0.586593	0.868306	lncRNA
ENSG0000(	CCL14	315.9647	0.116343	0.586802	0.86837	protein_coding
ENSG0000(	COL6A5	111.5158	0.167934	0.586861	0.86837	protein_coding
ENSG0000(	DNAJC21	1187.039	0.046091	0.586795	0.86837	protein_coding
ENSG0000(	ENSG0000(	221.5806	-0.05272	0.586889	0.86837	lncRNA
ENSG0000(	LINC02087	42.93824	-0.135	0.586887	0.86837	lncRNA
ENSG0000(	RPL10	3749.179	-0.07521	0.586732	0.86837	protein_coding
ENSG0000(	SNORA80A	43.60732	-0.11022	0.586878	0.86837	snoRNA
ENSG0000(	TRAJ22	21.82793	-0.23434	0.58688	0.86837	TR_J_gene
ENSG0000(	MAN2A1	2914.803	0.030428	0.586941	0.868401	protein_coding
ENSG0000(	7SK	862.8476	-0.15223	0.587318	0.868642	misc_RNA
ENSG0000(	AKR1B10	588.875	0.153215	0.587948	0.868642	protein_coding
ENSG0000(	AP2B1	2845.048	0.04343	0.587429	0.868642	protein_coding
ENSG0000(	CASQ2	26.63983	-0.20182	0.5875	0.868642	protein_coding
ENSG0000(	CD163L1	1478.719	0.123552	0.588077	0.868642	protein_coding
ENSG0000(	DLGAP1-AS	145.8485	0.068316	0.588132	0.868642	lncRNA
ENSG0000(	ENSG0000(	231.9091	0.05861	0.587426	0.868642	lncRNA
ENSG0000(	ENSG0000(	139.9754	0.073448	0.587645	0.868642	lncRNA
ENSG0000(	ENSG0000(	40.62197	0.121567	0.587465	0.868642	lncRNA
ENSG0000(	ENSG0000(	140.9164	-0.06046	0.587582	0.868642	lncRNA
ENSG0000(	ENSG0000(	100.3054	0.075412	0.587876	0.868642	lncRNA
ENSG0000(	ENSG0000(	43.18322	-0.11091	0.588013	0.868642	lncRNA
ENSG0000(	ENSG0000(	25.65638	-0.20986	0.587375	0.868642	lncRNA
ENSG0000(	ENSG0000(	247.931	-0.06336	0.588017	0.868642	lncRNA
ENSG0000(	FRMPD1	231.772	0.054827	0.588006	0.868642	protein_coding
ENSG0000(	KLHDC7A	40.60218	0.231903	0.58784	0.868642	protein_coding

ENSG0000(	LINC01531	390.5503	0.056008	0.587947	0.868642	lncRNA
ENSG0000(	LY6G5B	864.6543	-0.06165	0.587608	0.868642	protein_coding
ENSG0000(	ODCP	160.2454	0.063418	0.588139	0.868642	processed_pseudogene
ENSG0000(	PEX2	803.4137	0.059057	0.587517	0.868642	protein_coding
ENSG0000(	PFKFB1	19.56985	0.138797	0.587977	0.868642	protein_coding
ENSG0000(	POLR3F	178.5297	-0.06636	0.587895	0.868642	protein_coding
ENSG0000(	PPME1	695.3995	0.052085	0.587786	0.868642	protein_coding
ENSG0000(	PRADC1	89.37305	0.12144	0.587697	0.868642	protein_coding
ENSG0000(	PRR29-AS1	283.3625	-0.04667	0.587933	0.868642	lncRNA
ENSG0000(	RFFL	3128.876	-0.05671	0.587895	0.868642	protein_coding
ENSG0000(	RN7SL277F	30.71837	0.181866	0.588058	0.868642	misc_RNA
ENSG0000(	RN7SL526F	89.28055	0.065108	0.587517	0.868642	misc_RNA
ENSG0000(	RN7SL712F	19.51092	0.140832	0.587167	0.868642	misc_RNA
ENSG0000(	RPL15	3853.17	-0.07647	0.587678	0.868642	protein_coding
ENSG0000(	SYNCRIP	4003.686	-0.03126	0.588145	0.868642	protein_coding
ENSG0000(	WNK3	169.501	-0.07413	0.587369	0.868642	protein_coding
ENSG0000(	ZKSCAN2-D	1096.257	0.041043	0.587406	0.868642	lncRNA
ENSG0000(	KCNJ14	497.4063	-0.04787	0.588217	0.8687	protein_coding
ENSG0000(	ENSG0000(	27.47388	-0.37301	0.588281	0.868749	IG_V_gene
ENSG0000(	SCN7A	465.7826	0.058499	0.588327	0.868769	protein_coding
ENSG0000(	AMD1	2905.511	-0.03499	0.588579	0.868798	protein_coding
ENSG0000(	CKS2	54.84976	-0.10095	0.588579	0.868798	protein_coding
ENSG0000(	CWC15	254.4069	-0.07021	0.588613	0.868798	protein_coding
ENSG0000(	LINC00595	73.00916	0.107199	0.588511	0.868798	lncRNA
ENSG0000(	LINC01535	201.4796	-0.06761	0.588401	0.868798	lncRNA
ENSG0000(	LINC01998	31.14454	-0.10563	0.588594	0.868798	lncRNA
ENSG0000(	PPP1R3F	200.2554	-0.07624	0.588495	0.868798	protein_coding
ENSG0000(	PTH1R	53.64943	-0.16973	0.588521	0.868798	protein_coding
ENSG0000(	RAB37	297.8153	0.067964	0.58863	0.868798	protein_coding
ENSG0000(	RPH3AL	613.1764	0.088713	0.588811	0.869018	protein_coding
ENSG0000(	AURKAP1	55.42704	0.177699	0.588974	0.869184	transcribed_processed_pseudoge
ENSG0000(	ERGIC3	1640.653	0.040903	0.588986	0.869184	protein_coding
ENSG0000(	RN7SKP9	1290.052	0.082153	0.58907	0.869261	misc_RNA
ENSG0000(	PNLIPP1	42.02685	0.101686	0.589148	0.869329	unprocessed_pseudogene
ENSG0000(	ENSG0000(	145.2686	-0.05111	0.589211	0.869375	lncRNA
ENSG0000(	ENSG0000(	45.86656	0.090607	0.589313	0.869394	lncRNA
ENSG0000(	ENSG0000(	31.49713	-0.11398	0.589413	0.869394	lncRNA
ENSG0000(	KIF2C	153.5078	-0.1246	0.589332	0.869394	protein_coding
ENSG0000(	MKLN1-AS	685.5653	0.040608	0.58931	0.869394	lncRNA
ENSG0000(	MST1R	2180.673	0.098051	0.589401	0.869394	protein_coding
ENSG0000(	SHISA2	97.79803	-0.07049	0.589389	0.869394	protein_coding
ENSG0000(	LINC01511	276.4701	0.056609	0.589633	0.869527	lncRNA
ENSG0000(	MTHFS	156.6849	-0.07613	0.589593	0.869527	protein_coding
ENSG0000(	RAD1	855.6793	-0.05252	0.589661	0.869527	protein_coding
ENSG0000(	RNF7	654.8637	0.06801	0.58966	0.869527	protein_coding
ENSG0000(	SNORA7B	141.6064	0.08862	0.589619	0.869527	snoRNA
ENSG0000(	ENSG0000(	168.7263	-0.07399	0.589736	0.869537	lncRNA

ENSG0000(	HINT1	2401.517	0.053804	0.589763	0.869537	protein_coding
ENSG0000(	NQO1	695.6638	0.125358	0.589749	0.869537	protein_coding
ENSG0000(	DCAF8	3969.872	-0.03084	0.590364	0.869555	protein_coding
ENSG0000(	EIF2AK4	1519.324	-0.03316	0.590011	0.869555	protein_coding
ENSG0000(	ENSG0000(	60.94862	0.081217	0.589951	0.869555	lncRNA
ENSG0000(	ENSG0000(	36.32833	0.112249	0.590108	0.869555	lncRNA
ENSG0000(	ENSG0000(	110.9068	0.067748	0.590074	0.869555	lncRNA
ENSG0000(	ENSG0000(	154.1687	-0.06194	0.590291	0.869555	lncRNA
ENSG0000(	HLA-F-AS1	176.9984	-0.08508	0.590276	0.869555	lncRNA
ENSG0000(	LINC01499	82.10193	-0.07435	0.590305	0.869555	lncRNA
ENSG0000(	MON1B	2238.01	0.040073	0.590267	0.869555	protein_coding
ENSG0000(	NKRF	332.9122	0.058834	0.59038	0.869555	protein_coding
ENSG0000(	PLAAT2	40.90775	0.226659	0.589982	0.869555	protein_coding
ENSG0000(	REV1	1586.584	0.046423	0.590068	0.869555	protein_coding
ENSG0000(	RGS1	905.002	-0.20708	0.590049	0.869555	protein_coding
ENSG0000(	RILPL1	871.877	0.031309	0.590086	0.869555	protein_coding
ENSG0000(	SEC31B	1032.983	0.054069	0.590407	0.869555	protein_coding
ENSG0000(	TMEM203	219.0595	0.058033	0.590236	0.869555	protein_coding
ENSG0000(	USP33	2754.964	-0.03833	0.590306	0.869555	protein_coding
ENSG0000(	VPS25	339.3528	0.058995	0.590394	0.869555	protein_coding
ENSG0000(	ZDHHC17	1379.059	-0.06149	0.590316	0.869555	protein_coding
ENSG0000(	ZNF688	320.4368	0.052741	0.590162	0.869555	protein_coding
ENSG0000(	ECT2	610.8068	-0.04956	0.590552	0.869582	protein_coding
ENSG0000(	EDRF1-DT	44.471	-0.10056	0.590501	0.869582	lncRNA
ENSG0000(	ISLR2	125.295	-0.08065	0.590548	0.869582	protein_coding
ENSG0000(	PUS10	779.0218	0.056416	0.590537	0.869582	protein_coding
ENSG0000(	FAM78B-AS	78.84896	0.072042	0.59062	0.869636	lncRNA
ENSG0000(	LINC02367	61.75828	-0.13171	0.590728	0.869747	lncRNA
ENSG0000(	NOS2P3	129.037	0.054816	0.590858	0.869754	transcribed_unprocessed_pseudo
ENSG0000(	RAB3IL1	76.54894	-0.12773	0.590847	0.869754	protein_coding
ENSG0000(	SNHG12	822.5437	-0.03794	0.590823	0.869754	lncRNA
ENSG0000(	VASP	1580.271	0.056093	0.590774	0.869754	protein_coding
ENSG0000(	MUC20P1	183.6525	0.181283	0.590954	0.869847	unprocessed_pseudogene
ENSG0000(	KIAA0319	132.1429	0.107731	0.591039	0.869926	protein_coding
ENSG0000(	ENSG0000(	192.8424	0.048032	0.591122	0.870002	lncRNA
ENSG0000(	DGCR6L	86.30593	-0.09296	0.591235	0.870122	protein_coding
ENSG0000(	ANKRD20A	235.7506	0.153881	0.591485	0.870304	transcribed_unprocessed_pseudo
ENSG0000(	COA4	418.0825	-0.04246	0.59143	0.870304	protein_coding
ENSG0000(	ENSG0000(	266.9827	0.043295	0.591481	0.870304	lncRNA
ENSG0000(	ZNF667-AS	829.6135	0.031365	0.591446	0.870304	lncRNA
ENSG0000(	CSE1L-AS1	27.34147	-0.12069	0.591735	0.870625	lncRNA
ENSG0000(	PLGLA	59.18739	0.108385	0.591798	0.87067	transcribed_unprocessed_pseudo
ENSG0000(	KCTD18	1013.531	0.022647	0.591857	0.870712	protein_coding
ENSG0000(	NAV2	1366.712	0.067534	0.591938	0.870738	protein_coding
ENSG0000(	RPS6KC1	825.3253	-0.05864	0.591919	0.870738	protein_coding
ENSG0000(	ENSG0000(	65.64798	-0.08859	0.591996	0.870776	lncRNA
ENSG0000(	ENSG0000(	1166.54	0.02727	0.592032	0.870782	lncRNA

ENSG0000(	B3GLCT	162.1738	0.064853	0.592119	0.870863	protein_coding
ENSG0000(	RN7SL405F	215.6988	0.054595	0.592153	0.870867	misc_RNA
ENSG0000(	LINC02556	25.96112	0.130755	0.59225	0.870916	lncRNA
ENSG0000(	SCMH1	975.6408	0.052316	0.59222	0.870916	protein_coding
ENSG0000(	ENSG0000(	24.86834	-0.1449	0.592596	0.871243	lncRNA
ENSG0000(	ENSG0000(	5647.482	-0.04474	0.592548	0.871243	lncRNA
ENSG0000(	METTL21A	4776.409	0.04024	0.592645	0.871243	protein_coding
ENSG0000(	RPL3L	308.3324	-0.05848	0.592604	0.871243	protein_coding
ENSG0000(	SAMD5	1140.721	0.065188	0.592662	0.871243	protein_coding
ENSG0000(	XKR5	124.8001	-0.05487	0.592554	0.871243	protein_coding
ENSG0000(	ENSG0000(	24.51478	0.12875	0.592696	0.871246	lncRNA
ENSG0000(	ENSG0000(	154.3504	-0.06284	0.592764	0.871301	lncRNA
ENSG0000(	DAZAP2	2187.728	-0.05973	0.592867	0.871305	protein_coding
ENSG0000(	ENSG0000(	21.06482	-0.16985	0.592857	0.871305	lncRNA
ENSG0000(	HAS2	357.0817	0.109628	0.592839	0.871305	protein_coding
ENSG0000(	SFTPB	170.258	-0.07329	0.592894	0.871305	protein_coding
ENSG0000(	AGBL2	225.1661	0.064665	0.59307	0.871428	protein_coding
ENSG0000(	CYP27C1	406.2564	0.040965	0.593073	0.871428	protein_coding
ENSG0000(	ENSG0000(	47.66522	-0.17254	0.593023	0.871428	processed_pseudogene
ENSG0000(	MORC3	1573.398	0.036796	0.593126	0.87146	protein_coding
ENSG0000(	PCNX4	4116.945	-0.03606	0.593163	0.871467	protein_coding
ENSG0000(	ADH1B	1971.706	0.109127	0.593391	0.87171	protein_coding
ENSG0000(	CCDC30	2713.394	0.024812	0.593377	0.87171	protein_coding
ENSG0000(	ENSG0000(	607.714	-0.05843	0.593447	0.871745	lncRNA
ENSG0000(	PTPN20	91.30704	-0.17298	0.593514	0.871797	protein_coding
ENSG0000(	SH3GL1P2	34.5022	-0.29659	0.593593	0.871866	processed_pseudogene
ENSG0000(	ANXA2	6825.825	-0.06885	0.59366	0.871871	protein_coding
ENSG0000(	PNMA3	86.10991	0.181509	0.593628	0.871871	protein_coding
ENSG0000(	PIANP	22.99921	-0.21419	0.59372	0.871914	protein_coding
ENSG0000(	ENSG0000(	29.09354	0.120001	0.593785	0.871963	lncRNA
ENSG0000(	GOLGA8N	557.5802	0.079201	0.593859	0.871977	protein_coding
ENSG0000(	HDAC2	1235.706	-0.04779	0.593843	0.871977	protein_coding
ENSG0000(	NPIPP1	202.751	0.071482	0.593984	0.872115	transcribed_unprocessed_pseudo
ENSG0000(	SDHAF4	355.4993	0.046003	0.594029	0.872135	protein_coding
ENSG0000(	LINC02789	136.9349	0.056667	0.594202	0.872299	lncRNA
ENSG0000(	SLC51B	174.2943	0.183717	0.594204	0.872299	protein_coding
ENSG0000(	ARHGAP39	260.2551	0.106396	0.594367	0.87231	protein_coding
ENSG0000(	BRCC3P1	41.88104	0.106529	0.594434	0.87231	processed_pseudogene
ENSG0000(	EIF3E	5238.228	-0.03489	0.594368	0.87231	protein_coding
ENSG0000(	ENSG0000(	277.1649	0.044589	0.594612	0.87231	processed_pseudogene
ENSG0000(	ENSG0000(	385.9254	-0.04248	0.594966	0.87231	lncRNA
ENSG0000(	ENSG0000(	23.57321	0.205869	0.594777	0.87231	lncRNA
ENSG0000(	ENSG0000(	35.44826	0.155735	0.594592	0.87231	lncRNA
ENSG0000(	ENSG0000(	198.2722	0.049818	0.594836	0.87231	lncRNA
ENSG0000(	ENSG0000(	2523.363	-0.04348	0.594582	0.87231	lncRNA
ENSG0000(	ENSG0000(	31.45382	0.127231	0.594908	0.87231	lncRNA
ENSG0000(	ENSG0000(	147.7226	-0.07783	0.594723	0.87231	lncRNA

ENSG0000(	ENSG0000(	41.74678	0.114761	0.594711	0.87231	processed_pseudogene
ENSG0000(	ENSG0000(	503.2671	-0.04341	0.595163	0.87231	lncRNA
ENSG0000(	ENSG0000(	101.0323	-0.26895	0.595119	0.87231	protein_coding
ENSG0000(	FAM217B	637.1282	0.03936	0.594476	0.87231	protein_coding
ENSG0000(	FAR2P4	126.4517	0.093918	0.595121	0.87231	transcribed_unprocessed_pseudo
ENSG0000(	FHL1	877.6021	-0.08376	0.594614	0.87231	protein_coding
ENSG0000(	GIMAP2	620.3316	0.077888	0.59475	0.87231	protein_coding
ENSG0000(	H1-2	367.7759	0.08446	0.594956	0.87231	protein_coding
ENSG0000(	HIF3A	783.45	0.054589	0.595143	0.87231	protein_coding
ENSG0000(	LINC02306	256.7402	-0.08265	0.594347	0.87231	lncRNA
ENSG0000(	NHP2	187.2036	0.055946	0.594976	0.87231	protein_coding
ENSG0000(	PIK3CB	1837.316	0.046872	0.594757	0.87231	protein_coding
ENSG0000(	RN7SL57P	82.42791	-0.0803	0.59506	0.87231	misc_RNA
ENSG0000(	STIMATE-M	286.7833	-0.14269	0.594962	0.87231	protein_coding
ENSG0000(	THRAP3P3	22.93903	-0.17022	0.594962	0.87231	processed_pseudogene
ENSG0000(	TMEM200(	91.98422	-0.18103	0.595004	0.87231	protein_coding
ENSG0000(	TMEM92	128.5725	-0.13777	0.594408	0.87231	protein_coding
ENSG0000(	ZNF316	1018.432	-0.04885	0.594812	0.87231	protein_coding
ENSG0000(	ZNF512	979.6417	-0.05607	0.595127	0.87231	protein_coding
ENSG0000(	PINLYP	296.1418	-0.05946	0.595201	0.872319	protein_coding
ENSG0000(	B4GALT5	1768.506	0.073062	0.59541	0.872339	protein_coding
ENSG0000(	ENSG0000(	60.75848	-0.06731	0.595398	0.872339	lncRNA
ENSG0000(	ENSG0000(	110.8011	-0.1282	0.595308	0.872339	lncRNA
ENSG0000(	ENSG0000(	1015.332	-0.04759	0.595247	0.872339	lncRNA
ENSG0000(	FBXO9	1375.392	-0.04122	0.595436	0.872339	protein_coding
ENSG0000(	IL2RG	701.4729	-0.07844	0.595332	0.872339	protein_coding
ENSG0000(	TMEM38A	294.3665	0.061174	0.595424	0.872339	protein_coding
ENSG0000(	METAP2	600.2484	0.04917	0.595611	0.872548	protein_coding
ENSG0000(	RN7SKP17(	209.5313	0.105407	0.595708	0.872643	misc_RNA
ENSG0000(	CDK4P1	86.32669	-0.06369	0.595903	0.872651	processed_pseudogene
ENSG0000(	F13A1	396.4505	0.160129	0.595811	0.872651	protein_coding
ENSG0000(	MOSPD3	54.49144	-0.11716	0.595771	0.872651	protein_coding
ENSG0000(	SLC6A16	216.1625	-0.08442	0.595859	0.872651	protein_coding
ENSG0000(	SMAP1	888.9709	-0.06418	0.595876	0.872651	protein_coding
ENSG0000(	SSX2	53.09744	0.163427	0.595806	0.872651	protein_coding
ENSG0000(	ENSG0000(	38.36095	-0.09177	0.596247	0.872782	transcribed_processed_pseudoge
ENSG0000(	ENSG0000(	162.3814	0.063466	0.596241	0.872782	lncRNA
ENSG0000(	ENSG0000(	83.996	0.072974	0.5962	0.872782	lncRNA
ENSG0000(	GATD3A	349.6614	0.100039	0.596197	0.872782	protein_coding
ENSG0000(	GRM2	74.13103	0.123229	0.596147	0.872782	protein_coding
ENSG0000(	LINC00383	83.75299	0.104528	0.596152	0.872782	lncRNA
ENSG0000(	LINC02210	1702.947	0.042739	0.596194	0.872782	transcribed_unitary_pseudogene
ENSG0000(	PYGL	727.5934	-0.0654	0.596202	0.872782	protein_coding
ENSG0000(	TMPRSS11I	263.7225	0.063492	0.596294	0.872805	protein_coding
ENSG0000(	JAG2	214.4653	-0.1069	0.596337	0.872821	protein_coding
ENSG0000(	ENSG0000(	21.05286	0.17043	0.596373	0.872827	lncRNA
ENSG0000(	ENSG0000(	107.1135	0.09052	0.596436	0.872872	lncRNA

ENSG0000(	SARS1	905.9446	-0.04156	0.596467	0.872872	protein_coding
ENSG0000(	ATP6V1E2	90.71288	-0.08982	0.596522	0.872907	protein_coding
ENSG0000(	ENSG0000(	60.31955	-0.09107	0.596641	0.872988	lncRNA
ENSG0000(	IARS2	819.6943	-0.06103	0.59662	0.872988	protein_coding
ENSG0000(	ENSG0000(	124.1594	-0.08816	0.59669	0.873014	lncRNA
ENSG0000(	ENSG0000(	300.0987	-0.04101	0.596808	0.873139	lncRNA
ENSG0000(	BRD9	1364.159	0.039355	0.597179	0.873186	protein_coding
ENSG0000(	COX5A	718.6986	0.098295	0.597179	0.873186	protein_coding
ENSG0000(	CTRC	311.8777	0.04308	0.597067	0.873186	protein_coding
ENSG0000(	DNAJC18	612.0853	-0.0617	0.597189	0.873186	protein_coding
ENSG0000(	ENSG0000(	32.35657	-0.12273	0.596958	0.873186	lncRNA
ENSG0000(	ENSG0000(	26.48555	0.167706	0.597167	0.873186	transcribed_unitary_pseudogene
ENSG0000(	FXYD2	91.7538	0.129609	0.597101	0.873186	protein_coding
ENSG0000(	NR1H2	523.2472	0.055504	0.596972	0.873186	protein_coding
ENSG0000(	S100PBP	2407.113	0.039595	0.597104	0.873186	protein_coding
ENSG0000(	TBC1D24	839.3384	-0.03062	0.596906	0.873186	protein_coding
ENSG0000(	TMEM59	1975.054	0.068245	0.597106	0.873186	protein_coding
ENSG0000(	CDKN3	57.29852	0.134914	0.597283	0.87323	protein_coding
ENSG0000(	PUSL1	84.45847	0.133366	0.597282	0.87323	protein_coding
ENSG0000(	E2F5	496.4725	-0.0852	0.597371	0.873312	protein_coding
ENSG0000(	ENSG0000(	32.29199	-0.11084	0.597412	0.873319	lncRNA
ENSG0000(	NAP1L4	1906.881	0.033632	0.597439	0.873319	protein_coding
ENSG0000(	PLA2G12B	33.96357	0.283312	0.597481	0.873335	protein_coding
ENSG0000(	MRPS34	171.22	-0.07025	0.597534	0.873366	protein_coding
ENSG0000(	ZFAS1	1375.973	-0.03463	0.597587	0.873396	lncRNA
ENSG0000(	BEGAIN	100.5277	0.126947	0.597914	0.873611	protein_coding
ENSG0000(	CKAP5	1555.755	0.055995	0.597869	0.873611	protein_coding
ENSG0000(	ENSG0000(	47.4074	0.13338	0.597924	0.873611	processed_pseudogene
ENSG0000(	ENSG0000(	378.8498	0.041142	0.597875	0.873611	lncRNA
ENSG0000(	FAM74A6	185.0781	0.068017	0.597783	0.873611	transcribed_processed_pseudoge
ENSG0000(	ZWINT	200.3298	0.084359	0.597923	0.873611	protein_coding
ENSG0000(	HLA-W	20.74156	-0.19941	0.597959	0.873615	unprocessed_pseudogene
ENSG0000(	FBXO36-IT:	19.96861	0.13829	0.598133	0.873777	lncRNA
ENSG0000(	WASH3P	720.2167	-0.05907	0.598117	0.873777	transcribed_unprocessed_pseudo
ENSG0000(	MASTL	696.1258	0.049507	0.598259	0.873915	protein_coding
ENSG0000(	ENSG0000(	170.8276	0.047183	0.5983	0.873928	lncRNA
ENSG0000(	LINC00683	493.6832	0.072283	0.598406	0.874036	lncRNA
ENSG0000(	ENSG0000(	181.8739	0.08657	0.598573	0.874054	lncRNA
ENSG0000(	ENSG0000(	58.68278	0.140843	0.598577	0.874054	unprocessed_pseudogene
ENSG0000(	ENSG0000(	198.6863	0.065429	0.598497	0.874054	lncRNA
ENSG0000(	RBBP5	924.1684	0.031449	0.598493	0.874054	protein_coding
ENSG0000(	UPK3BP1	31.58371	0.245056	0.598525	0.874054	transcribed_unprocessed_pseudo
ENSG0000(	ENSG0000(	48.02542	-0.09763	0.598707	0.874106	lncRNA
ENSG0000(	ENSG0000(	84.86435	-0.08418	0.598708	0.874106	lncRNA
ENSG0000(	NUDT8	34.23636	-0.16821	0.598684	0.874106	protein_coding
ENSG0000(	BMP1	1251.003	0.059641	0.598768	0.874126	protein_coding
ENSG0000(	ENSG0000(	32.81733	-0.17743	0.598817	0.874126	lncRNA

ENSG0000(	MED8	506.0455	-0.0352	0.598807	0.874126	protein_coding
ENSG0000(	ENSG0000(	2269.816	-0.06205	0.598861	0.874145	lncRNA
ENSG0000(	ENSG0000(	65.70449	-0.08476	0.598965	0.874249	lncRNA
ENSG0000(	ADNP2	1051.162	0.103242	0.599029	0.874297	protein_coding
ENSG0000(	ENSG0000(	23.51379	0.134023	0.599091	0.874341	lncRNA
ENSG0000(	HCFC2	540.7656	-0.06895	0.599143	0.87437	protein_coding
ENSG0000(	NEK11	170.8448	0.093719	0.59923	0.874404	protein_coding
ENSG0000(	PLPBP	729.8626	0.055766	0.599208	0.874404	protein_coding
ENSG0000(	ENSG0000(	210.5346	0.07164	0.599355	0.874448	lncRNA
ENSG0000(	HNRNPA1	8016.946	-0.04091	0.599292	0.874448	protein_coding
ENSG0000(	PNPT1P1	59.92056	-0.13275	0.59935	0.874448	processed_pseudogene
ENSG0000(	ENSG0000(	32.53639	-0.1869	0.59951	0.874627	lncRNA
ENSG0000(	ANKS3	1022.686	0.035884	0.59966	0.874707	protein_coding
ENSG0000(	PLD1	2808.837	0.071529	0.599609	0.874707	protein_coding
ENSG0000(	RN7SKP115	37.99773	-0.1223	0.599654	0.874707	misc_RNA
ENSG0000(	FAM161A	590.6269	0.056568	0.599757	0.874803	protein_coding
ENSG0000(	CIB1	645.4929	0.082824	0.599927	0.874838	protein_coding
ENSG0000(	ENSG0000(	22.71431	0.140972	0.599963	0.874838	transcribed_processed_pseudoge
ENSG0000(	GCSIR	88.47704	-0.11615	0.599896	0.874838	lncRNA
ENSG0000(	HERC5	263.4277	0.145095	0.599972	0.874838	protein_coding
ENSG0000(	MRPL46	371.7443	0.047977	0.599935	0.874838	protein_coding
ENSG0000(	NANOGP1	199.7095	0.049375	0.599819	0.874838	transcribed_unprocessed_pseudo
ENSG0000(	MECR	482.2925	0.047102	0.600033	0.87488	protein_coding
ENSG0000(	GMEB2	372.4237	0.043802	0.600079	0.874901	protein_coding
ENSG0000(	OR1J1	27.18011	-0.12271	0.600123	0.874919	protein_coding
ENSG0000(	DYRK1B	200.1306	0.067223	0.600207	0.874994	protein_coding
ENSG0000(	MYADM	945.8425	-0.06489	0.600291	0.87507	protein_coding
ENSG0000(	TNK2-AS1	116.2222	-0.07957	0.600349	0.875109	lncRNA
ENSG0000(	ENSG0000(	30.33586	-0.13015	0.600416	0.875137	lncRNA
ENSG0000(	ENSG0000(	92.35254	0.067479	0.600462	0.875137	lncRNA
ENSG0000(	IPO5	1587.77	-0.04732	0.600528	0.875137	protein_coding
ENSG0000(	RPL18	1508.182	-0.07067	0.600526	0.875137	protein_coding
ENSG0000(	ZNF71	382.8402	-0.06242	0.60048	0.875137	protein_coding
ENSG0000(	SLC38A1	7058.133	0.045707	0.600617	0.875222	protein_coding
ENSG0000(	LINC02427	384.5914	0.040412	0.60071	0.875264	lncRNA
ENSG0000(	METTL8	1554.644	0.04417	0.600699	0.875264	protein_coding
ENSG0000(	PNKD	633.7768	-0.05019	0.60076	0.87529	protein_coding
ENSG0000(	ENSG0000(	250.402	-0.05095	0.600913	0.875327	lncRNA
ENSG0000(	LSM8	3125.693	0.02492	0.600889	0.875327	protein_coding
ENSG0000(	SGF29	159.7715	0.0638	0.600909	0.875327	protein_coding
ENSG0000(	USP46	926.8819	-0.05254	0.600885	0.875327	protein_coding
ENSG0000(	CHMP5	397.5136	-0.06769	0.601009	0.875376	protein_coding
ENSG0000(	ENSG0000(	23.08068	0.134957	0.600986	0.875376	lncRNA
ENSG0000(	HRH1	655.8388	0.103138	0.601073	0.875422	protein_coding
ENSG0000(	LINC02635	81.21316	0.093784	0.601175	0.875491	lncRNA
ENSG0000(	NCAM1	484.935	-0.08277	0.601184	0.875491	protein_coding
ENSG0000(	ENSG0000(	43.32539	-0.12378	0.601353	0.875599	lncRNA

ENSG0000(	ENSG0000(	498.1849	-0.04737	0.601302	0.875599	lncRNA
ENSG0000(	ENSG0000(	49.67328	0.085247	0.601333	0.875599	lncRNA
ENSG0000(	TRIM31	2134.372	-0.14101	0.60154	0.875824	protein_coding
ENSG0000(	ENSG0000(	122.326	0.061997	0.601636	0.875873	lncRNA
ENSG0000(	ZNF594	680.3489	0.059899	0.601638	0.875873	protein_coding
ENSG0000(	ZNF10	471.3323	0.064869	0.601681	0.87589	protein_coding
ENSG0000(	EEA1	1395.311	0.067557	0.601895	0.876155	protein_coding
ENSG0000(	EMC6	55.44497	-0.07421	0.602093	0.876308	protein_coding
ENSG0000(	ENSG0000(	157.4292	-0.06164	0.602106	0.876308	TEC
ENSG0000(	ENSG0000(	47.5353	-0.10634	0.602127	0.876308	lncRNA
ENSG0000(	ENSG0000(	114.1511	0.072394	0.602037	0.876308	lncRNA
ENSG0000(	LINC02419	97.95468	-0.07452	0.602169	0.876323	lncRNA
ENSG0000(	ENSG0000(	21.19956	-0.13523	0.602552	0.876409	lncRNA
ENSG0000(	ENSG0000(	118.9583	0.080568	0.60229	0.876409	lncRNA
ENSG0000(	ENSG0000(	51.69908	0.1028	0.602373	0.876409	lncRNA
ENSG0000(	ENSG0000(	248.7129	-0.0379	0.602523	0.876409	TEC
ENSG0000(	HAGH	826.2623	0.037412	0.602443	0.876409	protein_coding
ENSG0000(	LINC02631	60.18897	0.08125	0.602579	0.876409	lncRNA
ENSG0000(	RPL12P20	26.98878	-0.12826	0.602455	0.876409	processed_pseudogene
ENSG0000(	RPL7P7	74.04553	-0.09248	0.60253	0.876409	processed_pseudogene
ENSG0000(	RREB1	7454.561	0.040688	0.602452	0.876409	protein_coding
ENSG0000(	TAS2R14	240.5475	0.068602	0.602565	0.876409	protein_coding
ENSG0000(	YIPF3	577.1469	0.041257	0.602444	0.876409	protein_coding
ENSG0000(	ABCG5	75.44719	-0.07263	0.603057	0.87641	protein_coding
ENSG0000(	ARF3	3252.332	-0.06148	0.602619	0.87641	protein_coding
ENSG0000(	CELSR1	594.429	0.103478	0.602969	0.87641	protein_coding
ENSG0000(	CORO1B	1188.381	0.085752	0.602748	0.87641	protein_coding
ENSG0000(	DYRK1A	6062.826	0.026488	0.603025	0.87641	protein_coding
ENSG0000(	ENSG0000(	461.7951	-0.06265	0.602886	0.87641	lncRNA
ENSG0000(	ENSG0000(	20.52462	0.135965	0.602697	0.87641	lncRNA
ENSG0000(	ENSG0000(	79.2289	-0.08907	0.60286	0.87641	lncRNA
ENSG0000(	ENSG0000(	26.22925	-0.14802	0.602847	0.87641	lncRNA
ENSG0000(	FCMR	734.514	-0.19245	0.602936	0.87641	protein_coding
ENSG0000(	IL9R	85.24154	-0.18204	0.602987	0.87641	protein_coding
ENSG0000(	OGN	38.44471	0.594333	0.602776	0.87641	protein_coding
ENSG0000(	PAQR7	349.002	-0.04483	0.602725	0.87641	protein_coding
ENSG0000(	SLC22A10	142.0968	0.056982	0.603012	0.87641	protein_coding
ENSG0000(	UBXN6	549.4271	0.063234	0.60303	0.87641	protein_coding
ENSG0000(	GFI1B	467.6819	0.03967	0.603134	0.876474	protein_coding
ENSG0000(	TVP23C-CD	21.64728	-0.37254	0.603209	0.876538	protein_coding
ENSG0000(	DPCD	32.98922	-0.12273	0.603297	0.876573	protein_coding
ENSG0000(	ENSG0000(	278.5204	0.064517	0.603284	0.876573	lncRNA
ENSG0000(	EEF1A1P10	48.23326	-0.14379	0.60334	0.87659	processed_pseudogene
ENSG0000(	LINC00408	38.93998	0.109952	0.603405	0.876637	lncRNA
ENSG0000(	ENSG0000(	39.0548	-0.08706	0.603729	0.876783	lncRNA
ENSG0000(	ENSG0000(	23.98944	0.728754	0.603721	0.876783	protein_coding
ENSG0000(	RNF213-AS	313.8248	0.049205	0.603566	0.876783	lncRNA

ENSG0000(	RPRD2	1683.444	-0.03319	0.603604	0.876783	protein_coding
ENSG0000(	SOX6	1755.017	0.100801	0.603672	0.876783	protein_coding
ENSG0000(	TK2	1135.458	0.064506	0.603637	0.876783	protein_coding
ENSG0000(	TP53BP2	879.2469	0.065237	0.603603	0.876783	protein_coding
ENSG0000(	ENSG0000(	147.7452	0.082609	0.60378	0.876812	lncRNA
ENSG0000(	CATSPERE	64.44895	-0.13287	0.603861	0.876856	protein_coding
ENSG0000(	ENSG0000(	127.2867	-0.0688	0.603874	0.876856	lncRNA
ENSG0000(	RCN2	1643.972	0.036919	0.603912	0.876864	protein_coding
ENSG0000(	ACTR3B	630.0987	0.040324	0.604101	0.876996	protein_coding
ENSG0000(	COL4A3	602.537	0.093137	0.604073	0.876996	protein_coding
ENSG0000(	ENSG0000(	32.19707	-0.14039	0.604162	0.876996	lncRNA
ENSG0000(	INO80B	184.8621	-0.07494	0.604137	0.876996	protein_coding
ENSG0000(	QDPR	170.7686	-0.07794	0.604161	0.876996	protein_coding
ENSG0000(	ENSG0000(	813.4638	-0.09229	0.604227	0.877044	lncRNA
ENSG0000(	ANKHD1	6504.856	0.030225	0.604268	0.877057	protein_coding
ENSG0000(	HSPE1P26	100.2083	0.074879	0.604362	0.877064	processed_pseudogene
ENSG0000(	MTCO1P53	36.30892	-0.27159	0.604347	0.877064	processed_pseudogene
ENSG0000(	SF3A1	1228.295	-0.05155	0.604369	0.877064	protein_coding
ENSG0000(	GJA9	52.77983	0.114068	0.604483	0.877143	protein_coding
ENSG0000(	PRAMENP	19.79497	-0.1949	0.604486	0.877143	transcribed_unprocessed_pseudo
ENSG0000(	ENSG0000(	1212.731	0.030338	0.604577	0.877228	lncRNA
ENSG0000(	MUC21	71.04176	0.066851	0.604626	0.877253	protein_coding
ENSG0000(	ALKAL2	54.80784	-0.1846	0.60469	0.8773	protein_coding
ENSG0000(	ANKRD30B	228.157	-0.10573	0.604941	0.877525	transcribed_unprocessed_pseudo
ENSG0000(	ENSG0000(	45.95206	-0.09613	0.604902	0.877525	lncRNA
ENSG0000(	RNU6-466F	21.44055	-0.15349	0.604915	0.877525	snRNA
ENSG0000(	RNU6-82P	47.55232	-0.10626	0.605013	0.877583	snRNA
ENSG0000(	ENSG0000(	146.5787	-0.05648	0.605125	0.877699	lncRNA
ENSG0000(	ENSG0000(	231.7055	0.056741	0.605226	0.877712	lncRNA
ENSG0000(	TADA3	764.3155	0.039289	0.605229	0.877712	protein_coding
ENSG0000(	TMEM30BF	69.84291	0.091152	0.605185	0.877712	processed_pseudogene
ENSG0000(	PMM2	4536.76	0.040291	0.605277	0.877734	protein_coding
ENSG0000(	IRGQ	2001.667	-0.02536	0.605372	0.877827	protein_coding
ENSG0000(	DNAJA2	950.9318	0.027303	0.605565	0.877968	protein_coding
ENSG0000(	TRHDE-AS1	146.8462	0.093798	0.605538	0.877968	lncRNA
ENSG0000(	TRIM5	822.8863	-0.06029	0.605547	0.877968	protein_coding
ENSG0000(	ASNSD1	312.2442	-0.06137	0.60604	0.877991	protein_coding
ENSG0000(	BLOC1S4	108.5334	-0.07171	0.606082	0.877991	protein_coding
ENSG0000(	CD160	144.0286	-0.10771	0.60584	0.877991	protein_coding
ENSG0000(	DHRS7B	526.1922	0.036208	0.605794	0.877991	protein_coding
ENSG0000(	EGFR	2988.848	0.076336	0.605814	0.877991	protein_coding
ENSG0000(	ENSG0000(	598.2381	0.04336	0.605769	0.877991	lncRNA
ENSG0000(	ENSG0000(	21.05113	0.107829	0.605903	0.877991	lncRNA
ENSG0000(	ENSG0000(	86.40314	0.072162	0.606124	0.877991	lncRNA
ENSG0000(	ENSG0000(	163.0314	-0.07475	0.606106	0.877991	lncRNA
ENSG0000(	HSPD1P13	33.15866	-0.15276	0.606086	0.877991	processed_pseudogene
ENSG0000(	IGSF10	167.1345	-0.16356	0.605927	0.877991	protein_coding

ENSG0000(	KCNIP3	57.52299	0.110386	0.605819	0.877991	protein_coding
ENSG0000(	NCCRP1	199.441	0.059231	0.60595	0.877991	protein_coding
ENSG0000(	PGM3	1582.342	-0.0911	0.605894	0.877991	protein_coding
ENSG0000(	PKD1P1	993.1908	0.215252	0.605932	0.877991	unprocessed_pseudogene
ENSG0000(	TINCR	343.6409	0.053203	0.605902	0.877991	protein_coding
ENSG0000(	TMEM267	269.0353	0.051927	0.606005	0.877991	protein_coding
ENSG0000(	ARHGAP29	56.81137	-0.10602	0.606192	0.877997	lncRNA
ENSG0000(	CCNK	1354.673	-0.03921	0.606169	0.877997	protein_coding
ENSG0000(	CDKN2AIP	392.1903	0.061698	0.606262	0.878006	protein_coding
ENSG0000(	PROSER1	1116.148	0.057604	0.606249	0.878006	protein_coding
ENSG0000(	IQCF1	50.2345	0.084051	0.606323	0.878048	protein_coding
ENSG0000(	ENSG0000(	59.22063	0.083827	0.606366	0.878064	lncRNA
ENSG0000(	C10orf82	287.4145	0.056207	0.606508	0.878178	protein_coding
ENSG0000(	CHMP2B	969.1891	0.050149	0.606498	0.878178	protein_coding
ENSG0000(	ARRDC3	1671.012	-0.06549	0.606555	0.878199	protein_coding
ENSG0000(	ANXA8	24.62606	0.170406	0.606682	0.878222	protein_coding
ENSG0000(	CARD18	70.81718	0.090017	0.606677	0.878222	protein_coding
ENSG0000(	ENSG0000(	172.1206	0.062731	0.606699	0.878222	lncRNA
ENSG0000(	RUFY1	750.1707	0.032601	0.606623	0.878222	protein_coding
ENSG0000(	TATDN1	501.2498	-0.0405	0.606742	0.878238	protein_coding
ENSG0000(	EGLN2	2349.026	0.036296	0.606852	0.878352	protein_coding
ENSG0000(	SLC25A36	2399.039	0.04648	0.606925	0.878412	protein_coding
ENSG0000(	GALT	595.7725	0.048332	0.606966	0.878424	protein_coding
ENSG0000(	ENSG0000(	523.508	0.044	0.607009	0.878441	lncRNA
ENSG0000(	TBC1D20	1714.78	-0.0333	0.607051	0.878455	protein_coding
ENSG0000(	FHIP2B	1436.255	-0.03129	0.607093	0.87847	protein_coding
ENSG0000(	ARGFXP1	20.89462	-0.12589	0.607251	0.878488	processed_pseudogene
ENSG0000(	ENSG0000(	107.5993	0.095394	0.60724	0.878488	lncRNA
ENSG0000(	ENSG0000(	259.3576	-0.05977	0.607363	0.878488	lncRNA
ENSG0000(	ENSG0000(	38.3624	0.130756	0.607393	0.878488	TEC
ENSG0000(	ENSG0000(	237.9882	-0.04997	0.607336	0.878488	lncRNA
ENSG0000(	HCG15	146.7745	0.065418	0.607188	0.878488	lncRNA
ENSG0000(	PLCB4	2699.847	0.076488	0.607338	0.878488	protein_coding
ENSG0000(	RSKR	1659.605	0.031066	0.607148	0.878488	protein_coding
ENSG0000(	TRAPPC8	1873.56	0.032998	0.607288	0.878488	protein_coding
ENSG0000(	SLC35G1	591.4008	0.115135	0.607505	0.878604	protein_coding
ENSG0000(	DNAJC3-DT	325.9583	-0.04925	0.607588	0.878678	lncRNA
ENSG0000(	APBB2	1389.855	0.053409	0.607704	0.878707	protein_coding
ENSG0000(	ENSG0000(	37.811	0.1209	0.607702	0.878707	processed_pseudogene
ENSG0000(	ENSG0000(	247.5194	-0.04331	0.607689	0.878707	lncRNA
ENSG0000(	BIN1	728.7958	0.043445	0.607766	0.87875	protein_coding
ENSG0000(	ENSG0000(	61.61228	0.076523	0.607921	0.878883	lncRNA
ENSG0000(	TBL2	785.7573	-0.0374	0.607922	0.878883	protein_coding
ENSG0000(	CELSR2	287.358	0.108363	0.607967	0.878902	protein_coding
ENSG0000(	MAP7D3	421.1224	-0.05692	0.608004	0.87891	protein_coding
ENSG0000(	CYP39A1	62.92093	-0.15269	0.608244	0.879083	protein_coding
ENSG0000(	ENSG0000(	146.7832	0.050555	0.608219	0.879083	processed_pseudogene

ENSG0000(	FAM227B	252.6852	-0.05051	0.608276	0.879083	protein_coding
ENSG0000(	NPFFR1	1124.317	-0.03654	0.608284	0.879083	protein_coding
ENSG0000(	TMEM139-	146.3343	-0.05546	0.608213	0.879083	lncRNA
ENSG0000(	PCMTD2	1352.233	0.051943	0.608388	0.879159	protein_coding
ENSG0000(	SDHAP3	2142.181	0.048477	0.6084	0.879159	transcribed_unprocessed_pseudo
ENSG0000(	ENSG0000(	25.81831	0.252582	0.608447	0.87918	lncRNA
ENSG0000(	EREG	524.7475	-0.07183	0.608536	0.879216	protein_coding
ENSG0000(	RPL36AP16	59.39444	-0.07939	0.608525	0.879216	processed_pseudogene
ENSG0000(	CAMK1D	2211.777	0.046681	0.608767	0.879324	protein_coding
ENSG0000(	CENPM	154.4306	-0.07803	0.608925	0.879324	protein_coding
ENSG0000(	DPYD-AS1	30.26258	-0.11268	0.608899	0.879324	lncRNA
ENSG0000(	ENSG0000(	539.6893	0.0424	0.60898	0.879324	lncRNA
ENSG0000(	ENSG0000(	40.37351	-0.10004	0.608682	0.879324	lncRNA
ENSG0000(	ENSG0000(	211.3546	-0.05019	0.608797	0.879324	lncRNA
ENSG0000(	ENSG0000(	22.86975	-0.13822	0.608819	0.879324	lncRNA
ENSG0000(	GPKOW	143.2916	-0.05677	0.609025	0.879324	protein_coding
ENSG0000(	KHNYN	4515.935	0.042983	0.608999	0.879324	protein_coding
ENSG0000(	KRTCAP3	141.6253	0.102922	0.608943	0.879324	protein_coding
ENSG0000(	LINC01580	85.70526	0.066763	0.608998	0.879324	lncRNA
ENSG0000(	MIR1302-9	59.95989	0.177017	0.608903	0.879324	lncRNA
ENSG0000(	NT5C1A	480.3617	0.075292	0.608817	0.879324	protein_coding
ENSG0000(	AREG	217.5789	0.156354	0.609251	0.87934	protein_coding
ENSG0000(	ENSG0000(	68.65631	-0.08857	0.60929	0.87934	lncRNA
ENSG0000(	ENSG0000(	97.49794	0.07557	0.609159	0.87934	lncRNA
ENSG0000(	ENSG0000(	271.71	0.060717	0.609292	0.87934	TEC
ENSG0000(	HEXIM1	758.2374	-0.06454	0.609128	0.87934	protein_coding
ENSG0000(	PAXX	166.8592	0.07202	0.609087	0.87934	protein_coding
ENSG0000(	PRPF40A	2098.195	0.041602	0.609225	0.87934	protein_coding
ENSG0000(	YWHAB	4342.16	-0.04363	0.609195	0.87934	protein_coding
ENSG0000(	CBR1	266.9586	-0.10043	0.609589	0.879676	protein_coding
ENSG0000(	ENSG0000(	180.6521	0.071189	0.609558	0.879676	lncRNA
ENSG0000(	NIP7	482.8774	0.049476	0.609622	0.879677	protein_coding
ENSG0000(	ENSG0000(	144.1334	0.065611	0.609821	0.879726	transcribed_processed_pseudoge
ENSG0000(	MAGEA10	148.8185	0.050383	0.609764	0.879726	protein_coding
ENSG0000(	MKI67	3230.236	-0.08311	0.609694	0.879726	protein_coding
ENSG0000(	MUC3A	5508.278	0.101133	0.609848	0.879726	protein_coding
ENSG0000(	PPEF2	202.5718	0.118584	0.60982	0.879726	protein_coding
ENSG0000(	SMIM11A	814.8318	0.077527	0.609753	0.879726	protein_coding
ENSG0000(	KLF13	3269.598	-0.05494	0.609898	0.879726	protein_coding
ENSG0000(	MAP2K4	858.559	0.039847	0.609912	0.879726	protein_coding
ENSG0000(	FRAT1	148.7131	-0.08402	0.60995	0.879733	protein_coding
ENSG0000(	SEPTIN7P9	404.3829	0.03955	0.609981	0.879733	transcribed_unprocessed_pseudo
ENSG0000(	NLRX1	265.0043	-0.06524	0.610092	0.879847	protein_coding
ENSG0000(	SC5D	1129.134	-0.04604	0.610199	0.879956	protein_coding
ENSG0000(	CYP2C9	579.3101	0.102765	0.610381	0.880034	protein_coding
ENSG0000(	ENSG0000(	311.358	0.037183	0.610364	0.880034	lncRNA
ENSG0000(	SCAF11	6712.266	-0.02631	0.610377	0.880034	protein_coding

ENSG0000(	VSIG4	142.9337	-0.14122	0.610313	0.880034	protein_coding
ENSG0000(	CALCB	36.07386	-0.08956	0.610552	0.880142	protein_coding
ENSG0000(	DMRTA1	67.17278	-0.15793	0.6105	0.880142	protein_coding
ENSG0000(	ENSG0000(	46.85961	0.228016	0.610549	0.880142	lncRNA
ENSG0000(	SEPTIN7P2	961.1852	0.038768	0.610652	0.880148	transcribed_unprocessed_pseudo
ENSG0000(	TOPBP1	1266.329	0.048454	0.610644	0.880148	protein_coding
ENSG0000(	ZNF542P	495.5272	0.04211	0.610617	0.880148	transcribed_unprocessed_pseudo
ENSG0000(	ARSJ	171.1607	0.092169	0.610806	0.880268	protein_coding
ENSG0000(	ATXN7L3-A	105.0016	-0.10422	0.610966	0.880268	lncRNA
ENSG0000(	ENSG0000(	32.62489	-0.10721	0.610849	0.880268	lncRNA
ENSG0000(	ENSG0000(	10012.96	-0.13021	0.610941	0.880268	lncRNA
ENSG0000(	IQGAP1	6538.841	-0.02994	0.611016	0.880268	protein_coding
ENSG0000(	LLPH	1533.491	0.022729	0.611024	0.880268	protein_coding
ENSG0000(	PROX2	40.38017	-0.1176	0.611003	0.880268	protein_coding
ENSG0000(	ROMO1	77.97476	0.108539	0.610991	0.880268	protein_coding
ENSG0000(	SUCO	1258.73	-0.05175	0.610802	0.880268	protein_coding
ENSG0000(	POLR2A	2818.527	0.044794	0.611069	0.880287	protein_coding
ENSG0000(	ENSG0000(	52.3201	0.093789	0.611117	0.88031	lncRNA
ENSG0000(	ENSG0000(	443.2616	0.049296	0.611249	0.880363	lncRNA
ENSG0000(	LINC02762	487.8847	0.05914	0.611233	0.880363	lncRNA
ENSG0000(	UMAD1	478.9862	0.057768	0.611216	0.880363	protein_coding
ENSG0000(	OR2T12	1301.486	-0.03506	0.611326	0.880427	protein_coding
ENSG0000(	ENSG0000(	147.9868	0.067089	0.611493	0.880529	lncRNA
ENSG0000(	ESYT3	305.1397	0.06005	0.611475	0.880529	protein_coding
ENSG0000(	UBQLN4	533.411	-0.06613	0.611487	0.880529	protein_coding
ENSG0000(	ENSG0000(	280.457	-0.05094	0.611608	0.880571	lncRNA
ENSG0000(	ENSG0000(	151.2128	-0.04922	0.611573	0.880571	lncRNA
ENSG0000(	ENSG0000(	43.21472	0.105425	0.611649	0.880571	lncRNA
ENSG0000(	SLC18A1	70.08436	0.11612	0.61165	0.880571	protein_coding
ENSG0000(	RPS27	1981.606	-0.07607	0.611769	0.880697	protein_coding
ENSG0000(	ENSG0000(	23.73762	-0.12758	0.611845	0.88076	lncRNA
ENSG0000(	LRCH2	88.78854	-0.12029	0.611912	0.88081	protein_coding
ENSG0000(	RPS2P44	79.95536	0.104666	0.612062	0.880934	processed_pseudogene
ENSG0000(	USP14	979.2897	0.048497	0.612061	0.880934	protein_coding
ENSG0000(	SDR42E1	1233.374	0.116801	0.612201	0.881088	protein_coding
ENSG0000(	ENSG0000(	608.1189	-0.07385	0.612292	0.881126	TEC
ENSG0000(	MRPS12	158.7209	0.050543	0.612319	0.881126	protein_coding
ENSG0000(	PRR4	97.21038	-0.09542	0.612324	0.881126	protein_coding
ENSG0000(	ENSG0000(	43.21026	0.082051	0.612509	0.881209	lncRNA
ENSG0000(	ENSG0000(	25.88351	-0.1261	0.61261	0.881209	lncRNA
ENSG0000(	ENSG0000(	66.5473	-0.09188	0.612553	0.881209	TEC
ENSG0000(	ENSG0000(	112.3473	-0.07584	0.612638	0.881209	lncRNA
ENSG0000(	HIBCH	1273.561	0.044541	0.612583	0.881209	protein_coding
ENSG0000(	LINC00491	285.6749	-0.06676	0.612635	0.881209	lncRNA
ENSG0000(	LINC02871	171.1633	0.059259	0.612498	0.881209	lncRNA
ENSG0000(	SGMS2	836.3037	0.079232	0.612446	0.881209	protein_coding
ENSG0000(	NUDT4	5200.724	-0.07601	0.612674	0.881216	protein_coding

ENSG0000(	RPL4	4691.973	-0.06164	0.612722	0.881239	protein_coding
ENSG0000(	ENSG0000(	23.40543	0.131684	0.612884	0.881284	lncRNA
ENSG0000(	ENSG0000(	219.7117	-0.04796	0.613032	0.881284	lncRNA
ENSG0000(	FOSB	1131.069	0.277399	0.612937	0.881284	protein_coding
ENSG0000(	LINC02710	293.7605	-0.03721	0.613037	0.881284	lncRNA
ENSG0000(	LRRC38	53.73648	-0.08112	0.612824	0.881284	protein_coding
ENSG0000(	NCKIPSD	382.4762	0.084362	0.612977	0.881284	protein_coding
ENSG0000(	PKN1	599.9189	0.053272	0.612977	0.881284	protein_coding
ENSG0000(	RHBDF1	499.1099	0.081475	0.613042	0.881284	protein_coding
ENSG0000(	RN7SL472F	47.47623	-0.11534	0.61301	0.881284	misc_RNA
ENSG0000(	ENSG0000(	32.01234	-0.14659	0.613133	0.881369	lncRNA
ENSG0000(	EARS2	1094.263	0.031461	0.613196	0.881413	protein_coding
ENSG0000(	C9orf40	110.9443	0.099598	0.613279	0.881486	protein_coding
ENSG0000(	ZACN	27.7345	-0.25382	0.613354	0.881548	protein_coding
ENSG0000(	CADM2	45.17341	-0.19541	0.613609	0.881592	protein_coding
ENSG0000(	ENSG0000(	314.3524	-0.04386	0.613534	0.881592	TEC
ENSG0000(	HTR7P1	188.1605	0.059721	0.613603	0.881592	transcribed_processed_pseudoge
ENSG0000(	LINC00337	206.799	0.056918	0.61349	0.881592	lncRNA
ENSG0000(	SCARNA21	589.7318	-0.06853	0.613466	0.881592	scaRNA
ENSG0000(	SP2	485.8336	-0.06123	0.613424	0.881592	protein_coding
ENSG0000(	TTC7B	2108.376	-0.05772	0.61355	0.881592	protein_coding
ENSG0000(	ENSG0000(	330.3664	-0.04615	0.613717	0.881655	transcribed_processed_pseudoge
ENSG0000(	RAF1	5117.29	0.01778	0.613695	0.881655	protein_coding
ENSG0000(	ENSG0000(	40.00839	-0.11331	0.613835	0.881779	lncRNA
ENSG0000(	ENSG0000(	144.912	0.07565	0.613868	0.88178	processed_pseudogene
ENSG0000(	SLC5A12	276.4327	0.046227	0.613937	0.881833	protein_coding
ENSG0000(	PRR34-AS1	343.7856	0.074855	0.61399	0.881863	lncRNA
ENSG0000(	C1QBP	304.4791	-0.06555	0.614058	0.881915	protein_coding
ENSG0000(	HDHD5	467.5974	0.066092	0.614134	0.881979	protein_coding
ENSG0000(	AK6	168.569	-0.06454	0.614463	0.882012	protein_coding
ENSG0000(	DZIP3	1001.195	0.044604	0.614302	0.882012	protein_coding
ENSG0000(	ENSG0000(	65.06959	-0.14584	0.614362	0.882012	lncRNA
ENSG0000(	ENSG0000(	66.39928	1.852669	0.614478	0.882012	protein_coding
ENSG0000(	ENSG0000(	49.82695	0.10996	0.614228	0.882012	lncRNA
ENSG0000(	ENSG0000(	227.5545	-0.06037	0.614474	0.882012	lncRNA
ENSG0000(	ENSG0000(	170.4278	-0.06148	0.61438	0.882012	lncRNA
ENSG0000(	MGRN1	2260.143	0.028124	0.614402	0.882012	protein_coding
ENSG0000(	SSBP2	2657.609	0.029157	0.614436	0.882012	protein_coding
ENSG0000(	ZNF786	300.2367	-0.0587	0.614239	0.882012	protein_coding
ENSG0000(	ASH2L	1182.061	-0.03104	0.614523	0.882031	protein_coding
ENSG0000(	ENSG0000(	3764.679	0.023422	0.61461	0.882055	lncRNA
ENSG0000(	ENSG0000(	24.24162	0.12887	0.614668	0.882055	lncRNA
ENSG0000(	ENSG0000(	373.4447	-0.03601	0.614637	0.882055	lncRNA
ENSG0000(	HOXB8	136.1027	0.102255	0.614579	0.882055	protein_coding
ENSG0000(	MIR619	34.28336	0.133972	0.614764	0.882055	miRNA
ENSG0000(	PAXIP1	1152.034	0.049214	0.61474	0.882055	protein_coding
ENSG0000(	UPK3B	98.71311	-0.106	0.614747	0.882055	protein_coding

ENSG0000(	DDX59	641.3196	-0.05523	0.614832	0.88206	protein_coding
ENSG0000(	ENSG0000(	40.22381	-0.0925	0.61483	0.88206	lncRNA
ENSG0000(	ENSG0000(	20.18119	0.151806	0.614881	0.882085	lncRNA
ENSG0000(	ZFC3H1	3812.609	0.037721	0.614924	0.882099	protein_coding
ENSG0000(	LINC02484	47.49707	-0.14713	0.614975	0.882127	lncRNA
ENSG0000(	LINC01228	71.54108	-0.06479	0.615187	0.882384	lncRNA
ENSG0000(	PLEKHA5	3304.736	0.046344	0.615259	0.882442	protein_coding
ENSG0000(	ACTA2	1058.338	-0.07969	0.615979	0.882692	protein_coding
ENSG0000(	ALDOC	76.22548	-0.12661	0.616099	0.882692	protein_coding
ENSG0000(	AP3M2	552.5016	0.055316	0.616132	0.882692	protein_coding
ENSG0000(	AQP1	409.3722	-0.10087	0.615854	0.882692	protein_coding
ENSG0000(	ARMC3	50.23776	0.106315	0.6163	0.882692	protein_coding
ENSG0000(	BNIP3P4	212.3334	-0.18052	0.615997	0.882692	processed_pseudogene
ENSG0000(	ELF3-AS1	1214.183	0.062448	0.615608	0.882692	lncRNA
ENSG0000(	ENSG0000(	62.98602	-0.0856	0.615648	0.882692	lncRNA
ENSG0000(	ENSG0000(	112.0501	0.074195	0.61574	0.882692	lncRNA
ENSG0000(	ENSG0000(	83.32607	0.121525	0.616238	0.882692	lncRNA
ENSG0000(	ENSG0000(	95.25062	-0.12424	0.615708	0.882692	lncRNA
ENSG0000(	ENSG0000(	191.5279	0.044643	0.616058	0.882692	lncRNA
ENSG0000(	ENSG0000(	45.44706	0.116022	0.616176	0.882692	processed_pseudogene
ENSG0000(	ENSG0000(	219.7571	0.066449	0.616068	0.882692	lncRNA
ENSG0000(	ENSG0000(	29.32045	0.102408	0.616285	0.882692	lncRNA
ENSG0000(	ENSG0000(	166.4649	-0.06634	0.616047	0.882692	lncRNA
ENSG0000(	FUT2	2034.535	0.084601	0.615997	0.882692	protein_coding
ENSG0000(	H2BC4	222.559	-0.07651	0.616274	0.882692	protein_coding
ENSG0000(	KAT6A	4908.465	-0.02892	0.615767	0.882692	protein_coding
ENSG0000(	LINC00477	35.79273	-0.10669	0.616009	0.882692	lncRNA
ENSG0000(	LINC01361	356.7939	0.048839	0.616272	0.882692	lncRNA
ENSG0000(	LINC01612	239.3334	0.132554	0.616117	0.882692	lncRNA
ENSG0000(	LINC02082	350.3016	0.053452	0.615762	0.882692	lncRNA
ENSG0000(	PAK1	1737.14	0.041634	0.615927	0.882692	protein_coding
ENSG0000(	RBMY2EP	20.13986	-0.2127	0.616185	0.882692	transcribed_unprocessed_pseudo
ENSG0000(	RPS20	2460.21	-0.06457	0.616278	0.882692	protein_coding
ENSG0000(	TK1	120.3404	-0.09866	0.615775	0.882692	protein_coding
ENSG0000(	SCARNA10	5218.107	0.056984	0.616434	0.882801	snoRNA
ENSG0000(	SF3B3	2860.617	-0.03394	0.61644	0.882801	protein_coding
ENSG0000(	CENPU	209.4358	-0.08347	0.616611	0.882925	protein_coding
ENSG0000(	RAB1B	796.7349	0.058182	0.616603	0.882925	protein_coding
ENSG0000(	XAF1	7419.626	-0.13228	0.616623	0.882925	protein_coding
ENSG0000(	WDR64	23.49747	-0.19284	0.616684	0.882966	protein_coding
ENSG0000(	GTF2IRD1P	295.0525	0.037752	0.616877	0.883198	transcribed_unprocessed_pseudo
ENSG0000(	CARMAL	88.51805	0.06976	0.616984	0.883259	lncRNA
ENSG0000(	SNORD35A	27.14712	-0.15385	0.61697	0.883259	snoRNA
ENSG0000(	LINC01558	149.6836	0.060386	0.617024	0.88327	lncRNA
ENSG0000(	BMS1	1073.666	0.041707	0.617137	0.883385	protein_coding
ENSG0000(	ENSG0000(	25.66877	0.130535	0.61724	0.883431	lncRNA
ENSG0000(	ENSG0000(	20.83412	-0.16512	0.617332	0.883431	lncRNA

ENSG0000(	RFX4	124.9565	-0.09276	0.617341	0.883431	protein_coding
ENSG0000(	TSN	757.6533	-0.04301	0.617361	0.883431	protein_coding
ENSG0000(	XRCC1	232.9521	0.054602	0.617329	0.883431	protein_coding
ENSG0000(	ZGLP1	45.15321	0.139036	0.617305	0.883431	protein_coding
ENSG0000(	AP1AR	667.8327	-0.07679	0.617546	0.883596	protein_coding
ENSG0000(	ENSG0000(	58.3693	0.179299	0.617766	0.883596	lncRNA
ENSG0000(	ENSG0000(	98.99818	0.065818	0.617726	0.883596	lncRNA
ENSG0000(	ENSG0000(	394.7488	0.036417	0.617587	0.883596	lncRNA
ENSG0000(	ENSG0000(	118.1846	-0.05925	0.617742	0.883596	lncRNA
ENSG0000(	FAM120AC	3262.799	0.063615	0.617703	0.883596	protein_coding
ENSG0000(	GOLGA8K	123.3998	0.07594	0.617743	0.883596	protein_coding
ENSG0000(	SMC3	1991.97	0.031209	0.617646	0.883596	protein_coding
ENSG0000(	UTP14C	116.2606	-0.27477	0.617595	0.883596	protein_coding
ENSG0000(	PDS5A	3465.311	0.029882	0.618014	0.883905	protein_coding
ENSG0000(	CCL16	264.084	-0.04132	0.618204	0.883918	protein_coding
ENSG0000(	CCNT1	1532.671	0.039039	0.618151	0.883918	protein_coding
ENSG0000(	ENSG0000(	29.19128	-0.12253	0.6181	0.883918	lncRNA
ENSG0000(	ENSG0000(	102.444	0.072151	0.618161	0.883918	lncRNA
ENSG0000(	PTCSC2	206.1475	-0.06389	0.618215	0.883918	lncRNA
ENSG0000(	SLC26A11	496.5101	-0.05074	0.618182	0.883918	protein_coding
ENSG0000(	ALDH6A1	1576.99	0.038069	0.618769	0.883993	protein_coding
ENSG0000(	C16orf91	48.22806	0.096239	0.618767	0.883993	protein_coding
ENSG0000(	CLCN6	1356.179	-0.03107	0.618373	0.883993	protein_coding
ENSG0000(	DLEU2L	157.6408	0.066367	0.61854	0.883993	lncRNA
ENSG0000(	ENSG0000(	61.642	0.076518	0.618701	0.883993	lncRNA
ENSG0000(	ENSG0000(	57.67342	0.07363	0.618377	0.883993	lncRNA
ENSG0000(	ENSG0000(	111.5271	-0.06665	0.618424	0.883993	lncRNA
ENSG0000(	ENSG0000(	26.92455	0.244165	0.618748	0.883993	lncRNA
ENSG0000(	GLA	770.5514	0.036914	0.618694	0.883993	protein_coding
ENSG0000(	LINC02864	242.2416	-0.05432	0.618747	0.883993	lncRNA
ENSG0000(	LZTS2	815.9508	0.057002	0.618508	0.883993	protein_coding
ENSG0000(	MTND3P12	20.30623	-1.32153	0.618827	0.883993	processed_pseudogene
ENSG0000(	NUDT4B	528.6625	-0.08474	0.618846	0.883993	protein_coding
ENSG0000(	PGAM5	924.8001	0.031303	0.618475	0.883993	protein_coding
ENSG0000(	RNU6-125F	24.61072	-0.11128	0.61875	0.883993	snRNA
ENSG0000(	RPL5P4	102.7	-0.0822	0.618564	0.883993	processed_pseudogene
ENSG0000(	TLNRD1	621.3776	-0.04159	0.618788	0.883993	protein_coding
ENSG0000(	TOP1P1	23.26007	-0.14954	0.618454	0.883993	processed_pseudogene
ENSG0000(	MRE11	1588.474	0.047002	0.618939	0.884033	protein_coding
ENSG0000(	SMIM15-AS	105.4747	0.080853	0.618921	0.884033	lncRNA
ENSG0000(	ENSG0000(	121.831	0.060897	0.619041	0.884133	lncRNA
ENSG0000(	ENSG0000(	352.8247	-0.05097	0.619089	0.884135	lncRNA
ENSG0000(	FAM167B	24.82138	-0.13134	0.61914	0.884135	protein_coding
ENSG0000(	KCNH6	202.843	0.058414	0.619165	0.884135	protein_coding
ENSG0000(	TRIM49D1	349.98	-0.0542	0.619171	0.884135	protein_coding
ENSG0000(	EIF2AK2	3668.357	0.083016	0.619209	0.884144	protein_coding
ENSG0000(	AIMP1	1711.838	0.033281	0.619416	0.884286	protein_coding

ENSG0000(	JRKL	360.3958	0.051363	0.619463	0.884286	protein_coding
ENSG0000(	LMO3	186.132	0.146639	0.619466	0.884286	protein_coding
ENSG0000(	ST3GAL3-A	50.32267	0.097454	0.619469	0.884286	lncRNA
ENSG0000(	TSPYL1	1602.693	-0.03068	0.61947	0.884286	protein_coding
ENSG0000(	ENSG0000(	107.8307	-0.11525	0.619556	0.884362	protein_coding
ENSG0000(	RNU2-58P	119.051	-0.10502	0.619587	0.884362	snRNA
ENSG0000(	ENSG0000(	185.6155	0.047284	0.619682	0.884406	lncRNA
ENSG0000(	ENSG0000(	23.06394	-0.11952	0.619674	0.884406	lncRNA
ENSG0000(	LINC02908	365.1952	-0.06668	0.619752	0.88446	lncRNA
ENSG0000(	GS1-279B7	29.27073	0.121005	0.61982	0.884465	transcribed_processed_pseudoge
ENSG0000(	PRR19	279.5316	-0.04275	0.619788	0.884465	protein_coding
ENSG0000(	PCYOX1L	469.8686	-0.06955	0.619873	0.884495	protein_coding
ENSG0000(	TWF2	231.2949	-0.06526	0.619951	0.88456	protein_coding
ENSG0000(	CDC45	88.06456	-0.12988	0.620136	0.884776	protein_coding
ENSG0000(	ENSG0000(	118.2109	0.274234	0.620166	0.884776	protein_coding
ENSG0000(	ENSG0000(	89.84842	0.089804	0.620245	0.884842	lncRNA
ENSG0000(	CCDC15-DT	101.9465	0.064991	0.620292	0.884864	lncRNA
ENSG0000(	AFAP1	1564.992	0.03907	0.621398	0.885041	protein_coding
ENSG0000(	ANKRD13C	781.0334	-0.04171	0.621517	0.885041	protein_coding
ENSG0000(	AP1S1	216.6411	-0.08335	0.621306	0.885041	protein_coding
ENSG0000(	AP4M1	387.1891	0.047721	0.620997	0.885041	protein_coding
ENSG0000(	BAD	186.2627	0.06925	0.620836	0.885041	protein_coding
ENSG0000(	C5	204.9021	0.075375	0.621571	0.885041	protein_coding
ENSG0000(	CCDC60	53.00088	0.116718	0.621192	0.885041	protein_coding
ENSG0000(	CEACAM7	15973.18	0.177306	0.620551	0.885041	protein_coding
ENSG0000(	CTC-338M1	362.5681	0.05966	0.621313	0.885041	lncRNA
ENSG0000(	DNM3	221.3593	-0.05235	0.620821	0.885041	protein_coding
ENSG0000(	ENSG0000(	248.9687	-0.05237	0.621414	0.885041	lncRNA
ENSG0000(	ENSG0000(	33.9114	0.116581	0.621276	0.885041	processed_pseudogene
ENSG0000(	ENSG0000(	233.2715	0.04708	0.621242	0.885041	lncRNA
ENSG0000(	ENSG0000(	28.30398	0.095726	0.620667	0.885041	lncRNA
ENSG0000(	ENSG0000(	233.2334	0.050405	0.620551	0.885041	lncRNA
ENSG0000(	ENSG0000(	135.252	-0.05184	0.620679	0.885041	lncRNA
ENSG0000(	ENSG0000(	119.3884	-0.06571	0.621083	0.885041	lncRNA
ENSG0000(	ENSG0000(	315.8735	-0.04252	0.62151	0.885041	lncRNA
ENSG0000(	ENSG0000(	35.22765	-0.10559	0.620868	0.885041	lncRNA
ENSG0000(	FGD4	3507.275	0.056948	0.620532	0.885041	protein_coding
ENSG0000(	FZD3	675.3704	0.048518	0.621518	0.885041	protein_coding
ENSG0000(	LINC01255	57.05943	-0.07687	0.620718	0.885041	lncRNA
ENSG0000(	MPP7	852.6357	0.110443	0.620616	0.885041	protein_coding
ENSG0000(	NKAIN3	6939.31	-0.02786	0.621457	0.885041	protein_coding
ENSG0000(	PAAF1	1281.994	-0.02396	0.620796	0.885041	protein_coding
ENSG0000(	PLAC8	4126.247	-0.09395	0.621574	0.885041	protein_coding
ENSG0000(	PLCB2	1216.129	-0.06927	0.621099	0.885041	protein_coding
ENSG0000(	RN7SL735F	71.20619	-0.08029	0.620552	0.885041	misc_RNA
ENSG0000(	RNF32-AS1	129.7702	-0.08732	0.621488	0.885041	lncRNA
ENSG0000(	SAMD10	124.2086	0.118802	0.620803	0.885041	protein_coding

ENSG0000(	SCN3B	313.2681	0.052977	0.620984	0.885041	protein_coding
ENSG0000(	SOWAHA	225.3033	0.164437	0.62078	0.885041	protein_coding
ENSG0000(	THSD4-AS1	80.82247	0.06377	0.621315	0.885041	lncRNA
ENSG0000(	TP53	1144.342	-0.03214	0.621086	0.885041	protein_coding
ENSG0000(	ZNF205	109.9668	0.080875	0.621421	0.885041	protein_coding
ENSG0000(	ZNF429	1387.34	0.032727	0.62111	0.885041	protein_coding
ENSG0000(	ABCC6P2	79.67844	0.177678	0.621775	0.885109	transcribed_unprocessed_pseudo
ENSG0000(	ENSG0000(	777.938	-0.03174	0.621854	0.885109	lncRNA
ENSG0000(	ENSG0000(	26.40353	-0.12797	0.621929	0.885109	lncRNA
ENSG0000(	ENSG0000(	304.8896	0.045089	0.621944	0.885109	TEC
ENSG0000(	ENSG0000(	156.075	-0.06718	0.621904	0.885109	lncRNA
ENSG0000(	ENSG0000(	28.62103	-0.13346	0.621921	0.885109	lncRNA
ENSG0000(	ETFBKMT	542.9341	0.052057	0.621865	0.885109	protein_coding
ENSG0000(	FOXJ3	1959.287	0.039594	0.621714	0.885109	protein_coding
ENSG0000(	LINC02059	22.05553	0.168497	0.621939	0.885109	lncRNA
ENSG0000(	PAN2	1676.194	0.042059	0.621733	0.885109	protein_coding
ENSG0000(	MZT2B	151.844	0.070217	0.621999	0.885141	protein_coding
ENSG0000(	ENSG0000(	128.5073	0.074315	0.622102	0.885243	lncRNA
ENSG0000(	JPH2	554.8048	-0.0768	0.622241	0.885394	protein_coding
ENSG0000(	ALG10B	1248.559	-0.03063	0.62265	0.885525	protein_coding
ENSG0000(	DLGAP1-AS	172.9249	0.038151	0.62255	0.885525	lncRNA
ENSG0000(	ENSG0000(	37.4505	0.074712	0.622588	0.885525	lncRNA
ENSG0000(	HELZ2	2290.882	-0.09003	0.622577	0.885525	protein_coding
ENSG0000(	LINC00461	444.4447	-0.05794	0.622601	0.885525	lncRNA
ENSG0000(	PTPRZ1	162.2838	-0.11346	0.622542	0.885525	protein_coding
ENSG0000(	RAB3D	653.3581	-0.05134	0.622655	0.885525	protein_coding
ENSG0000(	SBK2	68.55654	0.08947	0.622587	0.885525	protein_coding
ENSG0000(	TM2D1	1725.808	0.034592	0.622579	0.885525	protein_coding
ENSG0000(	UCHL3	80.78346	-0.06667	0.622539	0.885525	protein_coding
ENSG0000(	ENSG0000(	103.1607	1.138126	0.622751	0.885615	unprocessed_pseudogene
ENSG0000(	ATP5MF	386.5868	-0.07742	0.622897	0.885636	protein_coding
ENSG0000(	CLCA1	5848.047	0.170173	0.622886	0.885636	protein_coding
ENSG0000(	ENSG0000(	75.85218	0.075359	0.622944	0.885636	lncRNA
ENSG0000(	MYOZ3	121.4809	0.08874	0.622958	0.885636	protein_coding
ENSG0000(	PRDX3	731.1168	-0.05583	0.622837	0.885636	protein_coding
ENSG0000(	SECISBP2L	2303.267	0.025428	0.622822	0.885636	protein_coding
ENSG0000(	RNU6-90P	35.18578	-0.101	0.623033	0.885697	snRNA
ENSG0000(	SH3TC2-DT	37.848	-0.19261	0.623078	0.885715	lncRNA
ENSG0000(	ENSG0000(	45.41208	0.694318	0.623155	0.885733	transcribed_unprocessed_pseudo
ENSG0000(	EXT2	2879.252	-0.03349	0.62313	0.885733	protein_coding
ENSG0000(	RNF34	1124.431	0.028858	0.62327	0.885851	protein_coding
ENSG0000(	ENSG0000(	44.56977	0.188753	0.623304	0.885853	lncRNA
ENSG0000(	APMAP	656.6203	-0.04453	0.623359	0.885886	protein_coding
ENSG0000(	ENSG0000(	188.1335	-0.07901	0.623468	0.885911	lncRNA
ENSG0000(	LINC02616	36.98311	0.175325	0.623502	0.885911	lncRNA
ENSG0000(	OOEP-AS1	26.26129	0.128724	0.623538	0.885911	lncRNA
ENSG0000(	RPL30	1964.442	-0.0537	0.623508	0.885911	protein_coding

ENSG0000(	STK17A	1257.854	-0.07086	0.623505	0.885911	protein_coding
ENSG0000(	RASAL2	1888.161	0.048141	0.623616	0.885976	protein_coding
ENSG0000(	ATP1B1P1	66.61084	-0.07263	0.623801	0.886045	unprocessed_pseudogene
ENSG0000(	ATP2B1	2809.016	0.061732	0.623786	0.886045	protein_coding
ENSG0000(	ENSG0000(	49.30024	0.313496	0.623859	0.886045	lncRNA
ENSG0000(	ENSG0000(	797.9836	0.032985	0.623758	0.886045	lncRNA
ENSG0000(	FAR2P2	176.4949	-0.10076	0.62375	0.886045	transcribed_unprocessed_pseudo
ENSG0000(	LINC00402	213.2742	0.142015	0.62389	0.886045	lncRNA
ENSG0000(	LINC02475	75.35065	-0.06514	0.623842	0.886045	lncRNA
ENSG0000(	BOD1	371.801	-0.04694	0.624055	0.886094	protein_coding
ENSG0000(	CEACAM8	181.7966	0.064808	0.623999	0.886094	protein_coding
ENSG0000(	CSMD3	459.7274	0.063732	0.62413	0.886094	protein_coding
ENSG0000(	ENSG0000(	23.98461	0.113525	0.624092	0.886094	lncRNA
ENSG0000(	GRIK5	43.03928	-0.13188	0.624141	0.886094	protein_coding
ENSG0000(	ITPR1-DT	42.7586	-0.11506	0.624095	0.886094	lncRNA
ENSG0000(	SELENOK	330.2605	0.069268	0.62415	0.886094	protein_coding
ENSG0000(	AGAP1-IT1	362.8208	0.0751	0.624319	0.886126	lncRNA
ENSG0000(	CDCA5	131.3193	-0.09375	0.624297	0.886126	protein_coding
ENSG0000(	ENSG0000(	83.0061	0.098124	0.624366	0.886126	lncRNA
ENSG0000(	ENSG0000(	112.6245	0.117927	0.624334	0.886126	lncRNA
ENSG0000(	LINC02010	21.79085	0.133085	0.624235	0.886126	lncRNA
ENSG0000(	SEPTIN9	4634.501	-0.03438	0.624328	0.886126	protein_coding
ENSG0000(	CHST12	3526.292	0.032928	0.624413	0.886143	protein_coding
ENSG0000(	PARP4	4408.718	-0.04972	0.624474	0.886143	protein_coding
ENSG0000(	TGFA-IT1	20.94769	-0.17184	0.624445	0.886143	lncRNA
ENSG0000(	ABHD17A	1563.417	0.030877	0.624665	0.886185	protein_coding
ENSG0000(	DHDDS-AS1	84.61014	-0.0874	0.624564	0.886185	lncRNA
ENSG0000(	ENSG0000(	143.7423	-0.04887	0.624616	0.886185	lncRNA
ENSG0000(	ENSG0000(	174.6919	0.066729	0.624663	0.886185	lncRNA
ENSG0000(	TMED7-TIC	74.7352	0.120672	0.624621	0.886185	protein_coding
ENSG0000(	C18orf15	381.0462	0.051064	0.624768	0.886245	TEC
ENSG0000(	HDHD2	691.5881	0.043275	0.624772	0.886245	protein_coding
ENSG0000(	ENSG0000(	51.75927	0.119857	0.624893	0.886371	lncRNA
ENSG0000(	GABRA2	518.7394	0.102196	0.624926	0.886372	protein_coding
ENSG0000(	MAGEA6-D	71.68037	-0.07261	0.625005	0.886438	lncRNA
ENSG0000(	CLINT1	2563.579	-0.04069	0.6255	0.886665	protein_coding
ENSG0000(	DYNC1I1	48.12148	0.097271	0.625515	0.886665	protein_coding
ENSG0000(	ENSG0000(	918.9602	-0.03232	0.625822	0.886665	lncRNA
ENSG0000(	ENSG0000(	126.2531	0.053692	0.625352	0.886665	lncRNA
ENSG0000(	ENSG0000(	44.91965	-0.10017	0.625623	0.886665	processed_pseudogene
ENSG0000(	ENSG0000(	33.32876	0.145843	0.625759	0.886665	lncRNA
ENSG0000(	ENSG0000(	66.54379	0.101421	0.62584	0.886665	lncRNA
ENSG0000(	ENSG0000(	64.50435	0.085856	0.625662	0.886665	lncRNA
ENSG0000(	GAS5	1576.355	-0.03883	0.625873	0.886665	lncRNA
ENSG0000(	GPR108	449.3473	0.050586	0.625831	0.886665	protein_coding
ENSG0000(	LINC00689	146.7581	-0.05629	0.625836	0.886665	lncRNA
ENSG0000(	LINC01363	26.50475	0.14088	0.625646	0.886665	lncRNA

ENSG0000(	LINC01622	41.88379	-0.1032	0.625251	0.886665	lncRNA
ENSG0000(	LINC02027	292.4927	0.065062	0.625672	0.886665	lncRNA
ENSG0000(	LINC02086	1302.589	0.089336	0.625497	0.886665	lncRNA
ENSG0000(	LUC7L	2532.81	0.030293	0.625856	0.886665	protein_coding
ENSG0000(	NUP98	2911.309	0.044246	0.625644	0.886665	protein_coding
ENSG0000(	RASL10A	37.72945	-0.11715	0.625742	0.886665	protein_coding
ENSG0000(	SKIDA1	191.6607	0.076388	0.625373	0.886665	protein_coding
ENSG0000(	SLC27A3	304.4134	0.082305	0.625753	0.886665	protein_coding
ENSG0000(	UNC119B	730.7029	-0.05956	0.625338	0.886665	protein_coding
ENSG0000(	XRCC2	1003.476	-0.02625	0.625559	0.886665	protein_coding
ENSG0000(	COIL	345.0683	-0.05422	0.625992	0.886695	protein_coding
ENSG0000(	GYPB	27.25714	-0.13305	0.625944	0.886695	protein_coding
ENSG0000(	KMT2D	10301.82	-0.02455	0.625981	0.886695	protein_coding
ENSG0000(	C19orf54	502.3512	0.059905	0.626078	0.886771	protein_coding
ENSG0000(	ENSG0000(	714.2416	-0.04184	0.626178	0.886868	lncRNA
ENSG0000(	BMS1P10	84.72443	-0.13243	0.626309	0.886887	transcribed_unprocessed_pseudo
ENSG0000(	CCDC28B	313.1613	-0.03711	0.626405	0.886887	protein_coding
ENSG0000(	ENSG0000(	83.30311	0.076481	0.62637	0.886887	lncRNA
ENSG0000(	ENSG0000(	50.9361	-0.07553	0.626266	0.886887	TEC
ENSG0000(	LINC00327	118.6599	0.063503	0.626363	0.886887	lncRNA
ENSG0000(	NSMCE2	549.9812	0.029287	0.626307	0.886887	protein_coding
ENSG0000(	SPATA31C1	112.5758	-0.04884	0.626417	0.886887	transcribed_unprocessed_pseudo
ENSG0000(	ENSG0000(	246.6779	0.044391	0.626651	0.886933	lncRNA
ENSG0000(	ENSG0000(	20.3312	0.146194	0.62653	0.886933	lncRNA
ENSG0000(	ENSG0000(	60.59862	-0.08676	0.626645	0.886933	lncRNA
ENSG0000(	OPCML	20.6732	-0.34884	0.626502	0.886933	protein_coding
ENSG0000(	PAK3	284.7107	-0.07585	0.626623	0.886933	protein_coding
ENSG0000(	SERHL2	52.75636	0.085862	0.626675	0.886933	protein_coding
ENSG0000(	TAB3-AS1	36.55051	0.087425	0.626592	0.886933	lncRNA
ENSG0000(	ADNP	3218.528	0.028649	0.626769	0.887	protein_coding
ENSG0000(	RBM44	124.8711	-0.11193	0.626787	0.887	protein_coding
ENSG0000(	NYAP2	135.306	0.069635	0.626871	0.887027	protein_coding
ENSG0000(	RN7SL709P	39.39477	-0.10168	0.626858	0.887027	misc_RNA
ENSG0000(	ARSL	340.2752	0.064734	0.627146	0.887148	protein_coding
ENSG0000(	ENSG0000(	72.98364	0.101554	0.627136	0.887148	processed_pseudogene
ENSG0000(	ENSG0000(	945.2986	0.031831	0.62715	0.887148	lncRNA
ENSG0000(	MUC6	339.474	-1.37578	0.627078	0.887148	protein_coding
ENSG0000(	RTL8A	131.0866	-0.08034	0.627067	0.887148	protein_coding
ENSG0000(	SPRYD4	2083.314	0.022004	0.627146	0.887148	protein_coding
ENSG0000(	LINC01560	75.56898	0.086246	0.627295	0.887307	lncRNA
ENSG0000(	ENSG0000(	28.73393	0.16338	0.627503	0.887543	processed_pseudogene
ENSG0000(	ENSG0000(	32.95877	0.089294	0.627558	0.887543	lncRNA
ENSG0000(	SPATA6	626.1827	-0.07532	0.627555	0.887543	protein_coding
ENSG0000(	LINC01141	72.86466	0.087423	0.627593	0.887547	lncRNA
ENSG0000(	RCE1	242.3547	0.081826	0.627679	0.887623	protein_coding
ENSG0000(	ENSG0000(	175.4167	0.186279	0.627782	0.887669	protein_coding
ENSG0000(	LINC02795	82.95105	-0.08791	0.627808	0.887669	lncRNA

ENSG0000(	PIK3C3	2563.51	-0.02655	0.627753	0.887669	protein_coding
ENSG0000(	TSC2	3369.887	0.037682	0.627862	0.8877	protein_coding
ENSG0000(	OR51B5	89.69367	-0.07547	0.628001	0.88785	protein_coding
ENSG0000(	DDX17	38402.6	-0.02203	0.628048	0.887871	protein_coding
ENSG0000(	LINC01278	1719.914	-0.02612	0.62819	0.887889	lncRNA
ENSG0000(	NOTCH2NL	31.75431	-0.28513	0.628171	0.887889	protein_coding
ENSG0000(	RFTN2	416.3496	-0.05755	0.628145	0.887889	protein_coding
ENSG0000(	TBC1D26	40.13336	-0.14713	0.628169	0.887889	protein_coding
ENSG0000(	RSF1-IT2	78.60521	-0.06489	0.628266	0.887923	lncRNA
ENSG0000(	TMEM105	227.0899	0.065559	0.628306	0.887923	lncRNA
ENSG0000(	TVP23CP2	68.50477	-0.07239	0.628311	0.887923	processed_pseudogene
ENSG0000(	ENSG0000(	20.85999	-0.14319	0.628363	0.887951	lncRNA
ENSG0000(	ADRA2B	24.68571	0.1483	0.628469	0.888036	protein_coding
ENSG0000(	ENSG0000(	29.13032	-0.10956	0.628487	0.888036	lncRNA
ENSG0000(	OSBP2	241.4518	0.059126	0.628566	0.888101	protein_coding
ENSG0000(	ENSG0000(	228.2575	-0.04719	0.628691	0.888208	lncRNA
ENSG0000(	REX1BD	667.8395	-0.03994	0.628706	0.888208	protein_coding
ENSG0000(	ENSG0000(	64.5741	-0.12105	0.62878	0.888266	lncRNA
ENSG0000(	RNU12	609.0872	-0.07268	0.628881	0.888364	snRNA
ENSG0000(	OGFOD2	302.4673	0.074121	0.628929	0.888386	protein_coding
ENSG0000(	CERS6-AS1	90.08397	0.067809	0.629134	0.888402	lncRNA
ENSG0000(	ENSG0000(	116.8666	0.060761	0.629084	0.888402	lncRNA
ENSG0000(	LINC01747	24.4537	-0.14511	0.629024	0.888402	lncRNA
ENSG0000(	LINC02315	140.7657	0.07101	0.629113	0.888402	lncRNA
ENSG0000(	MS4A1	2107.288	-0.23875	0.628984	0.888402	protein_coding
ENSG0000(	ZNF226	1128.58	0.046412	0.629053	0.888402	protein_coding
ENSG0000(	LINC02390	39.01003	-0.09305	0.629268	0.8885	lncRNA
ENSG0000(	MT-RNR2	41592.56	0.118667	0.62925	0.8885	Mt_rRNA
ENSG0000(	CSPG4P11	398.3339	-0.06722	0.629331	0.888515	transcribed_unprocessed_pseudo
ENSG0000(	ZNF595	633.324	0.046219	0.629343	0.888515	protein_coding
ENSG0000(	LINC01901	101.9918	0.06759	0.629401	0.888551	lncRNA
ENSG0000(	KIF20B	510.9337	-0.06993	0.629451	0.888576	protein_coding
ENSG0000(	FMR1-IT1	137.2492	0.07644	0.629487	0.888581	lncRNA
ENSG0000(	ENSG0000(	120.059	-0.0892	0.629609	0.888649	transcribed_unprocessed_pseudo
ENSG0000(	ENSG0000(	35.87174	0.1094	0.629664	0.888649	TEC
ENSG0000(	GATC	1064.343	-0.03147	0.629651	0.888649	protein_coding
ENSG0000(	OR2V1	51.19355	-0.1034	0.629615	0.888649	protein_coding
ENSG0000(	ENSG0000(	260.198	0.042549	0.629705	0.888661	lncRNA
ENSG0000(	DCST1-AS1	25.09975	-0.1596	0.629742	0.888667	lncRNA
ENSG0000(	ENSG0000(	31.80628	0.106214	0.629819	0.88873	lncRNA
ENSG0000(	MAML1	1625.546	0.027628	0.629946	0.888864	protein_coding
ENSG0000(	ADAM3A	65.61995	0.092598	0.630012	0.888911	transcribed_unitary_pseudogene
ENSG0000(	ENSG0000(	68.37042	0.066441	0.630052	0.888923	lncRNA
ENSG0000(	ENSG0000(	220.8388	-0.04493	0.630209	0.889098	lncRNA
ENSG0000(	ENSG0000(	34.07678	-0.10693	0.630276	0.889117	processed_pseudogene
ENSG0000(	GNAO1	356.2945	-0.07114	0.630287	0.889117	protein_coding
ENSG0000(	MXI1	1450.313	0.119316	0.630361	0.889176	protein_coding

ENSG0000(	TPSD1	19.79823	-0.2932	0.630416	0.889209	protein_coding
ENSG0000(	ADH4	202.3133	-0.05056	0.630461	0.889213	protein_coding
ENSG0000(	PCSK9	259.2965	-0.20339	0.630484	0.889213	protein_coding
ENSG0000(	ENSG0000(	58.58661	-0.08847	0.630603	0.88923	lncRNA
ENSG0000(	ENSG0000(	48.8004	-0.07261	0.630552	0.88923	lncRNA
ENSG0000(	FKBP2	174.6878	-0.11308	0.630569	0.88923	protein_coding
ENSG0000(	PLEKHA3	1642.701	0.04101	0.630625	0.88923	protein_coding
ENSG0000(	IFRD1	2021.252	-0.06744	0.630694	0.889253	protein_coding
ENSG0000(	LMF2	741.8276	-0.04711	0.630706	0.889253	protein_coding
ENSG0000(	RN7SKP186	167.3892	-0.09391	0.630838	0.889393	misc_RNA
ENSG0000(	YPEL1	378.8178	0.052812	0.630901	0.889436	protein_coding
ENSG0000(	PPP2R5A	1061.255	0.048042	0.630962	0.889477	protein_coding
ENSG0000(	SOCS7	1022.652	0.036134	0.631007	0.889494	protein_coding
ENSG0000(	SLC30A6	1010.637	-0.04403	0.631086	0.889561	protein_coding
ENSG0000(	GOLGA6B	23.67758	0.386166	0.631129	0.889575	protein_coding
ENSG0000(	DNAAF2	139.3964	0.050905	0.63131	0.889722	protein_coding
ENSG0000(	ENSG0000(	52.05863	0.118591	0.631326	0.889722	lncRNA
ENSG0000(	ENSG0000(	80.7669	-0.05928	0.631363	0.889722	TEC
ENSG0000(	FAM138C	23.43074	0.891742	0.63136	0.889722	lncRNA
ENSG0000(	ENSG0000(	47.81197	-0.08305	0.631408	0.88974	lncRNA
ENSG0000(	ADPRS	144.2783	-0.05799	0.631531	0.889796	protein_coding
ENSG0000(	CCDC126	116.5334	0.075561	0.631544	0.889796	protein_coding
ENSG0000(	GLOD4	855.556	0.038281	0.631525	0.889796	protein_coding
ENSG0000(	C1orf167-A	135.9078	0.060644	0.631836	0.889797	lncRNA
ENSG0000(	DYNC1LI1	552.7782	-0.0456	0.631965	0.889797	protein_coding
ENSG0000(	ENSG0000(	68.38197	0.064951	0.631819	0.889797	lncRNA
ENSG0000(	ENSG0000(	20.25318	-0.56132	0.631908	0.889797	protein_coding
ENSG0000(	ENSG0000(	45.02139	0.121726	0.631769	0.889797	processed_pseudogene
ENSG0000(	ENSG0000(	79.50945	-0.15042	0.631926	0.889797	protein_coding
ENSG0000(	LINC00670	1050.581	-0.05609	0.631842	0.889797	lncRNA
ENSG0000(	LINC01597	160.6336	0.076318	0.631966	0.889797	lncRNA
ENSG0000(	MGAT4A	4375.358	0.054946	0.63194	0.889797	protein_coding
ENSG0000(	ODF2	794.6322	-0.04032	0.631709	0.889797	protein_coding
ENSG0000(	PHKG1	270.682	0.051476	0.631931	0.889797	protein_coding
ENSG0000(	PYROXD2	273.6111	0.056418	0.631953	0.889797	protein_coding
ENSG0000(	RBM19	1546.496	0.037456	0.631902	0.889797	protein_coding
ENSG0000(	ENSG0000(	47.82012	0.158101	0.632107	0.889873	lncRNA
ENSG0000(	PAMR1	71.49417	-0.17252	0.632116	0.889873	protein_coding
ENSG0000(	PNPLA4	572.0231	0.054633	0.632064	0.889873	protein_coding
ENSG0000(	DCLRE1C	1649.013	0.034933	0.632285	0.889954	protein_coding
ENSG0000(	ENSG0000(	354.2874	0.03595	0.632257	0.889954	lncRNA
ENSG0000(	PUS1	877.8817	-0.03171	0.632302	0.889954	protein_coding
ENSG0000(	RNVU1-7	2968.148	0.206843	0.632303	0.889954	snRNA
ENSG0000(	ABTB1	740.6843	-0.05637	0.632503	0.889996	protein_coding
ENSG0000(	ADIPOQ	249.5178	0.049387	0.632881	0.889996	protein_coding
ENSG0000(	C19orf47	431.2963	-0.04819	0.632534	0.889996	protein_coding
ENSG0000(	CCN3	28.48625	-0.17206	0.633017	0.889996	protein_coding

ENSG0000(	CSRP1	2680.534	0.052501	0.632644	0.889996	protein_coding
ENSG0000(	CXCR4	909.0666	-0.17777	0.633107	0.889996	protein_coding
ENSG0000(	DRD5	45.29974	-0.2465	0.632739	0.889996	protein_coding
ENSG0000(	EIF5	3680.644	-0.02634	0.63306	0.889996	protein_coding
ENSG0000(	ELOC	2374.475	-0.0211	0.632753	0.889996	protein_coding
ENSG0000(	ENSG0000(	89.13049	0.064277	0.632539	0.889996	lncRNA
ENSG0000(	ENSG0000(	20.43052	0.176965	0.632485	0.889996	lncRNA
ENSG0000(	ENSG0000(	156.6533	0.058594	0.633142	0.889996	lncRNA
ENSG0000(	IGHV7-4-1	75.23907	-0.91899	0.632537	0.889996	IG_V_gene
ENSG0000(	PKIA	68.63965	-0.13871	0.632702	0.889996	protein_coding
ENSG0000(	PLGLB2	244.0779	-0.15666	0.632643	0.889996	protein_coding
ENSG0000(	PRSS53	198.7539	-0.05246	0.632558	0.889996	protein_coding
ENSG0000(	PSPN	392.2815	-0.03626	0.632892	0.889996	protein_coding
ENSG0000(	RAP2B	1613.475	-0.05237	0.63303	0.889996	protein_coding
ENSG0000(	RN7SKP227	373.4915	0.08631	0.633137	0.889996	misc_RNA
ENSG0000(	RPS20P9	22.60554	0.124204	0.632794	0.889996	processed_pseudogene
ENSG0000(	SNORA65	131.9739	0.069863	0.632956	0.889996	snoRNA
ENSG0000(	TAF6L	161.8116	-0.05671	0.632756	0.889996	protein_coding
ENSG0000(	TMEM231F	149.5415	0.09442	0.632924	0.889996	transcribed_unprocessed_pseudo
ENSG0000(	TMEM52B	146.8904	0.064046	0.632617	0.889996	protein_coding
ENSG0000(	TTC9C	464.5787	-0.05215	0.632944	0.889996	protein_coding
ENSG0000(	GBE1	368.3463	-0.06459	0.633195	0.890026	protein_coding
ENSG0000(	ENSG0000(	64.17071	-0.06752	0.633297	0.890058	lncRNA
ENSG0000(	IL6R-AS1	184.5365	-0.05294	0.633348	0.890058	lncRNA
ENSG0000(	LMBRD1	1228.627	0.03474	0.633258	0.890058	protein_coding
ENSG0000(	RPS4Y1	676.5945	-0.1881	0.633316	0.890058	protein_coding
ENSG0000(	RACK1	3645.773	-0.03923	0.633436	0.890137	protein_coding
ENSG0000(	ENSG0000(	551.3522	-0.03645	0.633573	0.890283	transcribed_processed_pseudoge
ENSG0000(	ENSG0000(	184.3375	-0.06472	0.633888	0.890287	lncRNA
ENSG0000(	FBXO28	924.4892	0.036682	0.633896	0.890287	protein_coding
ENSG0000(	GLRX3	828.5596	0.036629	0.633971	0.890287	protein_coding
ENSG0000(	HSD17B4	4917.137	-0.02748	0.633807	0.890287	protein_coding
ENSG0000(	HSPA4L	1104.88	0.054355	0.633706	0.890287	protein_coding
ENSG0000(	KLHL4	45.42656	0.094134	0.633734	0.890287	protein_coding
ENSG0000(	LMO7-AS1	180.6111	-0.05923	0.633997	0.890287	lncRNA
ENSG0000(	MRPL35P3	30.55472	-0.14702	0.63384	0.890287	processed_pseudogene
ENSG0000(	RPL29	1560.386	0.05413	0.633921	0.890287	protein_coding
ENSG0000(	RPS2P5	288.2107	-0.15015	0.633879	0.890287	processed_pseudogene
ENSG0000(	THBS1-IT1	540.2401	-0.14193	0.633978	0.890287	lncRNA
ENSG0000(	TMBIM6	6681.468	0.047312	0.633871	0.890287	protein_coding
ENSG0000(	ZNF677	757.4945	-0.03002	0.633631	0.890287	protein_coding
ENSG0000(	ADAMTS19	29.88166	-0.21324	0.63424	0.890448	lncRNA
ENSG0000(	ENSG0000(	389.2985	0.041345	0.634197	0.890448	lncRNA
ENSG0000(	LIMD1	3344.879	0.031285	0.634209	0.890448	protein_coding
ENSG0000(	SMIM27	248.8821	-0.05081	0.634231	0.890448	protein_coding
ENSG0000(	METTL26	124.3619	-0.0723	0.634275	0.890452	protein_coding
ENSG0000(	VSTM5	72.5789	-0.12611	0.634351	0.890513	protein_coding

ENSG0000(	MED13	5816.011	0.047911	0.634434	0.890583	protein_coding
ENSG0000(	ENSG0000(	677.8259	0.100937	0.634491	0.890607	transcribed_unprocessed_pseudo
ENSG0000(	HDAC3	588.4375	-0.03637	0.634629	0.890607	protein_coding
ENSG0000(	HERC4	5700.63	-0.03983	0.634542	0.890607	protein_coding
ENSG0000(	IL23R	222.8461	0.056162	0.634706	0.890607	protein_coding
ENSG0000(	RORA-AS1	82.44389	-0.07958	0.63464	0.890607	lncRNA
ENSG0000(	SNTG1	101.7347	0.078194	0.634704	0.890607	protein_coding
ENSG0000(	VPS72	176.2803	0.038918	0.634635	0.890607	protein_coding
ENSG0000(	WNT2	19.91626	-0.8631	0.63473	0.890607	protein_coding
ENSG0000(	ZNF180	400.9421	0.046769	0.634742	0.890607	protein_coding
ENSG0000(	ANKRD34C	270.5573	-0.0646	0.634825	0.890669	lncRNA
ENSG0000(	IFITM3	809.7237	-0.07634	0.634883	0.890669	protein_coding
ENSG0000(	RNU1-19P	95.31734	0.085681	0.634859	0.890669	snRNA
ENSG0000(	NEURL4	687.2713	0.055592	0.635	0.890787	protein_coding
ENSG0000(	ENSG0000(	229.1526	0.064134	0.635095	0.890813	TEC
ENSG0000(	ENSG0000(	25.56369	-0.13254	0.635081	0.890813	lncRNA
ENSG0000(	GABPA	654.655	0.041764	0.635119	0.890813	protein_coding
ENSG0000(	OR7E7P	62.8744	0.106498	0.635148	0.890813	unprocessed_pseudogene
ENSG0000(	ANKLE2	5068.57	-0.03384	0.635296	0.890936	protein_coding
ENSG0000(	OTUD4	2408.09	-0.03642	0.635319	0.890936	protein_coding
ENSG0000(	UQCRB	1034.15	-0.05939	0.635332	0.890936	protein_coding
ENSG0000(	ESPN	245.8367	0.138999	0.635413	0.891003	protein_coding
ENSG0000(	SFXN5	1290.488	-0.03538	0.635456	0.891018	protein_coding
ENSG0000(	LINC01284	378.7625	0.034042	0.635609	0.891096	lncRNA
ENSG0000(	TLCD2	2961.094	0.067603	0.635591	0.891096	protein_coding
ENSG0000(	TTC31	808.464	0.036155	0.635547	0.891096	protein_coding
ENSG0000(	CISD1	199.0093	-0.0689	0.635739	0.891142	protein_coding
ENSG0000(	ENSG0000(	312.7466	-0.12763	0.635734	0.891142	lncRNA
ENSG0000(	GEMIN2	245.1412	-0.05474	0.635723	0.891142	protein_coding
ENSG0000(	ENSG0000(	46.15704	0.082158	0.635802	0.891186	lncRNA
ENSG0000(	TSSK6	20.79911	0.136856	0.635865	0.891228	protein_coding
ENSG0000(	ENSG0000(	97.83671	0.090238	0.63596	0.891316	TEC
ENSG0000(	ENSG0000(	106.2004	-0.06097	0.636099	0.891394	lncRNA
ENSG0000(	ENSG0000(	71.72991	0.075287	0.636113	0.891394	lncRNA
ENSG0000(	IFI6	151.9308	-0.1929	0.636081	0.891394	protein_coding
ENSG0000(	ENSG0000(	218.4616	0.05607	0.636302	0.891477	lncRNA
ENSG0000(	ENSG0000(	54.98694	-0.07586	0.636261	0.891477	lncRNA
ENSG0000(	MIPEP	223.3145	0.068095	0.636264	0.891477	protein_coding
ENSG0000(	SPC25	545.6967	-0.04084	0.636289	0.891477	protein_coding
ENSG0000(	F11R	3318.625	0.060806	0.636416	0.8915	protein_coding
ENSG0000(	FOXK1	3402.194	-0.0301	0.636357	0.8915	protein_coding
ENSG0000(	LZTS3	158.8427	-0.08273	0.636409	0.8915	protein_coding
ENSG0000(	DUSP3	1293.955	-0.04036	0.636589	0.89161	protein_coding
ENSG0000(	EHD4	1708.574	-0.05827	0.636591	0.89161	protein_coding
ENSG0000(	MRPL3	1415.708	-0.03285	0.636544	0.89161	protein_coding
ENSG0000(	COPZ1	812.7713	-0.04875	0.636806	0.891692	protein_coding
ENSG0000(	ENSG0000(	407.0398	0.047341	0.636853	0.891692	lncRNA

ENSG0000(	ENSG0000(	85.86254	0.076442	0.636697	0.891692	lncRNA
ENSG0000(	LINC02569	77.58804	0.077217	0.636821	0.891692	lncRNA
ENSG0000(	LRRC37A4F	3643.941	-0.13532	0.636775	0.891692	transcribed_unprocessed_pseudo
ENSG0000(	LUZP1	2800.115	0.045939	0.636942	0.891692	protein_coding
ENSG0000(	MED24	1142.468	-0.02745	0.636933	0.891692	protein_coding
ENSG0000(	SCARB2	6790.19	-0.03146	0.636867	0.891692	protein_coding
ENSG0000(	ZNF749	721.0174	0.077125	0.636885	0.891692	protein_coding
ENSG0000(	CEBPG	1519.288	0.064465	0.637029	0.891769	protein_coding
ENSG0000(	PTGER4P2-	171.3565	-0.06552	0.637074	0.891787	lncRNA
ENSG0000(	EXO1	123.9789	-0.08952	0.63711	0.891792	protein_coding
ENSG0000(	ENSG0000(	51.91856	0.081935	0.637173	0.891835	lncRNA
ENSG0000(	ENSG0000(	50.71081	0.092764	0.637229	0.891868	TEC
ENSG0000(	SMIM14-D	722.8103	-0.03714	0.637379	0.892032	lncRNA
ENSG0000(	ACAP3	1249.391	0.051922	0.637449	0.89206	protein_coding
ENSG0000(	ELP2	1851.22	-0.04489	0.637598	0.89206	protein_coding
ENSG0000(	ENSG0000(	122.2942	0.1008	0.63762	0.89206	processed_pseudogene
ENSG0000(	ENSG0000(	266.0874	-0.04864	0.637658	0.89206	TEC
ENSG0000(	LRRC40	215.3414	0.057996	0.637576	0.89206	protein_coding
ENSG0000(	MIR647	21.08811	0.136118	0.637478	0.89206	miRNA
ENSG0000(	RPL29P14	130.4778	0.061017	0.637552	0.89206	processed_pseudogene
ENSG0000(	SFTPD-AS1	878.0956	-0.02462	0.637648	0.89206	lncRNA
ENSG0000(	CFAP74	641.6962	-0.03631	0.637777	0.892099	protein_coding
ENSG0000(	FAM151B-I	541.8992	0.033583	0.637783	0.892099	lncRNA
ENSG0000(	GBP6	381.8501	0.035504	0.637733	0.892099	protein_coding
ENSG0000(	PFDN4	80.76483	0.092274	0.637876	0.892183	protein_coding
ENSG0000(	ENSG0000(	69.71539	-0.08069	0.637954	0.892247	lncRNA
ENSG0000(	ENSG0000(	25.78949	0.226967	0.638026	0.89229	lncRNA
ENSG0000(	RPL21	2175.811	-0.05378	0.63805	0.89229	protein_coding
ENSG0000(	ENSG0000(	56.13858	0.067563	0.638131	0.892358	lncRNA
ENSG0000(	CFAP99	49.75384	0.202285	0.638229	0.892393	protein_coding
ENSG0000(	CROCCP2	1669.479	-0.05746	0.638322	0.892393	transcribed_unprocessed_pseudo
ENSG0000(	ENSG0000(	354.6063	0.033168	0.638337	0.892393	lncRNA
ENSG0000(	ENSG0000(	25.06714	0.125595	0.638351	0.892393	TEC
ENSG0000(	RN7SL322F	21.91126	-0.12211	0.638298	0.892393	misc_RNA
ENSG0000(	ZNF652	3113.922	-0.03532	0.638339	0.892393	protein_coding
ENSG0000(	CKMT2-AS1	882.6468	-0.03319	0.638454	0.892446	lncRNA
ENSG0000(	ENSG0000(	55.84317	-0.1128	0.638485	0.892446	lncRNA
ENSG0000(	SKIV2L	1000.935	0.043069	0.638423	0.892446	protein_coding
ENSG0000(	ENSG0000(	157.0841	0.046375	0.638564	0.892471	lncRNA
ENSG0000(	RAB12	931.9785	0.084635	0.638568	0.892471	protein_coding
ENSG0000(	CYP17A1	185.1258	0.040828	0.638669	0.892475	protein_coding
ENSG0000(	ENSG0000(	113.1902	0.113539	0.638628	0.892475	lncRNA
ENSG0000(	ENSG0000(	295.5971	-0.04756	0.638708	0.892475	lncRNA
ENSG0000(	HDGF	1651.731	-0.04951	0.638673	0.892475	protein_coding
ENSG0000(	NBPF20	226.3167	0.065292	0.638733	0.892475	protein_coding
ENSG0000(	ENSG0000(	440.2408	-0.13296	0.638877	0.892557	lncRNA
ENSG0000(	PKIB	780.251	0.096886	0.638848	0.892557	protein_coding

ENSG0000(	PLXNB3	98.23334	-0.16195	0.638889	0.892557	protein_coding
ENSG0000(	SRSF10	4708.066	0.024444	0.639023	0.892699	protein_coding
ENSG0000(	LINC02469	110.1121	-0.05605	0.63906	0.892704	lncRNA
ENSG0000(	CCND3	1121.381	-0.11429	0.639199	0.892822	protein_coding
ENSG0000(	ENSG0000(	72.73503	-0.07578	0.639209	0.892822	lncRNA
ENSG0000(	ENSG0000(	61.94284	0.087002	0.639256	0.892842	transcribed_unprocessed_pseudo
ENSG0000(	ENSG0000(	62.32879	-0.07186	0.639322	0.892859	lncRNA
ENSG0000(	RN7SKP187	65.74843	-0.18895	0.639332	0.892859	misc_RNA
ENSG0000(	EMP1	3924.049	0.133798	0.639418	0.89288	protein_coding
ENSG0000(	ENSG0000(	26.58025	0.113908	0.639466	0.89288	lncRNA
ENSG0000(	ENSG0000(	339.0024	-0.06344	0.639488	0.89288	lncRNA
ENSG0000(	LINC01516	23.6119	0.117146	0.639446	0.89288	lncRNA
ENSG0000(	PNKP	974.6433	0.041899	0.63951	0.89288	protein_coding
ENSG0000(	ENSG0000(	181.5063	0.053519	0.639616	0.892983	TEC
ENSG0000(	CCDC25	485.1215	-0.03659	0.639685	0.892984	protein_coding
ENSG0000(	ENSG0000(	75.45018	0.068479	0.639775	0.892984	lncRNA
ENSG0000(	ENSG0000(	113.0044	0.07664	0.639799	0.892984	lncRNA
ENSG0000(	ENSG0000(	33.9916	0.142167	0.639932	0.892984	processed_pseudogene
ENSG0000(	ENSG0000(	27.06448	0.196315	0.639866	0.892984	lncRNA
ENSG0000(	EZH1	2232.733	0.024029	0.639741	0.892984	protein_coding
ENSG0000(	NOC2L	1036.7	0.036387	0.639924	0.892984	protein_coding
ENSG0000(	PEBP1	1207.606	0.066989	0.639808	0.892984	protein_coding
ENSG0000(	PI3	173.73	-0.35936	0.639882	0.892984	protein_coding
ENSG0000(	RN7SL591F	27.84331	0.128461	0.639942	0.892984	misc_RNA
ENSG0000(	ENSG0000(	267.8334	0.033409	0.640039	0.893074	lncRNA
ENSG0000(	ENSG0000(	54.67136	-0.11186	0.640148	0.893085	protein_coding
ENSG0000(	PIGAP1	134.0663	0.096773	0.640119	0.893085	processed_pseudogene
ENSG0000(	TTC32	182.1535	-0.04606	0.640176	0.893085	protein_coding
ENSG0000(	ZHX1-C8orf	77.23488	-0.11344	0.640134	0.893085	protein_coding
ENSG0000(	ENSG0000(	3044.394	0.024795	0.640256	0.893105	lncRNA
ENSG0000(	KL	52.3812	-0.12652	0.640239	0.893105	protein_coding
ENSG0000(	ENSG0000(	19.51754	-0.22016	0.640295	0.893114	lncRNA
ENSG0000(	ENSG0000(	53.04248	-0.10188	0.640415	0.893206	lncRNA
ENSG0000(	ENSG0000(	480.4521	-0.02877	0.640426	0.893206	lncRNA
ENSG0000(	NBR2	720.016	0.040758	0.640522	0.893296	lncRNA
ENSG0000(	NIFKP4	420.3241	0.041257	0.64063	0.893401	processed_pseudogene
ENSG0000(	CSTF3-DT	286.8109	0.04671	0.640692	0.893442	lncRNA
ENSG0000(	RBKS	202.7729	-0.06712	0.640731	0.893451	protein_coding
ENSG0000(	ATP8A1	5490.663	0.077023	0.641018	0.893495	protein_coding
ENSG0000(	CABP5	47.31235	0.103015	0.641672	0.893495	protein_coding
ENSG0000(	CS	3125.155	0.04223	0.641396	0.893495	protein_coding
ENSG0000(	DAG1	1203.04	-0.04863	0.641439	0.893495	protein_coding
ENSG0000(	ECI2-DT	53.41499	0.12224	0.640966	0.893495	lncRNA
ENSG0000(	ENSG0000(	548.8803	-0.05177	0.641612	0.893495	lncRNA
ENSG0000(	ENSG0000(	183.835	0.049021	0.641569	0.893495	lncRNA
ENSG0000(	ENSG0000(	361.2381	0.032124	0.641628	0.893495	lncRNA
ENSG0000(	ENSG0000(	485.3803	0.025545	0.641083	0.893495	lncRNA

ENSG0000(	ENSG0000(	161.5693	-0.05119	0.641492	0.893495	TEC
ENSG0000(	ENSG0000(	59.64955	0.073536	0.641419	0.893495	lncRNA
ENSG0000(	ENSG0000(	37.62028	-0.10913	0.641243	0.893495	lncRNA
ENSG0000(	ENSG0000(	76.58909	-0.19243	0.641573	0.893495	lncRNA
ENSG0000(	ENSG0000(	38.18577	-0.09379	0.641498	0.893495	lncRNA
ENSG0000(	GALNT16	456.0199	-0.04507	0.641117	0.893495	protein_coding
ENSG0000(	GMEB1	1254.406	0.025181	0.641063	0.893495	protein_coding
ENSG0000(	HNRNPL	2977.119	-0.03641	0.6414	0.893495	protein_coding
ENSG0000(	HSPA9	2393.87	0.051913	0.641182	0.893495	protein_coding
ENSG0000(	KCNQ2	142.7898	1.429495	0.641669	0.893495	protein_coding
ENSG0000(	MTMR2	1031.024	-0.0408	0.640879	0.893495	protein_coding
ENSG0000(	MYRF	813.1579	-0.10297	0.641088	0.893495	protein_coding
ENSG0000(	NKPD1	81.88762	0.056019	0.641016	0.893495	protein_coding
ENSG0000(	R3HDM1	1422.135	0.025634	0.641156	0.893495	protein_coding
ENSG0000(	RECQL5	1031.623	-0.03665	0.641411	0.893495	protein_coding
ENSG0000(	RPL12P38	57.61857	0.079938	0.64124	0.893495	transcribed_processed_pseudoge
ENSG0000(	RXRB	905.3924	0.029814	0.641403	0.893495	protein_coding
ENSG0000(	SENP6	3427.415	-0.02767	0.640821	0.893495	protein_coding
ENSG0000(	THBS4	22.64075	0.158245	0.641034	0.893495	protein_coding
ENSG0000(	RNF6	863.1893	0.036437	0.641752	0.893561	protein_coding
ENSG0000(	ENSG0000(	42.08387	0.085269	0.641785	0.893562	lncRNA
ENSG0000(	CRX	498.3823	0.027318	0.641854	0.89357	protein_coding
ENSG0000(	RN7SL385F	22.31335	0.136781	0.641856	0.89357	misc_RNA
ENSG0000(	ENSG0000(	81.3231	0.097097	0.641935	0.89359	lncRNA
ENSG0000(	RHOD	24.56235	0.164336	0.641914	0.89359	protein_coding
ENSG0000(	STK4-AS1	53.31492	0.10222	0.641992	0.893624	lncRNA
ENSG0000(	ENSG0000(	104.8747	-0.06129	0.64208	0.893667	lncRNA
ENSG0000(	UBE2B	751.8737	-0.04245	0.642087	0.893667	protein_coding
ENSG0000(	BORCS6	95.22624	-0.06234	0.642143	0.893691	protein_coding
ENSG0000(	NDUFS4	187.3742	-0.05891	0.642234	0.893691	protein_coding
ENSG0000(	SAP30L	769.5081	-0.04518	0.642188	0.893691	protein_coding
ENSG0000(	SUGP1	500.5674	-0.03943	0.642235	0.893691	protein_coding
ENSG0000(	LINC01685	22.23269	0.172033	0.642301	0.893738	lncRNA
ENSG0000(	CCDC28A-A	58.08561	0.11037	0.642405	0.893837	lncRNA
ENSG0000(	COA3	414.4357	0.075653	0.642505	0.893884	protein_coding
ENSG0000(	DUSP8P5	101.1661	-0.0741	0.642537	0.893884	processed_pseudogene
ENSG0000(	ENSG0000(	31.36111	0.122852	0.642652	0.893884	lncRNA
ENSG0000(	ETFA	2797.63	0.039096	0.642661	0.893884	protein_coding
ENSG0000(	HTN1	844.5704	-0.03353	0.64252	0.893884	protein_coding
ENSG0000(	LMX1B	234.3636	0.058713	0.642666	0.893884	protein_coding
ENSG0000(	UBAP2L	2032.689	-0.0315	0.642647	0.893884	protein_coding
ENSG0000(	TOX	378.5562	0.069013	0.642703	0.893891	protein_coding
ENSG0000(	ENSG0000(	120.276	-0.08547	0.642901	0.893981	lncRNA
ENSG0000(	ENSG0000(	37.67262	0.103575	0.642962	0.893981	lncRNA
ENSG0000(	ENSG0000(	183.6461	0.044211	0.642826	0.893981	lncRNA
ENSG0000(	MAFF	1244.876	0.057365	0.642996	0.893981	protein_coding
ENSG0000(	MUC17	3216.021	0.158064	0.642974	0.893981	protein_coding

ENSG0000(	PYM1	364.2133	0.038396	0.642843	0.893981	protein_coding
ENSG0000(	SLC9A5	96.16094	0.096377	0.642893	0.893981	protein_coding
ENSG0000(	OOSP1	37.4096	0.075262	0.643185	0.894199	protein_coding
ENSG0000(	GPAT3	364.7147	-0.10734	0.643236	0.894225	protein_coding
ENSG0000(	ENSG0000(	622.6643	0.046602	0.643327	0.894306	lncRNA
ENSG0000(	BRI3	927.3552	-0.04421	0.643455	0.894362	protein_coding
ENSG0000(	CC2D2B	166.8044	-0.09908	0.643545	0.894362	protein_coding
ENSG0000(	ELOCP19	150.5508	0.066862	0.643475	0.894362	processed_pseudogene
ENSG0000(	ENSG0000(	96.41862	-0.06588	0.643529	0.894362	lncRNA
ENSG0000(	ENSG0000(	102.2305	-0.05645	0.643439	0.894362	lncRNA
ENSG0000(	RTTN	3551.655	0.029299	0.643562	0.894362	protein_coding
ENSG0000(	ARMC1	475.9512	-0.0417	0.643693	0.894386	protein_coding
ENSG0000(	ENSG0000(	849.9466	-0.03268	0.643631	0.894386	lncRNA
ENSG0000(	ENSG0000(	35.75257	0.111871	0.64371	0.894386	lncRNA
ENSG0000(	LINC01429	99.28687	0.073627	0.643705	0.894386	lncRNA
ENSG0000(	CDC26	84.62658	-0.10145	0.643744	0.894388	protein_coding
ENSG0000(	THRIL	127.5774	0.056788	0.643841	0.894478	lncRNA
ENSG0000(	RETREG3	1618.136	-0.04055	0.64388	0.894487	protein_coding
ENSG0000(	ENSG0000(	192.7467	0.042955	0.64408	0.894656	lncRNA
ENSG0000(	ENSG0000(	22.36734	-0.18764	0.644054	0.894656	protein_coding
ENSG0000(	HELQ	441.4925	0.054362	0.644124	0.894656	protein_coding
ENSG0000(	NDUFAF7	578.6362	-0.03738	0.644132	0.894656	protein_coding
ENSG0000(	ARL2	603.6261	0.029184	0.644178	0.894675	protein_coding
ENSG0000(	ENSG0000(	96.99536	0.080609	0.64426	0.894699	lncRNA
ENSG0000(	WAC-AS1	836.6644	0.040484	0.644245	0.894699	lncRNA
ENSG0000(	CDK3	68.18597	0.14872	0.644496	0.894713	protein_coding
ENSG0000(	CHCHD7	700.4541	0.038231	0.644636	0.894713	protein_coding
ENSG0000(	DDB2	903.922	-0.03793	0.644521	0.894713	protein_coding
ENSG0000(	ENSG0000(	43.21961	0.104818	0.644714	0.894713	TEC
ENSG0000(	LINC01442	36.2028	0.106129	0.644617	0.894713	lncRNA
ENSG0000(	MIRLET7A1	2064.755	0.028988	0.644392	0.894713	lncRNA
ENSG0000(	NUBP1	161.0136	-0.05853	0.644557	0.894713	protein_coding
ENSG0000(	PCDHGA1	37.44029	0.122388	0.644564	0.894713	protein_coding
ENSG0000(	PHF2	857.4038	0.030841	0.644691	0.894713	protein_coding
ENSG0000(	POC5	192.5002	-0.06788	0.644719	0.894713	protein_coding
ENSG0000(	PPP2R1B	1732.898	0.108706	0.644603	0.894713	protein_coding
ENSG0000(	PRMT9	260.7133	-0.06379	0.644692	0.894713	protein_coding
ENSG0000(	TMEM69	238.4011	-0.05322	0.644725	0.894713	protein_coding
ENSG0000(	ZG16B	147.0026	-0.13427	0.644615	0.894713	protein_coding
ENSG0000(	TMOD4	100.8934	-0.06017	0.644759	0.894715	protein_coding
ENSG0000(	RHEX	60.42701	-0.11763	0.644843	0.894786	protein_coding
ENSG0000(	ENSG0000(	86.67743	-0.07425	0.644901	0.894822	TEC
ENSG0000(	DDX60	5337.746	0.116148	0.645055	0.894945	protein_coding
ENSG0000(	PQBP1	350.8903	-0.04956	0.645029	0.894945	protein_coding
ENSG0000(	SYCE2	22.649	-0.13272	0.645103	0.894966	protein_coding
ENSG0000(	ENSG0000(	37.91148	0.095652	0.645199	0.895009	lncRNA
ENSG0000(	MAN1A2	2511.408	-0.03914	0.645173	0.895009	protein_coding

ENSG0000(	WASHC5	781.8491	0.049386	0.645311	0.89512	protein_coding
ENSG0000(	HAX1	311.9004	-0.05647	0.645513	0.895219	protein_coding
ENSG0000(	HDX	180.1825	-0.07375	0.645507	0.895219	protein_coding
ENSG0000(	ITGA2-AS1	54.35641	0.089939	0.645507	0.895219	lncRNA
ENSG0000(	MAFG-DT	99.55856	-0.0529	0.645424	0.895219	lncRNA
ENSG0000(	MYNN	479.3931	0.032336	0.645554	0.89523	protein_coding
ENSG0000(	ZNF658	253.5986	-0.07852	0.645608	0.89526	protein_coding
ENSG0000(	ANKEF1	582.4686	-0.05175	0.645686	0.89528	protein_coding
ENSG0000(	HPSE	576.9863	-0.03969	0.645687	0.89528	protein_coding
ENSG0000(	ENSG0000(	945.6735	0.036274	0.645752	0.895321	lncRNA
ENSG0000(	LINC01220	190.8244	-0.04765	0.645782	0.895321	lncRNA
ENSG0000(	VENTX	228.1017	0.053914	0.645825	0.895336	protein_coding
ENSG0000(	ENSG0000(	26.92624	-0.08696	0.645862	0.895342	lncRNA
ENSG0000(	ENSG0000(	99.46312	-0.0581	0.645907	0.895359	lncRNA
ENSG0000(	ENSG0000(	218.2589	-0.08827	0.645974	0.895379	lncRNA
ENSG0000(	MUC15	39.10619	0.107507	0.645986	0.895379	protein_coding
ENSG0000(	ATF4	2407.089	-0.03929	0.646155	0.895473	protein_coding
ENSG0000(	BMP2K	2664.384	0.043391	0.646112	0.895473	protein_coding
ENSG0000(	ENSG0000(	1470.787	0.02311	0.646315	0.895473	lncRNA
ENSG0000(	INAFM2	301.5827	0.052107	0.64622	0.895473	protein_coding
ENSG0000(	KCTD6	377.8125	0.043573	0.646237	0.895473	protein_coding
ENSG0000(	PGAM1P8	306.914	-0.03892	0.646164	0.895473	transcribed_processed_pseudoge
ENSG0000(	TMEM104	406.691	-0.06903	0.646289	0.895473	protein_coding
ENSG0000(	TTC26	271.707	-0.04924	0.646251	0.895473	protein_coding
ENSG0000(	ENSG0000(	378.6337	0.042006	0.646649	0.895709	lncRNA
ENSG0000(	ENSG0000(	29.23964	0.119802	0.646774	0.895709	lncRNA
ENSG0000(	ENSG0000(	518.5342	-0.03535	0.646729	0.895709	TEC
ENSG0000(	EPS15L1	1227.595	-0.02664	0.646875	0.895709	protein_coding
ENSG0000(	FANCC	952.1842	0.042117	0.646608	0.895709	protein_coding
ENSG0000(	LINC02609	418.7104	-0.03204	0.646908	0.895709	lncRNA
ENSG0000(	MTFR1	488.2462	-0.04848	0.646734	0.895709	protein_coding
ENSG0000(	NEK7	933.1041	-0.0442	0.646832	0.895709	protein_coding
ENSG0000(	OR10A3	108.5282	0.078882	0.646733	0.895709	protein_coding
ENSG0000(	PCID2	964.0338	0.039261	0.646669	0.895709	protein_coding
ENSG0000(	PIK3C2B	3751.497	0.039225	0.646523	0.895709	protein_coding
ENSG0000(	RAB3IP	1899.847	0.058616	0.646891	0.895709	protein_coding
ENSG0000(	WDR1	4323.706	0.040118	0.646819	0.895709	protein_coding
ENSG0000(	ENSG0000(	99.37499	-0.09194	0.646975	0.895712	transcribed_processed_pseudoge
ENSG0000(	RNF138P1	158.3	0.090011	0.64695	0.895712	processed_pseudogene
ENSG0000(	CRAT	1079.37	0.048129	0.647131	0.895728	protein_coding
ENSG0000(	MTCYBP18	23.04049	-0.27663	0.647141	0.895728	processed_pseudogene
ENSG0000(	PDHX	483.6466	0.045404	0.64715	0.895728	protein_coding
ENSG0000(	RALGAPA2	4942.506	-0.04365	0.647098	0.895728	protein_coding
ENSG0000(	RN7SL73P	168.8459	-0.04238	0.647133	0.895728	misc_RNA
ENSG0000(	GUCA1B	162.2447	-0.05328	0.647227	0.89579	protein_coding
ENSG0000(	RNGTTP1	34.95775	0.145127	0.647299	0.895844	processed_pseudogene
ENSG0000(	NKAPP1	443.227	-0.04903	0.64738	0.895911	transcribed_processed_pseudoge

ENSG0000(	ATP5IF1	819.7374	0.057537	0.647576	0.896006	protein_coding
ENSG0000(	ATRNL1	114.7324	0.093925	0.647578	0.896006	protein_coding
ENSG0000(	CLK1	2700.722	-0.03274	0.647559	0.896006	protein_coding
ENSG0000(	TYRO3	1259.304	0.030147	0.647498	0.896006	protein_coding
ENSG0000(	ZNF570	601.0606	0.050481	0.647639	0.896044	protein_coding
ENSG0000(	CRTC1	543.2372	0.044387	0.647724	0.896083	protein_coding
ENSG0000(	PKD1P6	961.1143	0.0388	0.647764	0.896083	transcribed_unprocessed_pseudo
ENSG0000(	SEPTIN8	1635.361	0.037891	0.647761	0.896083	protein_coding
ENSG0000(	LINC01545	26.85689	0.110255	0.64785	0.896156	lncRNA
ENSG0000(	LINC02006	225.1542	0.048011	0.64792	0.896208	lncRNA
ENSG0000(	ENSG0000(	85.502	0.076927	0.64803	0.896315	lncRNA
ENSG0000(	CDC40	828.9264	0.052764	0.648416	0.896354	protein_coding
ENSG0000(	CHMP4B	932.0395	0.074922	0.648255	0.896354	protein_coding
ENSG0000(	COLQ	1070.862	0.055653	0.64823	0.896354	protein_coding
ENSG0000(	CUL4A	1625.718	0.027795	0.648278	0.896354	protein_coding
ENSG0000(	FAM111A	3671.923	-0.02791	0.648203	0.896354	protein_coding
ENSG0000(	GTSE1	400.3333	0.050603	0.648276	0.896354	protein_coding
ENSG0000(	KMT5C	469.2773	0.050336	0.648397	0.896354	protein_coding
ENSG0000(	LINC01047	32.41059	0.114522	0.648203	0.896354	lncRNA
ENSG0000(	TCTN1	1552.666	-0.03105	0.64832	0.896354	protein_coding
ENSG0000(	XPO7	1479.894	-0.02753	0.648411	0.896354	protein_coding
ENSG0000(	ZNF460	2039.999	0.029053	0.648407	0.896354	protein_coding
ENSG0000(	ENSG0000(	25.63201	0.115584	0.648654	0.896623	lncRNA
ENSG0000(	ISG15	80.8312	-0.16794	0.648718	0.896623	protein_coding
ENSG0000(	SARNP	518.119	-0.04018	0.648702	0.896623	protein_coding
ENSG0000(	SMG6	2422.209	-0.02725	0.648742	0.896623	protein_coding
ENSG0000(	AURKC	22.16679	0.162321	0.649154	0.896833	protein_coding
ENSG0000(	ENSG0000(	91.48466	0.055868	0.649107	0.896833	lncRNA
ENSG0000(	ENSG0000(	246.0568	0.035961	0.64915	0.896833	lncRNA
ENSG0000(	MAOB	121.185	-0.11608	0.649093	0.896833	protein_coding
ENSG0000(	MCM8-AS1	65.46917	0.074247	0.648927	0.896833	lncRNA
ENSG0000(	NCBP2	1081.533	0.030801	0.64902	0.896833	protein_coding
ENSG0000(	SEMA4C	1106.88	-0.03669	0.649066	0.896833	protein_coding
ENSG0000(	ZSCAN30	1854.332	0.036117	0.649117	0.896833	protein_coding
ENSG0000(	TTTY10	8688.961	-0.02009	0.649238	0.896903	lncRNA
ENSG0000(	ENSG0000(	32.99238	0.227505	0.649283	0.896921	TEC
ENSG0000(	BNIP3P28	237.977	0.048713	0.649644	0.896946	processed_pseudogene
ENSG0000(	BRAP	576.9885	0.032331	0.649669	0.896946	protein_coding
ENSG0000(	ENSG0000(	26.37539	-0.12854	0.649453	0.896946	lncRNA
ENSG0000(	ENSG0000(	164.79	0.059004	0.649493	0.896946	TEC
ENSG0000(	ENSG0000(	353.2507	0.034456	0.649499	0.896946	transcribed_unprocessed_pseudo
ENSG0000(	GNAQ	2979.657	-0.04547	0.64935	0.896946	protein_coding
ENSG0000(	KIAA1671-/	219.1053	-0.04826	0.649682	0.896946	lncRNA
ENSG0000(	NOC4L	98.09246	-0.0679	0.649546	0.896946	protein_coding
ENSG0000(	PLBD1	354.0952	0.057528	0.649563	0.896946	protein_coding
ENSG0000(	QSOX2	705.3371	0.050609	0.649476	0.896946	protein_coding
ENSG0000(	SPG21	1488.146	0.023761	0.64957	0.896946	protein_coding

ENSG0000(	ZFHX2-AS1	683.8655	0.03555	0.649693	0.896946	lncRNA
ENSG0000(	ENSG0000(	101.6032	0.077324	0.64973	0.896952	lncRNA
ENSG0000(	GRIK4	55.5642	-0.20278	0.649765	0.896957	protein_coding
ENSG0000(	ATAD5	726.0009	0.048356	0.649815	0.89698	protein_coding
ENSG0000(	CHRAC1	262.2858	0.053035	0.649964	0.896995	protein_coding
ENSG0000(	ENSG0000(	180.5994	0.075072	0.650021	0.896995	lncRNA
ENSG0000(	IPO4	245.6784	-0.08046	0.649962	0.896995	protein_coding
ENSG0000(	LINC02885	30.72008	-0.10932	0.650009	0.896995	lncRNA
ENSG0000(	OR8G1	95.03025	0.060008	0.649946	0.896995	protein_coding
ENSG0000(	RN7SL378F	60.49443	0.076946	0.649993	0.896995	misc_RNA
ENSG0000(	EGFLAM-A!	25.32658	-0.13374	0.650059	0.897002	lncRNA
ENSG0000(	ENSG0000(	36.67328	0.110623	0.650101	0.897014	TEC
ENSG0000(	ENSG0000(	79.35611	0.070246	0.650348	0.89704	lncRNA
ENSG0000(	ENSG0000(	169.0899	-0.04951	0.650213	0.89704	lncRNA
ENSG0000(	ENSG0000(	788.0401	0.027632	0.650238	0.89704	lncRNA
ENSG0000(	PAPOLA	6245.303	-0.02456	0.650327	0.89704	protein_coding
ENSG0000(	POLR1E	191.4325	-0.06936	0.650286	0.89704	protein_coding
ENSG0000(	SNX18P9	93.91895	-0.20692	0.650254	0.89704	processed_pseudogene
ENSG0000(	TIMM23B	1059.708	-0.03146	0.650227	0.89704	protein_coding
ENSG0000(	ATP1A2	252.9379	0.068211	0.650424	0.897055	protein_coding
ENSG0000(	SNX11	530.7787	-0.03833	0.650398	0.897055	protein_coding
ENSG0000(	ENSG0000(	76.0547	0.068663	0.650461	0.897062	lncRNA
ENSG0000(	CCDC168	35.55212	-0.1415	0.650899	0.897074	protein_coding
ENSG0000(	ENSG0000(	59.28209	-0.08411	0.6508	0.897074	TEC
ENSG0000(	ENSG0000(	307.4695	-0.0495	0.650767	0.897074	protein_coding
ENSG0000(	ENSG0000(	20.36705	-0.13646	0.650784	0.897074	lncRNA
ENSG0000(	ENSG0000(	55.11541	-0.10964	0.650706	0.897074	lncRNA
ENSG0000(	FAM106C	61.93435	-0.19601	0.650666	0.897074	lncRNA
ENSG0000(	LINC00113	51.28801	-0.07595	0.650695	0.897074	lncRNA
ENSG0000(	LINC01170	60.89206	-0.09446	0.650926	0.897074	lncRNA
ENSG0000(	LINC01431	49.7284	-0.08086	0.650852	0.897074	lncRNA
ENSG0000(	LINC01806	118.4753	0.080423	0.650585	0.897074	lncRNA
ENSG0000(	RASD2	325.3793	0.117976	0.650888	0.897074	protein_coding
ENSG0000(	TAF10	693.1336	-0.06476	0.650843	0.897074	protein_coding
ENSG0000(	TAF1C	1259.449	0.035648	0.650896	0.897074	protein_coding
ENSG0000(	UMODL1	25.76582	0.163165	0.650677	0.897074	protein_coding
ENSG0000(	ENSG0000(	194.6283	-0.07092	0.651026	0.897166	lncRNA
ENSG0000(	ENSG0000(	26.91432	0.275013	0.651103	0.897191	lncRNA
ENSG0000(	RNF123	840.5158	-0.04586	0.651109	0.897191	protein_coding
ENSG0000(	ENSG0000(	33.26658	-0.11316	0.651182	0.897225	lncRNA
ENSG0000(	NDUFB3	212.8573	-0.07483	0.651199	0.897225	protein_coding
ENSG0000(	EPS15P1	68.03016	0.080183	0.651252	0.897227	transcribed_processed_pseudoge
ENSG0000(	LINC00426	793.7155	-0.03726	0.651266	0.897227	lncRNA
ENSG0000(	TXNDC17	274.4525	-0.08139	0.651299	0.897229	protein_coding
ENSG0000(	LINC01184	3103.119	-0.03711	0.651381	0.897251	lncRNA
ENSG0000(	PARP16	194.9872	0.06637	0.65135	0.897251	protein_coding
ENSG0000(	FOSL2	6240.823	-0.0712	0.65148	0.897343	protein_coding

ENSG0000(	ATG10	515.7138	0.047141	0.651524	0.897352	protein_coding
ENSG0000(	ENSG0000(	372.2377	0.04747	0.651555	0.897352	lncRNA
ENSG0000(	OR13K1P	54.2108	0.082989	0.651584	0.897352	unprocessed_pseudogene
ENSG0000(	ZNF548	990.9409	0.041309	0.651721	0.897495	protein_coding
ENSG0000(	CLASP2	2335.857	0.021019	0.651761	0.897505	protein_coding
ENSG0000(	C12orf75	191.0022	-0.08996	0.651965	0.897564	protein_coding
ENSG0000(	IFITM10	122.4502	-0.09241	0.651839	0.897564	protein_coding
ENSG0000(	LINC00507	904.1655	-0.03086	0.651957	0.897564	lncRNA
ENSG0000(	PCDHGA6	566.4393	0.030578	0.652	0.897564	protein_coding
ENSG0000(	PYCARD-AS	24.73325	-0.11154	0.651976	0.897564	lncRNA
ENSG0000(	YWHAQ	1485.061	0.052667	0.65199	0.897564	protein_coding
ENSG0000(	AK3P3	43.05631	0.108383	0.652166	0.897591	processed_pseudogene
ENSG0000(	H2BC19P	708.4373	0.045647	0.652182	0.897591	transcribed_unprocessed_pseudo
ENSG0000(	ITGA9	638.1765	0.05759	0.65218	0.897591	protein_coding
ENSG0000(	RAP2C	690.6115	-0.04298	0.652131	0.897591	protein_coding
ENSG0000(	RPP30	421.2892	0.038748	0.652103	0.897591	protein_coding
ENSG0000(	IPMK	612.8911	-0.04861	0.652309	0.897676	protein_coding
ENSG0000(	SUGT1P3	278.6796	-0.06946	0.652305	0.897676	transcribed_unprocessed_pseudo
ENSG0000(	BMP6	171.535	-0.09116	0.652537	0.897814	protein_coding
ENSG0000(	ENSG0000(	97.02346	-0.11071	0.65245	0.897814	protein_coding
ENSG0000(	FAM87A	28.30462	-0.13956	0.65254	0.897814	lncRNA
ENSG0000(	TGDS	197.4274	0.059507	0.652507	0.897814	protein_coding
ENSG0000(	CISH	722.1735	-0.05409	0.652662	0.89788	protein_coding
ENSG0000(	ENSG0000(	75.1773	0.085219	0.652741	0.89788	lncRNA
ENSG0000(	ENSG0000(	213.1759	0.045913	0.652641	0.89788	lncRNA
ENSG0000(	ENSG0000(	282.1473	0.035592	0.652757	0.89788	lncRNA
ENSG0000(	FAM86EP	151.8796	0.071035	0.652784	0.89788	transcribed_unprocessed_pseudo
ENSG0000(	GTPBP2	2089.084	0.045126	0.652783	0.89788	protein_coding
ENSG0000(	ENSG0000(	58.92669	0.133226	0.652867	0.897905	processed_pseudogene
ENSG0000(	ENSG0000(	24.14572	-0.11267	0.652848	0.897905	lncRNA
ENSG0000(	ENSG0000(	184.0354	-0.06139	0.652947	0.897923	protein_coding
ENSG0000(	TBR1	36.37188	-0.10155	0.652943	0.897923	protein_coding
ENSG0000(	TMPRSS15	28.60632	-0.61406	0.652979	0.897923	protein_coding
ENSG0000(	ENSG0000(	42.47179	0.089085	0.65305	0.897977	lncRNA
ENSG0000(	CYSLTR1	2389.039	-0.03868	0.653154	0.898073	protein_coding
ENSG0000(	RO60	1680.084	-0.03265	0.653186	0.898073	protein_coding
ENSG0000(	ACTR3-AS1	121.2626	-0.07371	0.653457	0.8981	lncRNA
ENSG0000(	CCDC200	210.0009	-0.05496	0.653499	0.8981	protein_coding
ENSG0000(	CERNA1	29.30357	0.128373	0.653339	0.8981	lncRNA
ENSG0000(	DPP3	428.6323	0.072503	0.653491	0.8981	protein_coding
ENSG0000(	JARID2	1045.878	-0.035	0.65325	0.8981	protein_coding
ENSG0000(	NPIPB2	199.2385	0.060997	0.653403	0.8981	protein_coding
ENSG0000(	RAD51D	1973.435	0.033017	0.65327	0.8981	protein_coding
ENSG0000(	RN7SL698F	40.10268	0.087187	0.653457	0.8981	misc_RNA
ENSG0000(	RPL35A	1069.309	-0.06256	0.653468	0.8981	protein_coding
ENSG0000(	ENSG0000(	126.676	-0.04749	0.653635	0.898153	lncRNA
ENSG0000(	ENSG0000(	44.70369	0.5841	0.653611	0.898153	protein_coding

ENSG0000(	KLHL12	759.6573	0.036698	0.653618	0.898153	protein_coding
ENSG0000(	SLC6A11	147.611	-0.04302	0.653685	0.898176	protein_coding
ENSG0000(	ACSM5	30.11454	-0.16045	0.653814	0.898219	protein_coding
ENSG0000(	ENSG0000(	27.19269	-0.14166	0.653808	0.898219	lncRNA
ENSG0000(	SLC25A13	572.4179	0.061876	0.65378	0.898219	protein_coding
ENSG0000(	ENSG0000(	187.0335	-0.04975	0.653863	0.898241	TEC
ENSG0000(	BET1	630.5574	-0.04265	0.654001	0.898317	protein_coding
ENSG0000(	LINC00692	24.79375	-0.11562	0.654049	0.898317	lncRNA
ENSG0000(	NPIPA1	6130.438	0.029993	0.654081	0.898317	protein_coding
ENSG0000(	POC1B-AS1	928.1419	-0.0498	0.653965	0.898317	lncRNA
ENSG0000(	SIDT1-AS1	21.02504	-0.12872	0.654067	0.898317	lncRNA
ENSG0000(	COX10-AS1	2026.433	-0.02597	0.654123	0.89833	lncRNA
ENSG0000(	FIGNL2	47.69141	-0.1042	0.654199	0.898361	protein_coding
ENSG0000(	KRT18P31	175.6852	-0.05859	0.654211	0.898361	processed_pseudogene
ENSG0000(	ENSG0000(	383.8704	-0.03712	0.654348	0.898414	lncRNA
ENSG0000(	ENSG0000(	1892.854	-0.02637	0.654348	0.898414	lncRNA
ENSG0000(	ENSG0000(	79.39165	0.074356	0.654329	0.898414	lncRNA
ENSG0000(	ENSG0000(	25.5516	-0.14259	0.654381	0.898415	processed_pseudogene
ENSG0000(	KAT8	1650.448	0.027449	0.654493	0.898524	protein_coding
ENSG0000(	CCL20	196.2587	-0.11251	0.654607	0.898609	protein_coding
ENSG0000(	ENSG0000(	70.71076	0.132631	0.654657	0.898609	processed_pseudogene
ENSG0000(	LINC00570	50.50741	0.112992	0.654719	0.898609	lncRNA
ENSG0000(	NENFP1	22.05379	0.099961	0.654712	0.898609	processed_pseudogene
ENSG0000(	PSMD7-DT	587.1586	-0.0334	0.654704	0.898609	lncRNA
ENSG0000(	ENSG0000(	247.358	-0.04187	0.654861	0.898683	lncRNA
ENSG0000(	RCAN3	1060.527	-0.04851	0.654871	0.898683	protein_coding
ENSG0000(	SDR39U1	546.3285	0.045732	0.65482	0.898683	protein_coding
ENSG0000(	BAAT	280.098	-0.04935	0.654908	0.89869	protein_coding
ENSG0000(	DLGAP1-AS	642.9453	-0.0315	0.654997	0.898722	lncRNA
ENSG0000(	TIMMDC1	487.1697	-0.03788	0.65498	0.898722	protein_coding
ENSG0000(	EEF1E1	161.6824	-0.06471	0.655055	0.898757	protein_coding
ENSG0000(	LINC01208	101.7252	-0.08213	0.655114	0.898793	lncRNA
ENSG0000(	AGXT	194.6169	-0.05479	0.655526	0.898836	protein_coding
ENSG0000(	APEX2	367.9262	0.044751	0.655335	0.898836	protein_coding
ENSG0000(	ARGFXP2	68.48875	0.061728	0.655255	0.898836	processed_pseudogene
ENSG0000(	C1QTNF6	1006.925	-0.04276	0.65564	0.898836	protein_coding
ENSG0000(	CNTNAP2-/	166.3027	0.049168	0.655397	0.898836	lncRNA
ENSG0000(	COMMD5	205.0743	-0.04347	0.655539	0.898836	protein_coding
ENSG0000(	CRELD1	5763.475	0.032825	0.655456	0.898836	protein_coding
ENSG0000(	CXCL14	3099.052	-0.08623	0.655797	0.898836	protein_coding
ENSG0000(	ENSG0000(	22.34177	-0.12059	0.655846	0.898836	lncRNA
ENSG0000(	ENSG0000(	55.14772	0.078801	0.655724	0.898836	lncRNA
ENSG0000(	ENSG0000(	79.52785	0.061685	0.65546	0.898836	lncRNA
ENSG0000(	ENSG0000(	312.4822	0.057274	0.655864	0.898836	lncRNA
ENSG0000(	EPCAM	3390.182	0.093733	0.655792	0.898836	protein_coding
ENSG0000(	IGF2R	9260.331	-0.0356	0.65568	0.898836	protein_coding
ENSG0000(	JMJD4	338.6574	0.052835	0.655577	0.898836	protein_coding

ENSG0000(	LINC00662	3022.258	0.028504	0.655533	0.898836	lncRNA
ENSG0000(	LINC00884	3299.019	-0.021	0.655847	0.898836	lncRNA
ENSG0000(	LINC00954	692.4432	0.047499	0.6552	0.898836	lncRNA
ENSG0000(	MTRNR2L4	280.7158	-0.04393	0.655533	0.898836	protein_coding
ENSG0000(	MZT2A	765.6397	-0.04538	0.655564	0.898836	protein_coding
ENSG0000(	SYNPO2	2649.884	-0.0611	0.655794	0.898836	protein_coding
ENSG0000(	TOMM7	443.5365	-0.04904	0.655251	0.898836	protein_coding
ENSG0000(	CCDC144A	2731.837	-0.07382	0.656069	0.898991	protein_coding
ENSG0000(	ENSG0000(	21.08637	0.114739	0.656108	0.898991	lncRNA
ENSG0000(	LINC01919	135.8287	-0.04389	0.656101	0.898991	lncRNA
ENSG0000(	PITPNM2	1219.249	0.048305	0.656029	0.898991	protein_coding
ENSG0000(	ANKS1A	1202.183	0.045702	0.656206	0.899081	protein_coding
ENSG0000(	CDH26	84.71937	-0.0903	0.656265	0.899116	protein_coding
ENSG0000(	COLGALT2	39.88549	-0.12908	0.656343	0.899125	protein_coding
ENSG0000(	ENSG0000(	1124.642	-0.02557	0.656304	0.899125	lncRNA
ENSG0000(	LINC01841	32.38255	0.098933	0.656369	0.899125	lncRNA
ENSG0000(	CCDC186	2280.41	-0.04121	0.656714	0.899159	protein_coding
ENSG0000(	CYP2AB1P	27.79528	-0.10186	0.65651	0.899159	transcribed_unitary_pseudogene
ENSG0000(	DDX3ILA1	322.407	0.093573	0.656795	0.899159	lncRNA
ENSG0000(	DUXA	33.51714	-0.09535	0.656474	0.899159	protein_coding
ENSG0000(	FAM222B	1193.2	0.045218	0.656799	0.899159	protein_coding
ENSG0000(	OCEL1	190.5494	0.073368	0.656588	0.899159	protein_coding
ENSG0000(	RN7SKP8	243.0525	0.081635	0.656494	0.899159	misc_RNA
ENSG0000(	RPLP0P2	445.3316	-0.07657	0.656771	0.899159	transcribed_processed_pseudoge
ENSG0000(	RPS4XP16	32.65583	0.117464	0.656633	0.899159	transcribed_processed_pseudoge
ENSG0000(	RPS6KA2-IT	33.66343	-0.10526	0.656536	0.899159	lncRNA
ENSG0000(	SLC4A2	696.1331	0.054526	0.656622	0.899159	protein_coding
ENSG0000(	SLX4	692.2957	0.031018	0.656819	0.899159	protein_coding
ENSG0000(	SRRT	1334.14	0.036474	0.656674	0.899159	protein_coding
ENSG0000(	CTTN	3627.522	0.041011	0.656912	0.899202	protein_coding
ENSG0000(	XK	818.196	0.117138	0.656916	0.899202	protein_coding
ENSG0000(	CROCC	1021.429	-0.04233	0.65695	0.899204	protein_coding
ENSG0000(	SKINT1L	775.7933	-0.05497	0.657032	0.899272	transcribed_unprocessed_pseudo
ENSG0000(	ENSG0000(	33.92986	0.174751	0.657123	0.899278	lncRNA
ENSG0000(	EXOC3-AS1	174.2164	0.052649	0.657135	0.899278	lncRNA
ENSG0000(	OPRM1	1421.899	-0.02554	0.657074	0.899278	protein_coding
ENSG0000(	ENSG0000(	195.9557	-0.04268	0.657216	0.899344	lncRNA
ENSG0000(	ELP5	201.167	-0.06578	0.657385	0.899487	protein_coding
ENSG0000(	ENSG0000(	250.4299	0.05634	0.657374	0.899487	lncRNA
ENSG0000(	AMMECR1	598.4932	0.042806	0.657688	0.899857	protein_coding
ENSG0000(	LINC02909	207.7704	0.041484	0.65781	0.899889	lncRNA
ENSG0000(	MDC1	1207.965	0.047427	0.65778	0.899889	protein_coding
ENSG0000(	UBA1	3382.932	-0.02048	0.657746	0.899889	protein_coding
ENSG0000(	CEP295	1556.572	-0.03713	0.657866	0.899921	protein_coding
ENSG0000(	ENSG0000(	41.07603	0.117831	0.65793	0.899941	lncRNA
ENSG0000(	LINC01694	996.5055	-0.04643	0.657964	0.899941	lncRNA
ENSG0000(	TPP2	2468.89	0.037597	0.657979	0.899941	protein_coding

ENSG0000(	CADM2-AS	133.2922	-0.04242	0.658128	0.900101	lncRNA
ENSG0000(	CSPG4P13	40.32251	-0.17936	0.65841	0.900173	transcribed_unprocessed_pseudo
ENSG0000(	ENSG0000(	101.879	0.057656	0.658389	0.900173	IG_V_pseudogene
ENSG0000(	ENSG0000(	51.25623	0.076412	0.658304	0.900173	lncRNA
ENSG0000(	FABP1	5527.732	0.162023	0.658307	0.900173	protein_coding
ENSG0000(	FANCL	351.921	0.043464	0.658364	0.900173	protein_coding
ENSG0000(	LMNTD2-A	115.2135	0.131485	0.658403	0.900173	lncRNA
ENSG0000(	RNMT	1235.872	0.050502	0.658328	0.900173	protein_coding
ENSG0000(	ACADL	483.6967	-0.03587	0.65966	0.90022	protein_coding
ENSG0000(	AFG3L1P	1377.546	-0.03005	0.659651	0.90022	transcribed_unitary_pseudogene
ENSG0000(	ARHGDIA	1695.291	0.05996	0.659445	0.90022	protein_coding
ENSG0000(	BIN3	735.5428	-0.03945	0.659023	0.90022	protein_coding
ENSG0000(	CA5B	1394.502	0.041106	0.659537	0.90022	protein_coding
ENSG0000(	CABYR	71.23076	-0.06616	0.659342	0.90022	protein_coding
ENSG0000(	COLEC11	100.5546	-0.07743	0.659067	0.90022	protein_coding
ENSG0000(	DDX23	1180.902	-0.02462	0.658544	0.90022	protein_coding
ENSG0000(	DNALI1	146.4919	0.06033	0.659041	0.90022	protein_coding
ENSG0000(	DNASE1L3	451.9662	-0.07802	0.659837	0.90022	protein_coding
ENSG0000(	ENSG0000(	75.69641	0.065478	0.659308	0.90022	lncRNA
ENSG0000(	ENSG0000(	44.11611	-0.13593	0.659072	0.90022	processed_pseudogene
ENSG0000(	ENSG0000(	95.82887	-0.05743	0.658478	0.90022	processed_pseudogene
ENSG0000(	ENSG0000(	87.36744	-0.07716	0.659476	0.90022	lncRNA
ENSG0000(	ENSG0000(	264.3171	-0.06068	0.659706	0.90022	lncRNA
ENSG0000(	ENSG0000(	41.66746	0.096579	0.659595	0.90022	lncRNA
ENSG0000(	ENSG0000(	379.6231	-0.0476	0.65865	0.90022	TEC
ENSG0000(	ENSG0000(	307.6655	-0.03707	0.65981	0.90022	lncRNA
ENSG0000(	GNL3L	3437.74	-0.02997	0.658547	0.90022	protein_coding
ENSG0000(	HNRNPH1	14267.9	-0.02949	0.659262	0.90022	protein_coding
ENSG0000(	LINC01492	292.9704	0.047823	0.659282	0.90022	lncRNA
ENSG0000(	MPP3	110.5256	-0.10556	0.658724	0.90022	protein_coding
ENSG0000(	NCOA2	2979.145	0.033676	0.659355	0.90022	protein_coding
ENSG0000(	NHEJ1	1203.378	0.082018	0.659852	0.90022	protein_coding
ENSG0000(	NPAS2	1546.194	0.067198	0.659611	0.90022	protein_coding
ENSG0000(	PARN	1519.303	-0.02163	0.658627	0.90022	protein_coding
ENSG0000(	PDCD2L	33.86443	0.115933	0.658591	0.90022	protein_coding
ENSG0000(	POLRMTP1	21.1658	0.213414	0.659828	0.90022	processed_pseudogene
ENSG0000(	PPFIBP1	1863.833	-0.04305	0.65874	0.90022	protein_coding
ENSG0000(	PRR5	823.325	0.029159	0.659735	0.90022	protein_coding
ENSG0000(	RASA3-IT1	32.83911	0.136643	0.658872	0.90022	lncRNA
ENSG0000(	RASA4	1030.822	-0.08576	0.659074	0.90022	protein_coding
ENSG0000(	RCC1L	375.0097	0.039885	0.659773	0.90022	protein_coding
ENSG0000(	RN7SL418F	19.88239	-0.12248	0.659545	0.90022	misc_RNA
ENSG0000(	RNU6-107F	39.9997	-0.09074	0.659453	0.90022	snRNA
ENSG0000(	RPL39	1363.736	-0.06354	0.659096	0.90022	protein_coding
ENSG0000(	THNSL1	170.2224	0.066274	0.659397	0.90022	protein_coding
ENSG0000(	TMEM132E	36.62285	-0.10365	0.659241	0.90022	lncRNA
ENSG0000(	TRAPPC13	534.595	-0.04272	0.659659	0.90022	protein_coding

ENSG0000(	TRPV1	967.6744	0.059863	0.659145	0.90022	protein_coding
ENSG0000(	TVP23B	1198.861	-0.03665	0.658879	0.90022	protein_coding
ENSG0000(	UBE2Q2P2	428.1819	0.038213	0.658928	0.90022	transcribed_unprocessed_pseudo
ENSG0000(	ZPLD2P	291.3058	-0.04479	0.658791	0.90022	transcribed_unprocessed_pseudo
ENSG0000(	ENSG0000(	324.3902	0.031542	0.660027	0.900236	lncRNA
ENSG0000(	ENSG0000(	49.42038	-0.07884	0.659964	0.900236	TEC
ENSG0000(	MRPL17	232.0358	-0.05931	0.659976	0.900236	protein_coding
ENSG0000(	RNU7-80P	46.18603	-0.11202	0.65994	0.900236	snRNA
ENSG0000(	TRBJ1-4	19.68627	0.768175	0.660013	0.900236	TR_J_gene
ENSG0000(	FDXACB1	180.513	0.051238	0.660061	0.900238	protein_coding
ENSG0000(	CGRRF1	157.7054	0.057406	0.660219	0.900409	protein_coding
ENSG0000(	ENSG0000(	200.2882	-0.04092	0.660295	0.900467	lncRNA
ENSG0000(	ENSG0000(	20.38598	-0.17316	0.660404	0.900483	lncRNA
ENSG0000(	LINC02471	84.55791	0.083589	0.660362	0.900483	lncRNA
ENSG0000(	TRIM59	768.5015	0.038231	0.660372	0.900483	protein_coding
ENSG0000(	MCM6	384.1948	-0.06288	0.660526	0.900604	protein_coding
ENSG0000(	ENSG0000(	598.0779	-0.06009	0.660572	0.900623	lncRNA
ENSG0000(	ATP6V1FNI	30.17345	-0.11039	0.660658	0.900627	protein_coding
ENSG0000(	CFAP206	39.22326	0.100484	0.660655	0.900627	protein_coding
ENSG0000(	RNU6-25P	21.6156	0.358176	0.660674	0.900627	snRNA
ENSG0000(	ENSG0000(	100.3096	0.058103	0.660741	0.90066	lncRNA
ENSG0000(	H3C1	35.47768	-0.10477	0.660821	0.90066	protein_coding
ENSG0000(	IGSF9B	534.9754	0.114086	0.660894	0.90066	protein_coding
ENSG0000(	NELFA	600.8563	-0.03421	0.660871	0.90066	protein_coding
ENSG0000(	PPFIA3	229.0777	0.100407	0.660838	0.90066	protein_coding
ENSG0000(	TASOR	2692.579	0.029624	0.660863	0.90066	protein_coding
ENSG0000(	EFHD2	1274.807	-0.04338	0.660967	0.900667	protein_coding
ENSG0000(	ENSG0000(	806.3831	-0.0413	0.660963	0.900667	TEC
ENSG0000(	LINC01480	130.8078	-0.08132	0.661013	0.900667	lncRNA
ENSG0000(	MMADHCP	214.4294	0.105266	0.66103	0.900667	processed_pseudogene
ENSG0000(	CADM4	37.18934	-0.1429	0.661218	0.900702	protein_coding
ENSG0000(	CCDC157	392.2149	0.03914	0.661253	0.900702	protein_coding
ENSG0000(	GAB3	639.4531	-0.06183	0.661194	0.900702	protein_coding
ENSG0000(	LDLR	2782.92	0.049006	0.661285	0.900702	protein_coding
ENSG0000(	PRR27	110.0421	0.069265	0.661188	0.900702	protein_coding
ENSG0000(	SNHG20	1308.17	0.033511	0.661269	0.900702	lncRNA
ENSG0000(	TANGO2	1302.823	0.065857	0.661269	0.900702	protein_coding
ENSG0000(	ENSG0000(	627.3106	0.033033	0.661342	0.900735	TEC
ENSG0000(	CBX7	1463.405	-0.03608	0.661488	0.900844	protein_coding
ENSG0000(	SORL1	6795.861	0.059039	0.661466	0.900844	protein_coding
ENSG0000(	ENSG0000(	221.5937	-0.13825	0.661524	0.900848	lncRNA
ENSG0000(	SNX32	46.46699	0.117285	0.6616	0.900908	protein_coding
ENSG0000(	ACTN4	6954.453	0.070364	0.661722	0.900946	protein_coding
ENSG0000(	RNU6-5P	1054.882	-0.10291	0.661727	0.900946	snRNA
ENSG0000(	ZFP69B	82.4647	-0.08953	0.661692	0.900946	protein_coding
ENSG0000(	ENSG0000(	728.1	-0.02841	0.66179	0.900988	lncRNA
ENSG0000(	ENSG0000(	540.1274	0.028947	0.661868	0.90105	lncRNA

ENSG0000(	ENSG0000(	40.68924	-0.1019	0.661917	0.901072	lncRNA
ENSG0000(	LINC01755	20.96041	0.141419	0.661974	0.901105	lncRNA
ENSG0000(	ENSG0000(	359.7077	-0.039	0.662065	0.901184	protein_coding
ENSG0000(	ENSG0000(	469.6858	-0.0516	0.662139	0.90124	processed_pseudogene
ENSG0000(	SAP30L-AS:	1045.487	-0.02891	0.662206	0.901287	lncRNA
ENSG0000(	ENSG0000(	84.79625	-0.07087	0.662402	0.901369	lncRNA
ENSG0000(	ENSG0000(	67.12204	0.059096	0.662391	0.901369	lncRNA
ENSG0000(	KCNT2	175.6426	0.095263	0.662408	0.901369	protein_coding
ENSG0000(	LINC02869	55.76155	0.08121	0.662367	0.901369	lncRNA
ENSG0000(	TTC3	8656.21	0.034472	0.66243	0.901369	protein_coding
ENSG0000(	CAPNS1	1479.95	0.051026	0.66253	0.901384	protein_coding
ENSG0000(	ENSG0000(	27.61257	-0.10581	0.66254	0.901384	lncRNA
ENSG0000(	GDF5-AS1	97.62918	-0.05283	0.662513	0.901384	lncRNA
ENSG0000(	ENSG0000(	45.57957	0.090149	0.662669	0.901426	lncRNA
ENSG0000(	ENSG0000(	187.2052	-0.06171	0.662647	0.901426	lncRNA
ENSG0000(	PPP1R15B	4027.756	-0.02442	0.662645	0.901426	protein_coding
ENSG0000(	APOA2	46.23624	0.118378	0.662822	0.90159	protein_coding
ENSG0000(	DDX1	1638.559	0.02091	0.662892	0.90164	protein_coding
ENSG0000(	CSNK2A1	6876.912	0.022183	0.66296	0.901644	protein_coding
ENSG0000(	VKORC1L1	1181.928	-0.03293	0.662953	0.901644	protein_coding
ENSG0000(	CUBN	444.8439	0.082293	0.66315	0.901684	protein_coding
ENSG0000(	DMBX1	97.44422	-0.05622	0.663241	0.901684	protein_coding
ENSG0000(	ENSG0000(	260.9786	0.036168	0.663239	0.901684	lncRNA
ENSG0000(	ENSG0000(	185.9425	0.075032	0.663287	0.901684	lncRNA
ENSG0000(	JADE1	1299.494	-0.05018	0.663131	0.901684	protein_coding
ENSG0000(	LRRC37A3	1257.549	-0.07138	0.663317	0.901684	protein_coding
ENSG0000(	MIR29B2CI	7591.655	-0.03439	0.663312	0.901684	lncRNA
ENSG0000(	OR2A42	120.4089	-0.06347	0.663187	0.901684	protein_coding
ENSG0000(	PGAM4	37.34772	0.10614	0.663287	0.901684	protein_coding
ENSG0000(	SNHG6	247.6563	-0.06239	0.663234	0.901684	lncRNA
ENSG0000(	ENSG0000(	58.84014	-0.14402	0.663353	0.901688	lncRNA
ENSG0000(	BORCS7	531.4905	-0.03644	0.663409	0.901715	protein_coding
ENSG0000(	KCNJ5	591.1167	0.038024	0.663439	0.901715	protein_coding
ENSG0000(	RTL8B	42.64499	-0.11148	0.663613	0.901908	protein_coding
ENSG0000(	ENSG0000(	28.29474	-0.16305	0.663746	0.901999	processed_pseudogene
ENSG0000(	ENSG0000(	208.0132	0.042327	0.663714	0.901999	lncRNA
ENSG0000(	CDKL3	330.669	-0.04071	0.663813	0.902046	protein_coding
ENSG0000(	ACOD1	44.6738	-1.3114	0.664109	0.902068	protein_coding
ENSG0000(	ATP6V0A1	3121.368	0.030508	0.664386	0.902068	protein_coding
ENSG0000(	CHIC1	445.0945	-0.04518	0.664369	0.902068	protein_coding
ENSG0000(	CIT	1647.762	-0.04359	0.663898	0.902068	protein_coding
ENSG0000(	CTC1	1722.943	0.04391	0.664467	0.902068	protein_coding
ENSG0000(	DHX8	1585.944	0.04772	0.664518	0.902068	protein_coding
ENSG0000(	DLG5-AS1	205.7467	0.04748	0.664369	0.902068	lncRNA
ENSG0000(	ENSG0000(	151.0459	-0.05215	0.664328	0.902068	lncRNA
ENSG0000(	ENSG0000(	243.5047	0.062162	0.66416	0.902068	lncRNA
ENSG0000(	FAM8A1	1113.826	0.047533	0.664341	0.902068	protein_coding

ENSG0000(	GFUS	402.7963	-0.06699	0.663978	0.902068	protein_coding
ENSG0000(	GSTM2	1422.436	0.040688	0.66401	0.902068	protein_coding
ENSG0000(	HNRNPH3	1900.413	0.024077	0.664254	0.902068	protein_coding
ENSG0000(	LEF1	463.2686	-0.12296	0.664387	0.902068	protein_coding
ENSG0000(	RGPD4	43.4102	0.208437	0.664286	0.902068	protein_coding
ENSG0000(	RNGTT	699.1581	-0.03069	0.66417	0.902068	protein_coding
ENSG0000(	SKA2	455.5704	-0.05764	0.664494	0.902068	protein_coding
ENSG0000(	TCOF1	1177.934	0.036901	0.663864	0.902068	protein_coding
ENSG0000(	UBE2V2	883.5427	-0.02977	0.664463	0.902068	protein_coding
ENSG0000(	WDR5B-DT	420.5058	0.030327	0.664211	0.902068	lncRNA
ENSG0000(	ZNF574	219.1336	0.055821	0.664449	0.902068	protein_coding
ENSG0000(	ALG5	1426.594	-0.03198	0.664745	0.902332	protein_coding
ENSG0000(	KTN1	6439.291	0.041028	0.664863	0.902447	protein_coding
ENSG0000(	NUDT6	457.8065	0.040937	0.664913	0.902472	protein_coding
ENSG0000(	ENSG0000(	230.3941	-0.05298	0.66495	0.902477	lncRNA
ENSG0000(	RPS12P16	149.5303	0.066867	0.664997	0.902495	processed_pseudogene
ENSG0000(	ENSG0000(	48.16751	-0.08906	0.665111	0.902562	lncRNA
ENSG0000(	KANTR	1818.005	-0.03478	0.665099	0.902562	protein_coding
ENSG0000(	ENSG0000(	67.41918	-0.09261	0.66524	0.902692	lncRNA
ENSG0000(	KCTD5	780.0817	-0.06082	0.665344	0.902788	protein_coding
ENSG0000(	ENSG0000(	416.9374	0.041841	0.665478	0.902925	lncRNA
ENSG0000(	CITED2	492.4089	-0.06315	0.66579	0.902945	protein_coding
ENSG0000(	ENSG0000(	228.3041	0.041744	0.665799	0.902945	TEC
ENSG0000(	ENSG0000(	215.2068	-0.0405	0.665606	0.902945	lncRNA
ENSG0000(	IQGAP3	765.12	-0.08347	0.665853	0.902945	protein_coding
ENSG0000(	NDEL1	2078.018	0.033359	0.665701	0.902945	protein_coding
ENSG0000(	PPP1R14C	300.3624	0.113051	0.665831	0.902945	protein_coding
ENSG0000(	RNU4-6P	28.2968	-0.09244	0.665615	0.902945	snRNA
ENSG0000(	RPH3A	161.9469	-0.06424	0.6657	0.902945	protein_coding
ENSG0000(	RPL22P1	145.7526	-0.18566	0.665847	0.902945	processed_pseudogene
ENSG0000(	ZNF197	980.6447	0.03748	0.665596	0.902945	protein_coding
ENSG0000(	ZNF761	1081.022	0.027537	0.665655	0.902945	protein_coding
ENSG0000(	DPM1	1707.618	0.018507	0.665913	0.902982	protein_coding
ENSG0000(	ENSG0000(	21.91295	0.162107	0.666045	0.90311	lncRNA
ENSG0000(	PPIAP46	416.5962	-0.04308	0.666106	0.90311	transcribed_processed_pseudoge
ENSG0000(	UBXN8	355.2446	0.041161	0.666078	0.90311	protein_coding
ENSG0000(	UBP1	2958.062	-0.03842	0.666149	0.903124	protein_coding
ENSG0000(	NUFIP1	94.73722	-0.07226	0.666302	0.903287	protein_coding
ENSG0000(	ENSG0000(	135.7566	0.054382	0.666376	0.903343	processed_pseudogene
ENSG0000(	ENSG0000(	70.09694	0.077042	0.666435	0.90335	lncRNA
ENSG0000(	PLA2G4C	740.9646	0.038824	0.666448	0.90335	protein_coding
ENSG0000(	ENSG0000(	41.0824	-0.07276	0.666577	0.903481	lncRNA
ENSG0000(	BRMS1L	83.60216	-0.09135	0.66666	0.903531	protein_coding
ENSG0000(	ITGA2B	79.29241	-0.08579	0.666679	0.903531	protein_coding
ENSG0000(	ENSG0000(	685.021	-0.02429	0.66676	0.903552	lncRNA
ENSG0000(	HPDL	28.4983	0.15085	0.66675	0.903552	protein_coding
ENSG0000(	BTAF1	3849.762	-0.03839	0.666806	0.903569	protein_coding

ENSG0000(	BPNT1	783.2284	0.047089	0.667299	0.903667	protein_coding
ENSG0000(	CASP14	147.1715	0.050579	0.667435	0.903667	protein_coding
ENSG0000(	EEF1A1P13	56.46496	-0.09435	0.66721	0.903667	processed_pseudogene
ENSG0000(	ENSG0000(	26.67436	0.113443	0.667147	0.903667	lncRNA
ENSG0000(	ENSG0000(	49.80505	-0.06296	0.666999	0.903667	lncRNA
ENSG0000(	ENSG0000(	54.92713	-1.00306	0.667437	0.903667	transcribed_unprocessed_pseudo
ENSG0000(	ENSG0000(	538.0527	-0.02683	0.667276	0.903667	lncRNA
ENSG0000(	ENSG0000(	663.3329	0.020964	0.667107	0.903667	protein_coding
ENSG0000(	FAM3D-AS:	363.5199	0.036916	0.667192	0.903667	lncRNA
ENSG0000(	GOLGA8G	39.92452	0.146208	0.667356	0.903667	protein_coding
ENSG0000(	MTRF1L	594.6642	0.049712	0.666957	0.903667	protein_coding
ENSG0000(	PDE8B	106.2412	0.08165	0.667224	0.903667	protein_coding
ENSG0000(	RN7SL788F	19.50077	0.130315	0.667341	0.903667	misc_RNA
ENSG0000(	SIVA1	575.3988	-0.04119	0.667248	0.903667	protein_coding
ENSG0000(	TMEM14EF	23.56126	-0.21056	0.667146	0.903667	transcribed_processed_pseudoge
ENSG0000(	TMPRSS11I	97.54525	0.07038	0.667086	0.903667	transcribed_unprocessed_pseudo
ENSG0000(	USP26	29.12103	-0.14523	0.66738	0.903667	protein_coding
ENSG0000(	TICRR	375.1185	-0.07294	0.667588	0.903828	protein_coding
ENSG0000(	ENSG0000(	97.54555	-0.08626	0.667829	0.903885	transcribed_unprocessed_pseudo
ENSG0000(	FIG4	3469.163	0.020226	0.667923	0.903885	protein_coding
ENSG0000(	HBEGF	446.7272	-0.12974	0.667705	0.903885	protein_coding
ENSG0000(	HTATIP2	421.5142	-0.07694	0.667856	0.903885	protein_coding
ENSG0000(	MUC20	2012.376	-0.07868	0.667869	0.903885	protein_coding
ENSG0000(	STAG3	1228.959	0.03876	0.667873	0.903885	protein_coding
ENSG0000(	SYNJ2BP	2810.326	0.03844	0.66795	0.903885	protein_coding
ENSG0000(	TAPT1	845.476	0.027781	0.667959	0.903885	protein_coding
ENSG0000(	TERB2	159.507	0.046937	0.667862	0.903885	protein_coding
ENSG0000(	ZNF500	1044.23	-0.03323	0.667866	0.903885	protein_coding
ENSG0000(	C17orf99	253.0575	-0.06336	0.668128	0.903981	protein_coding
ENSG0000(	ENSG0000(	28.86476	-0.09388	0.668081	0.903981	lncRNA
ENSG0000(	RPL23AP7	99.44016	-0.07993	0.668124	0.903981	transcribed_processed_pseudoge
ENSG0000(	ZNF333	1613.231	0.024015	0.668187	0.904015	protein_coding
ENSG0000(	ENSG0000(	535.3151	0.024412	0.66858	0.904114	lncRNA
ENSG0000(	ENSG0000(	55.10126	-0.06326	0.668546	0.904114	lncRNA
ENSG0000(	JAKMIP2-A	97.46397	-0.07302	0.668614	0.904114	lncRNA
ENSG0000(	LINC01672	469.187	0.041019	0.668445	0.904114	lncRNA
ENSG0000(	LINC02321	85.51864	-0.0725	0.66849	0.904114	lncRNA
ENSG0000(	NPHS1	355.6767	0.058347	0.668621	0.904114	protein_coding
ENSG0000(	TFAP2E	126.5489	0.076975	0.668532	0.904114	protein_coding
ENSG0000(	TRIM65	747.4037	0.027456	0.66862	0.904114	protein_coding
ENSG0000(	TSNAX-DIS(	29.67013	0.127815	0.668324	0.904114	protein_coding
ENSG0000(	USP51	58.33419	-0.10593	0.668347	0.904114	protein_coding
ENSG0000(	WIPI2	1058.291	0.027141	0.668367	0.904114	protein_coding
ENSG0000(	ENSG0000(	67.31323	-0.05977	0.668752	0.904157	lncRNA
ENSG0000(	MADCAM1	124.067	-0.05526	0.668748	0.904157	lncRNA
ENSG0000(	SOCS2	384.0308	0.062034	0.668735	0.904157	protein_coding
ENSG0000(	EBLN2	328.439	-0.0401	0.668896	0.904219	protein_coding

ENSG0000(	ENSG0000(	66.89161	-0.07175	0.668889	0.904219	lncRNA
ENSG0000(	TMEM272	145.8634	-0.12834	0.668878	0.904219	protein_coding
ENSG0000(	SAFB2	2034.377	0.022141	0.669127	0.904442	protein_coding
ENSG0000(	TRIL	131.9662	0.085595	0.669114	0.904442	protein_coding
ENSG0000(	CNOT6	1142.642	0.048961	0.669321	0.904616	protein_coding
ENSG0000(	IL6R	2576.295	-0.05207	0.669339	0.904616	protein_coding
ENSG0000(	ZNF653	119.7382	-0.06146	0.669354	0.904616	protein_coding
ENSG0000(	ZNF384	1081.122	-0.02534	0.669416	0.904655	protein_coding
ENSG0000(	CLTA	749.0735	-0.03921	0.669514	0.904743	protein_coding
ENSG0000(	CCDC9B	1660.493	0.088664	0.66957	0.904758	protein_coding
ENSG0000(	ENSG0000(	90.07797	0.048802	0.669591	0.904758	lncRNA
ENSG0000(	COX6C	611.0053	-0.04923	0.669785	0.904976	protein_coding
ENSG0000(	CRIP1	90.92969	-0.10939	0.670148	0.905111	protein_coding
ENSG0000(	ENSG0000(	57.58086	0.190266	0.66996	0.905111	lncRNA
ENSG0000(	ENSG0000(	51.69164	0.077776	0.670071	0.905111	lncRNA
ENSG0000(	FLOT2	762.6486	-0.05224	0.670142	0.905111	protein_coding
ENSG0000(	GRHL1	289.9763	-0.05079	0.670147	0.905111	protein_coding
ENSG0000(	LINC00687	152.9351	0.039943	0.670035	0.905111	lncRNA
ENSG0000(	MARK2	2795.837	0.032958	0.67002	0.905111	protein_coding
ENSG0000(	VWA7	171.08	-0.08323	0.66994	0.905111	protein_coding
ENSG0000(	ENSG0000(	297.5983	-0.07442	0.670298	0.90515	lncRNA
ENSG0000(	ENSG0000(	890.2879	0.059196	0.670291	0.90515	lncRNA
ENSG0000(	THUMPD2	388.3969	-0.05904	0.670309	0.90515	protein_coding
ENSG0000(	TTTY14	1893.203	-0.03136	0.670288	0.90515	lncRNA
ENSG0000(	LRFN5-DT	168.5425	0.08182	0.670515	0.905299	lncRNA
ENSG0000(	RBM38	336.9261	0.065709	0.67055	0.905299	protein_coding
ENSG0000(	WRAP53	110.9062	0.070909	0.670524	0.905299	protein_coding
ENSG0000(	ZC3H13	1661.716	-0.03112	0.670459	0.905299	protein_coding
ENSG0000(	CCDC92	671.9657	-0.03493	0.670671	0.905363	protein_coding
ENSG0000(	ENSG0000(	87.26629	0.147938	0.670755	0.905363	protein_coding
ENSG0000(	LINC02630	36.14661	0.094995	0.670763	0.905363	lncRNA
ENSG0000(	RN7SKP18(	333.7188	0.075019	0.670756	0.905363	misc_RNA
ENSG0000(	SLC66A3	207.931	0.068628	0.670675	0.905363	protein_coding
ENSG0000(	ANTXR2	2123.837	0.048943	0.671029	0.905445	protein_coding
ENSG0000(	CEMP1	21.93094	0.172666	0.671065	0.905445	protein_coding
ENSG0000(	ENSG0000(	158.1233	-0.05372	0.671034	0.905445	lncRNA
ENSG0000(	ENSG0000(	145.0358	0.041337	0.67121	0.905445	lncRNA
ENSG0000(	ENSG0000(	255.4972	0.107755	0.670949	0.905445	lncRNA
ENSG0000(	LINC02216	46.43271	0.076631	0.671014	0.905445	lncRNA
ENSG0000(	NDUFS2	1651.397	0.033058	0.670982	0.905445	protein_coding
ENSG0000(	NUCB2	1124.433	0.040092	0.671196	0.905445	protein_coding
ENSG0000(	RN7SL2	199438.6	-0.13632	0.671178	0.905445	misc_RNA
ENSG0000(	RPL27	901.529	-0.07298	0.671129	0.905445	protein_coding
ENSG0000(	RUNDC3B	239.0711	0.087123	0.67125	0.905445	protein_coding
ENSG0000(	SNORA36A	24.28622	-0.10674	0.671209	0.905445	snoRNA
ENSG0000(	TBC1D10A	808.7621	0.034349	0.671251	0.905445	protein_coding
ENSG0000(	GOT2	956.3663	0.033945	0.671385	0.905572	protein_coding

ENSG0000(	ING3	342.2105	0.045644	0.671411	0.905572	protein_coding
ENSG0000(	ABCC10	1606.85	-0.02538	0.671478	0.905618	protein_coding
ENSG0000(	MT1E	332.2861	-0.14239	0.671512	0.905619	protein_coding
ENSG0000(	NACA4P	72.45513	-0.05608	0.67162	0.905698	transcribed_processed_pseudoge
ENSG0000(	SPATA9	25.45626	0.101051	0.671636	0.905698	protein_coding
ENSG0000(	IGHV1-58	22.61496	-0.51417	0.671694	0.905731	IG_V_gene
ENSG0000(	ENSG0000(	2066.423	0.020045	0.671769	0.905744	lncRNA
ENSG0000(	NHLRC2	2657.394	0.02884	0.671755	0.905744	protein_coding
ENSG0000(	CCDC127	712.0722	0.058526	0.67201	0.905799	protein_coding
ENSG0000(	COL25A1	30.65641	0.176206	0.672222	0.905799	protein_coding
ENSG0000(	DGCR11	412.1654	0.039857	0.672445	0.905799	lncRNA
ENSG0000(	EID2	73.37479	-0.07606	0.672566	0.905799	protein_coding
ENSG0000(	ENSG0000(	53.15567	0.10617	0.672498	0.905799	lncRNA
ENSG0000(	ENSG0000(	42.42986	-0.10029	0.672659	0.905799	lncRNA
ENSG0000(	ENSG0000(	25.17855	-0.17414	0.67273	0.905799	lncRNA
ENSG0000(	ENSG0000(	24.77433	0.109573	0.672188	0.905799	lncRNA
ENSG0000(	FAM32A	620.3207	0.045759	0.672509	0.905799	protein_coding
ENSG0000(	FBXO16	69.3248	-0.07053	0.672611	0.905799	protein_coding
ENSG0000(	GALK2	1149.588	-0.02994	0.672261	0.905799	protein_coding
ENSG0000(	IQCN	1494.04	0.054303	0.672228	0.905799	protein_coding
ENSG0000(	LINC01018	153.1138	0.038742	0.672291	0.905799	lncRNA
ENSG0000(	LYRM4-AS1	154.2076	-0.04219	0.672639	0.905799	lncRNA
ENSG0000(	MALAT1	1418525	-0.04877	0.672641	0.905799	lncRNA
ENSG0000(	MAPRE2	835.585	-0.03374	0.672886	0.905799	protein_coding
ENSG0000(	MIR210HG	395.4423	-0.09824	0.671942	0.905799	lncRNA
ENSG0000(	NDUFB6	117.6416	-0.06934	0.672576	0.905799	protein_coding
ENSG0000(	NLRP10	292.9731	0.031614	0.672486	0.905799	protein_coding
ENSG0000(	PAIP2	681.638	-0.03167	0.672504	0.905799	protein_coding
ENSG0000(	PEX13	1342.457	0.031535	0.672732	0.905799	protein_coding
ENSG0000(	RANGAP1	1025.797	-0.03316	0.672383	0.905799	protein_coding
ENSG0000(	RBP7	64.18595	0.067582	0.672178	0.905799	protein_coding
ENSG0000(	RN7SL113P	53.8437	-0.08838	0.672113	0.905799	misc_RNA
ENSG0000(	RPL21P135	429.8975	0.035747	0.672771	0.905799	processed_pseudogene
ENSG0000(	RPL38	1274.676	-0.05636	0.67242	0.905799	protein_coding
ENSG0000(	RYK	1174.204	-0.03498	0.672822	0.905799	protein_coding
ENSG0000(	SNORA40	19.49961	0.258846	0.672929	0.905799	snoRNA
ENSG0000(	TBC1D30	412.4756	0.043312	0.672664	0.905799	protein_coding
ENSG0000(	TEFM	533.2576	0.026725	0.672482	0.905799	protein_coding
ENSG0000(	TMEM167E	792.828	-0.0262	0.672852	0.905799	protein_coding
ENSG0000(	WASF2	3831.227	-0.02689	0.672891	0.905799	protein_coding
ENSG0000(	ZBED5-AS1	188.3062	0.048029	0.672923	0.905799	lncRNA
ENSG0000(	ZNF286A-T	221.414	-0.09575	0.672765	0.905799	protein_coding
ENSG0000(	KIAA1191	1141.903	-0.03398	0.672997	0.905818	protein_coding
ENSG0000(	PRKAB2	1117.173	0.043877	0.673042	0.905818	protein_coding
ENSG0000(	SNRNP48	594.294	-0.03772	0.673026	0.905818	protein_coding
ENSG0000(	SNORA53	380.2262	0.088265	0.673078	0.905822	snoRNA
ENSG0000(	ENSG0000(	25.73373	0.096018	0.673246	0.90585	lncRNA

ENSG0000(	ENSG0000(	42.57411	0.074193	0.673168	0.90585	lncRNA
ENSG0000(	LINC01549	126.2002	-0.07335	0.673262	0.90585	lncRNA
ENSG0000(	OASL	898.6692	-0.07446	0.673289	0.90585	protein_coding
ENSG0000(	PAXIP1-AS2	771.258	0.038333	0.673212	0.90585	lncRNA
ENSG0000(	TRIM72	1250.435	0.024117	0.673297	0.90585	protein_coding
ENSG0000(	ENSG0000(	128.9288	-0.04585	0.67335	0.905879	lncRNA
ENSG0000(	ENSG0000(	121.3085	0.05142	0.673558	0.905998	lncRNA
ENSG0000(	ITIH5	1619.371	-0.05225	0.673481	0.905998	protein_coding
ENSG0000(	PDIK1L	420.1211	0.066538	0.673542	0.905998	protein_coding
ENSG0000(	RNU1-34P	50.0687	-0.11295	0.673571	0.905998	snRNA
ENSG0000(	DENND2C	760.5243	-0.03194	0.673611	0.906008	protein_coding
ENSG0000(	ENSG0000(	35.40289	-0.07355	0.673756	0.906092	lncRNA
ENSG0000(	ENSG0000(	402.6004	0.039157	0.673809	0.906092	TEC
ENSG0000(	FKBPL	35.62222	0.098717	0.673926	0.906092	protein_coding
ENSG0000(	GLB1L3	709.8528	-0.036	0.67396	0.906092	protein_coding
ENSG0000(	GSTM1	67.10184	0.282252	0.673709	0.906092	protein_coding
ENSG0000(	LIPC	106.5586	-0.05763	0.674003	0.906092	protein_coding
ENSG0000(	PCGF6	294.2729	0.051563	0.673979	0.906092	protein_coding
ENSG0000(	PKD1P4	743.0681	-0.06612	0.674002	0.906092	transcribed_unprocessed_pseudo
ENSG0000(	RYBP	1351.513	-0.03602	0.673961	0.906092	protein_coding
ENSG0000(	TOGARAM1	1766.399	0.0445	0.673992	0.906092	protein_coding
ENSG0000(	ATP6V0E2	287.7822	0.075031	0.674234	0.906205	protein_coding
ENSG0000(	ENSG0000(	477.2856	0.030094	0.67423	0.906205	lncRNA
ENSG0000(	ENSG0000(	219.1693	0.078928	0.674143	0.906205	lncRNA
ENSG0000(	FAM76B	508.3317	-0.04748	0.674187	0.906205	protein_coding
ENSG0000(	LINC01013	171.2453	-0.07468	0.674252	0.906205	lncRNA
ENSG0000(	CCDC144N	934.7027	-0.03528	0.674346	0.906214	lncRNA
ENSG0000(	CGGBP1	2145.305	0.033669	0.67441	0.906214	protein_coding
ENSG0000(	CT70	117.2183	-0.05128	0.674433	0.906214	lncRNA
ENSG0000(	ENSG0000(	137.0498	-0.04725	0.674376	0.906214	lncRNA
ENSG0000(	ENSG0000(	251.0144	0.039827	0.674489	0.906214	lncRNA
ENSG0000(	ENSG0000(	343.6444	-0.04466	0.674484	0.906214	lncRNA
ENSG0000(	ENSG0000(	143.7987	0.055875	0.674352	0.906214	lncRNA
ENSG0000(	ENSG0000(	43.27399	0.072855	0.674575	0.906285	transcribed_unprocessed_pseudo
ENSG0000(	VNN1	283.2223	-0.21082	0.674633	0.906318	protein_coding
ENSG0000(	RSRP1	6898.595	0.031114	0.674698	0.906362	protein_coding
ENSG0000(	GPR20	27.18648	-0.12015	0.674897	0.906585	protein_coding
ENSG0000(	ANKRD55	27.20849	0.133634	0.675477	0.906639	protein_coding
ENSG0000(	ATP5F1B	3421.431	0.068648	0.675449	0.906639	protein_coding
ENSG0000(	CASP7	1683.092	0.06718	0.675114	0.906639	protein_coding
ENSG0000(	CFAP43	32.08365	-0.11779	0.675734	0.906639	protein_coding
ENSG0000(	COQ4	1988.491	0.032668	0.675759	0.906639	protein_coding
ENSG0000(	CUL9	1615.366	-0.02717	0.675326	0.906639	protein_coding
ENSG0000(	DIPK2A	415.2714	-0.06053	0.675192	0.906639	protein_coding
ENSG0000(	ENSG0000(	157.8224	0.060751	0.675503	0.906639	lncRNA
ENSG0000(	ENSG0000(	72.76496	0.055068	0.675541	0.906639	TEC
ENSG0000(	ENSG0000(	48.87506	-0.08341	0.675255	0.906639	lncRNA

ENSG0000(	HHLA1	323.8779	-0.06478	0.675559	0.906639	protein_coding
ENSG0000(	HLA-J	251.875	-0.08968	0.675094	0.906639	transcribed_unprocessed_pseudo
ENSG0000(	INSL4	21.52318	0.112037	0.675565	0.906639	protein_coding
ENSG0000(	LINC00678	37.78804	0.091464	0.675165	0.906639	lncRNA
ENSG0000(	MRPL54	54.97239	-0.10758	0.675691	0.906639	protein_coding
ENSG0000(	PRRT2	215.2268	0.082	0.675761	0.906639	protein_coding
ENSG0000(	PTPRCAP	132.4991	-0.19578	0.675695	0.906639	protein_coding
ENSG0000(	RIT2	32.8143	-0.09056	0.675734	0.906639	protein_coding
ENSG0000(	RN7SKP268	32.98991	0.115457	0.675562	0.906639	misc_RNA
ENSG0000(	RRAGC	766.0008	-0.04071	0.675324	0.906639	protein_coding
ENSG0000(	SNORA2B	27.61438	0.077967	0.675098	0.906639	snoRNA
ENSG0000(	TMEM202-	155.1461	0.048803	0.675023	0.906639	lncRNA
ENSG0000(	TMEM253	85.61053	0.114236	0.675438	0.906639	protein_coding
ENSG0000(	TOMM40	635.0994	0.046471	0.674988	0.906639	protein_coding
ENSG0000(	VWA2	540.9819	-0.04857	0.67542	0.906639	protein_coding
ENSG0000(	AGAP5	703.5817	0.036809	0.675933	0.906693	protein_coding
ENSG0000(	KDM8	190.4856	-0.05483	0.675933	0.906693	protein_coding
ENSG0000(	PGR	161.8148	0.113857	0.675881	0.906693	protein_coding
ENSG0000(	TFIP11-DT	61.01396	-0.06761	0.675899	0.906693	lncRNA
ENSG0000(	ENSG0000(	69.95113	-0.08329	0.676018	0.906763	lncRNA
ENSG0000(	ZNF699	934.6824	0.031123	0.676058	0.906772	protein_coding
ENSG0000(	SIMC1	1031.366	0.038019	0.676227	0.906954	protein_coding
ENSG0000(	ENSG0000(	447.7054	-0.0674	0.676305	0.907014	lncRNA
ENSG0000(	ENSG0000(	34.63117	-0.11725	0.676348	0.907029	unprocessed_pseudogene
ENSG0000(	AOC2	83.0993	-0.07264	0.676392	0.907043	protein_coding
ENSG0000(	ADPGK-AS1	172.9588	-0.04694	0.676532	0.907157	lncRNA
ENSG0000(	CD46	5679.208	0.030912	0.676576	0.907157	protein_coding
ENSG0000(	RNU6-570F	104.4916	-0.09938	0.676553	0.907157	snRNA
ENSG0000(	ENSG0000(	22.23116	0.136778	0.676618	0.907169	lncRNA
ENSG0000(	ENSG0000(	275.0635	-0.04682	0.676705	0.907198	lncRNA
ENSG0000(	KLK7	62.3229	-0.11532	0.676698	0.907198	protein_coding
ENSG0000(	PRSS8	1738.322	0.086937	0.676768	0.907238	protein_coding
ENSG0000(	GOLGA8R	647.3062	0.031785	0.676833	0.907243	protein_coding
ENSG0000(	PDE11A-AS	36.83073	-0.15145	0.676871	0.907243	lncRNA
ENSG0000(	ZBTB20-AS	110.4473	-0.04817	0.676853	0.907243	lncRNA
ENSG0000(	BDKRB2	1054.544	-0.06849	0.67696	0.907318	protein_coding
ENSG0000(	ATPAF1	770.3782	-0.03751	0.677006	0.907336	protein_coding
ENSG0000(	ADH1C	1494.783	0.145311	0.677302	0.9076	protein_coding
ENSG0000(	ENSG0000(	49.27998	-0.09052	0.677299	0.9076	lncRNA
ENSG0000(	VAPB	1840.108	0.023638	0.677273	0.9076	protein_coding
ENSG0000(	MAGOH	141.7026	-0.04194	0.677398	0.907685	protein_coding
ENSG0000(	PTGFRN	1299.319	-0.0728	0.677432	0.907685	protein_coding
ENSG0000(	ANKRD18D	30.02211	-0.18299	0.677571	0.907739	transcribed_unprocessed_pseudo
ENSG0000(	R3HCC1	180.0313	0.053077	0.677568	0.907739	protein_coding
ENSG0000(	ZNF230	370.1061	0.031352	0.677549	0.907739	protein_coding
ENSG0000(	C10orf88	233.7076	0.059673	0.6779	0.907782	protein_coding
ENSG0000(	COPG1	1811.791	-0.0397	0.677704	0.907782	protein_coding

ENSG0000(	CTH	113.9547	0.067157	0.677729	0.907782	protein_coding
ENSG0000(	DMXL2	6173.91	-0.03529	0.677888	0.907782	protein_coding
ENSG0000(	ENSG0000(	22.13357	-0.12946	0.677785	0.907782	processed_pseudogene
ENSG0000(	ENSG0000(	1770.266	-0.02682	0.677706	0.907782	lncRNA
ENSG0000(	ERVH48-1	35.73301	0.098144	0.677754	0.907782	protein_coding
ENSG0000(	SEC14L6	509.1776	-0.02876	0.677867	0.907782	protein_coding
ENSG0000(	USP36	1499.881	0.039869	0.677868	0.907782	protein_coding
ENSG0000(	ART3	210.8061	-0.04858	0.678261	0.907845	protein_coding
ENSG0000(	DTWD2	853.9943	0.031395	0.678255	0.907845	protein_coding
ENSG0000(	ENSG0000(	277.2808	-0.04339	0.678093	0.907845	lncRNA
ENSG0000(	EYA2	414.8936	0.14898	0.678125	0.907845	protein_coding
ENSG0000(	GPBP1	2681.547	-0.04307	0.678061	0.907845	protein_coding
ENSG0000(	LHFPL3-AS:	577.9933	0.112755	0.678151	0.907845	lncRNA
ENSG0000(	LRRC37A	1110.264	0.083611	0.678277	0.907845	protein_coding
ENSG0000(	RAB33B-AS	2277.595	0.022306	0.678095	0.907845	lncRNA
ENSG0000(	RN7SL375F	27.28707	0.104517	0.678127	0.907845	misc_RNA
ENSG0000(	SEMA3F	188.2315	0.10478	0.678203	0.907845	protein_coding
ENSG0000(	GOLGA8IP	68.73415	-0.06444	0.678396	0.90796	transcribed_unprocessed_pseudo
ENSG0000(	MAPK7	654.5141	0.061165	0.678471	0.908016	protein_coding
ENSG0000(	SLCO5A1-A	42.86452	-0.09871	0.678511	0.908026	lncRNA
ENSG0000(	ENSG0000(	29.70435	-0.093	0.678566	0.908055	lncRNA
ENSG0000(	ENSG0000(	33.50516	-0.10525	0.678725	0.908224	lncRNA
ENSG0000(	SRP14-DT	490.9552	-0.04702	0.678845	0.90834	lncRNA
ENSG0000(	ASCC2	1382.815	0.035832	0.678939	0.908364	protein_coding
ENSG0000(	C18orf32	936.9421	0.031159	0.678947	0.908364	protein_coding
ENSG0000(	CTAGE15	95.08867	0.287229	0.679	0.908364	protein_coding
ENSG0000(	MKX-AS1	41.7326	-0.10196	0.679022	0.908364	lncRNA
ENSG0000(	RPL4P7	59.81657	-0.10652	0.679028	0.908364	processed_pseudogene
ENSG0000(	IMP3	388.2744	-0.03857	0.679063	0.908367	protein_coding
ENSG0000(	RBM33	4777.577	-0.02107	0.67938	0.908747	protein_coding
ENSG0000(	ENSG0000(	211.7059	0.058221	0.679549	0.908929	lncRNA
ENSG0000(	GIPC2	614.3688	0.104667	0.679591	0.908941	protein_coding
ENSG0000(	ERCC3	1223.455	0.025171	0.679625	0.908942	protein_coding
ENSG0000(	CAPNS2	19.68942	-0.14489	0.680171	0.909013	protein_coding
ENSG0000(	CYP4F35P	116.8285	-0.08445	0.680027	0.909013	transcribed_unprocessed_pseudo
ENSG0000(	DIAPH2-AS	57.08284	-0.07794	0.680187	0.909013	lncRNA
ENSG0000(	DNM1P47	740.5481	0.086994	0.680085	0.909013	transcribed_unprocessed_pseudo
ENSG0000(	ENSG0000(	543.7739	0.036166	0.679879	0.909013	lncRNA
ENSG0000(	ENSG0000(	134.1409	0.055522	0.679935	0.909013	lncRNA
ENSG0000(	ENSG0000(	83.51068	-0.12554	0.680169	0.909013	lncRNA
ENSG0000(	ENSG0000(	197.1978	-0.06539	0.680207	0.909013	TEC
ENSG0000(	ENSG0000(	32.32834	-0.10472	0.679803	0.909013	TEC
ENSG0000(	ENSG0000(	22.67618	0.108881	0.679987	0.909013	processed_pseudogene
ENSG0000(	LINC02552	561.6728	-0.02916	0.680152	0.909013	lncRNA
ENSG0000(	LINC02604	317.9483	0.046229	0.680105	0.909013	lncRNA
ENSG0000(	PRR12	1305.845	-0.05079	0.680168	0.909013	protein_coding
ENSG0000(	RN7SL582F	96.80605	-0.05581	0.679998	0.909013	misc_RNA

ENSG0000(	SOWAHC	1078.45	0.058015	0.680069	0.909013	protein_coding
ENSG0000(	ZNF726	668.5115	0.045487	0.680082	0.909013	protein_coding
ENSG0000(	DNAJC5G	34.74439	0.090563	0.680303	0.909058	protein_coding
ENSG0000(	FAM181B	118.3415	-0.04768	0.680336	0.909058	protein_coding
ENSG0000(	NDUFAF6	563.9847	0.039351	0.68034	0.909058	protein_coding
ENSG0000(	ENSG0000(	179.7668	0.051359	0.680455	0.909167	lncRNA
ENSG0000(	AFF3	1455.234	-0.08221	0.680596	0.909216	protein_coding
ENSG0000(	EEF1G	5002.895	0.053774	0.680726	0.909216	protein_coding
ENSG0000(	ENSG0000(	29.53955	0.089529	0.680647	0.909216	lncRNA
ENSG0000(	ENSG0000(	239.8797	0.074446	0.680605	0.909216	lncRNA
ENSG0000(	ENSG0000(	147.0727	-0.05837	0.680672	0.909216	lncRNA
ENSG0000(	FAM156A	284.6608	0.104877	0.680574	0.909216	protein_coding
ENSG0000(	TIMM10	55.97778	-0.09923	0.680755	0.909216	protein_coding
ENSG0000(	ZNF501	144.3609	-0.07092	0.68072	0.909216	protein_coding
ENSG0000(	C2orf49	823.3679	-0.02376	0.68086	0.90924	protein_coding
ENSG0000(	GORAB-AS1	167.4566	-0.05221	0.680844	0.90924	lncRNA
ENSG0000(	POLR2J2	1219.957	0.061678	0.680873	0.90924	protein_coding
ENSG0000(	SELENOP	4357.531	-0.10109	0.680931	0.909274	protein_coding
ENSG0000(	ANO3	50.87197	-0.07234	0.681192	0.90953	protein_coding
ENSG0000(	HMCES	329.7232	0.047563	0.681222	0.90953	protein_coding
ENSG0000(	MRPL20-DT	334.5661	0.035179	0.681209	0.90953	lncRNA
ENSG0000(	CLUAP1	863.1682	0.029734	0.681502	0.909809	protein_coding
ENSG0000(	ENSG0000(	120.65	-0.06226	0.681563	0.909809	lncRNA
ENSG0000(	ENSG0000(	46.48116	0.093949	0.681553	0.909809	lncRNA
ENSG0000(	ENSG0000(	68.46326	-0.17198	0.681477	0.909809	unprocessed_pseudogene
ENSG0000(	IER2	1224.017	-0.0494	0.681629	0.909809	protein_coding
ENSG0000(	U7	23.36737	-0.12253	0.681607	0.909809	snRNA
ENSG0000(	BCAM	453.6129	0.039357	0.681708	0.909811	protein_coding
ENSG0000(	FBXO36	271.5568	0.043016	0.6817	0.909811	protein_coding
ENSG0000(	RNU2-2P	18401.31	0.066729	0.68173	0.909811	snRNA
ENSG0000(	FLAD1	757.3419	-0.02722	0.681772	0.909823	protein_coding
ENSG0000(	LINC00365	32.67975	0.120357	0.681847	0.909878	lncRNA
ENSG0000(	SUN3	28.60399	-0.11881	0.681891	0.909893	protein_coding
ENSG0000(	HMGCL	899.5619	0.032761	0.681957	0.909938	protein_coding
ENSG0000(	BMX	40.47856	-0.12076	0.682036	0.909999	protein_coding
ENSG0000(	FSD2	194.9753	0.065974	0.682118	0.910017	protein_coding
ENSG0000(	KIF3C	117.4499	0.099101	0.682149	0.910017	protein_coding
ENSG0000(	RSF1	2562.85	0.02463	0.682106	0.910017	protein_coding
ENSG0000(	ACP6	1089.238	0.047173	0.682221	0.910069	protein_coding
ENSG0000(	CYP4F8	478.2029	-0.0289	0.682438	0.910204	protein_coding
ENSG0000(	GEMIN5	298.6427	0.082277	0.682455	0.910204	protein_coding
ENSG0000(	NEURL3	75.41978	-0.13817	0.6824	0.910204	protein_coding
ENSG0000(	RYR1	181.3207	0.074343	0.682401	0.910204	protein_coding
ENSG0000(	THAP9-AS1	1316.074	0.031804	0.682611	0.910369	lncRNA
ENSG0000(	ENSG0000(	107.3238	-0.05142	0.682646	0.910372	lncRNA
ENSG0000(	ENSG0000(	21.75457	0.136749	0.682718	0.910422	lncRNA
ENSG0000(	C19orf53	160.2589	-0.03736	0.68288	0.910527	protein_coding

ENSG0000(	ENSG0000(	48.37394	0.069085	0.682895	0.910527	lncRNA
ENSG0000(	ENSG0000(	55.8951	0.057362	0.68283	0.910527	lncRNA
ENSG0000(	EIF4A1	2400.197	-0.03608	0.68293	0.910529	protein_coding
ENSG0000(	ENSG0000(	91.58118	0.809202	0.683007	0.910544	protein_coding
ENSG0000(	TMEM100	60.39044	-0.11917	0.683007	0.910544	protein_coding
ENSG0000(	DNASE1L2	24.11677	0.09453	0.683197	0.9106	protein_coding
ENSG0000(	ENSG0000(	70.69445	0.083903	0.683248	0.9106	lncRNA
ENSG0000(	ENSG0000(	105.1366	0.065299	0.683098	0.9106	TEC
ENSG0000(	ENSG0000(	102.7359	0.073477	0.683217	0.9106	lncRNA
ENSG0000(	ENSG0000(	22.74383	0.100282	0.683184	0.9106	lncRNA
ENSG0000(	TRIM36-IT1	56.40351	0.147965	0.68313	0.9106	lncRNA
ENSG0000(	DCXR-DT	45.14312	-0.09211	0.683466	0.910626	lncRNA
ENSG0000(	DLD	1019.585	0.038497	0.683436	0.910626	protein_coding
ENSG0000(	LIN28B-AS1	493.9756	0.038148	0.683338	0.910626	lncRNA
ENSG0000(	MRM1	218.9197	0.064048	0.68341	0.910626	protein_coding
ENSG0000(	RNU2-65P	43.56998	-0.08339	0.683344	0.910626	snRNA
ENSG0000(	SLC35F4	40.41788	0.099257	0.683384	0.910626	protein_coding
ENSG0000(	AKAP10	1287.872	0.030426	0.683659	0.91073	protein_coding
ENSG0000(	ENSG0000(	37.18808	-0.27706	0.683614	0.91073	lncRNA
ENSG0000(	ENSG0000(	216.4109	0.048963	0.683705	0.91073	lncRNA
ENSG0000(	LAMTOR3	582.7254	0.05312	0.683739	0.91073	protein_coding
ENSG0000(	RN7SL689F	25.32408	-0.10238	0.683742	0.91073	misc_RNA
ENSG0000(	SNORA11B	80.05334	0.074809	0.683671	0.91073	snoRNA
ENSG0000(	ADGRF3	48.62317	0.17605	0.683853	0.910833	protein_coding
ENSG0000(	ENSG0000(	72.70243	0.051785	0.683945	0.91087	lncRNA
ENSG0000(	TTLL9	127.2196	-0.06327	0.683947	0.91087	protein_coding
ENSG0000(	CECR7	414.0205	-0.04932	0.684422	0.910929	transcribed_unprocessed_pseudo
ENSG0000(	ENSG0000(	21.39686	0.121276	0.684206	0.910929	lncRNA
ENSG0000(	ENSG0000(	74.30827	-0.08142	0.684421	0.910929	unprocessed_pseudogene
ENSG0000(	ENSG0000(	242.0296	0.062527	0.684236	0.910929	lncRNA
ENSG0000(	ENSG0000(	277.6459	0.043784	0.684291	0.910929	lncRNA
ENSG0000(	ENSG0000(	101.7989	0.053202	0.684404	0.910929	lncRNA
ENSG0000(	ENSG0000(	77.97256	-0.05869	0.684179	0.910929	lncRNA
ENSG0000(	ENSG0000(	40.46382	-0.15336	0.684327	0.910929	protein_coding
ENSG0000(	ENSG0000(	27.04262	0.108038	0.684054	0.910929	lncRNA
ENSG0000(	MUC4	41119.49	-0.09917	0.684109	0.910929	protein_coding
ENSG0000(	OR2AT4	1430.708	-0.028	0.684323	0.910929	protein_coding
ENSG0000(	SPECC1	4734.472	0.04284	0.68431	0.910929	protein_coding
ENSG0000(	UPF3A	1719.352	0.02346	0.684203	0.910929	protein_coding
ENSG0000(	ARAP2	2619.97	-0.05045	0.68453	0.910997	protein_coding
ENSG0000(	ATP2C2-AS	144.6106	-0.07026	0.684707	0.910997	lncRNA
ENSG0000(	ENSG0000(	27.08547	0.139022	0.684858	0.910997	processed_pseudogene
ENSG0000(	ENSG0000(	970.0793	-0.03133	0.684787	0.910997	TEC
ENSG0000(	ENSG0000(	102.6552	0.04303	0.684609	0.910997	lncRNA
ENSG0000(	GDF9	31.94225	0.104717	0.684903	0.910997	protein_coding
ENSG0000(	H4C5	288.8031	-0.06969	0.684745	0.910997	protein_coding
ENSG0000(	MTO1	2709.645	0.021856	0.684708	0.910997	protein_coding

ENSG0000(	NEURL2	56.60933	0.087476	0.684638	0.910997	protein_coding
ENSG0000(	OPTN	1657.887	0.047903	0.684877	0.910997	protein_coding
ENSG0000(	TBC1D8	1273.29	-0.02908	0.684699	0.910997	protein_coding
ENSG0000(	UNC80	1416.846	0.027004	0.684881	0.910997	protein_coding
ENSG0000(	YKT6	1259.014	0.034855	0.684795	0.910997	protein_coding
ENSG0000(	ENSG0000(	80.05458	-0.07301	0.684997	0.911034	lncRNA
ENSG0000(	TPTE2P5	288.8079	0.067071	0.684977	0.911034	transcribed_unprocessed_pseudo
ENSG0000(	MDH1B	36.52056	-0.09495	0.685051	0.911061	protein_coding
ENSG0000(	HAAO	242.4615	-0.0484	0.685102	0.911084	protein_coding
ENSG0000(	DENND4C	3270.775	0.045266	0.685137	0.911087	protein_coding
ENSG0000(	TDRD3	1035.628	0.031826	0.685191	0.911115	protein_coding
ENSG0000(	ENSG0000(	48.17077	-0.07632	0.685277	0.911142	lncRNA
ENSG0000(	STAU2	1408.68	0.026543	0.68527	0.911142	protein_coding
ENSG0000(	LINC01707	127.9186	-0.04382	0.685383	0.911238	lncRNA
ENSG0000(	CZIB-DT	632.8581	0.030613	0.685501	0.911351	lncRNA
ENSG0000(	HOXB2	96.51485	-0.07848	0.685747	0.911571	protein_coding
ENSG0000(	KLRC1	178.0744	0.056776	0.685823	0.911571	protein_coding
ENSG0000(	NOC3L	400.9575	-0.05779	0.685832	0.911571	protein_coding
ENSG0000(	SCAF8	2485.194	-0.02397	0.68583	0.911571	protein_coding
ENSG0000(	TACR2	85.66702	0.074376	0.685791	0.911571	protein_coding
ENSG0000(	BCL2L15	3708.934	0.063649	0.685881	0.911592	protein_coding
ENSG0000(	ENSG0000(	53.0927	0.087296	0.68597	0.911623	lncRNA
ENSG0000(	MRPL49	428.8272	0.035578	0.685961	0.911623	protein_coding
ENSG0000(	ENSG0000(	223.231	0.039605	0.686095	0.911744	lncRNA
ENSG0000(	ENSG0000(	432.9466	-0.03507	0.68615	0.911773	lncRNA
ENSG0000(	ENSG0000(	325.3387	0.097808	0.686222	0.911782	TEC
ENSG0000(	MSR1	270.4015	0.06641	0.686218	0.911782	protein_coding
ENSG0000(	ADAD2	39.04919	-0.07499	0.686297	0.911837	protein_coding
ENSG0000(	AGAP9	804.7882	0.075749	0.686376	0.911848	protein_coding
ENSG0000(	EDF1	728.8018	0.048444	0.686384	0.911848	protein_coding
ENSG0000(	ENSG0000(	24.25551	0.110667	0.686428	0.911848	lncRNA
ENSG0000(	RN7SKP74	92.66712	0.084935	0.686438	0.911848	misc_RNA
ENSG0000(	ENSG0000(	160.5551	-0.04082	0.6866	0.91187	lncRNA
ENSG0000(	IFI27L2	61.43581	-0.11121	0.686506	0.91187	protein_coding
ENSG0000(	RB1CC1	1895.965	-0.02593	0.686528	0.91187	protein_coding
ENSG0000(	SNORA7A	409.6923	0.055545	0.686561	0.91187	snoRNA
ENSG0000(	STX4	755.9339	-0.0271	0.686654	0.91187	protein_coding
ENSG0000(	TLCD3A	131.4572	-0.07319	0.686623	0.91187	protein_coding
ENSG0000(	ENSG0000(	104.4142	0.053258	0.686816	0.911919	protein_coding
ENSG0000(	GOLGA2P1	286.5472	-0.03756	0.686846	0.911919	transcribed_unprocessed_pseudo
ENSG0000(	LINC02474	31.30896	0.097946	0.686852	0.911919	lncRNA
ENSG0000(	SELE	112.4421	-0.76471	0.686856	0.911919	protein_coding
ENSG0000(	USPL1	902.2457	0.034168	0.686854	0.911919	protein_coding
ENSG0000(	KRCC1	518.955	-0.05535	0.686977	0.911992	protein_coding
ENSG0000(	MTMR1	833.9243	-0.03329	0.686962	0.911992	protein_coding
ENSG0000(	SELENOI	1837.308	0.034338	0.687029	0.912016	protein_coding
ENSG0000(	ENSG0000(	81.8595	0.080035	0.687148	0.912077	lncRNA

ENSG0000(	HAMP	324.3749	0.038308	0.687166	0.912077	protein_coding
ENSG0000(	ZNF282	495.0444	0.06218	0.687174	0.912077	protein_coding
ENSG0000(	COLEC10	24.57936	0.156308	0.687219	0.912093	protein_coding
ENSG0000(	PLXNB2	4413.378	0.051831	0.687259	0.912101	protein_coding
ENSG0000(	ENSG0000(	30.54682	0.111642	0.687367	0.912113	lncRNA
ENSG0000(	ENSG0000(	303.063	0.076924	0.687306	0.912113	lncRNA
ENSG0000(	ENSG0000(	111.0963	0.042616	0.687341	0.912113	lncRNA
ENSG0000(	AMZ1	174.3189	-0.06281	0.687465	0.912178	protein_coding
ENSG0000(	SLC35A4	768.03	0.040239	0.687482	0.912178	protein_coding
ENSG0000(	MUC5B	8539.298	0.140353	0.687534	0.912203	protein_coding
ENSG0000(	ENSG0000(	99.10855	-0.05401	0.687655	0.91232	lncRNA
ENSG0000(	LHX1-DT	20.29749	0.100645	0.687702	0.912338	lncRNA
ENSG0000(	LRPAP1	1954.583	0.030263	0.687804	0.912429	protein_coding
ENSG0000(	ANAPC11	197.5406	0.045496	0.687889	0.912474	protein_coding
ENSG0000(	QRICH2	248.6767	-0.04557	0.687914	0.912474	protein_coding
ENSG0000(	SLC25A5	1688.599	0.090824	0.687938	0.912474	protein_coding
ENSG0000(	APLF	215.9626	-0.04545	0.688013	0.912522	protein_coding
ENSG0000(	ENSG0000(	973.7491	0.033983	0.68808	0.912522	lncRNA
ENSG0000(	ENSG0000(	30.28234	-0.12631	0.688052	0.912522	lncRNA
ENSG0000(	RNU6-181F	21.17733	-0.13529	0.688129	0.912522	snRNA
ENSG0000(	SNORA27	52.72578	0.079198	0.688171	0.912522	snoRNA
ENSG0000(	TUBA3FP	306.1424	-0.03457	0.688173	0.912522	lncRNA
ENSG0000(	CASP8AP2	1452.111	-0.0298	0.688283	0.912624	protein_coding
ENSG0000(	GINS1	428.23	-0.04115	0.688373	0.912656	protein_coding
ENSG0000(	SLC8A3	49.9783	-0.11894	0.688358	0.912656	protein_coding
ENSG0000(	ENSG0000(	101.6638	-0.04112	0.688483	0.912661	lncRNA
ENSG0000(	LINC02015	33.37212	0.102624	0.68851	0.912661	lncRNA
ENSG0000(	RNU6-611F	36.91836	-0.07635	0.688504	0.912661	snRNA
ENSG0000(	SLC6A1	593.3943	-0.02802	0.688417	0.912661	protein_coding
ENSG0000(	PLEKHA7	3103.748	0.072938	0.688588	0.91272	protein_coding
ENSG0000(	ENSG0000(	156.1434	0.04942	0.689085	0.913335	lncRNA
ENSG0000(	ENSG0000(	160.8026	-0.04096	0.68914	0.913365	lncRNA
ENSG0000(	FKTN	1795.478	0.030802	0.689229	0.913438	protein_coding
ENSG0000(	KNL1	950.2599	-0.05894	0.689292	0.913479	protein_coding
ENSG0000(	MICU3	97.02269	-0.1073	0.689357	0.913519	protein_coding
ENSG0000(	ATP5F1C	1098.727	0.043964	0.689736	0.913645	protein_coding
ENSG0000(	ENSG0000(	20.69679	-0.10558	0.689668	0.913645	processed_pseudogene
ENSG0000(	ENSG0000(	81.56581	0.065413	0.689578	0.913645	protein_coding
ENSG0000(	ENSG0000(	25.0385	0.105166	0.689945	0.913645	lncRNA
ENSG0000(	ENSG0000(	191.4644	-0.03911	0.689801	0.913645	lncRNA
ENSG0000(	FBXW9	51.38613	0.068464	0.689816	0.913645	protein_coding
ENSG0000(	GTF2H2C	1770.371	-0.06125	0.689577	0.913645	protein_coding
ENSG0000(	H2AC8	99.16895	0.077042	0.689686	0.913645	protein_coding
ENSG0000(	LINC02759	144.8838	0.044915	0.689599	0.913645	lncRNA
ENSG0000(	LRRC36	246.362	0.043086	0.689905	0.913645	protein_coding
ENSG0000(	OR4F3	20.98776	0.233238	0.689711	0.913645	protein_coding
ENSG0000(	OXNAD1	1392.995	-0.04234	0.689877	0.913645	protein_coding

ENSG0000(	PIR	123.3633	-0.05827	0.68995	0.913645	protein_coding
ENSG0000(	PPP1R13B-	73.80286	-0.0711	0.68993	0.913645	lncRNA
ENSG0000(	SCARNA18	35.81465	-0.08331	0.689627	0.913645	snoRNA
ENSG0000(	ENSG0000(	115.3689	0.042876	0.689999	0.913667	lncRNA
ENSG0000(	RGS12	1538.253	-0.03223	0.690073	0.91372	protein_coding
ENSG0000(	BNIP2	2189.508	-0.03079	0.690224	0.913858	protein_coding
ENSG0000(	ENSG0000(	81.59978	0.065756	0.690267	0.913858	processed_pseudogene
ENSG0000(	LINC00211	74.43984	0.058529	0.69031	0.913858	lncRNA
ENSG0000(	NRAS	1384.382	-0.0341	0.690285	0.913858	protein_coding
ENSG0000(	C1QTNF3	320.2528	0.055068	0.690375	0.9139	protein_coding
ENSG0000(	ATP5PD	755.346	0.049724	0.69059	0.913977	protein_coding
ENSG0000(	BAZ2A	5226.452	-0.02727	0.690845	0.913977	protein_coding
ENSG0000(	BUD23	852.8191	0.038408	0.69092	0.913977	protein_coding
ENSG0000(	DENND2D	2728.342	-0.0444	0.690825	0.913977	protein_coding
ENSG0000(	ENSG0000(	133.7795	0.056989	0.690594	0.913977	lncRNA
ENSG0000(	ENSG0000(	71.36515	0.348493	0.690927	0.913977	lncRNA
ENSG0000(	ENSG0000(	87.12413	0.069327	0.690931	0.913977	lncRNA
ENSG0000(	ENSG0000(	124.9481	0.048069	0.69055	0.913977	TEC
ENSG0000(	ENSG0000(	309.5909	0.0447	0.690649	0.913977	lncRNA
ENSG0000(	LBX1-AS1	38.47617	0.08004	0.690608	0.913977	lncRNA
ENSG0000(	NAGA	600.1701	-0.03527	0.690846	0.913977	protein_coding
ENSG0000(	NET1	2230.411	0.062582	0.690749	0.913977	protein_coding
ENSG0000(	PTMS	166.9563	0.059154	0.690883	0.913977	protein_coding
ENSG0000(	SPAG16-DT	1005.27	-0.02415	0.69092	0.913977	lncRNA
ENSG0000(	TSEN34	233.4383	-0.04239	0.690643	0.913977	protein_coding
ENSG0000(	ASS1P1	51.8154	-0.13279	0.691138	0.914207	processed_pseudogene
ENSG0000(	CHMP4A	515.5015	0.027042	0.691683	0.914224	protein_coding
ENSG0000(	CRTC3-AS1	64.48806	-0.08219	0.691214	0.914224	lncRNA
ENSG0000(	DNAAF11	94.96702	0.091335	0.691638	0.914224	protein_coding
ENSG0000(	EGLN1	1173.653	0.039423	0.691764	0.914224	protein_coding
ENSG0000(	ENSG0000(	168.5963	-0.03809	0.691446	0.914224	lncRNA
ENSG0000(	ENSG0000(	94.43645	0.052395	0.691652	0.914224	lncRNA
ENSG0000(	ENSG0000(	74.58938	0.054867	0.691929	0.914224	lncRNA
ENSG0000(	ENSG0000(	312.8281	0.031534	0.691725	0.914224	TEC
ENSG0000(	ENSG0000(	43.41399	-0.07635	0.691844	0.914224	lncRNA
ENSG0000(	ENSG0000(	64.21446	0.069696	0.691894	0.914224	lncRNA
ENSG0000(	ENSG0000(	78.16356	-0.05435	0.691939	0.914224	lncRNA
ENSG0000(	ENSG0000(	245.0532	-0.031	0.691381	0.914224	lncRNA
ENSG0000(	ENSG0000(	48.84952	-0.06273	0.692015	0.914224	lncRNA
ENSG0000(	FUT9	125.0921	0.064099	0.691716	0.914224	protein_coding
ENSG0000(	GOLGA7	502.3797	-0.03933	0.691612	0.914224	protein_coding
ENSG0000(	GPX3	354.6651	-0.07379	0.691798	0.914224	protein_coding
ENSG0000(	LINC01448	45.61216	0.099043	0.691977	0.914224	lncRNA
ENSG0000(	MAP3K2	3744.474	-0.02392	0.691237	0.914224	protein_coding
ENSG0000(	METTL4	474.4862	0.055537	0.69164	0.914224	protein_coding
ENSG0000(	MYG1-AS1	95.22652	0.054056	0.691873	0.914224	lncRNA
ENSG0000(	NDUFA11	684.6662	-0.035	0.691875	0.914224	protein_coding

ENSG0000(	PPP2CA	1621.527	-0.02898	0.691303	0.914224	protein_coding
ENSG0000(	RB1-DT	42.14875	0.076129	0.691993	0.914224	lncRNA
ENSG0000(	SLC28A2-A:	324.3415	0.04618	0.691802	0.914224	lncRNA
ENSG0000(	SPIN2A	116.4797	0.062454	0.691417	0.914224	protein_coding
ENSG0000(	TNFAIP6	51.08725	-0.88416	0.691973	0.914224	protein_coding
ENSG0000(	ENSG0000(	67.87292	0.111339	0.692105	0.914255	lncRNA
ENSG0000(	ENSG0000(	144.2266	0.056892	0.692087	0.914255	lncRNA
ENSG0000(	RNASEH2A	253.2442	-0.03123	0.692258	0.914414	protein_coding
ENSG0000(	ARSA	7959.805	0.093841	0.692336	0.91445	protein_coding
ENSG0000(	ENSA	2544.256	0.023803	0.692352	0.91445	protein_coding
ENSG0000(	ECE1	2652.85	-0.04593	0.692484	0.914547	protein_coding
ENSG0000(	RNF148	32.92463	-0.09483	0.692492	0.914547	protein_coding
ENSG0000(	ENSG0000(	104.309	0.052771	0.692619	0.914639	lncRNA
ENSG0000(	ENSG0000(	26.39482	0.129341	0.692628	0.914639	lncRNA
ENSG0000(	ANKRA2	277.4945	0.03856	0.692691	0.91466	protein_coding
ENSG0000(	ENSG0000(	24.98639	0.11397	0.692711	0.91466	lncRNA
ENSG0000(	C1GALT1	1559.535	-0.04067	0.693018	0.914715	protein_coding
ENSG0000(	ENSG0000(	86.99656	-0.05375	0.693002	0.914715	processed_pseudogene
ENSG0000(	ENSG0000(	177.5236	0.034367	0.692857	0.914715	lncRNA
ENSG0000(	ENSG0000(	28.86546	0.091426	0.69299	0.914715	lncRNA
ENSG0000(	ENSG0000(	47.7714	-0.06838	0.692954	0.914715	lncRNA
ENSG0000(	MGAT4EP	221.3911	-0.02934	0.692972	0.914715	transcribed_unitary_pseudogene
ENSG0000(	POLR1B	1169.977	0.038944	0.692923	0.914715	protein_coding
ENSG0000(	TCEAL3-AS:	20.74483	0.114396	0.692817	0.914715	lncRNA
ENSG0000(	LINC01087	335.4707	-0.03013	0.69311	0.914793	lncRNA
ENSG0000(	ENSG0000(	147.4414	0.048068	0.69318	0.91484	lncRNA
ENSG0000(	ENSG0000(	227.3928	0.037599	0.693399	0.914866	lncRNA
ENSG0000(	ENSG0000(	470.8326	-0.03492	0.693396	0.914866	lncRNA
ENSG0000(	ENSG0000(	42.51132	-0.09337	0.693354	0.914866	lncRNA
ENSG0000(	SETD6	1033.044	0.033939	0.693397	0.914866	protein_coding
ENSG0000(	SLCO2B1	4029.304	-0.04663	0.693306	0.914866	protein_coding
ENSG0000(	SS18L1	786.3119	0.035621	0.693293	0.914866	protein_coding
ENSG0000(	FAM126B	2724.647	-0.03201	0.693542	0.914924	protein_coding
ENSG0000(	RN7SL491F	45.93375	0.06704	0.693498	0.914924	misc_RNA
ENSG0000(	WWP2	2157.005	0.02903	0.69353	0.914924	protein_coding
ENSG0000(	ADGRL3	1176.481	0.091335	0.693747	0.915064	protein_coding
ENSG0000(	ENSG0000(	113.3316	-1.45132	0.693815	0.915064	lncRNA
ENSG0000(	PRMT3	525.3055	0.034955	0.693772	0.915064	protein_coding
ENSG0000(	SNHG27	141.7972	0.061793	0.693687	0.915064	lncRNA
ENSG0000(	SPATA41	99.12861	-0.07505	0.693796	0.915064	lncRNA
ENSG0000(	CCT4P2	133.4845	0.078618	0.693987	0.915205	processed_pseudogene
ENSG0000(	ENSG0000(	55.22103	0.062431	0.693988	0.915205	lncRNA
ENSG0000(	ENSG0000(	20.42231	0.125286	0.69408	0.915282	lncRNA
ENSG0000(	ENSG0000(	112.4625	0.07116	0.694264	0.915349	lncRNA
ENSG0000(	IFT43	248.304	-0.03644	0.694176	0.915349	protein_coding
ENSG0000(	MET	1215.188	-0.07222	0.69425	0.915349	protein_coding
ENSG0000(	RNU6-1157	20.78768	0.100824	0.694251	0.915349	snRNA

ENSG0000(	ENSG0000(	176.1009	0.037141	0.694392	0.915461	lncRNA
ENSG0000(	ENSG0000(	93.87694	0.064424	0.694437	0.915461	TEC
ENSG0000(	RASSF9	168.5199	-0.07188	0.694449	0.915461	protein_coding
ENSG0000(	BIRC6	13329.39	-0.02699	0.694577	0.9155	protein_coding
ENSG0000(	ENSG0000(	140.1653	-0.08058	0.694578	0.9155	lncRNA
ENSG0000(	RTF1	920.5833	-0.03596	0.694519	0.9155	protein_coding
ENSG0000(	RN7SL1	254767.5	-0.11547	0.69466	0.915523	misc_RNA
ENSG0000(	TMEM37	774.222	0.107813	0.694662	0.915523	protein_coding
ENSG0000(	BMS1P8	79.79991	-0.08496	0.69477	0.915621	transcribed_unprocessed_pseudo
ENSG0000(	APOBEC3D	568.0858	0.034853	0.694997	0.915764	protein_coding
ENSG0000(	ASMT	46.21384	0.101114	0.694917	0.915764	protein_coding
ENSG0000(	C11orf58	2695.381	0.026811	0.695174	0.915764	protein_coding
ENSG0000(	ENSG0000(	200.8356	0.052554	0.695141	0.915764	lncRNA
ENSG0000(	LINC00604	30.86585	-0.08077	0.695139	0.915764	lncRNA
ENSG0000(	LINC02202	316.8194	-0.04689	0.69504	0.915764	lncRNA
ENSG0000(	PCTP	408.6805	0.043713	0.695178	0.915764	protein_coding
ENSG0000(	SDHAF2	388.1465	-0.03272	0.695062	0.915764	protein_coding
ENSG0000(	TAF4B	400.6453	-0.06028	0.695023	0.915764	protein_coding
ENSG0000(	COL11A1	28.59417	-0.16676	0.695399	0.915894	protein_coding
ENSG0000(	ENSG0000(	40.4802	0.083118	0.695439	0.915894	lncRNA
ENSG0000(	LYRM1	377.8856	-0.03714	0.695443	0.915894	protein_coding
ENSG0000(	MPHOSPH1	464.37	0.036813	0.695339	0.915894	protein_coding
ENSG0000(	PCNT	2226.854	0.040413	0.695401	0.915894	protein_coding
ENSG0000(	ACTA2-AS1	485.8918	-0.05313	0.695875	0.915928	lncRNA
ENSG0000(	AK4	610.8326	0.054908	0.696119	0.915928	protein_coding
ENSG0000(	C4orf46	113.3363	-0.08921	0.695668	0.915928	protein_coding
ENSG0000(	CCBE1	543.698	-0.05268	0.695755	0.915928	protein_coding
ENSG0000(	CCDC110	45.792	0.13417	0.695597	0.915928	protein_coding
ENSG0000(	CHRM3-AS1	619.0427	0.110381	0.695767	0.915928	lncRNA
ENSG0000(	DUS3L	272.2407	-0.05573	0.69605	0.915928	protein_coding
ENSG0000(	ENSG0000(	53.10964	0.080874	0.69583	0.915928	lncRNA
ENSG0000(	ENSG0000(	19.77644	-0.10076	0.695913	0.915928	lncRNA
ENSG0000(	ENSG0000(	142.1367	0.044404	0.695528	0.915928	TEC
ENSG0000(	GOLGA4	5722.448	-0.0323	0.696156	0.915928	protein_coding
ENSG0000(	GOLGA6C	36.70153	0.09458	0.696058	0.915928	protein_coding
ENSG0000(	HIC1	622.3495	0.057078	0.696043	0.915928	protein_coding
ENSG0000(	NEIL1	979.9766	0.038978	0.696017	0.915928	protein_coding
ENSG0000(	PASK	1026.324	0.045739	0.696147	0.915928	protein_coding
ENSG0000(	RN7SL5P	47.27121	0.120096	0.696046	0.915928	misc_RNA
ENSG0000(	SMDT1	288.5427	0.056544	0.696168	0.915928	protein_coding
ENSG0000(	SMIM10L2I	34.17169	-0.10934	0.695874	0.915928	lncRNA
ENSG0000(	SPEN	5050.973	-0.02606	0.695865	0.915928	protein_coding
ENSG0000(	VPS50	819.6067	0.03067	0.695902	0.915928	protein_coding
ENSG0000(	ZNF142	1416.518	0.033432	0.695822	0.915928	protein_coding
ENSG0000(	DNAJC25	193.3703	-0.06434	0.696251	0.91595	protein_coding
ENSG0000(	ENSG0000(	330.4172	0.042572	0.696224	0.91595	lncRNA
ENSG0000(	LINC00630	11073.15	-0.0262	0.696344	0.915984	lncRNA

ENSG0000(	PAQR3	636.4144	-0.04357	0.696312	0.915984	protein_coding
ENSG0000(	MTCH1	1532.233	-0.02935	0.696456	0.916088	protein_coding
ENSG0000(	AVL9	4503.467	-0.02144	0.696505	0.916108	protein_coding
ENSG0000(	ENSG0000(	33.09779	0.068814	0.696556	0.916132	processed_pseudogene
ENSG0000(	SMIM12	1791.06	0.022792	0.696633	0.91619	protein_coding
ENSG0000(	RNF14P1	60.91849	0.06446	0.696737	0.916197	processed_pseudogene
ENSG0000(	TBCAP3	34.53263	-0.09062	0.696772	0.916197	transcribed_processed_pseudoge
ENSG0000(	TMEM219	684.6773	0.039525	0.696772	0.916197	protein_coding
ENSG0000(	UBL4A	233.0939	-0.05896	0.696742	0.916197	protein_coding
ENSG0000(	LINC02837	173.6481	0.045487	0.696817	0.916212	lncRNA
ENSG0000(	CDAN1	556.5432	0.056433	0.696945	0.916293	protein_coding
ENSG0000(	NR4A1	1385.889	0.132172	0.696914	0.916293	protein_coding
ENSG0000(	DISP1	749.4885	0.029392	0.696984	0.916301	protein_coding
ENSG0000(	PKD2L2-DT	3174.372	0.022994	0.69702	0.916304	lncRNA
ENSG0000(	COX6CP1	55.09144	0.068815	0.697237	0.91637	processed_pseudogene
ENSG0000(	DIS3	2021.097	-0.02278	0.697153	0.91637	protein_coding
ENSG0000(	GUCA1A	163.2972	0.038606	0.697201	0.91637	protein_coding
ENSG0000(	NUP54	457.6713	0.039854	0.697151	0.91637	protein_coding
ENSG0000(	XRCC3	458.3216	0.039775	0.697209	0.91637	protein_coding
ENSG0000(	USP46-DT	95.83131	-0.05236	0.697293	0.9164	lncRNA
ENSG0000(	ENSG0000(	113.207	0.076016	0.697379	0.916427	lncRNA
ENSG0000(	ZNF594-DT	677.1125	-0.02195	0.69738	0.916427	lncRNA
ENSG0000(	ACTG1P25	100.716	-0.05284	0.697533	0.916569	transcribed_processed_pseudoge
ENSG0000(	ENSG0000(	23.96828	0.107235	0.697554	0.916569	lncRNA
ENSG0000(	ANAPC7	1199.368	0.024635	0.697796	0.916636	protein_coding
ENSG0000(	ASMTL	216.7249	-0.0464	0.697674	0.916636	protein_coding
ENSG0000(	ENSG0000(	532.8218	-0.08041	0.697777	0.916636	lncRNA
ENSG0000(	ENSG0000(	30.06426	-0.10886	0.697647	0.916636	lncRNA
ENSG0000(	ENSG0000(	219.2127	-0.04654	0.697782	0.916636	transcribed_unprocessed_pseudo
ENSG0000(	ENSG0000(	92.27991	0.061987	0.697806	0.916636	lncRNA
ENSG0000(	COL8A2	85.7305	-0.0836	0.697972	0.916697	protein_coding
ENSG0000(	DBH-AS1	78.56195	-0.13322	0.697985	0.916697	lncRNA
ENSG0000(	LIPT1	255.7255	0.031581	0.697918	0.916697	protein_coding
ENSG0000(	PROSER2	117.3703	0.091841	0.697984	0.916697	protein_coding
ENSG0000(	CIBAR1-DT	47.05659	0.133052	0.698086	0.916698	lncRNA
ENSG0000(	ENSG0000(	20.51863	0.10076	0.698037	0.916698	processed_pseudogene
ENSG0000(	TBX3	773.3379	-0.0679	0.698085	0.916698	protein_coding
ENSG0000(	CCDC90B-A	339.3828	-0.03666	0.698253	0.916701	lncRNA
ENSG0000(	CFAP418	209.9781	-0.0475	0.69819	0.916701	protein_coding
ENSG0000(	ENSG0000(	87.93808	0.052238	0.698284	0.916701	lncRNA
ENSG0000(	LINC01135	101.4752	-0.06393	0.698143	0.916701	lncRNA
ENSG0000(	MUTYH	840.3948	0.028146	0.698288	0.916701	protein_coding
ENSG0000(	ZC3H3	802.322	0.068866	0.698165	0.916701	protein_coding
ENSG0000(	LINC01355	1459.912	-0.03392	0.698477	0.916872	lncRNA
ENSG0000(	VPS18	338.0381	-0.03602	0.698485	0.916872	protein_coding
ENSG0000(	CACNA1F	130.3538	0.054354	0.698678	0.91695	protein_coding
ENSG0000(	DHX34	508.7228	0.044725	0.698678	0.91695	protein_coding

ENSG0000(	MT1X	208.183	0.113875	0.698629	0.91695	protein_coding
ENSG0000(	SRD5A3-AS	566.6883	0.023646	0.698657	0.91695	lncRNA
ENSG0000(	NPHP3-AS1	30.43869	0.10286	0.698726	0.91697	lncRNA
ENSG0000(	ENSG0000(	37.37302	-0.08664	0.698908	0.917121	lncRNA
ENSG0000(	ENSG0000(	116.5911	-0.05003	0.698886	0.917121	transcribed_unprocessed_pseudo
ENSG0000(	LAMA3	2868.683	-0.09658	0.698941	0.917121	protein_coding
ENSG0000(	PCDHGA9	176.6898	0.058281	0.699019	0.917139	protein_coding
ENSG0000(	SMYD2	673.7427	0.044409	0.699022	0.917139	protein_coding
ENSG0000(	RAB33B	455.7939	-0.03266	0.699063	0.91715	protein_coding
ENSG0000(	CYP2D7	330.3318	-0.06288	0.699207	0.917163	polymorphic_pseudogene
ENSG0000(	ENSG0000(	74.75914	-0.0527	0.699202	0.917163	lncRNA
ENSG0000(	IPO9	2697.908	-0.03778	0.699134	0.917163	protein_coding
ENSG0000(	TNFRSF14-/	2064.056	0.061941	0.699158	0.917163	lncRNA
ENSG0000(	RNU4-86P	47.59574	0.075365	0.699241	0.917164	snRNA
ENSG0000(	BTG1	2881.508	-0.04905	0.699312	0.91717	protein_coding
ENSG0000(	ENSG0000(	43.39785	0.081599	0.699304	0.91717	lncRNA
ENSG0000(	PARP15	3997.602	-0.06037	0.699351	0.917177	protein_coding
ENSG0000(	ENSG0000(	85.81089	-0.07501	0.699412	0.917213	processed_pseudogene
ENSG0000(	ENSG0000(	290.4996	0.101154	0.69946	0.917225	processed_pseudogene
ENSG0000(	XPC	729.9117	-0.03465	0.699487	0.917225	protein_coding
ENSG0000(	RALBP1	1579.773	0.028221	0.699661	0.917409	protein_coding
ENSG0000(	ANKIB1	1587.901	0.029499	0.69978	0.917521	protein_coding
ENSG0000(	MYO15A	391.7445	0.032055	0.699824	0.917536	protein_coding
ENSG0000(	ENSG0000(	430.6385	-0.03742	0.699919	0.917616	lncRNA
ENSG0000(	MGA	4442.505	0.02144	0.699964	0.917631	protein_coding
ENSG0000(	ZDHHC4	441.8642	-0.03204	0.700185	0.917837	protein_coding
ENSG0000(	ZNF664	3227.684	0.039727	0.700188	0.917837	protein_coding
ENSG0000(	BTBD8	342.4206	0.05354	0.700296	0.917856	protein_coding
ENSG0000(	MOG	89.38215	0.054199	0.700302	0.917856	protein_coding
ENSG0000(	SDCCAG8	1128.871	0.03314	0.700242	0.917856	protein_coding
ENSG0000(	ENSG0000(	87.40301	-0.0601	0.700404	0.917946	lncRNA
ENSG0000(	ENSG0000(	109.4095	0.054316	0.700488	0.918012	lncRNA
ENSG0000(	FIGNL1	418.7807	0.048209	0.70061	0.918041	protein_coding
ENSG0000(	LINC02611	444.3284	0.033568	0.700599	0.918041	lncRNA
ENSG0000(	USP3-AS1	176.4015	0.062573	0.700565	0.918041	lncRNA
ENSG0000(	LINC02712	23.55548	0.086962	0.70074	0.918168	lncRNA
ENSG0000(	ADRM1	263.2843	-0.05382	0.701168	0.918196	protein_coding
ENSG0000(	AVEN	297.6342	0.052597	0.701166	0.918196	protein_coding
ENSG0000(	CDPF1	73.26493	0.072733	0.700961	0.918196	protein_coding
ENSG0000(	DDX25	384.4943	0.031038	0.700938	0.918196	protein_coding
ENSG0000(	ENSG0000(	25.79249	-0.11322	0.701181	0.918196	lncRNA
ENSG0000(	ENSG0000(	269.0381	-0.04413	0.701196	0.918196	lncRNA
ENSG0000(	MYH9	15962.48	-0.02331	0.700953	0.918196	protein_coding
ENSG0000(	NSF	1281.608	0.053725	0.701052	0.918196	protein_coding
ENSG0000(	OGG1	1417.812	0.034632	0.700997	0.918196	protein_coding
ENSG0000(	OR11A1	560.4705	-0.04718	0.701168	0.918196	protein_coding
ENSG0000(	PCGF2	394.2171	-0.05856	0.700973	0.918196	protein_coding

ENSG0000(	RAB11FIP2	713.2446	0.038819	0.701166	0.918196	protein_coding
ENSG0000(	TRMT1L	389.5631	-0.04423	0.700886	0.918196	protein_coding
ENSG0000(	BCL2L13	1138.15	0.028587	0.701381	0.918263	protein_coding
ENSG0000(	ENSG0000(	71.71785	-0.07374	0.701414	0.918263	lncRNA
ENSG0000(	ENSG0000(	251.0416	0.036278	0.701394	0.918263	lncRNA
ENSG0000(	TIMD4	39.92552	-0.1345	0.701347	0.918263	protein_coding
ENSG0000(	VN1R1	63.68996	-0.06313	0.701405	0.918263	protein_coding
ENSG0000(	AKAP1-DT	299.3085	0.034169	0.701549	0.918353	lncRNA
ENSG0000(	ENSG0000(	122.3274	0.056728	0.701519	0.918353	lncRNA
ENSG0000(	FAM230H	52.21237	-0.18044	0.701673	0.918472	lncRNA
ENSG0000(	ACVRL1	1756.386	0.062218	0.70185	0.918476	protein_coding
ENSG0000(	ENSG0000(	105.2774	0.188801	0.701756	0.918476	TEC
ENSG0000(	ENSG0000(	117.2949	0.035252	0.70191	0.918476	lncRNA
ENSG0000(	LDB3	431.485	0.056938	0.701817	0.918476	protein_coding
ENSG0000(	PAIP2B	436.3177	0.045297	0.701834	0.918476	protein_coding
ENSG0000(	PDE6B	191.2851	0.084703	0.701905	0.918476	protein_coding
ENSG0000(	TM2D2	353.639	-0.04706	0.701772	0.918476	protein_coding
ENSG0000(	ENSG0000(	243.6037	0.040637	0.702047	0.918611	lncRNA
ENSG0000(	DIAPH3	376.3754	0.043216	0.702154	0.918682	protein_coding
ENSG0000(	H1-10-AS1	287.7004	-0.0499	0.702168	0.918682	lncRNA
ENSG0000(	ENSG0000(	21.33402	0.10542	0.702295	0.918793	lncRNA
ENSG0000(	TMEM144	1000.049	-0.0509	0.70232	0.918793	protein_coding
ENSG0000(	ENSG0000(	163.0019	-0.03938	0.702413	0.918872	lncRNA
ENSG0000(	FAM163A	67.8092	-0.0724	0.702578	0.918957	protein_coding
ENSG0000(	PRELID3B	974.5885	0.064348	0.702549	0.918957	protein_coding
ENSG0000(	SFRP1	66.04922	-0.13517	0.702531	0.918957	protein_coding
ENSG0000(	DMKN	242.1034	-0.06103	0.702661	0.918966	protein_coding
ENSG0000(	DNMBP-AS	325.5099	0.023609	0.702719	0.918966	lncRNA
ENSG0000(	ENSG0000(	247.3633	-0.03185	0.702644	0.918966	lncRNA
ENSG0000(	RNU6-272F	23.04697	0.124187	0.702709	0.918966	snRNA
ENSG0000(	ENSG0000(	239.3016	0.035076	0.702913	0.919001	lncRNA
ENSG0000(	ENSG0000(	25.46938	0.079752	0.702824	0.919001	lncRNA
ENSG0000(	NFIB	3136.816	0.033157	0.702904	0.919001	protein_coding
ENSG0000(	SPATA2P1	78.2498	-0.09206	0.702844	0.919001	processed_pseudogene
ENSG0000(	VTI1B	1297.65	0.038694	0.702886	0.919001	protein_coding
ENSG0000(	CDC37L1	256.0665	-0.04733	0.702953	0.91901	protein_coding
ENSG0000(	E2F2	693.6	0.058077	0.703051	0.919051	protein_coding
ENSG0000(	LINC00506	16955.7	-0.02748	0.703023	0.919051	lncRNA
ENSG0000(	ENSG0000(	70.89191	-0.05496	0.703236	0.919248	lncRNA
ENSG0000(	ENSG0000(	35.88522	-0.0858	0.703382	0.91931	lncRNA
ENSG0000(	ENSG0000(	91.78489	-0.04471	0.703383	0.91931	lncRNA
ENSG0000(	ENSG0000(	127.8042	0.056905	0.703336	0.91931	processed_pseudogene
ENSG0000(	ANXA7	635.1738	0.036664	0.703648	0.919569	protein_coding
ENSG0000(	OSBPL5	839.4125	0.040199	0.703633	0.919569	protein_coding
ENSG0000(	ENSG0000(	721.4725	0.04689	0.703762	0.919674	lncRNA
ENSG0000(	ENSG0000(	681.4139	-0.03184	0.703882	0.919761	TEC
ENSG0000(	NEK8	524.0518	0.029293	0.70396	0.919761	protein_coding

ENSG0000(	NPIPB4	6757.853	0.028639	0.703933	0.919761	protein_coding
ENSG0000(	UBQLN3	22.25815	0.11902	0.703962	0.919761	protein_coding
ENSG0000(	FBP1	161.2786	0.078113	0.704069	0.919814	protein_coding
ENSG0000(	TRIP11	2857.549	0.028576	0.704043	0.919814	protein_coding
ENSG0000(	ENSG0000(	162.7468	0.044231	0.704212	0.91993	lncRNA
ENSG0000(	TEKT4P2	815.3123	-0.11747	0.704226	0.91993	transcribed_unprocessed_pseudo
ENSG0000(	BICD1-AS1	34.04021	-0.08415	0.704315	0.91997	lncRNA
ENSG0000(	PSORS1C3	108.9807	0.124723	0.704323	0.91997	lncRNA
ENSG0000(	ENSG0000(	245.857	0.046403	0.704731	0.920137	lncRNA
ENSG0000(	ENSG0000(	297.1039	0.02837	0.704568	0.920137	lncRNA
ENSG0000(	ENSG0000(	64.48454	1.314091	0.704658	0.920137	protein_coding
ENSG0000(	ENSG0000(	173.6667	0.077319	0.704785	0.920137	TEC
ENSG0000(	ENSG0000(	46.01923	-0.06143	0.704552	0.920137	lncRNA
ENSG0000(	ENSG0000(	67.64801	-0.20013	0.704502	0.920137	protein_coding
ENSG0000(	LINC01752	127.9785	0.058919	0.704703	0.920137	lncRNA
ENSG0000(	MRPL23	203.3618	0.05966	0.704765	0.920137	protein_coding
ENSG0000(	MYH3	395.5514	0.035597	0.704709	0.920137	protein_coding
ENSG0000(	ZNF391	135.6977	-0.10355	0.704687	0.920137	protein_coding
ENSG0000(	ENSG0000(	75.3087	-0.05575	0.704891	0.920232	lncRNA
ENSG0000(	ARHGEF18	2106.503	0.049126	0.705216	0.920292	protein_coding
ENSG0000(	CDH19	194.6388	0.05881	0.704977	0.920292	protein_coding
ENSG0000(	ENSG0000(	38.94077	0.080502	0.705131	0.920292	lncRNA
ENSG0000(	ENSG0000(	843.2607	0.019777	0.70536	0.920292	lncRNA
ENSG0000(	ENSG0000(	22.49555	-0.61769	0.705223	0.920292	protein_coding
ENSG0000(	GPRASP1	901.6149	-0.04687	0.705368	0.920292	protein_coding
ENSG0000(	LINC00342	7330.204	0.04259	0.705197	0.920292	lncRNA
ENSG0000(	MAGOHB	505.3526	0.043393	0.705323	0.920292	protein_coding
ENSG0000(	MTFP1	78.70362	-0.07084	0.705149	0.920292	protein_coding
ENSG0000(	NTRK2	750.9086	0.070004	0.705051	0.920292	protein_coding
ENSG0000(	RASL10B	96.56977	0.065976	0.705402	0.920292	protein_coding
ENSG0000(	SYT15	531.8856	-0.04256	0.705365	0.920292	protein_coding
ENSG0000(	WDR3	938.3393	0.028678	0.705203	0.920292	protein_coding
ENSG0000(	ZNF626	504.7993	0.05232	0.705406	0.920292	protein_coding
ENSG0000(	ATP2A2	6669.741	-0.02266	0.705541	0.920337	protein_coding
ENSG0000(	ENSG0000(	63.05562	-0.05383	0.705528	0.920337	transcribed_processed_pseudoge
ENSG0000(	UBXN2A	1299.395	0.026462	0.7055	0.920337	protein_coding
ENSG0000(	CASC20	30.2338	0.084326	0.705672	0.920353	lncRNA
ENSG0000(	CH25H	79.04871	-0.10974	0.705715	0.920353	protein_coding
ENSG0000(	ENSG0000(	114.0639	0.048205	0.705616	0.920353	lncRNA
ENSG0000(	ENSG0000(	42.46024	0.078936	0.7057	0.920353	lncRNA
ENSG0000(	ENSG0000(	352.8837	0.026415	0.705734	0.920353	lncRNA
ENSG0000(	PHYHD1	209.523	-0.05987	0.705754	0.920353	protein_coding
ENSG0000(	PCDH10	27.46098	-0.09767	0.705793	0.920361	protein_coding
ENSG0000(	ENSG0000(	73.32679	0.059665	0.705924	0.920422	lncRNA
ENSG0000(	PIGV	746.4907	0.025428	0.706007	0.920422	protein_coding
ENSG0000(	RN7SL326F	32.65753	0.064204	0.706002	0.920422	misc_RNA
ENSG0000(	TAF15	5375.144	-0.02282	0.706001	0.920422	protein_coding

ENSG0000(	ZBTB49	189.0162	-0.07719	0.705901	0.920422	protein_coding
ENSG0000(	ENSG0000(	130.2753	0.058233	0.706149	0.92052	unprocessed_pseudogene
ENSG0000(	FGF14	78.09127	-0.13373	0.706129	0.92052	protein_coding
ENSG0000(	COX15	1094.336	0.034791	0.706394	0.920585	protein_coding
ENSG0000(	ENSG0000(	164.6969	0.042875	0.706255	0.920585	lncRNA
ENSG0000(	ENSG0000(	170.9982	-0.07789	0.706318	0.920585	lncRNA
ENSG0000(	LIX1	92.72448	0.043487	0.706336	0.920585	protein_coding
ENSG0000(	MED19	76.73987	-0.06338	0.706297	0.920585	protein_coding
ENSG0000(	PCDHAC2	37.6193	-0.18983	0.7064	0.920585	protein_coding
ENSG0000(	ENSG0000(	295.5953	-0.03802	0.706469	0.920631	lncRNA
ENSG0000(	ENSG0000(	51.16417	-0.07393	0.706623	0.920788	lncRNA
ENSG0000(	ENSG0000(	84.53669	-0.05267	0.70669	0.920832	lncRNA
ENSG0000(	EEF1A1P9	25.09844	0.098885	0.70708	0.921072	processed_pseudogene
ENSG0000(	ENSG0000(	83.9075	0.059467	0.707018	0.921072	processed_pseudogene
ENSG0000(	FBXO17	254.3764	0.050198	0.707108	0.921072	protein_coding
ENSG0000(	LINC01115	138.1888	0.038833	0.706978	0.921072	lncRNA
ENSG0000(	PRDM5	536.6833	0.027712	0.706944	0.921072	protein_coding
ENSG0000(	SACM1L	1434.596	-0.01964	0.707093	0.921072	protein_coding
ENSG0000(	SNORD22	526.0121	-0.05151	0.707065	0.921072	snoRNA
ENSG0000(	C11orf45	35.25089	-0.07379	0.707722	0.921186	protein_coding
ENSG0000(	COG4	1189.048	0.031636	0.707537	0.921186	protein_coding
ENSG0000(	CYB5D2	231.2701	0.034298	0.707696	0.921186	protein_coding
ENSG0000(	ENSG0000(	38.49469	-0.08537	0.707412	0.921186	lncRNA
ENSG0000(	ENSG0000(	234.8397	-0.0332	0.707408	0.921186	lncRNA
ENSG0000(	ENSG0000(	798.2501	0.047544	0.707913	0.921186	protein_coding
ENSG0000(	ENSG0000(	20.27904	-0.11693	0.707659	0.921186	transcribed_processed_pseudoge
ENSG0000(	ENSG0000(	46.47862	-0.08395	0.707755	0.921186	TEC
ENSG0000(	ENSG0000(	1066.004	0.026448	0.707888	0.921186	lncRNA
ENSG0000(	ENSG0000(	78.96053	-0.05423	0.707871	0.921186	lncRNA
ENSG0000(	FAM138D	50.19725	0.076477	0.707878	0.921186	lncRNA
ENSG0000(	FBXO32	1481.797	0.071845	0.707984	0.921186	protein_coding
ENSG0000(	HDAC1	2234.528	0.037437	0.70797	0.921186	protein_coding
ENSG0000(	KCNG4	342.9995	0.035167	0.707576	0.921186	protein_coding
ENSG0000(	LINC00410	79.02864	0.054617	0.707641	0.921186	lncRNA
ENSG0000(	LINC02582	93.53168	0.099495	0.707854	0.921186	lncRNA
ENSG0000(	LNPK	1326.858	0.046171	0.707258	0.921186	protein_coding
ENSG0000(	MSTO2P	38.16469	-0.09749	0.707914	0.921186	unprocessed_pseudogene
ENSG0000(	PLXNA3	2481.681	-0.06989	0.707999	0.921186	protein_coding
ENSG0000(	RCAN2	317.6679	-0.06347	0.707613	0.921186	protein_coding
ENSG0000(	RN7SL121F	431.188	0.040612	0.707481	0.921186	misc_RNA
ENSG0000(	RN7SL301F	78.54381	0.102202	0.707901	0.921186	misc_RNA
ENSG0000(	RPL36	984.4291	0.048461	0.707755	0.921186	protein_coding
ENSG0000(	TIGAR	1106.106	0.052585	0.707651	0.921186	protein_coding
ENSG0000(	CA3-AS1	71.86836	0.122635	0.708375	0.921196	lncRNA
ENSG0000(	ENSG0000(	41.45361	0.08015	0.708677	0.921196	lncRNA
ENSG0000(	ENSG0000(	210.1001	-0.1261	0.708184	0.921196	lncRNA
ENSG0000(	ENSG0000(	304.0478	0.034088	0.708078	0.921196	lncRNA

ENSG0000(	ENSG0000(	1331.826	-0.02258	0.708308	0.921196	lncRNA
ENSG0000(	ENSG0000(	143.733	0.041922	0.708542	0.921196	processed_pseudogene
ENSG0000(	ENSG0000(	38.21597	-0.0745	0.708278	0.921196	lncRNA
ENSG0000(	ENSG0000(	145.3704	0.048144	0.708408	0.921196	lncRNA
ENSG0000(	ENSG0000(	6795.129	0.019303	0.708536	0.921196	lncRNA
ENSG0000(	ENSG0000(	1558.341	0.034094	0.708328	0.921196	lncRNA
ENSG0000(	ENSG0000(	185.0174	0.044017	0.708351	0.921196	lncRNA
ENSG0000(	EPGN	112.2381	-0.0464	0.708426	0.921196	protein_coding
ENSG0000(	LINC01720	135.9401	0.062124	0.708164	0.921196	lncRNA
ENSG0000(	LRRC70	73.71346	-0.08206	0.708591	0.921196	protein_coding
ENSG0000(	MT1H	51.76142	-0.17365	0.708567	0.921196	protein_coding
ENSG0000(	MYCBP2	6868.618	-0.05811	0.708115	0.921196	protein_coding
ENSG0000(	PIP4K2B	1104.222	0.03054	0.708659	0.921196	protein_coding
ENSG0000(	SOWAHD	22.29727	-0.10853	0.708109	0.921196	protein_coding
ENSG0000(	SYS1	964.5237	-0.03498	0.708658	0.921196	protein_coding
ENSG0000(	ZCCHC14-D	28.27803	0.102294	0.708541	0.921196	lncRNA
ENSG0000(	APH1A	1032.859	0.039833	0.709204	0.921273	protein_coding
ENSG0000(	CRLF3	1019.811	-0.02481	0.709272	0.921273	protein_coding
ENSG0000(	DNAH12	105.365	0.06847	0.708967	0.921273	protein_coding
ENSG0000(	ENSG0000(	100.2932	-0.04311	0.708776	0.921273	processed_pseudogene
ENSG0000(	ENSG0000(	207.555	0.03981	0.7091	0.921273	lncRNA
ENSG0000(	ENSG0000(	337.0631	-0.05005	0.708965	0.921273	lncRNA
ENSG0000(	ENSG0000(	177.5532	0.02962	0.709247	0.921273	lncRNA
ENSG0000(	ENSG0000(	350.0606	0.030597	0.709241	0.921273	lncRNA
ENSG0000(	ENSG0000(	93.50101	0.061713	0.709082	0.921273	pseudogene
ENSG0000(	ENSG0000(	90.85984	0.054266	0.709022	0.921273	lncRNA
ENSG0000(	GOLGA8T	39.85142	-0.73301	0.708954	0.921273	protein_coding
ENSG0000(	HLTF-AS1	36.29313	-0.09903	0.709202	0.921273	lncRNA
ENSG0000(	LAGE3P1	44.68156	0.096385	0.709217	0.921273	processed_pseudogene
ENSG0000(	OAT	417.1543	-0.04725	0.70909	0.921273	protein_coding
ENSG0000(	RNF121	557.8158	0.027375	0.709095	0.921273	protein_coding
ENSG0000(	TMBIM4	1338.905	0.025987	0.708899	0.921273	protein_coding
ENSG0000(	ENSG0000(	86.2388	-0.06125	0.709319	0.92129	lncRNA
ENSG0000(	ZFP82	944.0593	-0.03571	0.709397	0.921349	protein_coding
ENSG0000(	ENSG0000(	449.268	-0.07708	0.709439	0.92136	protein_coding
ENSG0000(	DUX4L25	19.92971	0.765276	0.709686	0.921419	unprocessed_pseudogene
ENSG0000(	ENSG0000(	76.56254	0.084087	0.709673	0.921419	lncRNA
ENSG0000(	ENSG0000(	319.2282	0.027823	0.709521	0.921419	lncRNA
ENSG0000(	ENSG0000(	47.14768	0.073081	0.709595	0.921419	lncRNA
ENSG0000(	KRT20	6036.574	0.099332	0.709634	0.921419	protein_coding
ENSG0000(	ZFYVE28	999.0096	0.055036	0.709614	0.921419	protein_coding
ENSG0000(	ENSG0000(	220.6242	0.034663	0.710029	0.921741	lncRNA
ENSG0000(	RNU6-37P	66.12152	-0.97466	0.710034	0.921741	snRNA
ENSG0000(	ZNF566	743.6838	-0.02752	0.710014	0.921741	protein_coding
ENSG0000(	FLVCR1-DT	444.4933	0.031285	0.710109	0.921795	lncRNA
ENSG0000(	EEF1D	2623.587	-0.02516	0.710294	0.921992	protein_coding
ENSG0000(	HERC2	4744.142	0.022037	0.710358	0.922031	protein_coding

ENSG0000(	CDCA7L	584.3335	0.055549	0.710483	0.922087	protein_coding
ENSG0000(	CICP14	29.71097	-0.20399	0.710578	0.922087	processed_pseudogene
ENSG0000(	COX6A1	732.4651	0.072584	0.710523	0.922087	protein_coding
ENSG0000(	FREM2	156.9924	-0.08061	0.710552	0.922087	protein_coding
ENSG0000(	LINC02108	474.1918	0.028013	0.710602	0.922087	lncRNA
ENSG0000(	SLC6A4	246.7995	0.042002	0.710598	0.922087	protein_coding
ENSG0000(	CISD2	488.5835	0.034883	0.710636	0.922087	protein_coding
ENSG0000(	DCLRE1A	265.3911	0.068031	0.710792	0.922203	protein_coding
ENSG0000(	MYBPC2	26.19539	-0.1988	0.710782	0.922203	protein_coding
ENSG0000(	ACTR8	497.1999	-0.04236	0.715849	0.922703	protein_coding
ENSG0000(	ANKRD13B	66.80377	-0.08803	0.712816	0.922703	protein_coding
ENSG0000(	AP1B1	1566.631	-0.04119	0.713295	0.922703	protein_coding
ENSG0000(	ARID4A	1180.839	0.03151	0.714362	0.922703	protein_coding
ENSG0000(	B3GAT1	161.4723	-0.06884	0.715112	0.922703	protein_coding
ENSG0000(	BDH2	532.0071	-0.0343	0.716023	0.922703	protein_coding
ENSG0000(	BMP4	425.9035	-0.07626	0.711367	0.922703	protein_coding
ENSG0000(	C16orf54	342.7646	0.062081	0.712074	0.922703	protein_coding
ENSG0000(	C1QTNF9	48.92467	0.081334	0.713813	0.922703	protein_coding
ENSG0000(	C20orf96	110.7683	0.061853	0.714051	0.922703	protein_coding
ENSG0000(	CAPN7	1661.96	0.022462	0.713991	0.922703	protein_coding
ENSG0000(	CASC6	139.8342	0.034694	0.712409	0.922703	lncRNA
ENSG0000(	CCL15-CCL1	32.08552	-0.19788	0.71556	0.922703	protein_coding
ENSG0000(	CCNB3	20.83011	-0.16222	0.711504	0.922703	protein_coding
ENSG0000(	CEBPZ	677.0344	-0.04426	0.715843	0.922703	protein_coding
ENSG0000(	CRAT37	395.5839	0.041894	0.714425	0.922703	lncRNA
ENSG0000(	CRYBB3	61.89658	0.12634	0.715273	0.922703	protein_coding
ENSG0000(	CSF2RA	1034.239	-0.04824	0.715415	0.922703	protein_coding
ENSG0000(	CTAGE8	332.7359	0.27012	0.712774	0.922703	protein_coding
ENSG0000(	CTNNA1	4438.665	0.034719	0.714213	0.922703	protein_coding
ENSG0000(	DCTN6-DT	25.04489	0.076905	0.713202	0.922703	lncRNA
ENSG0000(	DDX59-AS1	139.7571	-0.05383	0.711254	0.922703	lncRNA
ENSG0000(	ENSG0000(	44.06073	-0.06279	0.712968	0.922703	processed_pseudogene
ENSG0000(	ENSG0000(	31.25647	0.069606	0.71439	0.922703	lncRNA
ENSG0000(	ENSG0000(	87.2191	-0.08741	0.711775	0.922703	processed_pseudogene
ENSG0000(	ENSG0000(	46.55189	0.09838	0.713259	0.922703	lncRNA
ENSG0000(	ENSG0000(	95.63114	0.050709	0.714324	0.922703	lncRNA
ENSG0000(	ENSG0000(	33.49995	0.078525	0.715029	0.922703	lncRNA
ENSG0000(	ENSG0000(	56.74557	0.063874	0.711544	0.922703	lncRNA
ENSG0000(	ENSG0000(	202.8134	0.033611	0.714785	0.922703	lncRNA
ENSG0000(	ENSG0000(	254.1938	-0.0366	0.712161	0.922703	lncRNA
ENSG0000(	ENSG0000(	136.4647	-0.0585	0.711854	0.922703	lncRNA
ENSG0000(	ENSG0000(	1269.75	0.061285	0.713534	0.922703	lncRNA
ENSG0000(	ENSG0000(	33.60583	-0.07404	0.71578	0.922703	protein_coding
ENSG0000(	ENSG0000(	67.72987	0.0608	0.714128	0.922703	lncRNA
ENSG0000(	ENSG0000(	150.057	0.041119	0.715154	0.922703	lncRNA
ENSG0000(	ENSG0000(	278.7531	-0.06391	0.713556	0.922703	lncRNA
ENSG0000(	ENSG0000(	40.52712	-0.05884	0.712894	0.922703	lncRNA

ENSG0000(	ENSG0000(	30.44332	0.085087	0.711434	0.922703	lncRNA
ENSG0000(	ENSG0000(	202.3517	0.03662	0.715462	0.922703	lncRNA
ENSG0000(	ENSG0000(	100.6177	0.04225	0.713951	0.922703	protein_coding
ENSG0000(	ENSG0000(	48.83248	-0.07789	0.714483	0.922703	lncRNA
ENSG0000(	ENSG0000(	88.32426	-0.08364	0.712996	0.922703	lncRNA
ENSG0000(	ENSG0000(	39.39897	-0.20651	0.715019	0.922703	lncRNA
ENSG0000(	ENSG0000(	267.083	-0.04019	0.713671	0.922703	lncRNA
ENSG0000(	ENSG0000(	720.0449	0.112287	0.714233	0.922703	lncRNA
ENSG0000(	ENSG0000(	25.31337	-0.09567	0.711816	0.922703	TEC
ENSG0000(	ENSG0000(	96.753	-0.04685	0.713958	0.922703	TEC
ENSG0000(	ENSG0000(	423.0512	-0.02848	0.71523	0.922703	lncRNA
ENSG0000(	ENSG0000(	33.13854	0.091722	0.712295	0.922703	lncRNA
ENSG0000(	ENSG0000(	168.7678	-0.04666	0.712448	0.922703	lncRNA
ENSG0000(	ENSG0000(	43.47972	0.08502	0.711768	0.922703	lncRNA
ENSG0000(	ENSG0000(	1827.207	0.024769	0.714062	0.922703	lncRNA
ENSG0000(	ENSG0000(	131.8049	-0.04264	0.714583	0.922703	lncRNA
ENSG0000(	ENSG0000(	111.7216	0.05004	0.715112	0.922703	lncRNA
ENSG0000(	ENSG0000(	54.83204	-0.1084	0.714846	0.922703	lncRNA
ENSG0000(	ENSG0000(	83.12726	-0.05191	0.715721	0.922703	lncRNA
ENSG0000(	ENSG0000(	24.11132	-0.13149	0.715981	0.922703	lncRNA
ENSG0000(	ENSG0000(	289.9136	0.052395	0.715396	0.922703	lncRNA
ENSG0000(	ENSG0000(	38.52604	-0.065	0.71345	0.922703	lncRNA
ENSG0000(	ENSG0000(	34.85395	-0.0881	0.711748	0.922703	lncRNA
ENSG0000(	ENSG0000(	263.4842	-0.03968	0.715568	0.922703	lncRNA
ENSG0000(	ERLIN1	860.9725	-0.04311	0.715417	0.922703	protein_coding
ENSG0000(	FAM133B	1489.853	0.022455	0.716041	0.922703	protein_coding
ENSG0000(	FAM53B	1827.299	-0.03478	0.71346	0.922703	protein_coding
ENSG0000(	FCRL4	82.87044	0.183389	0.712757	0.922703	protein_coding
ENSG0000(	FGF9	295.2347	0.067039	0.711953	0.922703	protein_coding
ENSG0000(	FNTB	238.4765	-0.04933	0.714295	0.922703	protein_coding
ENSG0000(	GACAT3	191.2856	0.038783	0.71544	0.922703	lncRNA
ENSG0000(	GAPDHP62	28.28024	-0.10999	0.712722	0.922703	processed_pseudogene
ENSG0000(	GDE1	1204.368	0.04982	0.713187	0.922703	protein_coding
ENSG0000(	GPC3	108.292	0.052324	0.713321	0.922703	protein_coding
ENSG0000(	GRASLND	667.2336	0.023108	0.712627	0.922703	lncRNA
ENSG0000(	HRAT17	406.7234	0.046102	0.714385	0.922703	lncRNA
ENSG0000(	IMPACT	426.9931	0.032042	0.715805	0.922703	protein_coding
ENSG0000(	ITGA10	314.5691	0.034709	0.712433	0.922703	protein_coding
ENSG0000(	JAGN1	264.2982	-0.03345	0.715533	0.922703	protein_coding
ENSG0000(	JPT1	677.4366	-0.04874	0.714084	0.922703	protein_coding
ENSG0000(	KLC1	4948.946	-0.02383	0.715802	0.922703	protein_coding
ENSG0000(	KPNA7	20.84595	0.138763	0.715839	0.922703	protein_coding
ENSG0000(	KRT18P59	37.03272	0.087849	0.711665	0.922703	transcribed_processed_pseudoge
ENSG0000(	LINC00499	543.9816	0.030619	0.714086	0.922703	lncRNA
ENSG0000(	LINC00608	427.3907	-0.03747	0.712939	0.922703	lncRNA
ENSG0000(	LINC00960	855.8531	-0.03318	0.715913	0.922703	lncRNA
ENSG0000(	LINC01151	623.5625	0.026214	0.711564	0.922703	lncRNA

ENSG0000(	LINC01727	647.1228	-0.02994	0.713022	0.922703	lncRNA
ENSG0000(	LINC01857	110.4529	0.058319	0.71138	0.922703	lncRNA
ENSG0000(	LINC02204	31.27829	-0.07282	0.713058	0.922703	lncRNA
ENSG0000(	LINC02211	40.39432	0.083964	0.715072	0.922703	lncRNA
ENSG0000(	LINC02451	34.76166	-0.05982	0.713513	0.922703	lncRNA
ENSG0000(	LINC02897	182.53	0.04612	0.713836	0.922703	lncRNA
ENSG0000(	LIPC-AS1	91.44761	0.060335	0.714128	0.922703	lncRNA
ENSG0000(	MAP3K9	980.0238	0.042592	0.714703	0.922703	protein_coding
ENSG0000(	MFSD8	1289.02	0.036865	0.713669	0.922703	protein_coding
ENSG0000(	MINDY2	1706.412	-0.02182	0.715997	0.922703	protein_coding
ENSG0000(	MORN1	684.201	0.032445	0.715367	0.922703	protein_coding
ENSG0000(	MT-TN	133.6381	-0.51805	0.715698	0.922703	Mt_tRNA
ENSG0000(	MTX3	1271.693	0.026073	0.712259	0.922703	protein_coding
ENSG0000(	NAT10	749.7026	0.036902	0.711551	0.922703	protein_coding
ENSG0000(	NCKAP1	3317.464	0.045453	0.715555	0.922703	protein_coding
ENSG0000(	NOB1	207.4139	-0.04976	0.712041	0.922703	protein_coding
ENSG0000(	NOTCH2NL	9126.523	-0.03658	0.712271	0.922703	protein_coding
ENSG0000(	ODAD2	35.88792	-0.09507	0.71203	0.922703	protein_coding
ENSG0000(	OR1AB1P	58.38306	0.080093	0.714472	0.922703	unprocessed_pseudogene
ENSG0000(	PDE12	1856.463	0.01605	0.714535	0.922703	protein_coding
ENSG0000(	PI4KB	1475.027	-0.02725	0.712223	0.922703	protein_coding
ENSG0000(	PIGC	343.0923	0.042114	0.712387	0.922703	protein_coding
ENSG0000(	PMCHL2	75.46776	0.072324	0.715939	0.922703	transcribed_unprocessed_pseudo
ENSG0000(	PRKAA1	2414.374	0.02345	0.712926	0.922703	protein_coding
ENSG0000(	PSG4	73.07309	0.104748	0.715469	0.922703	protein_coding
ENSG0000(	QPCTL	420.8552	0.03129	0.713625	0.922703	protein_coding
ENSG0000(	RBM12	3404.456	-0.03039	0.712744	0.922703	protein_coding
ENSG0000(	RN7SL538P	38.25561	0.080573	0.712828	0.922703	misc_RNA
ENSG0000(	RNU5E-1	358.6265	0.089245	0.712074	0.922703	snRNA
ENSG0000(	RPL8	2206.551	0.048978	0.713704	0.922703	protein_coding
ENSG0000(	RUNDC3A	201.5846	0.038728	0.711536	0.922703	protein_coding
ENSG0000(	SART1	530.4816	-0.04635	0.71384	0.922703	protein_coding
ENSG0000(	SATB1	2102.598	-0.0584	0.713525	0.922703	protein_coding
ENSG0000(	SETBP1	2030.868	-0.04222	0.711623	0.922703	protein_coding
ENSG0000(	SIGLEC1	972.1387	0.079266	0.711826	0.922703	protein_coding
ENSG0000(	SLC9A3R2	384.0429	0.045132	0.714777	0.922703	protein_coding
ENSG0000(	SLC9B1	88.5272	-0.05291	0.714299	0.922703	protein_coding
ENSG0000(	SMARCD2	726.8883	-0.02951	0.712983	0.922703	protein_coding
ENSG0000(	SNORA12	189.6399	-0.05151	0.715403	0.922703	snoRNA
ENSG0000(	SNORA50C	51.1839	-0.08791	0.712092	0.922703	snoRNA
ENSG0000(	SNORA55	90.27603	0.056606	0.714393	0.922703	snoRNA
ENSG0000(	SNRNP70	6138.36	0.023054	0.714577	0.922703	protein_coding
ENSG0000(	SNTG2	52.48375	-0.0977	0.715684	0.922703	protein_coding
ENSG0000(	SPATA2L	103.5531	0.053282	0.713063	0.922703	protein_coding
ENSG0000(	SPRTN	349.5211	-0.04038	0.713854	0.922703	protein_coding
ENSG0000(	SUV39H2	345.4418	-0.04759	0.713113	0.922703	protein_coding
ENSG0000(	TBC1D5	2081.554	-0.02894	0.714995	0.922703	protein_coding

ENSG0000(	TCP1	1274.747	0.031311	0.712397	0.922703	protein_coding
ENSG0000(	TGIF1	1181.185	0.031508	0.713822	0.922703	protein_coding
ENSG0000(	THAP7-AS1	49.71312	-0.06678	0.712307	0.922703	lncRNA
ENSG0000(	TIGD1	5264.954	-0.02083	0.715991	0.922703	protein_coding
ENSG0000(	TMED1	64.26315	0.071857	0.714092	0.922703	protein_coding
ENSG0000(	TMPRSS13	98.43983	-0.07104	0.711521	0.922703	protein_coding
ENSG0000(	TMSB15B	461.3621	-0.03633	0.713428	0.922703	protein_coding
ENSG0000(	TPM3P7	27.98475	-0.09232	0.715039	0.922703	processed_pseudogene
ENSG0000(	TPTEP1	1202.98	-0.02751	0.715144	0.922703	transcribed_unprocessed_pseudo
ENSG0000(	UBXN1	278.0842	0.04683	0.714477	0.922703	protein_coding
ENSG0000(	XIST	21617.97	0.06639	0.713009	0.922703	lncRNA
ENSG0000(	YEATS4	85.90255	-0.06091	0.714777	0.922703	protein_coding
ENSG0000(	ZSCAN5A-A	382.0324	-0.0395	0.714162	0.922703	lncRNA
ENSG0000(	RAB44	61.58744	-0.10943	0.716132	0.922778	protein_coding
ENSG0000(	MTHFD1	1690.41	-0.03028	0.716179	0.922795	protein_coding
ENSG0000(	ENSG0000(	2197.043	-0.02506	0.716253	0.922826	lncRNA
ENSG0000(	TMEM254-	158.6842	-0.04744	0.71627	0.922826	lncRNA
ENSG0000(	ITGB8	1868.695	0.050436	0.7164	0.922906	protein_coding
ENSG0000(	NEK10	273.91	-0.0463	0.716376	0.922906	protein_coding
ENSG0000(	DCP1B	323.87	-0.03332	0.716518	0.922938	protein_coding
ENSG0000(	FXN	950.5006	0.02588	0.716592	0.922938	protein_coding
ENSG0000(	LINC00939	1544.311	-0.01896	0.716461	0.922938	lncRNA
ENSG0000(	MYO18A	4160.777	-0.02995	0.716579	0.922938	protein_coding
ENSG0000(	PSD3	3009.935	0.050997	0.716558	0.922938	protein_coding
ENSG0000(	IQCD	62.54125	-0.07694	0.716795	0.923157	protein_coding
ENSG0000(	COA5	617.9333	0.023634	0.716875	0.923191	protein_coding
ENSG0000(	PTPN3	2477.819	0.030155	0.716889	0.923191	protein_coding
ENSG0000(	NAALAD2	113.8599	0.07559	0.716931	0.923202	protein_coding
ENSG0000(	ENSG0000(	158.0528	-0.03918	0.71701	0.923261	lncRNA
ENSG0000(	LINC01202	187.6105	0.047028	0.717175	0.923391	lncRNA
ENSG0000(	LINC01934	1697.723	-0.05617	0.717219	0.923391	lncRNA
ENSG0000(	SCARNA8	37.49061	0.066047	0.717246	0.923391	scaRNA
ENSG0000(	USF1	1051.115	0.045089	0.717218	0.923391	protein_coding
ENSG0000(	RRAS2	521.1809	0.037538	0.717317	0.923439	protein_coding
ENSG0000(	TMEM254	292.7188	-0.05462	0.717511	0.923646	protein_coding
ENSG0000(	ATP6V0D1	3025.178	0.04901	0.717552	0.923655	protein_coding
ENSG0000(	RN7SKP292	36.75644	-0.06545	0.717597	0.923671	misc_RNA
ENSG0000(	ENSG0000(	32.68249	-0.08565	0.717766	0.923789	lncRNA
ENSG0000(	ENSG0000(	34.07036	0.079465	0.717857	0.923789	lncRNA
ENSG0000(	ENSG0000(	129.1007	0.039785	0.717794	0.923789	lncRNA
ENSG0000(	PCSK2	147.0046	0.057719	0.717837	0.923789	protein_coding
ENSG0000(	ZNF213-AS	1613.167	0.024078	0.717801	0.923789	lncRNA
ENSG0000(	ENSG0000(	43.44106	0.075924	0.718027	0.923859	lncRNA
ENSG0000(	GHR	444.4203	0.03125	0.718054	0.923859	protein_coding
ENSG0000(	IP6K1	792.1759	0.042105	0.7181	0.923859	protein_coding
ENSG0000(	SLC9A4	312.9208	0.042529	0.718113	0.923859	protein_coding
ENSG0000(	UBE2T	44.68108	0.104023	0.717993	0.923859	protein_coding

ENSG0000(	UPRT	323.1637	0.033924	0.718053	0.923859	protein_coding
ENSG0000(	KLHL34	44.09022	0.091948	0.718161	0.923861	protein_coding
ENSG0000(	SLAMF6P1	48.21857	-0.13185	0.718182	0.923861	unprocessed_pseudogene
ENSG0000(	ITIH3	29.47584	-0.19713	0.718221	0.923869	protein_coding
ENSG0000(	LINC02172	135.6134	0.0539	0.718315	0.923946	lncRNA
ENSG0000(	BRD2	4770.836	-0.01884	0.718446	0.923948	protein_coding
ENSG0000(	DDB1	2745.022	-0.01718	0.718483	0.923948	protein_coding
ENSG0000(	ENSG0000(	20.97188	0.098783	0.718464	0.923948	lncRNA
ENSG0000(	ENSG0000(	83.40161	0.054757	0.718381	0.923948	lncRNA
ENSG0000(	TUBGCP2	2332.729	0.014887	0.718484	0.923948	protein_coding
ENSG0000(	ENSG0000(	21.27234	0.293202	0.718561	0.923951	lncRNA
ENSG0000(	ENSG0000(	42.72283	0.064629	0.718535	0.923951	lncRNA
ENSG0000(	SNX18P26	32.92952	-0.10926	0.71862	0.923951	processed_pseudogene
ENSG0000(	ZNF117	1388.899	-0.03442	0.718621	0.923951	protein_coding
ENSG0000(	ENSG0000(	78.76742	-0.04077	0.718696	0.923971	lncRNA
ENSG0000(	STAT6	6535.204	0.021407	0.718704	0.923971	protein_coding
ENSG0000(	A2ML1	283.3644	0.027717	0.719023	0.924019	protein_coding
ENSG0000(	BUD13	252.1098	-0.03717	0.718937	0.924019	protein_coding
ENSG0000(	ENSG0000(	23.00586	-0.09748	0.71883	0.924019	lncRNA
ENSG0000(	ENSG0000(	484.8773	-0.0293	0.719078	0.924019	lncRNA
ENSG0000(	ENSG0000(	52.74657	0.077005	0.718947	0.924019	lncRNA
ENSG0000(	FBXO44	557.8218	-0.04947	0.718851	0.924019	protein_coding
ENSG0000(	LINC00997	199.8685	-0.04887	0.719111	0.924019	lncRNA
ENSG0000(	OR52E4	151.9266	0.04734	0.718929	0.924019	protein_coding
ENSG0000(	TBC1D3L	1298.914	0.055239	0.719059	0.924019	protein_coding
ENSG0000(	WASH2P	2102.869	0.034141	0.719105	0.924019	transcribed_unprocessed_pseudo
ENSG0000(	ZC3H18	1094.508	0.029111	0.719011	0.924019	protein_coding
ENSG0000(	ENSG0000(	153.6285	0.048923	0.71918	0.924045	lncRNA
ENSG0000(	IPPK	372.1805	-0.03414	0.719198	0.924045	protein_coding
ENSG0000(	USP34-DT	340.0389	0.031717	0.719232	0.924046	lncRNA
ENSG0000(	RAB5B	3000.919	0.024523	0.719301	0.924048	protein_coding
ENSG0000(	SARAF	1622.175	-0.04339	0.719297	0.924048	protein_coding
ENSG0000(	ABCD4	1485.909	0.025148	0.719706	0.924055	protein_coding
ENSG0000(	AGL	934.5951	0.039593	0.719579	0.924055	protein_coding
ENSG0000(	ARL17B	1789.431	-0.05342	0.719347	0.924055	protein_coding
ENSG0000(	BMS1P23	2153.964	-0.03668	0.720192	0.924055	transcribed_unprocessed_pseudo
ENSG0000(	C20orf27	102.8692	-0.06774	0.719792	0.924055	protein_coding
ENSG0000(	CHTF8	428.6181	0.067071	0.720279	0.924055	protein_coding
ENSG0000(	CIB2	53.70802	0.107092	0.720246	0.924055	protein_coding
ENSG0000(	DNAH9	163.9038	-0.04973	0.719679	0.924055	protein_coding
ENSG0000(	ENSG0000(	45.62003	-0.0567	0.720065	0.924055	lncRNA
ENSG0000(	ENSG0000(	52.9184	0.069032	0.720031	0.924055	transcribed_unprocessed_pseudo
ENSG0000(	ENSG0000(	2105.837	0.071267	0.72	0.924055	lncRNA
ENSG0000(	ENSG0000(	64.0842	-0.05733	0.720138	0.924055	lncRNA
ENSG0000(	ENSG0000(	174.5844	-0.04944	0.719853	0.924055	lncRNA
ENSG0000(	ENSG0000(	20.22556	-0.12189	0.720216	0.924055	lncRNA
ENSG0000(	ENSG0000(	68.55493	0.218945	0.720265	0.924055	lncRNA

ENSG0000(	ENSG0000(	145.9009	0.056783	0.719773	0.924055	protein_coding
ENSG0000(	ENSG0000(	20.17912	0.105573	0.719637	0.924055	lncRNA
ENSG0000(	ENSG0000(	38.49562	0.065224	0.719792	0.924055	lncRNA
ENSG0000(	FBXO39	20.92894	-0.14102	0.719594	0.924055	protein_coding
ENSG0000(	GLIDR	497.7683	0.028151	0.720281	0.924055	lncRNA
ENSG0000(	LINC00092	181.5689	0.041208	0.7199	0.924055	lncRNA
ENSG0000(	MTRR	723.4661	-0.03473	0.719497	0.924055	protein_coding
ENSG0000(	NDUFA2	261.5188	0.035568	0.720261	0.924055	protein_coding
ENSG0000(	OR10K2	65.34125	-0.0622	0.719853	0.924055	protein_coding
ENSG0000(	PSD4	3806.345	-0.04062	0.719697	0.924055	protein_coding
ENSG0000(	RN7SL33P	55.50187	0.066339	0.720224	0.924055	misc_RNA
ENSG0000(	RN7SL667F	304.2013	0.037795	0.71973	0.924055	misc_RNA
ENSG0000(	SLC52A1	251.7995	0.037797	0.719415	0.924055	protein_coding
ENSG0000(	ZNF780B	2272.169	0.025325	0.71964	0.924055	protein_coding
ENSG0000(	RN7SKP62	49.30335	0.090984	0.720357	0.92411	misc_RNA
ENSG0000(	AEBP2	1163.938	0.026694	0.720502	0.924253	protein_coding
ENSG0000(	ENSG0000(	91.74855	-0.04833	0.7206	0.924311	lncRNA
ENSG0000(	FLG-AS1	478.9364	0.028833	0.720615	0.924311	lncRNA
ENSG0000(	APP	4023.728	0.034065	0.7213	0.924334	protein_coding
ENSG0000(	APPL1	1394.462	-0.02975	0.721962	0.924334	protein_coding
ENSG0000(	ARF1P2	80.01914	0.044541	0.721873	0.924334	processed_pseudogene
ENSG0000(	CARD10	876.0205	0.065419	0.72197	0.924334	protein_coding
ENSG0000(	CCND2-AS1	80.31787	-0.05124	0.72072	0.924334	lncRNA
ENSG0000(	CHMP1A	1381.538	0.036188	0.721988	0.924334	protein_coding
ENSG0000(	CUEDC2	84.10919	-0.0588	0.721111	0.924334	protein_coding
ENSG0000(	DDX6	5393.081	-0.01808	0.720925	0.924334	protein_coding
ENSG0000(	ENSG0000(	691.4401	-0.03104	0.721548	0.924334	transcribed_unprocessed_pseudo
ENSG0000(	ENSG0000(	70.13985	-0.06341	0.721505	0.924334	lncRNA
ENSG0000(	ENSG0000(	403.5363	-0.04418	0.722044	0.924334	lncRNA
ENSG0000(	ENSG0000(	128.1161	0.052579	0.721216	0.924334	lncRNA
ENSG0000(	ENSG0000(	50.23932	0.069201	0.721764	0.924334	lncRNA
ENSG0000(	ENSG0000(	133.3914	0.044051	0.721167	0.924334	lncRNA
ENSG0000(	ENSG0000(	30.10278	-0.07921	0.721798	0.924334	lncRNA
ENSG0000(	ENSG0000(	104.3263	-0.05122	0.72133	0.924334	lncRNA
ENSG0000(	ENSG0000(	357.9639	-0.04443	0.720792	0.924334	protein_coding
ENSG0000(	ENSG0000(	164.4087	-0.0406	0.721918	0.924334	lncRNA
ENSG0000(	ENSG0000(	207.4395	0.030818	0.72179	0.924334	lncRNA
ENSG0000(	FCAR	582.3276	-0.04138	0.722029	0.924334	protein_coding
ENSG0000(	HOOK3	3987.835	0.021551	0.721354	0.924334	protein_coding
ENSG0000(	INO80C	1303.345	0.024262	0.721429	0.924334	protein_coding
ENSG0000(	KCTD1	286.9801	0.041506	0.721604	0.924334	protein_coding
ENSG0000(	KLK10	255.0971	-0.05466	0.721467	0.924334	protein_coding
ENSG0000(	LAMTOR2	107.5144	-0.05175	0.721999	0.924334	protein_coding
ENSG0000(	LINC02576	267.1978	0.044115	0.721537	0.924334	lncRNA
ENSG0000(	MYOM1	191.6743	0.049743	0.721155	0.924334	protein_coding
ENSG0000(	PAFAH1B1	5902.991	0.01671	0.721844	0.924334	protein_coding
ENSG0000(	PARG	895.9174	-0.02972	0.721863	0.924334	protein_coding

ENSG0000(	PHF6	2595.211	-0.01981	0.721593	0.924334	protein_coding
ENSG0000(	PLAGL2	1013.724	-0.03406	0.720802	0.924334	protein_coding
ENSG0000(	RHOH	1527.835	-0.0829	0.721065	0.924334	protein_coding
ENSG0000(	RNU6-883F	24.07447	0.109877	0.721769	0.924334	snRNA
ENSG0000(	SMTN	1663.208	-0.0491	0.721782	0.924334	protein_coding
ENSG0000(	SNRPA	428.8216	0.037975	0.721828	0.924334	protein_coding
ENSG0000(	TF	2676.967	0.04185	0.721955	0.924334	protein_coding
ENSG0000(	TPRXL	184.618	0.036851	0.721748	0.924334	transcribed_processed_pseudoge
ENSG0000(	TTC23	584.0624	0.038955	0.721776	0.924334	protein_coding
ENSG0000(	TUBBP5	54.32677	0.15604	0.721644	0.924334	transcribed_unprocessed_pseudo
ENSG0000(	UBQLN1	1980.826	-0.02045	0.720834	0.924334	protein_coding
ENSG0000(	USP21	345.1198	0.035233	0.72095	0.924334	protein_coding
ENSG0000(	ZDHHC24	1043.975	-0.02582	0.721161	0.924334	protein_coding
ENSG0000(	C8orf17	88.20279	-0.04418	0.72214	0.924414	TEC
ENSG0000(	ENSG0000(	49.01229	0.086384	0.722259	0.924524	lncRNA
ENSG0000(	IRS2	1600.448	-0.05719	0.722328	0.924569	protein_coding
ENSG0000(	ATP1B1	10649.46	-0.06906	0.723117	0.924574	protein_coding
ENSG0000(	C8G	30.66948	0.105341	0.722543	0.924574	protein_coding
ENSG0000(	CCDC50	2374.451	0.03883	0.723036	0.924574	protein_coding
ENSG0000(	CNTN1	129.0544	0.051299	0.723197	0.924574	protein_coding
ENSG0000(	ENSG0000(	137.4388	0.043926	0.722436	0.924574	lncRNA
ENSG0000(	ENSG0000(	251.9263	0.028608	0.723223	0.924574	protein_coding
ENSG0000(	ENSG0000(	200.7564	-0.03418	0.722859	0.924574	lncRNA
ENSG0000(	ENSG0000(	1012.517	0.020015	0.723174	0.924574	lncRNA
ENSG0000(	ENSG0000(	68.02707	-0.08569	0.723302	0.924574	processed_pseudogene
ENSG0000(	ENSG0000(	109.5687	0.045295	0.722778	0.924574	lncRNA
ENSG0000(	ENSG0000(	134.4614	-0.04242	0.723306	0.924574	lncRNA
ENSG0000(	ENSG0000(	46.24492	0.085215	0.723159	0.924574	lncRNA
ENSG0000(	ENSG0000(	59.34853	0.051083	0.723294	0.924574	lncRNA
ENSG0000(	FAM222A	45.32692	0.102796	0.722618	0.924574	protein_coding
ENSG0000(	HNMT	789.1086	0.029062	0.723184	0.924574	protein_coding
ENSG0000(	KY	37.24439	-0.09834	0.722916	0.924574	protein_coding
ENSG0000(	LASP1	5024.698	0.031998	0.722506	0.924574	protein_coding
ENSG0000(	LINC00929	110.6844	0.044928	0.722445	0.924574	lncRNA
ENSG0000(	MAP2K2	1777.549	0.0421	0.722875	0.924574	protein_coding
ENSG0000(	PKIG	511.3471	0.042775	0.72321	0.924574	protein_coding
ENSG0000(	RBM23	2949.046	0.022122	0.723176	0.924574	protein_coding
ENSG0000(	RN7SL398F	76.92193	-0.06232	0.722963	0.924574	misc_RNA
ENSG0000(	RN7SL596F	112.842	0.039394	0.722628	0.924574	misc_RNA
ENSG0000(	SEH1L	1523.705	0.021619	0.722889	0.924574	protein_coding
ENSG0000(	SNRPB2	190.1895	-0.05659	0.72296	0.924574	protein_coding
ENSG0000(	SOX4	1081.344	0.050239	0.722762	0.924574	protein_coding
ENSG0000(	SPAG5-AS1	136.345	0.038109	0.722507	0.924574	lncRNA
ENSG0000(	SRSF7	1673.181	0.024958	0.723217	0.924574	protein_coding
ENSG0000(	WARS2-IT1	1440.637	0.019975	0.723283	0.924574	lncRNA
ENSG0000(	ATG2A	1052.472	-0.03062	0.723674	0.924697	protein_coding
ENSG0000(	CENATAC-[	392.8676	-0.02759	0.723738	0.924697	lncRNA

ENSG0000(	ENSG0000(	62.03311	-0.06742	0.7236	0.924697	transcribed_unprocessed_pseudo
ENSG0000(	ENSG0000(	39.97116	-0.06225	0.723541	0.924697	lncRNA
ENSG0000(	HLA-DQB2	147.2192	-0.07226	0.723565	0.924697	protein_coding
ENSG0000(	MIR7515H(	21.86325	-0.11842	0.723707	0.924697	lncRNA
ENSG0000(	OTOA	232.0884	0.047702	0.723544	0.924697	protein_coding
ENSG0000(	PDE9A	1772.911	0.117705	0.723633	0.924697	protein_coding
ENSG0000(	TLE5	2204.842	0.033405	0.723684	0.924697	protein_coding
ENSG0000(	TNKS2-AS1	64.33779	-0.05415	0.723703	0.924697	lncRNA
ENSG0000(	TENM2	52.28508	-0.17056	0.723784	0.924712	protein_coding
ENSG0000(	LL22NC01-8	88.96719	0.053947	0.723875	0.924785	lncRNA
ENSG0000(	ARNT2	460.5232	0.039022	0.724044	0.924787	protein_coding
ENSG0000(	ENSG0000(	55.37519	0.093936	0.724015	0.924787	lncRNA
ENSG0000(	ENSG0000(	153.8035	0.046841	0.724041	0.924787	lncRNA
ENSG0000(	NOPCHAP1	14683.22	-0.01379	0.724003	0.924787	protein_coding
ENSG0000(	NOTCH2	4404.697	0.033586	0.723961	0.924787	protein_coding
ENSG0000(	TNFSF8	307.55	-0.1216	0.72408	0.92479	protein_coding
ENSG0000(	ENSG0000(	144.293	-0.087	0.724195	0.924824	lncRNA
ENSG0000(	RAB11FIP4	1774.604	0.030461	0.724208	0.924824	protein_coding
ENSG0000(	RN7SL444F	85.87399	0.049695	0.724182	0.924824	misc_RNA
ENSG0000(	CEP250-AS	130.2714	-0.04965	0.724369	0.924858	lncRNA
ENSG0000(	DCTN3	323.7719	-0.04081	0.72431	0.924858	protein_coding
ENSG0000(	ENSG0000(	26.3419	-0.09961	0.724361	0.924858	lncRNA
ENSG0000(	FAM224A	84.94056	0.283781	0.724357	0.924858	lncRNA
ENSG0000(	ENSG0000(	57.51029	0.053021	0.724451	0.92492	lncRNA
ENSG0000(	ENSG0000(	1212.102	0.022846	0.724581	0.924968	lncRNA
ENSG0000(	ENSG0000(	155.2016	0.03516	0.72459	0.924968	lncRNA
ENSG0000(	ENSG0000(	459.6353	0.022359	0.724579	0.924968	lncRNA
ENSG0000(	CAND2	74.21938	-0.10751	0.724677	0.92499	protein_coding
ENSG0000(	CTSD	3951.202	0.058417	0.724664	0.92499	protein_coding
ENSG0000(	ENSG0000(	28.86812	-0.09928	0.724713	0.92499	lncRNA
ENSG0000(	PIAS3	546.6233	-0.0377	0.724775	0.92499	protein_coding
ENSG0000(	RPS6	4351.413	0.048048	0.724747	0.92499	protein_coding
ENSG0000(	MED10	220.4978	0.030863	0.724879	0.92508	protein_coding
ENSG0000(	ENSG0000(	25.43262	-0.07874	0.72495	0.925128	lncRNA
ENSG0000(	KIF17	41.08077	-0.08809	0.725066	0.925147	protein_coding
ENSG0000(	KMT5B	2094.06	0.027593	0.725063	0.925147	protein_coding
ENSG0000(	LINC00114	102.381	0.051456	0.72502	0.925147	lncRNA
ENSG0000(	FOXP1-AS1	186.678	0.032481	0.725114	0.925153	lncRNA
ENSG0000(	RN7SL677F	40.95197	-0.06349	0.725138	0.925153	misc_RNA
ENSG0000(	ENSG0000(	73.71561	-0.04623	0.725221	0.925207	lncRNA
ENSG0000(	REV3L	4316.978	-0.02508	0.725248	0.925207	protein_coding
ENSG0000(	CYP51A1P3	49.39495	-0.07624	0.72556	0.925262	processed_pseudogene
ENSG0000(	ENSG0000(	164.1067	-0.04053	0.725516	0.925262	lncRNA
ENSG0000(	ENSG0000(	124.9678	-0.05377	0.725572	0.925262	lncRNA
ENSG0000(	ENSG0000(	73.70776	0.053862	0.725593	0.925262	TEC
ENSG0000(	MAPK6	2197.158	-0.03675	0.725422	0.925262	protein_coding
ENSG0000(	MIR3159	26.98921	-0.08991	0.725465	0.925262	miRNA

ENSG0000(	PIP5K1A	1684.884	0.020476	0.72556	0.925262	protein_coding
ENSG0000(	RBP5	231.7285	-0.062	0.725525	0.925262	protein_coding
ENSG0000(	RN7SL744F	322.8407	0.035778	0.725497	0.925262	misc_RNA
ENSG0000(	HSF5	36.1253	-0.13364	0.725774	0.92545	protein_coding
ENSG0000(	SMIM6	37.50789	0.115063	0.725827	0.925474	protein_coding
ENSG0000(	TDRD10	56.11536	-0.08427	0.725939	0.925573	protein_coding
ENSG0000(	ENSG0000(	50.79877	-0.07202	0.726056	0.92568	processed_pseudogene
ENSG0000(	ENSG0000(	19.94695	0.093913	0.726093	0.925684	lncRNA
ENSG0000(	ENSG0000(	63.68542	-0.05805	0.726191	0.925767	lncRNA
ENSG0000(	ENSG0000(	26.84711	0.09236	0.726503	0.925993	lncRNA
ENSG0000(	JDP2	730.5881	0.04803	0.726406	0.925993	protein_coding
ENSG0000(	MEGF11	30.703	0.131163	0.726502	0.925993	protein_coding
ENSG0000(	MRPS28	364.6836	0.029819	0.726495	0.925993	protein_coding
ENSG0000(	ENSG0000(	296.7953	0.036182	0.726623	0.926103	lncRNA
ENSG0000(	TDG	829.2962	-0.02119	0.72667	0.926119	protein_coding
ENSG0000(	INTS10	1422.989	0.022372	0.726706	0.926123	protein_coding
ENSG0000(	PLS1	3389.71	0.074414	0.726851	0.926162	protein_coding
ENSG0000(	PSIP1	898.227	-0.03373	0.726822	0.926162	protein_coding
ENSG0000(	SNRPEP10	58.91325	-0.11883	0.726872	0.926162	processed_pseudogene
ENSG0000(	ZBTB18	844.8234	0.028895	0.726868	0.926162	protein_coding
ENSG0000(	PJVK	220.9497	-0.04188	0.726947	0.926215	protein_coding
ENSG0000(	DQX1	601.627	0.114975	0.727033	0.926239	protein_coding
ENSG0000(	PSG6	247.6732	-0.06961	0.727019	0.926239	protein_coding
ENSG0000(	GSG1L2	245.5935	-0.02652	0.727069	0.926242	protein_coding
ENSG0000(	CDC42EP3	1575.258	-0.02714	0.727218	0.926266	protein_coding
ENSG0000(	CHMP7	1061.445	0.047987	0.727344	0.926266	protein_coding
ENSG0000(	DBI	665.8648	-0.0509	0.727296	0.926266	protein_coding
ENSG0000(	EDARADD	28.3154	0.115973	0.7273	0.926266	protein_coding
ENSG0000(	ENSG0000(	42.78858	-0.06096	0.727418	0.926266	lncRNA
ENSG0000(	ENSG0000(	110.3167	-0.06791	0.727464	0.926266	lncRNA
ENSG0000(	ENSG0000(	266.7526	0.037345	0.727492	0.926266	lncRNA
ENSG0000(	ENSG0000(	43.84001	-0.06215	0.727409	0.926266	lncRNA
ENSG0000(	LINC02223	73.1361	-0.07793	0.727303	0.926266	lncRNA
ENSG0000(	NECAP1	1571.683	-0.02586	0.727183	0.926266	protein_coding
ENSG0000(	POR	491.7309	0.036298	0.727433	0.926266	protein_coding
ENSG0000(	RN7SL237F	55.63905	0.061764	0.727147	0.926266	misc_RNA
ENSG0000(	ENSG0000(	22.77927	0.103706	0.727591	0.926345	lncRNA
ENSG0000(	ZNF582	59.08375	-0.06938	0.727621	0.926345	protein_coding
ENSG0000(	MHENCR	211.9043	-0.04669	0.72776	0.92637	lncRNA
ENSG0000(	RECQL4	207.5126	-0.07127	0.727765	0.92637	protein_coding
ENSG0000(	SLC5A6	454.4188	0.033935	0.727776	0.92637	protein_coding
ENSG0000(	SMARCC2	3306.912	0.017925	0.727715	0.92637	protein_coding
ENSG0000(	SMURF2	1474.364	-0.03757	0.727809	0.92637	protein_coding
ENSG0000(	ENSG0000(	48.77017	-0.06007	0.727904	0.926405	processed_pseudogene
ENSG0000(	RPGRIP1L	629.6142	0.029437	0.727892	0.926405	protein_coding
ENSG0000(	ZNF473	235.7785	0.040648	0.727942	0.92641	protein_coding
ENSG0000(	LINC00352	306.8838	0.030745	0.728155	0.926621	lncRNA

ENSG0000(	OVGP1	100.2438	-0.06277	0.728175	0.926621	protein_coding
ENSG0000(	RPPH1	20443.01	0.06525	0.728246	0.926669	ribozyme
ENSG0000(	ADRA2A	706.8609	0.077194	0.728364	0.926692	protein_coding
ENSG0000(	ENSG0000(	1429.837	-0.03086	0.728365	0.926692	lncRNA
ENSG0000(	ENSG0000(	42.16094	-0.07704	0.728308	0.926692	lncRNA
ENSG0000(	ASS1P2	20.20642	-0.09907	0.728828	0.92681	processed_pseudogene
ENSG0000(	CUX1	3389.011	-0.02201	0.728631	0.92681	protein_coding
ENSG0000(	ENSG0000(	198.6188	-0.04649	0.728723	0.92681	lncRNA
ENSG0000(	ENSG0000(	172.0761	-0.13173	0.728612	0.92681	lncRNA
ENSG0000(	ENSG0000(	46.00629	0.063914	0.72866	0.92681	lncRNA
ENSG0000(	ENSG0000(	22.91511	-0.06382	0.728824	0.92681	lncRNA
ENSG0000(	H2AC19	167.0223	0.39829	0.728822	0.92681	protein_coding
ENSG0000(	MYRFL	58.55313	-0.14001	0.728771	0.92681	protein_coding
ENSG0000(	NOVA2	190.5538	-0.05656	0.728703	0.92681	protein_coding
ENSG0000(	PINK1-AS	601.6595	-0.02414	0.728722	0.92681	lncRNA
ENSG0000(	TLCD3B	276.6317	-0.02856	0.728588	0.92681	protein_coding
ENSG0000(	HASPIN	89.99138	-0.06096	0.728947	0.926875	protein_coding
ENSG0000(	HEATR5A-D	222.7227	0.042078	0.728921	0.926875	lncRNA
ENSG0000(	PLA2G2C	98.16157	-0.06321	0.729076	0.926996	protein_coding
ENSG0000(	CXXC5	559.3823	-0.03106	0.72915	0.927	protein_coding
ENSG0000(	DDX24	2083.795	0.029772	0.729144	0.927	protein_coding
ENSG0000(	DDX46	1411.596	-0.02284	0.729203	0.927	protein_coding
ENSG0000(	ENSG0000(	38.87884	0.070898	0.729247	0.927	lncRNA
ENSG0000(	HENMT1	207.0836	0.046269	0.729229	0.927	protein_coding
ENSG0000(	ENSG0000(	162.1705	-0.05493	0.729389	0.927094	lncRNA
ENSG0000(	SHCBP1L	38.09621	0.063198	0.729365	0.927094	protein_coding
ENSG0000(	ANKRD31	26.58258	-0.10778	0.730082	0.927149	protein_coding
ENSG0000(	BORCS5	1162.351	0.029765	0.729996	0.927149	protein_coding
ENSG0000(	BTBD6P1	61.73174	-0.1486	0.730049	0.927149	processed_pseudogene
ENSG0000(	CENPE	715.7848	0.058547	0.730073	0.927149	protein_coding
ENSG0000(	ENSG0000(	112.7366	-0.04755	0.730075	0.927149	processed_pseudogene
ENSG0000(	ENSG0000(	56.71535	-0.08557	0.729766	0.927149	lncRNA
ENSG0000(	ENSG0000(	27.2934	0.068297	0.729541	0.927149	lncRNA
ENSG0000(	ENSG0000(	84.32106	0.051534	0.730153	0.927149	lncRNA
ENSG0000(	ENSG0000(	427.2535	-0.02639	0.729654	0.927149	lncRNA
ENSG0000(	H4C12	144.2845	-0.10505	0.7299	0.927149	protein_coding
ENSG0000(	KIF20A	102.1484	0.075913	0.729849	0.927149	protein_coding
ENSG0000(	MDM4	9205.933	-0.03007	0.72988	0.927149	protein_coding
ENSG0000(	P2RX3	48.39328	0.067213	0.729848	0.927149	protein_coding
ENSG0000(	RAD21-AS1	182.8934	0.039228	0.729931	0.927149	lncRNA
ENSG0000(	RN7SL151F	426.7715	-0.08782	0.729823	0.927149	misc_RNA
ENSG0000(	RNVU1-15	315.2726	-0.07116	0.730003	0.927149	snRNA
ENSG0000(	RPS12	1504.158	-0.04976	0.729813	0.927149	protein_coding
ENSG0000(	RPS8	2716.164	-0.04681	0.730133	0.927149	protein_coding
ENSG0000(	RRBP1	10311.95	0.034254	0.730174	0.927149	protein_coding
ENSG0000(	SIGLEC11	226.1881	-0.03862	0.729553	0.927149	protein_coding
ENSG0000(	SNHG15	398.2855	-0.03856	0.729802	0.927149	lncRNA

ENSG0000(	SNORA66	118.588	0.057635	0.729542	0.927149	snoRNA
ENSG0000(	ENSG0000(	88.95244	-0.07738	0.730257	0.927212	lncRNA
ENSG0000(	FAM53A	41.06029	0.085478	0.7303	0.927221	protein_coding
ENSG0000(	MTREX	1483.566	0.024367	0.730331	0.927221	protein_coding
ENSG0000(	G6PD	225.7326	0.059879	0.730401	0.927267	protein_coding
ENSG0000(	ZBTB44	1777.142	0.02997	0.730441	0.927274	protein_coding
ENSG0000(	ENSG0000(	163.8198	0.036841	0.73057	0.927377	protein_coding
ENSG0000(	ST3GAL3	2220.11	-0.01882	0.730589	0.927377	protein_coding
ENSG0000(	ENSG0000(	80.98167	-0.04768	0.730664	0.927429	lncRNA
ENSG0000(	COX4I1	1650.122	0.059725	0.730741	0.927437	protein_coding
ENSG0000(	ENSG0000(	27.68	-0.43894	0.730801	0.927437	protein_coding
ENSG0000(	KRT10	56.34951	-0.05278	0.730805	0.927437	protein_coding
ENSG0000(	SDR16C5	61.54672	0.119344	0.73077	0.927437	protein_coding
ENSG0000(	LRRC37A11	85.87681	-0.05549	0.730886	0.927497	transcribed_unprocessed_pseudo
ENSG0000(	ENSG0000(	71.26577	0.100425	0.731036	0.927602	lncRNA
ENSG0000(	PLA2G4E	71.54069	-0.0949	0.73102	0.927602	protein_coding
ENSG0000(	CASC11	29.89019	-0.1044	0.731499	0.927628	lncRNA
ENSG0000(	ENSG0000(	21.01538	-0.14585	0.731253	0.927628	lncRNA
ENSG0000(	ENSG0000(	33.98584	0.065596	0.731441	0.927628	lncRNA
ENSG0000(	ENSG0000(	248.8167	-0.04701	0.731138	0.927628	lncRNA
ENSG0000(	ICE2	2455.527	0.021997	0.73139	0.927628	protein_coding
ENSG0000(	OR7E14P	305.7651	-0.04568	0.731425	0.927628	transcribed_unprocessed_pseudo
ENSG0000(	PRR14L	4850.871	-0.03801	0.731322	0.927628	protein_coding
ENSG0000(	SLC13A2	42.42641	0.206567	0.731197	0.927628	protein_coding
ENSG0000(	SLC35F2	501.7682	-0.04218	0.73146	0.927628	protein_coding
ENSG0000(	SPOUT1	186.6105	-0.05628	0.731515	0.927628	protein_coding
ENSG0000(	SYNPO2L-A	168.0977	0.044669	0.731511	0.927628	lncRNA
ENSG0000(	THADA	2825.094	-0.03465	0.73124	0.927628	protein_coding
ENSG0000(	TMEM127	2535.173	0.037068	0.731529	0.927628	protein_coding
ENSG0000(	VIPAS39	433.6676	-0.0307	0.731216	0.927628	protein_coding
ENSG0000(	ENSG0000(	109.1157	0.128382	0.731642	0.927692	lncRNA
ENSG0000(	ENSG0000(	60.14396	-0.05106	0.731647	0.927692	lncRNA
ENSG0000(	ENSG0000(	580.5161	0.037077	0.731761	0.927751	lncRNA
ENSG0000(	TSPAN14	3615.605	0.023906	0.731761	0.927751	protein_coding
ENSG0000(	ENSG0000(	20.31193	0.094199	0.73218	0.927798	lncRNA
ENSG0000(	ENSG0000(	337.8851	0.030642	0.732114	0.927798	lncRNA
ENSG0000(	ENSG0000(	164.5602	0.033391	0.732091	0.927798	lncRNA
ENSG0000(	ENSG0000(	20.21436	-0.10685	0.732175	0.927798	lncRNA
ENSG0000(	FDX1	493.1044	-0.03326	0.731879	0.927798	protein_coding
ENSG0000(	H2BC5	237.3181	-0.05851	0.732141	0.927798	protein_coding
ENSG0000(	IFT74	634.3278	0.035786	0.732016	0.927798	protein_coding
ENSG0000(	LINC00865	181.9637	0.051698	0.732198	0.927798	lncRNA
ENSG0000(	LINC02185	386.5076	-0.02204	0.731951	0.927798	lncRNA
ENSG0000(	PRKX	1861.188	0.030171	0.731844	0.927798	protein_coding
ENSG0000(	RPS2	2874.582	-0.04939	0.732202	0.927798	protein_coding
ENSG0000(	TNFSF11	72.74856	0.136814	0.732052	0.927798	protein_coding
ENSG0000(	DPP9	2171.679	0.023672	0.732379	0.927808	protein_coding

ENSG0000(	ENSG0000(	24.2488	0.086877	0.732307	0.927808	lncRNA
ENSG0000(	ENSG0000(	254.4495	-0.03464	0.732358	0.927808	lncRNA
ENSG0000(	PROSER3	1024.517	0.017638	0.73227	0.927808	protein_coding
ENSG0000(	SNTB1	556.7775	0.040564	0.732321	0.927808	protein_coding
ENSG0000(	MICALL2	2287.922	0.038296	0.732726	0.928205	protein_coding
ENSG0000(	NAIPP2	373.6571	0.089074	0.732792	0.928245	unprocessed_pseudogene
ENSG0000(	ART4	172.9309	0.048864	0.732987	0.928381	protein_coding
ENSG0000(	RHOXF1P1	37.6763	0.07293	0.733	0.928381	transcribed_unprocessed_pseudo
ENSG0000(	RN7SL81P	71.41688	-0.06796	0.732966	0.928381	misc_RNA
ENSG0000(	EAF1-AS1	233.495	-0.03149	0.733099	0.928421	lncRNA
ENSG0000(	ENSG0000(	22.54893	-0.10738	0.733089	0.928421	TEC
ENSG0000(	CHMP1B	1188.953	0.037961	0.733222	0.928463	protein_coding
ENSG0000(	ENSG0000(	82.82417	0.128029	0.733234	0.928463	lncRNA
ENSG0000(	WSB2	1313.117	0.038416	0.733211	0.928463	protein_coding
ENSG0000(	ENSG0000(	19.89825	-0.10767	0.733518	0.928738	processed_pseudogene
ENSG0000(	LINC01126	126.0401	0.049172	0.733516	0.928738	lncRNA
ENSG0000(	MOCS3	419.1562	-0.02937	0.73358	0.928774	protein_coding
ENSG0000(	DCTN2	2560.984	0.024686	0.733736	0.92888	protein_coding
ENSG0000(	ESPL1	530.3512	0.046234	0.733712	0.92888	protein_coding
ENSG0000(	HSPE1P11	173.62	-0.03933	0.733823	0.92888	processed_pseudogene
ENSG0000(	LIG1	566.387	0.052566	0.733803	0.92888	protein_coding
ENSG0000(	LINC00680	496.5462	-0.05481	0.733833	0.92888	transcribed_unprocessed_pseudo
ENSG0000(	NUP50-DT	904.3923	-0.02415	0.73393	0.92896	lncRNA
ENSG0000(	ADGRB2	77.98078	-0.10383	0.733981	0.928983	protein_coding
ENSG0000(	TRANK1	7281.023	-0.05587	0.734017	0.928985	protein_coding
ENSG0000(	ENSG0000(	85.47867	-0.05547	0.734141	0.929056	lncRNA
ENSG0000(	ENSG0000(	210.2515	0.030551	0.734127	0.929056	lncRNA
ENSG0000(	KATNBL1	1402.742	0.017605	0.734198	0.929067	protein_coding
ENSG0000(	WHAMM	1026.701	0.020209	0.734217	0.929067	protein_coding
ENSG0000(	ENSG0000(	411.6925	0.023566	0.73427	0.929091	lncRNA
ENSG0000(	POLR1H	113.0068	-0.0503	0.734353	0.929154	protein_coding
ENSG0000(	GRAMD2A	136.4261	-0.05303	0.734503	0.929275	protein_coding
ENSG0000(	ITGBL1	85.02043	-0.07794	0.734516	0.929275	protein_coding
ENSG0000(	PIP5KL1	115.4694	-0.03842	0.734678	0.929437	protein_coding
ENSG0000(	CCDC27	145.799	0.037642	0.735174	0.929512	protein_coding
ENSG0000(	DLG2	167.8933	0.060368	0.735189	0.929512	protein_coding
ENSG0000(	DPP3-DT	388.8802	0.027063	0.735021	0.929512	lncRNA
ENSG0000(	ENSG0000(	175.8058	-0.04488	0.734899	0.929512	lncRNA
ENSG0000(	ENSG0000(	20.1138	-0.11103	0.735049	0.929512	lncRNA
ENSG0000(	ENSG0000(	33.4389	-0.11581	0.735005	0.929512	lncRNA
ENSG0000(	ENSG0000(	71.18059	-0.07028	0.735092	0.929512	lncRNA
ENSG0000(	ENSG0000(	246.4782	0.053647	0.735073	0.929512	lncRNA
ENSG0000(	L3MBTL4	581.9908	-0.03812	0.735134	0.929512	protein_coding
ENSG0000(	LINC01915	258.7517	0.032822	0.735142	0.929512	lncRNA
ENSG0000(	MPL	243.1633	0.034243	0.735033	0.929512	protein_coding
ENSG0000(	POLD2	279.6824	-0.04101	0.735228	0.929512	protein_coding
ENSG0000(	RESF1	6152.206	-0.0528	0.735107	0.929512	protein_coding

ENSG0000(	USP11	422.3434	0.049956	0.735244	0.929512	protein_coding
ENSG0000(	ZNF766	1649.04	-0.02442	0.734835	0.929512	protein_coding
ENSG0000(	INTS2	768.4503	0.031608	0.735328	0.929575	protein_coding
ENSG0000(	ENSG0000(	21.6971	0.097296	0.7362	0.929738	lncRNA
ENSG0000(	ENSG0000(	146.4808	0.090492	0.735932	0.929738	lncRNA
ENSG0000(	ENSG0000(	266.7833	-0.03142	0.736006	0.929738	lncRNA
ENSG0000(	ENSG0000(	95.25922	-0.03965	0.735746	0.929738	TEC
ENSG0000(	ENSG0000(	199.0365	0.041085	0.736006	0.929738	lncRNA
ENSG0000(	ENSG0000(	131.1038	-0.04154	0.736113	0.929738	lncRNA
ENSG0000(	GTF3C1	1689.646	0.02353	0.73592	0.929738	protein_coding
ENSG0000(	H3C14	37.42894	-1.25234	0.735927	0.929738	protein_coding
ENSG0000(	HDDC3	275.1883	-0.03513	0.735526	0.929738	protein_coding
ENSG0000(	LRRC74B	649.5925	0.046479	0.736097	0.929738	protein_coding
ENSG0000(	NLE1	352.7298	0.035544	0.735752	0.929738	protein_coding
ENSG0000(	ODAD4	19.87902	-0.16108	0.735633	0.929738	protein_coding
ENSG0000(	PIK3R2	494.09	0.034389	0.735548	0.929738	protein_coding
ENSG0000(	PUS7	341.6024	0.048284	0.735608	0.929738	protein_coding
ENSG0000(	RALY	2281.825	0.031087	0.736193	0.929738	protein_coding
ENSG0000(	RNF216	1665.613	-0.0317	0.736138	0.929738	protein_coding
ENSG0000(	RPL7L1	2917.264	-0.01521	0.735829	0.929738	protein_coding
ENSG0000(	RPS6KL1	506.3575	-0.03636	0.73614	0.929738	protein_coding
ENSG0000(	TMIGD3	22.96878	0.160121	0.7361	0.929738	protein_coding
ENSG0000(	TNFRSF10D	467.8779	-0.03702	0.736037	0.929738	protein_coding
ENSG0000(	TRMT61B	184.95	-0.04606	0.736112	0.929738	protein_coding
ENSG0000(	ZNF561	1147.049	-0.02558	0.735827	0.929738	protein_coding
ENSG0000(	EME2	1436.446	0.032943	0.736317	0.929761	protein_coding
ENSG0000(	ENSG0000(	37.06431	0.08684	0.736354	0.929761	lncRNA
ENSG0000(	MRPS24	213.6299	-0.04295	0.736312	0.929761	protein_coding
ENSG0000(	SPACA6P-A	363.2533	-0.02222	0.736332	0.929761	lncRNA
ENSG0000(	ENSG0000(	79.79686	0.077782	0.736395	0.92977	TEC
ENSG0000(	CEP20	462.55	0.032842	0.737083	0.929828	protein_coding
ENSG0000(	COQ6	1085.819	0.017943	0.736484	0.929828	protein_coding
ENSG0000(	DHRS4L2	172.514	-0.05551	0.736863	0.929828	protein_coding
ENSG0000(	ENSG0000(	108.6069	0.062595	0.736681	0.929828	lncRNA
ENSG0000(	ENSG0000(	47.88761	0.053331	0.736596	0.929828	lncRNA
ENSG0000(	ENSG0000(	25.27094	-0.07967	0.737027	0.929828	lncRNA
ENSG0000(	ENSG0000(	8537.942	0.088391	0.736849	0.929828	lncRNA
ENSG0000(	GPC5	25.44616	-0.16268	0.736645	0.929828	protein_coding
ENSG0000(	HEXB	673.981	-0.03488	0.737053	0.929828	protein_coding
ENSG0000(	LINC01647	121.558	0.045404	0.737033	0.929828	lncRNA
ENSG0000(	LZTFL1	469.4088	-0.03812	0.736933	0.929828	protein_coding
ENSG0000(	MEIS2	915.8782	0.095102	0.73688	0.929828	protein_coding
ENSG0000(	MIR646HG	1061.755	0.045431	0.737008	0.929828	lncRNA
ENSG0000(	RPL9P16	121.5313	0.076429	0.737116	0.929828	processed_pseudogene
ENSG0000(	SLC10A6	27.49861	0.107133	0.736538	0.929828	protein_coding
ENSG0000(	STPG2-AS1	549.6161	0.030613	0.736694	0.929828	lncRNA
ENSG0000(	TET2-AS1	22.56362	-0.07876	0.736855	0.929828	lncRNA

ENSG0000(	TYW5	2210.291	0.017265	0.736867	0.929828	protein_coding
ENSG0000(	USP9X	5525.936	-0.02538	0.736803	0.929828	protein_coding
ENSG0000(	ZSCAN12P1	50.63411	-0.09591	0.736955	0.929828	transcribed_unprocessed_pseudo
ENSG0000(	ATF7IP2	890.1827	0.03195	0.737341	0.929869	protein_coding
ENSG0000(	CADM3	114.7185	-0.08987	0.737243	0.929869	protein_coding
ENSG0000(	DIXDC1	555.4724	0.046701	0.737352	0.929869	protein_coding
ENSG0000(	ENSG0000(	282.6423	-0.02363	0.737256	0.929869	lncRNA
ENSG0000(	LINC02591	28.94568	-0.49244	0.737309	0.929869	lncRNA
ENSG0000(	PC	392.0838	0.06955	0.73729	0.929869	protein_coding
ENSG0000(	SPATA17	378.0132	-0.03919	0.737398	0.929885	protein_coding
ENSG0000(	ENSG0000(	24.57958	-0.17554	0.737502	0.929961	lncRNA
ENSG0000(	ENSG0000(	198.0012	-0.03028	0.737526	0.929961	lncRNA
ENSG0000(	SCAMP1	763.144	-0.03075	0.737592	0.929991	protein_coding
ENSG0000(	UFSP1	42.49918	0.067827	0.737617	0.929991	protein_coding
ENSG0000(	ARL9	108.3026	-0.05207	0.737799	0.930034	protein_coding
ENSG0000(	CDC73	1843.988	-0.01232	0.73799	0.930034	protein_coding
ENSG0000(	ENSG0000(	286.5245	-0.0413	0.737904	0.930034	lncRNA
ENSG0000(	ENSG0000(	27.126	-0.09351	0.737905	0.930034	lncRNA
ENSG0000(	ENSG0000(	23.60853	-0.09244	0.737971	0.930034	processed_pseudogene
ENSG0000(	ENSG0000(	402.6242	-0.02141	0.737943	0.930034	lncRNA
ENSG0000(	EPB41L4A	955.2203	0.027888	0.737848	0.930034	protein_coding
ENSG0000(	MIATNB	1531.09	-0.03453	0.737947	0.930034	lncRNA
ENSG0000(	PIEZO2	1319.076	-0.02435	0.73779	0.930034	protein_coding
ENSG0000(	UQCRHL	225.4434	-0.02506	0.737956	0.930034	protein_coding
ENSG0000(	CHRNG	196.1865	-0.03823	0.738025	0.930036	protein_coding
ENSG0000(	DOT1L	2096.958	0.030819	0.738124	0.930069	protein_coding
ENSG0000(	ENSG0000(	47.95464	-0.08856	0.738153	0.930069	lncRNA
ENSG0000(	ENSG0000(	65.93408	0.043597	0.738165	0.930069	lncRNA
ENSG0000(	SNORD95	39.40445	-0.09058	0.738186	0.930069	snoRNA
ENSG0000(	LIX1-AS1	38.64236	-0.11518	0.738298	0.930167	lncRNA
ENSG0000(	ENSG0000(	131.9036	-0.04369	0.738402	0.930213	lncRNA
ENSG0000(	ENSG0000(	72.77454	-0.05788	0.738389	0.930213	lncRNA
ENSG0000(	BTBD7	2658.292	0.022998	0.73848	0.930264	protein_coding
ENSG0000(	ENSG0000(	41.72426	-0.08743	0.73851	0.930264	lncRNA
ENSG0000(	ADGRE4P	199.4521	0.060852	0.739061	0.930309	transcribed_unprocessed_pseudo
ENSG0000(	C1orf127	60.48185	-0.05311	0.73889	0.930309	protein_coding
ENSG0000(	CCDC160	65.91125	-0.06478	0.73885	0.930309	protein_coding
ENSG0000(	ENSG0000(	58.97821	0.083601	0.738998	0.930309	lncRNA
ENSG0000(	ENSG0000(	379.7503	0.024689	0.73899	0.930309	lncRNA
ENSG0000(	ENSG0000(	31.24501	-0.07206	0.739145	0.930309	lncRNA
ENSG0000(	ENSG0000(	201.1904	0.042803	0.739103	0.930309	lncRNA
ENSG0000(	FABP2	320.3114	0.08098	0.73882	0.930309	protein_coding
ENSG0000(	FAM149B1	666.1488	0.02546	0.738942	0.930309	protein_coding
ENSG0000(	FAM229B	141.4085	0.030562	0.739281	0.930309	protein_coding
ENSG0000(	JAKMIP3	81.98865	-0.07637	0.738843	0.930309	protein_coding
ENSG0000(	LEMD3	1212.888	0.030259	0.738977	0.930309	protein_coding
ENSG0000(	LINC01117	22.87083	-0.09676	0.739178	0.930309	lncRNA

ENSG0000(	LINC01118	116.4642	-0.03297	0.73929	0.930309	lncRNA
ENSG0000(	LURAP1L	621.6905	-0.04554	0.739119	0.930309	protein_coding
ENSG0000(	MRPL16	231.9494	-0.03798	0.7392	0.930309	protein_coding
ENSG0000(	NPIPB9	215.3784	-0.51211	0.739249	0.930309	protein_coding
ENSG0000(	RNU6-377F	31.78348	-0.09957	0.738854	0.930309	snRNA
ENSG0000(	SLC25A29	1321.404	-0.04676	0.738985	0.930309	protein_coding
ENSG0000(	SNX25P1	20.97263	-0.11811	0.738699	0.930309	unprocessed_pseudogene
ENSG0000(	TAX1BP1	1972.486	0.020895	0.739199	0.930309	protein_coding
ENSG0000(	UBE2Q2L	79.06402	-0.05966	0.738644	0.930309	transcribed_unprocessed_pseudo
ENSG0000(	CDK12	4293.793	0.018701	0.739475	0.930341	protein_coding
ENSG0000(	ENSG0000(	31.35887	0.153878	0.740241	0.930341	protein_coding
ENSG0000(	ENSG0000(	466.6535	0.075399	0.739726	0.930341	processed_pseudogene
ENSG0000(	ENSG0000(	94.22501	-0.05678	0.739664	0.930341	lncRNA
ENSG0000(	ENSG0000(	331.076	-0.02184	0.739736	0.930341	lncRNA
ENSG0000(	ENSG0000(	372.7211	-0.02523	0.740262	0.930341	lncRNA
ENSG0000(	ENSG0000(	155.0706	-0.03591	0.739885	0.930341	lncRNA
ENSG0000(	ENSG0000(	133.5977	0.084201	0.739687	0.930341	lncRNA
ENSG0000(	ENSG0000(	23.66876	-0.07721	0.740254	0.930341	lncRNA
ENSG0000(	ENSG0000(	59.50769	-0.05675	0.740245	0.930341	lncRNA
ENSG0000(	HSPD1P6	22.84382	0.092638	0.739932	0.930341	transcribed_processed_pseudoge
ENSG0000(	ITPKB-IT1	94.34873	-0.07405	0.740226	0.930341	lncRNA
ENSG0000(	LBX2	28.27892	-0.09299	0.740141	0.930341	protein_coding
ENSG0000(	LIFR	1687.155	0.070974	0.739742	0.930341	protein_coding
ENSG0000(	PABIR1	1704.942	0.042073	0.740221	0.930341	protein_coding
ENSG0000(	PFKM	747.9686	0.025756	0.740012	0.930341	protein_coding
ENSG0000(	PHOSPHO2	218.6111	0.060051	0.740145	0.930341	protein_coding
ENSG0000(	PLD4	37.63098	-0.13327	0.740087	0.930341	protein_coding
ENSG0000(	RPS15A	2123.556	-0.0481	0.740043	0.930341	protein_coding
ENSG0000(	S100A16	553.0811	-0.0701	0.740006	0.930341	protein_coding
ENSG0000(	SLC37A1	1849.106	-0.05231	0.73957	0.930341	protein_coding
ENSG0000(	SYAP1	2299.887	0.020075	0.73944	0.930341	protein_coding
ENSG0000(	TM9SF4	1377.992	0.030611	0.739453	0.930341	protein_coding
ENSG0000(	UBE2V1P1	37.94886	-0.06849	0.739418	0.930341	processed_pseudogene
ENSG0000(	ZNF133	642.9417	-0.02162	0.739808	0.930341	protein_coding
ENSG0000(	ZNF229	126.6808	0.059261	0.739747	0.930341	protein_coding
ENSG0000(	ZNF781	146.0087	-0.0556	0.739853	0.930341	protein_coding
ENSG0000(	ZNF85	481.2569	0.023531	0.740213	0.930341	protein_coding
ENSG0000(	TMA16	404.9651	-0.0307	0.740318	0.930369	protein_coding
ENSG0000(	LINC01360	66.29734	0.063798	0.740423	0.930416	lncRNA
ENSG0000(	NBPF15	1830.749	-0.03811	0.7404	0.930416	protein_coding
ENSG0000(	IGLV10-54	106.3422	-0.46867	0.740537	0.930474	IG_V_gene
ENSG0000(	POLR3C	783.1271	0.024739	0.740509	0.930474	protein_coding
ENSG0000(	ENSG0000(	36.01954	-0.07087	0.740628	0.930546	lncRNA
ENSG0000(	TMEM184(	96.69912	-0.07313	0.740679	0.930567	lncRNA
ENSG0000(	TTLL5	1361.637	-0.02412	0.740715	0.93057	protein_coding
ENSG0000(	PPP2R2C	714.629	0.084655	0.740784	0.930572	protein_coding
ENSG0000(	PRICKLE2-A	65.4902	-0.06574	0.740762	0.930572	lncRNA

ENSG0000(	CYP2D8P	43.99371	-0.07824	0.740841	0.930601	unprocessed_pseudogene
ENSG0000(	CPSF7	2105.659	0.01516	0.740901	0.930634	protein_coding
ENSG0000(	UBTFL11	62.81803	-0.05836	0.741021	0.930741	processed_pseudogene
ENSG0000(	DFFB	686.9633	0.023681	0.741158	0.930872	protein_coding
ENSG0000(	PSMD6-AS:	171.0629	-0.03307	0.74122	0.930897	lncRNA
ENSG0000(	SLC25A12	637.495	0.030336	0.741246	0.930897	protein_coding
ENSG0000(	STAMBP	3438.64	-0.01668	0.741315	0.930942	protein_coding
ENSG0000(	ENSG0000(	60.09733	0.068605	0.741447	0.931021	lncRNA
ENSG0000(	H2BP1	70.01267	0.124814	0.741429	0.931021	transcribed_unprocessed_pseudo
ENSG0000(	EFCAB12	477.8172	-0.02408	0.741537	0.931065	protein_coding
ENSG0000(	ENSG0000(	187.4832	0.050281	0.741549	0.931065	lncRNA
ENSG0000(	ATP13A4-A	62.54123	-0.05948	0.741728	0.931097	lncRNA
ENSG0000(	CLTRN	32.69986	-0.07613	0.741709	0.931097	protein_coding
ENSG0000(	ENSG0000(	188.2011	-0.03578	0.741677	0.931097	lncRNA
ENSG0000(	GAMT	180.6632	-0.02867	0.741744	0.931097	protein_coding
ENSG0000(	SLC4A7	1602.612	0.043368	0.741612	0.931097	protein_coding
ENSG0000(	AAAS	457.3552	-0.0261	0.742221	0.931102	protein_coding
ENSG0000(	ARMC10	1050.914	-0.02188	0.742224	0.931102	protein_coding
ENSG0000(	BRIX1	353.7948	-0.03436	0.741883	0.931102	protein_coding
ENSG0000(	CHRNB4	144.1678	-0.03787	0.742058	0.931102	protein_coding
ENSG0000(	ENSG0000(	42.51175	-0.14896	0.74237	0.931102	protein_coding
ENSG0000(	ENSG0000(	46.63599	0.053787	0.741925	0.931102	lncRNA
ENSG0000(	ENSG0000(	102.9135	0.536384	0.742077	0.931102	protein_coding
ENSG0000(	ENSG0000(	64.08972	-0.06276	0.742123	0.931102	lncRNA
ENSG0000(	ENSG0000(	122.1621	-0.04277	0.742308	0.931102	lncRNA
ENSG0000(	FIP1L1	814.5031	-0.02098	0.742083	0.931102	protein_coding
ENSG0000(	GLCE	926.2083	0.042338	0.742036	0.931102	protein_coding
ENSG0000(	IGSF3	819.7078	0.094163	0.742391	0.931102	protein_coding
ENSG0000(	LBX2-AS1	172.8298	0.060949	0.742216	0.931102	lncRNA
ENSG0000(	LINC02742	878.685	0.028842	0.742285	0.931102	lncRNA
ENSG0000(	LRP4-AS1	59.67947	-0.06456	0.742272	0.931102	lncRNA
ENSG0000(	RANBP3L	50.17009	-0.08258	0.742365	0.931102	protein_coding
ENSG0000(	SPAG16	315.5992	0.039778	0.741899	0.931102	protein_coding
ENSG0000(	ST8SIA6-AS	239.318	0.042895	0.741791	0.931102	lncRNA
ENSG0000(	ZGRF1	756.1008	0.030328	0.742234	0.931102	protein_coding
ENSG0000(	ENSG0000(	24.98274	0.080259	0.742446	0.931129	lncRNA
ENSG0000(	ENSG0000(	474.4207	-0.03426	0.742537	0.931158	lncRNA
ENSG0000(	NTRK3	659.0728	0.044753	0.742512	0.931158	protein_coding
ENSG0000(	ENSG0000(	31.91715	0.081041	0.742618	0.931185	lncRNA
ENSG0000(	LINC00702	563.9654	0.027493	0.742626	0.931185	lncRNA
ENSG0000(	ENSG0000(	168.8071	0.046869	0.742746	0.931213	lncRNA
ENSG0000(	ERP29	1072.255	-0.01981	0.74275	0.931213	protein_coding
ENSG0000(	MCCC1	611.574	-0.0391	0.742697	0.931213	protein_coding
ENSG0000(	ANKRD10	4743.636	-0.04458	0.742845	0.931235	protein_coding
ENSG0000(	ENSG0000(	342.0779	0.03615	0.742936	0.931235	transcribed_processed_pseudoge
ENSG0000(	ISCA1	496.1698	0.031098	0.7429	0.931235	protein_coding
ENSG0000(	MARCHF8	1543.2	0.028657	0.742869	0.931235	protein_coding

ENSG0000(	YY1	1811.646	-0.0194	0.742935	0.931235	protein_coding
ENSG0000(	THOC3	319.2183	-0.04746	0.742998	0.931269	protein_coding
ENSG0000(	C2orf42	271.1241	-0.03048	0.743522	0.931335	protein_coding
ENSG0000(	CD28	685.7134	-0.07781	0.743591	0.931335	protein_coding
ENSG0000(	CEP85	656.2275	-0.03242	0.743499	0.931335	protein_coding
ENSG0000(	CRLF1	47.5114	-0.07074	0.743694	0.931335	protein_coding
ENSG0000(	CTSLP3	68.94518	0.057565	0.743462	0.931335	transcribed_unprocessed_pseudo
ENSG0000(	ENSG0000(	3250.161	0.02386	0.743178	0.931335	lncRNA
ENSG0000(	ENSG0000(	26.6659	0.074644	0.743647	0.931335	processed_pseudogene
ENSG0000(	ENSG0000(	38.09174	0.067898	0.743596	0.931335	lncRNA
ENSG0000(	ENSG0000(	119.2599	0.047239	0.743583	0.931335	lncRNA
ENSG0000(	ENSG0000(	92.17322	-0.04392	0.743449	0.931335	lncRNA
ENSG0000(	KPNA1	1458.161	0.018887	0.743199	0.931335	protein_coding
ENSG0000(	LIF-AS1	28.31837	-0.0556	0.743356	0.931335	lncRNA
ENSG0000(	MNAT1	213.1359	0.041119	0.743499	0.931335	protein_coding
ENSG0000(	MUSK	263.8169	0.080888	0.743532	0.931335	protein_coding
ENSG0000(	NAA25	1852.446	-0.02112	0.743451	0.931335	protein_coding
ENSG0000(	POLR2J3	11213.19	0.032313	0.74369	0.931335	protein_coding
ENSG0000(	RNASE1	254.1495	0.06934	0.743611	0.931335	protein_coding
ENSG0000(	SNORA68	288.237	0.045254	0.743121	0.931335	snoRNA
ENSG0000(	TTLL7	795.299	0.030509	0.74315	0.931335	protein_coding
ENSG0000(	ENSG0000(	112.8543	-0.04363	0.743852	0.931491	lncRNA
ENSG0000(	CACNA2D3	39.4895	0.120842	0.744021	0.931529	protein_coding
ENSG0000(	ENSG0000(	59.77334	-0.06007	0.744119	0.931529	processed_pseudogene
ENSG0000(	ENSG0000(	20.57763	0.091848	0.744013	0.931529	misc_RNA
ENSG0000(	RN7SL279F	44.06271	-0.04387	0.744057	0.931529	misc_RNA
ENSG0000(	RPL19	2945.597	-0.04235	0.74397	0.931529	protein_coding
ENSG0000(	RPL5	2731.012	-0.02802	0.744116	0.931529	protein_coding
ENSG0000(	WRNIP1	432.9609	-0.03129	0.74404	0.931529	protein_coding
ENSG0000(	DSTN	1376.987	0.040486	0.744316	0.931599	protein_coding
ENSG0000(	ENSG0000(	57.53801	0.069766	0.744344	0.931599	protein_coding
ENSG0000(	LIPE-AS1	93.77317	0.057996	0.74433	0.931599	lncRNA
ENSG0000(	RBFOX1	785.8279	0.047964	0.744286	0.931599	protein_coding
ENSG0000(	ZSCAN25	1191.019	-0.0231	0.744293	0.931599	protein_coding
ENSG0000(	C14orf28	139.1126	-0.05467	0.744451	0.931605	protein_coding
ENSG0000(	FCF1	1485.936	0.021612	0.744407	0.931605	protein_coding
ENSG0000(	HMGCS2	2540.042	0.185211	0.744439	0.931605	protein_coding
ENSG0000(	ALKBH2	84.08006	-0.04719	0.744652	0.931624	protein_coding
ENSG0000(	ENSG0000(	27.97065	-0.08637	0.74464	0.931624	processed_pseudogene
ENSG0000(	ENSG0000(	645.1559	0.023363	0.744703	0.931624	lncRNA
ENSG0000(	GOLGA8O	275.4996	-0.14193	0.7446	0.931624	protein_coding
ENSG0000(	MRM2	263.1645	-0.03312	0.744688	0.931624	protein_coding
ENSG0000(	PRCP	2399.683	0.032492	0.744548	0.931624	protein_coding
ENSG0000(	TRAPPC6A	51.29228	0.059641	0.74452	0.931624	protein_coding
ENSG0000(	DCLK1	322.2431	0.050484	0.744853	0.931684	protein_coding
ENSG0000(	GLIPR1L2	699.687	0.025622	0.744818	0.931684	protein_coding
ENSG0000(	HNRNPUL2	2243.31	0.022164	0.744833	0.931684	protein_coding

ENSG0000(	RN7SL357F	19.51148	-0.09296	0.744895	0.931694	misc_RNA
ENSG0000(	VSIG10L	44.47367	-0.06602	0.74496	0.931733	protein_coding
ENSG0000(	CLPTM1L	1879.389	-0.05727	0.745103	0.931743	protein_coding
ENSG0000(	MRPS35	284.9439	0.048327	0.745086	0.931743	protein_coding
ENSG0000(	PRR23A	167.8393	0.038849	0.745014	0.931743	protein_coding
ENSG0000(	SBSPON	187.6631	0.029388	0.745069	0.931743	protein_coding
ENSG0000(	BCL2L11	1768.84	-0.04197	0.745464	0.931834	protein_coding
ENSG0000(	CAMK4	844.0289	-0.06113	0.745551	0.931834	protein_coding
ENSG0000(	CLTC	6439.681	-0.02533	0.745436	0.931834	protein_coding
ENSG0000(	DYNLRB1	438.8136	-0.03546	0.745631	0.931834	protein_coding
ENSG0000(	ENSG0000(	27.09629	0.074561	0.745737	0.931834	processed_pseudogene
ENSG0000(	ENSG0000(	120.4984	0.049995	0.745749	0.931834	lncRNA
ENSG0000(	ENSG0000(	296.6385	0.029303	0.745515	0.931834	lncRNA
ENSG0000(	ENSG0000(	40.35896	0.077594	0.745742	0.931834	lncRNA
ENSG0000(	ENSG0000(	248.8858	-0.0287	0.745693	0.931834	lncRNA
ENSG0000(	ENSG0000(	712.3595	-0.04377	0.745257	0.931834	lncRNA
ENSG0000(	FAM98B	1048.353	-0.01969	0.745494	0.931834	protein_coding
ENSG0000(	LINC00222	71.86456	0.059359	0.745565	0.931834	lncRNA
ENSG0000(	LINC01362	20.13132	-0.10971	0.745752	0.931834	lncRNA
ENSG0000(	RN7SKP36	67.00046	0.069477	0.745462	0.931834	misc_RNA
ENSG0000(	SNRNP35	1024.038	-0.01751	0.745518	0.931834	protein_coding
ENSG0000(	UHRF2	2557.015	0.023606	0.745748	0.931834	protein_coding
ENSG0000(	XPA	268.2495	-0.03789	0.745634	0.931834	protein_coding
ENSG0000(	ARHGAP27	1098.404	0.034831	0.74631	0.931854	lncRNA
ENSG0000(	ENSG0000(	49.75139	0.055067	0.746155	0.931854	lncRNA
ENSG0000(	ENSG0000(	136.9172	0.04138	0.746057	0.931854	lncRNA
ENSG0000(	ENSG0000(	226.7561	-0.03689	0.745978	0.931854	transcribed_unprocessed_pseudo
ENSG0000(	ENSG0000(	27.23655	-0.06315	0.746193	0.931854	lncRNA
ENSG0000(	ENSG0000(	578.0812	-0.0269	0.745928	0.931854	lncRNA
ENSG0000(	ENSG0000(	542.6328	0.023798	0.746214	0.931854	lncRNA
ENSG0000(	ENSG0000(	32.65091	-0.08523	0.745887	0.931854	lncRNA
ENSG0000(	FAHD1	367.5367	0.046178	0.746282	0.931854	protein_coding
ENSG0000(	GRAMD1A-	187.3708	-0.03598	0.746269	0.931854	lncRNA
ENSG0000(	HRC	27.76249	-0.13976	0.746212	0.931854	protein_coding
ENSG0000(	LINC02359	453.1337	0.022825	0.745933	0.931854	lncRNA
ENSG0000(	MAPKBP1	1076.145	-0.03268	0.746016	0.931854	protein_coding
ENSG0000(	MOCS2	326.3328	-0.03716	0.746302	0.931854	protein_coding
ENSG0000(	MRPL57	609.7266	-0.01967	0.746214	0.931854	protein_coding
ENSG0000(	REEP5	968.1553	0.026246	0.746046	0.931854	protein_coding
ENSG0000(	ENSG0000(	234.9285	0.029681	0.746382	0.931902	lncRNA
ENSG0000(	ENSG0000(	32.01164	-0.07466	0.746458	0.931954	lncRNA
ENSG0000(	ENSG0000(	44.22066	0.983362	0.746548	0.932017	lncRNA
ENSG0000(	NOP56	921.4221	-0.03761	0.746616	0.932017	protein_coding
ENSG0000(	POLI	1033.527	0.037135	0.746577	0.932017	protein_coding
ENSG0000(	SNORA74D	85.31481	0.058052	0.746644	0.932017	snoRNA
ENSG0000(	BMS1P1	2909.249	-0.02875	0.746756	0.932053	transcribed_unprocessed_pseudo
ENSG0000(	STAU1	1408.471	-0.02562	0.746731	0.932053	protein_coding

ENSG0000(	ZCWPW1	114.7844	0.050306	0.746774	0.932053	protein_coding
ENSG0000(	ENSG0000(	308.5058	0.027804	0.746972	0.932089	lncRNA
ENSG0000(	KRT8P33	77.44142	-0.06146	0.74688	0.932089	processed_pseudogene
ENSG0000(	MSL3P1	22.99296	-0.15342	0.746859	0.932089	transcribed_processed_pseudoge
ENSG0000(	POLR3DP1	20.4766	-0.09355	0.74691	0.932089	processed_pseudogene
ENSG0000(	ZFPM2	457.9229	-0.03415	0.74694	0.932089	protein_coding
ENSG0000(	MEAF6	1145.809	-0.0259	0.747013	0.932097	protein_coding
ENSG0000(	ENSG0000(	37.21664	0.081656	0.747161	0.932197	lncRNA
ENSG0000(	RPL37	3973.293	-0.04333	0.747153	0.932197	protein_coding
ENSG0000(	ACTR5	202.7564	0.026643	0.747946	0.932203	protein_coding
ENSG0000(	AKT3-IT1	90.38772	0.050095	0.749476	0.932203	lncRNA
ENSG0000(	ALKBH8	267.8277	-0.04411	0.749425	0.932203	protein_coding
ENSG0000(	ANKUB1	133.9919	-0.0358	0.74773	0.932203	protein_coding
ENSG0000(	AP5Z1	990.1859	0.025324	0.750316	0.932203	protein_coding
ENSG0000(	BAZ1B	2107.426	-0.01993	0.74741	0.932203	protein_coding
ENSG0000(	BNIP1	93.82627	-0.0593	0.748636	0.932203	protein_coding
ENSG0000(	CALM2	5539.419	0.02616	0.75001	0.932203	protein_coding
ENSG0000(	CARS2	1376.437	-0.02087	0.748994	0.932203	protein_coding
ENSG0000(	CD36	940.3591	-0.04465	0.748269	0.932203	protein_coding
ENSG0000(	CEACAM16	542.9492	0.056013	0.747907	0.932203	lncRNA
ENSG0000(	CIAPIN1	415.2175	0.027714	0.749371	0.932203	protein_coding
ENSG0000(	CSE1L	557.6849	-0.03238	0.7502	0.932203	protein_coding
ENSG0000(	DCAF10	1717.923	-0.01807	0.749379	0.932203	protein_coding
ENSG0000(	DCST1	27.9752	0.11084	0.750474	0.932203	protein_coding
ENSG0000(	DSG2	4930.705	0.058567	0.749211	0.932203	protein_coding
ENSG0000(	DYRK4	318.9138	-0.03617	0.748714	0.932203	protein_coding
ENSG0000(	EIF4ENIF1	1125.712	-0.01739	0.749252	0.932203	protein_coding
ENSG0000(	EML4-AS1	22.66527	-0.09517	0.74999	0.932203	lncRNA
ENSG0000(	ENSG0000(	21.34397	-0.0981	0.750307	0.932203	lncRNA
ENSG0000(	ENSG0000(	78.79596	-0.0389	0.749284	0.932203	lncRNA
ENSG0000(	ENSG0000(	179.2212	0.036064	0.749358	0.932203	lncRNA
ENSG0000(	ENSG0000(	75.51197	-0.09349	0.749581	0.932203	lncRNA
ENSG0000(	ENSG0000(	71.47637	-0.04515	0.749907	0.932203	lncRNA
ENSG0000(	ENSG0000(	31.62334	0.068976	0.747323	0.932203	lncRNA
ENSG0000(	ENSG0000(	333.099	-0.05323	0.747915	0.932203	lncRNA
ENSG0000(	ENSG0000(	178.8699	-0.03966	0.749485	0.932203	lncRNA
ENSG0000(	ENSG0000(	291.1182	-0.02977	0.747735	0.932203	lncRNA
ENSG0000(	ENSG0000(	257.0009	0.034476	0.749879	0.932203	lncRNA
ENSG0000(	ENSG0000(	35.24647	0.064154	0.747426	0.932203	lncRNA
ENSG0000(	ENSG0000(	32.57647	0.068451	0.747783	0.932203	lncRNA
ENSG0000(	ENSG0000(	28.93678	-0.08506	0.748538	0.932203	lncRNA
ENSG0000(	ENSG0000(	113.8829	0.043705	0.749979	0.932203	lncRNA
ENSG0000(	ENSG0000(	21.60543	0.084246	0.749733	0.932203	lncRNA
ENSG0000(	ENSG0000(	188.8013	0.068256	0.750219	0.932203	TEC
ENSG0000(	ENSG0000(	25.4328	-0.08295	0.747528	0.932203	lncRNA
ENSG0000(	ENSG0000(	34.51733	-0.07174	0.750531	0.932203	lncRNA
ENSG0000(	ENSG0000(	158.2961	-0.03836	0.750587	0.932203	lncRNA

ENSG0000(	ENSG0000(	29.38585	-0.08708	0.749763	0.932203	lncRNA
ENSG0000(	ENSG0000(	38.84687	-0.06479	0.748939	0.932203	lncRNA
ENSG0000(	ENSG0000(	857.5974	-0.01932	0.74847	0.932203	lncRNA
ENSG0000(	ENSG0000(	534.5523	0.023189	0.748261	0.932203	lncRNA
ENSG0000(	ENSG0000(	172.8979	-0.0475	0.749627	0.932203	lncRNA
ENSG0000(	ENSG0000(	21.57953	-0.10286	0.748736	0.932203	lncRNA
ENSG0000(	ENSG0000(	33.33145	-0.07401	0.748841	0.932203	lncRNA
ENSG0000(	ENSG0000(	520.7533	-0.0399	0.749633	0.932203	lncRNA
ENSG0000(	ENSG0000(	121.0494	-0.0405	0.747816	0.932203	lncRNA
ENSG0000(	ERCC8	3103.606	-0.01357	0.747471	0.932203	protein_coding
ENSG0000(	ERVK13-1	5326.496	0.028139	0.750436	0.932203	lncRNA
ENSG0000(	FAM138A	146.8022	0.078695	0.748465	0.932203	lncRNA
ENSG0000(	FKRP	511.5765	-0.02107	0.75002	0.932203	protein_coding
ENSG0000(	FLVCR2	773.2604	-0.06472	0.749599	0.932203	protein_coding
ENSG0000(	GALNT13	52.40553	0.398342	0.750072	0.932203	protein_coding
ENSG0000(	GLMP	378.1746	0.035284	0.748766	0.932203	protein_coding
ENSG0000(	GLYR1	1678.345	-0.01527	0.748651	0.932203	protein_coding
ENSG0000(	GNA13	2513.596	-0.03407	0.750143	0.932203	protein_coding
ENSG0000(	GTF2E1	332.4462	0.031391	0.748756	0.932203	protein_coding
ENSG0000(	HNF4A-AS1	25.85129	0.075811	0.748214	0.932203	lncRNA
ENSG0000(	ITCH	3199.398	0.029318	0.749313	0.932203	protein_coding
ENSG0000(	KCNH3	27.65543	-0.14535	0.749521	0.932203	protein_coding
ENSG0000(	KCTD19	284.5949	-0.04015	0.747932	0.932203	protein_coding
ENSG0000(	KLHDC3	370.413	-0.03	0.75053	0.932203	protein_coding
ENSG0000(	KRT8P52	23.64602	-0.08794	0.749731	0.932203	processed_pseudogene
ENSG0000(	LGALS2	42.84636	0.095683	0.750374	0.932203	protein_coding
ENSG0000(	LINC00293	22.65609	0.117626	0.750219	0.932203	lncRNA
ENSG0000(	LINC00863	685.6387	0.024863	0.748124	0.932203	lncRNA
ENSG0000(	LINC01951	68.60455	-0.0583	0.750317	0.932203	lncRNA
ENSG0000(	LINC02541	113.0446	-0.06564	0.750373	0.932203	lncRNA
ENSG0000(	LINC02805	151.3144	0.040118	0.748211	0.932203	lncRNA
ENSG0000(	MAP2K7	952.837	0.022519	0.749092	0.932203	protein_coding
ENSG0000(	MICA	1242.547	0.046122	0.749431	0.932203	protein_coding
ENSG0000(	MT-RNR1	9240.058	0.082378	0.748781	0.932203	Mt_rRNA
ENSG0000(	MTATP6P1	1623.632	0.073703	0.74945	0.932203	unprocessed_pseudogene
ENSG0000(	PER1	1159.777	0.077968	0.747245	0.932203	protein_coding
ENSG0000(	PRR34	75.81818	0.044146	0.748732	0.932203	lncRNA
ENSG0000(	RN7SKP16(	34.71802	0.06375	0.748054	0.932203	misc_RNA
ENSG0000(	RN7SKP22	36.59431	0.086462	0.749812	0.932203	misc_RNA
ENSG0000(	RN7SL386F	38.04098	0.066483	0.748952	0.932203	misc_RNA
ENSG0000(	RN7SL483F	34.56009	-0.06656	0.748815	0.932203	misc_RNA
ENSG0000(	RNF182	21.67209	0.096104	0.749179	0.932203	protein_coding
ENSG0000(	RNU5A-1	3319.758	0.058439	0.748321	0.932203	snRNA
ENSG0000(	SDSL	135.6607	-0.04858	0.7505	0.932203	protein_coding
ENSG0000(	SIRPG-AS1	125.2216	-0.04838	0.749549	0.932203	lncRNA
ENSG0000(	SLC22A13	111.8805	0.04715	0.747429	0.932203	protein_coding
ENSG0000(	SMAD2	11870.83	-0.01948	0.747982	0.932203	protein_coding

ENSG0000(	TAGLN	1265.586	-0.06608	0.748066	0.932203	protein_coding
ENSG0000(	TCEANC	376.0103	-0.02811	0.748528	0.932203	protein_coding
ENSG0000(	TCFL5	196.8159	0.027997	0.748208	0.932203	protein_coding
ENSG0000(	TESPA1	607.8682	-0.05786	0.749448	0.932203	protein_coding
ENSG0000(	TJAP1	1107.305	-0.02135	0.749298	0.932203	protein_coding
ENSG0000(	TLR10	709.5555	-0.08502	0.748378	0.932203	protein_coding
ENSG0000(	TMEM198E	958.5726	-0.02327	0.74773	0.932203	transcribed_unitary_pseudogene
ENSG0000(	TMEM68	635.6134	-0.02492	0.748809	0.932203	protein_coding
ENSG0000(	TOR1AIP2	6465.282	-0.01982	0.750253	0.932203	protein_coding
ENSG0000(	TRADD	371.6055	-0.02676	0.750042	0.932203	protein_coding
ENSG0000(	TRAF3IP2-A	1047.155	-0.0215	0.747526	0.932203	lncRNA
ENSG0000(	TTLL10	110.4879	-0.05502	0.750573	0.932203	protein_coding
ENSG0000(	UBE2O	699.242	-0.03138	0.750541	0.932203	protein_coding
ENSG0000(	UBE3A	4151.659	-0.01874	0.74835	0.932203	protein_coding
ENSG0000(	YLPM1	4272.409	-0.02487	0.74952	0.932203	protein_coding
ENSG0000(	ZBTB34	799.9364	0.021547	0.748348	0.932203	protein_coding
ENSG0000(	LINC01476	409.5154	0.026484	0.750695	0.932294	lncRNA
ENSG0000(	CDC27P3	47.96776	0.13298	0.750763	0.932329	processed_pseudogene
ENSG0000(	ENSG0000(	129.8967	0.031277	0.750791	0.932329	lncRNA
ENSG0000(	AGO3	5281.739	-0.01942	0.750967	0.932331	protein_coding
ENSG0000(	FIRRE	948.6731	0.022614	0.750988	0.932331	lncRNA
ENSG0000(	GLDC	349.3207	-0.03883	0.750992	0.932331	protein_coding
ENSG0000(	LIN7B	256.4413	-0.03363	0.750996	0.932331	protein_coding
ENSG0000(	LINC01381	21.44186	0.066551	0.750877	0.932331	lncRNA
ENSG0000(	RNF186	258.3091	-0.07232	0.750932	0.932331	protein_coding
ENSG0000(	LINC02891	47.92049	0.06392	0.751122	0.932446	lncRNA
ENSG0000(	H2BC12	203.1523	-0.05384	0.751237	0.932504	protein_coding
ENSG0000(	PSMD13	801.4111	-0.02211	0.751203	0.932504	protein_coding
ENSG0000(	DHX36	2321.129	-0.02254	0.751365	0.932558	protein_coding
ENSG0000(	ENSG0000(	78.49128	-0.12425	0.751328	0.932558	lncRNA
ENSG0000(	WBP1L	1420.943	-0.0254	0.751382	0.932558	protein_coding
ENSG0000(	ZNF257	268.4669	-0.04228	0.751415	0.932558	protein_coding
ENSG0000(	RN7SL296F	61.48238	-0.06384	0.75148	0.932588	misc_RNA
ENSG0000(	TOMM22	434.3334	-0.04824	0.751508	0.932588	protein_coding
ENSG0000(	ENSG0000(	348.9068	-0.04245	0.75169	0.932688	lncRNA
ENSG0000(	FAM184A	95.63217	-0.085	0.75168	0.932688	protein_coding
ENSG0000(	PLEKHG4B	356.0433	-0.03951	0.751646	0.932688	protein_coding
ENSG0000(	ENSG0000(	95.36009	0.052978	0.751745	0.932714	lncRNA
ENSG0000(	ARPC4-TTL	76.52801	-0.06626	0.751964	0.932827	protein_coding
ENSG0000(	DDX3X	9359.118	-0.01925	0.752295	0.932827	protein_coding
ENSG0000(	ENSG0000(	50.24159	-0.06484	0.752134	0.932827	lncRNA
ENSG0000(	ENSG0000(	91.31761	-0.05461	0.75229	0.932827	lncRNA
ENSG0000(	ENSG0000(	116.6081	-0.03746	0.751976	0.932827	lncRNA
ENSG0000(	ENSG0000(	220.2894	0.030136	0.752047	0.932827	lncRNA
ENSG0000(	ENSG0000(	297.3476	-0.0274	0.75224	0.932827	lncRNA
ENSG0000(	LPP	20933.86	-0.02959	0.752313	0.932827	protein_coding
ENSG0000(	PRKCSH	1194.749	0.026023	0.75227	0.932827	protein_coding

ENSG0000(	PTOV1-AS2	184.3601	0.036018	0.752124	0.932827	lncRNA
ENSG0000(	RN7SKP134	26.01835	0.078197	0.752345	0.932827	misc_RNA
ENSG0000(	RPL3	5049.36	0.035598	0.752313	0.932827	protein_coding
ENSG0000(	TBC1D27P	416.5996	0.041095	0.751918	0.932827	transcribed_unprocessed_pseudo
ENSG0000(	USP9Y	1564.648	-0.03461	0.752065	0.932827	protein_coding
ENSG0000(	ZNF431	2432.368	-0.02635	0.752169	0.932827	protein_coding
ENSG0000(	ATP6V0B	435.9332	-0.04004	0.752495	0.932872	protein_coding
ENSG0000(	ENSG0000(	55.46889	-0.05557	0.752514	0.932872	lncRNA
ENSG0000(	HSD3B7	299.6711	-0.03637	0.752494	0.932872	protein_coding
ENSG0000(	ZSCAN29	1345.509	-0.01958	0.752516	0.932872	protein_coding
ENSG0000(	COL6A6	54.29603	-0.48998	0.752636	0.932895	protein_coding
ENSG0000(	FEZF2	30.31478	-0.21467	0.752607	0.932895	protein_coding
ENSG0000(	MCMBP	886.0137	0.019836	0.75258	0.932895	protein_coding
ENSG0000(	RDM1P4	51.54284	0.102882	0.752732	0.932971	transcribed_processed_pseudoge
ENSG0000(	ENSG0000(	34.66814	-0.07459	0.753053	0.933228	lncRNA
ENSG0000(	ENSG0000(	367.9558	-0.06205	0.753075	0.933228	protein_coding
ENSG0000(	ENSG0000(	103.0198	0.044098	0.753049	0.933228	lncRNA
ENSG0000(	SCUBE3	171.7967	0.028444	0.752981	0.933228	protein_coding
ENSG0000(	DENND6B	333.0069	-0.03748	0.753283	0.933301	protein_coding
ENSG0000(	ENSG0000(	89.3935	-0.04322	0.753371	0.933301	processed_pseudogene
ENSG0000(	ENSG0000(	111.3638	-0.03921	0.753366	0.933301	lncRNA
ENSG0000(	ESD	417.2517	-0.03702	0.753315	0.933301	protein_coding
ENSG0000(	RN7SL198F	77.61697	-0.05397	0.753336	0.933301	misc_RNA
ENSG0000(	RN7SL403F	77.19071	0.037256	0.753336	0.933301	misc_RNA
ENSG0000(	RUSC1-AS1	316.8691	0.032994	0.753202	0.933301	lncRNA
ENSG0000(	HDAC5	689.3194	0.034997	0.753437	0.933341	protein_coding
ENSG0000(	EFHD1	86.47129	-0.04145	0.753554	0.933444	protein_coding
ENSG0000(	ENSG0000(	19.62312	-0.0995	0.753633	0.933457	lncRNA
ENSG0000(	LINC01104	99.22508	-0.04464	0.753621	0.933457	lncRNA
ENSG0000(	CSMD1	151.8563	-0.08643	0.753687	0.93346	protein_coding
ENSG0000(	PYURF	303.5595	-0.04238	0.753703	0.93346	protein_coding
ENSG0000(	CD99P1	782.6432	-0.02303	0.754002	0.933639	transcribed_unprocessed_pseudo
ENSG0000(	ENSG0000(	195.004	0.030348	0.753967	0.933639	TEC
ENSG0000(	IFT172	2086.773	-0.01845	0.754052	0.933639	protein_coding
ENSG0000(	MAGED4	41.63982	0.262744	0.753965	0.933639	protein_coding
ENSG0000(	PIGA	765.6776	0.025932	0.753967	0.933639	protein_coding
ENSG0000(	POLE4	189.9696	-0.03877	0.754026	0.933639	protein_coding
ENSG0000(	BAIAP2L1	2204.382	-0.05609	0.754596	0.933683	protein_coding
ENSG0000(	CCL21	342.0584	0.166805	0.754564	0.933683	protein_coding
ENSG0000(	EEF1A1	40316.47	-0.03829	0.754181	0.933683	protein_coding
ENSG0000(	EIF1AD	552.4551	0.02204	0.754539	0.933683	protein_coding
ENSG0000(	ENSG0000(	50.20708	0.069179	0.754511	0.933683	lncRNA
ENSG0000(	ENSG0000(	31.63393	-0.06991	0.754429	0.933683	processed_pseudogene
ENSG0000(	ENSG0000(	162.5776	0.039107	0.754396	0.933683	lncRNA
ENSG0000(	ENSG0000(	89.9108	-0.04564	0.754296	0.933683	lncRNA
ENSG0000(	ENSG0000(	50.71554	0.054501	0.754429	0.933683	TEC
ENSG0000(	ENSG0000(	118.5357	0.032199	0.754365	0.933683	lncRNA

ENSG0000(	LINC01205	717.9708	0.021042	0.754419	0.933683	lncRNA
ENSG0000(	MIR5687	35.88539	0.109275	0.75445	0.933683	miRNA
ENSG0000(	MRPS18A	216.1128	0.038387	0.754548	0.933683	protein_coding
ENSG0000(	SLC14A1	298.1653	0.036775	0.754254	0.933683	protein_coding
ENSG0000(	TRMT2B-A!	131.6043	-0.03515	0.754143	0.933683	lncRNA
ENSG0000(	IGLV1-41	26.48573	-0.23004	0.754652	0.93371	IG_V_pseudogene
ENSG0000(	AKAP7	348.5264	-0.03393	0.754908	0.933776	protein_coding
ENSG0000(	CNTNAP2	732.287	-0.08196	0.754883	0.933776	protein_coding
ENSG0000(	ENSG0000(	51.21194	-0.09286	0.754799	0.933776	snoRNA
ENSG0000(	FAM20B	1255.233	0.021133	0.75482	0.933776	protein_coding
ENSG0000(	PCNX2	1898.257	0.020996	0.754759	0.933776	protein_coding
ENSG0000(	TESC	53.69781	0.064206	0.754907	0.933776	protein_coding
ENSG0000(	ENSG0000(	68.24011	-0.0616	0.755053	0.933787	lncRNA
ENSG0000(	FAM214A	3474.664	-0.02433	0.754995	0.933787	protein_coding
ENSG0000(	GNAO1-DT	1072.958	-0.01654	0.754985	0.933787	lncRNA
ENSG0000(	UFSP2	206.9428	0.046914	0.755049	0.933787	protein_coding
ENSG0000(	CHRM3	801.3611	-0.06	0.755187	0.933839	protein_coding
ENSG0000(	ENSG0000(	235.6895	-0.03071	0.75524	0.933839	lncRNA
ENSG0000(	ENSG0000(	140.2477	0.049167	0.755255	0.933839	lncRNA
ENSG0000(	FASTKD3	219.8966	0.049966	0.755185	0.933839	protein_coding
ENSG0000(	RN7SL812F	23.20842	-0.06969	0.755282	0.933839	misc_RNA
ENSG0000(	ZNF567	430.9132	-0.03425	0.755299	0.933839	protein_coding
ENSG0000(	ANTKMT	66.63757	0.054246	0.755463	0.933874	protein_coding
ENSG0000(	CAMKK2	1769.103	-0.02763	0.755389	0.933874	protein_coding
ENSG0000(	ENSG0000(	79.03241	0.062014	0.755444	0.933874	lncRNA
ENSG0000(	ENSG0000(	141.619	-0.68548	0.755451	0.933874	protein_coding
ENSG0000(	ABT1	310.9586	-0.02213	0.756768	0.933929	protein_coding
ENSG0000(	ARMCX5	508.31	0.024335	0.756122	0.933929	protein_coding
ENSG0000(	C8orf76	290.0657	0.030584	0.756871	0.933929	protein_coding
ENSG0000(	CASTOR3	499.1904	-0.03345	0.756757	0.933929	transcribed_unprocessed_pseudo
ENSG0000(	CCHCR1	618.2663	0.036014	0.75571	0.933929	protein_coding
ENSG0000(	CCSER2	1972.629	0.02634	0.756715	0.933929	protein_coding
ENSG0000(	CLCA4	5170.895	-0.09457	0.756109	0.933929	protein_coding
ENSG0000(	DANCR	1472.844	0.029852	0.755851	0.933929	lncRNA
ENSG0000(	DEPDC1B	136.9149	-0.04841	0.756315	0.933929	protein_coding
ENSG0000(	ENOX2	227.8082	-0.04824	0.755852	0.933929	protein_coding
ENSG0000(	ENSG0000(	198.6646	-0.05628	0.756949	0.933929	transcribed_unprocessed_pseudo
ENSG0000(	ENSG0000(	197.1804	-0.03046	0.75692	0.933929	lncRNA
ENSG0000(	ENSG0000(	23.71366	0.085709	0.756261	0.933929	processed_pseudogene
ENSG0000(	ENSG0000(	175.241	-0.04683	0.755649	0.933929	lncRNA
ENSG0000(	ENSG0000(	25.31536	-0.60491	0.756399	0.933929	unprocessed_pseudogene
ENSG0000(	ENSG0000(	633.0975	0.028541	0.756457	0.933929	lncRNA
ENSG0000(	ENSG0000(	144.1704	-0.0571	0.755973	0.933929	protein_coding
ENSG0000(	ENSG0000(	74.34001	-0.05485	0.756832	0.933929	lncRNA
ENSG0000(	ENSG0000(	81.66034	0.049193	0.7563	0.933929	lncRNA
ENSG0000(	ENSG0000(	32.18438	-0.08112	0.756697	0.933929	lncRNA
ENSG0000(	ENSG0000(	190.5584	0.040496	0.75648	0.933929	TEC

ENSG0000(	ENSG0000(	115.4189	-0.0337	0.755971	0.933929	transcribed_unprocessed_pseudo
ENSG0000(	ENSG0000(	139.907	0.031976	0.75692	0.933929	lncRNA
ENSG0000(	FAM156B	1844.347	0.049517	0.755593	0.933929	protein_coding
ENSG0000(	GTPBP8	335.0828	-0.02599	0.755898	0.933929	protein_coding
ENSG0000(	HDAC2-AS2	104.9806	0.039435	0.755896	0.933929	lncRNA
ENSG0000(	HGS	1877.844	0.046557	0.756228	0.933929	protein_coding
ENSG0000(	HSPA1A	658.5057	0.078757	0.756092	0.933929	protein_coding
ENSG0000(	KCTD10	1884.392	-0.02767	0.756392	0.933929	protein_coding
ENSG0000(	LSM3	345.4909	0.023987	0.7569	0.933929	protein_coding
ENSG0000(	MSI1	157.9865	-0.04786	0.756605	0.933929	protein_coding
ENSG0000(	ONECUT1	22.65291	0.106136	0.75667	0.933929	protein_coding
ENSG0000(	OR1I1	3777.067	-0.07122	0.756484	0.933929	protein_coding
ENSG0000(	OR6C66P	122.9156	0.051665	0.756231	0.933929	transcribed_unprocessed_pseudo
ENSG0000(	OR7E100P	22.58609	-0.0805	0.756514	0.933929	unprocessed_pseudogene
ENSG0000(	PCMT1	291.6104	-0.03641	0.755674	0.933929	protein_coding
ENSG0000(	PPID	326.3875	0.023807	0.756728	0.933929	protein_coding
ENSG0000(	PSG10P	40.91173	-0.10309	0.755795	0.933929	transcribed_unprocessed_pseudo
ENSG0000(	RNU6ATAC	53.50359	0.06307	0.756967	0.933929	snRNA
ENSG0000(	SCAPER	1081.516	0.034221	0.755937	0.933929	protein_coding
ENSG0000(	SPART-AS1	121.5586	-0.05214	0.756115	0.933929	lncRNA
ENSG0000(	ZNF543	504.7276	-0.02902	0.75694	0.933929	protein_coding
ENSG0000(	ZNF829	1050.724	0.026748	0.756566	0.933929	protein_coding
ENSG0000(	BUD31	382.6354	0.027828	0.757019	0.933951	protein_coding
ENSG0000(	CSNK2B	776.6808	-0.02711	0.757128	0.934002	protein_coding
ENSG0000(	ESRG	473.5333	-0.05008	0.757102	0.934002	lncRNA
ENSG0000(	ADAP2	586.5065	-0.03155	0.757425	0.934314	protein_coding
ENSG0000(	FSD1	223.1375	0.026565	0.757449	0.934314	protein_coding
ENSG0000(	SLMAP	3724.941	0.026536	0.757577	0.93443	protein_coding
ENSG0000(	ENSG0000(	142.6695	0.03396	0.757654	0.934442	lncRNA
ENSG0000(	ENSG0000(	82.05754	0.054811	0.757686	0.934442	lncRNA
ENSG0000(	LINC00621	2470.817	-0.01558	0.757689	0.934442	lncRNA
ENSG0000(	DDX56	1011.836	0.030064	0.757835	0.934466	protein_coding
ENSG0000(	DGKA	3560.367	-0.03068	0.757878	0.934466	protein_coding
ENSG0000(	ENSG0000(	1081.636	0.015711	0.757748	0.934466	lncRNA
ENSG0000(	KCNV1	667.794	-0.02539	0.757833	0.934466	protein_coding
ENSG0000(	SLC26A8	308.5671	-0.03517	0.757844	0.934466	protein_coding
ENSG0000(	AMD1P3	20.44504	-0.09734	0.758173	0.934556	processed_pseudogene
ENSG0000(	DSN1	358.546	0.025243	0.758272	0.934556	protein_coding
ENSG0000(	ENSG0000(	167.1277	-0.03209	0.758298	0.934556	lncRNA
ENSG0000(	ENSG0000(	301.8486	-0.03207	0.758098	0.934556	lncRNA
ENSG0000(	ENSG0000(	41.69107	0.062129	0.758292	0.934556	lncRNA
ENSG0000(	ENSG0000(	31.71756	0.08317	0.758325	0.934556	lncRNA
ENSG0000(	MDS2	111.672	0.053933	0.758179	0.934556	lncRNA
ENSG0000(	PDE2A	173.0366	-0.06675	0.758226	0.934556	protein_coding
ENSG0000(	SASS6	170.769	0.049438	0.758024	0.934556	protein_coding
ENSG0000(	SLC46A3	729.7637	-0.04306	0.75812	0.934556	protein_coding
ENSG0000(	SRPK1	2316.424	-0.02938	0.758199	0.934556	protein_coding

ENSG0000(	HSPBAP1	695.9362	-0.02886	0.758382	0.934584	protein_coding
ENSG0000(	CCDC198	148.4736	0.03773	0.758657	0.934637	protein_coding
ENSG0000(	FBXO33	403.8155	-0.02849	0.758523	0.934637	protein_coding
ENSG0000(	HADHB	1322.325	-0.03307	0.758672	0.934637	protein_coding
ENSG0000(	LINC00578	475.7578	-0.04013	0.758644	0.934637	lncRNA
ENSG0000(	MT-ND1	11320.93	-0.06066	0.758696	0.934637	protein_coding
ENSG0000(	NDUFA9P1	86.46209	0.050968	0.758503	0.934637	processed_pseudogene
ENSG0000(	NUTM2B-A	5484.265	0.023268	0.758589	0.934637	lncRNA
ENSG0000(	RN7SL481F	46.10839	-0.05482	0.758651	0.934637	misc_RNA
ENSG0000(	SRPK3	99.88279	-0.0588	0.758746	0.934657	protein_coding
ENSG0000(	GPR89A	661.881	0.036388	0.758857	0.934668	protein_coding
ENSG0000(	MT-CO3	26439.28	0.090127	0.758796	0.934668	protein_coding
ENSG0000(	ZNF35	191.8268	-0.03507	0.758833	0.934668	protein_coding
ENSG0000(	ENSG0000(	23.06934	-0.11676	0.759395	0.93488	lncRNA
ENSG0000(	ENSG0000(	24.10401	0.088955	0.759185	0.93488	lncRNA
ENSG0000(	ENSG0000(	180.389	0.030294	0.759437	0.93488	TEC
ENSG0000(	FGF12	202.8032	0.04862	0.759283	0.93488	protein_coding
ENSG0000(	METTL7A	2028.542	-0.03661	0.759197	0.93488	protein_coding
ENSG0000(	PDCD6-AHI	19.62844	1.129188	0.759331	0.93488	protein_coding
ENSG0000(	PPFIA2-AS1	27.64565	0.073223	0.759101	0.93488	lncRNA
ENSG0000(	RETREG1-A	111.3422	-0.06701	0.759192	0.93488	lncRNA
ENSG0000(	TTPAL	1056.808	0.017086	0.759257	0.93488	protein_coding
ENSG0000(	VARS1	528.85	-0.05116	0.759295	0.93488	protein_coding
ENSG0000(	ZNF235	643.1108	-0.03255	0.759436	0.93488	protein_coding
ENSG0000(	ZNF433	201.5486	-0.04568	0.759419	0.93488	protein_coding
ENSG0000(	MTCO3P21	44.95873	0.063924	0.759595	0.935033	unprocessed_pseudogene
ENSG0000(	ENSG0000(	21.28913	0.064729	0.759663	0.935044	lncRNA
ENSG0000(	GALR1	528.8064	0.021871	0.759672	0.935044	protein_coding
ENSG0000(	LEXM	37.10554	0.078877	0.759793	0.93515	protein_coding
ENSG0000(	IQCF3	60.05358	0.110929	0.759833	0.935158	protein_coding
ENSG0000(	ANXA2R	87.95901	-0.08077	0.760285	0.935196	protein_coding
ENSG0000(	ARMH3	1152.658	0.027372	0.760956	0.935196	protein_coding
ENSG0000(	CCDC174	562.2177	-0.02689	0.761413	0.935196	protein_coding
ENSG0000(	ENSG0000(	48.91613	0.080098	0.761041	0.935196	processed_pseudogene
ENSG0000(	ENSG0000(	23.71091	-0.10824	0.761387	0.935196	lncRNA
ENSG0000(	ENSG0000(	679.9004	-0.02162	0.760868	0.935196	lncRNA
ENSG0000(	ENSG0000(	31.86358	0.060456	0.760248	0.935196	lncRNA
ENSG0000(	ENSG0000(	68.78854	-0.06131	0.760565	0.935196	lncRNA
ENSG0000(	ENSG0000(	83.88387	0.049759	0.760269	0.935196	transcribed_processed_pseudoge
ENSG0000(	ENSG0000(	59.94713	0.037898	0.761374	0.935196	lncRNA
ENSG0000(	ENSG0000(	25.93155	0.133093	0.76105	0.935196	lncRNA
ENSG0000(	ENSG0000(	546.7728	-0.02218	0.761364	0.935196	lncRNA
ENSG0000(	ENSG0000(	21.86616	-0.1079	0.760725	0.935196	lncRNA
ENSG0000(	ENSG0000(	1333.453	0.024577	0.760084	0.935196	lncRNA
ENSG0000(	ENSG0000(	462.5404	0.023254	0.76145	0.935196	lncRNA
ENSG0000(	ENSG0000(	54.91568	-0.04396	0.76053	0.935196	lncRNA
ENSG0000(	ENSG0000(	30.85742	-0.08688	0.761329	0.935196	lncRNA

ENSG0000(	ENSG0000(	178.0801	0.040048	0.760901	0.935196	lncRNA
ENSG0000(	ENSG0000(	83.63221	-0.04796	0.760267	0.935196	lncRNA
ENSG0000(	EXOSC8	1005.735	-0.02136	0.760412	0.935196	protein_coding
ENSG0000(	FAM89B	212.0337	-0.03172	0.760918	0.935196	protein_coding
ENSG0000(	FKBP9	1087.191	0.028982	0.760446	0.935196	protein_coding
ENSG0000(	GMPS	1535.547	0.031784	0.760334	0.935196	protein_coding
ENSG0000(	HAGHL	101.3262	-0.0559	0.759932	0.935196	protein_coding
ENSG0000(	IQCB2P	27.0318	-0.08841	0.760045	0.935196	processed_pseudogene
ENSG0000(	KIFC1	306.0616	0.052225	0.761389	0.935196	protein_coding
ENSG0000(	LCN10	77.17653	0.11096	0.760556	0.935196	protein_coding
ENSG0000(	MGMT	430.8703	-0.02929	0.761335	0.935196	protein_coding
ENSG0000(	MT-ND2	11003.29	-0.06191	0.760929	0.935196	protein_coding
ENSG0000(	NSRP1P1	39.91263	0.087088	0.761373	0.935196	processed_pseudogene
ENSG0000(	POLR2C	646.8677	-0.02215	0.760992	0.935196	protein_coding
ENSG0000(	PRKD2	1605.592	-0.0243	0.76106	0.935196	protein_coding
ENSG0000(	PRPF38A	1538.711	-0.02226	0.761206	0.935196	protein_coding
ENSG0000(	RICTOR	3513.166	0.016807	0.760329	0.935196	protein_coding
ENSG0000(	ROR1-AS1	2040.444	0.018764	0.759958	0.935196	lncRNA
ENSG0000(	RPS3A	3182.956	-0.04189	0.760806	0.935196	protein_coding
ENSG0000(	SMURF1	1703.324	0.024807	0.760709	0.935196	protein_coding
ENSG0000(	SRRM1	2923.681	0.013831	0.76042	0.935196	protein_coding
ENSG0000(	TC2N	2920.97	-0.04014	0.761392	0.935196	protein_coding
ENSG0000(	THBS2	481.2129	-0.68706	0.761461	0.935196	protein_coding
ENSG0000(	TRIAP1	92.85864	-0.05518	0.760558	0.935196	protein_coding
ENSG0000(	TTLL4	935.0104	-0.03068	0.760004	0.935196	protein_coding
ENSG0000(	UTY	2716.031	-0.03309	0.760198	0.935196	protein_coding
ENSG0000(	WDR5	461.7268	0.027105	0.761007	0.935196	protein_coding
ENSG0000(	ZMYM2	4817.048	-0.01585	0.760093	0.935196	protein_coding
ENSG0000(	ZNF337-AS	509.5457	0.039205	0.760843	0.935196	lncRNA
ENSG0000(	ZNF652-AS	502.5308	-0.02802	0.760692	0.935196	lncRNA
ENSG0000(	CYP2C8	30.02875	-0.08945	0.761518	0.935219	protein_coding
ENSG0000(	ENSG0000(	32.41805	0.13087	0.761582	0.935219	protein_coding
ENSG0000(	FOXJ2	709.268	0.020769	0.76157	0.935219	protein_coding
ENSG0000(	CAMKMT	297.9588	0.031633	0.76223	0.935221	protein_coding
ENSG0000(	CDC20	119.8865	0.069129	0.761873	0.935221	protein_coding
ENSG0000(	ENSG0000(	1912.909	0.019508	0.762047	0.935221	lncRNA
ENSG0000(	ENSG0000(	73.91054	-0.04922	0.762072	0.935221	lncRNA
ENSG0000(	ENSG0000(	38.81023	0.063636	0.76204	0.935221	lncRNA
ENSG0000(	ENSG0000(	893.3072	0.045268	0.762139	0.935221	lncRNA
ENSG0000(	GPANK1	323.2985	0.025721	0.762172	0.935221	protein_coding
ENSG0000(	HSD17B3	46.743	0.099578	0.76219	0.935221	protein_coding
ENSG0000(	PPP2R3B	458.8988	0.03076	0.761782	0.935221	protein_coding
ENSG0000(	RHOB	880.7146	-0.05867	0.761898	0.935221	protein_coding
ENSG0000(	RN7SL153F	26.61033	-0.06688	0.762042	0.935221	misc_RNA
ENSG0000(	RPGRIP1	113.5187	-0.04846	0.761651	0.935221	protein_coding
ENSG0000(	SLTM	2648.948	0.022505	0.762119	0.935221	protein_coding
ENSG0000(	STAMBPL1	1186.018	0.03267	0.762228	0.935221	protein_coding

ENSG0000(	TMOD1	87.50811	0.079041	0.762028	0.935221	protein_coding
ENSG0000(	UPP2	28.03201	0.081798	0.761789	0.935221	protein_coding
ENSG0000(	ZNF280C	281.3532	-0.03401	0.76212	0.935221	protein_coding
ENSG0000(	ZNF528-AS	633.7476	0.018792	0.762134	0.935221	lncRNA
ENSG0000(	ZNHIT2	31.7419	0.064179	0.762061	0.935221	protein_coding
ENSG0000(	TSNAXIP1	26.67474	-0.10852	0.762288	0.935251	protein_coding
ENSG0000(	MAPK8IP3	3584.579	0.020308	0.76238	0.935322	protein_coding
ENSG0000(	GPR135	265.489	0.046287	0.762501	0.935345	protein_coding
ENSG0000(	STX18-AS1	4192.534	-0.01349	0.762476	0.935345	lncRNA
ENSG0000(	ZFHX3	4041.701	0.03752	0.762461	0.935345	protein_coding
ENSG0000(	EOLA1	546.0982	0.031553	0.76256	0.935377	protein_coding
ENSG0000(	ADGRG5	362.6935	-0.0693	0.762834	0.935387	protein_coding
ENSG0000(	BPTF	6900.634	-0.02346	0.762714	0.935387	protein_coding
ENSG0000(	ENSG0000(	21.75122	-0.06778	0.762891	0.935387	lncRNA
ENSG0000(	ENSG0000(	40.89264	0.060694	0.762907	0.935387	unprocessed_pseudogene
ENSG0000(	HMGCS1	1565.565	0.041699	0.762704	0.935387	protein_coding
ENSG0000(	KPNA5	5854.554	0.037186	0.762861	0.935387	protein_coding
ENSG0000(	LINC01687	653.057	0.060903	0.76285	0.935387	lncRNA
ENSG0000(	LINC02024	22.31765	-0.12738	0.762788	0.935387	lncRNA
ENSG0000(	MTMR9	1268.383	0.027339	0.762909	0.935387	protein_coding
ENSG0000(	RAB5C	832.7635	-0.0282	0.762613	0.935387	protein_coding
ENSG0000(	MRTFA-AS1	278.453	-0.02673	0.762949	0.935395	lncRNA
ENSG0000(	ARFGEF1-D	39.28042	0.066218	0.763007	0.935423	lncRNA
ENSG0000(	ADAL	447.6929	-0.02617	0.763672	0.935531	protein_coding
ENSG0000(	ATG101	208.5616	-0.0298	0.763546	0.935531	protein_coding
ENSG0000(	CD24	17684.01	0.069792	0.763483	0.935531	protein_coding
ENSG0000(	ENSG0000(	422.6093	0.02287	0.7636	0.935531	lncRNA
ENSG0000(	ENSG0000(	1478.963	-0.01846	0.763583	0.935531	transcribed_processed_pseudoge
ENSG0000(	ERC1	3095.938	-0.01475	0.763457	0.935531	protein_coding
ENSG0000(	HOGA1	178.2712	-0.04408	0.763395	0.935531	protein_coding
ENSG0000(	LINC00556	81.02381	-0.05166	0.763592	0.935531	lncRNA
ENSG0000(	LINC01067	33.15037	0.07635	0.763386	0.935531	lncRNA
ENSG0000(	LINC02449	416.3779	-0.02973	0.763361	0.935531	lncRNA
ENSG0000(	MRPS15	445.3515	0.024528	0.763252	0.935531	protein_coding
ENSG0000(	NAIPP1	115.2682	-0.05168	0.763663	0.935531	transcribed_unprocessed_pseudo
ENSG0000(	RAD17	636.4082	0.029537	0.763194	0.935531	protein_coding
ENSG0000(	RAET1E	310.5105	-0.03707	0.763198	0.935531	protein_coding
ENSG0000(	RN7SL838F	45.16869	0.061586	0.763175	0.935531	misc_RNA
ENSG0000(	RPS6KB1	1383.682	-0.01974	0.763611	0.935531	protein_coding
ENSG0000(	TNFRSF13C	1136.074	-0.07328	0.763604	0.935531	protein_coding
ENSG0000(	BOP1	187.9236	0.044095	0.763878	0.935551	protein_coding
ENSG0000(	ENSG0000(	49.80327	0.084526	0.763883	0.935551	lncRNA
ENSG0000(	ENSG0000(	240.4486	0.029997	0.763889	0.935551	TEC
ENSG0000(	IWS1	1120.829	0.022809	0.76386	0.935551	protein_coding
ENSG0000(	PEX14	199.4777	0.046028	0.763893	0.935551	protein_coding
ENSG0000(	SLC25A25	642.9488	0.039863	0.763745	0.935551	protein_coding
ENSG0000(	LINC02064	145.6868	-0.04073	0.764039	0.935689	lncRNA

ENSG0000(	TBC1D25	291.7116	-0.03776	0.764087	0.935706	protein_coding
ENSG0000(	ENSG0000(	21.40507	0.079986	0.764195	0.935714	lncRNA
ENSG0000(	ENSG0000(	102.4609	0.037407	0.76418	0.935714	protein_coding
ENSG0000(	SMG7	2224.517	-0.02335	0.764196	0.935714	protein_coding
ENSG0000(	TMEM205	161.3853	-0.03493	0.764269	0.935762	protein_coding
ENSG0000(	RANBP1	388.1879	0.036756	0.764371	0.935845	protein_coding
ENSG0000(	ENSG0000(	75.13234	-0.04455	0.764475	0.935931	lncRNA
ENSG0000(	ENSG0000(	394.1748	-0.02103	0.764577	0.936012	lncRNA
ENSG0000(	KIAA0586	1915.596	0.02107	0.764609	0.936012	protein_coding
ENSG0000(	KDM1B	1058.119	0.031454	0.764683	0.936061	protein_coding
ENSG0000(	AAMP	554.9657	0.043138	0.765177	0.936097	protein_coding
ENSG0000(	ACKR2	564.24	0.062446	0.766101	0.936097	protein_coding
ENSG0000(	ASF1A	180.174	-0.054	0.765721	0.936097	protein_coding
ENSG0000(	CALN1	202.7727	0.037008	0.765844	0.936097	protein_coding
ENSG0000(	CLEC3B	29.14086	0.089775	0.76482	0.936097	protein_coding
ENSG0000(	COQ2	362.2895	-0.03288	0.766113	0.936097	protein_coding
ENSG0000(	DCAF5	2099.143	-0.0162	0.765431	0.936097	protein_coding
ENSG0000(	DPY19L1P1	197.3202	-0.03229	0.765321	0.936097	unprocessed_pseudogene
ENSG0000(	EFHC2	36.80024	-0.07014	0.765955	0.936097	protein_coding
ENSG0000(	ENSG0000(	175.8184	0.041558	0.766215	0.936097	lncRNA
ENSG0000(	ENSG0000(	31.09164	0.076591	0.766169	0.936097	lncRNA
ENSG0000(	ENSG0000(	80.32404	-0.05328	0.765867	0.936097	lncRNA
ENSG0000(	ENSG0000(	109.8041	0.040675	0.765121	0.936097	lncRNA
ENSG0000(	ENSG0000(	320.7584	0.021932	0.765181	0.936097	lncRNA
ENSG0000(	ENSG0000(	200.6146	0.041864	0.765752	0.936097	TEC
ENSG0000(	ENSG0000(	57.55528	0.063839	0.765256	0.936097	lncRNA
ENSG0000(	ENSG0000(	75.64666	-0.05725	0.765334	0.936097	lncRNA
ENSG0000(	ENSG0000(	273.3691	0.027414	0.765376	0.936097	lncRNA
ENSG0000(	ENSG0000(	37.37409	0.060851	0.766119	0.936097	lncRNA
ENSG0000(	FXR2	551.6241	0.025433	0.765811	0.936097	protein_coding
ENSG0000(	H3C4	97.43773	-0.06774	0.765404	0.936097	protein_coding
ENSG0000(	H3P31	66.70595	0.054097	0.765866	0.936097	processed_pseudogene
ENSG0000(	LINC01139	286.9961	0.034141	0.765852	0.936097	lncRNA
ENSG0000(	LINC01551	1037.861	0.044698	0.765033	0.936097	lncRNA
ENSG0000(	LINC02450	49.50602	0.070148	0.766142	0.936097	lncRNA
ENSG0000(	LINC02615	290.2307	-0.03268	0.765595	0.936097	lncRNA
ENSG0000(	NANOGP8	67.94392	0.052563	0.764989	0.936097	protein_coding
ENSG0000(	NCEH1	609.1443	-0.05275	0.765329	0.936097	protein_coding
ENSG0000(	OBSL1	526.7644	0.042768	0.766174	0.936097	protein_coding
ENSG0000(	PAX8	129.7074	0.093329	0.766251	0.936097	protein_coding
ENSG0000(	POLR3K	464.6909	0.021458	0.766004	0.936097	protein_coding
ENSG0000(	POT1	457.2839	-0.03461	0.764824	0.936097	protein_coding
ENSG0000(	PTBP2	1359.792	-0.03917	0.765998	0.936097	protein_coding
ENSG0000(	RETREG2	1297.533	0.034521	0.766238	0.936097	protein_coding
ENSG0000(	RN7SL470F	139.0877	-0.03359	0.766219	0.936097	misc_RNA
ENSG0000(	RN7SL546F	35.02677	-0.05785	0.765783	0.936097	misc_RNA
ENSG0000(	RPS4XP2	26.82568	-0.0686	0.766312	0.936097	transcribed_processed_pseudoge

ENSG0000(	RSPH10B2	77.2768	0.12803	0.766068	0.936097	protein_coding
ENSG0000(	SIK1B	595.2743	0.109019	0.766311	0.936097	protein_coding
ENSG0000(	TMEM132I	45.55578	-0.09549	0.765281	0.936097	lncRNA
ENSG0000(	TOMM6	249.0536	-0.03326	0.76565	0.936097	protein_coding
ENSG0000(	TUBA8	541.4693	-0.02962	0.765272	0.936097	protein_coding
ENSG0000(	UBAP1	637.0147	0.024341	0.765886	0.936097	protein_coding
ENSG0000(	USF1P1	47.80144	-0.05974	0.765901	0.936097	processed_pseudogene
ENSG0000(	YIPF2	245.7158	-0.04386	0.764769	0.936097	protein_coding
ENSG0000(	ZNF2	282.817	-0.03084	0.765932	0.936097	protein_coding
ENSG0000(	ZNF680	564.2399	0.031927	0.765339	0.936097	protein_coding
ENSG0000(	COASY	757.7601	-0.03146	0.766483	0.93613	protein_coding
ENSG0000(	CSRP3	32.13331	0.048854	0.766969	0.93613	protein_coding
ENSG0000(	CYP4F29P	33.85925	-0.12336	0.766611	0.93613	transcribed_unprocessed_pseudo
ENSG0000(	DNASE1	1841.13	-0.02267	0.766819	0.93613	protein_coding
ENSG0000(	DRAIC	1010.732	0.026552	0.766402	0.93613	lncRNA
ENSG0000(	ENSG0000(	165.018	0.039109	0.766845	0.93613	lncRNA
ENSG0000(	ENSG0000(	184.338	-0.0337	0.766942	0.93613	lncRNA
ENSG0000(	ENSG0000(	46.45952	0.064204	0.76703	0.93613	lncRNA
ENSG0000(	ENSG0000(	353.7933	0.022155	0.766828	0.93613	processed_pseudogene
ENSG0000(	ENSG0000(	57.55789	-0.05057	0.767079	0.93613	lncRNA
ENSG0000(	ENSG0000(	24.80916	-0.07977	0.766973	0.93613	lncRNA
ENSG0000(	ENSG0000(	53.41476	-0.05005	0.766699	0.93613	processed_pseudogene
ENSG0000(	ENSG0000(	62.25496	0.042078	0.766416	0.93613	TEC
ENSG0000(	ENSG0000(	137.808	0.035857	0.766974	0.93613	lncRNA
ENSG0000(	GNAS-AS1	162.3857	0.036076	0.767011	0.93613	lncRNA
ENSG0000(	MEF2C-AS2	24.23729	-0.0819	0.766634	0.93613	lncRNA
ENSG0000(	OR2F1	160.7098	-0.03643	0.76712	0.93613	protein_coding
ENSG0000(	RN7SL441F	52.87439	-0.05326	0.766468	0.93613	misc_RNA
ENSG0000(	SDHD	1320.171	0.034537	0.766746	0.93613	protein_coding
ENSG0000(	THAP8	44.78379	0.067288	0.766816	0.93613	protein_coding
ENSG0000(	TRAJ17	25.48084	0.110444	0.767122	0.93613	TR_J_gene
ENSG0000(	TRIM41	2380.797	-0.01343	0.766875	0.93613	protein_coding
ENSG0000(	ZNF530	475.4147	-0.02772	0.766926	0.93613	protein_coding
ENSG0000(	AHRR	357.887	-0.08454	0.767199	0.936155	protein_coding
ENSG0000(	C2orf49-DT	204.9798	-0.03371	0.767244	0.936155	lncRNA
ENSG0000(	NT5C3A	924.7765	-0.03769	0.767228	0.936155	protein_coding
ENSG0000(	ARF1	2649.632	-0.02583	0.767396	0.936198	protein_coding
ENSG0000(	ENSG0000(	73.85764	0.039063	0.767337	0.936198	lncRNA
ENSG0000(	ENSG0000(	70.57053	-0.05411	0.767416	0.936198	lncRNA
ENSG0000(	ENSG0000(	40.26565	-0.04566	0.767348	0.936198	lncRNA
ENSG0000(	EPHA2	818.9413	-0.06146	0.767478	0.936233	protein_coding
ENSG0000(	PYCARD	104.1676	-0.05668	0.767517	0.936239	protein_coding
ENSG0000(	EFNA2	1225.632	0.071131	0.76762	0.936281	protein_coding
ENSG0000(	ENSG0000(	125.6858	0.041764	0.767616	0.936281	lncRNA
ENSG0000(	FRG2C	40.60163	0.068745	0.767829	0.936453	protein_coding
ENSG0000(	RUFY1-AS1	43.89692	0.064186	0.767819	0.936453	lncRNA
ENSG0000(	LINC01193	52.44181	0.055323	0.767982	0.936515	lncRNA

ENSG0000(	MID2	207.9488	-0.06675	0.767969	0.936515	protein_coding
ENSG0000(	NUDT2	119.633	-0.03352	0.767915	0.936515	protein_coding
ENSG0000(	ENSG0000(	752.338	0.013792	0.768052	0.936518	lncRNA
ENSG0000(	RSPH9	31.60076	0.083404	0.768026	0.936518	protein_coding
ENSG0000(	CDC14C	259.321	-0.03181	0.768263	0.936586	protein_coding
ENSG0000(	ENSG0000(	116.0865	-0.03565	0.768345	0.936586	lncRNA
ENSG0000(	NUBP2	494.1355	0.019596	0.768381	0.936586	protein_coding
ENSG0000(	PHF7	300.1303	0.038337	0.76825	0.936586	protein_coding
ENSG0000(	PLP2	395.2846	-0.05546	0.768376	0.936586	protein_coding
ENSG0000(	RNU6-890I	27.90548	0.06527	0.768204	0.936586	snRNA
ENSG0000(	RNY1P9	23.29513	-0.10135	0.76837	0.936586	misc_RNA
ENSG0000(	SETP12	32.34478	0.072737	0.768295	0.936586	processed_pseudogene
ENSG0000(	C4A-AS1	31.59065	-0.06227	0.768646	0.936643	lncRNA
ENSG0000(	CD8B2	92.20475	0.056436	0.76867	0.936643	protein_coding
ENSG0000(	ELMOD3	1450.455	-0.01903	0.768772	0.936643	protein_coding
ENSG0000(	ENSG0000(	23.97599	-0.08742	0.768734	0.936643	transcribed_processed_pseudoge
ENSG0000(	ENSG0000(	23.06667	-0.07235	0.76849	0.936643	lncRNA
ENSG0000(	ENSG0000(	121.9335	0.04089	0.768506	0.936643	lncRNA
ENSG0000(	ENSG0000(	28.95834	0.114949	0.768619	0.936643	lncRNA
ENSG0000(	ENSG0000(	60.20156	0.061372	0.768868	0.936643	lncRNA
ENSG0000(	MPRIP	7270.034	0.022293	0.768818	0.936643	protein_coding
ENSG0000(	PCDHB5	20.54489	0.106045	0.76887	0.936643	protein_coding
ENSG0000(	RPLP0	3891.168	0.040963	0.768853	0.936643	protein_coding
ENSG0000(	WDR18	124.3292	0.053765	0.768648	0.936643	protein_coding
ENSG0000(	ZNF461	381.9887	-0.02701	0.768692	0.936643	protein_coding
ENSG0000(	ENSG0000(	88.74262	-0.04048	0.769033	0.936801	lncRNA
ENSG0000(	NAA50	1983.284	-0.02156	0.769238	0.936967	protein_coding
ENSG0000(	PDCD6IP-D	445.652	0.055498	0.769233	0.936967	lncRNA
ENSG0000(	BBOX1-AS1	65.03984	-0.04996	0.769703	0.937036	lncRNA
ENSG0000(	BEST3	417.6213	0.028045	0.769508	0.937036	protein_coding
ENSG0000(	ENSG0000(	36.48438	0.060017	0.769541	0.937036	lncRNA
ENSG0000(	ENSG0000(	202.9839	0.053526	0.769477	0.937036	TEC
ENSG0000(	ENSG0000(	56.22797	0.048847	0.769529	0.937036	lncRNA
ENSG0000(	ENSG0000(	40.36586	0.068767	0.769651	0.937036	lncRNA
ENSG0000(	KMT2A	9800.929	-0.02016	0.769695	0.937036	protein_coding
ENSG0000(	LINC01289	93.55728	-0.03786	0.769334	0.937036	lncRNA
ENSG0000(	LINC02808	23.48768	-0.08334	0.769631	0.937036	lncRNA
ENSG0000(	PARP6	2304.738	-0.01546	0.769371	0.937036	protein_coding
ENSG0000(	RNVU1-27	73.4081	-0.04923	0.769578	0.937036	snRNA
ENSG0000(	SHF	228.1541	0.057318	0.769398	0.937036	protein_coding
ENSG0000(	ENSG0000(	76.91553	0.035254	0.769888	0.937095	lncRNA
ENSG0000(	ENSG0000(	76.07466	-0.04989	0.769827	0.937095	lncRNA
ENSG0000(	NFILZ	340.1669	-0.02292	0.769877	0.937095	protein_coding
ENSG0000(	NOP16	214.9413	0.041719	0.769804	0.937095	protein_coding
ENSG0000(	ENSG0000(	29.10835	0.084216	0.770363	0.937113	lncRNA
ENSG0000(	ENSG0000(	550.2194	0.044714	0.770302	0.937113	lncRNA
ENSG0000(	ENSG0000(	291.9167	-0.02224	0.770046	0.937113	lncRNA

ENSG0000(	HDGFL3	1341.879	-0.03894	0.770003	0.937113	protein_coding
ENSG0000(	HTR3B	695.3868	0.01919	0.770162	0.937113	protein_coding
ENSG0000(	KANSL2	523.5766	0.026369	0.770358	0.937113	protein_coding
ENSG0000(	LINC02141	243.6165	-0.03239	0.770418	0.937113	lncRNA
ENSG0000(	MON1A	204.5566	-0.03647	0.770394	0.937113	protein_coding
ENSG0000(	NOS1	723.0346	0.01979	0.770323	0.937113	protein_coding
ENSG0000(	NRBF2P6	30.44126	0.083702	0.770232	0.937113	processed_pseudogene
ENSG0000(	NSUN4	1160.673	0.029816	0.770355	0.937113	protein_coding
ENSG0000(	PDE4DIPP6	208.7837	-0.03143	0.769967	0.937113	transcribed_unprocessed_pseudo
ENSG0000(	PRR13	2335.564	-0.03611	0.770448	0.937113	protein_coding
ENSG0000(	SLIT1	46.56807	-0.07474	0.770266	0.937113	protein_coding
ENSG0000(	WDR45B	1173.964	0.021768	0.770359	0.937113	protein_coding
ENSG0000(	ZNF136	866.6424	0.023321	0.770306	0.937113	protein_coding
ENSG0000(	CRYBB2P1	1847.739	-0.02766	0.770534	0.937135	transcribed_unprocessed_pseudo
ENSG0000(	ENSG0000(	40.00908	-0.06084	0.770502	0.937135	lncRNA
ENSG0000(	LINC01836	342.6769	-0.02556	0.770622	0.937201	lncRNA
ENSG0000(	ENSG0000(	34.64679	-0.06336	0.77079	0.937322	lncRNA
ENSG0000(	ENSG0000(	171.0655	0.029299	0.77076	0.937322	lncRNA
ENSG0000(	SLC12A7	2426.978	-0.04186	0.770858	0.937363	protein_coding
ENSG0000(	CTNNA2-AS	141.3952	0.044954	0.770959	0.937445	lncRNA
ENSG0000(	CTAGE4	409.0673	0.15222	0.770997	0.937449	protein_coding
ENSG0000(	ENSG0000(	26.31172	-0.06897	0.771115	0.937552	TEC
ENSG0000(	KLHL38	78.07521	0.042674	0.771214	0.937559	protein_coding
ENSG0000(	UBALD2	380.015	-0.03338	0.771223	0.937559	protein_coding
ENSG0000(	USP20	668.9085	0.039885	0.771182	0.937559	protein_coding
ENSG0000(	ENSG0000(	85.55832	-0.05973	0.771369	0.937653	lncRNA
ENSG0000(	NDUFC1	260.5645	-0.04866	0.771338	0.937653	protein_coding
ENSG0000(	NLGN3	579.0245	0.018758	0.77148	0.93767	protein_coding
ENSG0000(	SOD2-OT1	66.29855	0.04088	0.771485	0.93767	lncRNA
ENSG0000(	WDR33	3390.032	-0.01466	0.771475	0.93767	protein_coding
ENSG0000(	ENSG0000(	376.3343	0.025562	0.771563	0.937672	lncRNA
ENSG0000(	ENSG0000(	324.5	0.025963	0.771576	0.937672	lncRNA
ENSG0000(	RPS5	936.0175	-0.03944	0.771589	0.937672	protein_coding
ENSG0000(	ARL6IP6	3593.185	0.023679	0.771657	0.937713	protein_coding
ENSG0000(	ENSG0000(	120.4503	0.03008	0.771739	0.937771	lncRNA
ENSG0000(	ENSG0000(	25.49375	-0.08937	0.771841	0.937812	lncRNA
ENSG0000(	TRAF3IP1	498.4767	-0.02048	0.77184	0.937812	protein_coding
ENSG0000(	HAS3	563.7119	0.113319	0.771966	0.937845	protein_coding
ENSG0000(	MAEA	2035.121	0.017998	0.772038	0.937845	protein_coding
ENSG0000(	PMS2P7	39.33321	0.066256	0.771912	0.937845	unprocessed_pseudogene
ENSG0000(	PTGER3	93.71094	-0.08669	0.772007	0.937845	protein_coding
ENSG0000(	ZNF485	171.2525	0.037494	0.771975	0.937845	protein_coding
ENSG0000(	MAP3K20-/	31.74966	-0.12537	0.772182	0.937895	lncRNA
ENSG0000(	PGA3	55.10162	0.060976	0.772155	0.937895	protein_coding
ENSG0000(	SERTAD3	327.01	0.040685	0.772149	0.937895	protein_coding
ENSG0000(	ABAT	1081.014	-0.02196	0.772253	0.937899	protein_coding
ENSG0000(	ENSG0000(	61.52955	-0.05263	0.772246	0.937899	lncRNA

ENSG0000(	PGGT1B	1810.048	-0.02434	0.772371	0.938	protein_coding
ENSG0000(	BEX4	126.6541	0.042159	0.772839	0.938052	protein_coding
ENSG0000(	DNA2	379.5815	0.034257	0.77276	0.938052	protein_coding
ENSG0000(	ENSG0000(	22.56291	0.075672	0.773126	0.938052	lncRNA
ENSG0000(	ENSG0000(	139.6057	0.027752	0.772825	0.938052	lncRNA
ENSG0000(	ENSG0000(	116.168	0.040102	0.772974	0.938052	lncRNA
ENSG0000(	ENSG0000(	95.47289	-0.04019	0.772644	0.938052	lncRNA
ENSG0000(	ENSG0000(	86.0509	0.044599	0.773085	0.938052	lncRNA
ENSG0000(	ENSG0000(	1418.977	-0.01493	0.772589	0.938052	lncRNA
ENSG0000(	ENSG0000(	54.15383	-0.05771	0.772557	0.938052	lncRNA
ENSG0000(	FXYD6	245.6766	-0.05969	0.772788	0.938052	protein_coding
ENSG0000(	IGSF11	68.3129	0.054466	0.772933	0.938052	protein_coding
ENSG0000(	LINC01405	378.8056	-0.02804	0.772907	0.938052	lncRNA
ENSG0000(	LINC01840	213.032	0.029723	0.772645	0.938052	lncRNA
ENSG0000(	NEPRO	882.5828	0.02513	0.773129	0.938052	protein_coding
ENSG0000(	PICK1	449.3734	0.025679	0.772758	0.938052	protein_coding
ENSG0000(	PLEKHG5	545.2355	-0.04988	0.772486	0.938052	protein_coding
ENSG0000(	SENP1	601.9655	-0.02879	0.772518	0.938052	protein_coding
ENSG0000(	SEPTIN10	920.3313	-0.0356	0.772725	0.938052	protein_coding
ENSG0000(	SPOP	1463.218	-0.01352	0.773064	0.938052	protein_coding
ENSG0000(	TIPIN	267.9337	0.031337	0.773115	0.938052	protein_coding
ENSG0000(	ZNF559-ZN	156.6681	0.055974	0.772875	0.938052	protein_coding
ENSG0000(	ZNF45	427.2354	-0.02971	0.773213	0.938112	protein_coding
ENSG0000(	FAM111B	594.7761	-0.04077	0.773288	0.938162	protein_coding
ENSG0000(	NFYA	1364.841	-0.0219	0.773428	0.938265	protein_coding
ENSG0000(	RN7SL144P	58.10965	0.053919	0.773441	0.938265	misc_RNA
ENSG0000(	ENSG0000(	40.22456	0.072264	0.773536	0.938298	lncRNA
ENSG0000(	PERP	767.3415	-0.05177	0.773509	0.938298	protein_coding
ENSG0000(	AIP	159.9896	-0.03869	0.773854	0.938424	protein_coding
ENSG0000(	C2orf92	778.4355	-0.02036	0.773863	0.938424	protein_coding
ENSG0000(	ENSG0000(	225.4141	0.026875	0.773824	0.938424	lncRNA
ENSG0000(	ETFB	2104.032	-0.02109	0.773712	0.938424	protein_coding
ENSG0000(	GLIPR2	370.8778	-0.04066	0.773718	0.938424	protein_coding
ENSG0000(	HSD17B1-A	300.5497	-0.02482	0.773842	0.938424	lncRNA
ENSG0000(	LYPLA2	408.1293	-0.03446	0.773879	0.938424	protein_coding
ENSG0000(	AGBL5	285.5576	0.028975	0.774045	0.938471	protein_coding
ENSG0000(	ENSG0000(	4508.137	0.03081	0.774088	0.938471	lncRNA
ENSG0000(	ENSG0000(	294.2133	-0.03249	0.774055	0.938471	lncRNA
ENSG0000(	ERCC6L	88.00375	-0.04528	0.774058	0.938471	protein_coding
ENSG0000(	FOSL1P1	29.50764	0.073576	0.774032	0.938471	processed_pseudogene
ENSG0000(	DDX47	534.7689	0.019465	0.774201	0.93849	protein_coding
ENSG0000(	ENSG0000(	21.3746	-0.1437	0.774206	0.93849	lncRNA
ENSG0000(	PCGF1	138.1112	0.042456	0.77414	0.93849	protein_coding
ENSG0000(	SCG5	29.83185	-0.11204	0.774247	0.938497	protein_coding
ENSG0000(	ACER3	7176.192	-0.02193	0.774412	0.938533	protein_coding
ENSG0000(	ENSG0000(	42.74629	-0.04938	0.774389	0.938533	processed_pseudogene
ENSG0000(	ENSG0000(	334.4316	0.022605	0.774365	0.938533	lncRNA

ENSG0000(	TP53AIP1	67.76688	-0.05596	0.774387	0.938533	protein_coding
ENSG0000(	ENSG0000(	22.14893	-0.05787	0.774521	0.938623	lncRNA
ENSG0000(	ENSG0000(	1461.187	0.018975	0.774728	0.938708	lncRNA
ENSG0000(	ENSG0000(	99.9379	-0.05104	0.774627	0.938708	transcribed_processed_pseudoge
ENSG0000(	ENSG0000(	89.8607	-0.03382	0.774721	0.938708	lncRNA
ENSG0000(	USP10	1329.855	0.021273	0.774682	0.938708	protein_coding
ENSG0000(	LINC01352	27.94086	0.090375	0.774805	0.938719	lncRNA
ENSG0000(	TMEM266	343.5359	-0.02477	0.774798	0.938719	protein_coding
ENSG0000(	IL34	105.1699	-0.05587	0.774867	0.938753	protein_coding
ENSG0000(	RAPGEF2	2815.477	-0.02599	0.774926	0.938784	protein_coding
ENSG0000(	RPS12P5	36.805	-0.07977	0.77498	0.938808	processed_pseudogene
ENSG0000(	PWRN1	1147.22	-0.0666	0.775068	0.938872	lncRNA
ENSG0000(	RAD51C	715.7998	0.019724	0.775147	0.938872	protein_coding
ENSG0000(	RPL21P32	83.30479	0.048264	0.77517	0.938872	processed_pseudogene
ENSG0000(	SLC2A1-AS:	453.1861	-0.02133	0.775163	0.938872	lncRNA
ENSG0000(	GLRX5	261.778	-0.03989	0.775493	0.939181	protein_coding
ENSG0000(	RALA	734.9088	-0.03038	0.775489	0.939181	protein_coding
ENSG0000(	ENSG0000(	162.2957	-0.03328	0.775539	0.939195	lncRNA
ENSG0000(	VIPR2	140.7601	-0.04884	0.775619	0.93925	protein_coding
ENSG0000(	CFAP54	1982.418	-0.0315	0.7757	0.939252	protein_coding
ENSG0000(	ENSG0000(	87.04141	0.03809	0.775678	0.939252	lncRNA
ENSG0000(	RPL6	2795.486	-0.03159	0.775723	0.939252	protein_coding
ENSG0000(	DEPDC1	274.6947	-0.04335	0.77581	0.939262	protein_coding
ENSG0000(	MYPOP	144.8426	-0.03572	0.775785	0.939262	protein_coding
ENSG0000(	NRSN1	48.94179	0.058697	0.775834	0.939262	protein_coding
ENSG0000(	PLOD3	881.8435	0.020851	0.775912	0.939316	protein_coding
ENSG0000(	DPY19L2	147.1044	0.068185	0.775984	0.939362	protein_coding
ENSG0000(	NAIPP4	266.7323	-0.07217	0.776172	0.939506	unprocessed_pseudogene
ENSG0000(	RN7SL125F	33.93341	0.059333	0.776171	0.939506	misc_RNA
ENSG0000(	ENSG0000(	116.4457	-0.03259	0.776357	0.939596	transcribed_processed_pseudoge
ENSG0000(	ENSG0000(	299.9043	0.019199	0.776382	0.939596	lncRNA
ENSG0000(	KCNH2	101.8979	0.062851	0.776316	0.939596	protein_coding
ENSG0000(	RN7SL592F	116.0731	0.039247	0.776315	0.939596	misc_RNA
ENSG0000(	ABHD11-AS	89.40805	0.089461	0.776457	0.939613	transcribed_unitary_pseudogene
ENSG0000(	ENSG0000(	106.2102	0.032775	0.776476	0.939613	lncRNA
ENSG0000(	SYPL1P2	40.61517	-0.05169	0.776499	0.939613	processed_pseudogene
ENSG0000(	ENSG0000(	50.82662	0.043303	0.776548	0.939628	lncRNA
ENSG0000(	MICOS10	1100.451	-0.02596	0.77658	0.939628	protein_coding
ENSG0000(	ENSG0000(	285.4581	-0.03274	0.776774	0.939629	lncRNA
ENSG0000(	ENSG0000(	25.12578	0.075098	0.776796	0.939629	lncRNA
ENSG0000(	ENSG0000(	54.70443	0.057443	0.776798	0.939629	lncRNA
ENSG0000(	PAK1IP1	88.85165	-0.04789	0.77682	0.939629	protein_coding
ENSG0000(	STXBP5	2774.051	0.02443	0.776734	0.939629	protein_coding
ENSG0000(	TCF7L2	4194.815	0.052406	0.776761	0.939629	protein_coding
ENSG0000(	ZNF322	802.4054	0.029157	0.776656	0.939629	protein_coding
ENSG0000(	ENSG0000(	29.25355	0.087522	0.776857	0.939632	lncRNA
ENSG0000(	CTNNA3	181.9429	-0.03527	0.776901	0.939645	protein_coding

ENSG0000(	SPATA4	49.52793	0.050049	0.776939	0.93965	protein_coding
ENSG0000(	ENSG0000(	48.14329	-0.05424	0.777	0.939682	lncRNA
ENSG0000(	CHD8	4226.606	-0.01345	0.777117	0.939697	protein_coding
ENSG0000(	CTR9	607.5924	0.032984	0.777145	0.939697	protein_coding
ENSG0000(	ENY2	762.0853	0.021426	0.777252	0.939697	protein_coding
ENSG0000(	GAREM2	284.2134	-0.05022	0.777271	0.939697	protein_coding
ENSG0000(	GGTA1	82.8246	-0.0597	0.777312	0.939697	protein_coding
ENSG0000(	HOXD10	283.9092	-0.06126	0.777075	0.939697	protein_coding
ENSG0000(	KCNAB3	251.3443	-0.02927	0.777354	0.939697	protein_coding
ENSG0000(	TEN1-CDK3	539.3092	0.031677	0.777335	0.939697	protein_coding
ENSG0000(	ZBTB16	2134.574	-0.09828	0.777226	0.939697	protein_coding
ENSG0000(	ZBTB38	4624.084	0.021333	0.777156	0.939697	protein_coding
ENSG0000(	GJD2-DT	203.5265	0.020571	0.777441	0.939719	lncRNA
ENSG0000(	SLC46A1	940.3749	0.068334	0.777419	0.939719	protein_coding
ENSG0000(	H2AC7	73.82017	-0.06059	0.777571	0.939757	protein_coding
ENSG0000(	MAP1LC3B	49.99982	0.050102	0.777575	0.939757	protein_coding
ENSG0000(	ZDHHC2	915.5558	-0.04637	0.777542	0.939757	protein_coding
ENSG0000(	ENSG0000(	123.1237	-0.04555	0.777619	0.93977	lncRNA
ENSG0000(	ENSG0000(	67.59765	-0.04441	0.777789	0.939851	lncRNA
ENSG0000(	ENSG0000(	98.92106	0.036673	0.777727	0.939851	lncRNA
ENSG0000(	TRAJ36	26.44314	-0.08602	0.777781	0.939851	TR_J_gene
ENSG0000(	COPE	552.1305	-0.03906	0.777832	0.939862	protein_coding
ENSG0000(	C5AR1	416.5733	-0.03144	0.778015	0.939886	protein_coding
ENSG0000(	FAM167A-/	171.6134	-0.04921	0.777987	0.939886	lncRNA
ENSG0000(	KHK	70.14404	-0.06181	0.777945	0.939886	protein_coding
ENSG0000(	SNORA23	228.9667	0.041945	0.777918	0.939886	snoRNA
ENSG0000(	TMEM147-	656.8789	0.023908	0.778023	0.939886	lncRNA
ENSG0000(	EGR1	1744.759	-0.09809	0.778145	0.939947	protein_coding
ENSG0000(	ENSG0000(	120.7596	-0.03797	0.778227	0.939947	lncRNA
ENSG0000(	OR7A17	451.8489	0.022536	0.778234	0.939947	protein_coding
ENSG0000(	RPS21	554.1003	0.044861	0.778196	0.939947	protein_coding
ENSG0000(	STX17	2669.113	0.01799	0.778244	0.939947	protein_coding
ENSG0000(	ATP10B	10184.51	-0.05607	0.779222	0.940122	protein_coding
ENSG0000(	C6	23.63594	0.139914	0.779256	0.940122	protein_coding
ENSG0000(	CCNDBP1	1374.893	0.019129	0.779004	0.940122	protein_coding
ENSG0000(	CDR2	473.1186	-0.02721	0.779008	0.940122	protein_coding
ENSG0000(	CLEC2D	2410.004	-0.05451	0.7787	0.940122	protein_coding
ENSG0000(	DNAJA3	1339.589	-0.02321	0.778562	0.940122	protein_coding
ENSG0000(	ENSG0000(	67.32818	0.050856	0.779278	0.940122	lncRNA
ENSG0000(	ENSG0000(	20.39177	-0.08357	0.778857	0.940122	lncRNA
ENSG0000(	ENSG0000(	833.8812	0.026689	0.778762	0.940122	TEC
ENSG0000(	FOXN3	3433.242	0.021753	0.778552	0.940122	protein_coding
ENSG0000(	GAPT	181.5434	-0.07968	0.778651	0.940122	protein_coding
ENSG0000(	MAP2	84.03864	-0.08855	0.778672	0.940122	protein_coding
ENSG0000(	MAP3K15	251.6854	0.02403	0.778975	0.940122	protein_coding
ENSG0000(	MKRN2	1179.702	0.015131	0.778613	0.940122	protein_coding
ENSG0000(	NCOA5	617.8734	0.023934	0.778774	0.940122	protein_coding

ENSG0000(	NDC80	330.6405	0.026859	0.778819	0.940122	protein_coding
ENSG0000(	PINX1	104.3478	-0.05127	0.779174	0.940122	protein_coding
ENSG0000(	PRPF18	377.5904	0.02946	0.779221	0.940122	protein_coding
ENSG0000(	RELL1	1302.268	0.04044	0.779104	0.940122	protein_coding
ENSG0000(	RN7SL246F	49.81268	-0.04417	0.779114	0.940122	misc_RNA
ENSG0000(	RPAP1	643.1549	0.024096	0.779255	0.940122	protein_coding
ENSG0000(	SLC1A5	990.0496	-0.03777	0.778444	0.940122	protein_coding
ENSG0000(	SOSTDC1	80.04773	0.100896	0.779131	0.940122	protein_coding
ENSG0000(	TMED3	1634.831	-0.03096	0.778463	0.940122	protein_coding
ENSG0000(	TTC1	1477.671	0.013379	0.779231	0.940122	protein_coding
ENSG0000(	ZNF853	50.00641	0.060368	0.779061	0.940122	protein_coding
ENSG0000(	ABRACL	183.5749	0.039432	0.779492	0.940134	protein_coding
ENSG0000(	ENSG0000(	23.36454	0.078602	0.779467	0.940134	lncRNA
ENSG0000(	FAM83D	153.109	-0.04131	0.779413	0.940134	protein_coding
ENSG0000(	LINC02251	43.17072	0.095343	0.779375	0.940134	lncRNA
ENSG0000(	NXPH3	29.74326	-0.09505	0.779474	0.940134	protein_coding
ENSG0000(	TMEM251	325.0099	-0.0366	0.77949	0.940134	protein_coding
ENSG0000(	CHORDC1	2090.334	-0.02358	0.779565	0.940155	protein_coding
ENSG0000(	SEPTIN7-DT	202.8469	0.034469	0.779578	0.940155	lncRNA
ENSG0000(	DHX38	1010.08	-0.01941	0.779807	0.940184	protein_coding
ENSG0000(	ENSG0000(	23.29019	-0.07023	0.779778	0.940184	lncRNA
ENSG0000(	ENSG0000(	113.9281	-0.03104	0.779716	0.940184	lncRNA
ENSG0000(	ENSG0000(	216.4235	-0.03318	0.779665	0.940184	TEC
ENSG0000(	ENSG0000(	76.24074	0.038279	0.779784	0.940184	lncRNA
ENSG0000(	UFC1	485.7683	-0.02331	0.779797	0.940184	protein_coding
ENSG0000(	CHD1L	728.6911	-0.0261	0.779858	0.940188	protein_coding
ENSG0000(	MAPRE3	367.9181	0.05504	0.779947	0.940188	protein_coding
ENSG0000(	MED23	1328.122	0.02025	0.779891	0.940188	protein_coding
ENSG0000(	NELL1	41.28399	-0.10335	0.77992	0.940188	protein_coding
ENSG0000(	ELMOD1	310.9975	-0.02429	0.780051	0.940209	protein_coding
ENSG0000(	ENSG0000(	251.8843	-0.02863	0.78017	0.940209	lncRNA
ENSG0000(	ENSG0000(	20.46614	0.066027	0.78015	0.940209	lncRNA
ENSG0000(	LETM2	390.4474	0.020975	0.78008	0.940209	protein_coding
ENSG0000(	SMC2-DT	713.5077	0.020294	0.780086	0.940209	lncRNA
ENSG0000(	SUPT4H1	298.4404	-0.02763	0.780141	0.940209	protein_coding
ENSG0000(	PIGT	3269.746	0.013871	0.780285	0.940306	protein_coding
ENSG0000(	ROCK1	2887.825	-0.02593	0.780435	0.940446	protein_coding
ENSG0000(	ENSG0000(	179.5957	0.026543	0.780682	0.940456	lncRNA
ENSG0000(	ENSG0000(	726.4054	-0.0296	0.780848	0.940456	transcribed_unprocessed_pseudo
ENSG0000(	ENSG0000(	45.1949	-0.06045	0.780761	0.940456	lncRNA
ENSG0000(	GIGYF2	3225.876	0.014462	0.78082	0.940456	protein_coding
ENSG0000(	LINC00488	48.05164	0.055954	0.780765	0.940456	lncRNA
ENSG0000(	MT1F	125.5942	0.094011	0.780658	0.940456	protein_coding
ENSG0000(	NONO	3616.266	0.024521	0.780854	0.940456	protein_coding
ENSG0000(	RN7SL395F	26.06163	0.058618	0.780733	0.940456	misc_RNA
ENSG0000(	THAP12P7	137.4806	0.051221	0.780581	0.940456	processed_pseudogene
ENSG0000(	TMC8	1194.499	-0.05679	0.780487	0.940456	protein_coding

ENSG0000(	UBB	1269.763	-0.04408	0.780714	0.940456	protein_coding
ENSG0000(	ZFPL1	483.2755	-0.02675	0.780811	0.940456	protein_coding
ENSG0000(	ENSG0000(	51.71771	0.050851	0.780968	0.940553	lncRNA
ENSG0000(	PCDHGA5	230.9096	0.0256	0.781012	0.940565	protein_coding
ENSG0000(	ENSG0000(	37.55549	0.065537	0.781158	0.9406	lncRNA
ENSG0000(	KLRB1	66.79441	0.062583	0.78128	0.9406	protein_coding
ENSG0000(	MTHFR	2707.163	-0.02155	0.781128	0.9406	protein_coding
ENSG0000(	NCALD	962.655	-0.03146	0.781272	0.9406	protein_coding
ENSG0000(	NELL2	212.8076	-0.09016	0.781101	0.9406	protein_coding
ENSG0000(	RN7SL460F	301.9167	0.028194	0.781215	0.9406	misc_RNA
ENSG0000(	RPS13	1169.927	-0.03089	0.781203	0.9406	protein_coding
ENSG0000(	ENSG0000(	111.6538	0.043031	0.781351	0.940603	lncRNA
ENSG0000(	ENSG0000(	261.0639	-0.03075	0.781326	0.940603	TEC
ENSG0000(	C2orf15	231.2966	0.026646	0.781439	0.940635	protein_coding
ENSG0000(	ENSG0000(	205.2465	0.039746	0.781446	0.940635	TEC
ENSG0000(	MBP	1655.953	0.019046	0.781488	0.940644	protein_coding
ENSG0000(	ENSG0000(	218.9681	0.046826	0.781525	0.940647	lncRNA
ENSG0000(	ENSG0000(	77.89938	0.059165	0.781644	0.940749	lncRNA
ENSG0000(	DPRXP3	72.5038	0.043985	0.78175	0.940789	processed_pseudogene
ENSG0000(	ENSG0000(	671.2777	-0.06244	0.78174	0.940789	transcribed_unprocessed_pseudo
ENSG0000(	ENSG0000(	238.3964	0.064244	0.781814	0.940789	transcribed_unprocessed_pseudo
ENSG0000(	TFPT	70.42322	-0.04129	0.781812	0.940789	protein_coding
ENSG0000(	CBS	95.37701	-0.10032	0.781936	0.940854	protein_coding
ENSG0000(	PRKAG1	708.8873	0.015924	0.781933	0.940854	protein_coding
ENSG0000(	POLR3B	300.1994	0.036444	0.782011	0.940903	protein_coding
ENSG0000(	ENSG0000(	157.0775	-0.04049	0.782057	0.940917	unprocessed_pseudogene
ENSG0000(	LINC01148	71.001	-0.04729	0.782205	0.941055	lncRNA
ENSG0000(	CIART	247.4114	0.030082	0.782446	0.94122	protein_coding
ENSG0000(	ENSG0000(	41.86764	-0.06231	0.782397	0.94122	lncRNA
ENSG0000(	GCNT1P1	30.24147	0.060522	0.782434	0.94122	processed_pseudogene
ENSG0000(	ENSG0000(	95.63047	0.04902	0.782528	0.941222	lncRNA
ENSG0000(	ENSG0000(	53.61097	-0.15249	0.782534	0.941222	unprocessed_pseudogene
ENSG0000(	ENSG0000(	78.05042	-0.05029	0.782584	0.941222	lncRNA
ENSG0000(	GYPA	134.7819	0.032956	0.78258	0.941222	protein_coding
ENSG0000(	LINC01311	61.19477	0.05646	0.782649	0.941259	lncRNA
ENSG0000(	ENSG0000(	25.66824	0.079551	0.782734	0.941281	lncRNA
ENSG0000(	GPR63	582.7458	0.043979	0.782747	0.941281	protein_coding
ENSG0000(	LINC02886	164.7861	-0.04553	0.78277	0.941281	lncRNA
ENSG0000(	ENSG0000(	154.846	0.026762	0.782831	0.941314	lncRNA
ENSG0000(	ENSG0000(	584.459	-0.04961	0.782913	0.94137	transcribed_unprocessed_pseudo
ENSG0000(	ATP1B2	199.3728	0.027274	0.783129	0.941405	protein_coding
ENSG0000(	BANF1	227.1562	-0.0439	0.783327	0.941405	protein_coding
ENSG0000(	CAT	768.5234	0.032832	0.783603	0.941405	protein_coding
ENSG0000(	CCS	478.8901	-0.02303	0.783196	0.941405	protein_coding
ENSG0000(	ENSG0000(	4199.09	0.016679	0.783638	0.941405	lncRNA
ENSG0000(	ENSG0000(	28.86893	-0.0718	0.783361	0.941405	lncRNA
ENSG0000(	ENSG0000(	113.6588	-0.03681	0.783327	0.941405	lncRNA

ENSG0000(	ENSG0000(	27.86196	0.070189	0.783355	0.941405	lncRNA
ENSG0000(	ENSG0000(	457.8269	-0.01746	0.783694	0.941405	lncRNA
ENSG0000(	ENSG0000(	1928.363	-0.01789	0.783276	0.941405	protein_coding
ENSG0000(	ENSG0000(	24.77244	0.066809	0.783572	0.941405	lncRNA
ENSG0000(	ENSG0000(	450.4985	0.022979	0.78345	0.941405	lncRNA
ENSG0000(	KIF6	696.5786	-0.02762	0.783068	0.941405	protein_coding
ENSG0000(	LINC02507	43.47489	0.060484	0.783454	0.941405	lncRNA
ENSG0000(	MRPL9	246.9983	0.037779	0.783044	0.941405	protein_coding
ENSG0000(	PLPP1	684.3893	0.048076	0.78306	0.941405	protein_coding
ENSG0000(	PNO1	369.0484	0.02628	0.783607	0.941405	protein_coding
ENSG0000(	SLC39A10	709.0691	0.040269	0.783685	0.941405	protein_coding
ENSG0000(	SLC39A3	415.4895	-0.02161	0.783496	0.941405	protein_coding
ENSG0000(	UGGT2	1416.119	-0.01944	0.783352	0.941405	protein_coding
ENSG0000(	USP6	480.0984	0.043863	0.783584	0.941405	protein_coding
ENSG0000(	ZNF426-DT	46.47712	0.053434	0.783246	0.941405	lncRNA
ENSG0000(	PPP1CC	3130.713	-0.02493	0.783786	0.941474	protein_coding
ENSG0000(	ENSG0000(	96.78381	-0.05869	0.784149	0.941486	processed_pseudogene
ENSG0000(	ENSG0000(	24.38597	-0.06917	0.784138	0.941486	lncRNA
ENSG0000(	ENSG0000(	44.86548	0.066887	0.784032	0.941486	lncRNA
ENSG0000(	ENSG0000(	44.47546	-0.06886	0.784109	0.941486	lncRNA
ENSG0000(	ENSG0000(	209.191	-0.02922	0.783928	0.941486	lncRNA
ENSG0000(	HACD2	1576.05	-0.02407	0.78417	0.941486	protein_coding
ENSG0000(	MTG1	1081.557	0.021344	0.783985	0.941486	protein_coding
ENSG0000(	MTURN	693.2817	-0.02563	0.784172	0.941486	protein_coding
ENSG0000(	NREP	937.3272	-0.03598	0.784041	0.941486	protein_coding
ENSG0000(	TTC22	1248.495	0.043617	0.784085	0.941486	protein_coding
ENSG0000(	VGLL3	527.8648	-0.05085	0.784123	0.941486	protein_coding
ENSG0000(	CNKSR2	1136.531	-0.02341	0.784427	0.941545	protein_coding
ENSG0000(	ENSG0000(	156.5664	-0.03376	0.784377	0.941545	lncRNA
ENSG0000(	ENSG0000(	30.68556	-0.06131	0.784367	0.941545	lncRNA
ENSG0000(	EP400	3739.94	-0.0121	0.784287	0.941545	protein_coding
ENSG0000(	LINC01007	20.50834	0.100571	0.784423	0.941545	lncRNA
ENSG0000(	N4BP2	1716.56	-0.02177	0.784337	0.941545	protein_coding
ENSG0000(	UPF2	1511.695	0.01758	0.784557	0.94166	protein_coding
ENSG0000(	DCAF11	1575.361	0.019752	0.784641	0.941683	protein_coding
ENSG0000(	ENSG0000(	36.8351	0.243507	0.784645	0.941683	transcribed_unprocessed_pseudo
ENSG0000(	IGFL2	88.76334	-0.03199	0.78483	0.941865	protein_coding
ENSG0000(	C10orf95	20.66606	-0.06863	0.785119	0.941968	protein_coding
ENSG0000(	C13orf46	123.9612	-0.05872	0.785093	0.941968	protein_coding
ENSG0000(	CYP8B1	184.3156	0.027817	0.785233	0.941968	protein_coding
ENSG0000(	ENSG0000(	298.6711	-0.02698	0.785424	0.941968	lncRNA
ENSG0000(	ENSG0000(	27.92156	0.068795	0.785516	0.941968	processed_pseudogene
ENSG0000(	ENSG0000(	134.1016	-0.02902	0.785532	0.941968	lncRNA
ENSG0000(	ENSG0000(	551.7213	0.040117	0.785305	0.941968	protein_coding
ENSG0000(	ENSG0000(	30.4356	0.073613	0.785481	0.941968	lncRNA
ENSG0000(	ENSG0000(	64.12467	-0.09338	0.785441	0.941968	TEC
ENSG0000(	GCKR	48.9385	-0.05341	0.78499	0.941968	protein_coding

ENSG0000(	NR2C1	933.017	0.020729	0.785518	0.941968	protein_coding
ENSG0000(	NSFP1	268.4586	0.909785	0.785248	0.941968	unprocessed_pseudogene
ENSG0000(	NUP93	921.4453	0.025796	0.785008	0.941968	protein_coding
ENSG0000(	RAE1	477.6966	0.024715	0.785303	0.941968	protein_coding
ENSG0000(	REM2	21.03655	-0.08761	0.785426	0.941968	protein_coding
ENSG0000(	RNU4-24P	34.63671	-0.05894	0.785262	0.941968	snRNA
ENSG0000(	SMCR5	112.1489	-0.04019	0.785426	0.941968	lncRNA
ENSG0000(	ZNF706	1861.886	-0.01864	0.785373	0.941968	protein_coding
ENSG0000(	SLC25A14	159.9528	0.031351	0.785582	0.941986	protein_coding
ENSG0000(	ATP8A2P3	20.58124	0.077091	0.785691	0.942049	unprocessed_pseudogene
ENSG0000(	KCNIP1-OT	68.41302	0.03845	0.785703	0.942049	lncRNA
ENSG0000(	BCL11B	929.4739	-0.05367	0.78585	0.942103	protein_coding
ENSG0000(	HCG18	7196.905	0.014714	0.785838	0.942103	lncRNA
ENSG0000(	RARS1	840.3879	0.030696	0.785802	0.942103	protein_coding
ENSG0000(	CBWD6	567.1874	-0.03683	0.785925	0.942151	protein_coding
ENSG0000(	ENSG0000(	283.2722	-0.03495	0.786141	0.942164	transcribed_processed_pseudoge
ENSG0000(	ENSG0000(	22.72101	-0.07126	0.78609	0.942164	misc_RNA
ENSG0000(	ENSG0000(	59.743	0.107255	0.786121	0.942164	processed_pseudogene
ENSG0000(	GPR199P	104.3693	-0.0523	0.786052	0.942164	transcribed_unitary_pseudogene
ENSG0000(	KIF5C	2207.723	0.013439	0.786047	0.942164	protein_coding
ENSG0000(	NBPF2P	80.23667	-0.05405	0.786005	0.942164	unprocessed_pseudogene
ENSG0000(	C11orf49	867.4089	0.021324	0.78621	0.942205	protein_coding
ENSG0000(	ACTBP4	44.15205	-0.07237	0.786429	0.942382	processed_pseudogene
ENSG0000(	CYHR1	1342	0.019381	0.786401	0.942382	protein_coding
ENSG0000(	ENSG0000(	81.9337	0.034567	0.78646	0.942382	TEC
ENSG0000(	CCT6B	264.3925	-0.03525	0.787008	0.942664	protein_coding
ENSG0000(	CNDP1	228.482	0.030605	0.78717	0.942664	protein_coding
ENSG0000(	COL4A5	685.2769	0.035003	0.787167	0.942664	protein_coding
ENSG0000(	DHX29	686.6733	-0.02493	0.787091	0.942664	protein_coding
ENSG0000(	DNAL4	125.2599	-0.03537	0.787154	0.942664	protein_coding
ENSG0000(	ENSG0000(	153.2357	-0.02877	0.786817	0.942664	lncRNA
ENSG0000(	ENSG0000(	131.1827	0.036167	0.786995	0.942664	lncRNA
ENSG0000(	EVI5L	368.695	-0.02718	0.786827	0.942664	protein_coding
ENSG0000(	FKBP6	185.7235	-0.03024	0.786867	0.942664	protein_coding
ENSG0000(	HSPA4	1404.533	0.023618	0.786871	0.942664	protein_coding
ENSG0000(	LAMB2P1	39.14052	-0.05777	0.787175	0.942664	unprocessed_pseudogene
ENSG0000(	LINC01194	22.65976	0.080792	0.786809	0.942664	lncRNA
ENSG0000(	MCUR1P1	133.162	-0.02678	0.786994	0.942664	processed_pseudogene
ENSG0000(	NPIPA9	490.4153	0.026784	0.787095	0.942664	protein_coding
ENSG0000(	BTF3L4	927.463	0.024269	0.787215	0.942671	protein_coding
ENSG0000(	SRRM5	23.87568	0.068898	0.787289	0.942719	protein_coding
ENSG0000(	ENSG0000(	37.54681	0.056525	0.787611	0.943063	lncRNA
ENSG0000(	ENSG0000(	116.3405	-0.03954	0.787692	0.943077	processed_pseudogene
ENSG0000(	OR2AG1	132.7199	0.028536	0.787679	0.943077	protein_coding
ENSG0000(	NDUFA8	100.5952	-0.04795	0.787752	0.943109	protein_coding
ENSG0000(	ENSG0000(	629.0396	-0.02317	0.787861	0.943157	lncRNA
ENSG0000(	ZC3HAV1L	274.3849	0.023561	0.787838	0.943157	protein_coding

ENSG0000(	VASH2	303.4317	-0.028	0.787906	0.94317	protein_coding
ENSG0000(	HNRNPF	1747.83	-0.0226	0.787954	0.943186	protein_coding
ENSG0000(	ENSG0000(	29.90063	0.093234	0.78804	0.943248	misc_RNA
ENSG0000(	HP	119.5662	0.042371	0.788143	0.94333	protein_coding
ENSG0000(	TSBP1-AS1	9658.964	0.014287	0.78834	0.943525	lncRNA
ENSG0000(	CCDC149	514.1026	-0.0361	0.788531	0.943548	protein_coding
ENSG0000(	DHRS4L1	128.4145	-0.04046	0.788508	0.943548	transcribed_unprocessed_pseudo
ENSG0000(	ENSG0000(	178.7409	-0.03542	0.788414	0.943548	lncRNA
ENSG0000(	LINC00559	57.66811	-0.04657	0.788433	0.943548	lncRNA
ENSG0000(	POLQ	867.1652	0.025199	0.788516	0.943548	protein_coding
ENSG0000(	ENSG0000(	42.08852	0.05261	0.788677	0.943622	miRNA
ENSG0000(	ENSG0000(	71.4433	-0.04666	0.788707	0.943622	lncRNA
ENSG0000(	NECTIN3-A	37.66998	-0.0713	0.788741	0.943622	lncRNA
ENSG0000(	PCDHB9	203.0365	0.024547	0.788764	0.943622	protein_coding
ENSG0000(	PPIC	355.954	0.038647	0.788658	0.943622	protein_coding
ENSG0000(	JHY	516.7277	0.019719	0.788807	0.943632	protein_coding
ENSG0000(	ALS2	2921.632	-0.01454	0.789759	0.943664	protein_coding
ENSG0000(	C1orf43	1238.595	0.017353	0.7892	0.943664	protein_coding
ENSG0000(	CCDC47	1111.056	-0.02313	0.789286	0.943664	protein_coding
ENSG0000(	DAPK2	3115.517	0.035255	0.789485	0.943664	protein_coding
ENSG0000(	ECT2L	54.38492	-0.06769	0.789316	0.943664	protein_coding
ENSG0000(	ENSG0000(	1790.941	0.013303	0.789599	0.943664	lncRNA
ENSG0000(	ENSG0000(	166.5507	0.029998	0.789036	0.943664	lncRNA
ENSG0000(	ENSG0000(	39.06094	-0.05771	0.789623	0.943664	processed_pseudogene
ENSG0000(	ENSG0000(	198.9743	-0.04308	0.789754	0.943664	lncRNA
ENSG0000(	ENSG0000(	87.05995	0.043172	0.789511	0.943664	TEC
ENSG0000(	ENSG0000(	44.88318	0.083625	0.789539	0.943664	protein_coding
ENSG0000(	ENSG0000(	25.78826	-0.05778	0.789489	0.943664	lncRNA
ENSG0000(	NDNF	84.94778	0.061246	0.789736	0.943664	protein_coding
ENSG0000(	NOVA1	485.0789	-0.04496	0.789476	0.943664	protein_coding
ENSG0000(	NUP188	979.1019	0.027563	0.789445	0.943664	protein_coding
ENSG0000(	PHF5A	246.9886	-0.03326	0.789682	0.943664	protein_coding
ENSG0000(	POTEM	284.8548	0.04843	0.789692	0.943664	protein_coding
ENSG0000(	SARDH	156.9269	0.048937	0.78936	0.943664	protein_coding
ENSG0000(	SMPD4P1	55.26634	0.050746	0.789406	0.943664	transcribed_unprocessed_pseudo
ENSG0000(	SPC24	515.7195	-0.01725	0.789622	0.943664	protein_coding
ENSG0000(	ST7-AS1	25.31908	-0.08719	0.789251	0.943664	lncRNA
ENSG0000(	STX16	4533.92	-0.01416	0.789539	0.943664	protein_coding
ENSG0000(	TOMM34	127.2984	-0.03505	0.789337	0.943664	protein_coding
ENSG0000(	UCHL1-DT	27.6022	0.060813	0.789462	0.943664	lncRNA
ENSG0000(	XYLT2	662.4422	0.018336	0.789082	0.943664	protein_coding
ENSG0000(	ZFAND6	1801.764	0.019231	0.789648	0.943664	protein_coding
ENSG0000(	ZNF691	205.4507	-0.03301	0.789266	0.943664	protein_coding
ENSG0000(	ENSG0000(	28.29477	-0.06891	0.78981	0.943673	lncRNA
ENSG0000(	RDM1P5	298.7585	-0.02233	0.789835	0.943673	transcribed_processed_pseudoge
ENSG0000(	DNAH17	101.0376	0.042863	0.790137	0.943951	protein_coding
ENSG0000(	TAFAZZIN	946.2515	-0.01718	0.790107	0.943951	protein_coding

ENSG0000(	ENSG0000(	130.8352	0.03194	0.790233	0.944025	lncRNA
ENSG0000(	AASDH	472.033	0.035406	0.79062	0.944037	protein_coding
ENSG0000(	ATMIN	1129.966	0.021	0.790594	0.944037	protein_coding
ENSG0000(	CLDN15	480.195	0.0247	0.790377	0.944037	protein_coding
ENSG0000(	CMTM8	35.65081	-0.04702	0.791229	0.944037	protein_coding
ENSG0000(	CNTNAP3	681.601	-0.03113	0.790854	0.944037	protein_coding
ENSG0000(	DNAJC16	1210.876	-0.02317	0.79036	0.944037	protein_coding
ENSG0000(	ENSG0000(	31.85963	0.058167	0.790835	0.944037	lncRNA
ENSG0000(	ENSG0000(	1413.093	0.019768	0.791035	0.944037	lncRNA
ENSG0000(	ENSG0000(	20.91328	-0.07169	0.790548	0.944037	lncRNA
ENSG0000(	ENSG0000(	298.5554	0.027095	0.79123	0.944037	lncRNA
ENSG0000(	ENSG0000(	354.156	-0.02573	0.790689	0.944037	TEC
ENSG0000(	ENSG0000(	128.7012	-0.02699	0.790804	0.944037	lncRNA
ENSG0000(	FTH1P16	47.16805	0.05933	0.790817	0.944037	processed_pseudogene
ENSG0000(	IGF1R	2393.814	0.029739	0.79124	0.944037	protein_coding
ENSG0000(	KLHL9	1652.723	0.014534	0.790754	0.944037	protein_coding
ENSG0000(	LINC00847	514.4269	0.023465	0.791147	0.944037	lncRNA
ENSG0000(	LINC01098	28.45484	-0.071	0.791276	0.944037	lncRNA
ENSG0000(	LINC01719	710.5784	0.023613	0.790915	0.944037	lncRNA
ENSG0000(	LINC01895	35.55772	-0.05012	0.791246	0.944037	lncRNA
ENSG0000(	MADD	2343.456	-0.02445	0.790415	0.944037	protein_coding
ENSG0000(	MBLAC2	272.8328	0.037528	0.790696	0.944037	protein_coding
ENSG0000(	OR51L1	150.0318	0.032672	0.791237	0.944037	protein_coding
ENSG0000(	OR8D1	113.7062	0.031308	0.790627	0.944037	protein_coding
ENSG0000(	PITX2	778.1739	0.105725	0.791251	0.944037	protein_coding
ENSG0000(	RGS7	786.9148	0.014724	0.790826	0.944037	protein_coding
ENSG0000(	RNF38	1340.644	0.022463	0.791127	0.944037	protein_coding
ENSG0000(	SNX16	207.2551	0.030321	0.790763	0.944037	protein_coding
ENSG0000(	SZT2	7198.991	0.010917	0.790963	0.944037	protein_coding
ENSG0000(	TATDN2	1608.478	-0.02483	0.790622	0.944037	protein_coding
ENSG0000(	THOC5	1193.397	0.021468	0.790954	0.944037	protein_coding
ENSG0000(	UBE2A	989.2016	0.037504	0.791307	0.944037	protein_coding
ENSG0000(	LINC02756	27.24169	-0.07894	0.791355	0.944053	lncRNA
ENSG0000(	ACER1	83.05357	-0.04065	0.791664	0.944101	protein_coding
ENSG0000(	AREL1	1529.248	0.021872	0.791858	0.944101	protein_coding
ENSG0000(	ARNILA	273.3488	0.033872	0.791923	0.944101	lncRNA
ENSG0000(	C1orf220	617.8189	-0.02856	0.792663	0.944101	lncRNA
ENSG0000(	C3orf62	947.9904	0.021651	0.792176	0.944101	protein_coding
ENSG0000(	CCR9	22.53172	-0.11708	0.792225	0.944101	protein_coding
ENSG0000(	CEBPB-AS1	404.7501	-0.02673	0.792411	0.944101	lncRNA
ENSG0000(	CTDSP2	5116.219	-0.02647	0.792541	0.944101	protein_coding
ENSG0000(	DNAH17-A:	241.1263	-0.03133	0.791607	0.944101	lncRNA
ENSG0000(	ELAC1	364.9408	0.02347	0.792319	0.944101	protein_coding
ENSG0000(	ENSG0000(	36.81142	0.044957	0.791586	0.944101	unprocessed_pseudogene
ENSG0000(	ENSG0000(	128.9342	-0.03643	0.792808	0.944101	lncRNA
ENSG0000(	ENSG0000(	177.0813	0.05074	0.791914	0.944101	protein_coding
ENSG0000(	ENSG0000(	174.2153	-0.03825	0.792984	0.944101	lncRNA

ENSG0000(	ENSG0000(	183.8756	0.031292	0.793244	0.944101	lncRNA
ENSG0000(	ENSG0000(	26.97741	0.067316	0.791517	0.944101	lncRNA
ENSG0000(	ENSG0000(	126.2793	0.079047	0.791955	0.944101	protein_coding
ENSG0000(	ENSG0000(	242.0352	-0.02544	0.792894	0.944101	TEC
ENSG0000(	ENSG0000(	1138.51	0.040965	0.791724	0.944101	TEC
ENSG0000(	ENSG0000(	218.7825	-0.03025	0.793196	0.944101	TEC
ENSG0000(	ENSG0000(	70.73745	-0.03768	0.793052	0.944101	lncRNA
ENSG0000(	ENSG0000(	64.05388	0.054506	0.791897	0.944101	lncRNA
ENSG0000(	FAM230J	53.83809	0.083491	0.793237	0.944101	lncRNA
ENSG0000(	FRS3	156.4005	0.03869	0.792899	0.944101	protein_coding
ENSG0000(	GATAD2A	1845.919	-0.01898	0.792124	0.944101	protein_coding
ENSG0000(	H2BC21	280.4784	-0.03958	0.791434	0.944101	protein_coding
ENSG0000(	HNRNPA1L	59.32184	0.158067	0.793086	0.944101	protein_coding
ENSG0000(	KIFBP	532.9534	-0.01916	0.793248	0.944101	protein_coding
ENSG0000(	LINC01031	34.40167	0.04951	0.79324	0.944101	lncRNA
ENSG0000(	M1AP	68.25823	-0.03994	0.792052	0.944101	protein_coding
ENSG0000(	MED7	374.8888	0.019224	0.792774	0.944101	protein_coding
ENSG0000(	OR8J3	36.77409	-0.07411	0.793043	0.944101	protein_coding
ENSG0000(	PCSK1N	48.65355	-0.06094	0.792589	0.944101	protein_coding
ENSG0000(	PPM1G	702.2483	-0.02808	0.79222	0.944101	protein_coding
ENSG0000(	PTPRN	45.61466	0.3552	0.793195	0.944101	protein_coding
ENSG0000(	PYGO2	607.8544	0.018457	0.792655	0.944101	protein_coding
ENSG0000(	RAB21	3029.271	-0.02807	0.792161	0.944101	protein_coding
ENSG0000(	RN7SL50P	63.39512	-0.03898	0.791957	0.944101	misc_RNA
ENSG0000(	RNU2-24P	37.11812	-0.05588	0.792965	0.944101	snRNA
ENSG0000(	RXYLT1-AS:	95.06096	0.030978	0.792193	0.944101	lncRNA
ENSG0000(	SAMD3	213.7338	-0.04679	0.79259	0.944101	protein_coding
ENSG0000(	SCART1	835.1732	-0.02925	0.792039	0.944101	protein_coding
ENSG0000(	SLC22A25	2726.783	-0.00961	0.793092	0.944101	protein_coding
ENSG0000(	SNORA18	72.46508	-0.04467	0.792335	0.944101	snoRNA
ENSG0000(	SNU13	487.1979	-0.02245	0.792494	0.944101	protein_coding
ENSG0000(	SNX27	3894.317	0.028399	0.792676	0.944101	protein_coding
ENSG0000(	TBX3-AS1	49.6772	0.043416	0.793032	0.944101	lncRNA
ENSG0000(	TMEM67	1246.937	0.016462	0.793211	0.944101	protein_coding
ENSG0000(	TTC30A	182.5553	0.036046	0.791716	0.944101	protein_coding
ENSG0000(	VPS4A	1361.983	0.018416	0.79177	0.944101	protein_coding
ENSG0000(	WFS1	346.8629	-0.03174	0.791492	0.944101	protein_coding
ENSG0000(	WWP1	2088.867	-0.02837	0.791556	0.944101	protein_coding
ENSG0000(	ZDHHC6	1799.095	-0.01095	0.792931	0.944101	protein_coding
ENSG0000(	ZNF177	50.45947	-0.14436	0.792389	0.944101	protein_coding
ENSG0000(	GOLGA8Q	56.1103	-0.41503	0.793288	0.944108	protein_coding
ENSG0000(	FLJ12825	306.7223	-0.03188	0.79343	0.944176	lncRNA
ENSG0000(	MTND5P32	72.14769	-0.64606	0.793449	0.944176	processed_pseudogene
ENSG0000(	TRIR	572	-0.02182	0.793448	0.944176	protein_coding
ENSG0000(	ALOX12-AS	843.7929	0.016869	0.793664	0.944225	lncRNA
ENSG0000(	ARL11	505.4954	0.051995	0.79373	0.944225	protein_coding
ENSG0000(	ATRAID	295.5755	-0.03314	0.793614	0.944225	protein_coding

ENSG0000(	BEND2	129.3887	0.032322	0.793581	0.944225	protein_coding
ENSG0000(	ENSG0000(	107.7627	-0.02913	0.793702	0.944225	processed_pseudogene
ENSG0000(	LSINCT5	158.3439	-0.03396	0.793575	0.944225	lncRNA
ENSG0000(	TXN	298.5817	-0.03856	0.793699	0.944225	protein_coding
ENSG0000(	MICB-DT	120.4597	-0.03116	0.79377	0.944232	lncRNA
ENSG0000(	ITPKB-AS1	45.44525	0.056916	0.793819	0.94425	lncRNA
ENSG0000(	ENSG0000(	62.46721	0.041722	0.793998	0.944253	lncRNA
ENSG0000(	ENSG0000(	34.01605	-0.0527	0.794028	0.944253	lncRNA
ENSG0000(	HEBP1	249.0967	0.037862	0.793879	0.944253	protein_coding
ENSG0000(	LINC02176	87.90604	0.041195	0.793953	0.944253	lncRNA
ENSG0000(	NRIR	203.1417	0.043962	0.794003	0.944253	lncRNA
ENSG0000(	TUBA5P	228.8055	0.032518	0.793909	0.944253	transcribed_unprocessed_pseudo
ENSG0000(	ENSG0000(	23.19657	0.063806	0.79407	0.944263	lncRNA
ENSG0000(	CBLN2	50.76287	-0.085	0.794131	0.944294	protein_coding
ENSG0000(	LINC01553	214.5914	0.030704	0.794222	0.944362	lncRNA
ENSG0000(	ENSG0000(	302.6558	0.025681	0.794348	0.944432	TEC
ENSG0000(	HMGA1	844.0628	-0.03194	0.79435	0.944432	protein_coding
ENSG0000(	MTRNR2L8	33.79925	-0.17415	0.79442	0.944434	protein_coding
ENSG0000(	NUDT16-D'	1102.116	-0.03007	0.794413	0.944434	lncRNA
ENSG0000(	CCDC3	88.42571	-0.13162	0.794715	0.944439	protein_coding
ENSG0000(	CENPH	144.5393	-0.03933	0.794644	0.944439	protein_coding
ENSG0000(	COX7B	824.79	-0.03981	0.79494	0.944439	protein_coding
ENSG0000(	ENSG0000(	60.246	-0.05034	0.794784	0.944439	lncRNA
ENSG0000(	ENSG0000(	539.0073	0.019562	0.79493	0.944439	lncRNA
ENSG0000(	ENSG0000(	37.67316	0.055537	0.794803	0.944439	lncRNA
ENSG0000(	EPB41L3	1544.469	-0.03745	0.794713	0.944439	protein_coding
ENSG0000(	GLYCTK-AS	21.49659	-0.13373	0.794591	0.944439	lncRNA
ENSG0000(	LINC00838	76.78506	0.041196	0.79463	0.944439	lncRNA
ENSG0000(	MIR9-1HG	579.0044	-0.02497	0.794556	0.944439	lncRNA
ENSG0000(	MYBPC1	34.34796	0.140395	0.794895	0.944439	protein_coding
ENSG0000(	NAIF1	364.4189	0.019763	0.794865	0.944439	protein_coding
ENSG0000(	PSMB5	254.8851	-0.03567	0.794581	0.944439	protein_coding
ENSG0000(	UBL5	562.6216	0.032822	0.79478	0.944439	protein_coding
ENSG0000(	WT1	118.1375	-0.0465	0.794749	0.944439	protein_coding
ENSG0000(	BFAR	1030.008	0.018238	0.795013	0.944463	protein_coding
ENSG0000(	ENSG0000(	24.51041	0.089182	0.795166	0.944463	lncRNA
ENSG0000(	ENSG0000(	23.96002	-0.07191	0.795082	0.944463	TEC
ENSG0000(	LINP1	69.26438	-0.03867	0.795107	0.944463	lncRNA
ENSG0000(	RPL21P10	50.8248	-0.04938	0.795134	0.944463	processed_pseudogene
ENSG0000(	SRRM2-AS:	281.9381	0.022433	0.795098	0.944463	lncRNA
ENSG0000(	NIPA1	1097.201	0.021094	0.795204	0.944468	protein_coding
ENSG0000(	ASH1L	9148.618	0.012716	0.79544	0.944702	protein_coding
ENSG0000(	FAM174C	141.0257	0.03647	0.79547	0.944702	protein_coding
ENSG0000(	ENSG0000(	97.37326	0.035786	0.795534	0.944738	lncRNA
ENSG0000(	ENSG0000(	41.95952	0.043216	0.795744	0.944758	lncRNA
ENSG0000(	ENSG0000(	256.7749	0.026387	0.795664	0.944758	TEC
ENSG0000(	ENSG0000(	248.7605	0.026395	0.795722	0.944758	protein_coding

ENSG0000(	PBOV1	88.03911	-0.04106	0.795757	0.944758	protein_coding
ENSG0000(	RBMS3-AS2	126.4597	0.029847	0.795599	0.944758	lncRNA
ENSG0000(	STAG1	1907.092	-0.02154	0.795697	0.944758	protein_coding
ENSG0000(	LINC02099	360.5887	-0.02034	0.795942	0.944936	lncRNA
ENSG0000(	ADCY10P1	493.0461	0.027712	0.796047	0.944952	transcribed_unprocessed_pseudo
ENSG0000(	OR5H1	116.4386	0.03531	0.796117	0.944952	protein_coding
ENSG0000(	PES1	293.0781	0.023578	0.796095	0.944952	protein_coding
ENSG0000(	PEX11B	213.0608	0.028415	0.796018	0.944952	protein_coding
ENSG0000(	RN7SL156P	52.78187	0.052056	0.796127	0.944952	misc_RNA
ENSG0000(	NBL1	2138.564	0.035916	0.796238	0.945043	protein_coding
ENSG0000(	CHFR	3000.451	0.013849	0.796503	0.945121	protein_coding
ENSG0000(	ENSG0000(	180.3444	0.031295	0.796493	0.945121	lncRNA
ENSG0000(	ENSG0000(	115.356	0.029533	0.796491	0.945121	lncRNA
ENSG0000(	ENSG0000(	1251.072	-0.04012	0.796776	0.945121	lncRNA
ENSG0000(	ENSG0000(	363.0299	0.019813	0.79656	0.945121	lncRNA
ENSG0000(	ENSG0000(	192.8073	0.04124	0.796674	0.945121	lncRNA
ENSG0000(	FKBP3	394.398	0.032063	0.796429	0.945121	protein_coding
ENSG0000(	FRMPD2	54.8881	0.125414	0.796662	0.945121	protein_coding
ENSG0000(	MGST3	2419.276	0.027079	0.796784	0.945121	protein_coding
ENSG0000(	NIBAN2	1813.806	0.032922	0.796687	0.945121	protein_coding
ENSG0000(	RBMS3-AS3	701.979	0.027912	0.796819	0.945121	lncRNA
ENSG0000(	SCN5A	55.20942	-0.10337	0.796606	0.945121	protein_coding
ENSG0000(	SPAG9	4335.355	-0.0193	0.796803	0.945121	protein_coding
ENSG0000(	TGFA	599.3616	0.05905	0.796508	0.945121	protein_coding
ENSG0000(	ZPR1	1635.516	0.044374	0.796786	0.945121	protein_coding
ENSG0000(	ENSG0000(	42.27717	0.103951	0.796854	0.945122	lncRNA
ENSG0000(	DIPK1B	60.98721	-0.06834	0.797023	0.94514	protein_coding
ENSG0000(	ENSG0000(	260.25	-0.02286	0.796988	0.94514	lncRNA
ENSG0000(	LINC00102	69.1203	0.04144	0.797075	0.94514	lncRNA
ENSG0000(	PBX1	4793.728	0.031417	0.797055	0.94514	protein_coding
ENSG0000(	PRSS2	43.85316	-0.31503	0.797006	0.94514	protein_coding
ENSG0000(	TLE3	2075.133	-0.03055	0.797057	0.94514	protein_coding
ENSG0000(	LINC02880	21.08392	0.088714	0.797253	0.94531	lncRNA
ENSG0000(	AVIL	1203.636	-0.0451	0.797605	0.945334	protein_coding
ENSG0000(	C1orf56	33.90723	-0.07829	0.79766	0.945334	protein_coding
ENSG0000(	C3orf38	460.8077	-0.02662	0.797823	0.945334	protein_coding
ENSG0000(	CELF1	4998.747	-0.01604	0.797715	0.945334	protein_coding
ENSG0000(	CPT2	1128.725	-0.02353	0.797704	0.945334	protein_coding
ENSG0000(	ENSG0000(	556.042	-0.02394	0.797671	0.945334	transcribed_unprocessed_pseudo
ENSG0000(	ENSG0000(	30.47485	-0.07285	0.797594	0.945334	TEC
ENSG0000(	ENSG0000(	36.61216	0.057754	0.797552	0.945334	lncRNA
ENSG0000(	F8A2	89.55301	-0.79053	0.797696	0.945334	protein_coding
ENSG0000(	HMGN1	2891.179	-0.02058	0.797559	0.945334	protein_coding
ENSG0000(	IQCH	83.01521	0.047739	0.797582	0.945334	protein_coding
ENSG0000(	ISOC2	366.5794	0.032787	0.797796	0.945334	protein_coding
ENSG0000(	ITGB7	342.3295	0.044664	0.797791	0.945334	protein_coding
ENSG0000(	U2SURP	3121.243	0.016452	0.797795	0.945334	protein_coding

ENSG0000(	ZFYVE1	581.9729	0.032498	0.797573	0.945334	protein_coding
ENSG0000(	ZNF277	523.3124	0.019429	0.797421	0.945334	protein_coding
ENSG0000(	ITPK1-AS1	57.58423	-0.03699	0.797891	0.945374	lncRNA
ENSG0000(	DENR	1043.434	-0.01424	0.798026	0.945493	protein_coding
ENSG0000(	RPLP1	2098.587	0.036692	0.798067	0.945501	protein_coding
ENSG0000(	ENSG0000(	690.4068	0.02084	0.798285	0.945601	lncRNA
ENSG0000(	JAKMIP1-D	476.8171	-0.01847	0.798262	0.945601	transcribed_unprocessed_pseudo
ENSG0000(	RNU6-998F	26.91758	-0.07558	0.798206	0.945601	snRNA
ENSG0000(	SENP7	1559.257	-0.01858	0.798324	0.945601	protein_coding
ENSG0000(	SLC39A11	698.7872	0.028256	0.79829	0.945601	protein_coding
ENSG0000(	ENSG0000(	160.4919	-0.03013	0.798461	0.945723	lncRNA
ENSG0000(	LRGUK	81.75811	-0.07132	0.798591	0.945796	protein_coding
ENSG0000(	SMN1	666.3317	-0.04074	0.798582	0.945796	protein_coding
ENSG0000(	CHD3	3491.12	-0.03885	0.798644	0.945818	protein_coding
ENSG0000(	NPHP1	746.9708	-0.01737	0.798691	0.945832	protein_coding
ENSG0000(	PPP1R11	776.3665	-0.03183	0.798783	0.945901	protein_coding
ENSG0000(	RN7SL130F	51.95829	0.0448	0.798875	0.945969	misc_RNA
ENSG0000(	ENSG0000(	20.23245	0.100837	0.799092	0.946142	lncRNA
ENSG0000(	H3C3	76.98411	-0.06272	0.799156	0.946142	protein_coding
ENSG0000(	KTN1-AS1	640.8213	-0.01678	0.799087	0.946142	lncRNA
ENSG0000(	RN7SKP296	20.31662	-0.08384	0.799189	0.946142	misc_RNA
ENSG0000(	SDC1	1669.191	-0.03928	0.799193	0.946142	protein_coding
ENSG0000(	SMIM11B	213.9468	0.045065	0.79923	0.946145	protein_coding
ENSG0000(	CASC17	119.1542	-0.03832	0.799369	0.946147	lncRNA
ENSG0000(	ENSG0000(	98.5425	0.060038	0.799321	0.946147	lncRNA
ENSG0000(	LINC00377	61.74326	-0.03596	0.799339	0.946147	lncRNA
ENSG0000(	STRC	312.848	0.029228	0.799336	0.946147	protein_coding
ENSG0000(	ATRN	972.7114	-0.01865	0.799604	0.946314	protein_coding
ENSG0000(	DCTN5	3341.513	0.019021	0.799613	0.946314	protein_coding
ENSG0000(	ENSG0000(	31.28161	-0.06033	0.799583	0.946314	lncRNA
ENSG0000(	ACSL5	3087.177	-0.04142	0.799917	0.946628	protein_coding
ENSG0000(	EBLN3P	5066.8	0.023767	0.799965	0.946628	lncRNA
ENSG0000(	RARS2	3497.482	-0.00906	0.800016	0.946628	protein_coding
ENSG0000(	RPL21P5	56.35107	-0.0446	0.799992	0.946628	processed_pseudogene
ENSG0000(	MYO18B	30.18038	0.123678	0.800088	0.946673	protein_coding
ENSG0000(	CYP3A4	71.81363	0.088338	0.800274	0.946789	protein_coding
ENSG0000(	FAR2	1843.427	0.040004	0.80029	0.946789	protein_coding
ENSG0000(	SLC26A1	49.7839	0.094186	0.800289	0.946789	protein_coding
ENSG0000(	ENSG0000(	66.15695	-0.05343	0.800339	0.946806	lncRNA
ENSG0000(	NARF-IT1	102.0558	-0.03624	0.800389	0.946824	lncRNA
ENSG0000(	ENSG0000(	52.57299	-0.059	0.800423	0.946824	lncRNA
ENSG0000(	MAP3K13	2679.504	-0.02768	0.800478	0.946847	protein_coding
ENSG0000(	RAVER1	637.2943	-0.03495	0.800544	0.946847	protein_coding
ENSG0000(	RPL11	2435.067	0.030443	0.800545	0.946847	protein_coding
ENSG0000(	DDR1-DT	1665.954	-0.0153	0.800721	0.946872	lncRNA
ENSG0000(	DTYMK	258.4802	-0.02452	0.800792	0.946872	protein_coding
ENSG0000(	ENSG0000(	578.0502	-0.01538	0.800699	0.946872	lncRNA

ENSG0000(	ENSG0000(	165.1841	0.02889	0.800839	0.946872	lncRNA
ENSG0000(	FAM240B	47.99054	0.046463	0.800655	0.946872	protein_coding
ENSG0000(	OR2T7	63.78373	0.039047	0.800876	0.946872	polymorphic_pseudogene
ENSG0000(	POM121C	1668.944	0.01666	0.800876	0.946872	protein_coding
ENSG0000(	RNU6-341F	41.04978	-0.05059	0.800767	0.946872	snRNA
ENSG0000(	ZNF674	757.5758	-0.01866	0.800792	0.946872	protein_coding
ENSG0000(	COQ5	454.7542	0.019614	0.801174	0.946891	protein_coding
ENSG0000(	ENSG0000(	24.53416	-0.05968	0.801237	0.946891	processed_pseudogene
ENSG0000(	GSTM5	160.2355	0.061117	0.801084	0.946891	protein_coding
ENSG0000(	IDH1	1086.221	-0.03424	0.801	0.946891	protein_coding
ENSG0000(	LINC00970	335.0233	-0.01984	0.800942	0.946891	lncRNA
ENSG0000(	LRRC37BP1	1020.045	0.018796	0.801213	0.946891	transcribed_unprocessed_pseudo
ENSG0000(	MAP2K5-D1	87.24573	-0.04205	0.801138	0.946891	lncRNA
ENSG0000(	OVCA2	23.48713	-0.13669	0.801007	0.946891	protein_coding
ENSG0000(	PLAC4	144.4465	-0.0509	0.801057	0.946891	lncRNA
ENSG0000(	TAS2R6P	53.23522	0.044893	0.801187	0.946891	processed_pseudogene
ENSG0000(	RNU6-577F	21.83374	-0.07492	0.801323	0.946953	snRNA
ENSG0000(	GINM1	563.6673	0.021143	0.801406	0.94701	protein_coding
ENSG0000(	BCR	1401.342	0.035298	0.801491	0.947029	protein_coding
ENSG0000(	SFXN1	2001.149	0.027984	0.801476	0.947029	protein_coding
ENSG0000(	ENSG0000(	37.08012	0.044739	0.801574	0.947048	lncRNA
ENSG0000(	TMEM120A	289.1914	0.05067	0.801576	0.947048	protein_coding
ENSG0000(	ENSG0000(	162.8842	-0.03209	0.801667	0.947115	lncRNA
ENSG0000(	DNAJC4	370.5417	0.026416	0.801838	0.947169	protein_coding
ENSG0000(	DSC1	23.689	-0.07694	0.801798	0.947169	protein_coding
ENSG0000(	FAM114A1	1193.47	-0.0272	0.801885	0.947169	protein_coding
ENSG0000(	OTULINL	1708.953	-0.03699	0.80175	0.947169	protein_coding
ENSG0000(	SGK1	3475.574	-0.07323	0.801884	0.947169	protein_coding
ENSG0000(	ADAM10	3786.178	-0.01479	0.803079	0.947193	protein_coding
ENSG0000(	ADAM1B	81.94004	0.035466	0.80302	0.947193	unitary_pseudogene
ENSG0000(	BLZF1	863.4122	0.014289	0.803394	0.947193	protein_coding
ENSG0000(	CBLB	2882.922	-0.01893	0.80339	0.947193	protein_coding
ENSG0000(	DERA	474.9805	0.026786	0.80213	0.947193	protein_coding
ENSG0000(	EFHB	68.99834	0.06217	0.802771	0.947193	protein_coding
ENSG0000(	EIPR1-IT1	51.95336	0.051119	0.803276	0.947193	lncRNA
ENSG0000(	ENSG0000(	111.8308	-0.03955	0.802158	0.947193	lncRNA
ENSG0000(	ENSG0000(	688.5007	0.018872	0.803068	0.947193	lncRNA
ENSG0000(	ENSG0000(	478.5136	-0.01966	0.802983	0.947193	lncRNA
ENSG0000(	ENSG0000(	24.40054	0.065881	0.802605	0.947193	lncRNA
ENSG0000(	ENSG0000(	105.7185	0.036304	0.803309	0.947193	TEC
ENSG0000(	ENSG0000(	191.7665	-0.06685	0.802947	0.947193	lncRNA
ENSG0000(	FAM228B	1888.434	0.022745	0.802475	0.947193	protein_coding
ENSG0000(	FILNC1	115.815	0.035452	0.803256	0.947193	lncRNA
ENSG0000(	GCSAML	169.5261	0.026511	0.801958	0.947193	protein_coding
ENSG0000(	GTF2F1	486.3693	0.033363	0.802551	0.947193	protein_coding
ENSG0000(	KLHL24	2332.101	0.019577	0.80205	0.947193	protein_coding
ENSG0000(	LINC02043	487.446	-0.02512	0.802822	0.947193	lncRNA

ENSG0000(	LINC02142	310.6071	-0.02093	0.8033	0.947193	lncRNA
ENSG0000(	LINC02661	31.48973	-0.05809	0.802304	0.947193	lncRNA
ENSG0000(	LINC02700	36.1722	-0.05461	0.802224	0.947193	lncRNA
ENSG0000(	LMBRD2	771.3199	-0.02884	0.80342	0.947193	protein_coding
ENSG0000(	MARCHF9	338.5698	0.02637	0.803395	0.947193	protein_coding
ENSG0000(	MDGA2	39.92446	0.063064	0.802213	0.947193	protein_coding
ENSG0000(	MEIS3P2	19.70845	0.110048	0.80313	0.947193	processed_pseudogene
ENSG0000(	MTND4P3(	152.075	-0.02531	0.802636	0.947193	unprocessed_pseudogene
ENSG0000(	NAPRT	527.1782	0.040558	0.803242	0.947193	protein_coding
ENSG0000(	NUDT22	862.1844	0.025061	0.802479	0.947193	protein_coding
ENSG0000(	OAS2	1702.568	0.046489	0.802149	0.947193	protein_coding
ENSG0000(	PABIR3	1242.399	0.015144	0.802291	0.947193	protein_coding
ENSG0000(	PCF11-AS1	184.938	0.031345	0.8026	0.947193	lncRNA
ENSG0000(	PRH1	450.1802	0.03181	0.802795	0.947193	protein_coding
ENSG0000(	PTBP3	4388.011	-0.02095	0.802521	0.947193	protein_coding
ENSG0000(	RN7SKP90	48.1217	-0.07288	0.803368	0.947193	misc_RNA
ENSG0000(	RPS15AP29	284.3991	-0.04168	0.802169	0.947193	processed_pseudogene
ENSG0000(	SIM1	41.89495	0.042008	0.802118	0.947193	protein_coding
ENSG0000(	SNORD3A	44673.02	0.057407	0.802444	0.947193	snoRNA
ENSG0000(	SUPT5H	2170.999	-0.01675	0.802784	0.947193	protein_coding
ENSG0000(	TAF6	838.7934	-0.023	0.803402	0.947193	protein_coding
ENSG0000(	TECR	1009.737	0.017261	0.803311	0.947193	protein_coding
ENSG0000(	TPD52	3303.693	0.026404	0.803269	0.947193	protein_coding
ENSG0000(	UNG	287.4769	0.028734	0.802667	0.947193	protein_coding
ENSG0000(	ZKSCAN7	274.5663	-0.02653	0.802388	0.947193	protein_coding
ENSG0000(	RNU6-769F	24.17409	-0.07044	0.803481	0.947224	snRNA
ENSG0000(	ENSG0000(	34.22703	-0.0627	0.803545	0.947256	lncRNA
ENSG0000(	GNB3	64.46867	-0.06593	0.803595	0.947256	protein_coding
ENSG0000(	RN7SL811F	194.9142	-0.03757	0.803611	0.947256	misc_RNA
ENSG0000(	HMGN2	2725.042	-0.02365	0.803705	0.947264	protein_coding
ENSG0000(	TMCO3	1273.803	-0.02324	0.803722	0.947264	protein_coding
ENSG0000(	ZNRD2-DT	262.403	0.019646	0.803717	0.947264	lncRNA
ENSG0000(	ENSG0000(	36.28716	-0.05503	0.804068	0.947285	lncRNA
ENSG0000(	ENSG0000(	27.57671	-0.05805	0.804037	0.947285	lncRNA
ENSG0000(	ENSG0000(	362.5775	0.023587	0.804011	0.947285	lncRNA
ENSG0000(	ENSG0000(	70.45335	-0.03162	0.803964	0.947285	lncRNA
ENSG0000(	ENSG0000(	351.6192	-0.03172	0.804115	0.947285	lncRNA
ENSG0000(	FGD5-AS1	2531.145	0.018696	0.804118	0.947285	lncRNA
ENSG0000(	OR2A1	21.62231	-0.14691	0.804112	0.947285	protein_coding
ENSG0000(	RIPPLY3	302.5224	0.018016	0.80401	0.947285	protein_coding
ENSG0000(	SLC35G5	19.97693	0.066168	0.80408	0.947285	protein_coding
ENSG0000(	SREK1IP1	834.6222	0.01834	0.804003	0.947285	protein_coding
ENSG0000(	ZNF260	885.1562	0.030388	0.803965	0.947285	protein_coding
ENSG0000(	AGAP12P	99.00795	0.11376	0.804214	0.947298	transcribed_unprocessed_pseudo
ENSG0000(	DLGAP1-AS	385.5157	0.030721	0.804233	0.947298	lncRNA
ENSG0000(	ENSG0000(	21.31248	-0.05596	0.804219	0.947298	lncRNA
ENSG0000(	ENSG0000(	50.18154	0.060382	0.804267	0.947298	lncRNA

ENSG0000(	ENSG0000(	22.21185	-0.06875	0.804363	0.947309	lncRNA
ENSG0000(	ENSG0000(	169.8341	-0.03032	0.804446	0.947309	lncRNA
ENSG0000(	ENSG0000(	43.36369	0.067267	0.804344	0.947309	lncRNA
ENSG0000(	RNU6-202F	24.2524	-0.05582	0.804447	0.947309	snRNA
ENSG0000(	RSRC1	2062.287	0.012972	0.804474	0.947309	protein_coding
ENSG0000(	SMG1-DT	48.69689	0.043642	0.804483	0.947309	lncRNA
ENSG0000(	METTL23	206.4583	-0.02581	0.804655	0.947472	protein_coding
ENSG0000(	FAM91A2P	19.88709	-0.17052	0.804752	0.947505	transcribed_processed_pseudoge
ENSG0000(	SMIM35	73.10765	-0.06197	0.804737	0.947505	protein_coding
ENSG0000(	BNIPL	219.9259	0.023555	0.805094	0.947711	protein_coding
ENSG0000(	ENSG0000(	89.00073	-0.03611	0.805215	0.947711	lncRNA
ENSG0000(	ENSG0000(	90.43507	0.033496	0.805269	0.947711	lncRNA
ENSG0000(	GPRC5A	7177.255	0.054563	0.805324	0.947711	protein_coding
ENSG0000(	HMGXB4	709.7888	-0.0177	0.805129	0.947711	protein_coding
ENSG0000(	MRPS31P4	31.37398	0.076372	0.804973	0.947711	transcribed_unprocessed_pseudo
ENSG0000(	PNRC2	2185.714	-0.02104	0.805306	0.947711	protein_coding
ENSG0000(	QTRT2	1627.637	0.016351	0.805289	0.947711	protein_coding
ENSG0000(	SNORA44	106.2281	-0.05467	0.805134	0.947711	snoRNA
ENSG0000(	TRNAU1AP	620.0762	-0.02154	0.80534	0.947711	protein_coding
ENSG0000(	TTBK2-AS1	54.21939	-0.03549	0.805323	0.947711	lncRNA
ENSG0000(	TXN2	376.4458	0.028234	0.805016	0.947711	protein_coding
ENSG0000(	FAM210A	566.7274	-0.02314	0.8055	0.947779	protein_coding
ENSG0000(	POLDIP2	515.4284	-0.02971	0.805458	0.947779	protein_coding
ENSG0000(	SALL4	195.2523	-0.05647	0.805502	0.947779	protein_coding
ENSG0000(	ACCS	896.683	0.033849	0.805755	0.947848	protein_coding
ENSG0000(	ENSG0000(	328.1931	-0.0438	0.805595	0.947848	lncRNA
ENSG0000(	LINC01150	50.52004	0.039241	0.805717	0.947848	lncRNA
ENSG0000(	NRG2	29.84658	0.084917	0.805767	0.947848	protein_coding
ENSG0000(	SNTA1	52.34835	-0.04573	0.805707	0.947848	protein_coding
ENSG0000(	UROS	897.5313	-0.01907	0.805644	0.947848	protein_coding
ENSG0000(	RPS2P45	172.7082	0.025677	0.805853	0.947908	transcribed_processed_pseudoge
ENSG0000(	BMI1	551.4516	-0.03147	0.805951	0.947936	protein_coding
ENSG0000(	CICP27	149.0958	-0.05066	0.806275	0.947936	processed_pseudogene
ENSG0000(	COL21A1	70.62413	-0.08147	0.806328	0.947936	protein_coding
ENSG0000(	DBP	596.4511	-0.02359	0.80634	0.947936	protein_coding
ENSG0000(	ENSG0000(	476.4347	-0.01902	0.806238	0.947936	lncRNA
ENSG0000(	GALNT4	1639.975	0.030687	0.806247	0.947936	protein_coding
ENSG0000(	GMPPB	1208.308	-0.02173	0.805944	0.947936	protein_coding
ENSG0000(	KBTBD3	127.402	-0.03229	0.806393	0.947936	protein_coding
ENSG0000(	LINC01800	26.99539	0.089124	0.806171	0.947936	lncRNA
ENSG0000(	LINC02156	48.10083	-0.05367	0.806373	0.947936	lncRNA
ENSG0000(	OR2T33	324.3483	0.022538	0.806192	0.947936	protein_coding
ENSG0000(	PLEKHH2	2088.31	-0.02472	0.806163	0.947936	protein_coding
ENSG0000(	SWSAP1	370.4803	0.017268	0.80629	0.947936	protein_coding
ENSG0000(	ZBTB20-AS	83.59449	-0.06879	0.806247	0.947936	lncRNA
ENSG0000(	ZNF70	1405.202	-0.01238	0.806357	0.947936	protein_coding
ENSG0000(	ENSG0000(	27.59737	0.395832	0.806578	0.947981	lncRNA

ENSG0000(	ENSG0000(	33.33969	-0.05259	0.806531	0.947981	lncRNA
ENSG0000(	HDGFL3P1	105.7907	0.035604	0.806494	0.947981	processed_pseudogene
ENSG0000(	MSS51	406.546	0.016639	0.806559	0.947981	protein_coding
ENSG0000(	TDGF1	159.4553	0.027807	0.806604	0.947981	protein_coding
ENSG0000(	ENSG0000(	101.6861	-0.0455	0.806874	0.948177	lncRNA
ENSG0000(	ENSG0000(	50.57965	-0.05653	0.806838	0.948177	lncRNA
ENSG0000(	STK26	267.8715	0.02838	0.806862	0.948177	protein_coding
ENSG0000(	TDRD15	32.6962	-0.08266	0.806908	0.948177	protein_coding
ENSG0000(	GUF1	1000.635	0.022422	0.806968	0.948206	protein_coding
ENSG0000(	TNFRSF1A	1765.969	-0.02499	0.807048	0.94826	protein_coding
ENSG0000(	NDUFAF5	737.1636	0.027809	0.807099	0.948279	protein_coding
ENSG0000(	LINC02150	54.87523	-0.06959	0.80714	0.948283	lncRNA
ENSG0000(	RN7SKP273	21.74279	-0.06626	0.807171	0.948283	misc_RNA
ENSG0000(	LRRC37A15	61.58064	0.070396	0.807213	0.948288	processed_pseudogene
ENSG0000(	RAC1	2761.89	-0.02816	0.807244	0.948288	protein_coding
ENSG0000(	DMAC1	329.2321	0.038542	0.807531	0.948345	protein_coding
ENSG0000(	ENSG0000(	33.50141	-0.07079	0.807385	0.948345	lncRNA
ENSG0000(	ENSG0000(	222.6177	-0.02118	0.807397	0.948345	lncRNA
ENSG0000(	GLI1	209.0664	0.057137	0.807571	0.948345	protein_coding
ENSG0000(	LCMT1-AS2	129.2802	0.028328	0.807456	0.948345	lncRNA
ENSG0000(	RN7SL68P	28.01462	-0.05497	0.807603	0.948345	misc_RNA
ENSG0000(	SLC35A2	268.1881	0.030731	0.807378	0.948345	protein_coding
ENSG0000(	UNK	1199.819	-0.01675	0.807582	0.948345	protein_coding
ENSG0000(	VSIG1	381.2567	0.03155	0.807529	0.948345	protein_coding
ENSG0000(	RN7SL4P	102.0666	0.065418	0.807772	0.948502	misc_RNA
ENSG0000(	ENSG0000(	22.69533	0.064209	0.807876	0.948584	lncRNA
ENSG0000(	ENSG0000(	51.42247	-0.04553	0.808111	0.9487	lncRNA
ENSG0000(	MCUR1	1294.899	0.020198	0.808112	0.9487	protein_coding
ENSG0000(	TSC22D3	1541.049	-0.09605	0.808019	0.9487	protein_coding
ENSG0000(	YAF2	1622.713	0.014898	0.808054	0.9487	protein_coding
ENSG0000(	CLUL1	28.31228	0.080439	0.808274	0.948815	protein_coding
ENSG0000(	CRYL1	809.9711	0.046567	0.808502	0.948815	protein_coding
ENSG0000(	DEPDC7	116.6348	0.056958	0.808659	0.948815	protein_coding
ENSG0000(	ENSG0000(	1058.618	0.014871	0.808545	0.948815	lncRNA
ENSG0000(	ENSG0000(	43.1222	0.056523	0.808362	0.948815	lncRNA
ENSG0000(	ENSG0000(	135.2884	0.033042	0.808451	0.948815	lncRNA
ENSG0000(	ENSG0000(	40.20126	0.049129	0.808624	0.948815	lncRNA
ENSG0000(	ENSG0000(	467.6731	-0.01615	0.808469	0.948815	lncRNA
ENSG0000(	FUT10	853.3811	0.020618	0.80841	0.948815	protein_coding
ENSG0000(	GRIA4	307.0585	0.040224	0.808619	0.948815	protein_coding
ENSG0000(	MTND5P5	51.86639	-0.11917	0.808584	0.948815	processed_pseudogene
ENSG0000(	NUF2	84.04233	-0.06322	0.808645	0.948815	protein_coding
ENSG0000(	SAE1	604.9371	0.020751	0.808326	0.948815	protein_coding
ENSG0000(	BCORP1	153.2389	0.039433	0.808835	0.948845	transcribed_unprocessed_pseudo
ENSG0000(	ENSG0000(	186.0757	-0.02636	0.808856	0.948845	lncRNA
ENSG0000(	ENSG0000(	210.9561	0.027381	0.808763	0.948845	lncRNA
ENSG0000(	SPACA9	245.2747	0.023842	0.808827	0.948845	protein_coding

ENSG0000(	ST8SIA5-DT	166.7779	0.025249	0.808731	0.948845	lncRNA
ENSG0000(	AMH	31.60444	0.065135	0.808928	0.948888	protein_coding
ENSG0000(	DCTPP1	168.6974	0.047123	0.809	0.948932	protein_coding
ENSG0000(	ENSG0000(	104.9238	0.037575	0.809082	0.948988	lncRNA
ENSG0000(	EIF4A2	6071.217	-0.01315	0.809219	0.949068	protein_coding
ENSG0000(	ENSG0000(	27.90135	-0.0701	0.809292	0.949068	lncRNA
ENSG0000(	ENSG0000(	56.61558	0.057197	0.809437	0.949068	lncRNA
ENSG0000(	ENSG0000(	34.6056	-0.05183	0.809398	0.949068	lncRNA
ENSG0000(	PAG1	4556.422	-0.03207	0.8093	0.949068	protein_coding
ENSG0000(	RCOR3	1923.928	0.017459	0.809419	0.949068	protein_coding
ENSG0000(	ZNF208	492.935	0.027305	0.809378	0.949068	protein_coding
ENSG0000(	ZNF276	2017.332	0.017027	0.809461	0.949068	protein_coding
ENSG0000(	ZNF576	429.7895	-0.01783	0.809219	0.949068	protein_coding
ENSG0000(	ID1	743.7736	-0.04961	0.809562	0.949146	protein_coding
ENSG0000(	DENND1B	2313.053	-0.02857	0.809669	0.949187	protein_coding
ENSG0000(	LINC00957	777.6976	-0.01832	0.809693	0.949187	lncRNA
ENSG0000(	SYNRG	3587.814	0.017246	0.8097	0.949187	protein_coding
ENSG0000(	CFL1P5	33.66135	0.069393	0.809986	0.949286	processed_pseudogene
ENSG0000(	ENSG0000(	99.89652	-0.05013	0.810095	0.949286	lncRNA
ENSG0000(	ENSG0000(	121.2729	-0.03584	0.809993	0.949286	lncRNA
ENSG0000(	IFT46	193.7559	-0.0277	0.809967	0.949286	protein_coding
ENSG0000(	LRRC15	91.4942	-0.12042	0.810026	0.949286	protein_coding
ENSG0000(	PLGLB1	431.991	0.046324	0.810089	0.949286	protein_coding
ENSG0000(	SHISA6	19.69845	-0.14511	0.80993	0.949286	protein_coding
ENSG0000(	TBC1D17	857.5314	-0.01556	0.809942	0.949286	protein_coding
ENSG0000(	TGM1	52.69674	0.069227	0.809997	0.949286	protein_coding
ENSG0000(	ACE	791.2558	-0.03623	0.810384	0.949364	protein_coding
ENSG0000(	ASPM	1284.837	-0.03766	0.810282	0.949364	protein_coding
ENSG0000(	ENSG0000(	128.5389	0.033716	0.810438	0.949364	lncRNA
ENSG0000(	ENSG0000(	40.00867	-0.05081	0.810366	0.949364	lncRNA
ENSG0000(	GARS1-DT	10566.59	0.015016	0.810404	0.949364	lncRNA
ENSG0000(	HERPUD2-/	778.7017	0.013239	0.810335	0.949364	lncRNA
ENSG0000(	RPL13	2614.958	0.032405	0.810396	0.949364	protein_coding
ENSG0000(	TESMIN	236.3003	0.034893	0.810381	0.949364	protein_coding
ENSG0000(	ENSG0000(	112.0433	-0.02388	0.810495	0.94939	lncRNA
ENSG0000(	ENSG0000(	24.54772	0.051712	0.810626	0.949393	lncRNA
ENSG0000(	ENSG0000(	343.658	0.020939	0.810635	0.949393	lncRNA
ENSG0000(	PTH2R	27.05723	0.07302	0.810616	0.949393	protein_coding
ENSG0000(	SPRY3	593.8244	0.025059	0.810537	0.949393	protein_coding
ENSG0000(	ANKRD24	76.5868	-0.07098	0.810708	0.949438	protein_coding
ENSG0000(	KIF1B	4862.12	-0.03066	0.810855	0.949529	protein_coding
ENSG0000(	RHOC	1611.088	0.043014	0.810834	0.949529	protein_coding
ENSG0000(	ENSG0000(	74.19231	-0.03414	0.810915	0.94955	processed_pseudogene
ENSG0000(	ENSG0000(	22.98158	-0.12926	0.810941	0.94955	transcribed_processed_pseudoge
ENSG0000(	ENSG0000(	181.478	0.041525	0.810978	0.949552	lncRNA
ENSG0000(	ENSG0000(	75.85056	0.035631	0.811022	0.949563	TEC
ENSG0000(	DNAJA2-DT	27.87344	-0.04151	0.811263	0.949564	lncRNA

ENSG0000(	FGL1	20.23646	0.100252	0.811161	0.949564	protein_coding
ENSG0000(	KCNS3	199.0283	0.058121	0.8111	0.949564	protein_coding
ENSG0000(	NCOR1	9801.628	0.016249	0.81123	0.949564	protein_coding
ENSG0000(	POLR3H	655.2733	-0.01874	0.811265	0.949564	protein_coding
ENSG0000(	SPAG7	552.9364	0.015607	0.811251	0.949564	protein_coding
ENSG0000(	VAMP8	497.3668	0.042697	0.811256	0.949564	protein_coding
ENSG0000(	EIF2B3	336.1397	-0.02769	0.811349	0.949582	protein_coding
ENSG0000(	ENSG0000(	24.57577	0.060427	0.811315	0.949582	lncRNA
ENSG0000(	AKAP6	555.4615	-0.02386	0.811583	0.949673	protein_coding
ENSG0000(	CCDC13	337.1632	0.037334	0.811689	0.949673	protein_coding
ENSG0000(	CFAP91	135.9344	0.046485	0.811497	0.949673	protein_coding
ENSG0000(	ENSG0000(	3112.686	-0.02706	0.811675	0.949673	lncRNA
ENSG0000(	ENSG0000(	176.1459	-0.02564	0.811711	0.949673	lncRNA
ENSG0000(	ENSG0000(	81.59095	0.034328	0.811682	0.949673	lncRNA
ENSG0000(	ENSG0000(	244.8331	-0.02139	0.811632	0.949673	TEC
ENSG0000(	NLRP8	88.14511	-0.03447	0.811532	0.949673	protein_coding
ENSG0000(	ZBTB8OS	963.1365	0.014084	0.811737	0.949673	protein_coding
ENSG0000(	ENSG0000(	118.3244	0.023588	0.811828	0.949699	lncRNA
ENSG0000(	ENSG0000(	212.6505	-0.02307	0.811815	0.949699	lncRNA
ENSG0000(	ENSG0000(	74.96308	0.031088	0.811933	0.949756	lncRNA
ENSG0000(	TDH-AS1	317.3148	0.04476	0.811946	0.949756	lncRNA
ENSG0000(	SNORD46	34.10957	-0.04129	0.811989	0.949766	snoRNA
ENSG0000(	BSPRY	326.744	0.034863	0.812154	0.9499	protein_coding
ENSG0000(	SNORA67	70.83298	-0.04477	0.812173	0.9499	snoRNA
ENSG0000(	ENSG0000(	187.7874	-0.02906	0.812373	0.94996	lncRNA
ENSG0000(	ENSG0000(	203.3411	0.018475	0.812307	0.94996	lncRNA
ENSG0000(	PAX8-AS1	3481.799	-0.06039	0.812368	0.94996	lncRNA
ENSG0000(	RN7SL180P	64.55327	0.038096	0.812318	0.94996	misc_RNA
ENSG0000(	USP2-AS1	465.0605	-0.02948	0.812397	0.94996	lncRNA
ENSG0000(	ENSG0000(	39.50531	-0.03895	0.812542	0.950009	unprocessed_pseudogene
ENSG0000(	ENSG0000(	256.6531	-0.01408	0.812542	0.950009	lncRNA
ENSG0000(	VTI1A	1885.858	-0.00995	0.812535	0.950009	protein_coding
ENSG0000(	ENSG0000(	71.31314	-0.04781	0.812613	0.95002	lncRNA
ENSG0000(	ENSG0000(	62.13736	0.045851	0.812826	0.95002	lncRNA
ENSG0000(	LINC01585	83.23291	-0.0471	0.81282	0.95002	lncRNA
ENSG0000(	RN7SL269P	45.66103	-0.03869	0.812699	0.95002	misc_RNA
ENSG0000(	SLC6A19	216.5143	0.15212	0.812643	0.95002	protein_coding
ENSG0000(	TSPAN10	827.4946	-0.01594	0.81273	0.95002	protein_coding
ENSG0000(	USP12	1715.177	0.023026	0.812799	0.95002	protein_coding
ENSG0000(	ZCCHC8	1626.756	-0.0127	0.812828	0.95002	protein_coding
ENSG0000(	ENSG0000(	67.11612	-0.03901	0.812897	0.950021	lncRNA
ENSG0000(	RRP36	559.6989	0.016373	0.812867	0.950021	protein_coding
ENSG0000(	H1-0	1483.608	-0.04634	0.813073	0.950182	protein_coding
ENSG0000(	LCAL1	81.54942	-0.05265	0.813105	0.950182	lncRNA
ENSG0000(	BCAS2	139.2352	-0.02931	0.813192	0.950236	protein_coding
ENSG0000(	ENSG0000(	286.3067	0.021826	0.813358	0.950236	processed_pseudogene
ENSG0000(	ENSG0000(	21.09828	-0.09612	0.813272	0.950236	lncRNA

ENSG0000(	FRRS1	1776.402	0.023566	0.813332	0.950236	protein_coding
ENSG0000(	RPS17	1816.452	-0.03232	0.813297	0.950236	protein_coding
ENSG0000(	SLC41A3	428.5308	0.023231	0.81322	0.950236	protein_coding
ENSG0000(	ACTG1P3	44.88218	0.048656	0.813553	0.950299	processed_pseudogene
ENSG0000(	BNIP3P5	43.19493	-0.05917	0.813642	0.950299	processed_pseudogene
ENSG0000(	HIVEP1	3244.022	-0.01948	0.8136	0.950299	protein_coding
ENSG0000(	KCNQ1	447.7799	-0.03829	0.813504	0.950299	protein_coding
ENSG0000(	POGZ	4105.383	-0.01289	0.813564	0.950299	protein_coding
ENSG0000(	PRELID2	3854.898	0.047026	0.813464	0.950299	protein_coding
ENSG0000(	PXDNL	30.38802	-0.07893	0.813653	0.950299	protein_coding
ENSG0000(	YY2	118.5105	0.026704	0.813702	0.950315	protein_coding
ENSG0000(	LINC02361	24.08028	0.070594	0.813864	0.950343	lncRNA
ENSG0000(	NUP42	714.8078	0.017055	0.813812	0.950343	protein_coding
ENSG0000(	PSMD10P2	104.1294	-0.03895	0.813863	0.950343	transcribed_processed_pseudoge
ENSG0000(	RN7SL67P	43.04066	0.046802	0.81384	0.950343	misc_RNA
ENSG0000(	CCDC74B	45.88853	-0.0687	0.81392	0.950368	protein_coding
ENSG0000(	ENSG0000(	67.60323	0.465969	0.814086	0.950372	lncRNA
ENSG0000(	ENSG0000(	24.38118	-0.05931	0.813988	0.950372	lncRNA
ENSG0000(	NAGLU	404.7352	0.025837	0.814027	0.950372	protein_coding
ENSG0000(	PLEKHF2	650.99	0.025409	0.814011	0.950372	protein_coding
ENSG0000(	RN7SL827F	27.80401	-0.05859	0.814096	0.950372	misc_RNA
ENSG0000(	ATXN10	910.913	-0.01688	0.814205	0.950378	protein_coding
ENSG0000(	CARMIL1	1622.167	0.03262	0.814183	0.950378	protein_coding
ENSG0000(	MCTS2P	21.96552	0.200007	0.814149	0.950378	protein_coding
ENSG0000(	LINC01715	59.50577	-0.04408	0.81425	0.95039	lncRNA
ENSG0000(	ENSG0000(	819.1629	0.017603	0.814311	0.950421	lncRNA
ENSG0000(	LCMT2	624.3851	0.018718	0.814405	0.950491	protein_coding
ENSG0000(	EMC8	330.1446	-0.02094	0.81473	0.950518	protein_coding
ENSG0000(	ENSG0000(	613.5043	0.019375	0.814638	0.950518	lncRNA
ENSG0000(	ENSG0000(	220.913	-0.02029	0.814741	0.950518	lncRNA
ENSG0000(	ENSG0000(	35.30917	0.079027	0.81461	0.950518	lncRNA
ENSG0000(	ENSG0000(	64.98085	-0.03765	0.814663	0.950518	lncRNA
ENSG0000(	HCG4B	34.34412	-0.05767	0.814774	0.950518	unprocessed_pseudogene
ENSG0000(	OR7C1	865.7207	-0.02189	0.814774	0.950518	protein_coding
ENSG0000(	THBS3	504.6381	-0.02486	0.814581	0.950518	protein_coding
ENSG0000(	YRDC	116.1174	-0.03326	0.81473	0.950518	protein_coding
ENSG0000(	ZNF350	517.0447	-0.03268	0.814699	0.950518	protein_coding
ENSG0000(	CDK5RAP1	613.7963	0.015981	0.814893	0.950608	protein_coding
ENSG0000(	RPS28P7	140.9908	-0.05353	0.814921	0.950608	processed_pseudogene
ENSG0000(	AARS2	838.6364	0.023027	0.815153	0.950624	protein_coding
ENSG0000(	ARHGAP21	3896.355	0.022553	0.81521	0.950624	protein_coding
ENSG0000(	DKFZP434A	42.45407	-0.05899	0.815034	0.950624	lncRNA
ENSG0000(	ENSG0000(	58.88349	-0.03786	0.815326	0.950624	lncRNA
ENSG0000(	ENSG0000(	431.8898	-0.02276	0.815375	0.950624	lncRNA
ENSG0000(	FUS	10510.05	-0.02001	0.81505	0.950624	protein_coding
ENSG0000(	LRCH4	1479.58	0.019036	0.815147	0.950624	protein_coding
ENSG0000(	LSM5	479.6865	0.021644	0.815544	0.950624	protein_coding

ENSG0000(	LYPD6	91.40199	0.054443	0.815212	0.950624	protein_coding
ENSG0000(	MIR22HG	1426.095	0.040257	0.815498	0.950624	lncRNA
ENSG0000(	MRPS31P5	793.9731	0.027742	0.815556	0.950624	transcribed_unprocessed_pseudo
ENSG0000(	NMD3	643.1128	-0.02013	0.815022	0.950624	protein_coding
ENSG0000(	PIGBOS1	198.5444	0.026017	0.815285	0.950624	protein_coding
ENSG0000(	RPS23P6	37.2082	-0.04992	0.81539	0.950624	processed_pseudogene
ENSG0000(	TET2	5809.415	-0.01572	0.815449	0.950624	protein_coding
ENSG0000(	TLE6	21.54334	0.074725	0.815497	0.950624	protein_coding
ENSG0000(	TMEM199	813.7207	-0.01639	0.815467	0.950624	protein_coding
ENSG0000(	ZNF713	1182.986	0.012632	0.815355	0.950624	protein_coding
ENSG0000(	RPL13P5	77.51045	0.042755	0.815599	0.950634	transcribed_processed_pseudoge
ENSG0000(	ENSG0000(	24.88751	0.055683	0.815679	0.950647	lncRNA
ENSG0000(	LAS1L	963.1562	-0.03003	0.815672	0.950647	protein_coding
ENSG0000(	ABCB5	219.9278	0.038055	0.816803	0.950717	protein_coding
ENSG0000(	CNOT4	1386.565	-0.01577	0.816702	0.950717	protein_coding
ENSG0000(	CPS1	867.1397	-0.03146	0.81649	0.950717	protein_coding
ENSG0000(	DUBR	375.0044	-0.03421	0.816595	0.950717	lncRNA
ENSG0000(	ENSG0000(	550.4352	-0.0209	0.815887	0.950717	transcribed_processed_pseudoge
ENSG0000(	ENSG0000(	40.96131	0.046651	0.816135	0.950717	lncRNA
ENSG0000(	ENSG0000(	76.68534	-0.02858	0.816043	0.950717	lncRNA
ENSG0000(	ENSG0000(	26.27173	-0.05974	0.81641	0.950717	lncRNA
ENSG0000(	ENSG0000(	427.093	0.02297	0.816351	0.950717	lncRNA
ENSG0000(	ENSG0000(	19.67389	0.060779	0.816374	0.950717	lncRNA
ENSG0000(	ENSG0000(	103.0398	0.030121	0.815851	0.950717	lncRNA
ENSG0000(	ENSG0000(	494.1105	0.017165	0.816487	0.950717	lncRNA
ENSG0000(	ENSG0000(	20.31728	0.069086	0.816723	0.950717	lncRNA
ENSG0000(	ENSG0000(	203.2425	0.027762	0.816456	0.950717	lncRNA
ENSG0000(	ENSG0000(	36.48758	-0.06882	0.816159	0.950717	lncRNA
ENSG0000(	ESRRAP2	118.3237	0.03436	0.816811	0.950717	transcribed_processed_pseudoge
ENSG0000(	GOLIM4	1727.984	0.034592	0.816558	0.950717	protein_coding
ENSG0000(	HMCN1	431.9133	-0.06554	0.816728	0.950717	protein_coding
ENSG0000(	LARGE-IT1	49.94549	0.04593	0.816051	0.950717	lncRNA
ENSG0000(	LINC00658	32.11692	-0.0558	0.815997	0.950717	lncRNA
ENSG0000(	LL22NC03-(	546.204	-0.0157	0.816418	0.950717	lncRNA
ENSG0000(	MOGS	1050.029	0.019098	0.815832	0.950717	protein_coding
ENSG0000(	MPP2	45.37607	-0.0719	0.815916	0.950717	protein_coding
ENSG0000(	MYL6	3660.319	0.027611	0.816515	0.950717	protein_coding
ENSG0000(	PDS5B	1460.338	0.020432	0.815965	0.950717	protein_coding
ENSG0000(	RBM5-AS1	99.34623	0.036175	0.8165	0.950717	lncRNA
ENSG0000(	RPL23	2509.174	0.030591	0.816452	0.950717	protein_coding
ENSG0000(	SETDB2	1143.59	0.015914	0.816323	0.950717	protein_coding
ENSG0000(	SPAAR	144.5816	0.028787	0.816624	0.950717	protein_coding
ENSG0000(	TMSB4Y	60.25373	-0.05528	0.816703	0.950717	protein_coding
ENSG0000(	UBE2J2	865.0267	0.013443	0.816752	0.950717	protein_coding
ENSG0000(	ENSG0000(	77.8567	-0.03277	0.816941	0.950759	lncRNA
ENSG0000(	FGF1	38.79336	0.060858	0.816985	0.950759	protein_coding
ENSG0000(	KDELR1	1160.079	-0.02056	0.816929	0.950759	protein_coding

ENSG0000(	NCAPG2	595.6246	-0.0365	0.816985	0.950759	protein_coding
ENSG0000(	CFAP298-T(	908.0704	-0.01689	0.817098	0.950779	protein_coding
ENSG0000(	ENSG0000(	343.8129	-0.02252	0.81714	0.950779	transcribed_processed_pseudoge
ENSG0000(	ENSG0000(	1242.58	0.022642	0.817132	0.950779	TEC
ENSG0000(	ENSG0000(	24.68677	-0.06563	0.817093	0.950779	lncRNA
ENSG0000(	ENSG0000(	50.7578	-0.03104	0.817445	0.950811	lncRNA
ENSG0000(	ENSG0000(	502.336	-0.03371	0.817347	0.950811	lncRNA
ENSG0000(	LINC01238	665.2053	0.025542	0.817311	0.950811	lncRNA
ENSG0000(	OR10AH1P	282.7788	0.016429	0.81731	0.950811	transcribed_unprocessed_pseudo
ENSG0000(	SEMA6D	2139.54	0.043173	0.817281	0.950811	protein_coding
ENSG0000(	SNORA5A	77.06911	0.048275	0.817383	0.950811	snoRNA
ENSG0000(	TIAL1	2603.551	0.022228	0.817245	0.950811	protein_coding
ENSG0000(	ZNF470	1121.625	0.012231	0.817421	0.950811	protein_coding
ENSG0000(	LINC02352	233.4907	-0.02376	0.817504	0.950824	lncRNA
ENSG0000(	MATN1-AS	129.9427	0.043336	0.817524	0.950824	lncRNA
ENSG0000(	LATS1	2519.784	0.011959	0.817622	0.950897	protein_coding
ENSG0000(	B4GALT6	145.755	-0.03521	0.817784	0.950964	protein_coding
ENSG0000(	LINC00106	189.5558	0.028807	0.817752	0.950964	lncRNA
ENSG0000(	TIGD4	19.94943	0.069357	0.817783	0.950964	protein_coding
ENSG0000(	BCL10	1267.45	0.030962	0.81797	0.951061	protein_coding
ENSG0000(	LINC00623	143.5904	0.042404	0.817957	0.951061	lncRNA
ENSG0000(	ZNF324B	242.7822	0.02495	0.817919	0.951061	protein_coding
ENSG0000(	ABCC13	1028.227	0.063059	0.818053	0.951117	transcribed_unprocessed_pseudo
ENSG0000(	ANGPT4	97.0582	-0.0332	0.818201	0.951248	protein_coding
ENSG0000(	ADI1	2364.165	0.014621	0.818301	0.951286	protein_coding
ENSG0000(	ENSG0000(	40.412	0.050491	0.818302	0.951286	lncRNA
ENSG0000(	ENSG0000(	119.7208	-0.0292	0.81838	0.951318	lncRNA
ENSG0000(	HERC2P3	1145.16	-0.03445	0.818399	0.951318	transcribed_unprocessed_pseudo
ENSG0000(	ZBTB1	1787.105	-0.02123	0.818438	0.951324	protein_coding
ENSG0000(	CEP68	1161.021	0.015036	0.818528	0.951355	protein_coding
ENSG0000(	ENSG0000(	101.5597	-0.0263	0.818558	0.951355	lncRNA
ENSG0000(	MTRFR	3928.252	-0.01136	0.818604	0.951355	protein_coding
ENSG0000(	YBX2	1263.398	0.060469	0.818587	0.951355	protein_coding
ENSG0000(	COL19A1	518.2893	-0.0385	0.818884	0.951357	protein_coding
ENSG0000(	ENSG0000(	20.29874	-0.06599	0.818752	0.951357	lncRNA
ENSG0000(	GALM	599.98	-0.02271	0.818785	0.951357	protein_coding
ENSG0000(	MALRD1	150.3916	0.1252	0.81902	0.951357	protein_coding
ENSG0000(	MIR604	40.97841	-0.04542	0.818911	0.951357	miRNA
ENSG0000(	NELFCD	931.5263	0.014542	0.818946	0.951357	protein_coding
ENSG0000(	SSB	461.4665	-0.02373	0.818793	0.951357	protein_coding
ENSG0000(	STAB1	5398.81	-0.03389	0.819	0.951357	protein_coding
ENSG0000(	TCEA1P4	58.15146	-0.0328	0.818734	0.951357	processed_pseudogene
ENSG0000(	TEAD3	688.1838	0.023452	0.818955	0.951357	protein_coding
ENSG0000(	WDR77	502.2471	0.022917	0.818826	0.951357	protein_coding
ENSG0000(	ZNF362	402.7185	-0.05488	0.818752	0.951357	protein_coding
ENSG0000(	ABCA9	810.351	-0.02352	0.819364	0.951375	protein_coding
ENSG0000(	AGPAT3	2791.19	-0.01636	0.819189	0.951375	protein_coding

ENSG0000(	ENSG0000(	257.6807	-0.01476	0.819191	0.951375	lncRNA
ENSG0000(	ENSG0000(	34.00492	-0.04357	0.819395	0.951375	lncRNA
ENSG0000(	ENSG0000(	764.0551	0.014941	0.819164	0.951375	lncRNA
ENSG0000(	ENSG0000(	108.5813	0.033304	0.819103	0.951375	lncRNA
ENSG0000(	ENSG0000(	46.86467	-0.06258	0.819451	0.951375	protein_coding
ENSG0000(	PLS3-AS1	134.5524	0.032302	0.819429	0.951375	lncRNA
ENSG0000(	RABL3	619.4693	0.020054	0.819404	0.951375	protein_coding
ENSG0000(	SDHAF3	63.74042	-0.04981	0.819396	0.951375	protein_coding
ENSG0000(	SPATA1	75.83215	0.045116	0.819422	0.951375	protein_coding
ENSG0000(	SRBD1	678.679	-0.02491	0.819378	0.951375	protein_coding
ENSG0000(	SLC48A1	621.4351	0.02786	0.8195	0.951392	protein_coding
ENSG0000(	CXXC4-AS1	507.6403	0.023433	0.819732	0.95154	lncRNA
ENSG0000(	DND1	48.42237	-0.04911	0.819711	0.95154	protein_coding
ENSG0000(	SNORD3B-1	133.0434	-0.07267	0.819701	0.95154	snoRNA
ENSG0000(	RPS6KA3	4131.845	-0.01414	0.819855	0.951644	protein_coding
ENSG0000(	ANGEL2	1497.967	-0.01354	0.819913	0.951671	protein_coding
ENSG0000(	GPR75	173.4636	-0.03475	0.819984	0.951713	protein_coding
ENSG0000(	CD3G	532.6675	-0.0341	0.8201	0.951808	protein_coding
ENSG0000(	ENSG0000(	464.3827	-0.01871	0.82017	0.951821	TEC
ENSG0000(	ZNF225	618.8893	0.016897	0.820181	0.951821	protein_coding
ENSG0000(	HSDL1	397.3678	0.027499	0.820295	0.951834	protein_coding
ENSG0000(	TMPO	2046.302	0.021222	0.820296	0.951834	protein_coding
ENSG0000(	WDR75	808.5821	0.023097	0.820257	0.951834	protein_coding
ENSG0000(	ENSG0000(	90.0761	-0.03568	0.820403	0.951887	lncRNA
ENSG0000(	ENSG0000(	104.6288	0.02856	0.820411	0.951887	lncRNA
ENSG0000(	CLCA4-AS1	147.8331	0.036339	0.820472	0.951918	lncRNA
ENSG0000(	AQP11	57.96289	0.070457	0.820747	0.951943	protein_coding
ENSG0000(	ATE1	3706.743	0.011963	0.820719	0.951943	protein_coding
ENSG0000(	CCNL2P1	200.1163	-0.03835	0.820711	0.951943	processed_pseudogene
ENSG0000(	EIF3A	3750.853	-0.01618	0.82077	0.951943	protein_coding
ENSG0000(	ENSG0000(	951.5357	-0.02044	0.820751	0.951943	lncRNA
ENSG0000(	GHDC	255.0177	-0.02657	0.820656	0.951943	protein_coding
ENSG0000(	HNRNPU	17947.25	-0.01148	0.820666	0.951943	protein_coding
ENSG0000(	TMA7	439.3541	0.026026	0.820759	0.951943	protein_coding
ENSG0000(	ARHGEF7	2653.991	0.012848	0.820814	0.951954	protein_coding
ENSG0000(	BUB1B	294.6839	0.044746	0.820964	0.952039	protein_coding
ENSG0000(	ENSG0000(	23.72278	0.058738	0.821117	0.952039	lncRNA
ENSG0000(	ENSG0000(	85.29951	0.342311	0.821129	0.952039	lncRNA
ENSG0000(	ENSG0000(	58.20648	-0.04035	0.820947	0.952039	lncRNA
ENSG0000(	OR1D2	422.9324	0.017291	0.82113	0.952039	protein_coding
ENSG0000(	RN7SL734F	20.38066	0.056405	0.821054	0.952039	misc_RNA
ENSG0000(	ZNF683	28.2909	0.054541	0.821089	0.952039	protein_coding
ENSG0000(	CCDC24	334.0365	0.027605	0.821348	0.952042	protein_coding
ENSG0000(	ENSG0000(	61.11504	0.03158	0.821312	0.952042	lncRNA
ENSG0000(	ENSG0000(	108.5685	-0.0357	0.821375	0.952042	lncRNA
ENSG0000(	ENSG0000(	72.272	0.035316	0.821251	0.952042	lncRNA
ENSG0000(	PFDN2	65.28469	-0.04171	0.821304	0.952042	protein_coding

ENSG0000(	SNORD15A	84.43364	0.034279	0.821274	0.952042	snoRNA
ENSG0000(	TNFSF4	135.9519	0.048133	0.821176	0.952042	protein_coding
ENSG0000(	ENSG0000(	199.2542	0.03358	0.821463	0.952064	lncRNA
ENSG0000(	ENSG0000(	32.07131	0.066864	0.821434	0.952064	lncRNA
ENSG0000(	GOLGA1	956.5914	0.026032	0.821514	0.952083	protein_coding
ENSG0000(	F5	352.7368	-0.05861	0.821562	0.952098	protein_coding
ENSG0000(	ENSG0000(	31.02478	0.18417	0.821664	0.952177	protein_coding
ENSG0000(	PWWP2A	1437.151	0.024086	0.821782	0.952233	protein_coding
ENSG0000(	SLC22A11	103.7848	-0.03658	0.821755	0.952233	protein_coding
ENSG0000(	LINC00331	133.7588	0.032448	0.821871	0.952251	lncRNA
ENSG0000(	LRFN3	244.7147	-0.0465	0.82191	0.952251	protein_coding
ENSG0000(	NEBL	790.5969	0.039549	0.821966	0.952251	protein_coding
ENSG0000(	OSTCP8	72.10234	-0.04017	0.822005	0.952251	processed_pseudogene
ENSG0000(	RNVU1-2A	41.85872	0.059252	0.821978	0.952251	snRNA
ENSG0000(	STX10	341.7037	0.024322	0.821937	0.952251	protein_coding
ENSG0000(	RAB10	1671.368	-0.02416	0.822068	0.952283	protein_coding
ENSG0000(	ENSG0000(	569.1243	0.017704	0.822153	0.952301	lncRNA
ENSG0000(	ENSG0000(	25.39809	-0.05843	0.822138	0.952301	lncRNA
ENSG0000(	LRRC28	1002.472	0.021497	0.822313	0.952447	protein_coding
ENSG0000(	ENSG0000(	82.1761	-0.02844	0.822356	0.952457	lncRNA
ENSG0000(	AAK1	7198.435	0.017987	0.822467	0.9525	protein_coding
ENSG0000(	BCO1	89.2182	0.073461	0.822635	0.9525	protein_coding
ENSG0000(	DNAJC14	770.3603	-0.01315	0.822507	0.9525	protein_coding
ENSG0000(	ENSG0000(	149.3655	-0.03184	0.822613	0.9525	processed_pseudogene
ENSG0000(	ENSG0000(	532.6601	-0.01736	0.822442	0.9525	lncRNA
ENSG0000(	ENSG0000(	37.19405	-0.04483	0.822633	0.9525	lncRNA
ENSG0000(	FZD2	118.1961	0.045849	0.822636	0.9525	protein_coding
ENSG0000(	ENSG0000(	102.2212	0.042196	0.822682	0.952514	TEC
ENSG0000(	RPL27A	2847.54	-0.02644	0.822736	0.952536	protein_coding
ENSG0000(	AP1S3	1161.515	0.016788	0.822926	0.952562	protein_coding
ENSG0000(	ENSG0000(	55.34382	-0.03781	0.82283	0.952562	lncRNA
ENSG0000(	ENSG0000(	198.1861	0.024848	0.822931	0.952562	lncRNA
ENSG0000(	PCDHGC5	57.74633	-0.04945	0.822907	0.952562	protein_coding
ENSG0000(	TAF5L	721.8315	0.018771	0.822812	0.952562	protein_coding
ENSG0000(	LINC02817	352.7789	0.018521	0.823036	0.952643	lncRNA
ENSG0000(	C3orf70	691.9186	-0.02532	0.823224	0.952652	protein_coding
ENSG0000(	ENSG0000(	81.74749	0.028313	0.823224	0.952652	lncRNA
ENSG0000(	ENSG0000(	46.53943	-0.0437	0.823158	0.952652	lncRNA
ENSG0000(	LINC01963	257.8422	-0.03607	0.823217	0.952652	lncRNA
ENSG0000(	RALGAPB	4060.391	-0.01201	0.823185	0.952652	protein_coding
ENSG0000(	UBR2	3821.053	-0.01575	0.823252	0.952652	protein_coding
ENSG0000(	ATP6V0CP4	49.53725	0.04946	0.823317	0.952688	processed_pseudogene
ENSG0000(	ENSG0000(	493.9535	0.015803	0.823361	0.952699	lncRNA
ENSG0000(	ENSG0000(	100.295	-0.03642	0.823447	0.952701	lncRNA
ENSG0000(	ENSG0000(	42.76667	0.063113	0.823421	0.952701	TEC
ENSG0000(	YTHDF3	2167.236	-0.01872	0.823467	0.952701	protein_coding
ENSG0000(	ENSG0000(	76.93898	-0.0349	0.823666	0.952891	lncRNA

ENSG0000(	SFXN2	1281.206	0.018513	0.823721	0.952914	protein_coding
ENSG0000(	FAM27C	1202.377	-0.02279	0.823797	0.952963	lncRNA
ENSG0000(	CNIH2	21.40294	0.074728	0.823952	0.952988	protein_coding
ENSG0000(	ENSG0000(	96.38032	-0.13793	0.824062	0.952988	protein_coding
ENSG0000(	ENSG0000(	297.9581	0.01682	0.823881	0.952988	TEC
ENSG0000(	ENSG0000(	99.83389	-0.03418	0.824046	0.952988	lncRNA
ENSG0000(	ENSG0000(	116.7954	-0.02634	0.823912	0.952988	lncRNA
ENSG0000(	GATAD1	2609.069	0.010775	0.824036	0.952988	protein_coding
ENSG0000(	SEPHS2	786.7822	0.033836	0.823983	0.952988	protein_coding
ENSG0000(	CENATAC	1658.806	0.014898	0.824108	0.953002	protein_coding
ENSG0000(	ENSG0000(	48.82365	-0.0367	0.824224	0.953091	lncRNA
ENSG0000(	ENSG0000(	112.4918	0.02592	0.824255	0.953091	TEC
ENSG0000(	ASIC1	66.38148	-0.05536	0.824397	0.953091	protein_coding
ENSG0000(	ENSG0000(	306.8553	-0.01893	0.824416	0.953091	lncRNA
ENSG0000(	ENSG0000(	61.86644	0.04015	0.824428	0.953091	lncRNA
ENSG0000(	ENSG0000(	305.5733	0.015166	0.824351	0.953091	lncRNA
ENSG0000(	SVIL	3946.357	-0.02351	0.82432	0.953091	protein_coding
ENSG0000(	P4HA3-AS1	144.4793	0.023274	0.824482	0.953113	lncRNA
ENSG0000(	CCNYL6	47.6562	0.040444	0.824685	0.953188	transcribed_processed_pseudoge
ENSG0000(	ENSG0000(	598.4899	-0.01256	0.824655	0.953188	lncRNA
ENSG0000(	ENSG0000(	59.60654	-0.04315	0.824684	0.953188	lncRNA
ENSG0000(	MAPK8IP2	148.7353	-0.02661	0.824631	0.953188	protein_coding
ENSG0000(	LEP	40.09231	-0.034	0.824731	0.953201	protein_coding
ENSG0000(	SNORA72	127.3213	-0.04045	0.824765	0.953201	snoRNA
ENSG0000(	ENSG0000(	110.8181	0.041205	0.824951	0.953363	lncRNA
ENSG0000(	ENSG0000(	25.1473	-0.06697	0.824995	0.953363	lncRNA
ENSG0000(	ENSG0000(	45.33298	-0.03616	0.825044	0.953363	lncRNA
ENSG0000(	LINC02249	204.772	0.02133	0.825019	0.953363	lncRNA
ENSG0000(	ENSG0000(	41.67915	-0.03835	0.825162	0.953459	lncRNA
ENSG0000(	CDHR2	2345.728	-0.06336	0.825424	0.953461	protein_coding
ENSG0000(	CEP152	1361.924	0.018985	0.825395	0.953461	protein_coding
ENSG0000(	ENSG0000(	19.4746	0.067254	0.825498	0.953461	lncRNA
ENSG0000(	ENSG0000(	149.7289	-0.81337	0.825241	0.953461	lncRNA
ENSG0000(	ENSG0000(	115.2874	-0.02636	0.825272	0.953461	lncRNA
ENSG0000(	GMDS	1995.295	-0.03002	0.82551	0.953461	protein_coding
ENSG0000(	MORN4	278.5049	-0.01782	0.82522	0.953461	protein_coding
ENSG0000(	RNU6-171F	57.49258	-0.05088	0.825419	0.953461	snRNA
ENSG0000(	TBL3	357.7127	0.028667	0.825482	0.953461	protein_coding
ENSG0000(	ZNF416	112.1541	0.030387	0.825438	0.953461	protein_coding
ENSG0000(	ADAMTS3	59.71243	-0.07193	0.825701	0.953498	protein_coding
ENSG0000(	ENSG0000(	26.83189	-0.05667	0.825667	0.953498	lncRNA
ENSG0000(	LINC00200	152.108	-0.03413	0.825666	0.953498	lncRNA
ENSG0000(	MAIP1	90.31367	0.036331	0.825701	0.953498	protein_coding
ENSG0000(	TSPOAP1	808.1773	0.031786	0.825715	0.953498	protein_coding
ENSG0000(	ENSG0000(	28.41404	-0.07194	0.825863	0.953628	lncRNA
ENSG0000(	BABAM2	526.6709	0.020688	0.826391	0.953757	protein_coding
ENSG0000(	ENSG0000(	748.3795	0.020705	0.826227	0.953757	lncRNA

ENSG0000(	ENSG0000(	298.8467	0.031845	0.826365	0.953757	lncRNA
ENSG0000(	ENSG0000(	58.99498	0.044352	0.826119	0.953757	lncRNA
ENSG0000(	ENSG0000(	114.4453	-0.02988	0.826205	0.953757	lncRNA
ENSG0000(	FOS	3009.866	-0.08383	0.826099	0.953757	protein_coding
ENSG0000(	GTF2H2B	179.0388	-0.07294	0.826131	0.953757	transcribed_unprocessed_pseudo
ENSG0000(	MIR548AJ2	25.77483	-0.05197	0.826184	0.953757	miRNA
ENSG0000(	MRPL44	273.0398	-0.0228	0.826117	0.953757	protein_coding
ENSG0000(	VRK1	16437.35	-0.01129	0.826276	0.953757	protein_coding
ENSG0000(	ZNF579	71.64751	-0.04755	0.826351	0.953757	protein_coding
ENSG0000(	ZNF655	5602.473	0.01159	0.826383	0.953757	protein_coding
ENSG0000(	ISY1	506.0183	-0.02197	0.826443	0.953778	protein_coding
ENSG0000(	CDC42BPB	3057.745	0.020763	0.826589	0.953814	protein_coding
ENSG0000(	ENSG0000(	219.6025	-0.032	0.826875	0.953814	lncRNA
ENSG0000(	ENSG0000(	68.68921	-0.03555	0.826763	0.953814	lncRNA
ENSG0000(	ENSG0000(	194.7527	-0.0223	0.826826	0.953814	lncRNA
ENSG0000(	MOCS1	578.7295	0.048323	0.826713	0.953814	protein_coding
ENSG0000(	PROX1	1154.138	0.048186	0.826616	0.953814	protein_coding
ENSG0000(	RGS3	1558.9	0.026404	0.826903	0.953814	protein_coding
ENSG0000(	RN7SL670F	364.2235	0.016888	0.826925	0.953814	misc_RNA
ENSG0000(	RNASE4	231.9704	0.055797	0.826674	0.953814	protein_coding
ENSG0000(	SLC25A6P3	25.87367	-0.0554	0.826867	0.953814	processed_pseudogene
ENSG0000(	TAF12	2351.626	-0.01514	0.826727	0.953814	protein_coding
ENSG0000(	VDAC1P8	198.1809	0.020343	0.826753	0.953814	transcribed_processed_pseudoge
ENSG0000(	ZNF800	1301.857	-0.0147	0.826784	0.953814	protein_coding
ENSG0000(	ENSG0000(	136.7932	0.023967	0.827041	0.953867	lncRNA
ENSG0000(	ENSG0000(	121.8662	-0.04539	0.827017	0.953867	lncRNA
ENSG0000(	ENSG0000(	131.189	0.026122	0.827188	0.953996	lncRNA
ENSG0000(	ENSG0000(	131.5083	-0.02183	0.827239	0.954016	lncRNA
ENSG0000(	CD70	24.42647	-0.06858	0.827348	0.954021	protein_coding
ENSG0000(	SP100	10862.97	-0.03392	0.827346	0.954021	protein_coding
ENSG0000(	TTF2	1880.807	0.023092	0.827338	0.954021	protein_coding
ENSG0000(	ENSG0000(	35.53981	0.044974	0.827425	0.95407	lncRNA
ENSG0000(	DICER1	4544.811	-0.01162	0.82758	0.95417	protein_coding
ENSG0000(	HOXD9	139.2993	-0.04594	0.827581	0.95417	protein_coding
ENSG0000(	AGMO	135.9313	-0.04966	0.828769	0.954221	protein_coding
ENSG0000(	ALDH8A1	178.1338	-0.02376	0.828359	0.954221	protein_coding
ENSG0000(	ARPIN-AP3	95.15735	0.418024	0.82877	0.954221	protein_coding
ENSG0000(	COPS8	550.6805	-0.02023	0.827942	0.954221	protein_coding
ENSG0000(	ENSG0000(	66.13714	-0.03864	0.828199	0.954221	lncRNA
ENSG0000(	ENSG0000(	89.40527	0.043594	0.828186	0.954221	lncRNA
ENSG0000(	ENSG0000(	41.59877	0.035532	0.828709	0.954221	lncRNA
ENSG0000(	ENSG0000(	491.766	0.012925	0.827815	0.954221	lncRNA
ENSG0000(	ENSG0000(	1000.702	0.011436	0.828238	0.954221	transcribed_processed_pseudoge
ENSG0000(	ENSG0000(	27.12099	0.051406	0.828415	0.954221	lncRNA
ENSG0000(	ENSG0000(	92.32642	-0.03011	0.827892	0.954221	lncRNA
ENSG0000(	ENSG0000(	110.1821	0.029622	0.828459	0.954221	lncRNA
ENSG0000(	ENSG0000(	79.90855	0.069808	0.828194	0.954221	protein_coding

ENSG0000(	FOXH1	23.55836	-0.07828	0.828185	0.954221	protein_coding
ENSG0000(	LINC01513	291.0218	0.021623	0.828621	0.954221	lncRNA
ENSG0000(	MBNL1	7828.497	0.015274	0.827878	0.954221	protein_coding
ENSG0000(	MCM7	936.1565	-0.01614	0.828009	0.954221	protein_coding
ENSG0000(	MEOX2	26.17981	0.100462	0.827768	0.954221	protein_coding
ENSG0000(	MIF4GD-DT	108.7712	0.028882	0.828687	0.954221	lncRNA
ENSG0000(	MIR99AHG	2069.06	-0.01778	0.828094	0.954221	lncRNA
ENSG0000(	N4BP2L2	14357.25	0.015349	0.828715	0.954221	protein_coding
ENSG0000(	PMS2P2	26.07792	0.179024	0.827672	0.954221	translated_unprocessed_pseudog
ENSG0000(	POLR2D	1418.825	0.013741	0.828351	0.954221	protein_coding
ENSG0000(	RFX2	592.8497	-0.03243	0.828587	0.954221	protein_coding
ENSG0000(	RN7SKP228	68.39528	0.058099	0.82862	0.954221	misc_RNA
ENSG0000(	RPL34-DT	233.3138	0.024775	0.828568	0.954221	lncRNA
ENSG0000(	SLC30A5	992.1165	0.015961	0.827976	0.954221	protein_coding
ENSG0000(	SLC38A9	1133.115	0.015288	0.828519	0.954221	protein_coding
ENSG0000(	SMARCAL1	513.5775	-0.01696	0.827778	0.954221	protein_coding
ENSG0000(	SPATA6L	1325.843	0.021997	0.82839	0.954221	protein_coding
ENSG0000(	TARBP2	271.3026	0.023161	0.828732	0.954221	protein_coding
ENSG0000(	TAS2R64P	416.5303	0.037622	0.828463	0.954221	transcribed_unprocessed_pseudo
ENSG0000(	ZFP91	2167.42	-0.0166	0.828594	0.954221	protein_coding
ENSG0000(	SLC10A5P1	29.86186	-0.09342	0.828843	0.954227	unprocessed_pseudogene
ENSG0000(	SLC7A9	204.3437	-0.04013	0.828844	0.954227	protein_coding
ENSG0000(	ADCYAP1R1	99.69299	0.067268	0.829125	0.954235	protein_coding
ENSG0000(	ASIC3	27.07218	-0.06698	0.829116	0.954235	protein_coding
ENSG0000(	ENSG0000(	69.07545	-0.03919	0.829087	0.954235	lncRNA
ENSG0000(	LINC01299	215.9736	0.021188	0.829022	0.954235	lncRNA
ENSG0000(	MIR23AHG	5889.243	-0.04027	0.829069	0.954235	lncRNA
ENSG0000(	MYC	528.0881	0.031982	0.829128	0.954235	protein_coding
ENSG0000(	PACRG-AS1	154.5994	0.029265	0.828893	0.954235	lncRNA
ENSG0000(	RAB2A	993.3158	0.017297	0.829076	0.954235	protein_coding
ENSG0000(	ENSG0000(	313.4142	-0.02328	0.829253	0.954263	lncRNA
ENSG0000(	SLC5A5	206.5306	-0.02602	0.829257	0.954263	protein_coding
ENSG0000(	UBE2D2	1200.43	0.015591	0.829234	0.954263	protein_coding
ENSG0000(	DIRAS2	132.6294	-0.02915	0.8295	0.954422	protein_coding
ENSG0000(	ENSG0000(	42.76939	-0.04769	0.829489	0.954422	unprocessed_pseudogene
ENSG0000(	NHS	552.0037	0.035828	0.82947	0.954422	protein_coding
ENSG0000(	MRGBP	167.1352	-0.02977	0.829663	0.954571	protein_coding
ENSG0000(	NPAP1P2	34.8298	-0.06056	0.829968	0.954882	processed_pseudogene
ENSG0000(	NR2C2	3168.647	-0.01413	0.830013	0.954887	protein_coding
ENSG0000(	TRIM28	1112.825	-0.02157	0.830042	0.954887	protein_coding
ENSG0000(	COMMD10	341.2179	0.02052	0.830146	0.954967	protein_coding
ENSG0000(	ANKRD53	19.67048	-0.08408	0.830761	0.954992	protein_coding
ENSG0000(	ANP32B	565.1859	0.023028	0.830767	0.954992	protein_coding
ENSG0000(	C14orf178	63.36528	-0.03132	0.830833	0.954992	lncRNA
ENSG0000(	ENSG0000(	122.6873	0.036438	0.830861	0.954992	lncRNA
ENSG0000(	ENSG0000(	25.16413	0.044534	0.830537	0.954992	lncRNA
ENSG0000(	ENSG0000(	32.59771	-0.06489	0.830641	0.954992	lncRNA

ENSG0000(	ENSG0000(	63.83591	0.030867	0.830653	0.954992	lncRNA
ENSG0000(	ENSG0000(	62.85755	-0.03871	0.830989	0.954992	lncRNA
ENSG0000(	ENSG0000(	122.4335	0.024553	0.83065	0.954992	TEC
ENSG0000(	ENSG0000(	21.24427	-0.06866	0.830546	0.954992	lncRNA
ENSG0000(	ENSG0000(	268.199	-0.03424	0.830897	0.954992	lncRNA
ENSG0000(	HAND2-AS1	989.1906	0.016133	0.830483	0.954992	lncRNA
ENSG0000(	KCNMB2-A	1135.623	-0.0176	0.830724	0.954992	lncRNA
ENSG0000(	LINC02028	228.6706	-0.0249	0.830905	0.954992	lncRNA
ENSG0000(	LINC02765	20.63528	-0.07306	0.83077	0.954992	lncRNA
ENSG0000(	LSMEM1	318.7236	0.044804	0.830284	0.954992	protein_coding
ENSG0000(	N4BP1	1943.358	-0.0256	0.830506	0.954992	protein_coding
ENSG0000(	PROB1	64.94914	-0.03376	0.831002	0.954992	protein_coding
ENSG0000(	PRPSAP1	1386.273	-0.01245	0.830495	0.954992	protein_coding
ENSG0000(	PTENP1	26.762	-0.0657	0.830442	0.954992	transcribed_processed_pseudoge
ENSG0000(	RN7SL49P	35.49006	0.05166	0.830771	0.954992	misc_RNA
ENSG0000(	SBF1	3130.106	-0.01647	0.830984	0.954992	protein_coding
ENSG0000(	SMURF2P1	105.8848	0.027649	0.830912	0.954992	transcribed_unprocessed_pseudo
ENSG0000(	TTC23L	23.60917	0.099488	0.830869	0.954992	protein_coding
ENSG0000(	ENSG0000(	27.89424	-0.04832	0.831089	0.955053	snoRNA
ENSG0000(	TTC9-DT	26.2643	-0.09225	0.831129	0.955058	lncRNA
ENSG0000(	CORIN	450.632	-0.01754	0.831253	0.955134	protein_coding
ENSG0000(	RPS15	798.043	-0.02974	0.831263	0.955134	protein_coding
ENSG0000(	CEP57	1247.886	-0.01478	0.831484	0.955245	protein_coding
ENSG0000(	CLN8	2226.43	0.017022	0.831608	0.955245	protein_coding
ENSG0000(	ENSG0000(	84.64491	-0.03596	0.831638	0.955245	lncRNA
ENSG0000(	HERC2P9	3610.192	-0.01289	0.831471	0.955245	transcribed_unprocessed_pseudo
ENSG0000(	MIS18BP1	1662.145	-0.01768	0.831638	0.955245	protein_coding
ENSG0000(	SNAPIN	171.3238	0.022693	0.831506	0.955245	protein_coding
ENSG0000(	STARD13-IT	68.91816	-0.04807	0.831632	0.955245	lncRNA
ENSG0000(	ZNF826P	494.8258	0.015522	0.831561	0.955245	transcribed_unprocessed_pseudo
ENSG0000(	MBD3	956.6958	0.013841	0.831719	0.955298	protein_coding
ENSG0000(	ENSG0000(	189.4896	-0.06666	0.83178	0.955328	lncRNA
ENSG0000(	LINC00920	29.44922	-0.05722	0.832101	0.955657	lncRNA
ENSG0000(	RN7SL478F	22.63414	-0.05059	0.832184	0.955673	misc_RNA
ENSG0000(	ZNF444	1032.55	-0.02368	0.83218	0.955673	protein_coding
ENSG0000(	CAPRIN2	1317.782	0.0175	0.832301	0.955767	protein_coding
ENSG0000(	ENSG0000(	34.34362	0.040562	0.832338	0.955769	lncRNA
ENSG0000(	DDX43	53.08108	-0.05432	0.832473	0.955776	protein_coding
ENSG0000(	ENSG0000(	341.7332	0.014296	0.832517	0.955776	lncRNA
ENSG0000(	PRUNE2	3813.503	0.041275	0.832512	0.955776	protein_coding
ENSG0000(	TEX21P	449.5818	-0.01605	0.832505	0.955776	transcribed_unitary_pseudogene
ENSG0000(	ZNF385B	48.14471	0.215862	0.832449	0.955776	protein_coding
ENSG0000(	ALG3	230.5295	-0.03007	0.83281	0.956073	protein_coding
ENSG0000(	WNT4	219.4726	-0.06234	0.832867	0.956098	protein_coding
ENSG0000(	DDX27	976.0249	-0.01819	0.83294	0.956102	protein_coding
ENSG0000(	GDPD2	116.2384	0.048215	0.832963	0.956102	protein_coding
ENSG0000(	GON4L	3235.13	0.011186	0.833008	0.956102	protein_coding

ENSG0000(	MT1M	56.12714	-0.08617	0.833079	0.956102	protein_coding
ENSG0000(	RIPK3	634.446	0.030064	0.833044	0.956102	protein_coding
ENSG0000(	SGPP1	351.2712	0.025787	0.833054	0.956102	protein_coding
ENSG0000(	RN7SL20P	20.64375	0.051885	0.833126	0.956115	misc_RNA
ENSG0000(	AIRE	165.117	-0.03232	0.833266	0.956132	protein_coding
ENSG0000(	ENSG0000(	101.5396	-0.02947	0.833266	0.956132	lncRNA
ENSG0000(	GOLGA8H	66.61464	0.056262	0.833188	0.956132	protein_coding
ENSG0000(	UNC45A	670.0503	-0.01301	0.83328	0.956132	protein_coding
ENSG0000(	ADGRF5P1	105.4402	0.02849	0.833378	0.956206	transcribed_processed_pseudoge
ENSG0000(	ACBD5	1919.782	0.011463	0.834346	0.956333	protein_coding
ENSG0000(	ACOT7	247.6362	-0.03394	0.834226	0.956333	protein_coding
ENSG0000(	ANKRD13A	2522.382	-0.01154	0.834239	0.956333	protein_coding
ENSG0000(	BBS12	118.7137	-0.03706	0.834484	0.956333	protein_coding
ENSG0000(	C16orf95	145.048	0.021931	0.834329	0.956333	protein_coding
ENSG0000(	CCDC78	24.14946	-0.09487	0.833994	0.956333	protein_coding
ENSG0000(	CLSPN	1176.919	-0.01567	0.834082	0.956333	protein_coding
ENSG0000(	DDOST	849.3212	-0.02183	0.833791	0.956333	protein_coding
ENSG0000(	EFS	147.1583	-0.03039	0.833768	0.956333	protein_coding
ENSG0000(	ENSG0000(	258.2679	0.019206	0.834177	0.956333	lncRNA
ENSG0000(	ENSG0000(	104.5503	0.02706	0.833527	0.956333	lncRNA
ENSG0000(	ENSG0000(	22.41957	-0.05735	0.834416	0.956333	processed_pseudogene
ENSG0000(	ENSG0000(	102.9213	0.021935	0.833796	0.956333	lncRNA
ENSG0000(	ENSG0000(	100.5151	0.032208	0.834193	0.956333	lncRNA
ENSG0000(	ENSG0000(	113.7662	-0.02479	0.834495	0.956333	lncRNA
ENSG0000(	ENSG0000(	71.67383	0.025457	0.8337	0.956333	lncRNA
ENSG0000(	ENSG0000(	174.1659	-0.0427	0.833625	0.956333	lncRNA
ENSG0000(	ENSG0000(	62.90519	0.034819	0.83438	0.956333	lncRNA
ENSG0000(	HSCB	320.6606	0.016645	0.834182	0.956333	protein_coding
ENSG0000(	JMJD7-PLA	1139.475	-0.01519	0.8339	0.956333	protein_coding
ENSG0000(	LINC02240	77.3179	-0.03215	0.833607	0.956333	lncRNA
ENSG0000(	PPIAP41	150.5694	0.021943	0.834395	0.956333	processed_pseudogene
ENSG0000(	PRMT8	59.63924	0.040138	0.834008	0.956333	protein_coding
ENSG0000(	SERP2	31.00337	0.048747	0.834567	0.956333	protein_coding
ENSG0000(	SLC9A1	1482.164	-0.03361	0.834528	0.956333	protein_coding
ENSG0000(	SNW1	437.5327	-0.02192	0.834562	0.956333	protein_coding
ENSG0000(	TMEM128	181.1265	0.026439	0.834111	0.956333	protein_coding
ENSG0000(	UTS2B	186.6322	-0.02677	0.833934	0.956333	protein_coding
ENSG0000(	YWHAH	825.9847	-0.02404	0.833938	0.956333	protein_coding
ENSG0000(	ZBTB40	2173.725	0.014534	0.834492	0.956333	protein_coding
ENSG0000(	ZNF546	829.0943	0.015958	0.833641	0.956333	protein_coding
ENSG0000(	AGPAT5	671.6276	0.029139	0.834708	0.956375	protein_coding
ENSG0000(	AKR1C2	203.2527	0.046497	0.834689	0.956375	protein_coding
ENSG0000(	IGLV5-52	27.83834	0.084112	0.834682	0.956375	IG_V_gene
ENSG0000(	ENSG0000(	264.0624	0.021074	0.834828	0.95642	lncRNA
ENSG0000(	ENSG0000(	156.8673	0.028066	0.834881	0.95642	lncRNA
ENSG0000(	LINC01285	1195.077	-0.01149	0.834886	0.95642	lncRNA
ENSG0000(	RNU11	110.1312	0.419302	0.834818	0.95642	snRNA

ENSG0000(	ENSG0000(	48.37444	-0.04607	0.834998	0.956508	lncRNA
ENSG0000(	CXXC1	605.6623	0.022359	0.835107	0.956514	protein_coding
ENSG0000(	ENSG0000(	33.03299	0.064259	0.835078	0.956514	lncRNA
ENSG0000(	PHACTR4	2561.671	0.013187	0.835106	0.956514	protein_coding
ENSG0000(	CTTNBP2NI	3379.553	0.028007	0.835281	0.956534	protein_coding
ENSG0000(	ENSG0000(	21.57121	-0.11984	0.835299	0.956534	lncRNA
ENSG0000(	ENSG0000(	97.62117	0.03191	0.835199	0.956534	lncRNA
ENSG0000(	ERICH3	23.0679	-0.10813	0.835245	0.956534	protein_coding
ENSG0000(	SPG11	7007.711	-0.0111	0.835194	0.956534	protein_coding
ENSG0000(	ENSG0000(	59.77465	0.032955	0.835464	0.956599	lncRNA
ENSG0000(	ENSG0000(	126.7646	0.033206	0.835542	0.956599	unprocessed_pseudogene
ENSG0000(	ENSG0000(	476.2532	0.024096	0.835599	0.956599	lncRNA
[truncated: 716,685 more chars]
